# Supplementary material for: The maintenance of oocytes in the mammalian ovary involves extreme protein longevity
Source: Nat Cell Biol. 2024 Jun 20;26(7):1124–38. doi: 10.1038/s41556-024-01442-7 (PMC11252011; doi:10.1038/s41556-024-01442-7)
Supplement: Supplementary file 1 — Supplementary information. [file 41556_2024_1442_MOESM1_ESM.pdf]

nature cell biology

Supplementary information

https://doi.org/10.1038/s41556-024-01442-7

The maintenance of oocytes in the mammalian ovary involves extreme protein longevity

In the format provided by the authors and unedited

Supplementary Note 1

Oocyte data analysis

Pulse-chase mass spectrometry data analysis

All data processing was performed in *R*[1].

Fractions of $^{13}C_6$-Lys labeled proteins, *F*, were computed for each technical replicate and each biological replicate as $F = 100 \cdot H/(H + L)$, where H is the DDA intensity of $^{13}C_6$-Lys labeled proteins and *L* is the DDA intensity of $^{12}C_6$-Lys labeled proteins. Per biological replicate, medians over technical replicates were only computed, if *H* was detected in at least 2 out of 4 technical replicates, otherwise this data point was omitted (set *n.a.*). Finally, means and standard deviations of *F* over biological replicates were computed, if *F* was detected in at least one of the two biological replicates.

Enrichment analysis of very long-lived oocyte proteins

A subset of $^{13}C_6$-Lys-positive proteins was used to perform over-representation analysis with "fora" algorithm[2]. Multiple testing correction was done using Benjamini-Hochberg method[3] and a threshold of 0.01 adjusted p-value was used for defining significantly enriched sets. A background set of all proteins detected in ovary DIA and all genes expressed in human ovary in Human Protein Atlas (https://www.proteinatlas.org/)[4] was combined using mouse gene symbols.

Ovary data analysis

Overview of modeling strategy

Protein turnover can be estimated from experimental pulse-chase mass spectrometry experiments. Commonly, an exponential-decay protein turnover model is calibrated to experimental data, resulting in estimation of protein turnover rates and protein half-lives. However, several factors impinge upon the applicability of the simple exponential decay model:

(i) In living animals, in contrast to cell culture experiments, the pulse isotope is not immediately removed from the circulating free amino acid pool upon switch from pulse to chase, which could lead to protein synthesis using amino acids containing the pulse label, rather than the chase label.

(ii) In developing organs, the concentration of individual proteins can vary strongly over time and, hence, impacts the observed pulse-chase dynamics.

(iii) Growth of the ovary results in strong dilution of pulse isotopes through the chase isotopes independent to protein turnover dynamics.

We developed a protein-centric exponential decay modeling framework (Fig. 2e,f, Extended Data Fig. 3a-b), which incorporates aspects (i-iii) in order to estimate protein turnover rates. Latter were in addition validated with a peptide-centric turnover modeling approach (Extended Data Fig. 3b-e). Resulting protein turnover rates were subject to further analysis to identify slow

turning over proteins that are relevant during ovarian development. The following sections provide details of the modeling strategy. All data processing, modeling and subsequent model downstream analysis was performed in *R*[1].

Data sets to model protein turnover

Two pulse-chase mass spectrometry data sets were used to model protein turnover during ovarian development (Fig. 2e,f and Extended Data Fig. 3a,b). For generation of the first data set, we fed pregnant mice $^{13}C_6$-Lys chow until they gave birth (pulse), and the pups were subsequently raised on $^{12}C_6$-Lys (chase). In the following we refer to this data set as short-pulse data. For generation of the second data set, we fed another cohort of pregnant mice $^{13}C_6$-Lys chow until the progeny were weaned (pulse). Progeny were then fed $^{12}C_6$-Lys chow (chase). We refer to this data set as long-pulse data. Firstly, we computed ratios of heavy ($^{13}C_6$-labeled) over light labeled ($^{12}C_6$-labeled) proteins (see below) for each biological replicate and time point. If ratios in less than two biological replicates of a given time point were detected, this time point was not considered in the respective data set. Furthermore, only proteins that had ratios detected in at least 3 time points in either the short- or long-pulse data were considered for modeling. Finally, only proteins also detected and quantified in the DIA mass spectrometry data set were considered. This resulted in 3,078 modeled proteins.

In addition to short- and long-pulse data, three other data sets were used in order to model protein turnover throughout ovarian development (Extended Data Fig. 3a):

(i) Data-independent acquisition (DIA) MS data of the ovary over mouse age to determine individual protein abundance changes throughout ovarian development. DIA signal from ovary measurements was aggregated as a mean across biological replicates per day per protein.

(ii) Morphology data to estimate volume changes of the ovary over mouse age. Volume changes were approximated via volume computation of an ellipsoid, derived from length and width measurements of the microscopy data.

(iii) Total protein amount measurements of the ovary over mouse age by BCA to estimate dilution factors of heavy labeled proteins over time.

The two data sets (ii) and (iii) show good agreement in terms of volume changes and total protein abundance changes over time (Extended Data Fig. 3f), indicating a constant total protein concentration during ovarian development.

2Lys-peptide-centric turnover model to determine dynamics of free $^{13}C_6$-Lys labeled amino acids

In the short-pulse experiment, all proteins are exclusively $^{13}C_6$-Lys labeled (heavy labeled) at birth of the mouse. Upon onset of the chase, heavy labeled proteins (P_H) are degraded with a turnover rate k, while newly synthesized proteins are $^{12}C_6$-Lys labeled (light labeled, P_L). While this holds true for most cell culture-based pulse-chase experiments, but not for *in vivo* animals-based pulse-chase experiments, because the assumption that upon initiation of the chase, all heavy labeled amino acids are removed from the animal and that no heavy labeled amino acids are reincorporated into newly synthesized proteins is violated. Recycling of heavy labeled

amino acids needs to be considered, depending on the experimental design. Shenheng Guan *et al.* 2012[5] proposed a three-compartment protein turnover model that allows to derive protein turnover rates under consideration of recycling of heavy labeled amino acids. In their model, the first compartment describes the overall turnover of all proteins in the system that contribute to the free heavy labeled amino acid pool and, hence, describing the dynamics of free heavy labeled amino acid pool. The second compartment describes the external space of the studied organ/tissue, from which labeled free amino acids are taken up and secreted to. The third compartment describes the turnover of a single protein of interest, which does not contribute to the free heavy amino acid pool, but its dynamics are dependent on the free heavy amino acid pool in the first compartment. Therefore, model parameters related to the first and second compartment are global parameters, which do not differ across proteins of interest, while model parameters related to the third compartment are protein of interest specific and describe the actual turnover rates. While this approach has already been successfully applied to various experimental systems[6, 7], it relies on the assumption that the overall system is not growing so that steady-state approximations can be applied. Latter fact, however, prohibits the direct application of the two-compartment model to derive protein turnover rates during ovarian developments, since the ovary strongly increases in size over the time course of our experiments. The three-compartment model could be adapted to incorporate ovary growth, which however, would result in a system of ordinary differential equations that can only be solved numerically, thereby increasing drastically computational demands during parameter inference. Because the two-compartment model relies on global parameter estimation across (at least) the most abundant proteins and due to the high computational cost, this approach became impractical.

Instead, we developed an alternative modeling approach to derive the dynamics of the free heavy labeled amino acids to estimate accurate turnover rates, which is peptide-centric and exploits the observation of peptides with missed tryptic cleavages in our MS data set. Specifically, we analyzed the MS data allowing for the detection of peptides that carry two lysines (2Lys-peptides) with different labeling status: exclusively heavy labeled peptides ($^{13}C_6$-Lys-$^{13}C_6$-Lys peptides), exclusively light labeled peptides ($^{12}C_6$-Lys-$^{12}C_6$-Lys peptides) as well as 'mixed' labelled peptides ($^{13}C_6$-Lys-$^{12}C_6$-Lys and $^{12}C_6$-Lys-$^{13}C_6$-Lys peptides) (Extended Data Fig. 3b-e). MS1 intensities of peptides of a given labelling status were extracted over time. Mean intensities over all 2Lys-peptides were extracted for each labelling status (exclusively heavy, exclusively light and mixed labelled) and time point. Latter were normalized, so that the total intensity was set to 1. Normalized MS1 intensities were used to inform the 2Lys-peptide model illustrated in Extended Data Fig. 3b. The 2Lys-peptide model consists of the ovary compartment, which grows over time, and an external compartment, from which heavy labelled lysines are taken up via feeding (influx) as well as secreted (efflux). Inside the ovary, heavy and light labelled free lysines can be incorporated with rate k_{on} into 2Lys-peptides, which can get degraded with rate k thereby releasing heavy and light labelled lysines into the ovary compartment. We describe this model with a set of ordinary differential equations:

$$\frac{dHH}{dt} = kon * h * h - koff * HH$$

$$\frac{dLL}{dt} = kon * l * l - koff * LL$$

$$\frac{dHL}{dt} = -kon * h * l - koff * HL$$

$$\frac{dl}{dt} = -2 * l * l * kon + 2 * LL * koff - l * h * kon + HL * koff - l * efflux + influx$$

$$\frac{dh}{dt} = -2 * h * h * kon + 2 * HH * koff - l * h * kon + HL * koff - h * efflux$$

$$\frac{dT}{dt} = -T * koff + p * p * kon,$$

$$influx(t) = a/(1 + exp(-b * (t - c))) + d$$

$$p(t) = l(t)+h(t),$$

where *HH* and *LL* indicate exclusively heavy and light labelled 2Lys-peptides, respectively, *HL* indicates 'mixed' labelled 2Lys-peptides, *h* and *l* indicate heavy and light labelled free lysines, respectively, *T* corresponds to the total protein amount in the ovary. This system of ordinary differential equations was solved numerically using the 'ode' function with the method 'lsoda' from the R package deSolve[8].

<u>*Estimation of free lysine pool using Bayesian inference*</u>

The 2Lys-peptide model has a set of parameters $\theta = (k, k_{on}, efflux, a, b, c, d)$. Parameters were estimated applying a Bayesian approach as originally proposed by Bayes and Price *et al.* 1958[9]. Briefly, the posterior distribution $p(\theta|D)$ of the parameter vector θ is defined as

$$p(\theta|D) = \frac{p(D|\theta) \cdot p(\theta)}{p(D)},$$

where $p(\theta)$ is the prior distribution of the parameters θ and $p(D|\theta)$ is the likelihood of the data *D* given the parameters θ.The aim is to find a set of parameters θ that maximize the likelihood $p(D|\theta)$.

In this study the log-likelihood was defined as

$$\ln(p(D|\theta)) = \sum_t \ln(L_{1,t}) + \ln(L_{2,t}),$$

with

$$L_{1,t} = p(x_{s/l}(t)|\theta) \sim \mathcal{N}(\mu = x^*_{s/l}(t), \sigma = sd \cdot x^*_{s/l}(t)),$$

where $\mathcal{N}$ indicates the probability density of the normal distribution with mean μ and standard deviation σ, x indicates model outputs for *HH*, *HL* and *LL* for the chase ($^{12}C_6$) from birth and from weaning experiment data sets, respectively, and x^* indicates experimental data for *HH*, *HL* and *LL* for the chase ($^{12}C_6$) from birth and from weaning experiment data sets, respectively. $L_{2,t}$ was defined as:

$$L_{2,t} = p(v(t)|\theta) \sim \mathcal{N}(\mu = v^*(t), \sigma = sd \cdot v^*(t)),$$

where v is the model output describing the ovary growth ($T_t/T_{t=1}$ and v^* indicates the experimentally measured fold changes of total protein across time.

Inference was realized using the *BayesianTools R* package[10]. A truncated normal prior distribution $p(\theta) \sim N_t(location, scale, [0, Inf])$ with parameters displayed in Supplementary Table 12 was used to infer the model parameters. Differential-Evolution Markov Chain Monte Carlo (DE-MCMC) with *Z* past steps and Snooker update (*zs*) sampler implemented in *R* was applied[11]. Parameters were inferred using three start values, a Snooker update probability of 1e-03, a thinning parameter of 10 and a multiplicative error of 0.2. The scaling factor γ was kept at 2.38, setting it to one with a probability of 0.1. The posterior distribution was saved and diagnostic plots were obtained. Inference was run for 10^6 iterations. Convergence was manually inspected for all proteins.

Inference results and model fits to data are shown in Extended Data Fig. c-e. Sampling 500 particles from the posterior parameter distribution, followed by model simulation with each particle allowed us to obtain the dynamics of the percentage of heavy-labeled free lysines compared to all free lysines.

Protein-centric turnover model in ovarian development

In the short-pulse experiment, all proteins are exclusively $^{13}C_6$-Lys labeled (heavy labeled) at birth of the mouse. Upon onset of the chase, heavy labeled proteins (P_H) are degraded with a turnover rate *k*, while newly synthetized proteins are $^{12}C_6$-Lys labeled (light labeled, P_L). Hence, at any time point the total amount of an individual protein ($P_{tot}(t)$) is the sum of heavy and light labeled proteins, *i.e.*

$$P_L(t) = P_{tot}(t) - P_H(t).$$

The total amount of the individual protein $P_{tot}(t)$ can be determined from the DIA MS data set (see above - i) via

$$P_{tot}(t) = P_0 \cdot G(t) = P_H(t=0) \cdot G(t),$$

where P_0 is the initial protein amount and $G(t)$ denotes the individual protein abundance change relative to *t=0*, *i.e.,* relative to the birth of the mouse, and is defined as

$$G(t) = \frac{DIA(t)}{G(t=0)}.$$

Hence, we obtain a description for the light labeled proteins over time

$$P_L(t) = P_H(t=0) \cdot G(t) - P_H(t).$$

The turnover of the heavy labeled protein pool $P_H(t)$ is commonly modeled as exponential decay, resulting from the differential equation

$$\frac{dP_H}{dt} = -k \cdot P_H(t) \qquad \text{(\textbf{\textit{equ. 1}})}$$

However, *equation 1* assumes that upon initiation of the chase, all heavy labeled amino acids are removed from the ovary and that no heavy labeled amino acids are reincorporated into

newly synthesized proteins. To consider recycling of heavy labeled amino acids, we describe the turnover of heavy labeled proteins in the chase ($^{12}C_6$) from birth experiment, $P_{H_s}(t)$, as

$$\frac{dP_{H_s}}{dt} = -k \cdot P_{H_s}(t) + k_{syn} \cdot \frac{Lys_{H_s}(t)}{Lys_{H_s}(t)+Lys_{L_s}(t)}, \qquad \textbf{(\textit{equ. 2})}$$

where k_{syn} indicates the protein synthesis rate, $Lys_{H_s}(t)$ is the free heavy labeled amino acid pool and $Lys_{L_s}(t)$ is the free light labeled amino acid pool. Therefore, synthesis of heavy labeled proteins is proportional to the fraction of free heavy labeled lysines compared to all free lysines at time *t* (Extended Data Fig. 3c). Latter was determined based on the *2Lys-peptide-centric model* described above.

Numeric integration of *equation 2* allows to obtain $P_{H_s}(t)$, describing the abundance of heavy labeled proteins over time in the ovary. Our experimental pulse-chase MS data, however, described the concentration of heavy labeled protein over time (*i.e.,* abundance of heavy labeled proteins in 1 μg total ovary organ). Because the total volume of the ovary strongly increases during ovarian development, and newly synthesized proteins that contribute to ovary growth are light labeled, the concentration of heavy labeled proteins over time $[H_s](t)$ was described as:

$$[H_s](t) = \frac{P_{H_s}(t)}{V(t)}, \qquad \textbf{(\textit{equ. 3})}$$

where *V(t)* is the ovary volume at time *t*. The concentration of light labeled proteins was described as:

$$[L_s](t) = P_{H_s}(t=0) \cdot G(t) - P_{H_s}(t). \qquad \textbf{(\textit{equ. 4})}$$

In the long-pulse data set all proteins are exclusively heavy labeled until 3 weeks after birth (ΔT) of the mouse, and only then the chase starts. Therefore, equations 2-4 were adapted, resulting in:

$$\frac{dP_{H_l}}{dt} = -k \cdot P_{H_l}(t) + k_{syn}, \text{ for } t < \Delta T$$

$$\frac{dP_{H_l}}{dt} = -k \cdot P_{H_l}(t) + k_{syn} \cdot \frac{Lys_{H_l}(t)}{Lys_{H_l}(t)+Lys_{L_l}(t)}, \text{ for } t \geq \Delta T \qquad \textbf{(\textit{equ. 5})}$$

$$[H_l](t) = \frac{P_{H_l}(t)}{V(t)/V(\Delta T)} \qquad \textbf{(\textit{equ. 6})}$$

$$[L_l](t) = P_{H_l}(t=0) \cdot G(t)/G(\Delta T) - P_{H_l}(t) \qquad \textbf{(\textit{equ. 7})}$$

The fraction of heavy labeled free lysines, $\frac{Lys_{H_l}(t)}{Lys_{H_l}(t)+Lys_{L_l}(t)}$, was determined from the 2Lys-peptide-centric turnover model described above. A double sigmoidal function was fitted to the modeling results and subsequently used in *equation 5*.

To learn turnover rates from mass spectrometry measurements of $[H](t)$ and $[L](t)$ their ratio $R(t)$ is commonly used and was defined as

$$R_s(t) = \ln\left(\frac{[L_s](t)}{[H_s](t)} + 1\right), \qquad \textbf{(}\textit{\textbf{equ. 8}}\textbf{)}$$

for the chase ($^{12}C_6$) from birth experiment MS data and

$$R_l(t) = \ln\left(\frac{[L_l](t)}{[H_l](t)} + 1\right), \qquad \textbf{(}\textit{\textbf{equ. 9}}\textbf{)}$$

for the chase ($^{12}C_6$) from weaning experiment MS data.

Protein turnover estimation using Bayesian inference

The protein turnover model results in a description of $R(t)$ for short-pulse and long-pulse data, subsequently termed $R_s(t)$ (equation 8) and $R_l(t)$ (equation 9), respectively. Accordingly, $R_s^*(t)$ and $R_l^*(t)$ denote the experimentally determined ratios of heavy and light labeled proteins $R(t)$.The models has a set of parameters, θ, with

$\theta = (k, k_{syn}, V(t)/V(t=0), G(t) \cdot G(t=0))$. Parameters were estimated as described above applying a Bayesian approach.

The log-likelihood was defined as

$$\ln\big(p(D|\theta)\big) = \sum_t \ln\big(L_{1,t}\big) + \ln\big(L_{2,t}\big) + \ln\big(L_{3,t}\big),$$

with

$$L_{1,t} = p(R_s(t)|\theta) \sim \mathcal{N}(\mu = R_s^*(t), \sigma = sd \cdot R_s^*(t)),$$
$$L_{2,t} = p(R_l(t)|\theta) \sim \mathcal{N}(\mu = R_l^*(t), \sigma = sd \cdot R_l^*(t))$$

and

$$L_{3,t} = p(G(t) \cdot G(t=0)|\theta) \sim \mathcal{N}(\mu = DIA(t), \sigma = sd \cdot DIA(t)),$$

where $\mathcal{N}$ indicates the probability density of the normal distribution with mean μ and standard deviation σ.

Inference was realized using the *BayesianTools R* package[10]. A uniform prior $p(\theta) \sim \mathcal{U}([min, max])$ was used to infer the model parameters. Uniform prior ranges (min, max) are displayed in Supplementary Table 13. Differential-Evolution Markov Chain Monte Carlo (DE-MCMC) with *Z* past steps and Snooker update (*zs*) sampler implemented in *R* was applied[11]. Parameters were inferred using three start values, a Snooker update probability of 1e-03, a thinning parameter of 10 and a multiplicative error of 0.2. The scaling factor γ was kept at 2.38, setting it to one with a probability of 0.1. The posterior distribution for each protein was saved and diagnostic plots were obtained. Inference was run for 10^6 iterations. Convergence was manually inspected for all proteins.

Quality of protein model fit

The mean squared error (*MSE*) between the experimentally determined $R_s^*(t)$, $R_l^*(t)$ and $DIA(t)$ as well as the corresponding median of the model simulations $R_s(t)$, $R_l(t)$and $G(t)$ derived from the estimated posterior distributions (see below for Analysis of posterior parameter distributions) were computed as

$$MSE = \mu((x^* - x)^2),$$

where μ indicates the mean, *x* = $R_s(t)$, $R_l(t)$ and $G(t)$, respectively, and x^*=$R_s^*(t)$, $R_l^*(t)$ and $DIA(t)$, respectively. The time points and biological replicates of the pulse-chase data are derived from individual mice, and are subject to noise. To determine if a median model fit is sufficient to interpret the corresponding posterior parameter distributions, we only consider proteins with *MSE*<0.1 or *MSE*<$3 \cdot \tau$, where τ is the mean square estimate between experimental data points of biological replicates. Furthermore, proteins with τ>0.5 were not further considered. This resulted in 2691 proteins with inferred parameters out of 3078 modeled proteins.

Analysis of posterior parameter distributions

Applying the Bayesian framework, we obtained posterior parameter distributions for all modeled proteins. Marginal posterior parameter distributions are visualized as density plots (Supplementary Data 2) upon burn-in (30%) removal and sampling 1000 parameter combinations from the corresponding posterior. Protein $H_{1/2}$ values in days were defined as:

$$H_{1/2} = \frac{\ln(2)}{k/7}. \qquad \textbf{(\textit{equ. 10})}$$

Median and 5%- and 95%-quantiles can be used to summarize marginal posterior distributions. No correlation between parameters was detected (data not shown). Low quantile ranges indicate good inference of the corresponding parameter and, hence, less uncertainty about that parameter.

Posterior parameter samples were used to compute the turnover rate $k(t)$ and $H_{\frac{1}{2}}(t)$ over time. Medians, 5%- and 95%-quantiles were computed (Supplementary Table 3). Accordingly, posterior parameter samples as well as *equations 2* and *3* were used to derive the percentage of heavy labeled proteins over time, defined as

$$H_{percent} = 100 \cdot \frac{[H](t)}{[H](t)+[L](t)}.$$

Estimates of half-lives of proteins, for which the marginal posterior distributions of the turnover rate, *k,* cover ranges that are very low (*i.e.,* close to zero), is extremely inaccurate, indicated also by very large confidence ranges for $H_{1/2}$ (Extended Data Fig. 4e).

Comparison to protein turnover model without consideration of heavy labeled free lysine pool

We compared the protein turnover model with a peptide-centric modeling approach, only considering 2Lys-peptides that are exclusively heavy or light labeled. For the latter, we

employed the equations 1-9, aggregating intensities of all 2Lys-peptides derived from the same protein as means over time. Parameter inference (as described above) and subsequent $H_{1/2}$ computation allowed us to correlate the estimated $H_{1/2}$ values between both approaches (Extended Data Fig. 4a). We found good agreement between determined $H_{1/2}$.

Furthermore, we compared the protein turnover model considering the heavy labeled free lysine pool with the 'classical' modeling approach, not considering incorporation of heavy labeled free lysines during protein synthesis (Extended Data Fig. 4b). In the latter, the parameter k_{syn} was set to k_{syn}=0, i.e., not allowing synthesis of heavy labeled proteins. Both approaches agreed in their $H_{1/2}$ values for long lived proteins ($H_{1/2}$>100 days). However, shorter lived proteins showed a bias towards higher $H_{1/2}$ values when not considering incorporation of heavy labeled amino acids into newly synthetized proteins. Finally, we repeated the latter modeling approach, only considering chase time points larger than 3 weeks and 6 weeks, respectively. At this time, the heavy labeled free lysine pool was estimated to be nearly removed and, hence, should not bias the estimation of $H_{1/2}$ with the 'classical' modeling approach. Indeed, we observed good agreement between the resulting estimated $H_{1/2}$ values with the full protein turnover modeling approach (Extended Data Fig. 4c-d).

Modeling protein turnover in liver, cartilage and skeletal muscle from Rolfs et al. 2021[6]

The 2Lys-peptide model and the protein-centric turnover model was applied to MS data published by Rolfs *et al.* (2021)[6] for comparison. Specifically, we downloaded the MS data for liver, cartilage (CC) and skeletal muscle (SM) and searched them with MaxQuant version 1.6.0.1, using the same protein sequence database as for the analysis of our SILAC oocyte and ovary data, and the following settings: enzyme, trypsin/P; multiplicity, 2; heavy labels, Lys8; fixed modifications, carbamidomethyl (C); variable modifications (included in protein quantification), oxidation (M), acetylation (protein N-term). For mixed peptide analysis, settings were the same except for multiplicity was set to 1 and Lys8 (^{13}C8-K) was set as variable modification. Proteins were modelled only if the MS1 signal for heavy and light isotopes were detected in at least three of five time points in at least two biological replicates.

Identification and enrichment analysis of protein clusters with $^{13}C_6$-Lys levels in the aging ovaries

The modeling employed in this study inferred distributions of $^{13}C_6$-Lys percentages left in a given protein at a given time point. Medians of these distributions per protein were used as point estimates of $^{13}C_6$-Lys percentage. All values in each time point from 42 to 350 days were log10-transformed. All values smaller than -10 after transformation were set to -10. A matrix of normalized values was clustered using "Ward.D" method as implemented in R stats::hclust function[12] using euclidean distance as a metric. This approach aimed to minimize variance within clusters. After clustering we observed that three groups of proteins emerged – those with next to no signal after 42 days ("short-lived" cluster), those with high signal ("long-lived" cluster) and those with small amounts of residual $^{13}C_6$-Lys percentage. Plotting was done with "pheatmap" R package[13]. Clusters were tested for gene over-representation using "fora" algorithm[2] based on hypergeometric test. Multiple testing correction was performed across all

p-values in all clusters using Benjamini-Hochberg method[3] and a threshold of 0.05 adjusted p-value was used for defining significantly enriched sets.

For over-representation analysis, a background set of all proteins detected in ovary DIA and all genes expressed in human ovary in Human Protein Atlas[14] was combined using mouse gene symbols. This background gene set aimed to get rid of tissue-specific expression bias, as compared to default choices of a full genome annotation as a background gene set.

In order to perform over-representation analysis and gene set enrichment analysis (GSEA) a custom gene set list was constructed. We used the following STRING ontology categories: "Protein Domains and Features (InterPro)", "Protein Domains (SMART)", "Protein Domains (Pfam)", "Reactome Pathways", "Subcellular localization (COMPARTMENTS)", "Local Network Cluster (STRING)", "Biological Process (Gene Ontology)", "Annotated Keywords (UniProt)". These genes were augmented with Aging Atlas[15], MsigDB[16] ovary-related sets from Fan *et al.* 2019[17], CORUM complex database[18], in-house made scRNA-seq cell type signatures (see '*Analysis of single-cell RNA sequencing data*') and sets manually constructed from the following Uniprot[19] keywords: "cohesin", "nucleosome", "lamin", "nucleoporin".

Mass spectrometry DIA data normalization to determine protein abundance profiles

Data-independent acquisition (DIA) signal from ovary measurements was aggregated as a mean across biological replicates per day per protein and log10-transformed. In order to obtain the relative protein changes between timepoints, the DIA intensity was further scaled to the mean of 0 and standard deviation of 1 on per-protein basis. This normalization resulted in protein-wise abundance relative to time and was used for GSEA[2].

Clustering and GSEA of protein abundance profiles

Mean relative abundancies per gene symbol in each time point were used as input for "fgseaMultilevel" analysis with p-value calculation, boundary set as 1e-100 and minimal gene set size of 4. Background gene set in GSEA was determined by a presence of protein signal – all proteins quantified in DIA ovary dataset were used. A significance threshold of 0.01 of p-value corrected by Benjamini-Hochberg method[3] was used.

In order to identify clusters of protein dynamics for late time points (64-350 days), the corresponding DIA intensities were aggregated as a mean across biological replicates per day per protein and log10-transformed. DIA intensity was further scaled to the mean of 0 and standard deviation of 1 on per-protein basis. This resulted in relative protein abundancies for late time points only. In order to identify groups of protein with distinct behavior with regards to abundance changes, clustering with "Ward.D" method in euclidean distance space was applied and resulted in 6 clusters. These clusters were tested for ontology over-representation using hypergeometric test using "fora" algorithm. Same as in other applications of over-representation testing, all proteins detected in ovary DIA and all genes expressed in human ovary in Human Protein Atlas were used as a background set. A significance threshold of 0.05 Benjamini-Hochberg p-adjusted was chosen. We focused on investigating clusters 2 and 5, as these protein groups either gradually go down with age or increase only at 350 days.

Analysis of single-cell RNA sequencing data

The scRNA-seq data was aligned and quantified using the cellranger software (version 3.0.2, 10x Genomics). Low-quality cells that were either apoptotic (>10% mitochondrial counts) or with fewer than 750 detected genes and 1000 unique molecular identifier counts were excluded from the analysis. Doublets were detected using DoubletFinder v2.0.3[20]. All downstream analyses, including data normalization to graph-based clustering, were carried out in R[1] using Seurat v4.05[21]. Following cluster identification based on the specific expression of known markers, the in-house scRNA-seq cell type signatures were generated with the following cutoffs: avglog2FC ≥ 0.25 and %cellular expression > 10%. Mapping of the transcripts to specific or multiple cellular compartments were based on an avglog2FC ≥ 1.0 cutoff in a one-vs-all analysis.

References

1. Team, R. R: A language and environment for statistical computing. R Foundation for Statistical Computing. (2021).
2. Korotkevich, G. *et al.* Fast gene set enrichment analysis. *bioRxiv* (2021).
3. Benjamini, Y. & Hochberg, Y. Controlling the False Discovery Rate - a Practical and Powerful Approach to Multiple Testing. *J R Stat Soc B* **57**, 289-300 (1995).
4. Uhlén, M. *et al.* Tissue-based map of the human proteome. *Science* **347** (2015).
5. Guan, S., Price, J.C., Ghaemmaghami, S., Prusiner, S.B. & Burlingame, A.L. Compartment modeling for mammalian protein turnover studies by stable isotope metabolic labeling. *Analytical Chemistry* **84**, 4014-4021 (2012).
6. Rolfs, Z. *et al.* An atlas of protein turnover rates in mouse tissues. *Nature Communications* **12** (2021).
7. Fornasiero, E.F. *et al.* Precisely measured protein lifetimes in the mouse brain reveal differences across tissues and subcellular fractions. *Nature Communications* **9** (2018).
8. Soetaert, K., Petzoldt, T. & Setzer, R.W. Solving Differential Equations in R: Package deSolve. *Journal of Statistical Software* **33**, 1-25 (2010).
9. Bayes, T. An Essay Towards Solving a Problem in the Doctrine of Chances. *Biometrika* **45**, 296-315 (1958).
10. Hartig, F., Calabrese, J.M., Reineking, B., Wiegand, T. & Huth, A. Statistical inference for stochastic simulation models - theory and application. *Ecol Lett* **14**, 816-827 (2011).
11. ter Braak, C.J.F. & Vrugt, J.A. Differential Evolution Markov Chain with snooker updater and fewer chains. *Stat Comput* **18**, 435-446 (2008).
12. Murtagh, F. & Legendre, P. Ward's Hierarchical Agglomerative Clustering Method: Which Algorithms Implement Ward's Criterion? *J Classif* **31**, 274-295 (2014).
13. Kolde, R. Pheatmap: pretty heatmaps. (2012).
14. Uhlen, M. *et al.* Tissue-based map of the human proteome. *Science* **347** (2015).
15. Liu, G.H. *et al.* Aging Atlas: a multi-omics database for aging biology. *Nucleic Acids Research* **49**, D825-D830 (2021).

16. Liberzon, A. *et al.* Molecular signatures database (MSigDB) 3.0. *Bioinformatics* **27**, 1739-1740 (2011).
17. Fan, X. *et al.* Single-cell reconstruction of follicular remodeling in the human adult ovary. *Nature Communications* (2019).
18. Giurgiu, M. *et al.* CORUM: the comprehensive resource of mammalian protein complexes-2019. *Nucleic acids research* **47**, D559-D563 (2019).
19. Bateman, A. *et al.* UniProt: a worldwide hub of protein knowledge. *Nucleic Acids Research* **47**, D506-D515 (2019).
20. McGinnis, C.S., Murrow, L.M. & Gartner, Z.J. DoubletFinder: Doublet Detection in Single-Cell RNA Sequencing Data Using Artificial Nearest Neighbors. *Cell Systems* **8**, 329-337.e324 (2019).
21. Stuart, T. *et al.* Comprehensive Integration of Single-Cell Data. *Cell* **177**, 1888-1902.e1821 (2019).
