# Supplementary material for: The maintenance of oocytes in the mammalian ovary involves extreme protein longevity
Source: Nat Cell Biol. 2024 Jun 20;26(7):1124–38. doi: 10.1038/s41556-024-01442-7 (PMC11252011; doi:10.1038/s41556-024-01442-7)
Supplement: Supplementary file 2 — Reporting Summary [file 41556_2024_1442_MOESM2_ESM.pdf]

nature portfolio

Corresponding author(s): Prof Melina Schuh
Prof Henning Urlaub
Dr Juliane Liepe

Last updated by author(s): Apr 17, 2024

Reporting Summary

Nature Portfolio wishes to improve the reproducibility of the work that we publish. This form provides structure for consistency and transparency in reporting. For further information on Nature Portfolio policies, see our Editorial Policies and the Editorial Policy Checklist.

Statistics

For all statistical analyses, confirm that the following items are present in the figure legend, table legend, main text, or Methods section.

n/a	Confirmed	
☐	☒	The exact sample size (*n*) for each experimental group/condition, given as a discrete number and unit of measurement
☐	☒	A statement on whether measurements were taken from distinct samples or whether the same sample was measured repeatedly
☐	☒	The statistical test(s) used AND whether they are one- or two-sided<br>*Only common tests should be described solely by name; describe more complex techniques in the Methods section.*
☒	☐	A description of all covariates tested
☐	☒	A description of any assumptions or corrections, such as tests of normality and adjustment for multiple comparisons
☐	☒	A full description of the statistical parameters including central tendency (e.g. means) or other basic estimates (e.g. regression coefficient) AND variation (e.g. standard deviation) or associated estimates of uncertainty (e.g. confidence intervals)
☐	☒	For null hypothesis testing, the test statistic (e.g. *F*, *t*, *r*) with confidence intervals, effect sizes, degrees of freedom and *P* value noted<br>*Give P values as exact values whenever suitable.*
☐	☒	For Bayesian analysis, information on the choice of priors and Markov chain Monte Carlo settings
☒	☐	For hierarchical and complex designs, identification of the appropriate level for tests and full reporting of outcomes
☒	☐	Estimates of effect sizes (e.g. Cohen's *d*, Pearson's *r*), indicating how they were calculated

Our web collection on statistics for biologists contains articles on many of the points above.

Software and code

Policy information about availability of computer code

Data collection	For mass spectrometry data acquisition: Thermo Fisher Scientific software: Thermo Xcalibur Instrument Setup, Thermo Scientific Xcalibur, Version 4.4.16.14, Tune Application, Version 4.0.309.28 (for monitoring detection). For confocal microscopy image acquisition: Zen Blue 3.2 and Zen Black 2.1.
Data analysis	R (version 4.1.2); MaxQuant software versions 1.6.0.1 (for analysis of DDA mass spectrometry data) and 1.5.2.8 (for analysis of blood samples); Spectronaut version 15.7.220308.50606 (for analysis of DIA-MS data).<br>R scripts for data processing and visualization of proteomics and single-cell RNA sequencing data are available on figshare (https://doi.org/10.6084/m9.figshare.25604232.v1).<br>No custom algorithm or software were generated in this study.

For manuscripts utilizing custom algorithms or software that are central to the research but not yet described in published literature, software must be made available to editors and reviewers. We strongly encourage code deposition in a community repository (e.g. GitHub). See the Nature Portfolio guidelines for submitting code & software for further information.

Data

Policy information about availability of data

All manuscripts must include a data availability statement. This statement should provide the following information, where applicable:

- Accession codes, unique identifiers, or web links for publicly available datasets
- A description of any restrictions on data availability
- For clinical datasets or third party data, please ensure that the statement adheres to our policy

The scRNAseq datasets are deposited in the NCBI Gene Expression Omnibus database under accession number GSE237012. The proteomics data are deposited to the ProteomeXchange Consortium via the UCSD MassIVE repository with the dataset identifier MSV000092528.

Research involving human participants, their data, or biological material

Policy information about studies with human participants or human data. See also policy information about sex, gender (identity/presentation), and sexual orientation and race, ethnicity and racism.

Reporting on sex and gender	N/A
Reporting on race, ethnicity, or other socially relevant groupings	N/A
Population characteristics	N/A
Recruitment	N/A
Ethics oversight	N/A

Note that full information on the approval of the study protocol must also be provided in the manuscript.

Field-specific reporting

Please select the one below that is the best fit for your research. If you are not sure, read the appropriate sections before making your selection.

☒ Life sciences ☐ Behavioural & social sciences ☐ Ecological, evolutionary & environmental sciences

For a reference copy of the document with all sections, see nature.com/documents/nr-reporting-summary-flat.pdf

Life sciences study design

All studies must disclose on these points even when the disclosure is negative.

Sample size	scRNA sequencing and proteomics data: no groups of conditions were compared to each other, and therefore, no sample-size calculation was performed.<br>Figures 6 and 7: No statistical methods were used to predetermine sample size. In retrospective, achieved sample sizes were determined to be adequate based on the magnitude and consistency of measurable differences between groups. Most importantly, sample size per experiment was dictated by the number of oocytes that could be processed (microinjection and live imaging) within a reasonable time by the researcher without affecting oocyte quality.
Data exclusions	Proteomics data:<br>In the oocyte protein turnover experiments (data-dependent acquisition [DDA] mass spectrometry [MS]), median over technical replicates were only computed if the DDA intensity of 13C6-Lys labeled protein was detected in at least 2 out of 4 technical replicates, otherwise the data point is omitted (set as n.a.). The mean and standard deviation of F, fractions of heavy (13C6-Lys) proteins left, were calculated if F was detected in at least one of the two biological replicates.<br><br>Figures 6 and 7:<br>No data points were excluded.
Replication	1. In the oocyte protein turnover experiments (data-dependent acquisition [DDA] mass spectrometry [MS]), two sets of oocytes (n=2,475, n= 2,473) were obtained from a total of 8-week old mice (12C6-Lys chow after birth until they were 8-weeks old) born from 13C6-Lys-labeled females. Oocytes from multiple animals were pooled for a single replicate to reach the minimum input required for the MS processing. Each DDA oocyte fraction was measured in quadruplicates.<br><br>2. In the ovary protein turnover experiments (DDA MS) with 13C6-Lys pulse until birth and 12C6-Lys chase from birth, ovaries were collected from the female progeny at 11 time points (24 hours, 48 hours, 1, 2, 3, 6, 9, 12, 30, 50, and 65 weeks). For each time point, three biological replicates were collected, each containing two ovaries from a single animal.

	3. In the ovary protein turnover experiments (DDA MS) with 13C6-Lys pulse until birth and 12C6-Lys chase from weaning, ovaries were collected from the female progeny at 6 time points (6, 9, 12, 30, 50, and 65 weeks). For each time point, three biological replicates were collected, each containing two ovaries from a single animal.<br><br>4. In the protein abundance experiments (data-independent acquisition [DIA] MS), ovaries were collected from unlabeled FVB/N female mice at 8 time points (24 hours, 1, 2, 3, 5, 9, 12, and 50 weeks). For each time point, three biological replicates were collected, each containing two ovaries from a single animal.<br><br>5. In the single-cell RNA sequencing experiments, each library was generated from 6 ovaries collected from 3 different pups in a single pregnant CD1 female mouse.<br><br>6. Figures 6 and 7: All data are from at least two independent experiments / multiple biological replicates. All attempts at replication were successful.
Randomization	Protein turnover and scRNA sequencing experiments did not have different treatments or conditions that could be randomized.<br><br>Figures 6 and 7: Mouse oocytes were collected from multiple ovaries/animals and then pooled. They were then randomly assigned to different experimental groups.
Blinding	scRNA sequencing and proteomics data:<br>No groups of conditions were compared to each other, and therefore, these experiments did not require blinding.<br><br>Figures 6 and 7: Investigators were not blinded to allocation during experiments and outcome assessment, as each experiment was performed by one researcher alone. Thus, blinding during group allocation was not possible to ensure samples received the right treatment/ manipulation during experiment. Blinding was also not possible during data analysis, as data analysis was performed by the same researcher that conducted the experiment.

Reporting for specific materials, systems and methods

We require information from authors about some types of materials, experimental systems and methods used in many studies. Here, indicate whether each material, system or method listed is relevant to your study. If you are not sure if a list item applies to your research, read the appropriate section before selecting a response.

Materials & experimental systems

n/a	Involved in the study
☒	☐ Antibodies
☒	☐ Eukaryotic cell lines
☒	☐ Palaeontology and archaeology
☐	☒ Animals and other organisms
☒	☐ Clinical data
☒	☐ Dual use research of concern
☒	☐ Plants

Methods

n/a	Involved in the study
☒	☐ ChIP-seq
☒	☐ Flow cytometry
☒	☐ MRI-based neuroimaging

Animals and other research organisms

Policy information about studies involving animals; ARRIVE guidelines recommended for reporting animal research, and Sex and Gender in Research

Laboratory animals	Mus musculus, FVB/N and CD1 strains. All mice were kept in rooms with constant temperature of 21°C and the humidity of 55%. The light/dark rhythm was 12:12 hours, from 5 am to 5 pm. Health monitoring was carried out in accordance with FELASA recommendations with large annual examinations in January and smaller scale in May and September.<br>We used animals of different ages, between 1 day and 65 weeks old. Age of animals is clearly indicated in each figure.
Wild animals	The study did not involve wild animals.
Reporting on sex	In this study we investigated protein turnover and abundance in ovaries and oocytes. Hence, only animals of female sex were analysed.
Field-collected samples	This study did not involve field-collected samples.
Ethics oversight	The maintenance and handling of all FVB/N mice was performed according to international animal welfare rules (Federation for Laboratory Animal Science Associations guidelines and recommendations). Requirements of formal control of the German national authorities and funding organizations were satisfied, and the study received approval by the Niedersächsisches Landesamt für Verbraucherschutz und Lebensmittelsicherheit (LAVES).

Note that full information on the approval of the study protocol must also be provided in the manuscript.

Plants

Seed stocks	N/A
Novel plant genotypes	N/A
Authentication	N/A
