# Supplementary material for: The maintenance of oocytes in the mammalian ovary involves extreme protein longevity
Source: Nat Cell Biol. 2024 Jun 20;26(7):1124–38. doi: 10.1038/s41556-024-01442-7 (PMC11252011; doi:10.1038/s41556-024-01442-7)
Supplement: Supplementary file 7 — Heatmaps of half-lives of different proteins belonging to different protein complexes and enriched GO terms in the ovary and organs from other studies19–21. [file 41556_2024_1442_MOESM7_ESM.pdf]

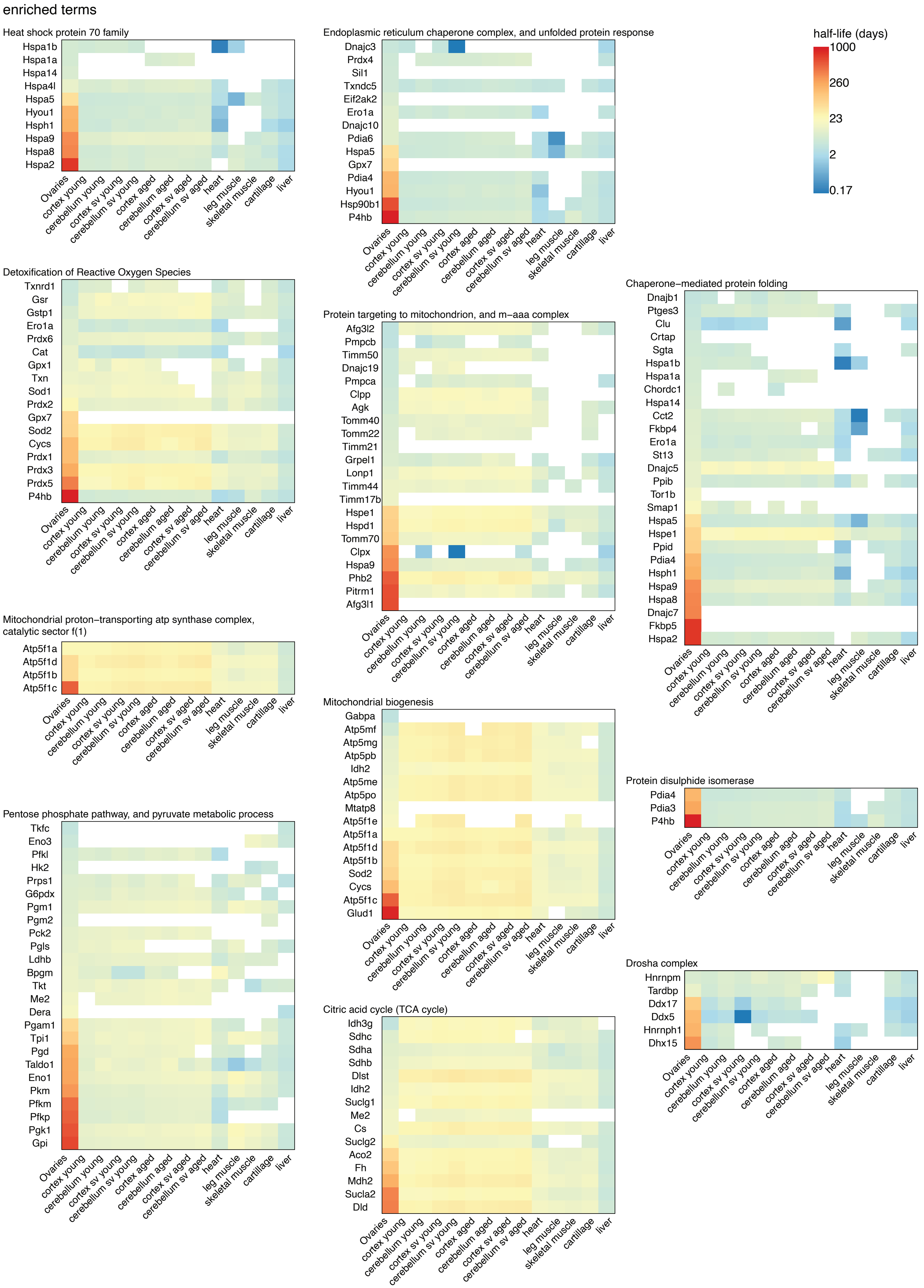

enriched terms
half-life (days)
1000
260
23
2
0.17
Heat shock protein 70 family
Hspa1b
Hspa1a
Hspa14
Hspa4l
Hspa5
Hyou1
Hsph1
Hspa9
Hspa8
Hspa2
Endoplasmic reticulum chaperone complex, and unfolded protein response
Dnajc3
Prdx4
Sil1
Txndc5
Eif2ak2
Ero1a
Dnajc10
Pdia6
Hspa5
Gpx7
Pdia4
Hyou1
Hsp90b1
P4hb
Detoxification of Reactive Oxygen Species
Txnrd1
Gsr
Gstp1
Ero1a
Prdx6
Cat
Gpx1
Txn
Sod1
Prdx2
Gpx7
Sod2
Cycs
Prdx1
Prdx3
Prdx5
P4hb
Protein targeting to mitochondrion, and m−aaa complex
Afg3l2
Pmpcb
Timm50
Dnajc19
Pmpca
Clpp
Agk
Tomm40
Tomm22
Timm21
Grpel1
Lonp1
Timm44
Timm17b
Hspe1
Hspd1
Tomm70
Clpx
Hspa9
Phb2
Pitrm1
Afg3l1
Chaperone−mediated protein folding
Dnajb1
Ptges3
Clu
Crtap
Sgta
Hspa1b
Hspa1a
Chordc1
Hspa14
Cct2
Fkbp4
Ero1a
St13
Dnajc5
Ppib
Tor1b
Smap1
Hspa5
Hspe1
Ppid
Pdia4
Hsph1
Hspa9
Hspa8
Dnajc7
Fkbp5
Hspa2
Mitochondrial proton−transporting atp synthase complex, catalytic sector f(1)
Atp5f1a
Atp5f1d
Atp5f1b
Atp5f1c
Mitochondrial biogenesis
Gabpa
Atp5mf
Atp5mg
Atp5pb
Idh2
Atp5me
Atp5po
Mtatp8
Atp5f1e
Atp5f1a
Atp5f1d
Atp5f1b
Sod2
Cycs
Atp5f1c
Glud1
Protein disulphide isomerase
Pdia4
Pdia3
P4hb
Pentose phosphate pathway, and pyruvate metabolic process
Tkfc
Eno3
Pfkl
Hk2
Prps1
G6pdx
Pgm1
Pgm2
Pck2
Pgls
Ldhb
Bpgm
Tkt
Me2
Dera
Pgam1
Tpi1
Pgd
Taldo1
Eno1
Pkm
Pfkm
Pfkp
Pgk1
Gpi
Citric acid cycle (TCA cycle)
Idh3g
Sdhc
Sdha
Sdhb
Dlst
Idh2
Suclg1
Me2
Cs
Suclg2
Aco2
Fh
Mdh2
Sucla2
Dld
Drosha complex
Hnrnpm
Tardbp
Ddx17
Ddx5
Hnrnph1
Dhx15
Ovaries
cortex young
cerebellum young
cortex sv young
cerebellum sv young
cortex aged
cerebellum aged
cortex sv aged
cerebellum sv aged
heart
leg muscle
skeletal muscle
cartilage
liver

enriched terms (p2)

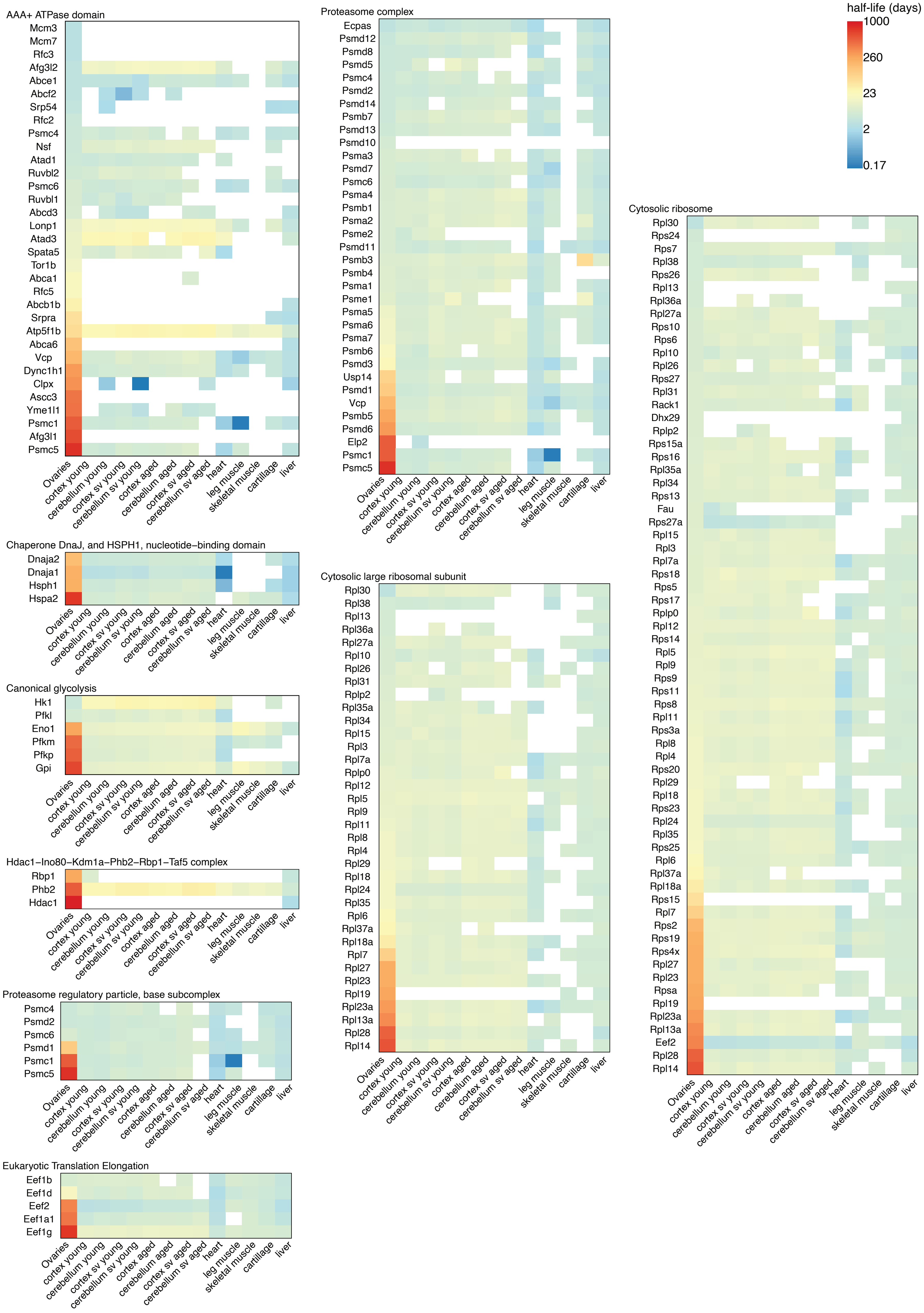

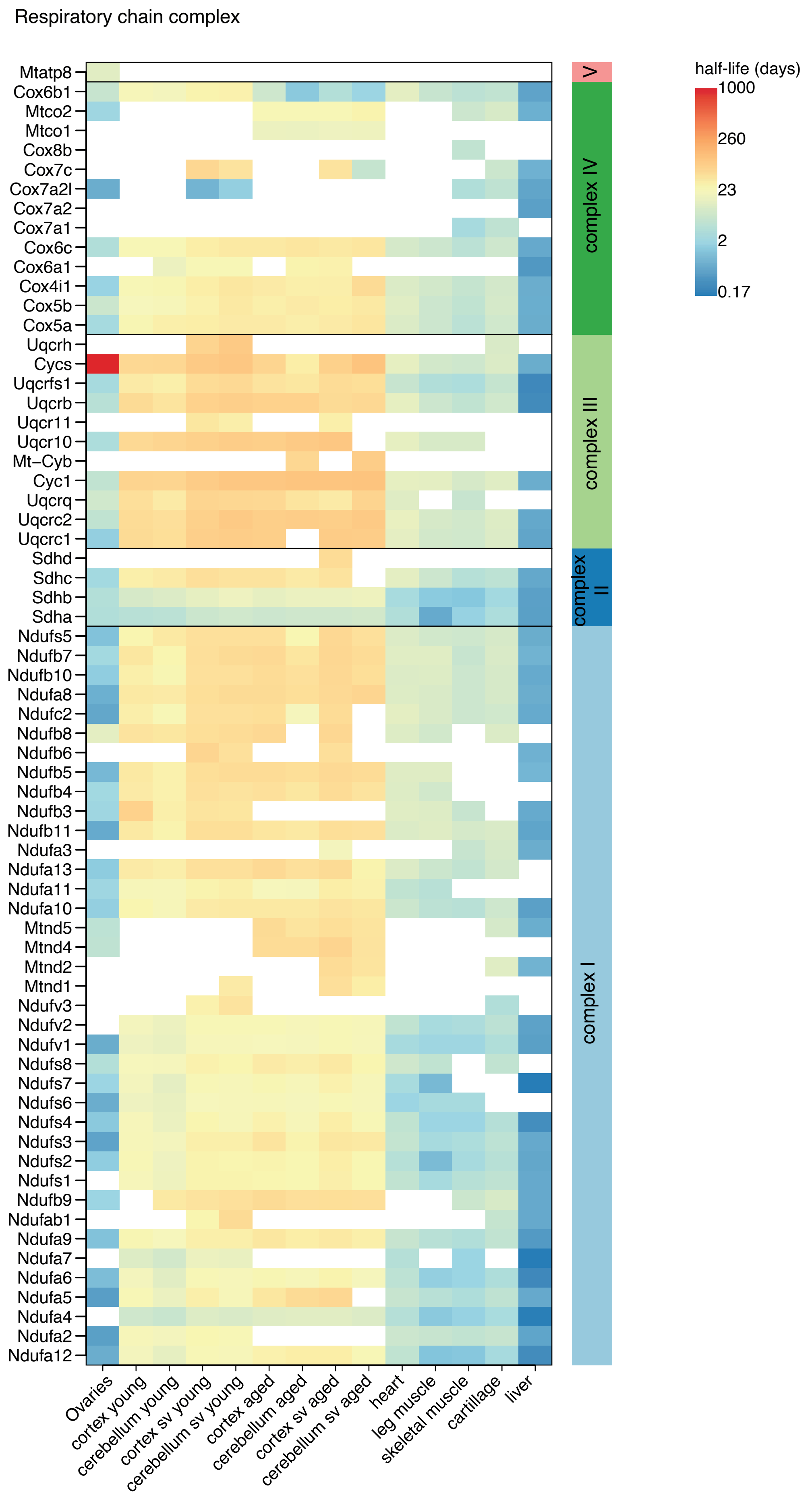
Respiratory chain complex
half-life (days)
1000
260
23
2
0.17
Mtatp8
Cox6b1
Mtco2
Mtco1
Cox8b
Cox7c
Cox7a2l
Cox7a2
Cox7a1
Cox6c
Cox6a1
Cox4i1
Cox5b
Cox5a
Uqcrh
Cycs
Uqcrfs1
Uqcrb
Uqcr11
Uqcr10
Mt−Cyb
Cyc1
Uqcrq
Uqcrc2
Uqcrc1
Sdhd
Sdhc
Sdhb
Sdha
Ndufs5
Ndufb7
Ndufb10
Ndufa8
Ndufc2
Ndufb8
Ndufb6
Ndufb5
Ndufb4
Ndufb3
Ndufb11
Ndufa3
Ndufa13
Ndufa11
Ndufa10
Mtnd5
Mtnd4
Mtnd2
Mtnd1
Ndufv3
Ndufv2
Ndufv1
Ndufs8
Ndufs7
Ndufs6
Ndufs4
Ndufs3
Ndufs2
Ndufs1
Ndufb9
Ndufab1
Ndufa9
Ndufa7
Ndufa6
Ndufa5
Ndufa4
Ndufa2
Ndufa12
Ovaries
cortex young
cerebellum young
cortex sv young
cerebellum sv young
cortex aged
cerebellum aged
cortex sv aged
cerebellum sv aged
heart
leg muscle
skeletal muscle
cartillage
liver
V
complex IV
complex III
complex II
complex I

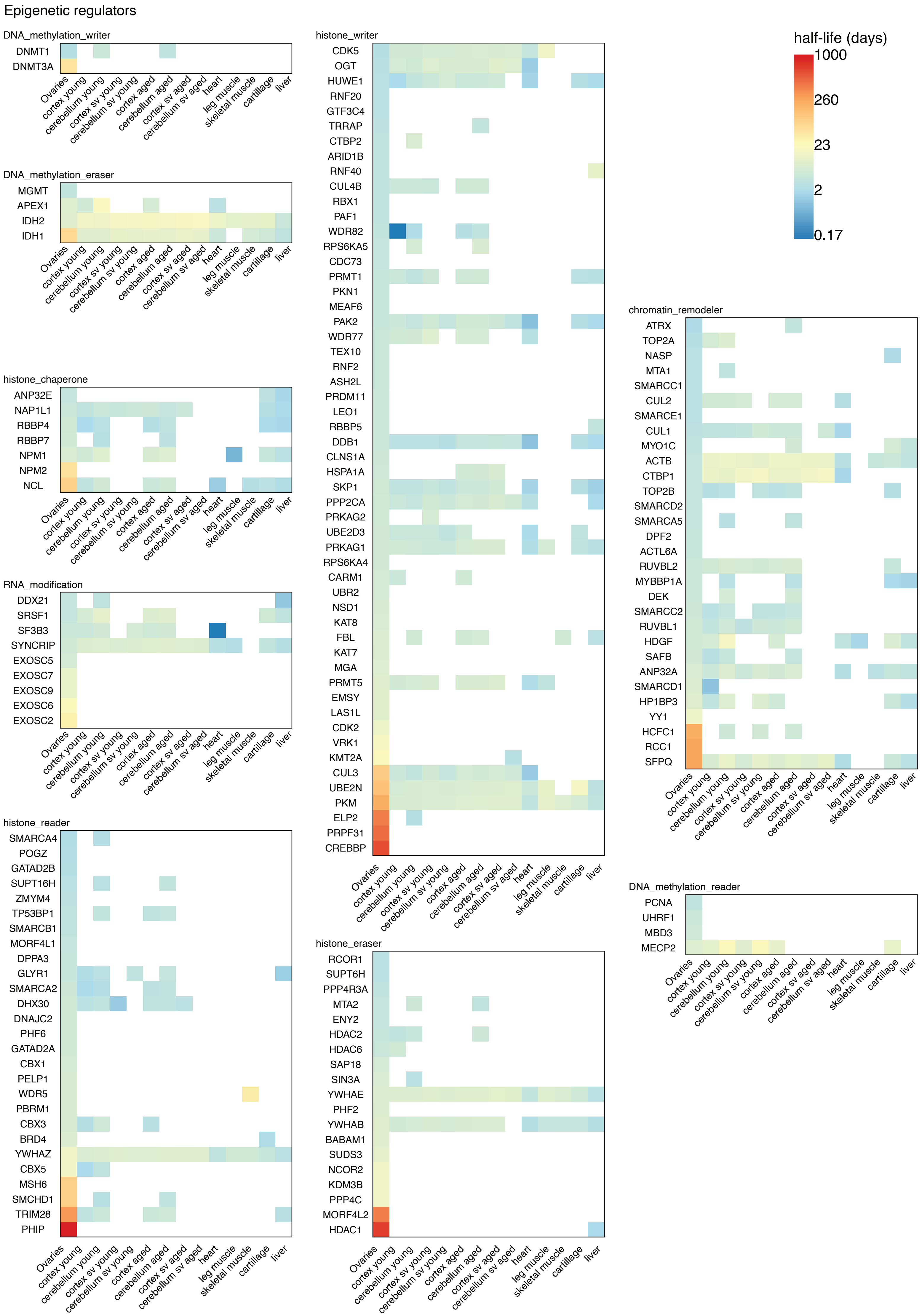

Epigenetic regulators
half-life (days)
1000
260
23
2
0.17
DNA_methylation_writer
DNMT1
DNMT3A
DNA_methylation_eraser
MGMT
APEX1
IDH2
IDH1
histone_chaperone
ANP32E
NAP1L1
RBBP4
RBBP7
NPM1
NPM2
NCL
RNA_modification
DDX21
SRSF1
SF3B3
SYNCRIP
EXOSC5
EXOSC7
EXOSC9
EXOSC6
EXOSC2
histone_reader
SMARCA4
POGZ
GATAD2B
SUPT16H
ZMYM4
TP53BP1
SMARCB1
MORF4L1
DPPA3
GLYR1
SMARCA2
DHX30
DNAJC2
PHF6
GATAD2A
CBX1
PELP1
WDR5
PBRM1
CBX3
BRD4
YWHAZ
CBX5
MSH6
SMCHD1
TRIM28
PHIP
histone_writer
CDK5
OGT
HUWE1
RNF20
GTF3C4
TRRAP
CTBP2
ARID1B
RNF40
CUL4B
RBX1
PAF1
WDR82
RPS6KA5
CDC73
PRMT1
PKN1
MEAF6
PAK2
WDR77
TEX10
RNF2
ASH2L
PRDM11
LEO1
RBBP5
DDB1
CLNS1A
HSPA1A
SKP1
PPP2CA
PRKAG2
UBE2D3
PRKAG1
RPS6KA4
CARM1
UBR2
NSD1
KAT8
FBL
KAT7
MGA
PRMT5
EMSY
LAS1L
CDK2
VRK1
KMT2A
CUL3
UBE2N
PKM
ELP2
PRPF31
CREBBP
histone_eraser
RCOR1
SUPT6H
PPP4R3A
MTA2
ENY2
HDAC2
HDAC6
SAP18
SIN3A
YWHAE
PHF2
YWHAB
BABAM1
SUDS3
NCOR2
KDM3B
PPP4C
MORF4L2
HDAC1
chromatin_remodeler
ATRX
TOP2A
NASP
MTA1
SMARCC1
CUL2
SMARCE1
CUL1
MYO1C
ACTB
CTBP1
TOP2B
SMARCD2
SMARCA5
DPF2
ACTL6A
RUVBL2
MYBBP1A
DEK
SMARCC2
RUVBL1
HDGF
SAFB
ANP32A
SMARCD1
HP1BP3
YY1
HCFC1
RCC1
SFPQ
DNA_methylation_reader
PCNA
UHRF1
MBD3
MECP2
Ovaries
cortex young
cerebellum young
cortex sv young
cerebellum sv young
cortex aged
cerebellum aged
cortex sv aged
cerebellum sv aged
heart
leg muscle
skeletal muscle
cartillage
liver

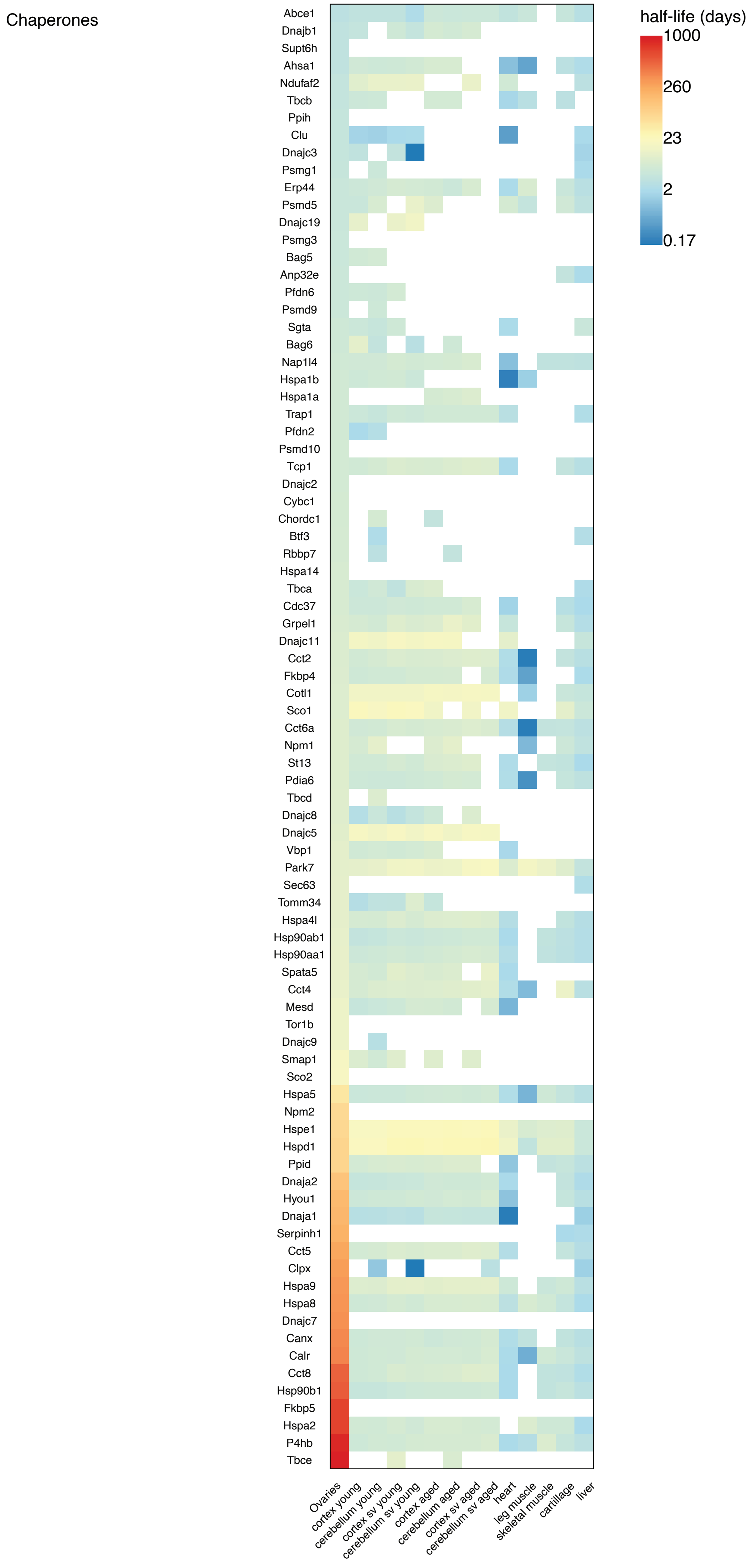
Chaperones
half-life (days)
1000
260
23
2
0.17
Abce1
Dnajb1
Supt6h
Ahsa1
Ndufaf2
Tbcb
Ppih
Clu
Dnajc3
Psmg1
Erp44
Psmd5
Dnajc19
Psmg3
Bag5
Anp32e
Pfdn6
Psmd9
Sgta
Bag6
Nap1l4
Hspa1b
Hspa1a
Trap1
Pfdn2
Psmd10
Tcp1
Dnajc2
Cybc1
Chordc1
Btf3
Rbbp7
Hspa14
Tbca
Cdc37
Grpel1
Dnajc11
Cct2
Fkbp4
Cotl1
Sco1
Cct6a
Npm1
St13
Pdia6
Tbcd
Dnajc8
Dnajc5
Vbp1
Park7
Sec63
Tomm34
Hspa4l
Hsp90ab1
Hsp90aa1
Spata5
Cct4
Mesd
Tor1b
Dnajc9
Smap1
Sco2
Hspa5
Npm2
Hspe1
Hspd1
Ppid
Dnaja2
Hyou1
Dnaja1
Serpinh1
Cct5
Clpx
Hspa9
Hspa8
Dnajc7
Canx
Calr
Cct8
Hsp90b1
Fkbp5
Hspa2
P4hb
Tbce
Ovaries
cortex young
cerebellum young
cortex sv young
cerebellum sv young
cortex aged
cerebellum aged
cortex sv aged
cerebellum sv aged
heart
leg muscle
skeletal muscle
cartilage
liver
