# Supplementary figures and images for: The maintenance of oocytes in the mammalian ovary involves extreme protein longevity
Source: Nat Cell Biol. 2024 Jun 20;26(7):1124–38. doi: 10.1038/s41556-024-01442-7 (PMC11252011; doi:10.1038/s41556-024-01442-7)

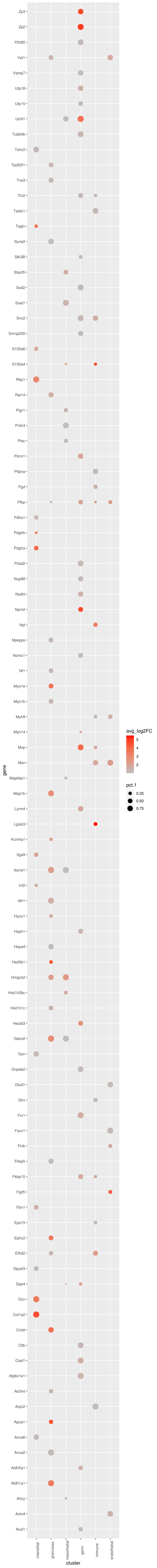

Supplement: Supplementary file 8 — Characterization of the localization of the transcripts encoding the proteins from the high protein longevity in the ovary using single-cell RNA-sequencing of postnatal day 2 ovaries. [file 41556_2024_1442_MOESM8_ESM.pdf]
