# Supplementary material for: The maintenance of oocytes in the mammalian ovary involves extreme protein longevity
Source: Nat Cell Biol. 2024 Jun 20;26(7):1124–38. doi: 10.1038/s41556-024-01442-7 (PMC11252011; doi:10.1038/s41556-024-01442-7)
Supplement: Supplementary file 5 — Plots of raw abundance values of proteins in unlabelled ovaries at eight different ages (1 day; 1, 2, 3, 5, 9, 12 and 50 weeks) measured using DIA mass spectrometry. [file 41556_2024_1442_MOESM5_ESM.pdf]

A0A087WPF7
GN=Auts2
autism susceptibility candidate 2

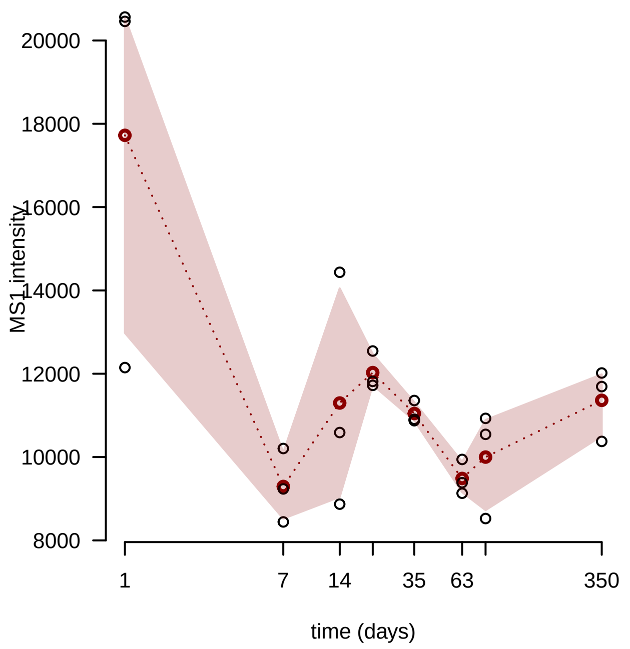

A0A0M3U1B0
GN=Sycp2l
synaptonemal complex protein 2−like

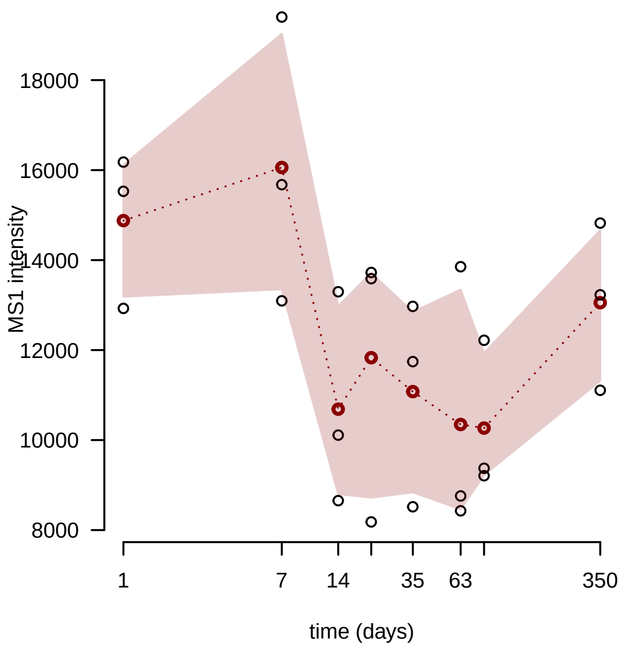

A1A5B6
GN=Tbc1d25
TBC1 domain family, member 25

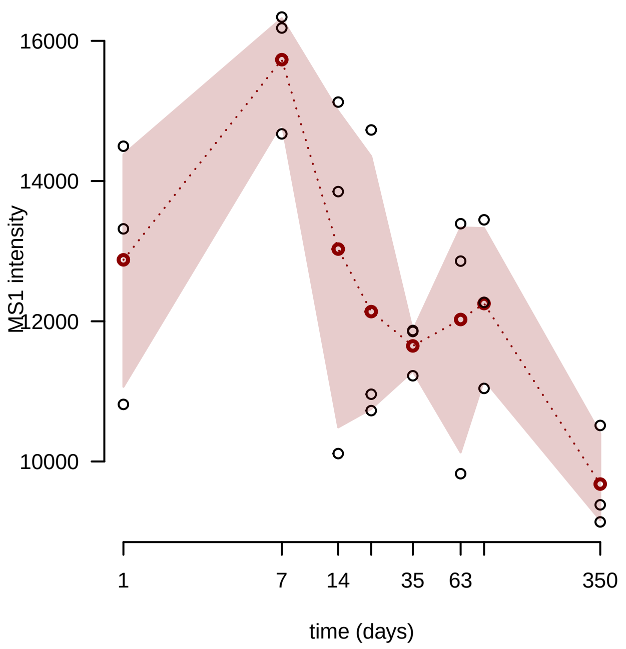

A1L314
GN=Mpeg1
macrophage expressed gene 1

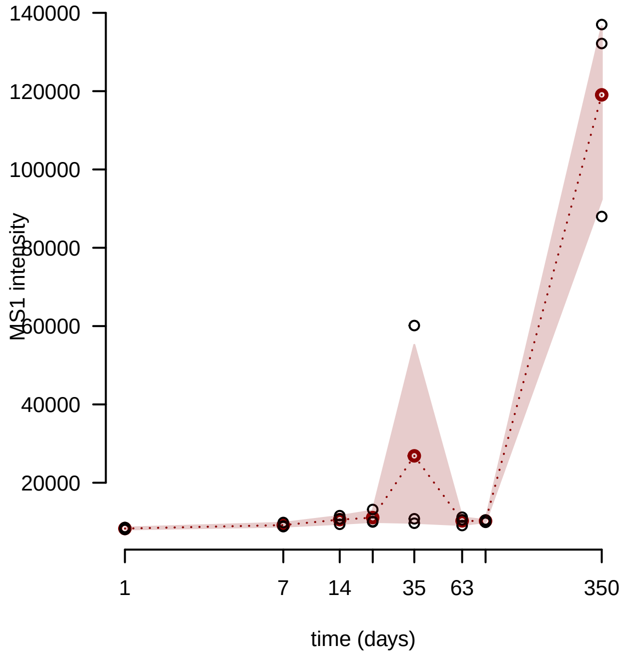

A1L317
GN=Krt24
keratin 24

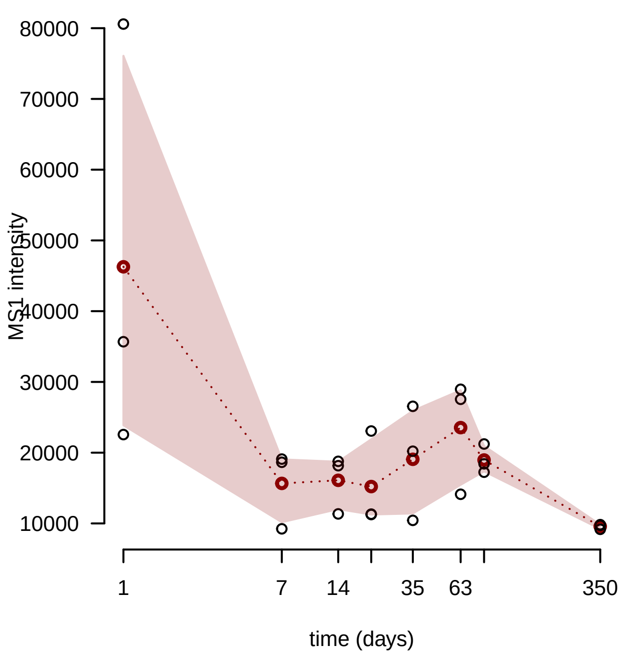

A2A3V1
GN=Akap17b
A kinase (PRKA) anchor protein 17B

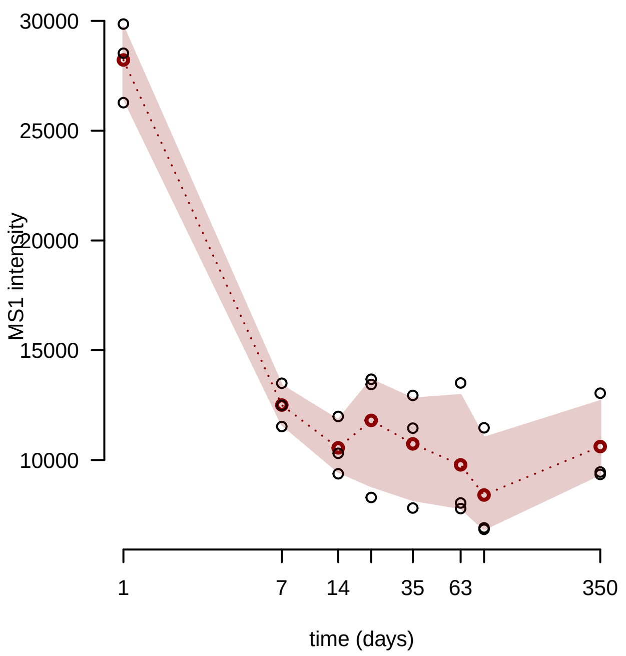

A2A432
GN=Cul4b
cullin 4B

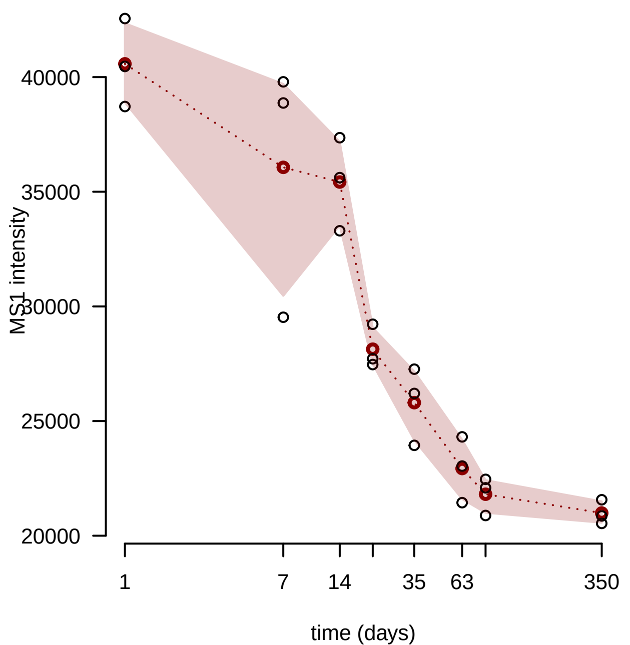

A2A4P0
GN=Dhx8
DEAH (Asp−Glu−Ala−His) box polypeptide 8

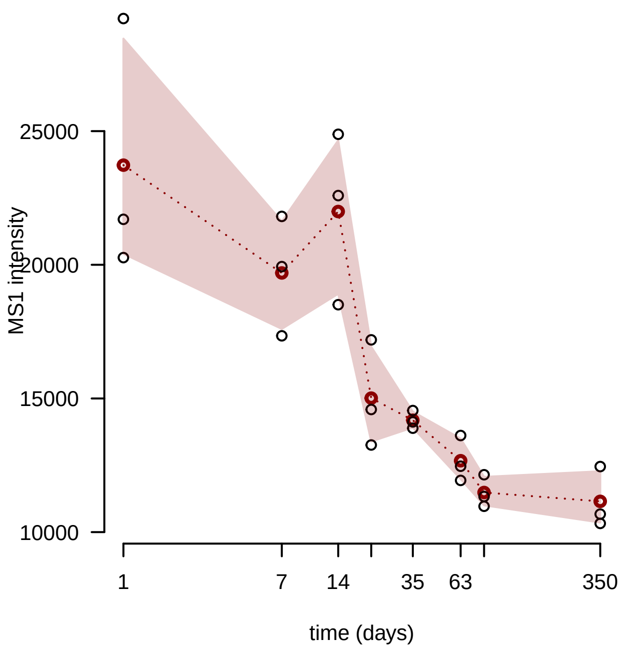

A2A5R2
GN=Arfgef2
ADP−ribosylation factor guanine nucleoti ...

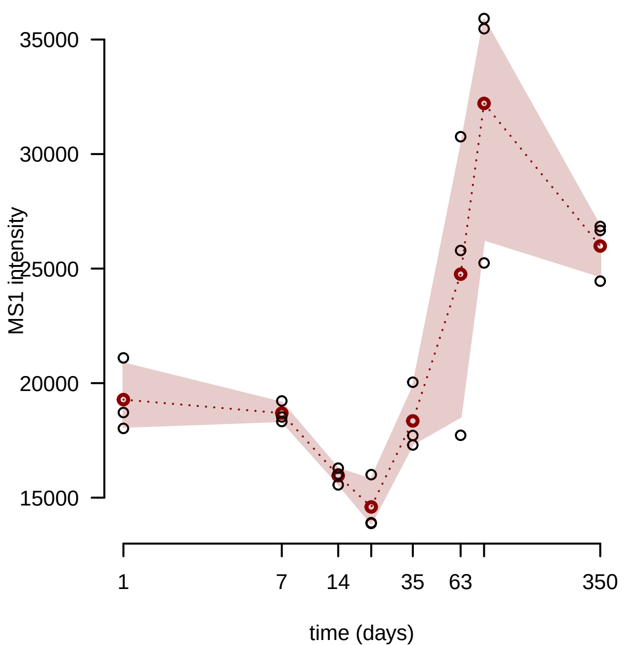

A2A5Z6
GN=Smurf2
SMAD specific E3 ubiquitin protein ligas ...

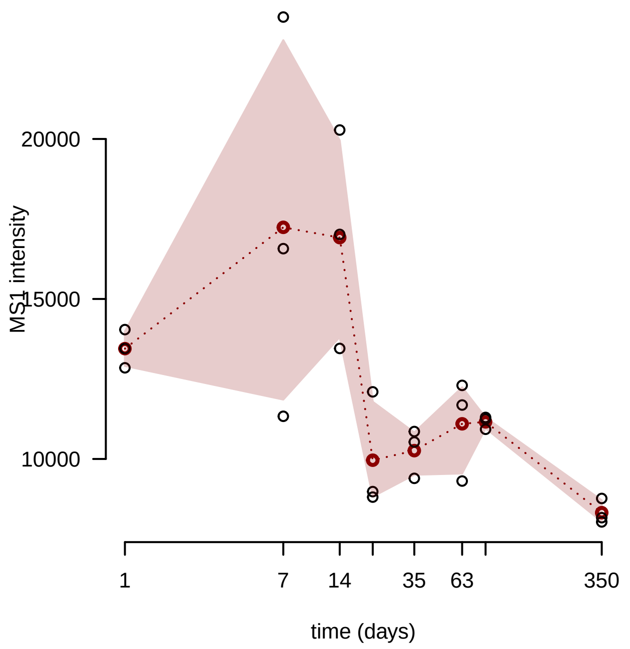

A2A690
GN=Tanc2
tetratricopeptide repeat, ankyrin repeat ...

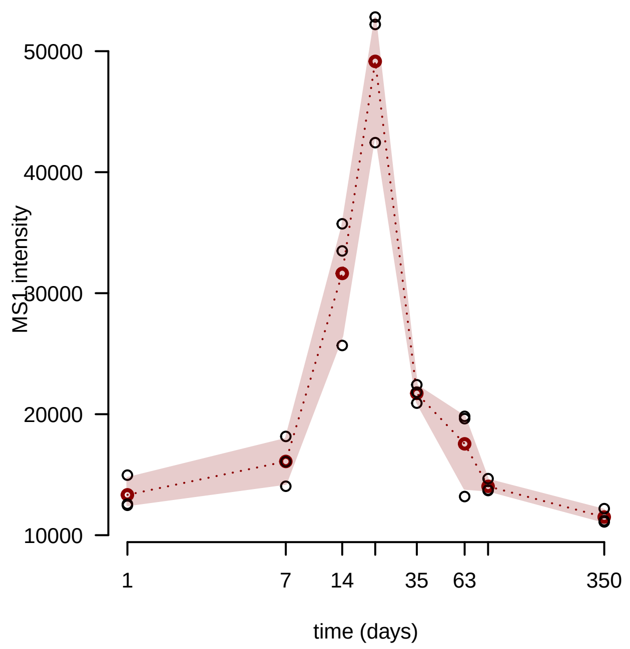

A2A699
GN=Fam171a2
family with sequence similarity 171, mem ...

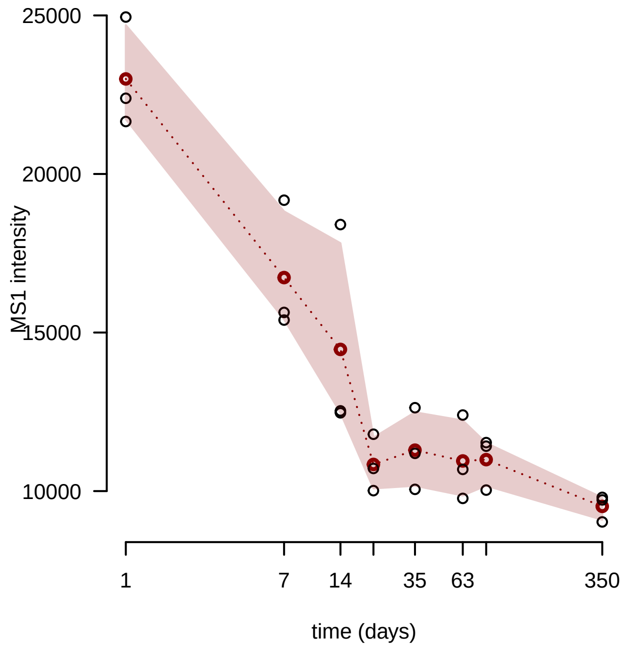

A2A6A1
GN=Gpatch8
G patch domain containing 8

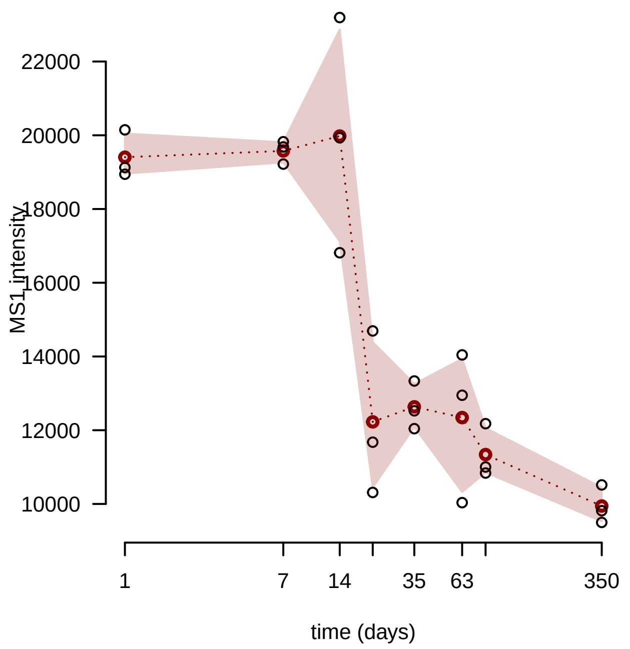

A2A6Q5
GN=Cdc27
cell division cycle 27

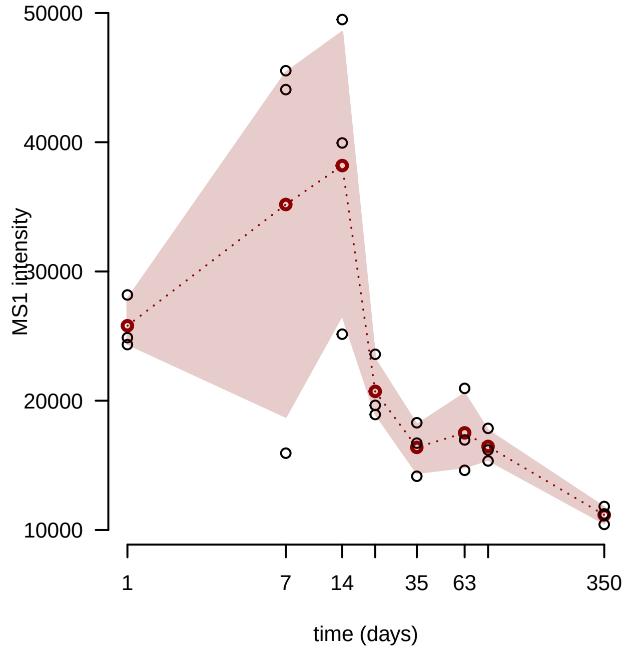

A2A6Q5
GN=Cdc27
cell division cycle 27

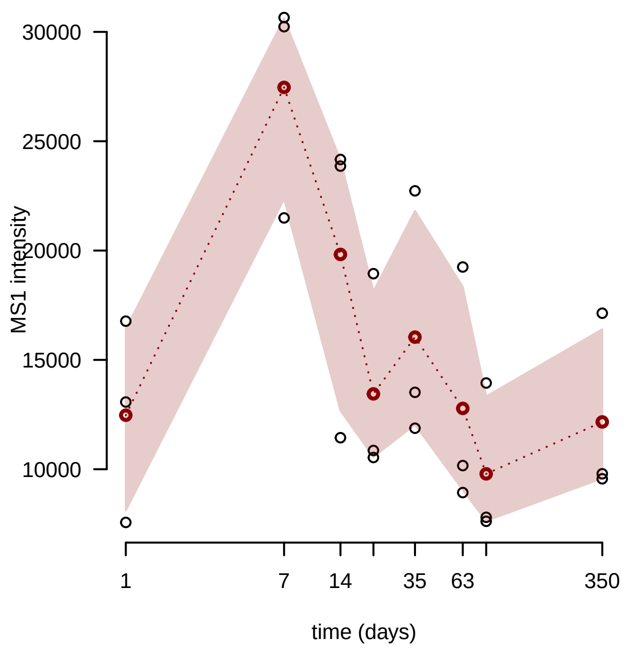

A2A6T1
GN=Cdr2l
cerebellar degeneration−related protein ...

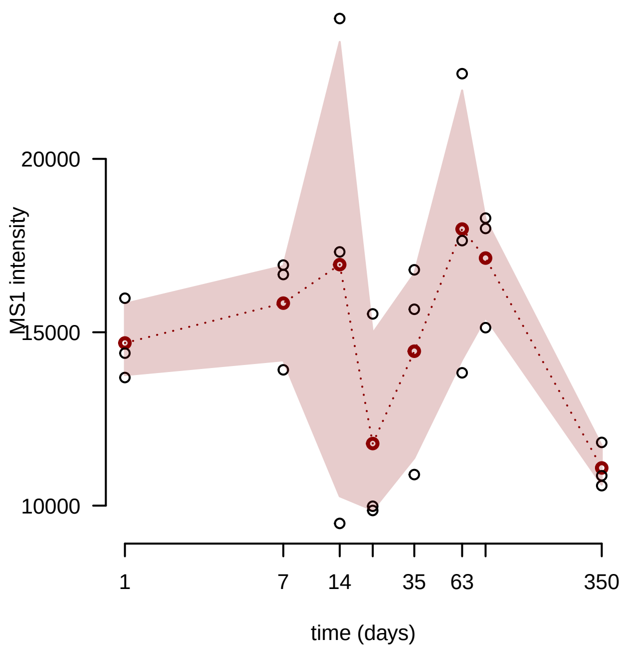

A2A791
GN=Zmym4
zinc finger, MYM−type 4

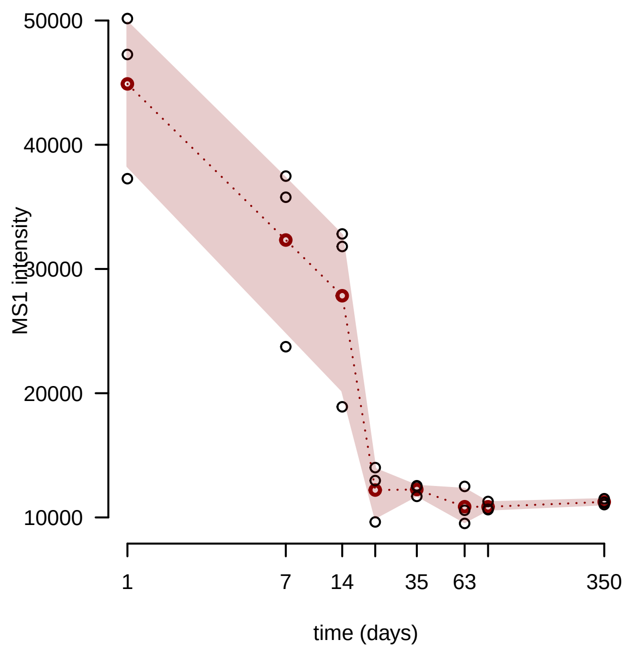

A2A7S8
GN=Kiaa1522

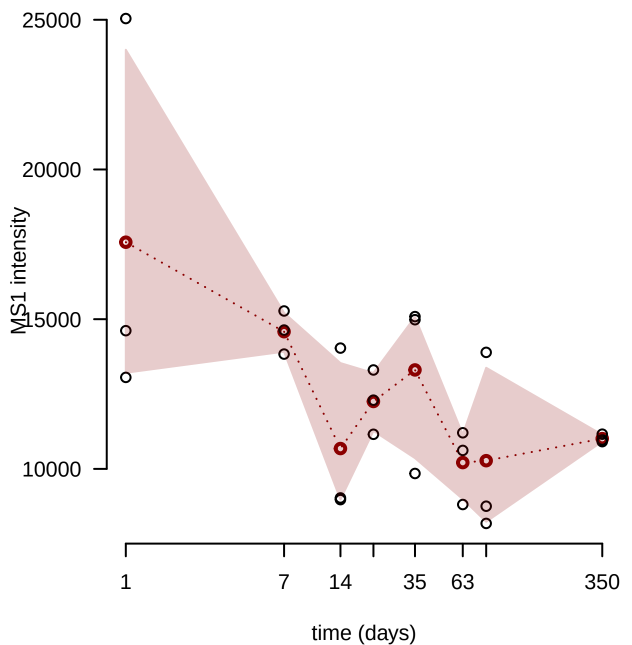

A2A863
GN=Itgb4
integrin beta 4

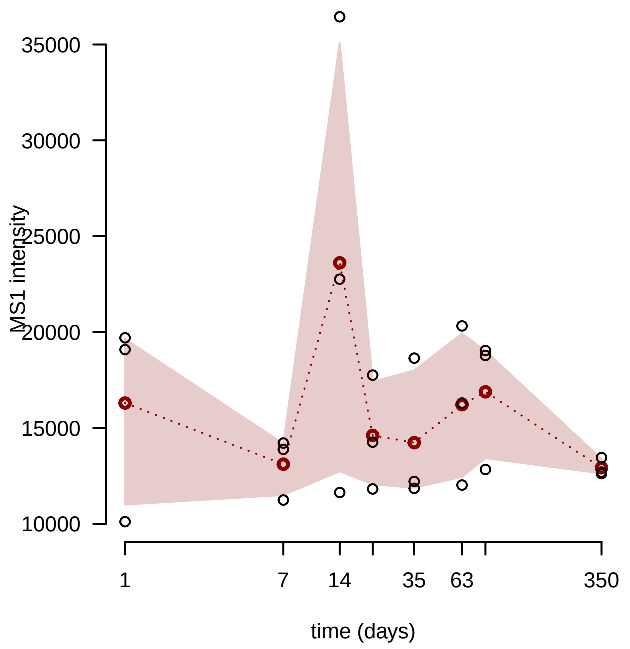

A2A8L1
GN=Chd5
chromodomain helicase DNA binding protei ...

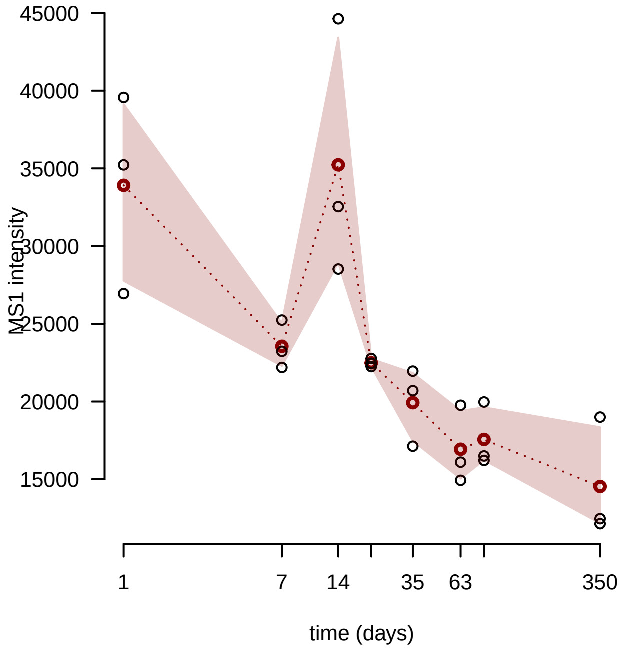

A2A8L5
GN=Ptprf
protein tyrosine phosphatase, receptor t ...

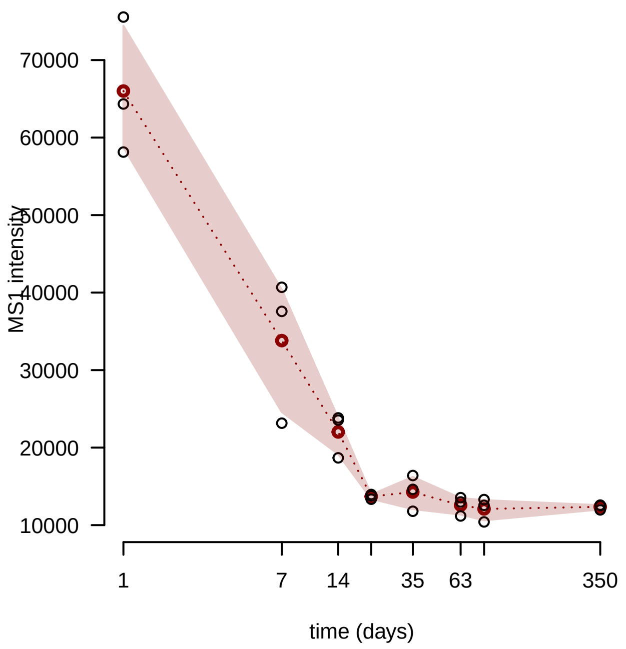

A2A8U2
GN=Tmem201
transmembrane protein 201

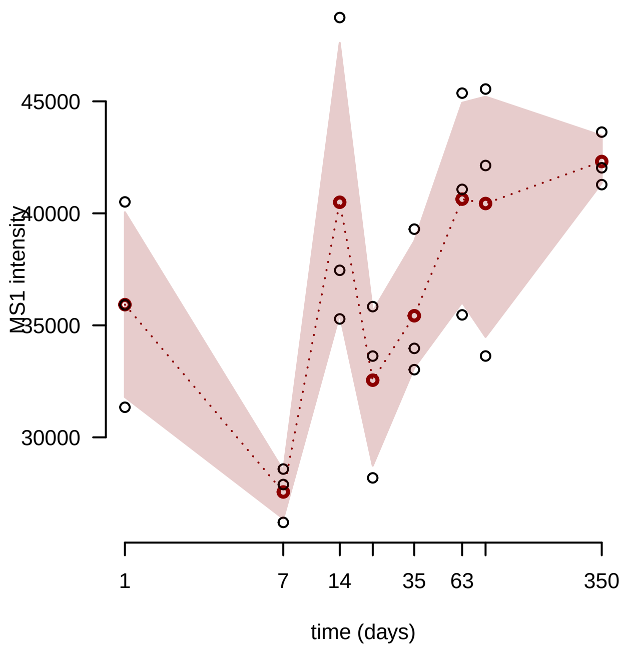

A2A8Z1
GN=Osbpl9
oxysterol binding protein−like 9

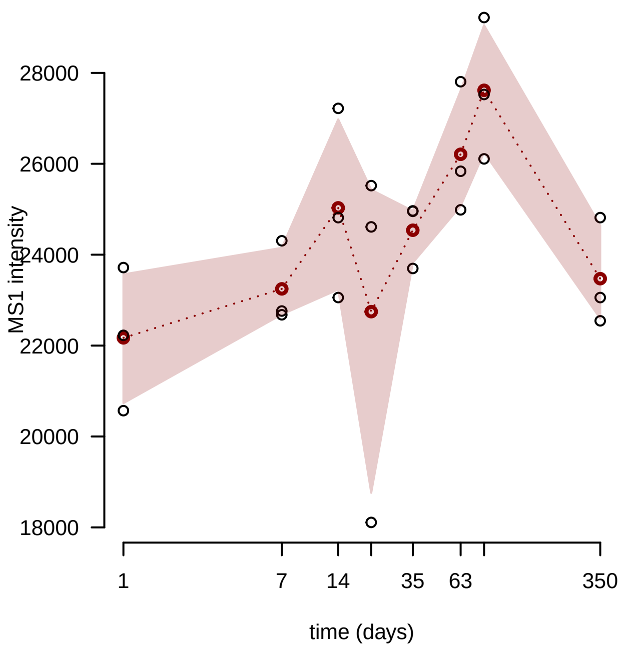

A2A9C3
GN=Szt2
SZT2 subunit of KICSTOR complex

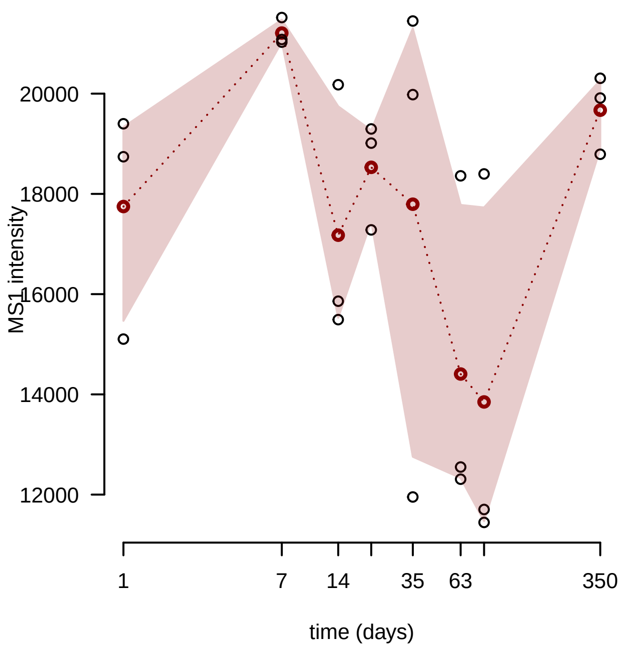

A2A9R3
GN=Mageb4
MAGE family member B4

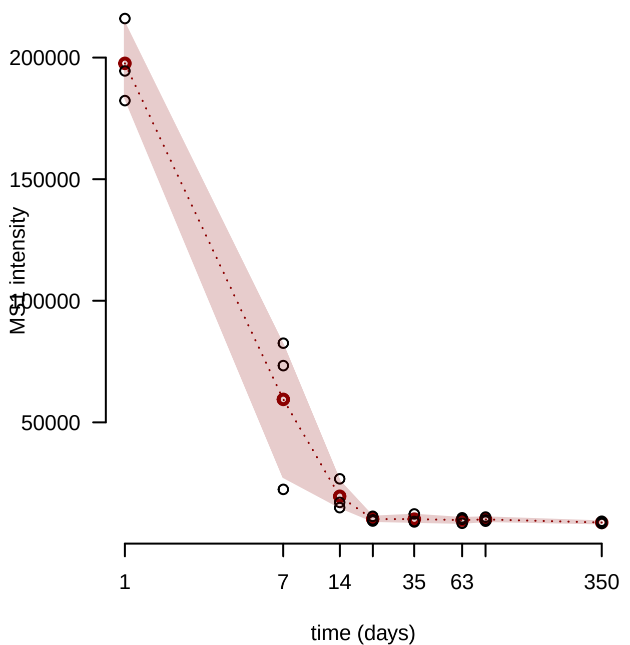

A2AAE1
GN=Kiaa1109

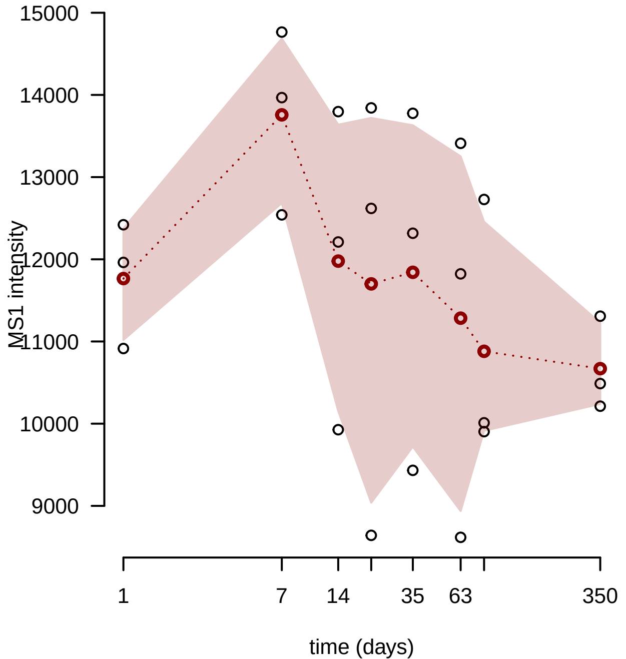

A2AAX3
GN=Klhl15
kelch−like 15

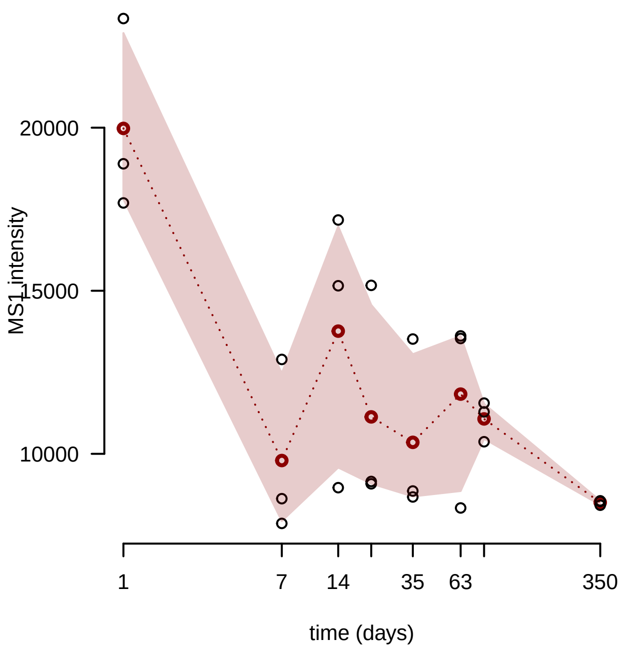

A2AAY5
GN=Sh3pxd2b
SH3 and PX domains 2B

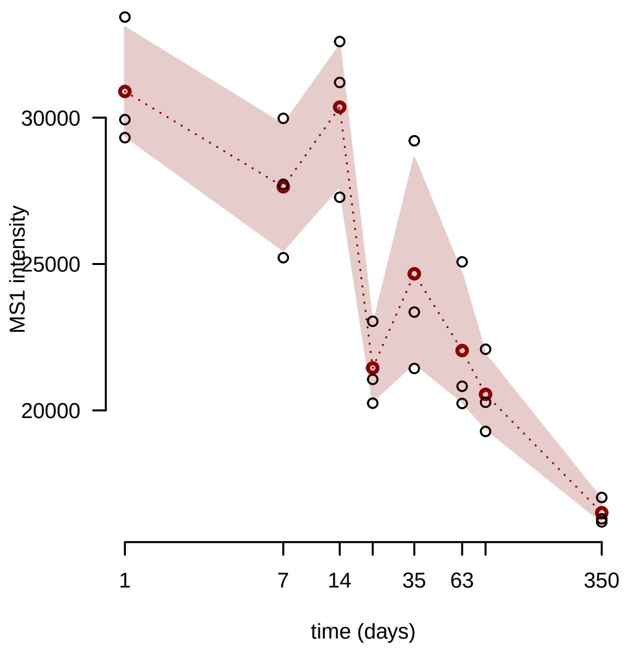

A2ABU4
GN=Myom3
myomesin family, member 3

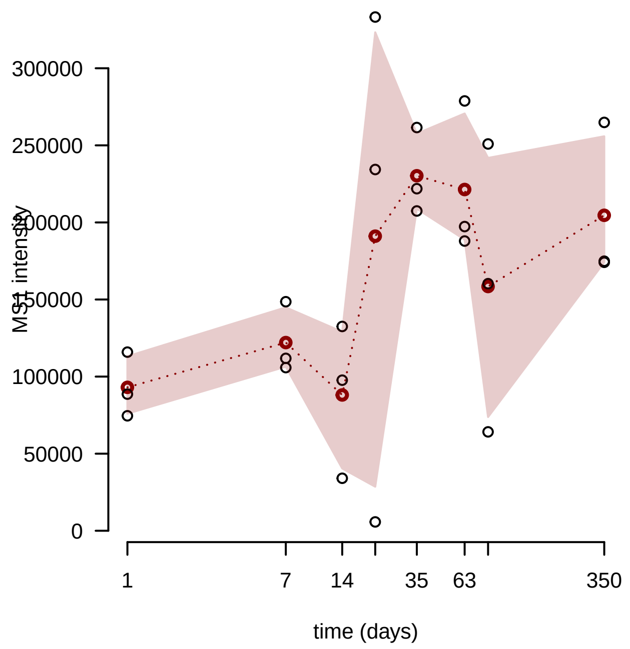

A2ABV5
GN=Med14
mediator complex subunit 14

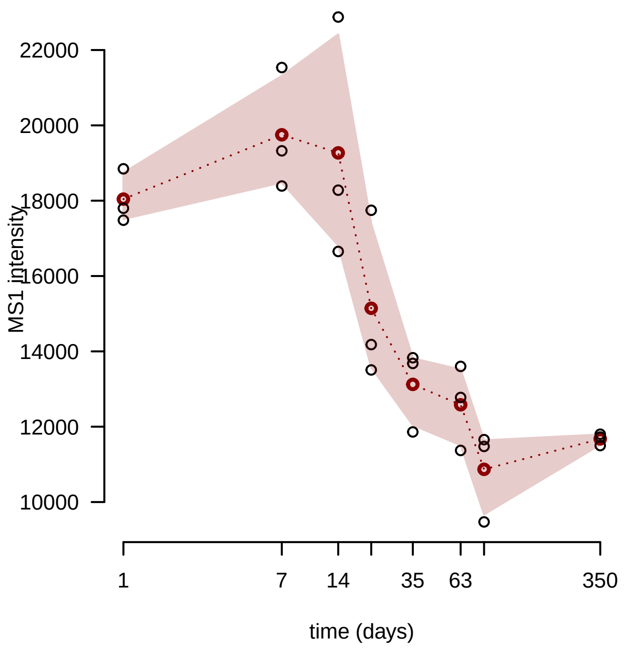

A2ACJ2
GN=Faap100
Fanconi anemia core complex associated p ...

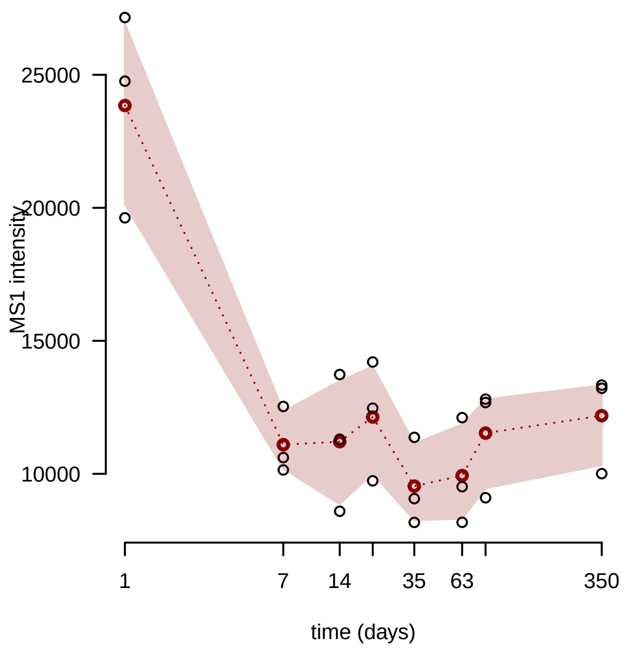

A2ACP1
GN=Ttc39a
tetratricopeptide repeat domain 39A

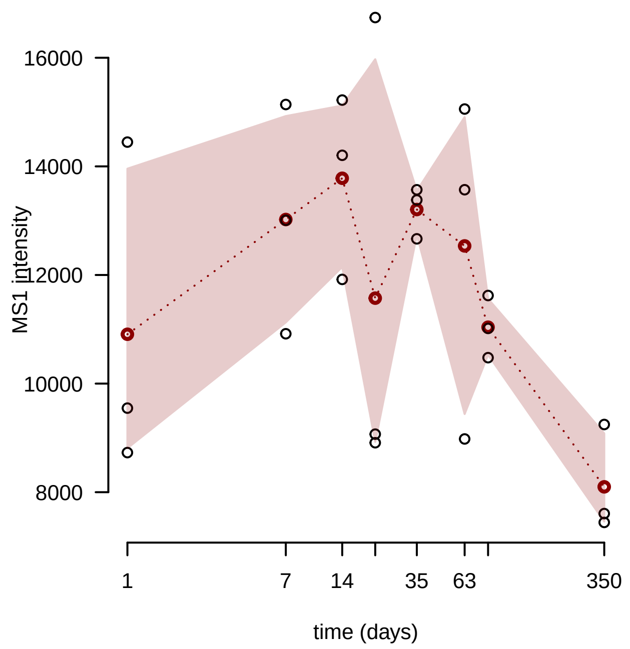

A2ADA5
GN=Pusl1
pseudouridylate synthase−like 1

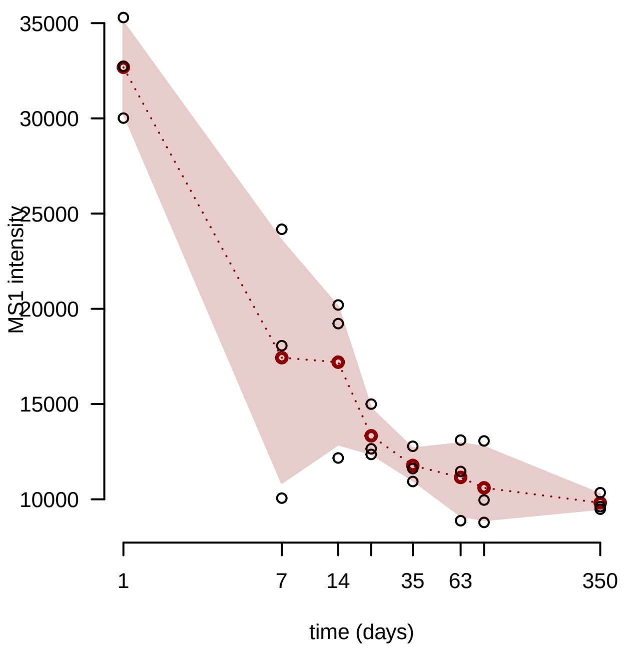

A2ADF7
GN=Slc25a34
solute carrier family 25, member 34

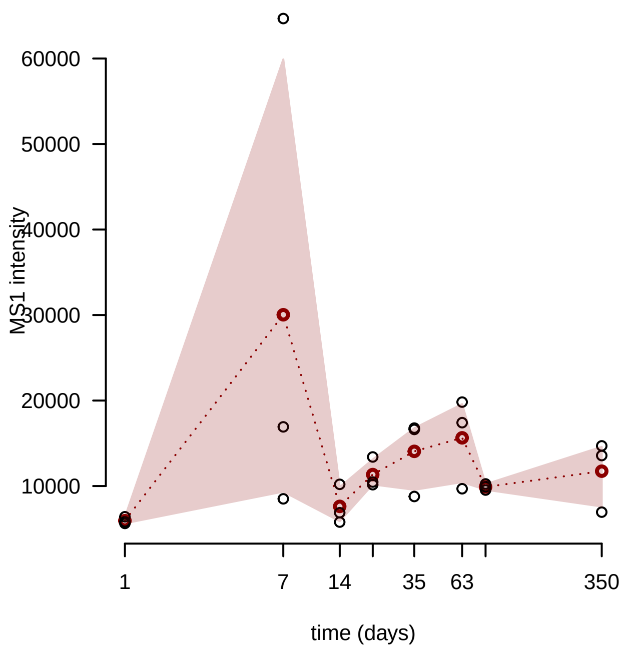

A2ADY9
GN=Ddi2
DNA−damage inducible protein 2

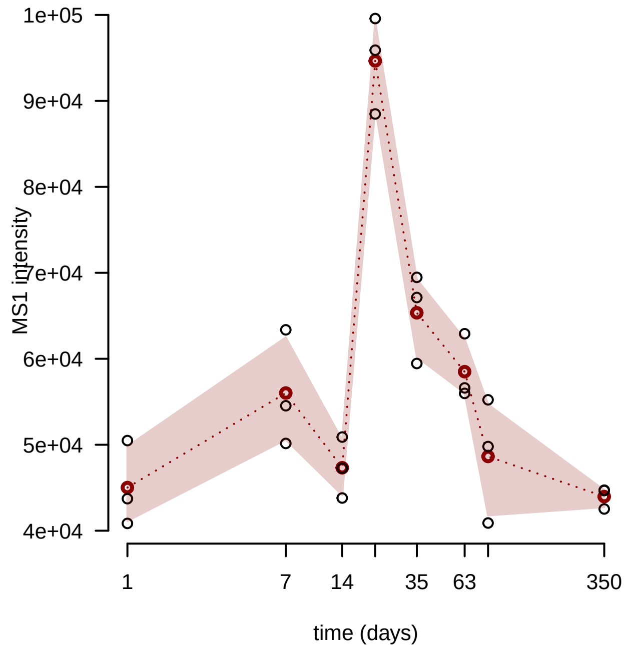

A2AEV7
GN=Ccdc120
coiled−coil domain containing 120

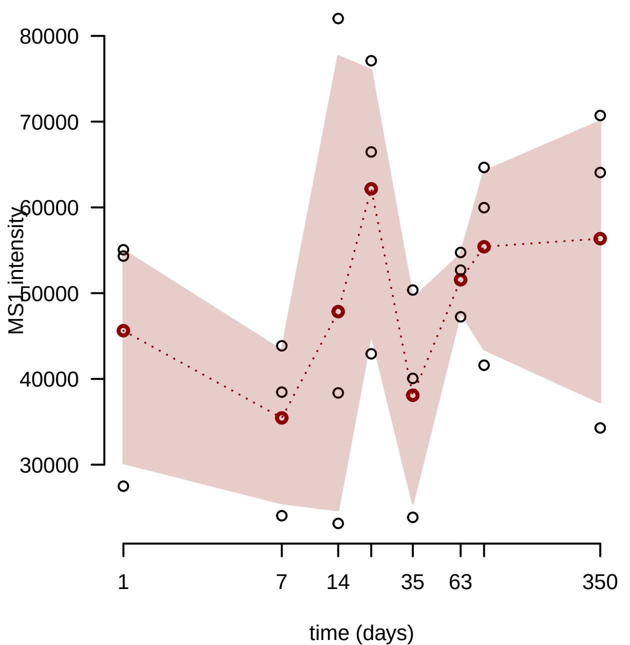

A2AEY4
GN=Map7d3
MAP7 domain containing 3

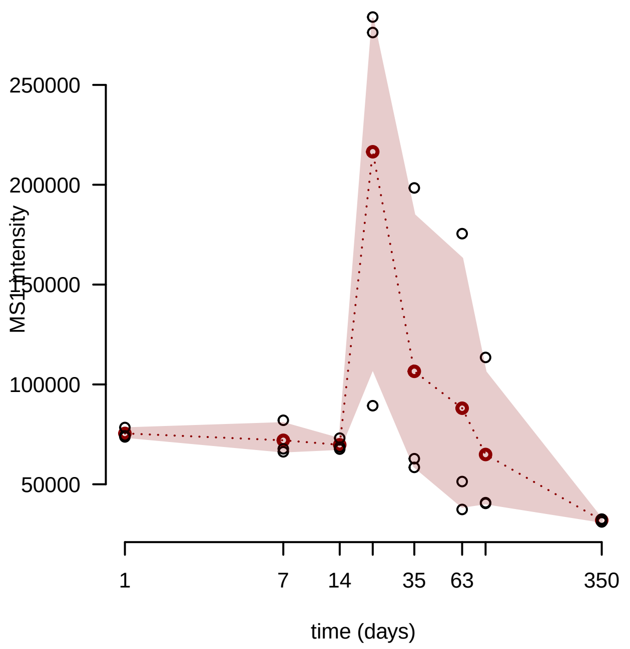

A2AF47
GN=Dock11
dedicator of cytokinesis 11

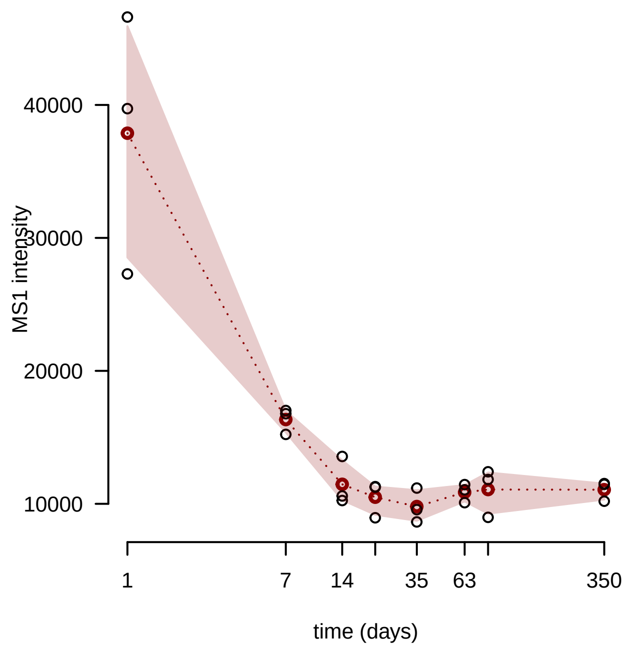

A2AG06
GN=Meioc
meiosis specific with coiled−coil domain

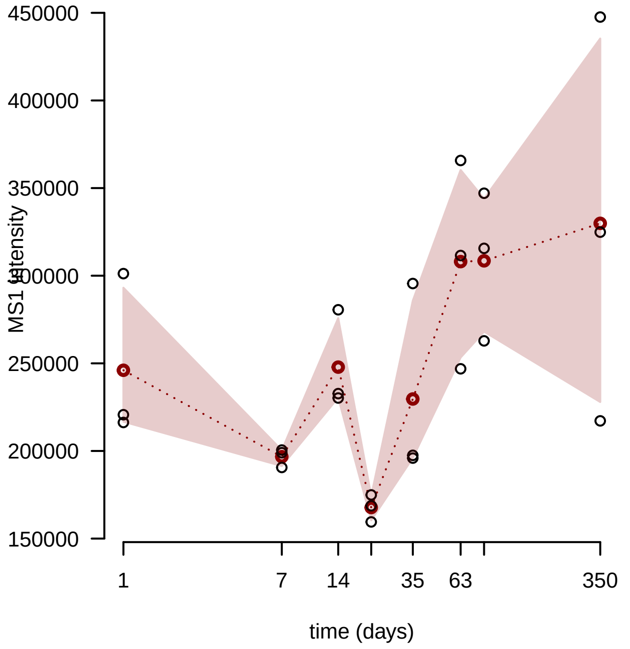

A2AG50
GN=Map7d2
MAP7 domain containing 2

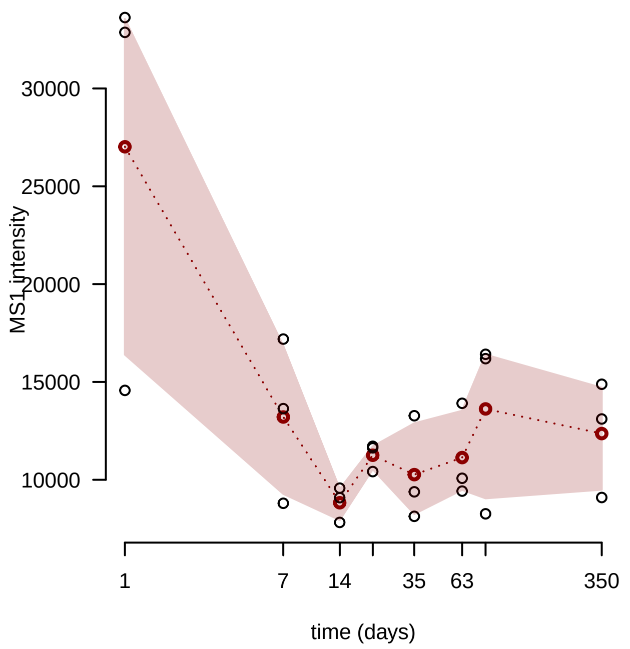

A2AG58
GN=Bclaf3
Bclaf1 and Thrap3 family member 3

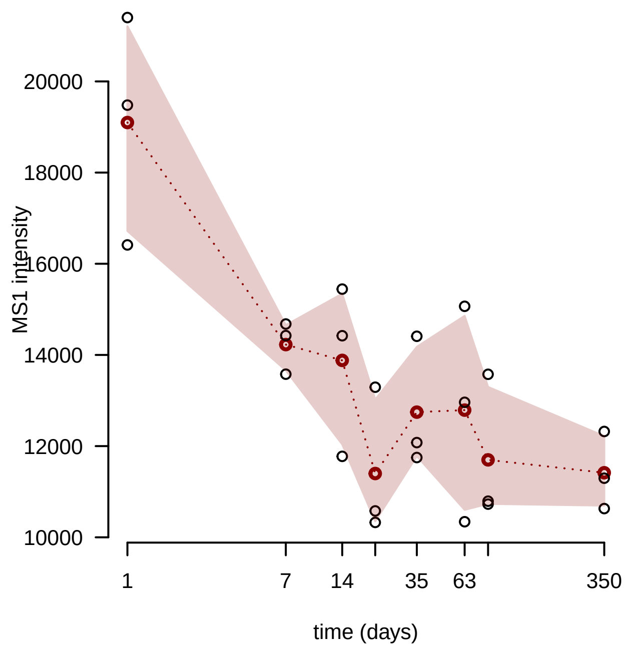

A2AGH6
GN=Med12
mediator complex subunit 12

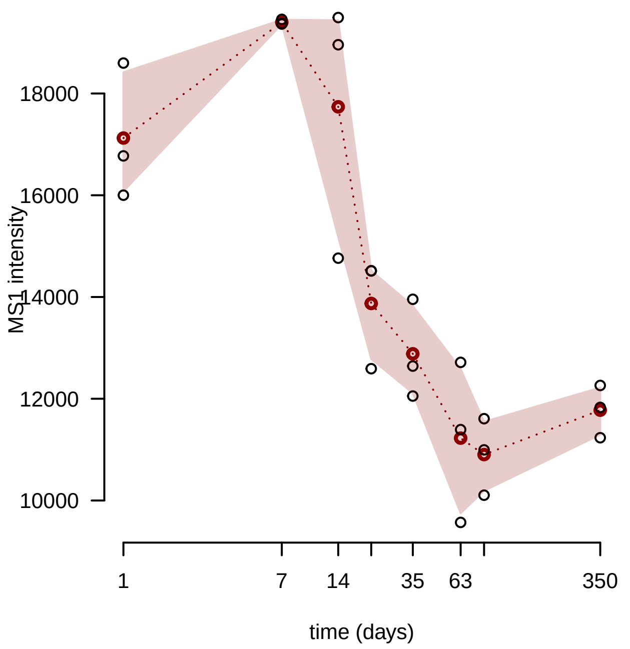

A2AGT5
GN=Ckap5
cytoskeleton associated protein 5

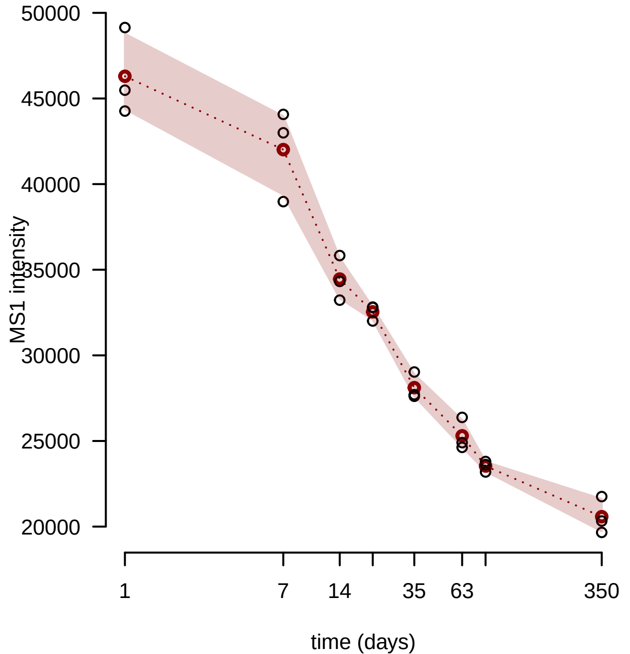

A2AGX3
GN=Prdm11
PR domain containing 11

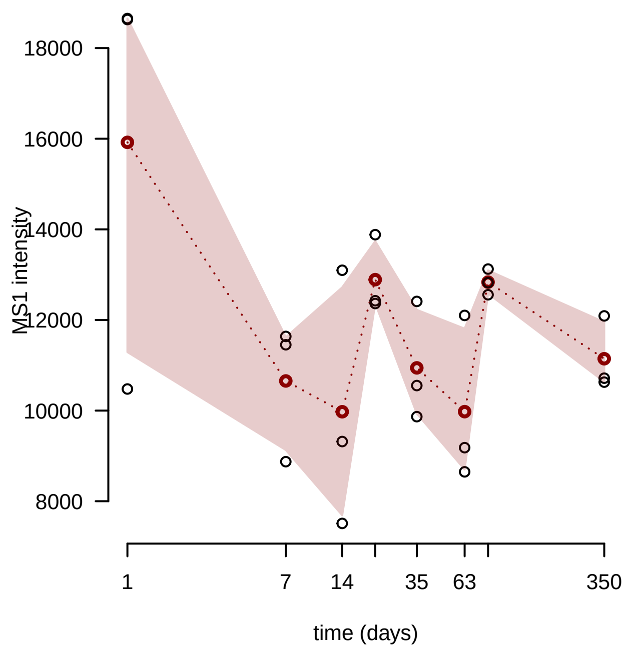

A2AH22
GN=Ambra1
autophagy/beclin 1 regulator 1

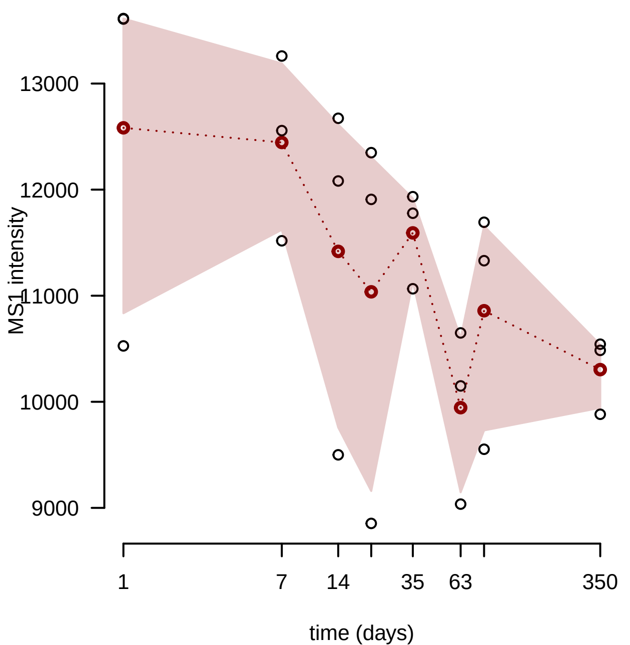

A2AHC3
GN=Camsap1
calmodulin regulated spectrin−associated ...

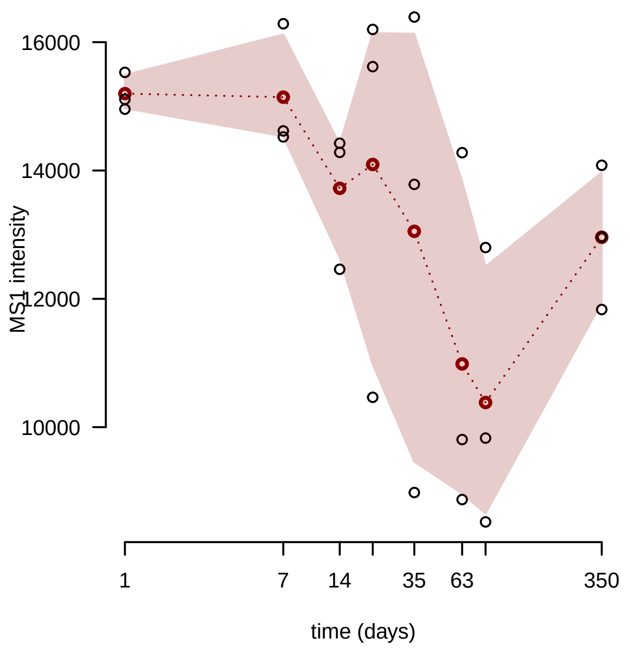

A2AHJ4
GN=Brwd3
bromodomain and WD repeat domain contain ...

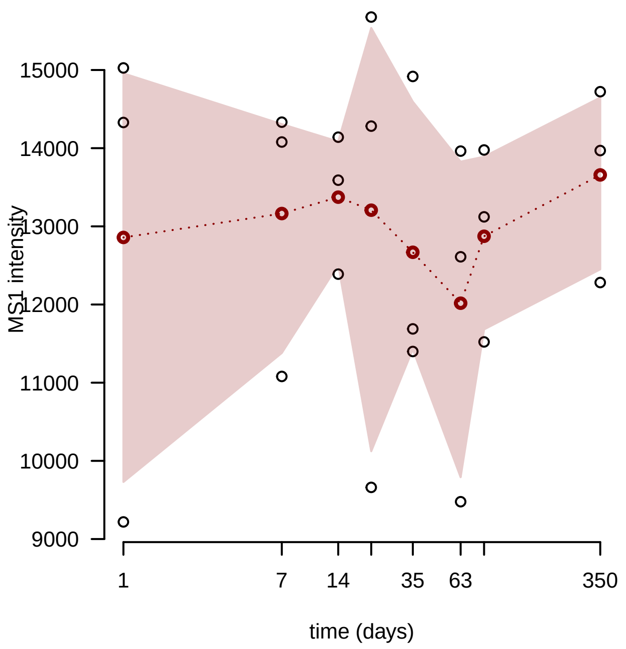

A2AI05
GN=Ndor1
NADPH dependent diflavin oxidoreductase ...

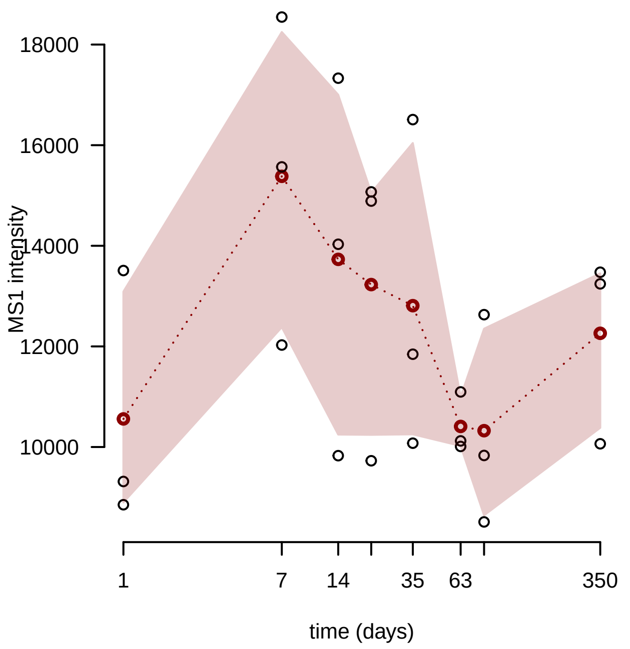

A2AI08
GN=Tprn
taperin

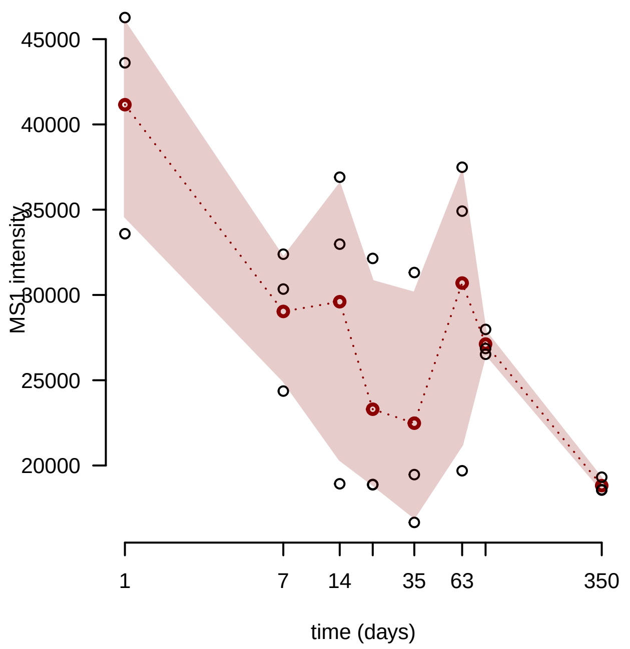

A2AIG8
GN=Accs
1−aminocyclopropane−1−carboxylate syntha ...

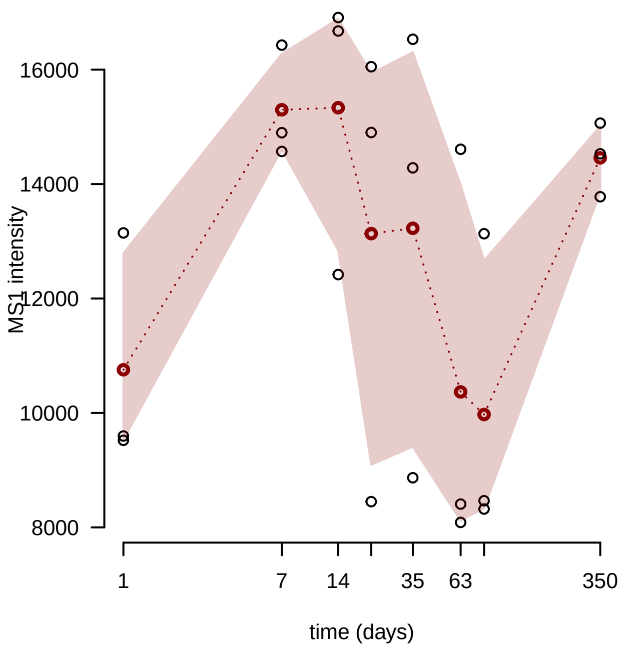

A2AIL4
GN=Ndufaf6
NADH:ubiquinone oxidoreductase complex a ...

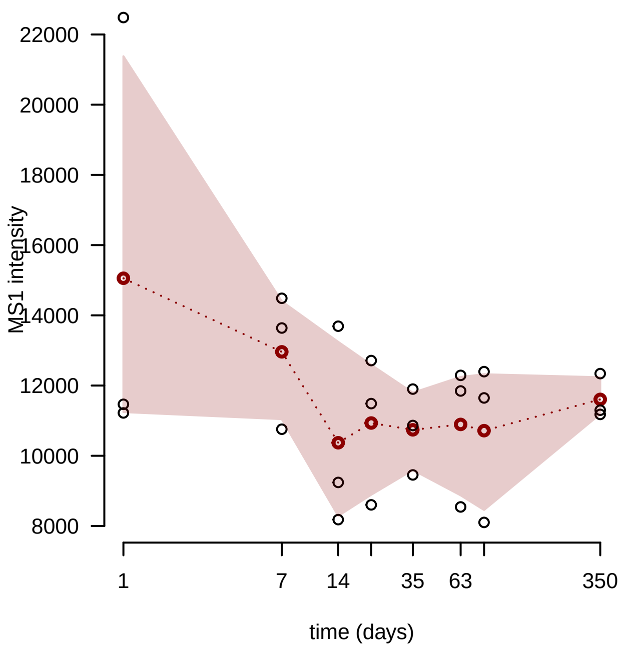

A2AIV2
GN=Virma
vir like m6A methyltransferase associate ...

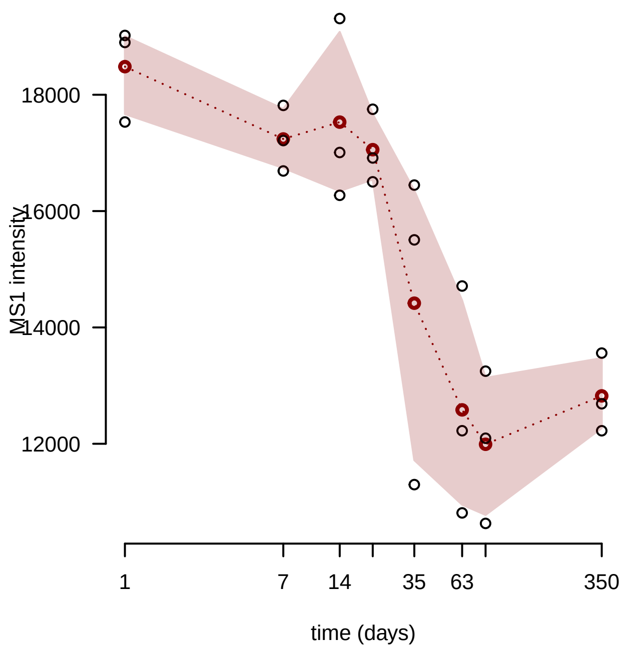

A2AJ15
GN=Man1b1
mannosidase, alpha, class 1B, member 1

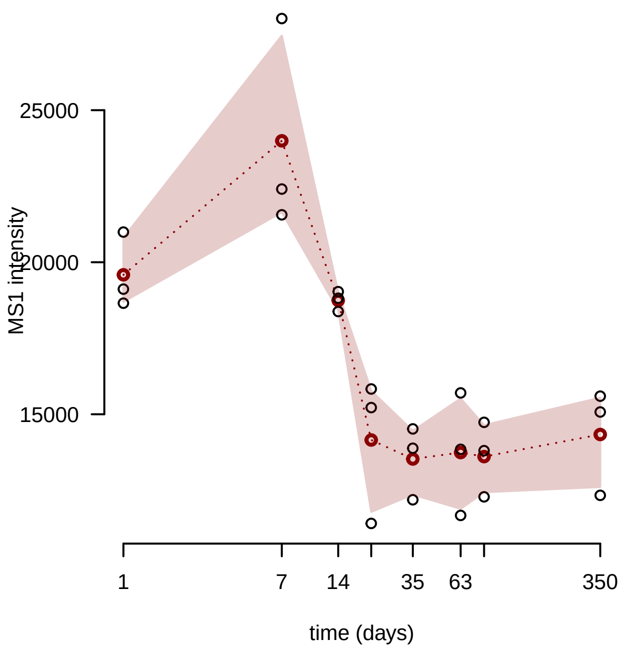

A2AJ88
GN=Pnpla7
patatin−like phospholipase domain contai ...

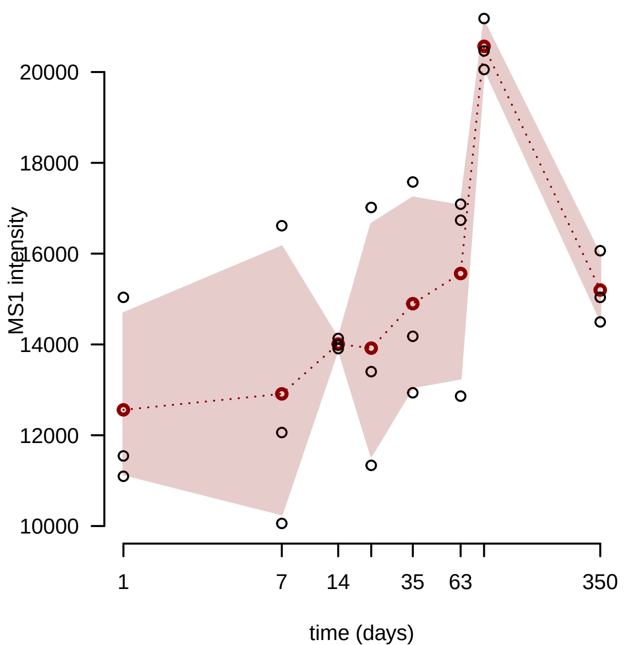

A2AJA7
GN=Mamdc4
MAM domain containing 4

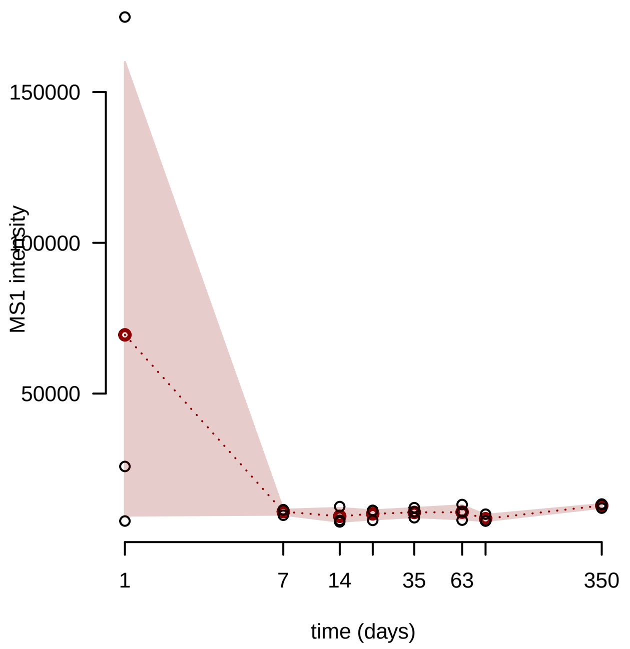

A2AJI0
GN=Map7d1
MAP7 domain containing 1

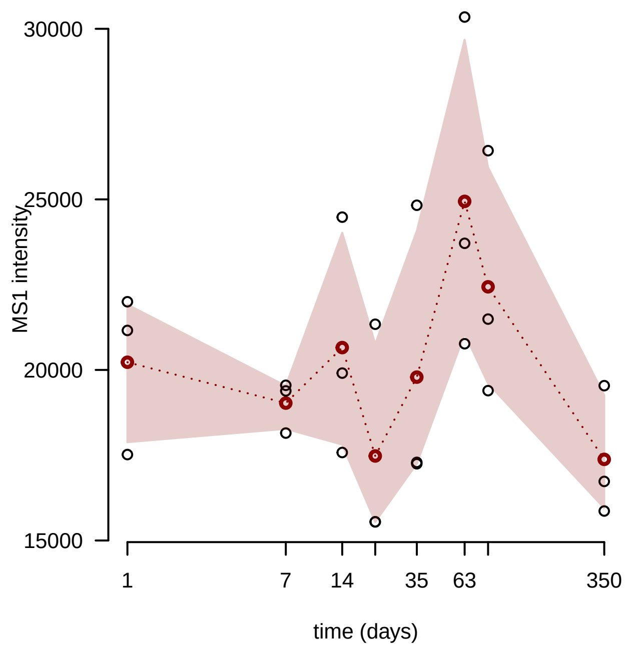

A2AJK6
GN=Chd7
chromodomain helicase DNA binding protei ...

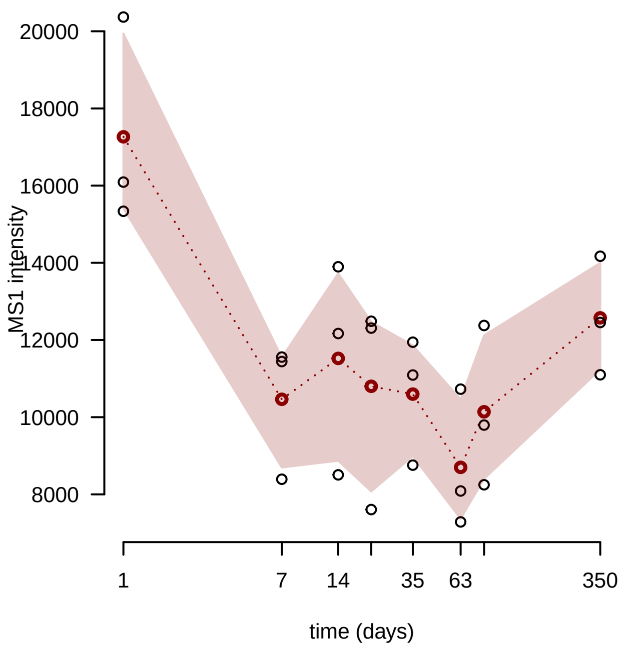

A2AJL3
GN=Fggy
FGGY carbohydrate kinase domain containi ...

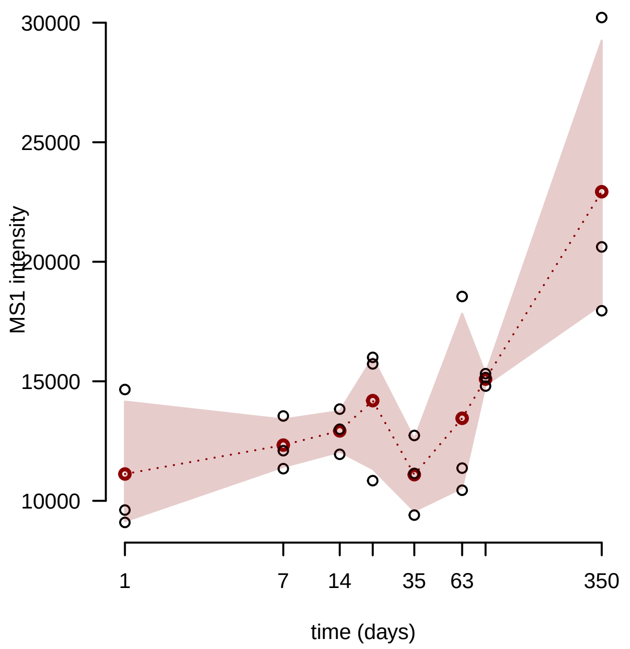

A2AJQ3
GN=Dpy19l4
dpy−19−like 4 (C. elegans)

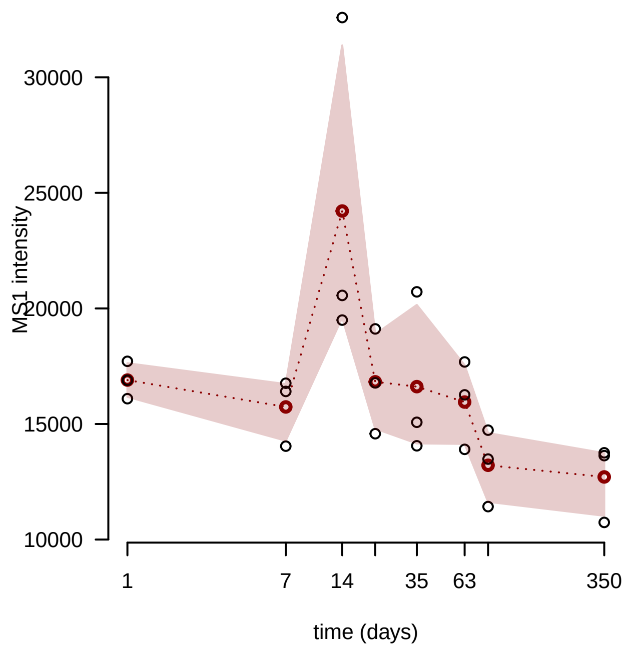

A2AJT4
GN=Pnisr
PNN interacting serine/arginine−rich

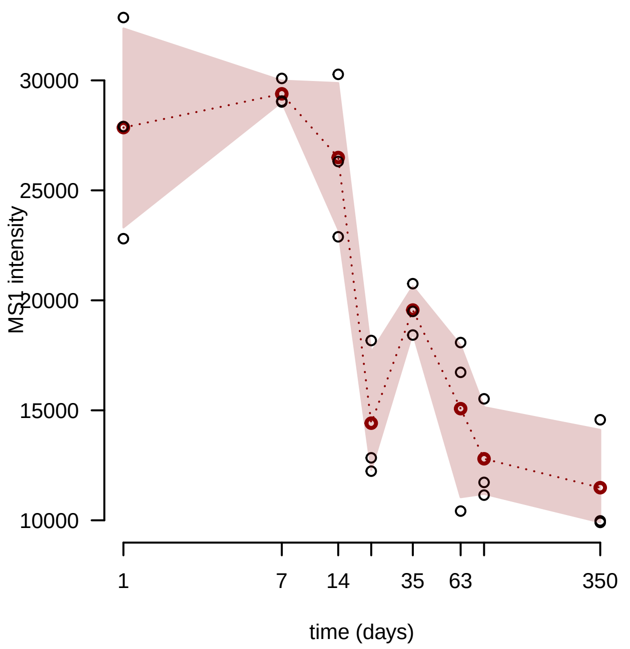

A2AKB9
GN=Dcaf10
DDB1 and CUL4 associated factor 10

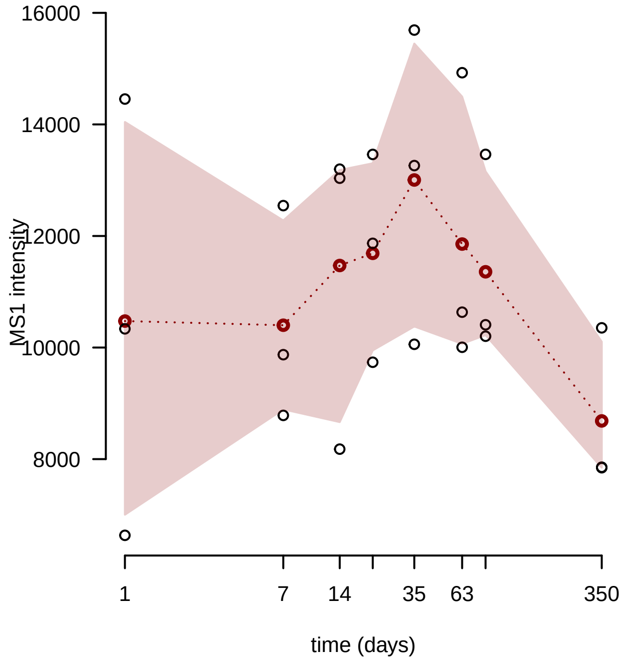

A2AKG8
GN=Focad
focadhesin

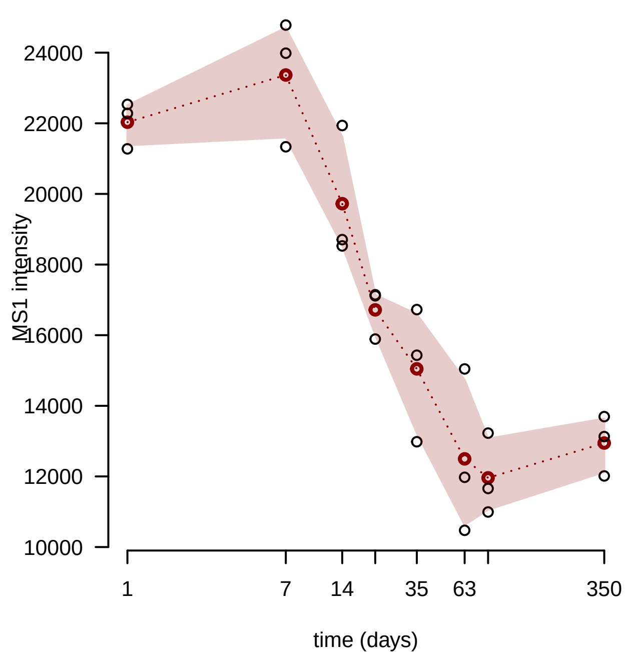

A2AKQ0
GN=Slc35d1
solute carrier family 35 (UDP−glucuronic ...

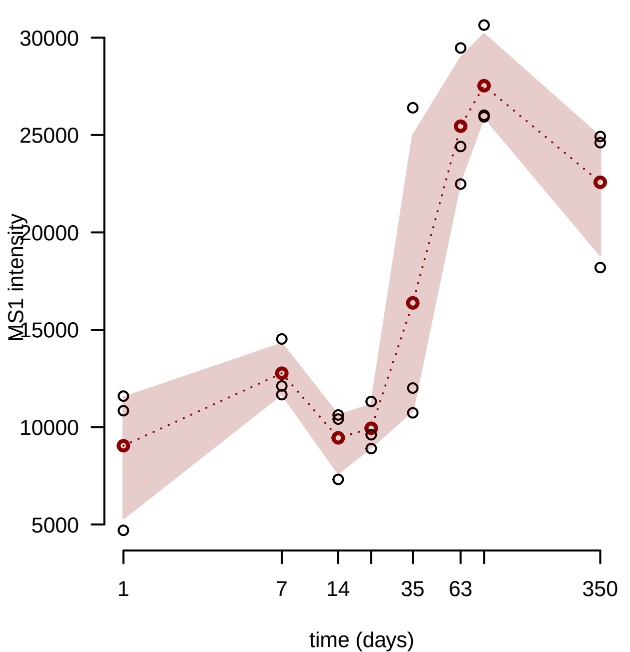

A2AKX3
GN=Setx
senataxin

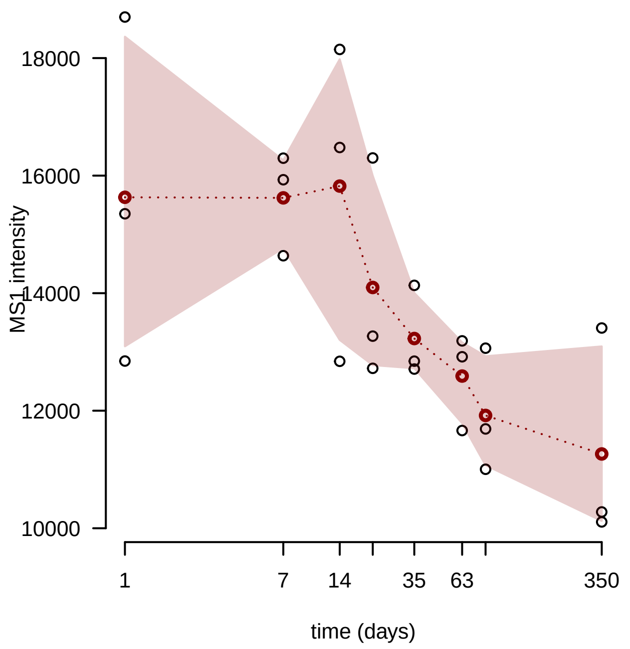

A2AL36
GN=Cntrl
centriolin

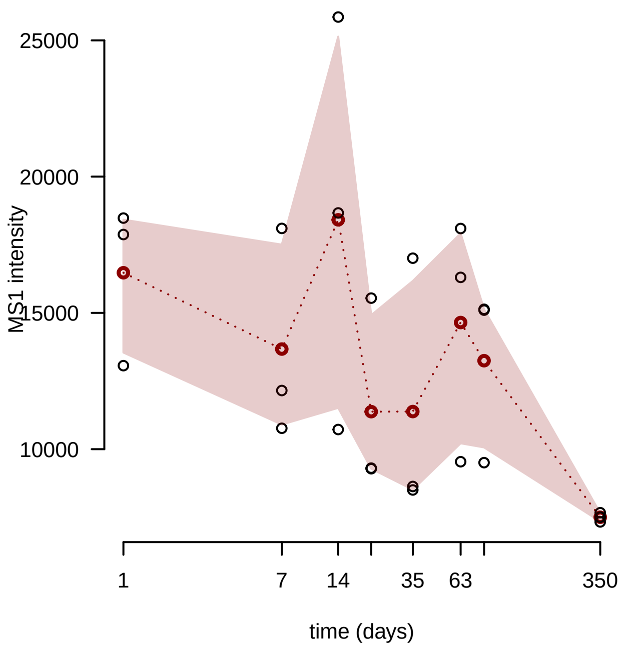

A2ALK8
GN=Ptpn3
protein tyrosine phosphatase, non−recept ...

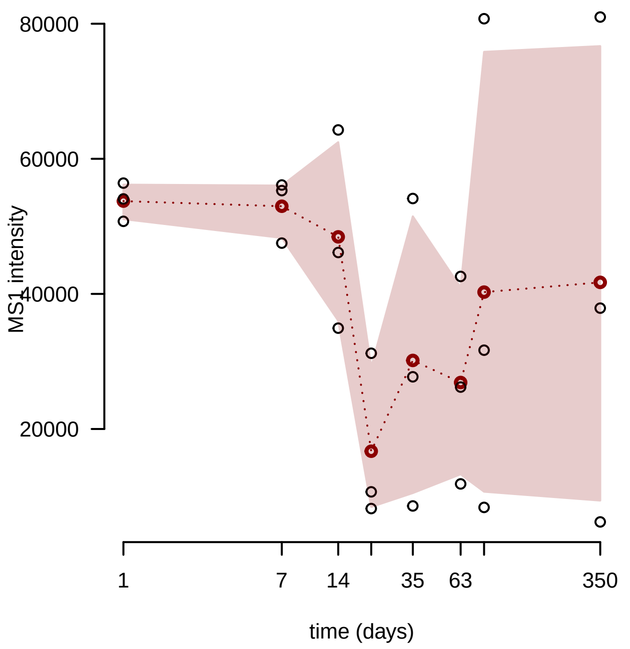

A2ALS5
GN=Rap1gap
Rap1 GTPase−activating protein

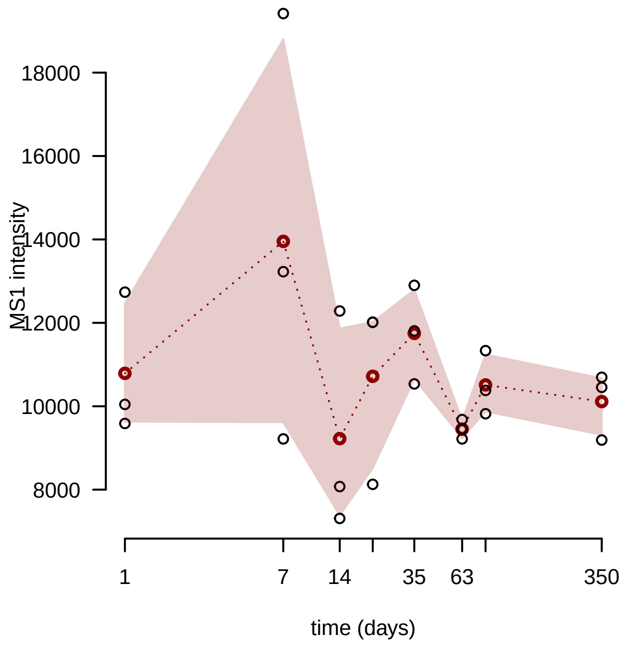

A2ALU4
GN=Shroom2
shroom family member 2

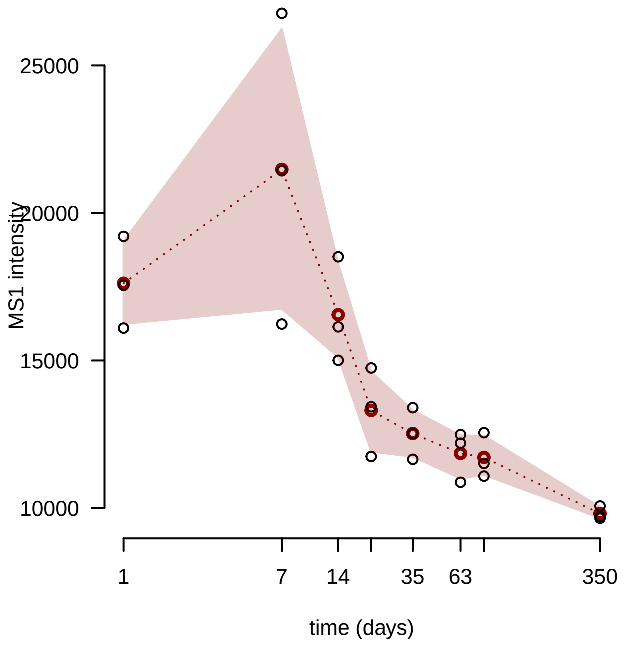

A2ALW5
GN=Dnajc25
DnaJ heat shock protein family (Hsp40) m ...

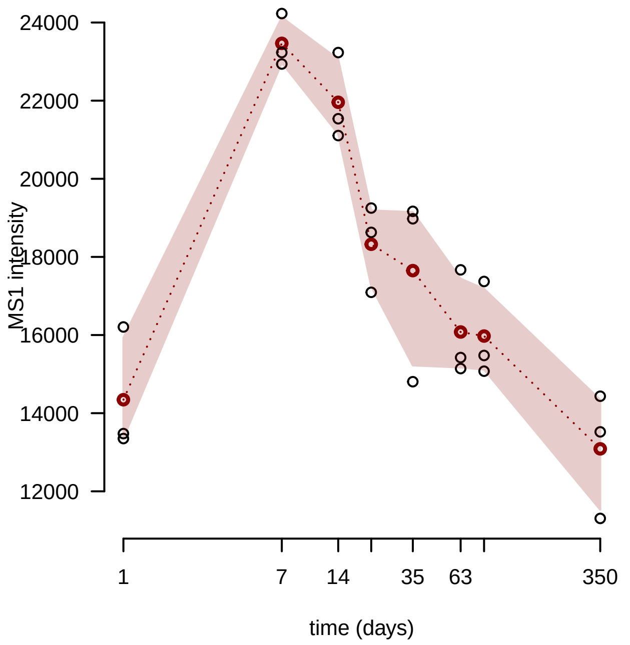

A2AM05
GN=Cntln
centlein, centrosomal protein

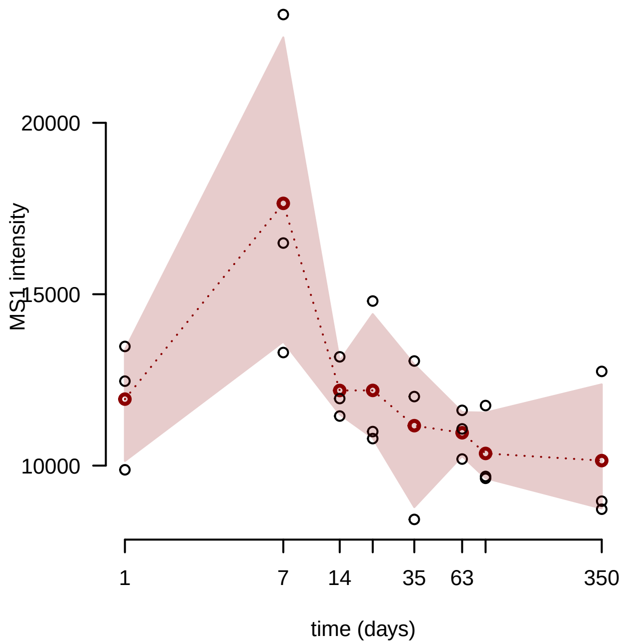

A2AM29
GN=Mllt3
myeloid/lymphoid or mixed−lineage leukem ...

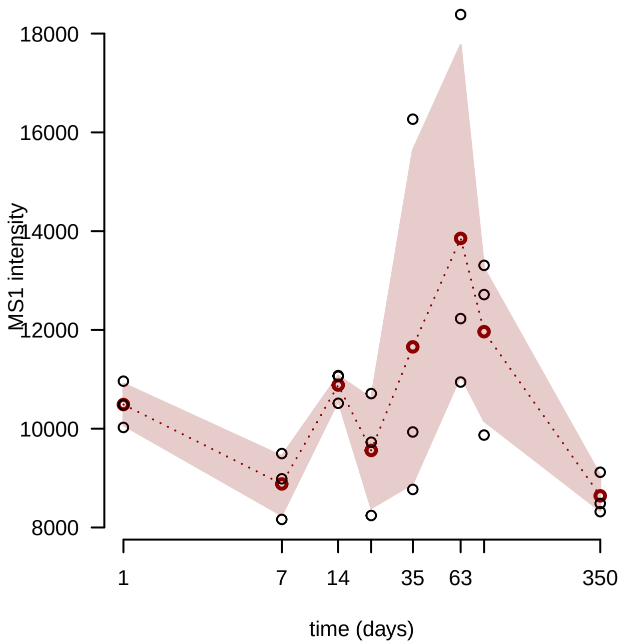

A2AN08
GN=Ubr4
ubiquitin protein ligase E3 component n− ...

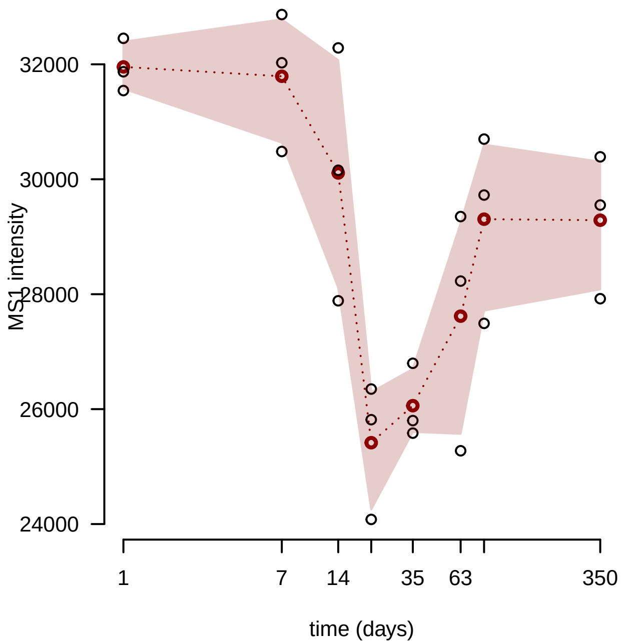

A2APB8
GN=Tpx2
TPX2, microtubule−associated

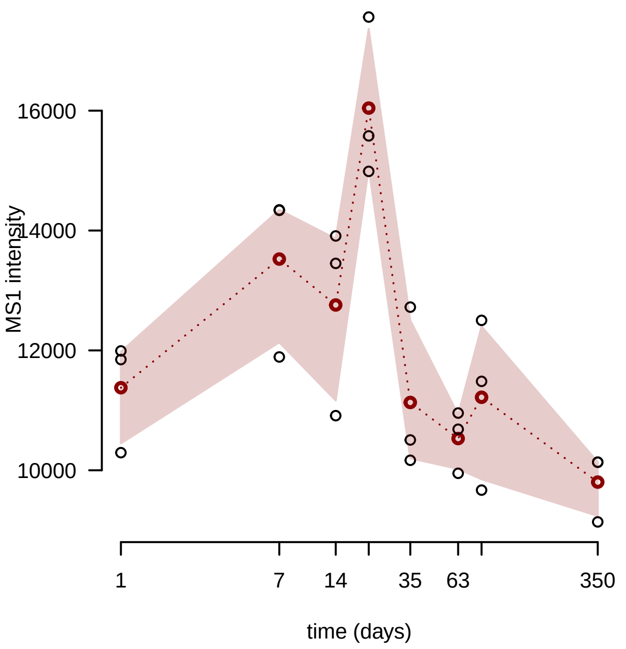

A2APT9
GN=Klhdc7a
kelch domain containing 7A

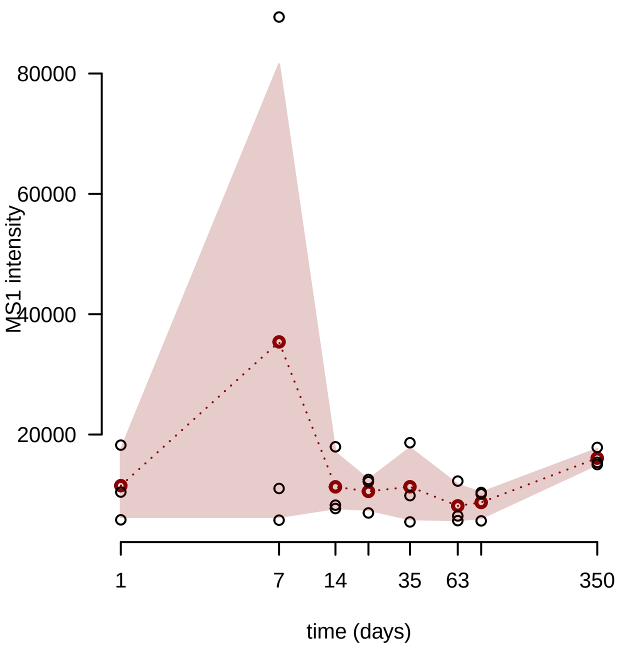

A2APV2
GN=Fmnl2
formin−like 2

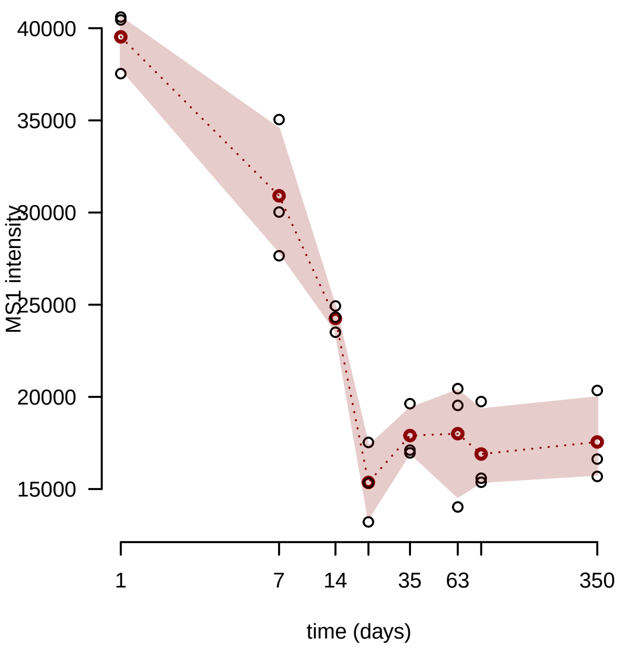

A2APY7
GN=Ndufaf5
NADH:ubiquinone oxidoreductase complex a ...

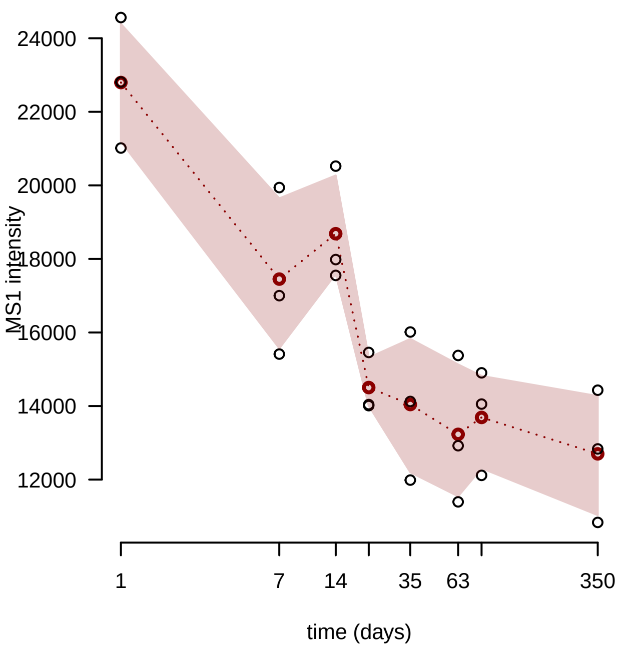

A2AQ07
GN=Tubb1
tubulin, beta 1 class VI

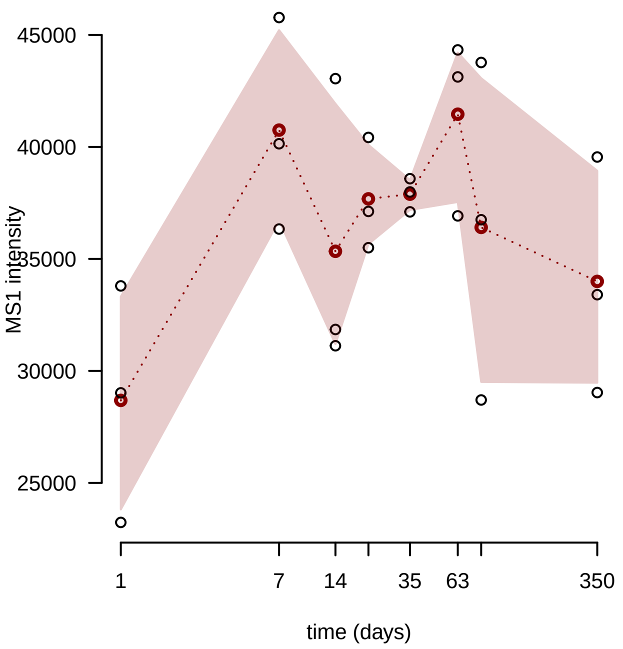

A2AQ19
GN=Rtf1
RTF1, Paf1/RNA polymerase II complex com ...

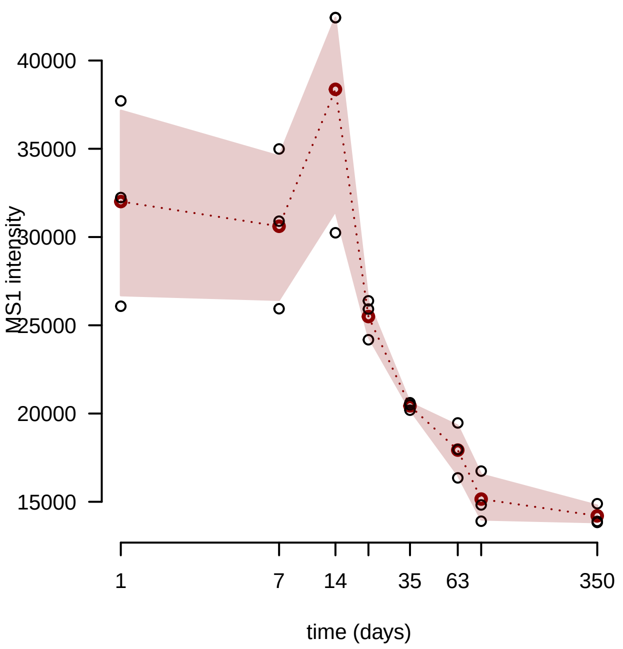

A2AQ25
GN=Skt

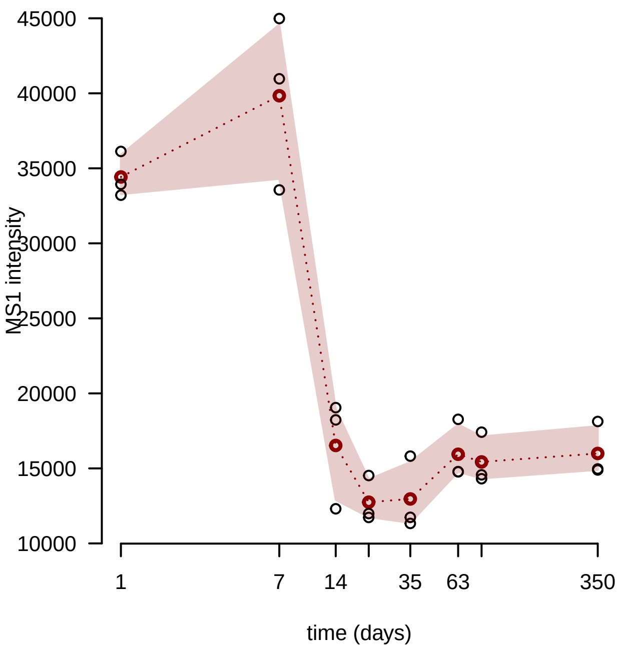

A2AR02
GN=Ppig
peptidyl−prolyl isomerase G (cyclophilin ...

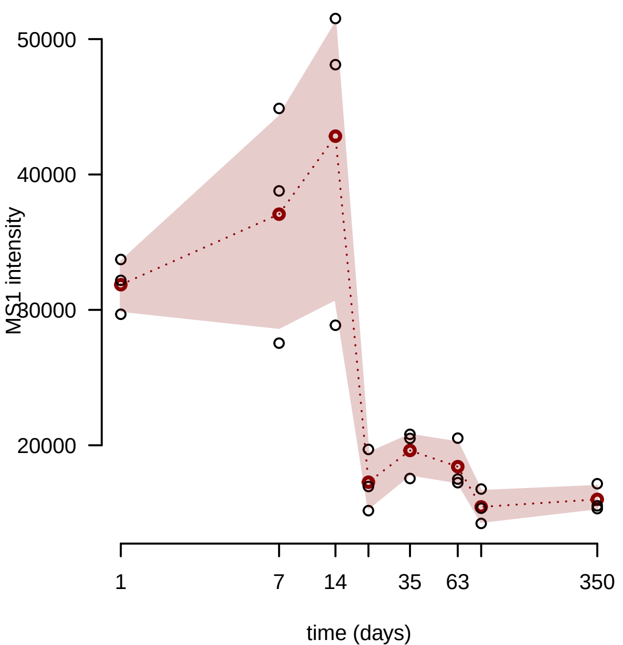

A2ARA8
GN=Itga8
integrin alpha 8

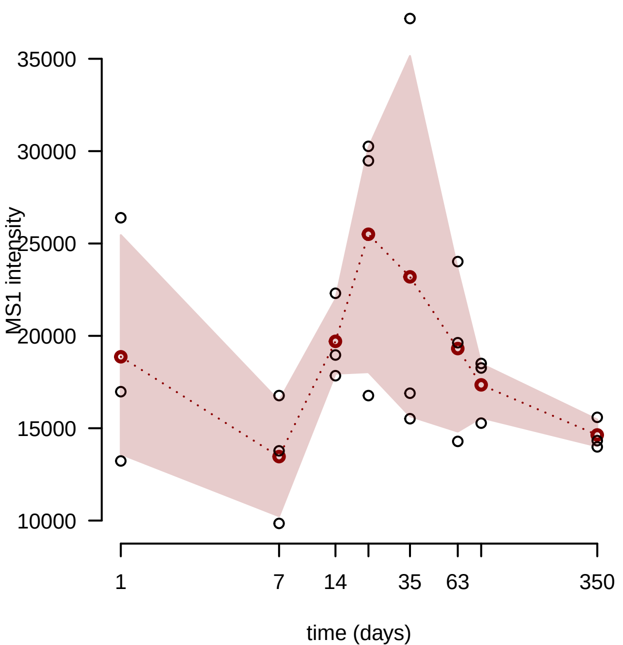

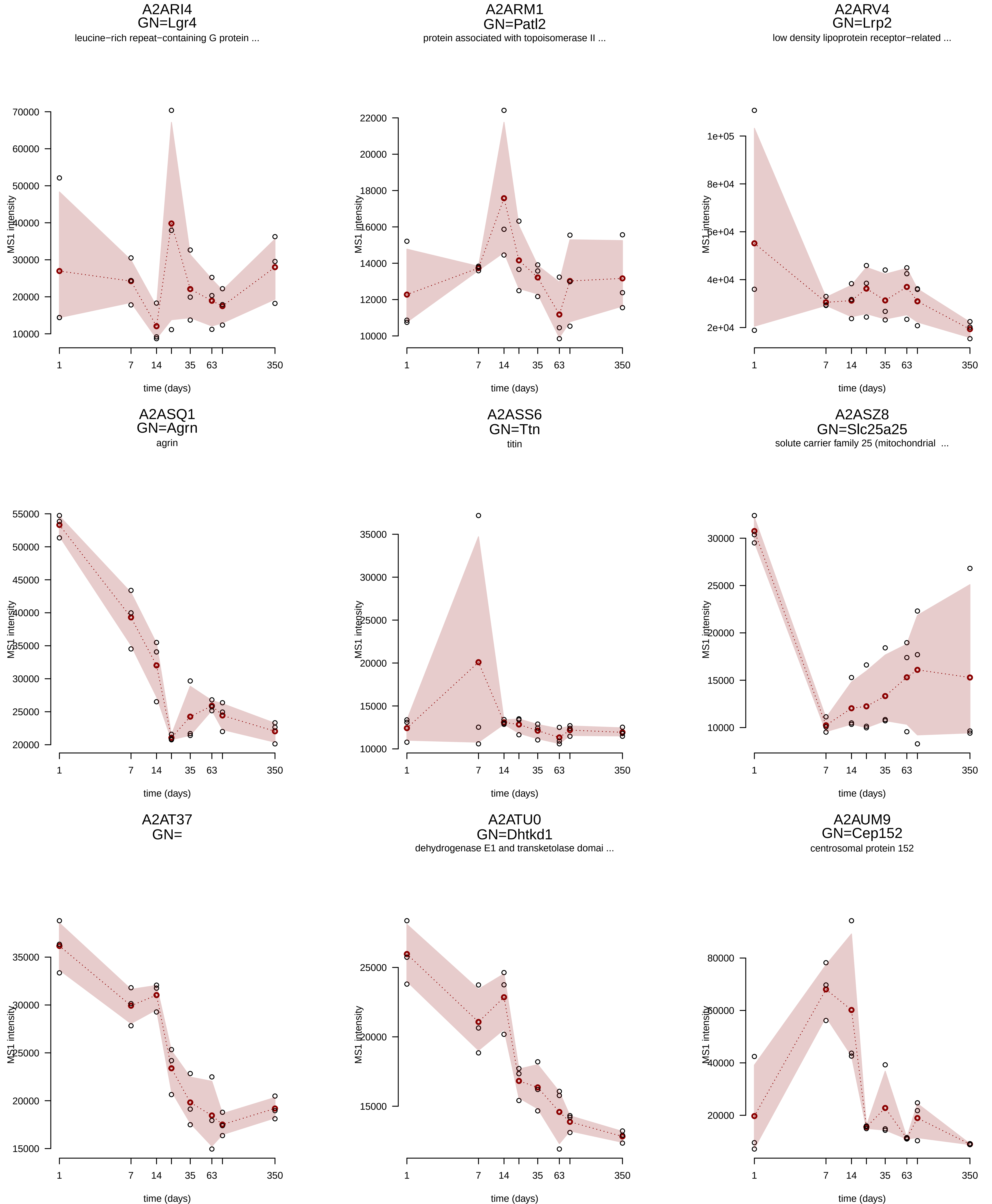
A2ARI4
GN=Lgr4
leucine−rich repeat−containing G protein ...
A2ARM1
GN=Patl2
protein associated with topoisomerase II ...
A2ARV4
GN=Lrp2
low density lipoprotein receptor−related ...
A2ASQ1
GN=Agrn
agrin
A2ASS6
GN=Ttn
titin
A2ASZ8
GN=Slc25a25
solute carrier family 25 (mitochondrial ...
A2AT37
GN=
A2ATU0
GN=Dhtkd1
dehydrogenase E1 and transketolase domai ...
A2AUM9
GN=Cep152
centrosomal protein 152
MS1 intensity
time (days)
1
7
14
35
63
350

A2AUU0
GN=Mettl8
methyltransferase like 8

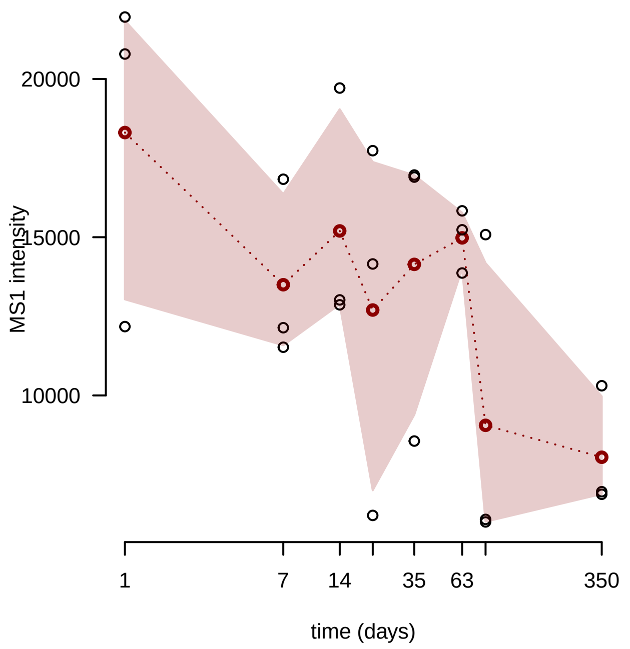

A2AUY4
GN=

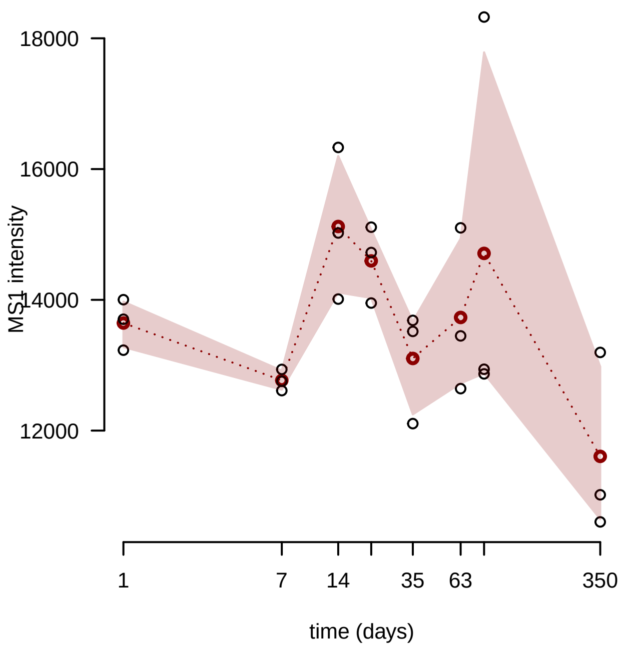

A2AVZ9
GN=Slc43a3
solute carrier family 43, member 3

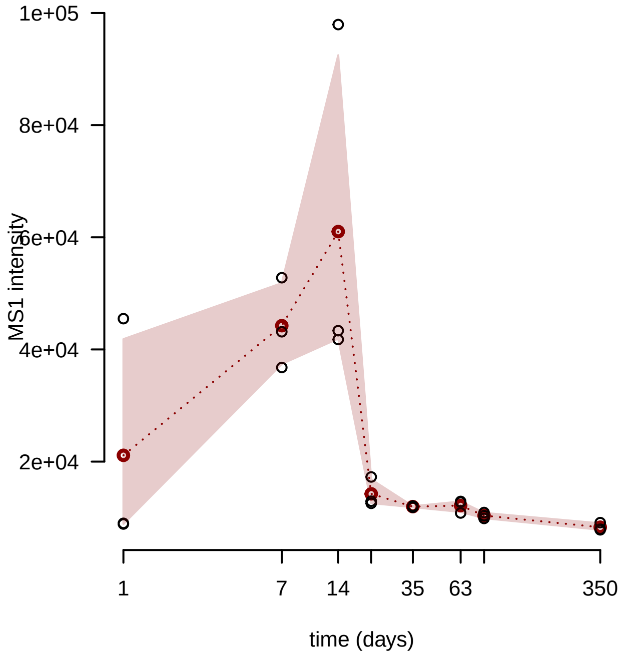

A2AWA9
GN=Rabgap1
RAB GTPase activating protein 1

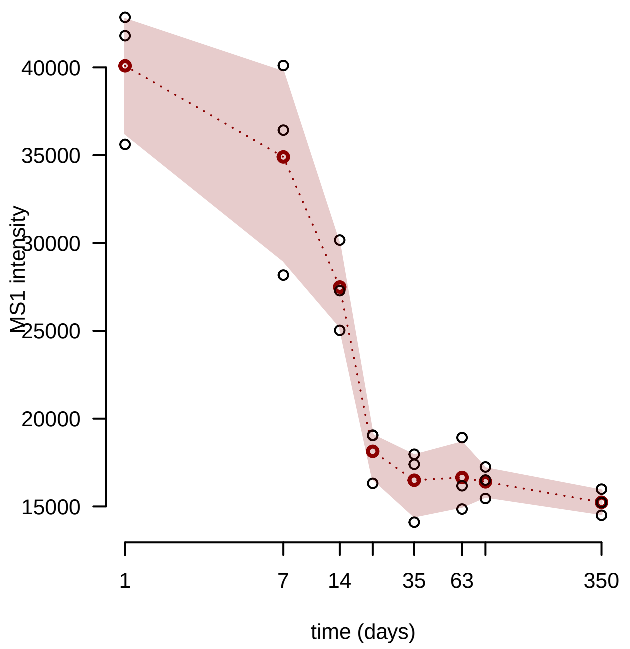

A2AWL7
GN=Mga
MAX gene associated

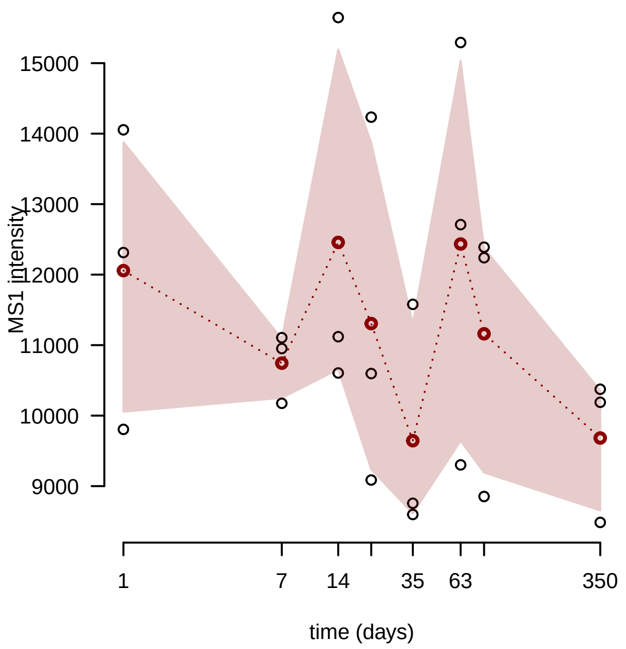

A2AX52
GN=Col6a4
collagen, type VI, alpha 4

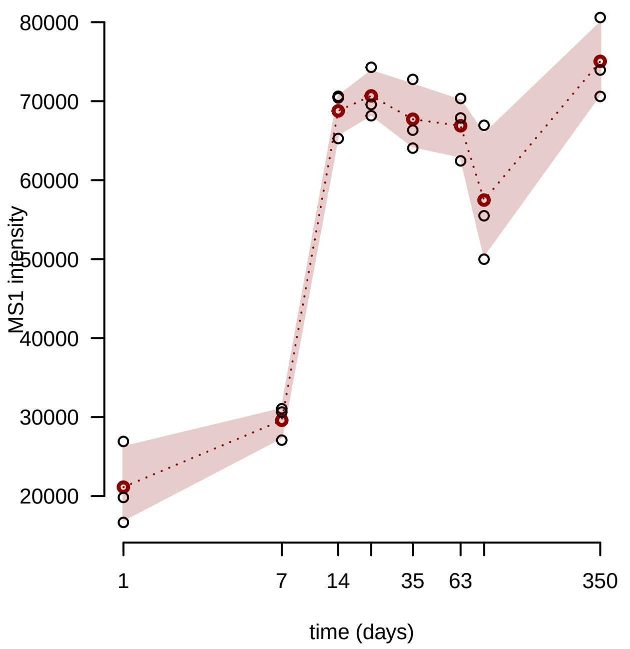

A2BDX3
GN=Mocs3
molybdenum cofactor synthesis 3

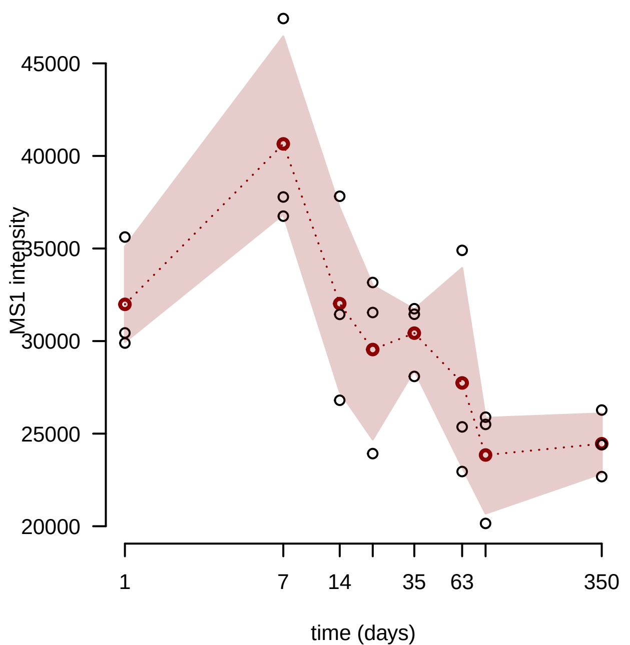

A2BE28
GN=Las1l
LAS1−like (S. cerevisiae)

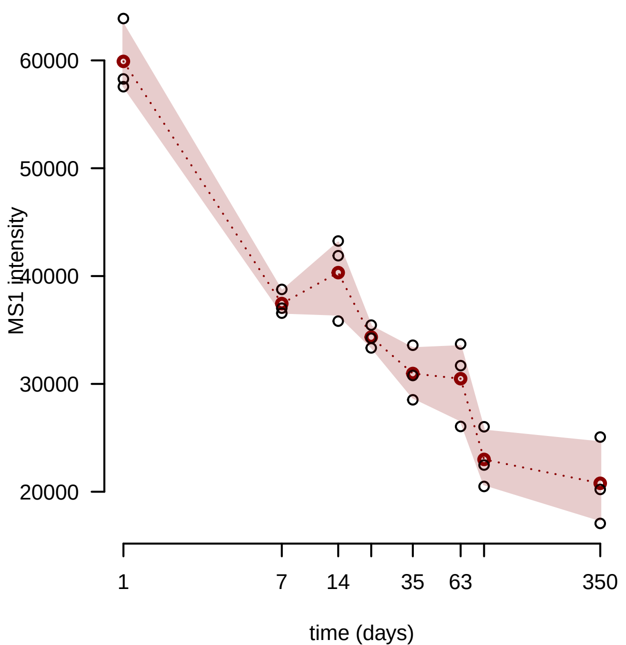

A2BH40
GN=Arid1a
AT rich interactive domain 1A (SWI−like)

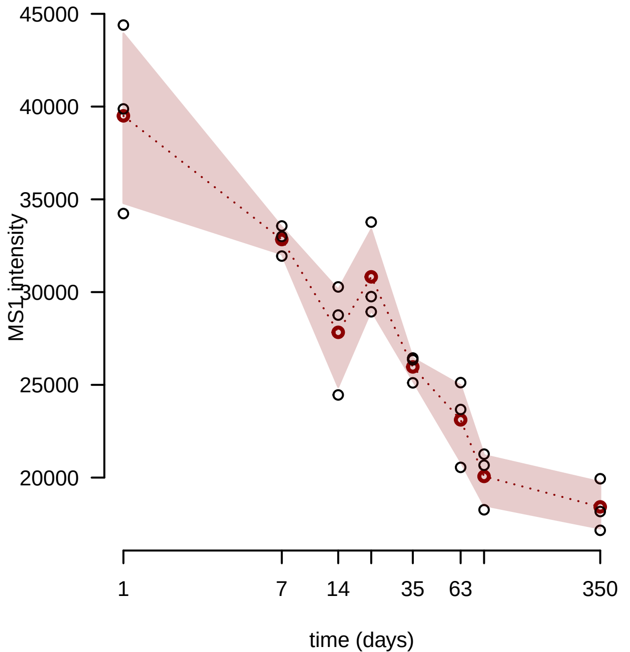

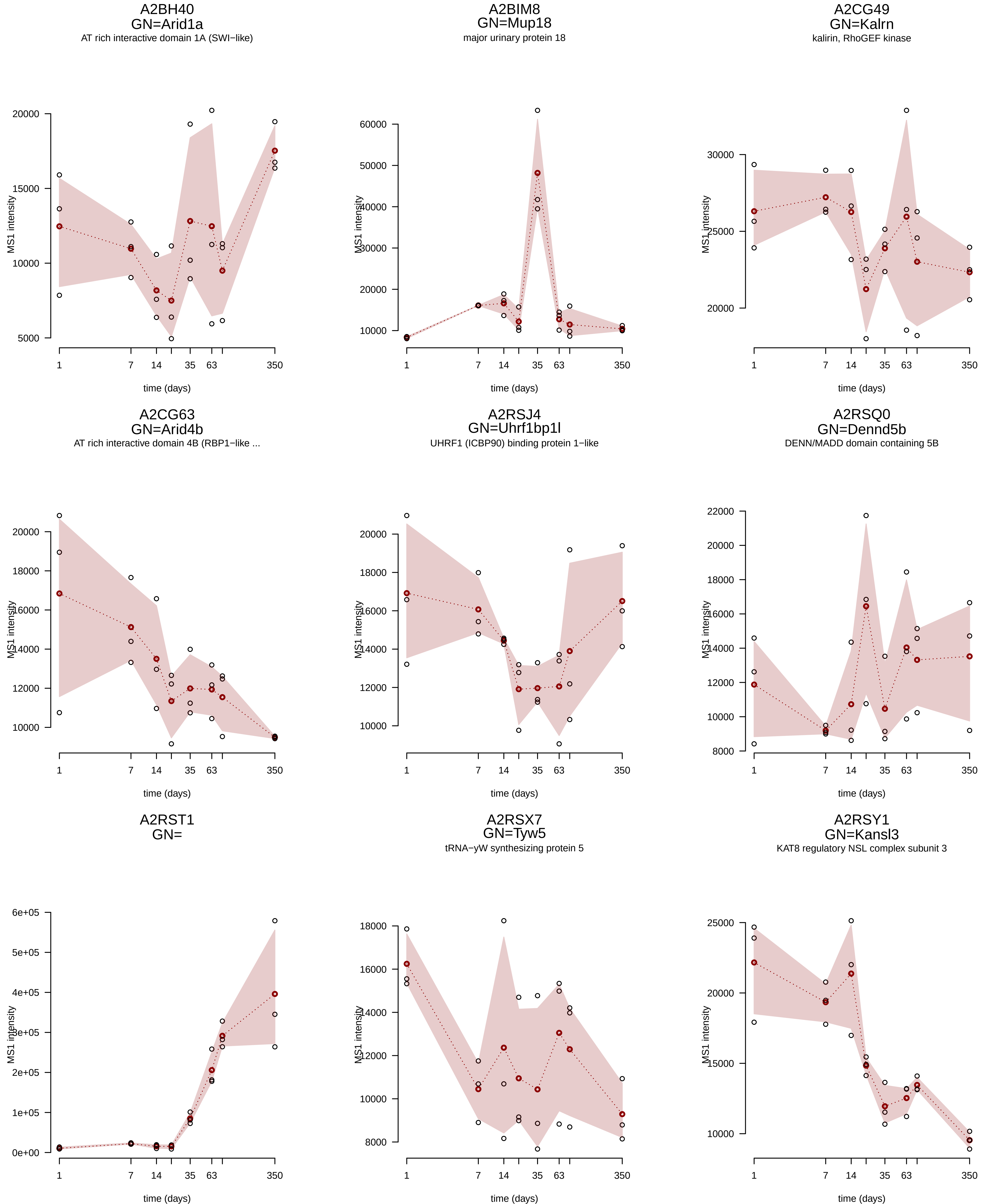
A2BH40
GN=Arid1a
AT rich interactive domain 1A (SWI−like)
MS1 intensity
20000
15000
10000
5000
1
7
14
35
63
350
time (days)
A2BIM8
GN=Mup18
major urinary protein 18
MS1 intensity
60000
50000
40000
30000
20000
10000
time (days)
A2CG49
GN=Kalrn
kalirin, RhoGEF kinase
MS1 intensity
30000
25000
20000
time (days)
A2CG63
GN=Arid4b
AT rich interactive domain 4B (RBP1−like ...
MS1 intensity
20000
18000
16000
14000
12000
10000
time (days)
A2RSJ4
GN=Uhrf1bp1l
UHRF1 (ICBP90) binding protein 1−like
MS1 intensity
20000
18000
16000
14000
12000
10000
time (days)
A2RSQ0
GN=Dennd5b
DENN/MADD domain containing 5B
MS1 intensity
22000
20000
18000
16000
14000
12000
10000
8000
time (days)
A2RST1
GN=
MS1 intensity
6e+05
5e+05
4e+05
3e+05
2e+05
1e+05
0e+00
time (days)
A2RSX7
GN=Tyw5
tRNA−yW synthesizing protein 5
MS1 intensity
18000
16000
14000
12000
10000
8000
time (days)
A2RSY1
GN=Kansl3
KAT8 regulatory NSL complex subunit 3
MS1 intensity
25000
20000
15000
10000
time (days)

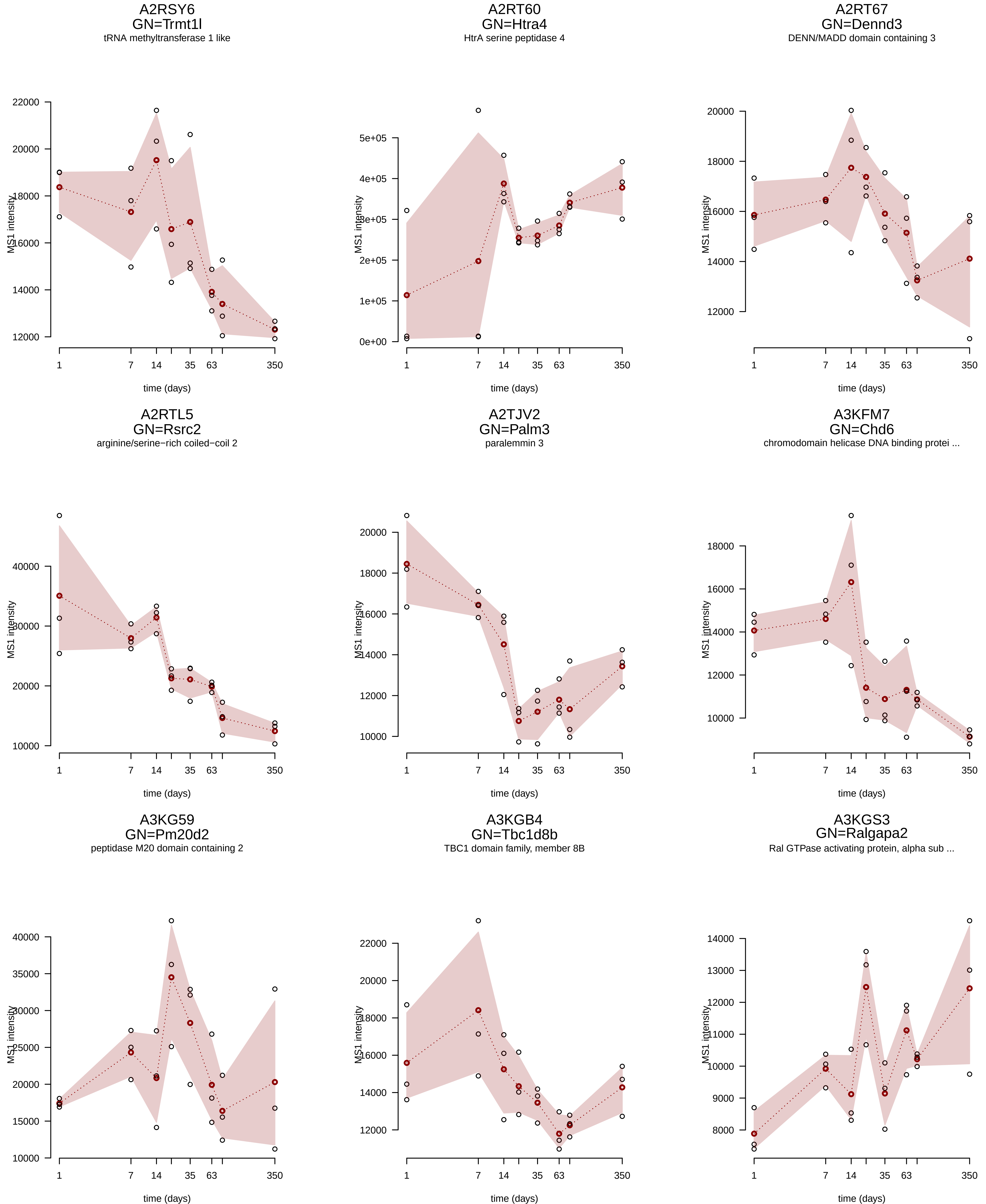
A2RSY6
GN=Trmt1l
tRNA methyltransferase 1 like
A2RT60
GN=Htra4
HtrA serine peptidase 4
A2RT67
GN=Dennd3
DENN/MADD domain containing 3
A2RTL5
GN=Rsrc2
arginine/serine−rich coiled−coil 2
A2TJV2
GN=Palm3
paralemmin 3
A3KFM7
GN=Chd6
chromodomain helicase DNA binding protei ...
A3KG59
GN=Pm20d2
peptidase M20 domain containing 2
A3KGB4
GN=Tbc1d8b
TBC1 domain family, member 8B
A3KGS3
GN=Ralgapa2
Ral GTPase activating protein, alpha sub ...
MS1 intensity
time (days)
1
7
14
35
63
350

A3KGV1
GN=Odf2
outer dense fiber of sperm tails 2

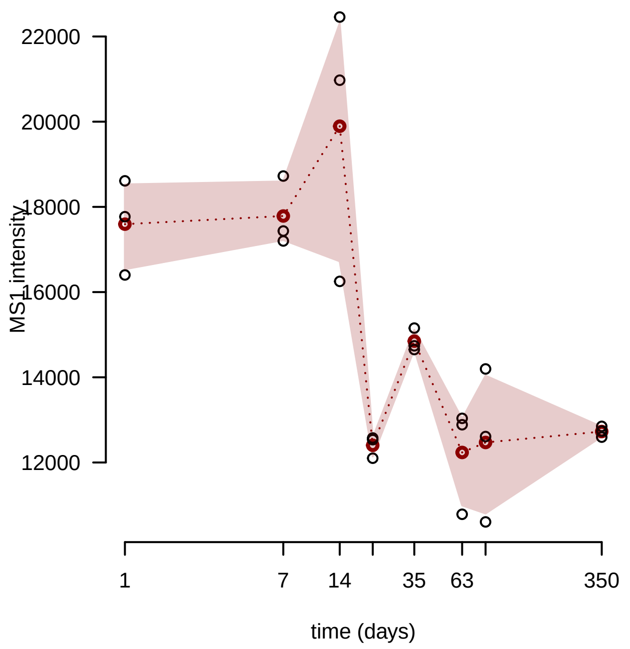

A3KGW5
GN=Cercam
cerebral endothelial cell adhesion molec ...

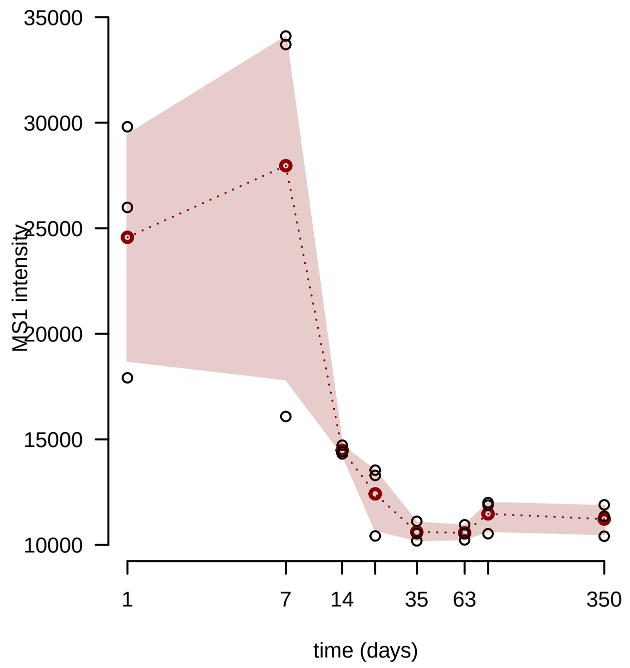

A3KMP2
GN=Ttc38
tetratricopeptide repeat domain 38

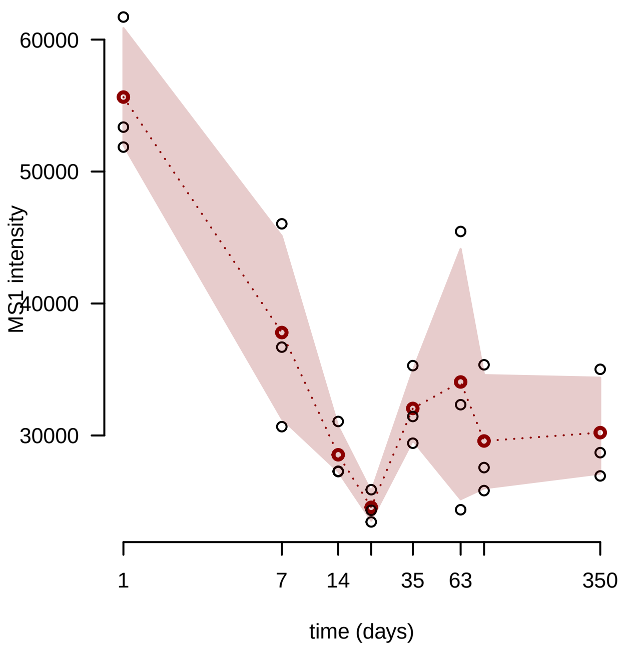

A6H584
GN=Col6a5
collagen, type VI, alpha 5

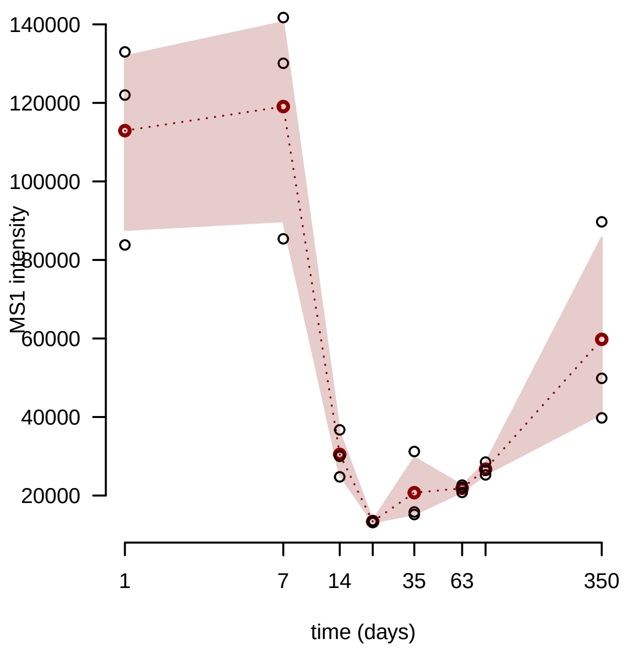

A6H5X4
GN=Phf11

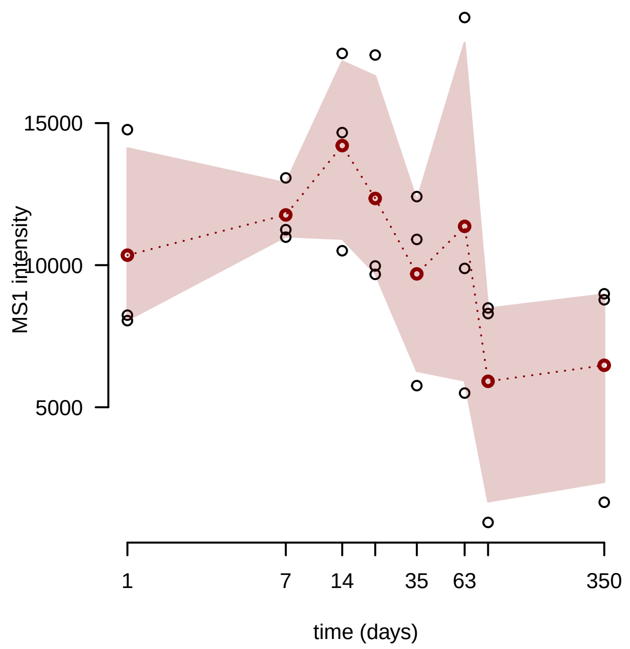

A6H5Y3
GN=Mtr
5−methyltetrahydrofolate−homocysteine me ...

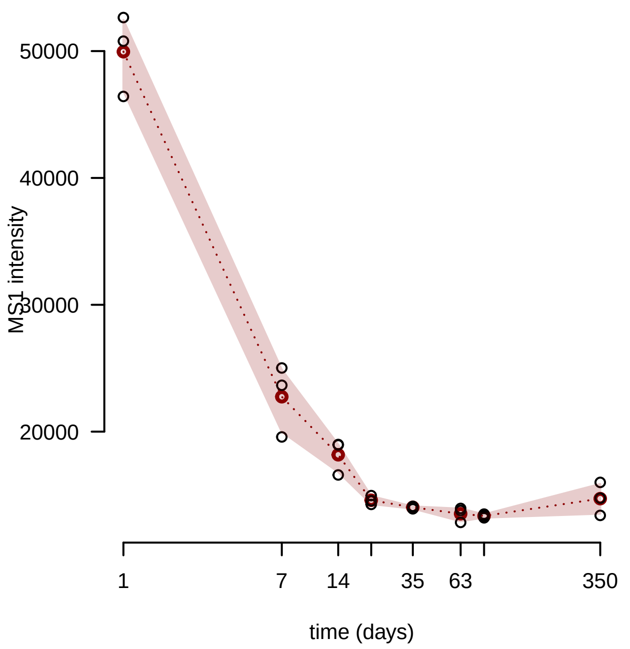

A6H5Z3
GN=Exoc6b
exocyst complex component 6B

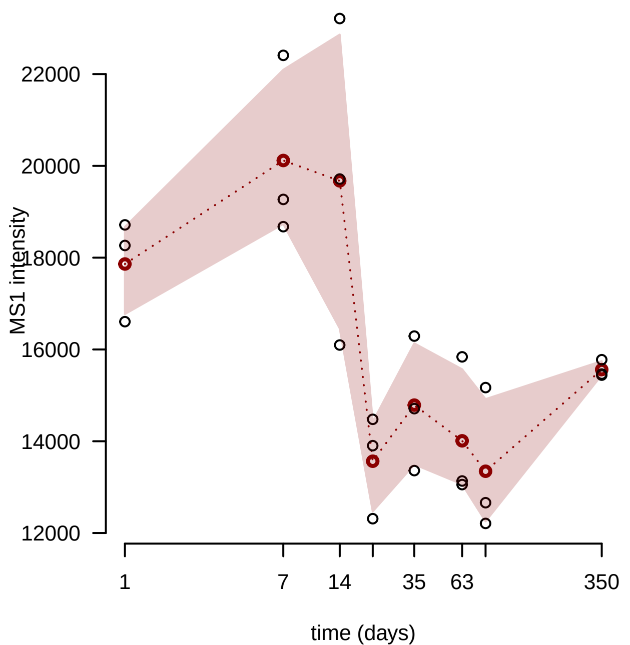

A6H611
GN=Mipep
mitochondrial intermediate peptidase

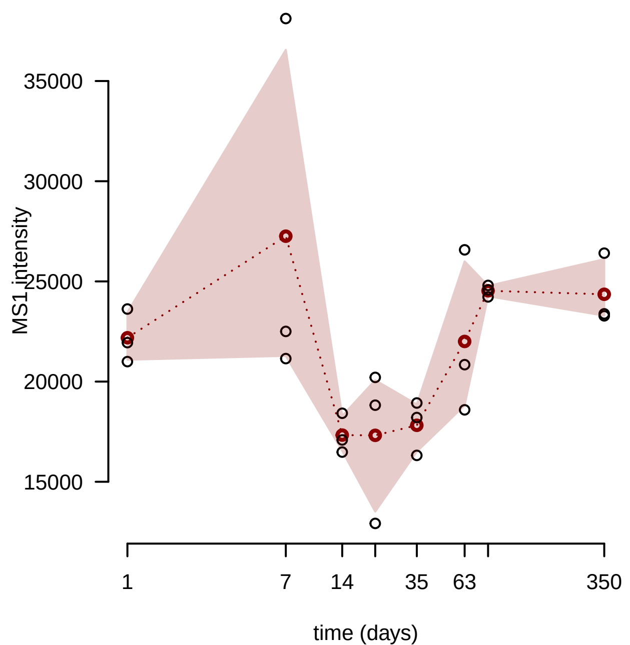

A6H619
GN=Phrf1
PHD and ring finger domains 1

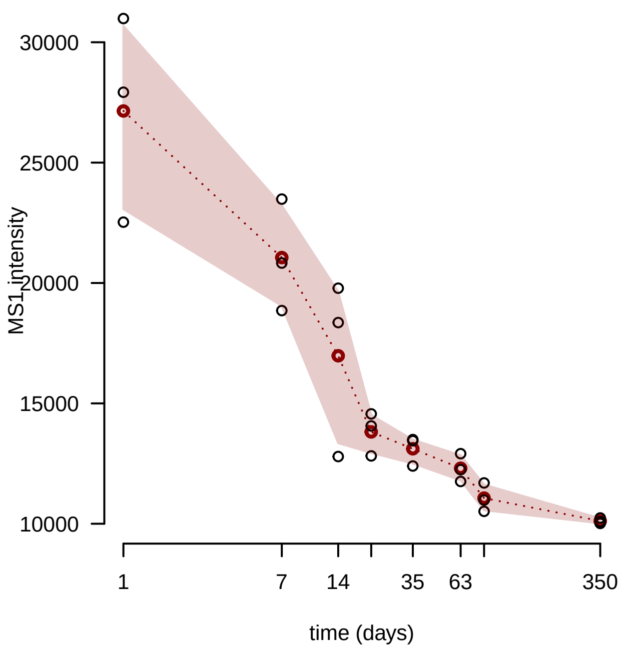

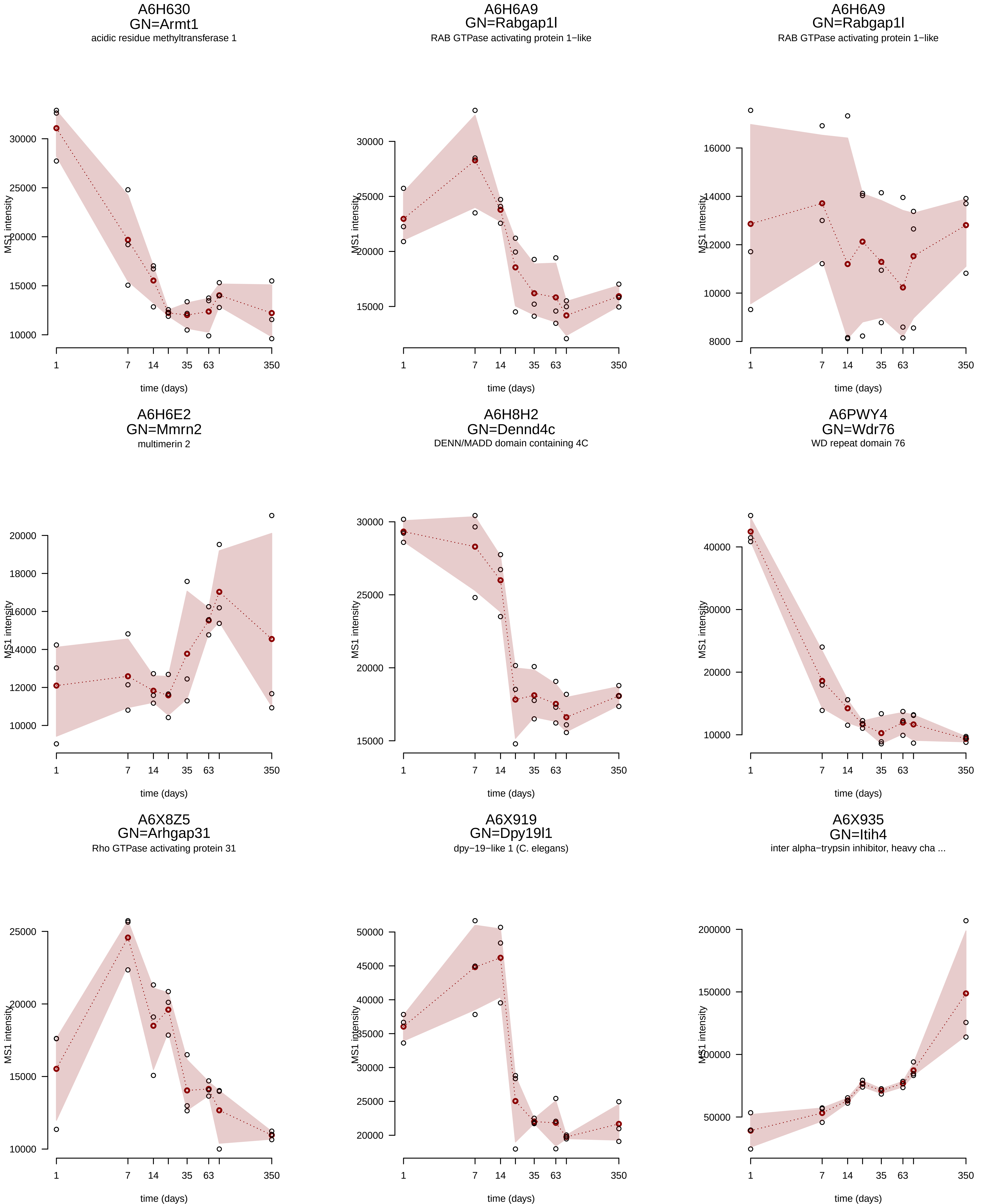
A6H630
GN=Armt1
acidic residue methyltransferase 1
A6H6A9
GN=Rabgap1l
RAB GTPase activating protein 1−like
A6H6A9
GN=Rabgap1l
RAB GTPase activating protein 1−like
A6H6E2
GN=Mmrn2
multimerin 2
A6H8H2
GN=Dennd4c
DENN/MADD domain containing 4C
A6PWY4
GN=Wdr76
WD repeat domain 76
A6X8Z5
GN=Arhgap31
Rho GTPase activating protein 31
A6X919
GN=Dpy19l1
dpy−19−like 1 (C. elegans)
A6X935
GN=Itih4
inter alpha−trypsin inhibitor, heavy cha ...
MS1 intensity
time (days)
1
7
14
35
63
350

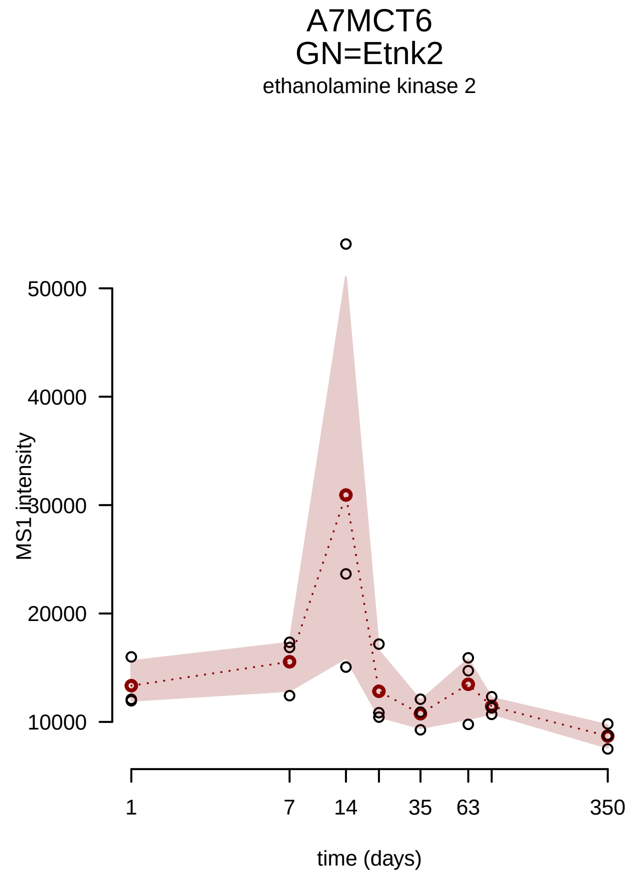
A7MCT6
GN=Etnk2
ethanolamine kinase 2
MS1 intensity
10000
20000
30000
40000
50000
time (days)
1
7
14
35
63
350
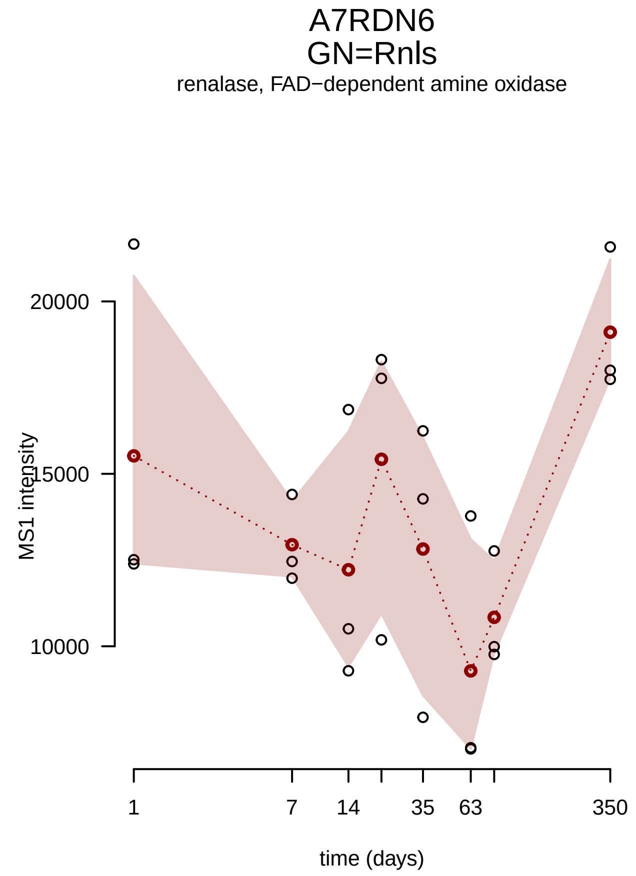
A7RDN6
GN=Rnls
renalase, FAD−dependent amine oxidase
MS1 intensity
10000
15000
20000
time (days)
1
7
14
35
63
350
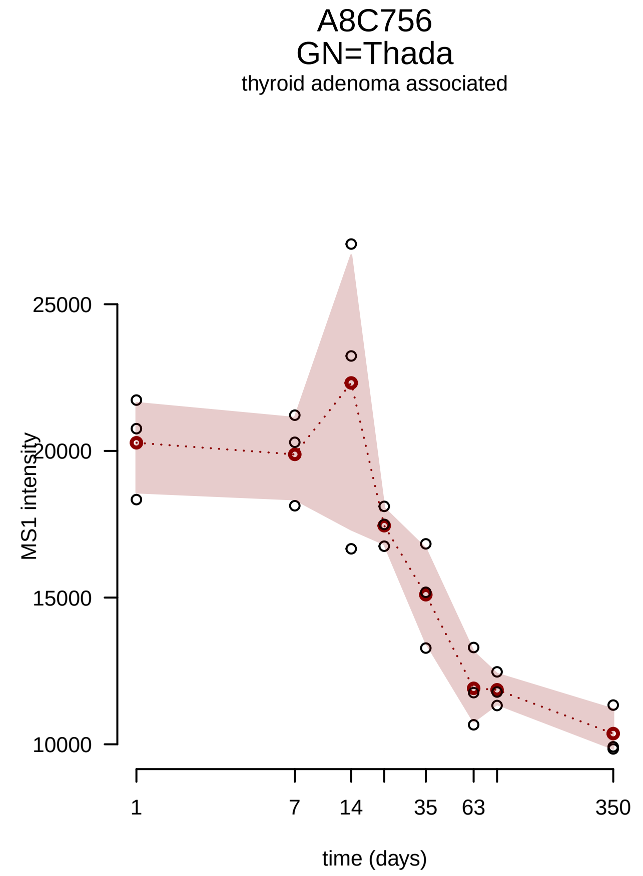
A8C756
GN=Thada
thyroid adenoma associated
MS1 intensity
10000
15000
20000
25000
time (days)
1
7
14
35
63
350
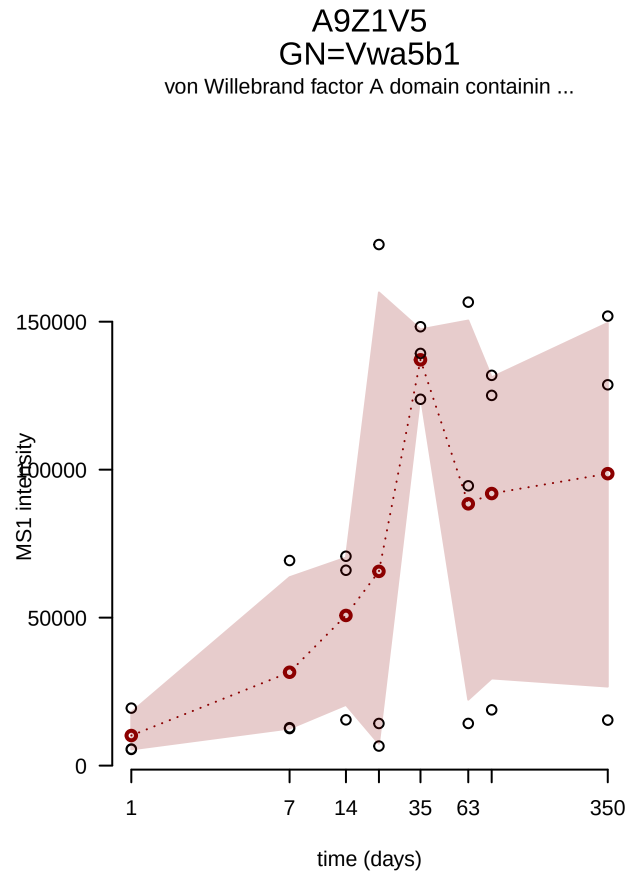
A9Z1V5
GN=Vwa5b1
von Willebrand factor A domain containin ...
MS1 intensity
0
50000
100000
150000
time (days)
1
7
14
35
63
350
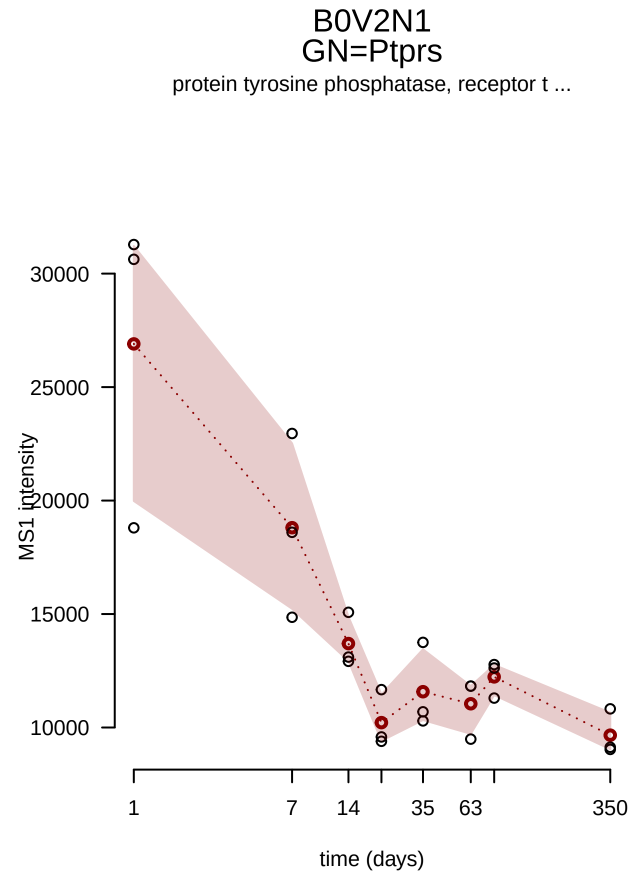
B0V2N1
GN=Ptprs
protein tyrosine phosphatase, receptor t ...
MS1 intensity
10000
15000
20000
25000
30000
time (days)
1
7
14
35
63
350
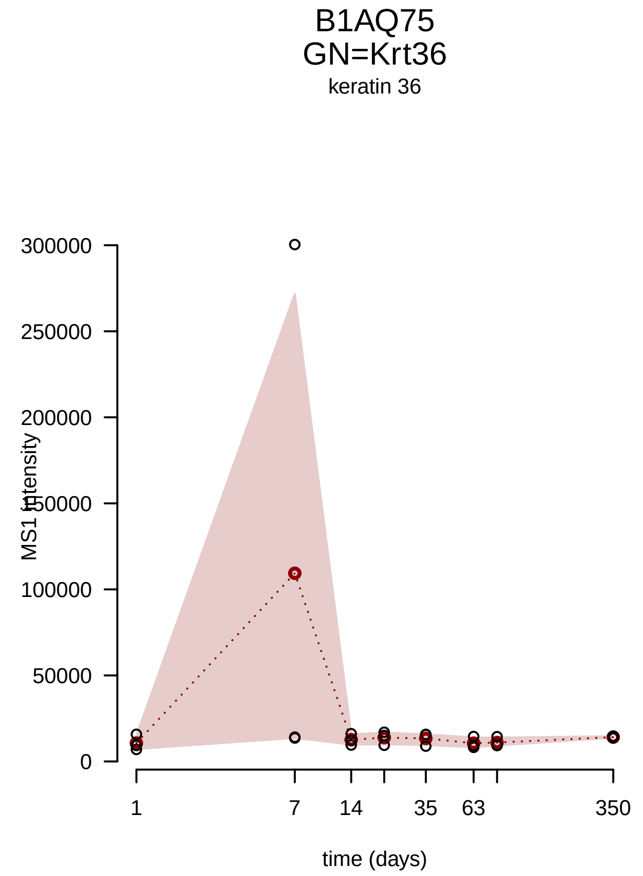
B1AQ75
GN=Krt36
keratin 36
MS1 intensity
0
50000
100000
150000
200000
250000
300000
time (days)
1
7
14
35
63
350
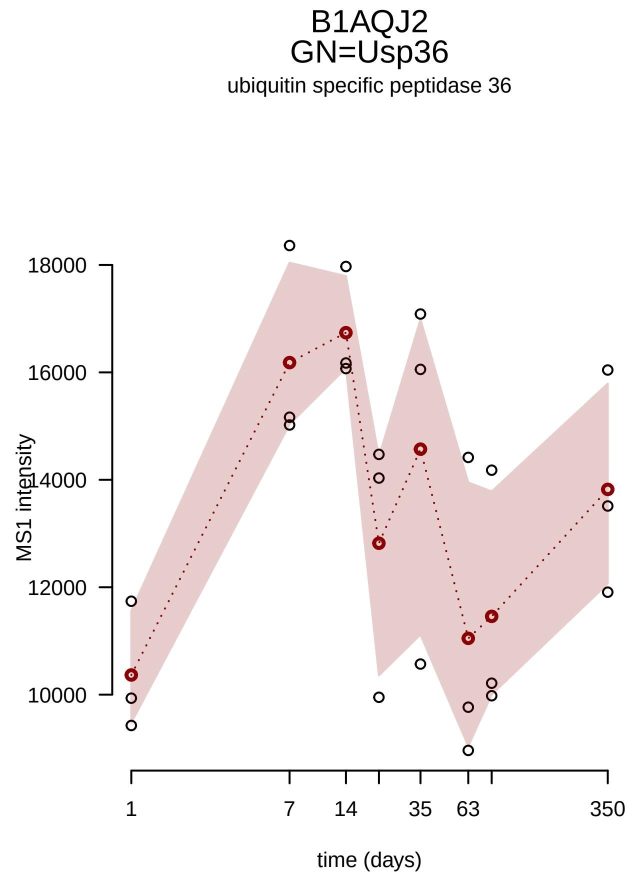
B1AQJ2
GN=Usp36
ubiquitin specific peptidase 36
MS1 intensity
10000
12000
14000
16000
18000
time (days)
1
7
14
35
63
350
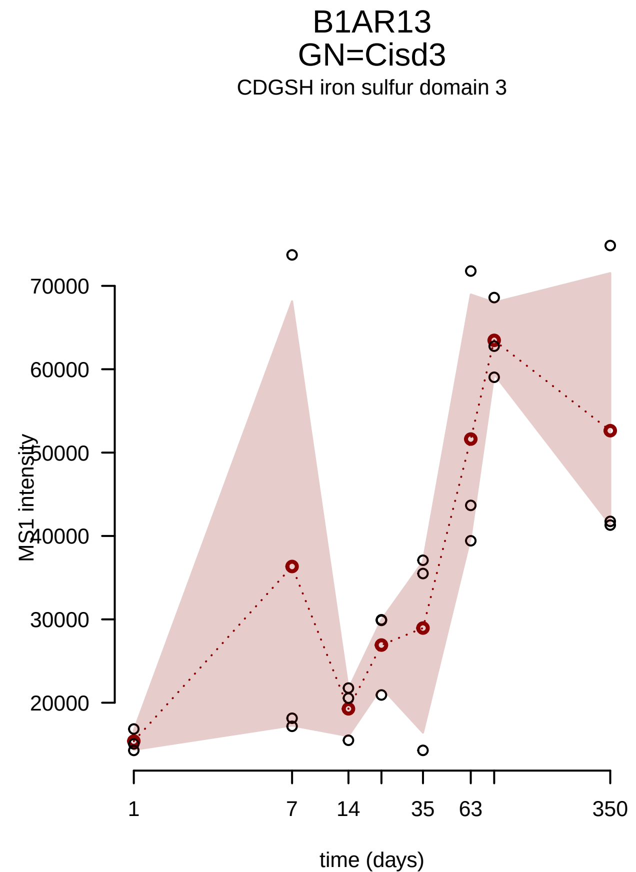
B1AR13
GN=Cisd3
CDGSH iron sulfur domain 3
MS1 intensity
20000
30000
40000
50000
60000
70000
time (days)
1
7
14
35
63
350
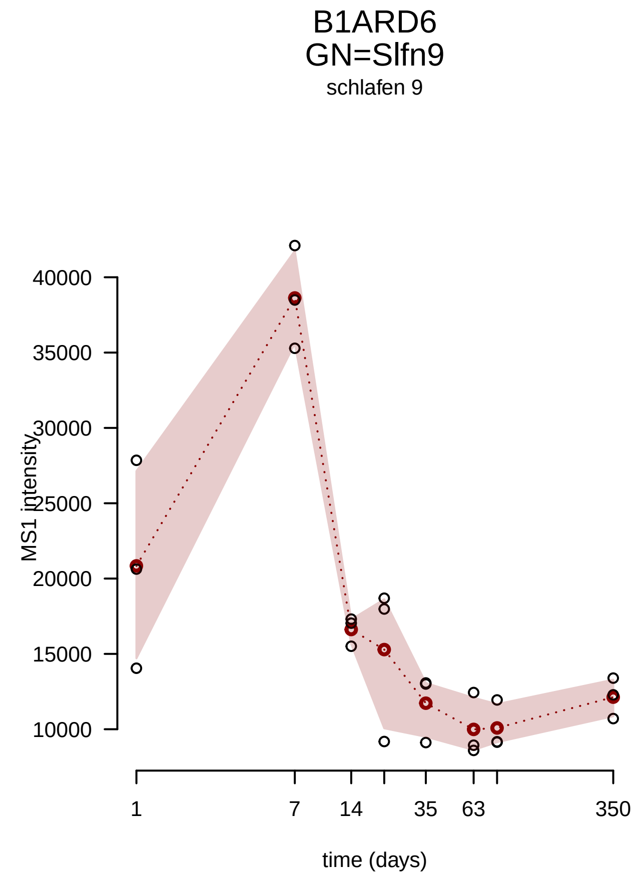
B1ARD6
GN=Slfn9
schlafen 9
MS1 intensity
10000
15000
20000
25000
30000
35000
40000
time (days)
1
7
14
35
63
350

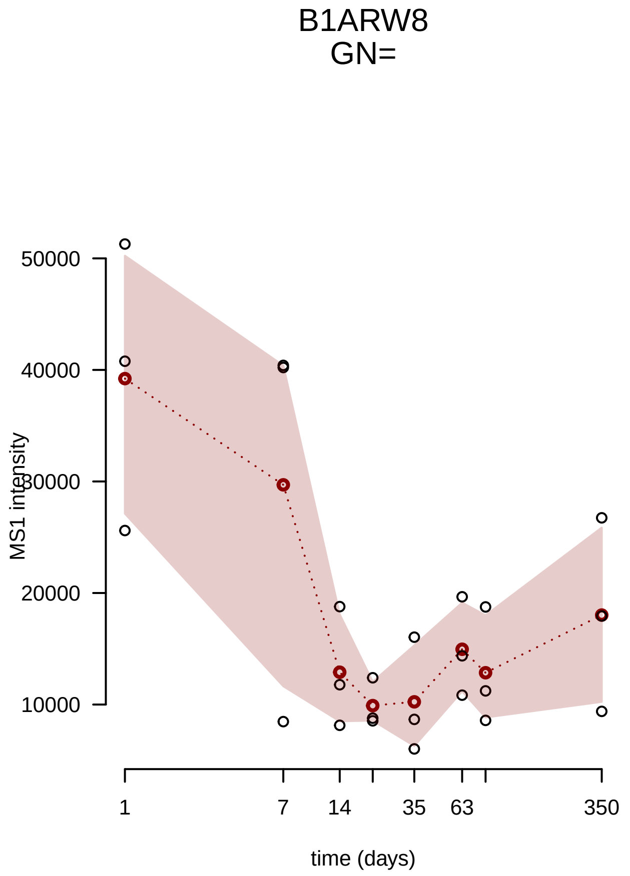
B1ARW8
GN=
50000
40000
30000
20000
10000
MS1 intensity
1
7
14
35
63
350
time (days)

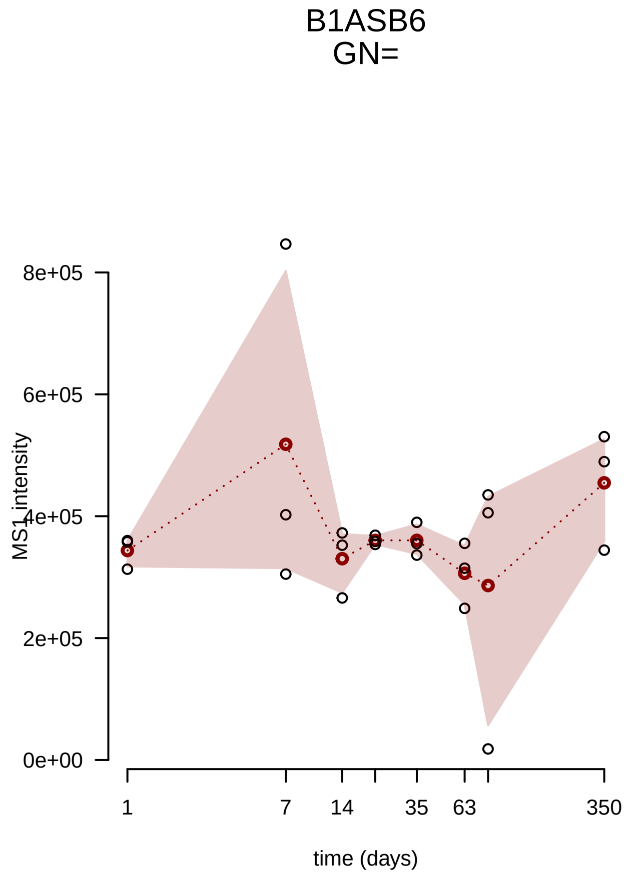
B1ASB6
GN=
8e+05
6e+05
4e+05
2e+05
0e+00
MS1 intensity
1
7
14
35
63
350
time (days)

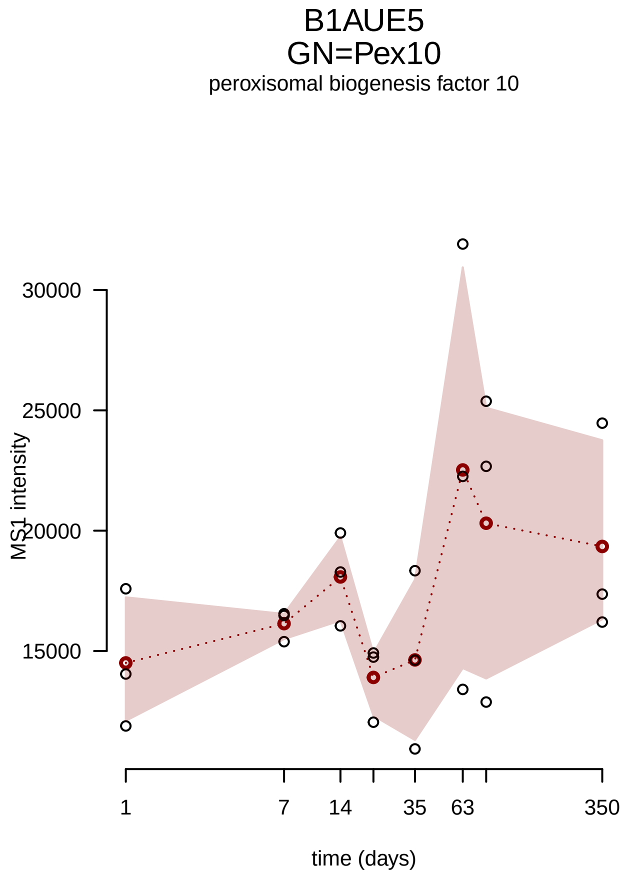
B1AUE5
GN=Pex10
peroxisomal biogenesis factor 10
30000
25000
20000
15000
MS1 intensity
1
7
14
35
63
350
time (days)

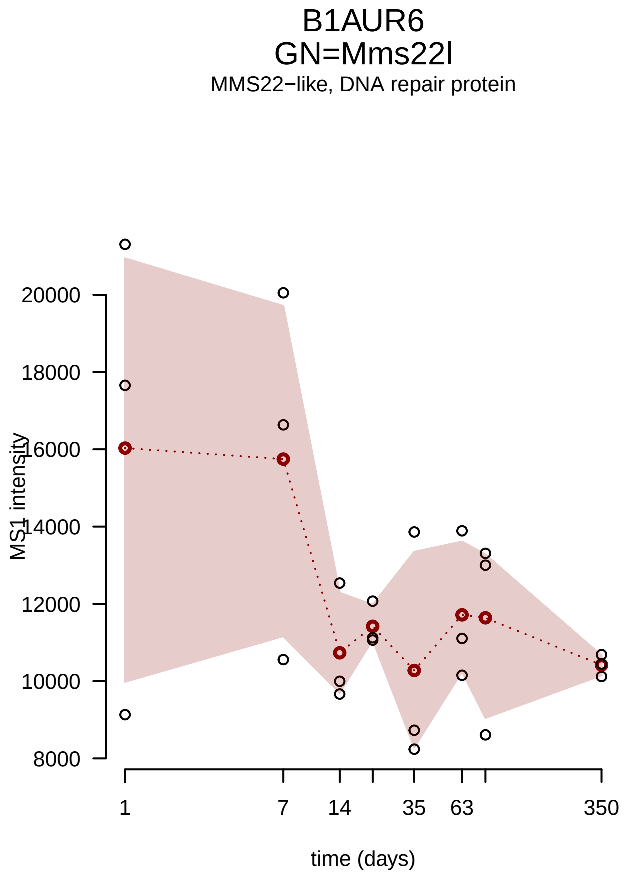
B1AUR6
GN=Mms22l
MMS22−like, DNA repair protein
20000
18000
16000
14000
12000
10000
8000
MS1 intensity
1
7
14
35
63
350
time (days)

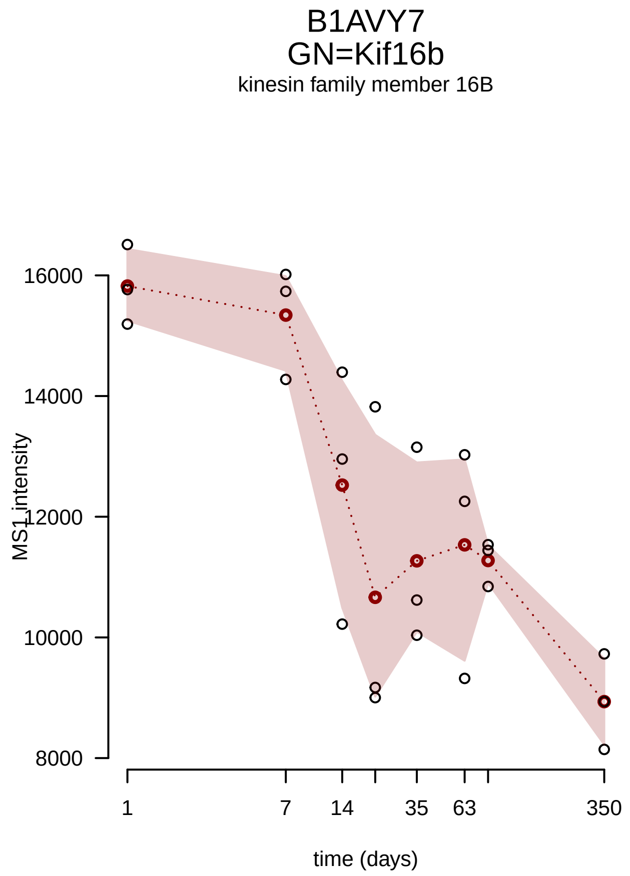
B1AVY7
GN=Kif16b
kinesin family member 16B
16000
14000
12000
10000
8000
MS1 intensity
1
7
14
35
63
350
time (days)

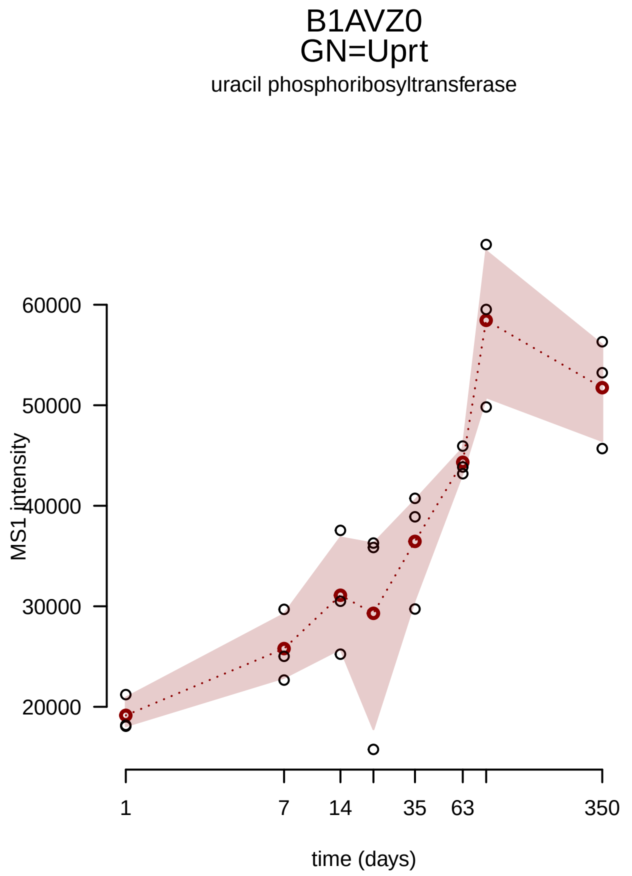
B1AVZ0
GN=Uprt
uracil phosphoribosyltransferase
60000
50000
40000
30000
20000
MS1 intensity
1
7
14
35
63
350
time (days)

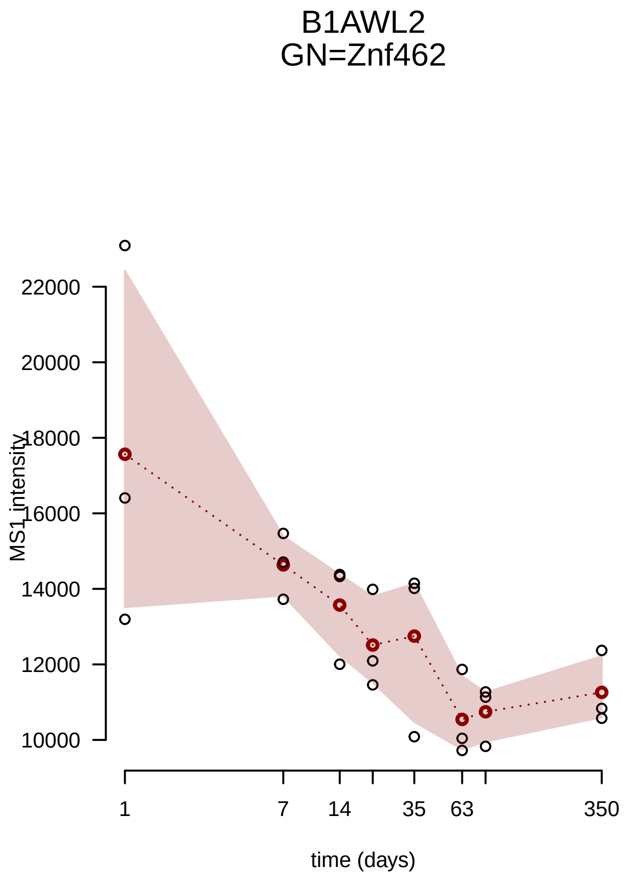
B1AWL2
GN=Znf462
22000
20000
18000
16000
14000
12000
10000
MS1 intensity
1
7
14
35
63
350
time (days)

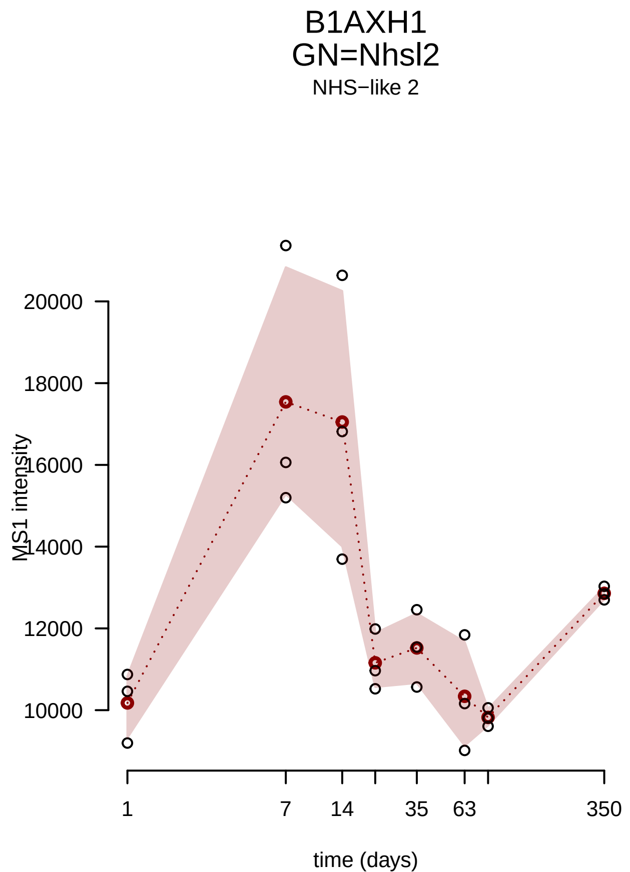
B1AXH1
GN=Nhsl2
NHS−like 2
20000
18000
16000
14000
12000
10000
MS1 intensity
1
7
14
35
63
350
time (days)

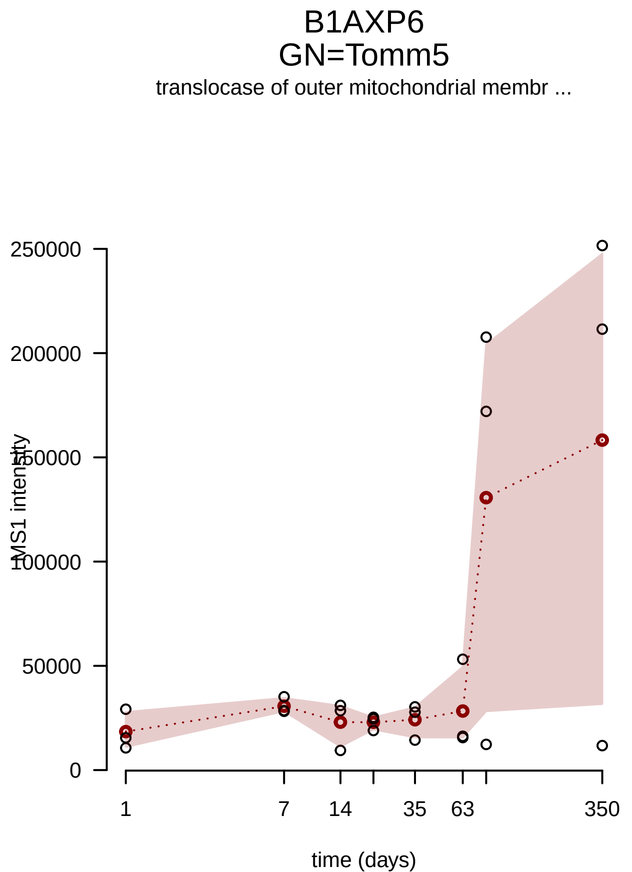
B1AXP6
GN=Tomm5
translocase of outer mitochondrial membr ...
250000
200000
150000
100000
50000
0
MS1 intensity
1
7
14
35
63
350
time (days)

B1AY10
GN=Nfx1
nuclear transcription factor, X−box bind ...

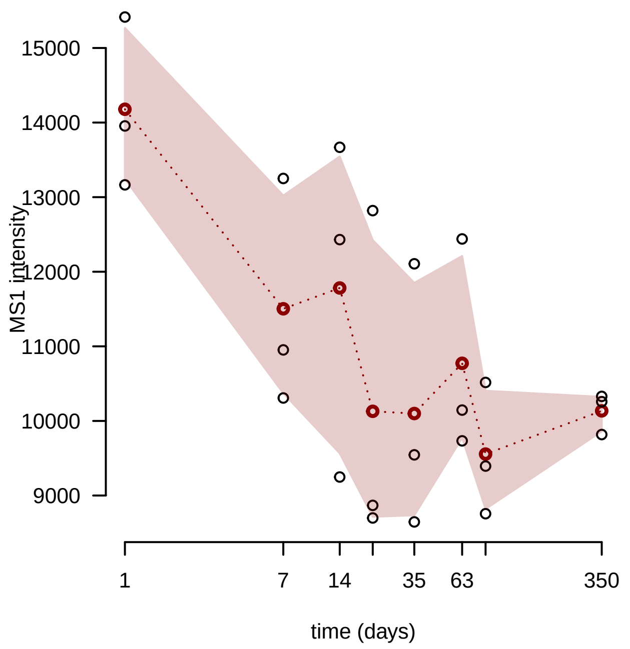

B1AY13
GN=Usp24
ubiquitin specific peptidase 24

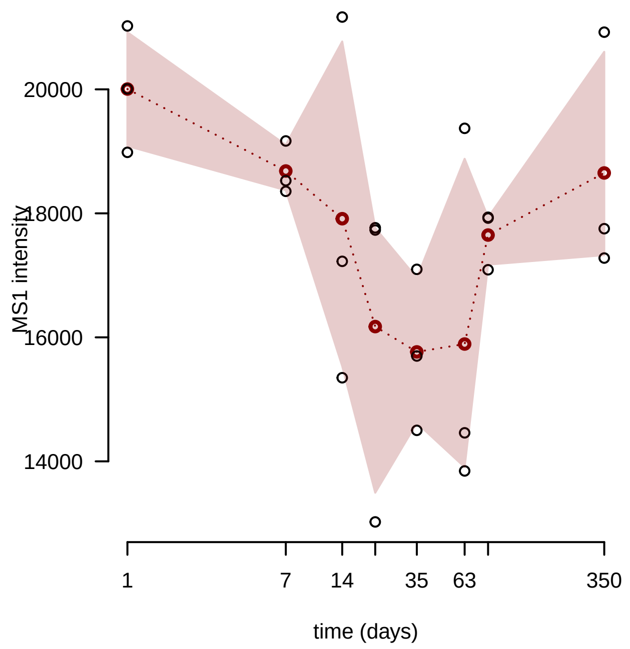

B1AZA5
GN=Tmem245
transmembrane protein 245

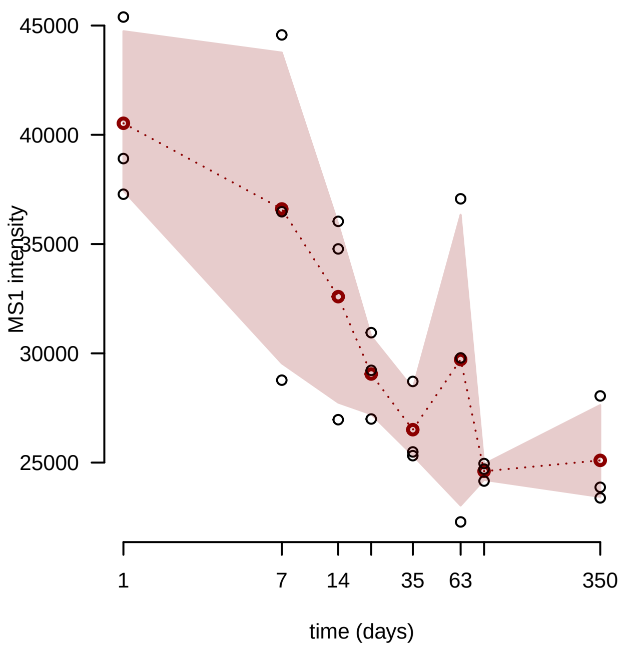

B1AZI6
GN=Thoc2
THO complex 2

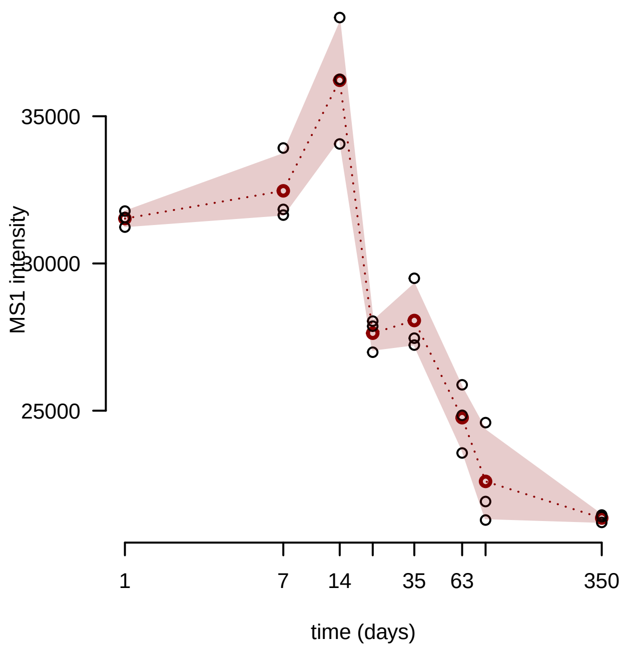

B1AZP2
GN=Dlgap4
DLG associated protein 4

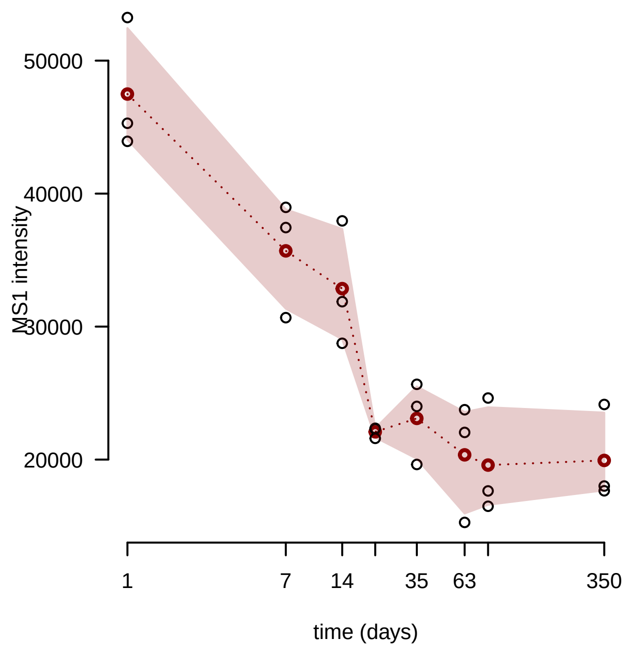

B1B0V2
GN=

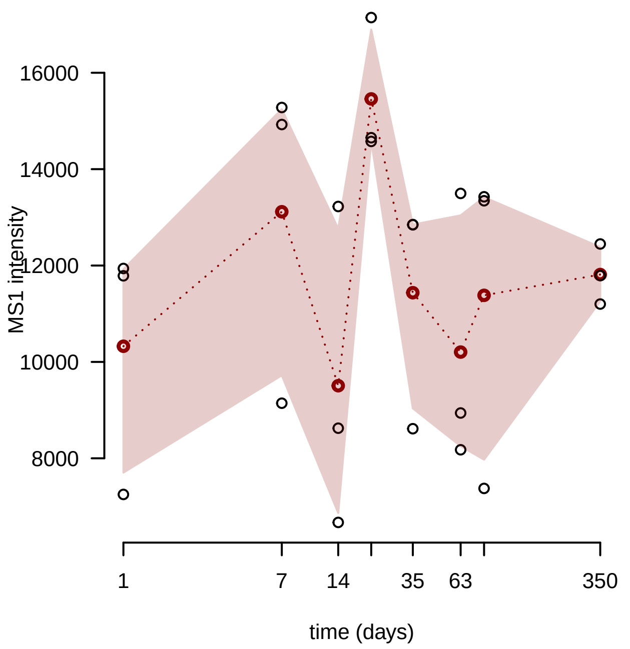

B2B9E1
GN=Triqk
triple QxxK/R motif containing

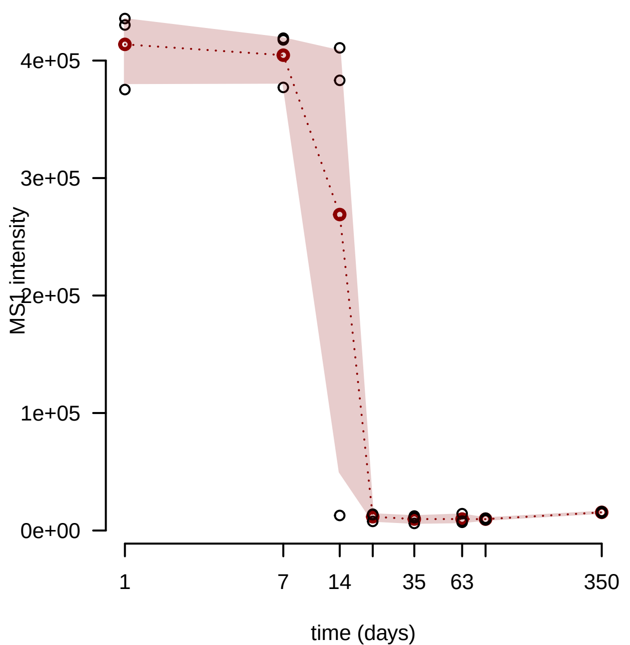

B2KF05
GN=

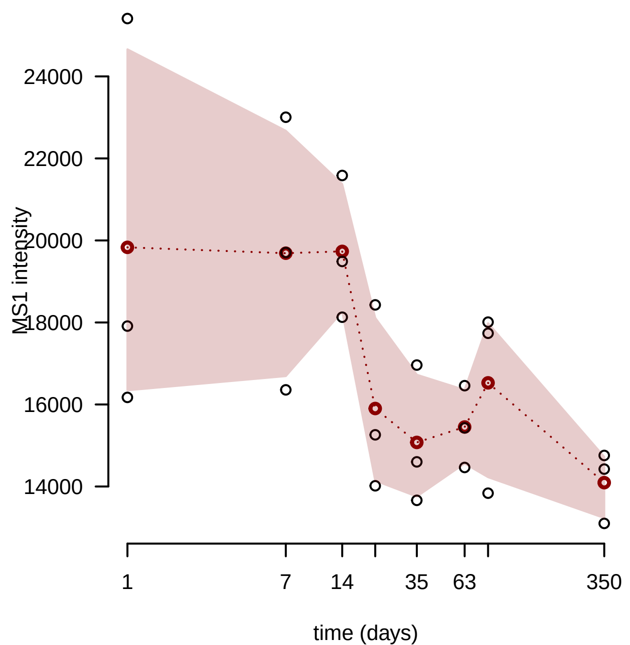

B2RQC2
GN=Usp42
ubiquitin specific peptidase 42

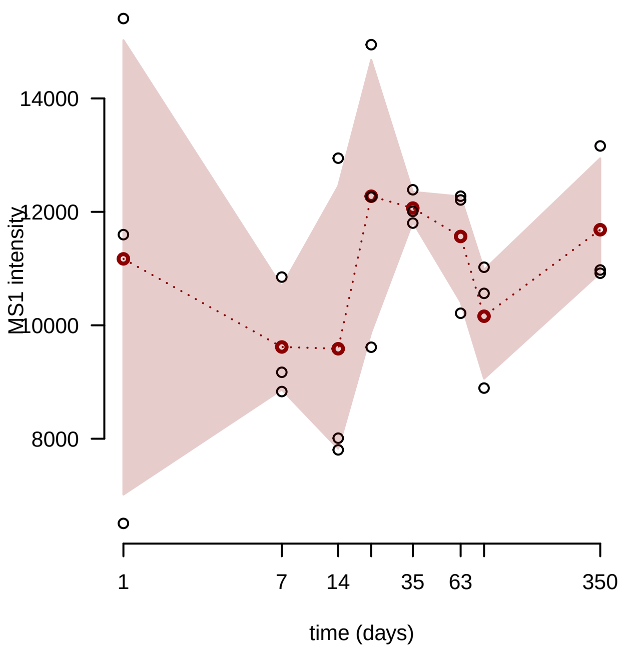

B2RQC6
GN=Cad
carbamoyl−phosphate synthetase 2, aspart ...

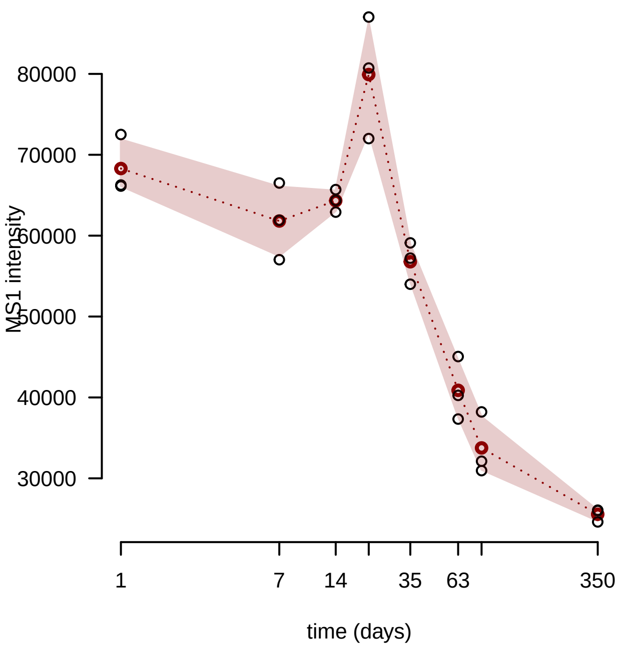

B2RQE8
GN=Arhgap42
Rho GTPase activating protein 42

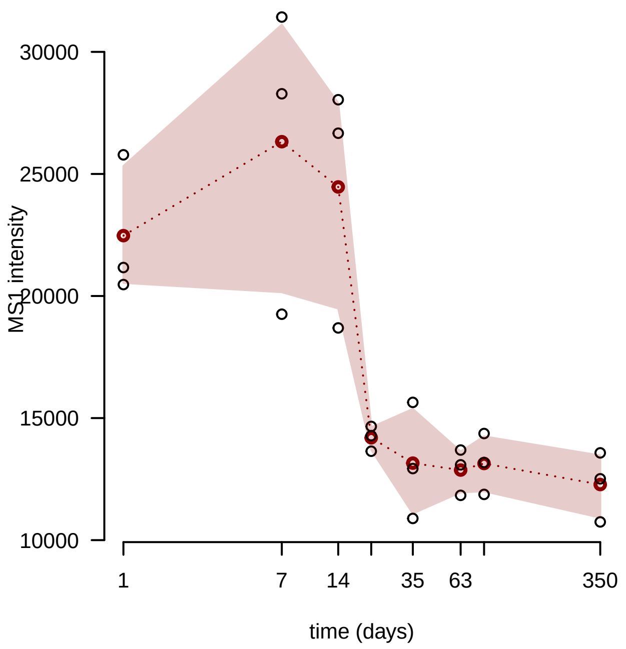

B2RR83
GN=Ythdc2
YTH domain containing 2

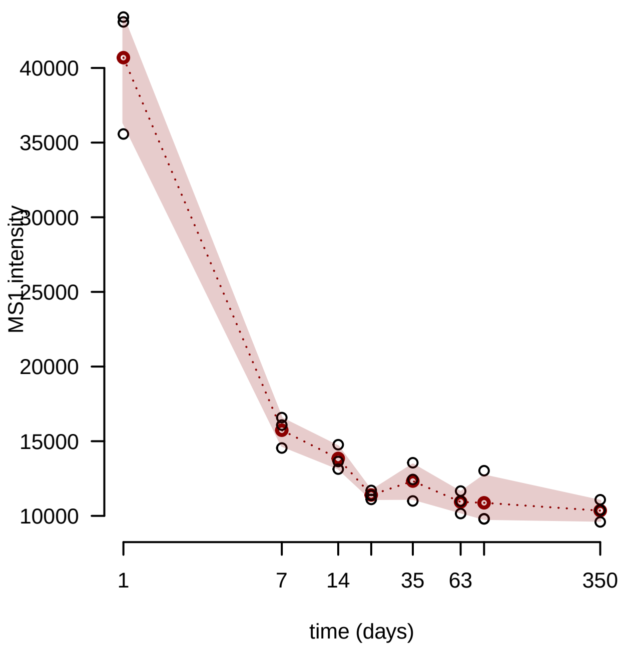

B2RRD7
GN=Brpf1
bromodomain and PHD finger containing, 1

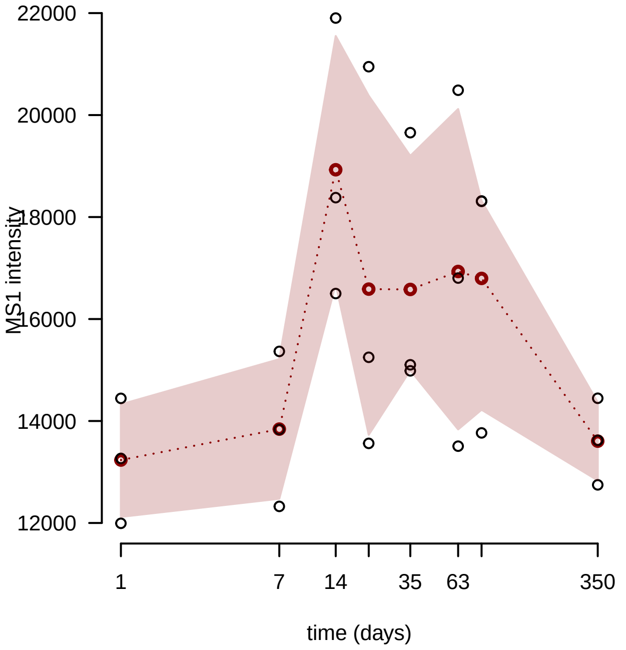

B2RRE7
GN=Otud4
OTU domain containing 4

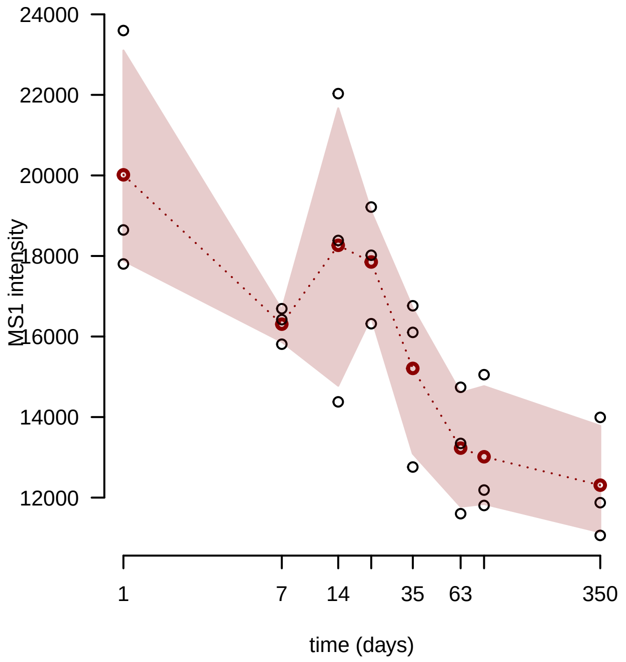

B2RRF6
GN=Znf518a

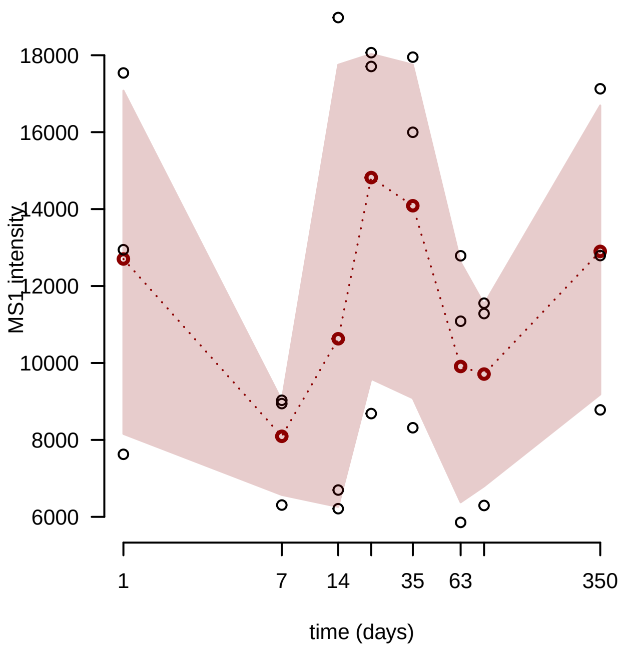

B2RS91
GN=Rrn3
RRN3 RNA polymerase I transcription fact ...

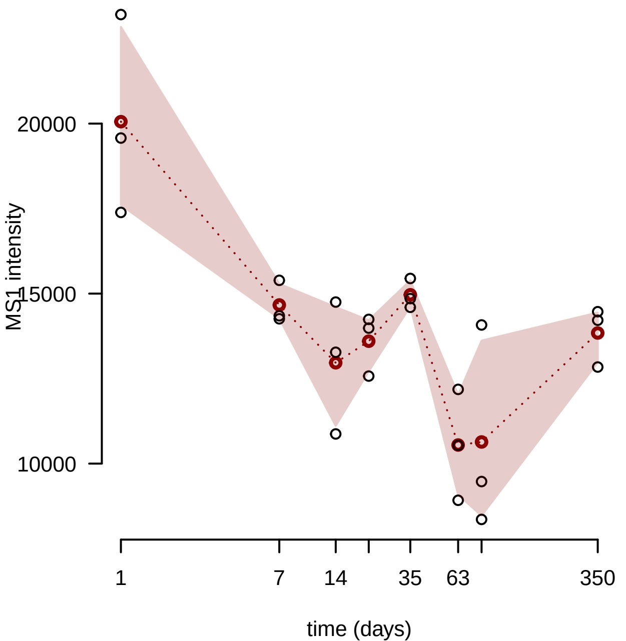

B2RSH2
GN=Gnai1
guanine nucleotide binding protein (G pr ...

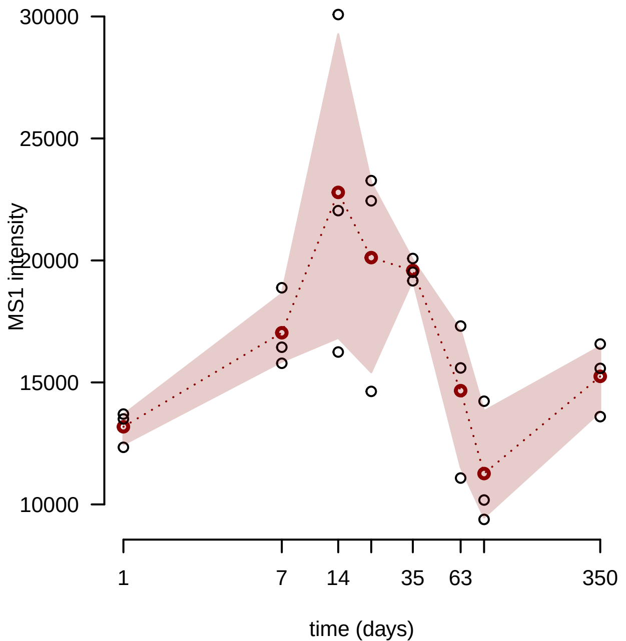

B2RUP2
GN=Unc13d
unc−13 homolog D

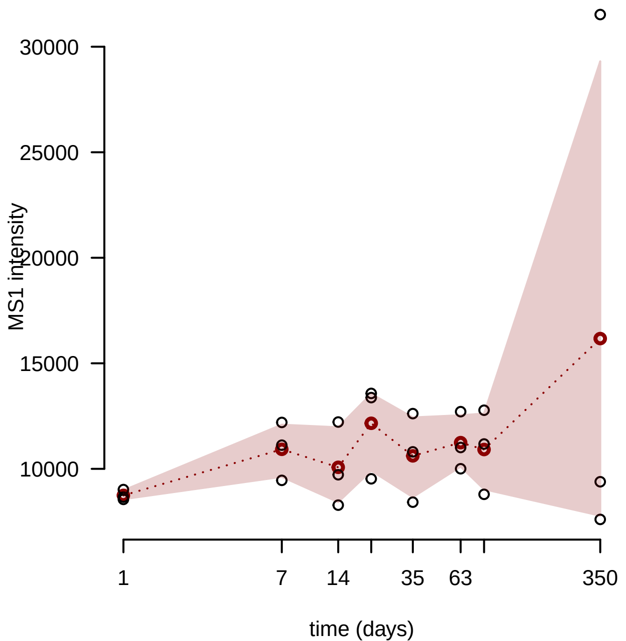

B2RUR8
GN=Otud7b
OTU domain containing 7B

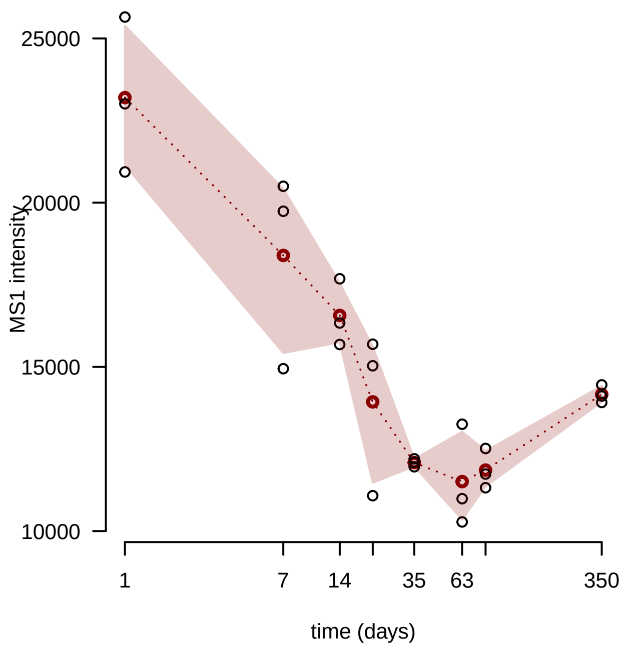

B2RVL6
GN=Zcchc24
zinc finger, CCHC domain containing 24

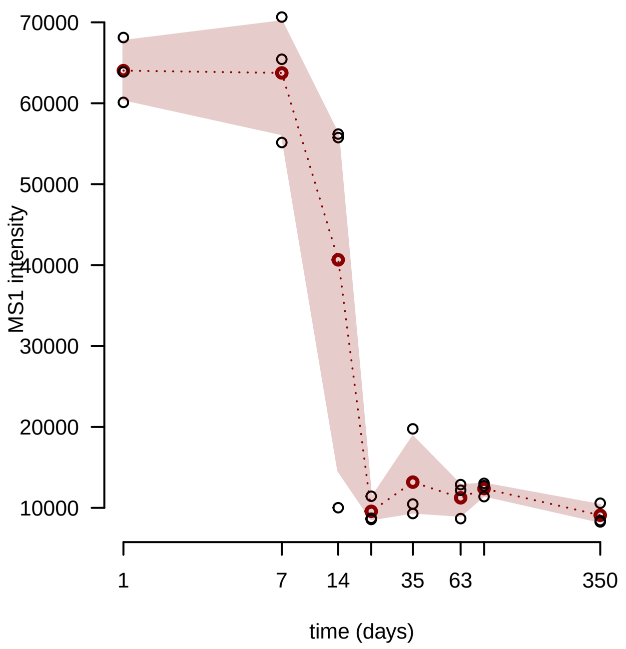

B2RWS6
GN=Ep300
E1A binding protein p300

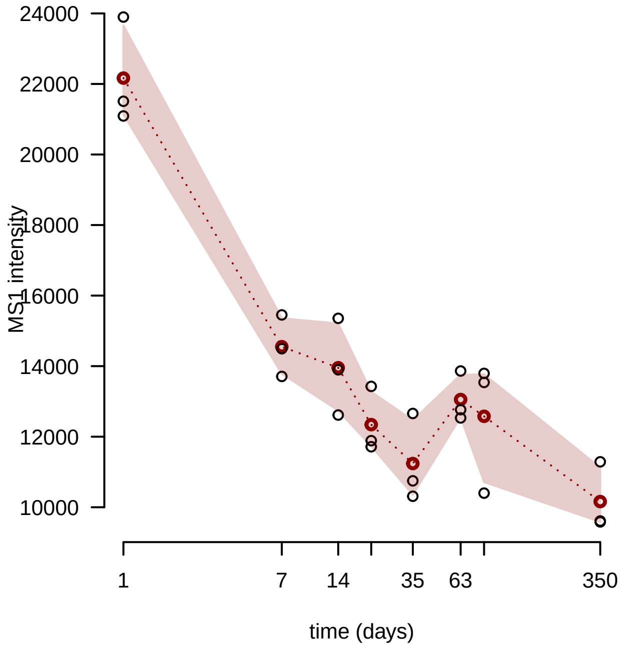

B2RX14
GN=Tut4
terminal uridylyl transferase 4

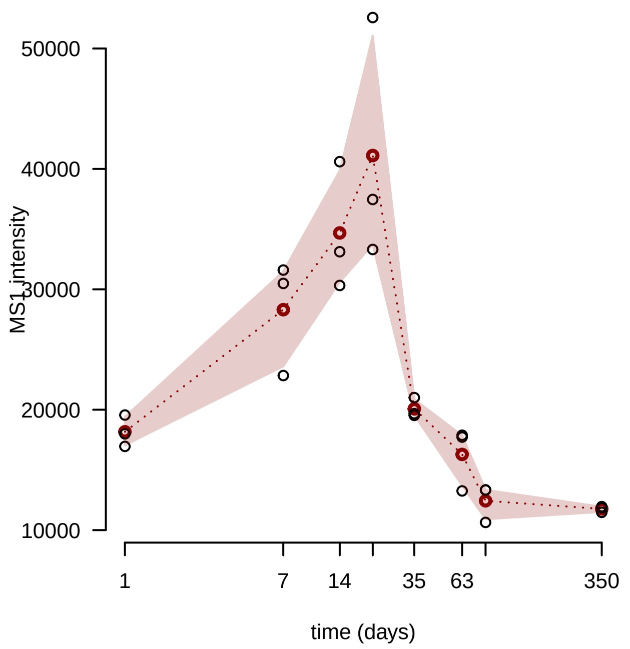

B2RXC1
GN=Trappc11
trafficking protein particle complex 11

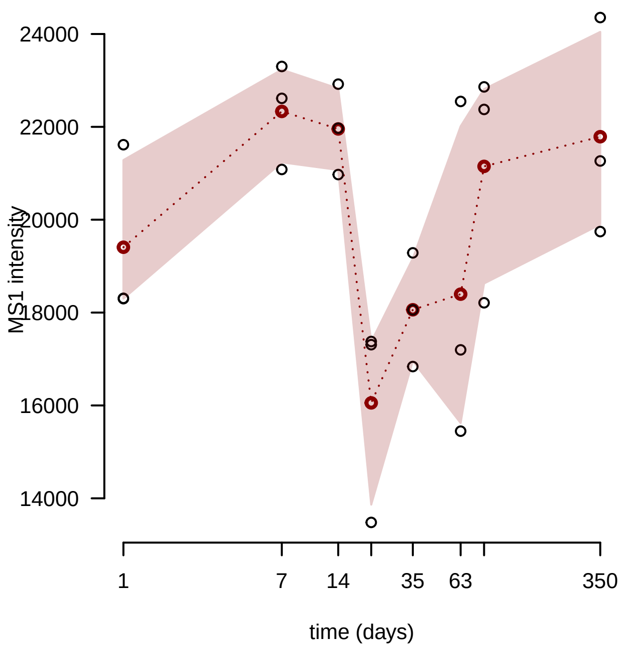

B2RXR6
GN=Ankrd44
ankyrin repeat domain 44

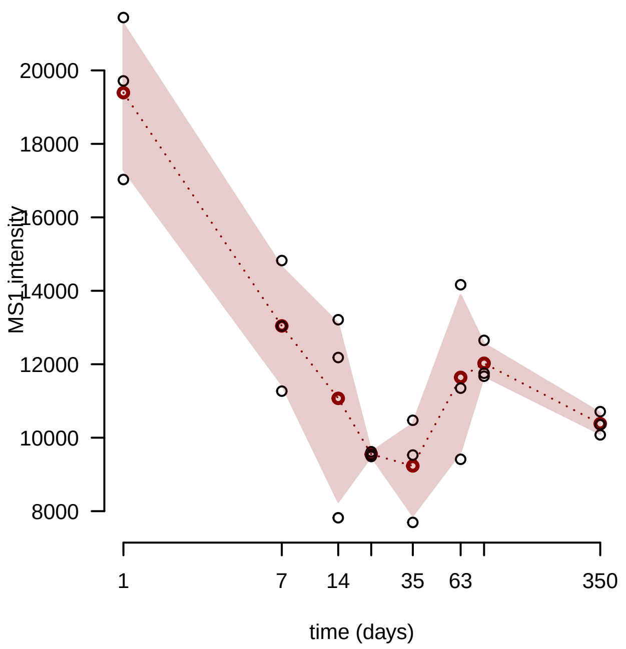

B2RXS4
GN=Plxnb2
plexin B2

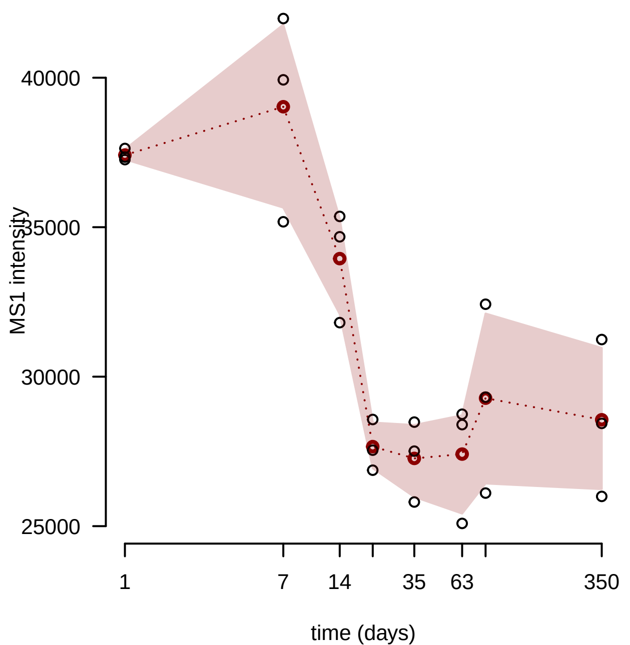

B2RY04
GN=Dock5
dedicator of cytokinesis 5

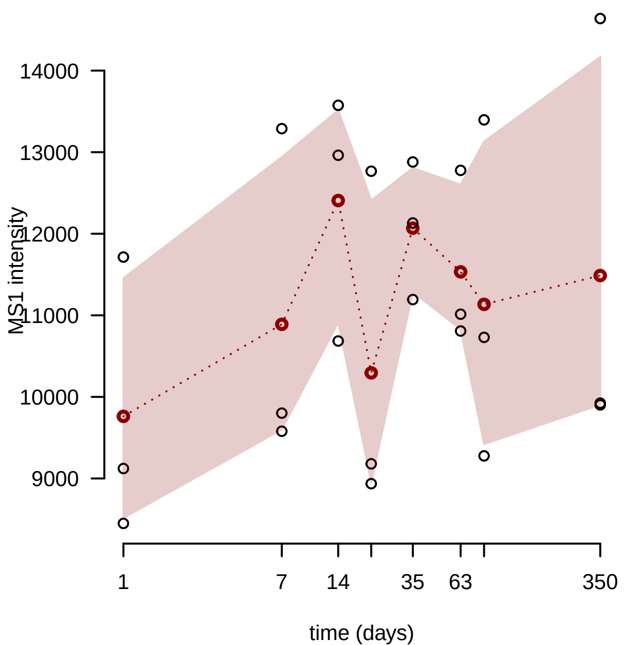

B2RY56
GN=Rbm25
RNA binding motif protein 25

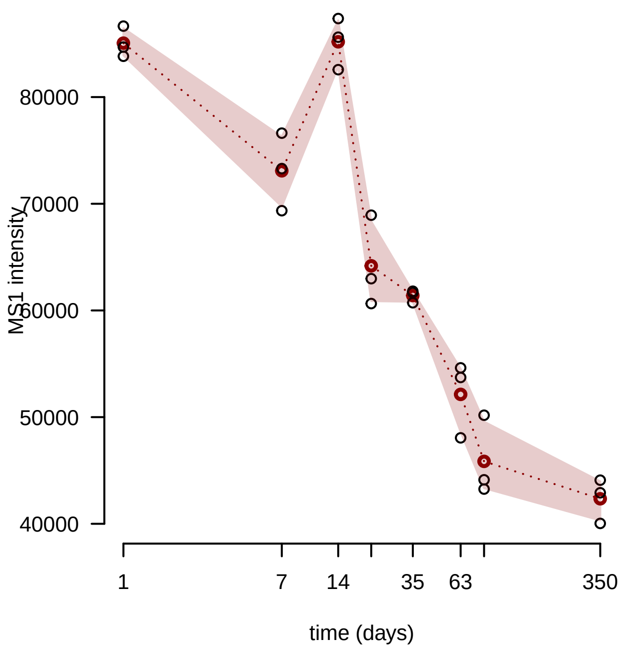

B7ZMP1
GN=Xpnpep3
X−prolyl aminopeptidase 3, mitochondrial

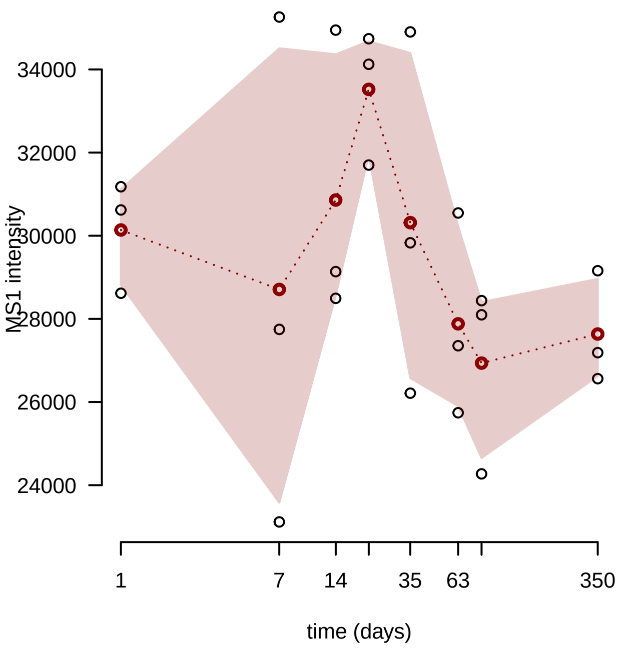

B7ZNG0
GN=Kif7
kinesin family member 7

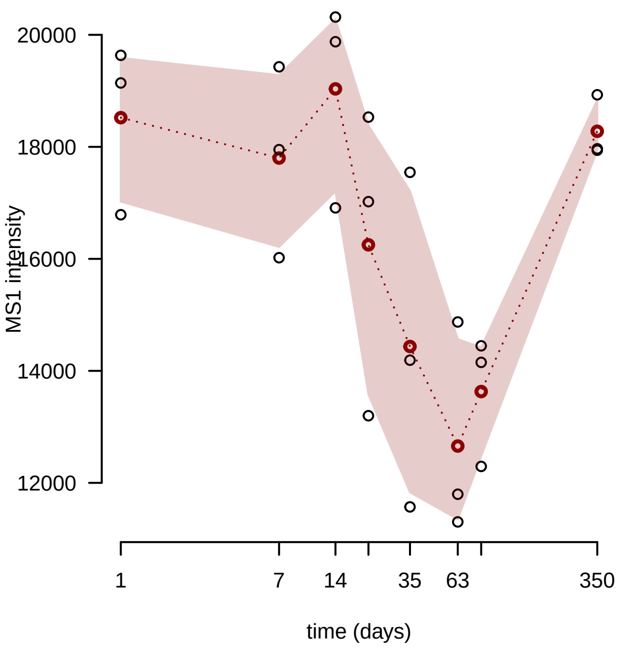

B8JK39
GN=Itga9
integrin alpha 9

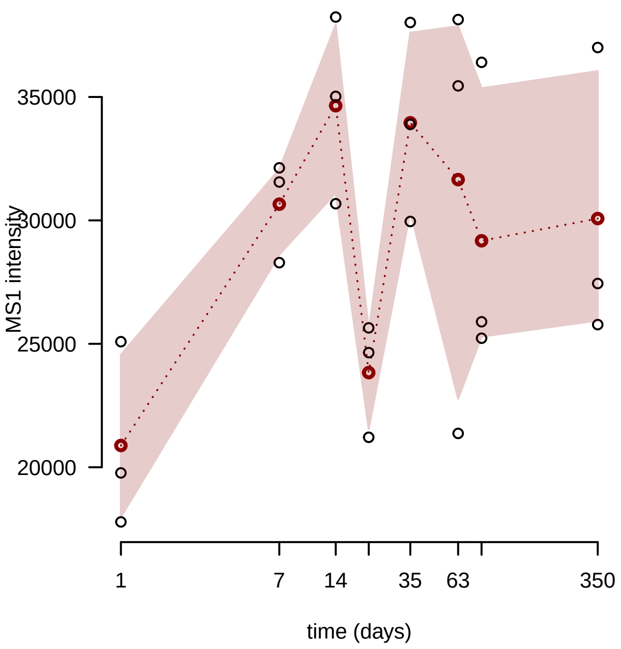

B8ZXI1
GN=Qtrt2
queuine tRNA−ribosyltransferase accessor ...

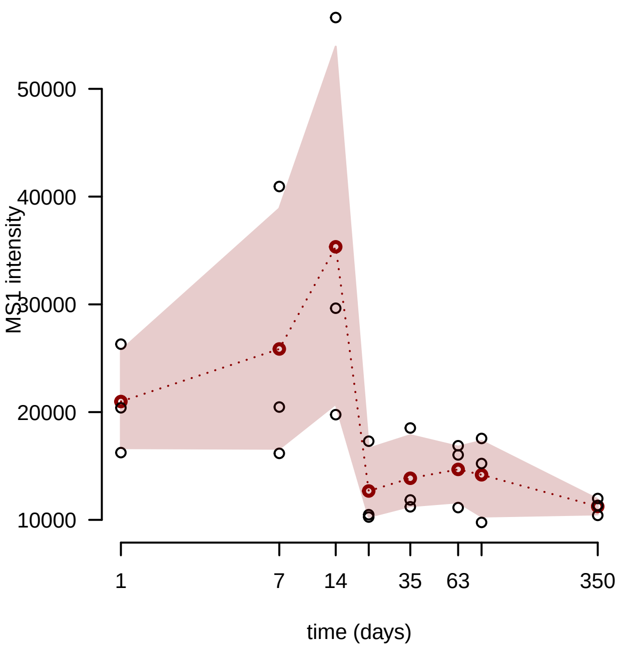

B9EJ80
GN=Pdzd8
PDZ domain containing 8

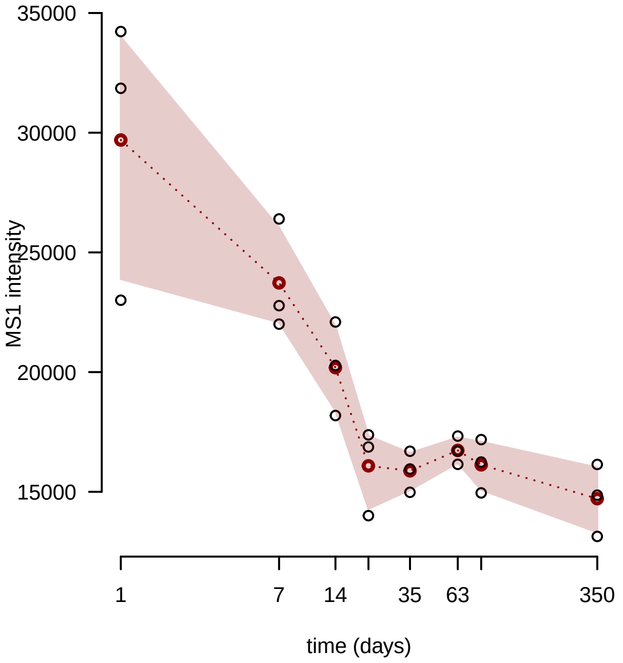

B9EJ86
GN=Osbpl8
oxysterol binding protein−like 8

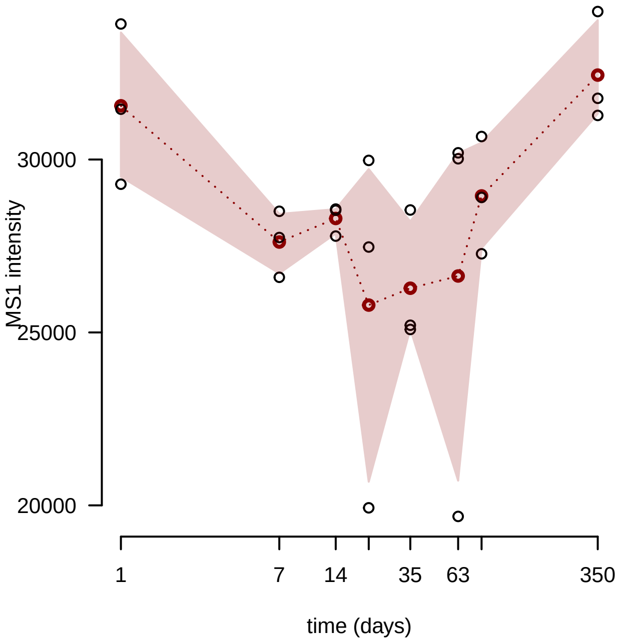

B9EJA2
GN=Cttnbp2
cortactin binding protein 2

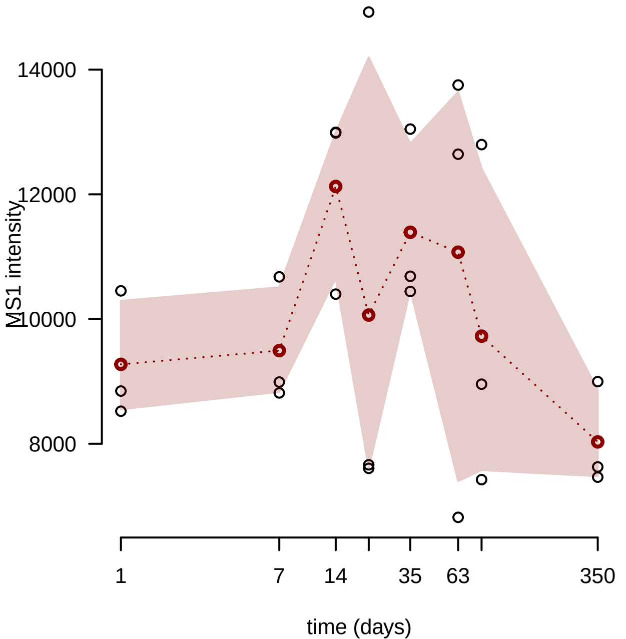

B9EJR8
GN=Dnaaf5
dynein, axonemal assembly factor 5

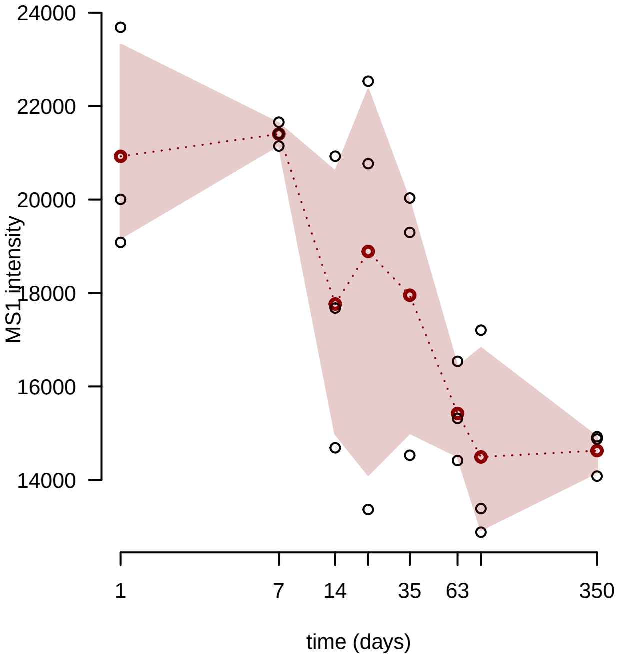

B9EJV3
GN=Greb1l
growth regulation by estrogen in breast ...

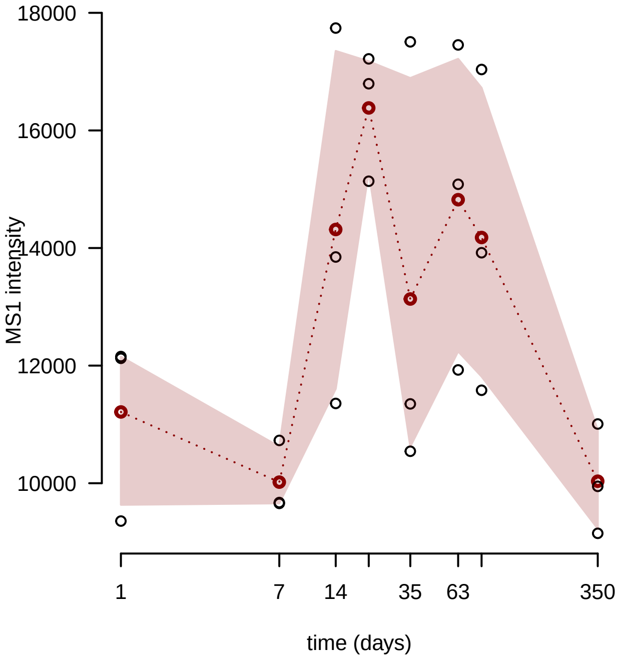

B9EKI3
GN=Tmf1
TATA element modulatory factor 1

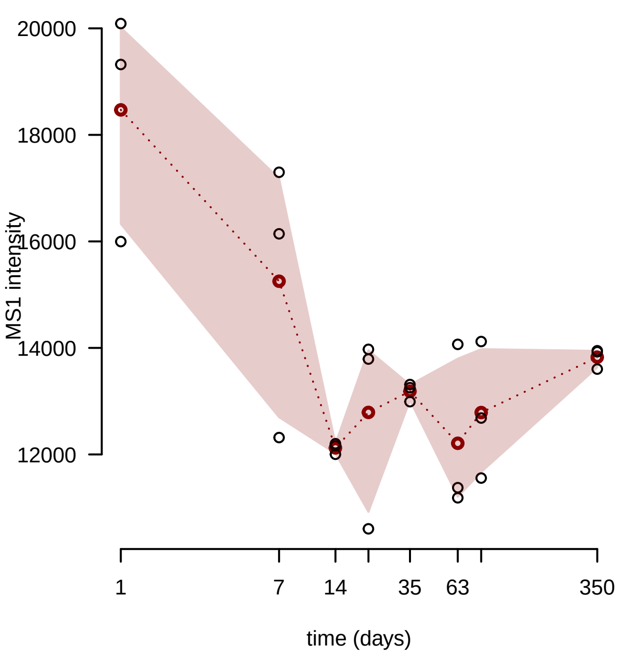

C0HK79
GN=Arxes1
adipocyte−related X−chromosome expressed ...

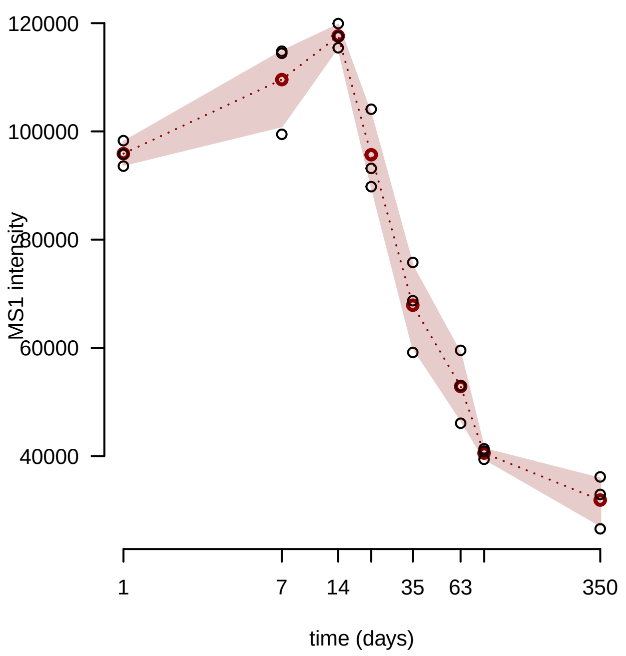

C0HKD8
GN=Mfap1a
microfibrillar−associated protein 1A

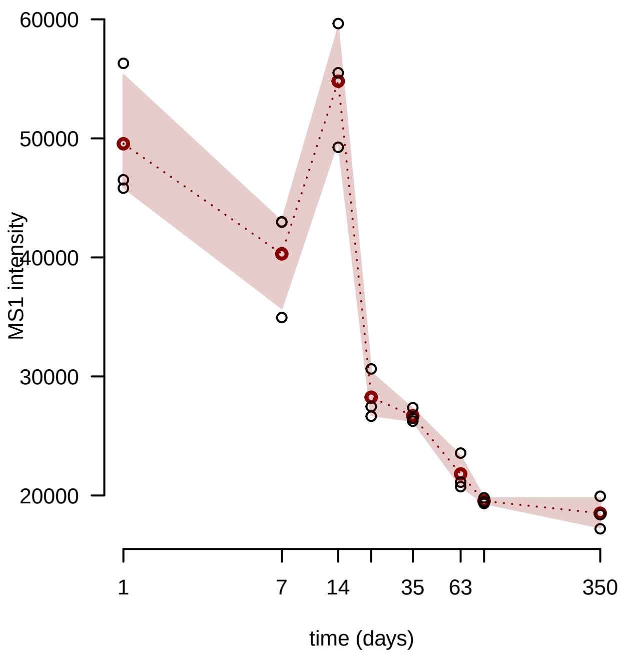

C0HKE1
GN=Hist1h2ab

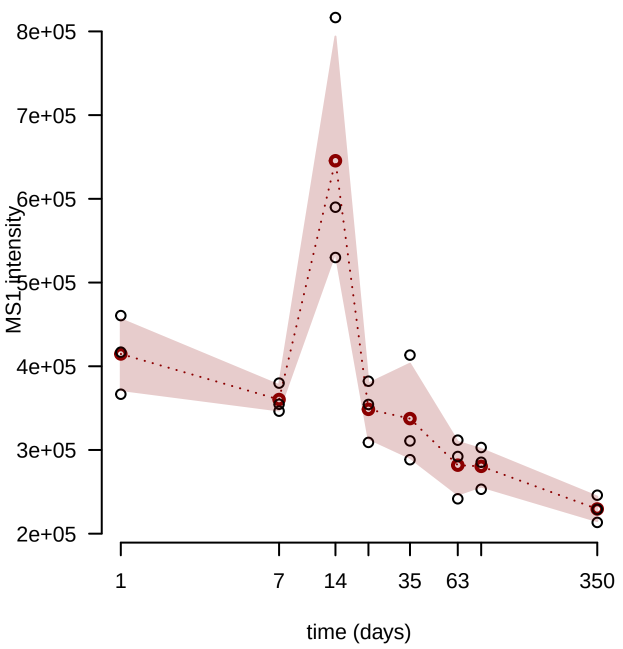

C0HKG5
GN=Rnaset2a
ribonuclease T2A

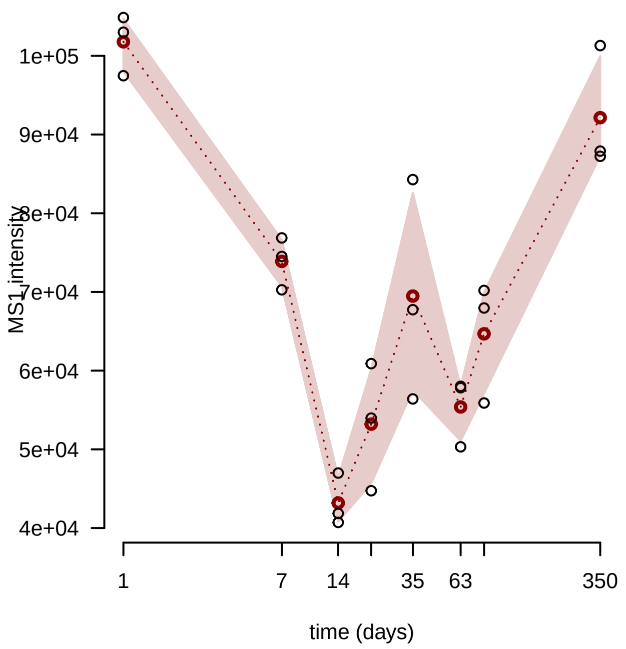

C0LLJ0
GN=Kpna7
karyopherin alpha 7 (importin alpha 8)

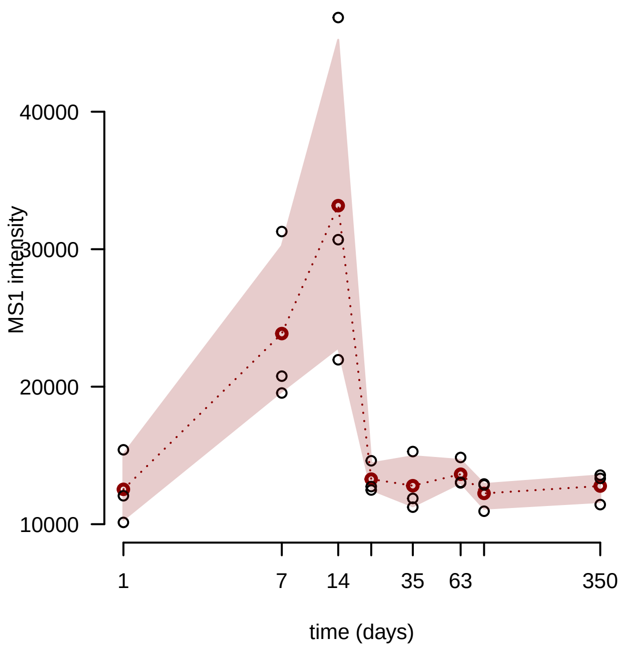

D2EAC2
GN=Zbed6
zinc finger, BED type containing 6

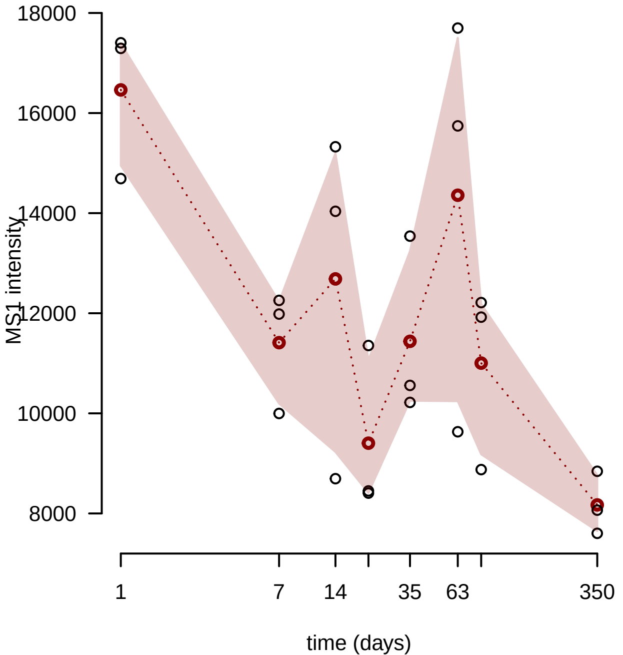

D3YVF0
GN=Akap5
A kinase (PRKA) anchor protein 5

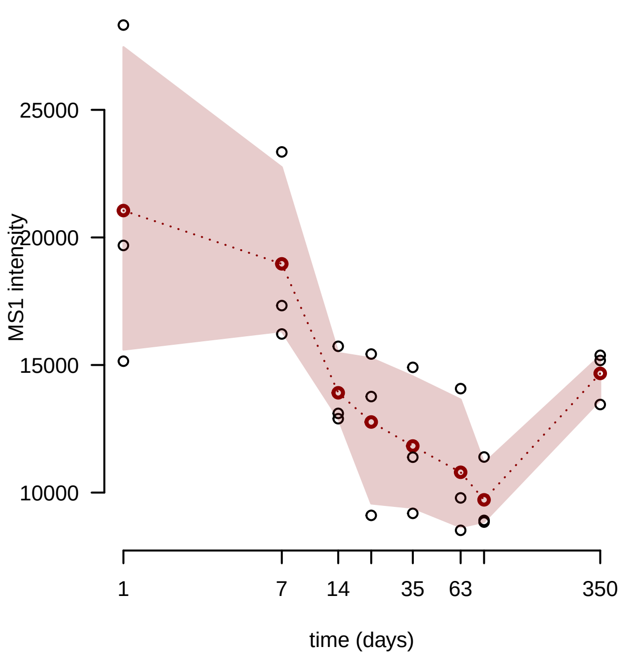

D3YX43
GN=Vsig10
V−set and immunoglobulin domain containi ...

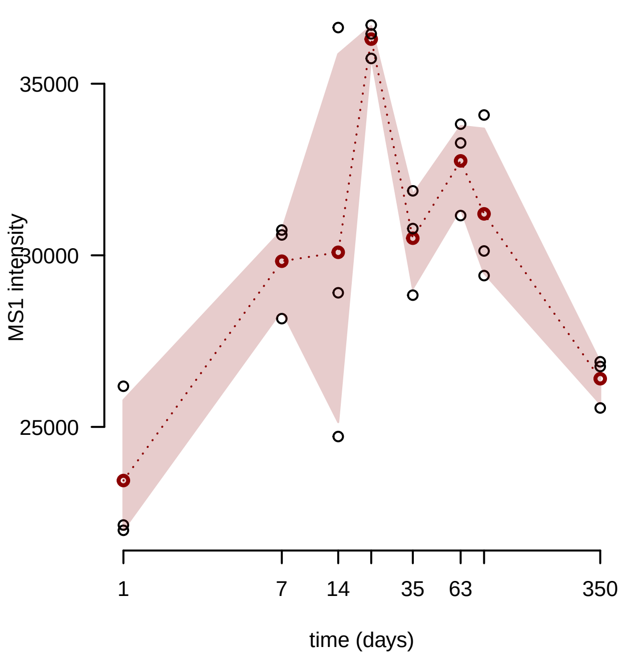

D3YXG0
GN=Hmcn1
hemicentin 1

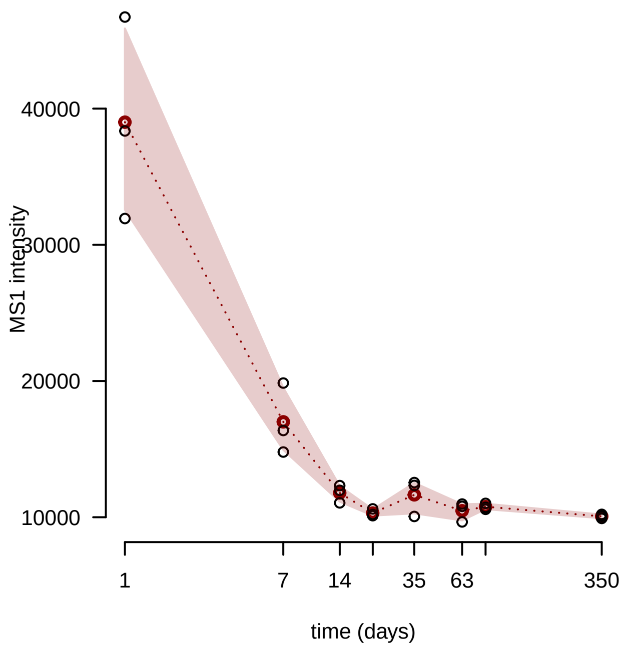

D3YXK1
GN=Samd1
sterile alpha motif domain containing 1

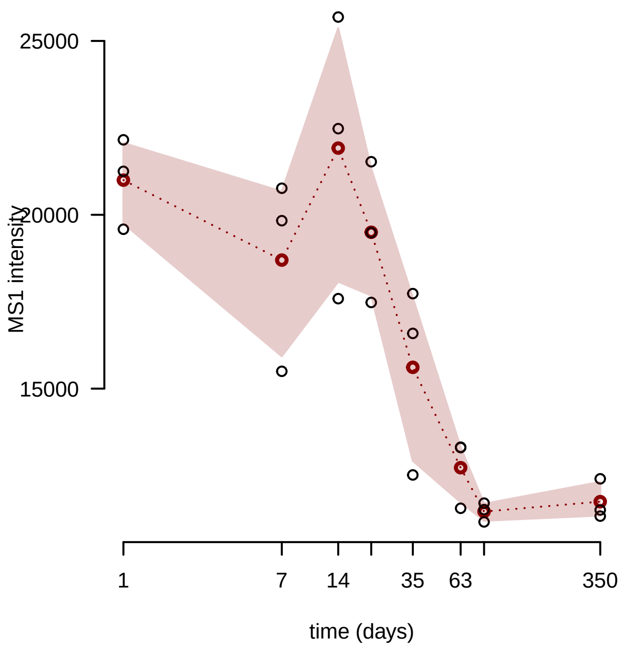

D3YXK2
GN=Safb
scaffold attachment factor B

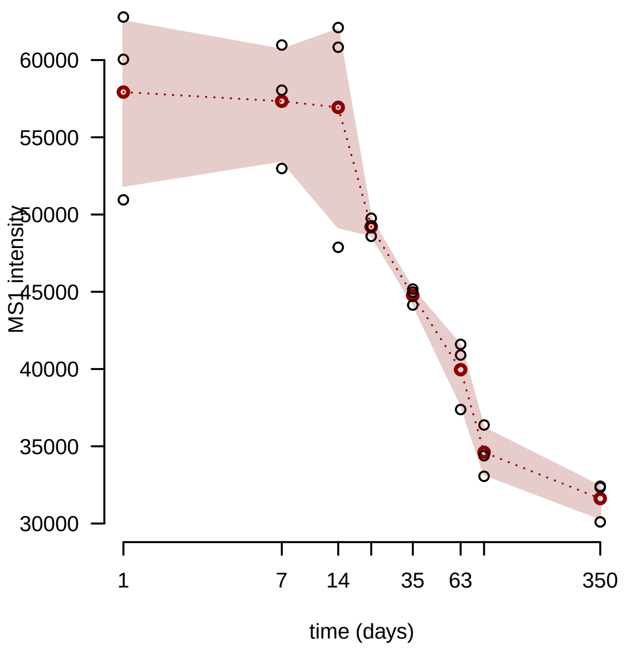

D3YYU8
GN=Obsl1
obscurin−like 1

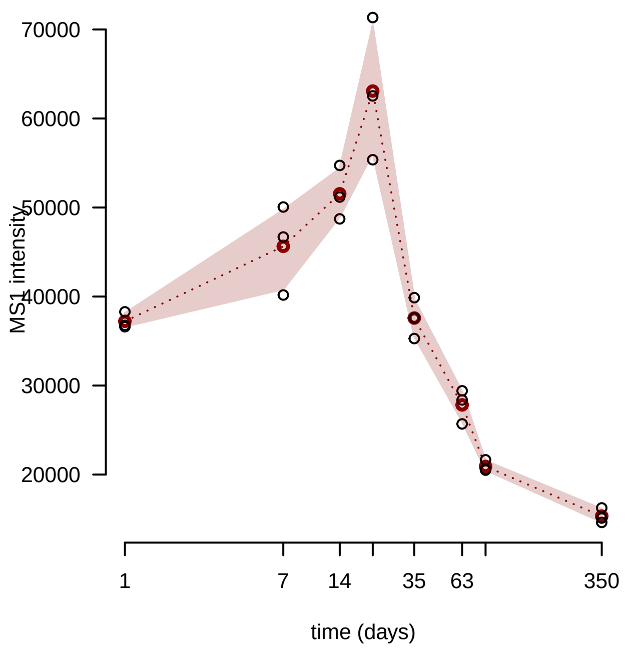

D3YYU8
GN=Obsl1
obscurin−like 1

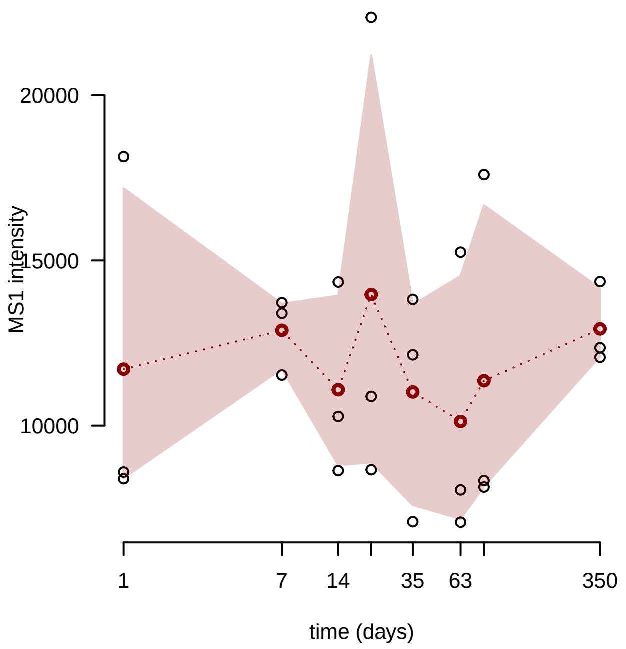

D3YZG8
GN=Mthfd2l
methylenetetrahydrofolate dehydrogenase ...

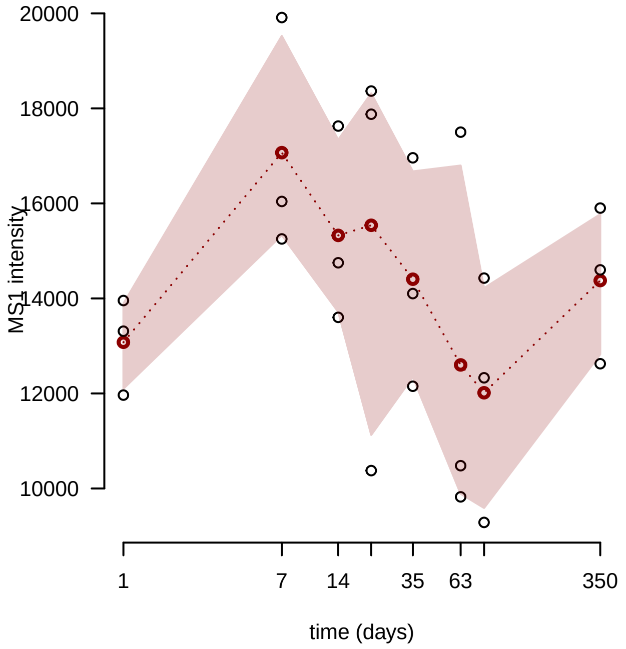

D3YZP9
GN=Ccdc6
coiled−coil domain containing 6

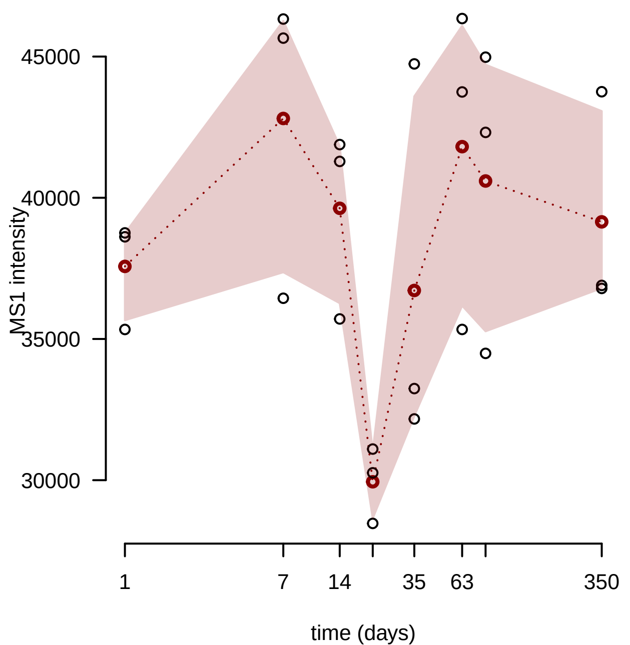

D3YZV8
GN=Ccdc8
coiled−coil domain containing 8

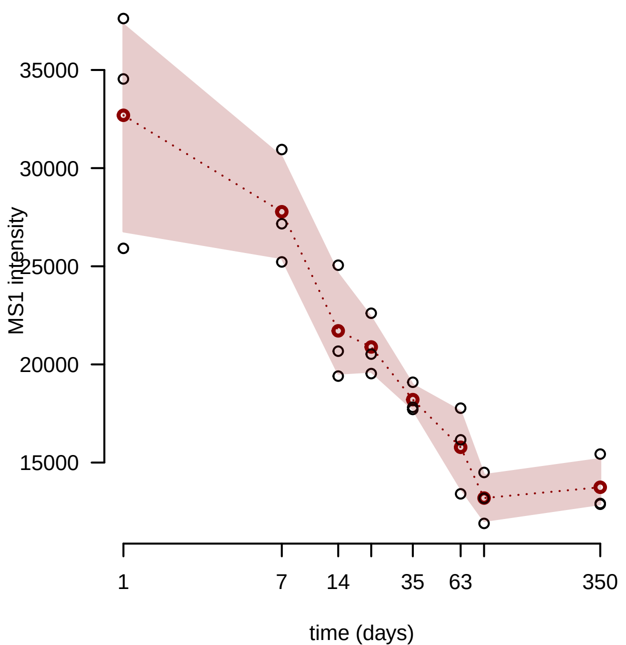

D3Z2R5
GN=Selenon
selenoprotein N

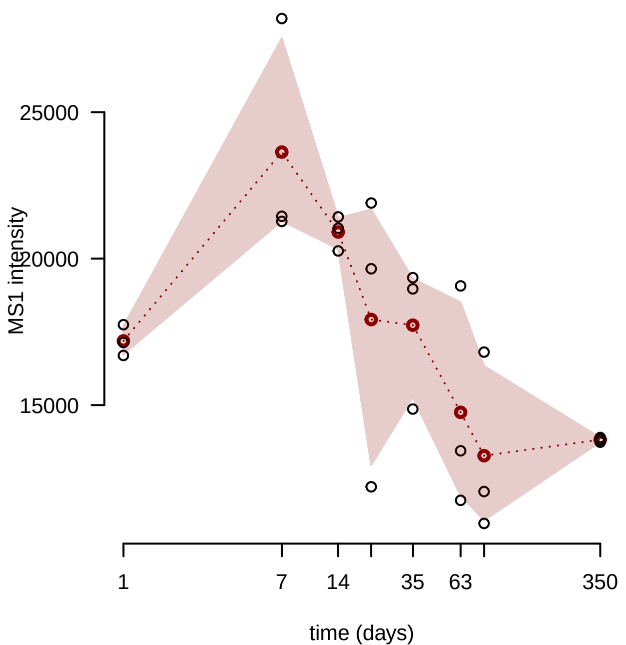

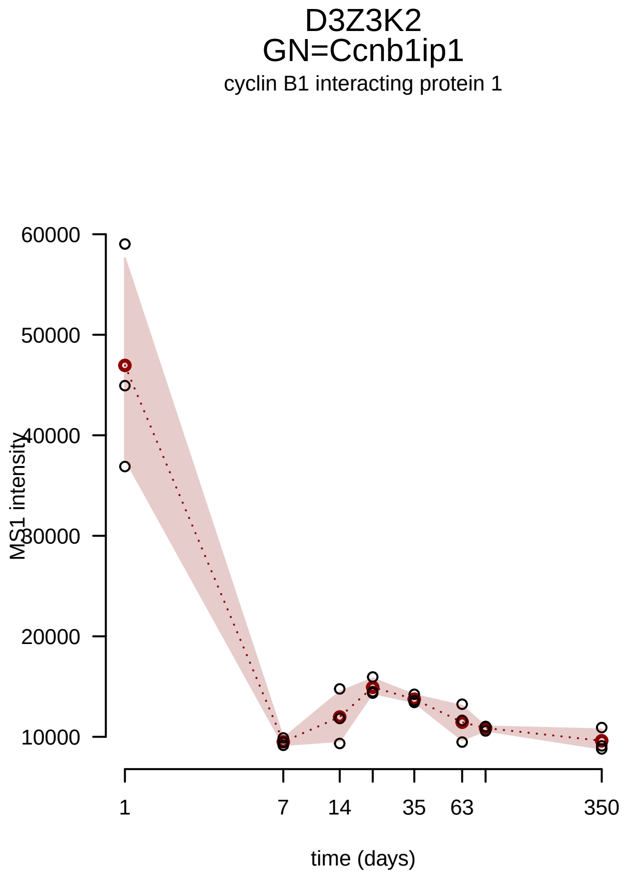
D3Z3K2
GN=Ccnb1ip1
cyclin B1 interacting protein 1
MS1 intensity
60000
50000
40000
30000
20000
10000
1
7
14
35
63
350
time (days)

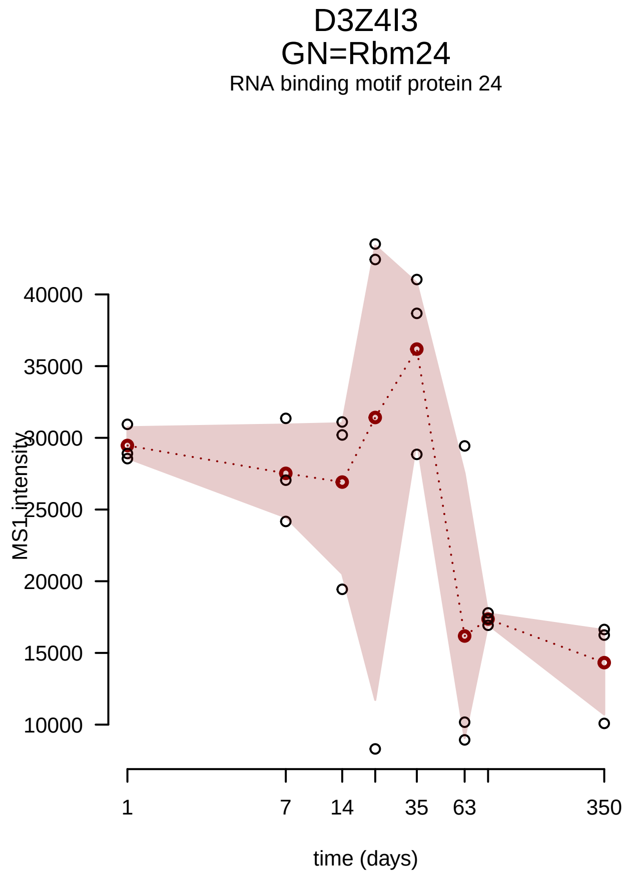
D3Z4I3
GN=Rbm24
RNA binding motif protein 24
MS1 intensity
40000
35000
30000
25000
20000
15000
10000
1
7
14
35
63
350
time (days)

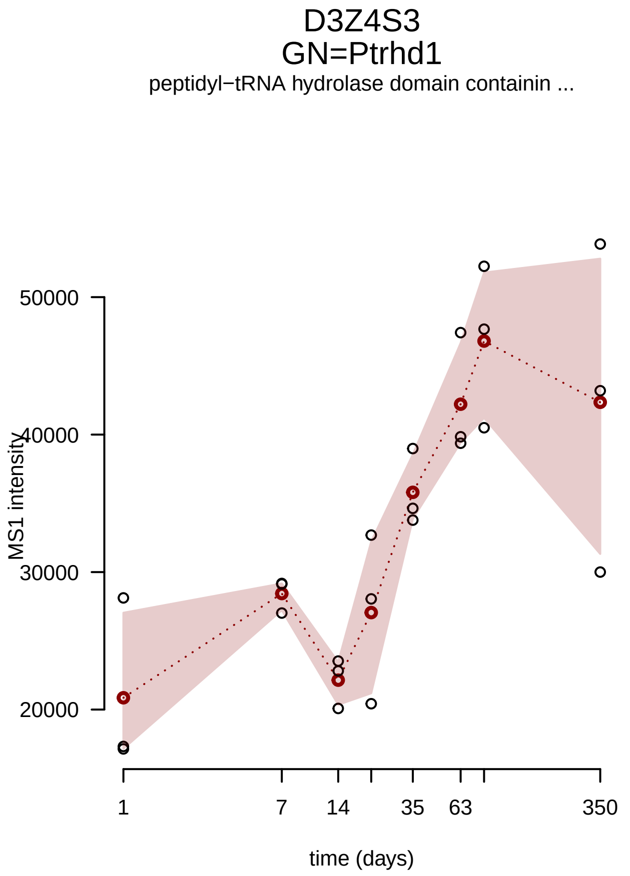
D3Z4S3
GN=Ptrhd1
peptidyl−tRNA hydrolase domain containin ...
MS1 intensity
50000
40000
30000
20000
1
7
14
35
63
350
time (days)

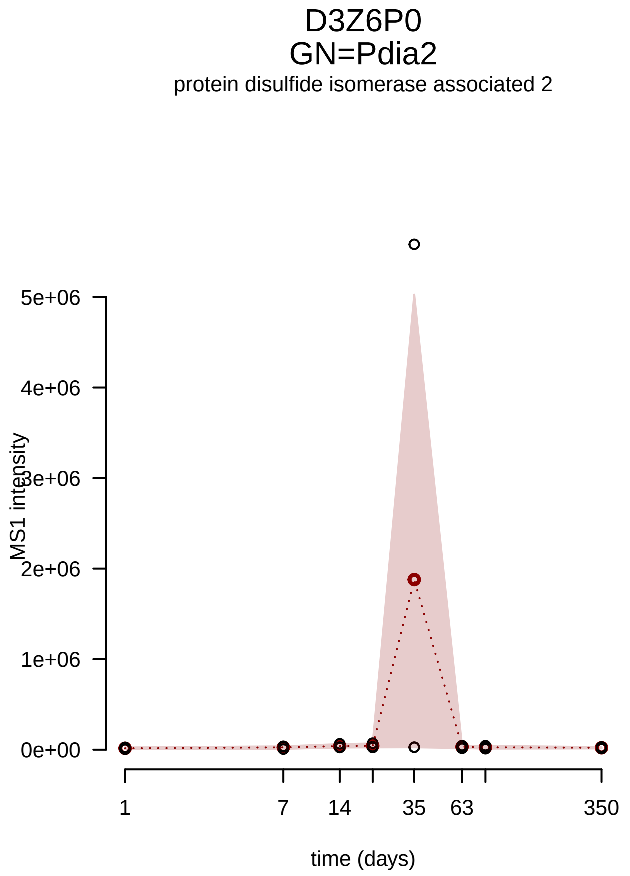
D3Z6P0
GN=Pdia2
protein disulfide isomerase associated 2
MS1 intensity
5e+06
4e+06
3e+06
2e+06
1e+06
0e+00
1
7
14
35
63
350
time (days)

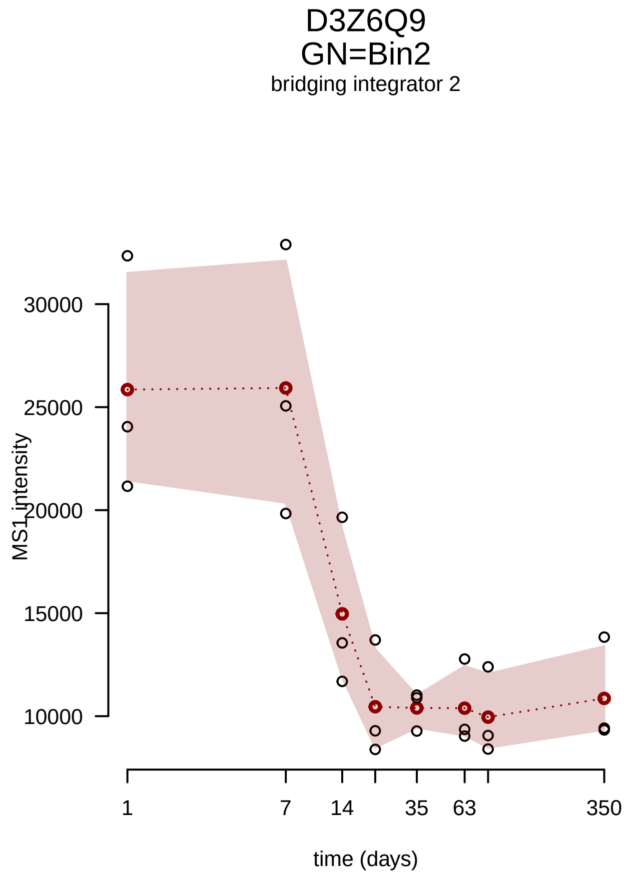
D3Z6Q9
GN=Bin2
bridging integrator 2
MS1 intensity
30000
25000
20000
15000
10000
1
7
14
35
63
350
time (days)

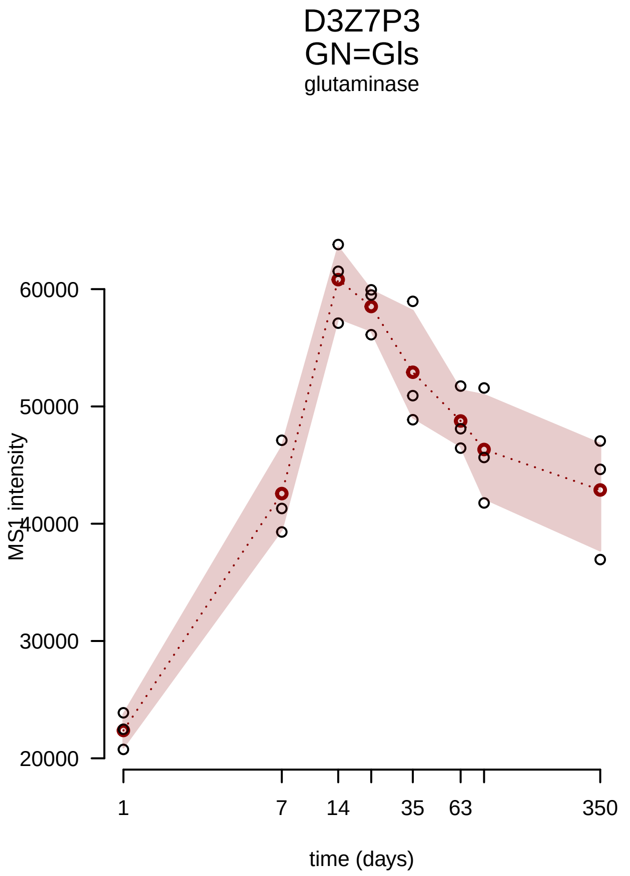
D3Z7P3
GN=Gls
glutaminase
MS1 intensity
60000
50000
40000
30000
20000
1
7
14
35
63
350
time (days)

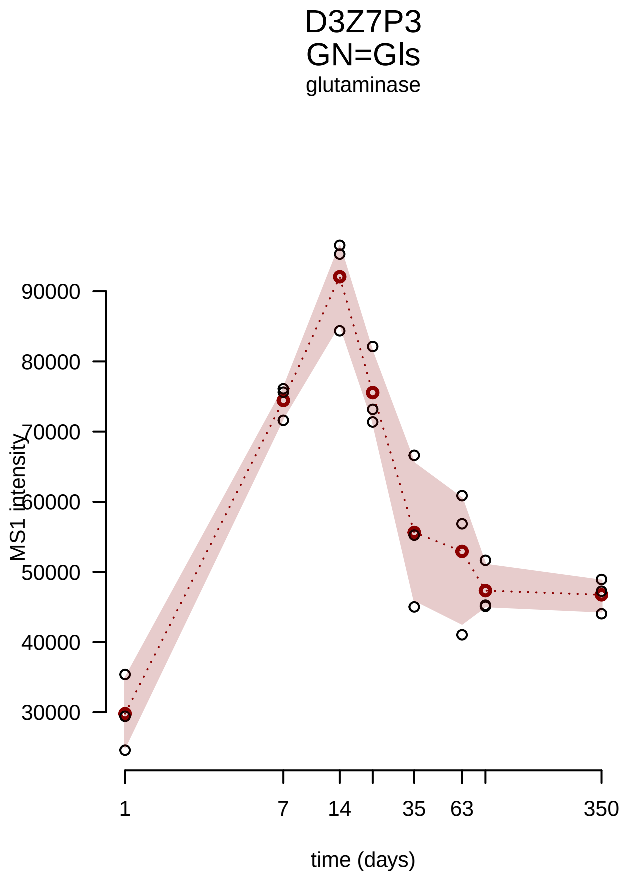
D3Z7P3
GN=Gls
glutaminase
MS1 intensity
90000
80000
70000
60000
50000
40000
30000
1
7
14
35
63
350
time (days)

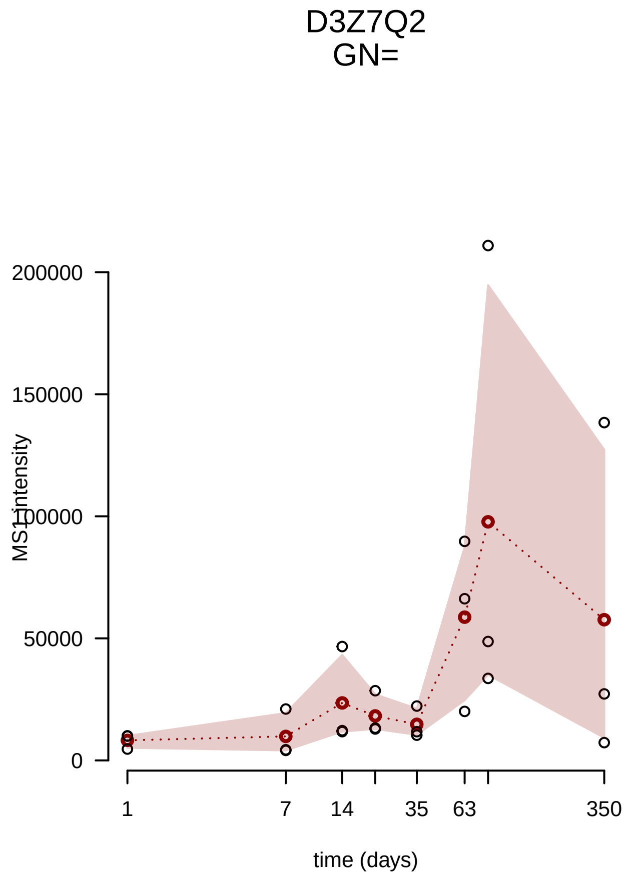
D3Z7Q2
GN=
MS1 intensity
200000
150000
100000
50000
0
1
7
14
35
63
350
time (days)

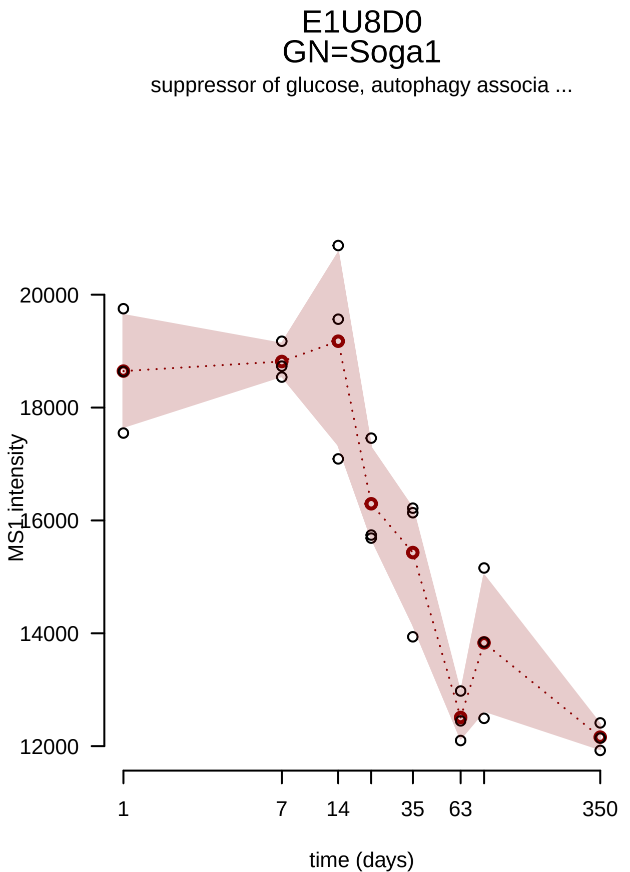
E1U8D0
GN=Soga1
suppressor of glucose, autophagy associa ...
MS1 intensity
20000
18000
16000
14000
12000
1
7
14
35
63
350
time (days)

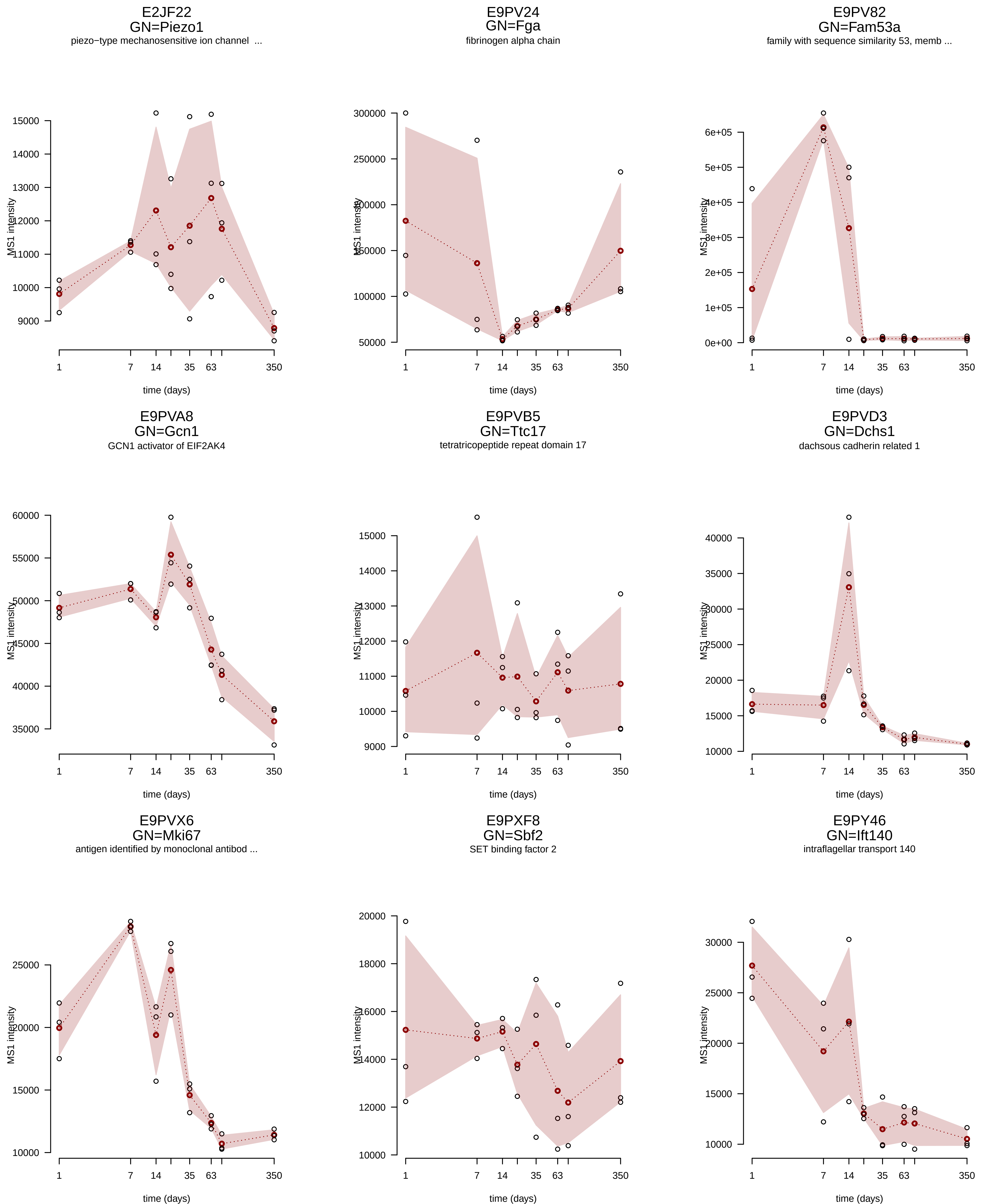

E2JF22
GN=Piezo1
piezo−type mechanosensitive ion channel ...
E9PV24
GN=Fga
fibrinogen alpha chain
E9PV82
GN=Fam53a
family with sequence similarity 53, memb ...
E9PVA8
GN=Gcn1
GCN1 activator of EIF2AK4
E9PVB5
GN=Ttc17
tetratricopeptide repeat domain 17
E9PVD3
GN=Dchs1
dachsous cadherin related 1
E9PVX6
GN=Mki67
antigen identified by monoclonal antibod ...
E9PXF8
GN=Sbf2
SET binding factor 2
E9PY46
GN=Ift140
intraflagellar transport 140
MS1 intensity
time (days)
1
7
14
35
63
350

E9PYH6
GN=Setd1a
SET domain containing 1A

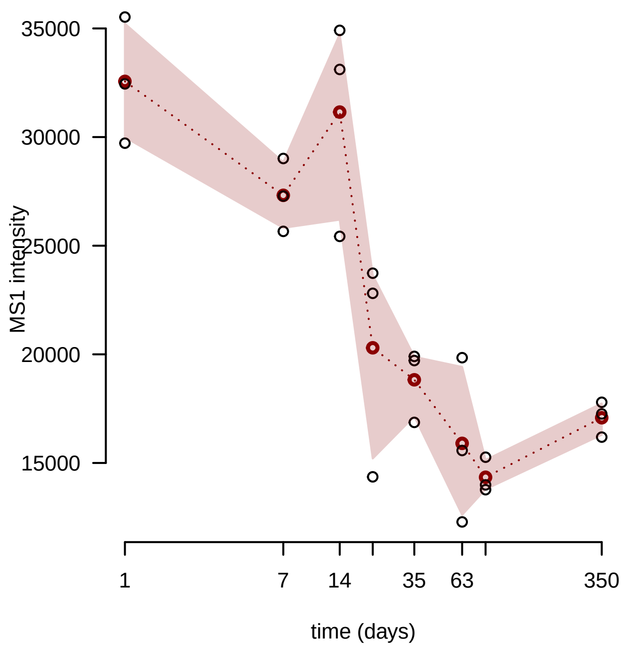

E9PYI1
GN=Znf568

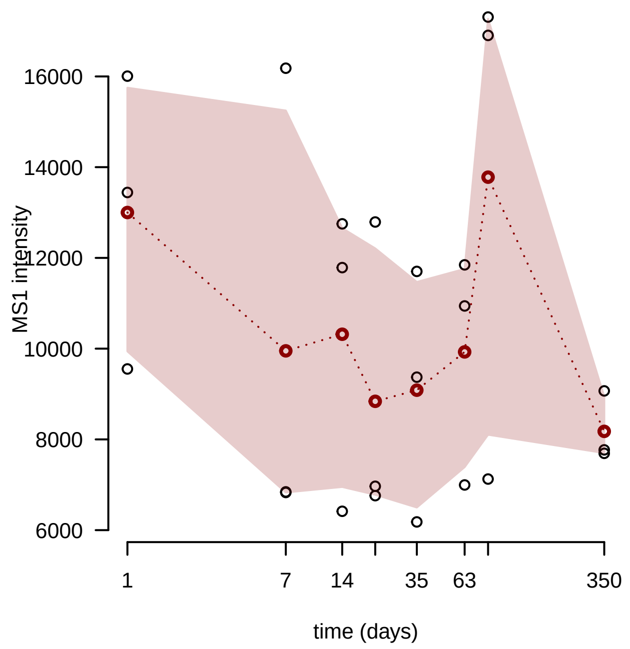

E9PYK3
GN=Parp4
poly (ADP−ribose) polymerase family, mem ...

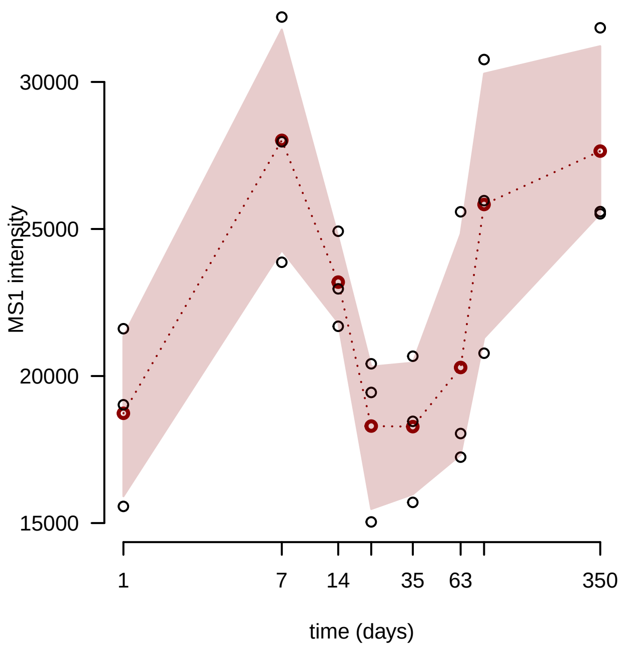

E9PYL2
GN=Prr12
proline rich 12

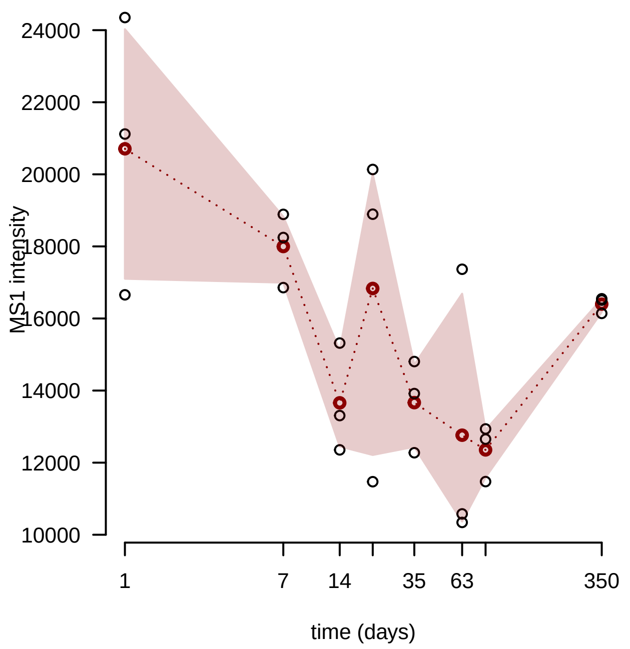

E9PZ36
GN=

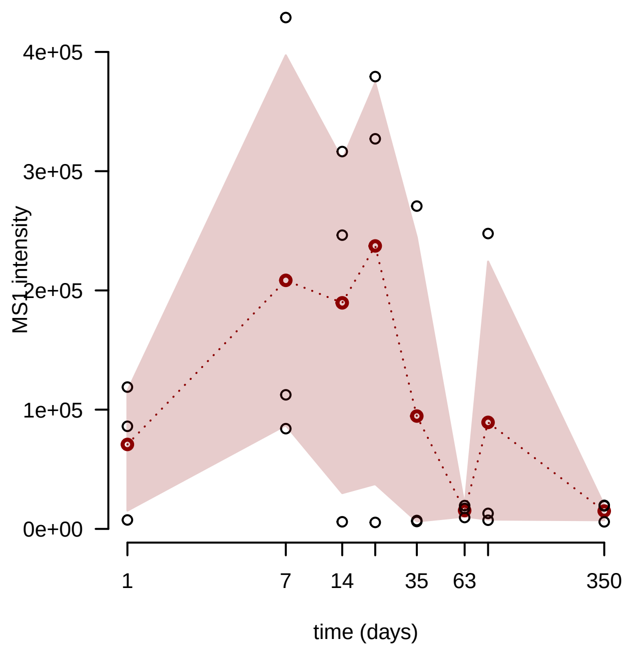

E9PZJ8
GN=Ascc3
activating signal cointegrator 1 complex ...

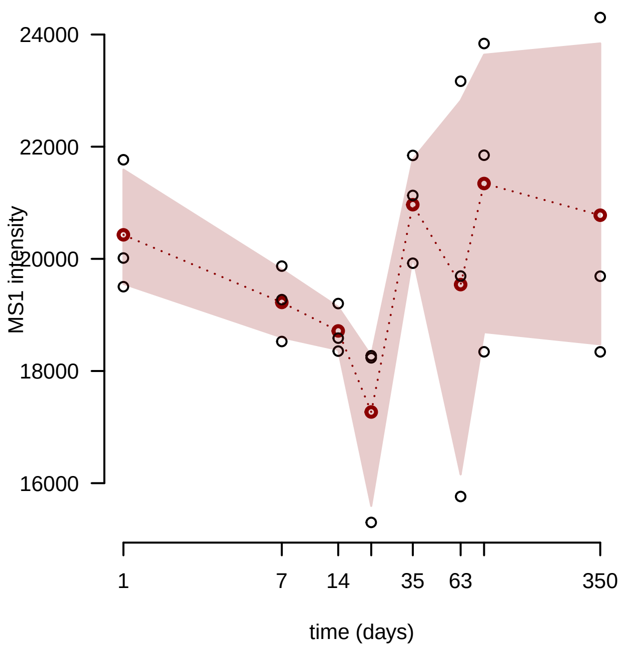

E9PZM4
GN=Chd2
chromodomain helicase DNA binding protei ...

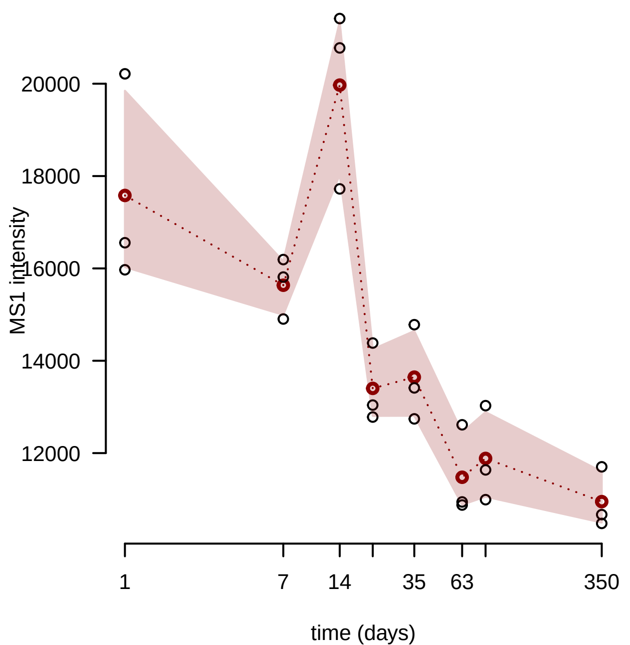

E9Q137
GN=

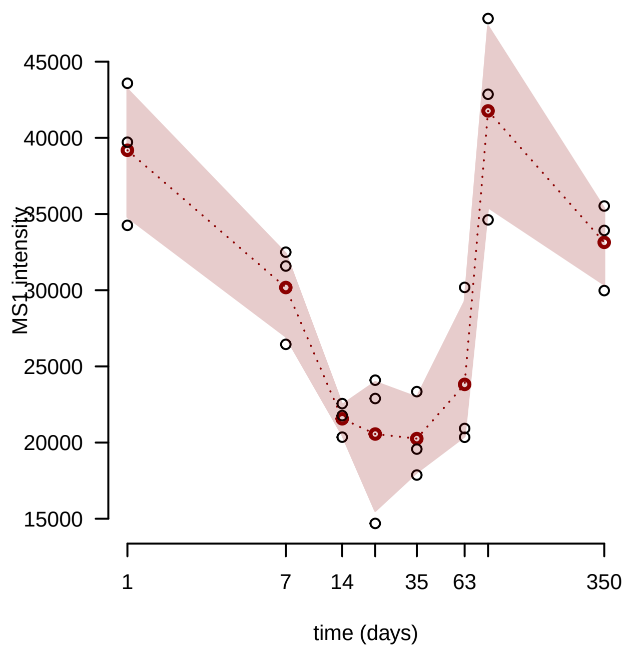

E9Q1P8
GN=Irf2bp2
interferon regulatory factor 2 binding p ...

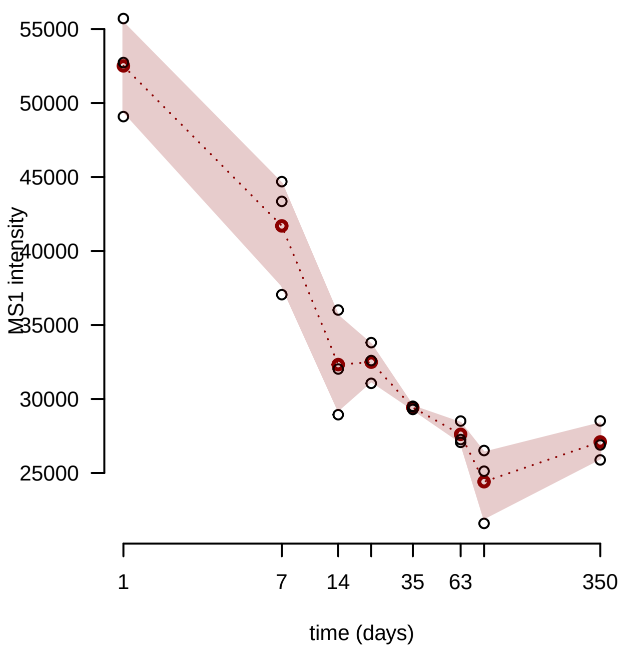

E9Q236
GN=

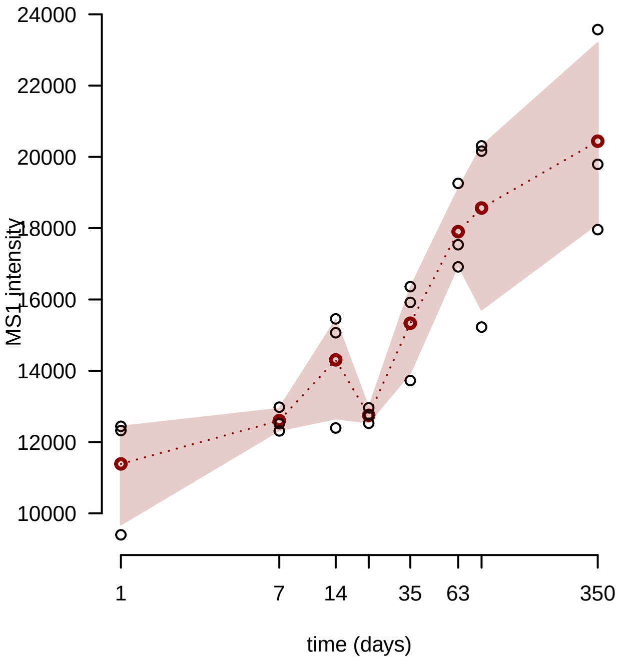

E9Q286
GN=Ice1
interactor of little elongation complex ...

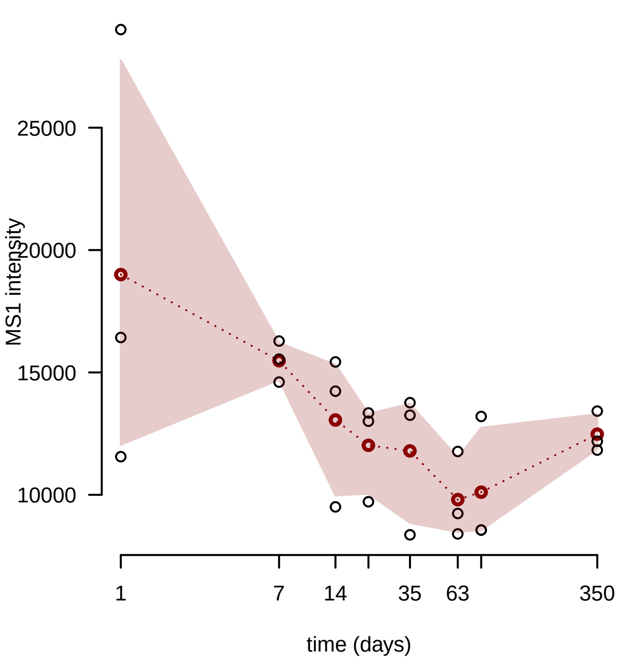

E9Q2M9
GN=

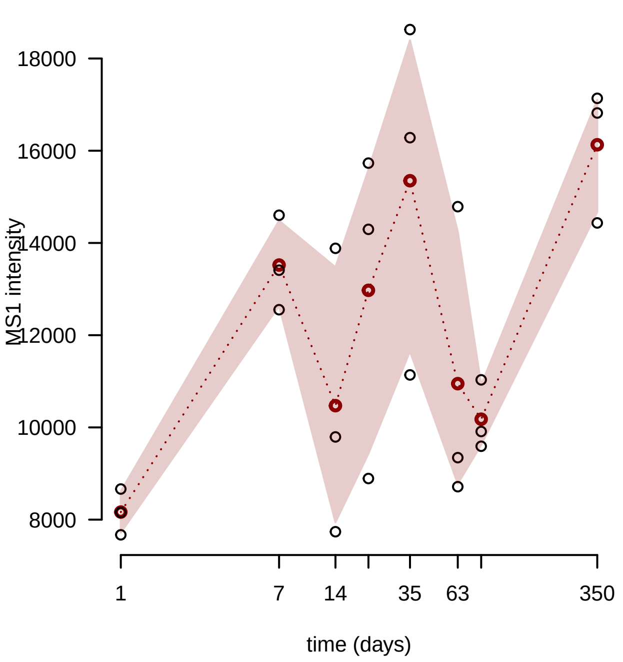

E9Q394
GN=Akap13
A kinase (PRKA) anchor protein 13

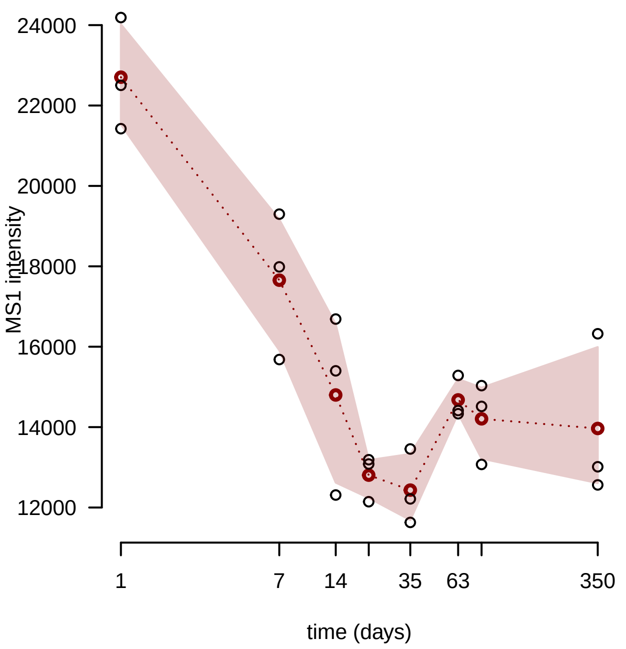

E9Q3C1
GN=C2cd2
C2 calcium−dependent domain containing 2

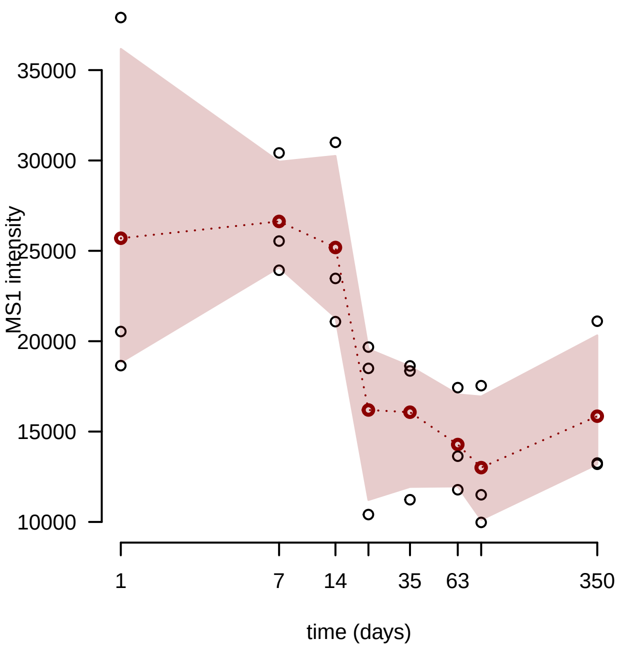

E9Q3E1
GN=Aldh3b2
aldehyde dehydrogenase 3 family, member ...

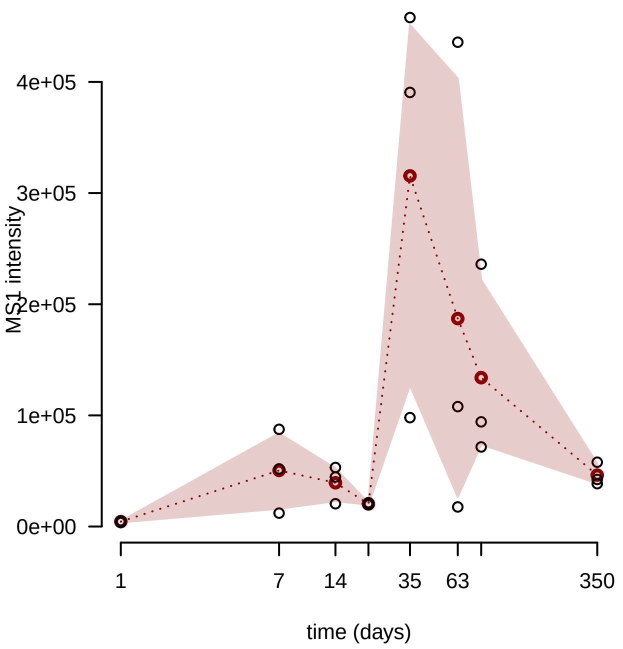

E9Q3L2
GN=Pi4ka
phosphatidylinositol 4−kinase alpha

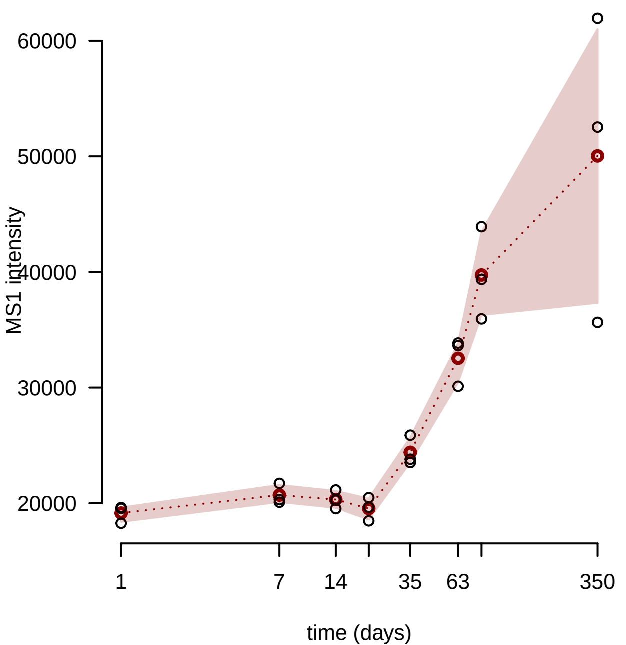

E9Q3S4
GN=Map3k19
mitogen−activated protein kinase kinase ...

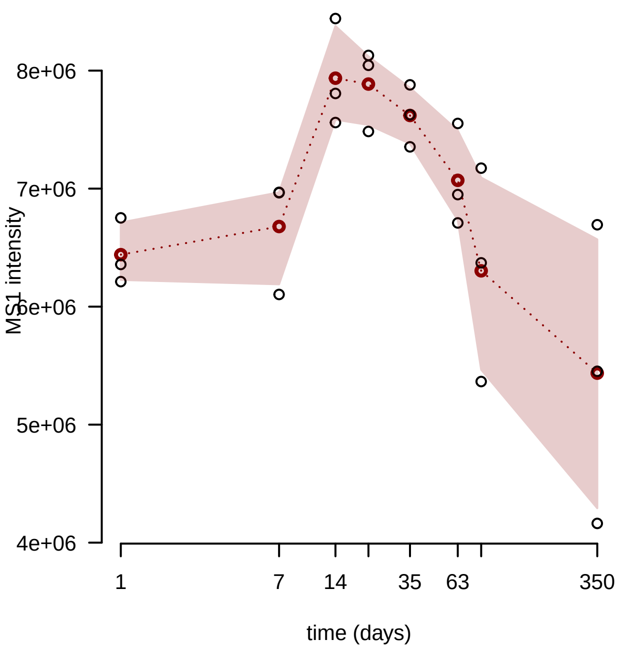

E9Q414
GN=Apob
apolipoprotein B

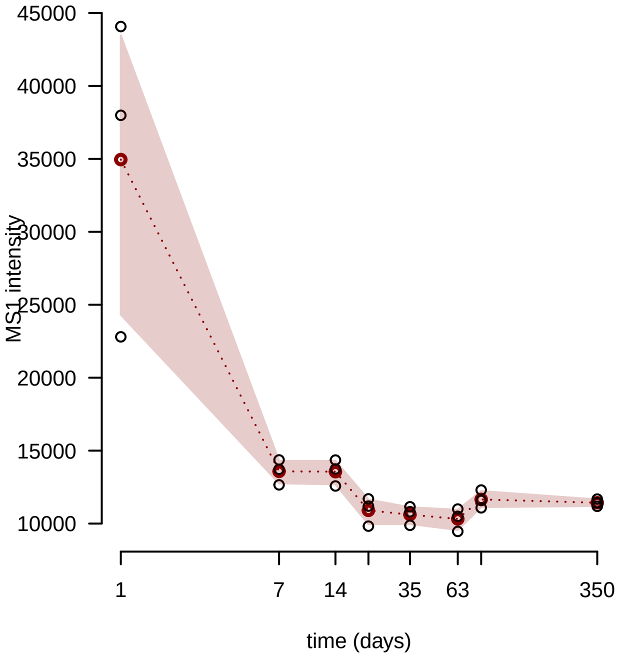

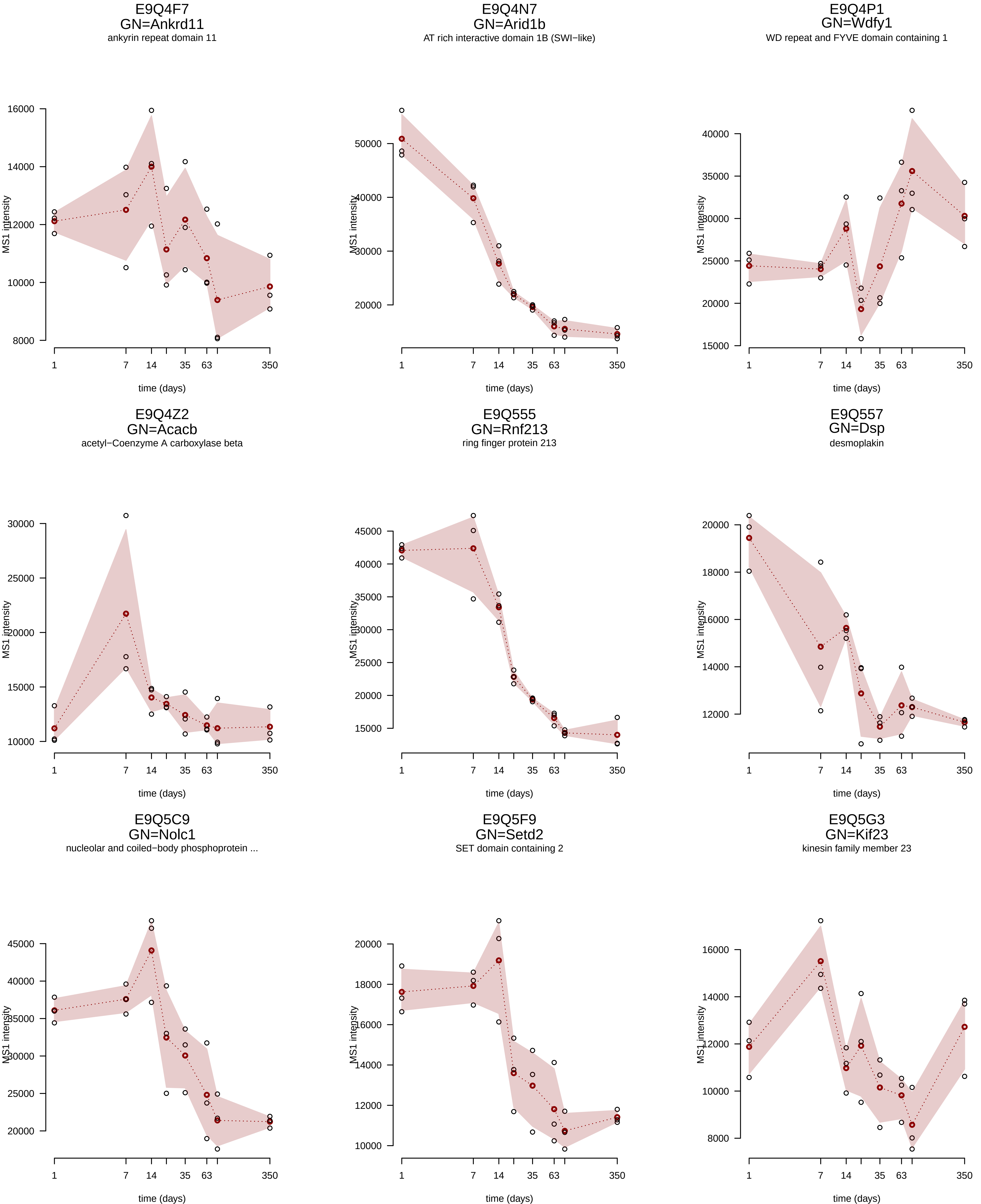
E9Q4F7
GN=Ankrd11
ankyrin repeat domain 11
MS1 intensity
16000
14000
12000
10000
8000
1
7
14
35
63
350
time (days)
E9Q4N7
GN=Arid1b
AT rich interactive domain 1B (SWI−like)
MS1 intensity
50000
40000
30000
20000
1
7
14
35
63
350
time (days)
E9Q4P1
GN=Wdfy1
WD repeat and FYVE domain containing 1
MS1 intensity
40000
35000
30000
25000
20000
15000
1
7
14
35
63
350
time (days)
E9Q4Z2
GN=Acacb
acetyl−Coenzyme A carboxylase beta
MS1 intensity
30000
25000
20000
15000
10000
1
7
14
35
63
350
time (days)
E9Q555
GN=Rnf213
ring finger protein 213
MS1 intensity
45000
40000
35000
30000
25000
20000
15000
1
7
14
35
63
350
time (days)
E9Q557
GN=Dsp
desmoplakin
MS1 intensity
20000
18000
16000
14000
12000
1
7
14
35
63
350
time (days)
E9Q5C9
GN=Nolc1
nucleolar and coiled−body phosphoprotein ...
MS1 intensity
45000
40000
35000
30000
25000
20000
1
7
14
35
63
350
time (days)
E9Q5F9
GN=Setd2
SET domain containing 2
MS1 intensity
20000
18000
16000
14000
12000
10000
1
7
14
35
63
350
time (days)
E9Q5G3
GN=Kif23
kinesin family member 23
MS1 intensity
16000
14000
12000
10000
8000
1
7
14
35
63
350
time (days)

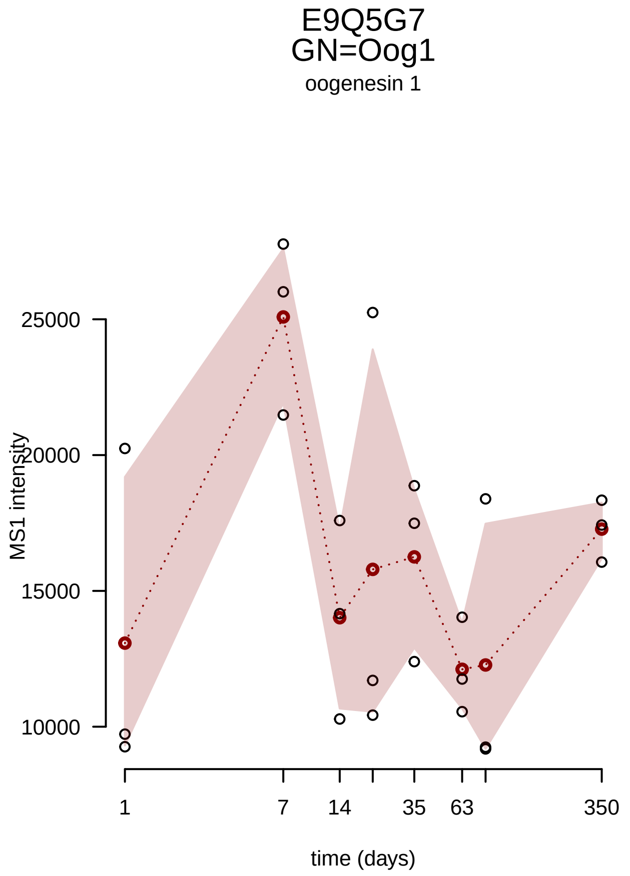
E9Q5G7
GN=Oog1
oogenesin 1
MS1 intensity
10000
15000
20000
25000
1
7
14
35
63
350
time (days)

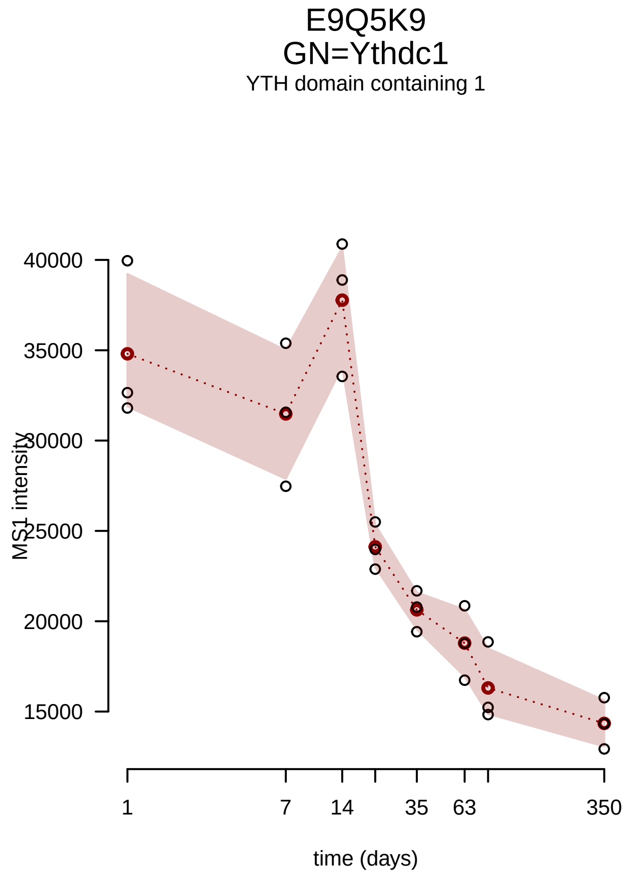
E9Q5K9
GN=Ythdc1
YTH domain containing 1
MS1 intensity
15000
20000
25000
30000
35000
40000
1
7
14
35
63
350
time (days)

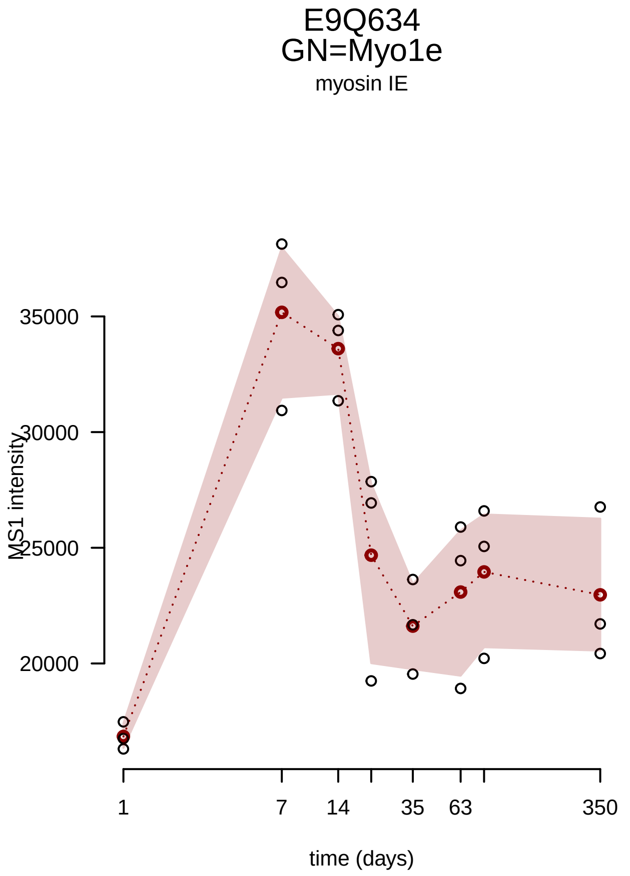
E9Q634
GN=Myo1e
myosin IE
MS1 intensity
20000
25000
30000
35000
1
7
14
35
63
350
time (days)

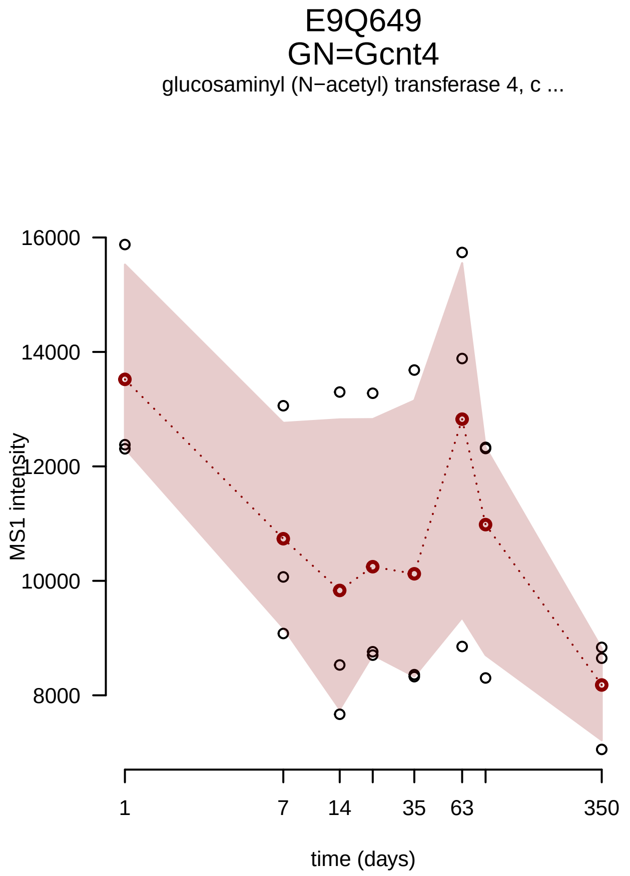
E9Q649
GN=Gcnt4
glucosaminyl (N−acetyl) transferase 4, c ...
MS1 intensity
8000
10000
12000
14000
16000
1
7
14
35
63
350
time (days)

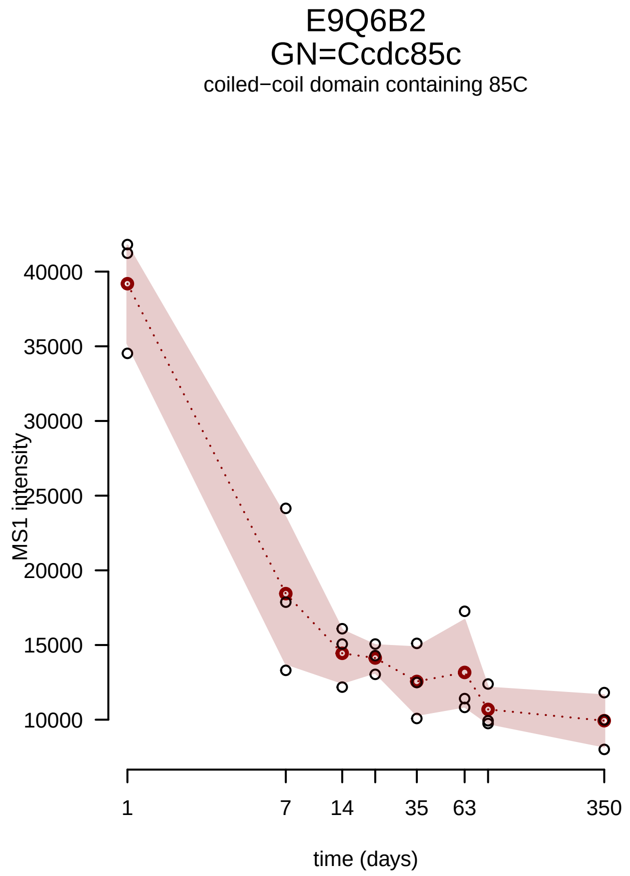
E9Q6B2
GN=Ccdc85c
coiled−coil domain containing 85C
MS1 intensity
10000
15000
20000
25000
30000
35000
40000
1
7
14
35
63
350
time (days)

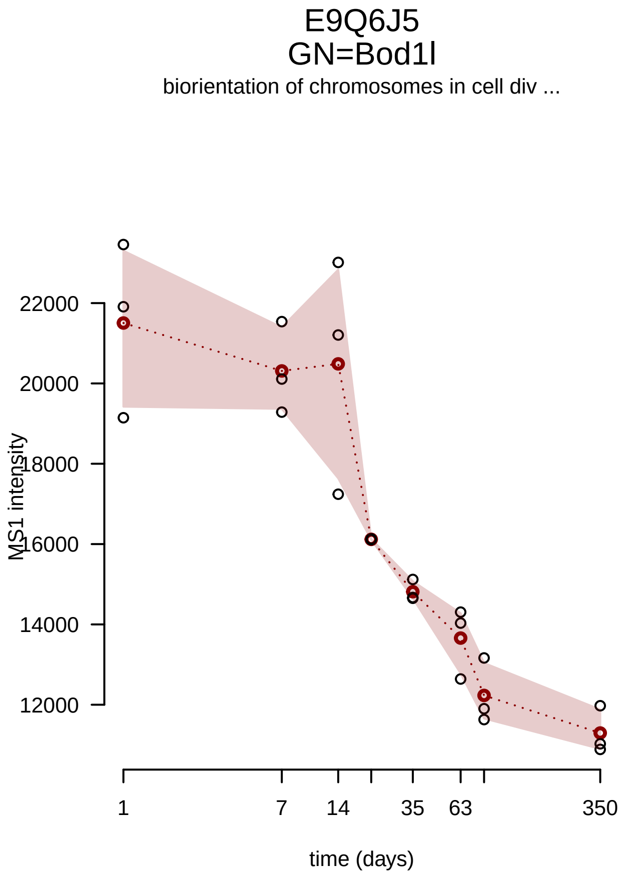
E9Q6J5
GN=Bod1l
biorientation of chromosomes in cell div ...
MS1 intensity
12000
14000
16000
18000
20000
22000
1
7
14
35
63
350
time (days)

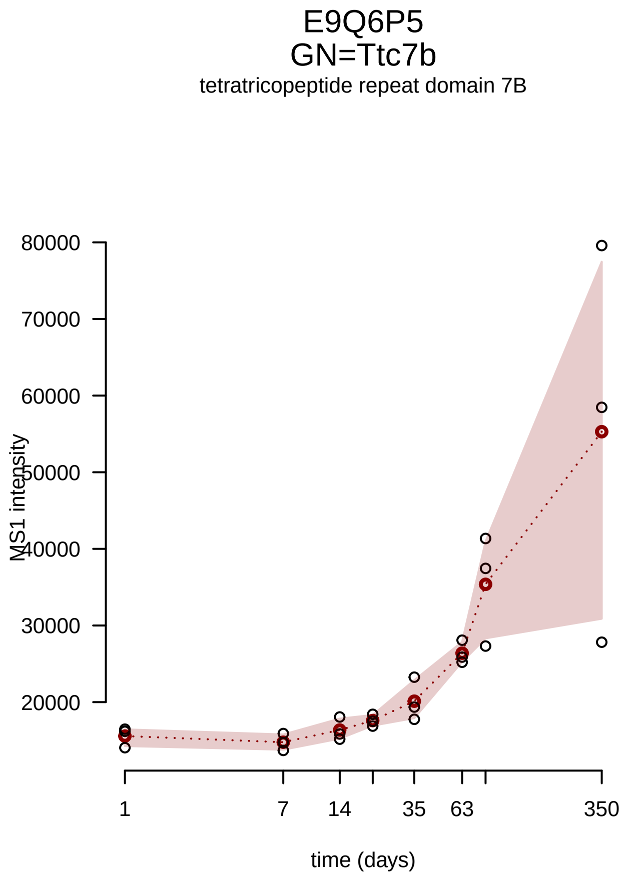
E9Q6P5
GN=Ttc7b
tetratricopeptide repeat domain 7B
MS1 intensity
20000
30000
40000
50000
60000
70000
80000
1
7
14
35
63
350
time (days)

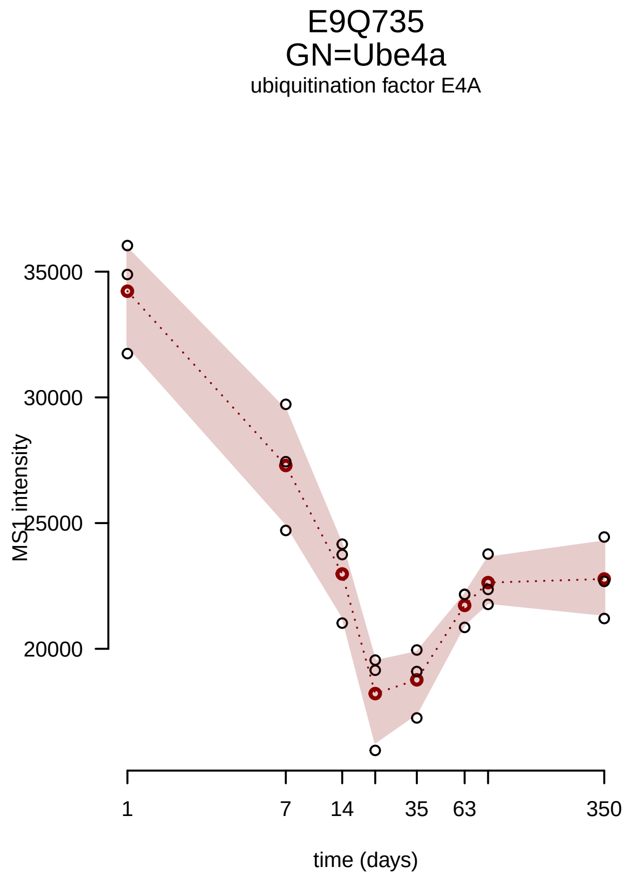
E9Q735
GN=Ube4a
ubiquitination factor E4A
MS1 intensity
20000
25000
30000
35000
1
7
14
35
63
350
time (days)

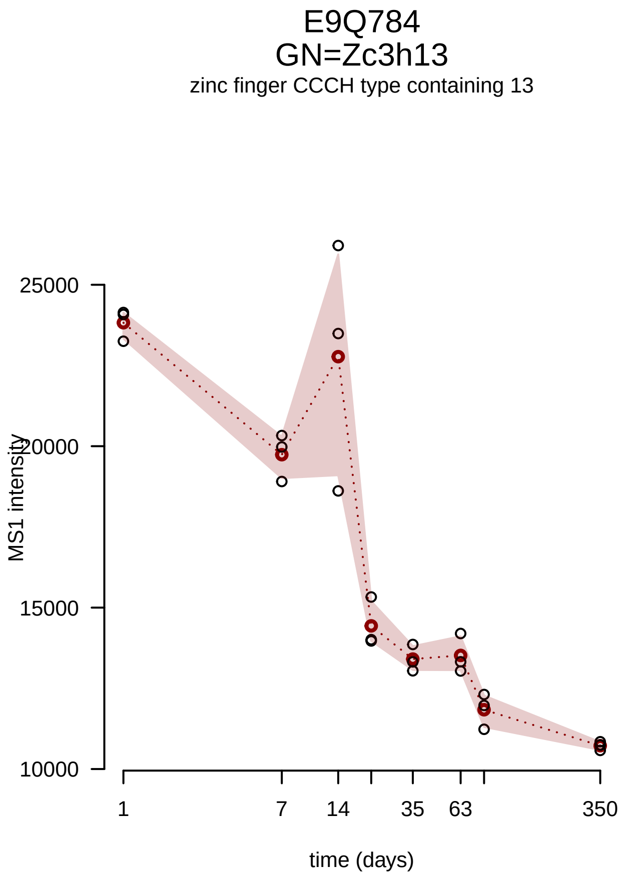
E9Q784
GN=Zc3h13
zinc finger CCCH type containing 13
MS1 intensity
10000
15000
20000
25000
1
7
14
35
63
350
time (days)

E9Q7D5
GN=Arhgef5
Rho guanine nucleotide exchange factor (...

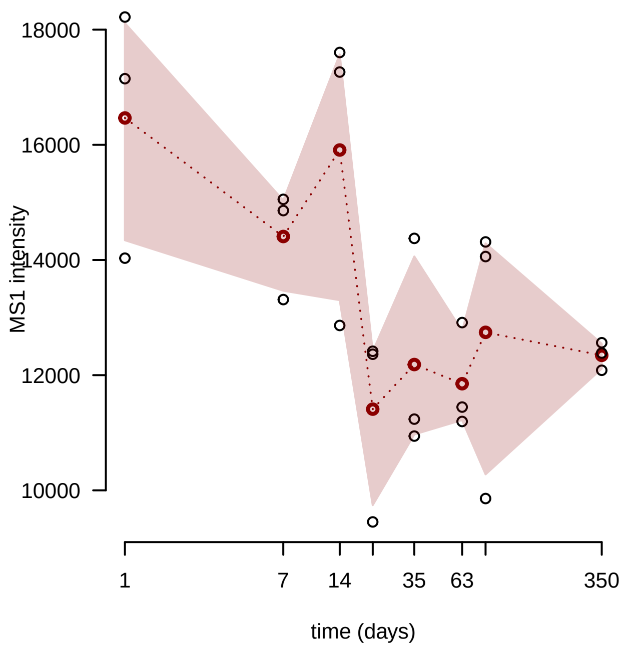

E9Q7E2
GN=Arid2
AT rich interactive domain 2 (ARID, RFX− ...

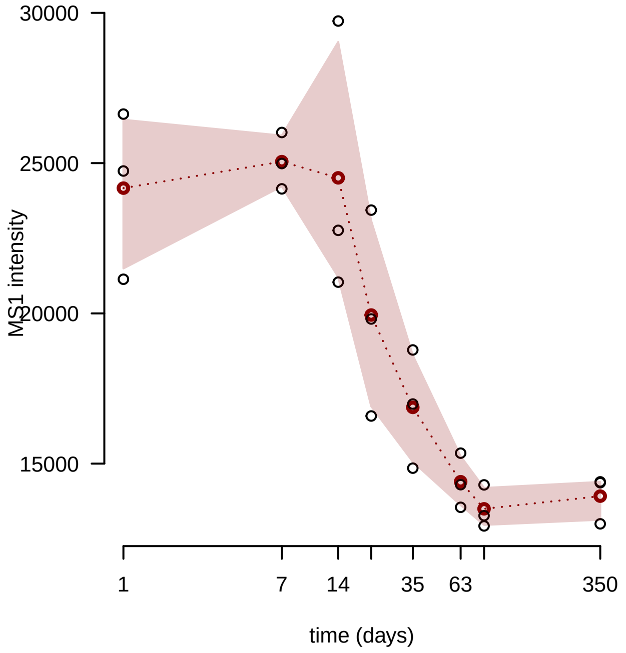

E9Q7F2
GN=Rnf169
ring finger protein 169

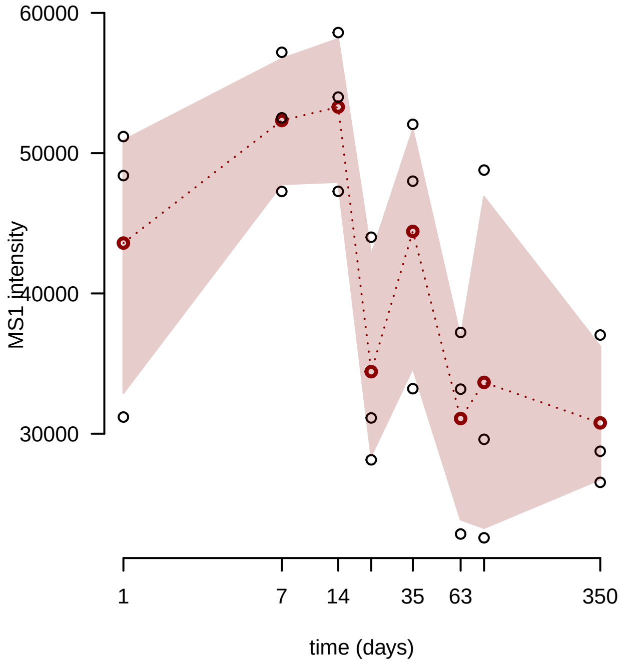

E9Q7G0
GN=Numa1
nuclear mitotic apparatus protein 1

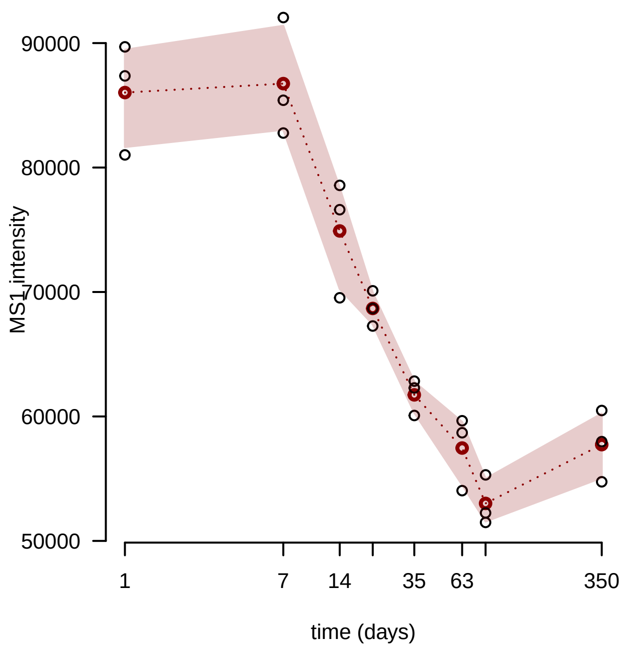

E9Q7X6
GN=Heg1
heart development protein with EGF−like ...

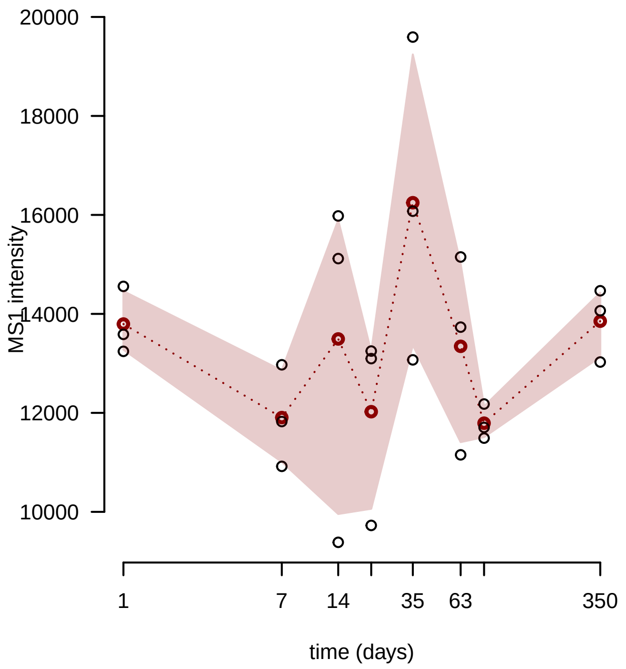

E9Q8D0
GN=Dnajc21
DnaJ heat shock protein family (Hsp40) m ...

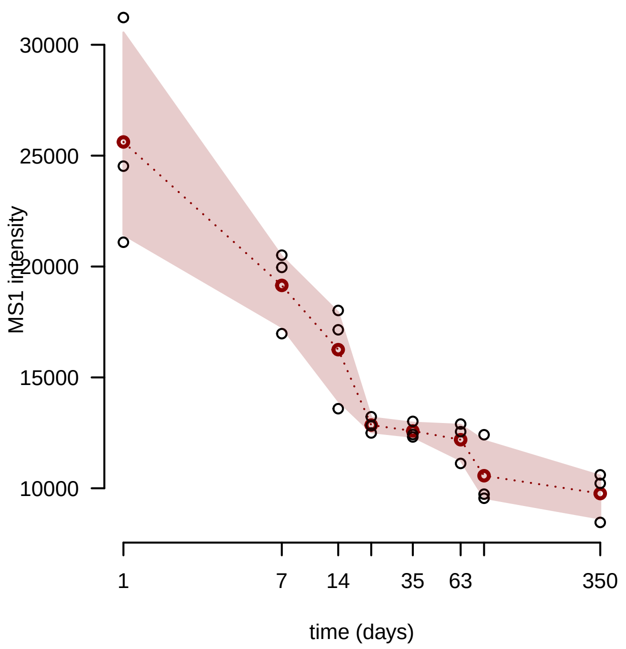

E9Q8I9
GN=Fry
FRY microtubule binding protein

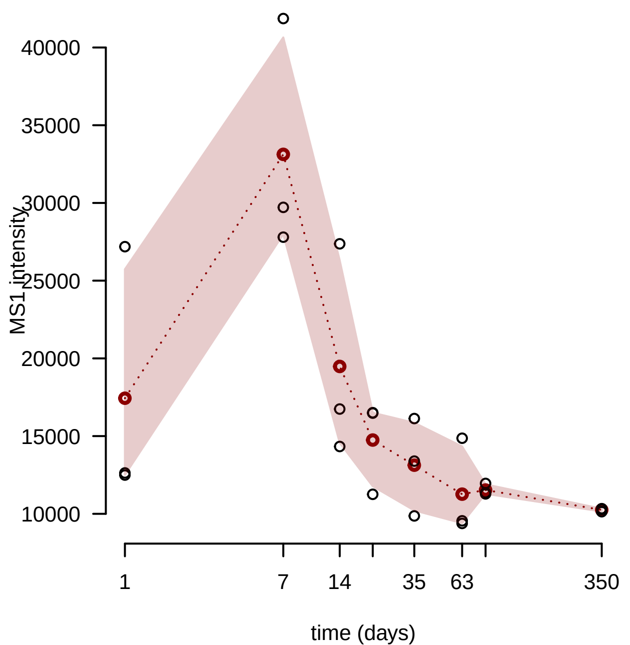

E9Q912
GN=

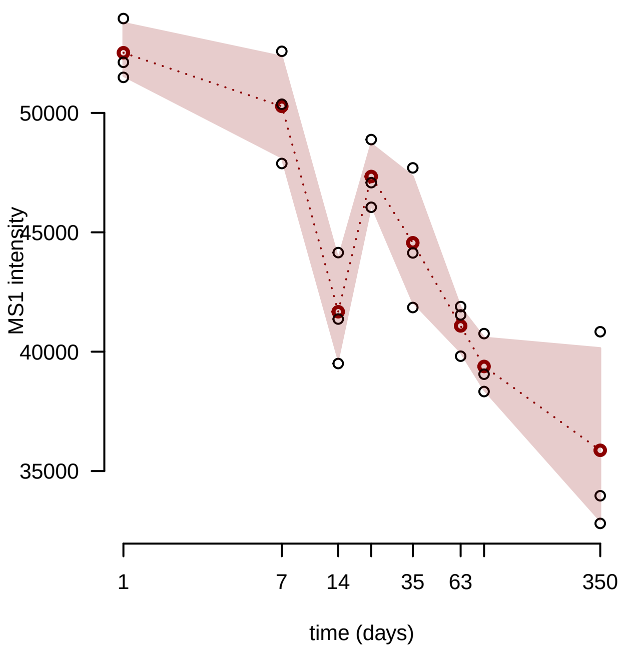

E9Q942
GN=Smim13
small integral membrane protein 13

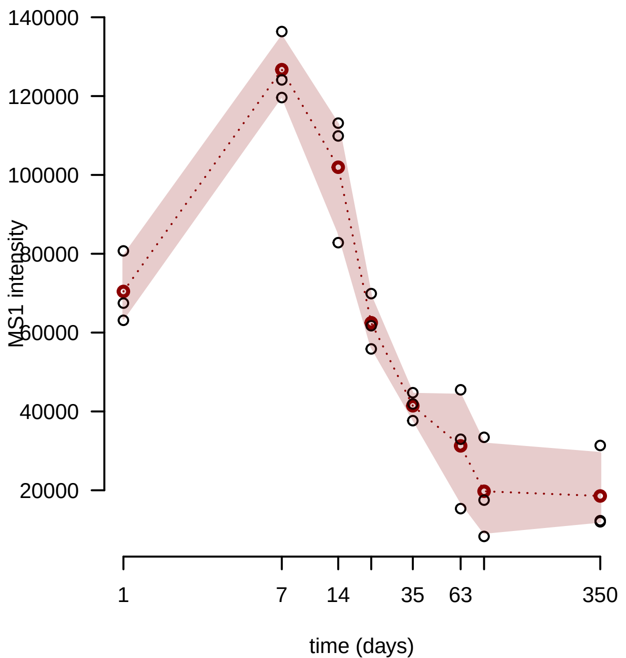

E9Q9W4
GN=

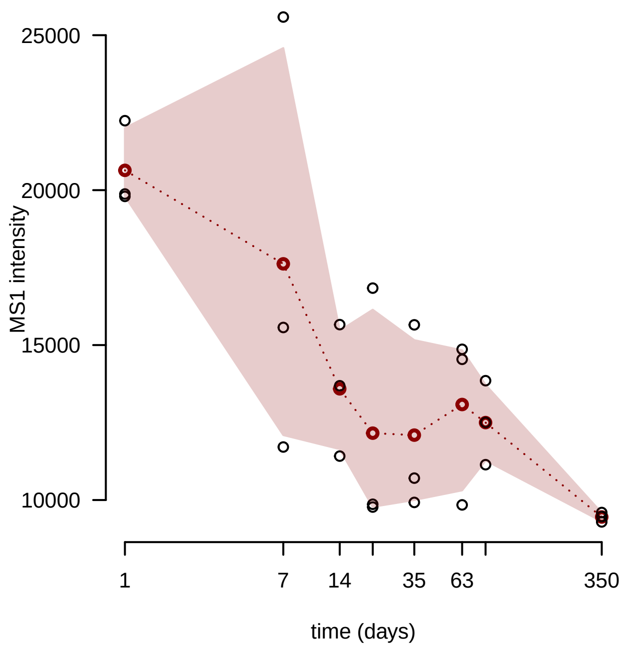

E9QAG8
GN=Znf431

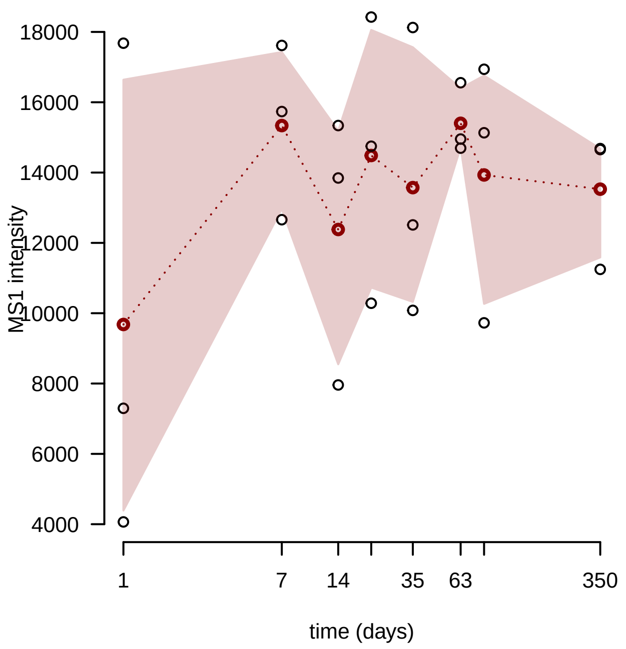

E9QAM5
GN=Helz2
helicase with zinc finger 2, transcripti ...

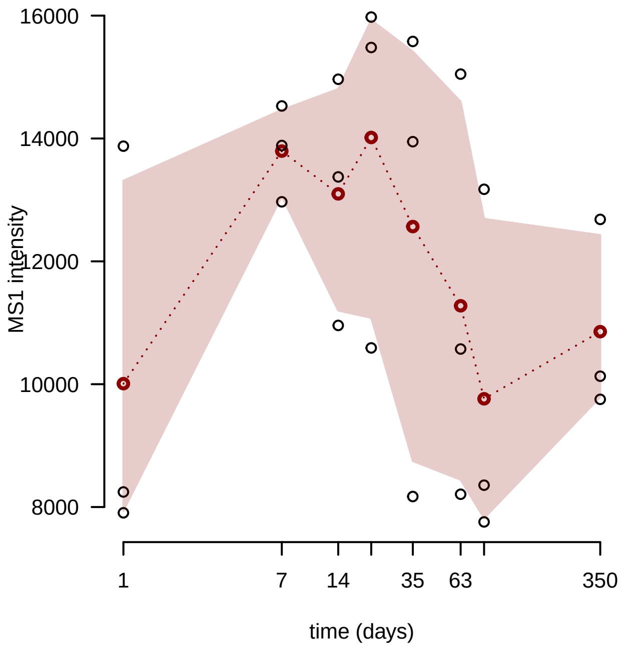

E9QAT4
GN=Sec16a
SEC16 homolog A, endoplasmic reticulum e ...

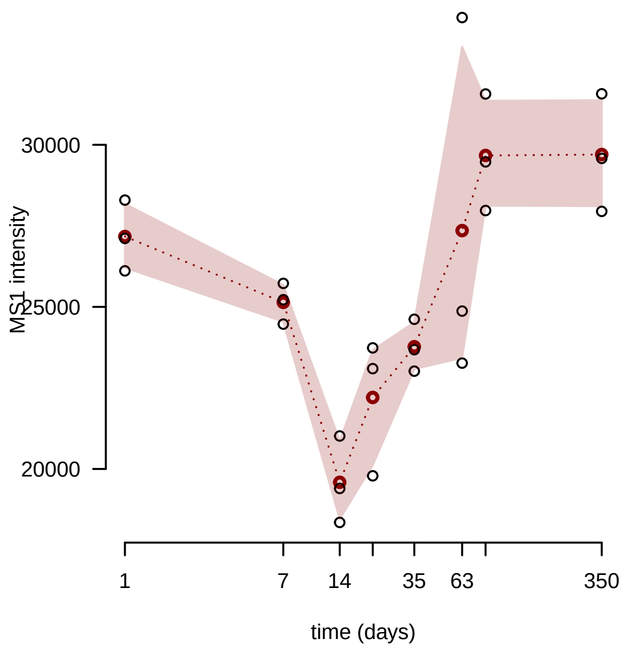

F2Z461
GN=Herc6
hect domain and RLD 6

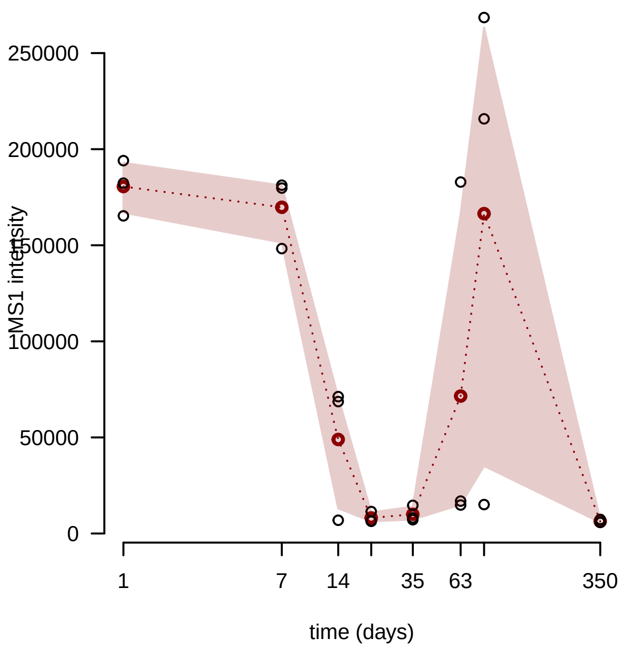

F6XLV1
GN=

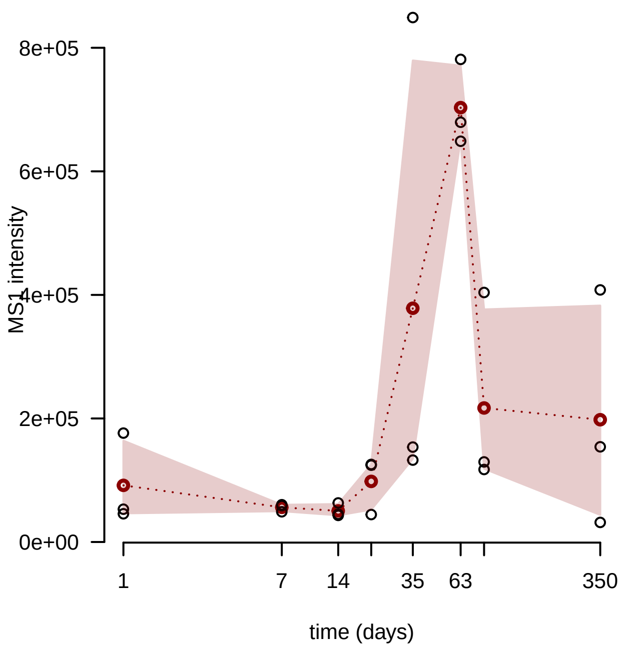

F6ZDS4
GN=Tpr
translocated promoter region, nuclear ba ...

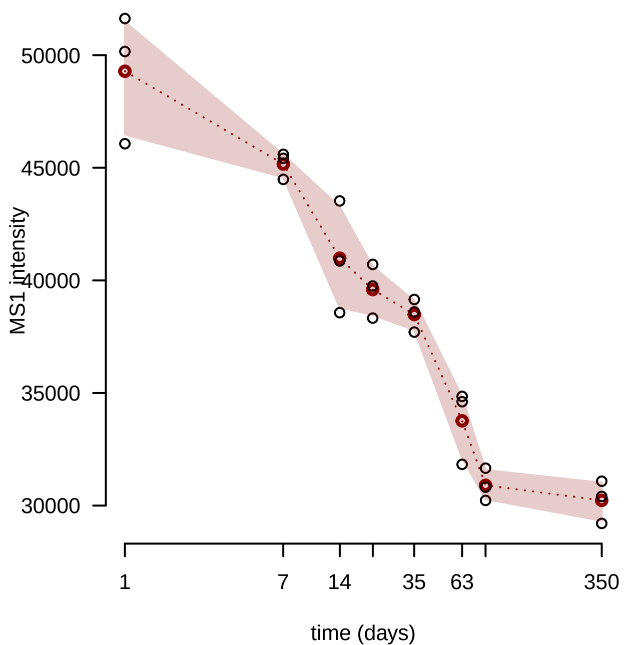

F7BJB9
GN=Morc3
microrchidia 3

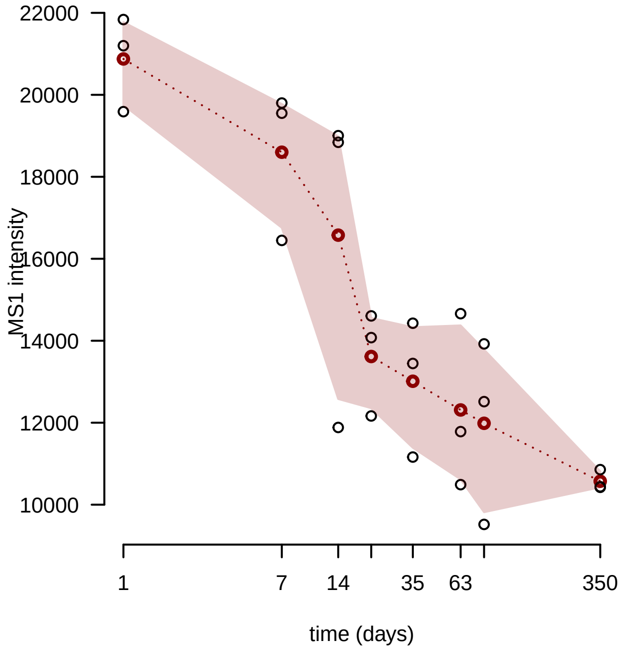

F7BWT7
GN=Tspan15
tetraspanin 15

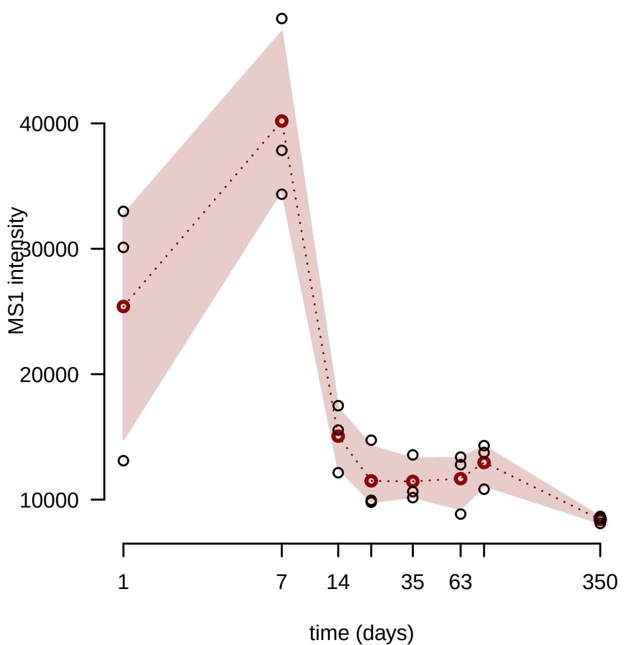

F8VPQ2
GN=Arid4a
AT rich interactive domain 4A (RBP1−like ...

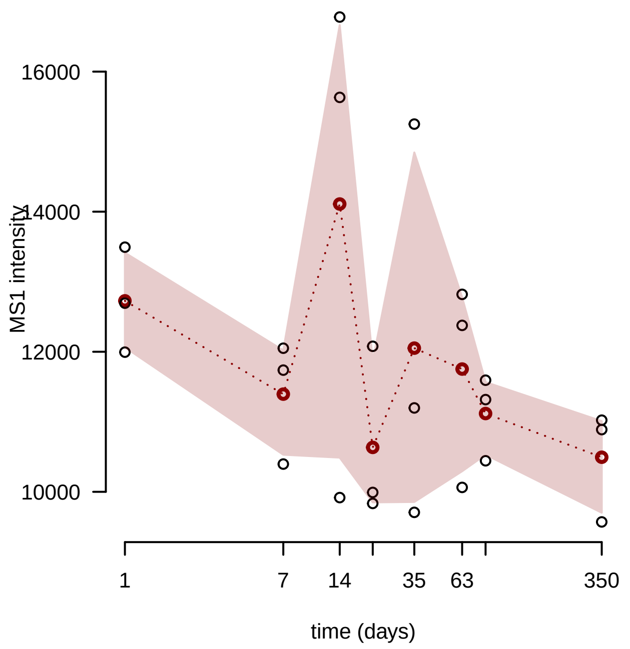

F8VPU2
GN=Farp1
FERM, RhoGEF (Arhgef) and pleckstrin dom ...

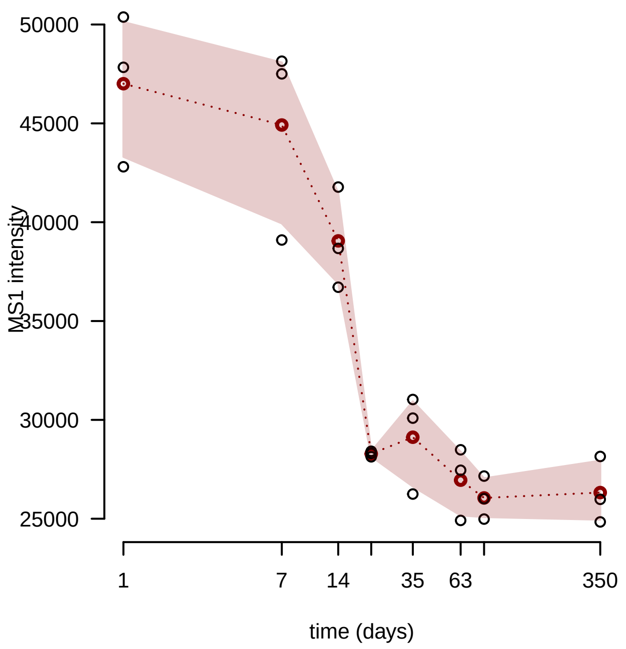

F8VPZ5
GN=

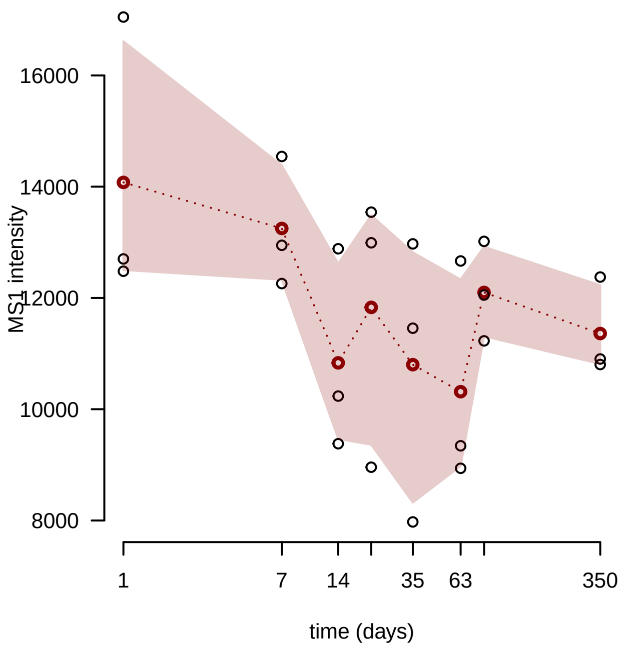

F8VPZ9
GN=Bicra
BRD4 interacting chromatin remodeling co ...

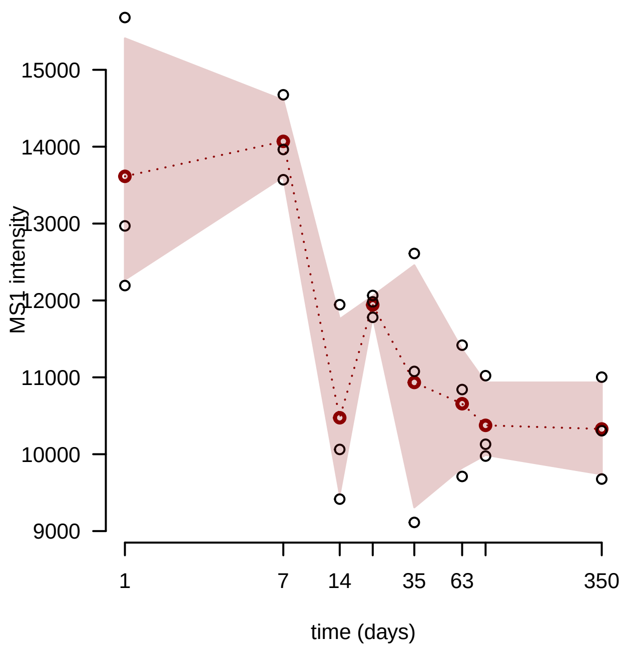

F8VQB6
GN=Myo10
myosin X

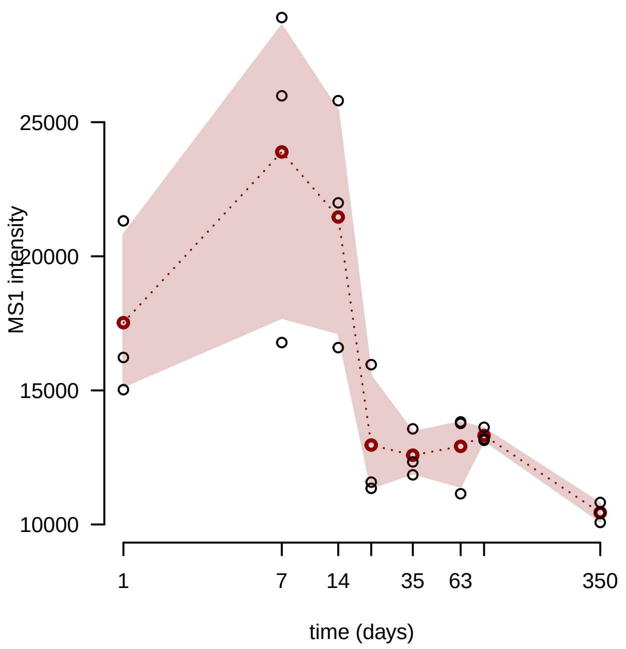

G3X982
GN=Aox3
aldehyde oxidase 3

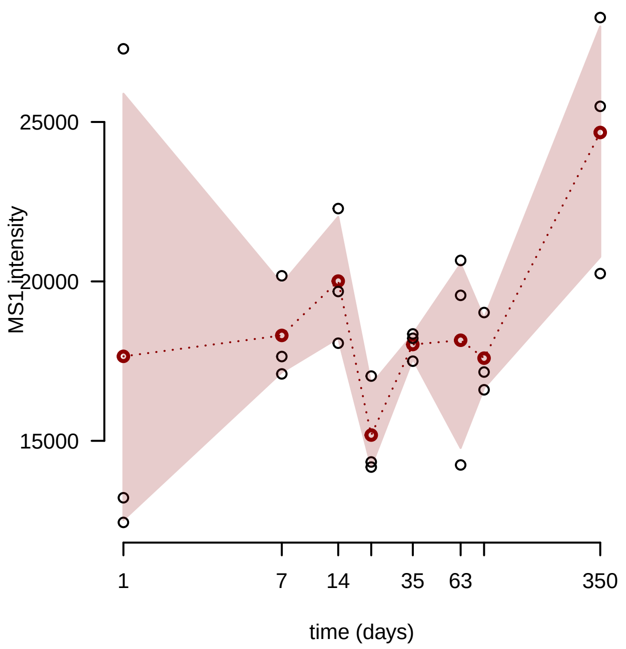

G3X9C2
GN=Nccrp1
non−specific cytotoxic cell receptor pro ...

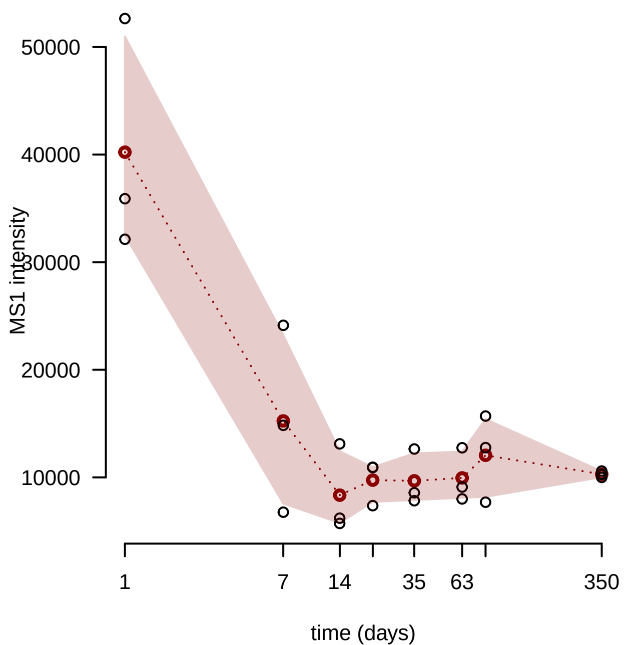

G3X9J0
GN=Sipa1l3
signal−induced proliferation−associated ...

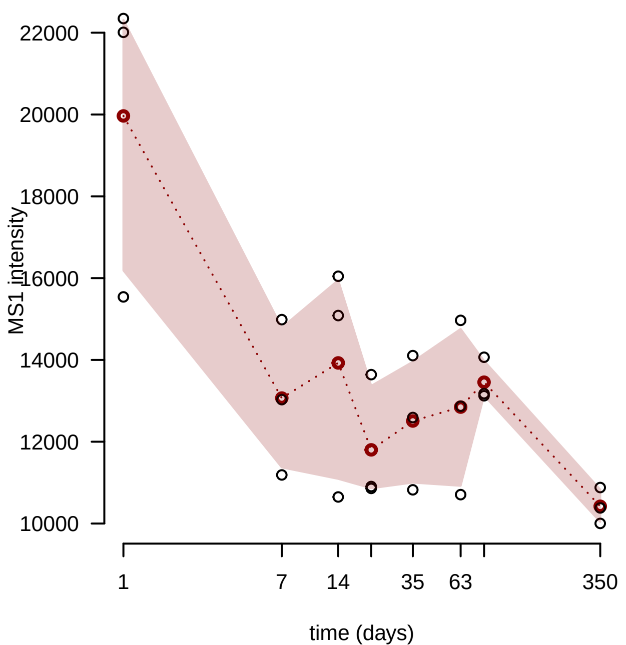

G3X9K3
GN=Arfgef1
ADP−ribosylation factor guanine nucleoti ...

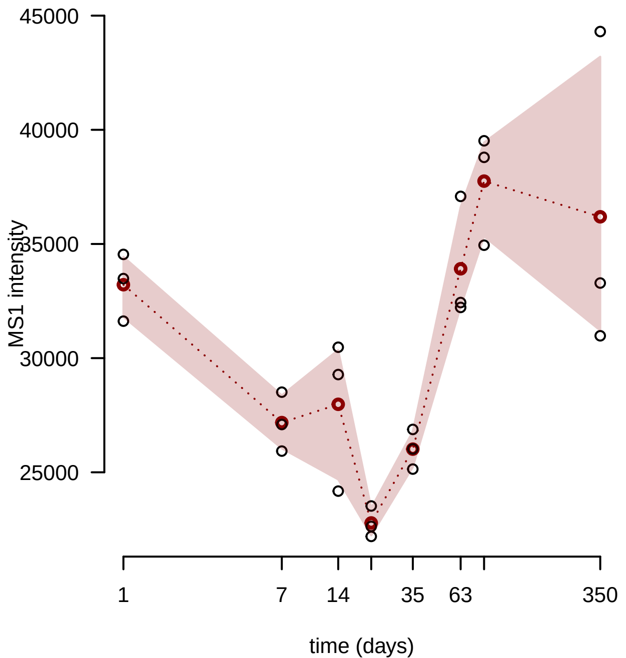

G3XA57
GN=Rab11fip2
RAB11 family interacting protein 2 (clas ...

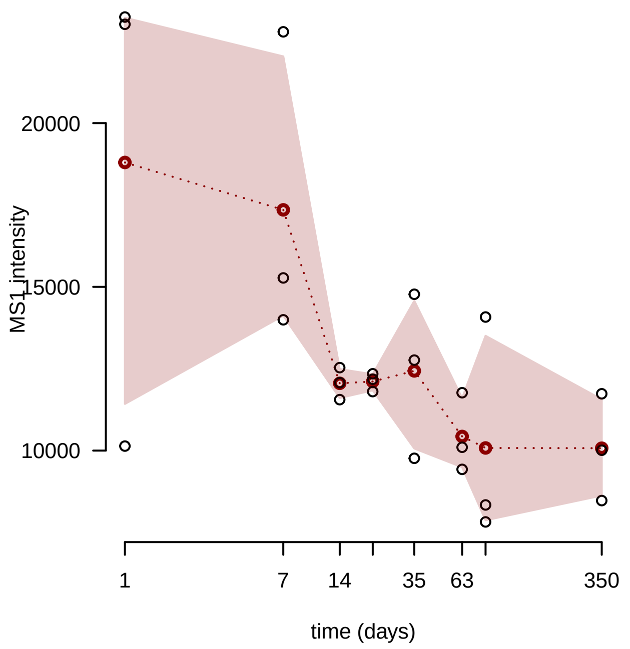

G3XA59
GN=Lrrc32
leucine rich repeat containing 32

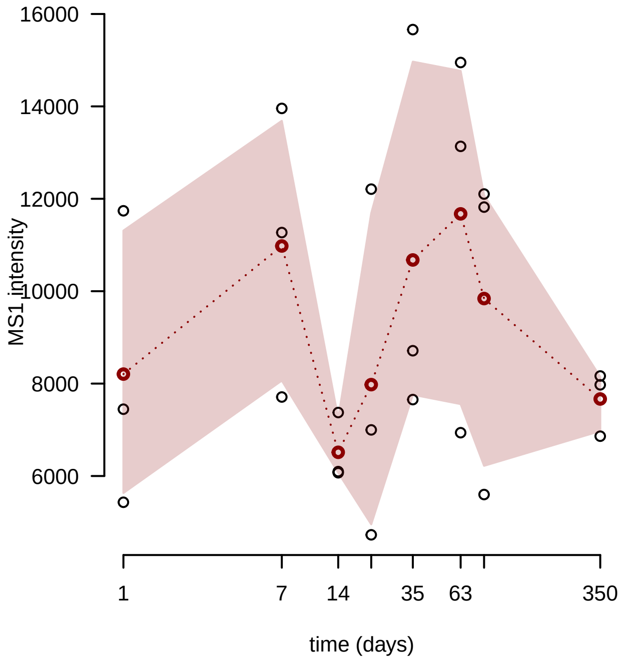

G5E829
GN=Atp2b1
ATPase, Ca++ transporting, plasma membra ...

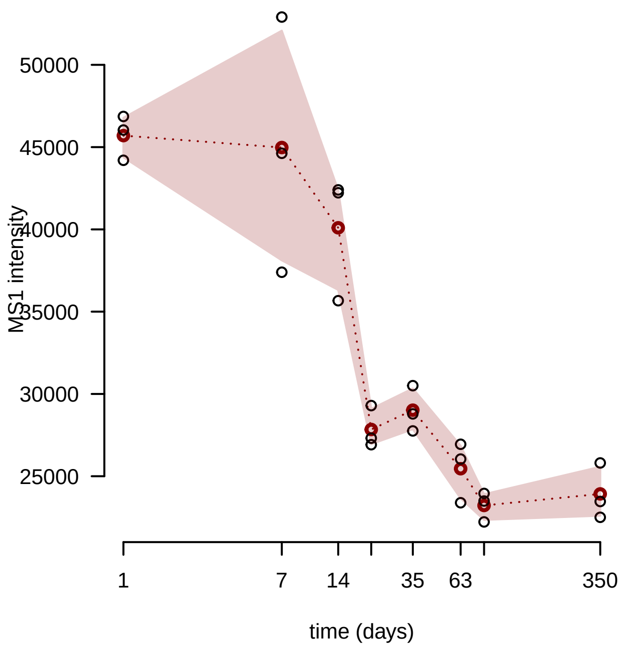

G5E861
GN=Sclt1
sodium channel and clathrin linker 1

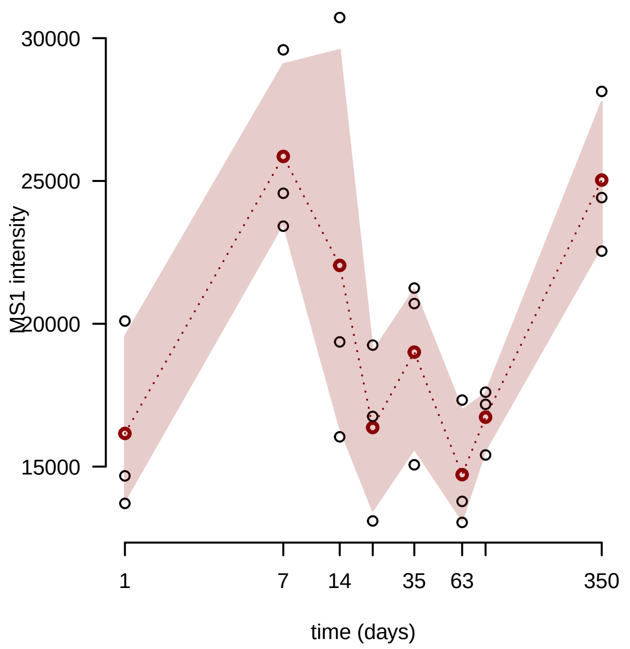

G5E870
GN=Trip12
thyroid hormone receptor interactor 12

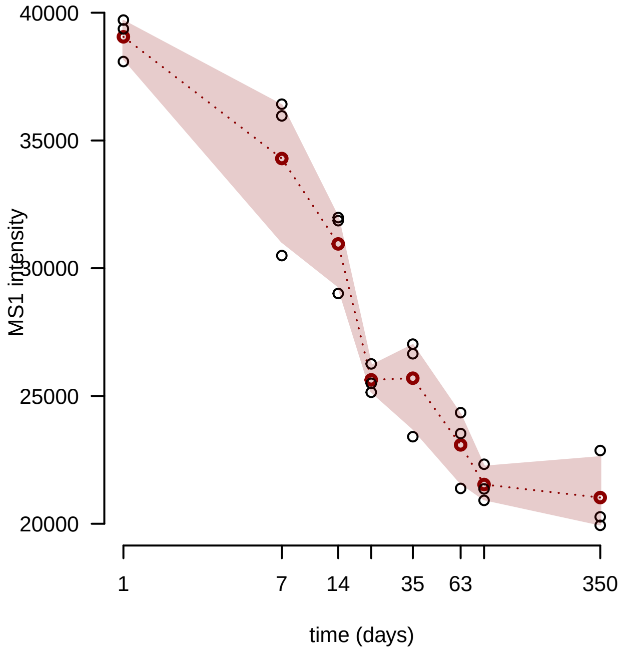

G5E897
GN=Poglut3
protein O−glucosyltransferase 3

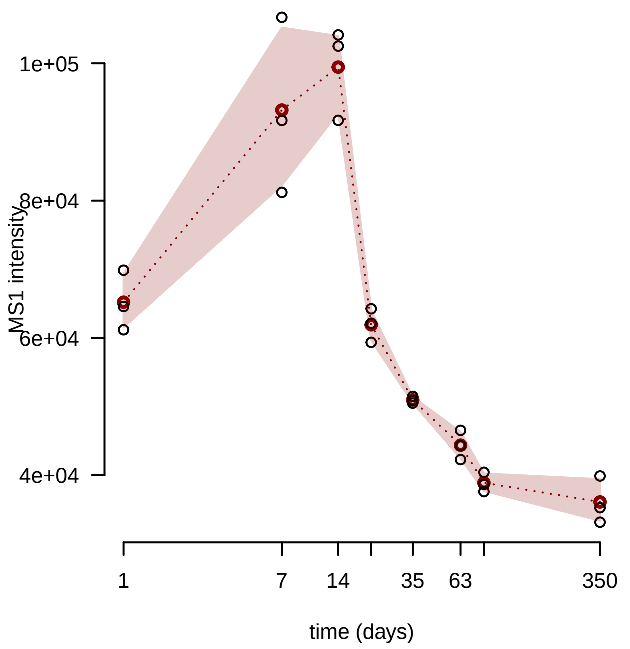

G5E8F4
GN=Fpgt
fucose−1−phosphate guanylyltransferase

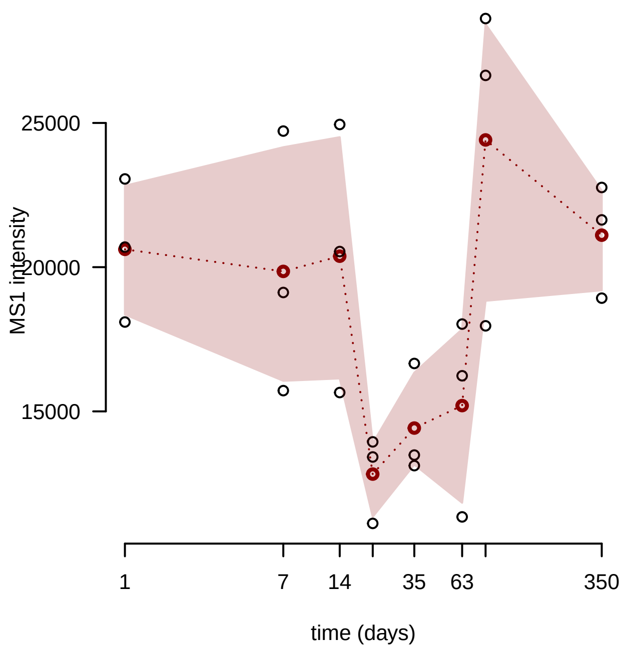

G5E8K5
GN=Ank3
ankyrin 3, epithelial

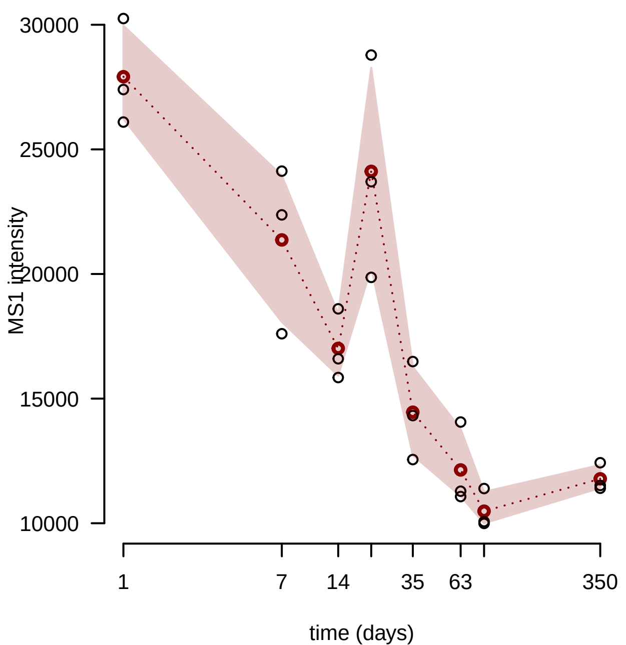

G5E8P0
GN=Tubgcp6
tubulin, gamma complex associated protei ...

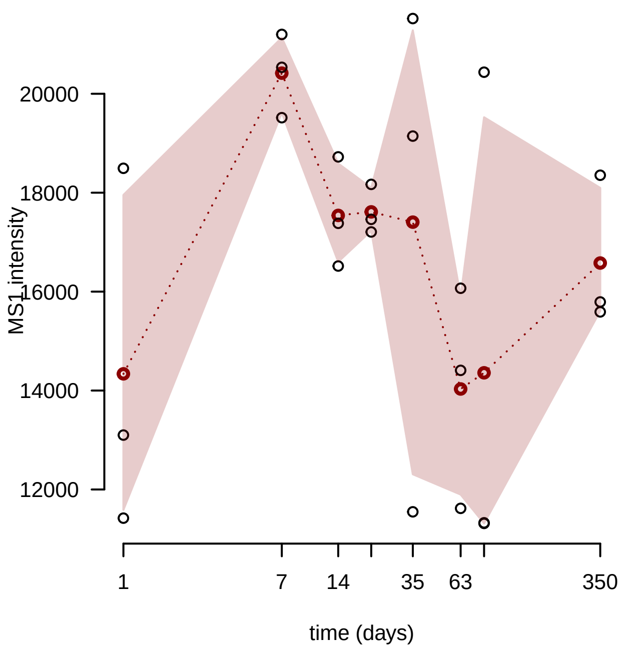

G5E8P1
GN=

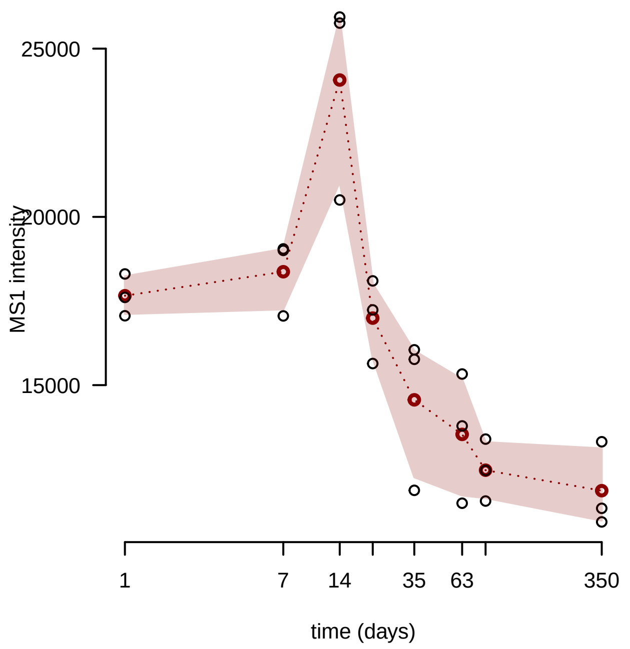

G5E8Q8
GN=Adgrf5
adhesion G protein−coupled receptor F5

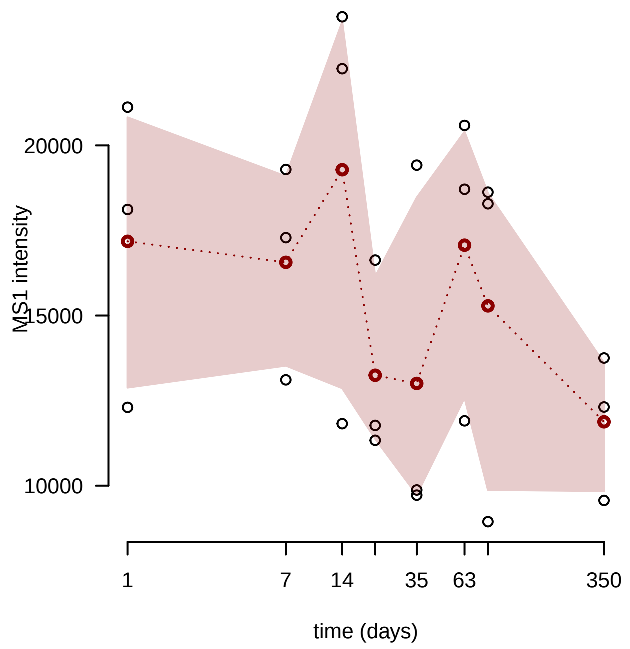

G5E8Z2
GN=Taf4b
TATA−box binding protein associated fact ...

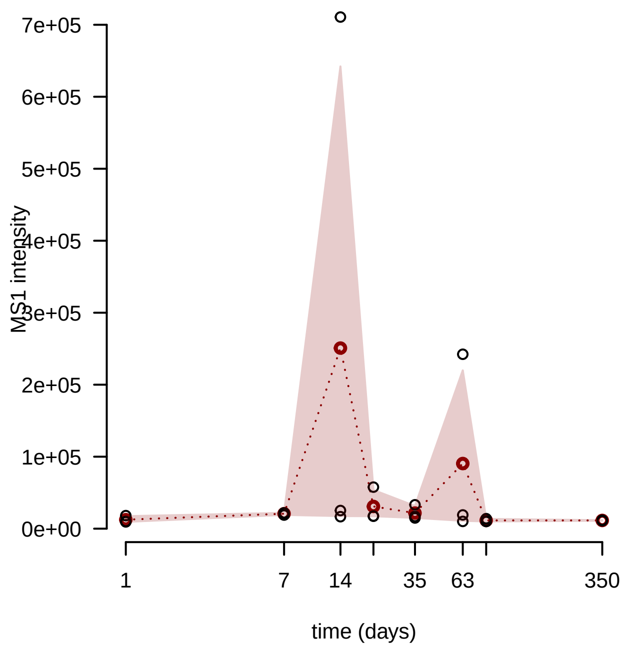

J3QMK6
GN=Aldh3b3
aldehyde dehydrogenase 3 family, member ...

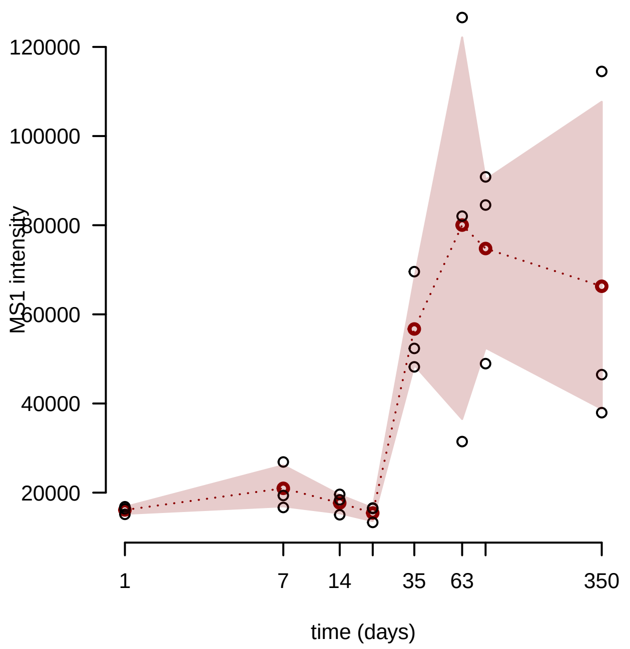

L0N7N1
GN=Kif14
kinesin family member 14

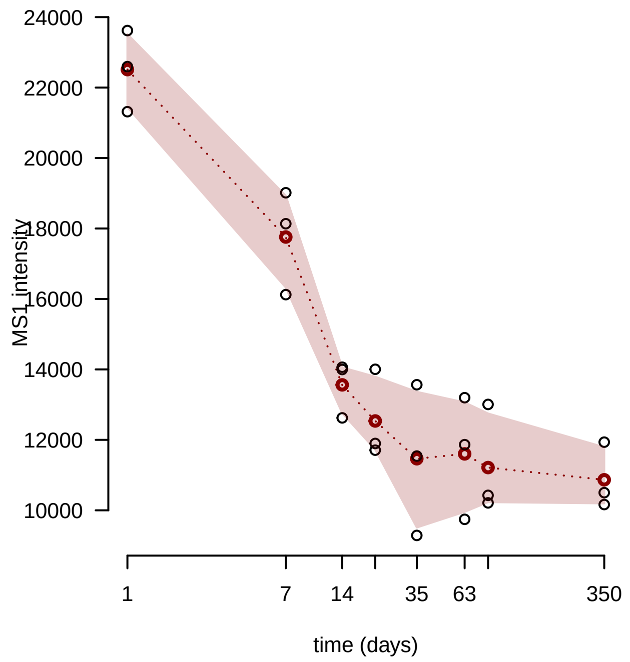

L7N1X6
GN=Fbxw15
F−box and WD−40 domain protein 15

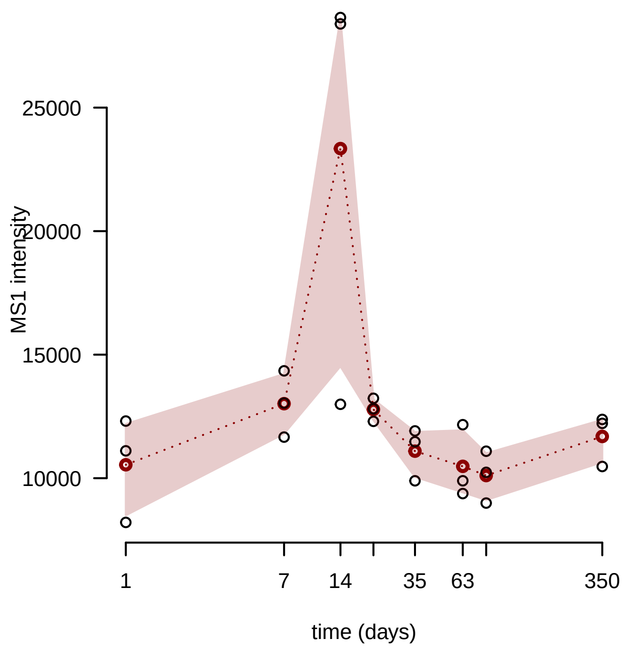

O08528
GN=Hk2
hexokinase 2

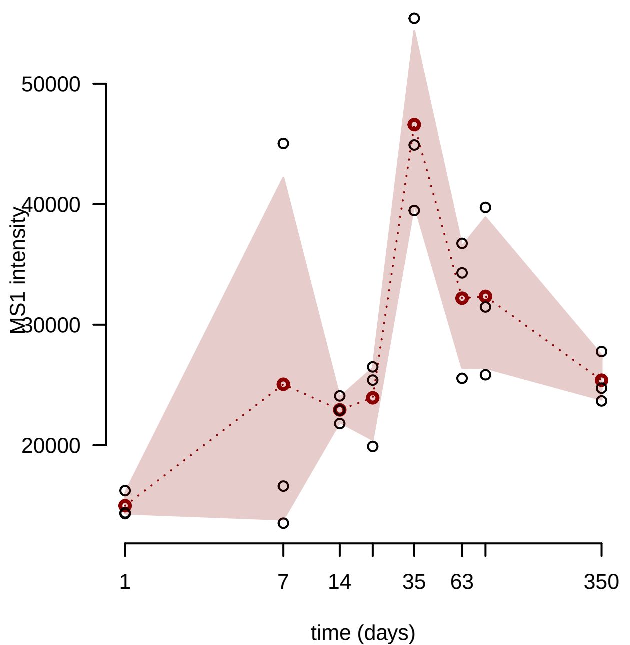

O08529
GN=Capn2
calpain 2

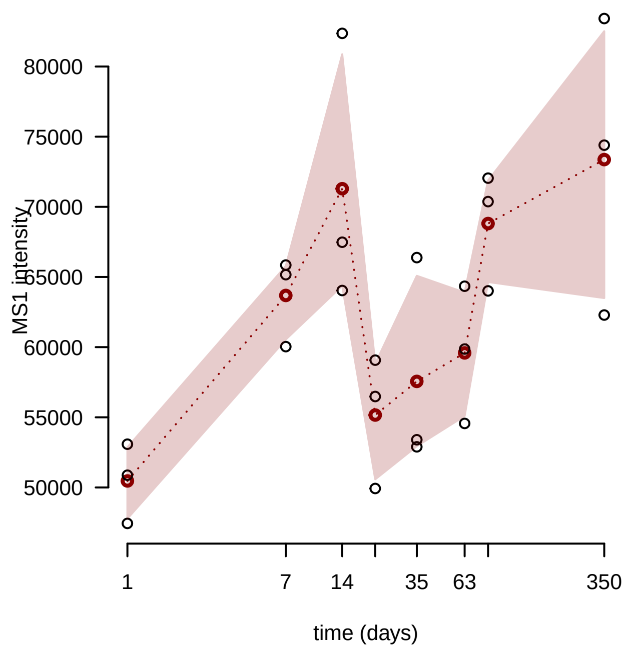

O08530
GN=S1pr1
sphingosine−1−phosphate receptor 1

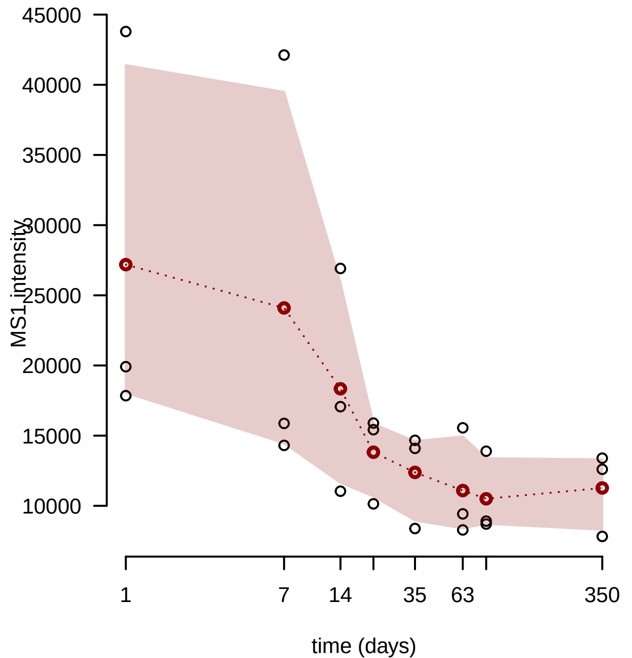

O08532
GN=Cacna2d1
calcium channel, voltage−dependent, alph ...

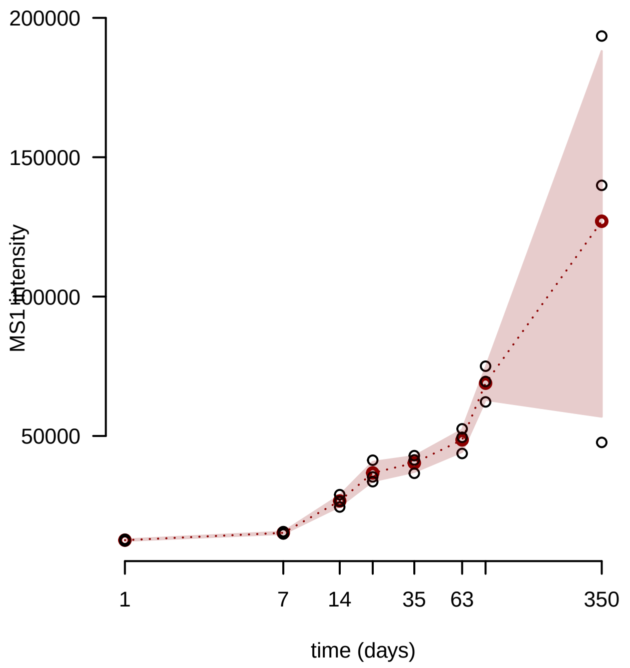

O08532
GN=Cacna2d1
calcium channel, voltage−dependent, alph ...

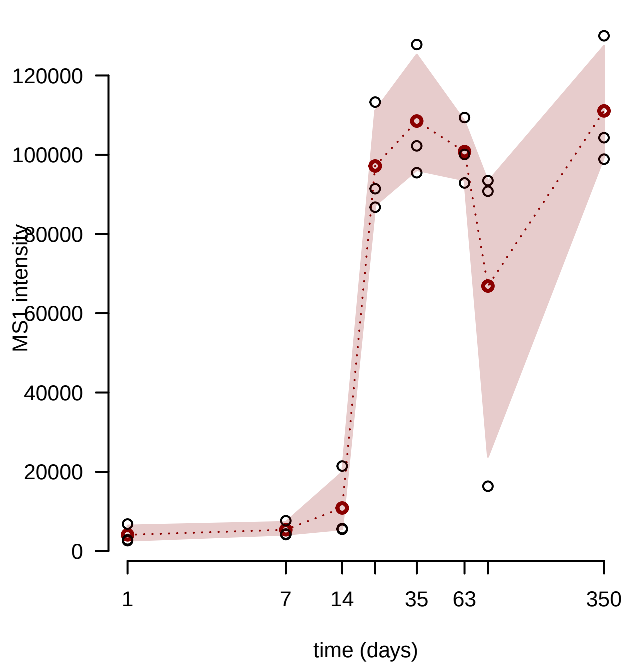

O08537
GN=Esr2
estrogen receptor 2 (beta)

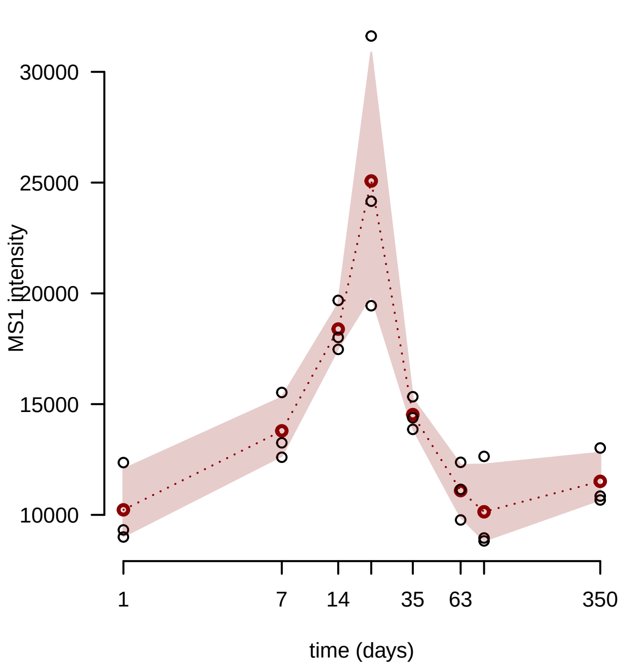

O08539
GN=Bin1
bridging integrator 1

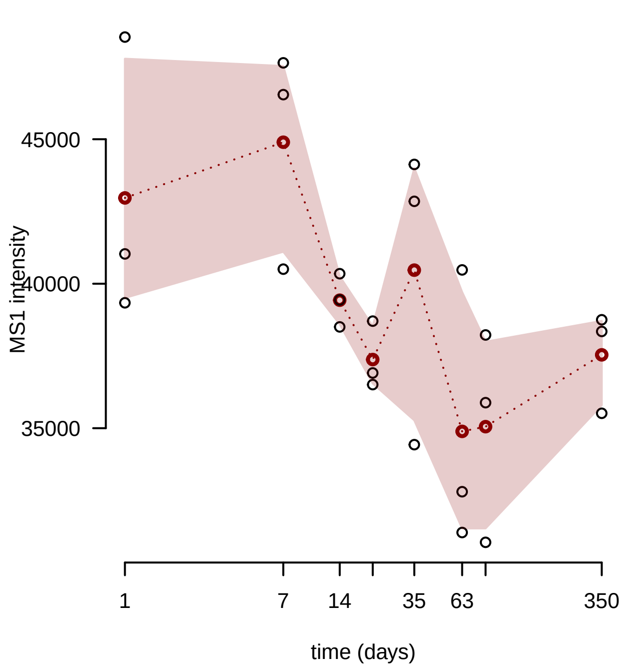

O08539
GN=Bin1
bridging integrator 1

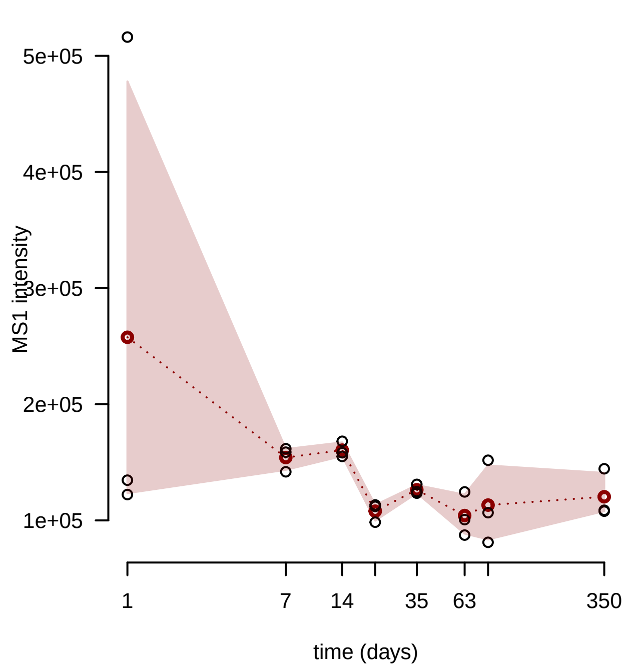

O08547
GN=Sec22b
SEC22 homolog B, vesicle trafficking pro ...

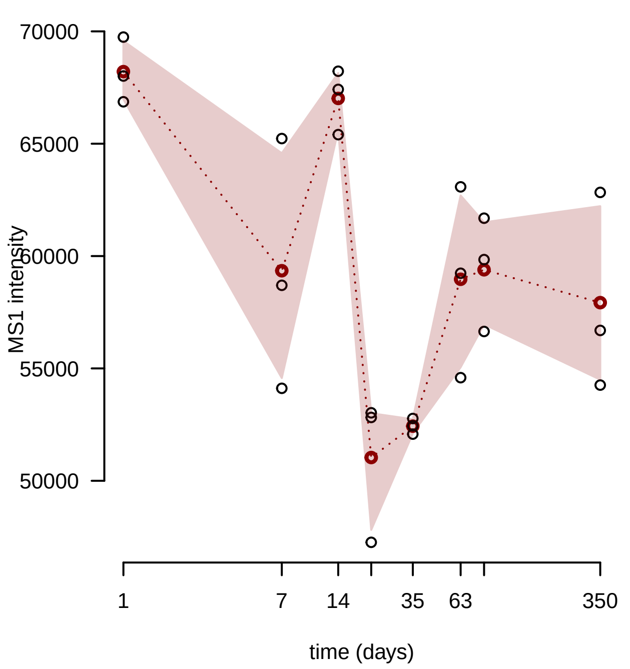

O08550
GN=Kmt2b
lysine (K)−specific methyltransferase 2B

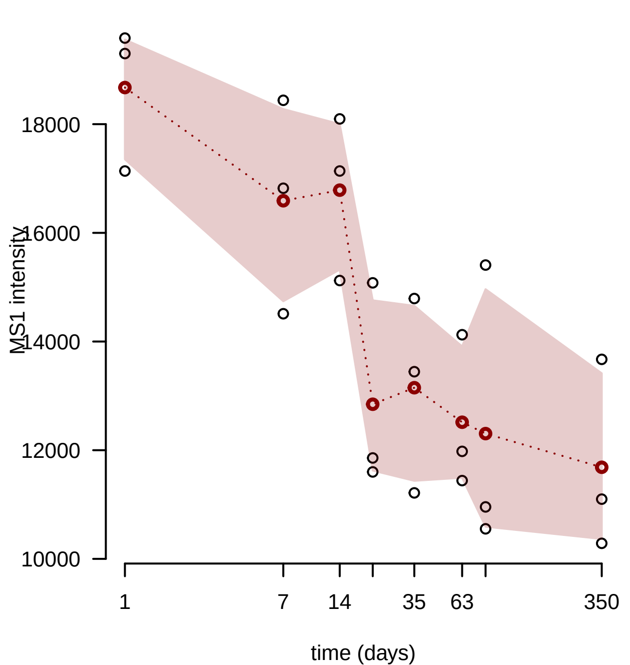

O08553
GN=Dpysl2
dihydropyrimidinase−like 2

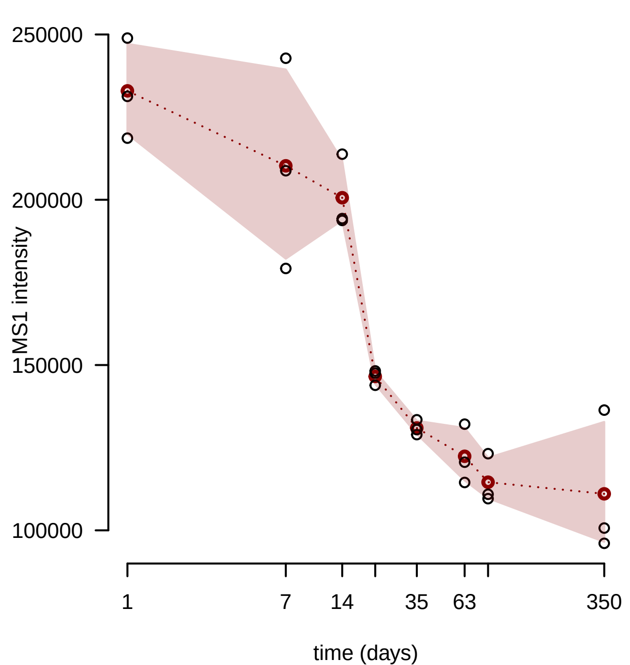

O08573
GN=Lgals9
lectin, galactose binding, soluble 9

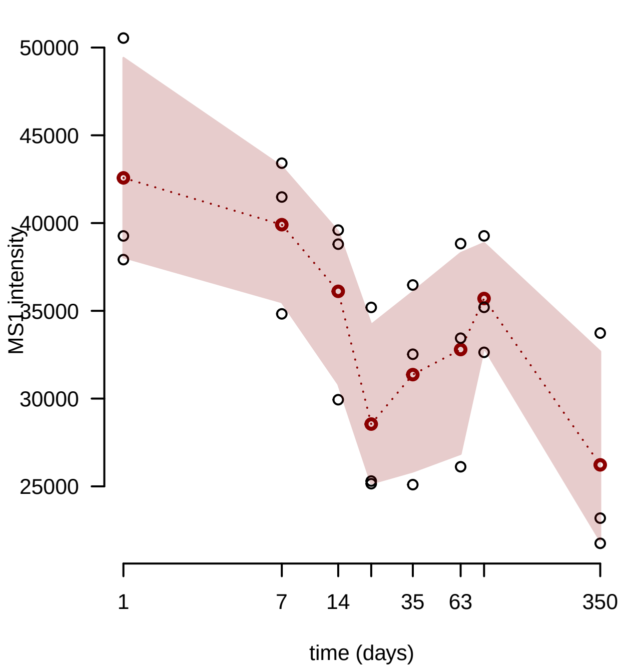

O08579
GN=Emd
emerin

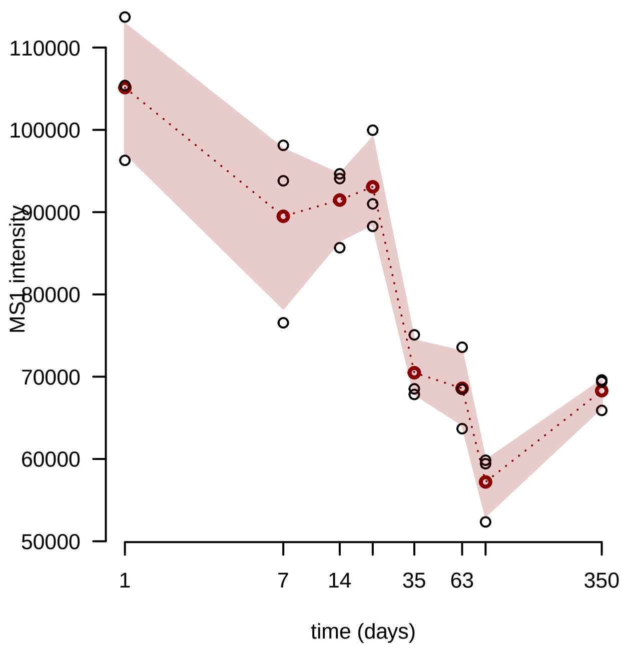

O08580
GN=Esrra
estrogen related receptor, alpha

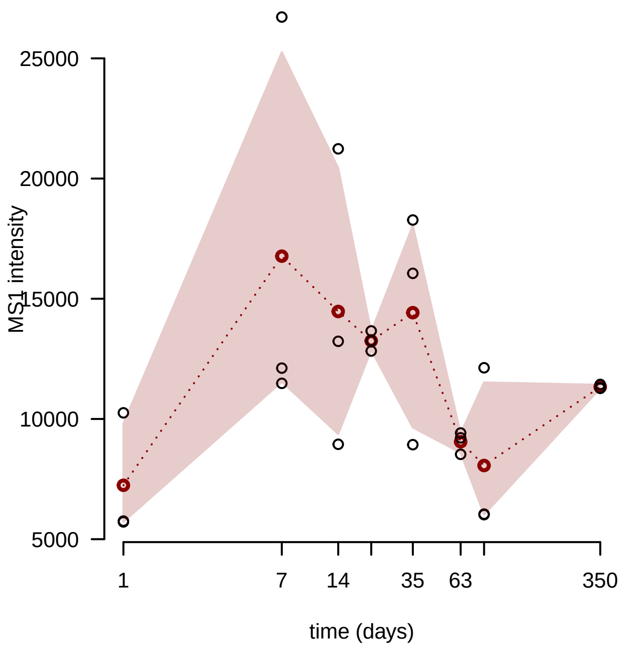

O08582
GN=Gtpbp1
GTP binding protein 1

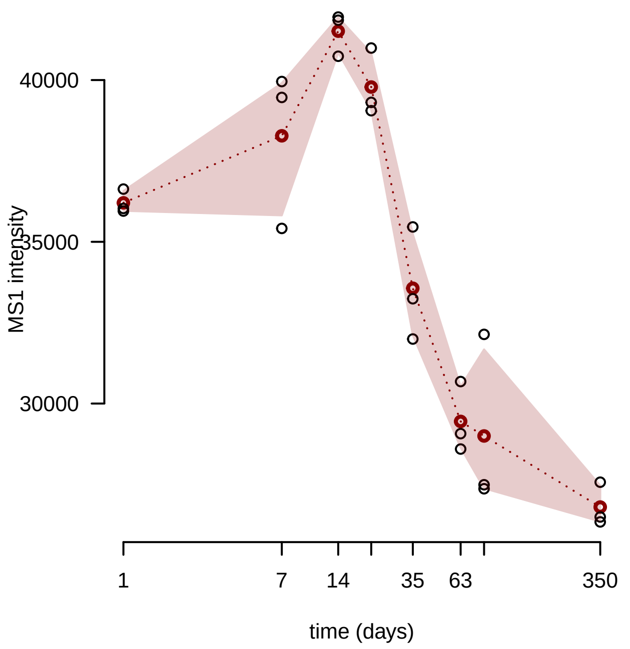

O08583
GN=Alyref
Aly/REF export factor

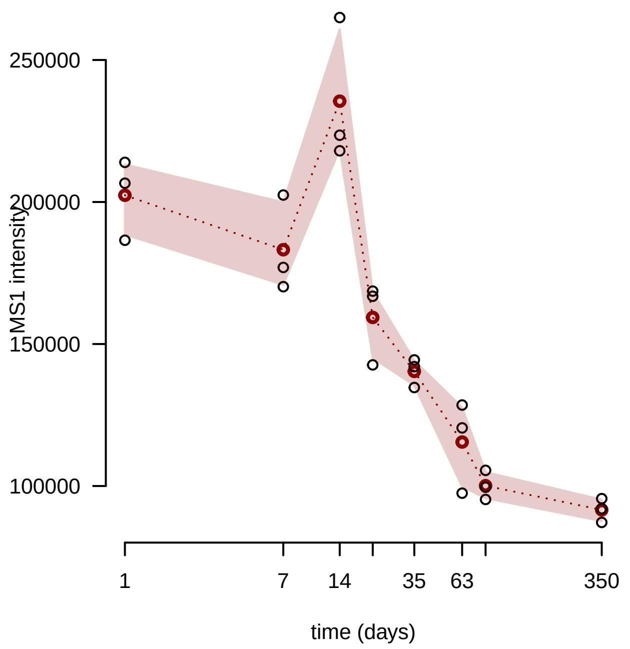

O08585
GN=Clta
clathrin, light polypeptide (Lca)

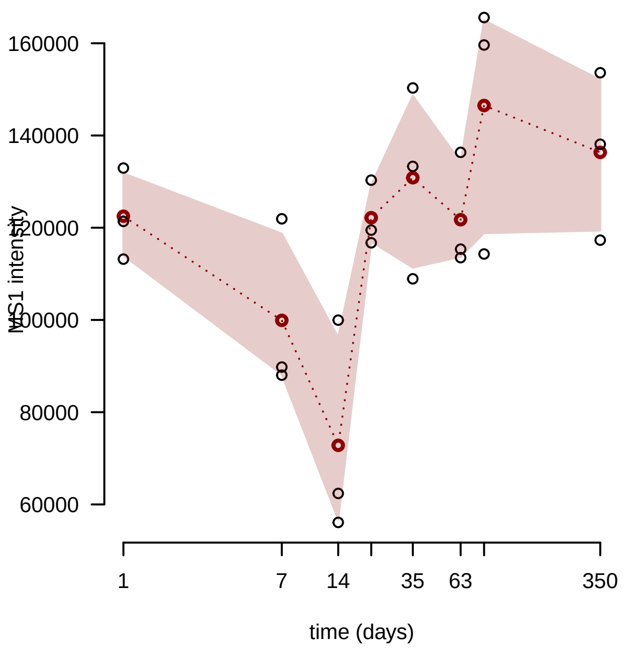

O08586
GN=Pten
phosphatase and tensin homolog

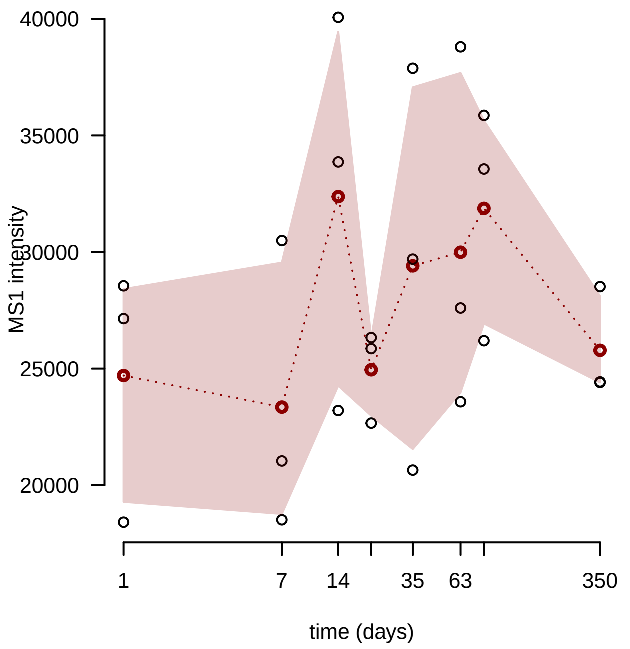

O08599
GN=Stxbp1
syntaxin binding protein 1

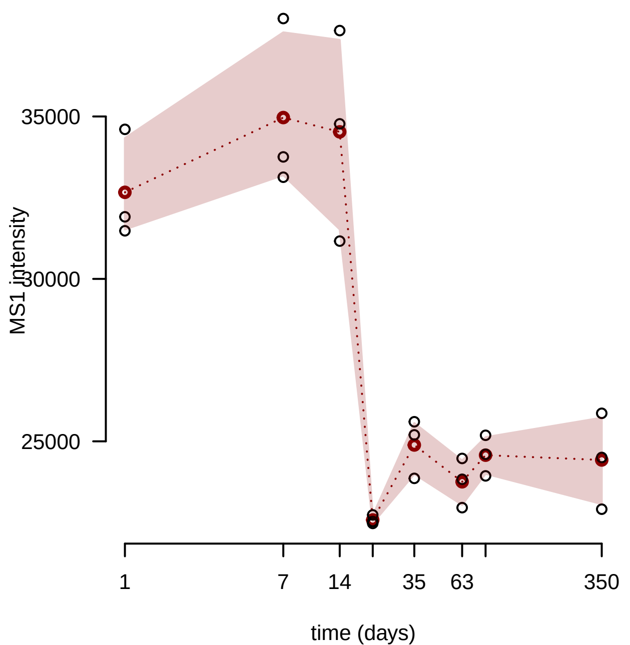

O08600
GN=Endog
endonuclease G

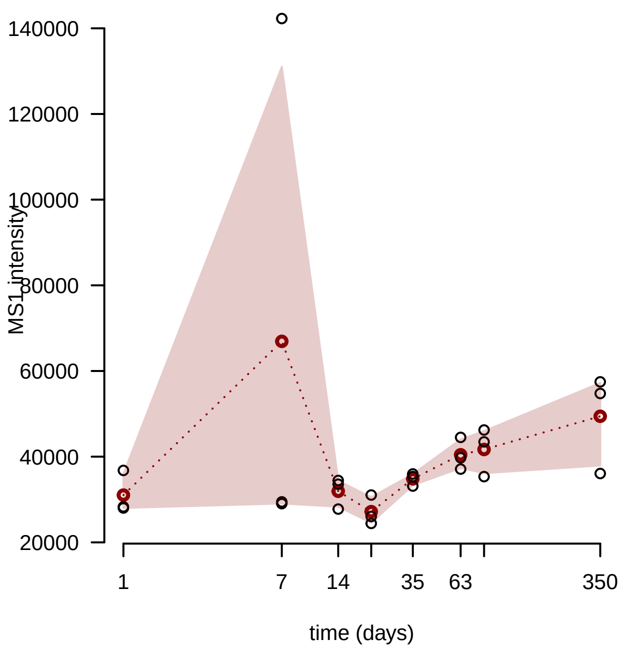

O08601
GN=Mttp
microsomal triglyceride transfer protein

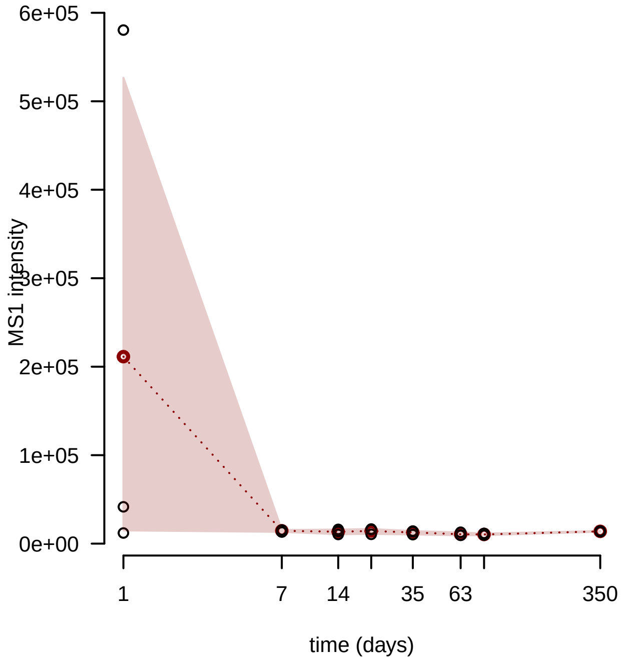

O08609
GN=Mlx
MAX−like protein X

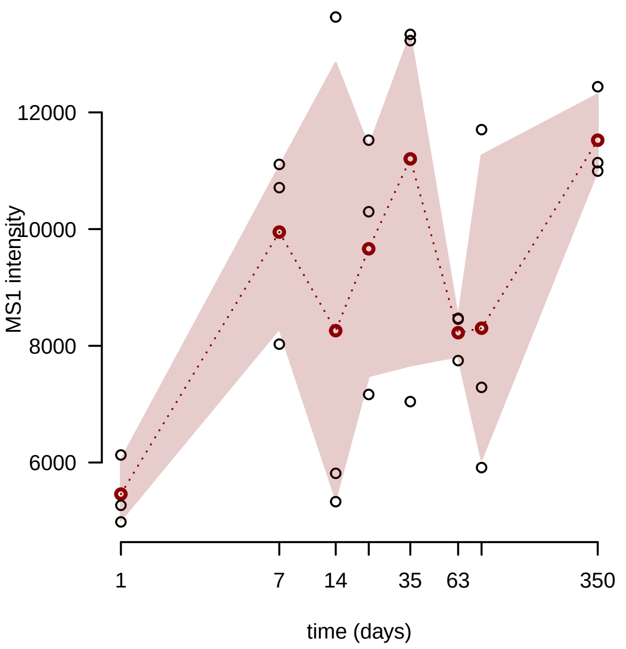

O08638
GN=Myh11
myosin, heavy polypeptide 11, smooth mus ...

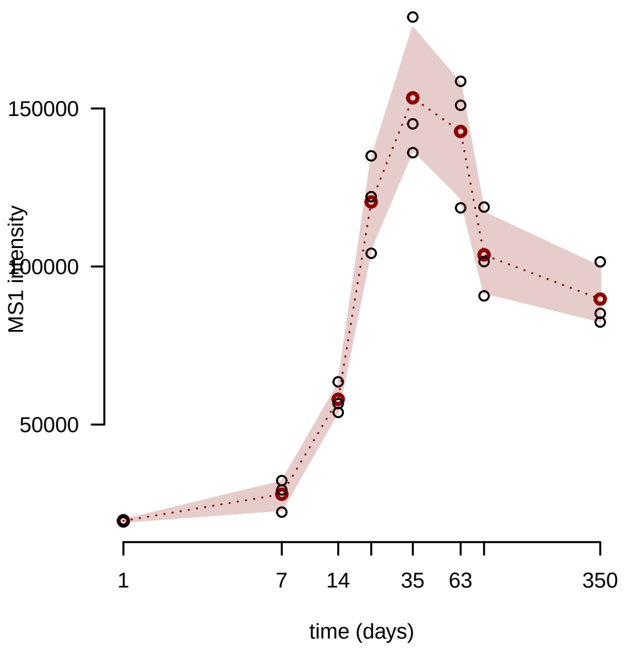

O08638
GN=Myh11
myosin, heavy polypeptide 11, smooth mus ...

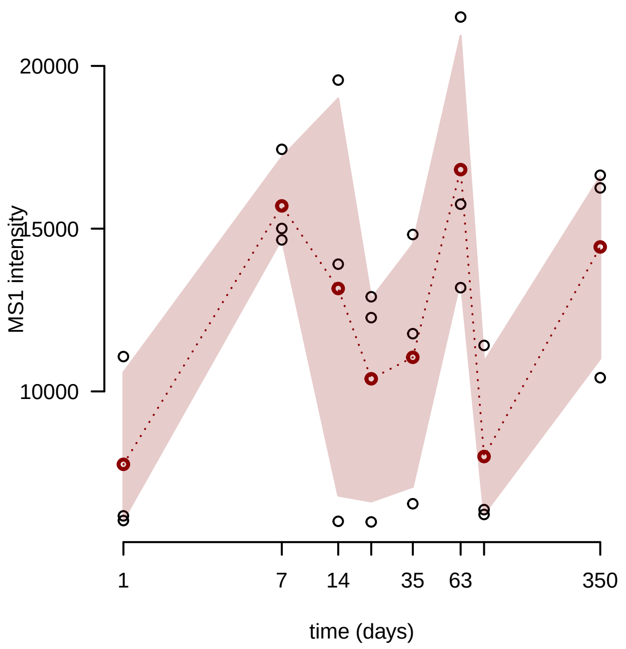

O08644
GN=Ephb6
Eph receptor B6

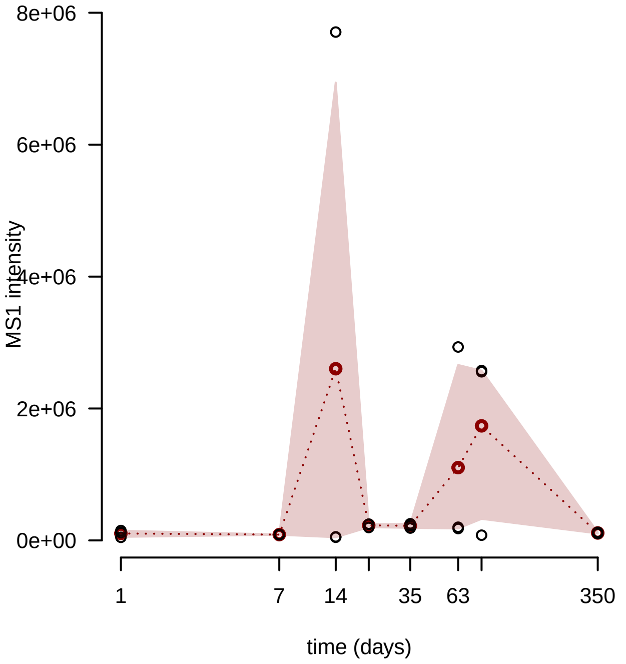

O08648
GN=Map3k4
mitogen−activated protein kinase kinase ...

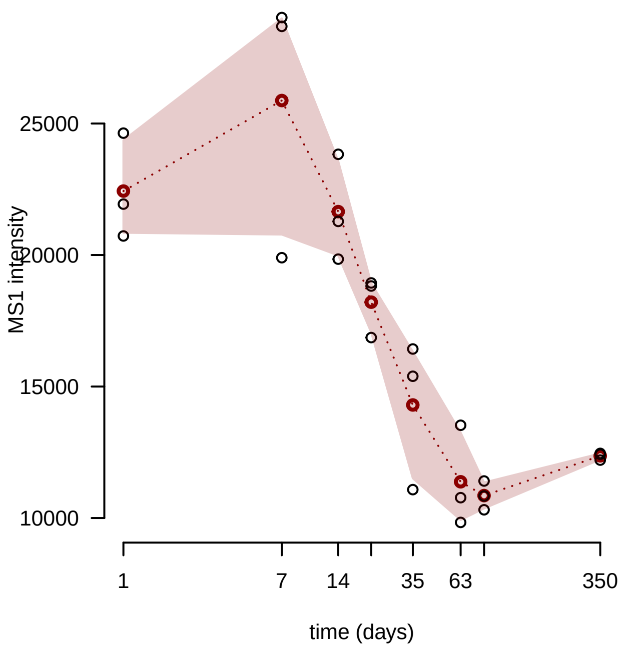

O08663
GN=Metap2
methionine aminopeptidase 2

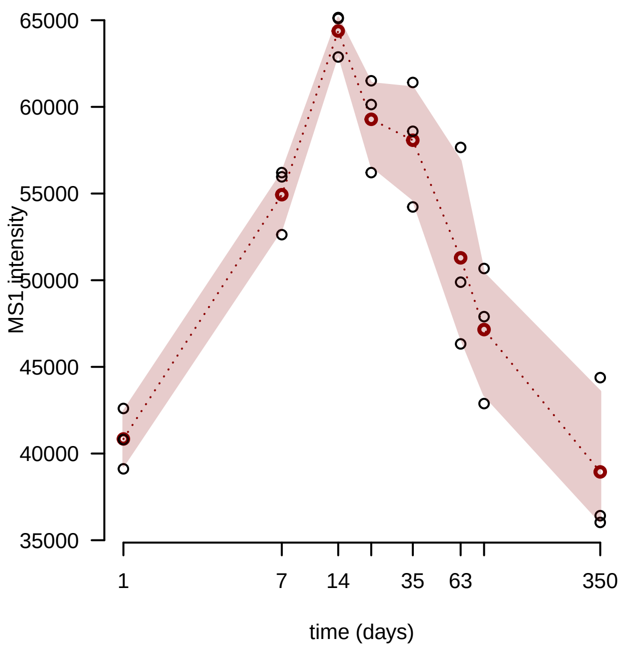

O08664
GN=Bcl7c
B cell CLL/lymphoma 7C

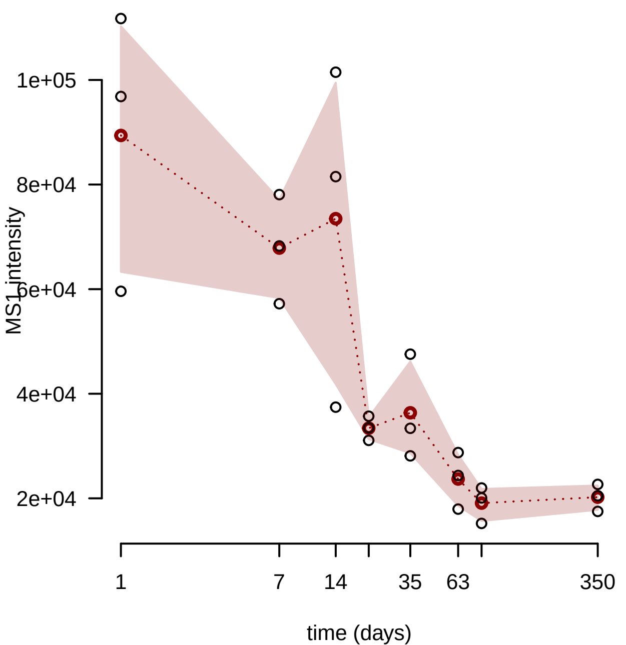

O08677
GN=Kng1
kininogen 1

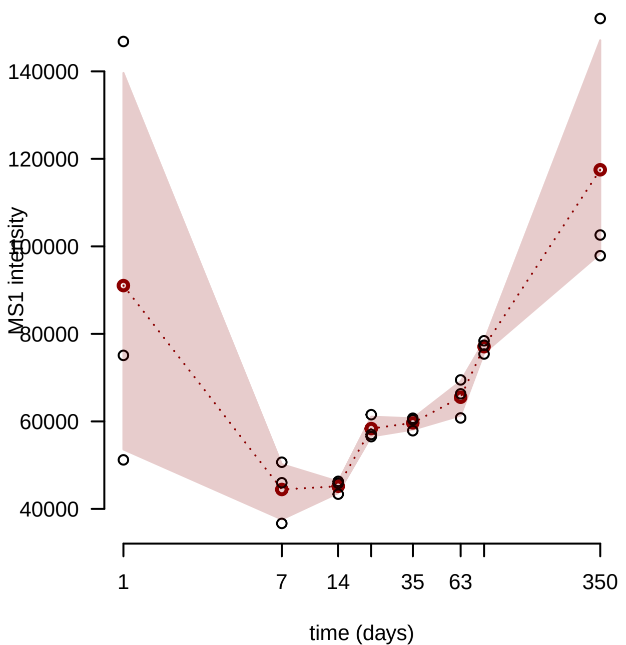

O08688
GN=Capn5
calpain 5

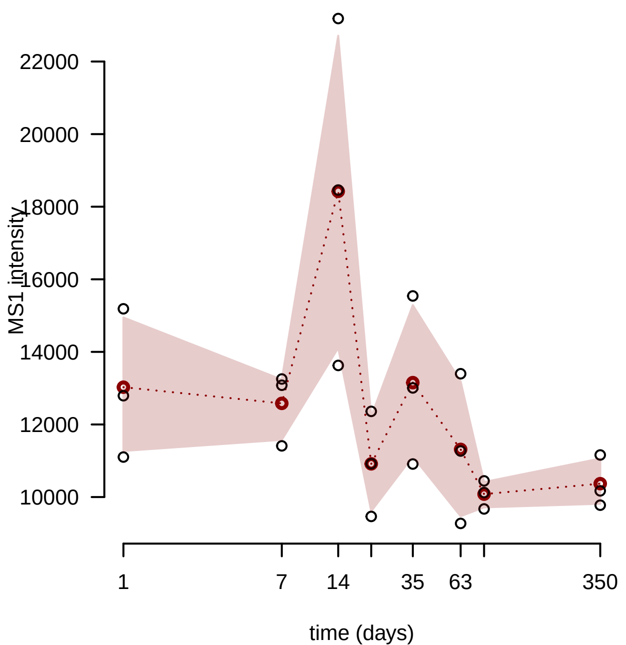

O08691
GN=Arg2
arginase type II

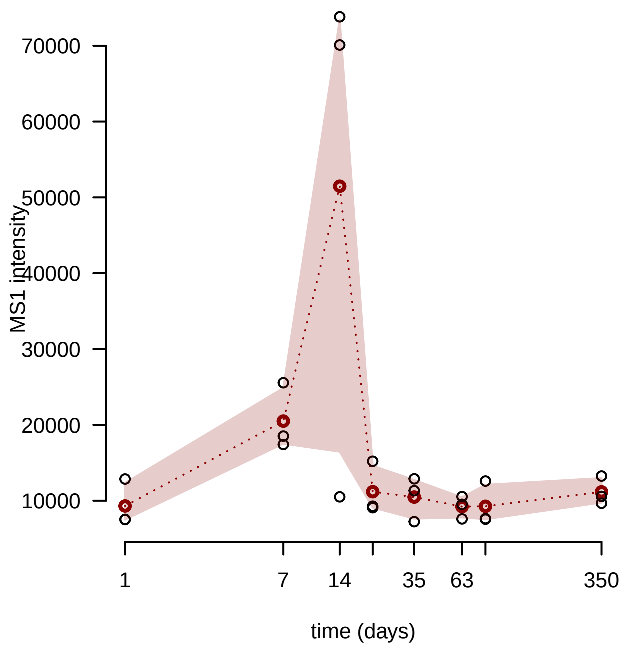

O08692
GN=Ngp
neutrophilic granule protein

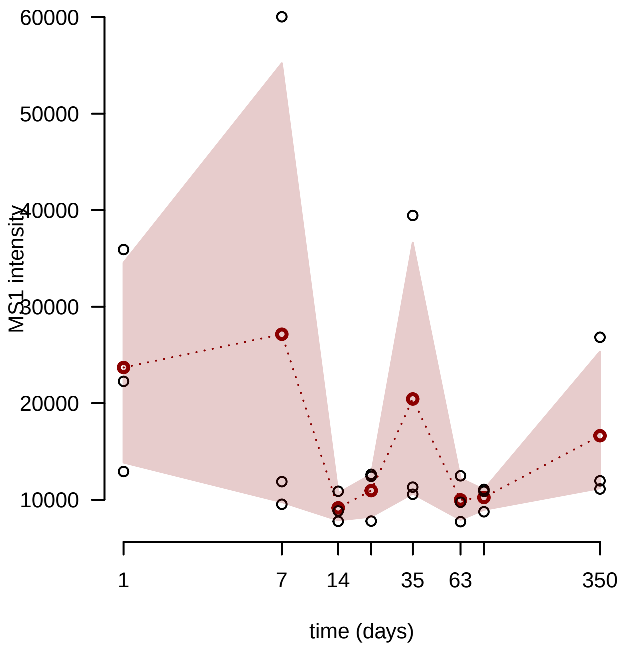

O08709
GN=Prdx6
peroxiredoxin 6

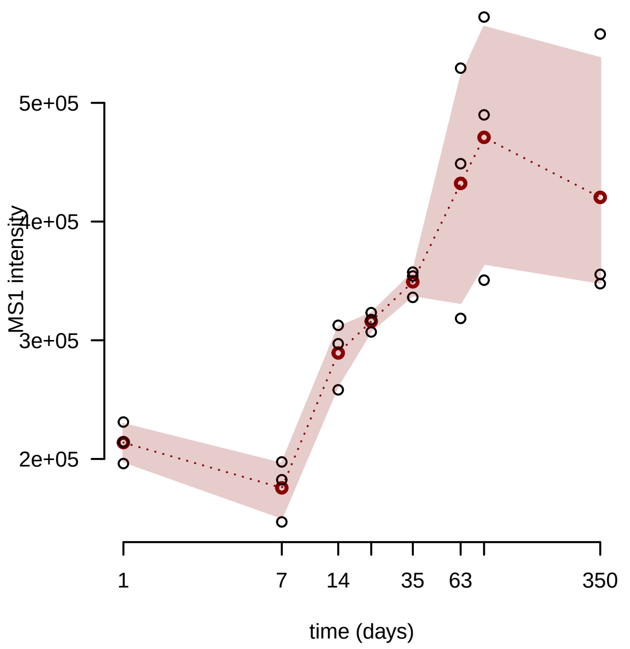

O08715
GN=Akap1
A kinase (PRKA) anchor protein 1

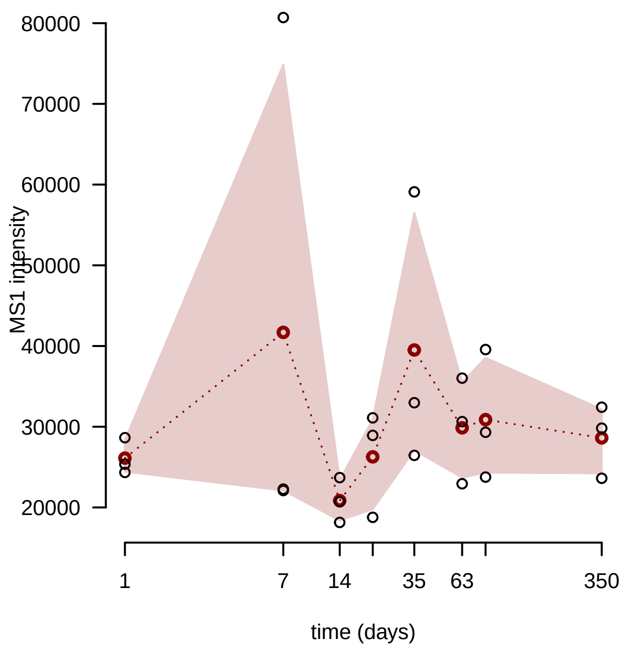

O08734
GN=Bak1
BCL2−antagonist/killer 1

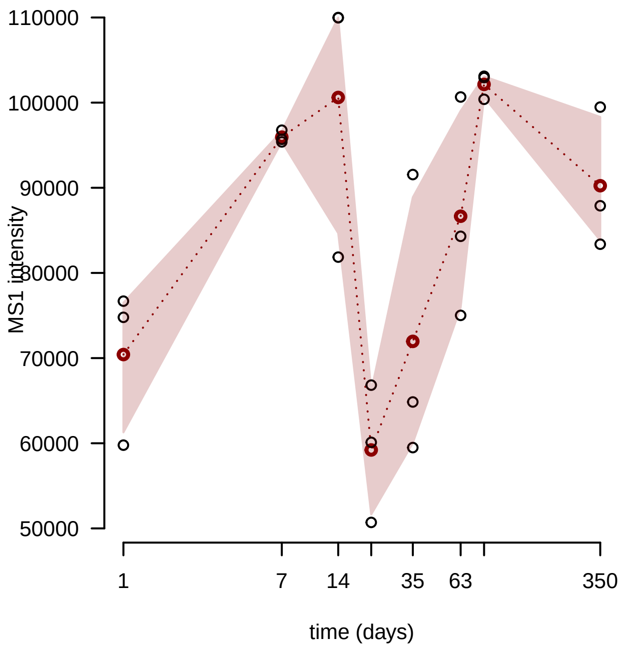

O08736
GN=Casp12
caspase 12

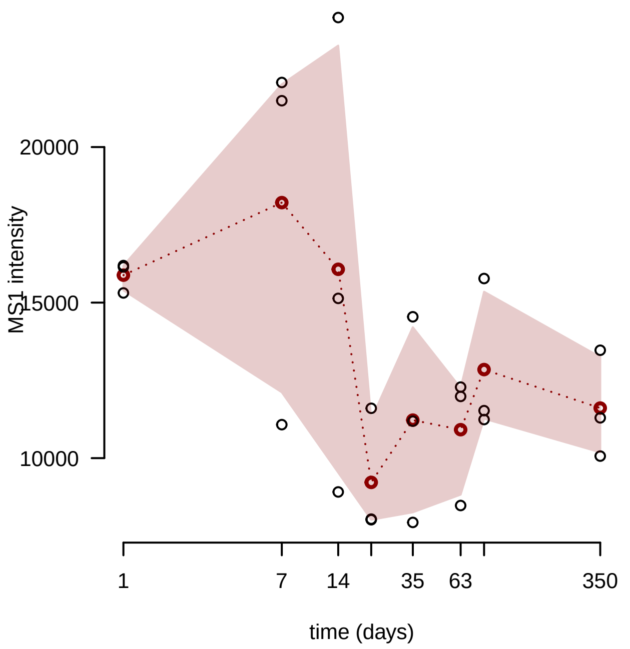

O08738
GN=Casp6
caspase 6

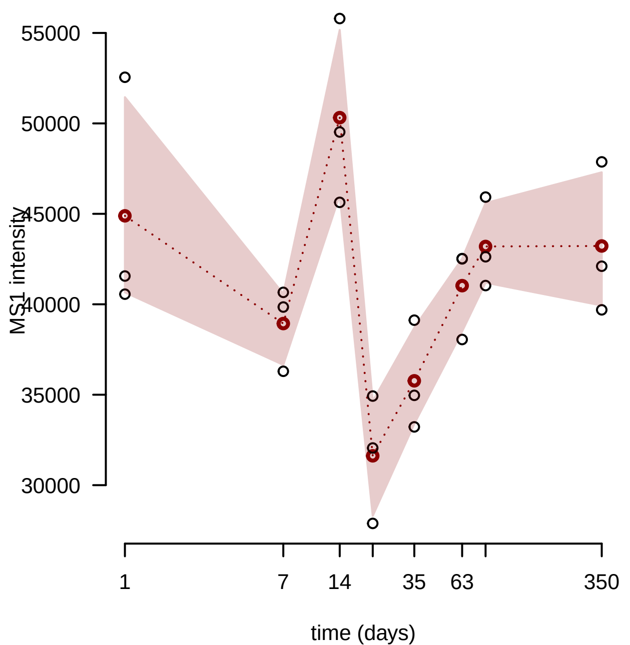

O08739
GN=Ampd3
adenosine monophosphate deaminase 3

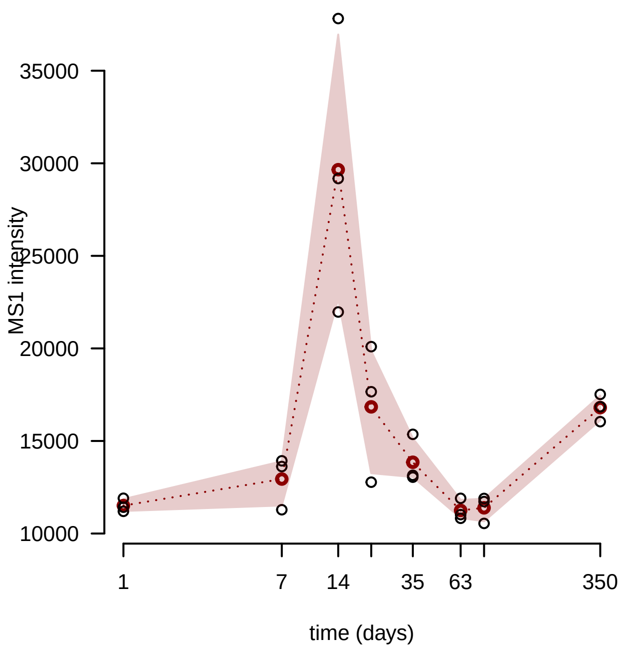

O08740
GN=Polr2j
polymerase (RNA) II (DNA directed) polyp ...

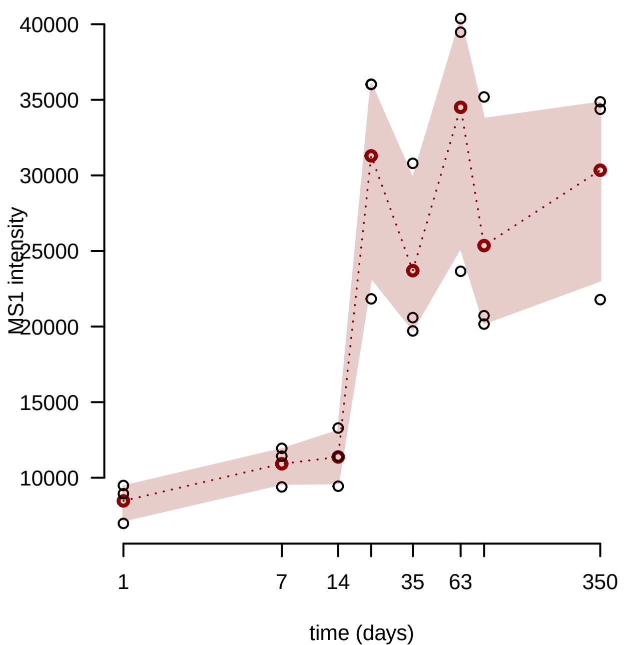

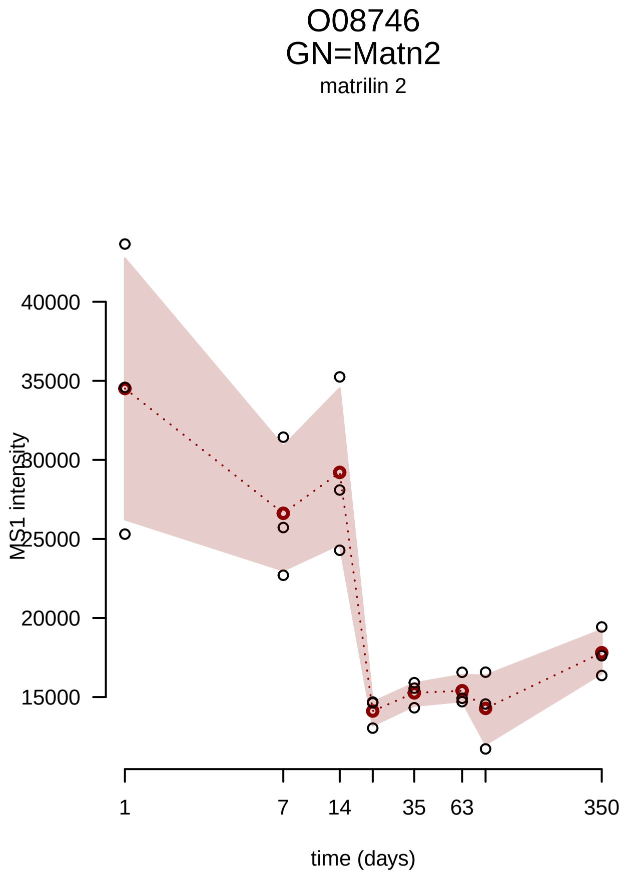
O08746
GN=Matn2
matrilin 2
40000
35000
30000
25000
20000
15000
MS1 intensity
1
7
14
35
63
350
time (days)

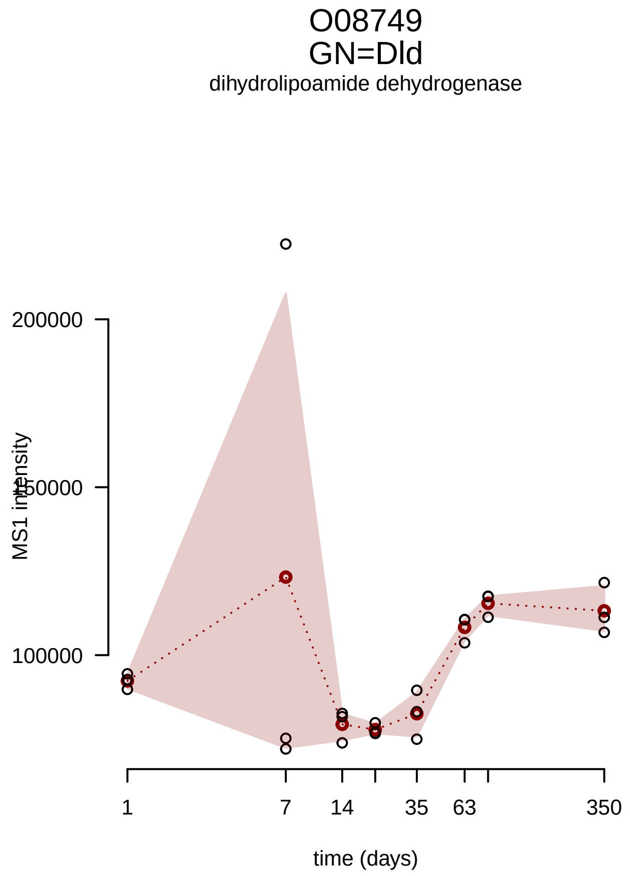
O08749
GN=Dld
dihydrolipoamide dehydrogenase
200000
150000
100000
MS1 intensity
1
7
14
35
63
350
time (days)

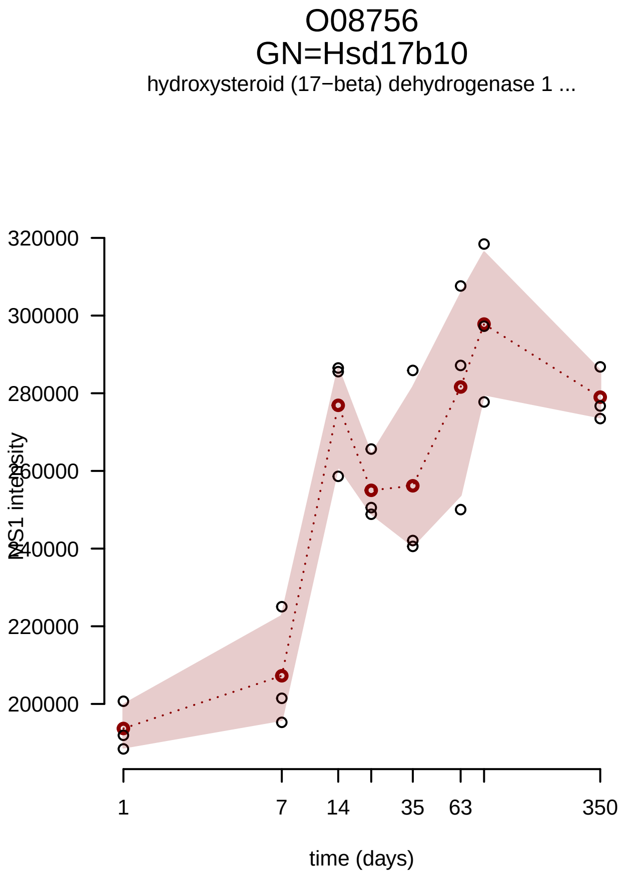
O08756
GN=Hsd17b10
hydroxysteroid (17−beta) dehydrogenase 1 ...
320000
300000
280000
260000
240000
220000
200000
MS1 intensity
1
7
14
35
63
350
time (days)

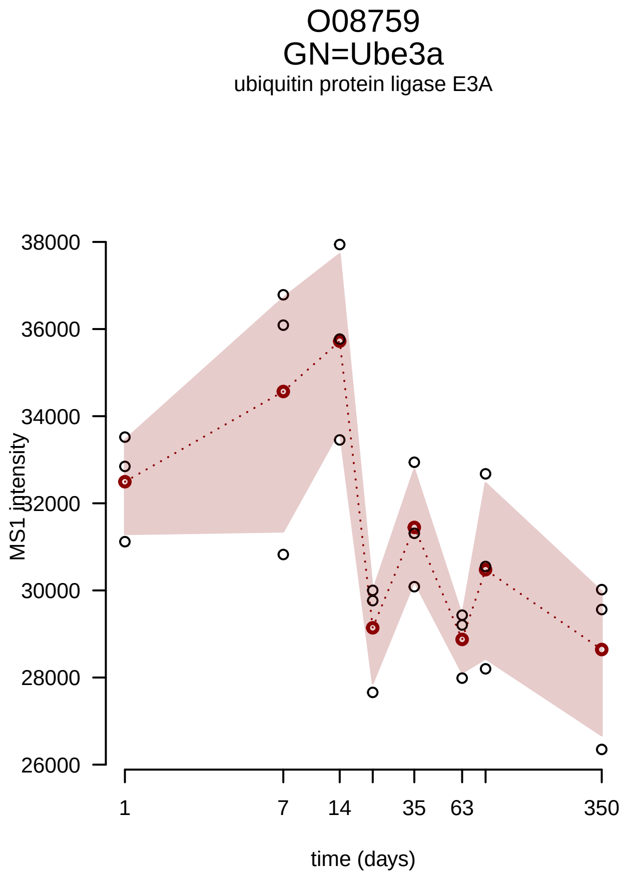
O08759
GN=Ube3a
ubiquitin protein ligase E3A
38000
36000
34000
32000
30000
28000
26000
MS1 intensity
1
7
14
35
63
350
time (days)

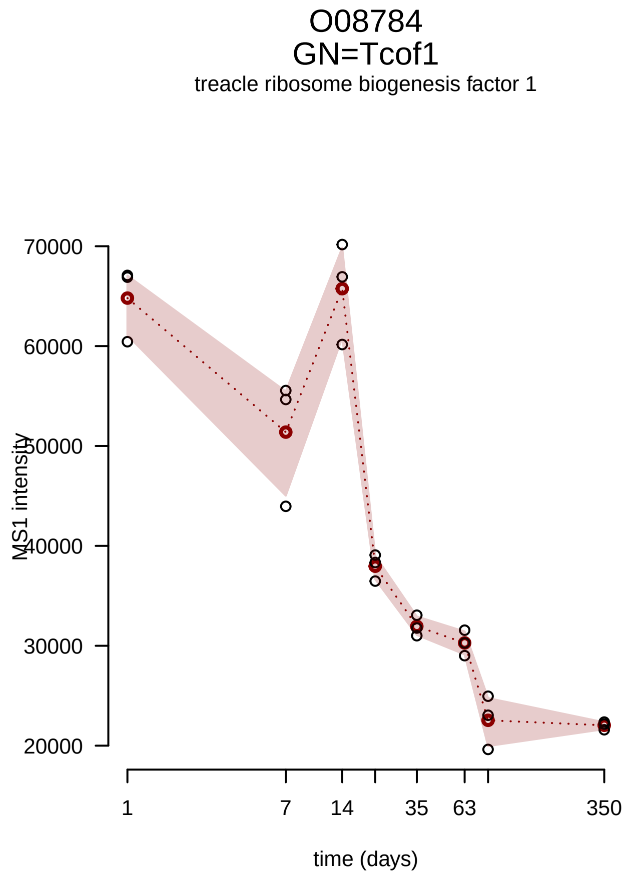
O08784
GN=Tcof1
treacle ribosome biogenesis factor 1
70000
60000
50000
40000
30000
20000
MS1 intensity
1
7
14
35
63
350
time (days)

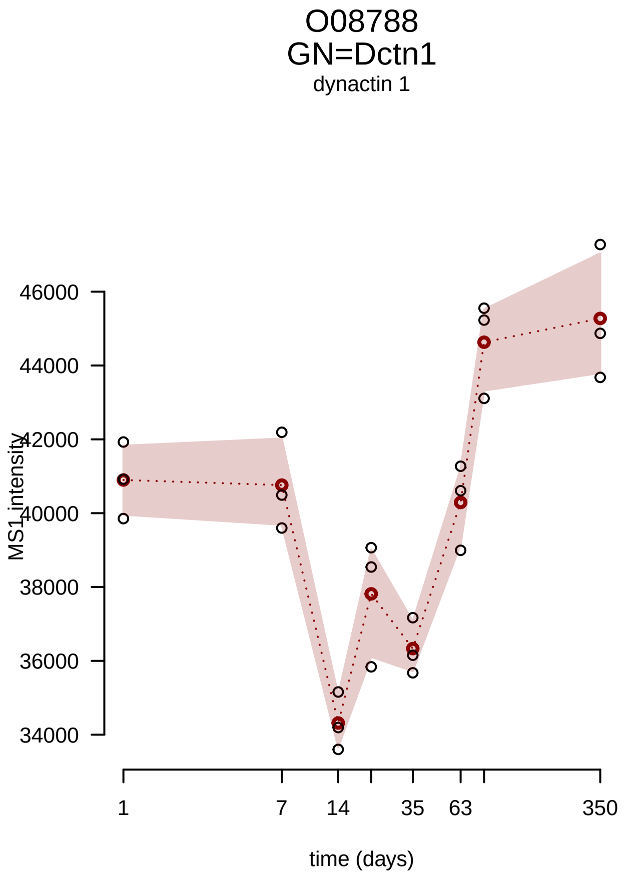
O08788
GN=Dctn1
dynactin 1
46000
44000
42000
40000
38000
36000
34000
MS1 intensity
1
7
14
35
63
350
time (days)

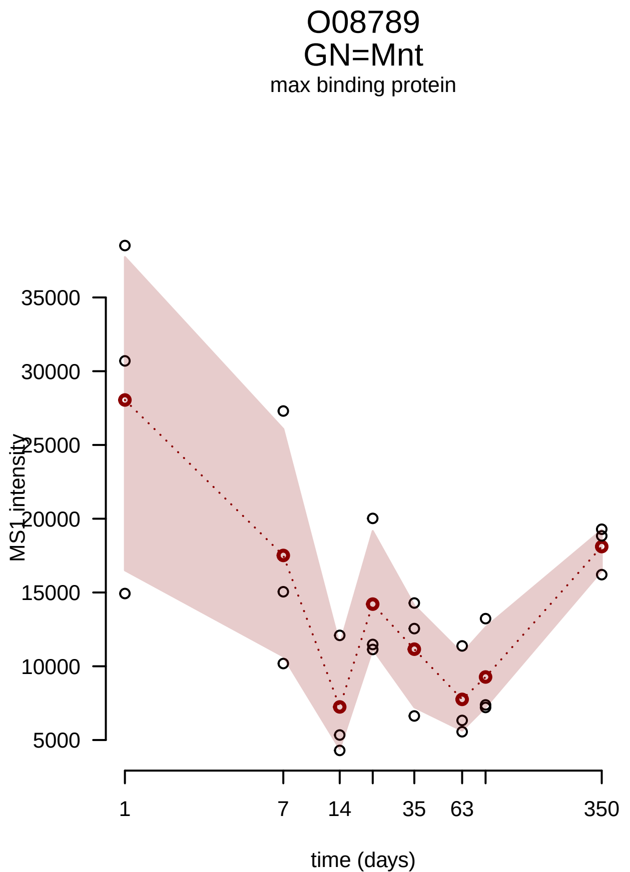
O08789
GN=Mnt
max binding protein
35000
30000
25000
20000
15000
10000
5000
MS1 intensity
1
7
14
35
63
350
time (days)

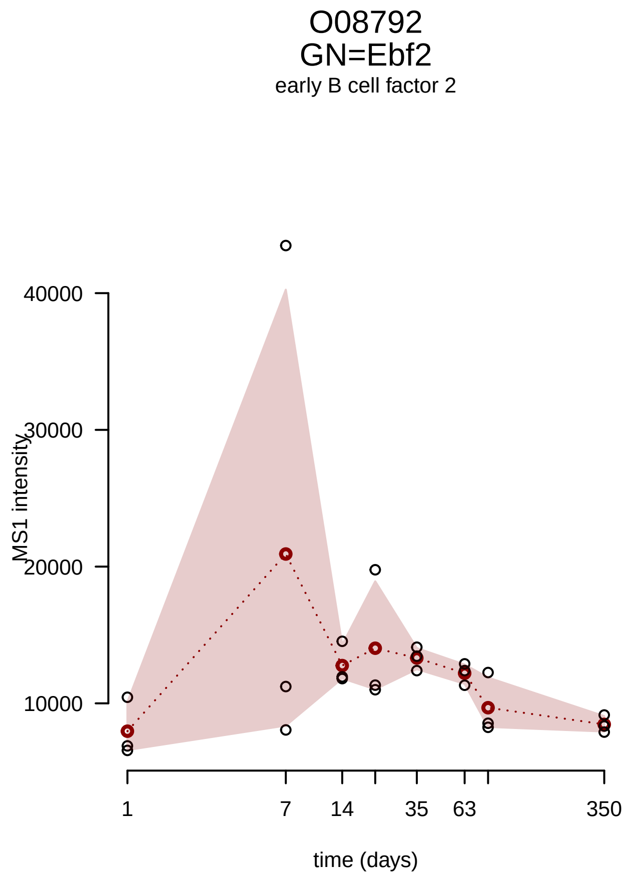
O08792
GN=Ebf2
early B cell factor 2
40000
30000
20000
10000
MS1 intensity
1
7
14
35
63
350
time (days)

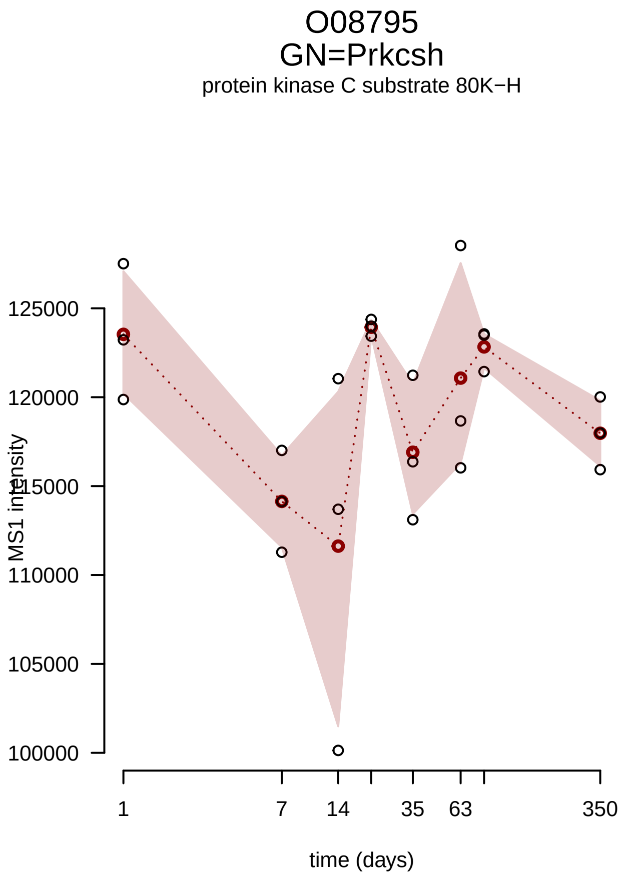
O08795
GN=Prkcsh
protein kinase C substrate 80K−H
125000
120000
115000
110000
105000
100000
MS1 intensity
1
7
14
35
63
350
time (days)

O08796
GN=Eef2k
eukaryotic elongation factor−2 kinase

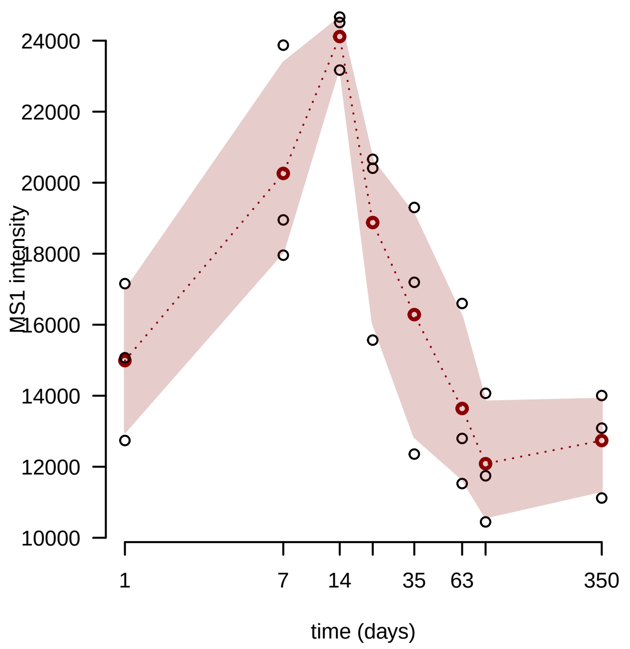

O08807
GN=Prdx4
peroxiredoxin 4

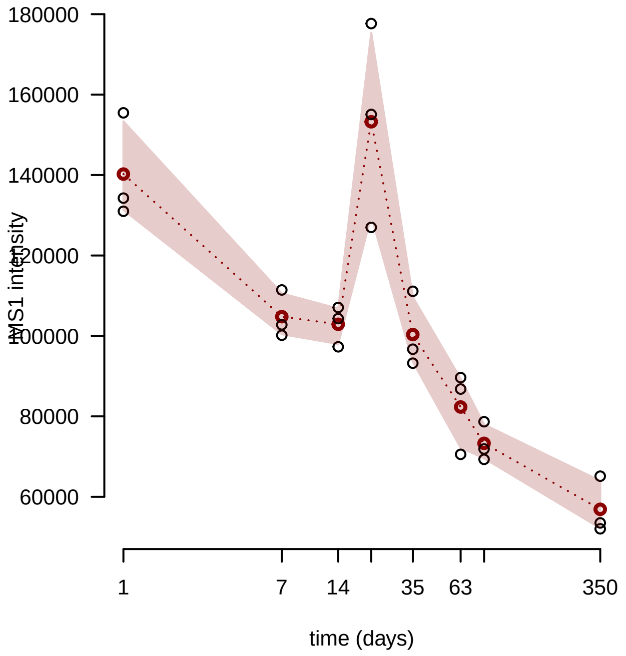

O08808
GN=Diaph1
diaphanous related formin 1

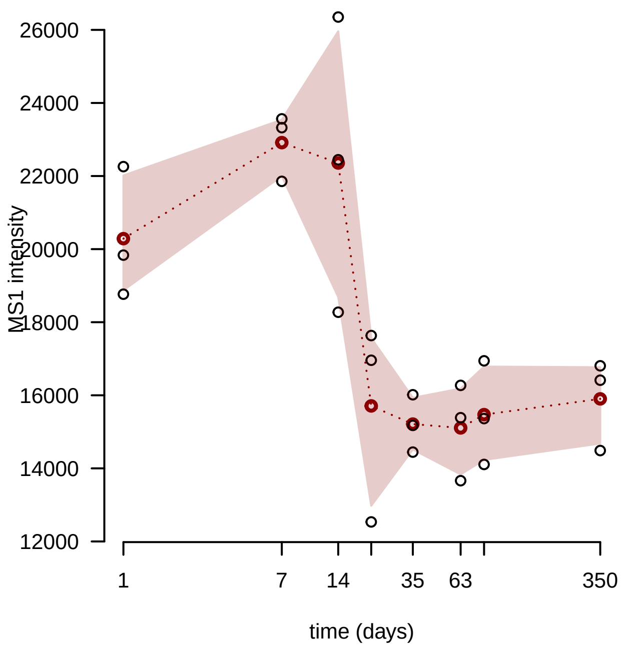

O08810
GN=Eftud2
elongation factor Tu GTP binding domain ...

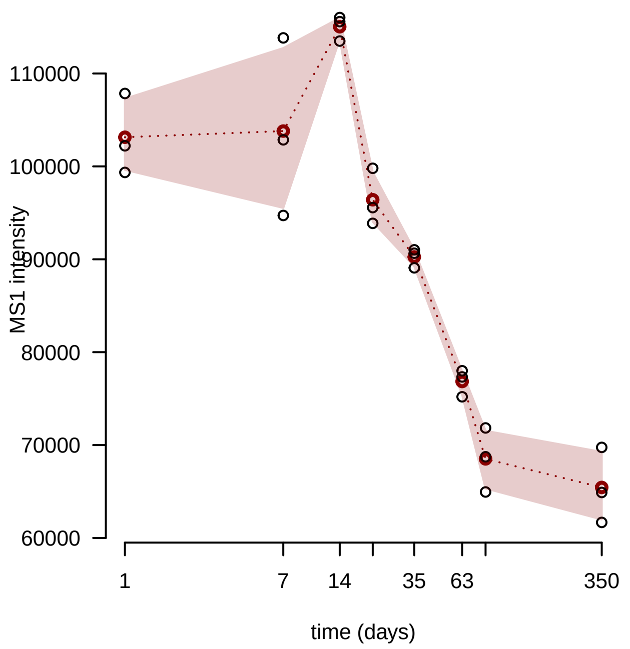

O08811
GN=Ercc2
excision repair cross−complementing rode ...

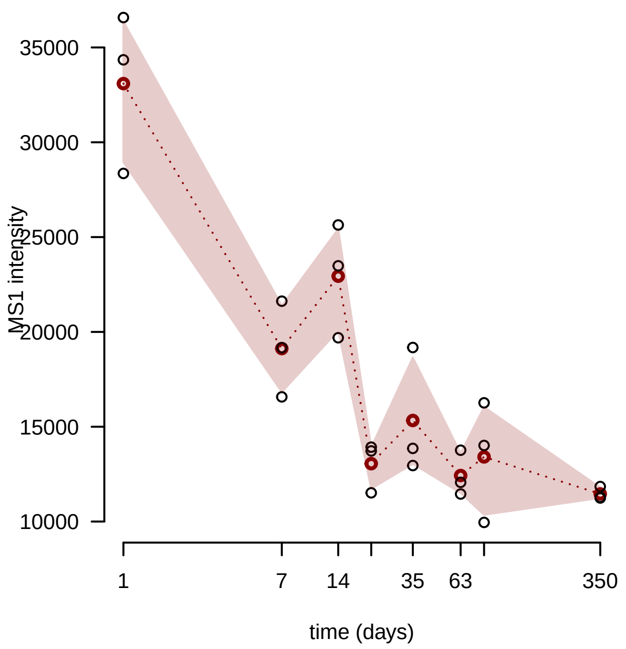

O08832
GN=Galnt4
polypeptide N−acetylgalactosaminyltransf ...

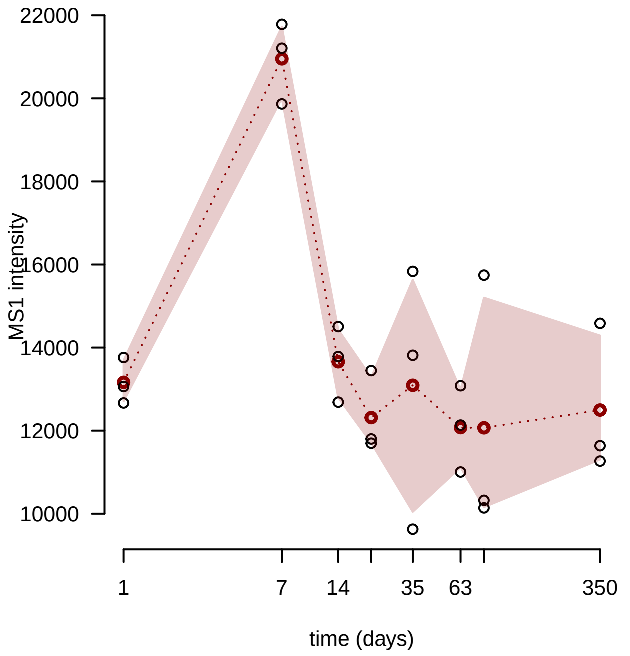

O08842
GN=Gfra2
glial cell line derived neurotrophic fac ...

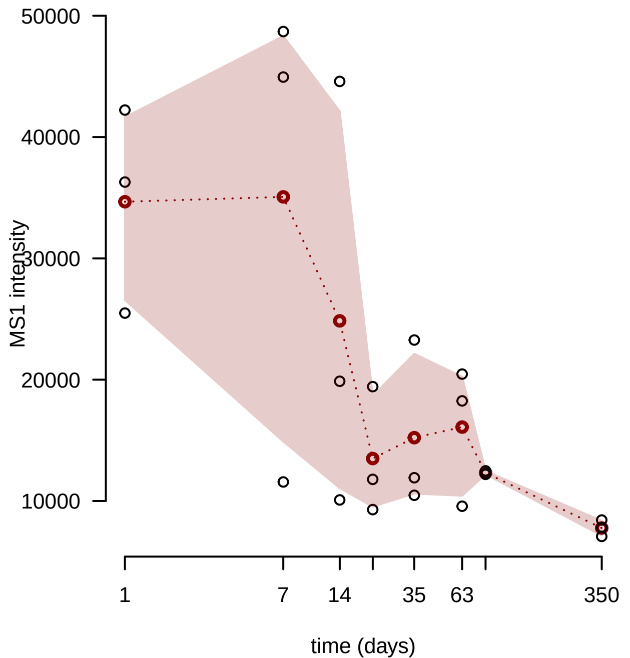

O08848
GN=RO60

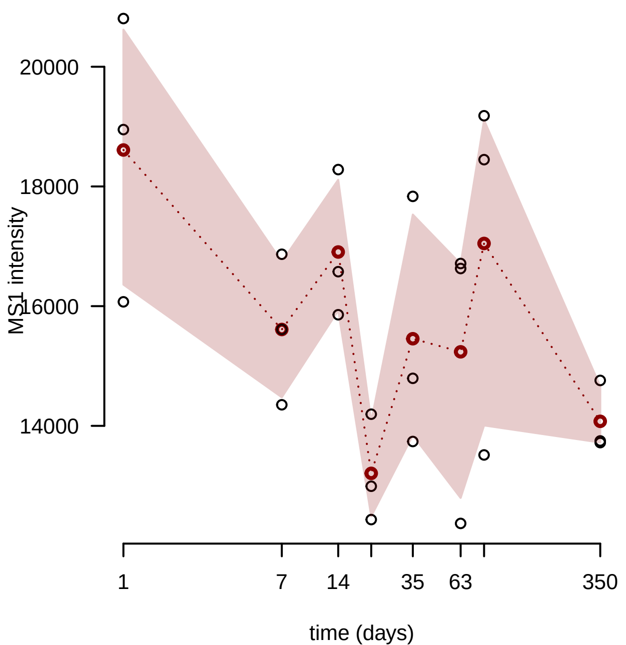

O08849
GN=Rgs2
regulator of G−protein signaling 2

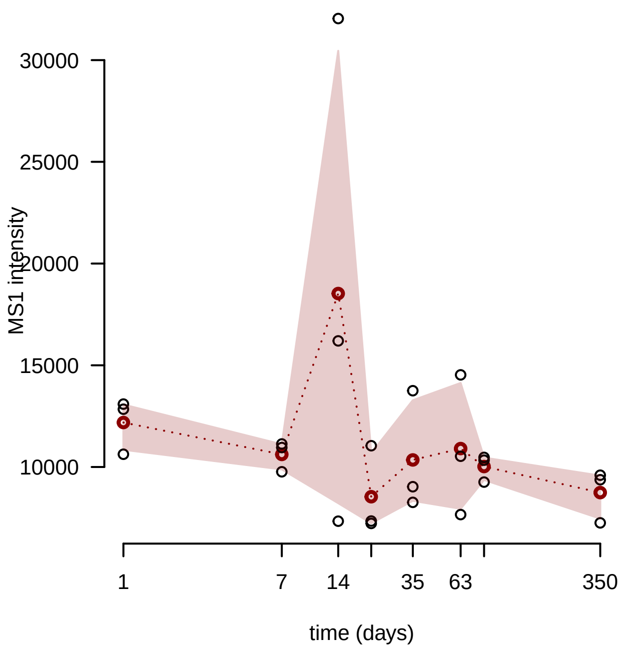

O08856
GN=Ell
elongation factor RNA polymerase II

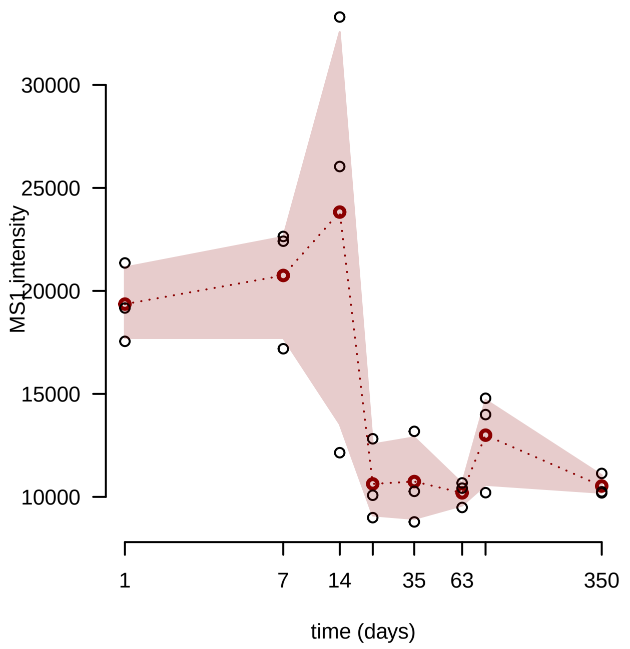

O08901
GN=Bub1
BUB1, mitotic checkpoint serine/threonin ...

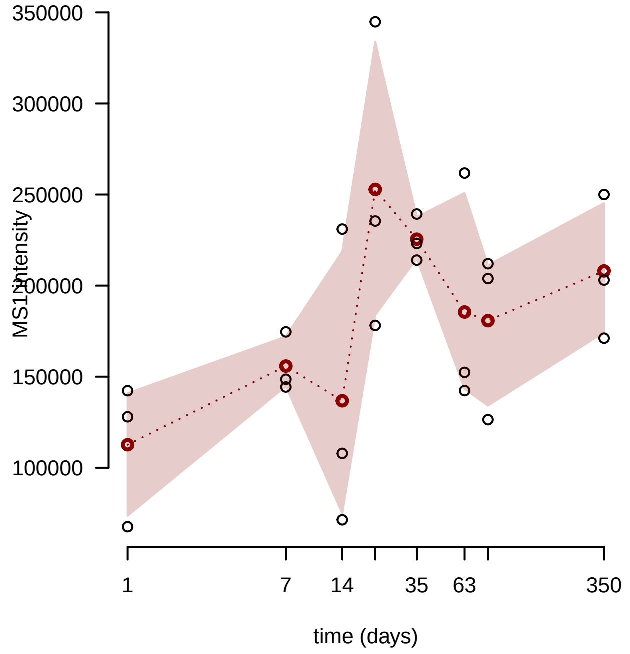

O08908
GN=Pik3r2
phosphoinositide−3−kinase regulatory sub ...

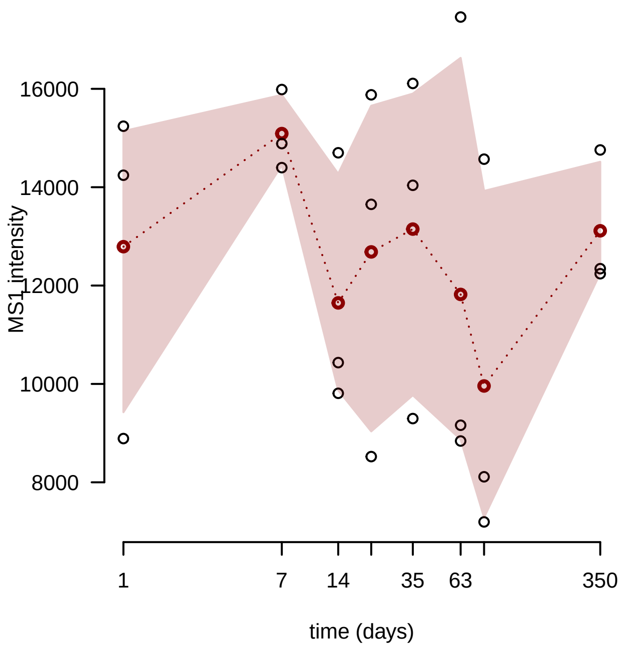

O08912
GN=Galnt1
polypeptide N−acetylgalactosaminyltransf ...

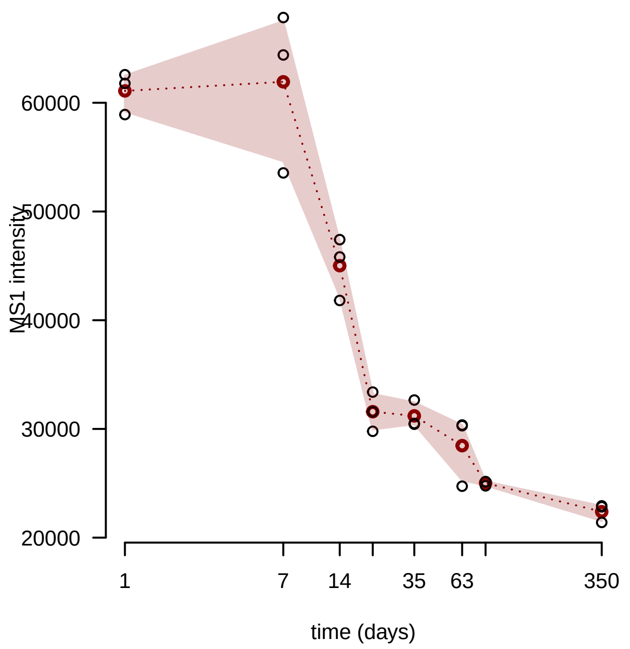

O08914
GN=Faah
fatty acid amide hydrolase

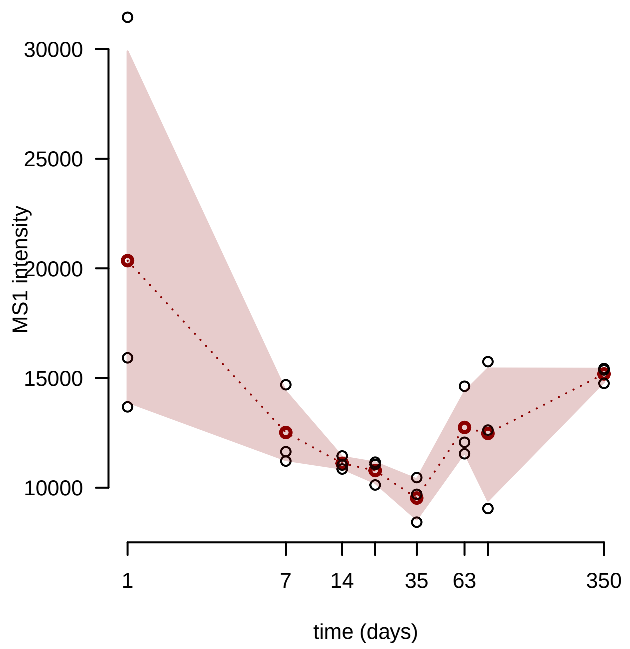

O08915
GN=Aip
aryl−hydrocarbon receptor−interacting pr ...

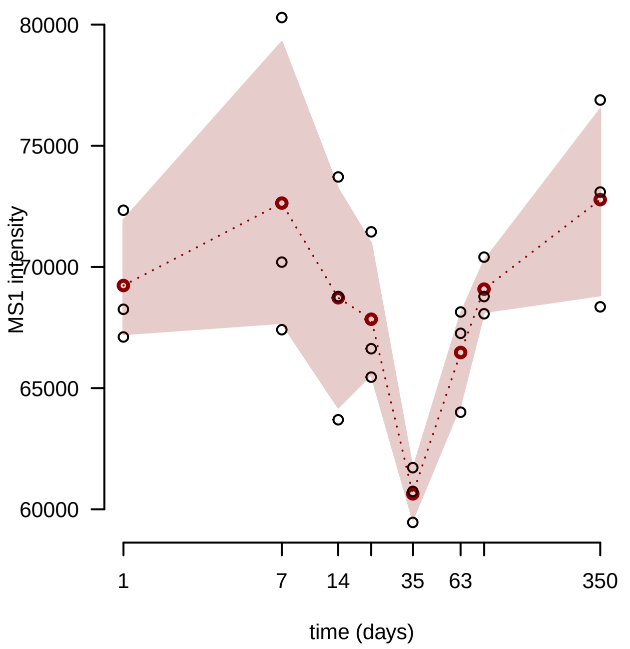

O08917
GN=Flot1
flotillin 1

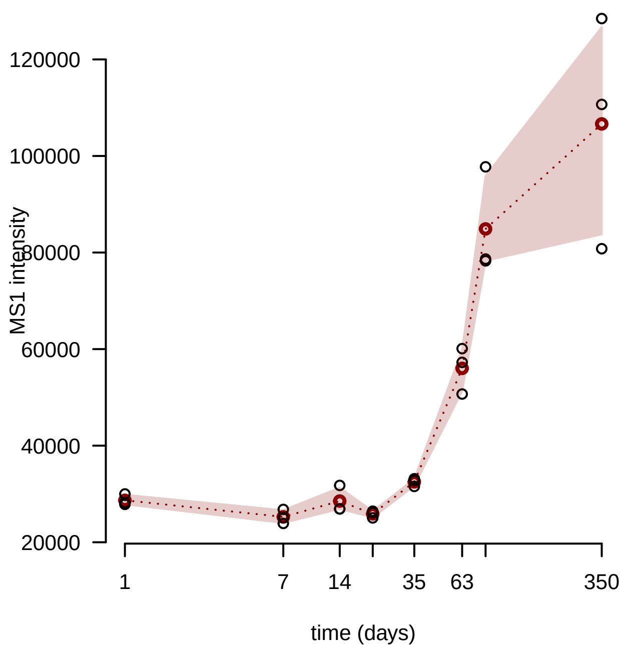

O08919
GN=Numbl
numb−like

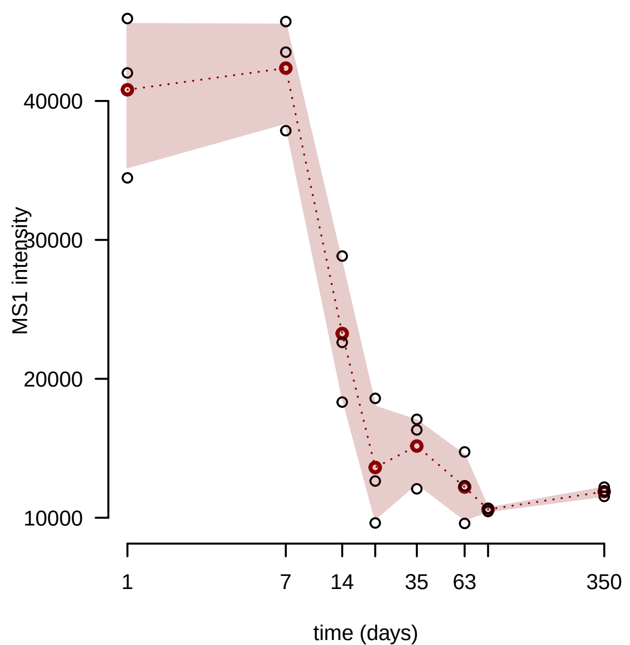

O08966
GN=Slc22a1
solute carrier family 22 (organic cation ...

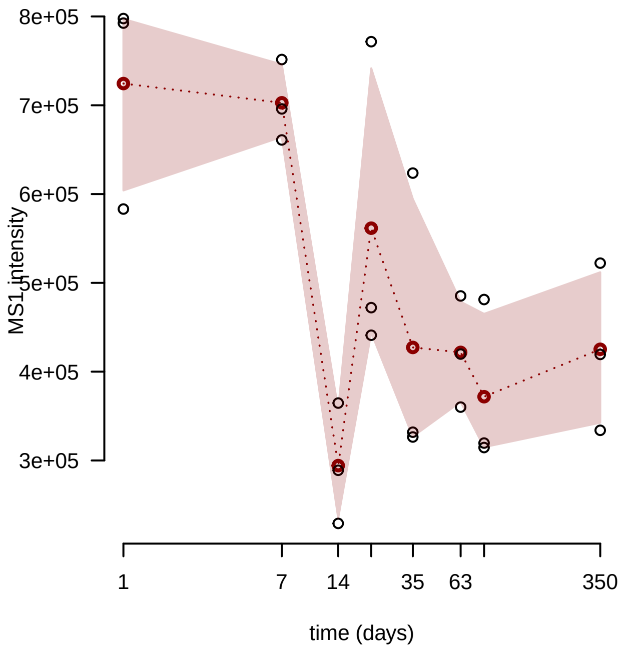

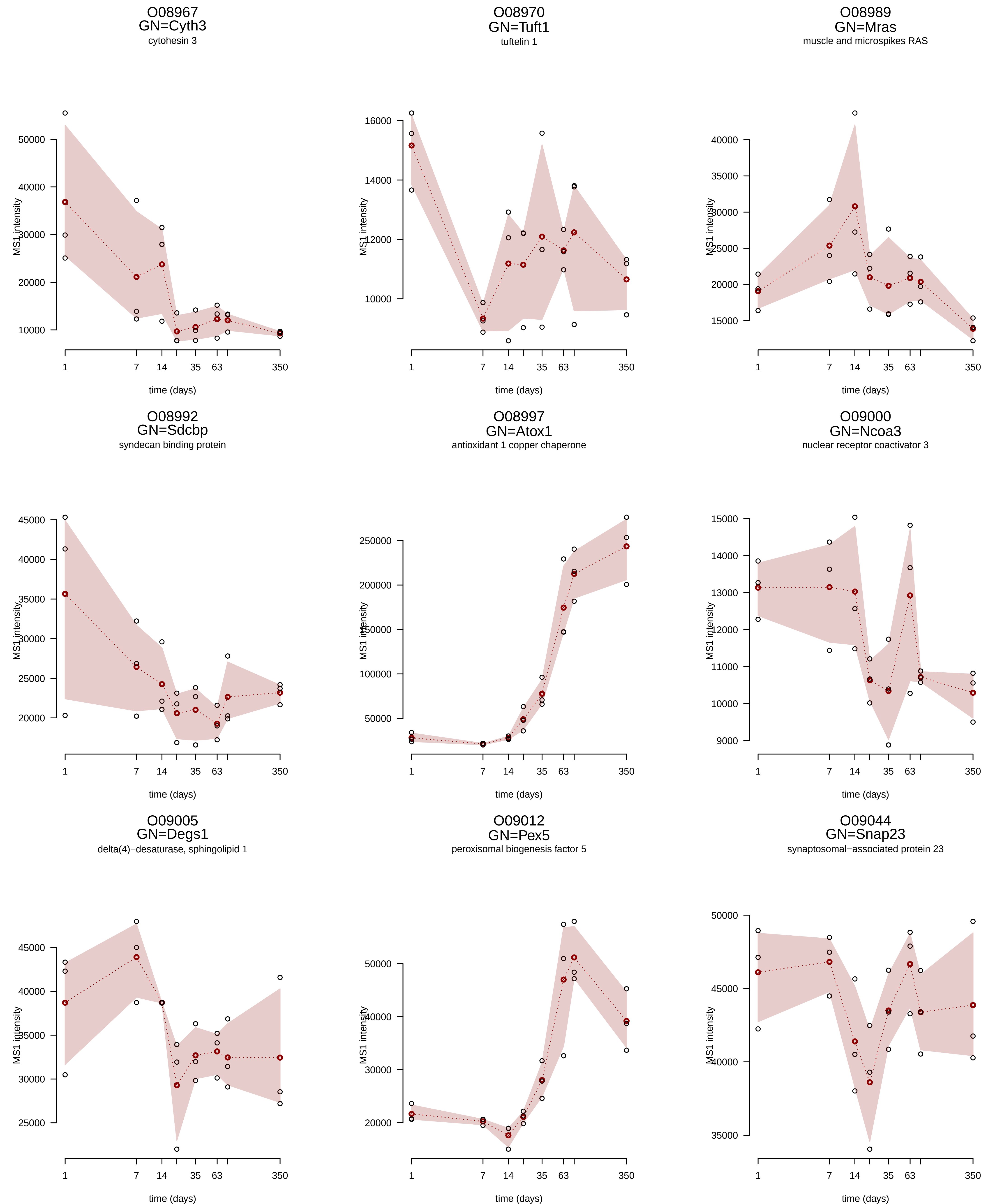
O08967
GN=Cyth3
cytohesin 3
O08970
GN=Tuft1
tuftelin 1
O08989
GN=Mras
muscle and microspikes RAS
O08992
GN=Sdcbp
syndecan binding protein
O08997
GN=Atox1
antioxidant 1 copper chaperone
O09000
GN=Ncoa3
nuclear receptor coactivator 3
O09005
GN=Degs1
delta(4)−desaturase, sphingolipid 1
O09012
GN=Pex5
peroxisomal biogenesis factor 5
O09044
GN=Snap23
synaptosomal−associated protein 23
MS1 intensity
time (days)
1
7
14
35
63
350

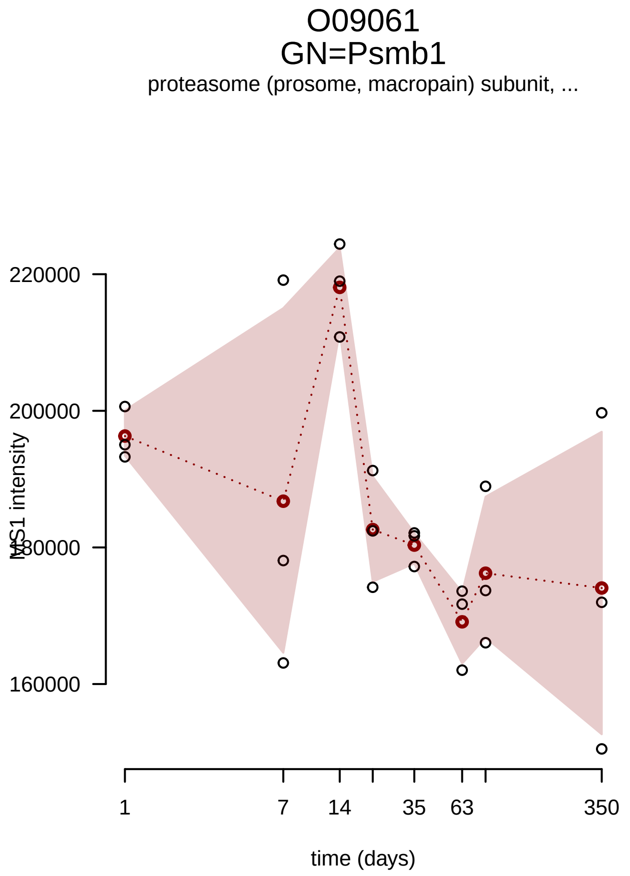
O09061
GN=Psmb1
proteasome (prosome, macropain) subunit, ...
220000
200000
180000
160000
MS1 intensity
1
7
14
35
63
350
time (days)

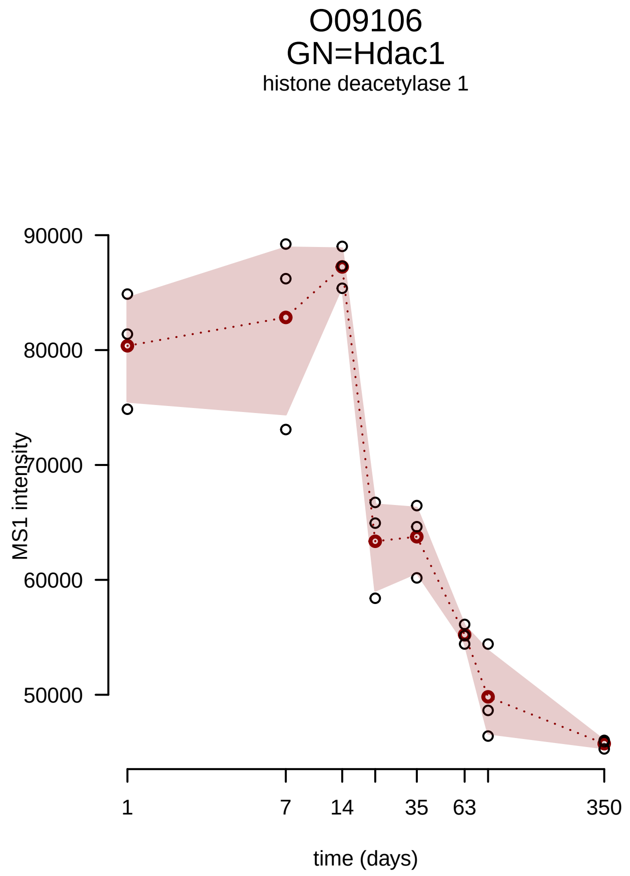
O09106
GN=Hdac1
histone deacetylase 1
90000
80000
70000
60000
50000
MS1 intensity
1
7
14
35
63
350
time (days)

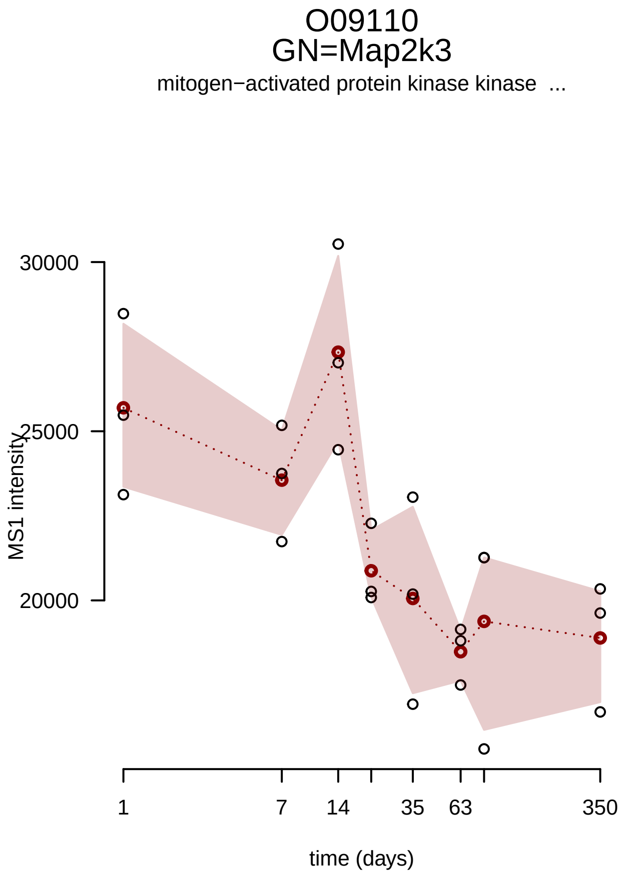
O09110
GN=Map2k3
mitogen−activated protein kinase kinase ...
30000
25000
20000
MS1 intensity
1
7
14
35
63
350
time (days)

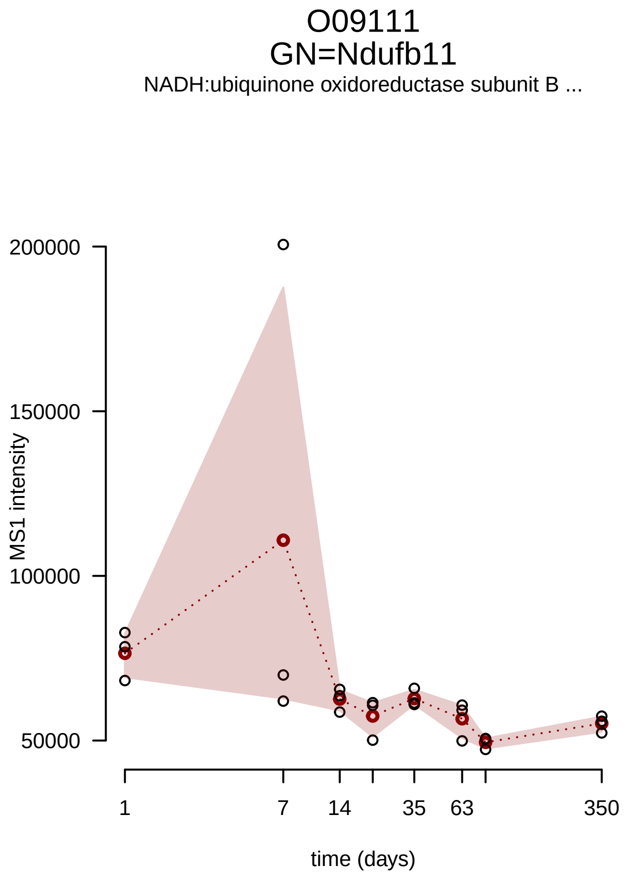
O09111
GN=Ndufb11
NADH:ubiquinone oxidoreductase subunit B ...
200000
150000
100000
50000
MS1 intensity
1
7
14
35
63
350
time (days)

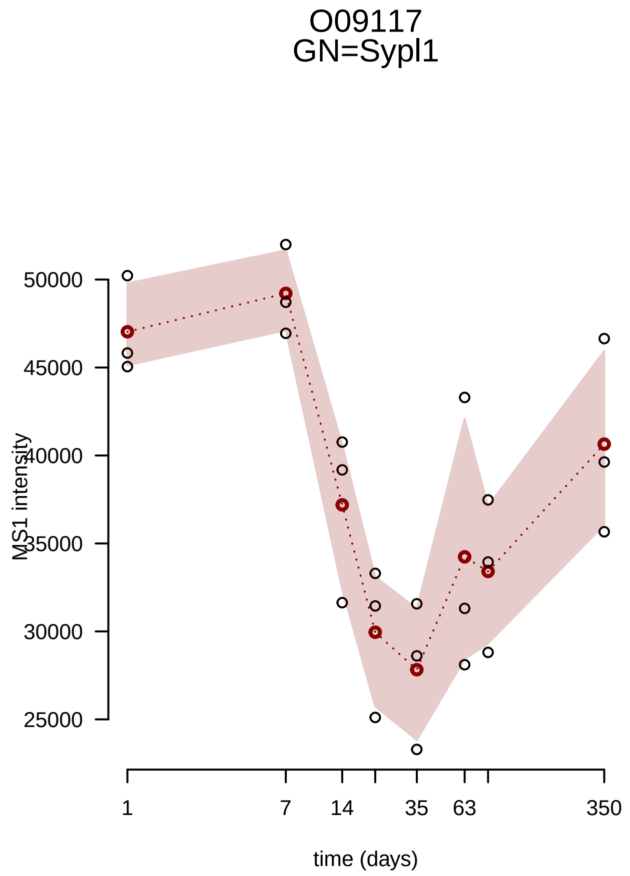
O09117
GN=Sypl1
50000
45000
40000
35000
30000
25000
MS1 intensity
1
7
14
35
63
350
time (days)

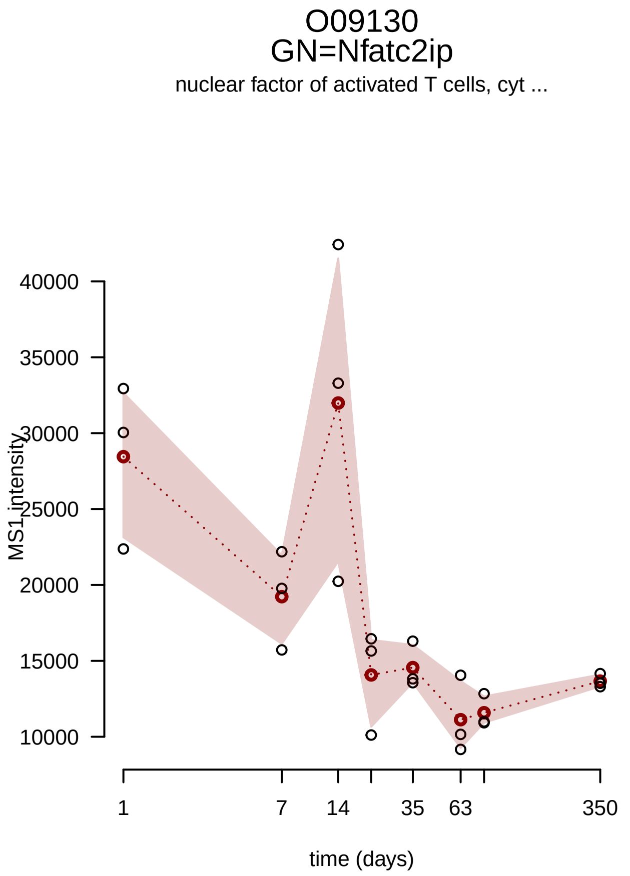
O09130
GN=Nfatc2ip
nuclear factor of activated T cells, cyt ...
40000
35000
30000
25000
20000
15000
10000
MS1 intensity
1
7
14
35
63
350
time (days)

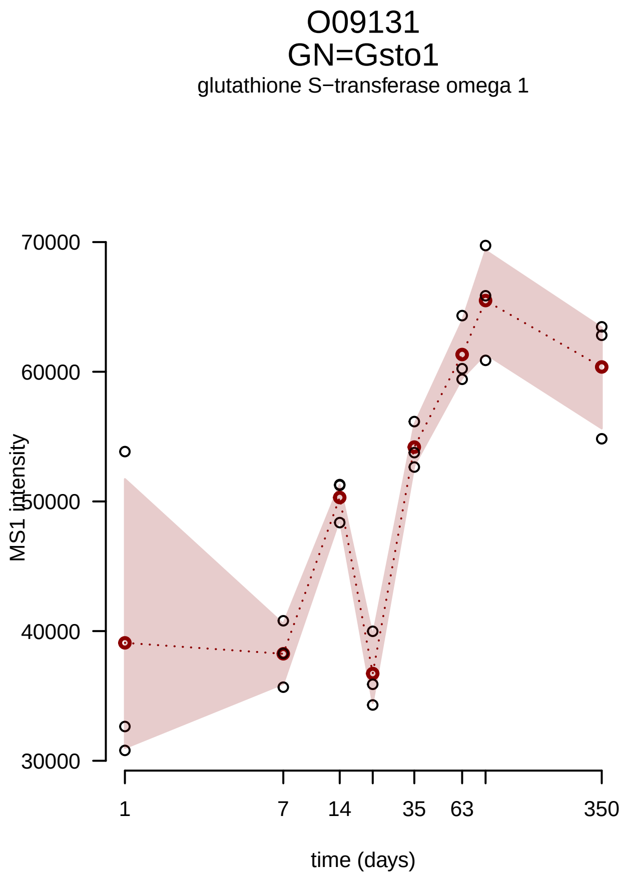
O09131
GN=Gsto1
glutathione S−transferase omega 1
70000
60000
50000
40000
30000
MS1 intensity
1
7
14
35
63
350
time (days)

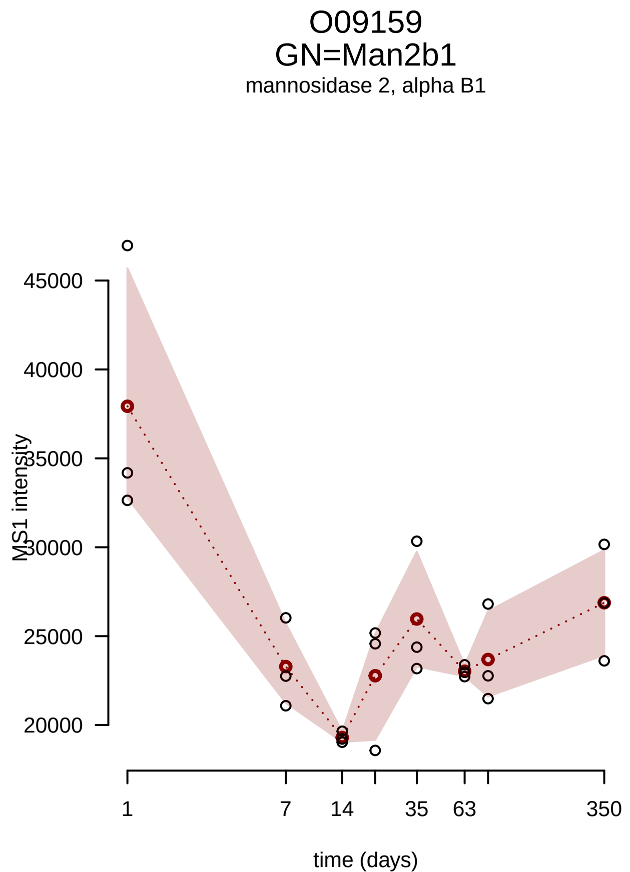
O09159
GN=Man2b1
mannosidase 2, alpha B1
45000
40000
35000
30000
25000
20000
MS1 intensity
1
7
14
35
63
350
time (days)

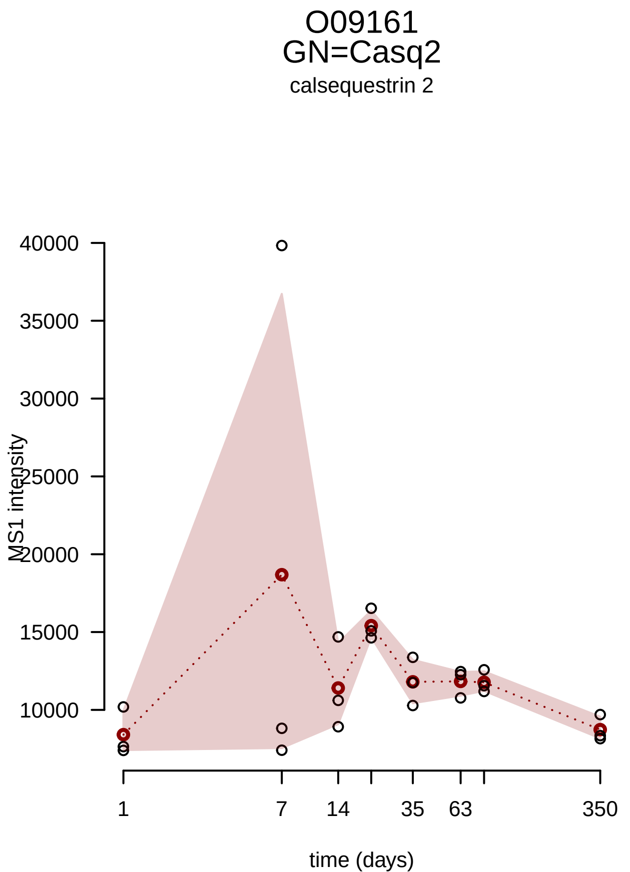
O09161
GN=Casq2
calsequestrin 2
40000
35000
30000
25000
20000
15000
10000
MS1 intensity
1
7
14
35
63
350
time (days)

O09164
GN=Sod3
superoxide dismutase 3, extracellular

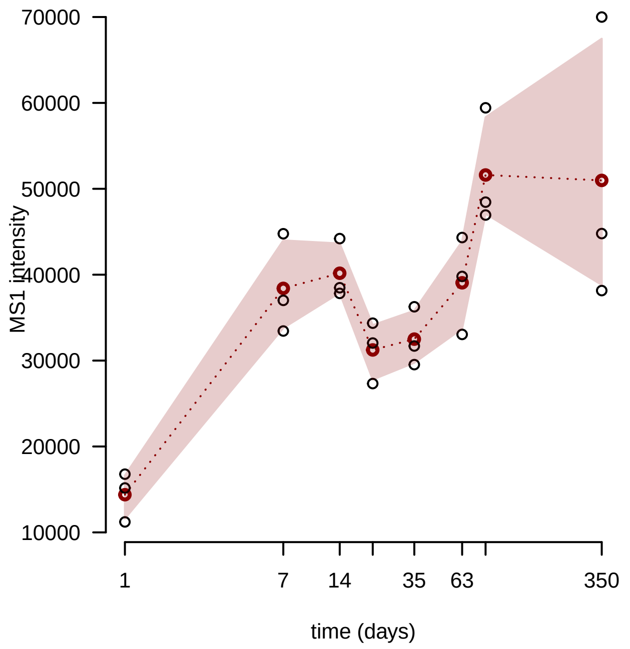

O09167
GN=Rpl21
ribosomal protein L21

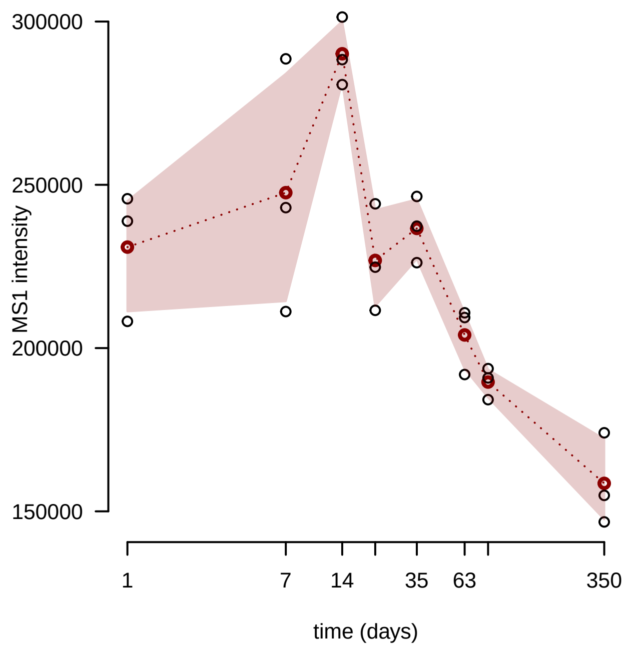

O09172
GN=Gclm
glutamate−cysteine ligase, modifier subu ...

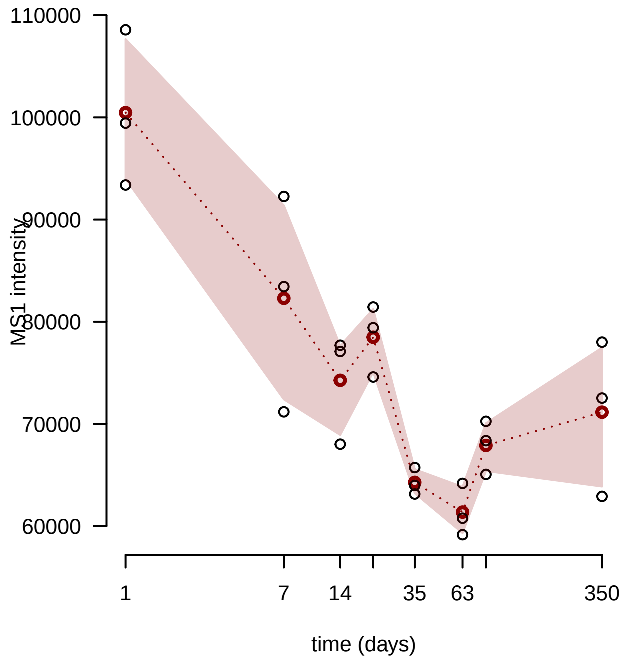

O09174
GN=Amacr
alpha−methylacyl−CoA racemase

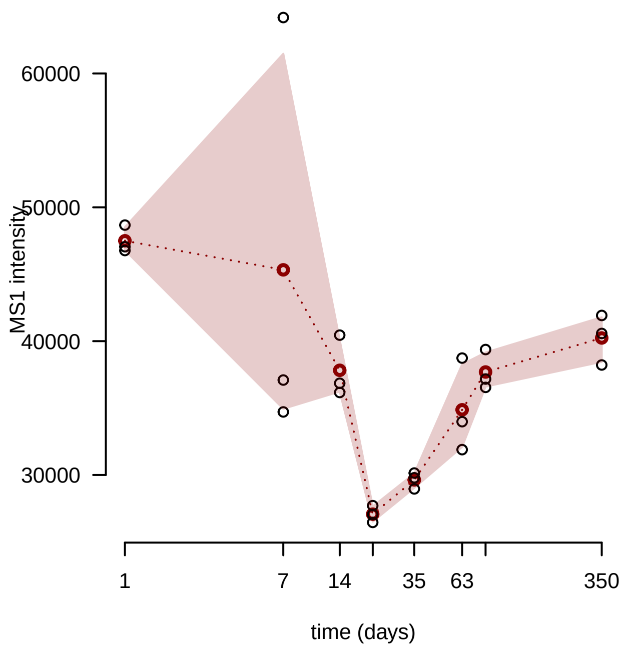

O35047
GN=Psmc3ip
proteasome (prosome, macropain) 26S subu ...

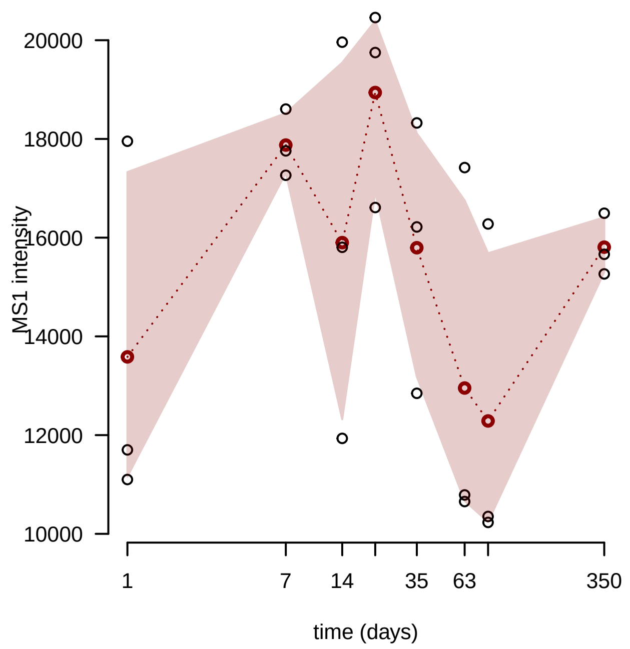

O35071
GN=Kif1c
kinesin family member 1C

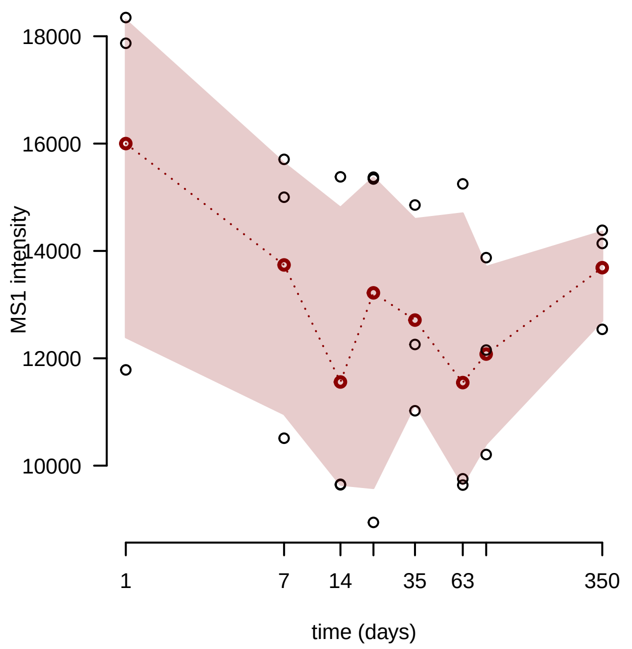

O35074
GN=Ptgis
prostaglandin I2 (prostacyclin) synthase

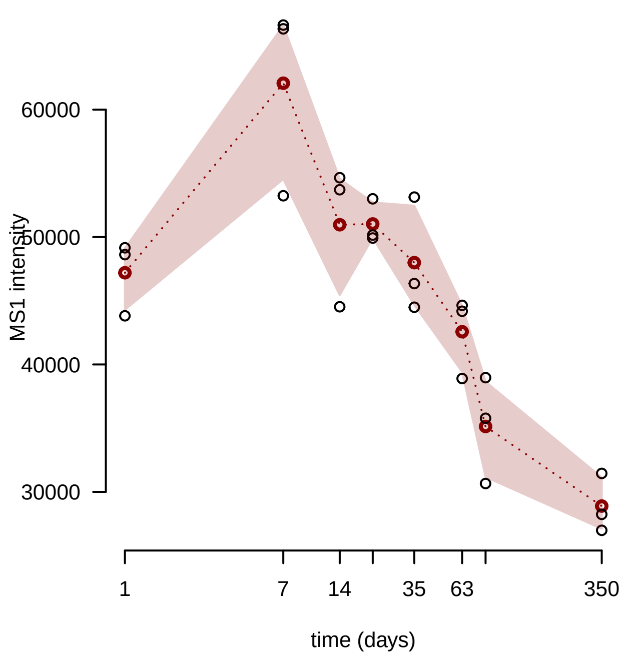

O35075
GN=Vps26c
VPS26 endosomal protein sorting factor C

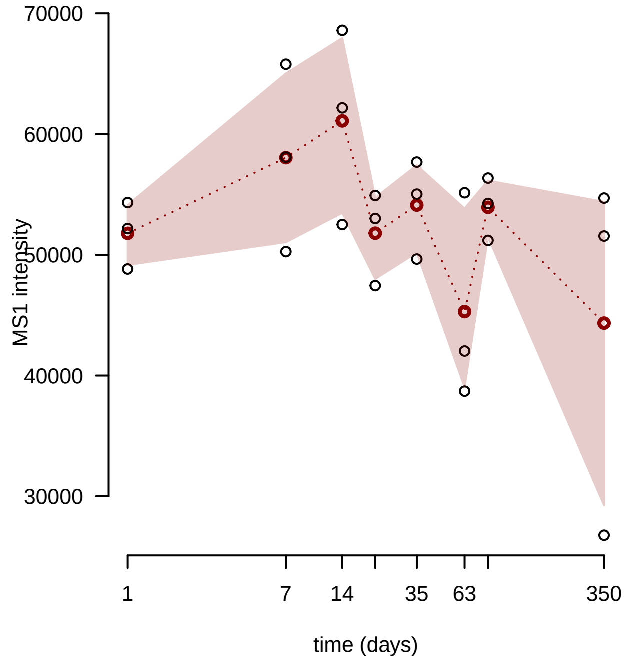

O35083
GN=Agpat1
1−acylglycerol−3−phosphate O−acyltransfe ...

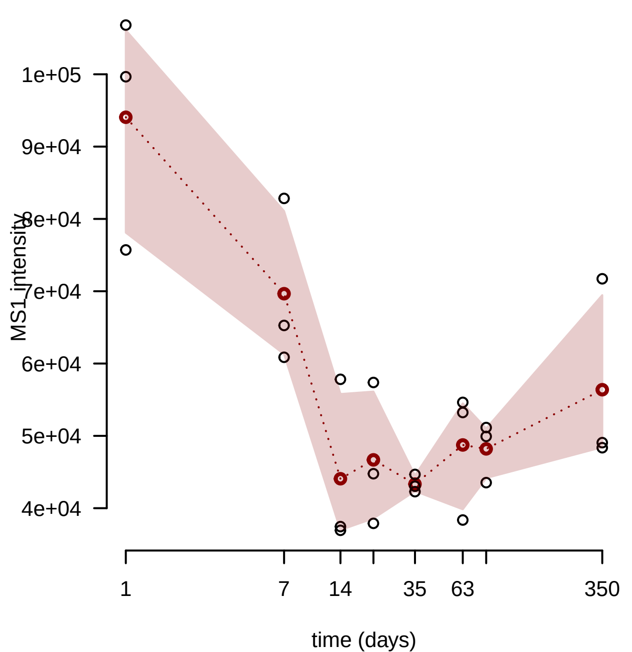

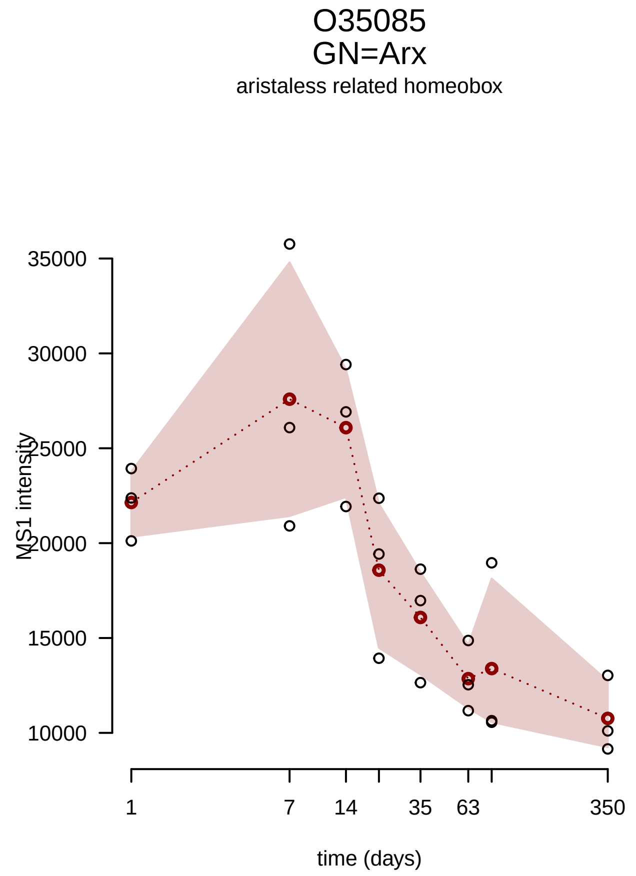
O35085
GN=Arx
aristaless related homeobox
MS1 intensity
35000
30000
25000
20000
15000
10000
1
7
14
35
63
350
time (days)
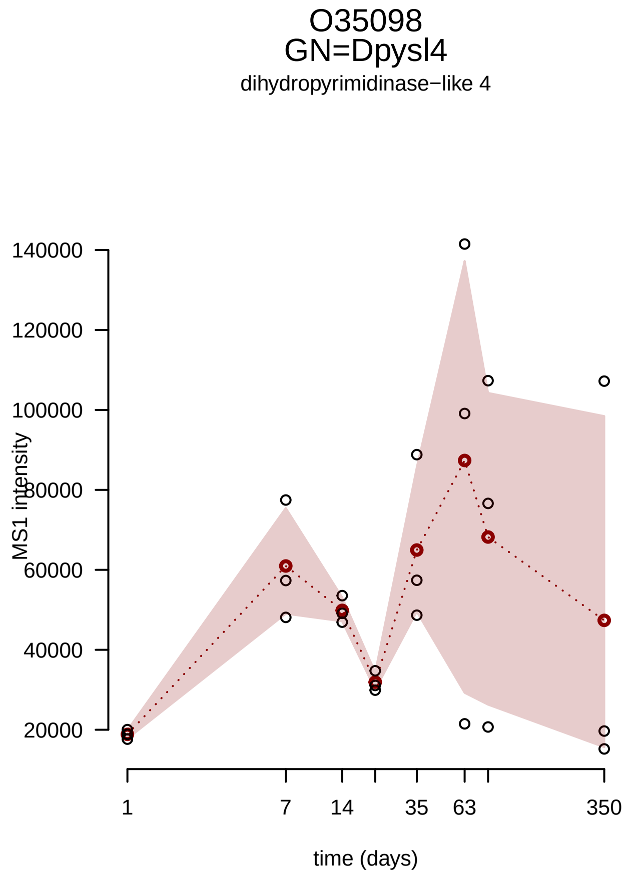
O35098
GN=Dpysl4
dihydropyrimidinase−like 4
MS1 intensity
140000
120000
100000
80000
60000
40000
20000
1
7
14
35
63
350
time (days)
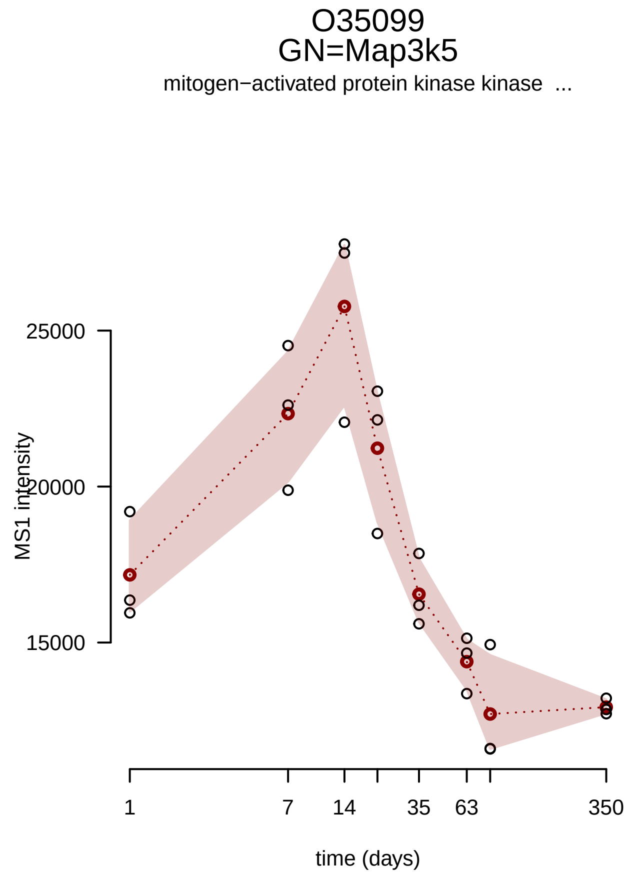
O35099
GN=Map3k5
mitogen−activated protein kinase kinase ...
MS1 intensity
25000
20000
15000
1
7
14
35
63
350
time (days)
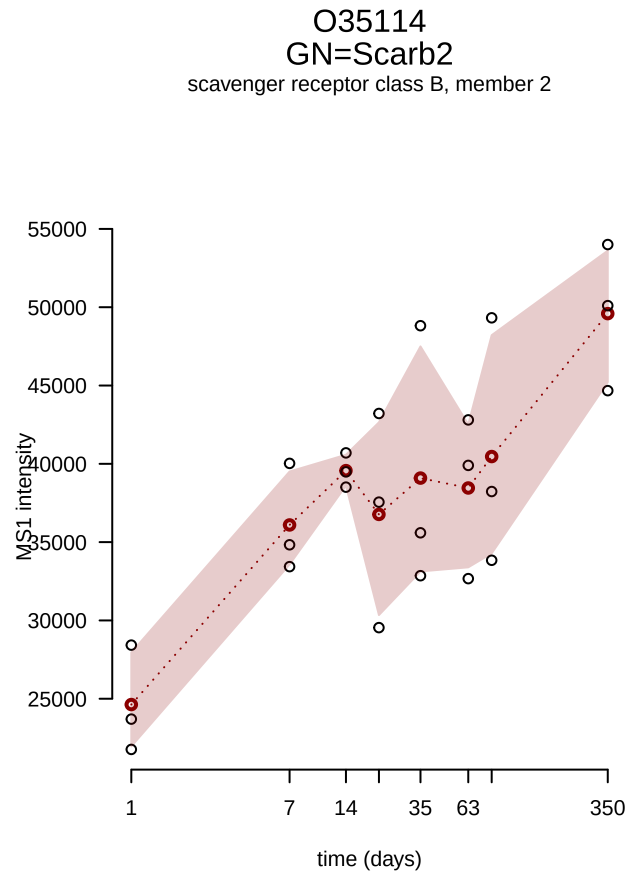
O35114
GN=Scarb2
scavenger receptor class B, member 2
MS1 intensity
55000
50000
45000
40000
35000
30000
25000
1
7
14
35
63
350
time (days)
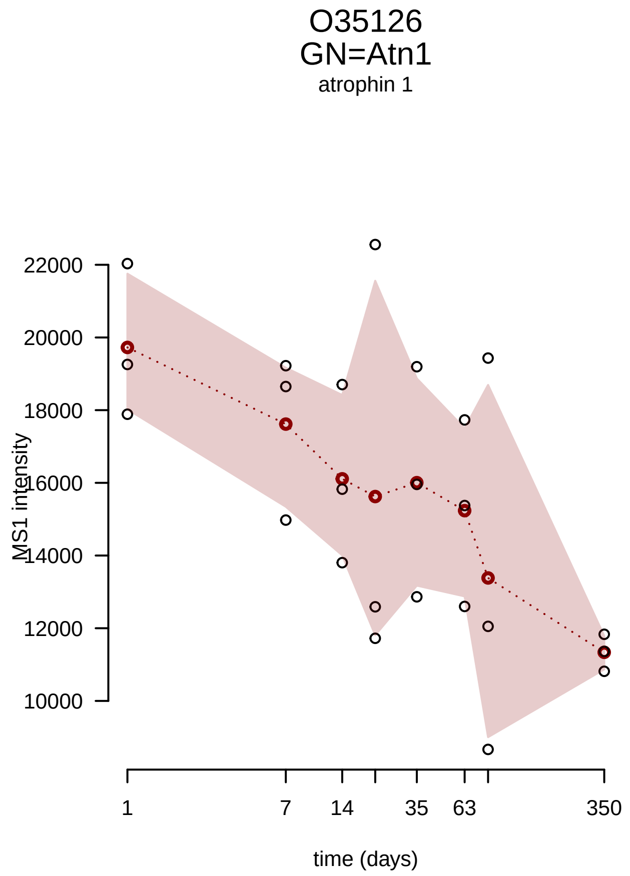
O35126
GN=Atn1
atrophin 1
MS1 intensity
22000
20000
18000
16000
14000
12000
10000
1
7
14
35
63
350
time (days)
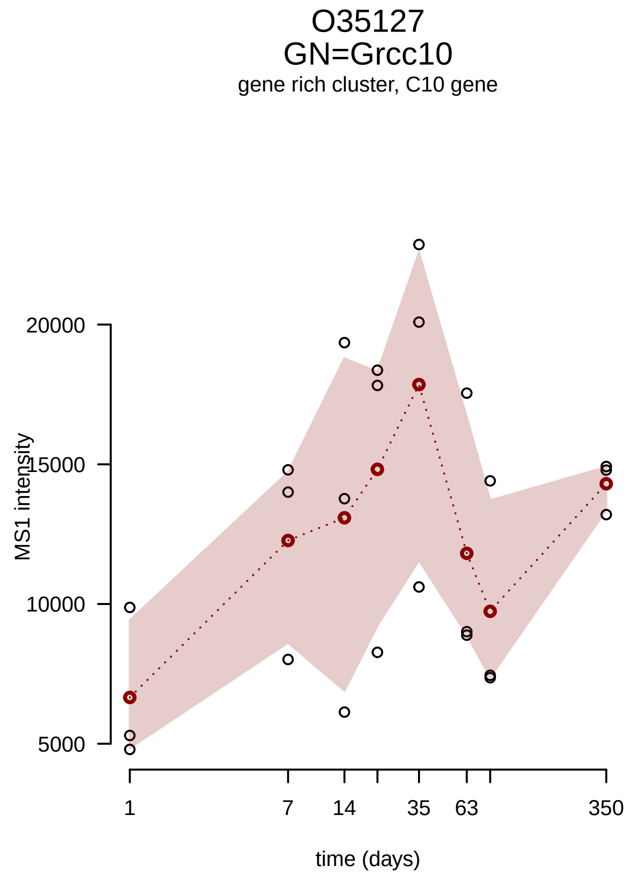
O35127
GN=Grcc10
gene rich cluster, C10 gene
MS1 intensity
20000
15000
10000
5000
1
7
14
35
63
350
time (days)
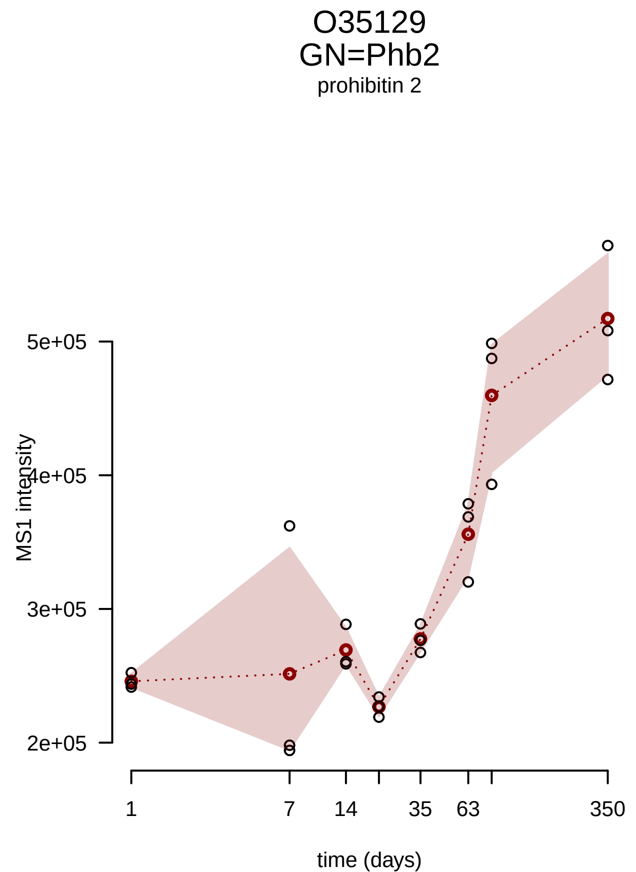
O35129
GN=Phb2
prohibitin 2
MS1 intensity
5e+05
4e+05
3e+05
2e+05
1
7
14
35
63
350
time (days)
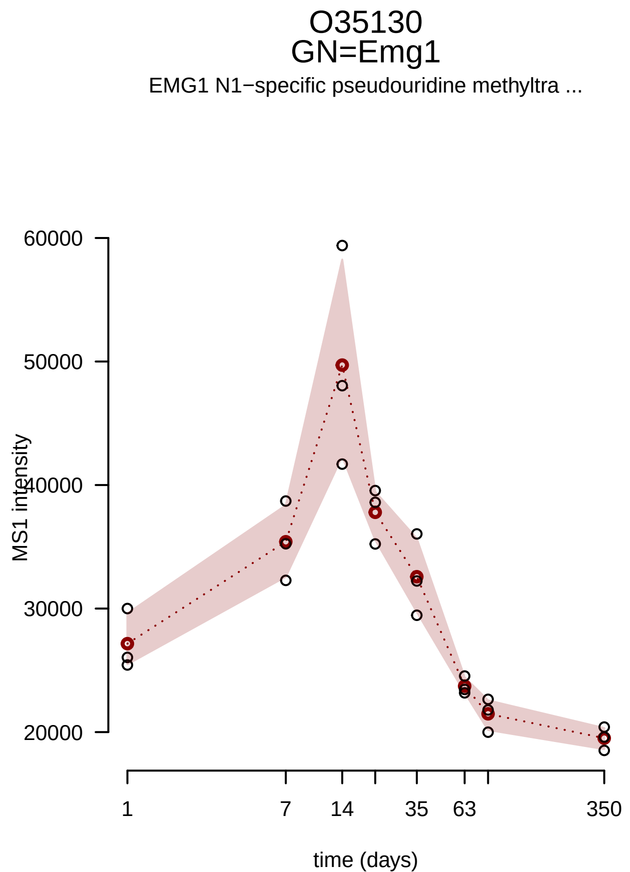
O35130
GN=Emg1
EMG1 N1−specific pseudouridine methyltra ...
MS1 intensity
60000
50000
40000
30000
20000
1
7
14
35
63
350
time (days)
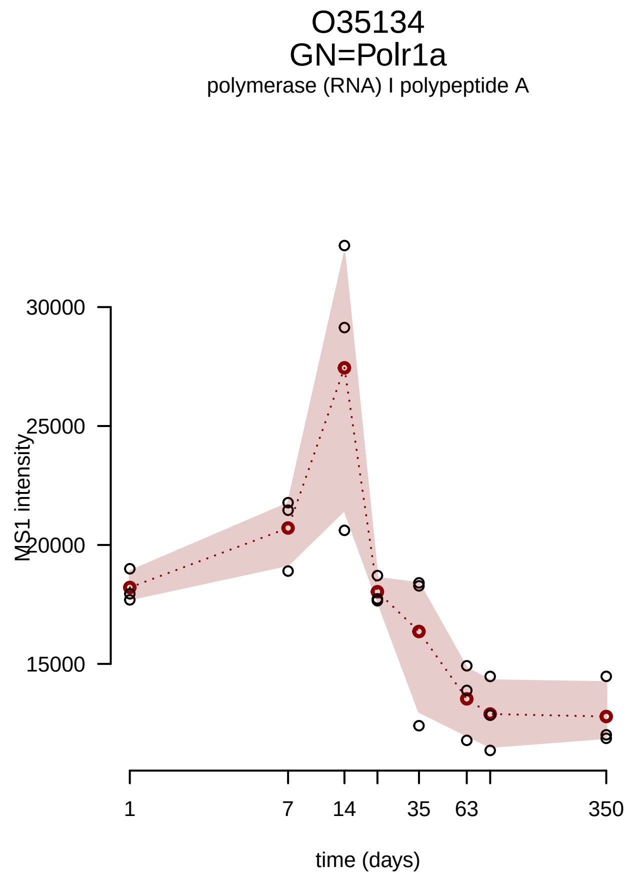
O35134
GN=Polr1a
polymerase (RNA) I polypeptide A
MS1 intensity
30000
25000
20000
15000
1
7
14
35
63
350
time (days)

O35143
GN=ATP5IF1

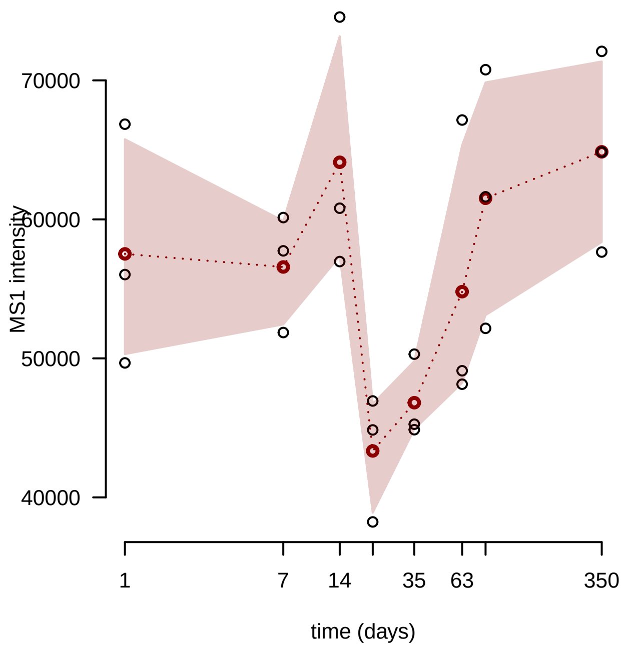

O35144
GN=Terf2
telomeric repeat binding factor 2

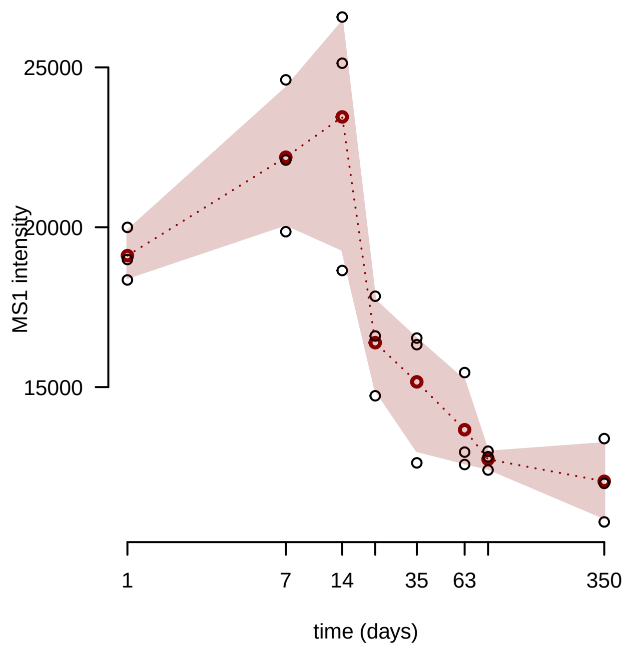

O35153
GN=Bet1l
Bet1 golgi vesicular membrane traffickin ...

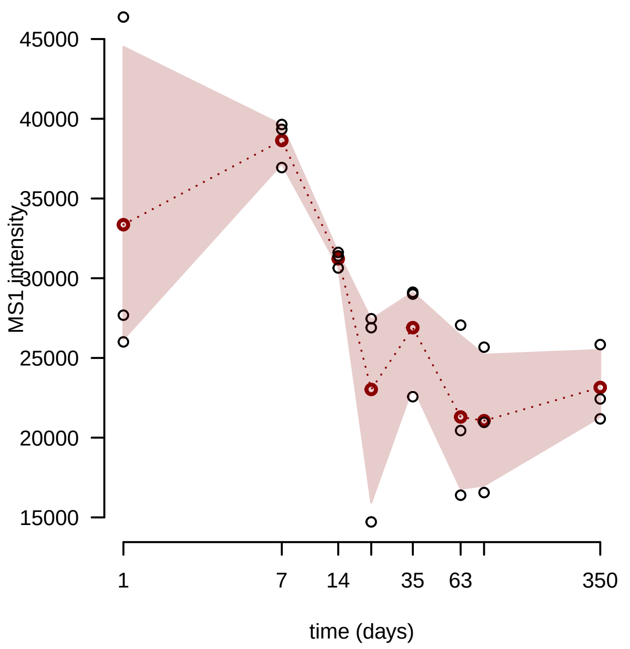

O35161
GN=Celsr1
cadherin, EGF LAG seven−pass G−type rece ...

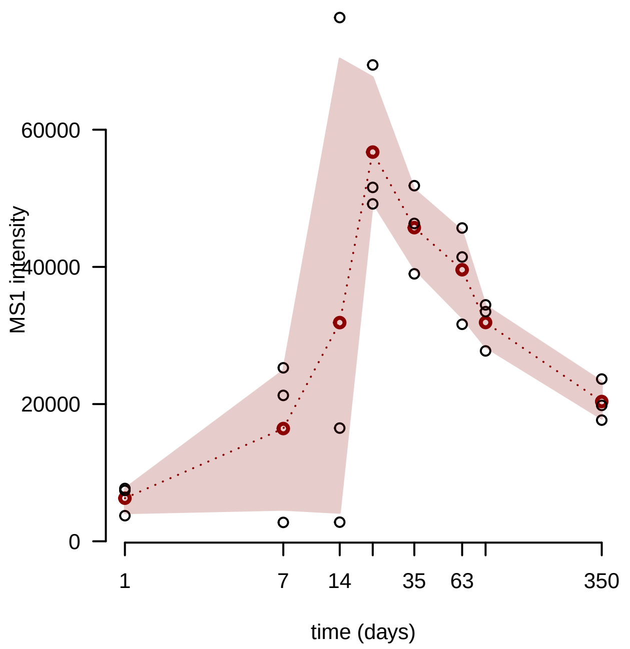

O35166
GN=Gosr2
golgi SNAP receptor complex member 2

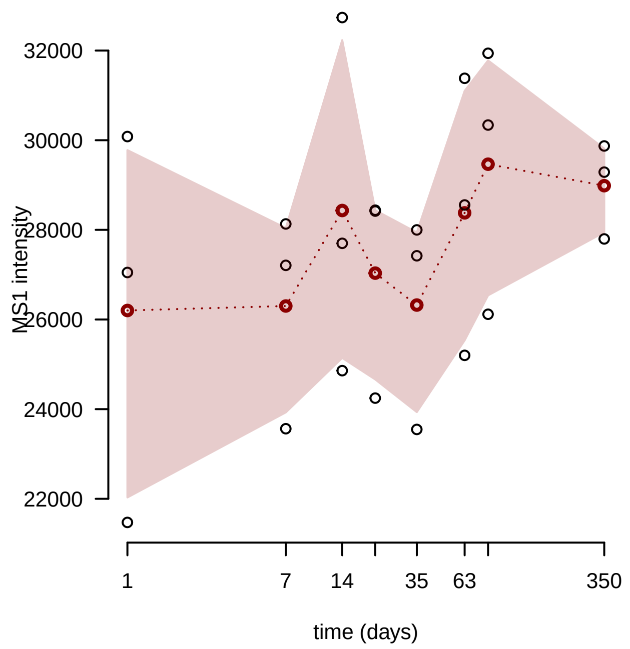

O35188
GN=Cx3cl1
chemokine (C−X3−C motif) ligand 1

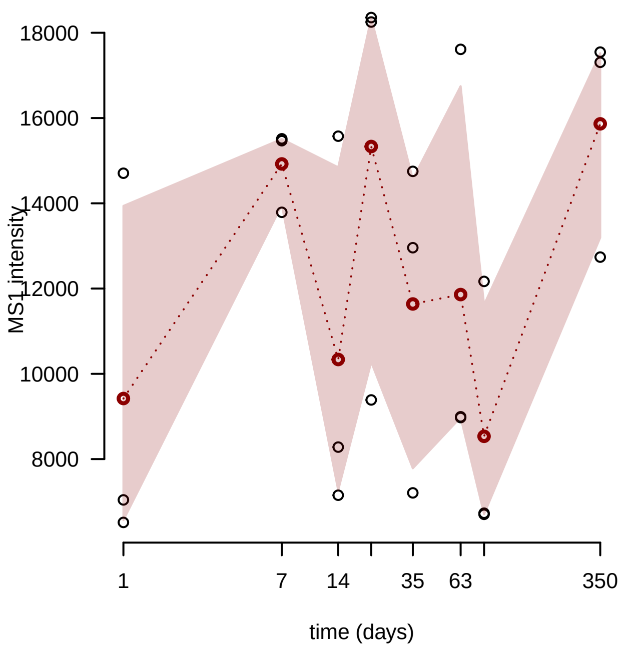

O35206
GN=Col15a1
collagen, type XV, alpha 1

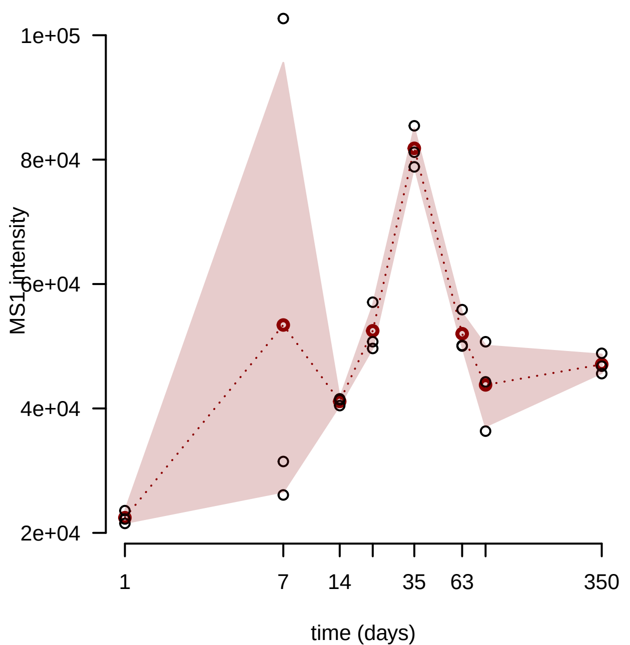

O35207
GN=Cdk2ap1
CDK2 (cyclin−dependent kinase 2)−associa ...

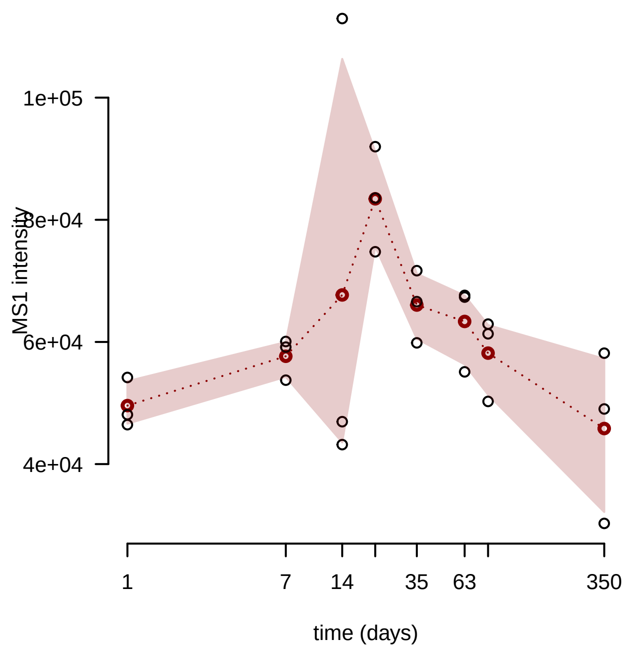

O35215
GN=Ddt
D−dopachrome tautomerase

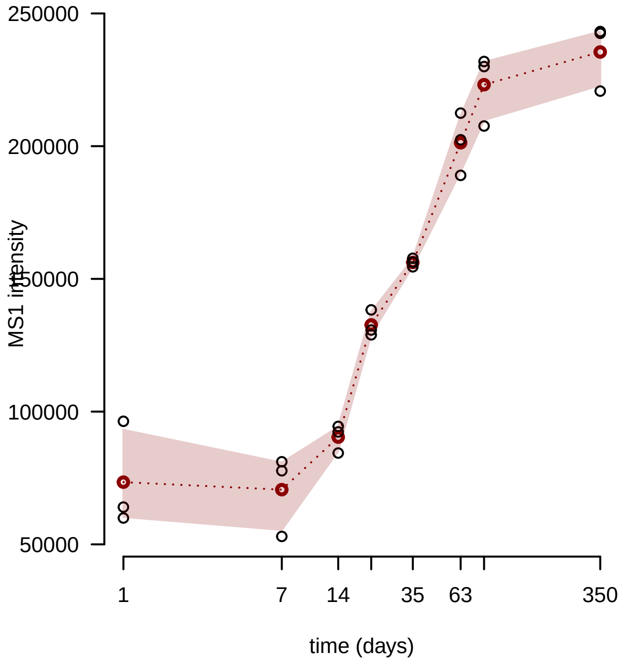

O35216
GN=Cenpa
centromere protein A

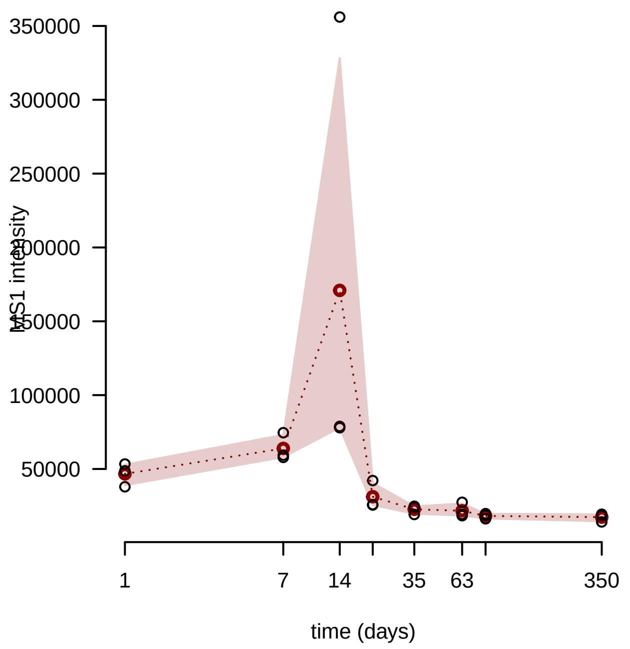

O35218
GN=Cpsf2
cleavage and polyadenylation specific fa ...

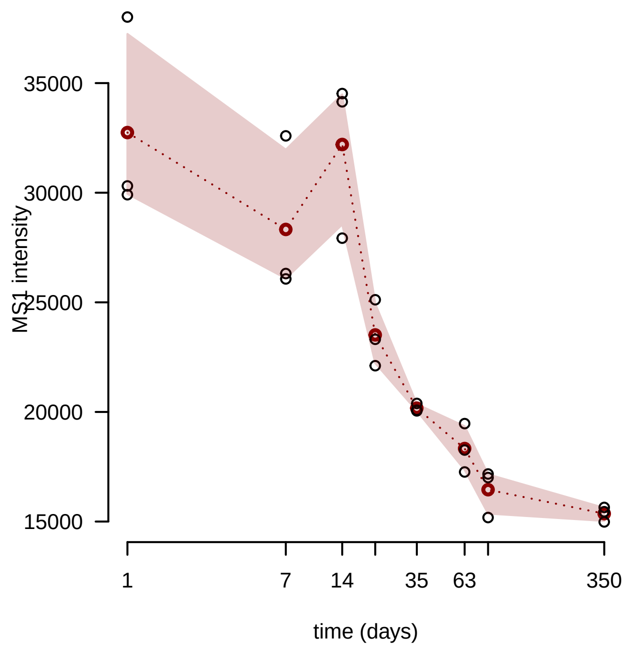

O35226
GN=Psmd4
proteasome (prosome, macropain) 26S subu ...

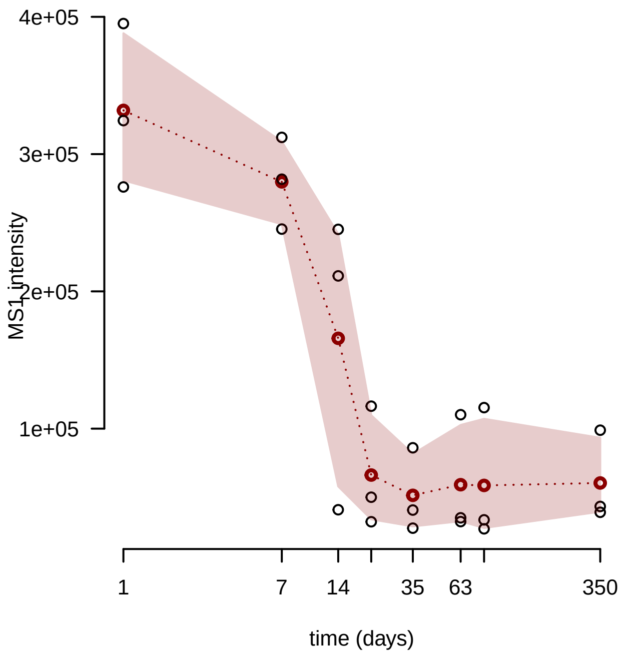

O35226
GN=Psmd4
proteasome (prosome, macropain) 26S subu ...

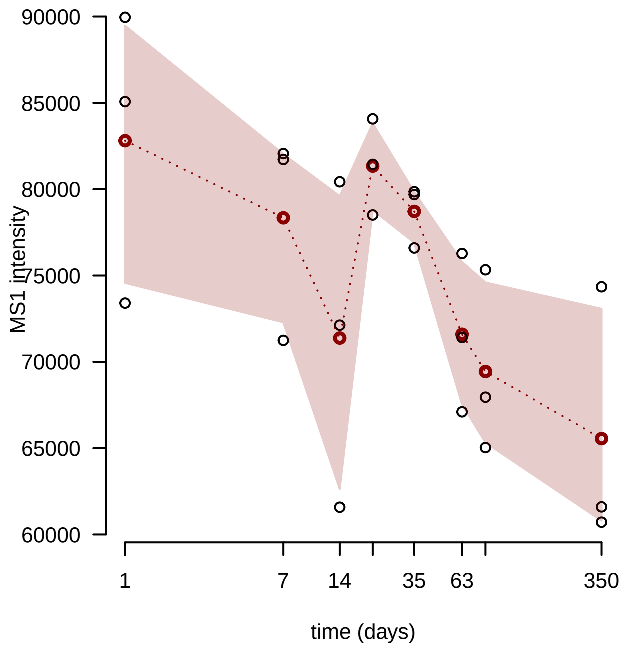

O35231
GN=Kifc3
kinesin family member C3

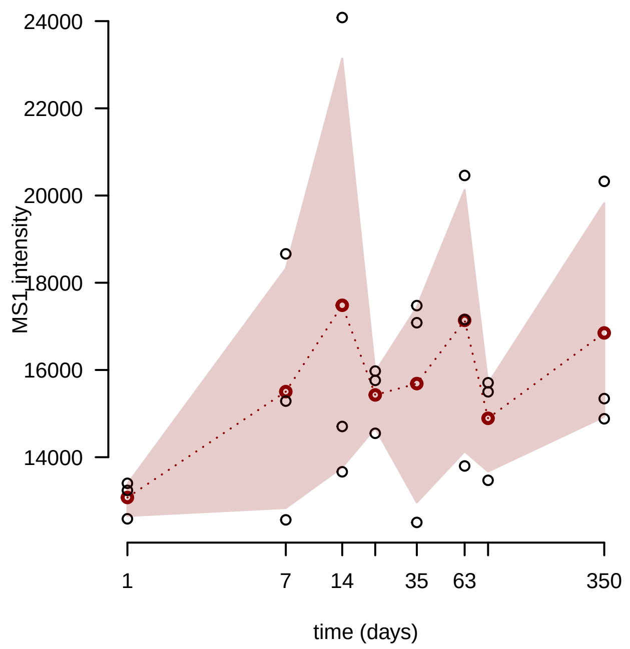

O35239
GN=Ptpn9
protein tyrosine phosphatase, non−recept ...

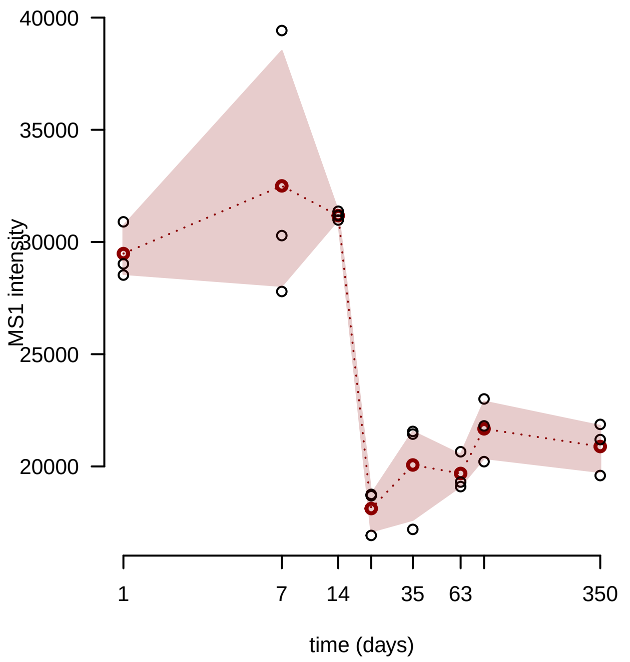

O35242
GN=Nsmaf
neutral sphingomyelinase (N−SMase) activ ...

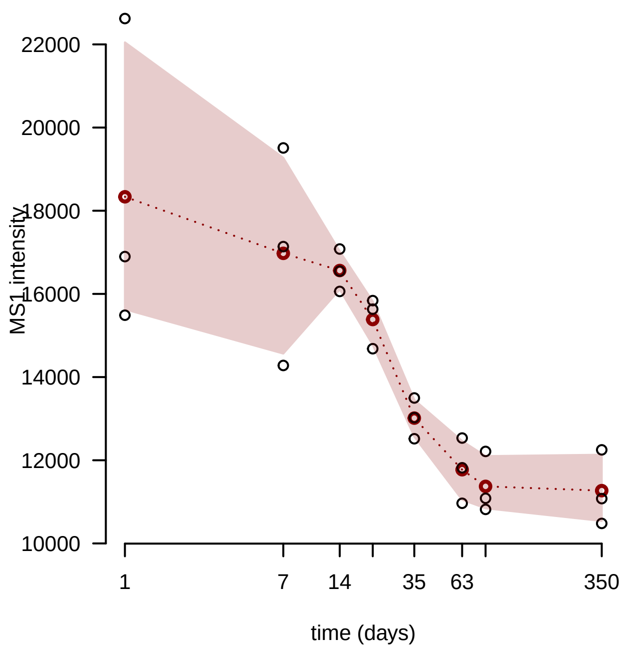

O35245
GN=Pkd2
polycystin 2, transient receptor potenti ...

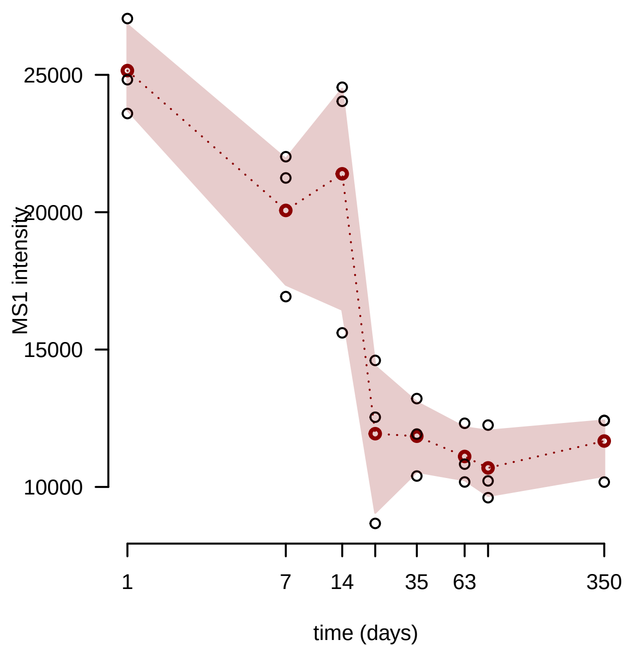

O35250
GN=Exoc7
exocyst complex component 7

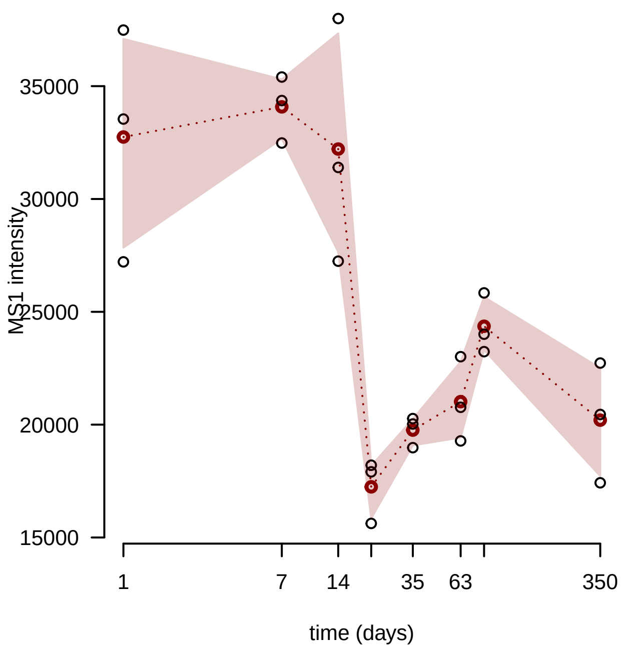

O35261
GN=E2f3
E2F transcription factor 3

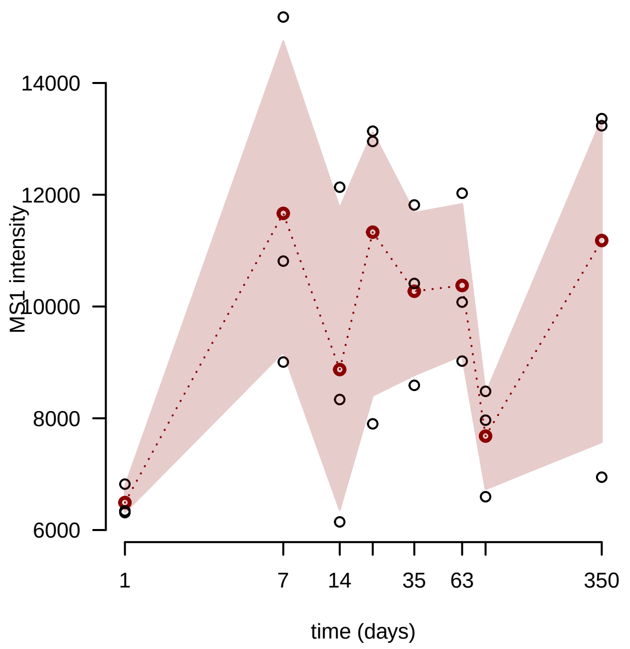

O35280
GN=Chek1
checkpoint kinase 1

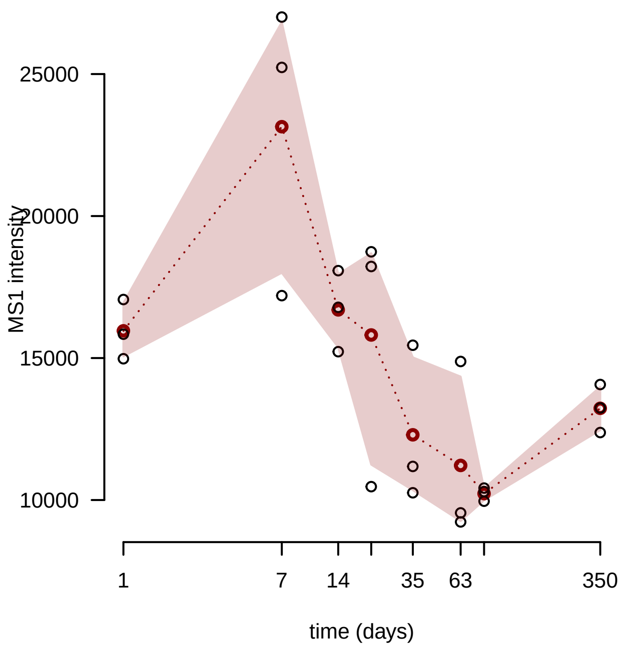

O35286
GN=Dhx15
DEAH (Asp−Glu−Ala−His) box polypeptide 1 ...

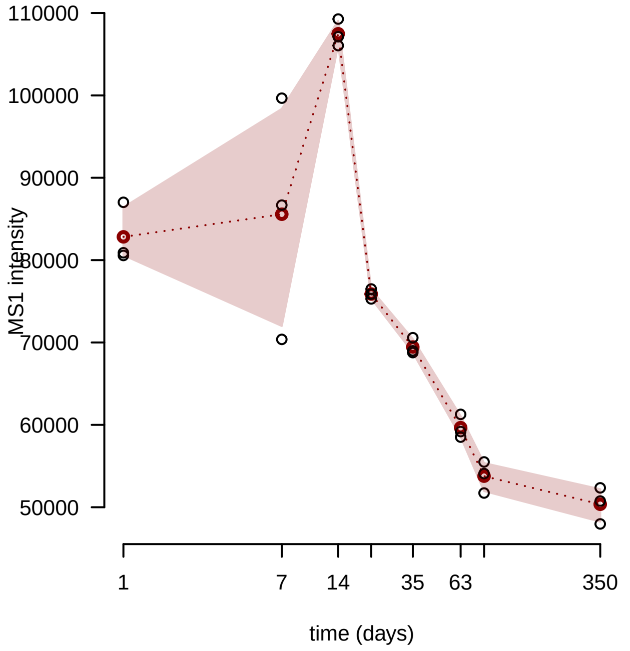

O35295
GN=Purb
purine rich element binding protein B

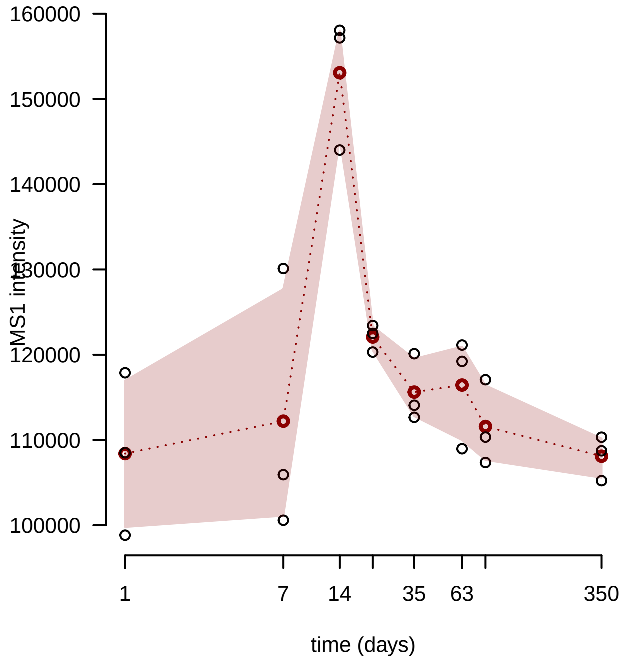

O35309
GN=Nmi
N−myc (and STAT) interactor

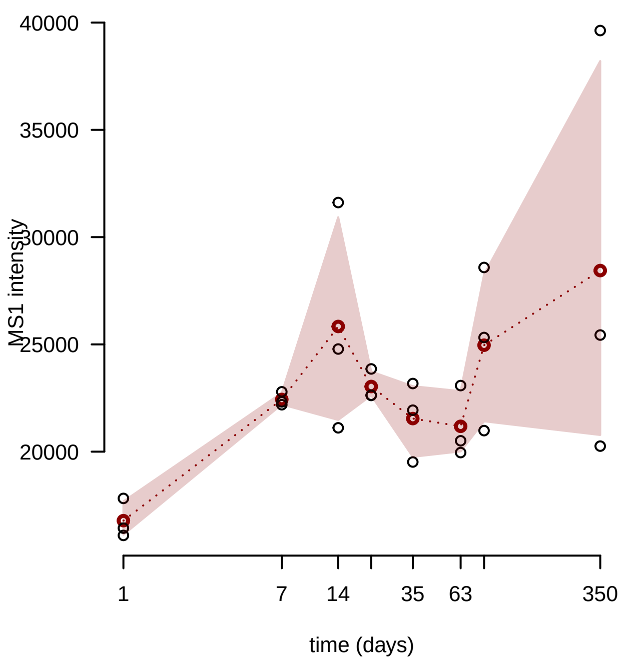

O35316
GN=Slc6a6
solute carrier family 6 (neurotransmitte ...

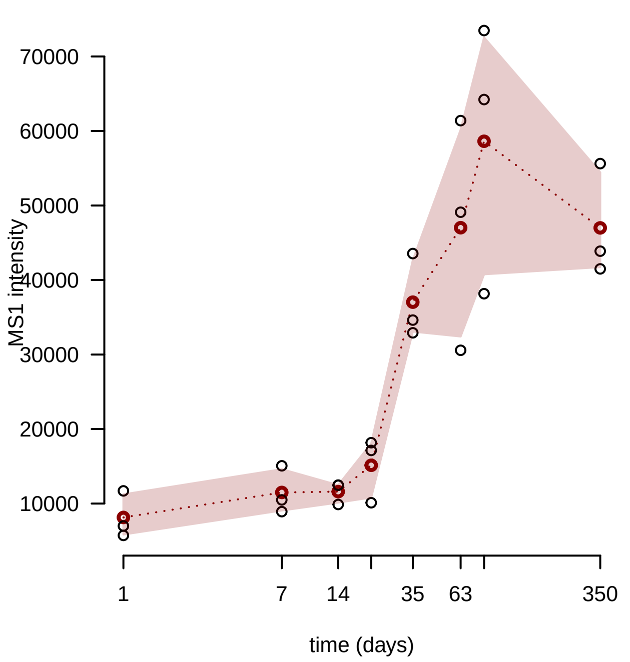

O35317
GN=Pbx3
pre B cell leukemia homeobox 3

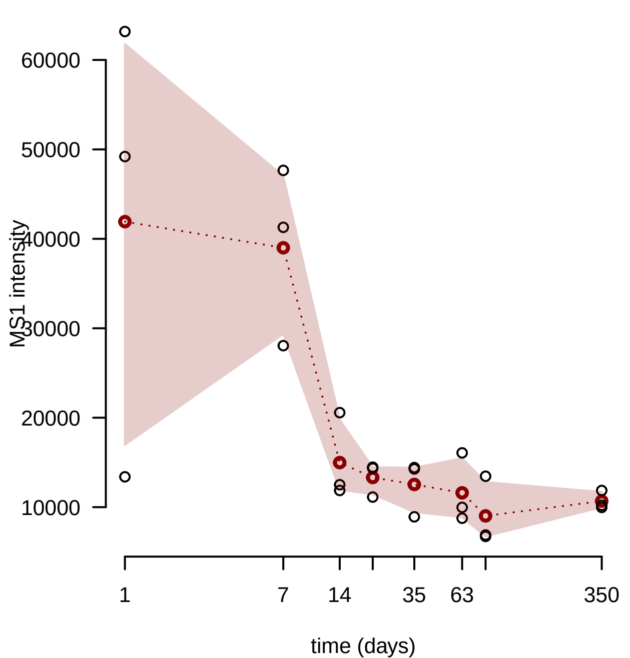

O35326
GN=Srsf5
serine and arginine−rich splicing factor ...

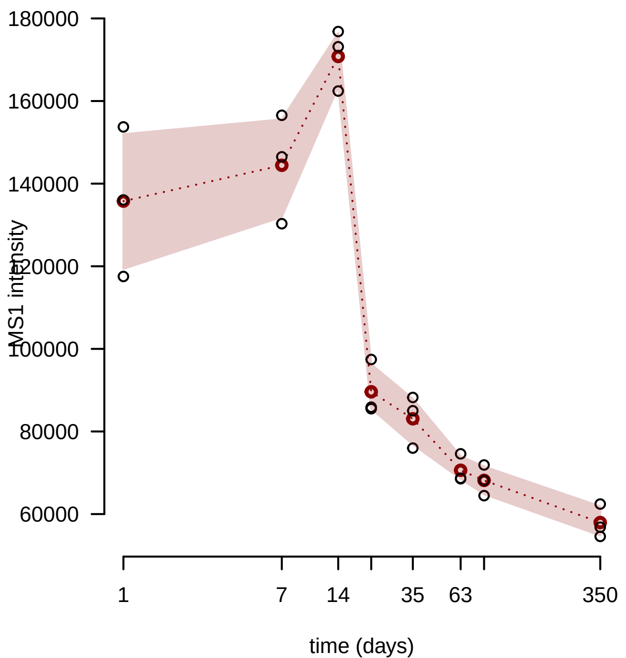

O35343
GN=Kpna4
karyopherin (importin) alpha 4

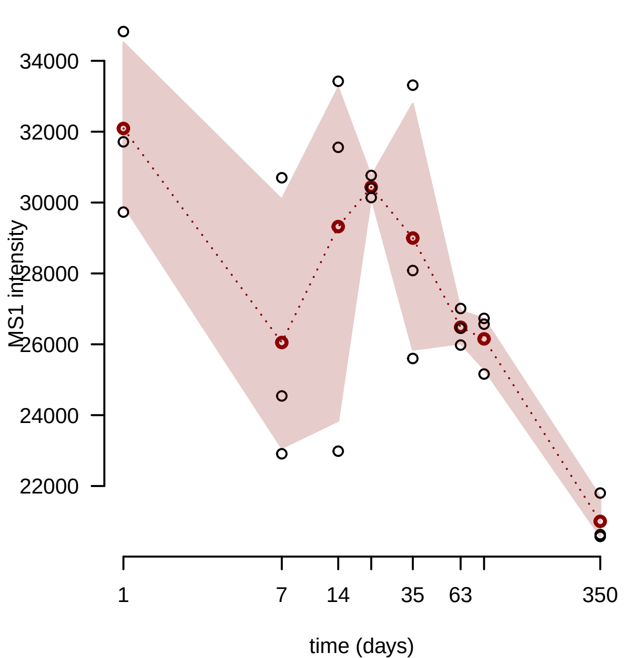

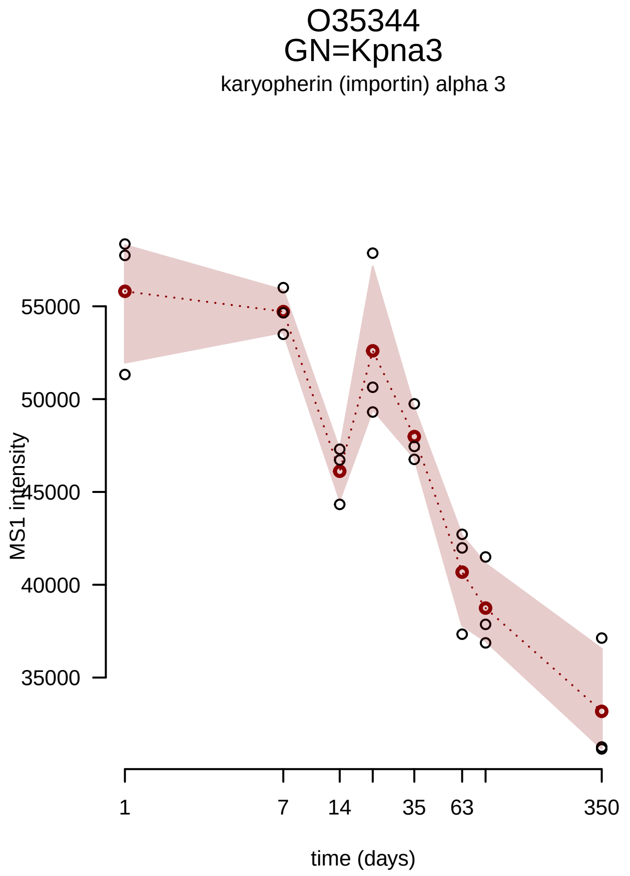
O35344
GN=Kpna3
karyopherin (importin) alpha 3
55000
50000
45000
40000
35000
MS1 intensity
1
7
14
35
63
350
time (days)

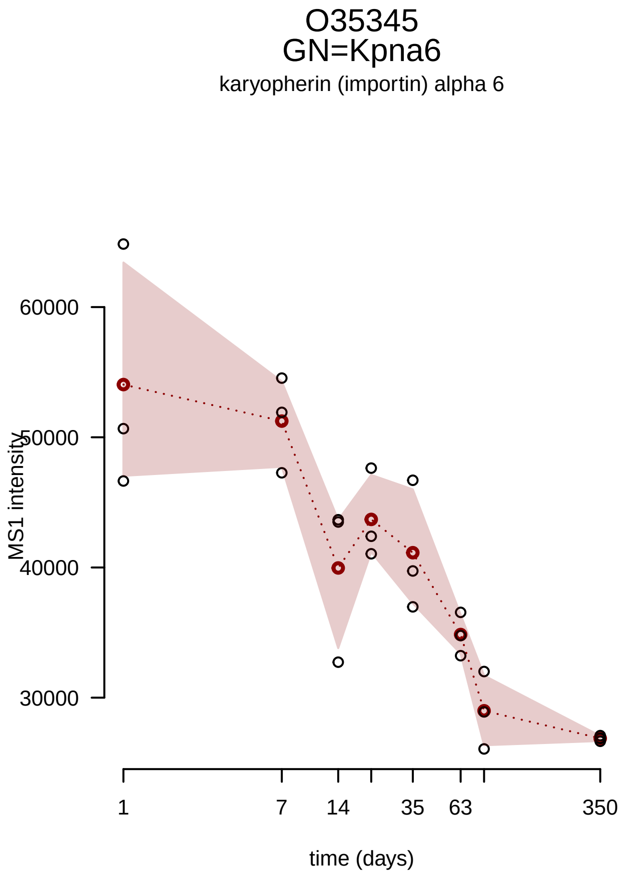
O35345
GN=Kpna6
karyopherin (importin) alpha 6
60000
50000
40000
30000
MS1 intensity
1
7
14
35
63
350
time (days)

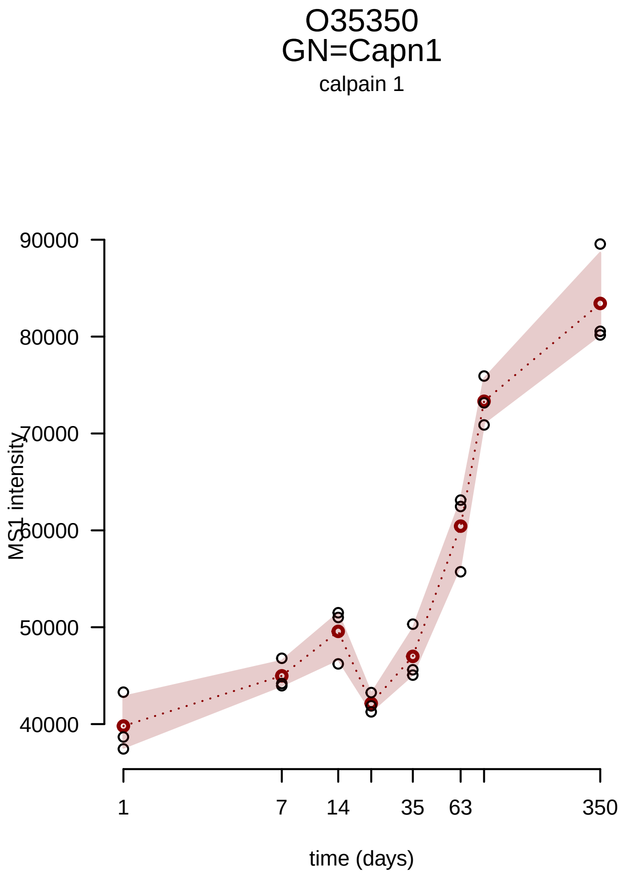
O35350
GN=Capn1
calpain 1
90000
80000
70000
60000
50000
40000
MS1 intensity
1
7
14
35
63
350
time (days)

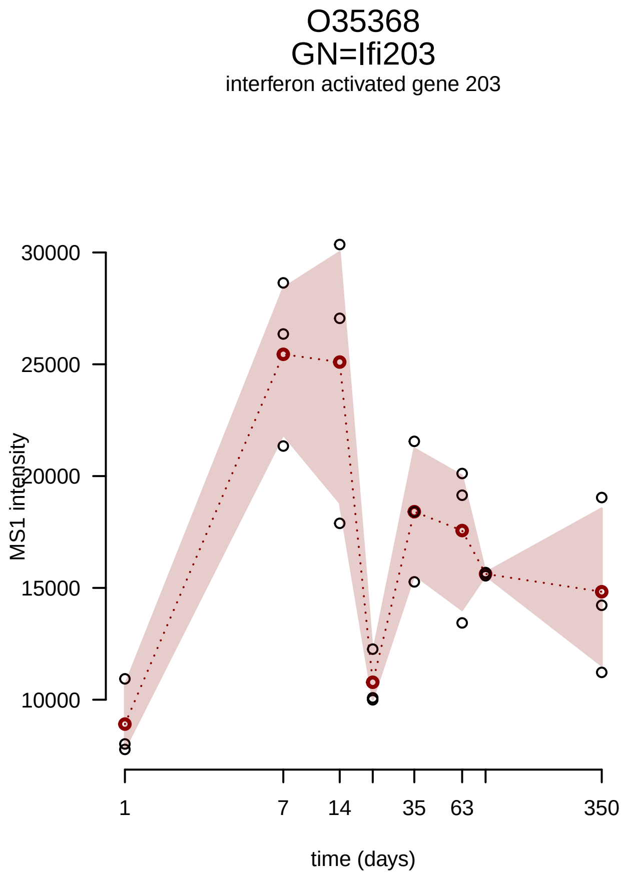
O35368
GN=Ifi203
interferon activated gene 203
30000
25000
20000
15000
10000
MS1 intensity
1
7
14
35
63
350
time (days)

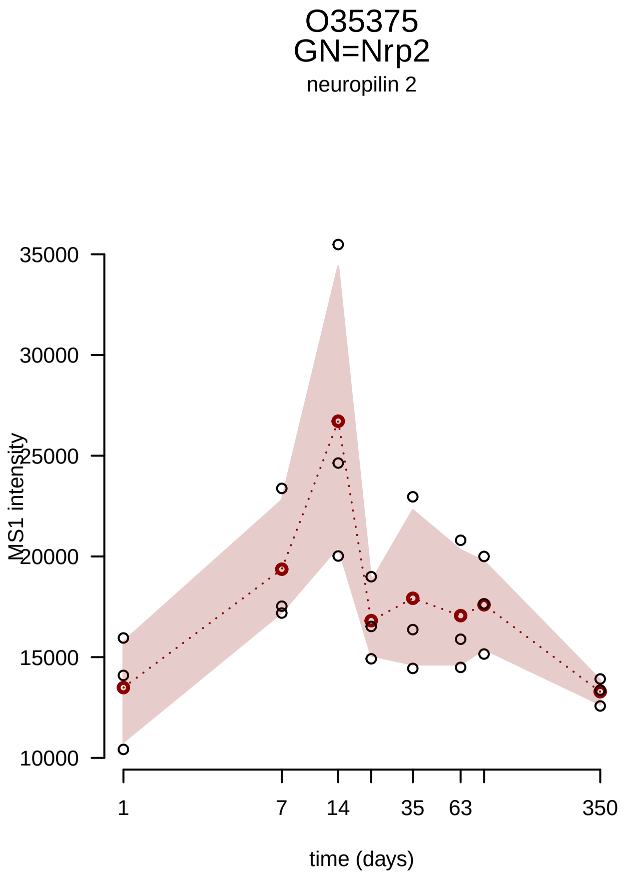
O35375
GN=Nrp2
neuropilin 2
35000
30000
25000
20000
15000
10000
MS1 intensity
1
7
14
35
63
350
time (days)

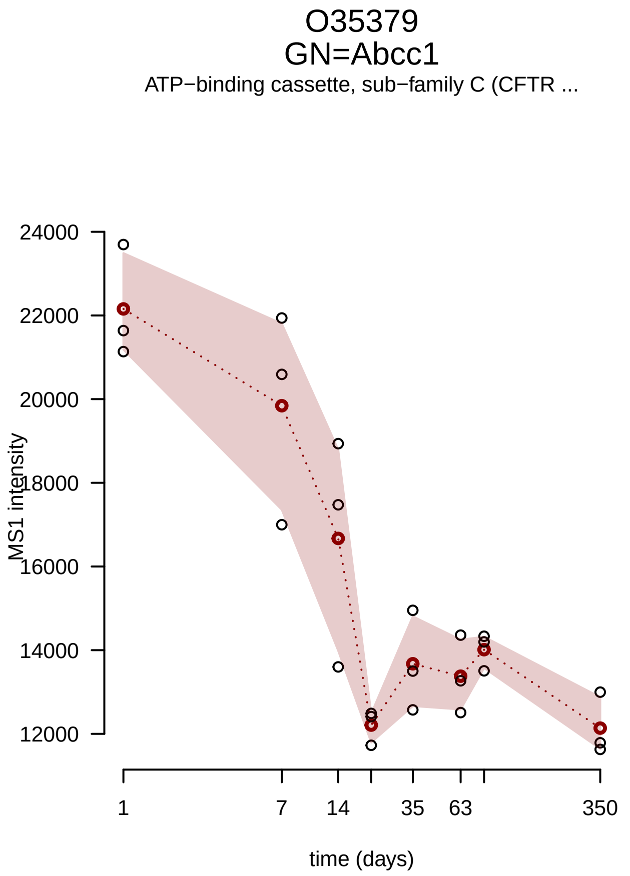
O35379
GN=Abcc1
ATP−binding cassette, sub−family C (CFTR ...
24000
22000
20000
18000
16000
14000
12000
MS1 intensity
1
7
14
35
63
350
time (days)

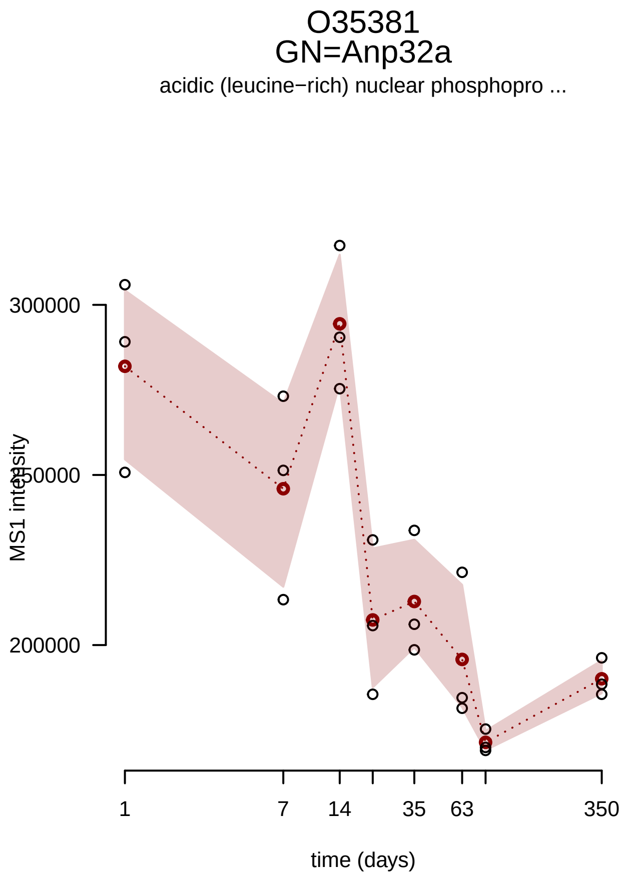
O35381
GN=Anp32a
acidic (leucine−rich) nuclear phosphopro ...
300000
250000
200000
MS1 intensity
1
7
14
35
63
350
time (days)

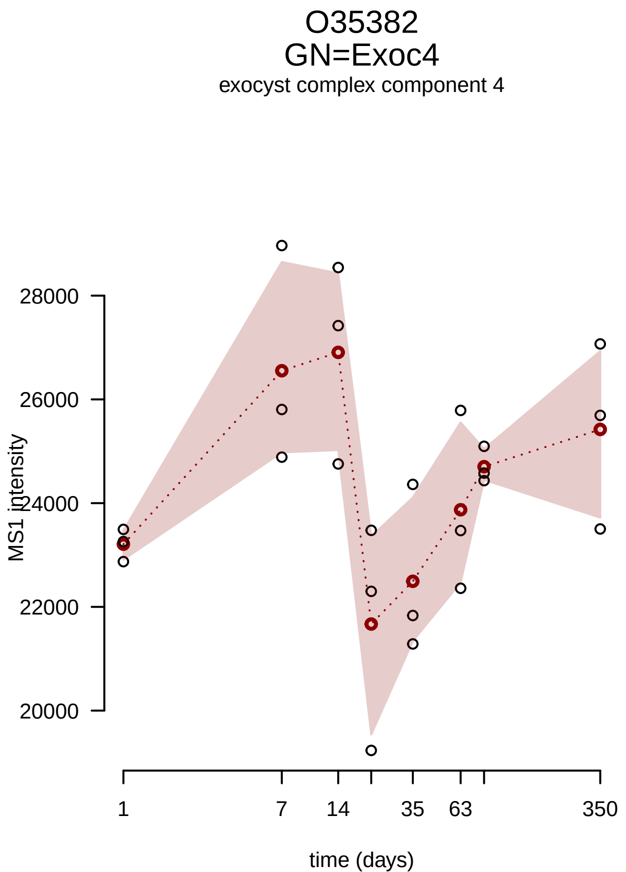
O35382
GN=Exoc4
exocyst complex component 4
28000
26000
24000
22000
20000
MS1 intensity
1
7
14
35
63
350
time (days)

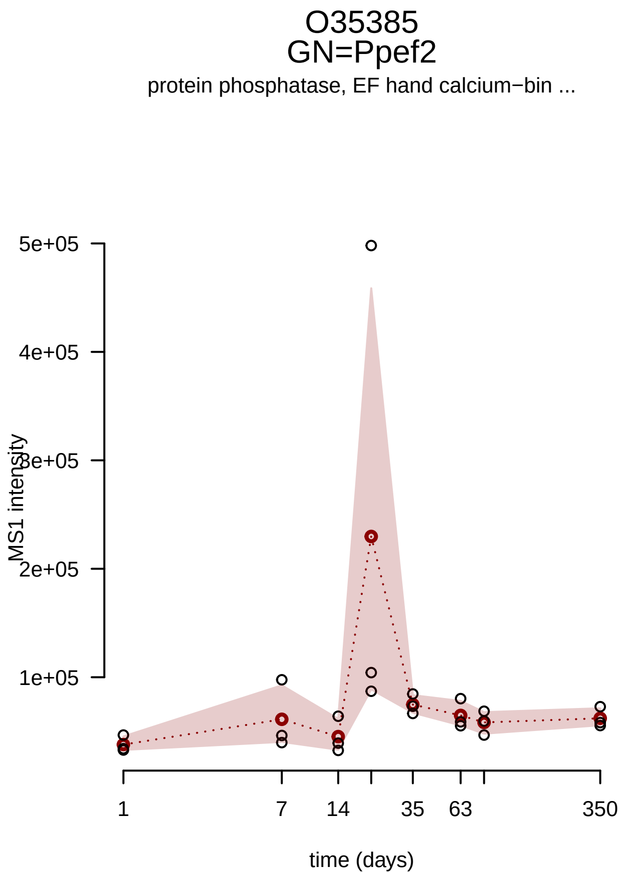
O35385
GN=Ppef2
protein phosphatase, EF hand calcium−bin ...
5e+05
4e+05
3e+05
2e+05
1e+05
MS1 intensity
1
7
14
35
63
350
time (days)

O35386
GN=Phyh
phytanoyl−CoA hydroxylase

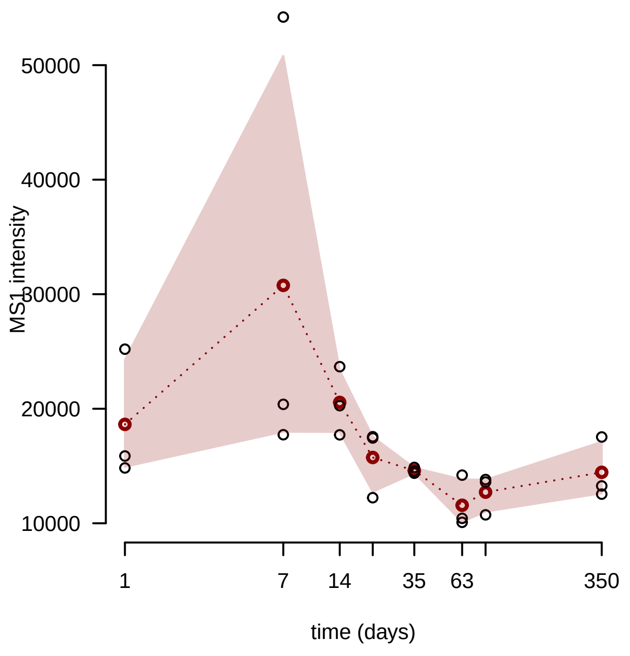

O35387
GN=Hax1
HCLS1 associated X−1

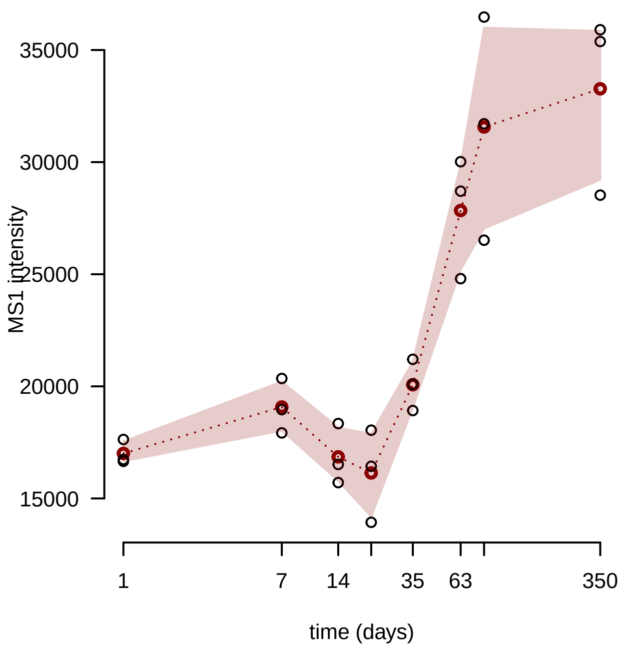

O35393
GN=Efnb3
ephrin B3

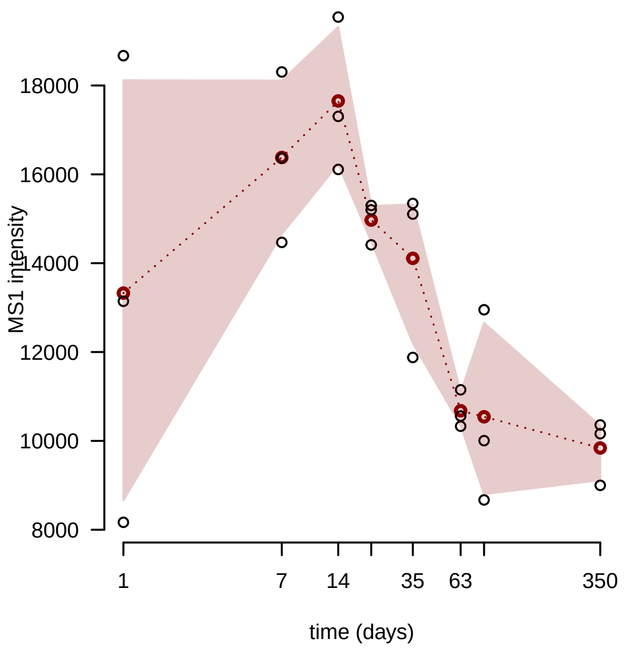

O35405
GN=Pld3
phospholipase D family, member 3

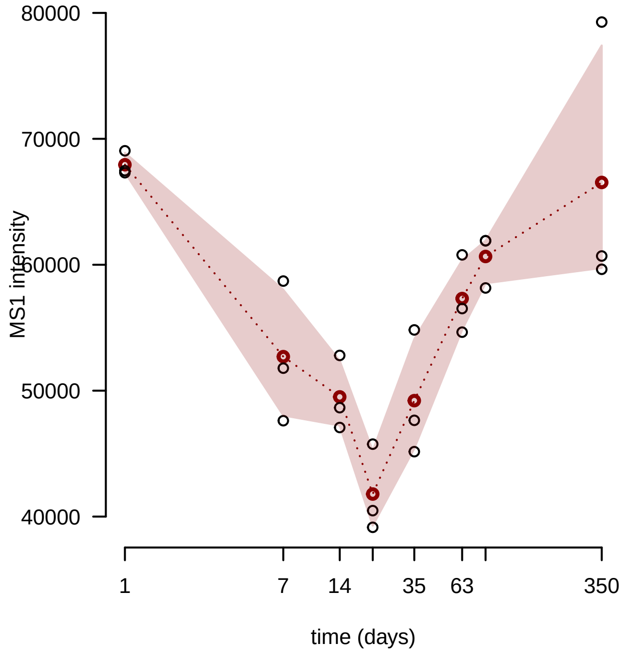

O35425
GN=Bok
BCL2−related ovarian killer

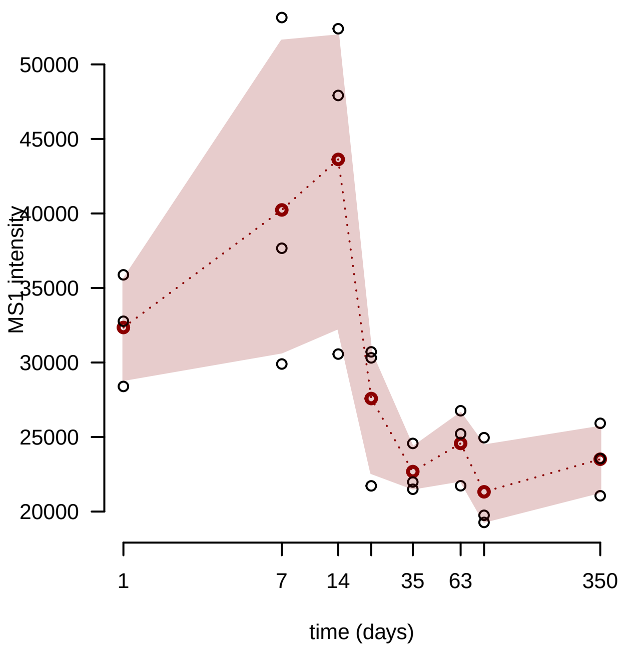

O35427
GN=Crcp
calcitonin gene−related peptide−receptor ...

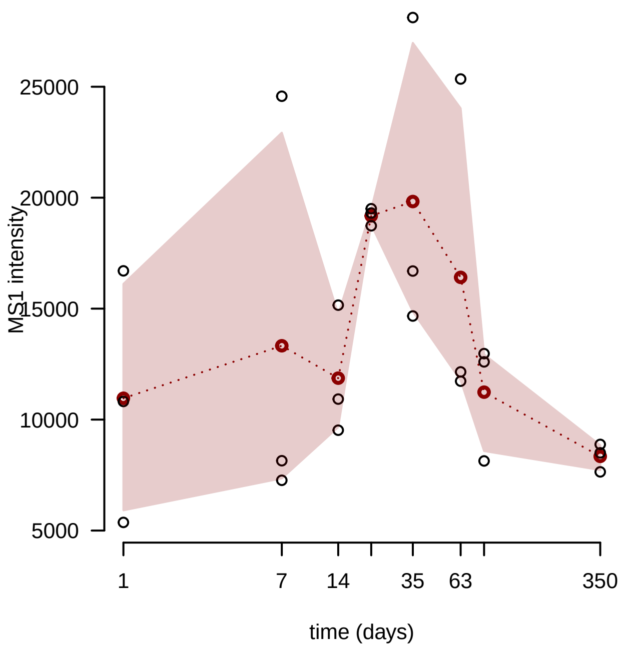

O35435
GN=Dhodh
dihydroorotate dehydrogenase

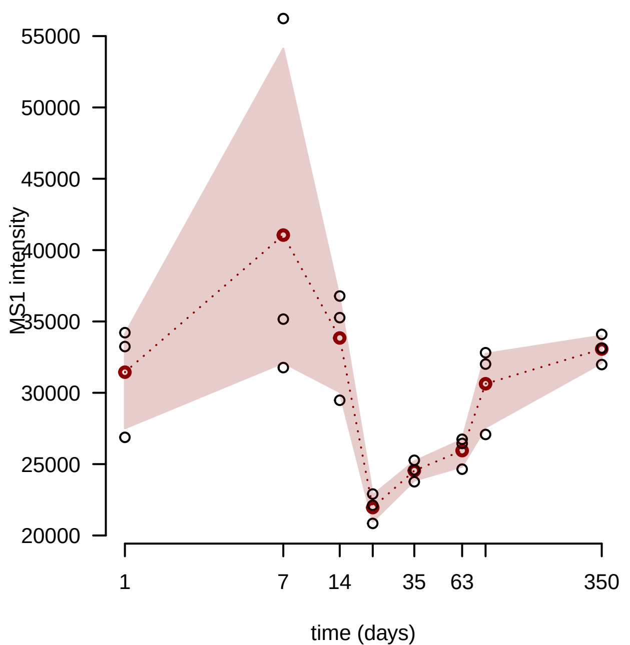

O35437
GN=Tcf21
transcription factor 21

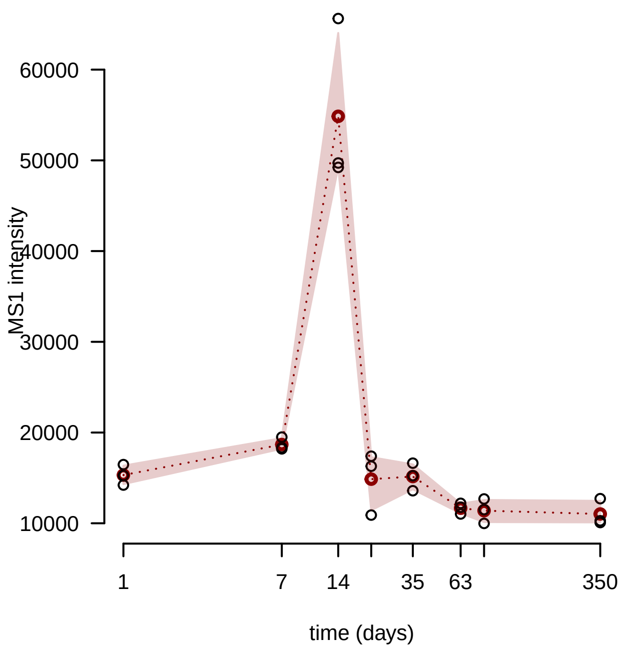

O35445
GN=Rnf5
ring finger protein 5

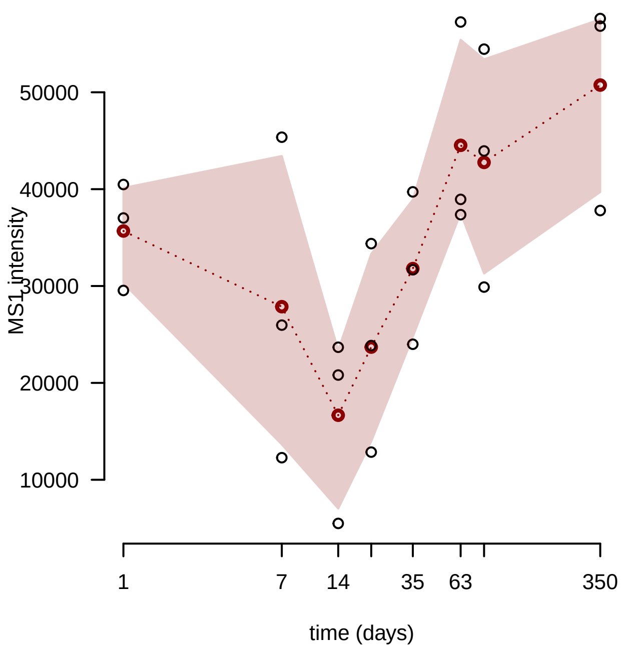

O35448
GN=Ppt2
palmitoyl−protein thioesterase 2

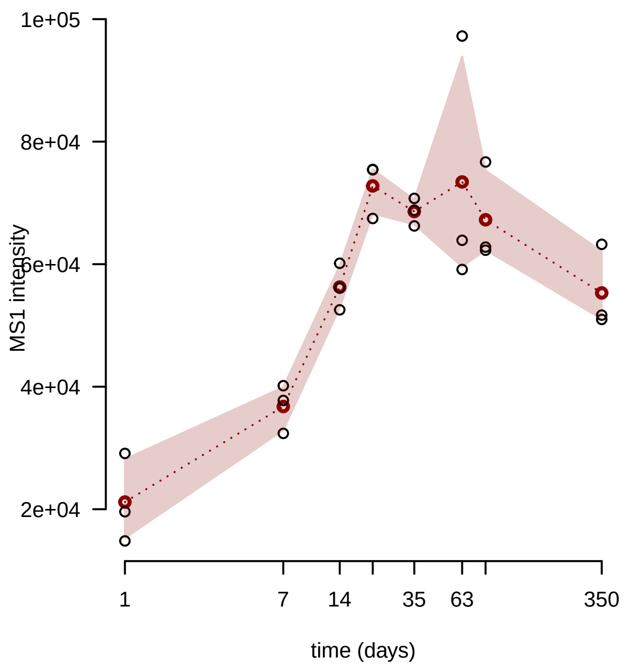

O35450
GN=Fkbpl
FK506 binding protein−like

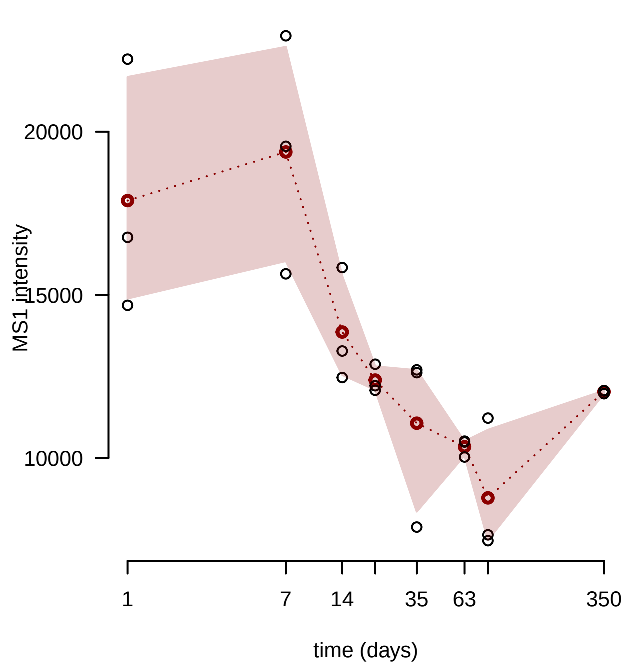

O35451
GN=Atf6b
activating transcription factor 6 beta

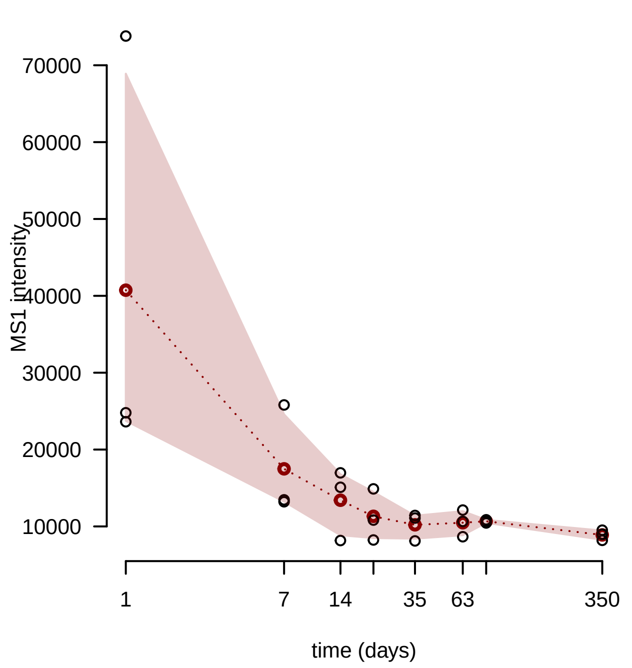

O35454
GN=Clcn6
chloride channel, voltage−sensitive 6

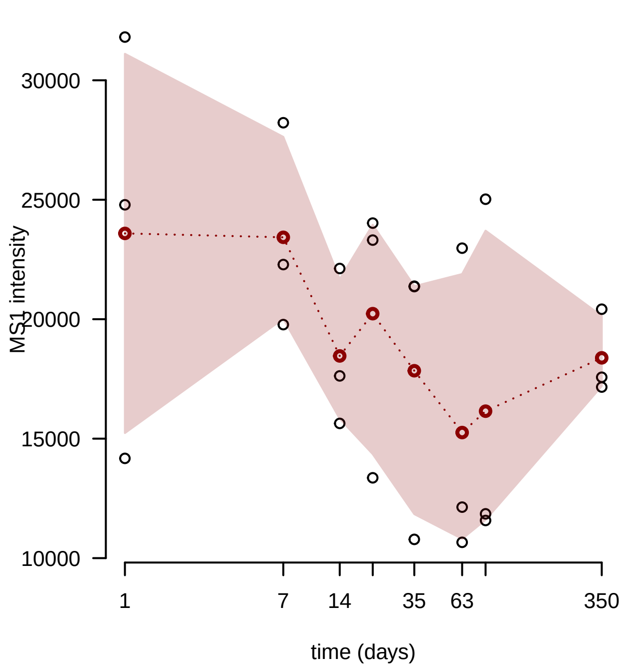

O35459
GN=Ech1
enoyl coenzyme A hydratase 1, peroxisoma ...

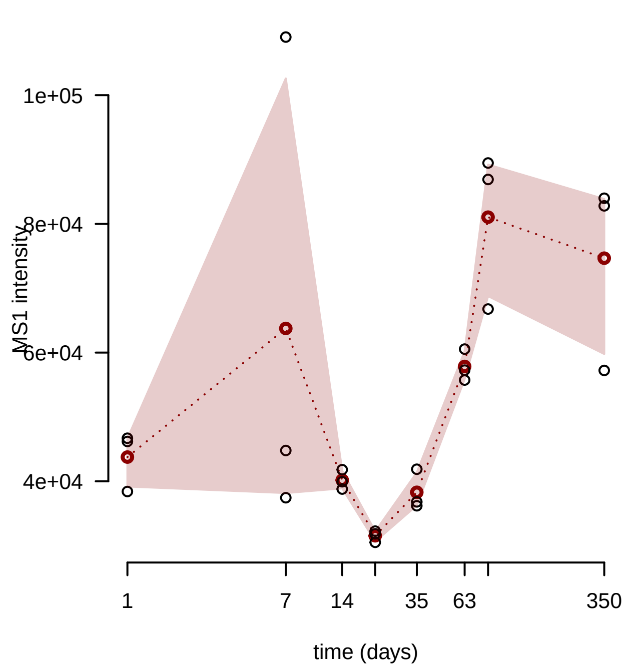

O35465
GN=Fkbp8
FK506 binding protein 8

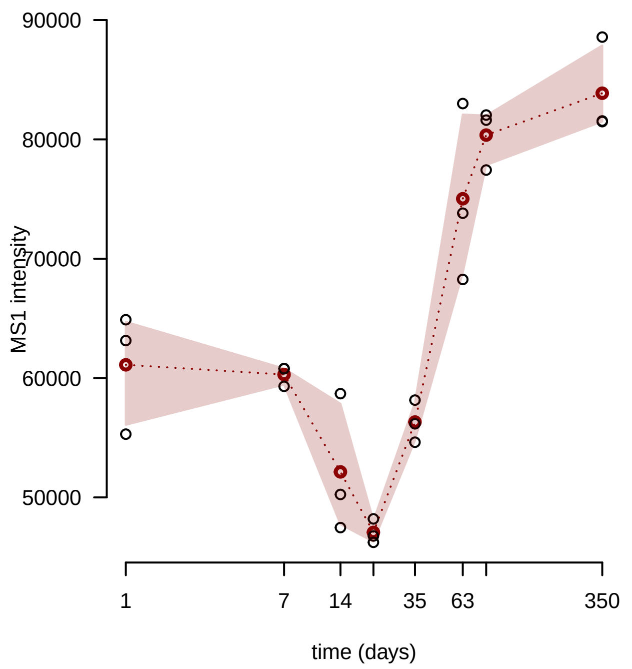

O35469
GN=Hsd3b6
hydroxy−delta−5−steroid dehydrogenase, 3 ...

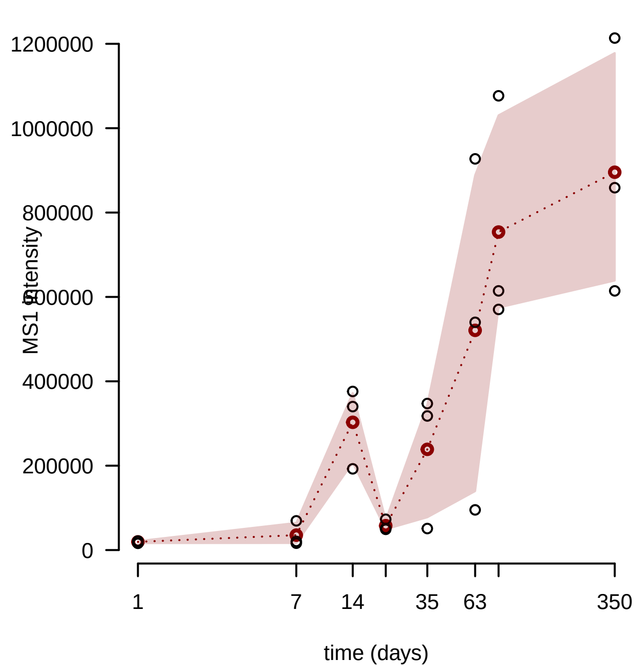

O35473
GN=C1d
C1D nuclear receptor co−repressor

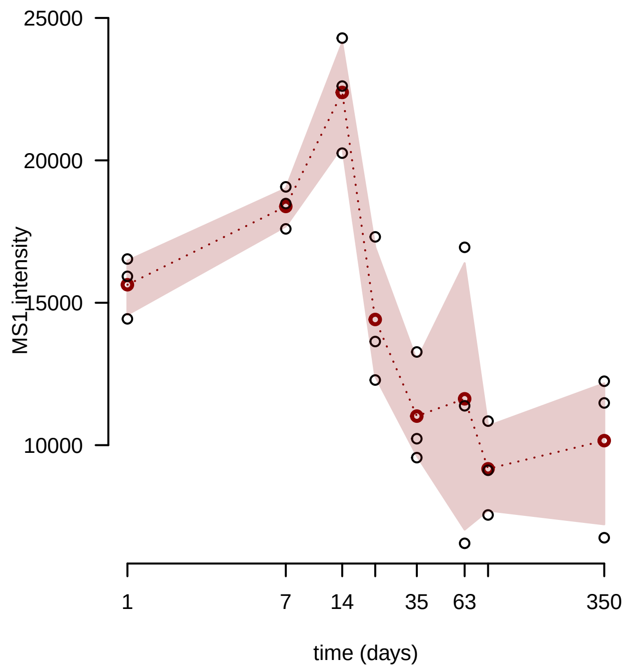

O35488
GN=Slc27a2
solute carrier family 27 (fatty acid tra ...

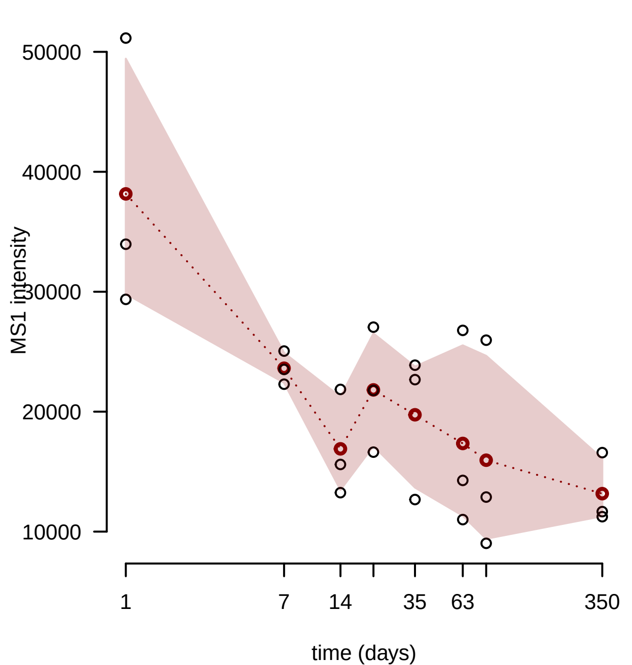

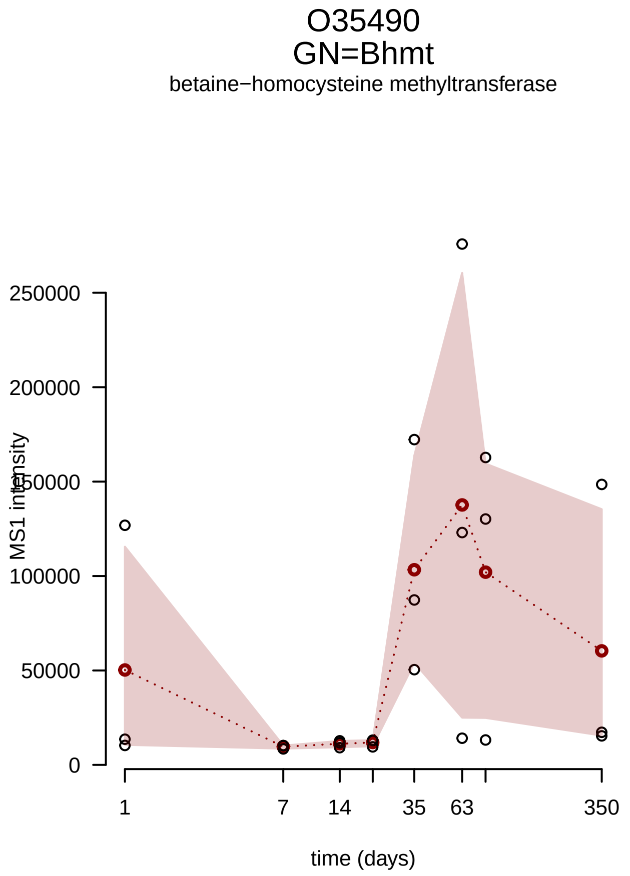
O35490
GN=Bhmt
betaine−homocysteine methyltransferase
MS1 intensity
0
50000
100000
150000
200000
250000
1
7
14
35
63
350
time (days)

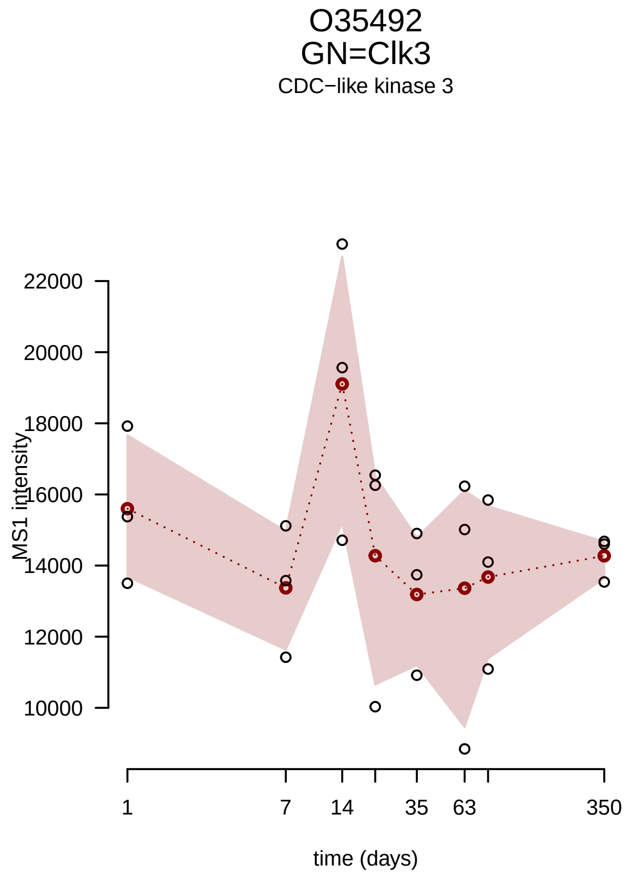
O35492
GN=Clk3
CDC−like kinase 3
MS1 intensity
10000
12000
14000
16000
18000
20000
22000
1
7
14
35
63
350
time (days)

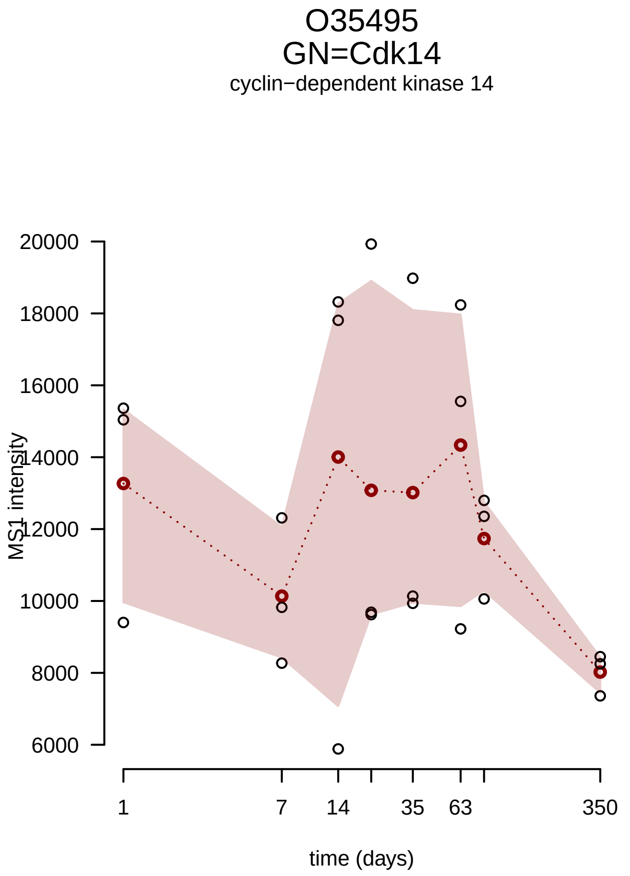
O35495
GN=Cdk14
cyclin−dependent kinase 14
MS1 intensity
6000
8000
10000
12000
14000
16000
18000
20000
1
7
14
35
63
350
time (days)

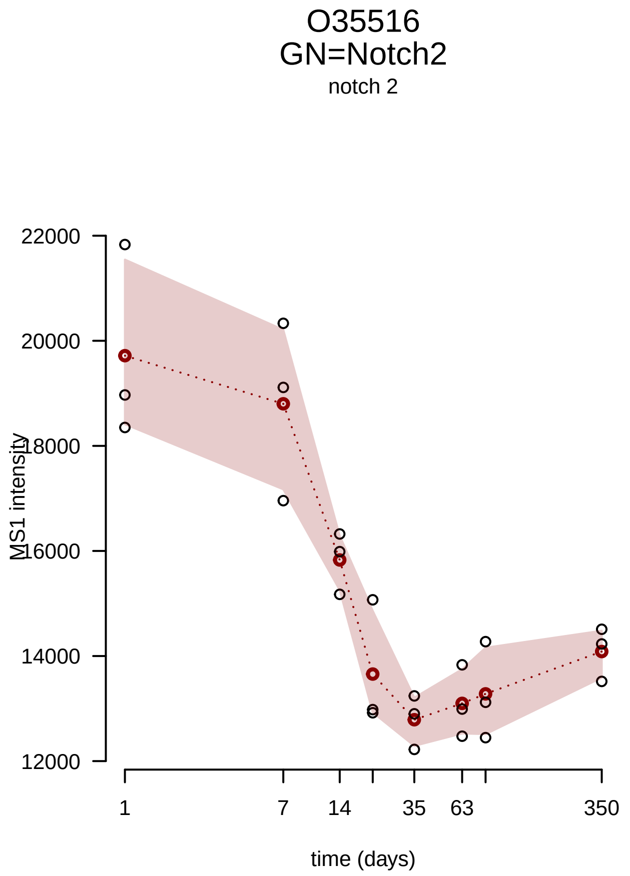
O35516
GN=Notch2
notch 2
MS1 intensity
12000
14000
16000
18000
20000
22000
1
7
14
35
63
350
time (days)

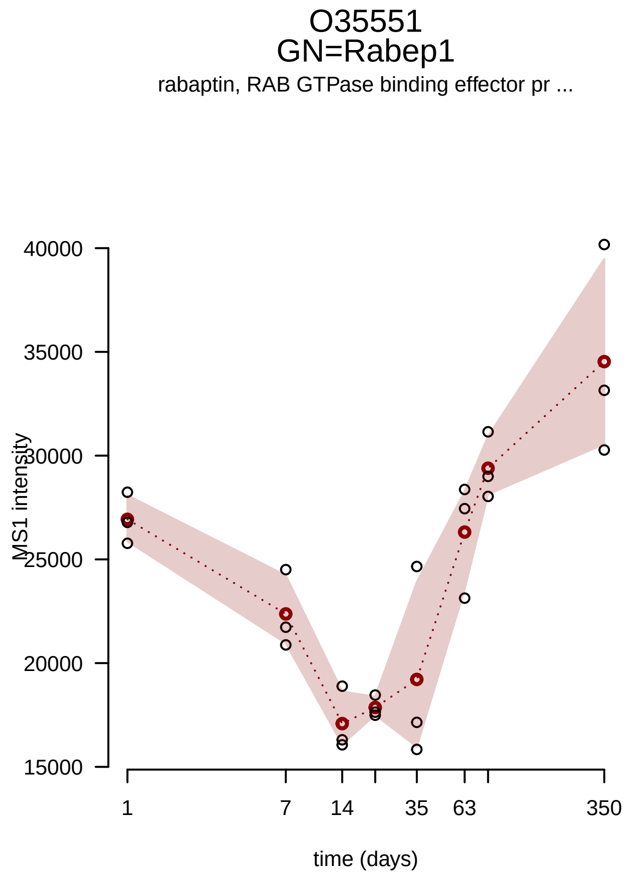
O35551
GN=Rabep1
rabaptin, RAB GTPase binding effector pr ...
MS1 intensity
15000
20000
25000
30000
35000
40000
1
7
14
35
63
350
time (days)

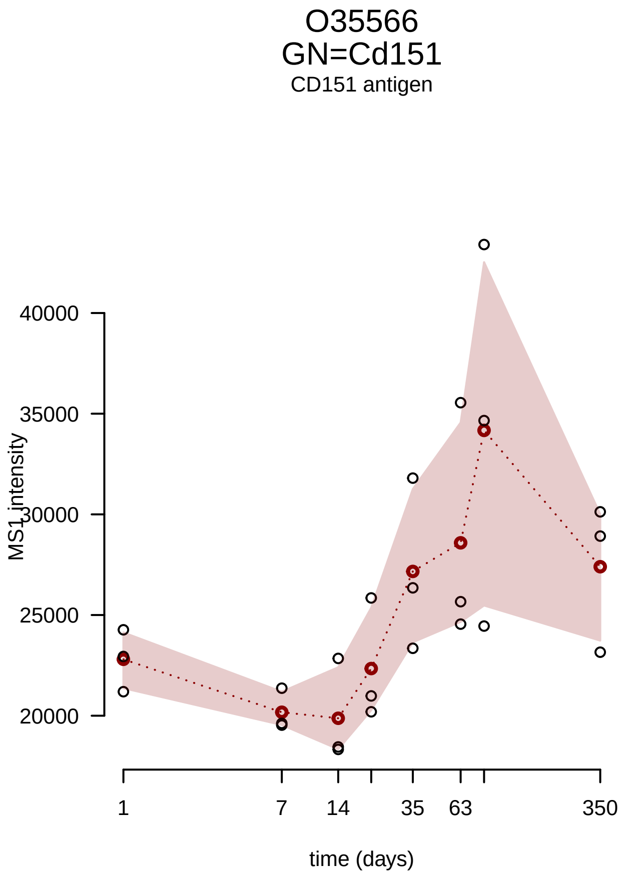
O35566
GN=Cd151
CD151 antigen
MS1 intensity
20000
25000
30000
35000
40000
1
7
14
35
63
350
time (days)

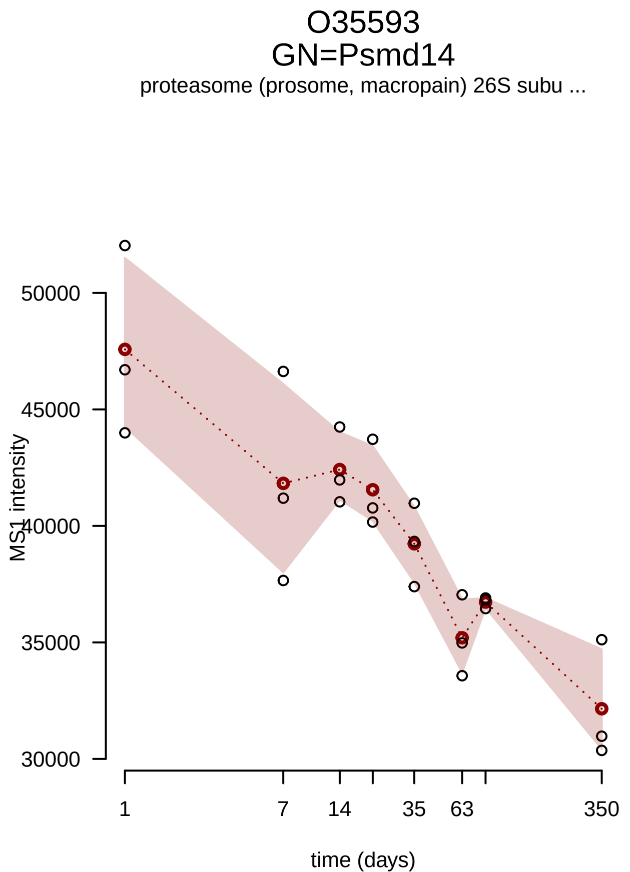
O35593
GN=Psmd14
proteasome (prosome, macropain) 26S subu ...
MS1 intensity
30000
35000
40000
45000
50000
1
7
14
35
63
350
time (days)

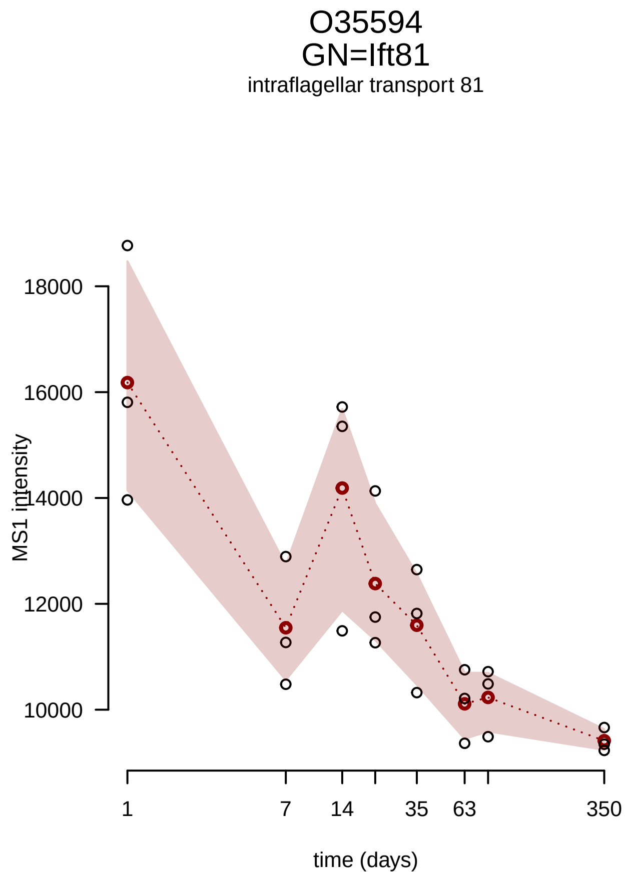
O35594
GN=Ift81
intraflagellar transport 81
MS1 intensity
10000
12000
14000
16000
18000
1
7
14
35
63
350
time (days)

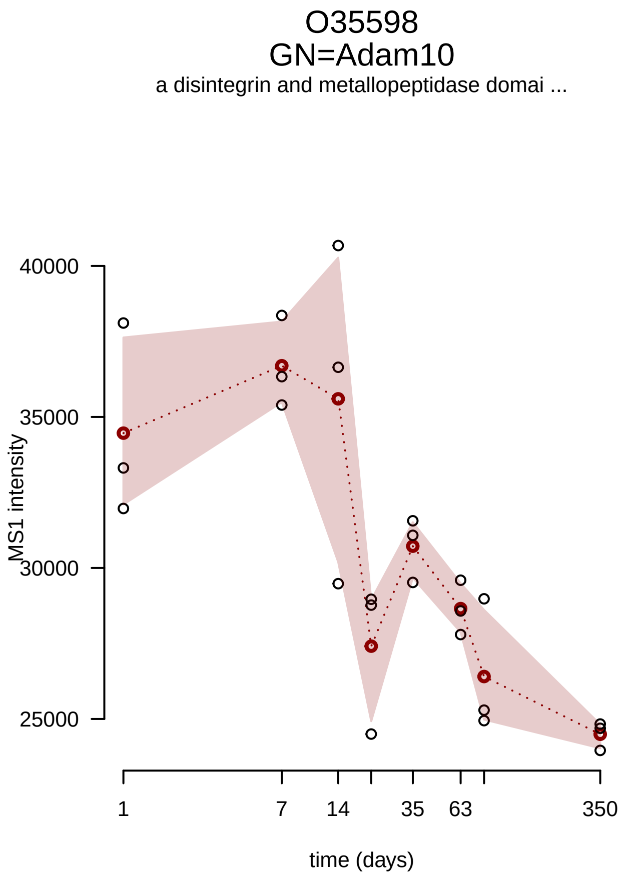
O35598
GN=Adam10
a disintegrin and metallopeptidase domai ...
MS1 intensity
25000
30000
35000
40000
1
7
14
35
63
350
time (days)

O35601
GN=Fyb1

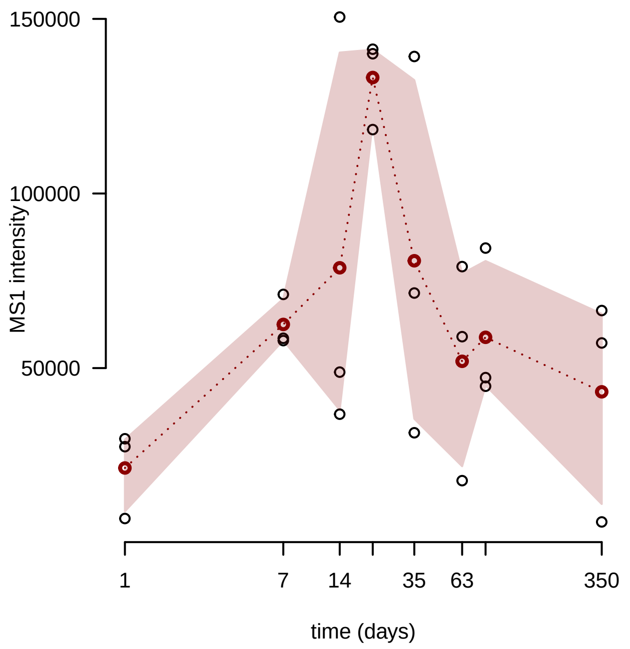

O35604
GN=Npc1
NPC intracellular cholesterol transporte ...

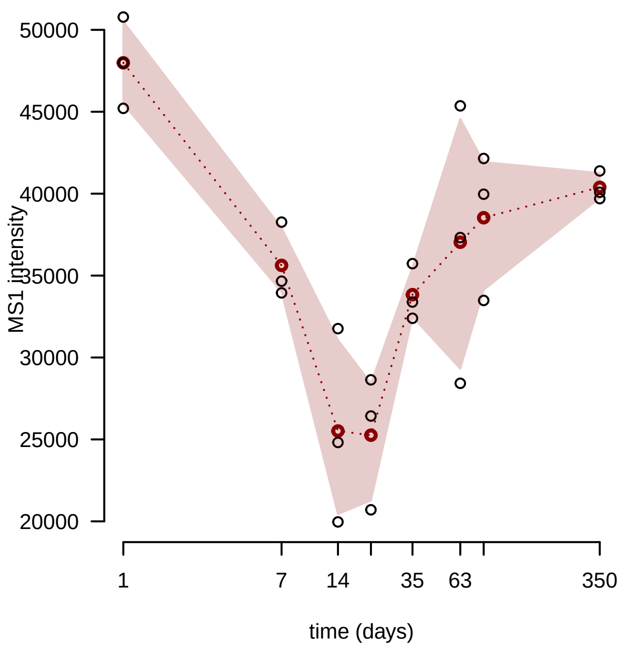

O35607
GN=Bmpr2
bone morphogenetic protein receptor, typ ...

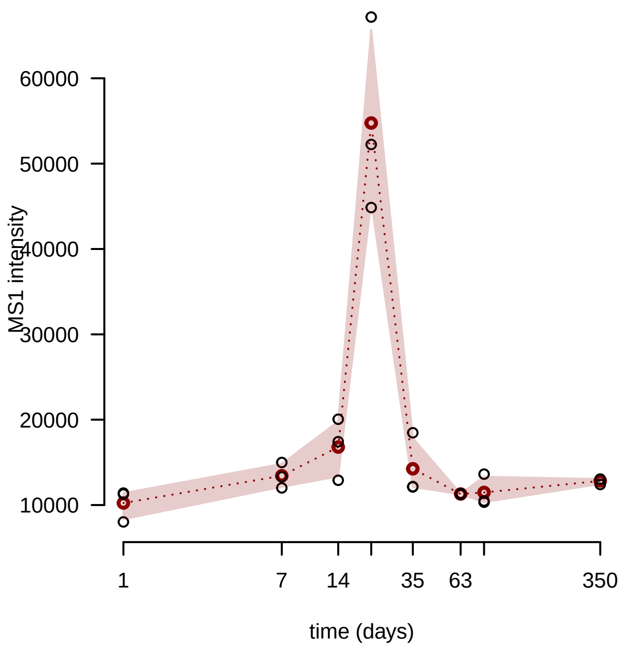

O35609
GN=Scamp3
secretory carrier membrane protein 3

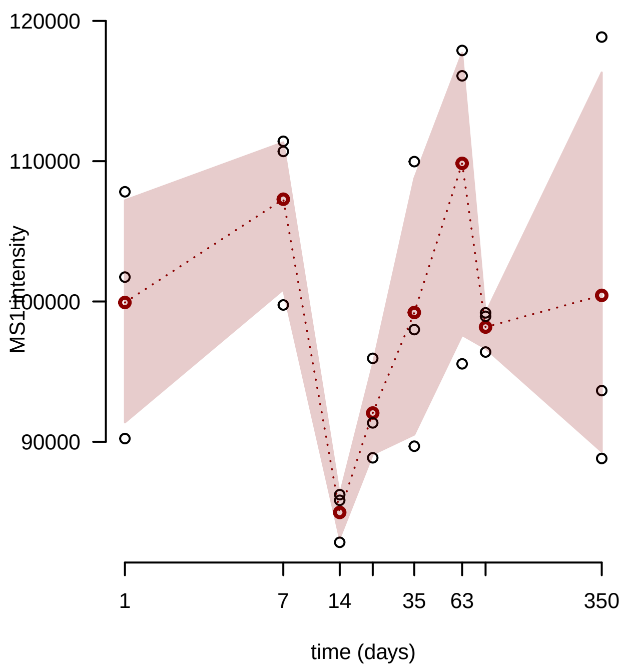

O35613
GN=Daxx
Fas death domain−associated protein

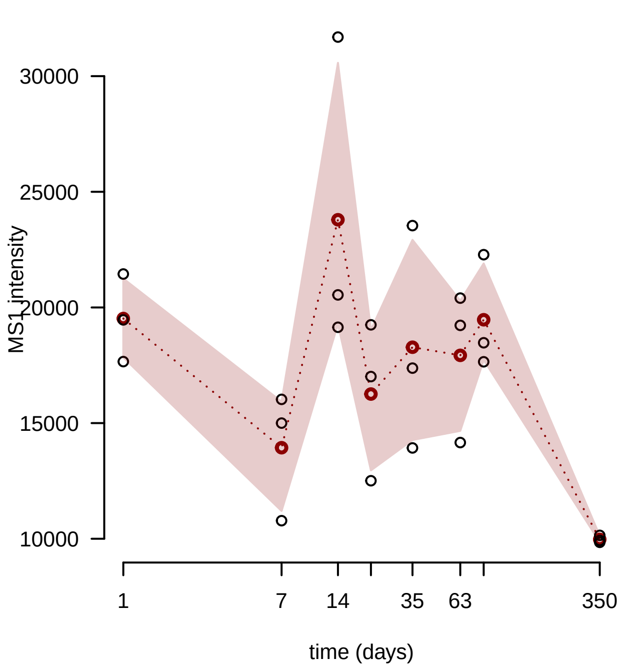

O35615
GN=Zfpm1
zinc finger protein, multitype 1

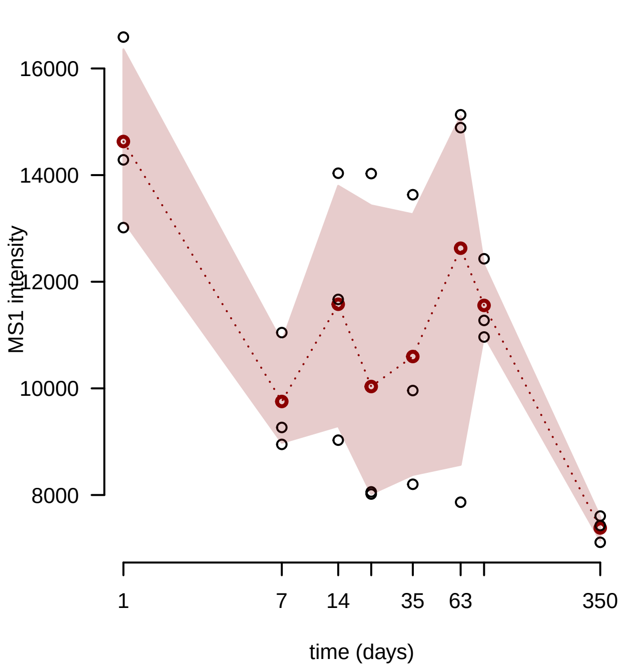

O35623
GN=Bet1
Bet1 golgi vesicular membrane traffickin ...

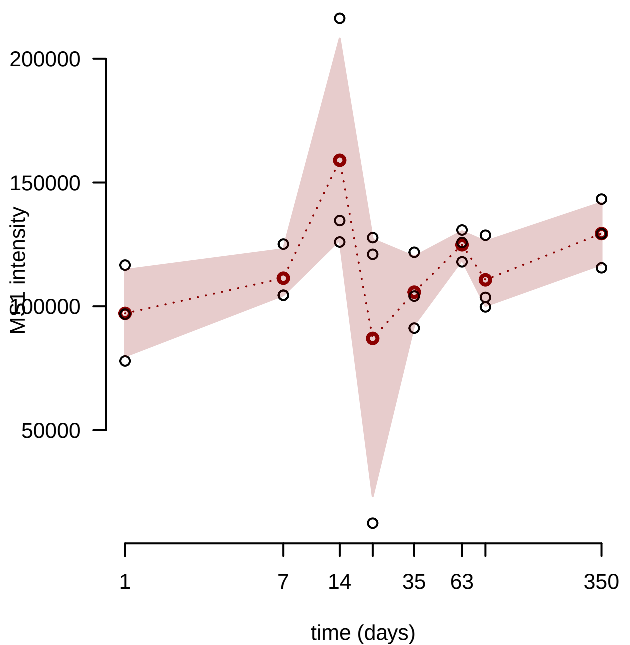

O35625
GN=Axin1
axin 1

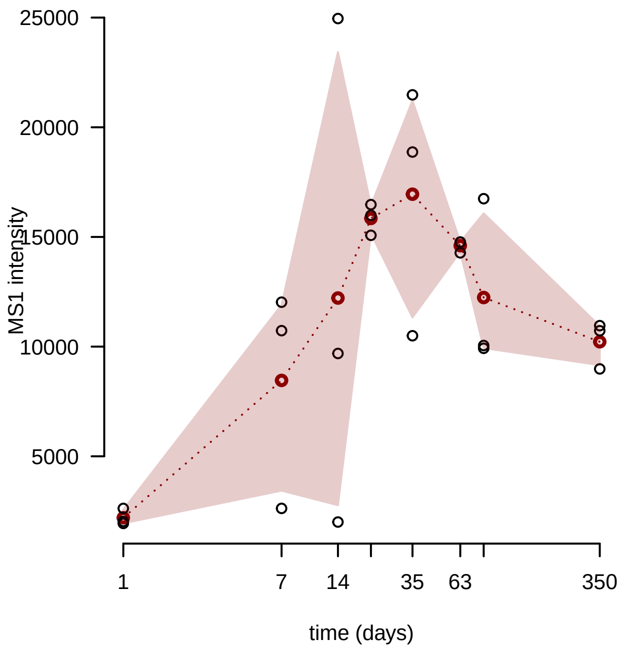

O35632
GN=Hyal2
hyaluronoglucosaminidase 2

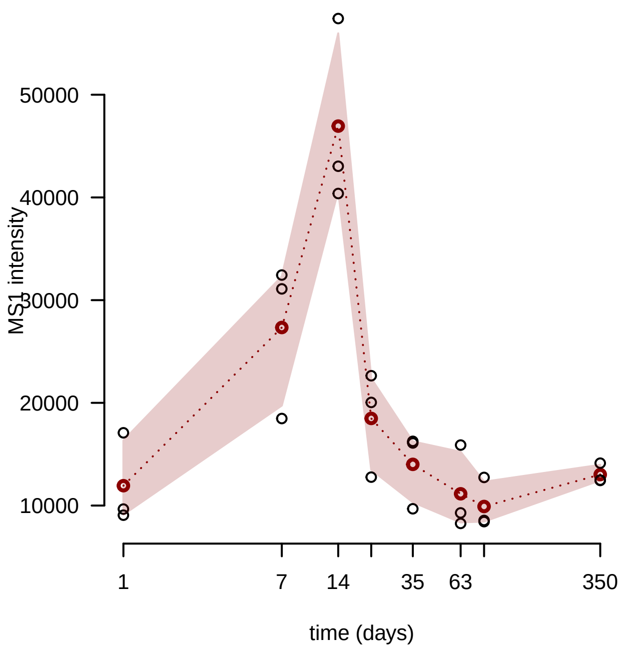

O35638
GN=Stag2
stromal antigen 2

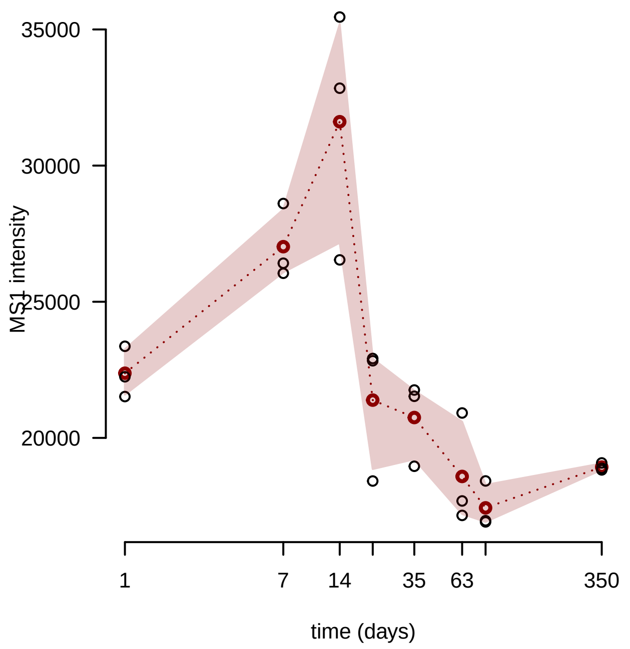

O35639
GN=Anxa3
annexin A3

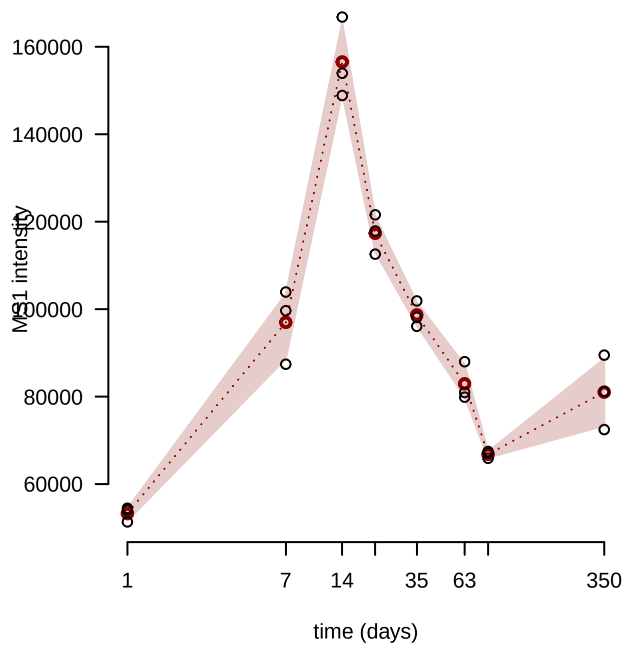

O35643
GN=Ap1b1
adaptor protein complex AP−1, beta 1 sub ...

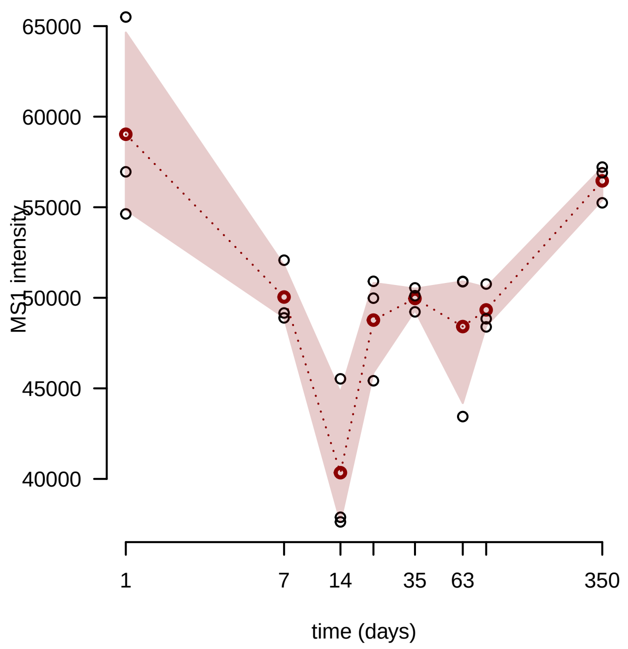

O35646
GN=Capn6
calpain 6

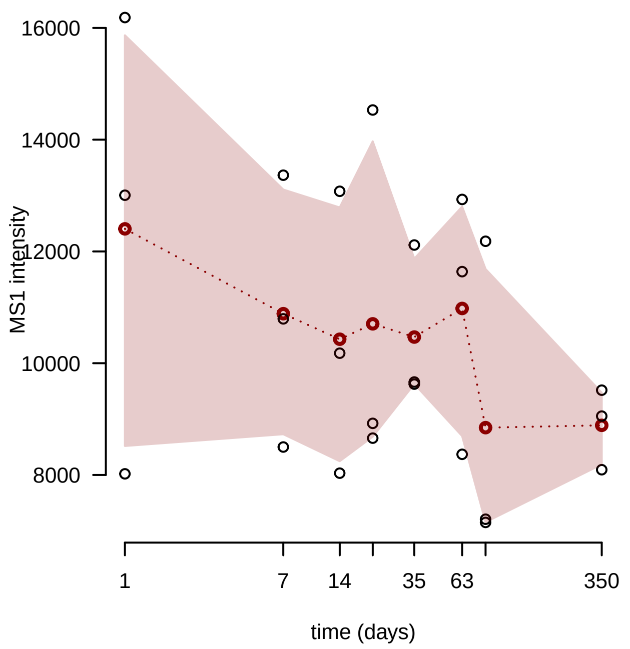

O35648
GN=Cetn3
centrin 3

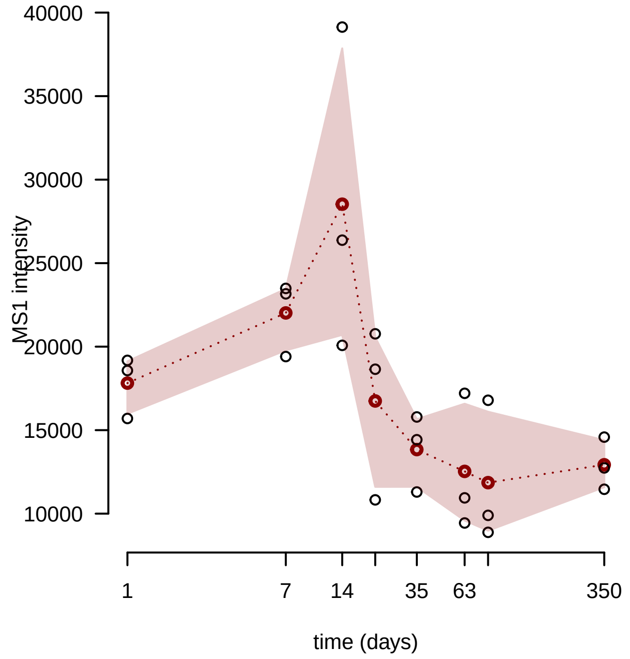

O35652
GN=Lhx8
LIM homeobox protein 8

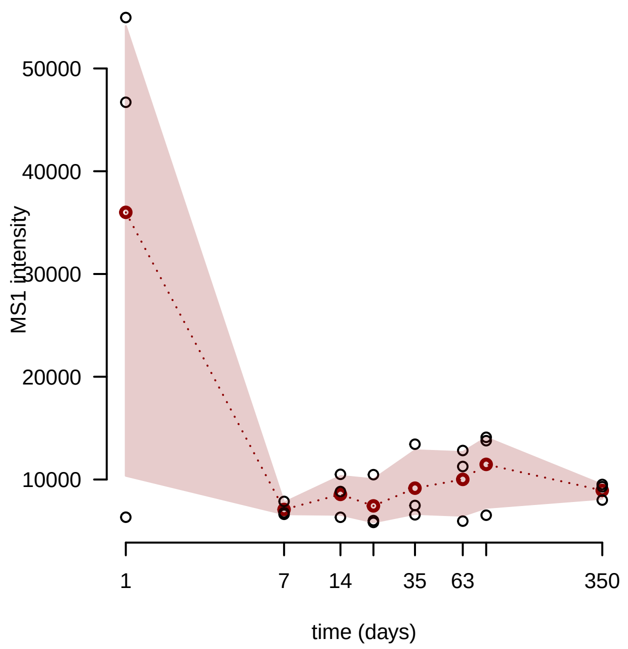

O35654
GN=Pold2
polymerase (DNA directed), delta 2, regu ...

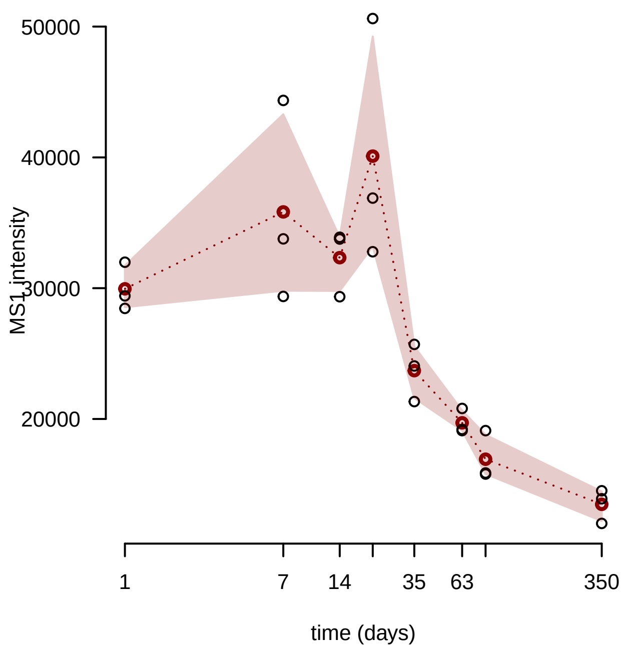

O35657
GN=Neu1
neuraminidase 1

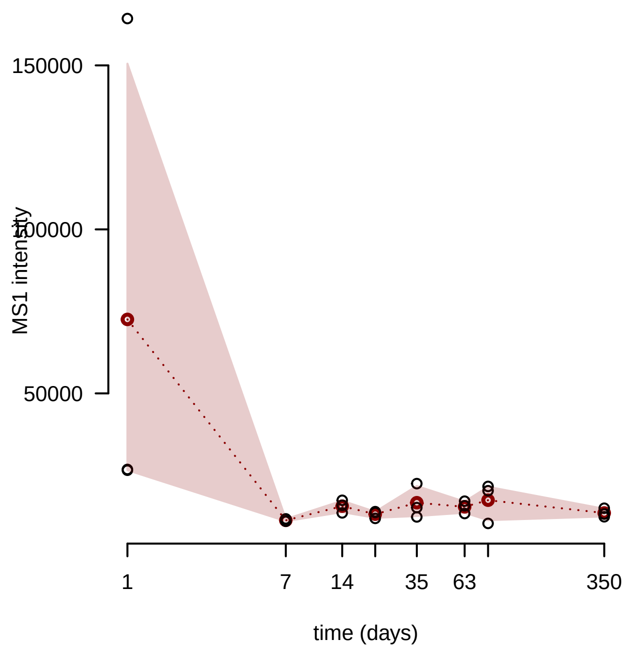

O35658
GN=C1qbp
complement component 1, q subcomponent b ...

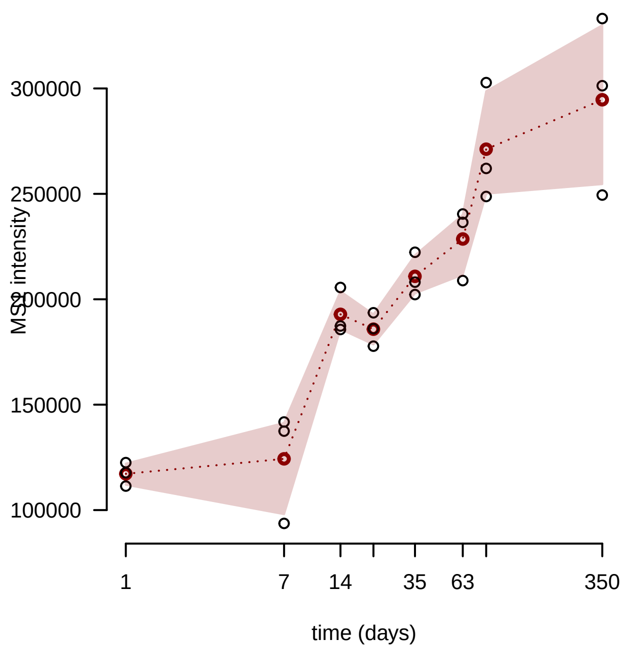

O35660
GN=Gstm6
glutathione S−transferase, mu 6

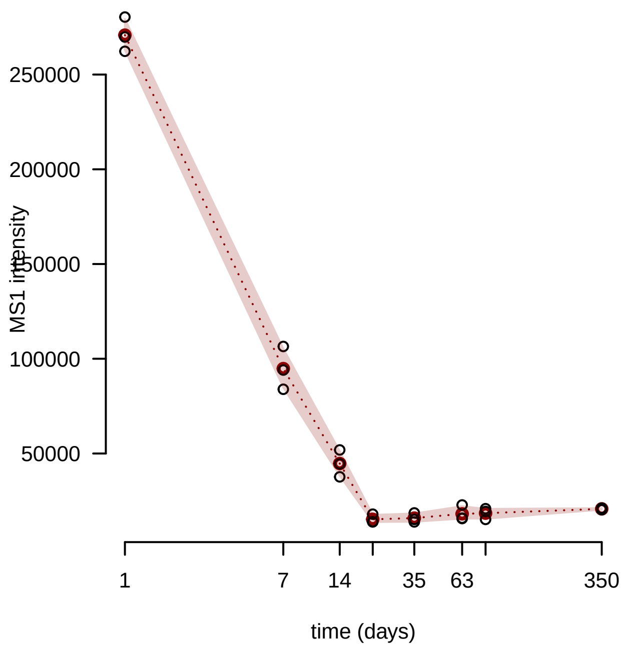

O35678
GN=Mgll
monoglyceride lipase

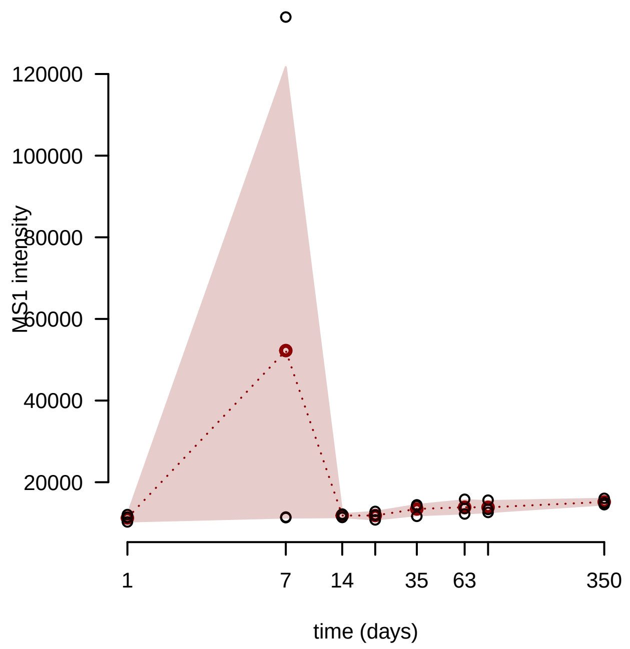

O35680
GN=Mrps12
mitochondrial ribosomal protein S12

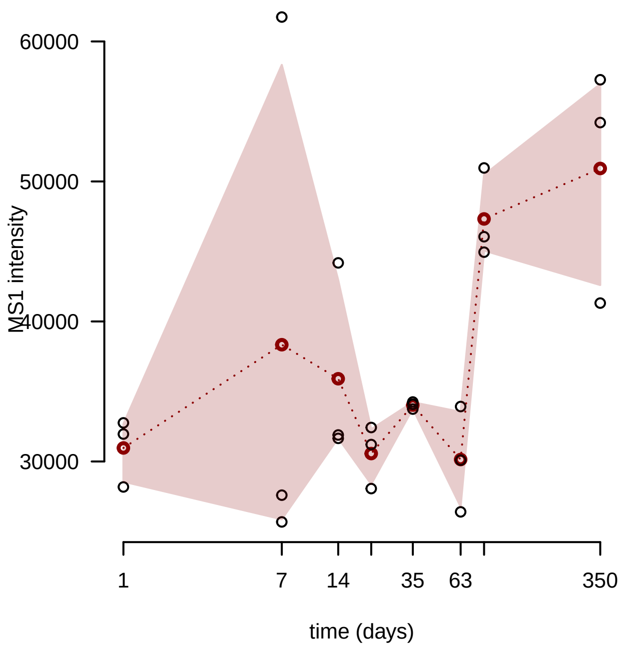

O35682
GN=Myadm
myeloid−associated differentiation marke ...

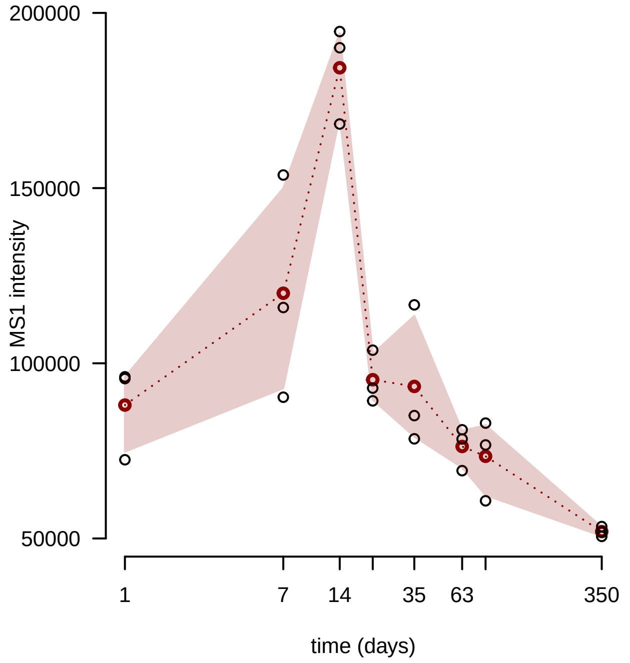

O35683
GN=Ndufa1
NADH:ubiquinone oxidoreductase subunit A ...

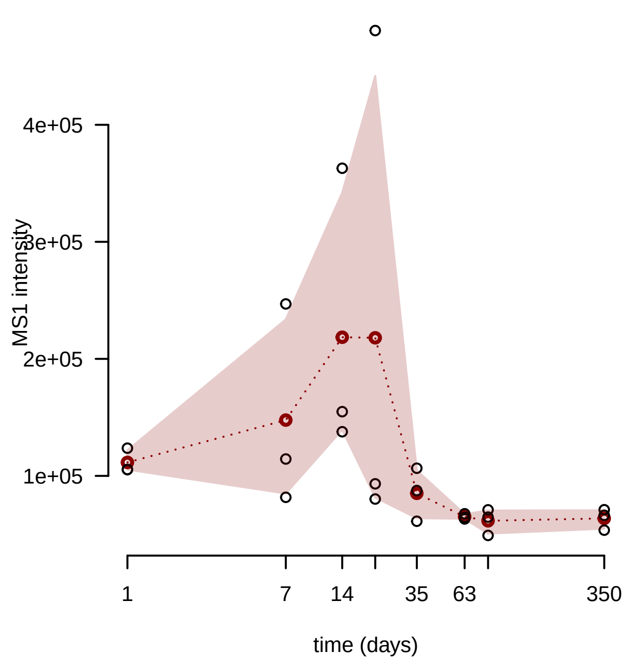

O35685
GN=Nudc
nudC nuclear distribution protein

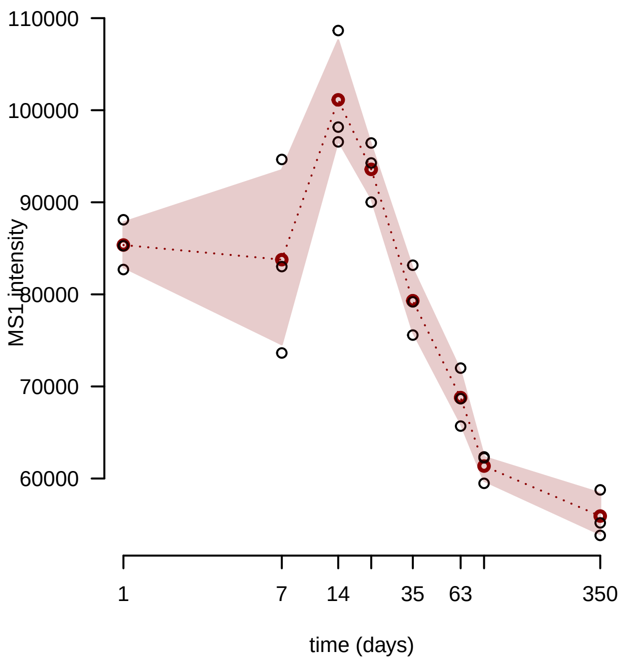

O35690
GN=Phox2b
paired−like homeobox 2b

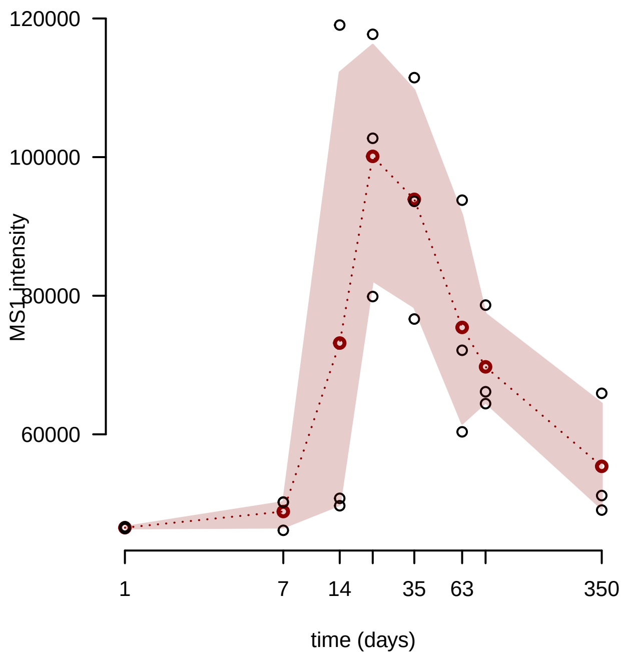

O35691
GN=Pnn
pinin

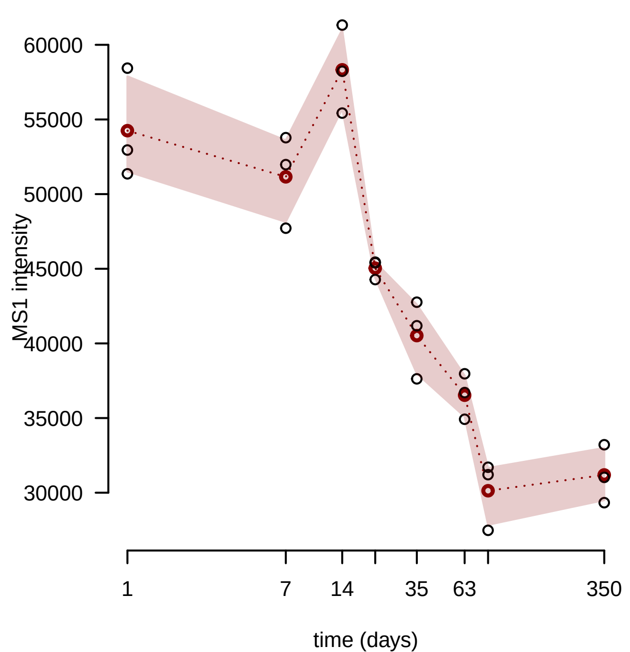

O35704
GN=Sptlc1
serine palmitoyltransferase, long chain ...

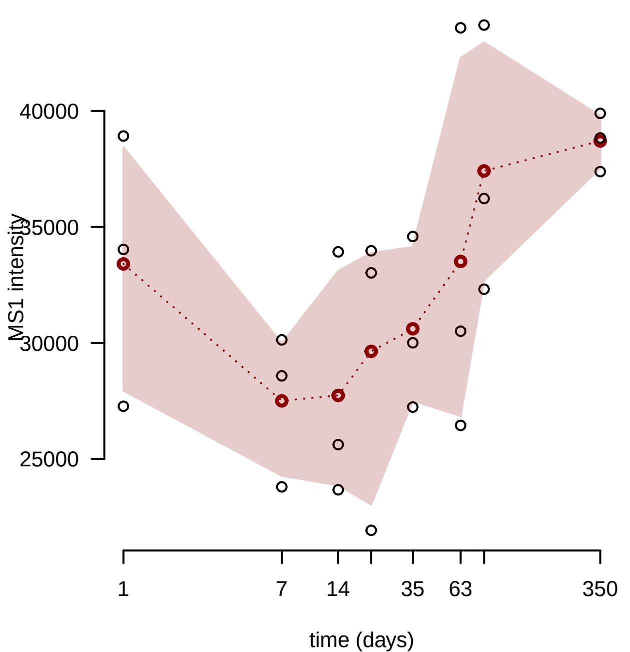

O35711
GN=Ppfibp2
PTPRF interacting protein, binding prote ...

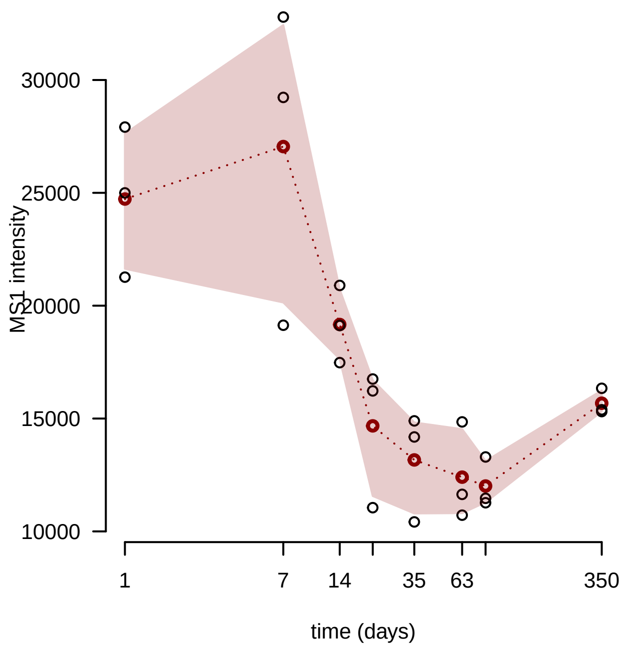

O35717
GN=Socs2
suppressor of cytokine signaling 2

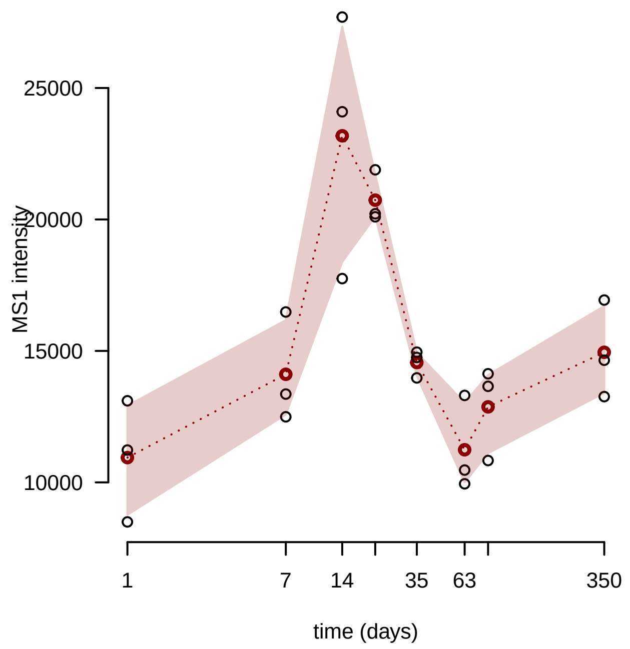

O35719
GN=Rad51b
RAD51 paralog B

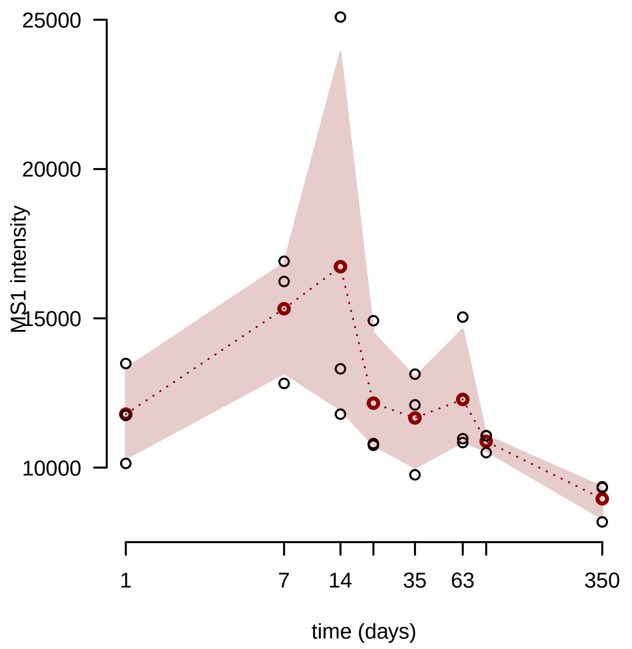

O35730
GN=Ring1
ring finger protein 1

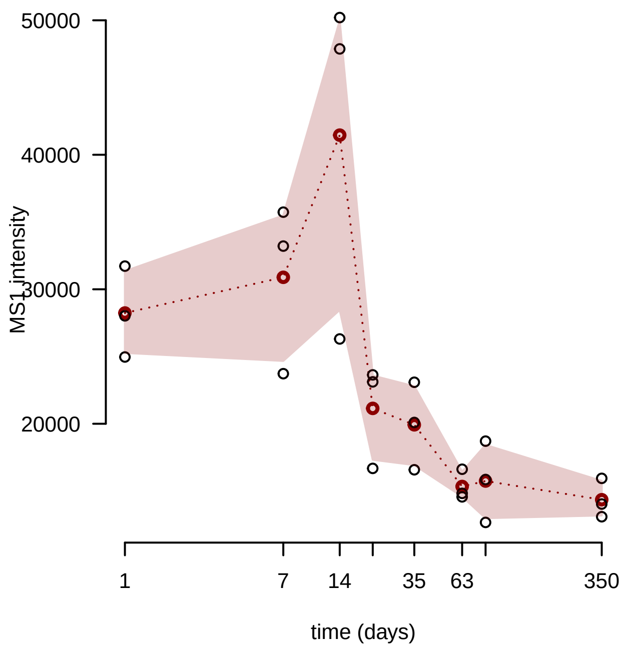

O35737
GN=Hnrnph1
heterogeneous nuclear ribonucleoprotein ...

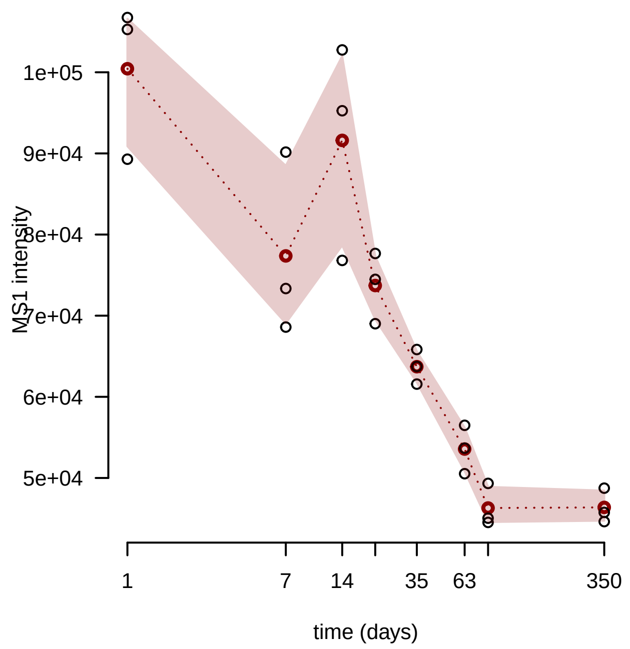

O35738
GN=Klf12
Kruppel−like factor 12

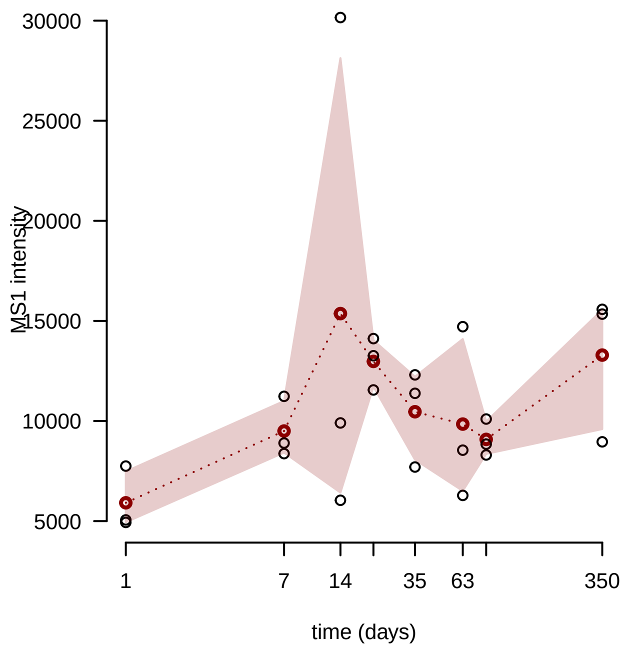

O35744
GN=Chil3
chitinase−like 3

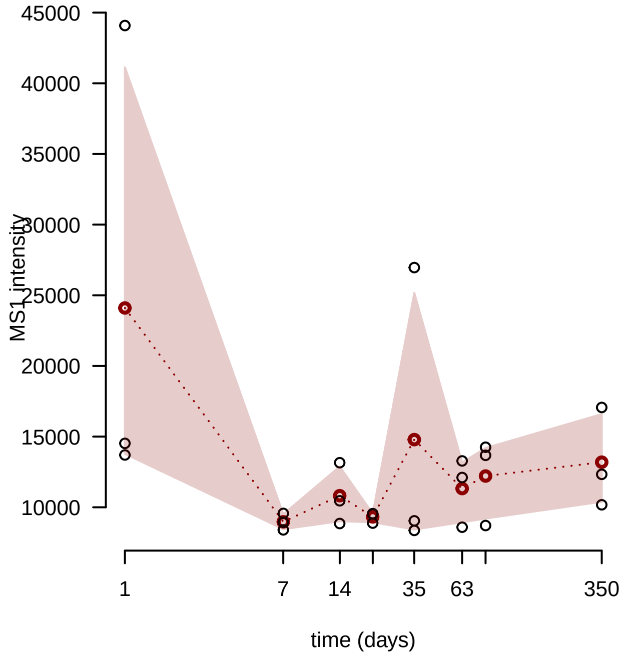

O35841
GN=Api5
apoptosis inhibitor 5

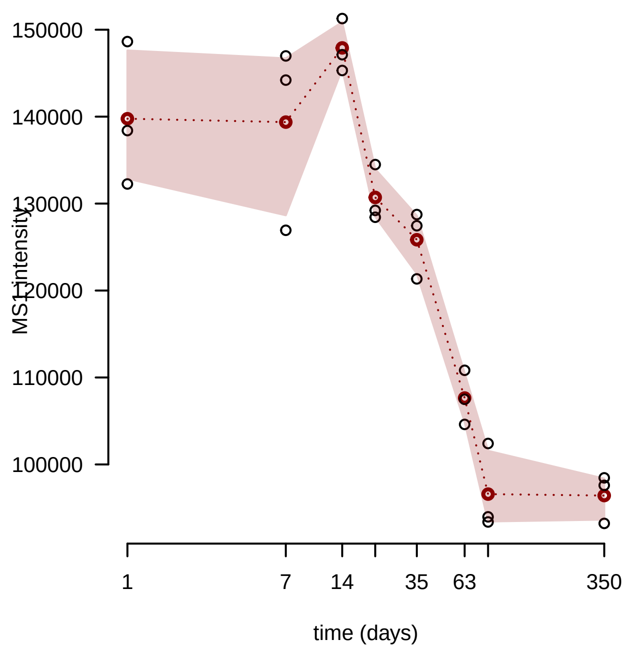

O35855
GN=Bcat2
branched chain aminotransferase 2, mitoc ...

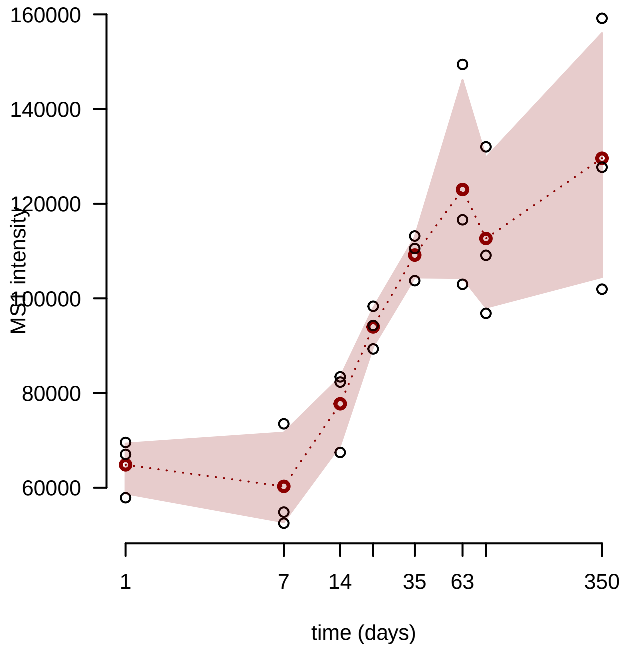

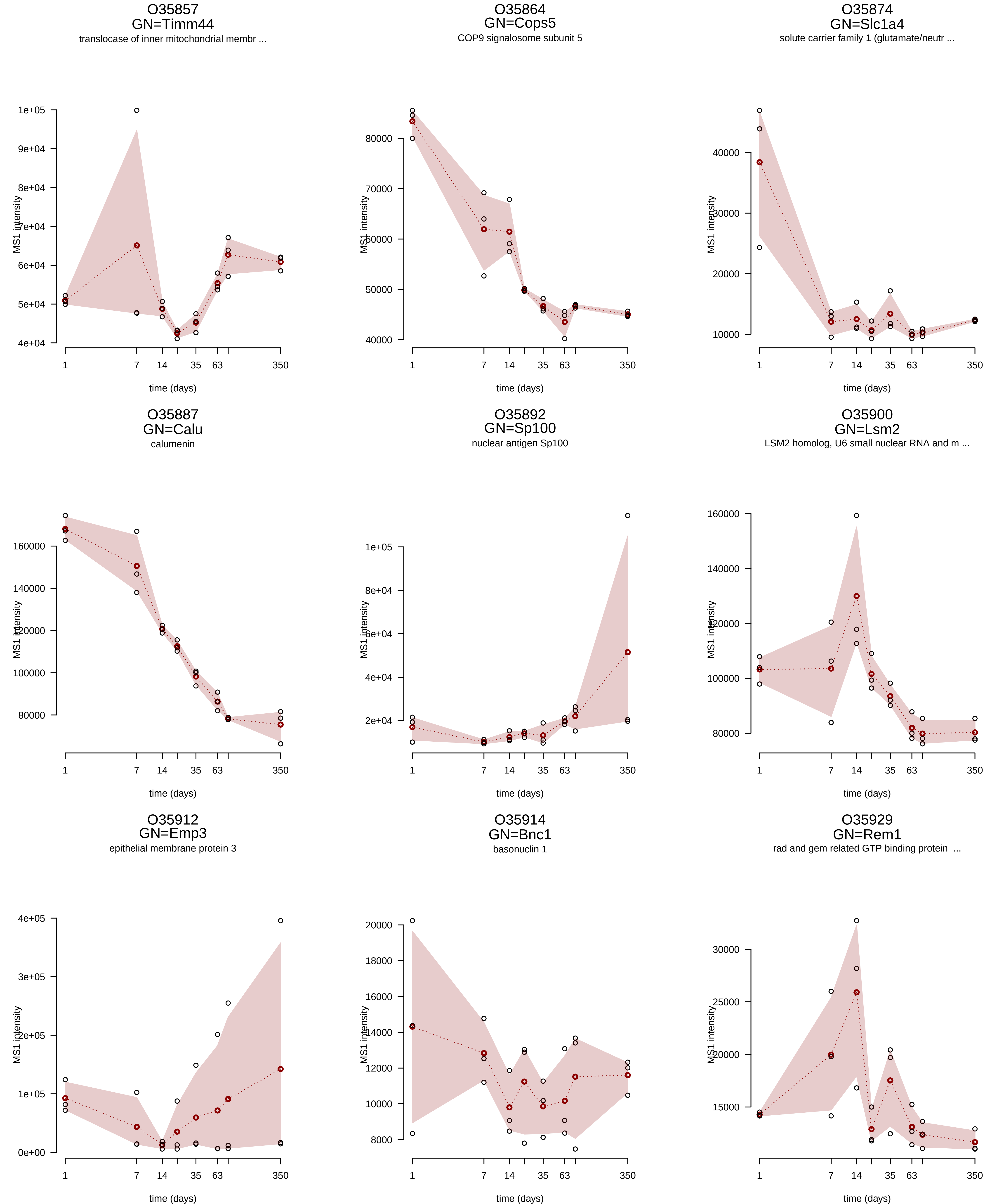
O35857
GN=Timm44
translocase of inner mitochondrial membr ...
MS1 intensity
1e+05
9e+04
8e+04
7e+04
6e+04
5e+04
4e+04
1
7
14
35
63
350
time (days)
O35864
GN=Cops5
COP9 signalosome subunit 5
MS1 intensity
80000
70000
60000
50000
40000
time (days)
O35874
GN=Slc1a4
solute carrier family 1 (glutamate/neutr ...
MS1 intensity
40000
30000
20000
10000
time (days)
O35887
GN=Calu
calumenin
MS1 intensity
160000
140000
120000
100000
80000
time (days)
O35892
GN=Sp100
nuclear antigen Sp100
MS1 intensity
1e+05
8e+04
6e+04
4e+04
2e+04
time (days)
O35900
GN=Lsm2
LSM2 homolog, U6 small nuclear RNA and m ...
MS1 intensity
160000
140000
120000
100000
80000
time (days)
O35912
GN=Emp3
epithelial membrane protein 3
MS1 intensity
4e+05
3e+05
2e+05
1e+05
0e+00
time (days)
O35914
GN=Bnc1
basonuclin 1
MS1 intensity
20000
18000
16000
14000
12000
10000
8000
time (days)
O35929
GN=Rem1
rad and gem related GTP binding protein ...
MS1 intensity
30000
25000
20000
15000
time (days)

O35930
GN=Gp1ba
glycoprotein 1b, alpha polypeptide

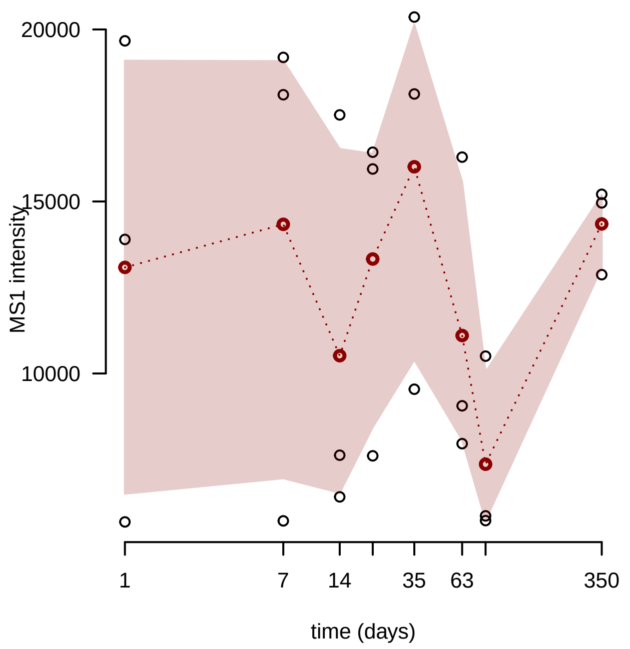

O35943
GN=Fxn
frataxin

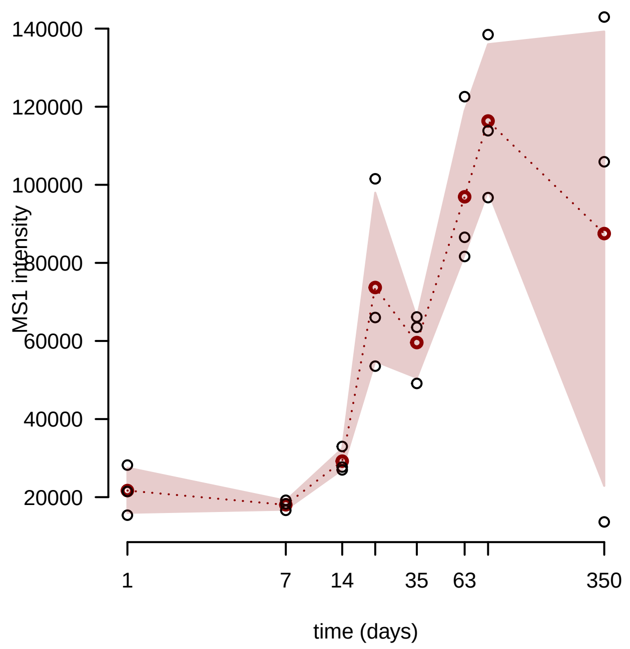

O35945
GN=Aldh1a7
aldehyde dehydrogenase family 1, subfami ...

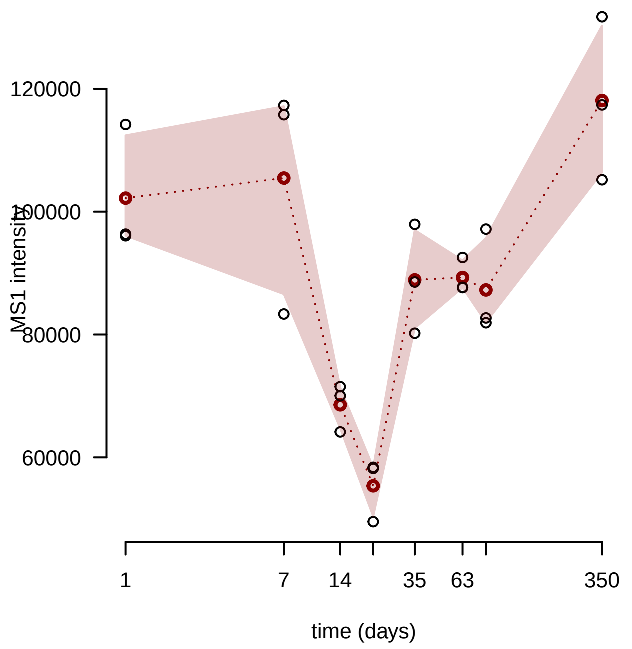

O35954
GN=Pitpnm1
phosphatidylinositol transfer protein, m ...

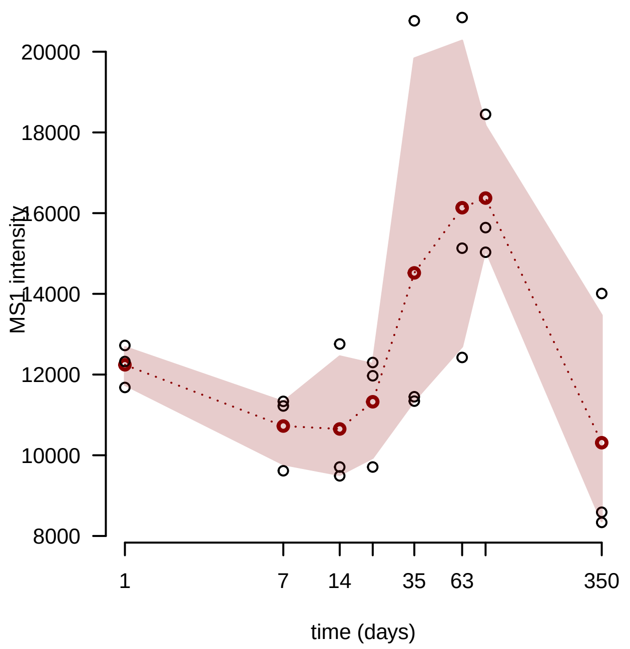

O35955
GN=Psmb10
proteasome (prosome, macropain) subunit, ...

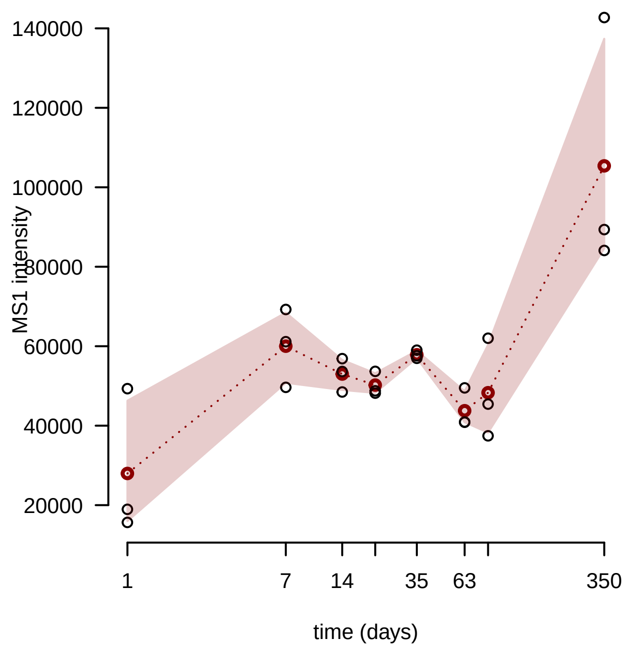

O35963
GN=Rab33b
RAB33B, member RAS oncogene family

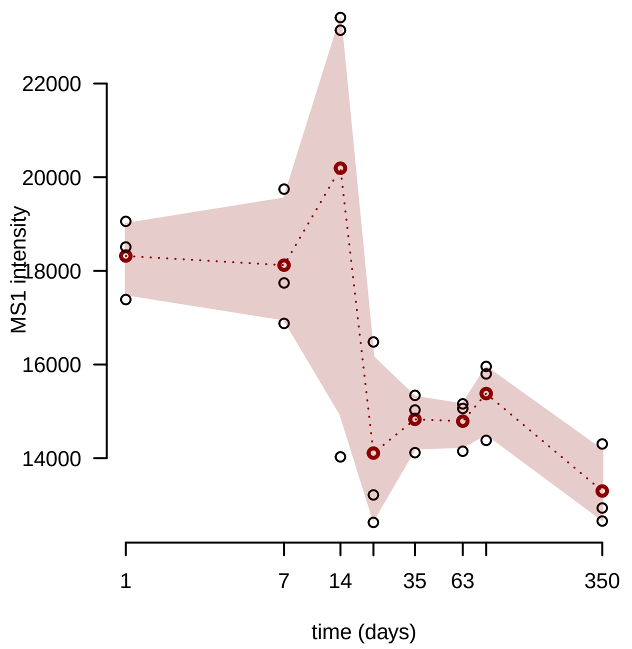

O35969
GN=Gamt
guanidinoacetate methyltransferase

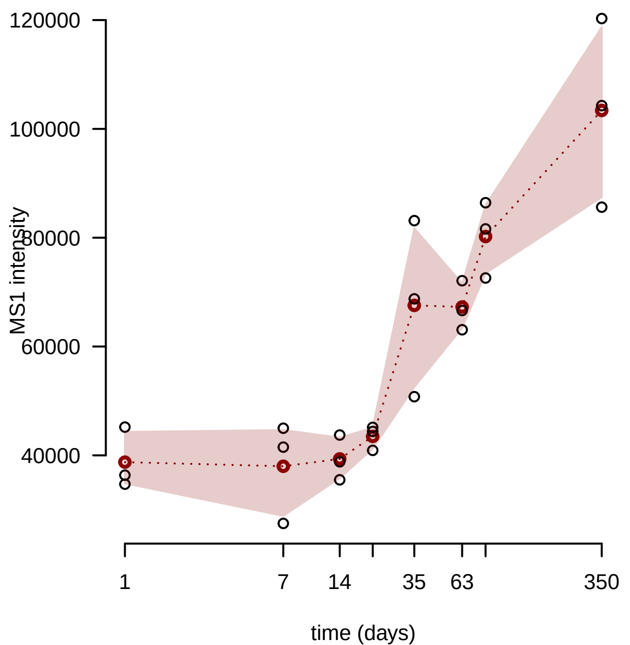

O35972
GN=Mrpl23
mitochondrial ribosomal protein L23

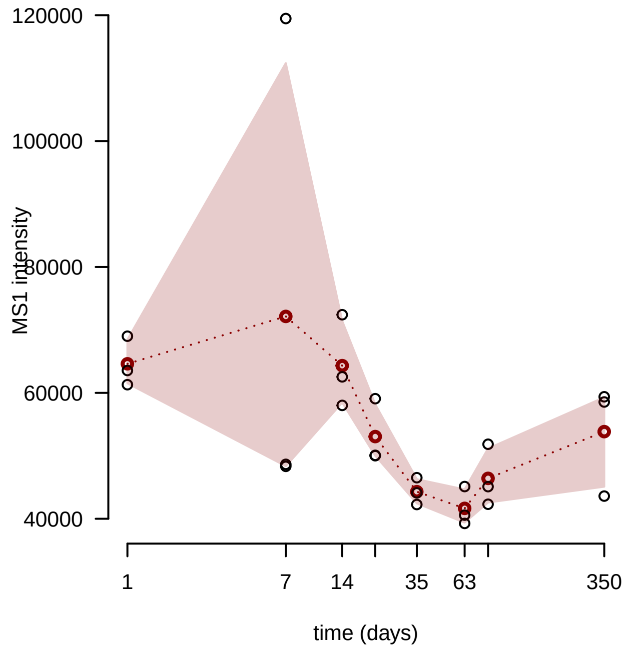

O35980
GN=Nthl1
nth (endonuclease III)−like 1 (E.coli)

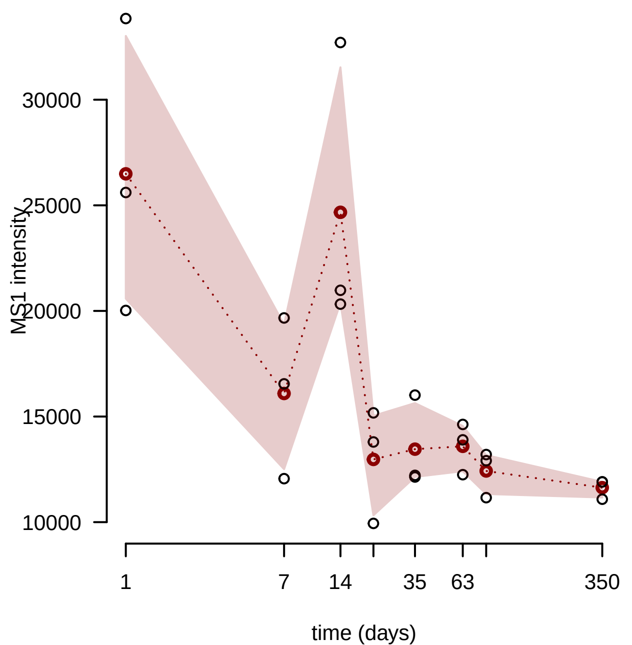

O35984
GN=Pbx2
pre B cell leukemia homeobox 2

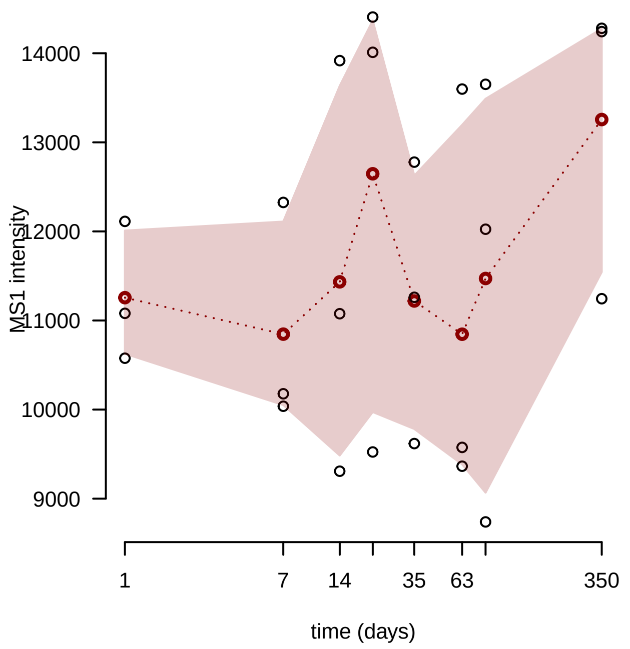

O35988
GN=Sdc4
syndecan 4

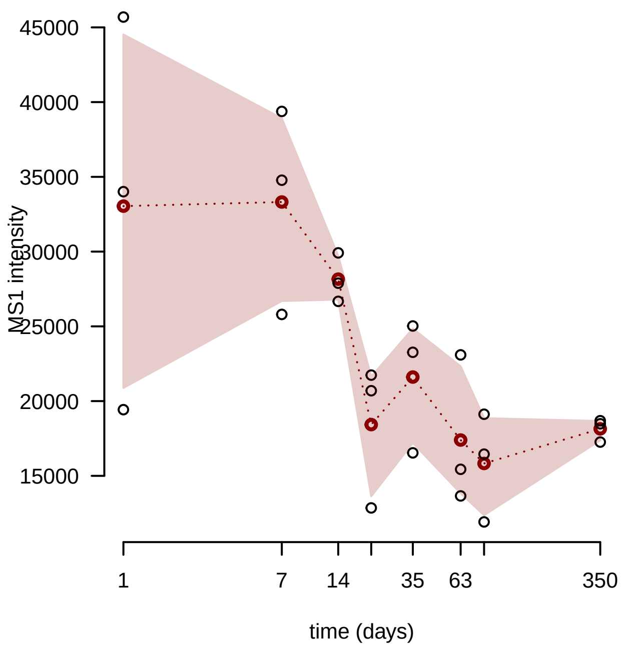

O54692
GN=Zw10
zw10 kinetochore protein

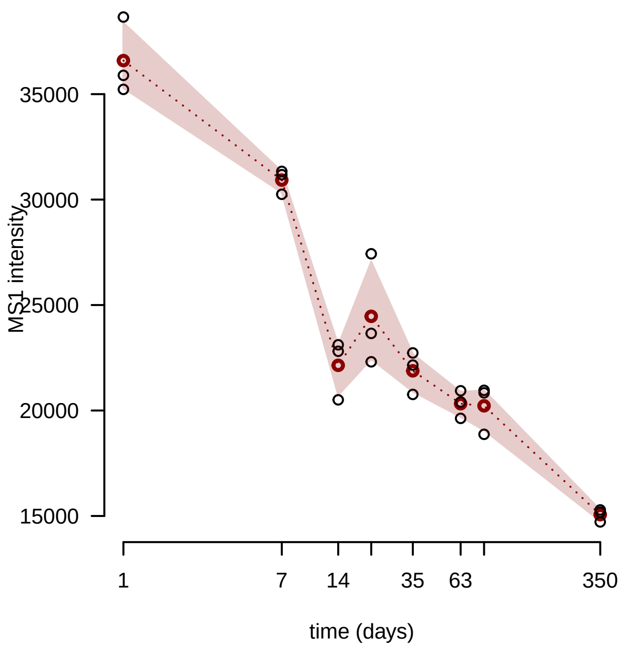

O54724
GN=Cavin1
caveolae associated 1

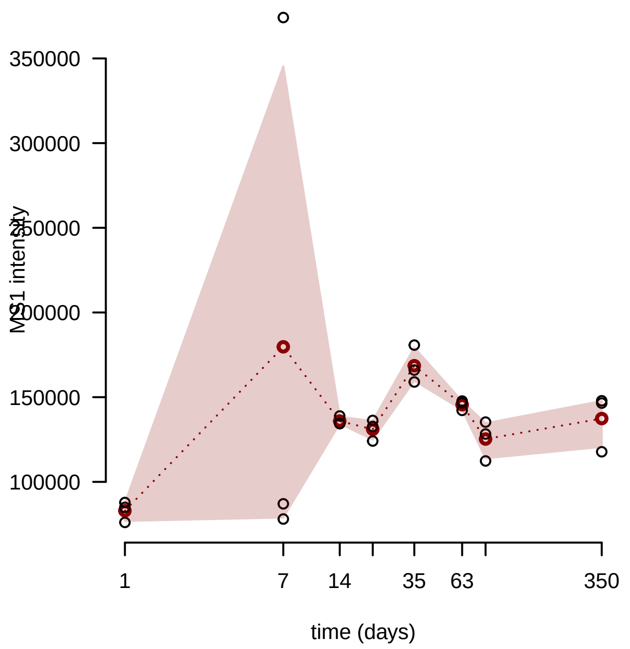

O54734
GN=Ddost
dolichyl−di−phosphooligosaccharide−prote ...

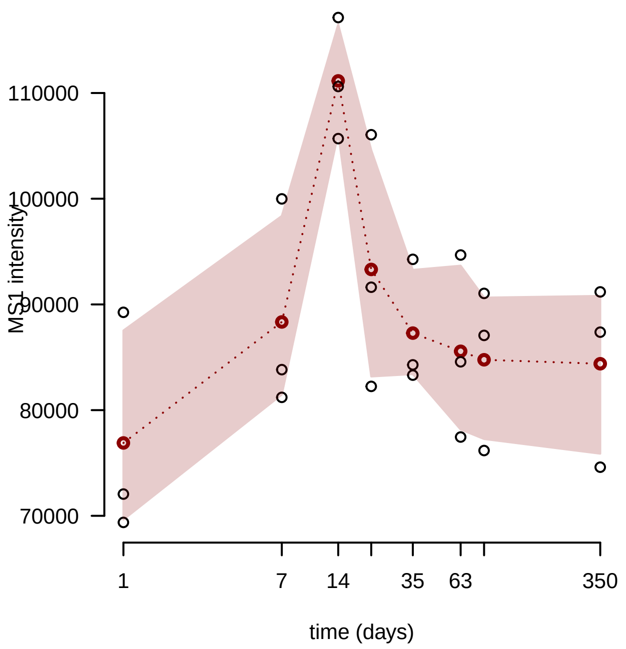

O54750
GN=Cyp2j6
cytochrome P450, family 2, subfamily j, ...

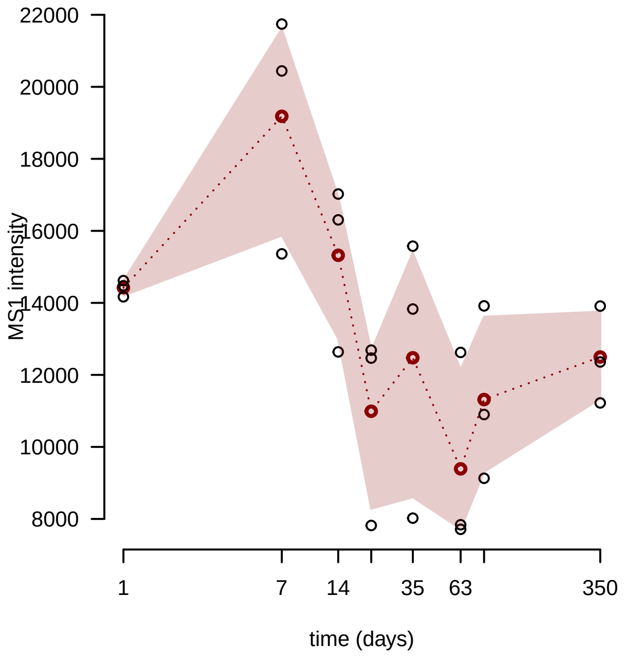

O54774
GN=Ap3d1
adaptor−related protein complex 3, delta ...

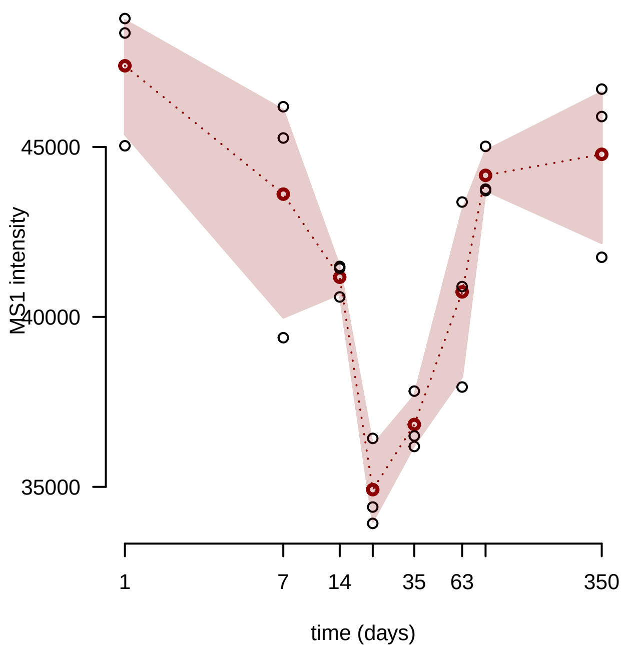

O54781
GN=Srpk2
serine/arginine−rich protein specific ki ...

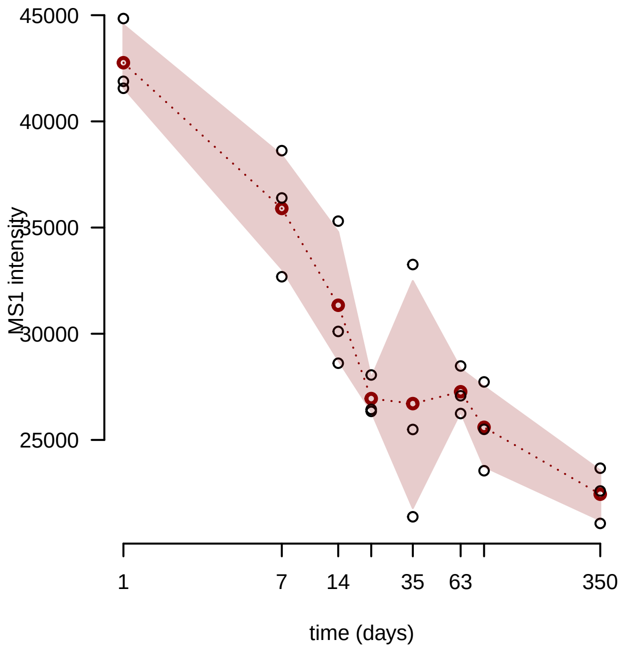

O54782
GN=Man2b2
mannosidase 2, alpha B2

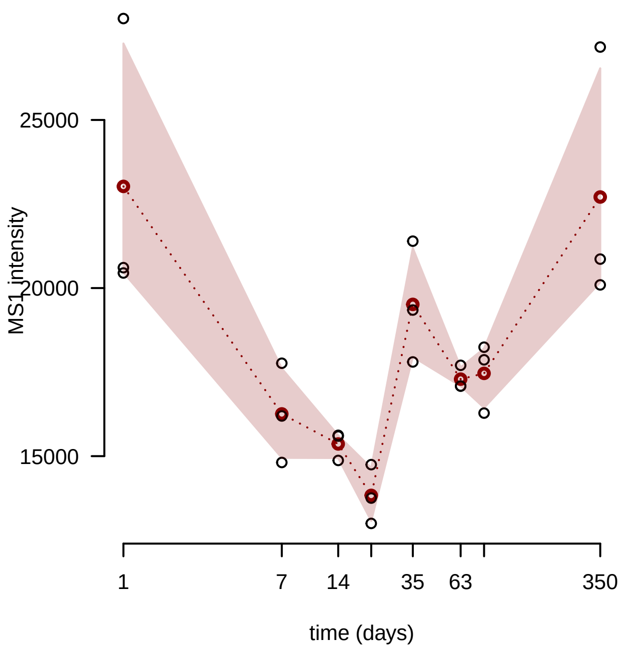

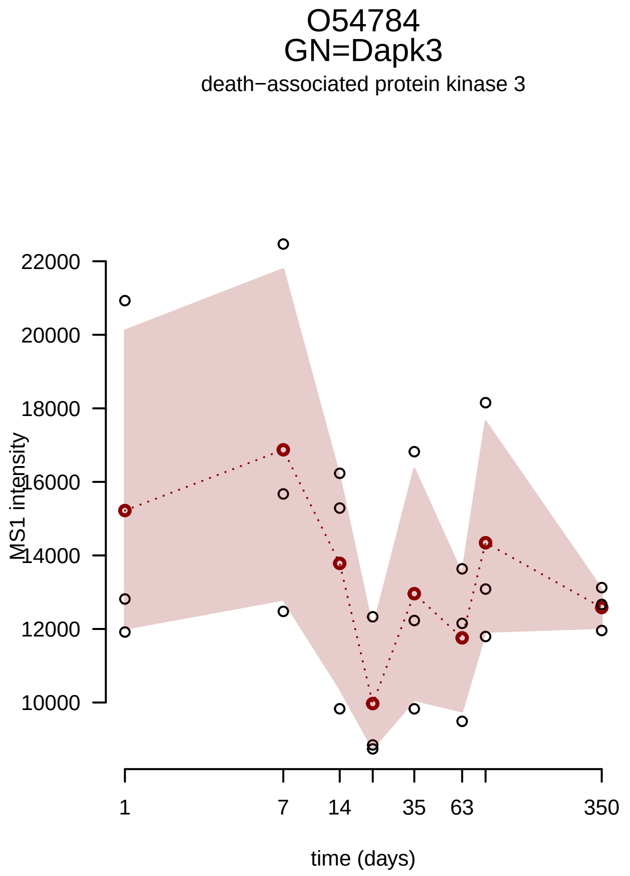
O54784
GN=Dapk3
death−associated protein kinase 3
MS1 intensity
22000
20000
18000
16000
14000
12000
10000
1
7
14
35
63
350
time (days)

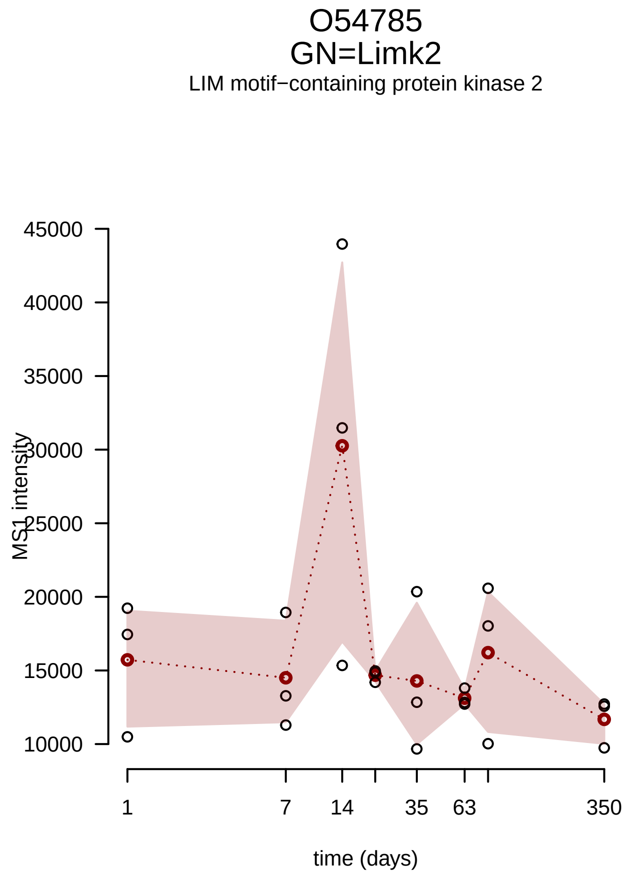
O54785
GN=Limk2
LIM motif−containing protein kinase 2
MS1 intensity
45000
40000
35000
30000
25000
20000
15000
10000
1
7
14
35
63
350
time (days)

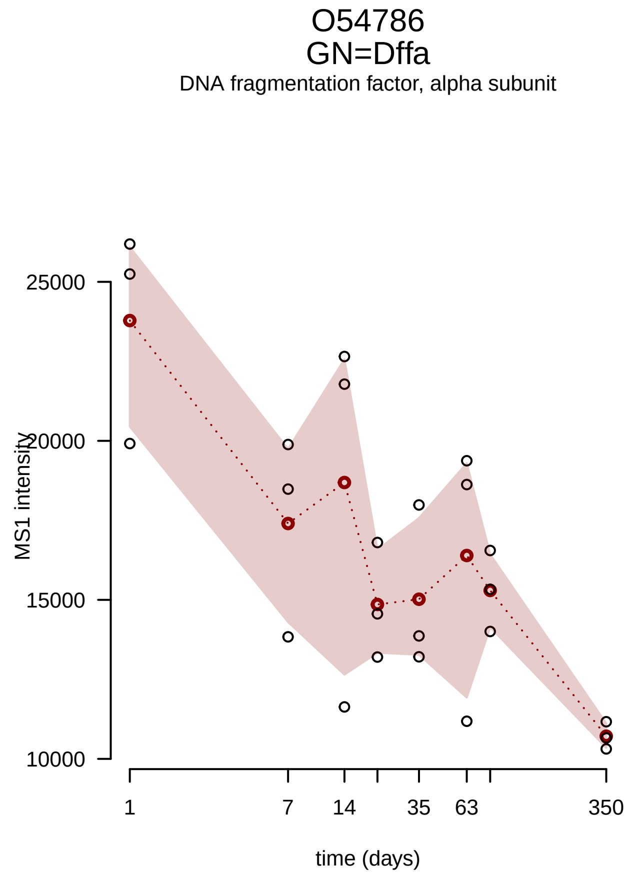
O54786
GN=Dffa
DNA fragmentation factor, alpha subunit
MS1 intensity
25000
20000
15000
10000
1
7
14
35
63
350
time (days)

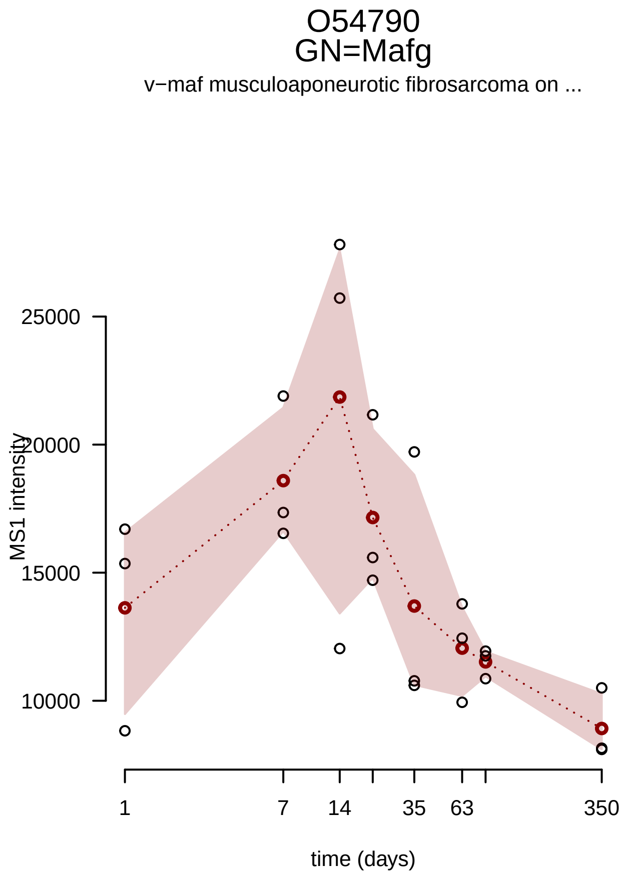
O54790
GN=Mafg
v−maf musculoaponeurotic fibrosarcoma on ...
MS1 intensity
25000
20000
15000
10000
1
7
14
35
63
350
time (days)

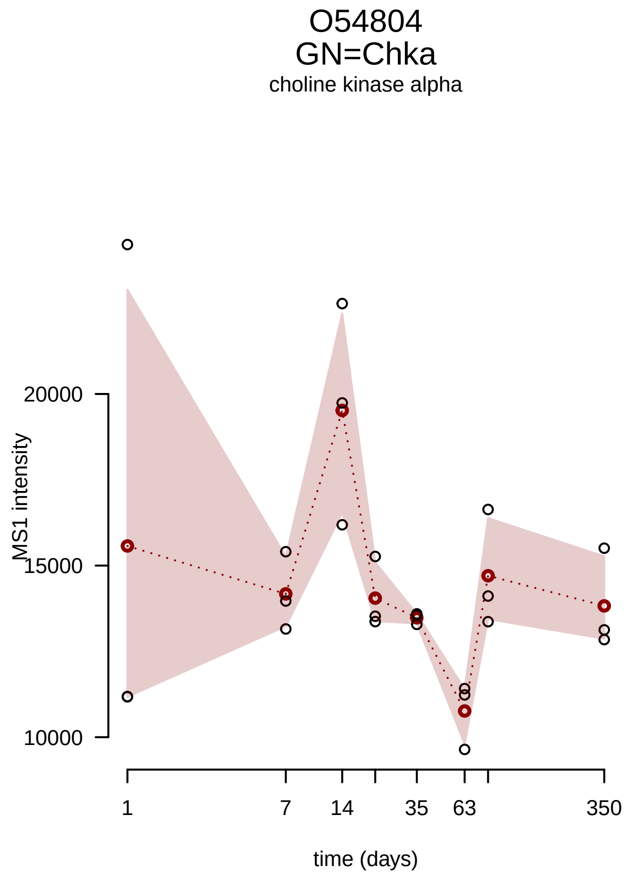
O54804
GN=Chka
choline kinase alpha
MS1 intensity
20000
15000
10000
1
7
14
35
63
350
time (days)

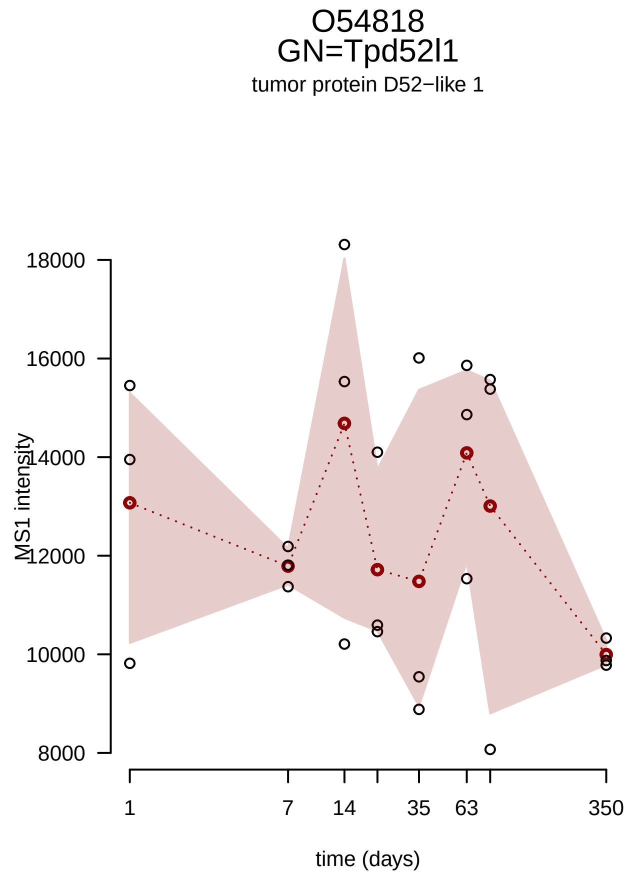
O54818
GN=Tpd52l1
tumor protein D52−like 1
MS1 intensity
18000
16000
14000
12000
10000
8000
1
7
14
35
63
350
time (days)

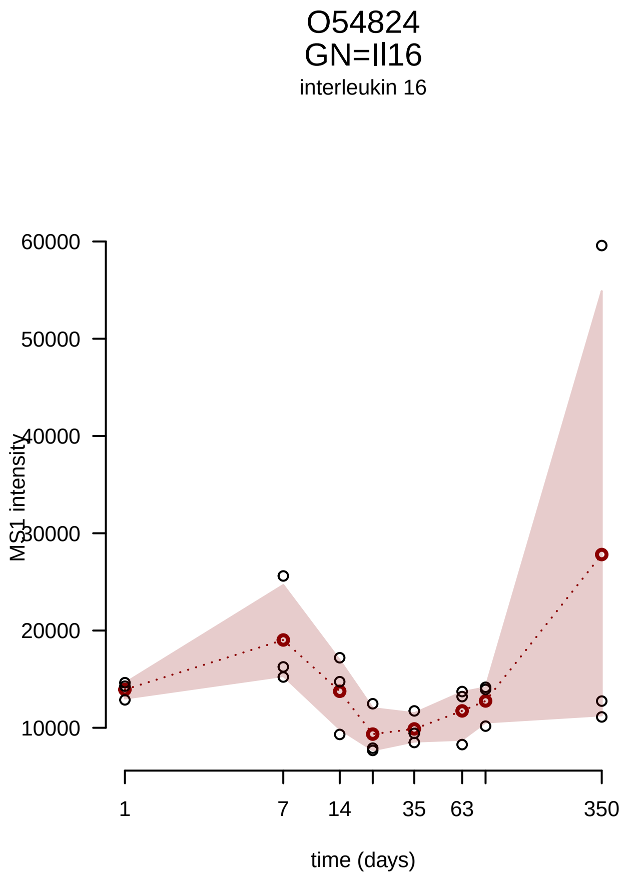
O54824
GN=Il16
interleukin 16
MS1 intensity
60000
50000
40000
30000
20000
10000
1
7
14
35
63
350
time (days)

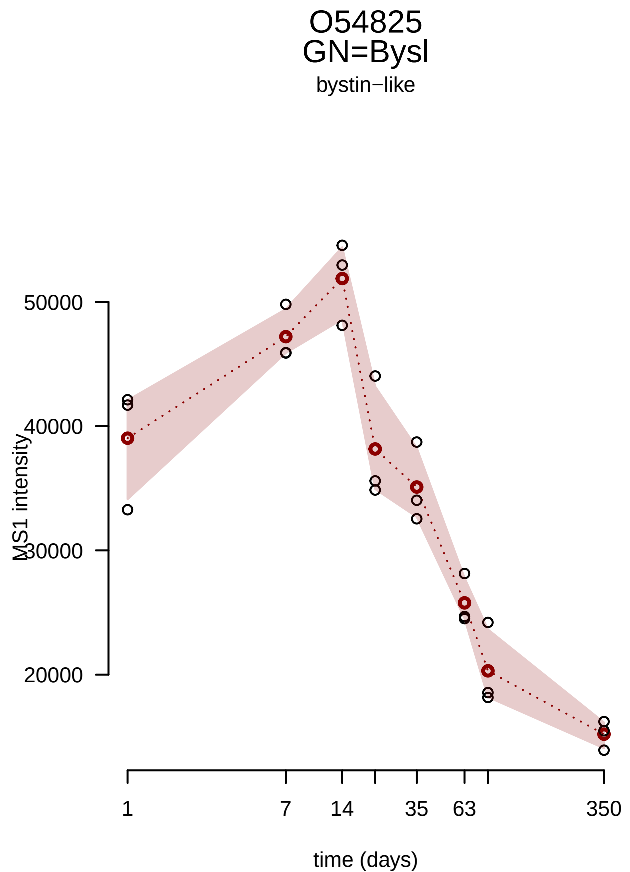
O54825
GN=Bysl
bystin−like
MS1 intensity
50000
40000
30000
20000
1
7
14
35
63
350
time (days)

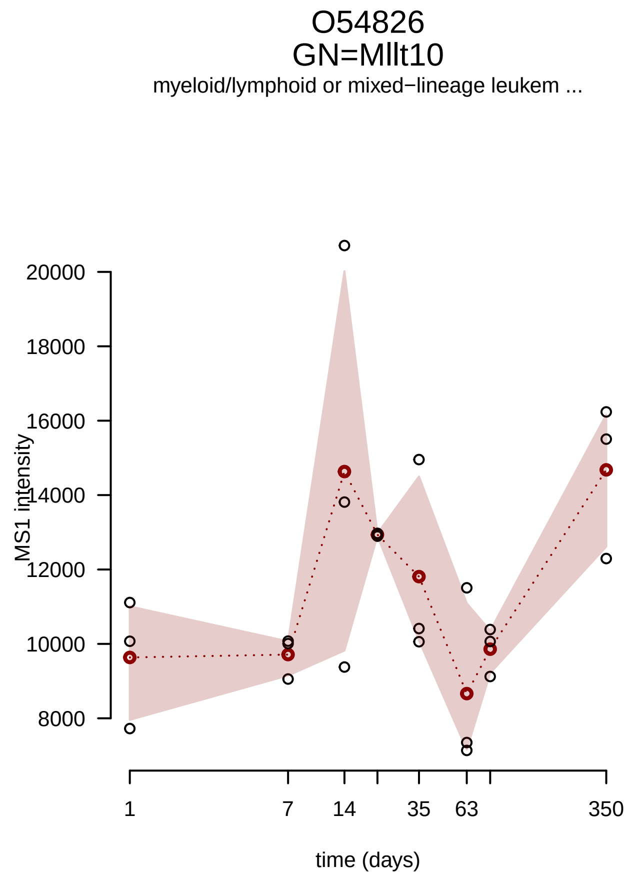
O54826
GN=Mllt10
myeloid/lymphoid or mixed−lineage leukem ...
MS1 intensity
20000
18000
16000
14000
12000
10000
8000
1
7
14
35
63
350
time (days)

O54833
GN=Csnk2a2
casein kinase 2, alpha prime polypeptide

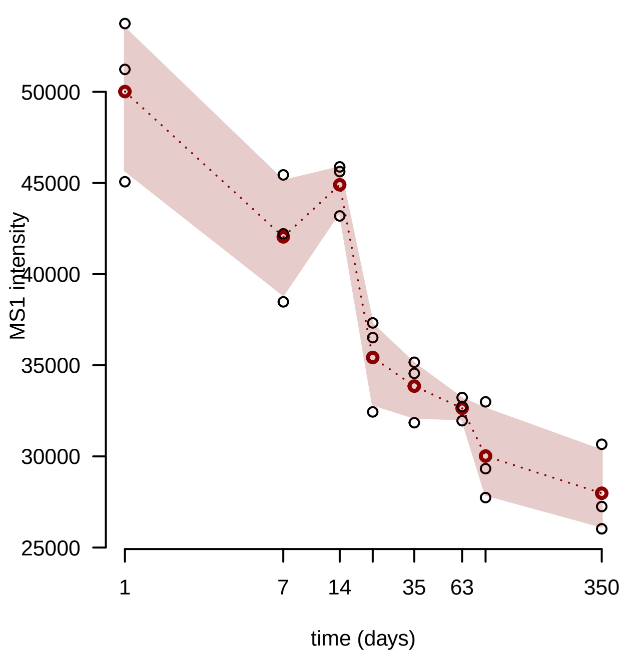

O54834
GN=Arhgap6
Rho GTPase activating protein 6

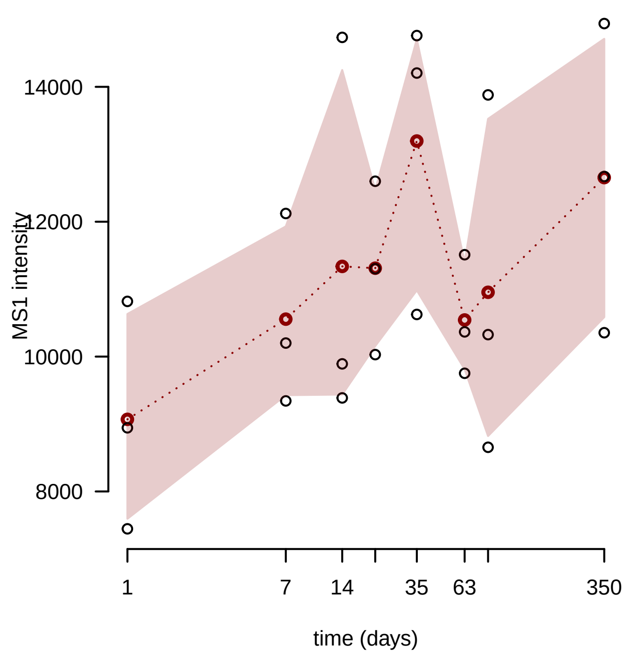

O54864
GN=Suv39h1
suppressor of variegation 3−9 1

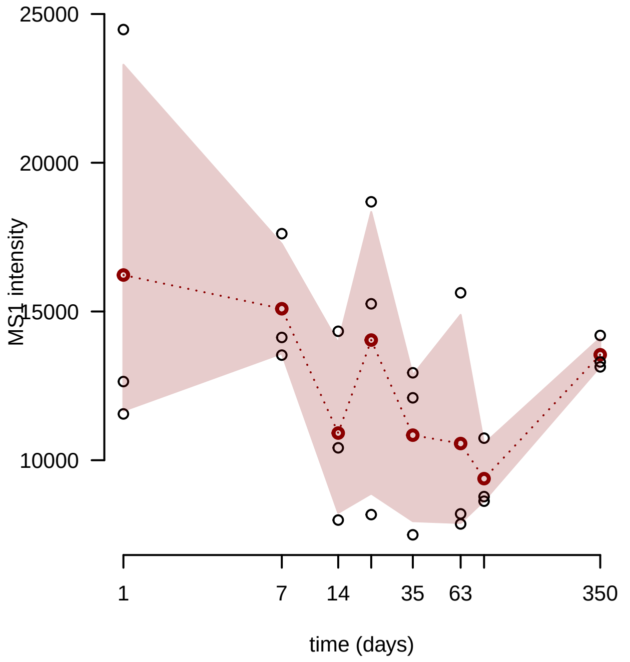

O54865
GN=Gucy1b1
guanylate cyclase 1, soluble, beta 1

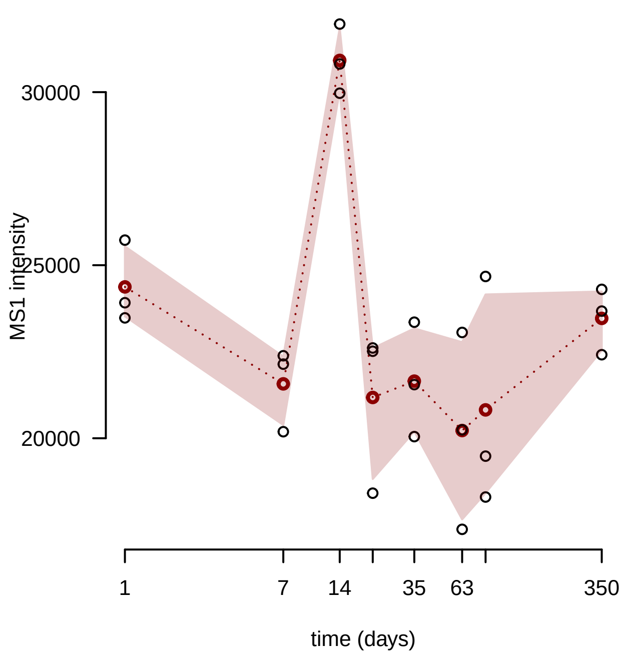

O54879
GN=Hmgb3
high mobility group box 3

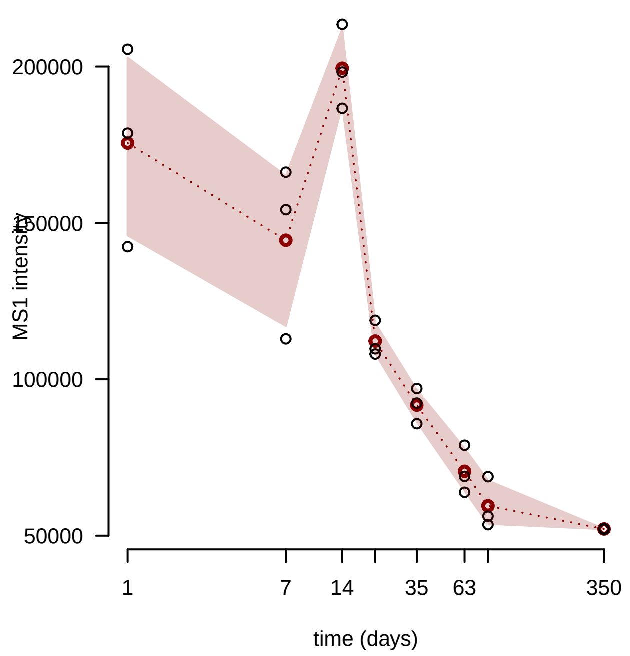

O54890
GN=Itgb3
integrin beta 3

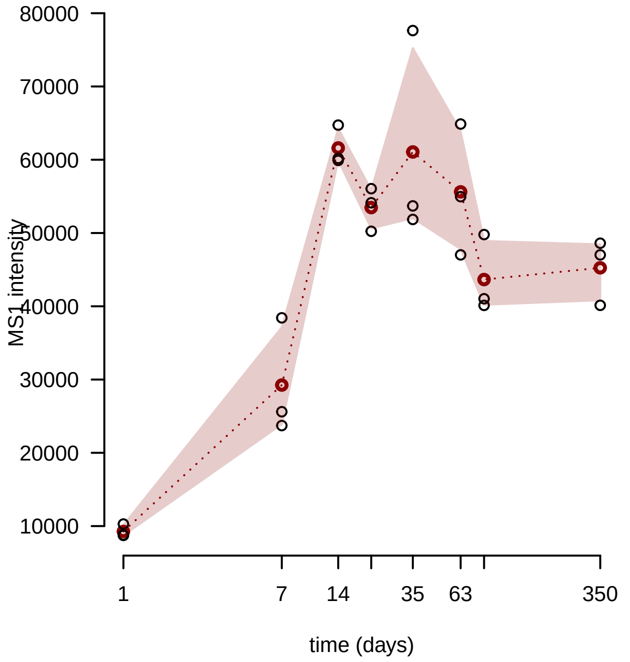

O54901
GN=Cd200
CD200 antigen

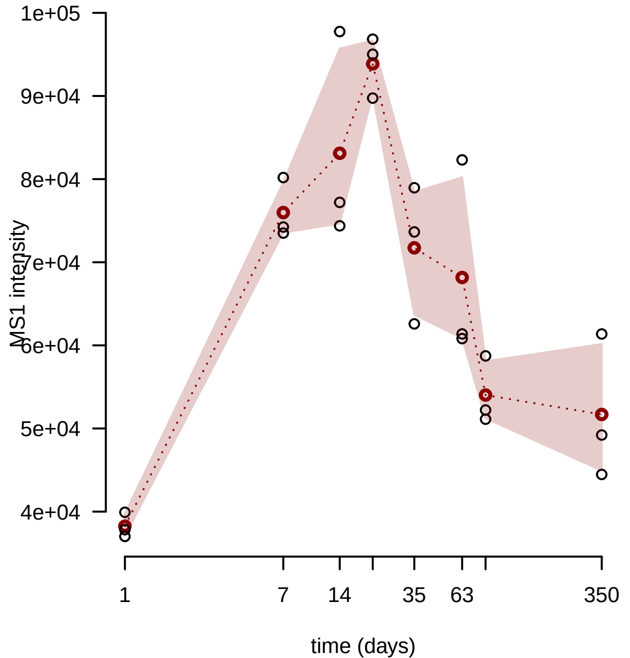

O54909
GN=Rdh16
retinol dehydrogenase 16

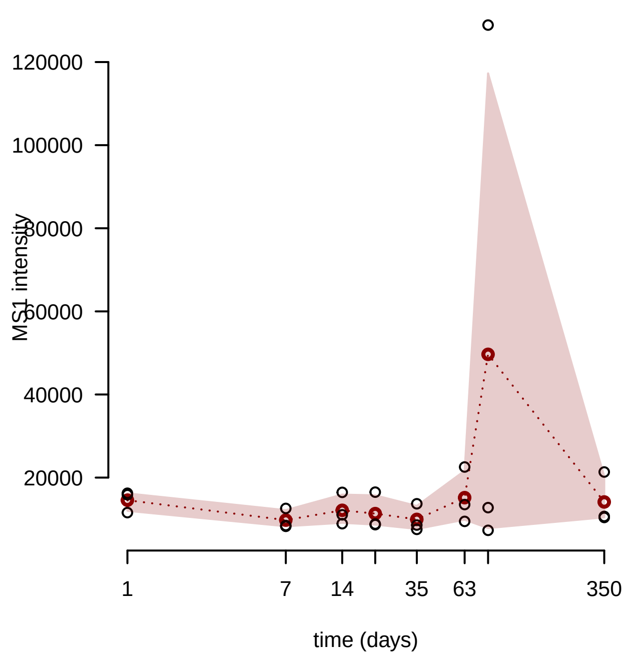

O54916
GN=Reps1
RalBP1 associated Eps domain containing ...

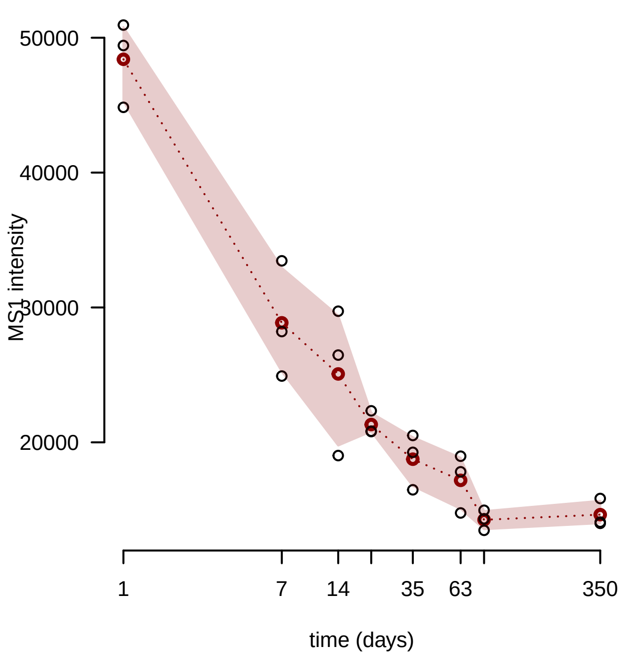

O54918
GN=Bcl2l11
BCL2−like 11 (apoptosis facilitator)

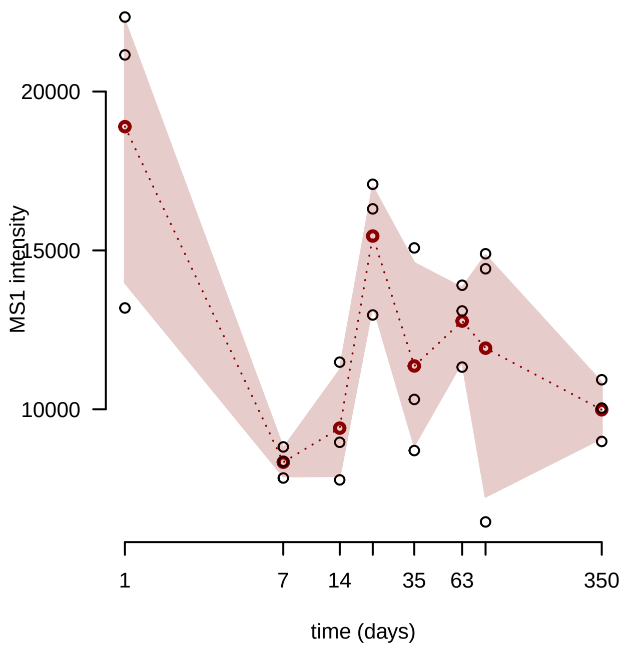

O54931
GN=Akap2

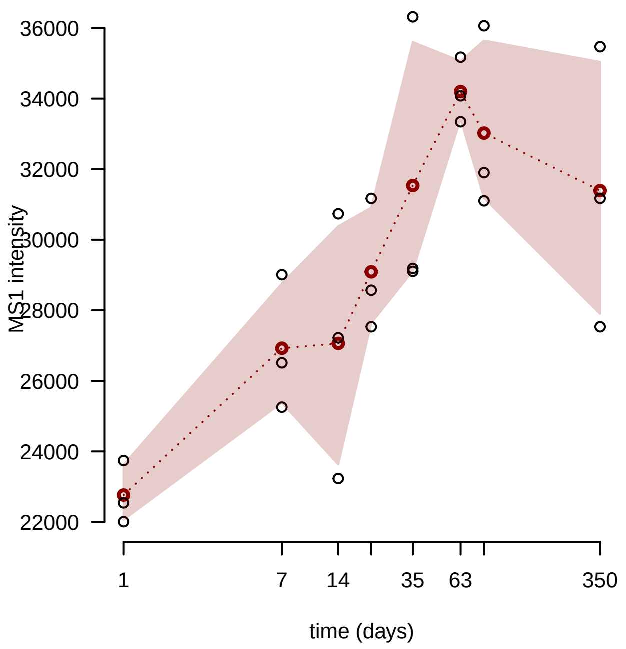

O54940
GN=Bnip2
BCL2/adenovirus E1B interacting protein ...

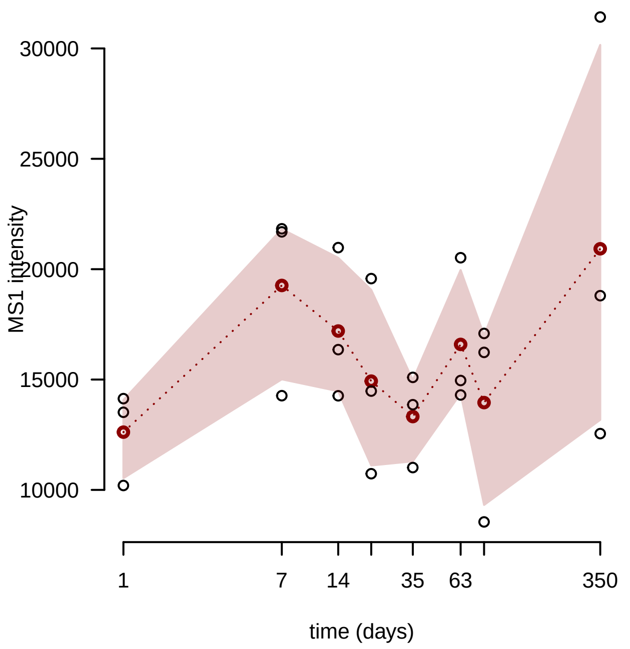

O54941
GN=Smarce1
SWI/SNF related, matrix associated, acti ...

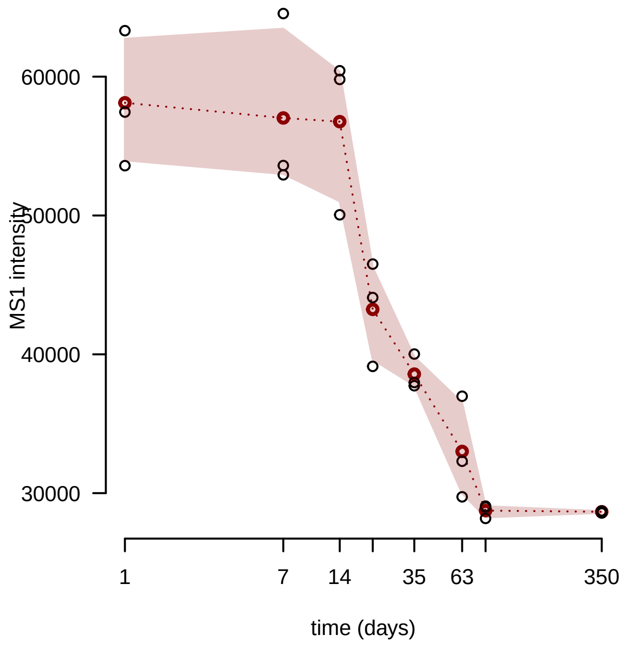

O54942
GN=Cldn5
claudin 5

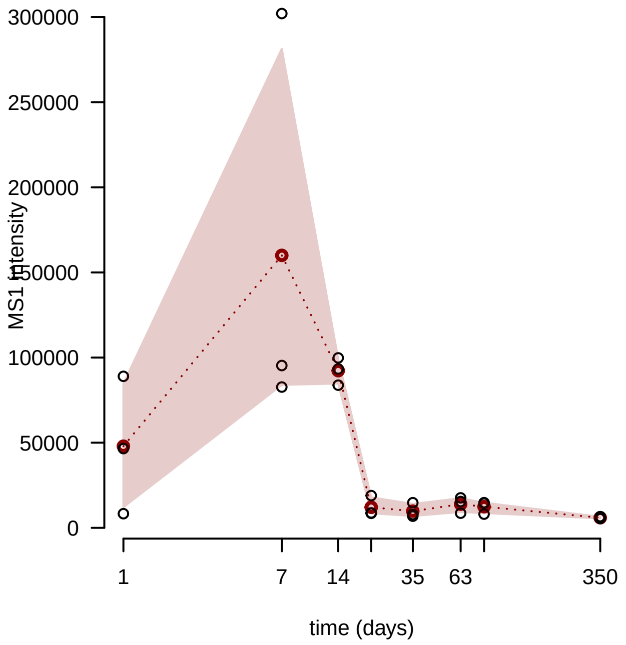

O54946
GN=Dnajb6
DnaJ heat shock protein family (Hsp40) m ...

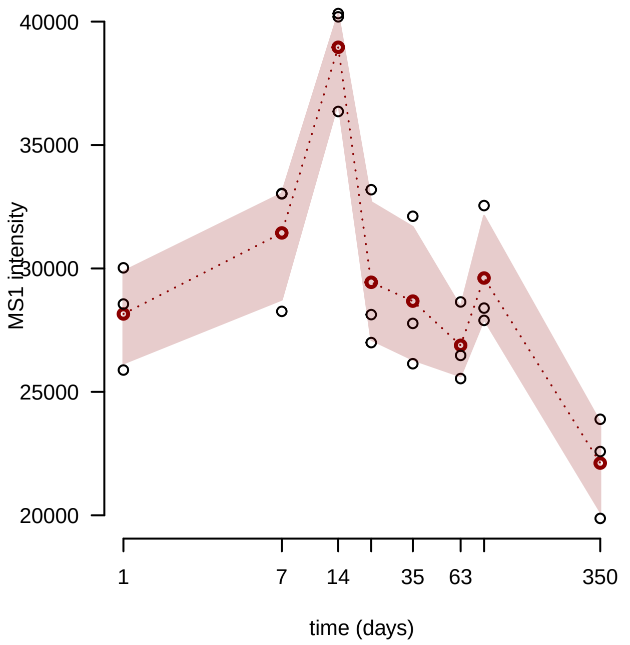

O54950
GN=Prkag1
protein kinase, AMP−activated, gamma 1 n ...

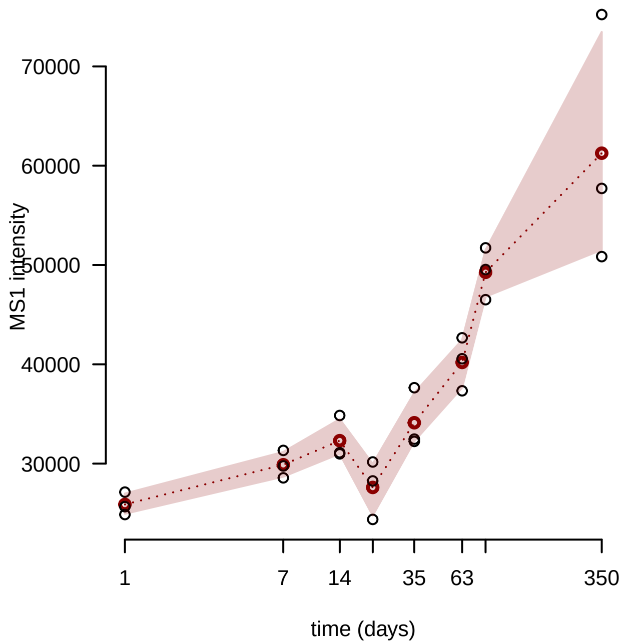

O54962
GN=Banf1
BAF nuclear assembly factor 1

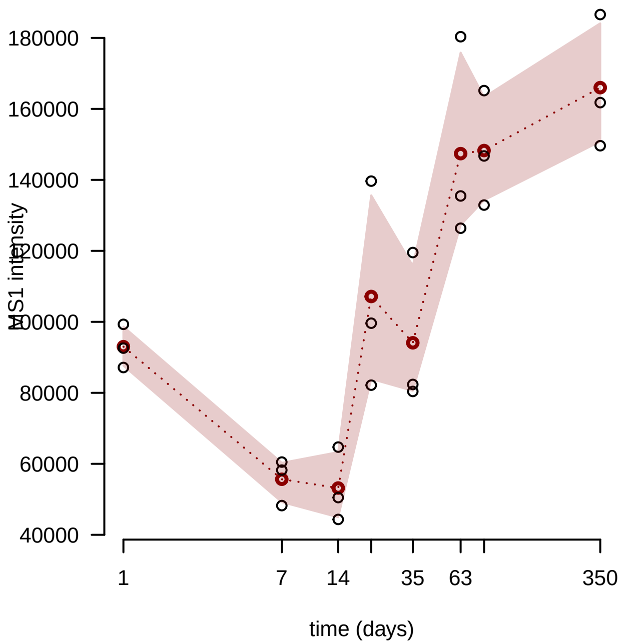

O54967
GN=Tnk2
tyrosine kinase, non−receptor, 2

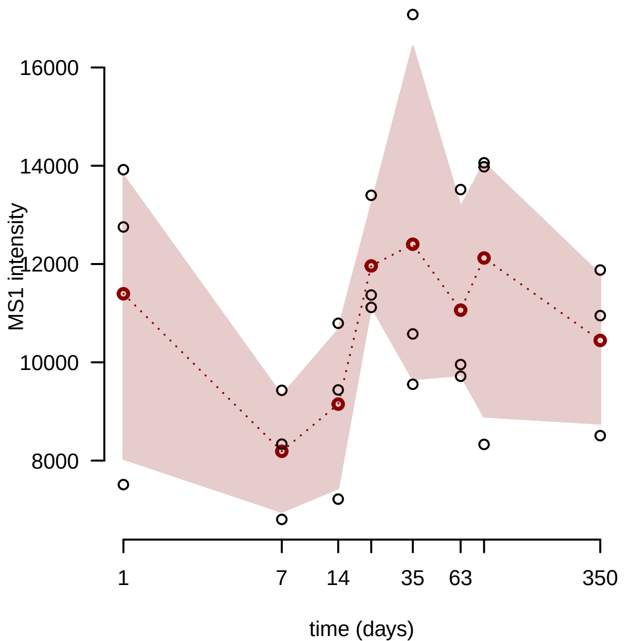

O54974
GN=Lgals7
lectin, galactose binding, soluble 7

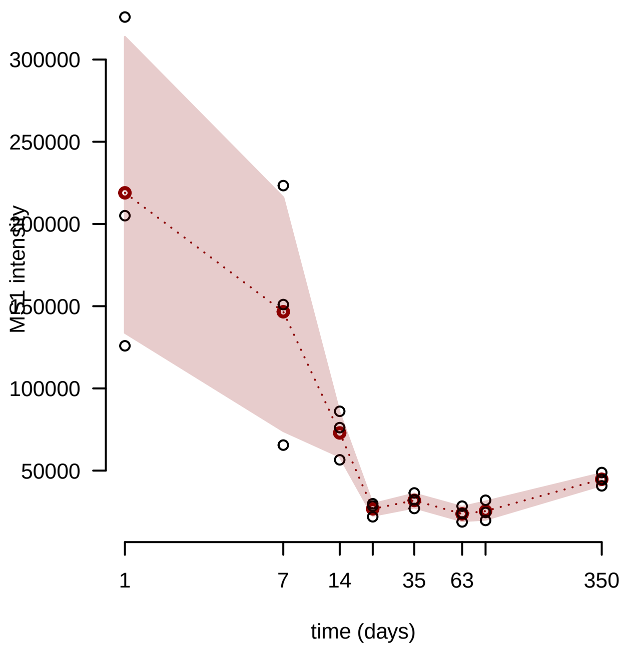

O54984
GN=Asna1

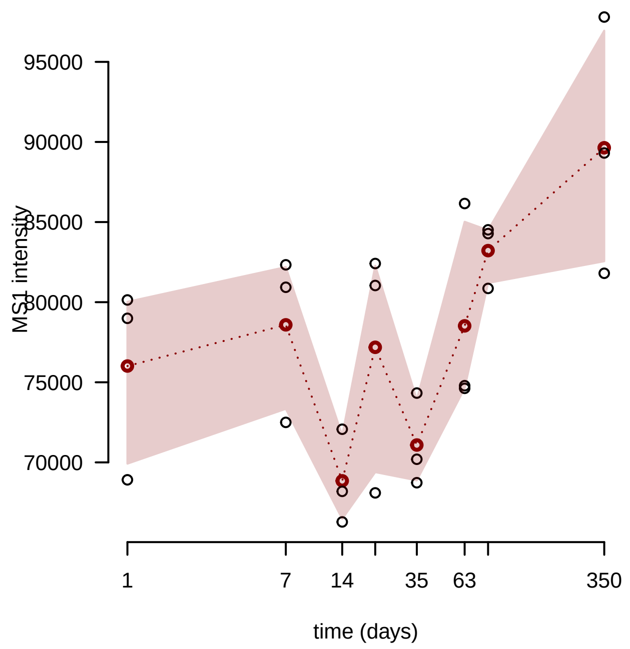

O54988
GN=Slk
STE20−like kinase

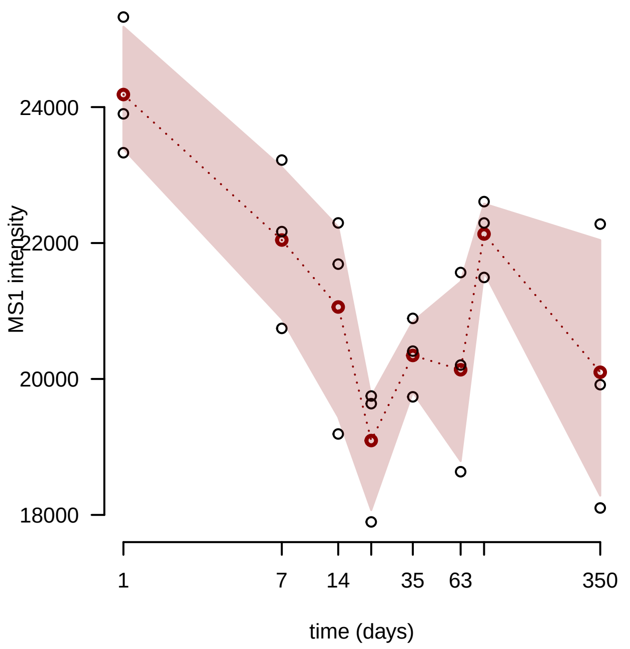

O54991
GN=Cntnap1
contactin associated protein−like 1

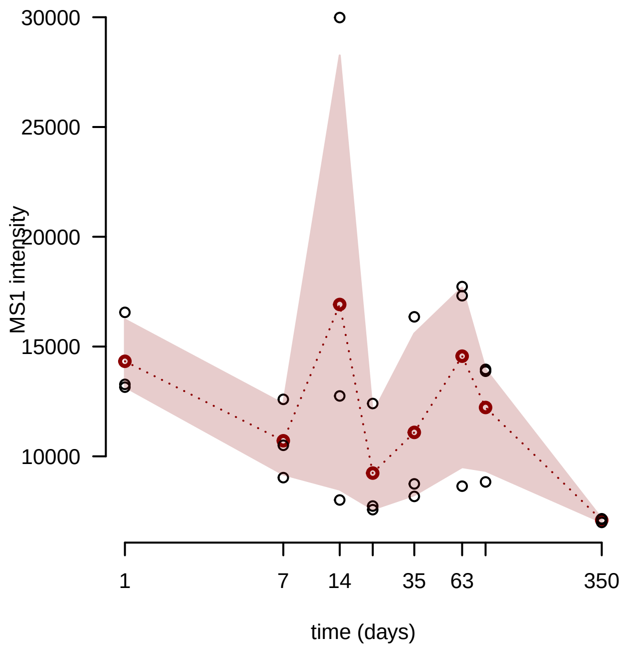

O54998
GN=Fkbp7
FK506 binding protein 7

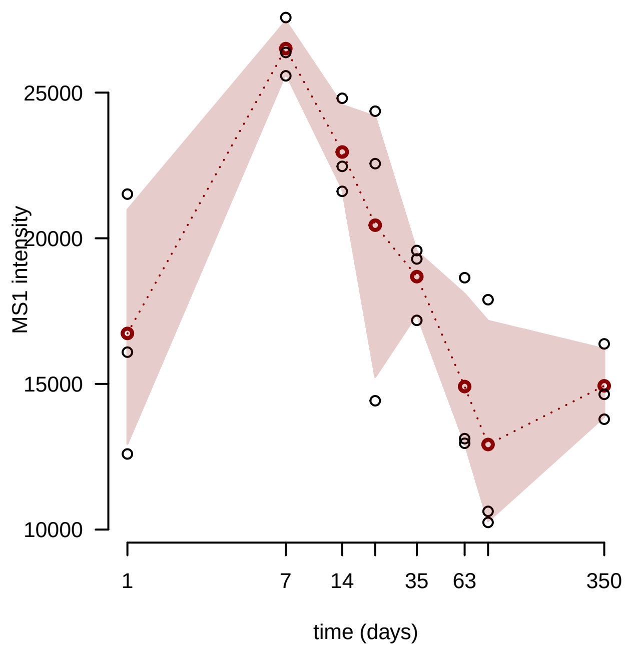

O55003
GN=Bnip3
BCL2/adenovirus E1B interacting protein ...

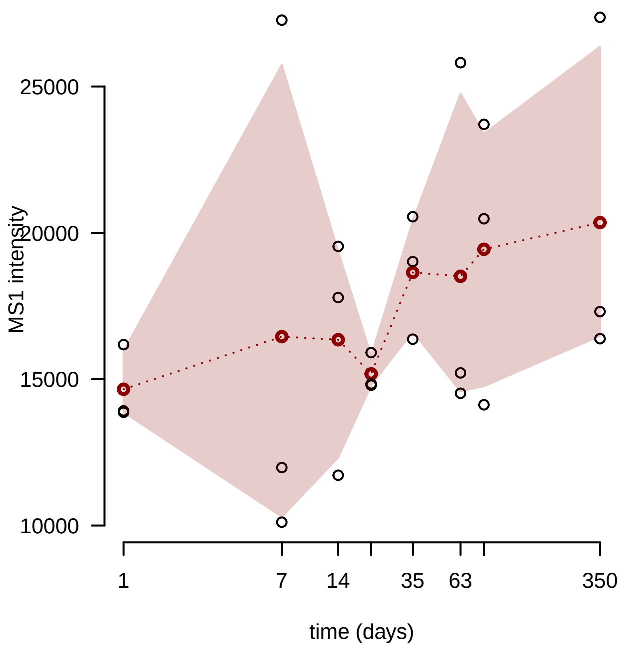

O55013
GN=Trappc3
trafficking protein particle complex 3

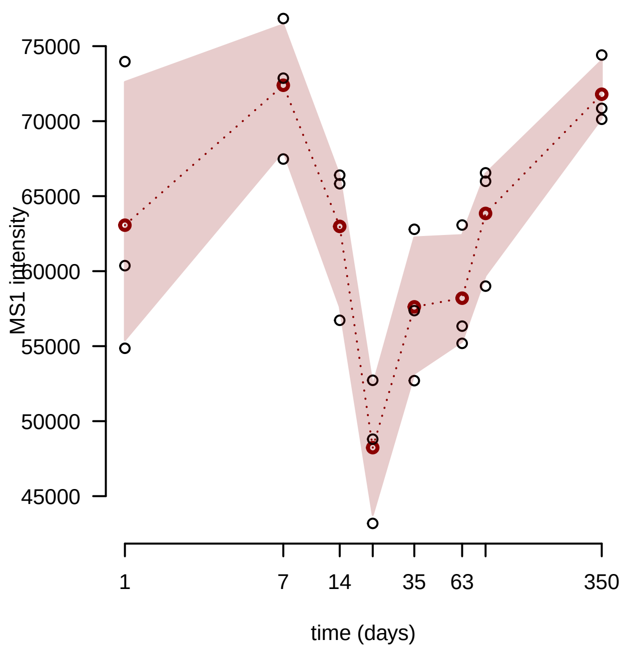

O55022
GN=Pgrmc1
progesterone receptor membrane component ...

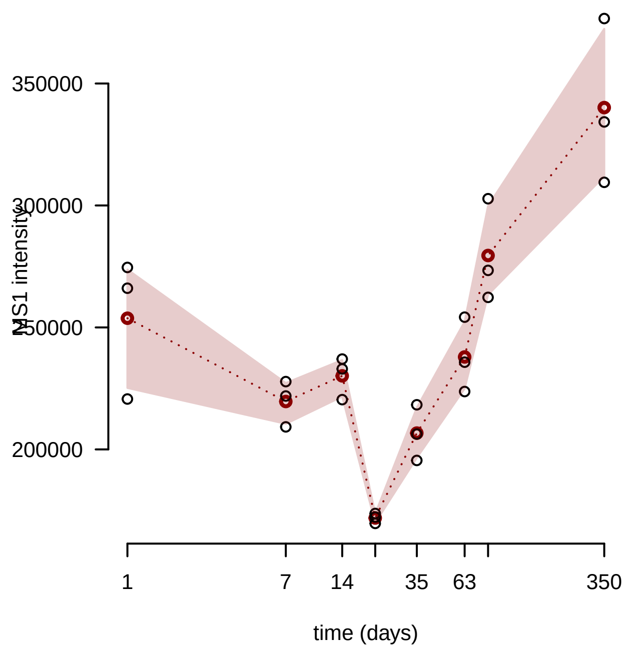

O55023
GN=Impa1
inositol (myo)−1(or 4)−monophosphatase 1

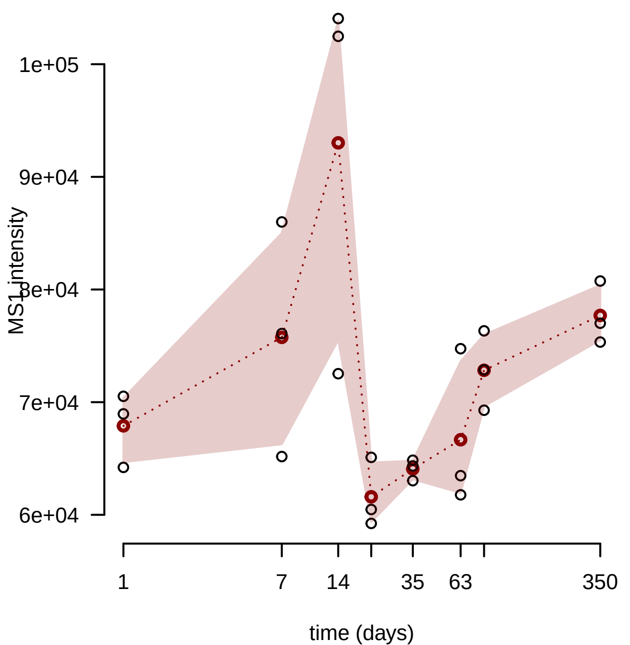

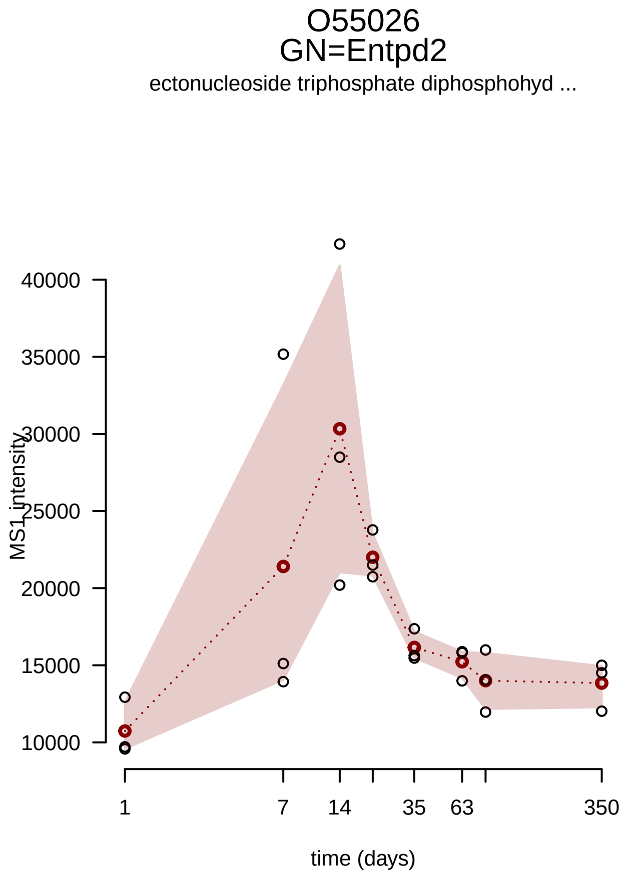
O55026
GN=Entpd2
ectonucleoside triphosphate diphosphohyd ...
MS1 intensity
10000
15000
20000
25000
30000
35000
40000
1
7
14
35
63
350
time (days)

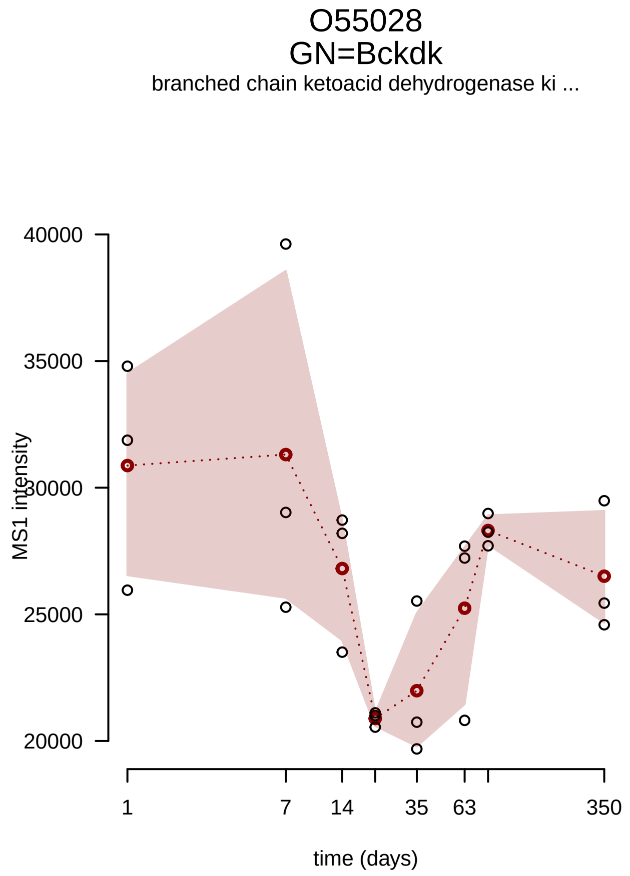
O55028
GN=Bckdk
branched chain ketoacid dehydrogenase ki ...
MS1 intensity
20000
25000
30000
35000
40000
1
7
14
35
63
350
time (days)

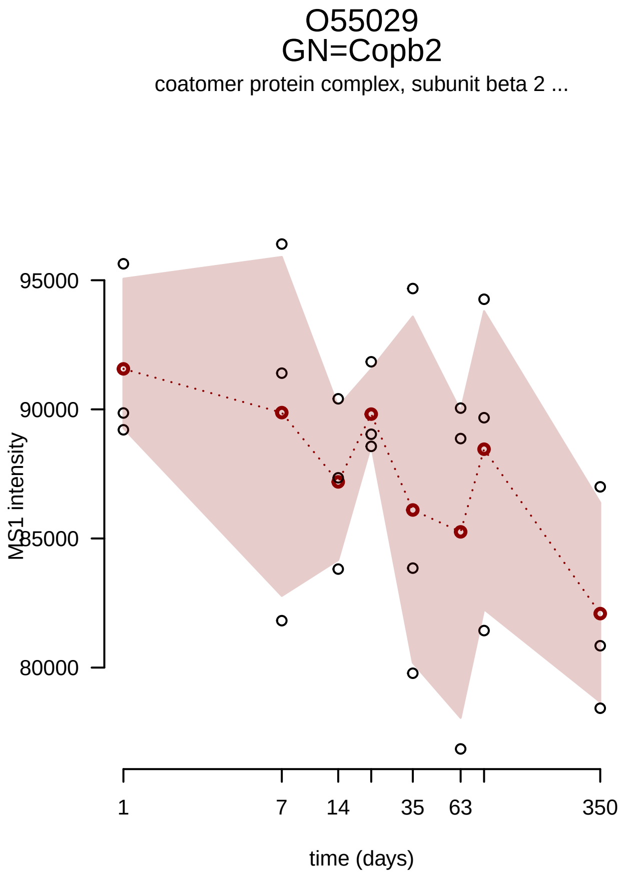
O55029
GN=Copb2
coatomer protein complex, subunit beta 2 ...
MS1 intensity
80000
85000
90000
95000
1
7
14
35
63
350
time (days)

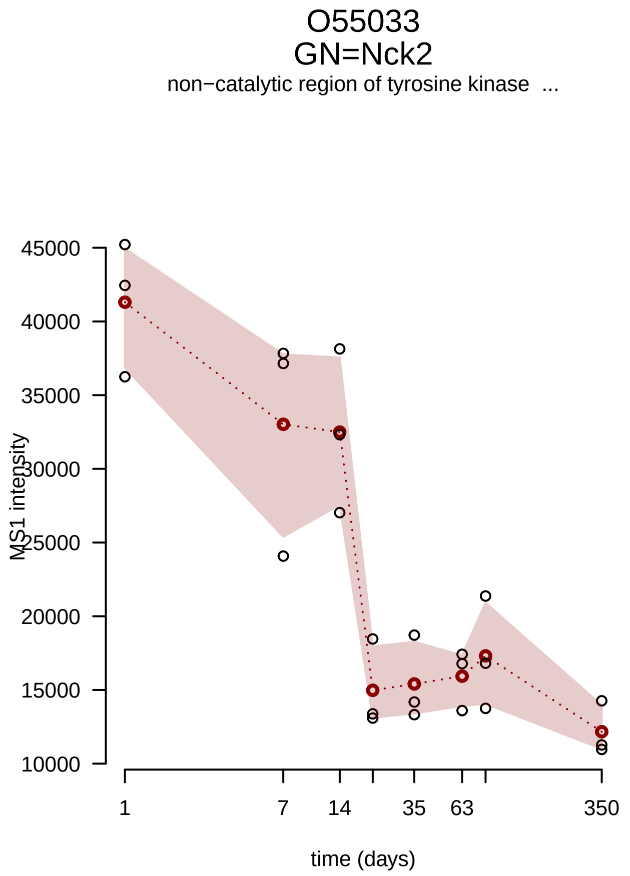
O55033
GN=Nck2
non−catalytic region of tyrosine kinase ...
MS1 intensity
10000
15000
20000
25000
30000
35000
40000
45000
1
7
14
35
63
350
time (days)

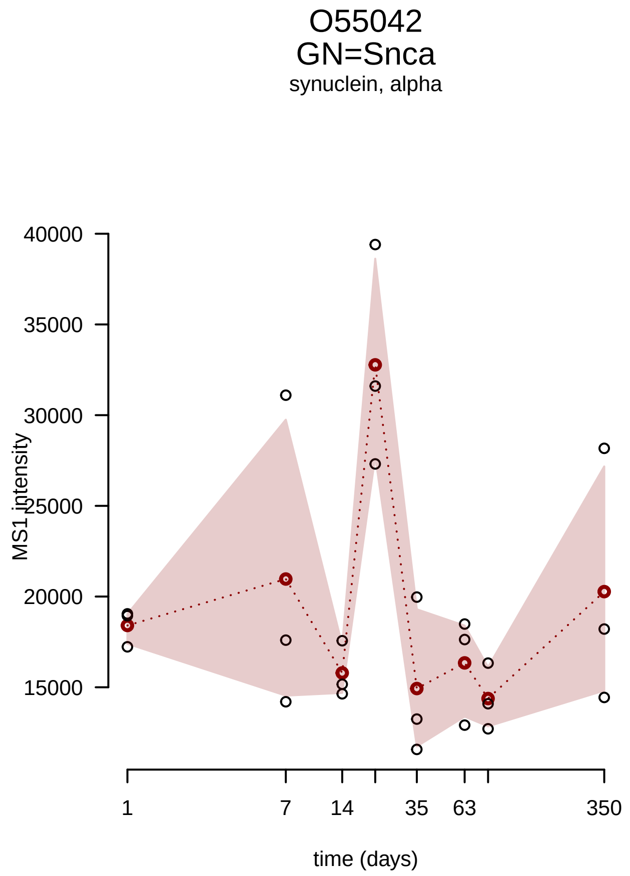
O55042
GN=Snca
synuclein, alpha
MS1 intensity
15000
20000
25000
30000
35000
40000
1
7
14
35
63
350
time (days)

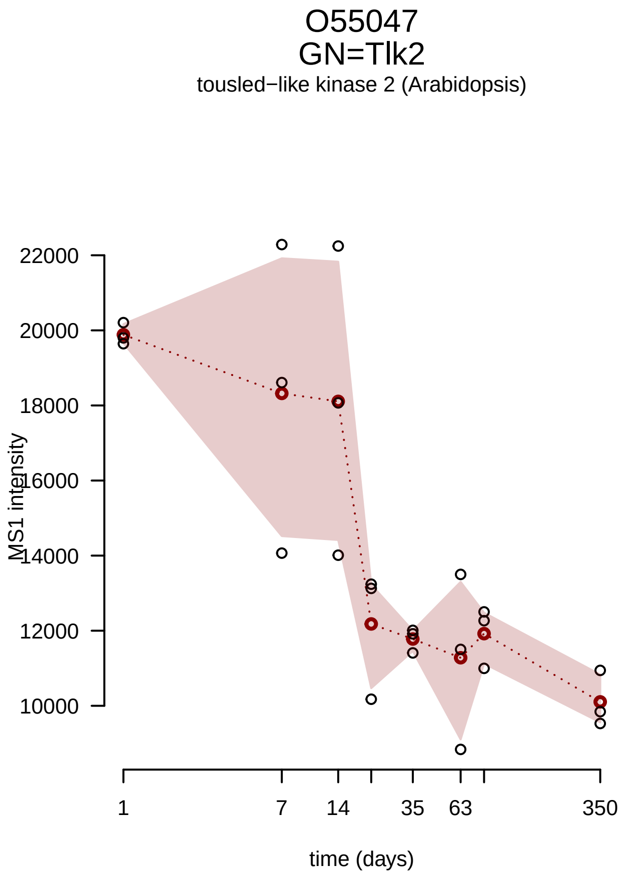
O55047
GN=Tlk2
tousled−like kinase 2 (Arabidopsis)
MS1 intensity
10000
12000
14000
16000
18000
20000
22000
1
7
14
35
63
350
time (days)

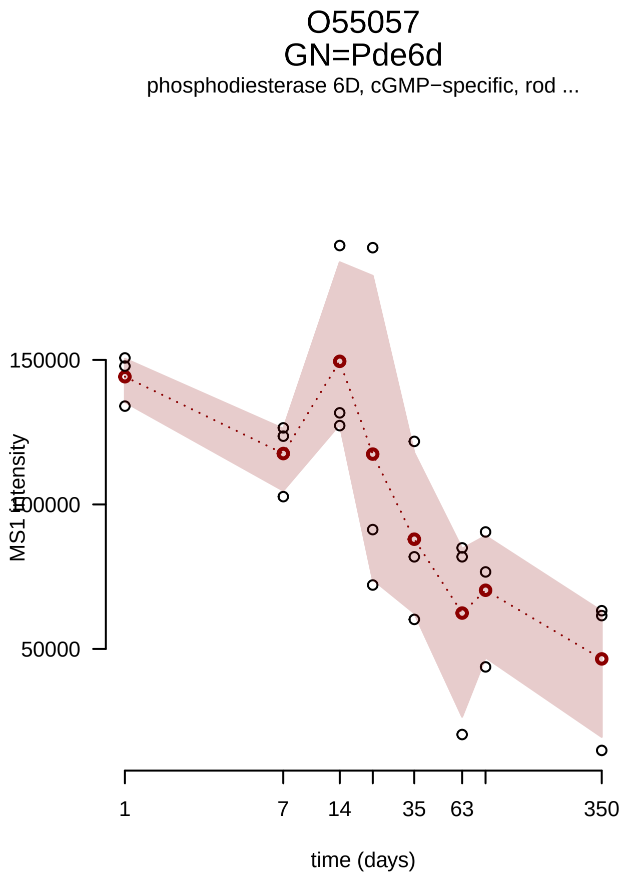
O55057
GN=Pde6d
phosphodiesterase 6D, cGMP−specific, rod ...
MS1 intensity
50000
100000
150000
1
7
14
35
63
350
time (days)

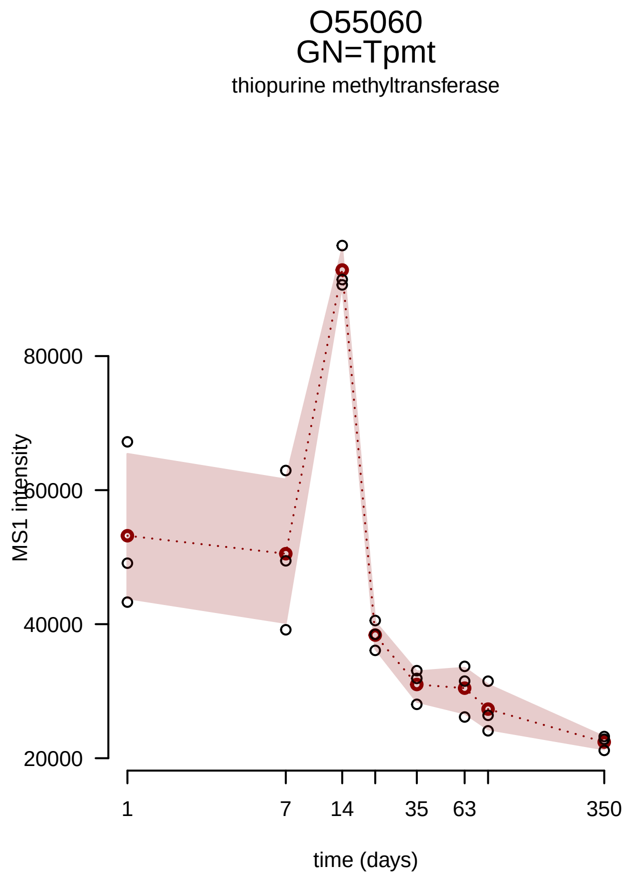
O55060
GN=Tpmt
thiopurine methyltransferase
MS1 intensity
20000
40000
60000
80000
1
7
14
35
63
350
time (days)

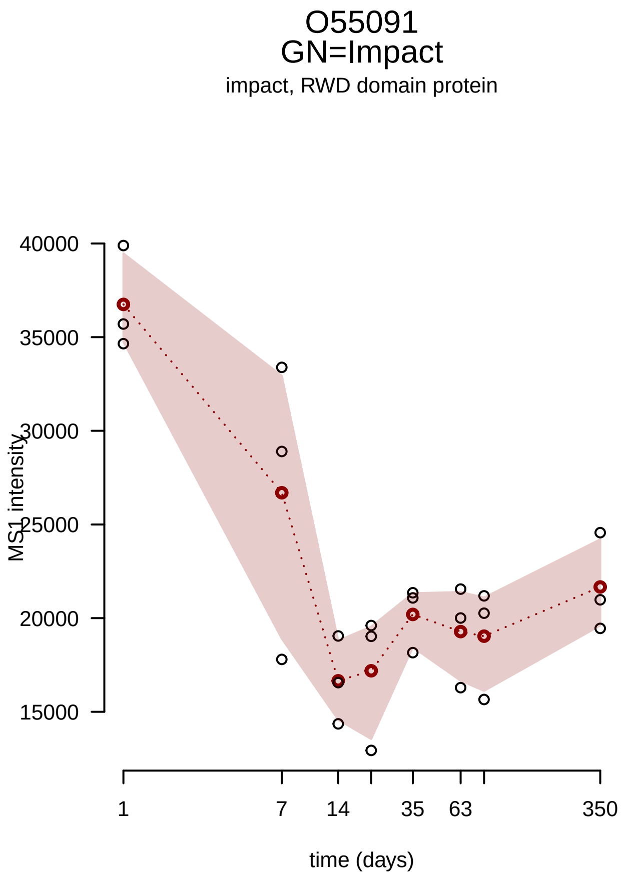
O55091
GN=Impact
impact, RWD domain protein
MS1 intensity
15000
20000
25000
30000
35000
40000
1
7
14
35
63
350
time (days)

O55098
GN=Stk10
serine/threonine kinase 10

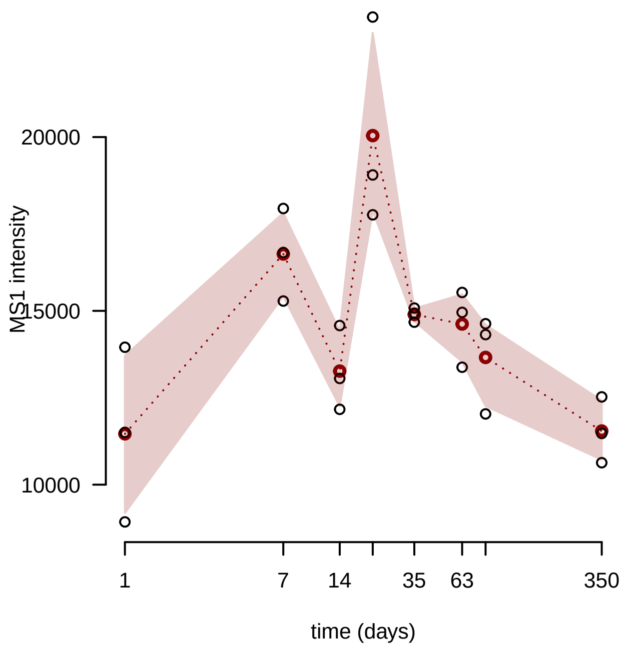

O55100
GN=Syngr1
synaptogyrin 1

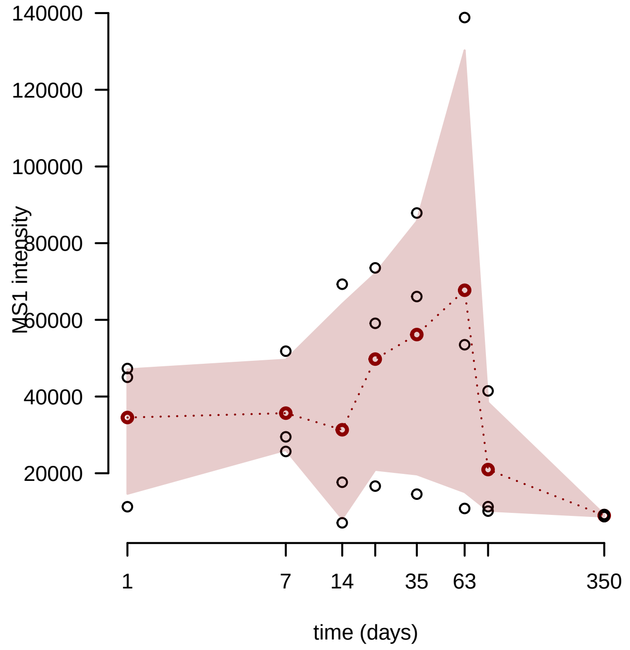

O55101
GN=Syngr2
synaptogyrin 2

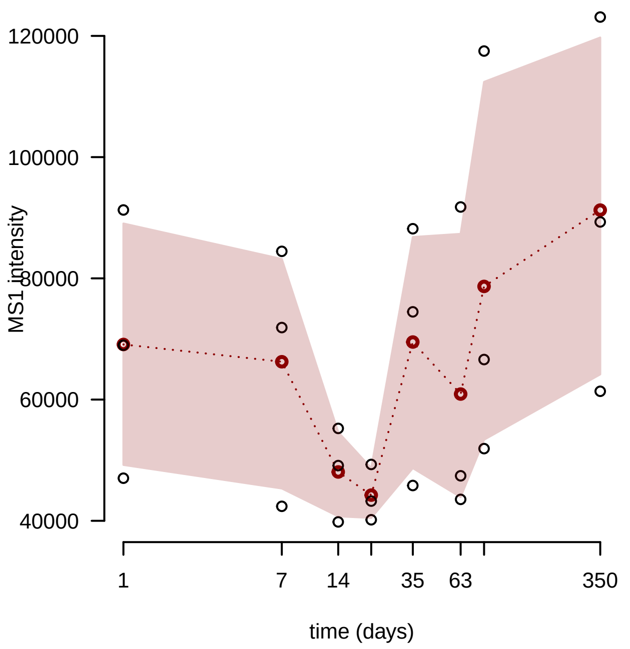

O55102
GN=Bloc1s1
biogenesis of lysosomal organelles compl ...

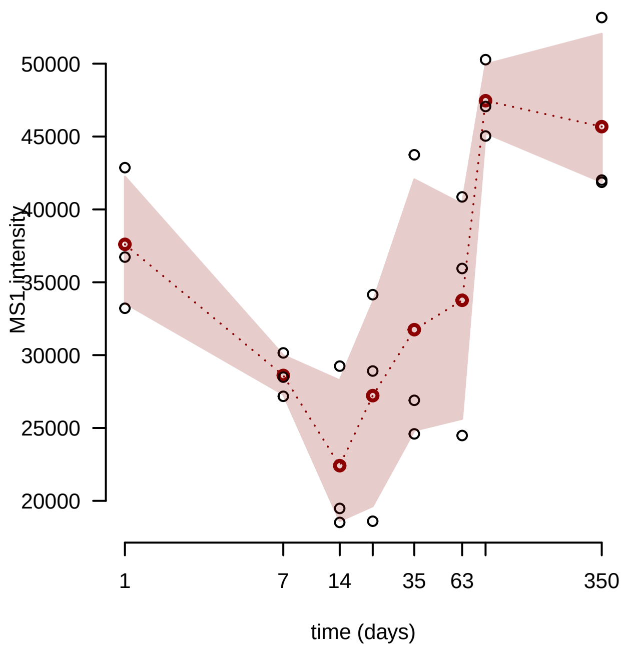

O55106
GN=Strn
striatin, calmodulin binding protein

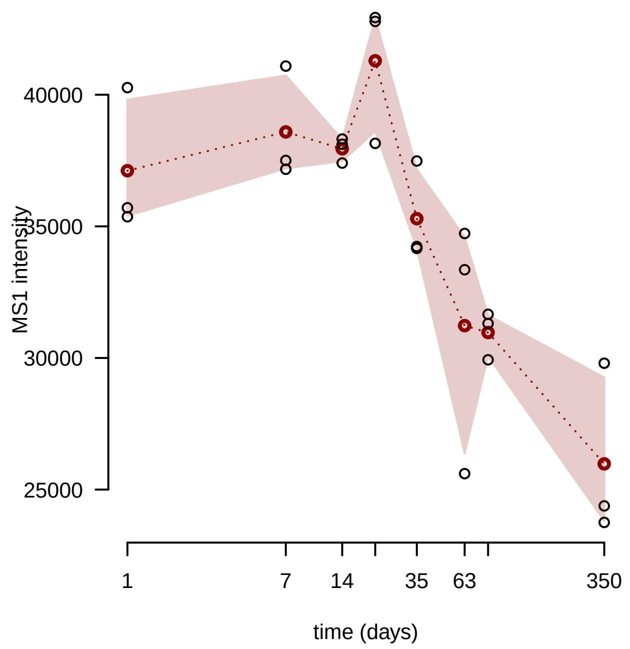

O55111
GN=Dsg2
desmoglein 2

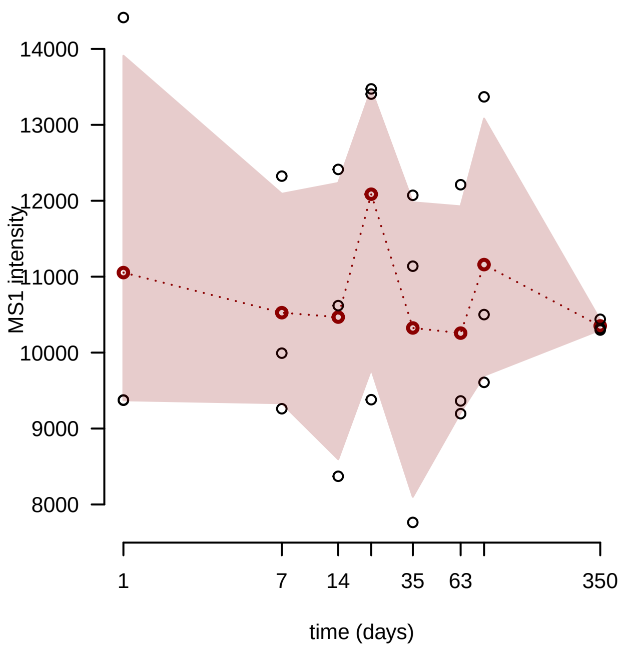

O55125
GN=Nipsnap1
nipsnap homolog 1

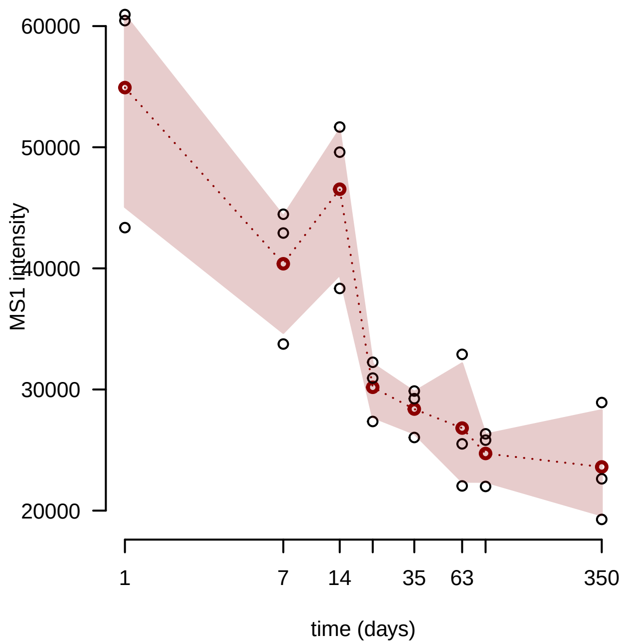

O55126
GN=Nipsnap2
nipsnap homolog 2

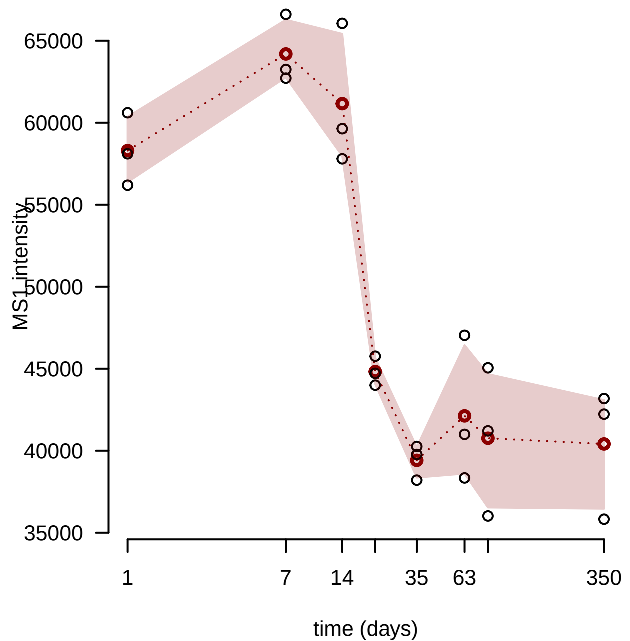

O55128
GN=Sap18
Sin3−associated polypeptide 18

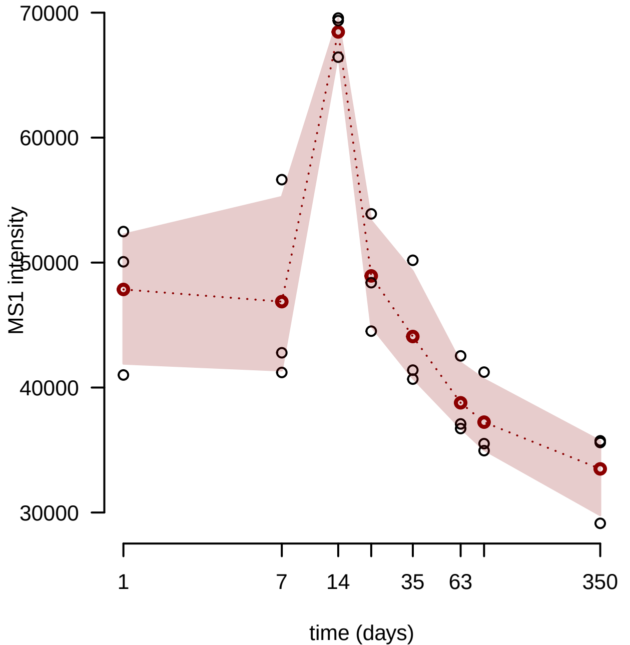

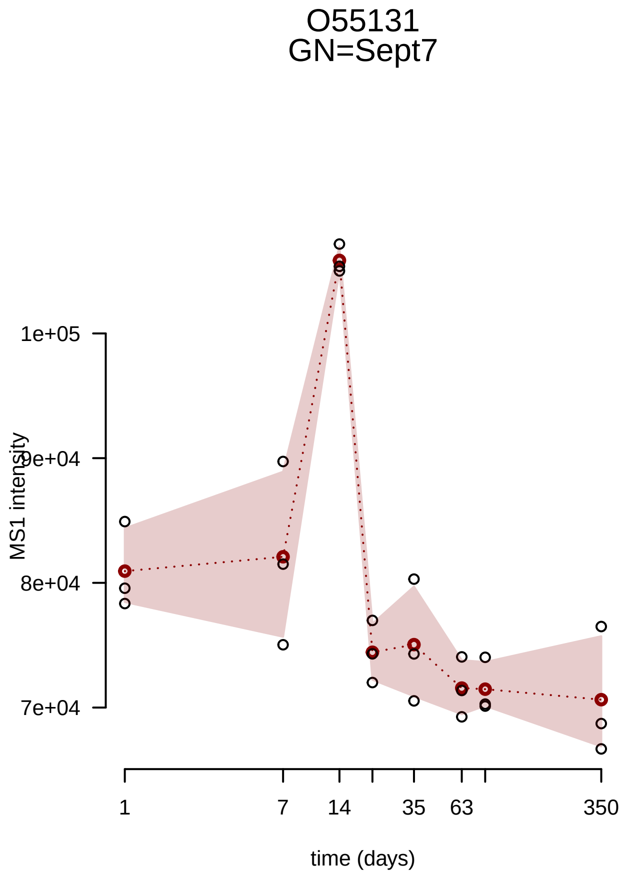
O55131
GN=Sept7
1e+05
9e+04
8e+04
7e+04
MS1 intensity
1
7
14
35
63
350
time (days)

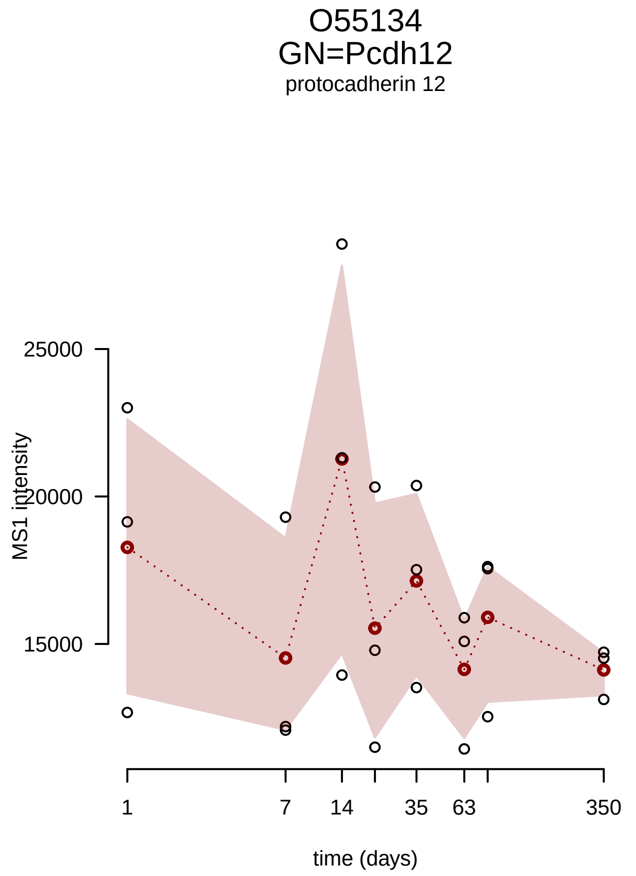
O55134
GN=Pcdh12
protocadherin 12
25000
20000
15000
MS1 intensity
1
7
14
35
63
350
time (days)

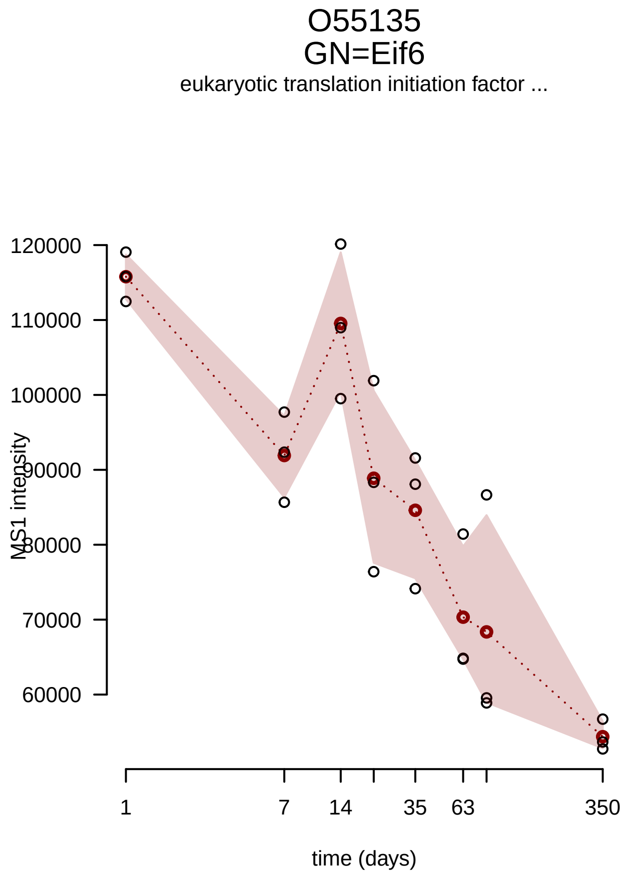
O55135
GN=Eif6
eukaryotic translation initiation factor ...
120000
110000
100000
90000
80000
70000
60000
MS1 intensity
1
7
14
35
63
350
time (days)

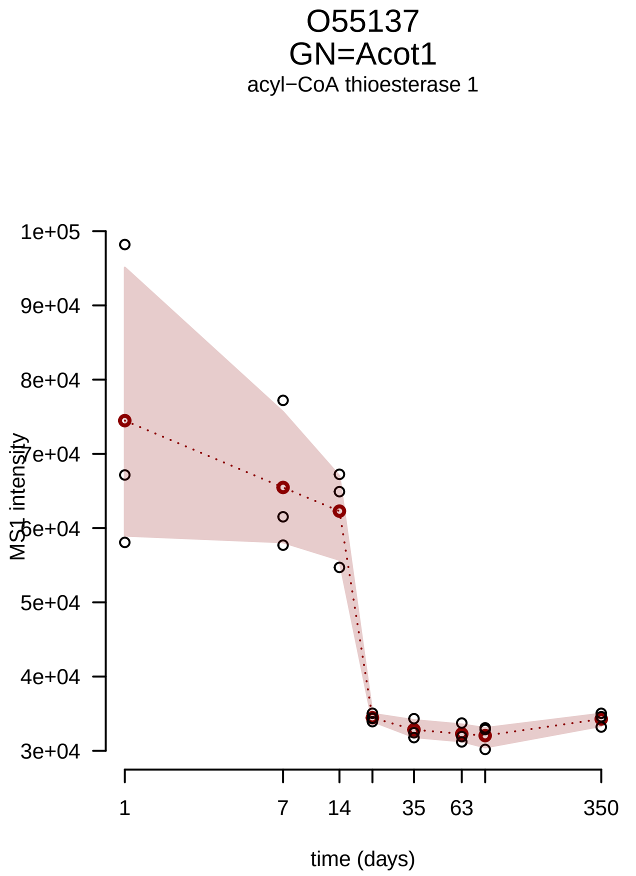
O55137
GN=Acot1
acyl−CoA thioesterase 1
1e+05
9e+04
8e+04
7e+04
6e+04
5e+04
4e+04
3e+04
MS1 intensity
1
7
14
35
63
350
time (days)

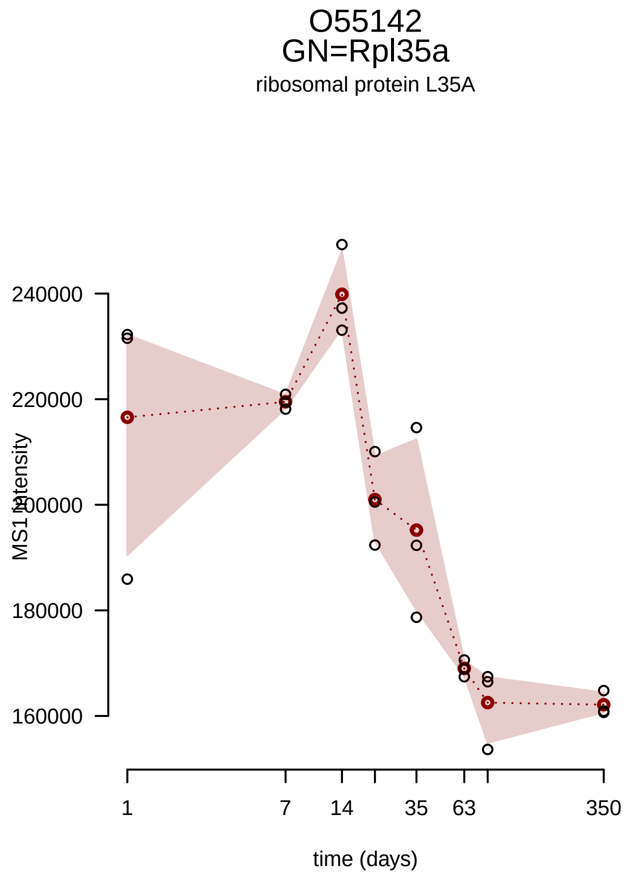
O55142
GN=Rpl35a
ribosomal protein L35A
240000
220000
200000
180000
160000
MS1 intensity
1
7
14
35
63
350
time (days)

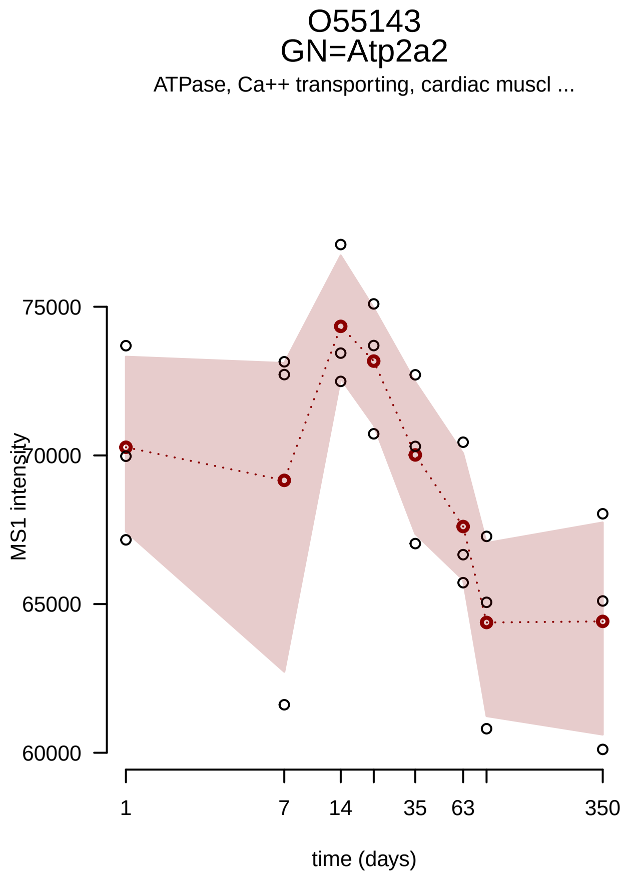
O55143
GN=Atp2a2
ATPase, Ca++ transporting, cardiac muscl ...
75000
70000
65000
60000
MS1 intensity
1
7
14
35
63
350
time (days)

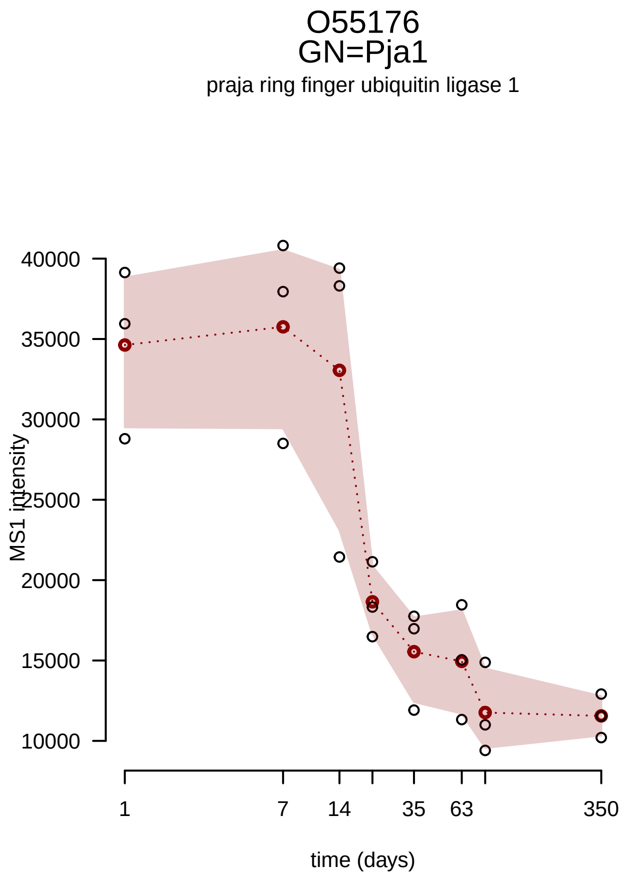
O55176
GN=Pja1
praja ring finger ubiquitin ligase 1
40000
35000
30000
25000
20000
15000
10000
MS1 intensity
1
7
14
35
63
350
time (days)

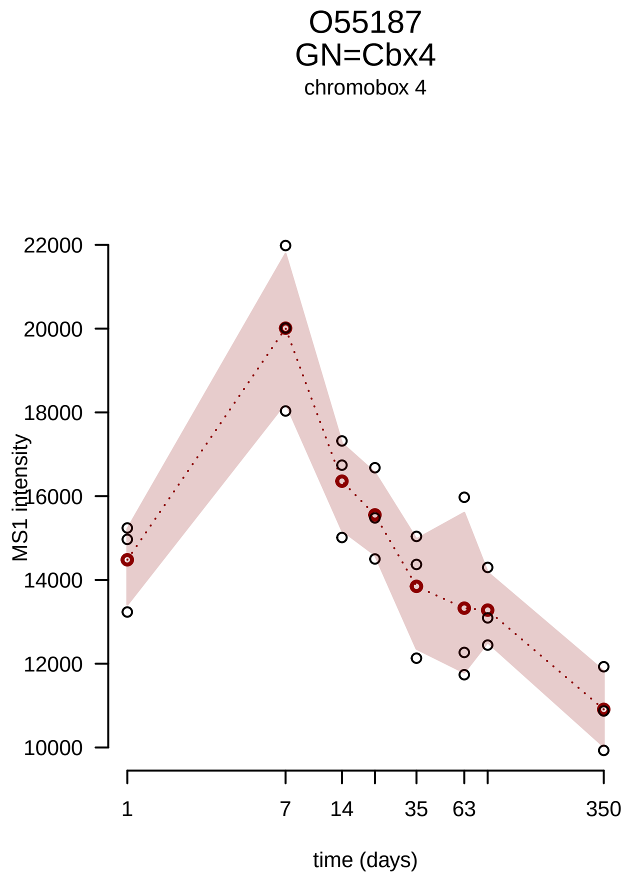
O55187
GN=Cbx4
chromobox 4
22000
20000
18000
16000
14000
12000
10000
MS1 intensity
1
7
14
35
63
350
time (days)

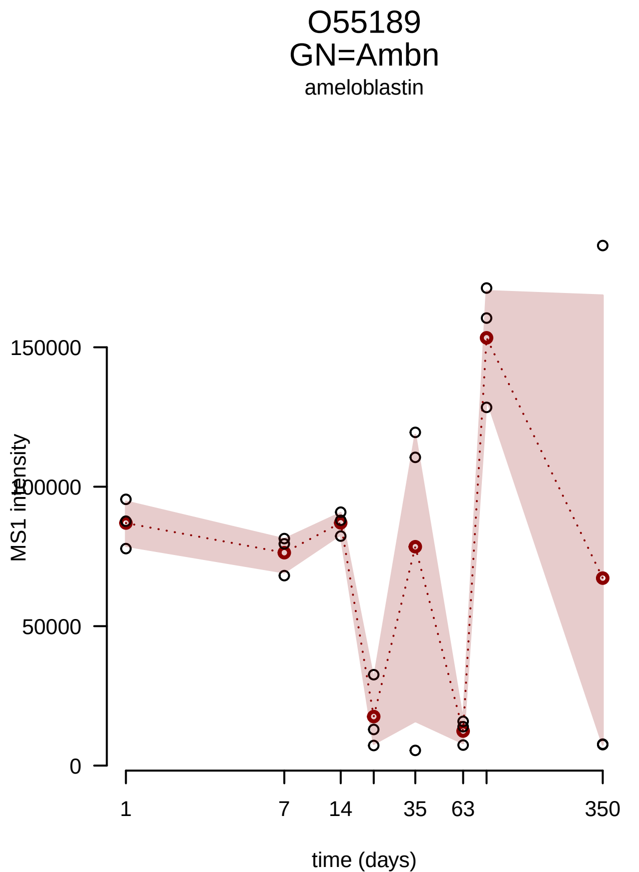
O55189
GN=Ambn
ameloblastin
150000
100000
50000
0
MS1 intensity
1
7
14
35
63
350
time (days)

O55201
GN=Supt5h

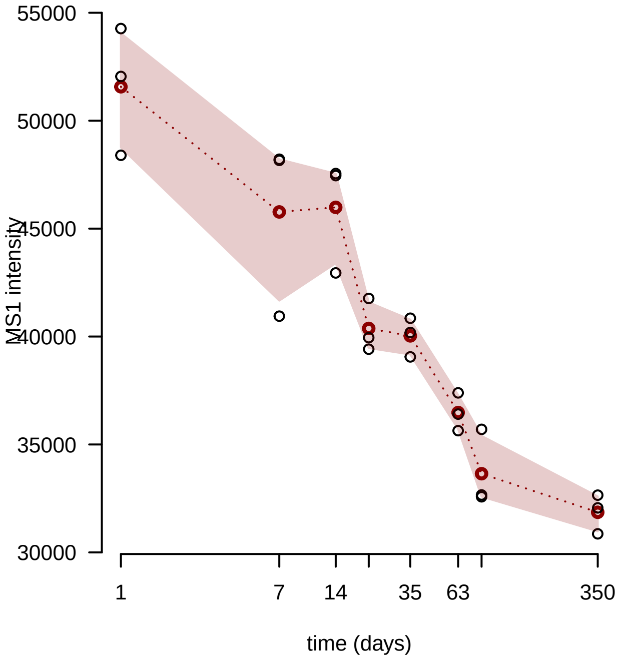

O55208
GN=Figla
folliculogenesis specific basic helix−lo ...

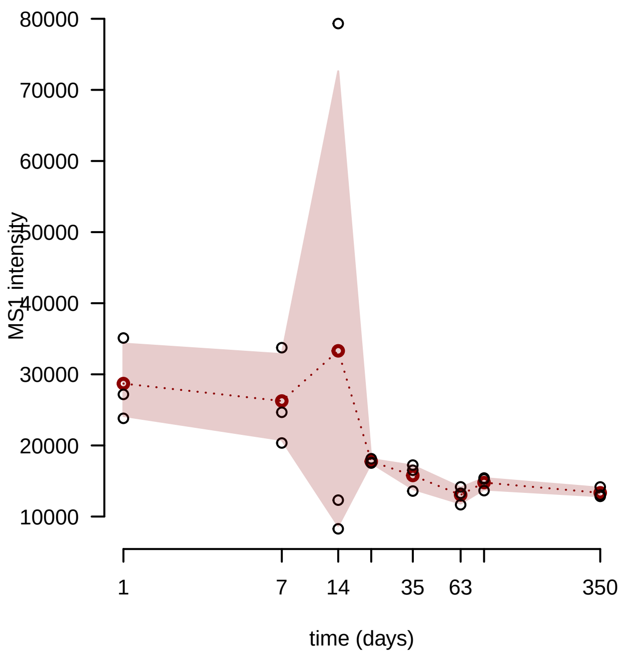

O55222
GN=Ilk
integrin linked kinase

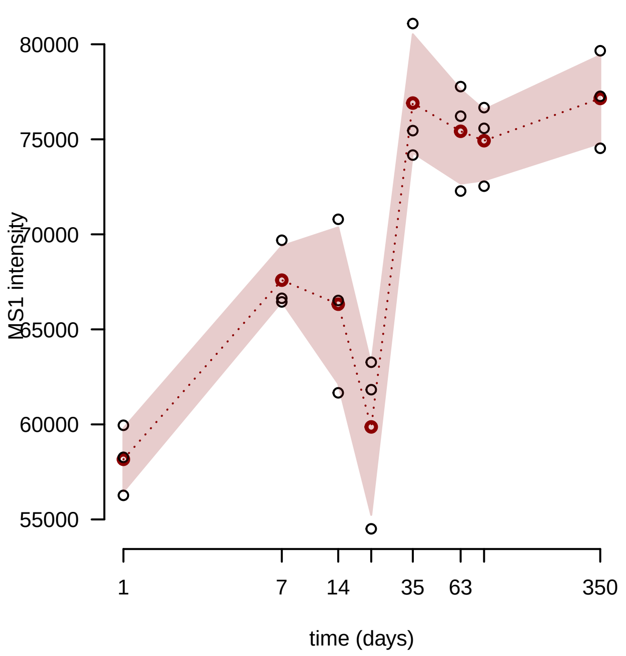

O55229
GN=Chkb
choline kinase beta

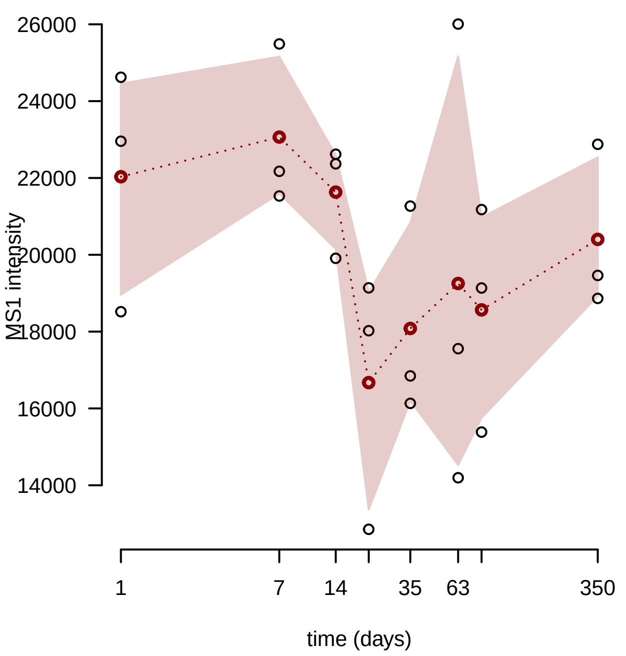

O55234
GN=Psmb5
proteasome (prosome, macropain) subunit, ...

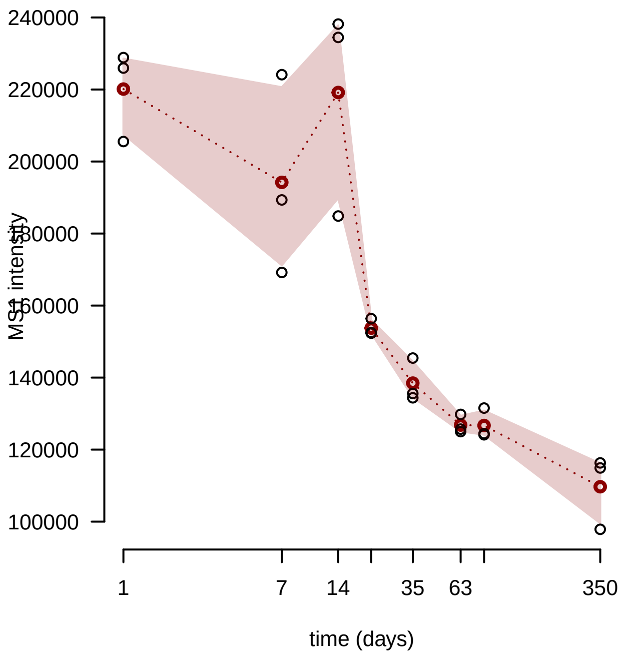

O55236
GN=Rngtt
RNA guanylyltransferase and 5'−phosphata ...

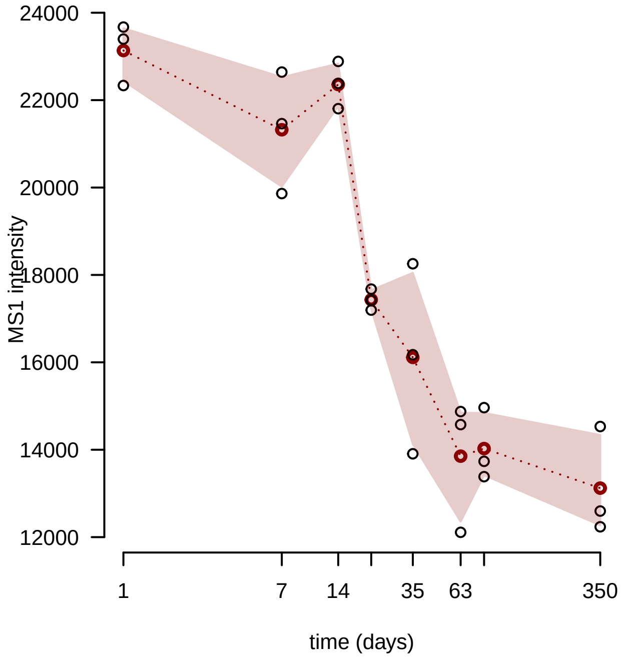

O55242
GN=Sigmar1
sigma non−opioid intracellular receptor ...

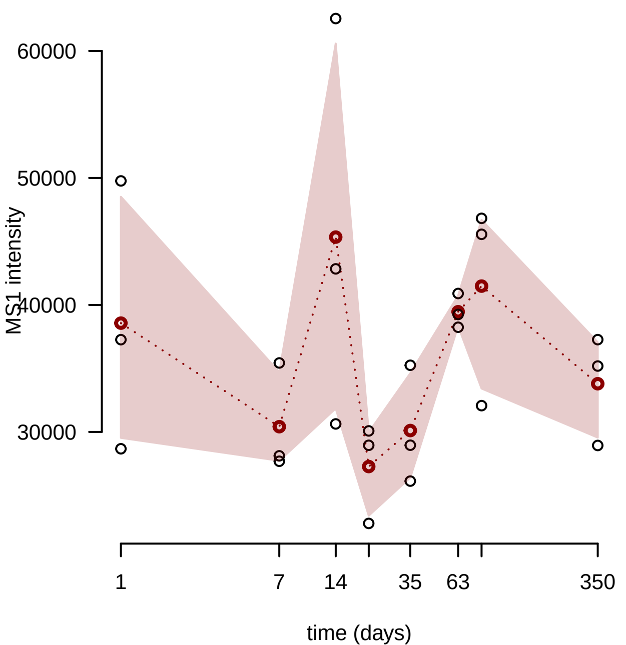

O70126
GN=Aurkb
aurora kinase B

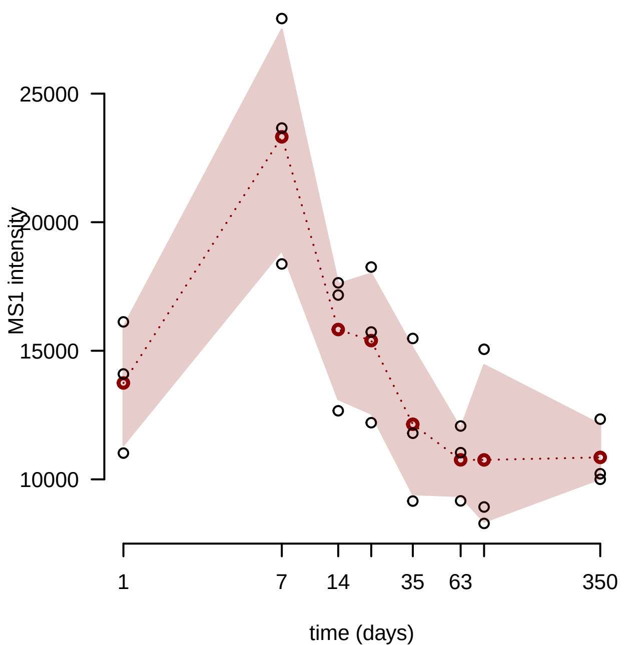

O70131
GN=Ninj1
ninjurin 1

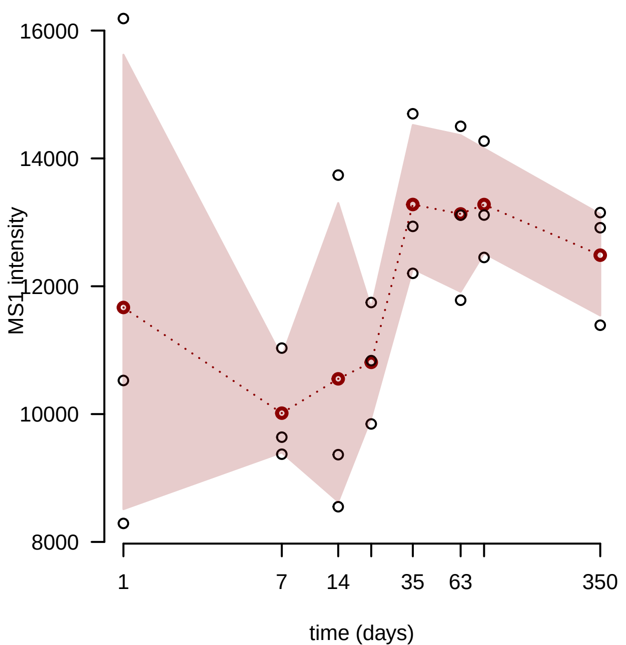

O70133
GN=Dhx9
DEAH (Asp−Glu−Ala−His) box polypeptide 9

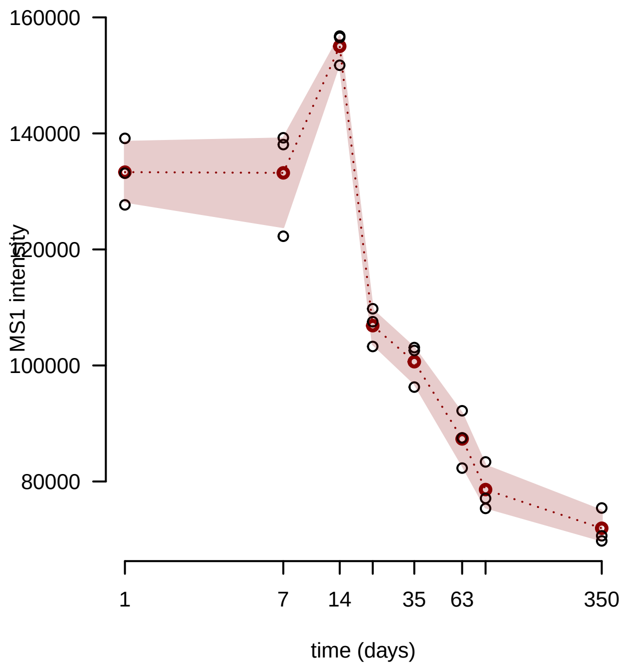

O70133
GN=Dhx9
DEAH (Asp−Glu−Ala−His) box polypeptide 9

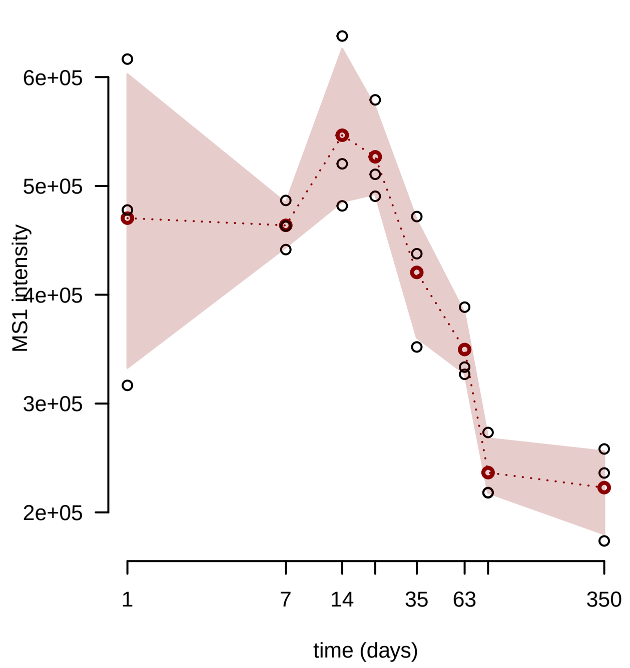

O70145
GN=Ncf2
neutrophil cytosolic factor 2

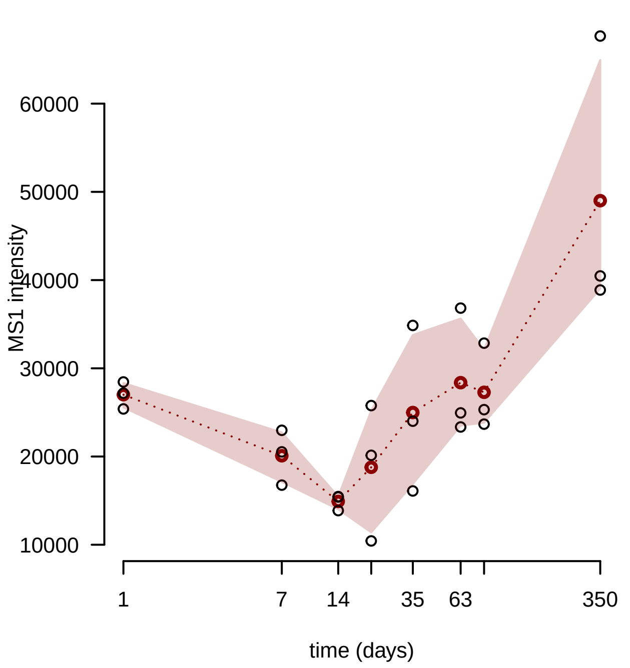

O70152
GN=Dpm1
dolichol−phosphate (beta−D) mannosyltran ...

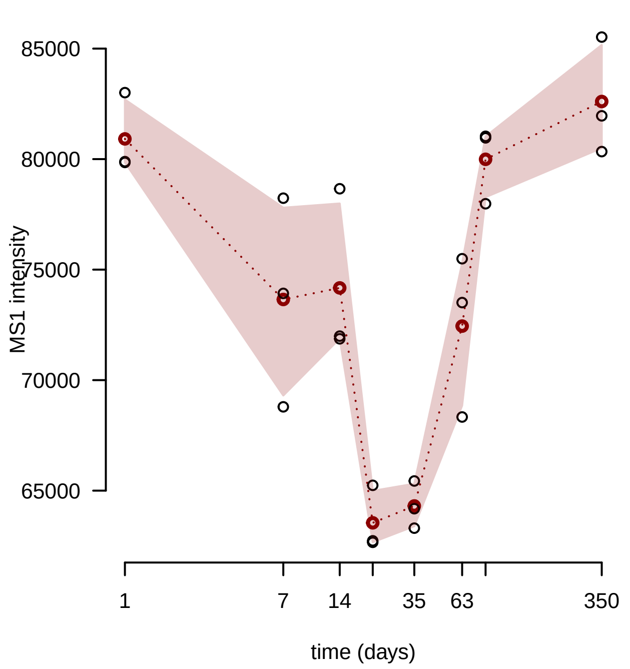

O70157
GN=Top3a
topoisomerase (DNA) III alpha

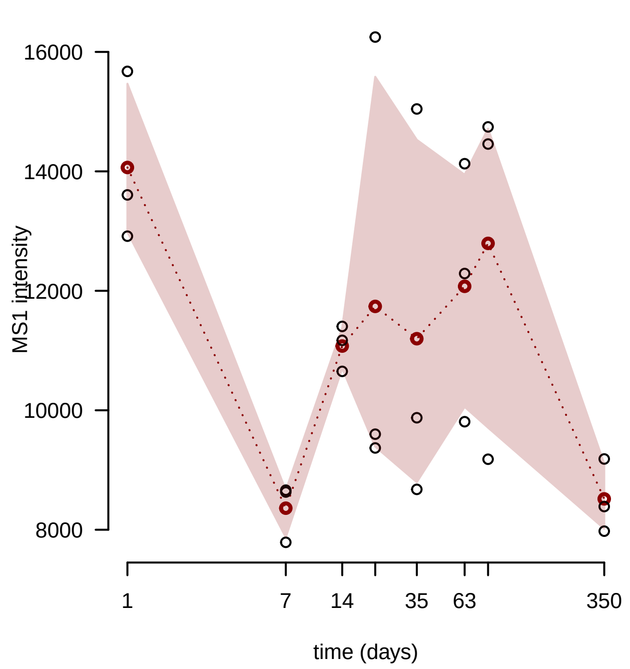

O70161
GN=Pip5k1c
phosphatidylinositol−4−phosphate 5−kinas ...

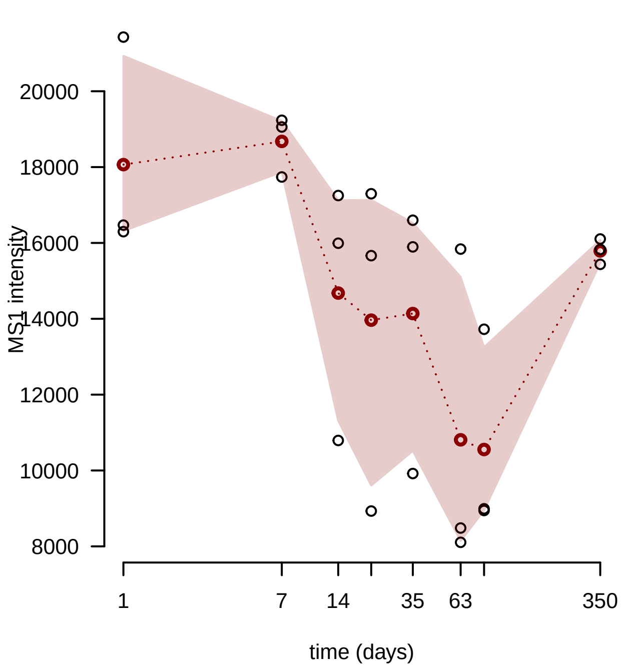

O70165
GN=Fcn1

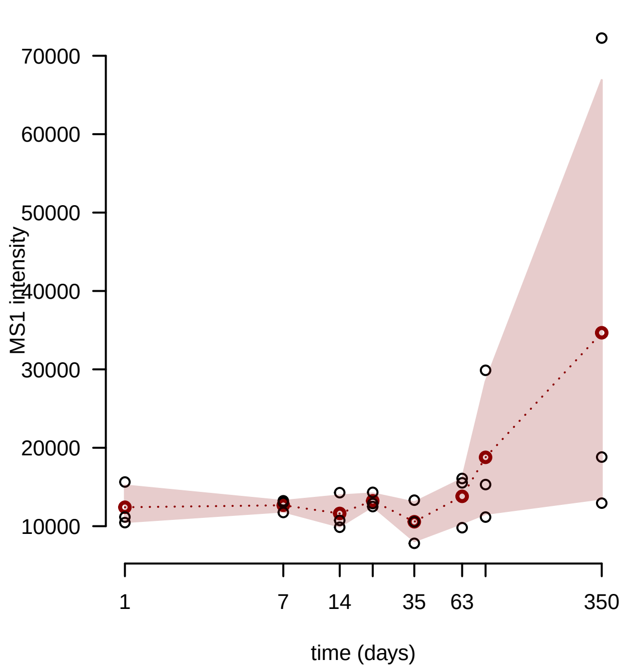

O70172
GN=Pip4k2a
phosphatidylinositol−5−phosphate 4−kinas ...

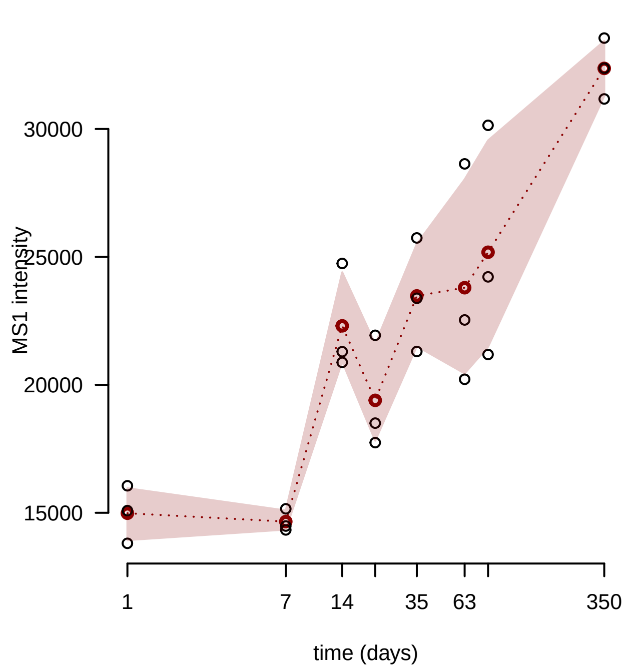

O70194
GN=Eif3d
eukaryotic translation initiation factor ...

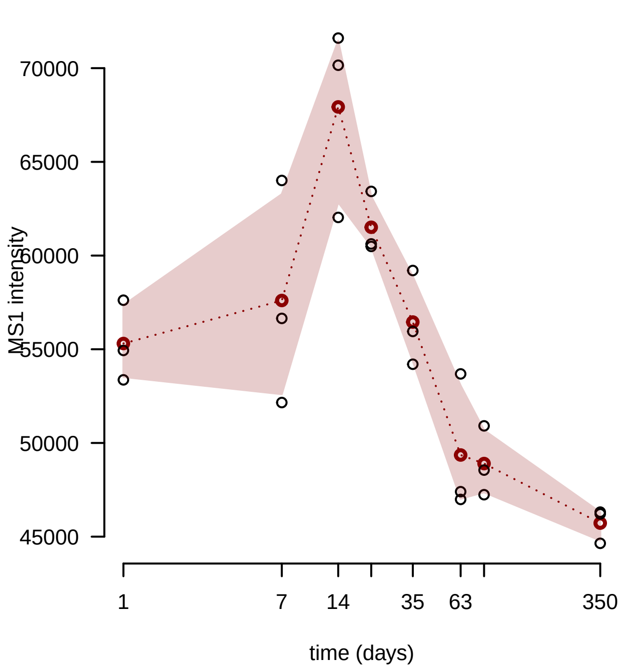

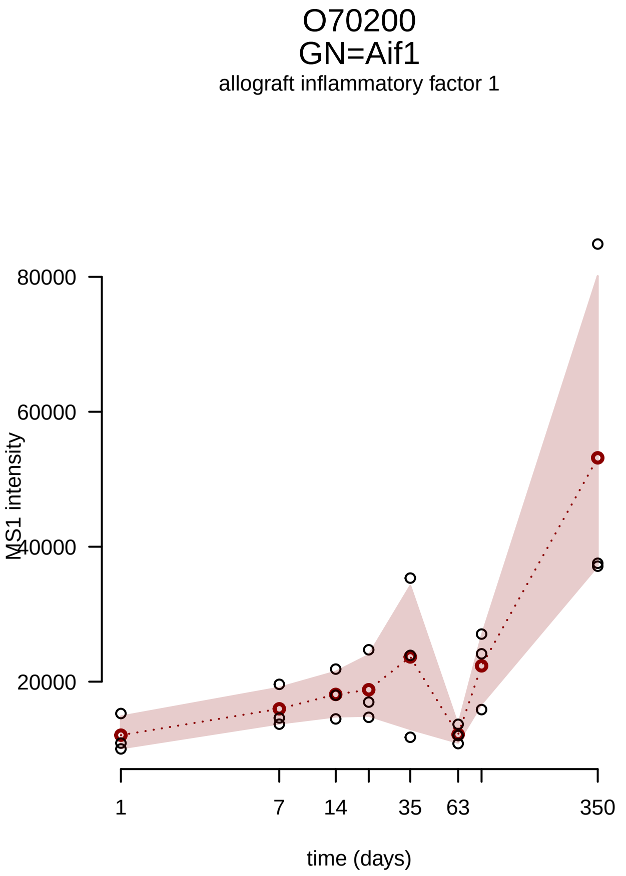
O70200
GN=Aif1
allograft inflammatory factor 1
80000
60000
40000
20000
MS1 intensity
1
7
14
35
63
350
time (days)

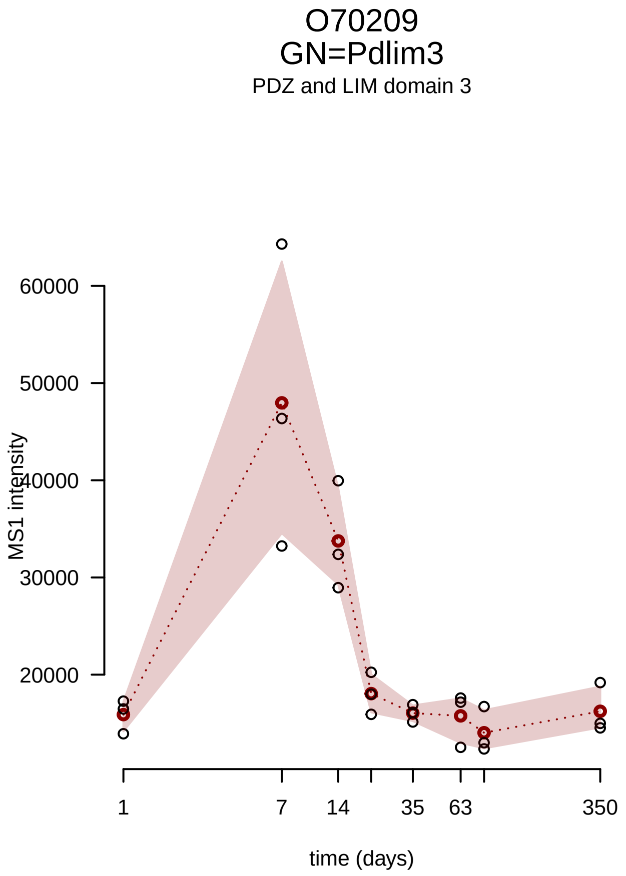
O70209
GN=Pdlim3
PDZ and LIM domain 3
60000
50000
40000
30000
20000
MS1 intensity
1
7
14
35
63
350
time (days)

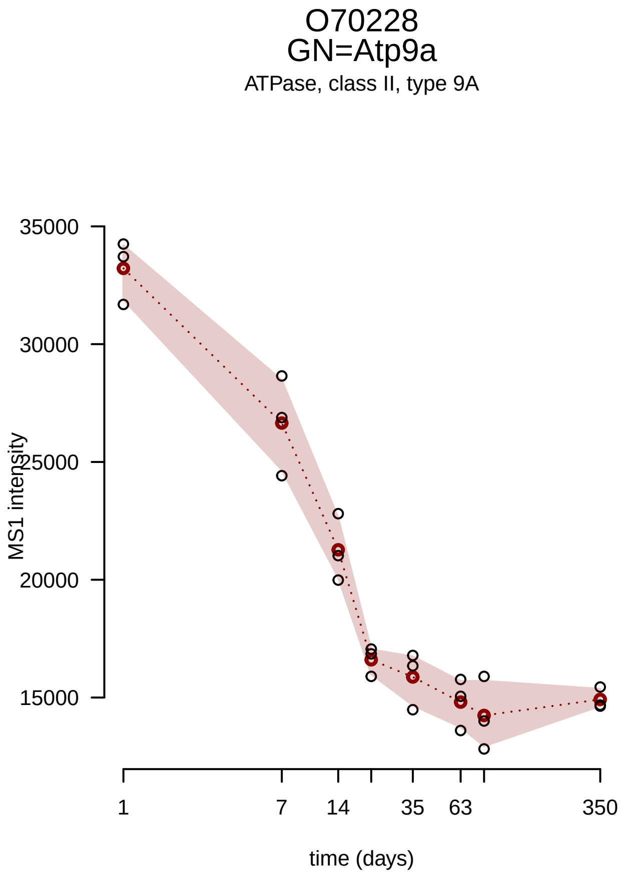
O70228
GN=Atp9a
ATPase, class II, type 9A
35000
30000
25000
20000
15000
MS1 intensity
1
7
14
35
63
350
time (days)

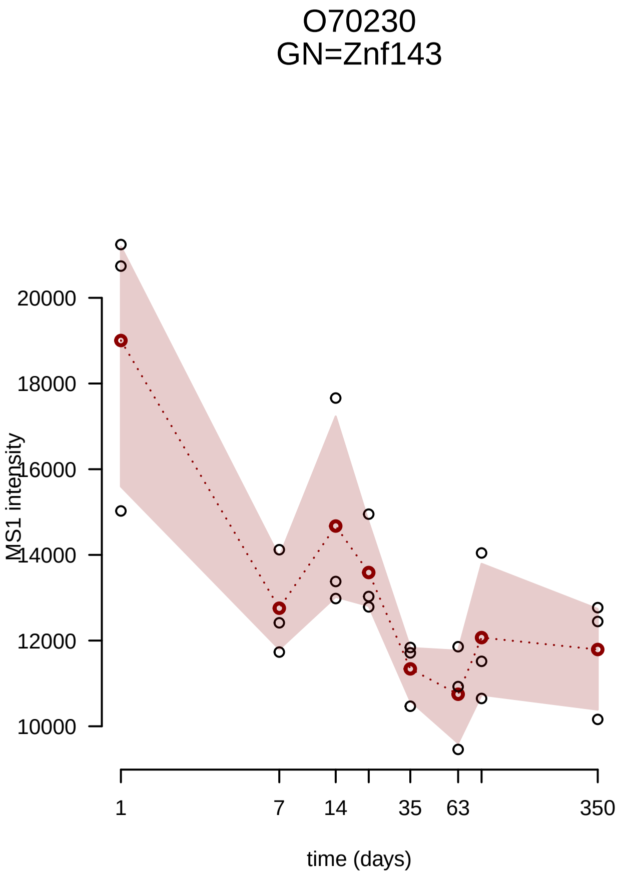
O70230
GN=Znf143
20000
18000
16000
14000
12000
10000
MS1 intensity
1
7
14
35
63
350
time (days)

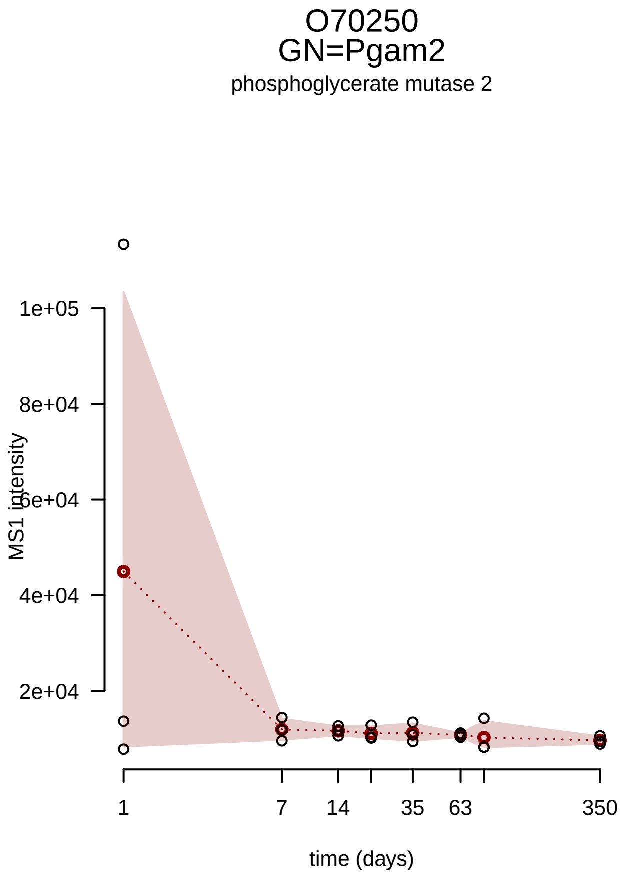
O70250
GN=Pgam2
phosphoglycerate mutase 2
1e+05
8e+04
6e+04
4e+04
2e+04
MS1 intensity
1
7
14
35
63
350
time (days)

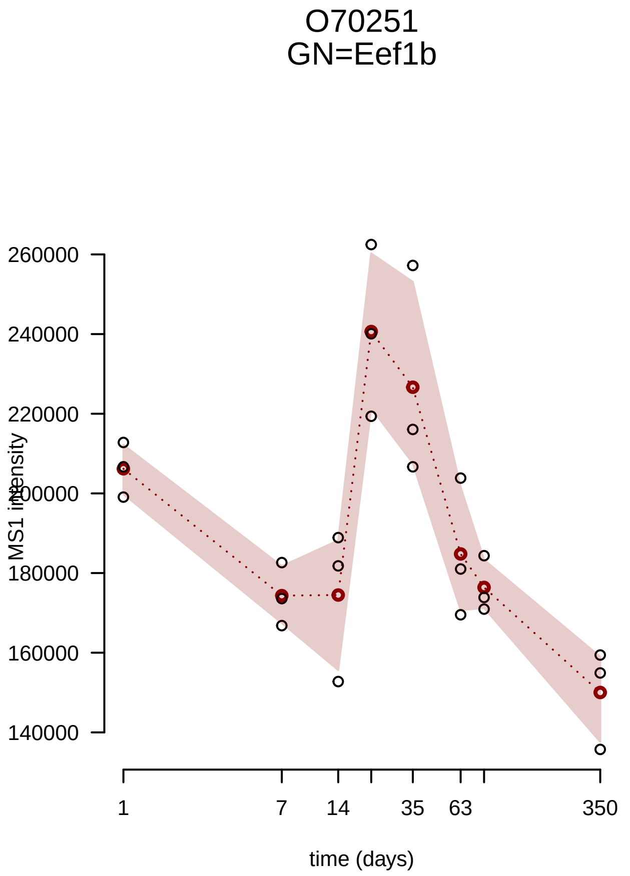
O70251
GN=Eef1b
260000
240000
220000
200000
180000
160000
140000
MS1 intensity
1
7
14
35
63
350
time (days)

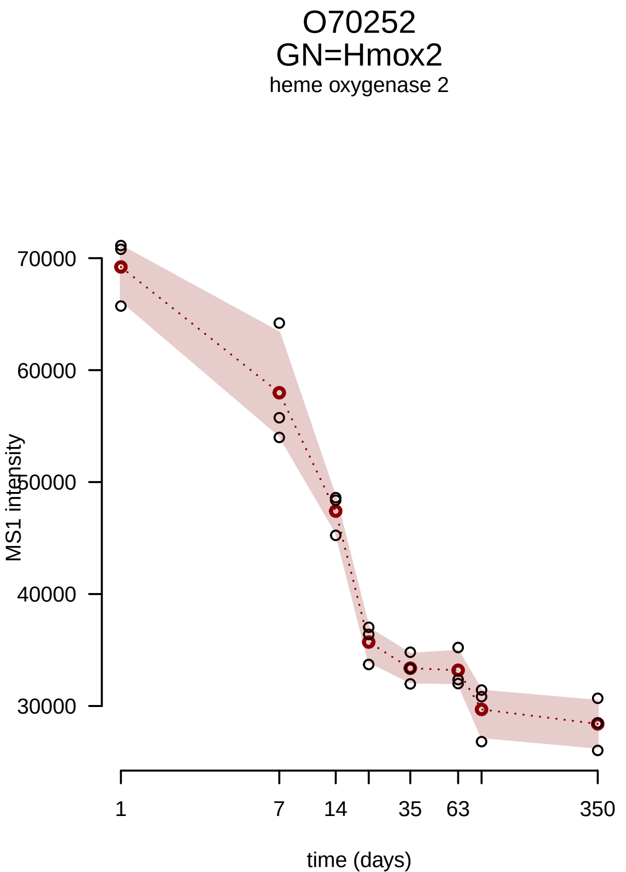
O70252
GN=Hmox2
heme oxygenase 2
70000
60000
50000
40000
30000
MS1 intensity
1
7
14
35
63
350
time (days)

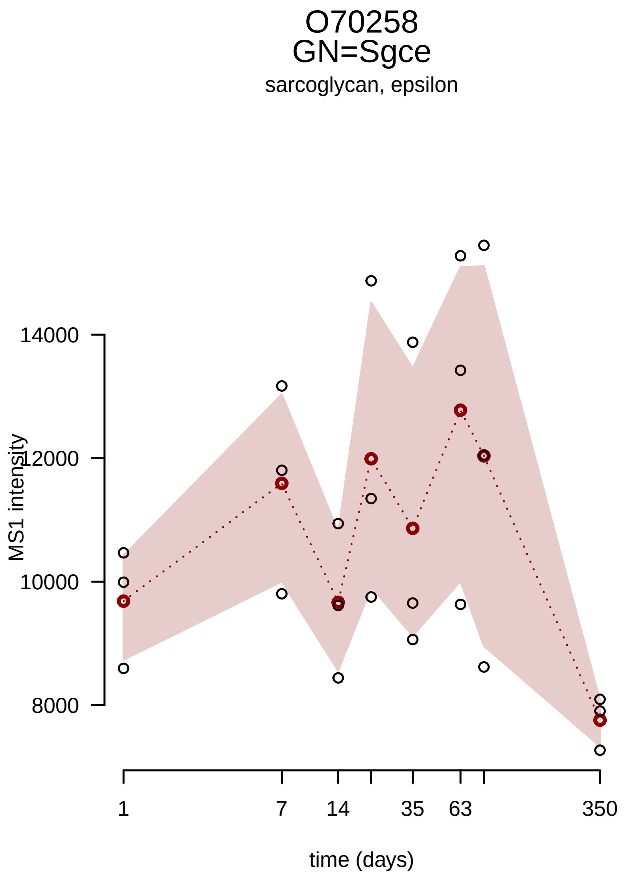
O70258
GN=Sgce
sarcoglycan, epsilon
14000
12000
10000
8000
MS1 intensity
1
7
14
35
63
350
time (days)

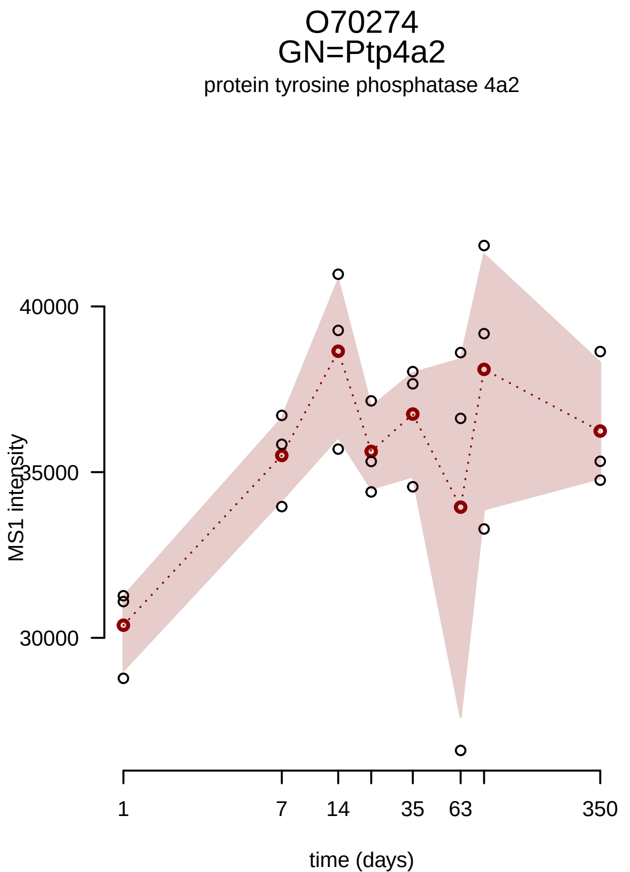
O70274
GN=Ptp4a2
protein tyrosine phosphatase 4a2
40000
35000
30000
MS1 intensity
1
7
14
35
63
350
time (days)

O70279
GN=Ess2
ess−2 splicing factor

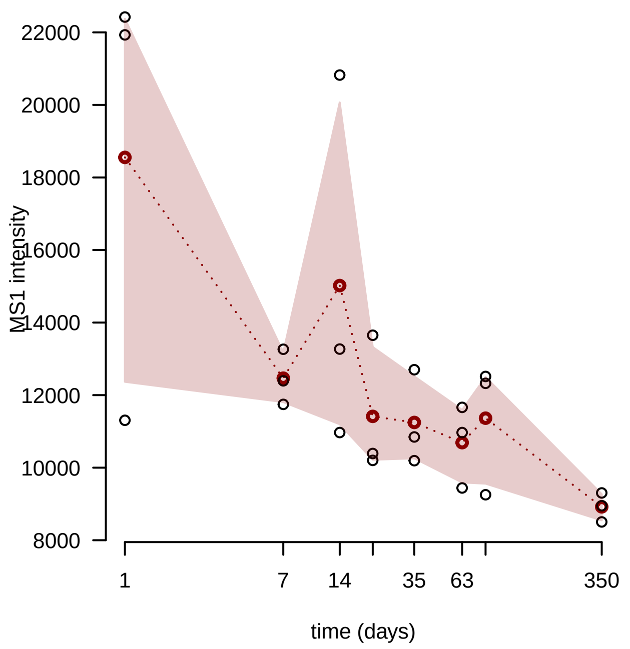

O70281
GN=Tpst1
protein−tyrosine sulfotransferase 1

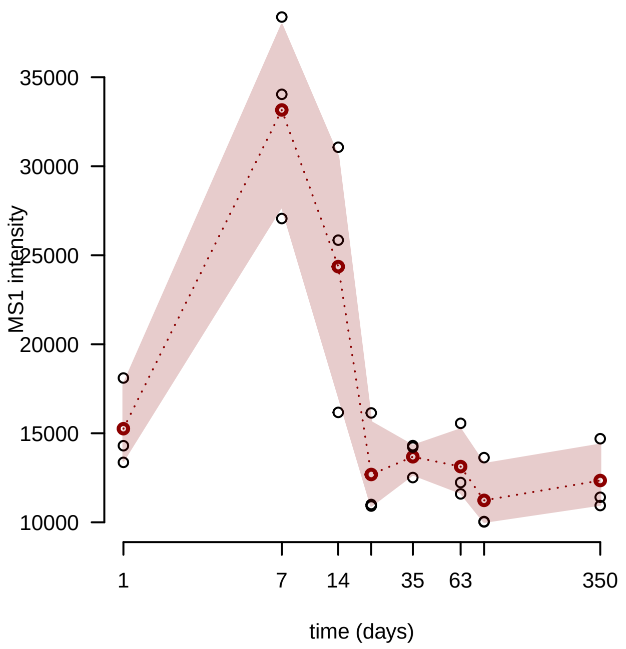

O70283
GN=Wnt2b
wingless−type MMTV integration site fami ...

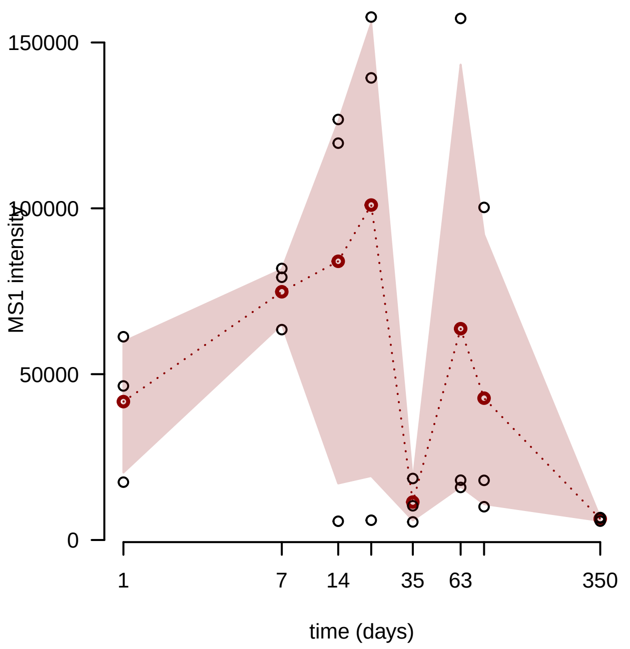

O70293
GN=Grk6
G protein−coupled receptor kinase 6

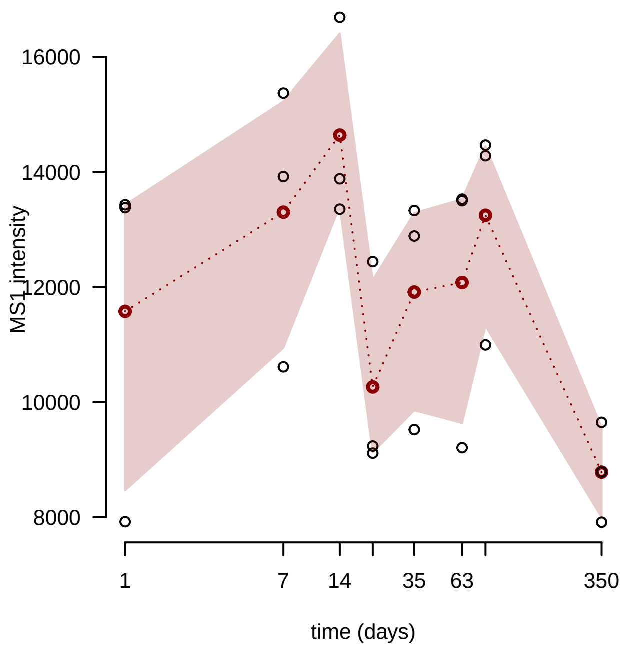

O70305
GN=Atxn2
ataxin 2

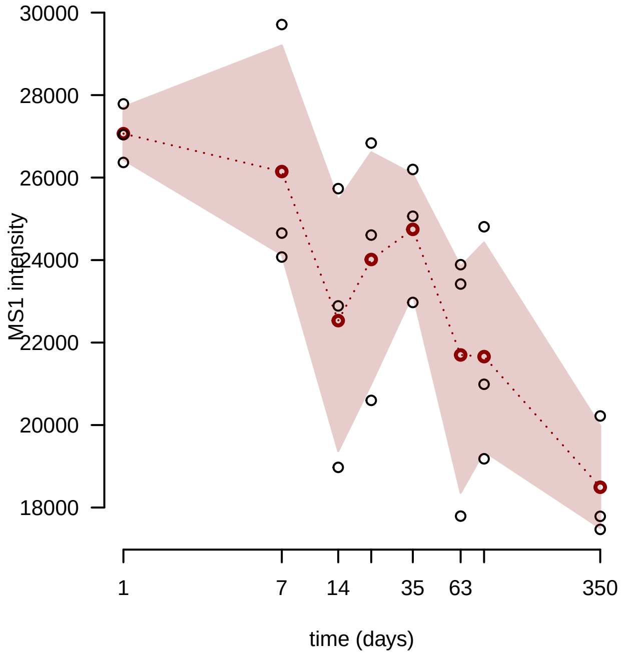

O70309
GN=Itgb5
integrin beta 5

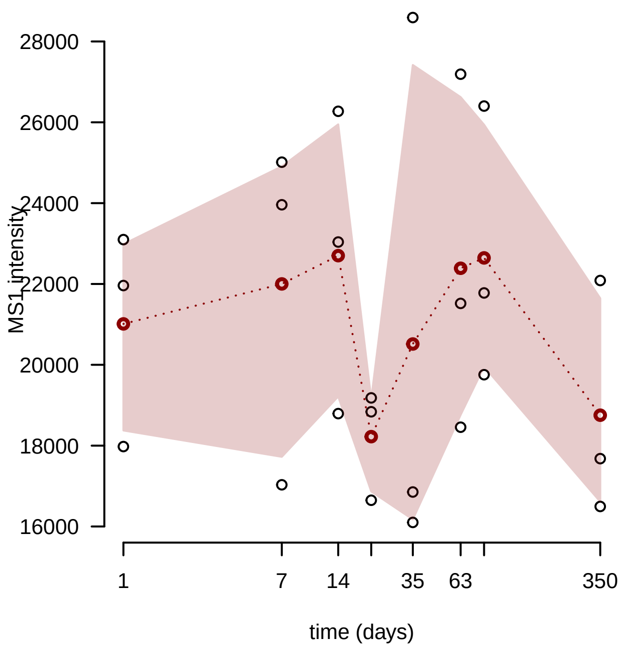

O70310
GN=Nmt1
N−myristoyltransferase 1

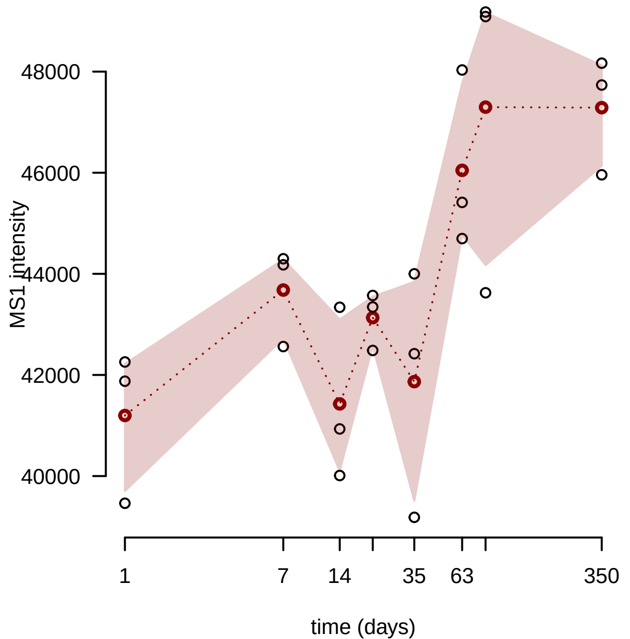

O70311
GN=Nmt2
N−myristoyltransferase 2

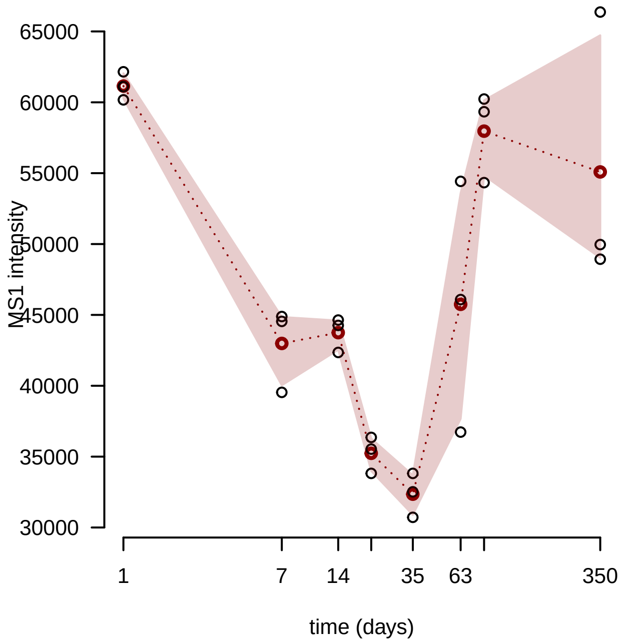

O70318
GN=Epb41l2
erythrocyte membrane protein band 4.1 li ...

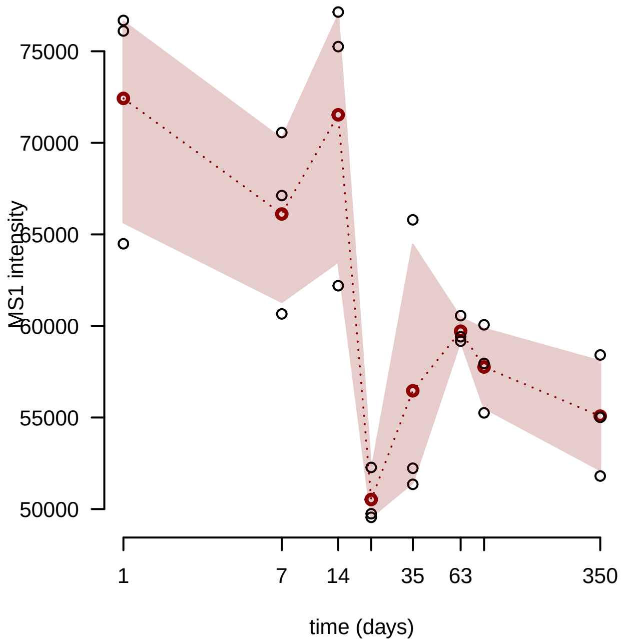

O70324
GN=Slc16a2
solute carrier family 16 (monocarboxylic ...

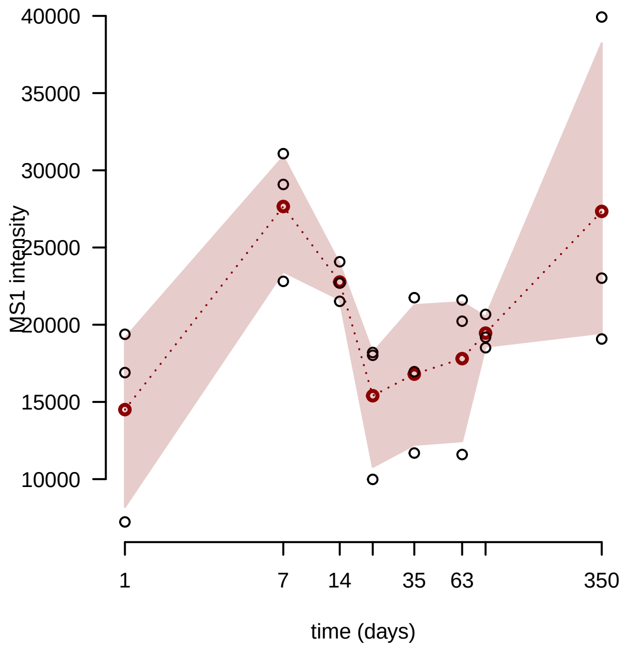

O70325
GN=Gpx4
glutathione peroxidase 4

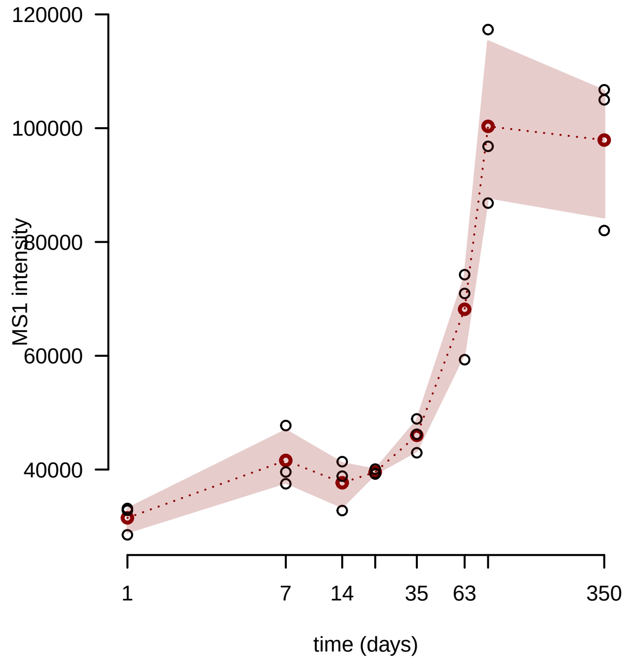

O70326
GN=Grem1
gremlin 1, DAN family BMP antagonist

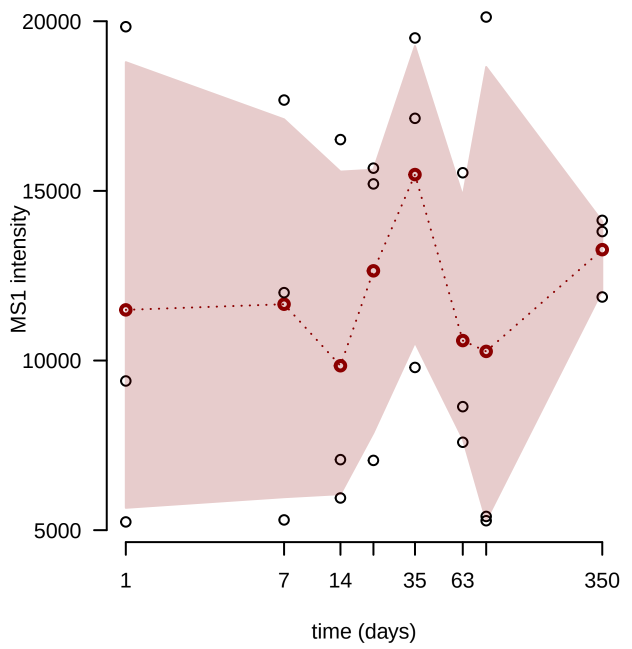

O70338
GN=Rnaseh1
ribonuclease H1

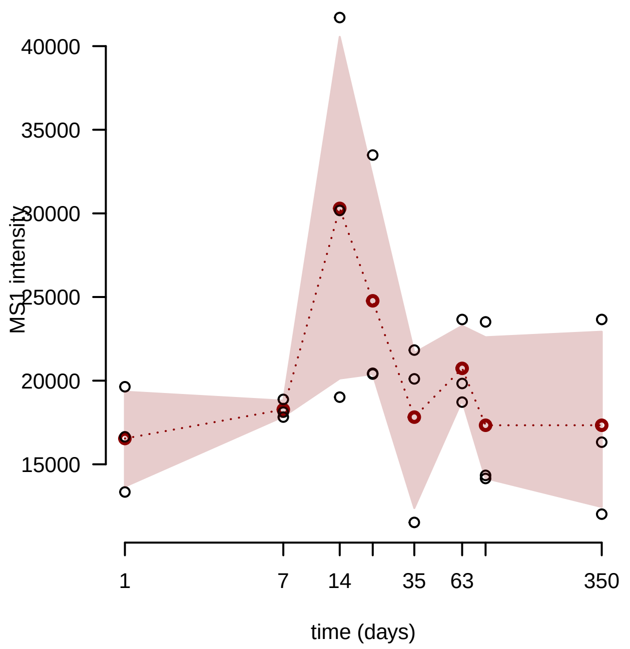

O70348
GN=Dxo
decapping exoribonuclease

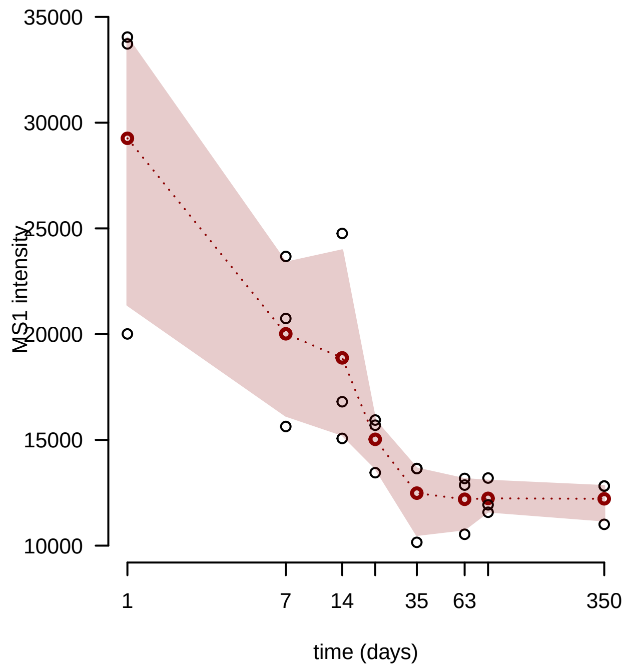

O70362
GN=Gpld1
glycosylphosphatidylinositol specific ph ...

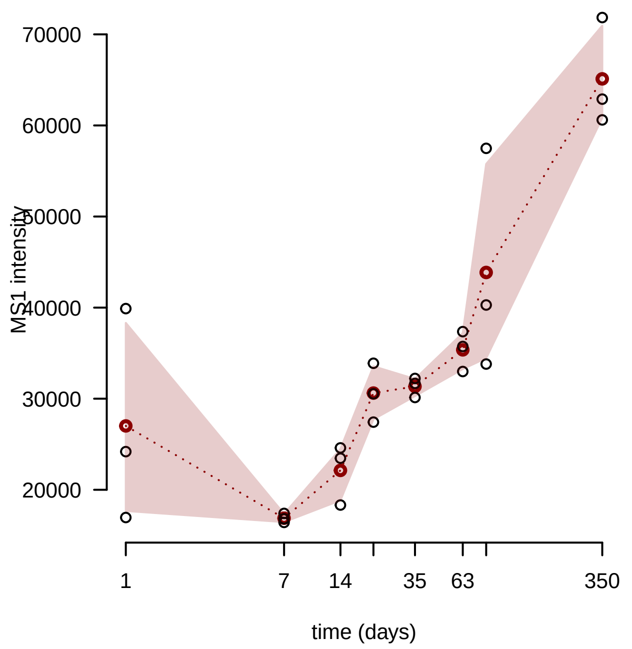

O70370
GN=Ctss
cathepsin S

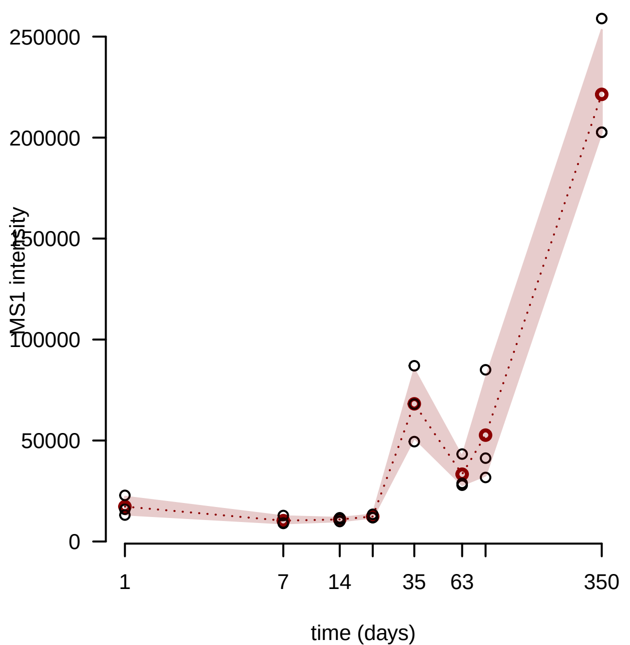

O70374
GN=Cbfa2t2
CBFA2/RUNX1 translocation partner 2

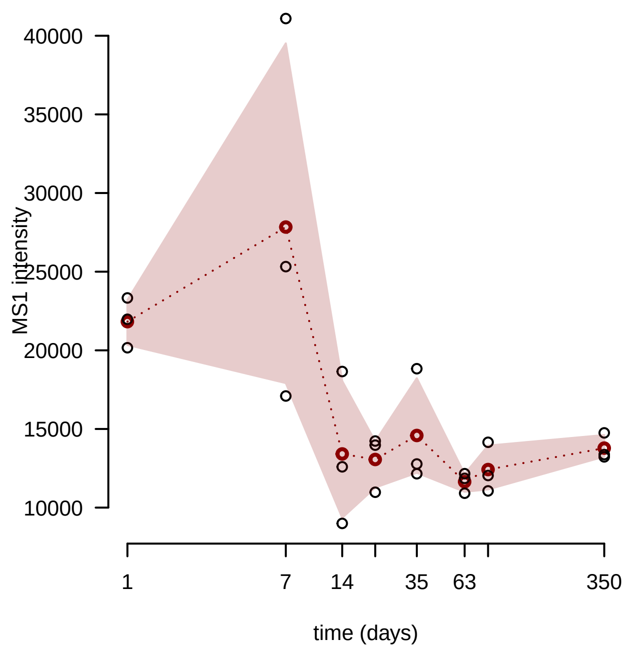

O70378
GN=Emc8
ER membrane protein complex subunit 8

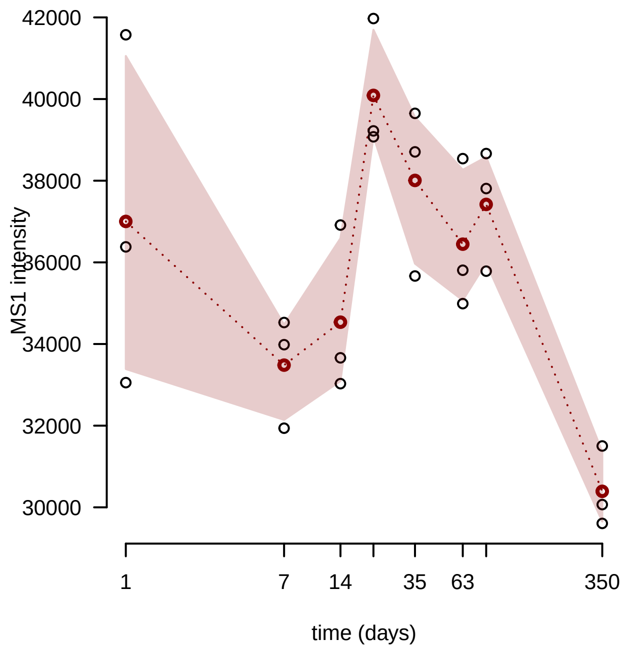

O70400
GN=Pdlim1
PDZ and LIM domain 1 (elfin)

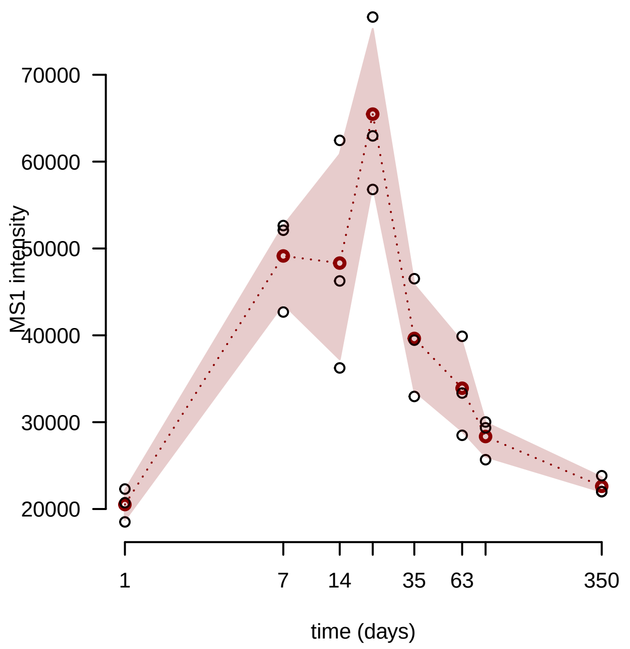

O70401
GN=Tspan6
tetraspanin 6

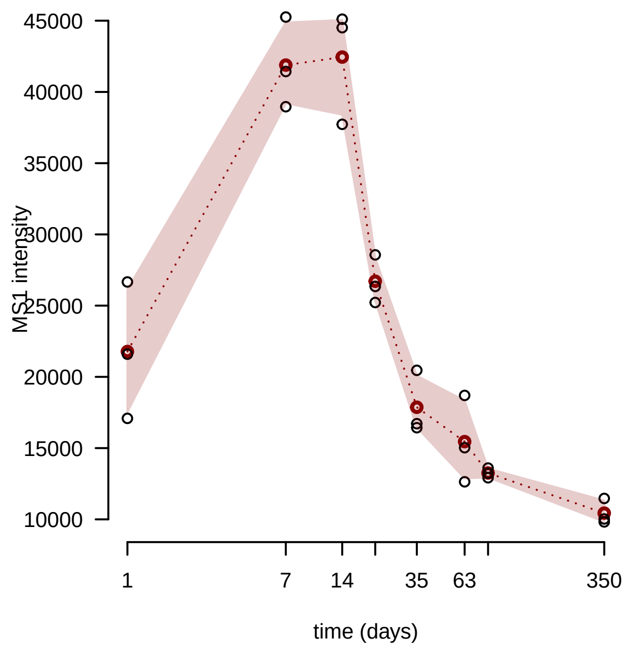

O70404
GN=Vamp8
vesicle−associated membrane protein 8

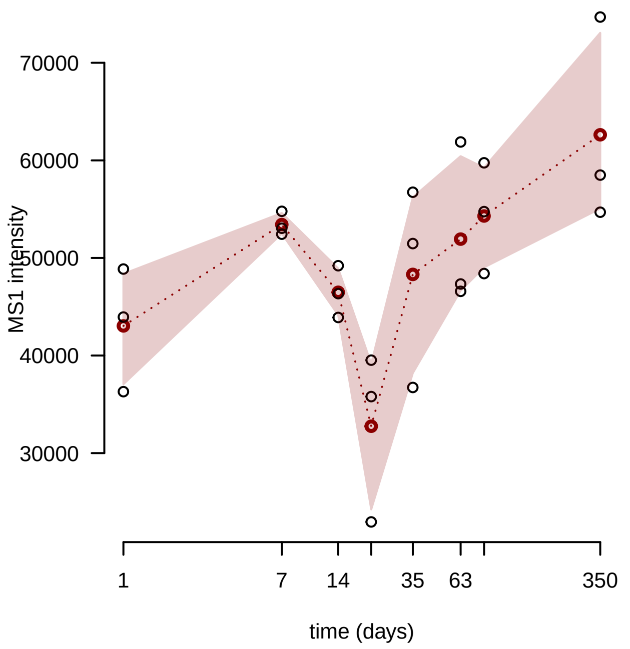

O70405
GN=Ulk1
unc−51 like kinase 1

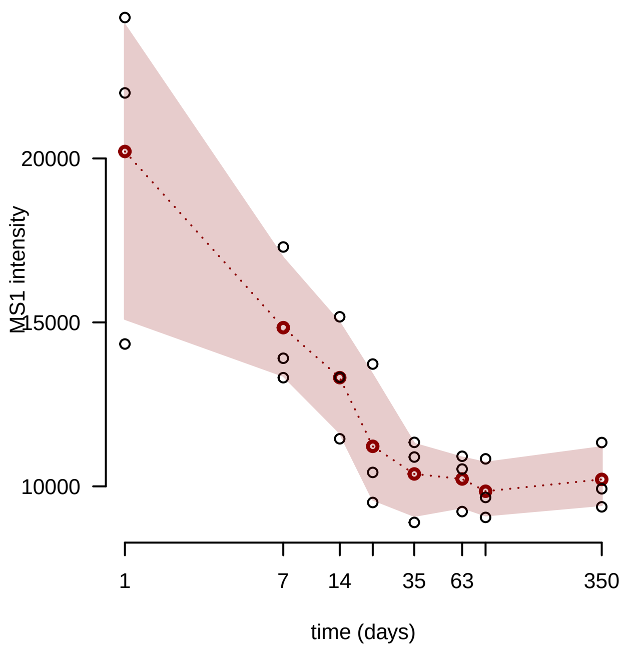

O70410
GN=Vmn2r1
vomeronasal 2, receptor 1

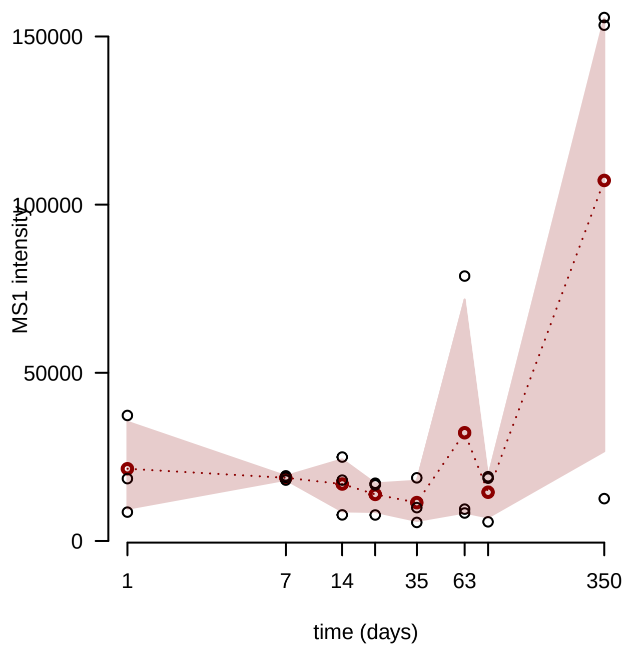

O70421
GN=Fzd1
frizzled class receptor 1

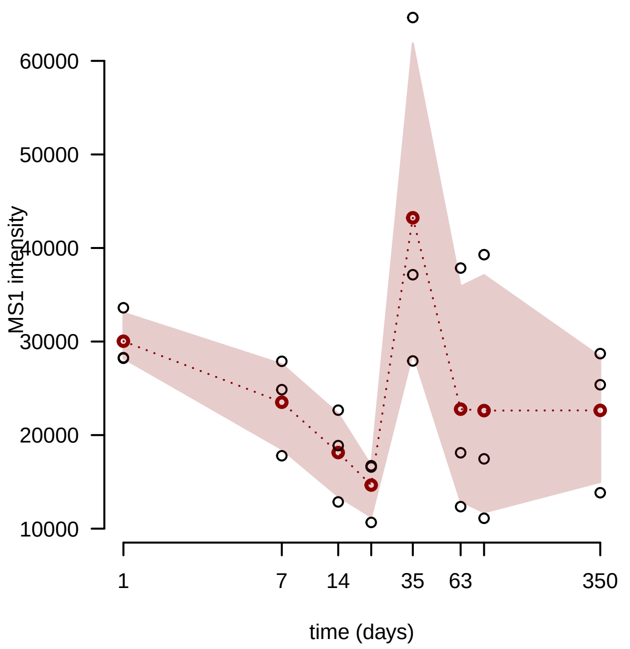

O70422
GN=Gtf2h4
general transcription factor II H, polyp ...

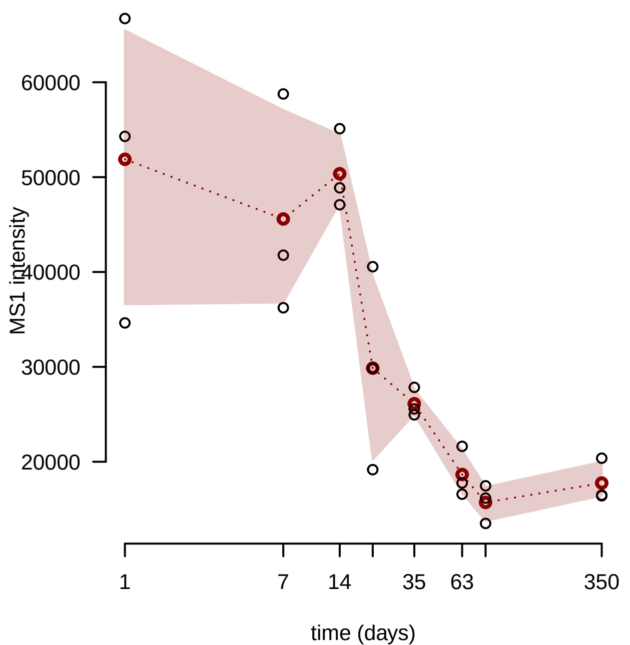

O70423
GN=Aoc3
amine oxidase, copper containing 3

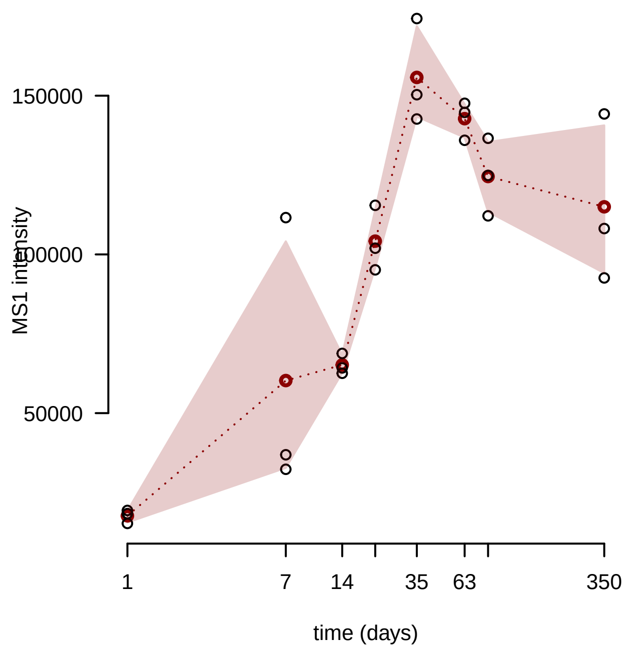

O70433
GN=Fhl2
four and a half LIM domains 2

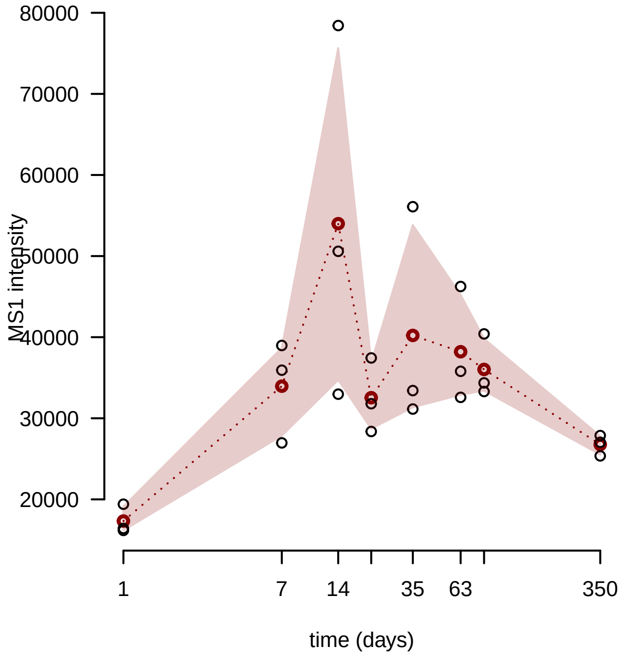

O70435
GN=Psma3
proteasome subunit alpha 3

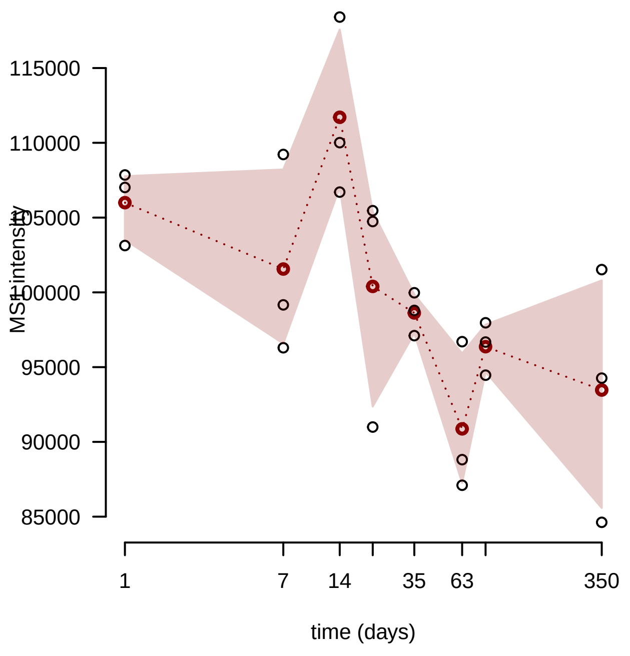

O70439
GN=Stx7
syntaxin 7

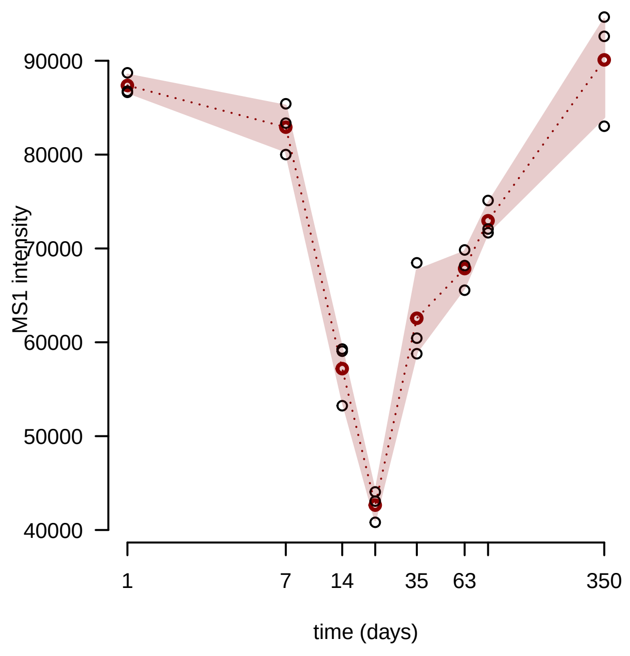

O70443
GN=Gnaz
guanine nucleotide binding protein, alph ...

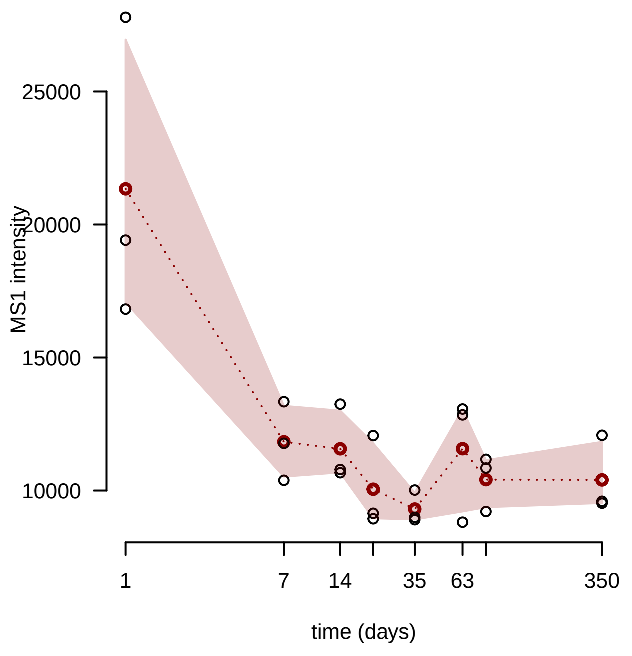

O70445
GN=Bard1
BRCA1 associated RING domain 1

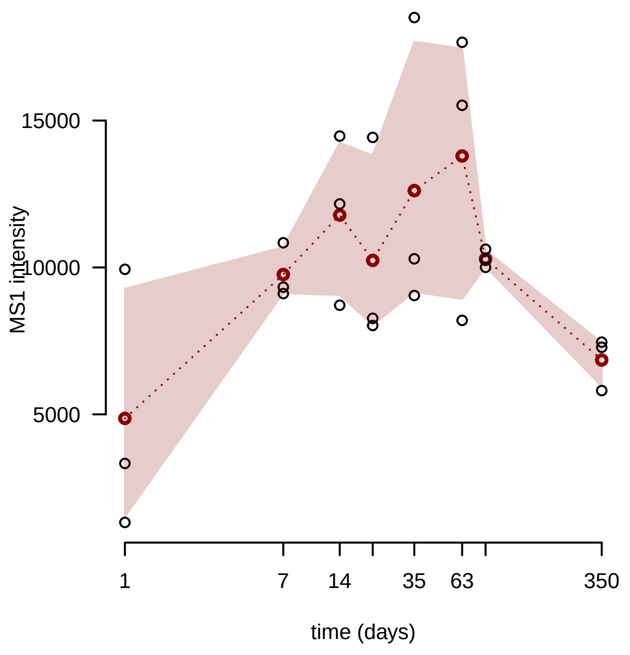

O70456
GN=Sfn
stratifin

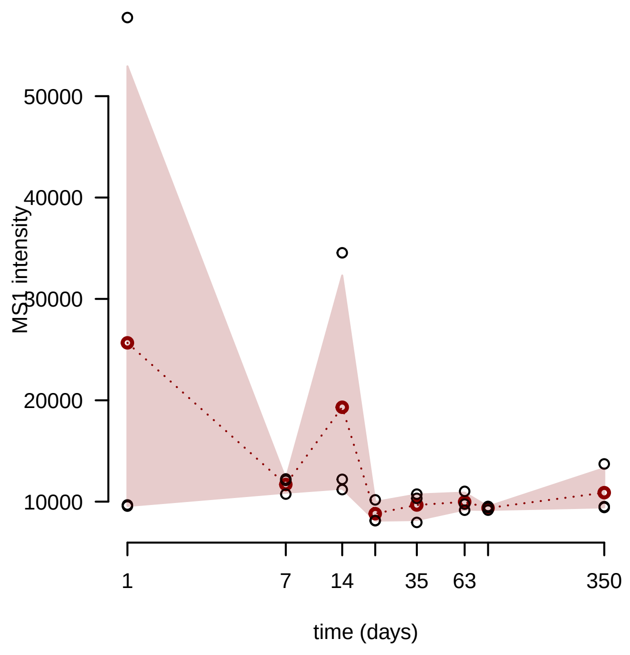

O70472
GN=Tmem131
transmembrane protein 131

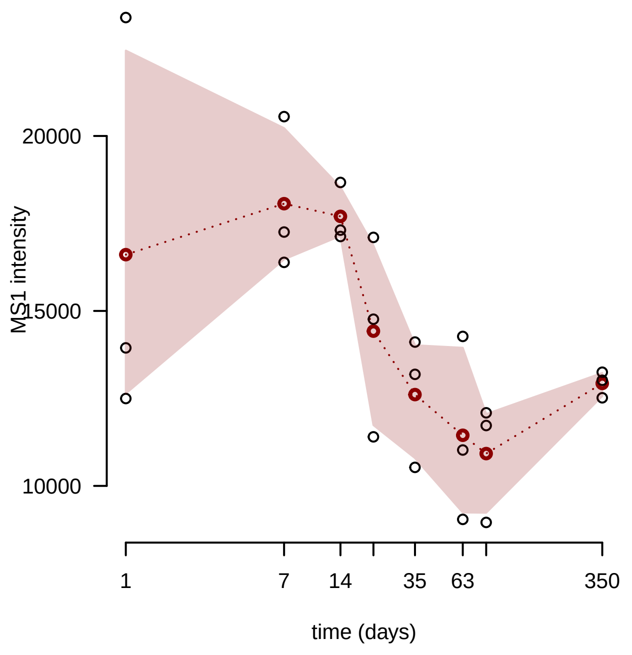

O70475
GN=Ugdh
UDP−glucose dehydrogenase

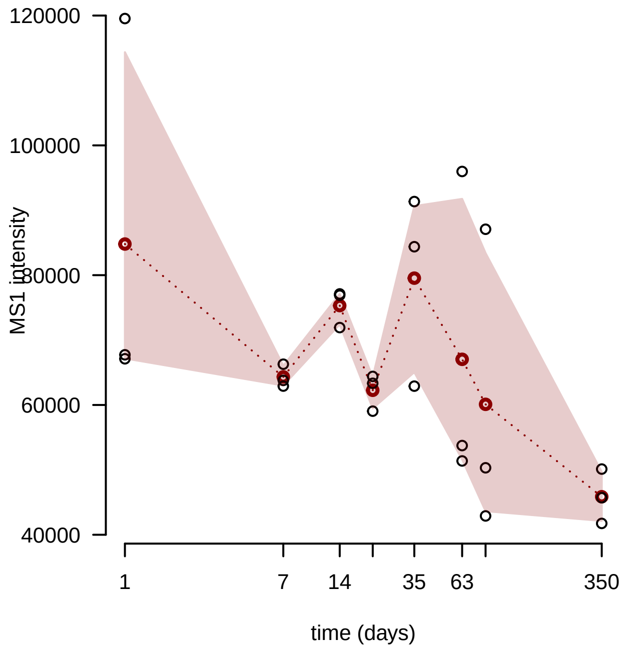

O70479
GN=Tnfaip1
tumor necrosis factor, alpha−induced pro ...

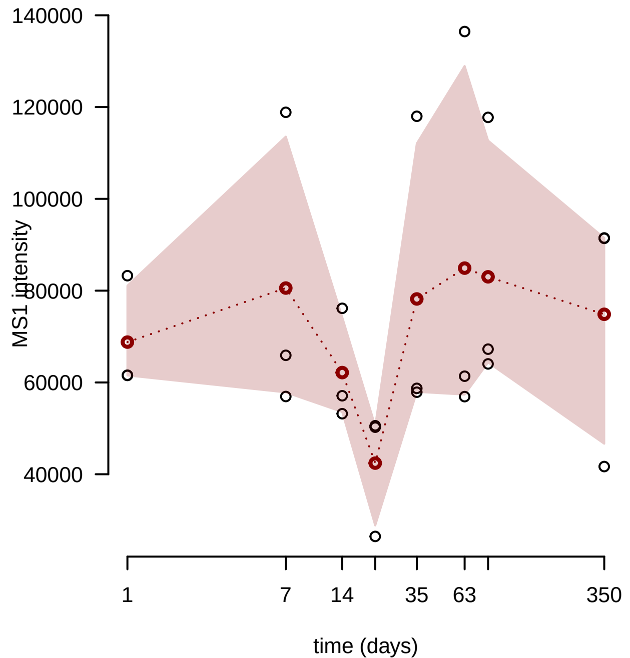

O70480
GN=Vamp4
vesicle−associated membrane protein 4

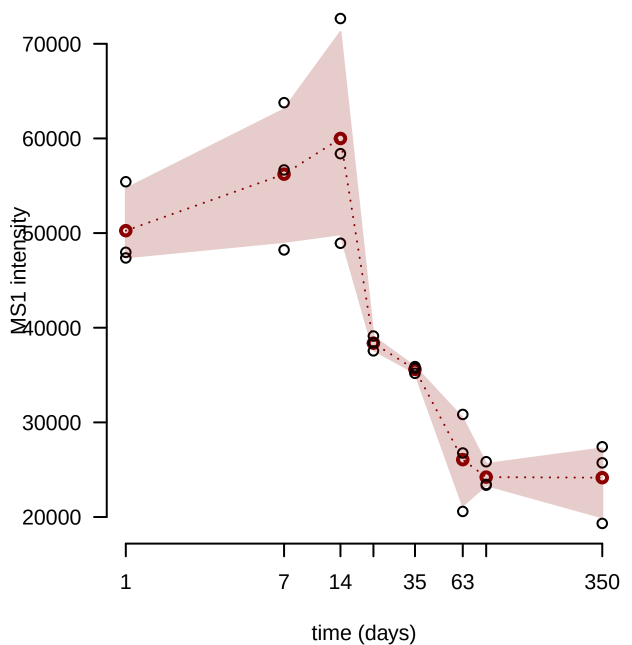

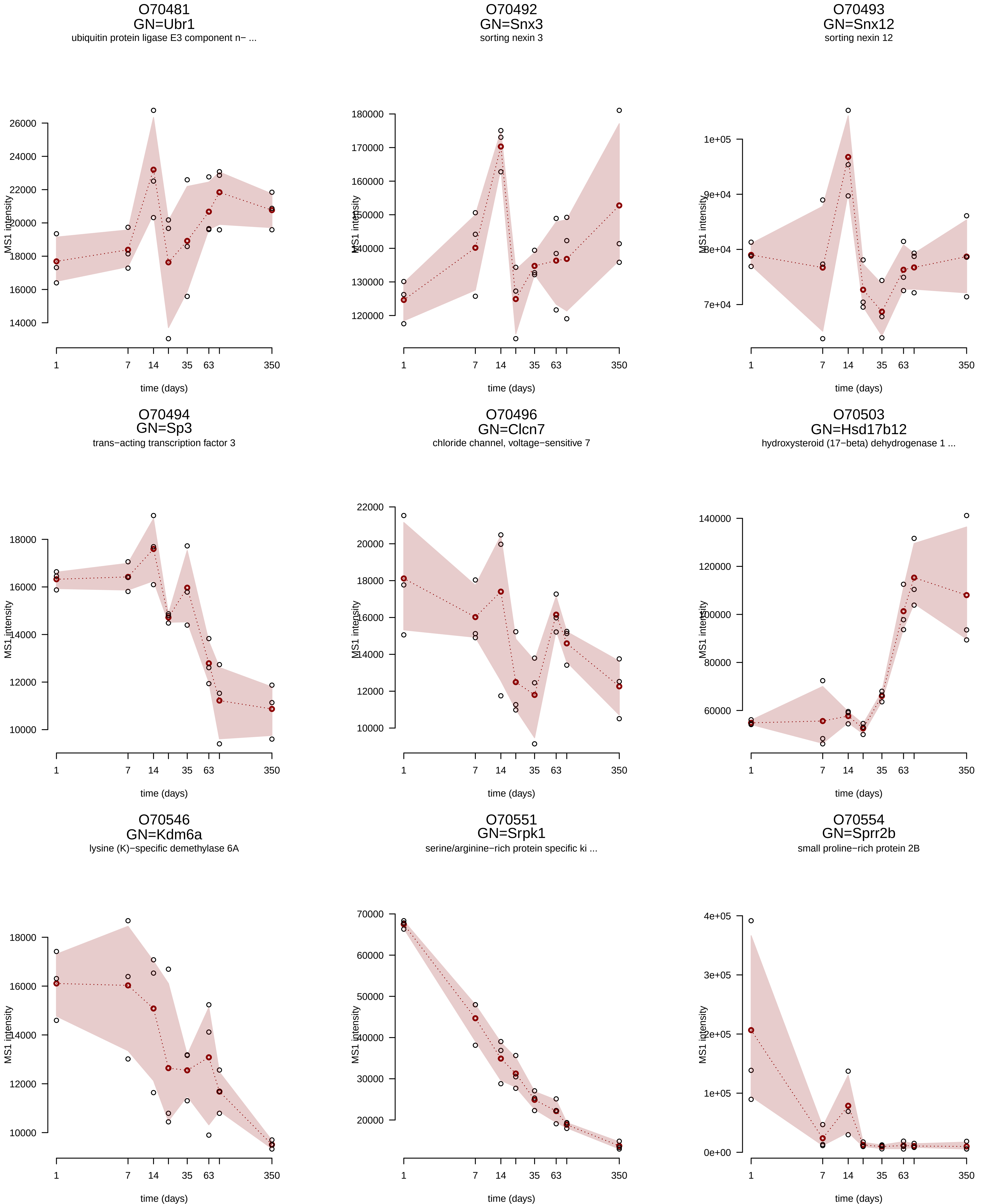
O70481
GN=Ubr1
ubiquitin protein ligase E3 component n− ...
O70492
GN=Snx3
sorting nexin 3
O70493
GN=Snx12
sorting nexin 12
O70494
GN=Sp3
trans−acting transcription factor 3
O70496
GN=Clcn7
chloride channel, voltage−sensitive 7
O70503
GN=Hsd17b12
hydroxysteroid (17−beta) dehydrogenase 1 ...
O70546
GN=Kdm6a
lysine (K)−specific demethylase 6A
O70551
GN=Srpk1
serine/arginine−rich protein specific ki ...
O70554
GN=Sprr2b
small proline−rich protein 2B
MS1 intensity
time (days)
1
7
14
35
63
350

O70566
GN=Diaph2
diaphanous related formin 2

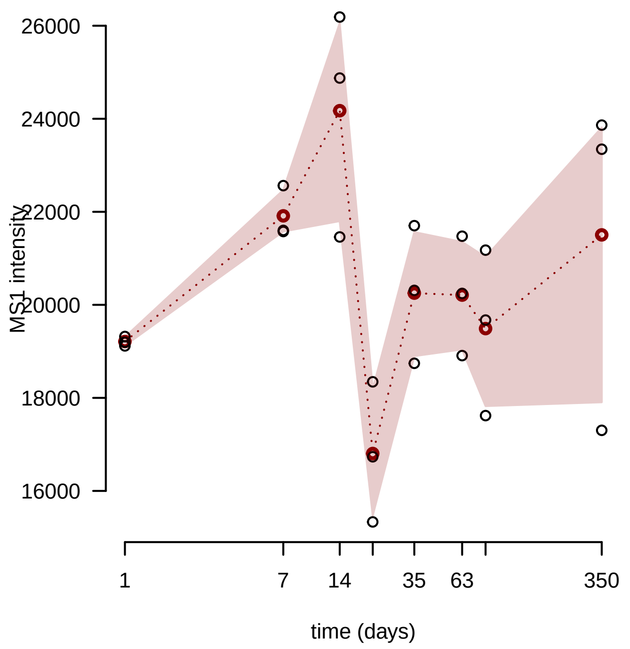

O70570
GN=Pigr
polymeric immunoglobulin receptor

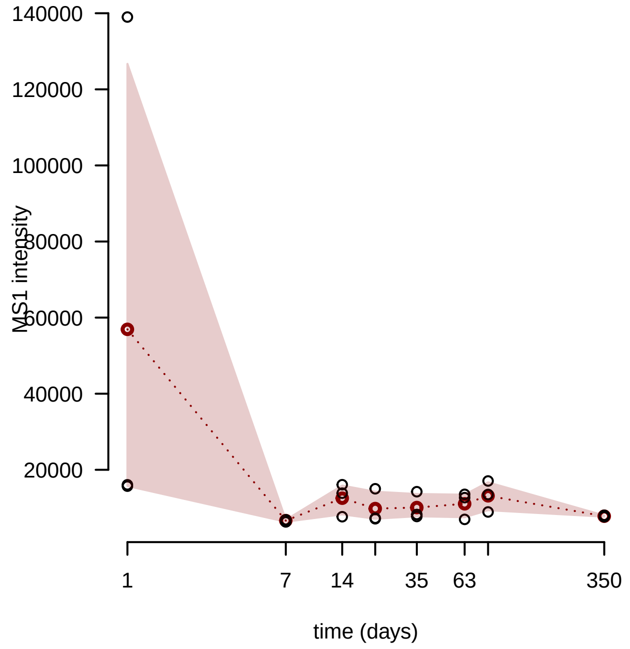

O70571
GN=Pdk4
pyruvate dehydrogenase kinase, isoenzyme ...

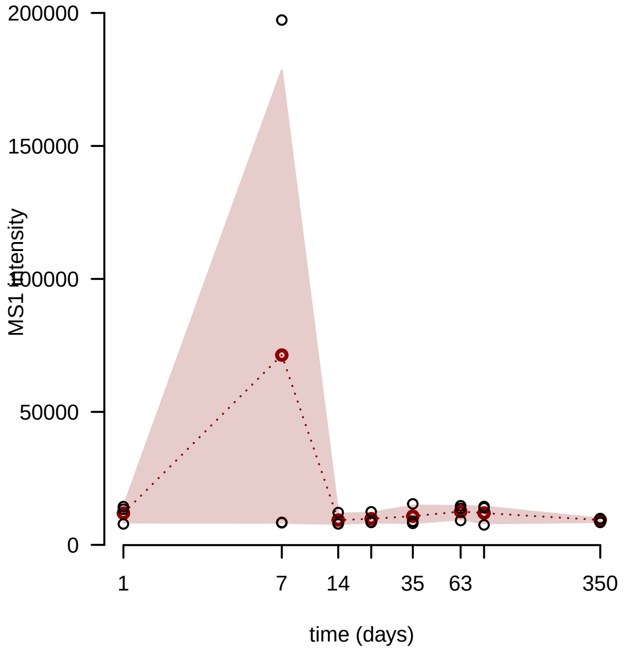

O70572
GN=Smpd2
sphingomyelin phosphodiesterase 2, neutr ...

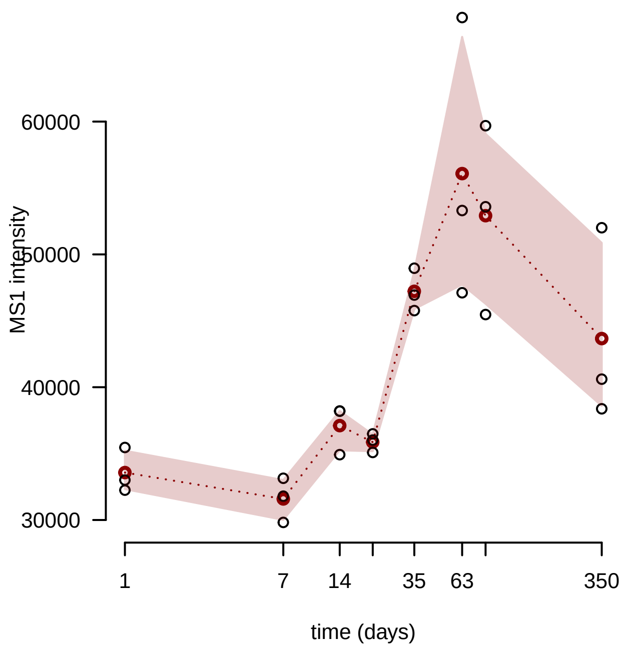

O70576
GN=Stag3
stromal antigen 3

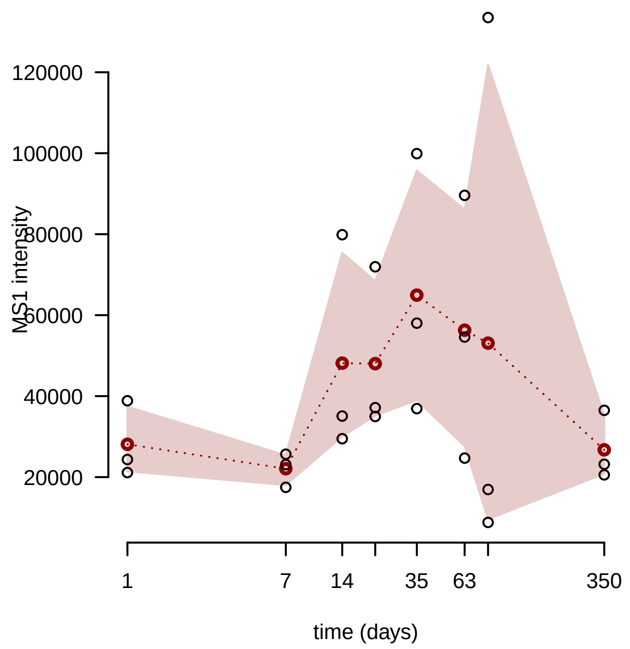

O70579
GN=Slc25a17
solute carrier family 25 (mitochondrial ...

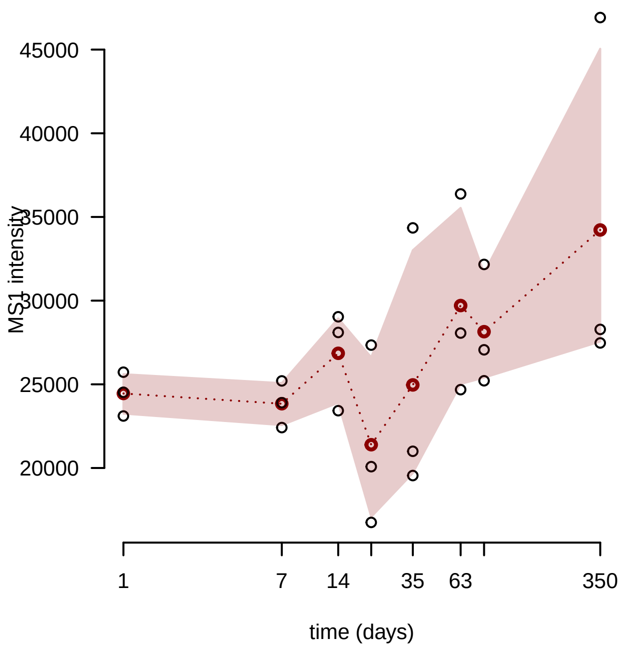

O70583
GN=Mid1
midline 1

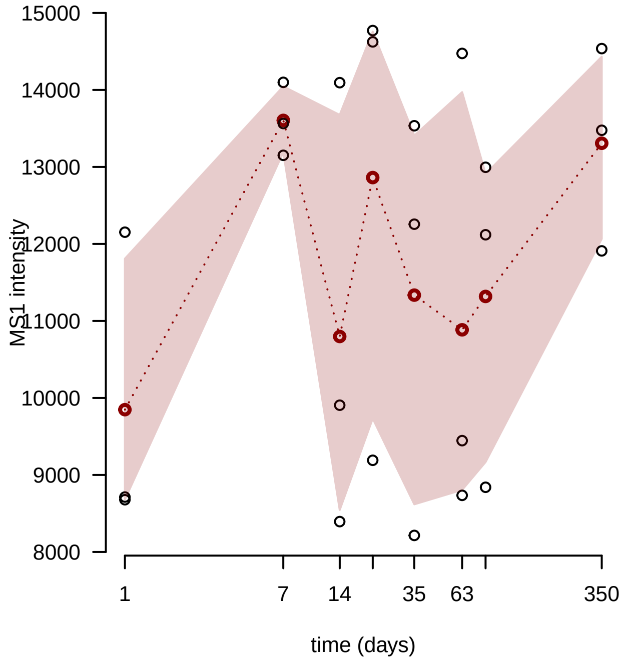

O70585
GN=Dtnb
dystrobrevin, beta

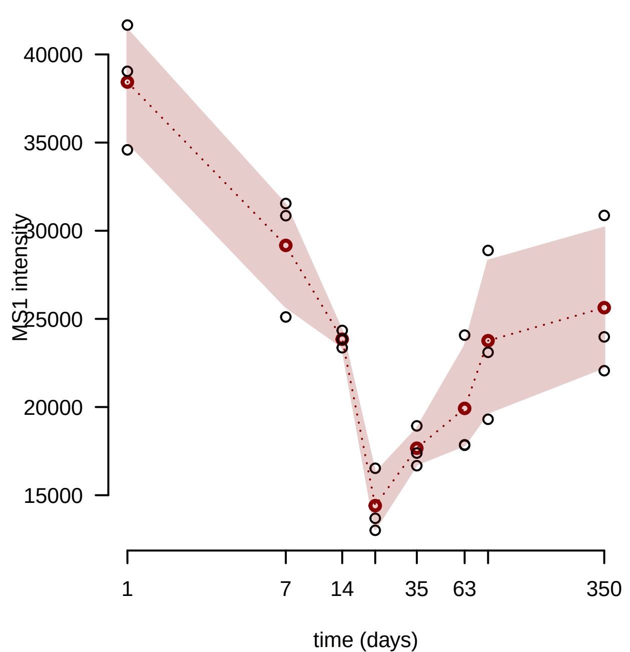

O70589
GN=Cask
calcium/calmodulin−dependent serine prot ...

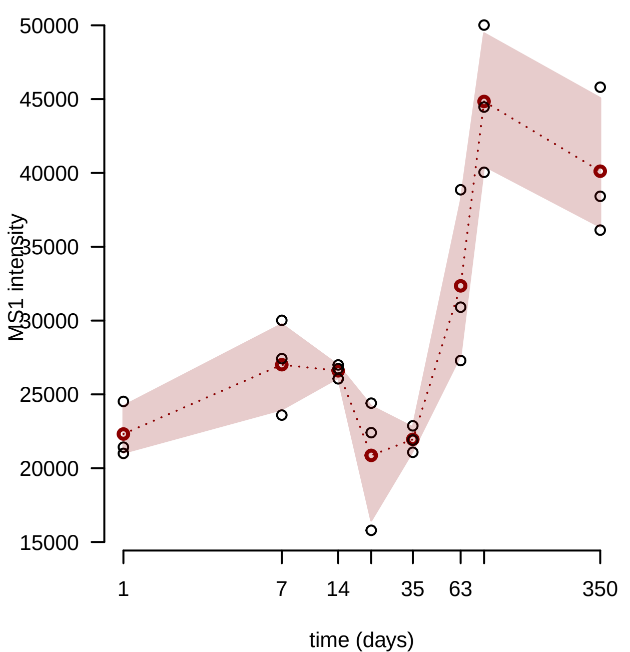

O70591
GN=Pfdn2
prefoldin 2

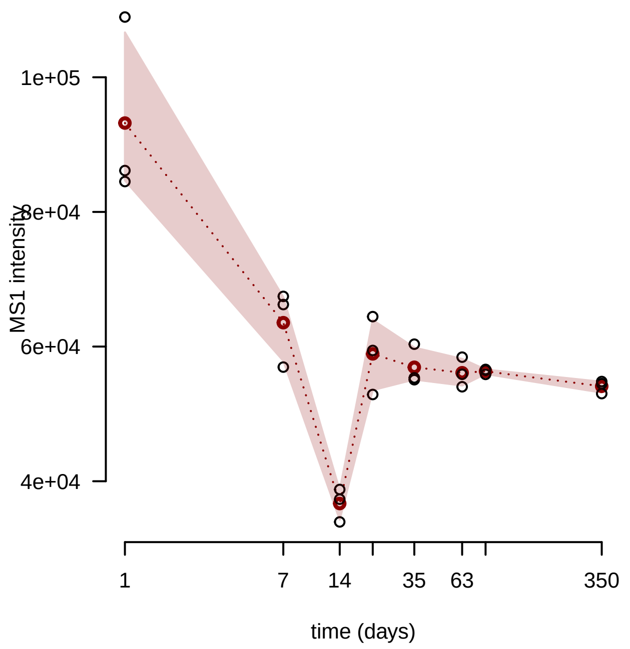

O70622
GN=Rtn2
reticulon 2 (Z−band associated protein)

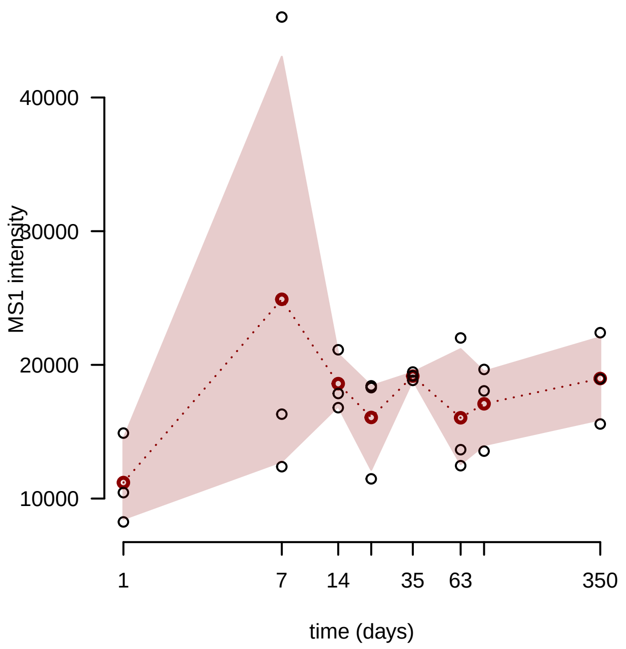

O70628
GN=Pde9a
phosphodiesterase 9A

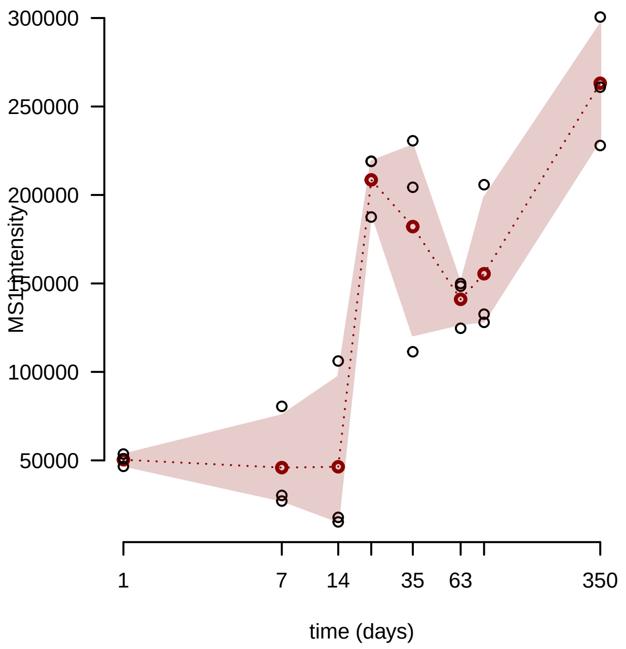

O88196
GN=Ttc3
tetratricopeptide repeat domain 3

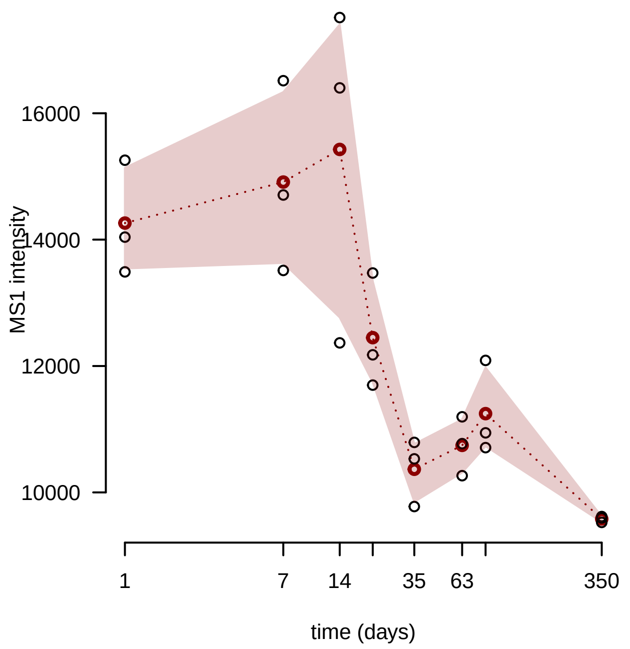

O88200
GN=Clec11a
C−type lectin domain family 11, member a

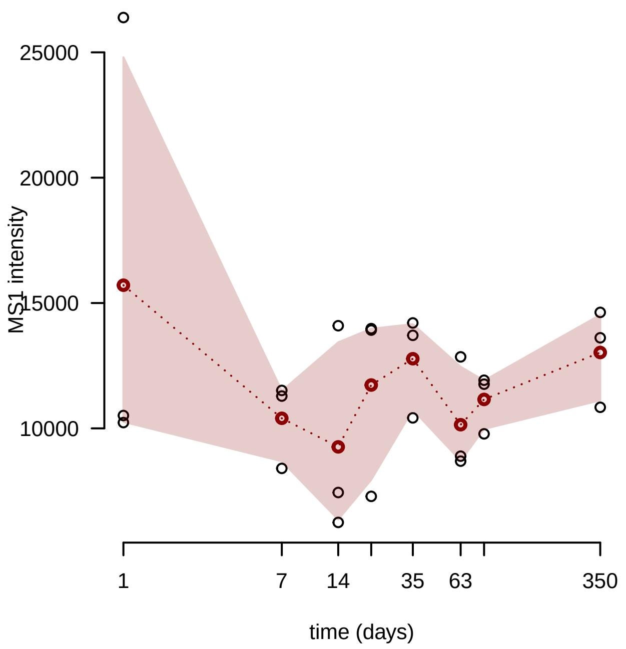

O88207
GN=Col5a1
collagen, type V, alpha 1

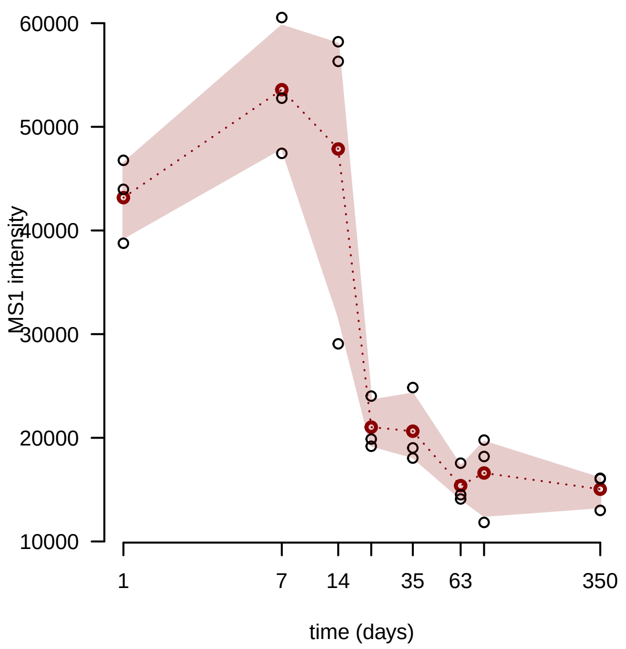

O88271
GN=Cfdp1
craniofacial development protein 1

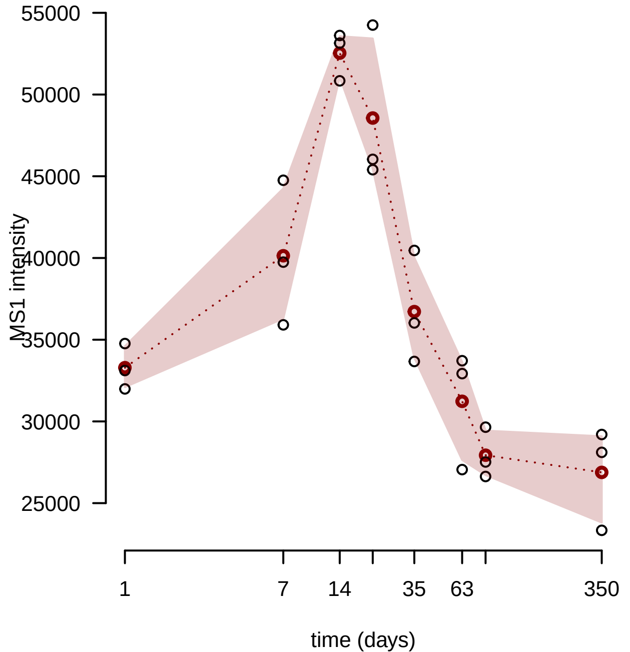

O88286
GN=Wiz
widely−interspaced zinc finger motifs

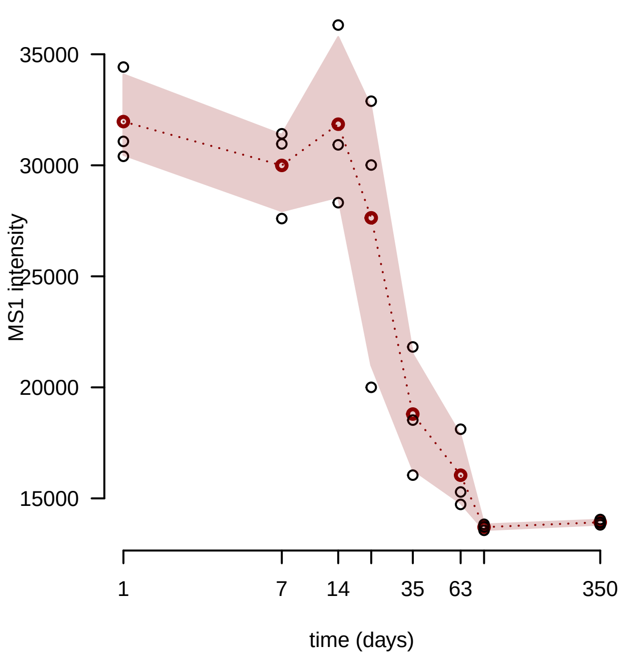

O88291
GN=Znf326

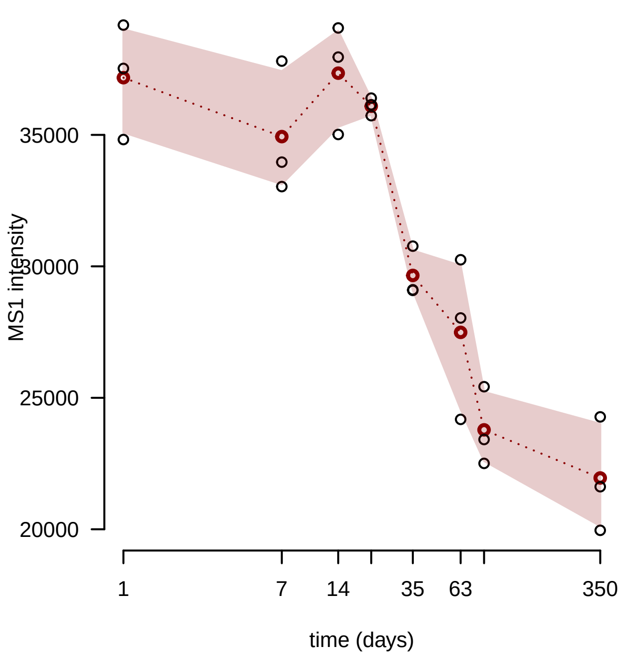

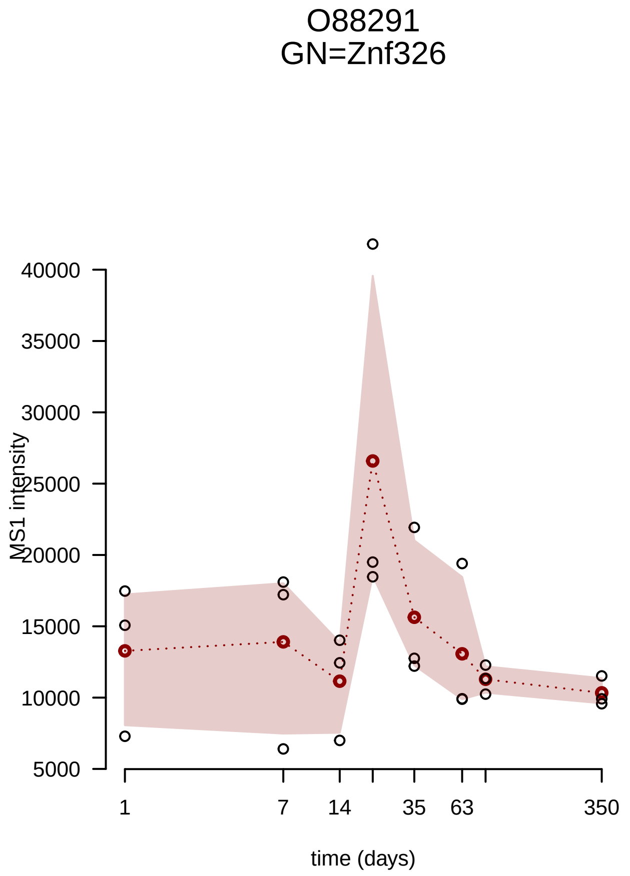
O88291
GN=Znf326
MS1 intensity
40000
35000
30000
25000
20000
15000
10000
5000
1
7
14
35
63
350
time (days)

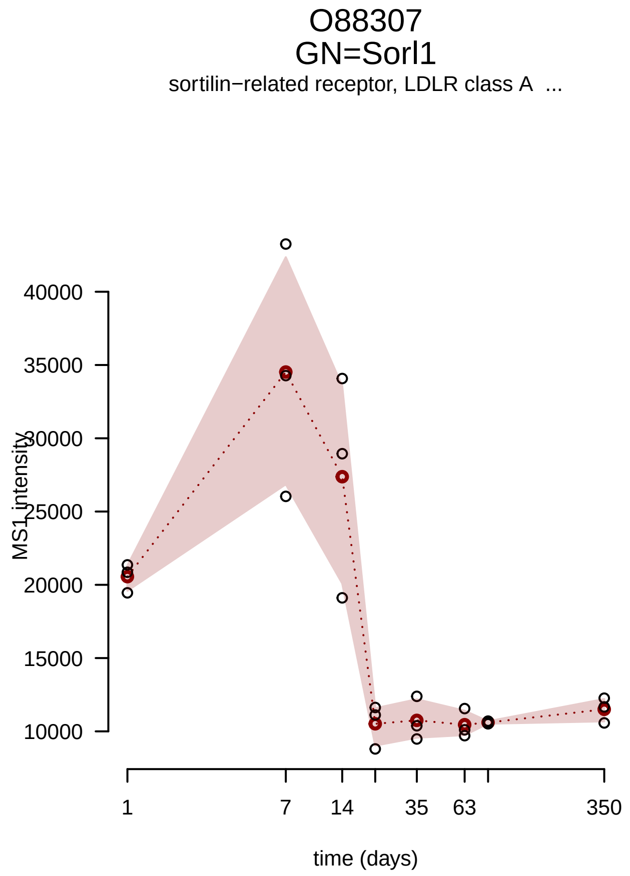
O88307
GN=Sorl1
sortilin−related receptor, LDLR class A ...
MS1 intensity
40000
35000
30000
25000
20000
15000
10000
1
7
14
35
63
350
time (days)

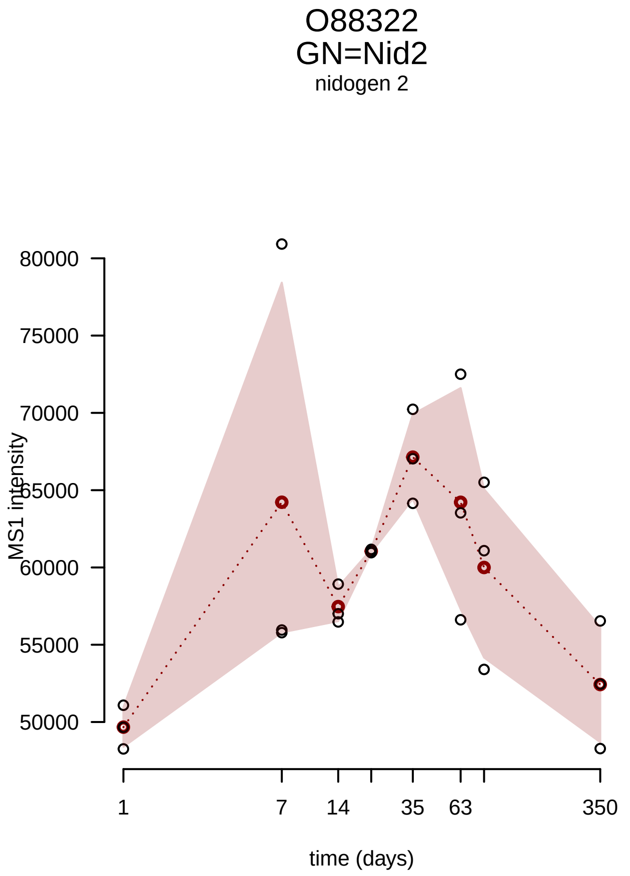
O88322
GN=Nid2
nidogen 2
MS1 intensity
80000
75000
70000
65000
60000
55000
50000
1
7
14
35
63
350
time (days)

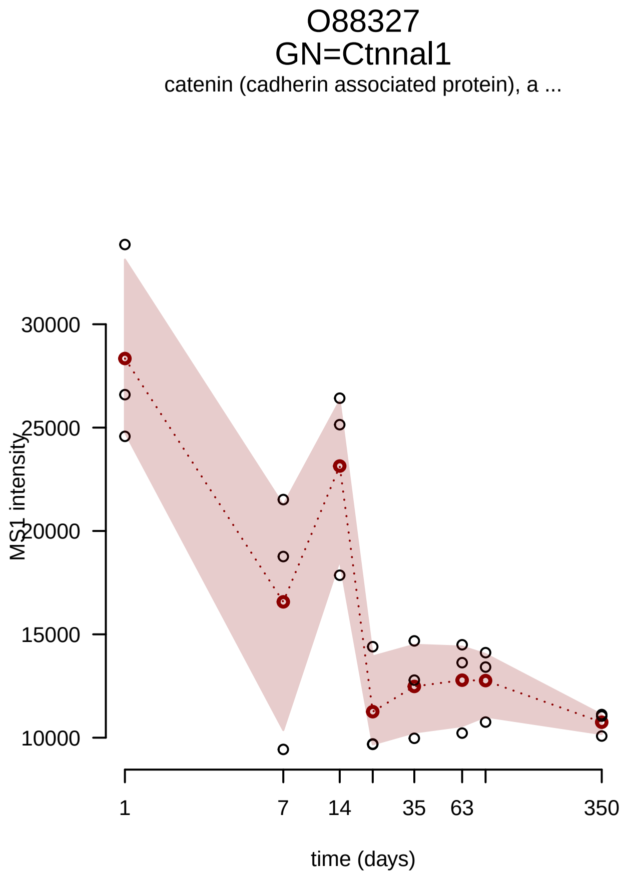
O88327
GN=Ctnnal1
catenin (cadherin associated protein), a ...
MS1 intensity
30000
25000
20000
15000
10000
1
7
14
35
63
350
time (days)

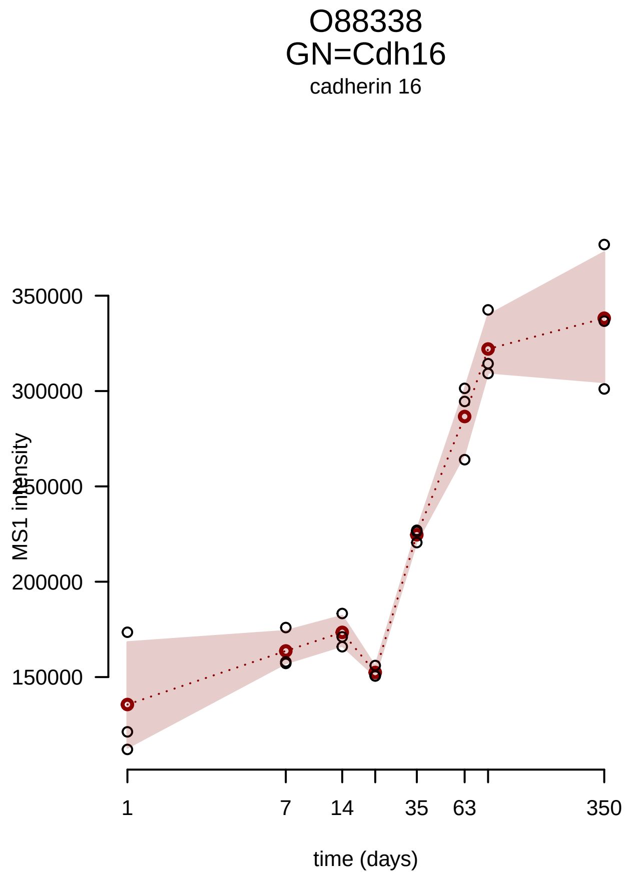
O88338
GN=Cdh16
cadherin 16
MS1 intensity
350000
300000
250000
200000
150000
1
7
14
35
63
350
time (days)

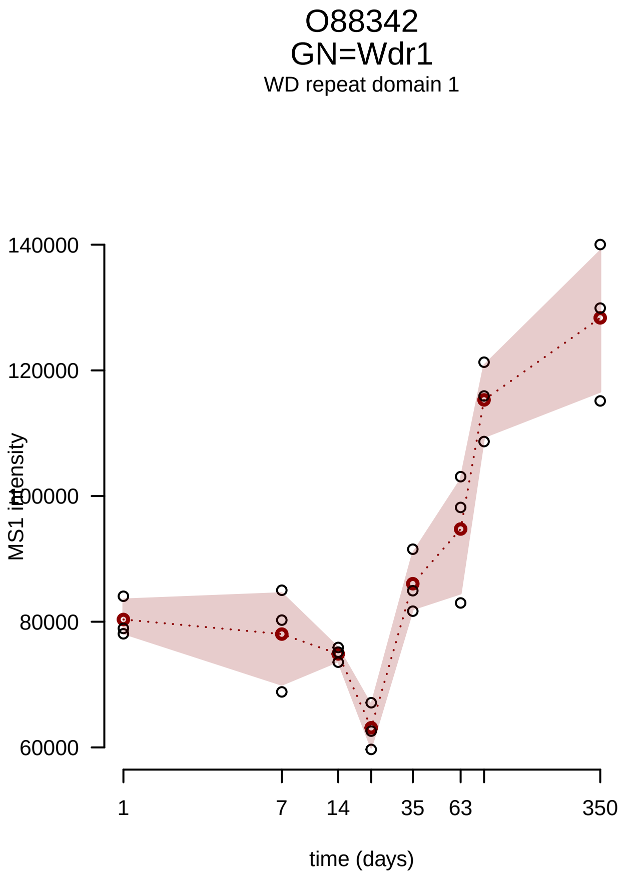
O88342
GN=Wdr1
WD repeat domain 1
MS1 intensity
140000
120000
100000
80000
60000
1
7
14
35
63
350
time (days)

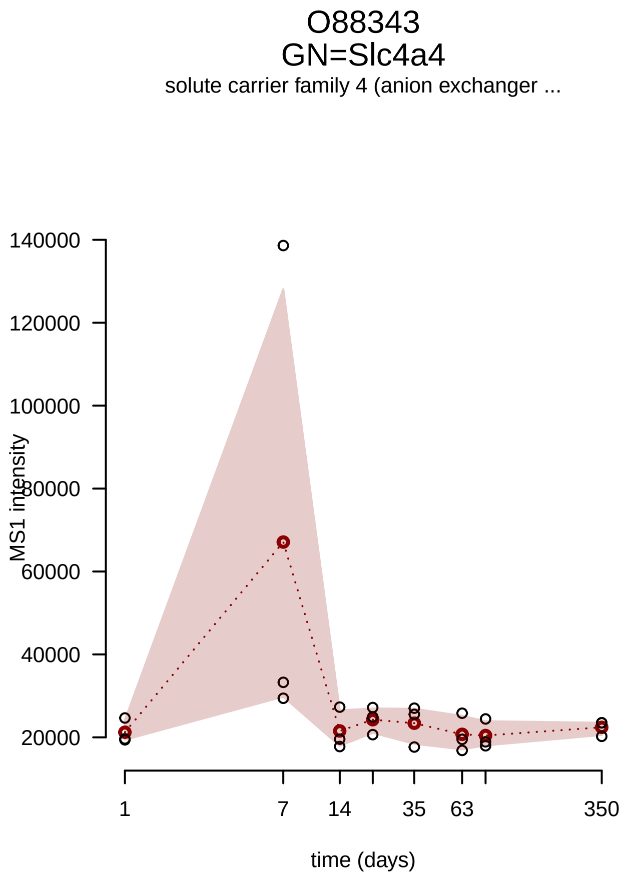
O88343
GN=Slc4a4
solute carrier family 4 (anion exchanger ...
MS1 intensity
140000
120000
100000
80000
60000
40000
20000
1
7
14
35
63
350
time (days)

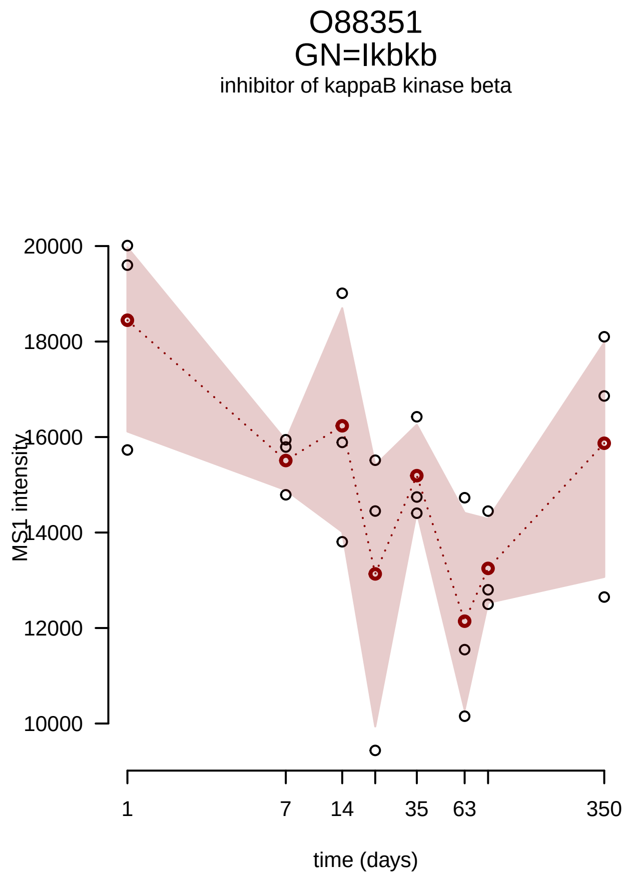
O88351
GN=Ikbkb
inhibitor of kappaB kinase beta
MS1 intensity
20000
18000
16000
14000
12000
10000
1
7
14
35
63
350
time (days)

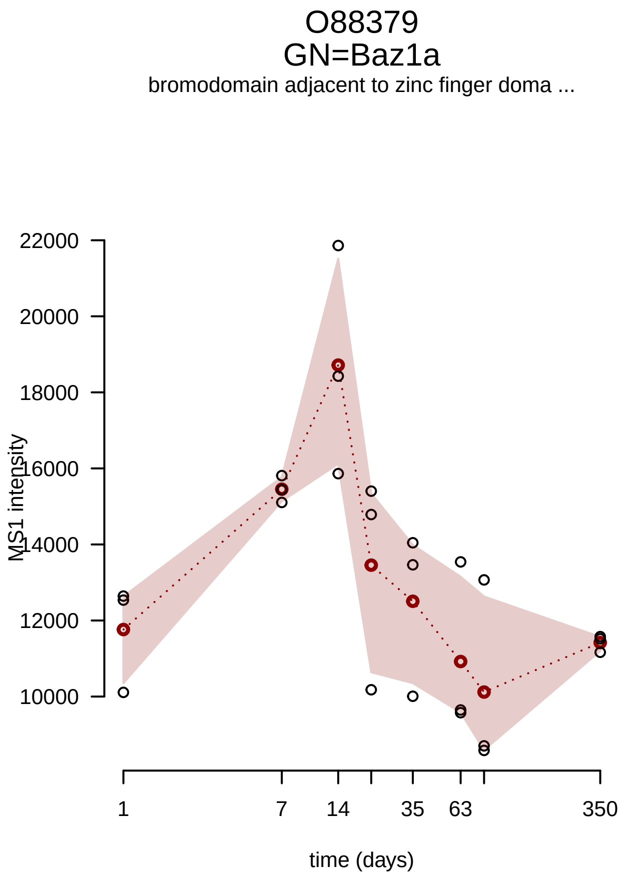
O88379
GN=Baz1a
bromodomain adjacent to zinc finger doma ...
MS1 intensity
22000
20000
18000
16000
14000
12000
10000
1
7
14
35
63
350
time (days)

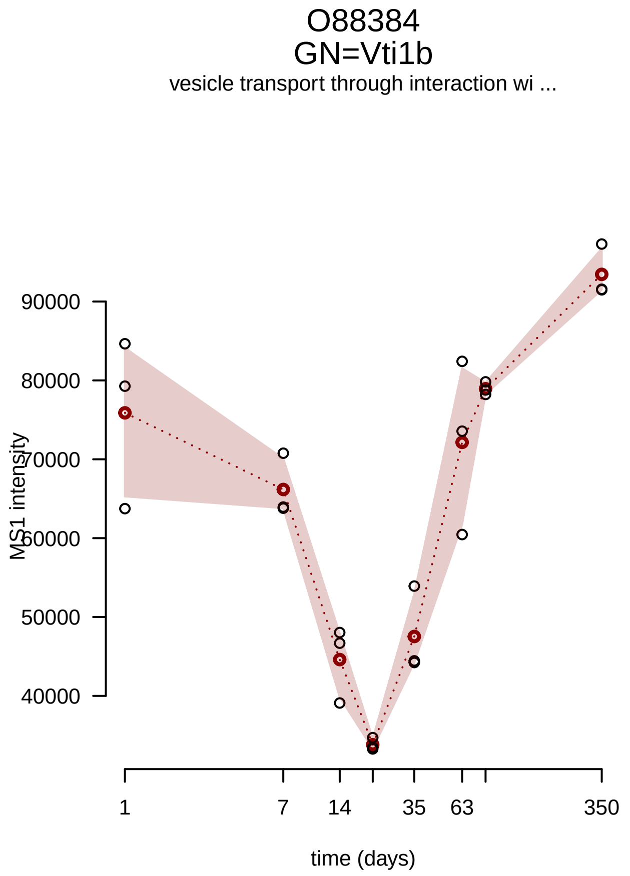
O88384
GN=Vti1b
vesicle transport through interaction wi ...
MS1 intensity
90000
80000
70000
60000
50000
40000
1
7
14
35
63
350
time (days)

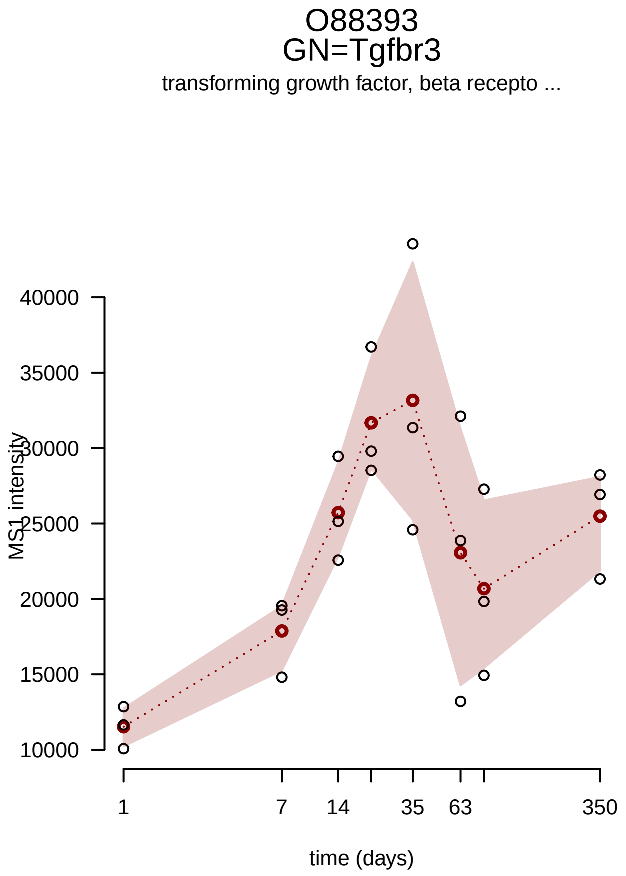
O88393
GN=Tgfbr3
transforming growth factor, beta recepto ...
MS1 intensity
40000
35000
30000
25000
20000
15000
10000
1
7
14
35
63
350
time (days)

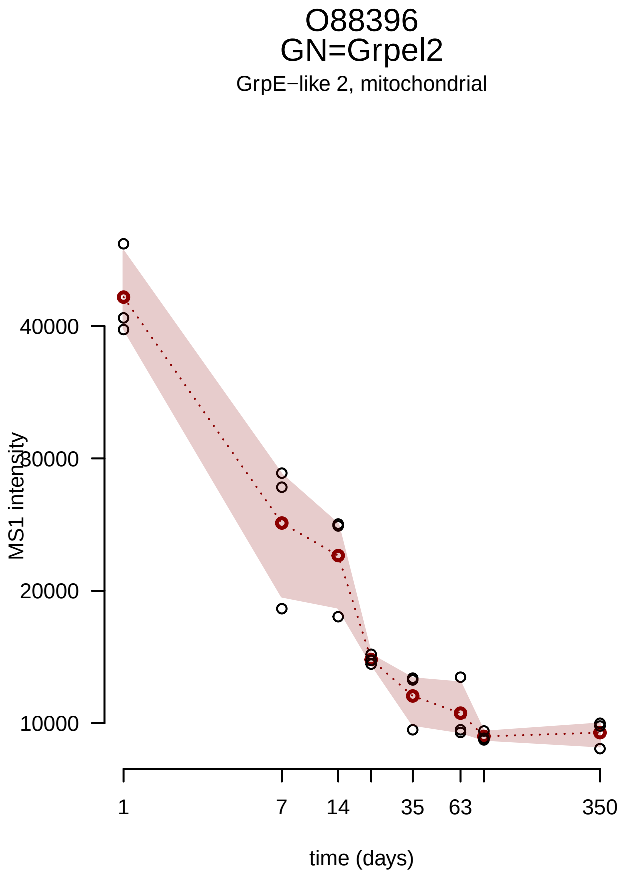
O88396
GN=Grpel2
GrpE−like 2, mitochondrial
MS1 intensity
40000
30000
20000
10000
1
7
14
35
63
350
time (days)

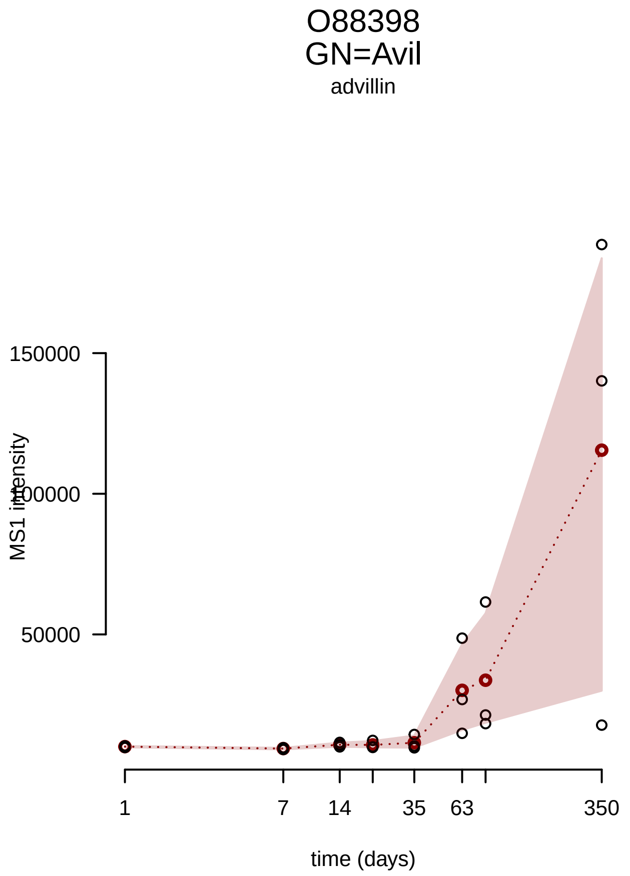
O88398
GN=Avil
advillin
MS1 intensity
150000
100000
50000
1
7
14
35
63
350
time (days)

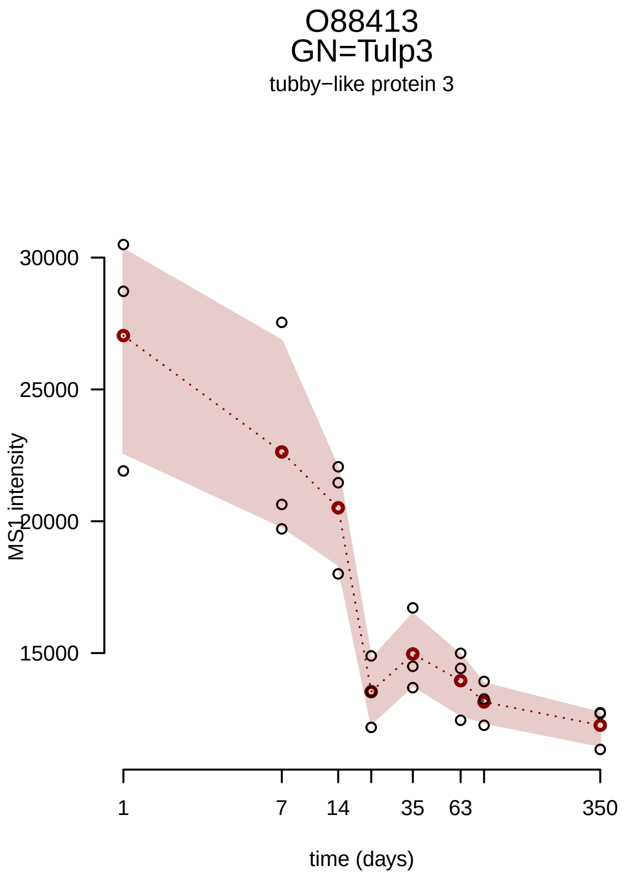
O88413
GN=Tulp3
tubby−like protein 3
MS1 intensity
30000
25000
20000
15000
1
7
14
35
63
350
time (days)

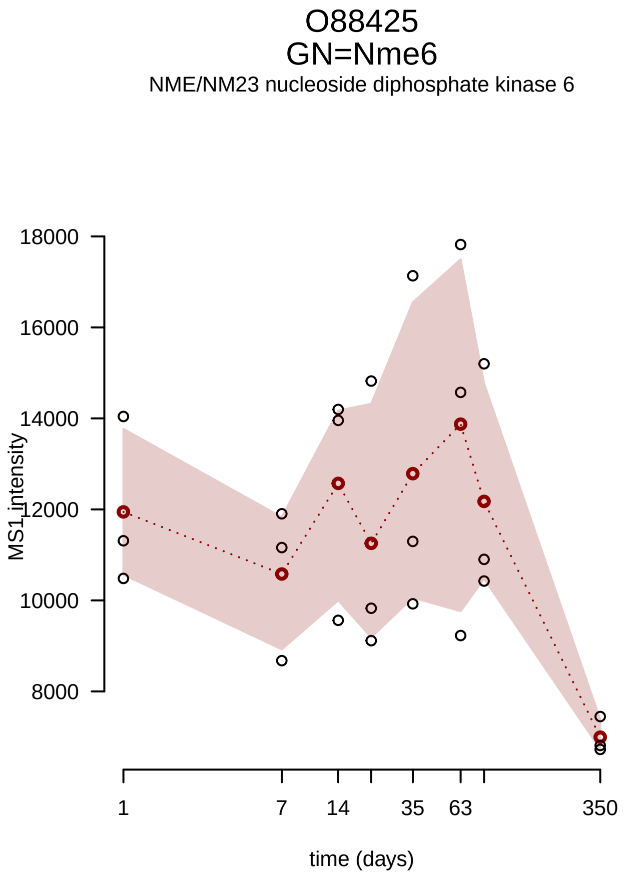
O88425
GN=Nme6
NME/NM23 nucleoside diphosphate kinase 6
MS1 intensity
18000
16000
14000
12000
10000
8000
1
7
14
35
63
350
time (days)

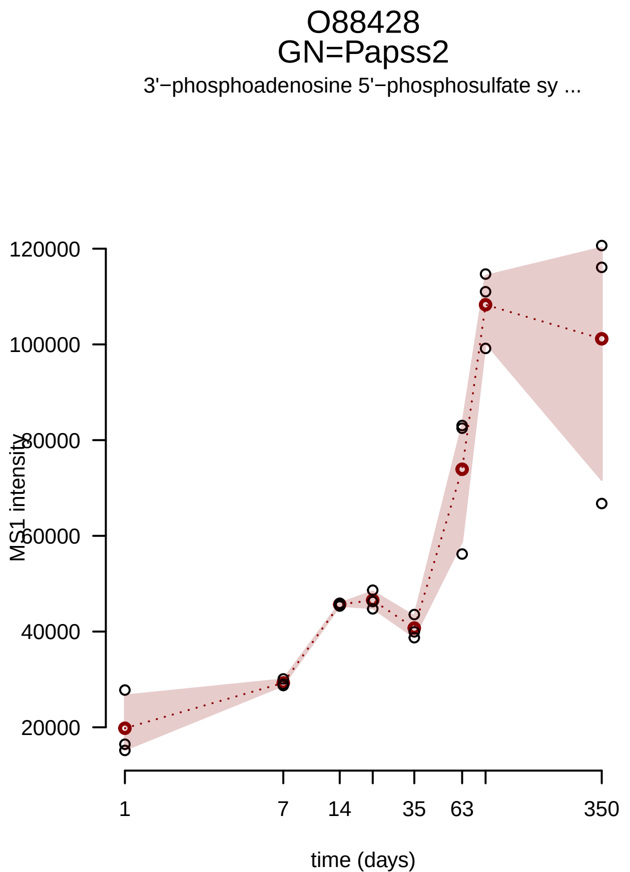
O88428
GN=Papss2
3'−phosphoadenosine 5'−phosphosulfate sy ...
MS1 intensity
120000
100000
80000
60000
40000
20000
1
7
14
35
63
350
time (days)

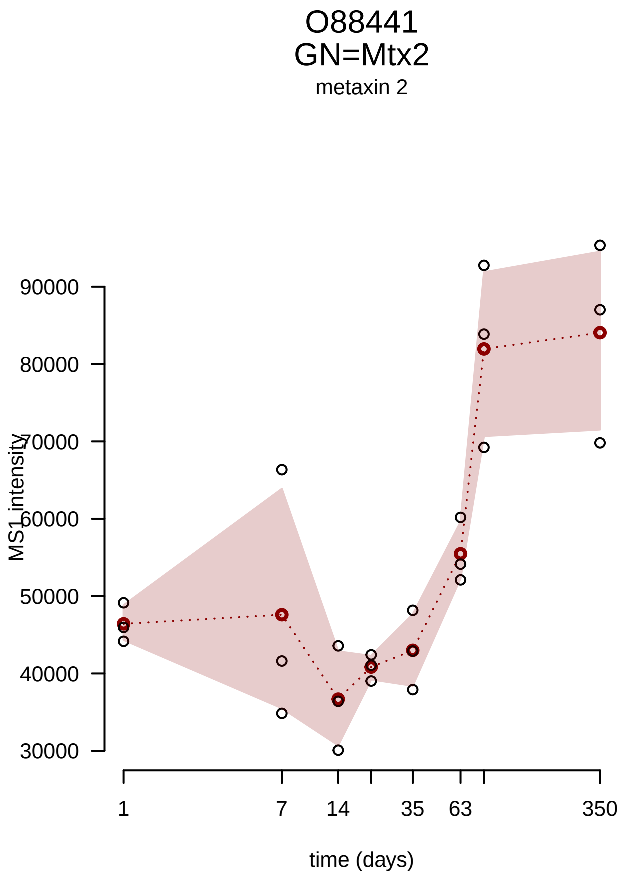
O88441
GN=Mtx2
metaxin 2
MS1 intensity
90000
80000
70000
60000
50000
40000
30000
1
7
14
35
63
350
time (days)

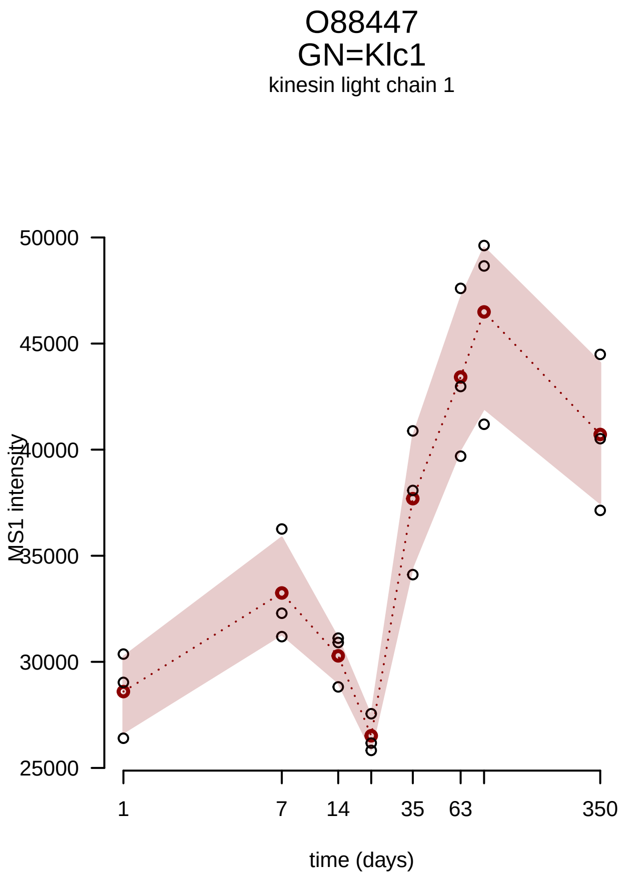
O88447
GN=Klc1
kinesin light chain 1
MS1 intensity
50000
45000
40000
35000
30000
25000
1
7
14
35
63
350
time (days)

O88448
GN=Klc2
kinesin light chain 2

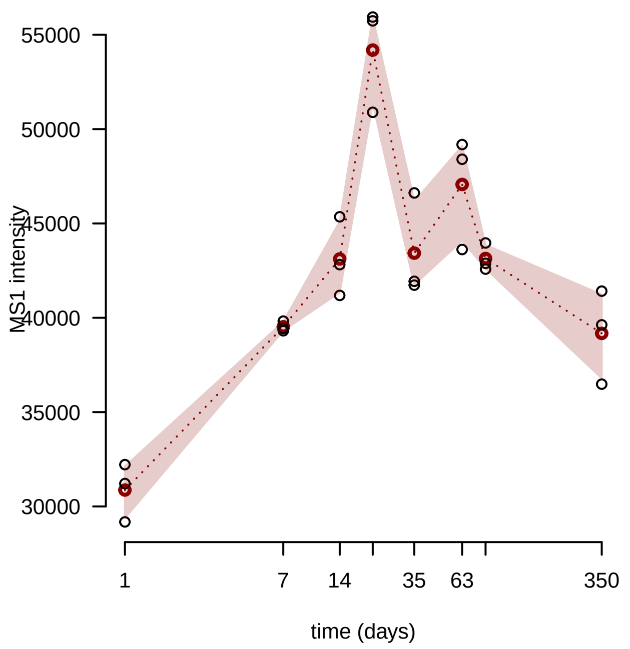

O88451
GN=Rdh7
retinol dehydrogenase 7

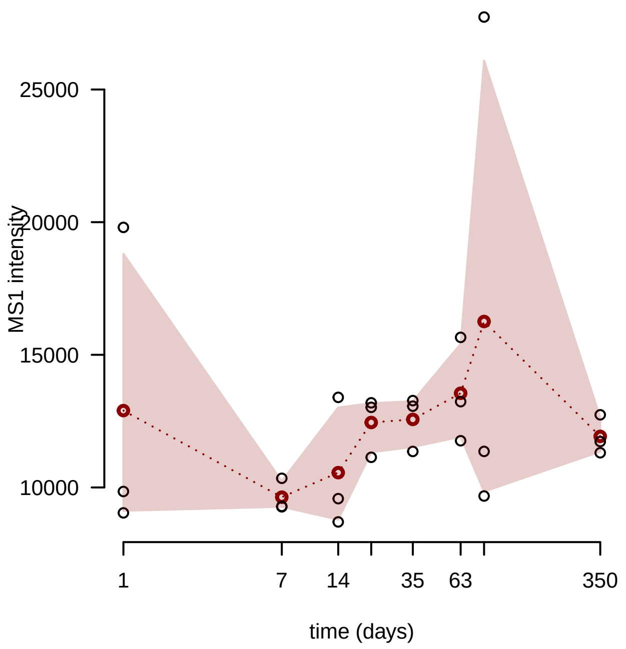

O88455
GN=Dhcr7
7−dehydrocholesterol reductase

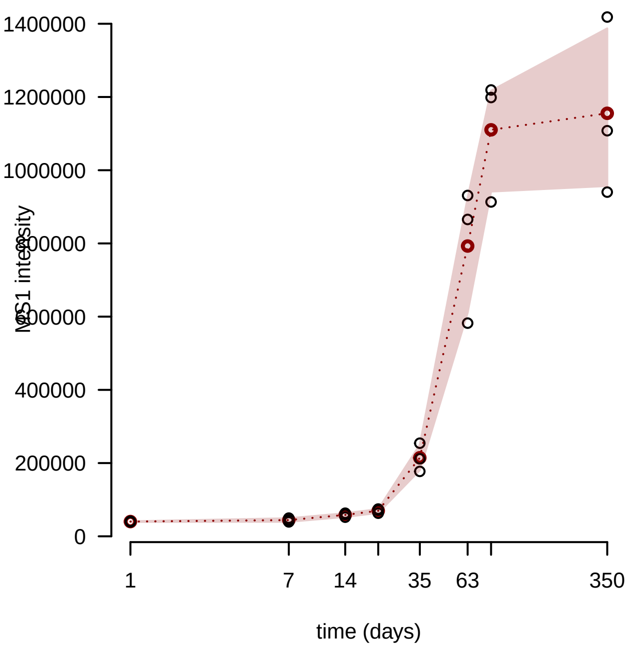

O88456
GN=Capns1
calpain, small subunit 1

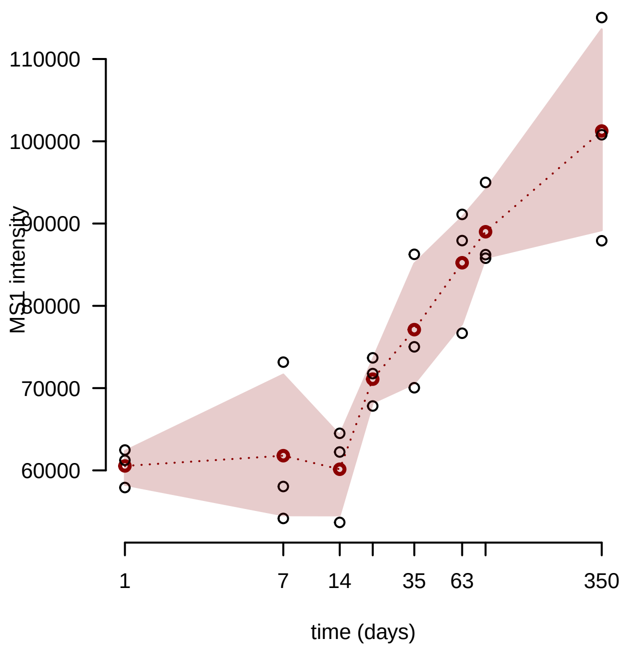

O88466
GN=Znf106

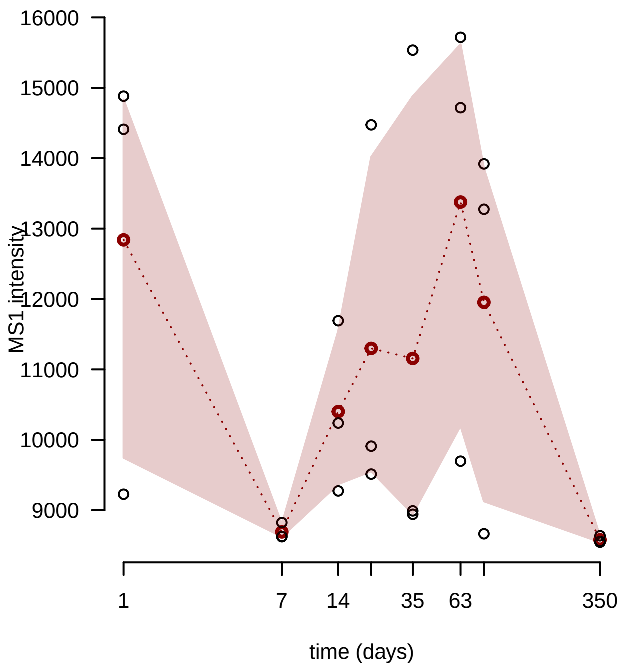

O88470
GN=Foxl2
forkhead box L2

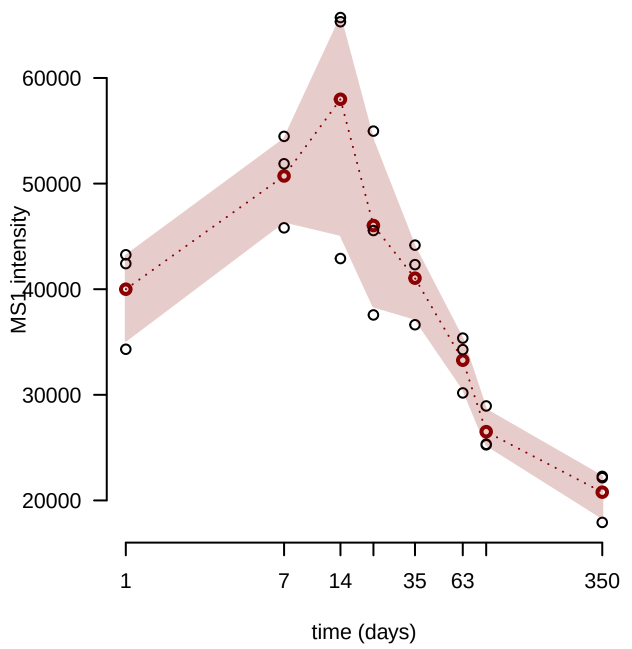

O88477
GN=Igf2bp1
insulin−like growth factor 2 mRNA bindin ...

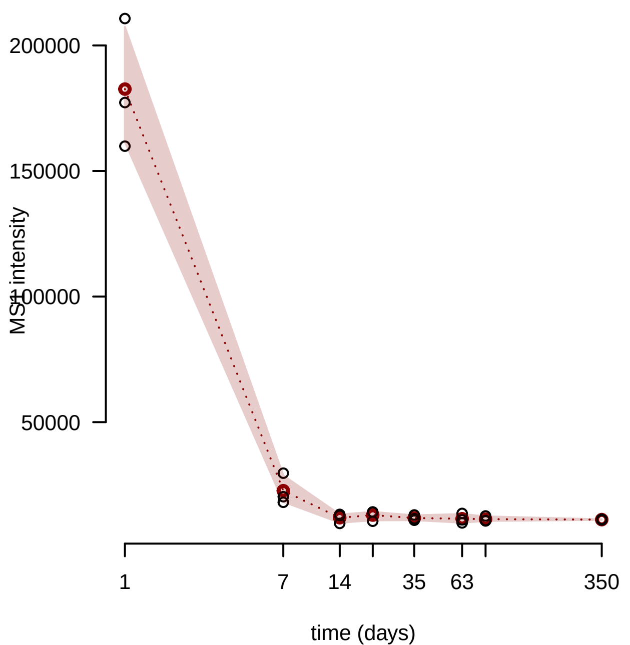

O88485
GN=Dync1i1
dynein cytoplasmic 1 intermediate chain ...

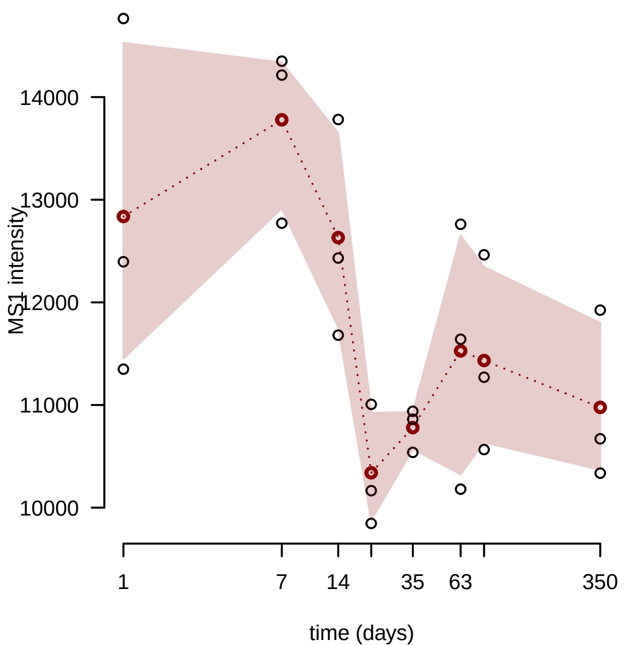

O88487
GN=Dync1i2
dynein cytoplasmic 1 intermediate chain ...

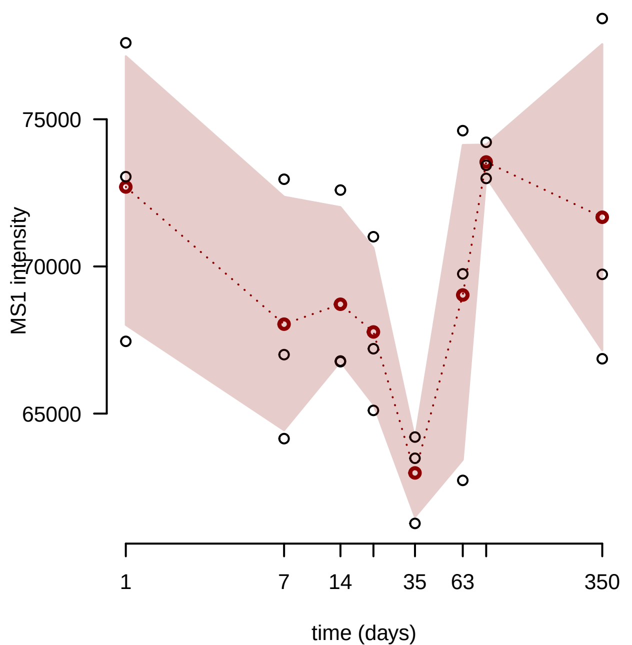

O88491
GN=Nsd1
nuclear receptor−binding SET−domain prot ...

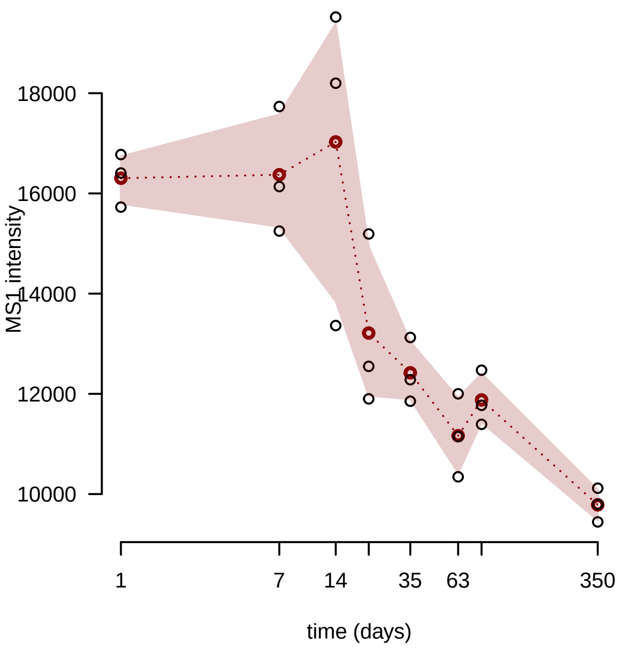

O88492
GN=Plin4
perilipin 4

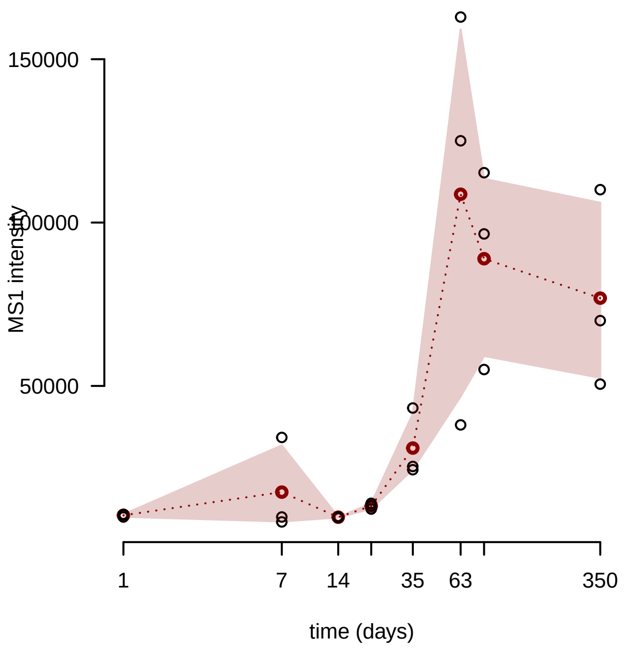

O88502
GN=Pde8a
phosphodiesterase 8A

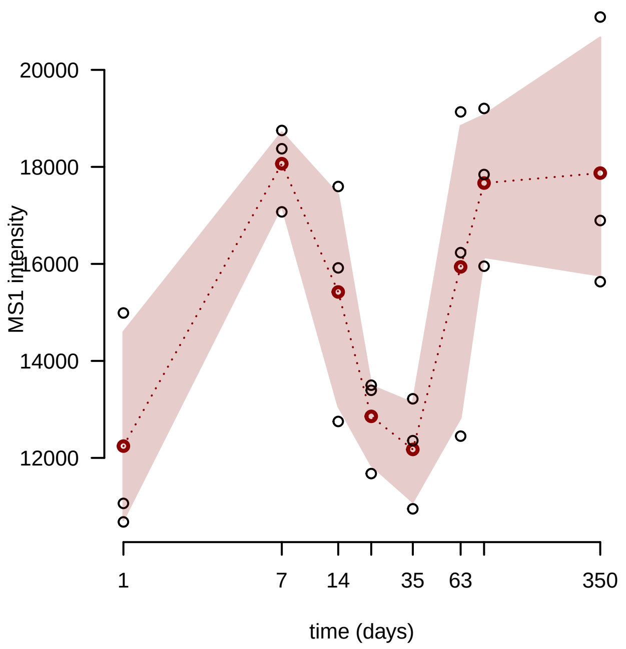

O88507
GN=Cntfr
ciliary neurotrophic factor receptor

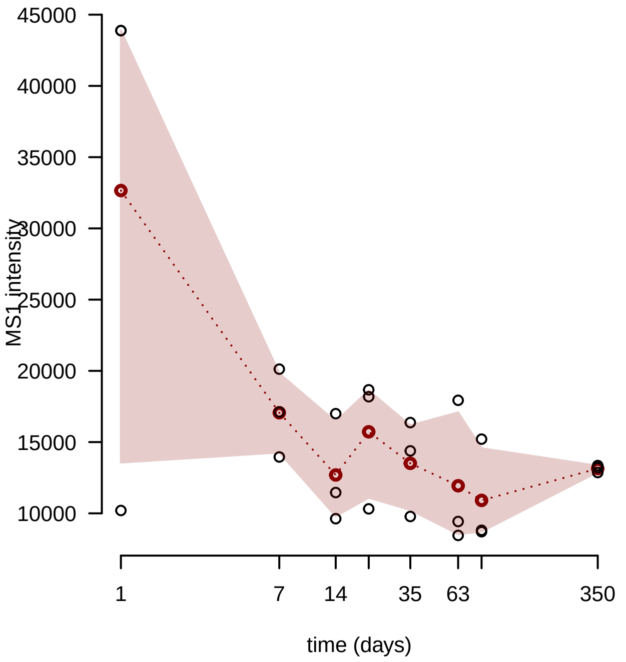

O88508
GN=Dnmt3a
DNA methyltransferase 3A

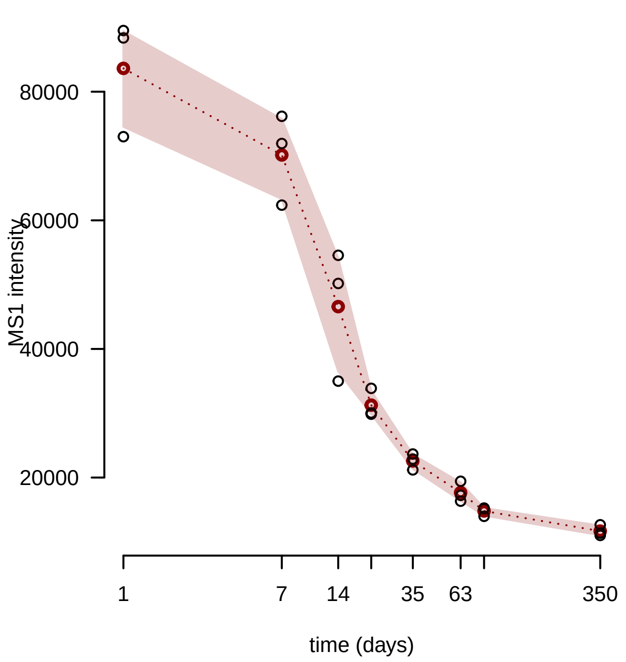

O88512
GN=Ap1g2
adaptor protein complex AP−1, gamma 2 su ...

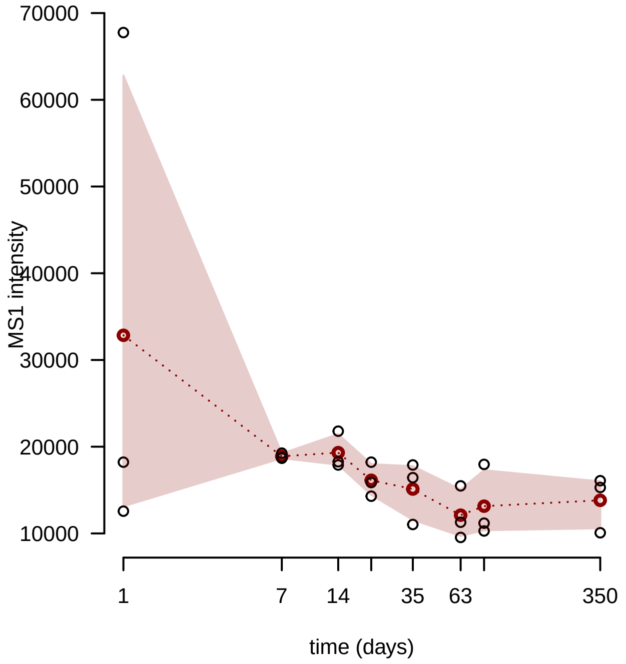

O88520
GN=Shoc2
Shoc2, leucine rich repeat scaffold prot ...

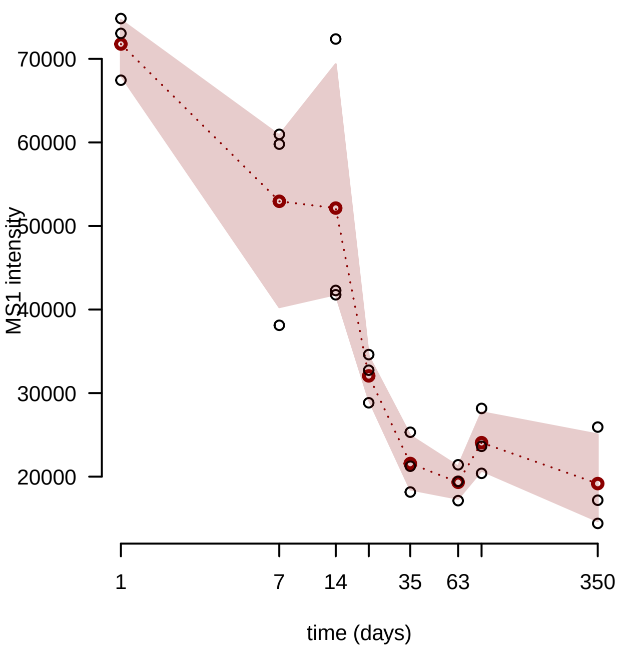

O88522
GN=Ikbkg
inhibitor of kappaB kinase gamma

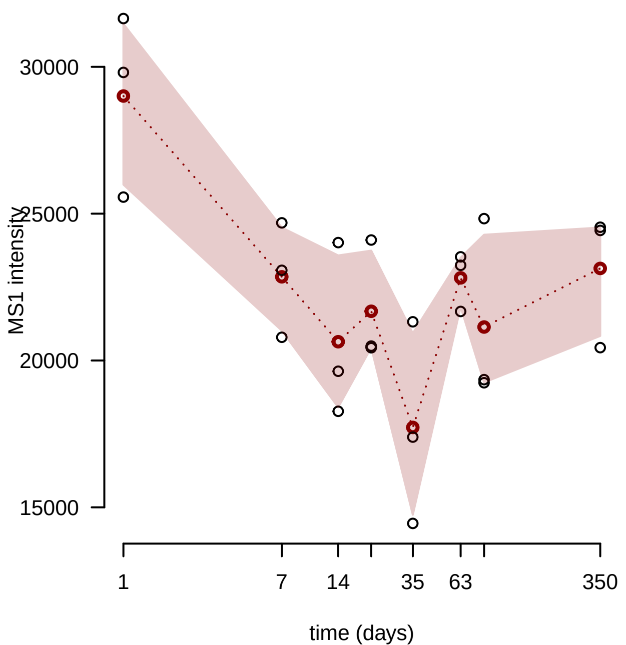

O88531
GN=Ppt1
palmitoyl−protein thioesterase 1

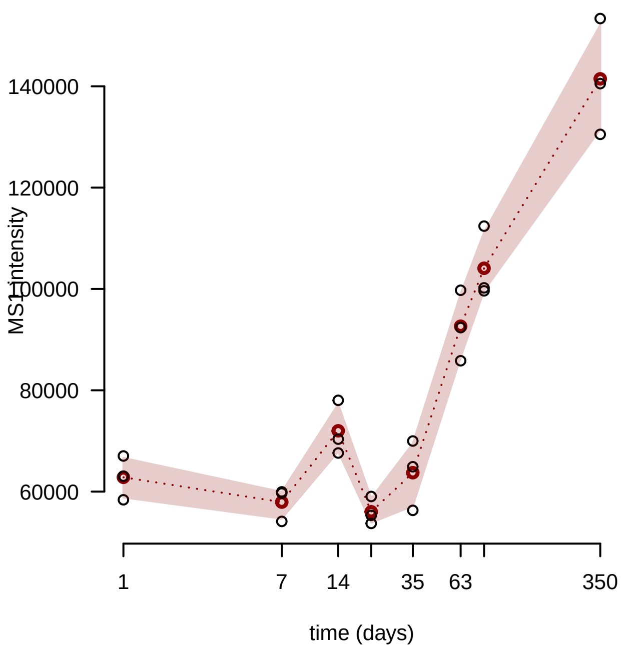

O88532
GN=Zfr
zinc finger RNA binding protein

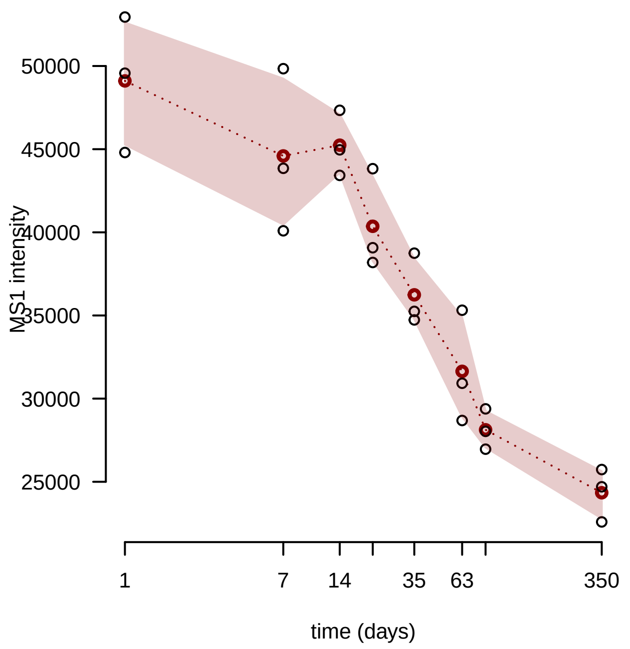

O88533
GN=Ddc
dopa decarboxylase

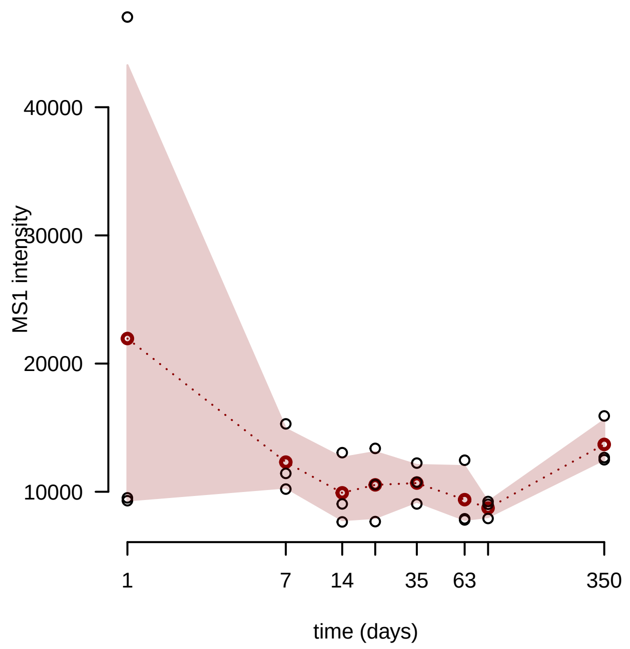

O88543
GN=Cops3
COP9 signalosome subunit 3

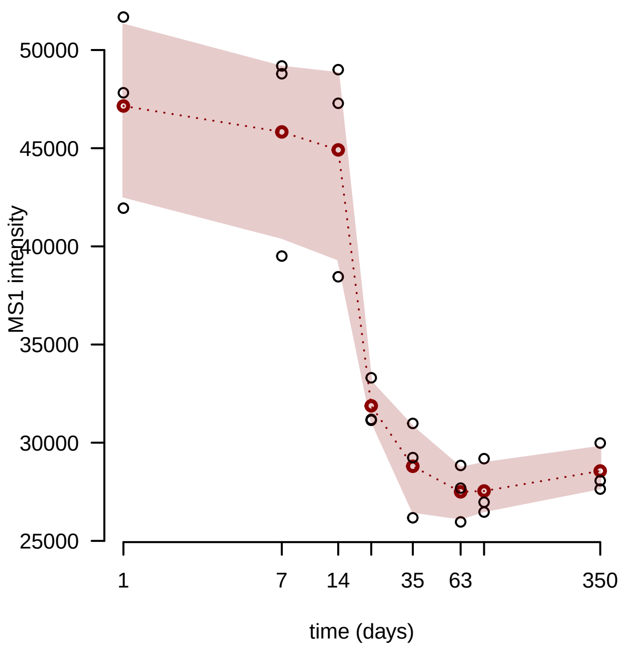

O88544
GN=Cops4
COP9 signalosome subunit 4

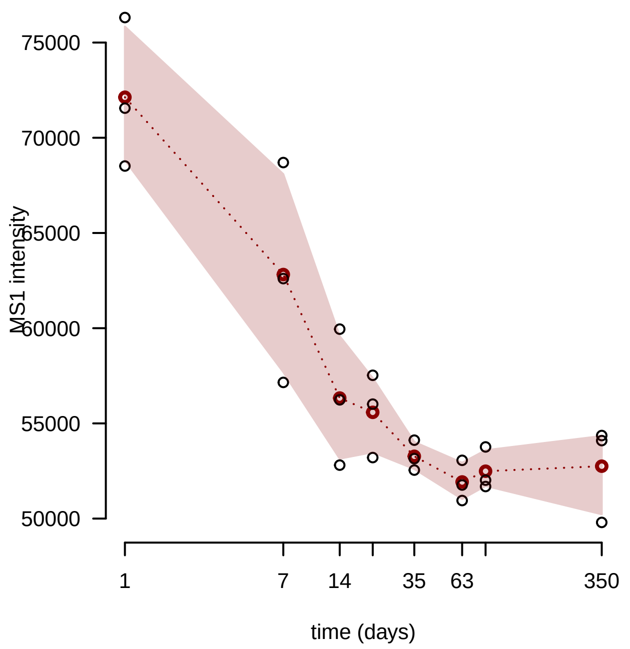

O88545
GN=Cops6
COP9 signalosome subunit 6

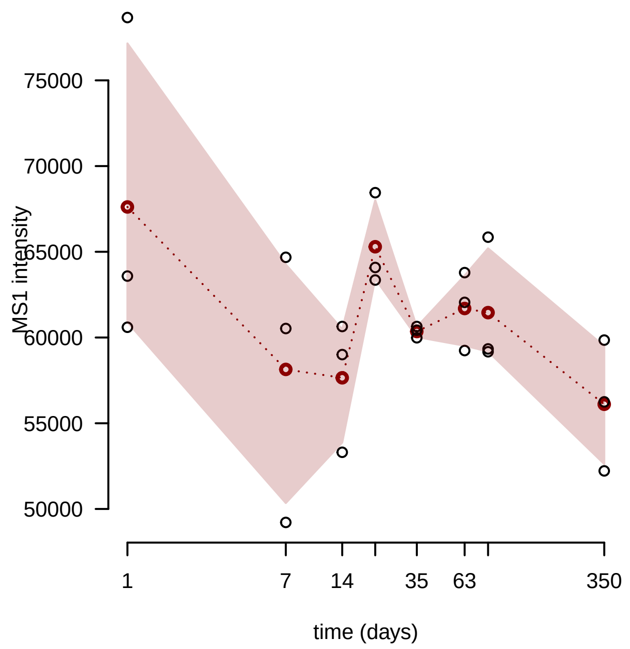

O88554
GN=Parp2
poly (ADP−ribose) polymerase family, mem ...

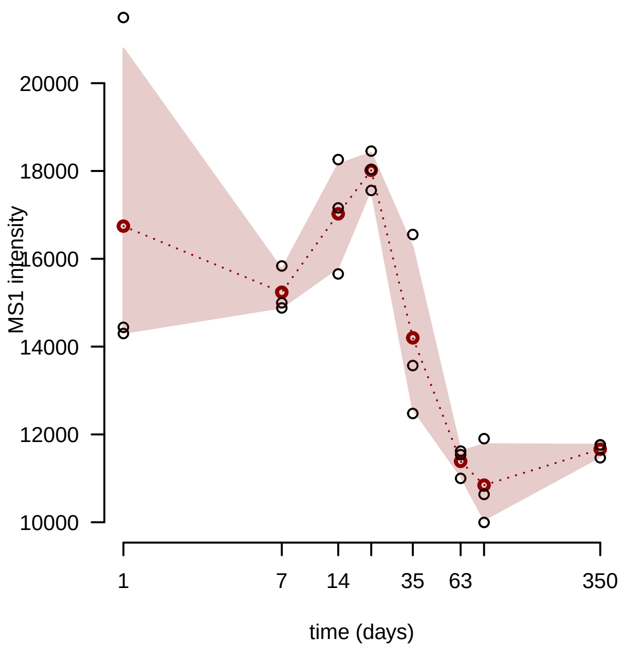

O88559
GN=Men1
multiple endocrine neoplasia 1

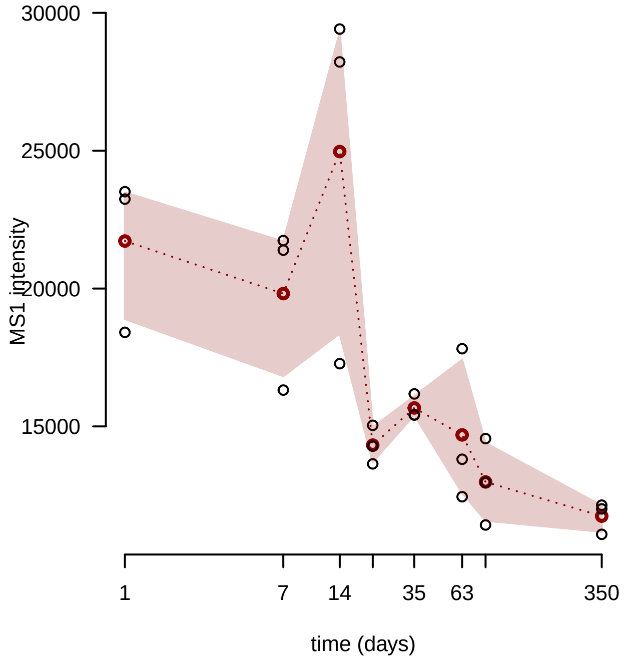

O88561
GN=Slc27a3
solute carrier family 27 (fatty acid tra ...

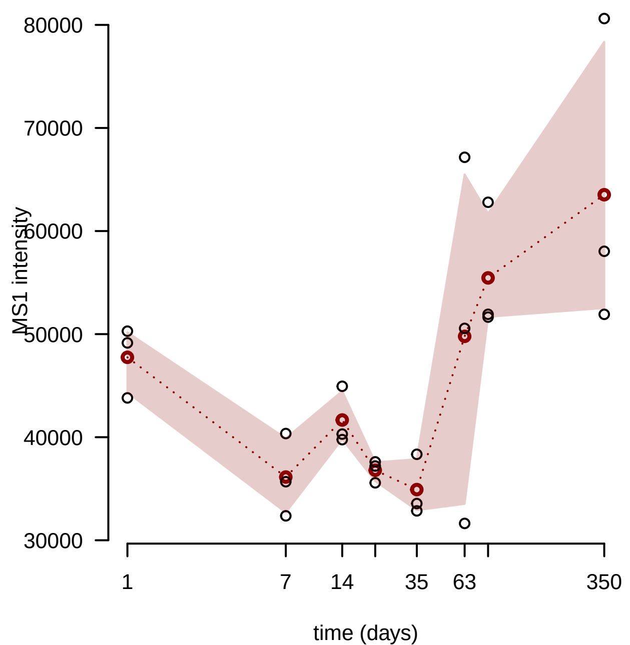

O88569
GN=Hnrnpa2b1
heterogeneous nuclear ribonucleoprotein ...

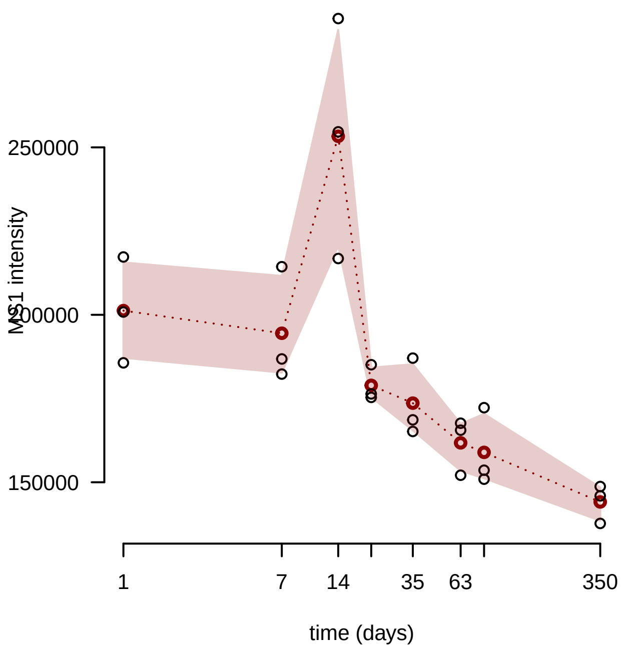

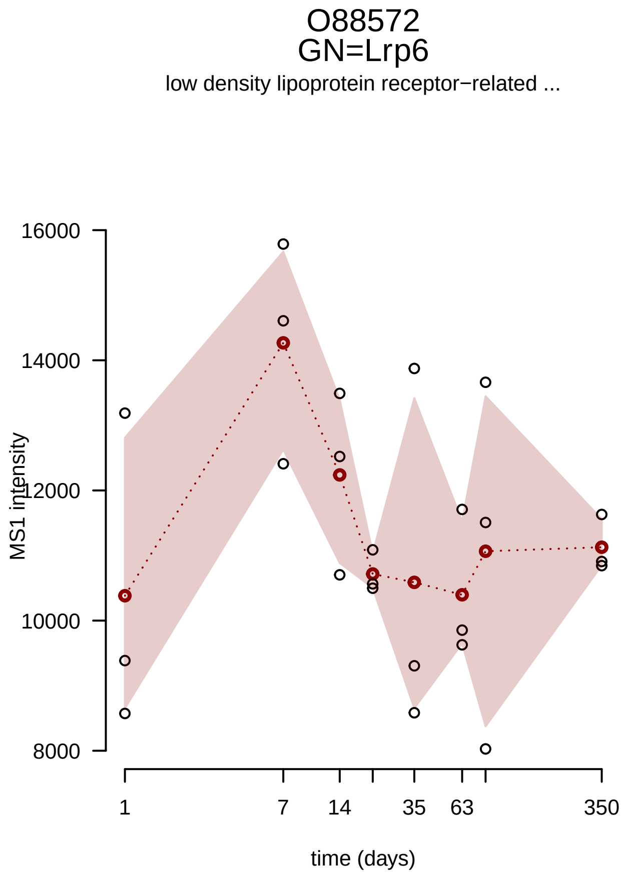
O88572
GN=Lrp6
low density lipoprotein receptor−related ...
16000
14000
12000
10000
8000
MS1 intensity
1
7
14
35
63
350
time (days)
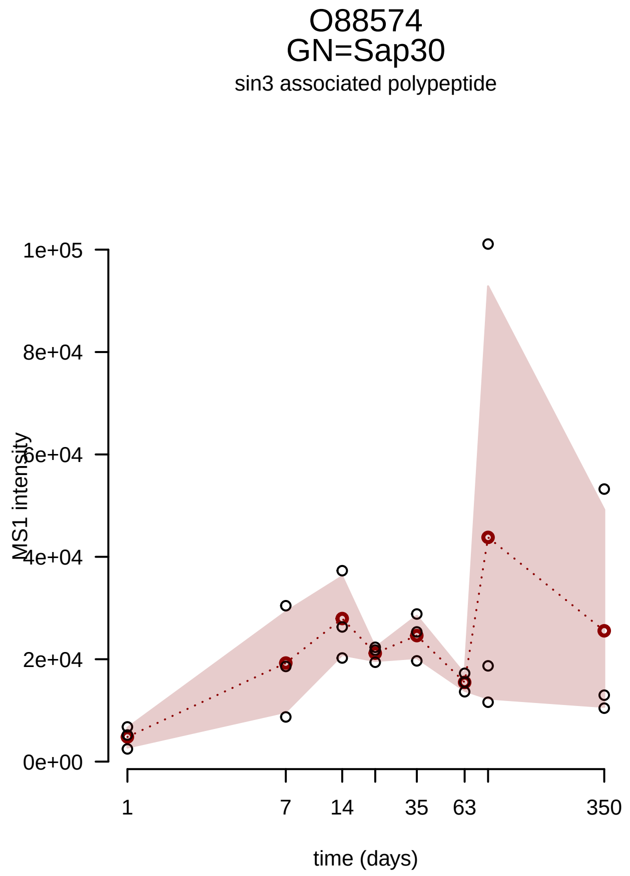
O88574
GN=Sap30
sin3 associated polypeptide
1e+05
8e+04
6e+04
4e+04
2e+04
0e+00
MS1 intensity
1
7
14
35
63
350
time (days)
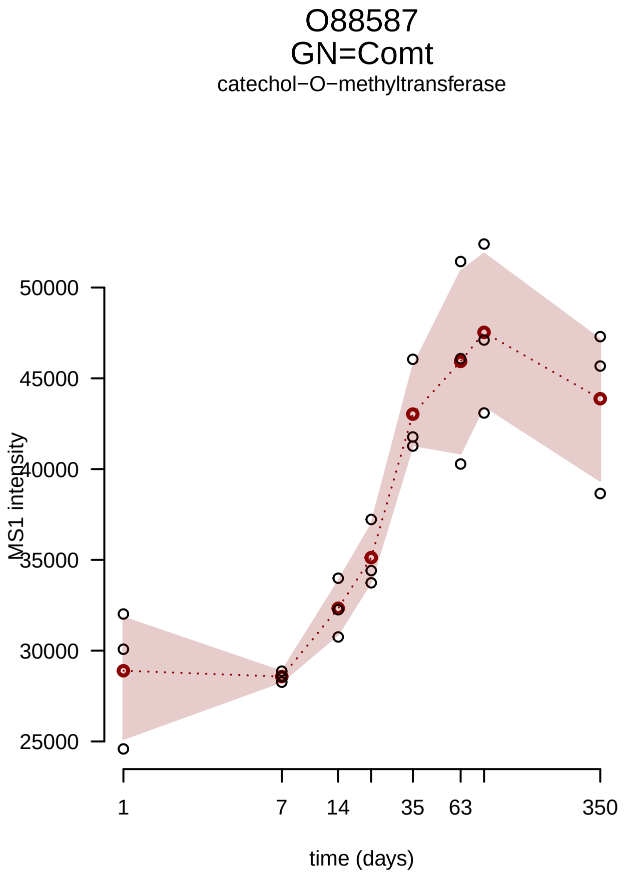
O88587
GN=Comt
catechol−O−methyltransferase
50000
45000
40000
35000
30000
25000
MS1 intensity
1
7
14
35
63
350
time (days)
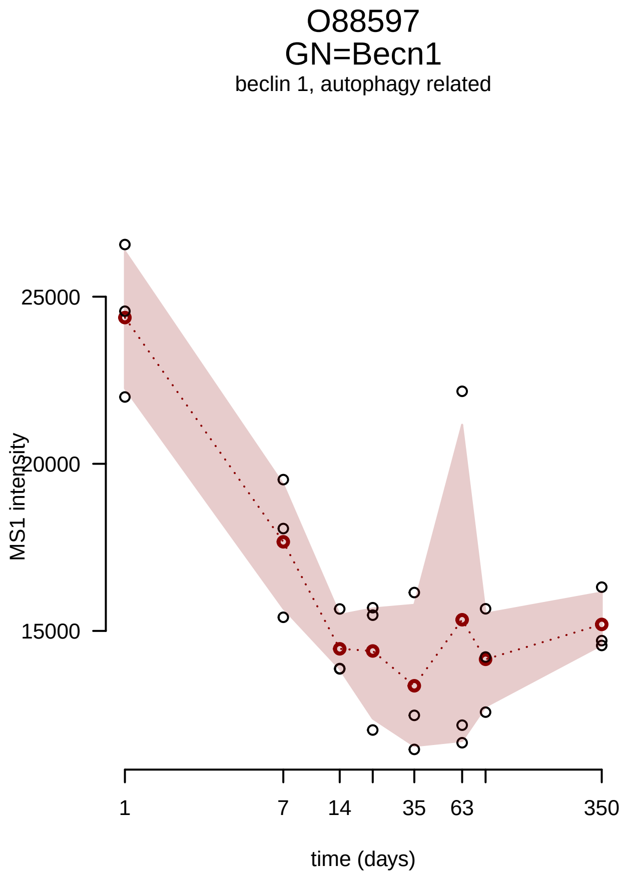
O88597
GN=Becn1
beclin 1, autophagy related
25000
20000
15000
MS1 intensity
1
7
14
35
63
350
time (days)
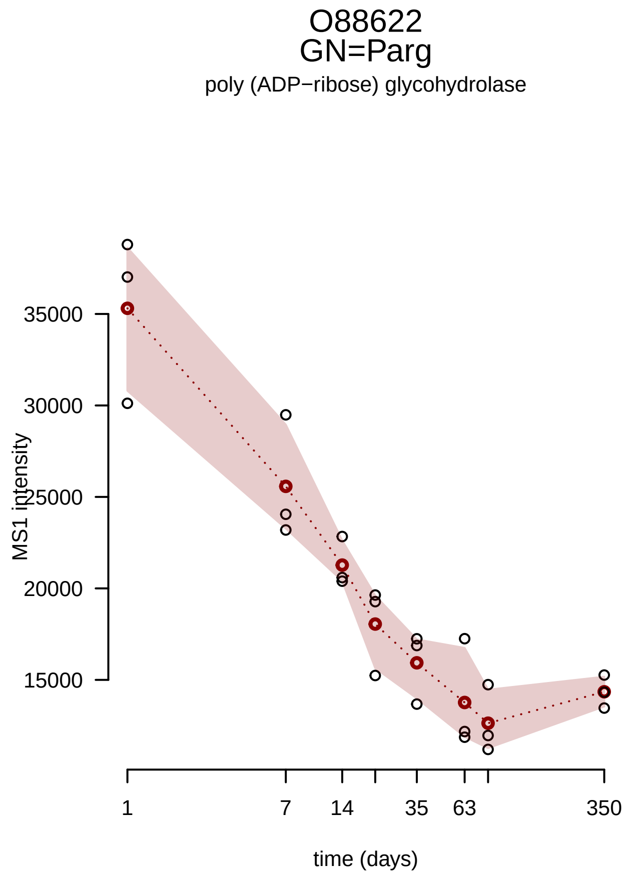
O88622
GN=Parg
poly (ADP−ribose) glycohydrolase
35000
30000
25000
20000
15000
MS1 intensity
1
7
14
35
63
350
time (days)
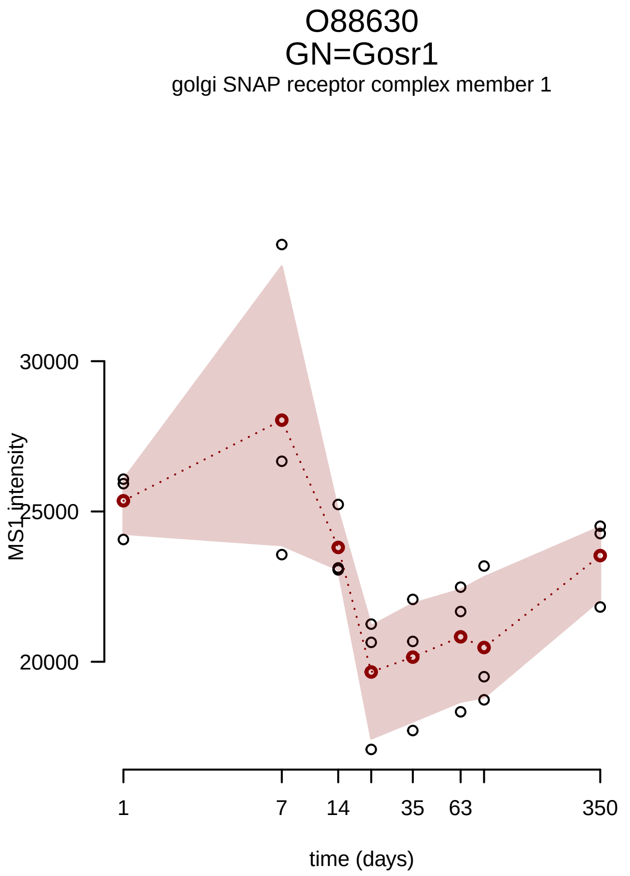
O88630
GN=Gosr1
golgi SNAP receptor complex member 1
30000
25000
20000
MS1 intensity
1
7
14
35
63
350
time (days)
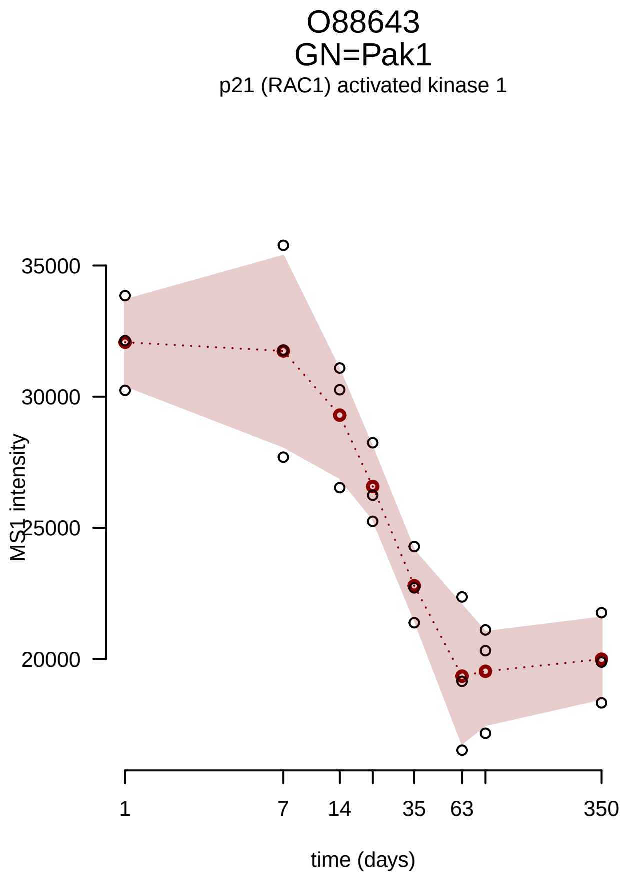
O88643
GN=Pak1
p21 (RAC1) activated kinase 1
35000
30000
25000
20000
MS1 intensity
1
7
14
35
63
350
time (days)
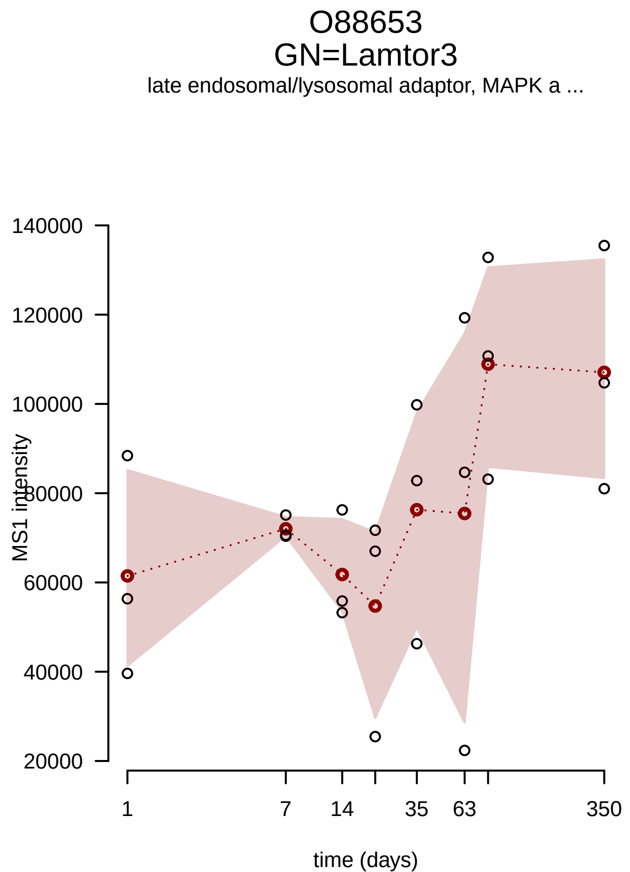
O88653
GN=Lamtor3
late endosomal/lysosomal adaptor, MAPK a ...
140000
120000
100000
80000
60000
40000
20000
MS1 intensity
1
7
14
35
63
350
time (days)
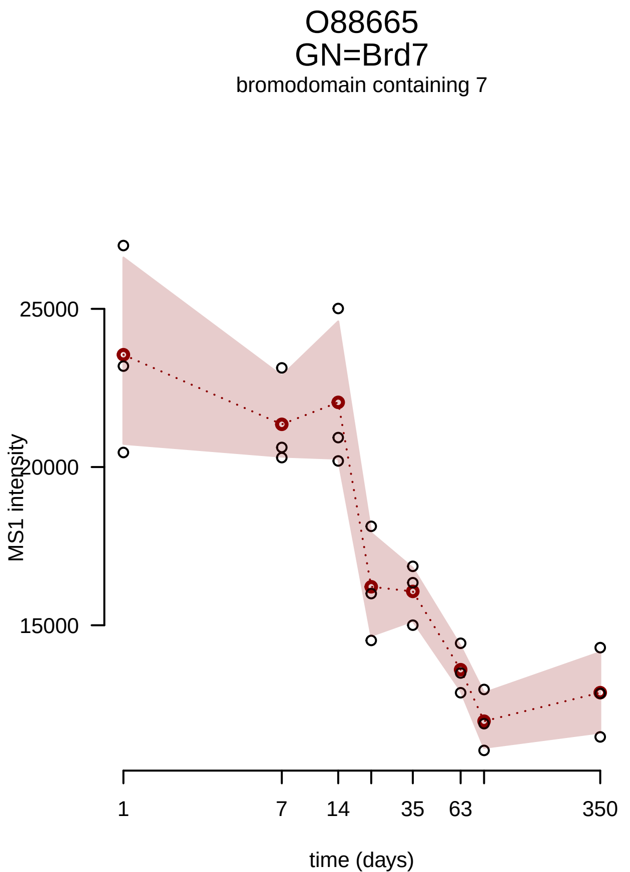
O88665
GN=Brd7
bromodomain containing 7
25000
20000
15000
MS1 intensity
1
7
14
35
63
350
time (days)

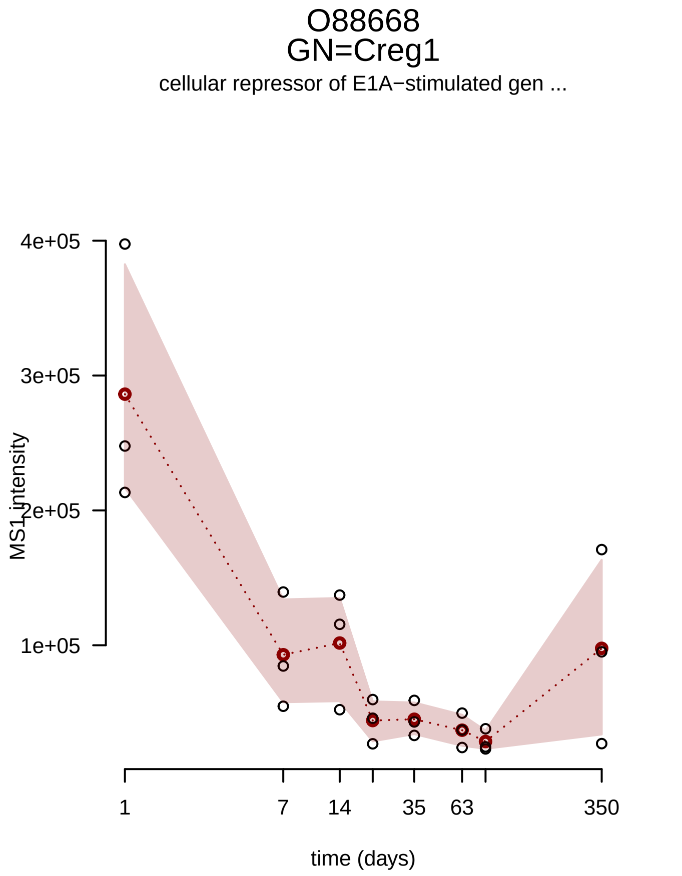
O88668
GN=Creg1
cellular repressor of E1A−stimulated gen ...
MS1 intensity
4e+05
3e+05
2e+05
1e+05
1
7
14
35
63
350
time (days)

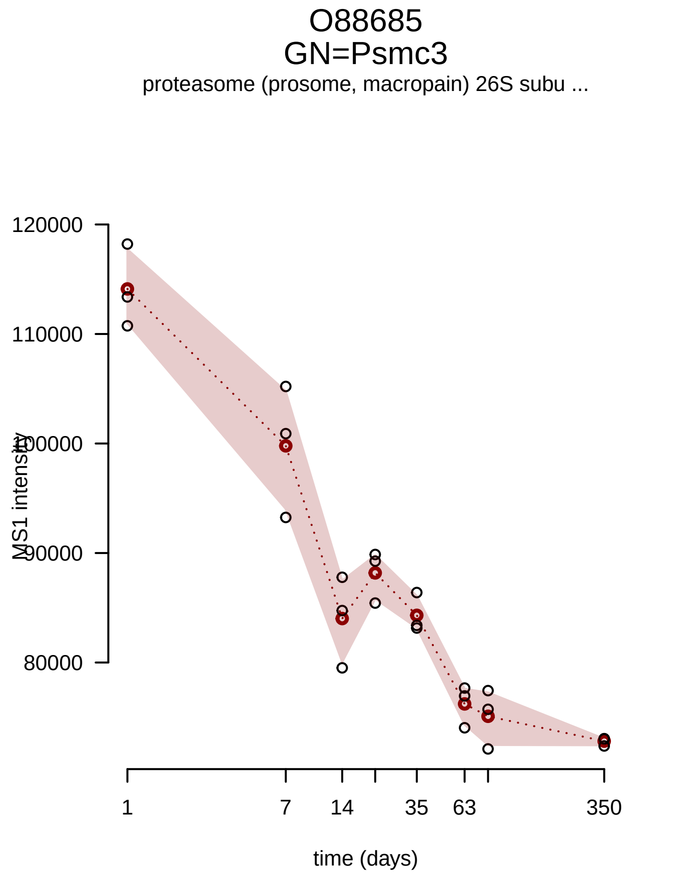
O88685
GN=Psmc3
proteasome (prosome, macropain) 26S subu ...
MS1 intensity
120000
110000
100000
90000
80000
1
7
14
35
63
350
time (days)

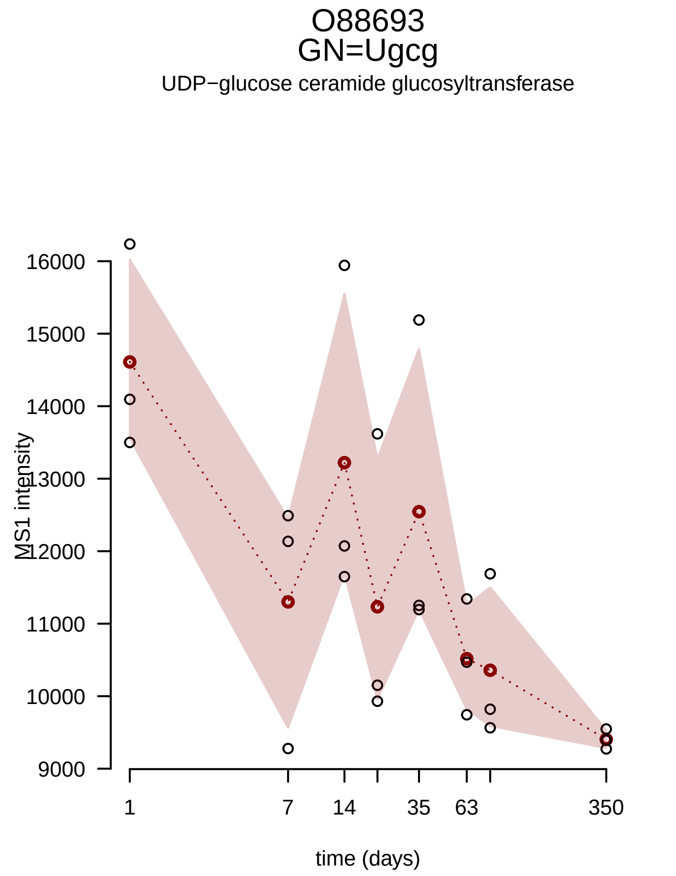
O88693
GN=Ugcg
UDP−glucose ceramide glucosyltransferase
MS1 intensity
16000
15000
14000
13000
12000
11000
10000
9000
1
7
14
35
63
350
time (days)

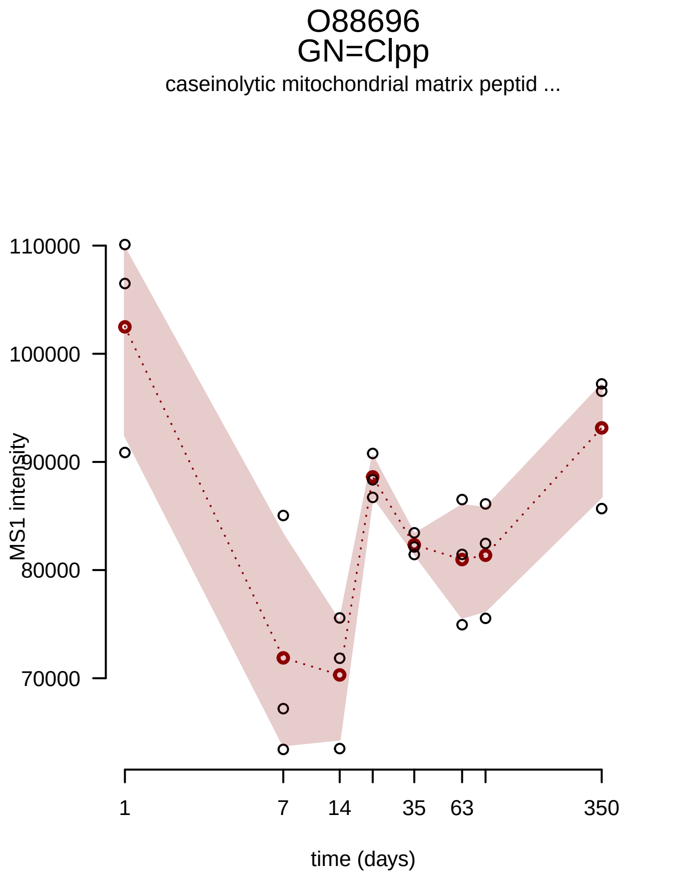
O88696
GN=Clpp
caseinolytic mitochondrial matrix peptid ...
MS1 intensity
110000
100000
90000
80000
70000
1
7
14
35
63
350
time (days)

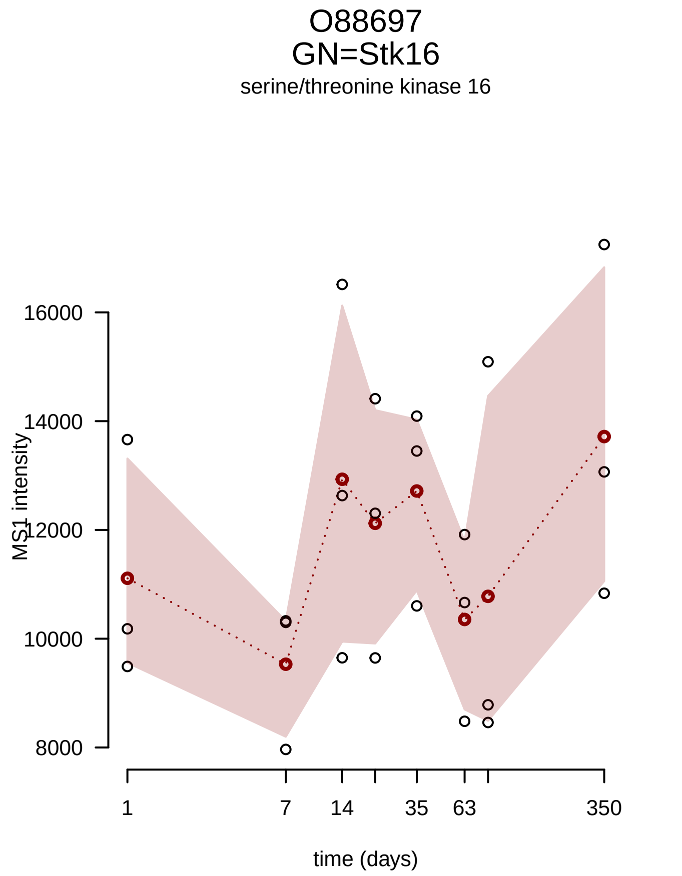
O88697
GN=Stk16
serine/threonine kinase 16
MS1 intensity
16000
14000
12000
10000
8000
1
7
14
35
63
350
time (days)

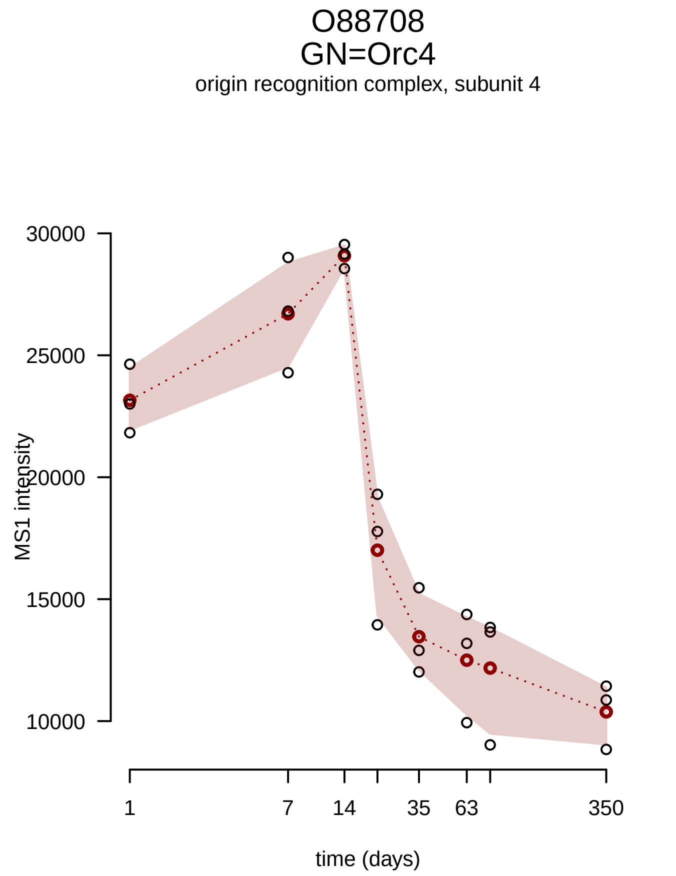
O88708
GN=Orc4
origin recognition complex, subunit 4
MS1 intensity
30000
25000
20000
15000
10000
1
7
14
35
63
350
time (days)

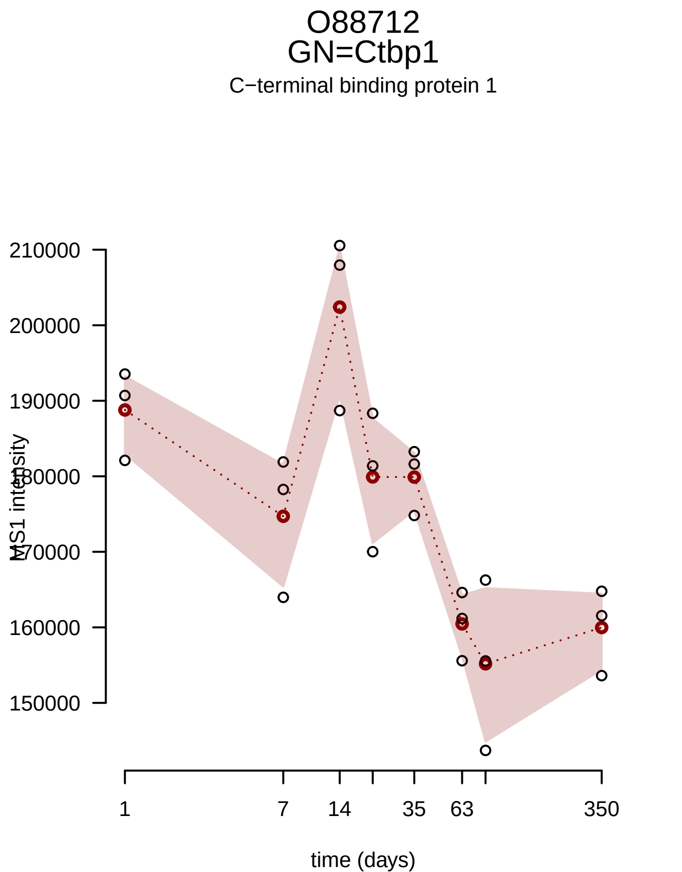
O88712
GN=Ctbp1
C−terminal binding protein 1
MS1 intensity
210000
200000
190000
180000
170000
160000
150000
1
7
14
35
63
350
time (days)

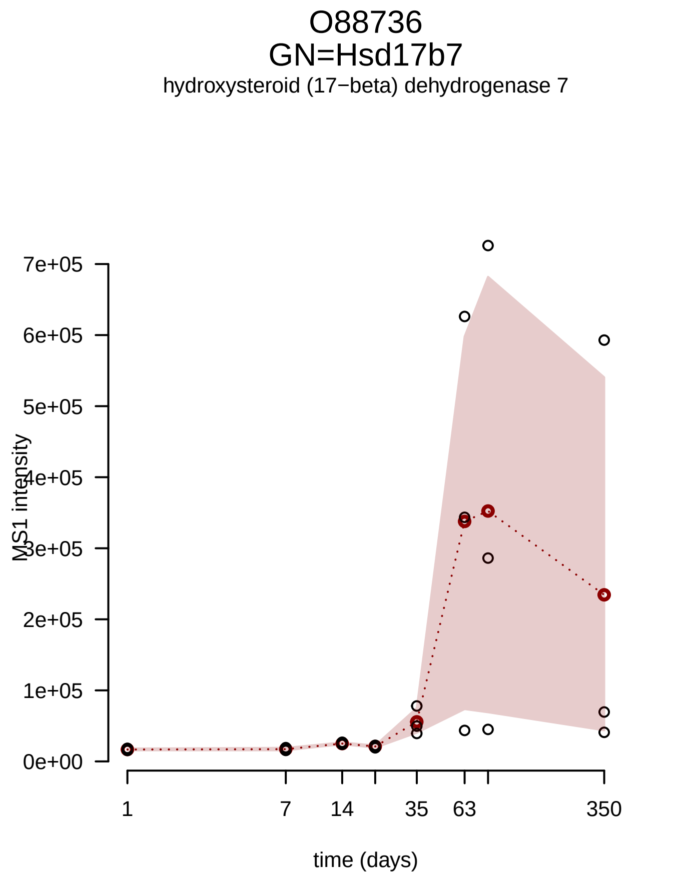
O88736
GN=Hsd17b7
hydroxysteroid (17−beta) dehydrogenase 7
MS1 intensity
7e+05
6e+05
5e+05
4e+05
3e+05
2e+05
1e+05
0e+00
1
7
14
35
63
350
time (days)

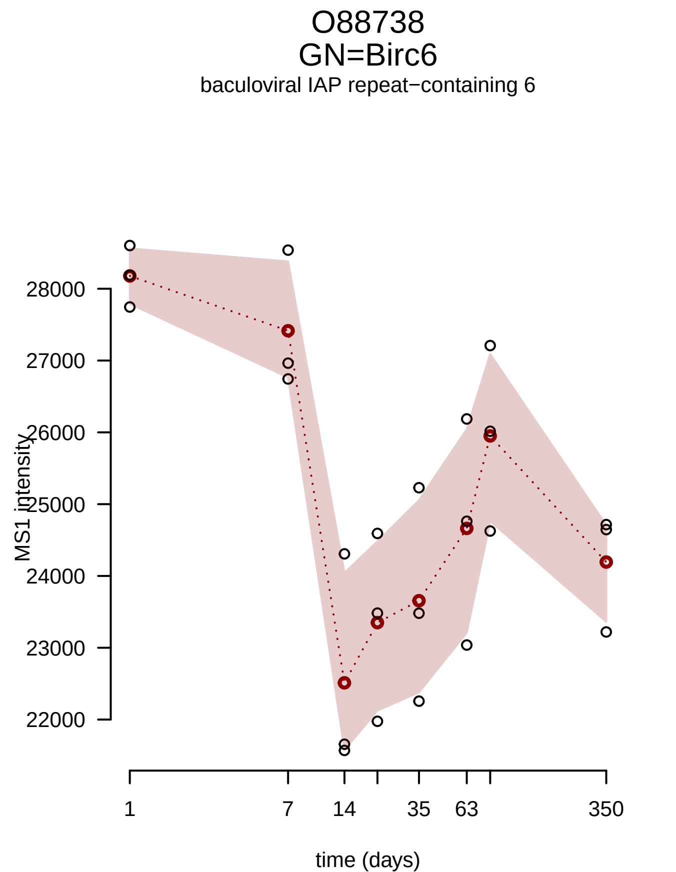
O88738
GN=Birc6
baculoviral IAP repeat−containing 6
MS1 intensity
28000
27000
26000
25000
24000
23000
22000
1
7
14
35
63
350
time (days)

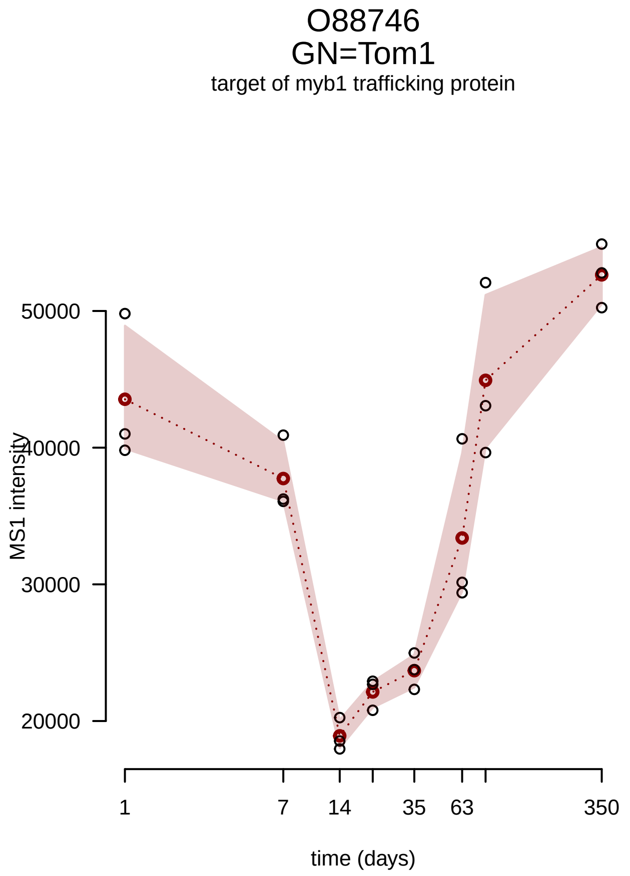
O88746
GN=Tom1
target of myb1 trafficking protein
MS1 intensity
20000
30000
40000
50000
1
7
14
35
63
350
time (days)

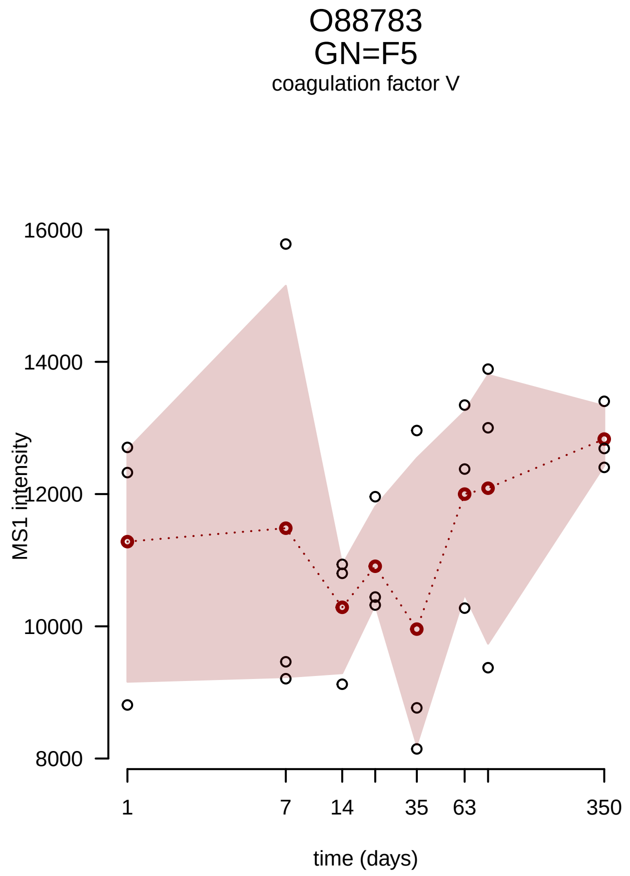
O88783
GN=F5
coagulation factor V
MS1 intensity
8000
10000
12000
14000
16000
1
7
14
35
63
350
time (days)

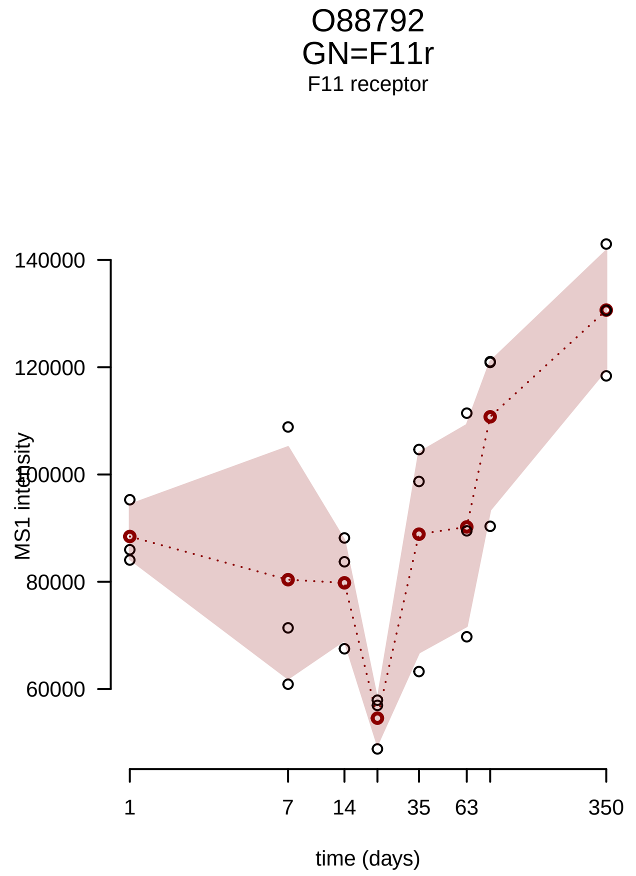
O88792
GN=F11r
F11 receptor
MS1 intensity
60000
80000
100000
120000
140000
1
7
14
35
63
350
time (days)

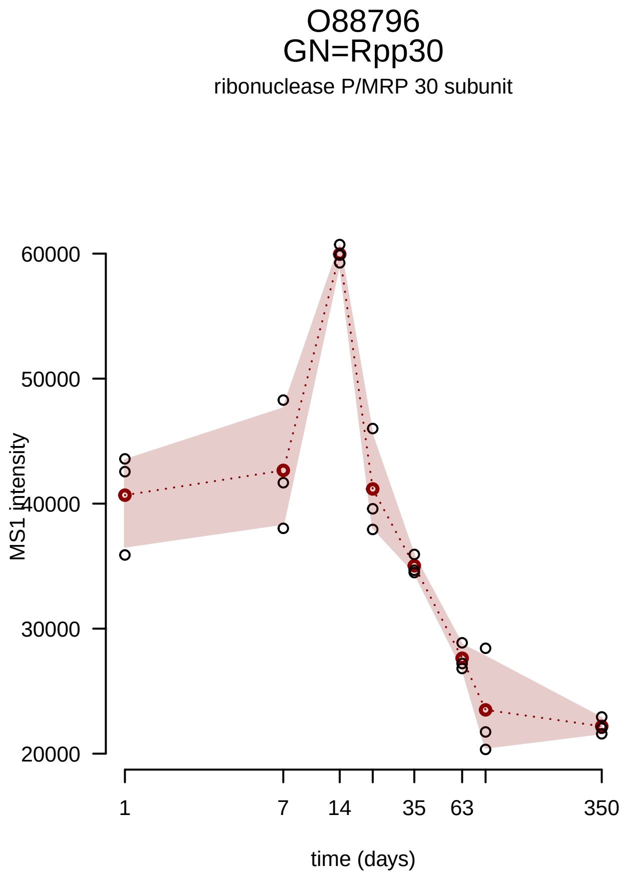
O88796
GN=Rpp30
ribonuclease P/MRP 30 subunit
MS1 intensity
20000
30000
40000
50000
60000
1
7
14
35
63
350
time (days)

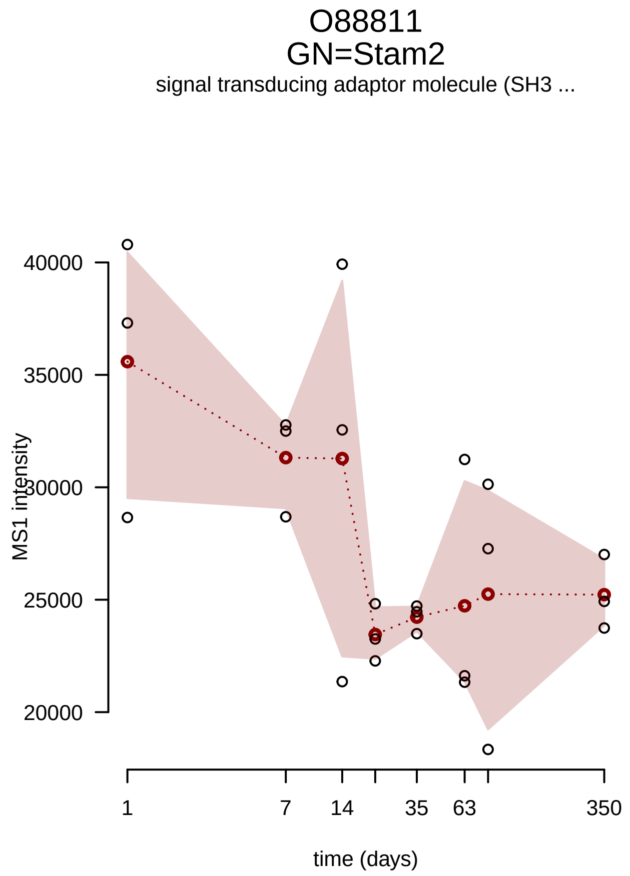
O88811
GN=Stam2
signal transducing adaptor molecule (SH3 ...
MS1 intensity
20000
25000
30000
35000
40000
1
7
14
35
63
350
time (days)

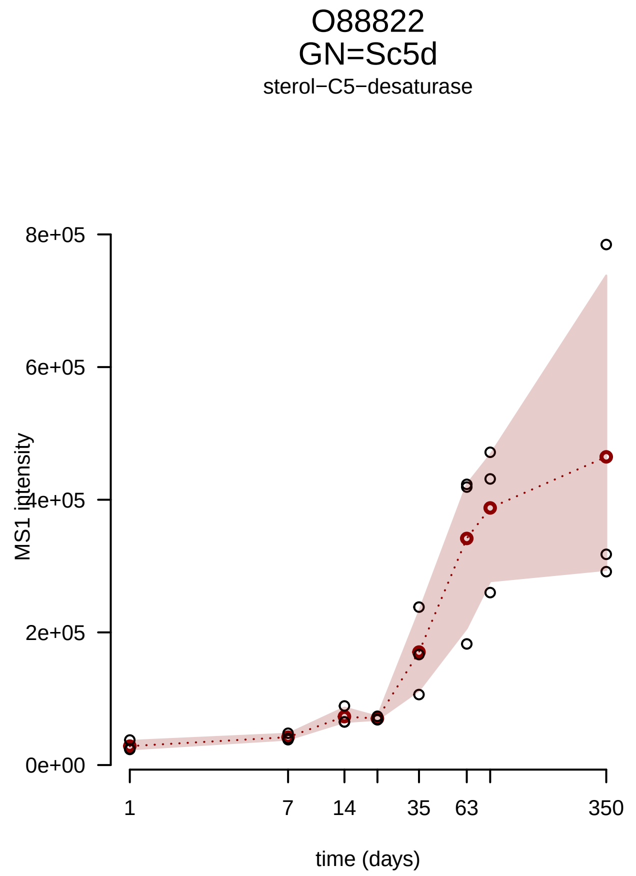
O88822
GN=Sc5d
sterol−C5−desaturase
MS1 intensity
0e+00
2e+05
4e+05
6e+05
8e+05
1
7
14
35
63
350
time (days)

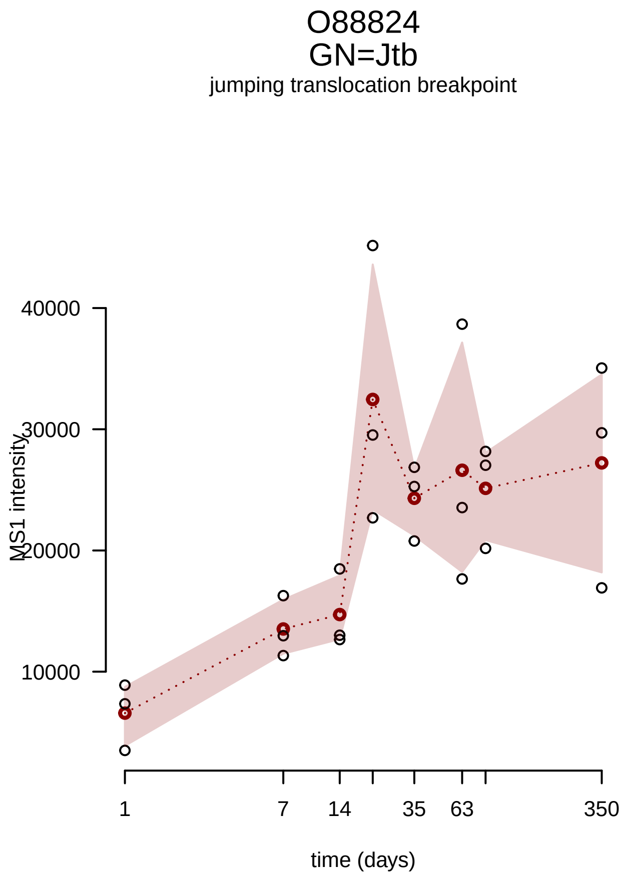
O88824
GN=Jtb
jumping translocation breakpoint
MS1 intensity
10000
20000
30000
40000
1
7
14
35
63
350
time (days)

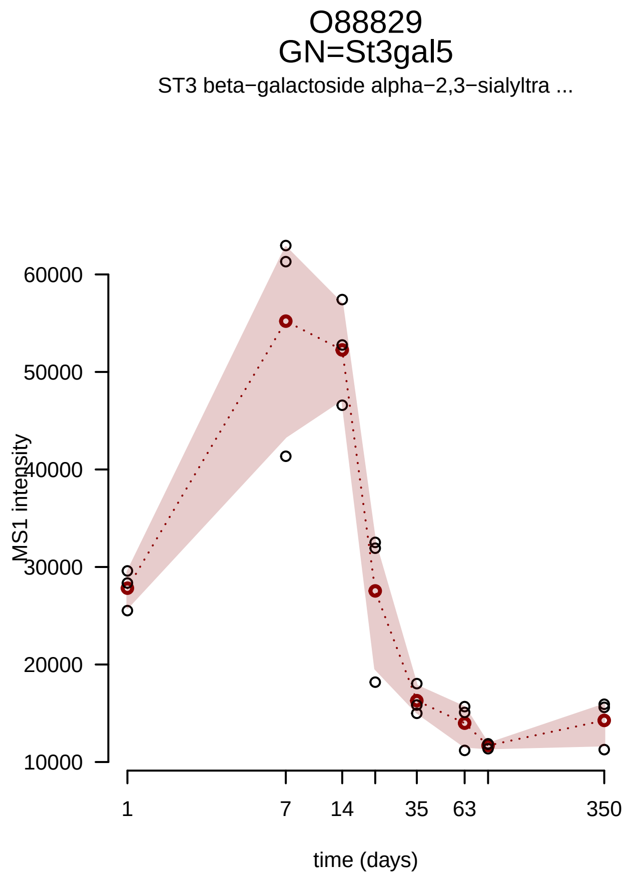
O88829
GN=St3gal5
ST3 beta−galactoside alpha−2,3−sialyltra ...
MS1 intensity
10000
20000
30000
40000
50000
60000
1
7
14
35
63
350
time (days)

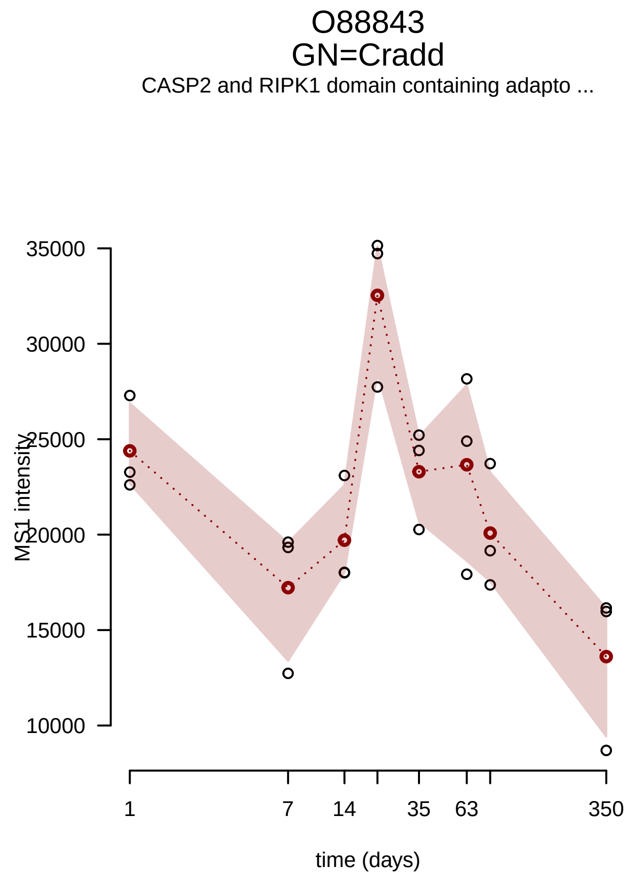
O88843
GN=Cradd
CASP2 and RIPK1 domain containing adapto ...
MS1 intensity
10000
15000
20000
25000
30000
35000
1
7
14
35
63
350
time (days)

O88844
GN=Idh1
isocitrate dehydrogenase 1 (NADP+), solu ...

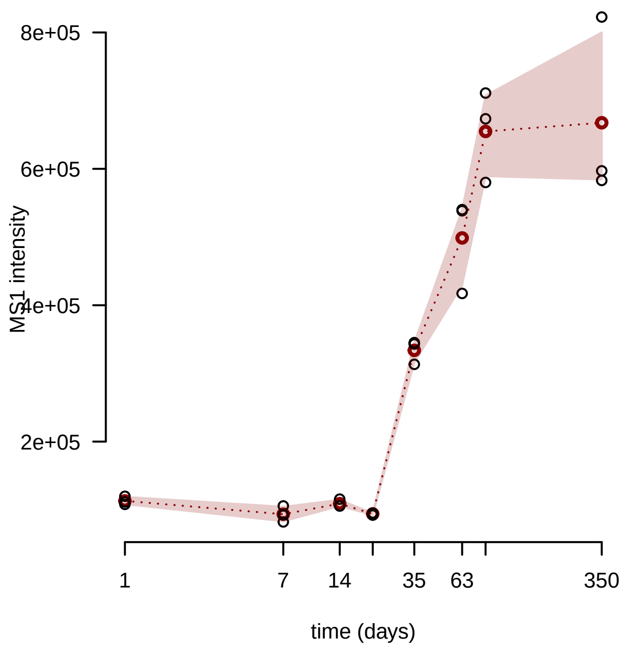

O88845
GN=Akap10
A kinase (PRKA) anchor protein 10

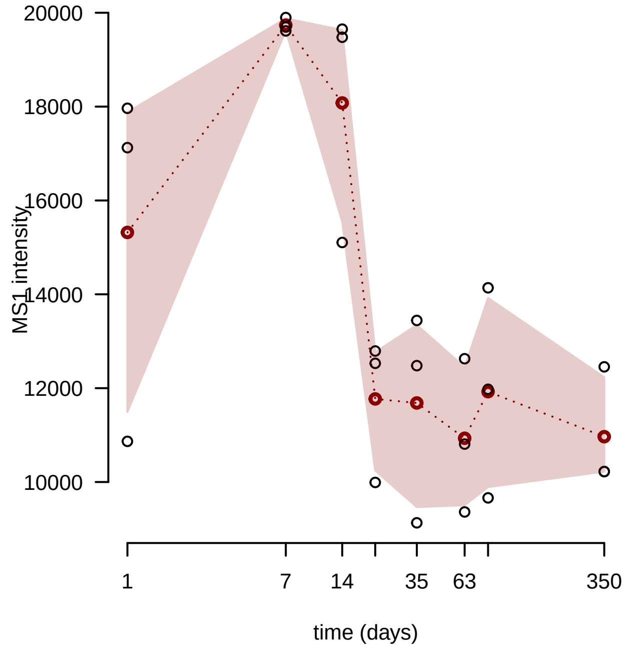

O88848
GN=Arl6
ADP−ribosylation factor−like 6

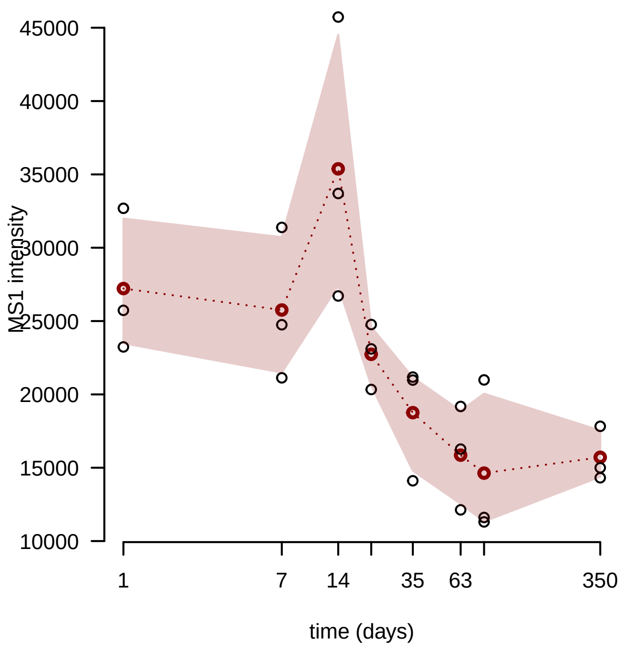

O88851
GN=Rbbp9
retinoblastoma binding protein 9, serine ...

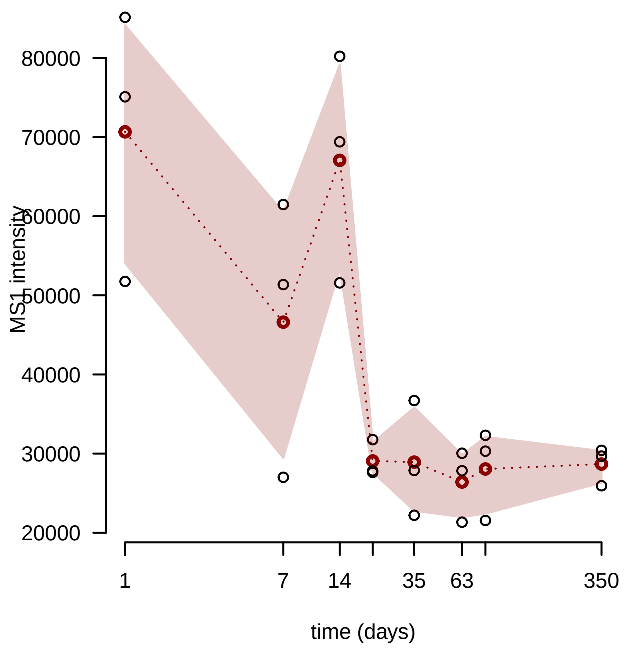

O88852
GN=Tspyl1
testis−specific protein, Y−encoded−like ...

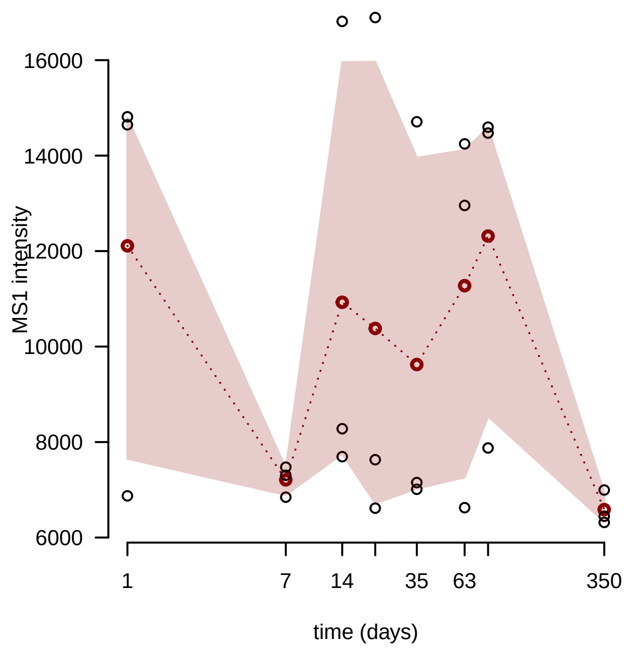

O88856
GN=Tpst2
protein−tyrosine sulfotransferase 2

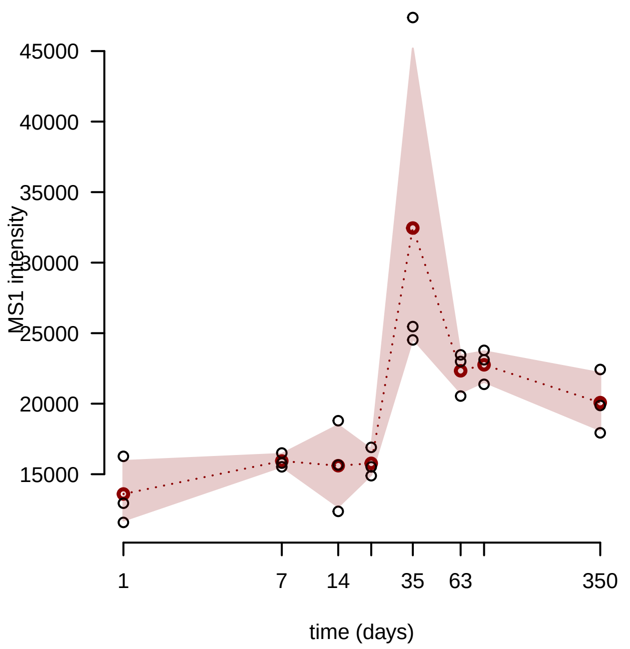

O88874
GN=Ccnk
cyclin K

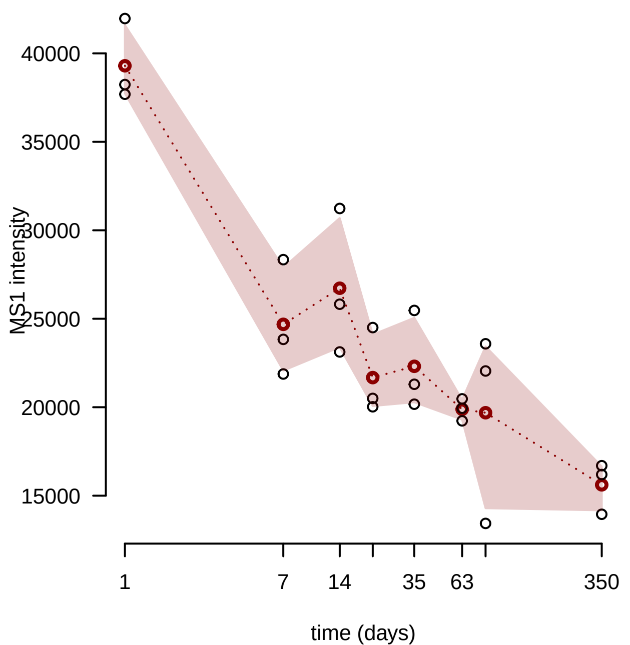

O88876
GN=Dhrs3
dehydrogenase/reductase (SDR family) mem ...

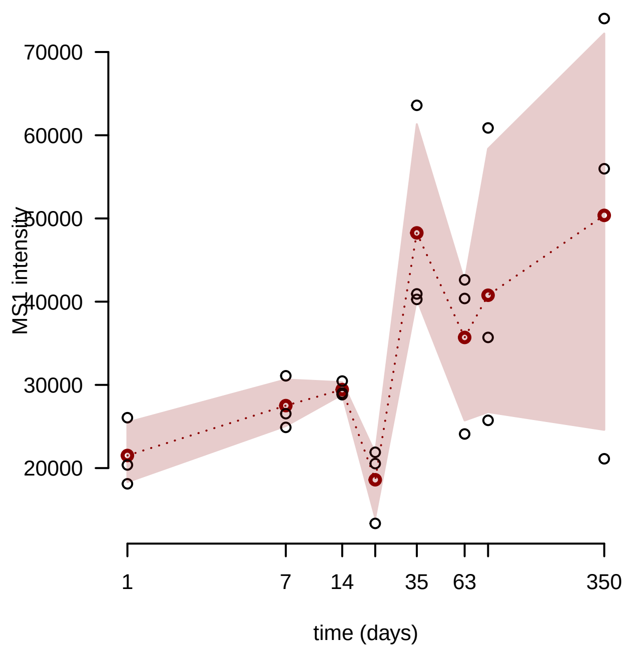

O88878
GN=Zfand5
zinc finger, AN1−type domain 5

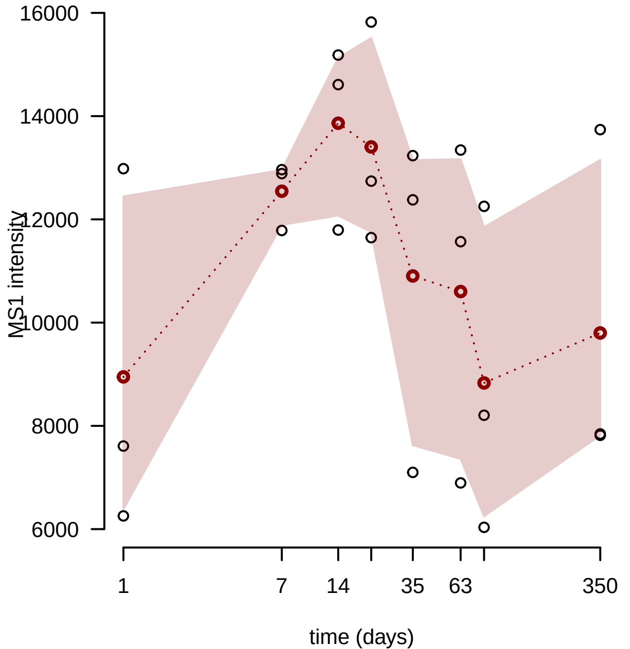

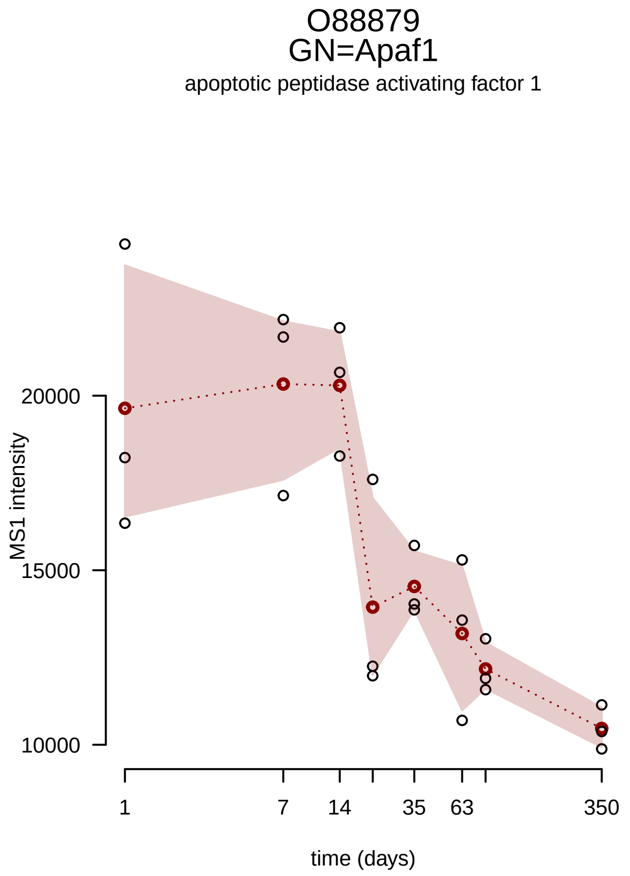
O88879
GN=Apaf1
apoptotic peptidase activating factor 1
MS1 intensity
10000
15000
20000
1
7
14
35
63
350
time (days)
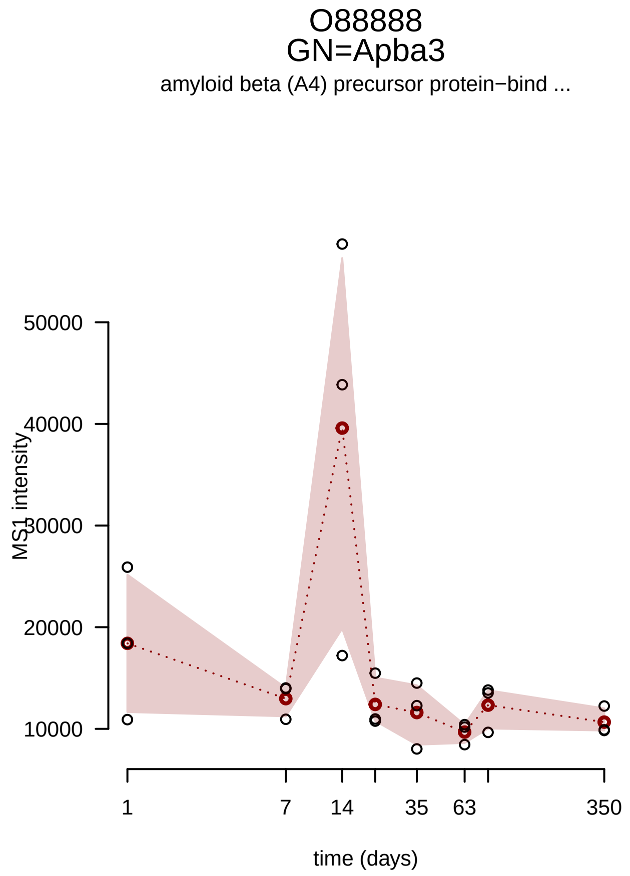
O88888
GN=Apba3
amyloid beta (A4) precursor protein−bind ...
MS1 intensity
10000
20000
30000
40000
50000
1
7
14
35
63
350
time (days)
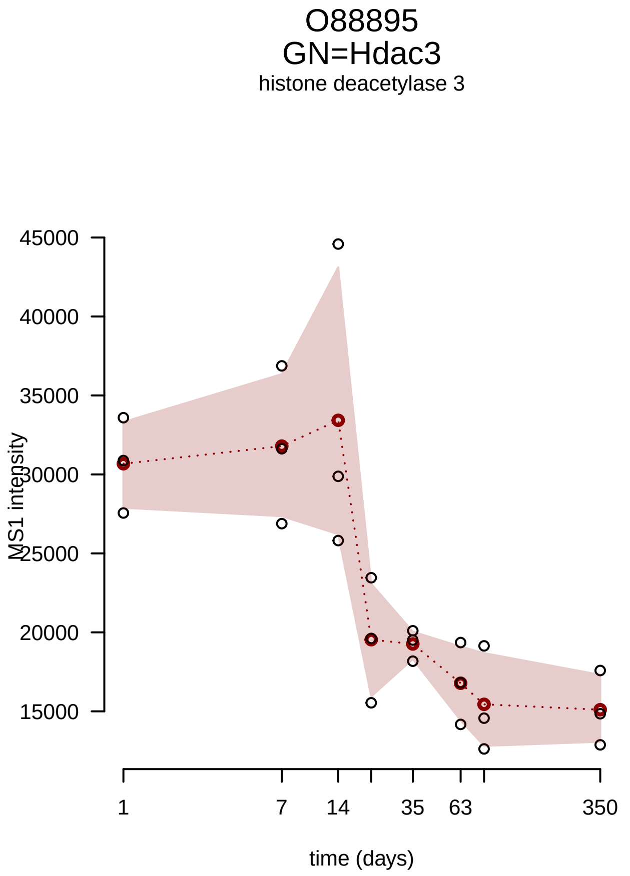
O88895
GN=Hdac3
histone deacetylase 3
MS1 intensity
15000
20000
25000
30000
35000
40000
45000
1
7
14
35
63
350
time (days)
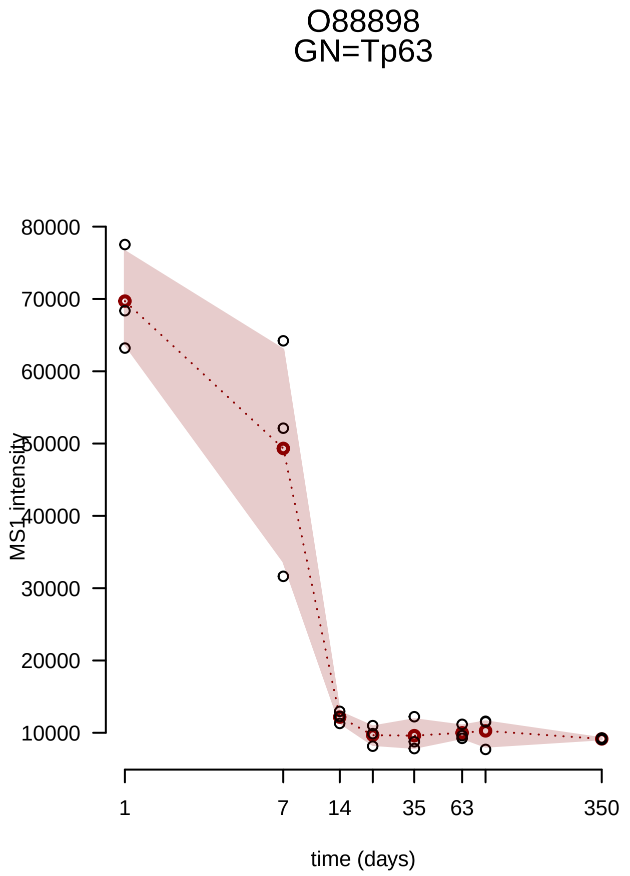
O88898
GN=Tp63
MS1 intensity
10000
20000
30000
40000
50000
60000
70000
80000
1
7
14
35
63
350
time (days)
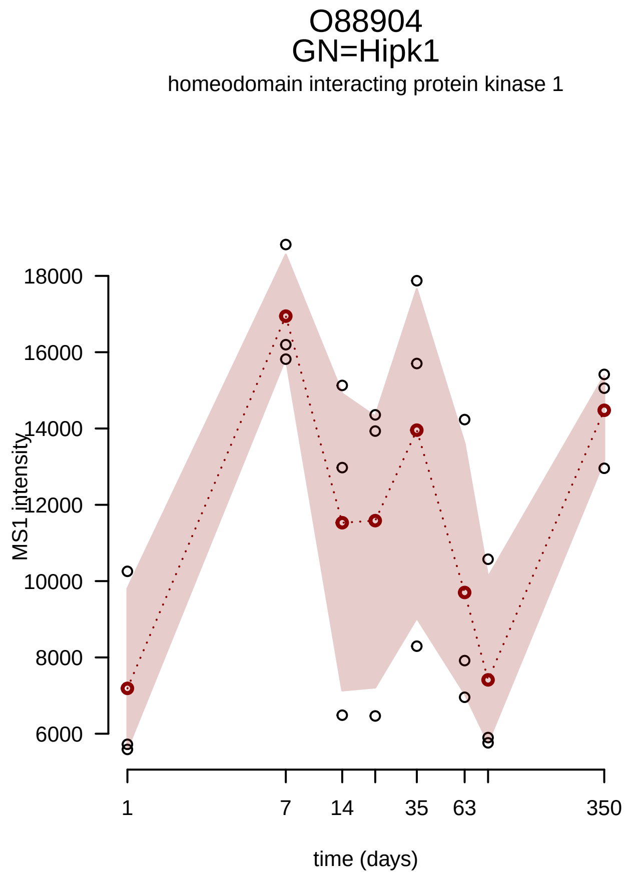
O88904
GN=Hipk1
homeodomain interacting protein kinase 1
MS1 intensity
6000
8000
10000
12000
14000
16000
18000
1
7
14
35
63
350
time (days)
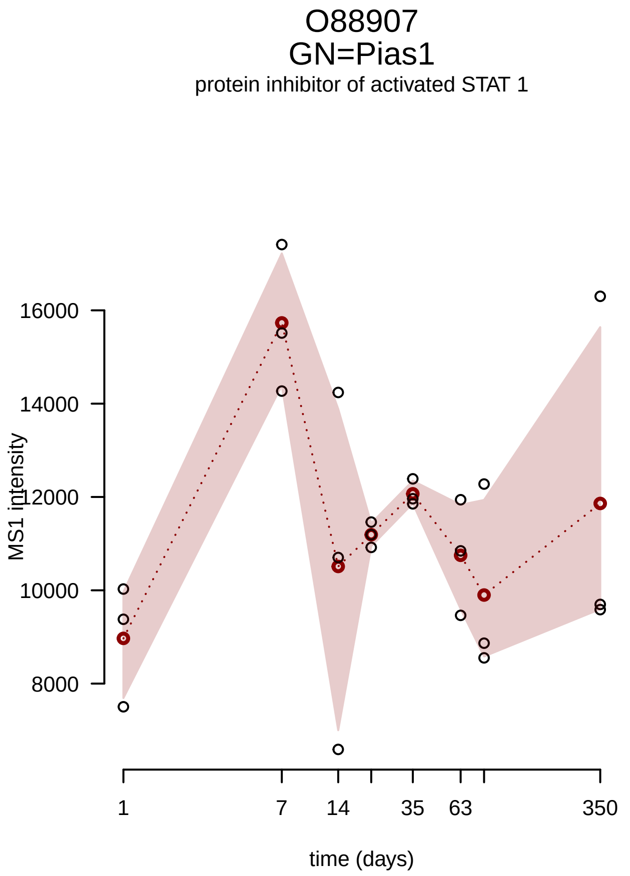
O88907
GN=Pias1
protein inhibitor of activated STAT 1
MS1 intensity
8000
10000
12000
14000
16000
1
7
14
35
63
350
time (days)
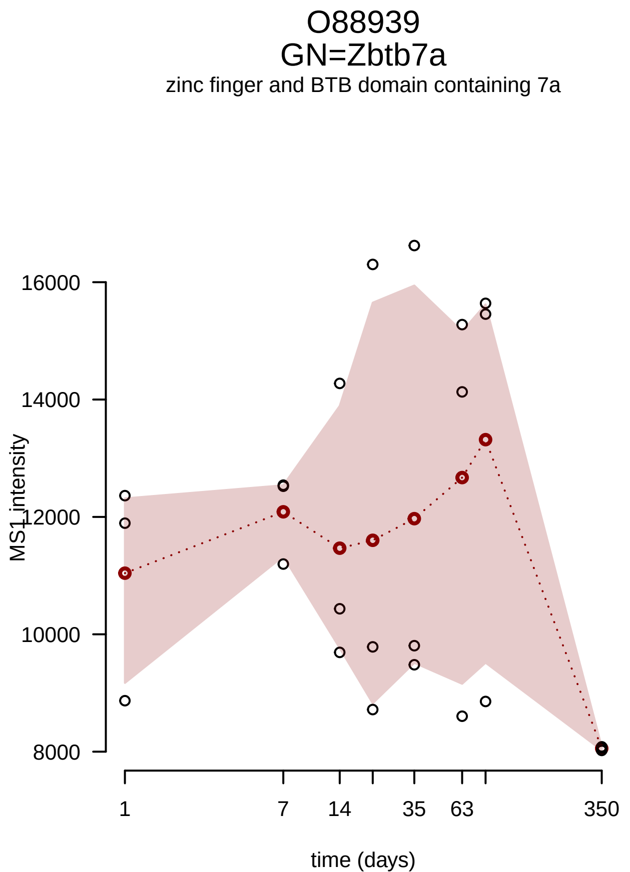
O88939
GN=Zbtb7a
zinc finger and BTB domain containing 7a
MS1 intensity
8000
10000
12000
14000
16000
1
7
14
35
63
350
time (days)
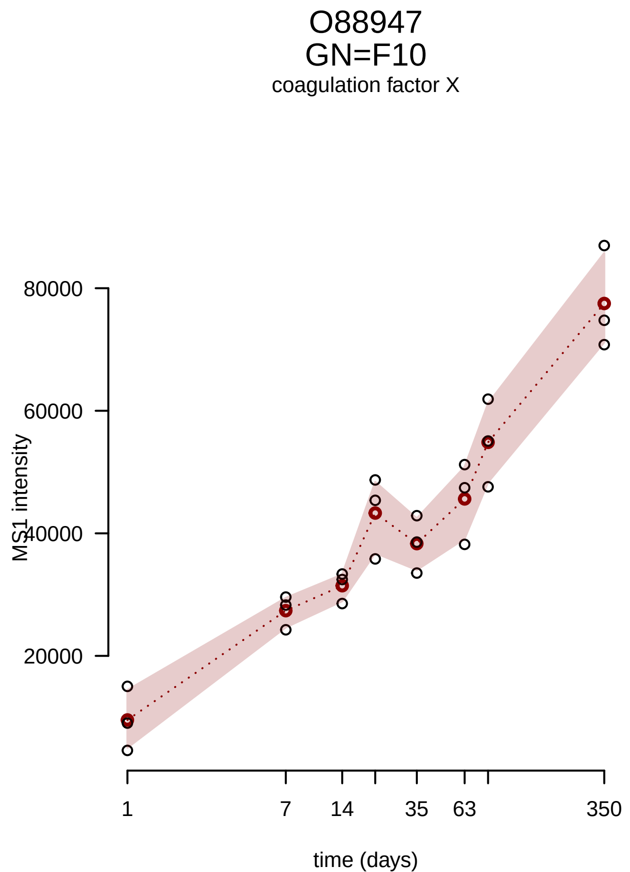
O88947
GN=F10
coagulation factor X
MS1 intensity
20000
40000
60000
80000
1
7
14
35
63
350
time (days)
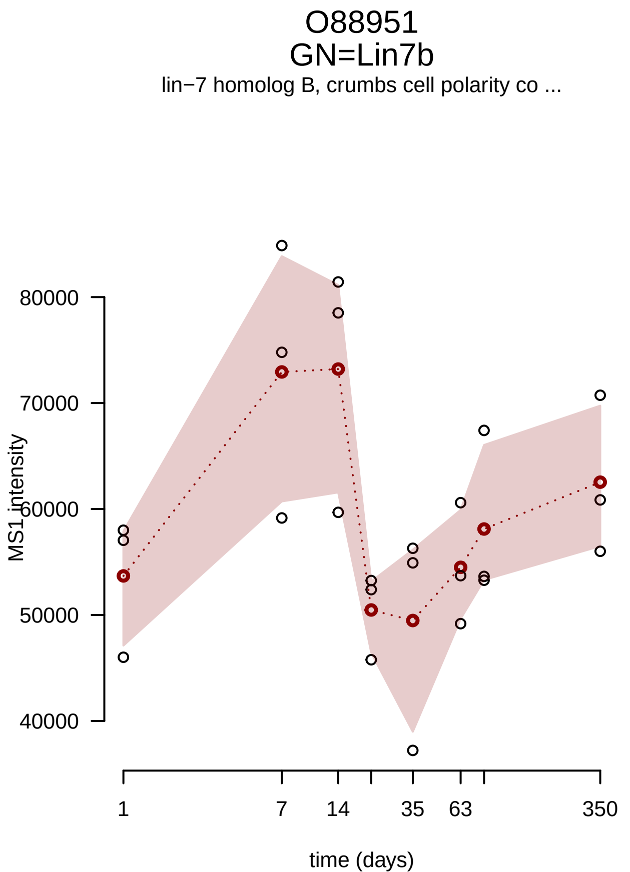
O88951
GN=Lin7b
lin−7 homolog B, crumbs cell polarity co ...
MS1 intensity
40000
50000
60000
70000
80000
1
7
14
35
63
350
time (days)

O88952
GN=Lin7c
lin−7 homolog C, crumbs cell polarity co ...

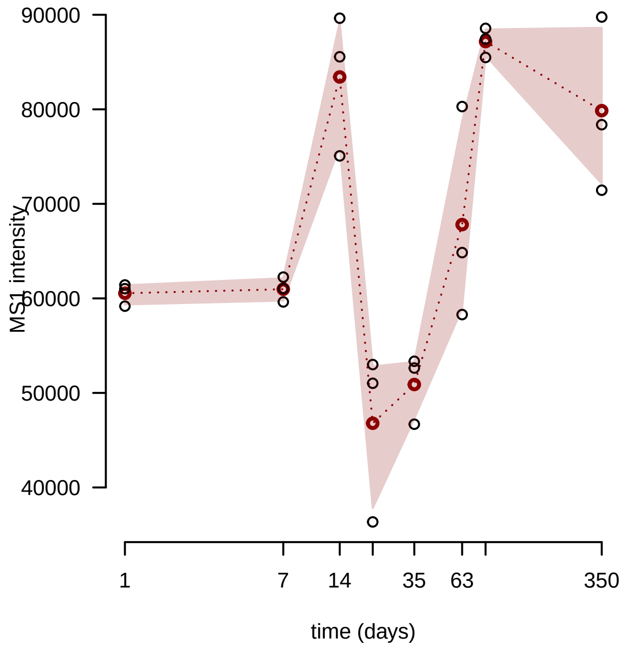

O88958
GN=Gnpda1
glucosamine−6−phosphate deaminase 1

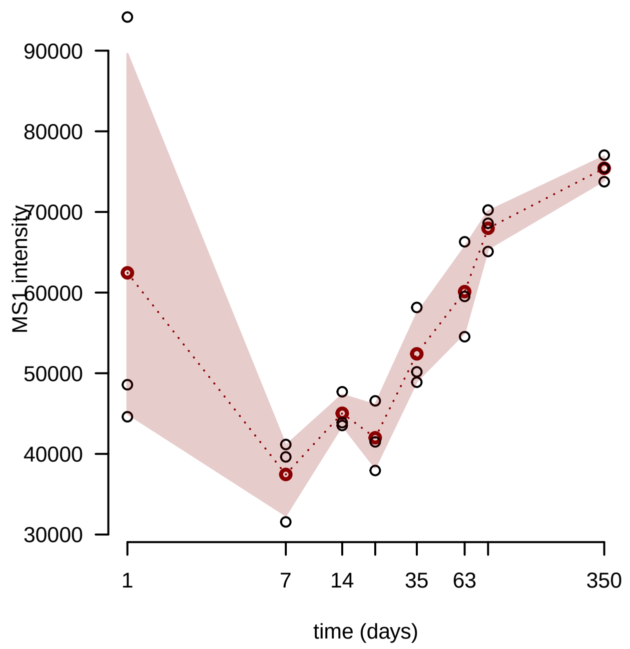

O88967
GN=Yme1l1
YME1−like 1 (S. cerevisiae)

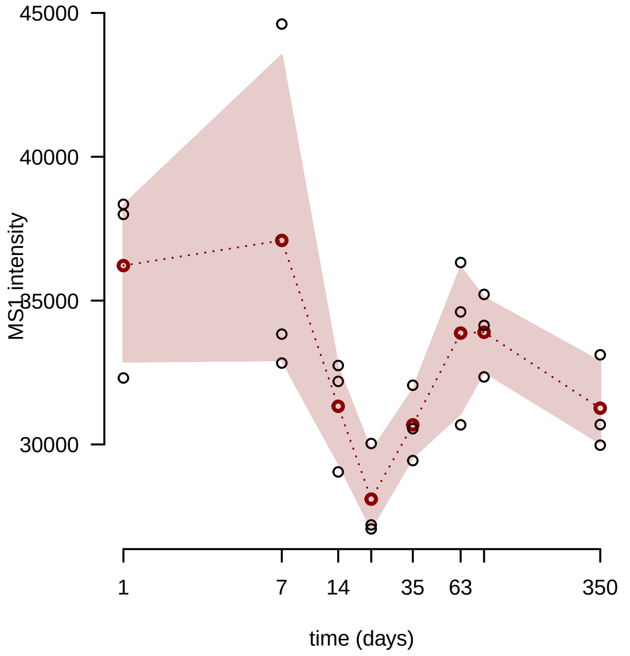

O88968
GN=Tcn2
transcobalamin 2

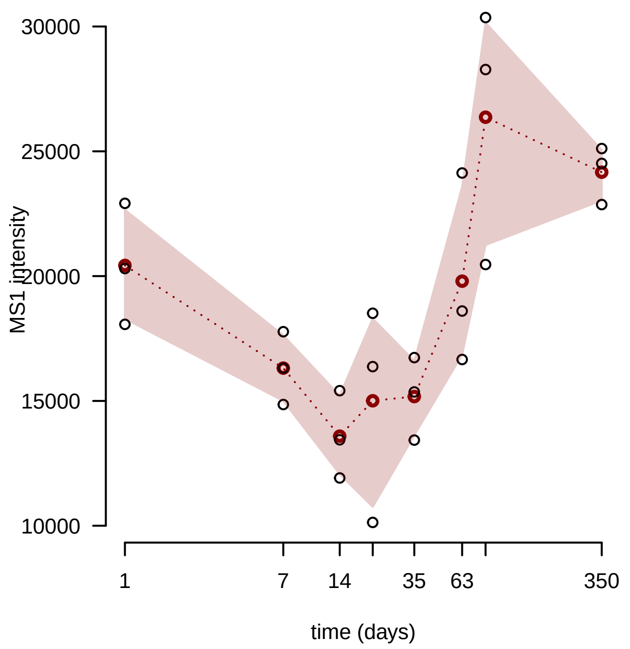

O88974
GN=Setdb1
SET domain, bifurcated 1

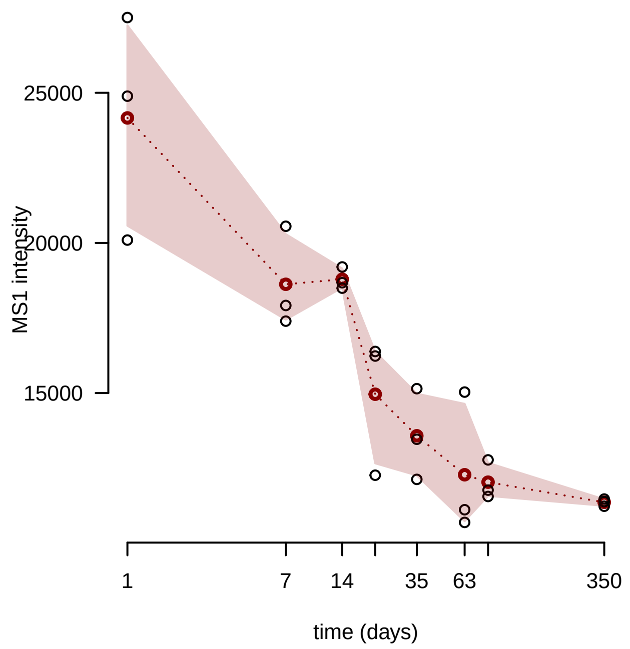

O88983
GN=Stx8
syntaxin 8

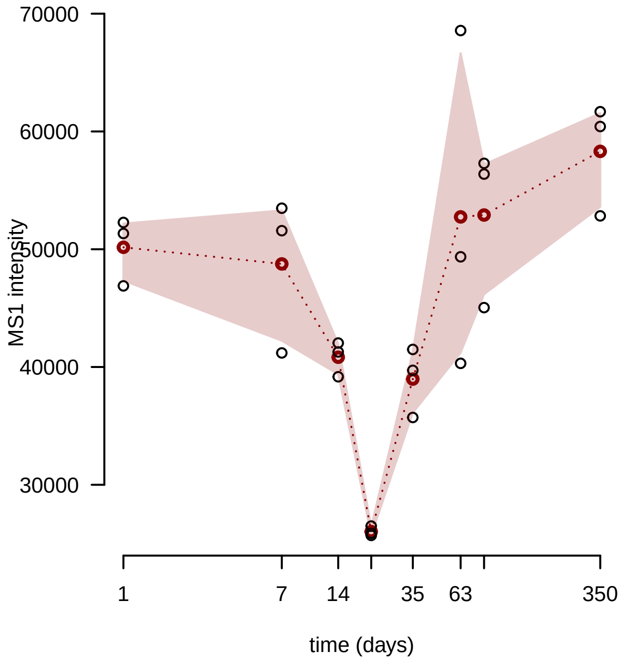

O88986
GN=Gcat
glycine C−acetyltransferase (2−amino−3−k ...

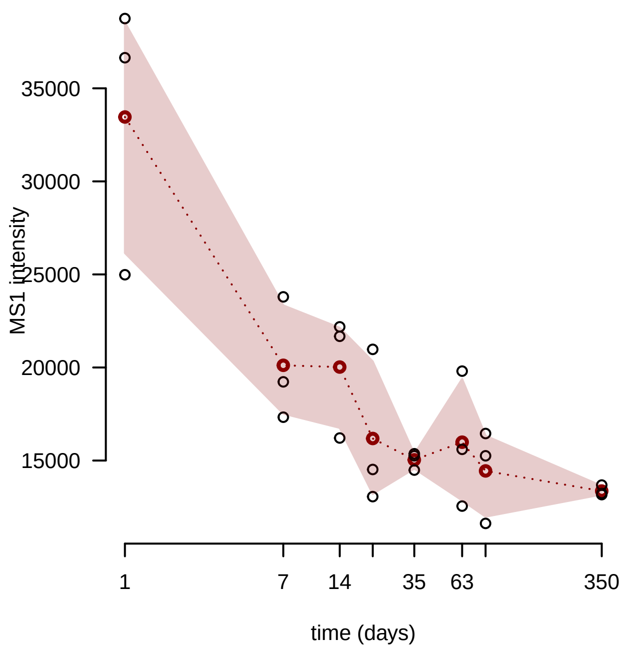

O88990
GN=Actn3
actinin alpha 3

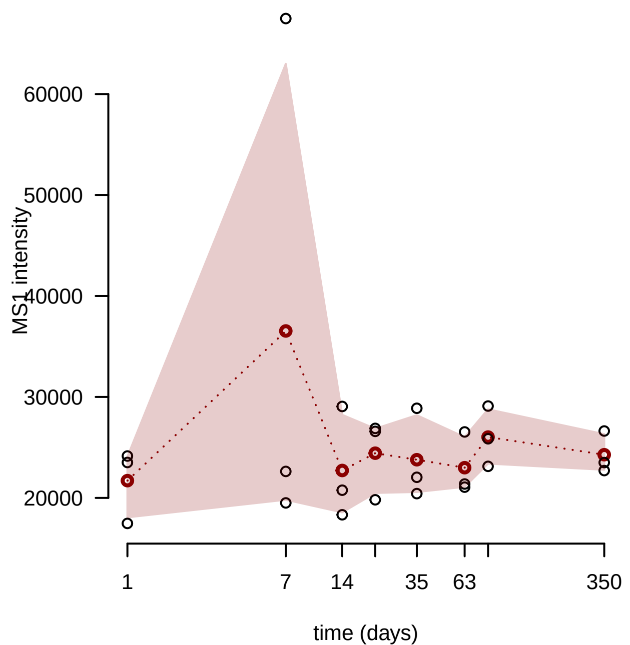

O88998
GN=Olfm1
olfactomedin 1

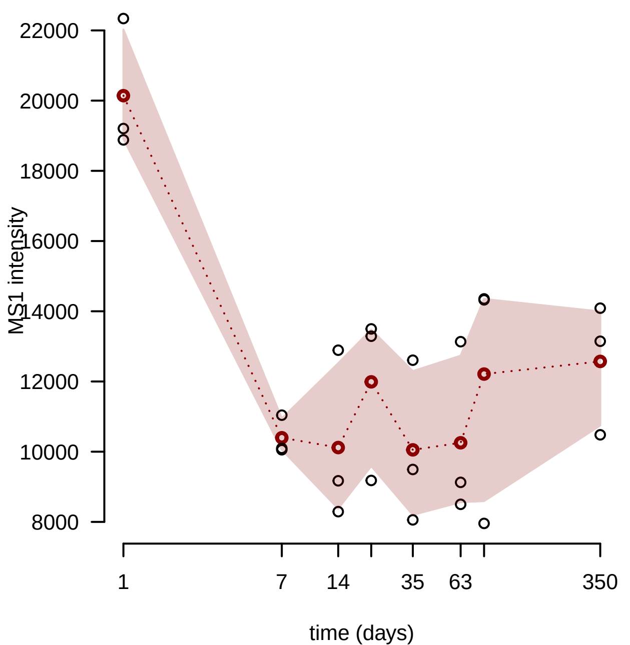

O89001
GN=Cpd
carboxypeptidase D

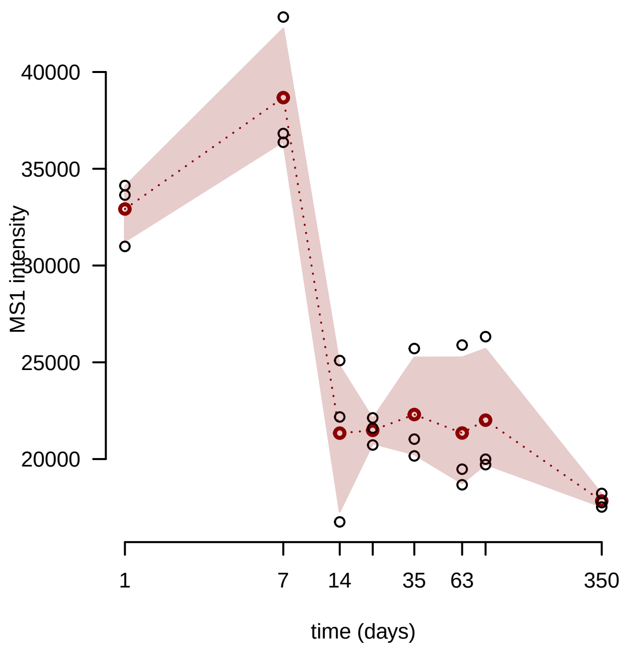

O89013
GN=Leprot
leptin receptor overlapping transcript

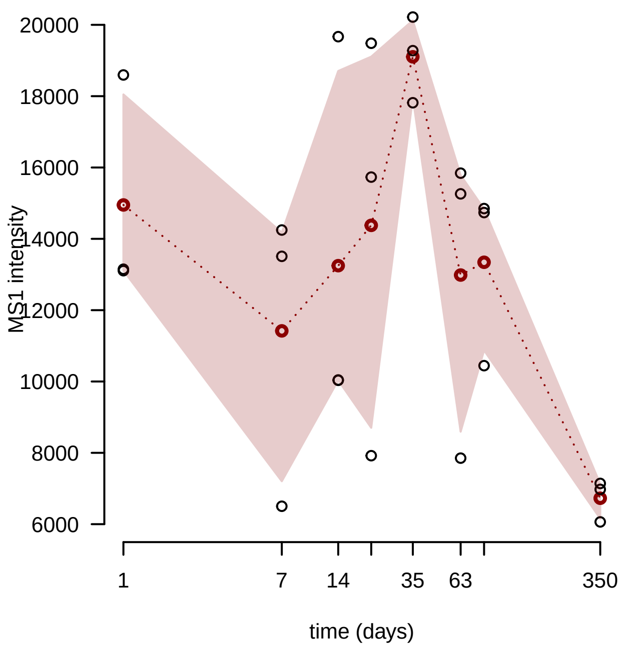

O89016
GN=Abcd4
ATP−binding cassette, sub−family D (ALD) ...

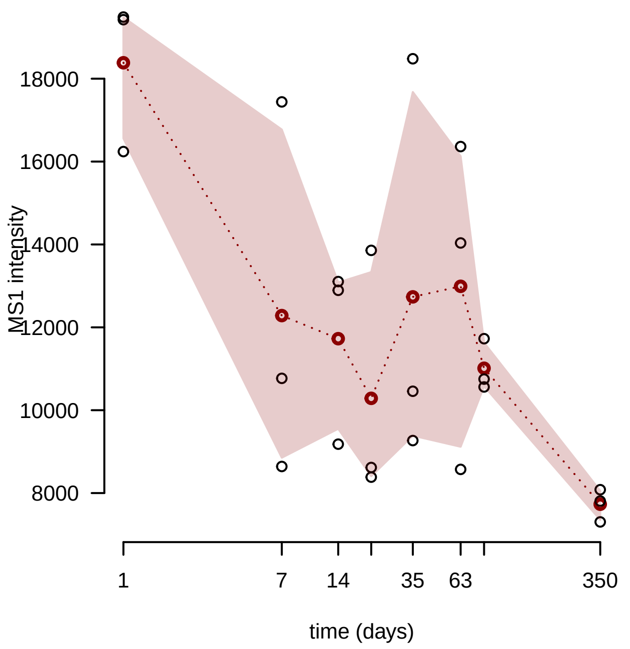

O89017
GN=Lgmn
legumain

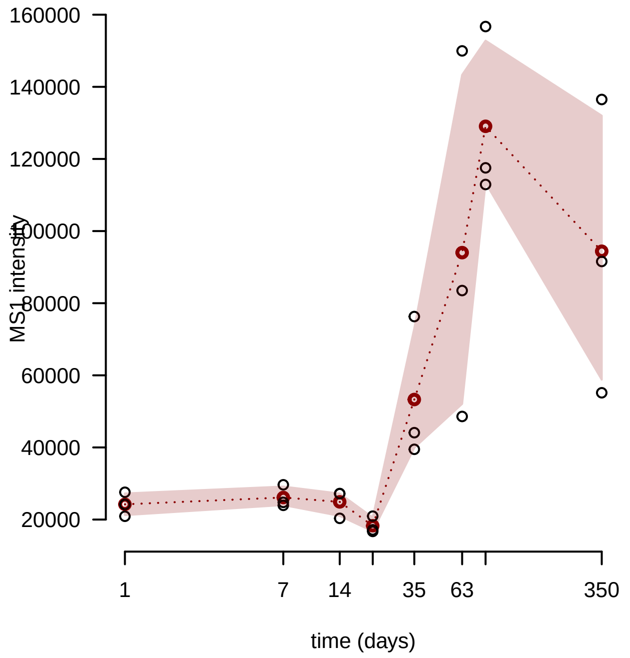

O89020
GN=Afm
afamin

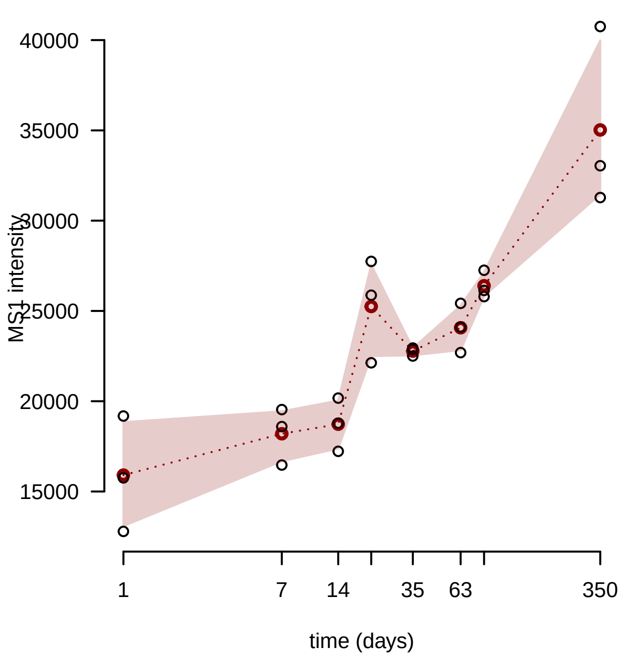

O89023
GN=Tpp1
tripeptidyl peptidase I

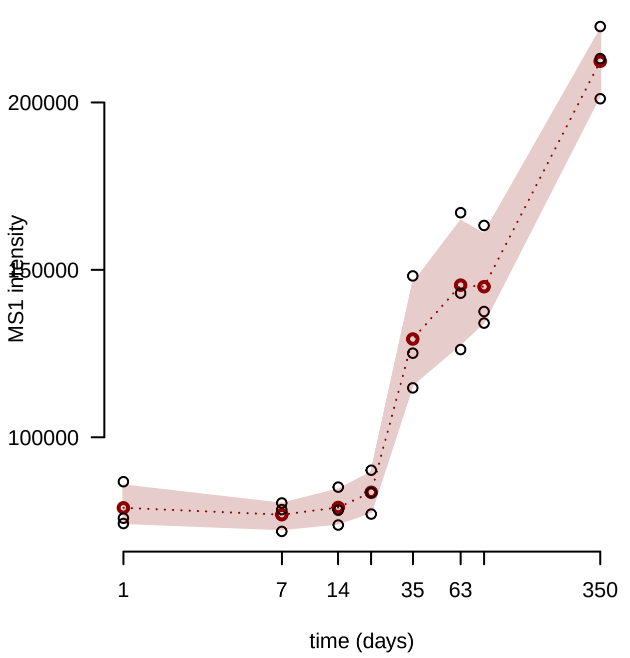

O89026
GN=Robo1
roundabout guidance receptor 1

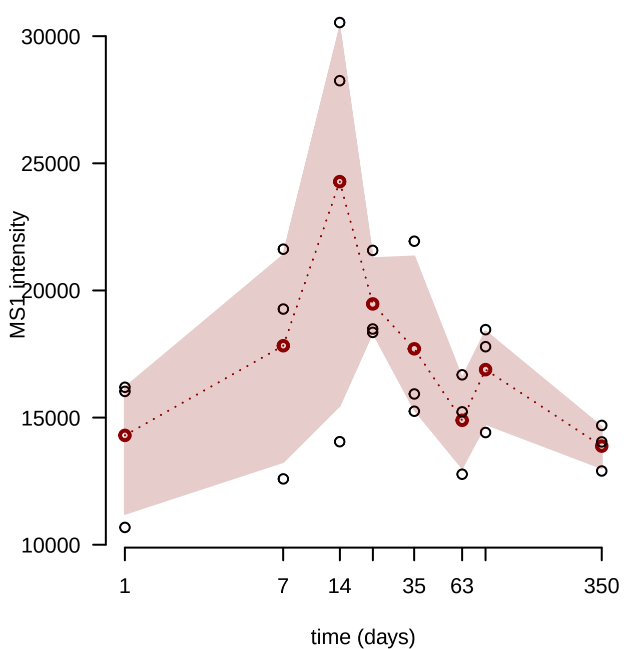

O89032
GN=Sh3pxd2a
SH3 and PX domains 2A

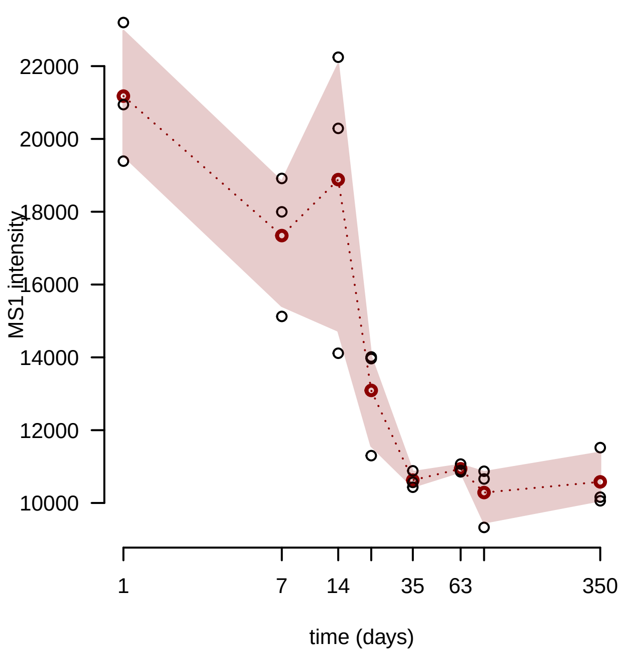

O89050
GN=Mkln1
muskelin 1, intracellular mediator conta ...

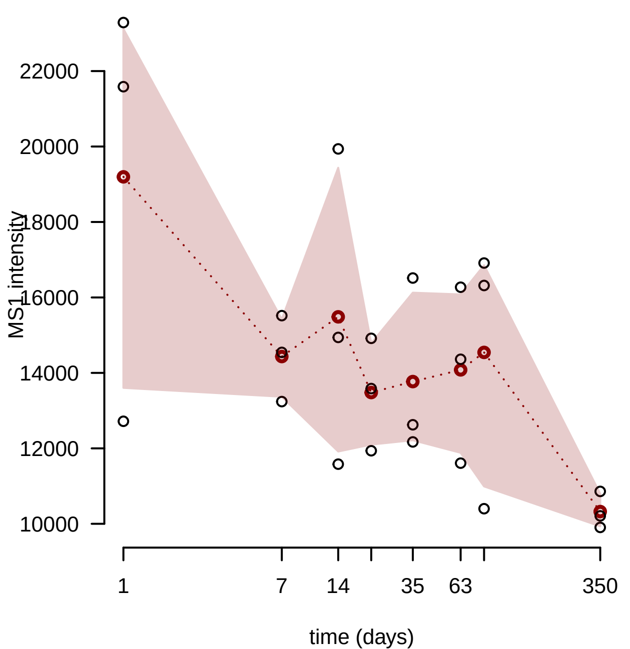

O089051
GN=Itm2b
integral membrane protein 2B

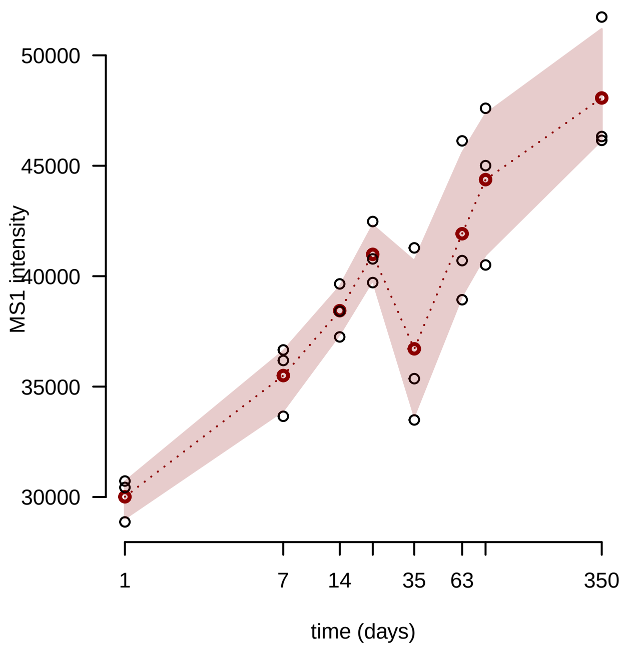

O089053
GN=Coro1a
coronin, actin binding protein 1A

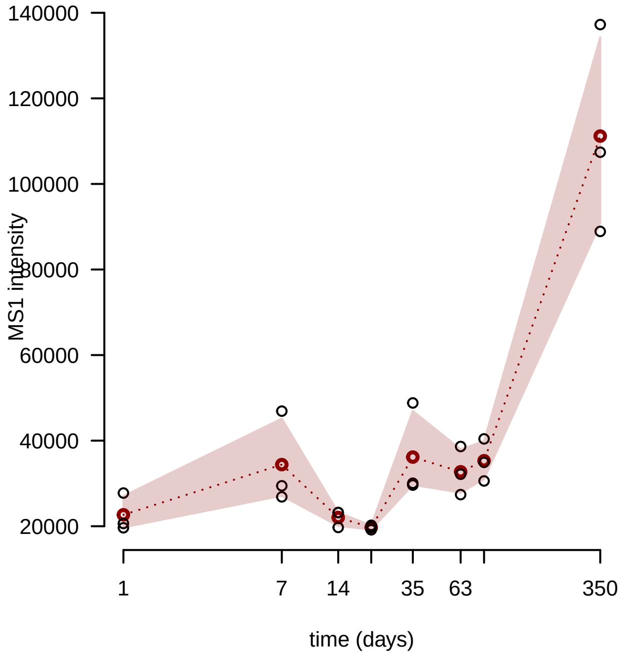

O089079
GN=Cope
coatomer protein complex, subunit epsilo ...

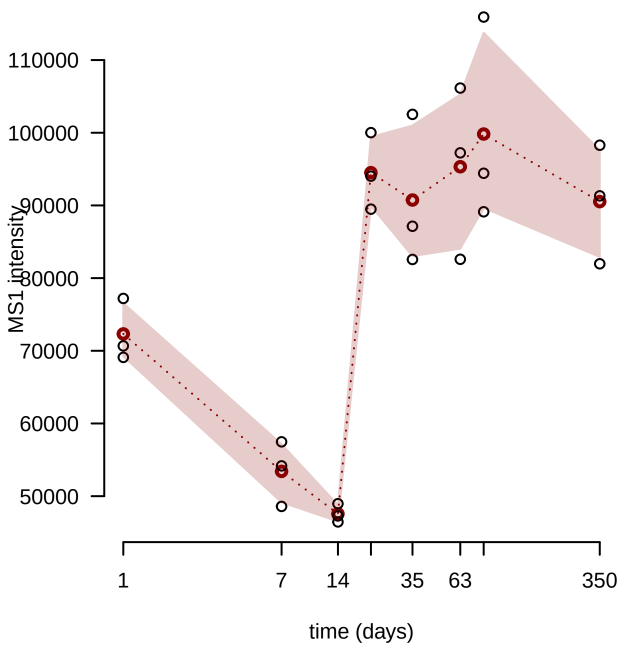

O089086
GN=Rbm3
RNA binding motif (RNP1, RRM) protein 3

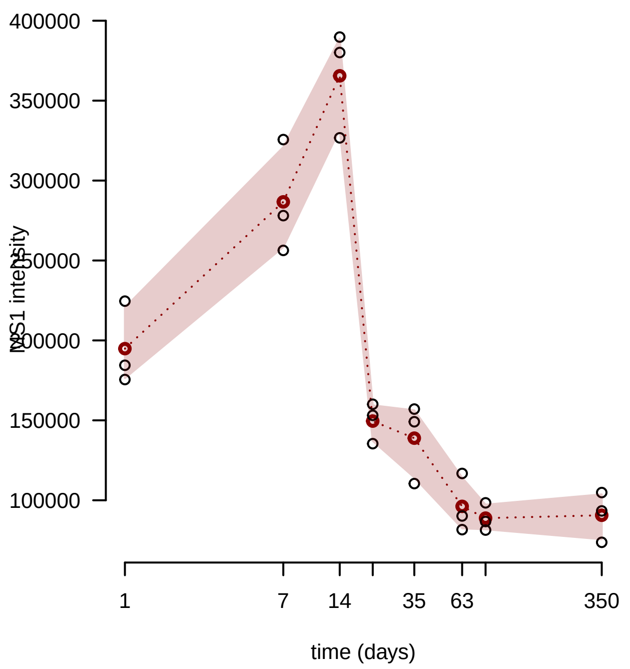

O089090
GN=Sp1
trans−acting transcription factor 1

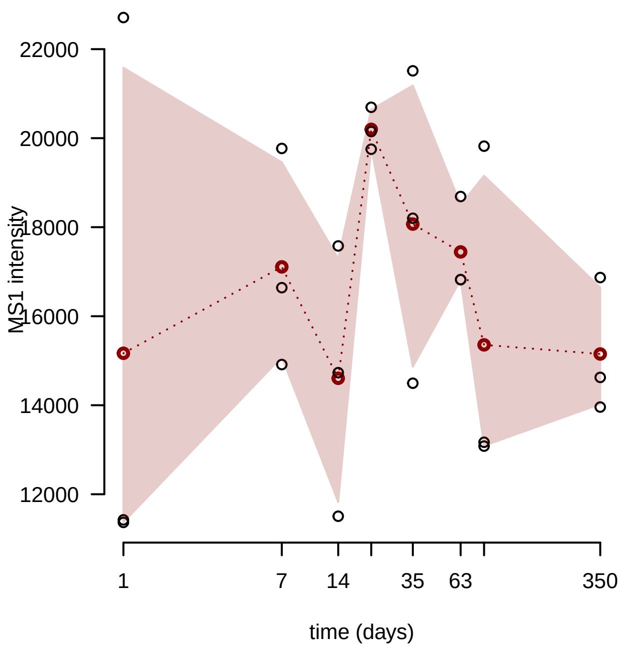

O089103
GN=Cd93
CD93 antigen

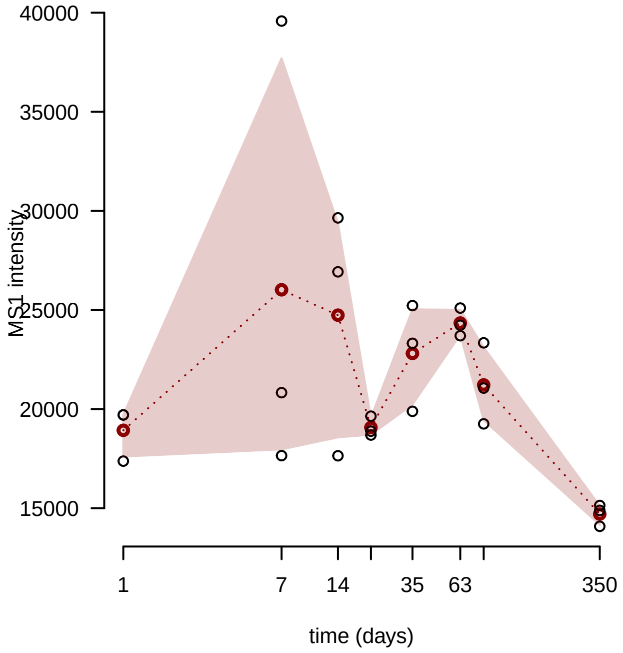

O089106
GN=Fhit
fragile histidine triad gene

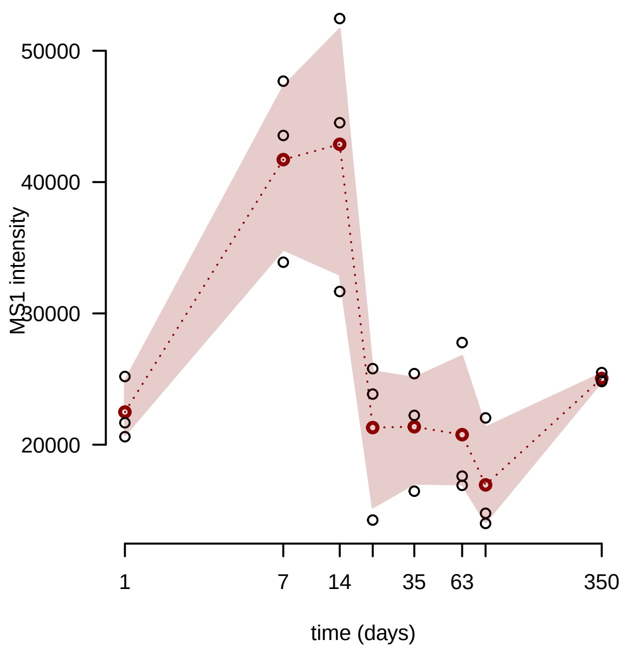

O089110
GN=Casp8
caspase 8

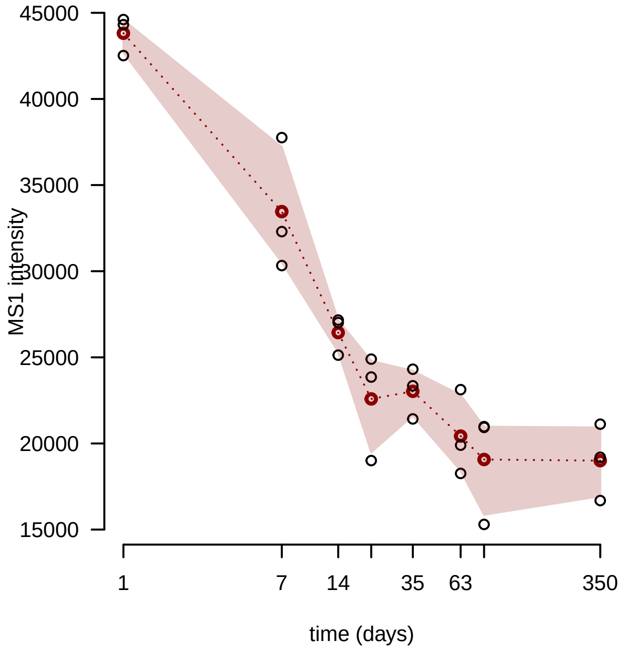

O089112
GN=Lancl1
LanC (bacterial lantibiotic synthetase c ...

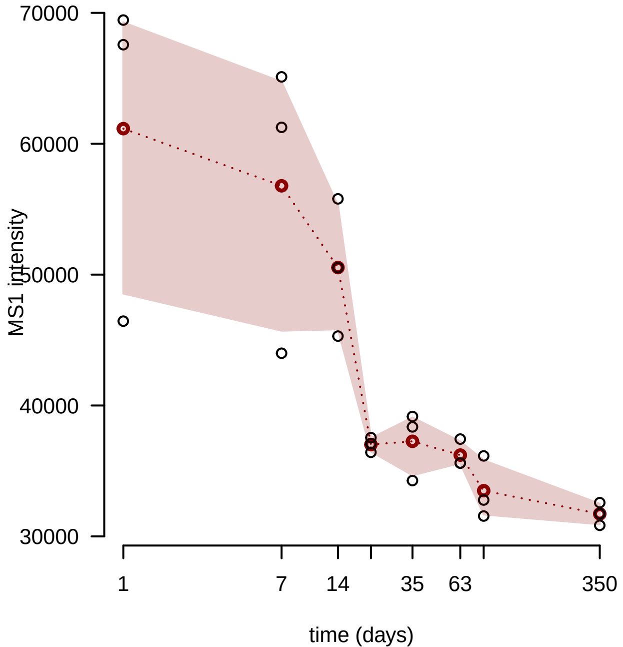

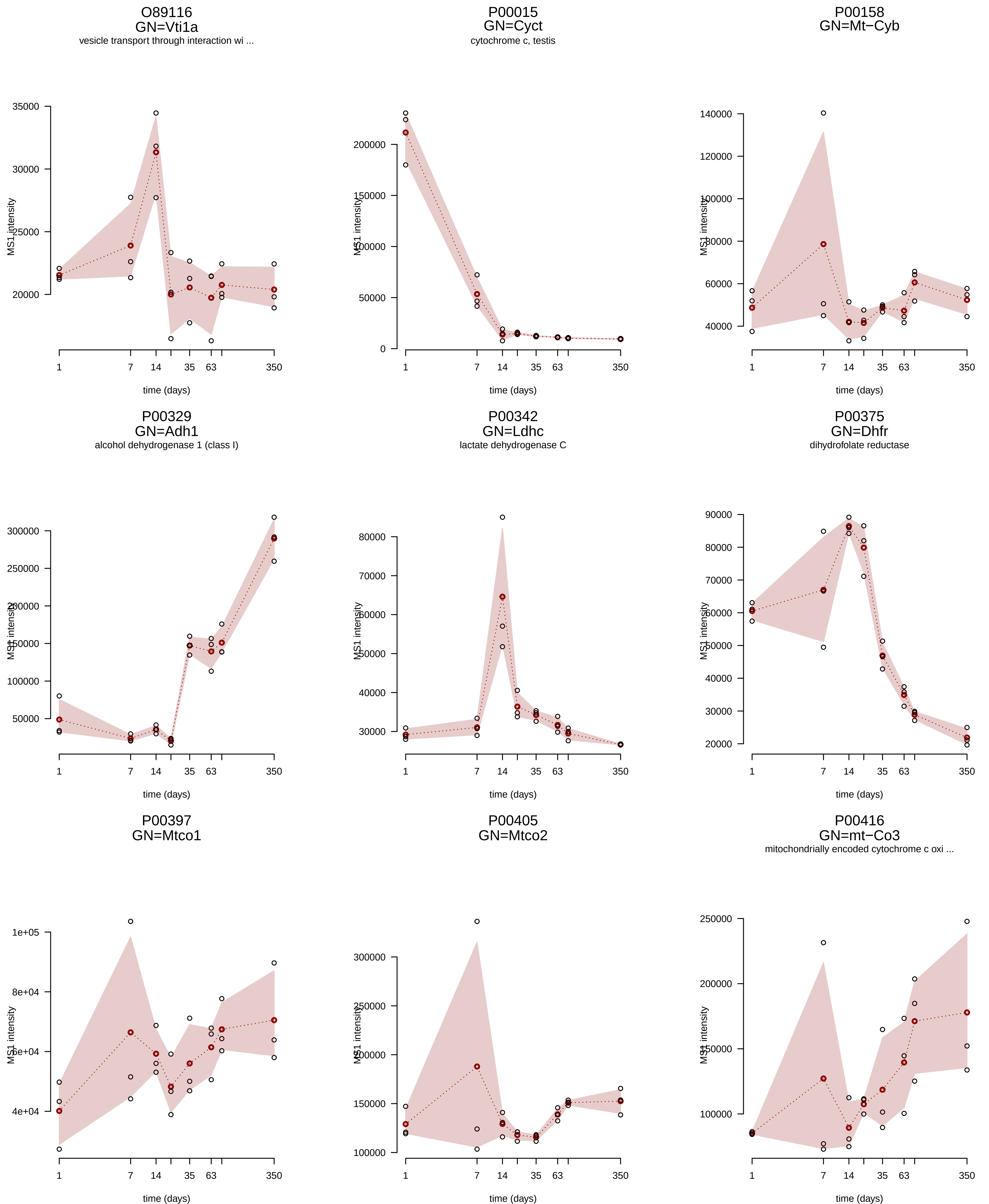
O89116
GN=Vti1a
vesicle transport through interaction wi ...
MS1 intensity
time (days)
P00015
GN=Cyct
cytochrome c, testis
MS1 intensity
time (days)
P00158
GN=Mt−Cyb
MS1 intensity
time (days)
P00329
GN=Adh1
alcohol dehydrogenase 1 (class I)
MS1 intensity
time (days)
P00342
GN=Ldhc
lactate dehydrogenase C
MS1 intensity
time (days)
P00375
GN=Dhfr
dihydrofolate reductase
MS1 intensity
time (days)
P00397
GN=Mtco1
MS1 intensity
time (days)
P00405
GN=Mtco2
MS1 intensity
time (days)
P00416
GN=mt−Co3
mitochondrially encoded cytochrome c oxi ...
MS1 intensity
time (days)
1
7
14
35
63
350

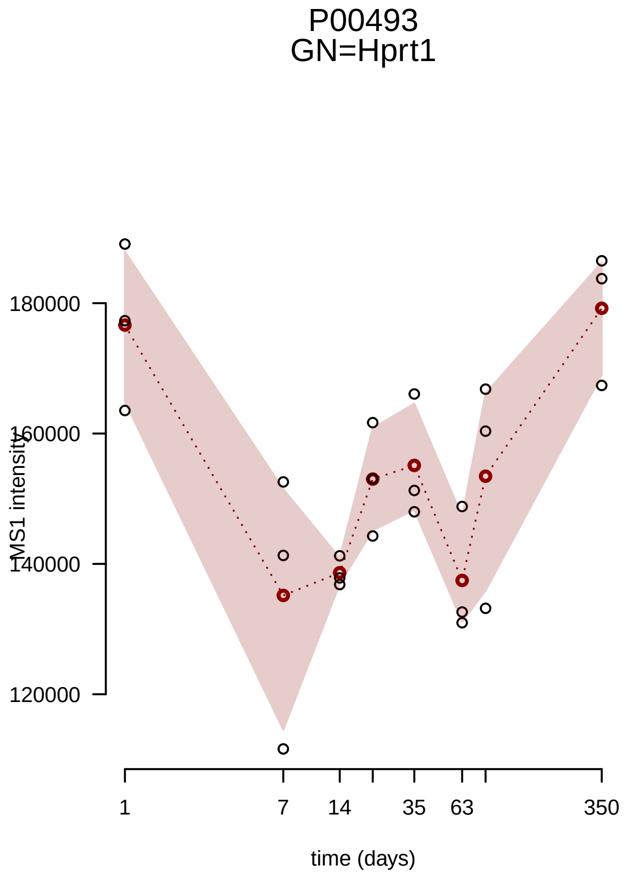
P00493
GN=Hprt1
180000
160000
140000
120000
MS1 intensity
1
7
14
35
63
350
time (days)

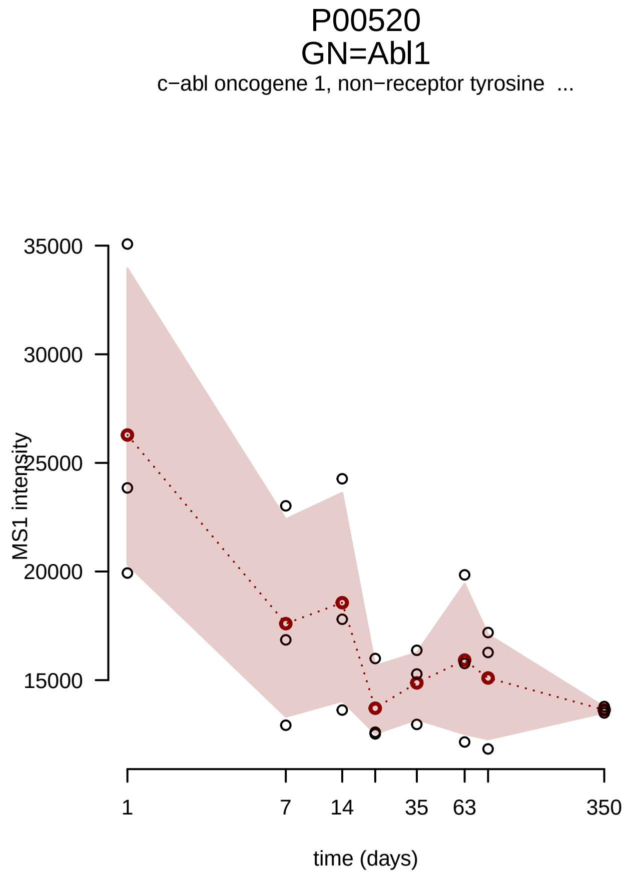
P00520
GN=Abl1
c−abl oncogene 1, non−receptor tyrosine ...
35000
30000
25000
20000
15000
MS1 intensity
1
7
14
35
63
350
time (days)

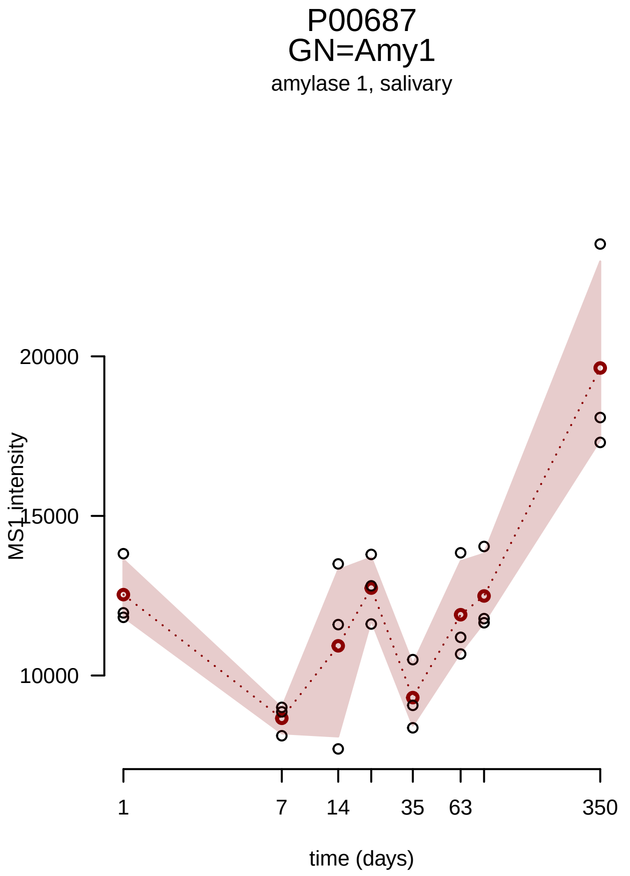
P00687
GN=Amy1
amylase 1, salivary
20000
15000
10000
MS1 intensity
1
7
14
35
63
350
time (days)

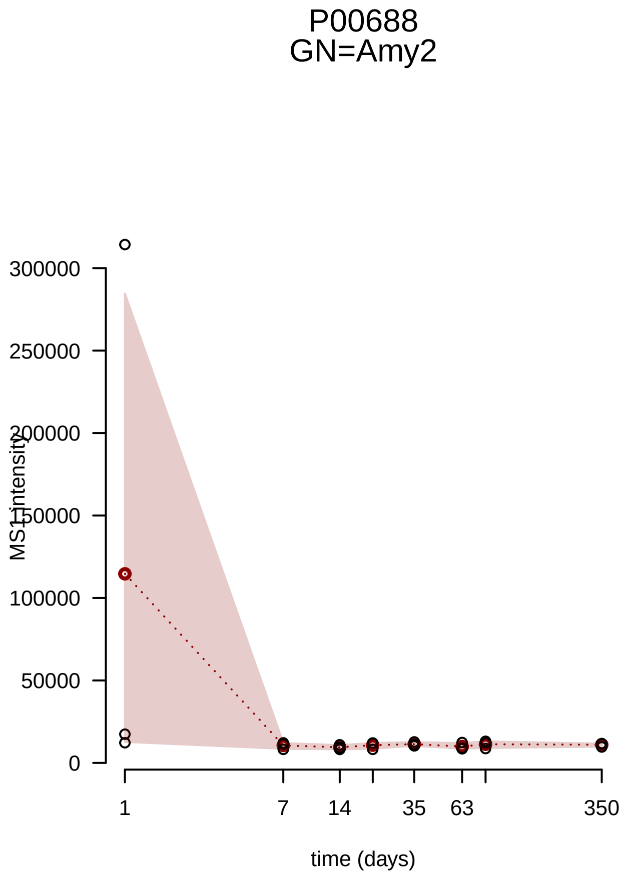
P00688
GN=Amy2
300000
250000
200000
150000
100000
50000
0
MS1 intensity
1
7
14
35
63
350
time (days)

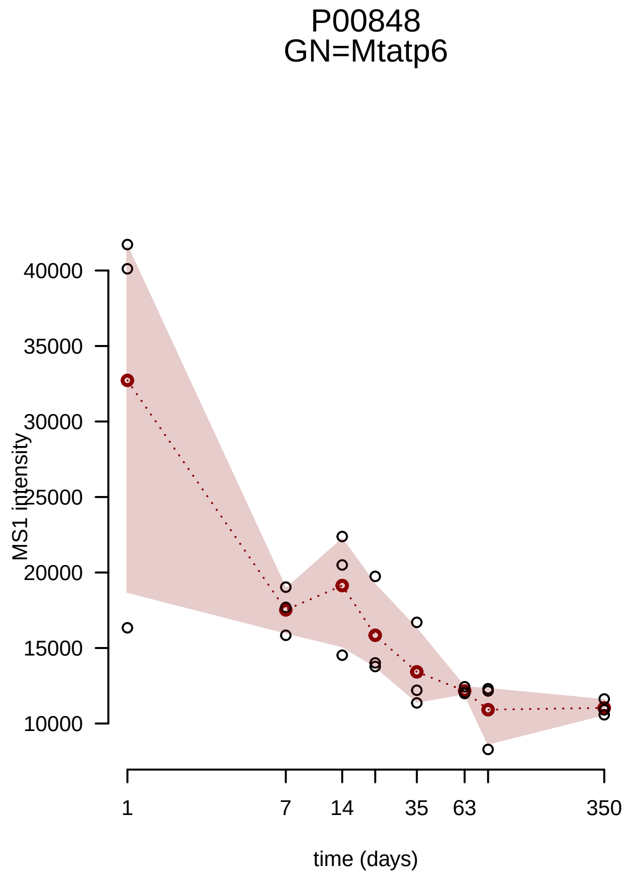
P00848
GN=Mtatp6
40000
35000
30000
25000
20000
15000
10000
MS1 intensity
1
7
14
35
63
350
time (days)

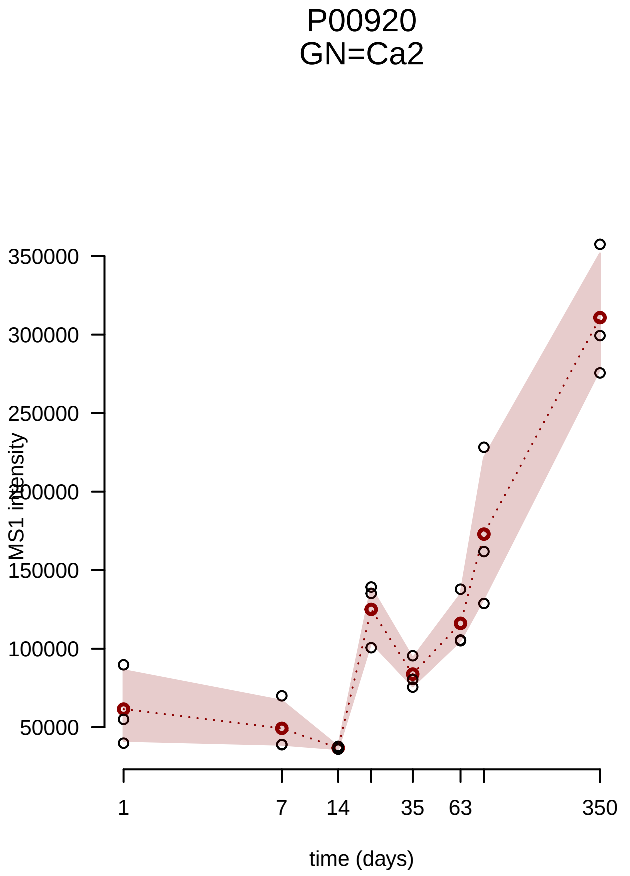
P00920
GN=Ca2
350000
300000
250000
200000
150000
100000
50000
MS1 intensity
1
7
14
35
63
350
time (days)

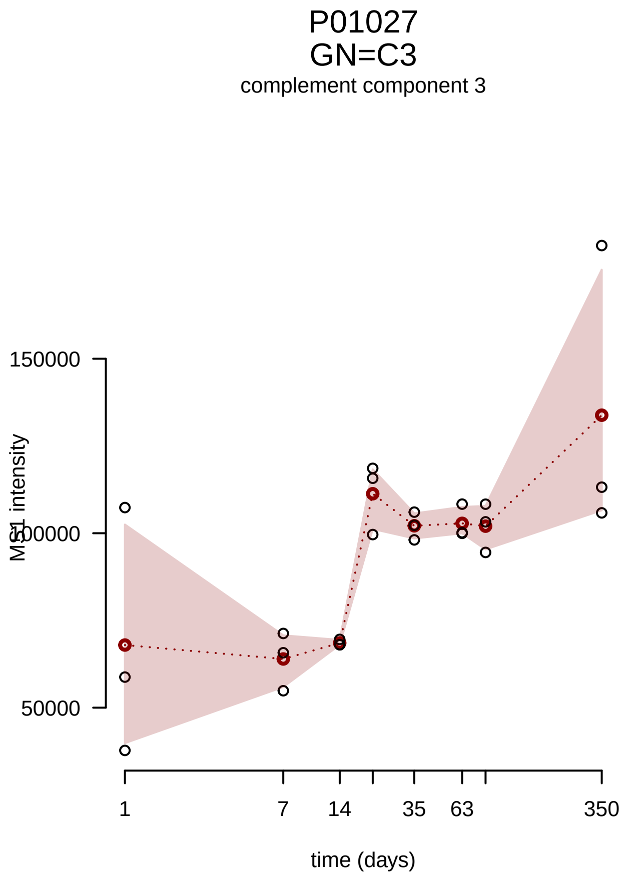
P01027
GN=C3
complement component 3
150000
100000
50000
MS1 intensity
1
7
14
35
63
350
time (days)

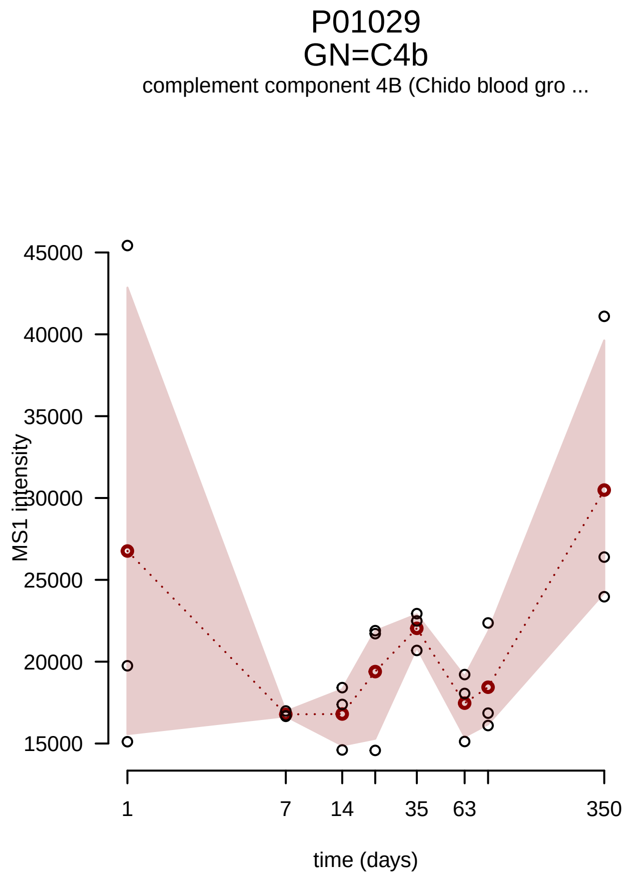
P01029
GN=C4b
complement component 4B (Chido blood gro ...
45000
40000
35000
30000
25000
20000
15000
MS1 intensity
1
7
14
35
63
350
time (days)

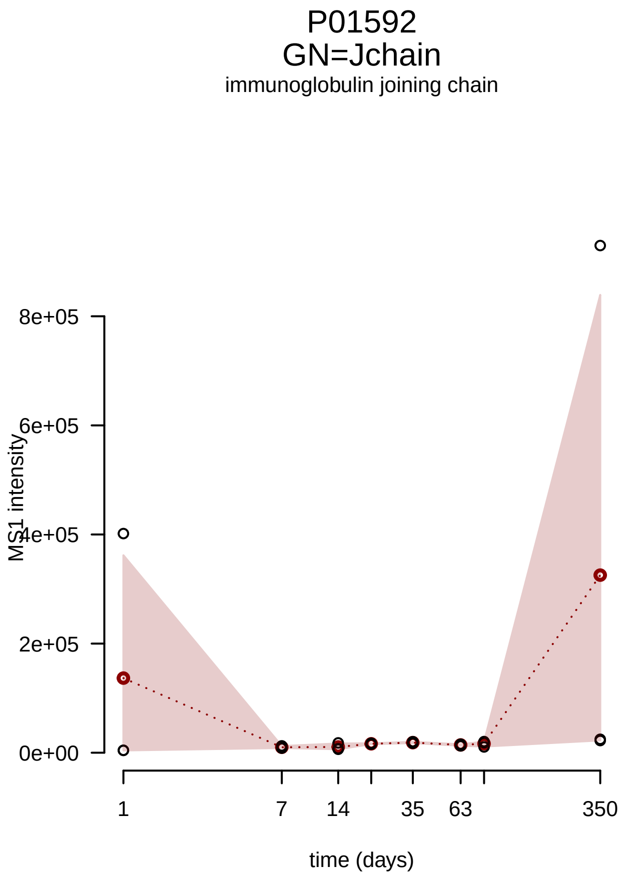
P01592
GN=Jchain
immunoglobulin joining chain
8e+05
6e+05
4e+05
2e+05
0e+00
MS1 intensity
1
7
14
35
63
350
time (days)

P01630
GN=

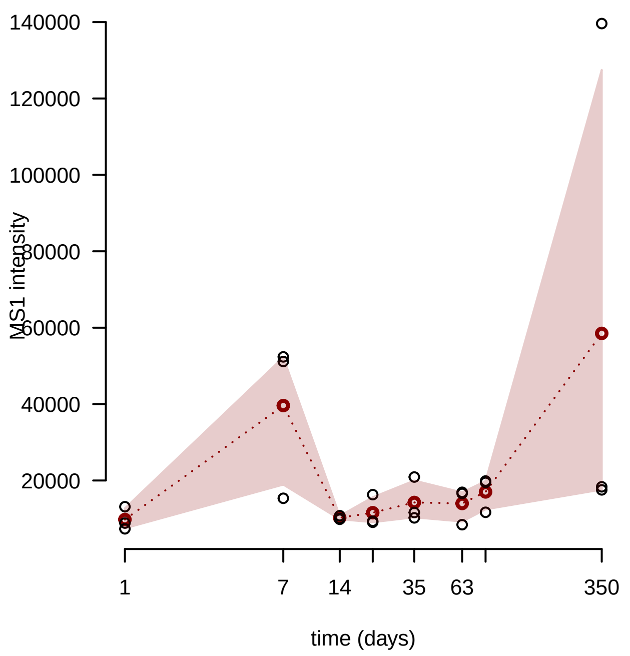

P01631
GN=

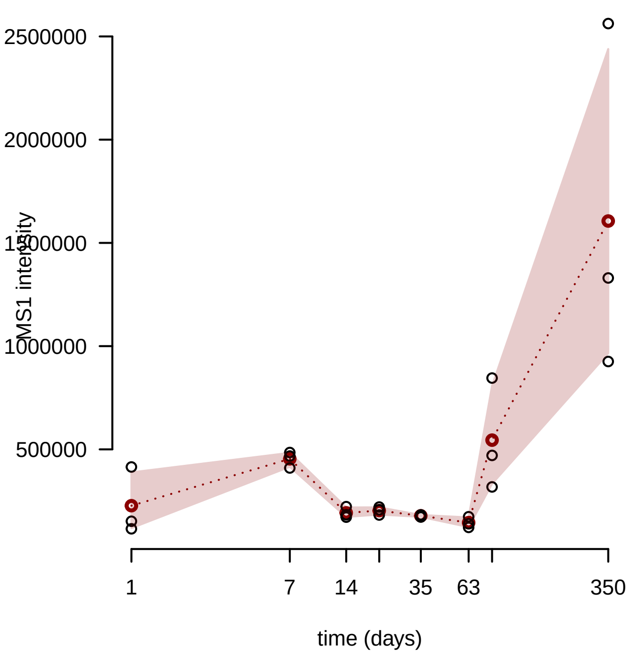

P01633
GN=Igk−V19−17

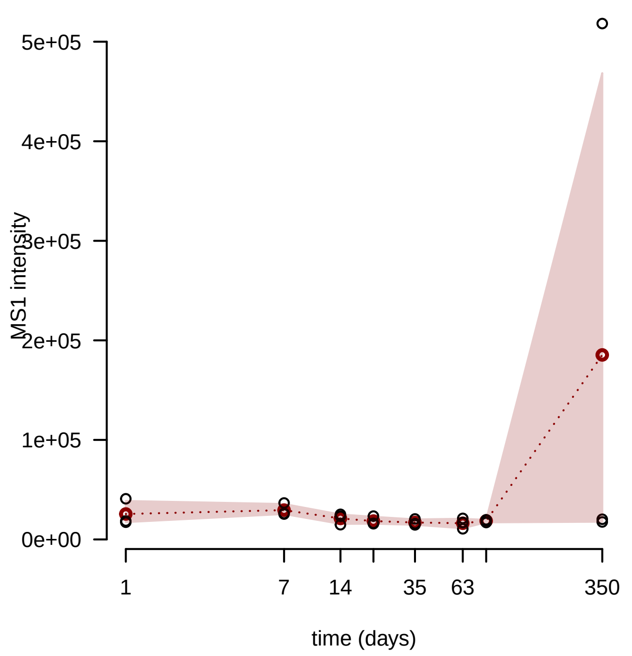

P01635
GN=

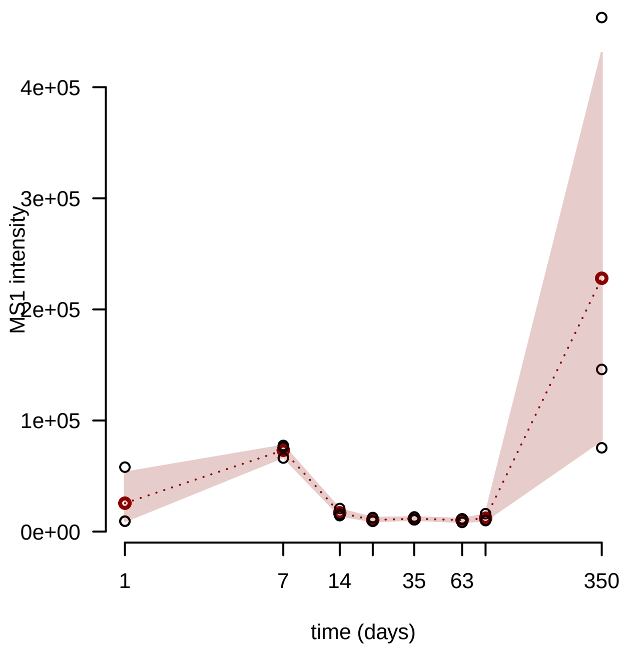

P01636
GN=

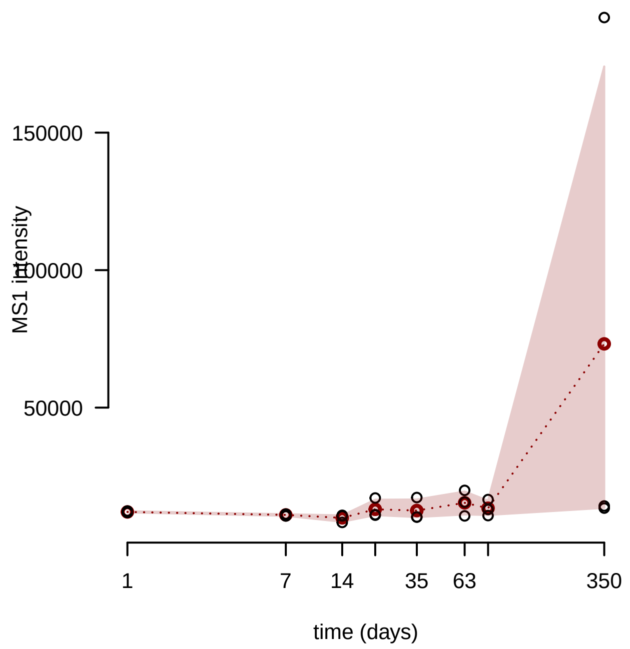

P01638
GN=

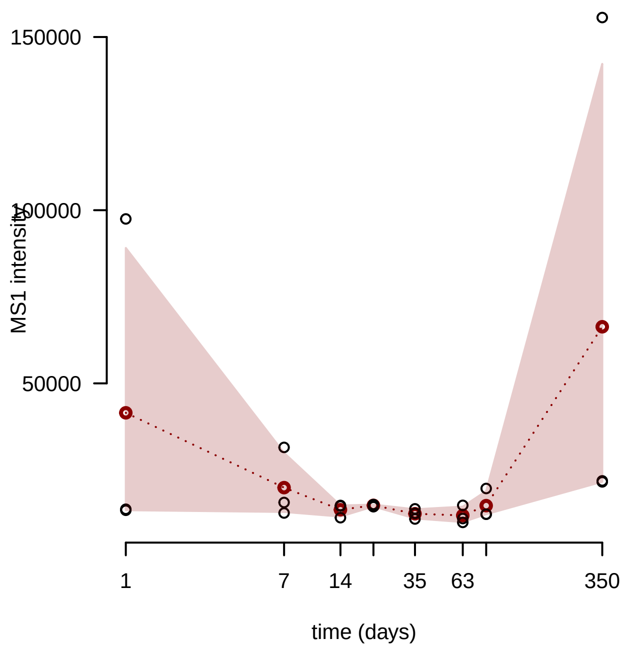

P01639
GN=Gm5571

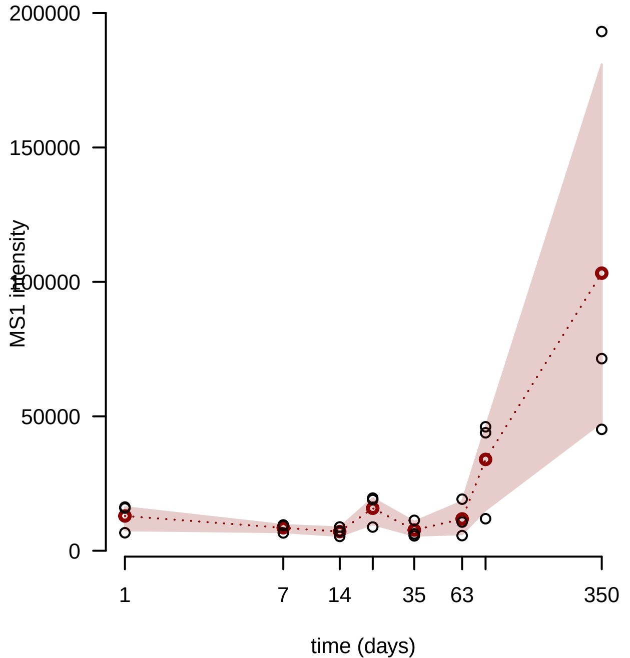

P01642
GN=Gm10881

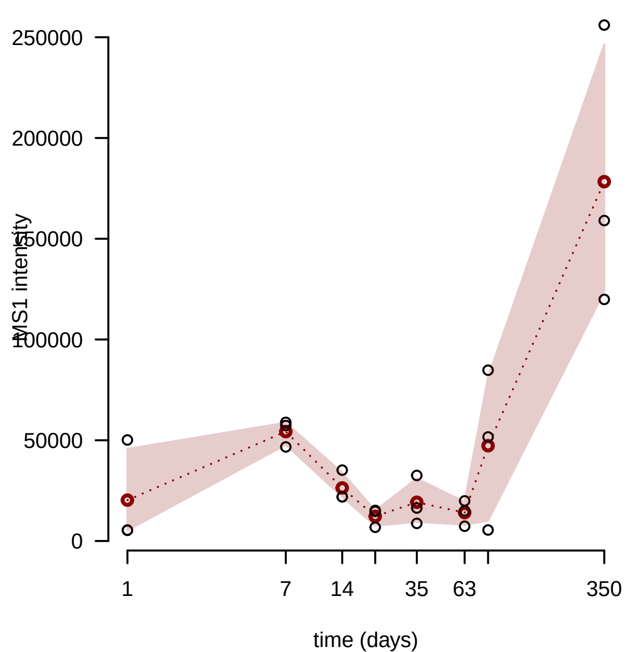

P01644
GN=

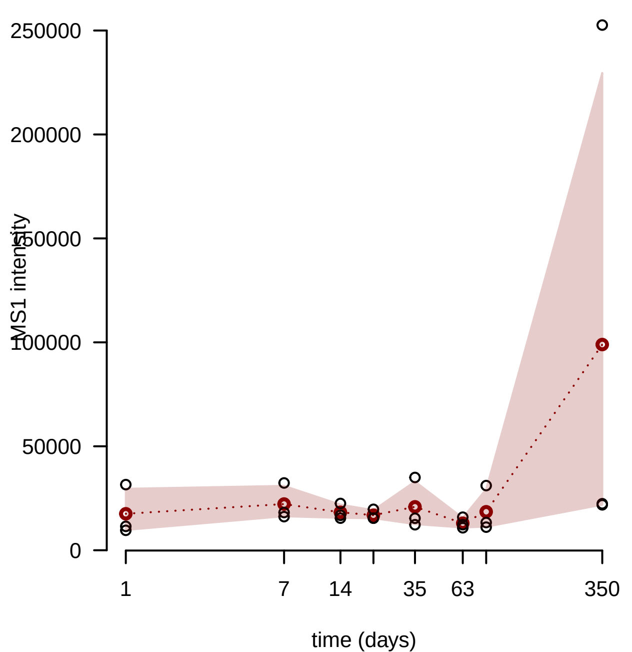

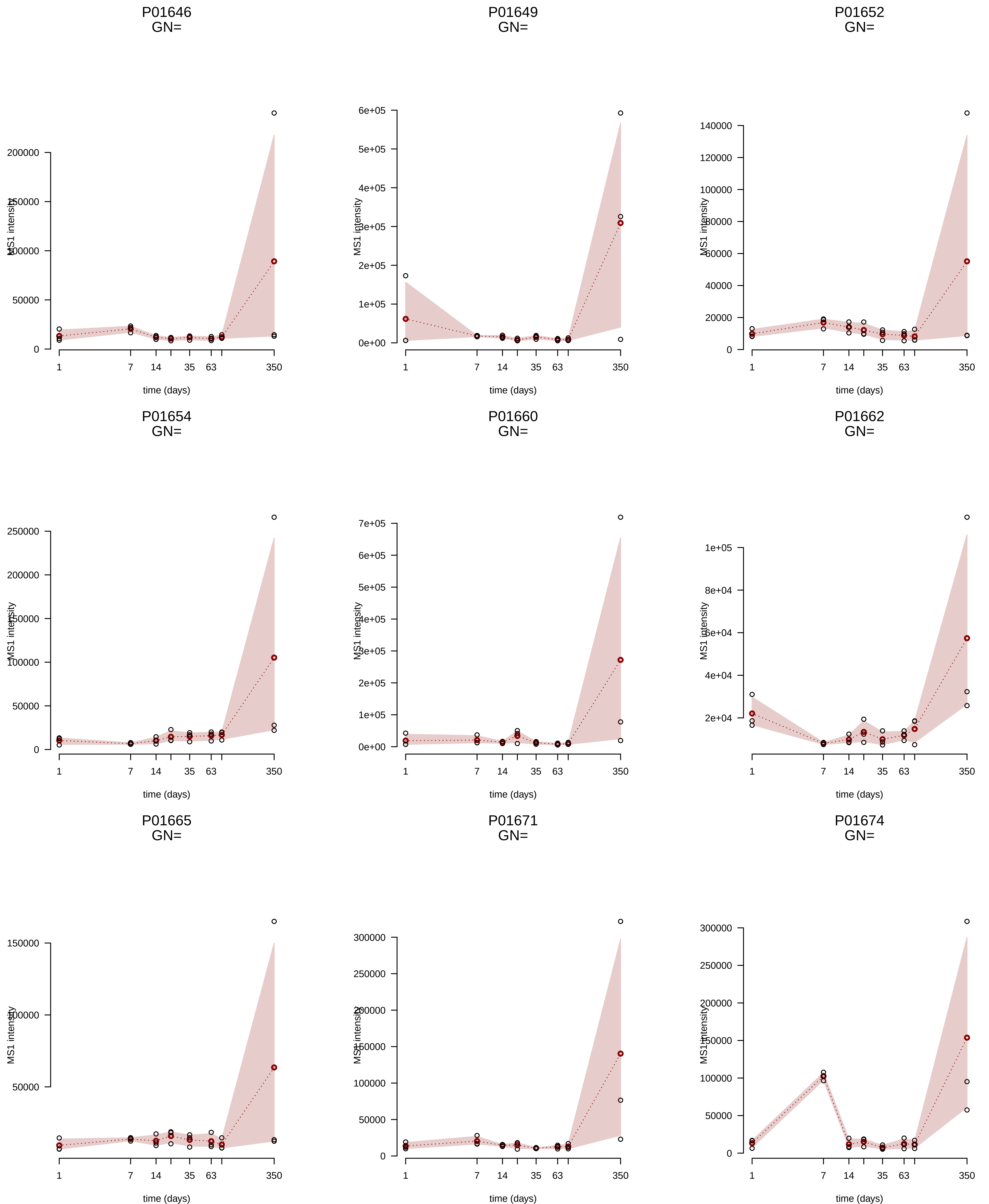
P01646
GN=
MS1 intensity
0
50000
100000
150000
200000
time (days)
1
7
14
35
63
350
P01649
GN=
MS1 intensity
0e+00
1e+05
2e+05
3e+05
4e+05
5e+05
6e+05
time (days)
1
7
14
35
63
350
P01652
GN=
MS1 intensity
0
20000
40000
60000
80000
100000
120000
140000
time (days)
1
7
14
35
63
350
P01654
GN=
MS1 intensity
0
50000
100000
150000
200000
250000
time (days)
1
7
14
35
63
350
P01660
GN=
MS1 intensity
0e+00
1e+05
2e+05
3e+05
4e+05
5e+05
6e+05
7e+05
time (days)
1
7
14
35
63
350
P01662
GN=
MS1 intensity
2e+04
4e+04
6e+04
8e+04
1e+05
time (days)
1
7
14
35
63
350
P01665
GN=
MS1 intensity
50000
100000
150000
time (days)
1
7
14
35
63
350
P01671
GN=
MS1 intensity
0
50000
100000
150000
200000
250000
300000
time (days)
1
7
14
35
63
350
P01674
GN=
MS1 intensity
0
50000
100000
150000
200000
250000
300000
time (days)
1
7
14
35
63
350

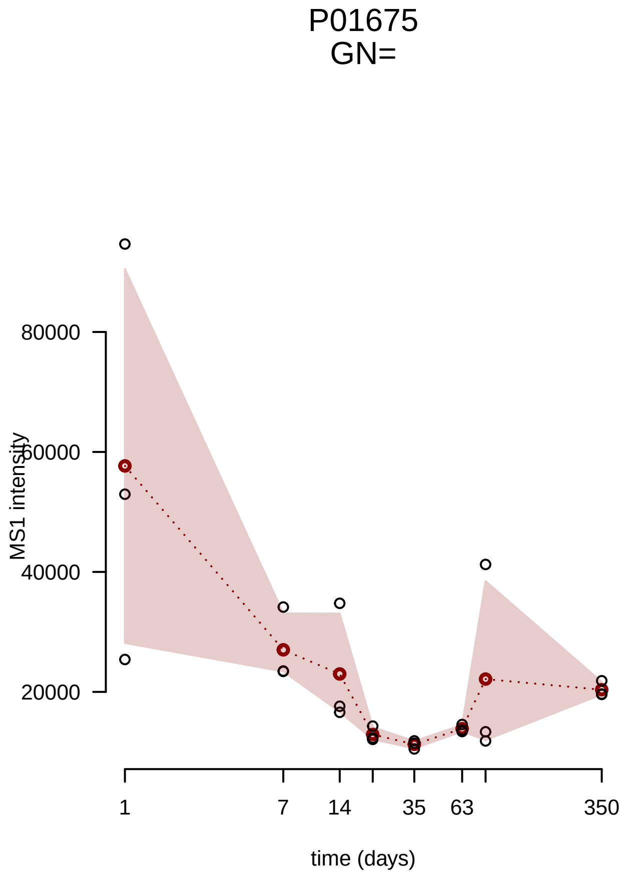
P01675
GN=
MS1 intensity
20000
40000
60000
80000
1
7
14
35
63
350
time (days)

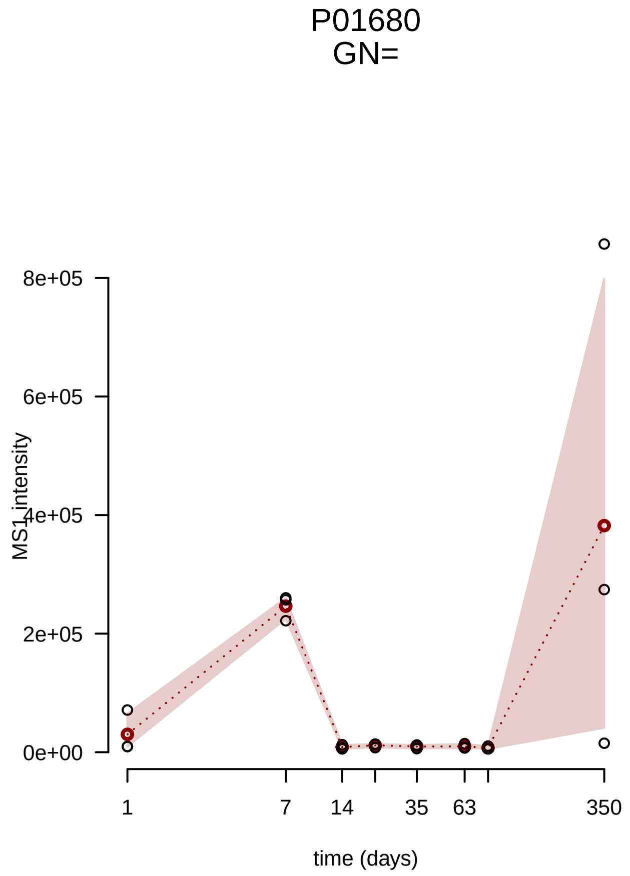
P01680
GN=
MS1 intensity
0e+00
2e+05
4e+05
6e+05
8e+05
1
7
14
35
63
350
time (days)

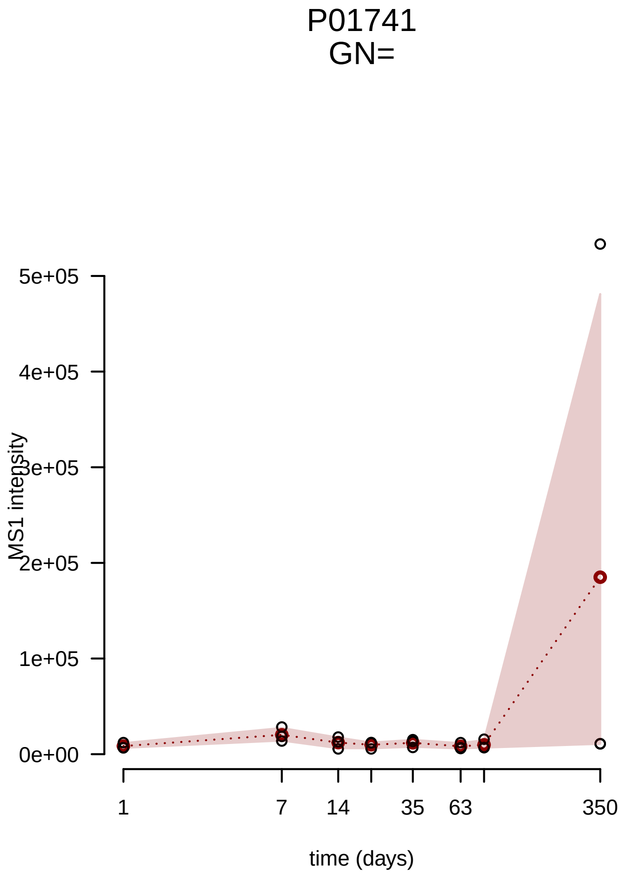
P01741
GN=
MS1 intensity
0e+00
1e+05
2e+05
3e+05
4e+05
5e+05
1
7
14
35
63
350
time (days)

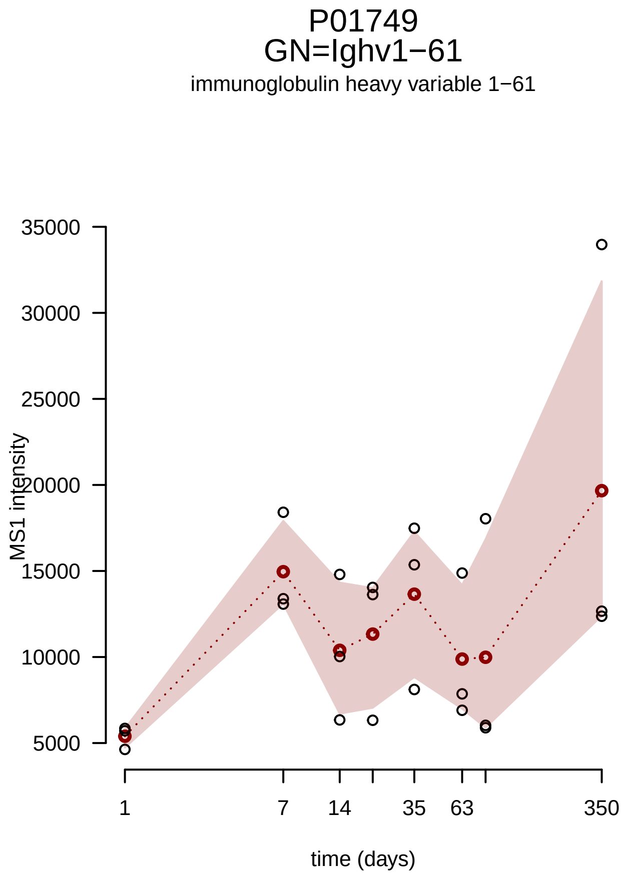
P01749
GN=Ighv1−61
immunoglobulin heavy variable 1−61
MS1 intensity
5000
10000
15000
20000
25000
30000
35000
1
7
14
35
63
350
time (days)

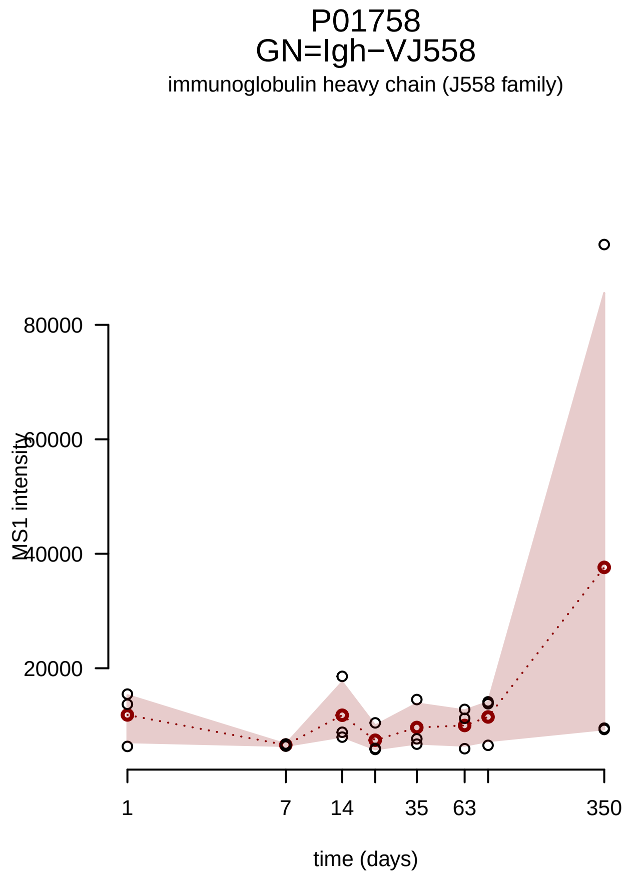
P01758
GN=Igh−VJ558
immunoglobulin heavy chain (J558 family)
MS1 intensity
20000
40000
60000
80000
1
7
14
35
63
350
time (days)

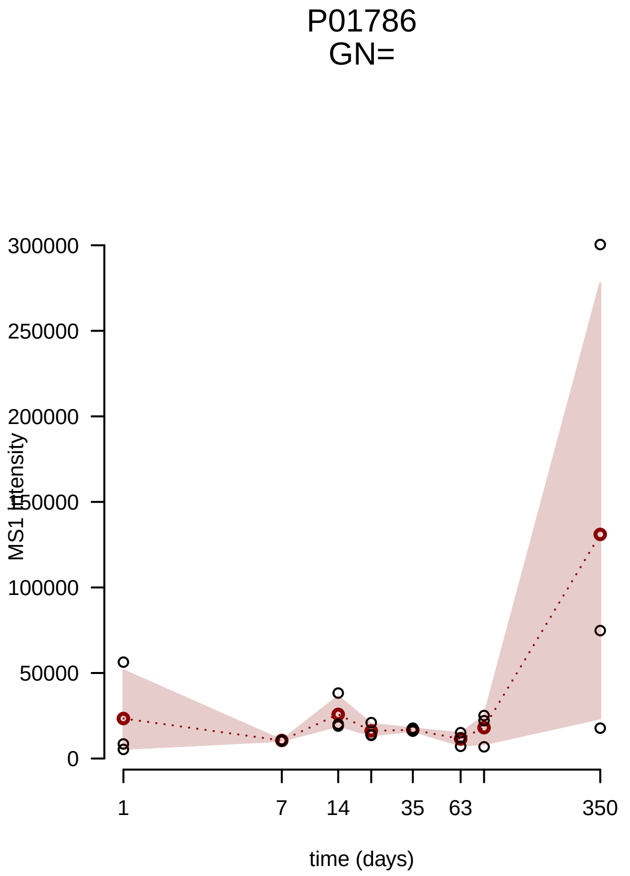
P01786
GN=
MS1 intensity
0
50000
100000
150000
200000
250000
300000
1
7
14
35
63
350
time (days)

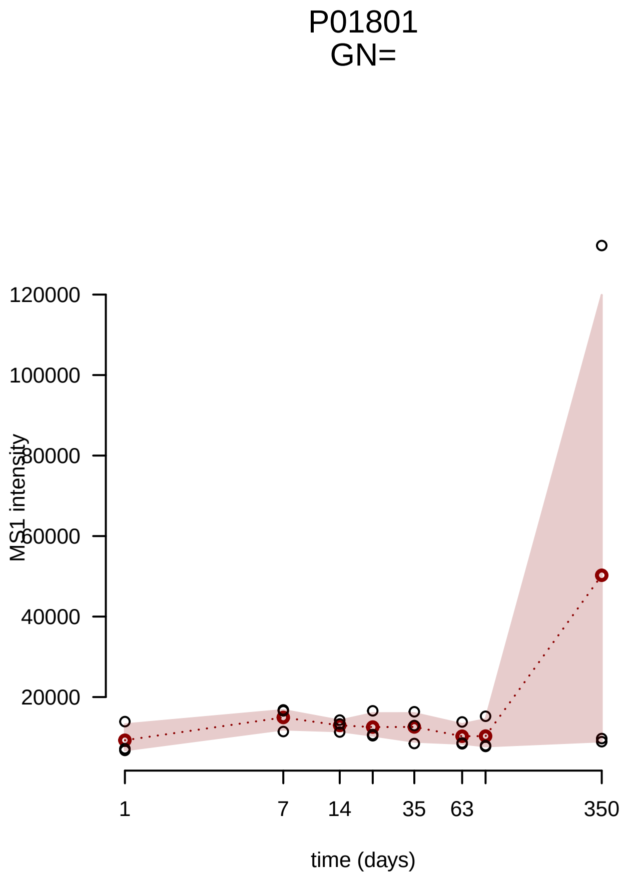
P01801
GN=
MS1 intensity
20000
40000
60000
80000
100000
120000
1
7
14
35
63
350
time (days)

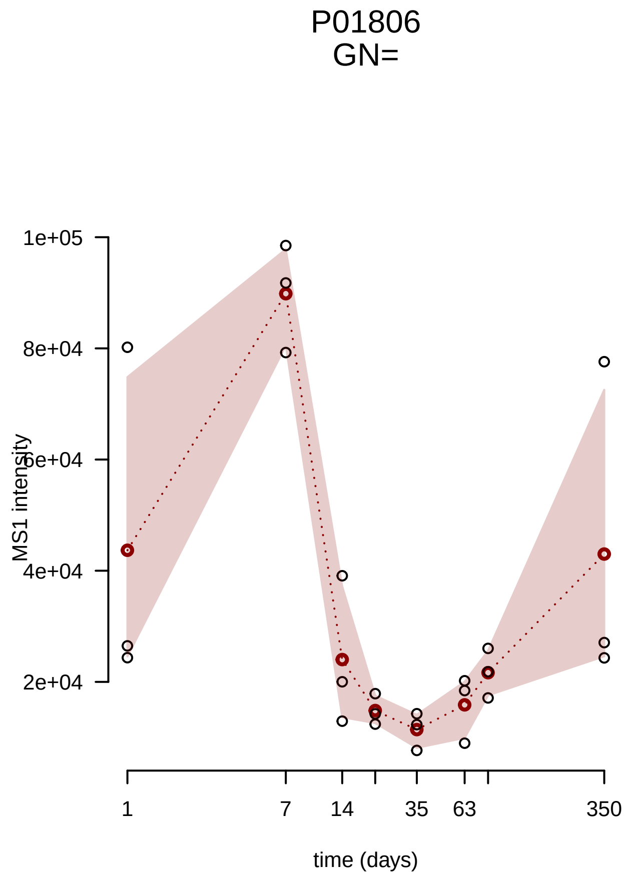
P01806
GN=
MS1 intensity
2e+04
4e+04
6e+04
8e+04
1e+05
1
7
14
35
63
350
time (days)

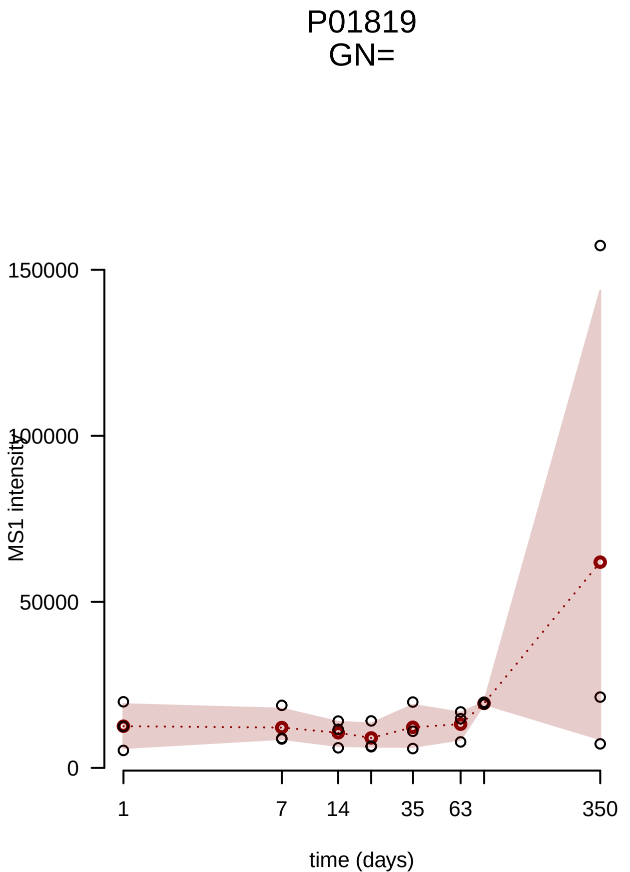
P01819
GN=
MS1 intensity
0
50000
100000
150000
1
7
14
35
63
350
time (days)

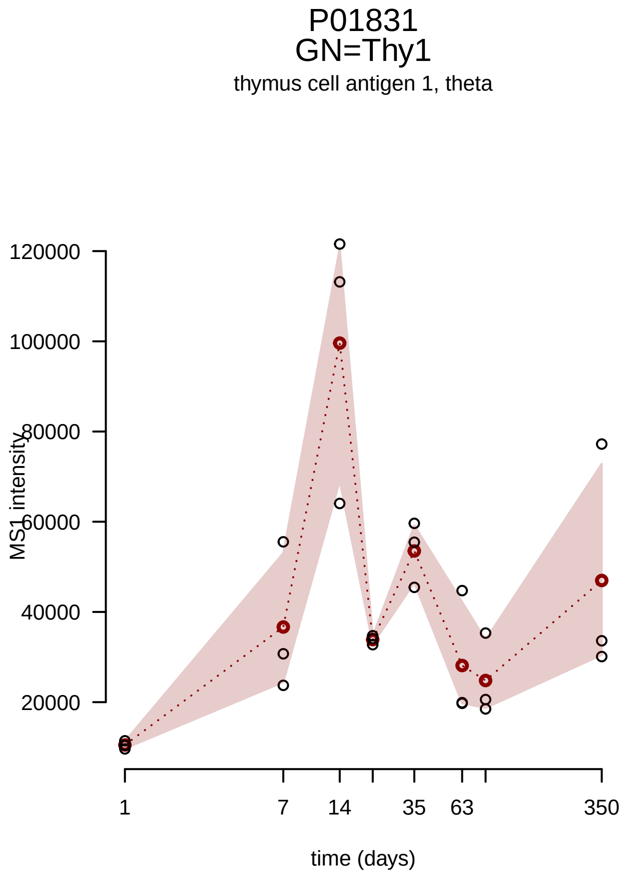
P01831
GN=Thy1
thymus cell antigen 1, theta
MS1 intensity
20000
40000
60000
80000
100000
120000
1
7
14
35
63
350
time (days)

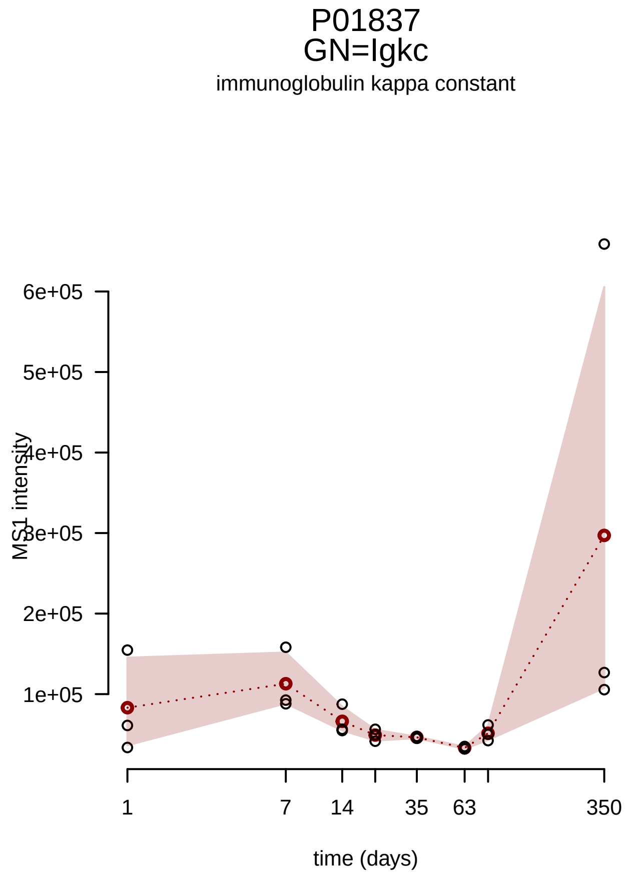
P01837
GN=Igkc
immunoglobulin kappa constant
MS1 intensity
1e+05
2e+05
3e+05
4e+05
5e+05
6e+05
1
7
14
35
63
350
time (days)

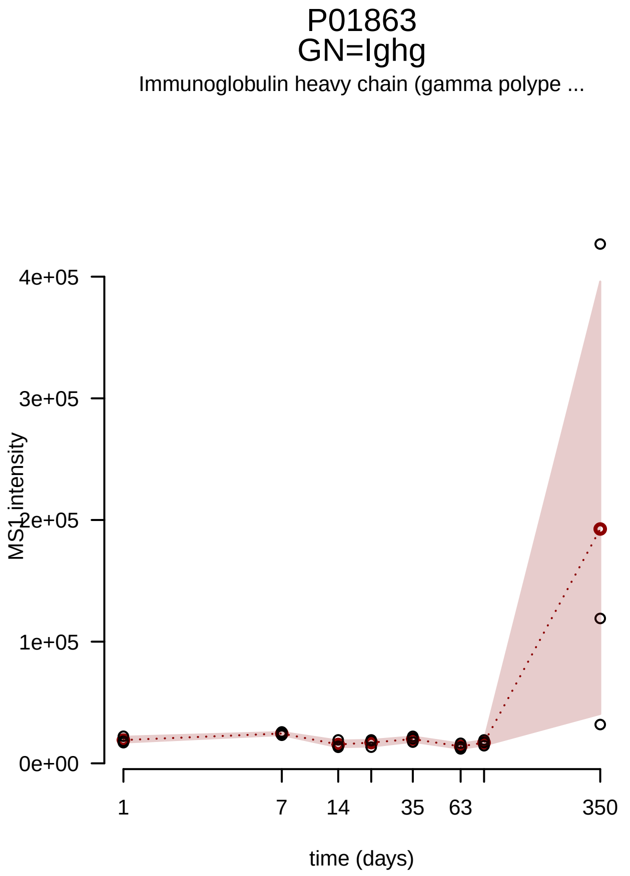
P01863
GN=Ighg
Immunoglobulin heavy chain (gamma polype ...
MS1 intensity
0e+00
1e+05
2e+05
3e+05
4e+05
1
7
14
35
63
350
time (days)

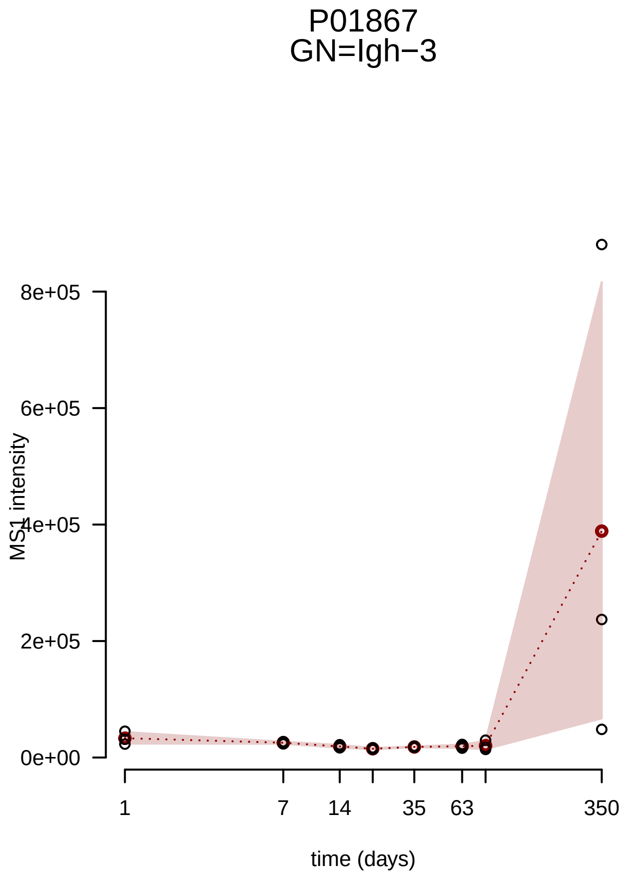
P01867
GN=Igh−3
MS1 intensity
0e+00
2e+05
4e+05
6e+05
8e+05
1
7
14
35
63
350
time (days)

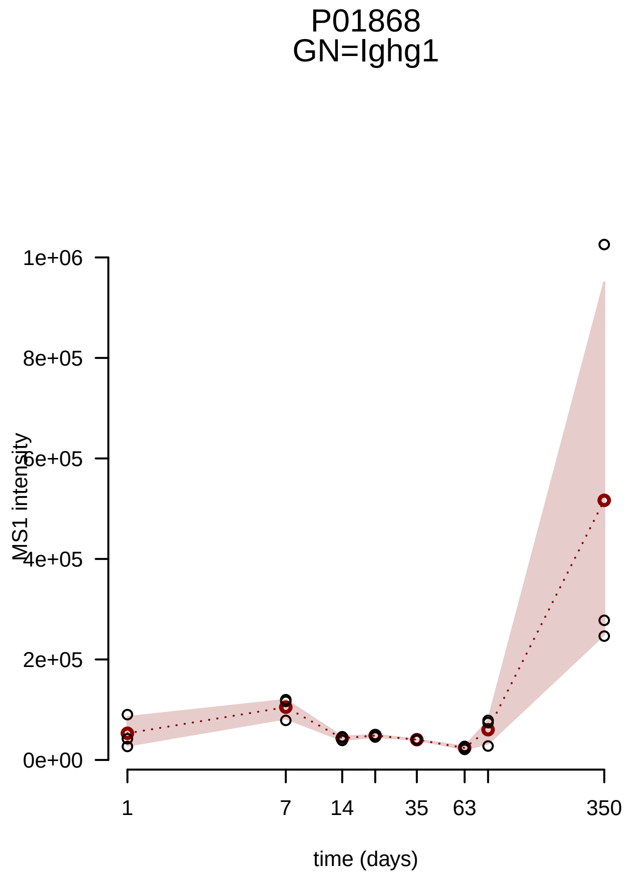
P01868
GN=Ighg1
MS1 intensity
0e+00
2e+05
4e+05
6e+05
8e+05
1e+06
1
7
14
35
63
350
time (days)

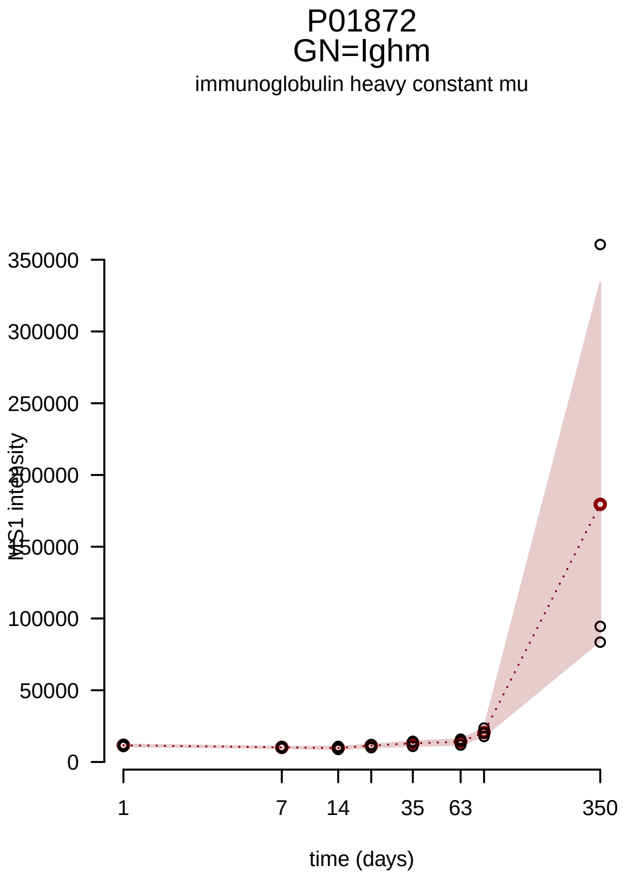
P01872
GN=Ighm
immunoglobulin heavy constant mu
MS1 intensity
0
50000
100000
150000
200000
250000
300000
350000
1
7
14
35
63
350
time (days)

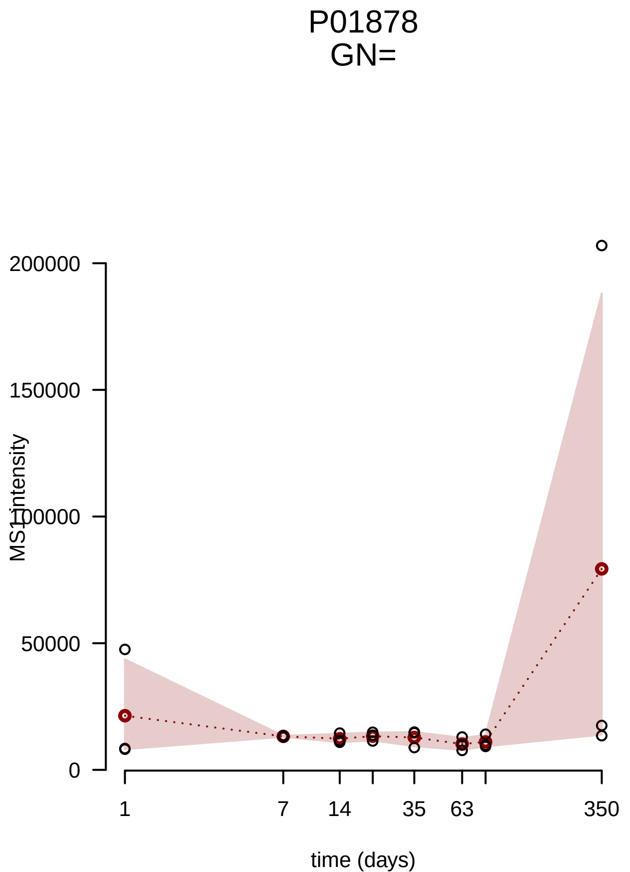
P01878
GN=
MS1 intensity
0
50000
100000
150000
200000
1
7
14
35
63
350
time (days)

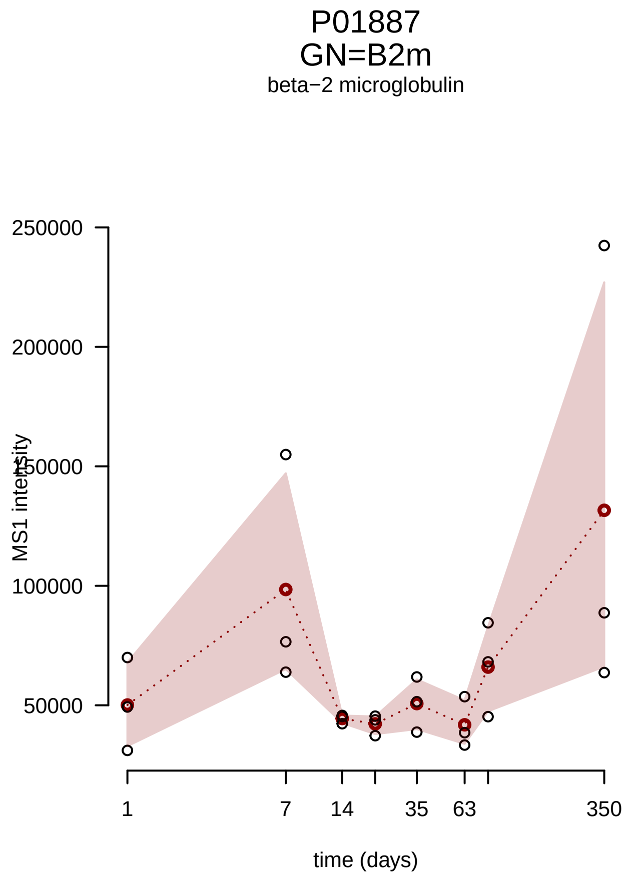
P01887
GN=B2m
beta−2 microglobulin
MS1 intensity
50000
100000
150000
200000
250000
1
7
14
35
63
350
time (days)

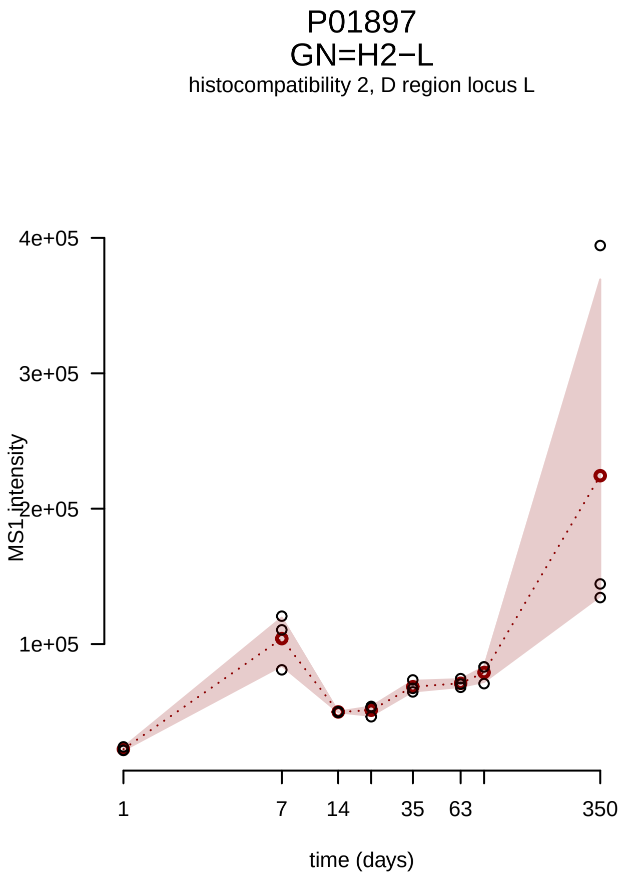
P01897
GN=H2−L
histocompatibility 2, D region locus L
MS1 intensity
1e+05
2e+05
3e+05
4e+05
1
7
14
35
63
350
time (days)

P01898
GN=H2−Q10
histocompatibility 2, Q region locus 10

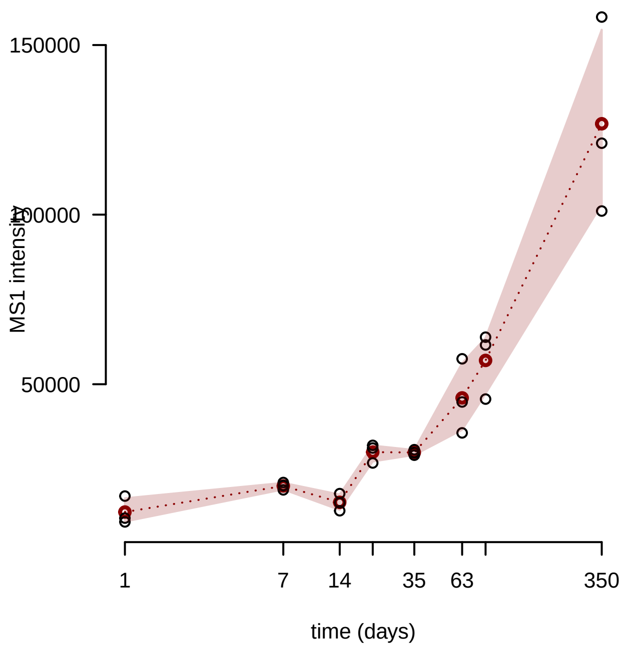

P01902
GN=H2−K1

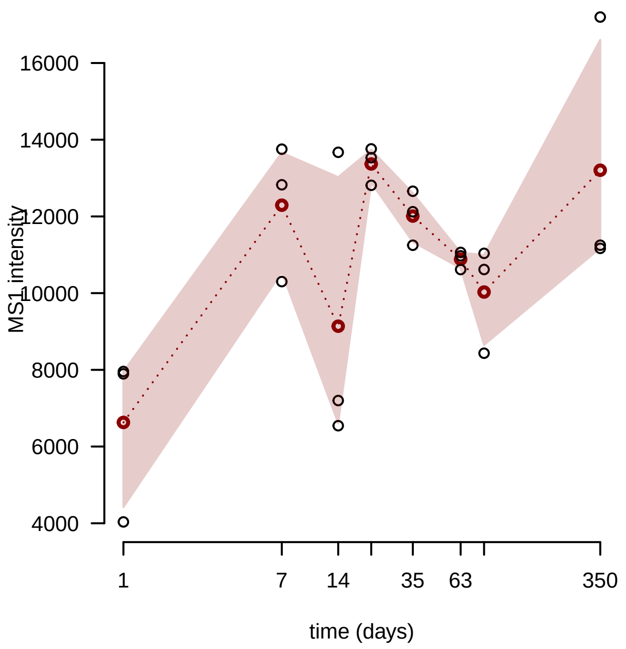

P01942
GN=Hba

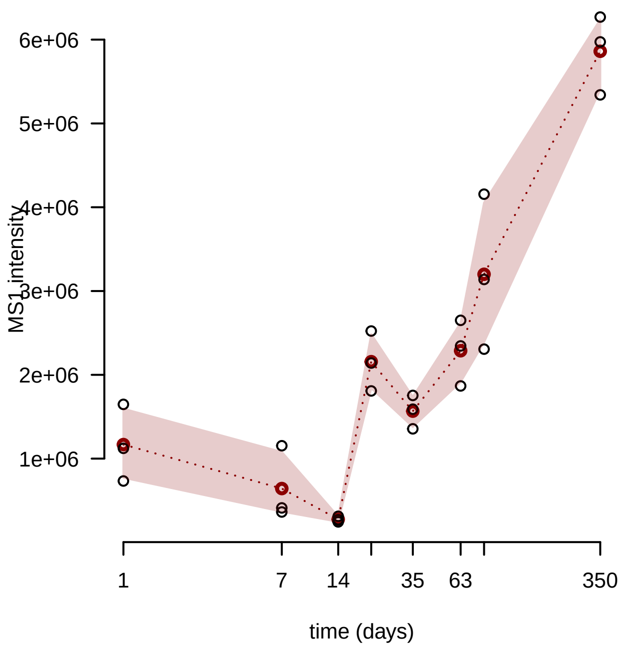

P02088
GN=Hbb−b1
hemoglobin, beta adult major chain

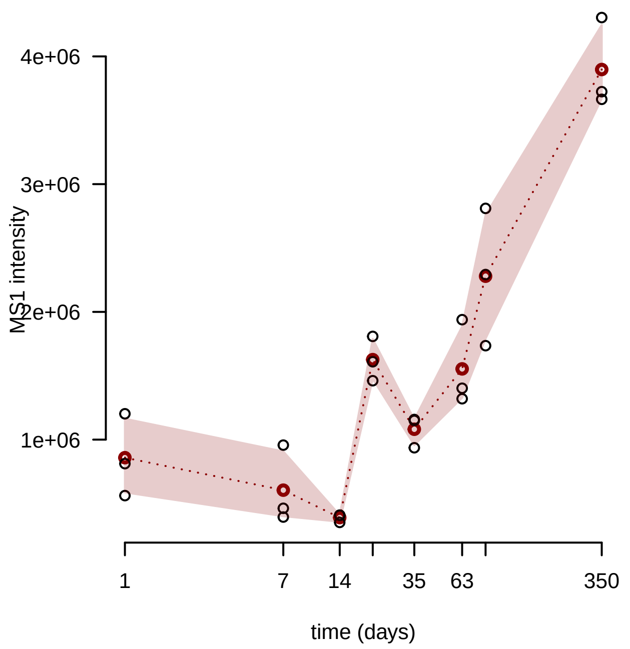

P02089
GN=Hbb−b2
hemoglobin, beta adult minor chain

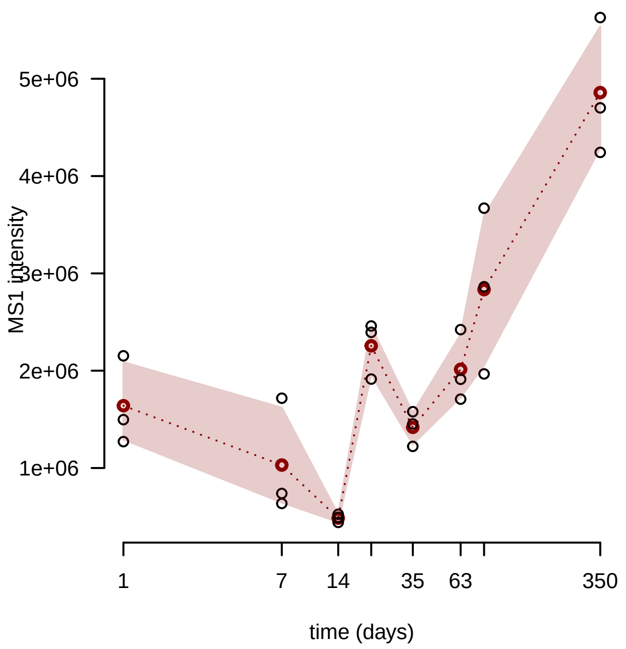

P02104
GN=Hbb−y
hemoglobin Y, beta−like embryonic chain

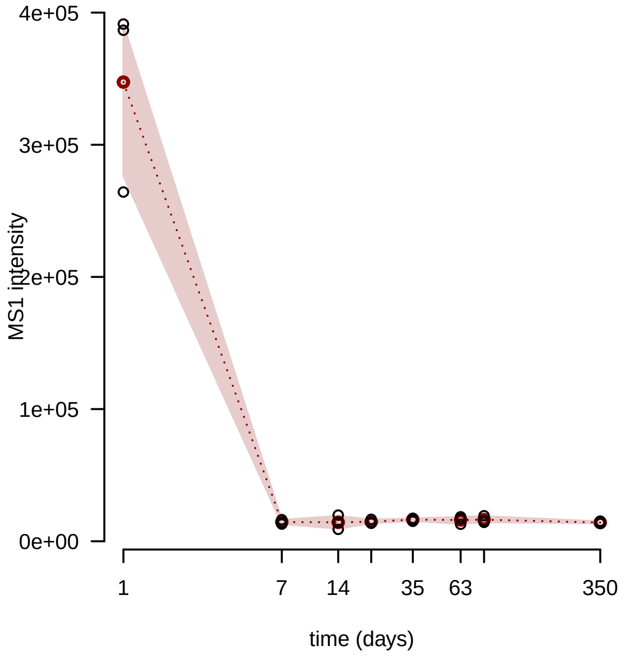

P02301
GN=H3f3c
H3 histone, family 3C

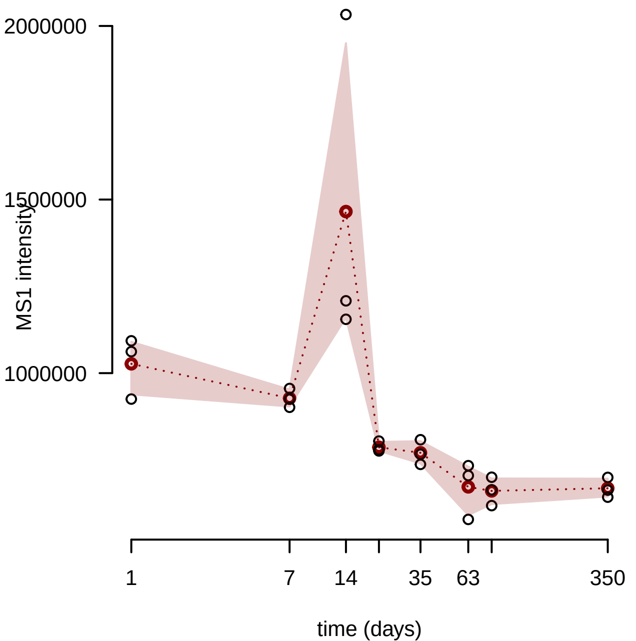

P02463
GN=Col4a1
collagen, type IV, alpha 1

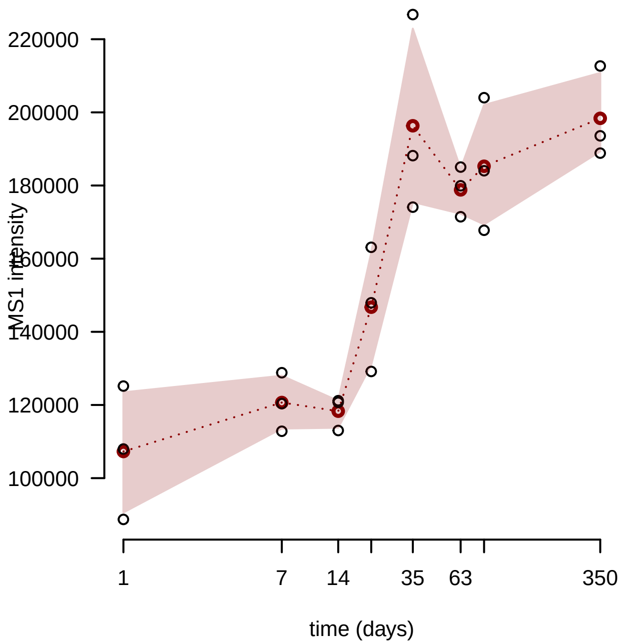

P02468
GN=Lamc1
laminin, gamma 1

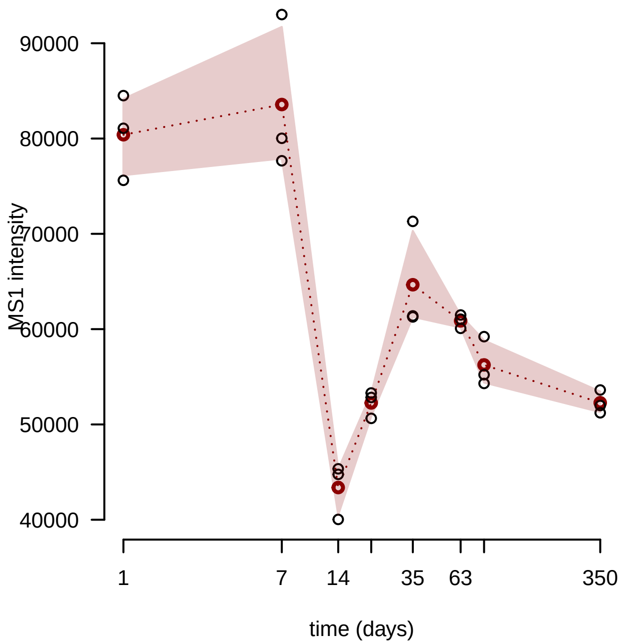

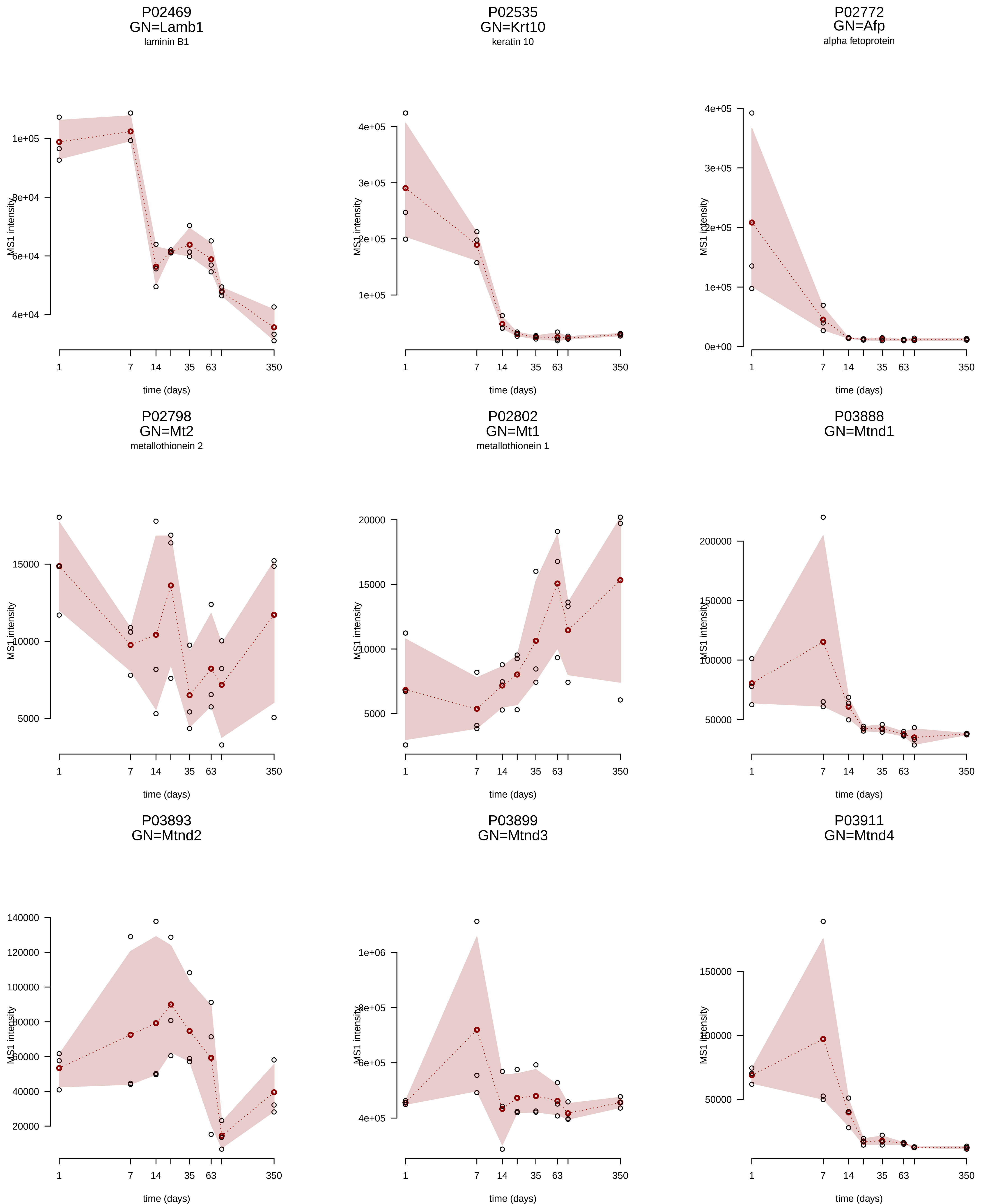
P02469
GN=Lamb1
laminin B1
P02535
GN=Krt10
keratin 10
P02772
GN=Afp
alpha fetoprotein
P02798
GN=Mt2
metallothionein 2
P02802
GN=Mt1
metallothionein 1
P03888
GN=Mtnd1
P03893
GN=Mtnd2
P03899
GN=Mtnd3
P03911
GN=Mtnd4
MS1 intensity
time (days)
1
7
14
35
63
350

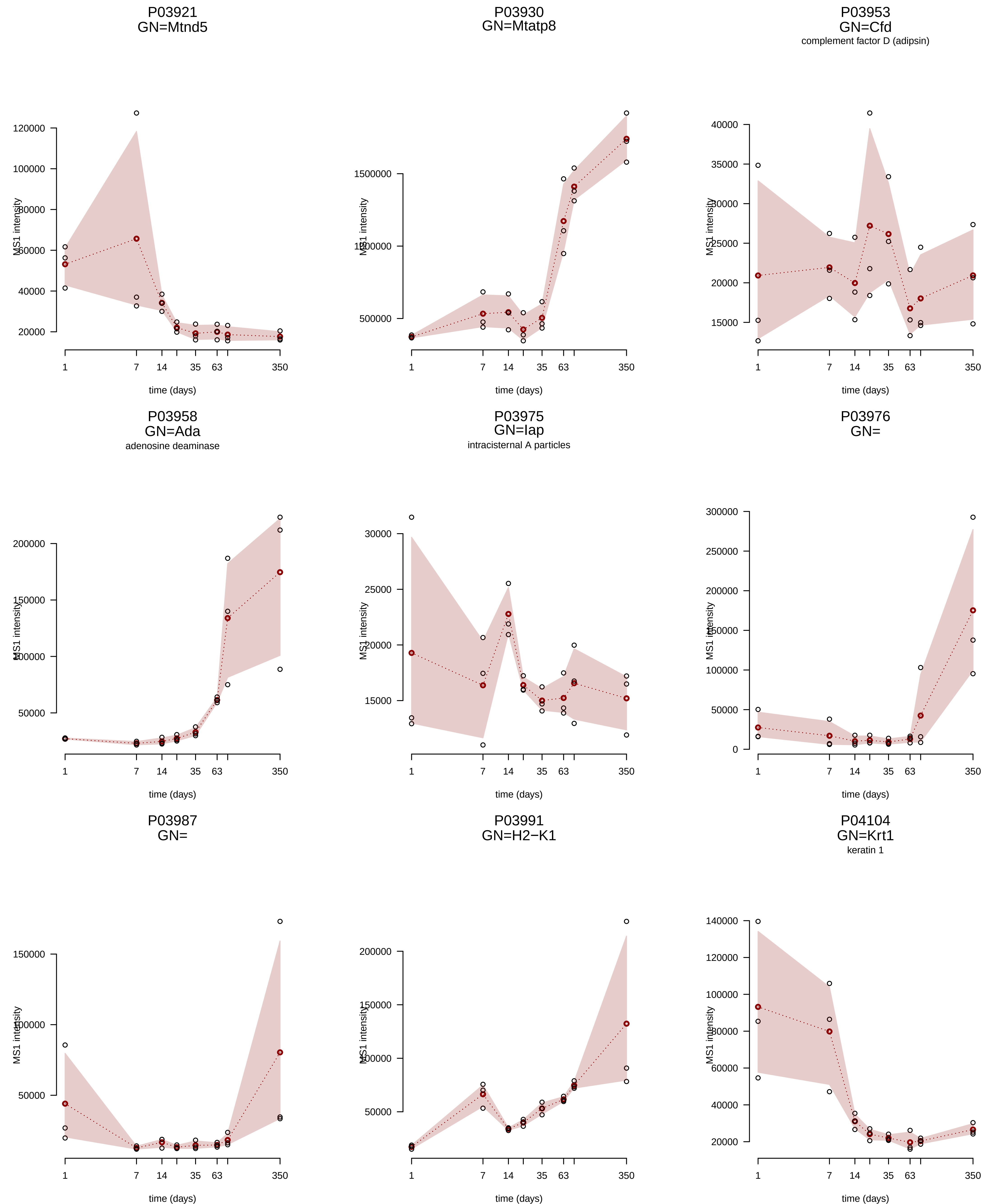
P03921
GN=Mtnd5
P03930
GN=Mtatp8
P03953
GN=Cfd
complement factor D (adipsin)
P03958
GN=Ada
adenosine deaminase
P03975
GN=Iap
intracisternal A particles
P03976
GN=
P03987
GN=
P03991
GN=H2−K1
P04104
GN=Krt1
keratin 1
MS1 intensity
time (days)
1
7
14
35
63
350

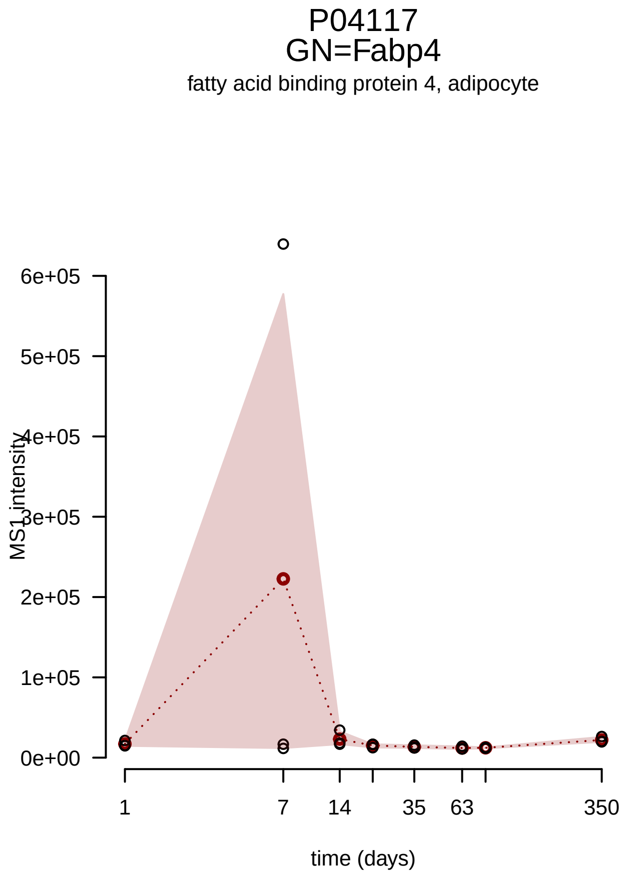
P04117
GN=Fabp4
fatty acid binding protein 4, adipocyte
MS1 intensity
0e+00
1e+05
2e+05
3e+05
4e+05
5e+05
6e+05
1
7
14
35
63
350
time (days)

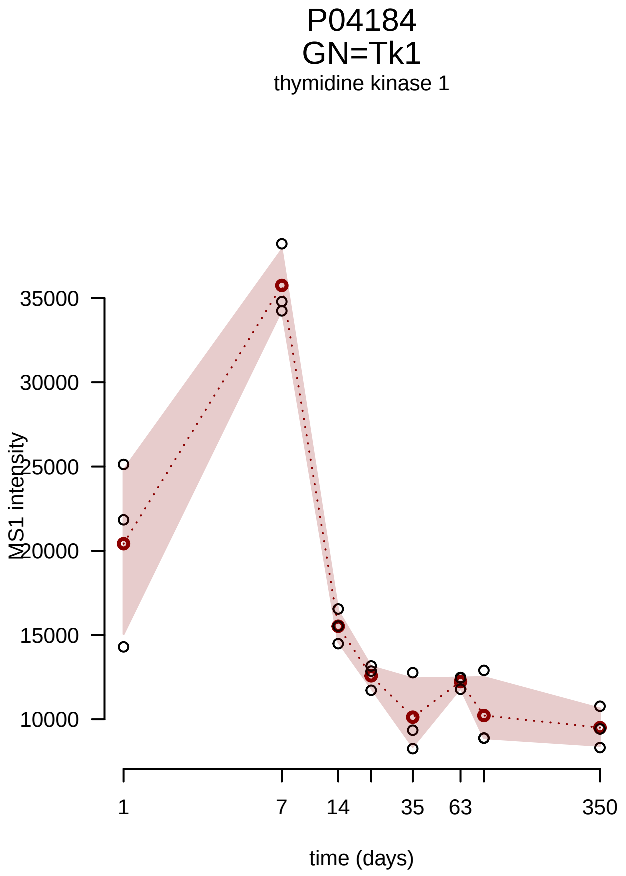
P04184
GN=Tk1
thymidine kinase 1
MS1 intensity
10000
15000
20000
25000
30000
35000
1
7
14
35
63
350
time (days)

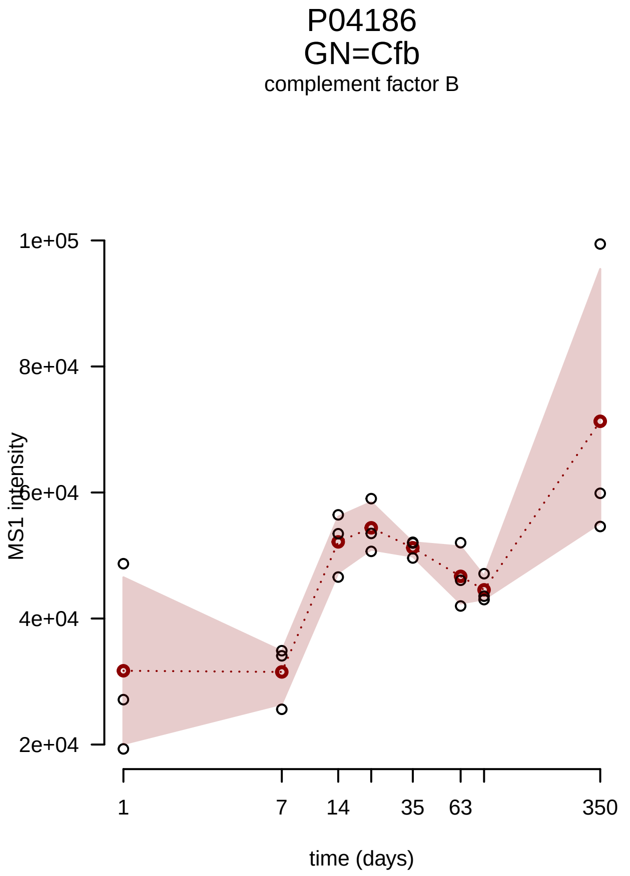
P04186
GN=Cfb
complement factor B
MS1 intensity
2e+04
4e+04
6e+04
8e+04
1e+05
1
7
14
35
63
350
time (days)

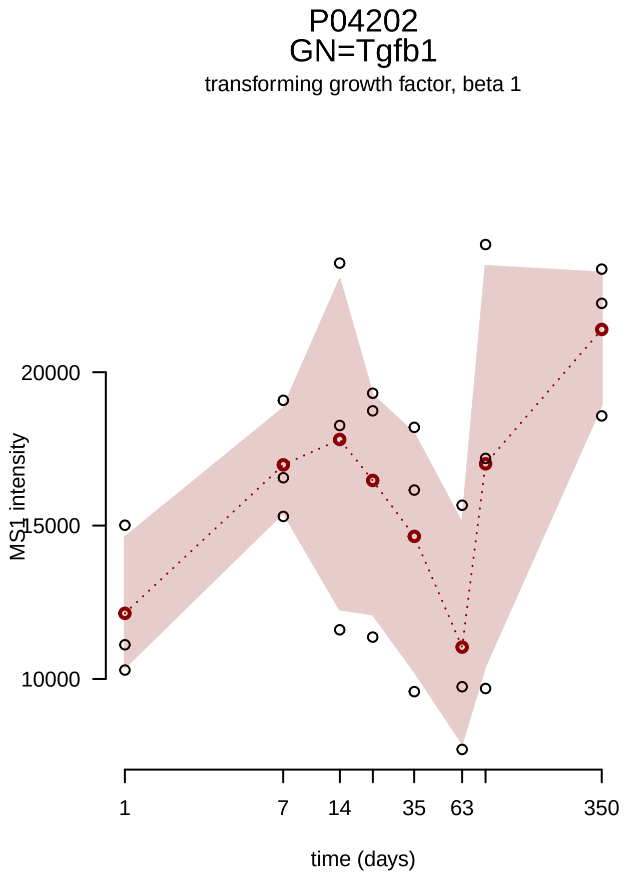
P04202
GN=Tgfb1
transforming growth factor, beta 1
MS1 intensity
10000
15000
20000
1
7
14
35
63
350
time (days)

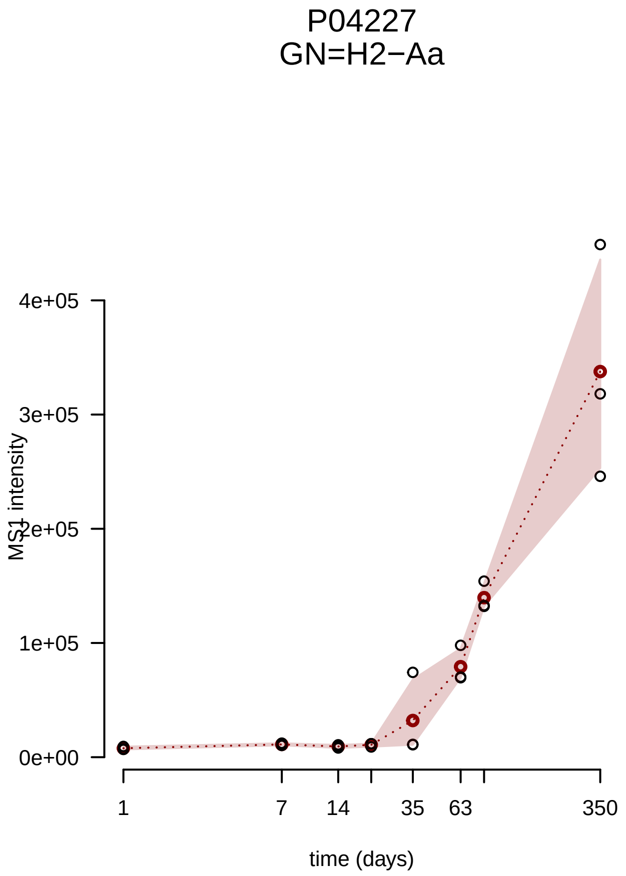
P04227
GN=H2−Aa
MS1 intensity
0e+00
1e+05
2e+05
3e+05
4e+05
1
7
14
35
63
350
time (days)

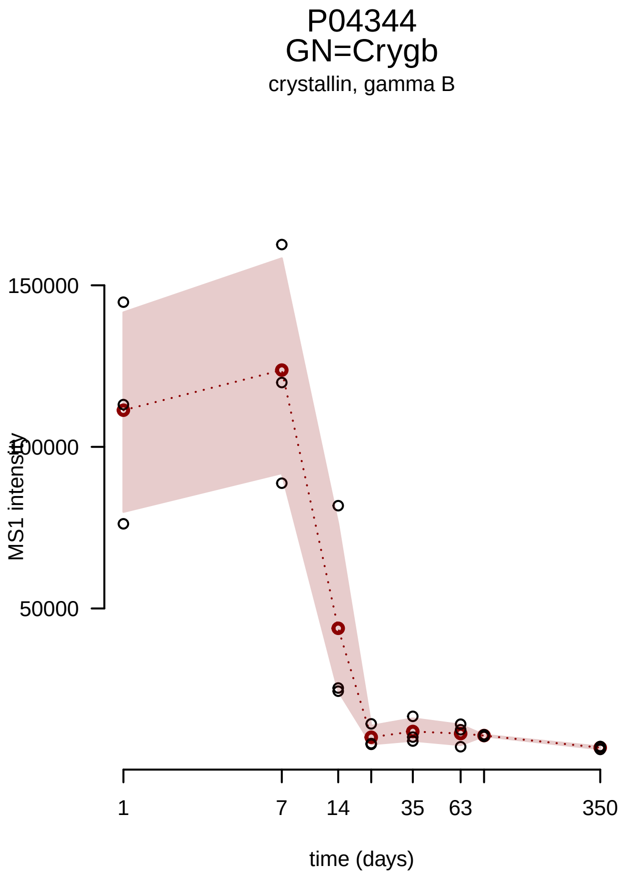
P04344
GN=Crygb
crystallin, gamma B
MS1 intensity
50000
100000
150000
1
7
14
35
63
350
time (days)

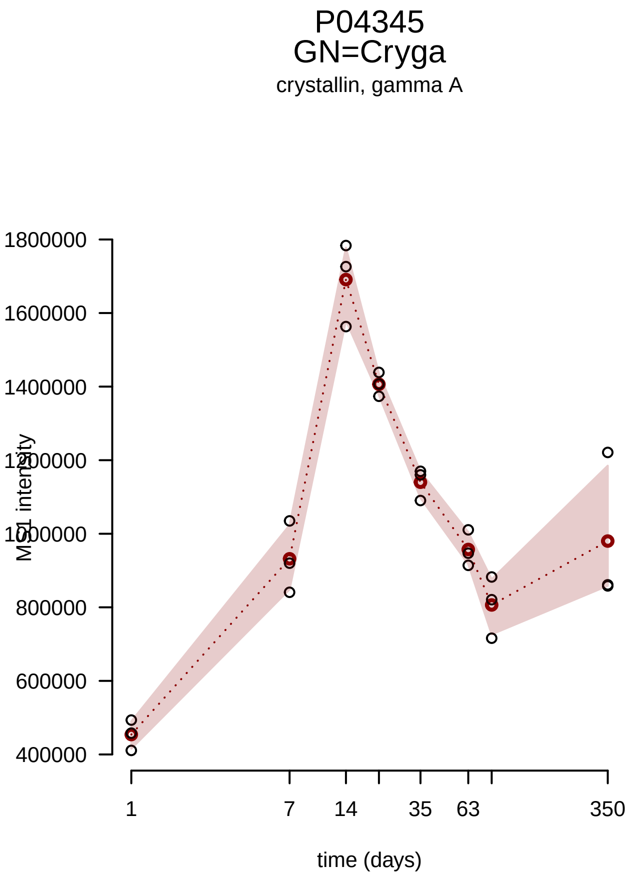
P04345
GN=Cryga
crystallin, gamma A
MS1 intensity
400000
600000
800000
1000000
1200000
1400000
1600000
1800000
1
7
14
35
63
350
time (days)

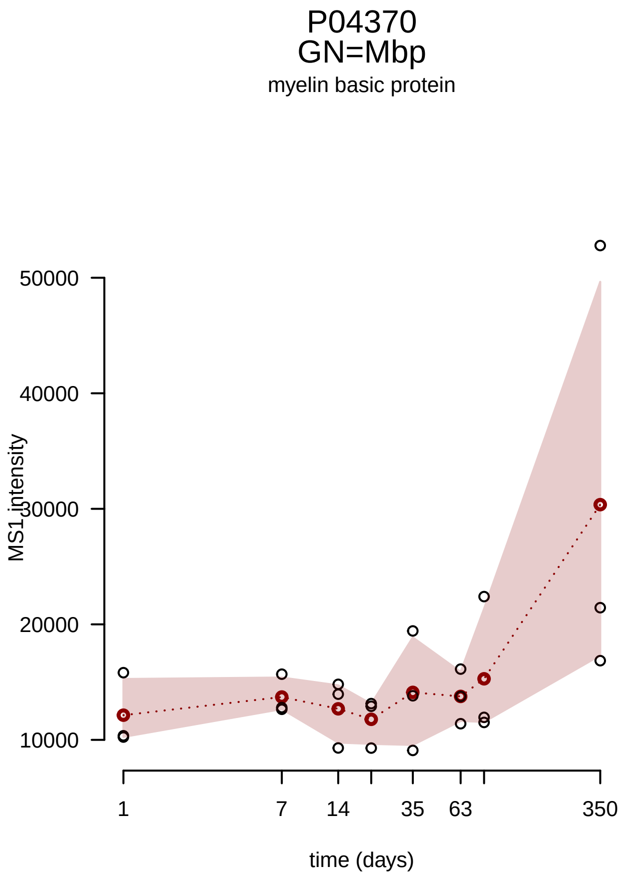
P04370
GN=Mbp
myelin basic protein
MS1 intensity
10000
20000
30000
40000
50000
1
7
14
35
63
350
time (days)

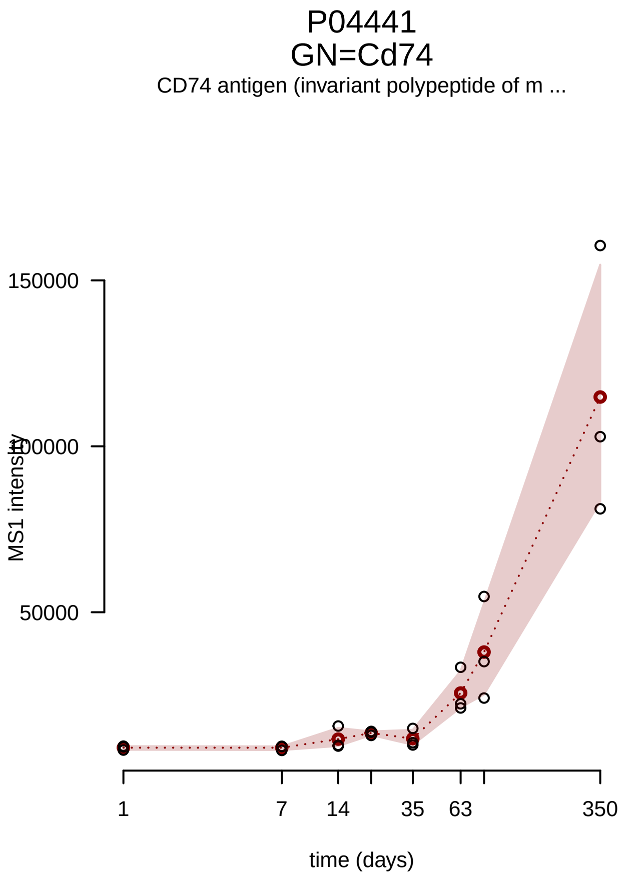
P04441
GN=Cd74
CD74 antigen (invariant polypeptide of m ...
MS1 intensity
50000
100000
150000
1
7
14
35
63
350
time (days)

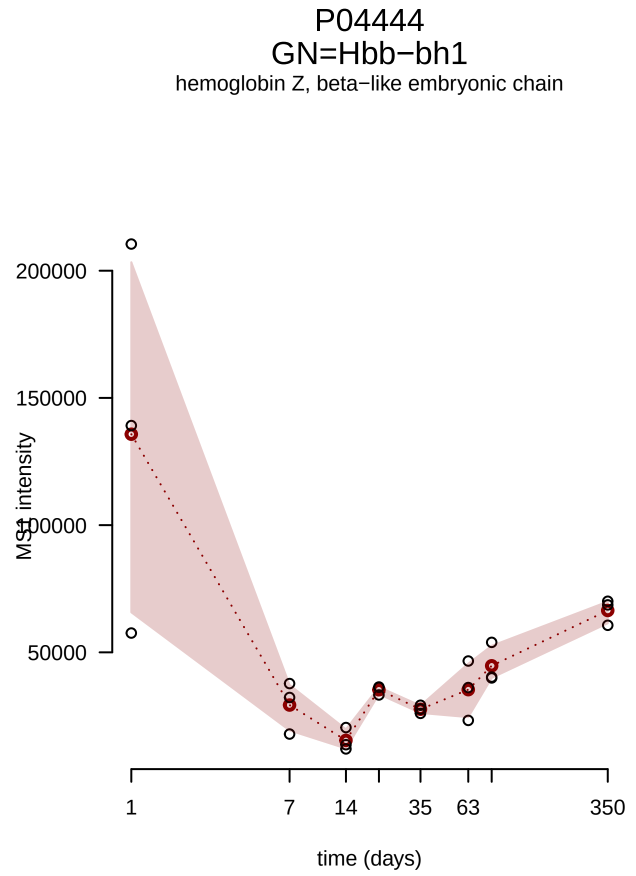
P04444
GN=Hbb−bh1
hemoglobin Z, beta−like embryonic chain
MS1 intensity
200000
150000
100000
50000
1
7
14
35
63
350
time (days)

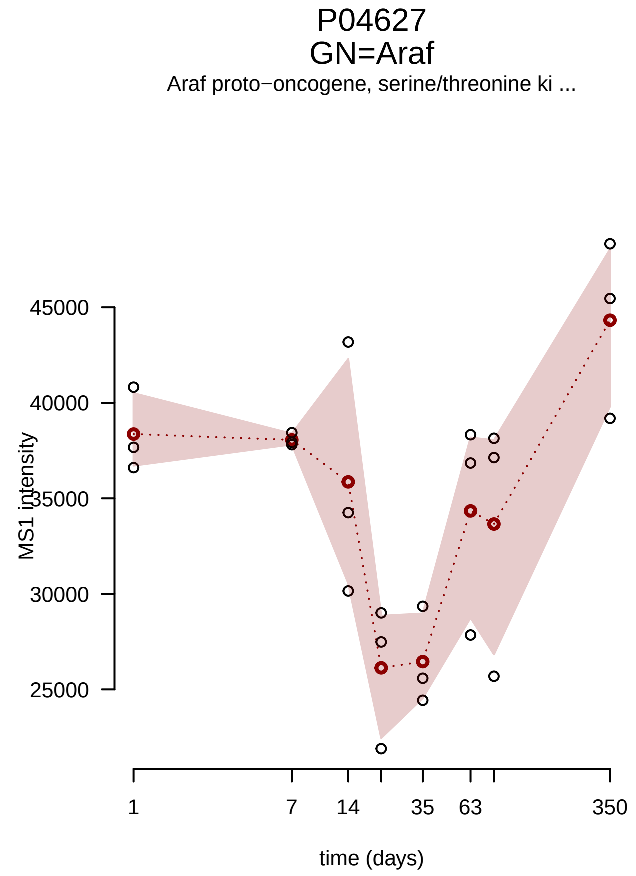
P04627
GN=Araf
Araf proto−oncogene, serine/threonine ki ...
MS1 intensity
45000
40000
35000
30000
25000
1
7
14
35
63
350
time (days)

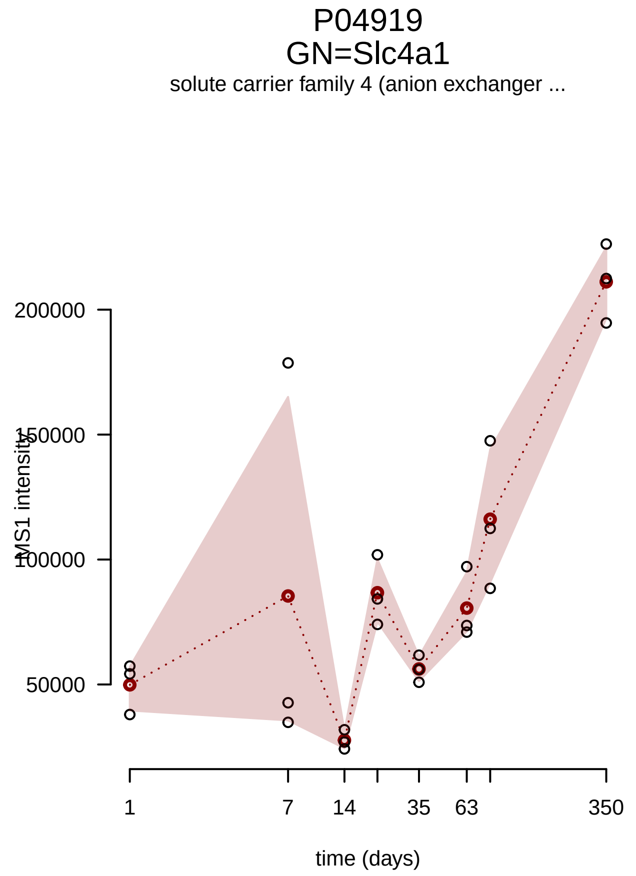
P04919
GN=Slc4a1
solute carrier family 4 (anion exchanger ...
MS1 intensity
200000
150000
100000
50000
1
7
14
35
63
350
time (days)

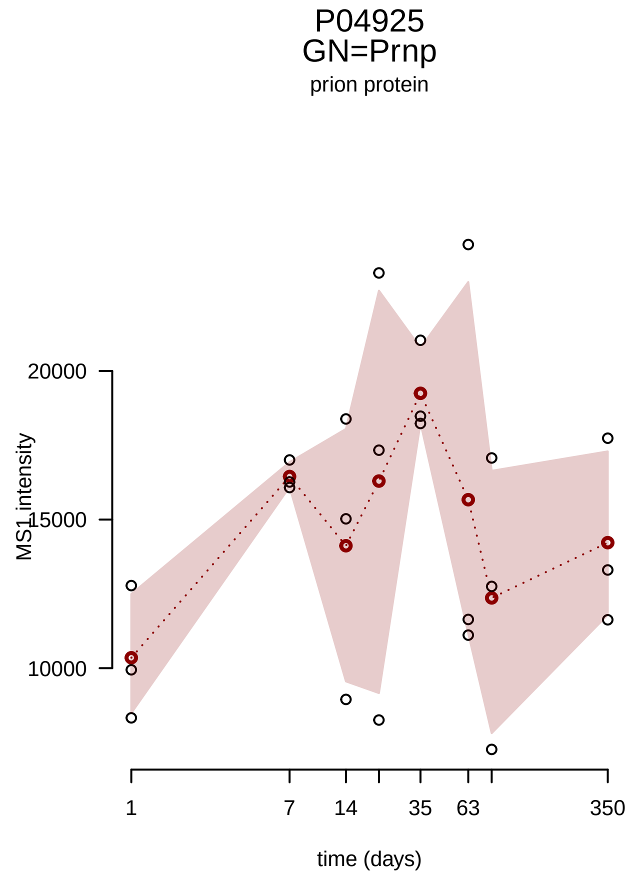
P04925
GN=Prnp
prion protein
MS1 intensity
20000
15000
10000
1
7
14
35
63
350
time (days)

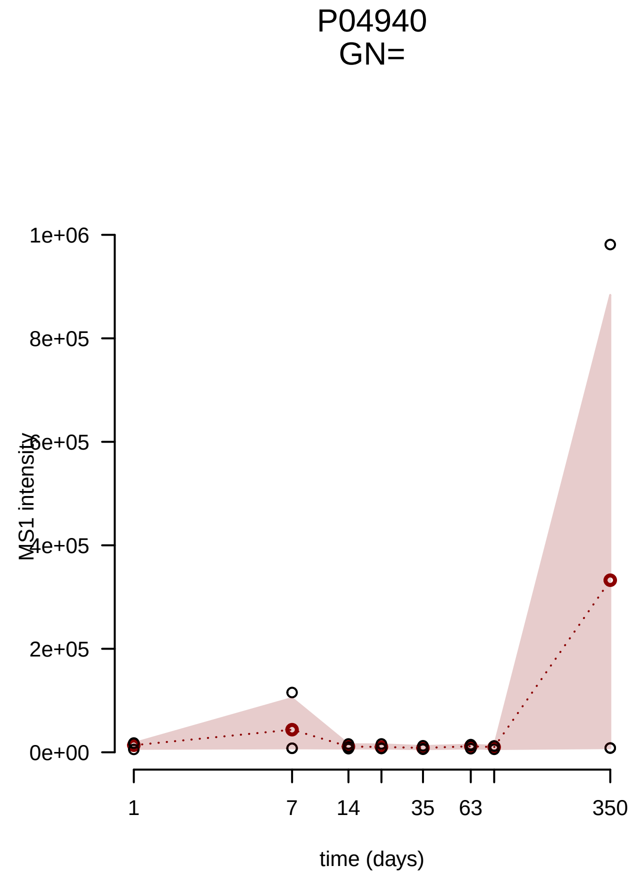
P04940
GN=
MS1 intensity
1e+06
8e+05
6e+05
4e+05
2e+05
0e+00
1
7
14
35
63
350
time (days)

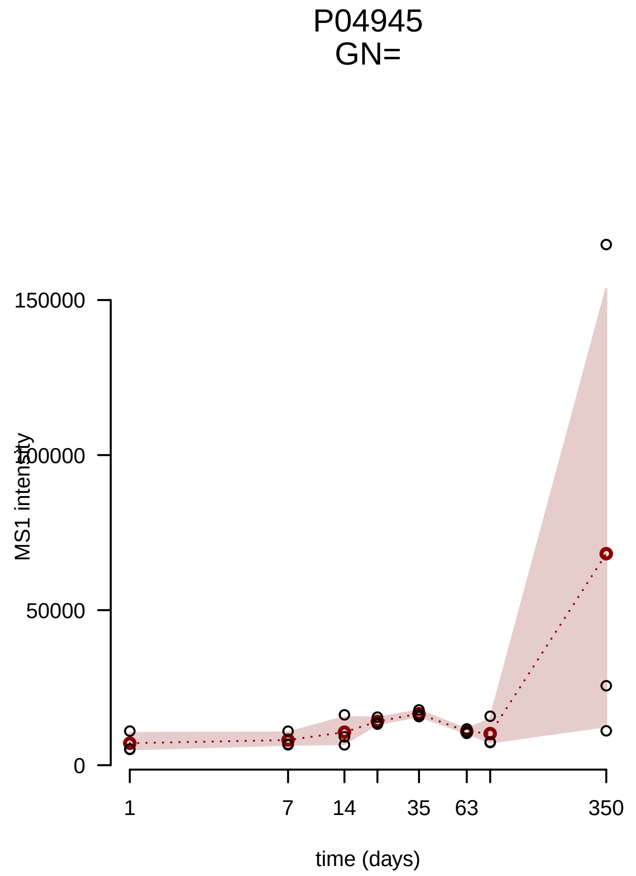
P04945
GN=
MS1 intensity
150000
100000
50000
0
1
7
14
35
63
350
time (days)

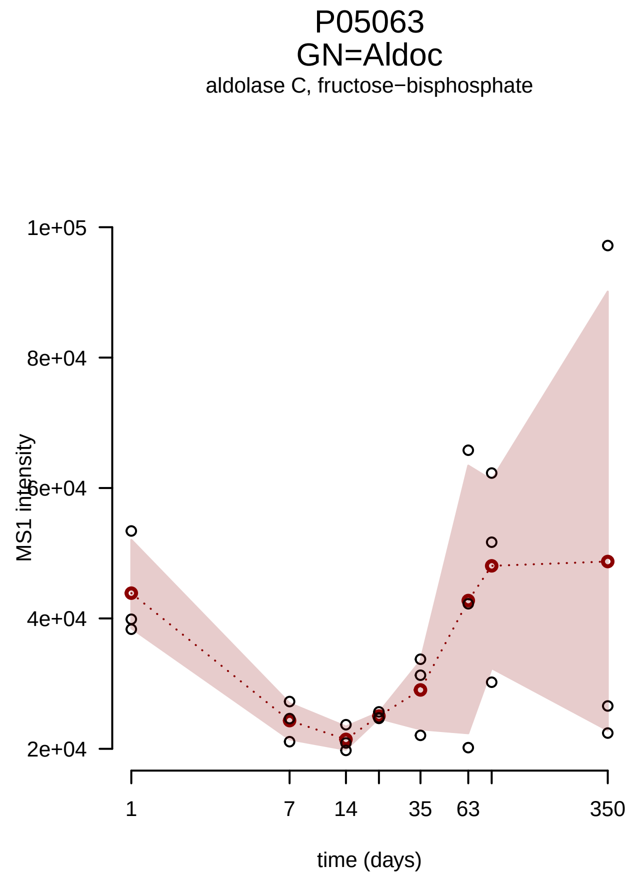
P05063
GN=Aldoc
aldolase C, fructose−bisphosphate
MS1 intensity
1e+05
8e+04
6e+04
4e+04
2e+04
1
7
14
35
63
350
time (days)

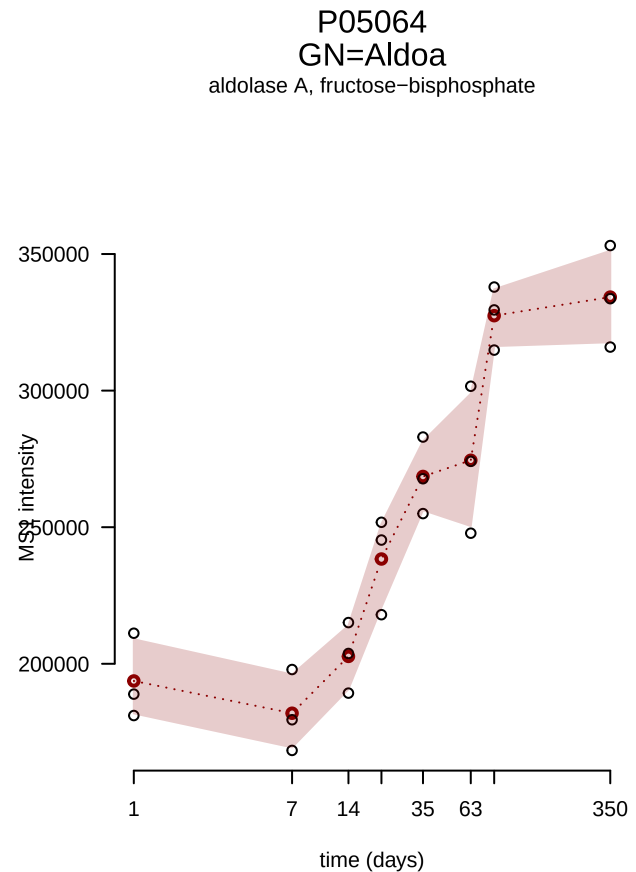
P05064
GN=Aldoa
aldolase A, fructose−bisphosphate
MS1 intensity
350000
300000
250000
200000
1
7
14
35
63
350
time (days)

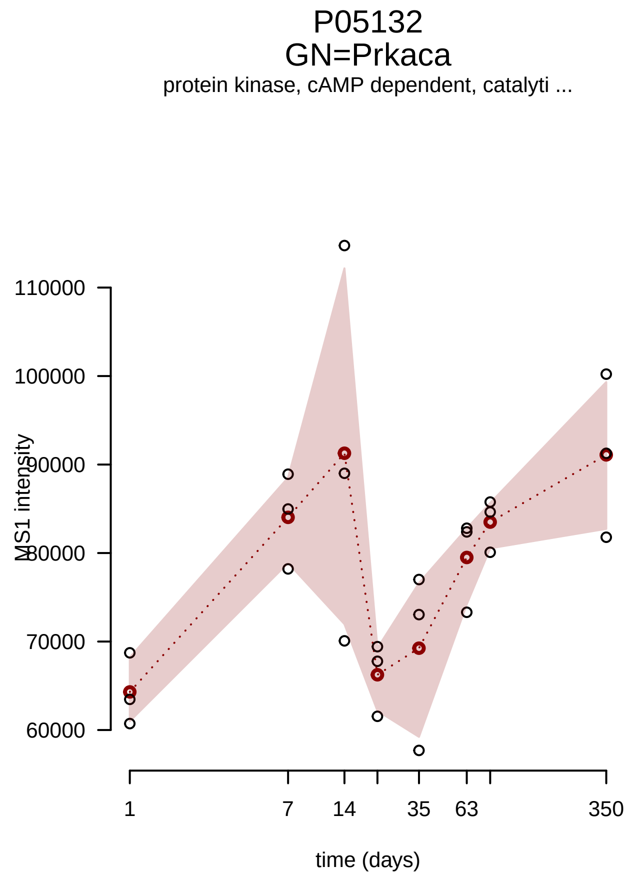
P05132
GN=Prkaca
protein kinase, cAMP dependent, catalyti ...
MS1 intensity
110000
100000
90000
80000
70000
60000
1
7
14
35
63
350
time (days)

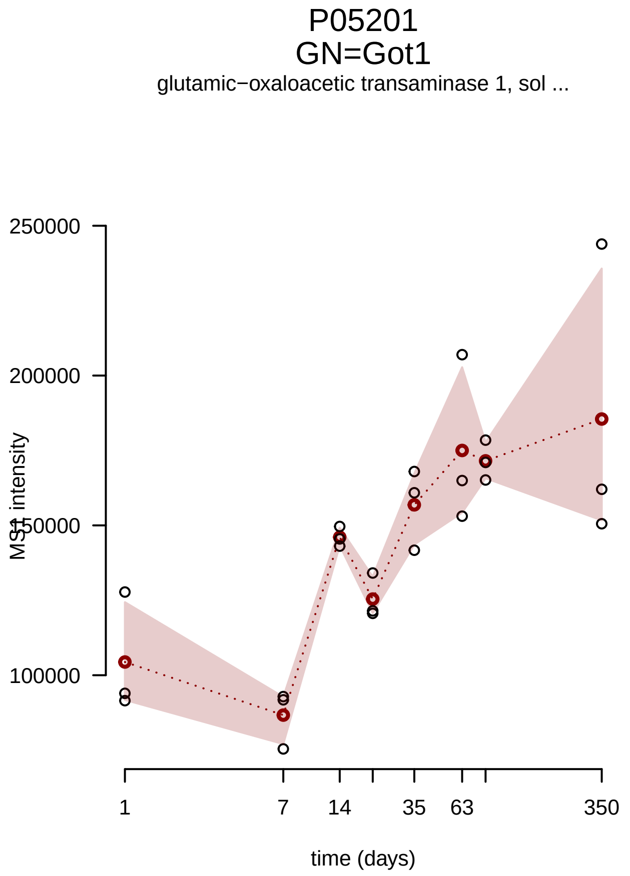
P05201
GN=Got1
glutamic−oxaloacetic transaminase 1, sol ...
MS1 intensity
250000
200000
150000
100000
1
7
14
35
63
350
time (days)

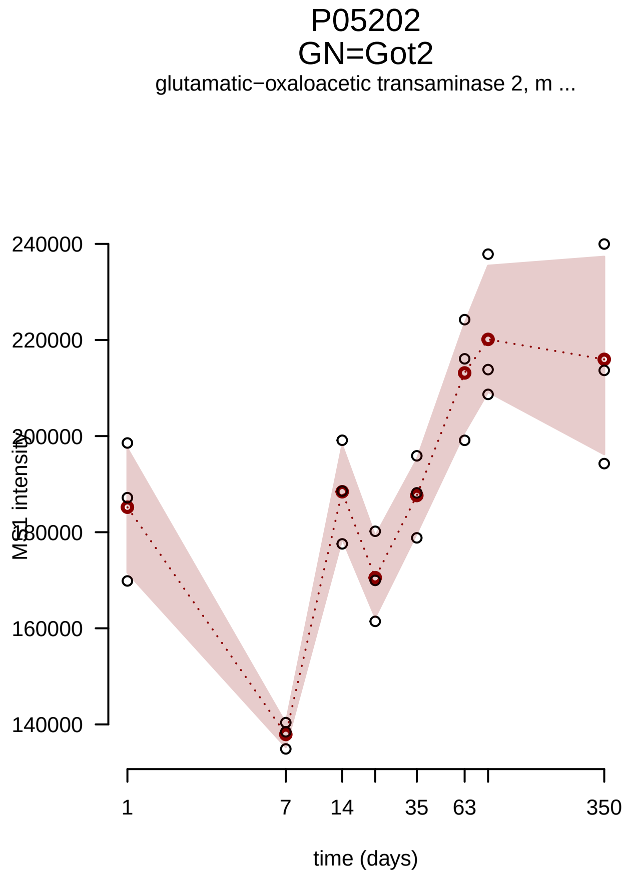
P05202
GN=Got2
glutamatic−oxaloacetic transaminase 2, m ...
MS1 intensity
240000
220000
200000
180000
160000
140000
1
7
14
35
63
350
time (days)

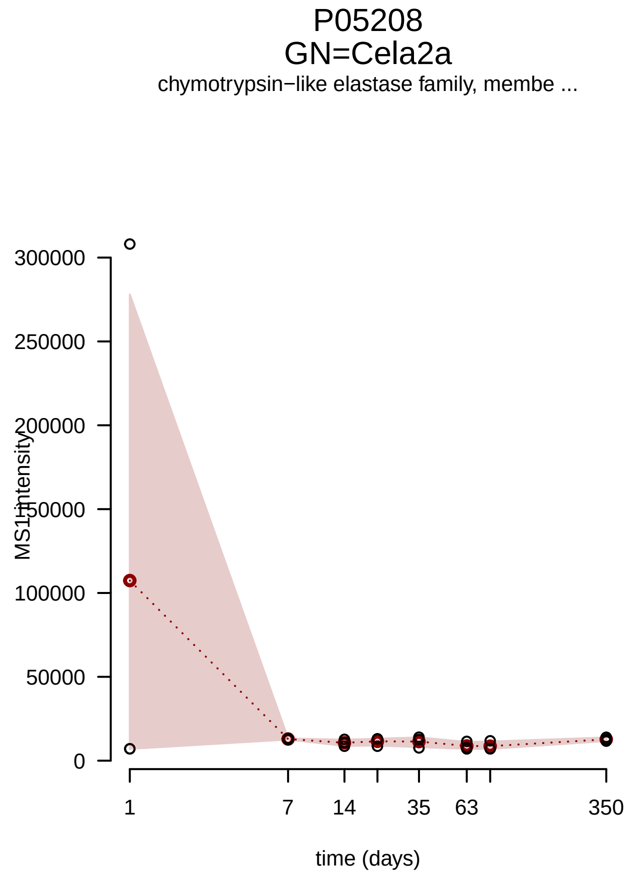
P05208
GN=Cela2a
chymotrypsin−like elastase family, membe ...
MS1 intensity
300000
250000
200000
150000
100000
50000
0
1
7
14
35
63
350
time (days)

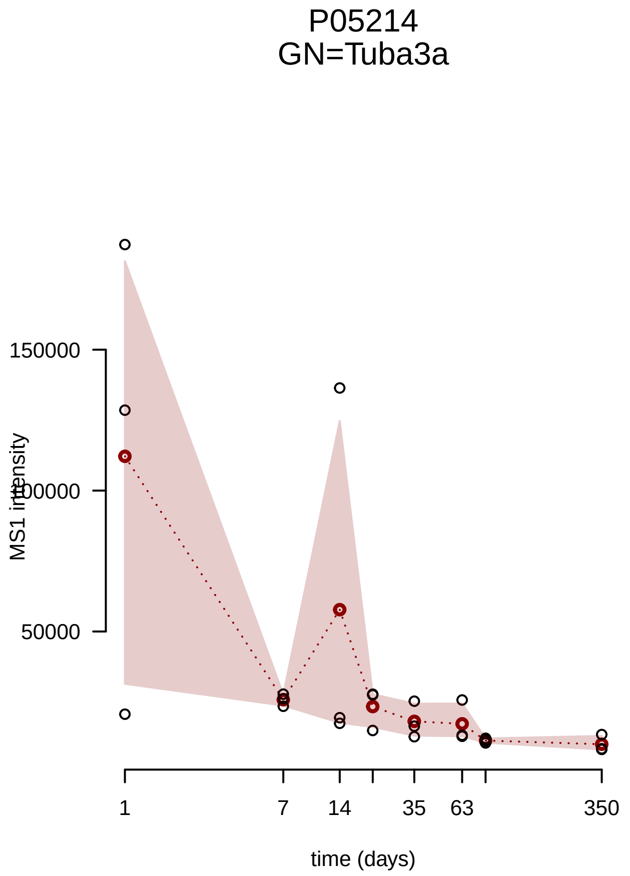
P05214
GN=Tuba3a
MS1 intensity
150000
100000
50000
1
7
14
35
63
350
time (days)

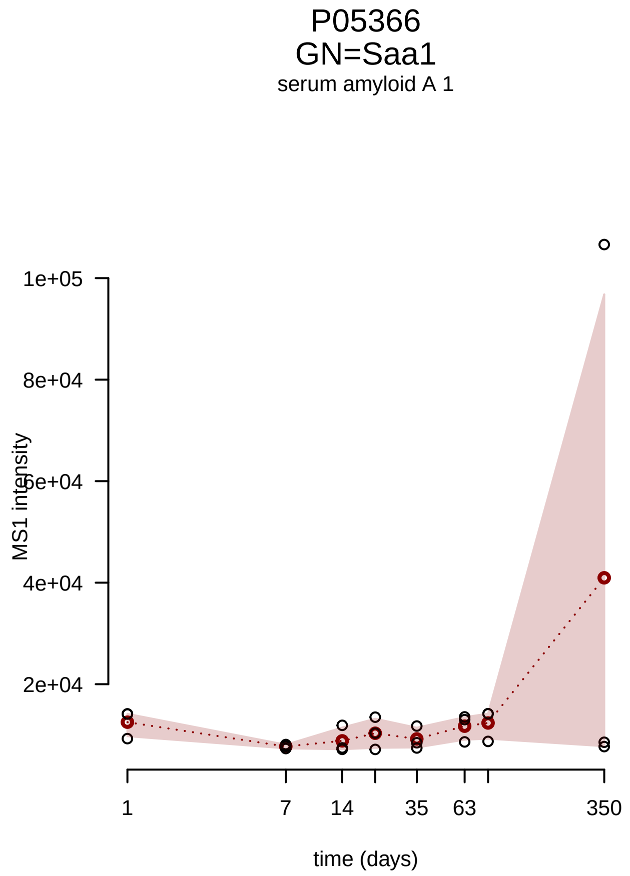
P05366
GN=Saa1
serum amyloid A 1
MS1 intensity
1e+05
8e+04
6e+04
4e+04
2e+04
1
7
14
35
63
350
time (days)

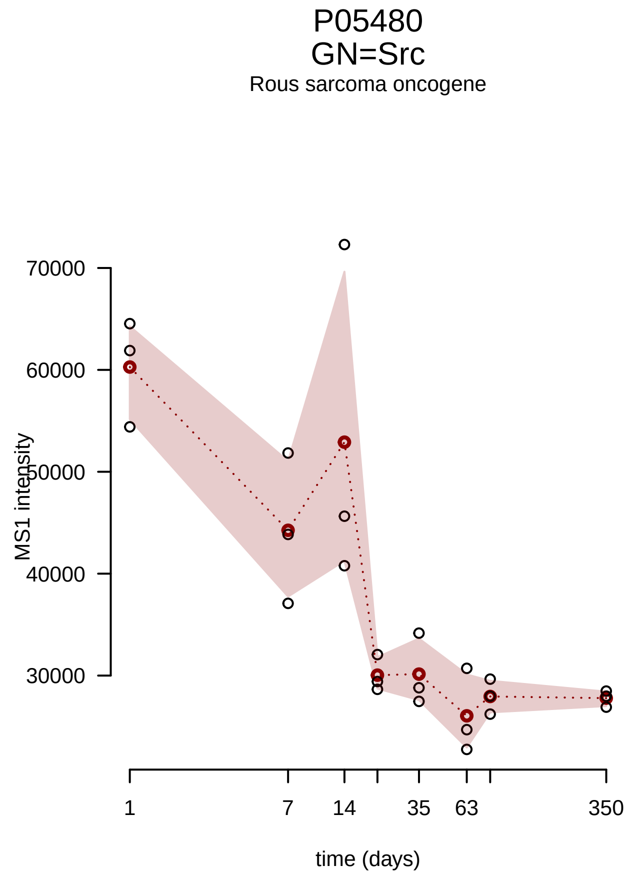
P05480
GN=Src
Rous sarcoma oncogene
MS1 intensity
70000
60000
50000
40000
30000
1
7
14
35
63
350
time (days)

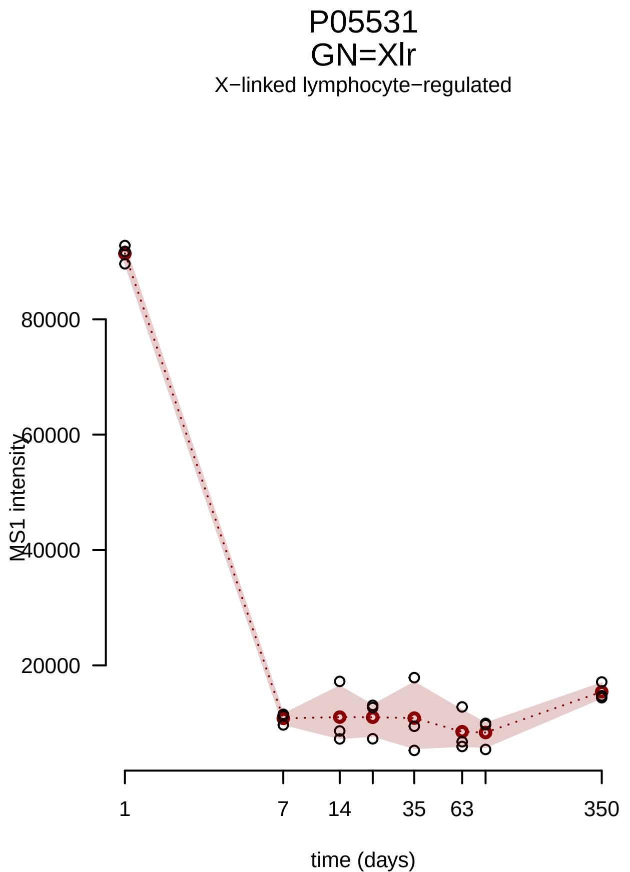
P05531
GN=Xlr
X−linked lymphocyte−regulated
MS1 intensity
80000
60000
40000
20000
1
7
14
35
63
350
time (days)

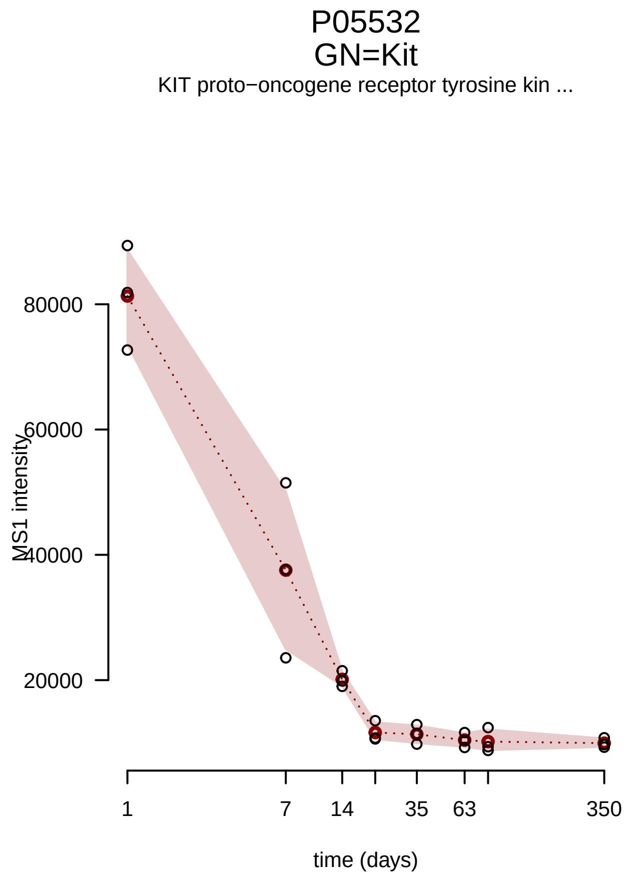
P05532
GN=Kit
KIT proto−oncogene receptor tyrosine kin ...
MS1 intensity
80000
60000
40000
20000
1
7
14
35
63
350
time (days)

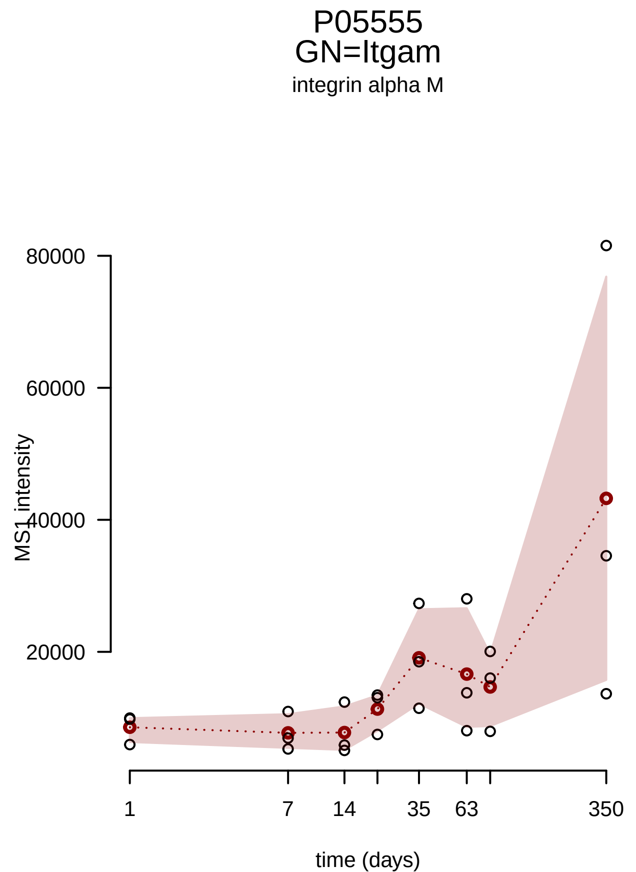
P05555
GN=Itgam
integrin alpha M
MS1 intensity
80000
60000
40000
20000
1
7
14
35
63
350
time (days)

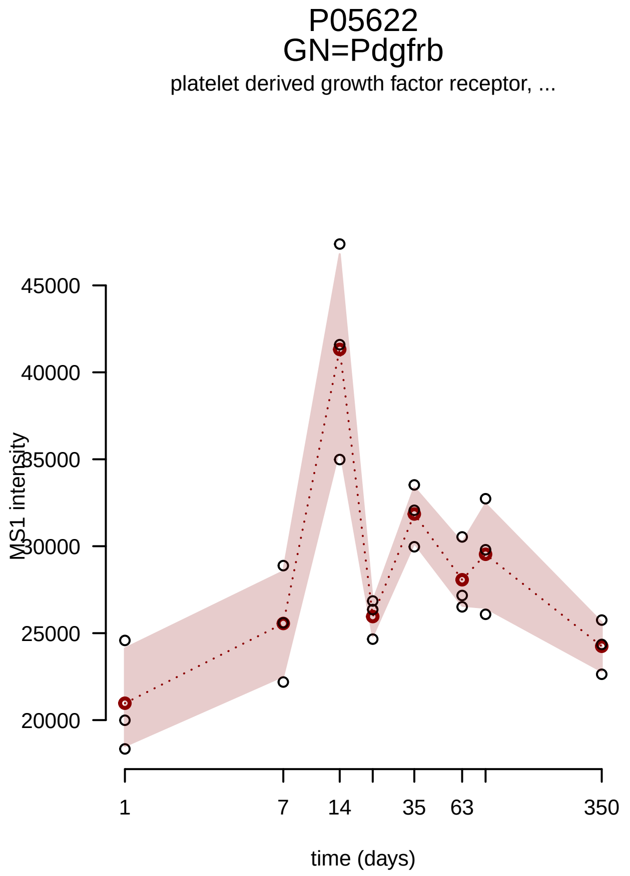
P05622
GN=Pdgfrb
platelet derived growth factor receptor, ...
45000
40000
35000
30000
25000
20000
MS1 intensity
1
7
14
35
63
350
time (days)

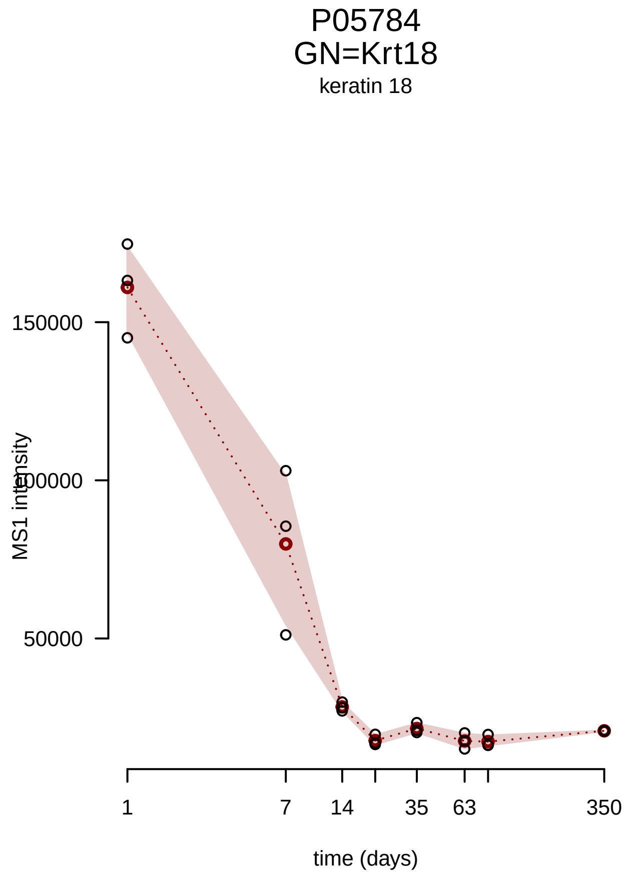
P05784
GN=Krt18
keratin 18
150000
100000
50000
MS1 intensity
1
7
14
35
63
350
time (days)

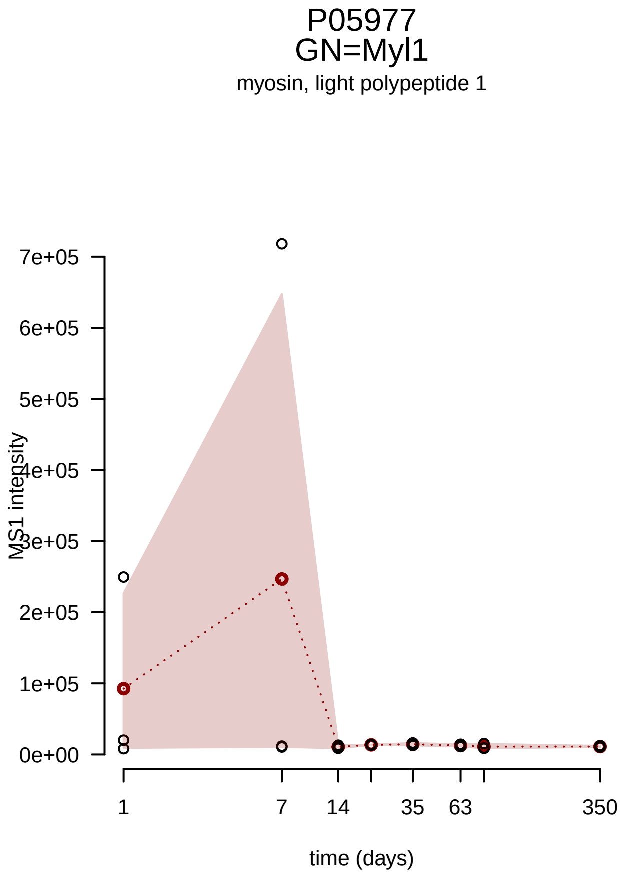
P05977
GN=Myl1
myosin, light polypeptide 1
7e+05
6e+05
5e+05
4e+05
3e+05
2e+05
1e+05
0e+00
MS1 intensity
1
7
14
35
63
350
time (days)

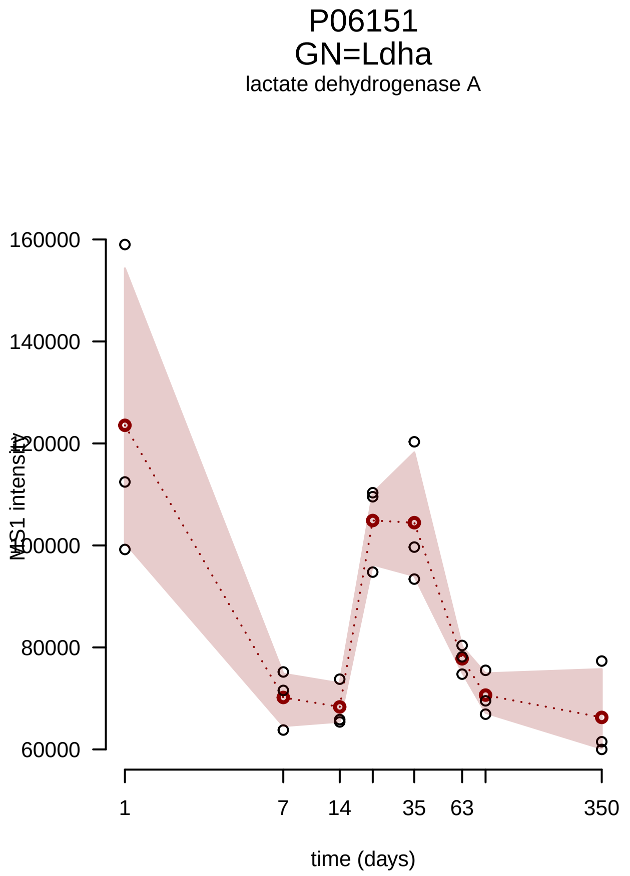
P06151
GN=Ldha
lactate dehydrogenase A
160000
140000
120000
100000
80000
60000
MS1 intensity
1
7
14
35
63
350
time (days)

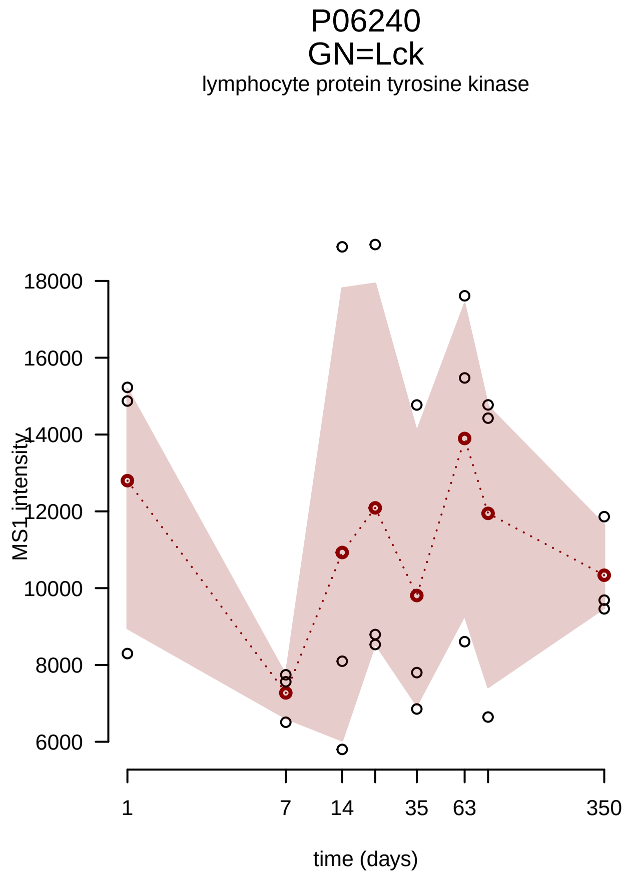
P06240
GN=Lck
lymphocyte protein tyrosine kinase
18000
16000
14000
12000
10000
8000
6000
MS1 intensity
1
7
14
35
63
350
time (days)

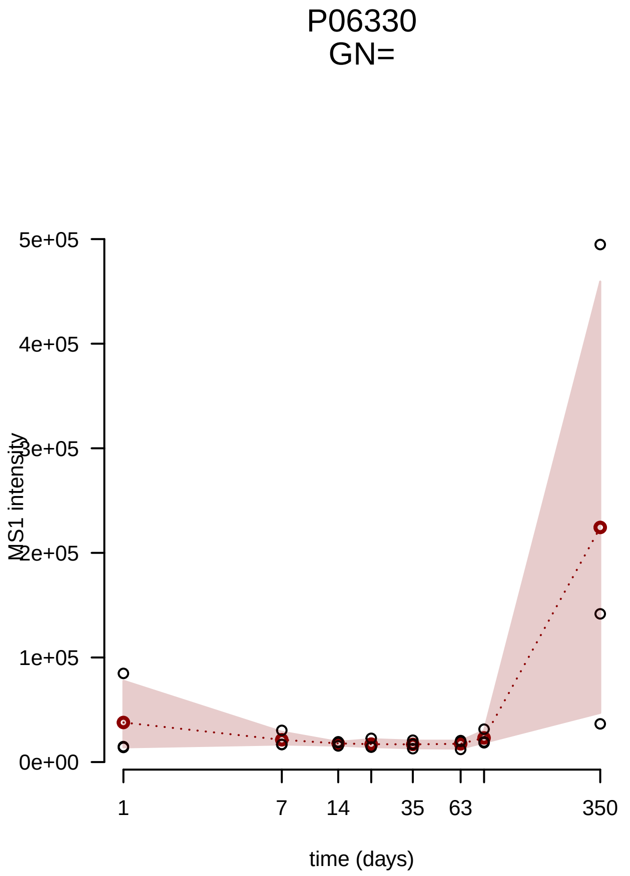
P06330
GN=
5e+05
4e+05
3e+05
2e+05
1e+05
0e+00
MS1 intensity
1
7
14
35
63
350
time (days)

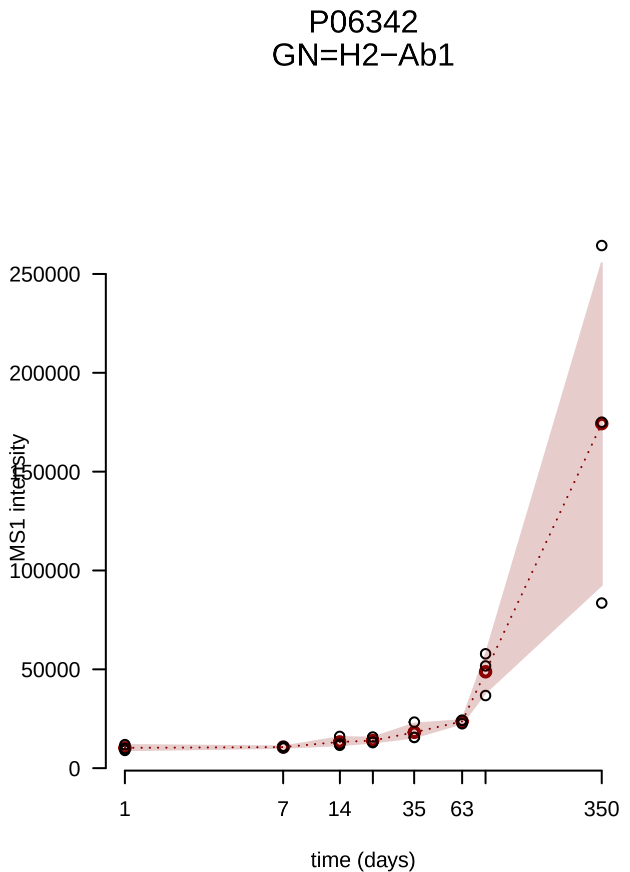
P06342
GN=H2−Ab1
250000
200000
150000
100000
50000
0
MS1 intensity
1
7
14
35
63
350
time (days)

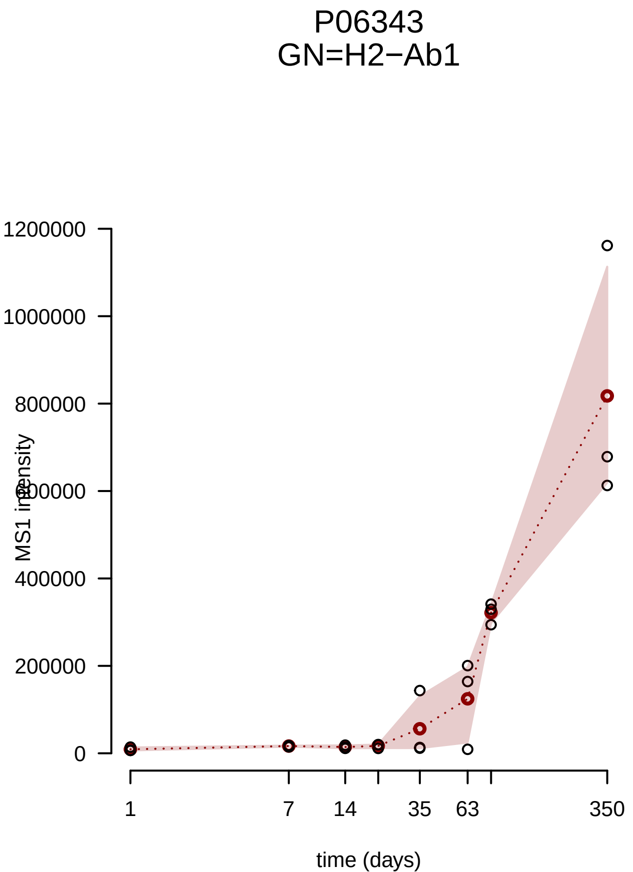
P06343
GN=H2−Ab1
1200000
1000000
800000
600000
400000
200000
0
MS1 intensity
1
7
14
35
63
350
time (days)

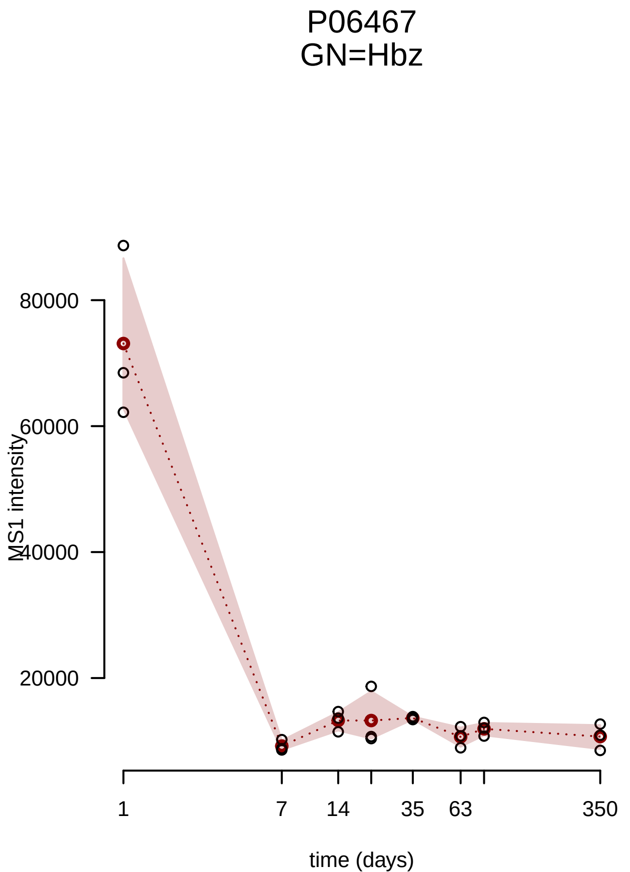
P06467
GN=Hbz
80000
60000
40000
20000
MS1 intensity
1
7
14
35
63
350
time (days)

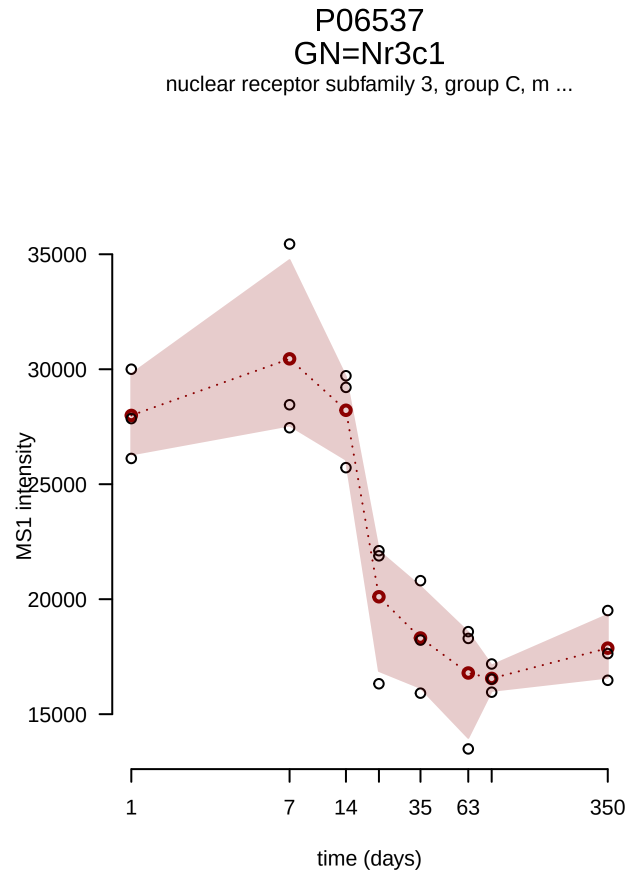
P06537
GN=Nr3c1
nuclear receptor subfamily 3, group C, m ...
MS1 intensity
time (days)
35000
30000
25000
20000
15000
1
7
14
35
63
350

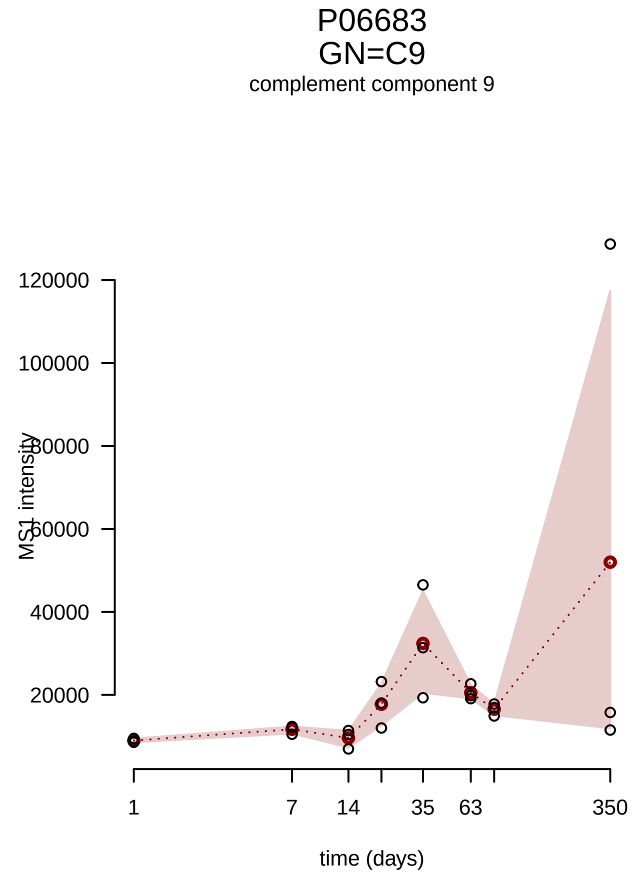
P06683
GN=C9
complement component 9
MS1 intensity
time (days)
120000
100000
20000
1
7
14
35
63
350

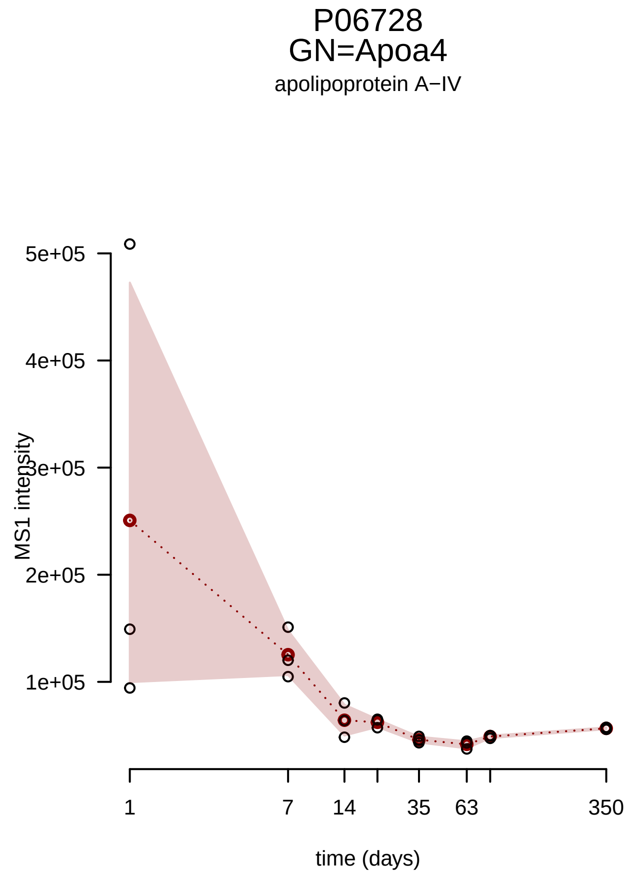
P06728
GN=Apoa4
apolipoprotein A−IV
MS1 intensity
time (days)
5e+05
4e+05
2e+05
1e+05
1
7
14
35
63
350

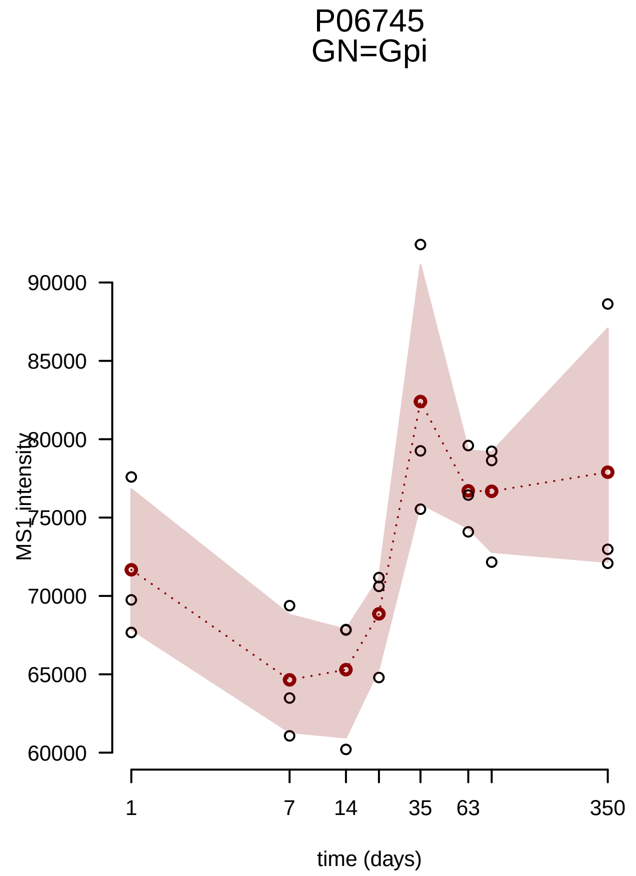
P06745
GN=Gpi
MS1 intensity
time (days)
90000
85000
75000
70000
65000
60000
1
7
14
35
63
350

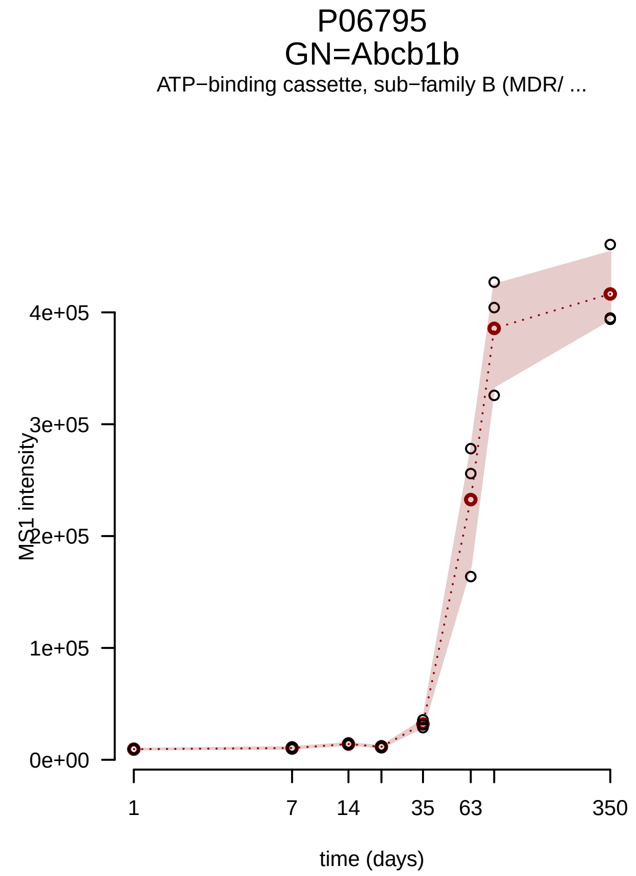
P06795
GN=Abcb1b
ATP−binding cassette, sub−family B (MDR/ ...
MS1 intensity
time (days)
4e+05
3e+05
1e+05
0e+00
1
7
14
35
63
350

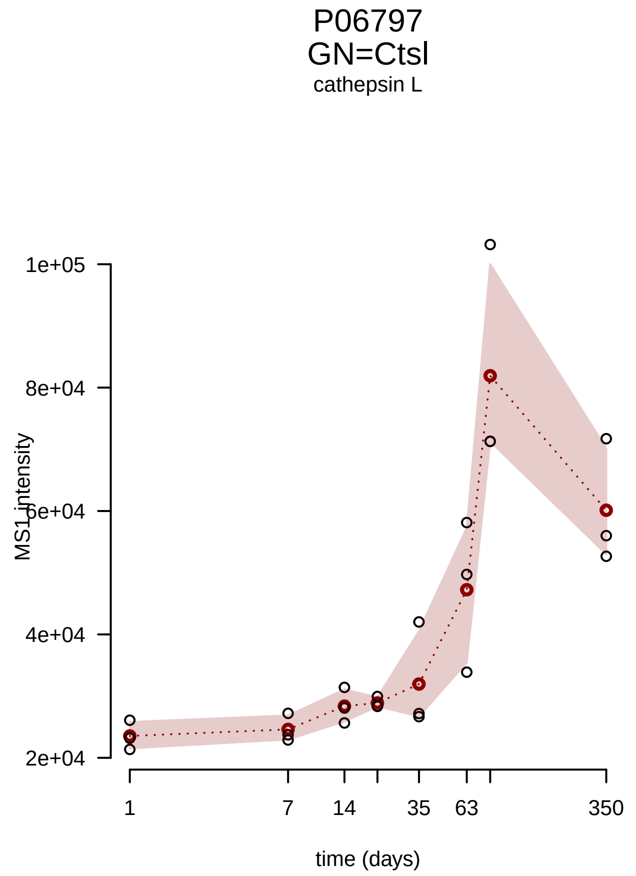
P06797
GN=Ctsl
cathepsin L
MS1 intensity
time (days)
1e+05
8e+04
4e+04
2e+04
1
7
14
35
63
350

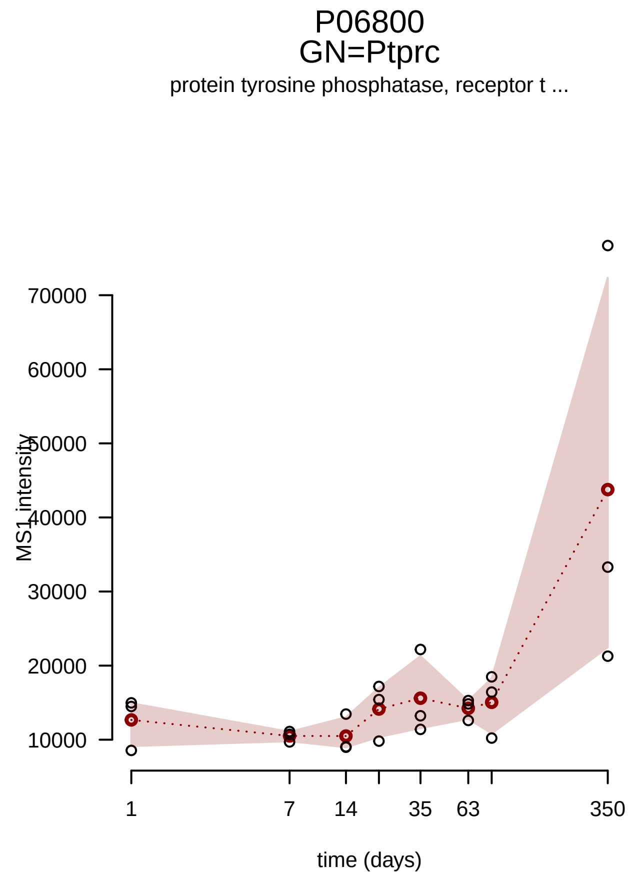
P06800
GN=Ptprc
protein tyrosine phosphatase, receptor t ...
MS1 intensity
time (days)
70000
60000
30000
20000
10000
1
7
14
35
63
350

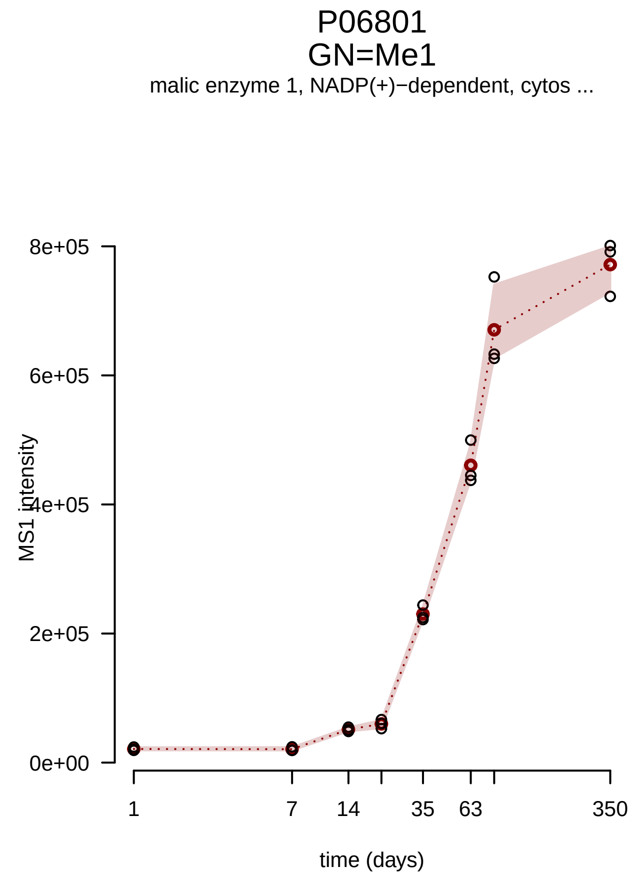
P06801
GN=Me1
malic enzyme 1, NADP(+)−dependent, cytos ...
MS1 intensity
time (days)
8e+05
6e+05
2e+05
0e+00
1
7
14
35
63
350

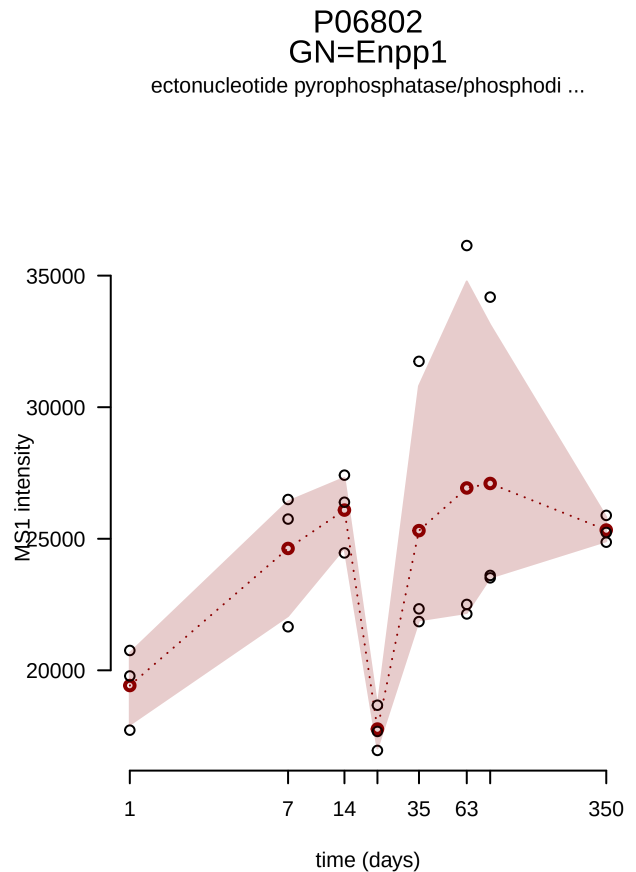
P06802
GN=Enpp1
ectonucleotide pyrophosphatase/phosphodi ...
MS1 intensity
time (days)
35000
30000
20000
1
7
14
35
63
350

P06837
GN=Gap43
growth associated protein 43

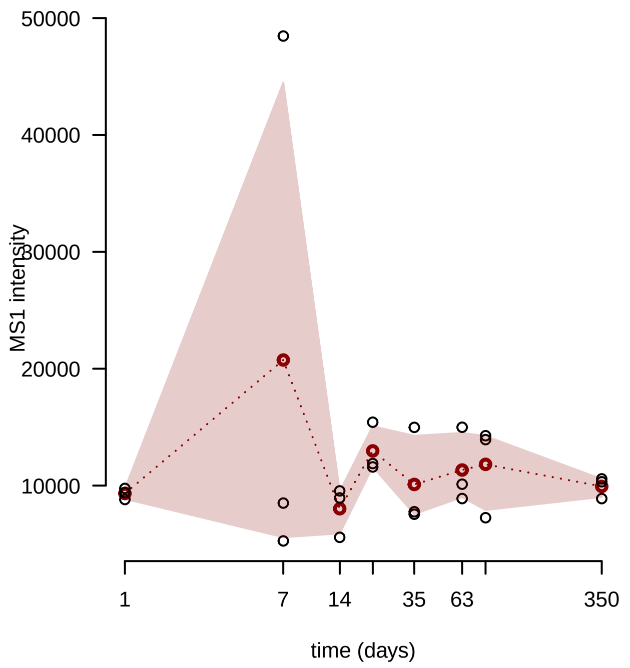

P06869
GN=Plau
plasminogen activator, urokinase

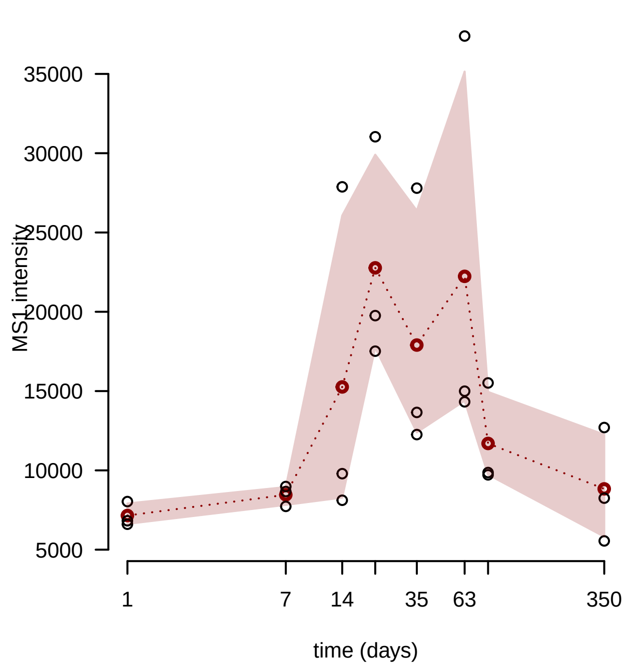

P06909
GN=Cfh
complement component factor h

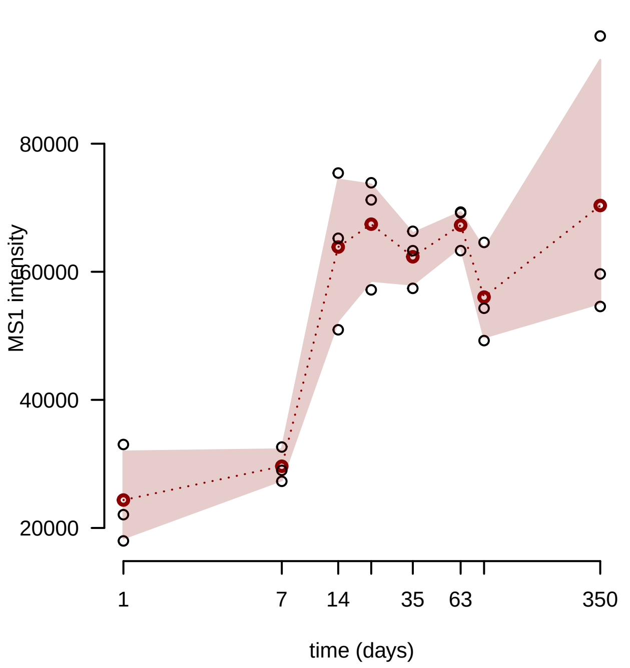

P07091
GN=S100a4
S100 calcium binding protein A4

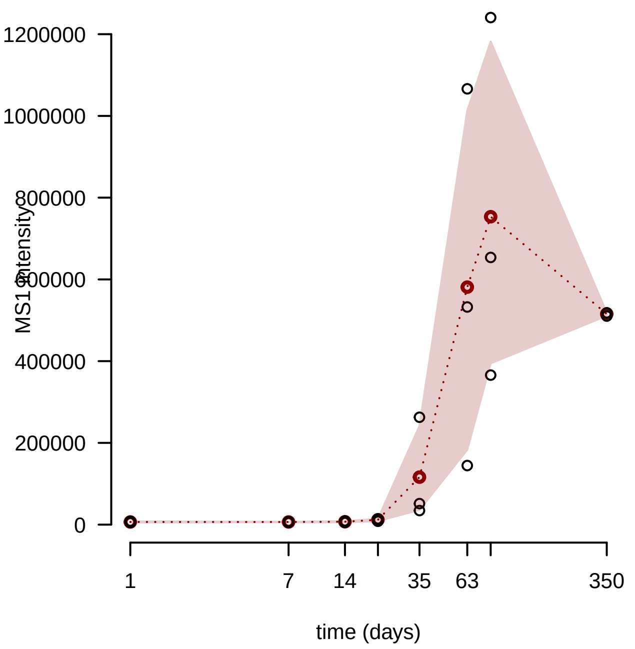

P07146
GN=Prss2
protease, serine 2

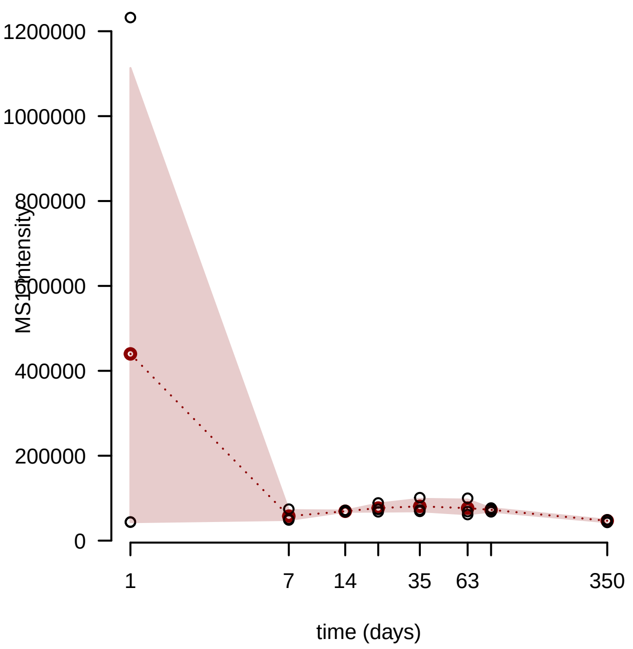

P07214
GN=Sparc
secreted acidic cysteine rich glycoprote ...

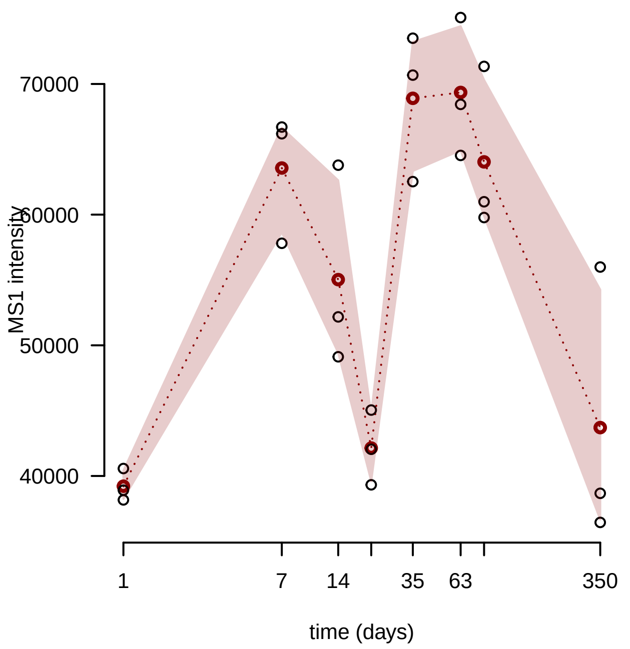

P07309
GN=Ttr
transthyretin

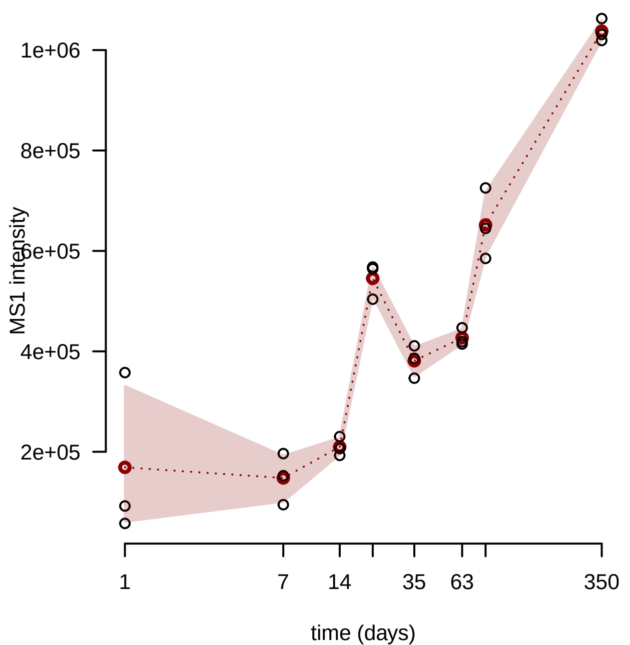

P07310
GN=Ckm
creatine kinase, muscle

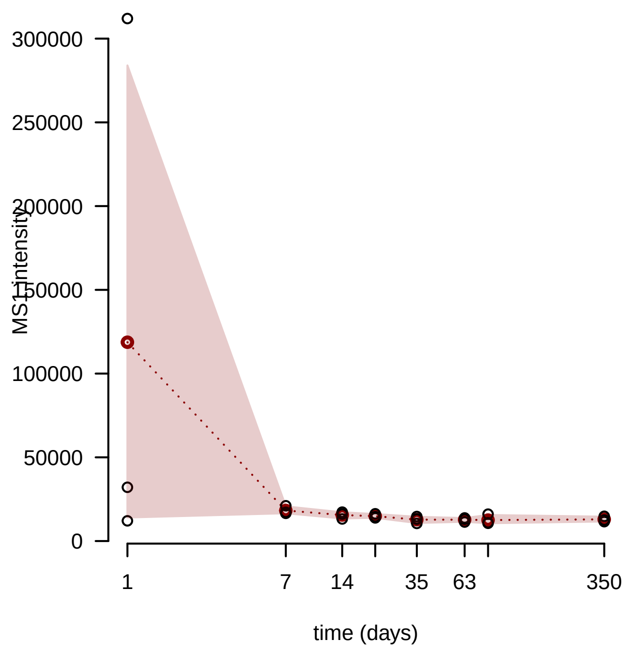

P07356
GN=Anxa2
annexin A2

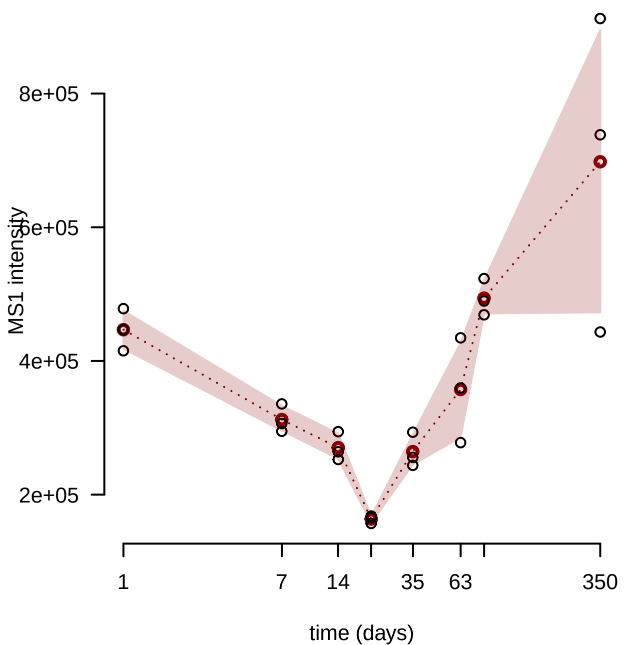

P07361
GN=Orm2
orosomucoid 2

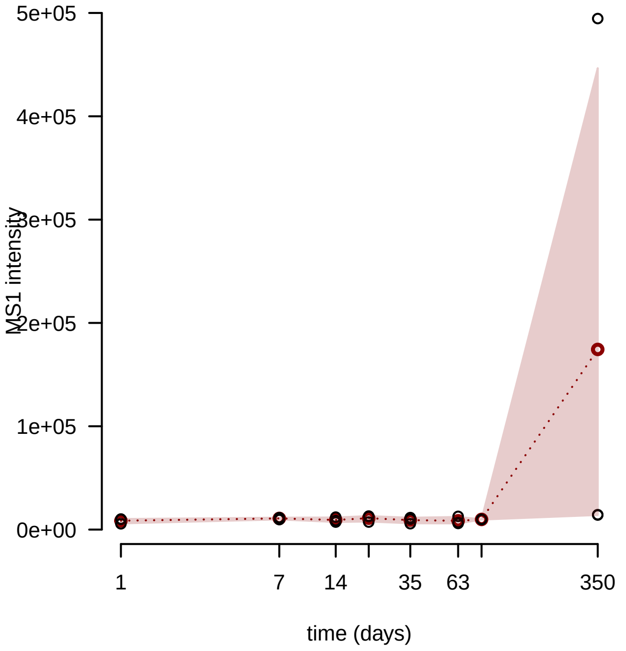

P07607
GN=Tyms
thymidylate synthase

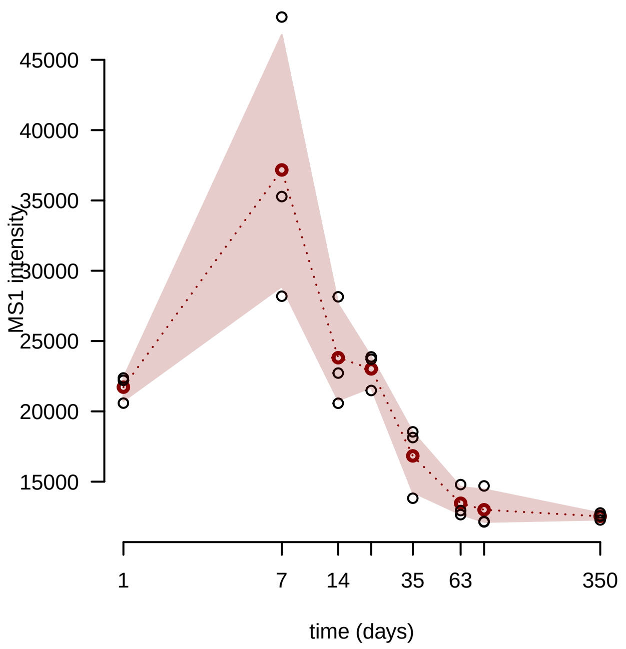

P07724
GN=Alb
albumin

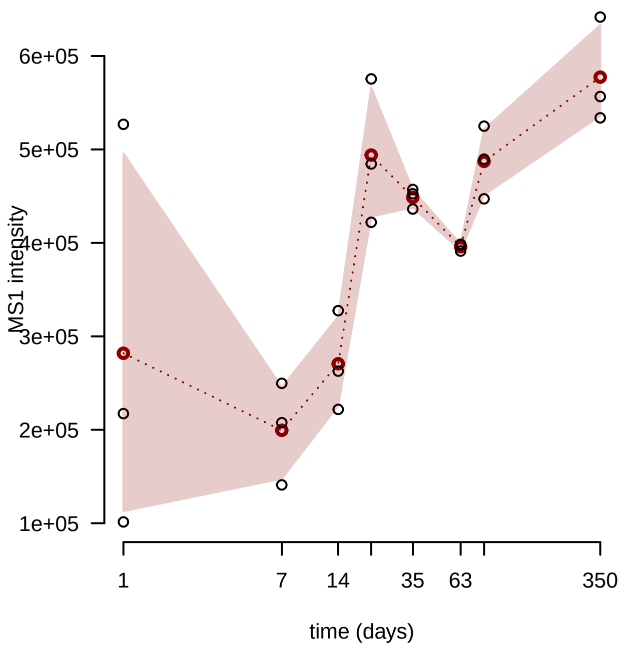

P07742
GN=Rrm1
ribonucleotide reductase M1

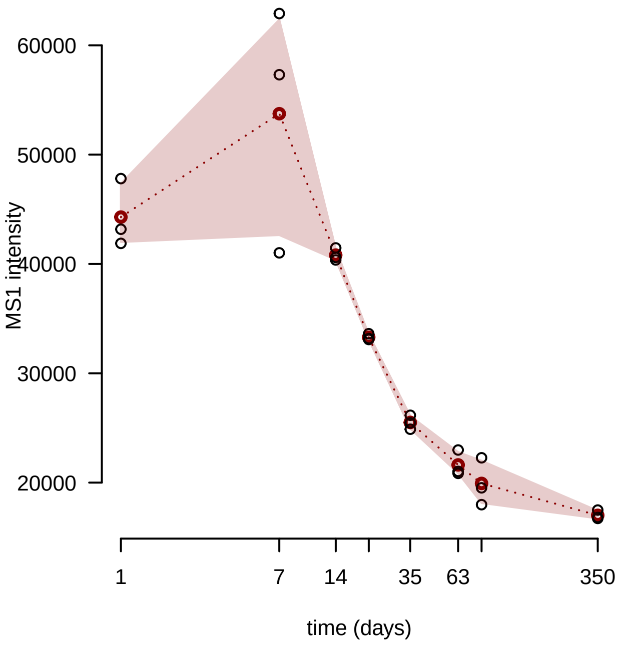

P07743
GN=Bpifa2
BPI fold containing family A, member 2

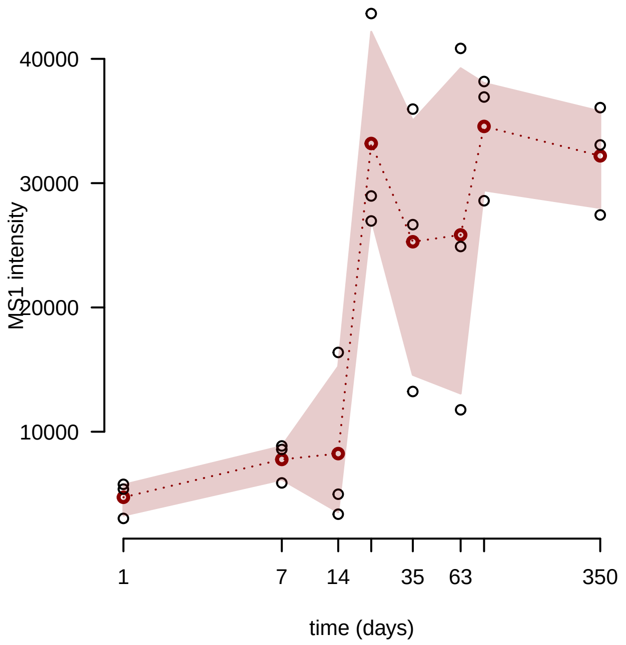

P07744
GN=Krt4
keratin 4

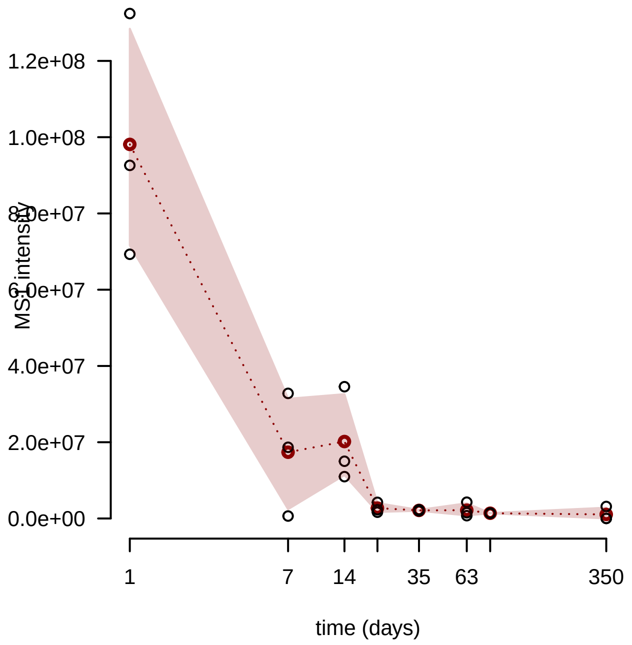

P07758
GN=Serpina1a
serine (or cysteine) peptidase inhibitor ...

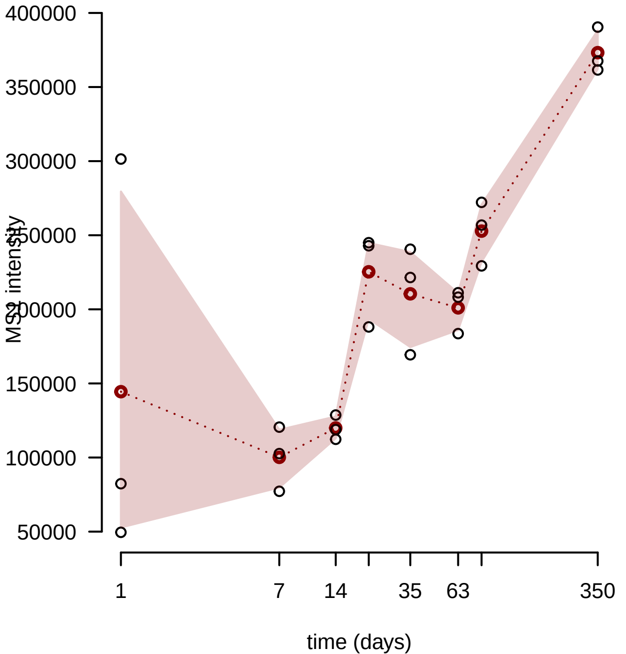

P07759
GN=Serpina3k
serine (or cysteine) peptidase inhibitor ...

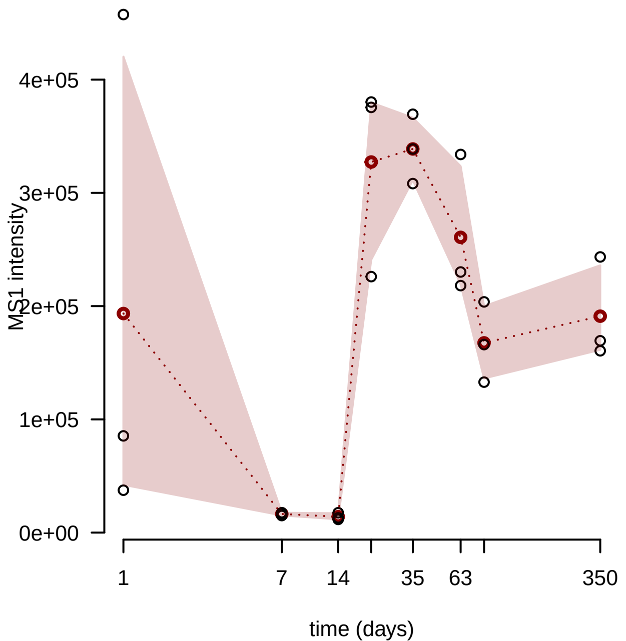

P07901
GN=Hsp90aa1
heat shock protein 90, alpha (cytosolic) ...

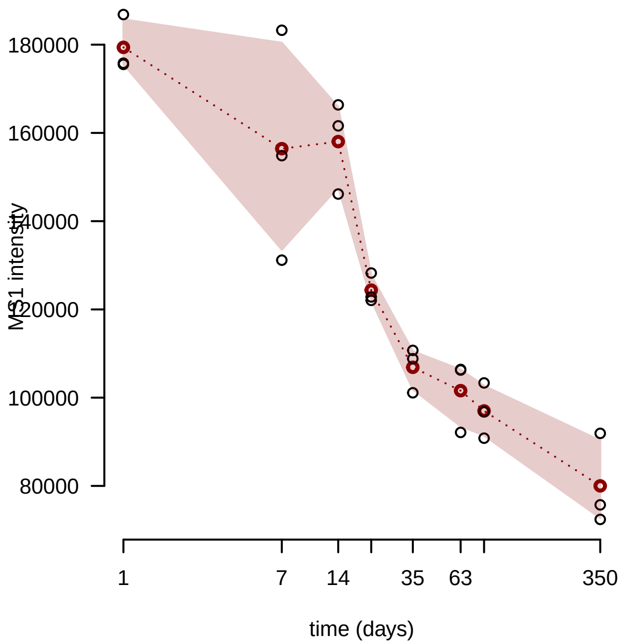

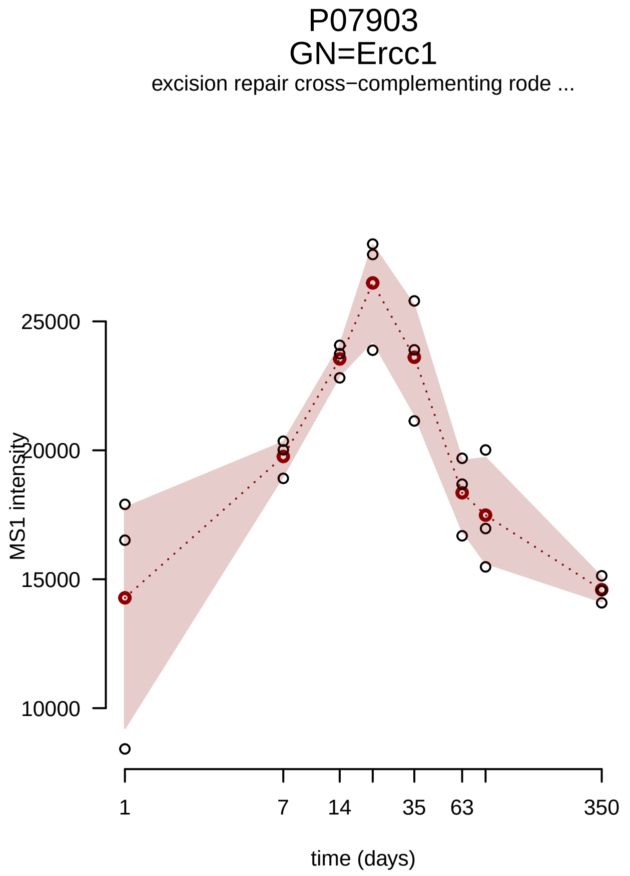
P07903
GN=Ercc1
excision repair cross−complementing rode ...
MS1 intensity
25000
20000
15000
10000
1
7
14
35
63
350
time (days)
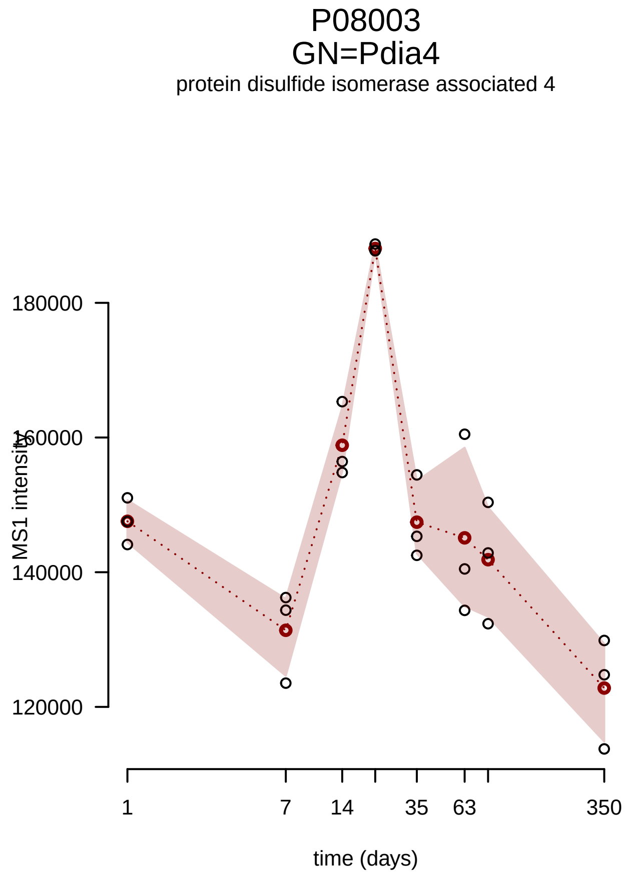
P08003
GN=Pdia4
protein disulfide isomerase associated 4
MS1 intensity
180000
160000
140000
120000
1
7
14
35
63
350
time (days)
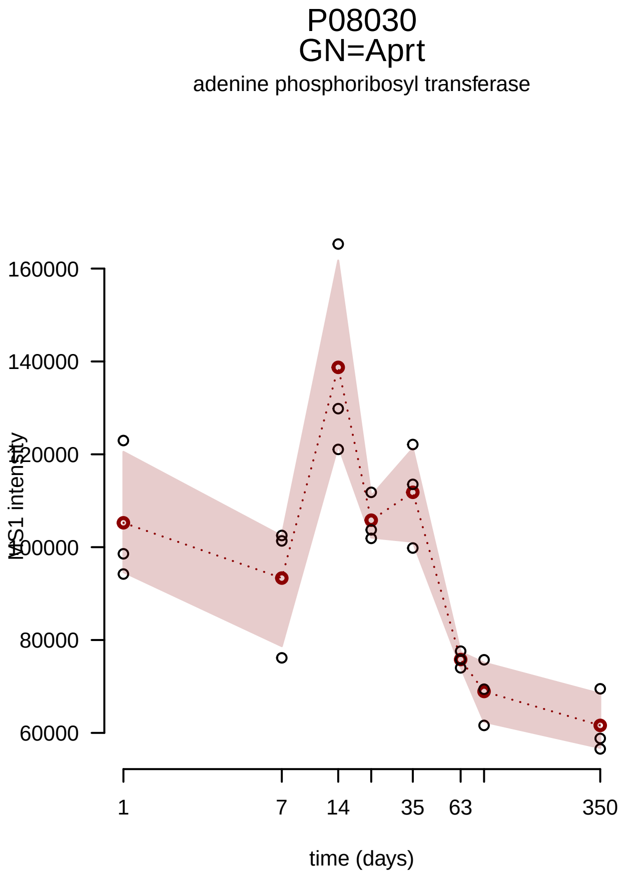
P08030
GN=Aprt
adenine phosphoribosyl transferase
MS1 intensity
160000
140000
120000
100000
80000
60000
1
7
14
35
63
350
time (days)
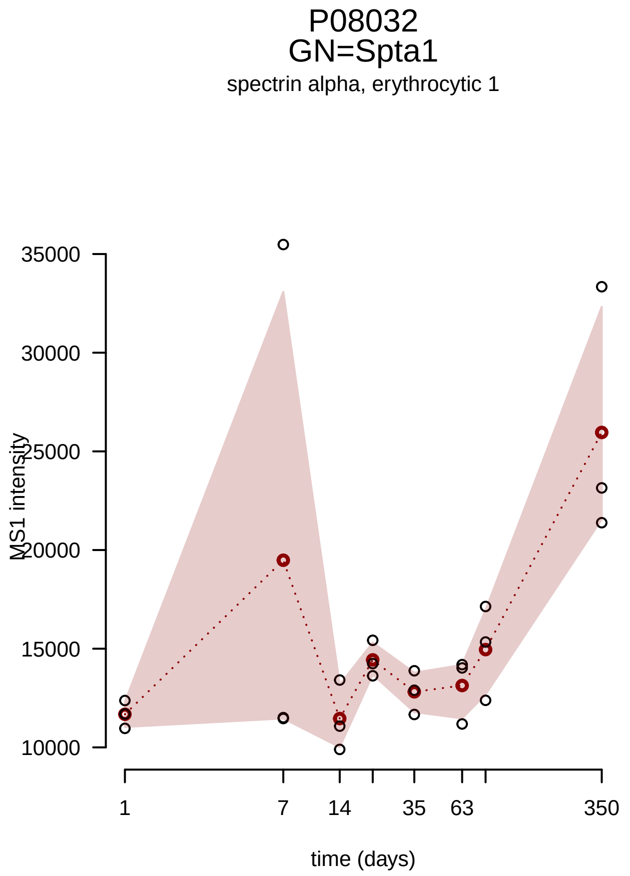
P08032
GN=Spta1
spectrin alpha, erythrocytic 1
MS1 intensity
35000
30000
25000
20000
15000
10000
1
7
14
35
63
350
time (days)
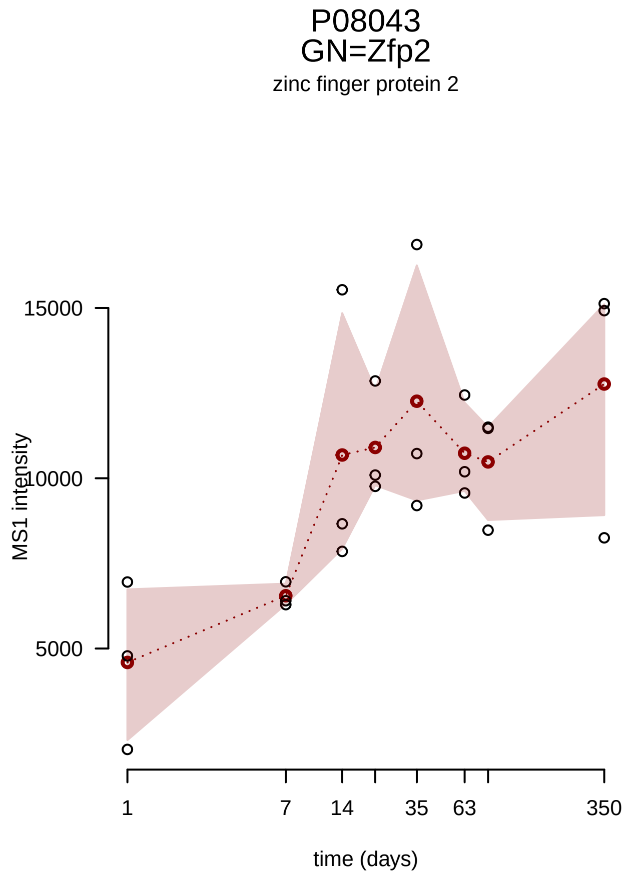
P08043
GN=Zfp2
zinc finger protein 2
MS1 intensity
15000
10000
5000
1
7
14
35
63
350
time (days)
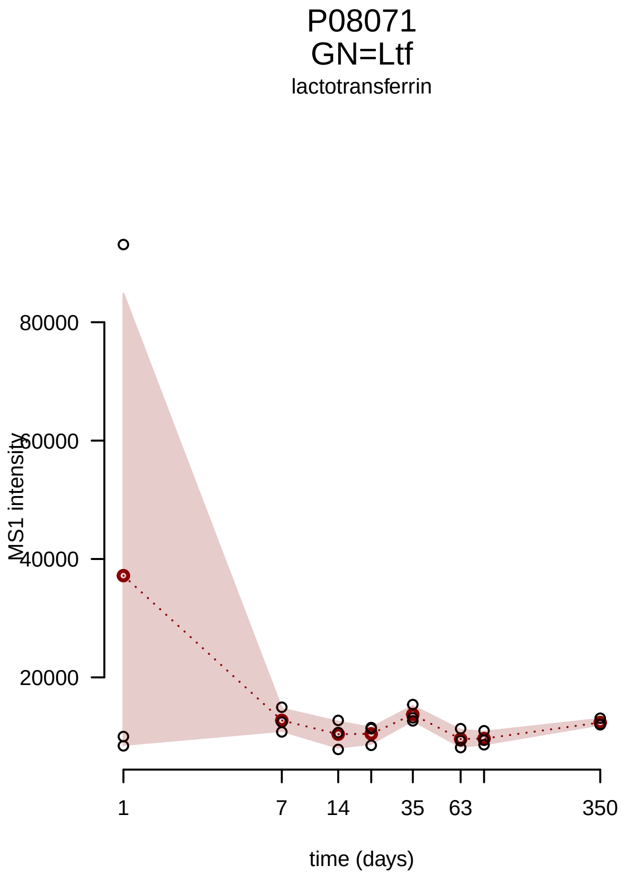
P08071
GN=Ltf
lactotransferrin
MS1 intensity
80000
60000
40000
20000
1
7
14
35
63
350
time (days)
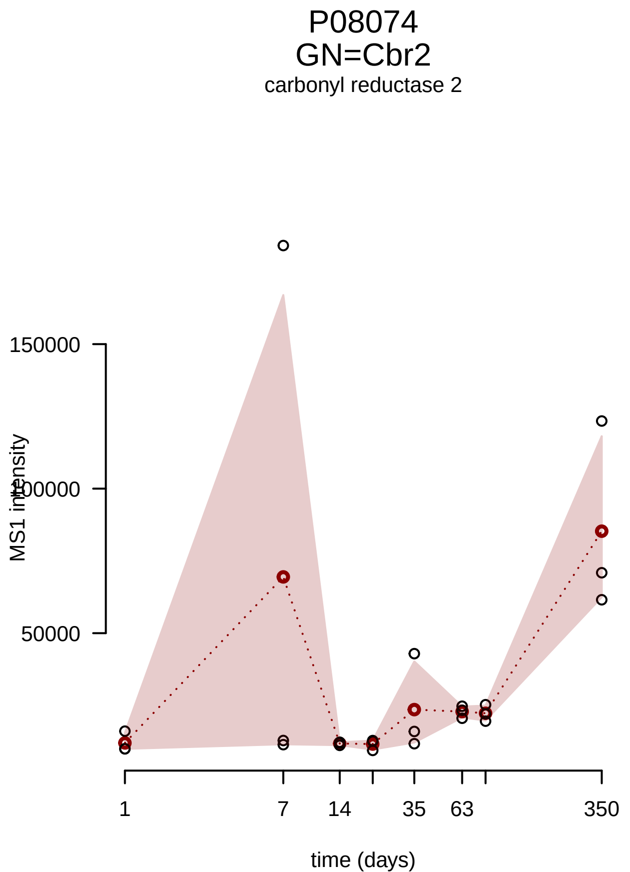
P08074
GN=Cbr2
carbonyl reductase 2
MS1 intensity
150000
100000
50000
1
7
14
35
63
350
time (days)
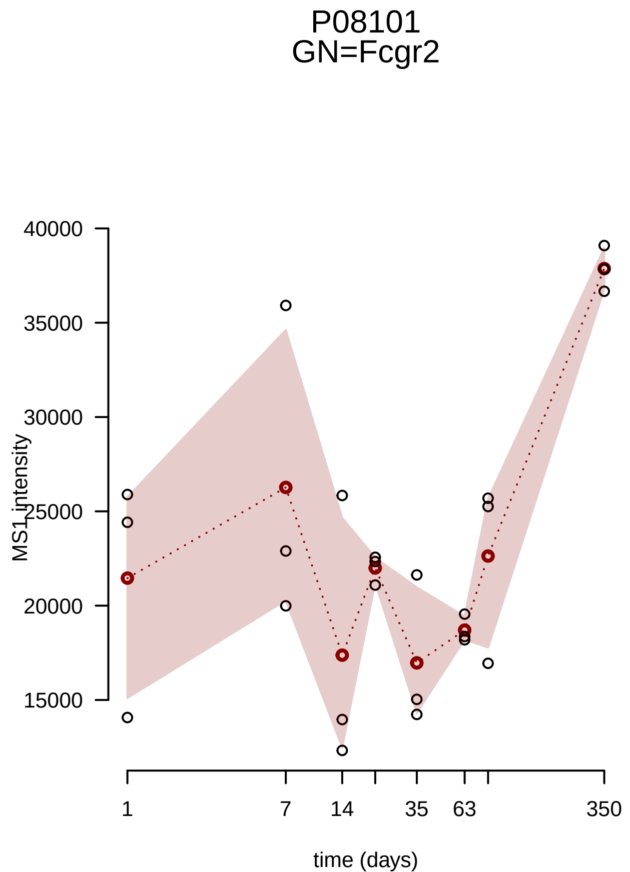
P08101
GN=Fcgr2
MS1 intensity
40000
35000
30000
25000
20000
15000
1
7
14
35
63
350
time (days)
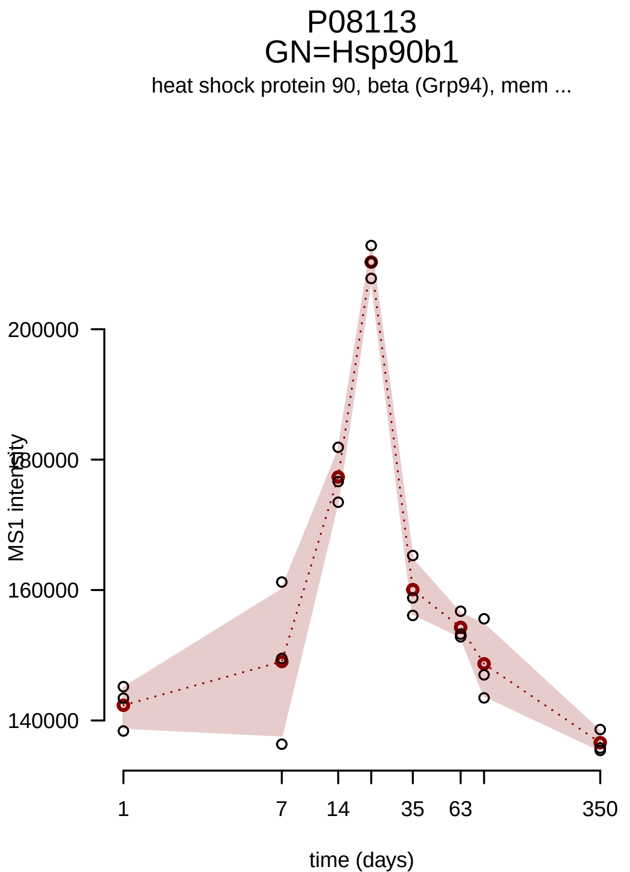
P08113
GN=Hsp90b1
heat shock protein 90, beta (Grp94), mem ...
MS1 intensity
200000
180000
160000
140000
1
7
14
35
63
350
time (days)

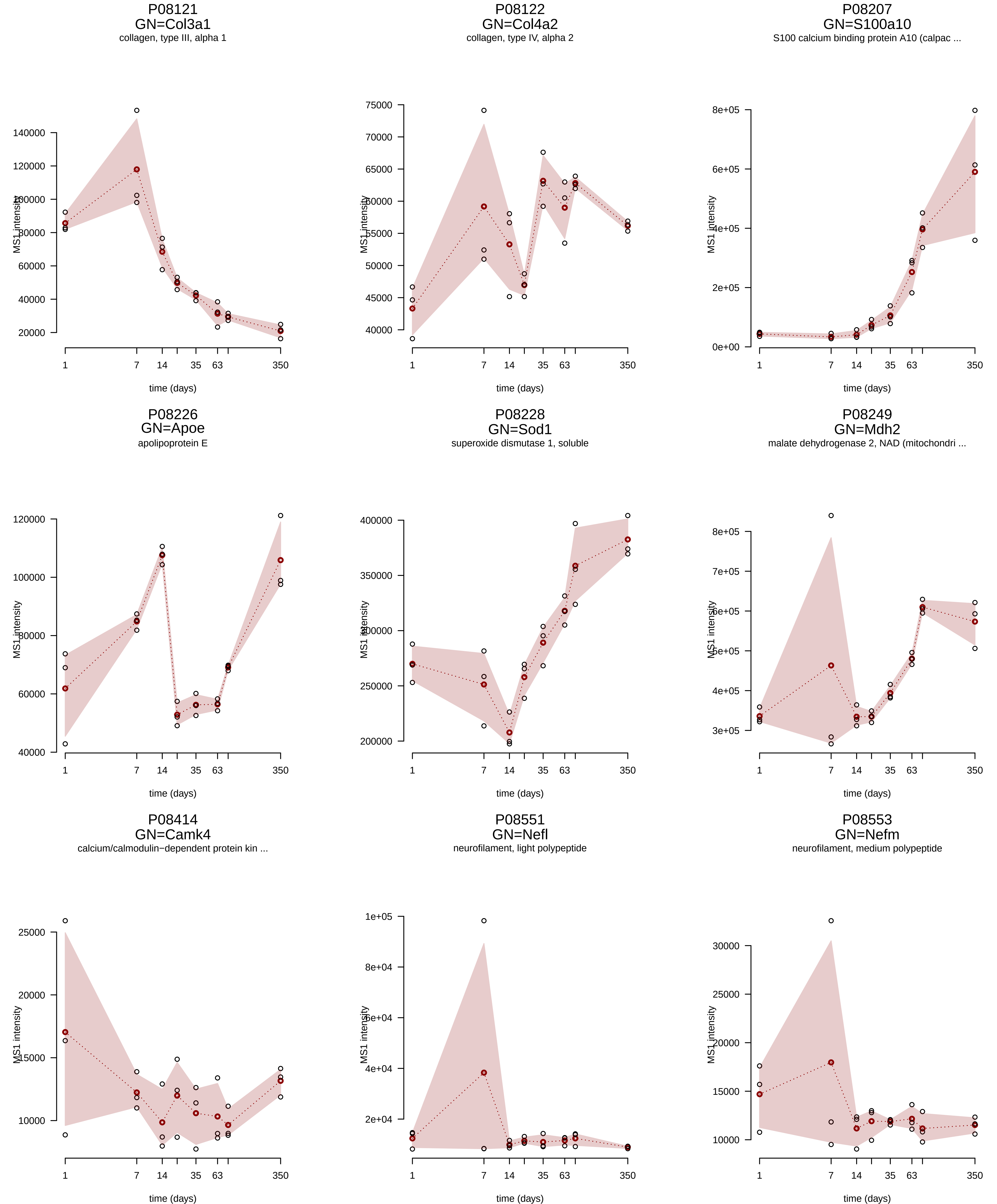
P08121
GN=Col3a1
collagen, type III, alpha 1
P08122
GN=Col4a2
collagen, type IV, alpha 2
P08207
GN=S100a10
S100 calcium binding protein A10 (calpac ...
P08226
GN=Apoe
apolipoprotein E
P08228
GN=Sod1
superoxide dismutase 1, soluble
P08249
GN=Mdh2
malate dehydrogenase 2, NAD (mitochondri ...
P08414
GN=Camk4
calcium/calmodulin−dependent protein kin ...
P08551
GN=Nefl
neurofilament, light polypeptide
P08553
GN=Nefm
neurofilament, medium polypeptide
MS1 intensity
time (days)
1
7
14
35
63
350

P08556
GN=Nras
neuroblastoma ras oncogene

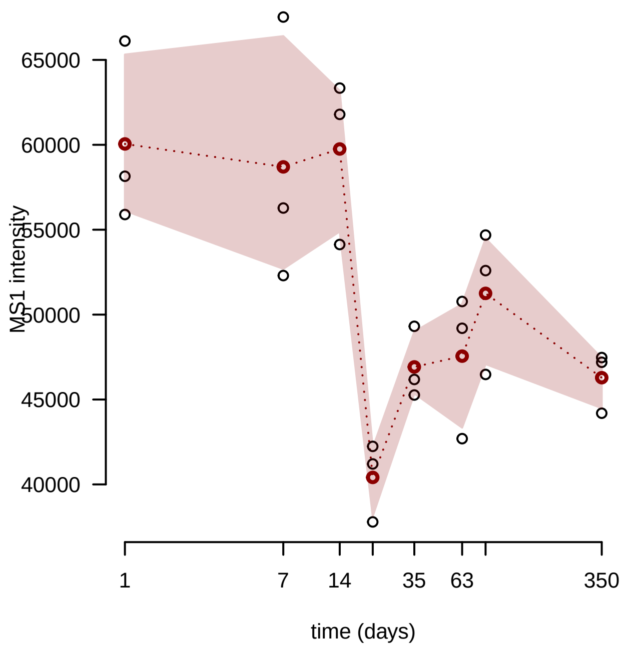

P08607
GN=C4bpa

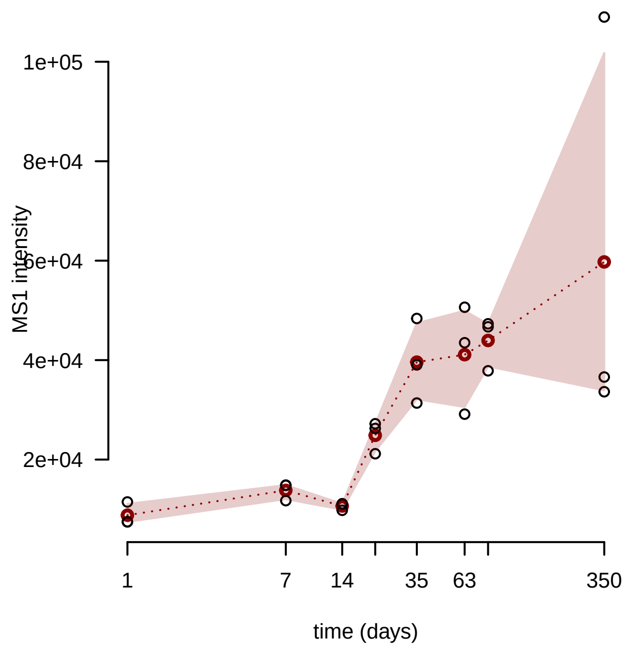

P08730
GN=Krt13
keratin 13

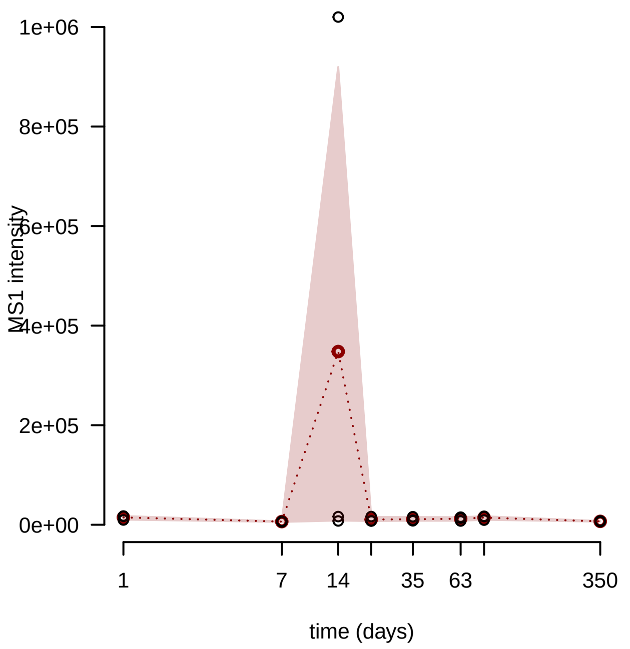

P08752
GN=Gnai2
guanine nucleotide binding protein (G pr ...

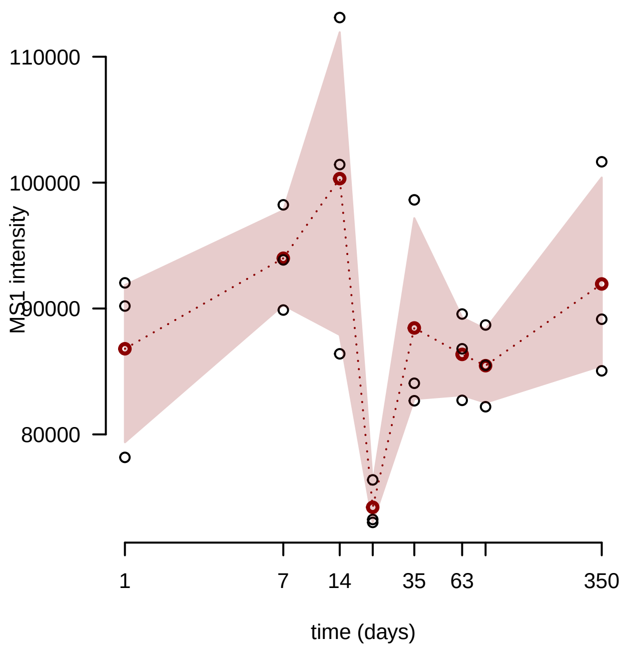

P08775
GN=Polr2a
polymerase (RNA) II (DNA directed) polyp ...

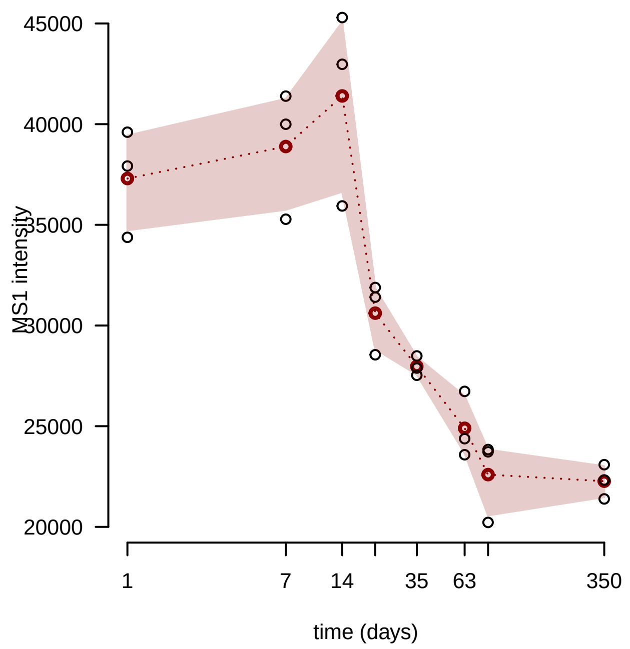

P08905
GN=Lyz2
lysozyme 2

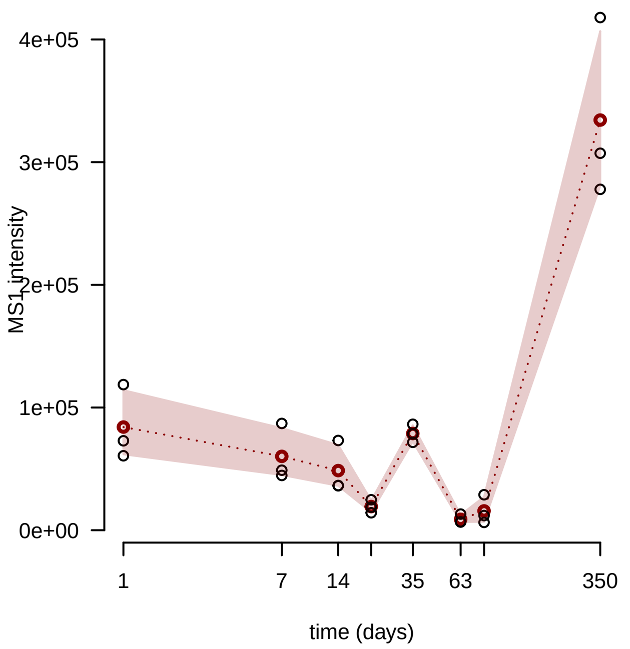

P09025
GN=Hoxc8
homeobox C8

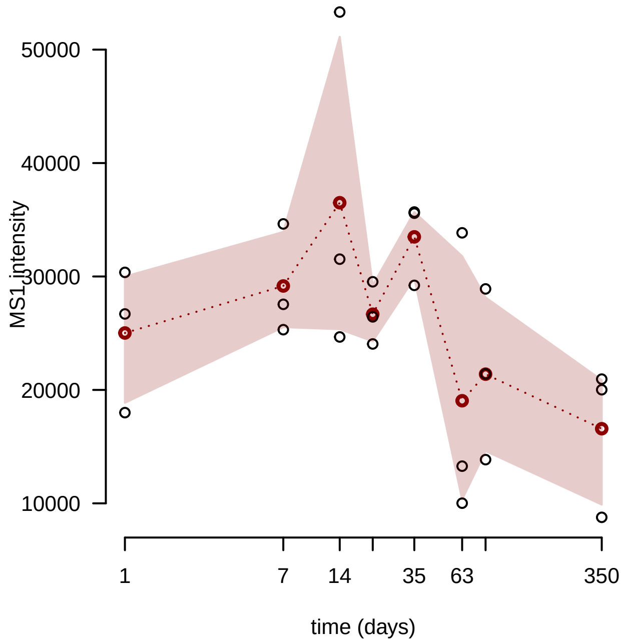

P09041
GN=Pgk2
phosphoglycerate kinase 2

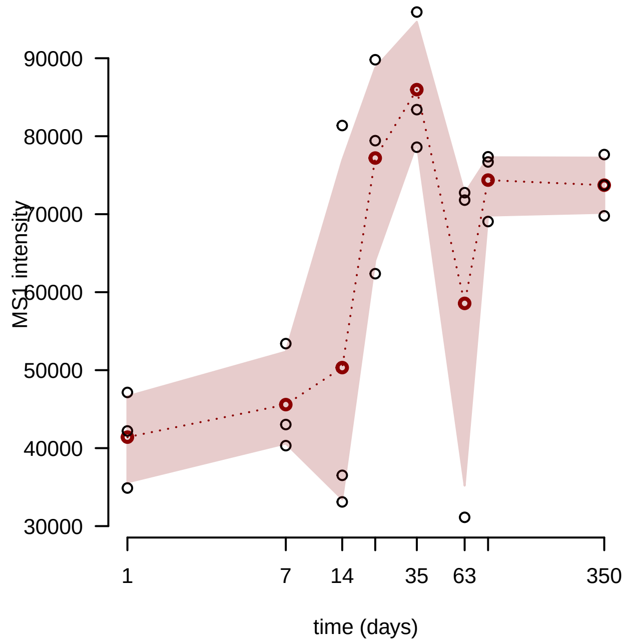

P09055
GN=Itgb1
integrin beta 1 (fibronectin receptor be ...

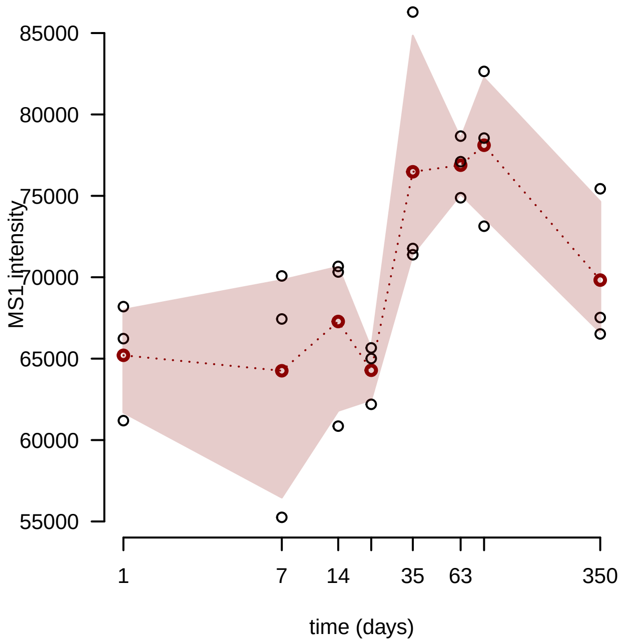

P09103
GN=P4hb
prolyl 4−hydroxylase, beta polypeptide

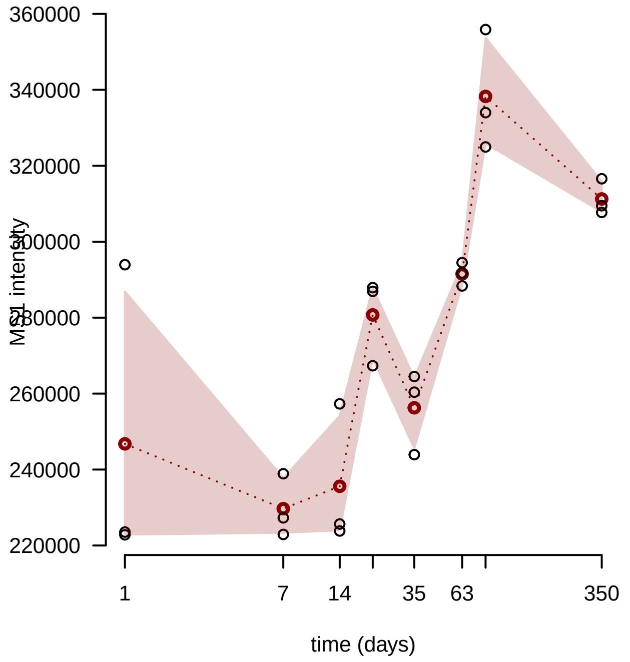

P09242
GN=Alpl
alkaline phosphatase, liver/bone/kidney

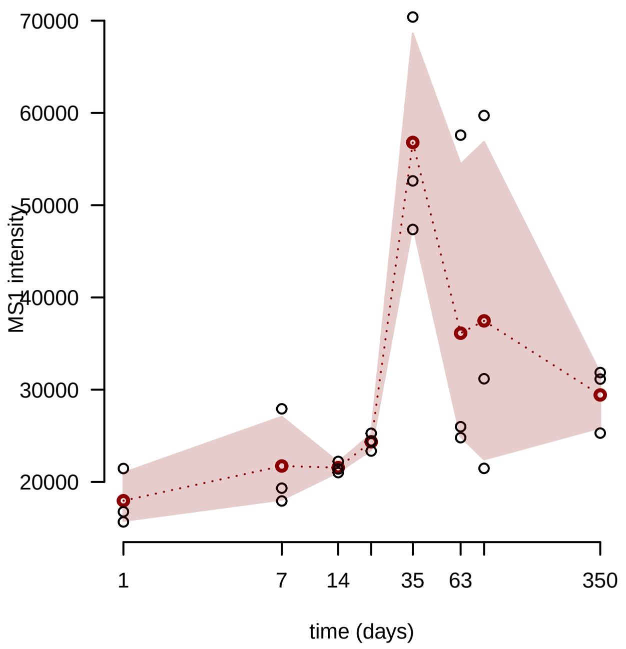

P09405
GN=Ncl
nucleolin

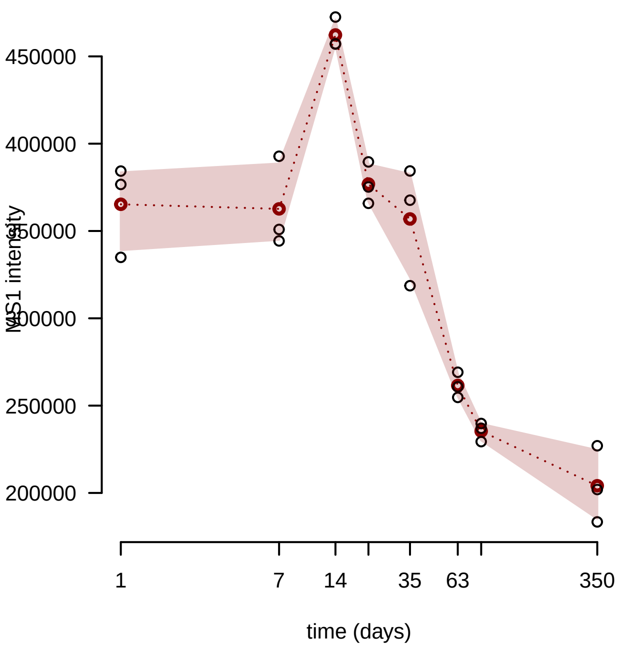

P09411
GN=Pgk1
phosphoglycerate kinase 1

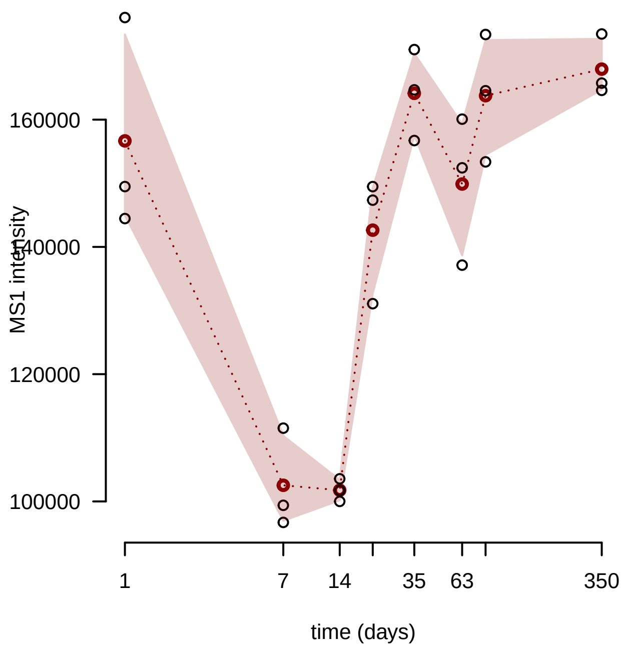

P09470
GN=Ace
angiotensin I converting enzyme (peptidy ...

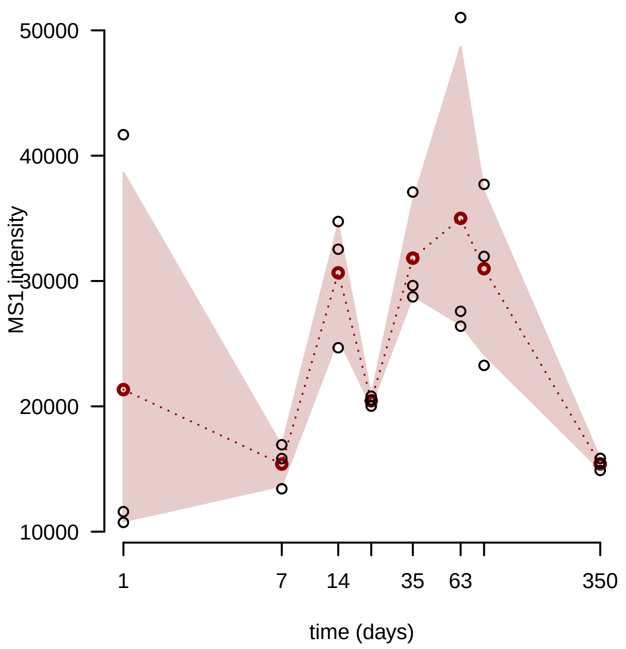

P09528
GN=Fth1
ferritin heavy polypeptide 1

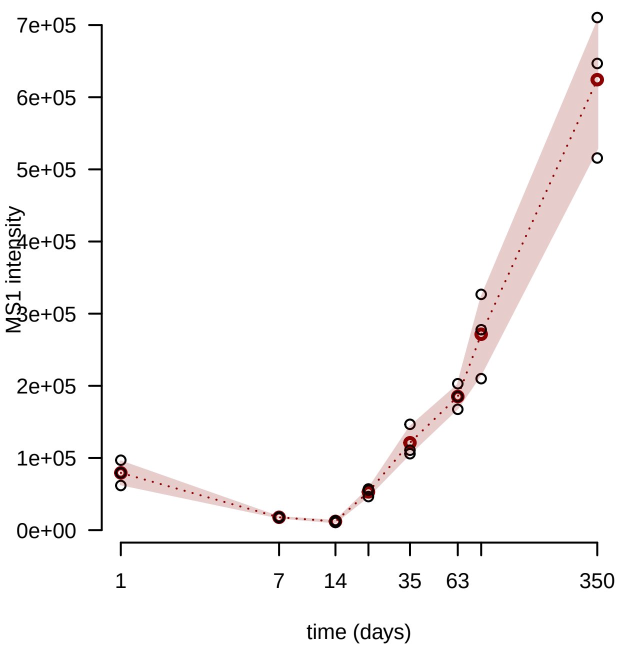

P09535
GN=Igf2
insulin−like growth factor 2

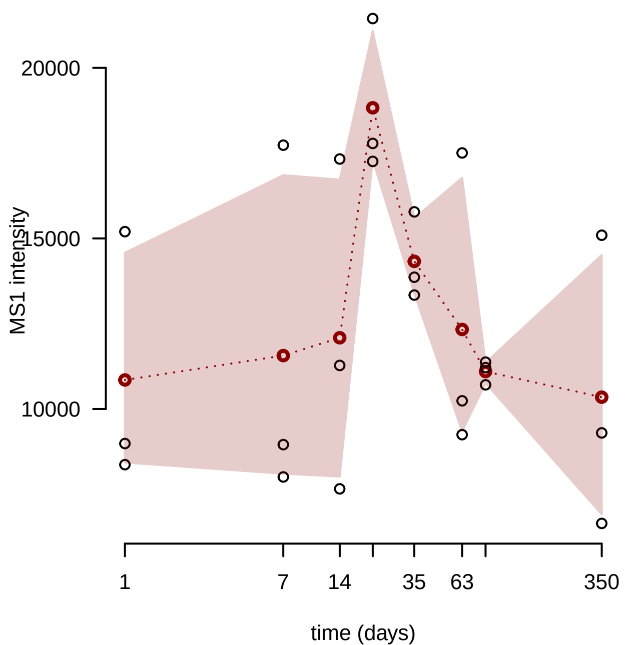

P09541
GN=Myl4
myosin, light polypeptide 4

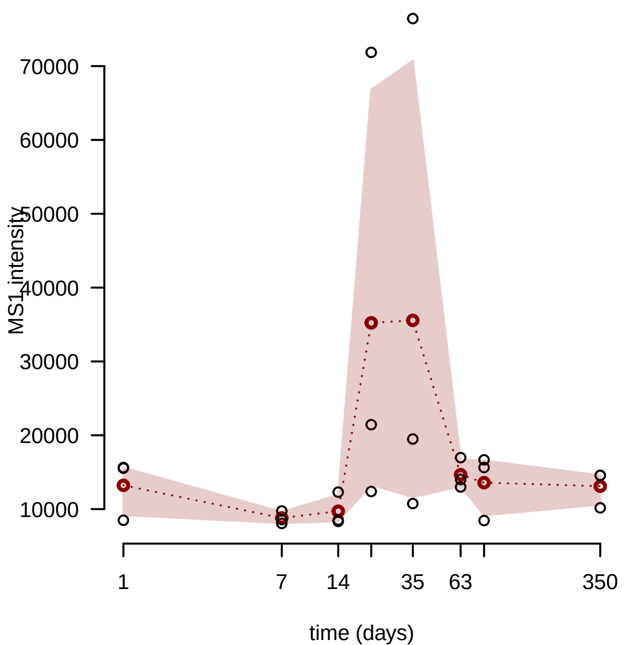

P09581
GN=Csf1r
colony stimulating factor 1 receptor

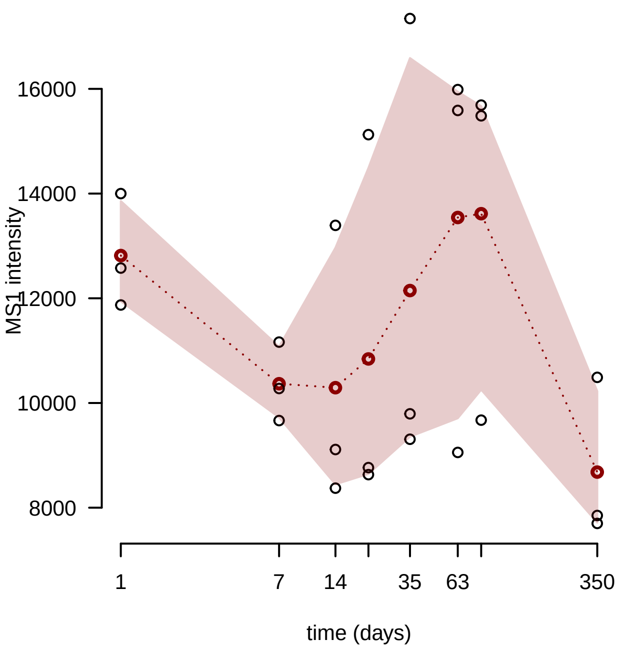

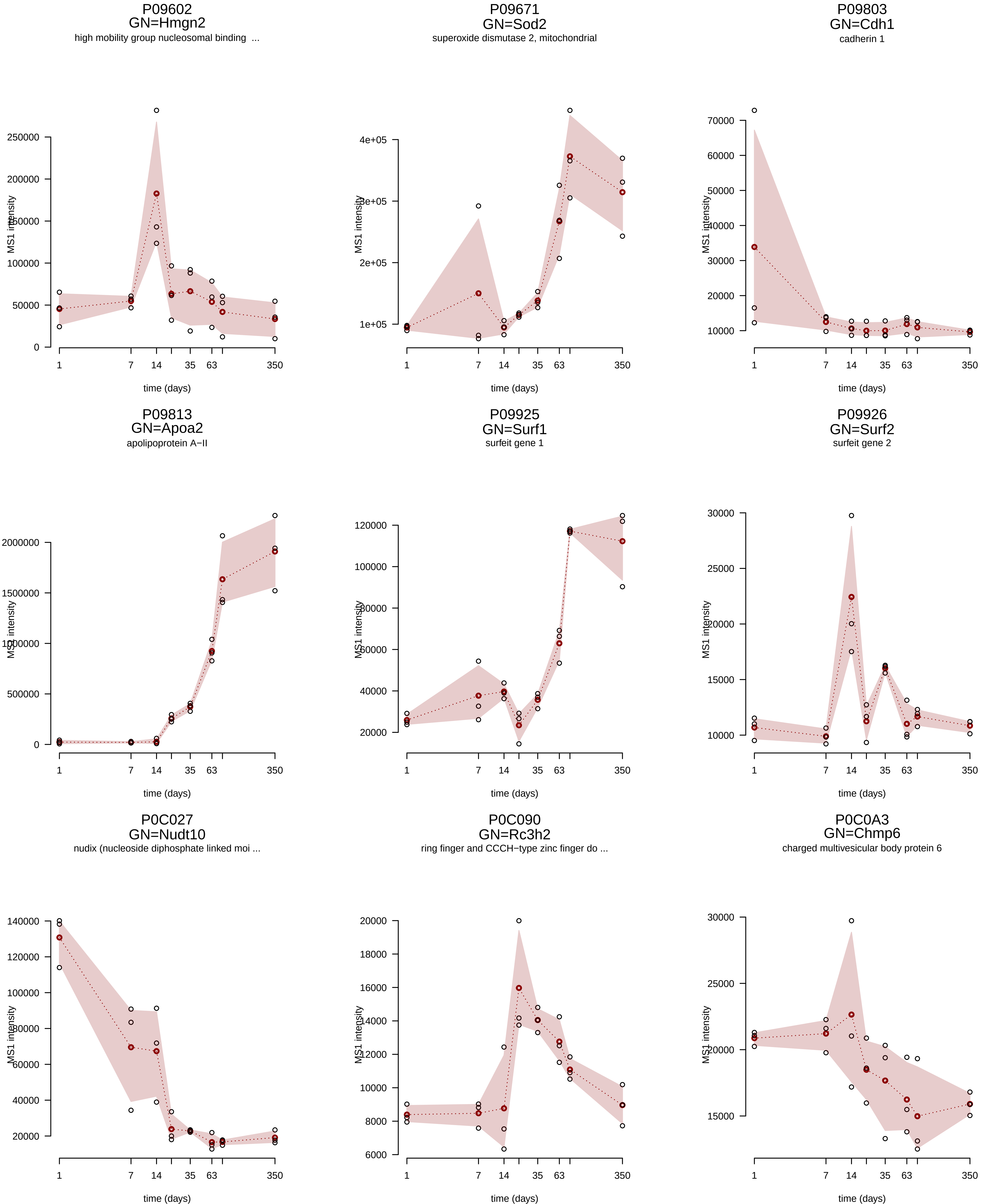
P09602
GN=Hmgn2
high mobility group nucleosomal binding ...
MS1 intensity
time (days)
P09671
GN=Sod2
superoxide dismutase 2, mitochondrial
MS1 intensity
time (days)
P09803
GN=Cdh1
cadherin 1
MS1 intensity
time (days)
P09813
GN=Apoa2
apolipoprotein A−II
MS1 intensity
time (days)
P09925
GN=Surf1
surfeit gene 1
MS1 intensity
time (days)
P09926
GN=Surf2
surfeit gene 2
MS1 intensity
time (days)
P0C027
GN=Nudt10
nudix (nucleoside diphosphate linked moi ...
MS1 intensity
time (days)
P0C090
GN=Rc3h2
ring finger and CCCH−type zinc finger do ...
MS1 intensity
time (days)
P0C0A3
GN=Chmp6
charged multivesicular body protein 6
MS1 intensity
time (days)

P0C0S6
GN=H2afz

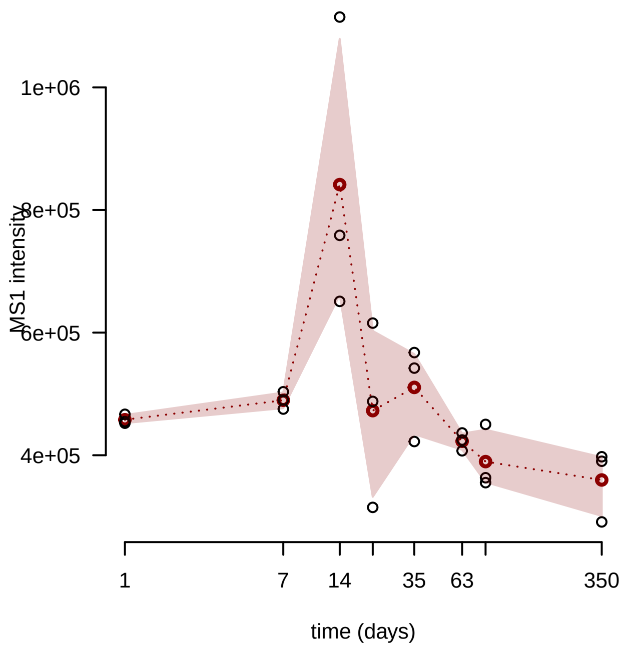

P0C673
GN=Igsf11
immunoglobulin superfamily, member 11

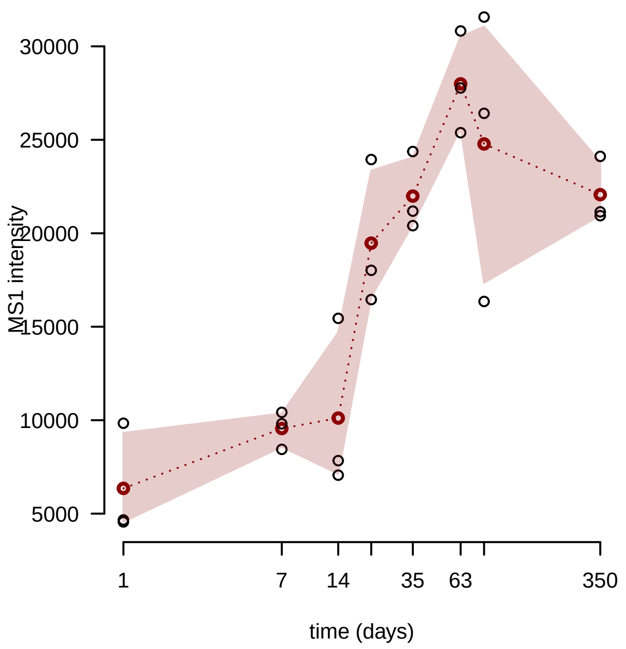

P0C6A2
GN=Mamld1
mastermind−like domain containing 1

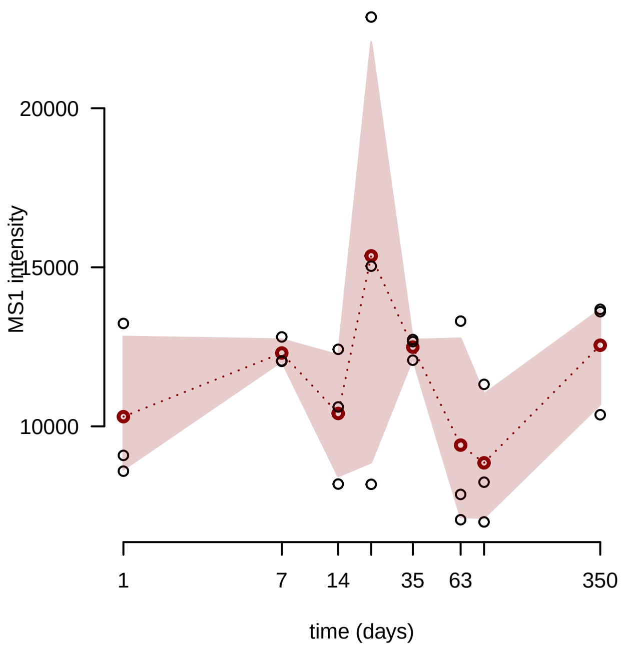

P0C7L0
GN=Wipf3
WAS/WASL interacting protein family, mem ...

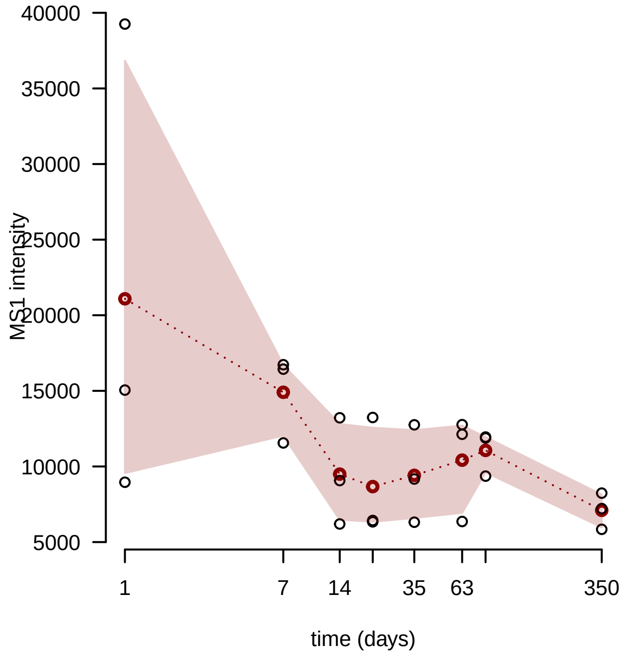

P0C7N9
GN=Psmg4
proteasome (prosome, macropain) assembly ...

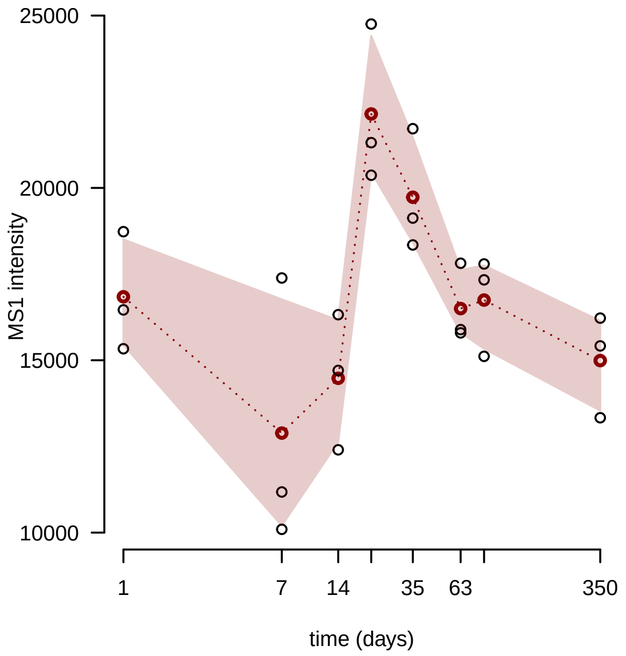

P0C7T6
GN=Atxn1l
ataxin 1−like

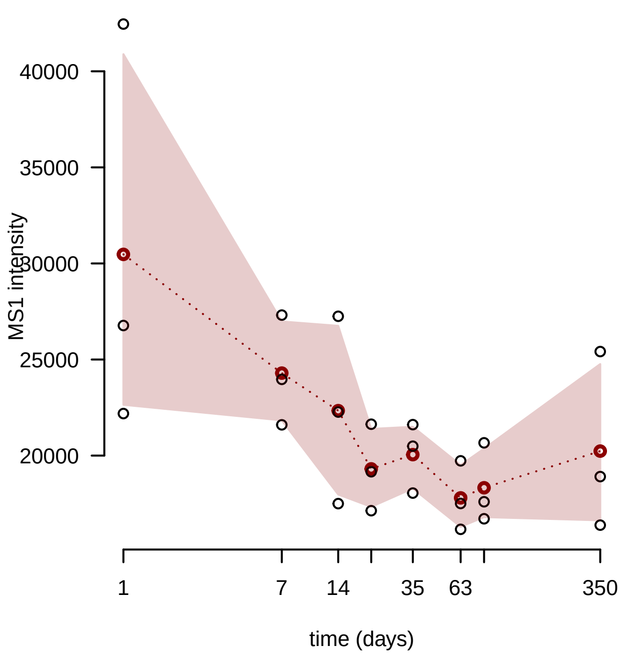

P0C872
GN=Jmjd7
jumonji domain containing 7

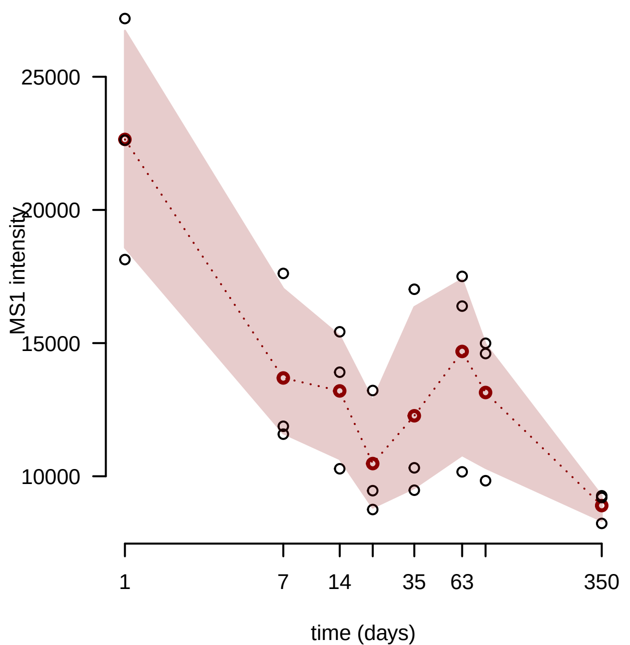

P0C8K7
GN=Smim1
small integral membrane protein 1

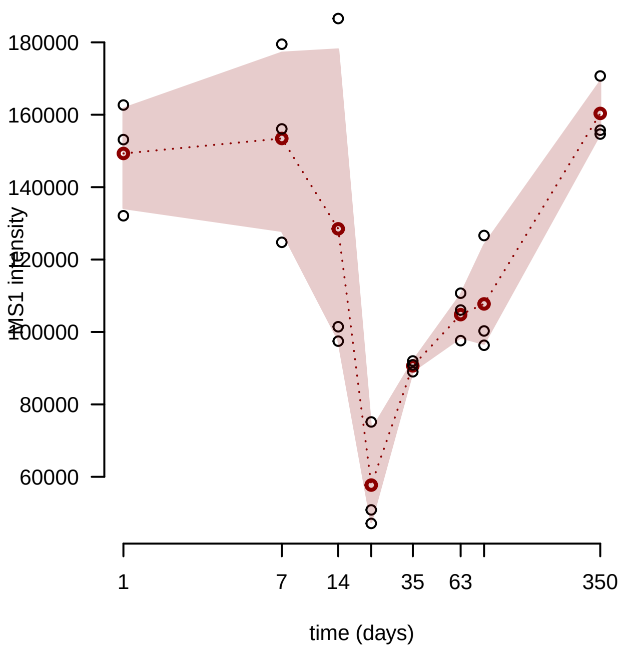

P0CG14
GN=Derpc

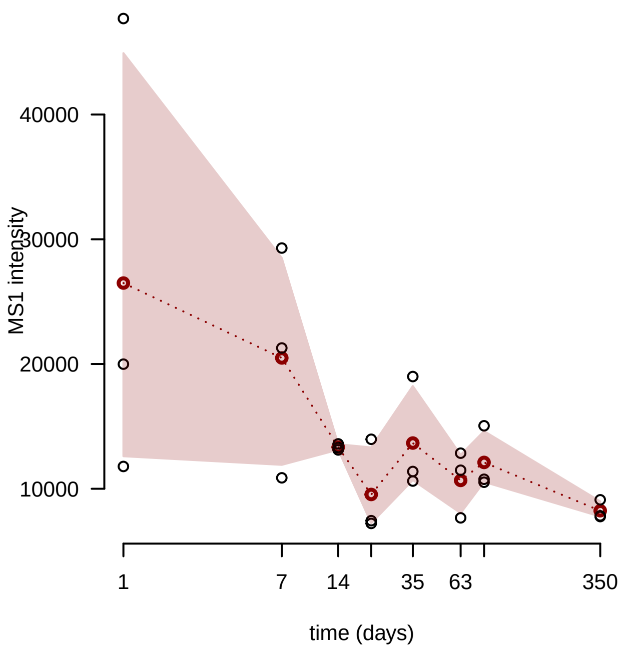

P0CG15
GN=Chtf8
CTF8, chromosome transmission fidelity f ...

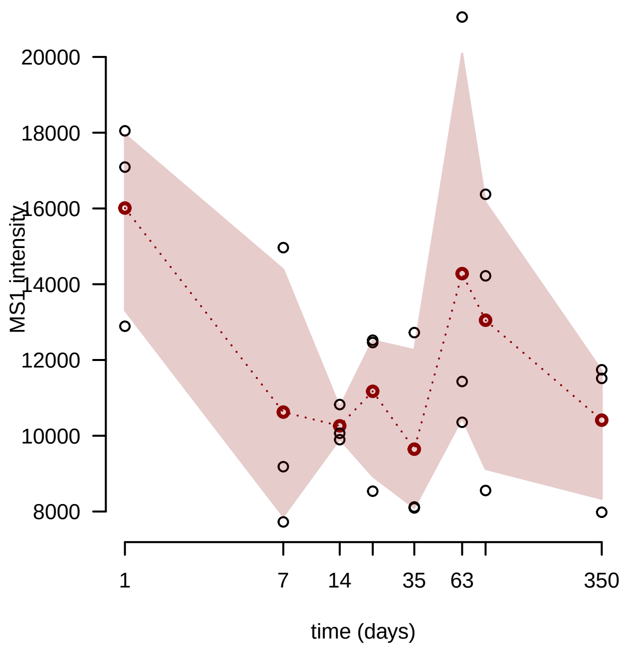

P0CL69
GN=Znf703

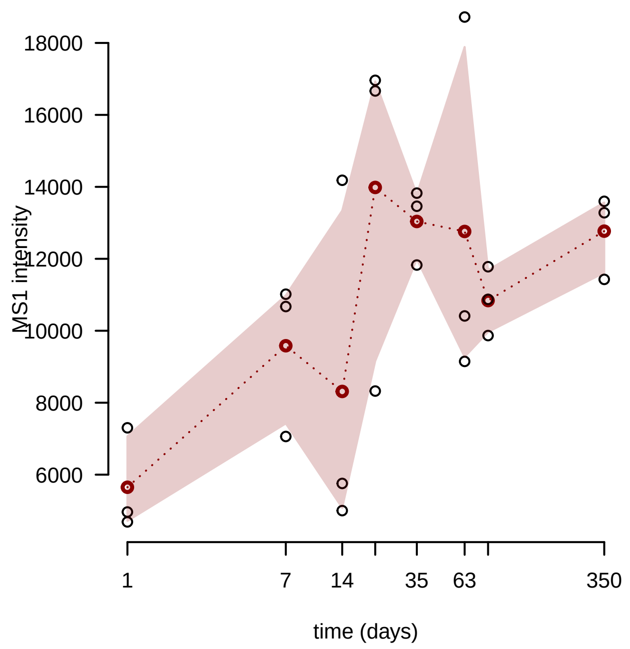

P0DJF2
GN=Pet117
PET117 homolog

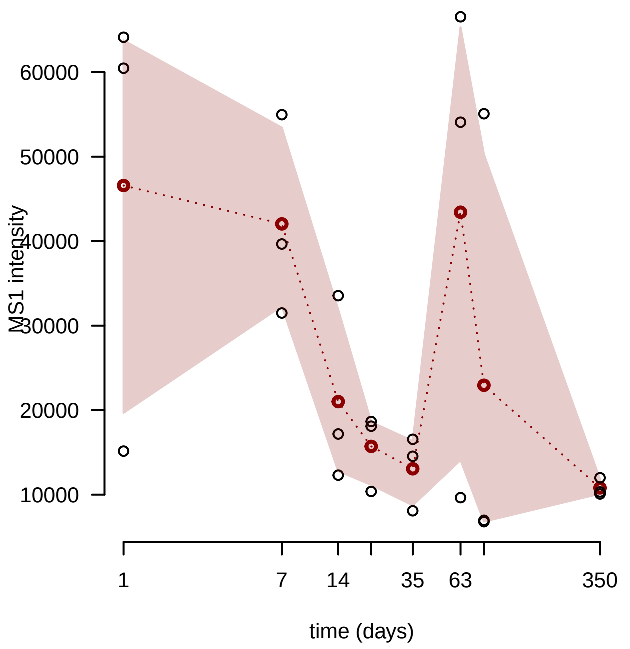

P0DN34
GN=Ndufb1
NADH:ubiquinone oxidoreductase subunit B ...

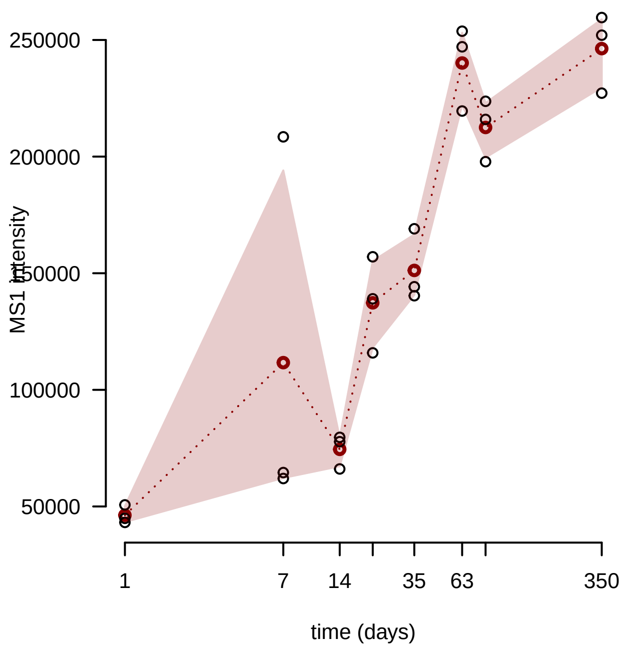

P0DN89
GN=Tmem254a

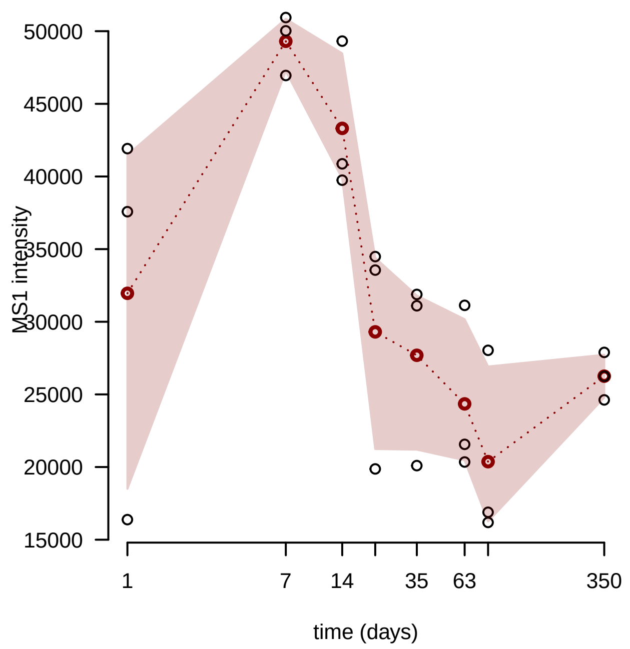

P0DOV2
GN=Ifi204
interferon activated gene 204

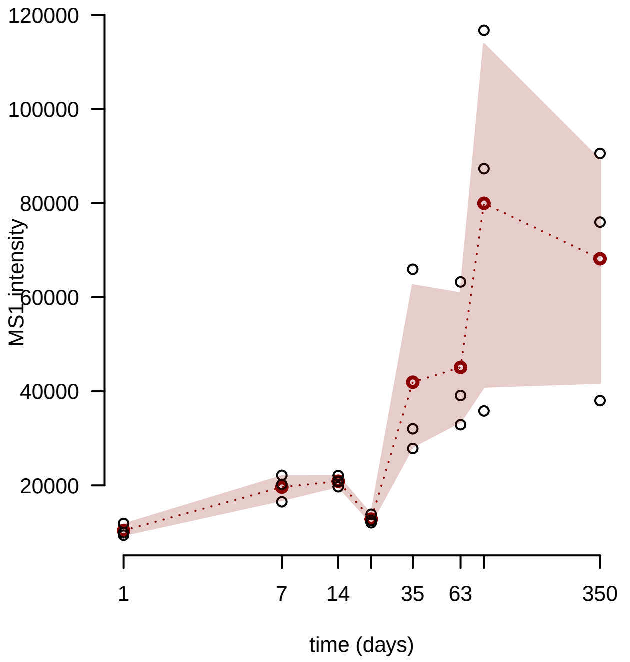

P0DP26
GN=Calm1
calmodulin 1

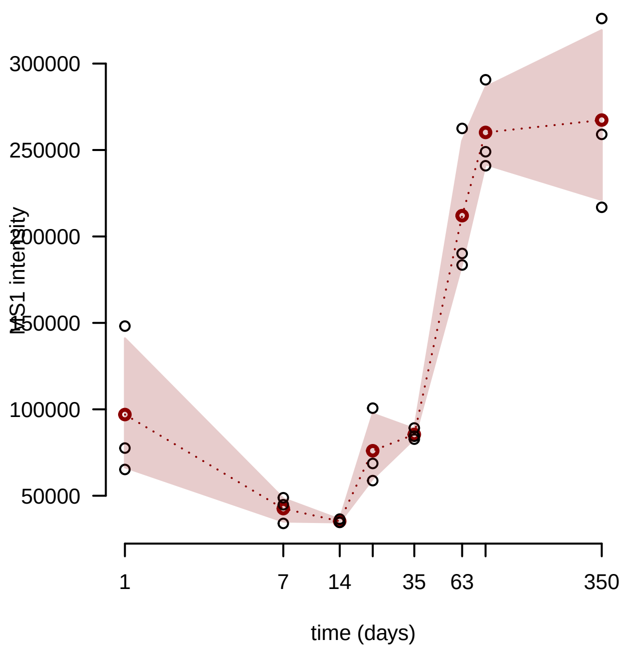

P10107
GN=Anxa1
annexin A1

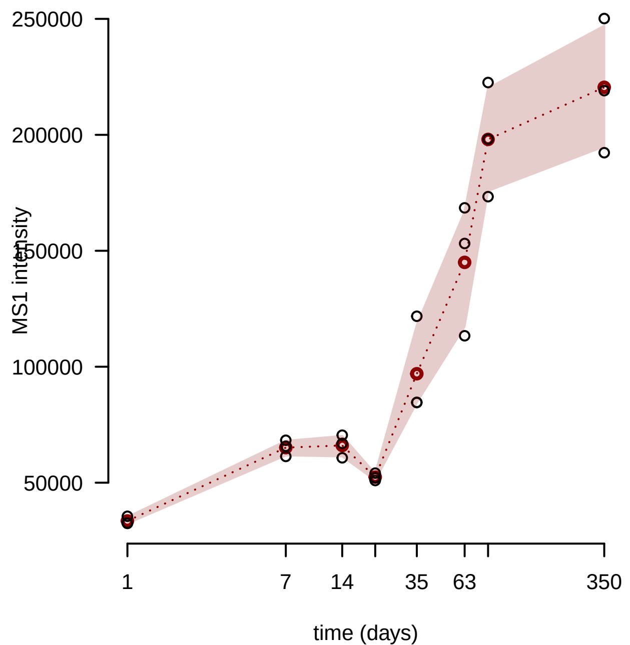

P10126
GN=Eef1a1
eukaryotic translation elongation factor ...

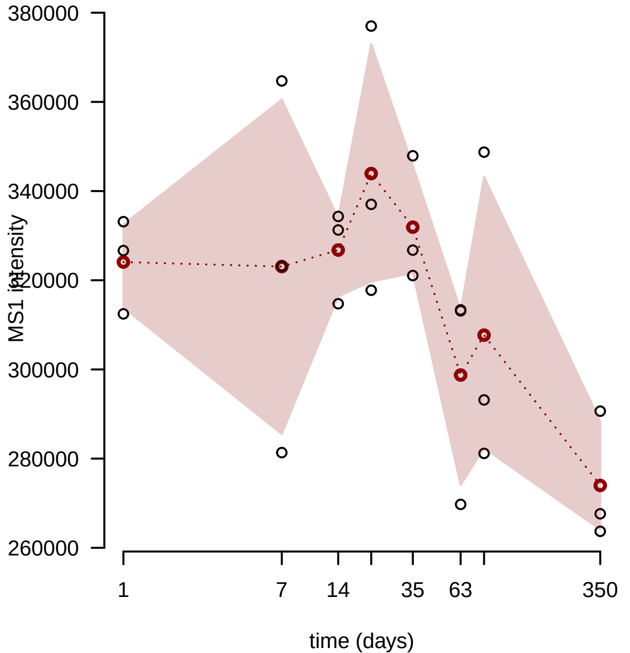

P10287
GN=Cdh3
cadherin 3

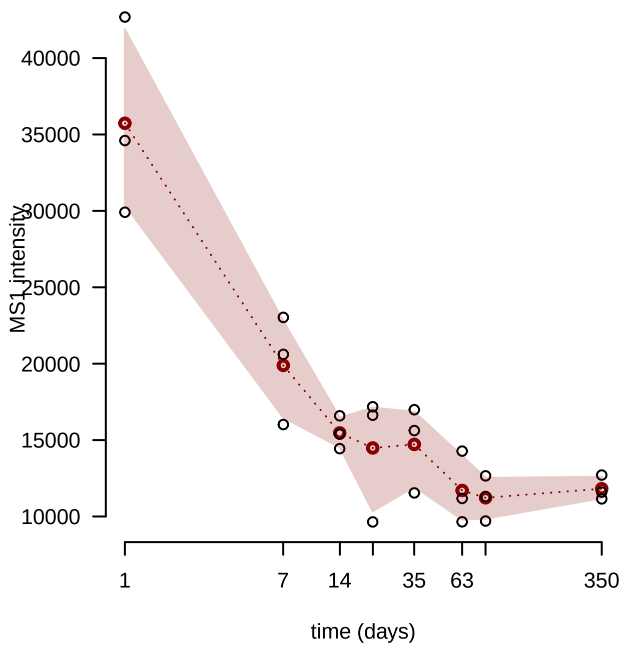

P10404
GN=

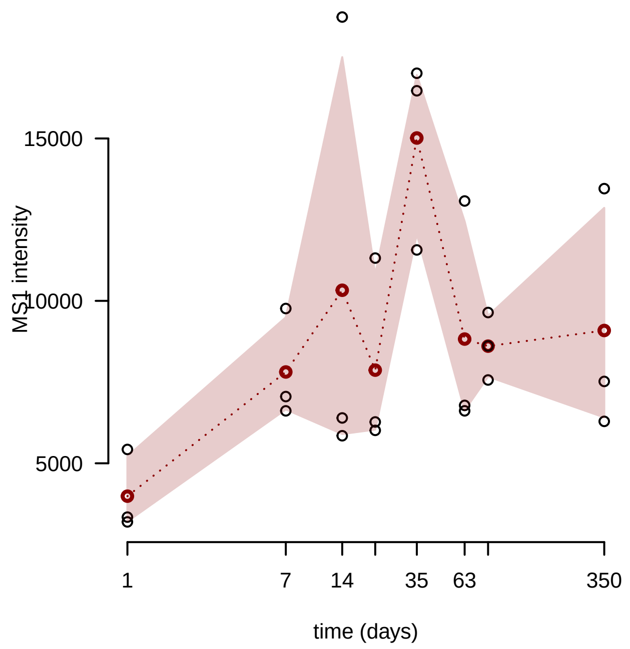

P10417
GN=Bcl2
B cell leukemia/lymphoma 2

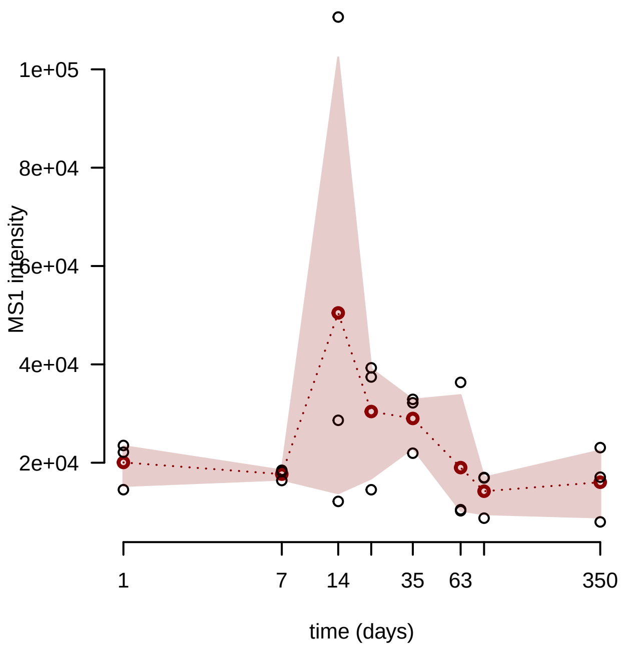

P10493
GN=Nid1
nidogen 1

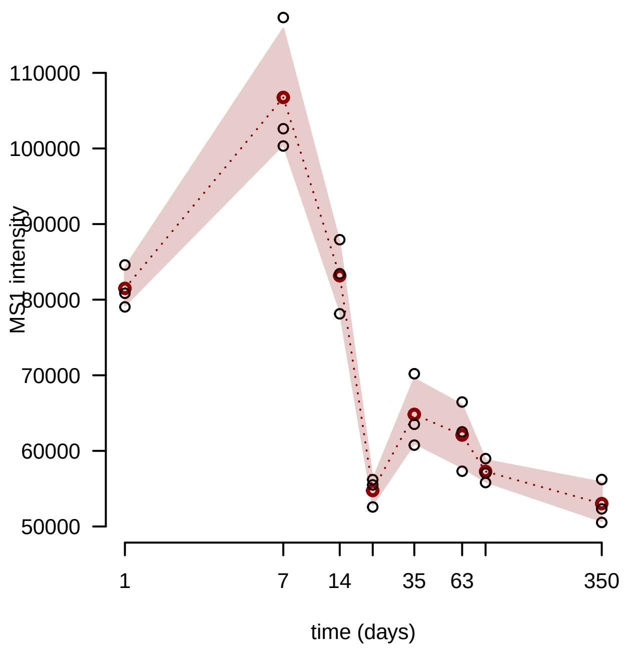

P10518
GN=Alad
aminolevulinate, delta−, dehydratase

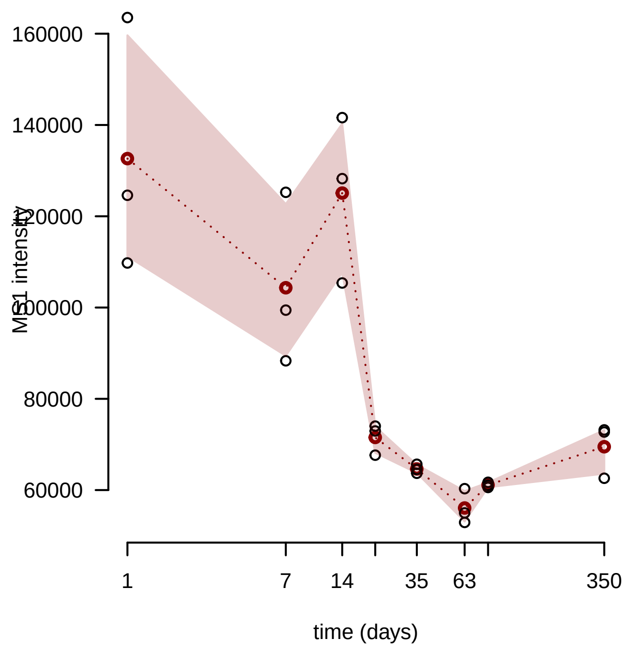

P10605
GN=Ctsb
cathepsin B

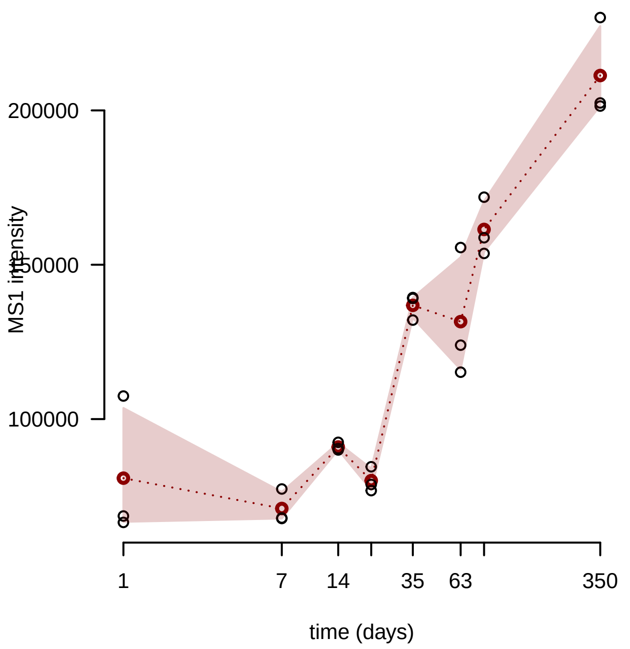

P10629
GN=Hoxc6
homeobox C6

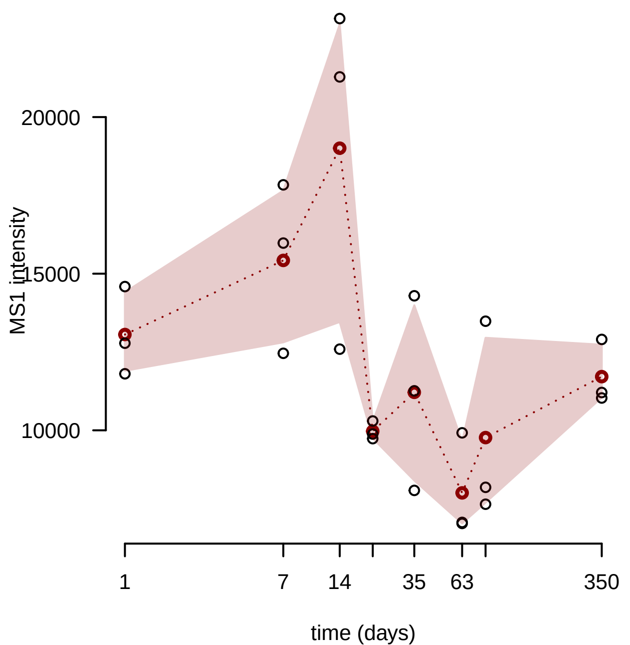

P10630
GN=Eif4a2
eukaryotic translation initiation factor ...

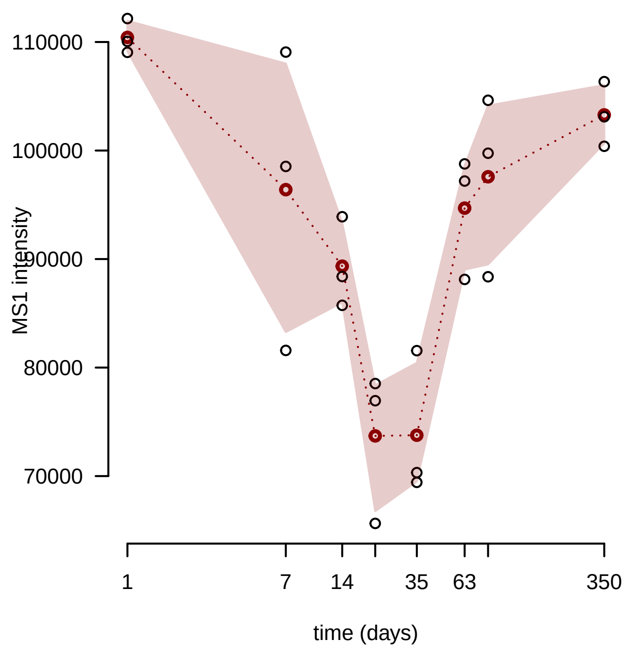

P10637
GN=Mapt
microtubule−associated protein tau

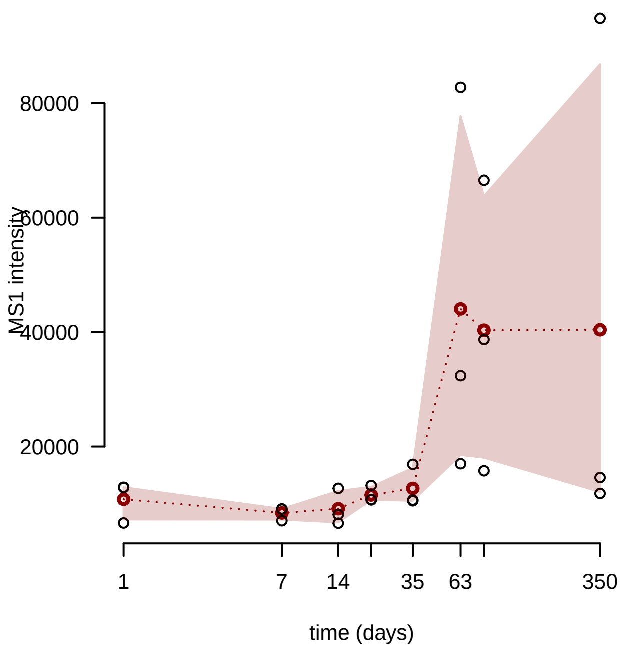

P10639
GN=Txn

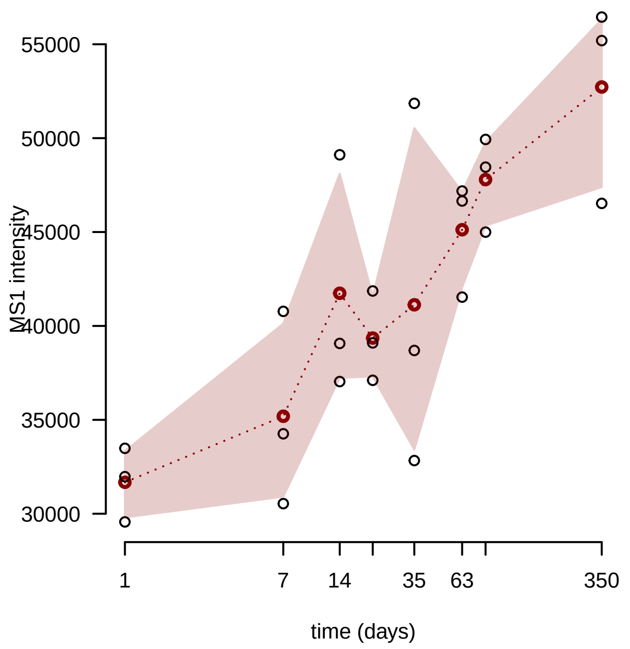

P10649
GN=Gstm1
glutathione S−transferase, mu 1

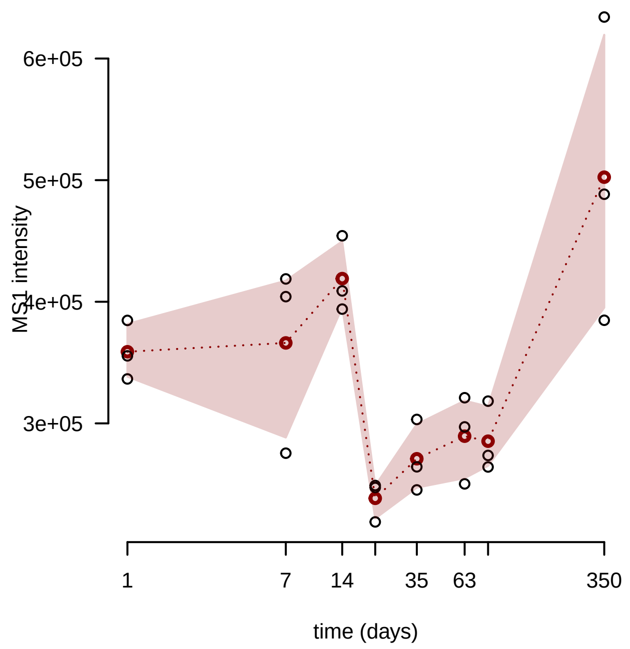

P10711
GN=Tcea1
transcription elongation factor A (SII) ...

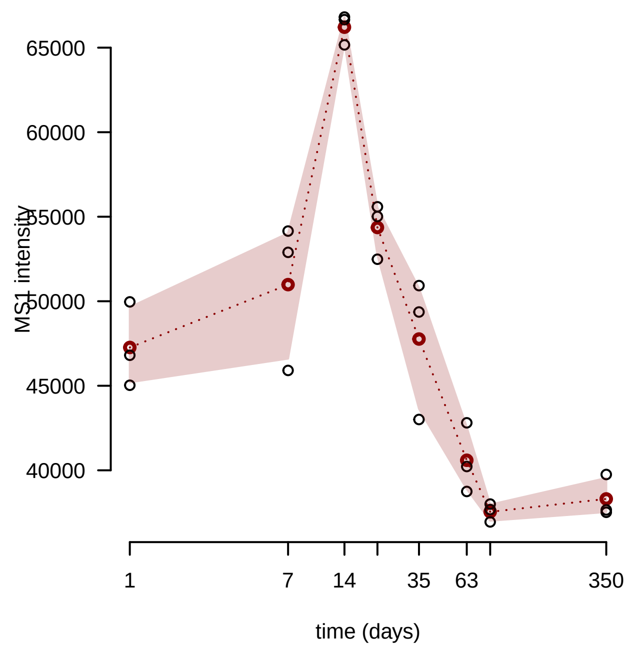

P10761
GN=Zp3
zona pellucida glycoprotein 3

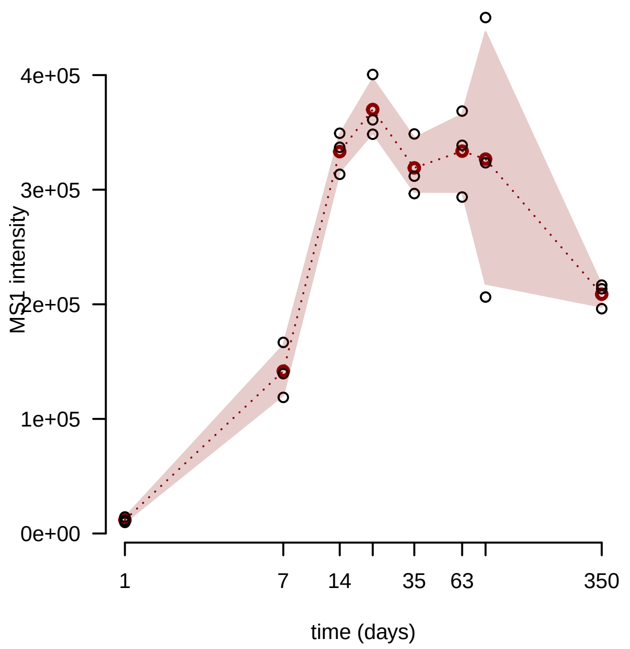

P10810
GN=Cd14
CD14 antigen

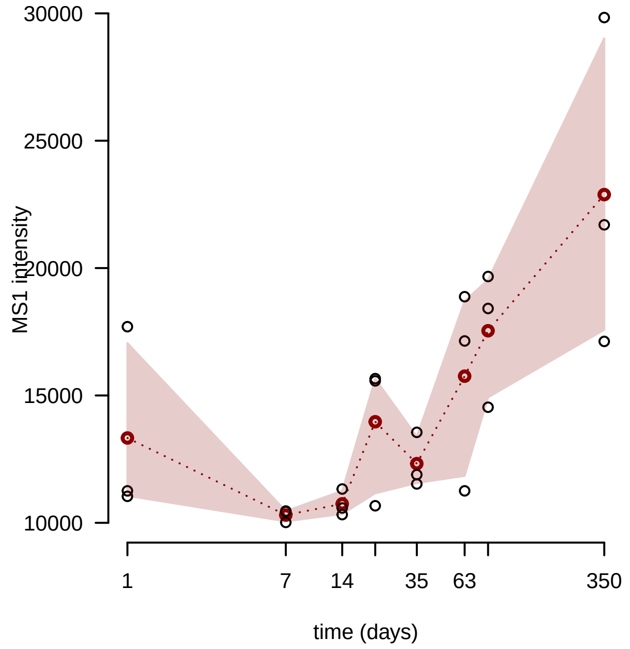

P10833
GN=Rras
related RAS viral (r−ras) oncogene

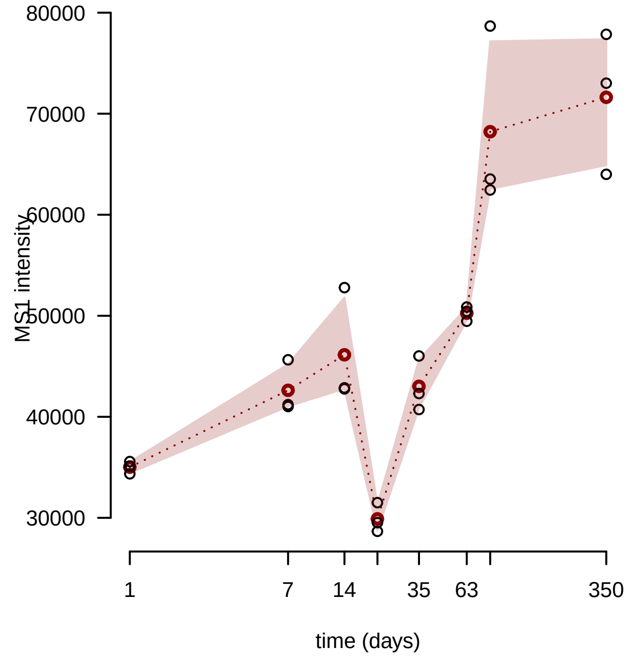

P10852
GN=Slc3a2
solute carrier family 3 (activators of d ...

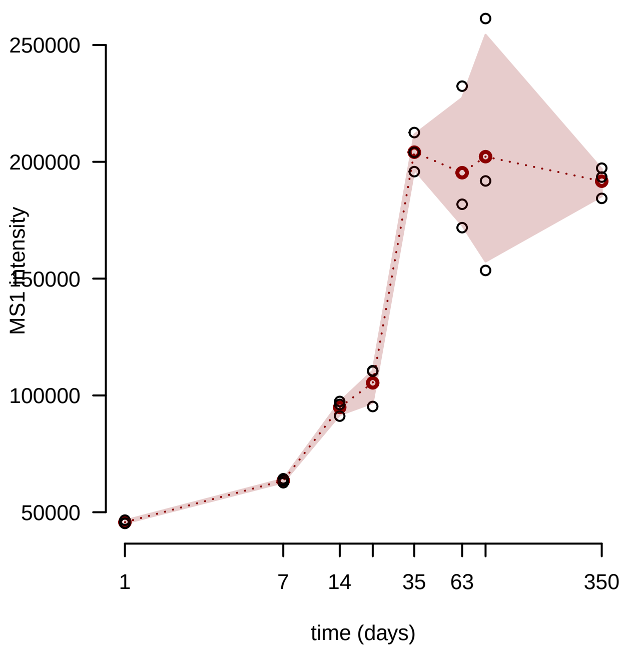

P10852
GN=Slc3a2
solute carrier family 3 (activators of d ...

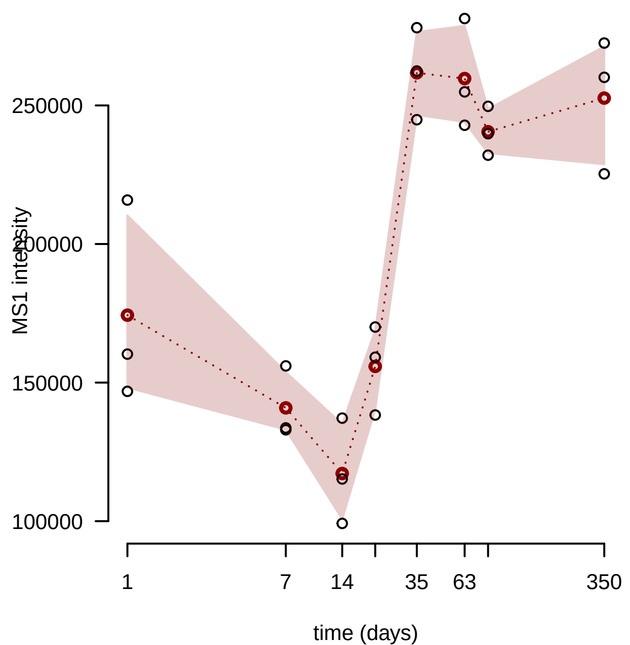

P10922
GN=H1f0
H1.0 linker histone

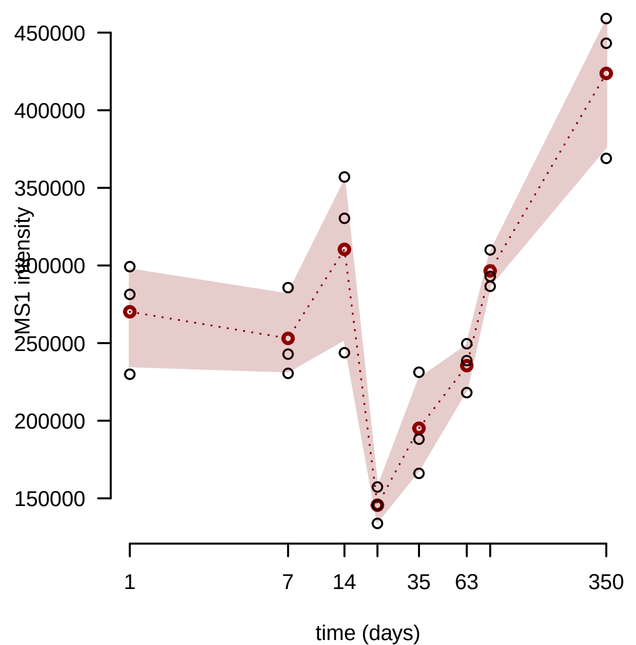

P11031
GN=Sub1
SUB1 homolog, transcriptional regulator

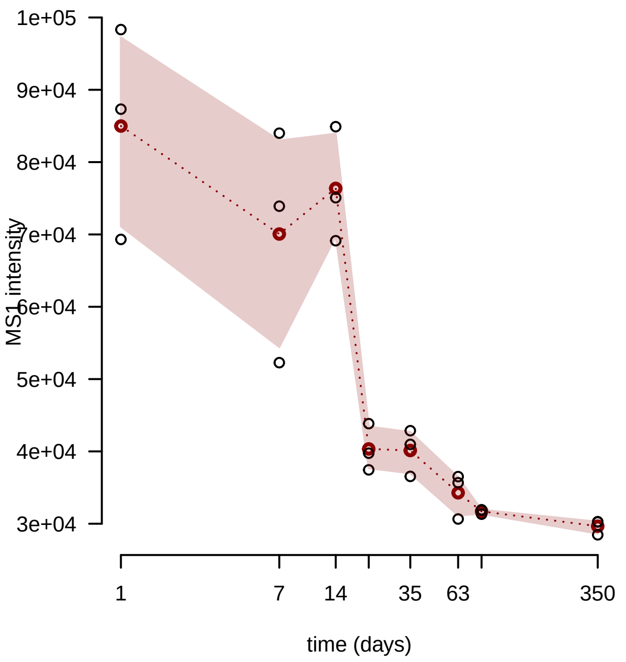

P11087
GN=Col1a1
collagen, type I, alpha 1

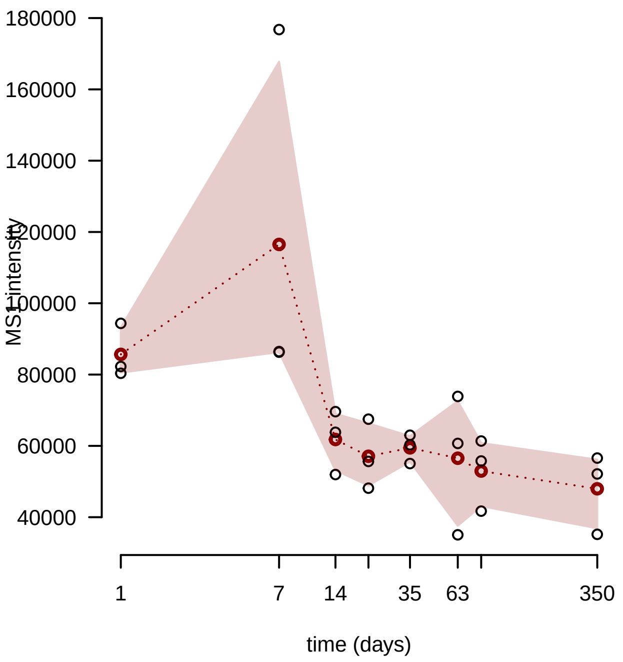

P11088
GN=Flg
filaggrin

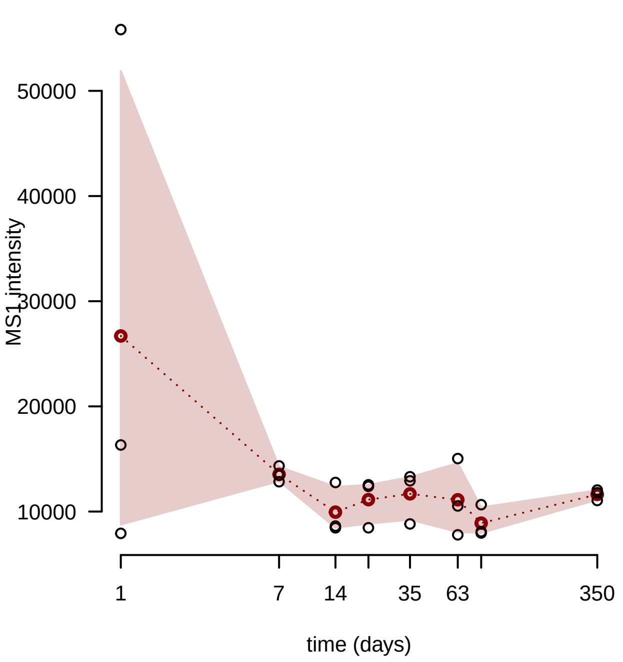

P11103
GN=Parp1
poly (ADP−ribose) polymerase family, mem ...

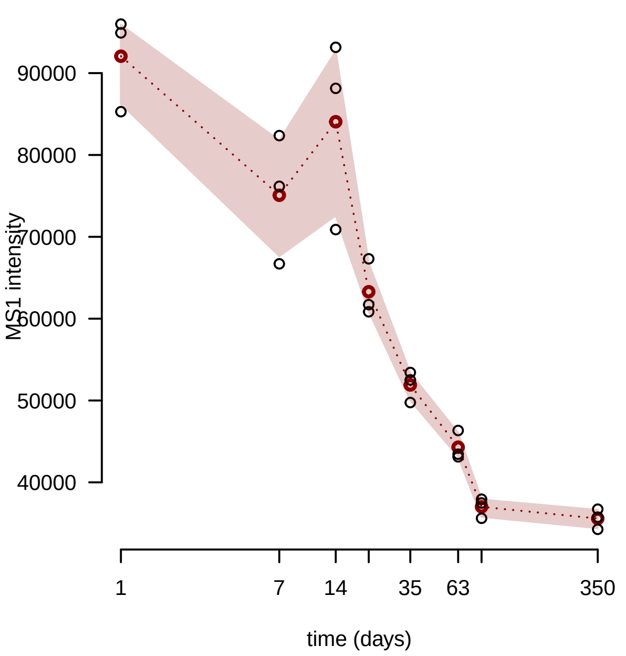

P11152
GN=Lpl
lipoprotein lipase

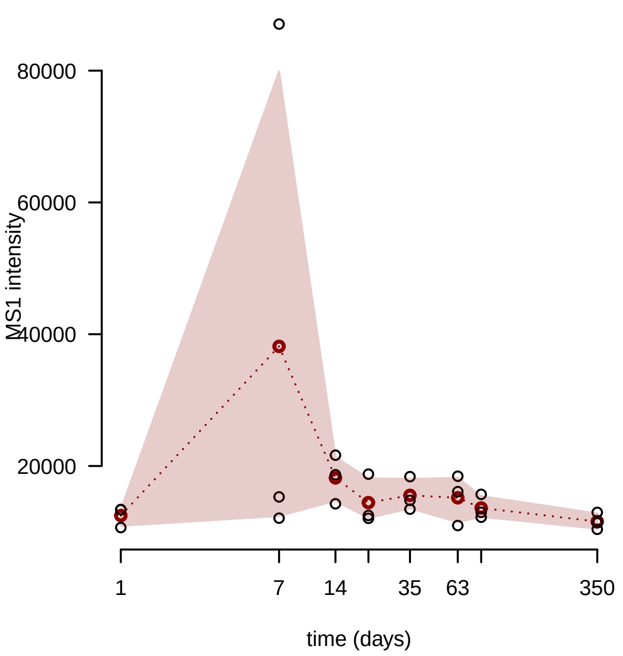

P11157
GN=Rrm2
ribonucleotide reductase M2

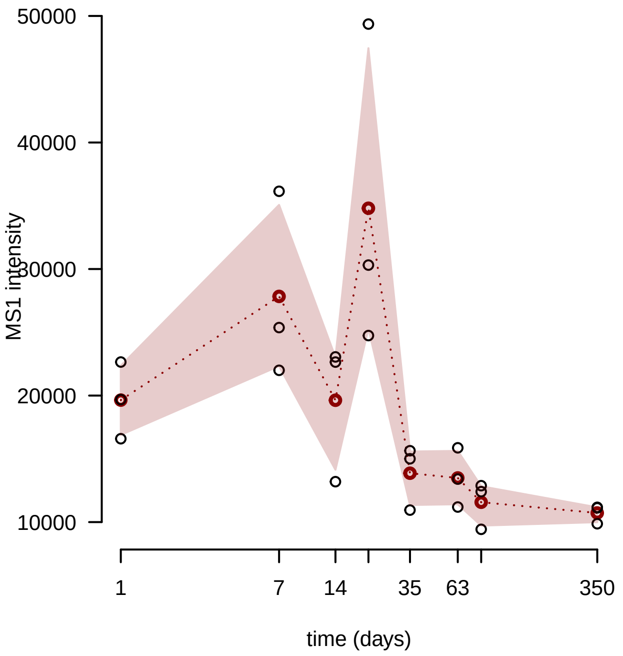

P11247
GN=Mpo
myeloperoxidase

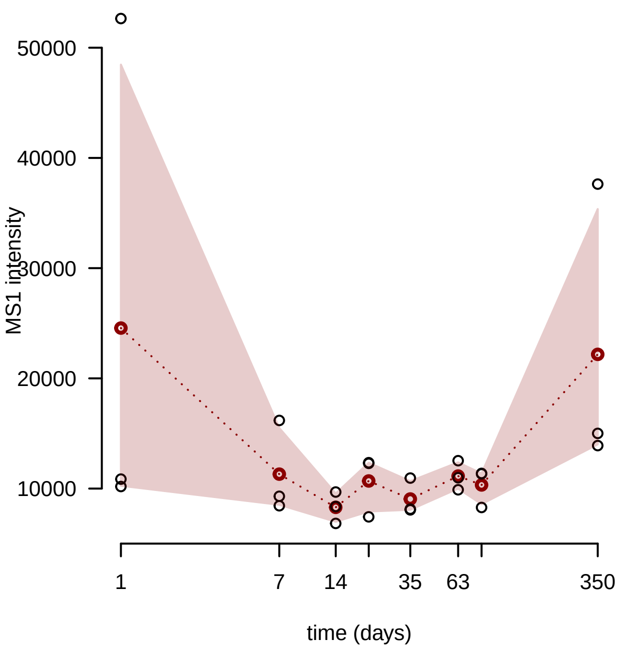

P11276
GN=Fn1
fibronectin 1

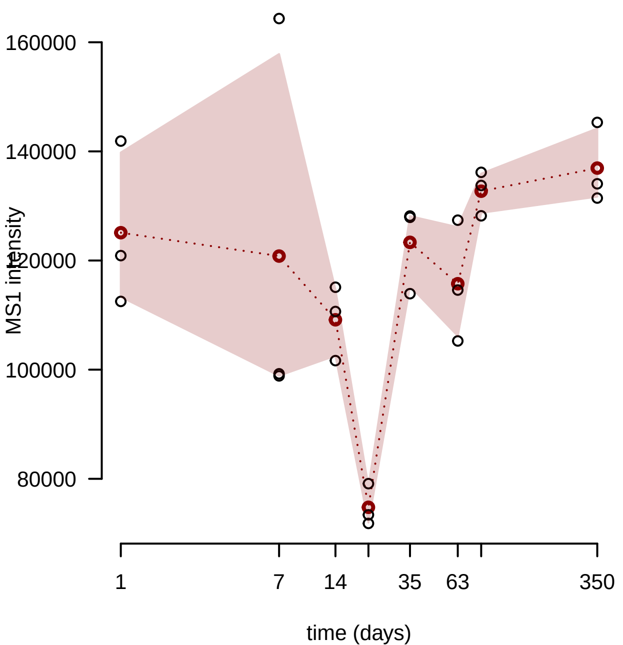

P11352
GN=Gpx1
glutathione peroxidase 1

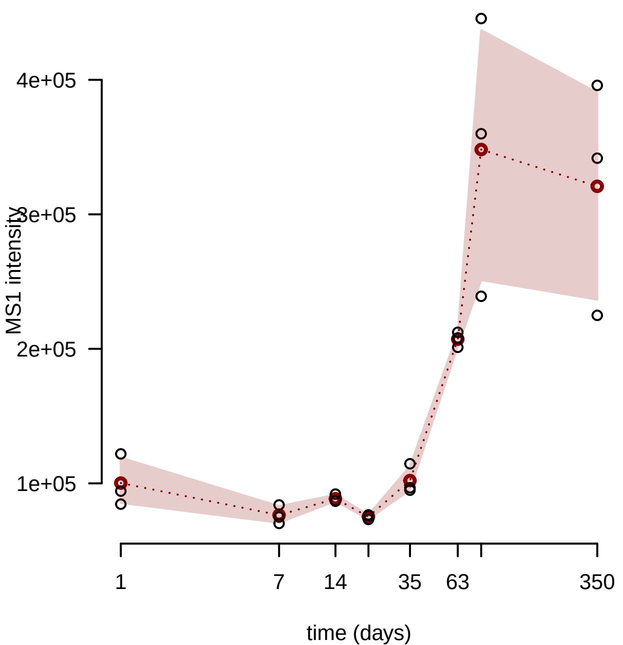

P11404
GN=Fabp3
fatty acid binding protein 3, muscle and ...

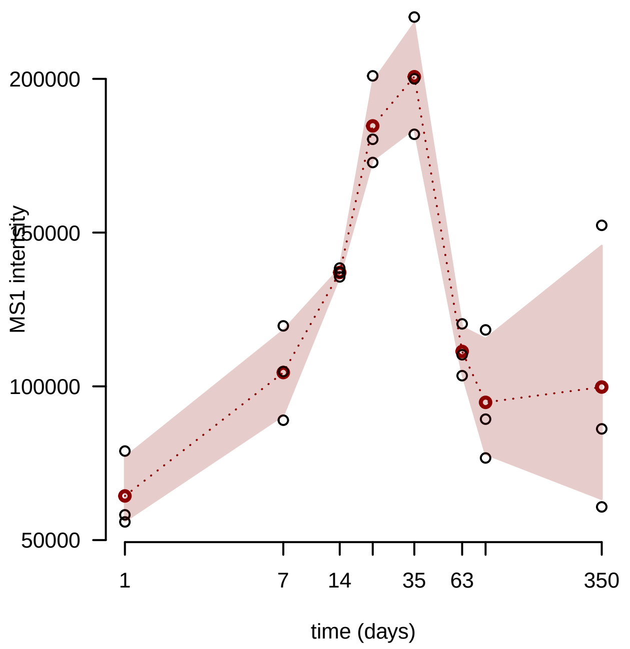

P11438
GN=Lamp1
lysosomal−associated membrane protein 1

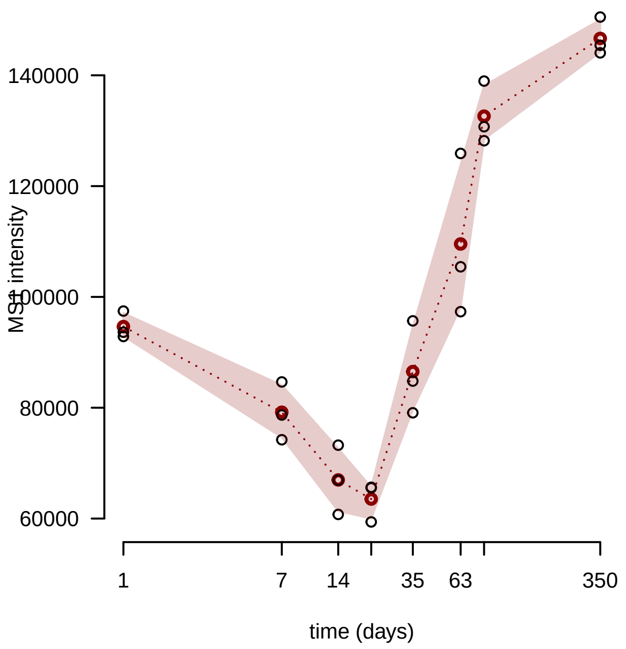

P11440
GN=Cdk1
cyclin−dependent kinase 1

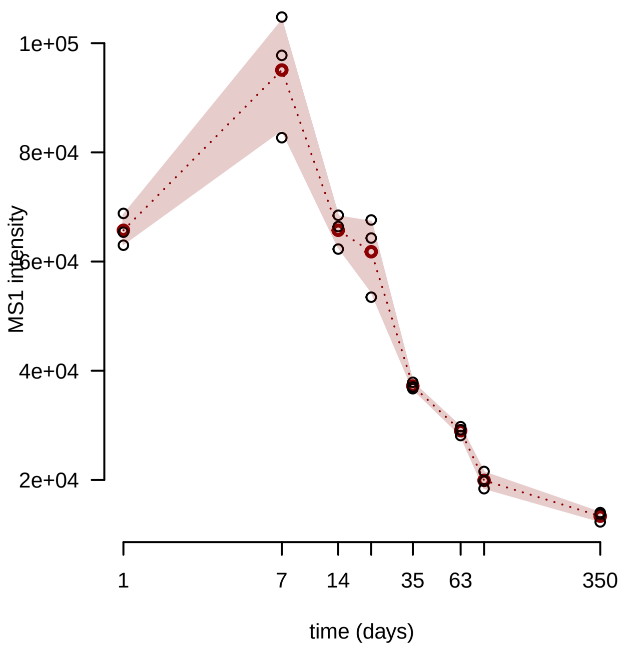

P11499
GN=Hsp90ab1
heat shock protein 90 alpha (cytosolic), ...

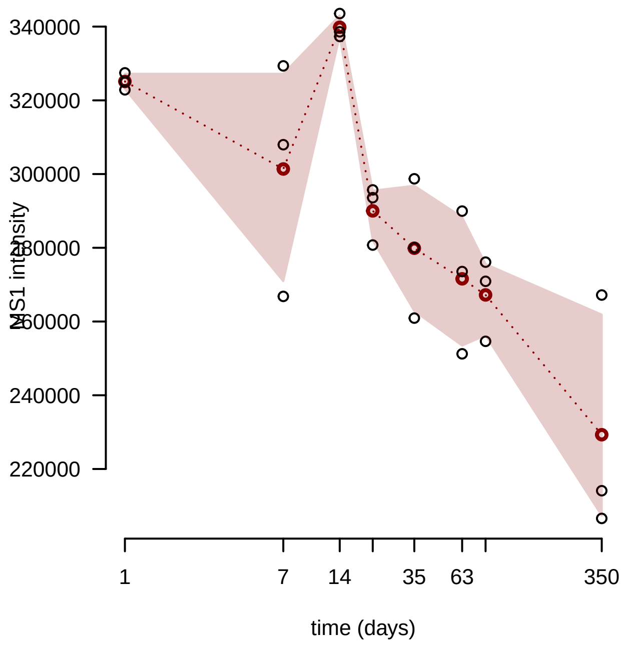

P11531
GN=Dmd
dystrophin, muscular dystrophy

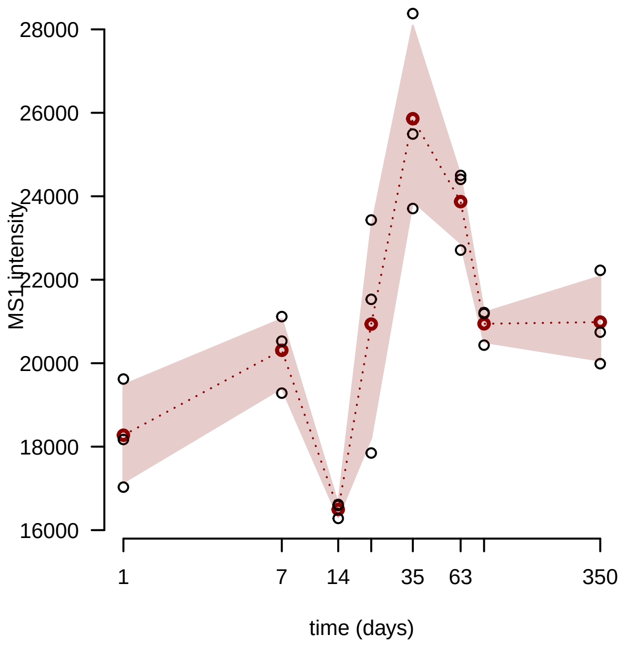

P11589
GN=Mup2
major urinary protein 2

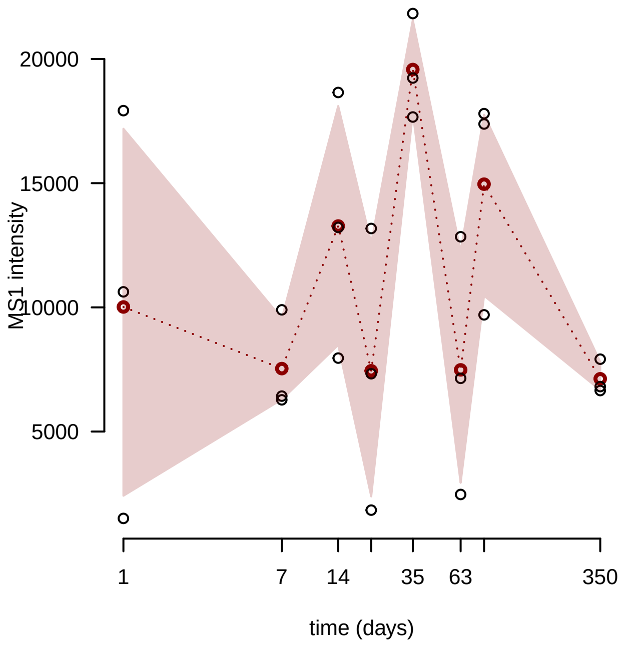

P11609
GN=Cd1d1
CD1d1 antigen

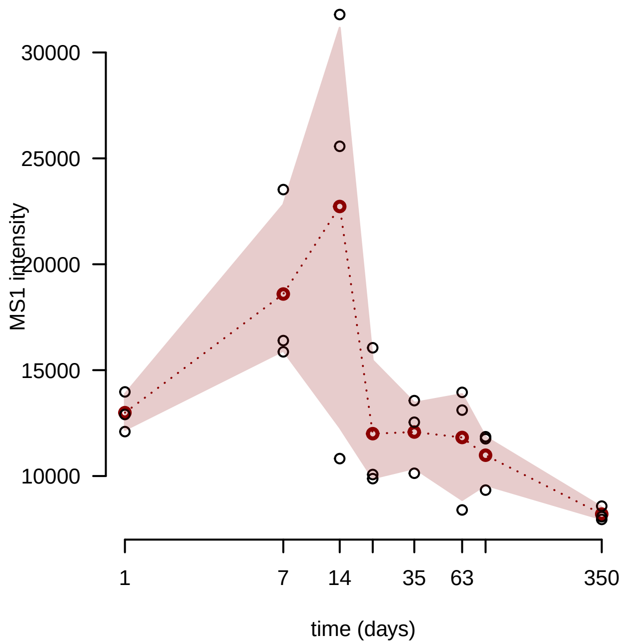

P11672
GN=Lcn2
lipocalin 2

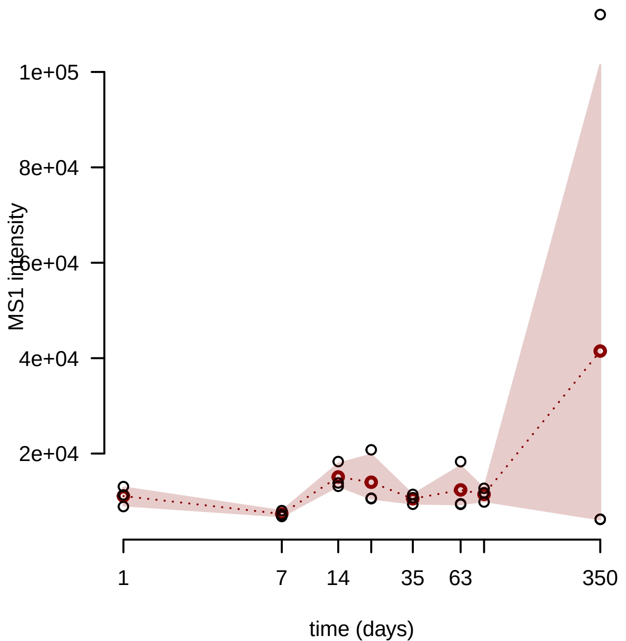

P11679
GN=Krt8
keratin 8

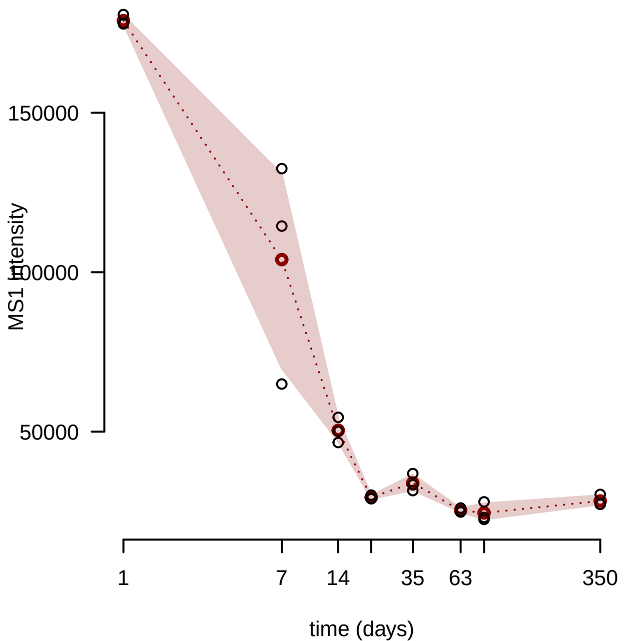

P11680
GN=Cfp
complement factor properdin

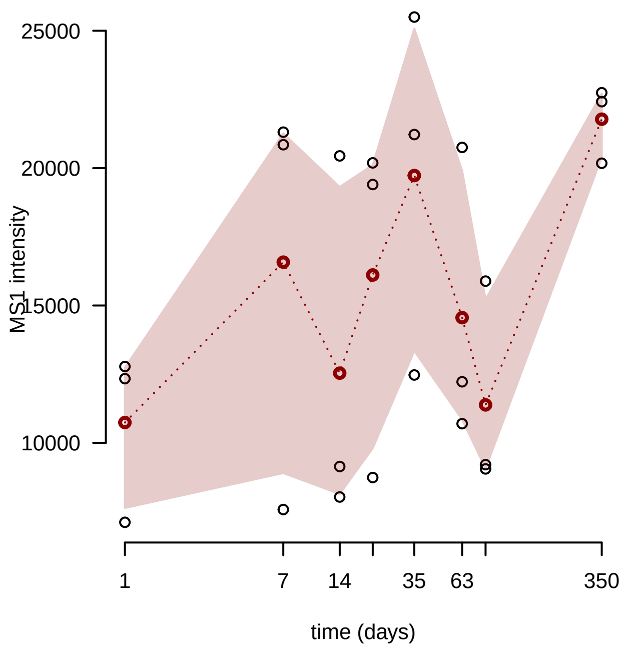

P11688
GN=Itga5
integrin alpha 5 (fibronectin receptor a ...

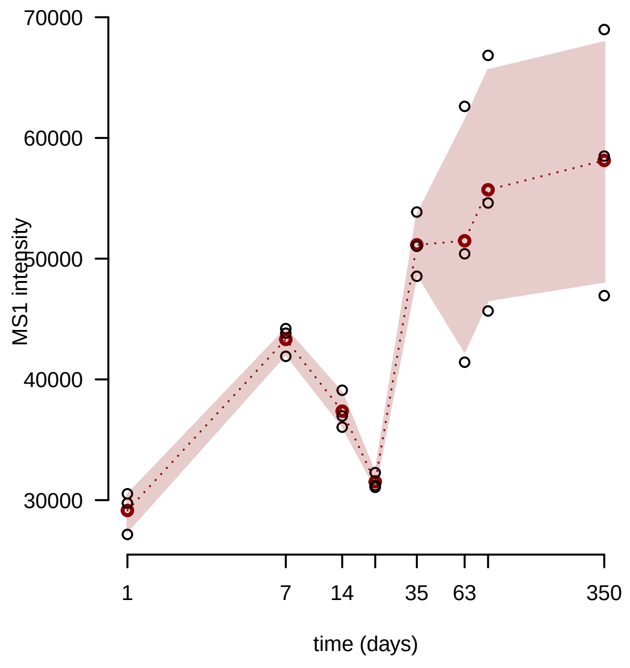

P11725
GN=Otc
ornithine transcarbamylase

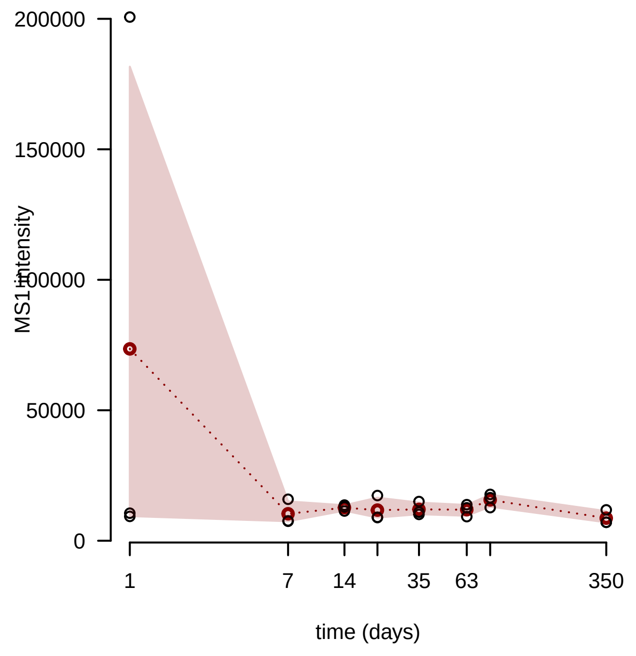

P11835
GN=Itgb2
integrin beta 2

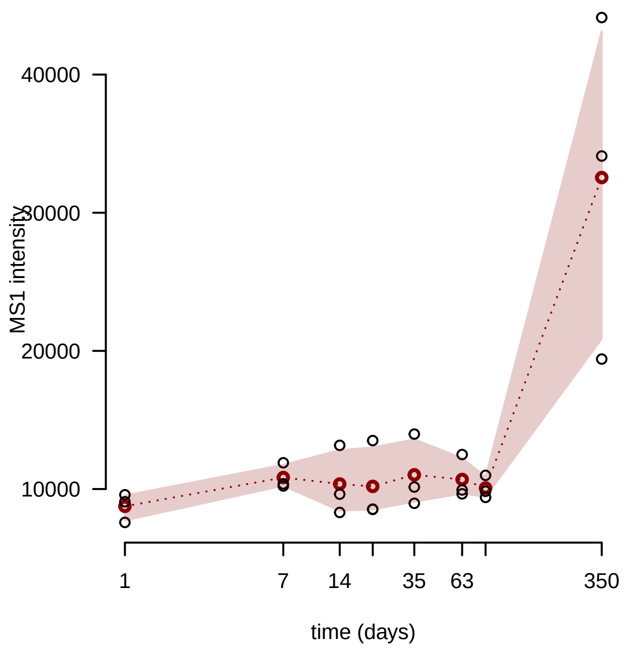

P11859
GN=Agt
angiotensinogen (serpin peptidase inhibi ...

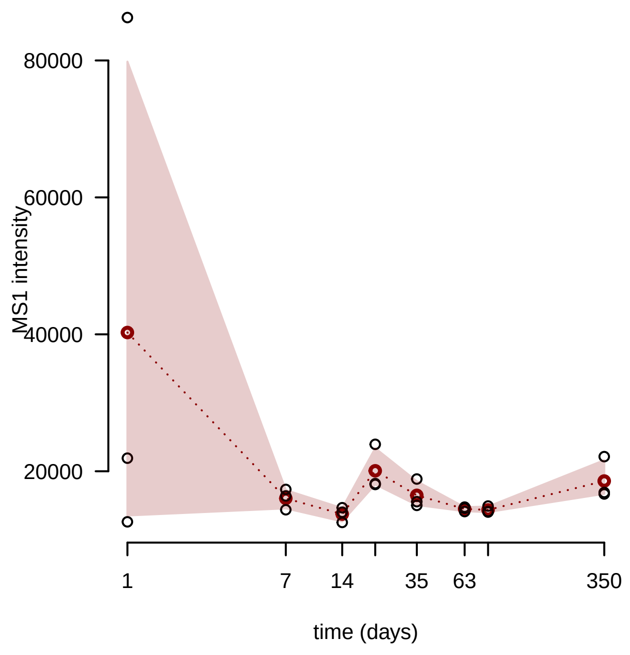

P11862
GN=Gas2
growth arrest specific 2

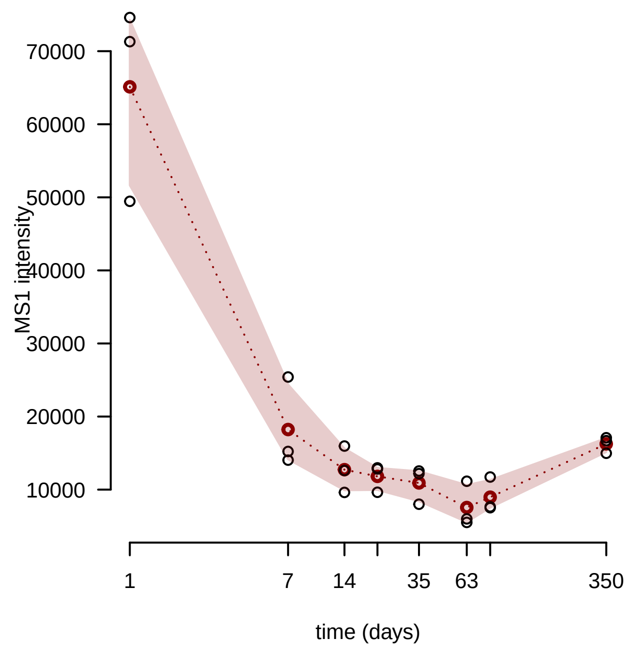

P11881
GN=Itpr1
inositol 1,4,5−trisphosphate receptor 1

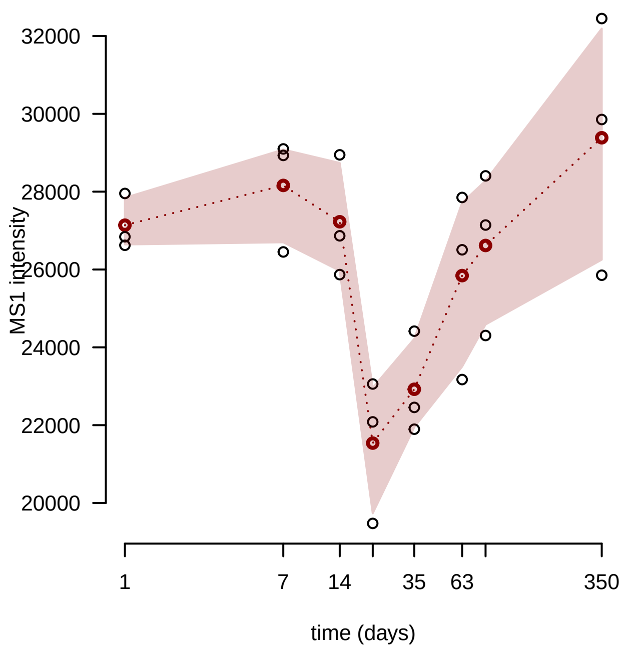

P11930
GN=Nudt19
nudix (nucleoside diphosphate linked moi ...

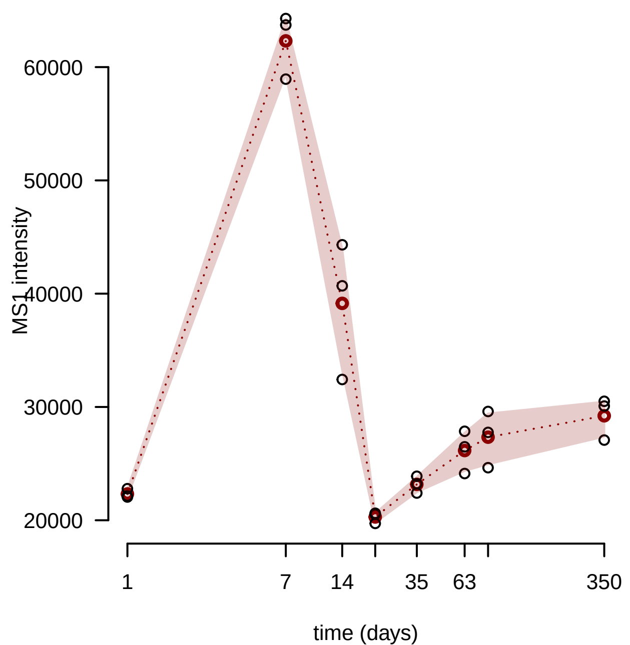

P11983
GN=Tcp1
t−complex protein 1

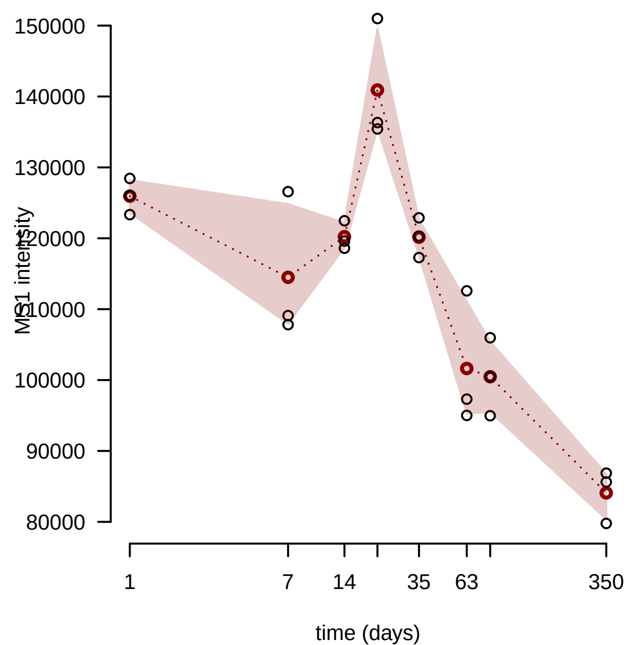

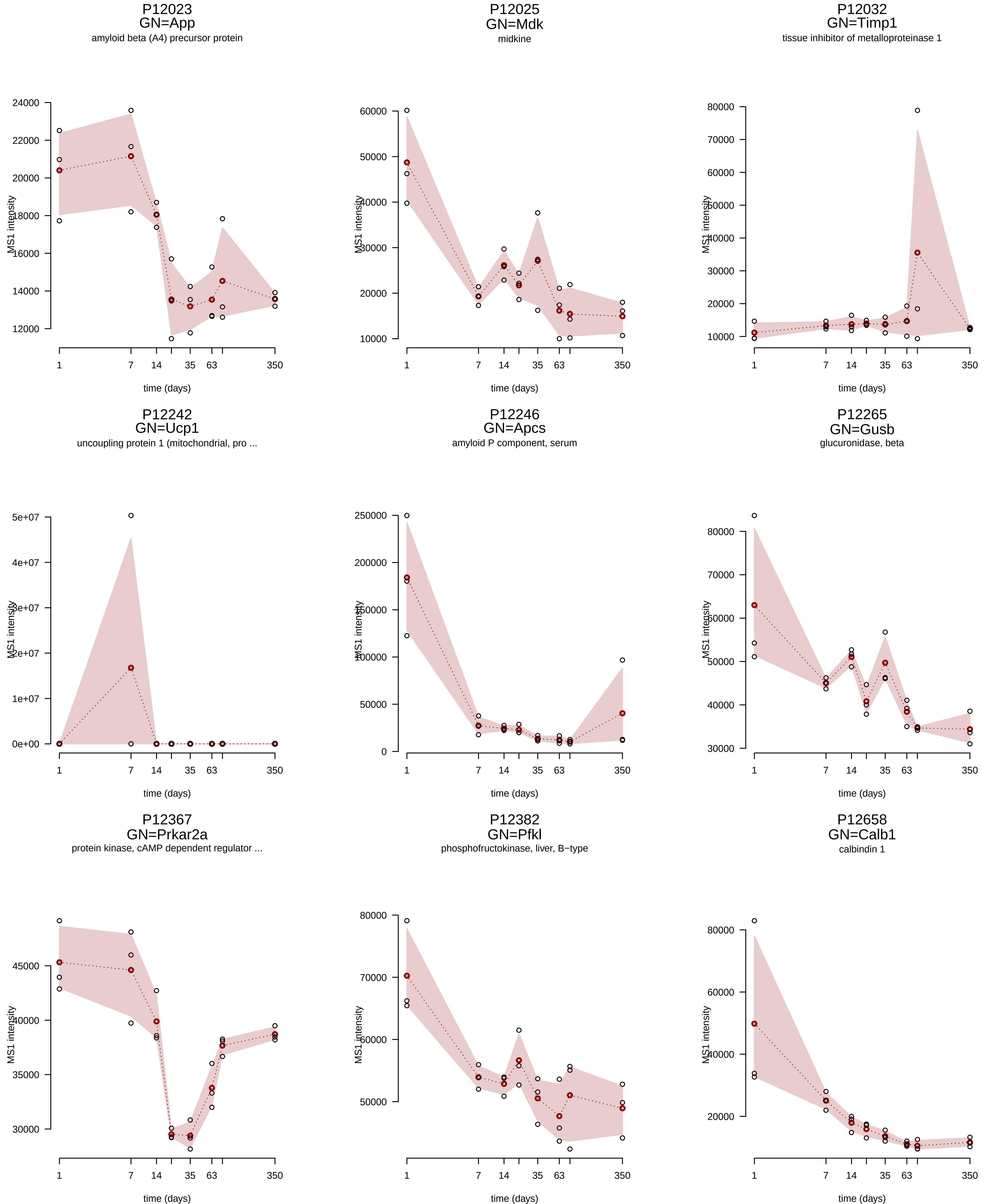
P12023
GN=App
amyloid beta (A4) precursor protein
24000
22000
20000
18000
16000
14000
12000
MS1 intensity
1
7
14
35
63
350
time (days)
P12025
GN=Mdk
midkine
60000
50000
40000
30000
20000
10000
MS1 intensity
1
7
14
35
63
350
time (days)
P12032
GN=Timp1
tissue inhibitor of metalloproteinase 1
80000
70000
60000
50000
40000
30000
20000
10000
MS1 intensity
1
7
14
35
63
350
time (days)
P12242
GN=Ucp1
uncoupling protein 1 (mitochondrial, pro ...
5e+07
4e+07
3e+07
2e+07
1e+07
0e+00
MS1 intensity
1
7
14
35
63
350
time (days)
P12246
GN=Apcs
amyloid P component, serum
250000
200000
150000
100000
50000
0
MS1 intensity
1
7
14
35
63
350
time (days)
P12265
GN=Gusb
glucuronidase, beta
80000
70000
60000
50000
40000
30000
MS1 intensity
1
7
14
35
63
350
time (days)
P12367
GN=Prkar2a
protein kinase, cAMP dependent regulator ...
45000
40000
35000
30000
MS1 intensity
1
7
14
35
63
350
time (days)
P12382
GN=Pfkl
phosphofructokinase, liver, B−type
80000
70000
60000
50000
MS1 intensity
1
7
14
35
63
350
time (days)
P12658
GN=Calb1
calbindin 1
80000
60000
40000
20000
MS1 intensity
1
7
14
35
63
350
time (days)

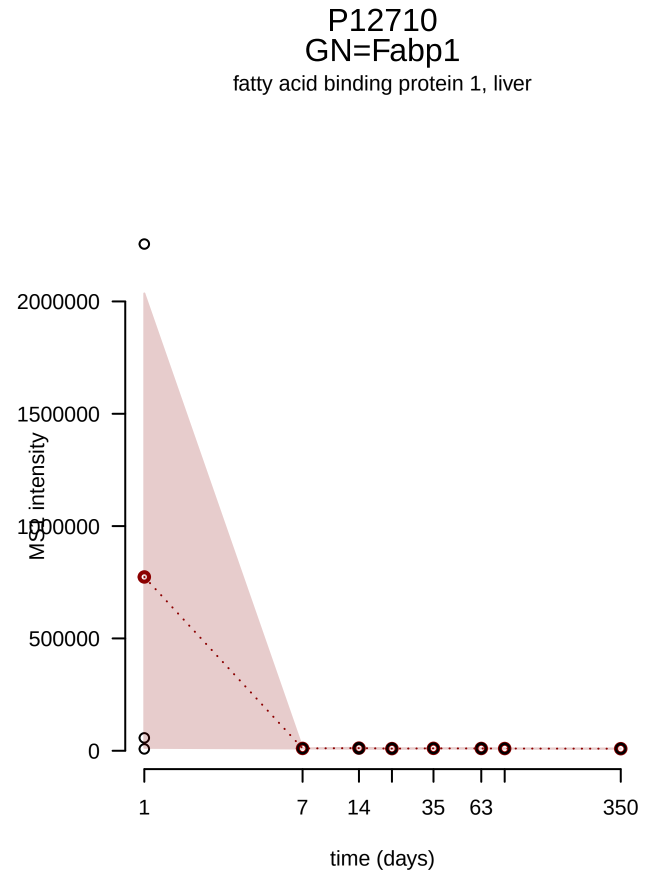
P12710
GN=Fabp1
fatty acid binding protein 1, liver
MS1 intensity
2000000
1500000
1000000
500000
0
1
7
14
35
63
350
time (days)

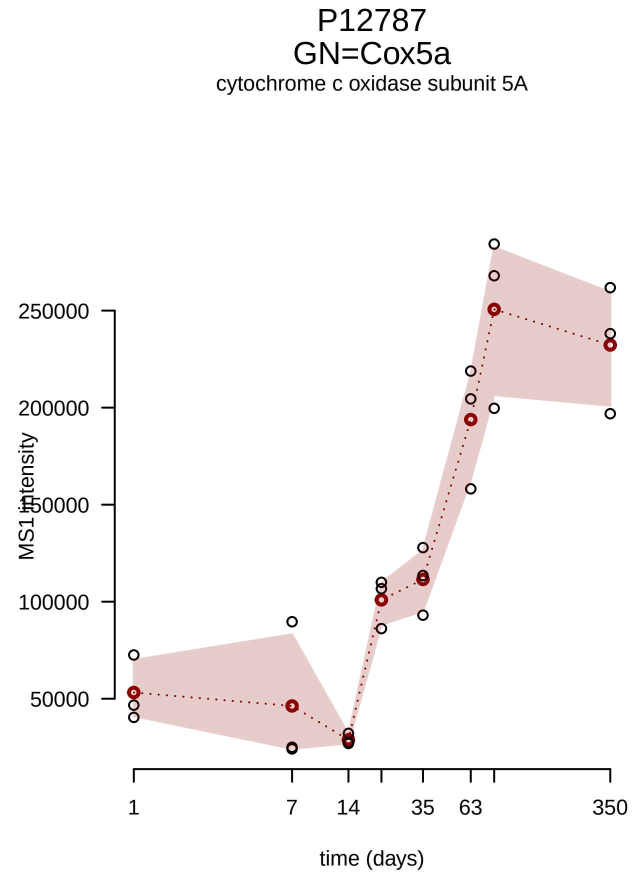
P12787
GN=Cox5a
cytochrome c oxidase subunit 5A
MS1 intensity
250000
200000
150000
100000
50000
1
7
14
35
63
350
time (days)

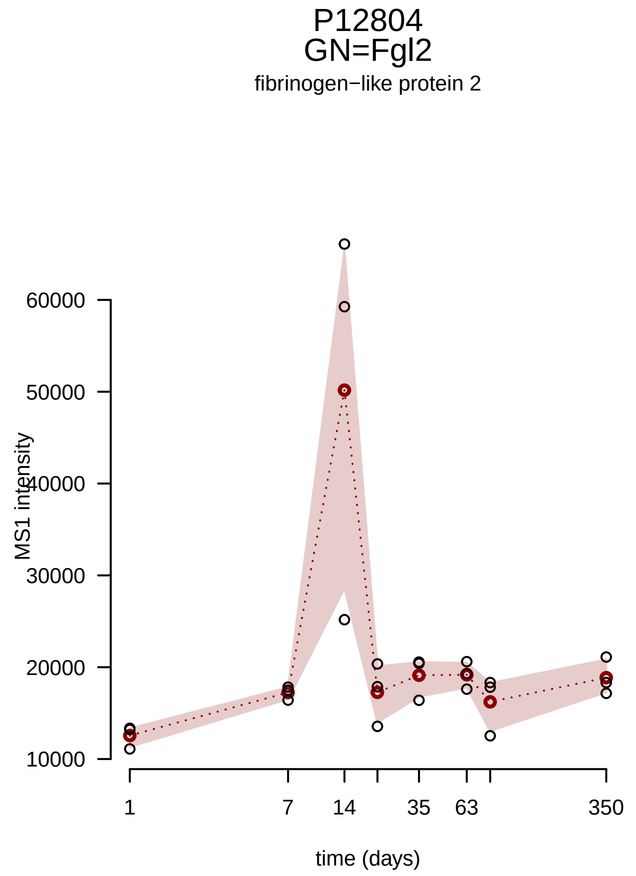
P12804
GN=Fgl2
fibrinogen−like protein 2
MS1 intensity
60000
50000
40000
30000
20000
10000
1
7
14
35
63
350
time (days)

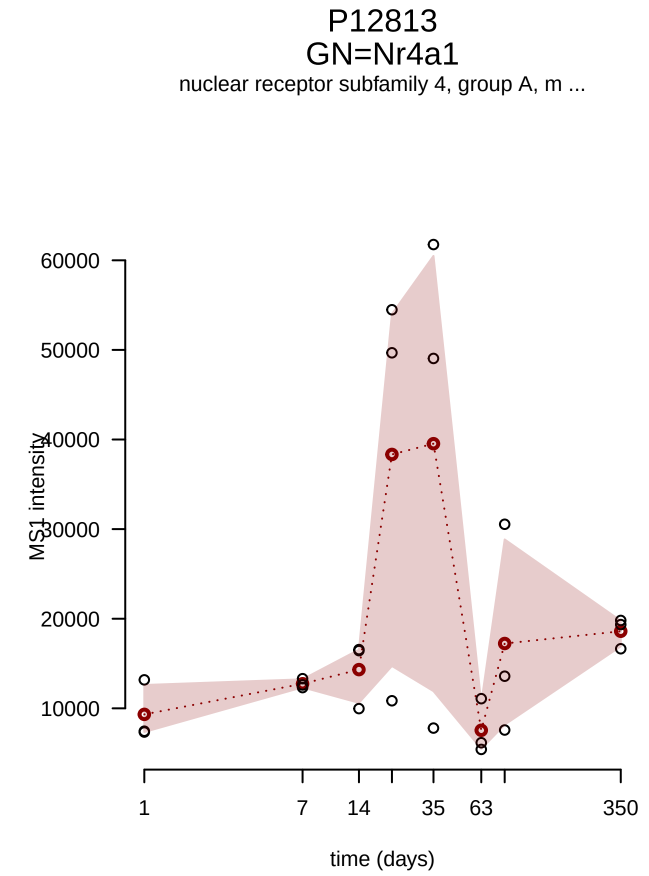
P12813
GN=Nr4a1
nuclear receptor subfamily 4, group A, m ...
MS1 intensity
60000
50000
40000
30000
20000
10000
1
7
14
35
63
350
time (days)

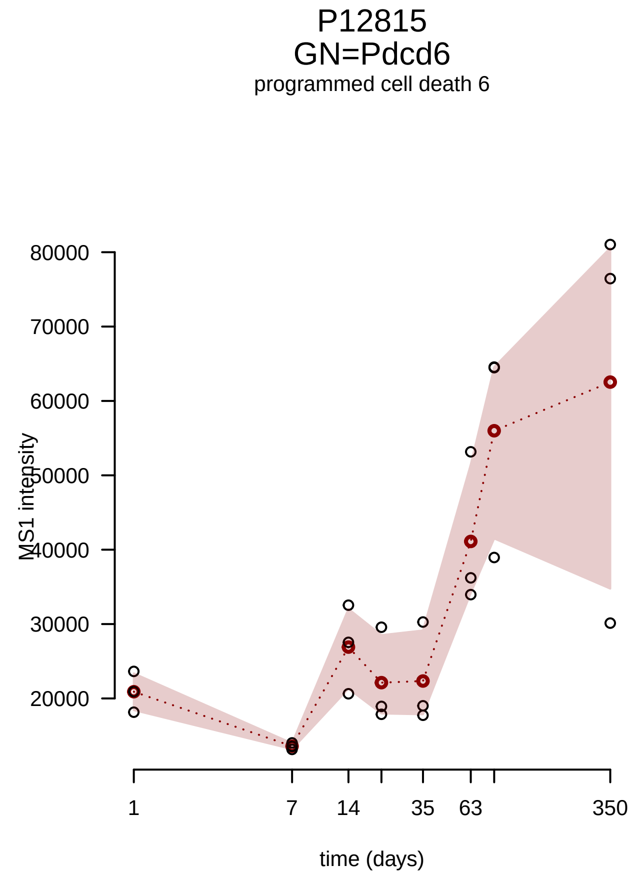
P12815
GN=Pdcd6
programmed cell death 6
MS1 intensity
80000
70000
60000
50000
40000
30000
20000
1
7
14
35
63
350
time (days)

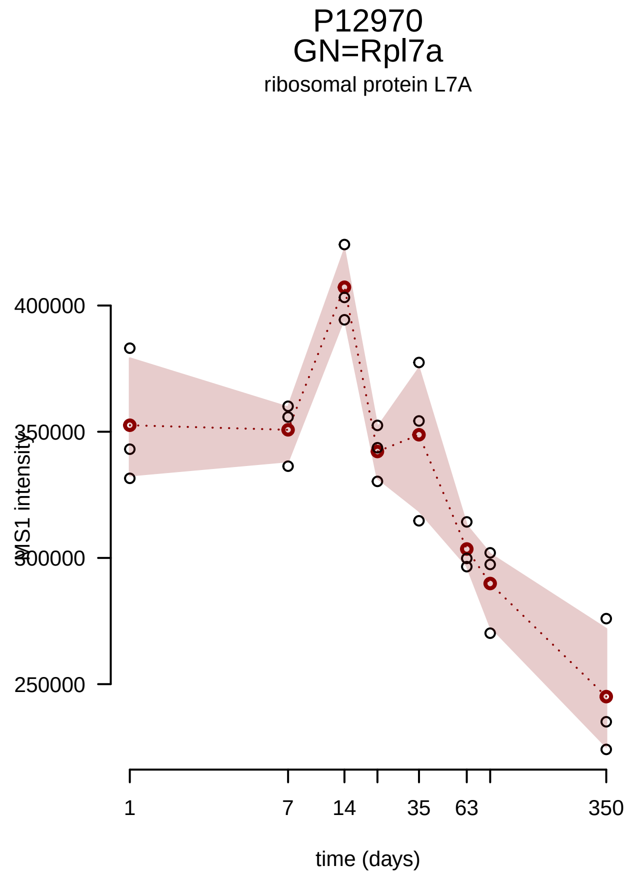
P12970
GN=Rpl7a
ribosomal protein L7A
MS1 intensity
400000
350000
300000
250000
1
7
14
35
63
350
time (days)

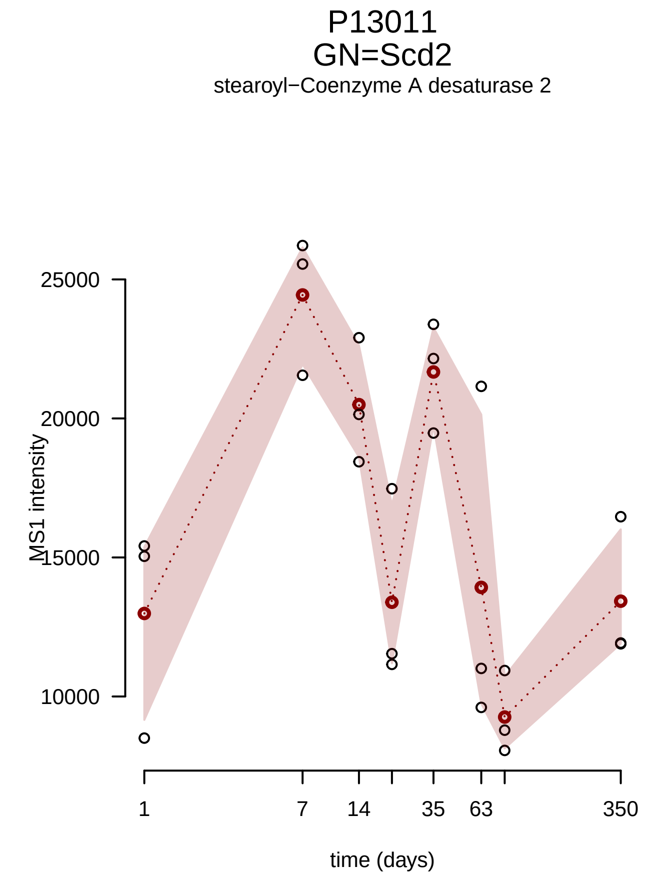
P13011
GN=Scd2
stearoyl−Coenzyme A desaturase 2
MS1 intensity
25000
20000
15000
10000
1
7
14
35
63
350
time (days)

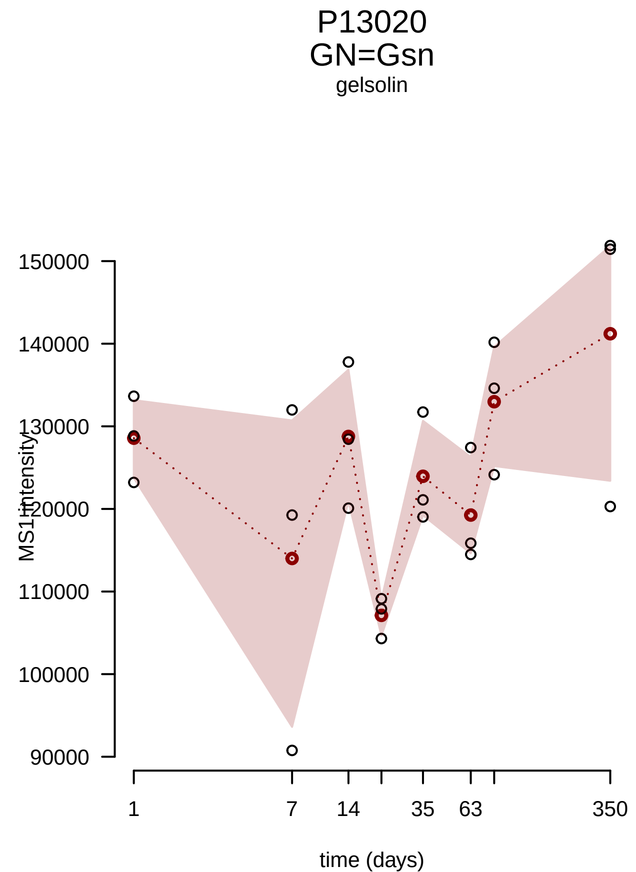
P13020
GN=Gsn
gelsolin
MS1 intensity
150000
140000
130000
120000
110000
100000
90000
1
7
14
35
63
350
time (days)

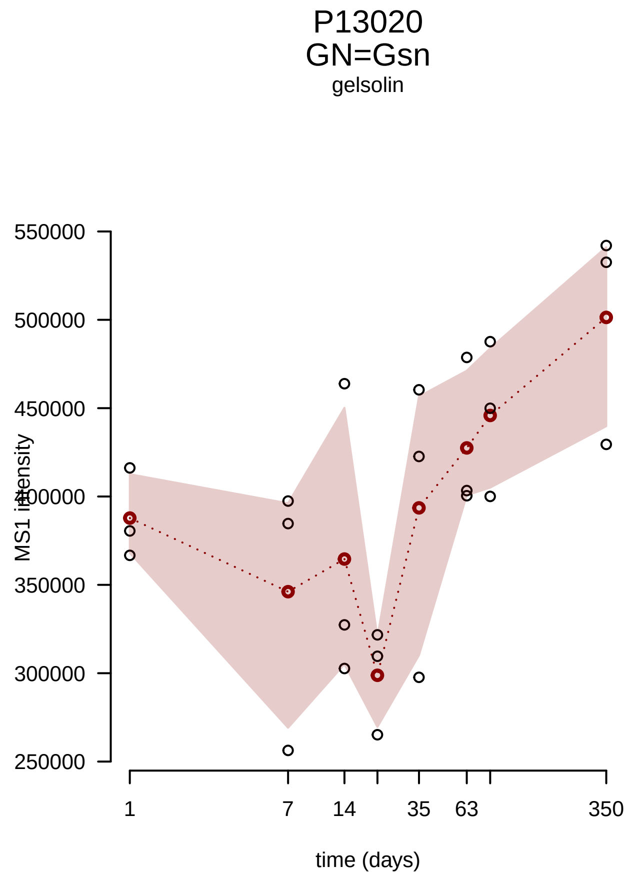
P13020
GN=Gsn
gelsolin
MS1 intensity
550000
500000
450000
400000
350000
300000
250000
1
7
14
35
63
350
time (days)

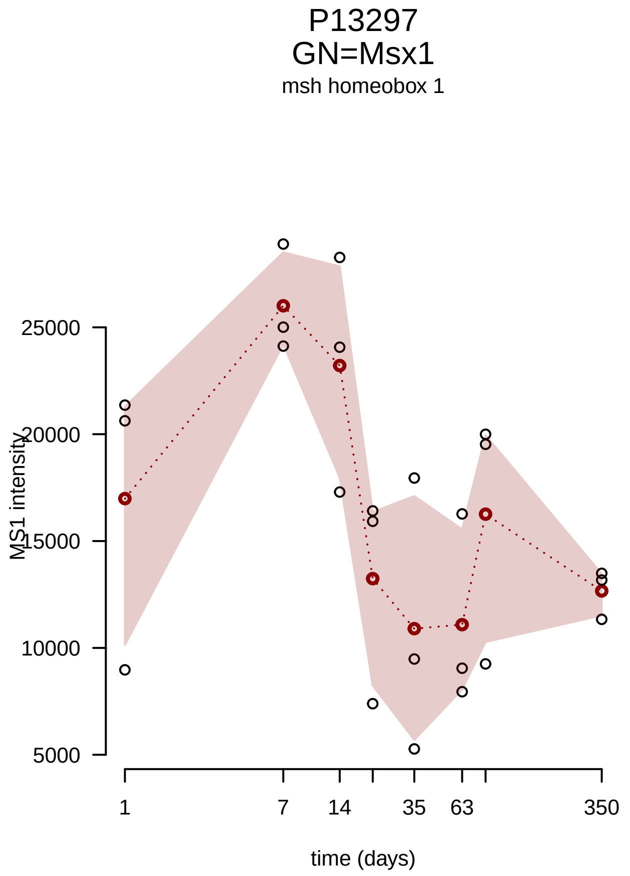
P13297
GN=Msx1
msh homeobox 1
5000
10000
15000
20000
25000
MS1 intensity
1
7
14
35
63
350
time (days)

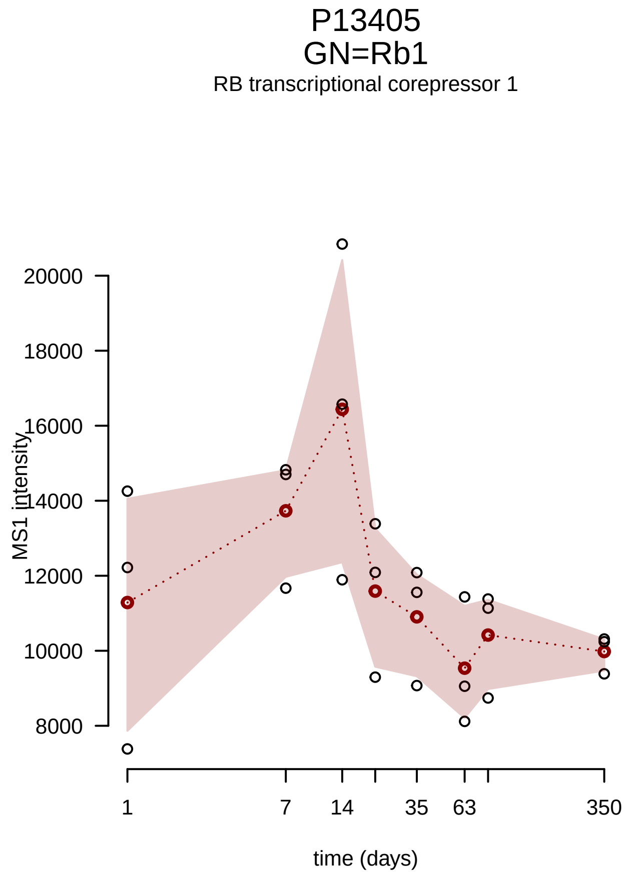
P13405
GN=Rb1
RB transcriptional corepressor 1
8000
10000
12000
14000
16000
18000
20000
MS1 intensity
1
7
14
35
63
350
time (days)

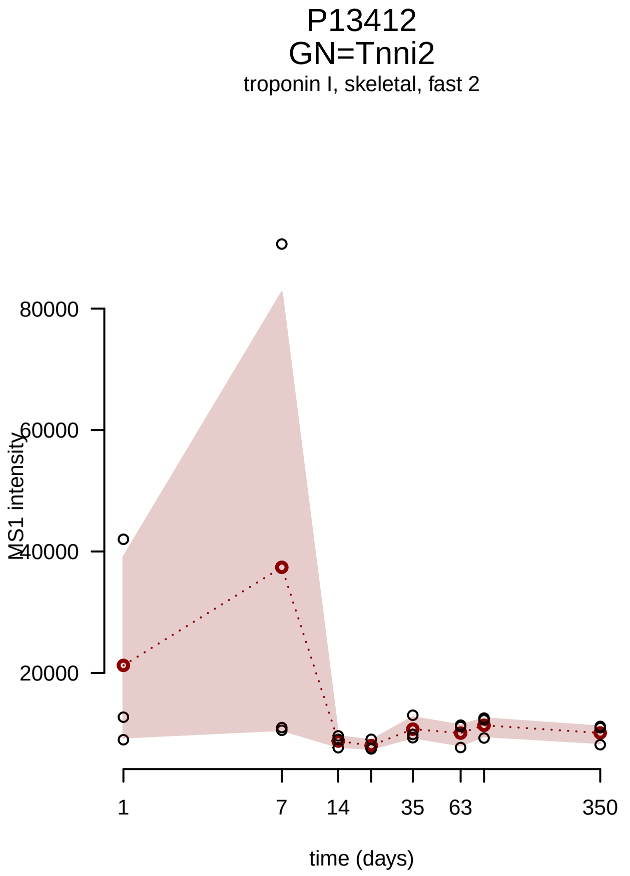
P13412
GN=Tnni2
troponin I, skeletal, fast 2
20000
40000
60000
80000
MS1 intensity
1
7
14
35
63
350
time (days)

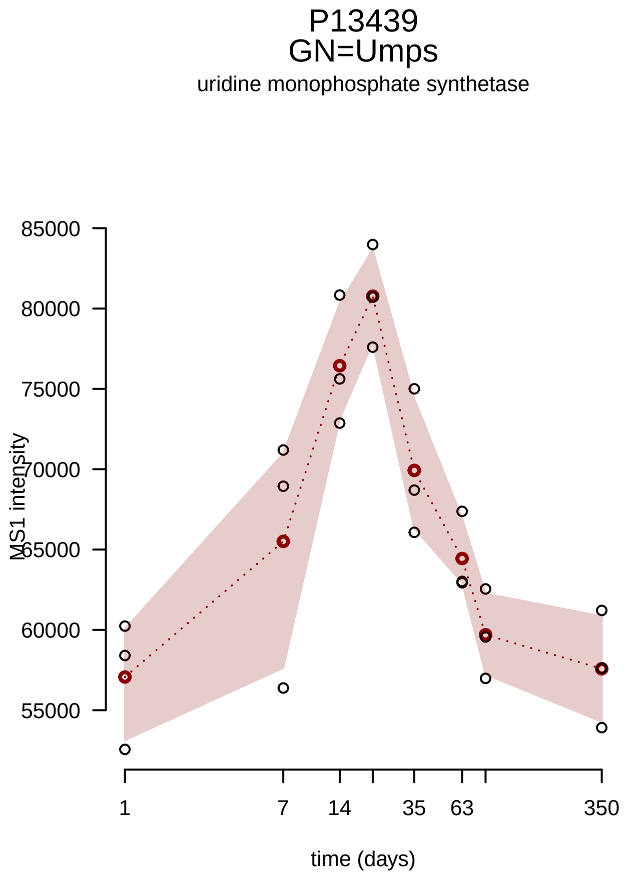
P13439
GN=Umps
uridine monophosphate synthetase
55000
60000
65000
70000
75000
80000
85000
MS1 intensity
1
7
14
35
63
350
time (days)

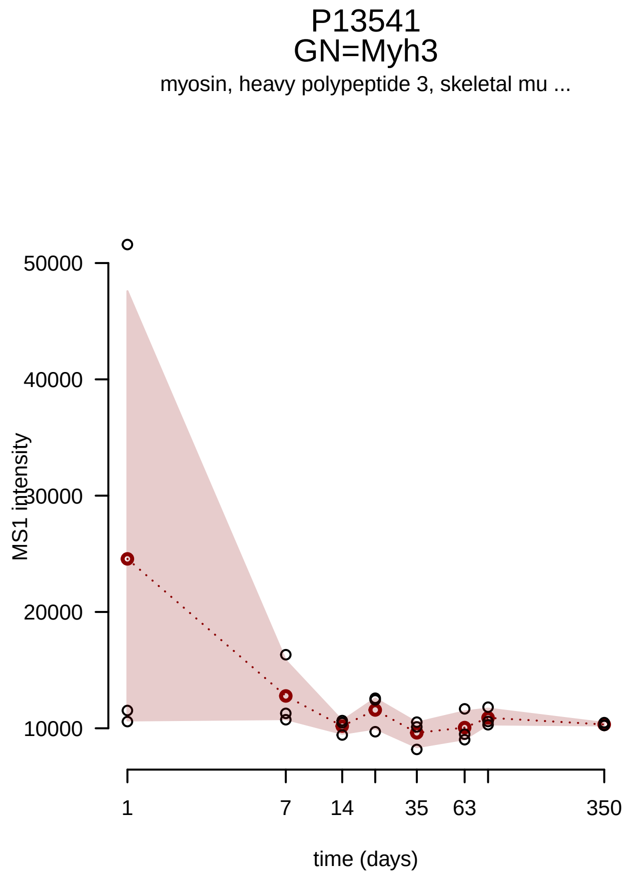
P13541
GN=Myh3
myosin, heavy polypeptide 3, skeletal mu ...
10000
20000
30000
40000
50000
MS1 intensity
1
7
14
35
63
350
time (days)

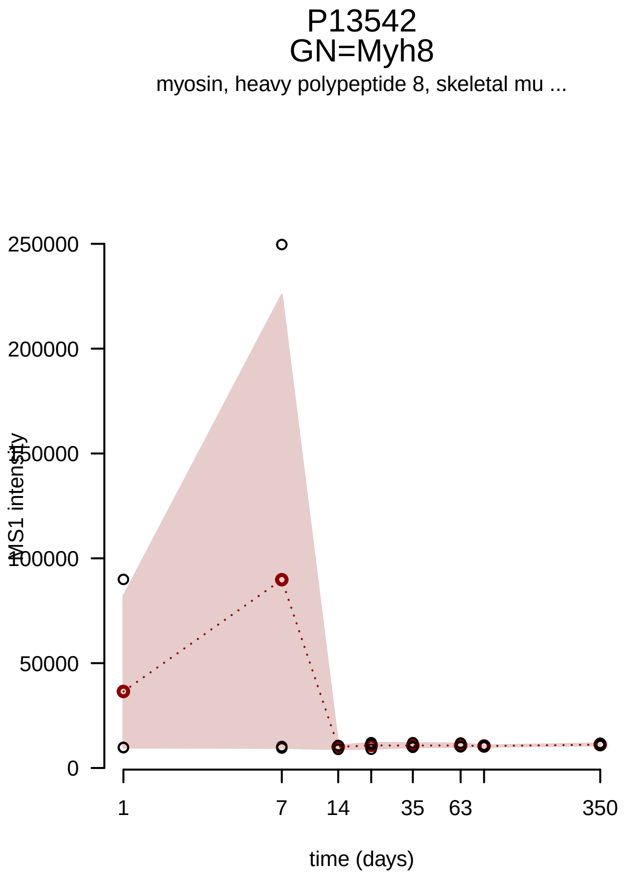
P13542
GN=Myh8
myosin, heavy polypeptide 8, skeletal mu ...
0
50000
100000
150000
200000
250000
MS1 intensity
1
7
14
35
63
350
time (days)

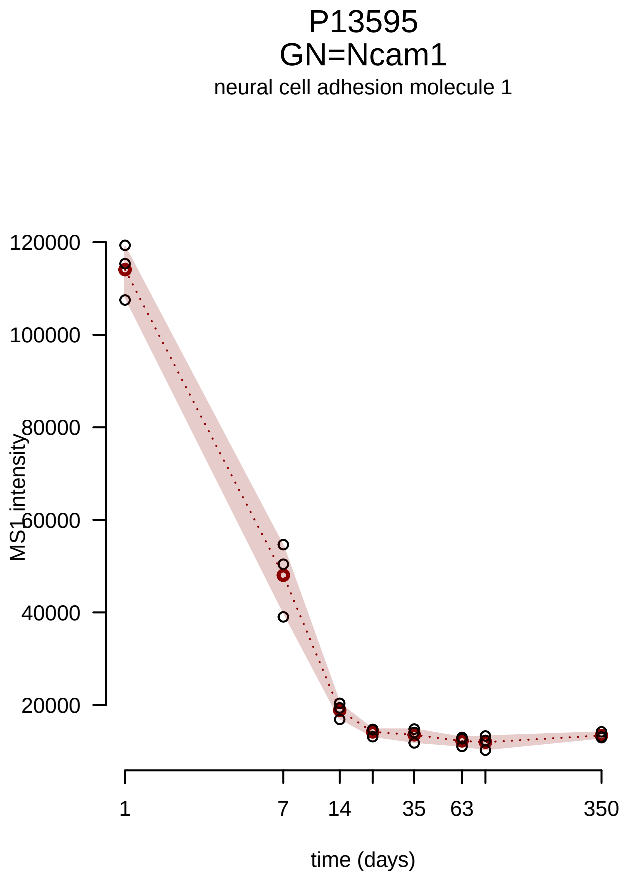
P13595
GN=Ncam1
neural cell adhesion molecule 1
20000
40000
60000
80000
100000
120000
MS1 intensity
1
7
14
35
63
350
time (days)

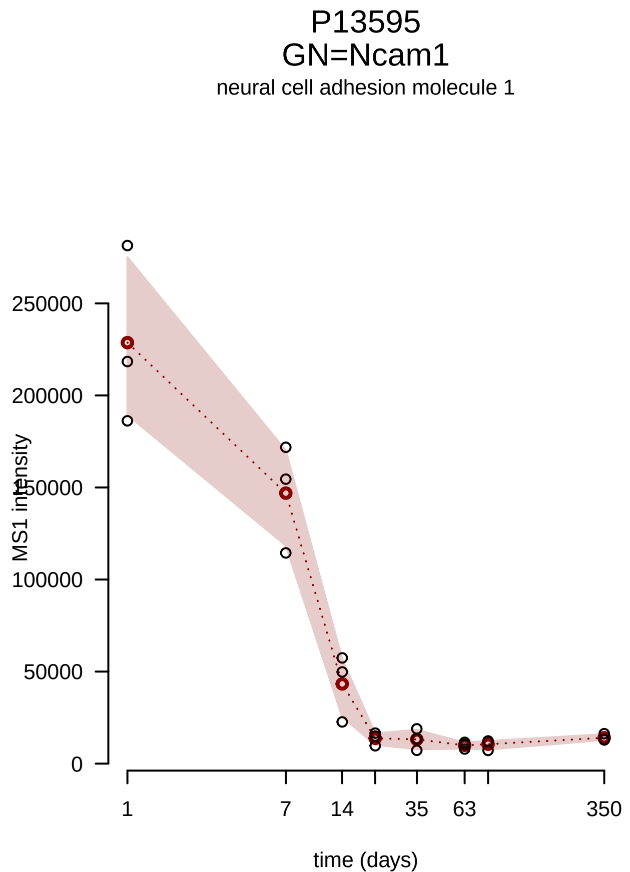
P13595
GN=Ncam1
neural cell adhesion molecule 1
0
50000
100000
150000
200000
250000
MS1 intensity
1
7
14
35
63
350
time (days)

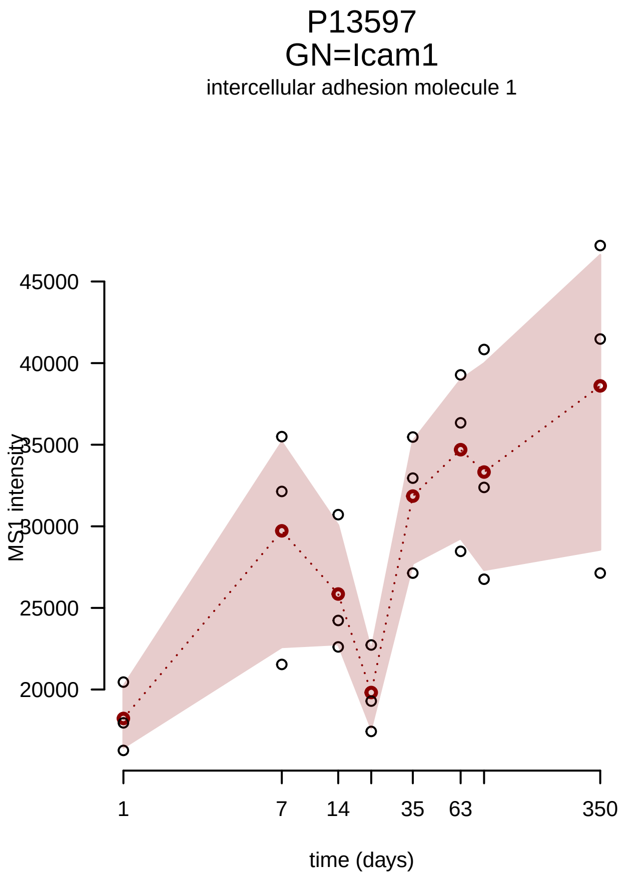
P13597
GN=Icam1
intercellular adhesion molecule 1
20000
25000
30000
35000
40000
45000
MS1 intensity
1
7
14
35
63
350
time (days)

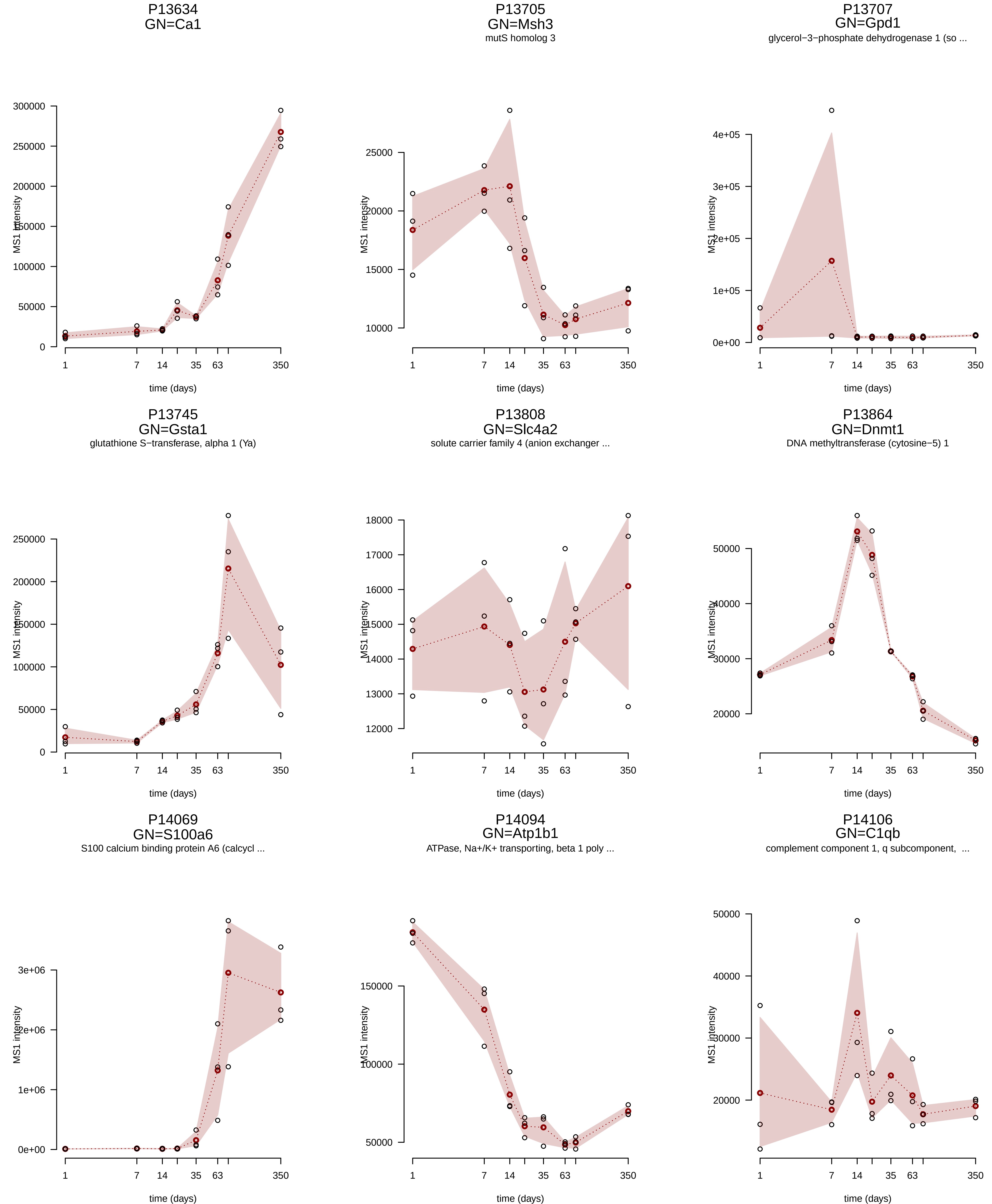

P13634
GN=Ca1
MS1 intensity
time (days)
P13705
GN=Msh3
mutS homolog 3
MS1 intensity
time (days)
P13707
GN=Gpd1
glycerol−3−phosphate dehydrogenase 1 (so ...
MS1 intensity
time (days)
P13745
GN=Gsta1
glutathione S−transferase, alpha 1 (Ya)
MS1 intensity
time (days)
P13808
GN=Slc4a2
solute carrier family 4 (anion exchanger ...
MS1 intensity
time (days)
P13864
GN=Dnmt1
DNA methyltransferase (cytosine−5) 1
MS1 intensity
time (days)
P14069
GN=S100a6
S100 calcium binding protein A6 (calcycl ...
MS1 intensity
time (days)
P14094
GN=Atp1b1
ATPase, Na+/K+ transporting, beta 1 poly ...
MS1 intensity
time (days)
P14106
GN=C1qb
complement component 1, q subcomponent, ...
MS1 intensity
time (days)

P14115
GN=Rpl27a
ribosomal protein L27A

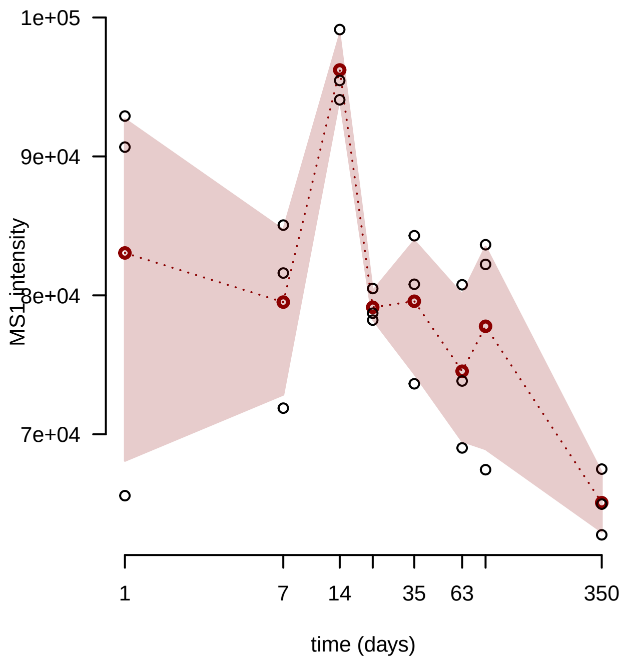

P14131
GN=Rps16
ribosomal protein S16

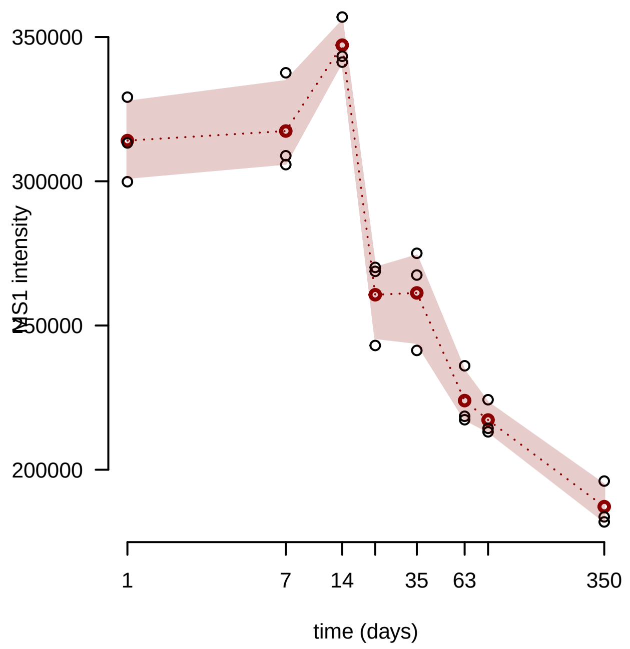

P14142
GN=Slc2a4
solute carrier family 2 (facilitated glu ...

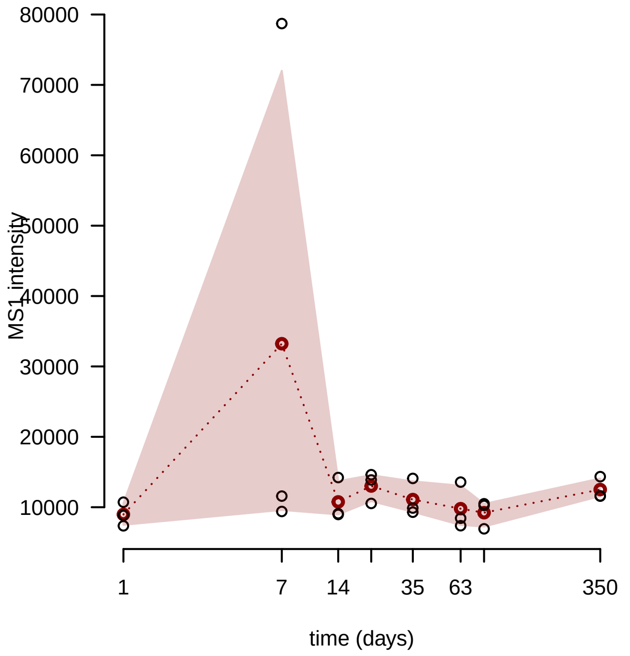

P14148
GN=Rpl7
ribosomal protein L7

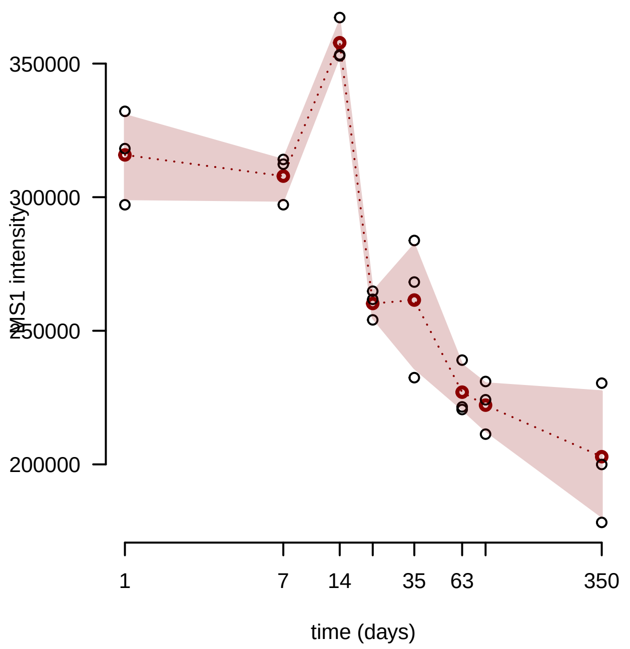

P14152
GN=Mdh1
malate dehydrogenase 1, NAD (soluble)

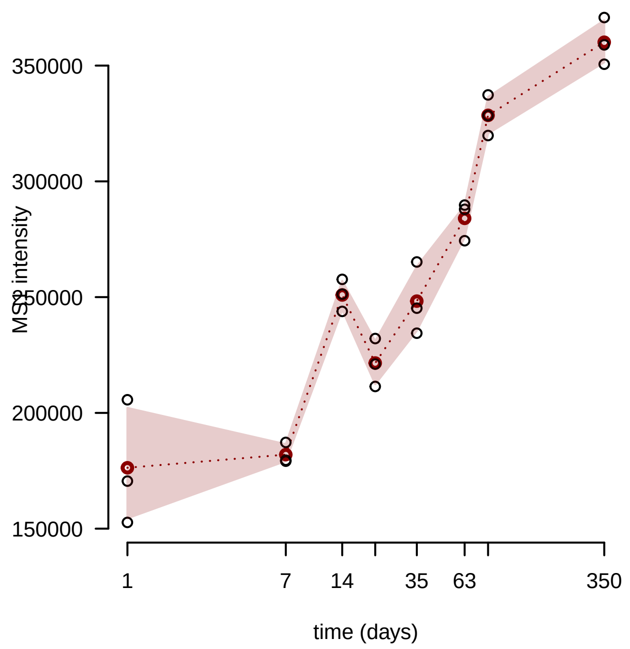

P14206
GN=Rpsa
ribosomal protein SA

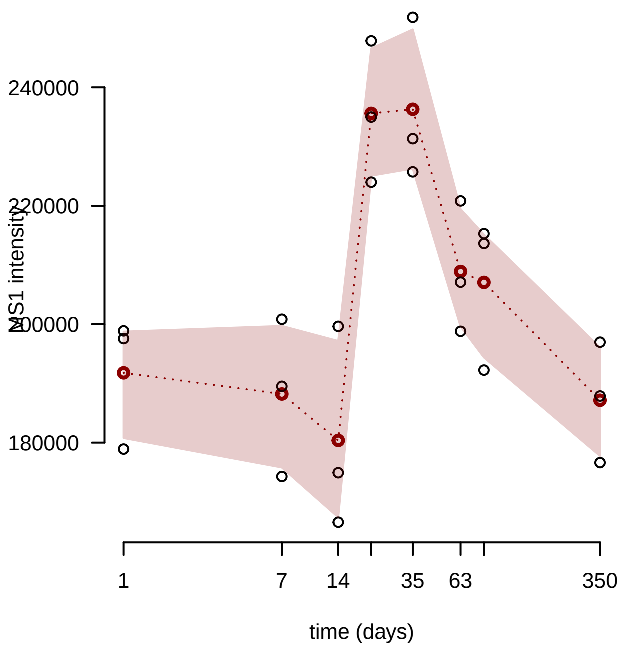

P14211
GN=Calr
calreticulin

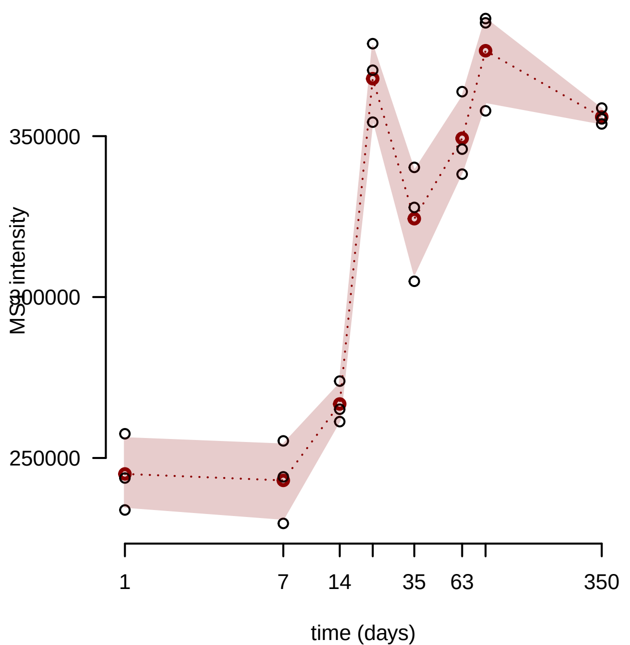

P14231
GN=Atp1b2
ATPase, Na+/K+ transporting, beta 2 poly ...

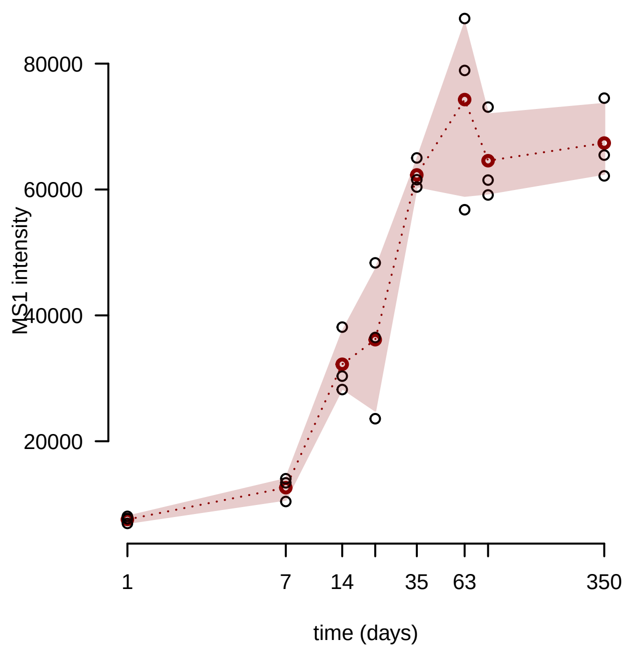

P14426
GN=H2−D1

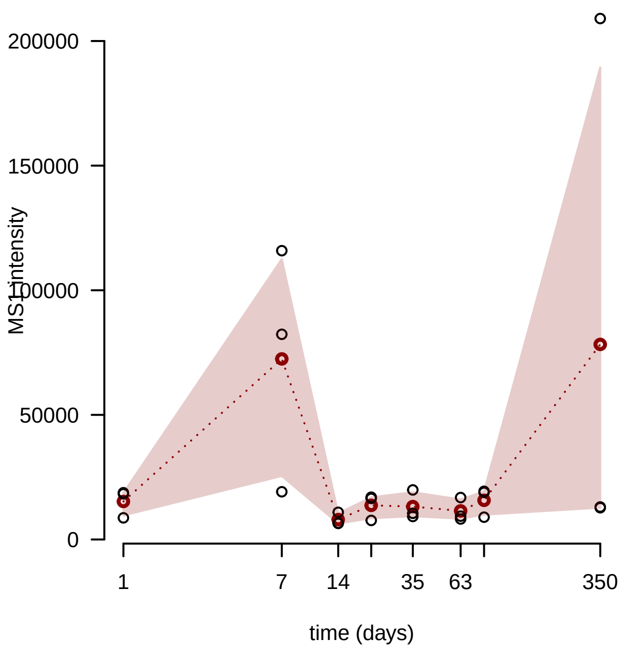

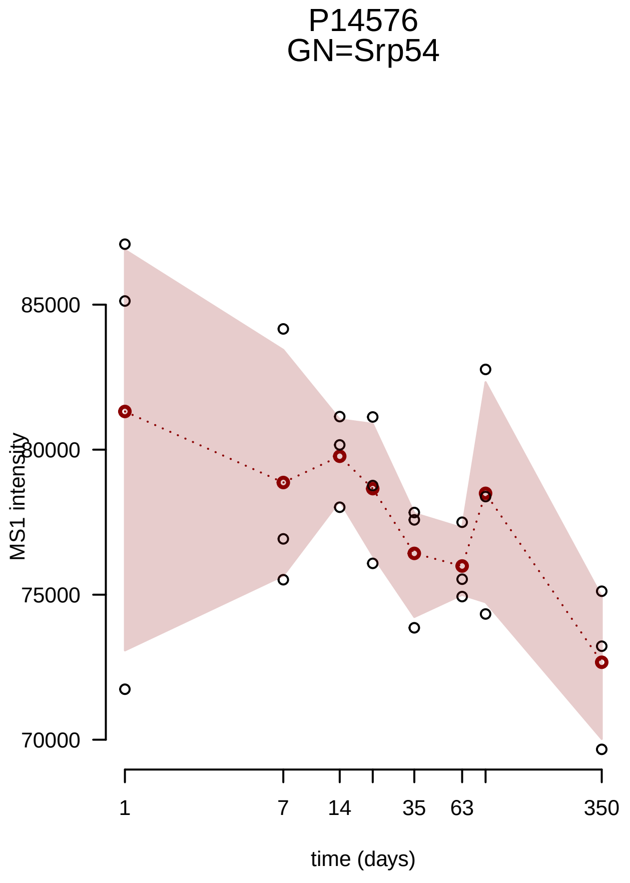
P14576
GN=Srp54
MS1 intensity
85000
80000
75000
70000
1
7
14
35
63
350
time (days)

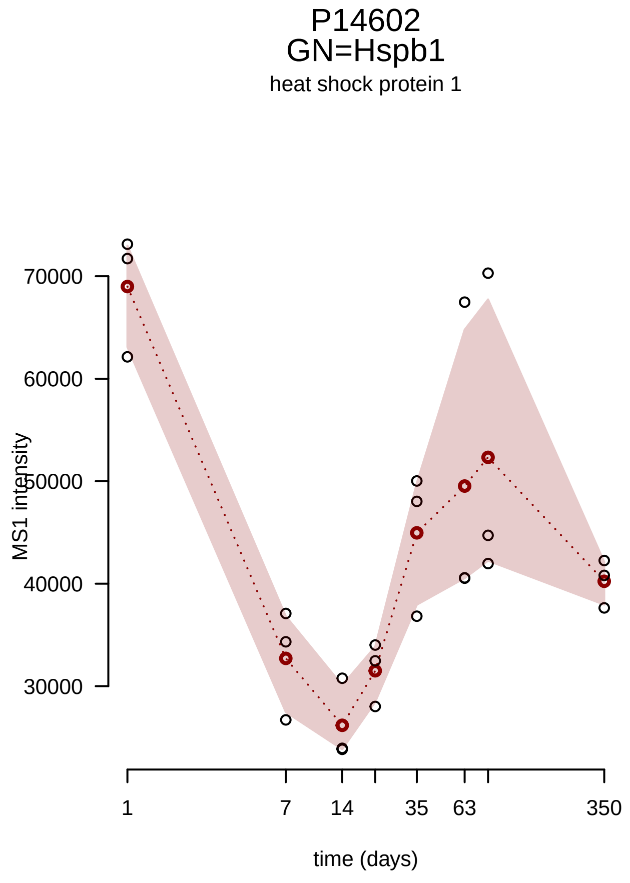
P14602
GN=Hspb1
heat shock protein 1
MS1 intensity
70000
60000
50000
40000
30000
1
7
14
35
63
350
time (days)

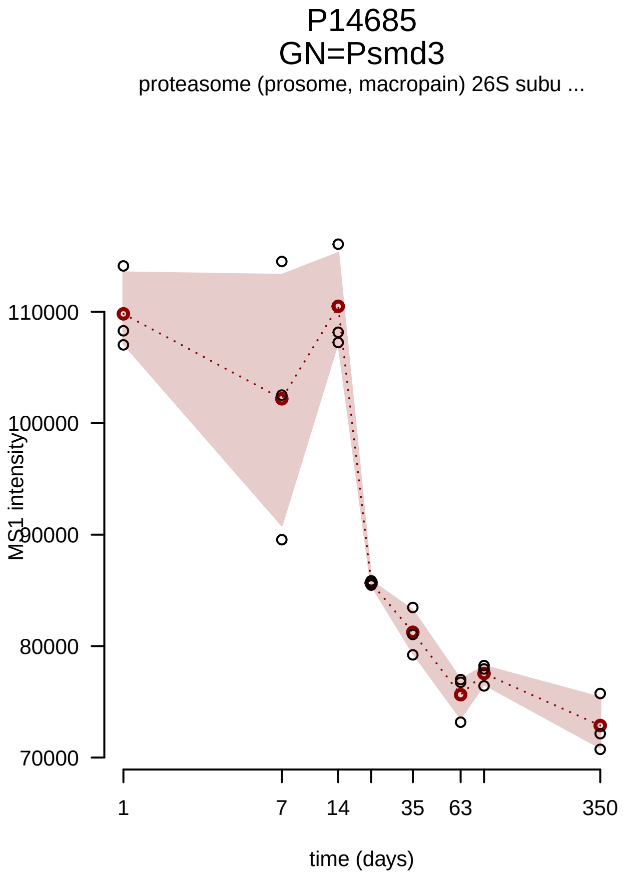
P14685
GN=Psmd3
proteasome (prosome, macropain) 26S subu ...
MS1 intensity
110000
100000
90000
80000
70000
1
7
14
35
63
350
time (days)

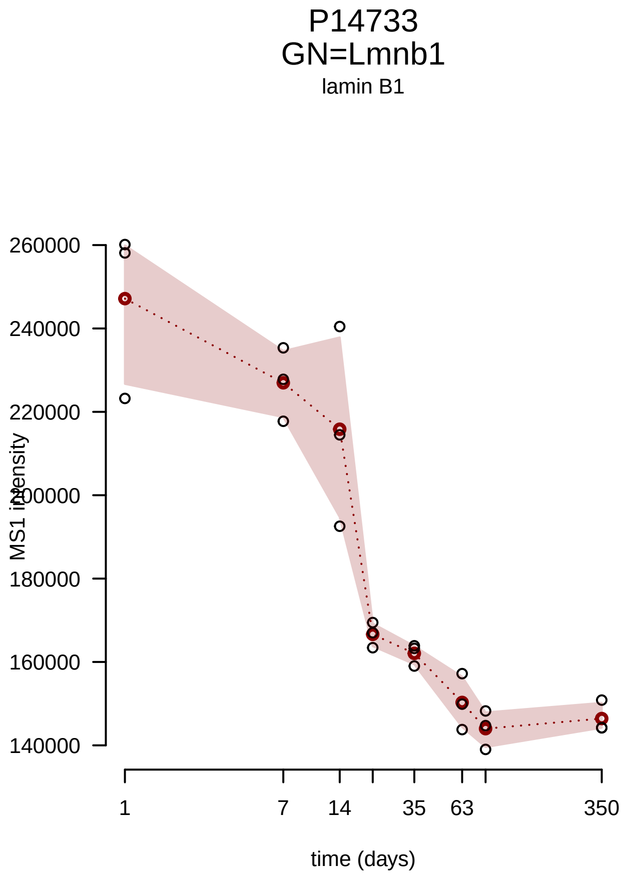
P14733
GN=Lmnb1
lamin B1
MS1 intensity
260000
240000
220000
200000
180000
160000
140000
1
7
14
35
63
350
time (days)

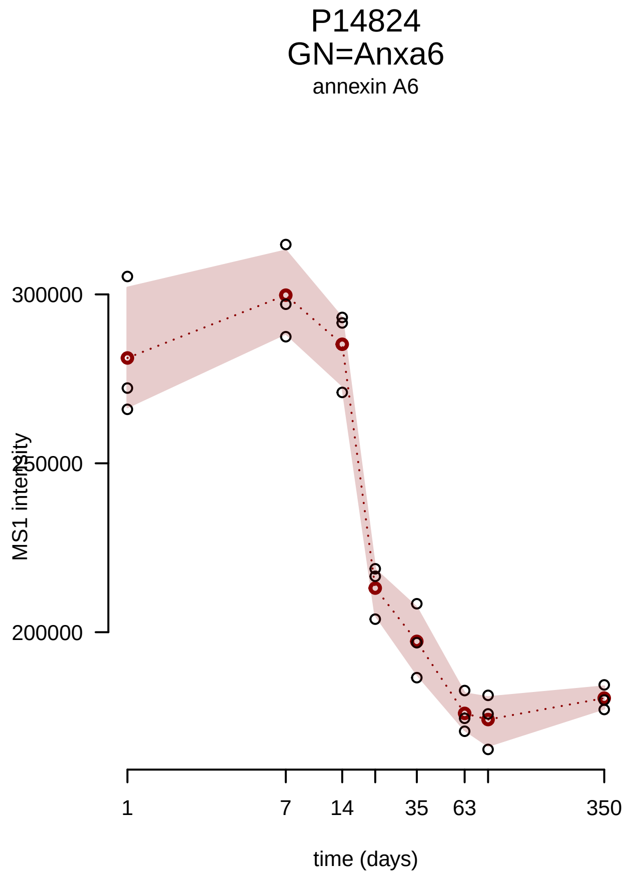
P14824
GN=Anxa6
annexin A6
MS1 intensity
300000
250000
200000
1
7
14
35
63
350
time (days)

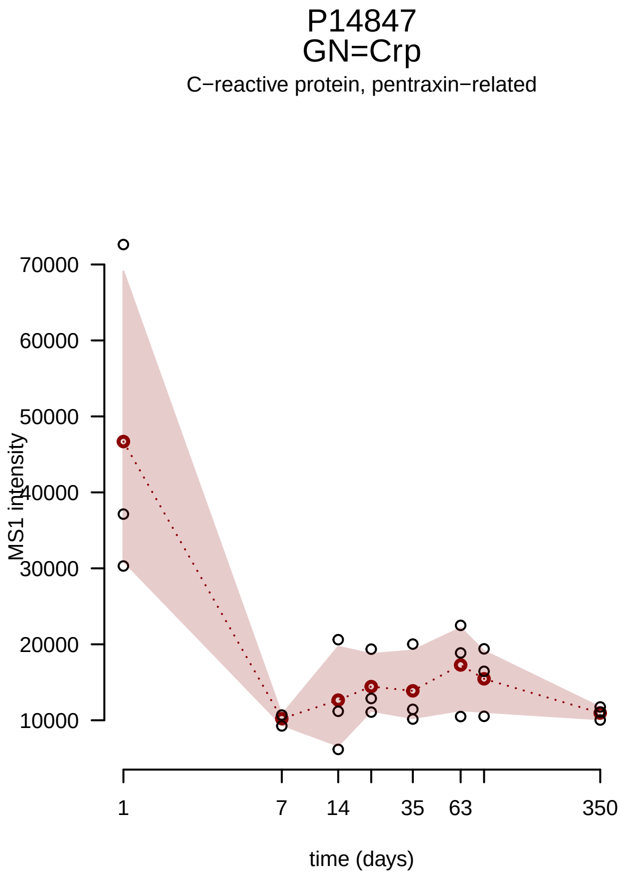
P14847
GN=Crp
C−reactive protein, pentraxin−related
MS1 intensity
70000
60000
50000
40000
30000
20000
10000
1
7
14
35
63
350
time (days)

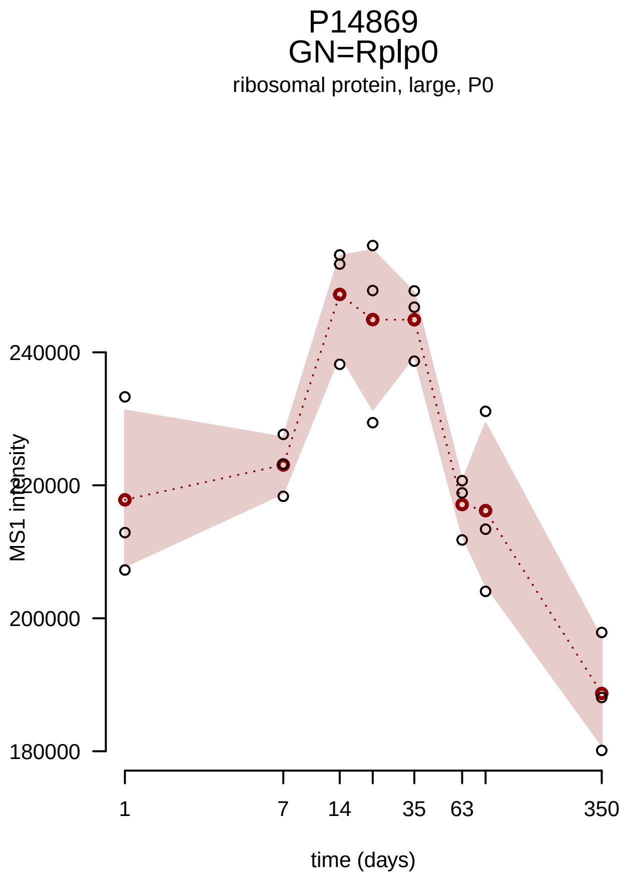
P14869
GN=Rplp0
ribosomal protein, large, P0
MS1 intensity
240000
220000
200000
180000
1
7
14
35
63
350
time (days)

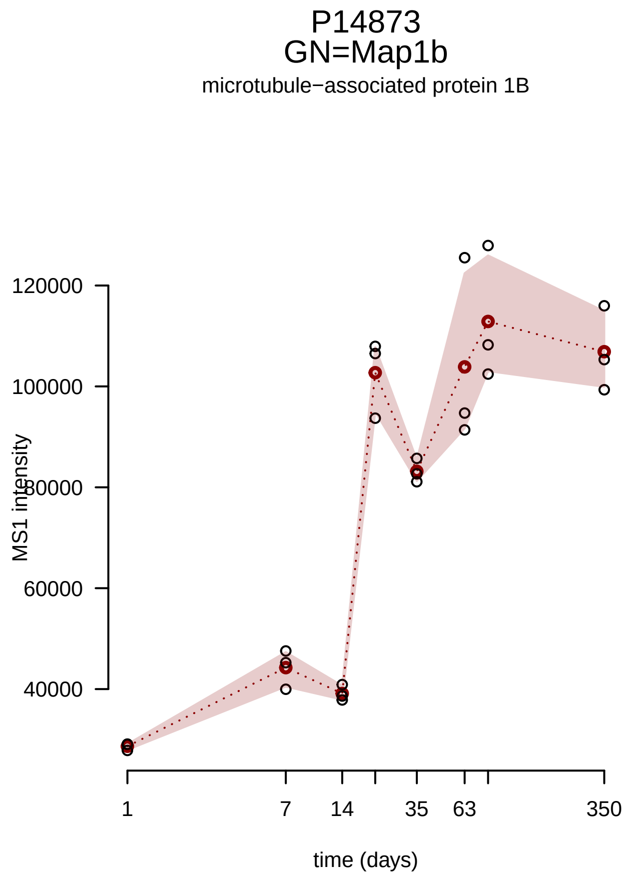
P14873
GN=Map1b
microtubule−associated protein 1B
MS1 intensity
120000
100000
80000
60000
40000
1
7
14
35
63
350
time (days)

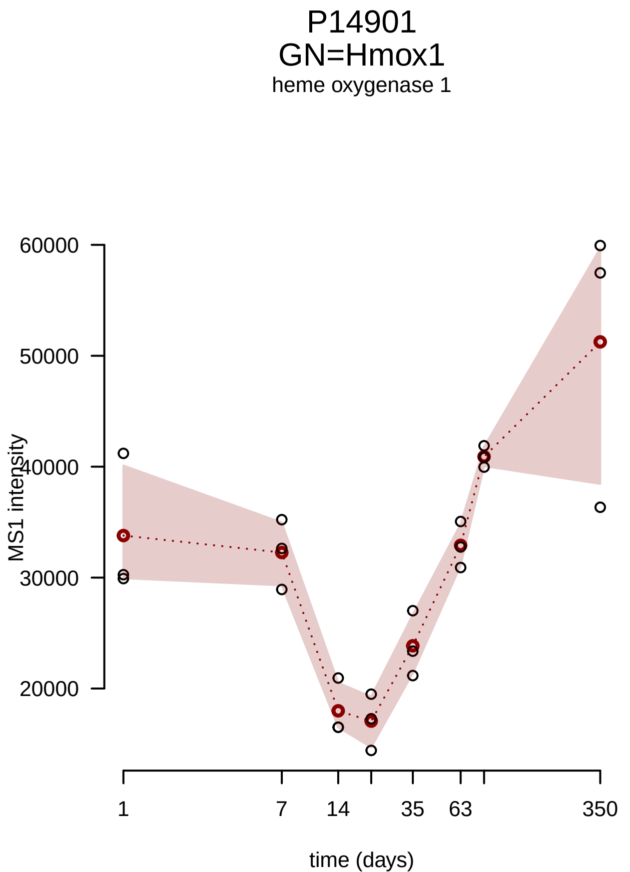
P14901
GN=Hmox1
heme oxygenase 1
MS1 intensity
60000
50000
40000
30000
20000
1
7
14
35
63
350
time (days)

P15066
GN=Jund
jun D proto−oncogene

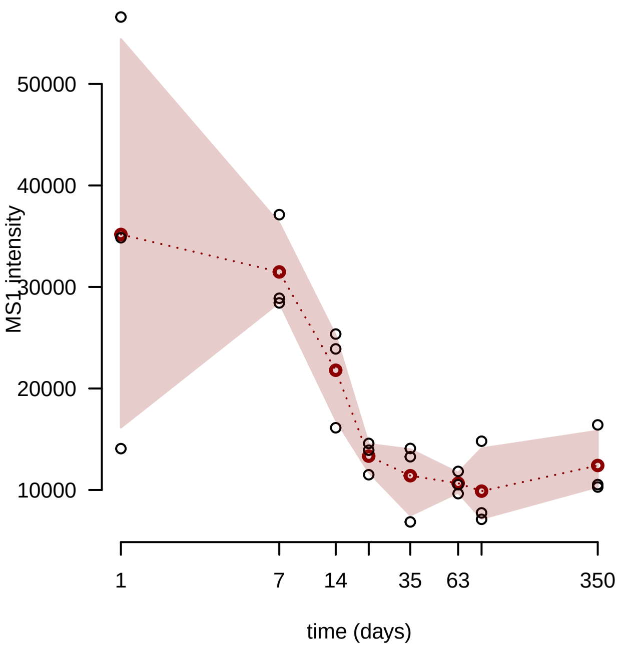

P15089
GN=Cpa3
carboxypeptidase A3, mast cell

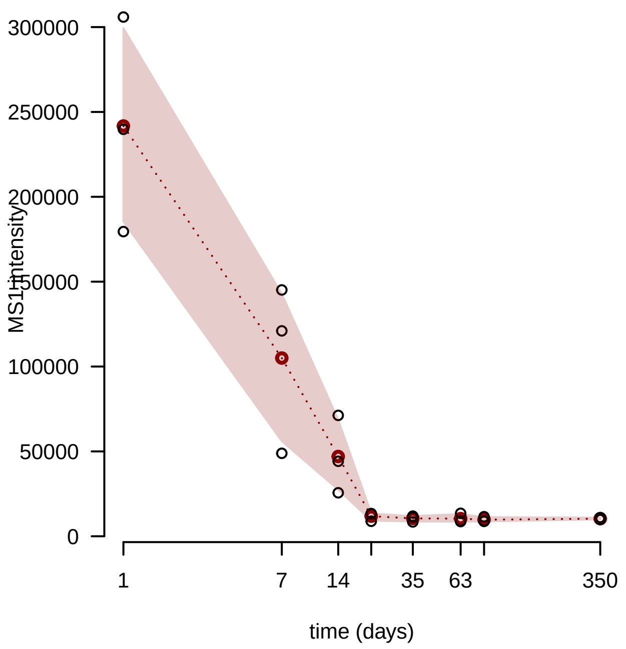

P15105
GN=Glul
glutamate−ammonia ligase (glutamine synt ...

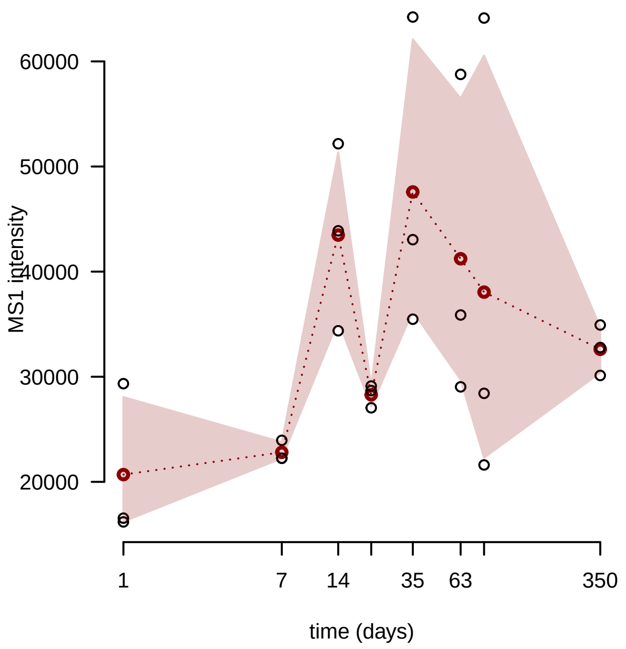

P15116
GN=Cdh2
cadherin 2

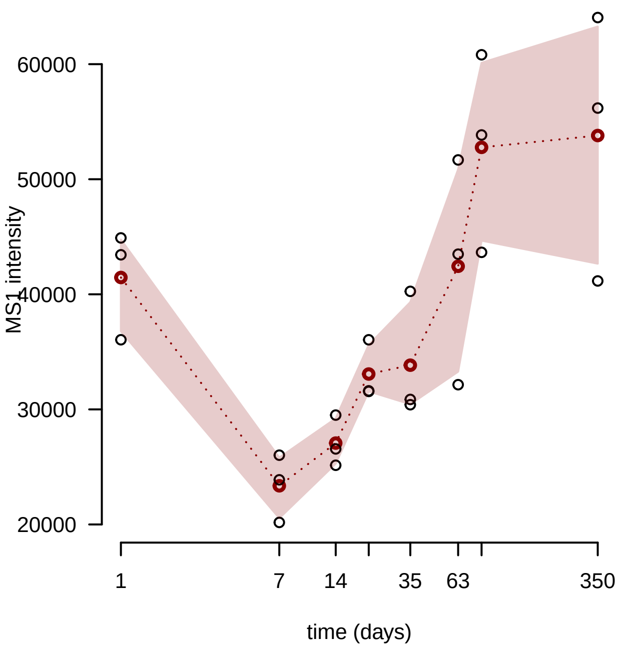

P15208
GN=Insr
insulin receptor

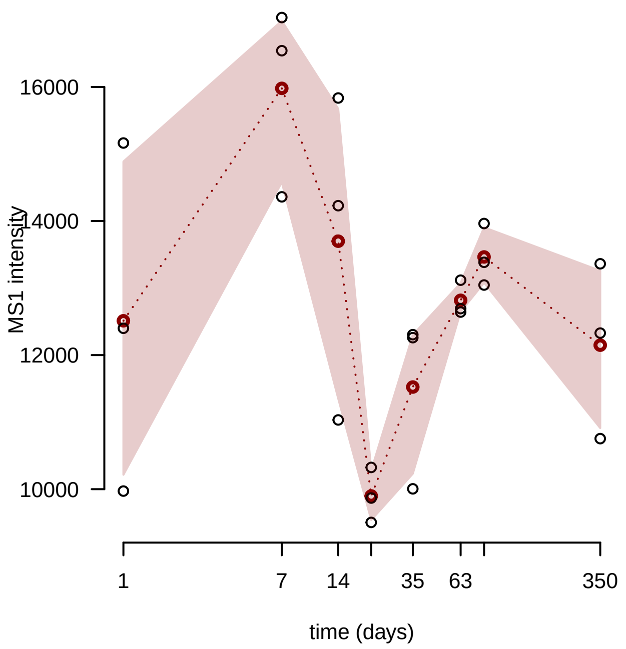

P15261
GN=Ifngr1
interferon gamma receptor 1

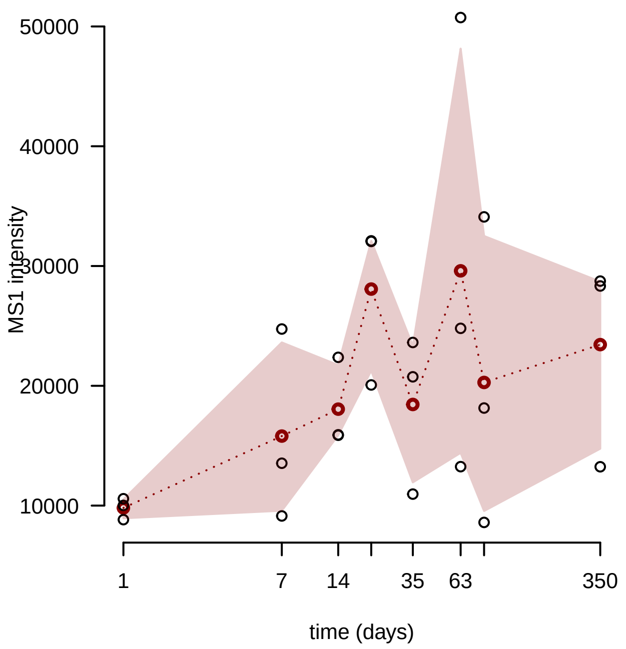

P15306
GN=Thbd
thrombomodulin

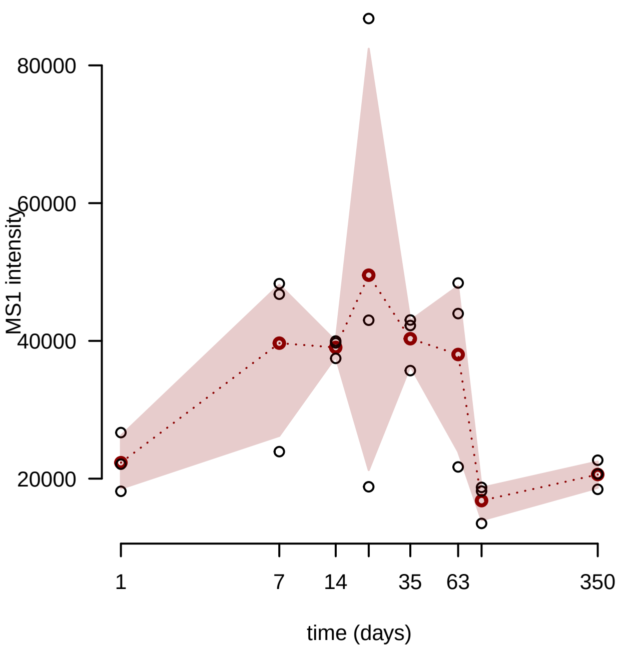

P15307
GN=Rel
reticuloendotheliosis oncogene

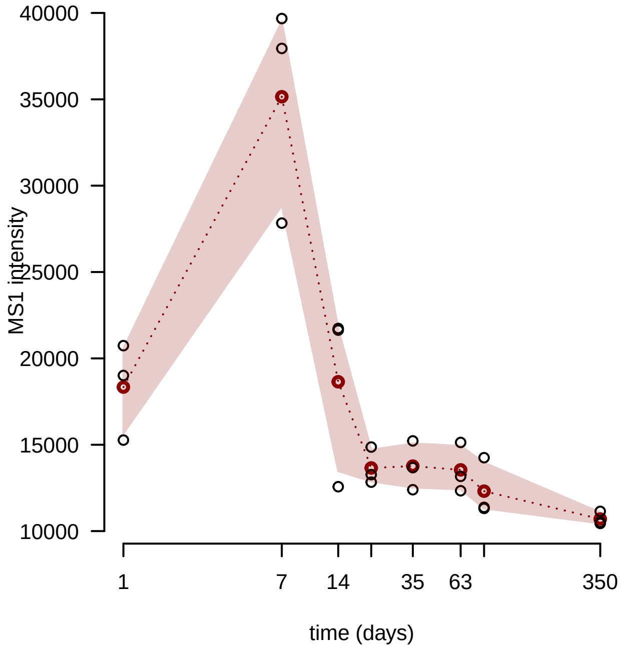

P15327
GN=Bpgm
2,3−bisphosphoglycerate mutase

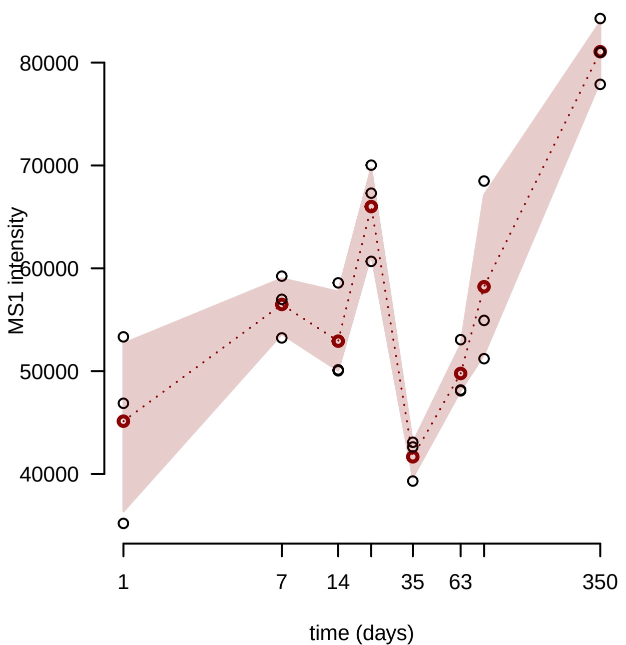

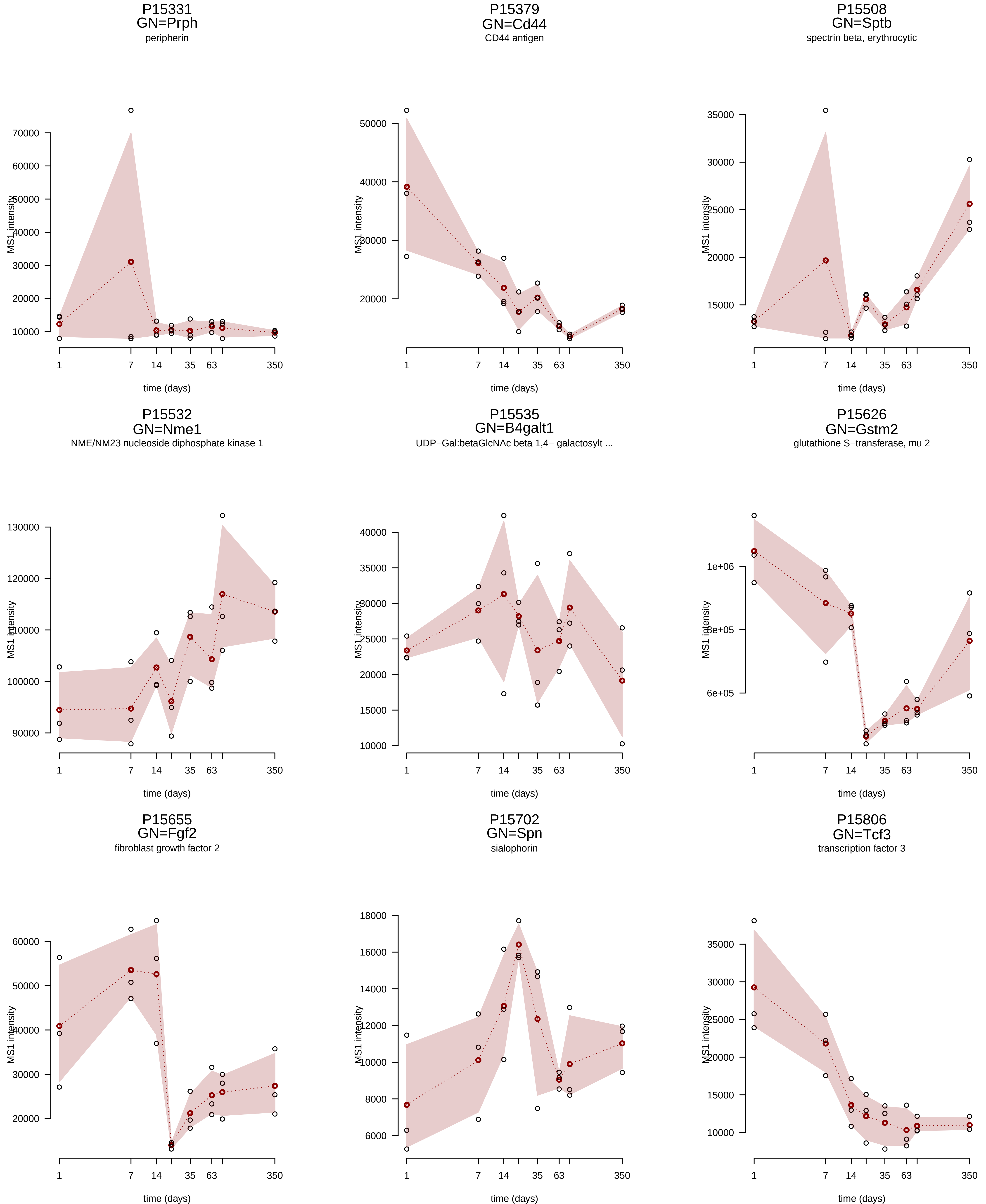
P15331
GN=Prph
peripherin
70000
60000
50000
40000
30000
20000
10000
MS1 intensity
1
7
14
35
63
350
time (days)
P15379
GN=Cd44
CD44 antigen
50000
40000
30000
20000
MS1 intensity
1
7
14
35
63
350
time (days)
P15508
GN=Sptb
spectrin beta, erythrocytic
35000
30000
25000
20000
15000
MS1 intensity
1
7
14
35
63
350
time (days)
P15532
GN=Nme1
NME/NM23 nucleoside diphosphate kinase 1
130000
120000
110000
100000
90000
MS1 intensity
1
7
14
35
63
350
time (days)
P15535
GN=B4galt1
UDP−Gal:betaGlcNAc beta 1,4− galactosylt ...
40000
35000
30000
25000
20000
15000
10000
MS1 intensity
1
7
14
35
63
350
time (days)
P15626
GN=Gstm2
glutathione S−transferase, mu 2
1e+06
8e+05
6e+05
MS1 intensity
1
7
14
35
63
350
time (days)
P15655
GN=Fgf2
fibroblast growth factor 2
60000
50000
40000
30000
20000
MS1 intensity
1
7
14
35
63
350
time (days)
P15702
GN=Spn
sialophorin
18000
16000
14000
12000
10000
8000
6000
MS1 intensity
1
7
14
35
63
350
time (days)
P15806
GN=Tcf3
transcription factor 3
35000
30000
25000
20000
15000
10000
MS1 intensity
1
7
14
35
63
350
time (days)

P15864
GN=Hist1h1c

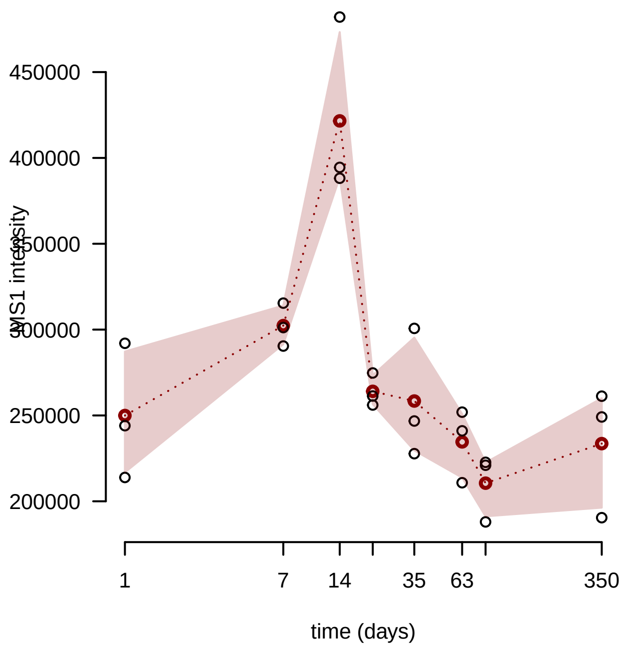

P15920
GN=Atp6v0a2
ATPase, H+ transporting, lysosomal V0 su ...

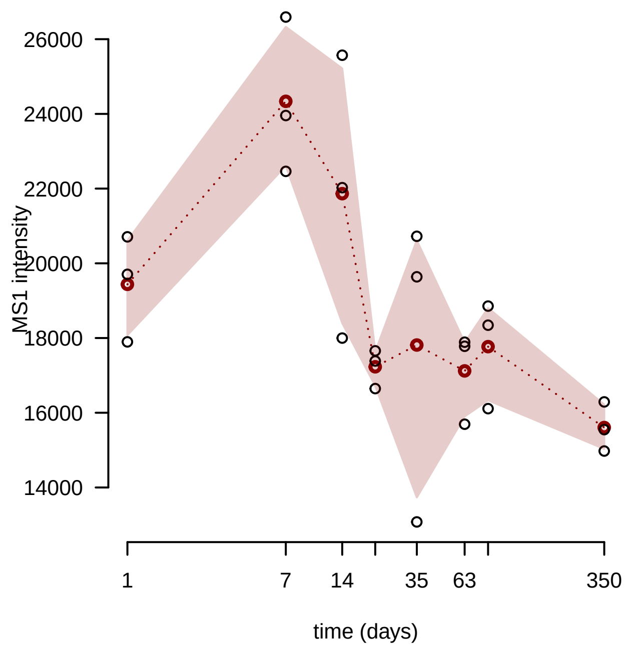

P16015
GN=Ca3

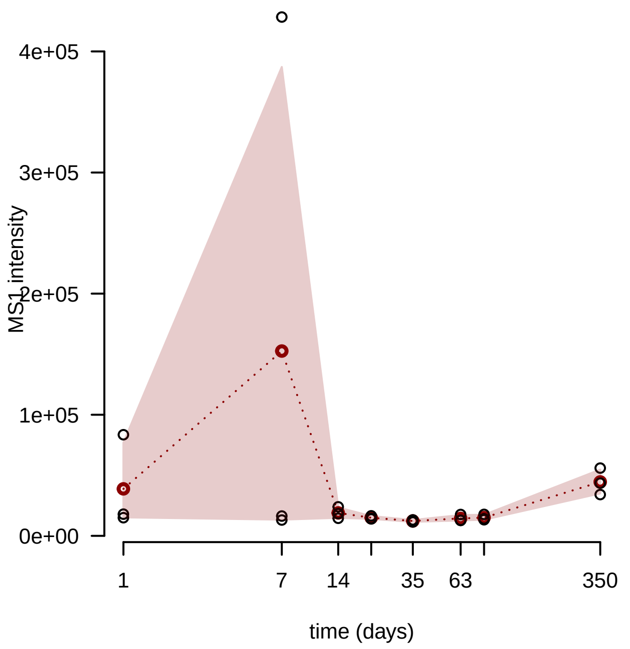

P16045
GN=Lgals1
lectin, galactose binding, soluble 1

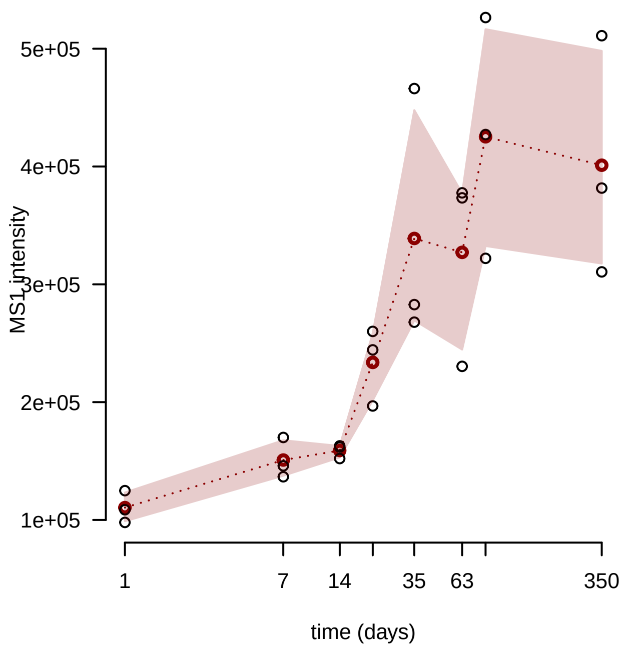

P16054
GN=Prkce
protein kinase C, epsilon

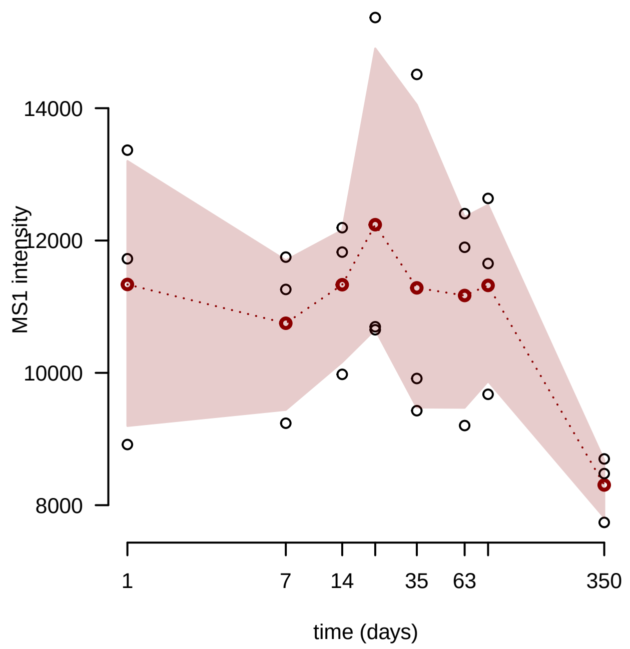

P16110
GN=Lgals3
lectin, galactose binding, soluble 3

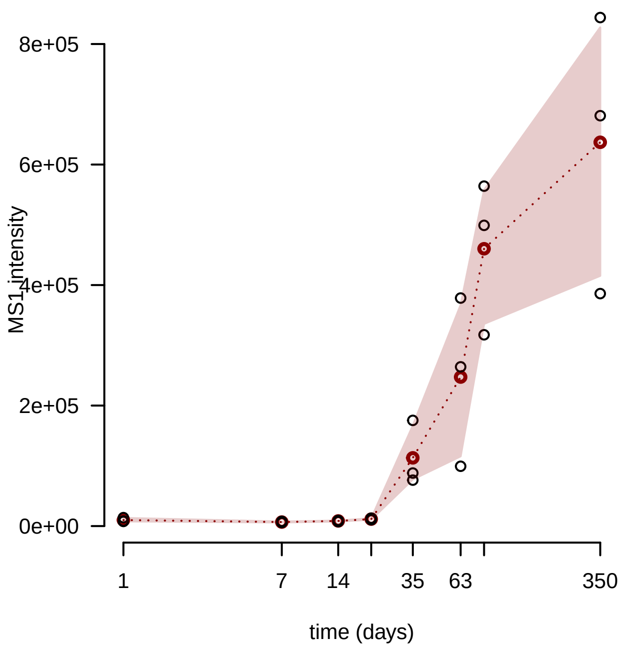

P16125
GN=Ldhb
lactate dehydrogenase B

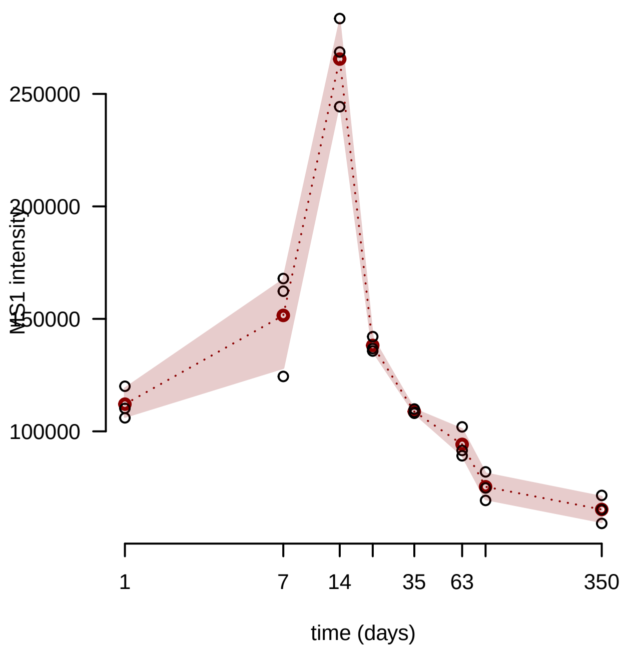

P16254
GN=Srp14
signal recognition particle 14

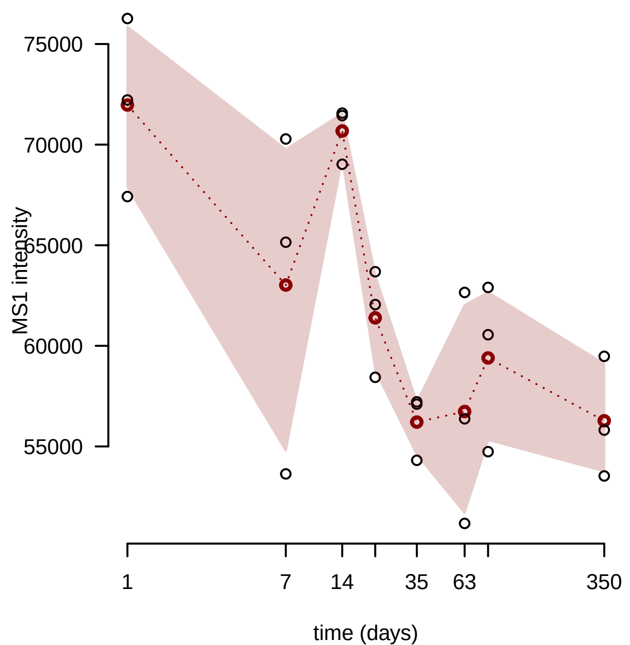

P16294
GN=F9
coagulation factor IX

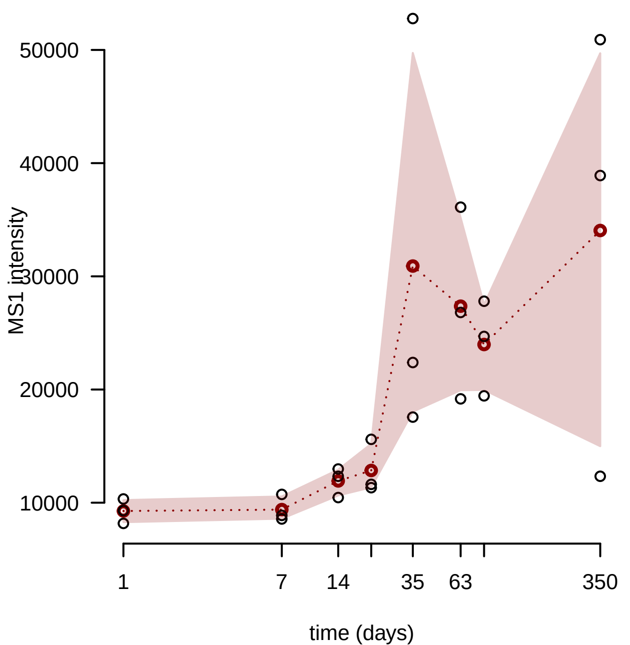

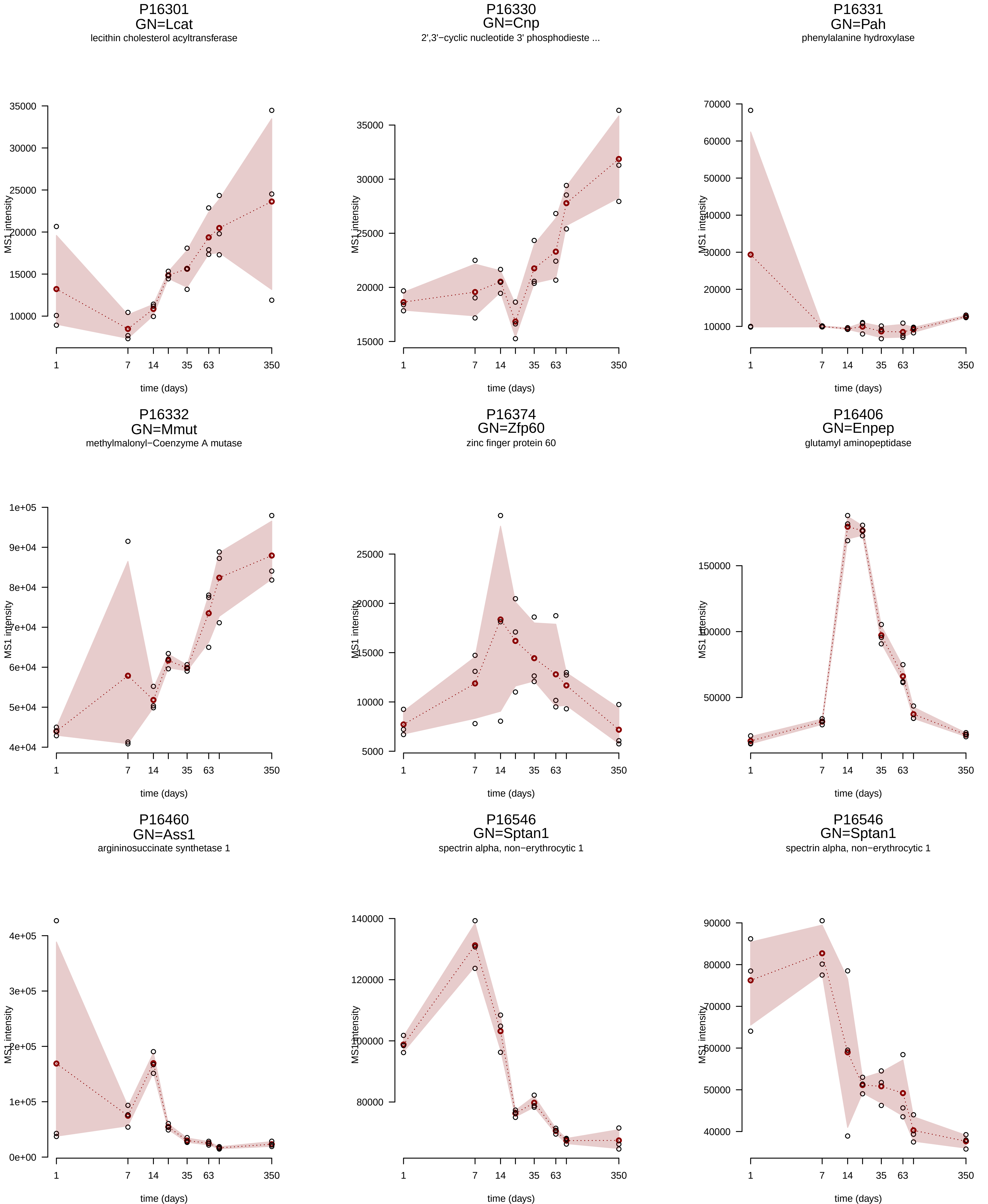
P16301
GN=Lcat
lecithin cholesterol acyltransferase
MS1 intensity
35000
30000
25000
20000
15000
10000
1
7
14
35
63
350
time (days)
P16330
GN=Cnp
2',3'−cyclic nucleotide 3' phosphodieste ...
MS1 intensity
35000
30000
25000
20000
15000
time (days)
P16331
GN=Pah
phenylalanine hydroxylase
MS1 intensity
70000
60000
50000
40000
30000
20000
10000
time (days)
P16332
GN=Mmut
methylmalonyl−Coenzyme A mutase
MS1 intensity
1e+05
9e+04
8e+04
7e+04
6e+04
5e+04
4e+04
time (days)
P16374
GN=Zfp60
zinc finger protein 60
MS1 intensity
25000
20000
15000
10000
5000
time (days)
P16406
GN=Enpep
glutamyl aminopeptidase
MS1 intensity
150000
100000
50000
time (days)
P16460
GN=Ass1
argininosuccinate synthetase 1
MS1 intensity
4e+05
3e+05
2e+05
1e+05
0e+00
time (days)
P16546
GN=Sptan1
spectrin alpha, non−erythrocytic 1
MS1 intensity
140000
120000
100000
80000
time (days)
P16546
GN=Sptan1
spectrin alpha, non−erythrocytic 1
MS1 intensity
90000
80000
70000
60000
50000
40000
time (days)

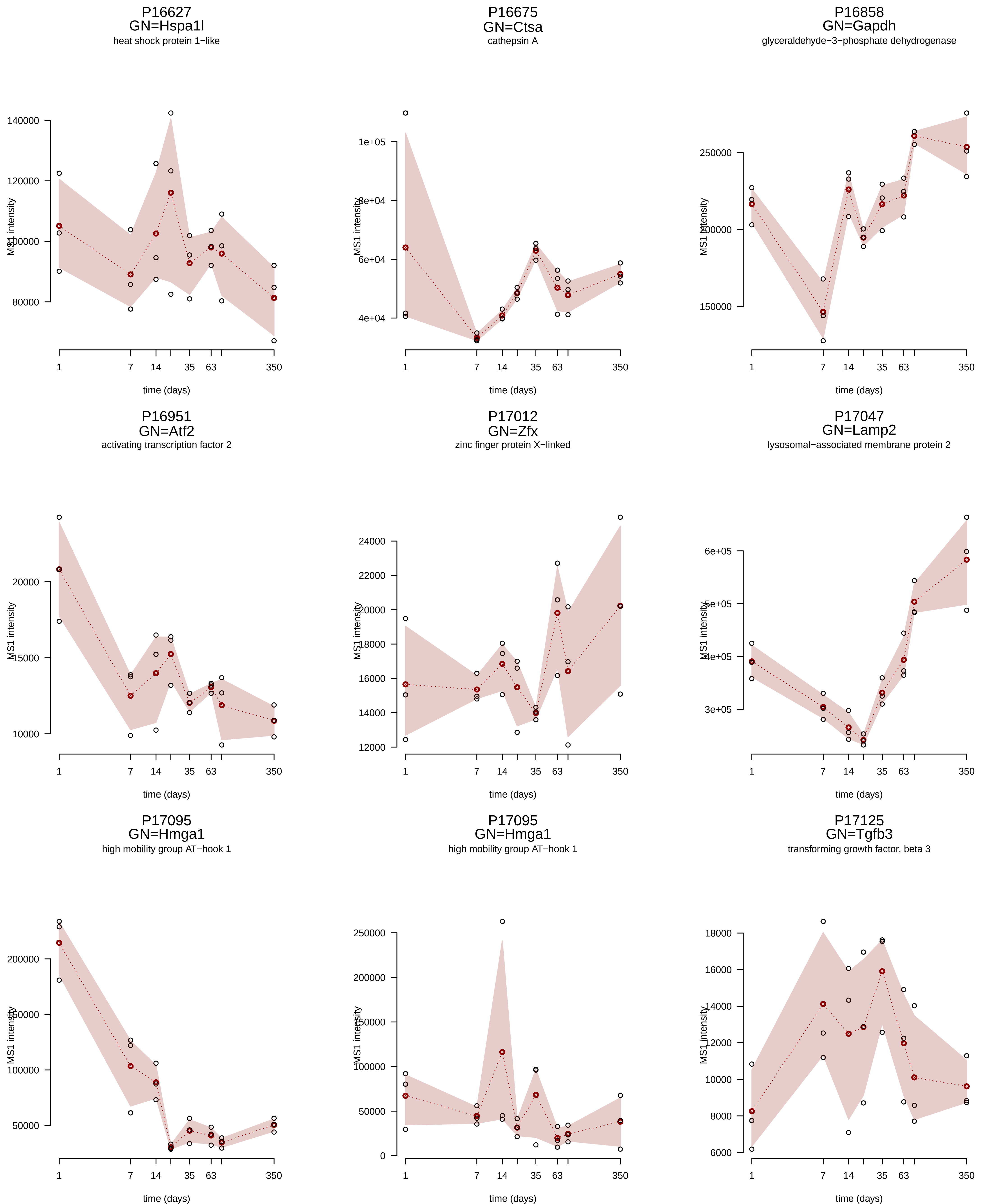
P16627
GN=Hspa1l
heat shock protein 1−like
MS1 intensity
140000
120000
100000
80000
1
7
14
35
63
350
time (days)
P16675
GN=Ctsa
cathepsin A
MS1 intensity
1e+05
8e+04
6e+04
4e+04
1
7
14
35
63
350
time (days)
P16858
GN=Gapdh
glyceraldehyde−3−phosphate dehydrogenase
MS1 intensity
250000
200000
150000
1
7
14
35
63
350
time (days)
P16951
GN=Atf2
activating transcription factor 2
MS1 intensity
20000
15000
10000
1
7
14
35
63
350
time (days)
P17012
GN=Zfx
zinc finger protein X−linked
MS1 intensity
24000
22000
20000
18000
16000
14000
12000
1
7
14
35
63
350
time (days)
P17047
GN=Lamp2
lysosomal−associated membrane protein 2
MS1 intensity
6e+05
5e+05
4e+05
3e+05
1
7
14
35
63
350
time (days)
P17095
GN=Hmga1
high mobility group AT−hook 1
MS1 intensity
200000
150000
100000
50000
1
7
14
35
63
350
time (days)
P17095
GN=Hmga1
high mobility group AT−hook 1
MS1 intensity
250000
200000
150000
100000
50000
0
1
7
14
35
63
350
time (days)
P17125
GN=Tgfb3
transforming growth factor, beta 3
MS1 intensity
18000
16000
14000
12000
10000
8000
6000
1
7
14
35
63
350
time (days)

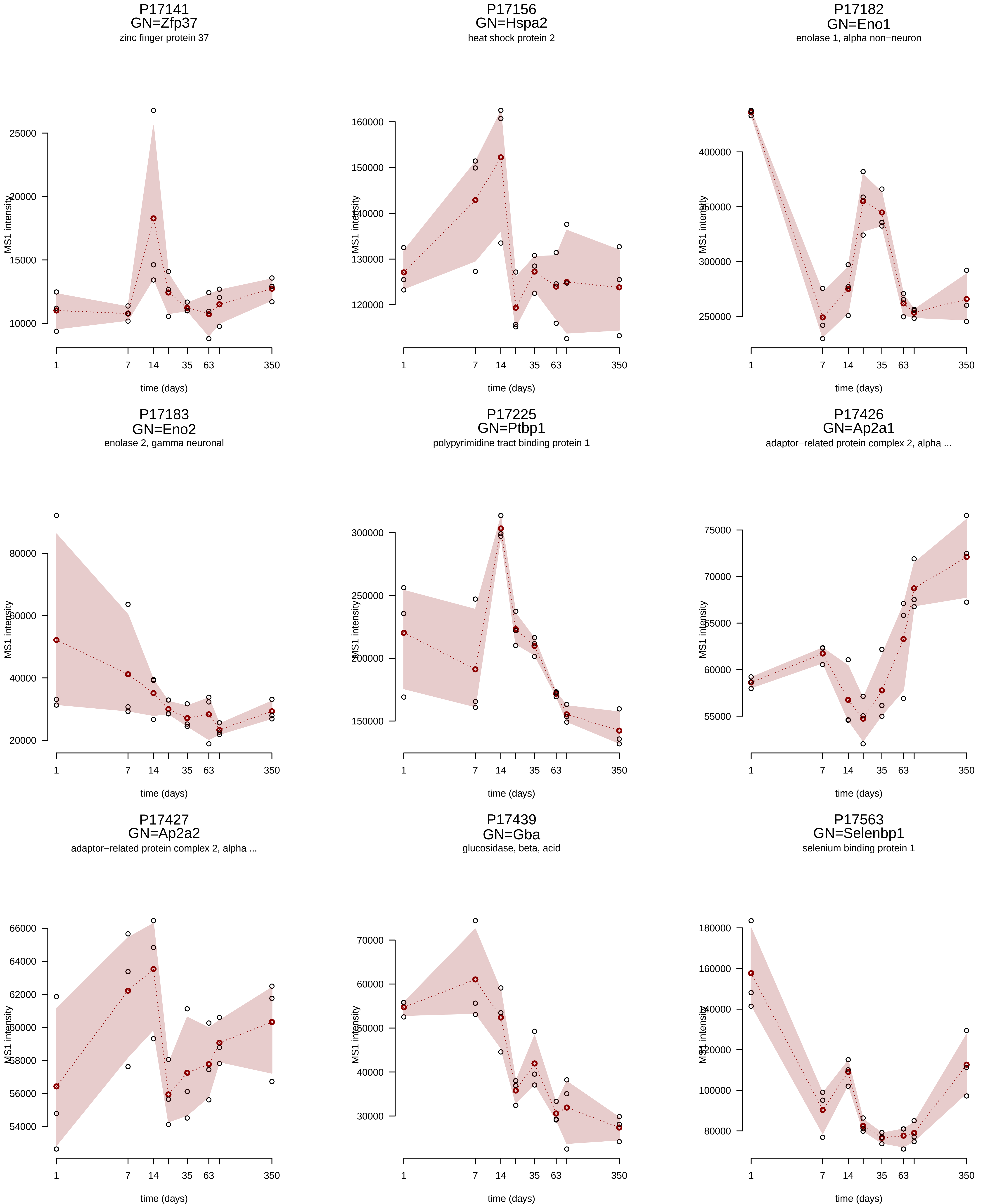
P17141
GN=Zfp37
zinc finger protein 37
MS1 intensity
25000
20000
15000
10000
1
7
14
35
63
350
time (days)
P17156
GN=Hspa2
heat shock protein 2
MS1 intensity
160000
150000
140000
130000
120000
time (days)
P17182
GN=Eno1
enolase 1, alpha non−neuron
MS1 intensity
400000
350000
300000
250000
time (days)
P17183
GN=Eno2
enolase 2, gamma neuronal
MS1 intensity
80000
60000
40000
20000
time (days)
P17225
GN=Ptbp1
polypyrimidine tract binding protein 1
MS1 intensity
300000
250000
200000
150000
time (days)
P17426
GN=Ap2a1
adaptor−related protein complex 2, alpha ...
MS1 intensity
75000
70000
65000
60000
55000
time (days)
P17427
GN=Ap2a2
adaptor−related protein complex 2, alpha ...
MS1 intensity
66000
64000
62000
60000
58000
56000
54000
time (days)
P17439
GN=Gba
glucosidase, beta, acid
MS1 intensity
70000
60000
50000
40000
30000
time (days)
P17563
GN=Selenbp1
selenium binding protein 1
MS1 intensity
180000
160000
140000
120000
100000
80000
time (days)

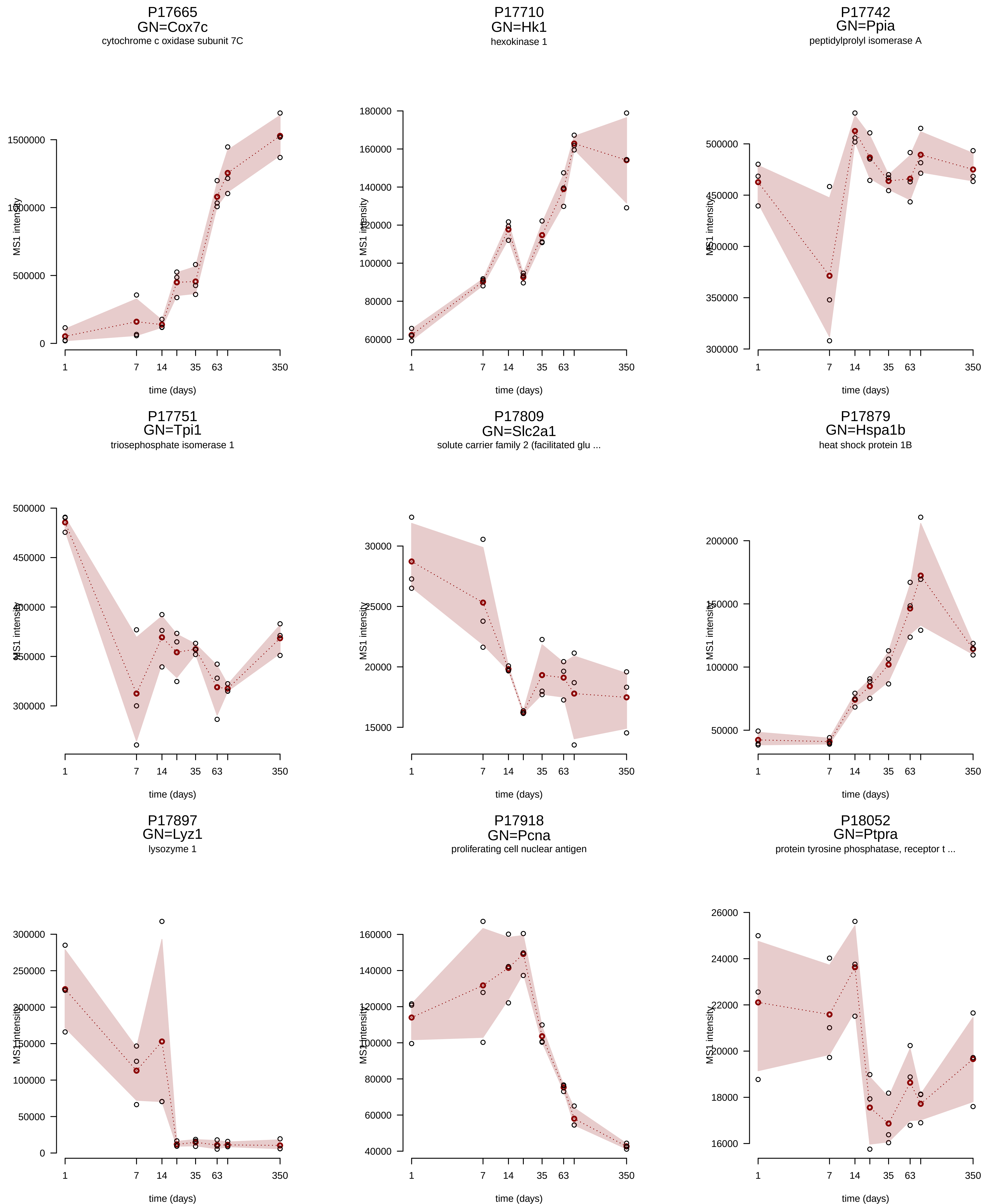
P17665
GN=Cox7c
cytochrome c oxidase subunit 7C
P17710
GN=Hk1
hexokinase 1
P17742
GN=Ppia
peptidylprolyl isomerase A
P17751
GN=Tpi1
triosephosphate isomerase 1
P17809
GN=Slc2a1
solute carrier family 2 (facilitated glu ...
P17879
GN=Hspa1b
heat shock protein 1B
P17897
GN=Lyz1
lysozyme 1
P17918
GN=Pcna
proliferating cell nuclear antigen
P18052
GN=Ptpra
protein tyrosine phosphatase, receptor t ...
MS1 intensity
time (days)
1
7
14
35
63
350

P18155
GN=Mthfd2
methylenetetrahydrofolate dehydrogenase ...

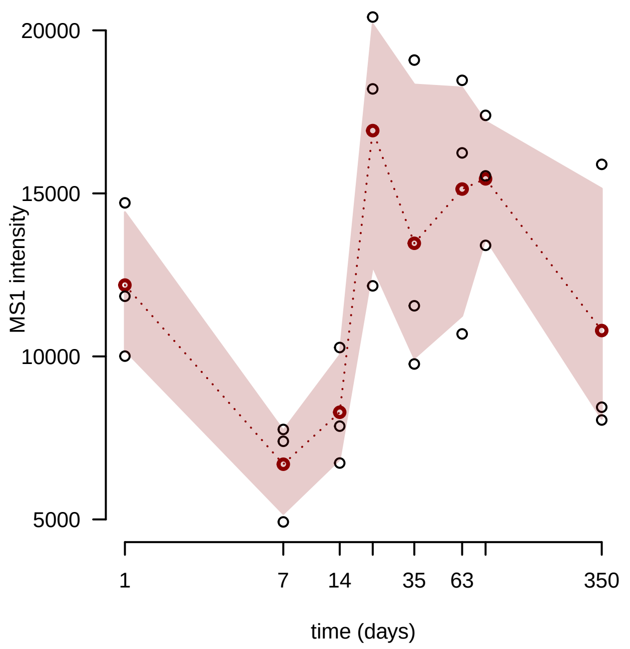

P18242
GN=Ctsd
cathepsin D

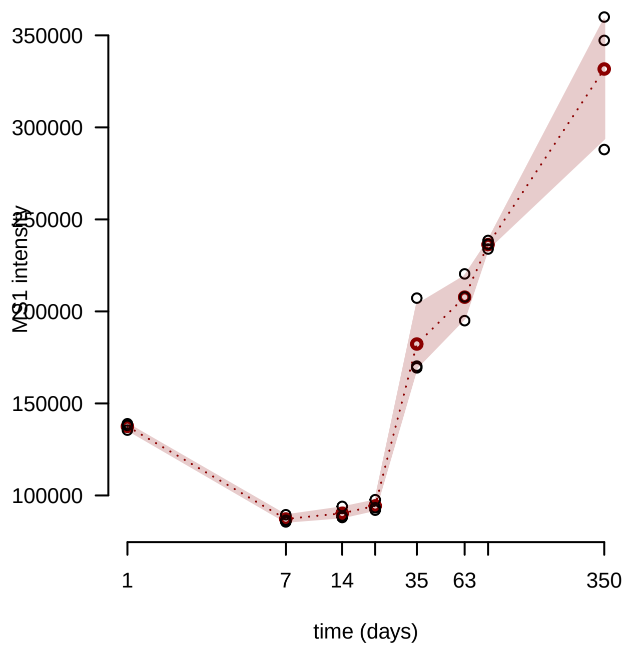

P18293
GN=Npr1
natriuretic peptide receptor 1

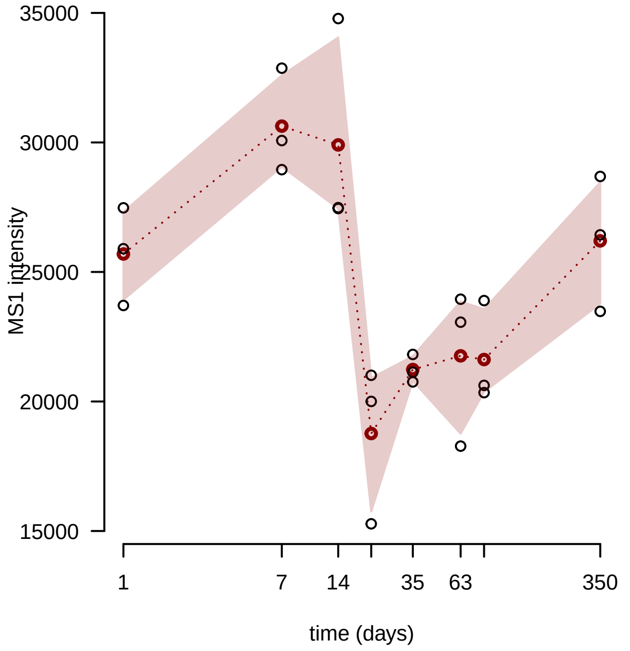

P18527
GN=

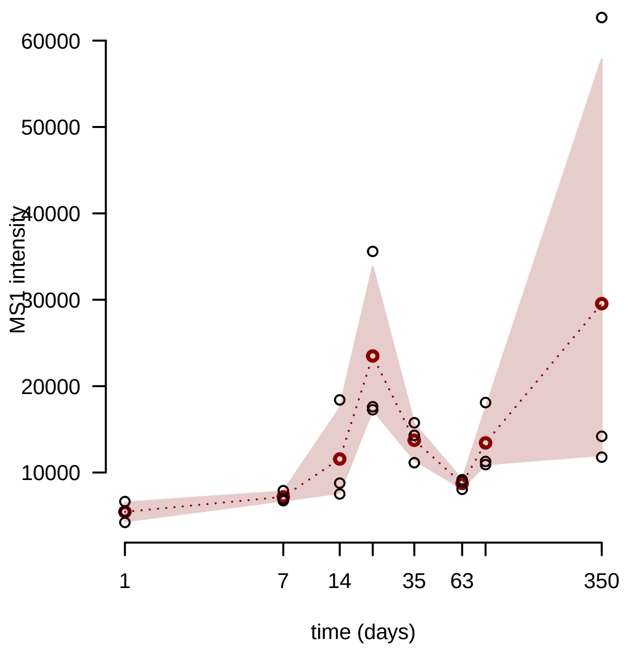

P18528
GN=

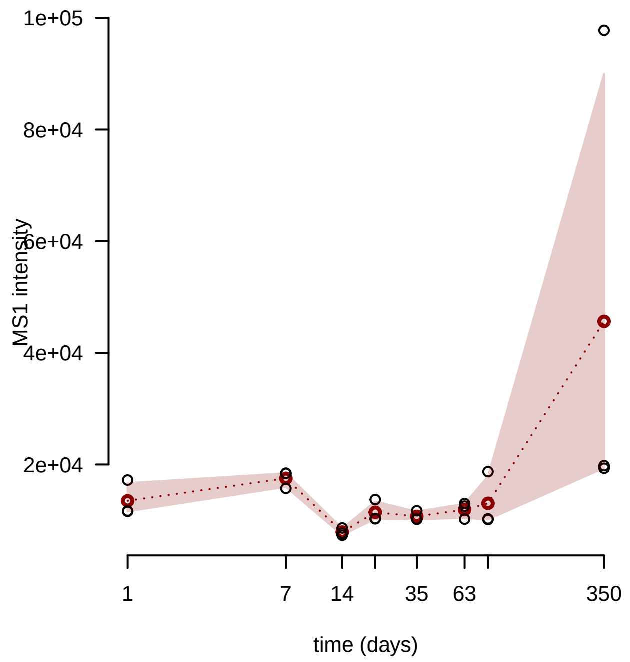

P18572
GN=Bsg
basigin

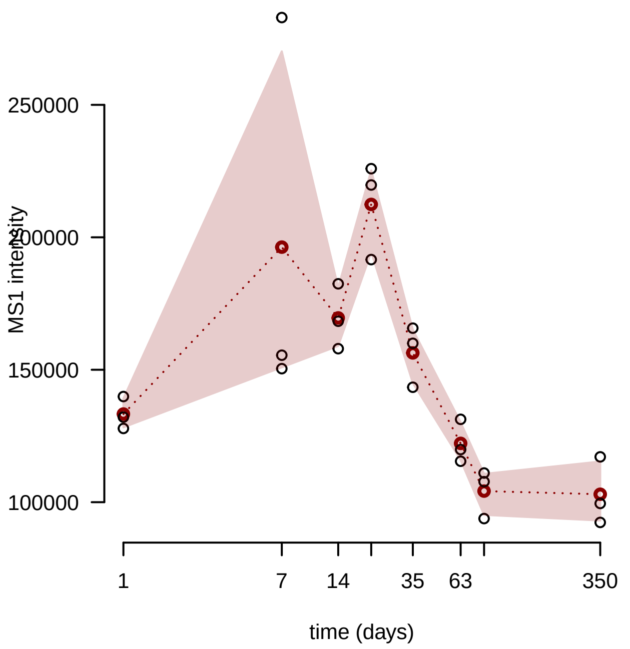

P18581
GN=Slc7a2
solute carrier family 7 (cationic amino ...

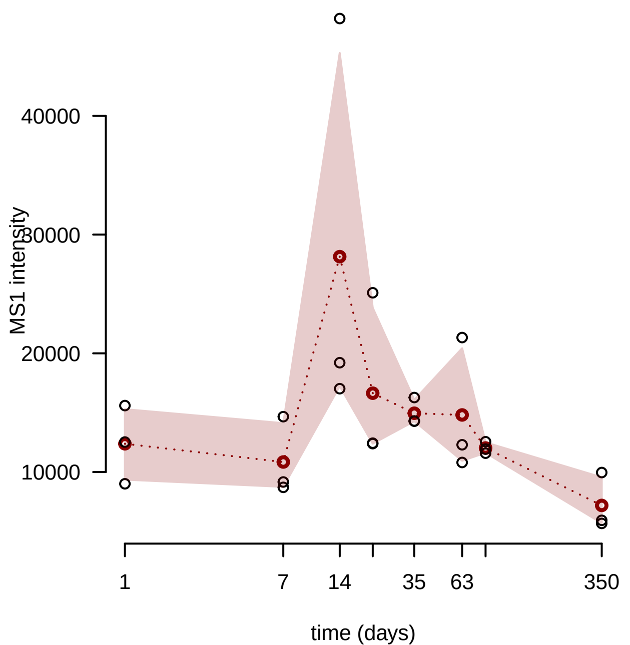

P18608
GN=Hmgn1
high mobility group nucleosomal binding ...

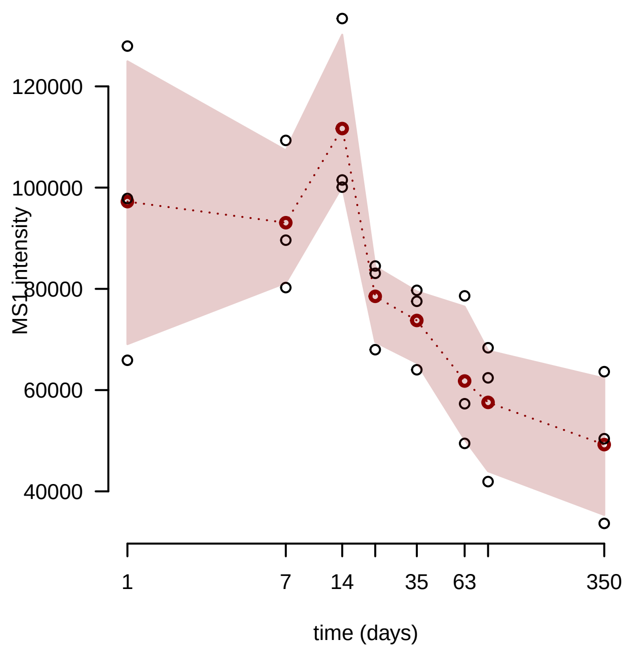

P18653
GN=Rps6ka1
ribosomal protein S6 kinase polypeptide ...

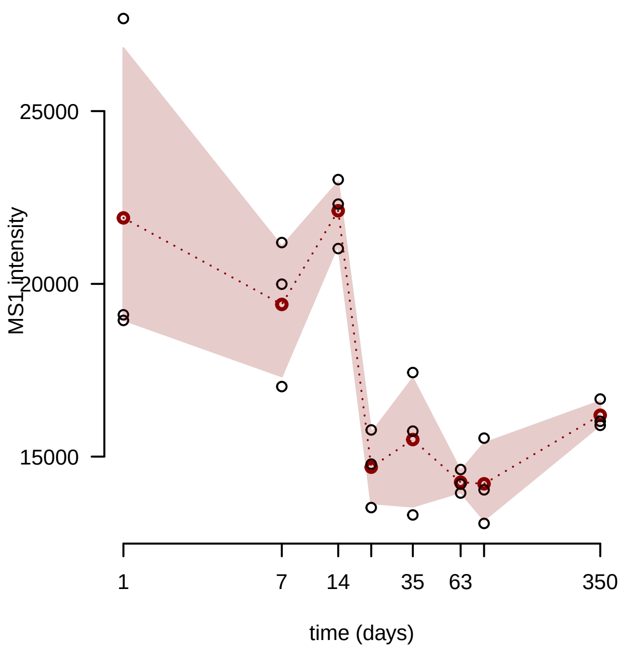

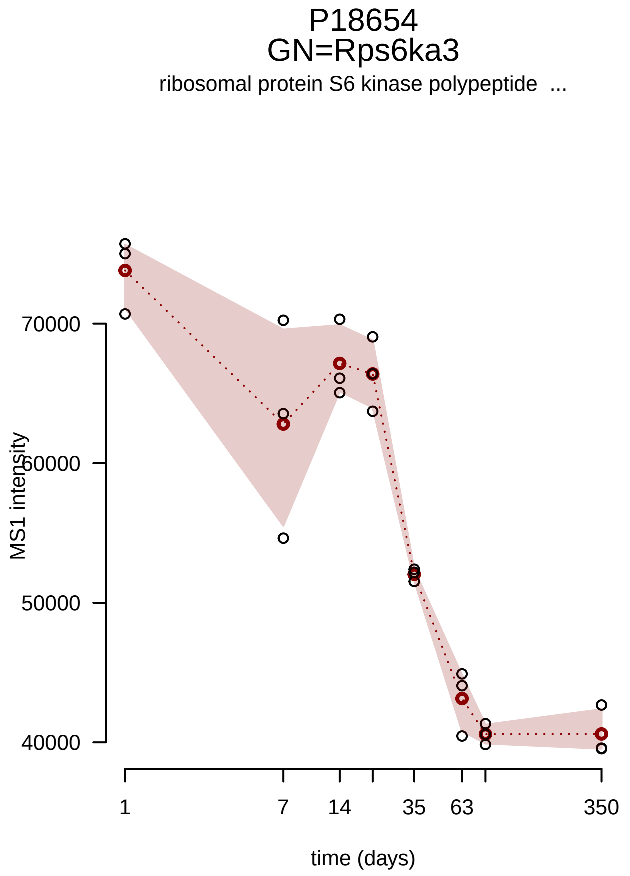
P18654
GN=Rps6ka3
ribosomal protein S6 kinase polypeptide ...
MS1 intensity
70000
60000
50000
40000
1
7
14
35
63
350
time (days)

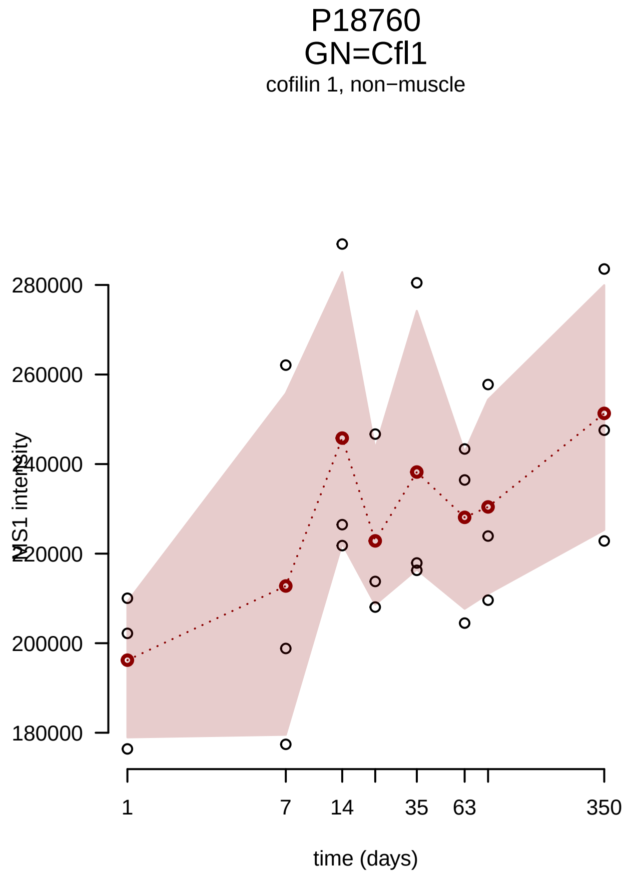
P18760
GN=Cfl1
cofilin 1, non−muscle
MS1 intensity
280000
260000
240000
220000
200000
180000
1
7
14
35
63
350
time (days)

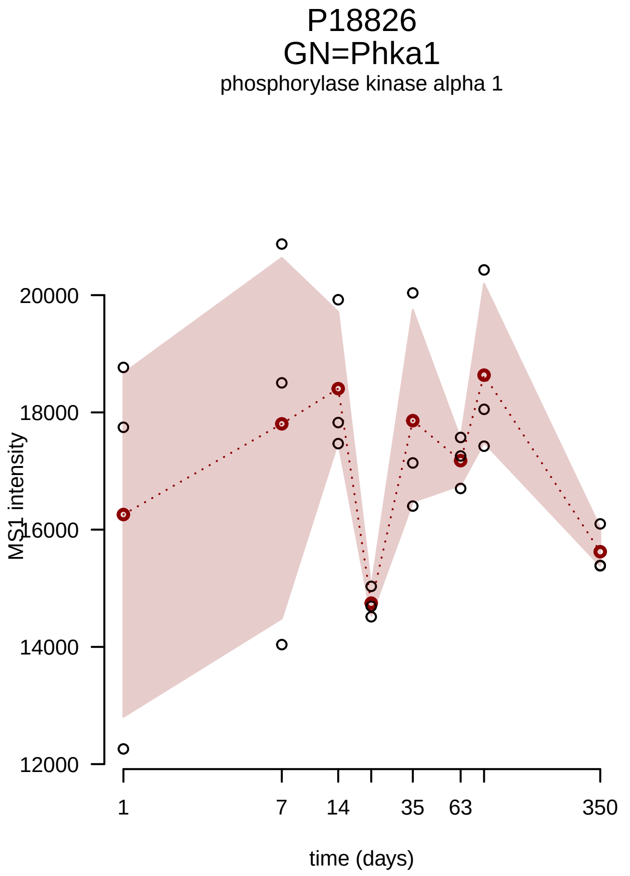
P18826
GN=Phka1
phosphorylase kinase alpha 1
MS1 intensity
20000
18000
16000
14000
12000
1
7
14
35
63
350
time (days)

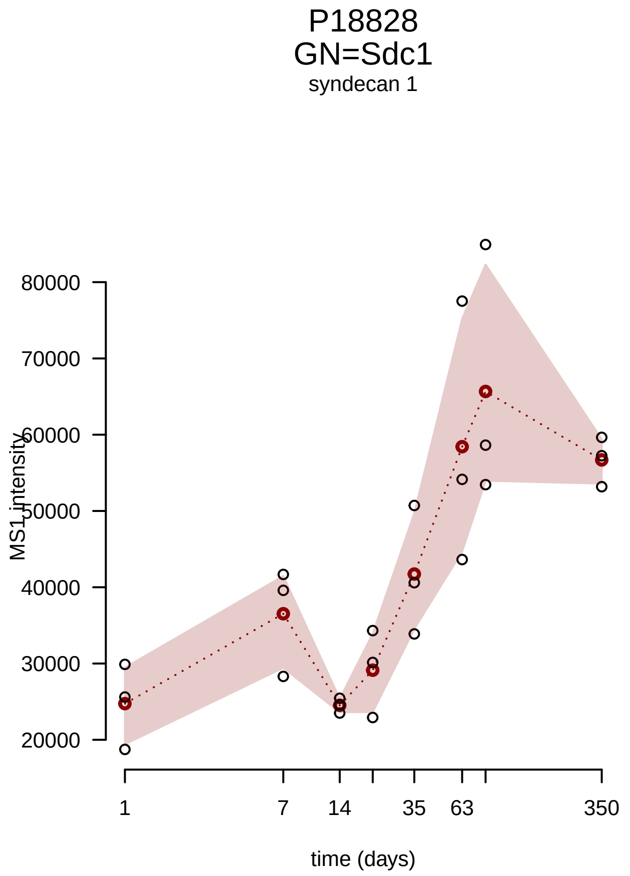
P18828
GN=Sdc1
syndecan 1
MS1 intensity
80000
70000
60000
50000
40000
30000
20000
1
7
14
35
63
350
time (days)

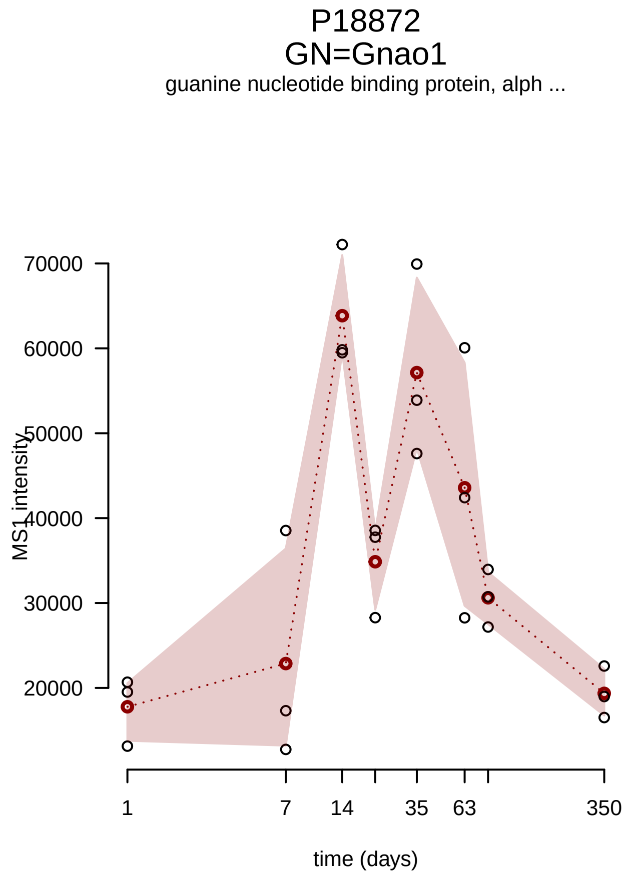
P18872
GN=Gnao1
guanine nucleotide binding protein, alph ...
MS1 intensity
70000
60000
50000
40000
30000
20000
1
7
14
35
63
350
time (days)

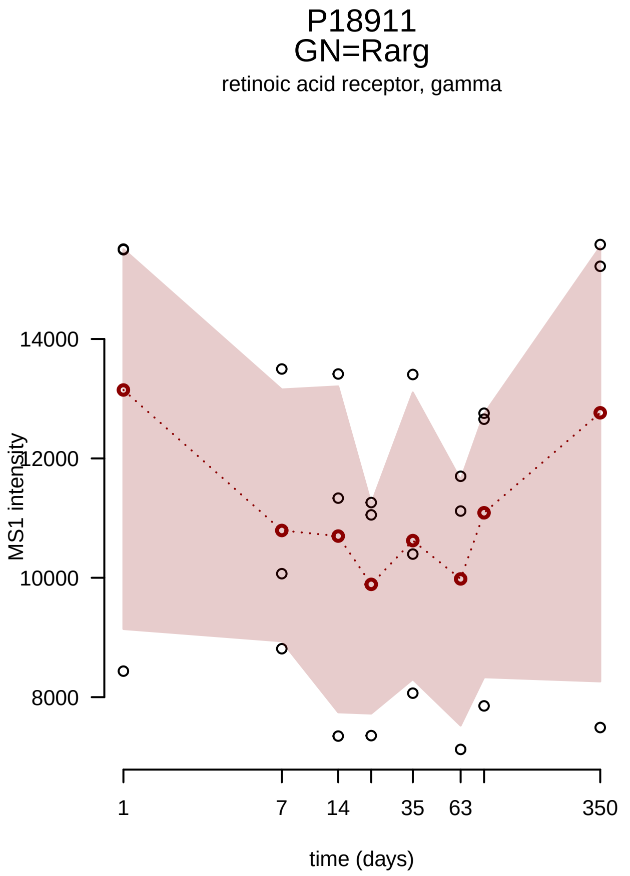
P18911
GN=Rarg
retinoic acid receptor, gamma
MS1 intensity
14000
12000
10000
8000
1
7
14
35
63
350
time (days)

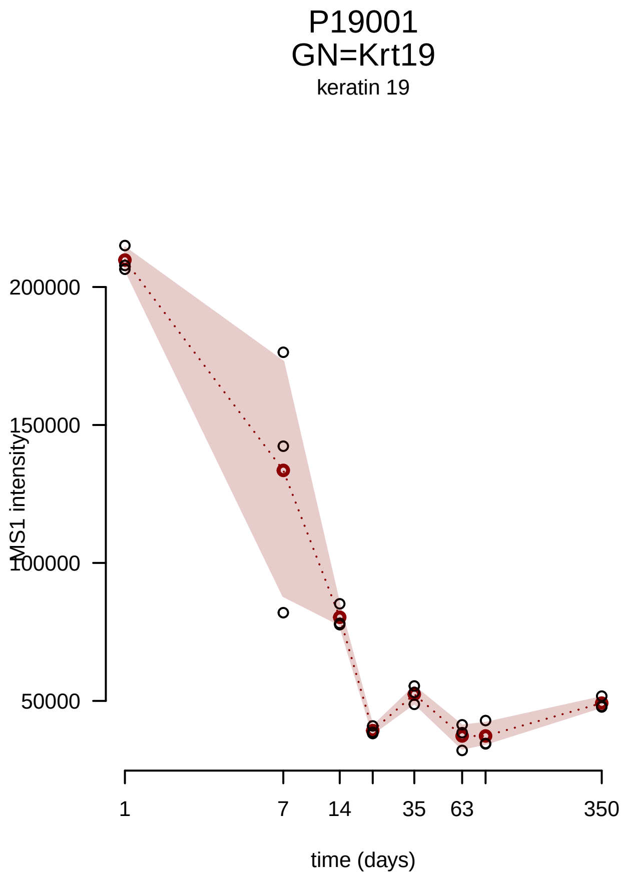
P19001
GN=Krt19
keratin 19
MS1 intensity
200000
150000
100000
50000
1
7
14
35
63
350
time (days)

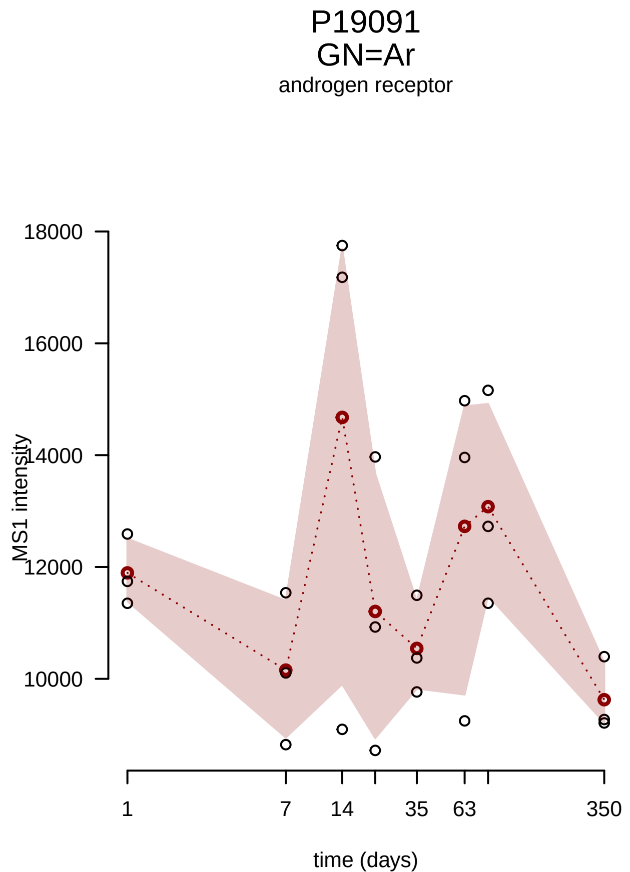
P19091
GN=Ar
androgen receptor
MS1 intensity
18000
16000
14000
12000
10000
1
7
14
35
63
350
time (days)

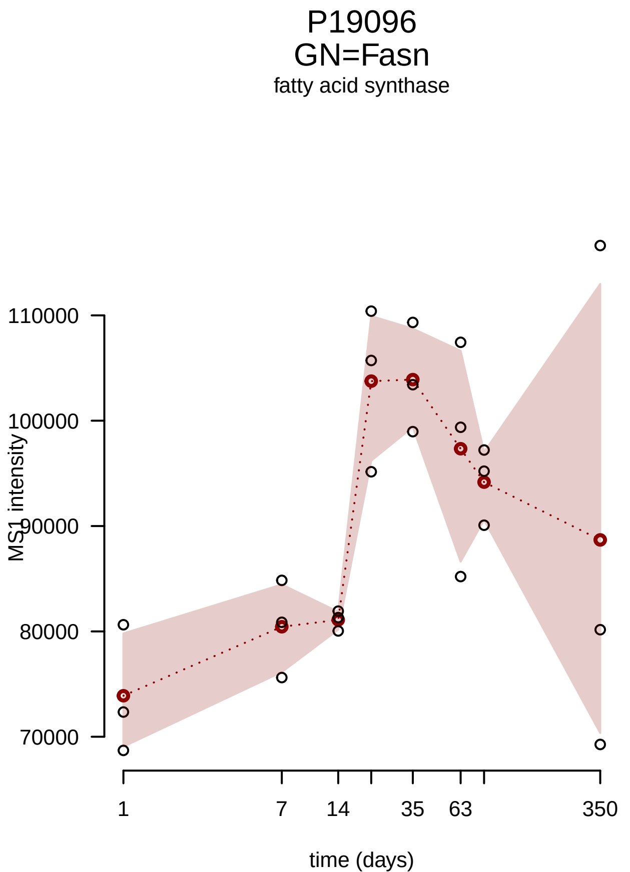
P19096
GN=Fasn
fatty acid synthase
MS1 intensity
110000
100000
90000
80000
70000
1
7
14
35
63
350
time (days)

P19137
GN=Lama1
laminin, alpha 1

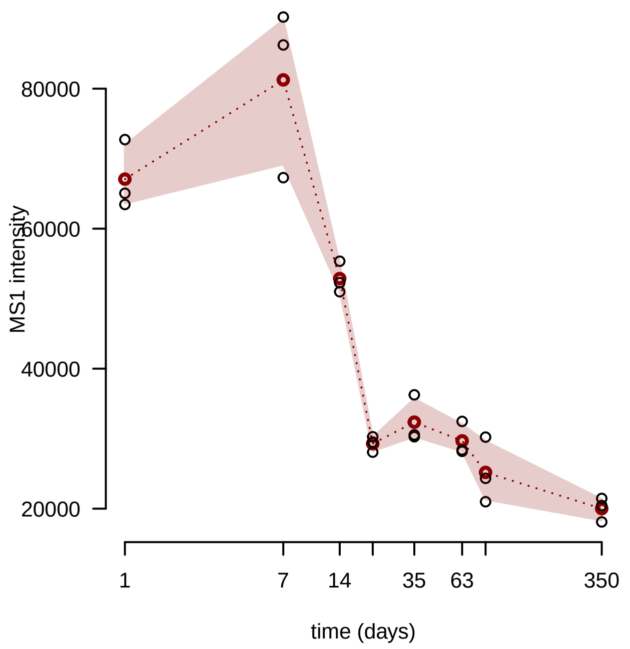

P19157
GN=Gstp1
glutathione S−transferase, pi 1

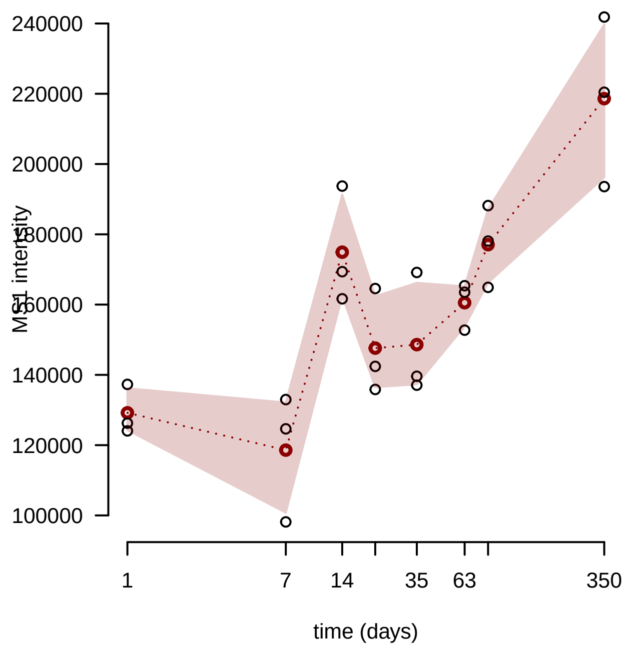

P19182
GN=Ifrd1
interferon−related developmental regulat ...

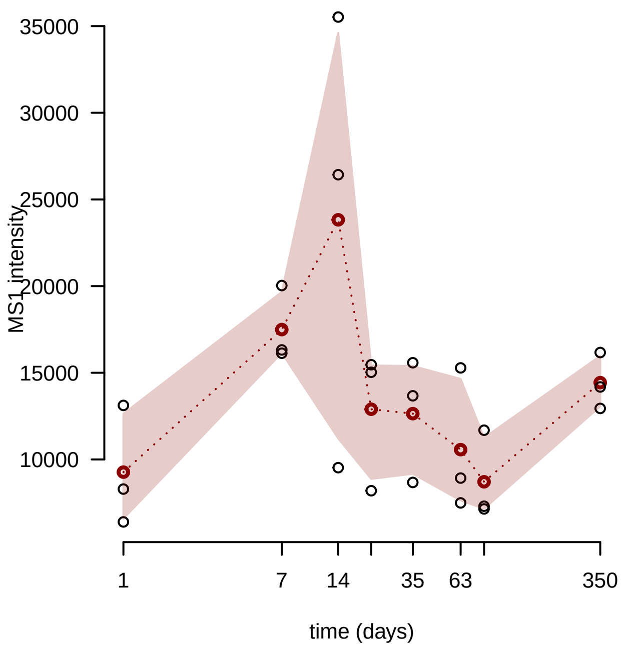

P19221
GN=F2
coagulation factor II

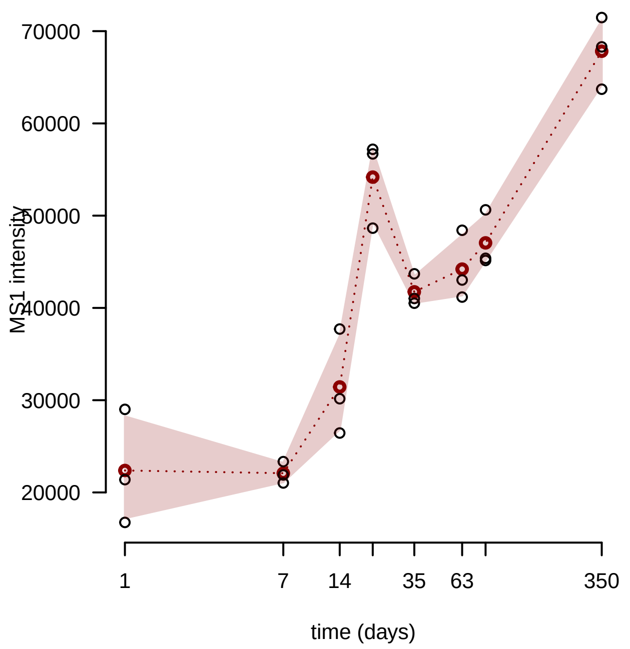

P19253
GN=Rpl13a
ribosomal protein L13A

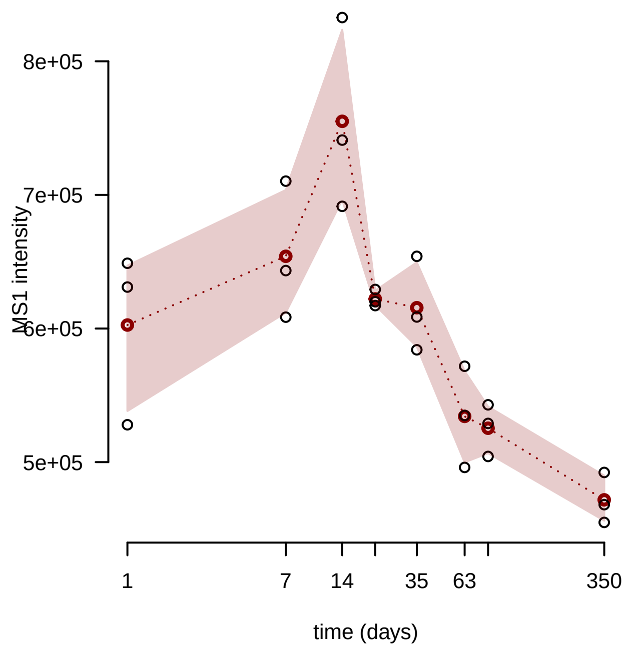

P19258
GN=Mpv17
MpV17 mitochondrial inner membrane prote ...

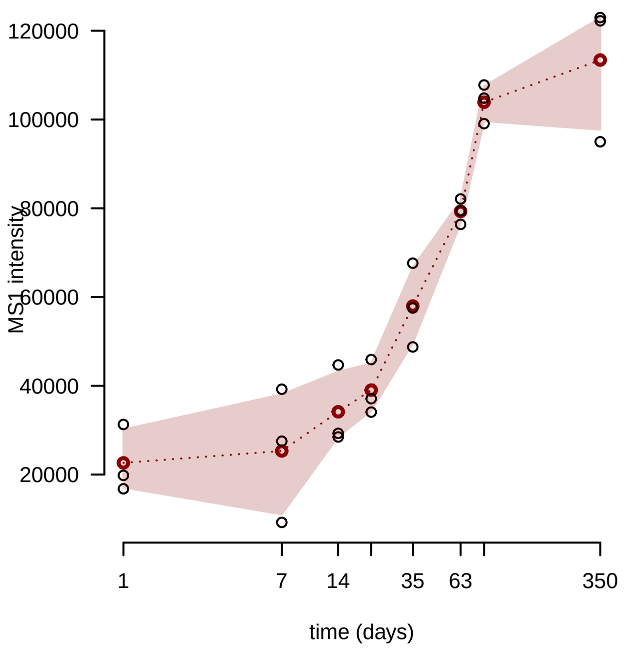

P19324
GN=Serpinh1
serine (or cysteine) peptidase inhibitor ...

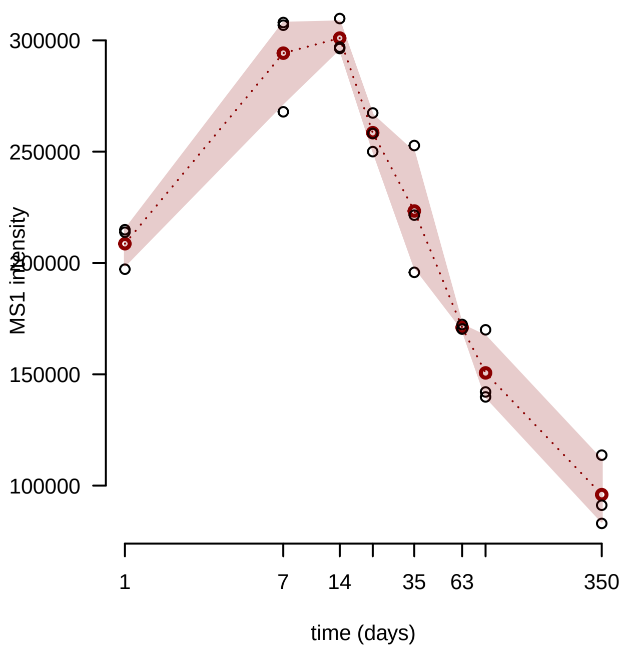

P19426
GN=Nelfe
negative elongation factor complex membe ...

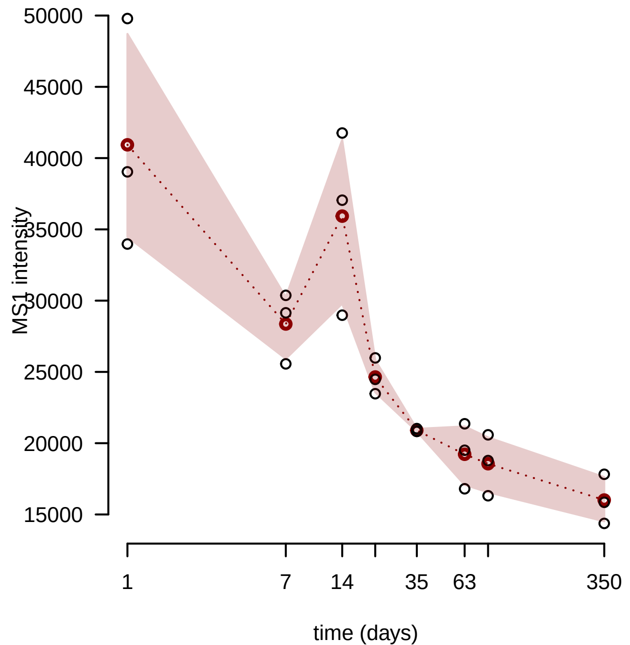

P19467
GN=Muc13
mucin 13, epithelial transmembrane

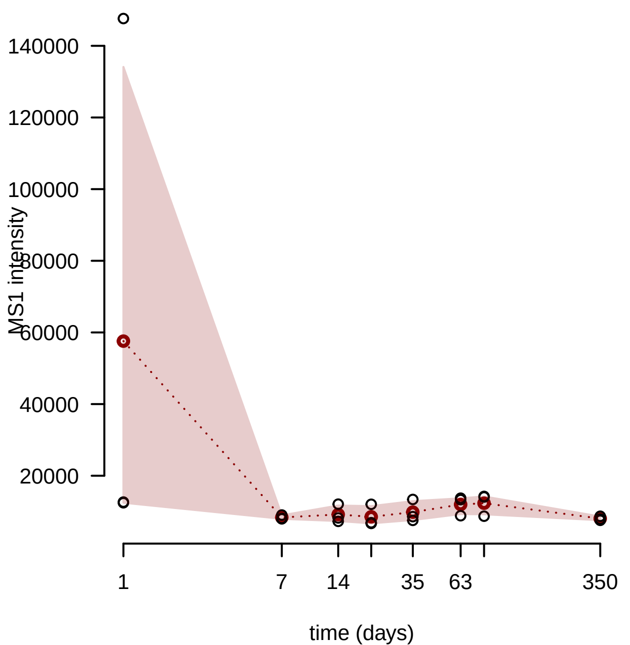

P19536
GN=Cox5b
cytochrome c oxidase subunit 5B

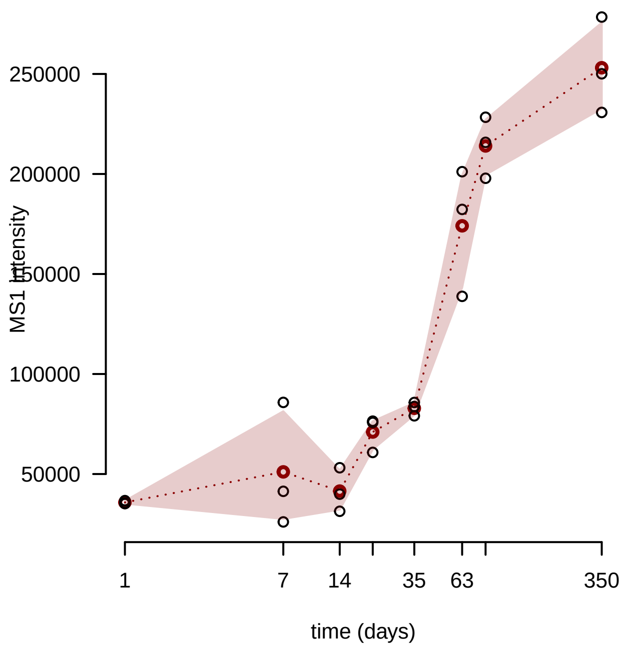

P19783
GN=Cox4i1
cytochrome c oxidase subunit 4I1

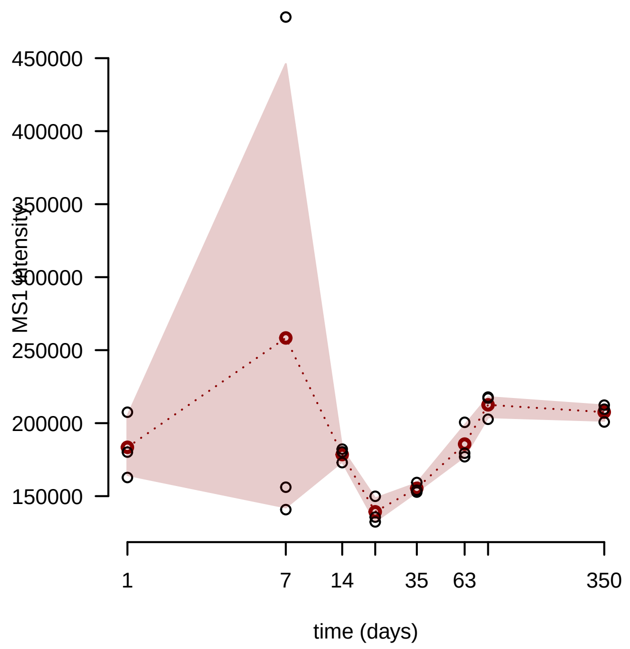

P19785
GN=Esr1
estrogen receptor 1 (alpha)

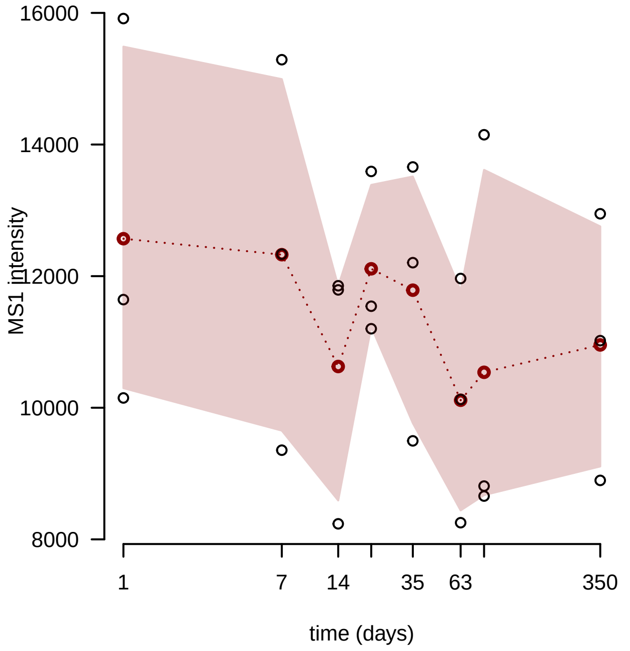

P19973
GN=Lsp1
lymphocyte specific 1

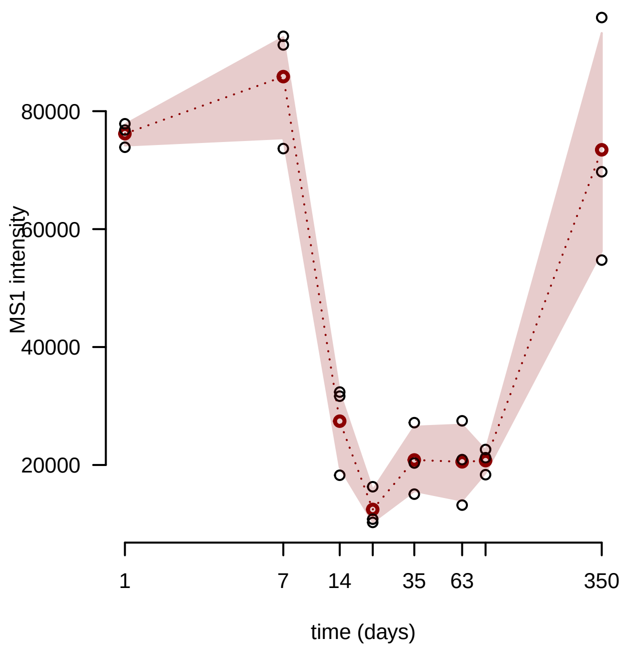

P20029
GN=Hspa5
heat shock protein 5

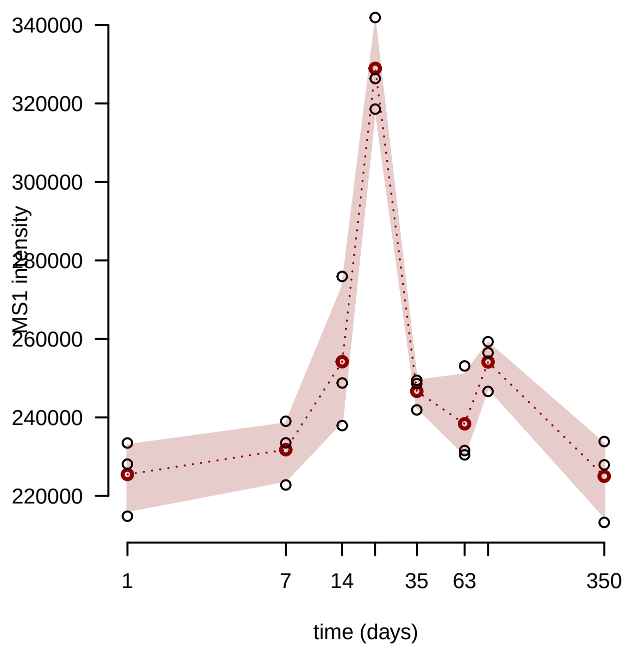

P20060
GN=Hexb
hexosaminidase B

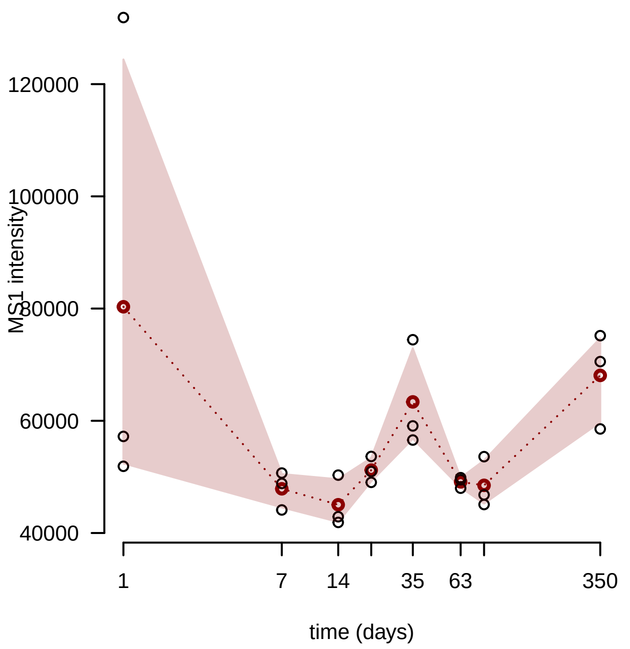

P20065
GN=Tmsb4x
thymosin, beta 4, X chromosome

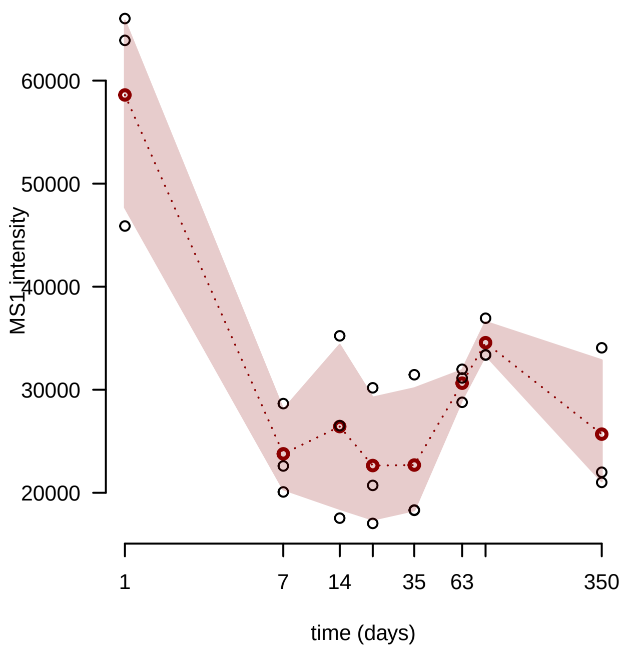

P20108
GN=Prdx3
peroxiredoxin 3

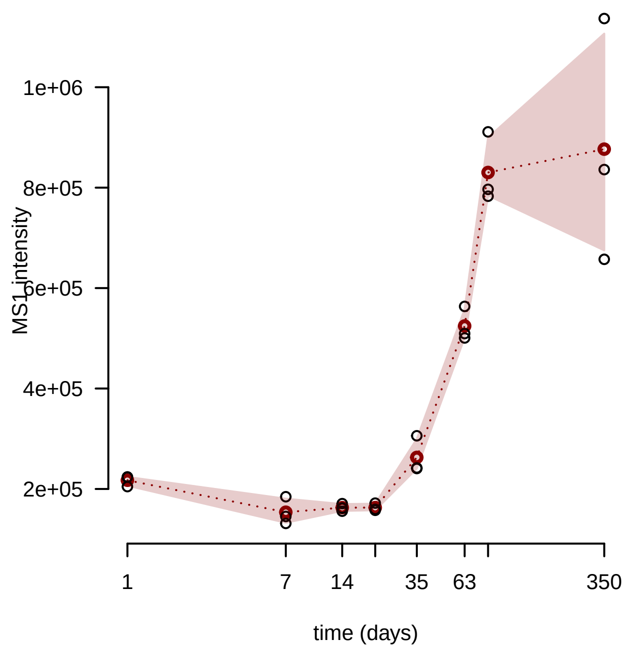

P20152
GN=Vim
vimentin

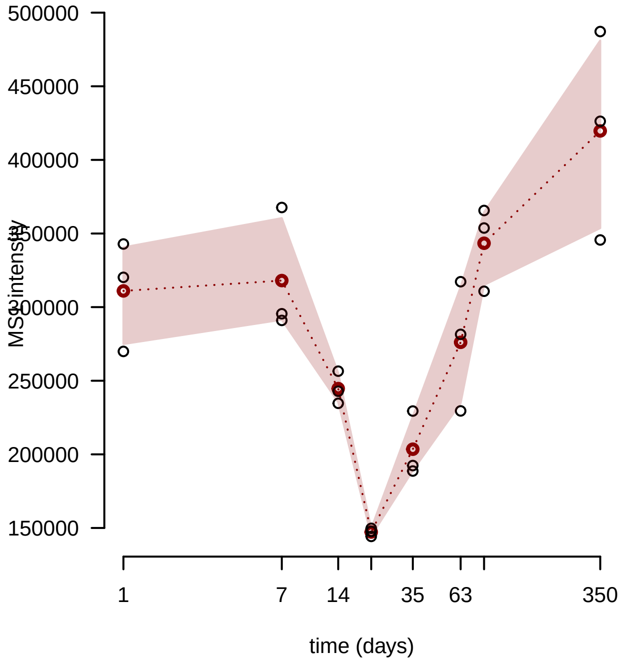

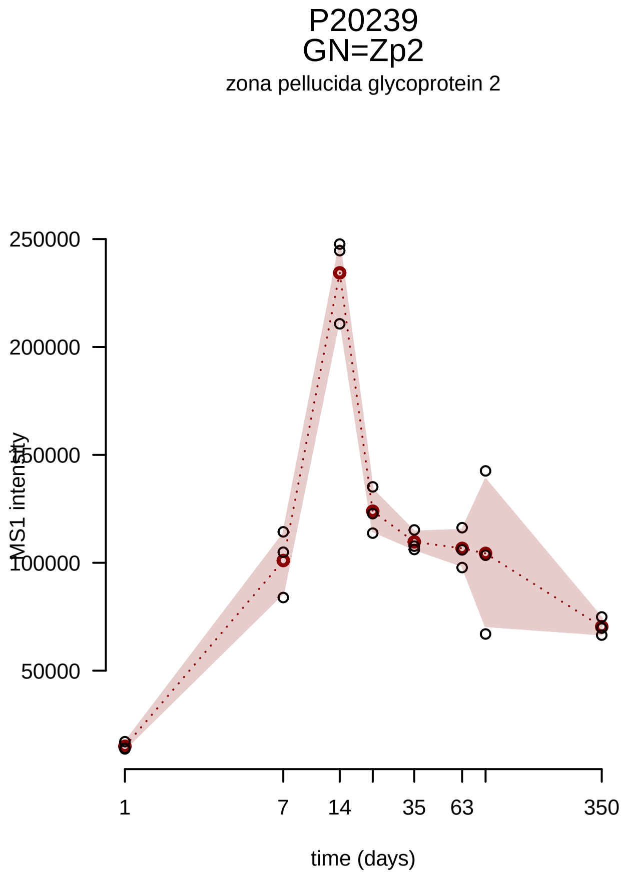
P20239
GN=Zp2
zona pellucida glycoprotein 2
250000
200000
150000
100000
50000
MS1 intensity
1
7
14
35
63
350
time (days)

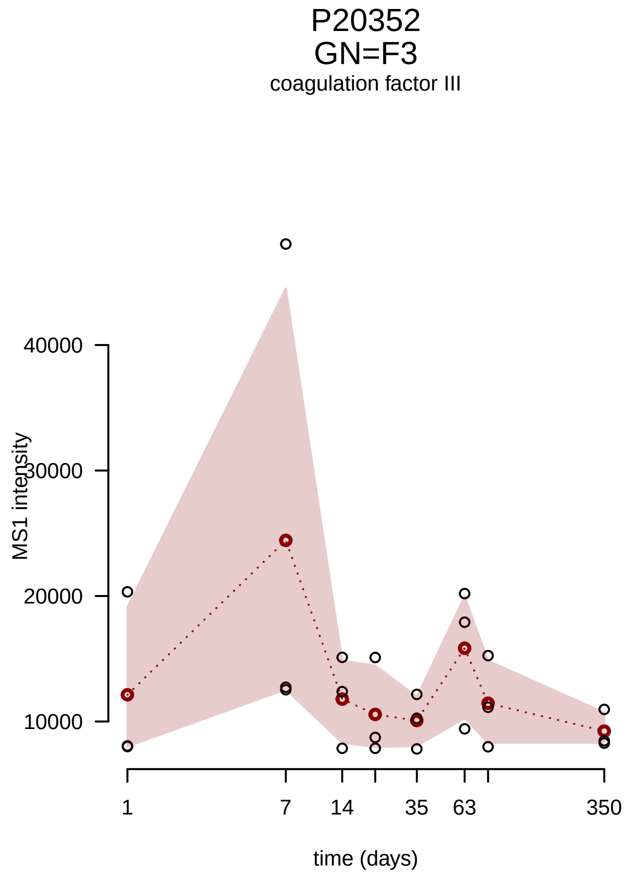
P20352
GN=F3
coagulation factor III
40000
30000
20000
10000
MS1 intensity
1
7
14
35
63
350
time (days)

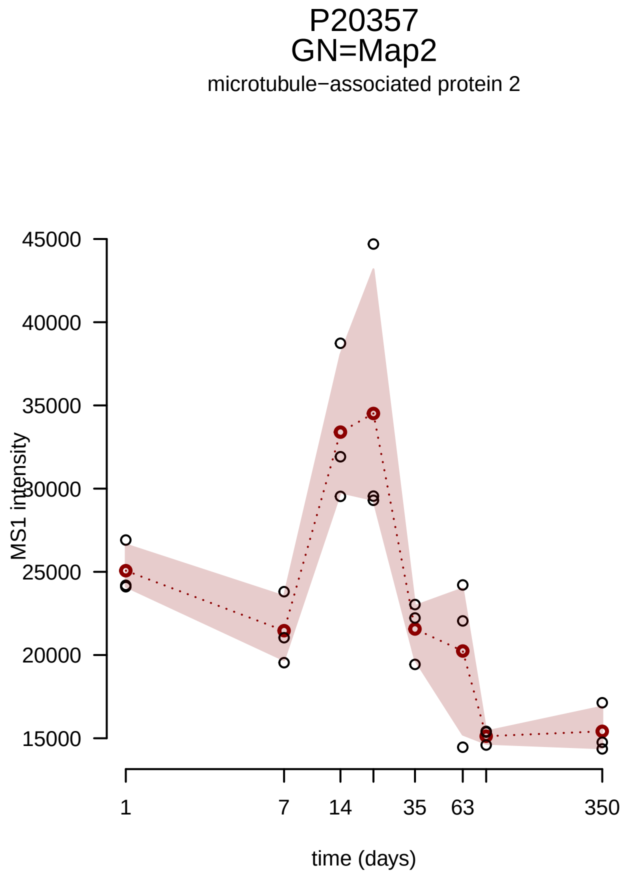
P20357
GN=Map2
microtubule−associated protein 2
45000
40000
35000
30000
25000
20000
15000
MS1 intensity
1
7
14
35
63
350
time (days)

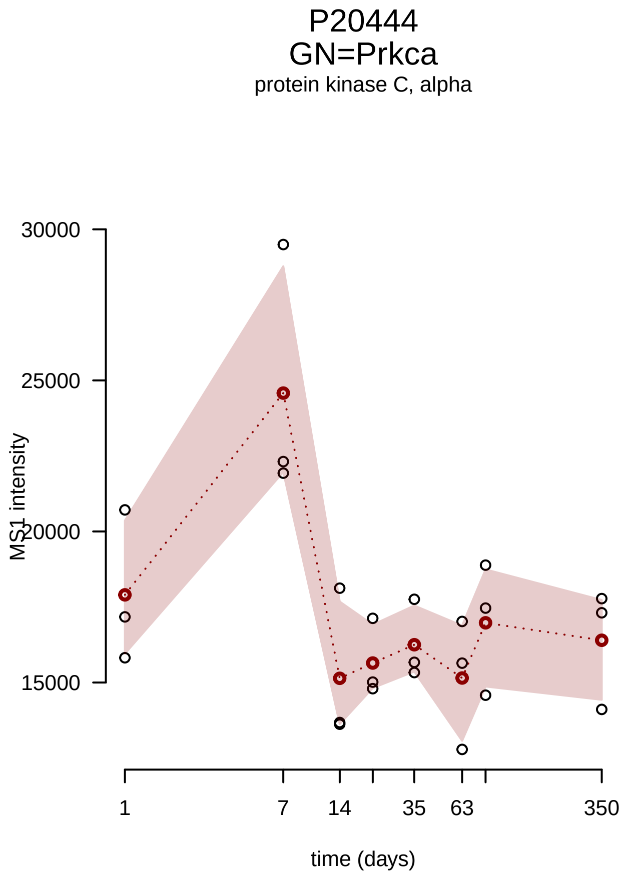
P20444
GN=Prkca
protein kinase C, alpha
30000
25000
20000
15000
MS1 intensity
1
7
14
35
63
350
time (days)

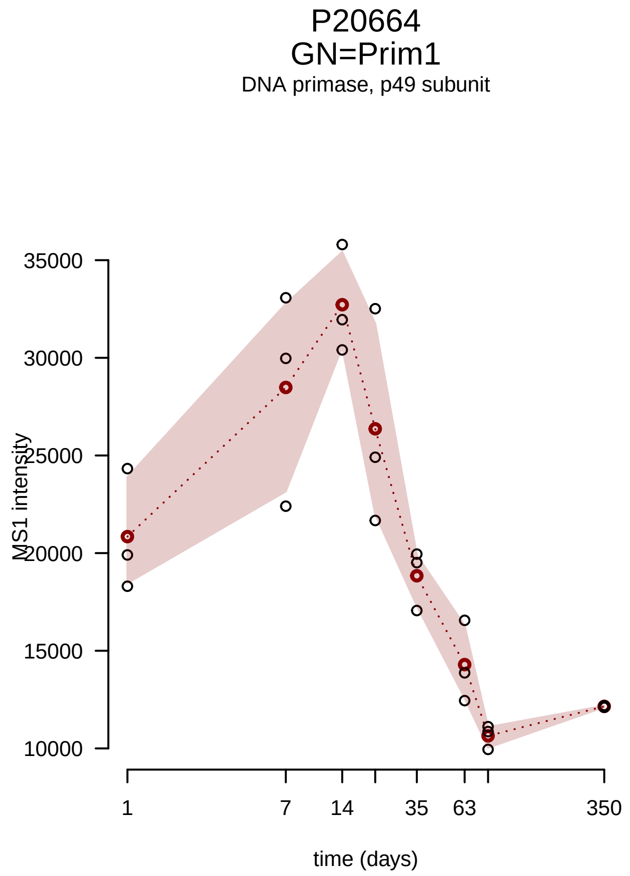
P20664
GN=Prim1
DNA primase, p49 subunit
35000
30000
25000
20000
15000
10000
MS1 intensity
1
7
14
35
63
350
time (days)

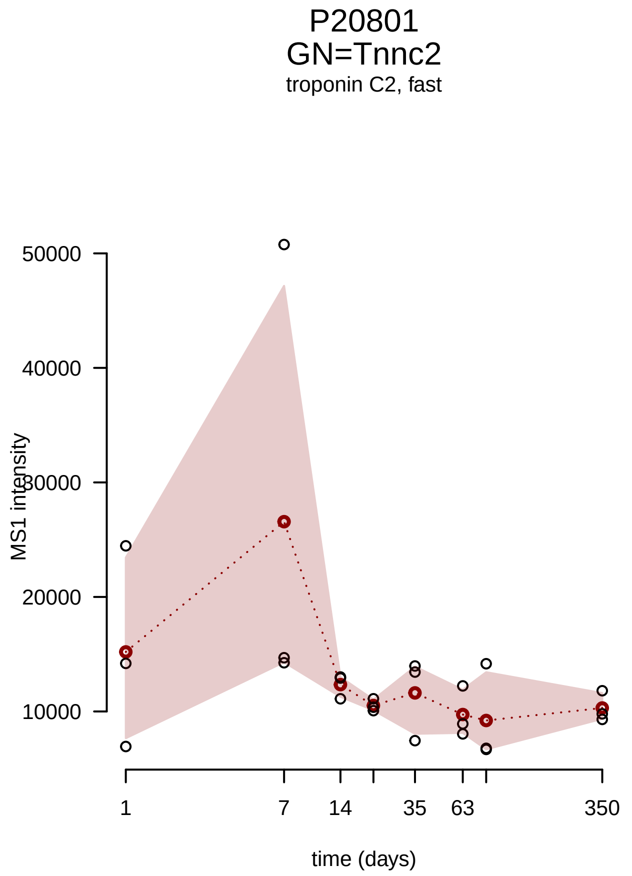
P20801
GN=Tnnc2
troponin C2, fast
50000
40000
30000
20000
10000
MS1 intensity
1
7
14
35
63
350
time (days)

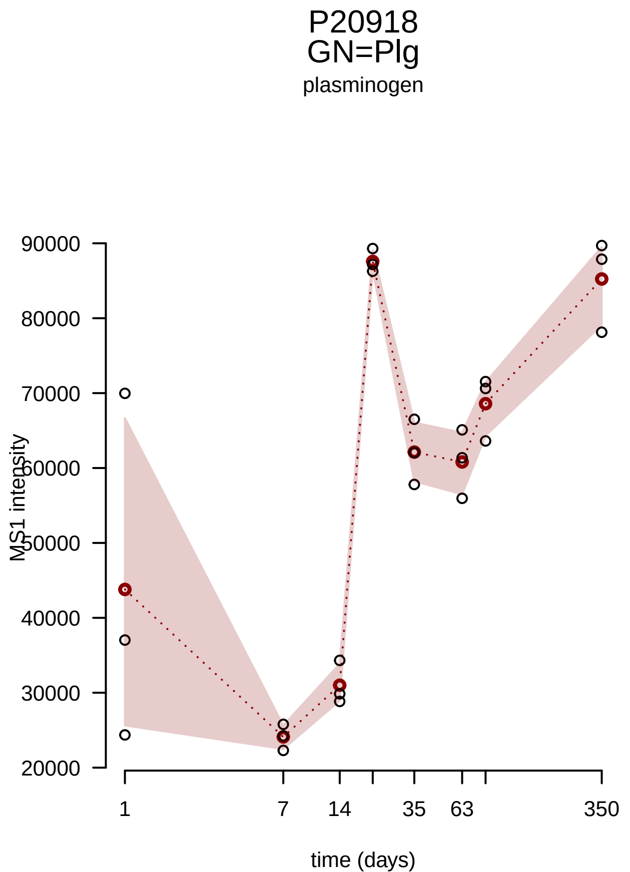
P20918
GN=Plg
plasminogen
90000
80000
70000
60000
50000
40000
30000
20000
MS1 intensity
1
7
14
35
63
350
time (days)

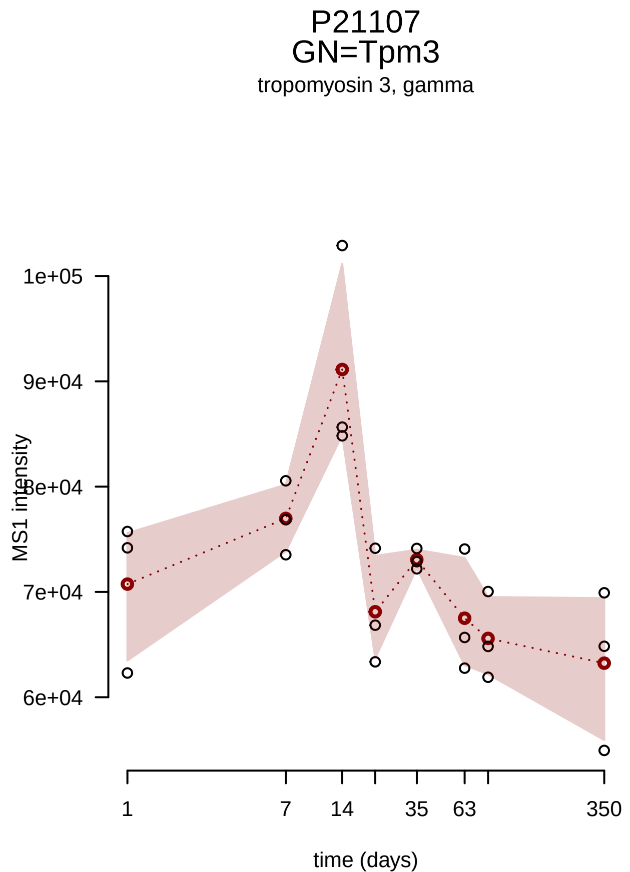
P21107
GN=Tpm3
tropomyosin 3, gamma
1e+05
9e+04
8e+04
7e+04
6e+04
MS1 intensity
1
7
14
35
63
350
time (days)

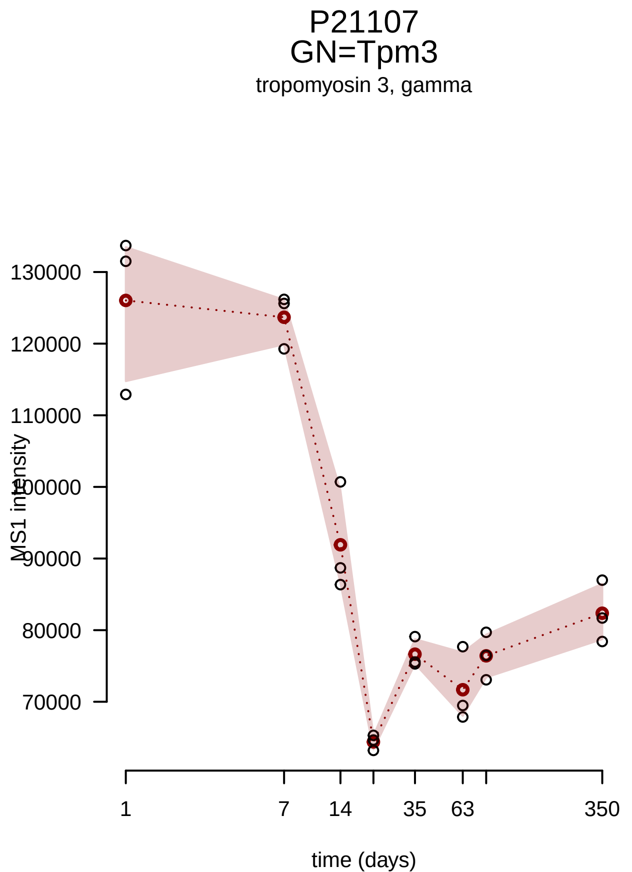
P21107
GN=Tpm3
tropomyosin 3, gamma
130000
120000
110000
100000
90000
80000
70000
MS1 intensity
1
7
14
35
63
350
time (days)

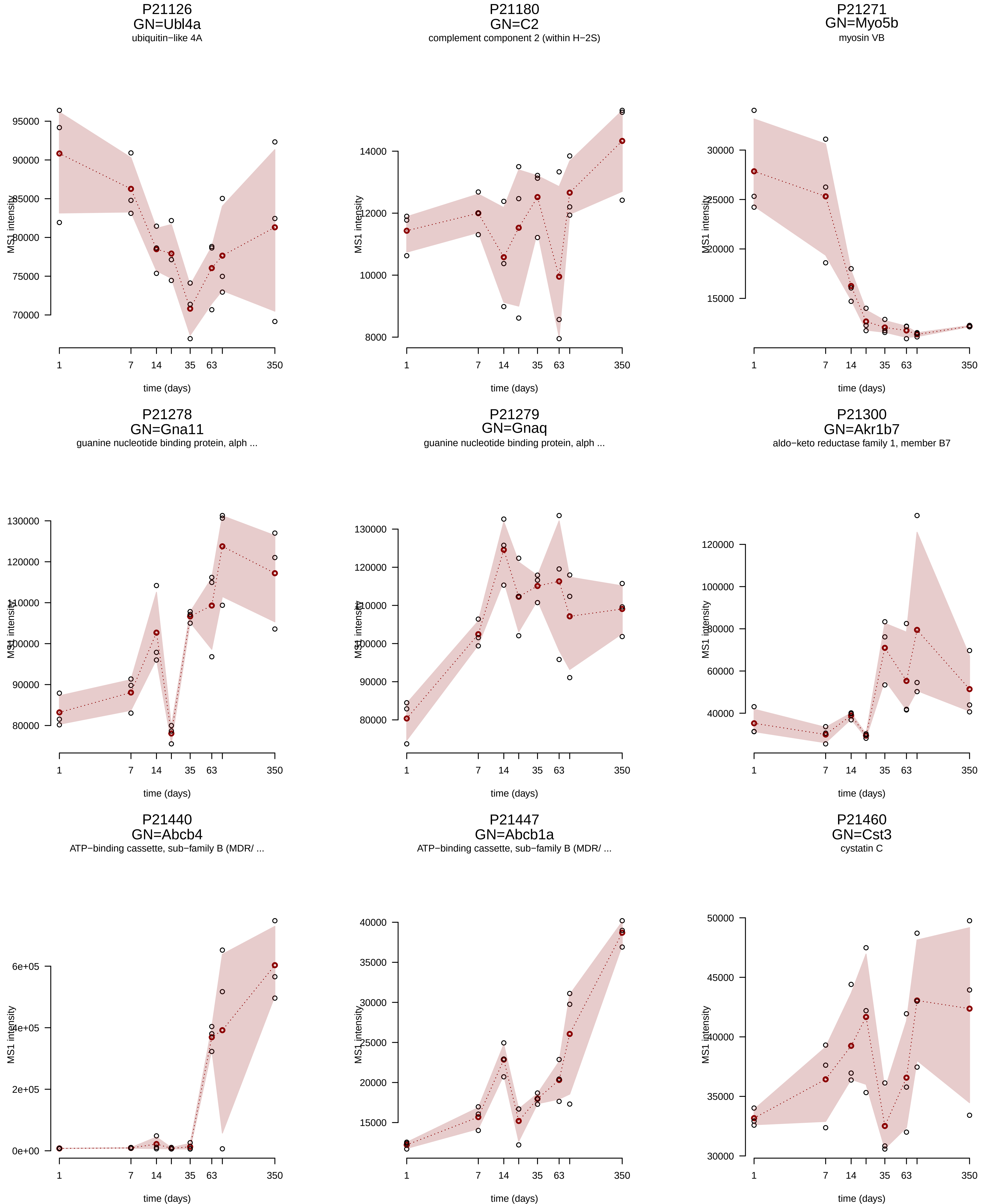
P21126
GN=Ubl4a
ubiquitin−like 4A
P21180
GN=C2
complement component 2 (within H−2S)
P21271
GN=Myo5b
myosin VB
P21278
GN=Gna11
guanine nucleotide binding protein, alph ...
P21279
GN=Gnaq
guanine nucleotide binding protein, alph ...
P21300
GN=Akr1b7
aldo−keto reductase family 1, member B7
P21440
GN=Abcb4
ATP−binding cassette, sub−family B (MDR/ ...
P21447
GN=Abcb1a
ATP−binding cassette, sub−family B (MDR/ ...
P21460
GN=Cst3
cystatin C
MS1 intensity
time (days)
1
7
14
35
63
350

P21550
GN=Eno3
enolase 3, beta muscle

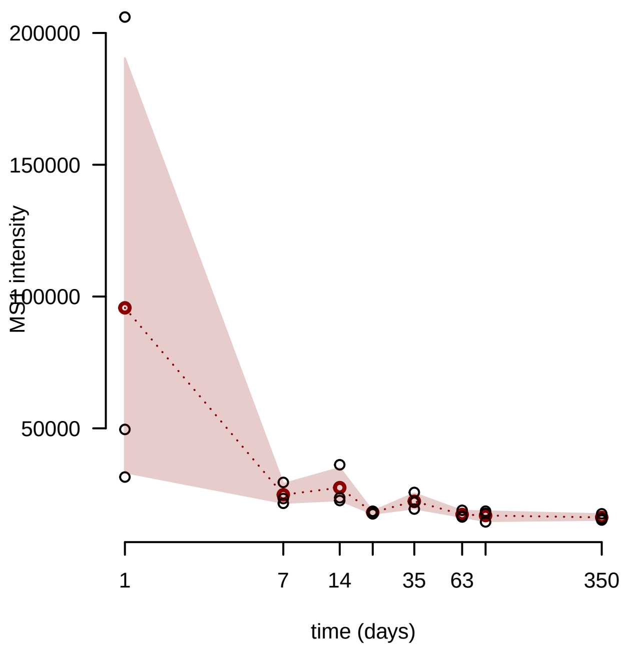

P21570
GN=Ang
angiogenin, ribonuclease, RNase A family ...

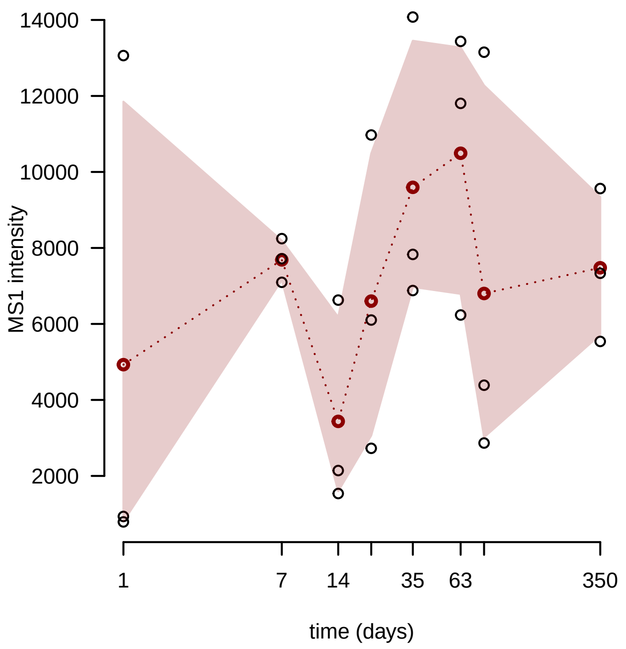

P21614
GN=Gc
vitamin D binding protein

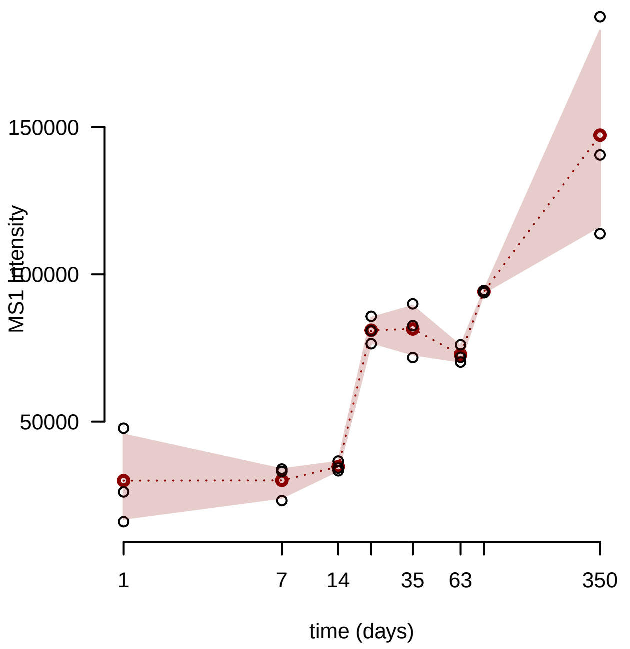

P21619
GN=Lmnb2
lamin B2

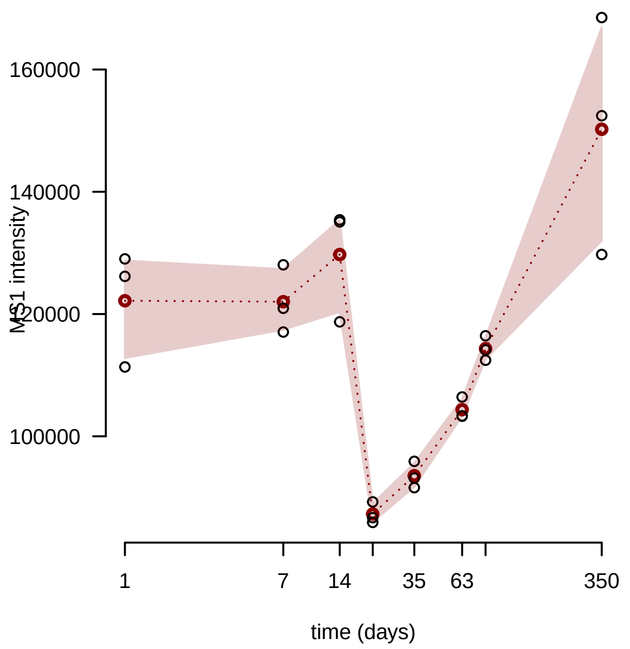

P21812
GN=Mcpt4
mast cell protease 4

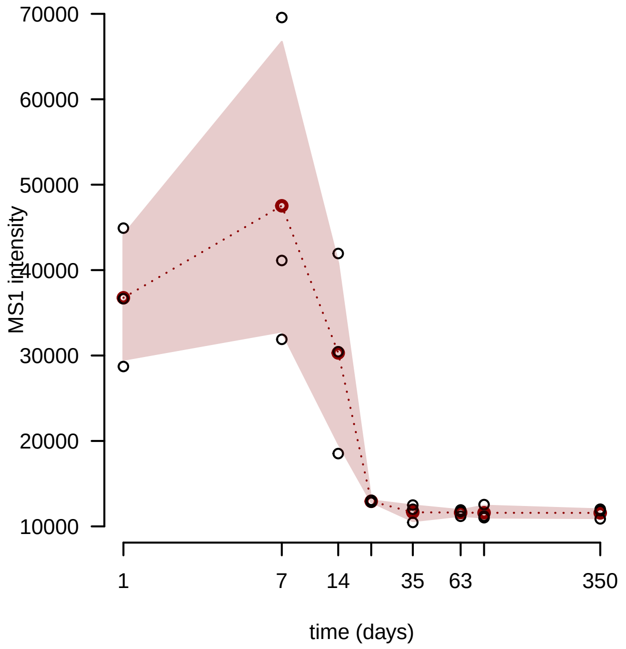

P21843
GN=Mcpt3

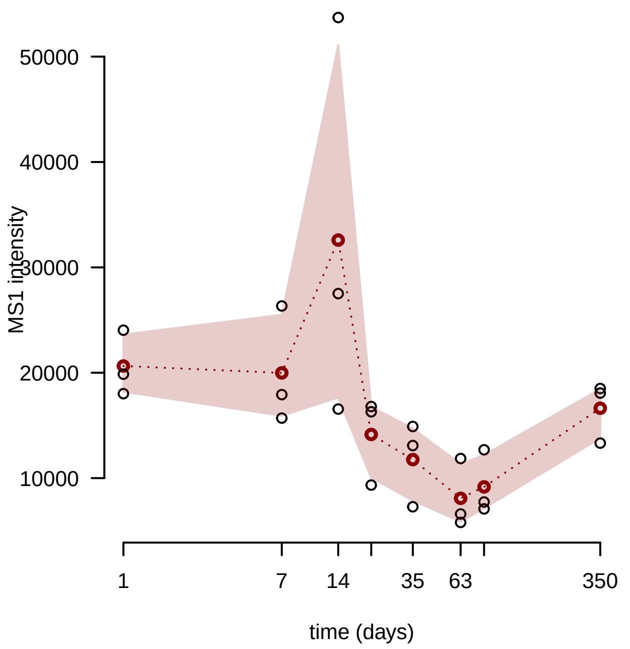

P21844
GN=Cma1
chymase 1, mast cell

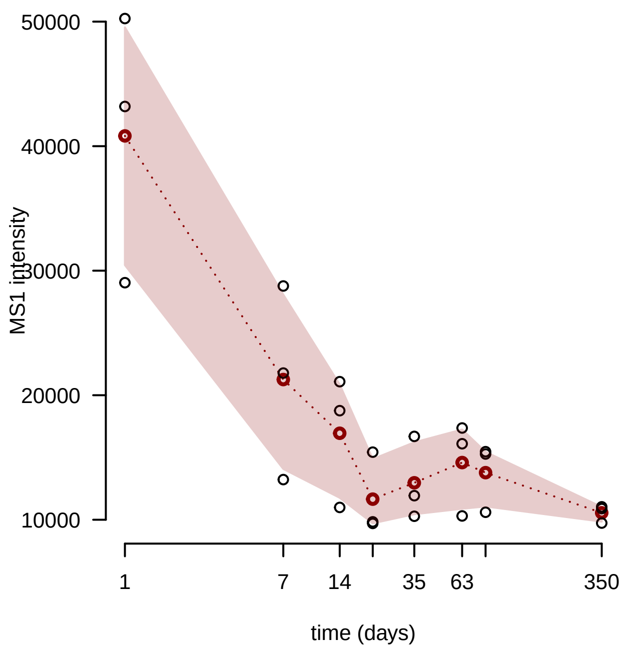

P21845
GN=Tpsb2
tryptase beta 2

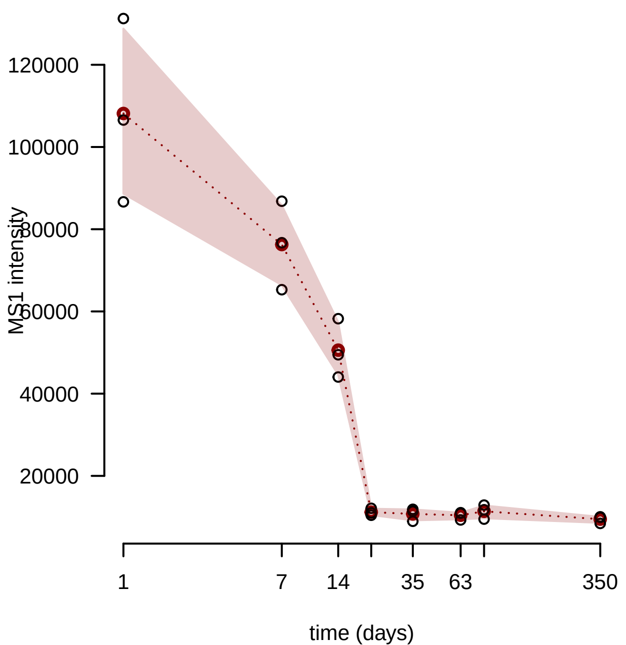

P21956
GN=Mfge8
milk fat globule EGF and factor V/VIII d ...

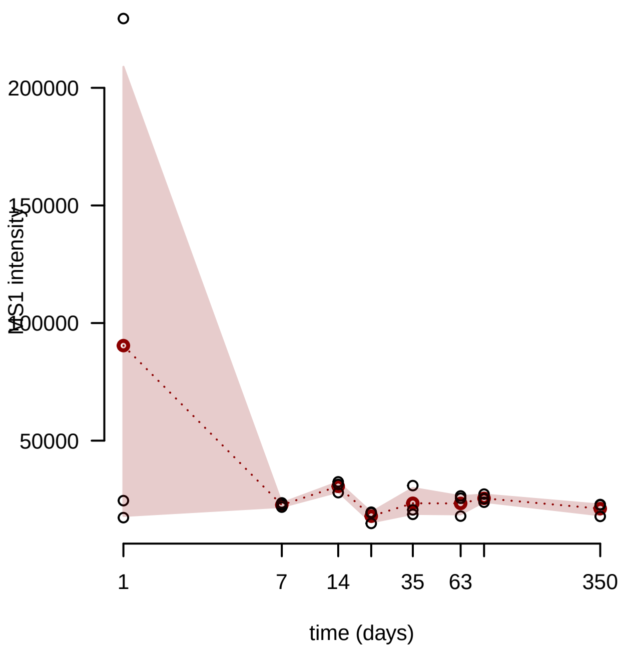

P21958
GN=Tap1
transporter 1, ATP−binding cassette, sub ...

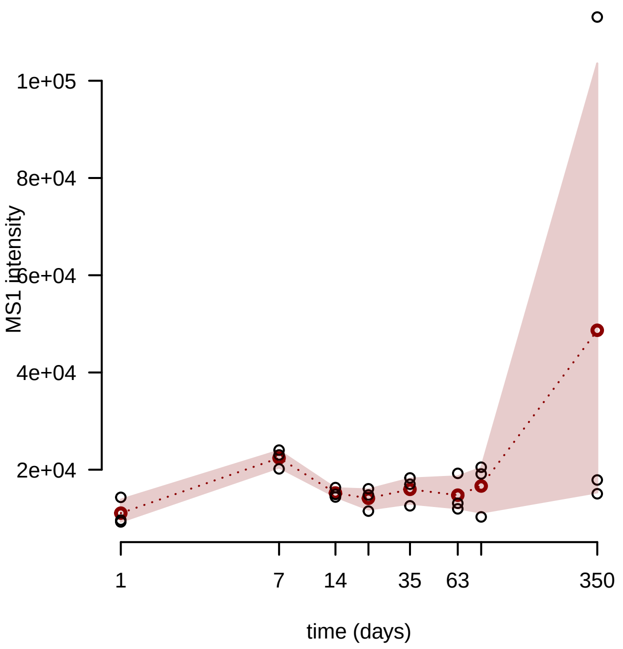

P21981
GN=Tgm2
transglutaminase 2, C polypeptide

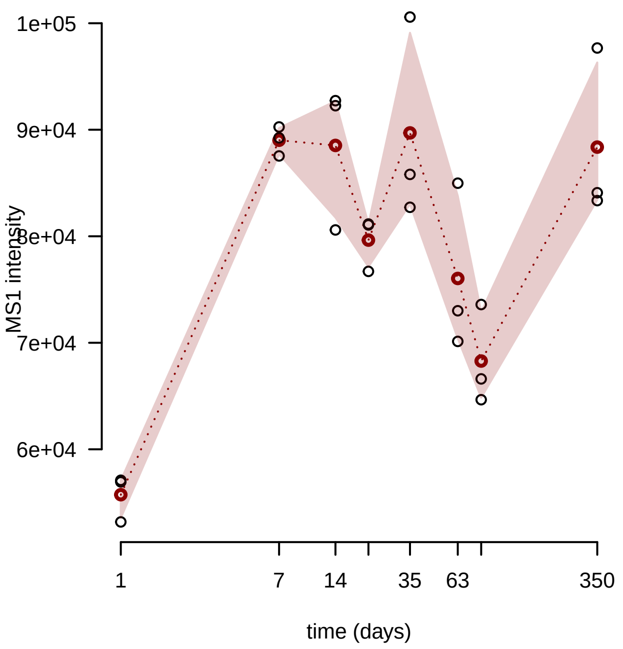

P21995
GN=Emb
embigin

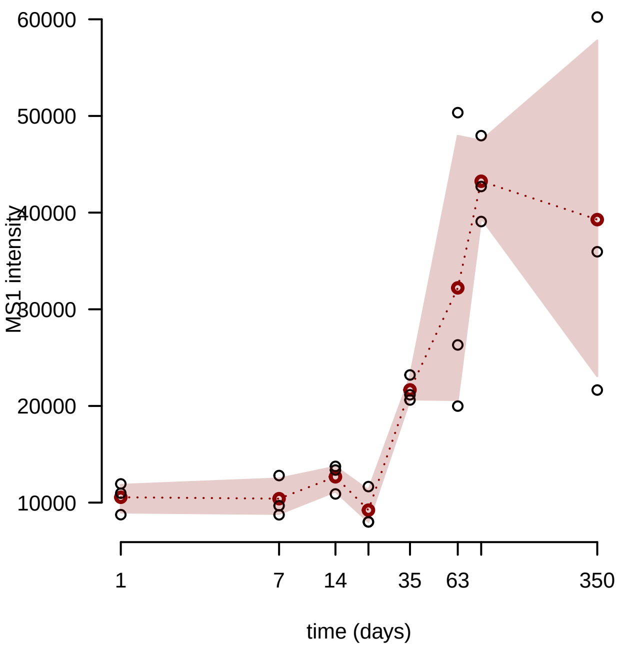

P22315
GN=Fech
ferrochelatase

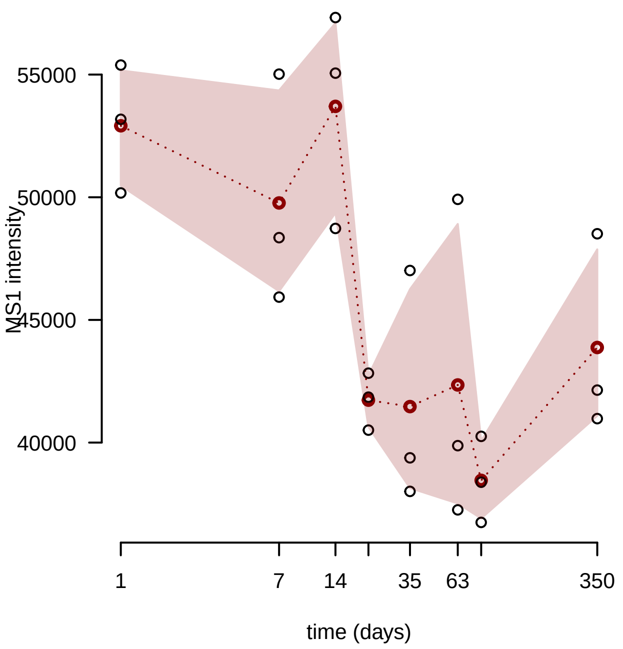

P22366
GN=Myd88
myeloid differentiation primary response ...

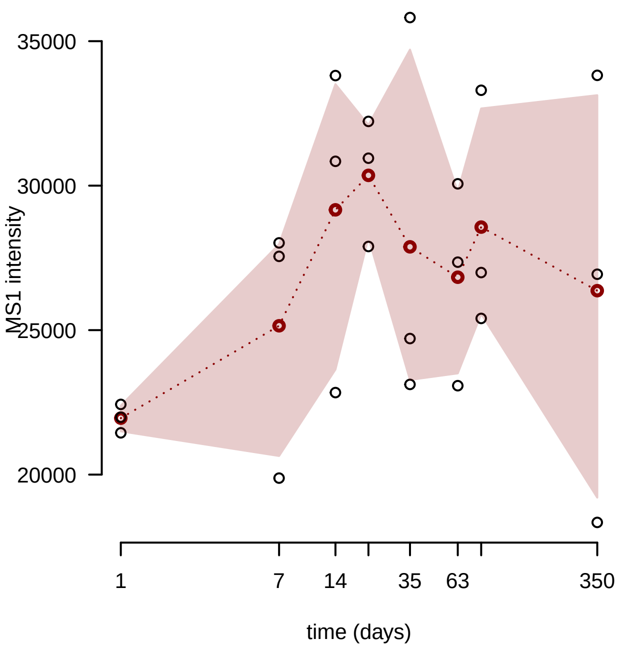

P22437
GN=Ptgs1
prostaglandin−endoperoxide synthase 1

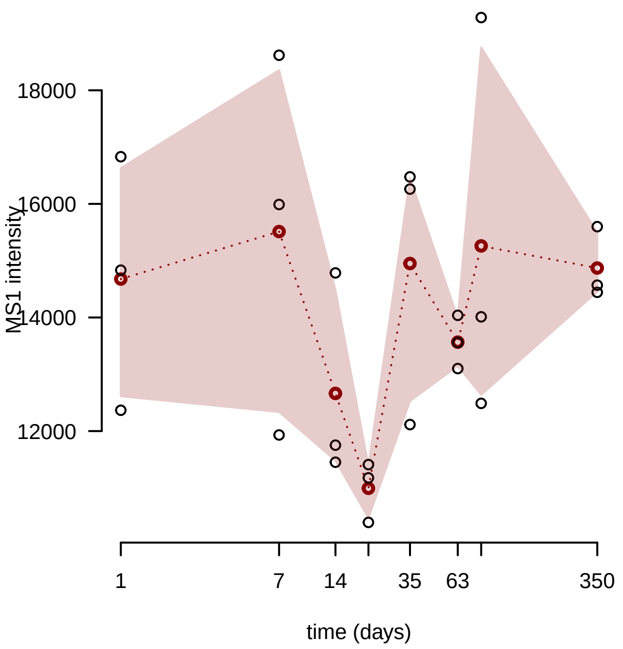

P22518
GN=Clk1
CDC−like kinase 1

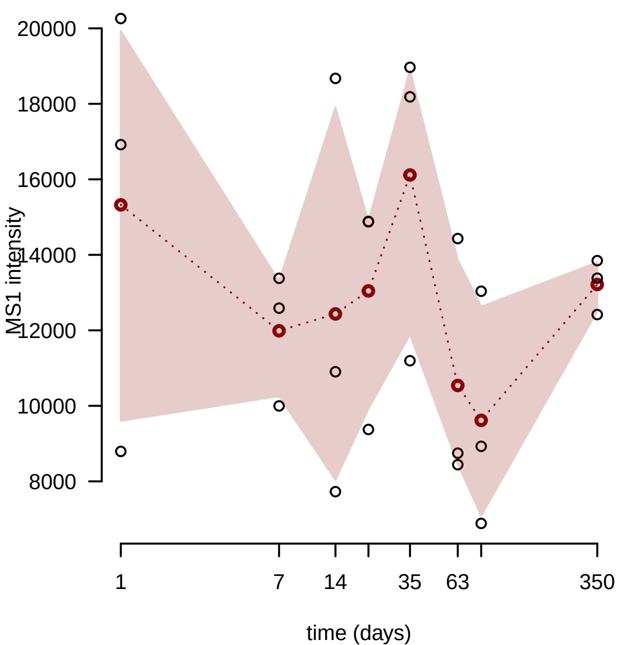

P22561
GN=Wt1
WT1 transcription factor

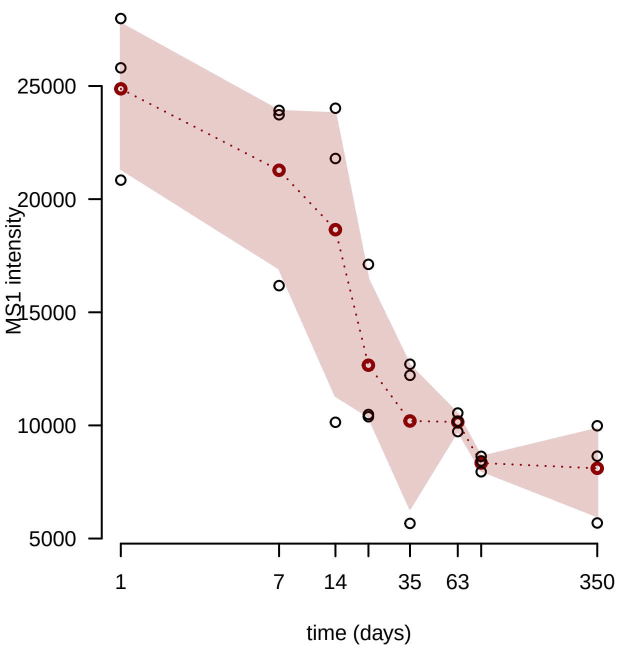

P22561
GN=Wt1
WT1 transcription factor

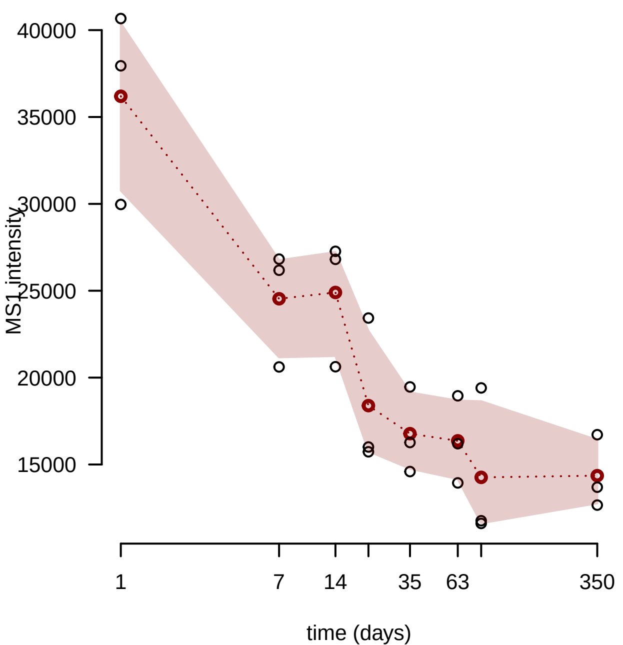

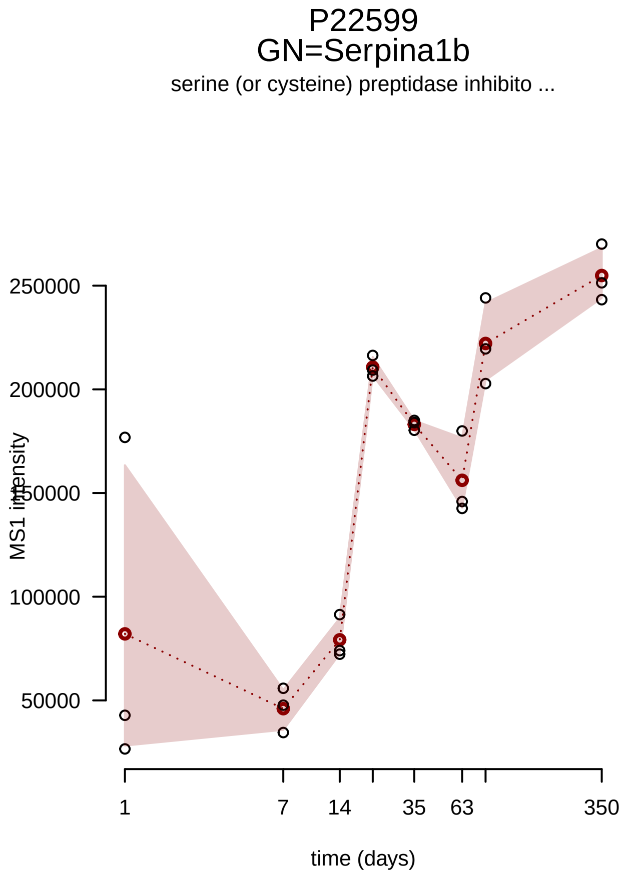
P22599
GN=Serpina1b
serine (or cysteine) peptidase inhibito ...
MS1 intensity
250000
200000
150000
100000
50000
1
7
14
35
63
350
time (days)

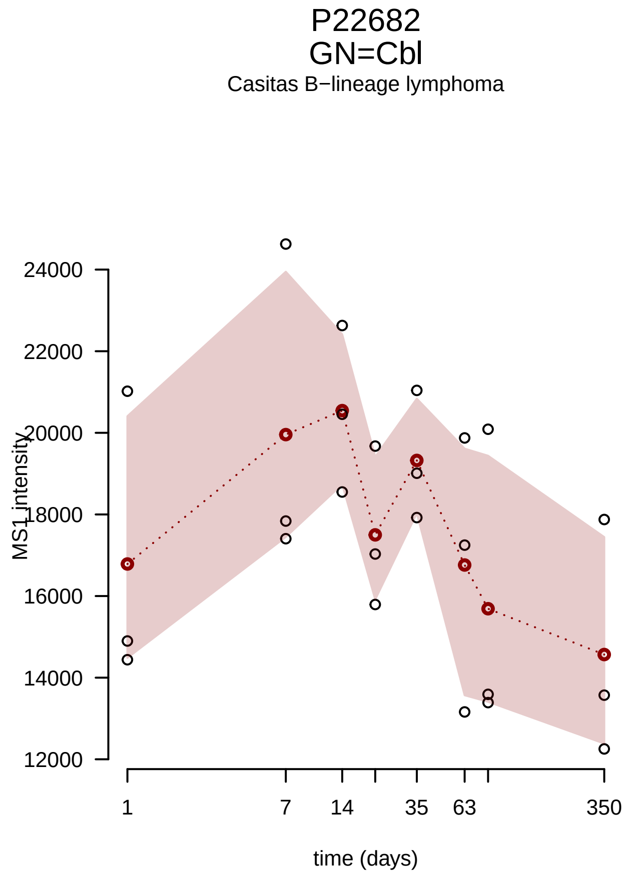
P22682
GN=Cbl
Casitas B−lineage lymphoma
MS1 intensity
24000
22000
20000
18000
16000
14000
12000
1
7
14
35
63
350
time (days)

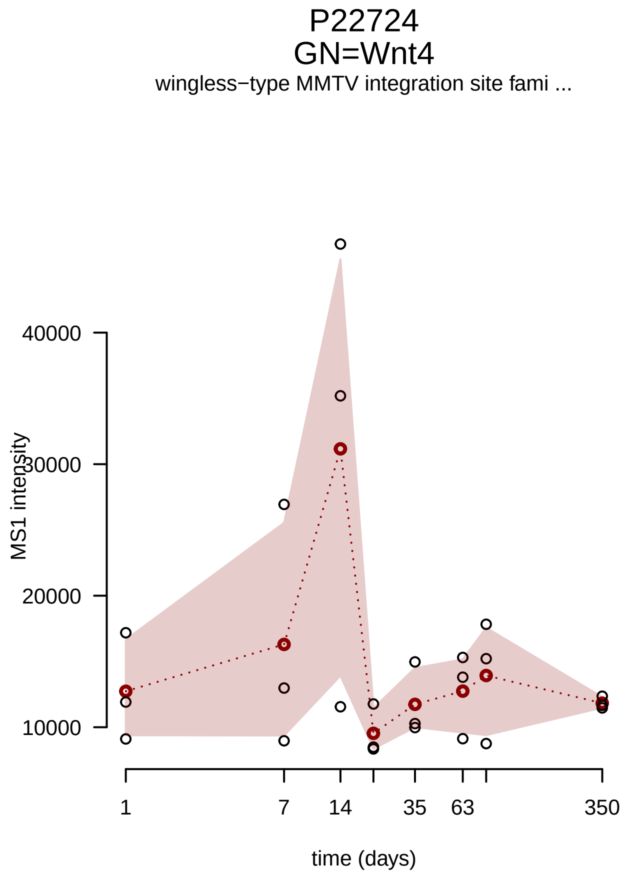
P22724
GN=Wnt4
wingless−type MMTV integration site fami ...
MS1 intensity
40000
30000
20000
10000
1
7
14
35
63
350
time (days)

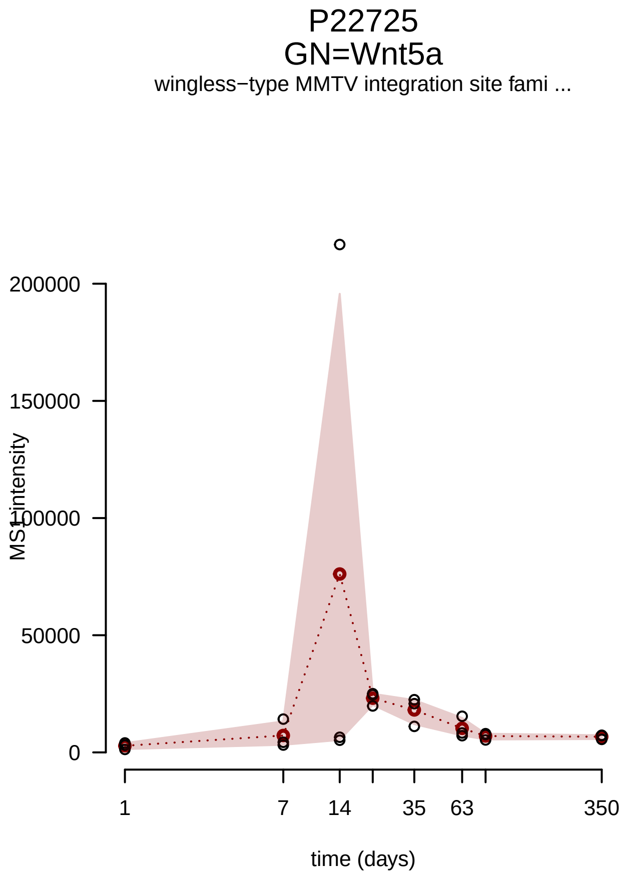
P22725
GN=Wnt5a
wingless−type MMTV integration site fami ...
MS1 intensity
200000
150000
100000
50000
0
1
7
14
35
63
350
time (days)

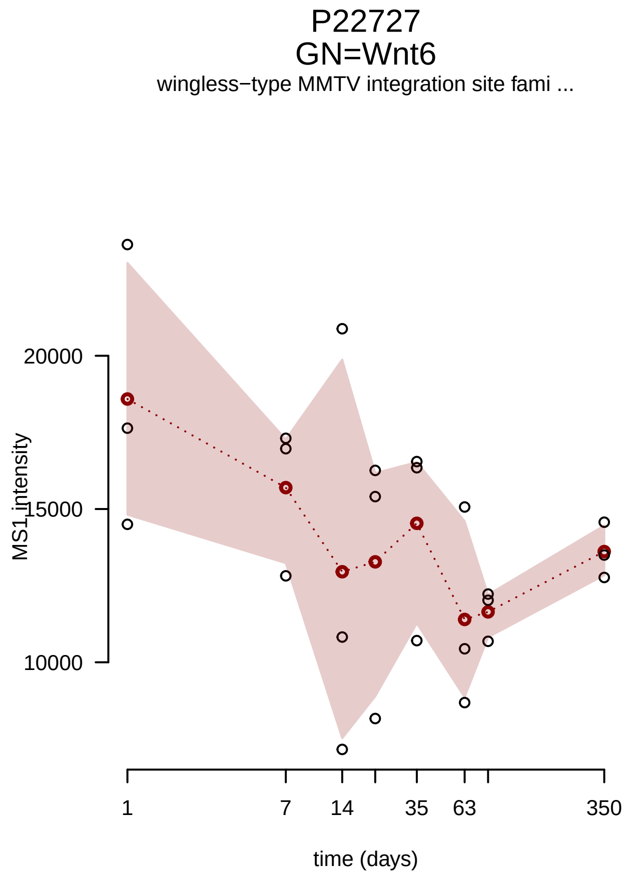
P22727
GN=Wnt6
wingless−type MMTV integration site fami ...
MS1 intensity
20000
15000
10000
1
7
14
35
63
350
time (days)

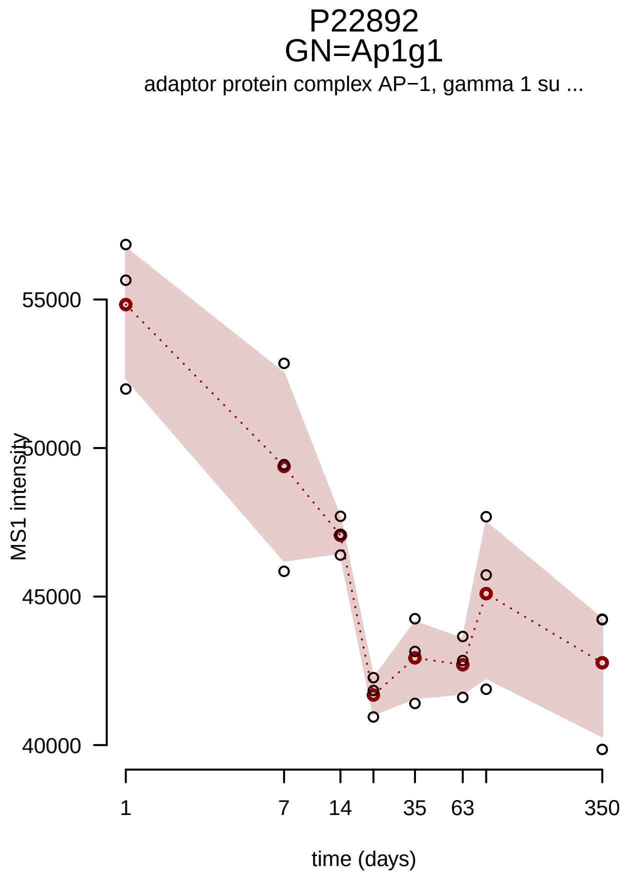
P22892
GN=Ap1g1
adaptor protein complex AP−1, gamma 1 su ...
MS1 intensity
55000
50000
45000
40000
1
7
14
35
63
350
time (days)

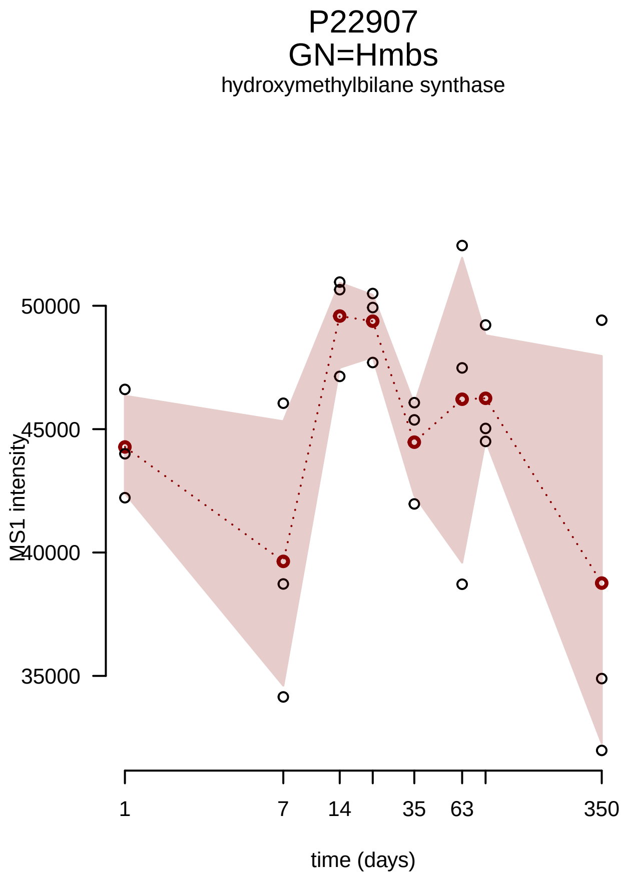
P22907
GN=Hmbs
hydroxymethylbilane synthase
MS1 intensity
50000
45000
40000
35000
1
7
14
35
63
350
time (days)

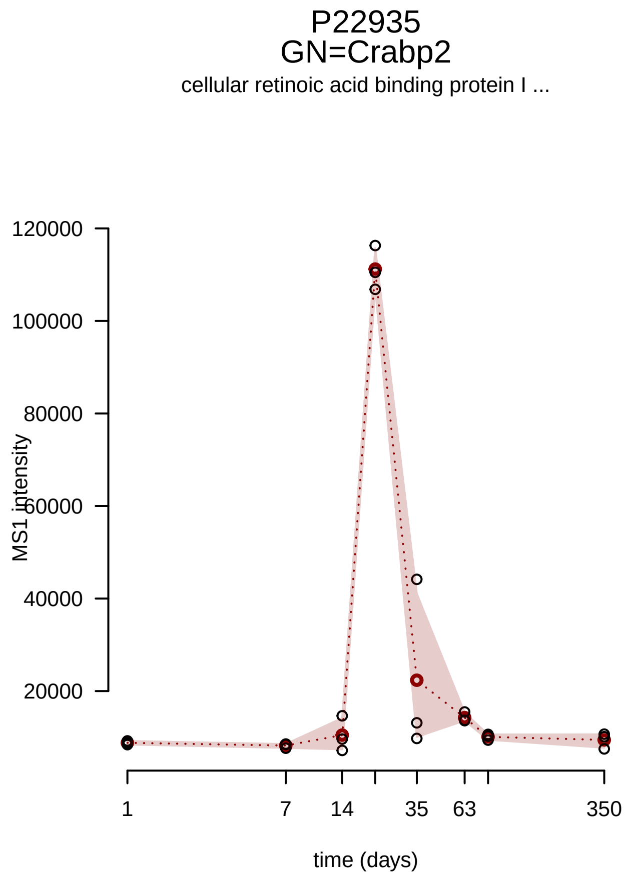
P22935
GN=Crabp2
cellular retinoic acid binding protein I ...
MS1 intensity
120000
100000
80000
60000
40000
20000
1
7
14
35
63
350
time (days)

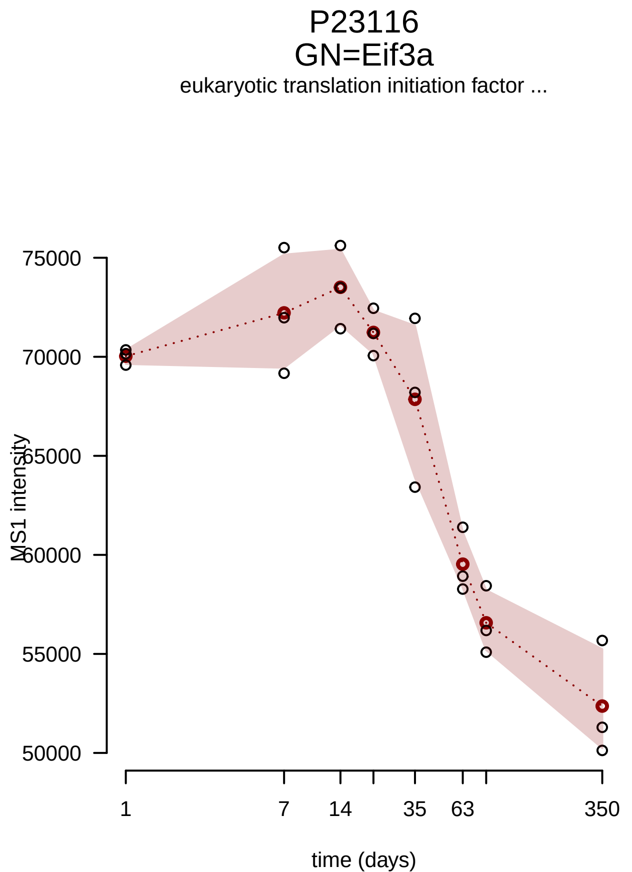
P23116
GN=Eif3a
eukaryotic translation initiation factor ...
MS1 intensity
75000
70000
65000
60000
55000
50000
1
7
14
35
63
350
time (days)

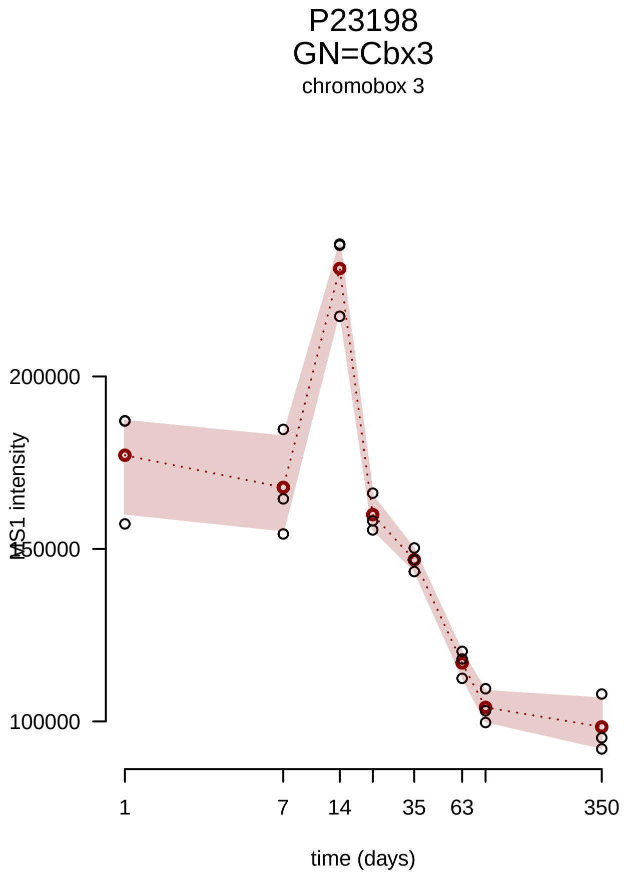
P23198
GN=Cbx3
chromobox 3
MS1 intensity
100000
150000
200000
1
7
14
35
63
350
time (days)

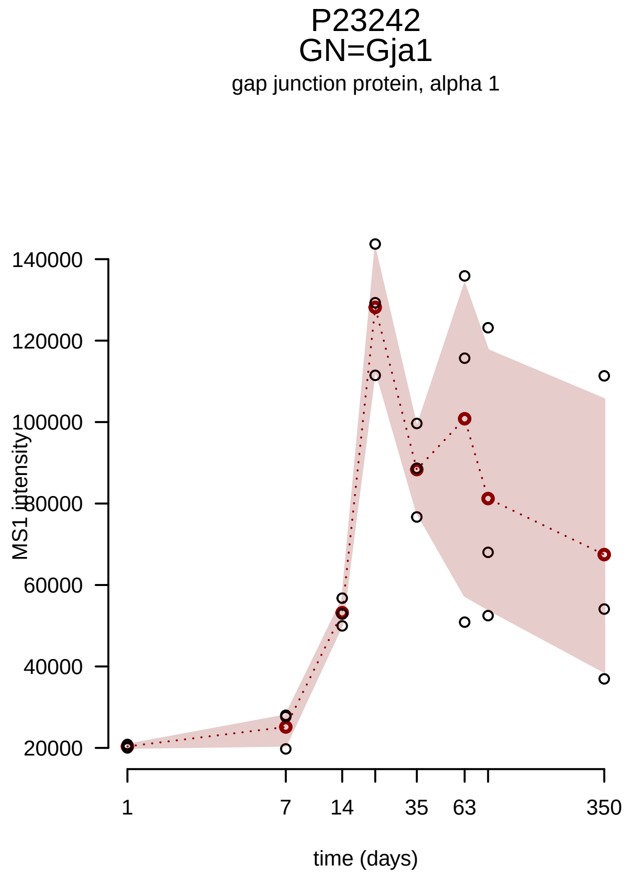
P23242
GN=Gja1
gap junction protein, alpha 1
MS1 intensity
20000
40000
60000
80000
100000
120000
140000
1
7
14
35
63
350
time (days)

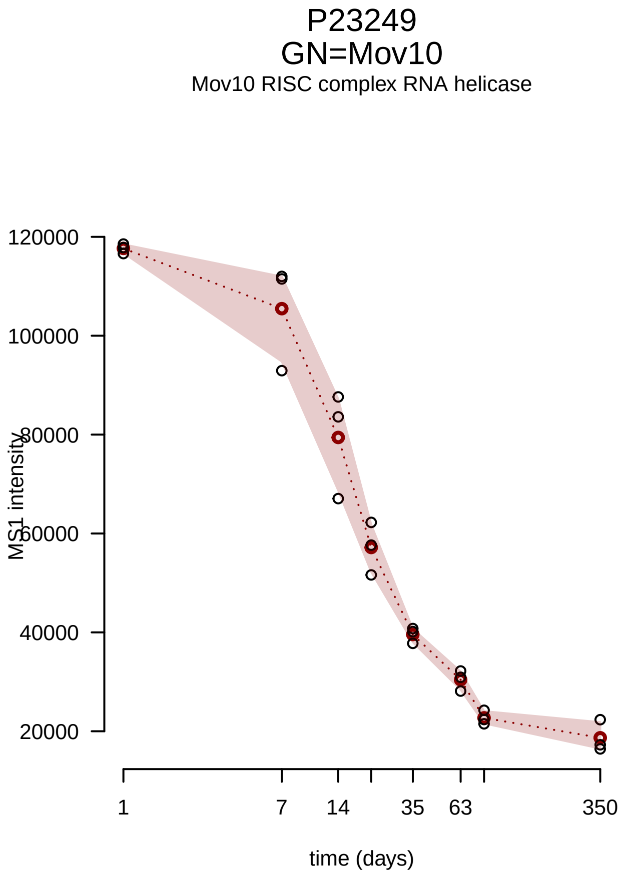
P23249
GN=Mov10
Mov10 RISC complex RNA helicase
MS1 intensity
20000
40000
60000
80000
100000
120000
1
7
14
35
63
350
time (days)

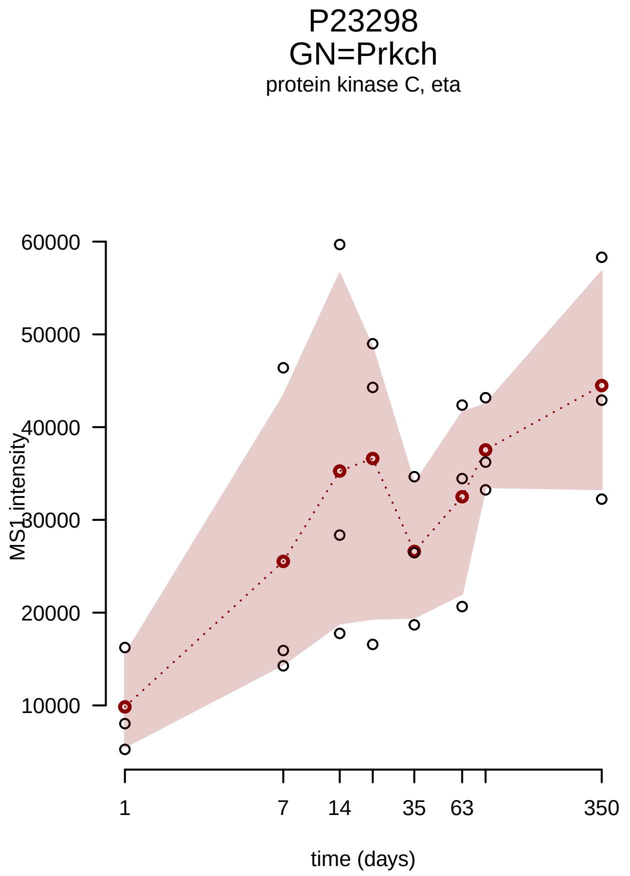
P23298
GN=Prkch
protein kinase C, eta
MS1 intensity
10000
20000
30000
40000
50000
60000
1
7
14
35
63
350
time (days)

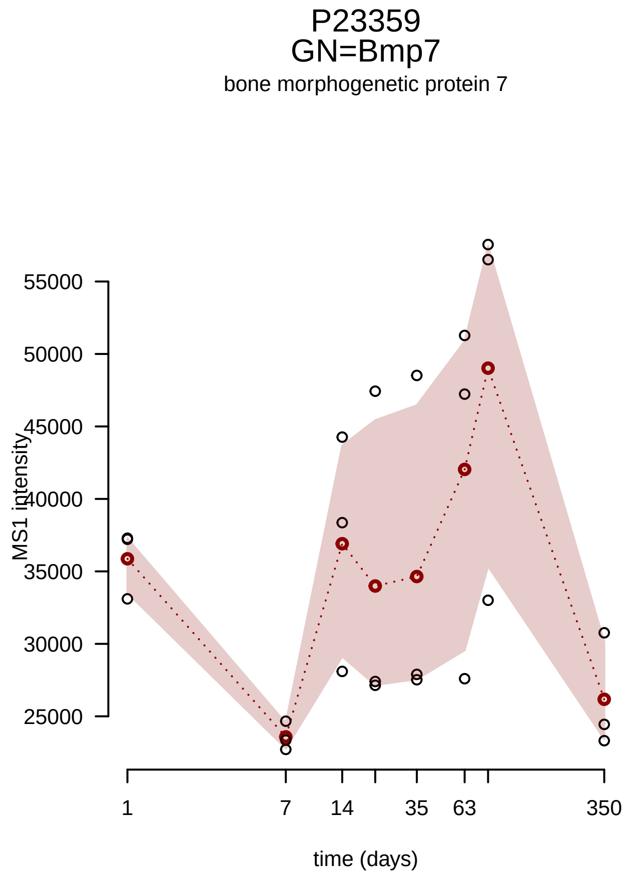
P23359
GN=Bmp7
bone morphogenetic protein 7
MS1 intensity
25000
30000
35000
40000
45000
50000
55000
1
7
14
35
63
350
time (days)

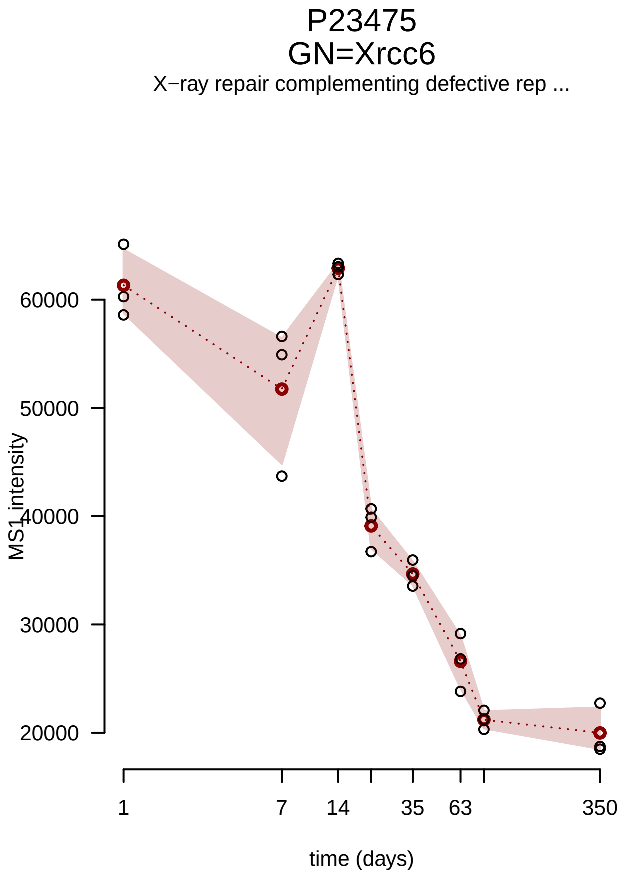
P23475
GN=Xrcc6
X−ray repair complementing defective rep ...
MS1 intensity
20000
30000
40000
50000
60000
1
7
14
35
63
350
time (days)

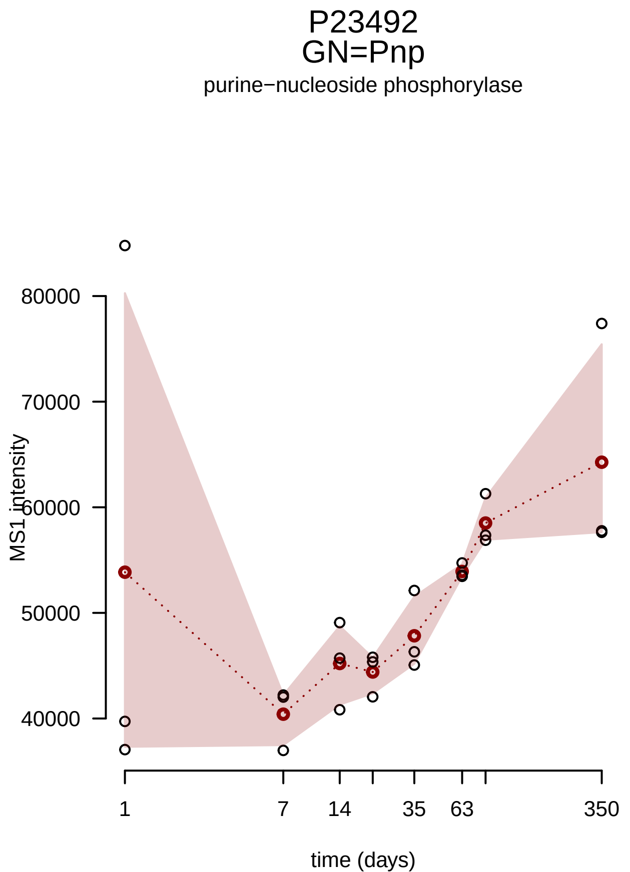
P23492
GN=Pnp
purine−nucleoside phosphorylase
MS1 intensity
40000
50000
60000
70000
80000
1
7
14
35
63
350
time (days)

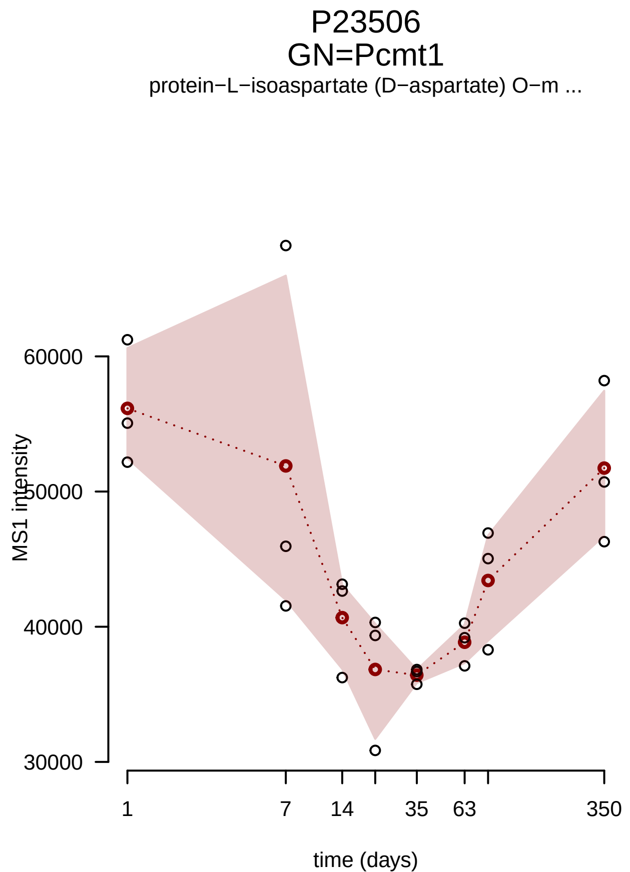
P23506
GN=Pcmt1
protein−L−isoaspartate (D−aspartate) O−m ...
MS1 intensity
30000
40000
50000
60000
1
7
14
35
63
350
time (days)

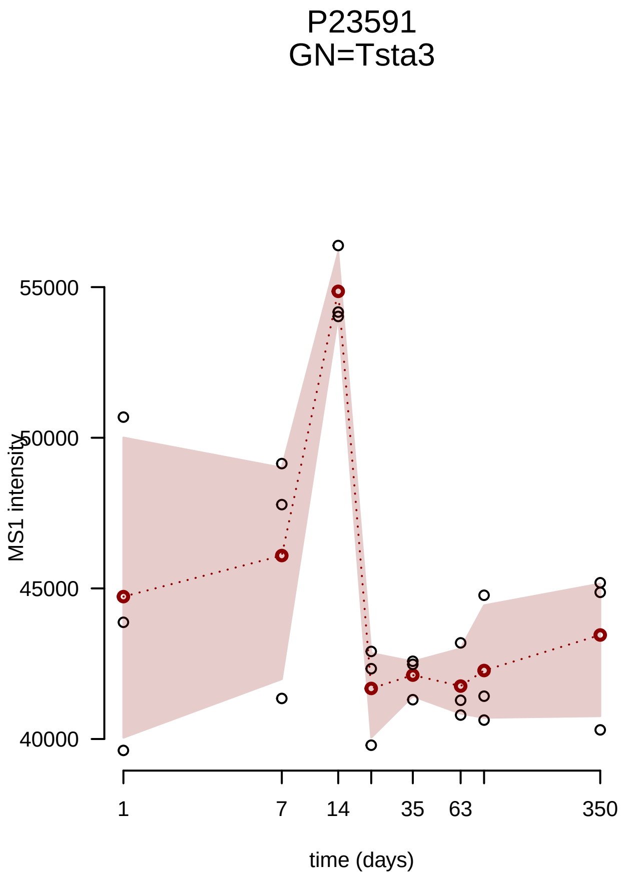
P23591
GN=Tsta3
MS1 intensity
40000
45000
50000
55000
1
7
14
35
63
350
time (days)

P23708
GN=Nfya
nuclear transcription factor−Y alpha

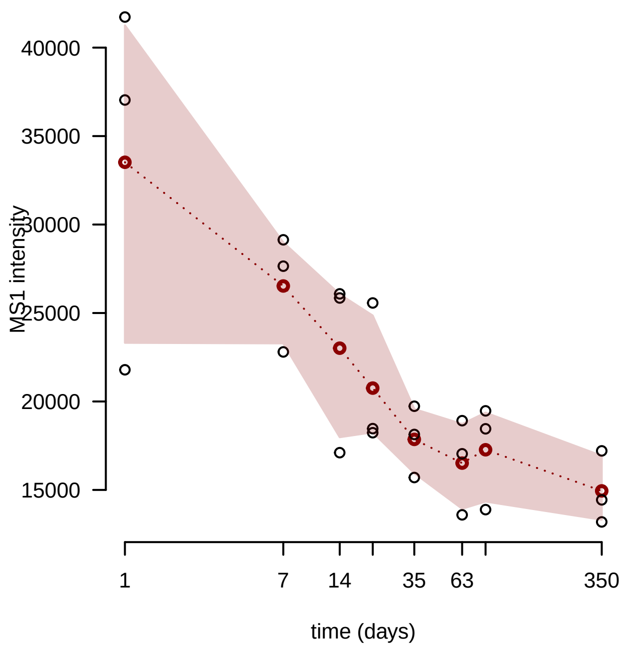

P23780
GN=Glb1
galactosidase, beta 1

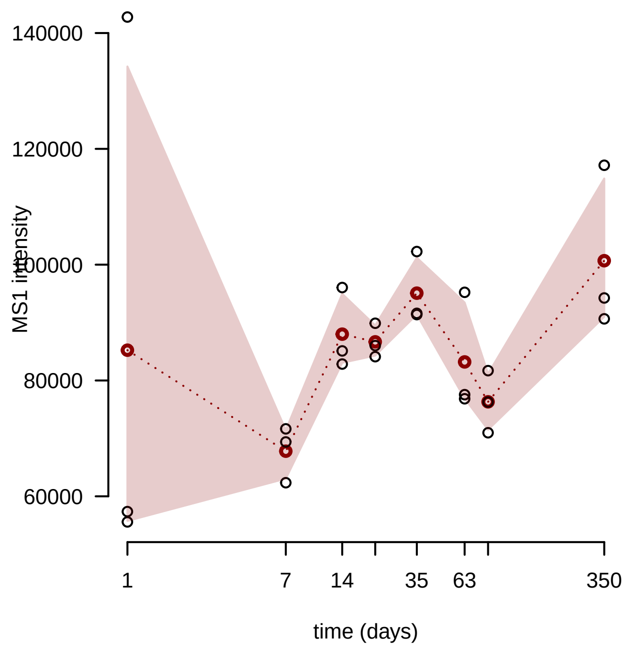

P23798
GN=Pcgf2
polycomb group ring finger 2

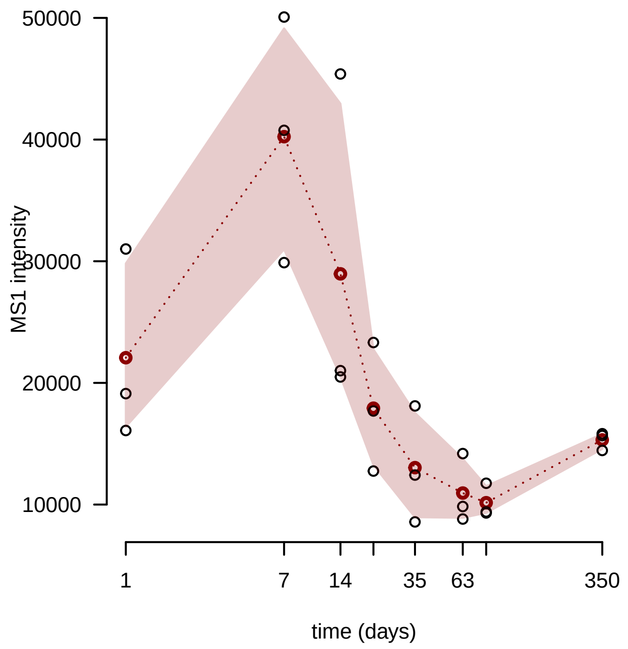

P23881
GN=Tcea3
transcription elongation factor A (SII), ...

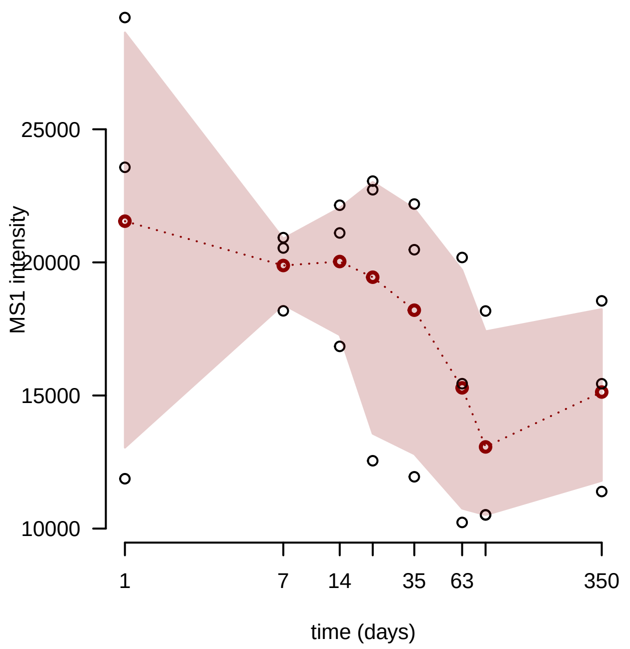

P23906
GN=Irf2
interferon regulatory factor 2

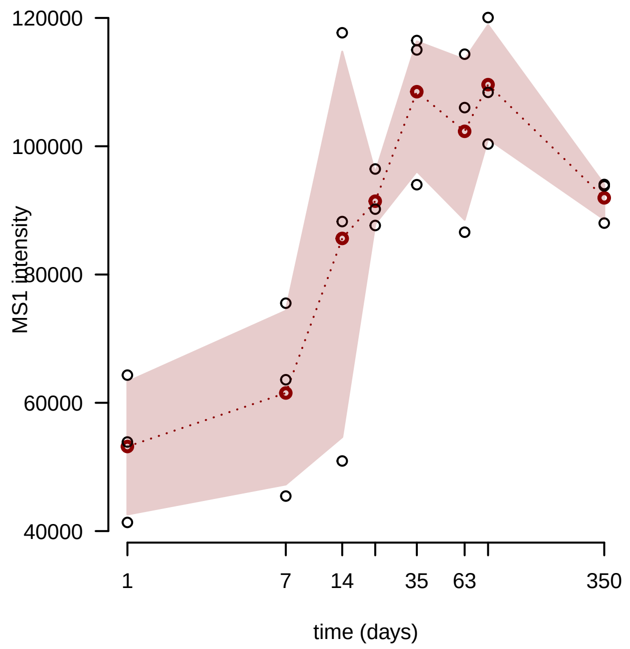

P23949
GN=Zfp36l2
zinc finger protein 36, C3H type−like 2

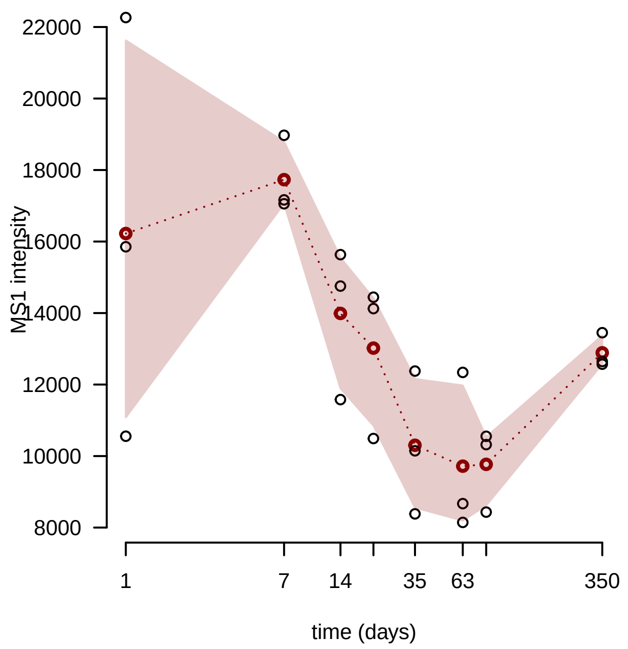

P23950
GN=Zfp36l1
zinc finger protein 36, C3H type−like 1

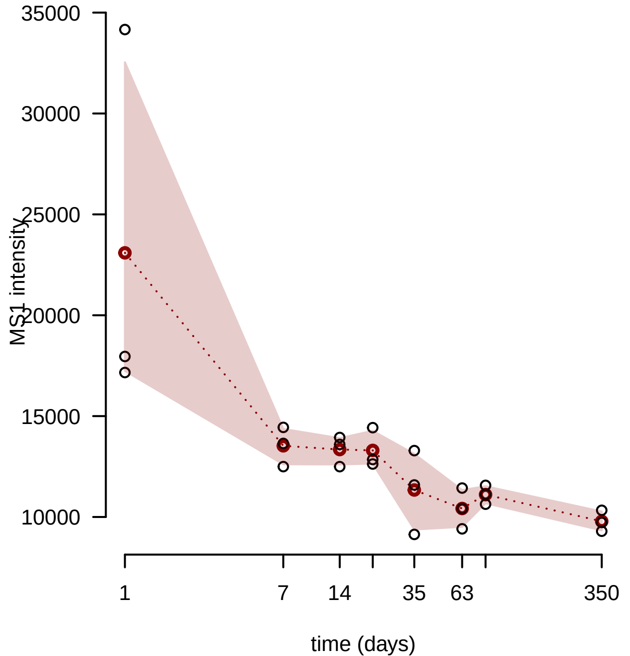

P23953
GN=Ces1c
carboxylesterase 1C

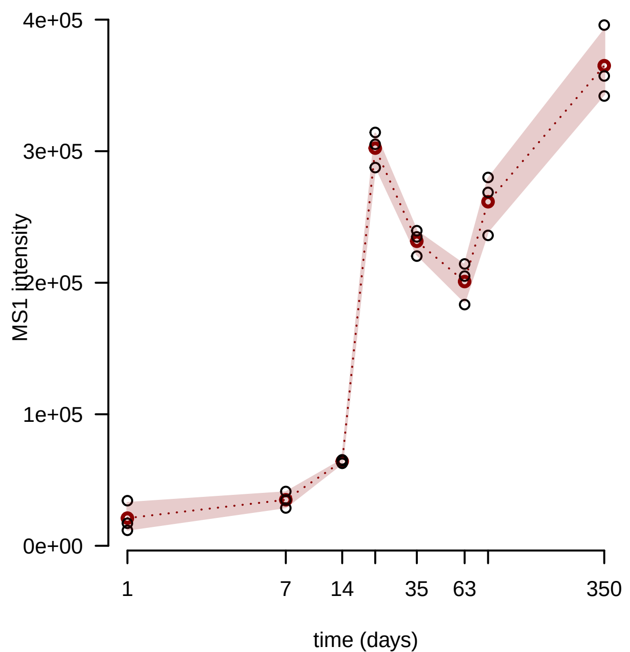

P24063
GN=Itgal
integrin alpha L

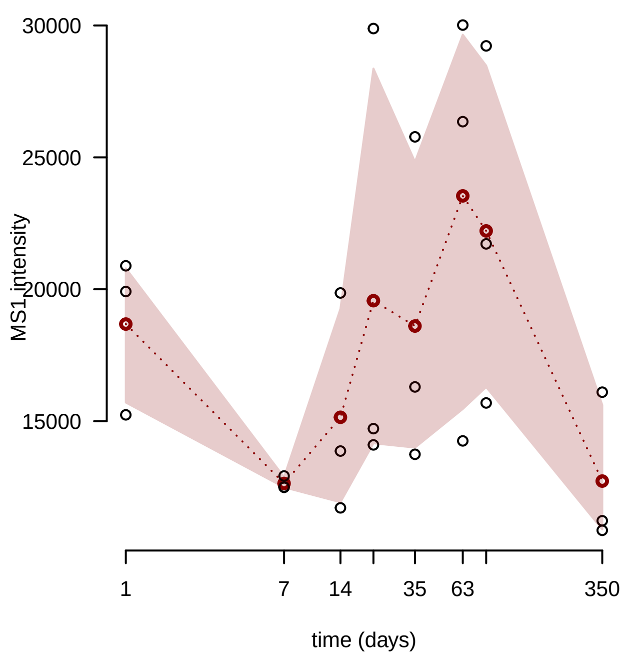

P24270
GN=Cat
catalase

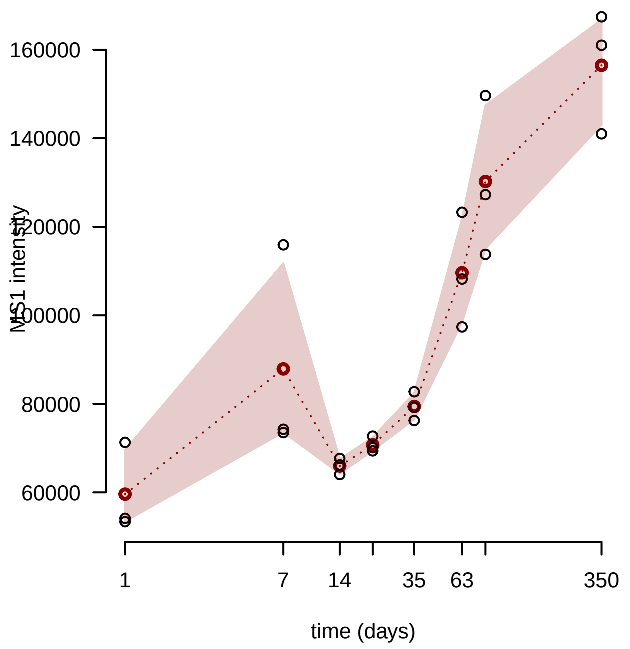

P24288
GN=Bcat1
branched chain aminotransferase 1, cytos ...

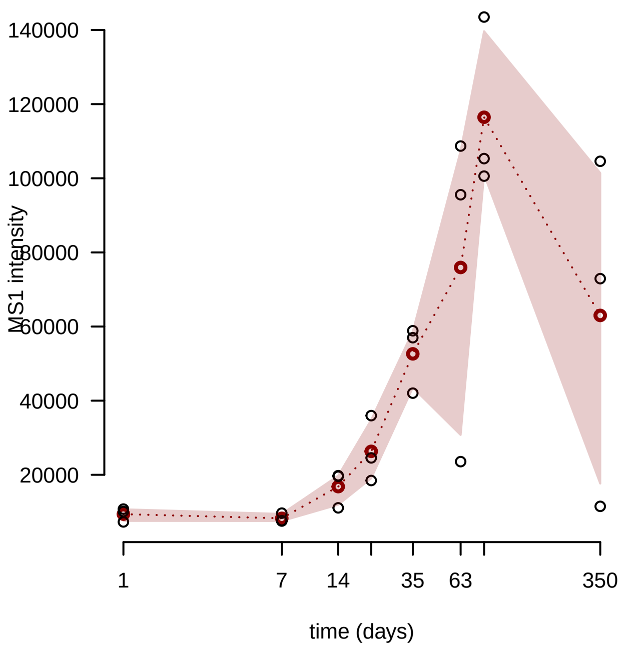

P24369
GN=Ppib
peptidylprolyl isomerase B

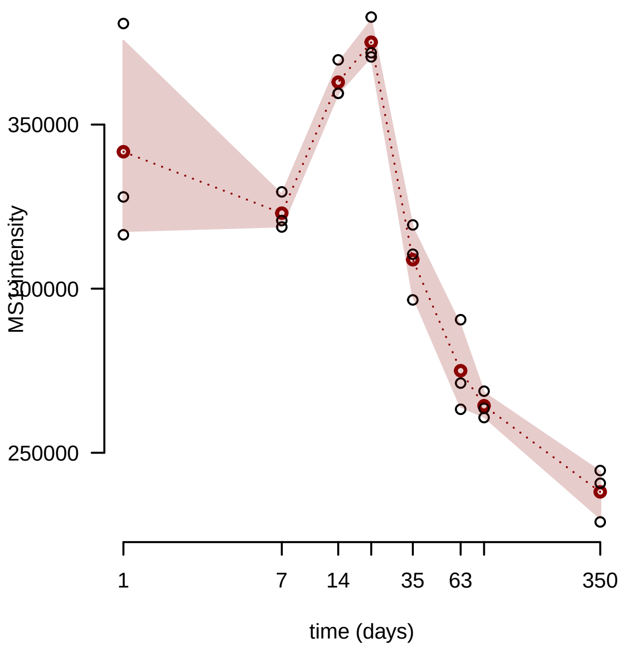

P24452
GN=Capg
capping protein (actin filament), gelsol ...

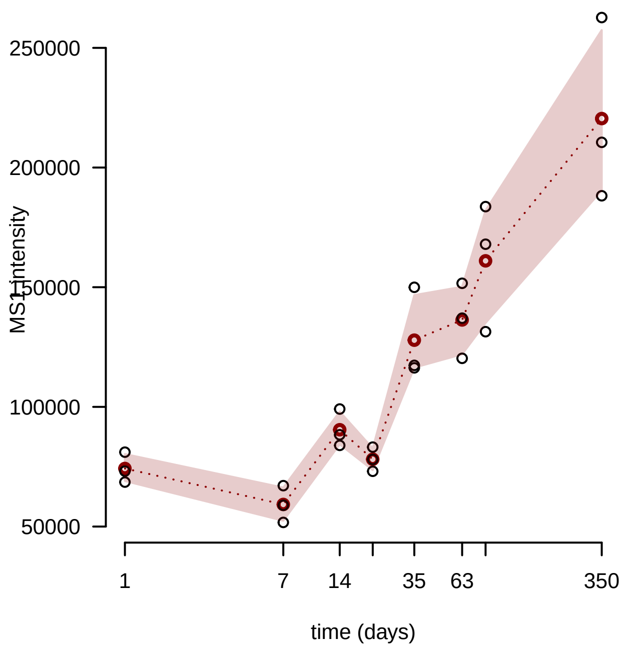

P24457
GN=Cyp2d11
cytochrome P450, family 2, subfamily d, ...

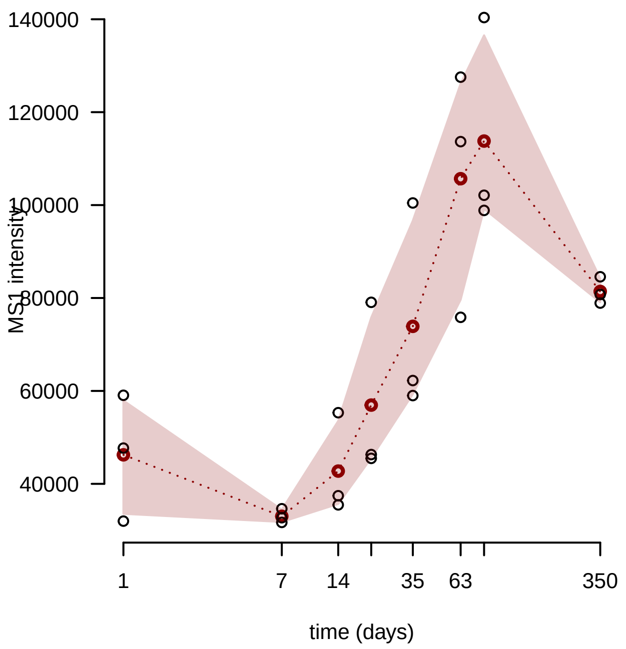

P24472
GN=Gsta4
glutathione S−transferase, alpha 4

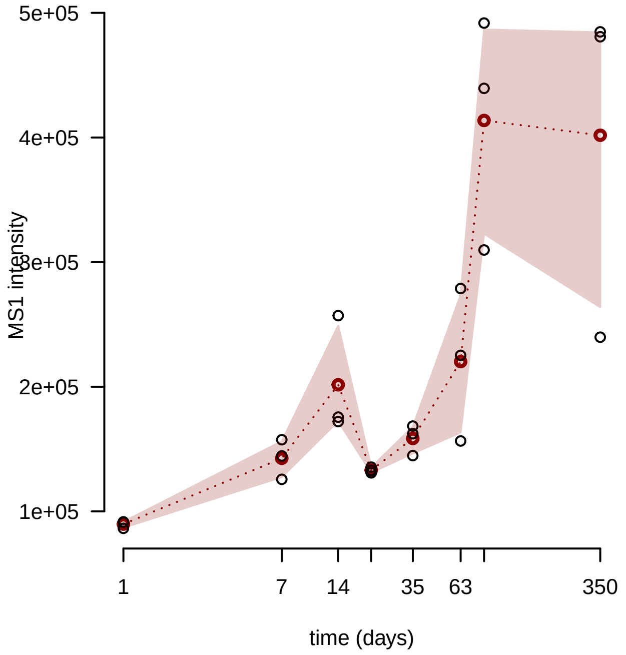

P24527
GN=Lta4h
leukotriene A4 hydrolase

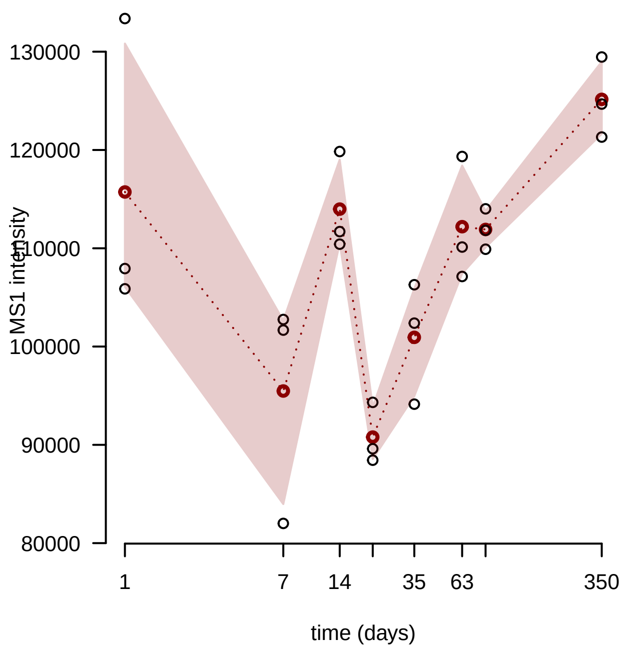

P24547
GN=Impdh2
inosine monophosphate dehydrogenase 2

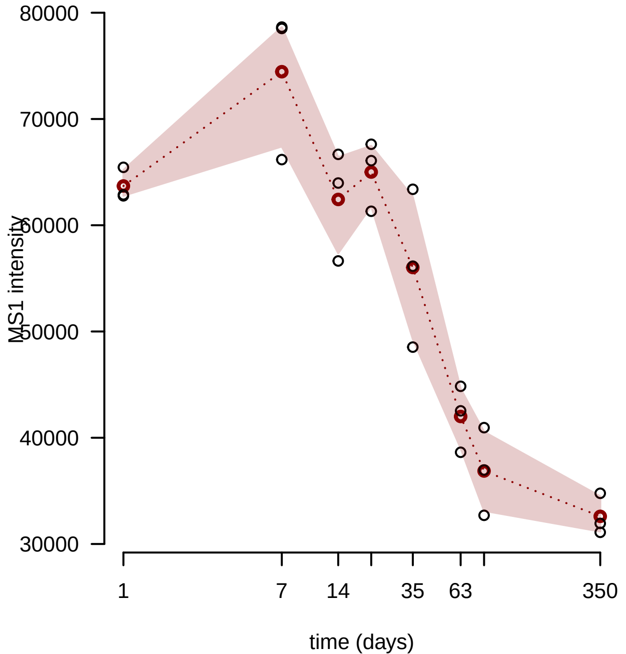

P24549
GN=Aldh1a1
aldehyde dehydrogenase family 1, subfami ...

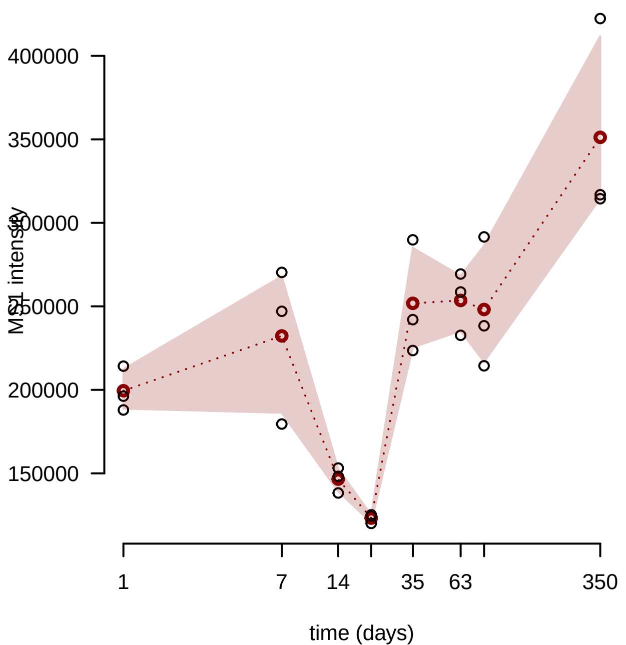

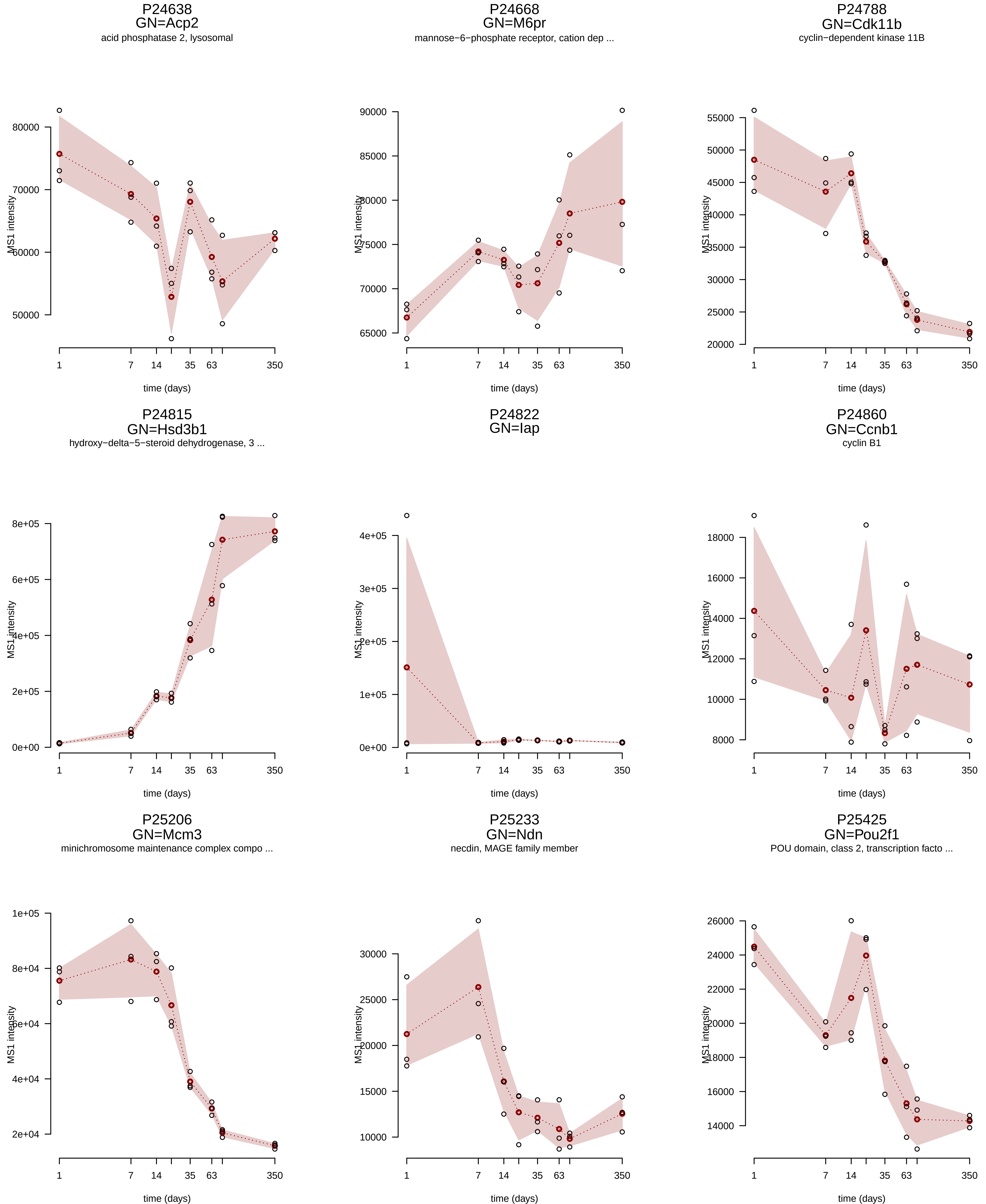
P24638
GN=Acp2
acid phosphatase 2, lysosomal
80000
70000
60000
50000
MS1 intensity
1
7
14
35
63
350
time (days)
P24668
GN=M6pr
mannose−6−phosphate receptor, cation dep ...
90000
85000
80000
75000
70000
65000
MS1 intensity
1
7
14
35
63
350
time (days)
P24788
GN=Cdk11b
cyclin−dependent kinase 11B
55000
50000
45000
40000
35000
30000
25000
20000
MS1 intensity
1
7
14
35
63
350
time (days)
P24815
GN=Hsd3b1
hydroxy−delta−5−steroid dehydrogenase, 3 ...
8e+05
6e+05
4e+05
2e+05
0e+00
MS1 intensity
1
7
14
35
63
350
time (days)
P24822
GN=Iap
4e+05
3e+05
2e+05
1e+05
0e+00
MS1 intensity
1
7
14
35
63
350
time (days)
P24860
GN=Ccnb1
cyclin B1
18000
16000
14000
12000
10000
8000
MS1 intensity
1
7
14
35
63
350
time (days)
P25206
GN=Mcm3
minichromosome maintenance complex compo ...
1e+05
8e+04
6e+04
4e+04
2e+04
MS1 intensity
1
7
14
35
63
350
time (days)
P25233
GN=Ndn
necdin, MAGE family member
30000
25000
20000
15000
10000
MS1 intensity
1
7
14
35
63
350
time (days)
P25425
GN=Pou2f1
POU domain, class 2, transcription facto ...
26000
24000
22000
20000
18000
16000
14000
MS1 intensity
1
7
14
35
63
350
time (days)

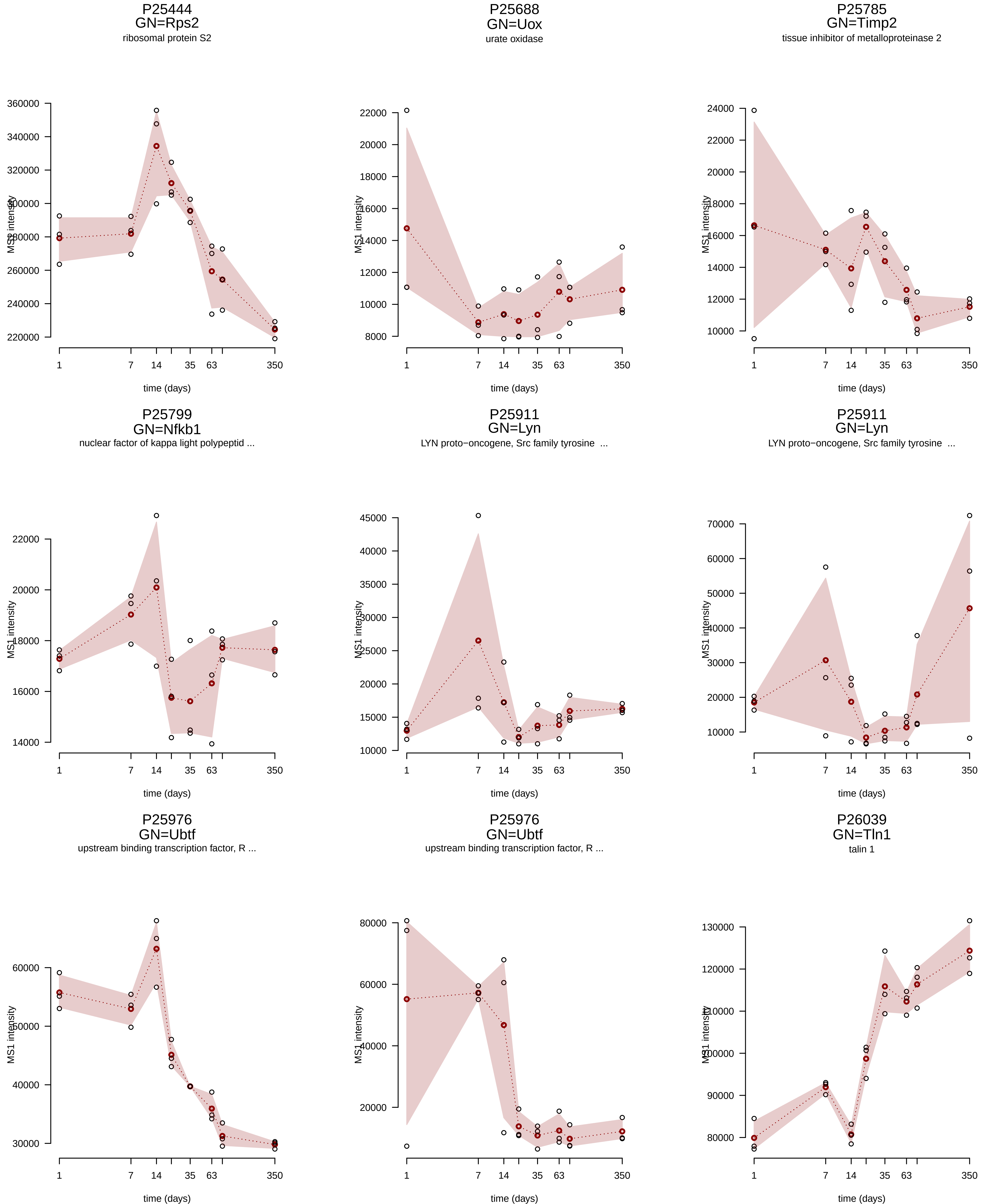
P25444
GN=Rps2
ribosomal protein S2
MS1 intensity
220000 240000 260000 280000 300000 320000 340000 360000
1 7 14 35 63 350
time (days)
P25688
GN=Uox
urate oxidase
MS1 intensity
8000 10000 12000 14000 16000 18000 20000 22000
1 7 14 35 63 350
time (days)
P25785
GN=Timp2
tissue inhibitor of metalloproteinase 2
MS1 intensity
10000 12000 14000 16000 18000 20000 22000 24000
1 7 14 35 63 350
time (days)
P25799
GN=Nfkb1
nuclear factor of kappa light polypeptid ...
MS1 intensity
14000 16000 18000 20000 22000
1 7 14 35 63 350
time (days)
P25911
GN=Lyn
LYN proto−oncogene, Src family tyrosine ...
MS1 intensity
10000 15000 20000 25000 30000 35000 40000 45000
1 7 14 35 63 350
time (days)
P25911
GN=Lyn
LYN proto−oncogene, Src family tyrosine ...
MS1 intensity
10000 20000 30000 40000 50000 60000 70000
1 7 14 35 63 350
time (days)
P25976
GN=Ubtf
upstream binding transcription factor, R ...
MS1 intensity
30000 40000 50000 60000
1 7 14 35 63 350
time (days)
P25976
GN=Ubtf
upstream binding transcription factor, R ...
MS1 intensity
20000 40000 60000 80000
1 7 14 35 63 350
time (days)
P26039
GN=Tln1
talin 1
MS1 intensity
80000 90000 100000 110000 120000 130000
1 7 14 35 63 350
time (days)

P26040
GN=Ezr
ezrin

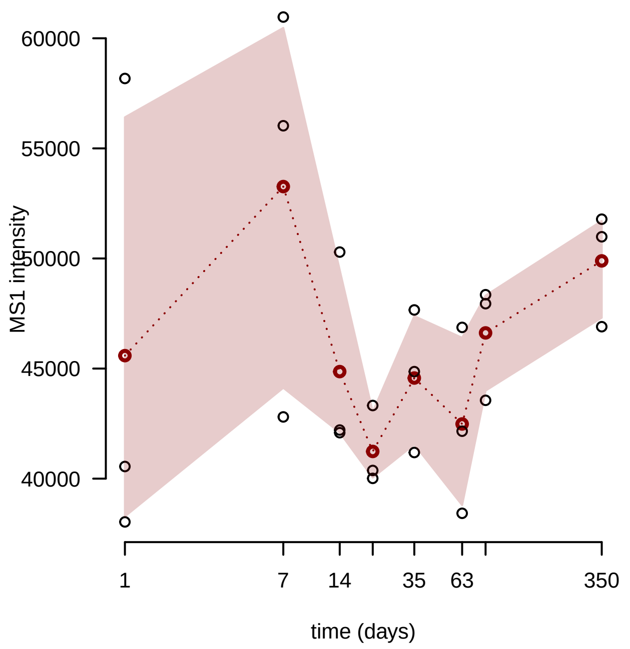

P26041
GN=Msn
moesin

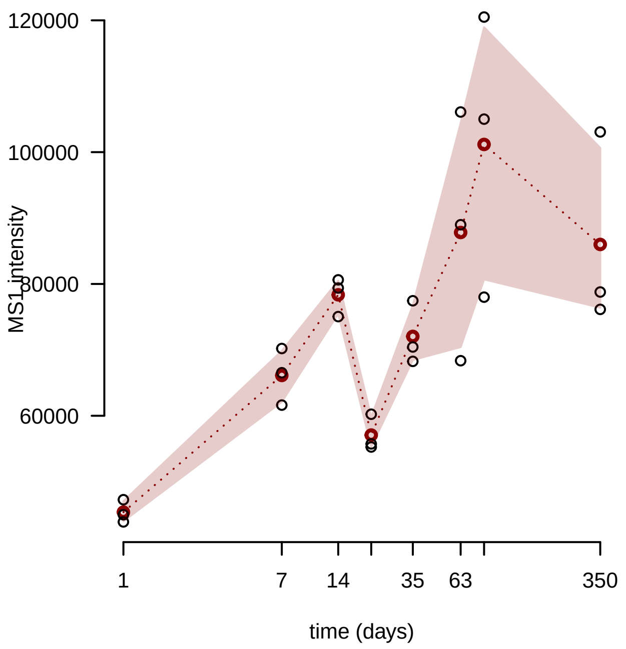

P26043
GN=Rdx
radixin

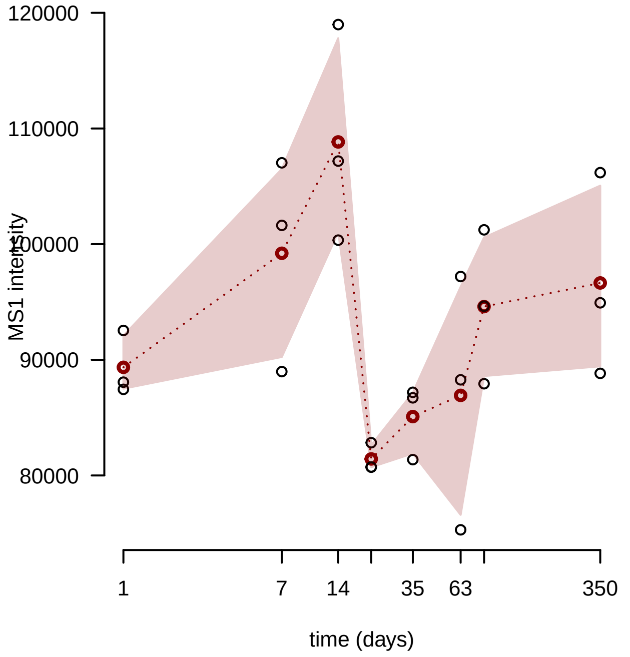

P26187
GN=Mgmt
O−6−methylguanine−DNA methyltransferase

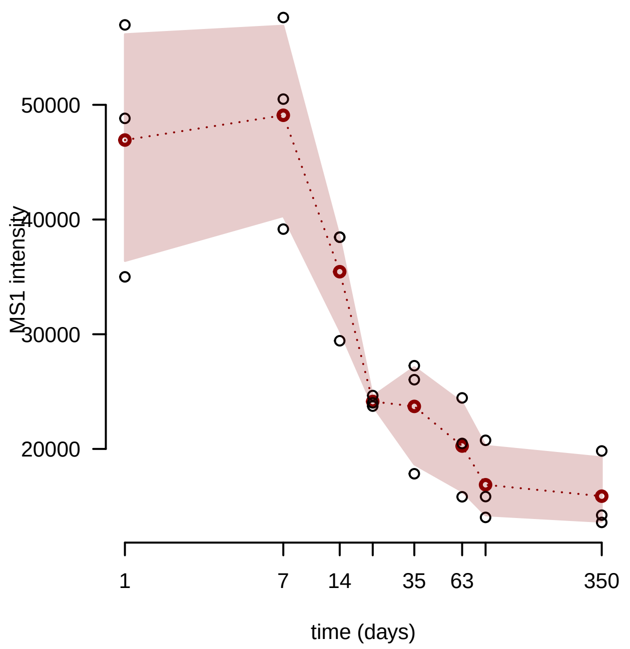

P26231
GN=Ctnna1
catenin (cadherin associated protein), a ...

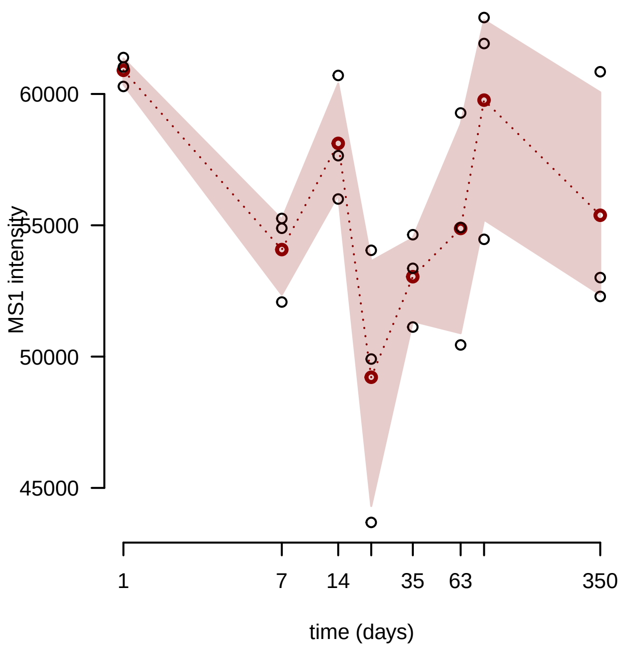

P26262
GN=Klkb1
kallikrein B, plasma 1

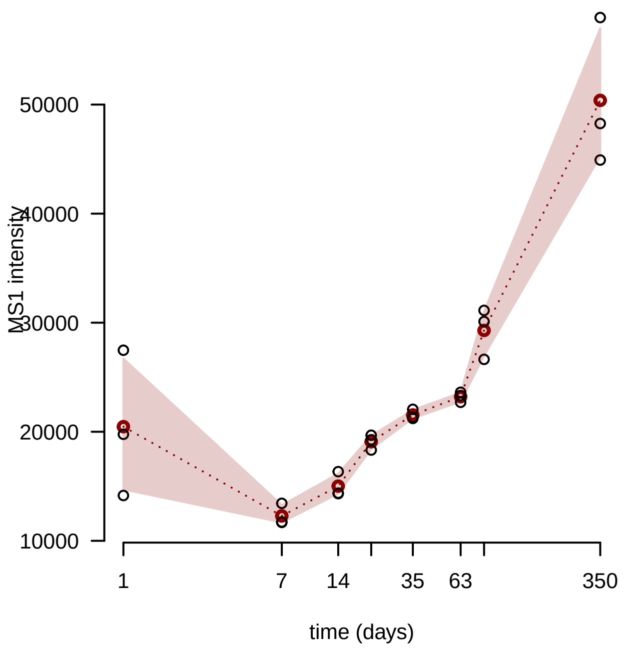

P26323
GN=Fli1
Friend leukemia integration 1

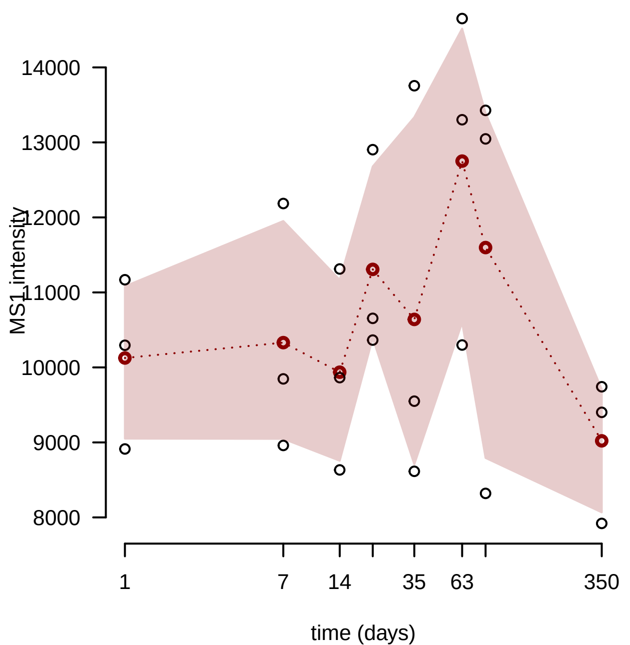

P26339
GN=Chga
chromogranin A

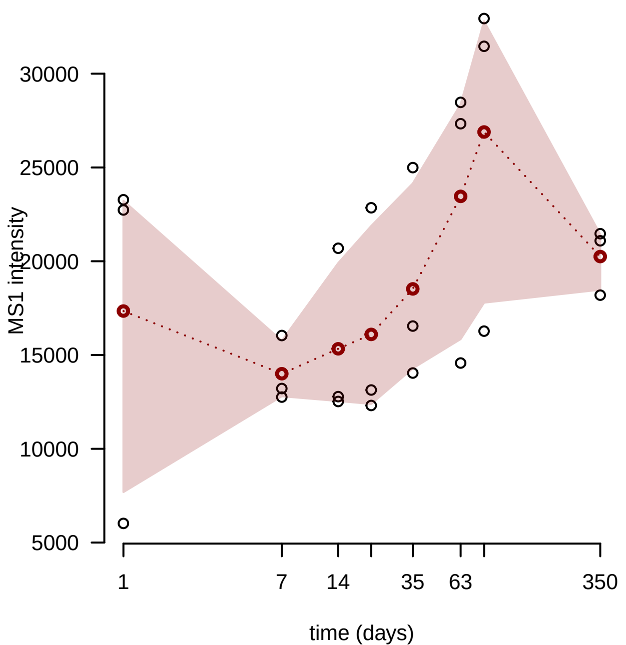

P26350
GN=Ptma
prothymosin alpha

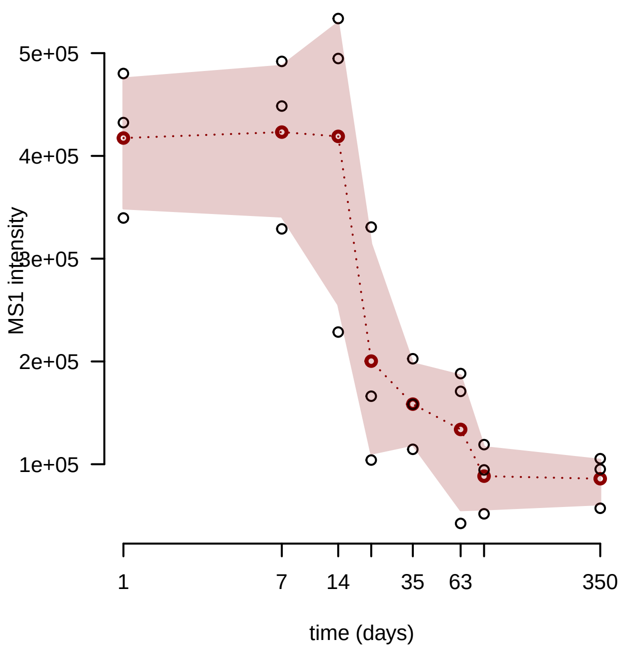

P26369
GN=U2af2
U2 small nuclear ribonucleoprotein auxil ...

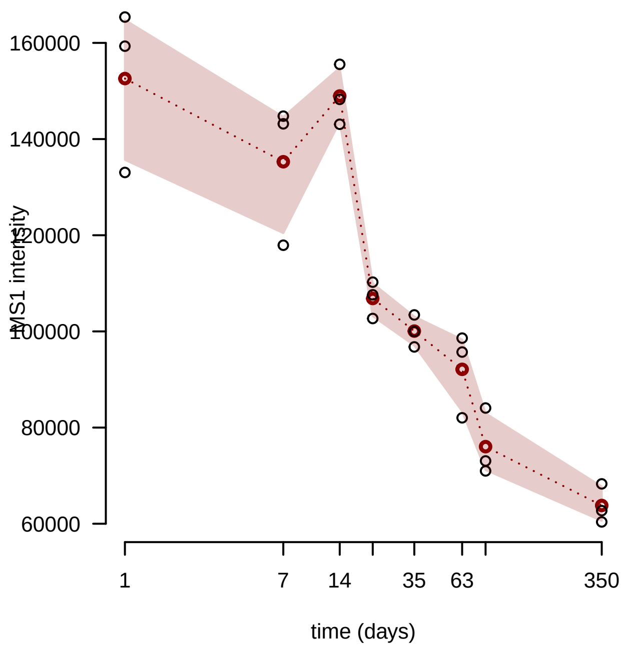

P26443
GN=Glud1
glutamate dehydrogenase 1

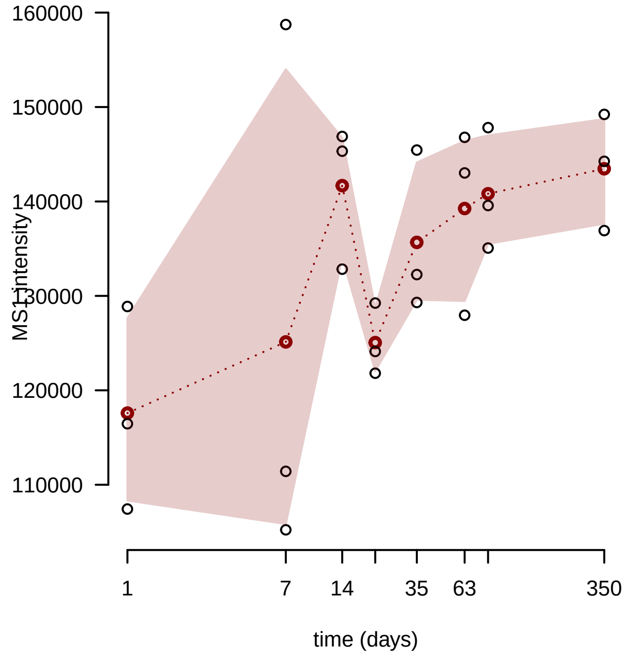

P26450
GN=Pik3r1
phosphoinositide−3−kinase regulatory sub ...

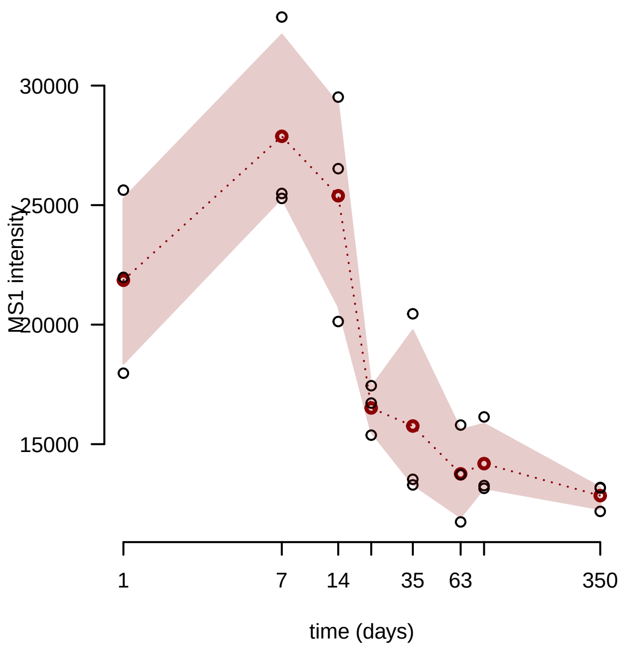

P26516
GN=Psmd7
proteasome (prosome, macropain) 26S subu ...

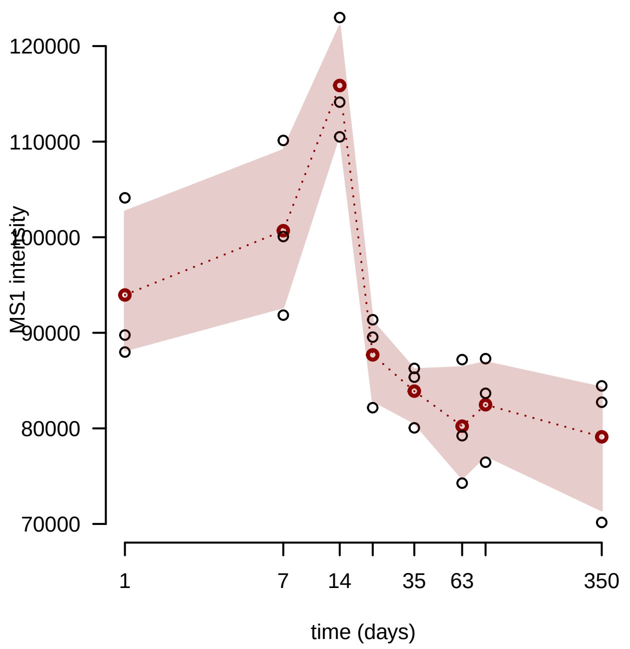

P26618
GN=Pdgfra
platelet derived growth factor receptor, ...

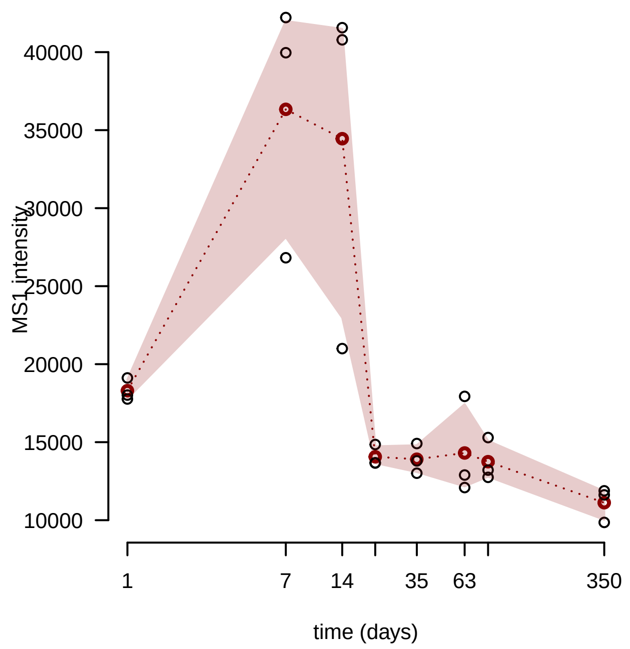

P26638
GN=Sars
seryl−aminoacyl−tRNA synthetase

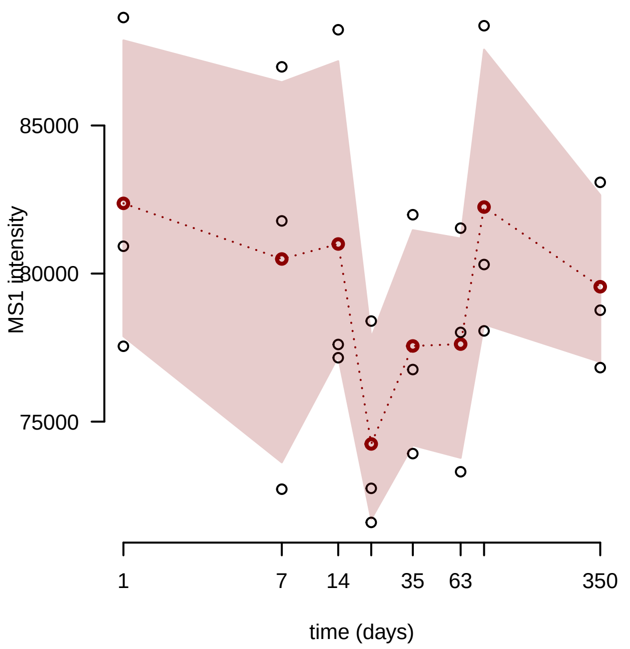

P26645
GN=Marcks
myristoylated alanine rich protein kinas ...

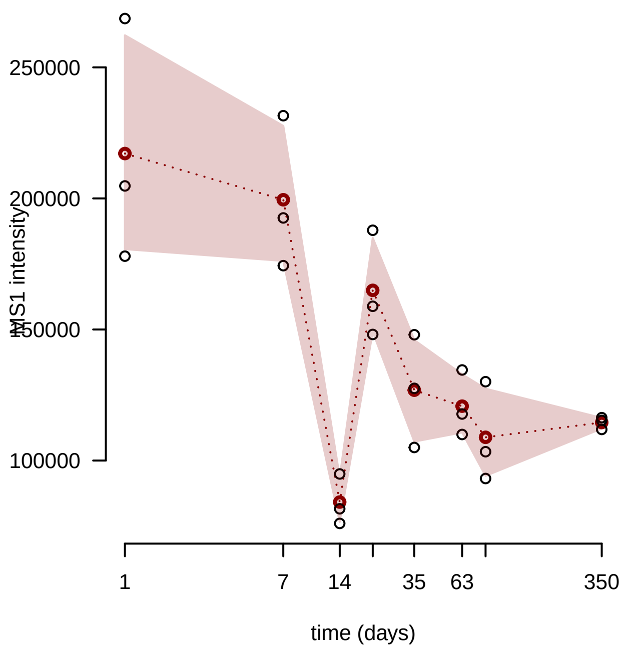

P26883
GN=Fkbp1a
FK506 binding protein 1a

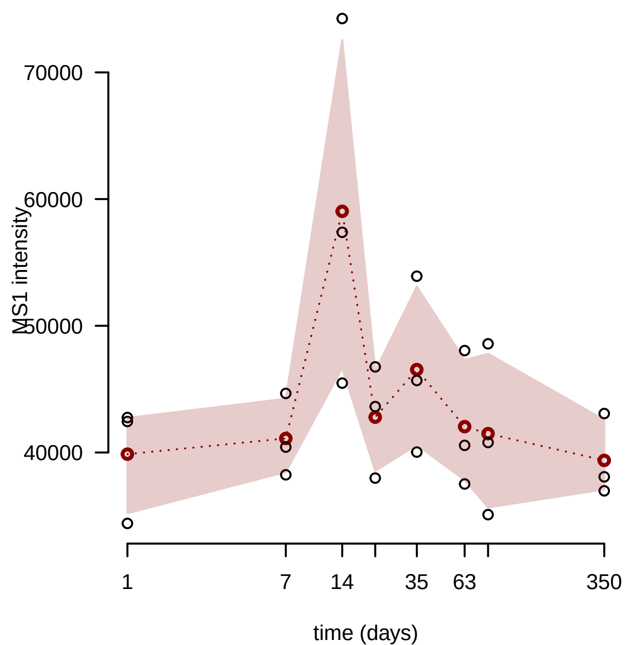

P26952
GN=Il3ra
interleukin 3 receptor, alpha chain

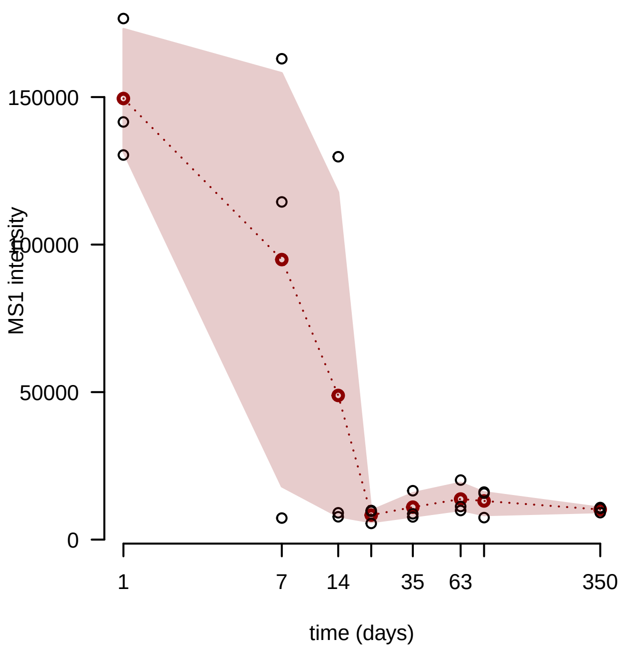

P27046
GN=Man2a1
mannosidase 2, alpha 1

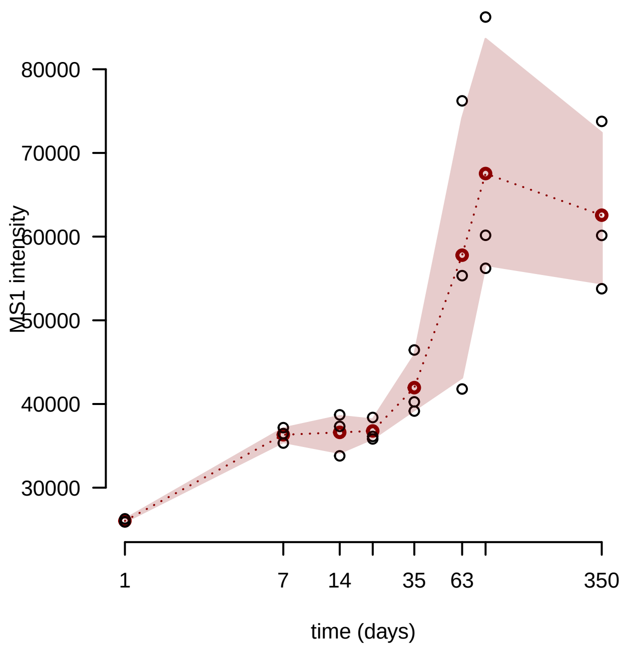

P27048
GN=Snrpb
small nuclear ribonucleoprotein B

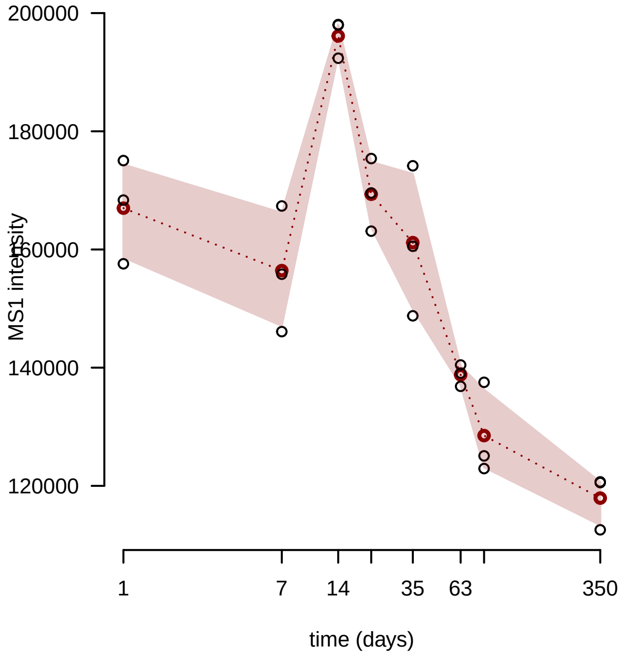

P27090
GN=Tgfb2
transforming growth factor, beta 2

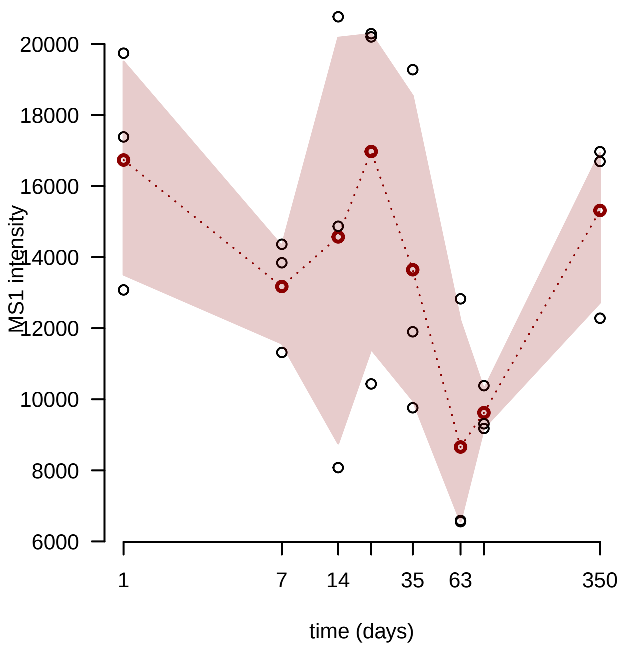

P27106
GN=Amh
anti−Mullerian hormone

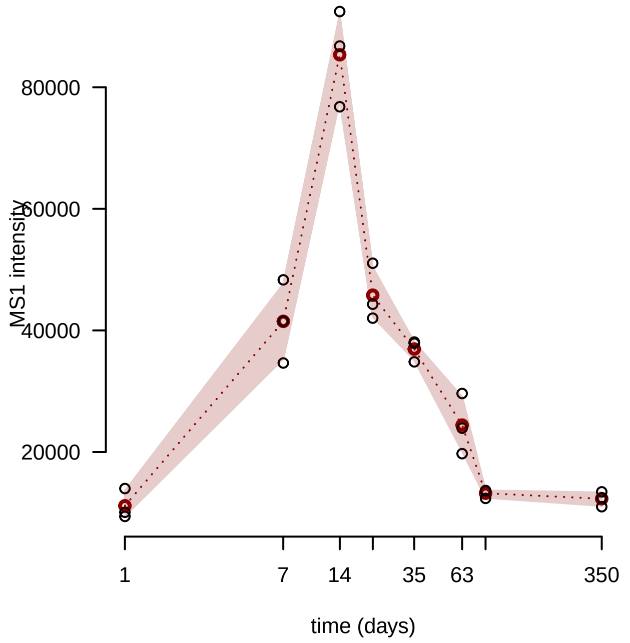

P27546
GN=Map4
microtubule−associated protein 4

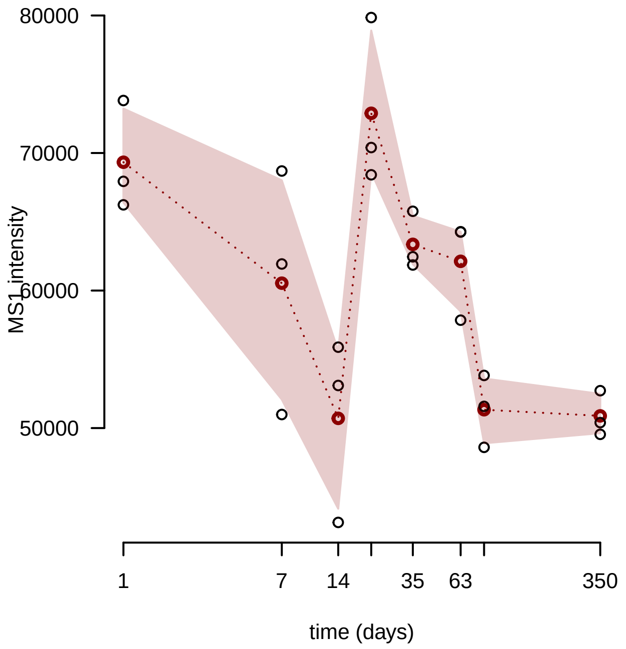

P27600
GN=Gna12
guanine nucleotide binding protein, alph ...

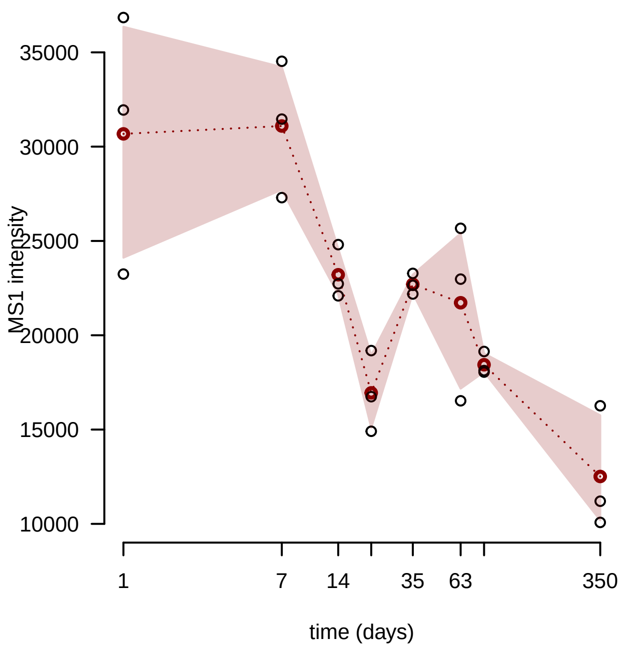

P27601
GN=Gna13
guanine nucleotide binding protein, alph ...

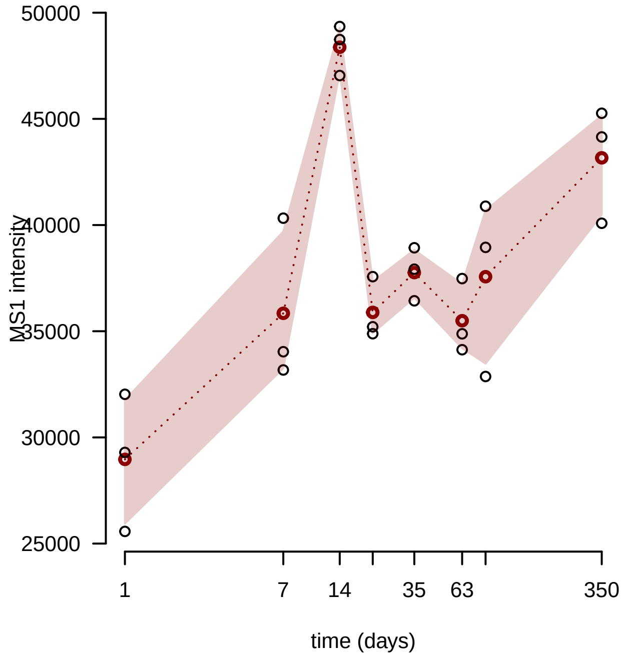

P27612
GN=Plaa
phospholipase A2, activating protein

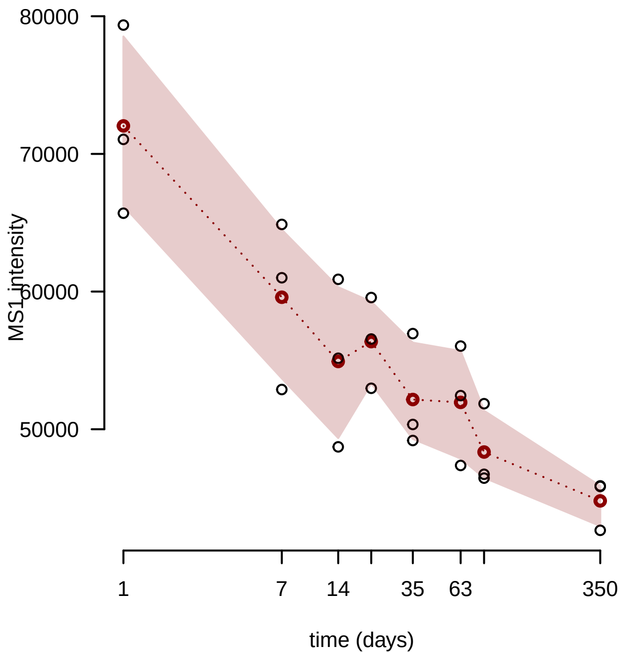

P27641
GN=Xrcc5
X−ray repair complementing defective rep ...

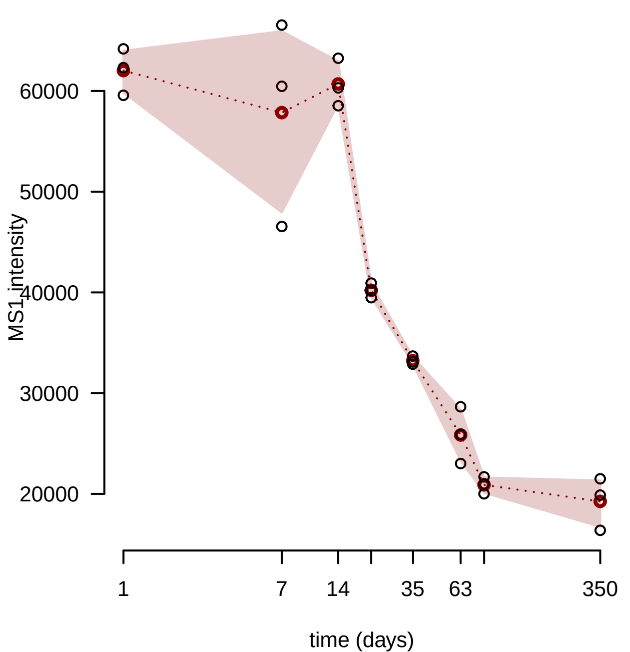

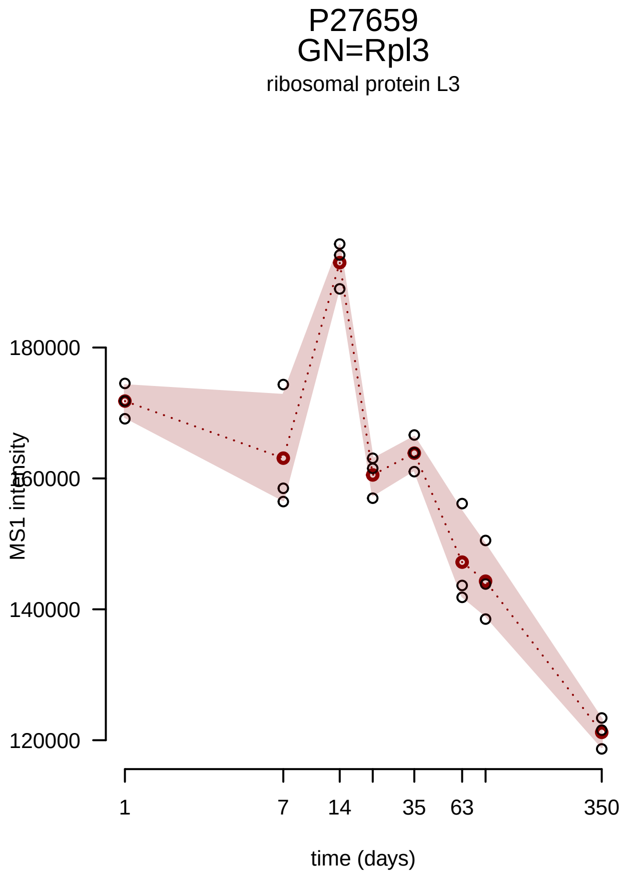
P27659
GN=Rpl3
ribosomal protein L3
MS1 intensity
120000
140000
160000
180000
1
7
14
35
63
350
time (days)

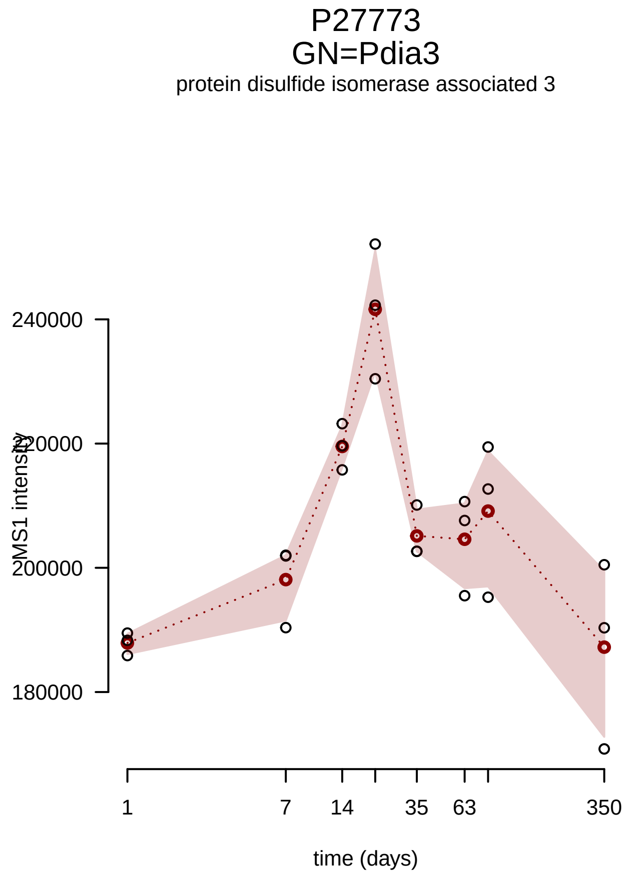
P27773
GN=Pdia3
protein disulfide isomerase associated 3
MS1 intensity
180000
200000
220000
240000
1
7
14
35
63
350
time (days)

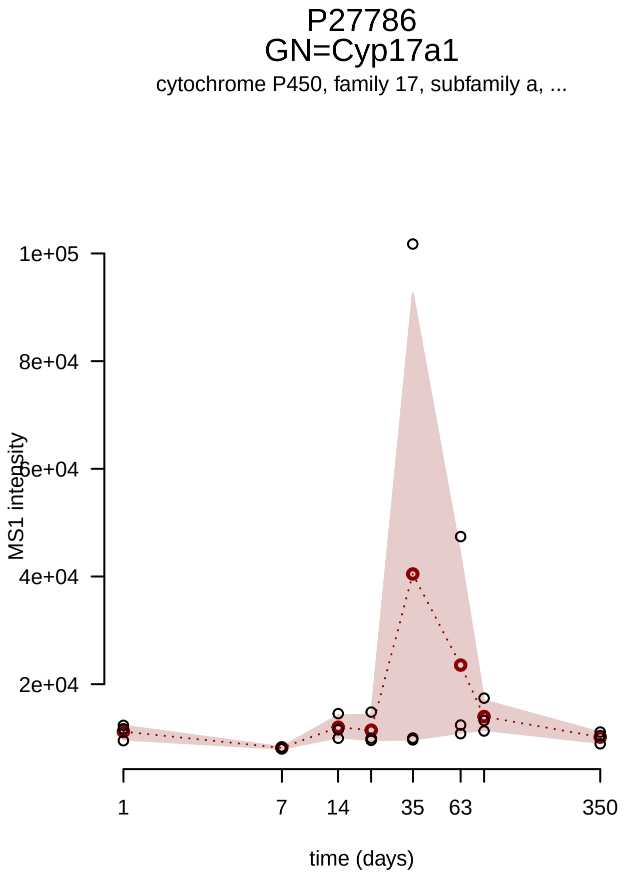
P27786
GN=Cyp17a1
cytochrome P450, family 17, subfamily a, ...
MS1 intensity
2e+04
4e+04
6e+04
8e+04
1e+05
1
7
14
35
63
350
time (days)

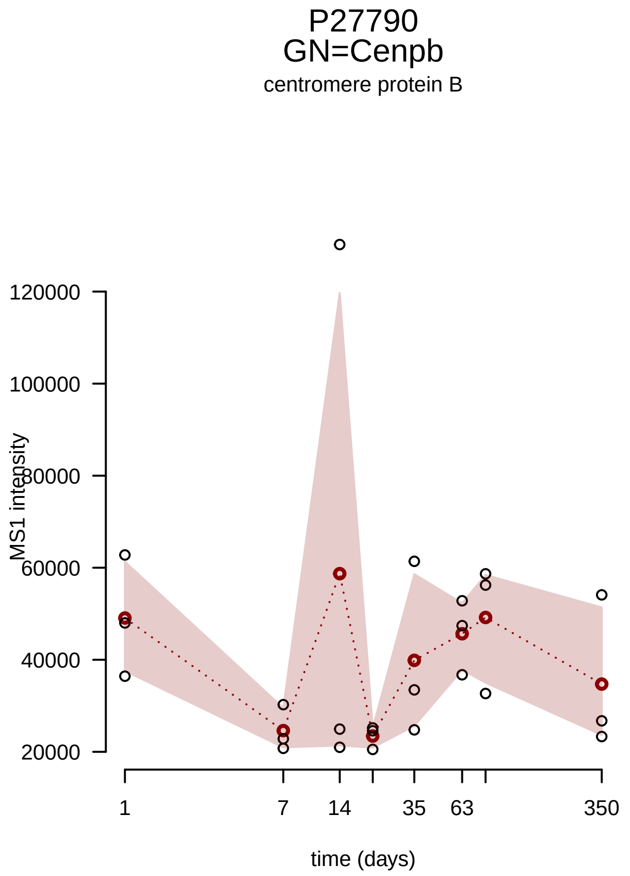
P27790
GN=Cenpb
centromere protein B
MS1 intensity
20000
40000
60000
80000
100000
120000
1
7
14
35
63
350
time (days)

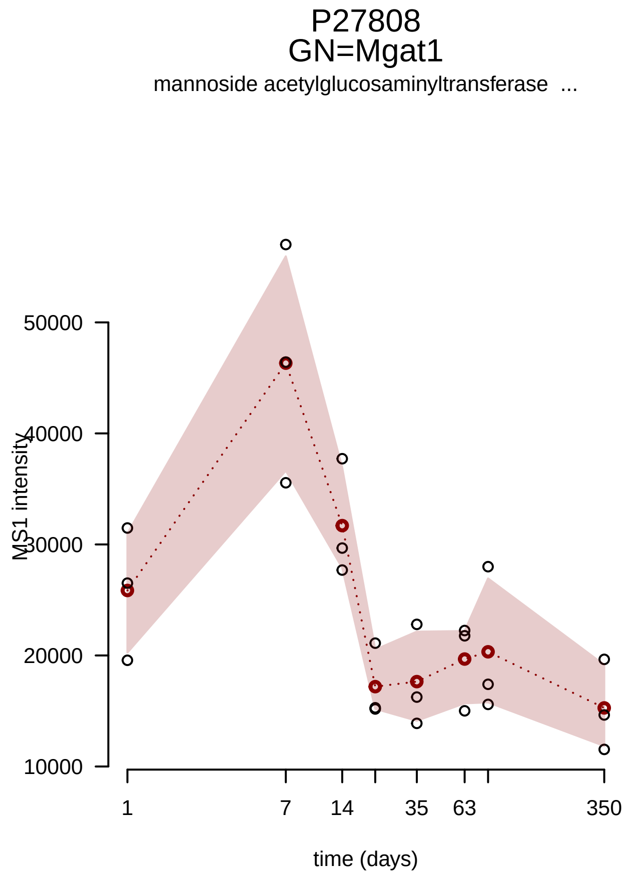
P27808
GN=Mgat1
mannoside acetylglucosaminyltransferase ...
MS1 intensity
10000
20000
30000
40000
50000
1
7
14
35
63
350
time (days)

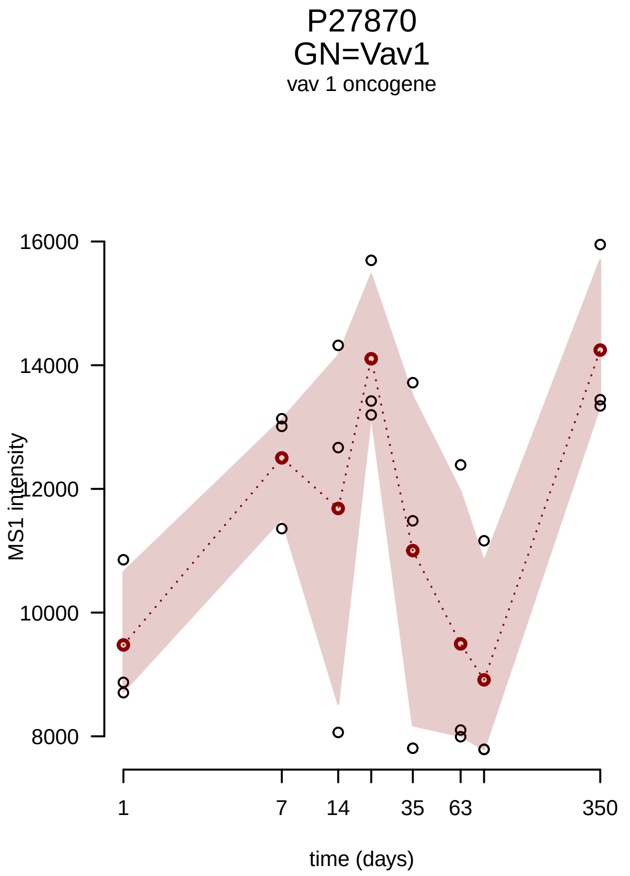
P27870
GN=Vav1
vav 1 oncogene
MS1 intensity
8000
10000
12000
14000
16000
1
7
14
35
63
350
time (days)

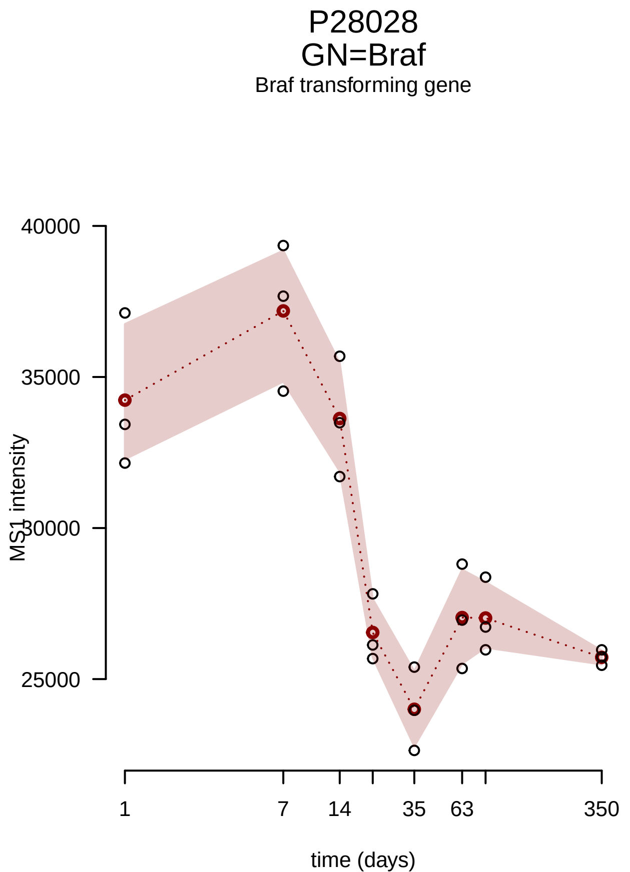
P28028
GN=Braf
Braf transforming gene
MS1 intensity
25000
30000
35000
40000
1
7
14
35
63
350
time (days)

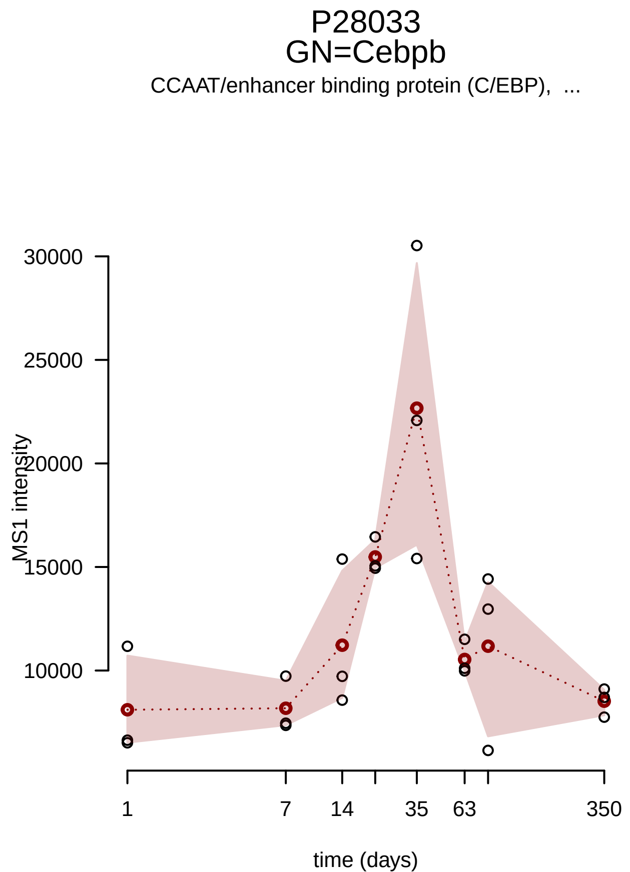
P28033
GN=Cebpb
CCAAT/enhancer binding protein (C/EBP), ...
MS1 intensity
10000
15000
20000
25000
30000
1
7
14
35
63
350
time (days)

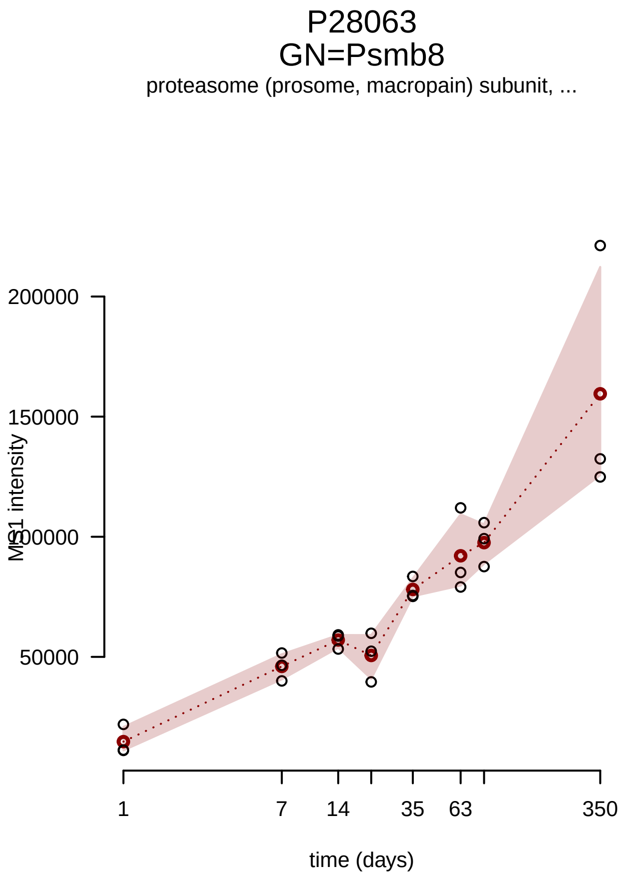
P28063
GN=Psmb8
proteasome (prosome, macropain) subunit, ...
MS1 intensity
50000
100000
150000
200000
1
7
14
35
63
350
time (days)

P28076
GN=Psmb9

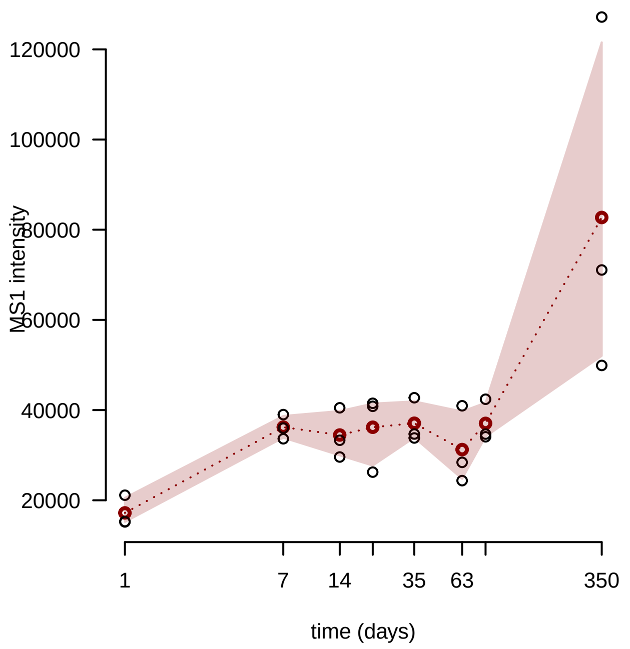

P28271
GN=Aco1
aconitase 1

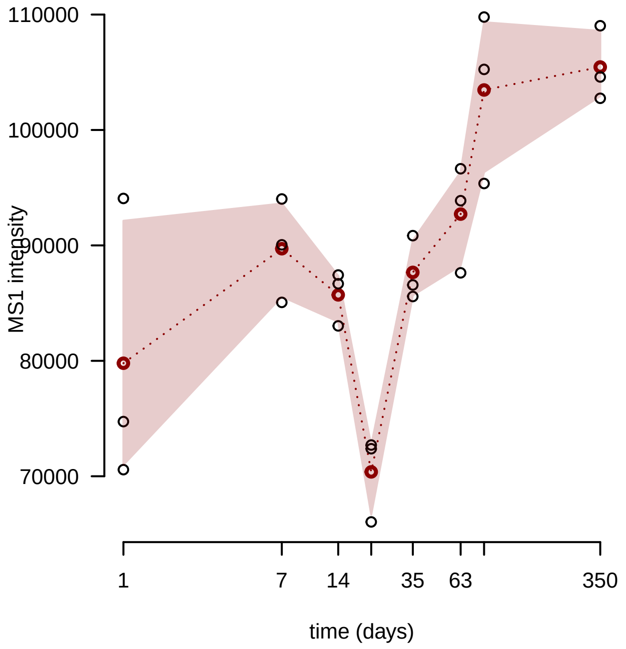

P28301
GN=Lox
lysyl oxidase

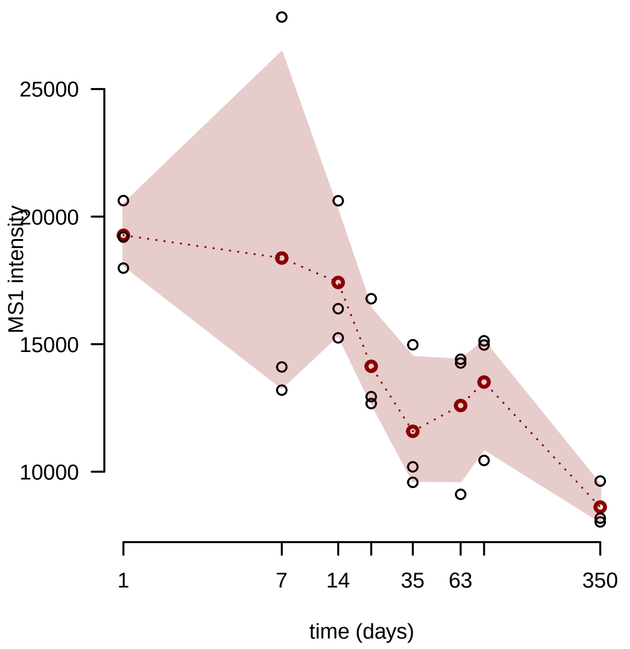

P28352
GN=Apex1
apurinic/apyrimidinic endonuclease 1

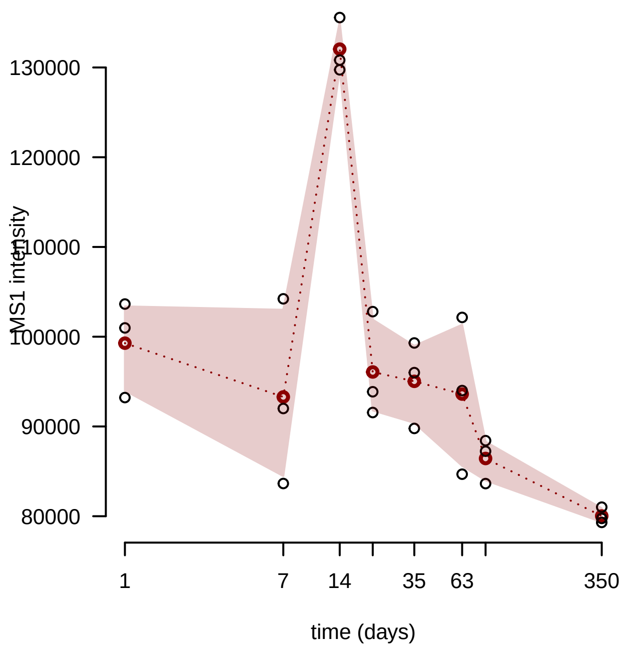

P28357
GN=Hoxd9
homeobox D9

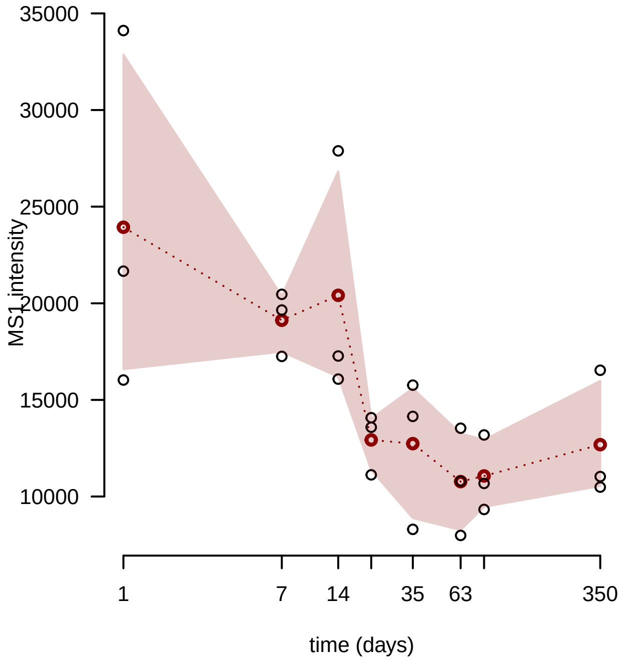

P28474
GN=Adh5
alcohol dehydrogenase 5 (class III), chi ...

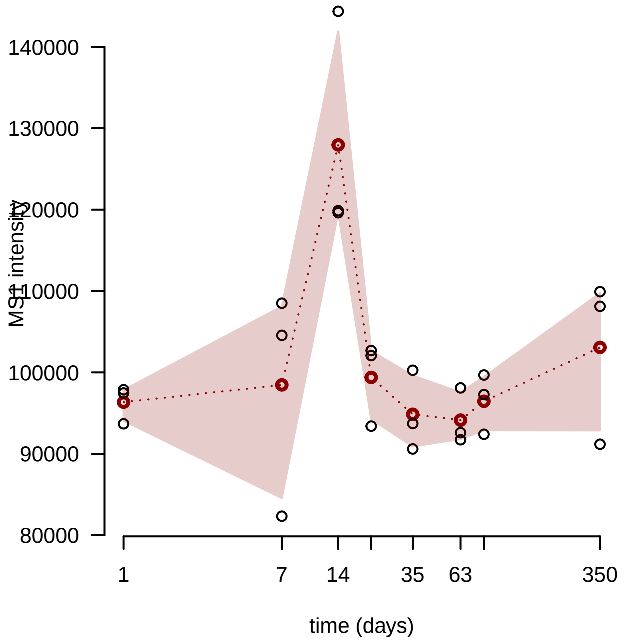

P28571
GN=Slc6a9
solute carrier family 6 (neurotransmitte ...

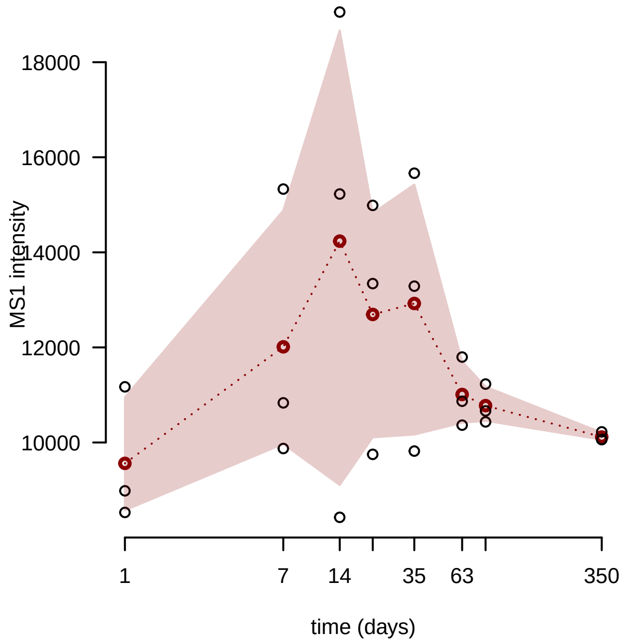

P28574
GN=Max
Max protein

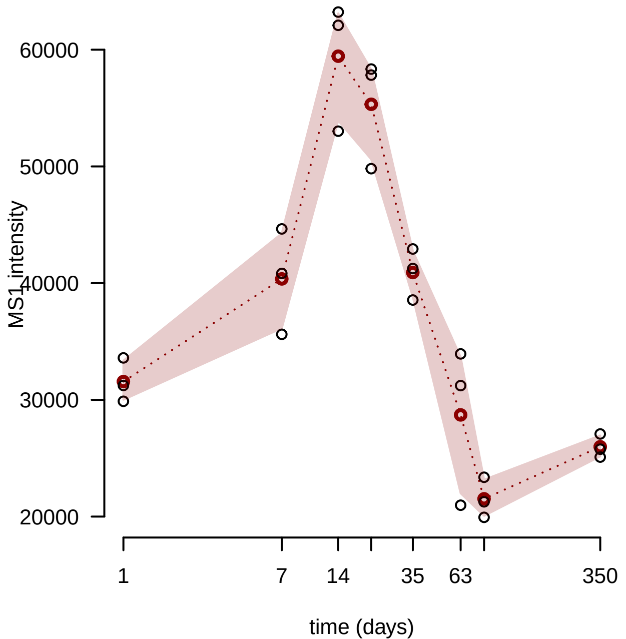

P28649
GN=Cyp19a1
cytochrome P450, family 19, subfamily a, ...

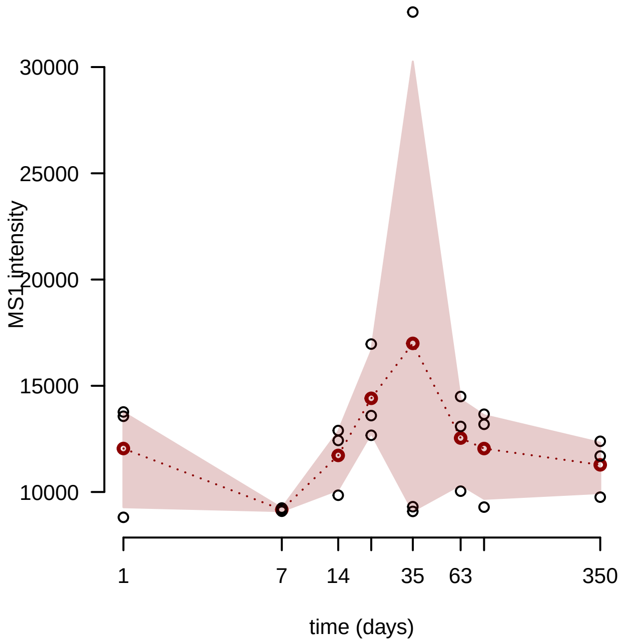

P28650
GN=Adssl1
adenylosuccinate synthetase like 1

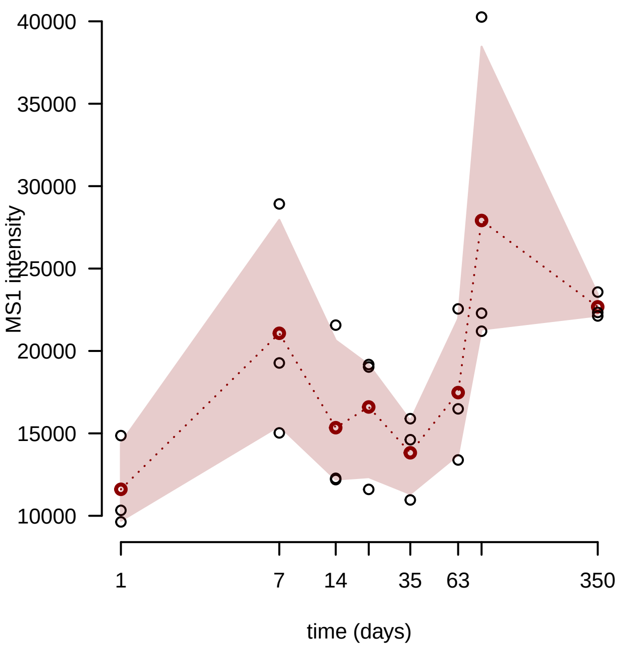

P28652
GN=Camk2b
calcium/calmodulin−dependent protein kin ...

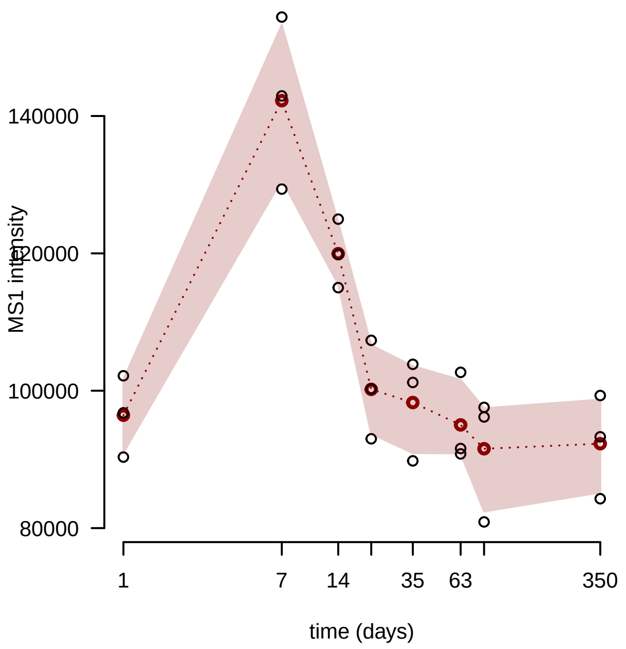

P28653
GN=Bgn
biglycan

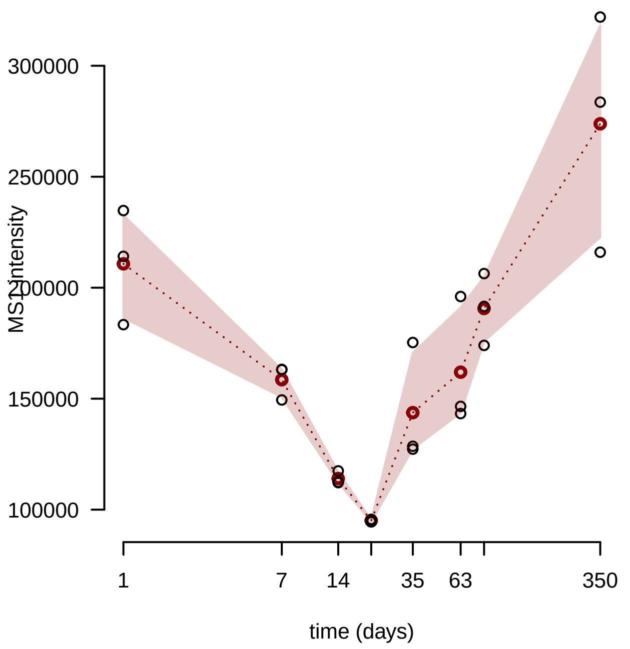

P28654
GN=Dcn
decorin

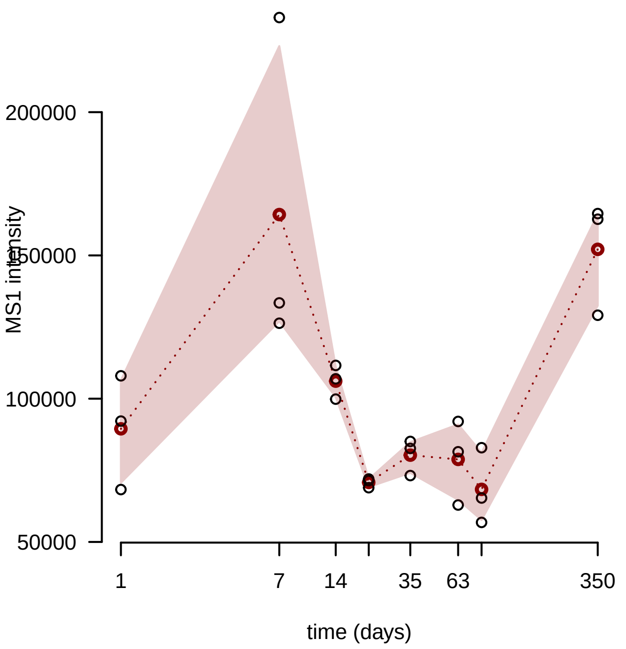

P28656
GN=Nap1l1
nucleosome assembly protein 1−like 1

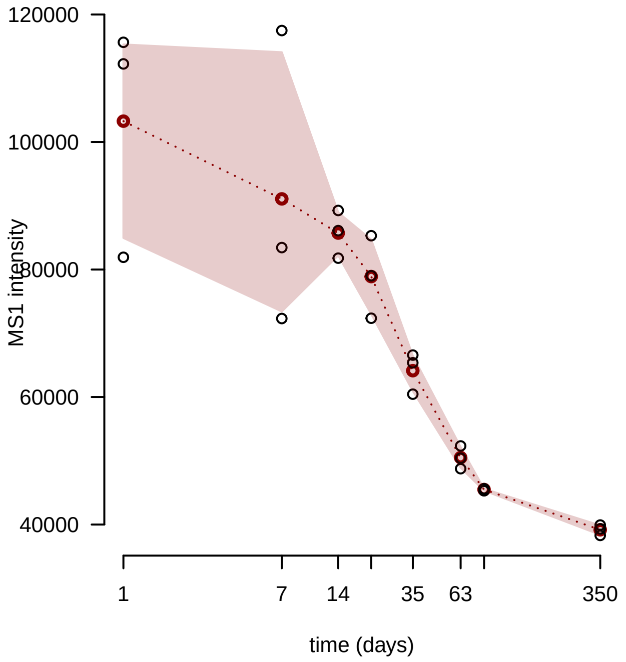

P28658
GN=Atxn10
ataxin 10

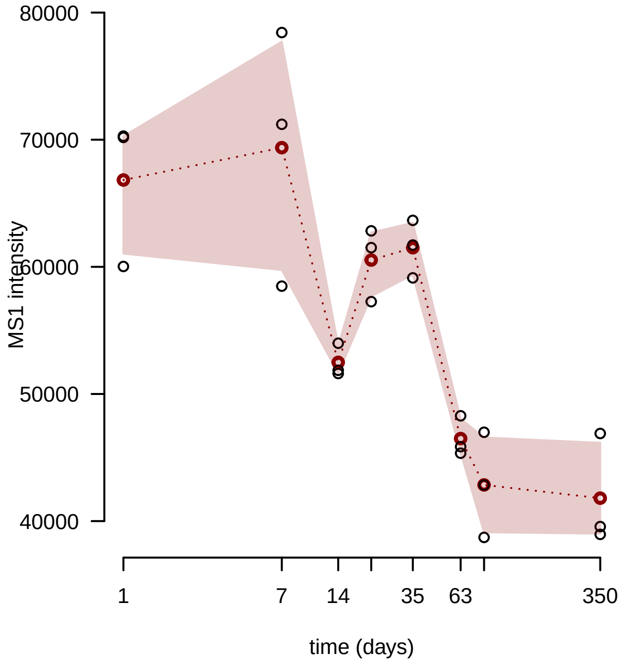

P28659
GN=Celf1
CUGBP, Elav−like family member 1

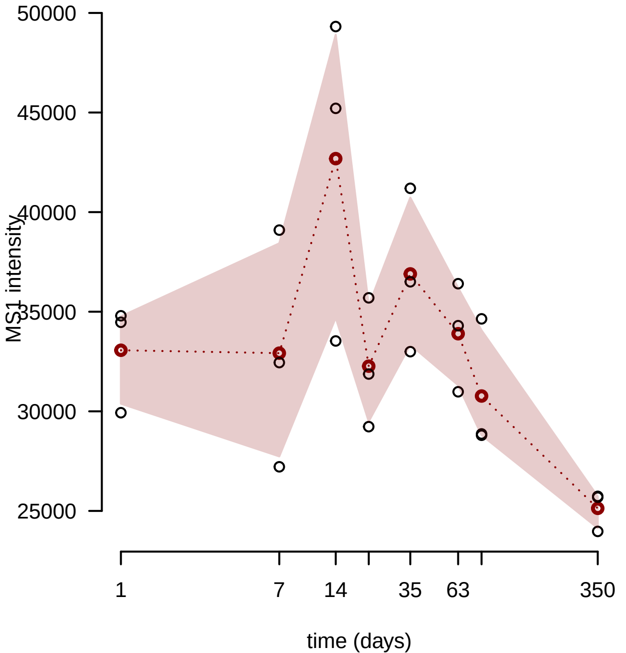

P28660
GN=Nckap1
NCK−associated protein 1

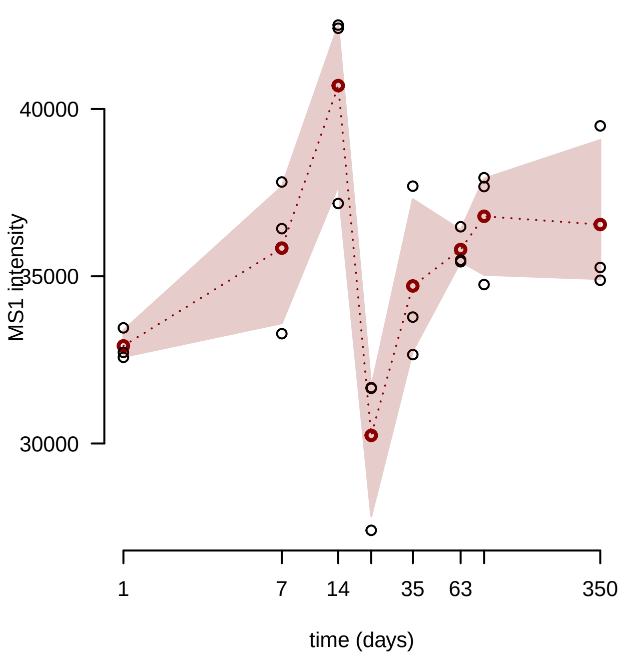

P28661
GN=Sept4

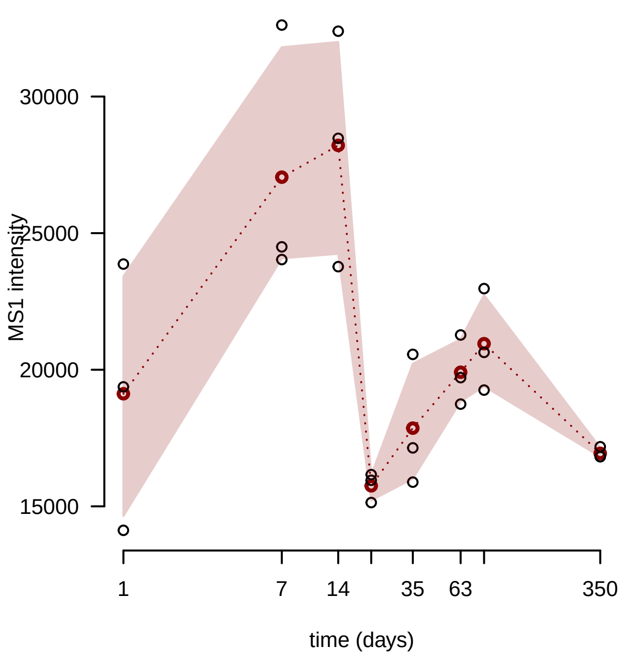

P28661
GN=Sept4

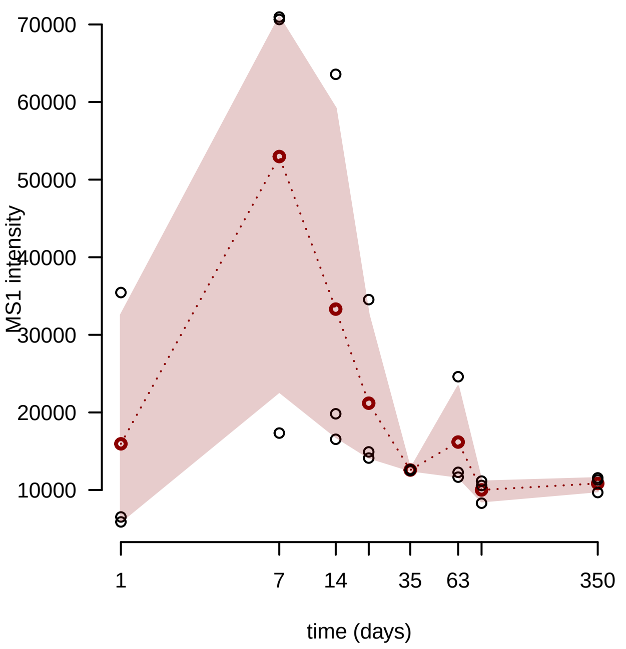

P28665
GN=Mug1
murinoglobulin 1

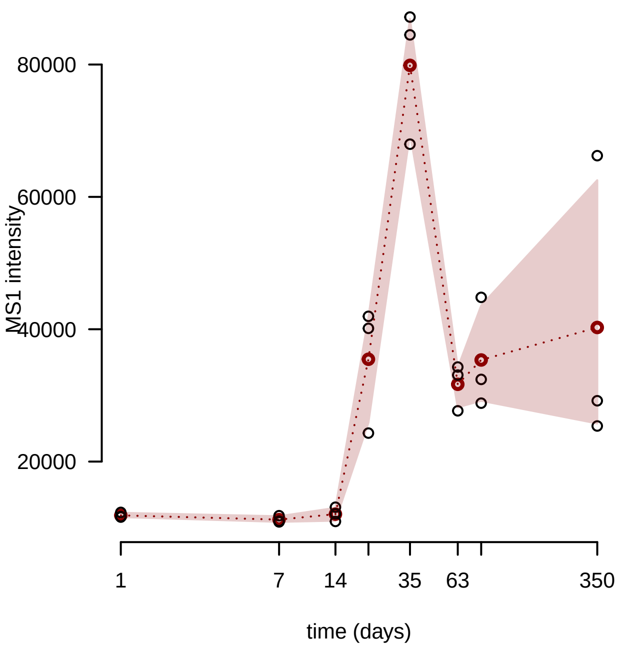

P28667
GN=Marcksl1
MARCKS−like 1

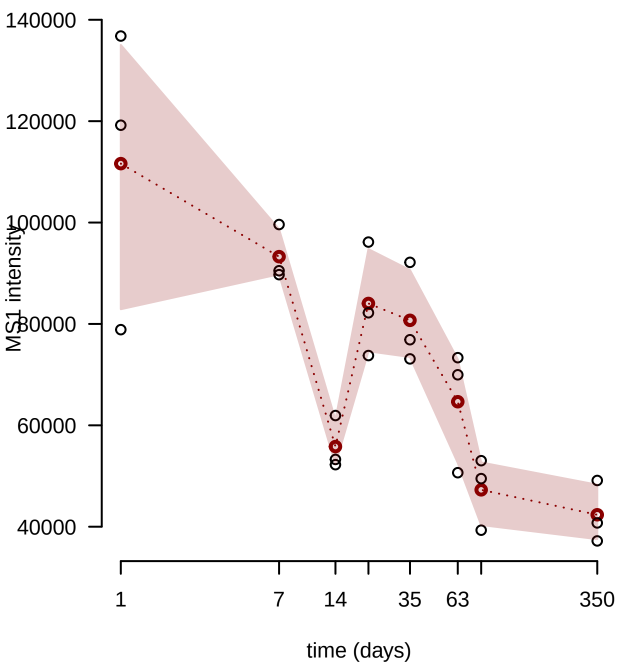

P28700
GN=Rxra
retinoid X receptor alpha

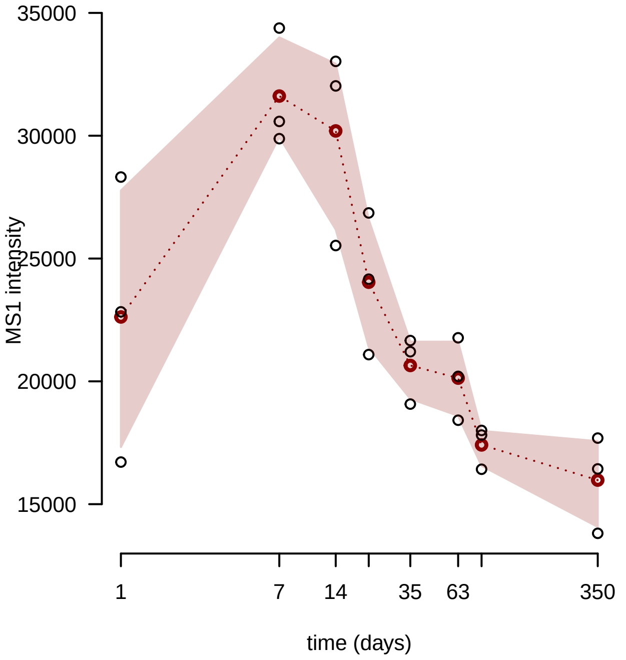

P28704
GN=Rxrb
retinoid X receptor beta

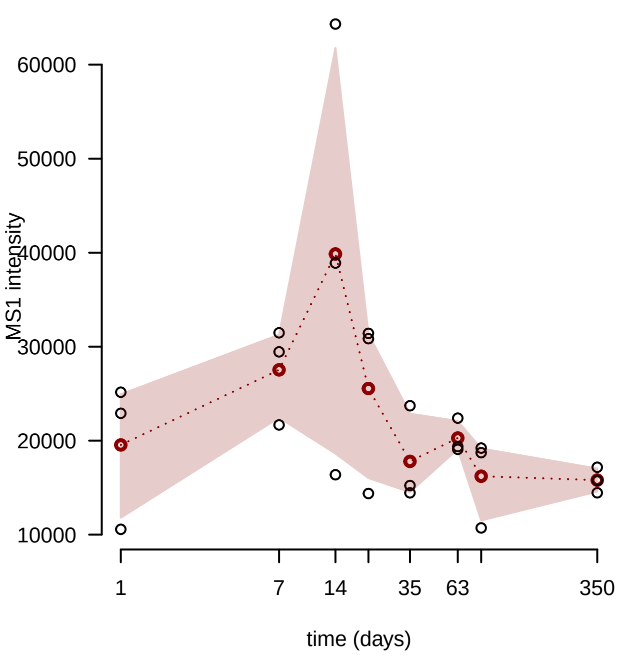

P28738
GN=Kif5c
kinesin family member 5C

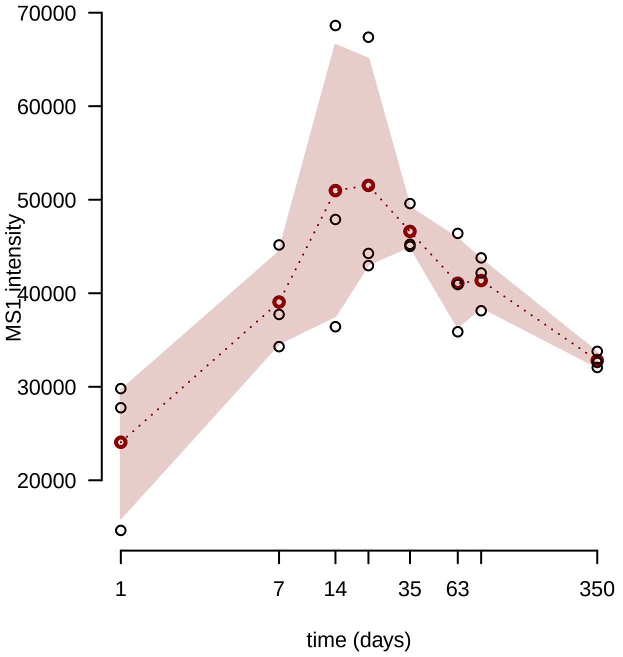

P28740
GN=Kif2a
kinesin family member 2A

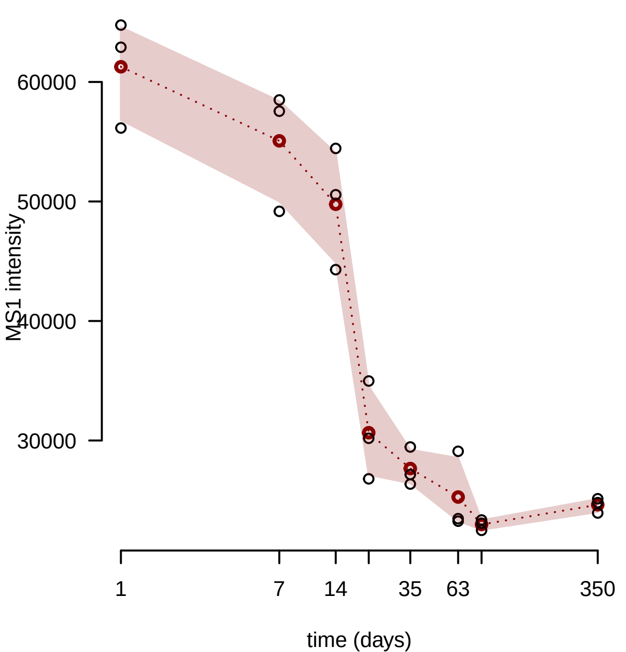

P28740
GN=Kif2a
kinesin family member 2A

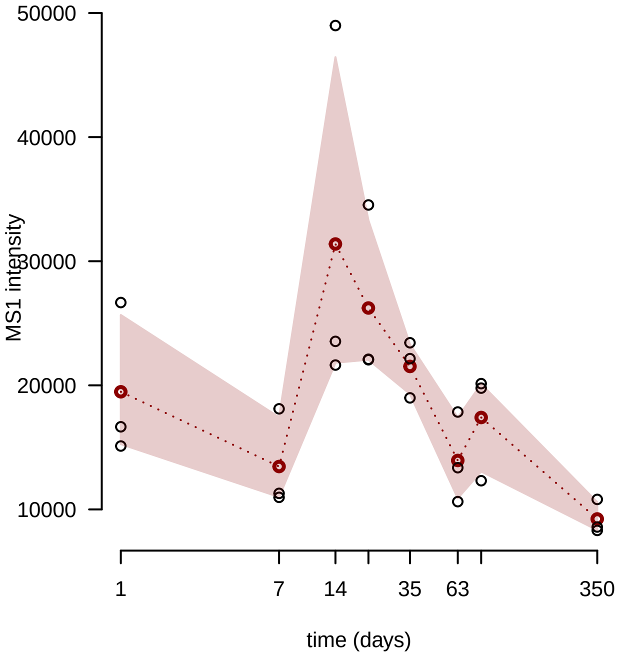

P28741
GN=Kif3a
kinesin family member 3A

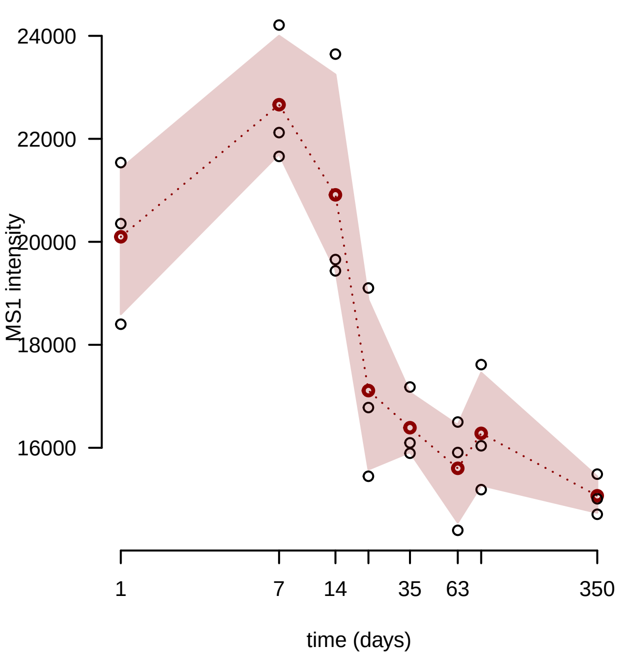

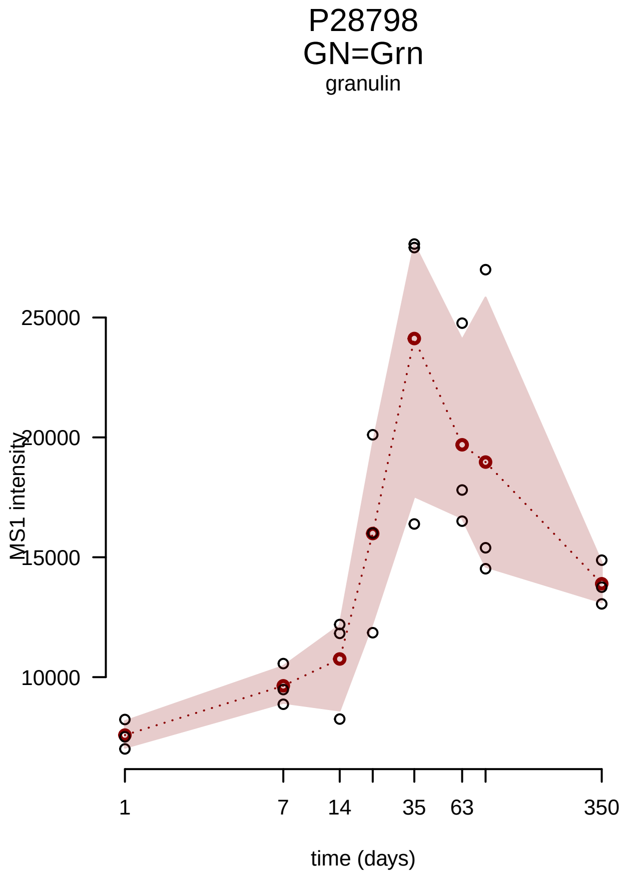
P28798
GN=Grn
granulin
MS1 intensity
25000
20000
15000
10000
1
7
14
35
63
350
time (days)

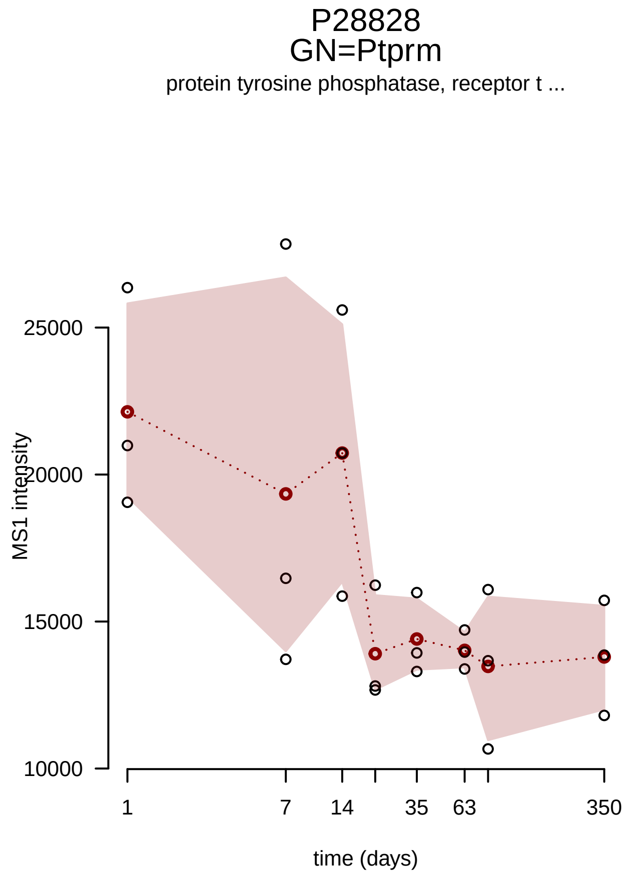
P28828
GN=Ptprm
protein tyrosine phosphatase, receptor t ...
MS1 intensity
25000
20000
15000
10000
1
7
14
35
63
350
time (days)

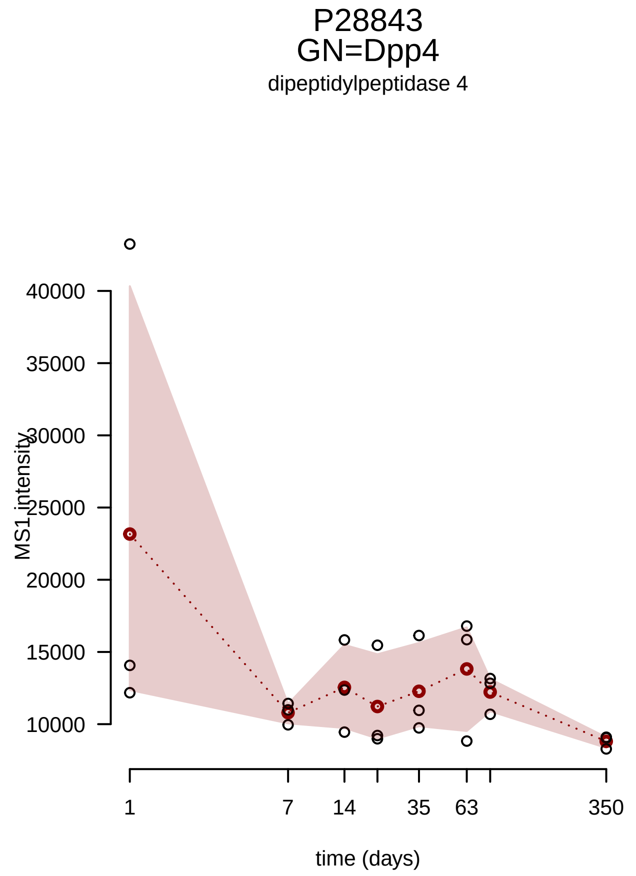
P28843
GN=Dpp4
dipeptidylpeptidase 4
MS1 intensity
40000
35000
30000
25000
20000
15000
10000
1
7
14
35
63
350
time (days)

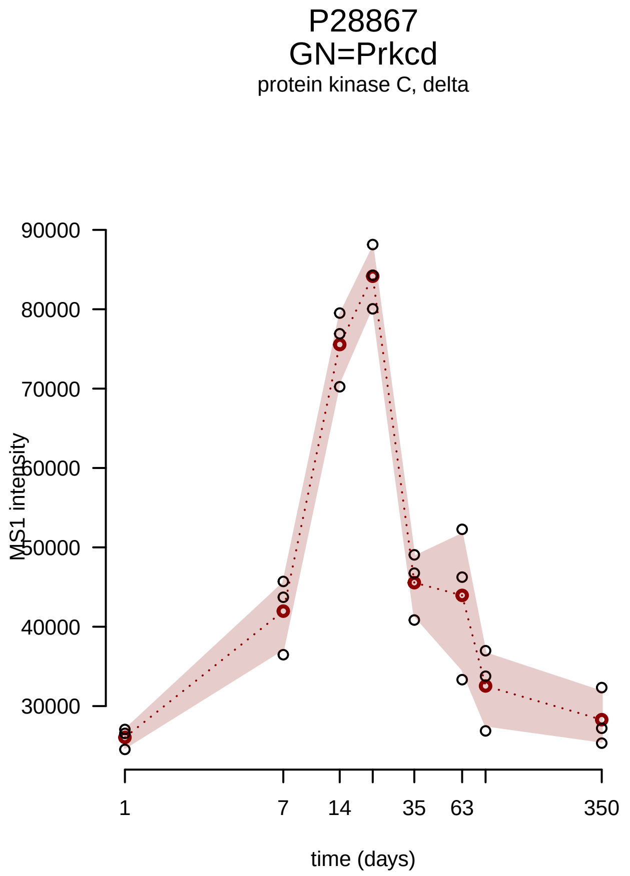
P28867
GN=Prkcd
protein kinase C, delta
MS1 intensity
90000
80000
70000
60000
50000
40000
30000
1
7
14
35
63
350
time (days)

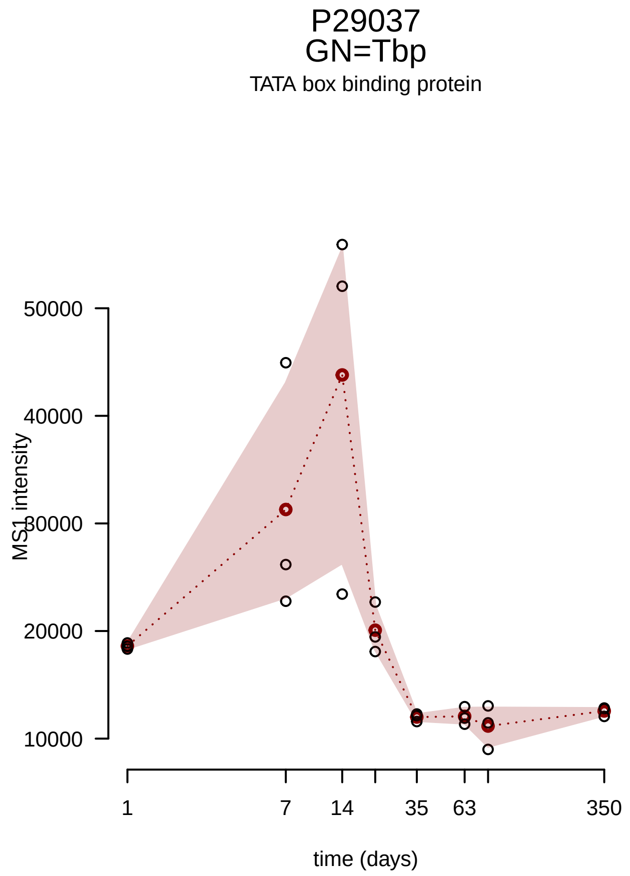
P29037
GN=Tbp
TATA box binding protein
MS1 intensity
50000
40000
30000
20000
10000
1
7
14
35
63
350
time (days)

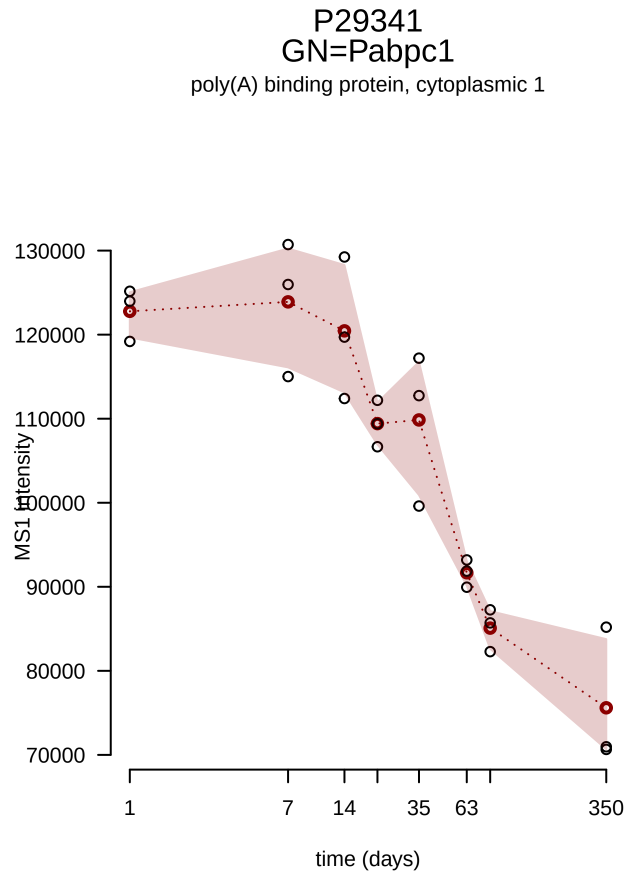
P29341
GN=Pabpc1
poly(A) binding protein, cytoplasmic 1
MS1 intensity
130000
120000
110000
100000
90000
80000
70000
1
7
14
35
63
350
time (days)

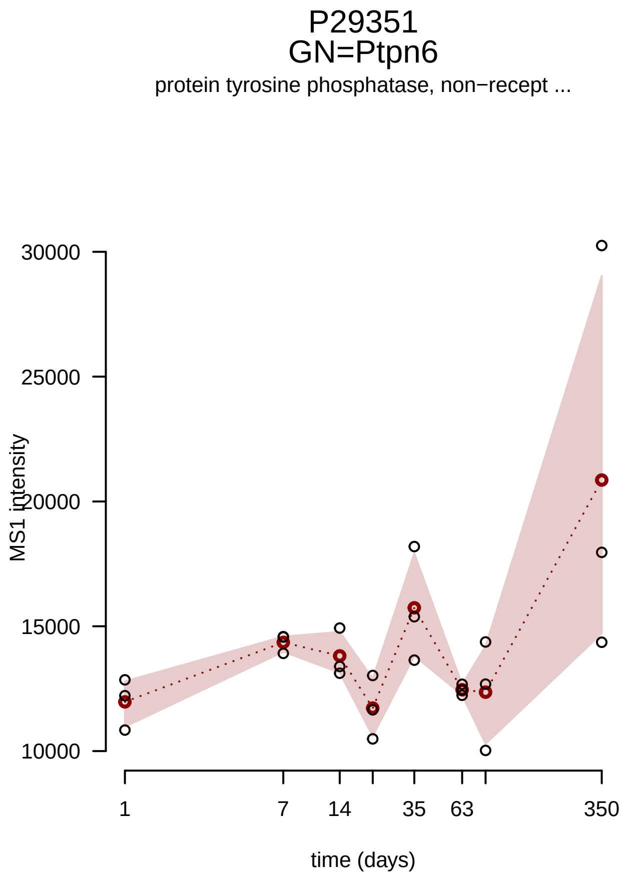
P29351
GN=Ptpn6
protein tyrosine phosphatase, non−recept ...
MS1 intensity
30000
25000
20000
15000
10000
1
7
14
35
63
350
time (days)

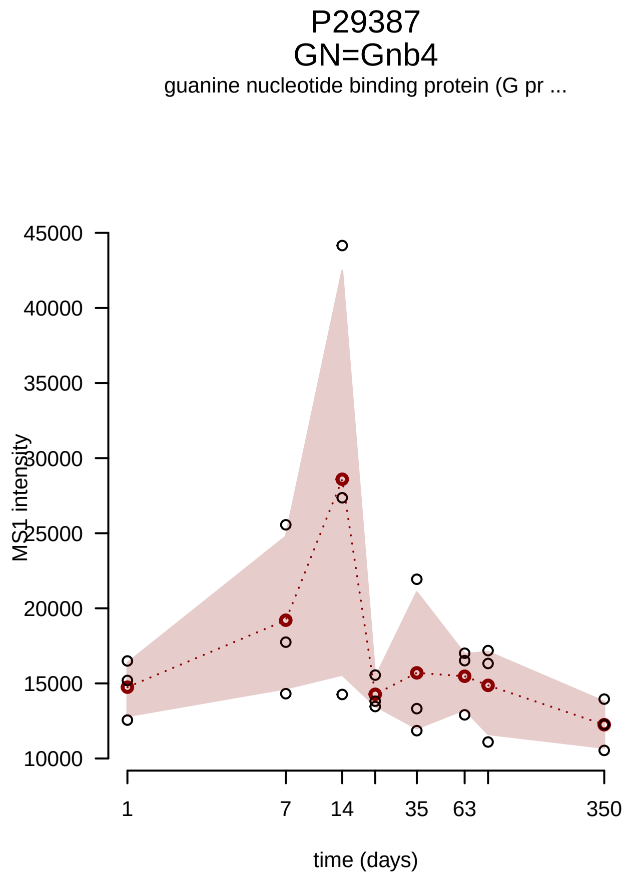
P29387
GN=Gnb4
guanine nucleotide binding protein (G pr ...
MS1 intensity
45000
40000
35000
30000
25000
20000
15000
10000
1
7
14
35
63
350
time (days)

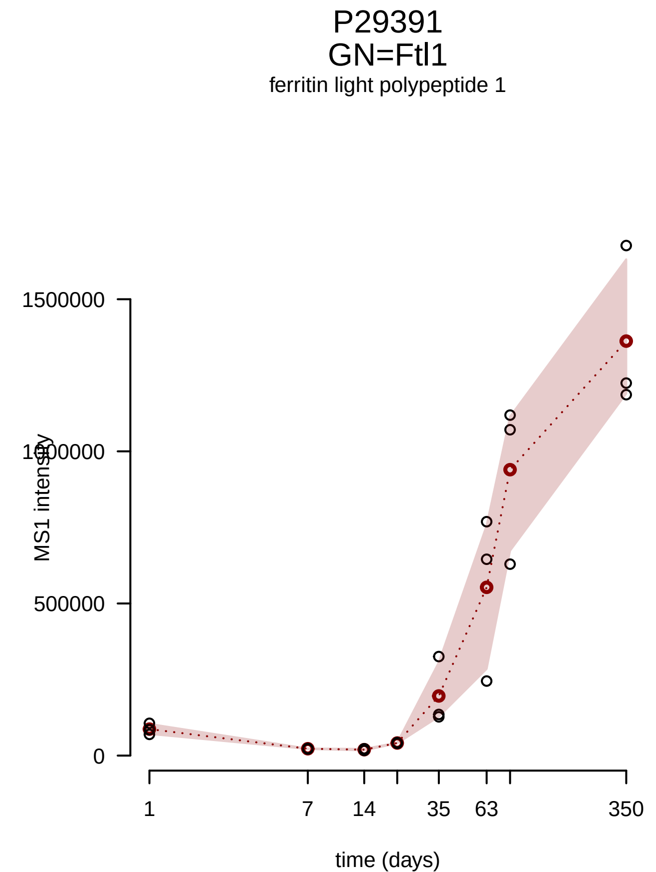
P29391
GN=Ftl1
ferritin light polypeptide 1
MS1 intensity
1500000
1000000
500000
0
1
7
14
35
63
350
time (days)

P29416
GN=Hexa
hexosaminidase A

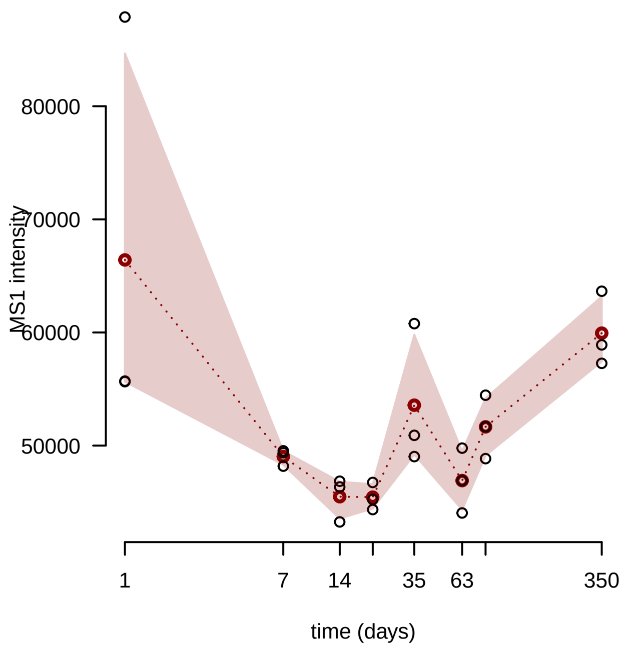

P29452
GN=Casp1
caspase 1

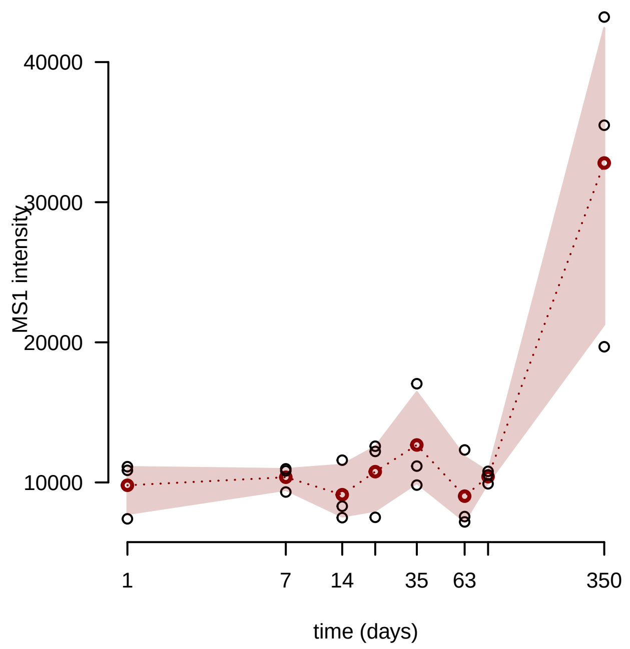

P29533
GN=Vcam1
vascular cell adhesion molecule 1

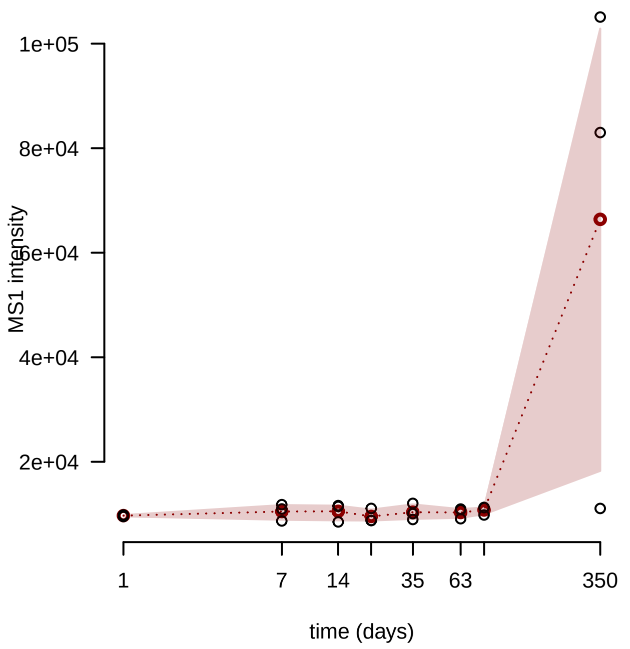

P29594
GN=Casp2
caspase 2

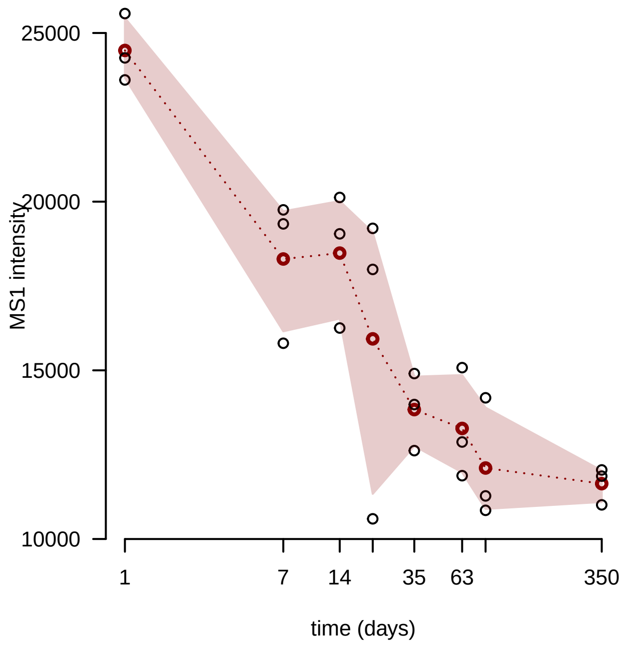

P29595
GN=Nedd8
neural precursor cell expressed, develop ...

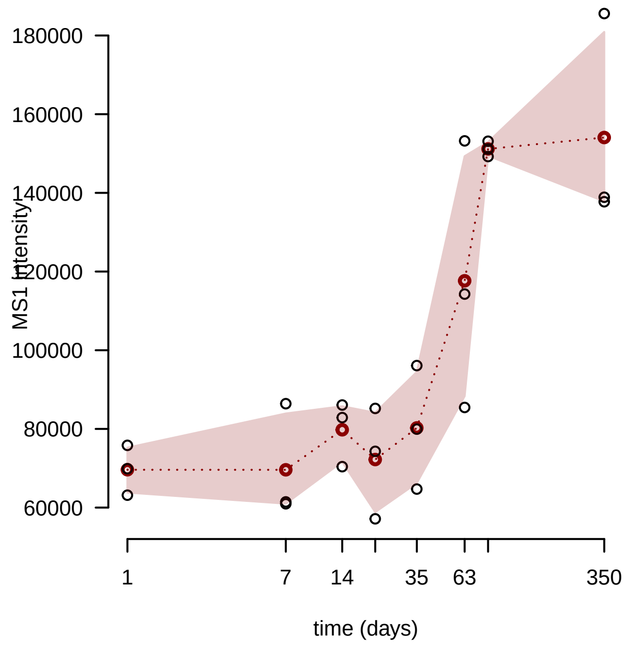

P29699
GN=Ahsg
alpha−2−HS−glycoprotein

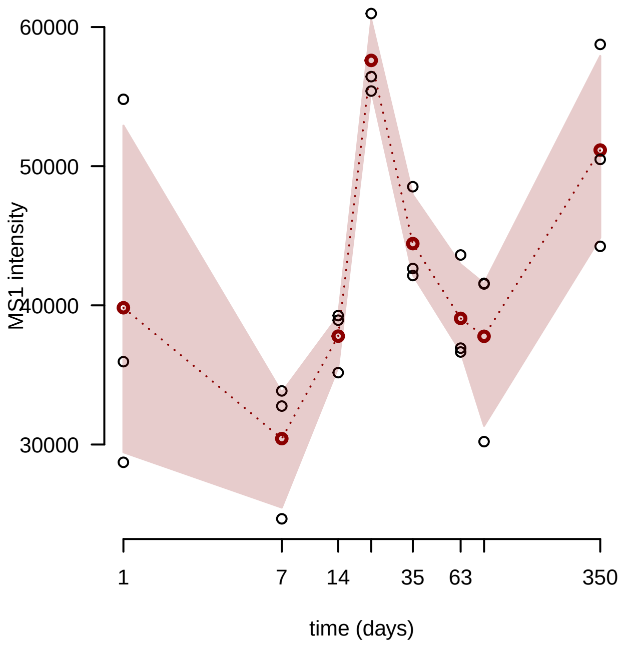

P29758
GN=Oat
ornithine aminotransferase

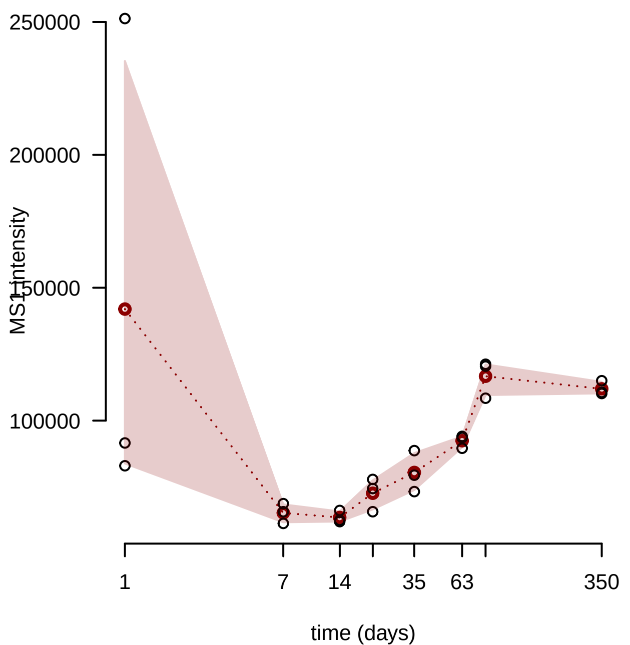

P29788
GN=Vtn
vitronectin

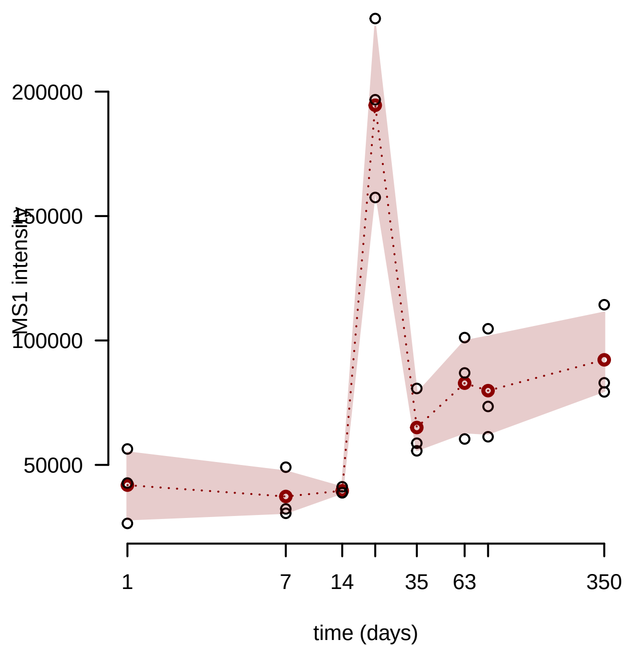

P30051
GN=Tead1
TEA domain family member 1

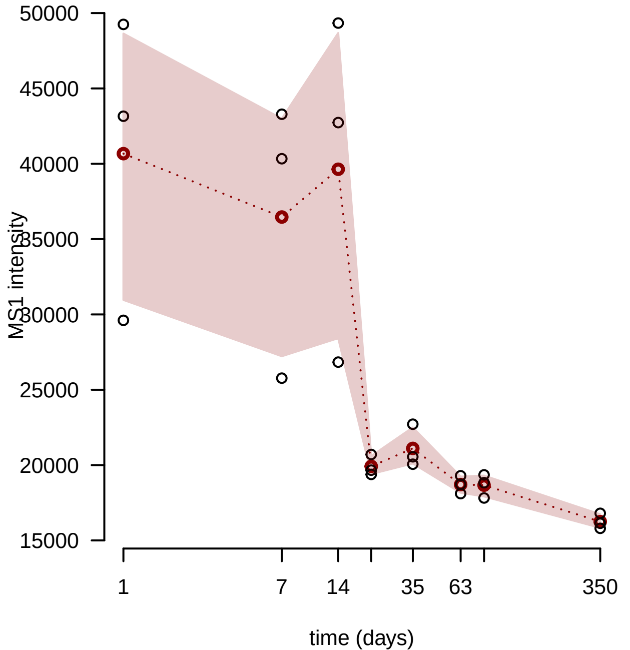

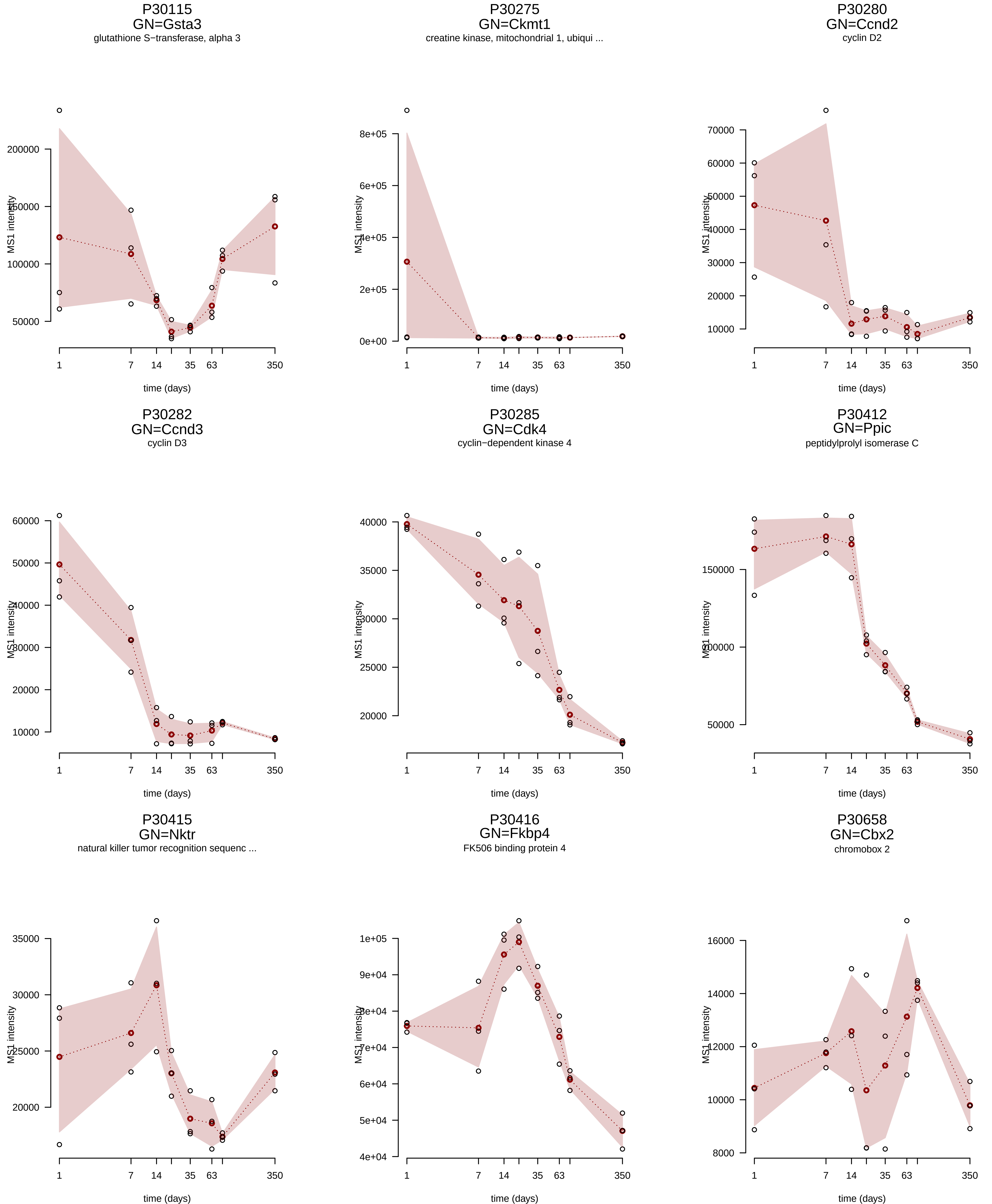
P30115
GN=Gsta3
glutathione S−transferase, alpha 3
MS1 intensity
200000
150000
100000
50000
time (days)
1
7
14
35
63
350
P30275
GN=Ckmt1
creatine kinase, mitochondrial 1, ubiqui ...
MS1 intensity
8e+05
6e+05
4e+05
2e+05
0e+00
time (days)
P30280
GN=Ccnd2
cyclin D2
MS1 intensity
70000
60000
50000
40000
30000
20000
10000
time (days)
P30282
GN=Ccnd3
cyclin D3
MS1 intensity
60000
50000
40000
30000
20000
10000
time (days)
P30285
GN=Cdk4
cyclin−dependent kinase 4
MS1 intensity
40000
35000
30000
25000
20000
time (days)
P30412
GN=Ppic
peptidylprolyl isomerase C
MS1 intensity
150000
100000
50000
time (days)
P30415
GN=Nktr
natural killer tumor recognition sequenc ...
MS1 intensity
35000
30000
25000
20000
time (days)
P30416
GN=Fkbp4
FK506 binding protein 4
MS1 intensity
1e+05
9e+04
8e+04
7e+04
6e+04
5e+04
4e+04
time (days)
P30658
GN=Cbx2
chromobox 2
MS1 intensity
16000
14000
12000
10000
8000
time (days)

P30677
GN=Gna14
guanine nucleotide binding protein, alph ...

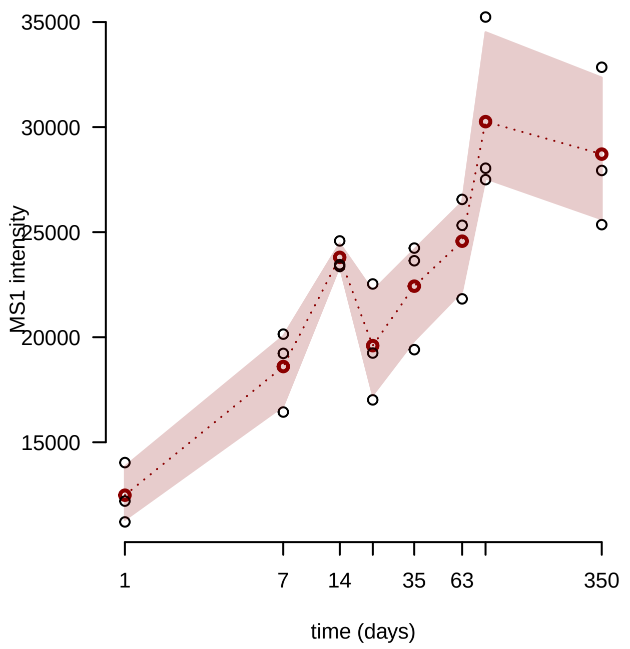

P30681
GN=Hmgb2
high mobility group box 2

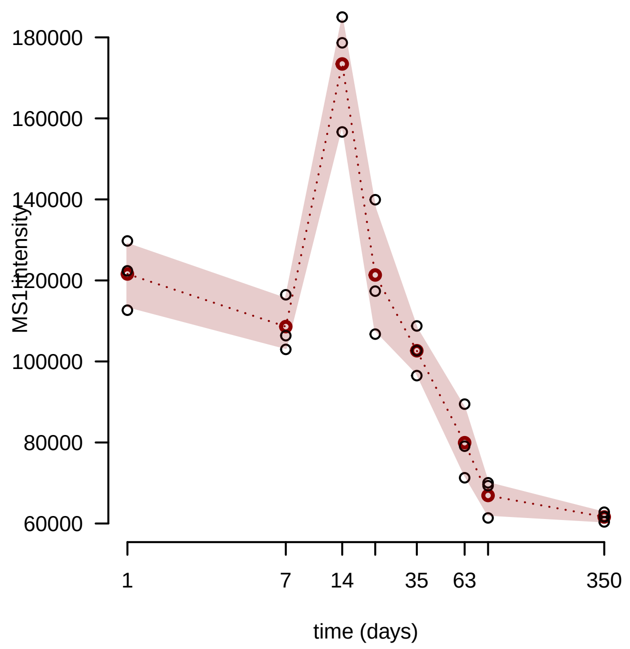

P30730
GN=Lhcgr
luteinizing hormone/choriogonadotropin r ...

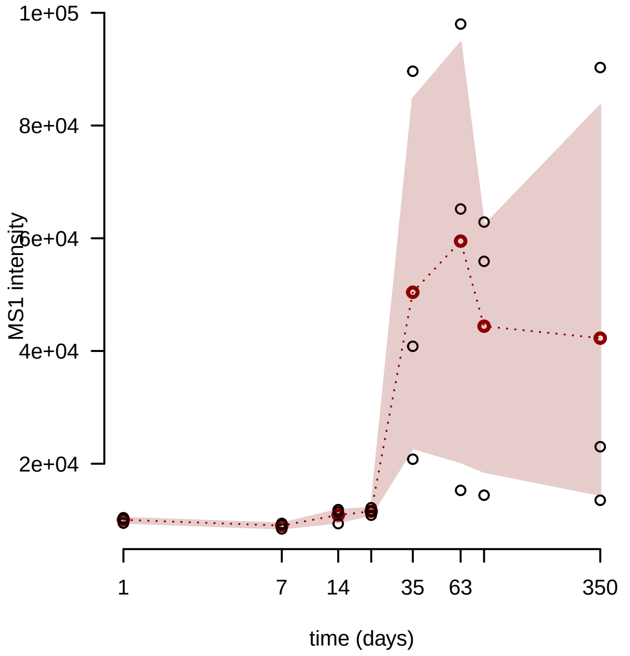

P30999
GN=Ctnnd1
catenin (cadherin associated protein), d ...

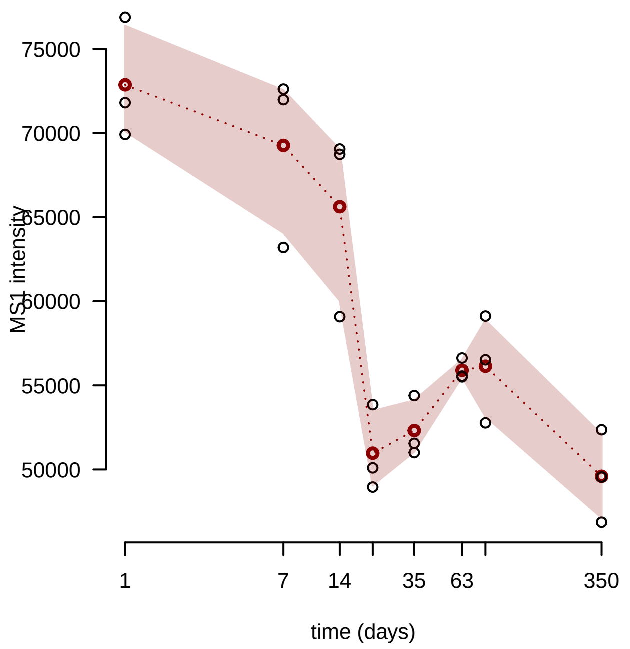

P30999
GN=Ctnnd1
catenin (cadherin associated protein), d ...

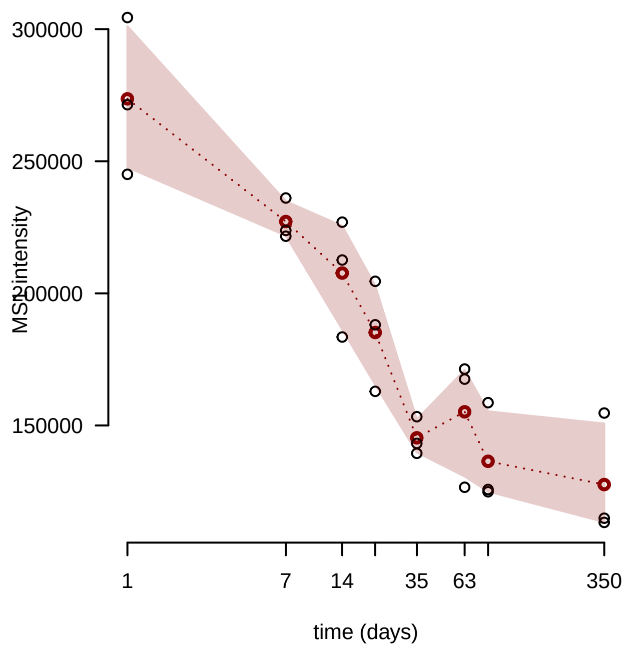

P31001
GN=Des
desmin

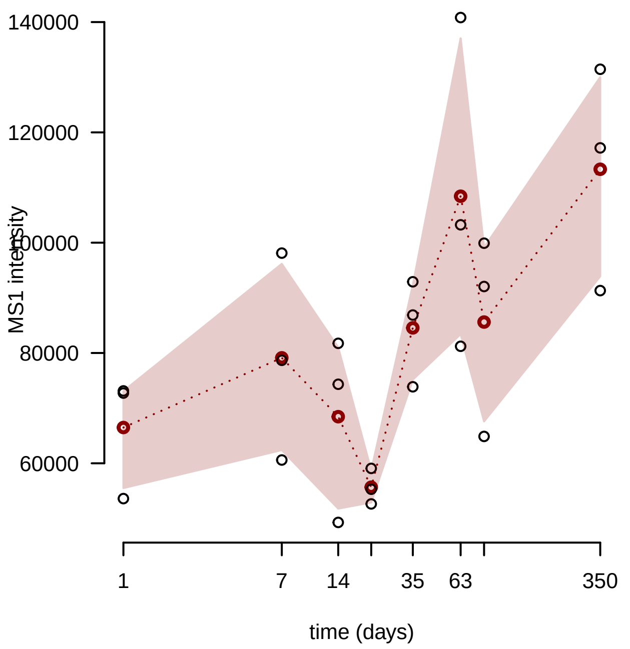

P31230
GN=Aimp1
aminoacyl tRNA synthetase complex−intera ...

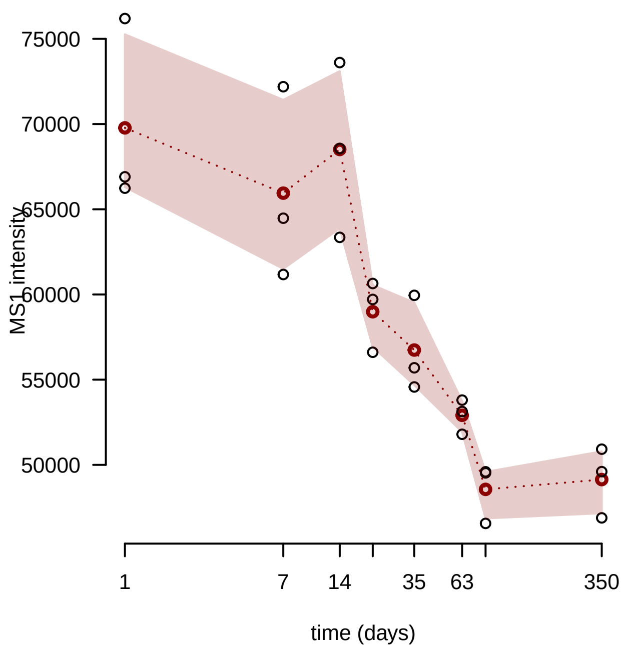

P31266
GN=Rbpj
recombination signal binding protein for ...

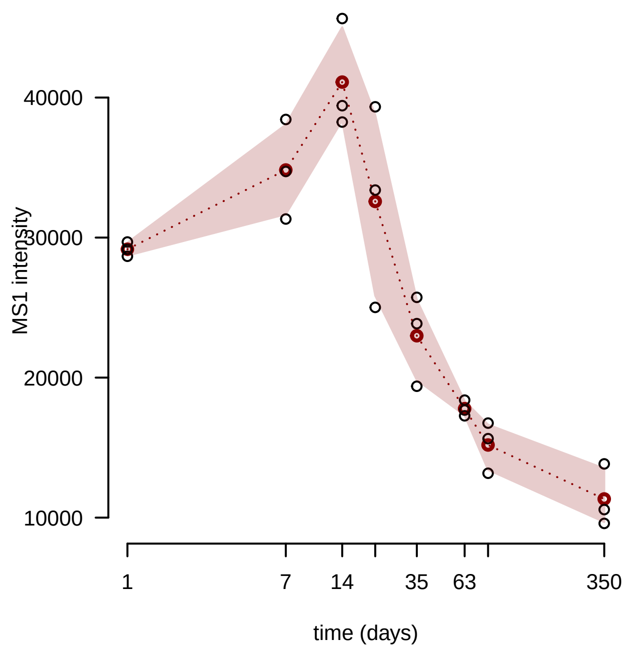

P31324
GN=Prkar2b
protein kinase, cAMP dependent regulator ...

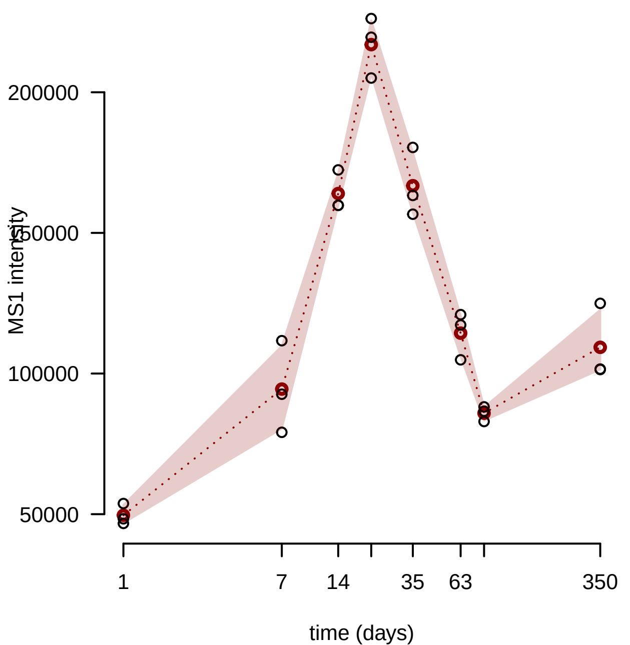

P31428
GN=Dpep1
dipeptidase 1

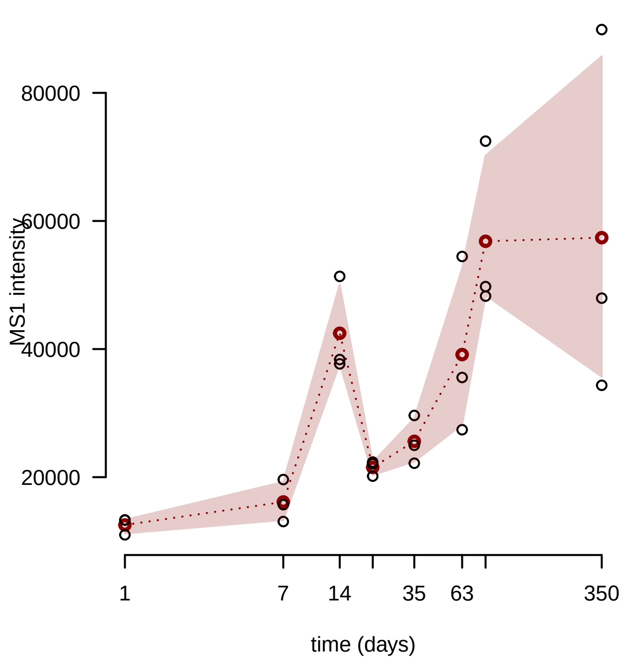

P31532
GN=Saa4
serum amyloid A 4

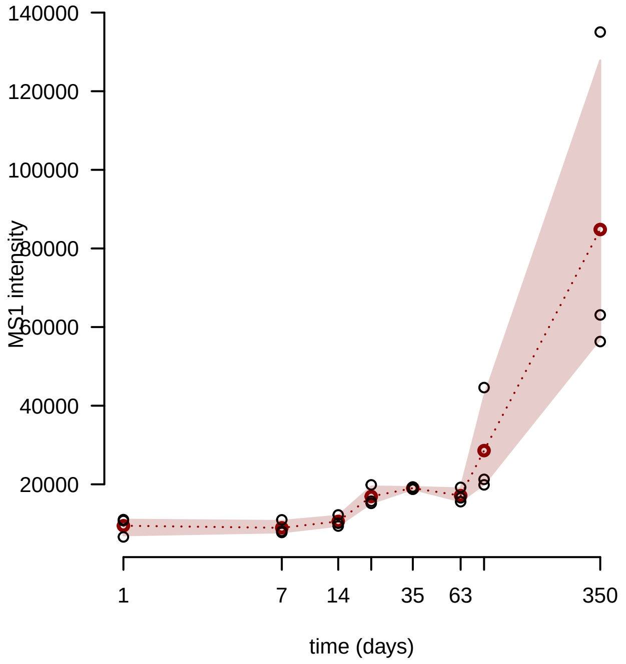

P31650
GN=Slc6a11
solute carrier family 6 (neurotransmitte ...

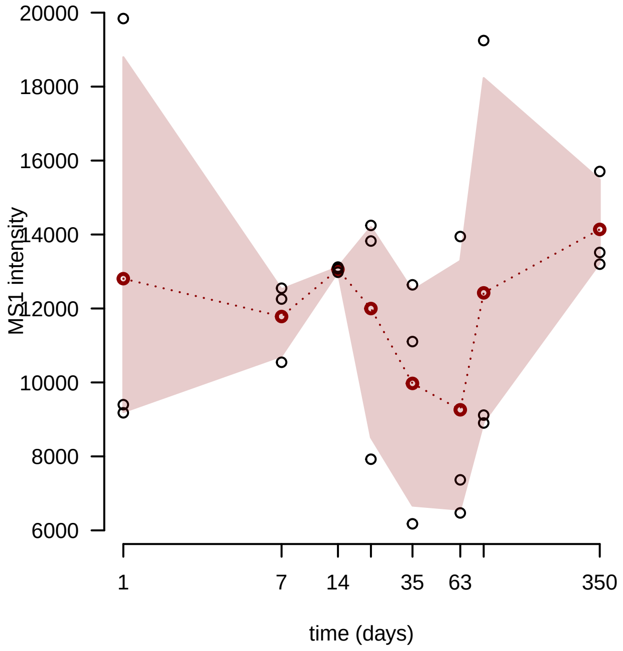

P31725
GN=S100a9
S100 calcium binding protein A9 (calgran ...

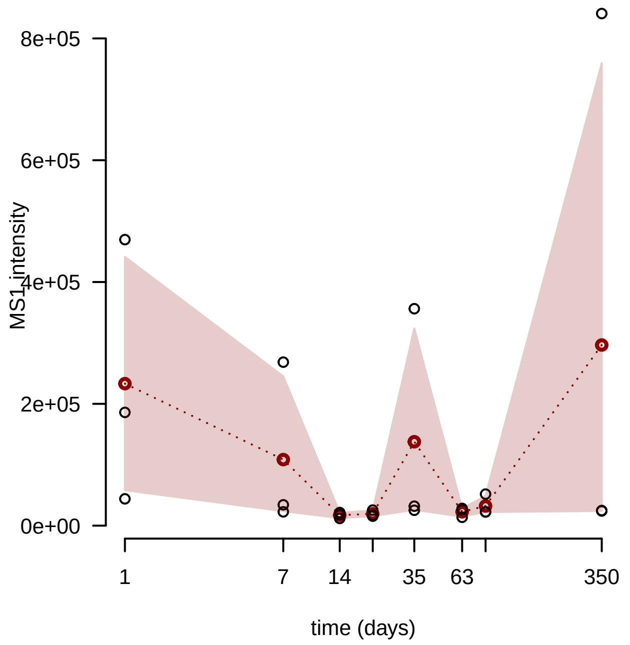

P31750
GN=Akt1
thymoma viral proto−oncogene 1

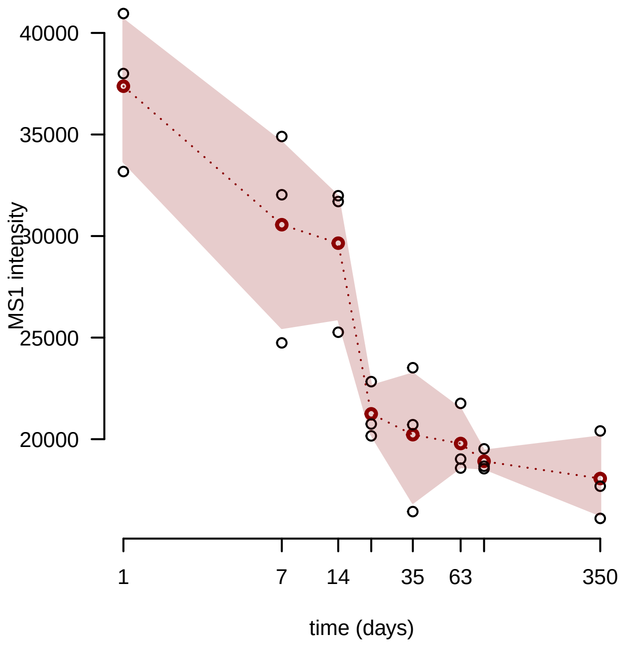

P31786
GN=Dbi
diazepam binding inhibitor

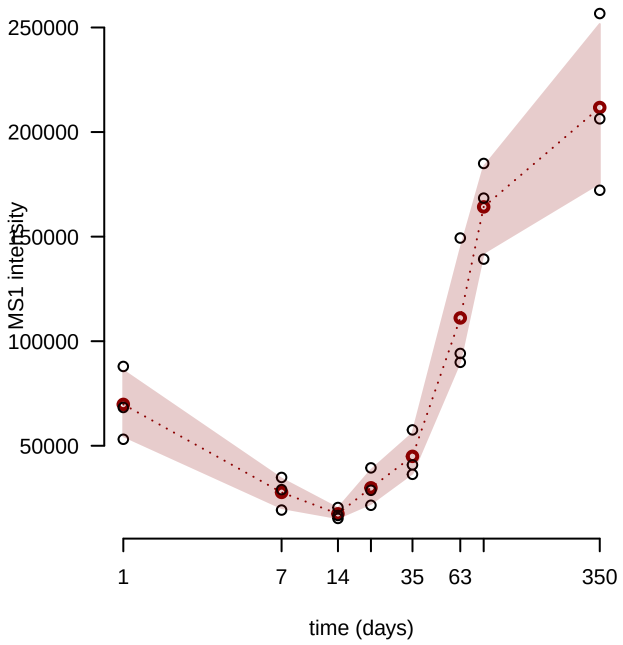

P31938
GN=Map2k1
mitogen−activated protein kinase kinase ...

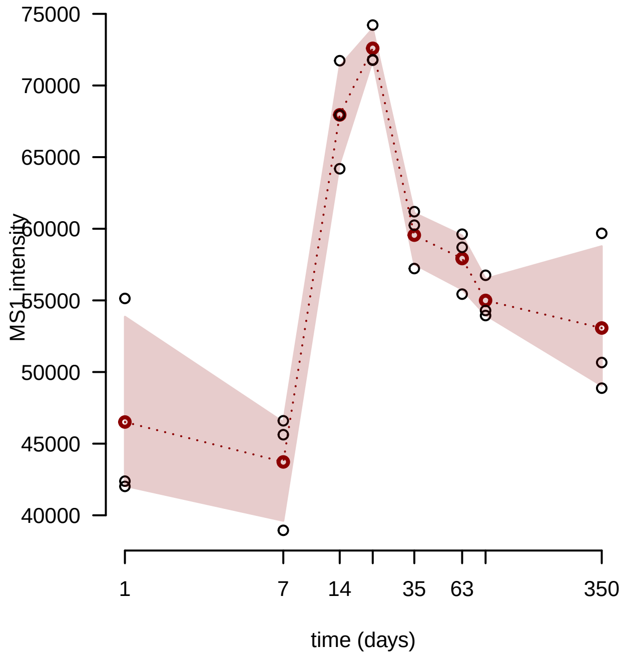

P31996
GN=Cd68
CD68 antigen

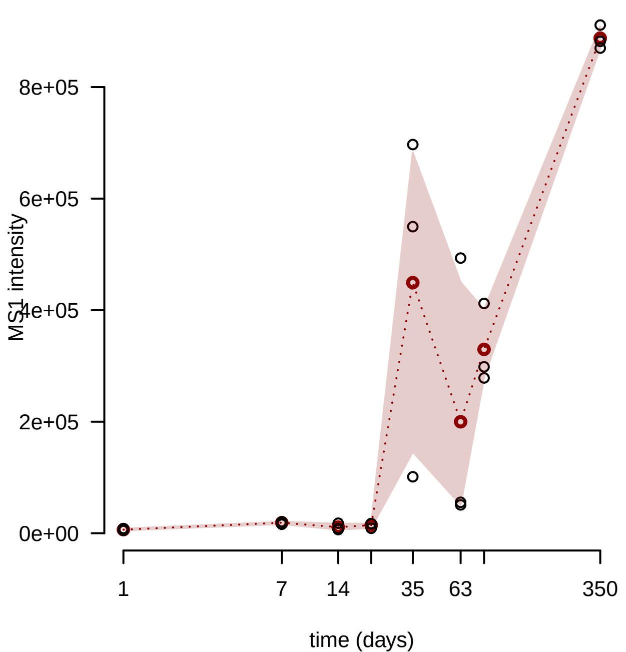

P32020
GN=Scp2
sterol carrier protein 2, liver

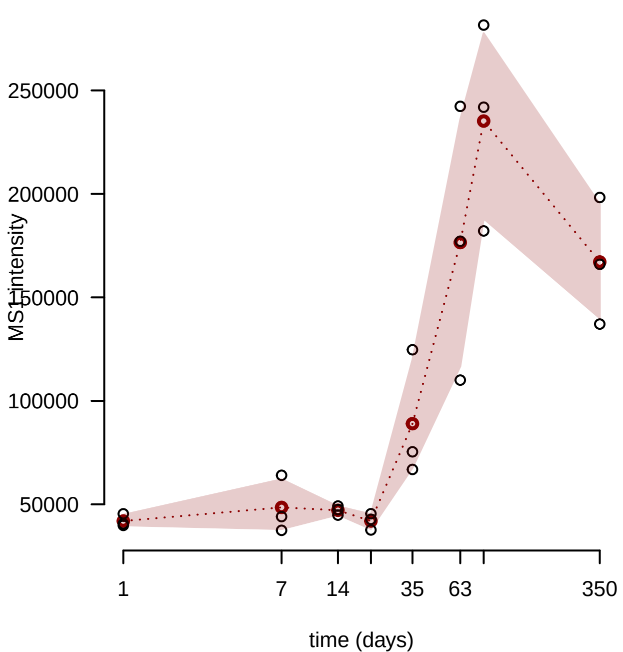

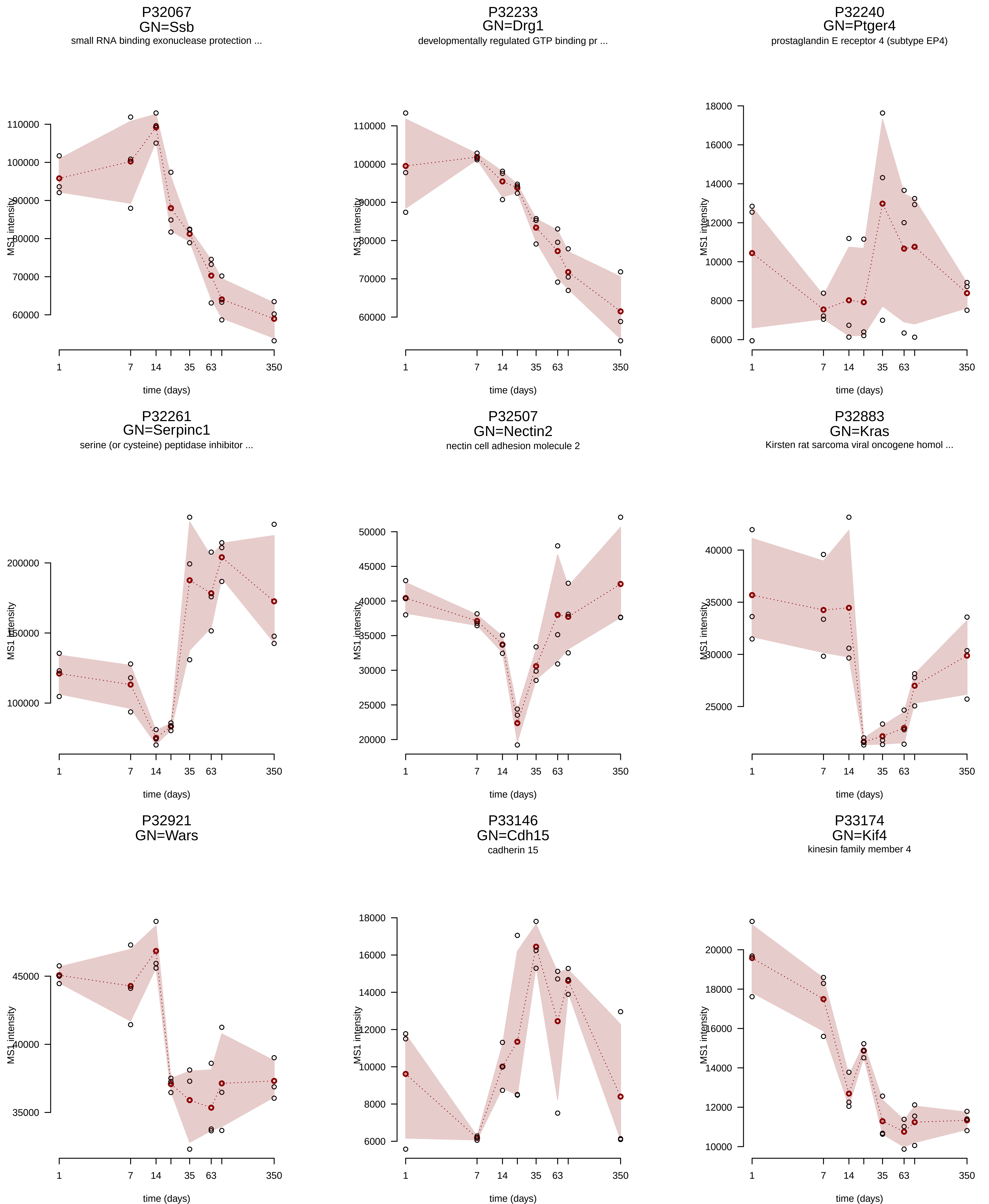

P32067
GN=Ssb
small RNA binding exonuclease protection ...
MS1 intensity
time (days)
P32233
GN=Drg1
developmentally regulated GTP binding pr ...
MS1 intensity
time (days)
P32240
GN=Ptger4
prostaglandin E receptor 4 (subtype EP4)
MS1 intensity
time (days)
P32261
GN=Serpinc1
serine (or cysteine) peptidase inhibitor ...
MS1 intensity
time (days)
P32507
GN=Nectin2
nectin cell adhesion molecule 2
MS1 intensity
time (days)
P32883
GN=Kras
Kirsten rat sarcoma viral oncogene homol ...
MS1 intensity
time (days)
P32921
GN=Wars
MS1 intensity
time (days)
P33146
GN=Cdh15
cadherin 15
MS1 intensity
time (days)
P33174
GN=Kif4
kinesin family member 4
MS1 intensity
time (days)

P33215
GN=Nedd1
neural precursor cell expressed, develop ...

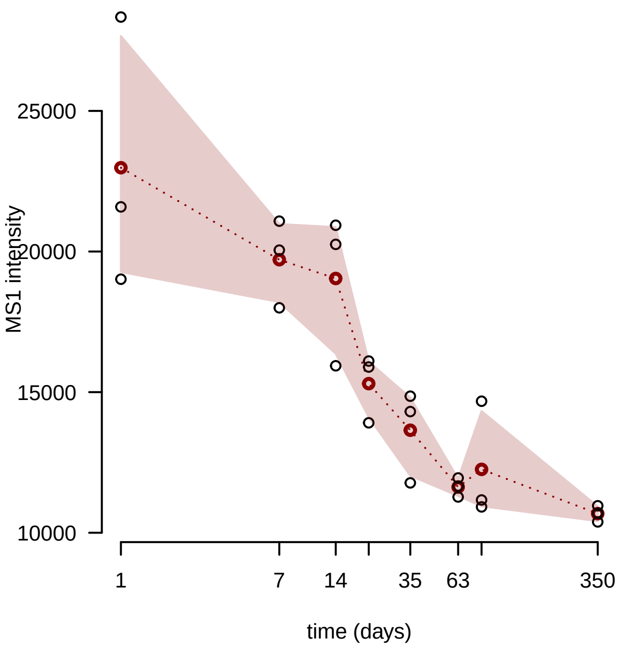

P33242
GN=Nr5a1
nuclear receptor subfamily 5, group A, m ...

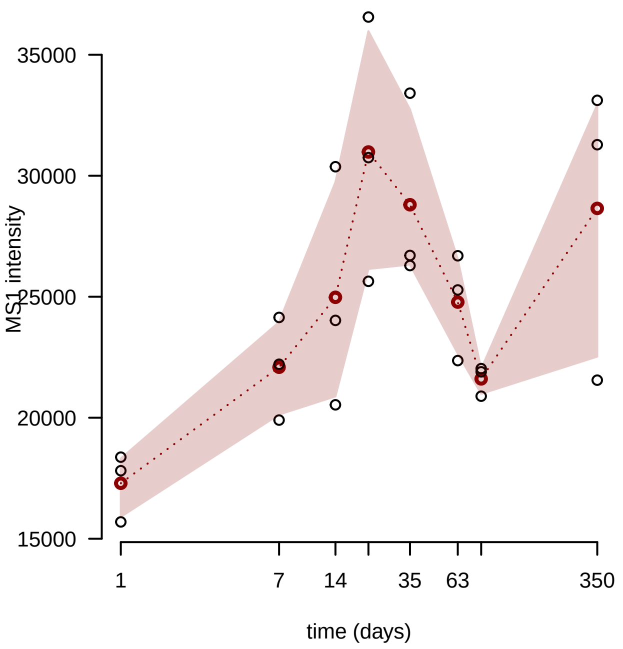

P33434
GN=Mmp2
matrix metallopeptidase 2

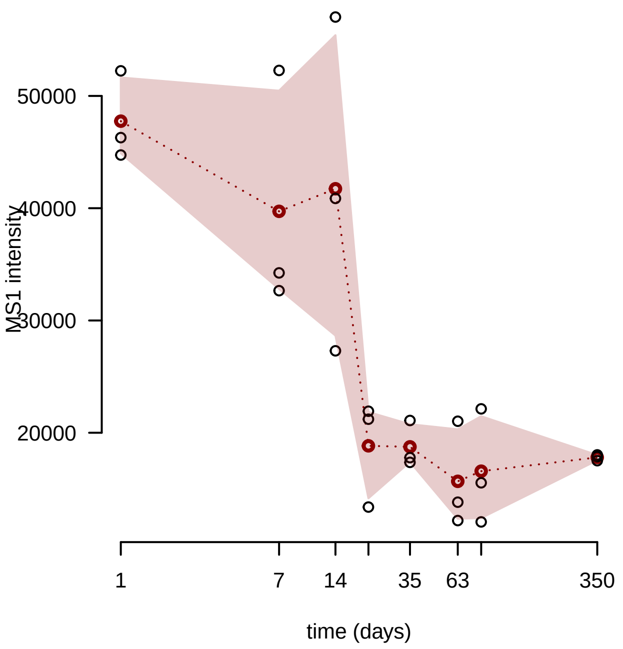

P33587
GN=Proc
protein C

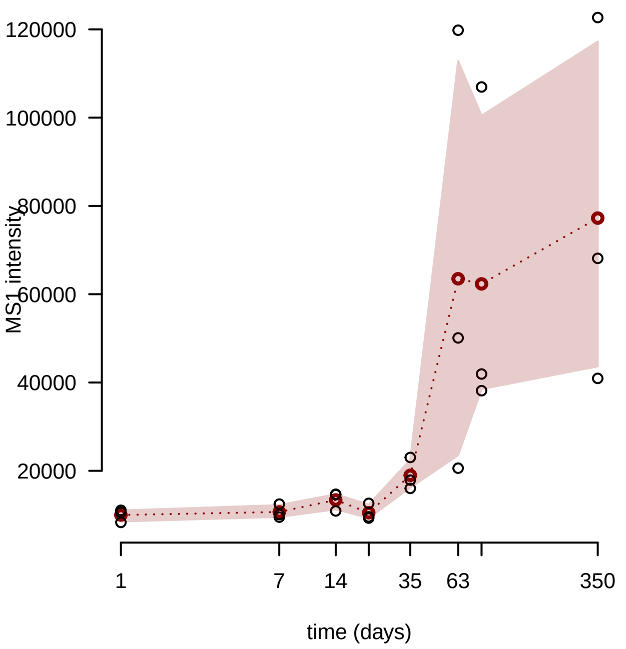

P33609
GN=Pola1
polymerase (DNA directed), alpha 1

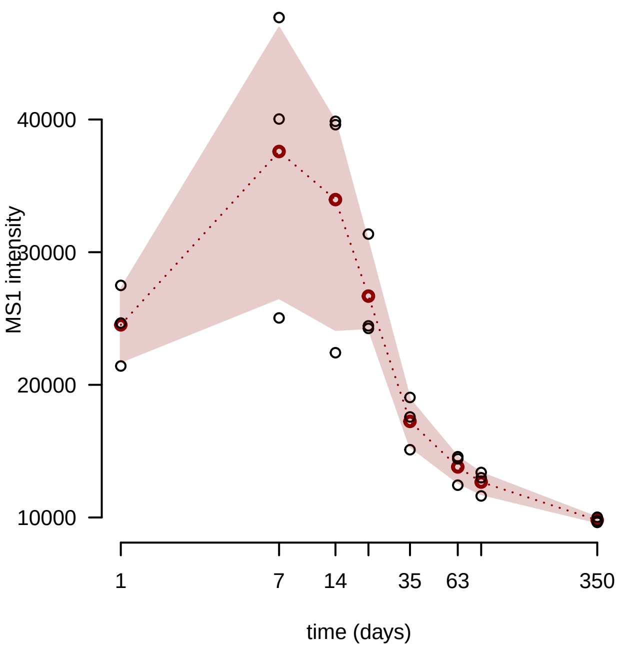

P33610
GN=Prim2
DNA primase, p58 subunit

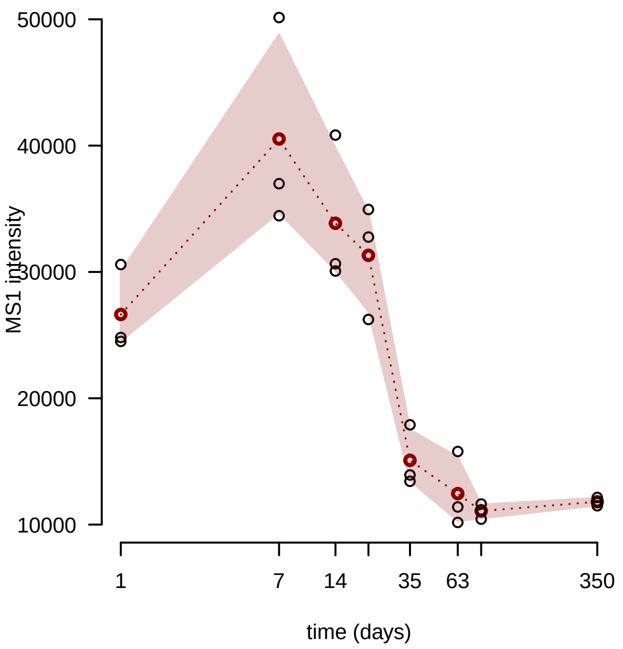

P33611
GN=Pola2
polymerase (DNA directed), alpha 2

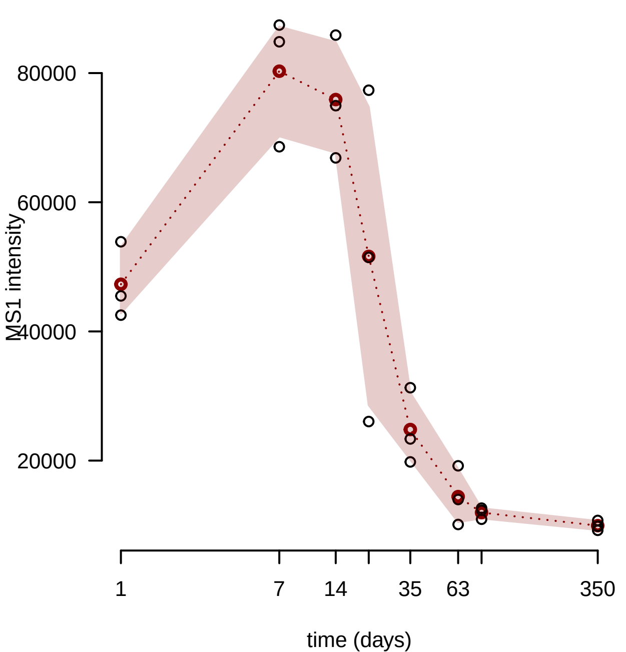

P33622
GN=Apoc3
apolipoprotein C−III

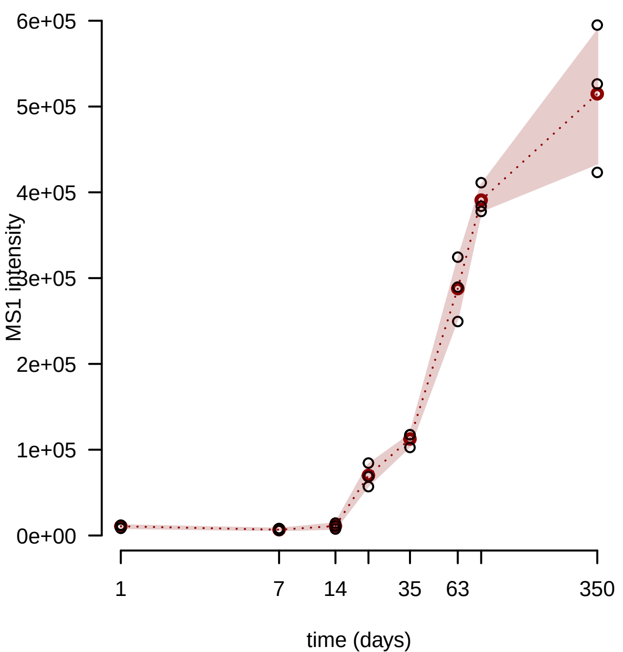

P34022
GN=Ranbp1
RAN binding protein 1

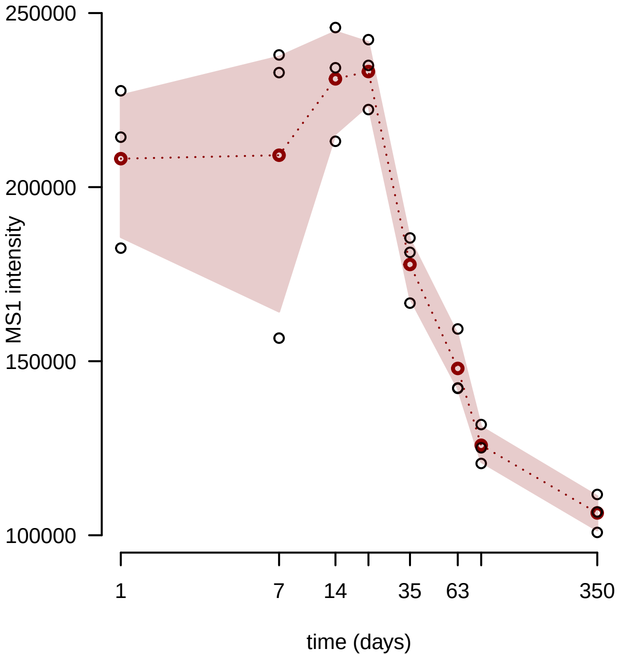

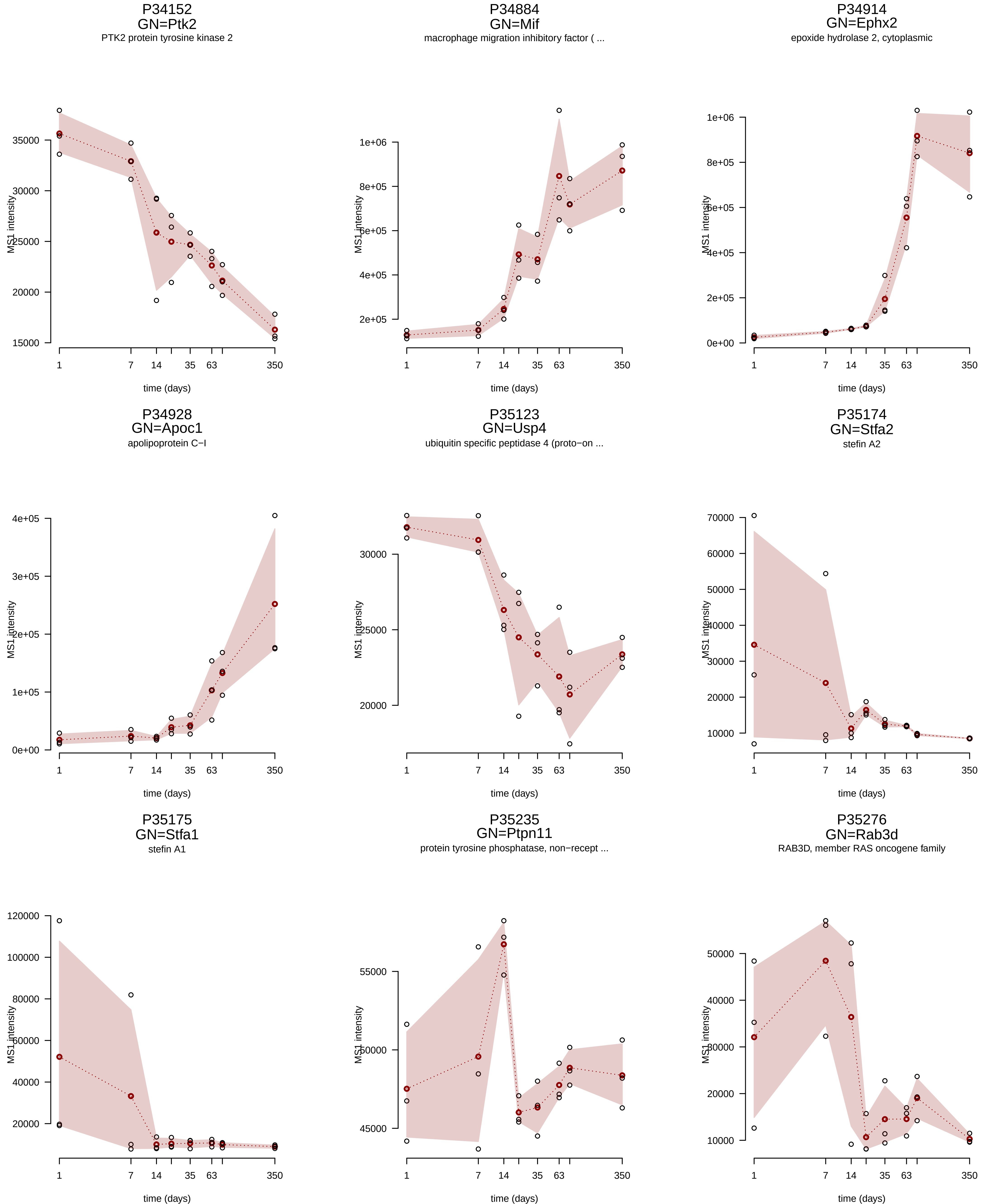

P34152
GN=Ptk2
PTK2 protein tyrosine kinase 2
MS1 intensity
15000
20000
25000
30000
35000
1
7
14
35
63
350
time (days)
P34884
GN=Mif
macrophage migration inhibitory factor (...
MS1 intensity
2e+05
4e+05
6e+05
8e+05
1e+06
time (days)
P34914
GN=Ephx2
epoxide hydrolase 2, cytoplasmic
MS1 intensity
0e+00
2e+05
4e+05
6e+05
8e+05
1e+06
time (days)
P34928
GN=Apoc1
apolipoprotein C−I
MS1 intensity
0e+00
1e+05
2e+05
3e+05
4e+05
time (days)
P35123
GN=Usp4
ubiquitin specific peptidase 4 (proto−on ...
MS1 intensity
20000
25000
30000
time (days)
P35174
GN=Stfa2
stefin A2
MS1 intensity
10000
20000
30000
40000
50000
60000
70000
time (days)
P35175
GN=Stfa1
stefin A1
MS1 intensity
20000
40000
60000
80000
100000
120000
time (days)
P35235
GN=Ptpn11
protein tyrosine phosphatase, non−recept ...
MS1 intensity
45000
50000
55000
time (days)
P35276
GN=Rab3d
RAB3D, member RAS oncogene family
MS1 intensity
10000
20000
30000
40000
50000
time (days)

P35278
GN=Rab5c
RAB5C, member RAS oncogene family

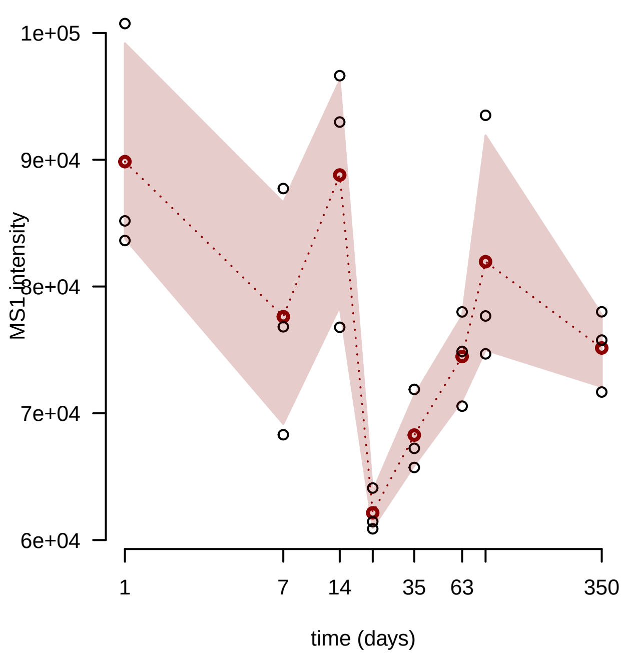

P35279
GN=Rab6a
RAB6A, member RAS oncogene family

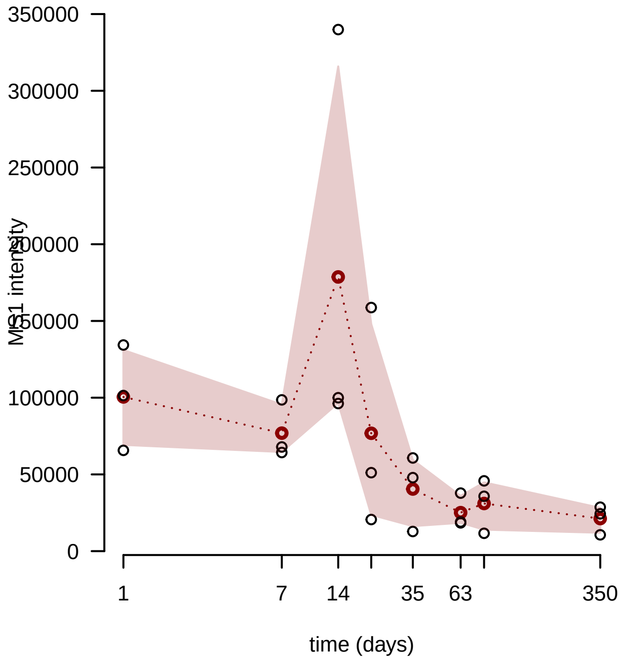

P35279
GN=Rab6a
RAB6A, member RAS oncogene family

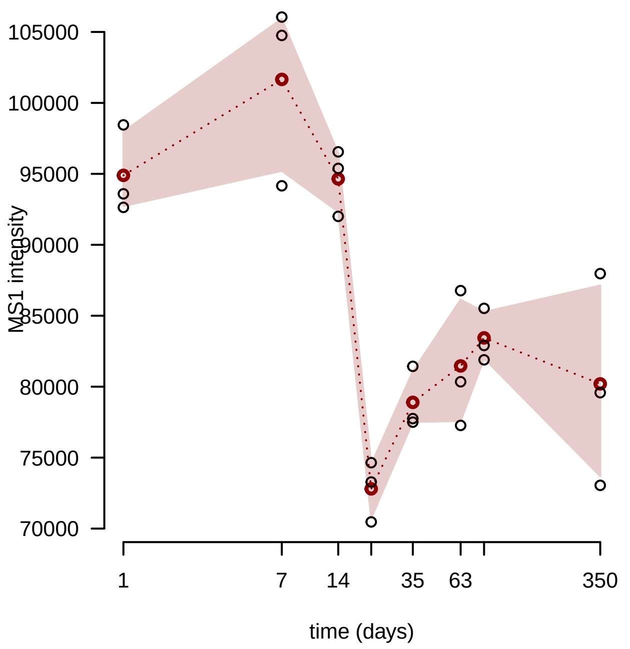

P35282
GN=Rab21
RAB21, member RAS oncogene family

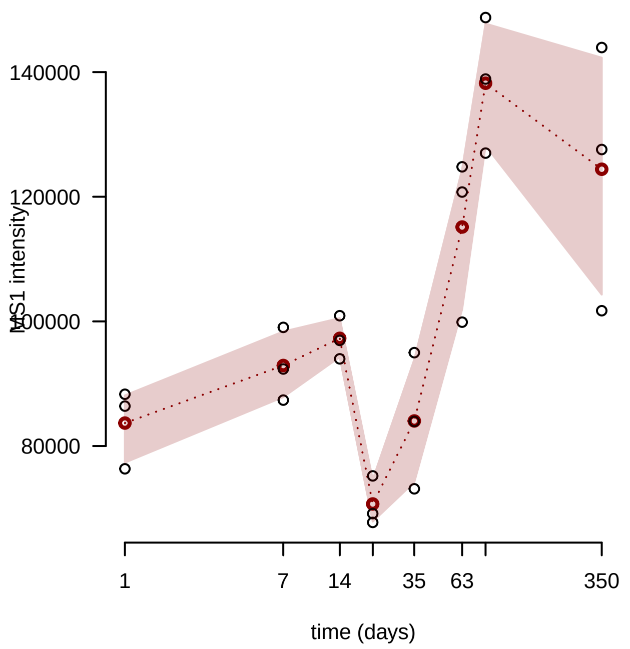

P35283
GN=Rab12
RAB12, member RAS oncogene family

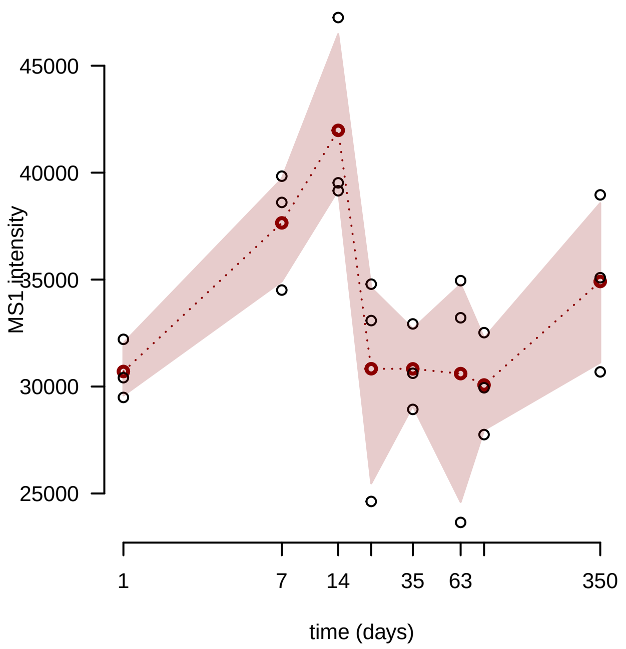

P35285
GN=Rab22a
RAB22A, member RAS oncogene family

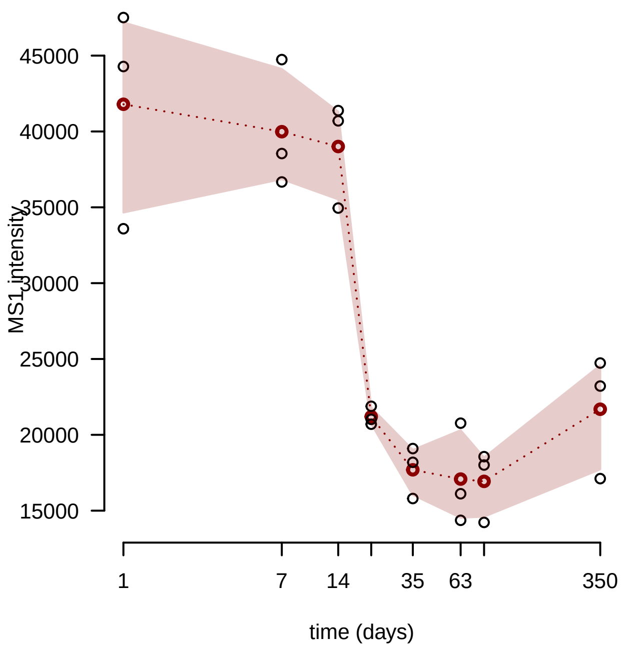

P35288
GN=Rab23
RAB23, member RAS oncogene family

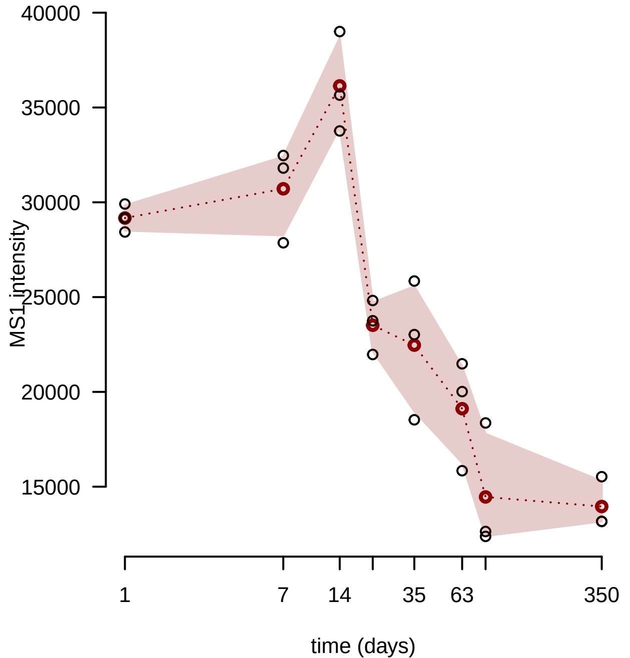

P35290
GN=Rab24
RAB24, member RAS oncogene family

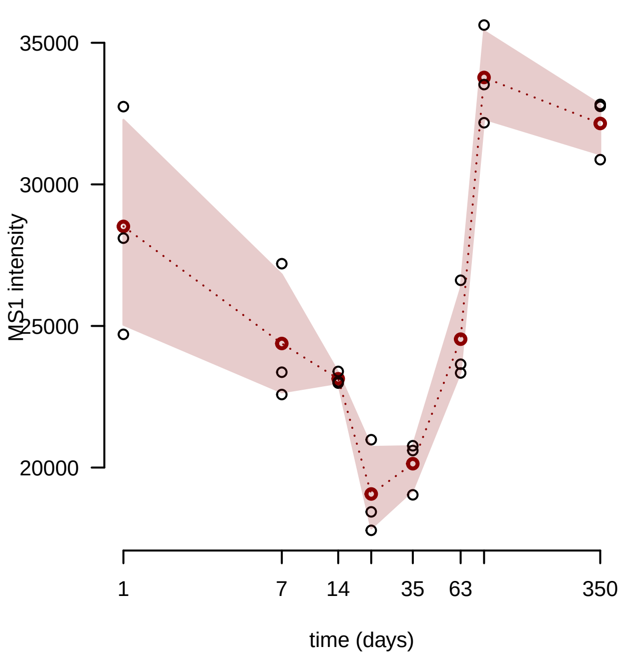

P35293
GN=Rab18
RAB18, member RAS oncogene family

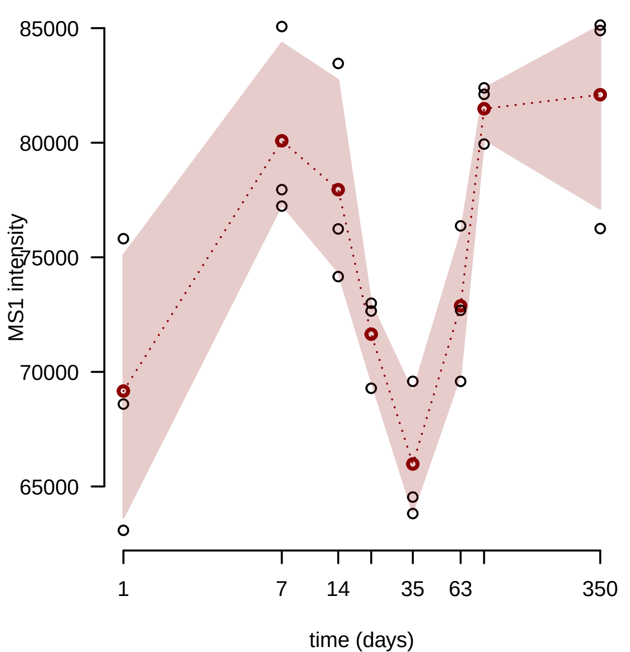

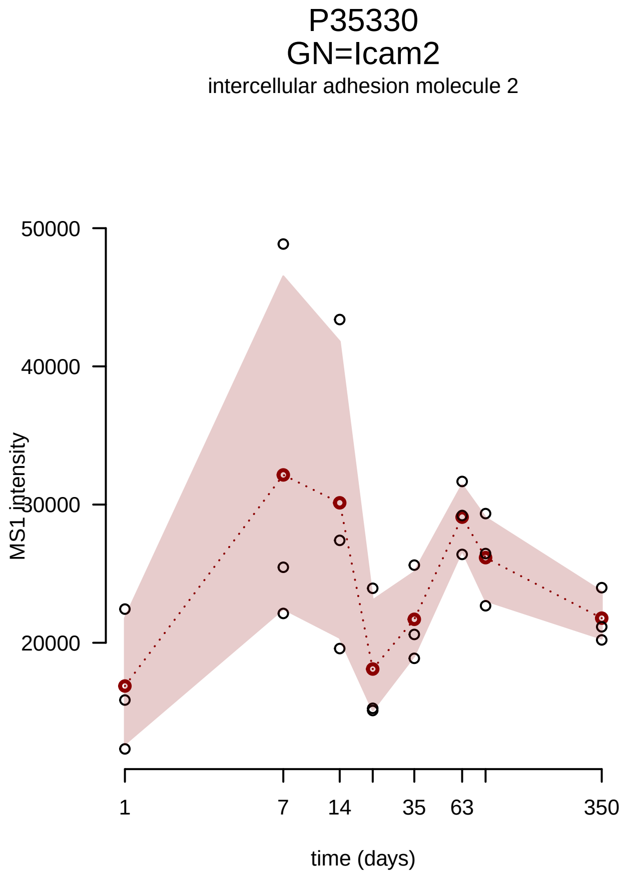
P35330
GN=Icam2
intercellular adhesion molecule 2
MS1 intensity
20000
30000
40000
50000
1
7
14
35
63
350
time (days)

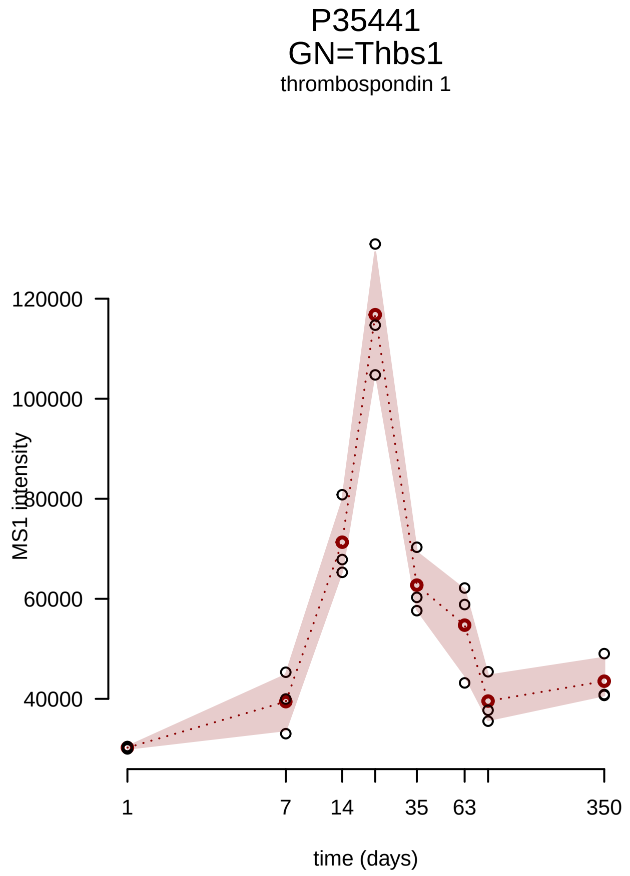
P35441
GN=Thbs1
thrombospondin 1
MS1 intensity
40000
60000
80000
100000
120000
1
7
14
35
63
350
time (days)

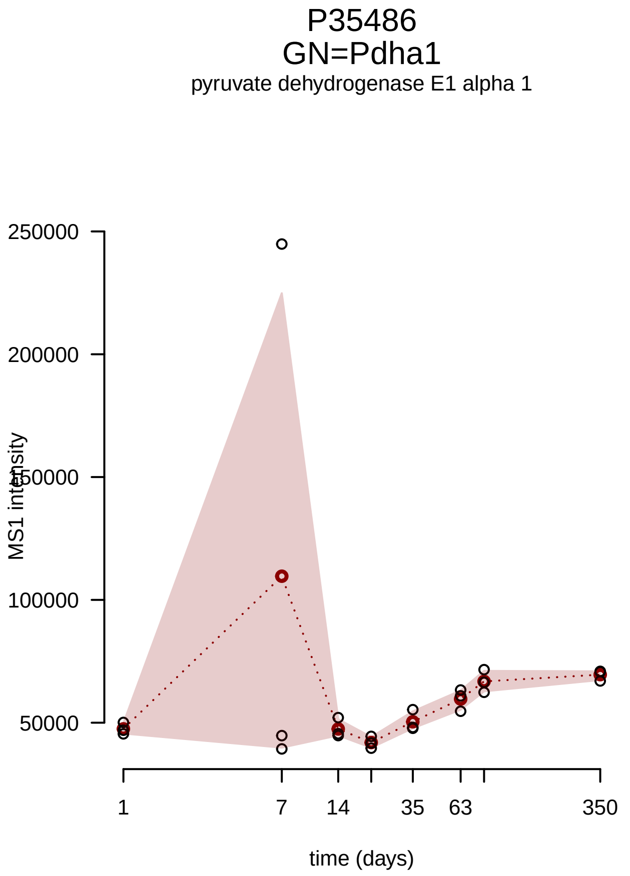
P35486
GN=Pdha1
pyruvate dehydrogenase E1 alpha 1
MS1 intensity
50000
100000
150000
200000
250000
1
7
14
35
63
350
time (days)

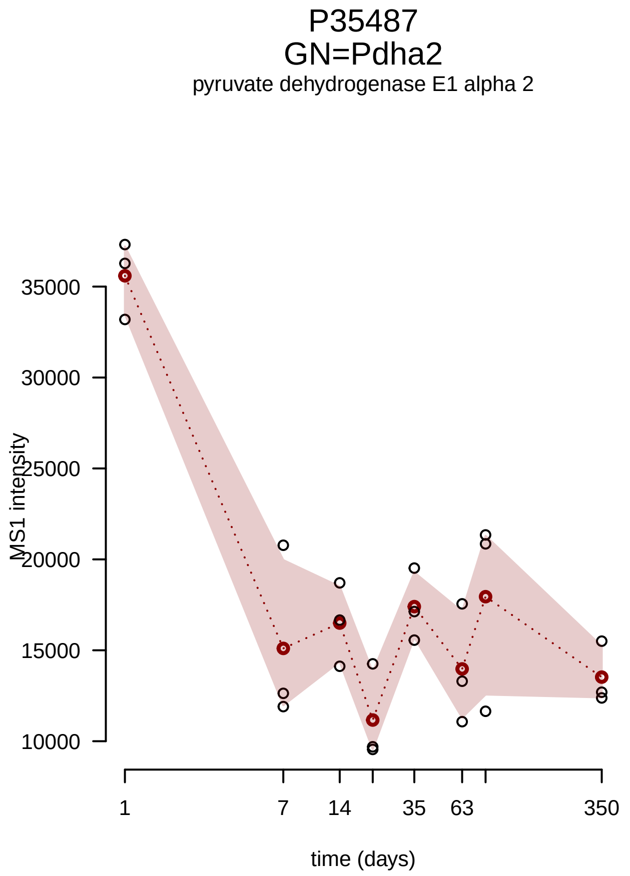
P35487
GN=Pdha2
pyruvate dehydrogenase E1 alpha 2
MS1 intensity
10000
15000
20000
25000
30000
35000
1
7
14
35
63
350
time (days)

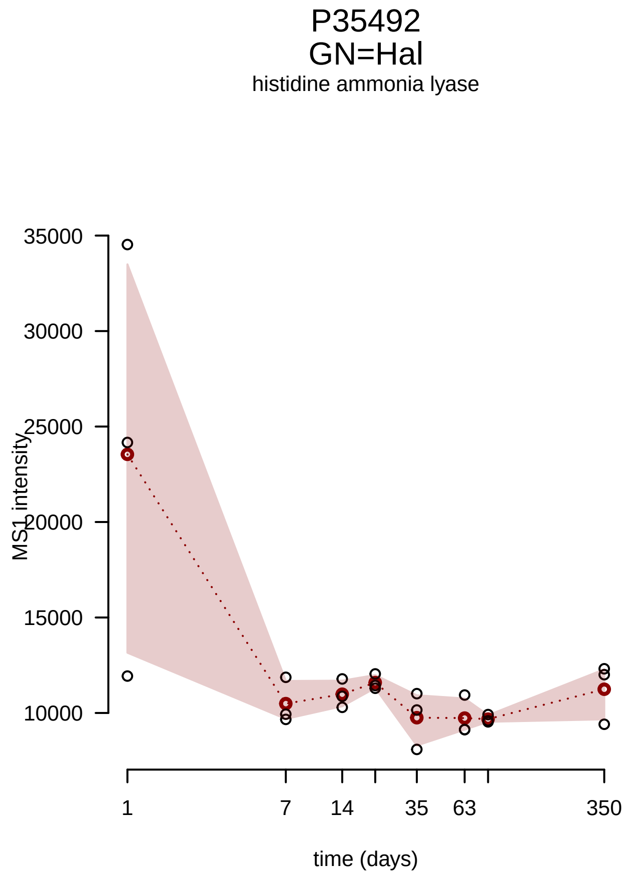
P35492
GN=Hal
histidine ammonia lyase
MS1 intensity
10000
15000
20000
25000
30000
35000
1
7
14
35
63
350
time (days)

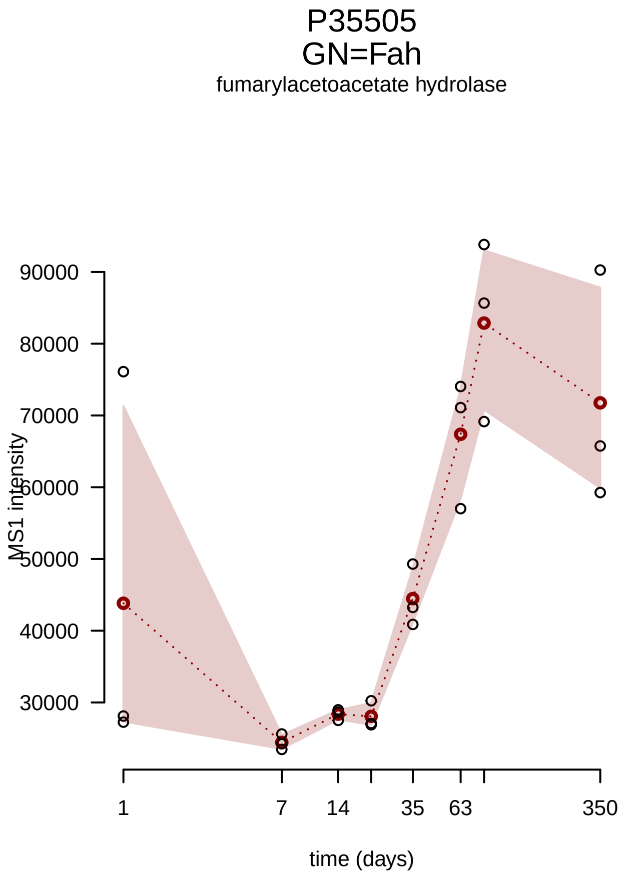
P35505
GN=Fah
fumarylacetoacetate hydrolase
MS1 intensity
30000
40000
50000
60000
70000
80000
90000
1
7
14
35
63
350
time (days)

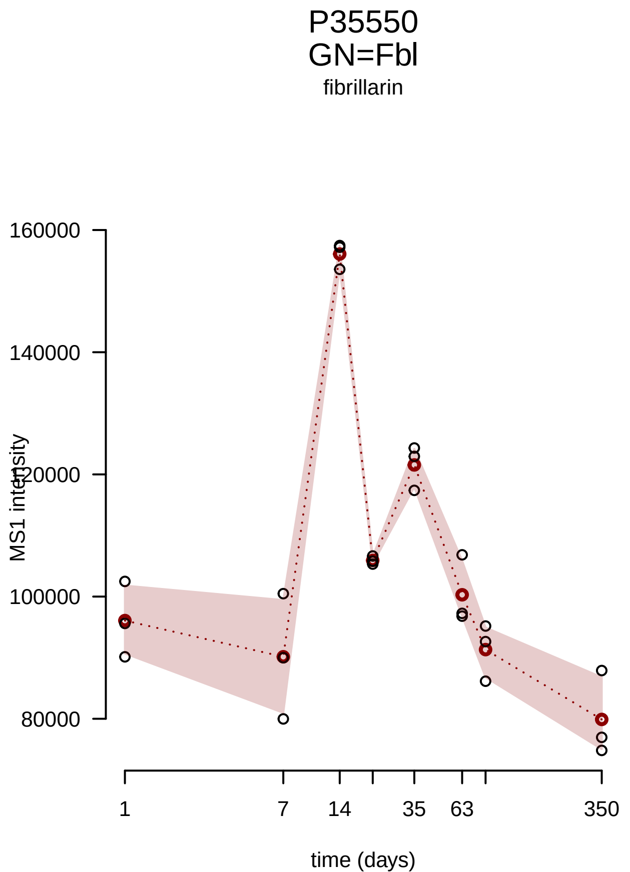
P35550
GN=Fbl
fibrillarin
MS1 intensity
80000
100000
120000
140000
160000
1
7
14
35
63
350
time (days)

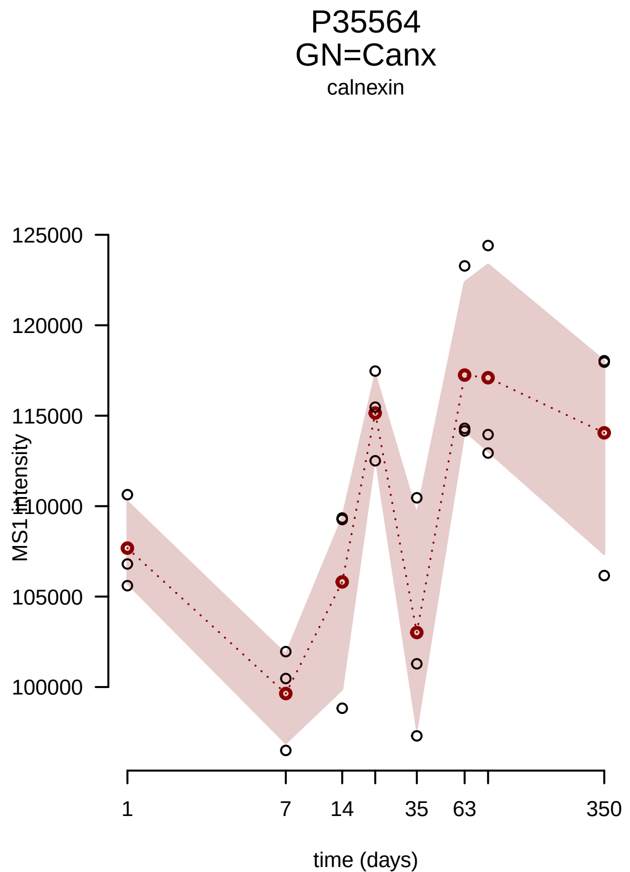
P35564
GN=Canx
calnexin
MS1 intensity
100000
105000
110000
115000
120000
125000
1
7
14
35
63
350
time (days)

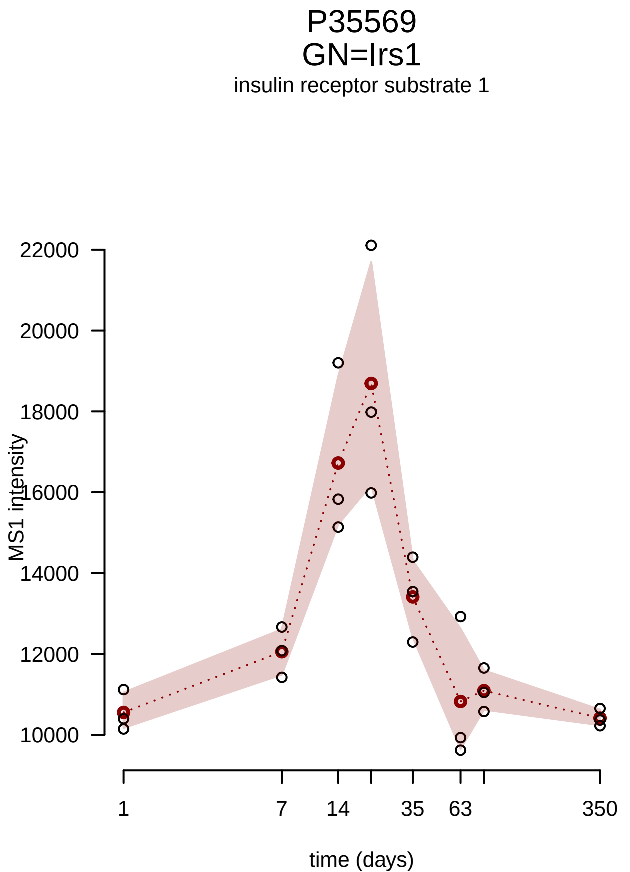
P35569
GN=Irs1
insulin receptor substrate 1
MS1 intensity
10000
12000
14000
16000
18000
20000
22000
1
7
14
35
63
350
time (days)

P35585
GN=Ap1m1
adaptor−related protein complex AP−1, mu ...

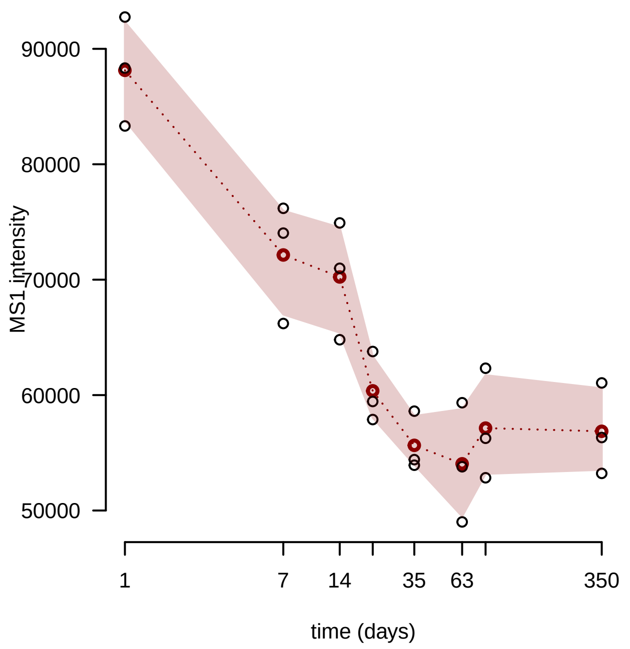

P35601
GN=Rfc1
replication factor C (activator 1) 1

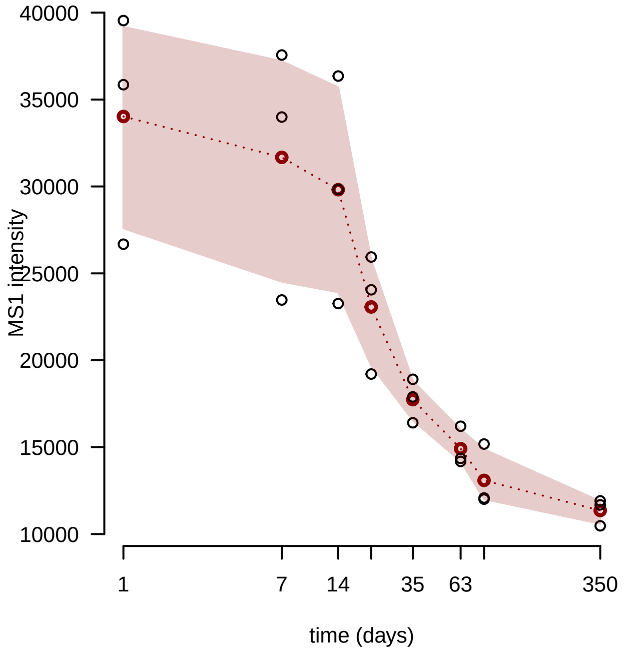

P35689
GN=Ercc5
excision repair cross−complementing rode ...

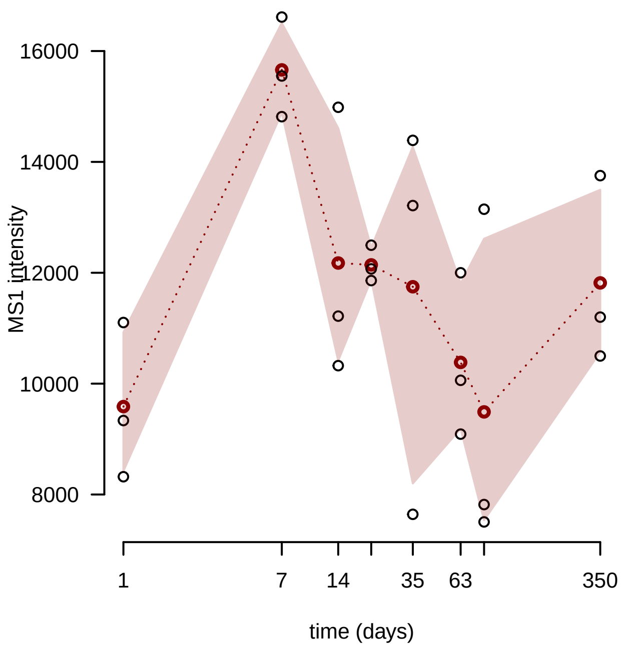

P35700
GN=Prdx1
peroxiredoxin 1

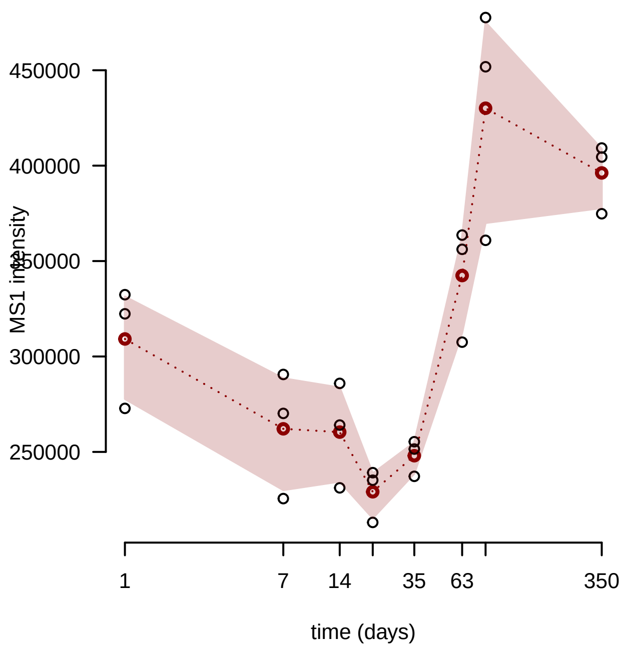

P35710
GN=Sox5
SRY (sex determining region Y)−box 5

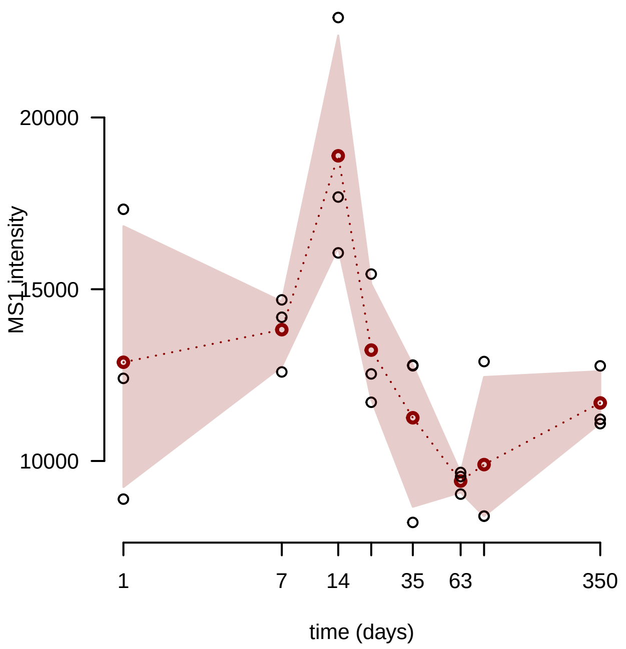

P35737
GN=H2−DMb1
histocompatibility 2, class II, locus Mb ...

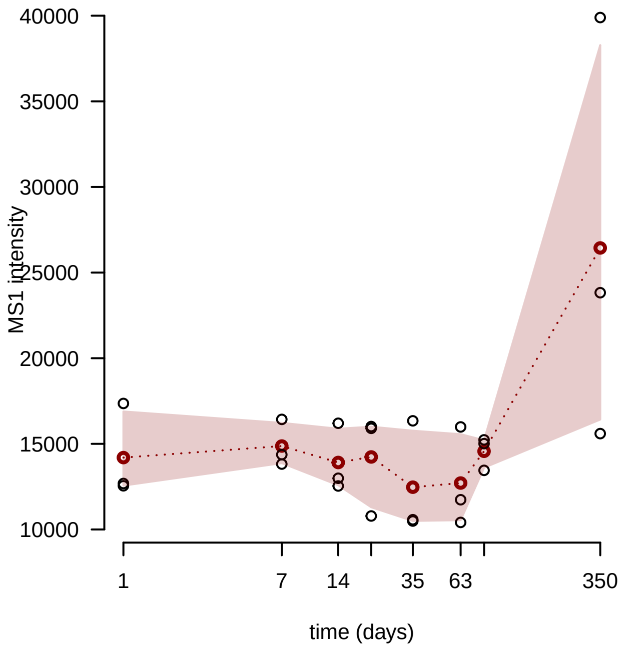

P35761
GN=Ttk
Ttk protein kinase

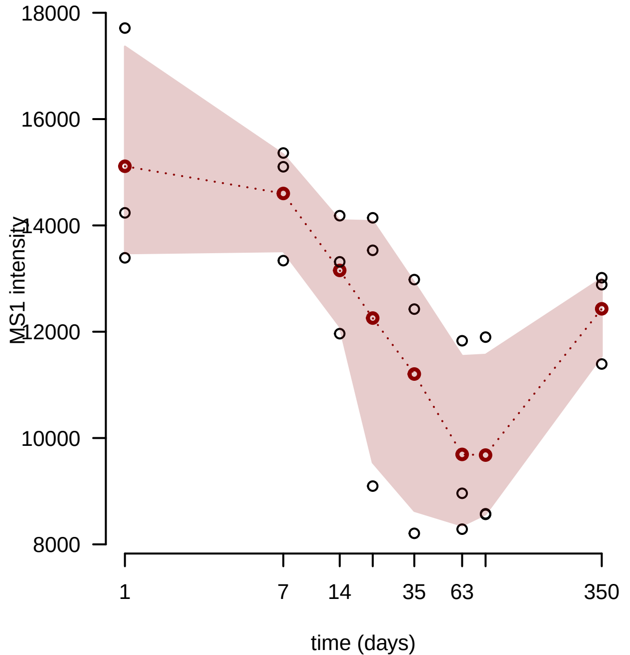

P35762
GN=Cd81
CD81 antigen

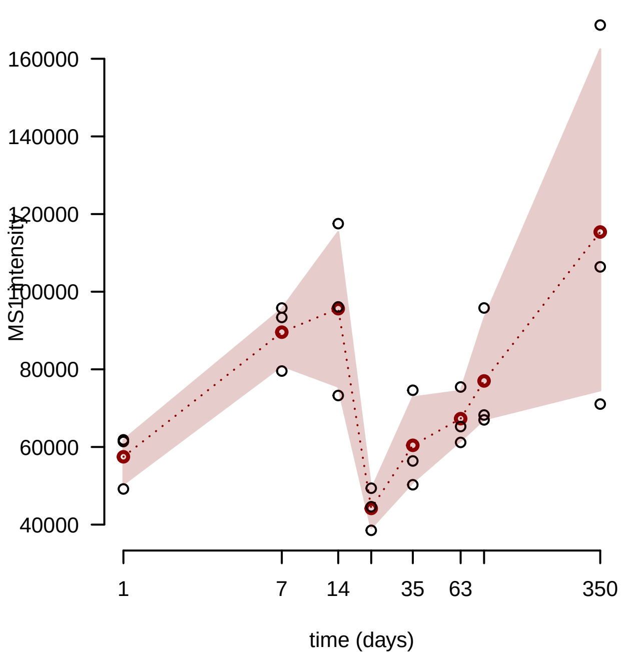

P35802
GN=Gpm6a
glycoprotein m6a

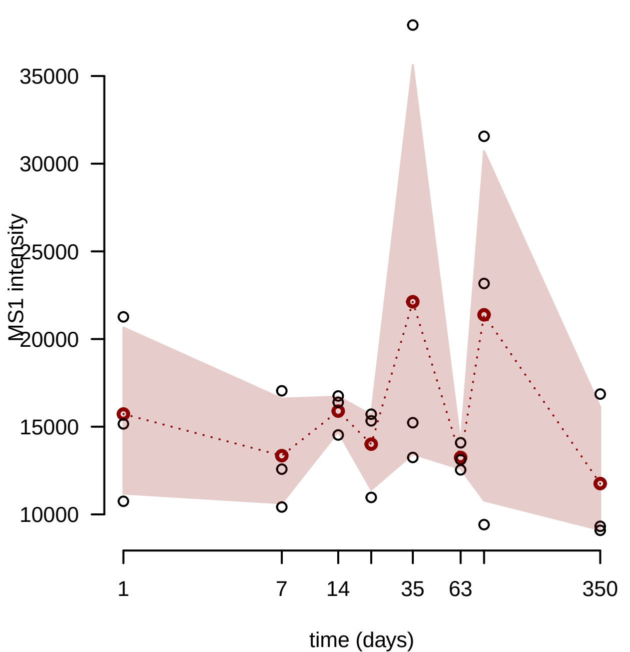

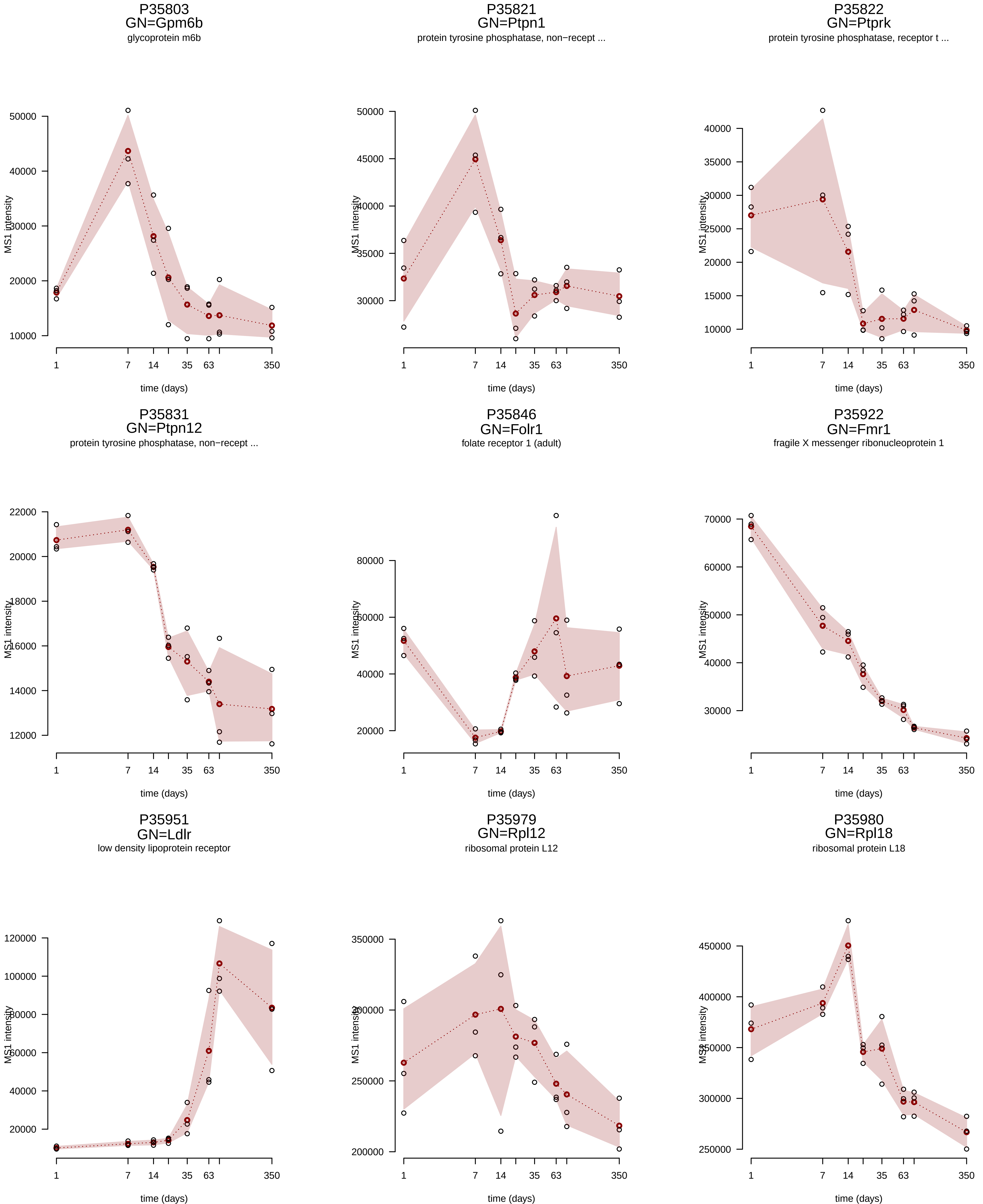
P35803
GN=Gpm6b
glycoprotein m6b
MS1 intensity
50000
40000
30000
20000
10000
1
7
14
35
63
350
time (days)
P35821
GN=Ptpn1
protein tyrosine phosphatase, non−recept ...
MS1 intensity
50000
45000
40000
35000
30000
1
7
14
35
63
350
time (days)
P35822
GN=Ptprk
protein tyrosine phosphatase, receptor t ...
MS1 intensity
40000
35000
30000
25000
20000
15000
10000
1
7
14
35
63
350
time (days)
P35831
GN=Ptpn12
protein tyrosine phosphatase, non−recept ...
MS1 intensity
22000
20000
18000
16000
14000
12000
1
7
14
35
63
350
time (days)
P35846
GN=Folr1
folate receptor 1 (adult)
MS1 intensity
80000
60000
40000
20000
1
7
14
35
63
350
time (days)
P35922
GN=Fmr1
fragile X messenger ribonucleoprotein 1
MS1 intensity
70000
60000
50000
40000
30000
1
7
14
35
63
350
time (days)
P35951
GN=Ldlr
low density lipoprotein receptor
MS1 intensity
120000
100000
80000
60000
40000
20000
1
7
14
35
63
350
time (days)
P35979
GN=Rpl12
ribosomal protein L12
MS1 intensity
350000
300000
250000
200000
1
7
14
35
63
350
time (days)
P35980
GN=Rpl18
ribosomal protein L18
MS1 intensity
450000
400000
350000
300000
250000
1
7
14
35
63
350
time (days)

P36371
GN=Tap2
transporter 2, ATP−binding cassette, sub ...

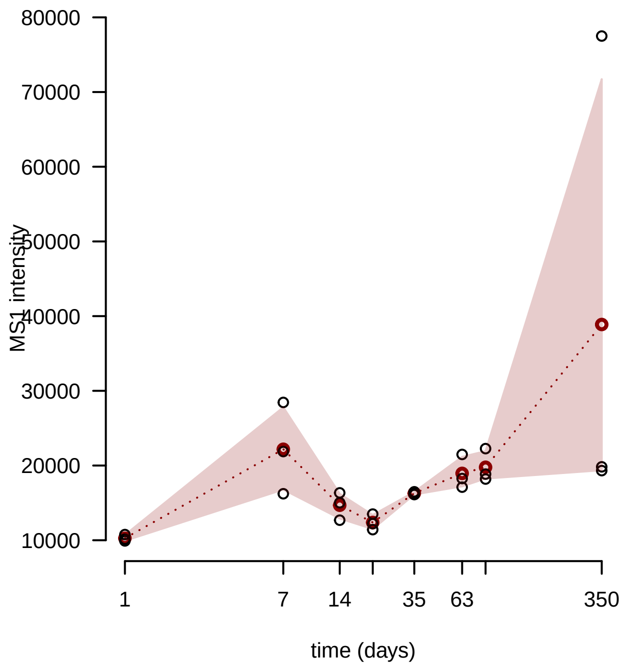

P36423
GN=Tbxas1
thromboxane A synthase 1, platelet

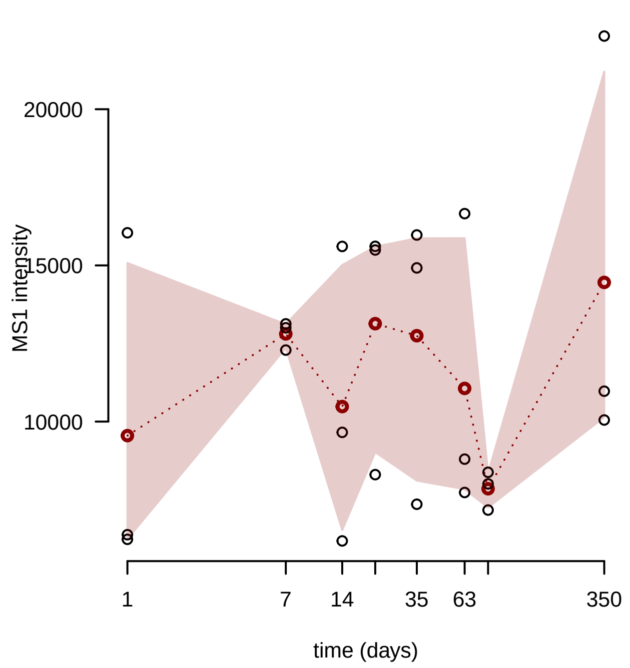

P36536
GN=Sar1a
secretion associated Ras related GTPase ...

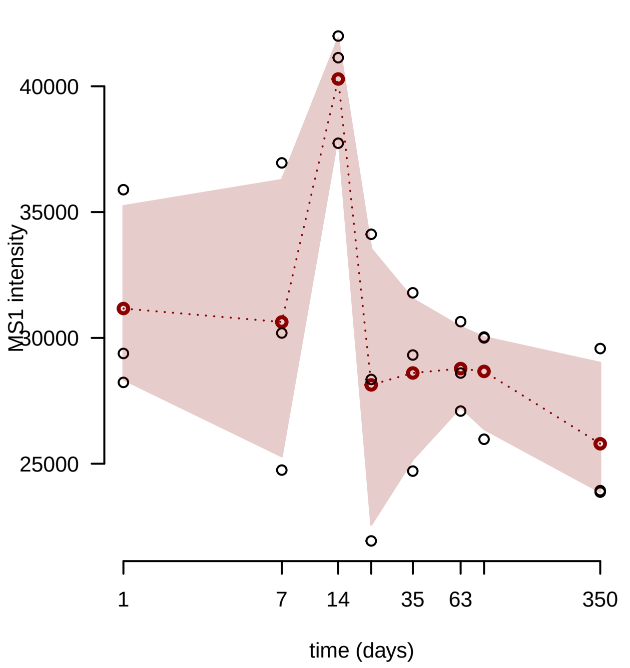

P36552
GN=Cpox
coproporphyrinogen oxidase

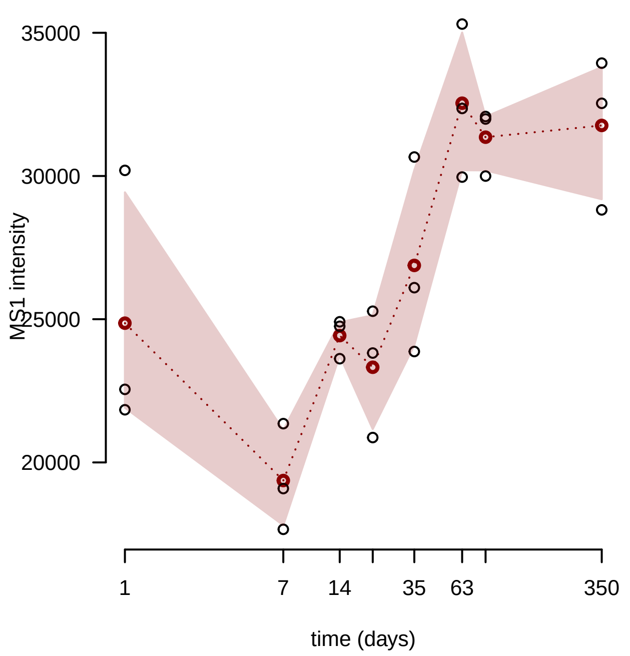

P36895
GN=Bmpr1a
bone morphogenetic protein receptor, typ ...

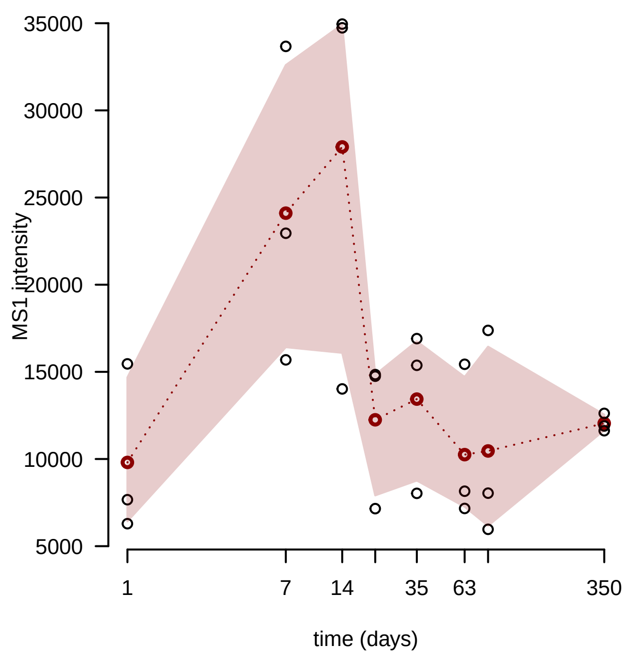

P36916
GN=Gnl1
guanine nucleotide binding protein−like ...

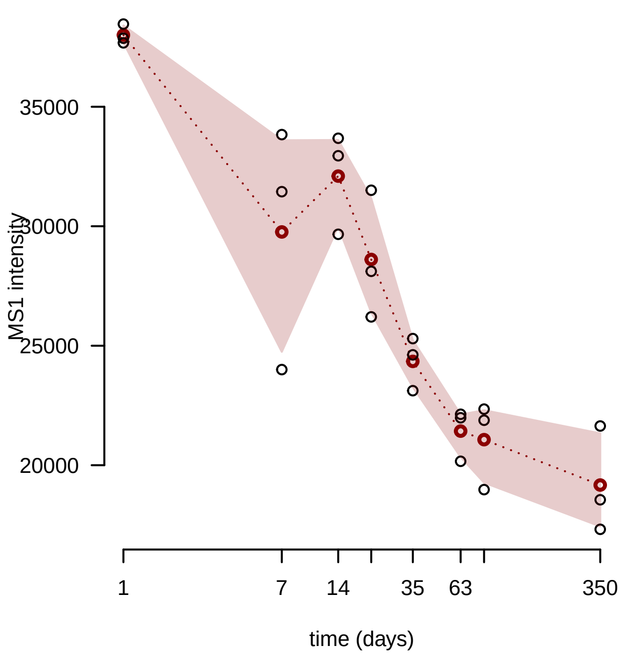

P36993
GN=Ppm1b
protein phosphatase 1B, magnesium depend ...

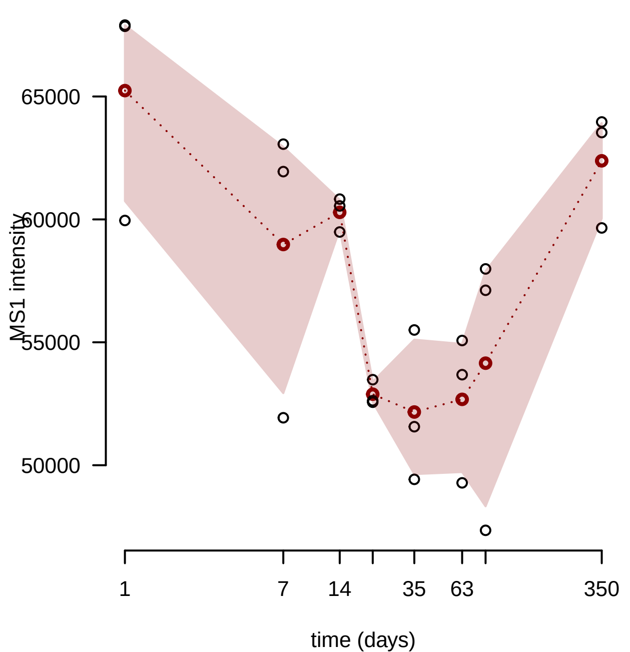

P37040
GN=Por
cytochrome p450 oxidoreductase

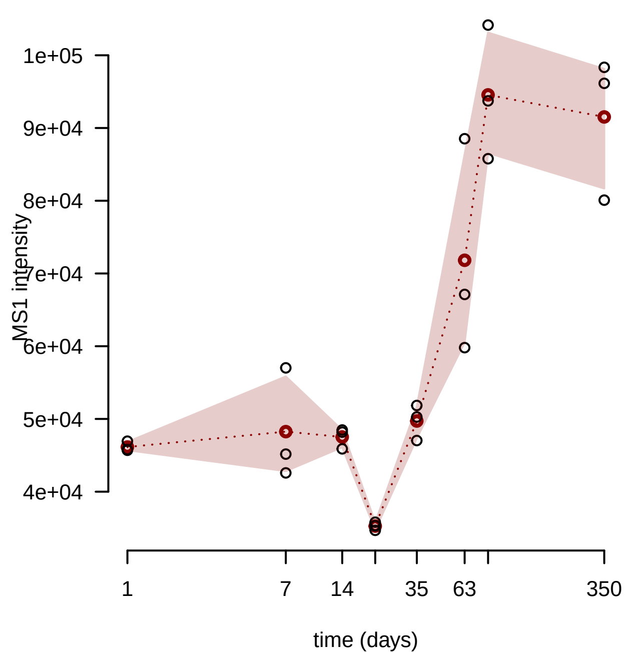

P37172
GN=Acvr1
activin A receptor, type 1

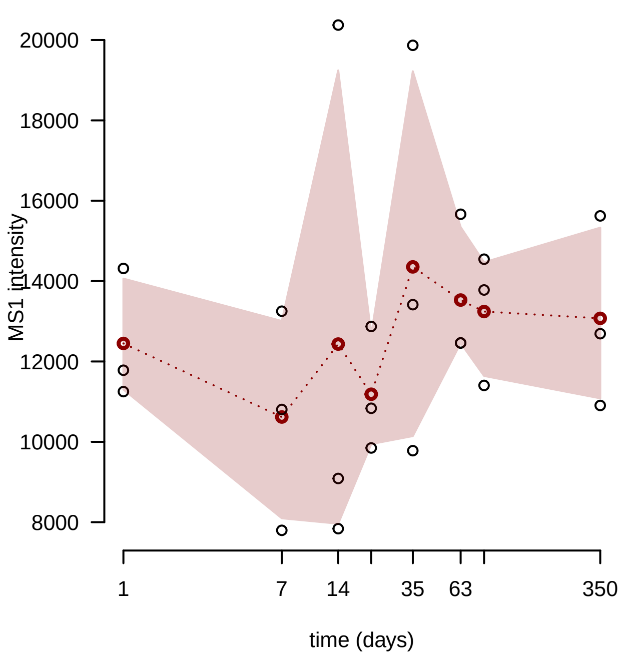

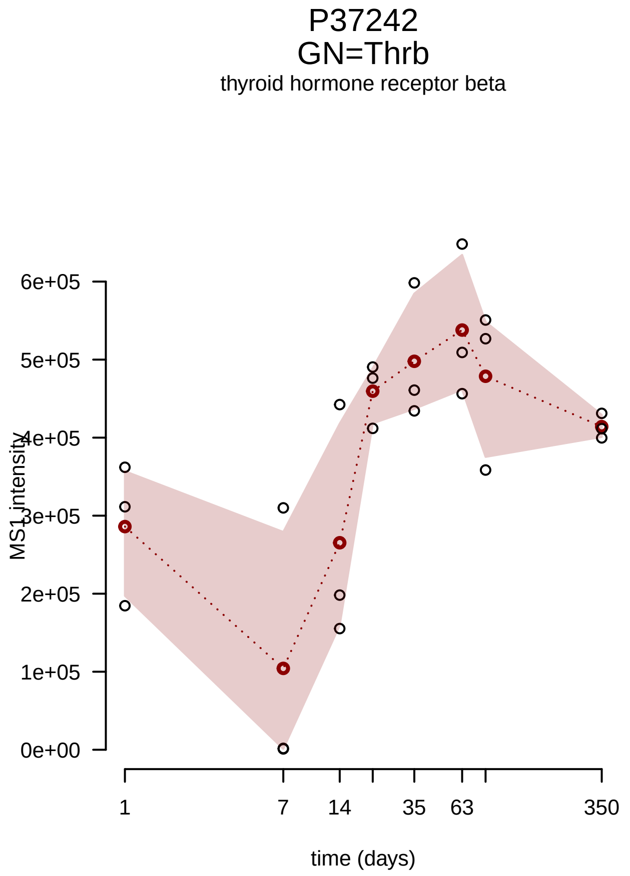
P37242
GN=Thrb
thyroid hormone receptor beta
MS1 intensity
0e+00
1e+05
2e+05
3e+05
4e+05
5e+05
6e+05
1
7
14
35
63
350
time (days)

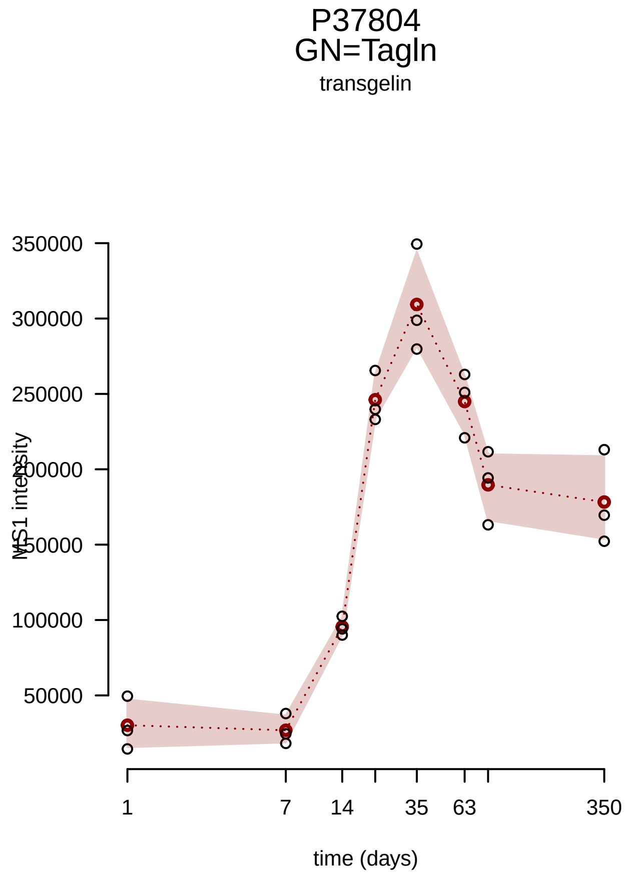
P37804
GN=Tagln
transgelin
MS1 intensity
50000
100000
150000
200000
250000
300000
350000
1
7
14
35
63
350
time (days)

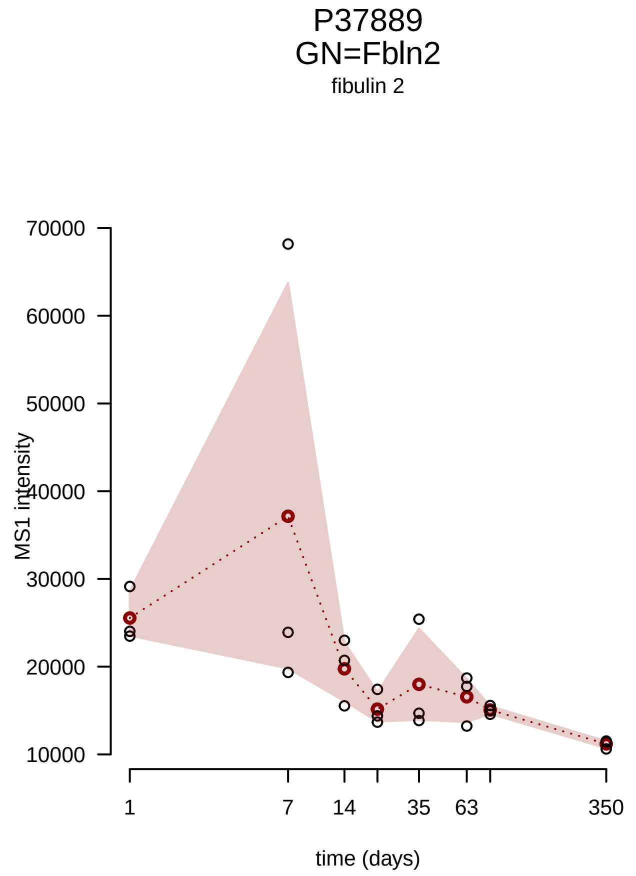
P37889
GN=Fbln2
fibulin 2
MS1 intensity
10000
20000
30000
40000
50000
60000
70000
1
7
14
35
63
350
time (days)

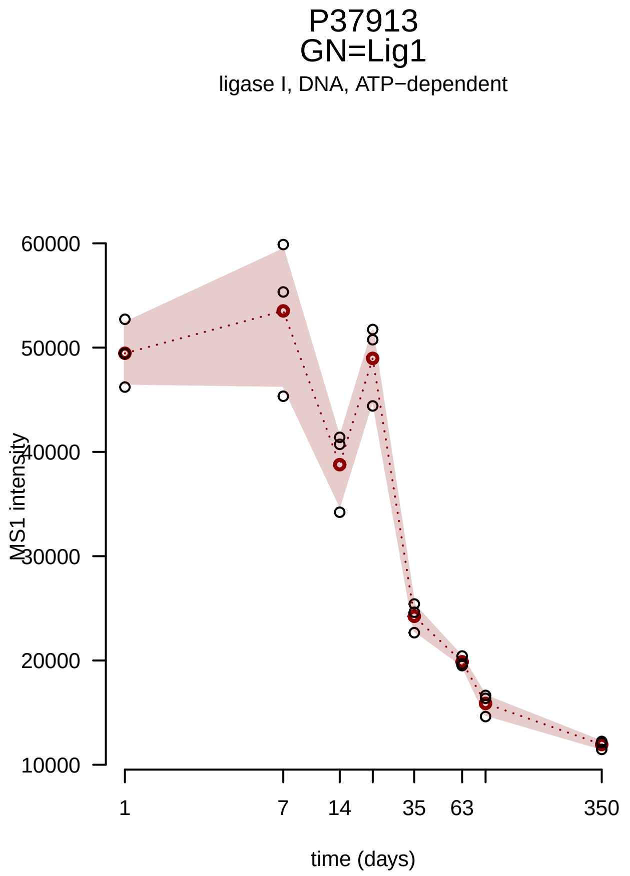
P37913
GN=Lig1
ligase I, DNA, ATP−dependent
MS1 intensity
10000
20000
30000
40000
50000
60000
1
7
14
35
63
350
time (days)

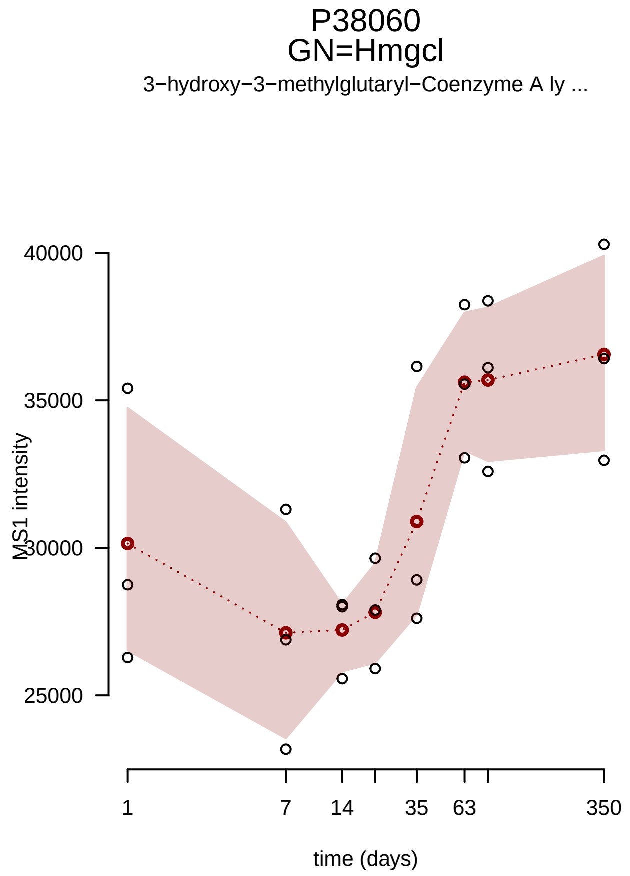
P38060
GN=Hmgcl
3−hydroxy−3−methylglutaryl−Coenzyme A ly ...
MS1 intensity
25000
30000
35000
40000
1
7
14
35
63
350
time (days)

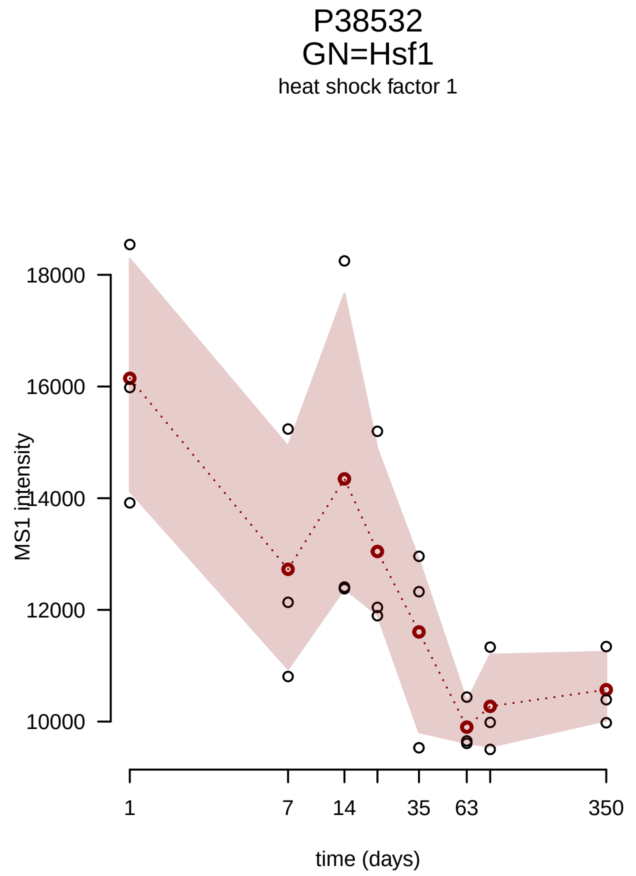
P38532
GN=Hsf1
heat shock factor 1
MS1 intensity
10000
12000
14000
16000
18000
1
7
14
35
63
350
time (days)

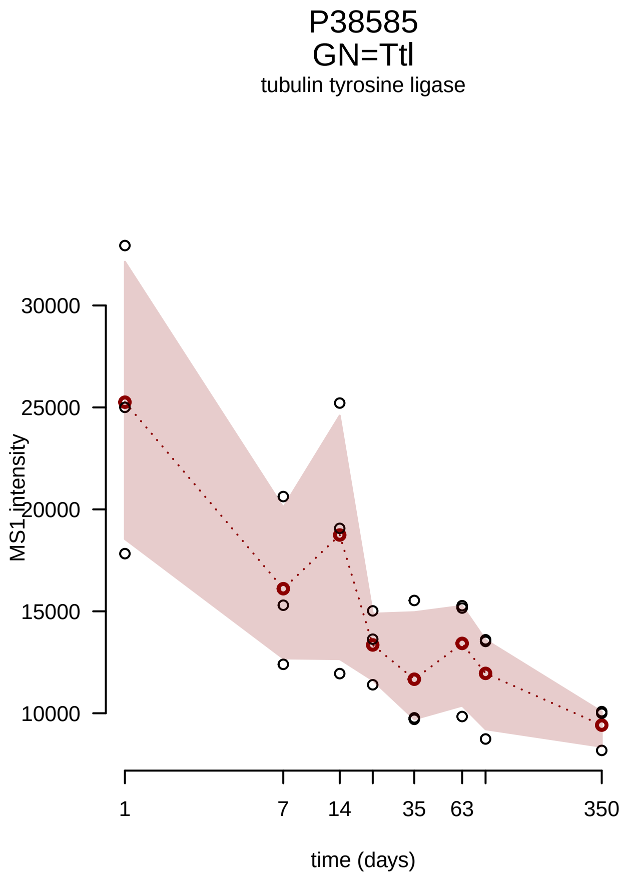
P38585
GN=Ttl
tubulin tyrosine ligase
MS1 intensity
10000
15000
20000
25000
30000
1
7
14
35
63
350
time (days)

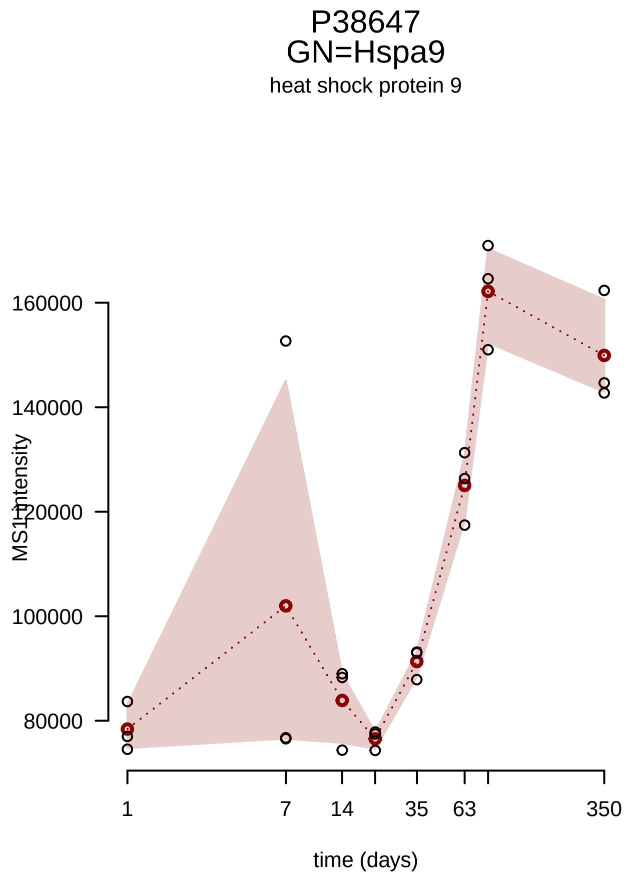
P38647
GN=Hspa9
heat shock protein 9
MS1 intensity
80000
100000
120000
140000
160000
1
7
14
35
63
350
time (days)

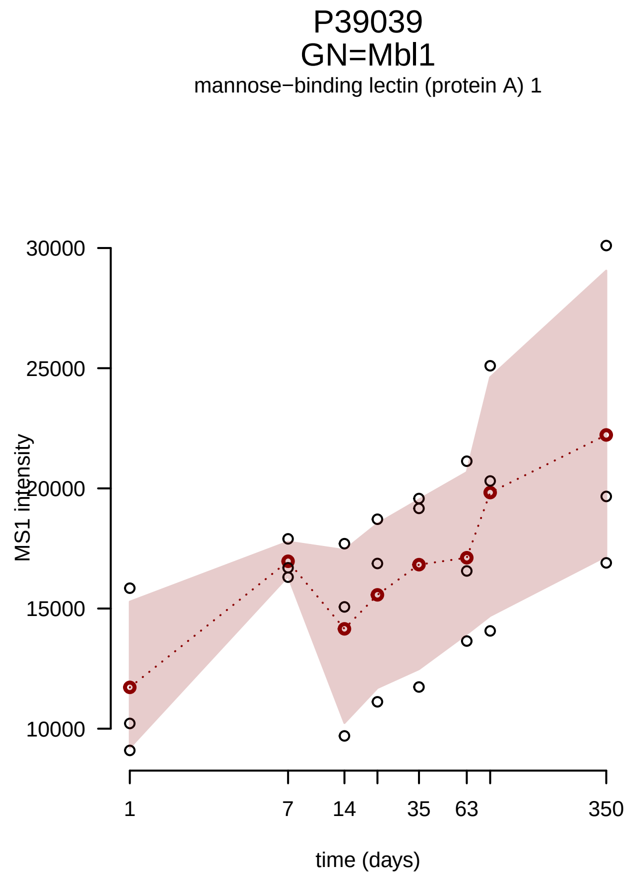
P39039
GN=Mbl1
mannose−binding lectin (protein A) 1
MS1 intensity
10000
15000
20000
25000
30000
1
7
14
35
63
350
time (days)

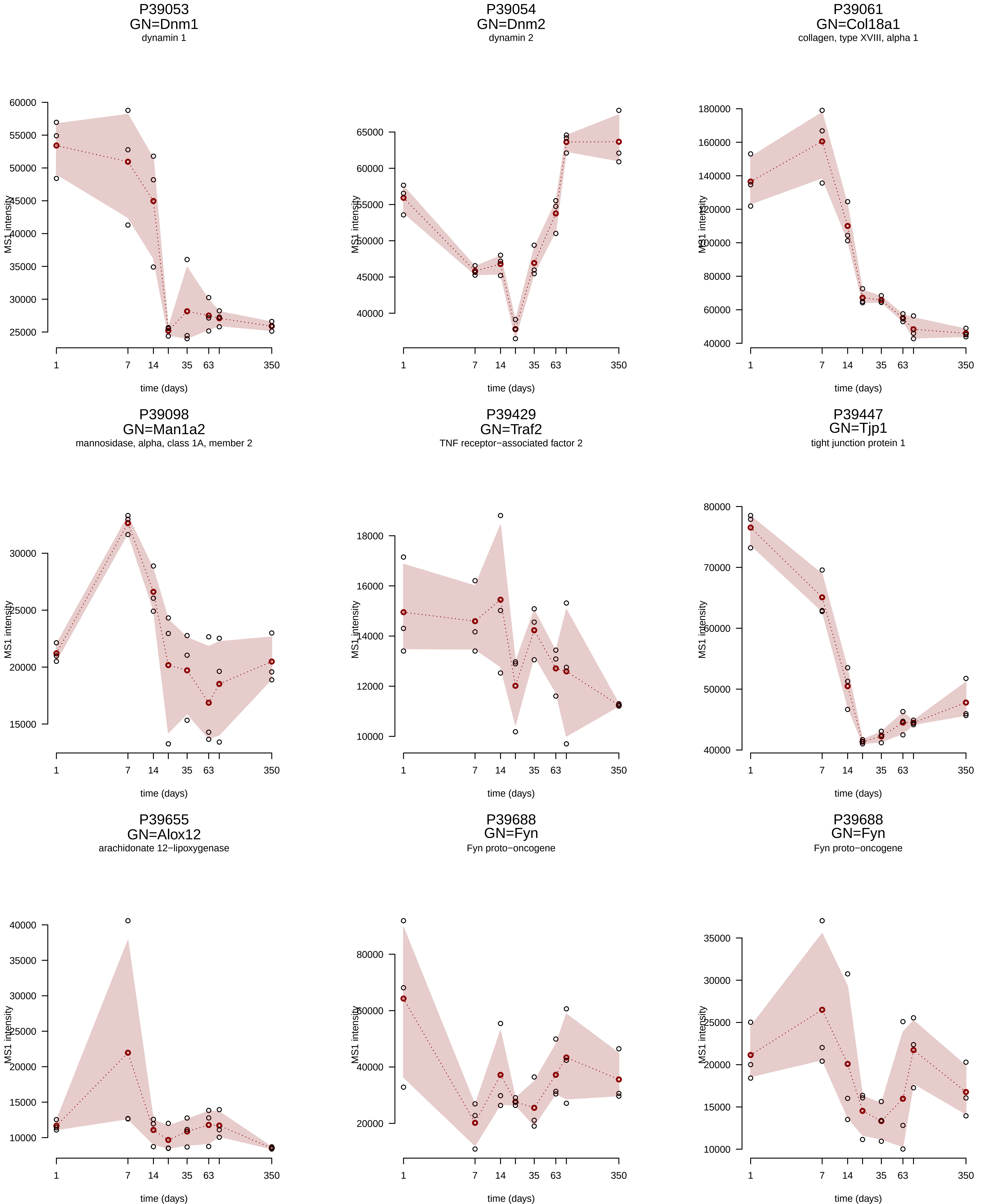
P39053
GN=Dnm1
dynamin 1
60000
55000
50000
45000
40000
35000
30000
25000
MS1 intensity
1
7
14
35
63
350
time (days)
P39054
GN=Dnm2
dynamin 2
65000
60000
55000
50000
45000
40000
MS1 intensity
1
7
14
35
63
350
time (days)
P39061
GN=Col18a1
collagen, type XVIII, alpha 1
180000
160000
140000
120000
100000
80000
60000
40000
MS1 intensity
1
7
14
35
63
350
time (days)
P39098
GN=Man1a2
mannosidase, alpha, class 1A, member 2
30000
25000
20000
15000
MS1 intensity
1
7
14
35
63
350
time (days)
P39429
GN=Traf2
TNF receptor−associated factor 2
18000
16000
14000
12000
10000
MS1 intensity
1
7
14
35
63
350
time (days)
P39447
GN=Tjp1
tight junction protein 1
80000
70000
60000
50000
40000
MS1 intensity
1
7
14
35
63
350
time (days)
P39655
GN=Alox12
arachidonate 12−lipoxygenase
40000
35000
30000
25000
20000
15000
10000
MS1 intensity
1
7
14
35
63
350
time (days)
P39688
GN=Fyn
Fyn proto−oncogene
80000
60000
40000
20000
MS1 intensity
1
7
14
35
63
350
time (days)
P39688
GN=Fyn
Fyn proto−oncogene
35000
30000
25000
20000
15000
10000
MS1 intensity
1
7
14
35
63
350
time (days)

P39749
GN=Fen1
flap structure specific endonuclease 1

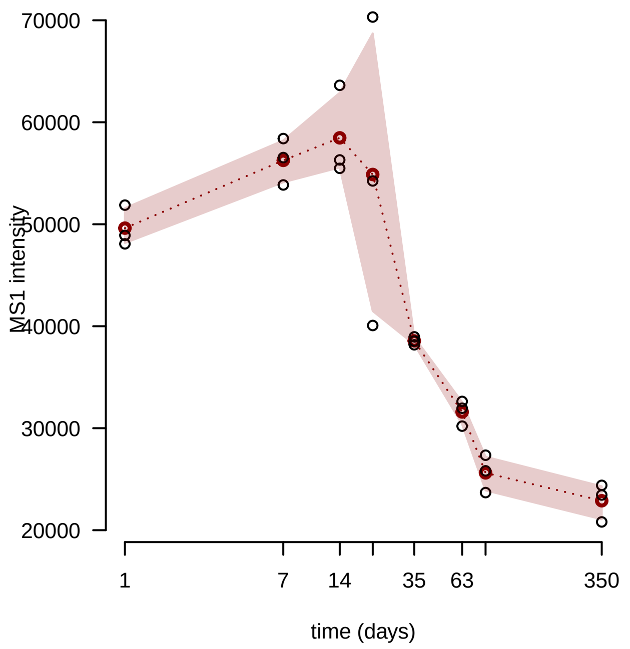

P39876
GN=Timp3
tissue inhibitor of metalloproteinase 3

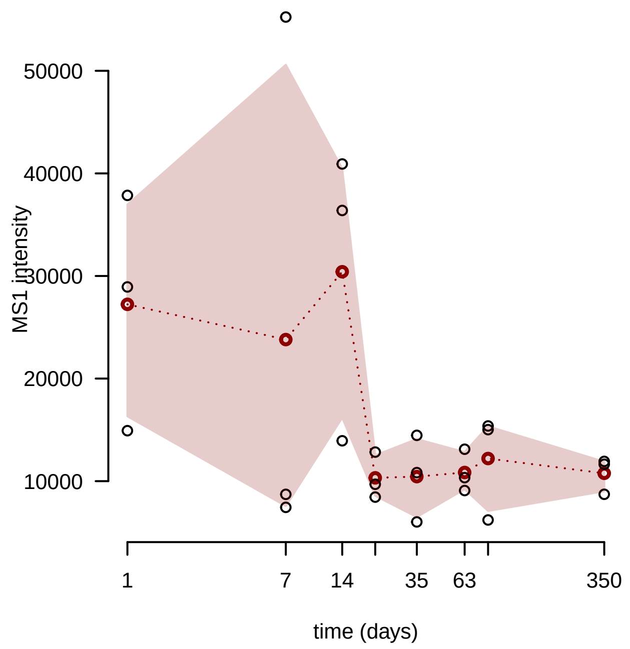

P40124
GN=Cap1
CAP, adenylate cyclase−associated protei ...

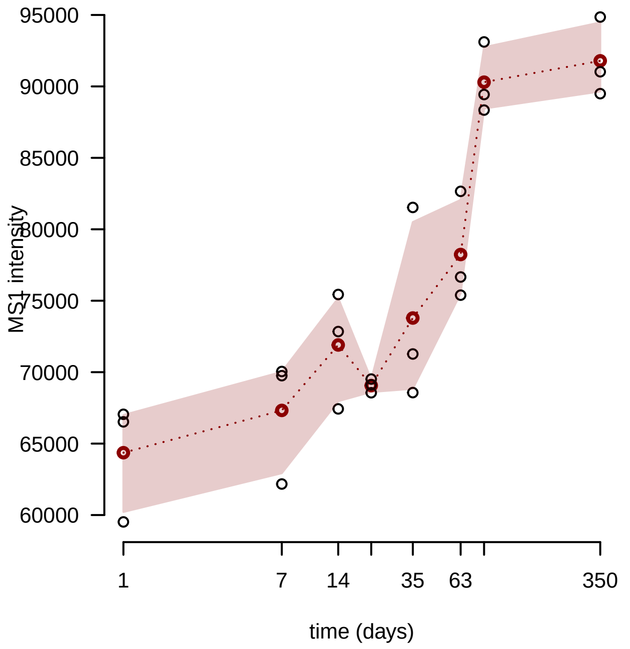

P40142
GN=Tkt
transketolase

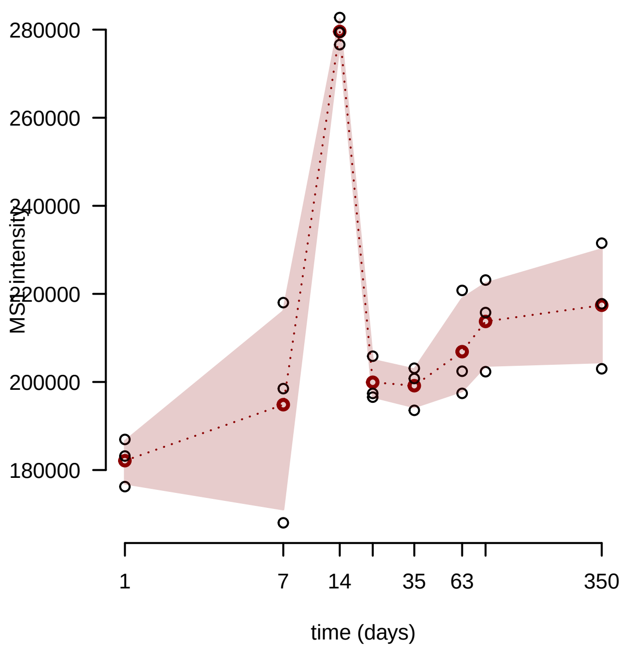

P40201
GN=Chd1
chromodomain helicase DNA binding protei ...

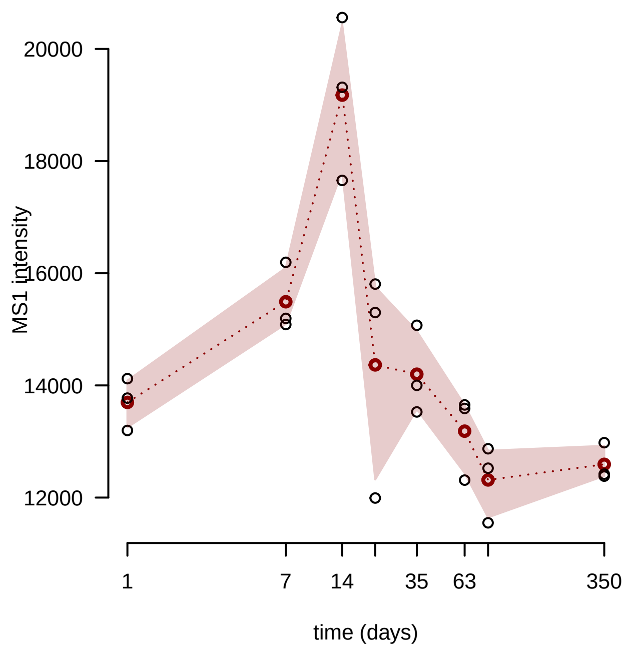

P40237
GN=Cd82
CD82 antigen

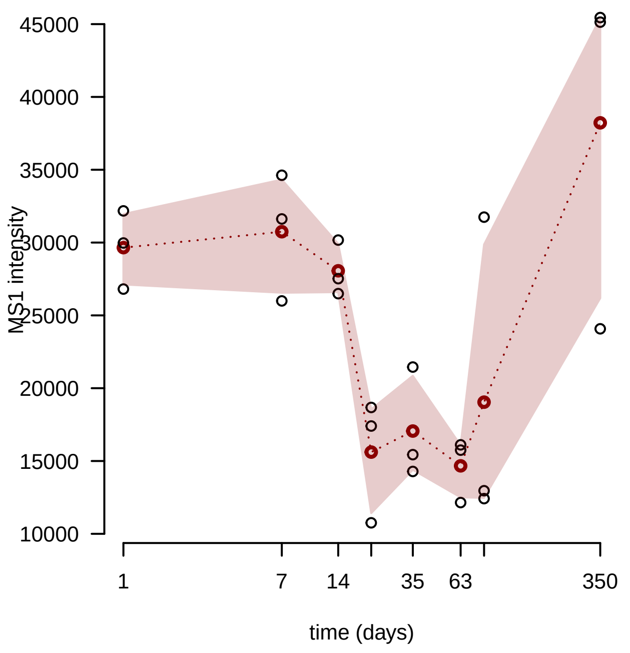

P40240
GN=Cd9
CD9 antigen

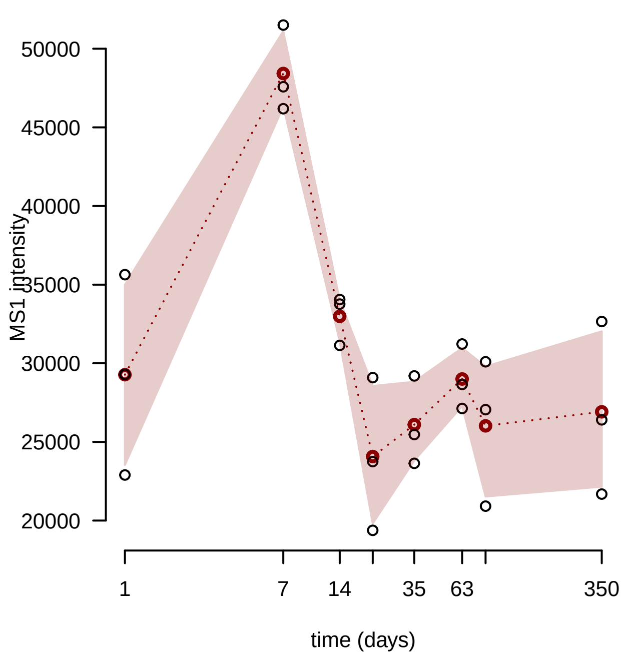

P40336
GN=Vps26a
VPS26 retromer complex component A

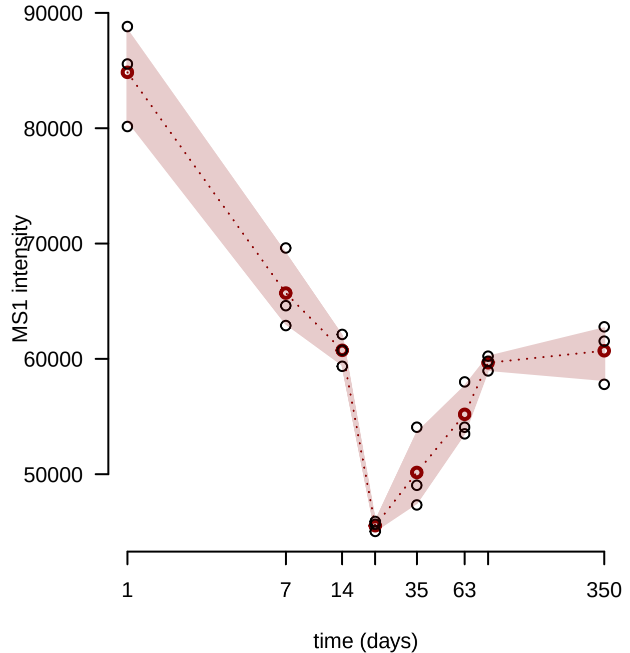

P40338
GN=Vhl
von Hippel−Lindau tumor suppressor

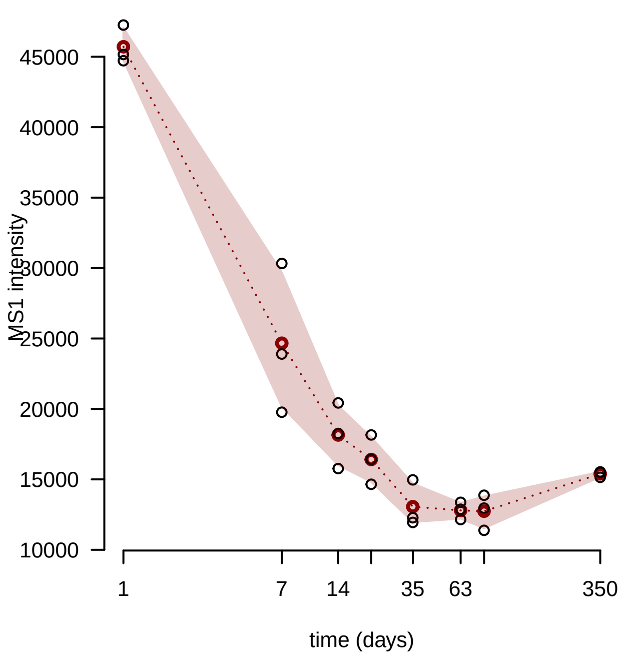

P40630
GN=Tfam
transcription factor A, mitochondrial

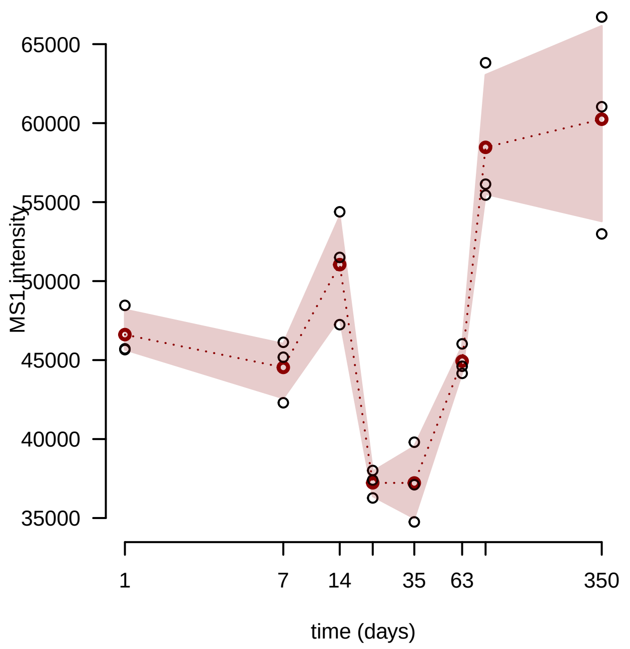

P40694
GN=Ighmbp2
immunoglobulin mu binding protein 2

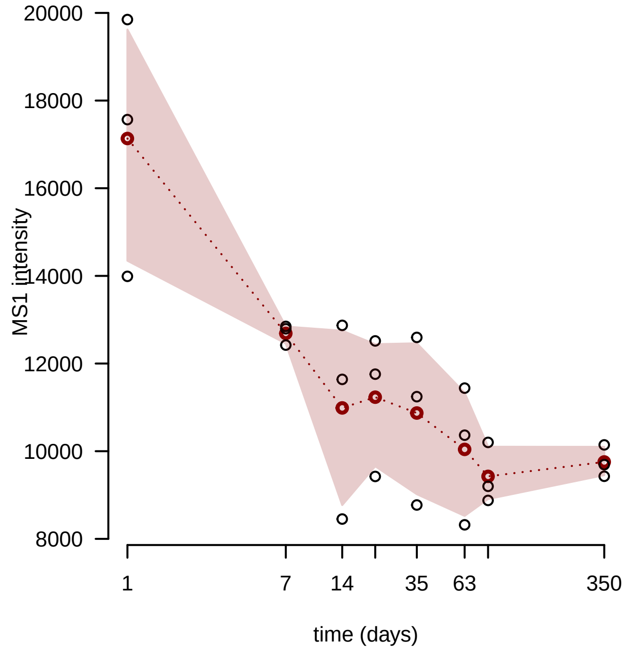

P40935
GN=Pnmt
phenylethanolamine−N−methyltransferase

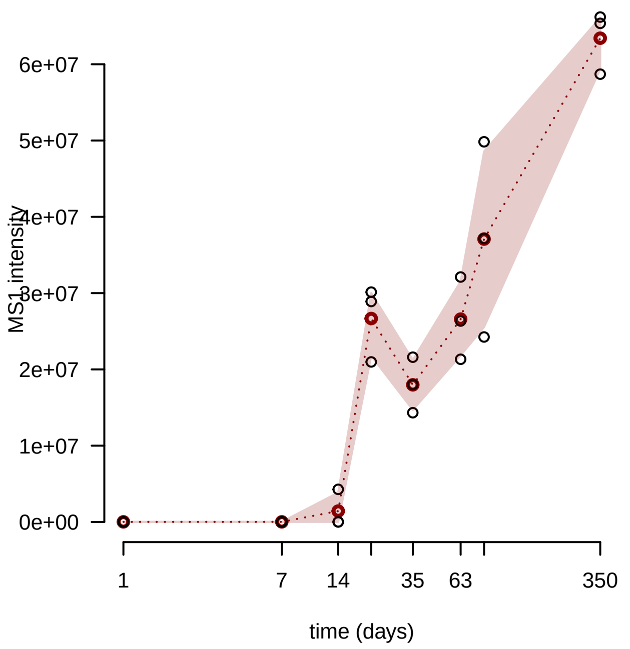

P41105
GN=Rpl28
ribosomal protein L28

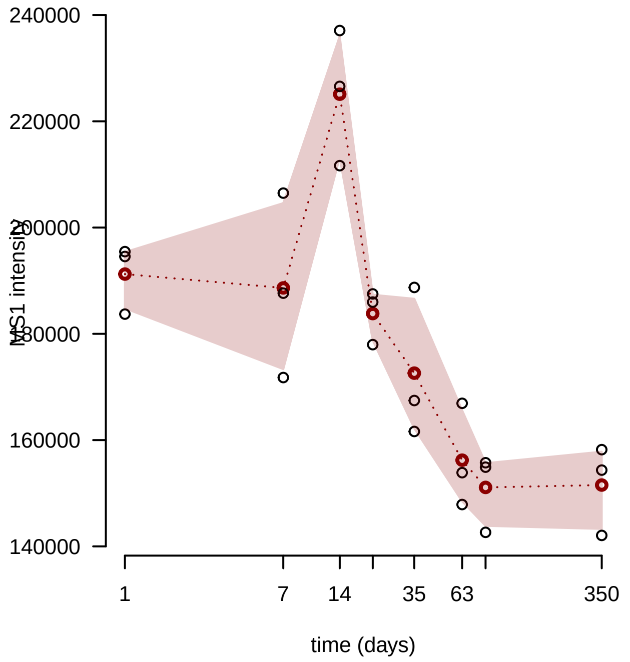

P41216
GN=Acsl1
acyl−CoA synthetase long−chain family me ...

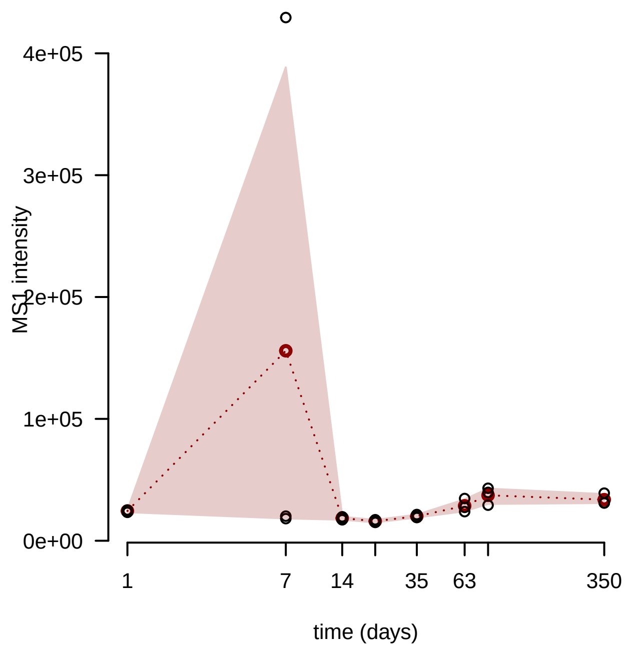

P41230
GN=Kdm5c
lysine (K)−specific demethylase 5C

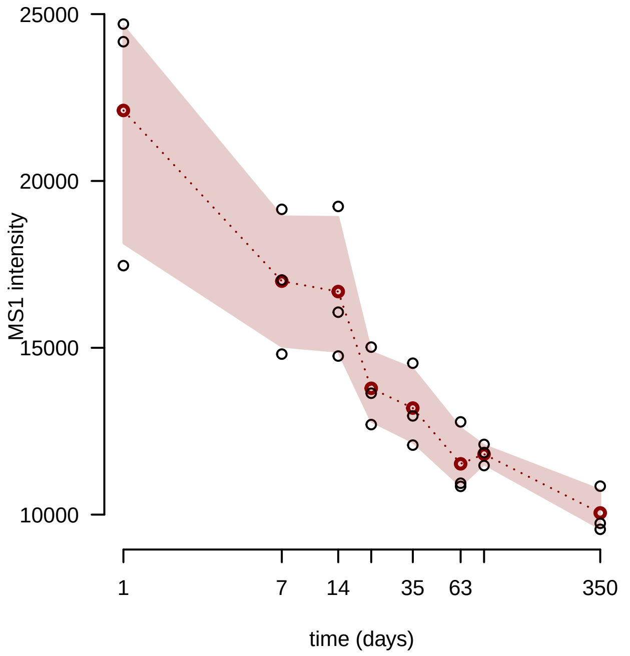

P41233
GN=Abca1
ATP−binding cassette, sub−family A (ABC1 ...

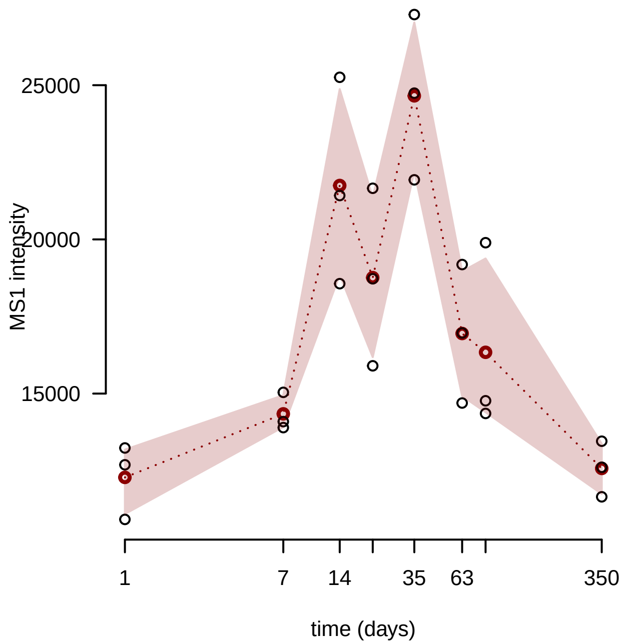

P41234
GN=Abca2
ATP−binding cassette, sub−family A (ABC1 ...

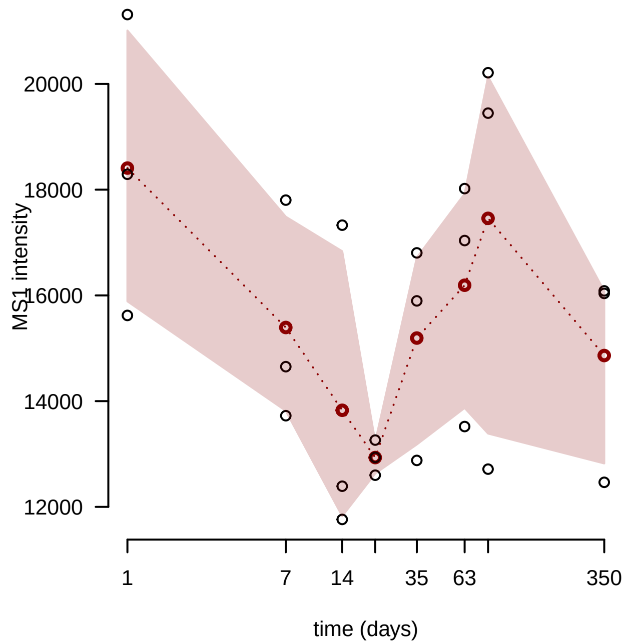

P41241
GN=Csk
c−src tyrosine kinase

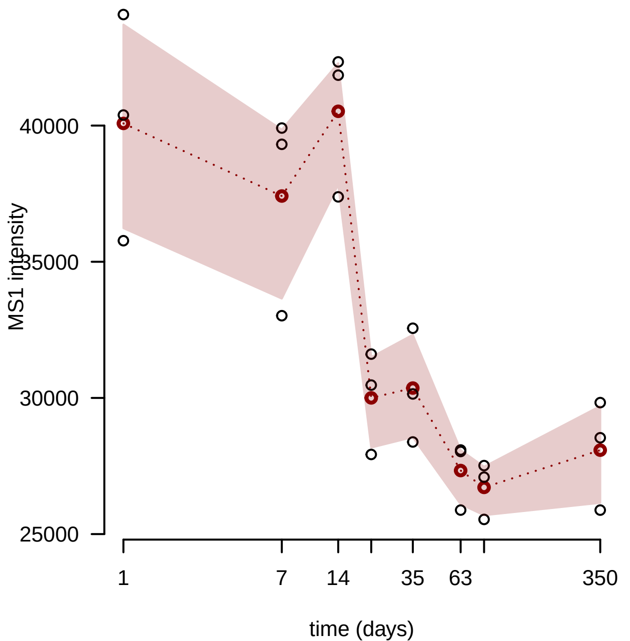

P41317
GN=Mbl2
mannose−binding lectin (protein C) 2

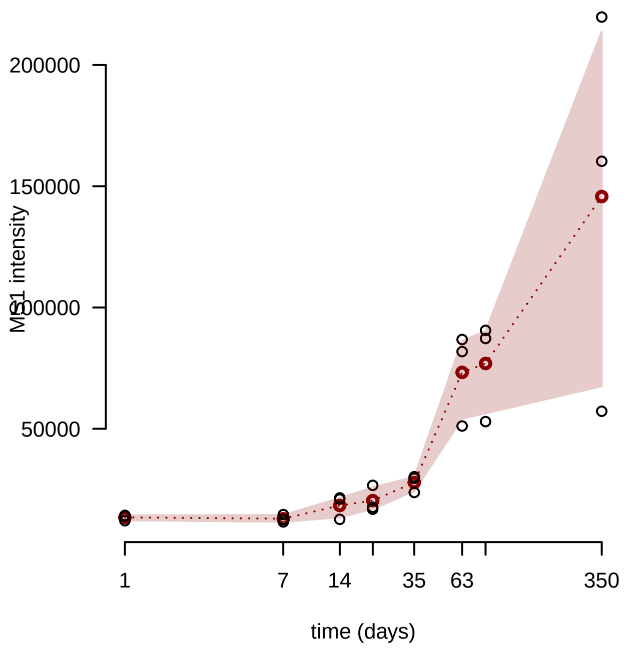

P41731
GN=Cd63
CD63 antigen

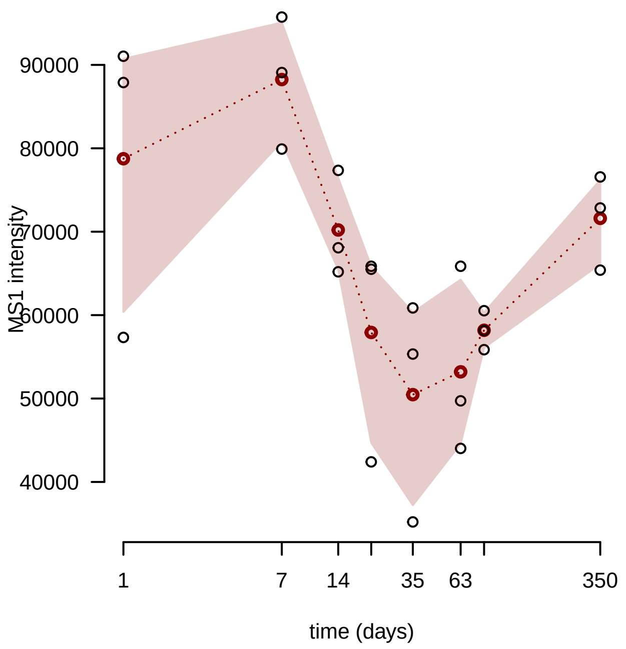

P41778
GN=Pbx1
pre B cell leukemia homeobox 1

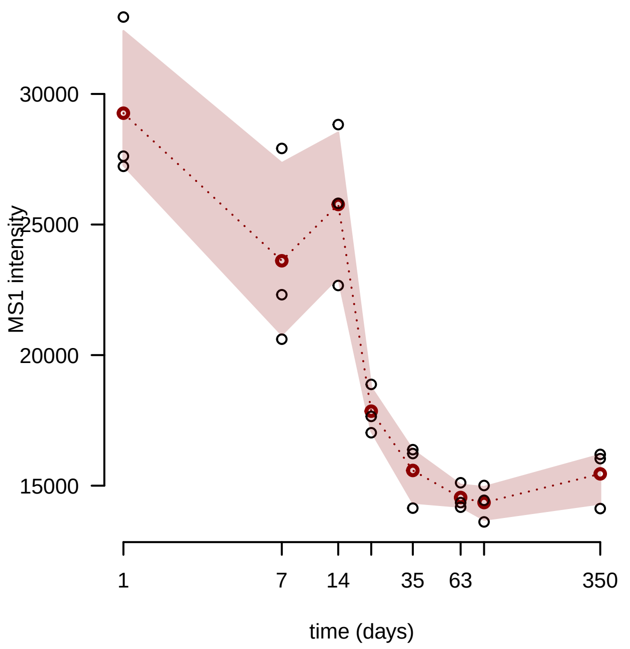

P41969
GN=Elk1
ELK1, member of ETS oncogene family

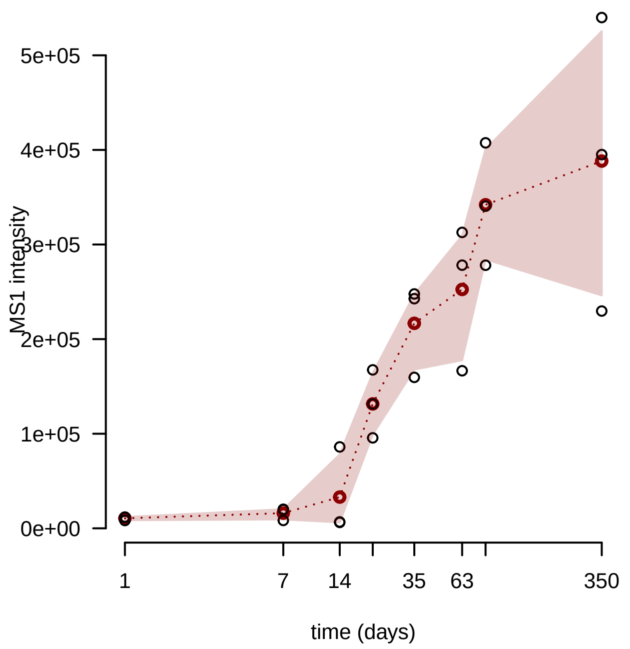

P41971
GN=Elk3
ELK3, member of ETS oncogene family

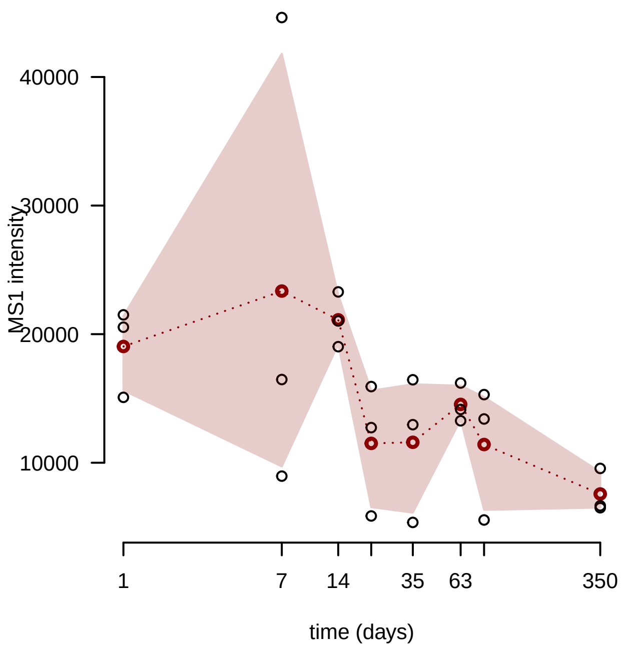

P42125
GN=Eci1
enoyl−Coenzyme A delta isomerase 1

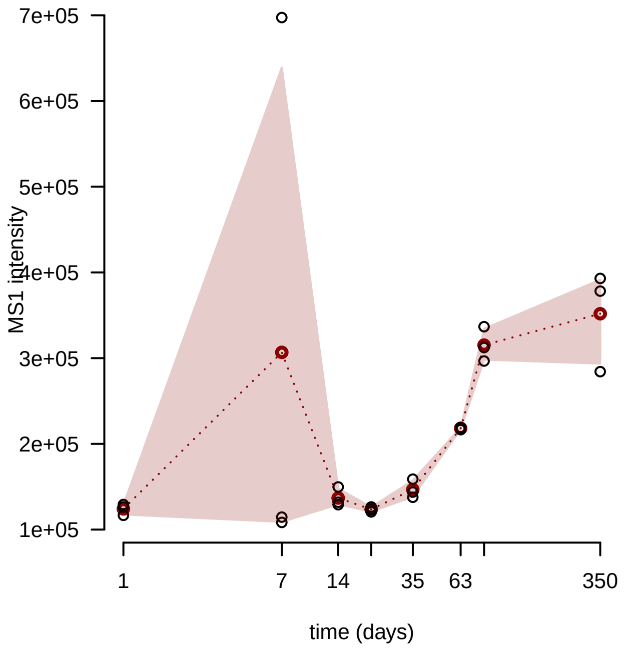

P42128
GN=Foxk1
forkhead box K1

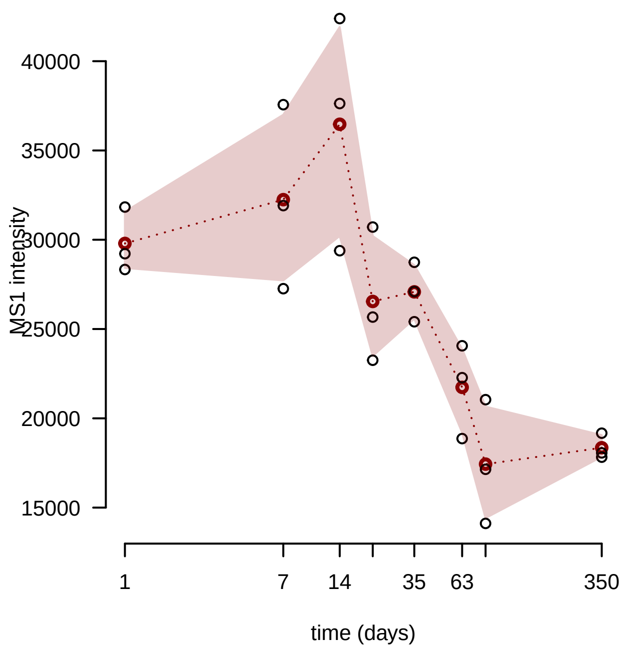

P42208
GN=Sept2

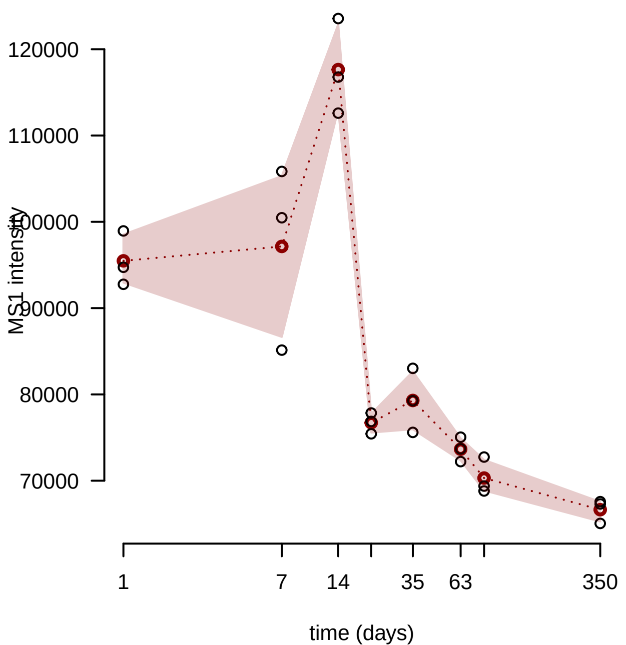

P42209
GN=Sept1

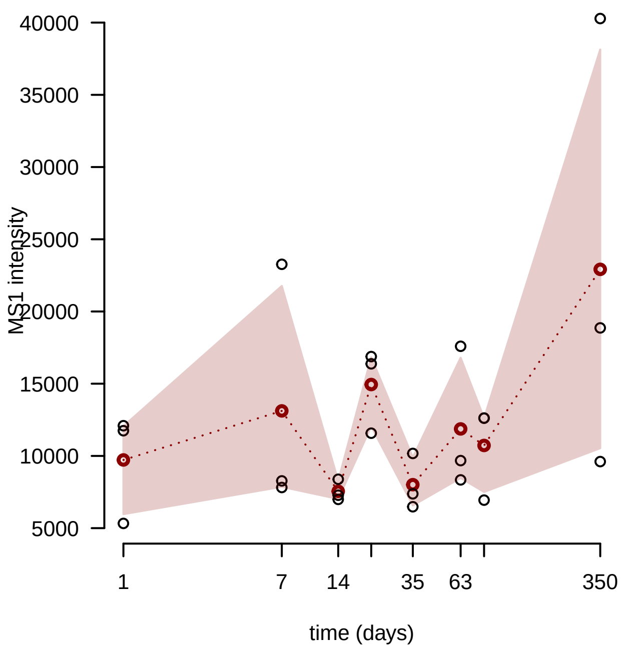

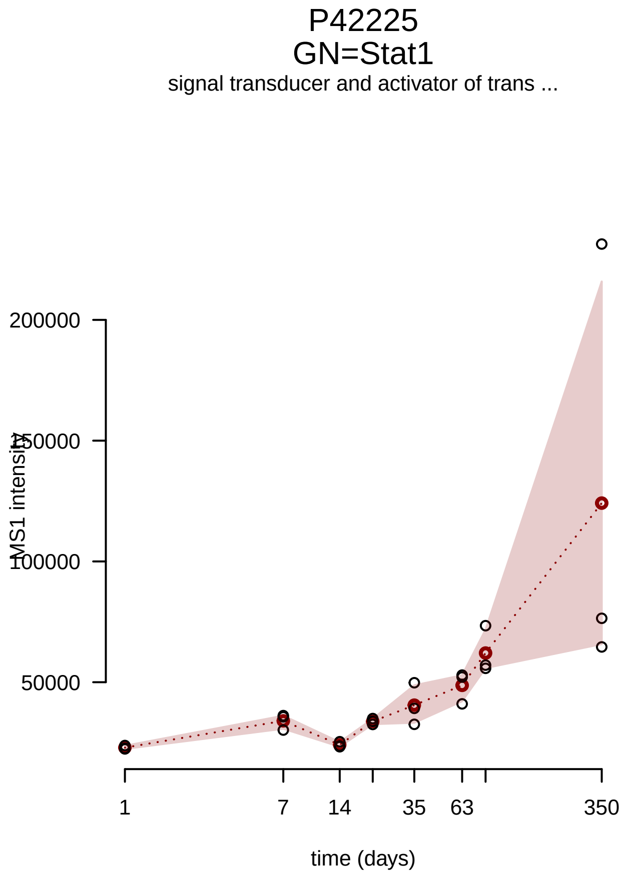
P42225
GN=Stat1
signal transducer and activator of trans ...
MS1 intensity
200000
150000
100000
50000
1
7
14
35
63
350
time (days)

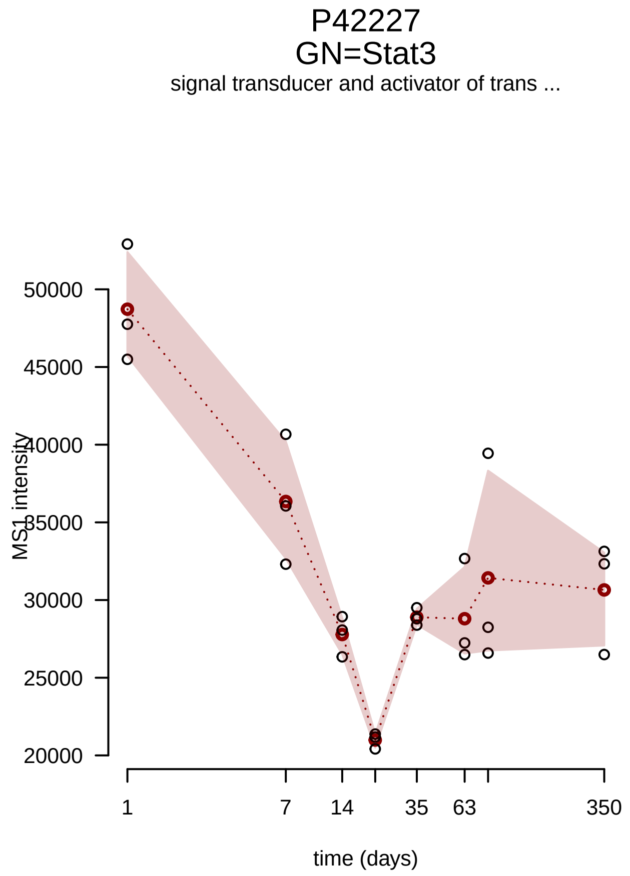
P42227
GN=Stat3
signal transducer and activator of trans ...
MS1 intensity
50000
45000
40000
35000
30000
25000
20000
1
7
14
35
63
350
time (days)

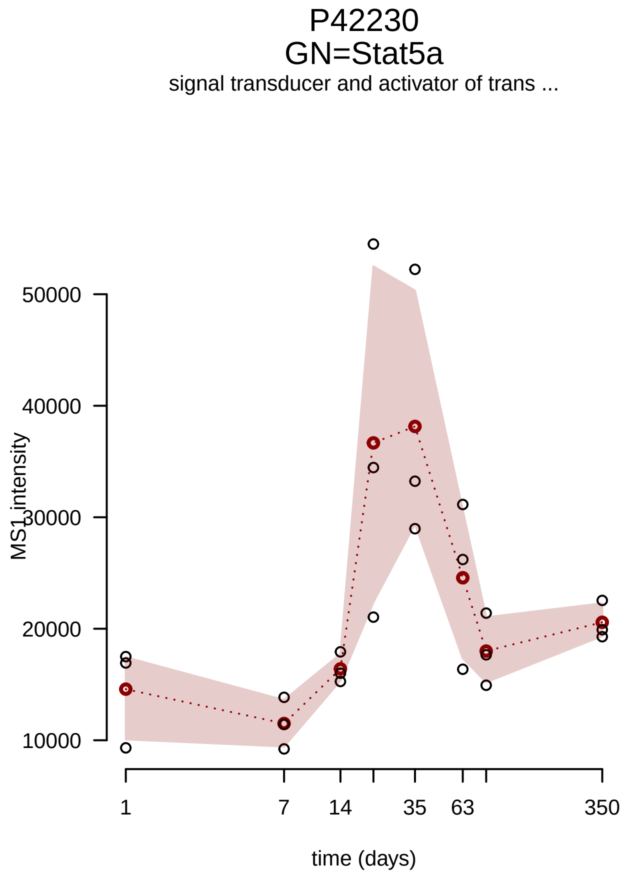
P42230
GN=Stat5a
signal transducer and activator of trans ...
MS1 intensity
50000
40000
30000
20000
10000
1
7
14
35
63
350
time (days)

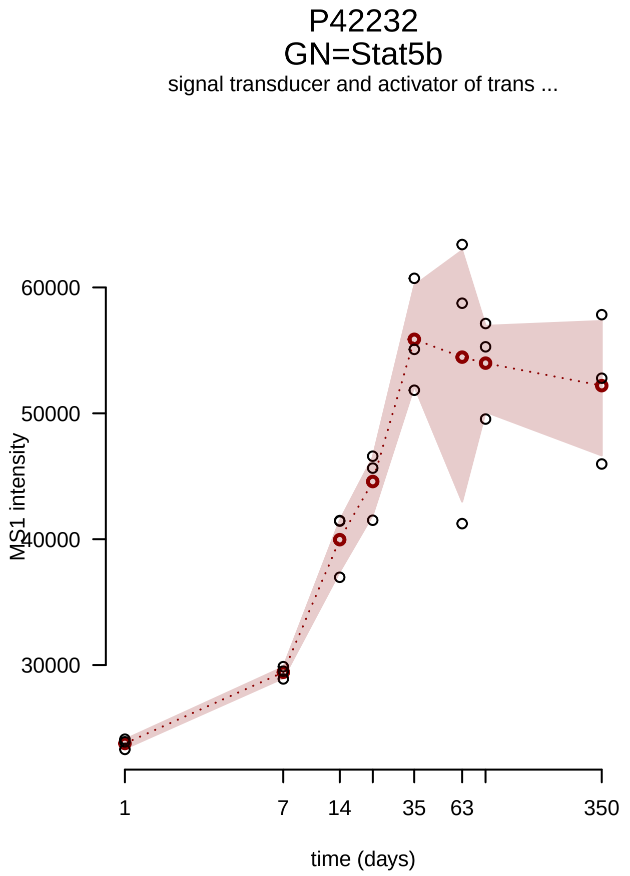
P42232
GN=Stat5b
signal transducer and activator of trans ...
MS1 intensity
60000
50000
40000
30000
1
7
14
35
63
350
time (days)

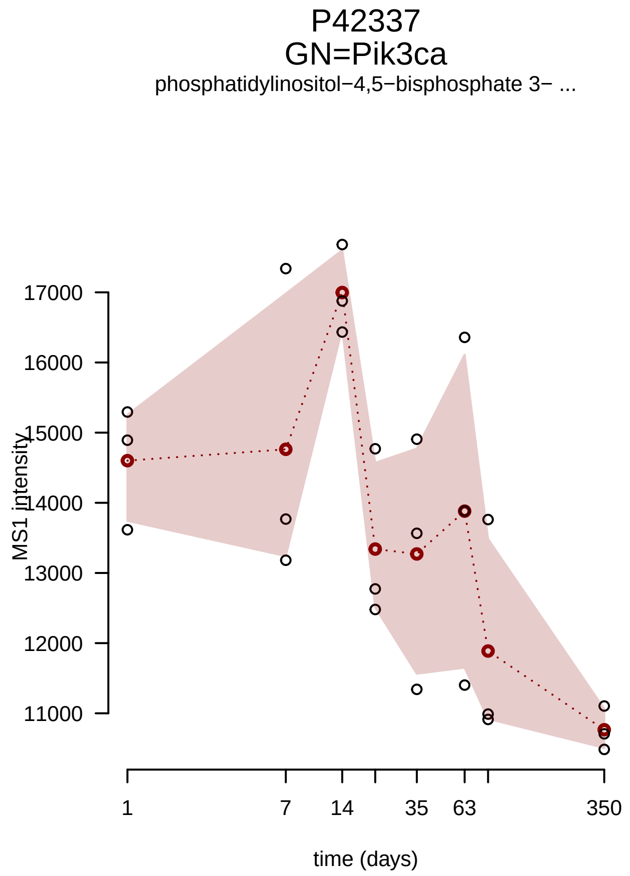
P42337
GN=Pik3ca
phosphatidylinositol−4,5−bisphosphate 3− ...
MS1 intensity
17000
16000
15000
14000
13000
12000
11000
1
7
14
35
63
350
time (days)

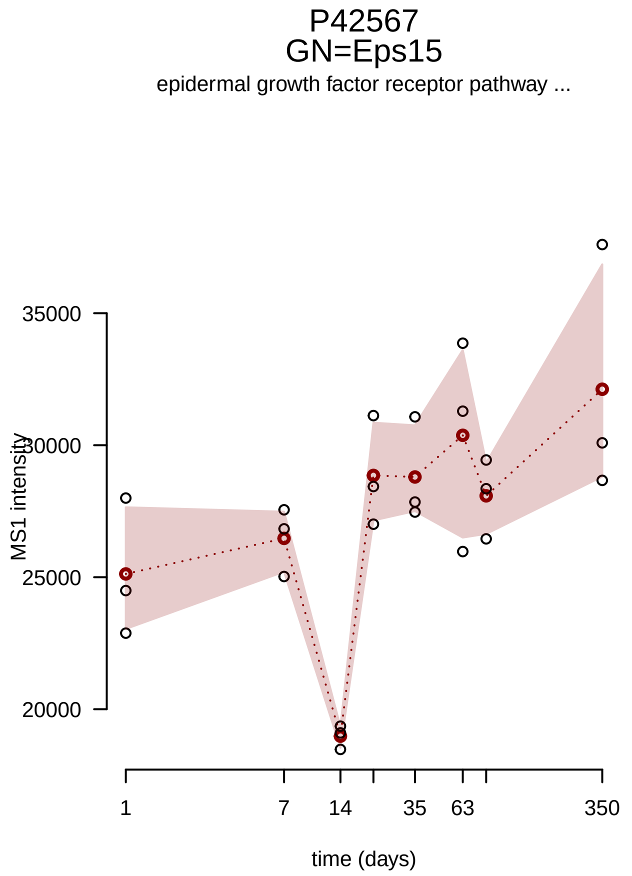
P42567
GN=Eps15
epidermal growth factor receptor pathway ...
MS1 intensity
35000
30000
25000
20000
1
7
14
35
63
350
time (days)

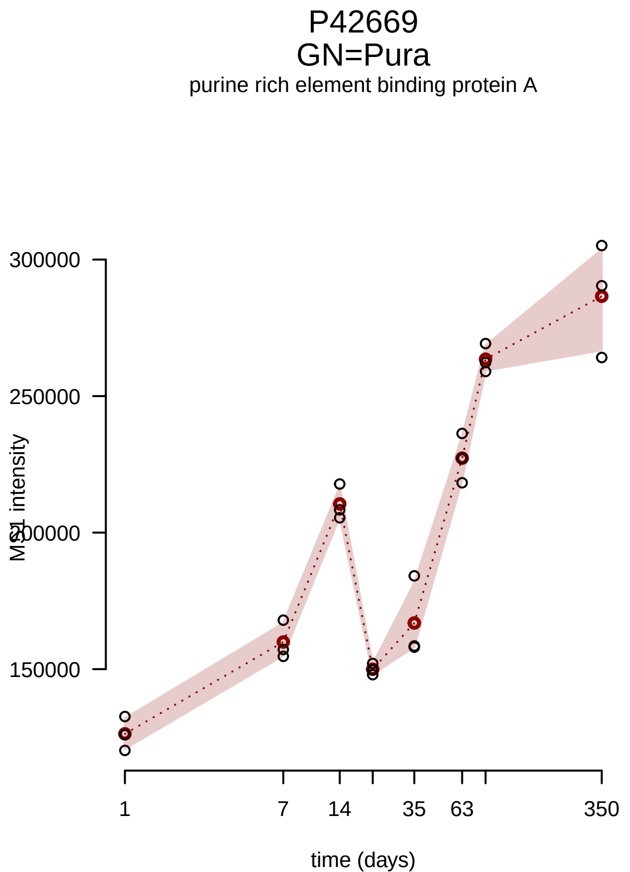
P42669
GN=Pura
purine rich element binding protein A
MS1 intensity
300000
250000
200000
150000
1
7
14
35
63
350
time (days)

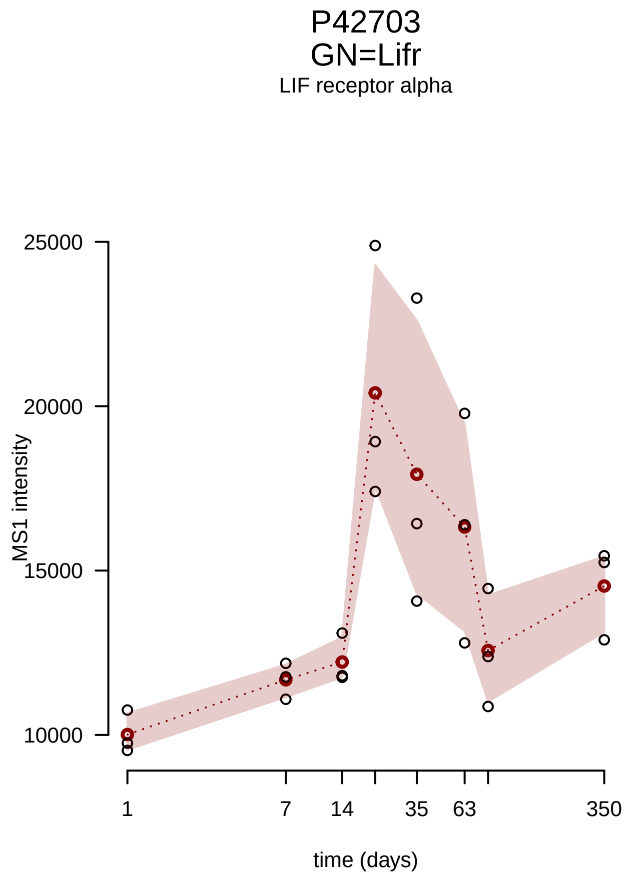
P42703
GN=Lifr
LIF receptor alpha
MS1 intensity
25000
20000
15000
10000
1
7
14
35
63
350
time (days)

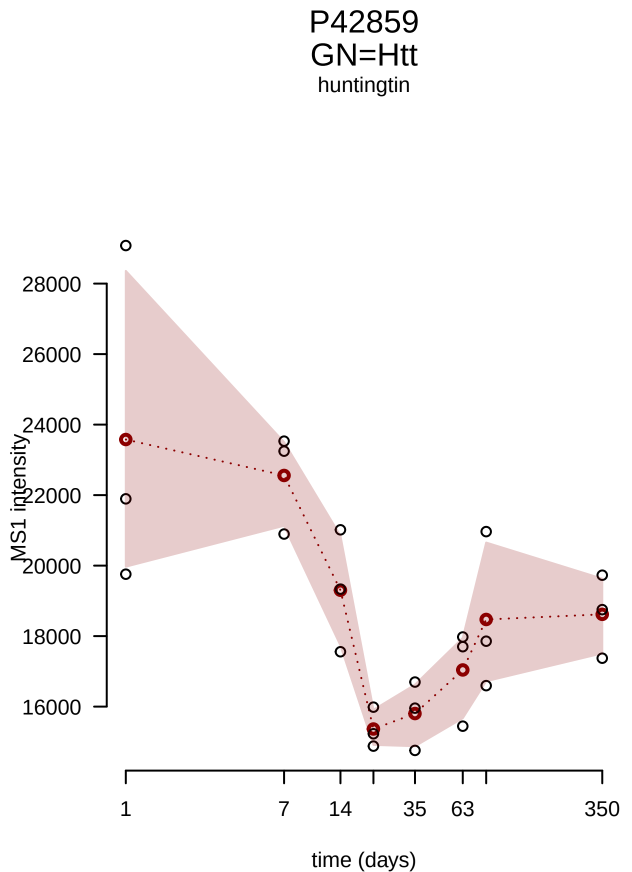
P42859
GN=Htt
huntingtin
MS1 intensity
28000
26000
24000
22000
20000
18000
16000
1
7
14
35
63
350
time (days)

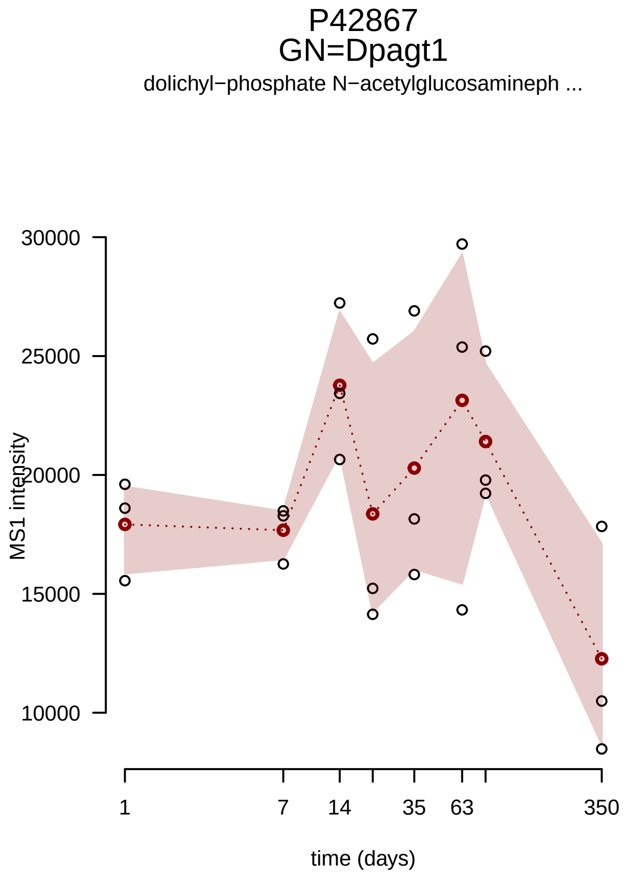
P42867
GN=Dpagt1
dolichyl−phosphate N−acetylglucosamineph ...
MS1 intensity
10000
15000
20000
25000
30000
1
7
14
35
63
350
time (days)

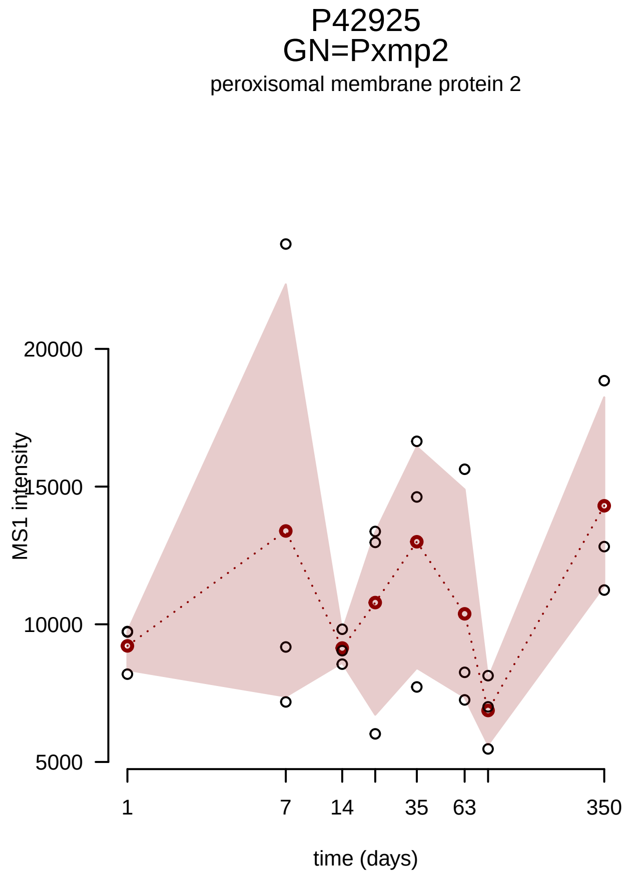
P42925
GN=Pxmp2
peroxisomal membrane protein 2
MS1 intensity
5000
10000
15000
20000
1
7
14
35
63
350
time (days)

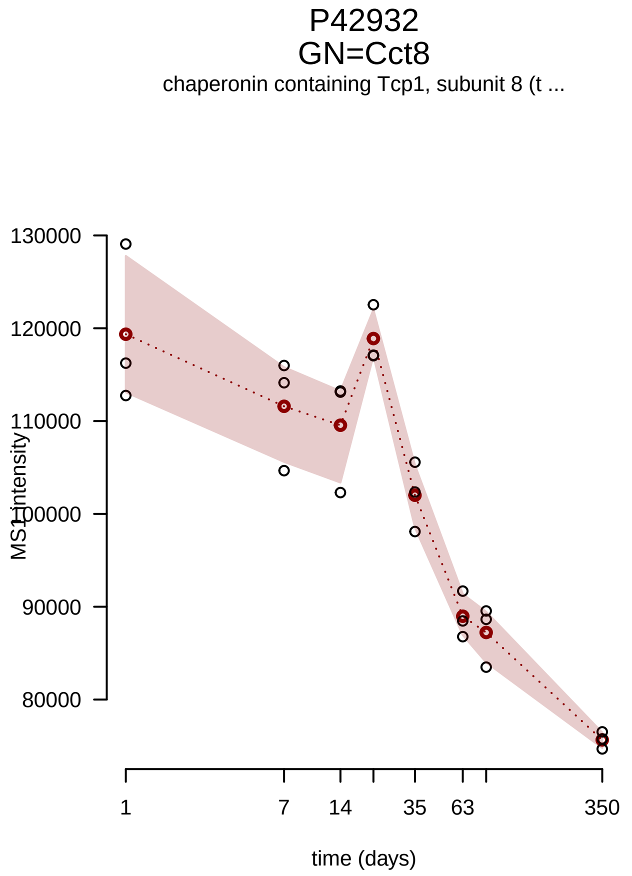
P42932
GN=Cct8
chaperonin containing Tcp1, subunit 8 (t ...
MS1 intensity
80000
90000
100000
110000
120000
130000
1
7
14
35
63
350
time (days)

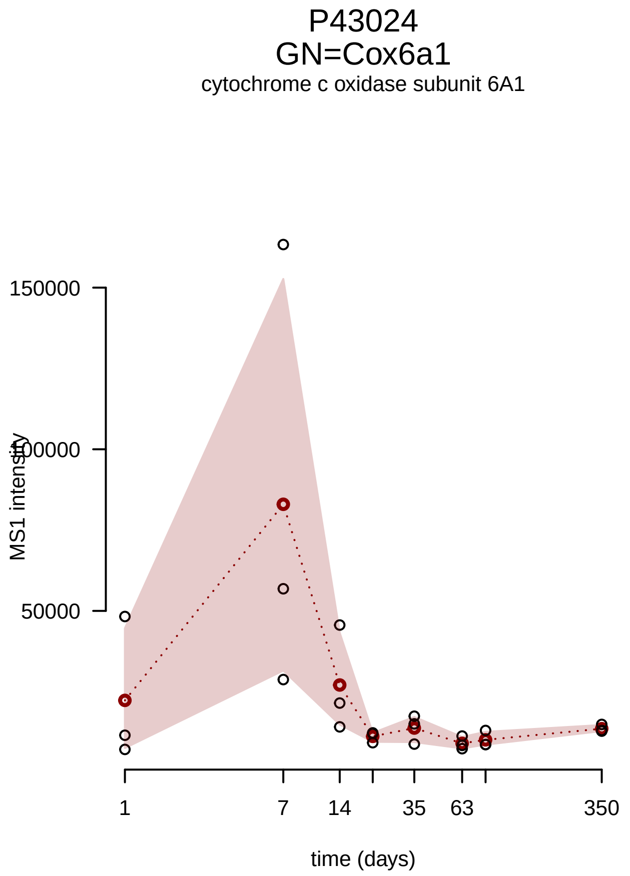
P43024
GN=Cox6a1
cytochrome c oxidase subunit 6A1
MS1 intensity
50000
100000
150000
1
7
14
35
63
350
time (days)

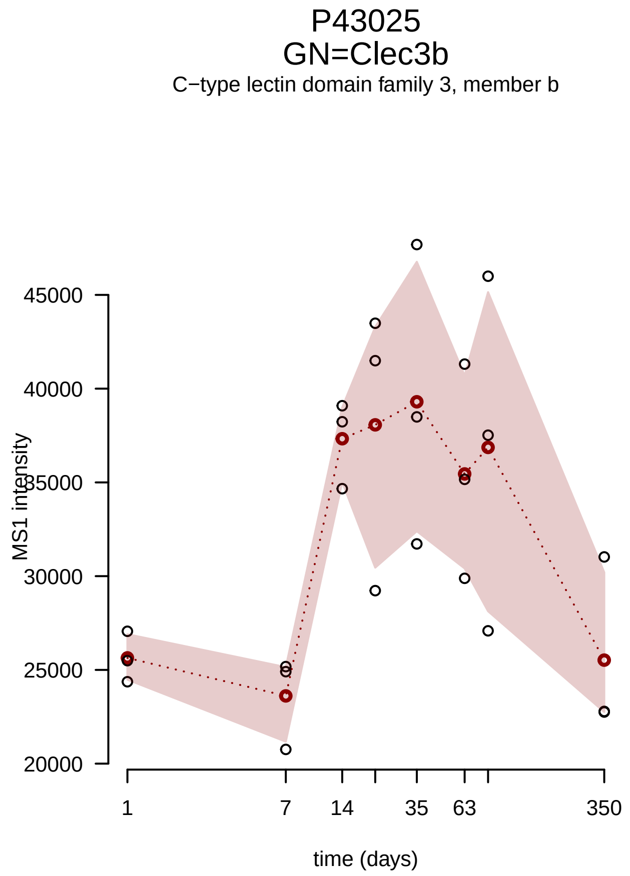
P43025
GN=Clec3b
C−type lectin domain family 3, member b
MS1 intensity
20000
25000
30000
35000
40000
45000
1
7
14
35
63
350
time (days)

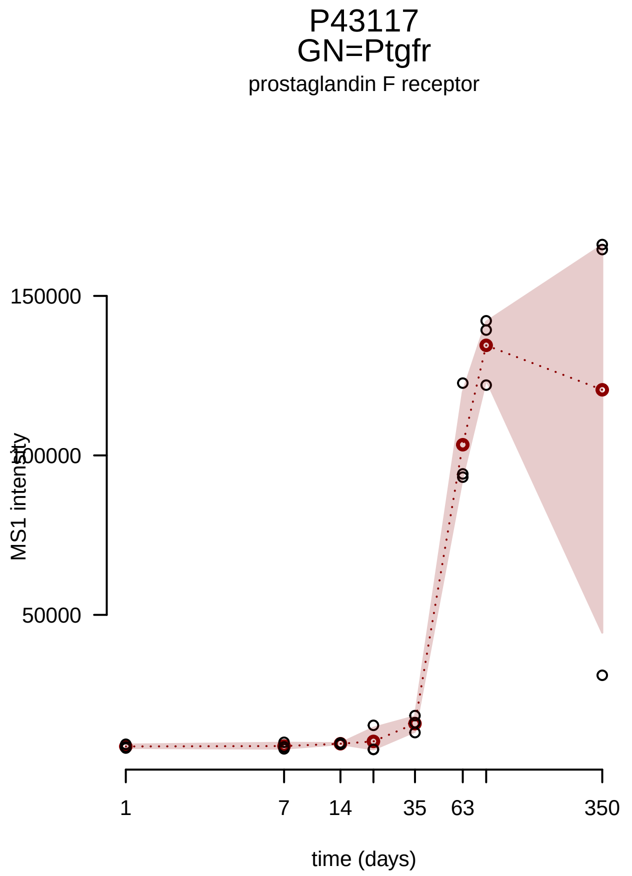
P43117
GN=Ptgfr
prostaglandin F receptor
MS1 intensity
50000
100000
150000
1
7
14
35
63
350
time (days)

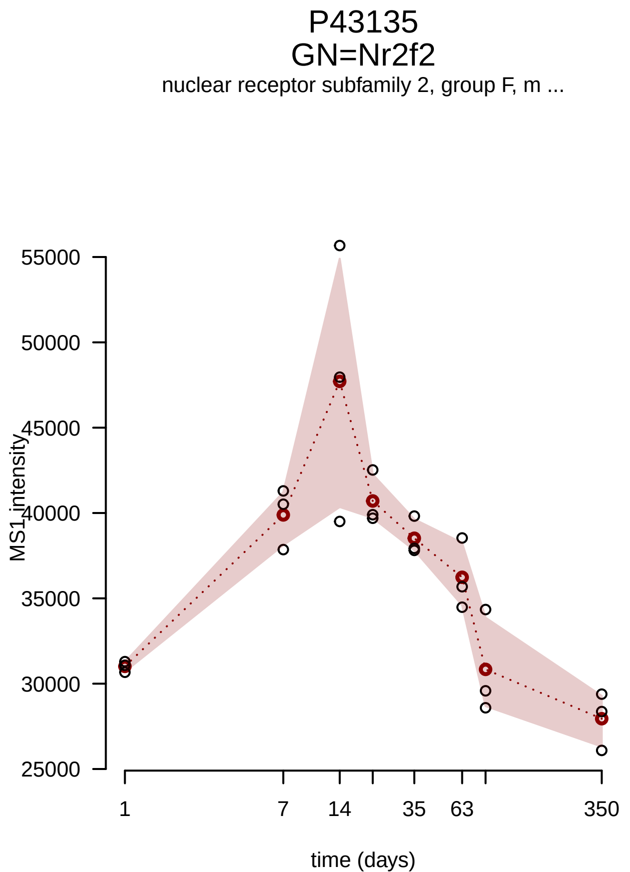
P43135
GN=Nr2f2
nuclear receptor subfamily 2, group F, m ...
MS1 intensity
25000
30000
35000
40000
45000
50000
55000
1
7
14
35
63
350
time (days)

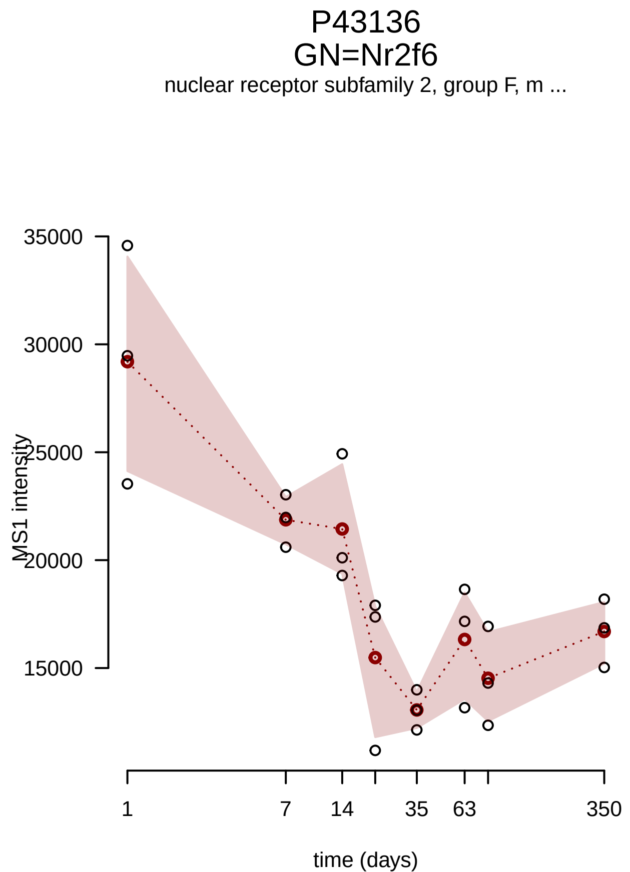
P43136
GN=Nr2f6
nuclear receptor subfamily 2, group F, m ...
MS1 intensity
15000
20000
25000
30000
35000
1
7
14
35
63
350
time (days)

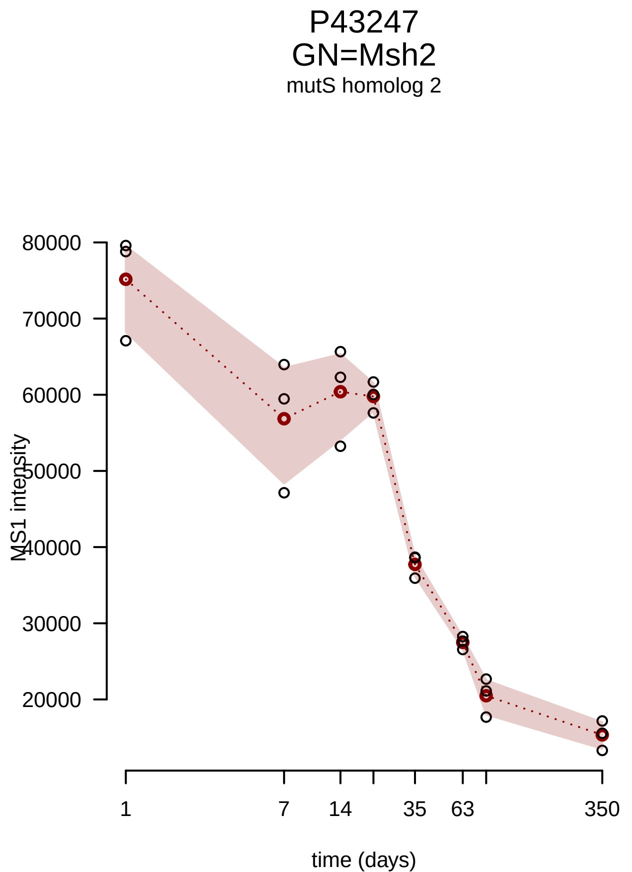
P43247
GN=Msh2
mutS homolog 2
MS1 intensity
20000
30000
40000
50000
60000
70000
80000
1
7
14
35
63
350
time (days)

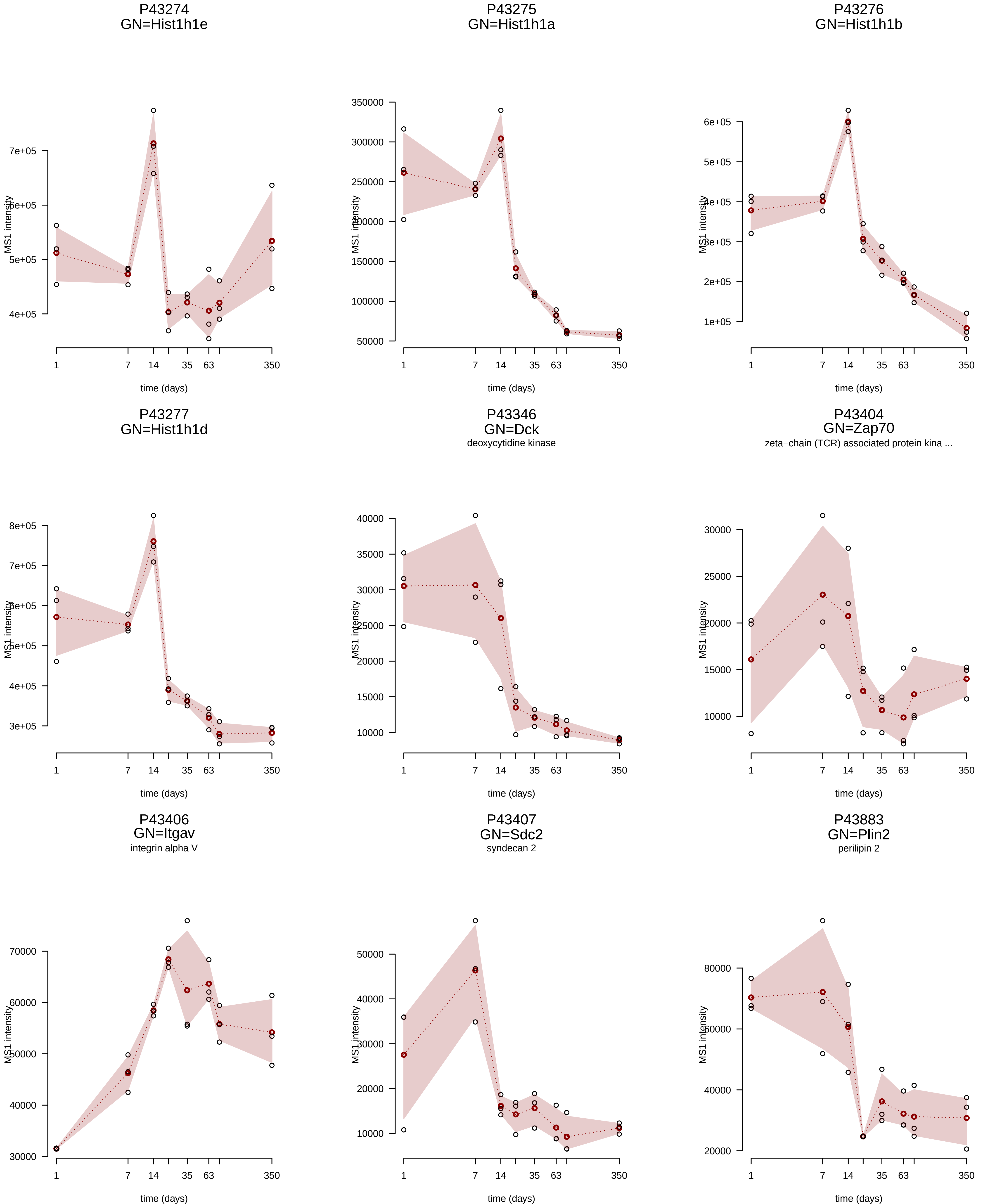
P43274
GN=Hist1h1e
MS1 intensity
7e+05
6e+05
5e+05
4e+05
time (days)
1
7
14
35
63
350
P43275
GN=Hist1h1a
MS1 intensity
350000
300000
250000
200000
150000
100000
50000
time (days)
P43276
GN=Hist1h1b
MS1 intensity
6e+05
5e+05
4e+05
3e+05
2e+05
1e+05
time (days)
P43277
GN=Hist1h1d
MS1 intensity
8e+05
7e+05
6e+05
5e+05
4e+05
3e+05
time (days)
P43346
GN=Dck
deoxycytidine kinase
MS1 intensity
40000
35000
30000
25000
20000
15000
10000
time (days)
P43404
GN=Zap70
zeta−chain (TCR) associated protein kina ...
MS1 intensity
30000
25000
20000
15000
10000
time (days)
P43406
GN=Itgav
integrin alpha V
MS1 intensity
70000
60000
50000
40000
30000
time (days)
P43407
GN=Sdc2
syndecan 2
MS1 intensity
50000
40000
30000
20000
10000
time (days)
P43883
GN=Plin2
perilipin 2
MS1 intensity
80000
60000
40000
20000
time (days)

P45376
GN=Akr1b1

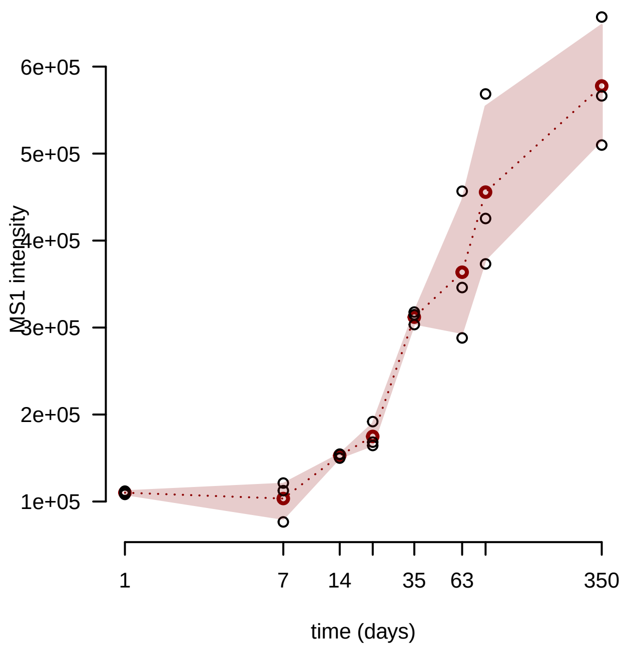

P45377
GN=Akr1b8
aldo−keto reductase family 1, member B8

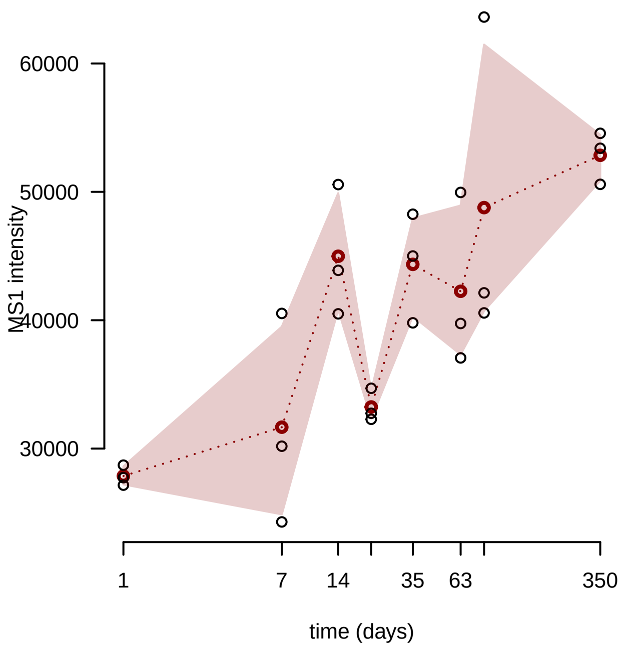

P45448
GN=Nr5a2
nuclear receptor subfamily 5, group A, m ...

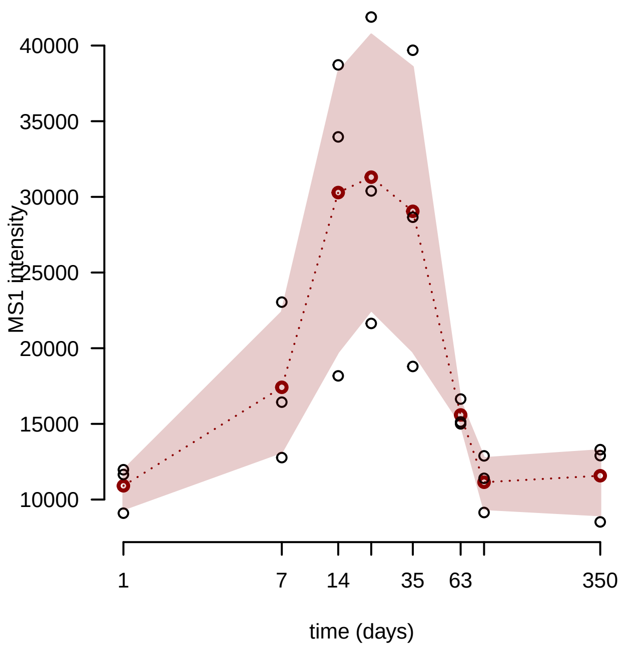

P45481
GN=Crebbp
CREB binding protein

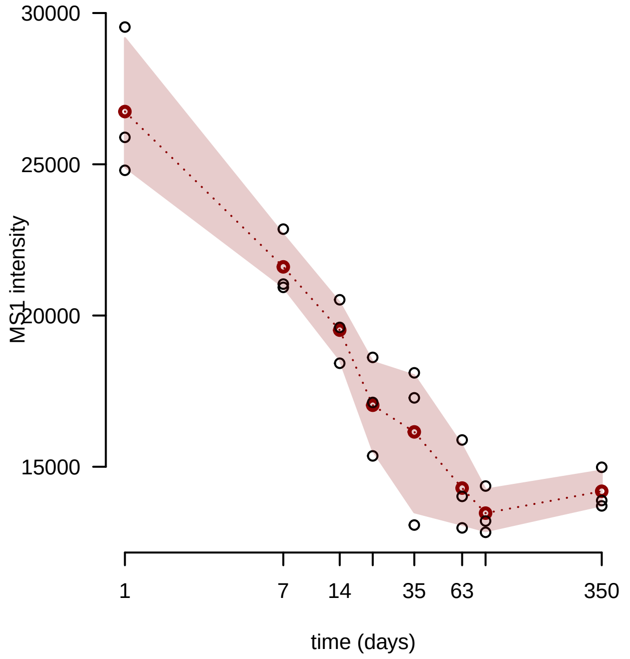

P45591
GN=Cfl2
cofilin 2, muscle

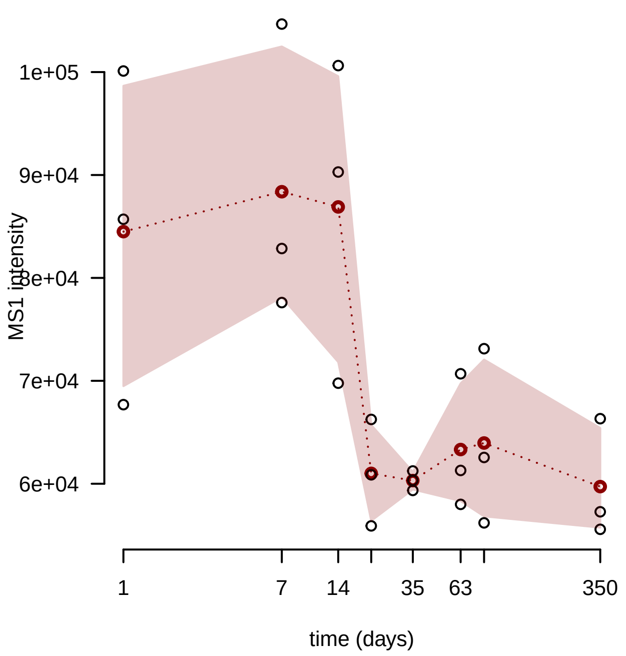

P45700
GN=Man1a1

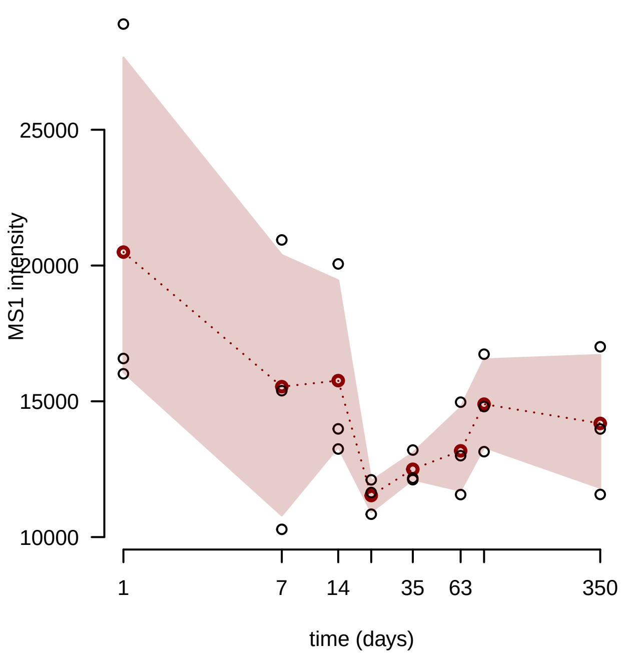

P45878
GN=Fkbp2
FK506 binding protein 2

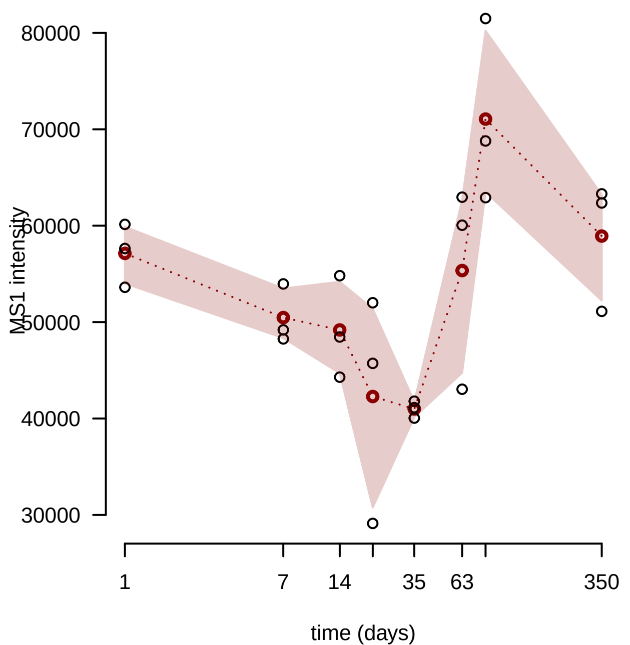

P45952
GN=Acadm
acyl−Coenzyme A dehydrogenase, medium ch ...

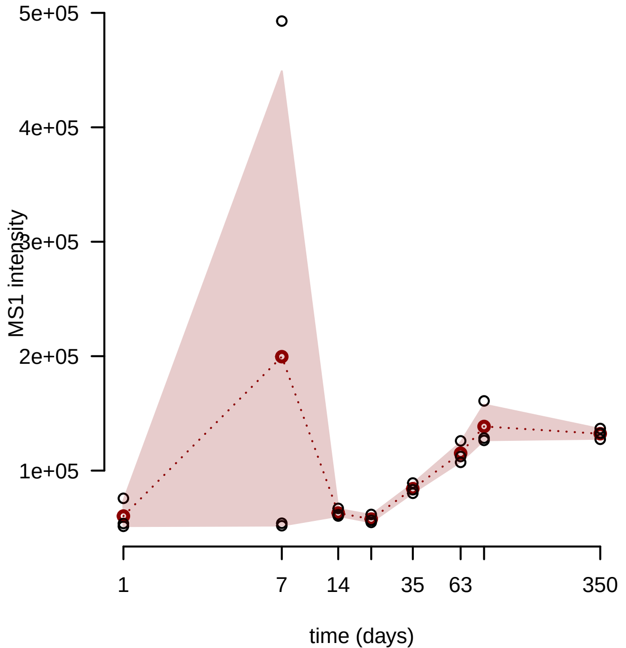

P46061
GN=Rangap1
RAN GTPase activating protein 1

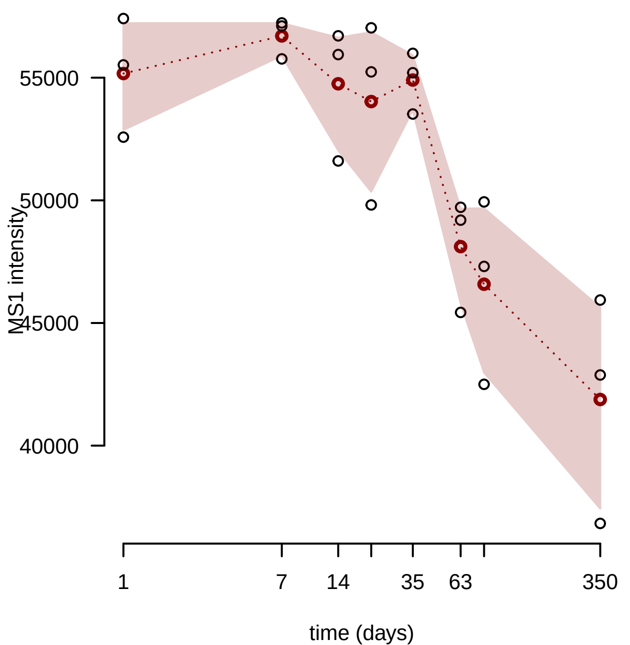

P46062
GN=Sipa1
signal−induced proliferation associated ...

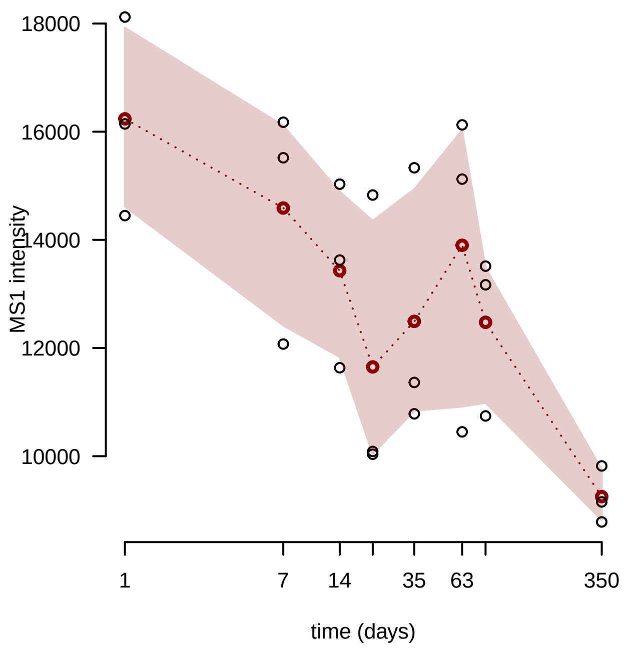

P46096
GN=Syt1
synaptotagmin I

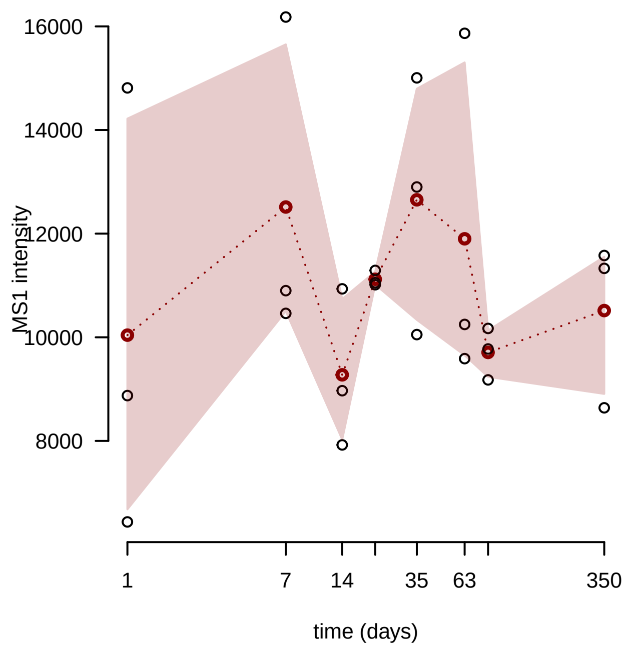

P46412
GN=Gpx3
glutathione peroxidase 3

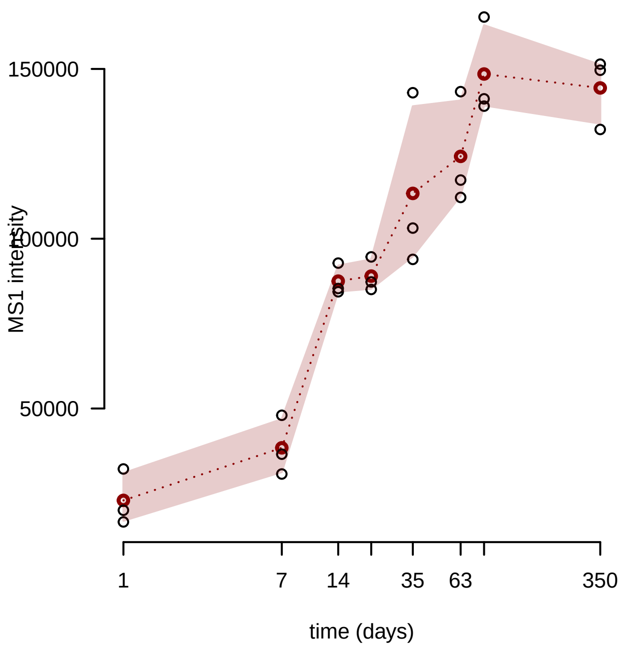

P46414
GN=Cdkn1b
cyclin−dependent kinase inhibitor 1B

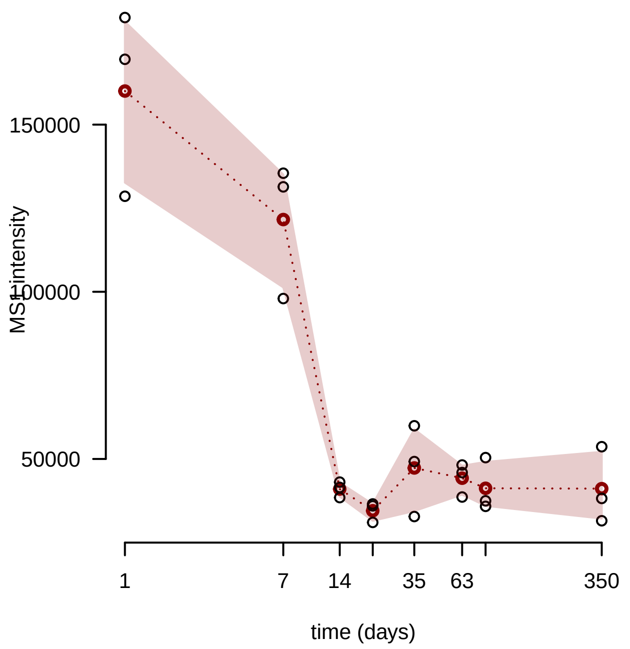

P46425
GN=Gstp2
glutathione S−transferase, pi 2

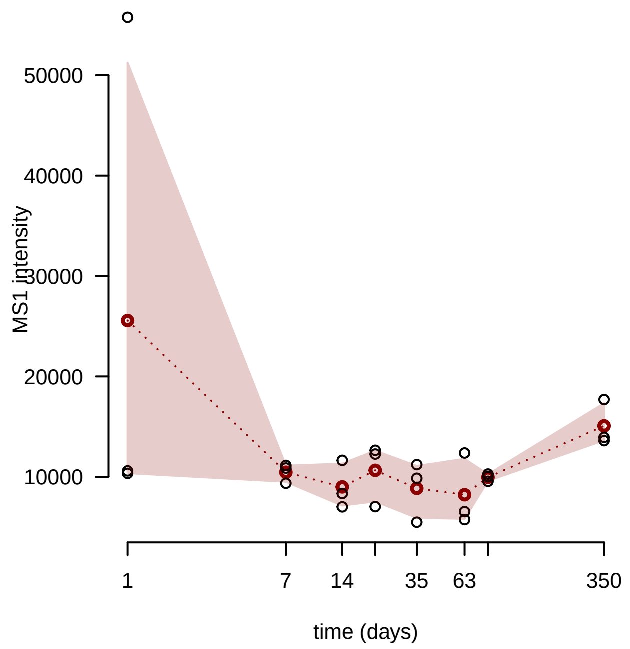

P46460
GN=Nsf
N−ethylmaleimide sensitive fusion protei ...

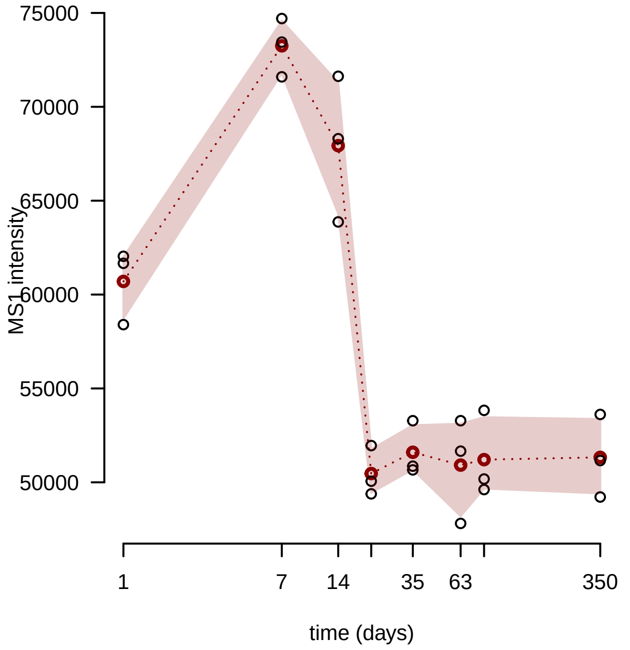

P46467
GN=Vps4b
vacuolar protein sorting 4B

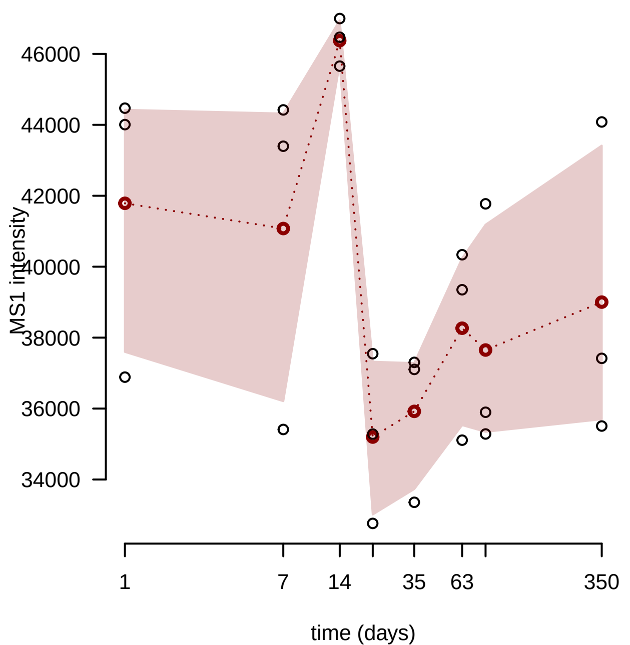

P46471
GN=Psmc2
proteasome (prosome, macropain) 26S subu ...

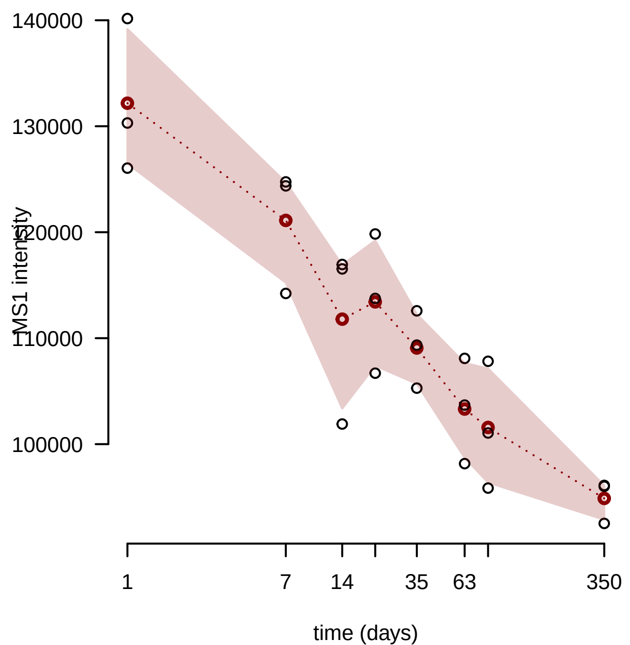

P46638
GN=Rab11b
RAB11B, member RAS oncogene family

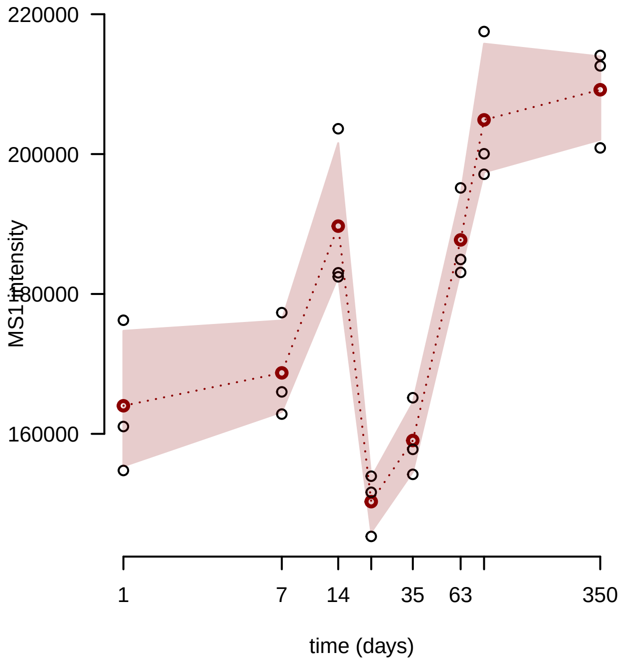

P46656
GN=Fdx1
ferredoxin 1

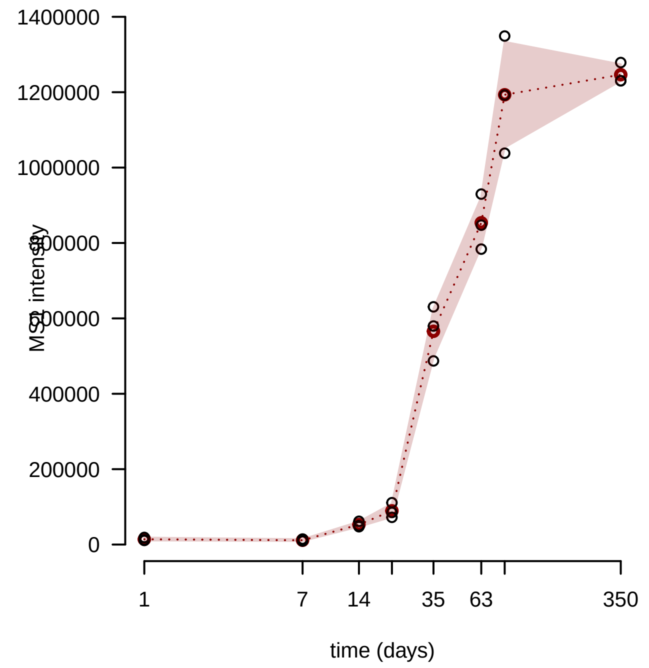

P46660
GN=Ina
internexin neuronal intermediate filamen ...

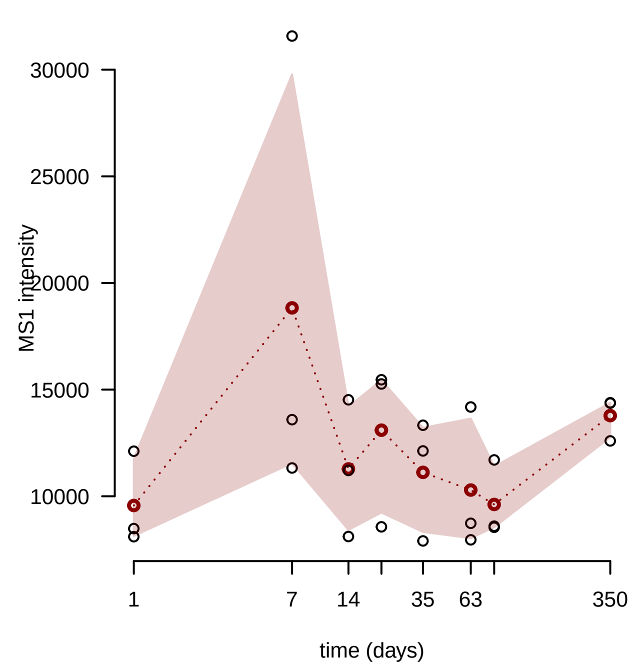

P46662
GN=Nf2
neurofibromin 2

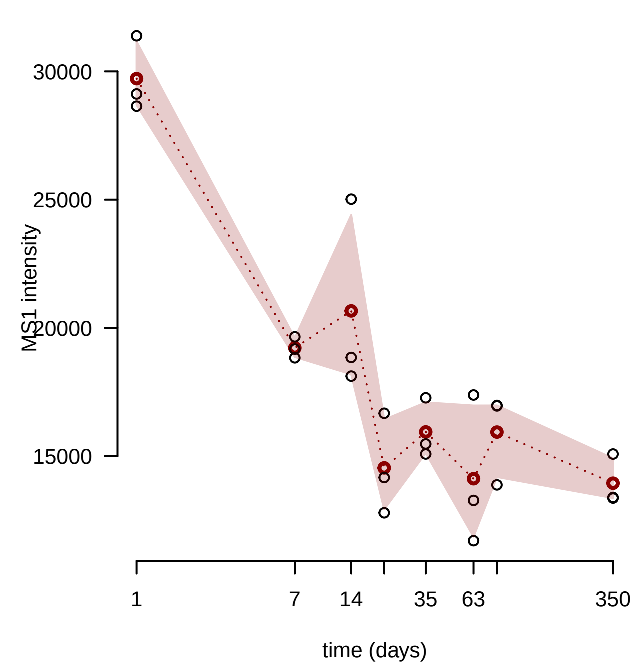

P46664
GN=Adss
adenylosuccinate synthetase, non muscle

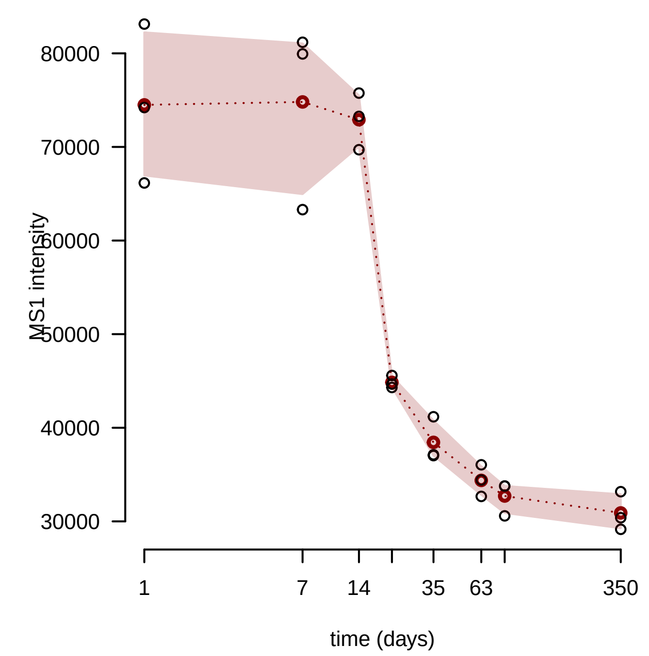

P46718
GN=Pdcd2
programmed cell death 2

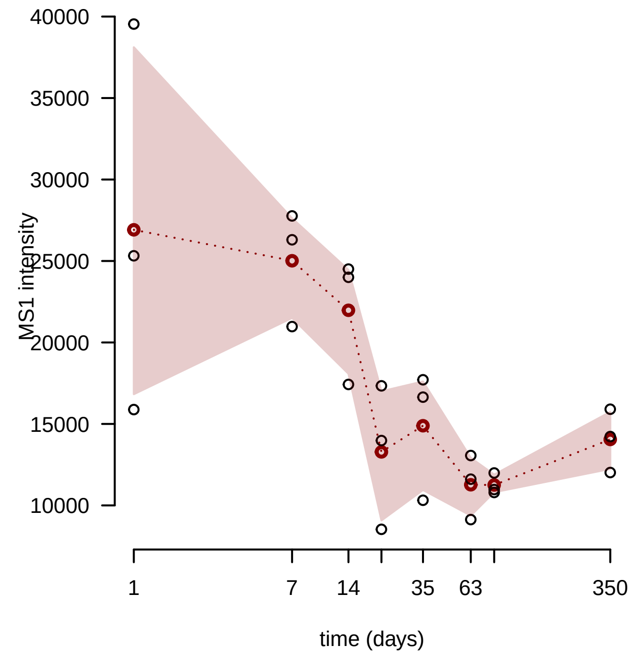

P46735
GN=Myo1b
myosin IB

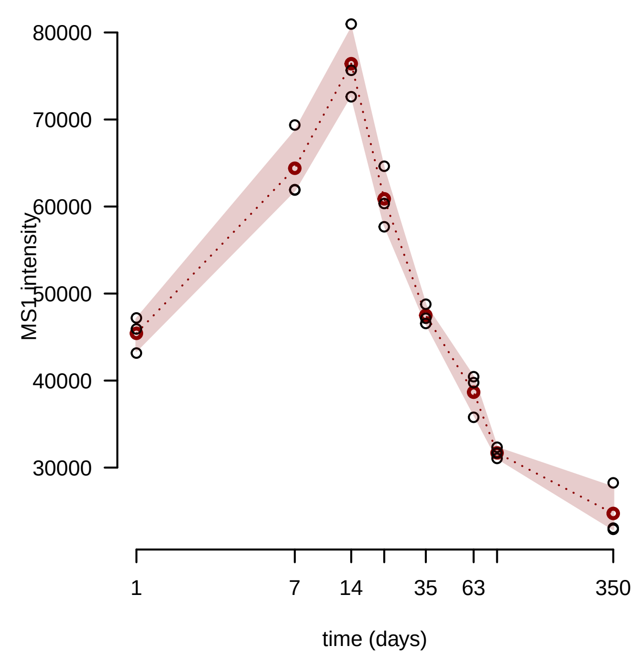

P46737
GN=Brcc3
BRCA1/BRCA2−containing complex, subunit ...

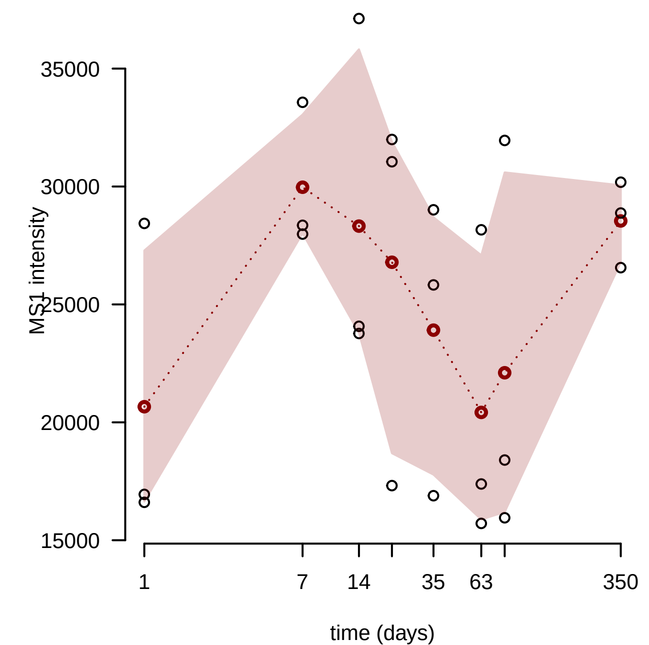

P46935
GN=Nedd4
neural precursor cell expressed, develop ...

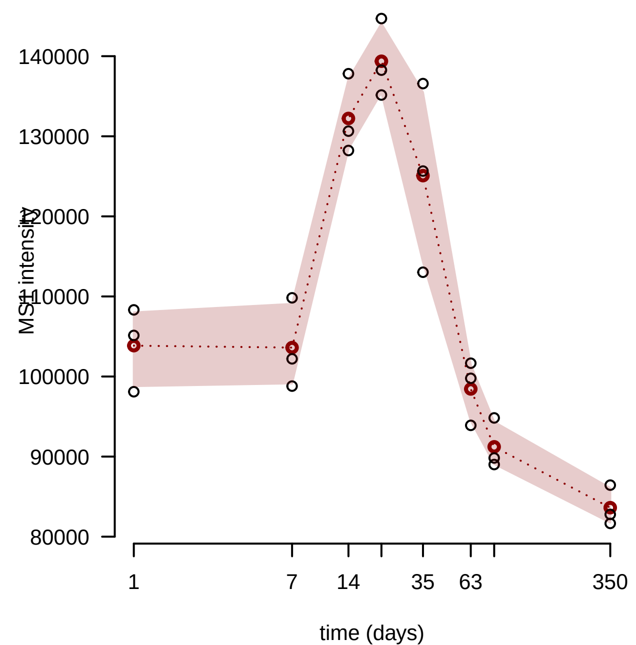

P46938
GN=Yap1
yes−associated protein 1

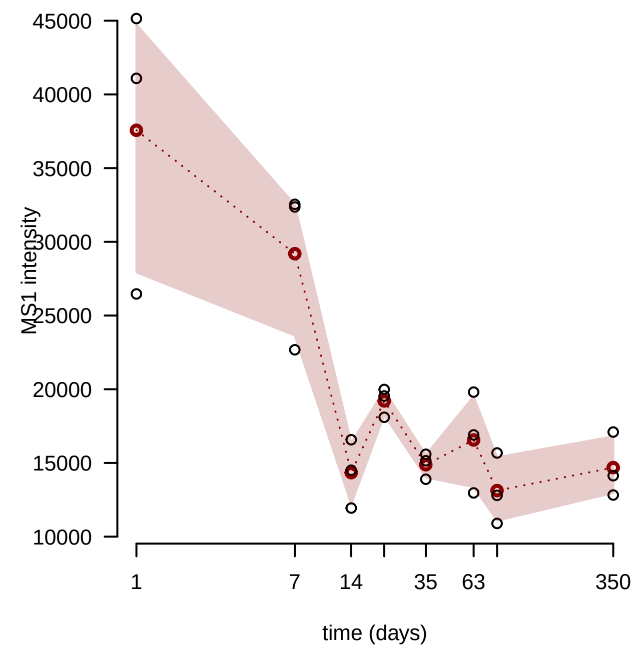

P46978
GN=Stt3a
STT3, subunit of the oligosaccharyltrans ...

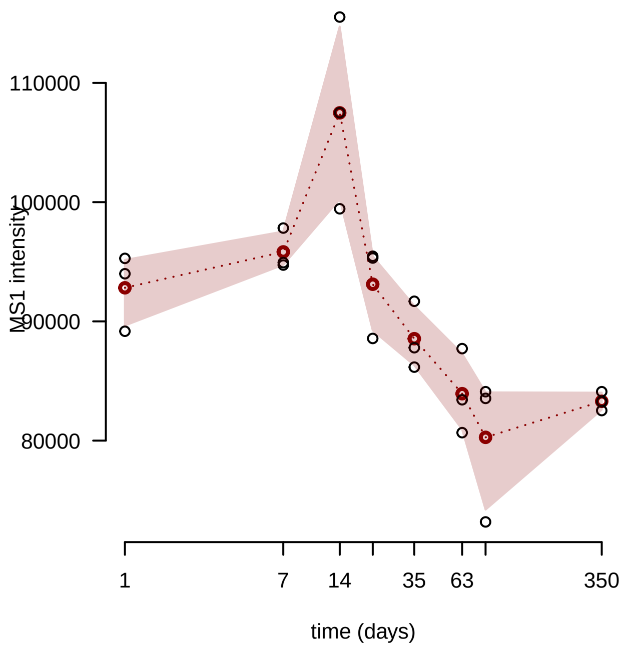

P47199
GN=Cryz
crystallin, zeta

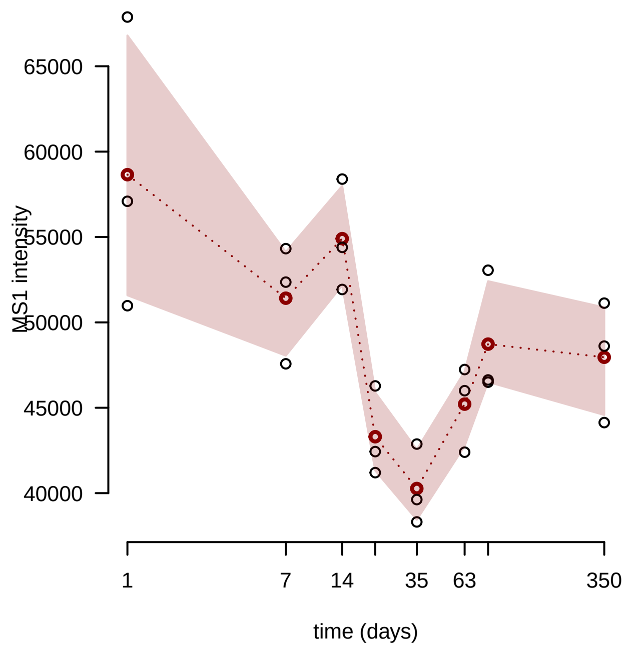

P47226
GN=Tes
testin LIM domain protein

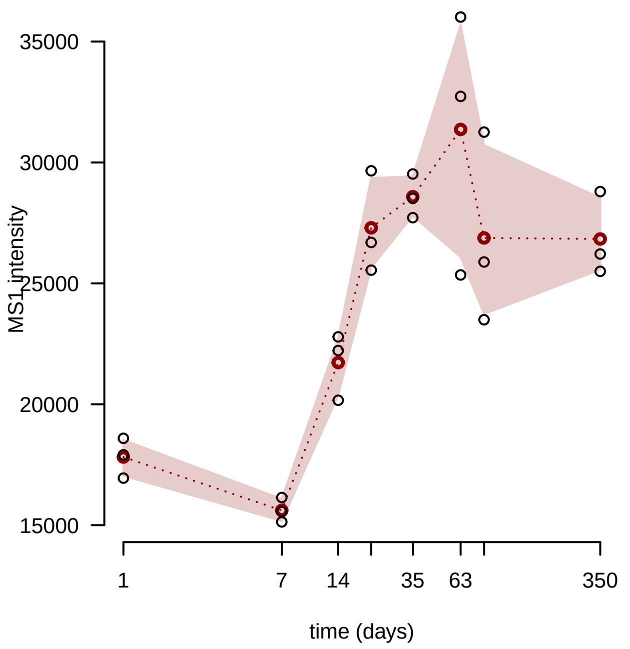

P47713
GN=Pla2g4a
phospholipase A2, group IVA (cytosolic, ...

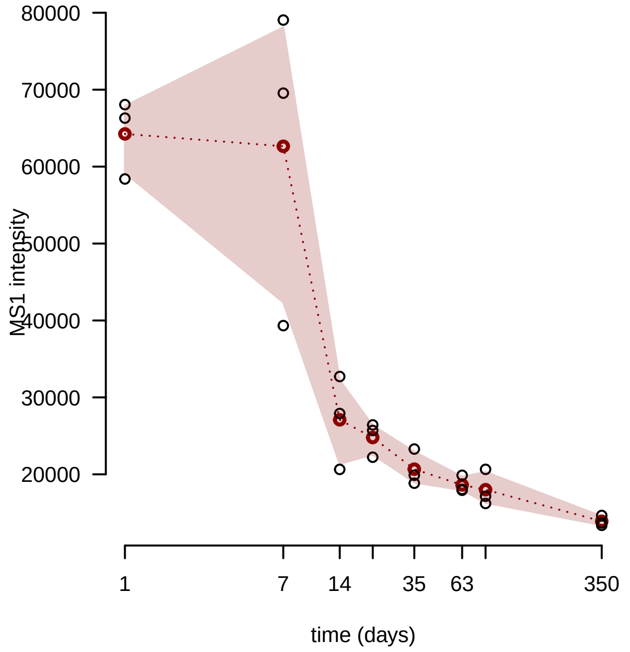

P47738
GN=Aldh2
aldehyde dehydrogenase 2, mitochondrial

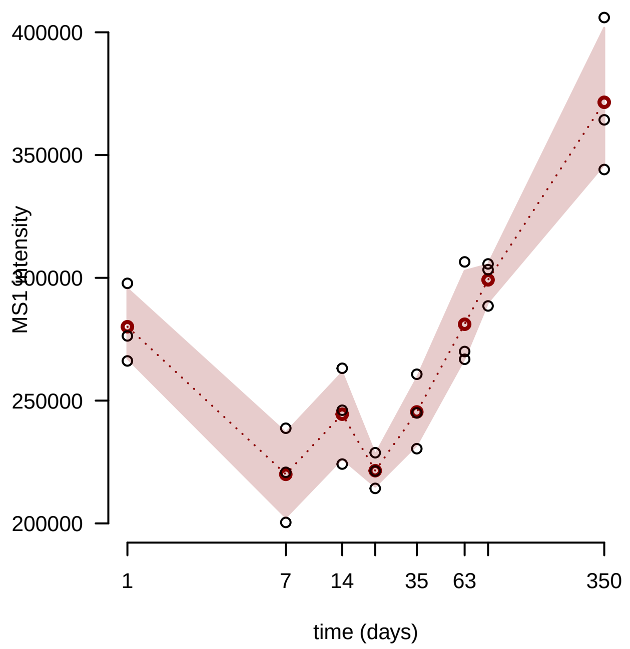

P47740
GN=Aldh3a2
aldehyde dehydrogenase family 3, subfami ...

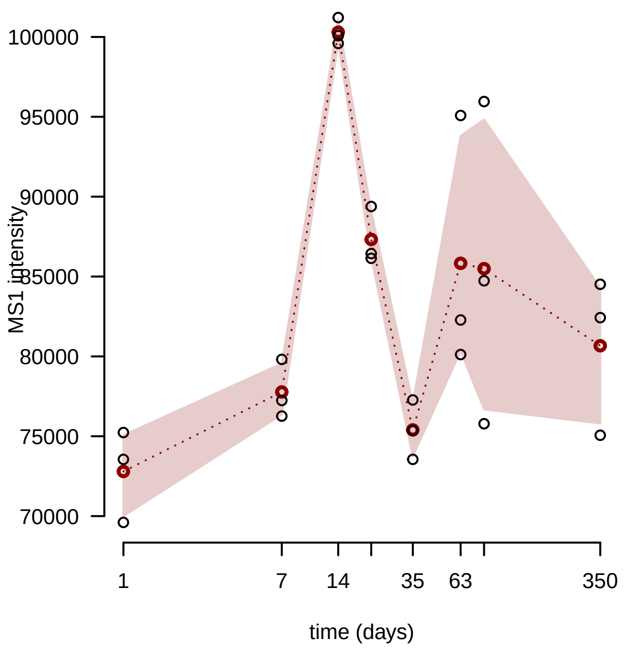

P47753
GN=Capza1
capping actin protein of muscle Z−line s ...

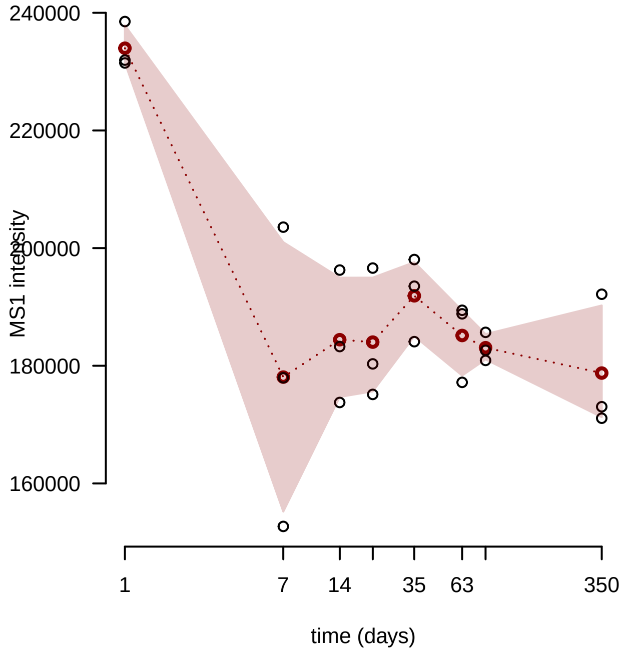

P47754
GN=Capza2
capping actin protein of muscle Z−line s ...

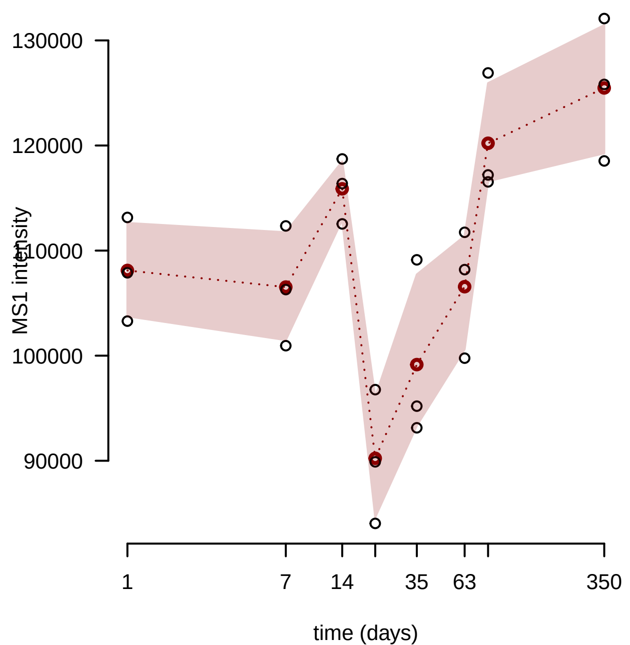

P47757
GN=Capzb
capping actin protein of muscle Z−line s ...

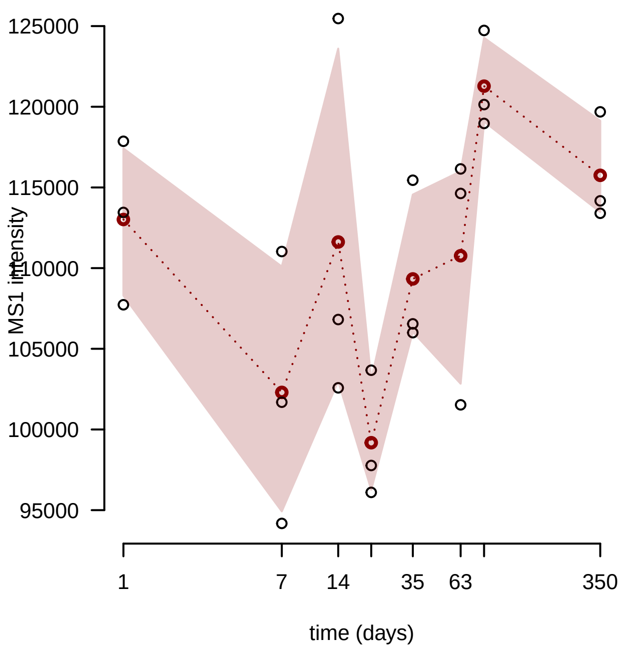

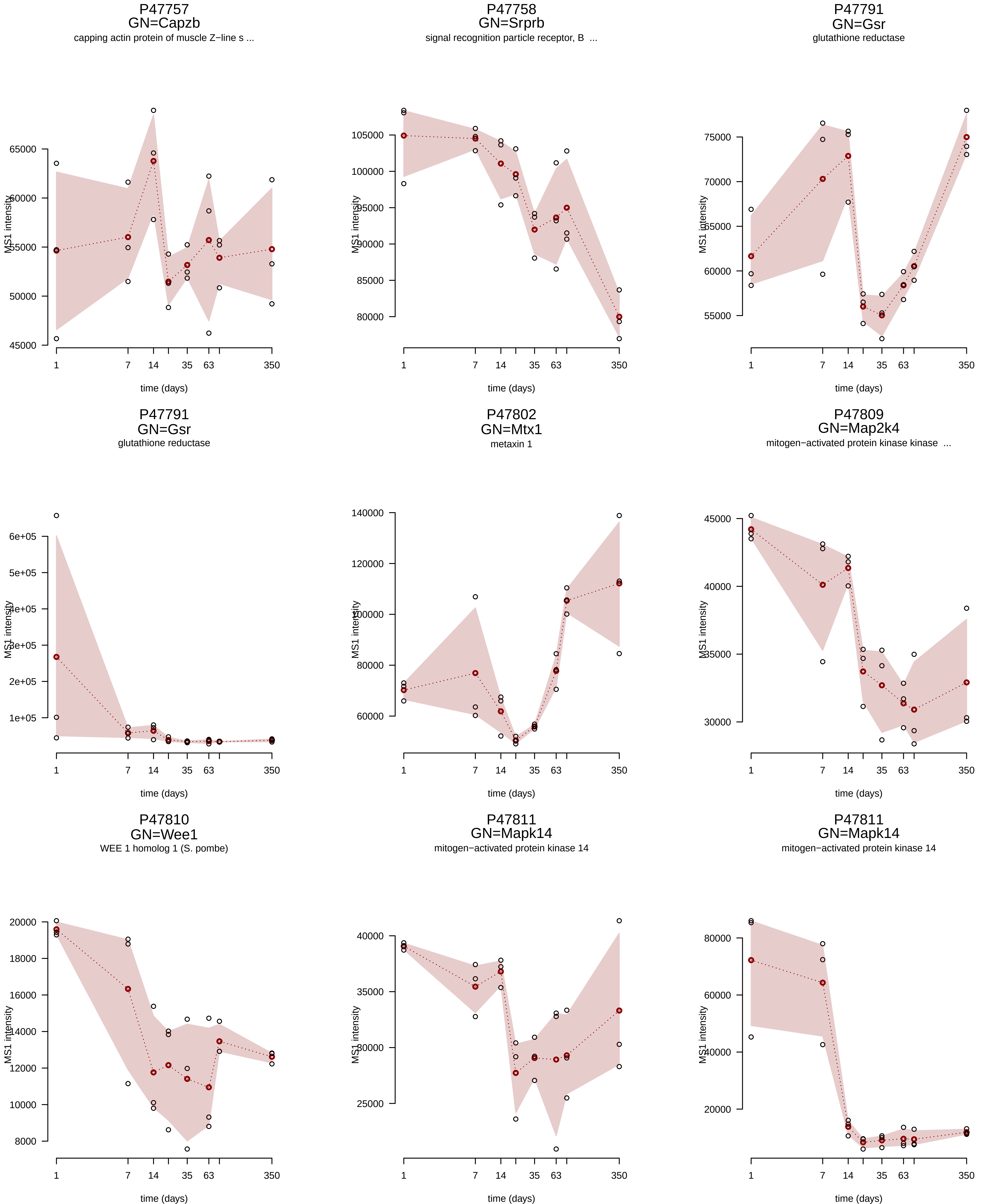
P47757
GN=Capzb
capping actin protein of muscle Z−line s ...
P47758
GN=Srprb
signal recognition particle receptor, B ...
P47791
GN=Gsr
glutathione reductase
P47791
GN=Gsr
glutathione reductase
P47802
GN=Mtx1
metaxin 1
P47809
GN=Map2k4
mitogen−activated protein kinase kinase ...
P47810
GN=Wee1
WEE 1 homolog 1 (S. pombe)
P47811
GN=Mapk14
mitogen−activated protein kinase 14
P47811
GN=Mapk14
mitogen−activated protein kinase 14
MS1 intensity
time (days)

P47856
GN=Gfpt1
glutamine fructose−6−phosphate transamin ...

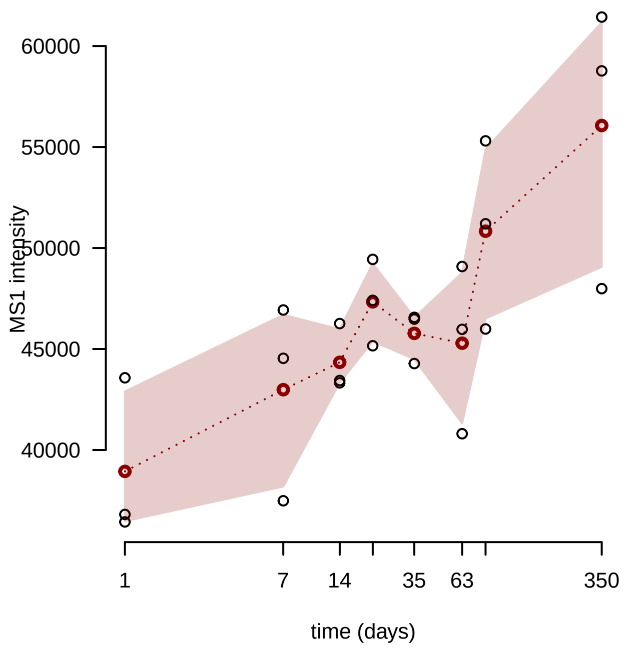

P47857
GN=Pfkm
phosphofructokinase, muscle

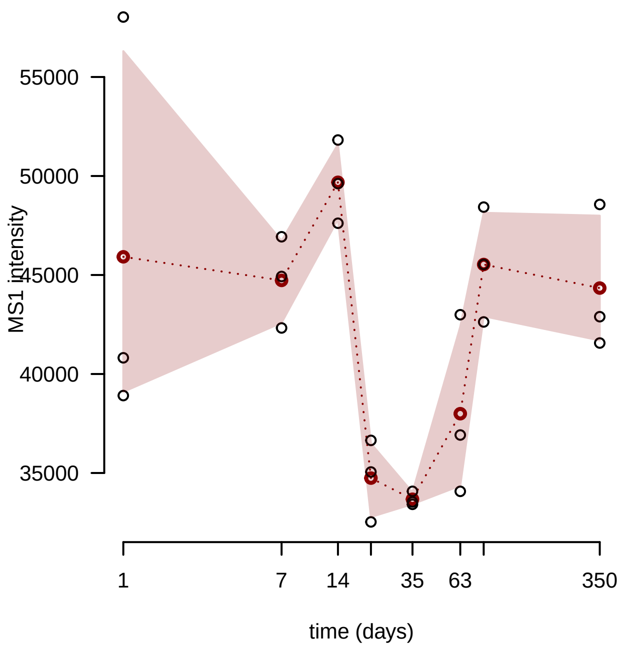

P47877
GN=Igfbp2
insulin−like growth factor binding prote ...

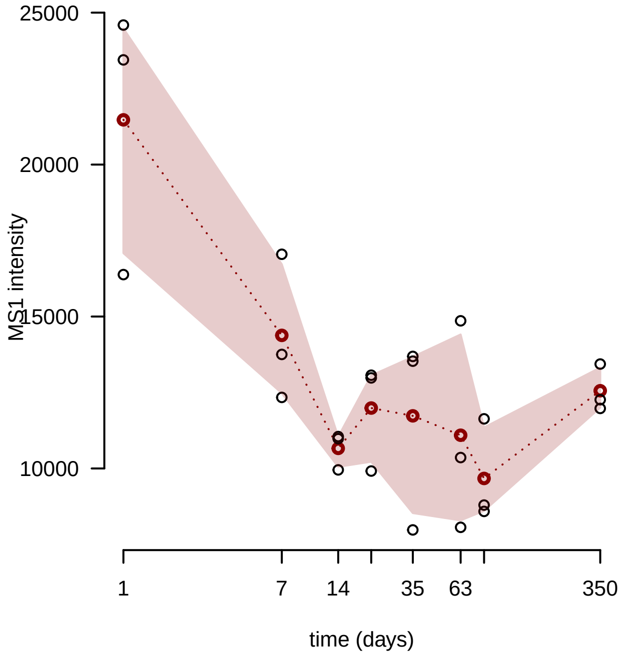

P47878
GN=Igfbp3
insulin−like growth factor binding prote ...

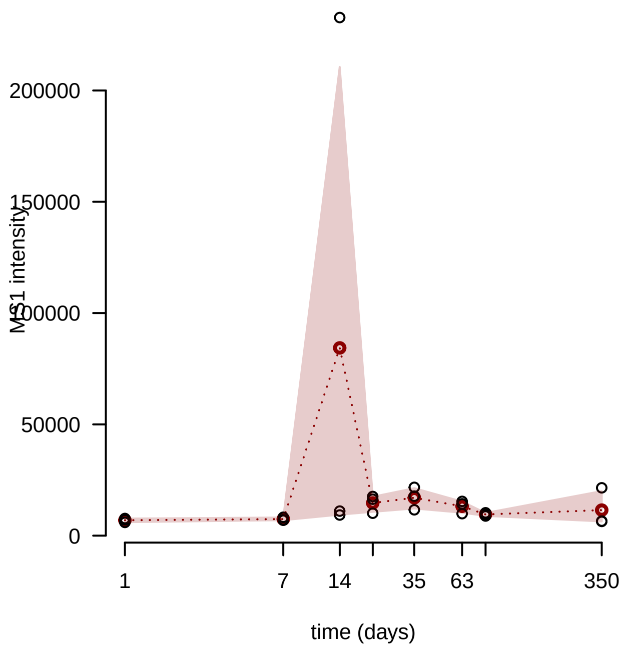

P47879
GN=Igfbp4
insulin−like growth factor binding prote ...

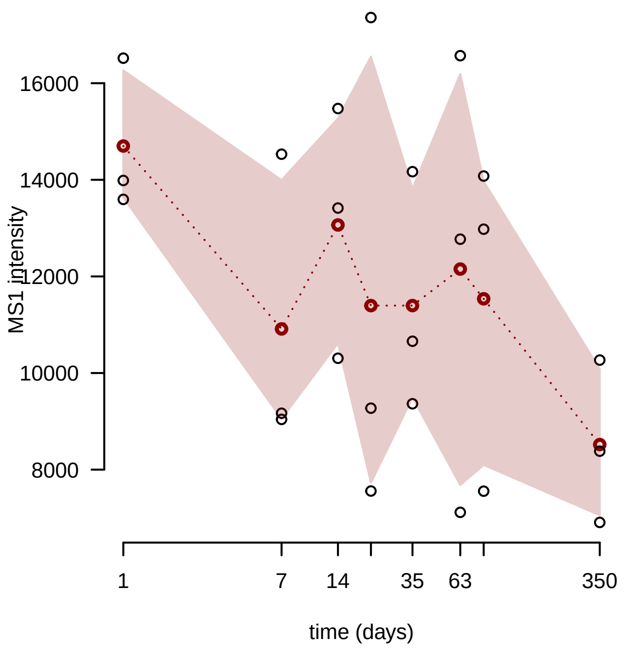

P47911
GN=Rpl6
ribosomal protein L6

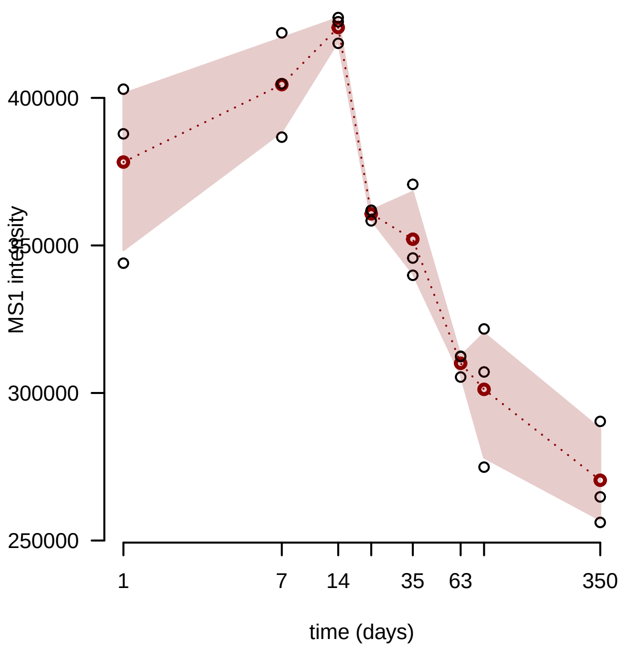

P47915
GN=Rpl29
ribosomal protein L29

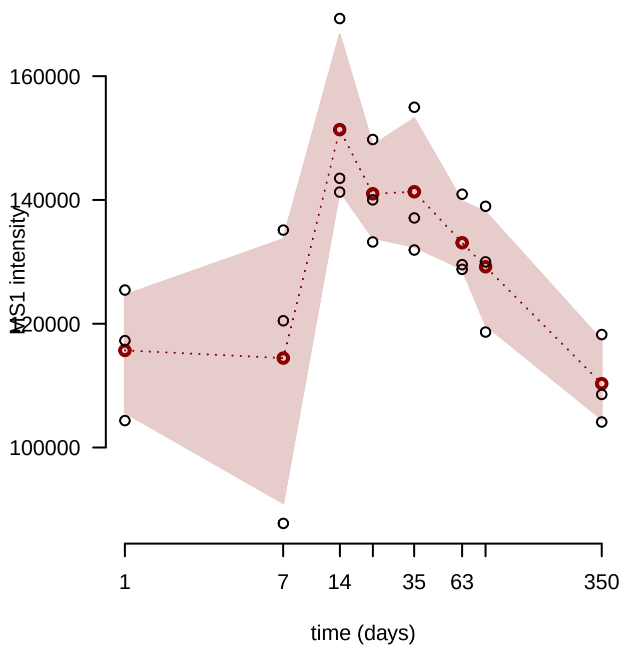

P47930
GN=Fosl2
fos−like antigen 2

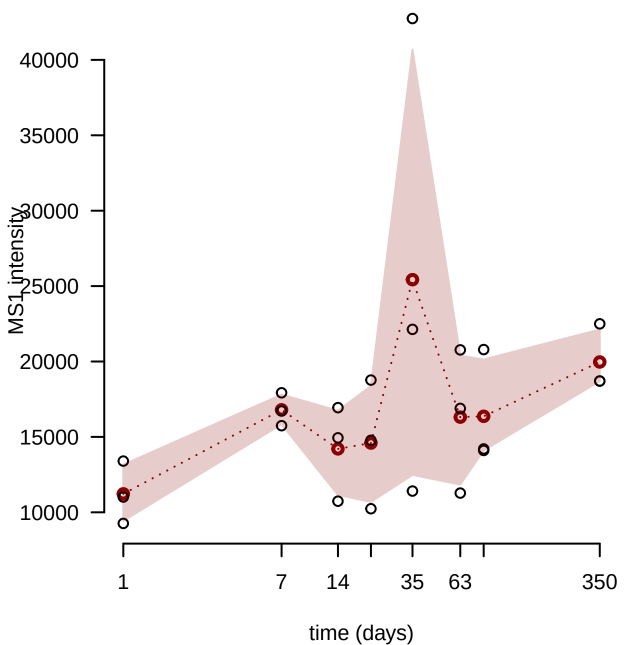

P47931
GN=Fst
follistatin

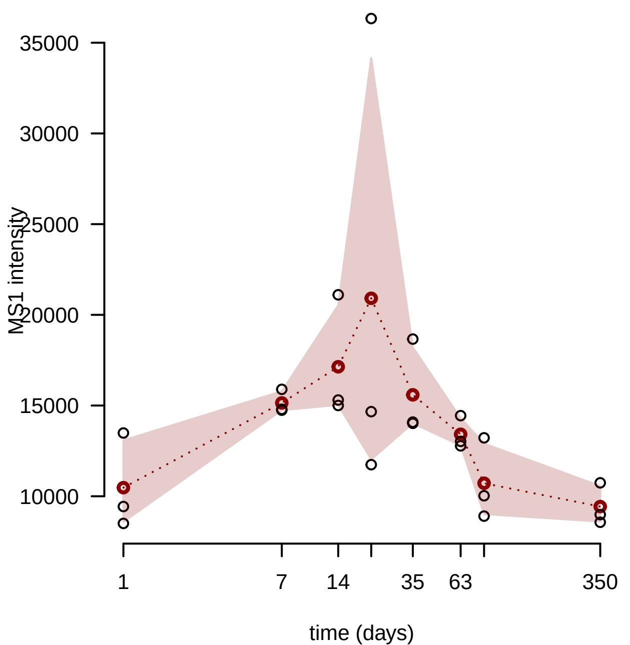

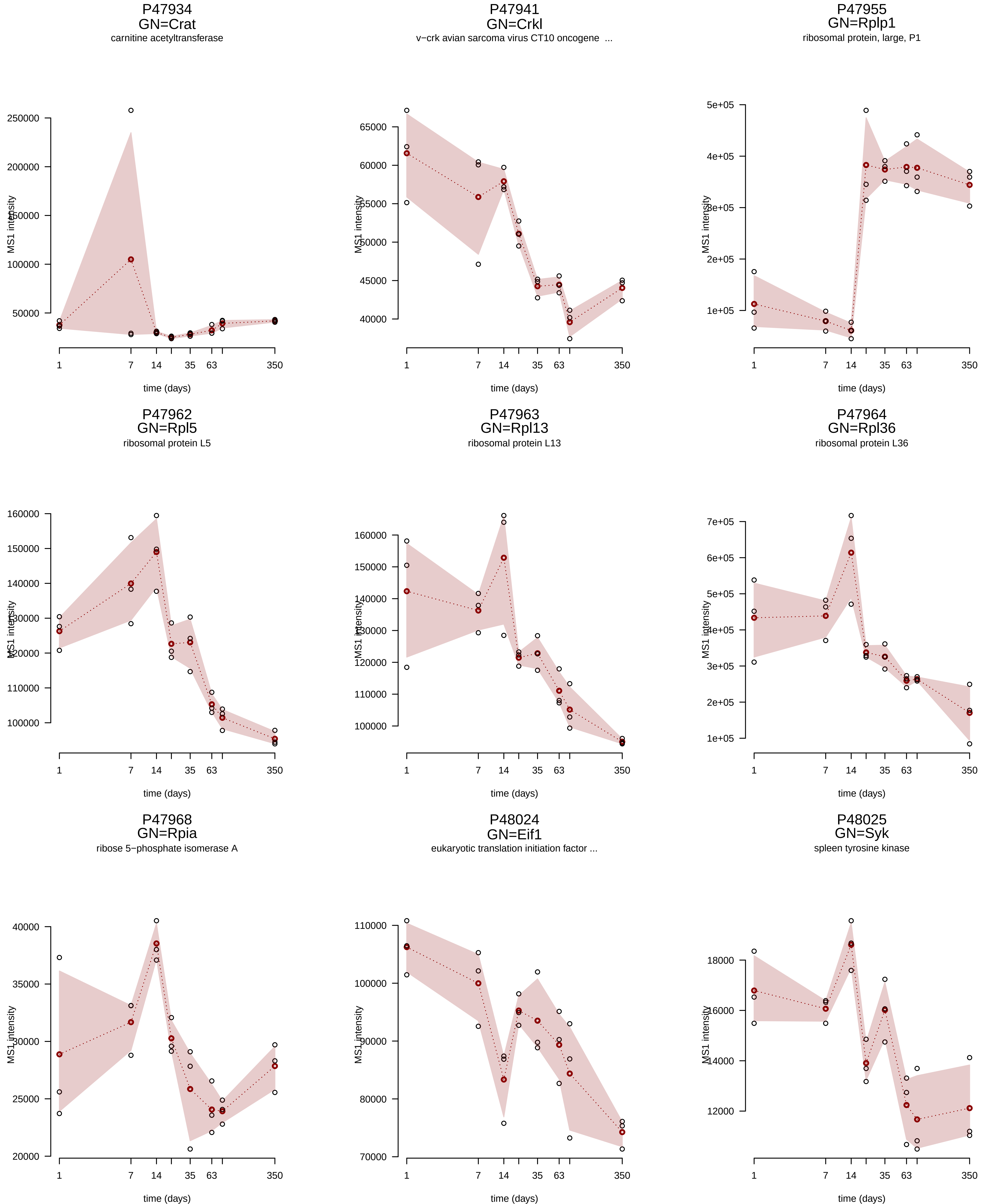
P47934
GN=Crat
carnitine acetyltransferase
MS1 intensity
time (days)
P47941
GN=Crkl
v−crk avian sarcoma virus CT10 oncogene ...
MS1 intensity
time (days)
P47955
GN=Rplp1
ribosomal protein, large, P1
MS1 intensity
time (days)
P47962
GN=Rpl5
ribosomal protein L5
MS1 intensity
time (days)
P47963
GN=Rpl13
ribosomal protein L13
MS1 intensity
time (days)
P47964
GN=Rpl36
ribosomal protein L36
MS1 intensity
time (days)
P47968
GN=Rpia
ribose 5−phosphate isomerase A
MS1 intensity
time (days)
P48024
GN=Eif1
eukaryotic translation initiation factor ...
MS1 intensity
time (days)
P48025
GN=Syk
spleen tyrosine kinase
MS1 intensity
time (days)
1
7
14
35
63
350

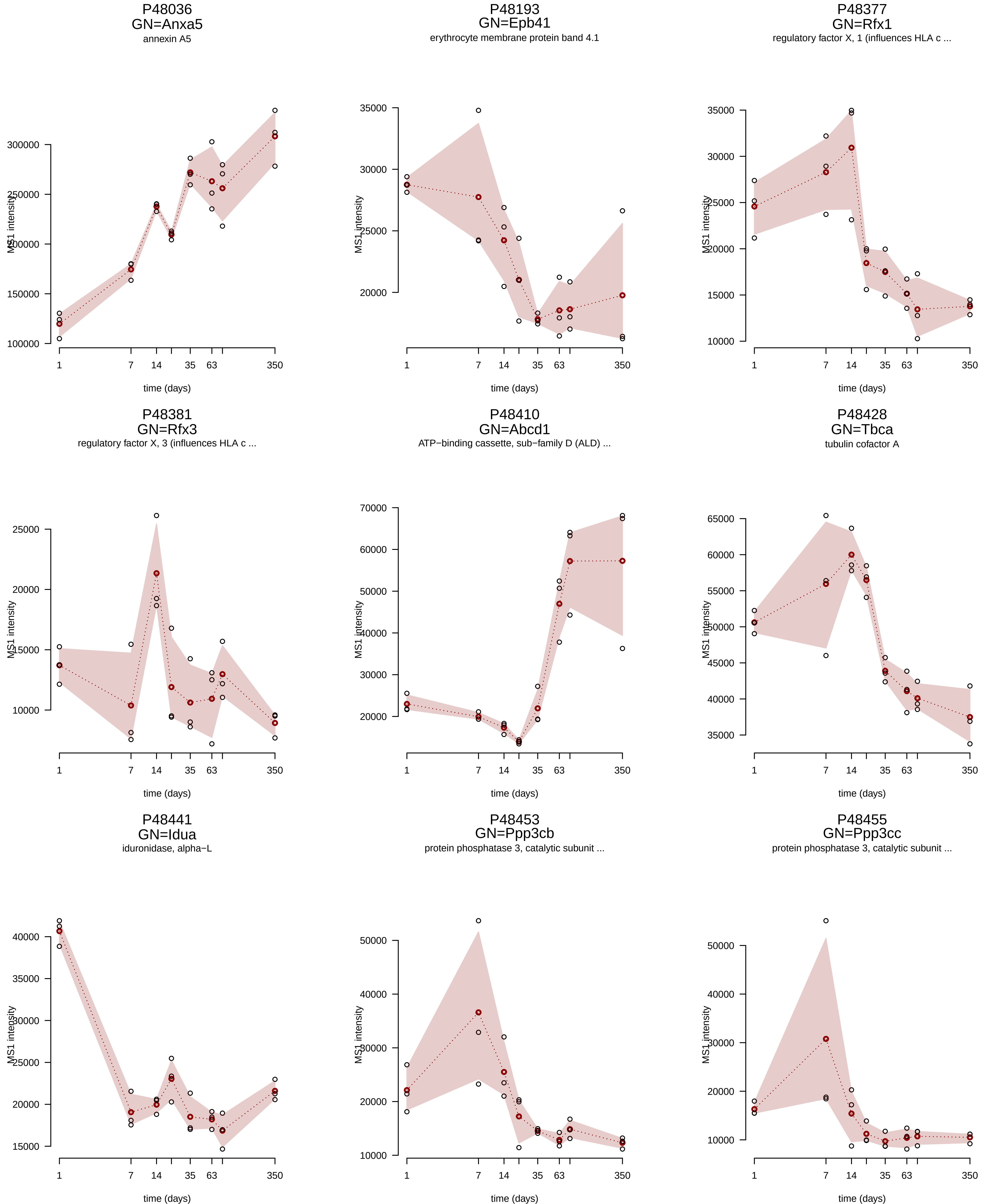
P48036
GN=Anxa5
annexin A5
P48193
GN=Epb41
erythrocyte membrane protein band 4.1
P48377
GN=Rfx1
regulatory factor X, 1 (influences HLA c ...
P48381
GN=Rfx3
regulatory factor X, 3 (influences HLA c ...
P48410
GN=Abcd1
ATP−binding cassette, sub−family D (ALD) ...
P48428
GN=Tbca
tubulin cofactor A
P48441
GN=Idua
iduronidase, alpha−L
P48453
GN=Ppp3cb
protein phosphatase 3, catalytic subunit ...
P48455
GN=Ppp3cc
protein phosphatase 3, catalytic subunit ...
MS1 intensity
time (days)
1
7
14
35
63
350

P48678
GN=Lmna
lamin A

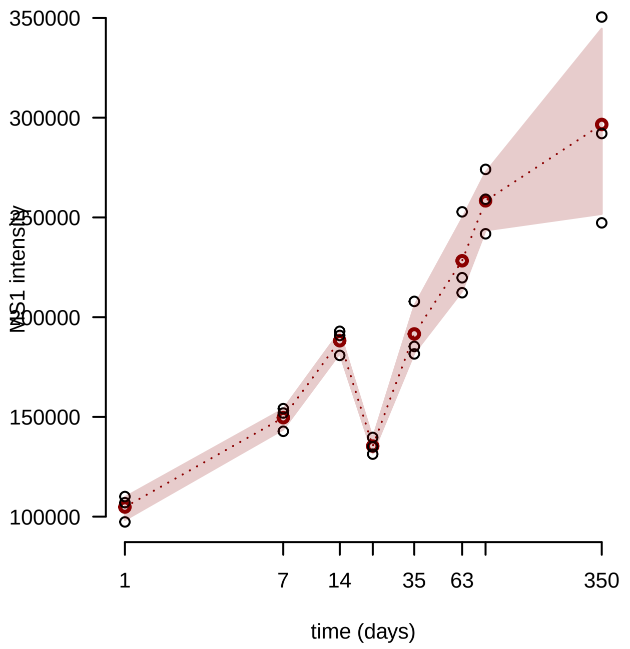

P48722
GN=Hspa4l
heat shock protein 4 like

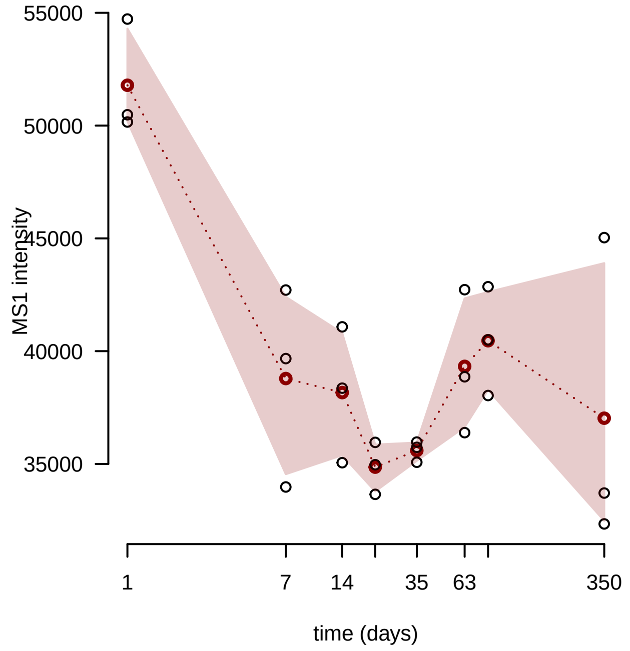

P48725
GN=Pcnt
pericentrin (kendrin)

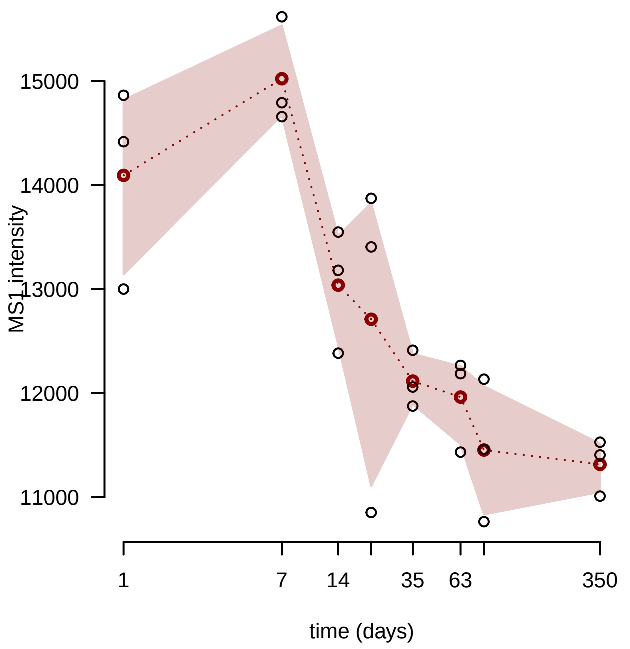

P48758
GN=Cbr1
carbonyl reductase 1

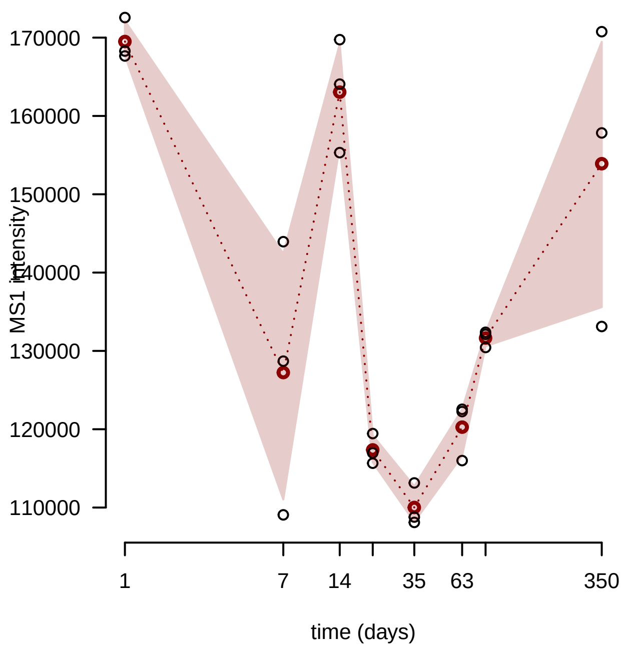

P48760
GN=Fpgs
folylpolyglutamyl synthetase

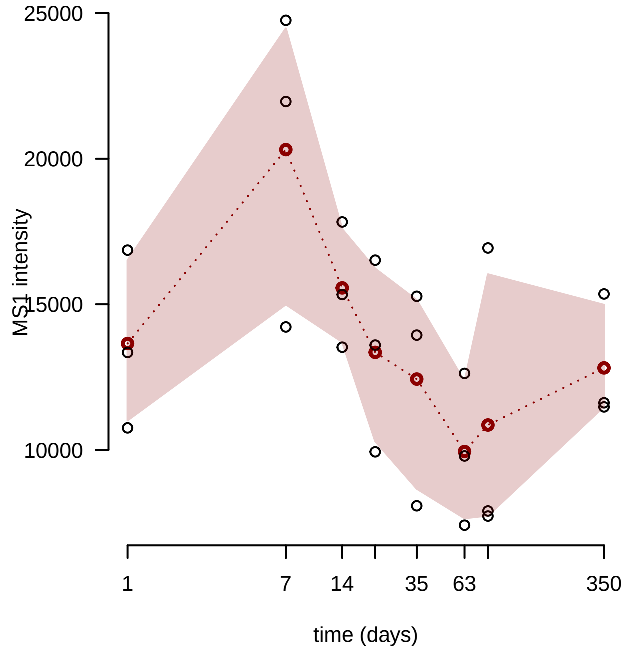

P48771
GN=Cox7a2
cytochrome c oxidase subunit 7A2

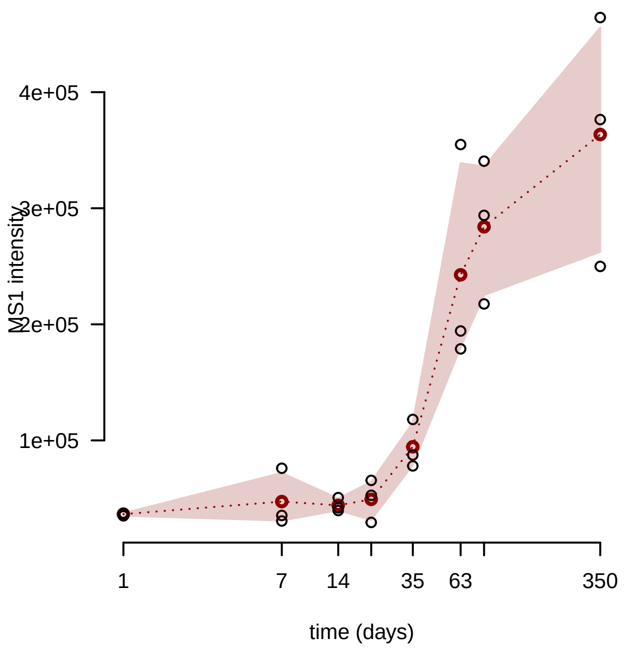

P48774
GN=Gstm5
glutathione S−transferase, mu 5

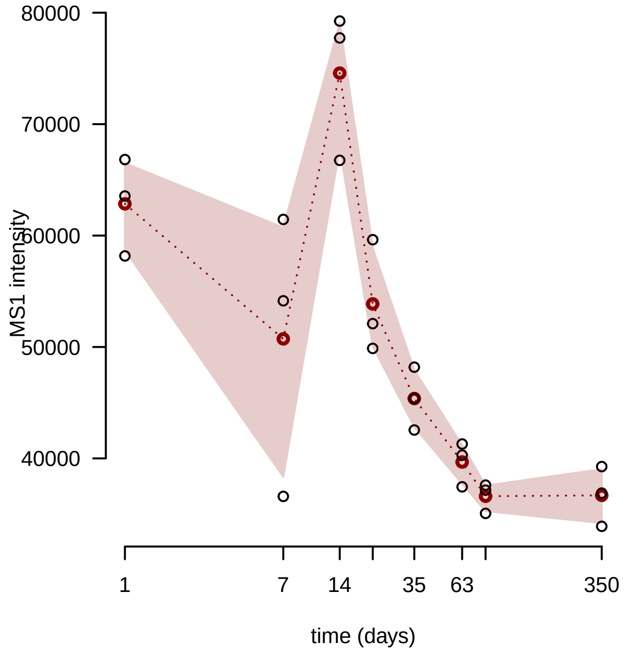

P48962
GN=Slc25a4
solute carrier family 25 (mitochondrial ...

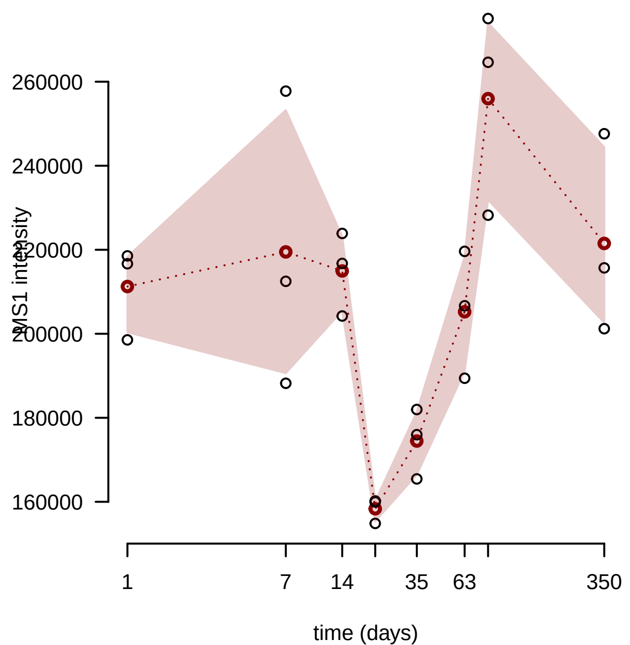

P49025
GN=Cit
citron

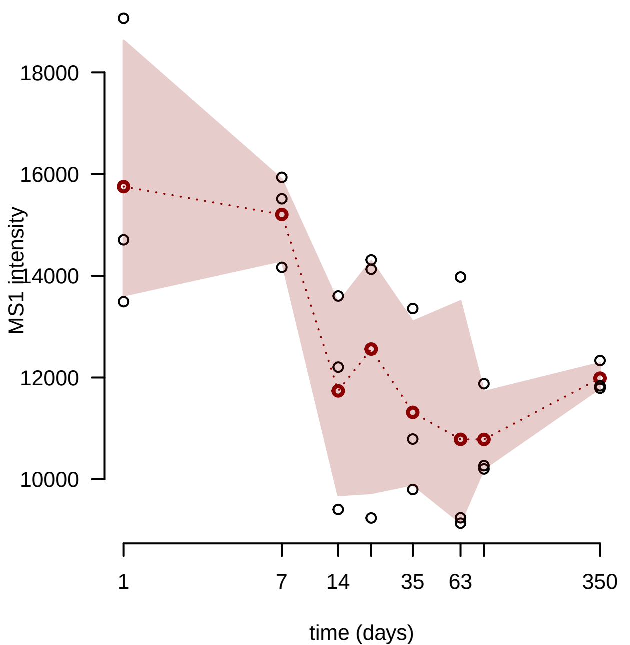

P49070
GN=Camlg

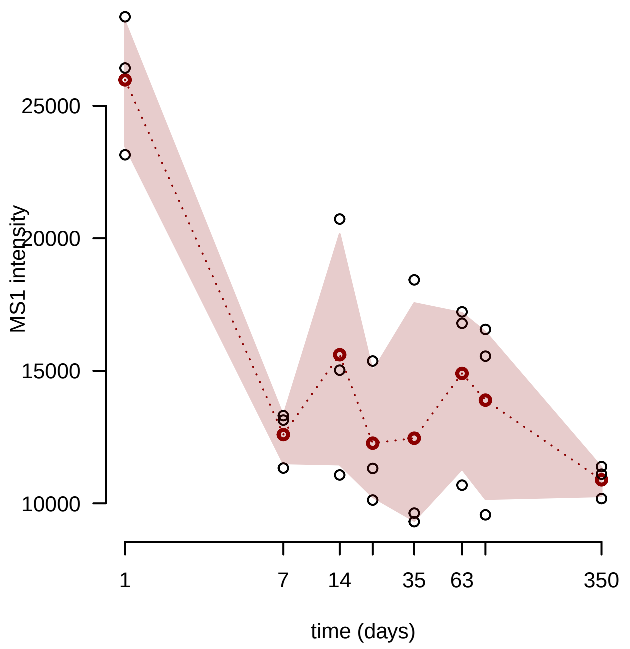

P49117
GN=Nr2c2
nuclear receptor subfamily 2, group C, m ...

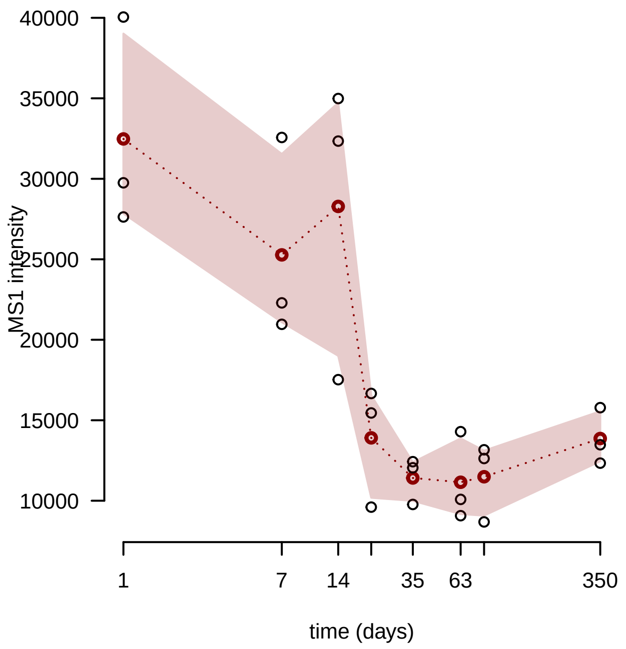

P49135
GN=Ercc3
excision repair cross−complementing rode ...

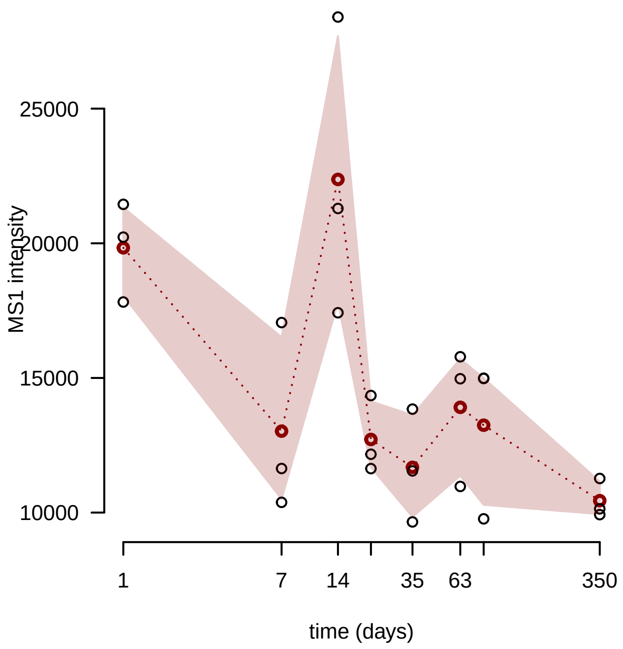

P49138
GN=Mapkapk2
MAP kinase−activated protein kinase 2

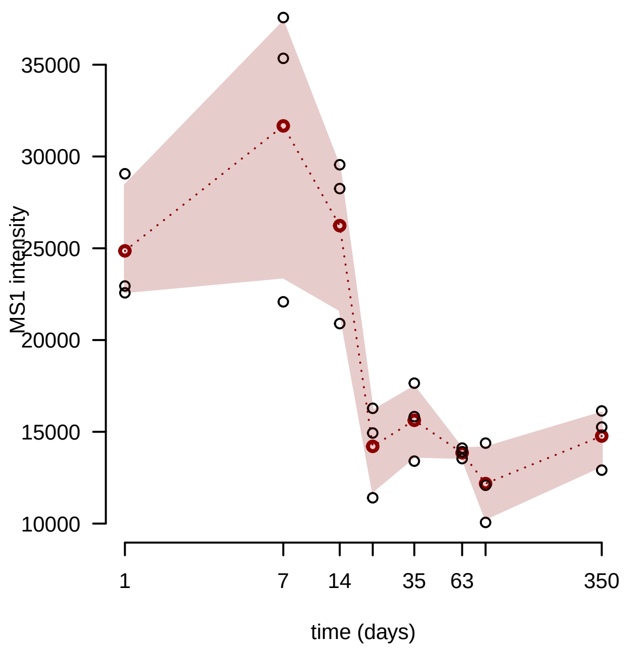

P49182
GN=Serpind1
serine (or cysteine) peptidase inhibitor ...

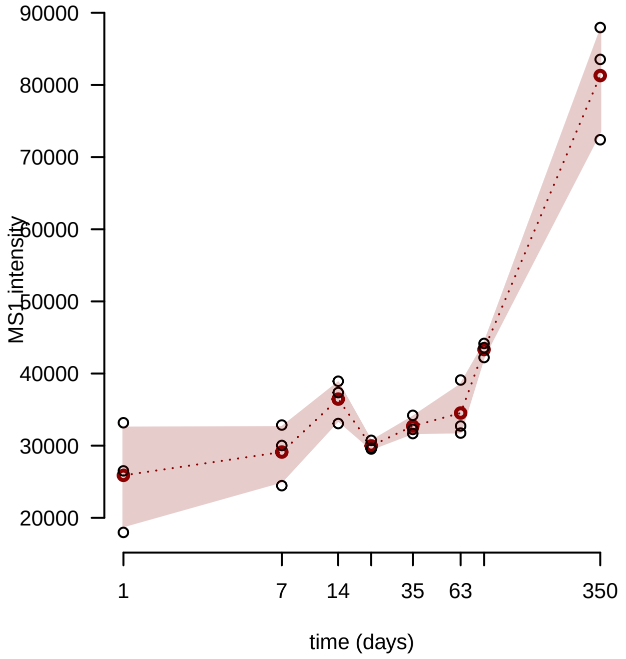

P49222
GN=Epb42
erythrocyte membrane protein band 4.2

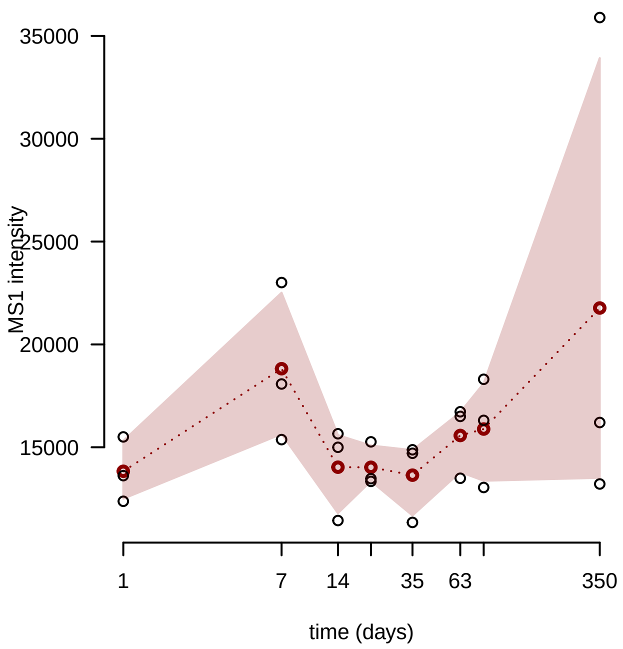

P49290
GN=Epx
eosinophil peroxidase

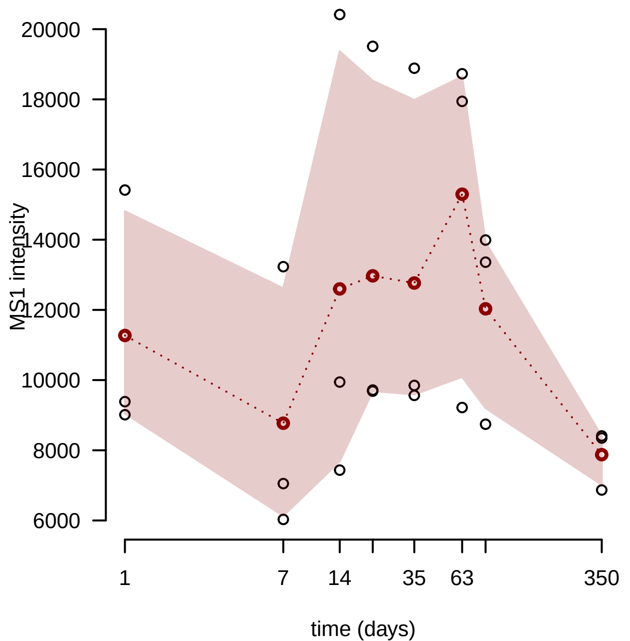

P49300
GN=Clec10a
C−type lectin domain family 10, member A

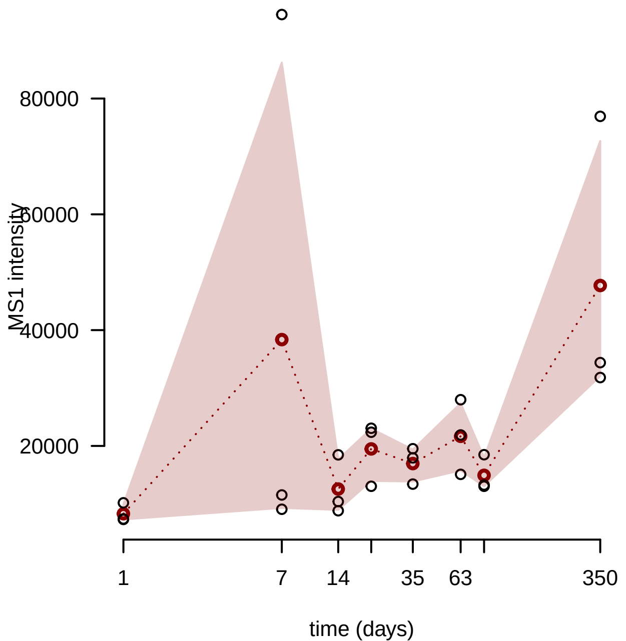

P49312
GN=Hnrnpa1
heterogeneous nuclear ribonucleoprotein ...

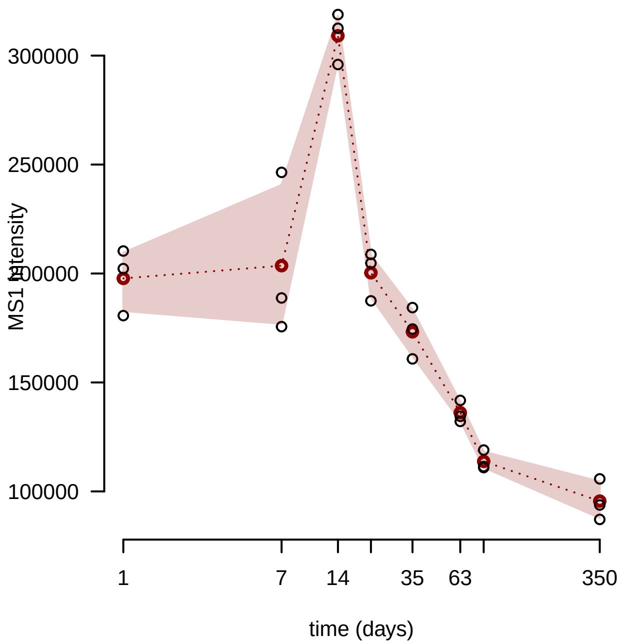

P49429
GN=Hpd
4−hydroxyphenylpyruvic acid dioxygenase

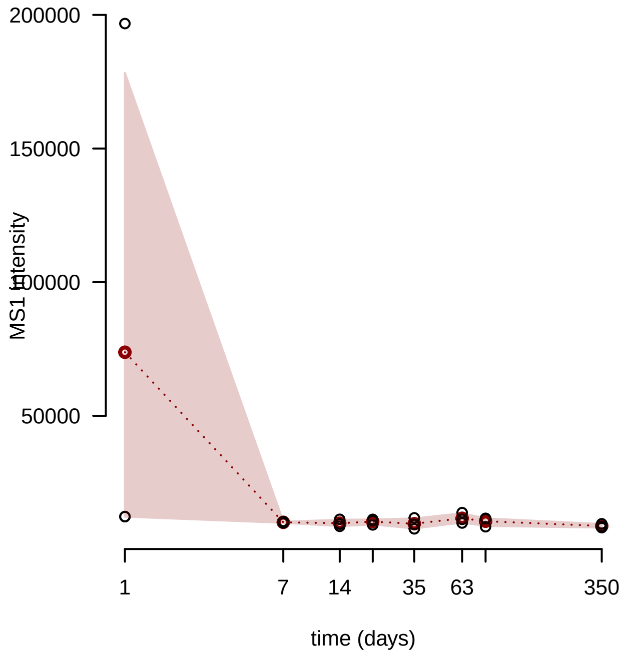

P49442
GN=Inpp1
inositol polyphosphate−1−phosphatase

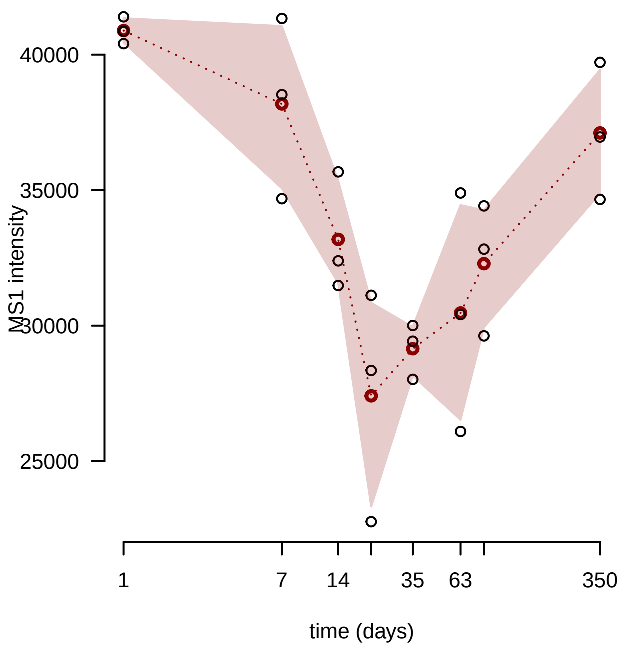

P49443
GN=Ppm1a
protein phosphatase 1A, magnesium depend ...

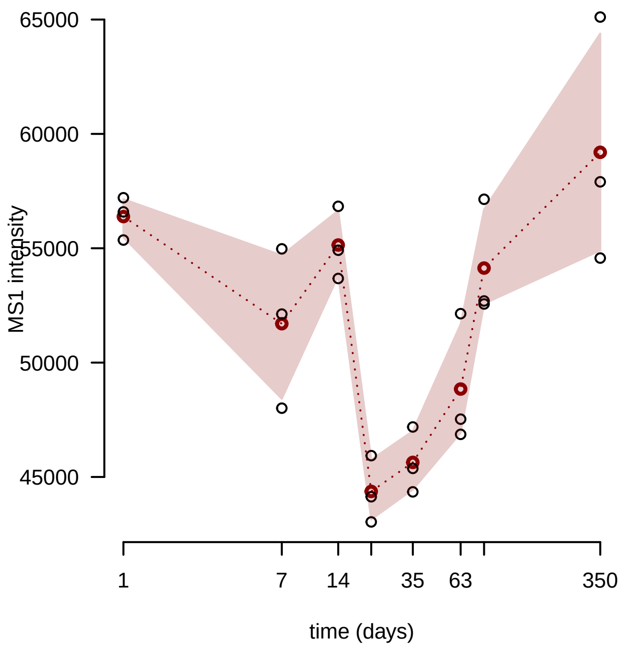

P49446
GN=Ptpre
protein tyrosine phosphatase, receptor t ...

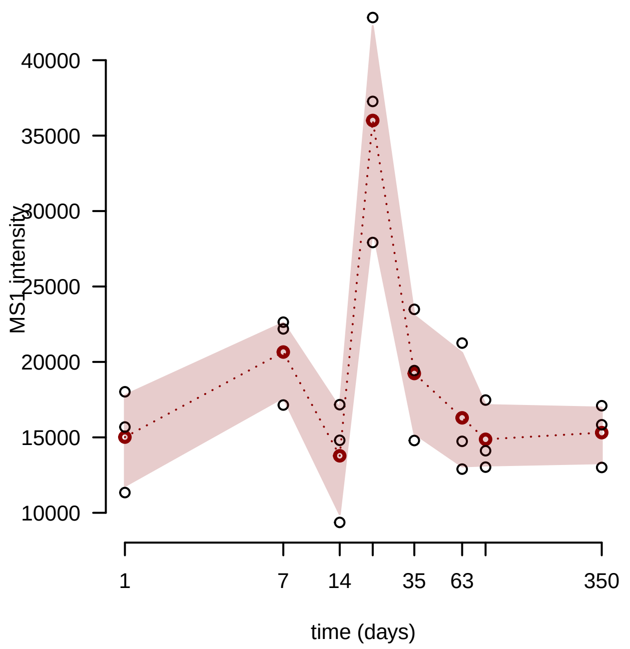

P49452
GN=Cenpc

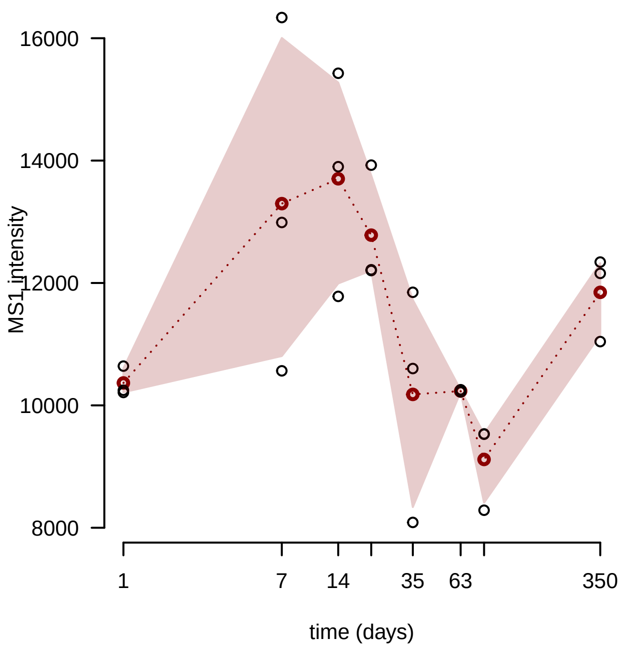

P49586
GN=Pcyt1a
phosphate cytidylyltransferase 1, cholin ...

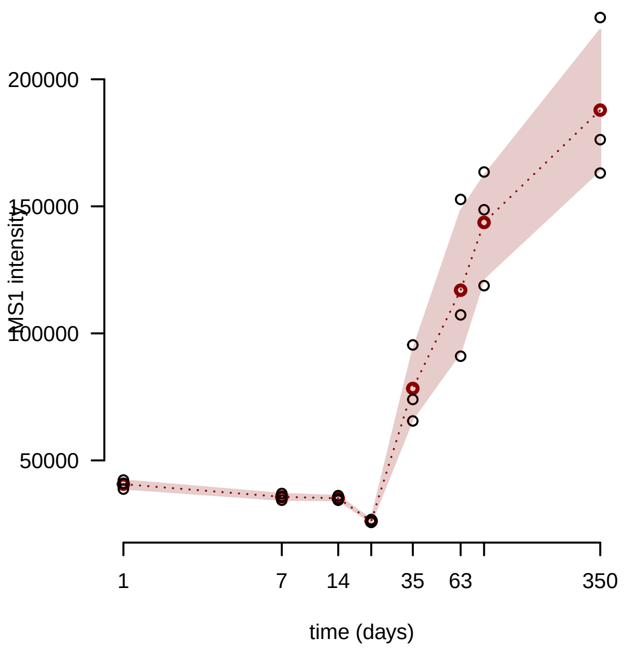

P49615
GN=Cdk5
cyclin−dependent kinase 5

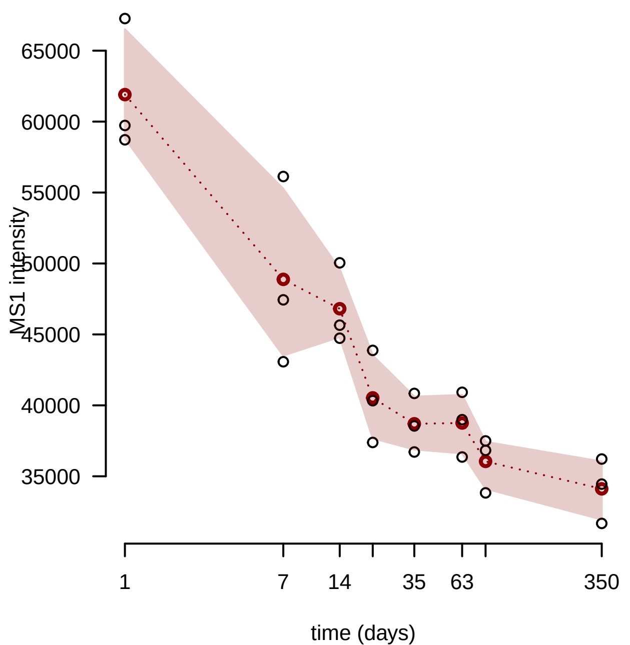

P49710
GN=Hcls1
hematopoietic cell specific Lyn substrat ...

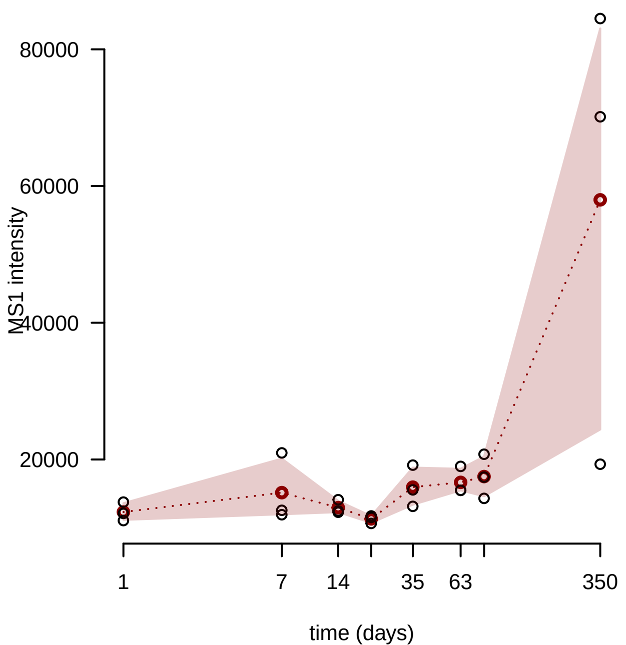

P49717
GN=Mcm4
minichromosome maintenance complex compo ...

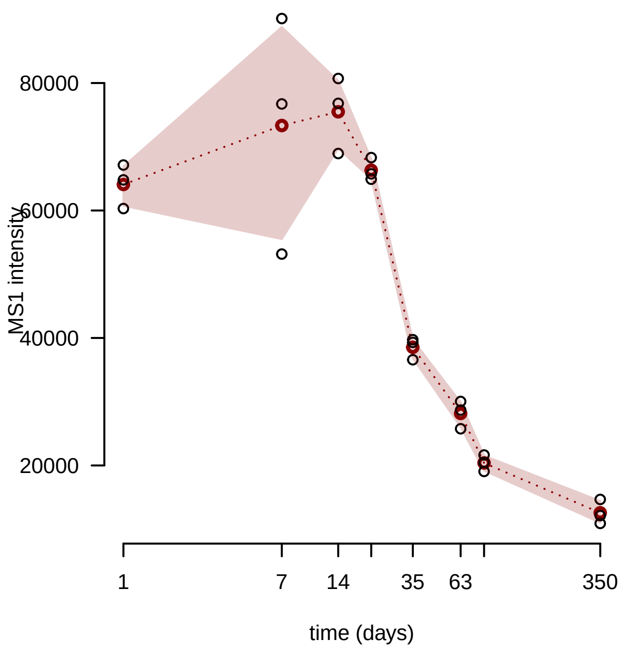

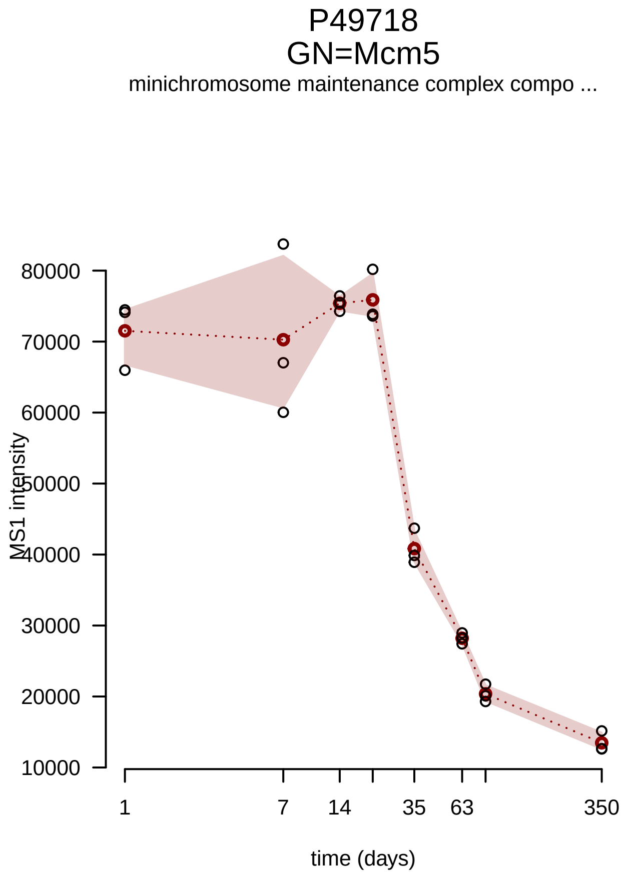
P49718
GN=Mcm5
minichromosome maintenance complex compo ...
MS1 intensity
80000
70000
60000
50000
40000
30000
20000
10000
1
7
14
35
63
350
time (days)

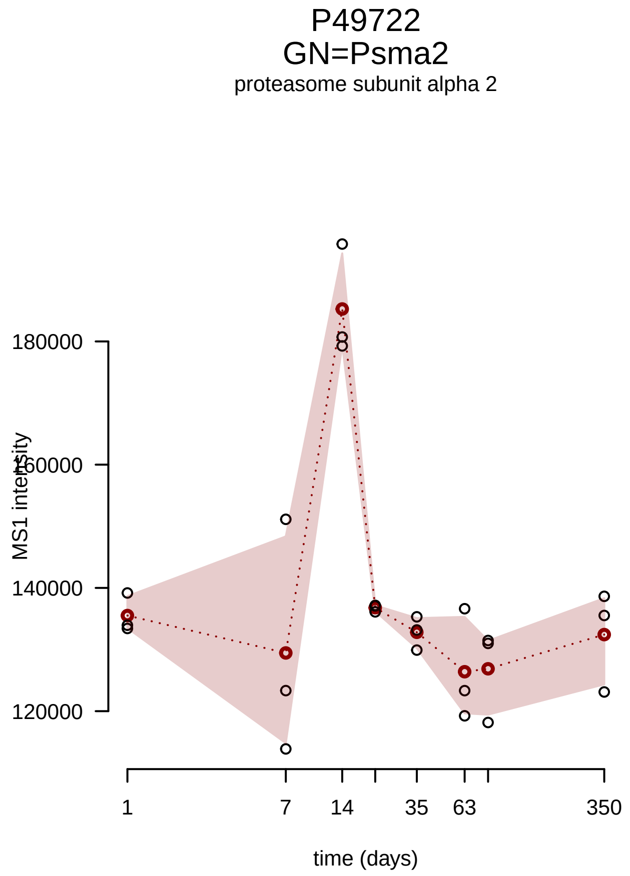
P49722
GN=Psma2
proteasome subunit alpha 2
MS1 intensity
180000
160000
140000
120000
1
7
14
35
63
350
time (days)

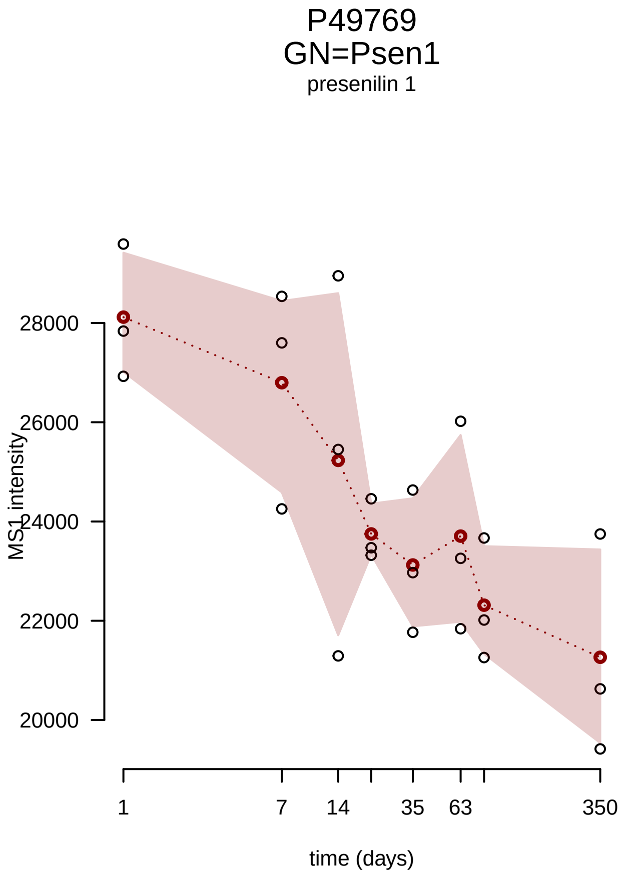
P49769
GN=Psen1
presenilin 1
MS1 intensity
28000
26000
24000
22000
20000
1
7
14
35
63
350
time (days)

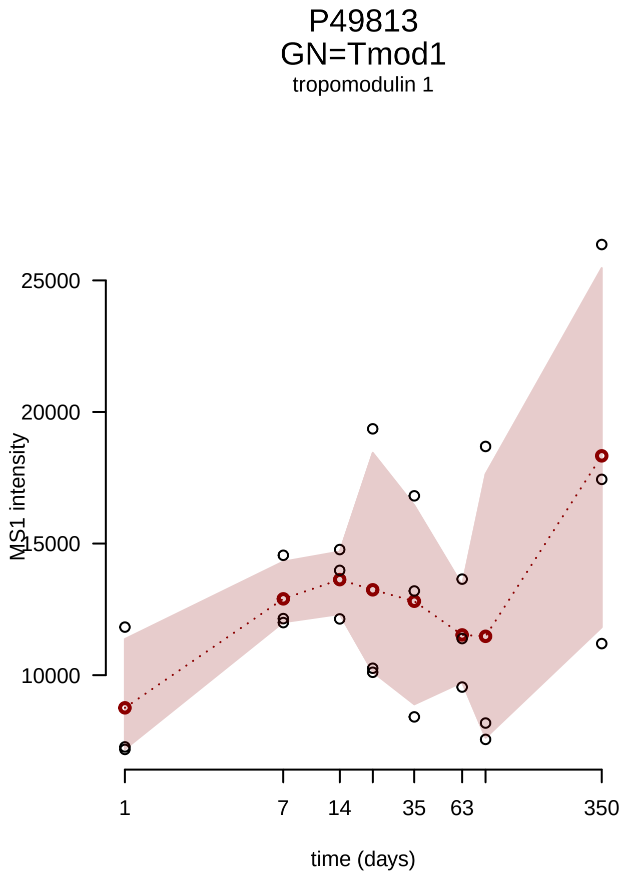
P49813
GN=Tmod1
tropomodulin 1
MS1 intensity
25000
20000
15000
10000
1
7
14
35
63
350
time (days)

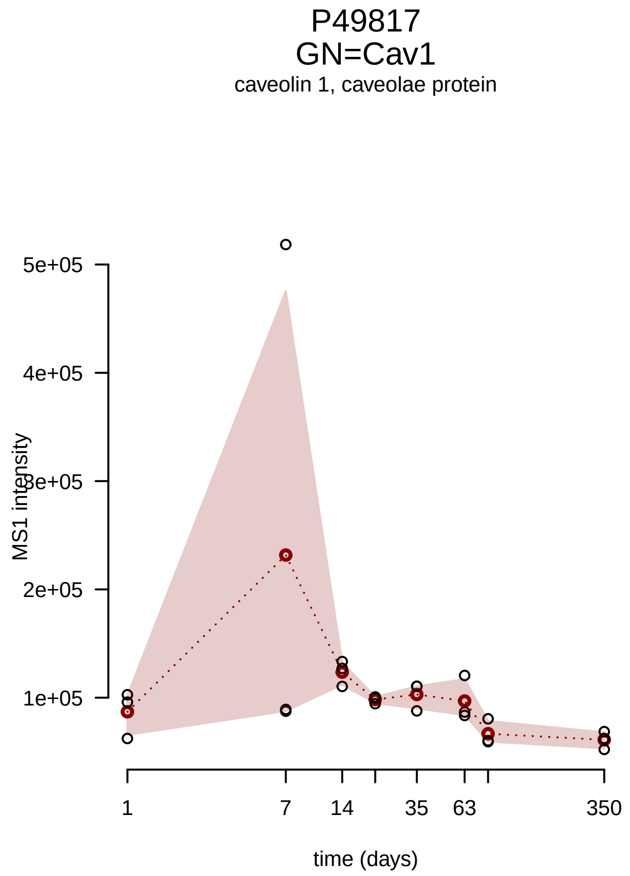
P49817
GN=Cav1
caveolin 1, caveolae protein
MS1 intensity
5e+05
4e+05
3e+05
2e+05
1e+05
1
7
14
35
63
350
time (days)

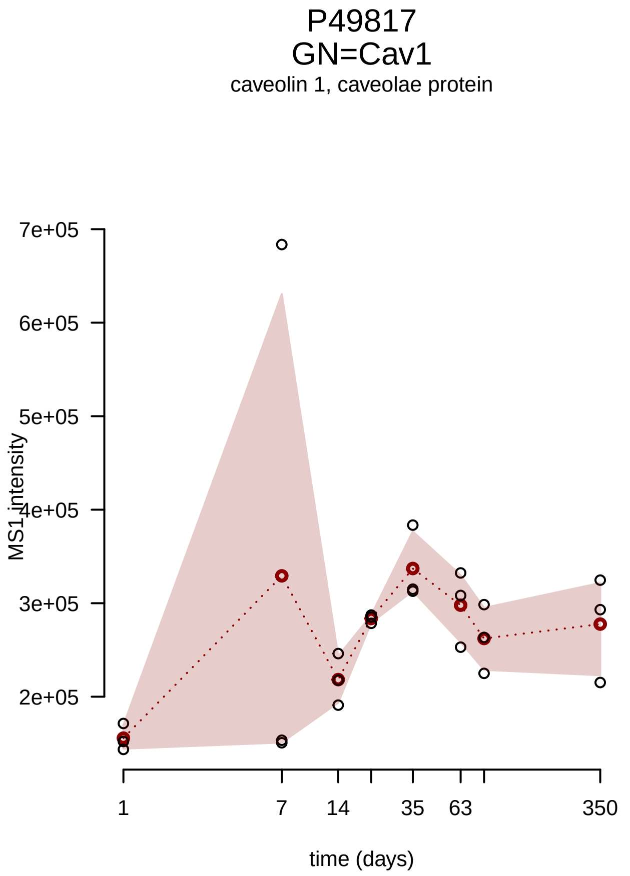
P49817
GN=Cav1
caveolin 1, caveolae protein
MS1 intensity
7e+05
6e+05
5e+05
4e+05
3e+05
2e+05
1
7
14
35
63
350
time (days)

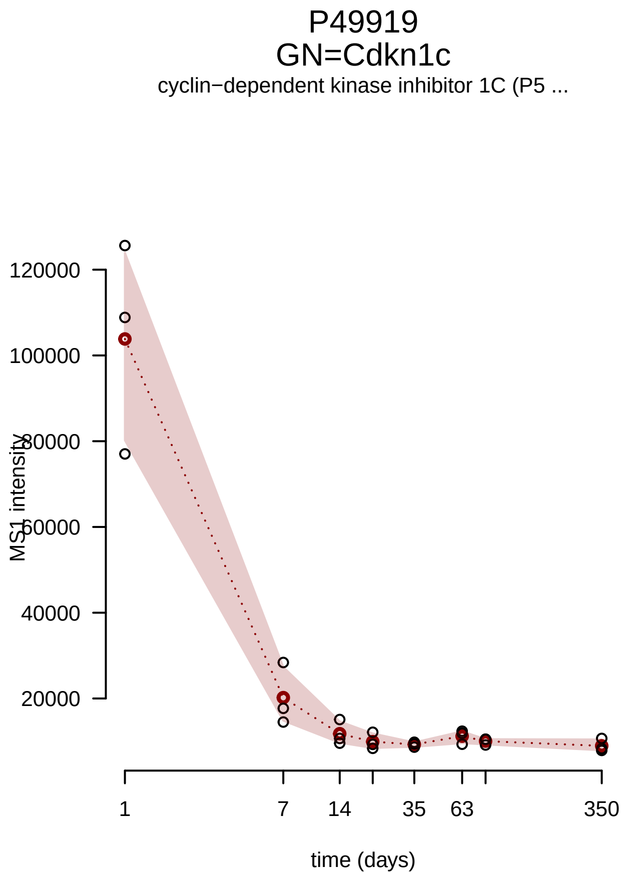
P49919
GN=Cdkn1c
cyclin−dependent kinase inhibitor 1C (P5 ...
MS1 intensity
120000
100000
80000
60000
40000
20000
1
7
14
35
63
350
time (days)

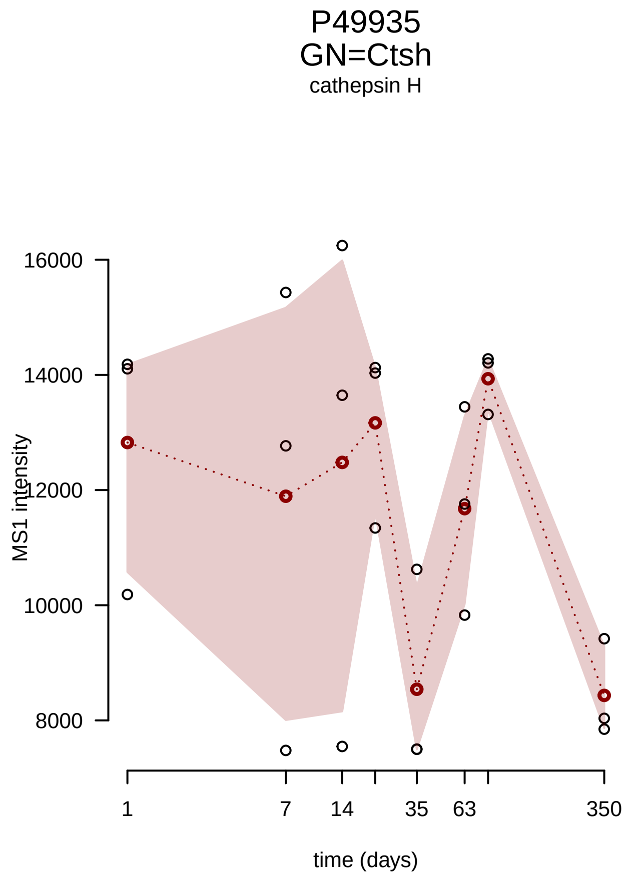
P49935
GN=Ctsh
cathepsin H
MS1 intensity
16000
14000
12000
10000
8000
1
7
14
35
63
350
time (days)

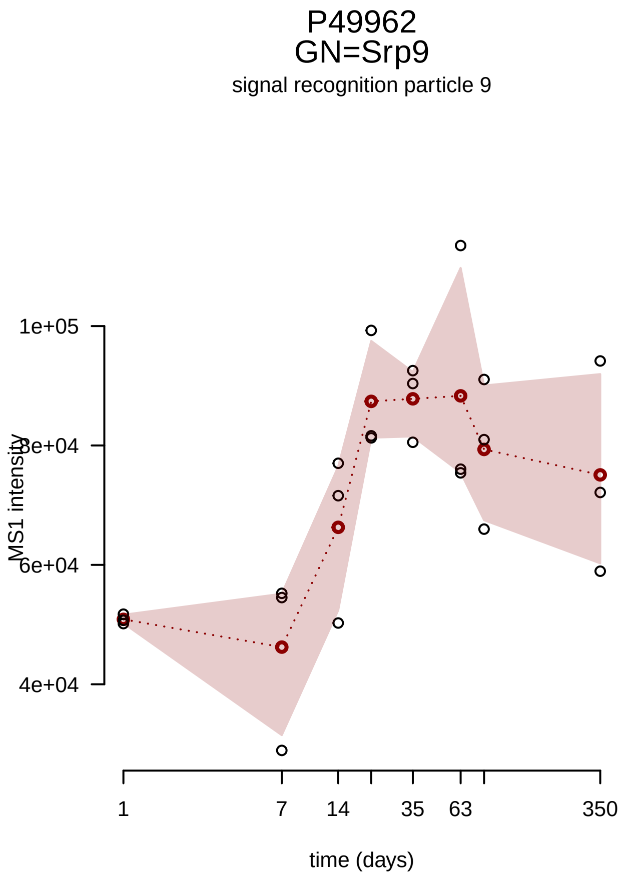
P49962
GN=Srp9
signal recognition particle 9
MS1 intensity
1e+05
8e+04
6e+04
4e+04
1
7
14
35
63
350
time (days)

P50096
GN=Impdh1
inosine monophosphate dehydrogenase 1

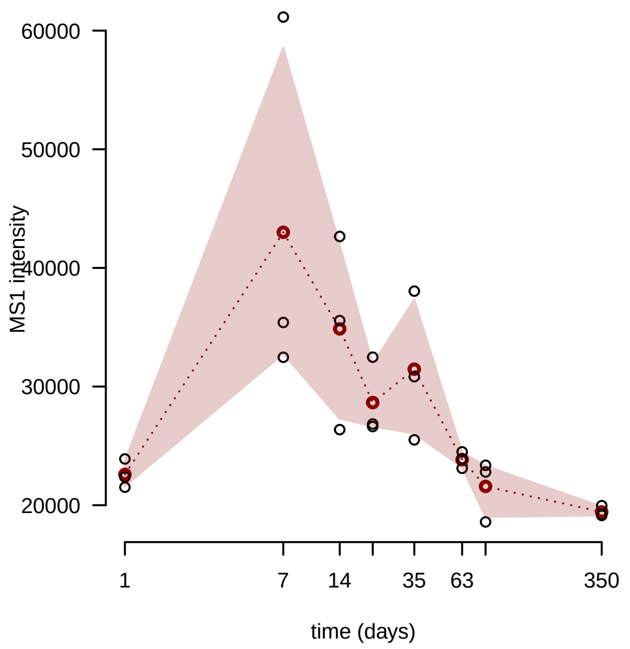

P50114
GN=S100b
S100 protein, beta polypeptide, neural

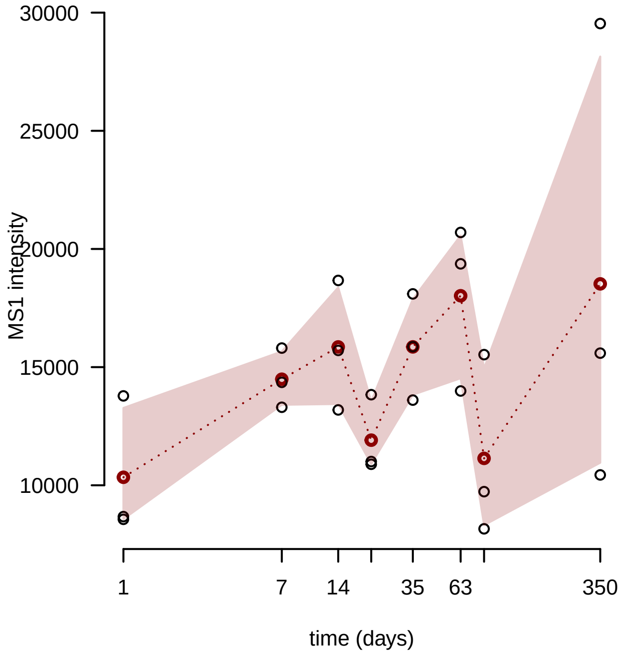

P50136
GN=Bckdha
branched chain ketoacid dehydrogenase E1 ...

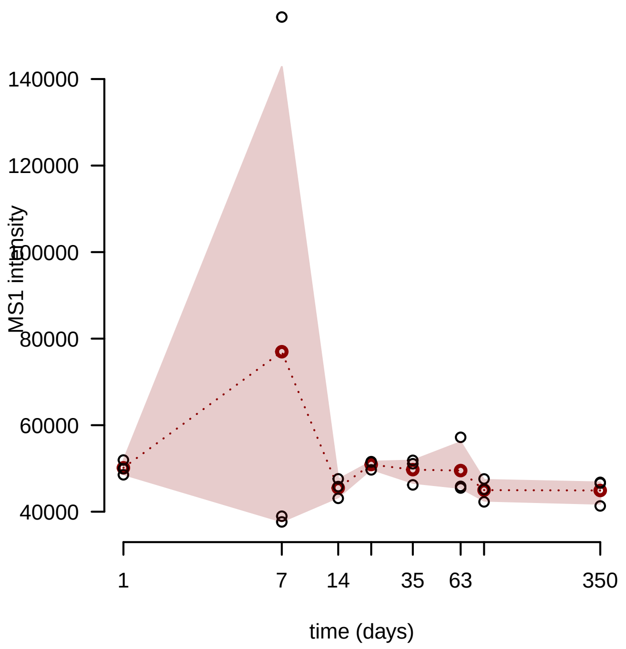

P50171
GN=Hsd17b8

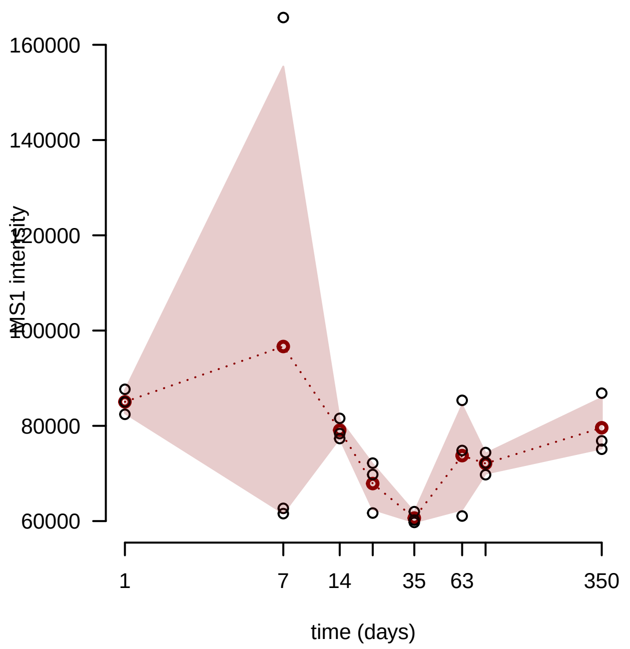

P50172
GN=Hsd11b1
hydroxysteroid 11−beta dehydrogenase 1

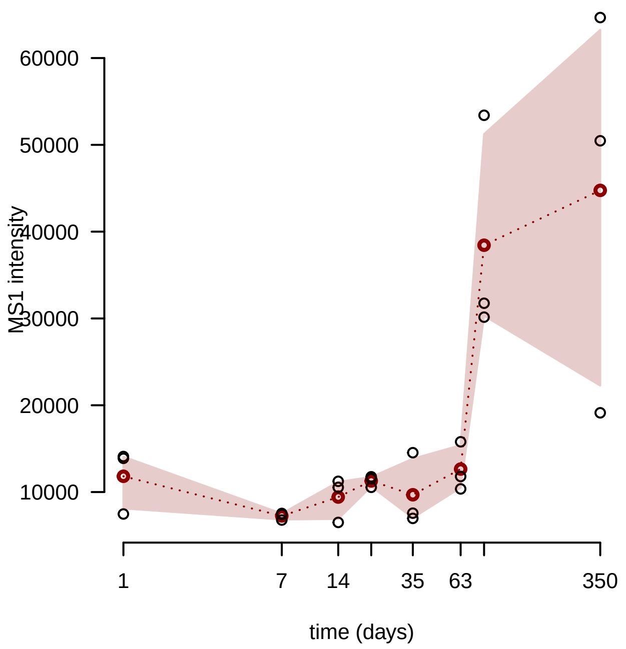

P50247
GN=Ahcy
S−adenosylhomocysteine hydrolase

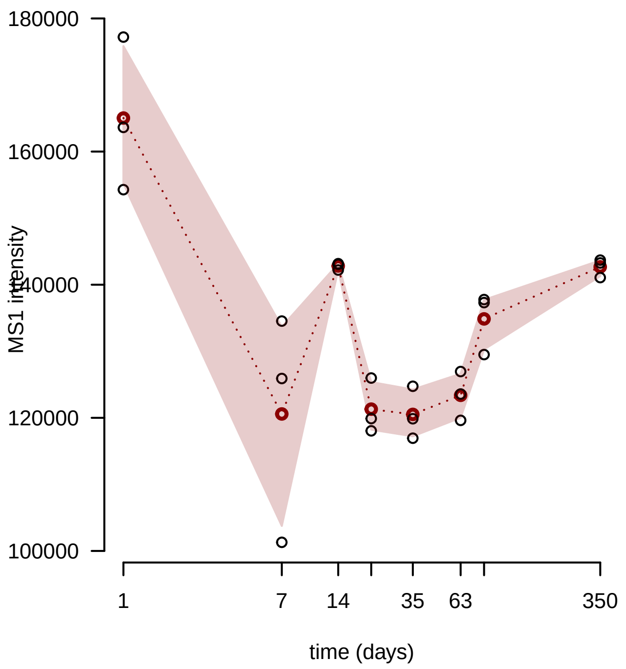

P50285
GN=Fmo1
flavin containing monooxygenase 1

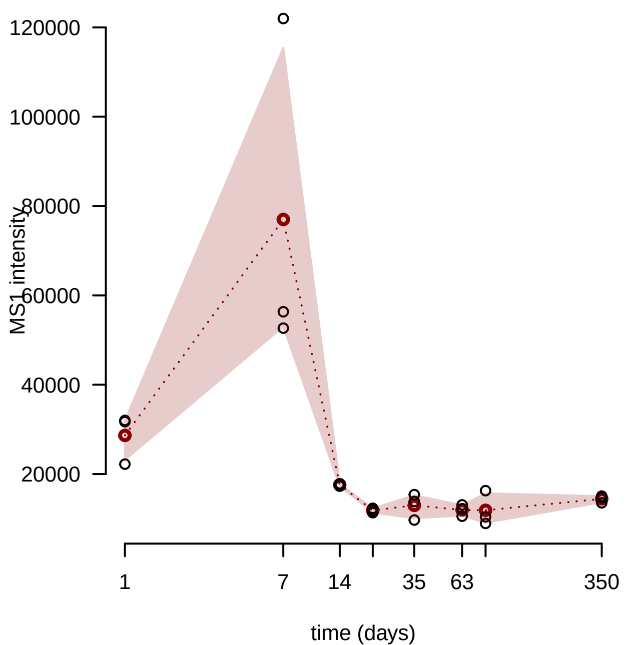

P50295
GN=Nat2
N−acetyltransferase 2 (arylamine N−acety ...

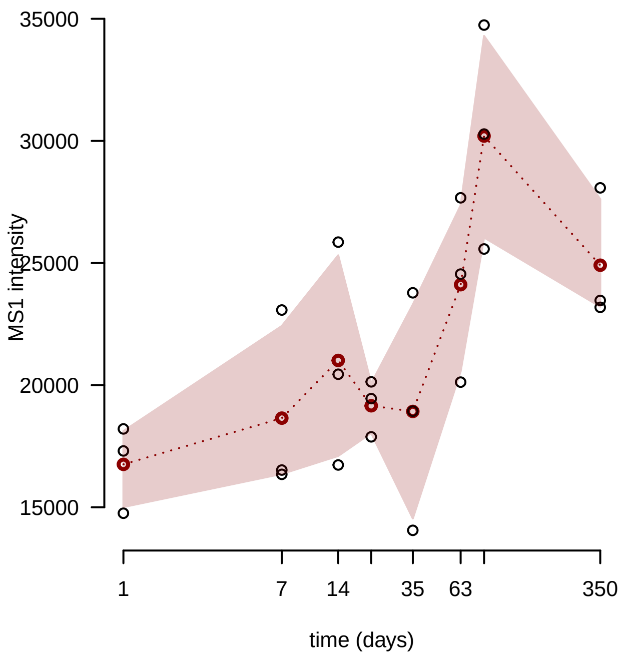

P50396
GN=Gdi1
guanosine diphosphate (GDP) dissociation ...

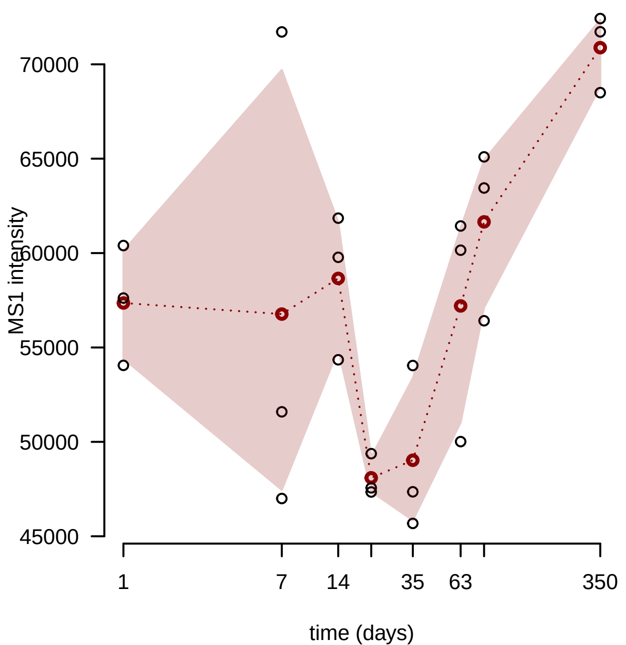

P50427
GN=Sts
steroid sulfatase

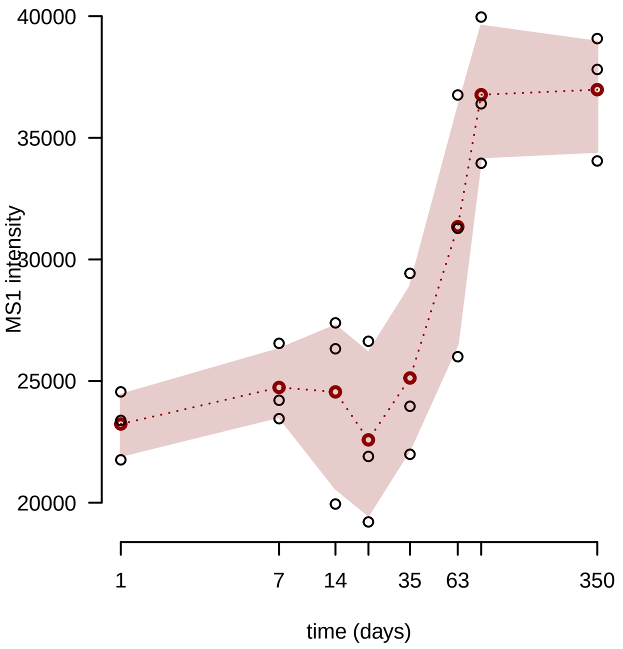

P50428
GN=Arsa
arylsulfatase A

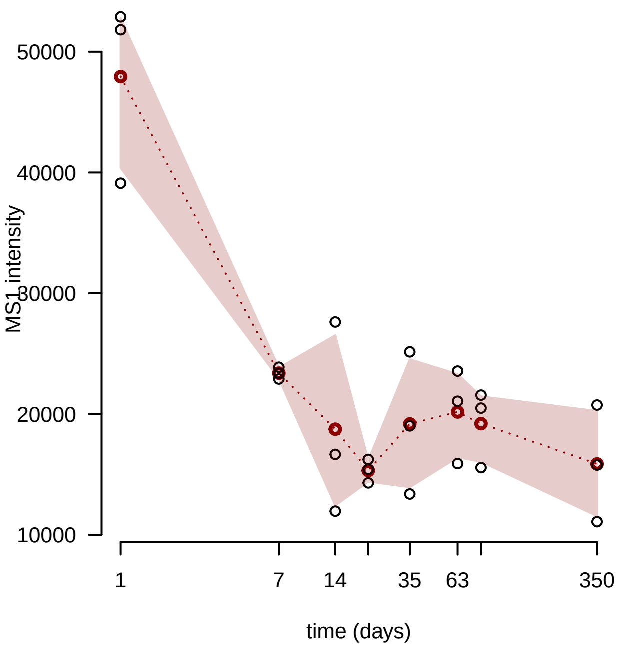

P50429
GN=Arsb
arylsulfatase B

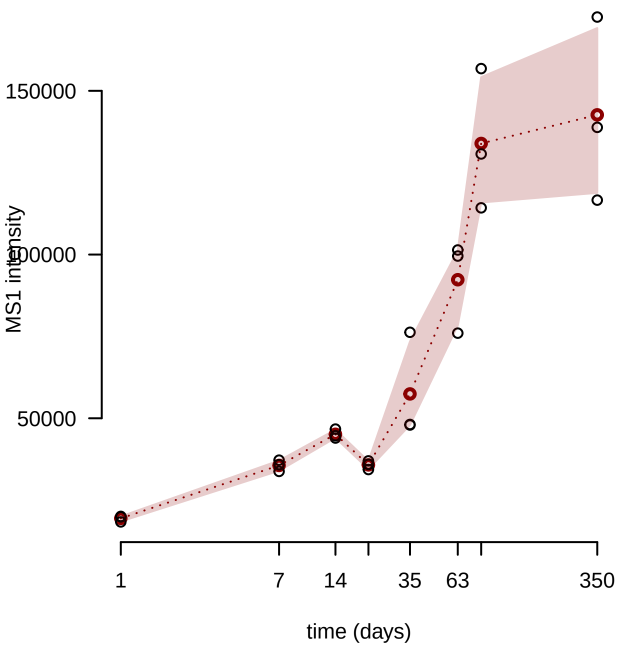

P50431
GN=Shmt1
serine hydroxymethyltransferase 1 (solub ...

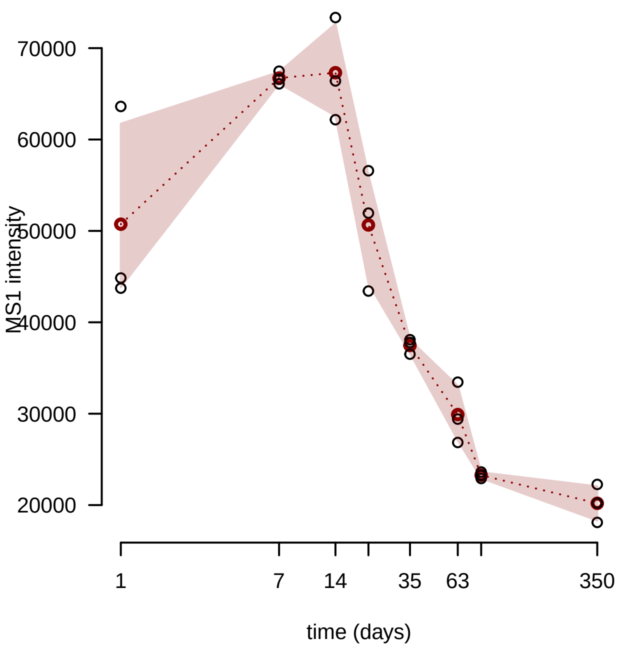

P50446
GN=Krt6a
keratin 6A

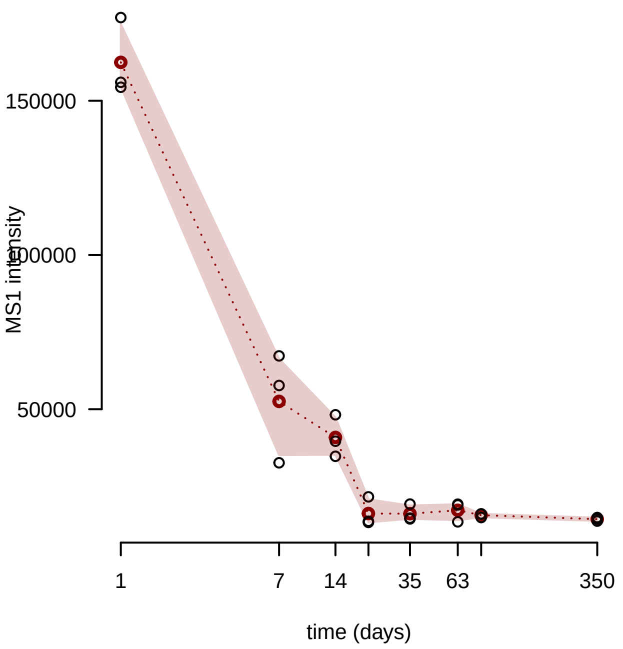

P50516
GN=Atp6v1a
ATPase, H+ transporting, lysosomal V1 su ...

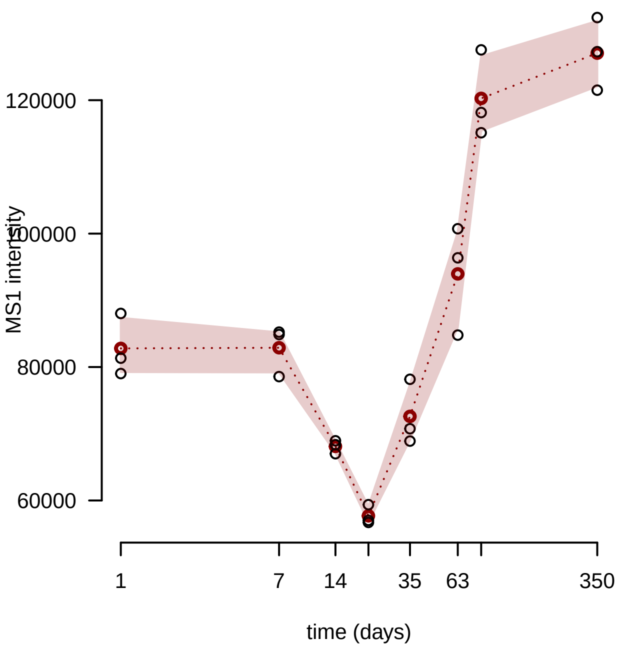

P50518
GN=Atp6v1e1
ATPase, H+ transporting, lysosomal V1 su ...

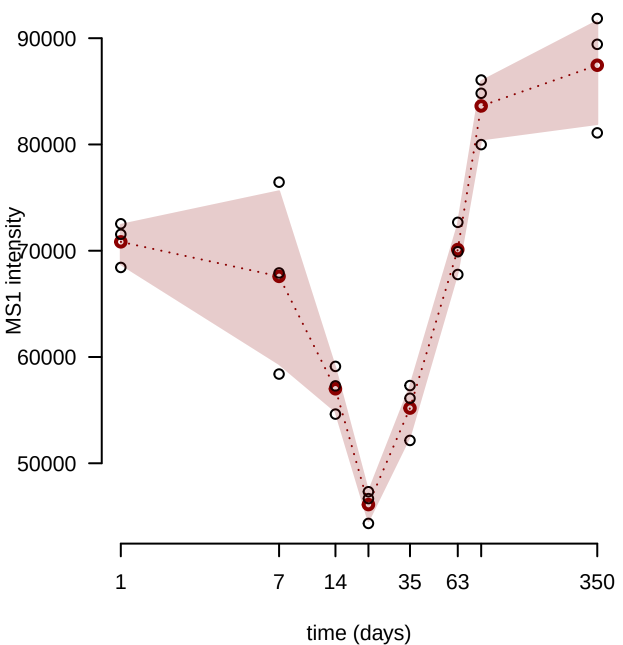

P50543
GN=S100a11
S100 calcium binding protein A11

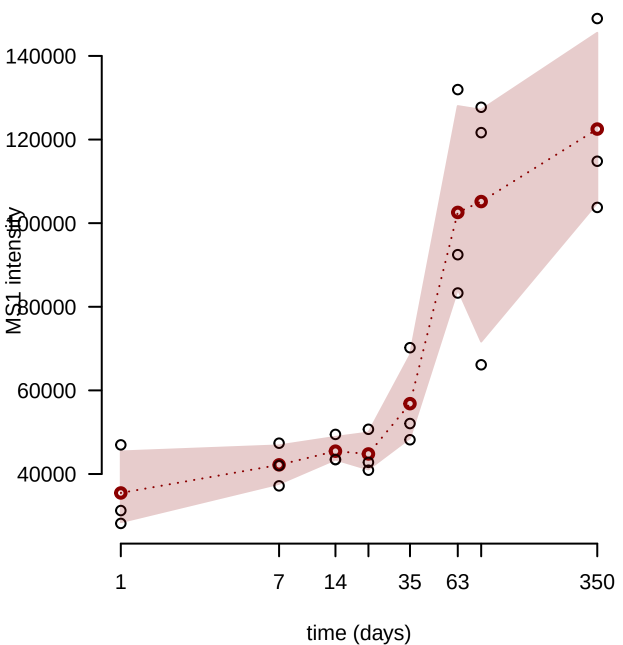

P50544
GN=Acadvl
acyl−Coenzyme A dehydrogenase, very long ...

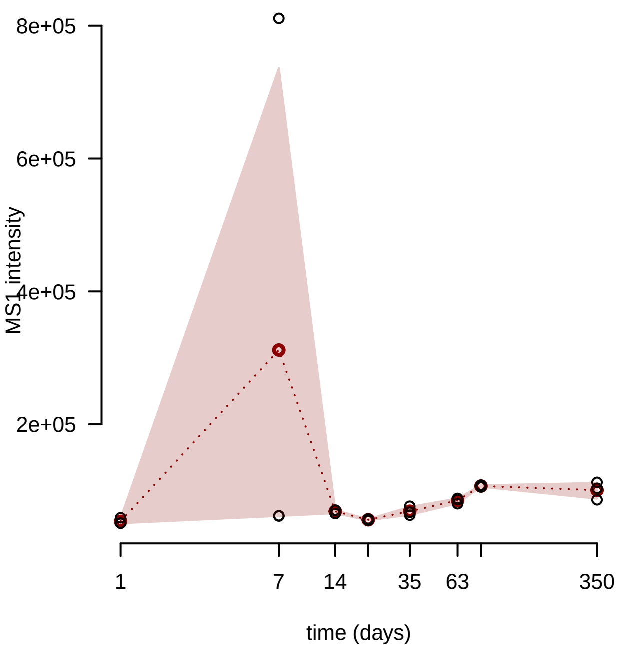

P50580
GN=Pa2g4
proliferation−associated 2G4

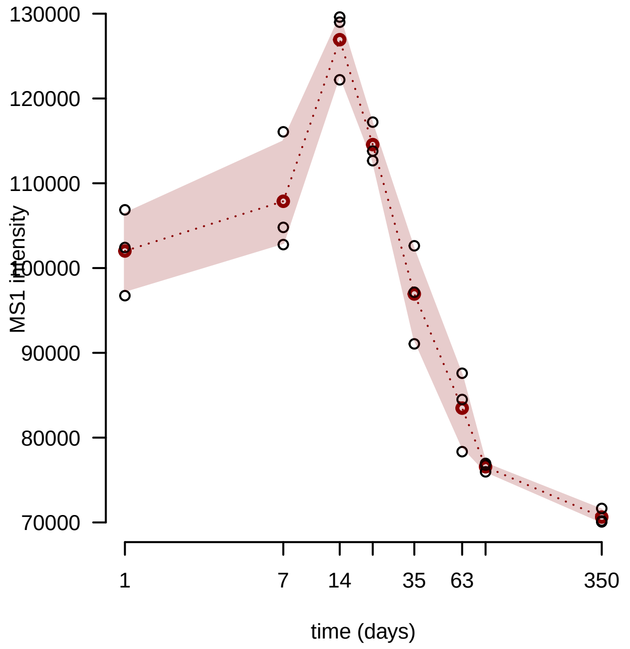

P50608
GN=Fmod
fibromodulin

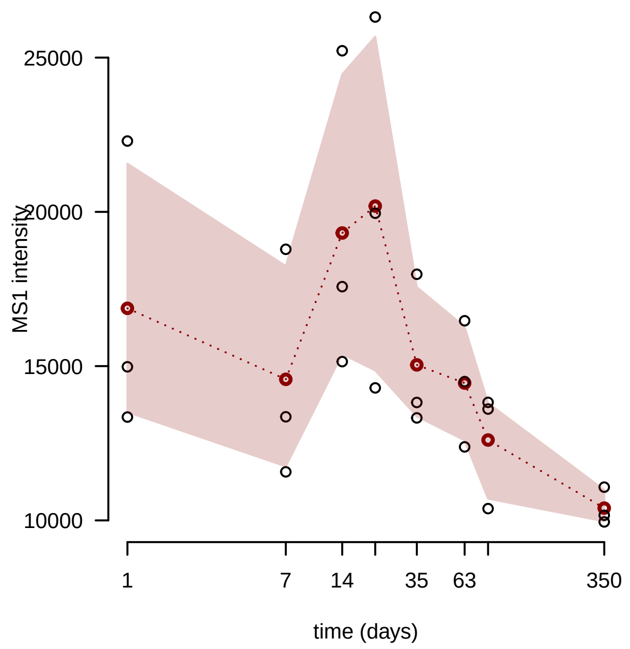

P50615
GN=Btg3
BTG anti−proliferation factor 3

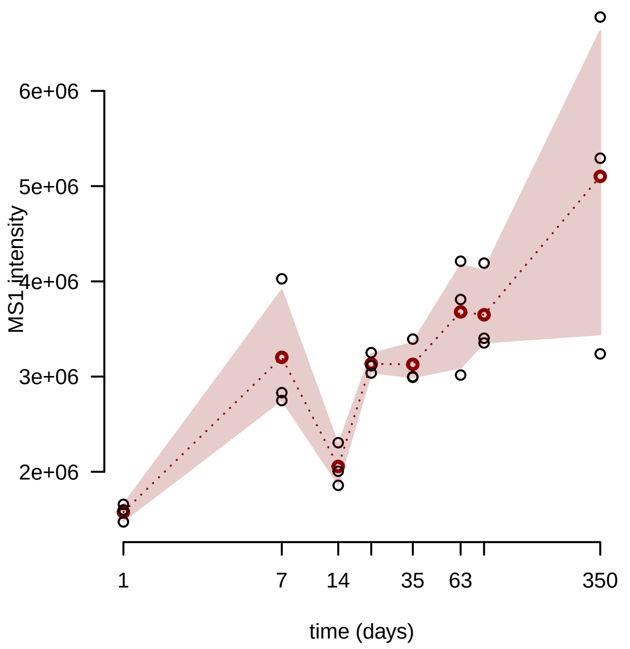

P50637
GN=Tspo
translocator protein

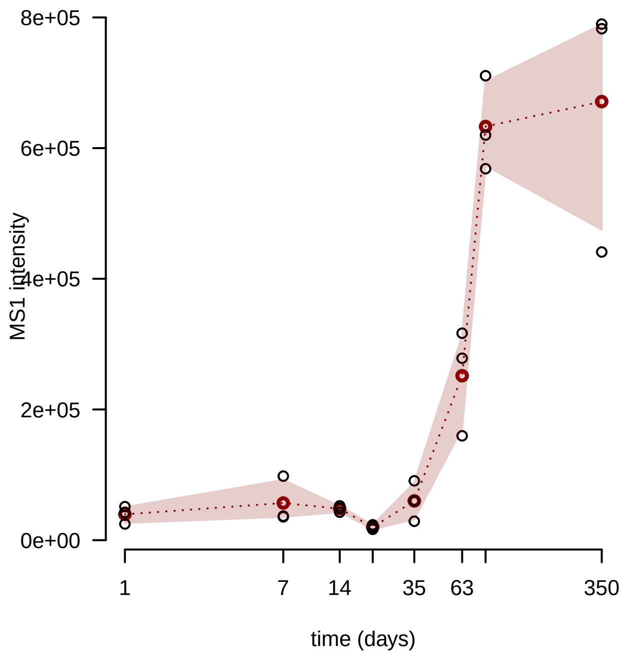

P51125
GN=Cast
calpastatin

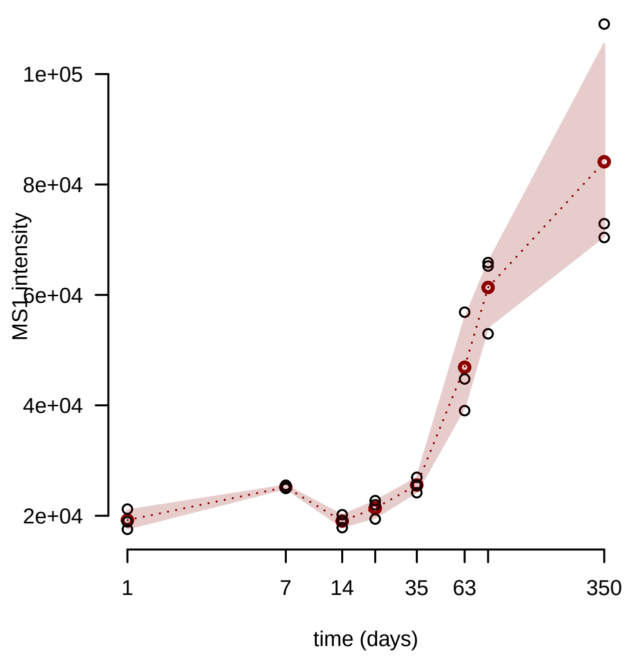

P51150
GN=Rab7a

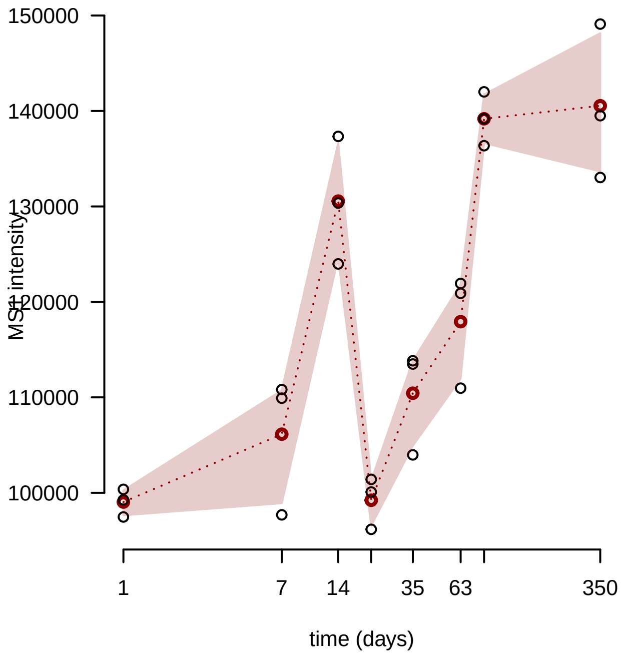

P51162
GN=Fabp6
fatty acid binding protein 6

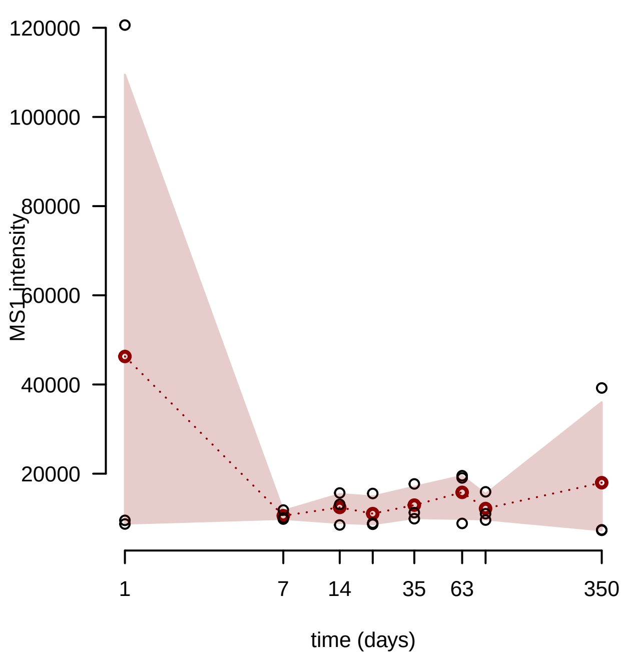

P51163
GN=Uros
uroporphyrinogen III synthase

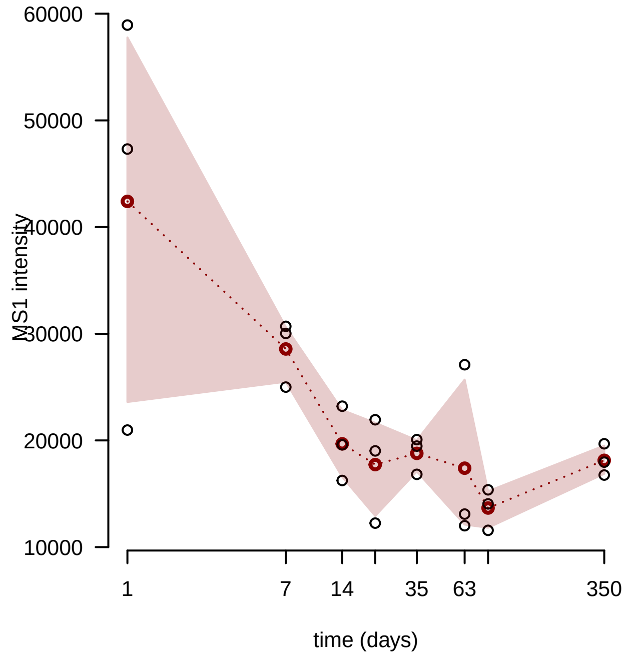

P51174
GN=Acadl
acyl−Coenzyme A dehydrogenase, long−chai ...

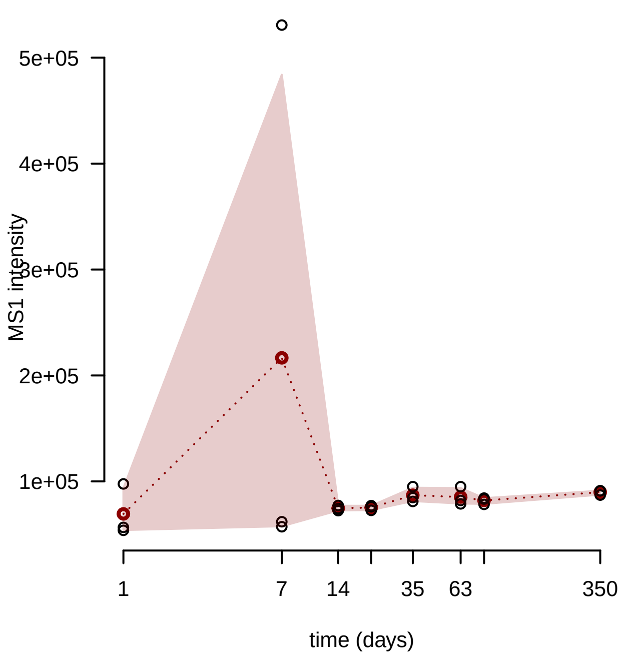

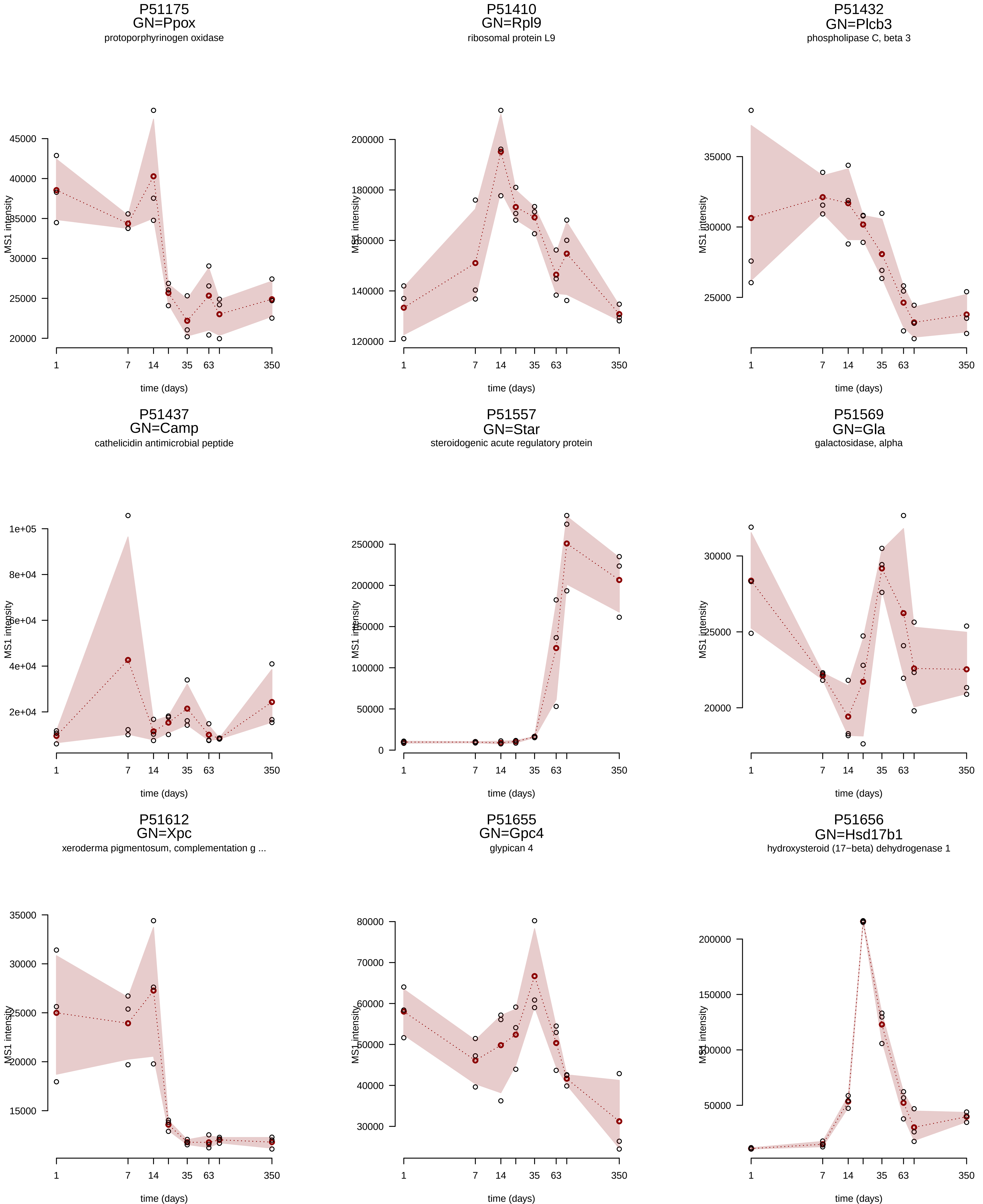
P51175
GN=Ppox
protoporphyrinogen oxidase
MS1 intensity
45000
40000
35000
30000
25000
20000
1
7
14
35
63
350
time (days)
P51410
GN=Rpl9
ribosomal protein L9
MS1 intensity
200000
180000
160000
140000
120000
1
7
14
35
63
350
time (days)
P51432
GN=Plcb3
phospholipase C, beta 3
MS1 intensity
35000
30000
25000
1
7
14
35
63
350
time (days)
P51437
GN=Camp
cathelicidin antimicrobial peptide
MS1 intensity
1e+05
8e+04
6e+04
4e+04
2e+04
1
7
14
35
63
350
time (days)
P51557
GN=Star
steroidogenic acute regulatory protein
MS1 intensity
250000
200000
150000
100000
50000
0
1
7
14
35
63
350
time (days)
P51569
GN=Gla
galactosidase, alpha
MS1 intensity
30000
25000
20000
1
7
14
35
63
350
time (days)
P51612
GN=Xpc
xeroderma pigmentosum, complementation g ...
MS1 intensity
35000
30000
25000
20000
15000
1
7
14
35
63
350
time (days)
P51655
GN=Gpc4
glypican 4
MS1 intensity
80000
70000
60000
50000
40000
30000
1
7
14
35
63
350
time (days)
P51656
GN=Hsd17b1
hydroxysteroid (17−beta) dehydrogenase 1
MS1 intensity
200000
150000
100000
50000
1
7
14
35
63
350
time (days)

P51658
GN=Hsd17b2
hydroxysteroid (17−beta) dehydrogenase 2

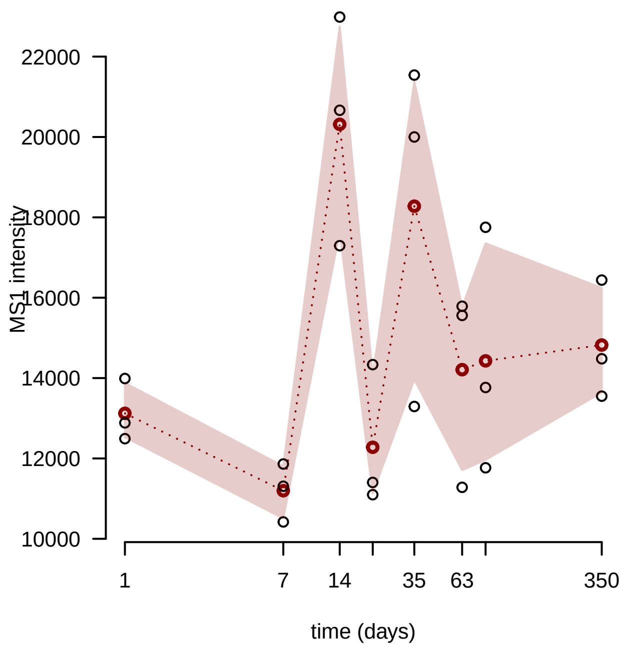

P51660
GN=Hsd17b4
hydroxysteroid (17−beta) dehydrogenase 4

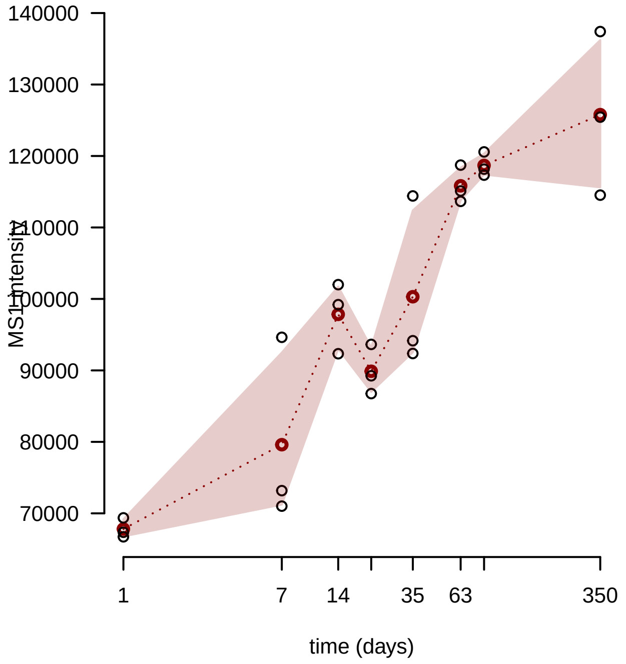

P51661
GN=Hsd11b2
hydroxysteroid 11−beta dehydrogenase 2

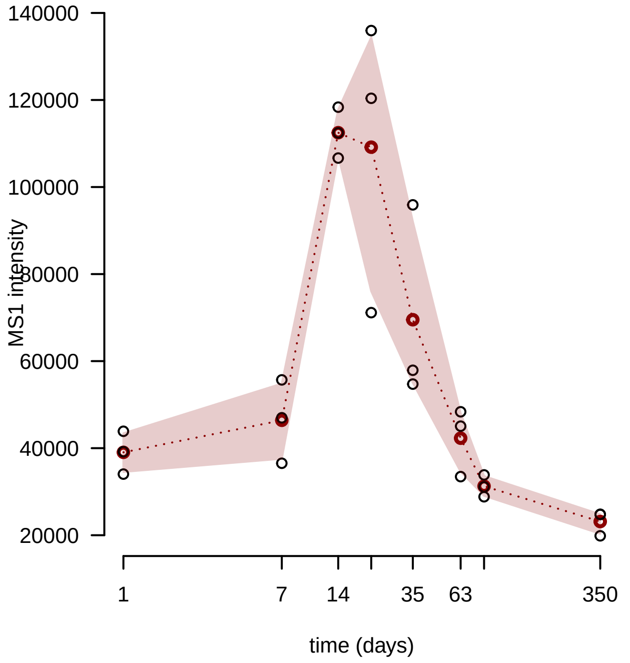

P51667
GN=Myl2
myosin, light polypeptide 2, regulatory, ...

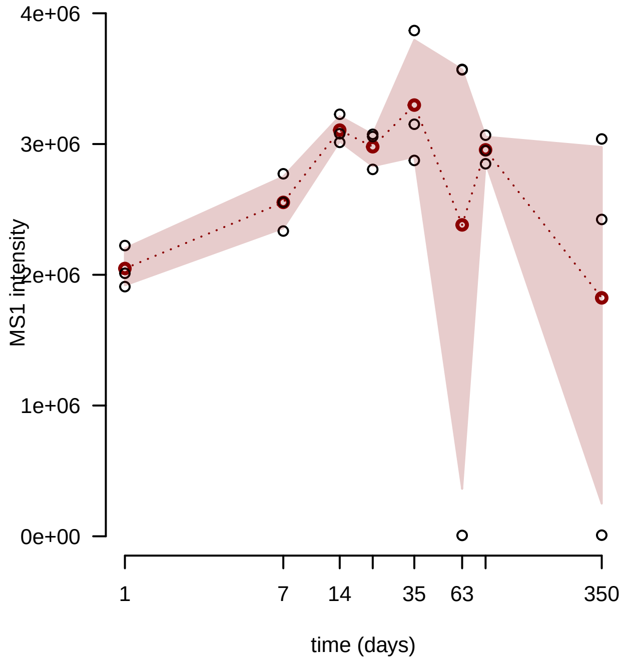

P51791
GN=Clcn3
chloride channel, voltage−sensitive 3

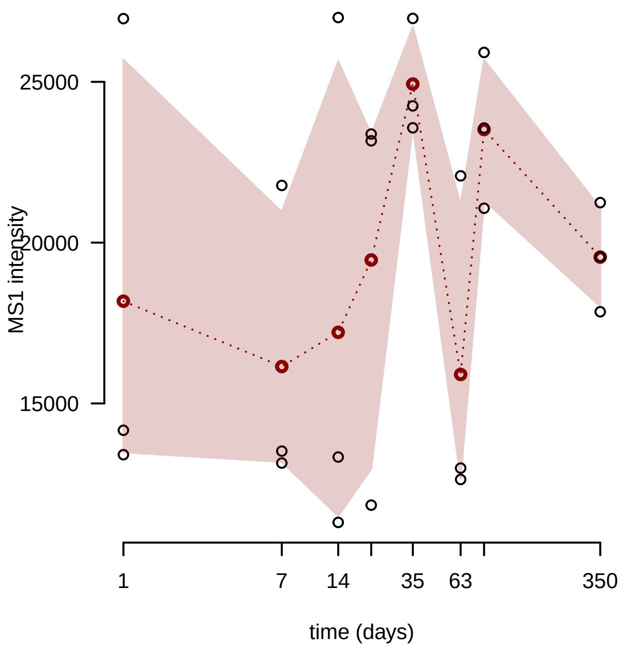

P51807
GN=Dynlt1

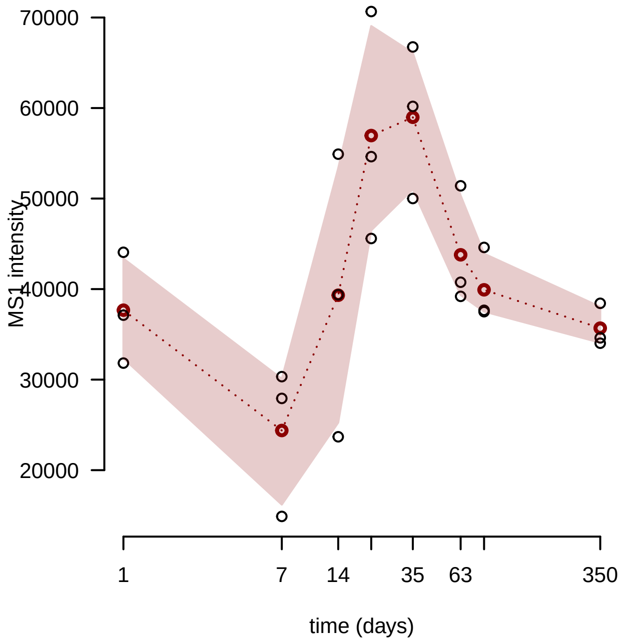

P51827
GN=Aff3
AF4/FMR2 family, member 3

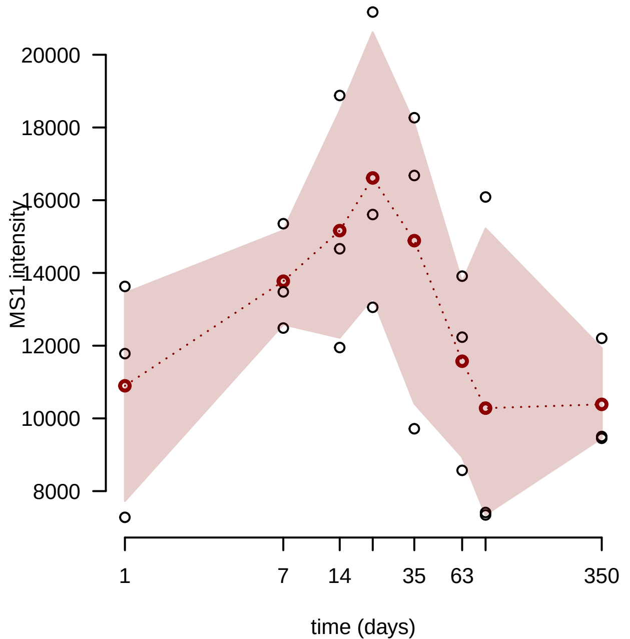

P51829
GN=Adcy7
adenylate cyclase 7

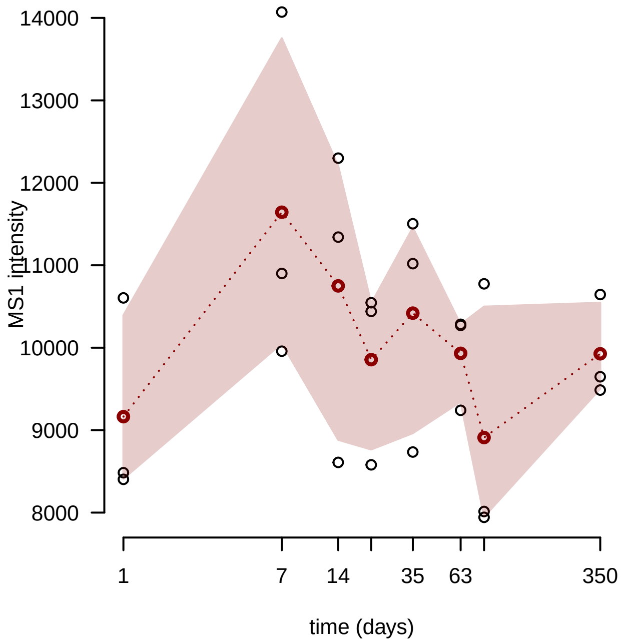

P51830
GN=Adcy9
adenylate cyclase 9

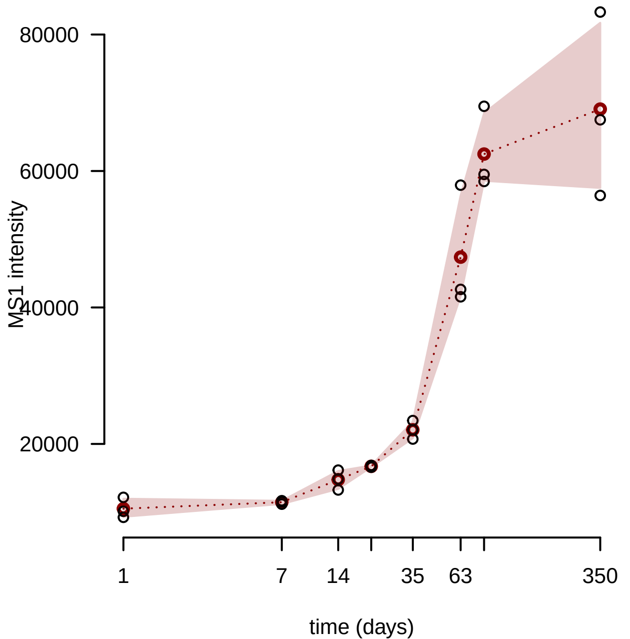

P51855
GN=Gss
glutathione synthetase

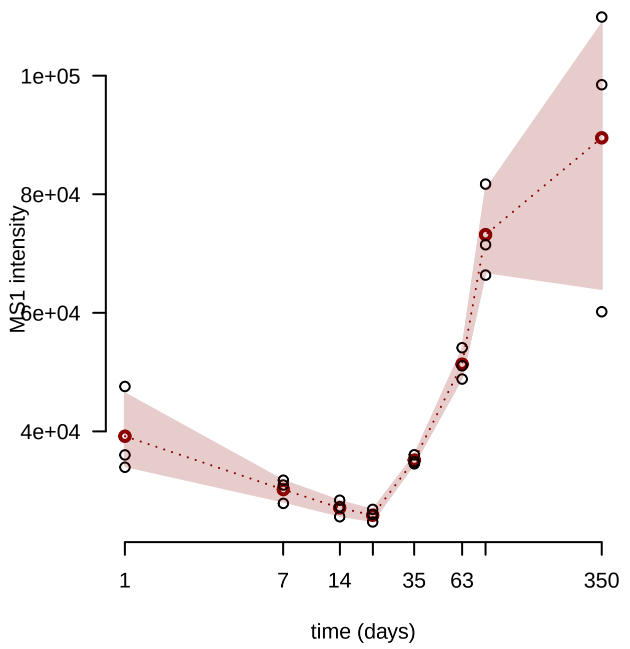

P51859
GN=Hdgf
heparin binding growth factor

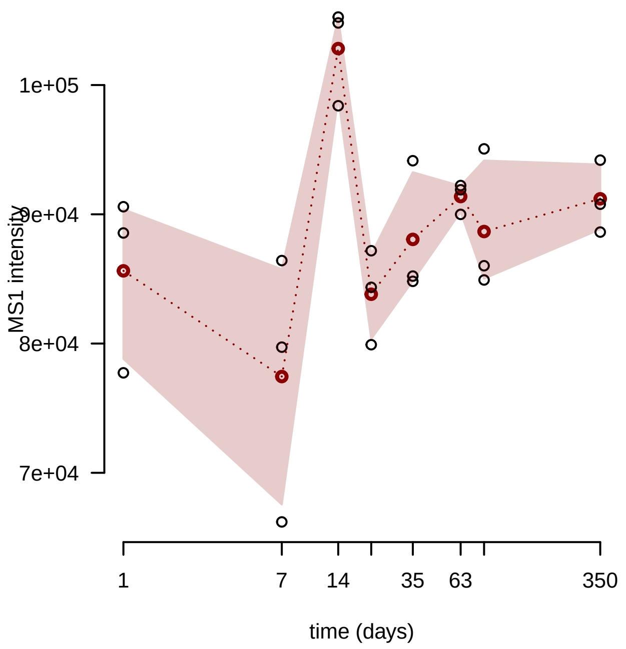

P51863
GN=Atp6v0d1
ATPase, H+ transporting, lysosomal V0 su ...

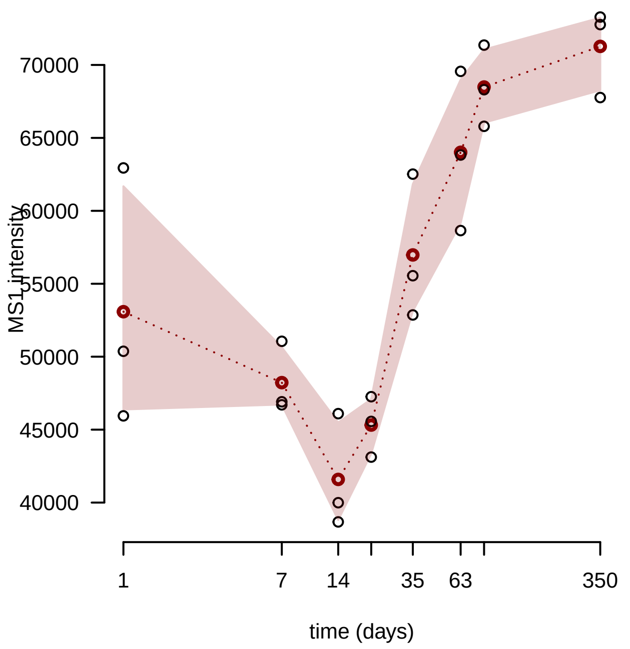

P51880
GN=Fabp7
fatty acid binding protein 7, brain

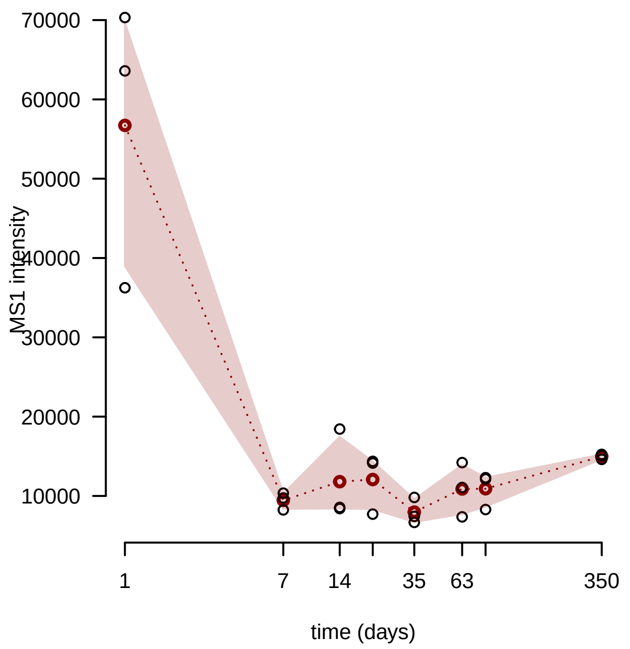

P51881
GN=Slc25a5
solute carrier family 25 (mitochondrial ...

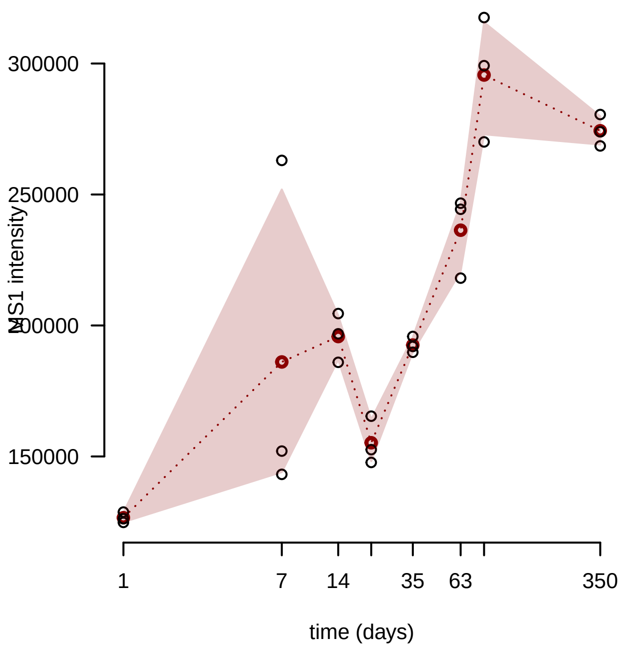

P51885
GN=Lum
lumican

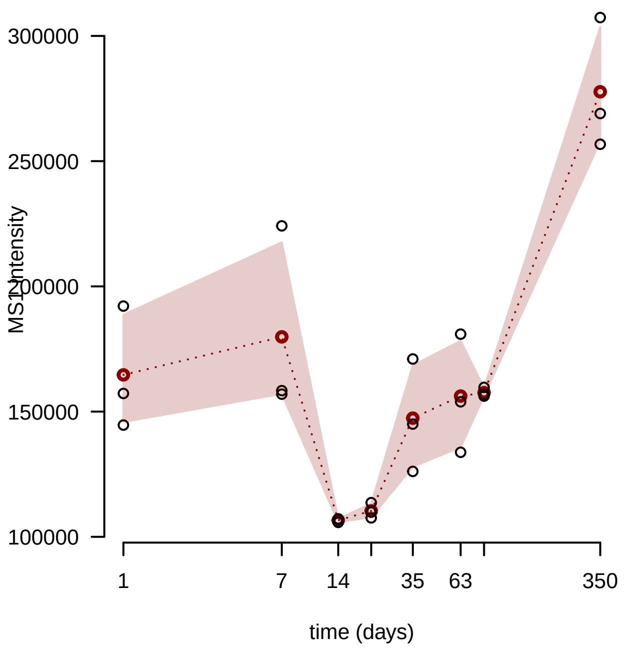

P51912
GN=Slc1a5
solute carrier family 1 (neutral amino a ...

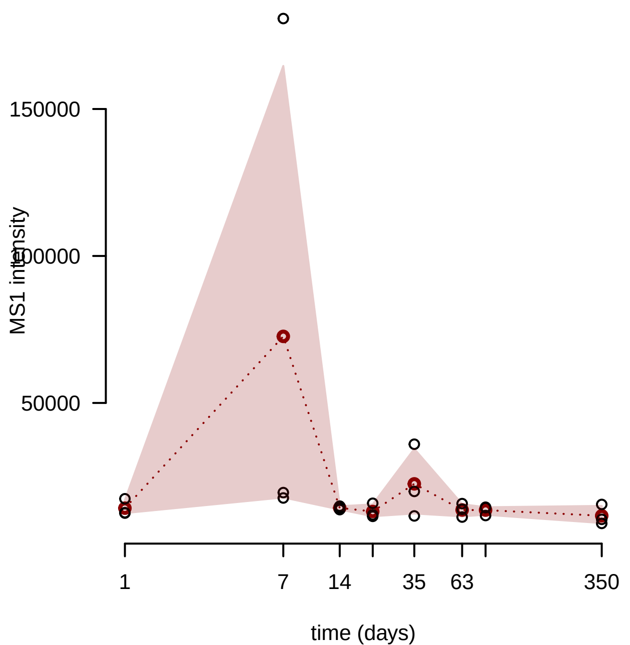

P51949
GN=Mnat1
menage a trois 1

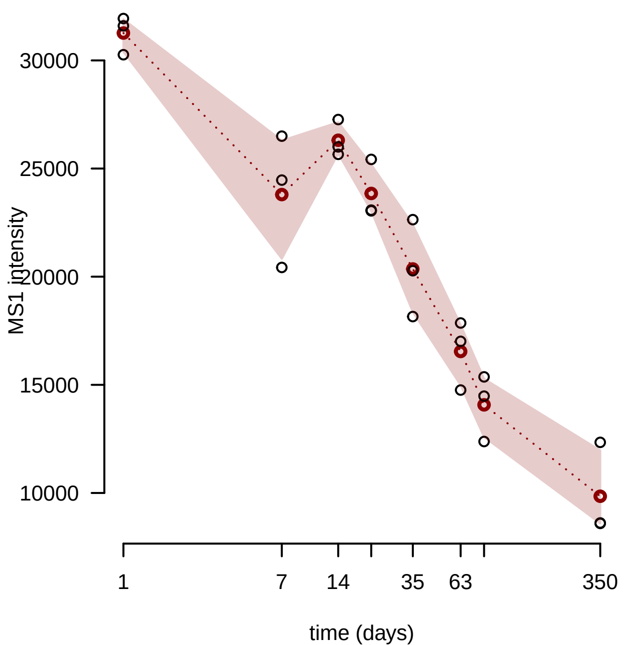

P51954
GN=Nek1
NIMA (never in mitosis gene a)−related e ...

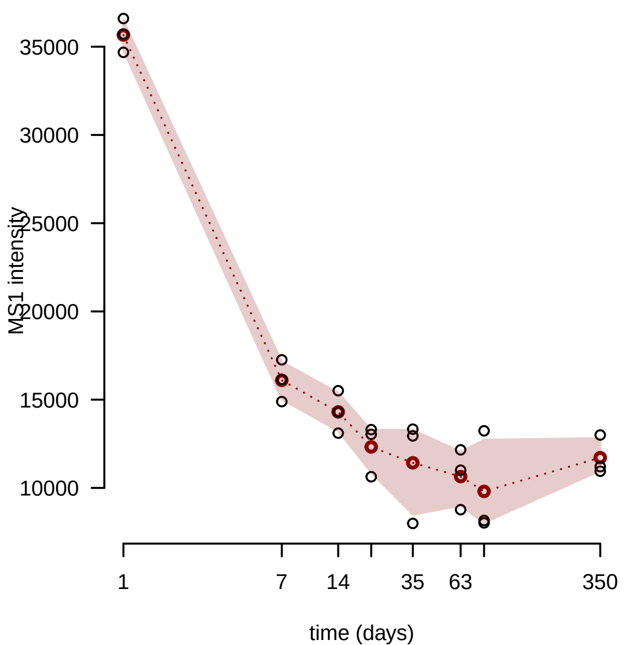

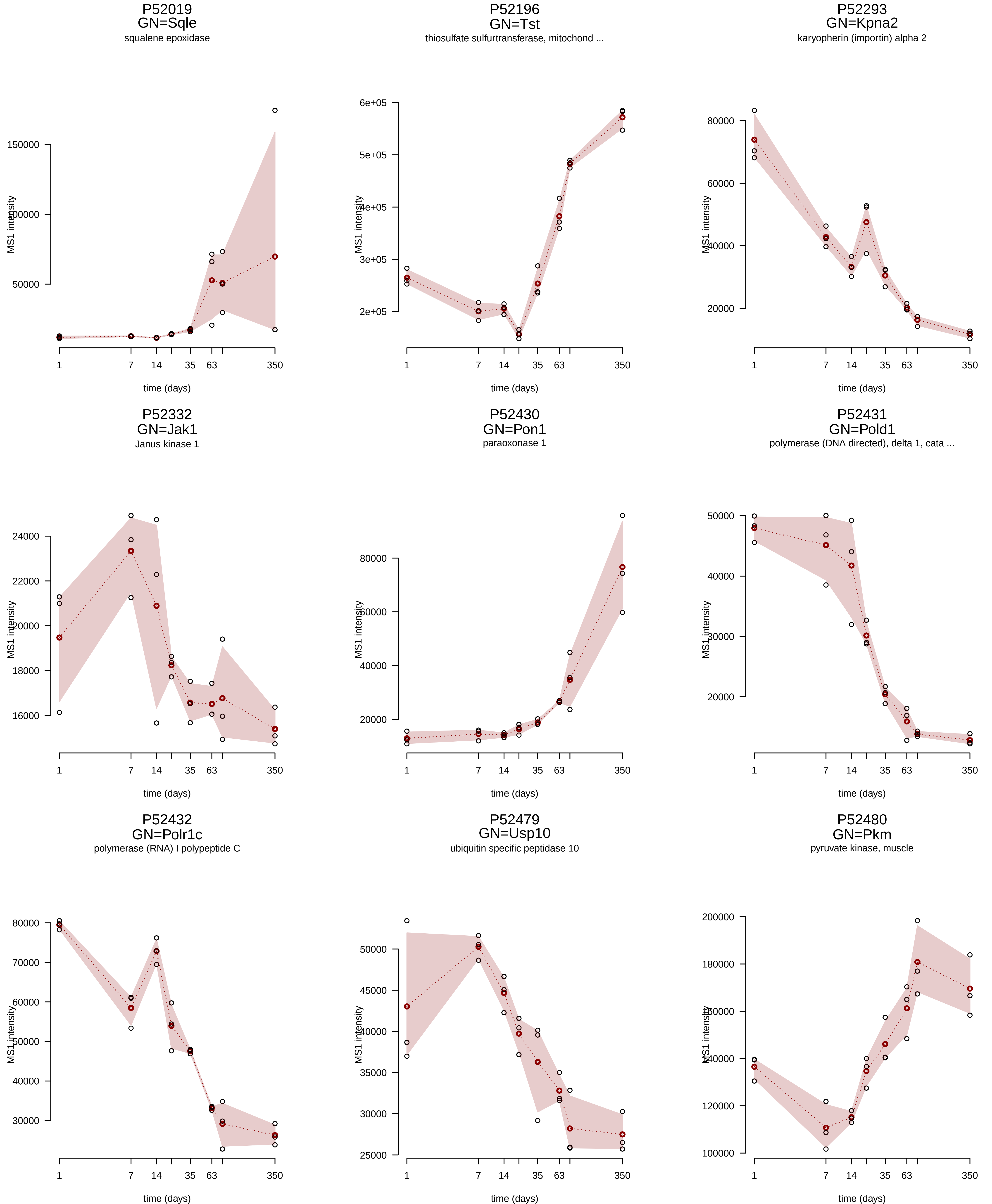
P52019
GN=Sqle
squalene epoxidase
150000
100000
50000
MS1 intensity
1
7
14
35
63
350
time (days)
P52196
GN=Tst
thiosulfate sulfurtransferase, mitochond ...
6e+05
5e+05
4e+05
3e+05
2e+05
MS1 intensity
1
7
14
35
63
350
time (days)
P52293
GN=Kpna2
karyopherin (importin) alpha 2
80000
60000
40000
20000
MS1 intensity
1
7
14
35
63
350
time (days)
P52332
GN=Jak1
Janus kinase 1
24000
22000
20000
18000
16000
MS1 intensity
1
7
14
35
63
350
time (days)
P52430
GN=Pon1
paraoxonase 1
80000
60000
40000
20000
MS1 intensity
1
7
14
35
63
350
time (days)
P52431
GN=Pold1
polymerase (DNA directed), delta 1, cata ...
50000
40000
30000
20000
MS1 intensity
1
7
14
35
63
350
time (days)
P52432
GN=Polr1c
polymerase (RNA) I polypeptide C
80000
70000
60000
50000
40000
30000
MS1 intensity
1
7
14
35
63
350
time (days)
P52479
GN=Usp10
ubiquitin specific peptidase 10
50000
45000
40000
35000
30000
25000
MS1 intensity
1
7
14
35
63
350
time (days)
P52480
GN=Pkm
pyruvate kinase, muscle
200000
180000
160000
140000
120000
100000
MS1 intensity
1
7
14
35
63
350
time (days)

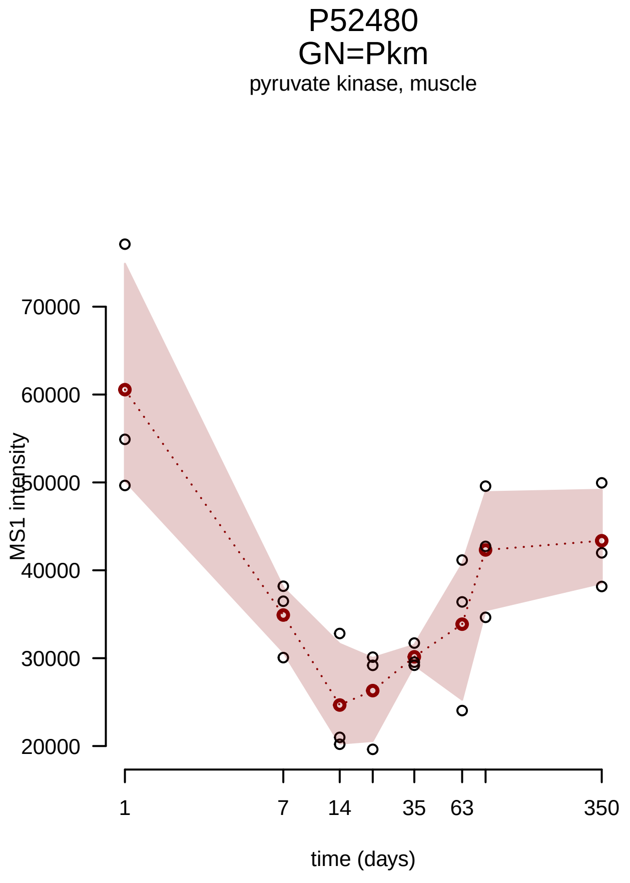
P52480
GN=Pkm
pyruvate kinase, muscle
MS1 intensity
20000
30000
40000
50000
60000
70000
1
7
14
35
63
350
time (days)

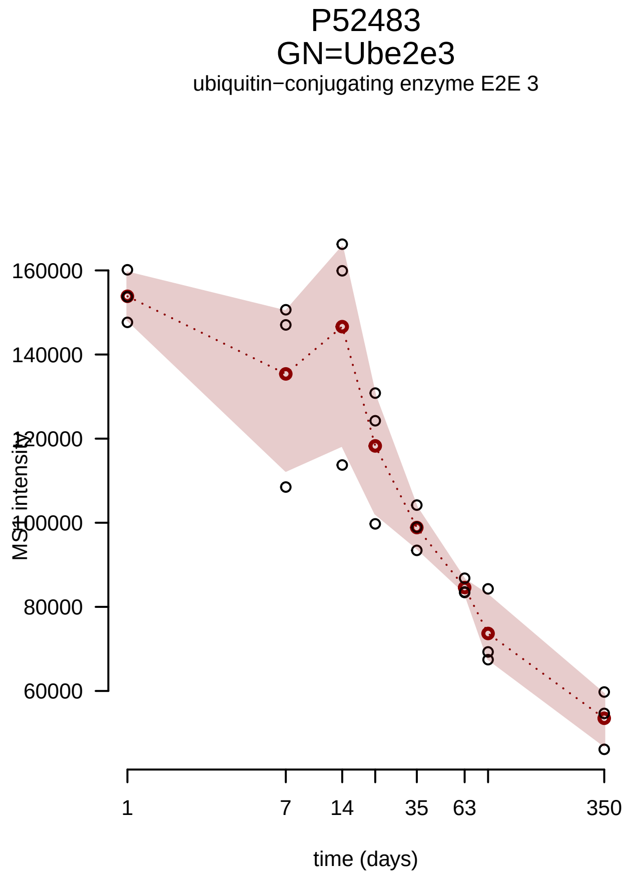
P52483
GN=Ube2e3
ubiquitin−conjugating enzyme E2E 3
MS1 intensity
60000
80000
100000
120000
140000
160000
1
7
14
35
63
350
time (days)

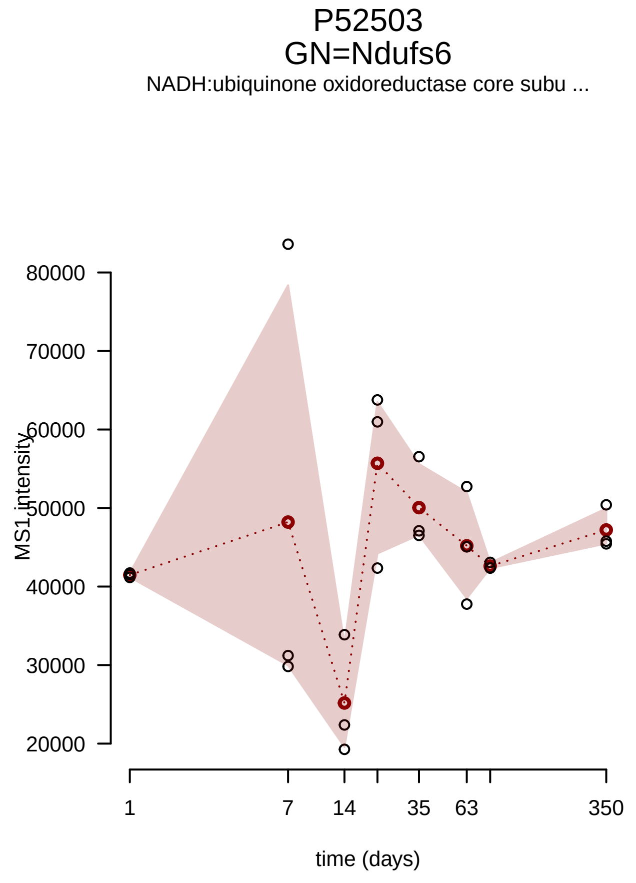
P52503
GN=Ndufs6
NADH:ubiquinone oxidoreductase core subu ...
MS1 intensity
20000
30000
40000
50000
60000
70000
80000
1
7
14
35
63
350
time (days)

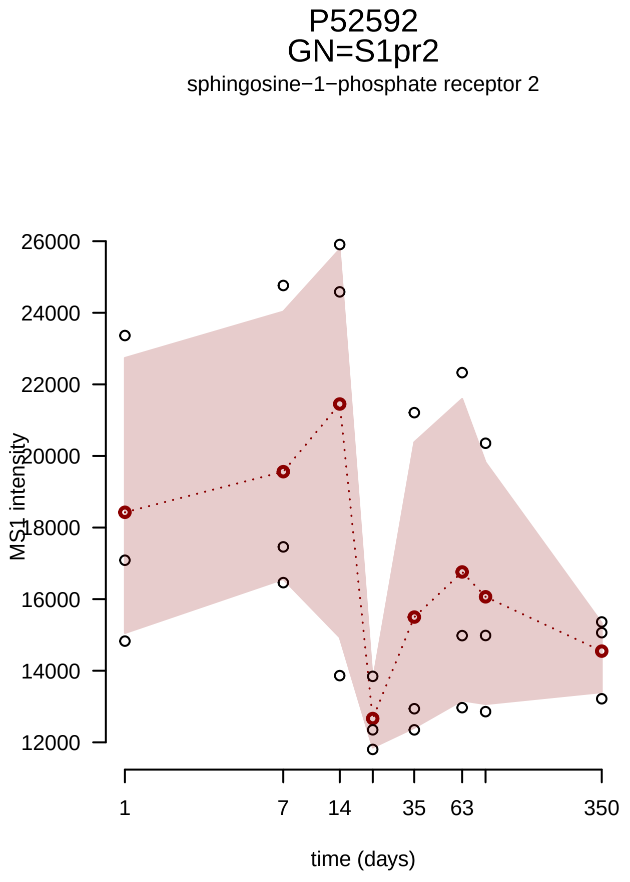
P52592
GN=S1pr2
sphingosine−1−phosphate receptor 2
MS1 intensity
12000
14000
16000
18000
20000
22000
24000
26000
1
7
14
35
63
350
time (days)

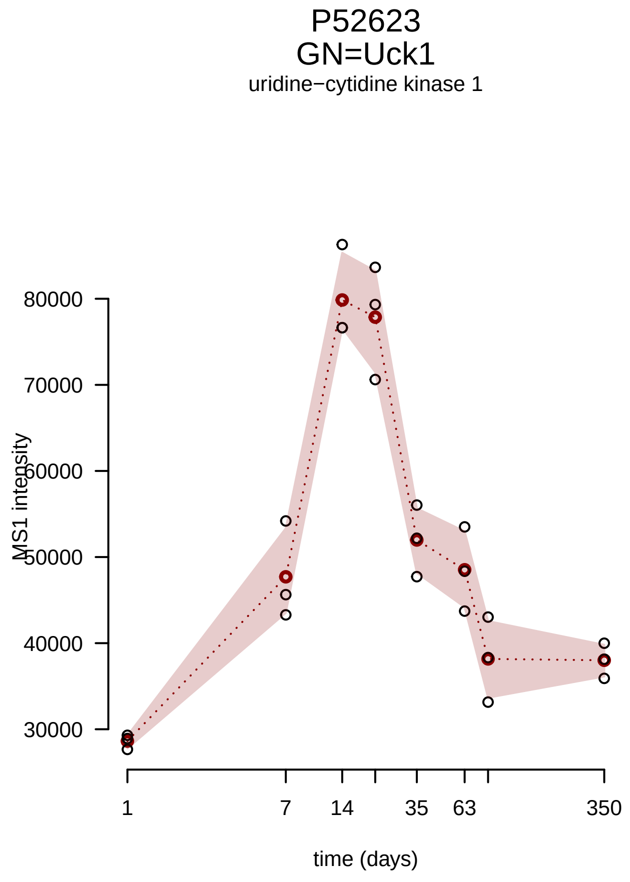
P52623
GN=Uck1
uridine−cytidine kinase 1
MS1 intensity
30000
40000
50000
60000
70000
80000
1
7
14
35
63
350
time (days)

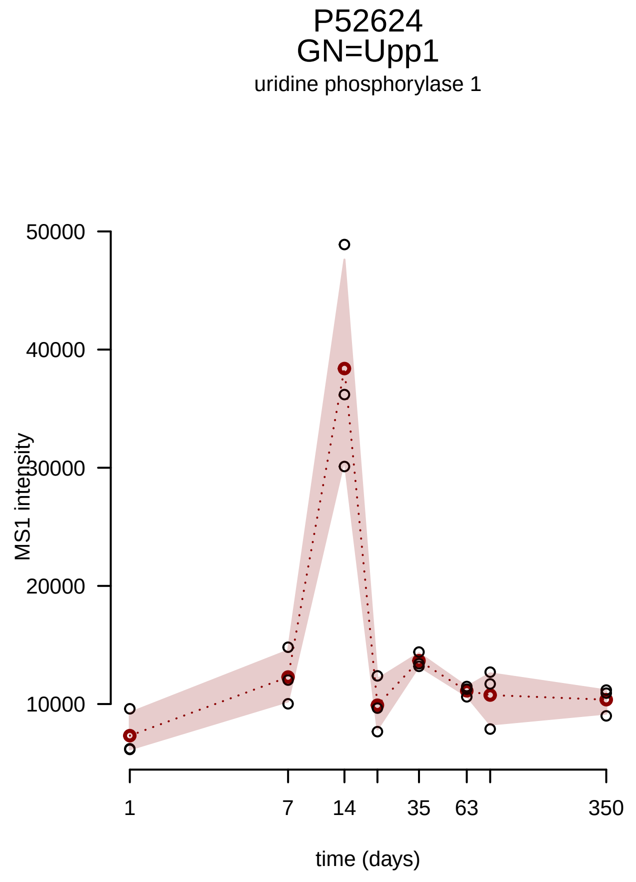
P52624
GN=Upp1
uridine phosphorylase 1
MS1 intensity
10000
20000
30000
40000
50000
1
7
14
35
63
350
time (days)

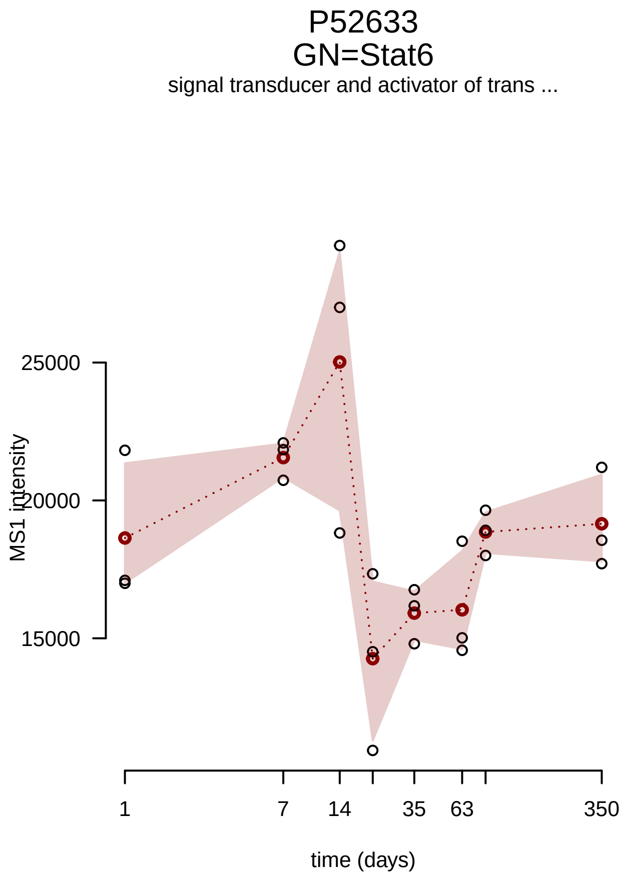
P52633
GN=Stat6
signal transducer and activator of trans ...
MS1 intensity
15000
20000
25000
1
7
14
35
63
350
time (days)

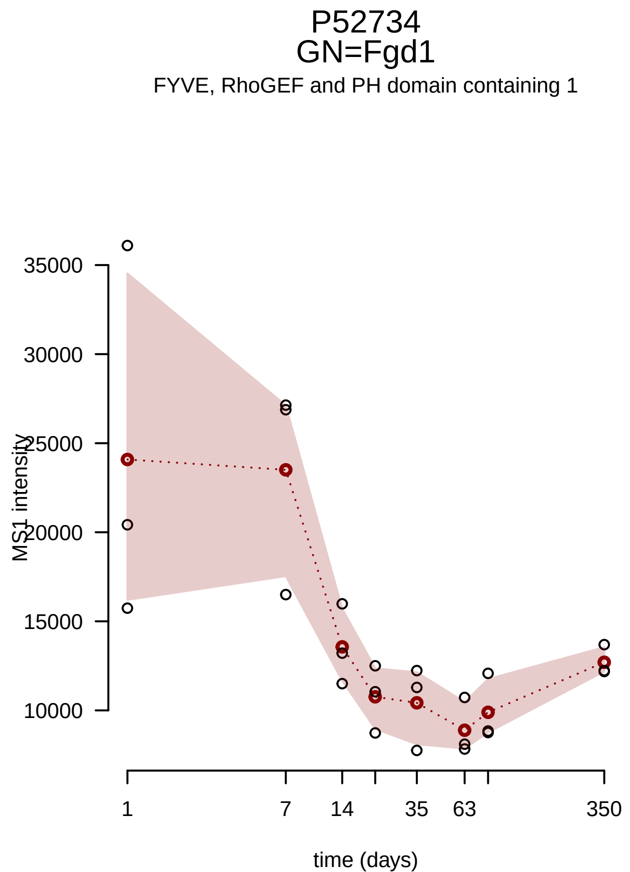
P52734
GN=Fgd1
FYVE, RhoGEF and PH domain containing 1
MS1 intensity
10000
15000
20000
25000
30000
35000
1
7
14
35
63
350
time (days)

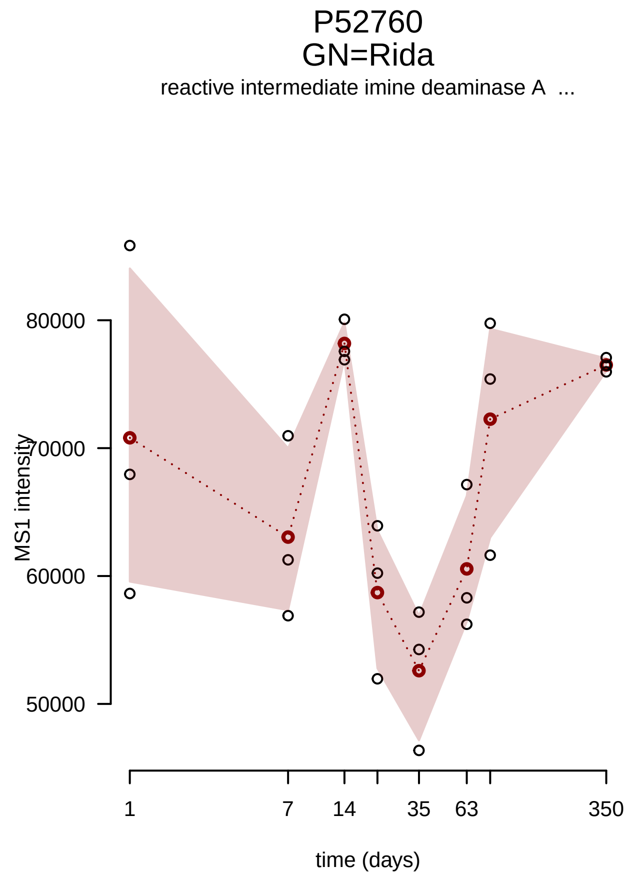
P52760
GN=Rida
reactive intermediate imine deaminase A ...
MS1 intensity
50000
60000
70000
80000
1
7
14
35
63
350
time (days)

P52795
GN=Efnb1
ephrin B1

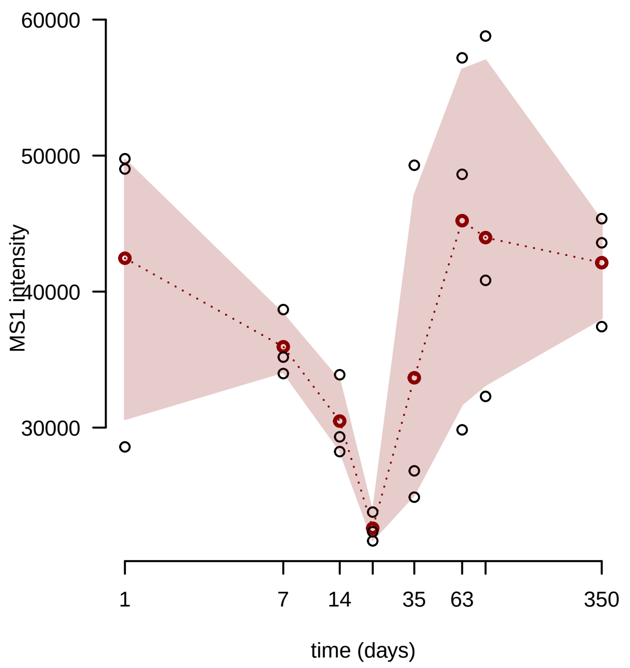

P52800
GN=Efnb2
ephrin B2

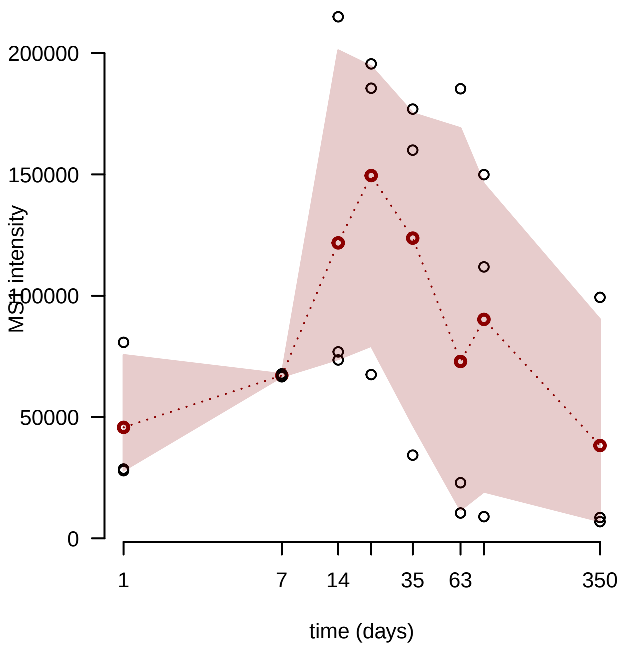

P52825
GN=Cpt2
carnitine palmitoyltransferase 2

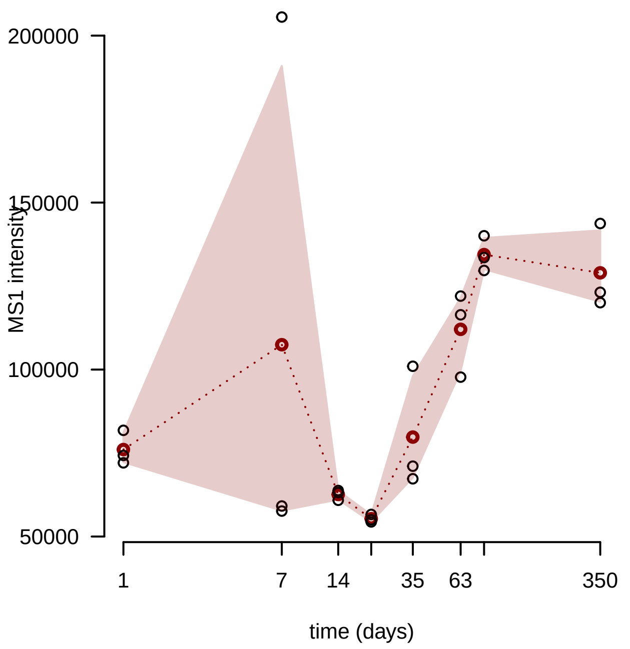

P52840
GN=Sult1a1
sulfotransferase family 1A, phenol−prefe ...

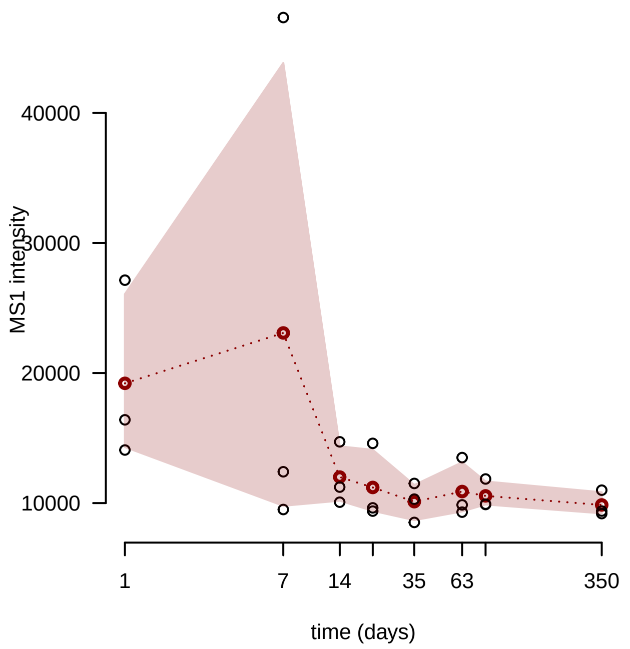

P52850
GN=Ndst2
N−deacetylase/N−sulfotransferase (hepara ...

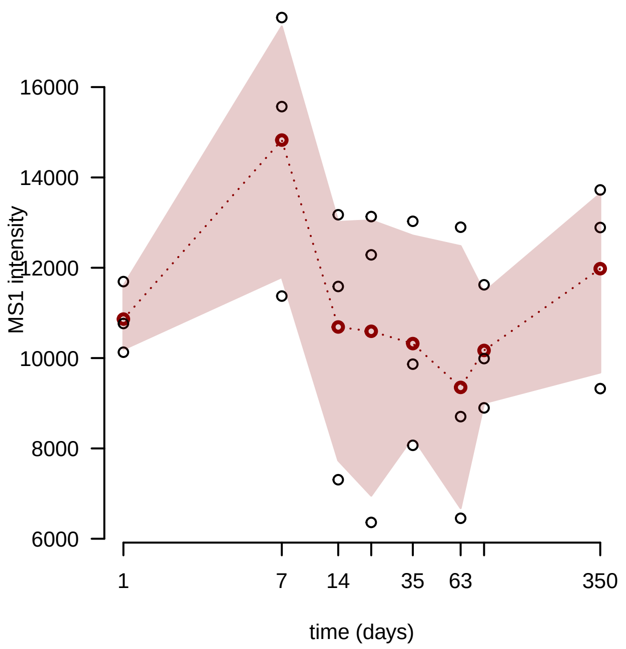

P52875
GN=Tmem165
transmembrane protein 165

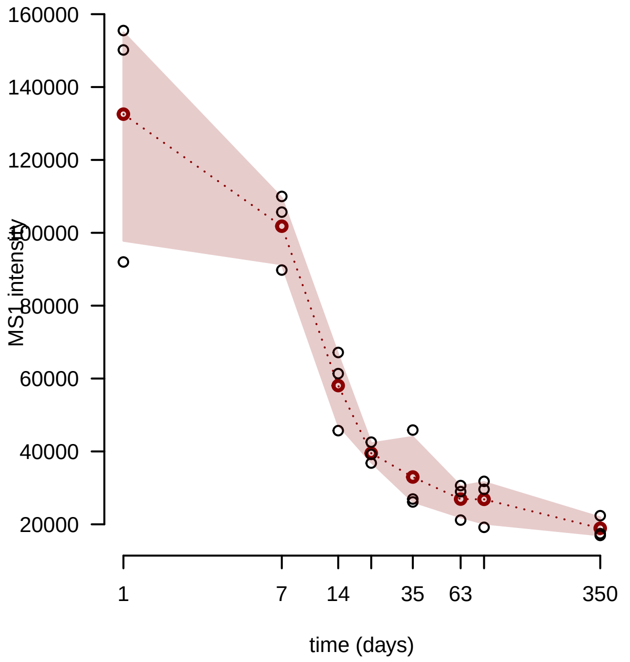

P52912
GN=Tia1
cytotoxic granule−associated RNA binding ...

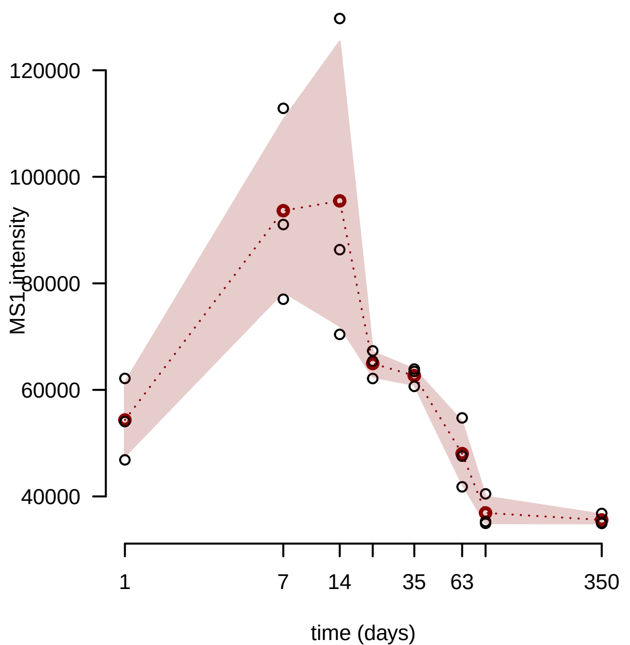

P52927
GN=Hmga2
high mobility group AT−hook 2

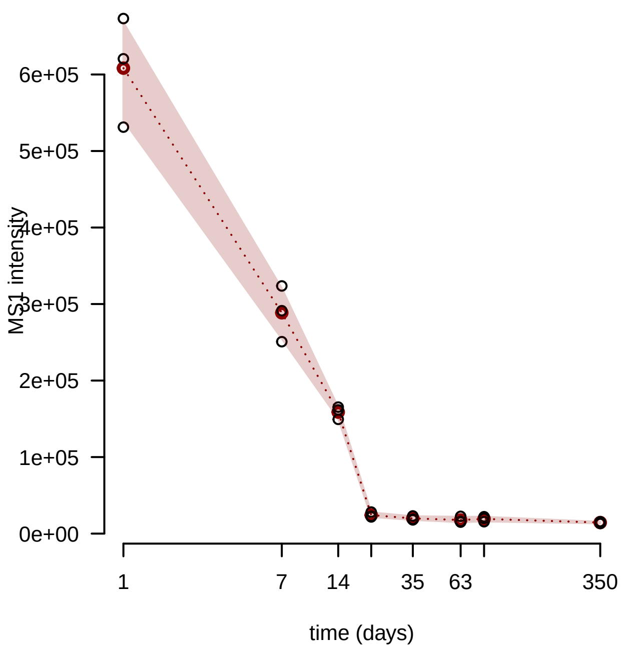

P53026
GN=Rpl10a
ribosomal protein L10A

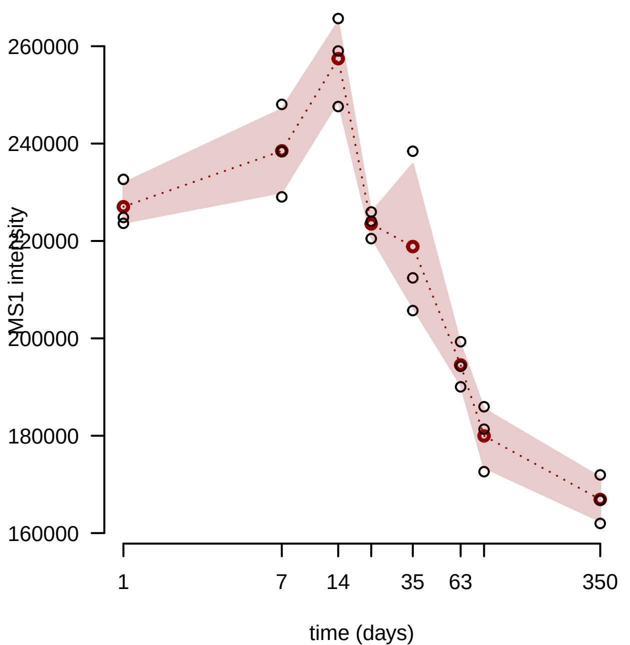

P53349
GN=Map3k1
mitogen−activated protein kinase kinase ...

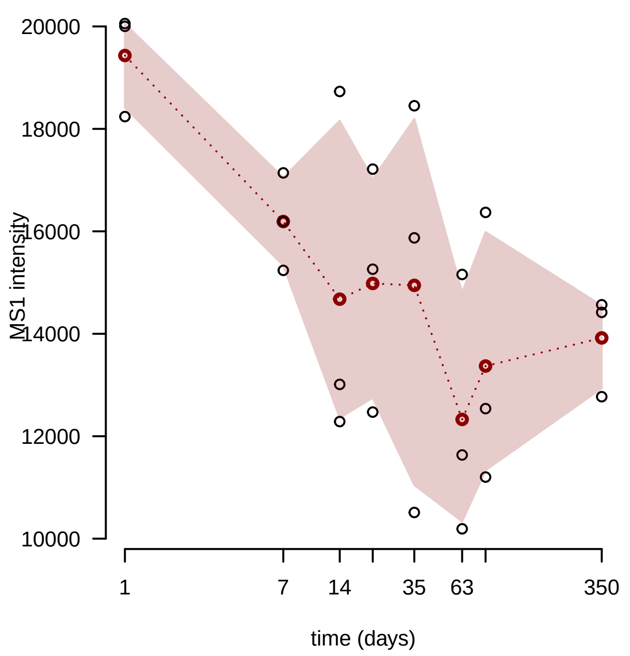

P53395
GN=Dbt
dihydrolipoamide branched chain transacy ...

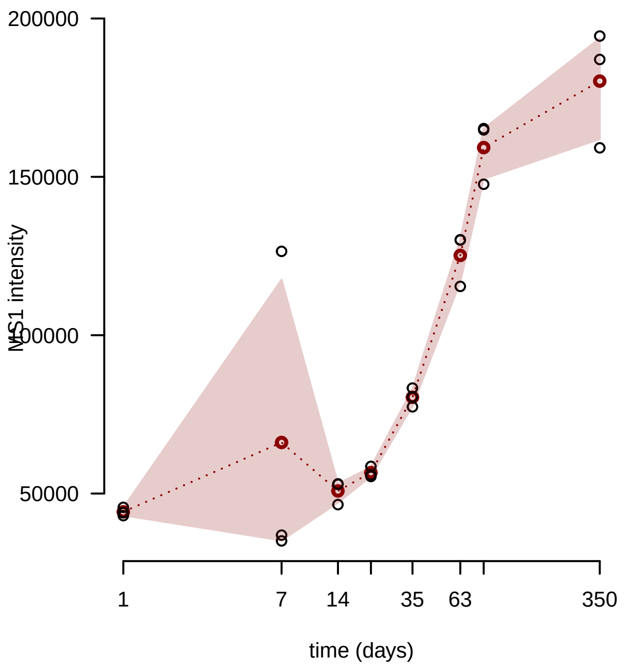

P53564
GN=Cux1
cut−like homeobox 1

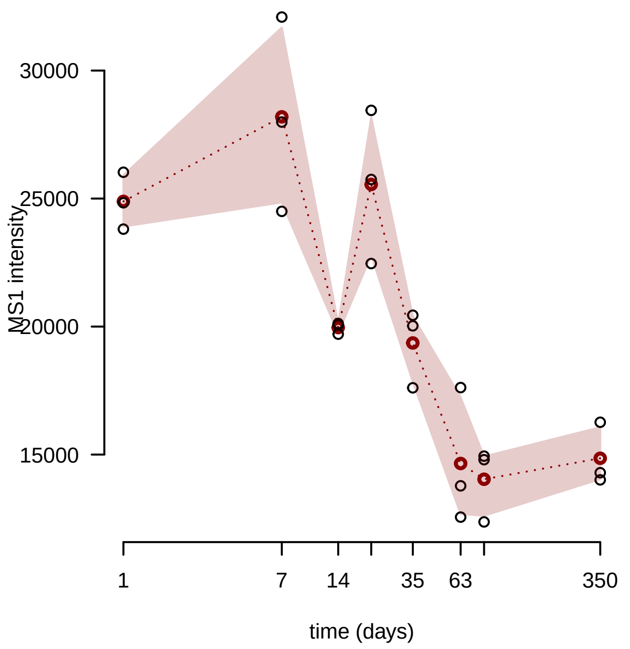

P53569
GN=Cebpz
CCAAT/enhancer binding protein zeta

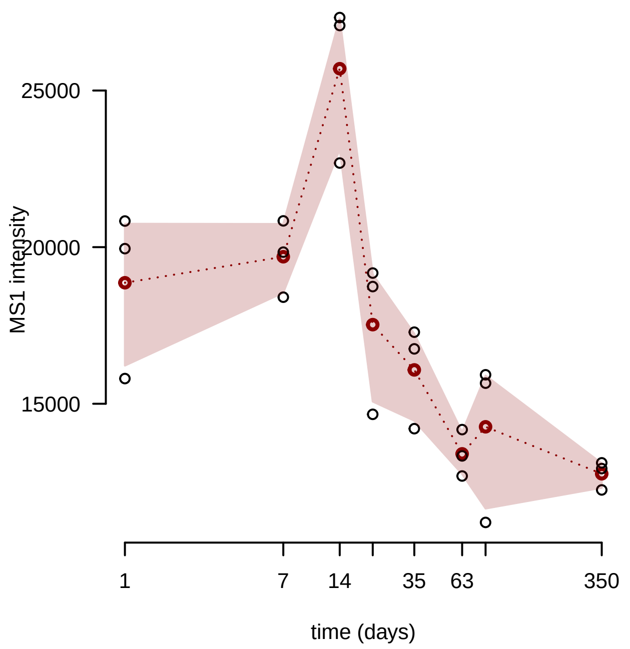

P53612
GN=Rabggtb
Rab geranylgeranyl transferase, b subuni ...

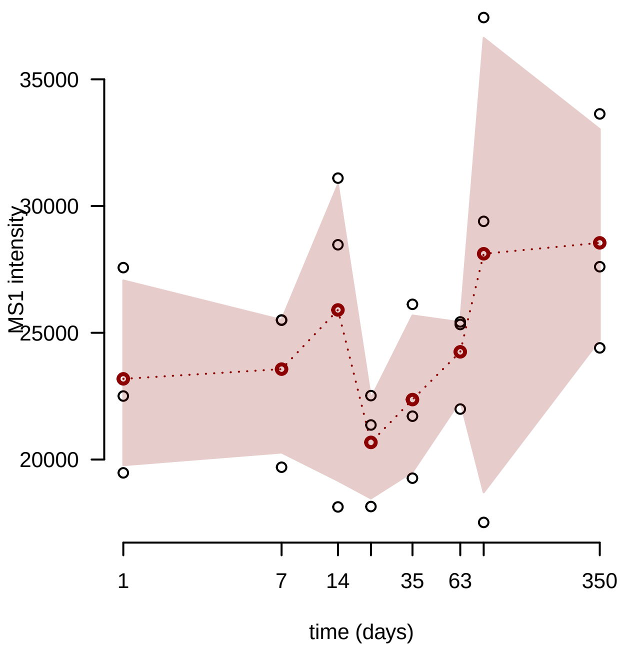

P53657
GN=Pklr
pyruvate kinase liver and red blood cell

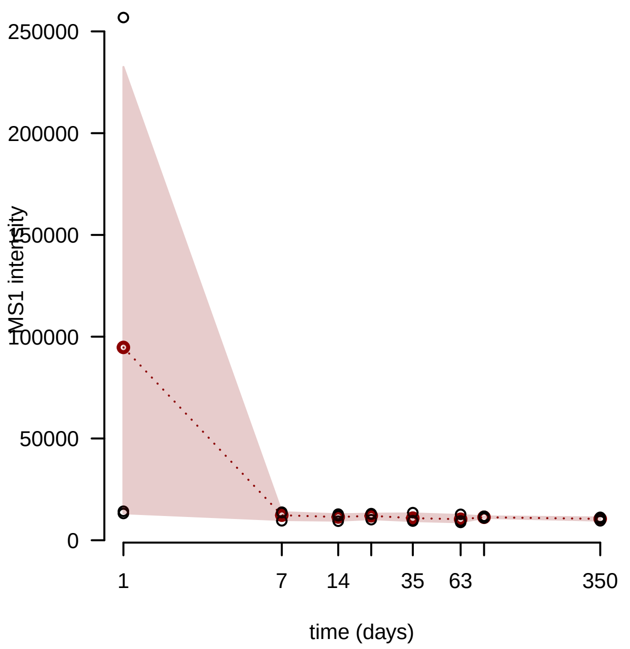

P53668
GN=Limk1
LIM−domain containing, protein kinase

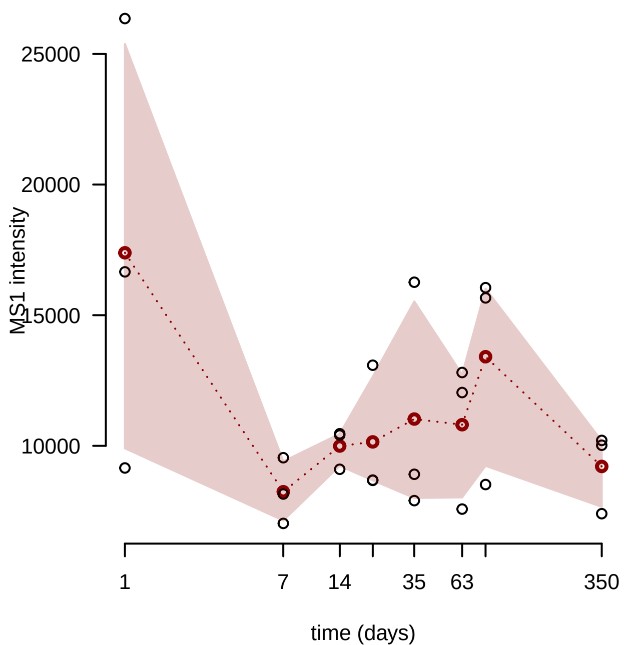

P53690
GN=Mmp14
matrix metallopeptidase 14 (membrane−ins ...

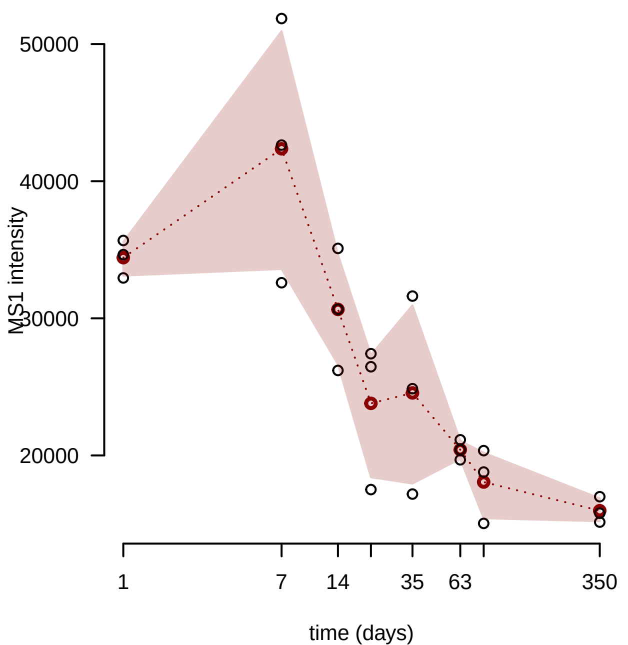

P53702
GN=Hccs
holocytochrome c synthetase

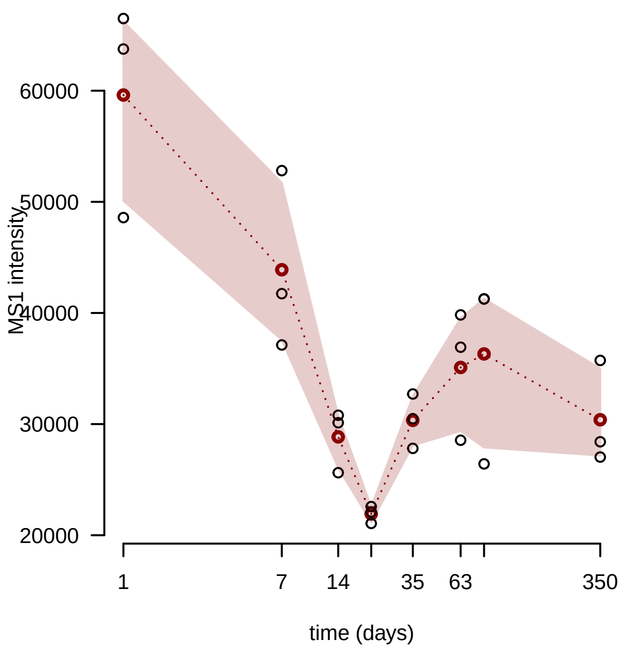

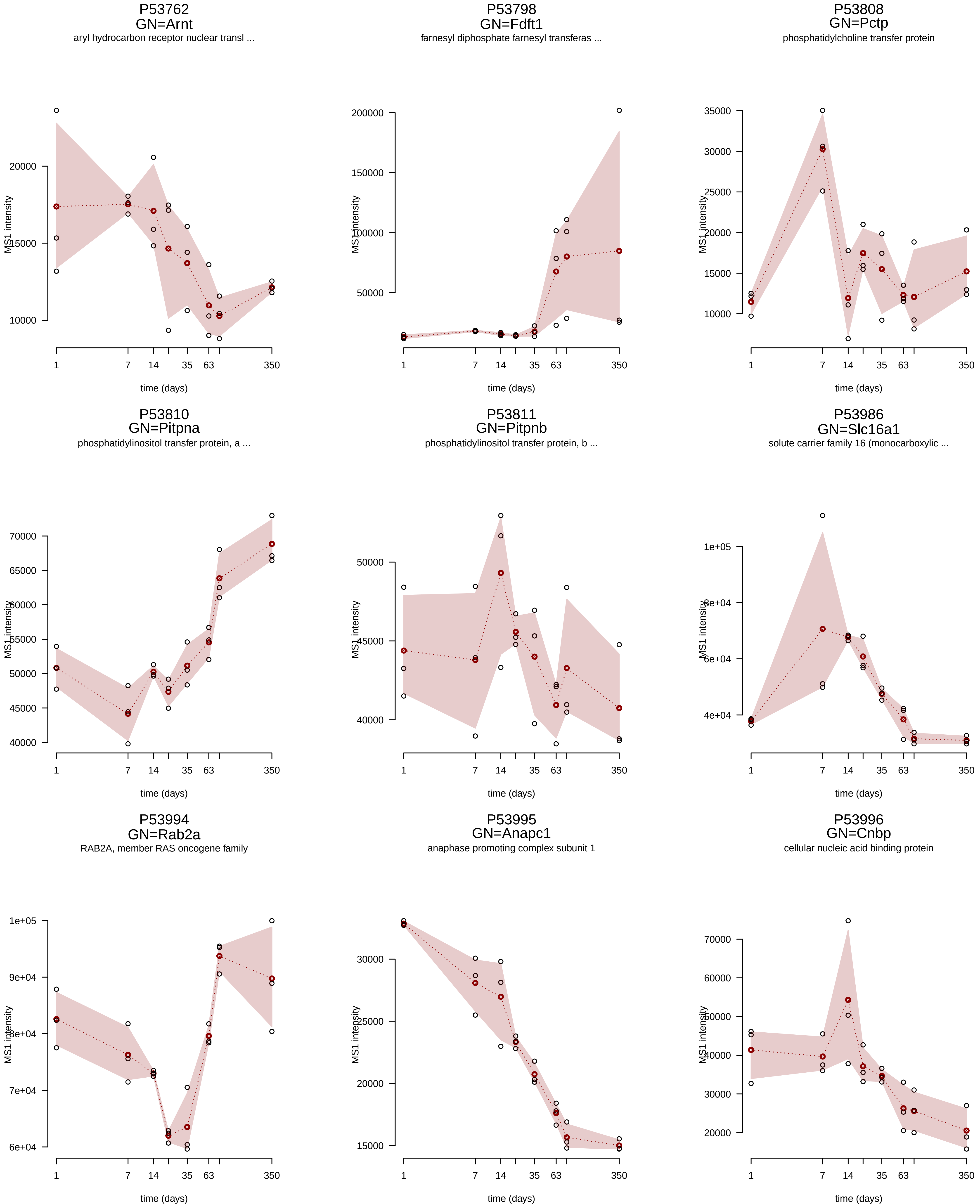
P53762
GN=Arnt
aryl hydrocarbon receptor nuclear transl ...
MS1 intensity
time (days)
P53798
GN=Fdft1
farnesyl diphosphate farnesyl transferas ...
MS1 intensity
time (days)
P53808
GN=Pctp
phosphatidylcholine transfer protein
MS1 intensity
time (days)
P53810
GN=Pitpna
phosphatidylinositol transfer protein, a ...
MS1 intensity
time (days)
P53811
GN=Pitpnb
phosphatidylinositol transfer protein, b ...
MS1 intensity
time (days)
P53986
GN=Slc16a1
solute carrier family 16 (monocarboxylic ...
MS1 intensity
time (days)
P53994
GN=Rab2a
RAB2A, member RAS oncogene family
MS1 intensity
time (days)
P53995
GN=Anapc1
anaphase promoting complex subunit 1
MS1 intensity
time (days)
P53996
GN=Cnbp
cellular nucleic acid binding protein
MS1 intensity
time (days)

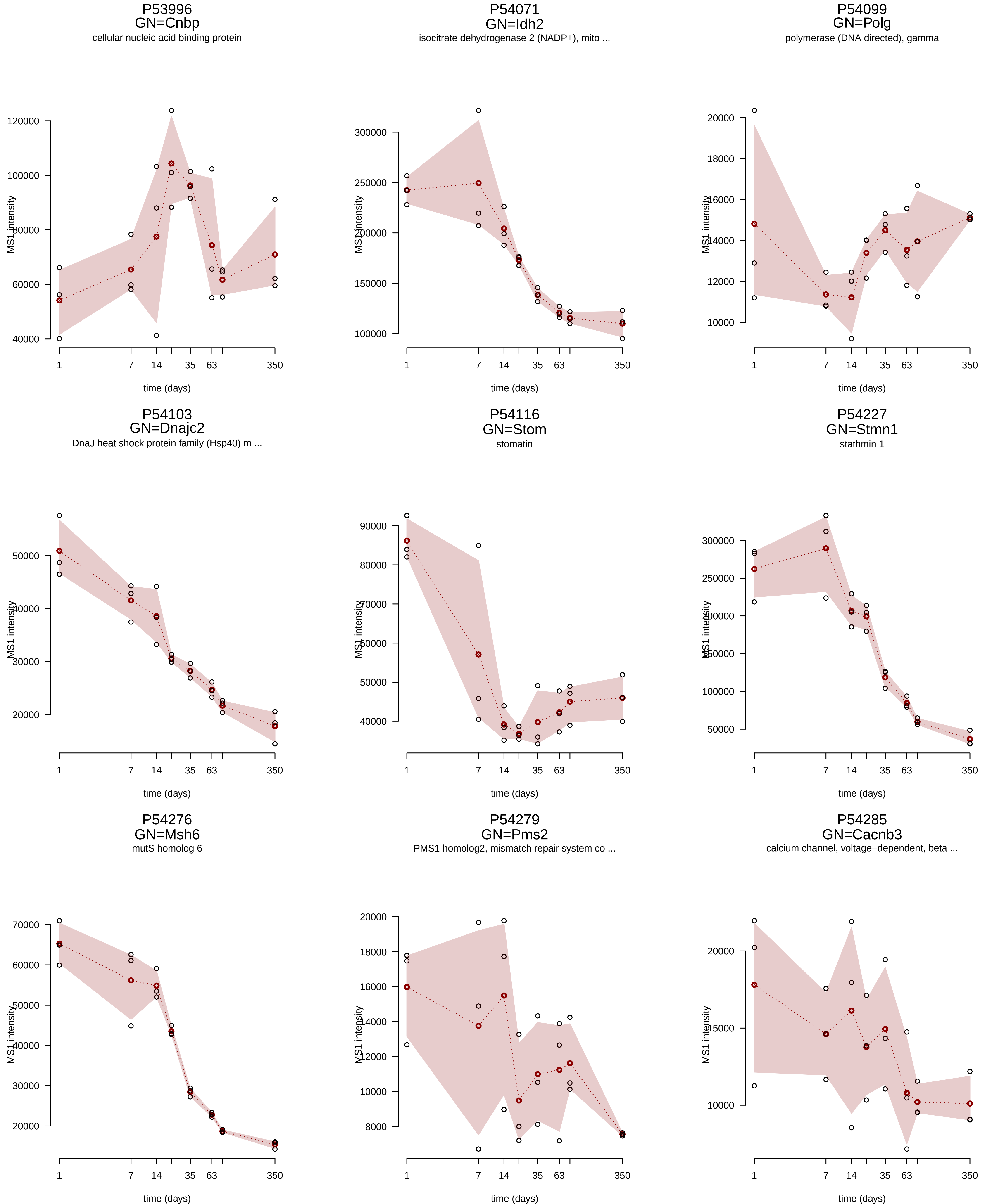
P53996
GN=Cnbp
cellular nucleic acid binding protein
MS1 intensity
time (days)
P54071
GN=Idh2
isocitrate dehydrogenase 2 (NADP+), mito ...
MS1 intensity
time (days)
P54099
GN=Polg
polymerase (DNA directed), gamma
MS1 intensity
time (days)
P54103
GN=Dnajc2
DnaJ heat shock protein family (Hsp40) m ...
MS1 intensity
time (days)
P54116
GN=Stom
stomatin
MS1 intensity
time (days)
P54227
GN=Stmn1
stathmin 1
MS1 intensity
time (days)
P54276
GN=Msh6
mutS homolog 6
MS1 intensity
time (days)
P54279
GN=Pms2
PMS1 homolog2, mismatch repair system co ...
MS1 intensity
time (days)
P54285
GN=Cacnb3
calcium channel, voltage−dependent, beta ...
MS1 intensity
time (days)
1
7
14
35
63
350

P54310
GN=Lipe
lipase, hormone sensitive

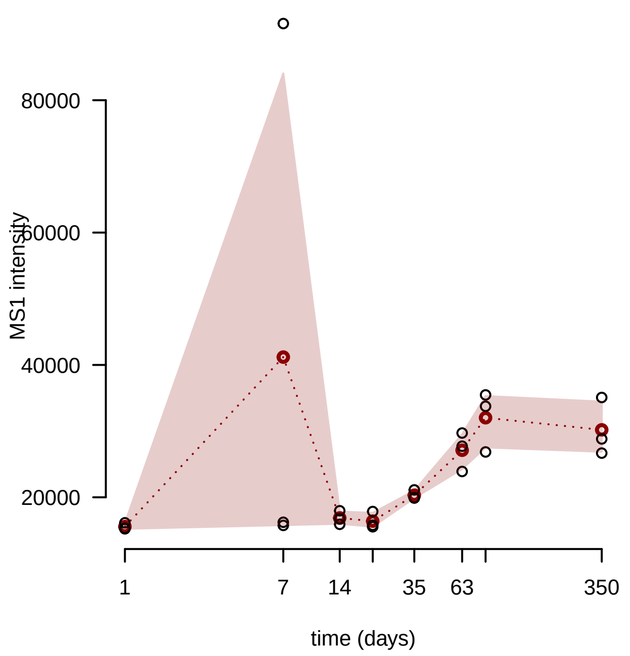

P54320
GN=Eln
elastin

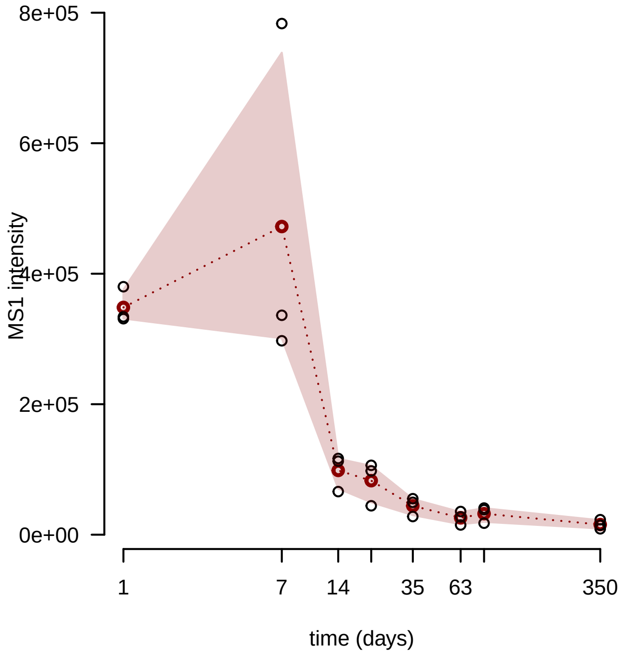

P54726
GN=Rad23a
RAD23 homolog A, nucleotide excision rep ...

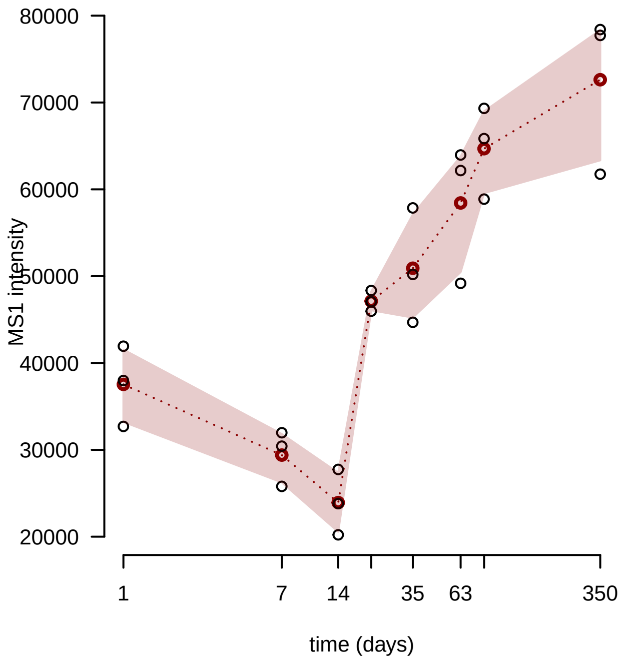

P54728
GN=Rad23b
RAD23 homolog B, nucleotide excision rep ...

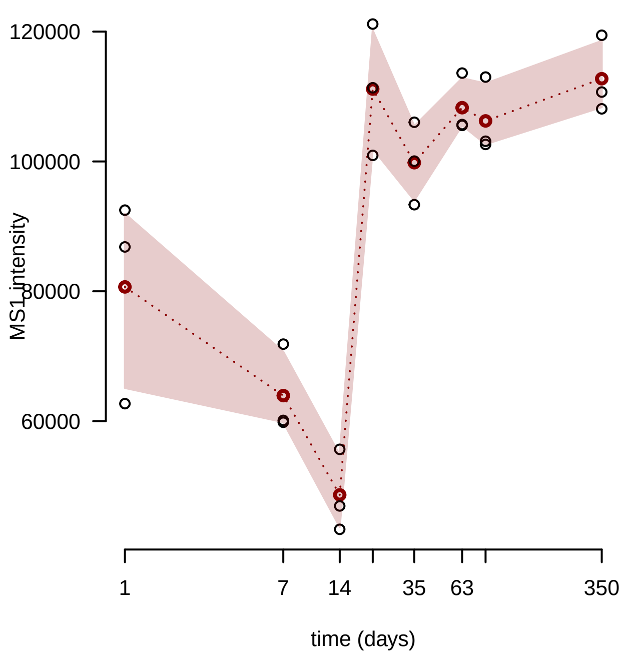

P54729
GN=Nub1
negative regulator of ubiquitin−like pro ...

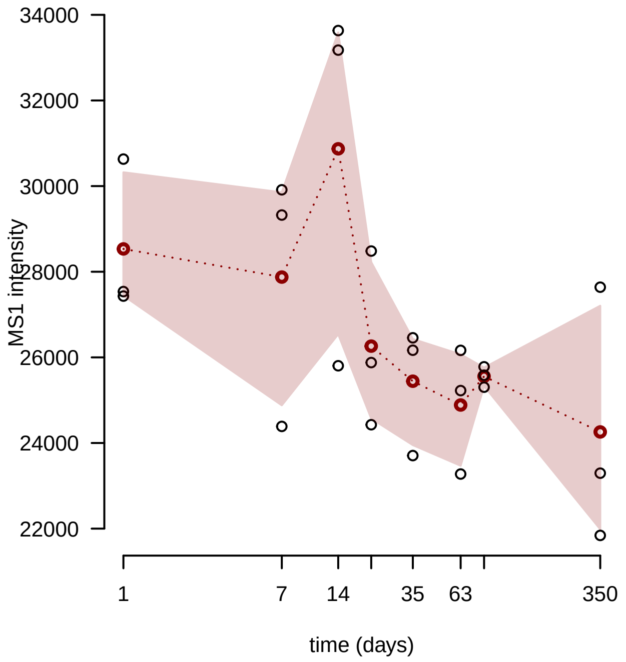

P54731
GN=Faf1
Fas−associated factor 1

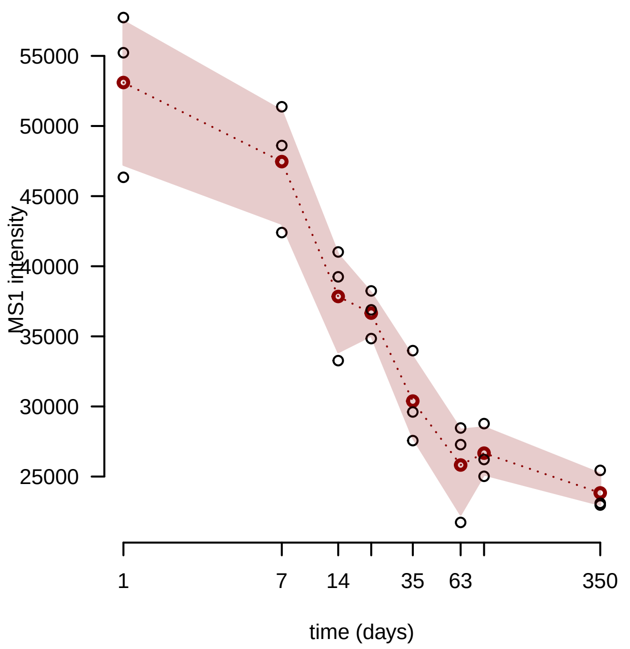

P54751
GN=St3gal1
ST3 beta−galactoside alpha−2,3−sialyltra ...

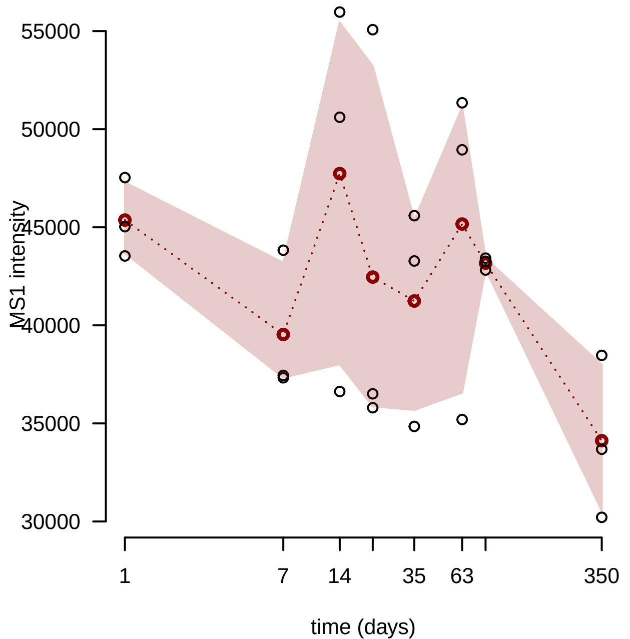

P54754
GN=Ephb3
Eph receptor B3

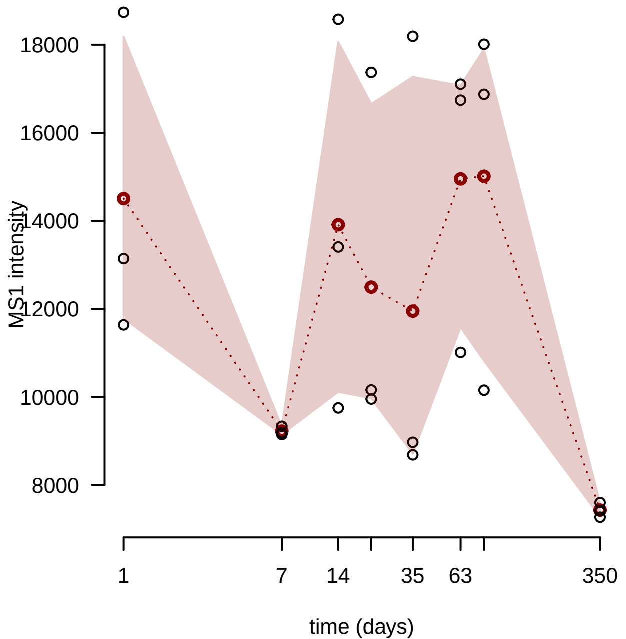

P54761
GN=Ephb4
Eph receptor B4

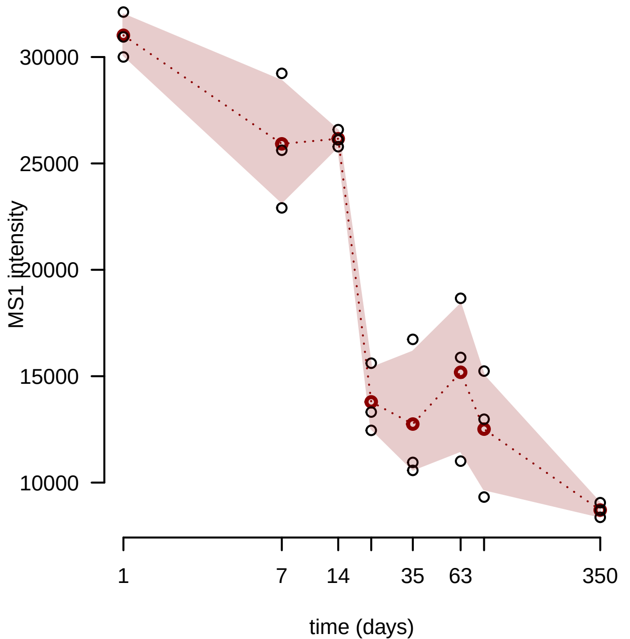

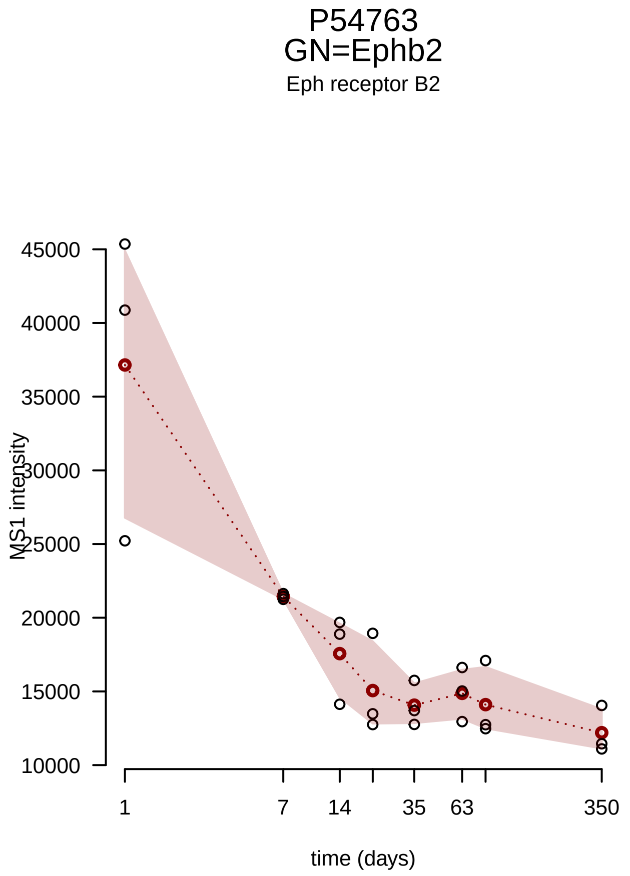
P54763
GN=Ephb2
Eph receptor B2
MS1 intensity
45000
40000
35000
30000
25000
20000
15000
10000
1
7
14
35
63
350
time (days)

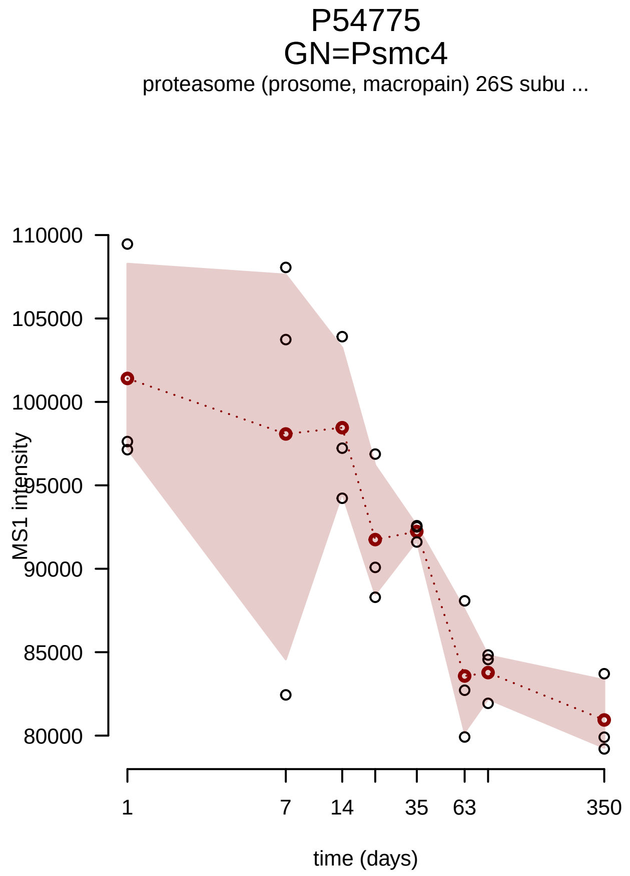
P54775
GN=Psmc4
proteasome (prosome, macropain) 26S subu ...
MS1 intensity
110000
105000
100000
95000
90000
85000
80000
1
7
14
35
63
350
time (days)

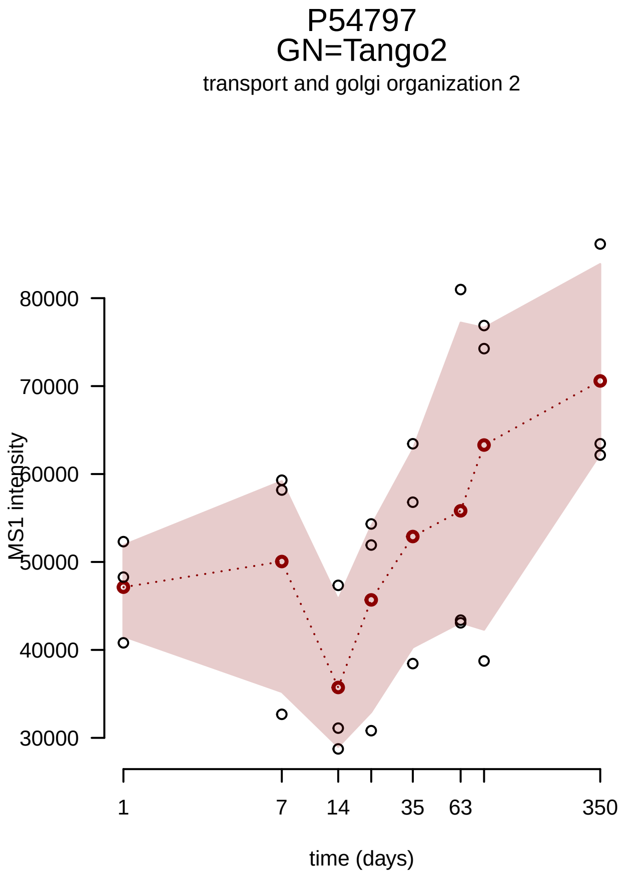
P54797
GN=Tango2
transport and golgi organization 2
MS1 intensity
80000
70000
60000
50000
40000
30000
1
7
14
35
63
350
time (days)

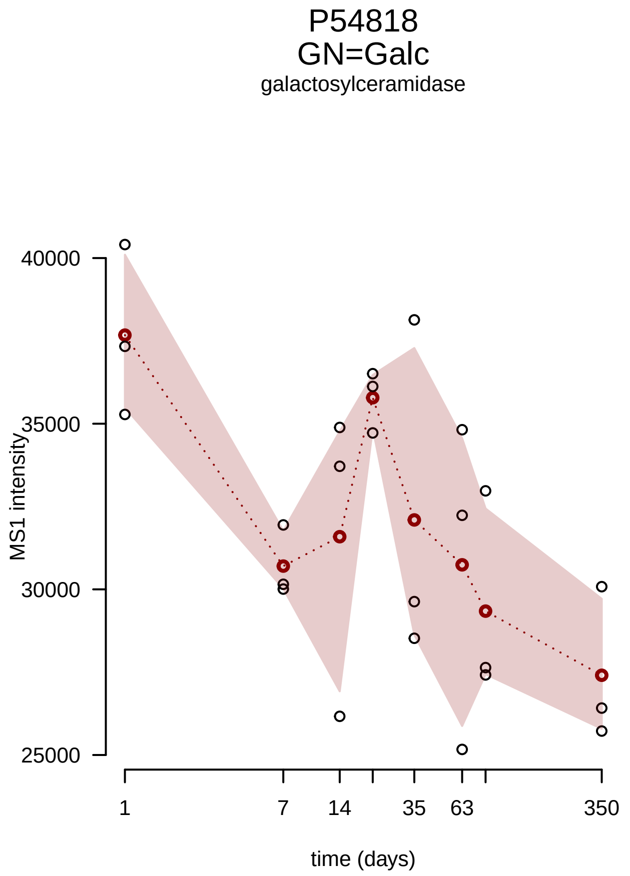
P54818
GN=Galc
galactosylceramidase
MS1 intensity
40000
35000
30000
25000
1
7
14
35
63
350
time (days)

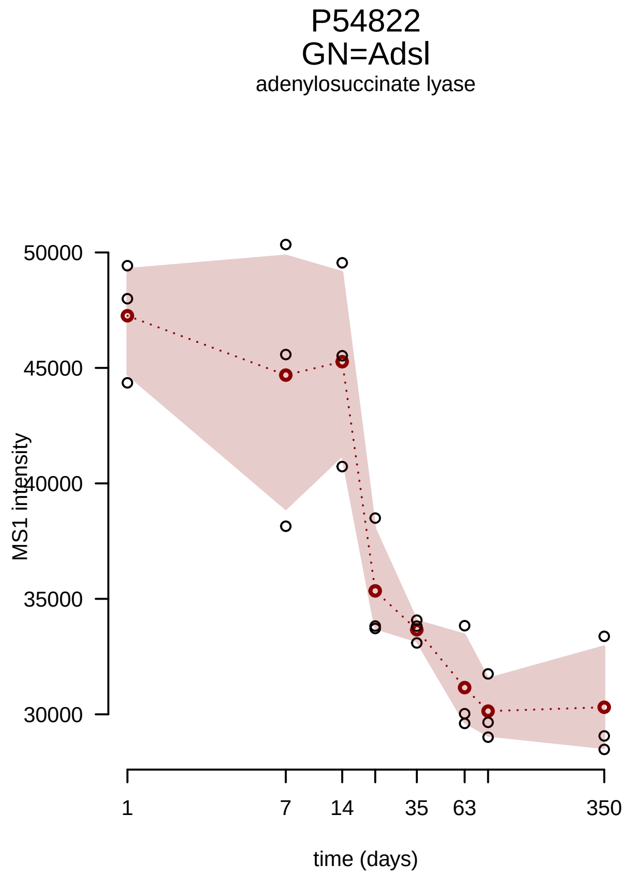
P54822
GN=Adsl
adenylosuccinate lyase
MS1 intensity
50000
45000
40000
35000
30000
1
7
14
35
63
350
time (days)

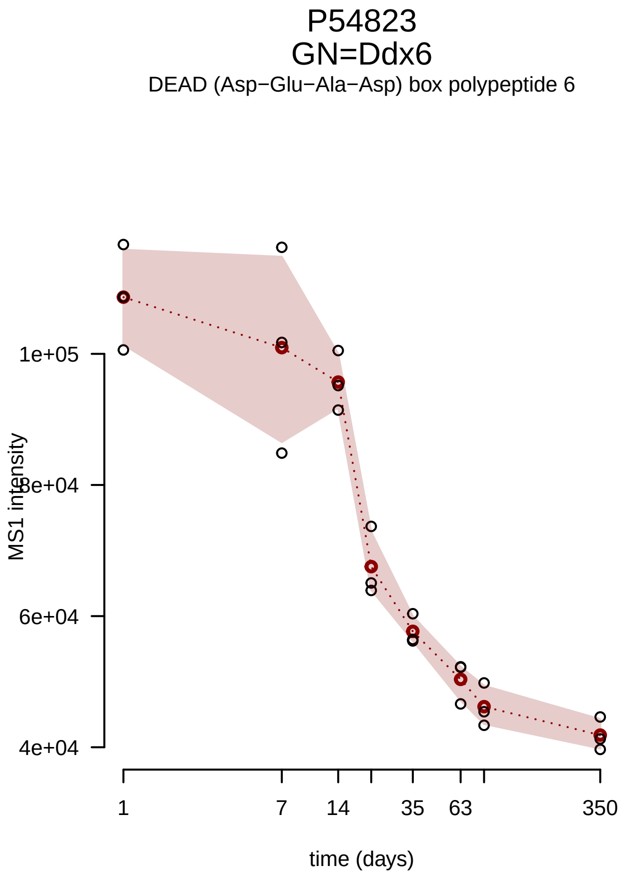
P54823
GN=Ddx6
DEAD (Asp−Glu−Ala−Asp) box polypeptide 6
MS1 intensity
1e+05
8e+04
6e+04
4e+04
1
7
14
35
63
350
time (days)

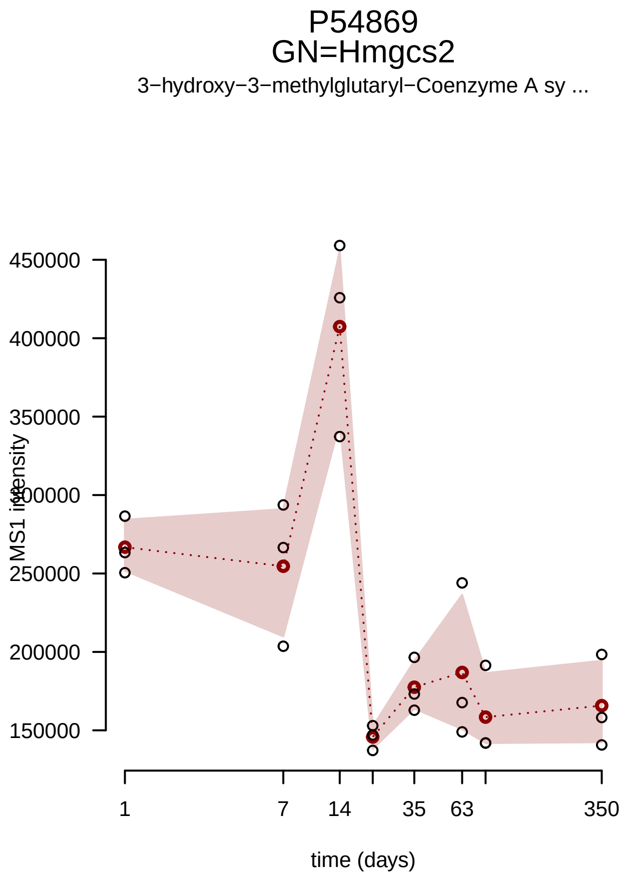
P54869
GN=Hmgcs2
3−hydroxy−3−methylglutaryl−Coenzyme A sy ...
MS1 intensity
450000
400000
350000
300000
250000
200000
150000
1
7
14
35
63
350
time (days)

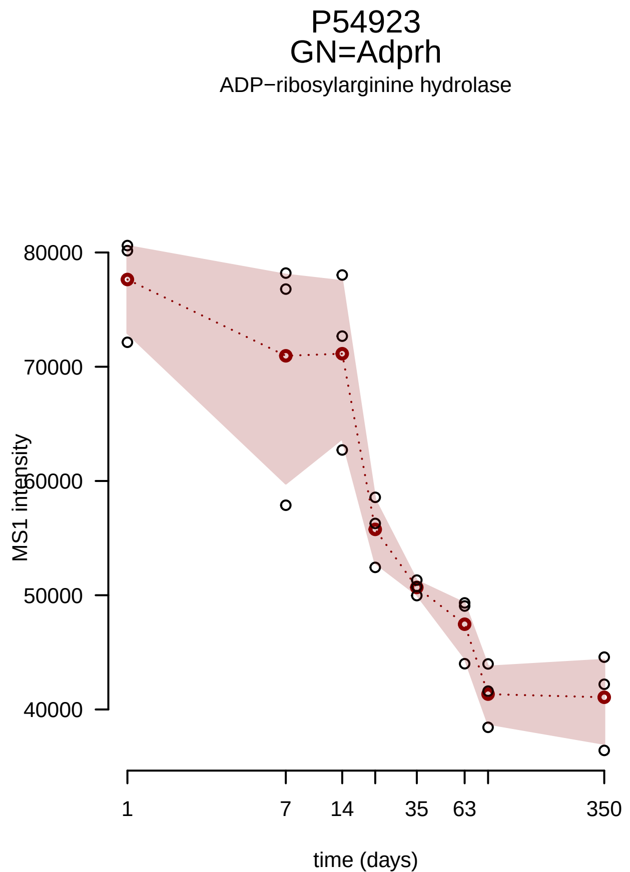
P54923
GN=Adprh
ADP−ribosylarginine hydrolase
MS1 intensity
80000
70000
60000
50000
40000
1
7
14
35
63
350
time (days)

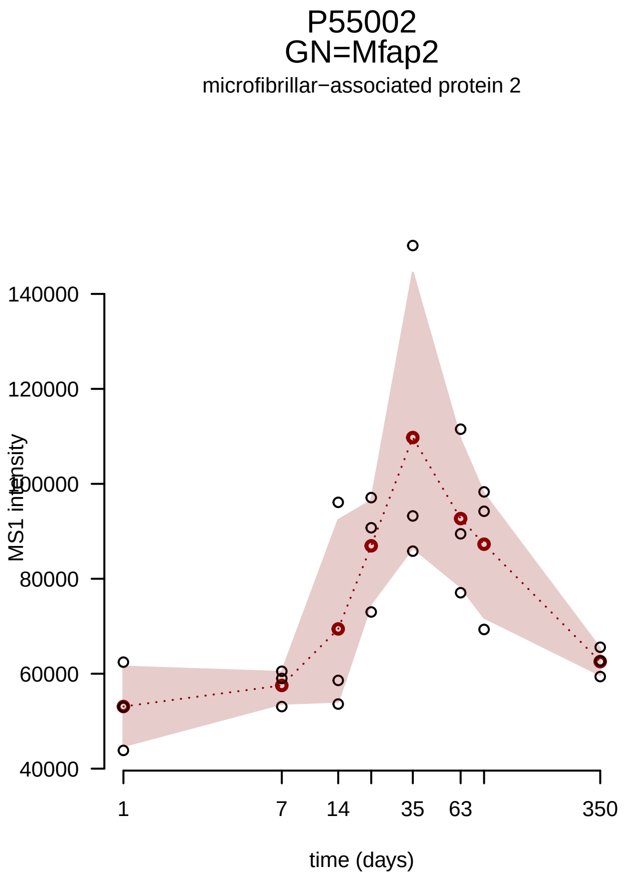
P55002
GN=Mfap2
microfibrillar−associated protein 2
MS1 intensity
140000
120000
100000
80000
60000
40000
1
7
14
35
63
350
time (days)

P55012
GN=Slc12a2
solute carrier family 12, member 2

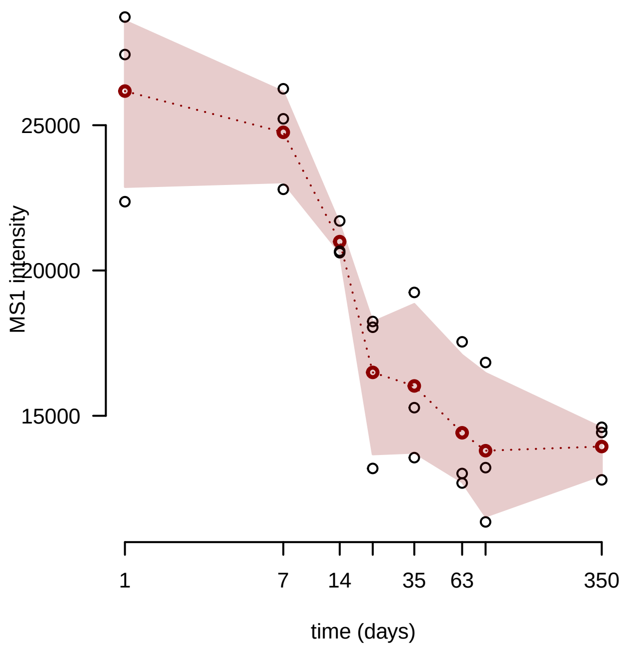

P55050
GN=Fabp2
fatty acid binding protein 2, intestinal

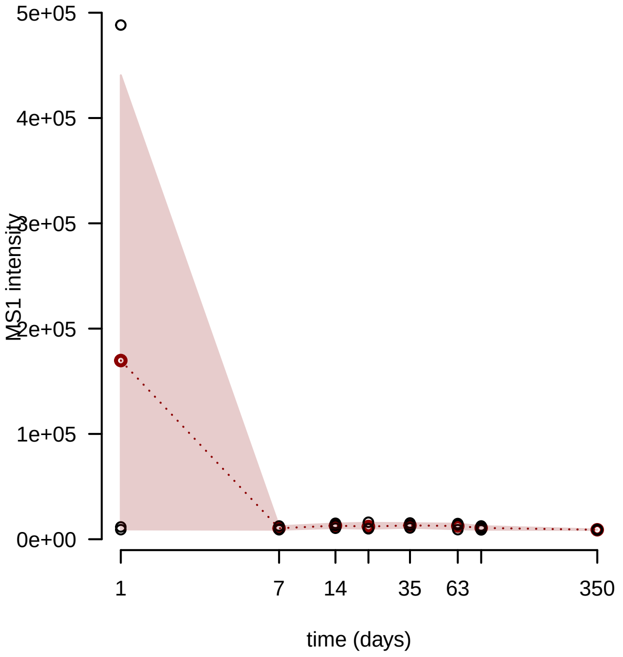

P55065
GN=Pltp
phospholipid transfer protein

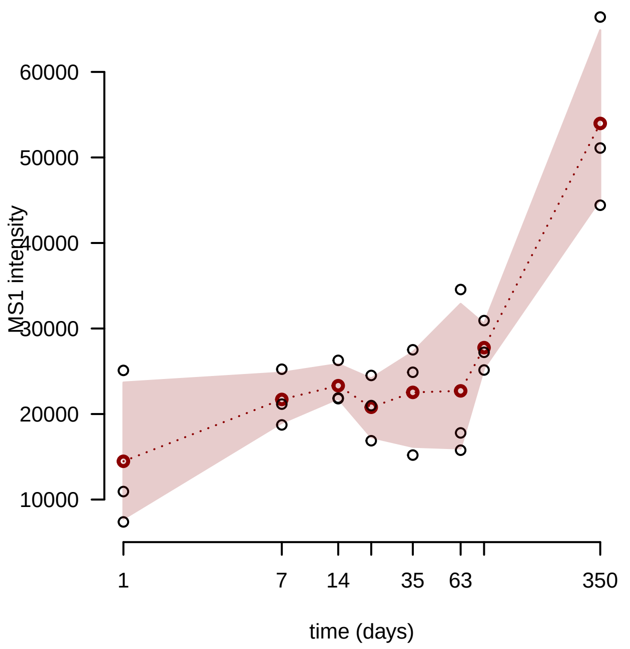

P55096
GN=Abcd3
ATP−binding cassette, sub−family D (ALD) ...

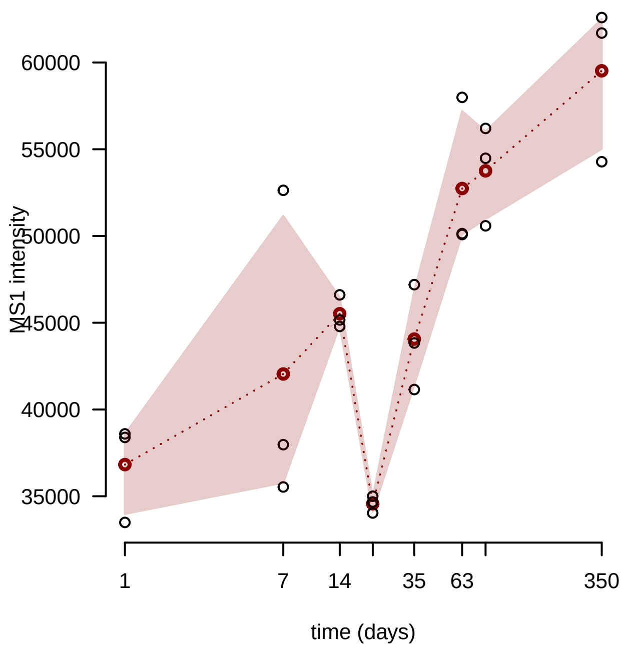

P55097
GN=Ctsk
cathepsin K

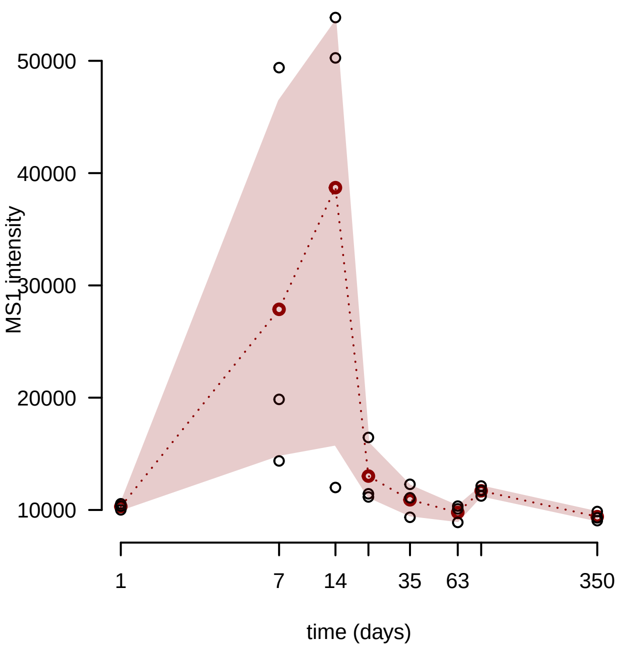

P55144
GN=Tyro3
TYRO3 protein tyrosine kinase 3

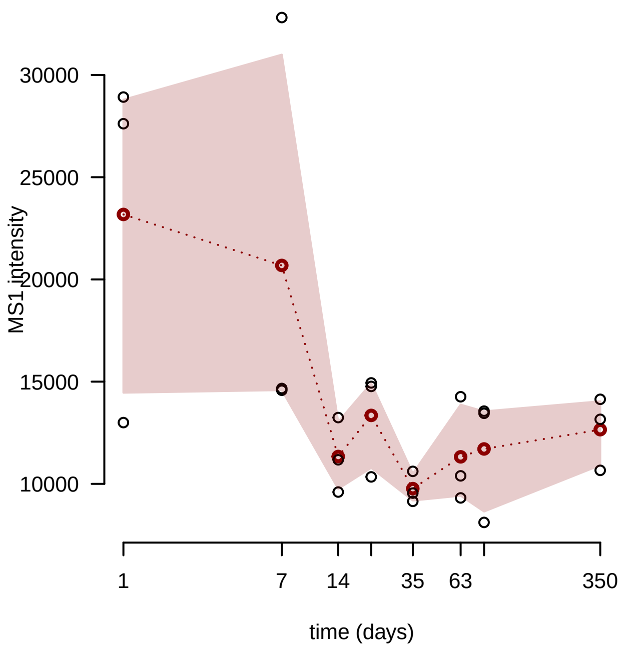

P55194
GN=Sh3bp1
SH3−domain binding protein 1

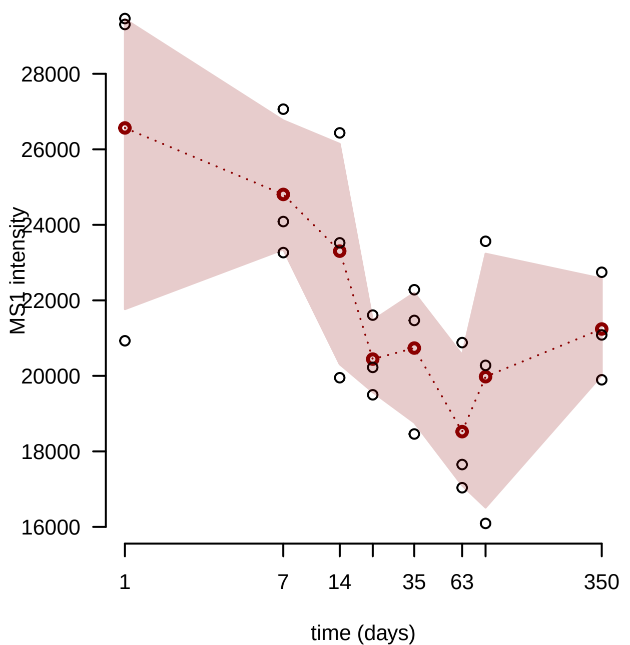

P55200
GN=Kmt2a
lysine (K)−specific methyltransferase 2A

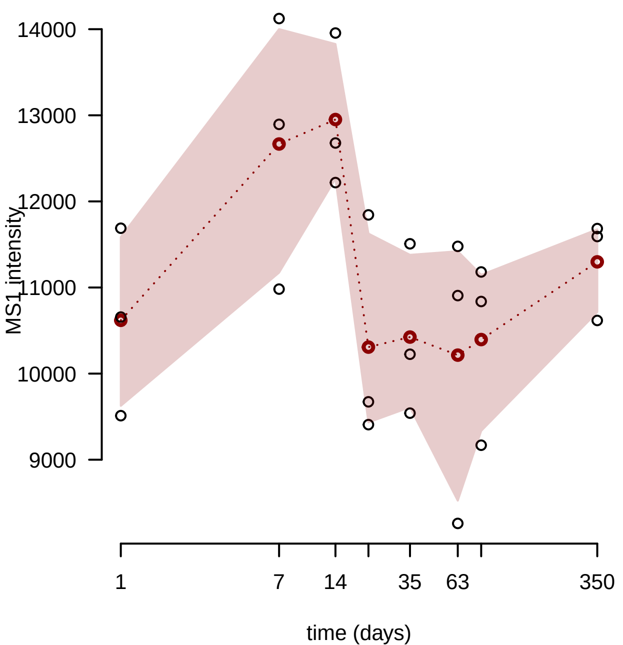

P55258
GN=Rab8a
RAB8A, member RAS oncogene family

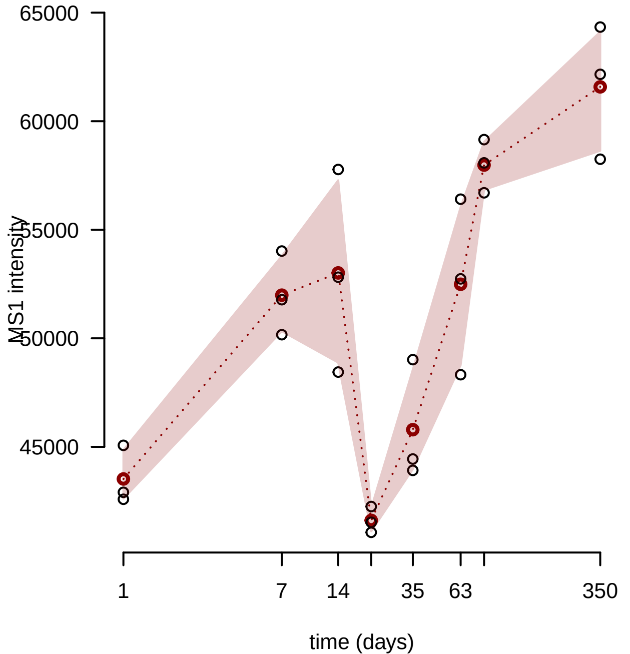

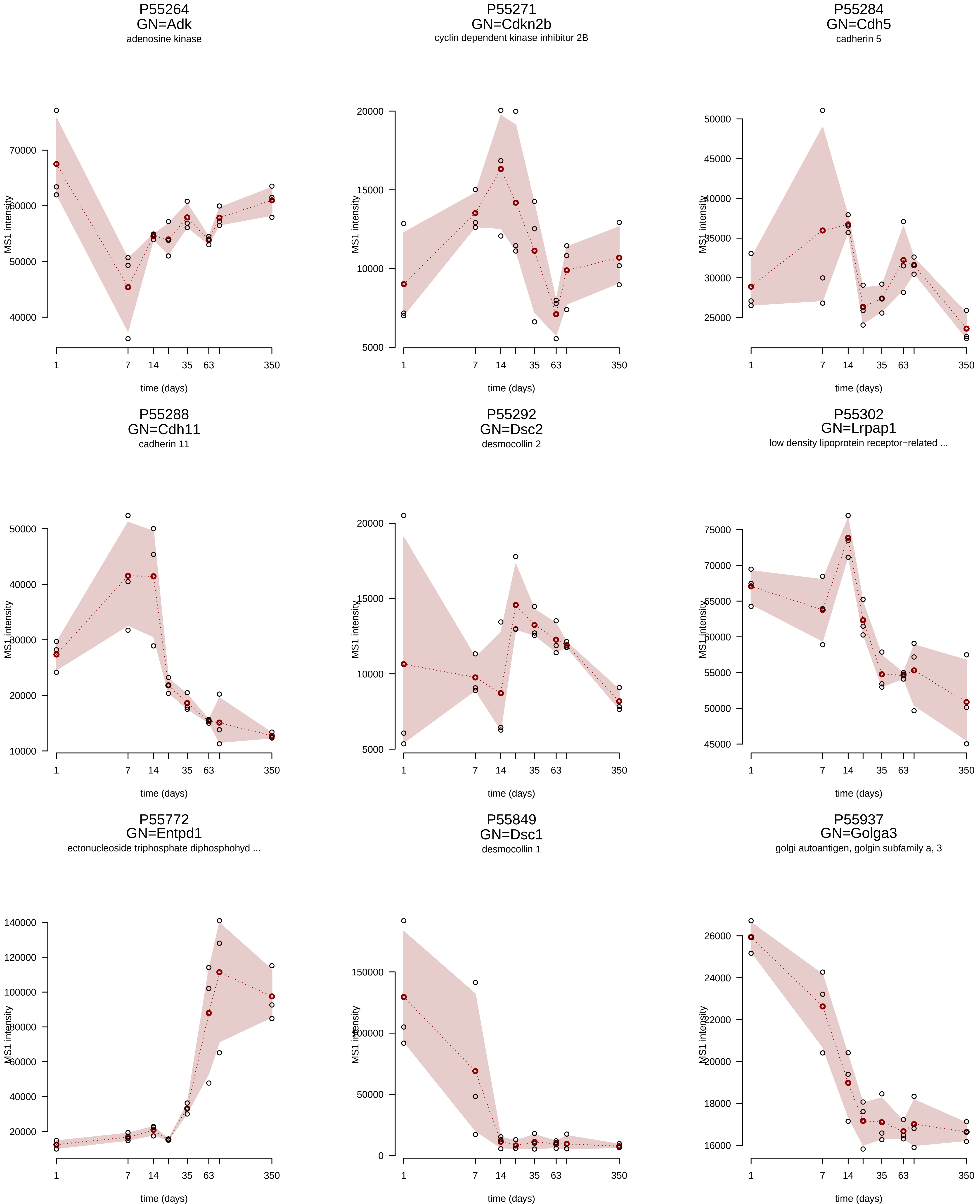
P55264
GN=Adk
adenosine kinase
P55271
GN=Cdkn2b
cyclin dependent kinase inhibitor 2B
P55284
GN=Cdh5
cadherin 5
P55288
GN=Cdh11
cadherin 11
P55292
GN=Dsc2
desmocollin 2
P55302
GN=Lrpap1
low density lipoprotein receptor−related ...
P55772
GN=Entpd1
ectonucleoside triphosphate diphosphohyd ...
P55849
GN=Dsc1
desmocollin 1
P55937
GN=Golga3
golgi autoantigen, golgin subfamily a, 3
MS1 intensity
time (days)
1
7
14
35
63
350

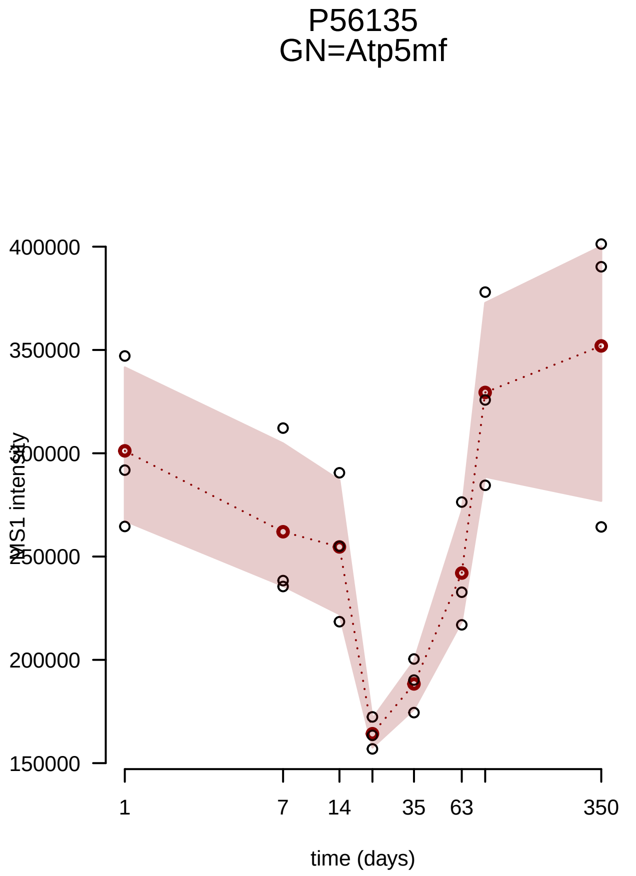
P56135
GN=Atp5mf
400000
350000
300000
250000
200000
150000
MS1 intensity
1
7
14
35
63
350
time (days)
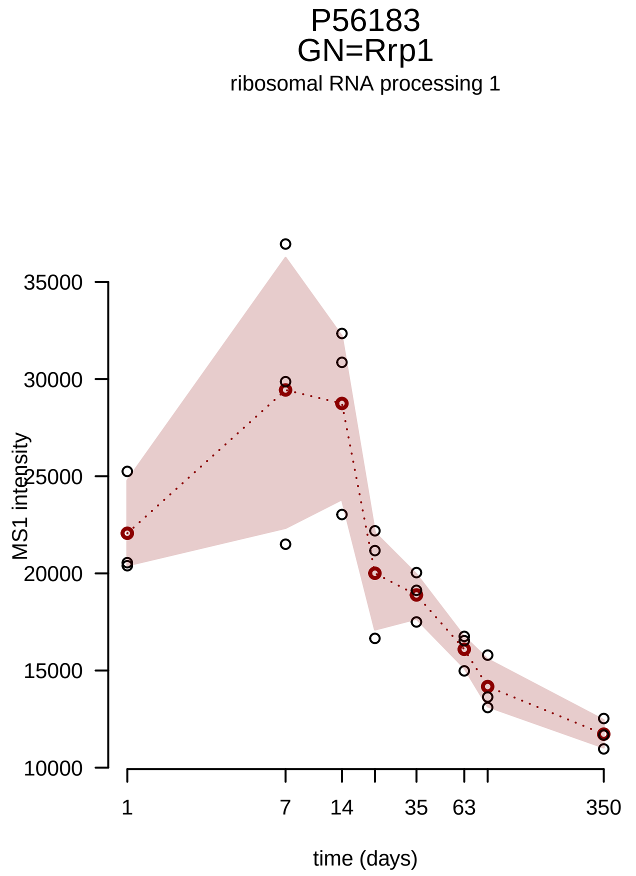
P56183
GN=Rrp1
ribosomal RNA processing 1
35000
30000
25000
20000
15000
10000
MS1 intensity
1
7
14
35
63
350
time (days)
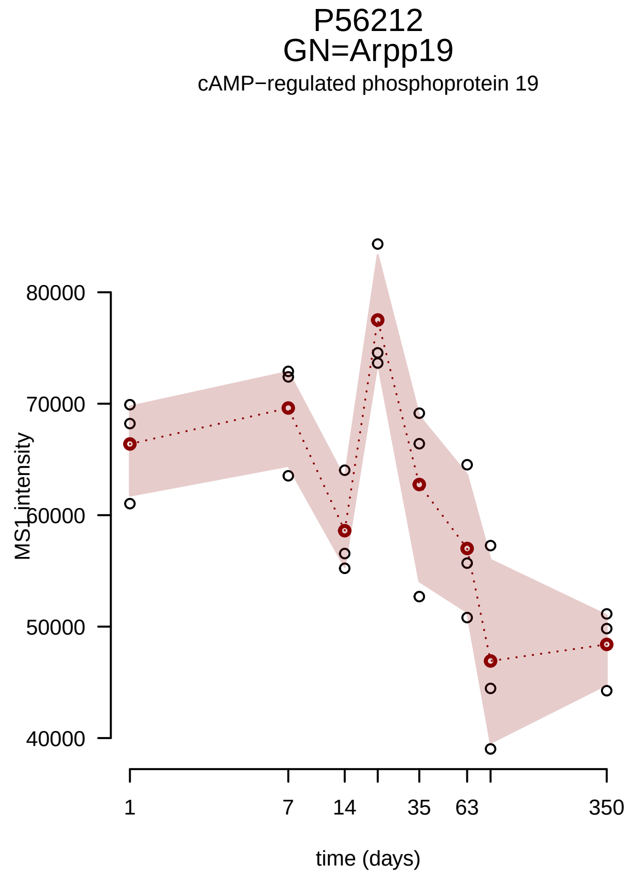
P56212
GN=Arpp19
cAMP−regulated phosphoprotein 19
80000
70000
60000
50000
40000
MS1 intensity
1
7
14
35
63
350
time (days)
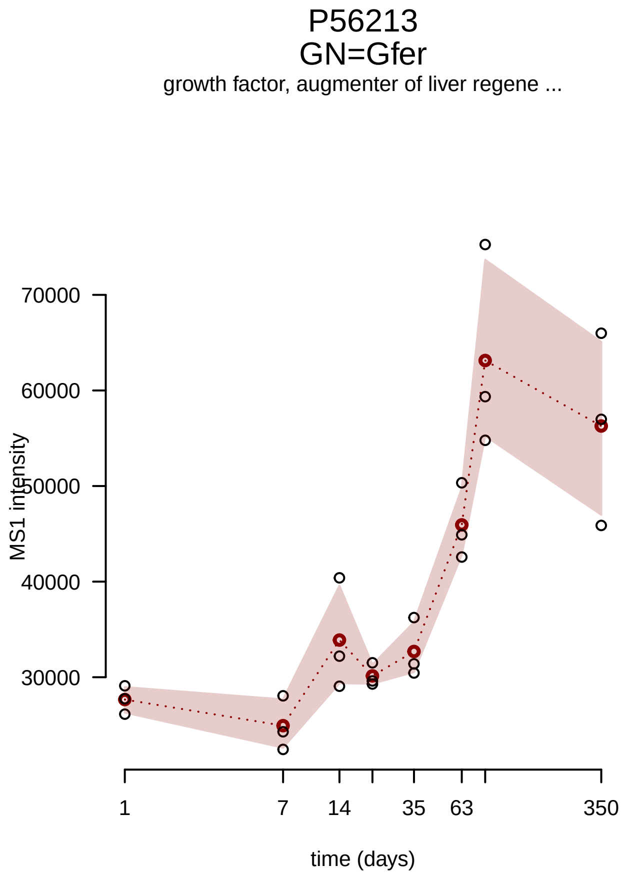
P56213
GN=Gfer
growth factor, augmenter of liver regene ...
70000
60000
50000
40000
30000
MS1 intensity
1
7
14
35
63
350
time (days)
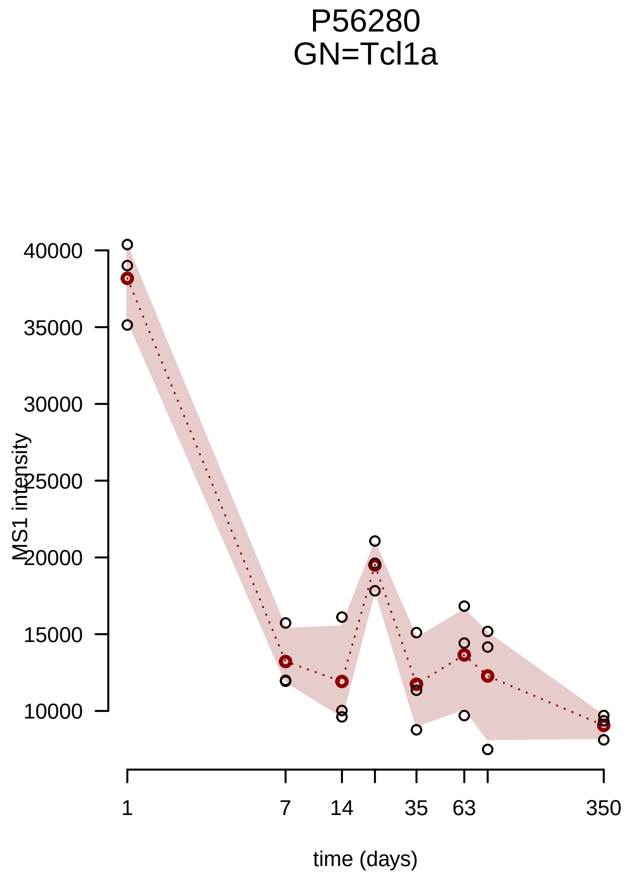
P56280
GN=Tcl1a
40000
35000
30000
25000
20000
15000
10000
MS1 intensity
1
7
14
35
63
350
time (days)
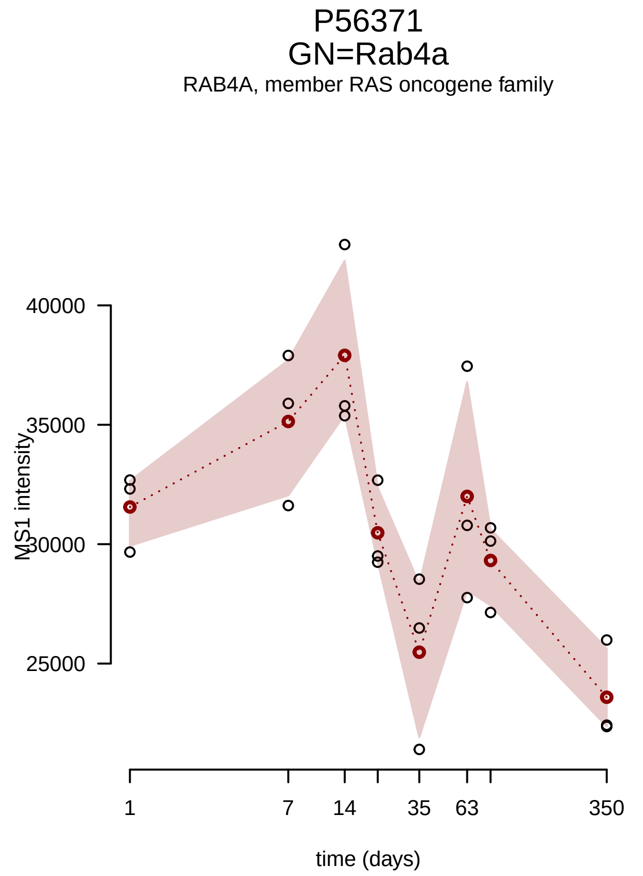
P56371
GN=Rab4a
RAB4A, member RAS oncogene family
40000
35000
30000
25000
MS1 intensity
1
7
14
35
63
350
time (days)
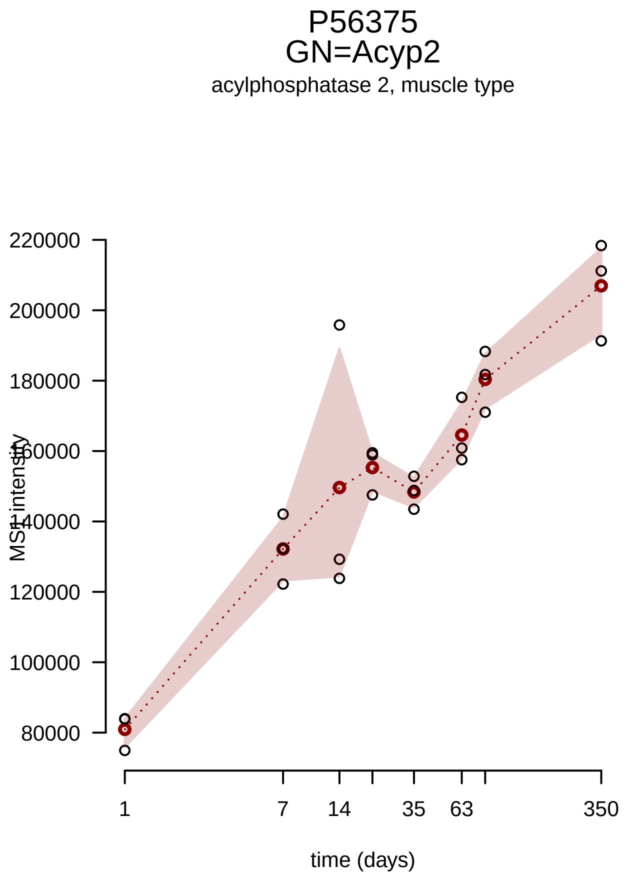
P56375
GN=Acyp2
acylphosphatase 2, muscle type
220000
200000
180000
160000
140000
120000
100000
80000
MS1 intensity
1
7
14
35
63
350
time (days)
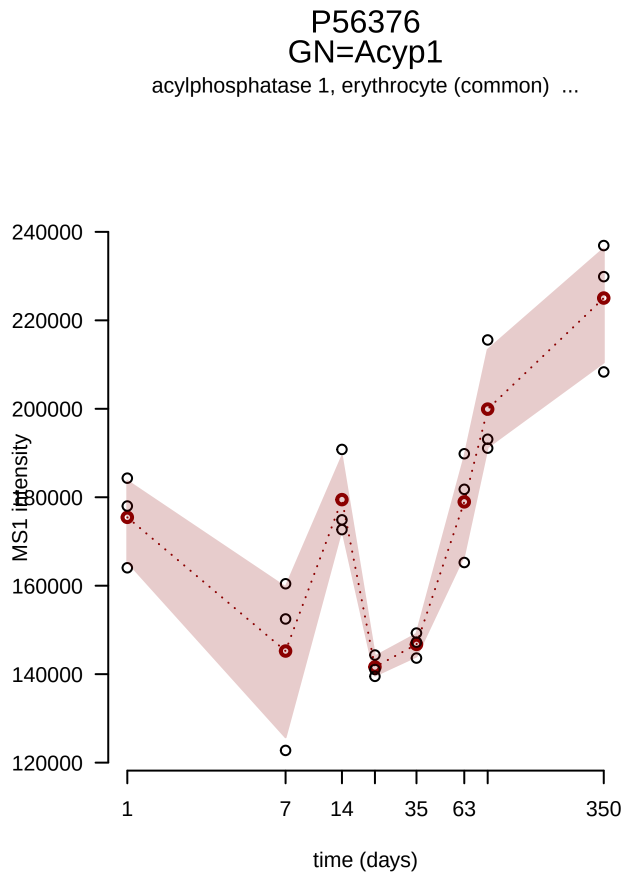
P56376
GN=Acyp1
acylphosphatase 1, erythrocyte (common) ...
240000
220000
200000
180000
160000
140000
120000
MS1 intensity
1
7
14
35
63
350
time (days)
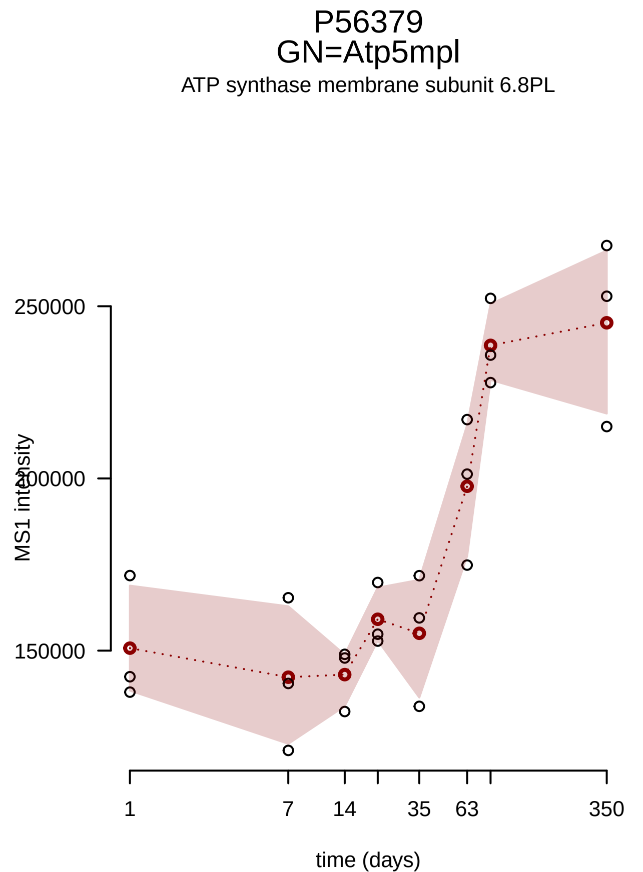
P56379
GN=Atp5mpl
ATP synthase membrane subunit 6.8PL
250000
200000
150000
MS1 intensity
1
7
14
35
63
350
time (days)

P56380
GN=Nudt2
nudix (nucleoside diphosphate linked moi ...

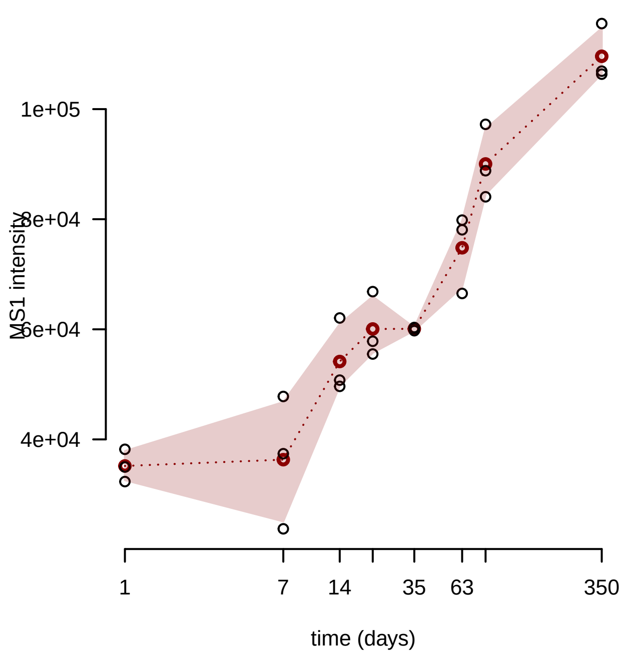

P56382
GN=Atp5f1e

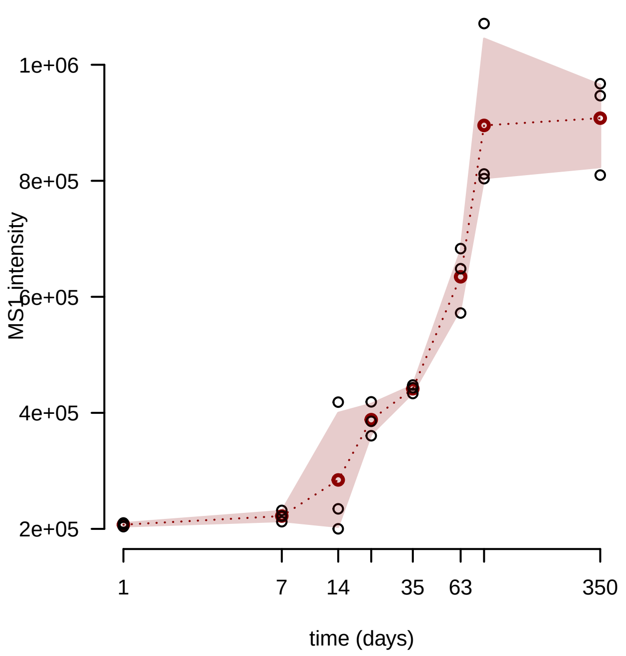

P56383
GN=Atp5mc2

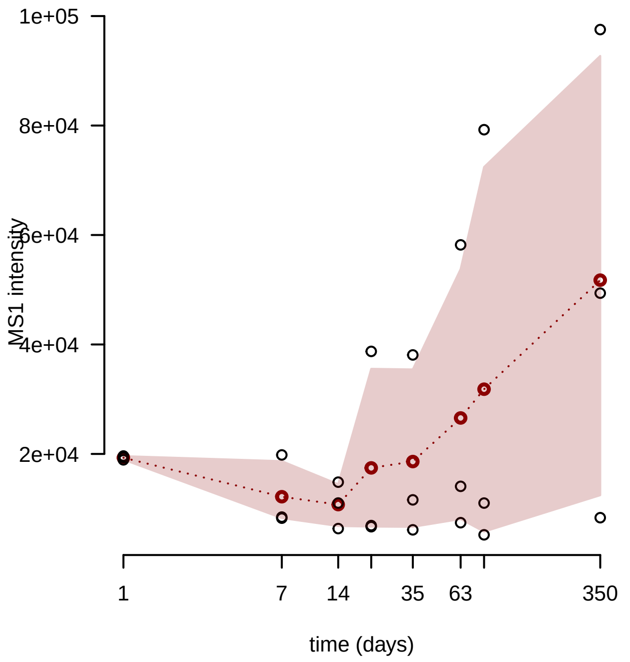

P56390
GN=Cks2
CDC28 protein kinase regulatory subunit ...

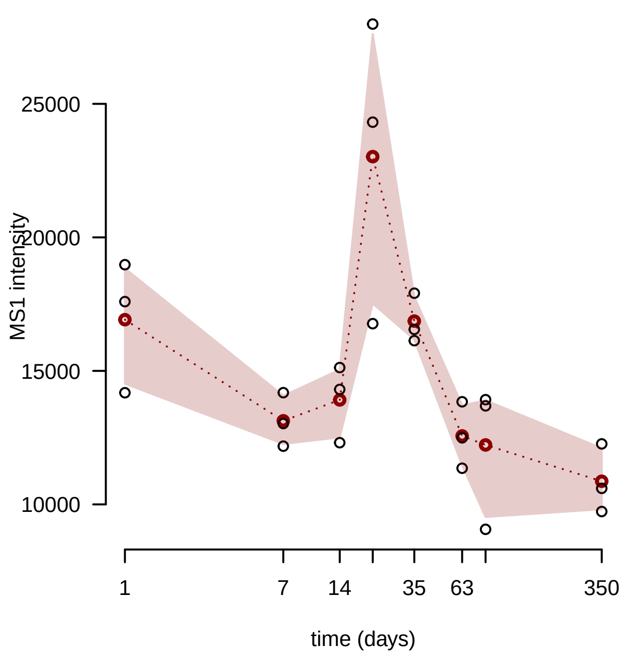

P56391
GN=Cox6b1
cytochrome c oxidase, subunit 6B1

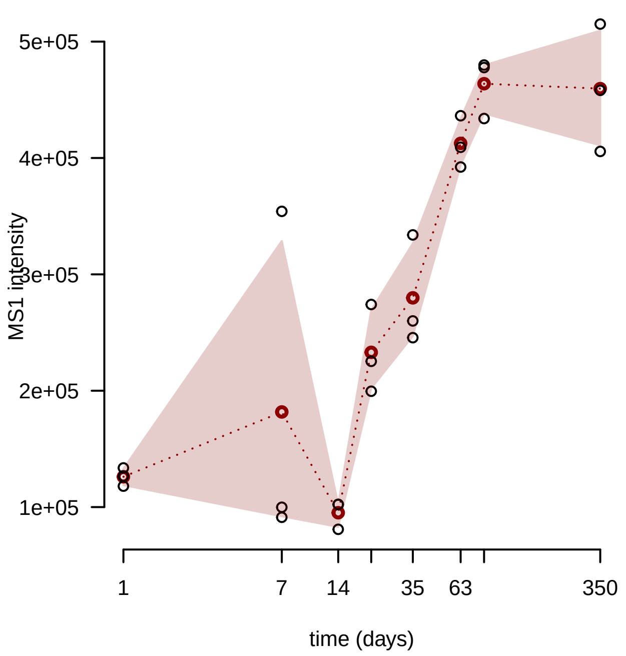

P56392
GN=Cox7a1
cytochrome c oxidase subunit 7A1

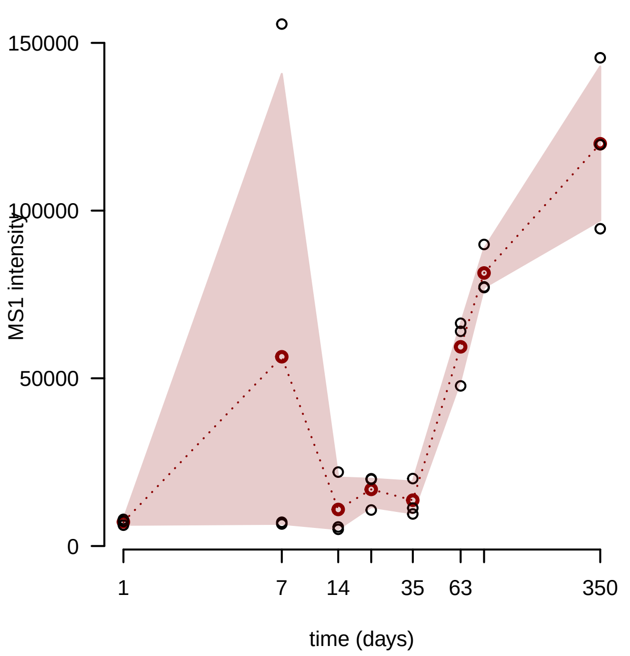

P56394
GN=Cox17
cytochrome c oxidase assembly protein 17 ...

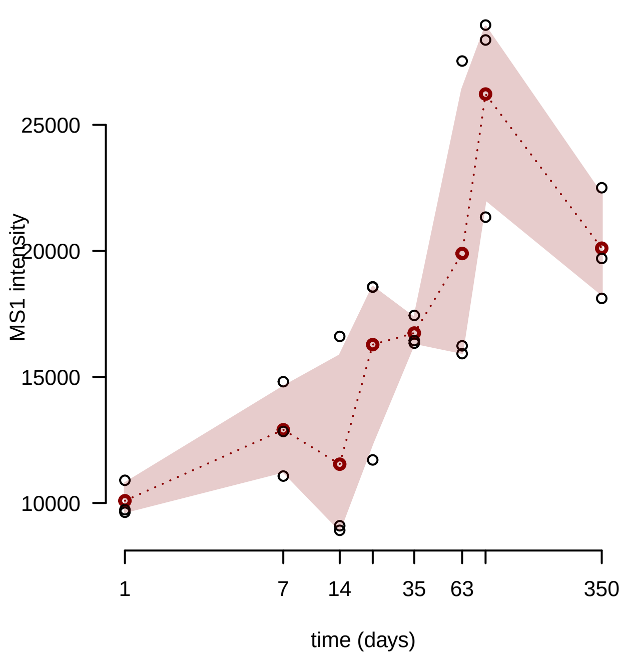

P56395
GN=Cyb5a
cytochrome b5 type A (microsomal)

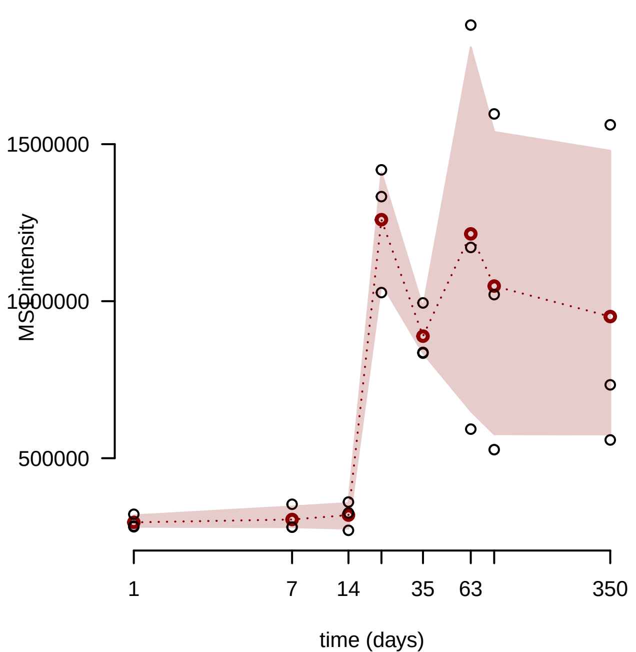

P56399
GN=Usp5
ubiquitin specific peptidase 5 (isopepti ...

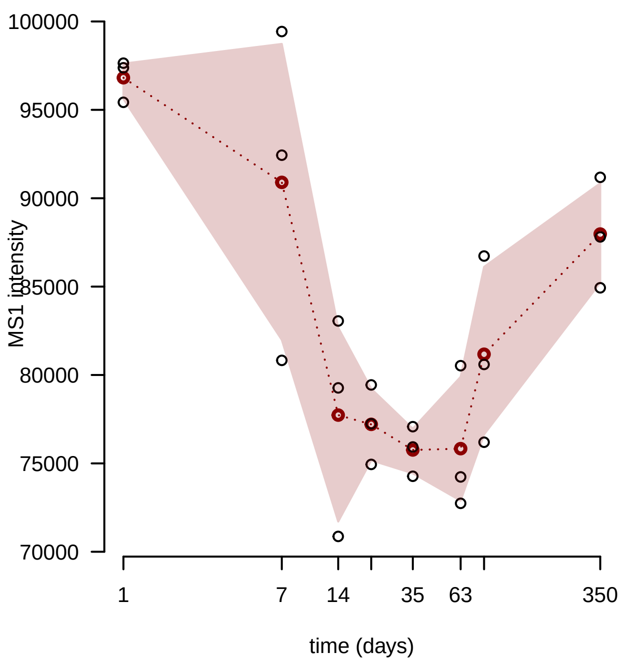

P56480
GN=Atp5f1b

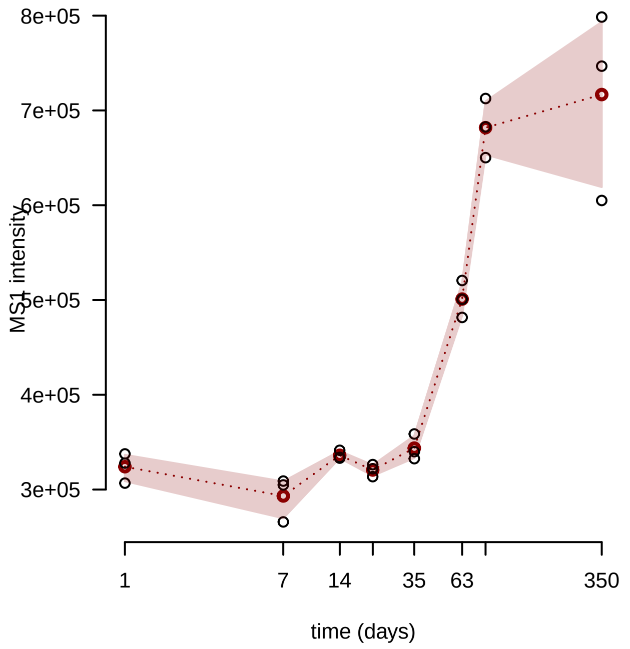

P56528
GN=Cd38
CD38 antigen

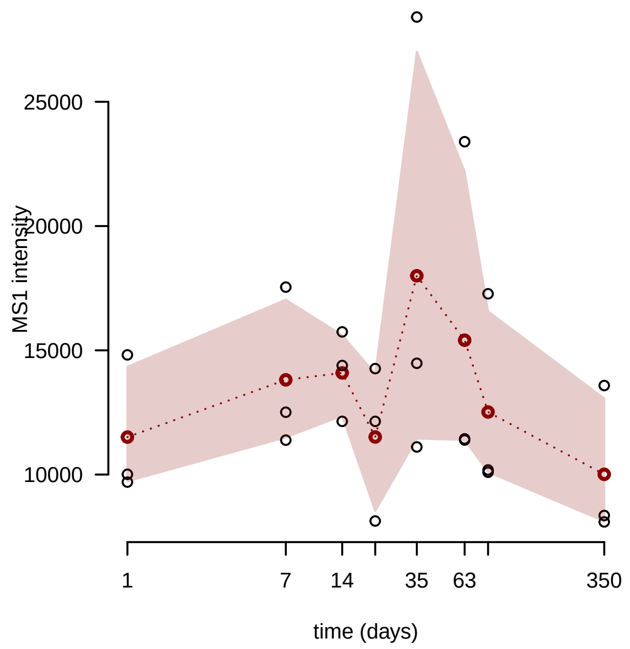

P56542
GN=Dnase2

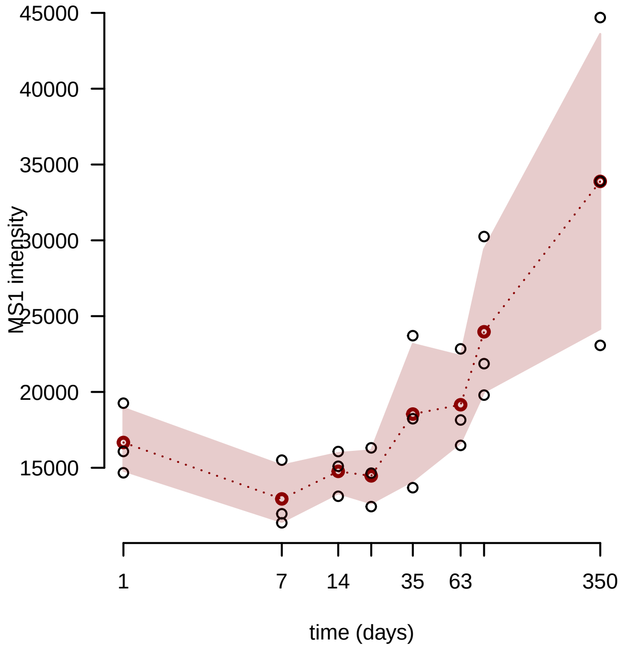

P56546
GN=Ctbp2
C−terminal binding protein 2

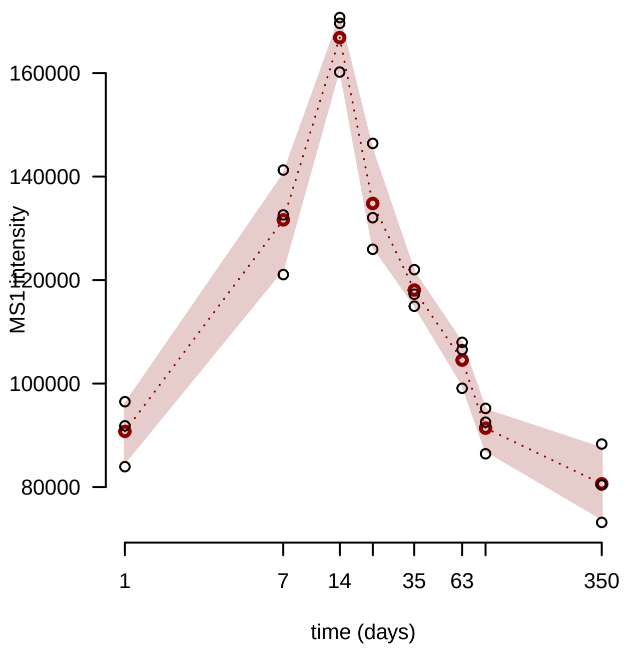

P56565
GN=S100a1
S100 calcium binding protein A1

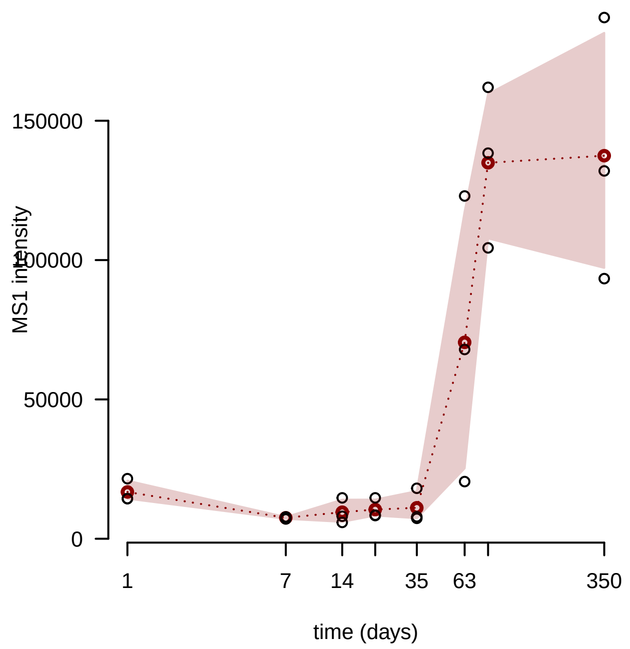

P56581
GN=Tdg
thymine DNA glycosylase

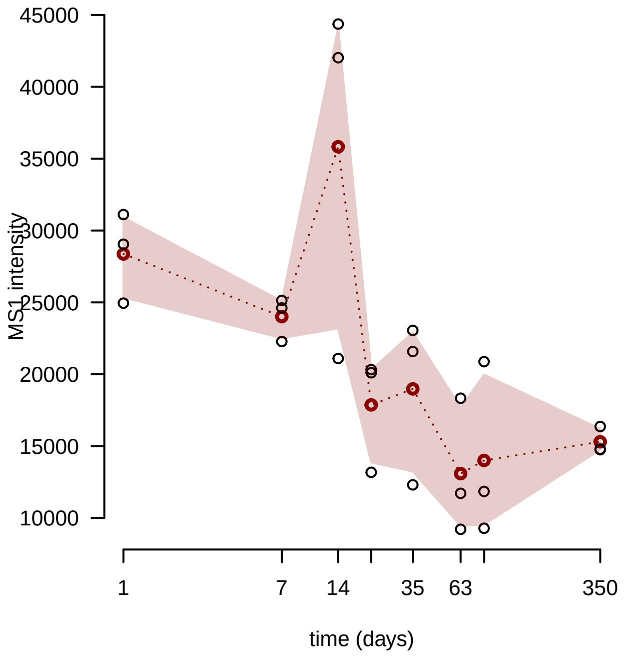

P56671
GN=Maz
MYC−associated zinc finger protein (puri ...

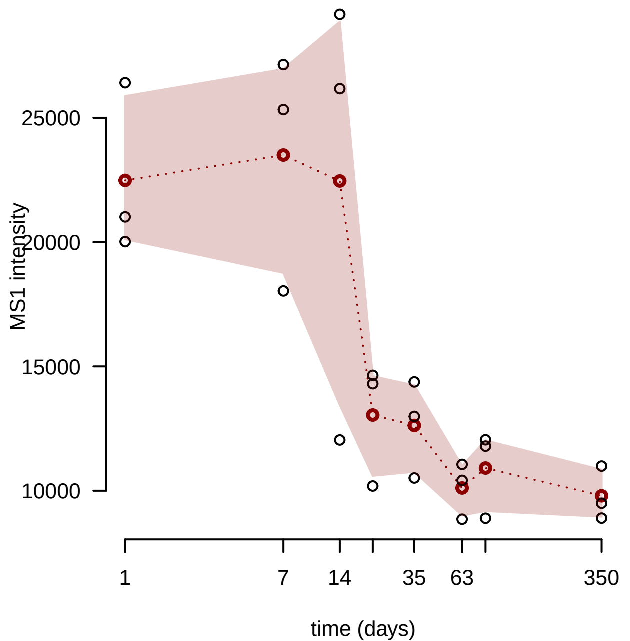

P56695
GN=Wfs1
wolframin ER transmembrane glycoprotein

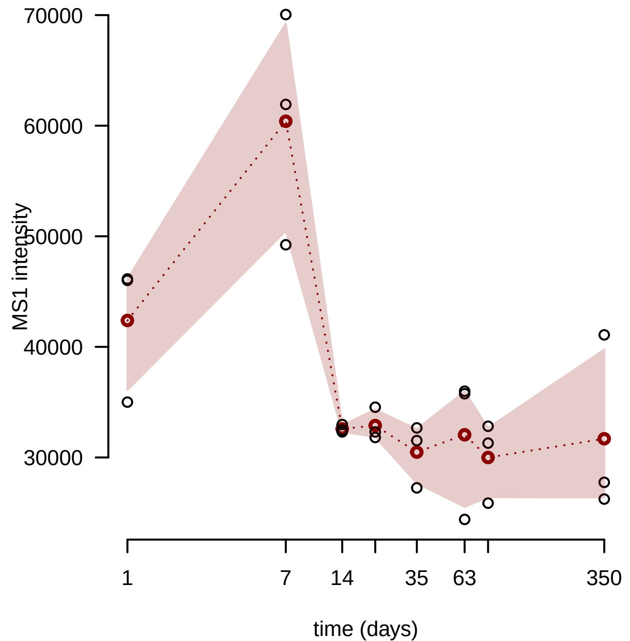

P56812
GN=Pdcd5
programmed cell death 5

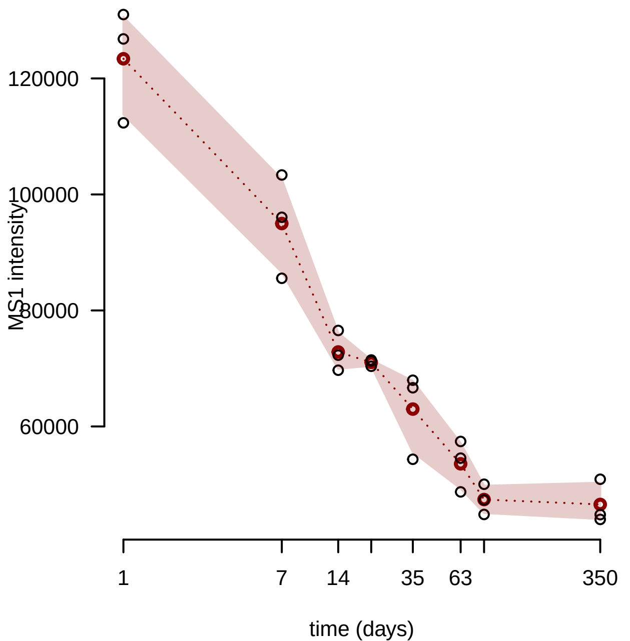

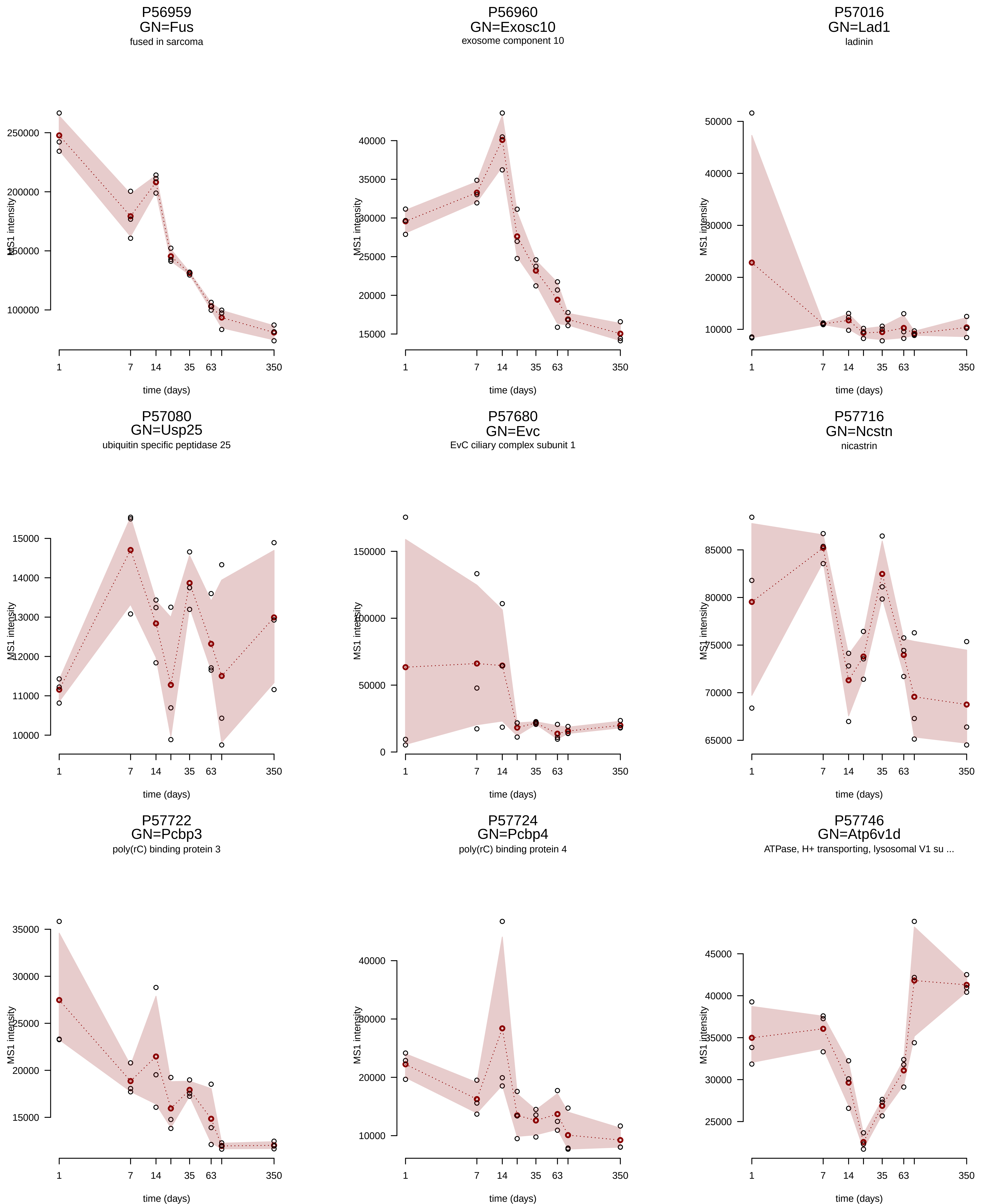
P56959
GN=Fus
fused in sarcoma
MS1 intensity
250000
200000
150000
100000
1
7
14
35
63
350
time (days)
P56960
GN=Exosc10
exosome component 10
MS1 intensity
40000
35000
30000
25000
20000
15000
time (days)
P57016
GN=Lad1
ladinin
MS1 intensity
50000
40000
30000
20000
10000
time (days)
P57080
GN=Usp25
ubiquitin specific peptidase 25
MS1 intensity
15000
14000
13000
12000
11000
10000
time (days)
P57680
GN=Evc
EvC ciliary complex subunit 1
MS1 intensity
150000
100000
50000
0
time (days)
P57716
GN=Ncstn
nicastrin
MS1 intensity
85000
80000
75000
70000
65000
time (days)
P57722
GN=Pcbp3
poly(rC) binding protein 3
MS1 intensity
35000
30000
25000
20000
15000
time (days)
P57724
GN=Pcbp4
poly(rC) binding protein 4
MS1 intensity
40000
30000
20000
10000
time (days)
P57746
GN=Atp6v1d
ATPase, H+ transporting, lysosomal V1 su ...
MS1 intensity
45000
40000
35000
30000
25000
time (days)

P57759
GN=Erp29
endoplasmic reticulum protein 29

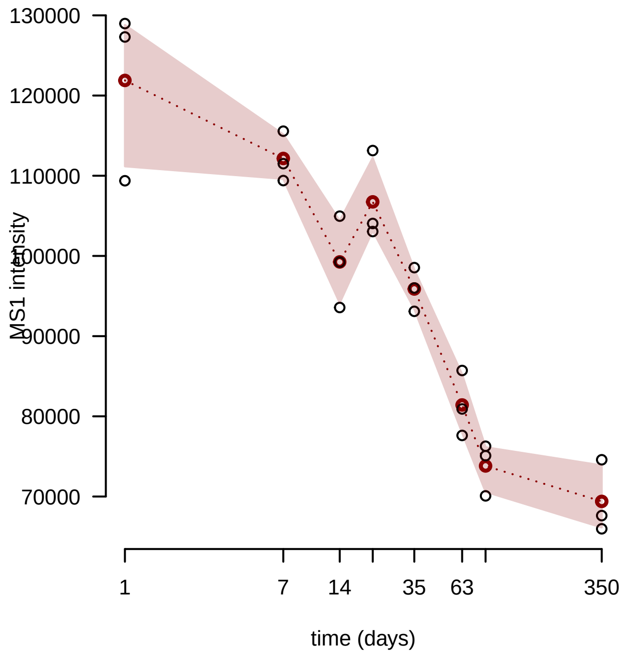

P57776
GN=Eef1d
eukaryotic translation elongation factor ...

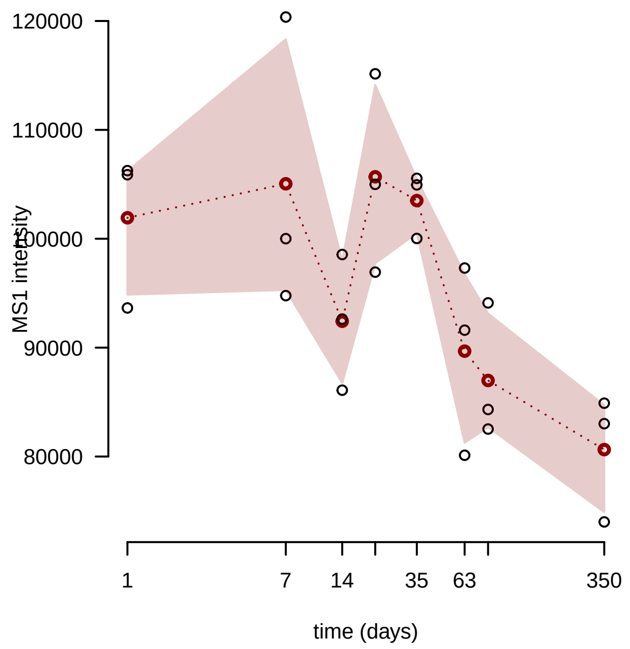

P57776
GN=Eef1d
eukaryotic translation elongation factor ...

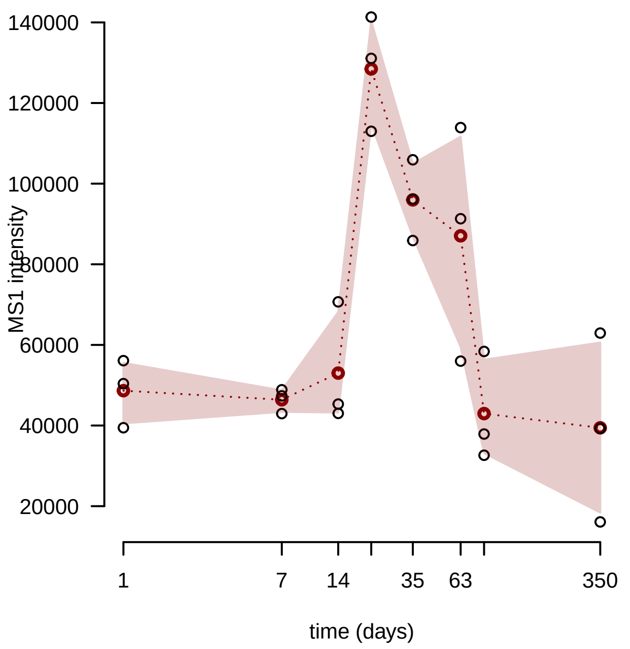

P57780
GN=Actn4
actinin alpha 4

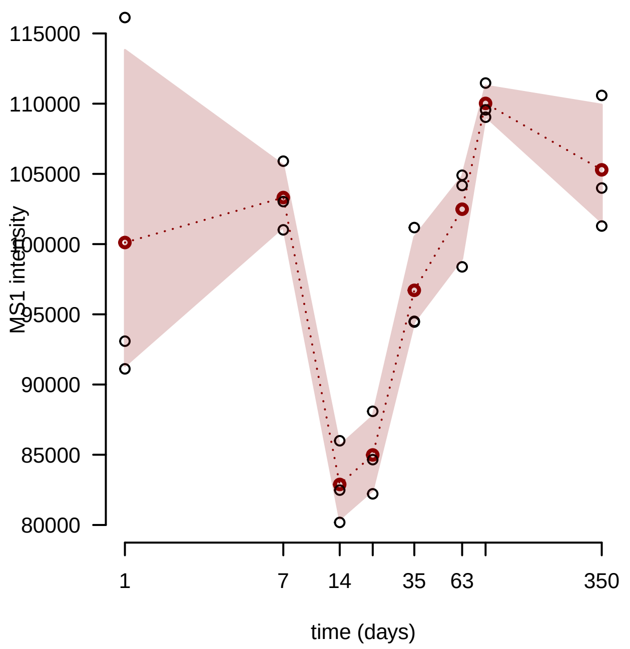

P57784
GN=Snrpa1
small nuclear ribonucleoprotein polypept ...

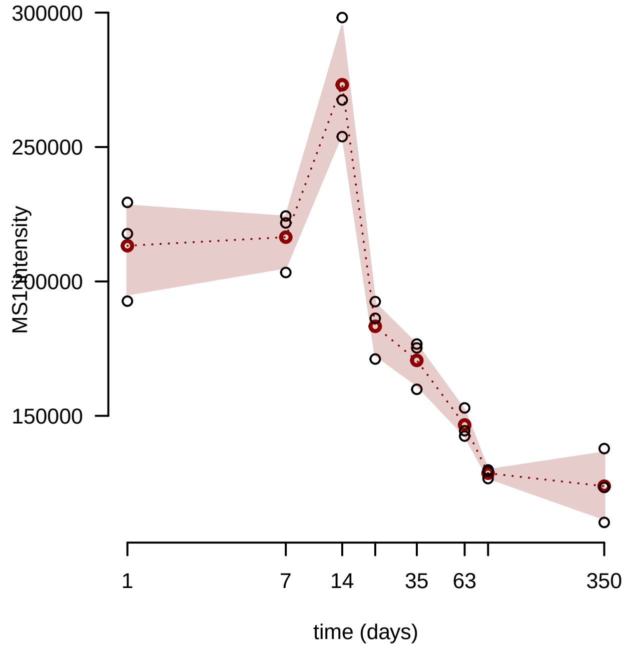

P57787
GN=Slc16a3
solute carrier family 16 (monocarboxylic ...

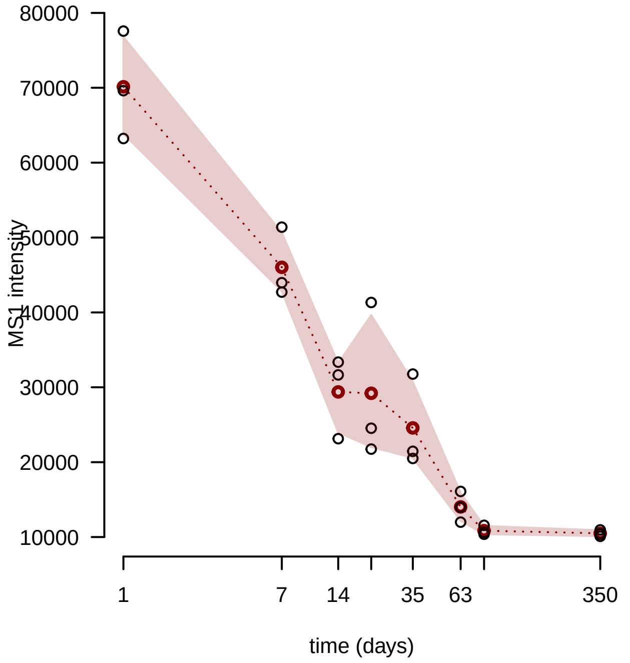

P57791
GN=Rce1
Ras converting CAAX endopeptidase 1

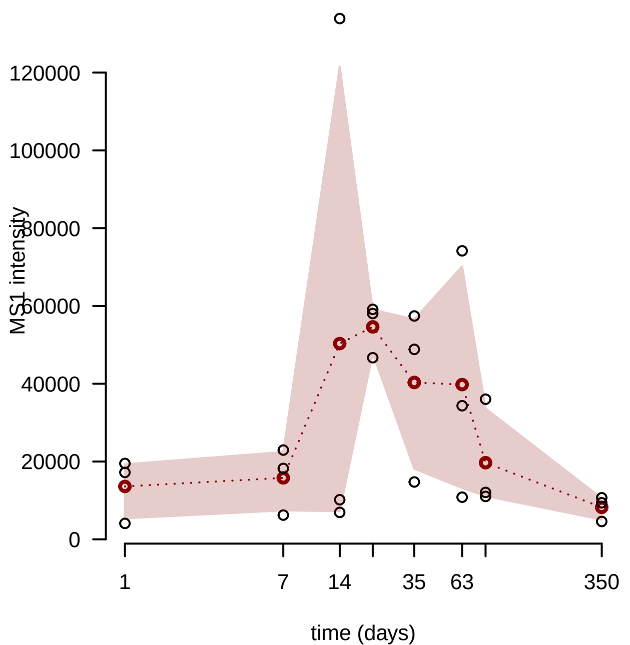

P58006
GN=Sesn1
sestrin 1

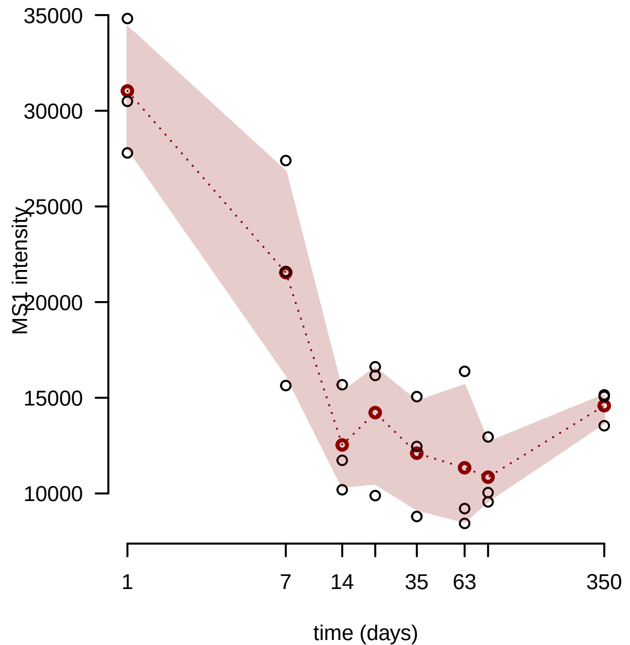

P58021
GN=Tm9sf2
transmembrane 9 superfamily member 2

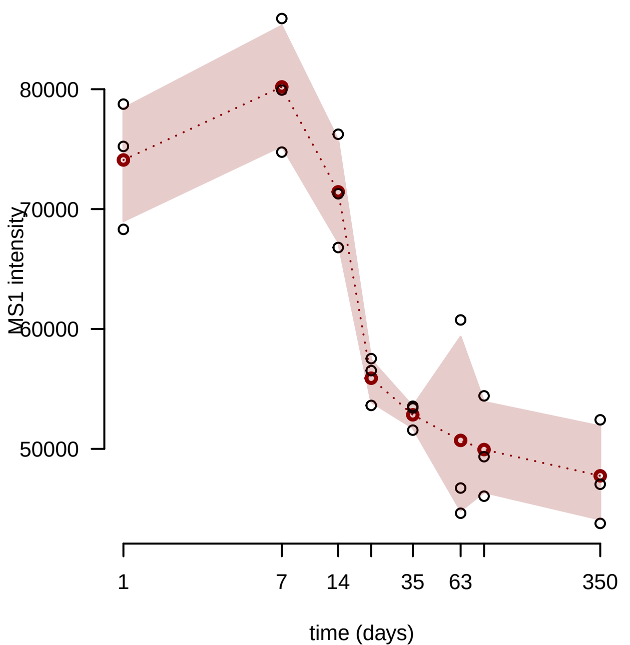

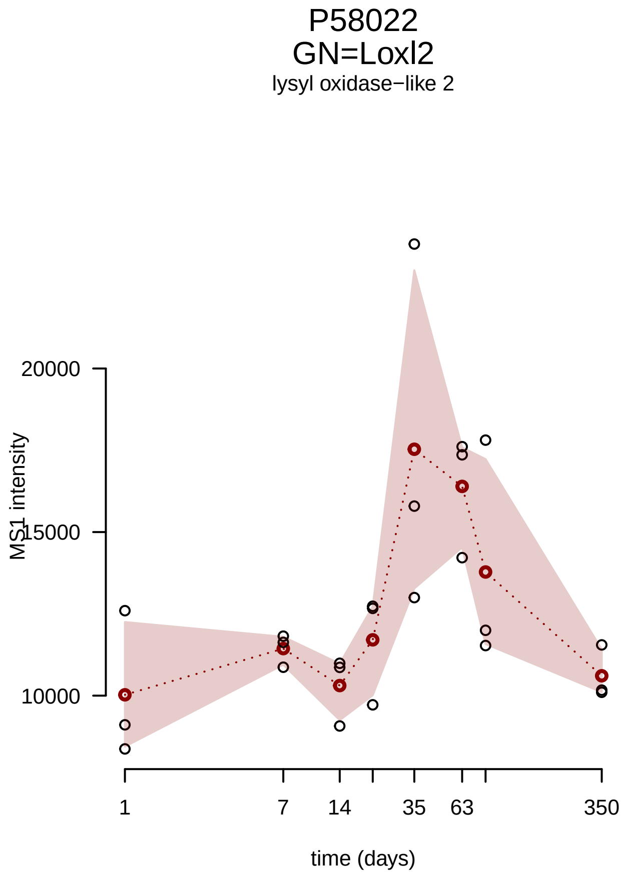
P58022
GN=Loxl2
lysyl oxidase−like 2
MS1 intensity
20000
15000
10000
1
7
14
35
63
350
time (days)

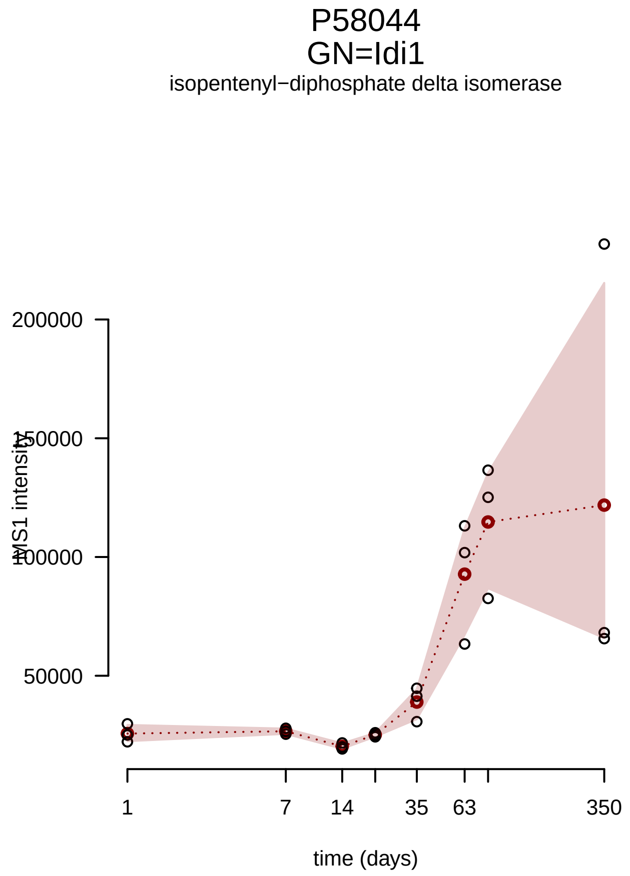
P58044
GN=Idi1
isopentenyl−diphosphate delta isomerase
MS1 intensity
200000
150000
100000
50000
1
7
14
35
63
350
time (days)

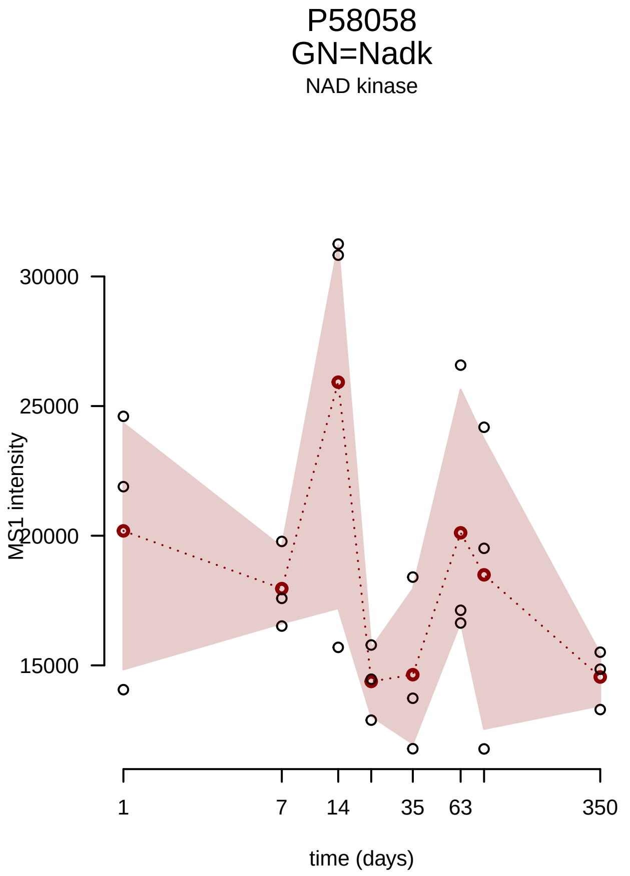
P58058
GN=Nadk
NAD kinase
MS1 intensity
30000
25000
20000
15000
1
7
14
35
63
350
time (days)

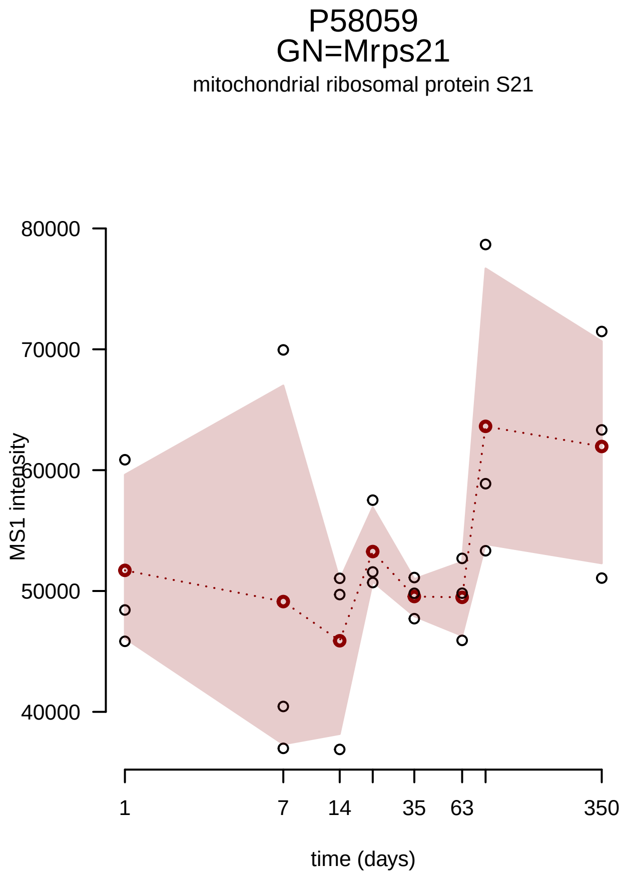
P58059
GN=Mrps21
mitochondrial ribosomal protein S21
MS1 intensity
80000
70000
60000
50000
40000
1
7
14
35
63
350
time (days)

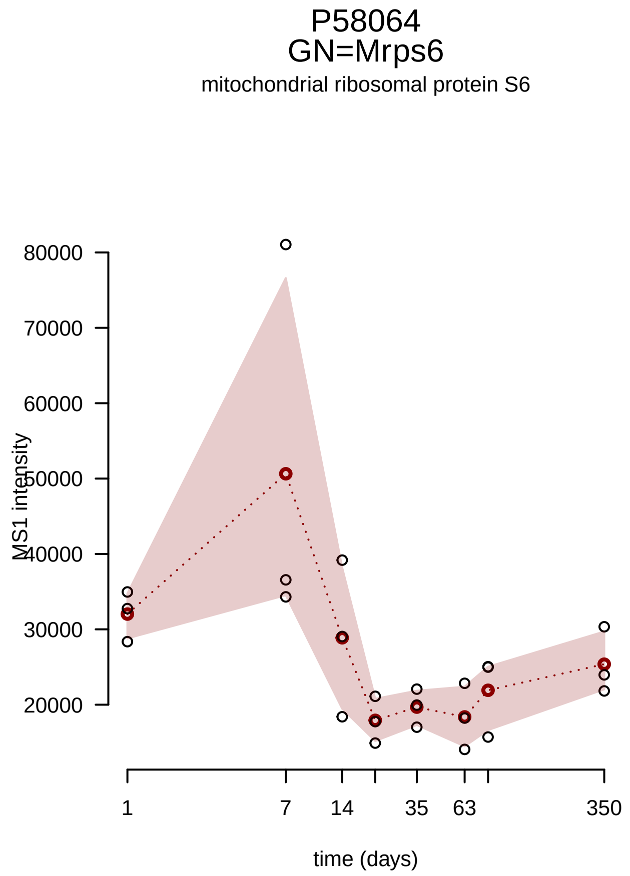
P58064
GN=Mrps6
mitochondrial ribosomal protein S6
MS1 intensity
80000
70000
60000
50000
40000
30000
20000
1
7
14
35
63
350
time (days)

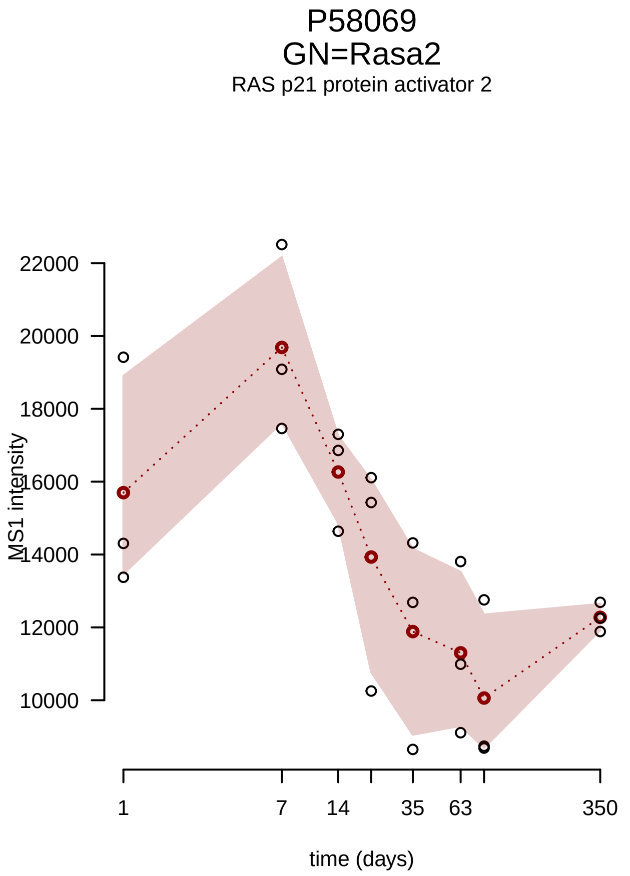
P58069
GN=Rasa2
RAS p21 protein activator 2
MS1 intensity
22000
20000
18000
16000
14000
12000
10000
1
7
14
35
63
350
time (days)

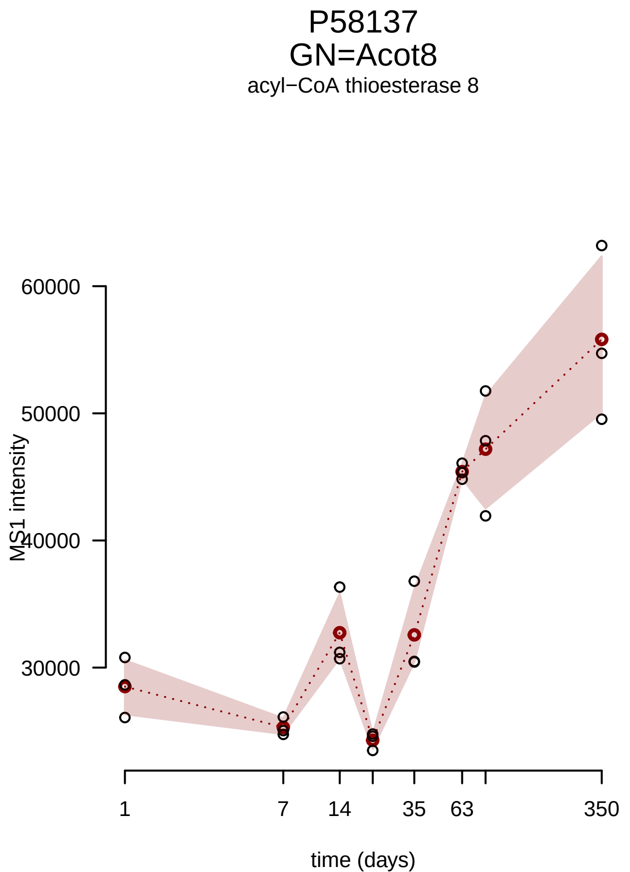
P58137
GN=Acot8
acyl−CoA thioesterase 8
MS1 intensity
60000
50000
40000
30000
1
7
14
35
63
350
time (days)

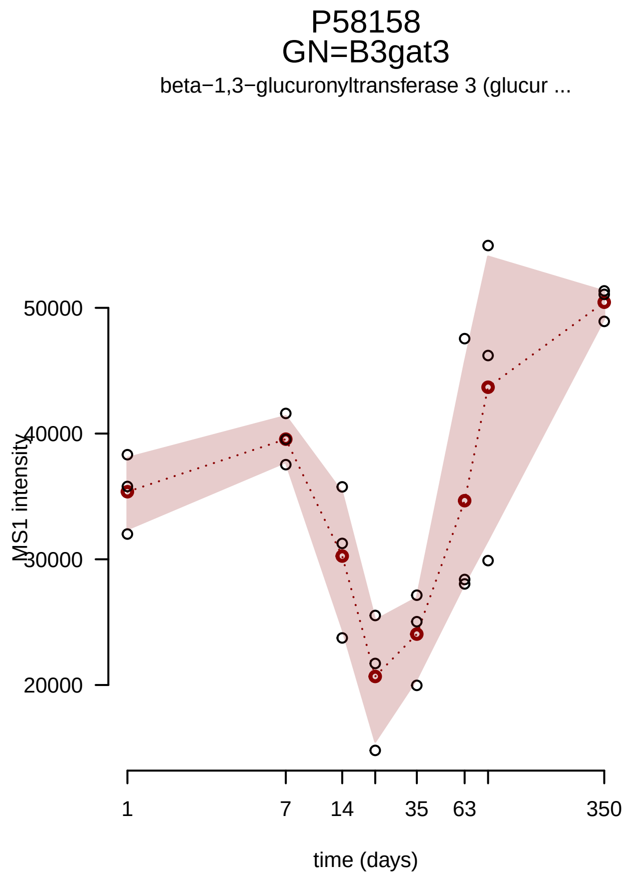
P58158
GN=B3gat3
beta−1,3−glucuronyltransferase 3 (glucur ...
MS1 intensity
50000
40000
30000
20000
1
7
14
35
63
350
time (days)

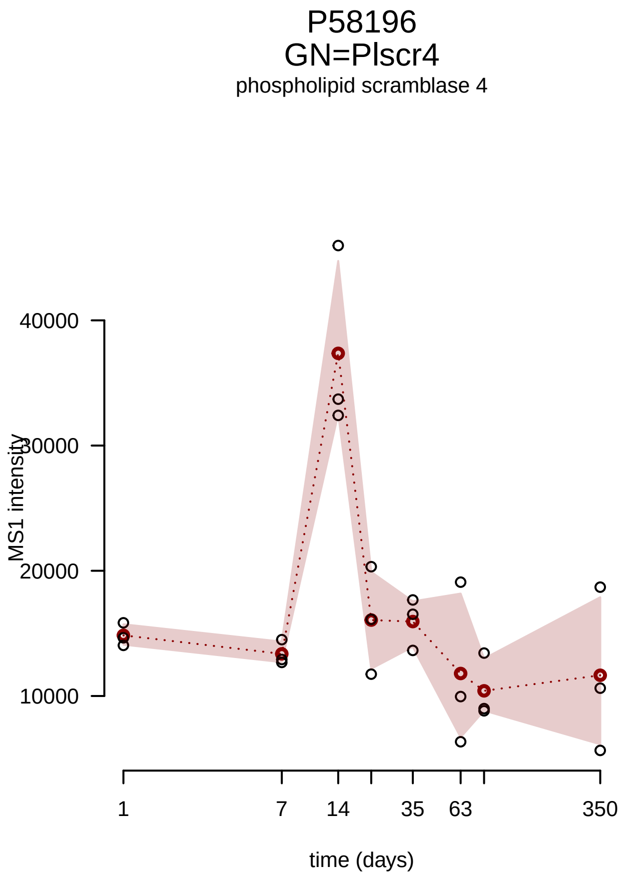
P58196
GN=Plscr4
phospholipid scramblase 4
MS1 intensity
40000
30000
20000
10000
1
7
14
35
63
350
time (days)

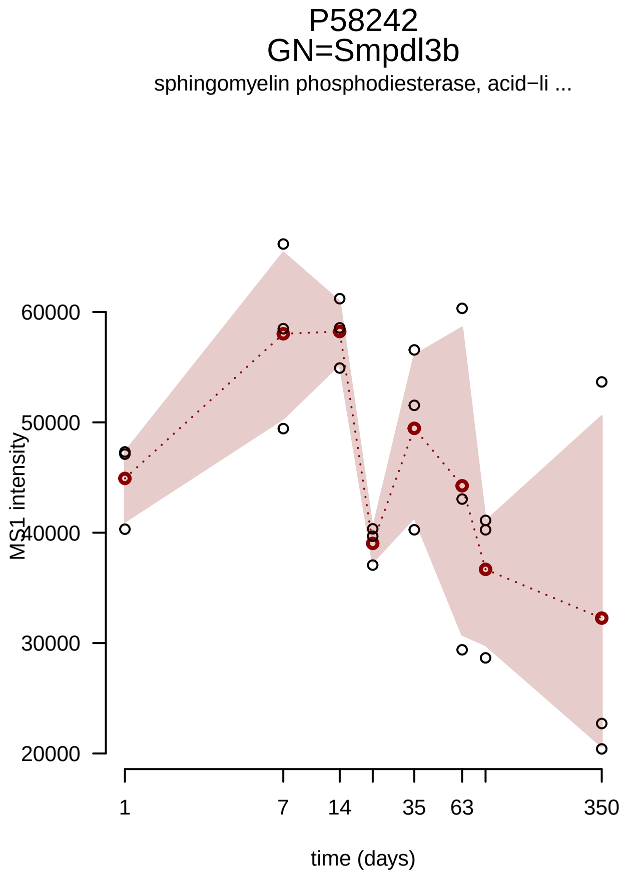
P58242
GN=Smpdl3b
sphingomyelin phosphodiesterase, acid−li ...
MS1 intensity
20000
30000
40000
50000
60000
1
7
14
35
63
350
time (days)

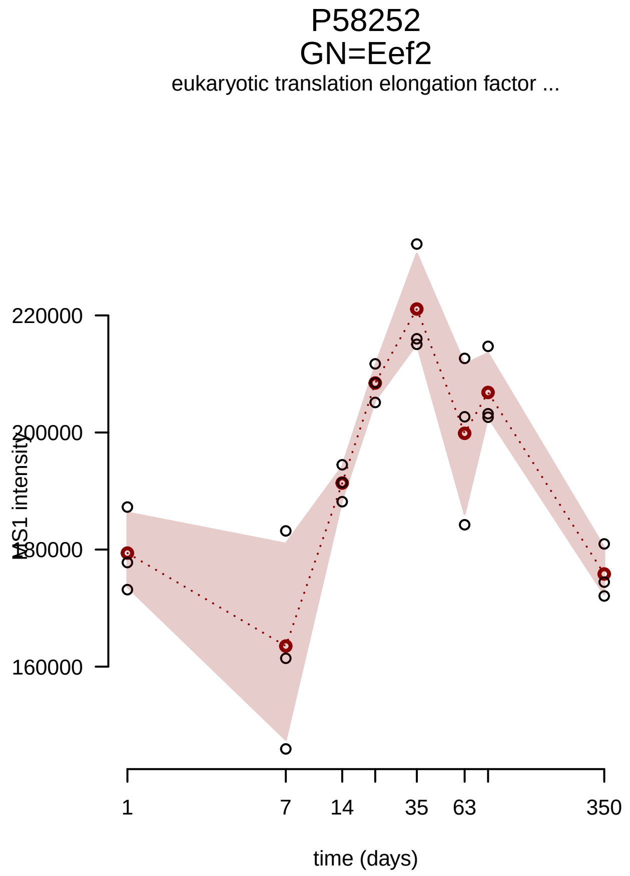
P58252
GN=Eef2
eukaryotic translation elongation factor ...
MS1 intensity
160000
180000
200000
220000
1
7
14
35
63
350
time (days)

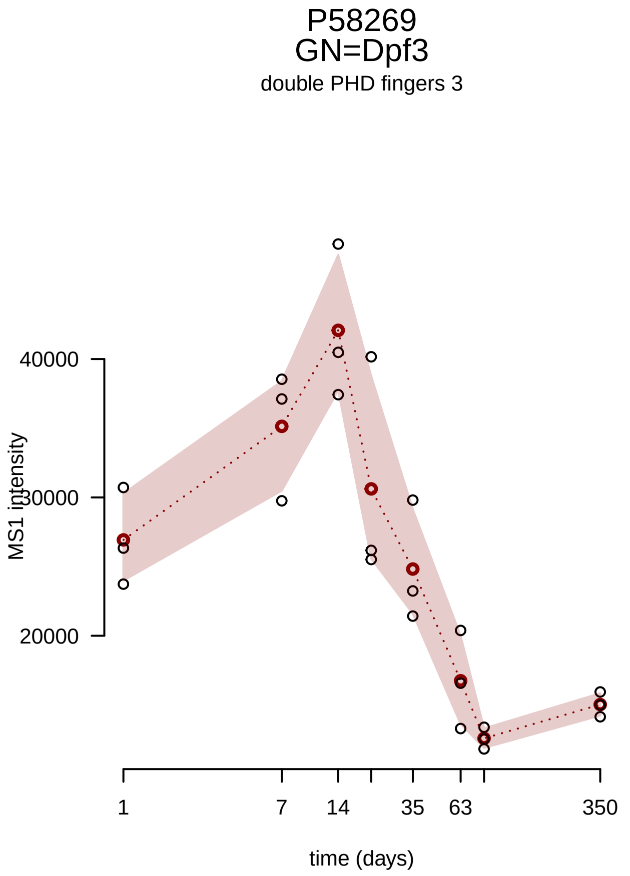
P58269
GN=Dpf3
double PHD fingers 3
MS1 intensity
20000
30000
40000
1
7
14
35
63
350
time (days)

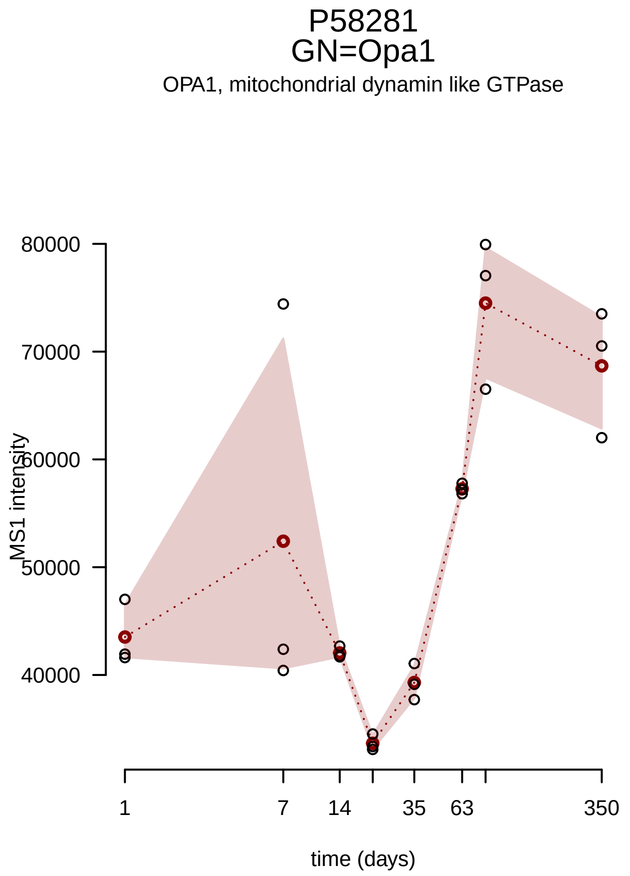
P58281
GN=Opa1
OPA1, mitochondrial dynamin like GTPase
MS1 intensity
40000
50000
60000
70000
80000
1
7
14
35
63
350
time (days)

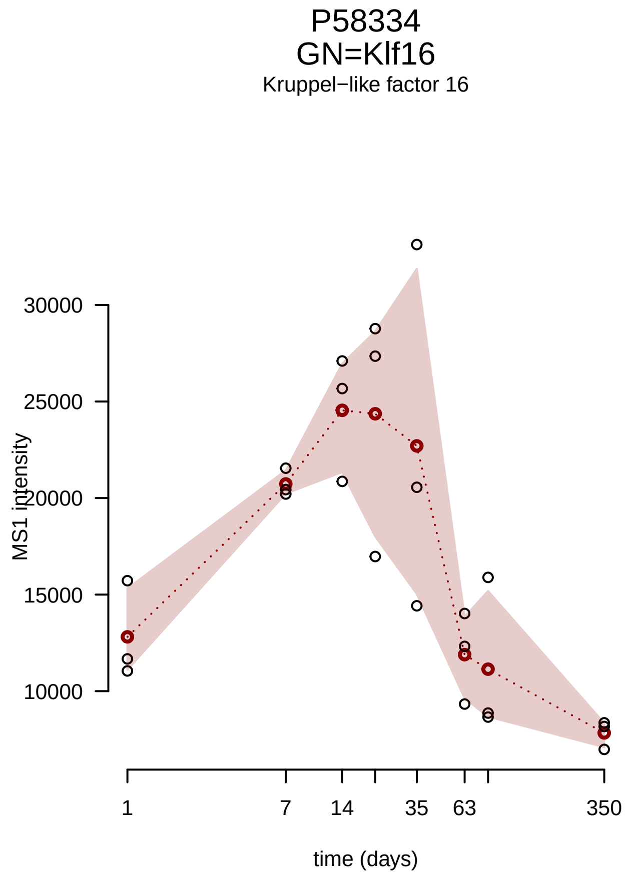
P58334
GN=Klf16
Kruppel−like factor 16
MS1 intensity
10000
15000
20000
25000
30000
1
7
14
35
63
350
time (days)

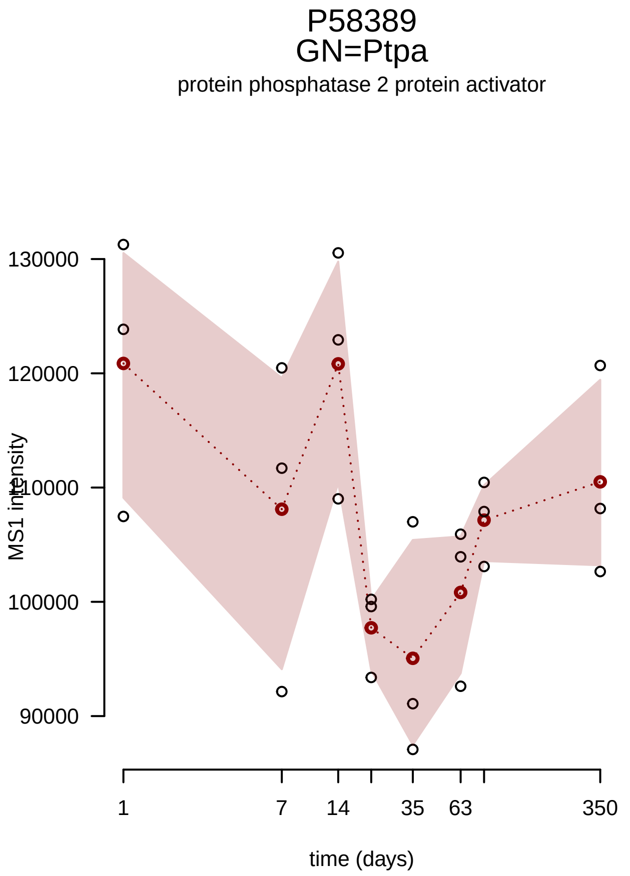
P58389
GN=Ptpa
protein phosphatase 2 protein activator
MS1 intensity
90000
100000
110000
120000
130000
1
7
14
35
63
350
time (days)

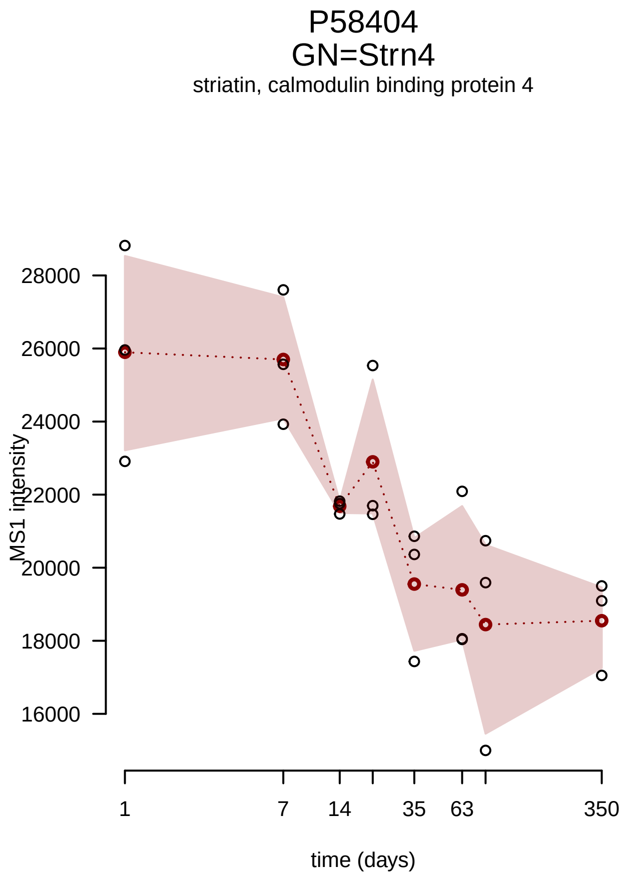
P58404
GN=Strn4
striatin, calmodulin binding protein 4
MS1 intensity
16000
18000
20000
22000
24000
26000
28000
1
7
14
35
63
350
time (days)

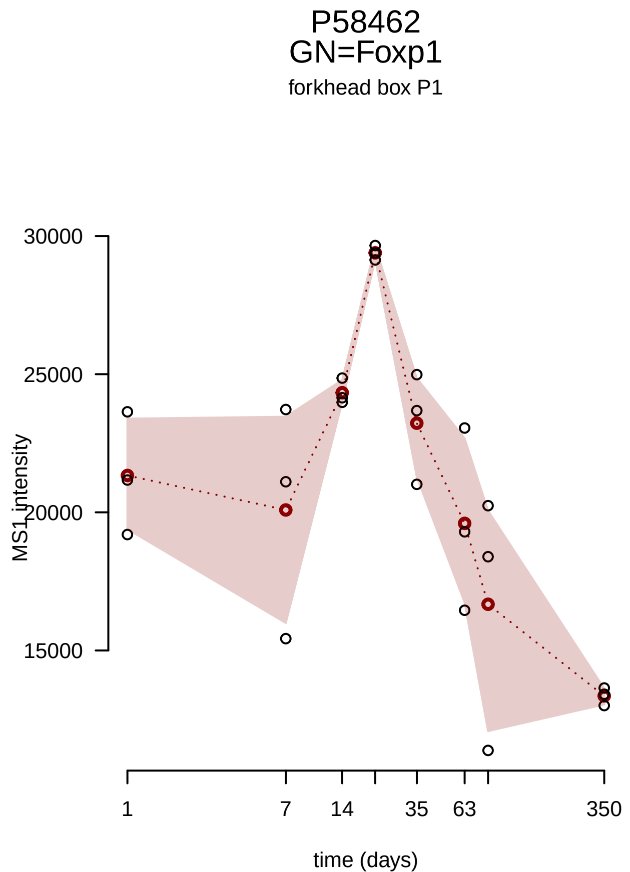
P58462
GN=Foxp1
forkhead box P1
MS1 intensity
15000
20000
25000
30000
1
7
14
35
63
350
time (days)

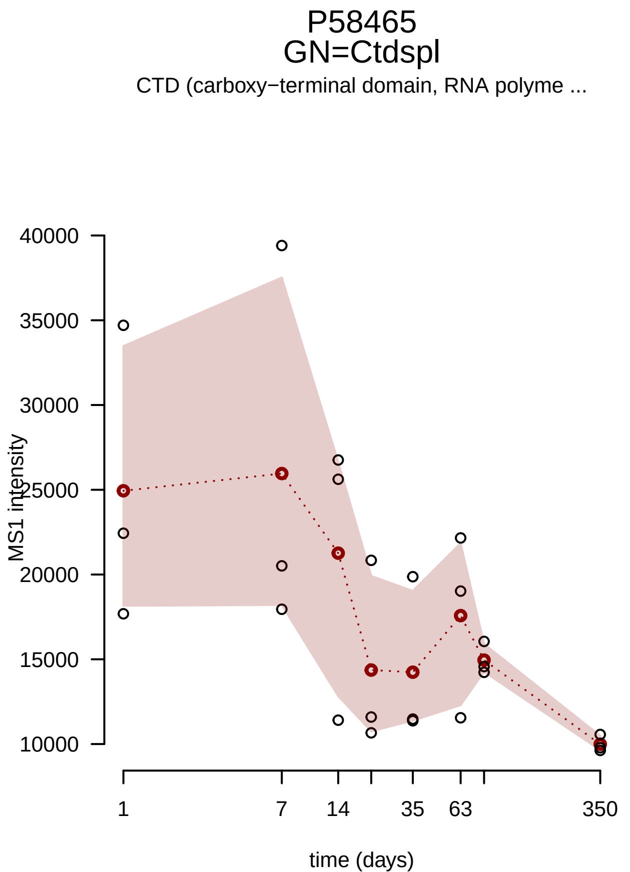
P58465
GN=Ctdspl
CTD (carboxy−terminal domain, RNA polyme ...
MS1 intensity
10000
15000
20000
25000
30000
35000
40000
1
7
14
35
63
350
time (days)

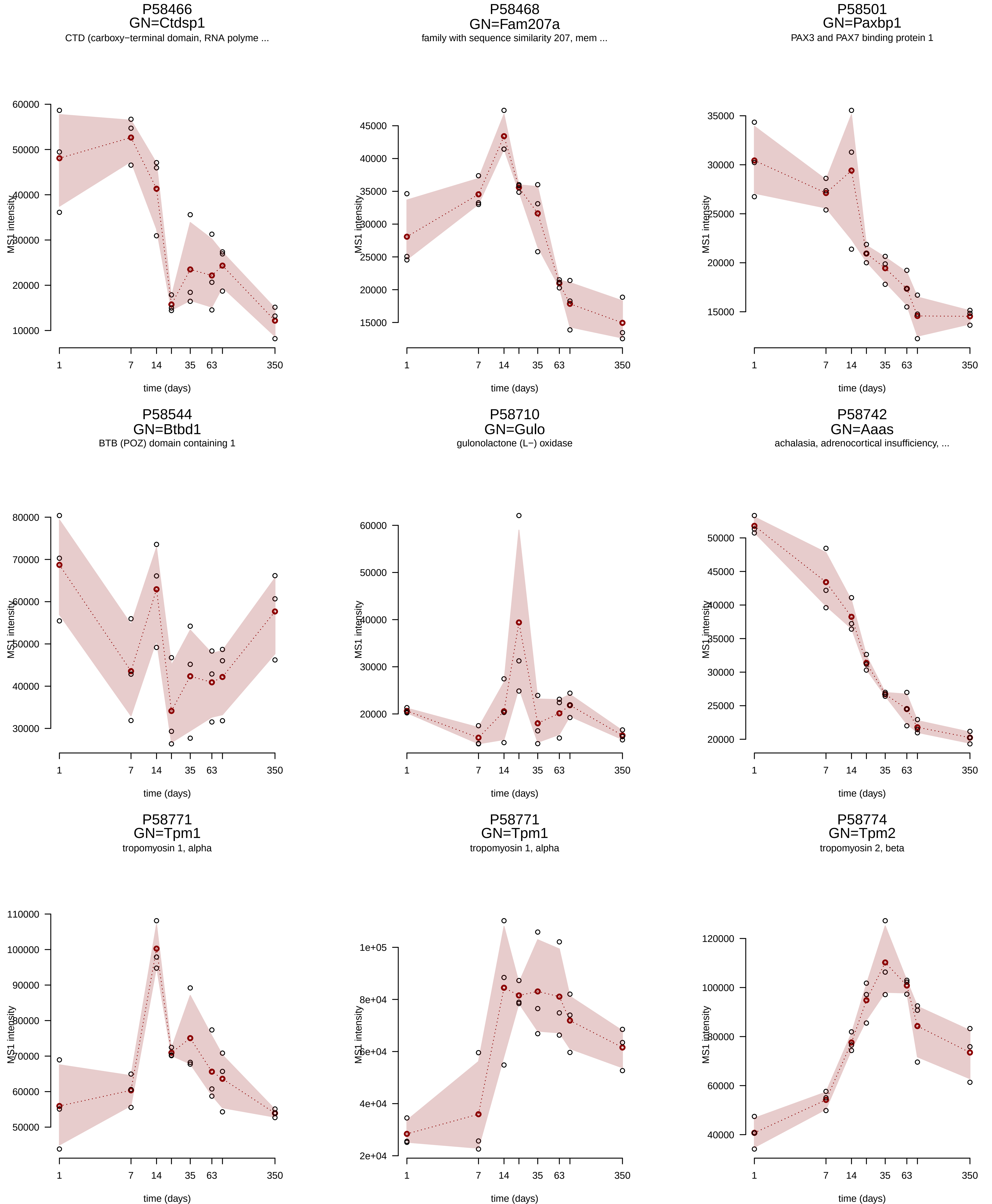
P58466
GN=Ctdsp1
CTD (carboxy−terminal domain, RNA polyme ...
P58468
GN=Fam207a
family with sequence similarity 207, mem ...
P58501
GN=Paxbp1
PAX3 and PAX7 binding protein 1
P58544
GN=Btbd1
BTB (POZ) domain containing 1
P58710
GN=Gulo
gulonolactone (L−) oxidase
P58742
GN=Aaas
achalasia, adrenocortical insufficiency, ...
P58771
GN=Tpm1
tropomyosin 1, alpha
P58771
GN=Tpm1
tropomyosin 1, alpha
P58774
GN=Tpm2
tropomyosin 2, beta
MS1 intensity
time (days)
1
7
14
35
63
350

P58774
GN=Tpm2
tropomyosin 2, beta

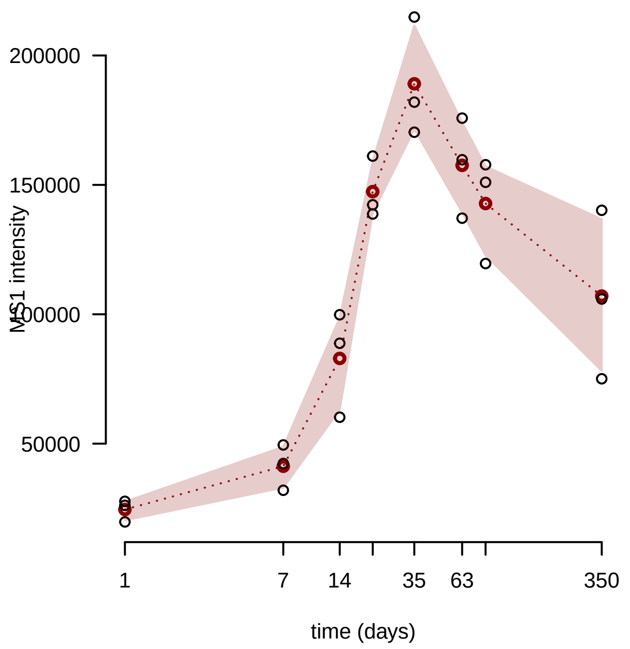

P58801
GN=Ripk2
receptor (TNFRSF)−interacting serine−thr ...

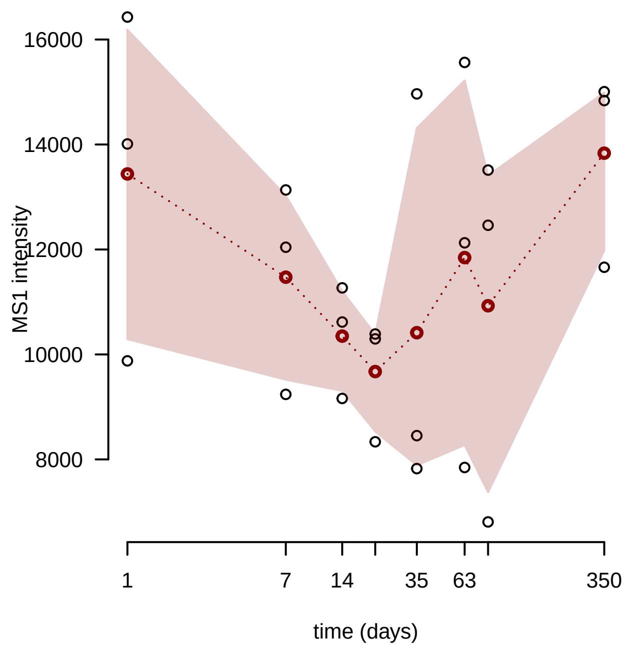

P58802
GN=Tbc1d10a
TBC1 domain family, member 10a

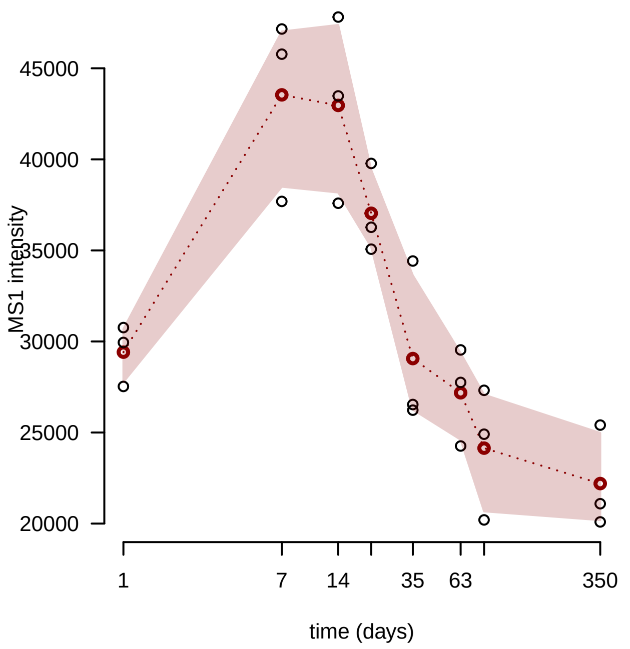

P58854
GN=Tubgcp3
tubulin, gamma complex associated protei ...

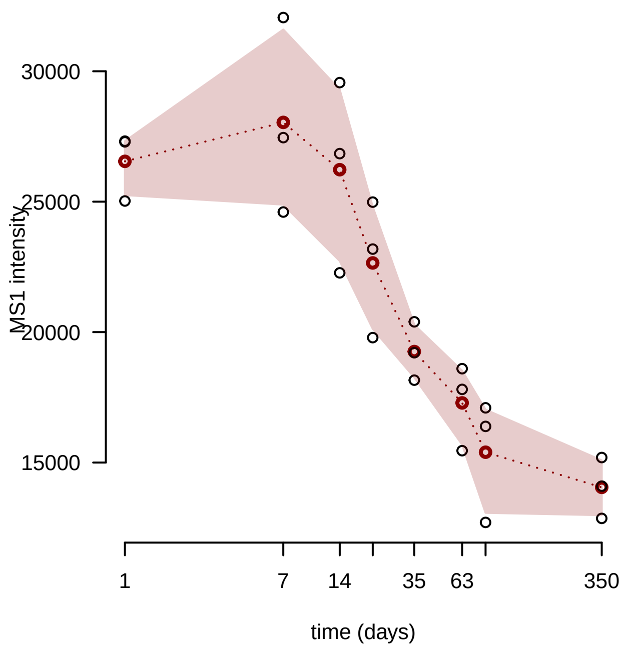

P58871
GN=Tnks1bp1
tankyrase 1 binding protein 1

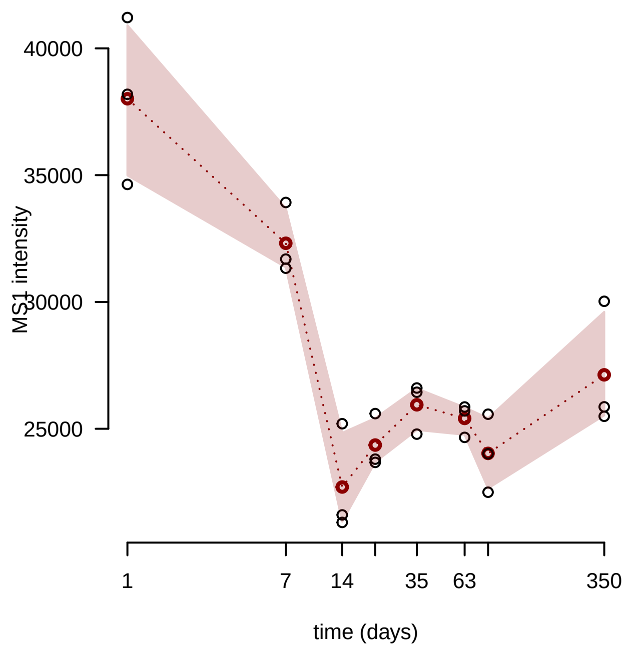

P58929
GN=Gmeb2
glucocorticoid modulatory element bindin ...

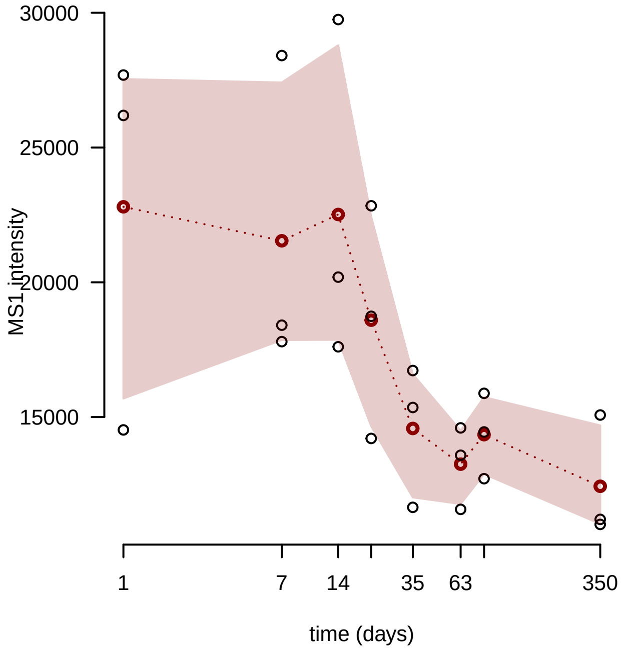

P59016
GN=Vps33b
vacuolar protein sorting 33B

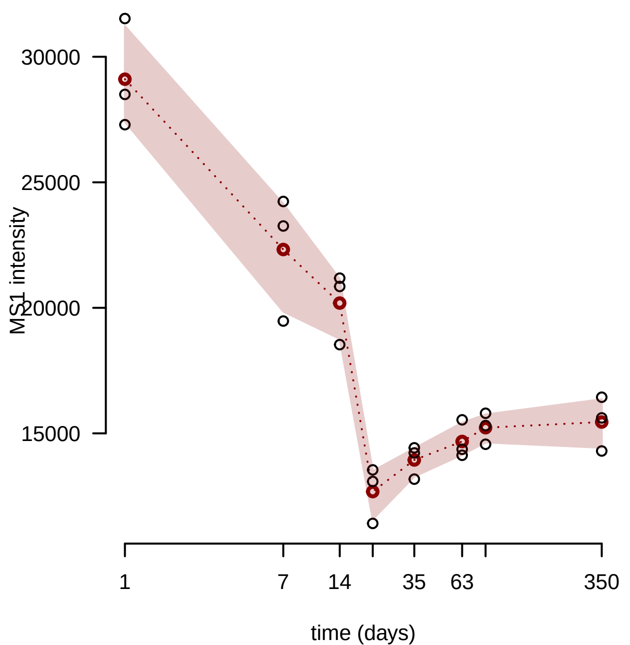

P59017
GN=Bcl2l13
BCL2−like 13 (apoptosis facilitator)

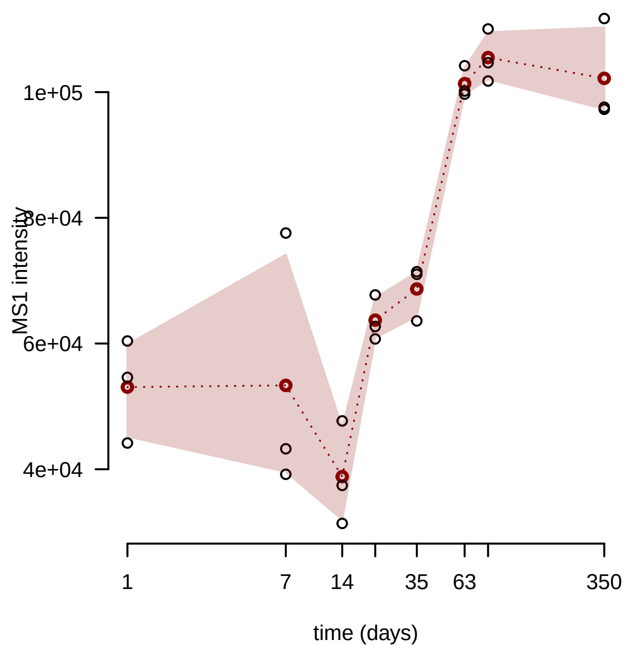

P59024
GN=Fkbp14
FK506 binding protein 14

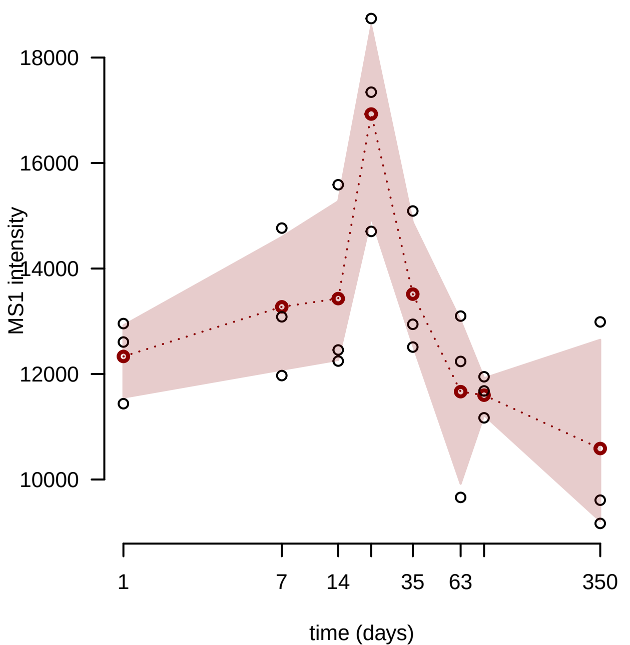

P59108
GN=Cpne2
copine II

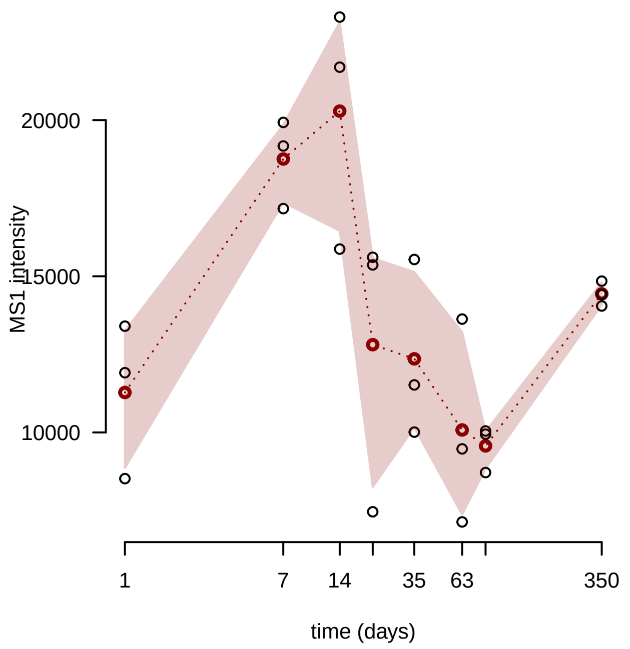

P59110
GN=Senp1
SUMO1/sentrin specific peptidase 1

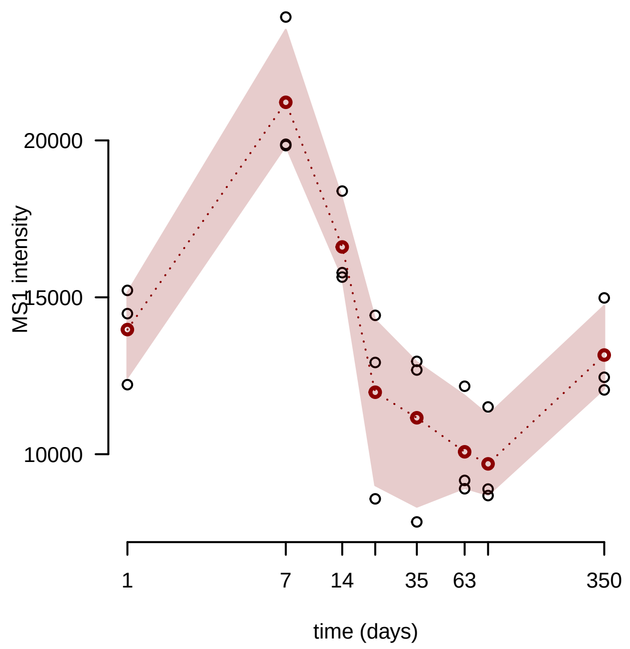

P59114
GN=Pcif1
phosphorylated CTD interacting factor 1

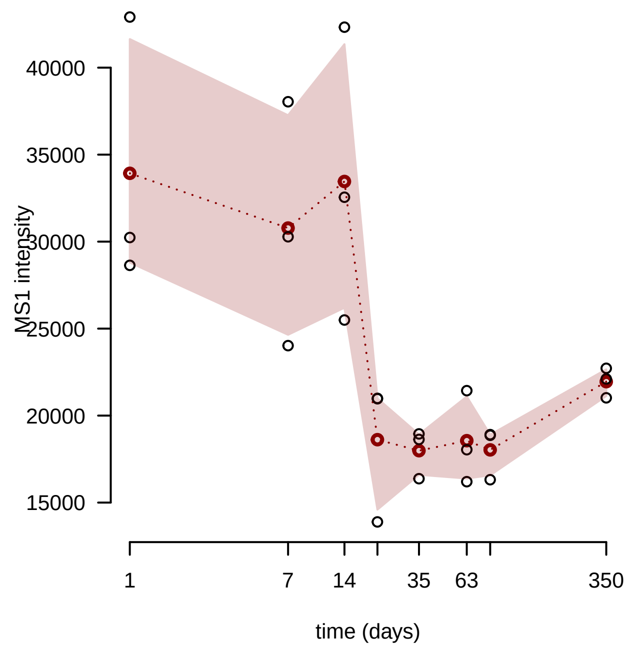

P59222
GN=Scarf2
scavenger receptor class F, member 2

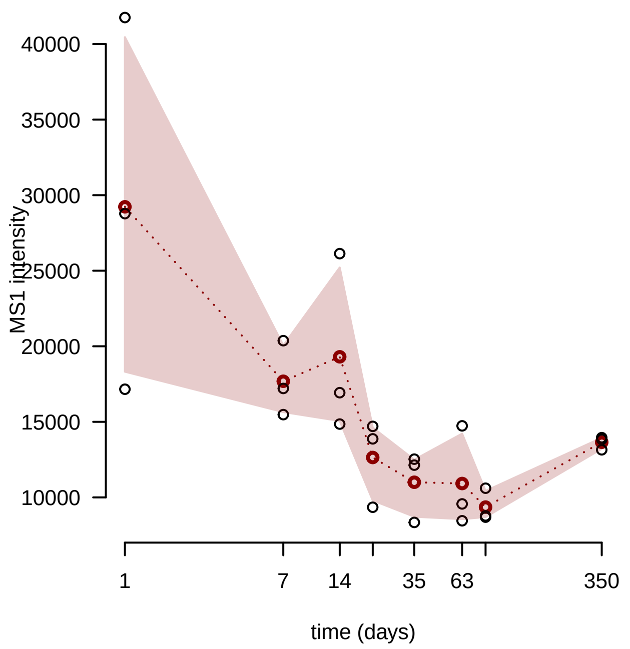

P59235
GN=Nup43
nucleoporin 43

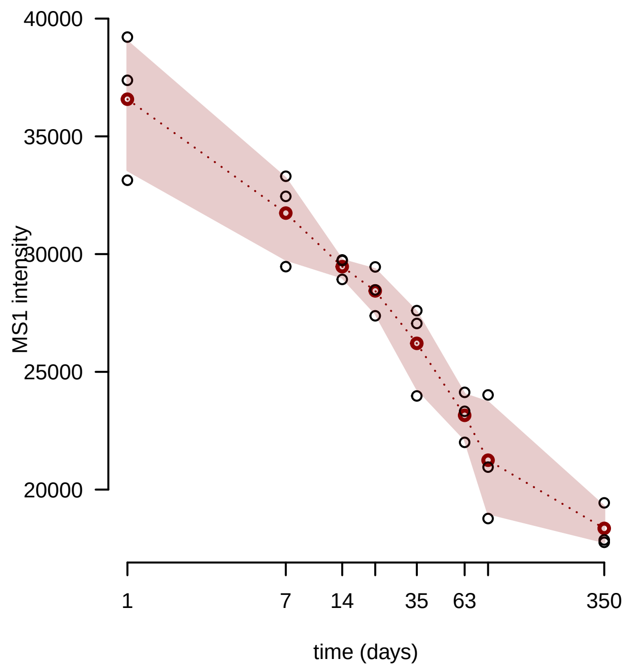

P59242
GN=Cgn
cingulin

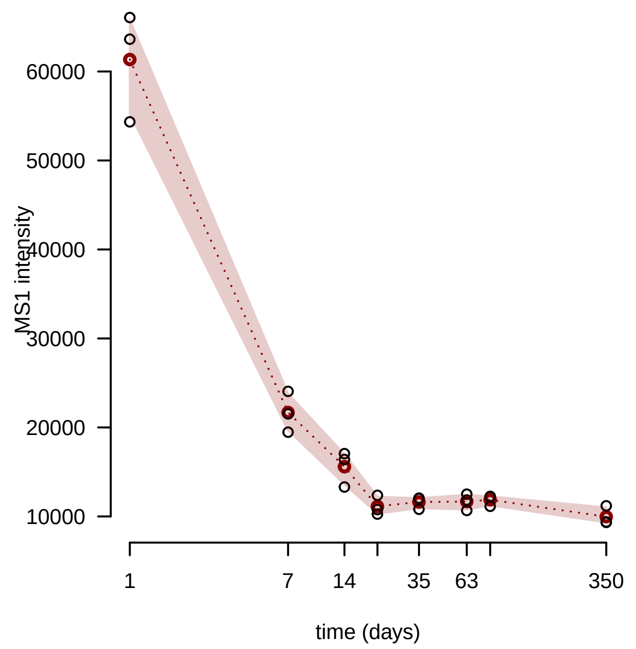

P59266
GN=Fitm2
fat storage−inducing transmembrane prote ...

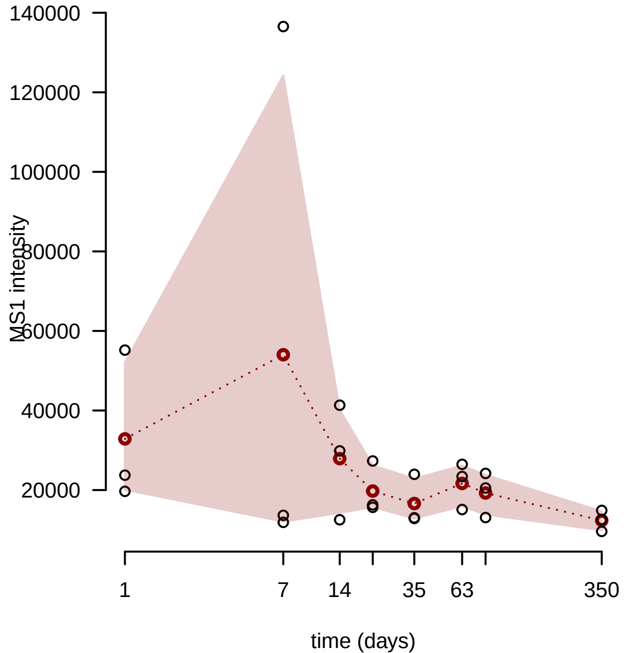

P59279
GN=Rab2b
RAB2B, member RAS oncogene family

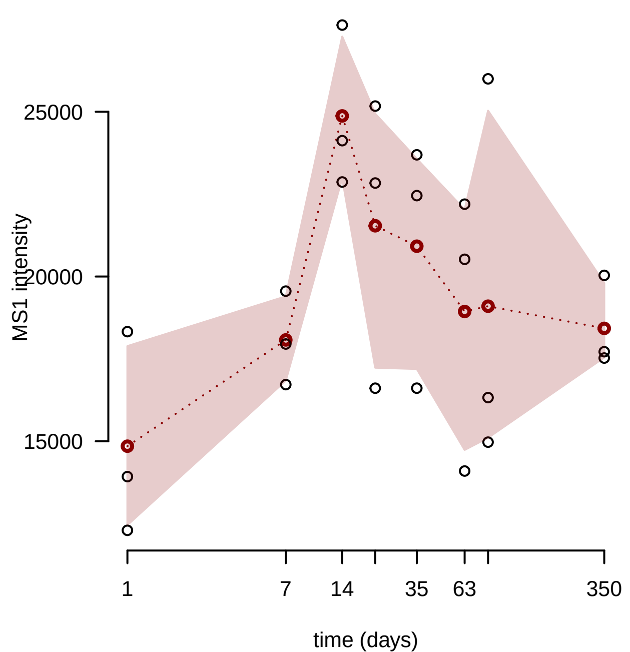

P59281
GN=Arhgap39
Rho GTPase activating protein 39

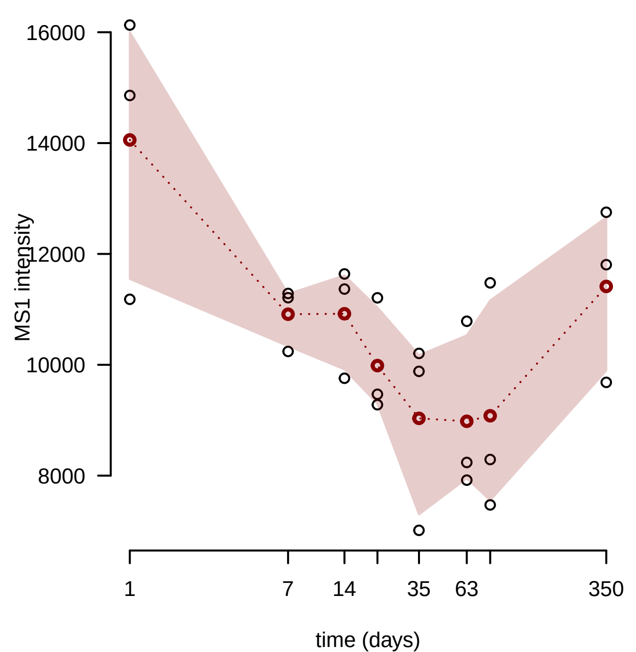

P59325
GN=Eif5
eukaryotic translation initiation factor ...

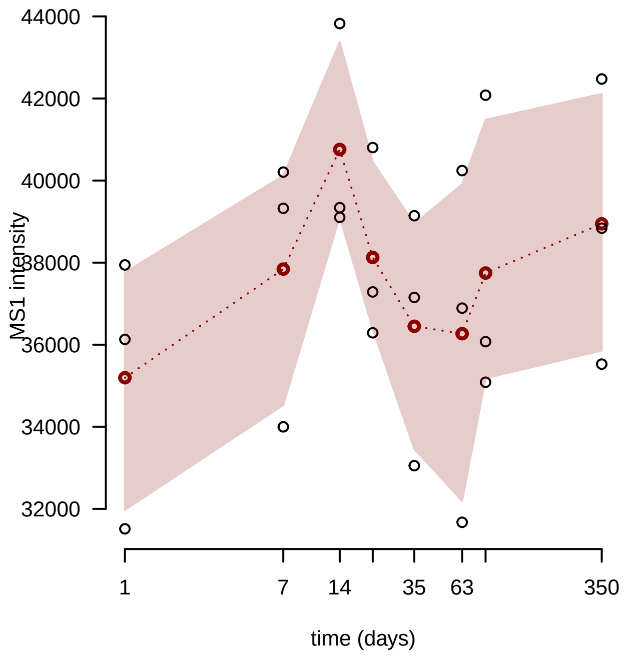

P59326
GN=Ythdf1
YTH N6−methyladenosine RNA binding prote ...

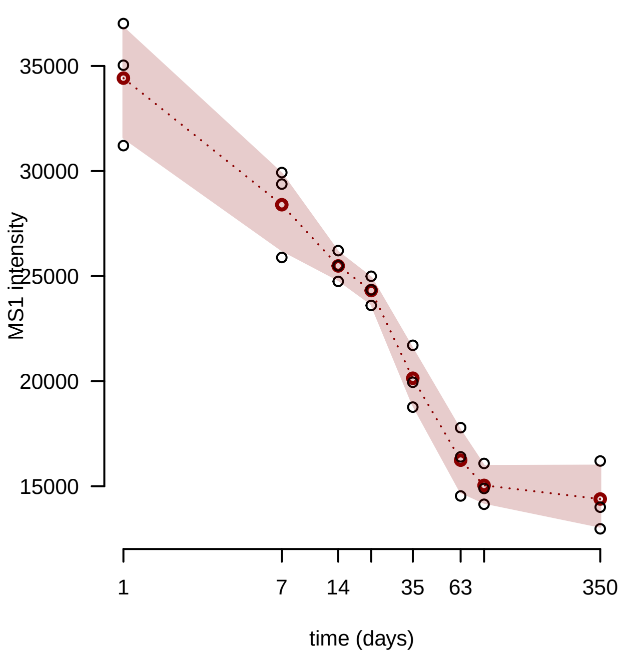

P59328
GN=Wdhd1
WD repeat and HMG−box DNA binding protei ...

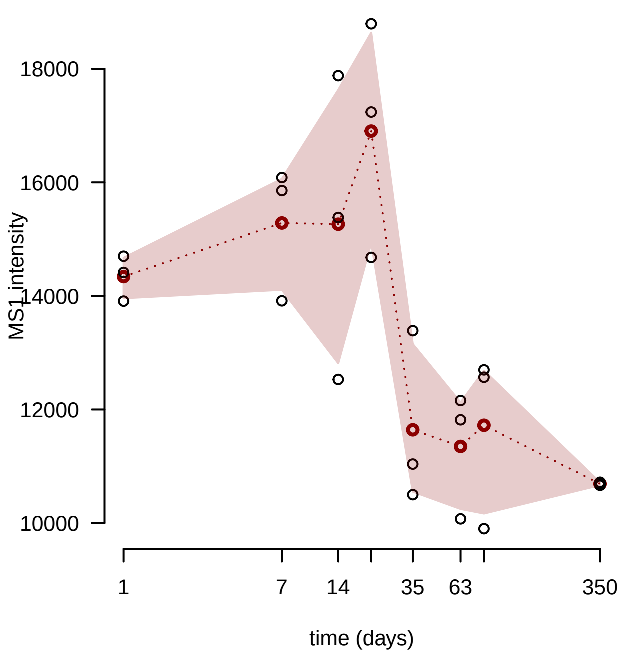

P59383
GN=Lrrn4
leucine rich repeat neuronal 4

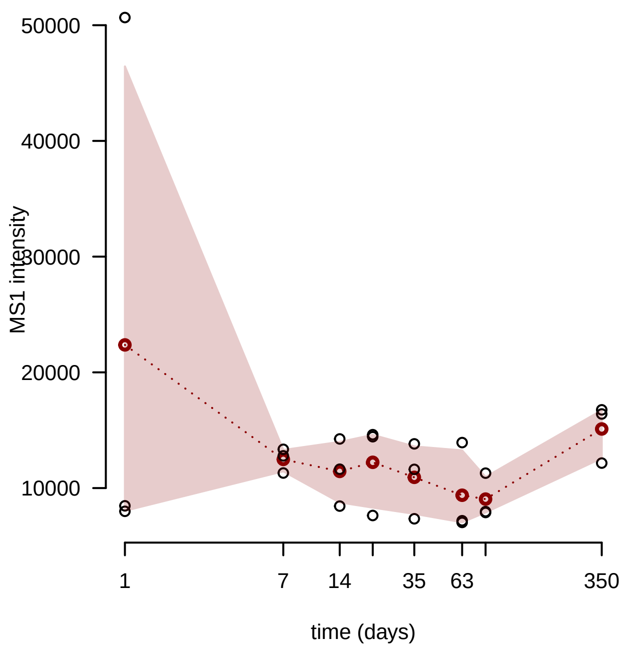

P59438
GN=Hps5
HPS5, biogenesis of lysosomal organelles ...

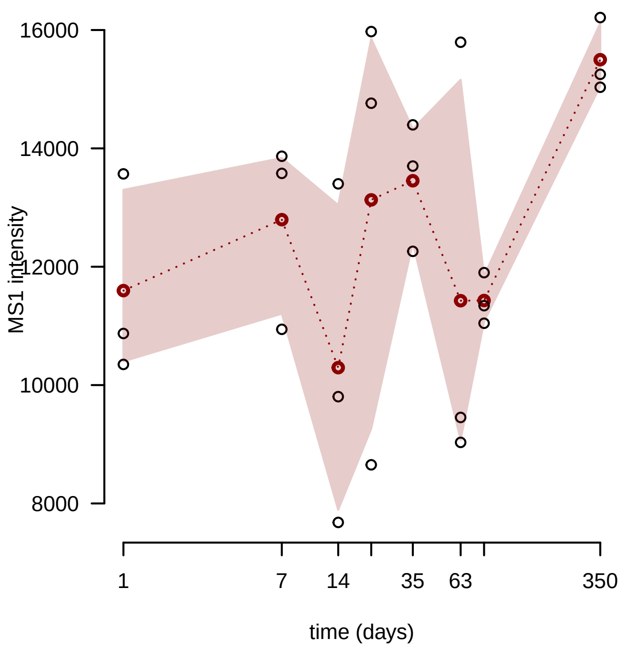

P59470
GN=Polr3b
polymerase (RNA) III (DNA directed) poly ...

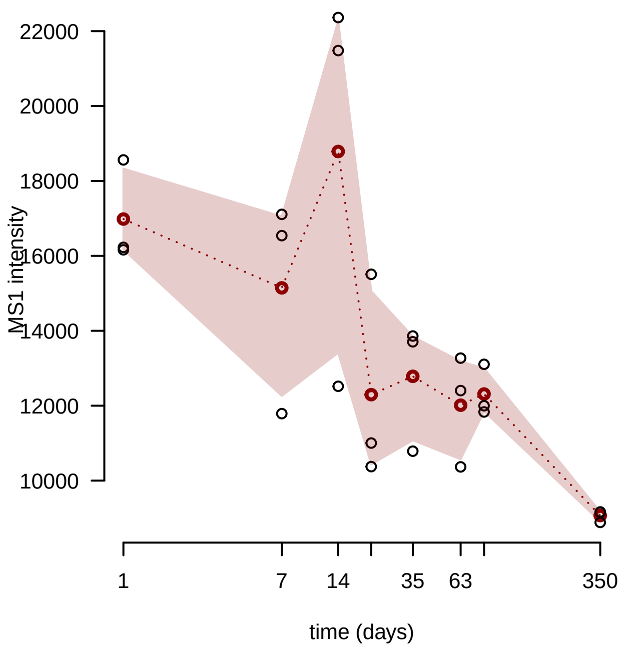

P59481
GN=Lman2l
lectin, mannose−binding 2−like

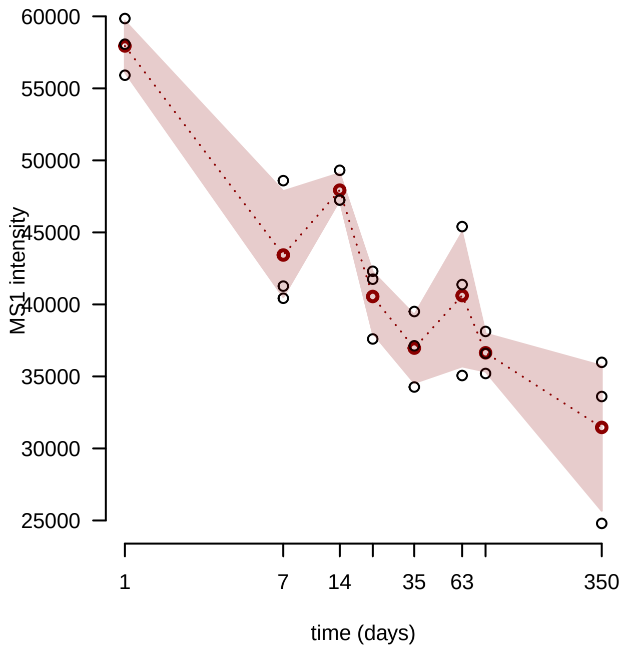

P59644
GN=Inpp5j
inositol polyphosphate 5−phosphatase J

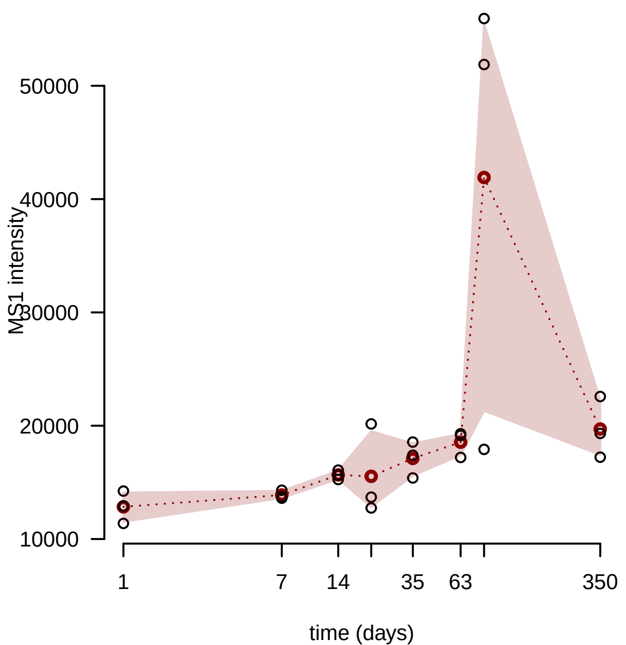

P59672
GN=Anks1a

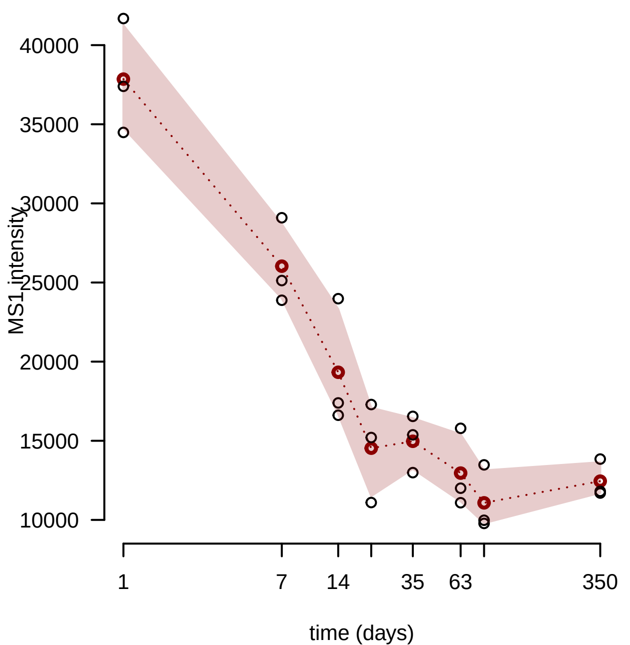

P59708
GN=Sf3b6
splicing factor 3B, subunit 6

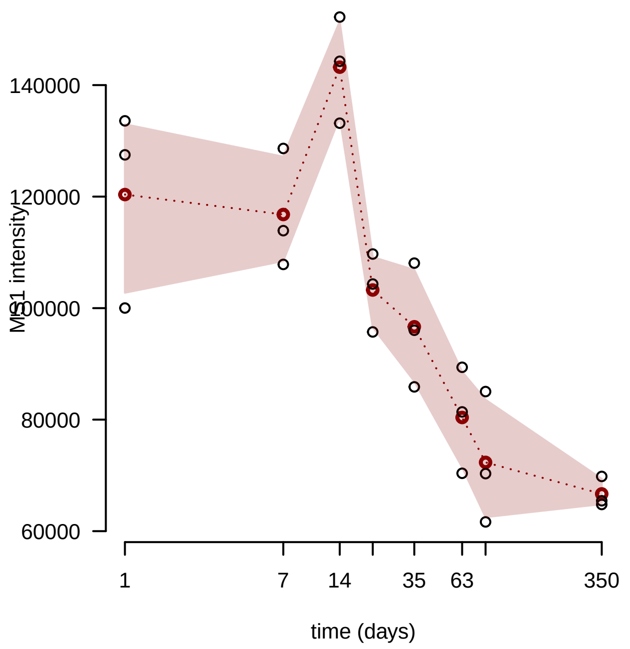

P59729
GN=Rin3
Ras and Rab interactor 3

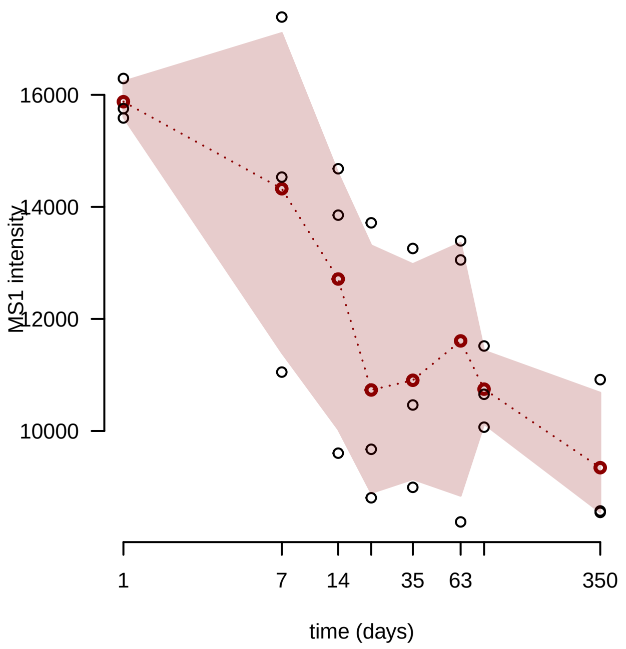

P59759
GN=Mrtfb
myocardin related transcription factor B

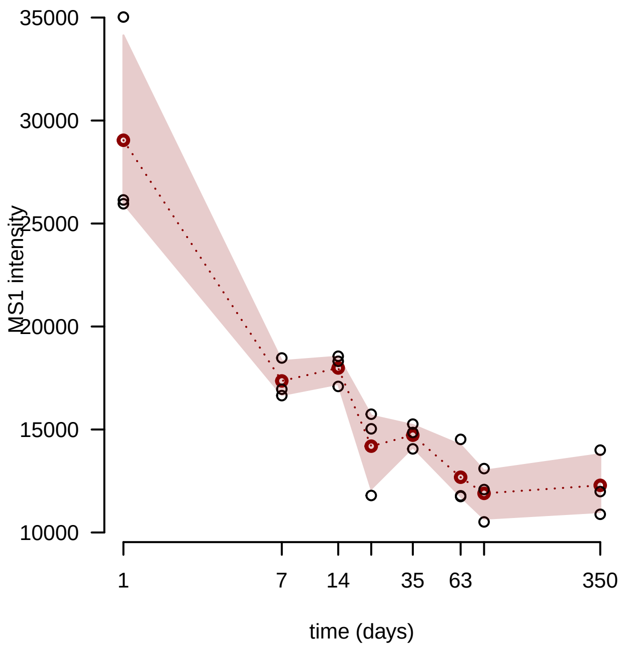

P59764
GN=Dock4
dedicator of cytokinesis 4

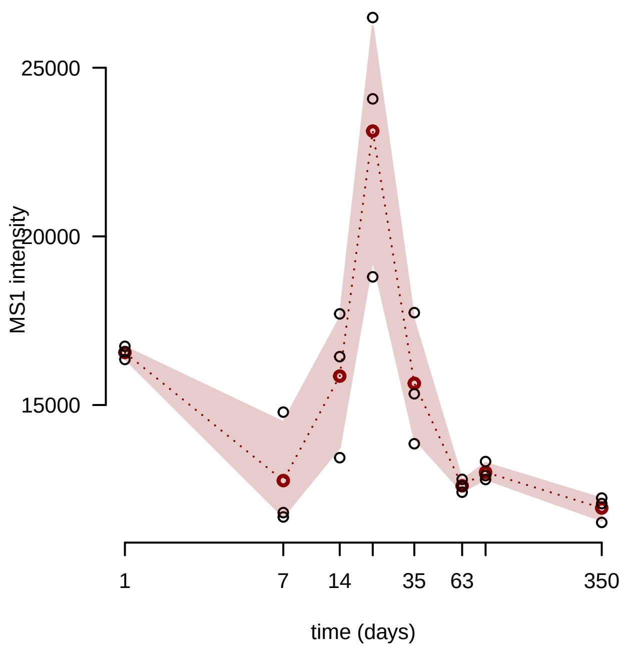

P59808
GN=Sash1
SAM and SH3 domain containing 1

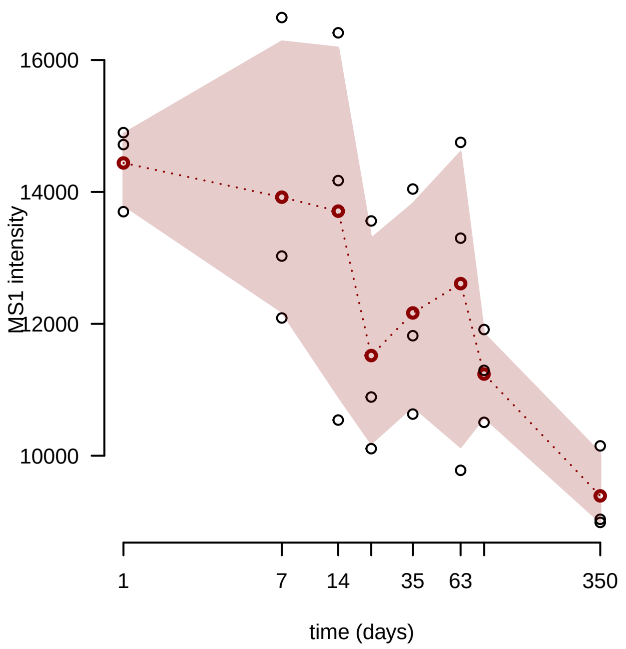

P59900
GN=Emilin3
elastin microfibril interfacer 3

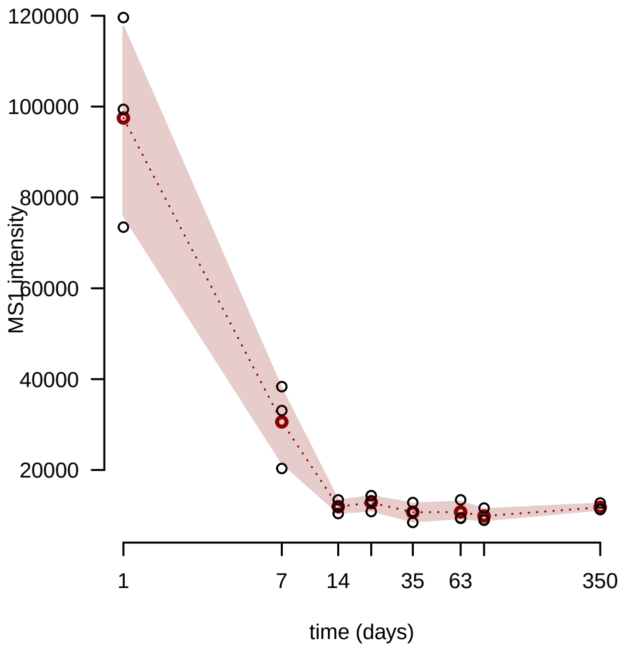

P59913
GN=Pcmtd1
protein−L−isoaspartate (D−aspartate) O−m ...

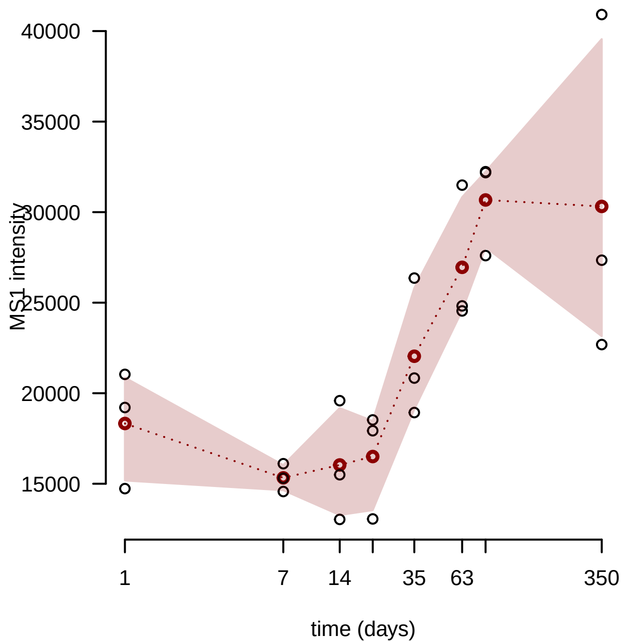

P59941
GN=Sirt6
sirtuin 6

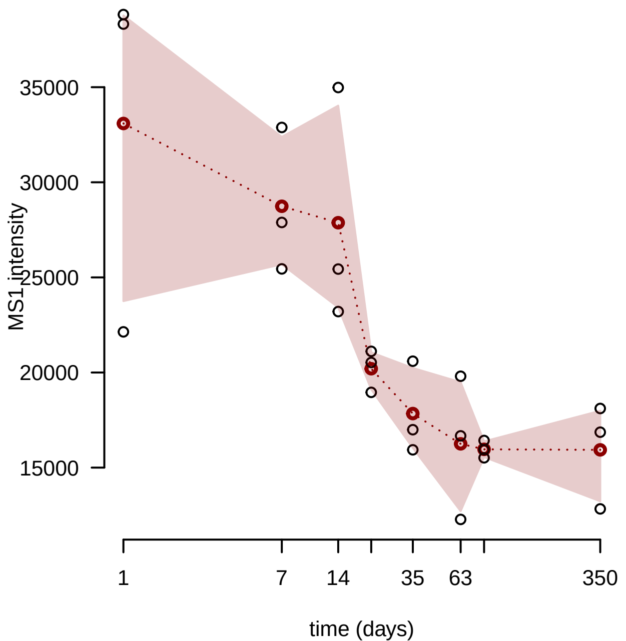

P59997
GN=Kdm2a
lysine (K)−specific demethylase 2A

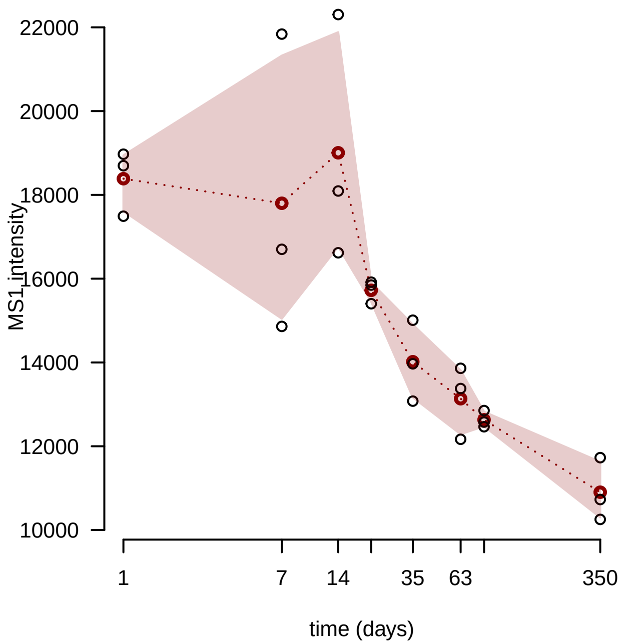

P59999
GN=Arpc4
actin related protein 2/3 complex, subun ...

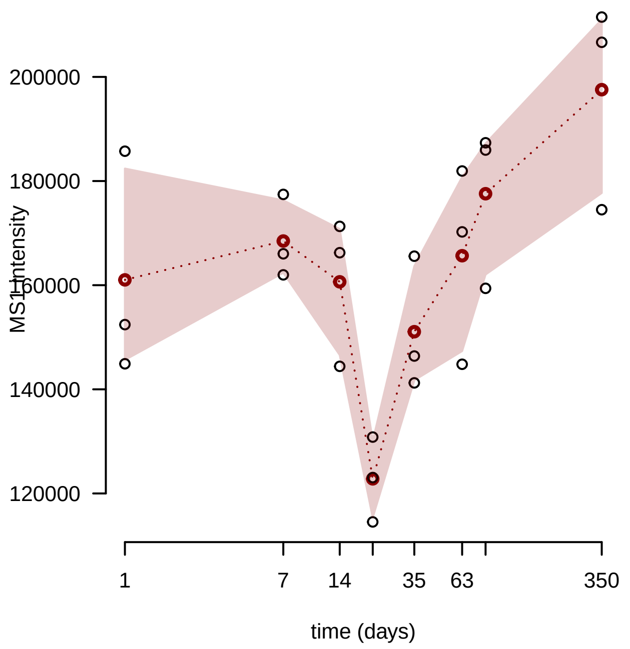

P60060
GN=Sec61g
SEC61, gamma subunit

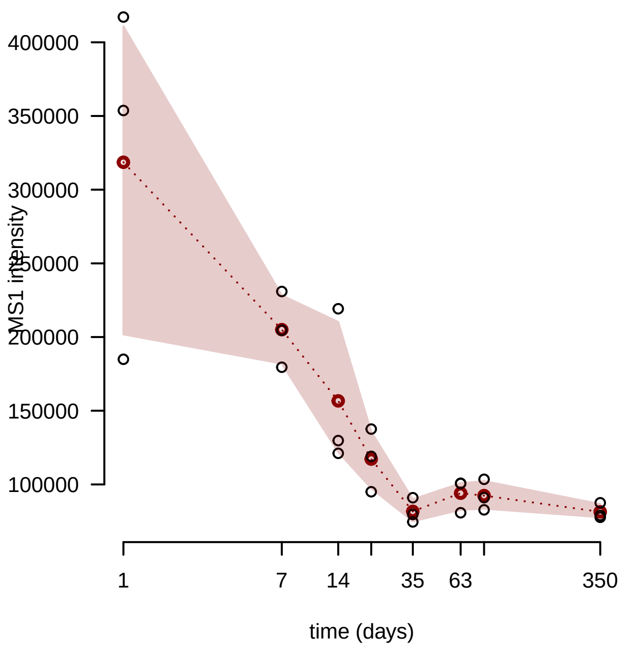

P60122
GN=Ruvbl1
RuvB−like protein 1

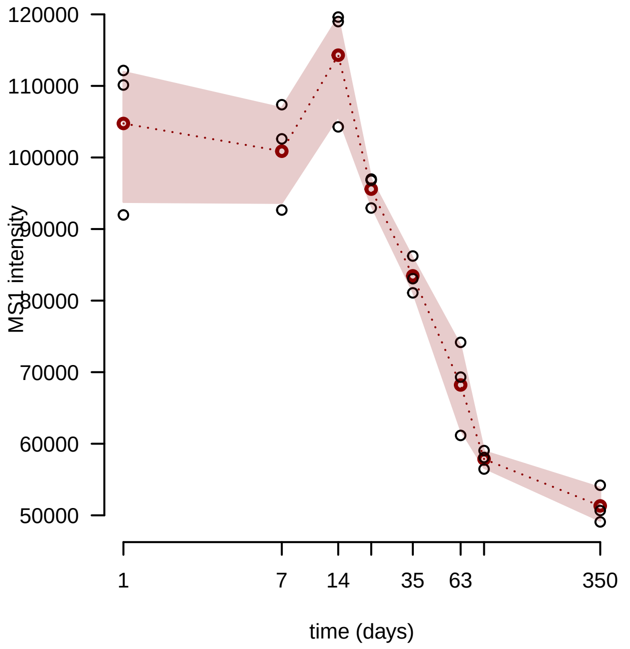

P60202
GN=Plp1
proteolipid protein (myelin) 1

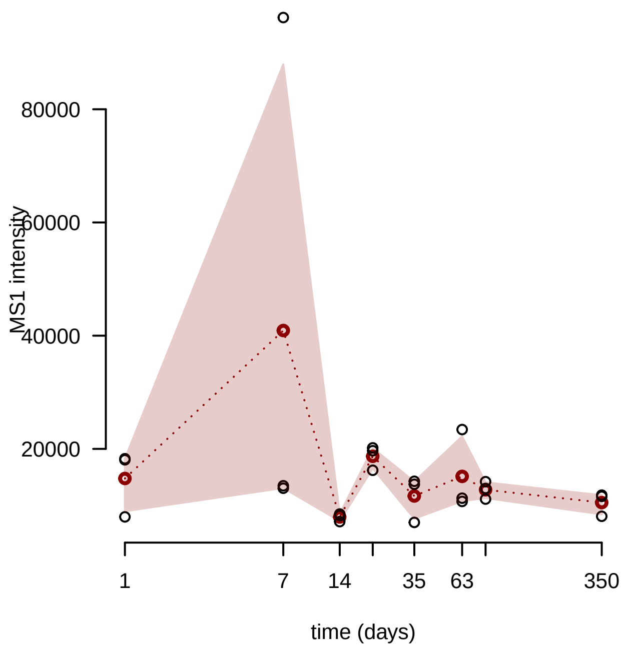

P60229
GN=Eif3e
eukaryotic translation initiation factor ...

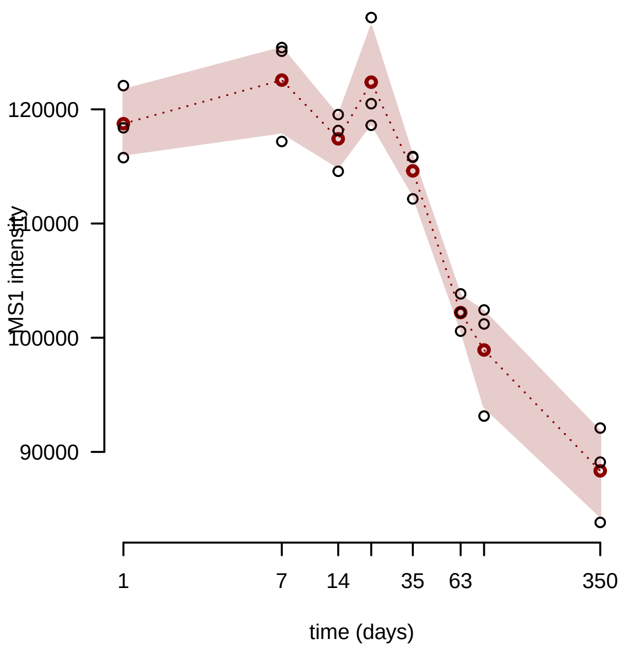

P60330
GN=Espl1
extra spindle pole bodies 1, separase

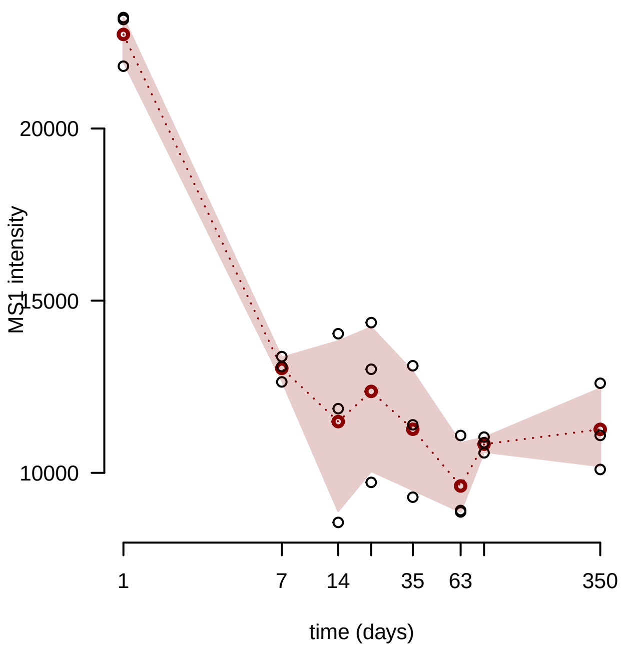

P60335
GN=Pcbp1
poly(rC) binding protein 1

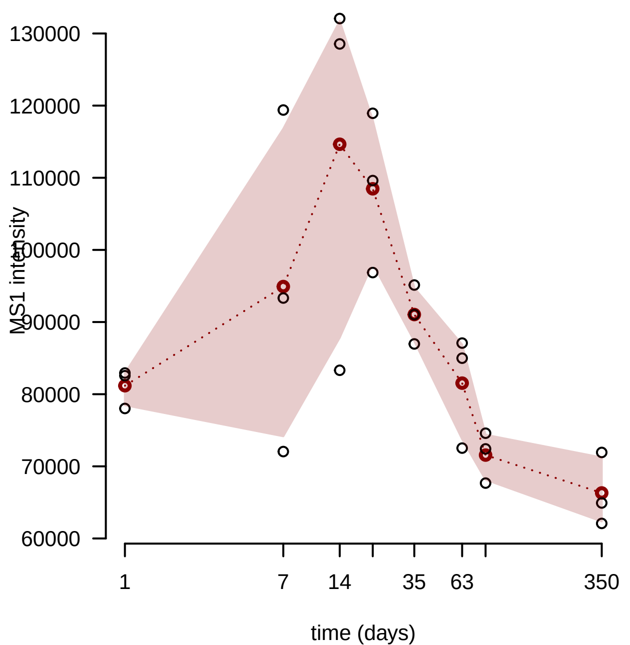

P60469
GN=Ppfia3
protein tyrosine phosphatase, receptor t ...

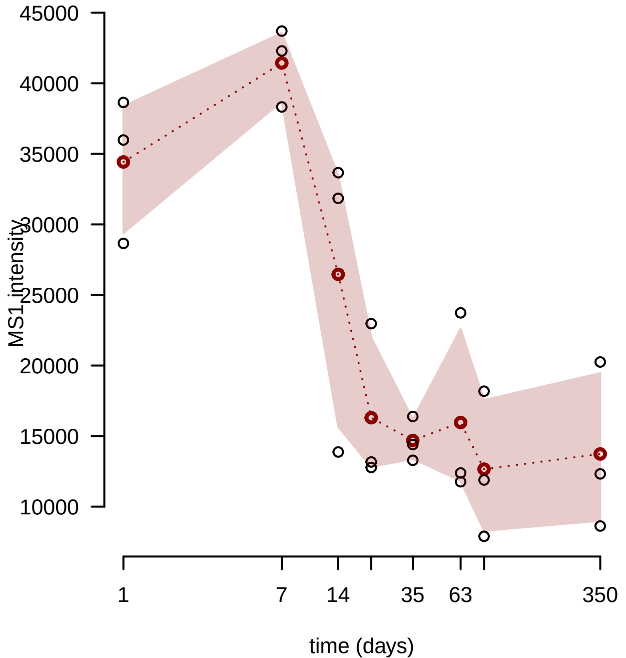

P60487
GN=Pdxp
pyridoxal (pyridoxine, vitamin B6) phosp ...

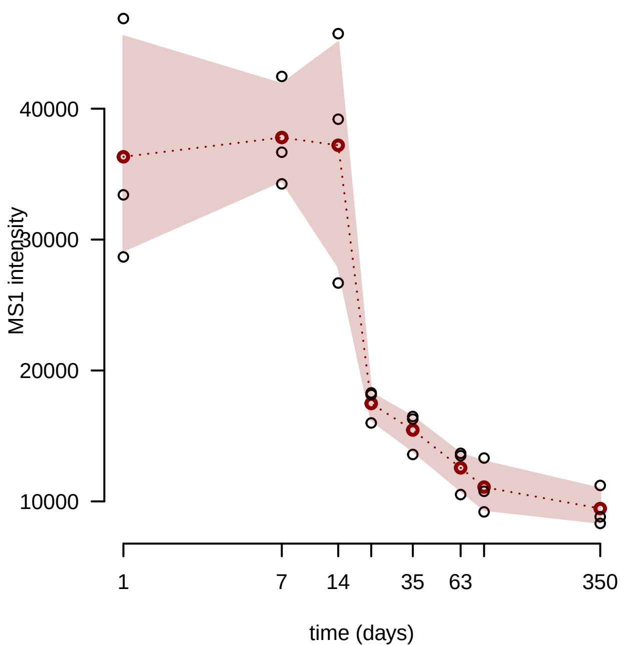

P60521
GN=Gabarapl2
gamma−aminobutyric acid (GABA) A recepto ...

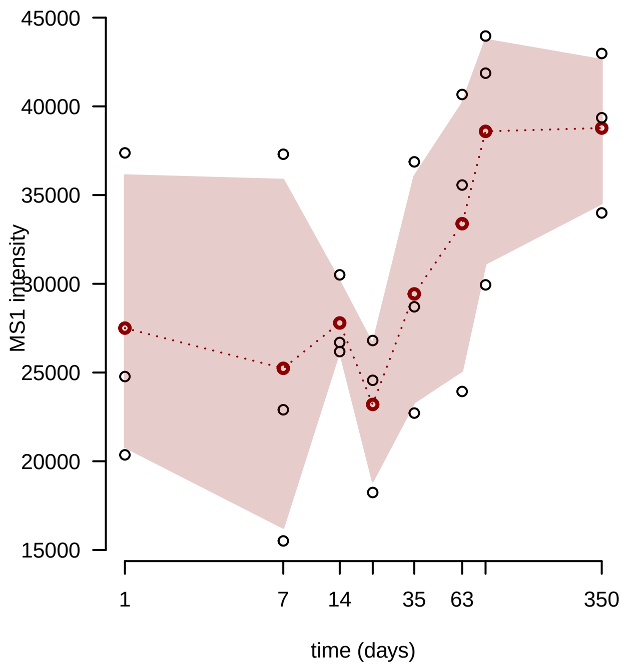

P60603
GN=Romo1
reactive oxygen species modulator 1

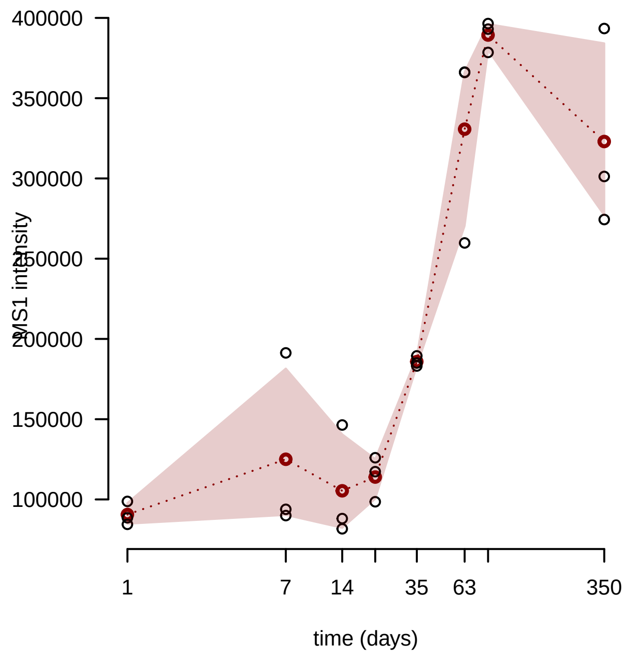

P60670
GN=Nploc4
NPL4 homolog, ubiquitin recognition fact ...

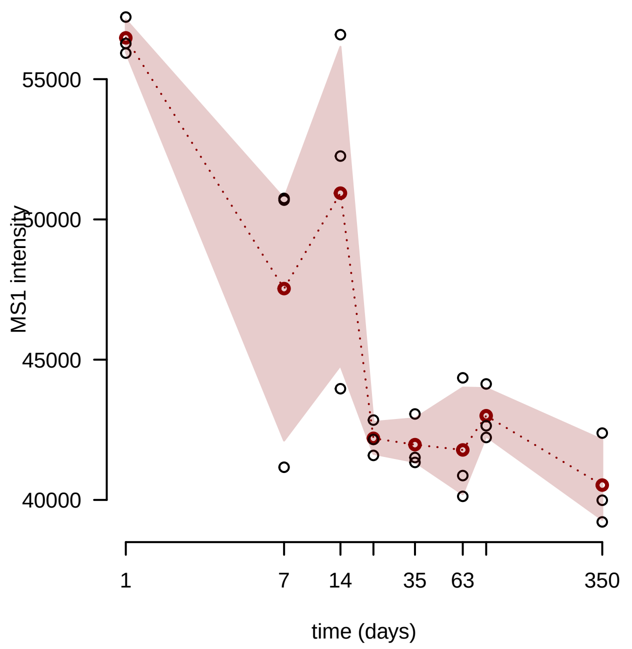

P60710
GN=Actb
actin, beta

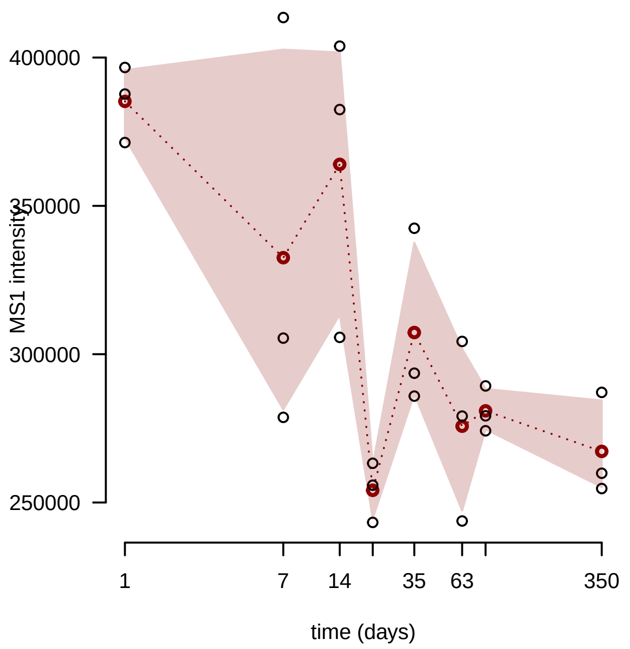

P60762
GN=Morf4l1
mortality factor 4 like 1

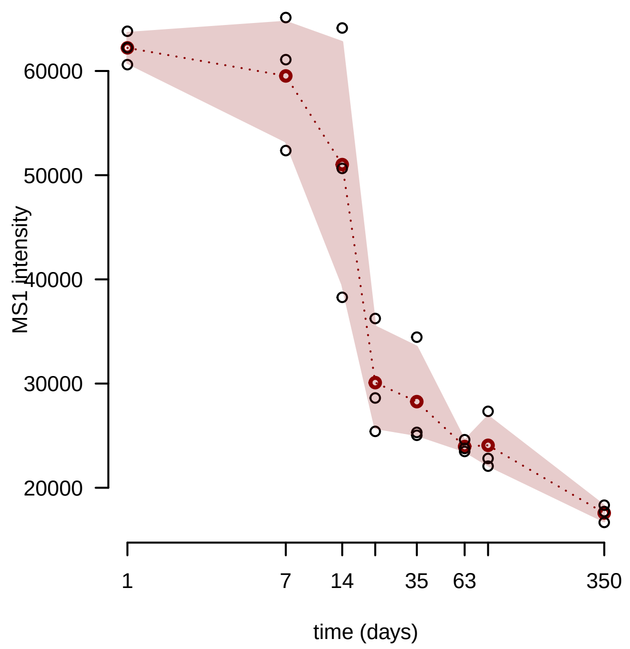

P60764
GN=Rac3
Rac family small GTPase 3

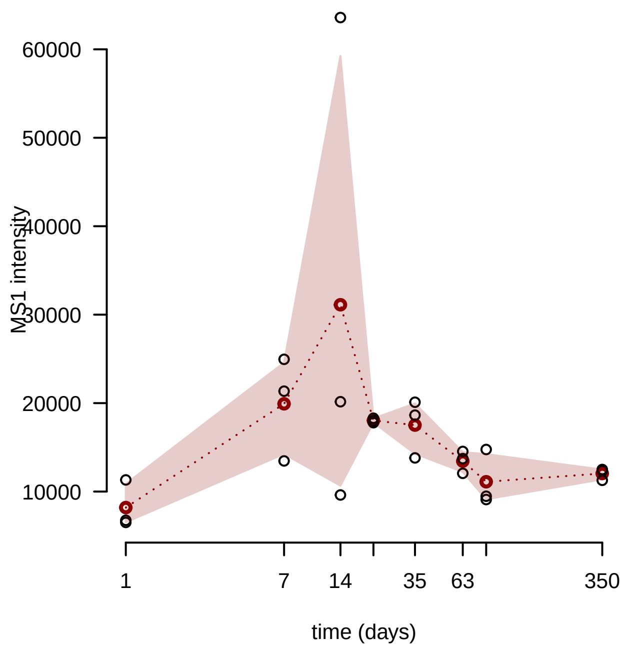

P60766
GN=Cdc42
cell division cycle 42

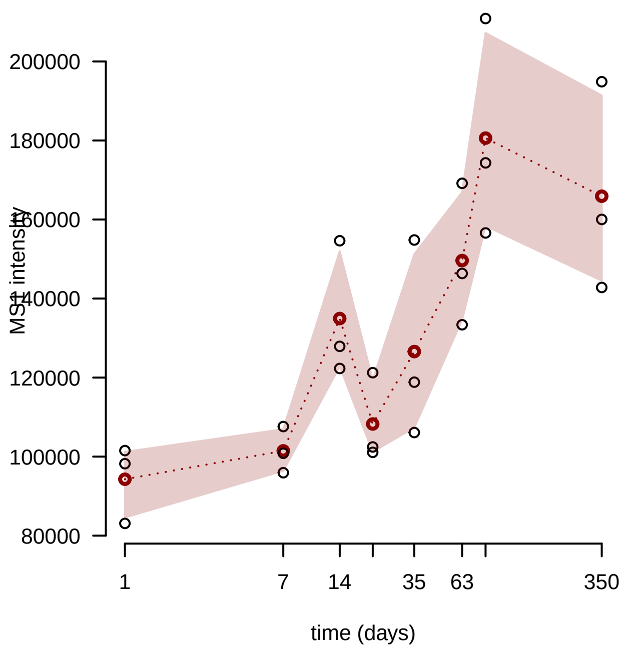

P60766
GN=Cdc42
cell division cycle 42

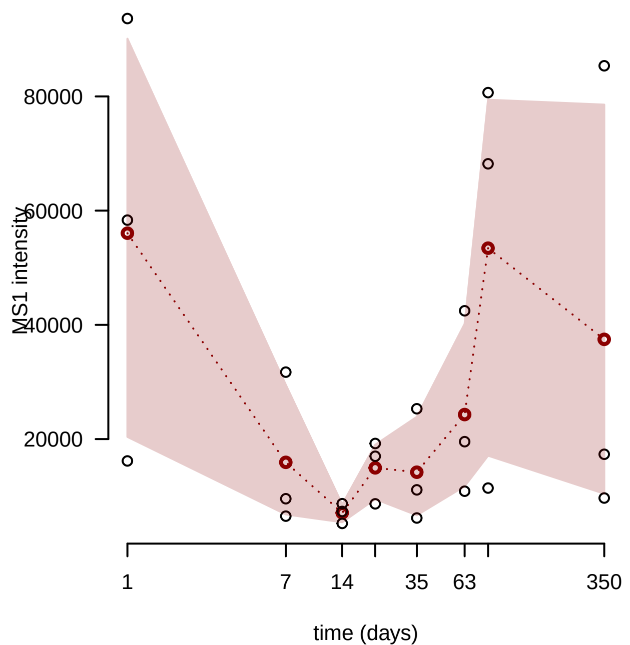

P60824
GN=Cirbp
cold inducible RNA binding protein

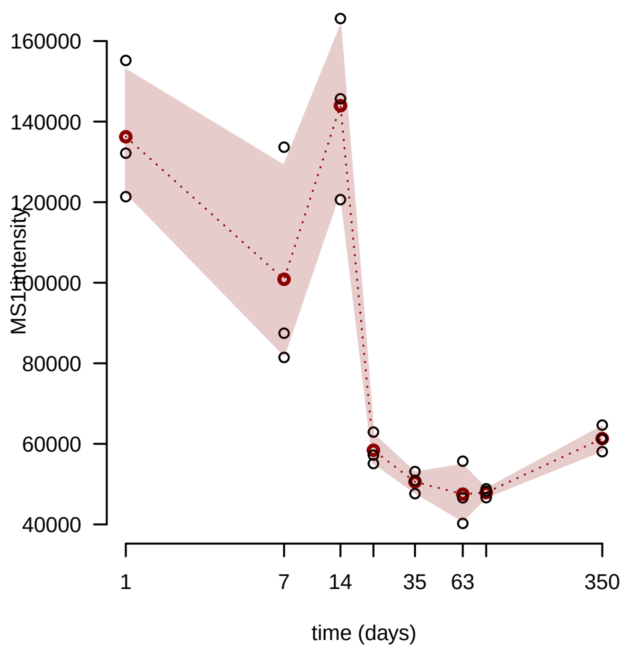

P60840
GN=Ensa
endosulfine alpha

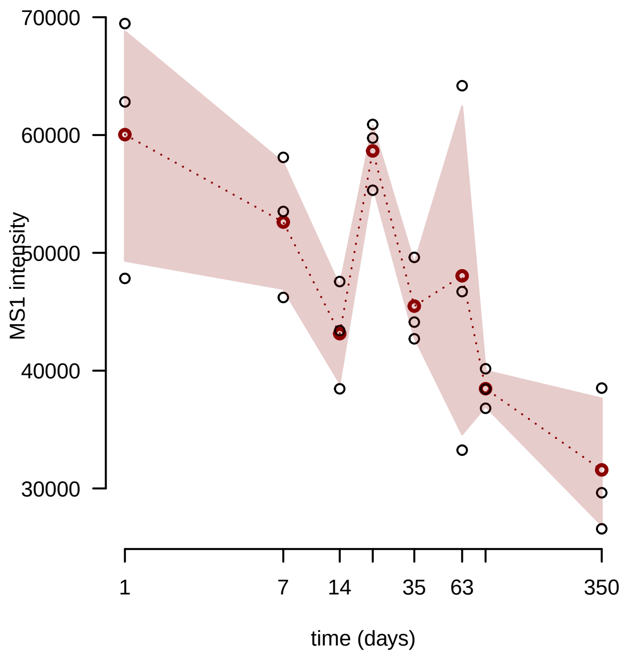

P60843
GN=Eif4a1
eukaryotic translation initiation factor ...

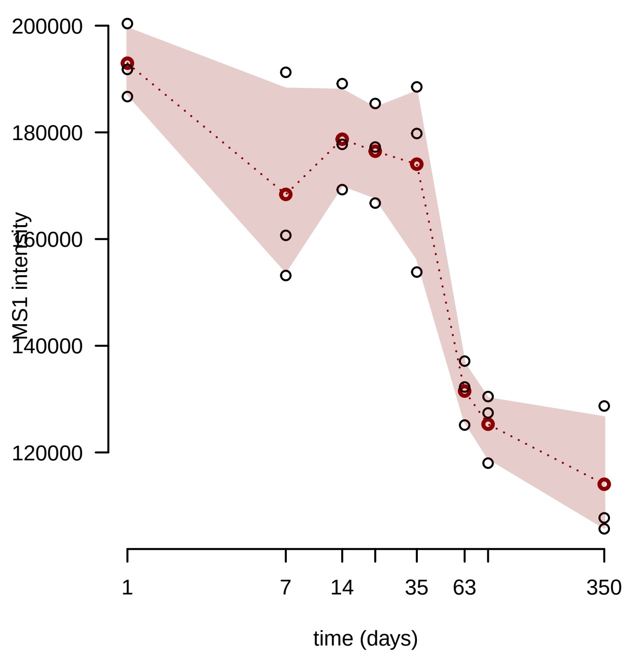

P60853
GN=Lzts1
leucine zipper, putative tumor suppresso ...

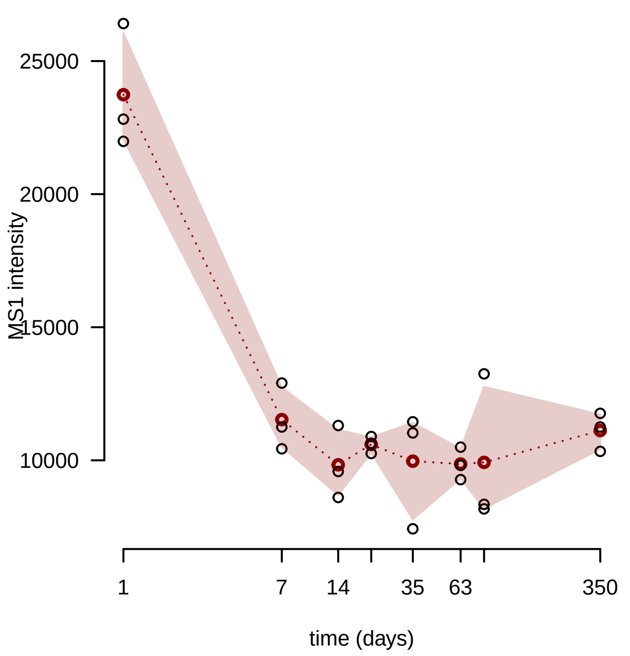

P60867
GN=Rps20
ribosomal protein S20

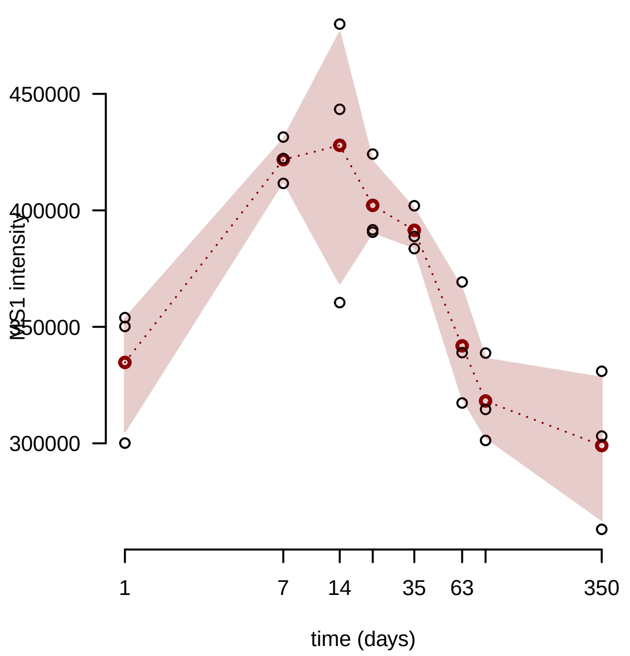

P60898
GN=Polr2i
polymerase (RNA) II (DNA directed) polyp ...

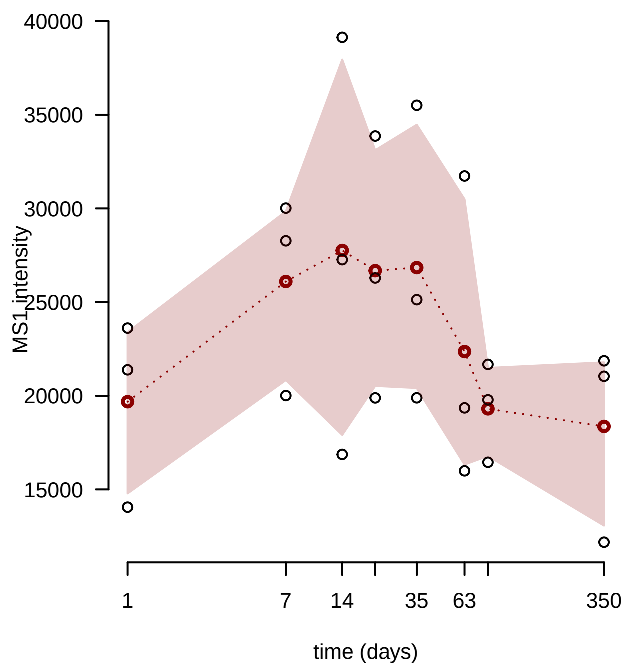

P60904
GN=Dnajc5
DnaJ heat shock protein family (Hsp40) m ...

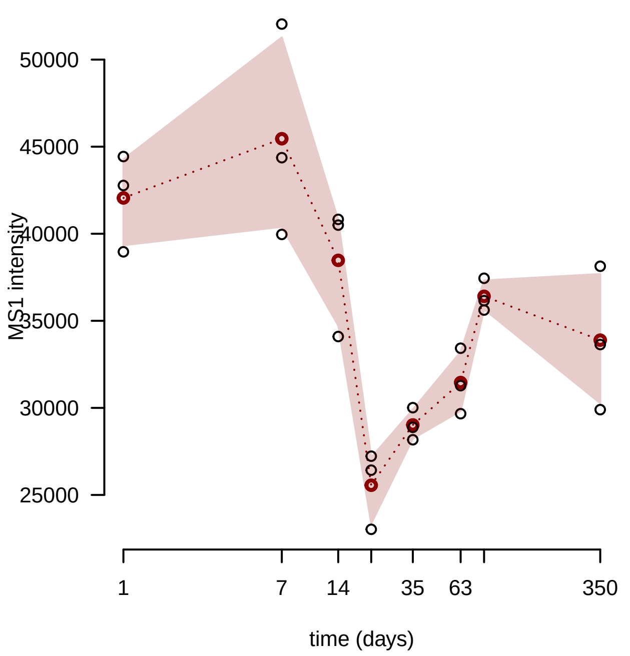

P61014
GN=Pln
phospholamban

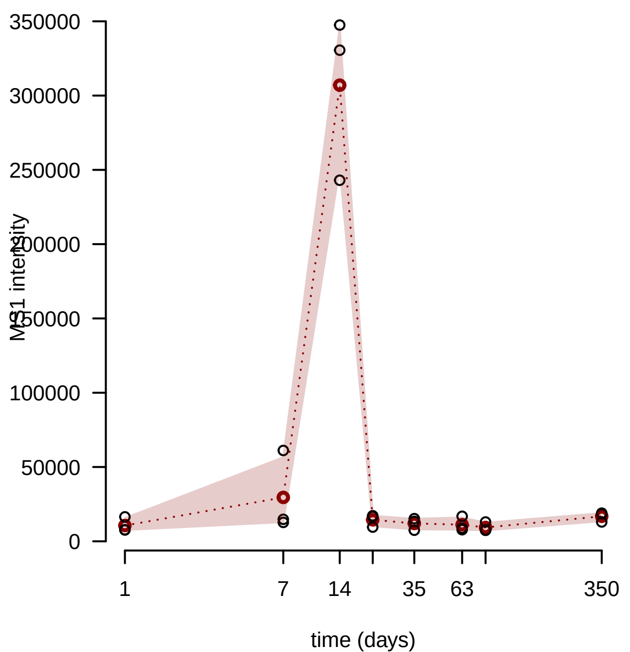

P61021
GN=Rab5b
RAB5B, member RAS oncogene family

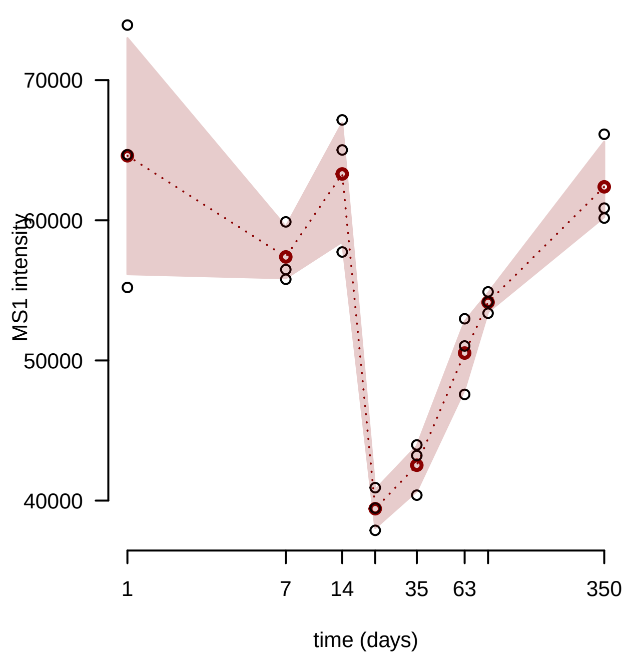

P61022
GN=Chp1
calcineurin−like EF hand protein 1

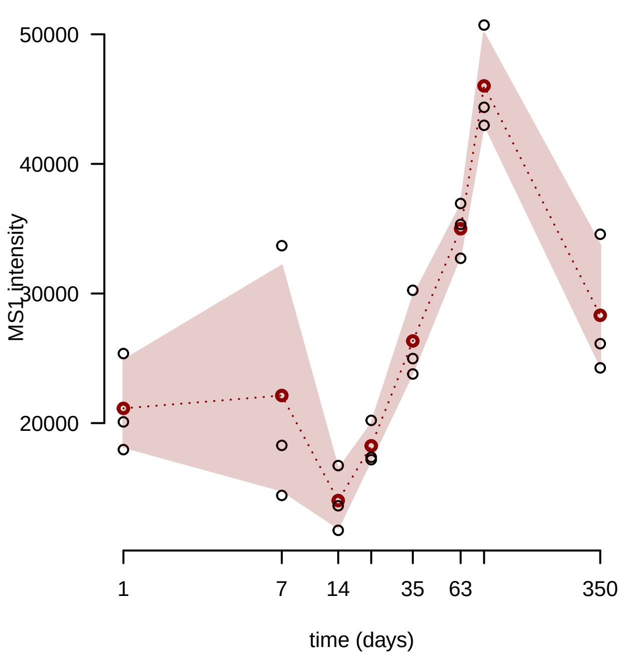

P61025
GN=Cks1b
CDC28 protein kinase 1b

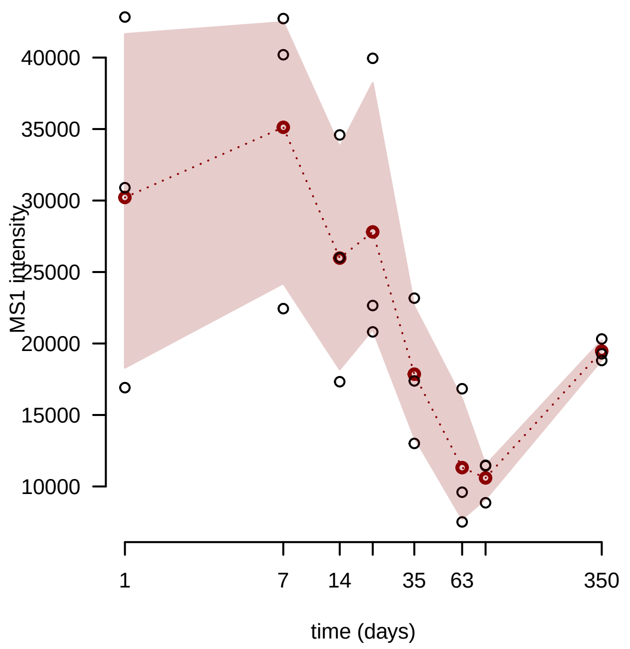

P61027
GN=Rab10
RAB10, member RAS oncogene family

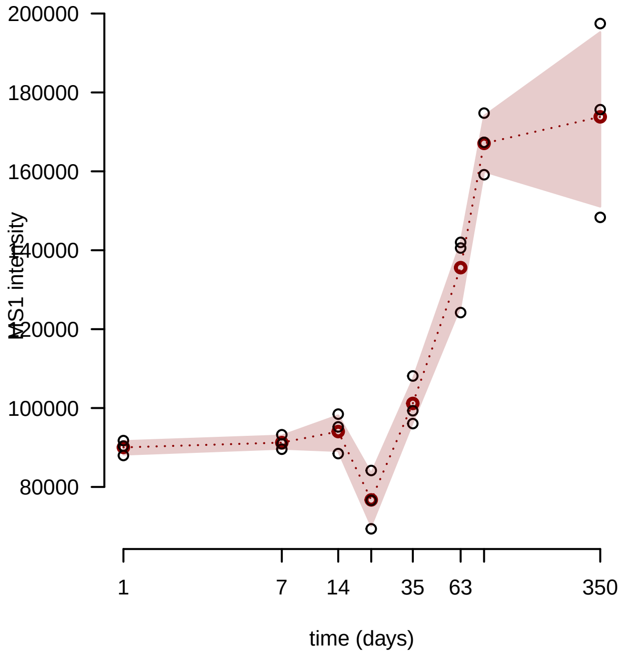

P61028
GN=Rab8b
RAB8B, member RAS oncogene family

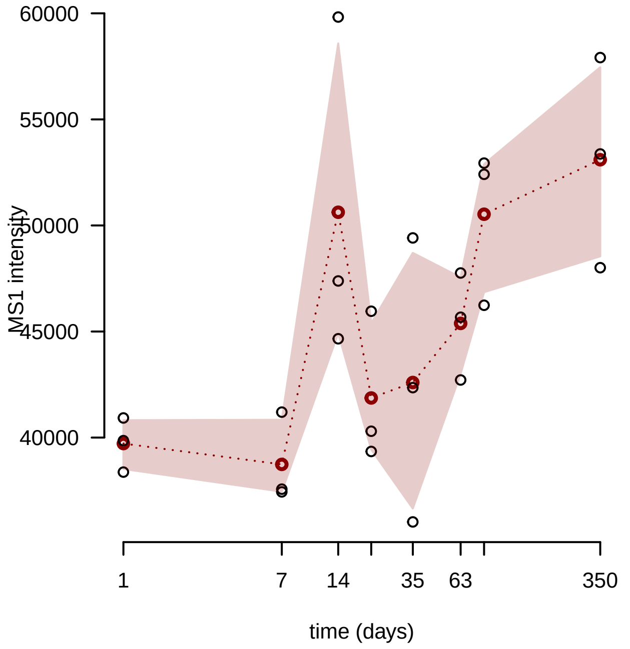

P61079
GN=Ube2d3
ubiquitin−conjugating enzyme E2D 3

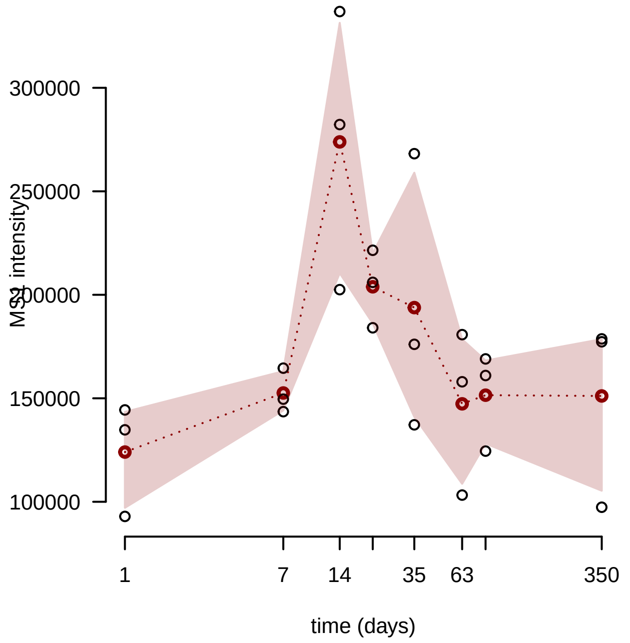

P61080
GN=Ube2d1
ubiquitin−conjugating enzyme E2D 1

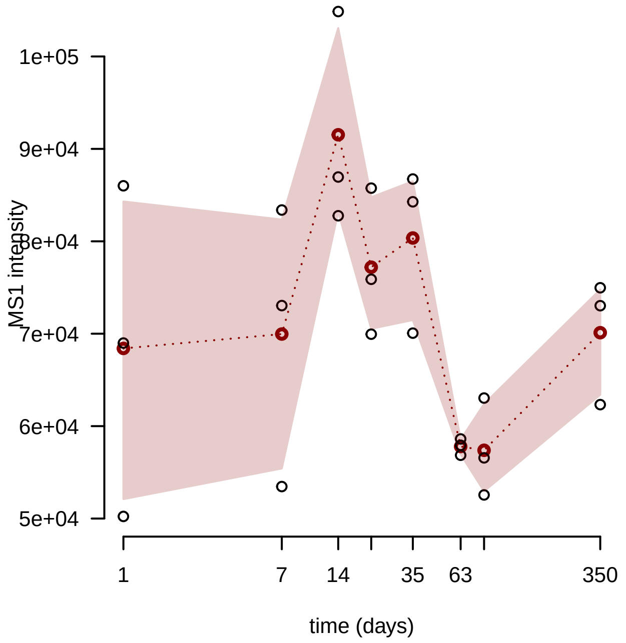

P61082
GN=Ube2m
ubiquitin−conjugating enzyme E2M

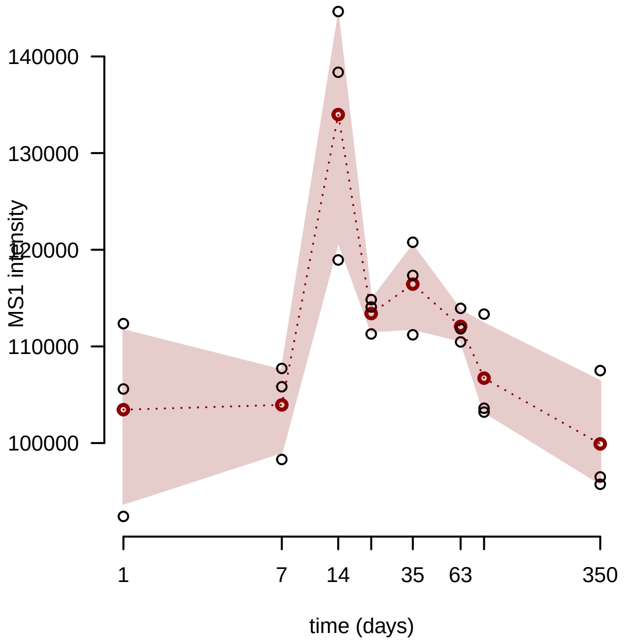

P61087
GN=Ube2k
ubiquitin−conjugating enzyme E2K

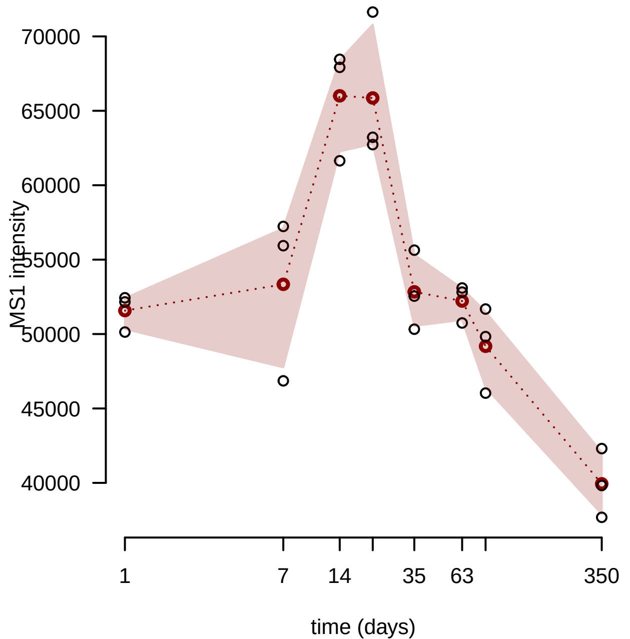

P61089
GN=Ube2n
ubiquitin−conjugating enzyme E2N

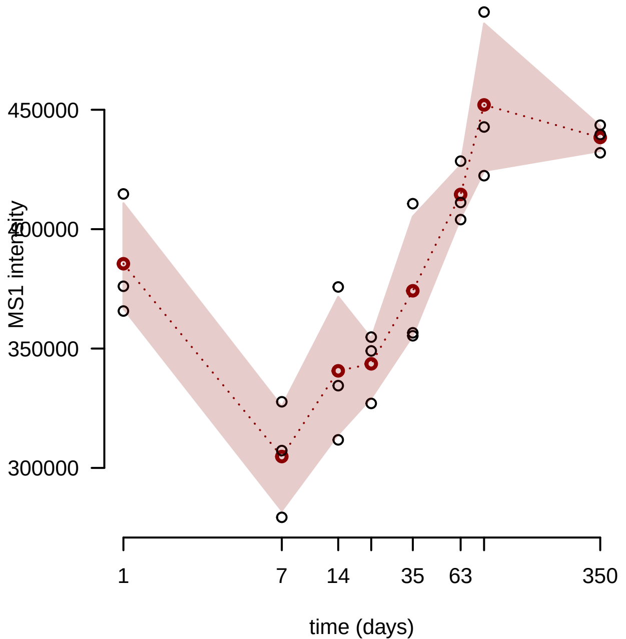

P61161
GN=Actr2
ARP2 actin−related protein 2

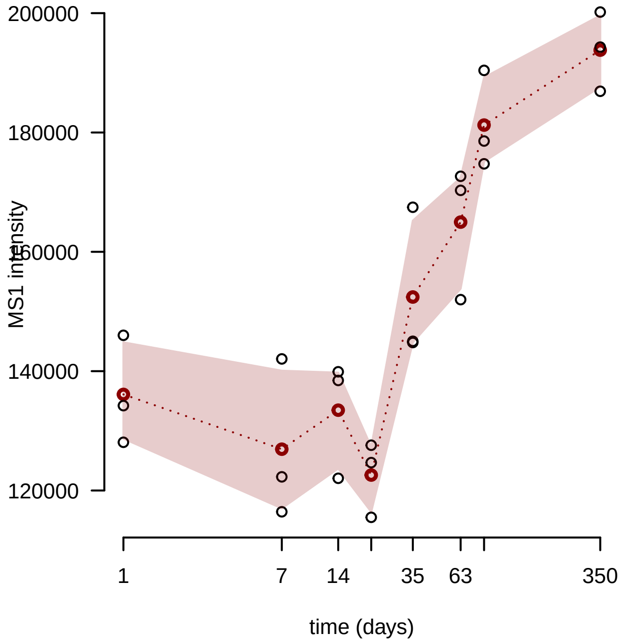

P61164
GN=Actr1a
ARP1 actin−related protein 1A, centracti ...

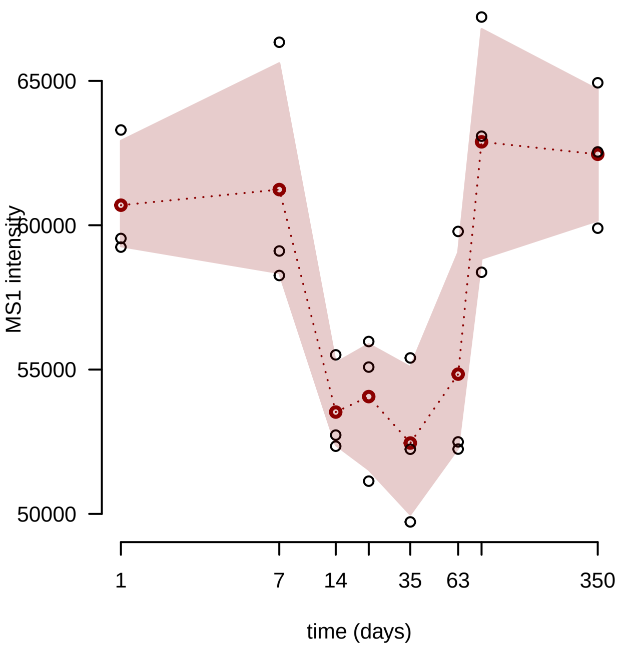

P61166
GN=Tmem258
transmembrane protein 258

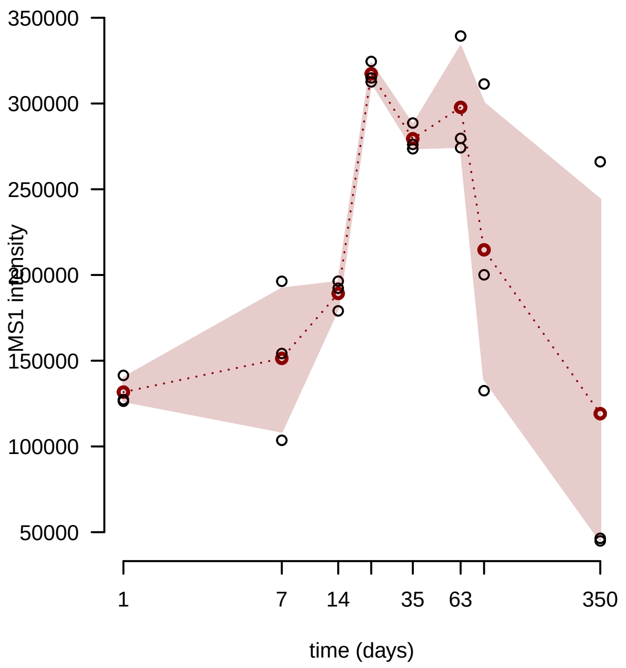

P61202
GN=Cops2
COP9 signalosome subunit 2

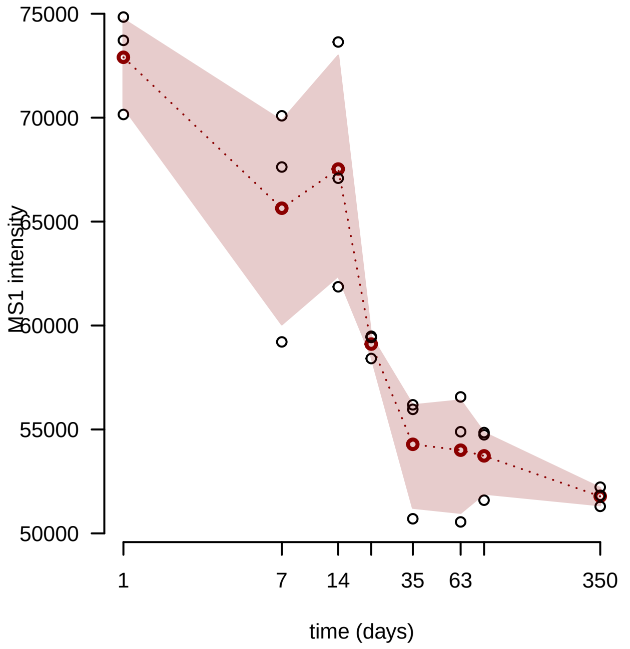

P61211
GN=Arl1
ADP−ribosylation factor−like 1

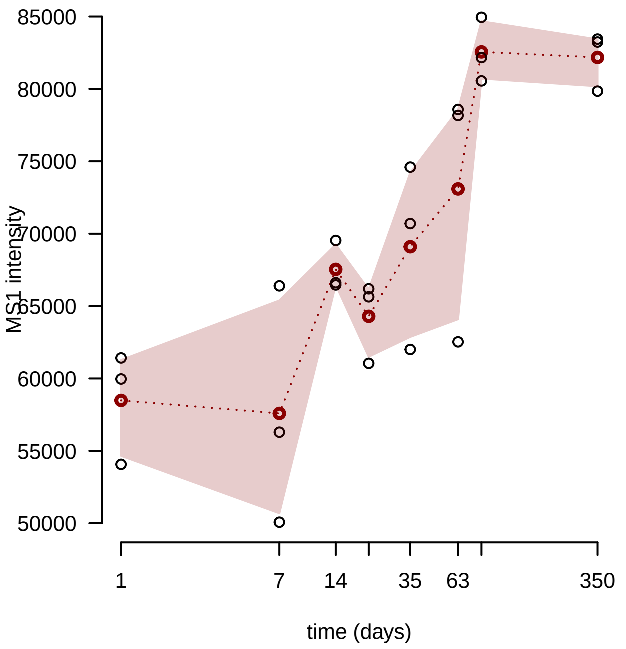

P61216
GN=Taf13
TATA−box binding protein associated fact ...

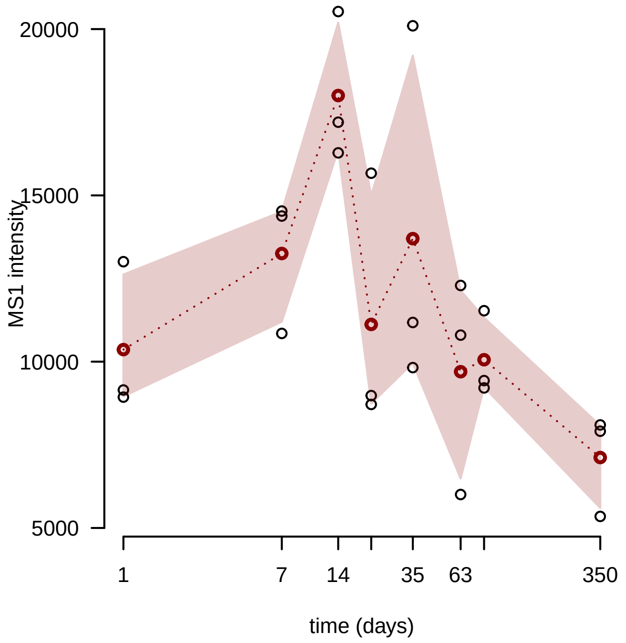

P61219
GN=Polr2f
polymerase (RNA) II (DNA directed) polyp ...

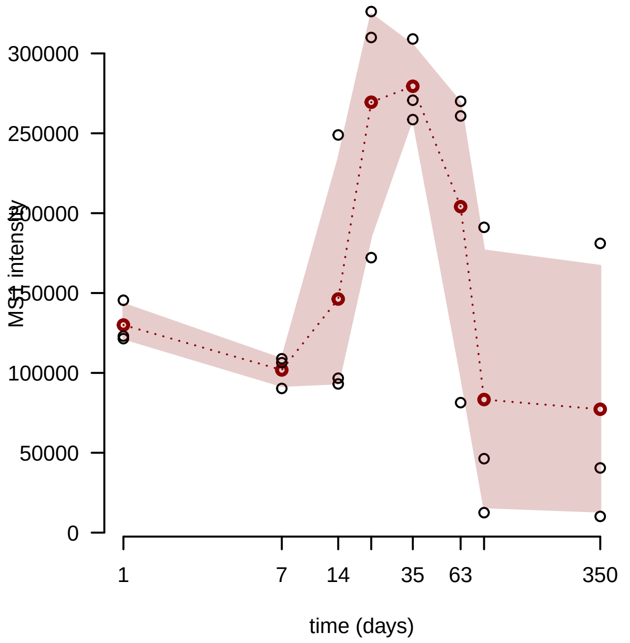

P61222
GN=Abce1
ATP−binding cassette, sub−family E (OABP ...

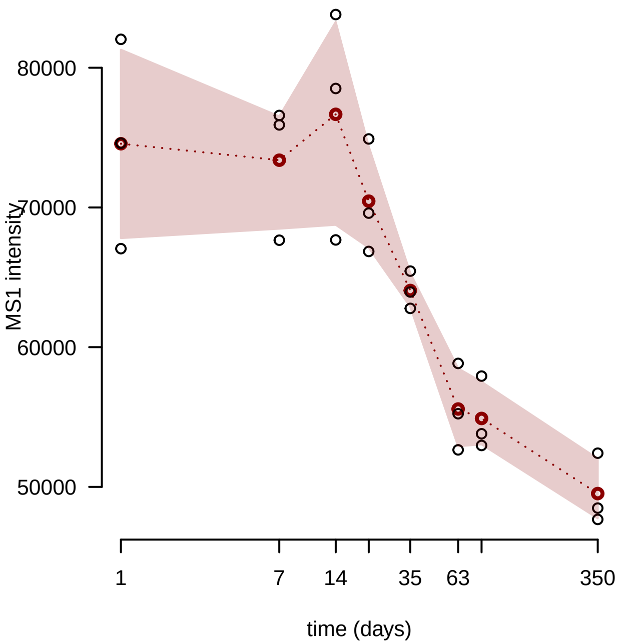

P61226
GN=Rap2b
RAP2B, member of RAS oncogene family

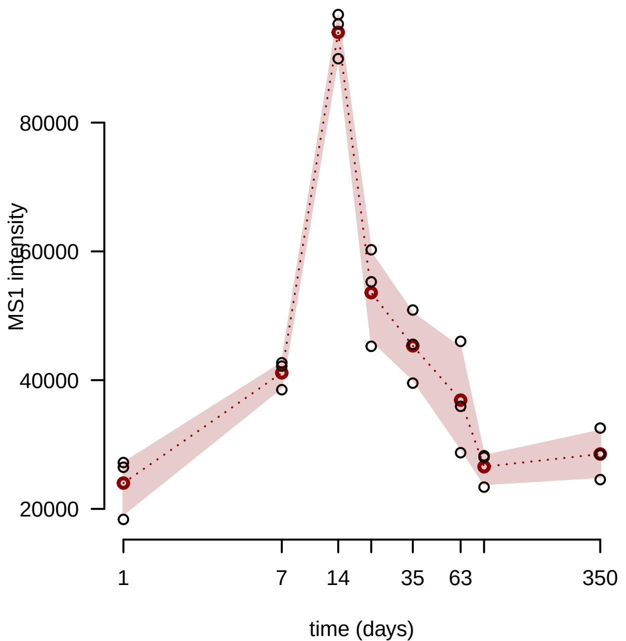

P61255
GN=Rpl26
ribosomal protein L26

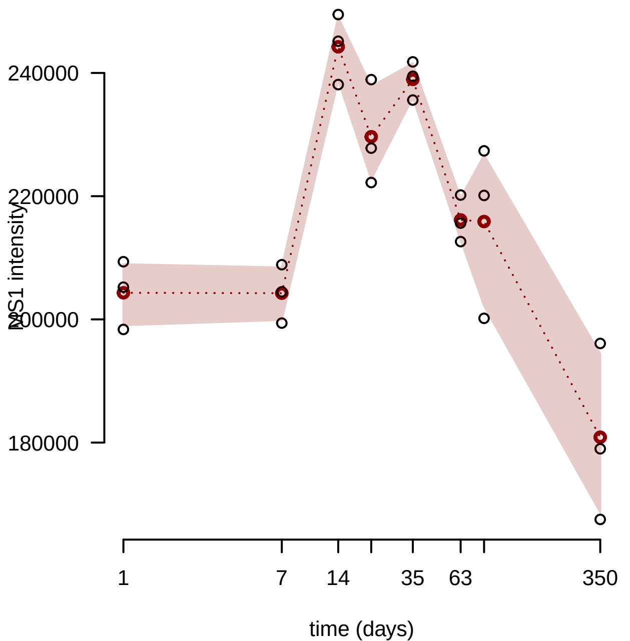

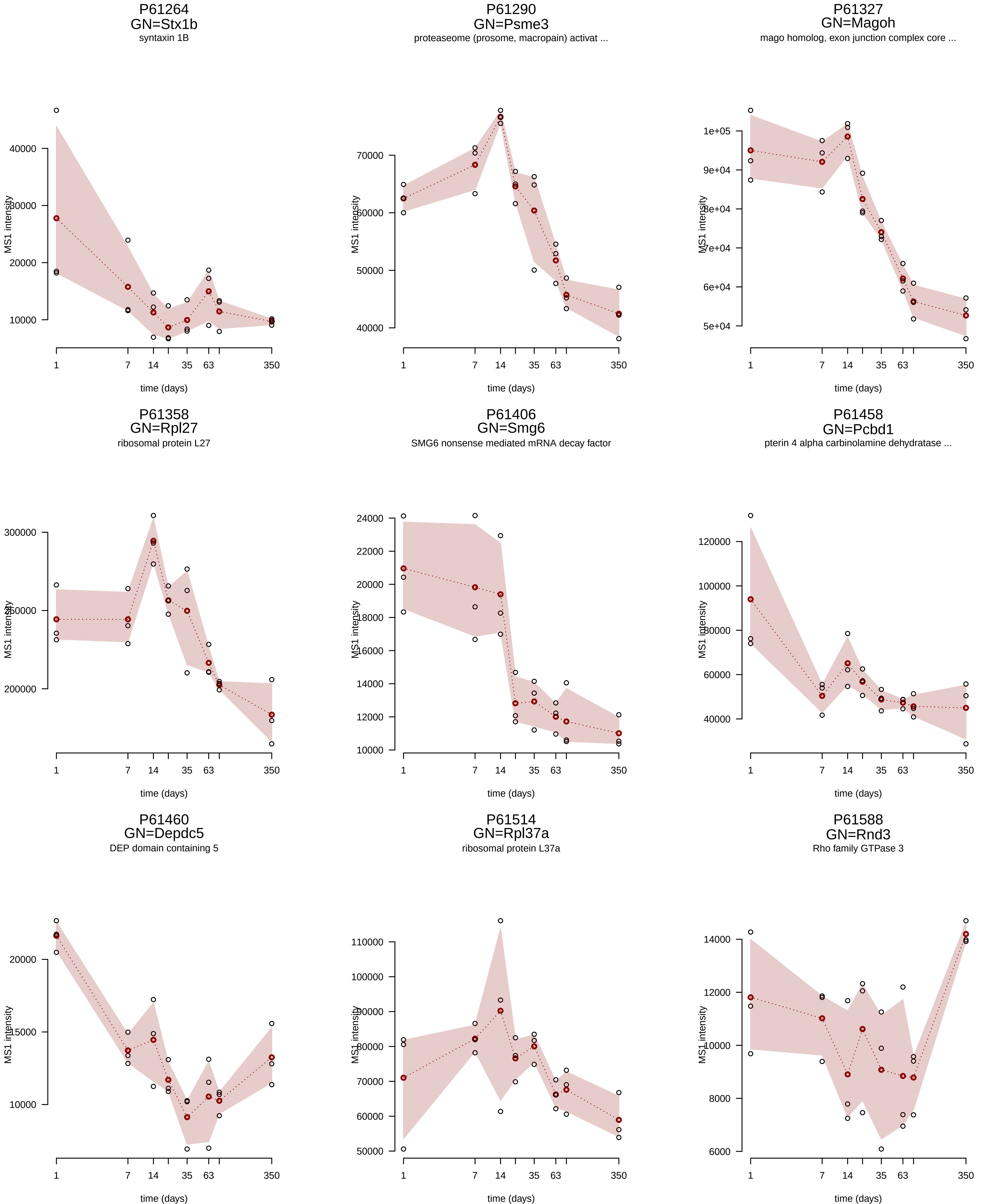
P61264
GN=Stx1b
syntaxin 1B
40000
30000
20000
10000
MS1 intensity
1 7 14 35 63 350
time (days)
P61290
GN=Psme3
proteaseome (prosome, macropain) activat ...
70000
60000
50000
40000
MS1 intensity
1 7 14 35 63 350
time (days)
P61327
GN=Magoh
mago homolog, exon junction complex core ...
1e+05
9e+04
8e+04
7e+04
6e+04
5e+04
MS1 intensity
1 7 14 35 63 350
time (days)
P61358
GN=Rpl27
ribosomal protein L27
300000
250000
200000
MS1 intensity
1 7 14 35 63 350
time (days)
P61406
GN=Smg6
SMG6 nonsense mediated mRNA decay factor
24000
22000
20000
18000
16000
14000
12000
10000
MS1 intensity
1 7 14 35 63 350
time (days)
P61458
GN=Pcbd1
pterin 4 alpha carbinolamine dehydratase ...
120000
100000
80000
60000
40000
MS1 intensity
1 7 14 35 63 350
time (days)
P61460
GN=Depdc5
DEP domain containing 5
20000
15000
10000
MS1 intensity
1 7 14 35 63 350
time (days)
P61514
GN=Rpl37a
ribosomal protein L37a
110000
100000
90000
80000
70000
60000
50000
MS1 intensity
1 7 14 35 63 350
time (days)
P61588
GN=Rnd3
Rho family GTPase 3
14000
12000
10000
8000
6000
MS1 intensity
1 7 14 35 63 350
time (days)

P61600
GN=Naa20
N(alpha)−acetyltransferase 20, NatB cata ...

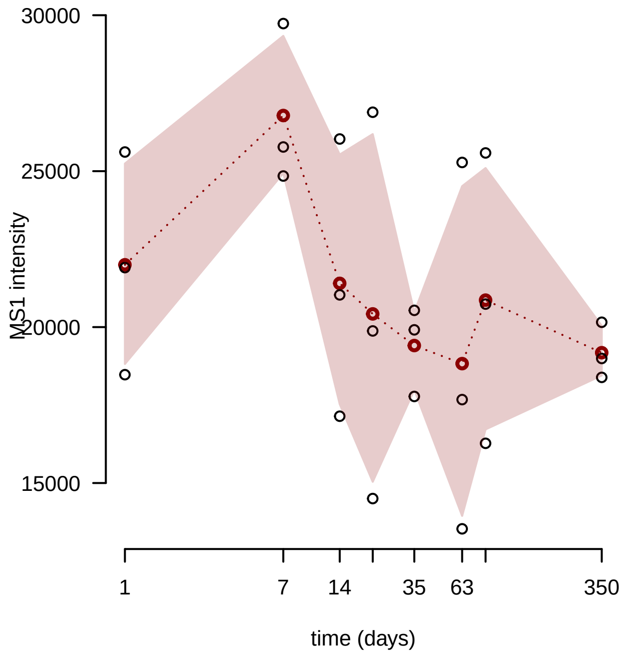

P61620
GN=Sec61a1
Sec61 alpha 1 subunit (S. cerevisiae)

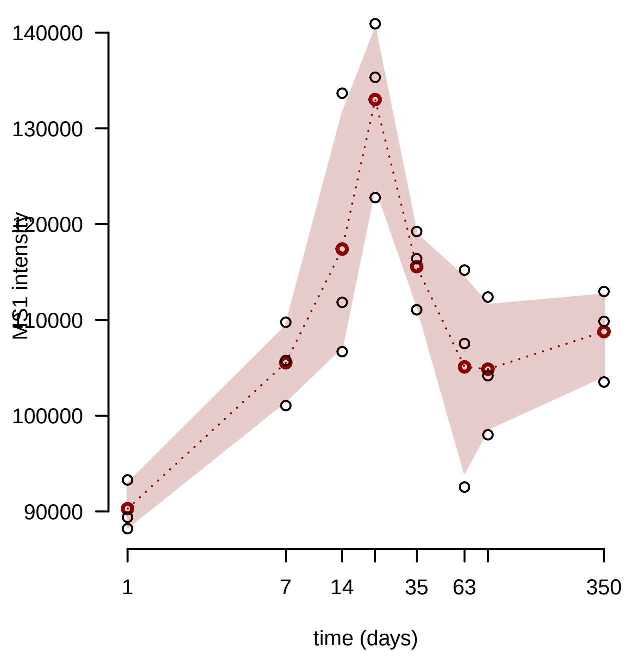

P61622
GN=Itga11
integrin alpha 11

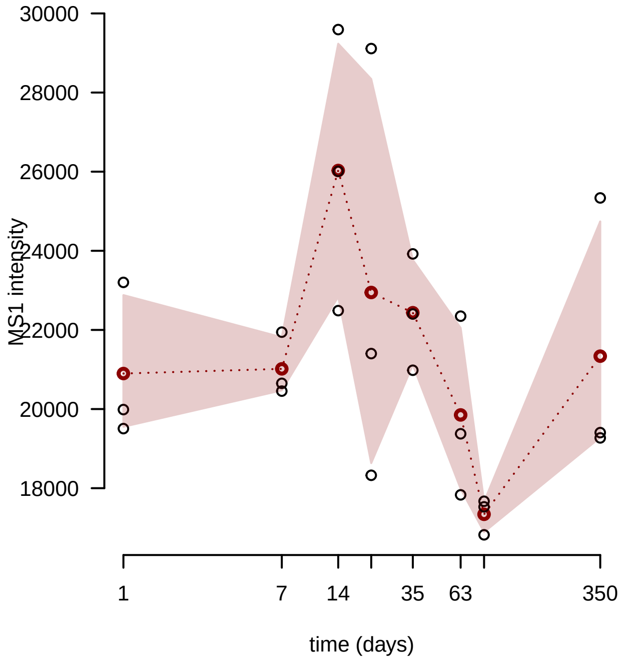

P61750
GN=Arf4
ADP−ribosylation factor 4

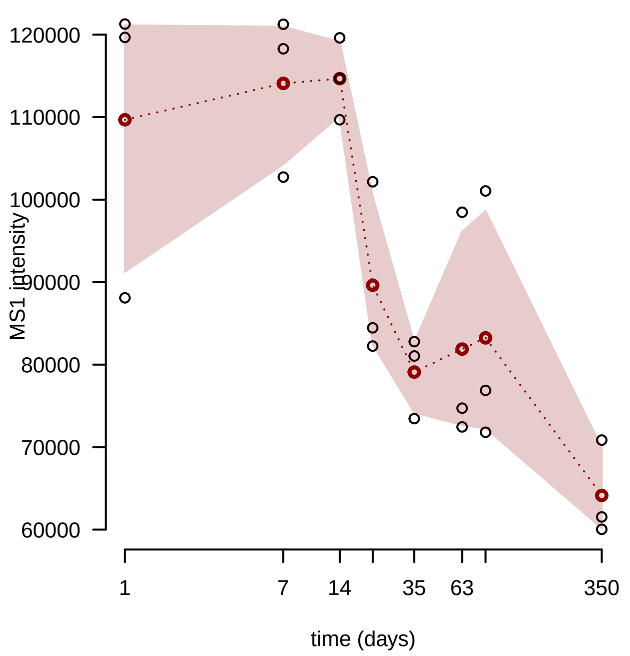

P61759
GN=Vbp1
von Hippel−Lindau binding protein 1

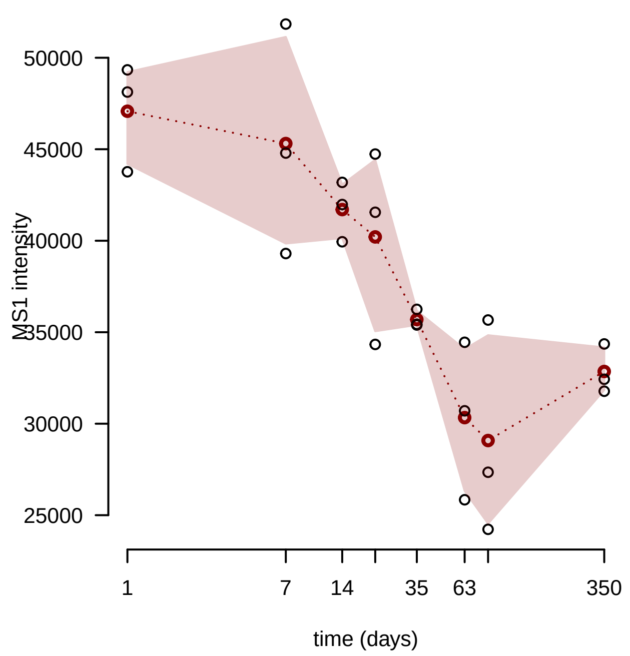

P61793
GN=Lpar1
lysophosphatidic acid receptor 1

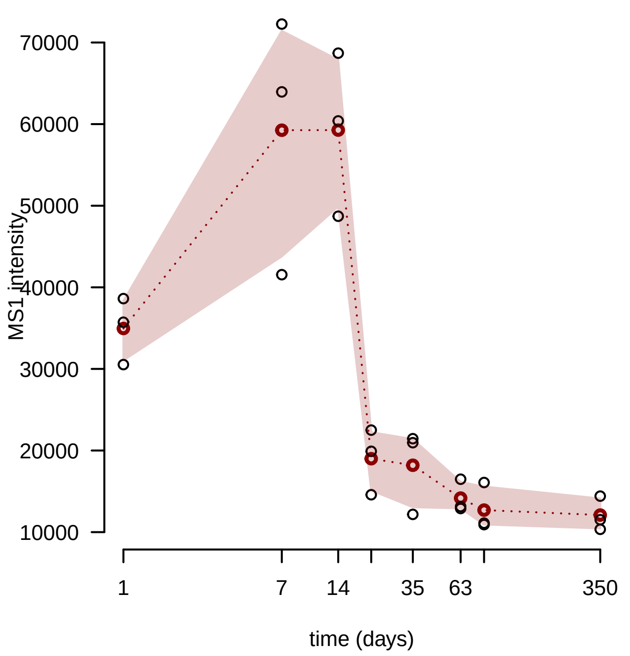

P61804
GN=Dad1
defender against cell death 1

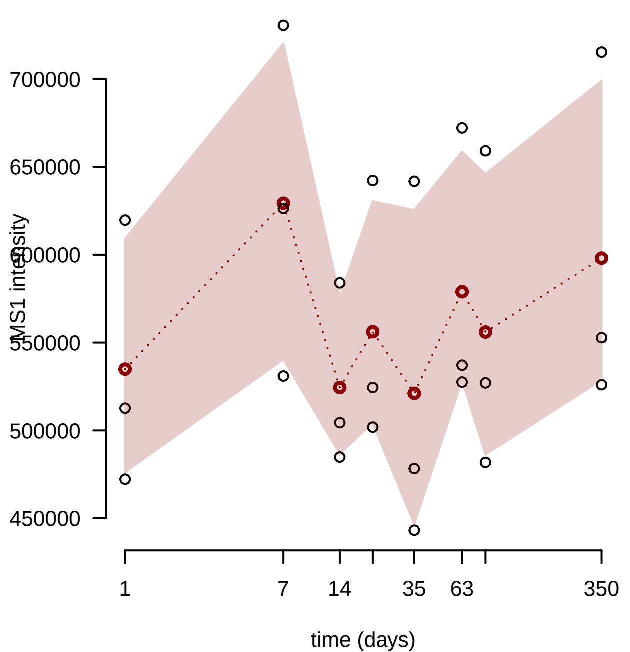

P61922
GN=Abat
4−aminobutyrate aminotransferase

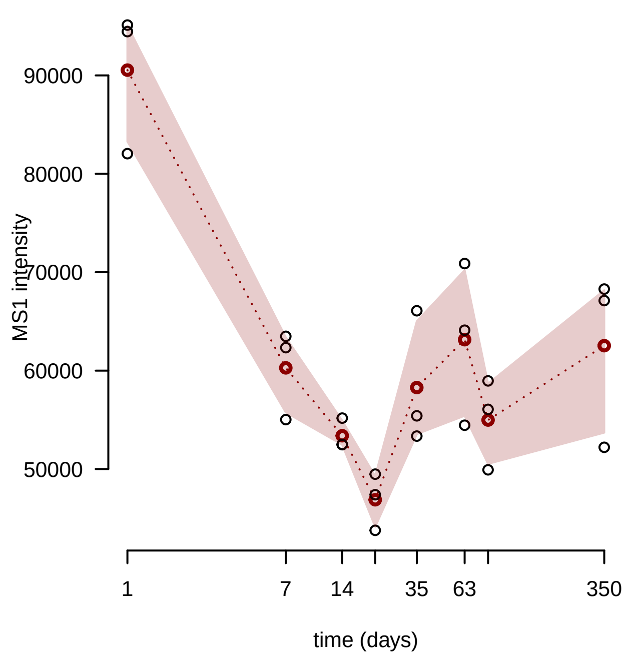

P61924
GN=Copz1
coatomer protein complex, subunit zeta 1

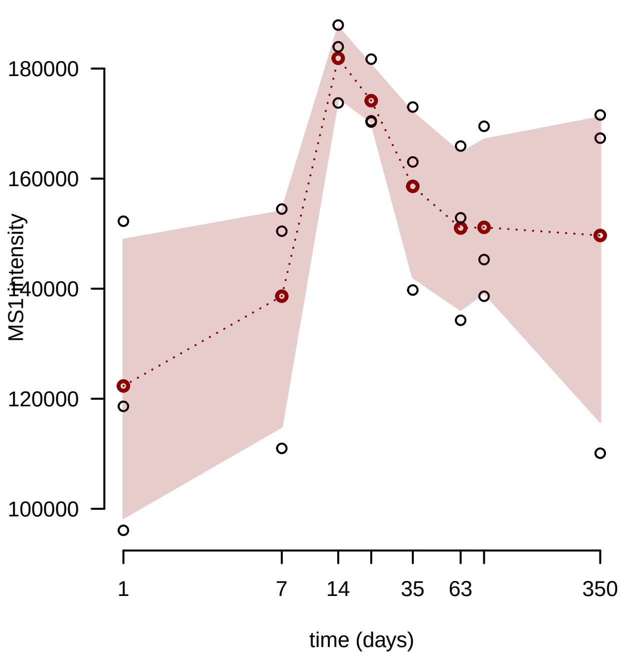

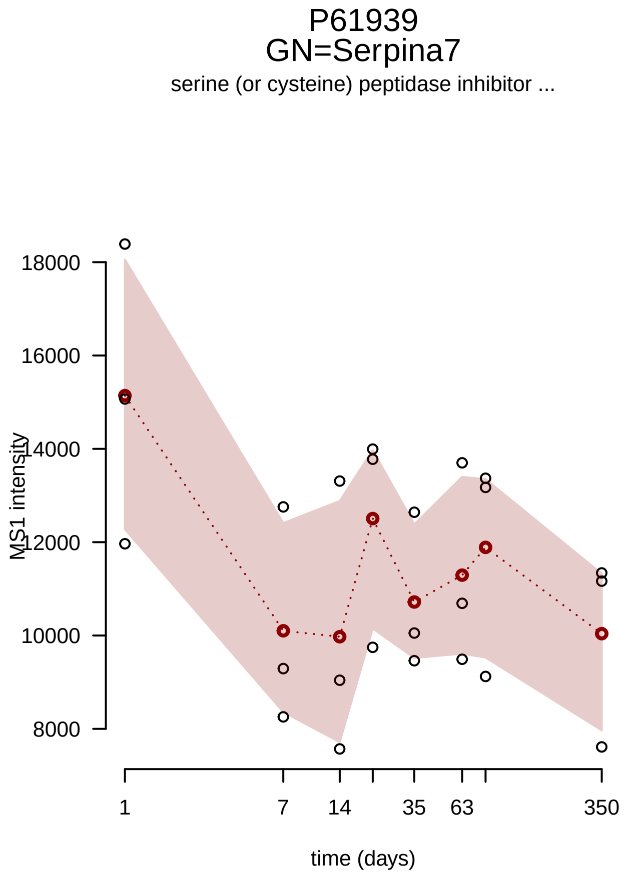
P61939
GN=Serpina7
serine (or cysteine) peptidase inhibitor ...
MS1 intensity
18000
16000
14000
12000
10000
8000
1
7
14
35
63
350
time (days)

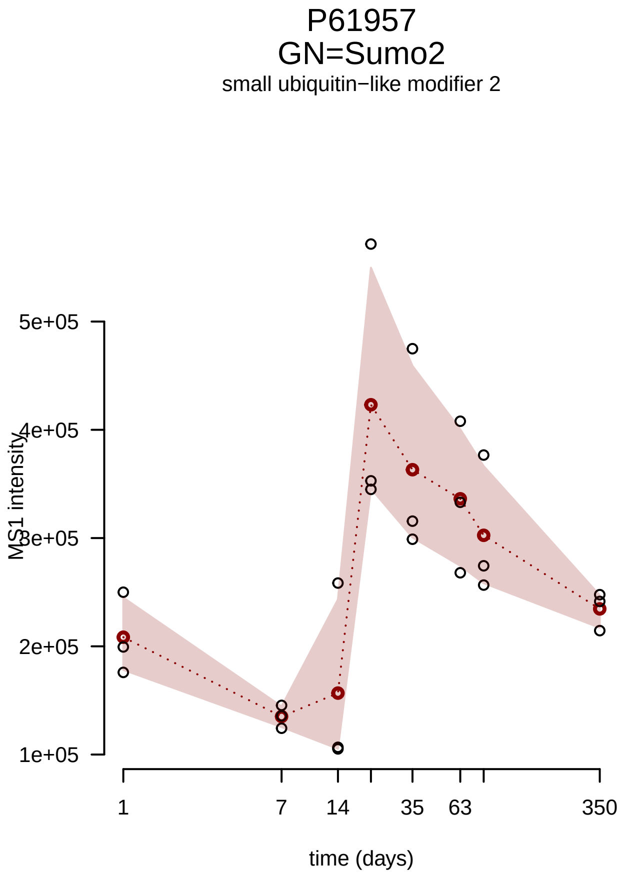
P61957
GN=Sumo2
small ubiquitin−like modifier 2
MS1 intensity
5e+05
4e+05
3e+05
2e+05
1e+05
1
7
14
35
63
350
time (days)

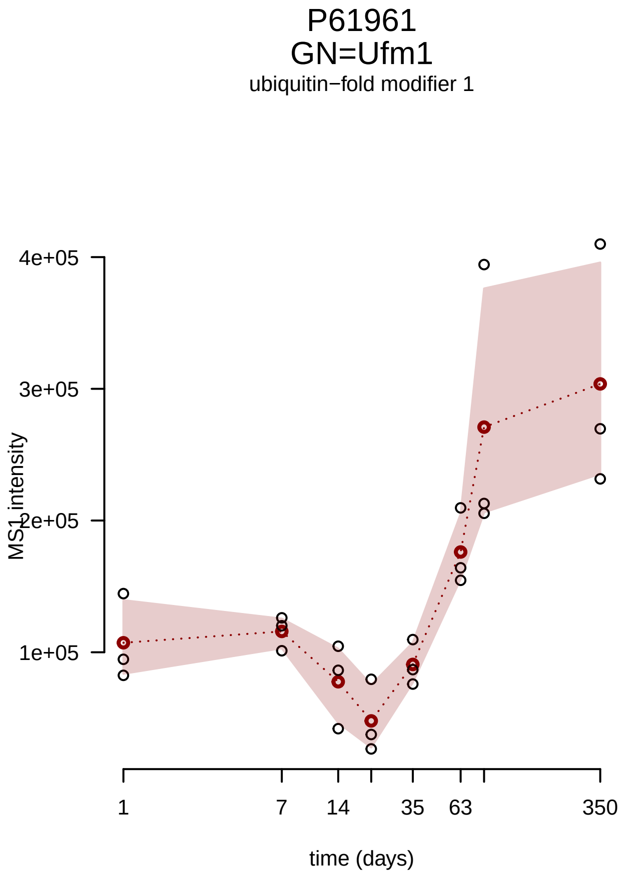
P61961
GN=Ufm1
ubiquitin−fold modifier 1
MS1 intensity
4e+05
3e+05
2e+05
1e+05
1
7
14
35
63
350
time (days)

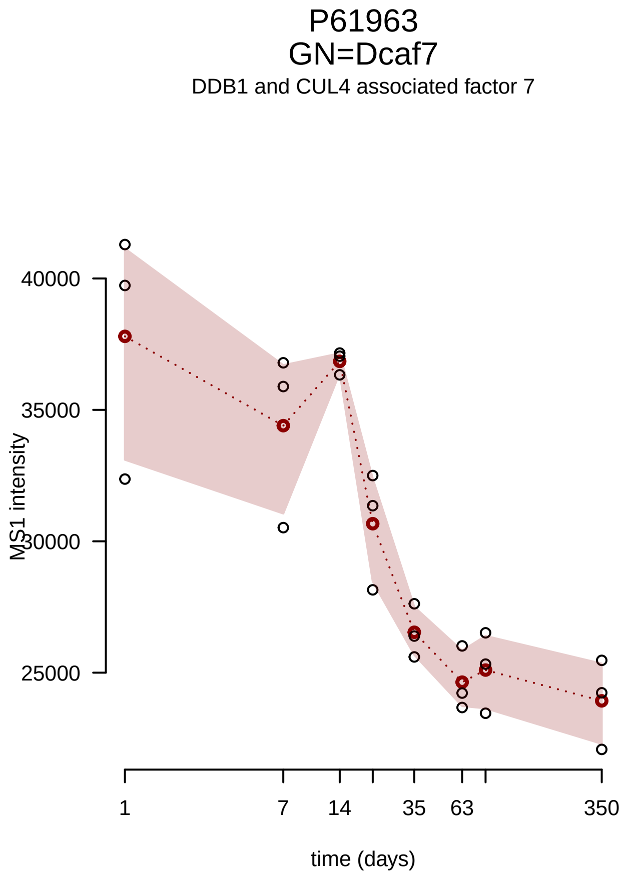
P61963
GN=Dcaf7
DDB1 and CUL4 associated factor 7
MS1 intensity
40000
35000
30000
25000
1
7
14
35
63
350
time (days)

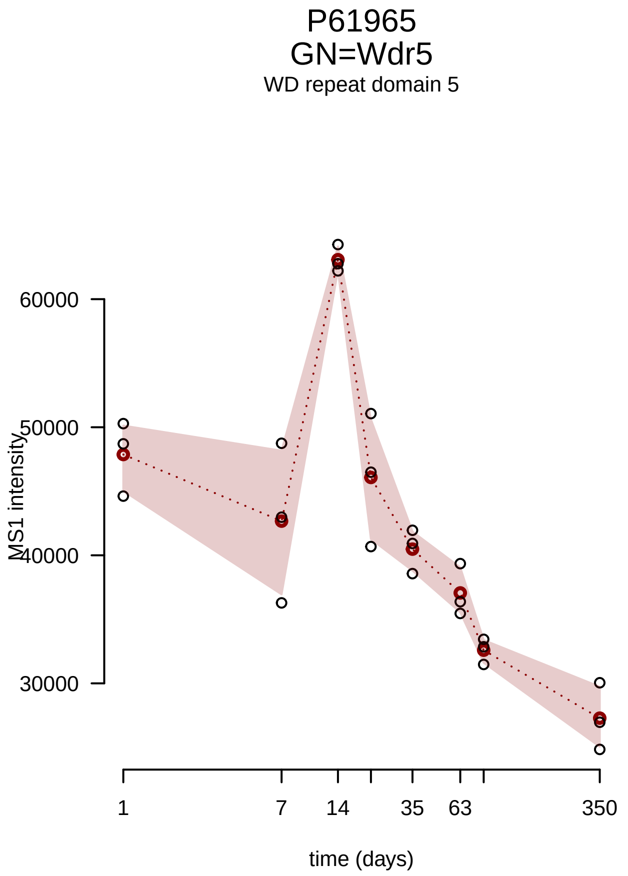
P61965
GN=Wdr5
WD repeat domain 5
MS1 intensity
60000
50000
40000
30000
1
7
14
35
63
350
time (days)

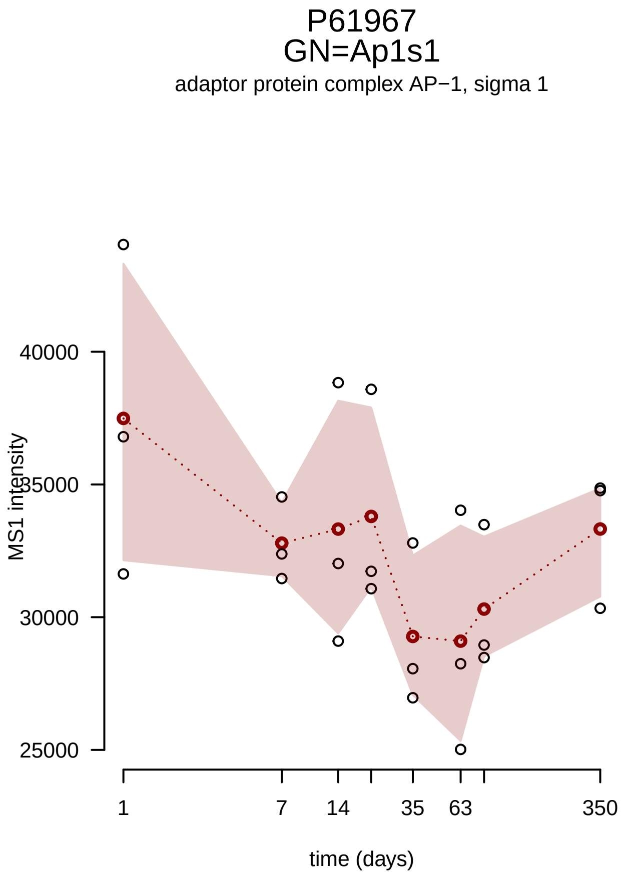
P61967
GN=Ap1s1
adaptor protein complex AP−1, sigma 1
MS1 intensity
40000
35000
30000
25000
1
7
14
35
63
350
time (days)

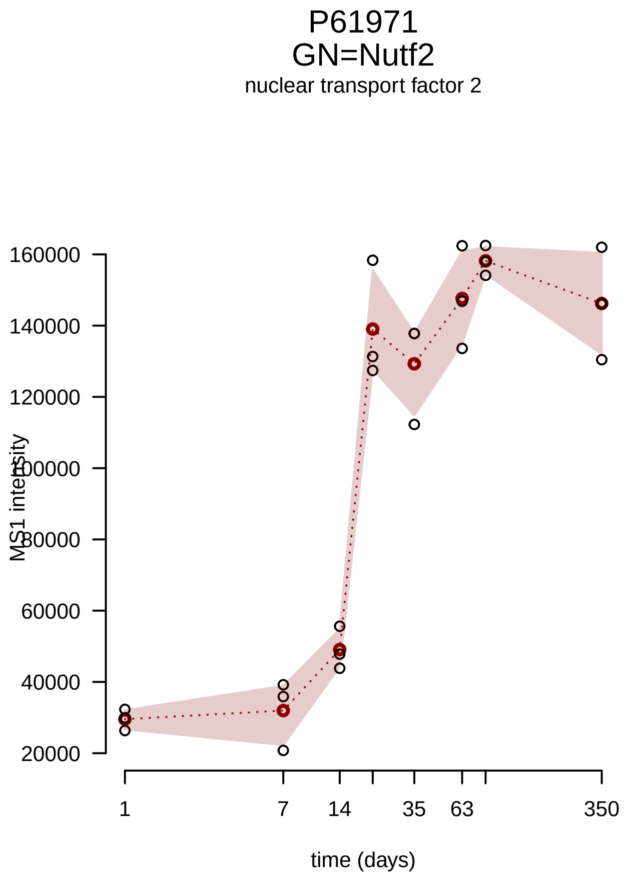
P61971
GN=Nutf2
nuclear transport factor 2
MS1 intensity
160000
140000
120000
100000
80000
60000
40000
20000
1
7
14
35
63
350
time (days)

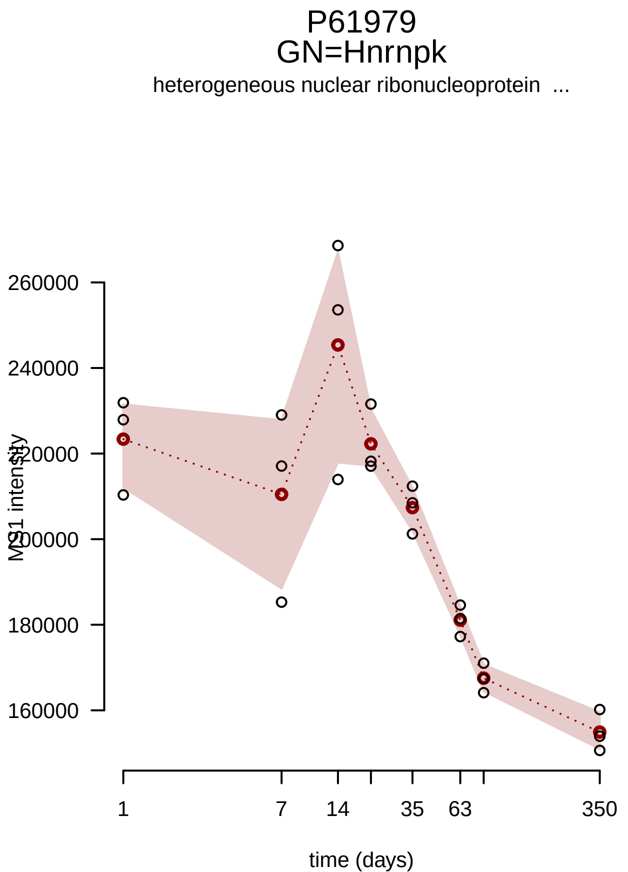
P61979
GN=Hnrnpk
heterogeneous nuclear ribonucleoprotein ...
MS1 intensity
260000
240000
220000
200000
180000
160000
1
7
14
35
63
350
time (days)

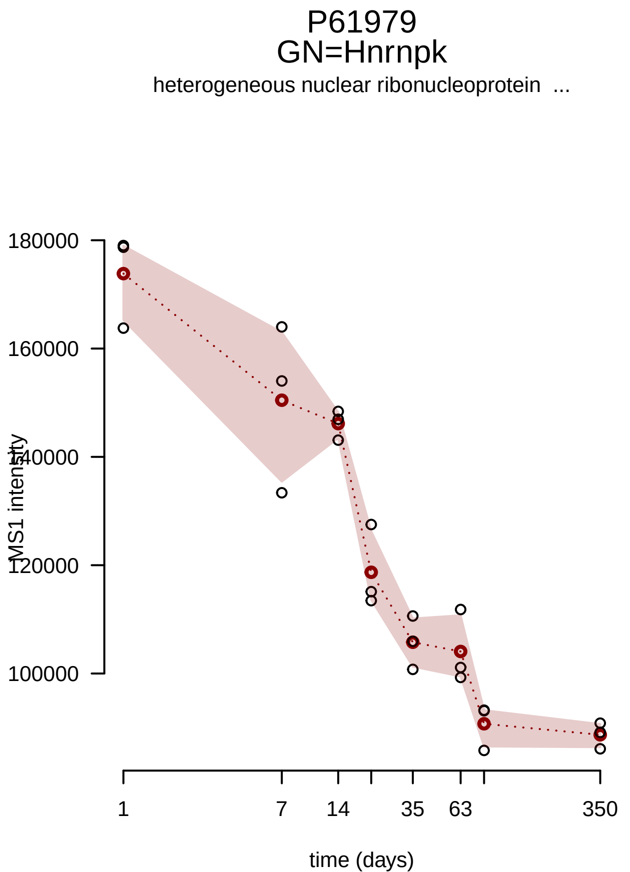
P61979
GN=Hnrnpk
heterogeneous nuclear ribonucleoprotein ...
MS1 intensity
180000
160000
140000
120000
100000
1
7
14
35
63
350
time (days)

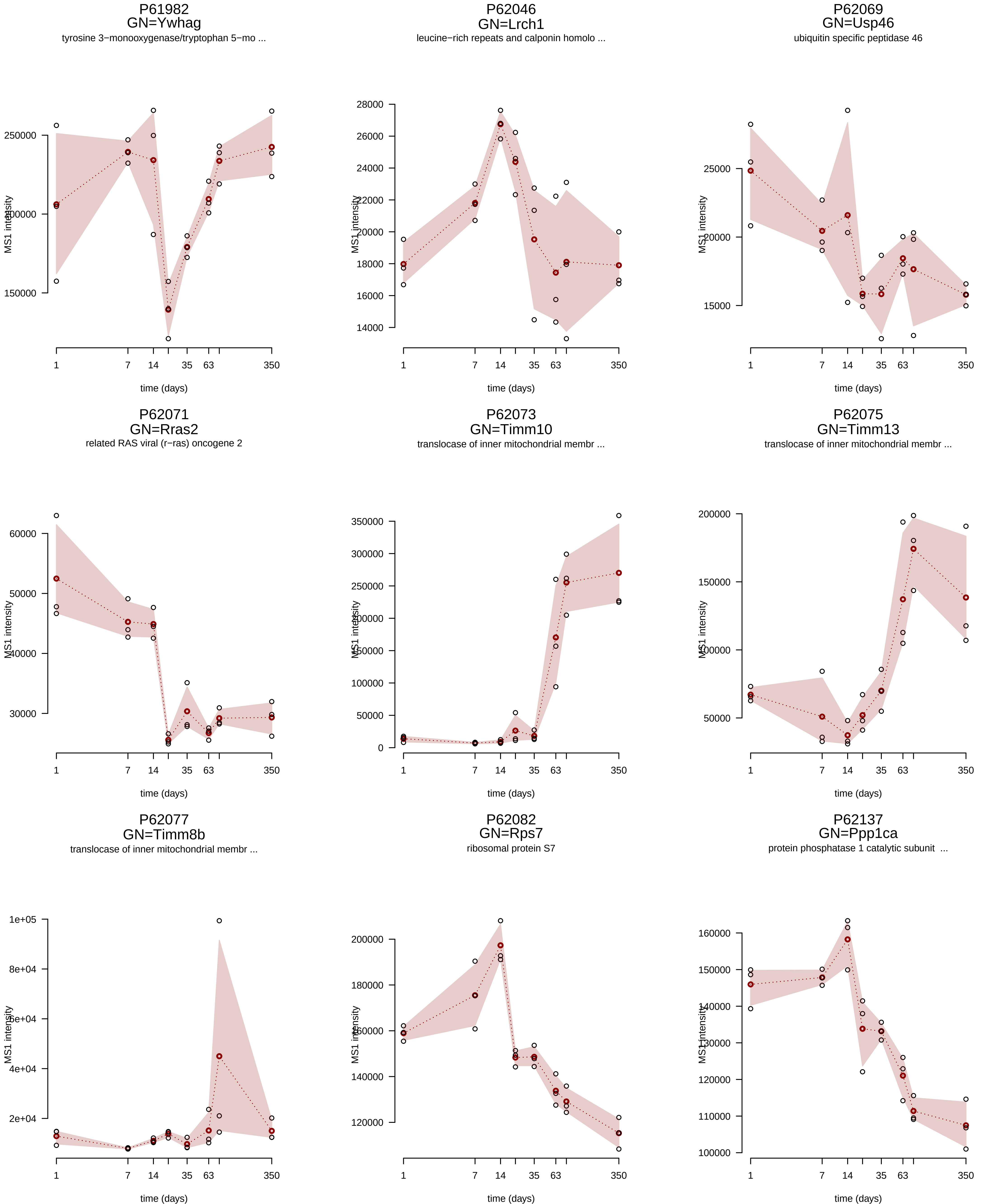

P61982
GN=Ywhag
tyrosine 3−monooxygenase/tryptophan 5−mo ...
MS1 intensity
time (days)
P62046
GN=Lrch1
leucine−rich repeats and calponin homolo ...
MS1 intensity
time (days)
P62069
GN=Usp46
ubiquitin specific peptidase 46
MS1 intensity
time (days)
P62071
GN=Rras2
related RAS viral (r−ras) oncogene 2
MS1 intensity
time (days)
P62073
GN=Timm10
translocase of inner mitochondrial membr ...
MS1 intensity
time (days)
P62075
GN=Timm13
translocase of inner mitochondrial membr ...
MS1 intensity
time (days)
P62077
GN=Timm8b
translocase of inner mitochondrial membr ...
MS1 intensity
time (days)
P62082
GN=Rps7
ribosomal protein S7
MS1 intensity
time (days)
P62137
GN=Ppp1ca
protein phosphatase 1 catalytic subunit ...
MS1 intensity
time (days)

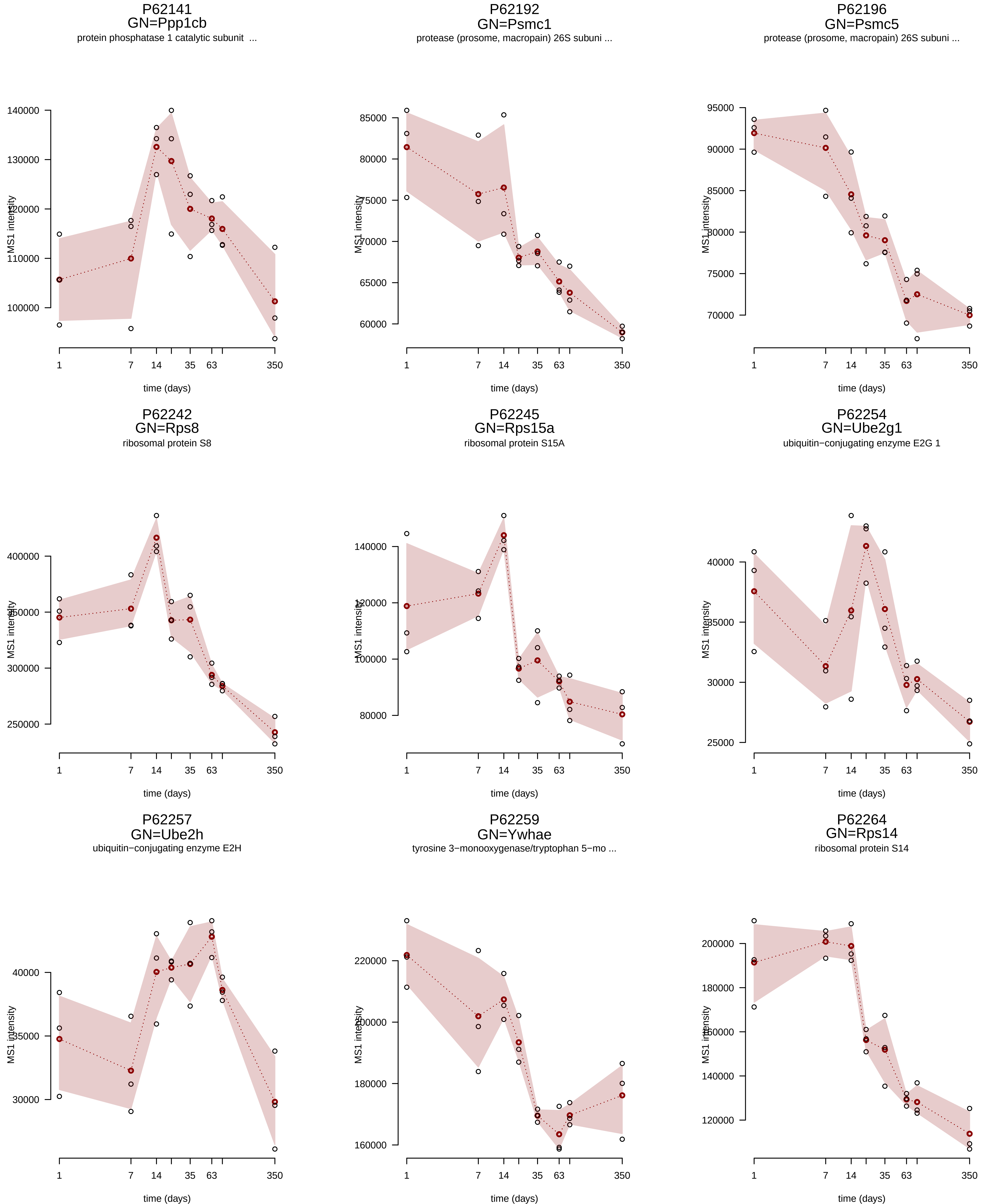
P62141
GN=Ppp1cb
protein phosphatase 1 catalytic subunit ...
140000
130000
120000
110000
100000
MS1 intensity
1
7
14
35
63
350
time (days)
P62192
GN=Psmc1
protease (prosome, macropain) 26S subuni ...
85000
80000
75000
70000
65000
60000
MS1 intensity
1
7
14
35
63
350
time (days)
P62196
GN=Psmc5
protease (prosome, macropain) 26S subuni ...
95000
90000
85000
80000
75000
70000
MS1 intensity
1
7
14
35
63
350
time (days)
P62242
GN=Rps8
ribosomal protein S8
400000
350000
300000
250000
MS1 intensity
1
7
14
35
63
350
time (days)
P62245
GN=Rps15a
ribosomal protein S15A
140000
120000
100000
80000
MS1 intensity
1
7
14
35
63
350
time (days)
P62254
GN=Ube2g1
ubiquitin−conjugating enzyme E2G 1
40000
35000
30000
25000
MS1 intensity
1
7
14
35
63
350
time (days)
P62257
GN=Ube2h
ubiquitin−conjugating enzyme E2H
40000
35000
30000
MS1 intensity
1
7
14
35
63
350
time (days)
P62259
GN=Ywhae
tyrosine 3−monooxygenase/tryptophan 5−mo ...
220000
200000
180000
160000
MS1 intensity
1
7
14
35
63
350
time (days)
P62264
GN=Rps14
ribosomal protein S14
200000
180000
160000
140000
120000
MS1 intensity
1
7
14
35
63
350
time (days)

P62267
GN=Rps23
ribosomal protein S23

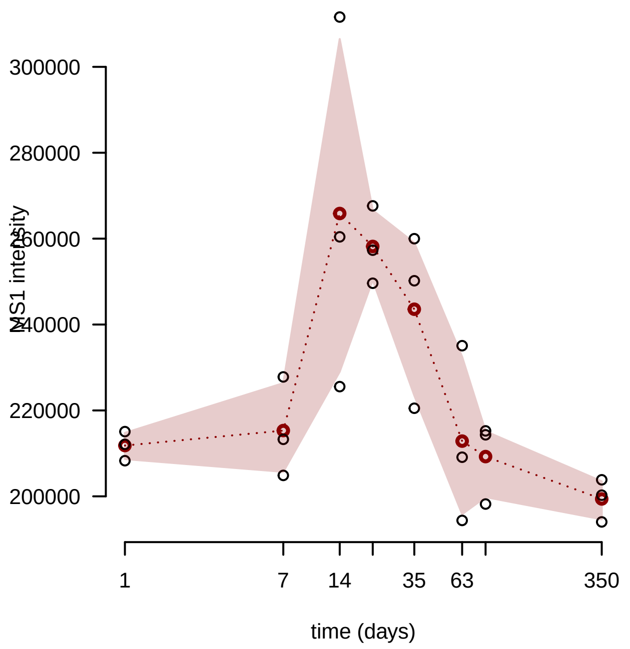

P62270
GN=Rps18
ribosomal protein S18

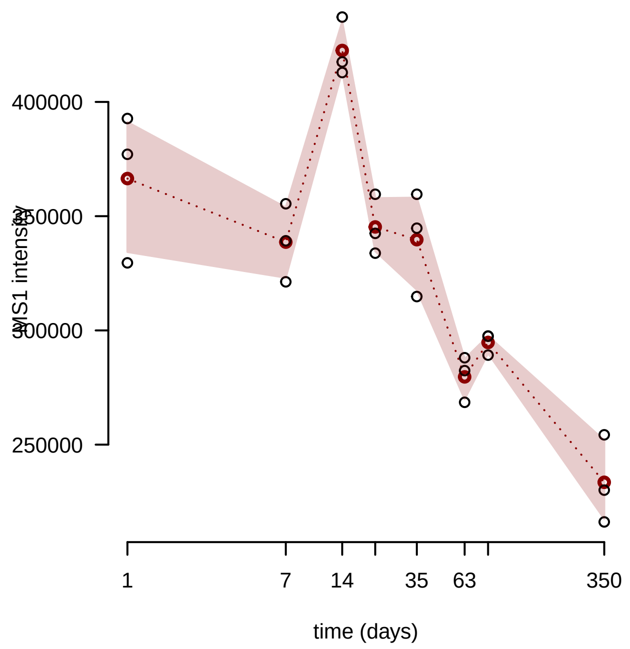

P62274
GN=Rps29
ribosomal protein S29

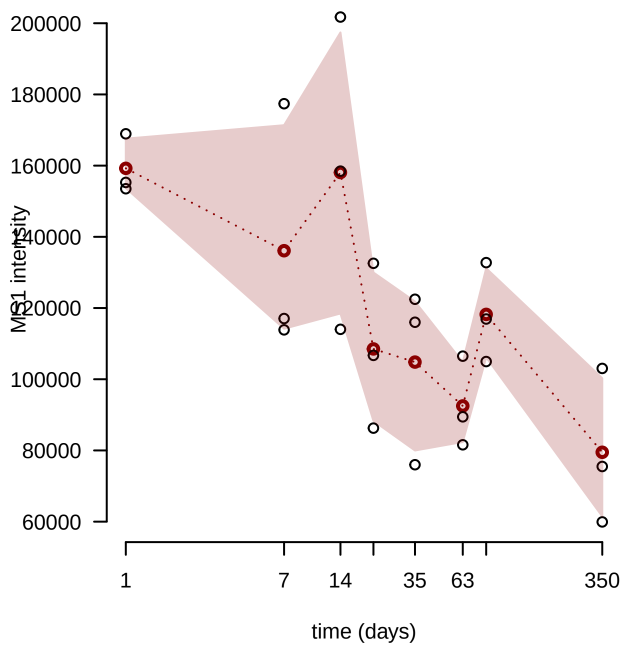

P62281
GN=Rps11
ribosomal protein S11

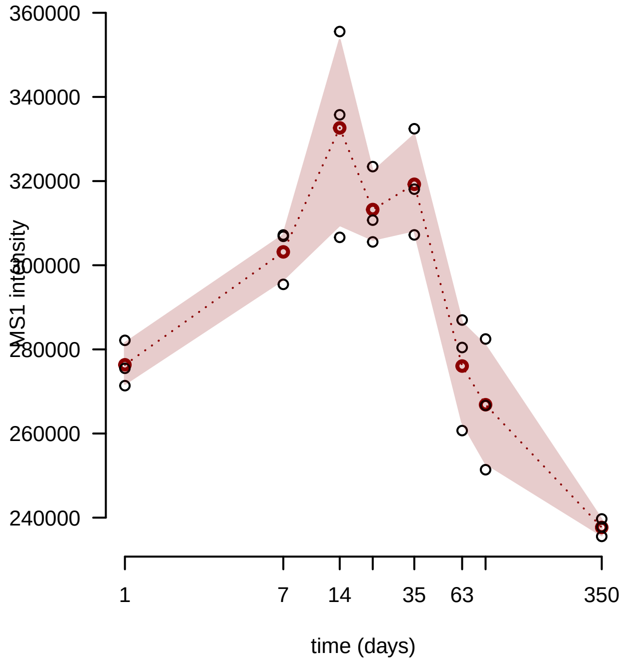

P62301
GN=Rps13
ribosomal protein S13

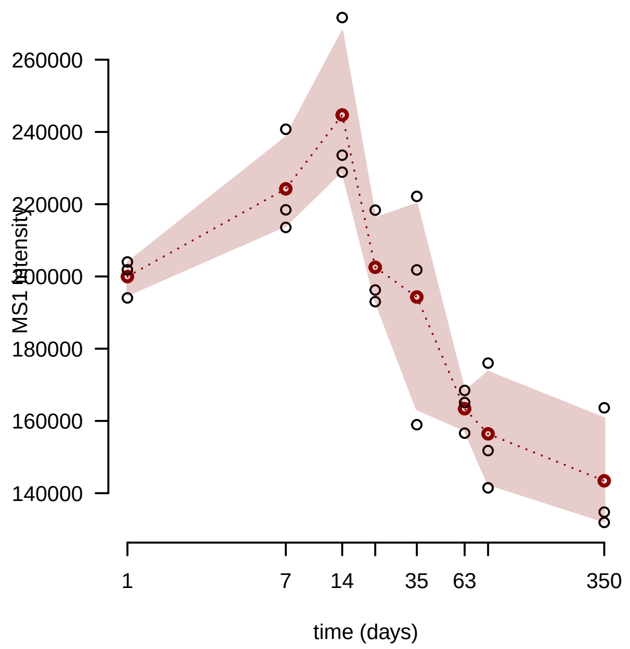

P62305
GN=Snrpe
small nuclear ribonucleoprotein E

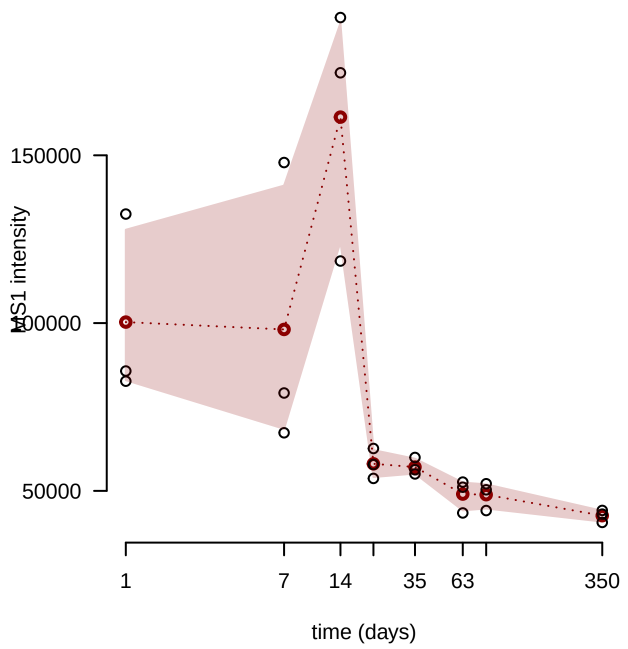

P62307
GN=Snrpf
small nuclear ribonucleoprotein polypept ...

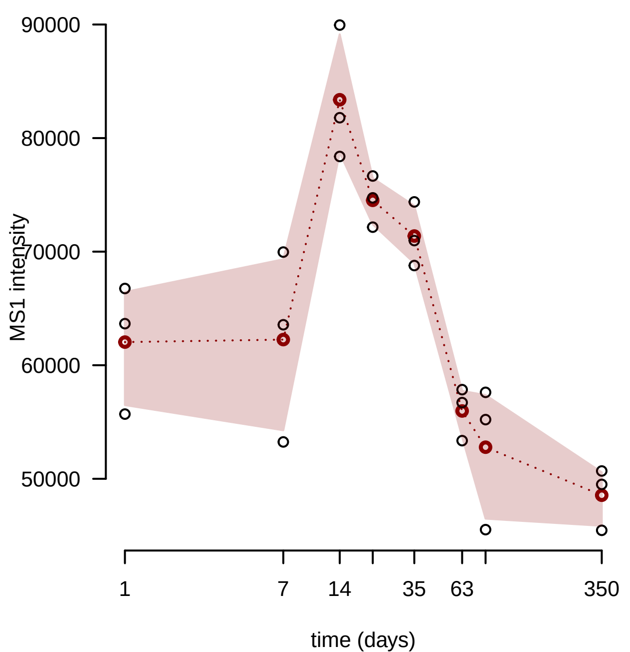

P62309
GN=Snrpg
small nuclear ribonucleoprotein polypept ...

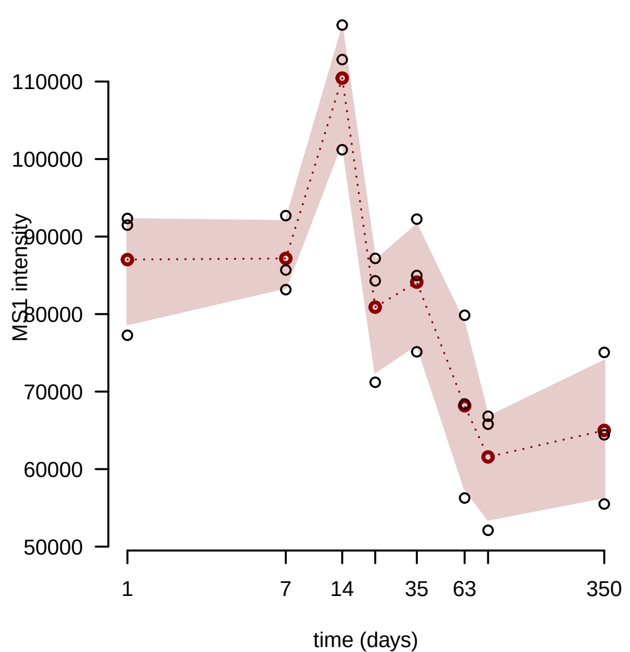

P62311
GN=Lsm3
LSM3 homolog, U6 small nuclear RNA and m ...

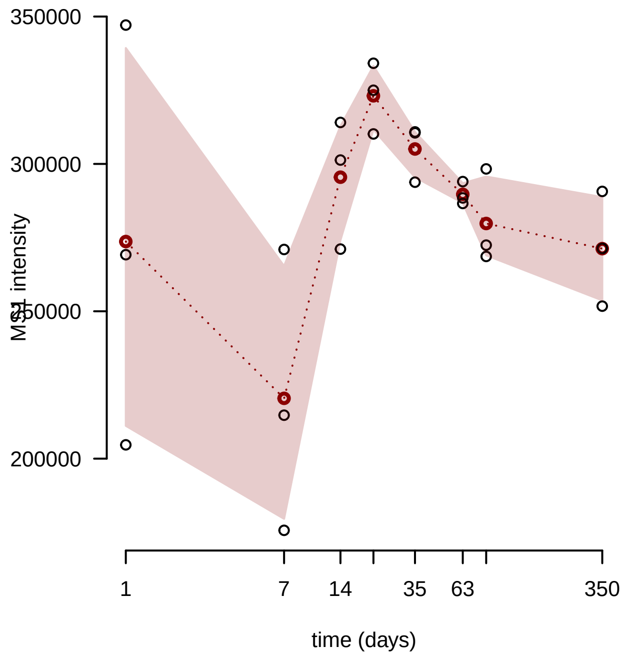

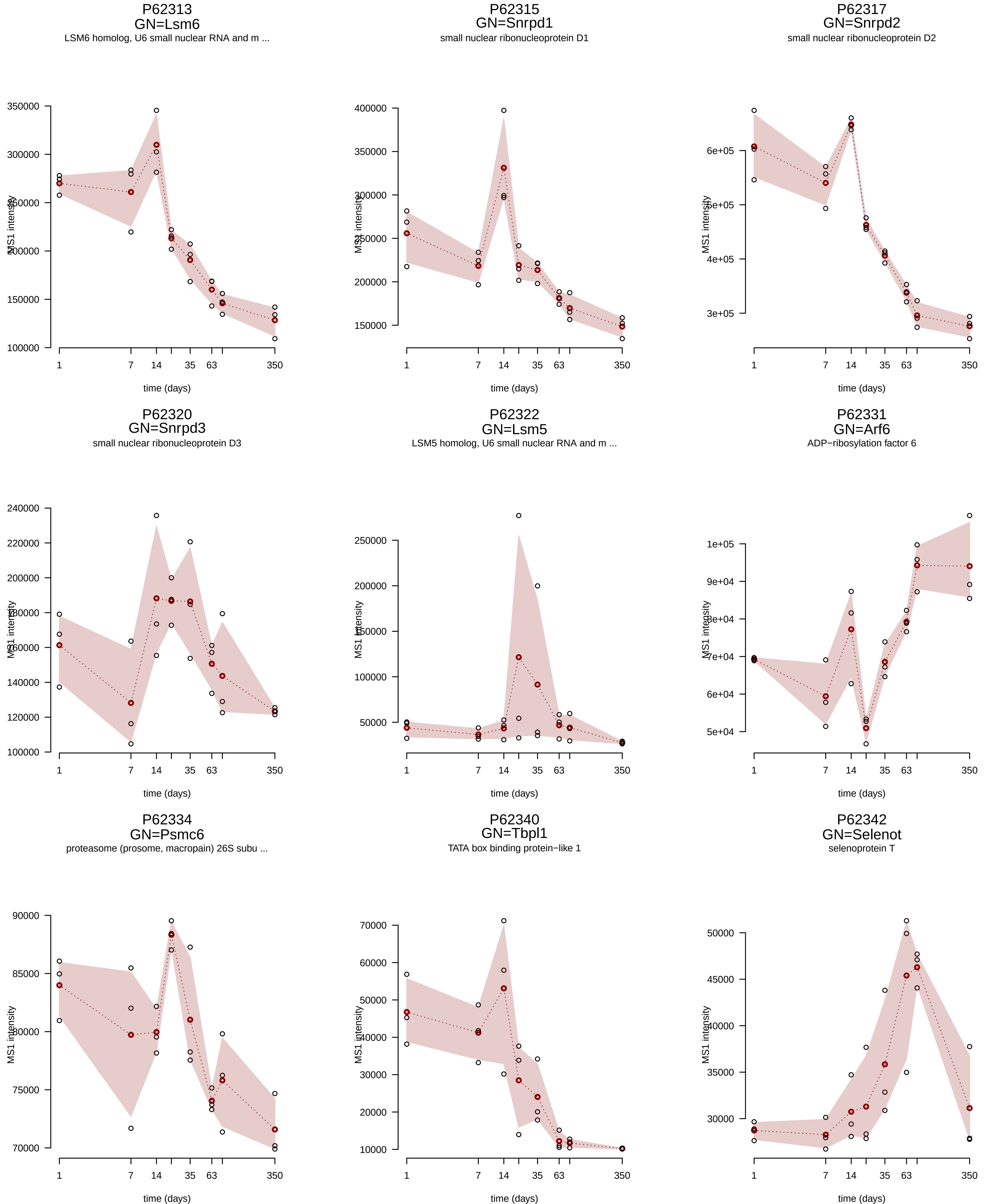
P62313
GN=Lsm6
LSM6 homolog, U6 small nuclear RNA and m ...
MS1 intensity
350000
300000
250000
200000
150000
100000
1
7
14
35
63
350
time (days)
P62315
GN=Snrpd1
small nuclear ribonucleoprotein D1
MS1 intensity
400000
350000
300000
250000
200000
150000
1
7
14
35
63
350
time (days)
P62317
GN=Snrpd2
small nuclear ribonucleoprotein D2
MS1 intensity
6e+05
5e+05
4e+05
3e+05
1
7
14
35
63
350
time (days)
P62320
GN=Snrpd3
small nuclear ribonucleoprotein D3
MS1 intensity
240000
220000
200000
180000
160000
140000
120000
100000
1
7
14
35
63
350
time (days)
P62322
GN=Lsm5
LSM5 homolog, U6 small nuclear RNA and m ...
MS1 intensity
250000
200000
150000
100000
50000
1
7
14
35
63
350
time (days)
P62331
GN=Arf6
ADP−ribosylation factor 6
MS1 intensity
1e+05
9e+04
8e+04
7e+04
6e+04
5e+04
1
7
14
35
63
350
time (days)
P62334
GN=Psmc6
proteasome (prosome, macropain) 26S subu ...
MS1 intensity
90000
85000
80000
75000
70000
1
7
14
35
63
350
time (days)
P62340
GN=Tbpl1
TATA box binding protein−like 1
MS1 intensity
70000
60000
50000
40000
30000
20000
10000
1
7
14
35
63
350
time (days)
P62342
GN=Selenot
selenoprotein T
MS1 intensity
50000
45000
40000
35000
30000
1
7
14
35
63
350
time (days)

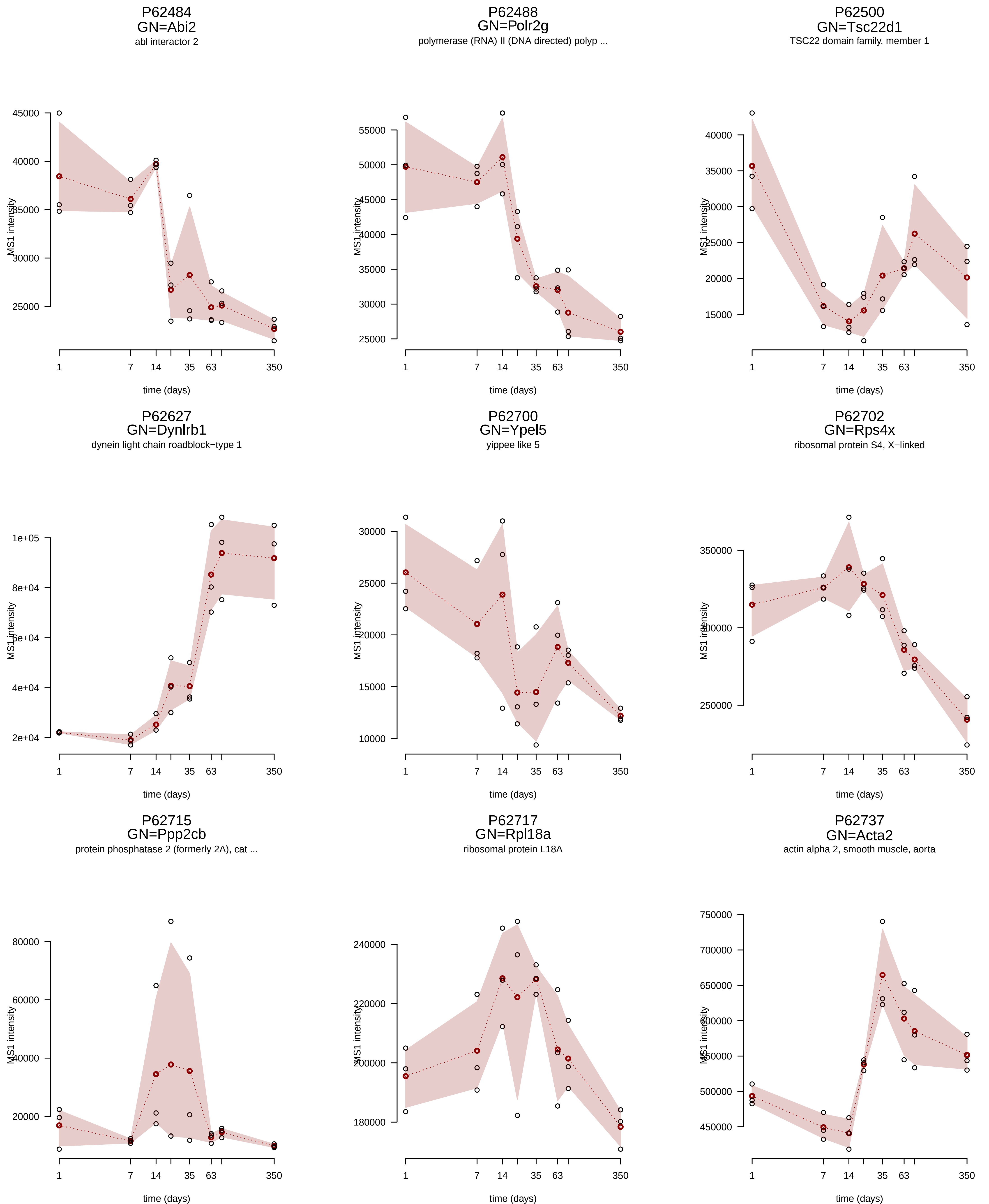
P62484
GN=Abi2
abl interactor 2
P62488
GN=Polr2g
polymerase (RNA) II (DNA directed) polyp ...
P62500
GN=Tsc22d1
TSC22 domain family, member 1
P62627
GN=Dynlrb1
dynein light chain roadblock−type 1
P62700
GN=Ypel5
yippee like 5
P62702
GN=Rps4x
ribosomal protein S4, X−linked
P62715
GN=Ppp2cb
protein phosphatase 2 (formerly 2A), cat ...
P62717
GN=Rpl18a
ribosomal protein L18A
P62737
GN=Acta2
actin alpha 2, smooth muscle, aorta
MS1 intensity
time (days)
1
7
14
35
63
350

P62743
GN=Ap2s1
adaptor−related protein complex 2, sigma ...

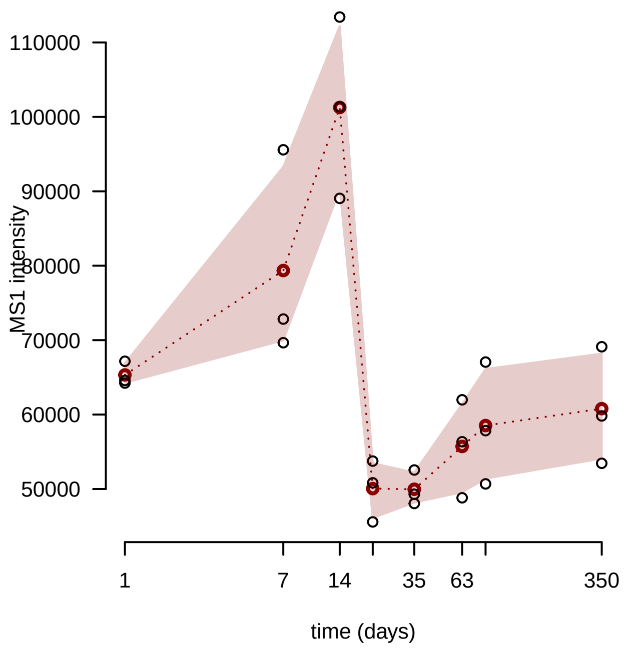

P62746
GN=Rhob
ras homolog family member B

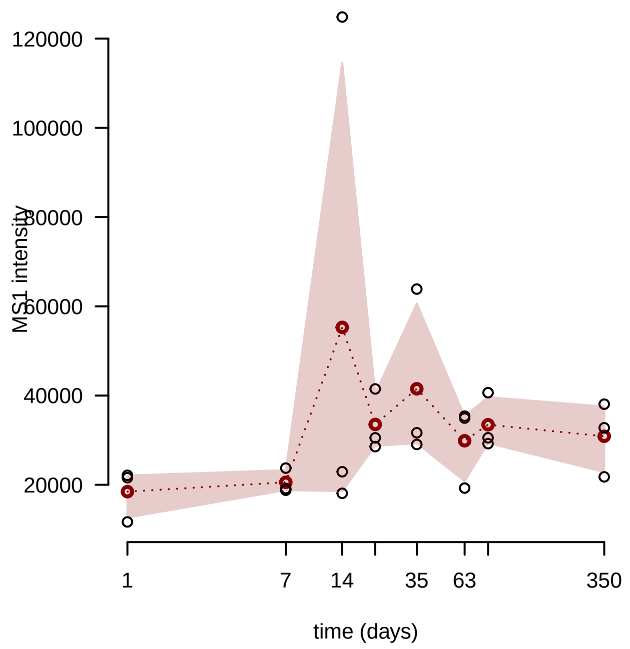

P62748
GN=Hpcal1
hippocalcin−like 1

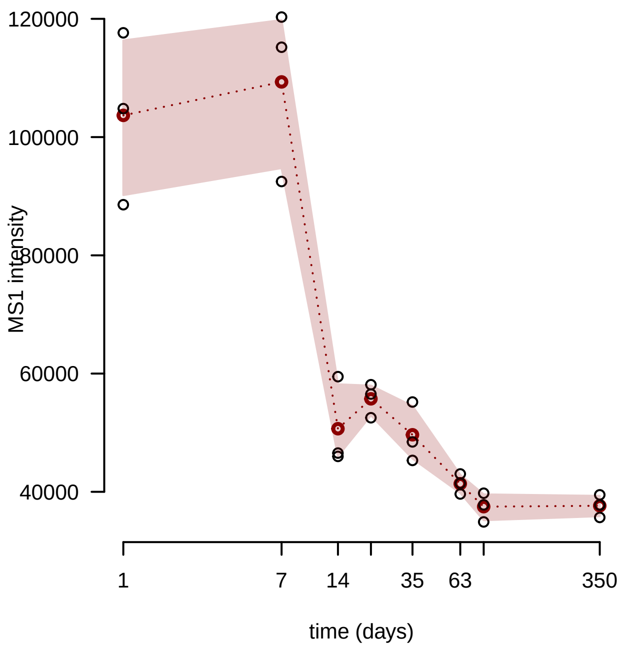

P62751
GN=Rpl23a
ribosomal protein L23A

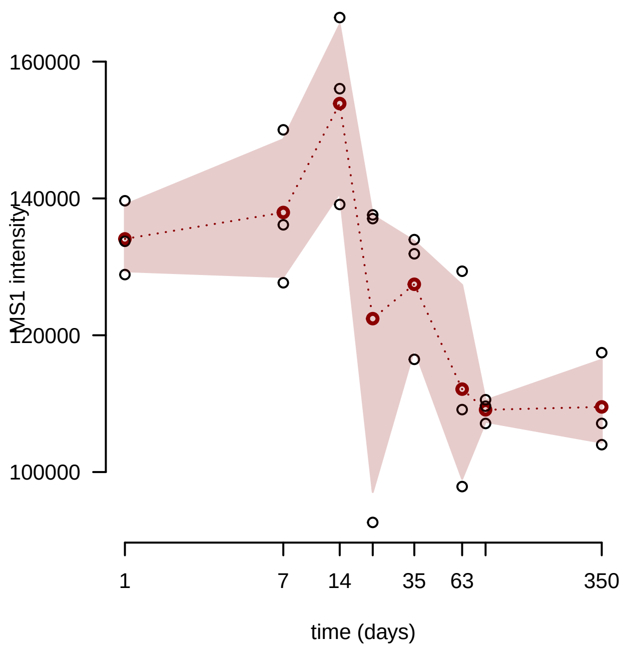

P62754
GN=Rps6
ribosomal protein S6

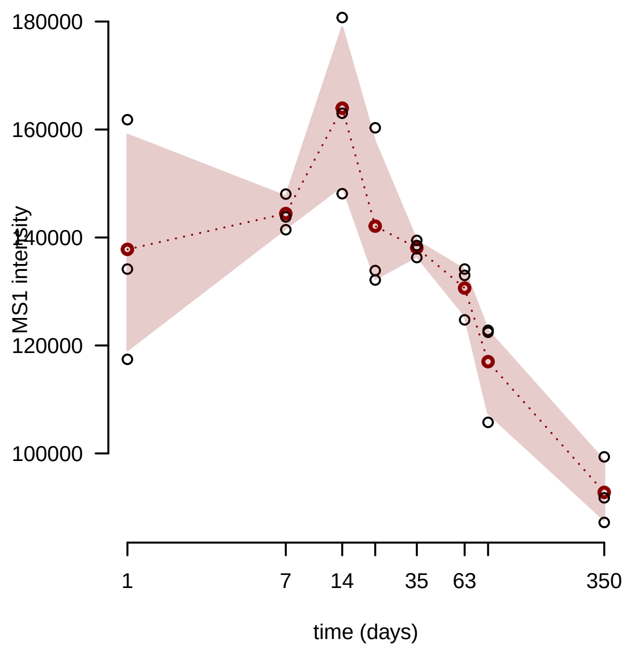

P62761
GN=Vsnl1
visinin−like 1

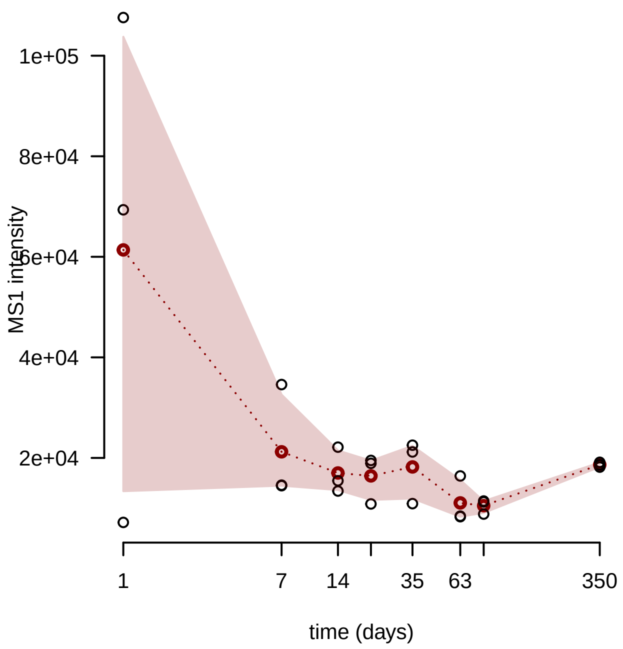

P62774
GN=Mtpn
myotrophin

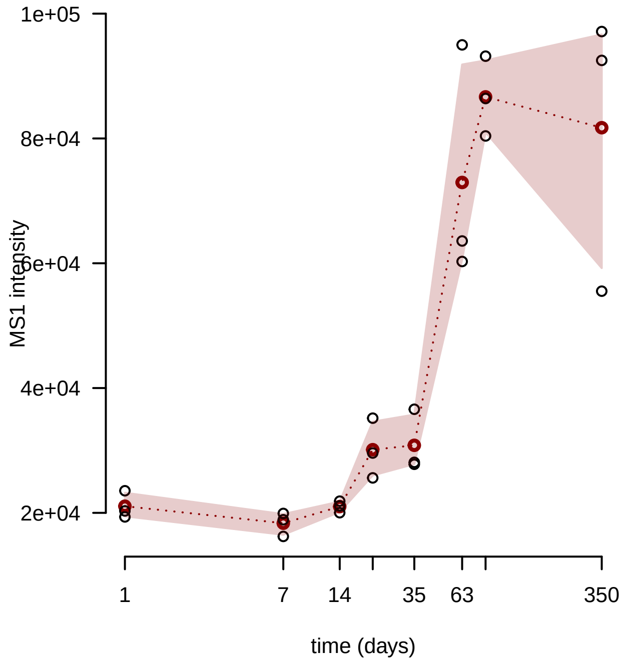

P62806
GN=Hist1h4a

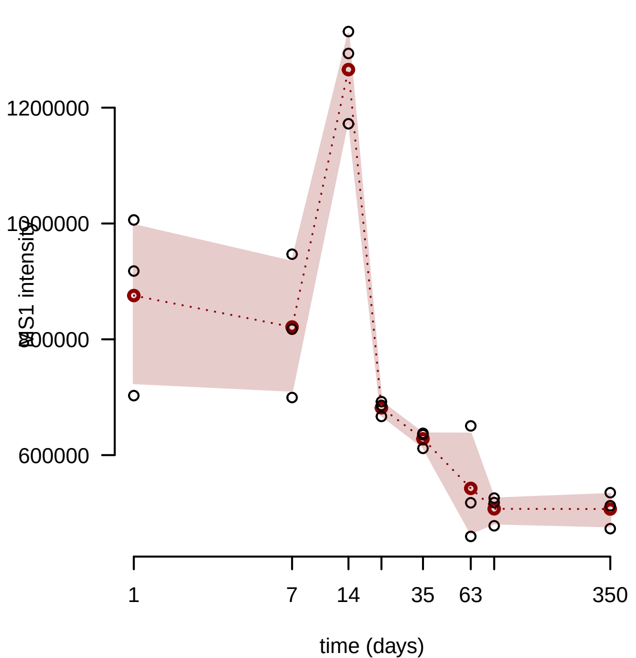

P62814
GN=Atp6v1b2
ATPase, H+ transporting, lysosomal V1 su ...

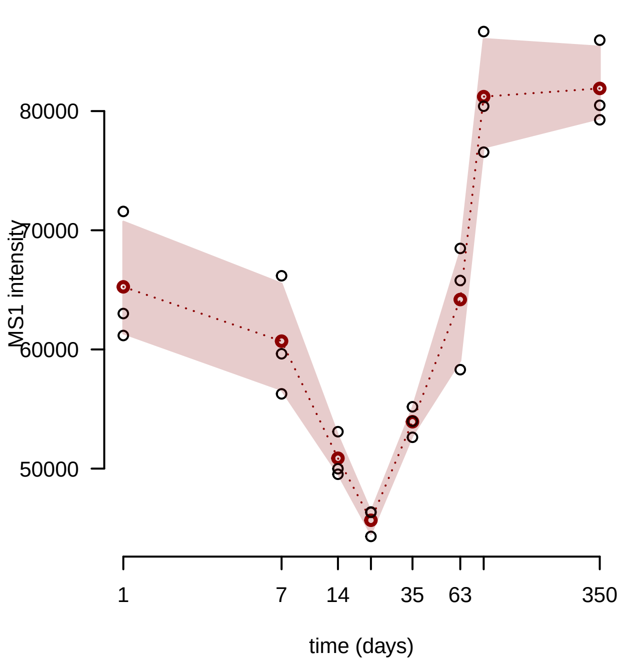

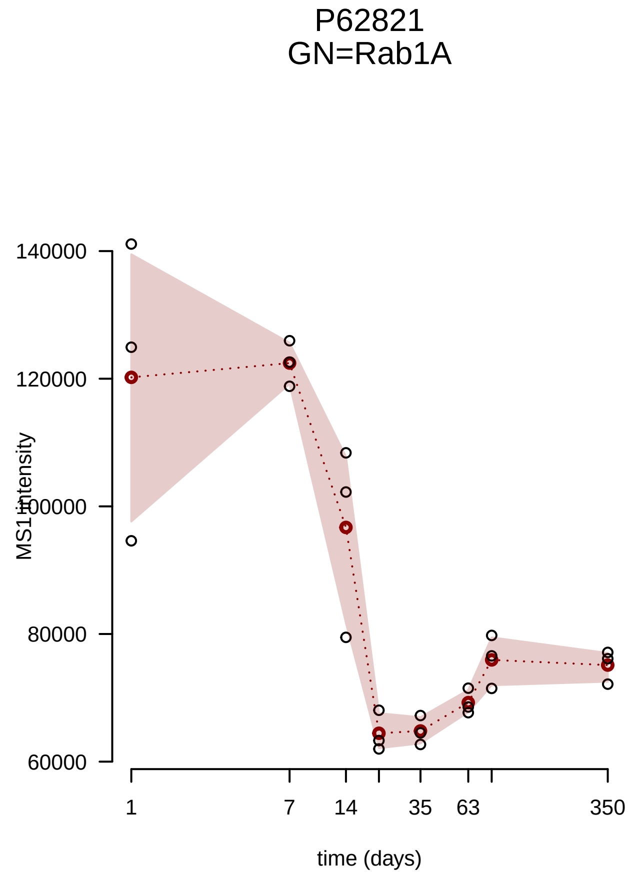
P62821
GN=Rab1A
MS1 intensity
140000
120000
100000
80000
60000
1
7
14
35
63
350
time (days)

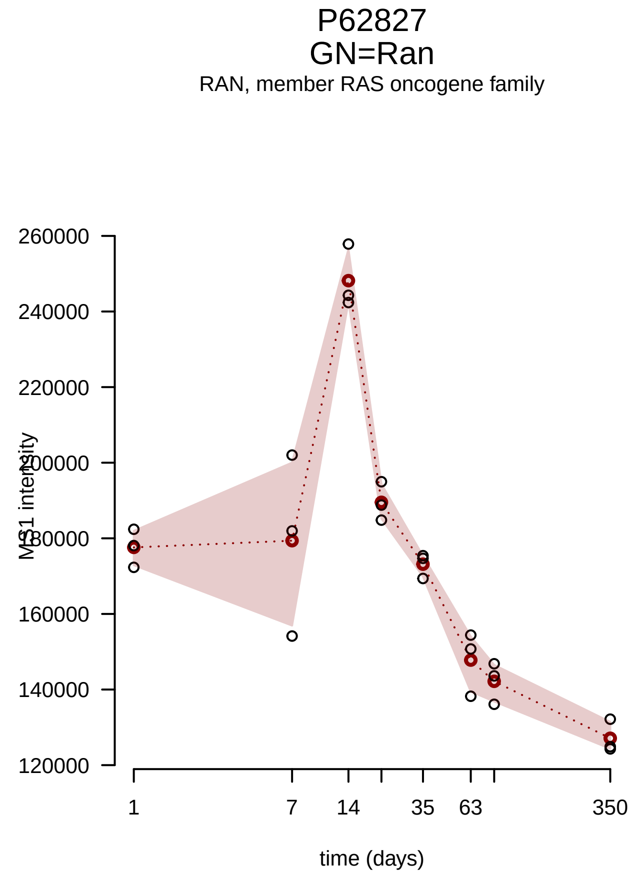
P62827
GN=Ran
RAN, member RAS oncogene family
MS1 intensity
260000
240000
220000
200000
180000
160000
140000
120000
1
7
14
35
63
350
time (days)

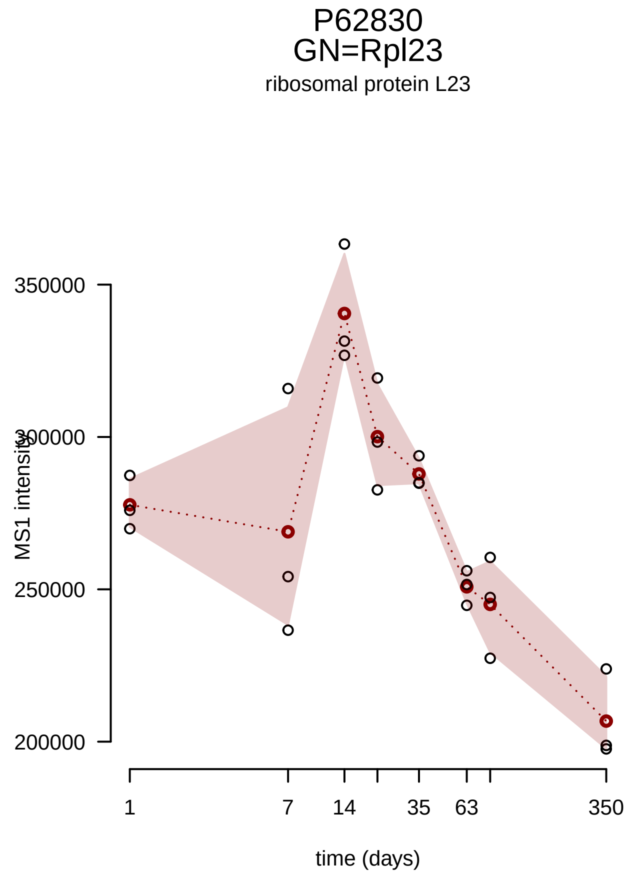
P62830
GN=Rpl23
ribosomal protein L23
MS1 intensity
350000
300000
250000
200000
1
7
14
35
63
350
time (days)

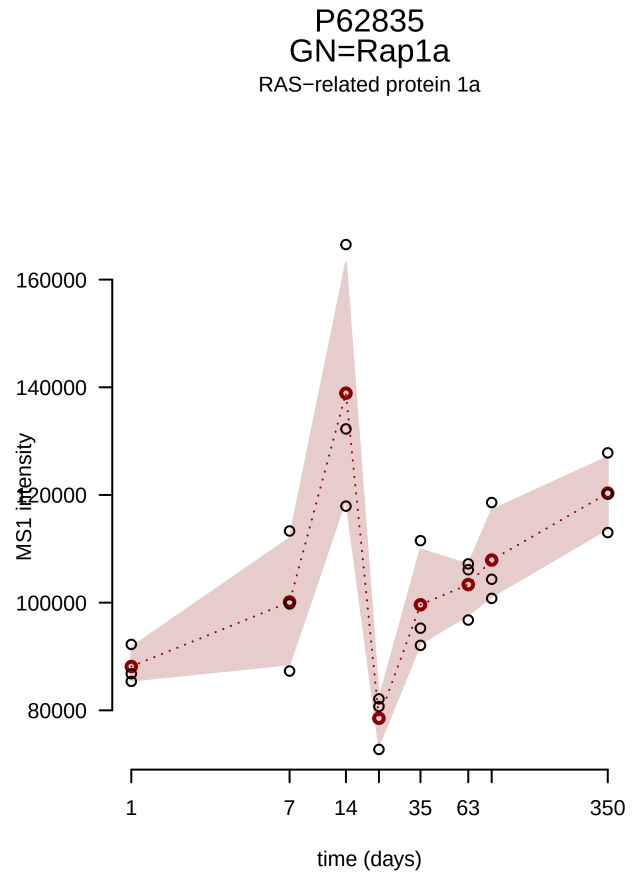
P62835
GN=Rap1a
RAS−related protein 1a
MS1 intensity
160000
140000
120000
100000
80000
1
7
14
35
63
350
time (days)

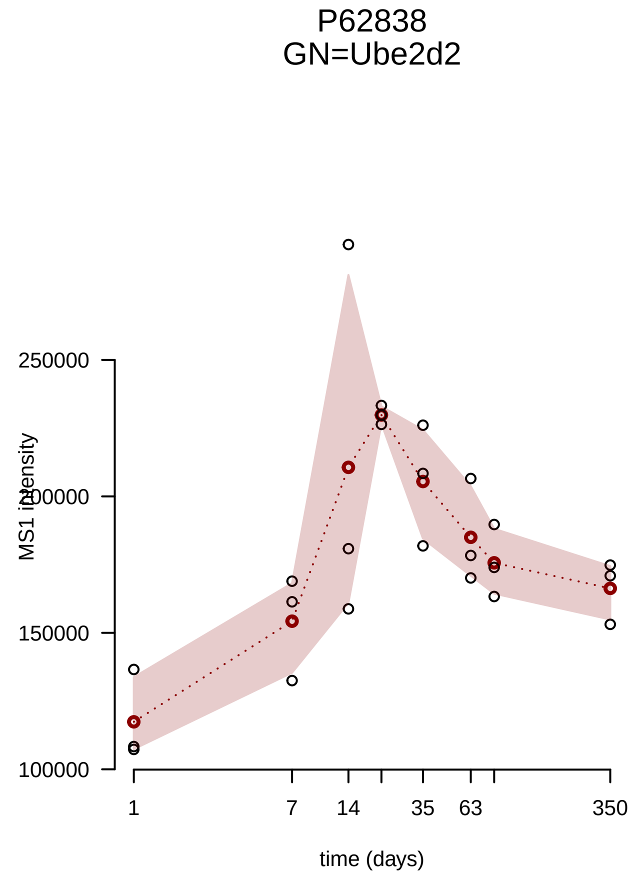
P62838
GN=Ube2d2
MS1 intensity
250000
200000
150000
100000
1
7
14
35
63
350
time (days)

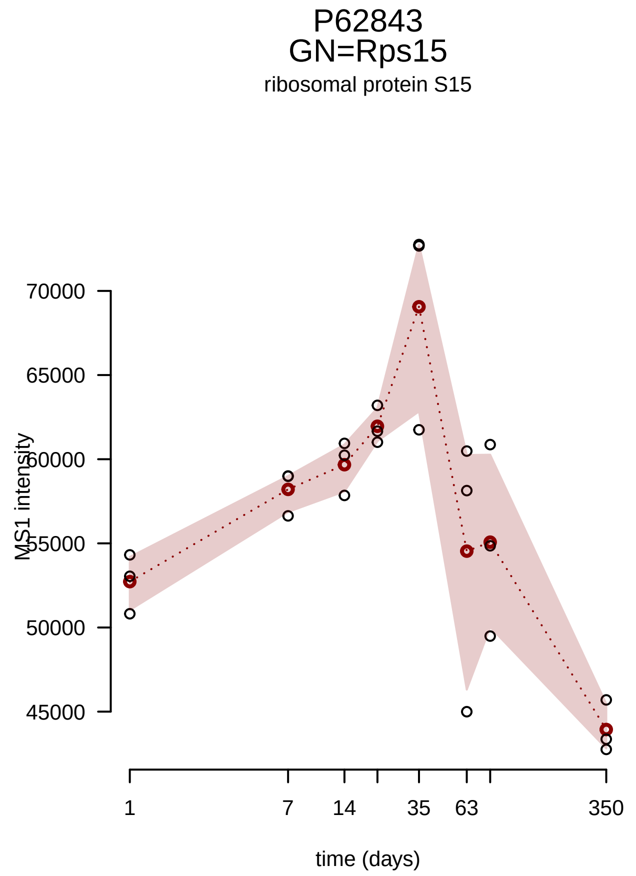
P62843
GN=Rps15
ribosomal protein S15
MS1 intensity
70000
65000
60000
55000
50000
45000
1
7
14
35
63
350
time (days)

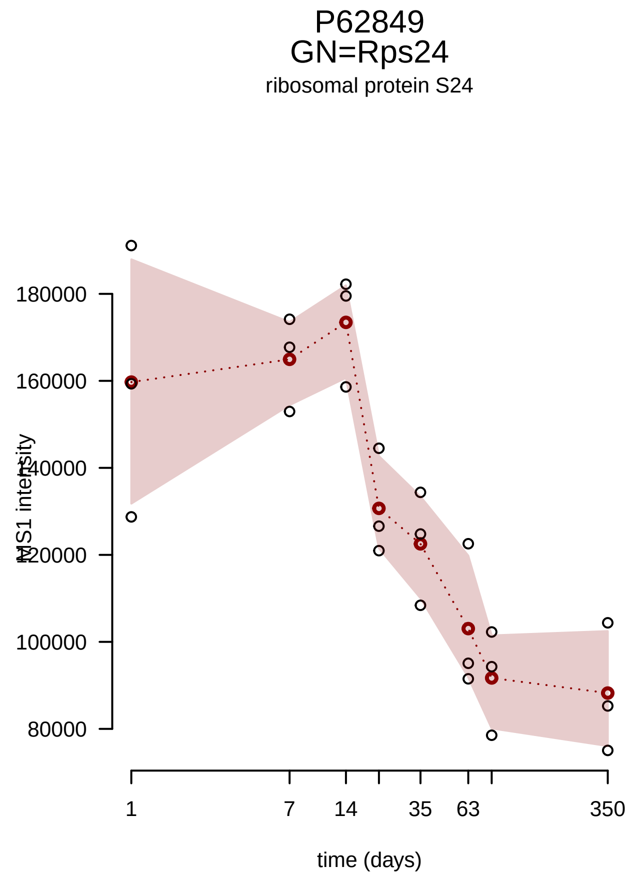
P62849
GN=Rps24
ribosomal protein S24
MS1 intensity
180000
160000
140000
120000
100000
80000
1
7
14
35
63
350
time (days)

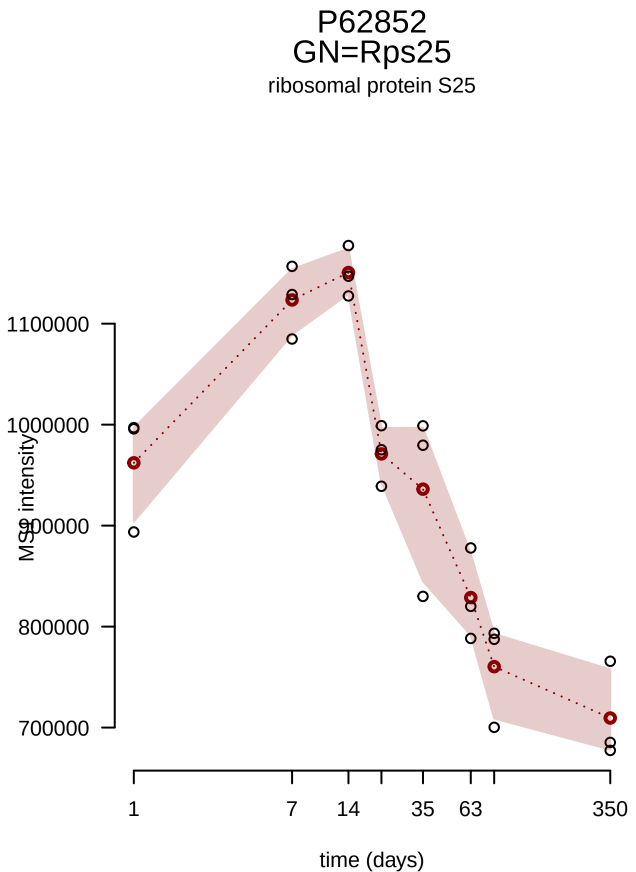
P62852
GN=Rps25
ribosomal protein S25
MS1 intensity
1100000
1000000
900000
800000
700000
1
7
14
35
63
350
time (days)

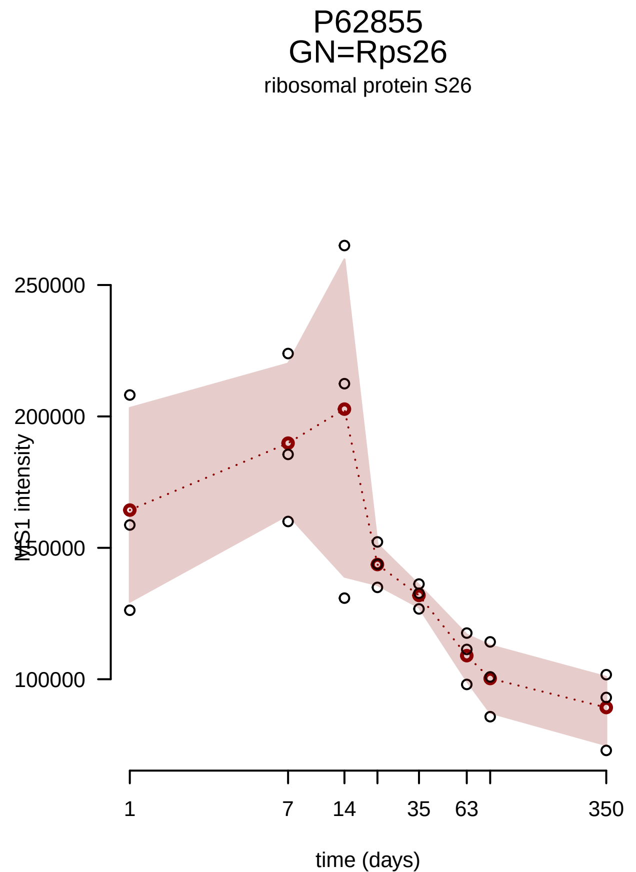
P62855
GN=Rps26
ribosomal protein S26
MS1 intensity
250000
200000
150000
100000
1
7
14
35
63
350
time (days)

P62858
GN=Rps28
ribosomal protein S28

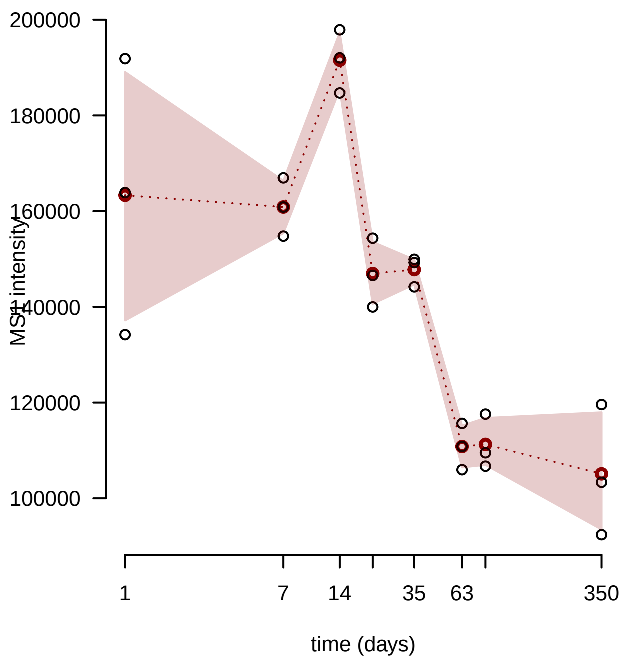

P62862
GN=Fau

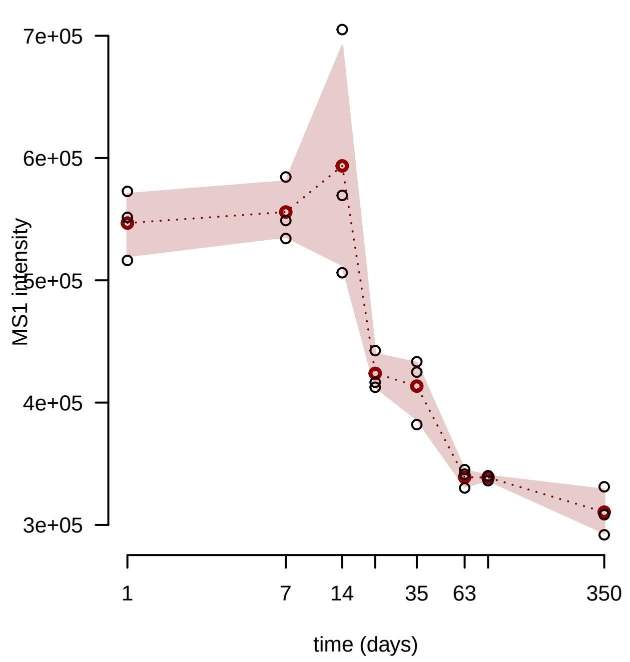

P62869
GN=Elob
elongin B

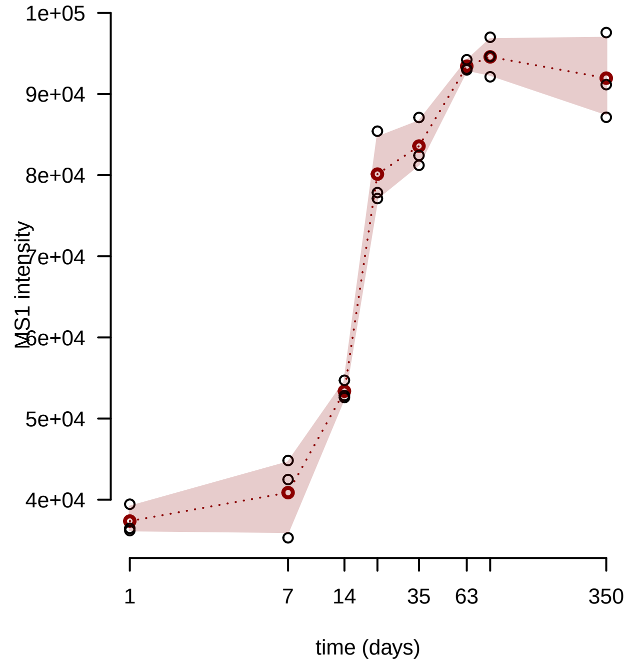

P62874
GN=Gnb1
guanine nucleotide binding protein (G pr ...

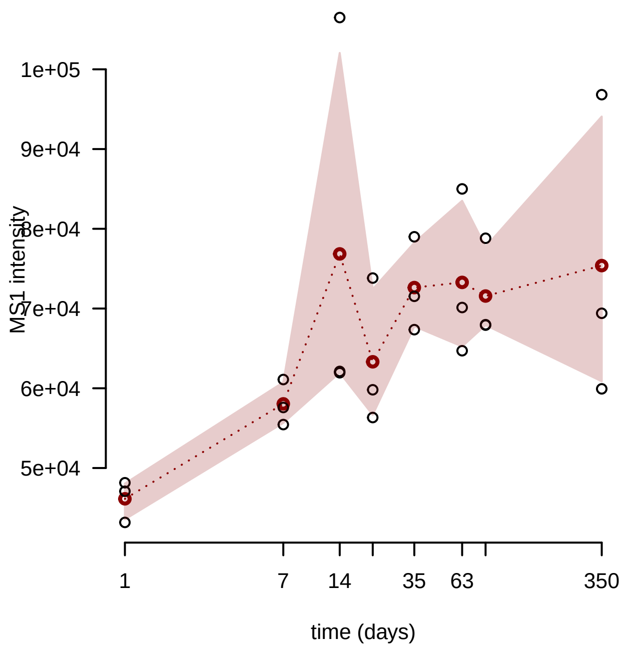

P62876
GN=Polr2l
polymerase (RNA) II (DNA directed) polyp ...

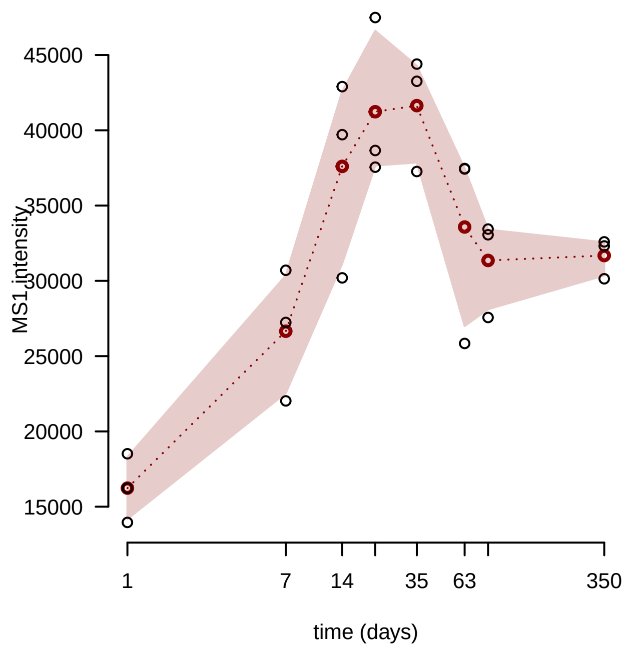

P62878
GN=Rbx1
ring−box 1

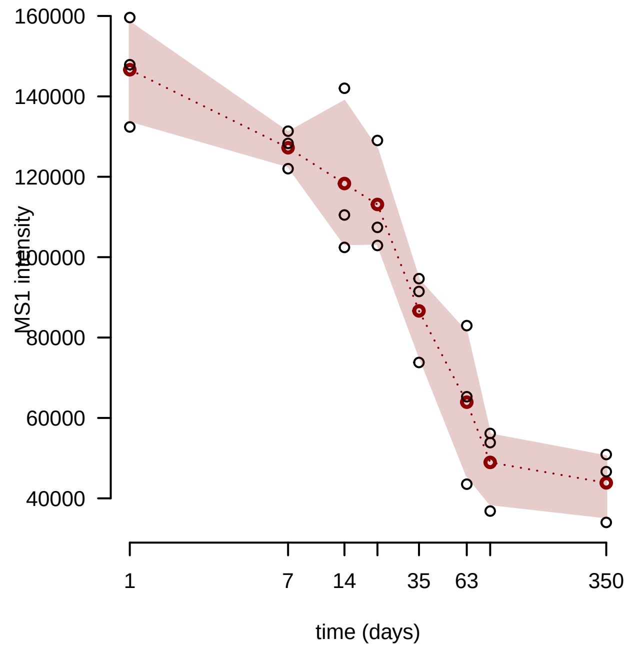

P62880
GN=Gnb2
guanine nucleotide binding protein (G pr ...

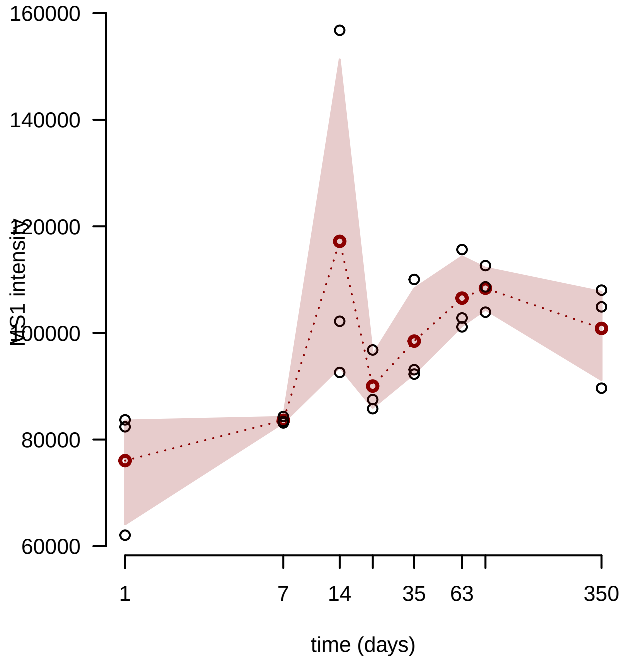

P62889
GN=Rpl30
ribosomal protein L30

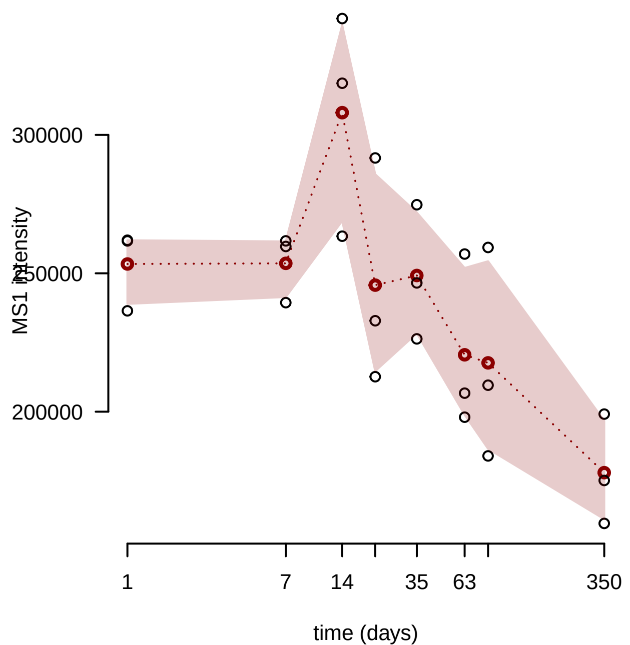

P62892
GN=Rpl39
ribosomal protein L39

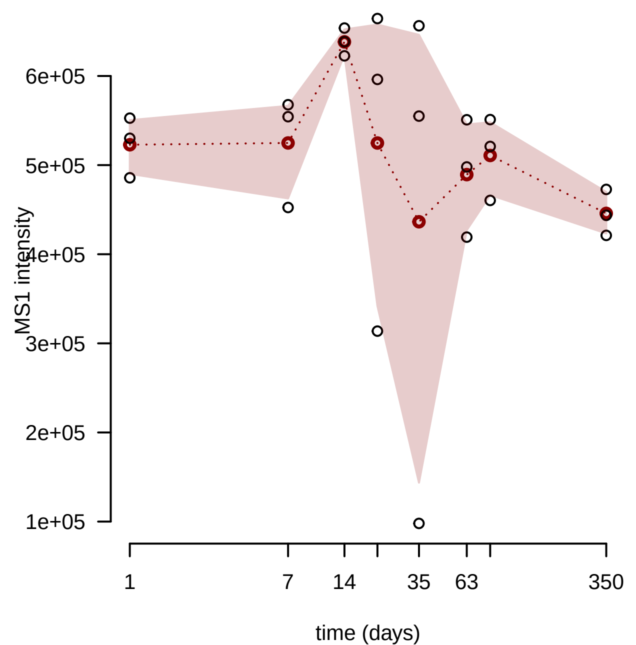

P62897
GN=Cycs
cytochrome c, somatic

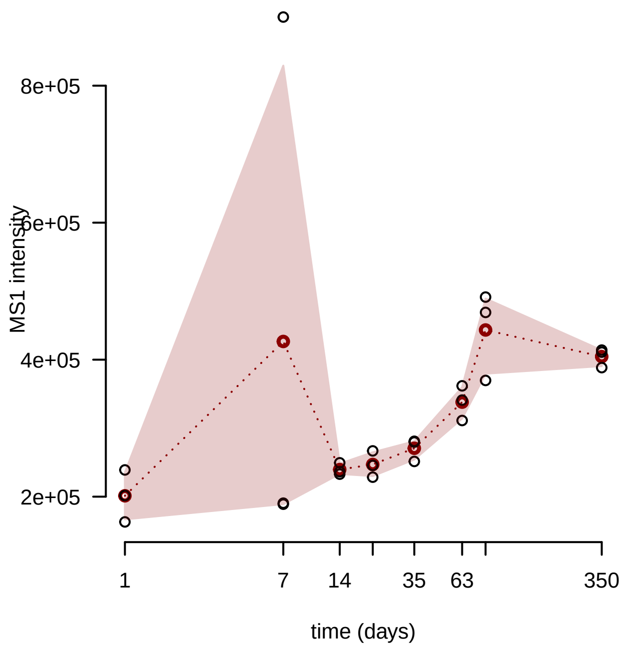

P62900
GN=Rpl31
ribosomal protein L31

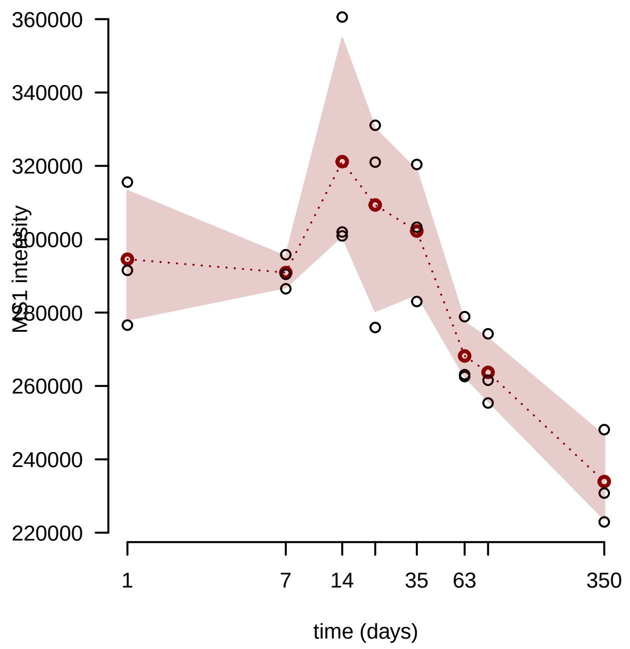

P62908
GN=Rps3
ribosomal protein S3

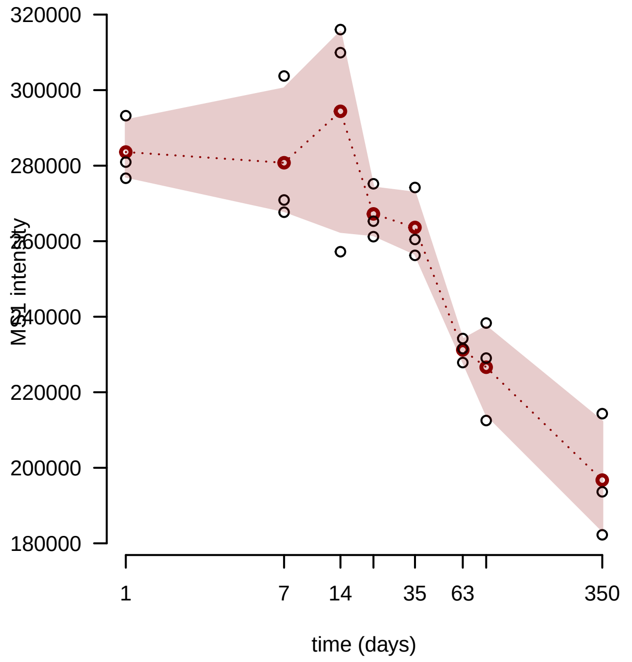

P62911
GN=Rpl32
ribosomal protein L32

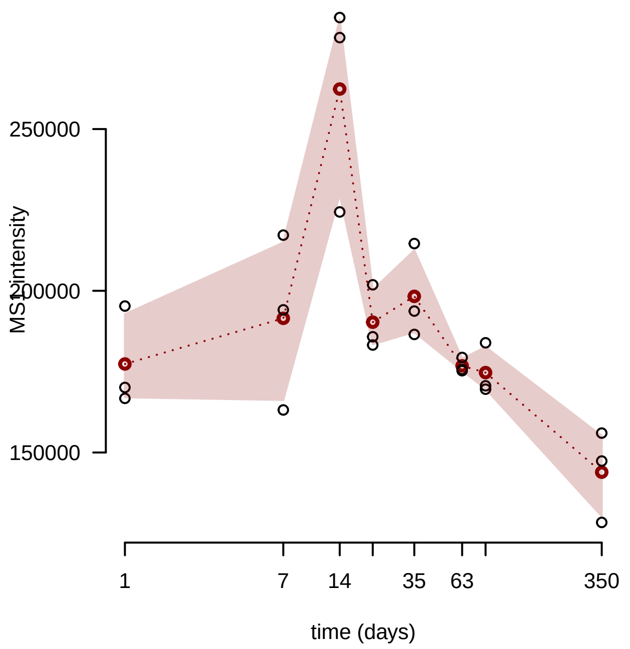

P62915
GN=Gtf2b
general transcription factor IIB

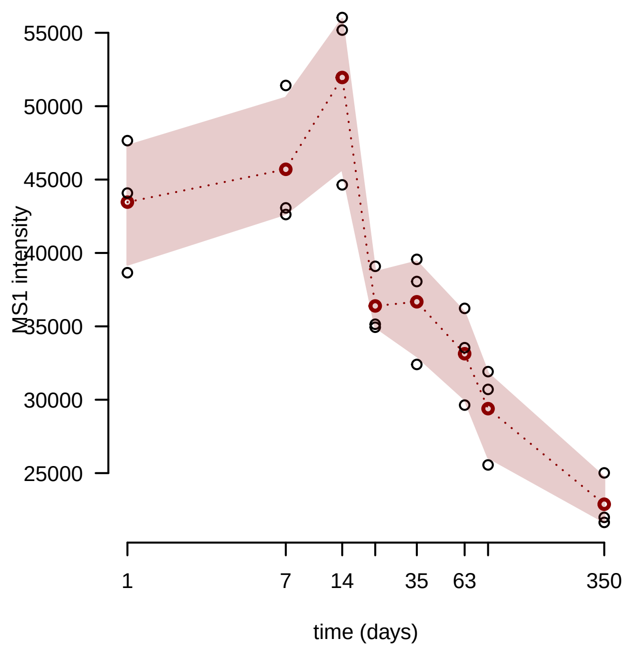

P62918
GN=Rpl8
ribosomal protein L8

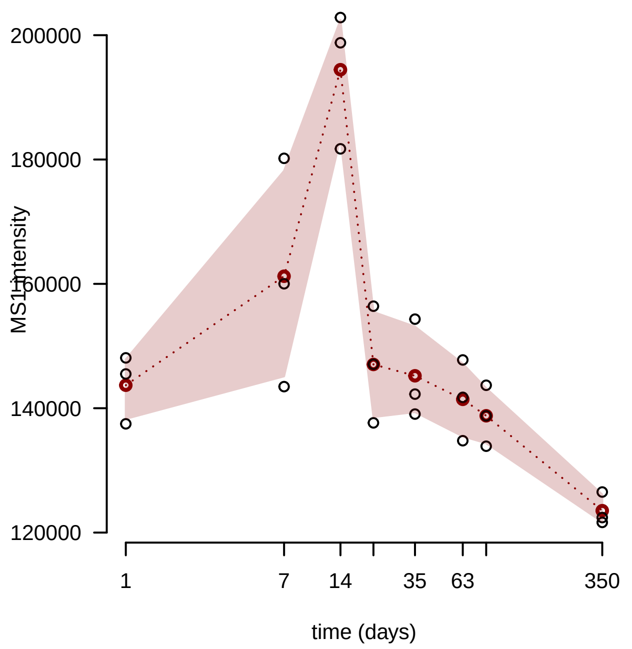

P62932
GN=Fbxo40
F−box protein 40

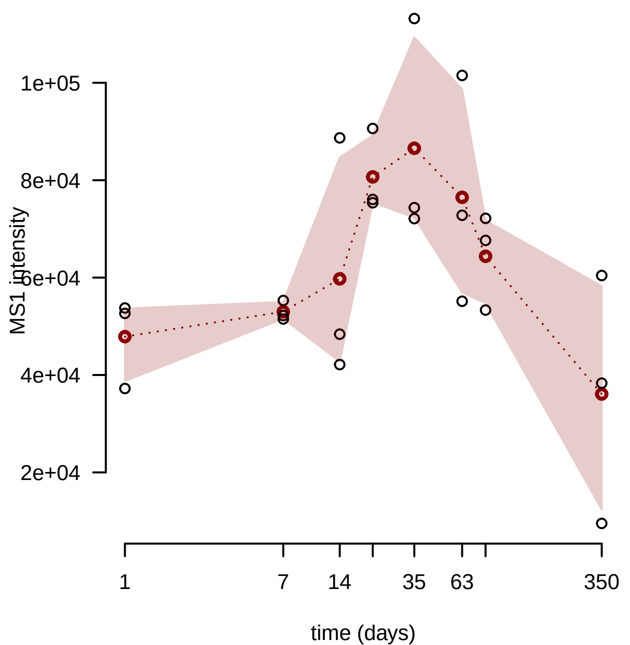

P62960
GN=Ybx1
Y box protein 1

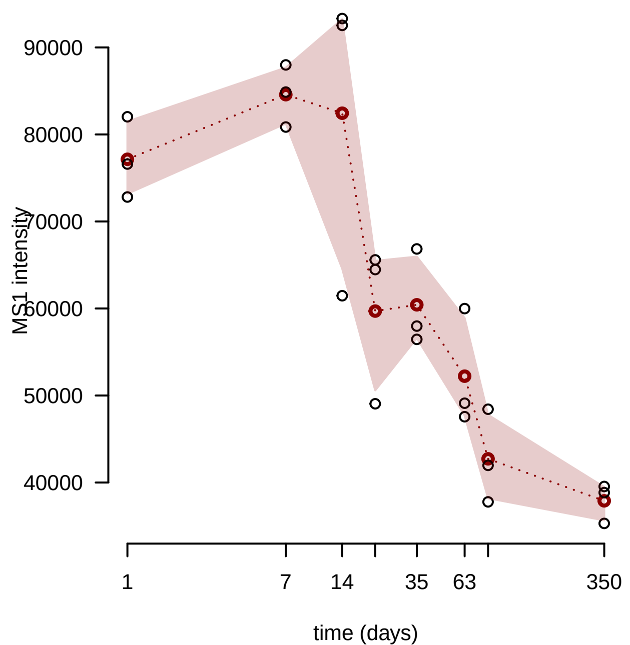

P62962
GN=Pfn1
profilin 1

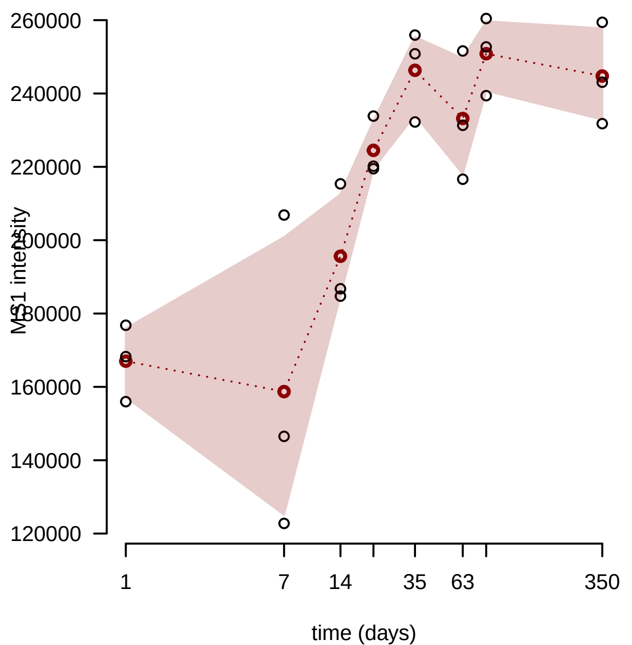

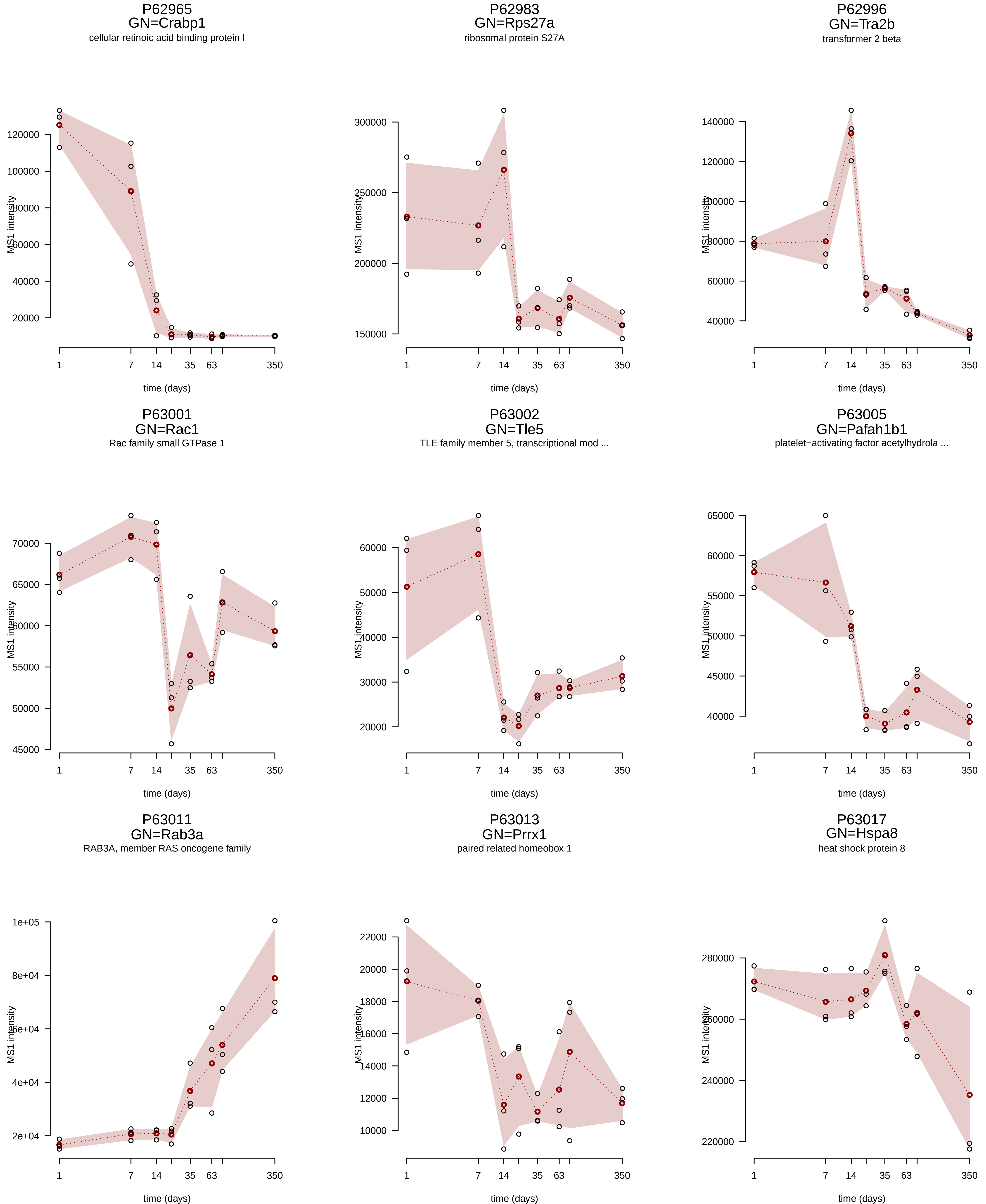
P62965
GN=Crabp1
cellular retinoic acid binding protein I
120000
100000
80000
60000
40000
20000
MS1 intensity
1
7
14
35
63
350
time (days)
P62983
GN=Rps27a
ribosomal protein S27A
300000
250000
200000
150000
MS1 intensity
1
7
14
35
63
350
time (days)
P62996
GN=Tra2b
transformer 2 beta
140000
120000
100000
80000
60000
40000
MS1 intensity
1
7
14
35
63
350
time (days)
P63001
GN=Rac1
Rac family small GTPase 1
70000
65000
60000
55000
50000
45000
MS1 intensity
1
7
14
35
63
350
time (days)
P63002
GN=Tle5
TLE family member 5, transcriptional mod ...
60000
50000
40000
30000
20000
MS1 intensity
1
7
14
35
63
350
time (days)
P63005
GN=Pafah1b1
platelet−activating factor acetylhydrola ...
65000
60000
55000
50000
45000
40000
MS1 intensity
1
7
14
35
63
350
time (days)
P63011
GN=Rab3a
RAB3A, member RAS oncogene family
1e+05
8e+04
6e+04
4e+04
2e+04
MS1 intensity
1
7
14
35
63
350
time (days)
P63013
GN=Prrx1
paired related homeobox 1
22000
20000
18000
16000
14000
12000
10000
MS1 intensity
1
7
14
35
63
350
time (days)
P63017
GN=Hspa8
heat shock protein 8
280000
260000
240000
220000
MS1 intensity
1
7
14
35
63
350
time (days)

P63024
GN=Vamp3
vesicle−associated membrane protein 3

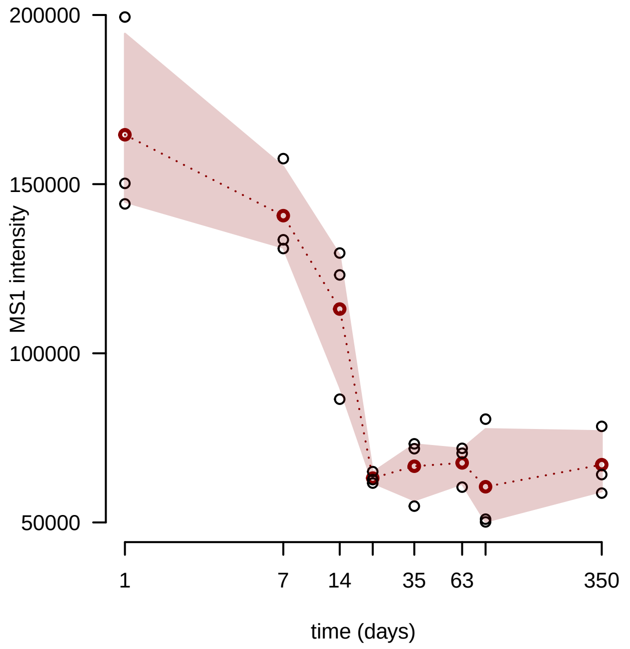

P63028
GN=Tpt1
tumor protein, translationally−controlle ...

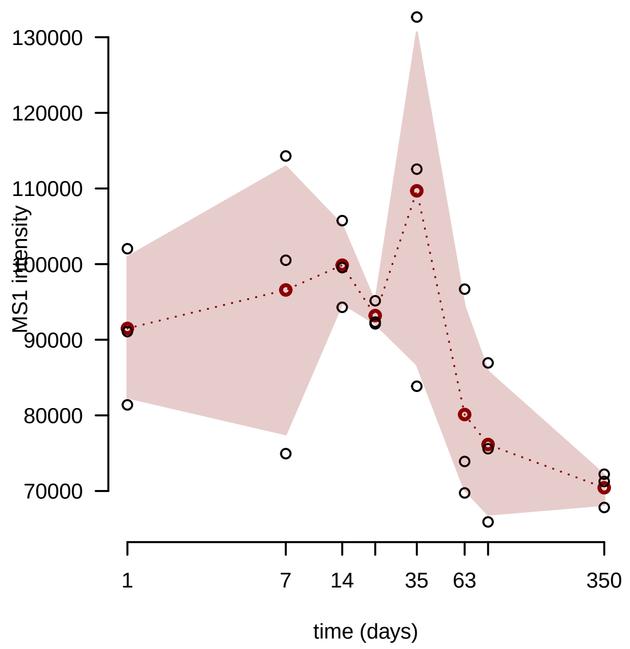

P63030
GN=Mpc1
mitochondrial pyruvate carrier 1

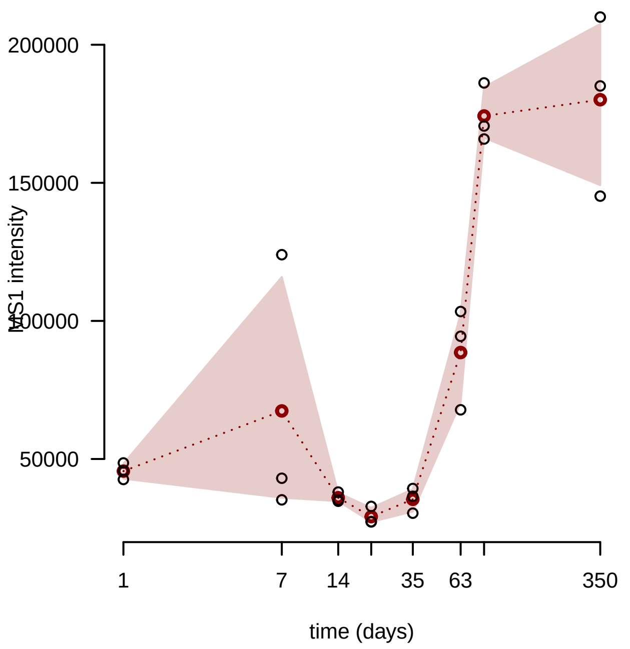

P63037
GN=Dnaja1
DnaJ heat shock protein family (Hsp40) m ...

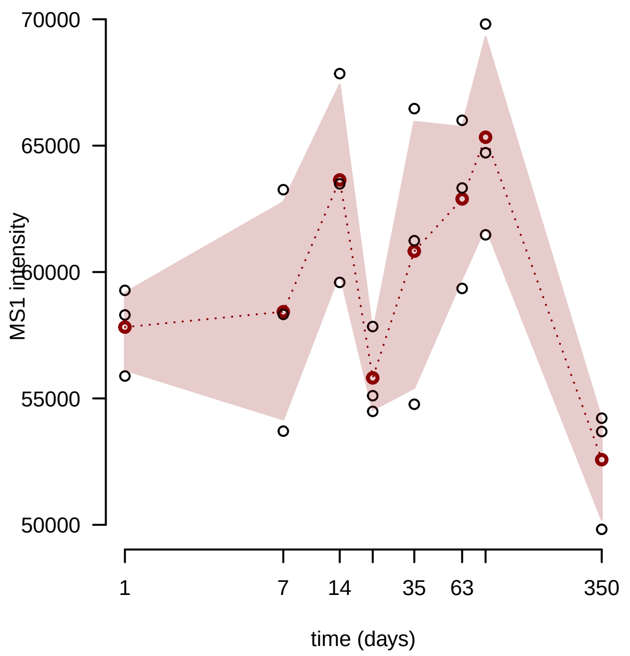

P63038
GN=Hspd1
heat shock protein 1 (chaperonin)

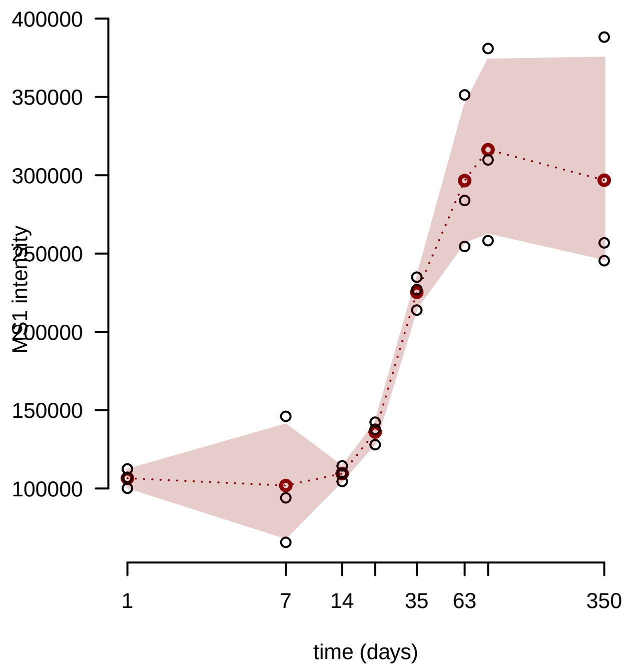

P63040
GN=Cplx1
complexin 1

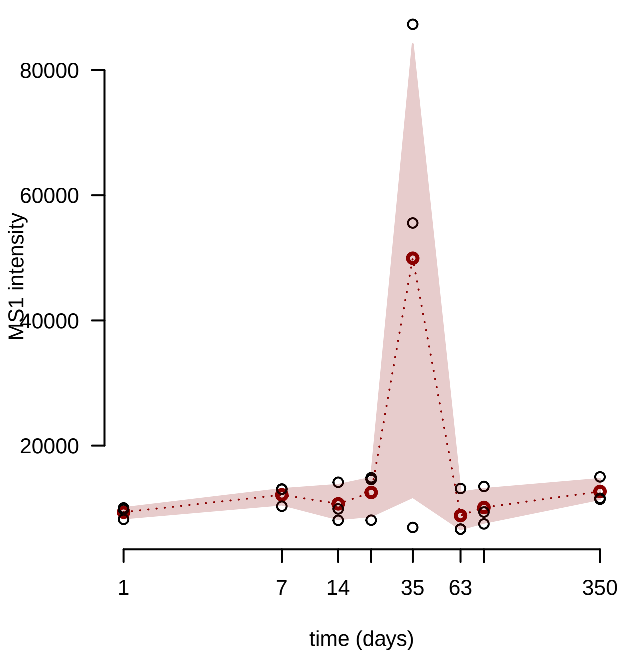

P63044
GN=Vamp2
vesicle−associated membrane protein 2

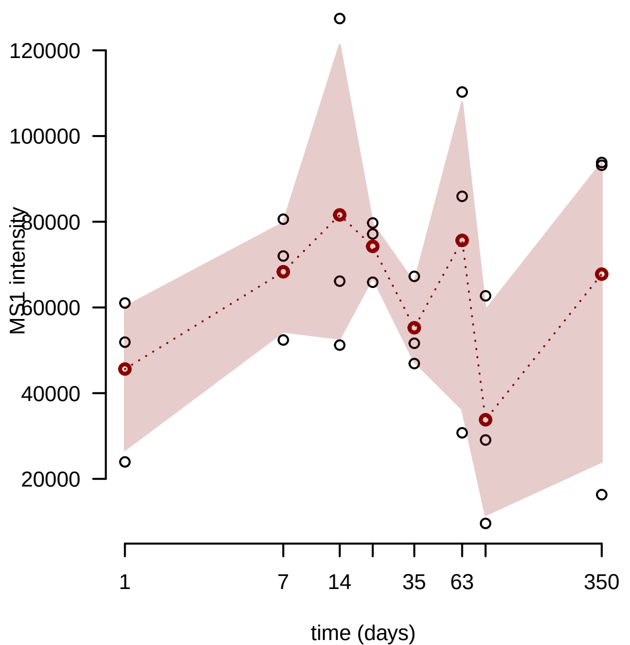

P63073
GN=Eif4e
eukaryotic translation initiation factor ...

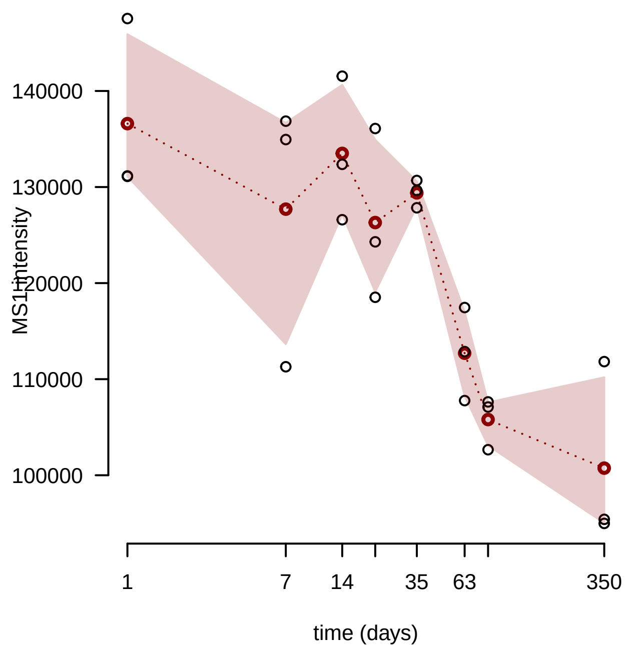

P63082
GN=Atp6v0c
ATPase, H+ transporting, lysosomal V0 su ...

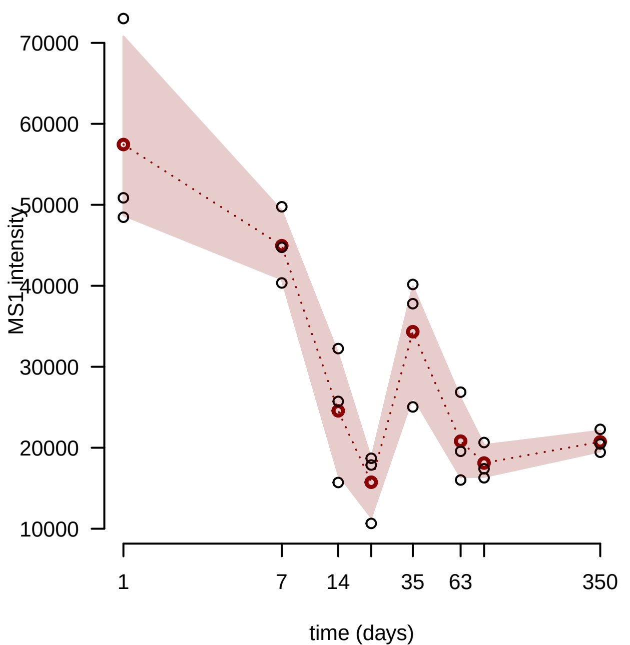

P63085
GN=Mapk1
mitogen−activated protein kinase 1

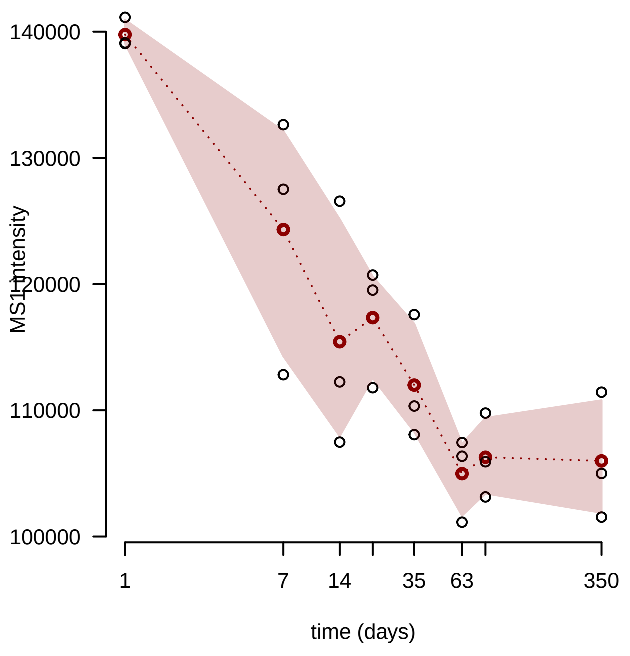

P63087
GN=Ppp1cc
protein phosphatase 1 catalytic subunit ...

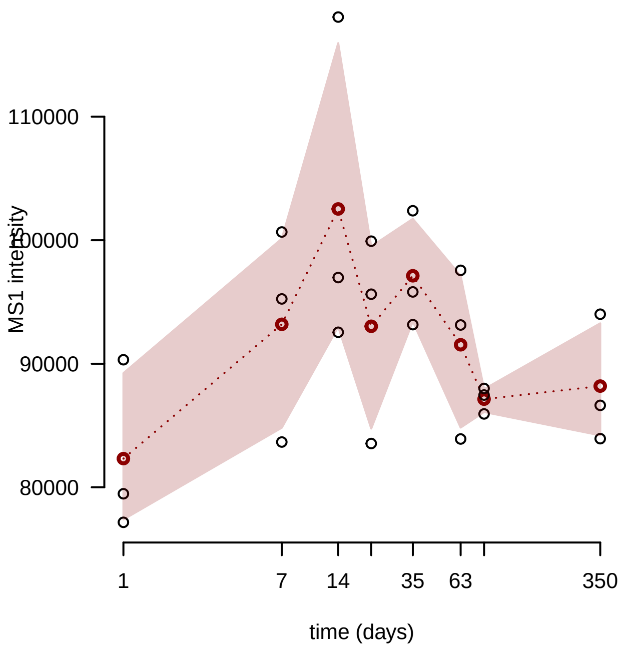

P63089
GN=Ptn
pleiotrophin

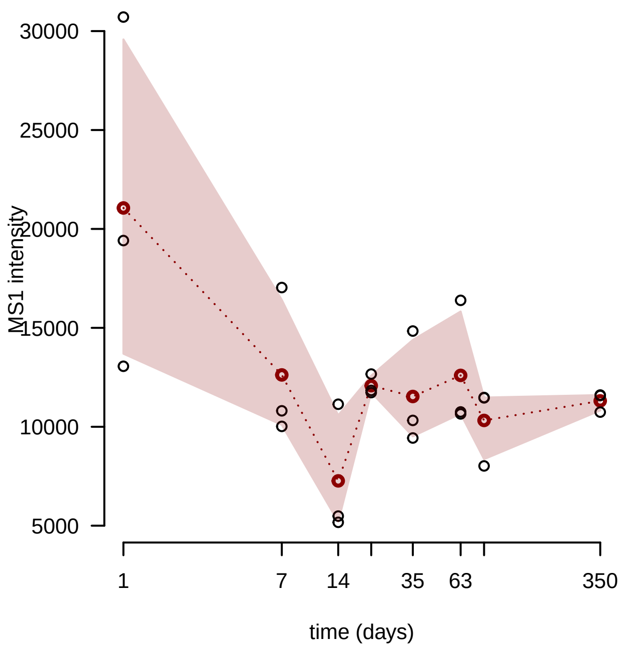

P63094
GN=Gnas
GNAS (guanine nucleotide binding protein ...

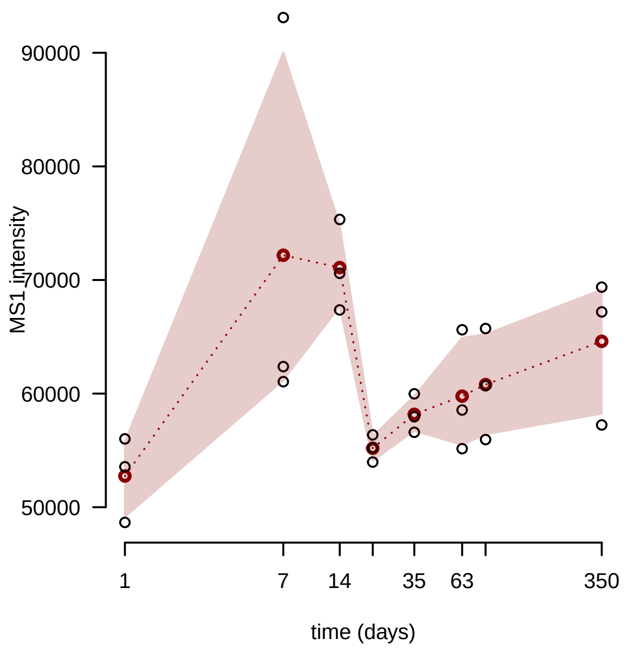

P63101
GN=Ywhaz
tyrosine 3−monooxygenase/tryptophan 5−mo ...

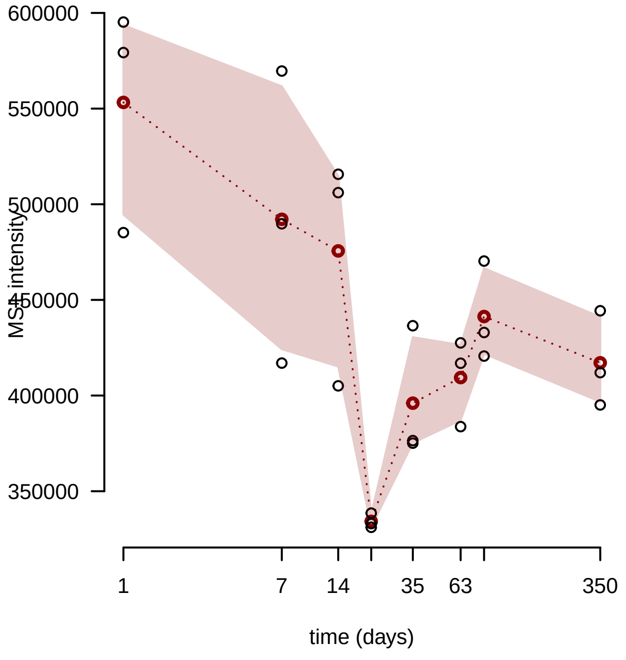

P63139
GN=Nfyb
nuclear transcription factor−Y beta

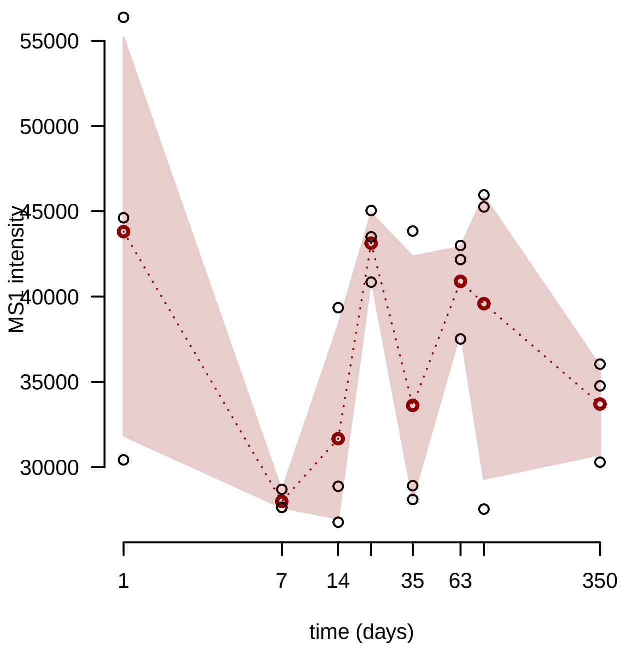

P63147
GN=Ube2b
ubiquitin−conjugating enzyme E2B

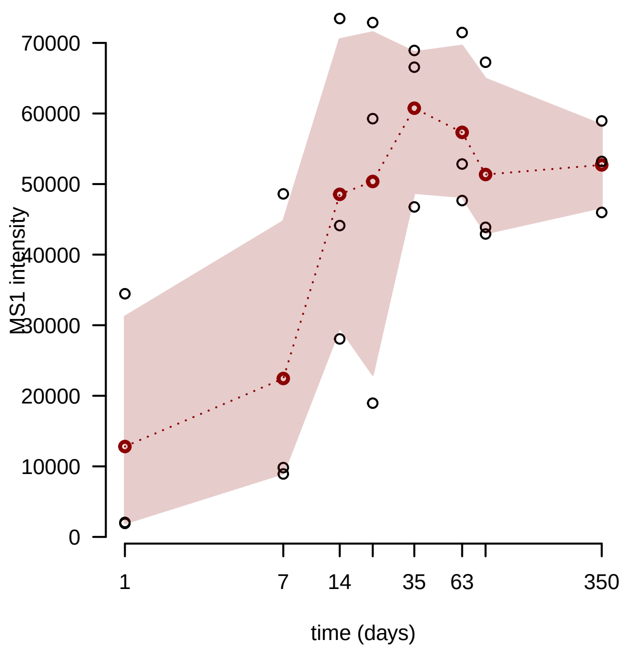

P63154
GN=Crnkl1
crooked neck pre−mRNA splicing factor 1

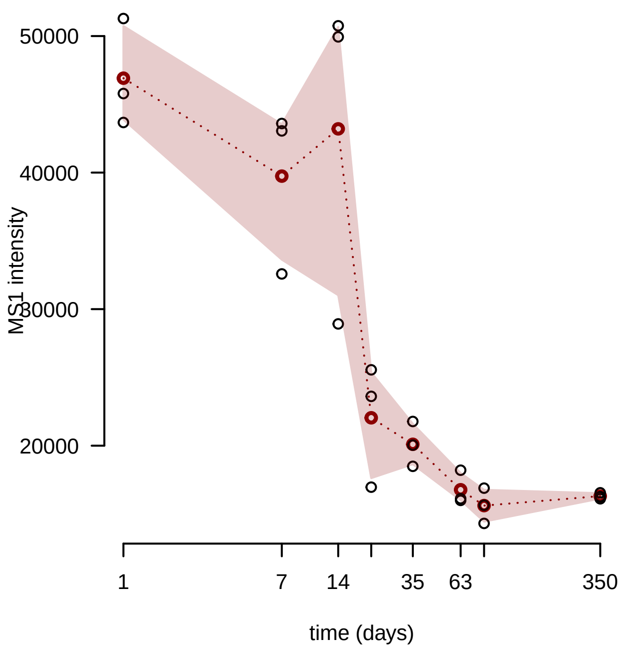

P63158
GN=Hmgb1
high mobility group box 1

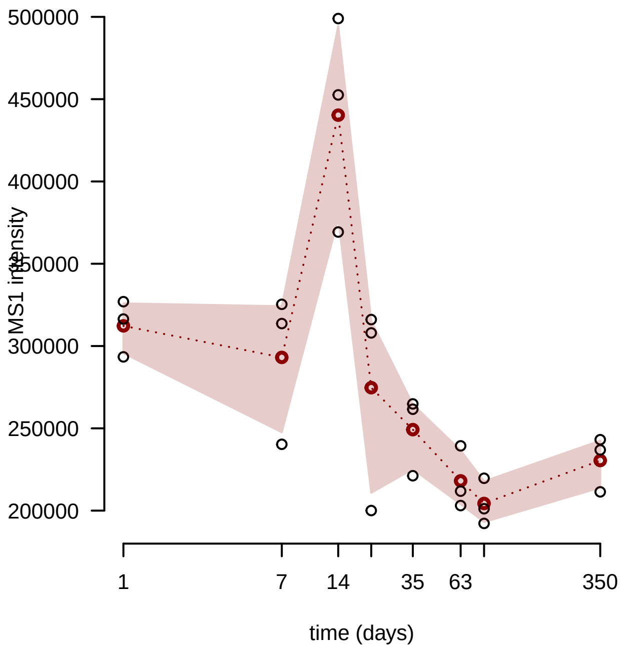

P63166
GN=Sumo1
small ubiquitin−like modifier 1

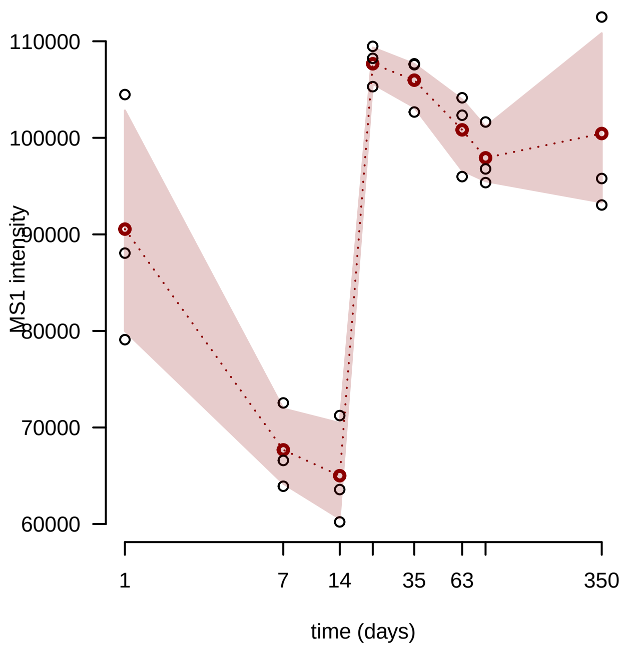

P63168
GN=Dynll1
dynein light chain LC8−type 1

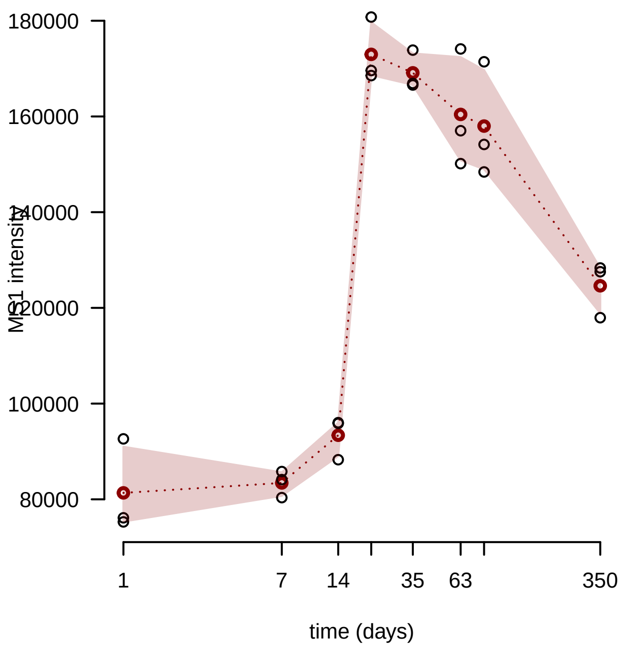

P63213
GN=Gng2
guanine nucleotide binding protein (G pr ...

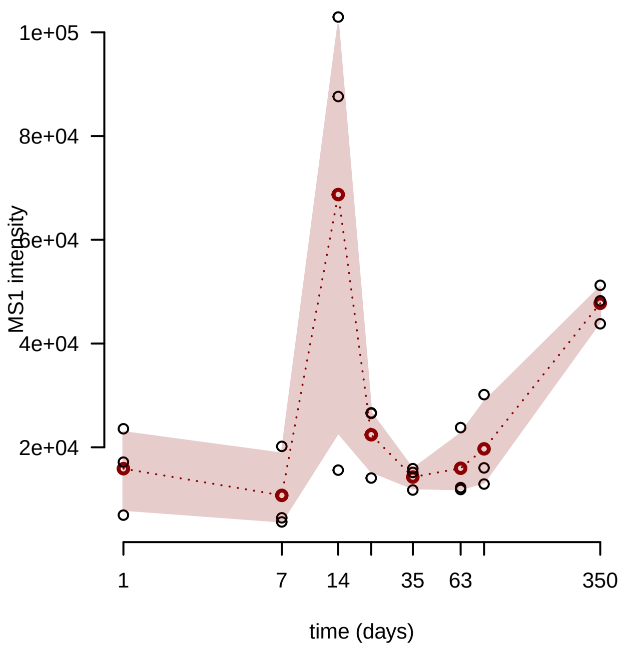

P63242
GN=Eif5a
eukaryotic translation initiation factor ...

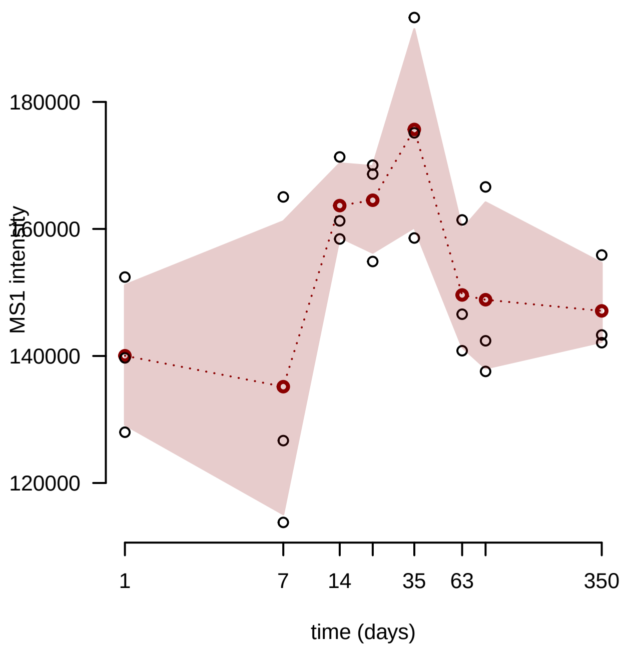

P63254
GN=Crip1
cysteine−rich protein 1 (intestinal)

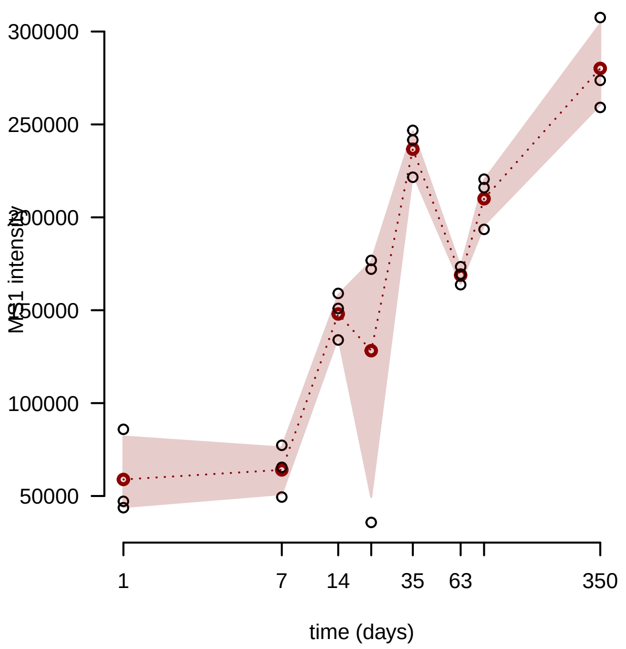

P63260
GN=Actg1
actin, gamma, cytoplasmic 1

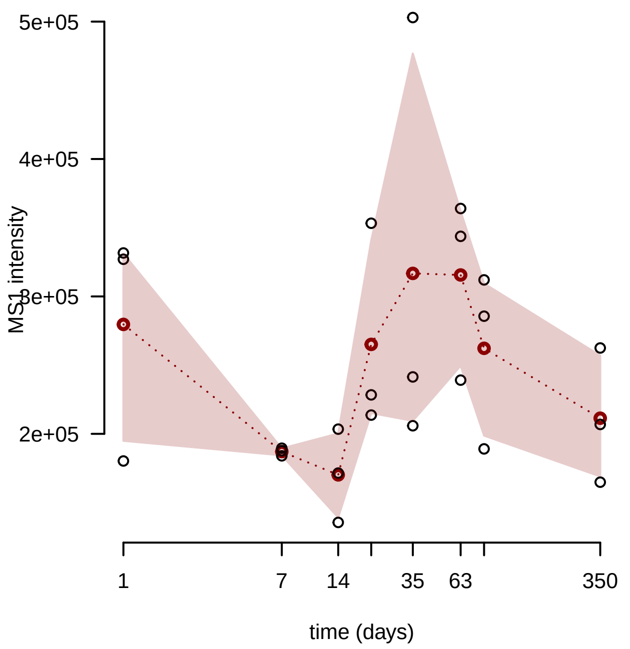

P63268
GN=Actg2
actin, gamma 2, smooth muscle, enteric

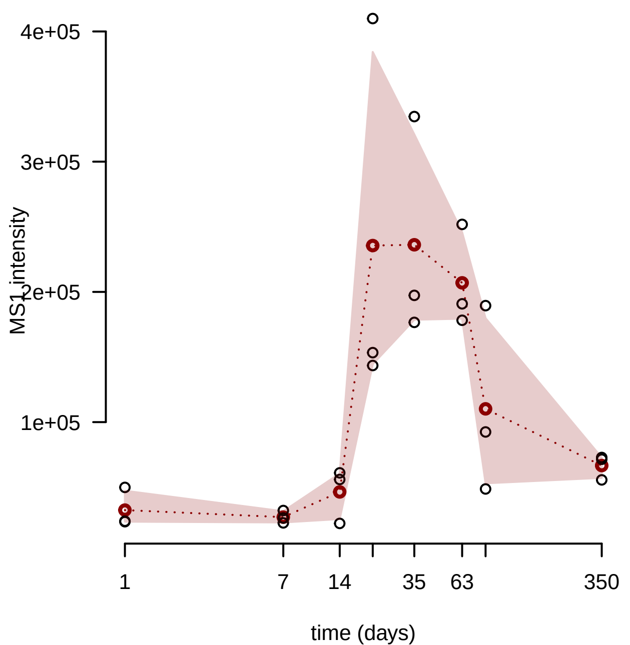

P63271
GN=Supt4h1a

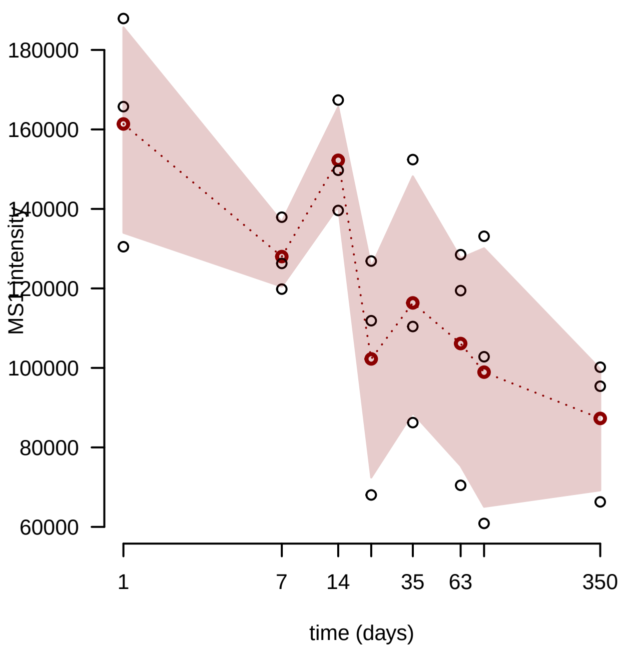

P63276
GN=Rps17
ribosomal protein S17

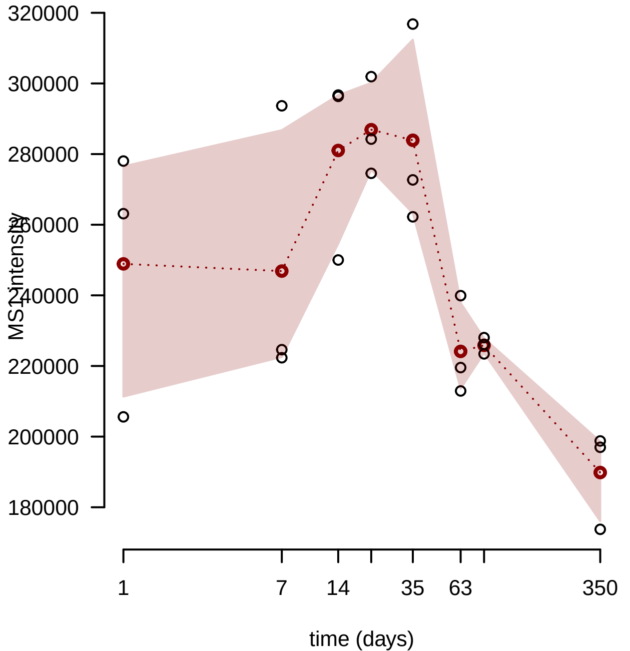

P63280
GN=Ube2i
ubiquitin−conjugating enzyme E2I

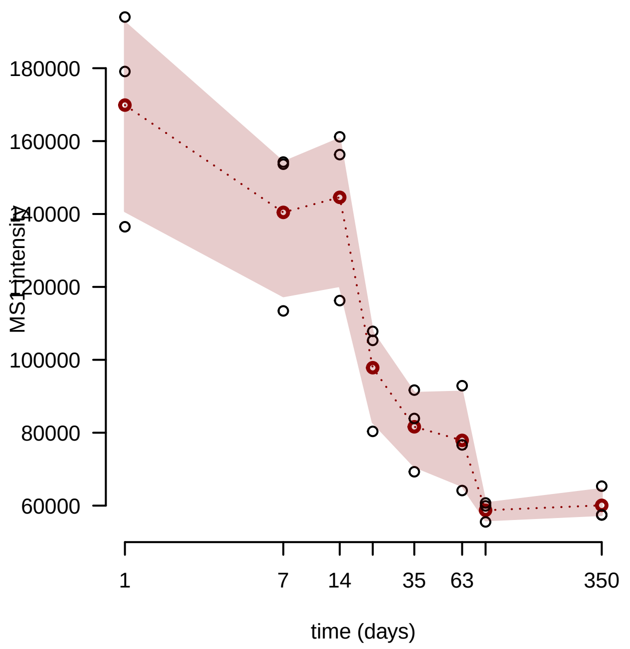

P63300
GN=Selenow
selenoprotein W

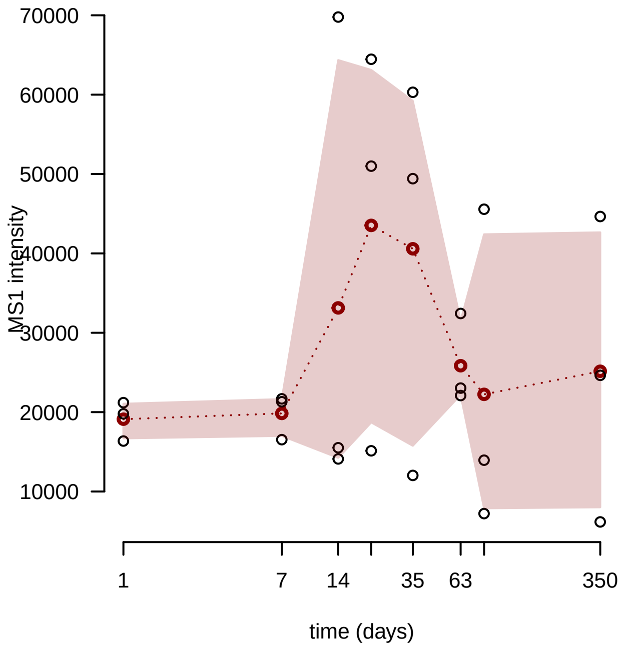

P63321
GN=Rala
v−ral simian leukemia viral oncogene A (...

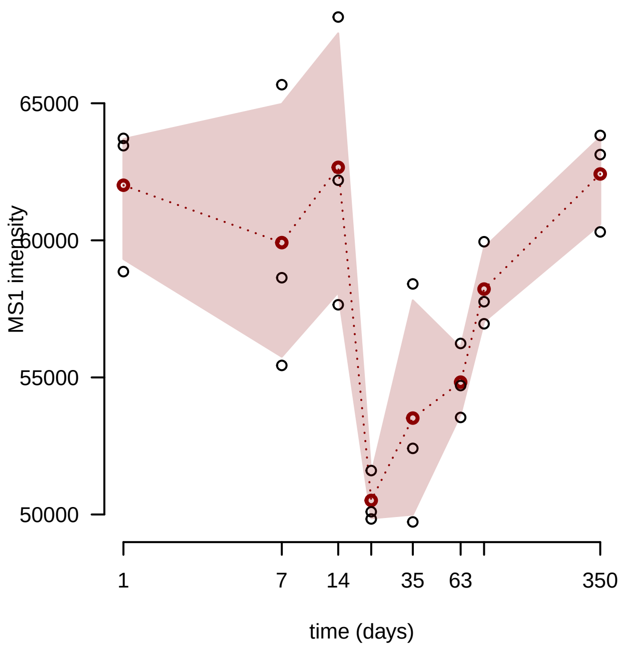

P63323
GN=Rps12
ribosomal protein S12

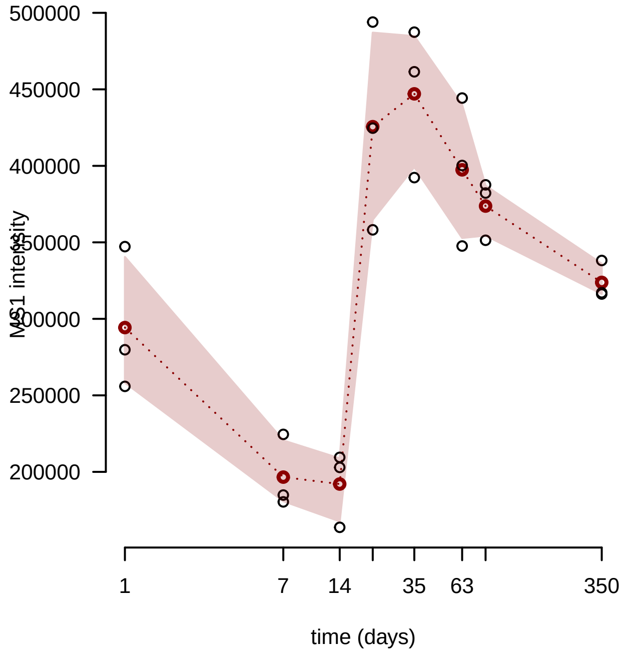

P63325
GN=Rps10
ribosomal protein S10

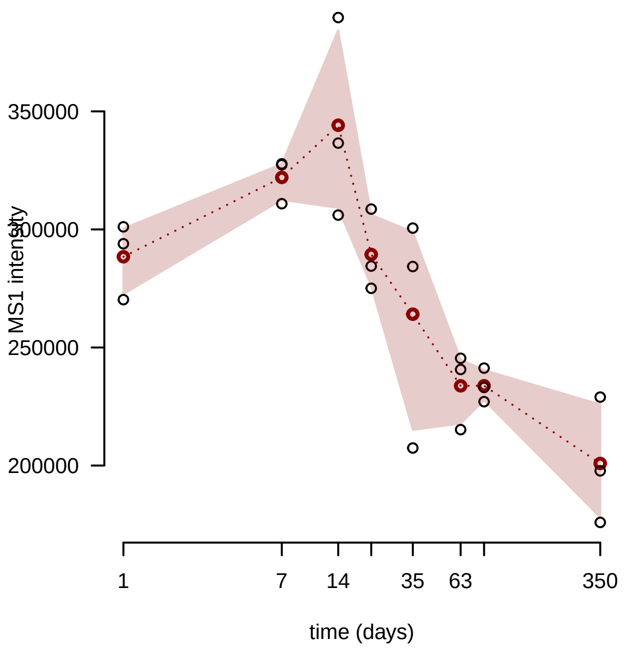

P63328
GN=Ppp3ca
protein phosphatase 3, catalytic subunit ...

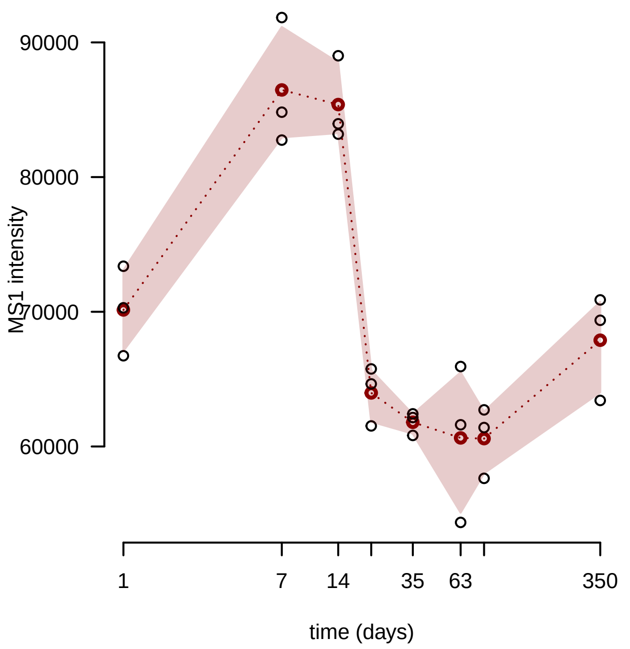

P63330
GN=Ppp2ca
protein phosphatase 2 (formerly 2A), cat ...

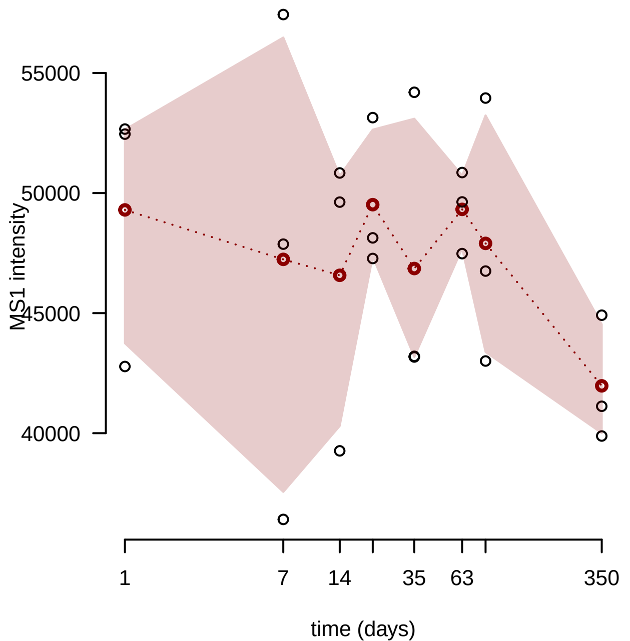

P67778
GN=Phb
prohibitin

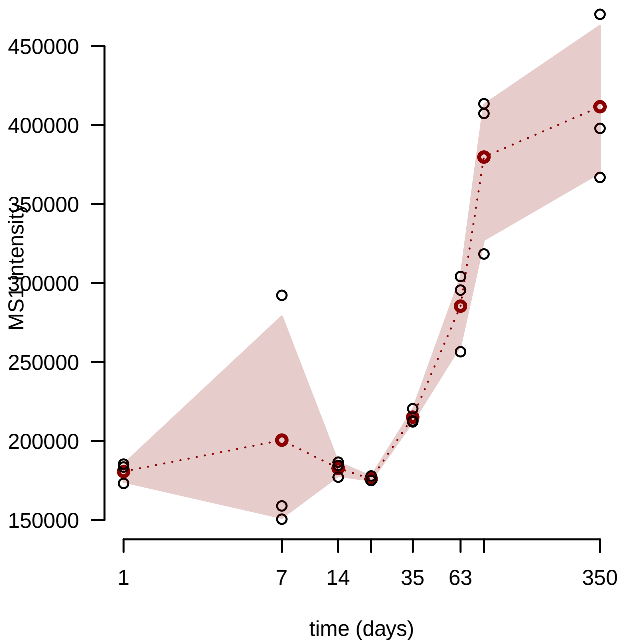

P67871
GN=Csnk2b
casein kinase 2, beta polypeptide

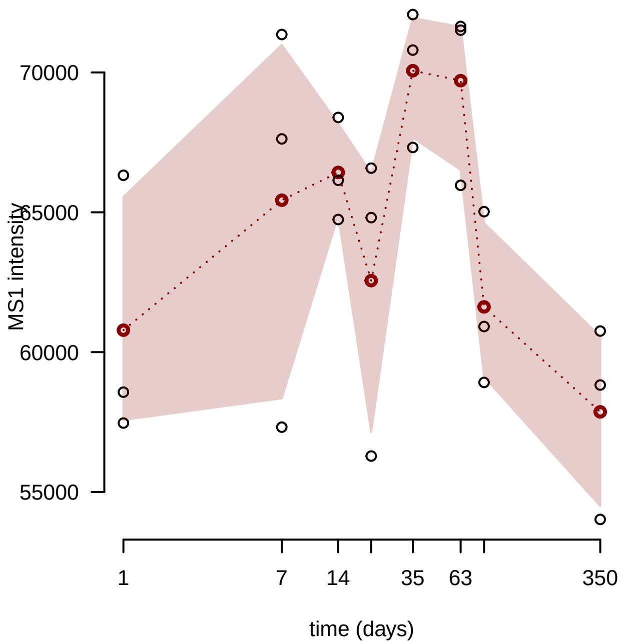

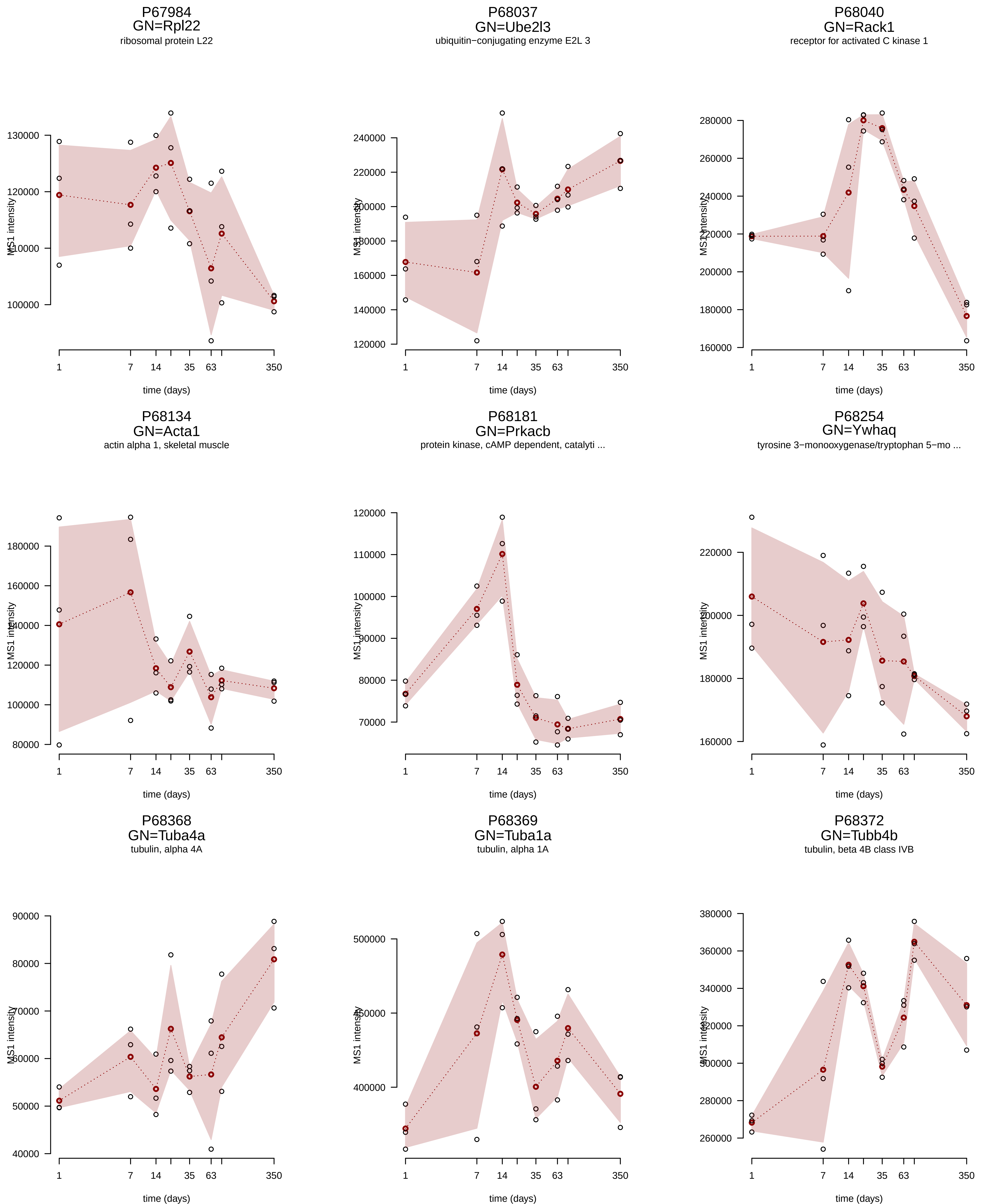
P67984
GN=Rpl22
ribosomal protein L22
MS1 intensity
100000
110000
120000
130000
time (days)
1
7
14
35
63
350
P68037
GN=Ube2l3
ubiquitin−conjugating enzyme E2L 3
MS1 intensity
120000
140000
160000
180000
200000
220000
240000
time (days)
1
7
14
35
63
350
P68040
GN=Rack1
receptor for activated C kinase 1
MS1 intensity
160000
180000
200000
220000
240000
260000
280000
time (days)
1
7
14
35
63
350
P68134
GN=Acta1
actin alpha 1, skeletal muscle
MS1 intensity
80000
100000
120000
140000
160000
180000
time (days)
1
7
14
35
63
350
P68181
GN=Prkacb
protein kinase, cAMP dependent, catalyti ...
MS1 intensity
70000
80000
90000
100000
110000
120000
time (days)
1
7
14
35
63
350
P68254
GN=Ywhaq
tyrosine 3−monooxygenase/tryptophan 5−mo ...
MS1 intensity
160000
180000
200000
220000
time (days)
1
7
14
35
63
350
P68368
GN=Tuba4a
tubulin, alpha 4A
MS1 intensity
40000
50000
60000
70000
80000
90000
time (days)
1
7
14
35
63
350
P68369
GN=Tuba1a
tubulin, alpha 1A
MS1 intensity
400000
450000
500000
time (days)
1
7
14
35
63
350
P68372
GN=Tubb4b
tubulin, beta 4B class IVB
MS1 intensity
260000
280000
300000
320000
340000
360000
380000
time (days)
1
7
14
35
63
350

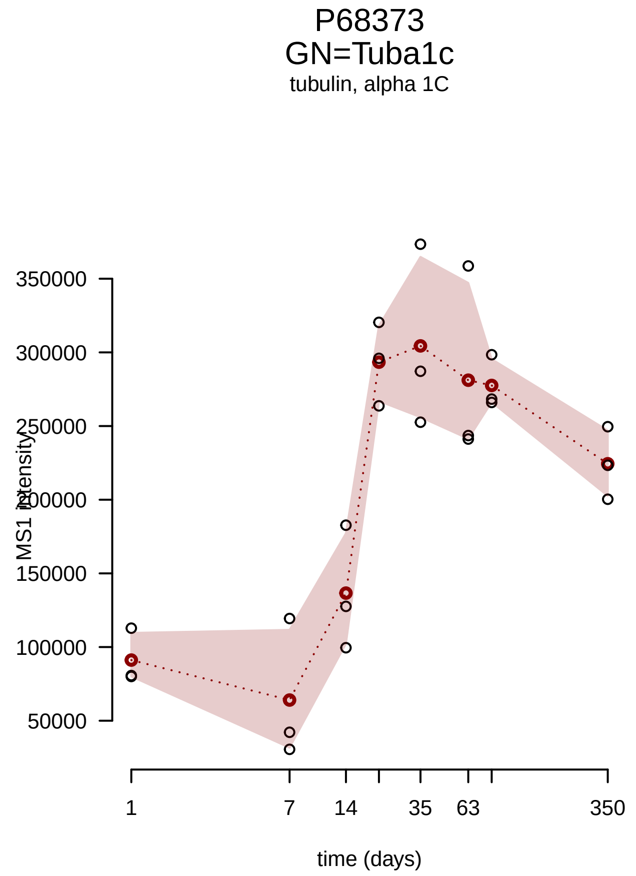
P68373
GN=Tuba1c
tubulin, alpha 1C
MS1 intensity
50000
100000
150000
200000
250000
300000
350000
1
7
14
35
63
350
time (days)

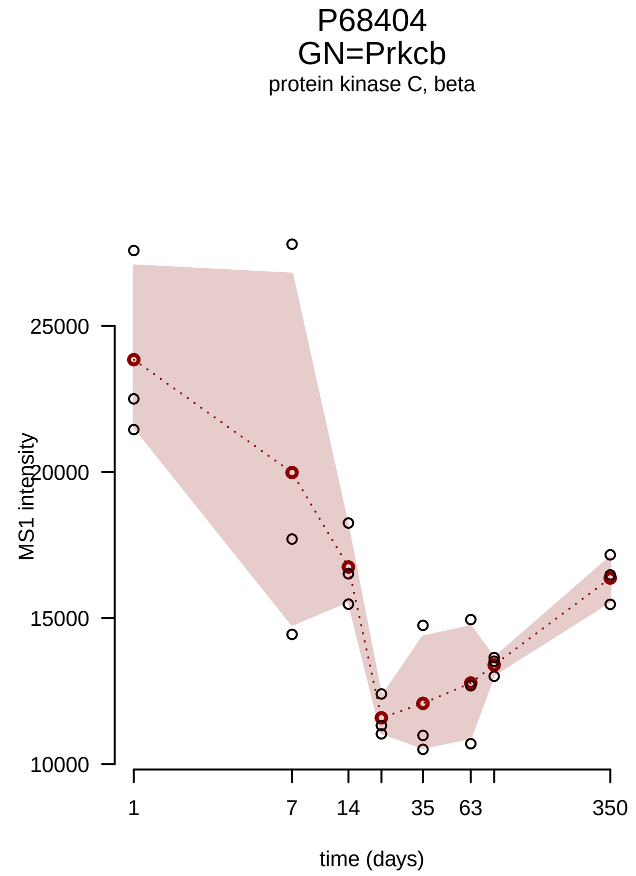
P68404
GN=Prkcb
protein kinase C, beta
MS1 intensity
10000
15000
20000
25000
1
7
14
35
63
350
time (days)

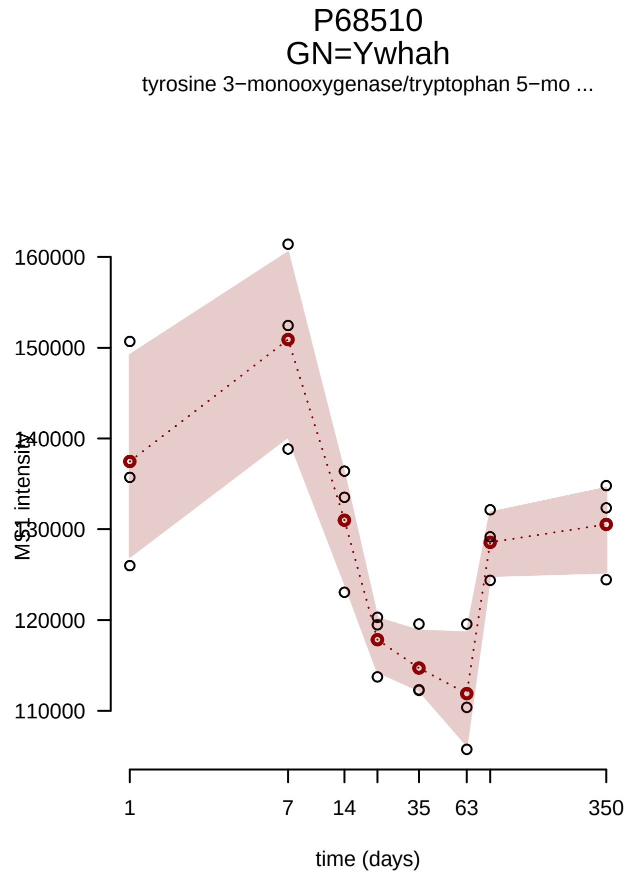
P68510
GN=Ywhah
tyrosine 3−monooxygenase/tryptophan 5−mo ...
MS1 intensity
110000
120000
130000
140000
150000
160000
1
7
14
35
63
350
time (days)

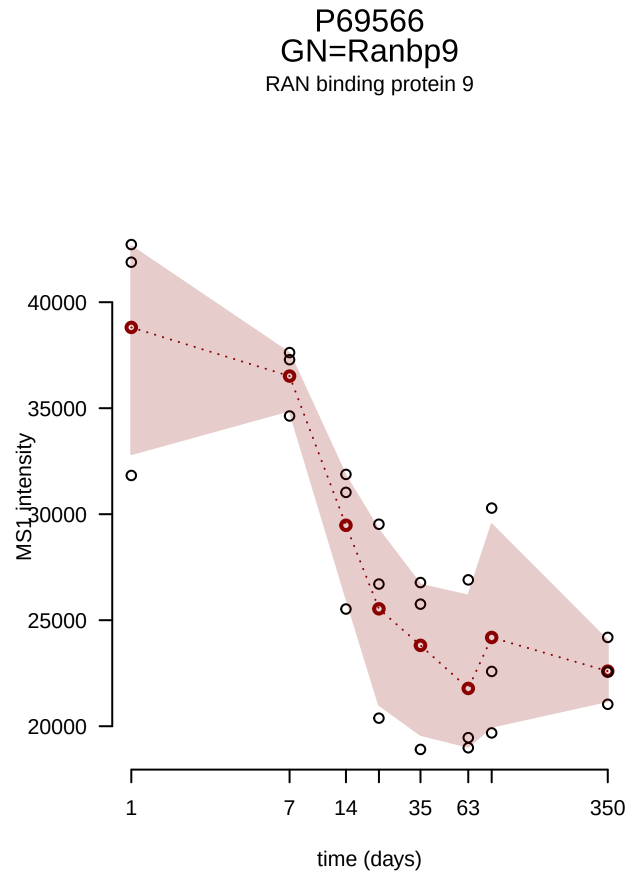
P69566
GN=Ranbp9
RAN binding protein 9
MS1 intensity
20000
25000
30000
35000
40000
1
7
14
35
63
350
time (days)

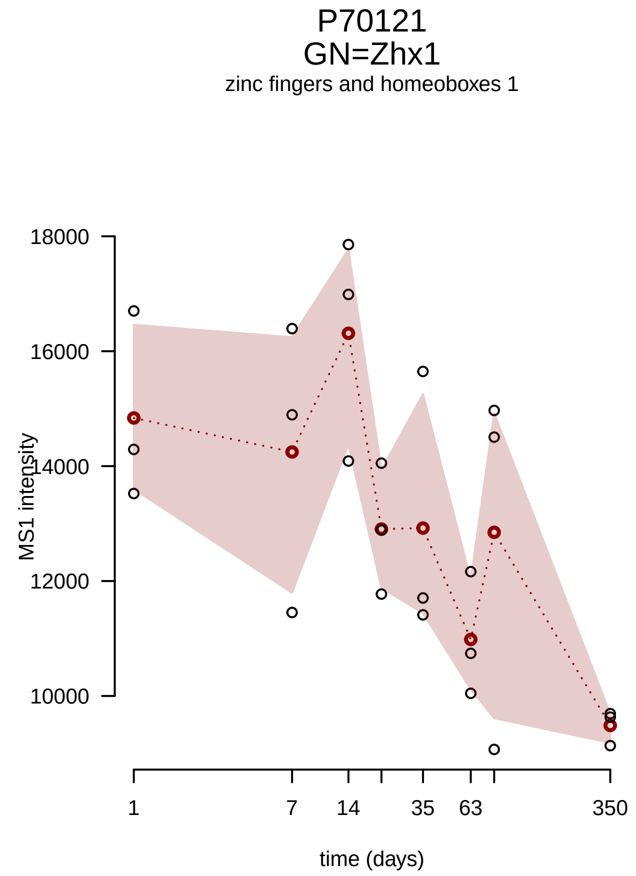
P70121
GN=Zhx1
zinc fingers and homeoboxes 1
MS1 intensity
10000
12000
14000
16000
18000
1
7
14
35
63
350
time (days)

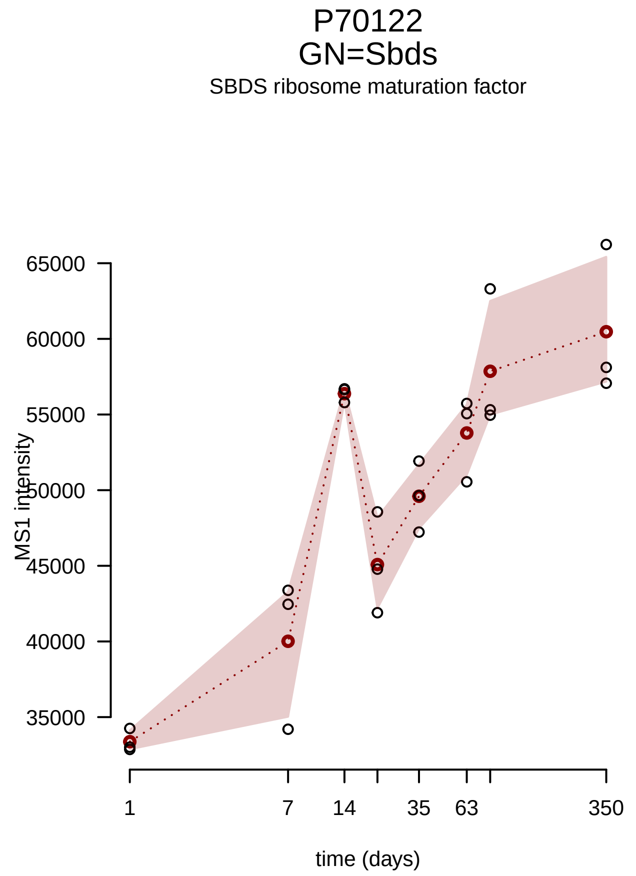
P70122
GN=Sbds
SBDS ribosome maturation factor
MS1 intensity
35000
40000
45000
50000
55000
60000
65000
1
7
14
35
63
350
time (days)

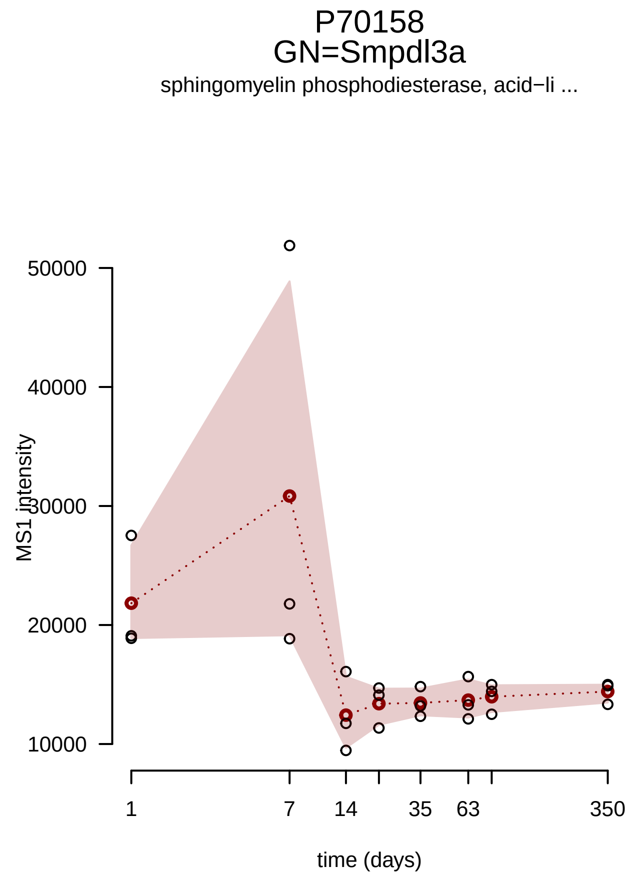
P70158
GN=Smpdl3a
sphingomyelin phosphodiesterase, acid−li ...
MS1 intensity
10000
20000
30000
40000
50000
1
7
14
35
63
350
time (days)

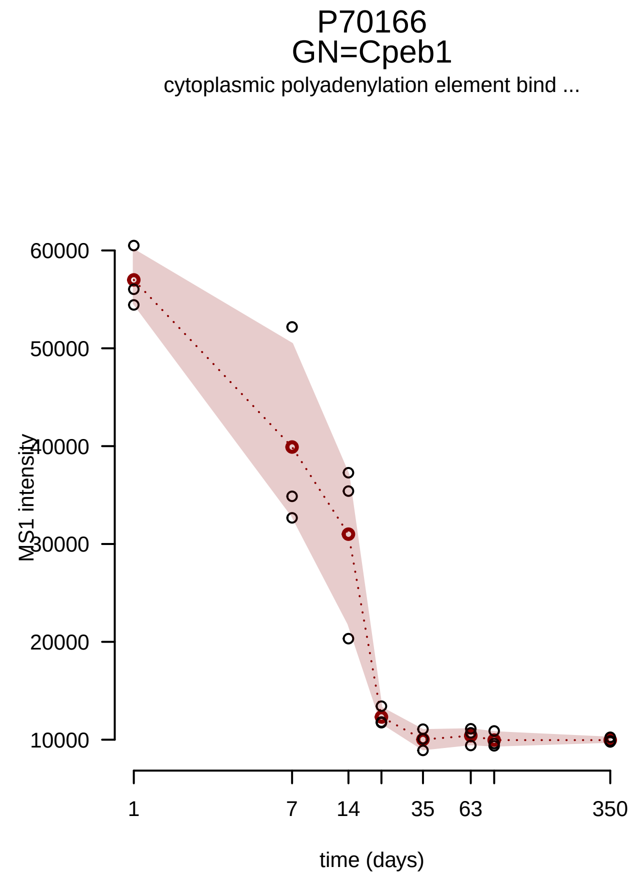
P70166
GN=Cpeb1
cytoplasmic polyadenylation element bind ...
MS1 intensity
10000
20000
30000
40000
50000
60000
1
7
14
35
63
350
time (days)

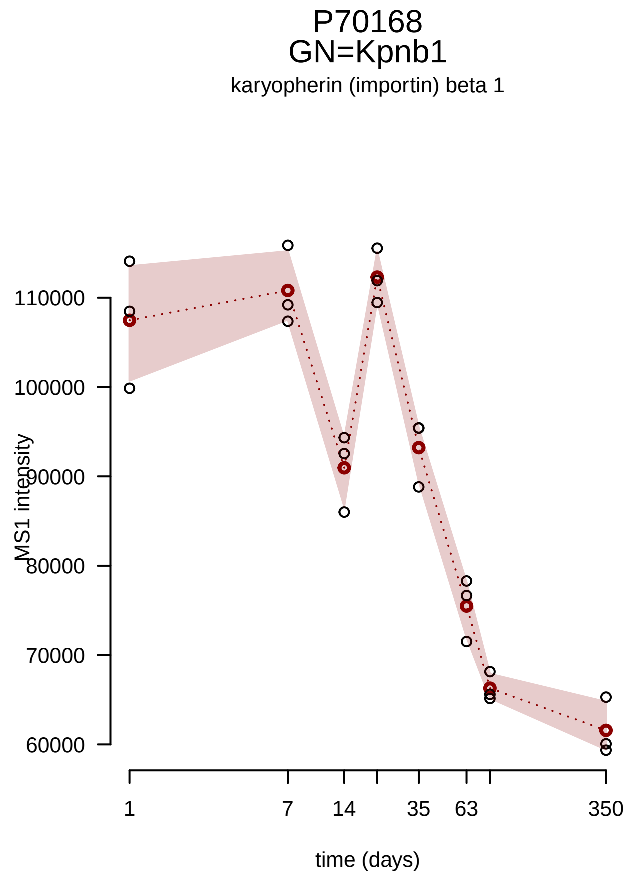
P70168
GN=Kpnb1
karyopherin (importin) beta 1
MS1 intensity
60000
70000
80000
90000
100000
110000
1
7
14
35
63
350
time (days)

P70169
GN=Doc2b
double C2, beta

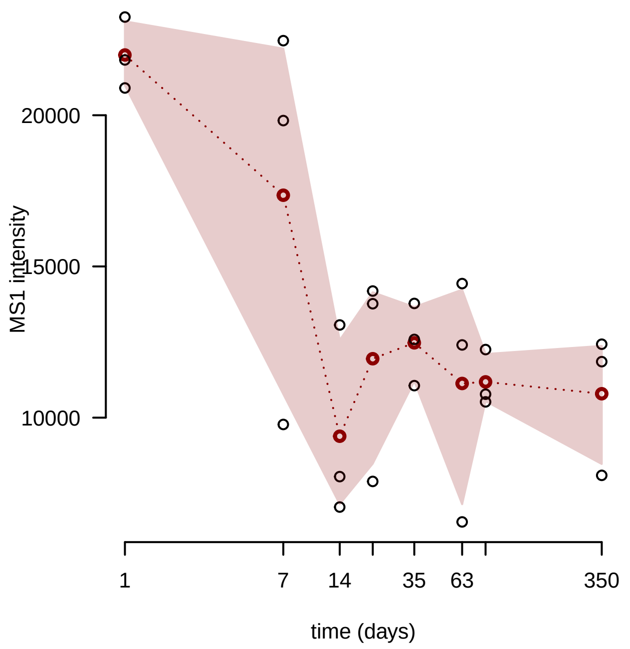

P70170
GN=Abcc9
ATP−binding cassette, sub−family C (CFTR ...

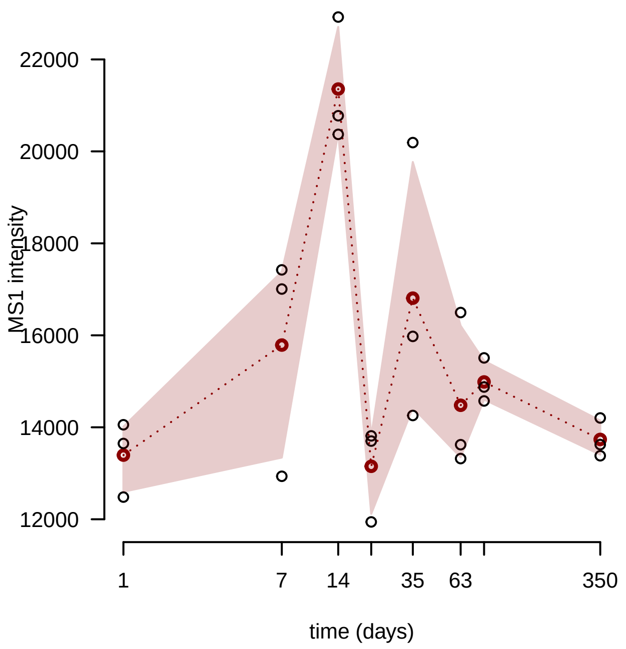

P70175
GN=Dlg3
discs large MAGUK scaffold protein 3

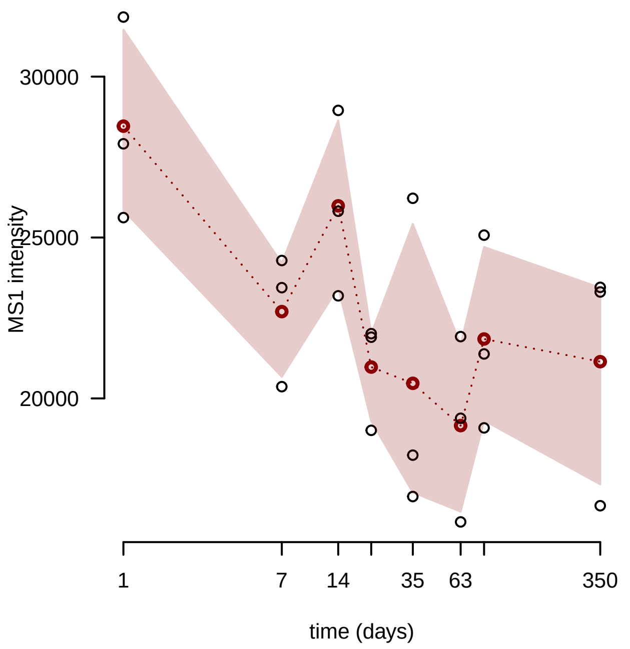

P70178
GN=Six5
sine oculis−related homeobox 5

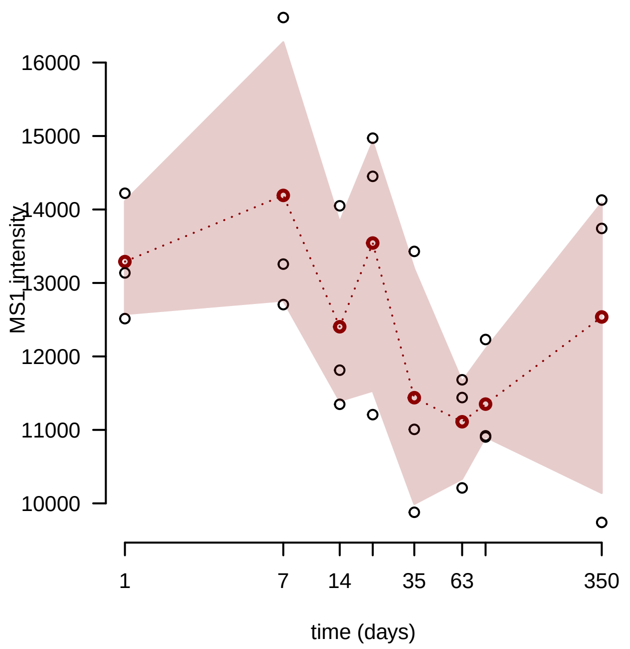

P70180
GN=Npr3
natriuretic peptide receptor 3

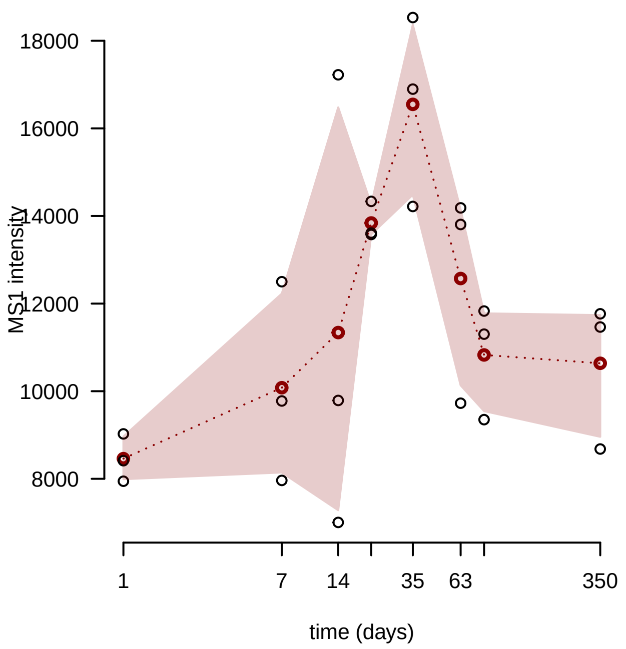

P70182
GN=Pip5k1a
phosphatidylinositol−4−phosphate 5−kinas ...

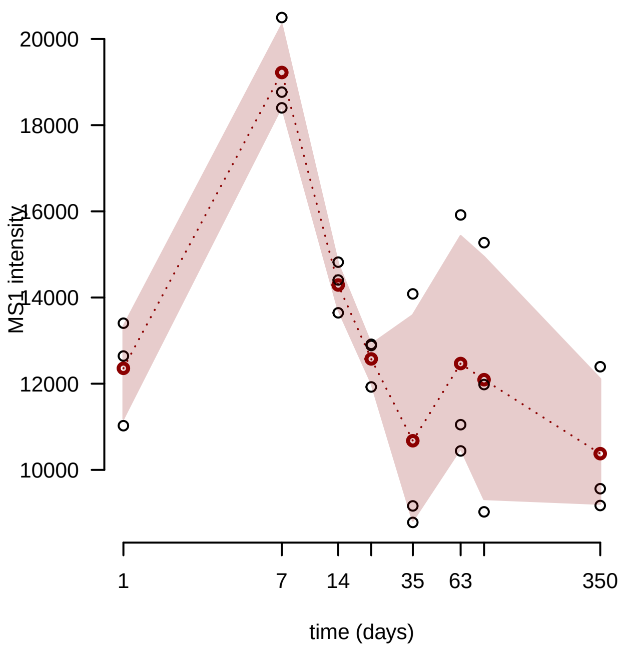

P70188
GN=Kifap3
kinesin−associated protein 3

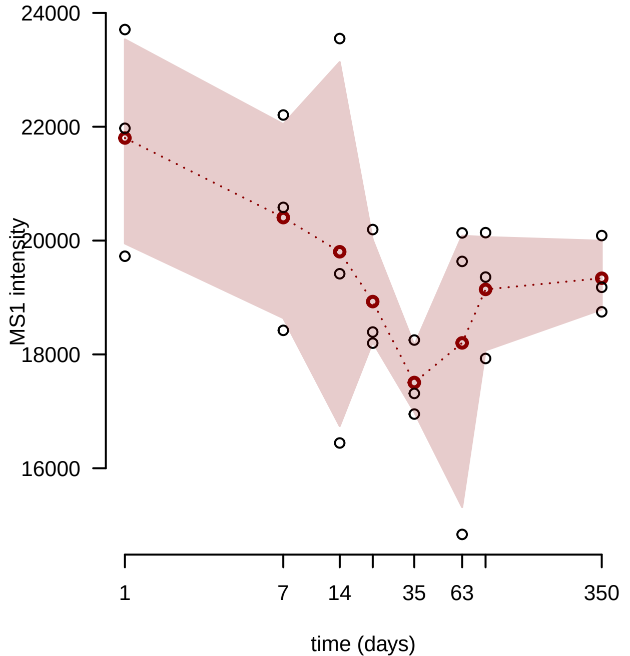

P70195
GN=Psmb7
proteasome (prosome, macropain) subunit, ...

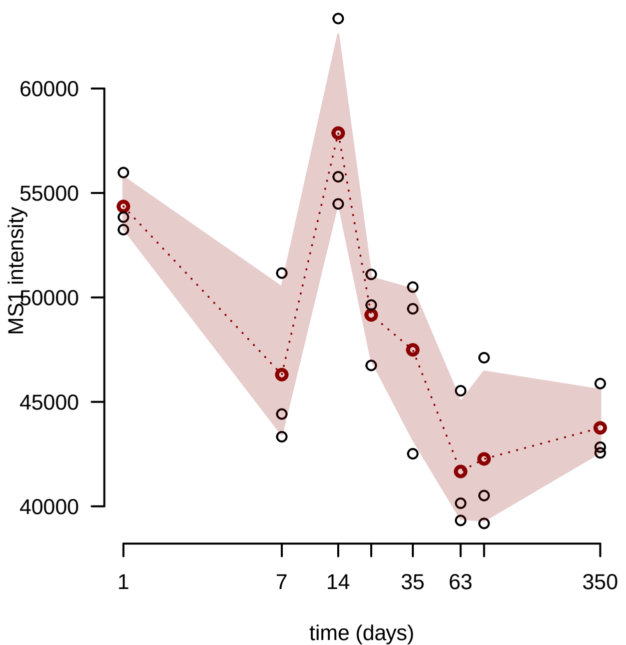

P70196
GN=Traf6
TNF receptor−associated factor 6

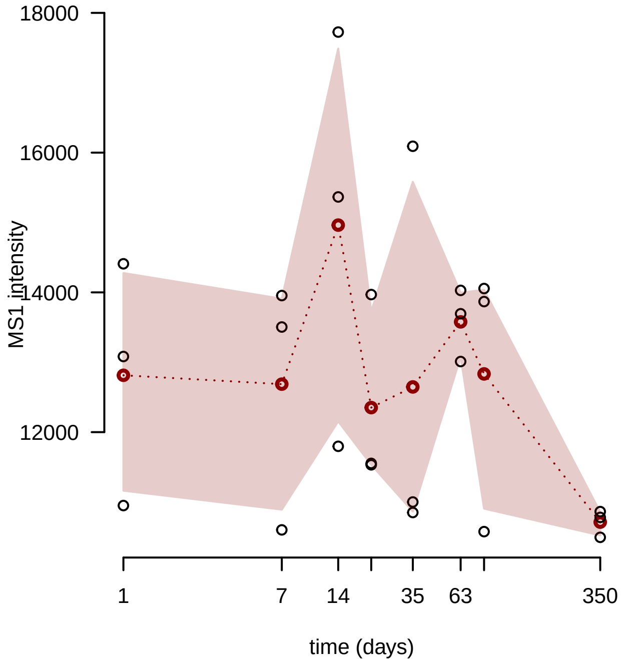

P70202
GN=Lxn
latexin

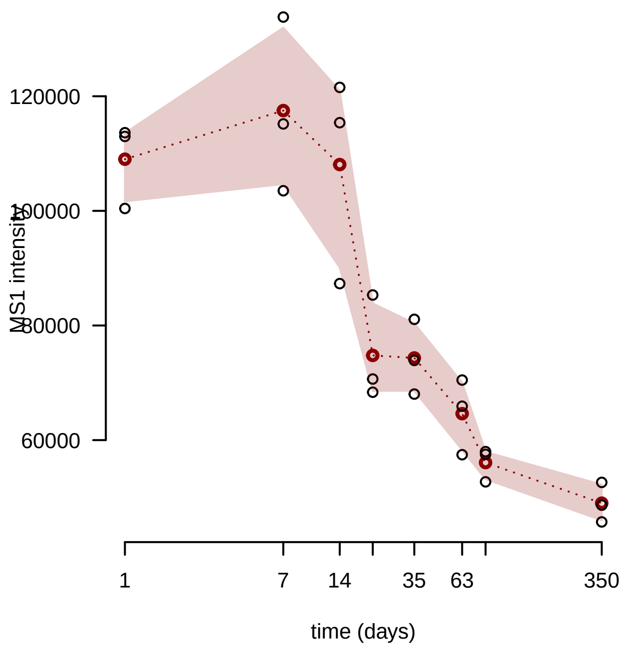

P70206
GN=Plxna1
plexin A1

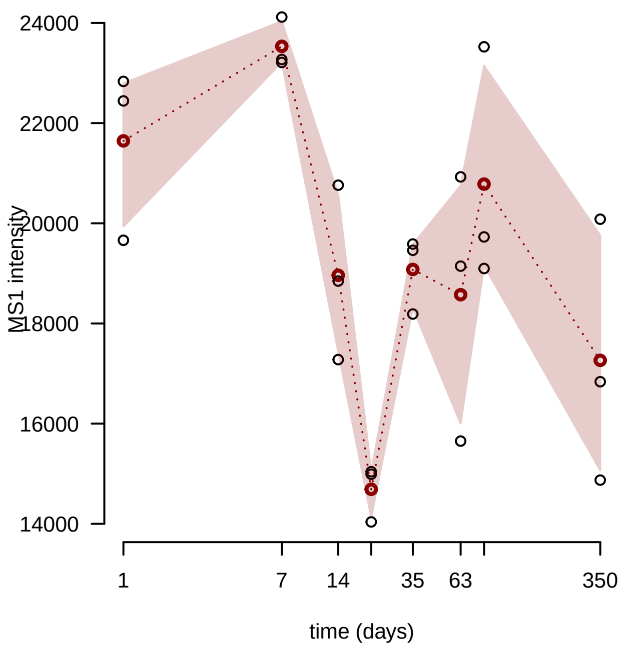

P70207
GN=Plxna2
plexin A2

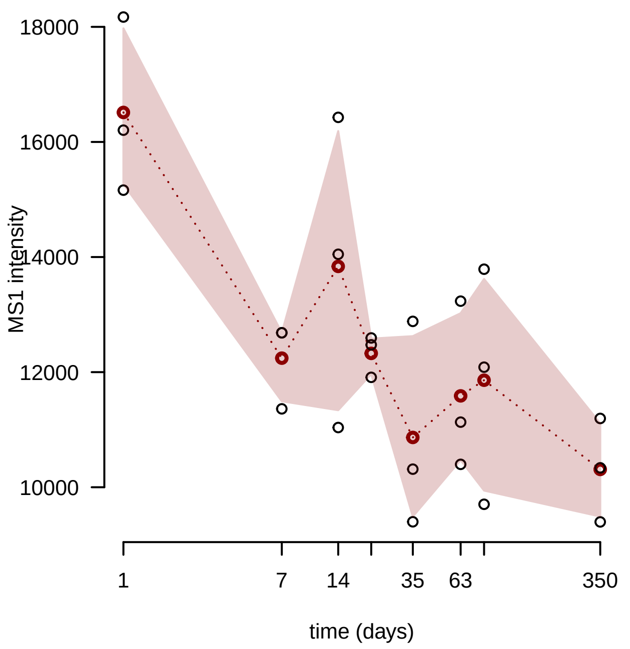

P70208
GN=Plxna3
plexin A3

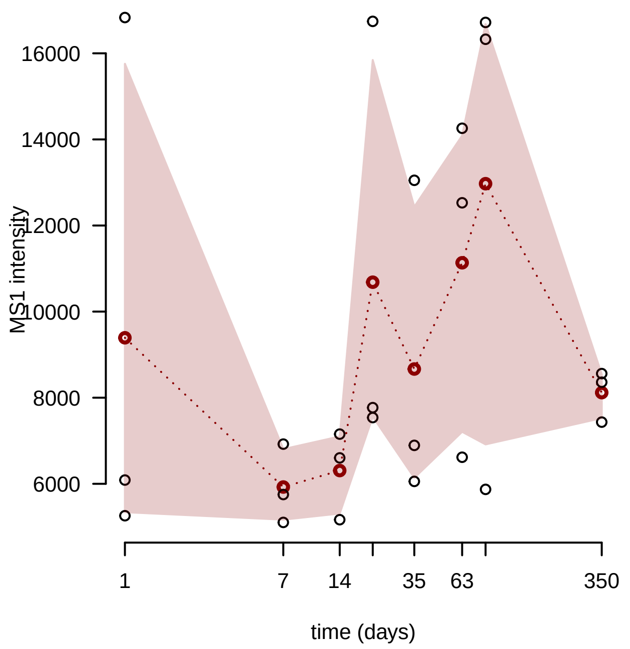

P70210
GN=Tead3
TEA domain family member 3

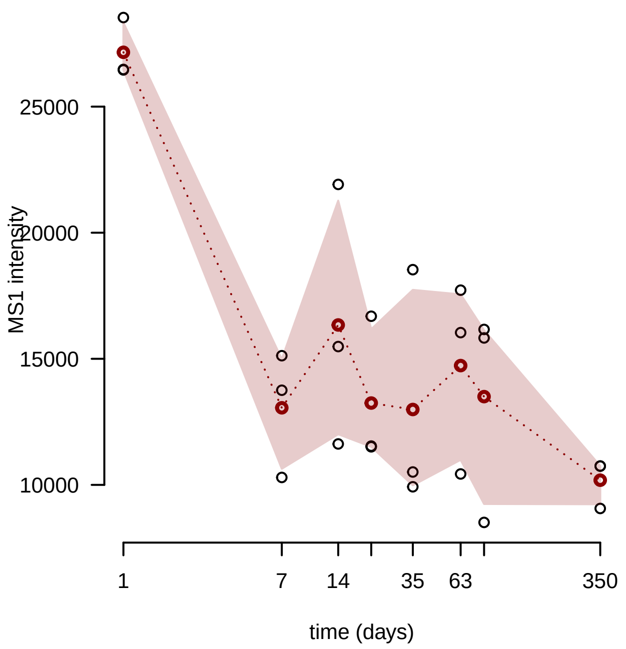

P70218
GN=Map4k1
mitogen−activated protein kinase kinase ...

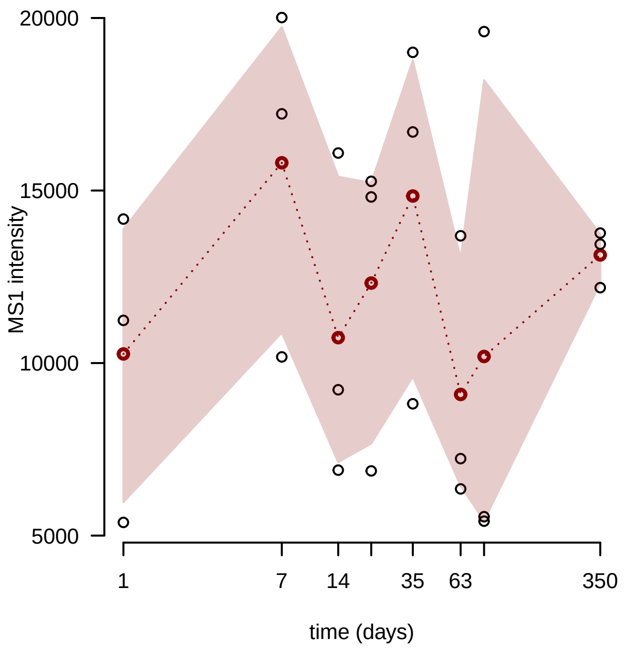

P70224
GN=Gimap1
GTPase, IMAP family member 1

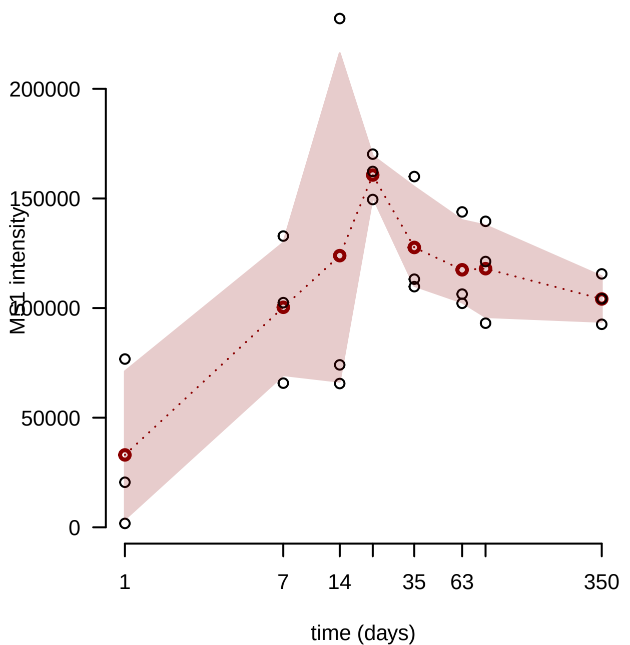

P70227
GN=Itpr3
inositol 1,4,5−triphosphate receptor 3

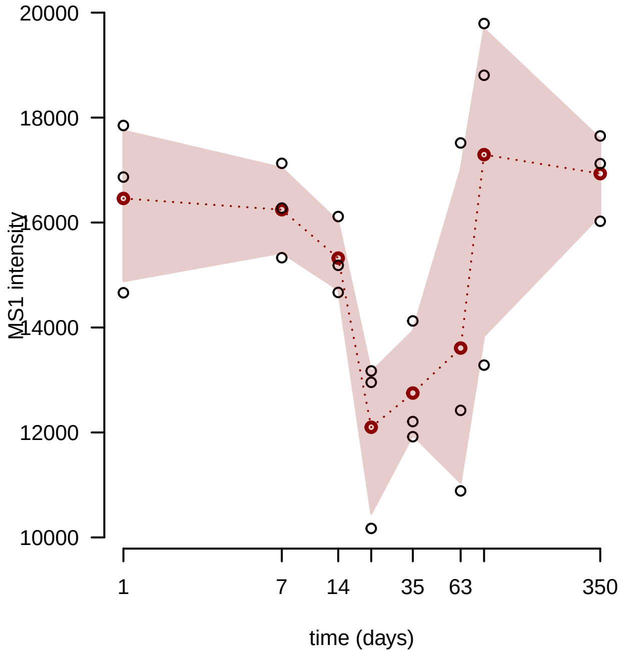

P70236
GN=Map2k6
mitogen−activated protein kinase kinase ...

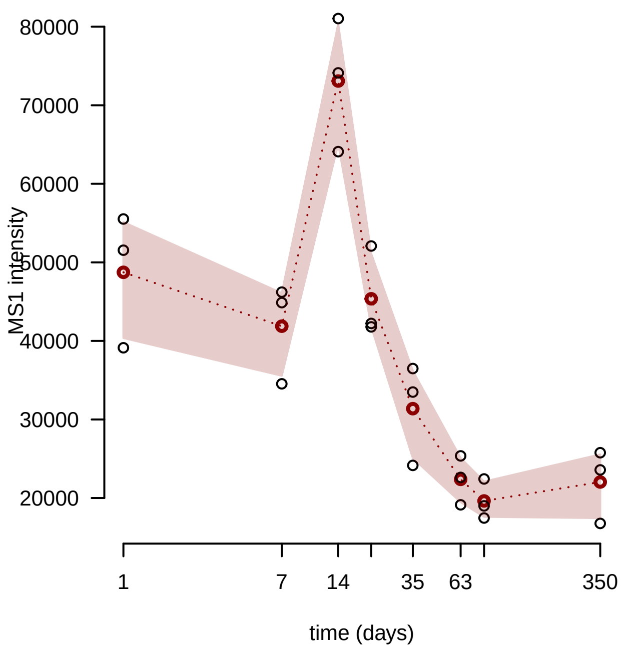

P70245
GN=Ebp
phenylalkylamine Ca2+ antagonist (emopam ...

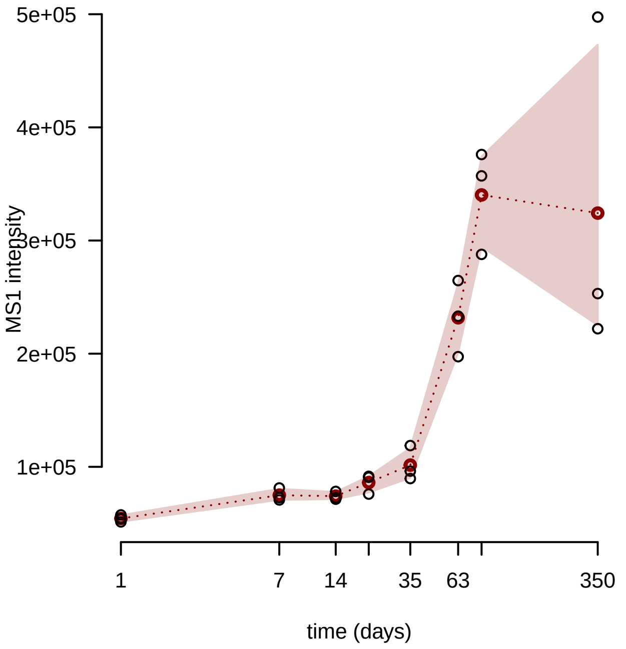

P70255
GN=Nfic
nuclear factor I/C

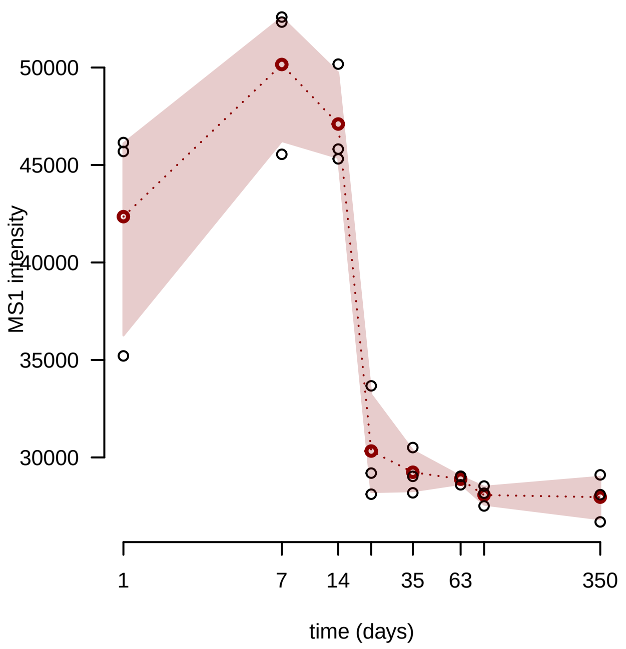

P70257
GN=Nfix
nuclear factor I/X

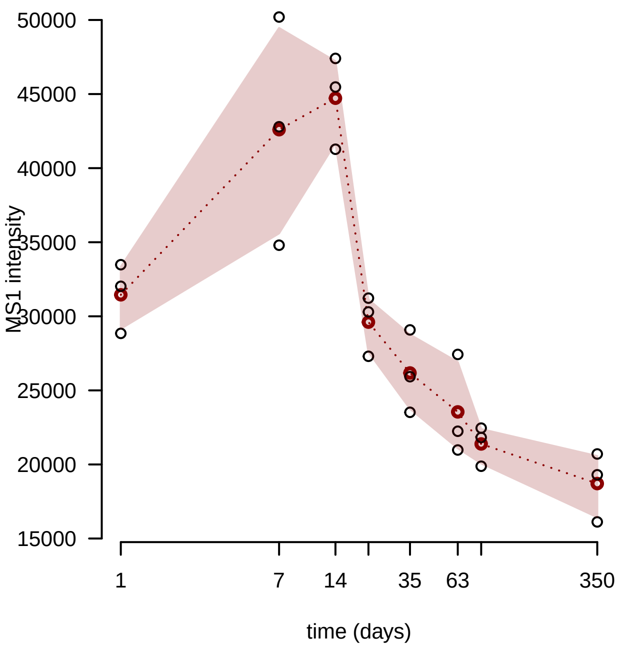

P70257
GN=Nfix
nuclear factor I/X

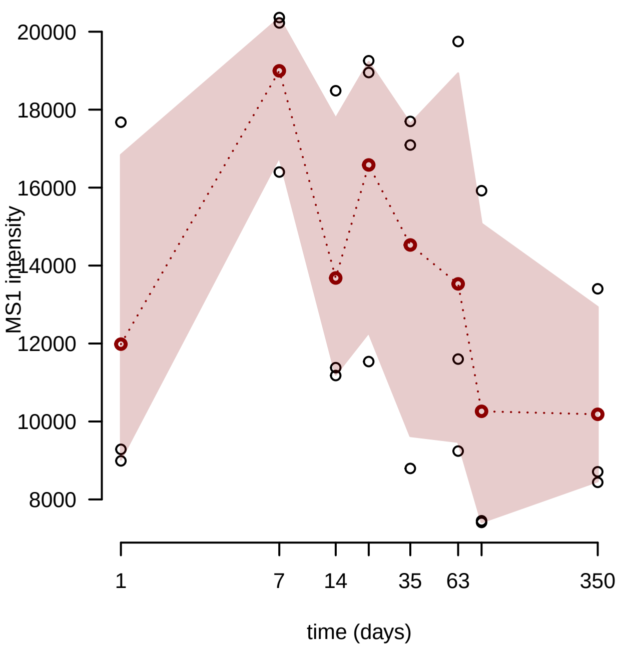

P70261
GN=Pald1
phosphatase domain containing, paladin 1

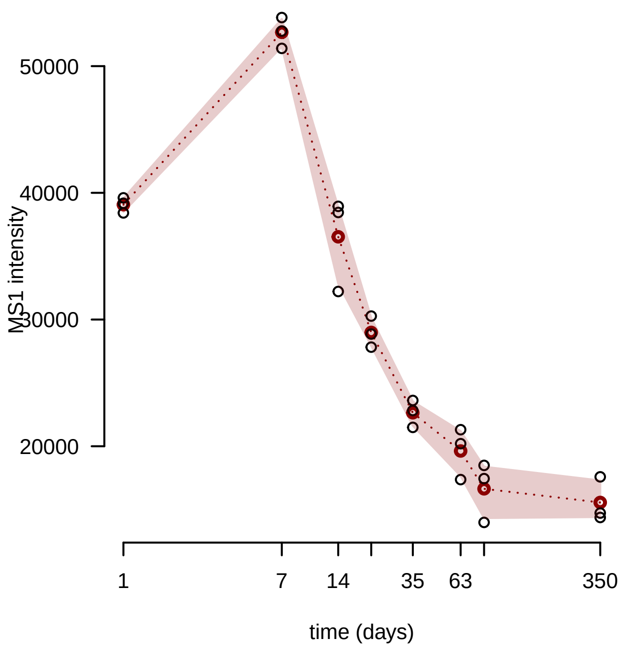

P70265
GN=Pfkfb2
6−phosphofructo−2−kinase/fructose−2,6−bi ...

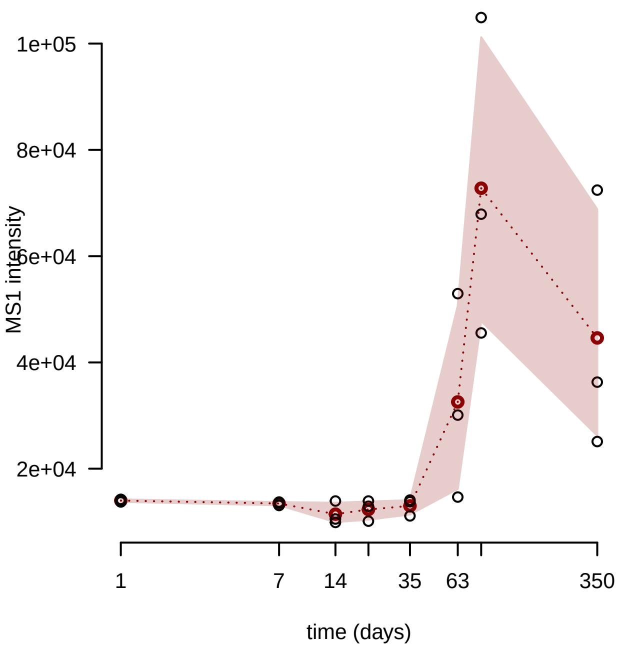

P70266
GN=Pfkfb1
6−phosphofructo−2−kinase/fructose−2,6−bi ...

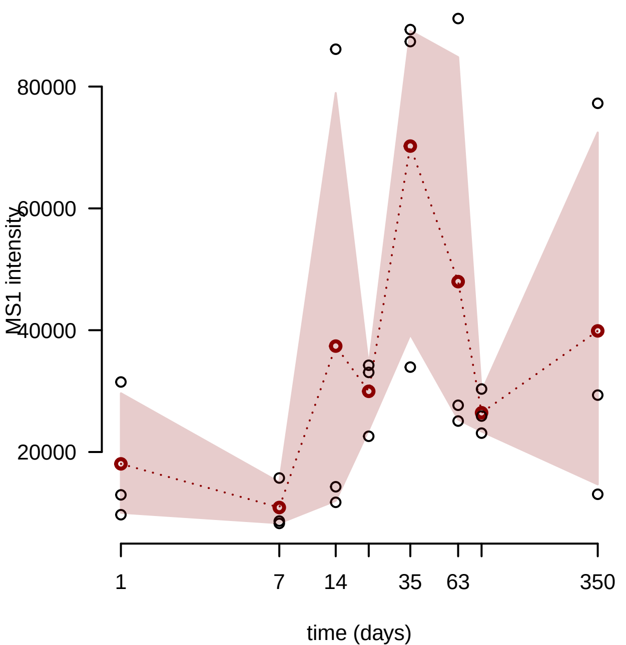

P70268
GN=Pkn1
protein kinase N1

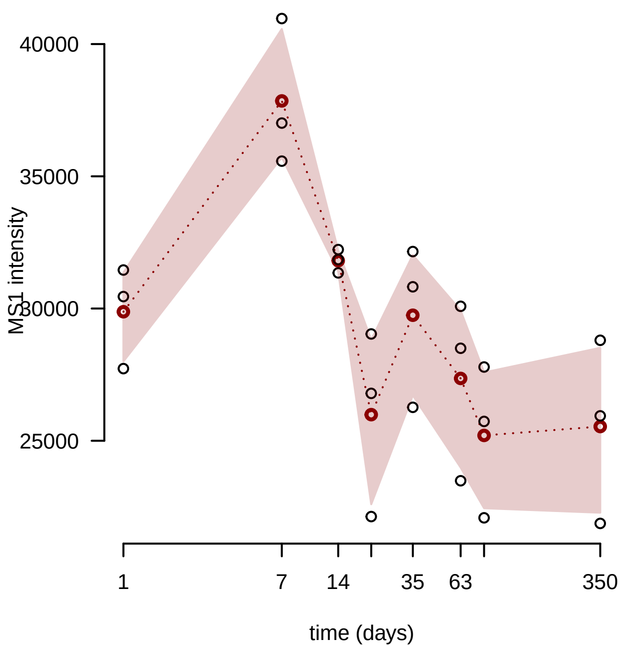

P70269
GN=Ctse
cathepsin E

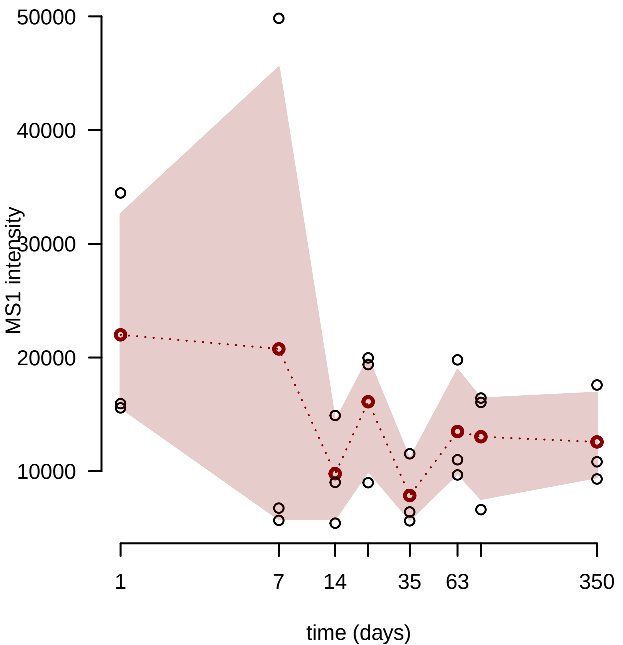

P70271
GN=Pdlim4
PDZ and LIM domain 4

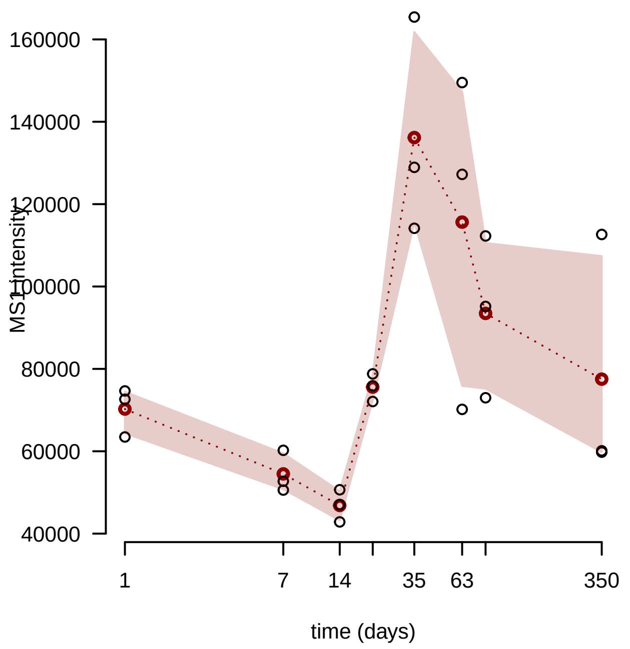

P70274
GN=Selenop
selenoprotein P

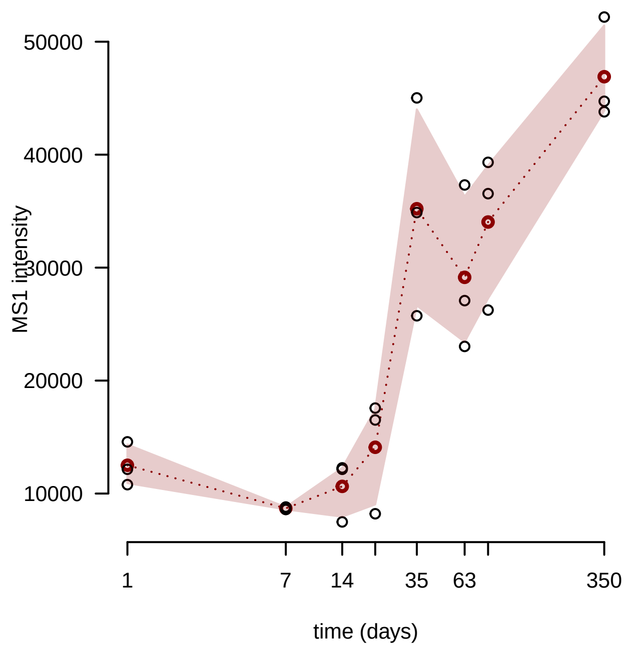

P70279
GN=Surf6
surfeit gene 6

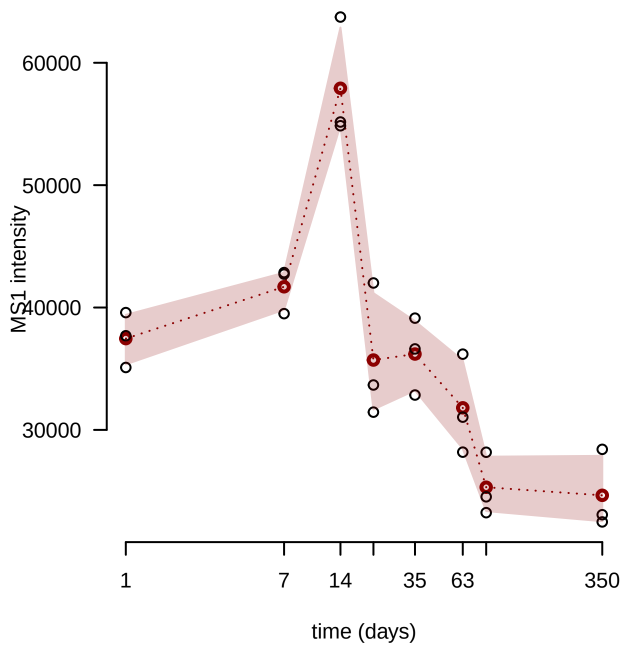

P70280
GN=Vamp7
vesicle−associated membrane protein 7

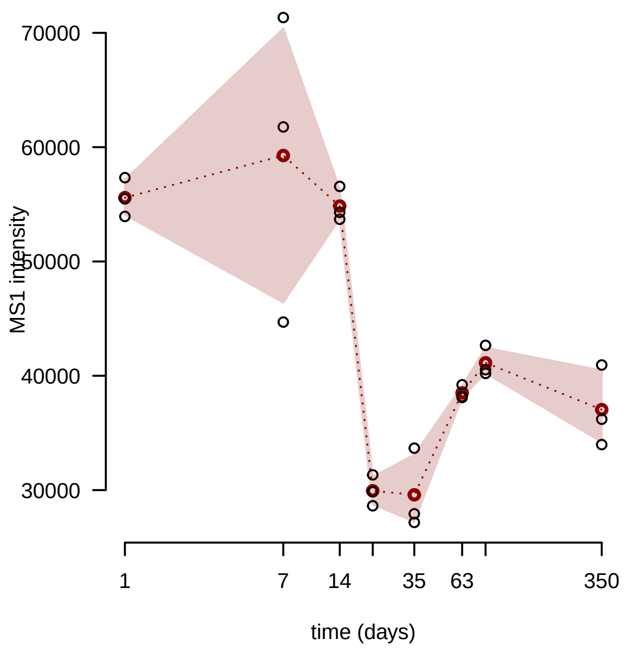

P70281
GN=Sycp3
synaptonemal complex protein 3

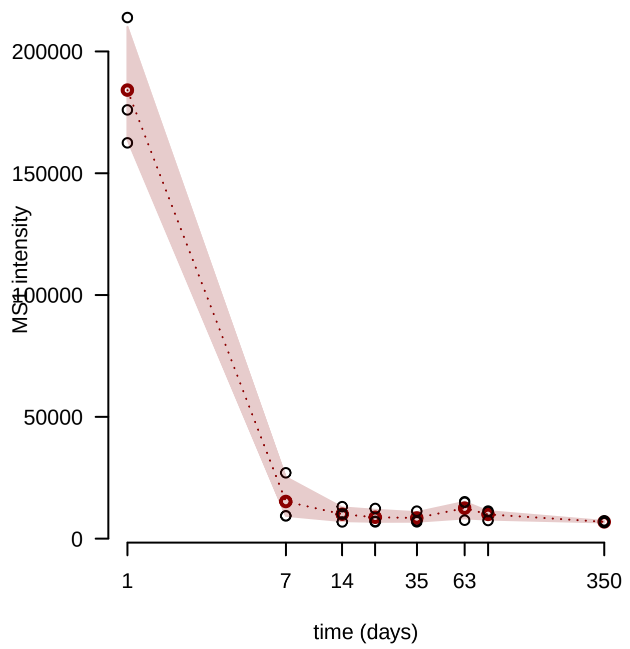

P70284
GN=Tgif1
TGFB−induced factor homeobox 1

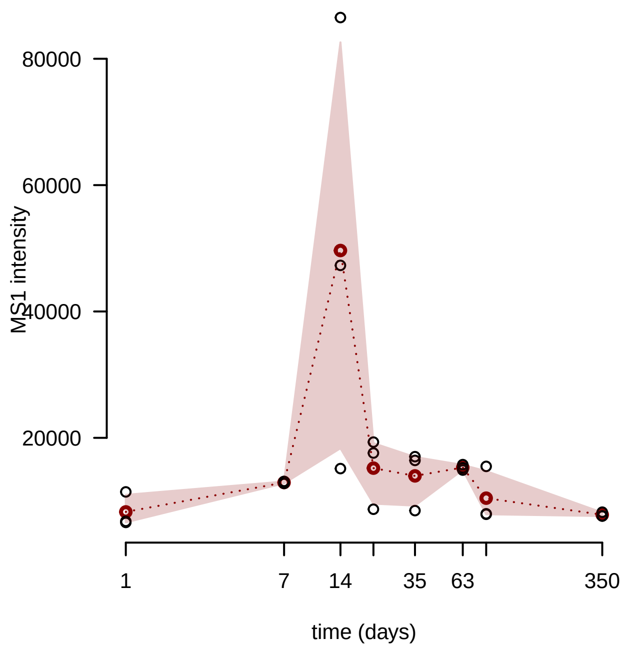

P70288
GN=Hdac2
histone deacetylase 2

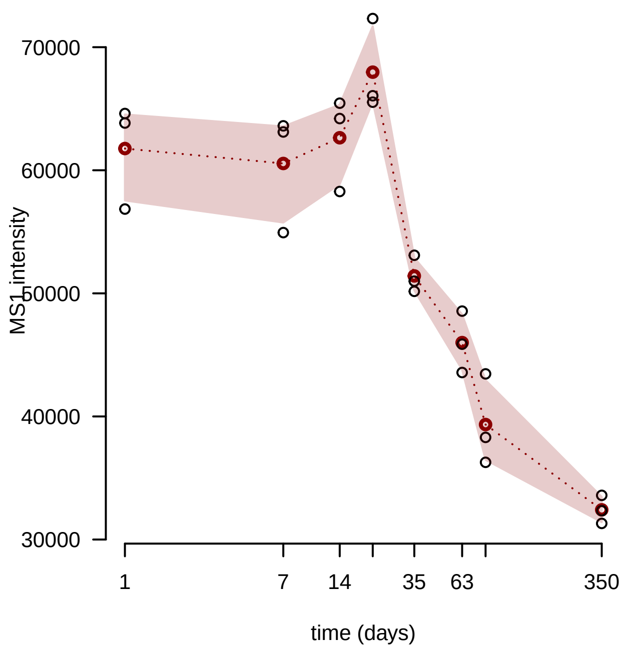

P70290
GN=Mpp1
membrane protein, palmitoylated

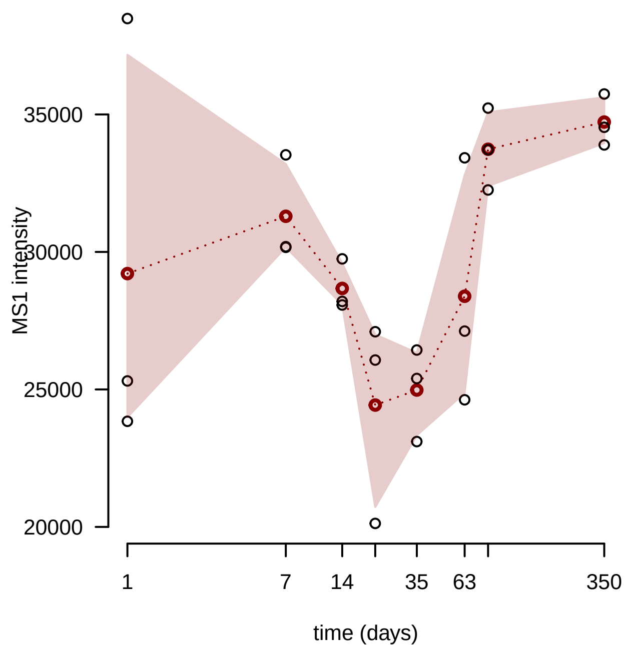

P70295
GN=Aup1
ancient ubiquitous protein 1

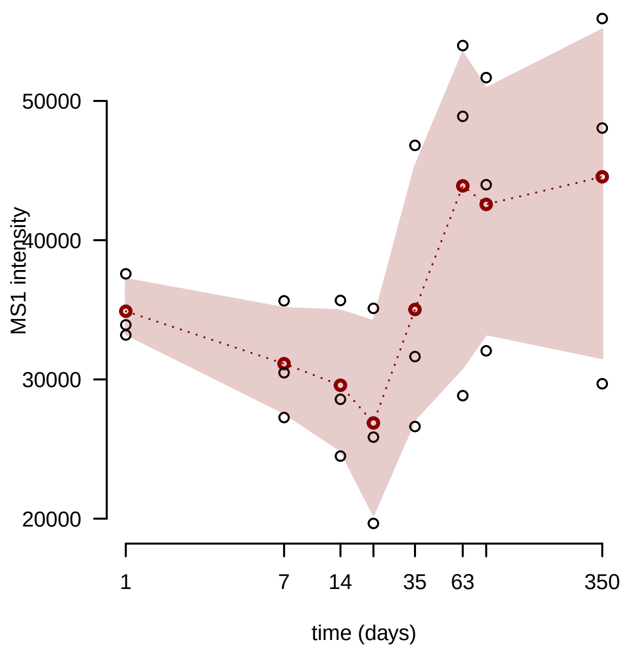

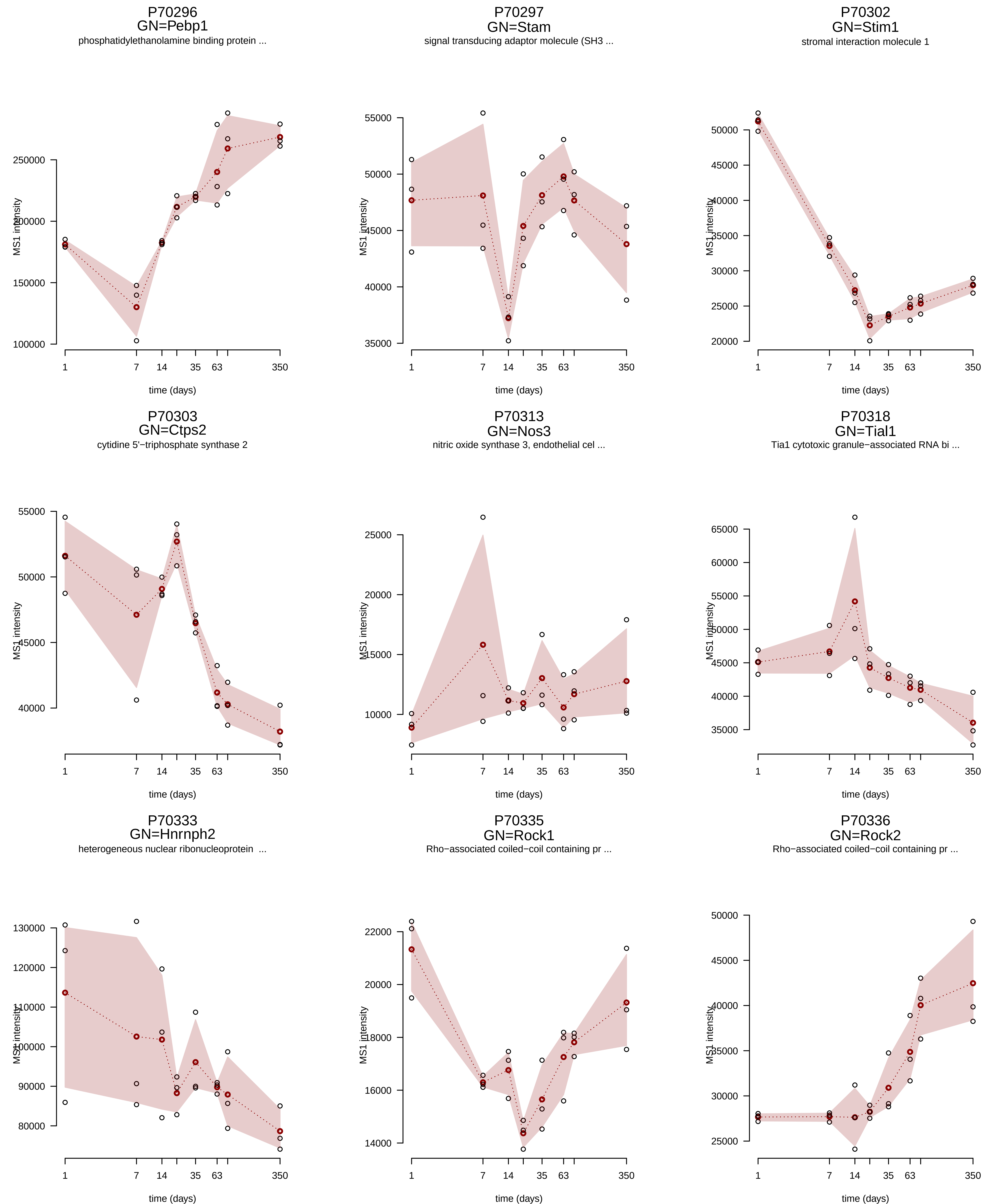
P70296
GN=Pebp1
phosphatidylethanolamine binding protein ...
MS1 intensity
100000
150000
200000
250000
time (days)
1
7
14
35
63
350
P70297
GN=Stam
signal transducing adaptor molecule (SH3 ...
MS1 intensity
35000
40000
45000
50000
55000
time (days)
P70302
GN=Stim1
stromal interaction molecule 1
MS1 intensity
20000
25000
30000
35000
40000
45000
50000
time (days)
P70303
GN=Ctps2
cytidine 5'−triphosphate synthase 2
MS1 intensity
40000
45000
50000
55000
time (days)
P70313
GN=Nos3
nitric oxide synthase 3, endothelial cel ...
MS1 intensity
10000
15000
20000
25000
time (days)
P70318
GN=Tial1
Tia1 cytotoxic granule−associated RNA bi ...
MS1 intensity
35000
40000
45000
50000
55000
60000
65000
time (days)
P70333
GN=Hnrnph2
heterogeneous nuclear ribonucleoprotein ...
MS1 intensity
80000
90000
100000
110000
120000
130000
time (days)
P70335
GN=Rock1
Rho−associated coiled−coil containing pr ...
MS1 intensity
14000
16000
18000
20000
22000
time (days)
P70336
GN=Rock2
Rho−associated coiled−coil containing pr ...
MS1 intensity
25000
30000
35000
40000
45000
50000
time (days)

P70340
GN=Smad1
SMAD family member 1

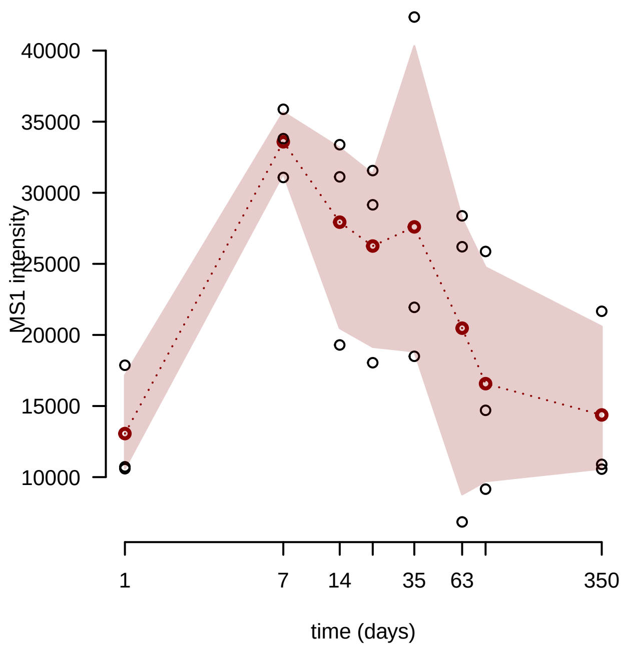

P70345
GN=Bcl2l2
BCL2−like 2

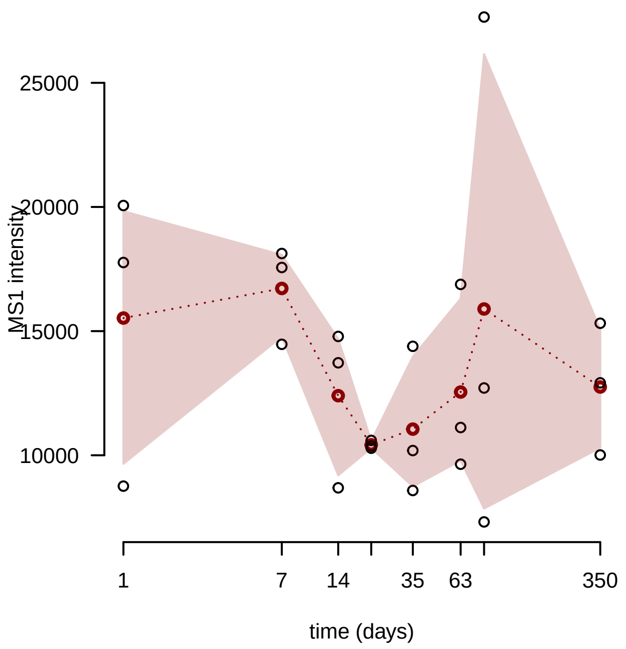

P70349
GN=Hint1
histidine triad nucleotide binding prote ...

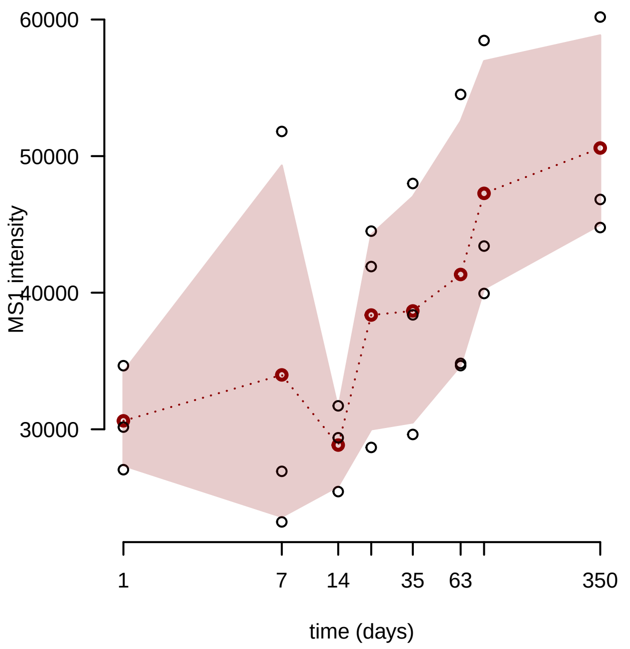

P70351
GN=Ezh1
enhancer of zeste 1 polycomb repressive ...

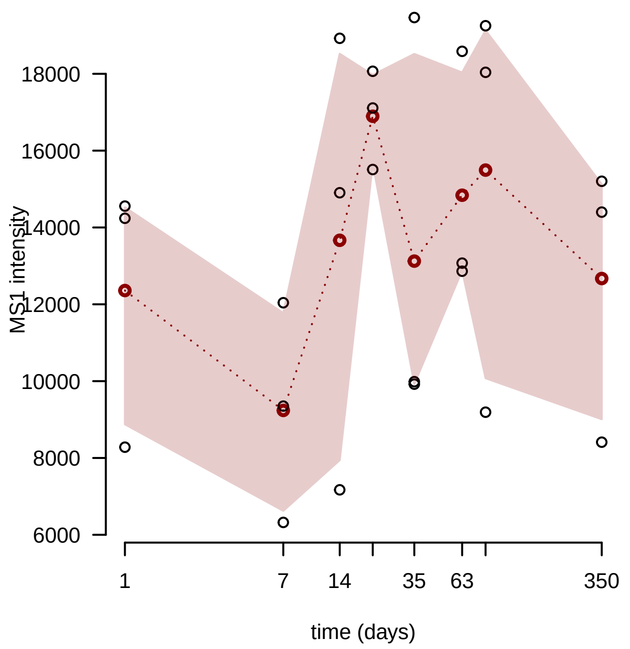

P70353
GN=Nfyc
nuclear transcription factor−Y gamma

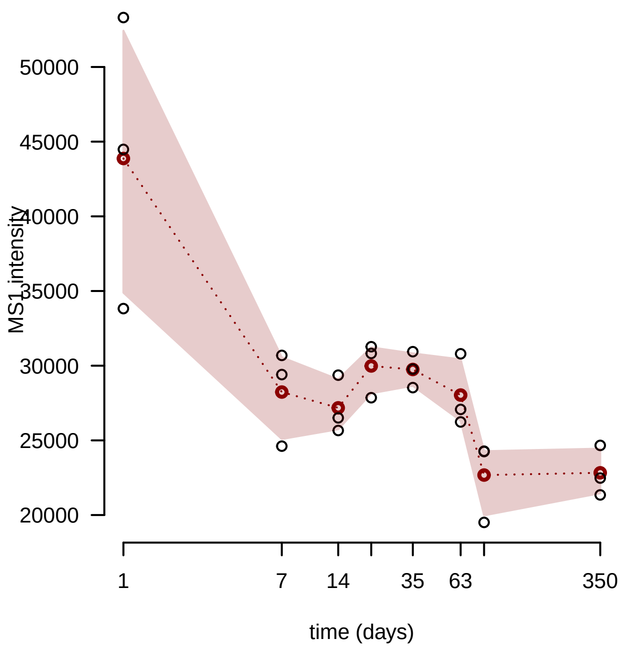

P70362
GN=Ufd1
ubiquitin recognition factor in ER−assoc ...

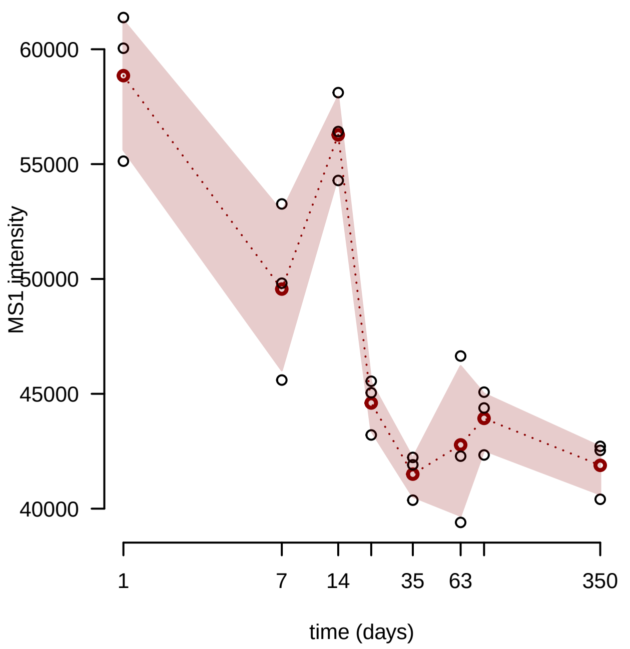

P70365
GN=Ncoa1
nuclear receptor coactivator 1

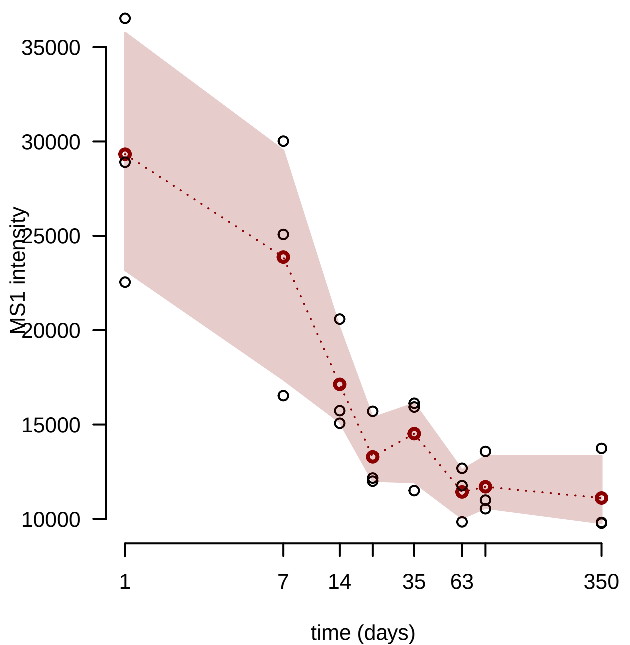

P70371
GN=Terf1
telomeric repeat binding factor 1

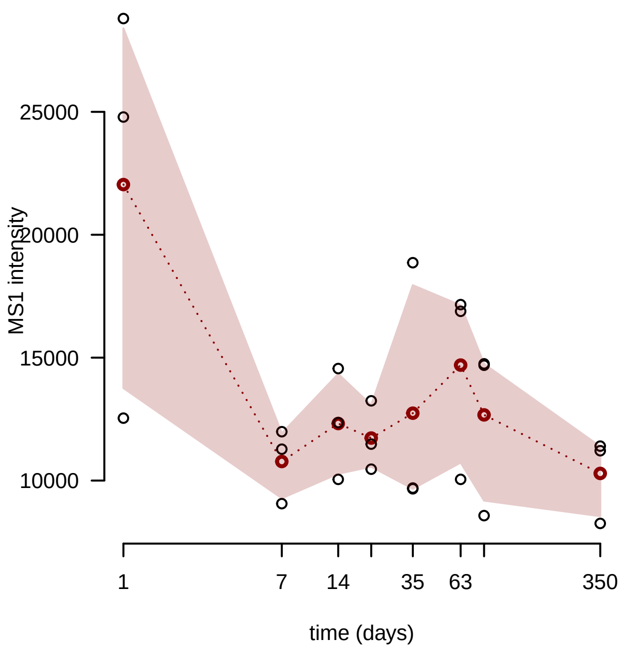

P70372
GN=Elavl1
ELAV (embryonic lethal, abnormal vision) ...

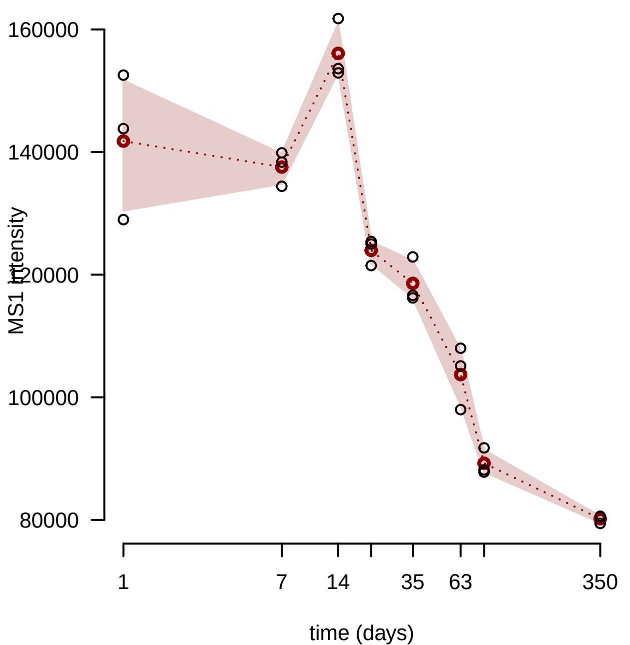

P70387
GN=Hfe
homeostatic iron regulator

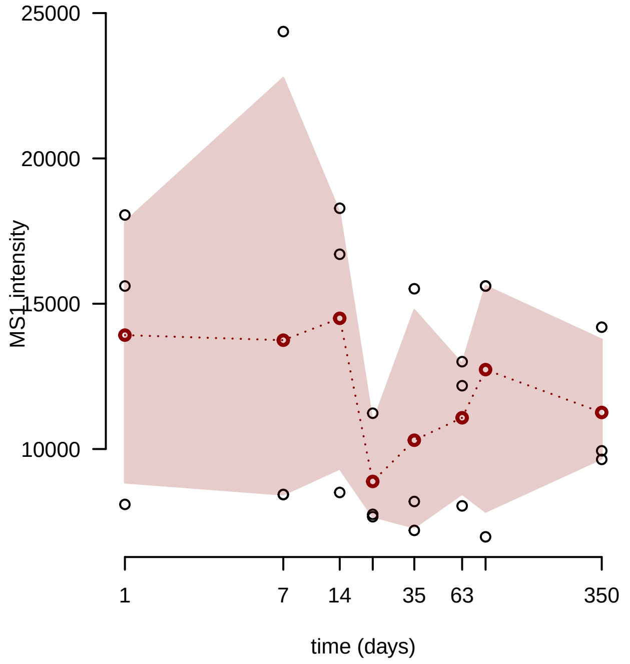

P70388
GN=Rad50
RAD50 double strand break repair protein

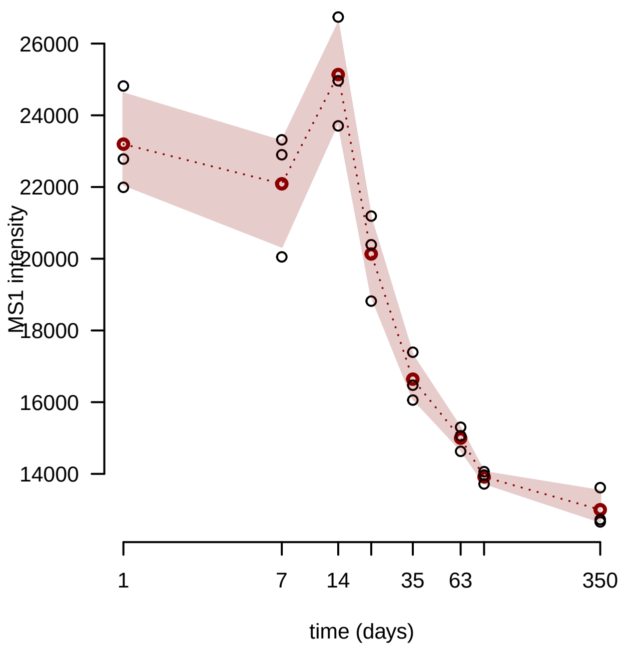

P70389
GN=Igfals
insulin−like growth factor binding prote ...

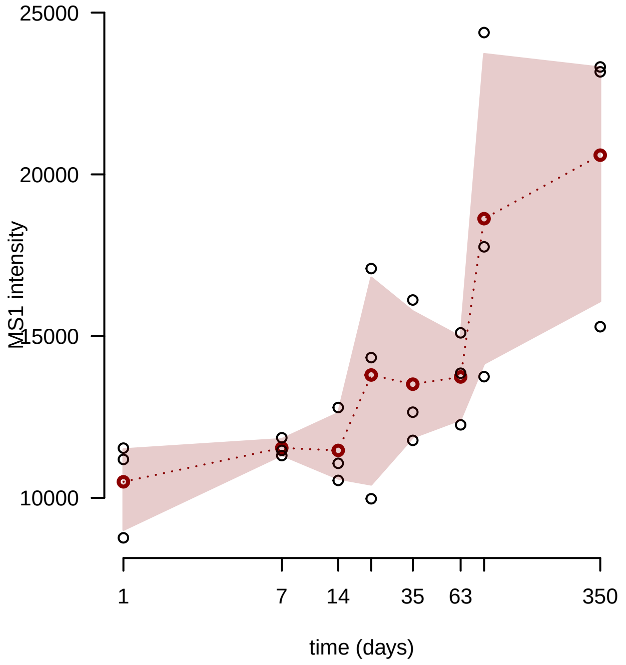

P70398
GN=Usp9x
ubiquitin specific peptidase 9, X chromo ...

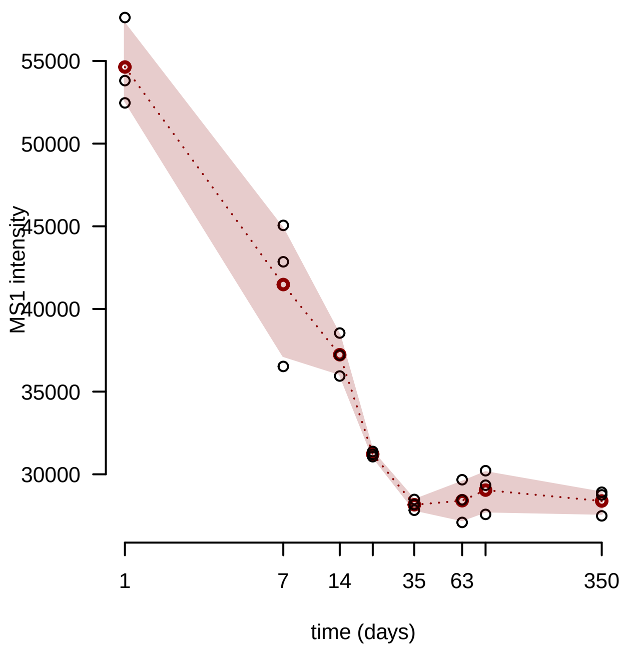

P70399
GN=Tp53bp1

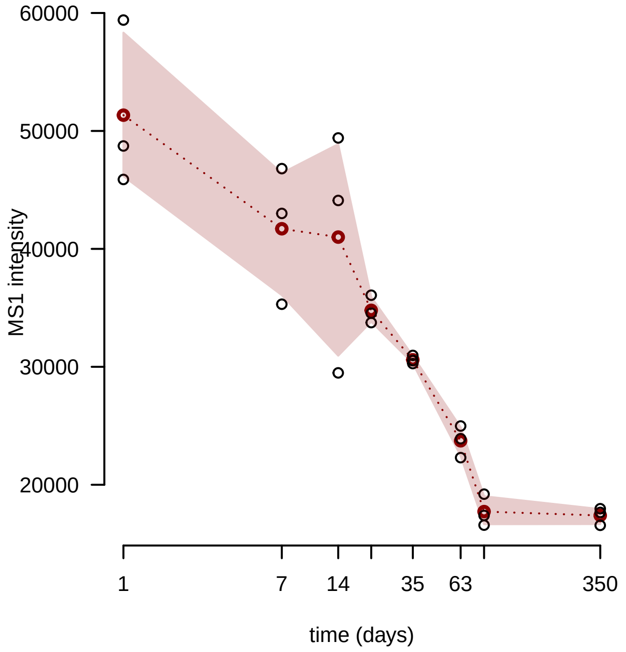

P70403
GN=Cux1

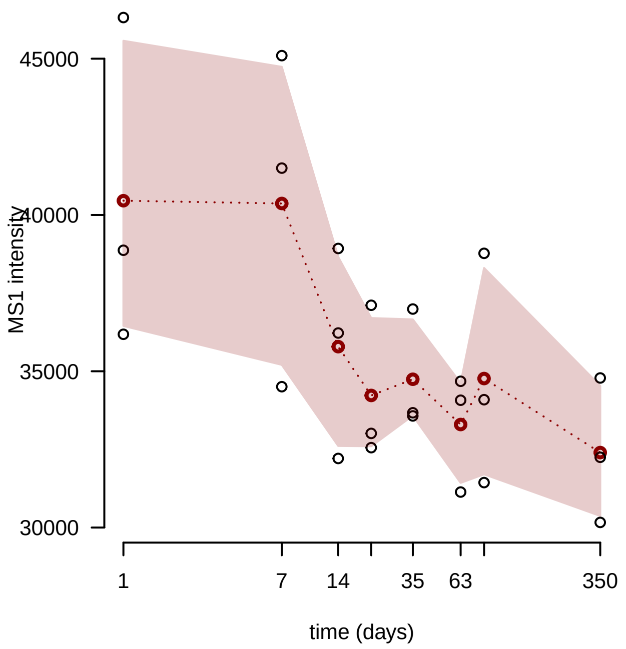

P70404
GN=Idh3g
isocitrate dehydrogenase 3 (NAD+), gamma

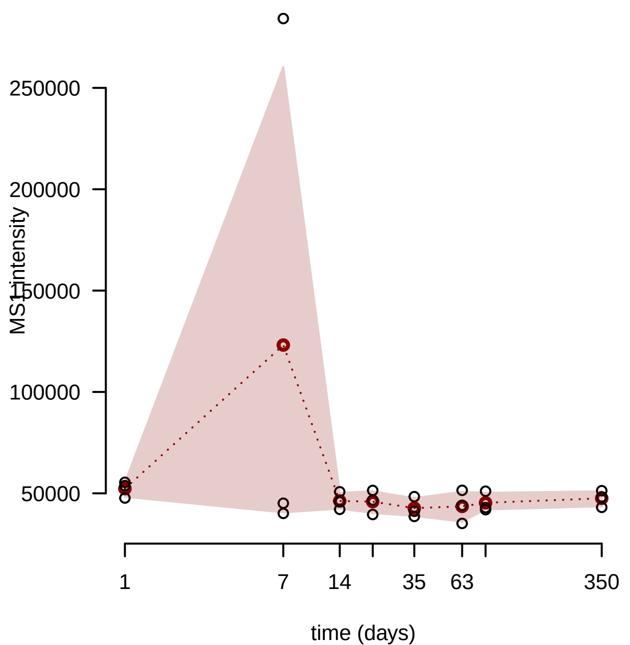

P70406
GN=Ucp2
uncoupling protein 2 (mitochondrial, pro ...

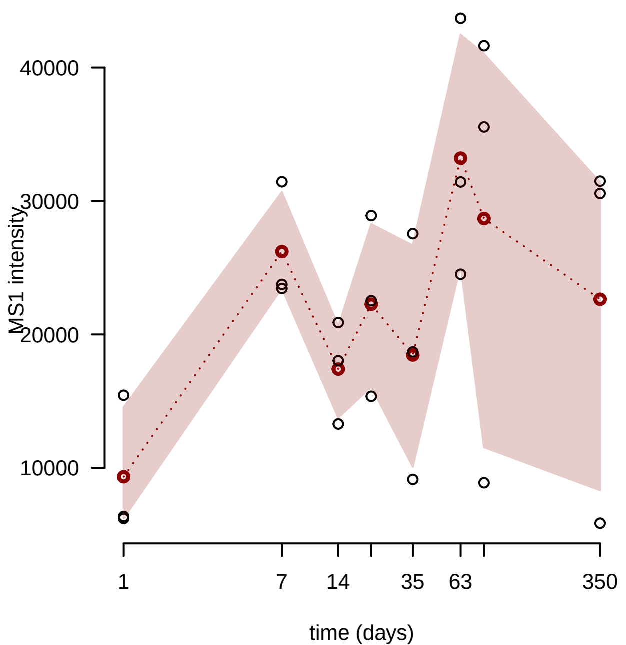

P70414
GN=Slc8a1
solute carrier family 8 (sodium/calcium ...

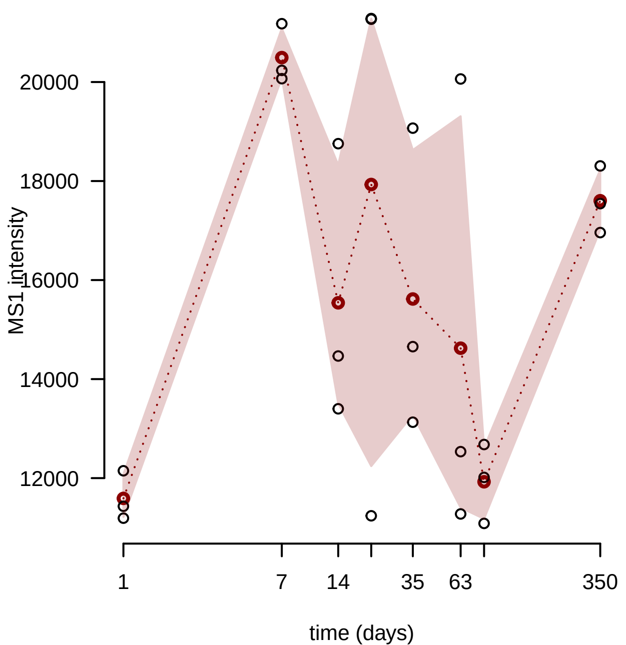

P70424
GN=Erbb2
erb−b2 receptor tyrosine kinase 2

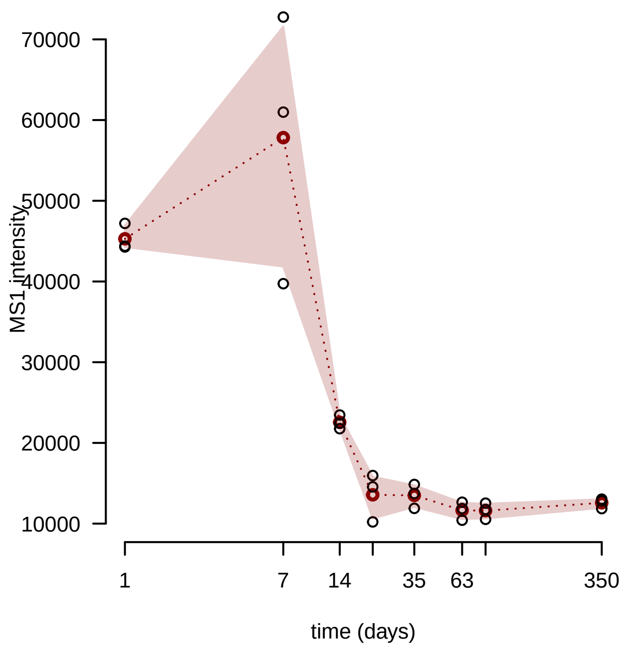

P70426
GN=Rit1
Ras−like without CAAX 1

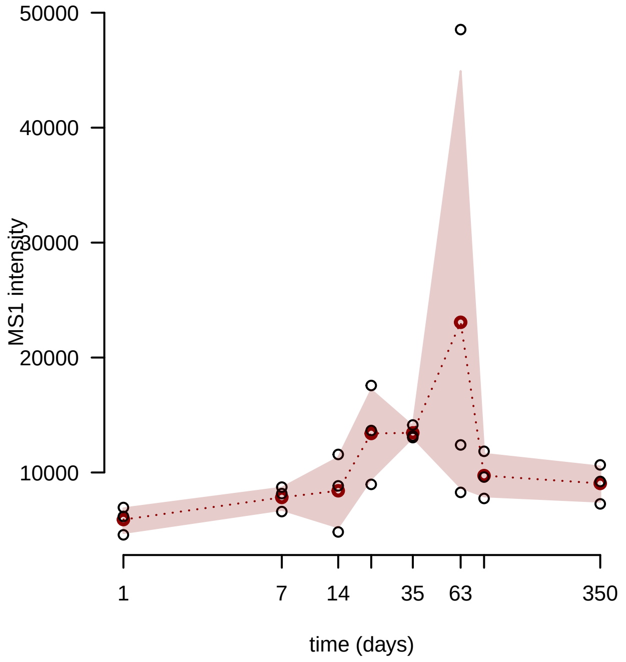

P70428
GN=Ext2
exostosin glycosyltransferase 2

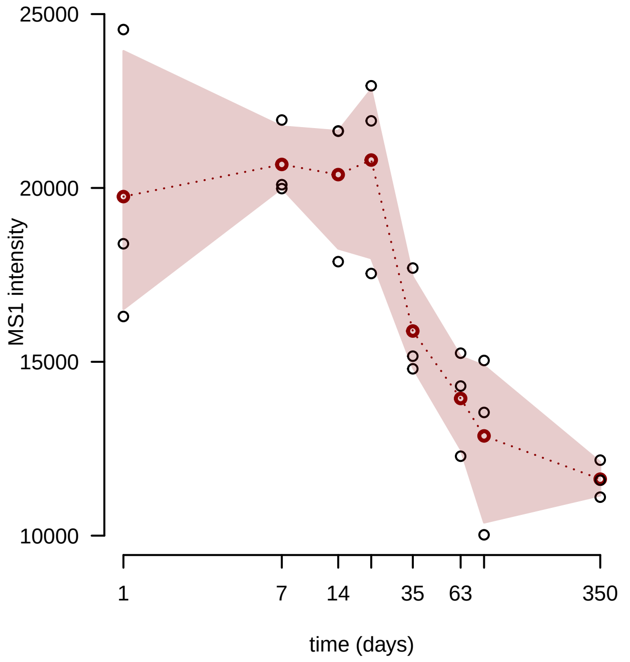

P70429
GN=Evl
Ena−vasodilator stimulated phosphoprotei ...

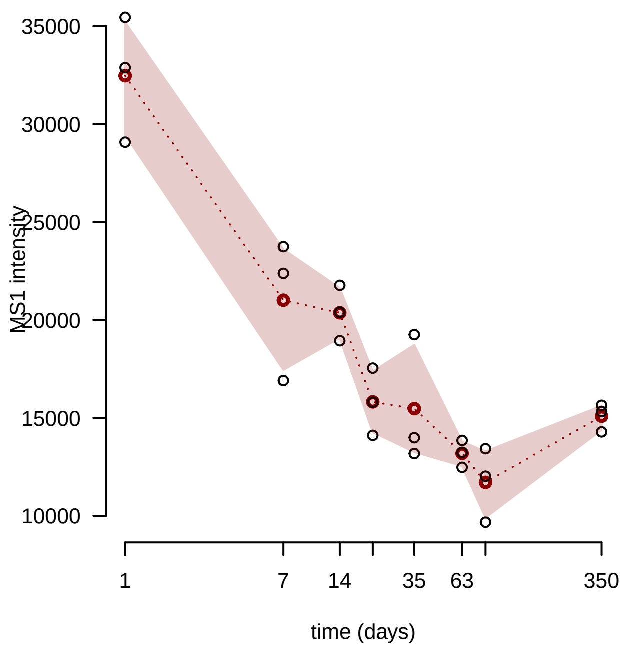

P70441
GN=Slc9a3r1
solute carrier family 9 (sodium/hydrogen ...

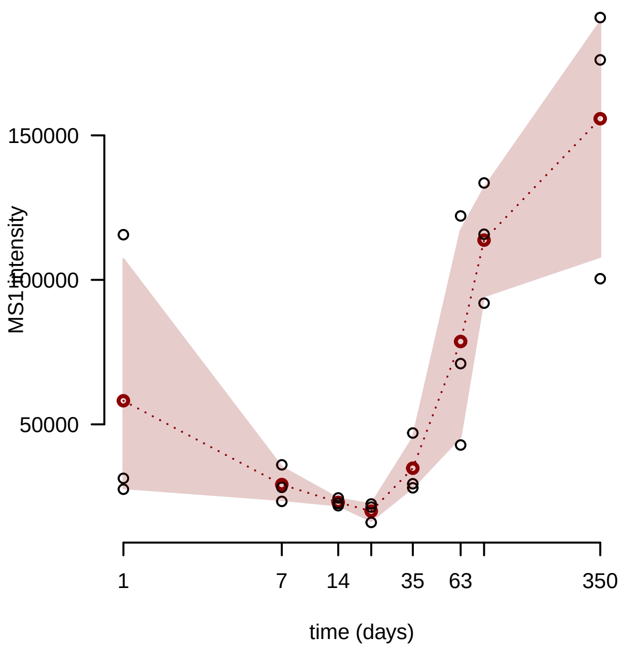

P70444
GN=Bid
BH3 interacting domain death agonist

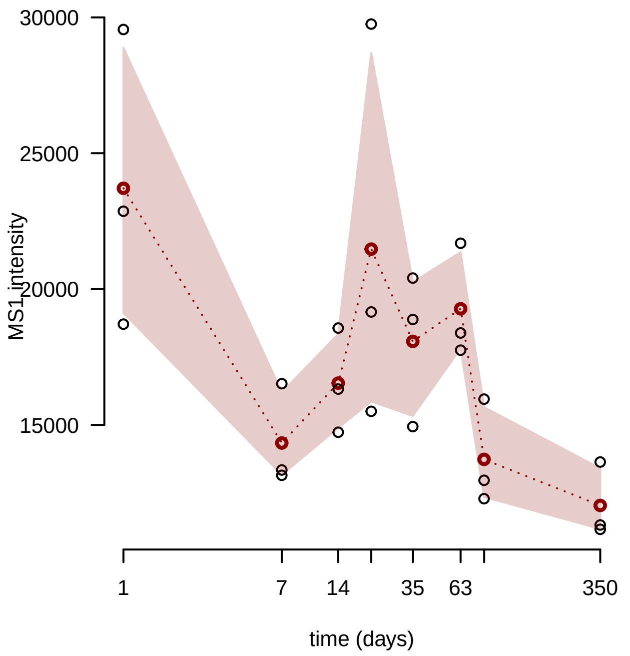

P70451
GN=Fer
fer (fms/fps related) protein kinase

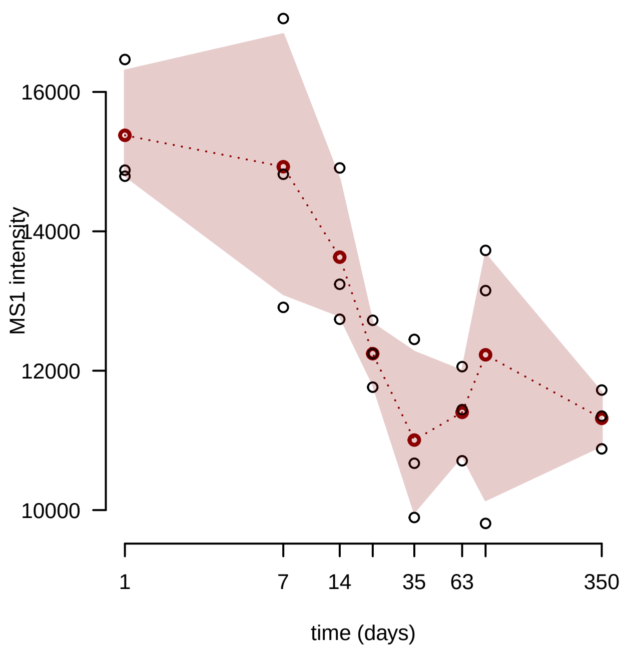

P70452
GN=Stx4

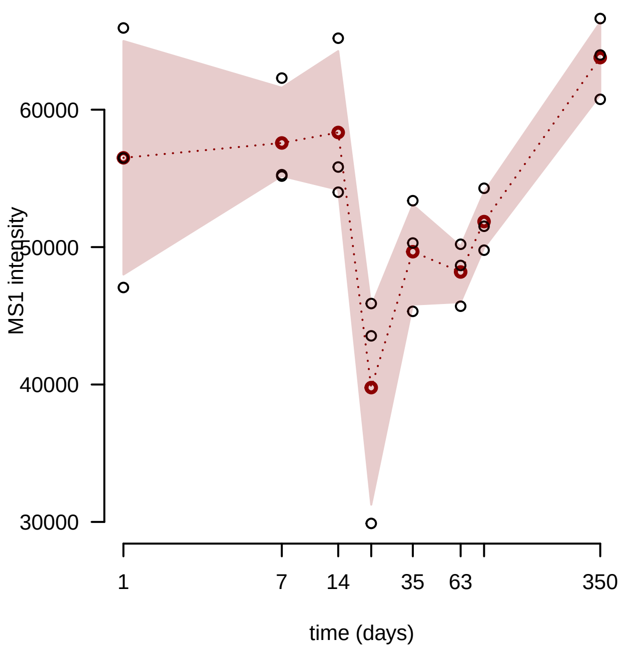

P70459
GN=Erf
Ets2 repressor factor

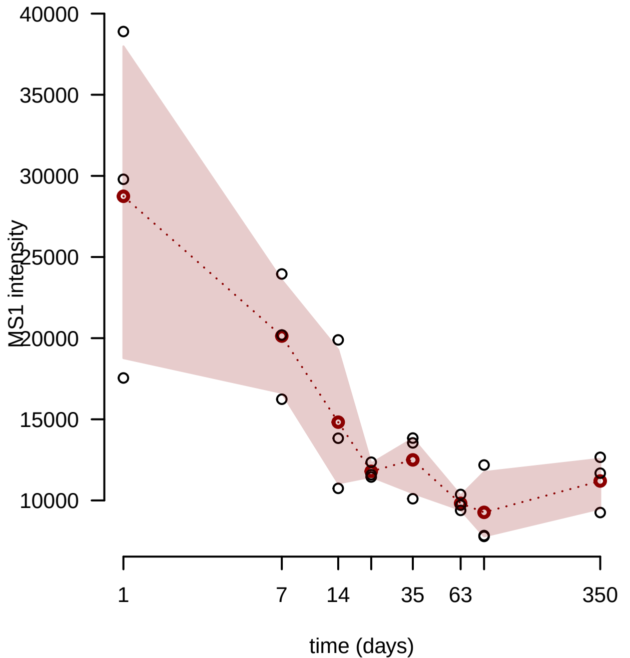

P70460
GN=Vasp
vasodilator−stimulated phosphoprotein

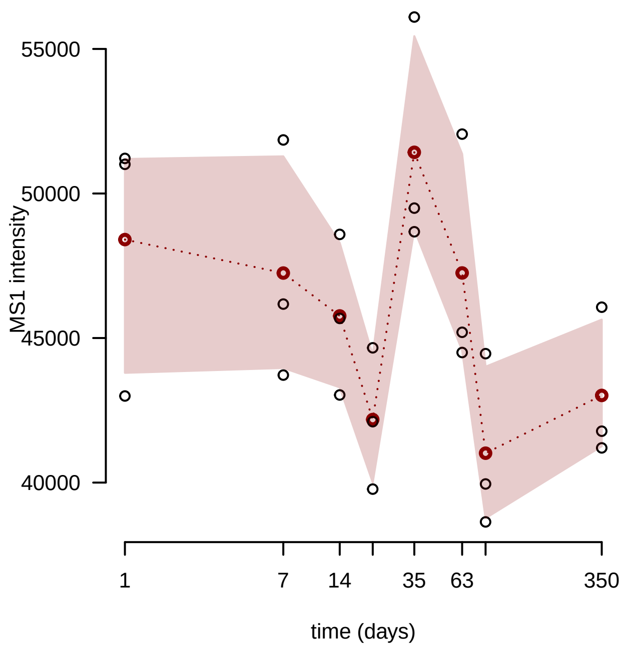

P70662
GN=Ldb1
LIM domain binding 1

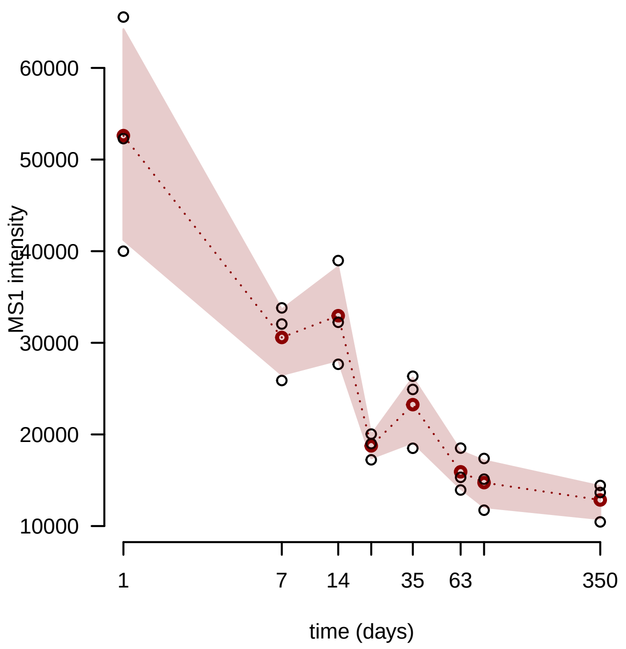

P70663
GN=Sparcl1
SPARC−like 1

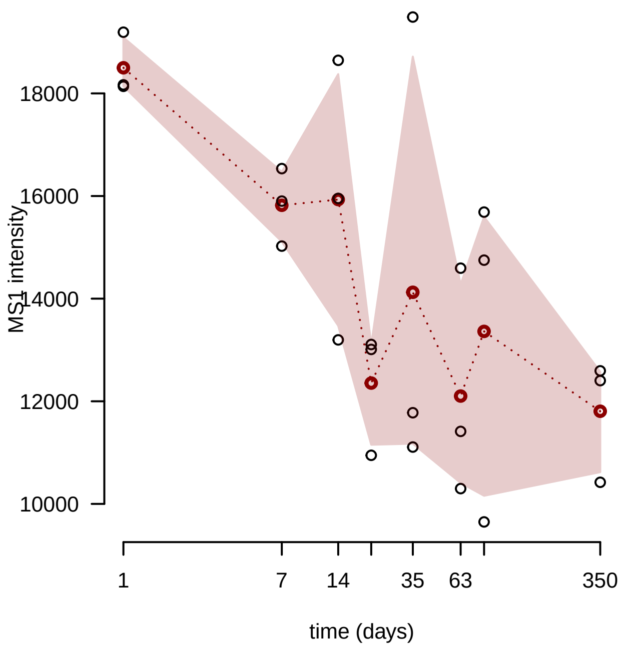

P70665
GN=Siae
sialic acid acetylesterase

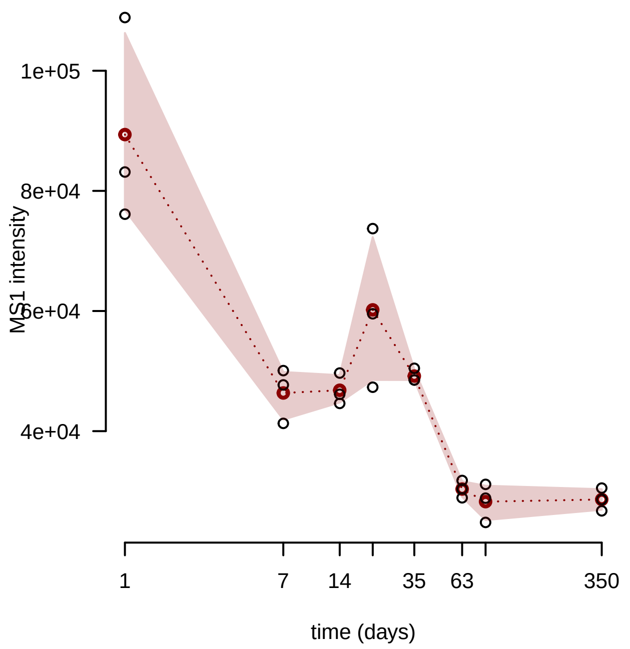

P70669
GN=Phex
phosphate regulating endopeptidase homol ...

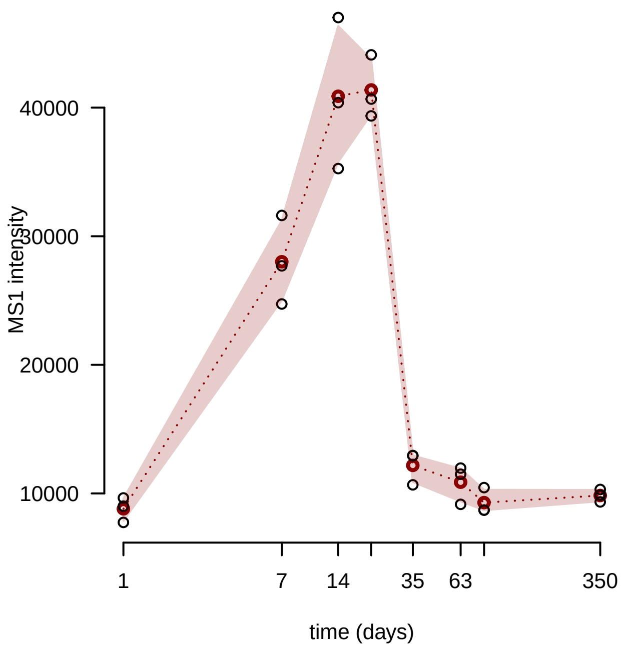

P70670
GN=Naca
nascent polypeptide−associated complex a ...

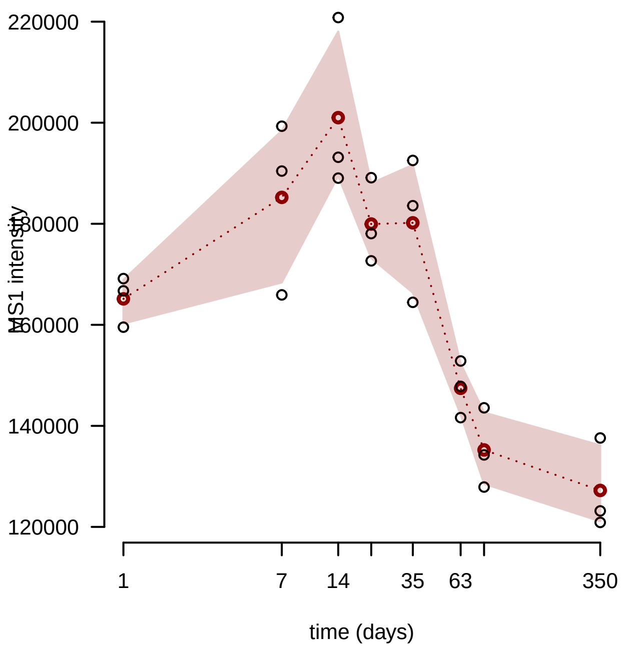

P70671
GN=Irf3
interferon regulatory factor 3

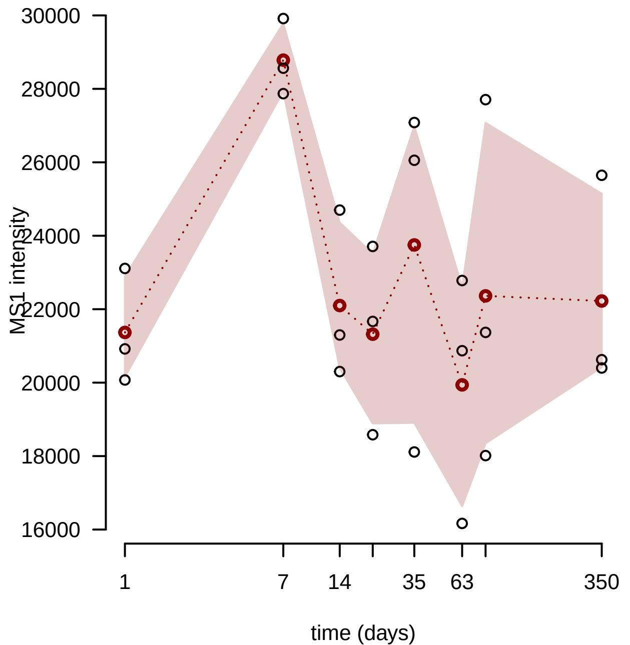

P70677
GN=Casp3
caspase 3

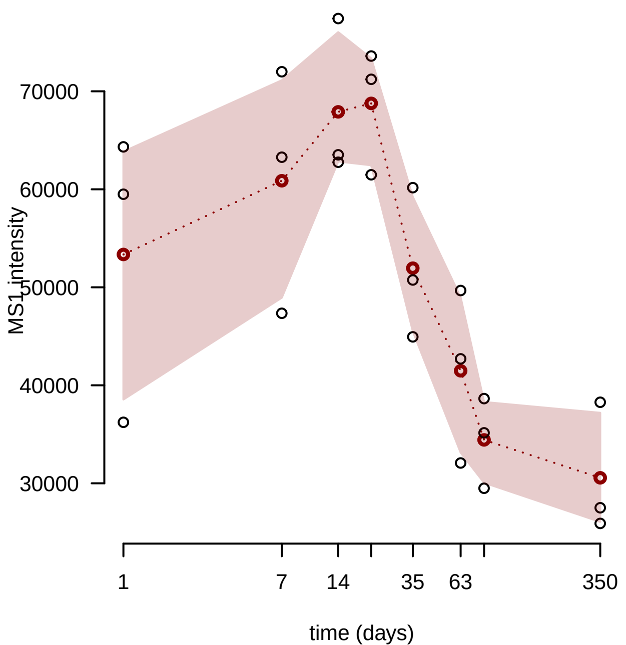

P70694
GN=Akr1c6
aldo−keto reductase family 1, member C6

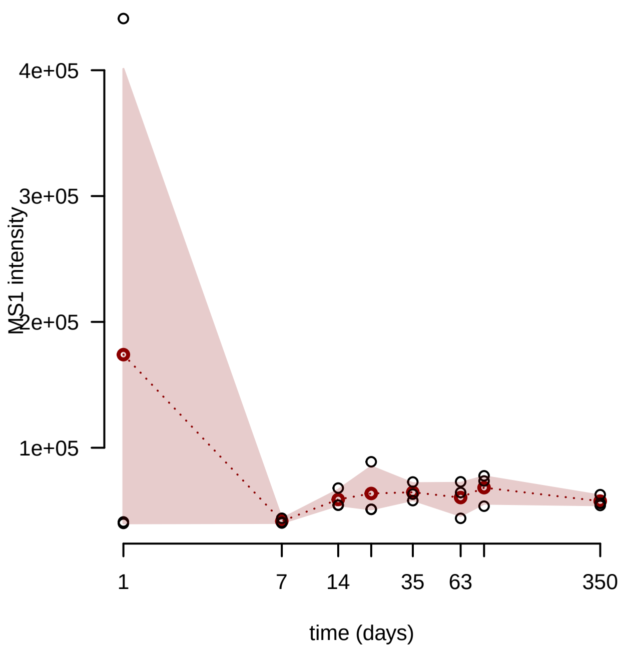

P70695
GN=Fbp2
fructose bisphosphatase 2

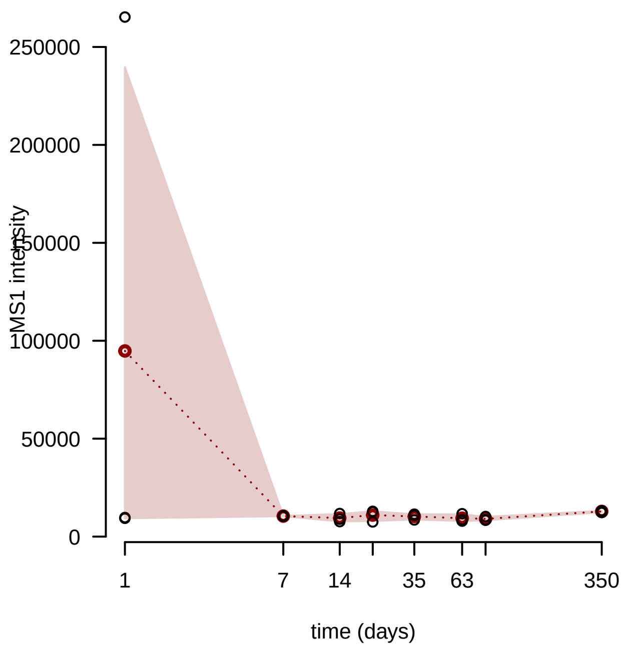

P70696
GN=Hist1h2ba

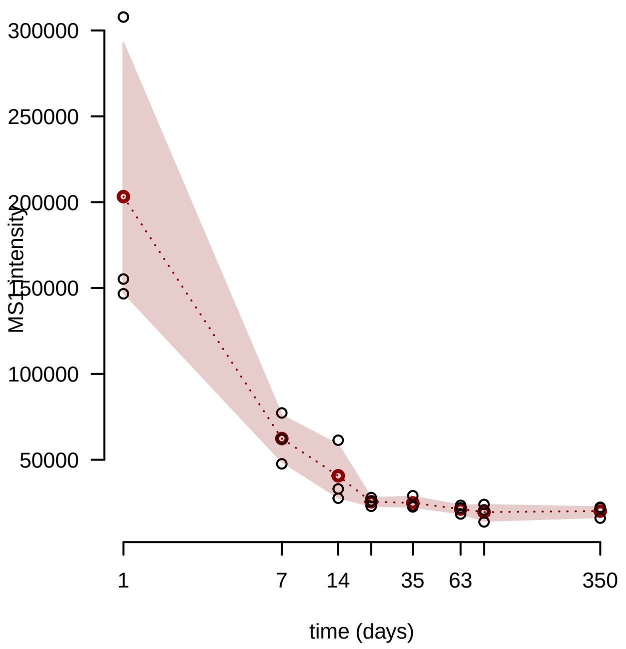

P70697
GN=Urod
uroporphyrinogen decarboxylase

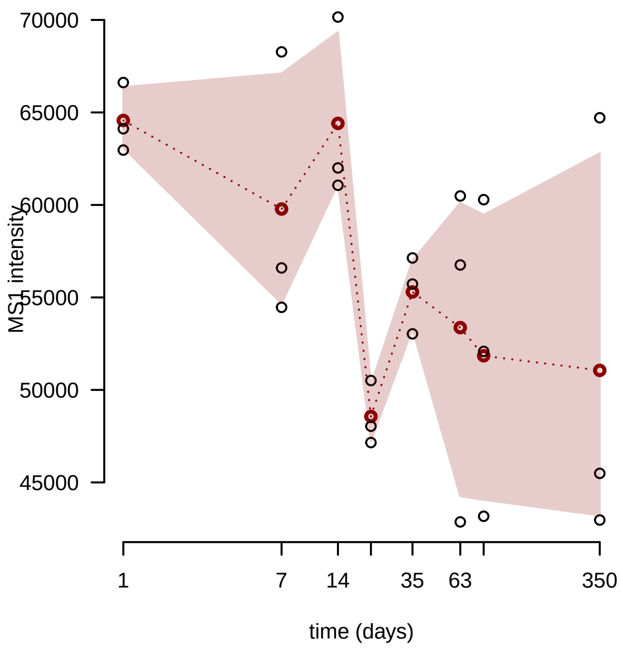

P70698
GN=Ctps1

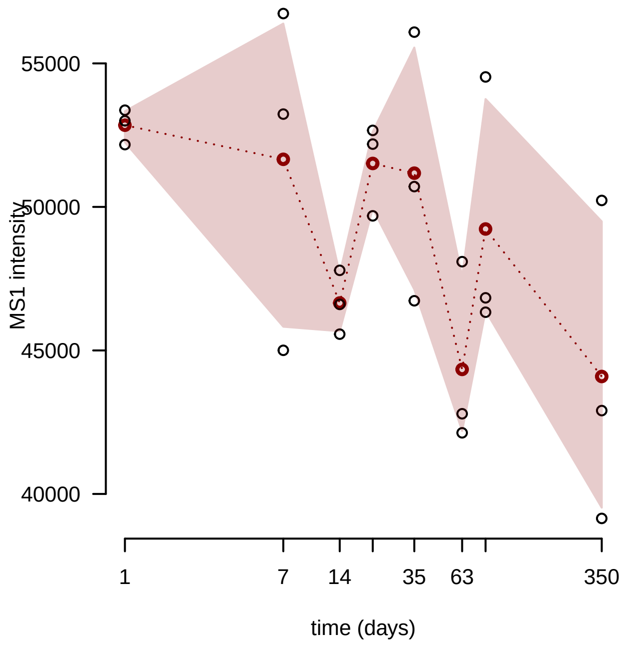

P70699
GN=Gaa
glucosidase, alpha, acid

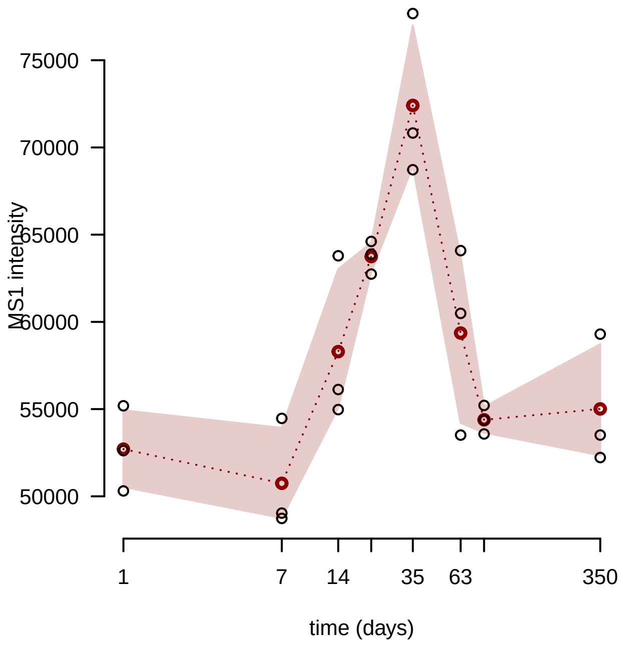

P70700
GN=Polr1b
polymerase (RNA) I polypeptide B

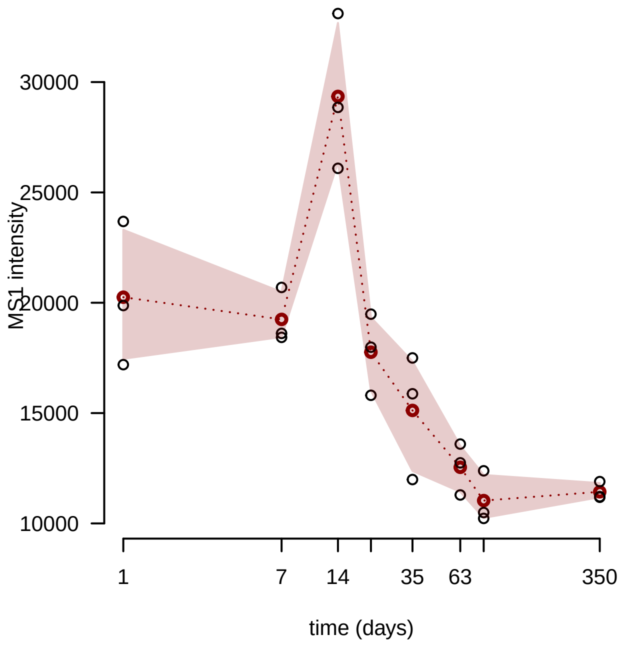

P70704
GN=Atp8a1
ATPase, aminophospholipid transporter (A ...

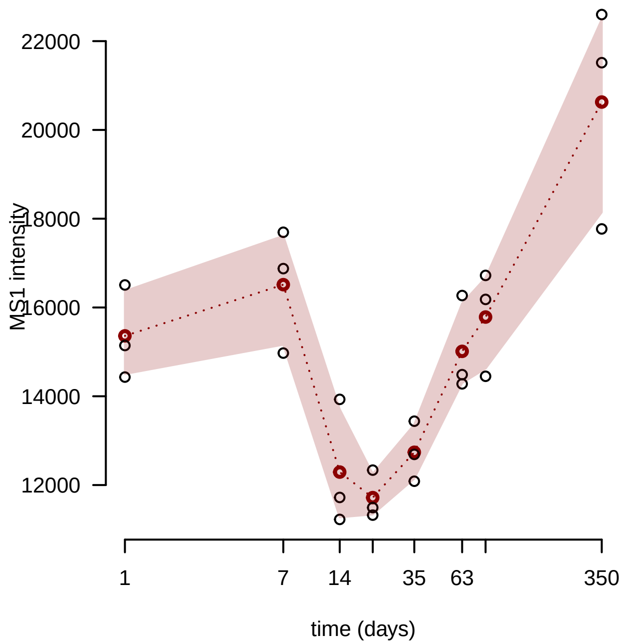

P80313
GN=Cct7
chaperonin containing Tcp1, subunit 7 (e ...

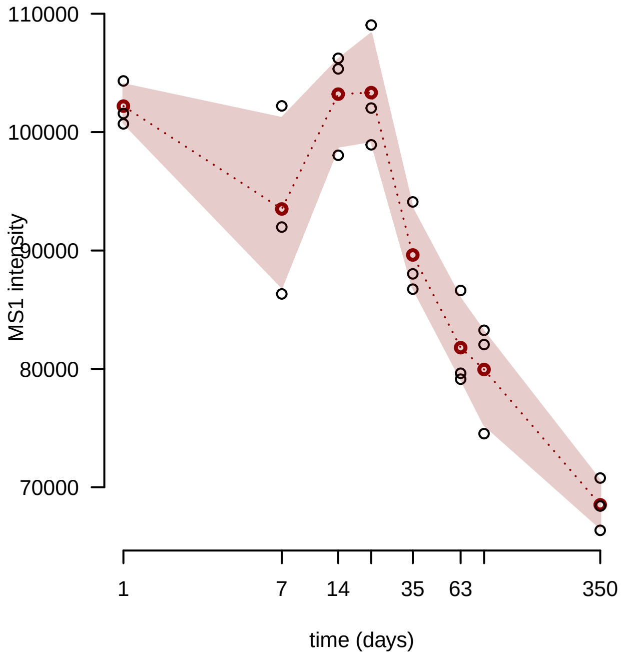

P80314
GN=Cct2
chaperonin containing Tcp1, subunit 2 (b ...

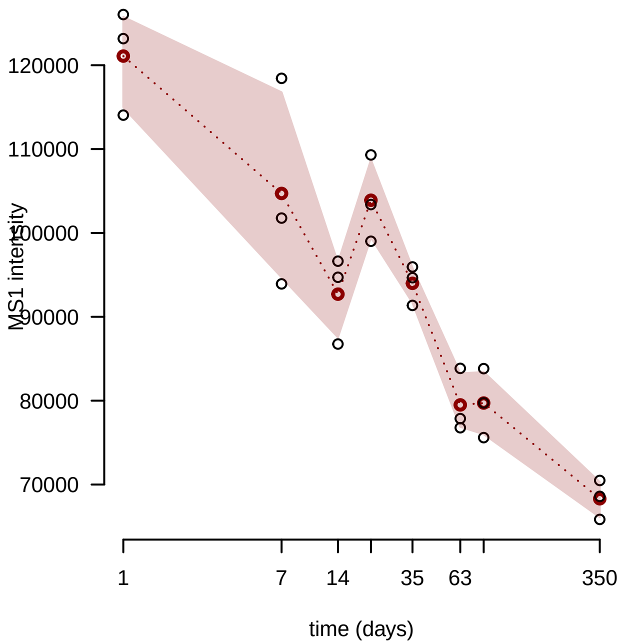

P80315
GN=Cct4
chaperonin containing Tcp1, subunit 4 (d ...

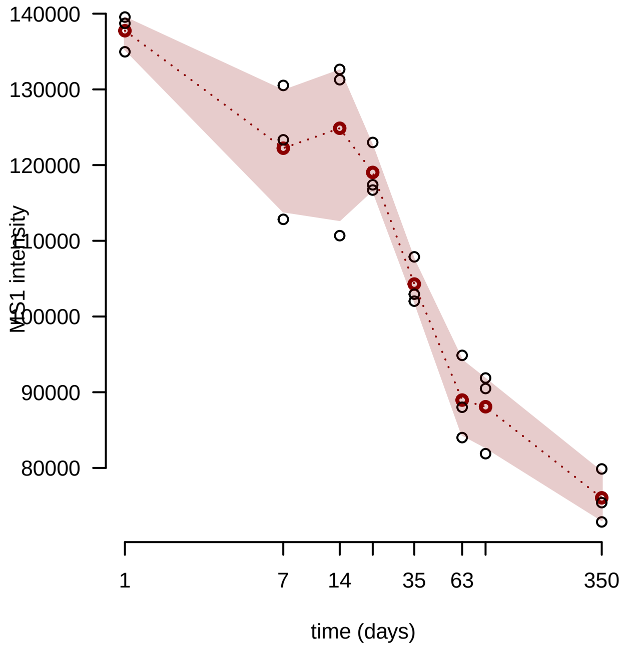

P80316
GN=Cct5
chaperonin containing Tcp1, subunit 5 (e ...

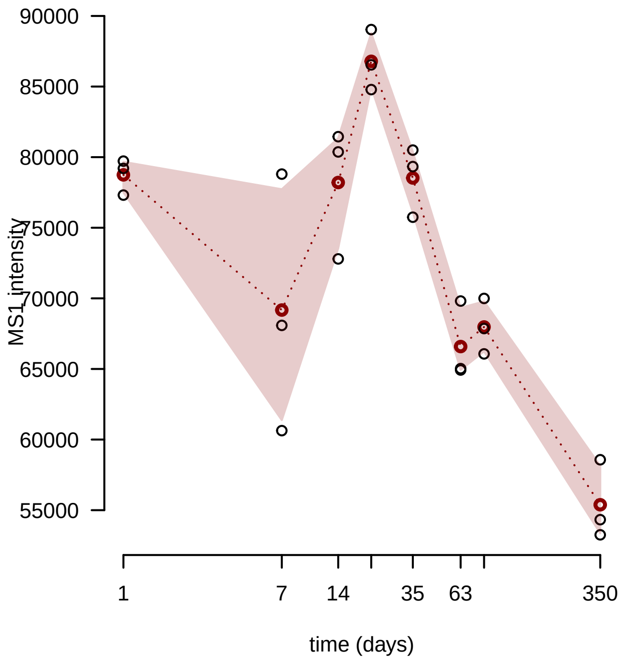

P80317
GN=Cct6a
chaperonin containing Tcp1, subunit 6a (...

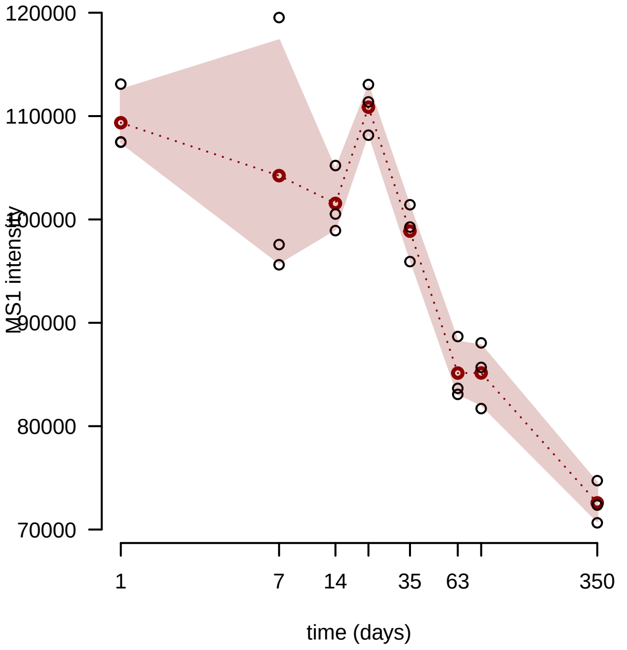

P80318
GN=Cct3
chaperonin containing Tcp1, subunit 3 (g ...

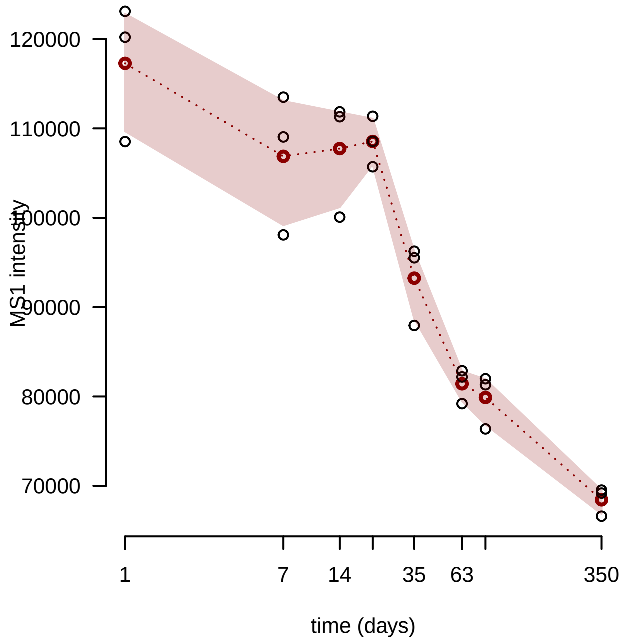

P81069
GN=Gabpb2
GA repeat binding protein, beta 2

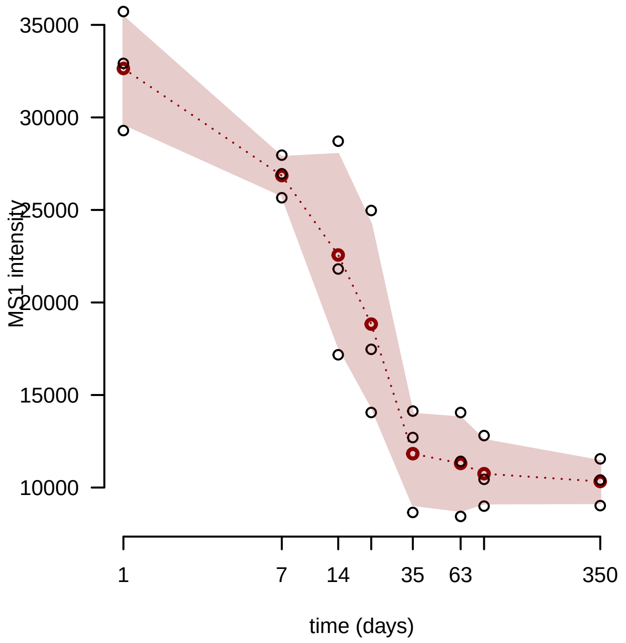

P81117
GN=Nucb2
nucleobindin 2

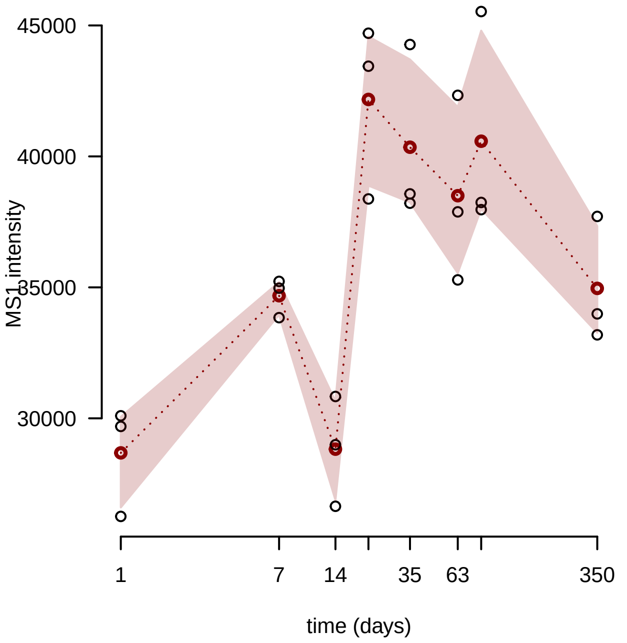

P81269
GN=Atf1
activating transcription factor 1

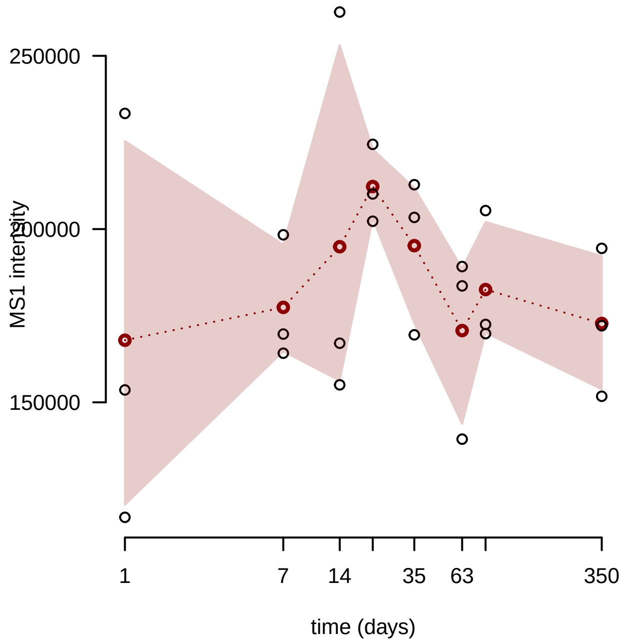

P81270
GN=Erg
ETS transcription factor

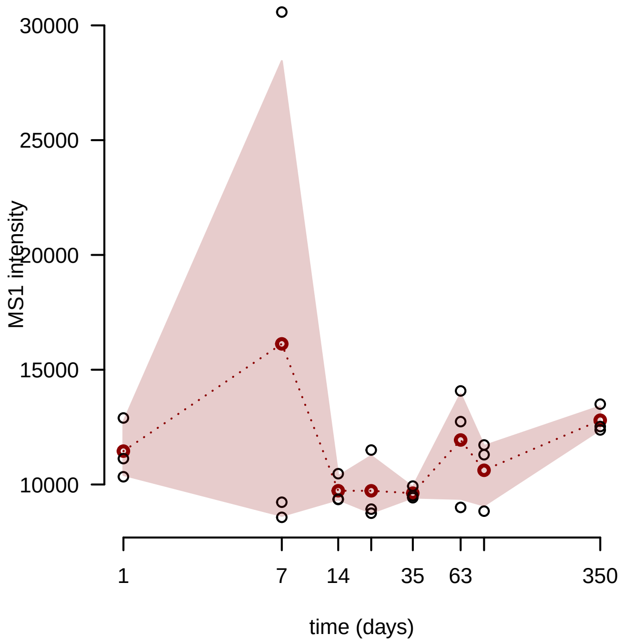

P82198
GN=Tgfbi
transforming growth factor, beta induced

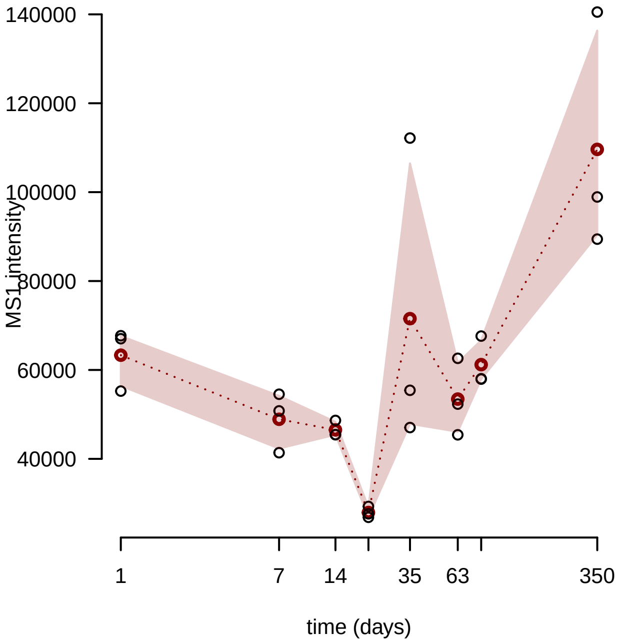

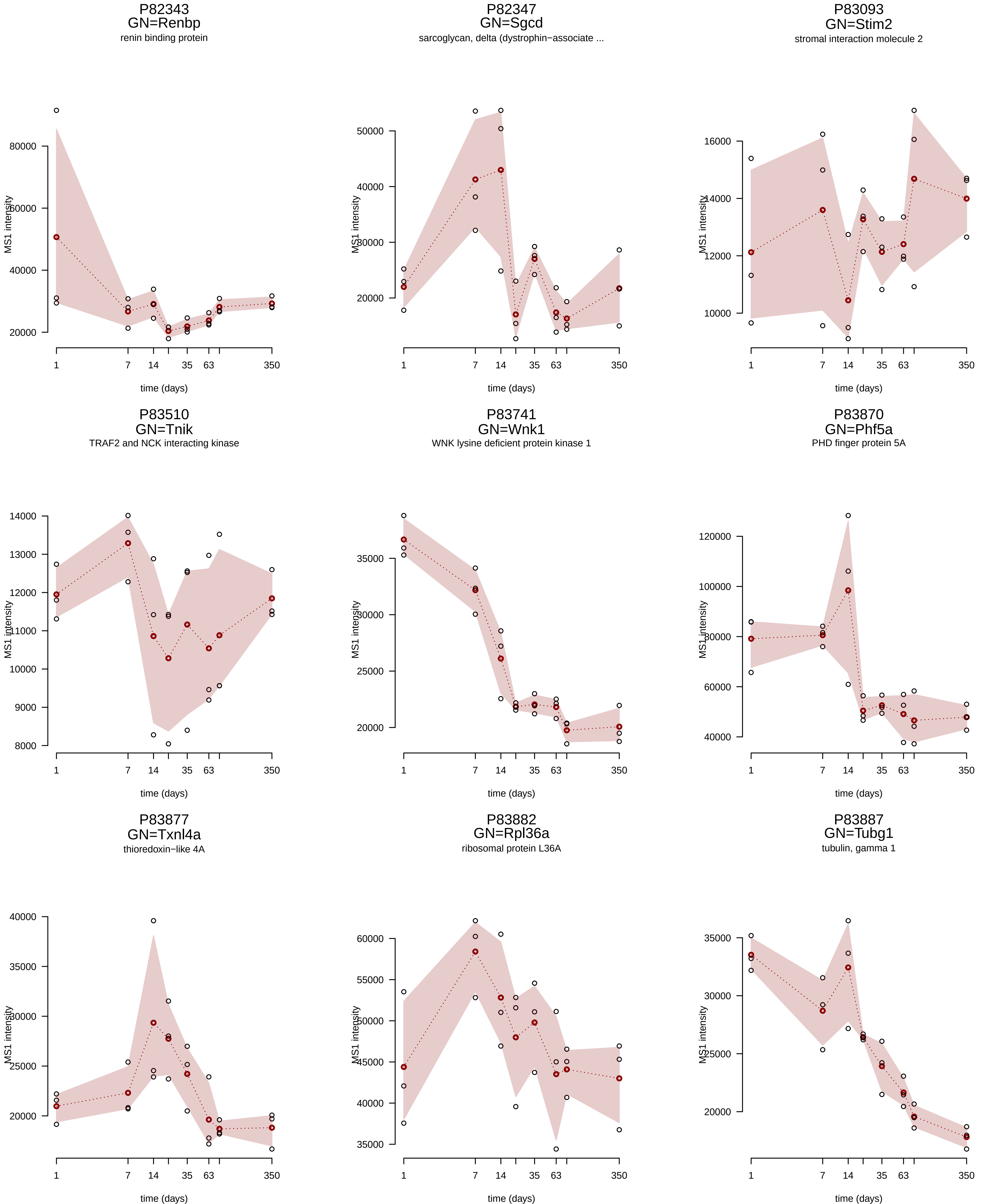
P82343
GN=Renbp
renin binding protein
P82347
GN=Sgcd
sarcoglycan, delta (dystrophin−associate ...
P83093
GN=Stim2
stromal interaction molecule 2
P83510
GN=Tnik
TRAF2 and NCK interacting kinase
P83741
GN=Wnk1
WNK lysine deficient protein kinase 1
P83870
GN=Phf5a
PHD finger protein 5A
P83877
GN=Txnl4a
thioredoxin−like 4A
P83882
GN=Rpl36a
ribosomal protein L36A
P83887
GN=Tubg1
tubulin, gamma 1
MS1 intensity
time (days)
1
7
14
35
63
350

P83917
GN=Cbx1
chromobox 1

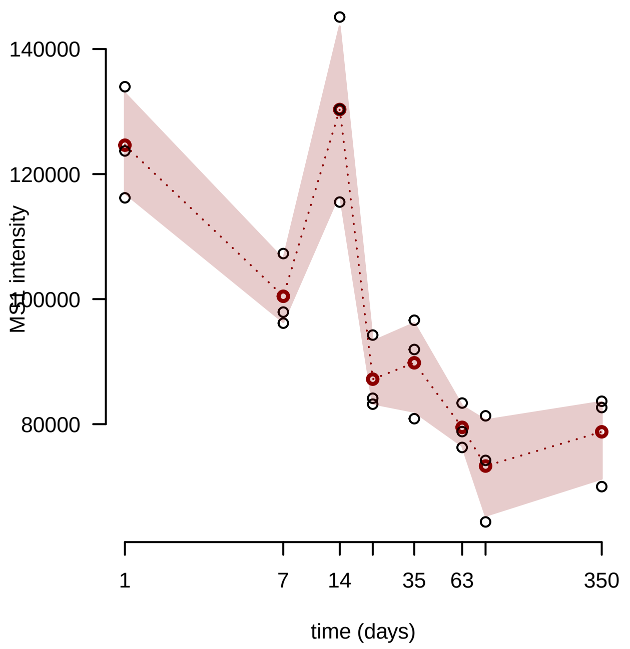

P83940
GN=Eloc
elongin C

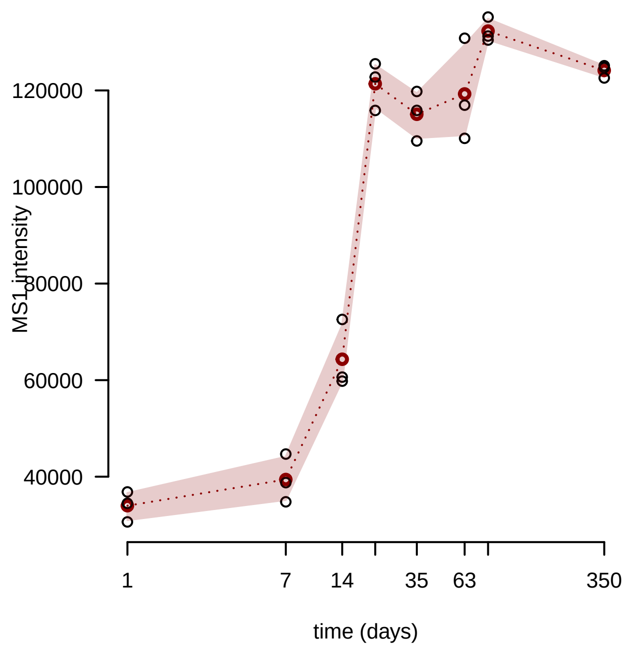

P84078
GN=Arf1
ADP−ribosylation factor 1

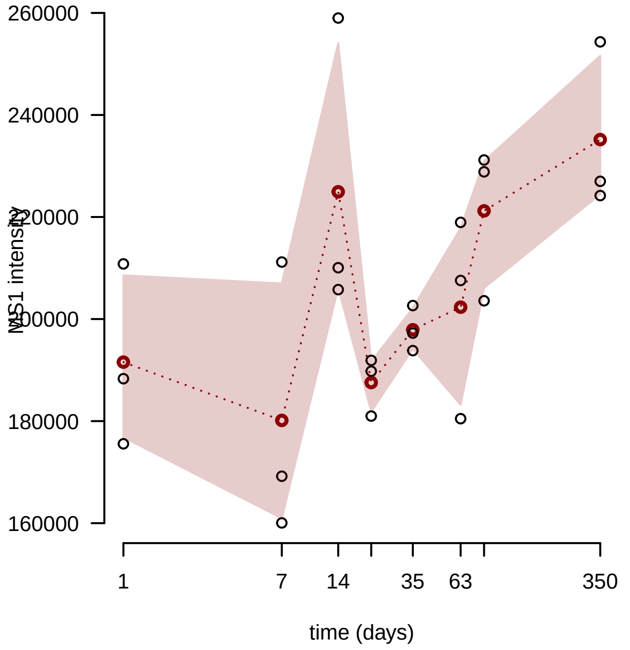

P84084
GN=Arf5
ADP−ribosylation factor 5

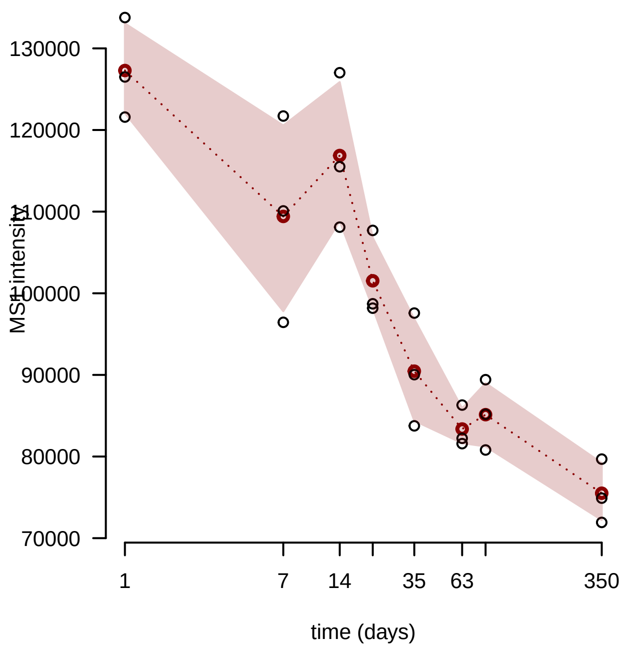

P84086
GN=Cplx2
complexin 2

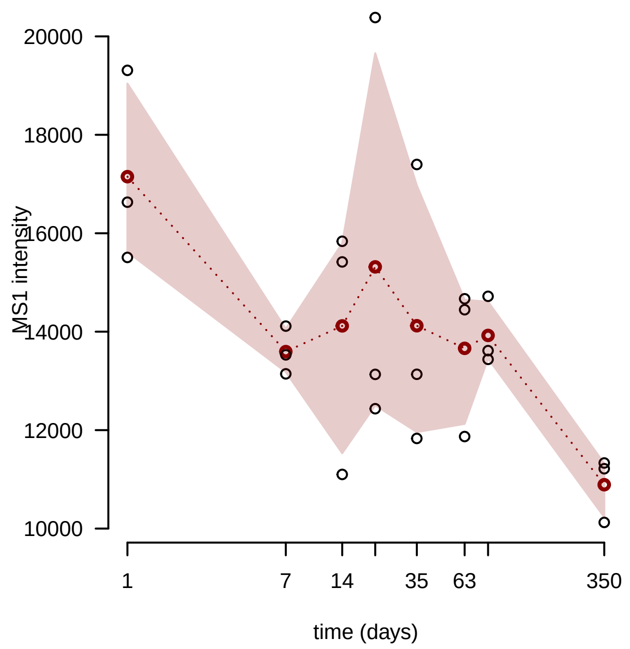

P84089
GN=Erh
ERH mRNA splicing and mitosis factor

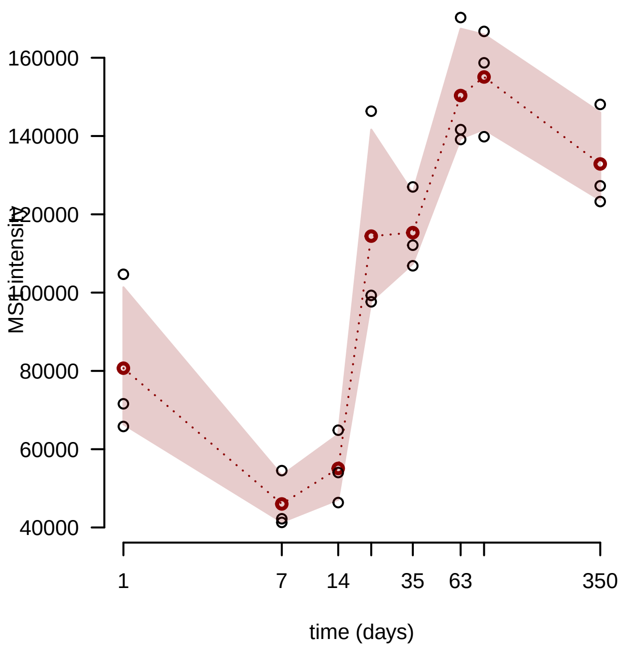

P84091
GN=Ap2m1
adaptor−related protein complex 2, mu 1 ...

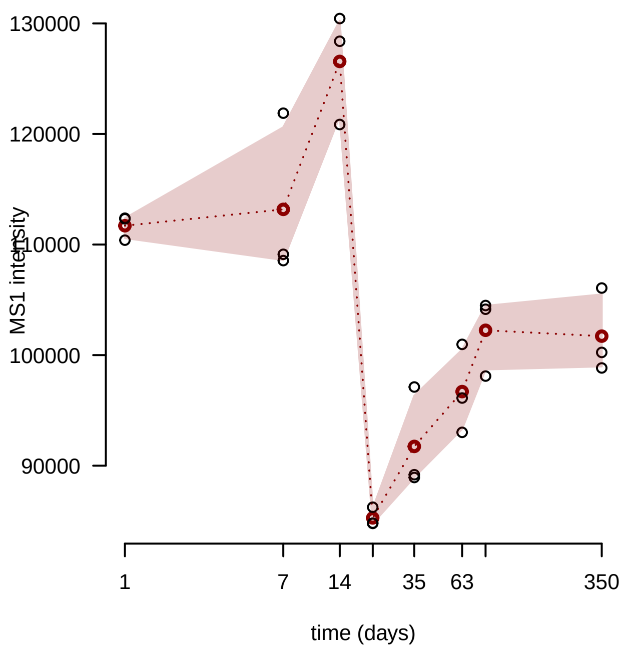

P84096
GN=Rhog
ras homolog family member G

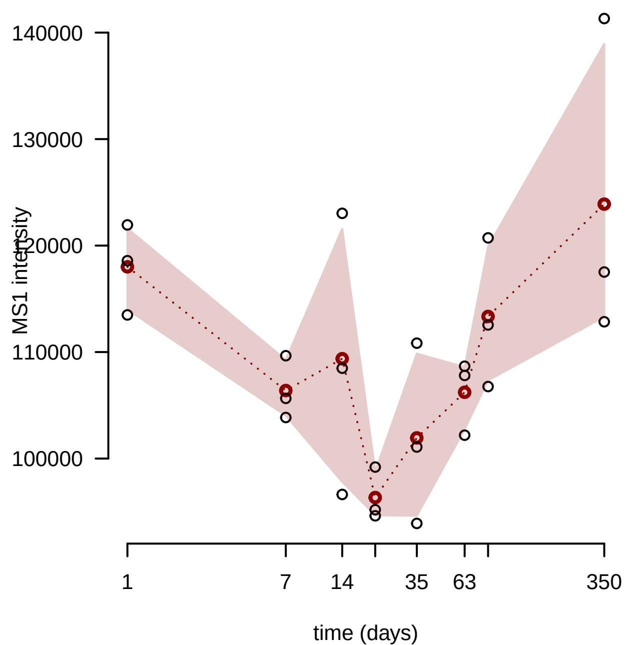

P84099
GN=Rpl19
ribosomal protein L19

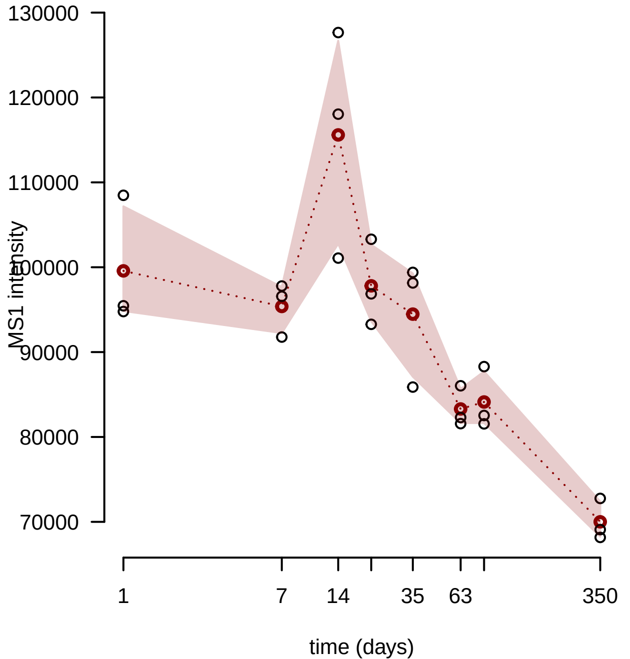

P84102
GN=Serf2
small EDRK−rich factor 2

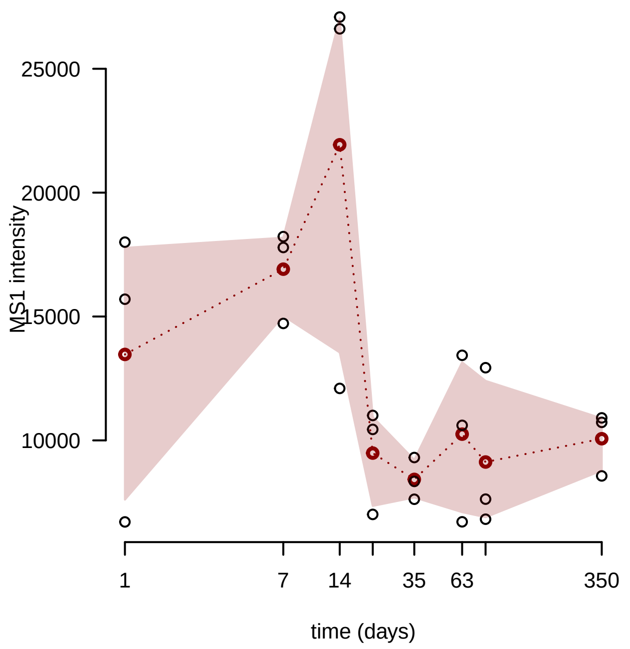

P84104
GN=Srsf3
serine and arginine−rich splicing factor ...

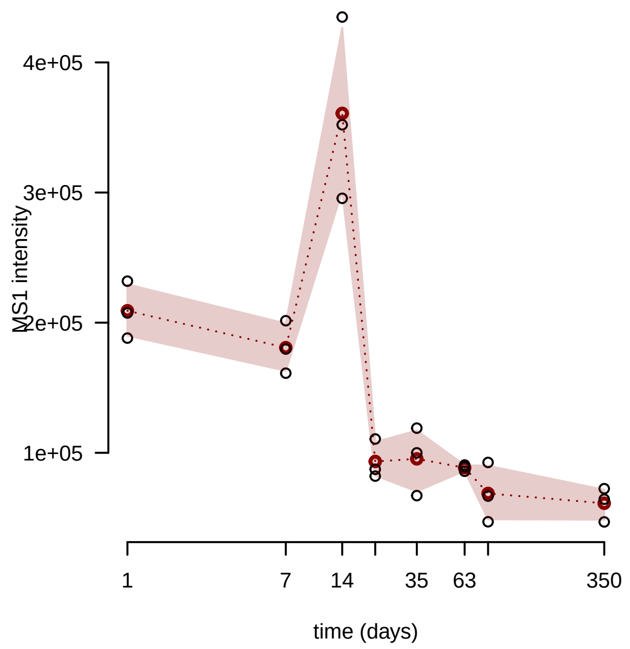

P84228
GN=Hist1h3b

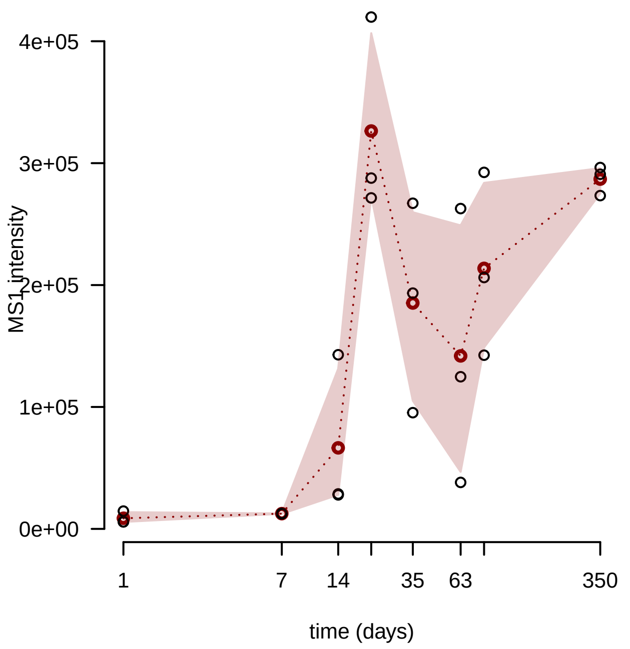

P84309
GN=Adcy5
adenylate cyclase 5

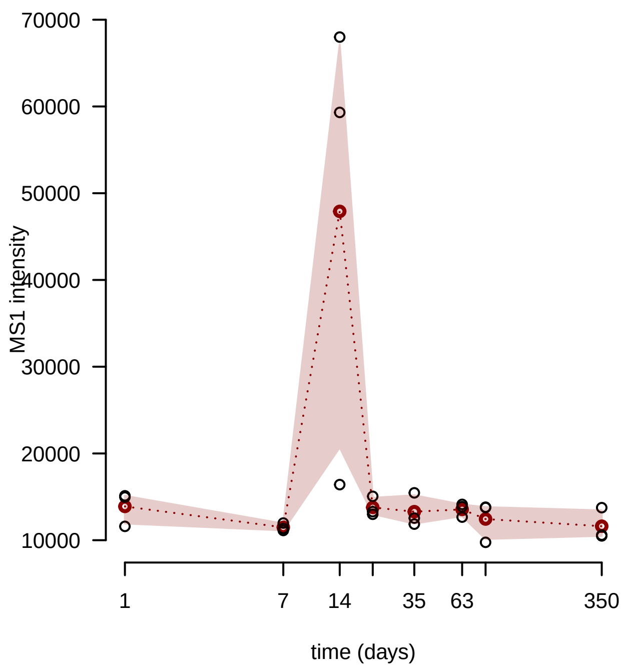

P84444
GN=Ccl21a
chemokine (C−C motif) ligand 21A (serine ...

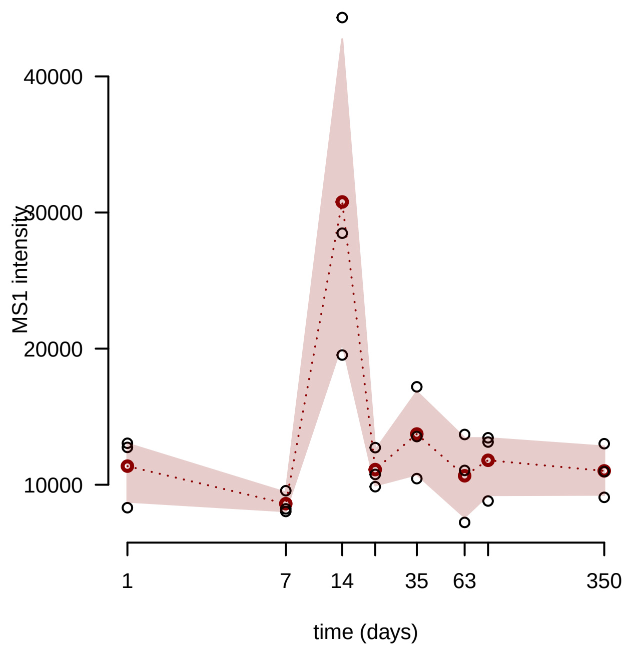

P85094
GN=Isoc2a
isochorismatase domain containing 2a

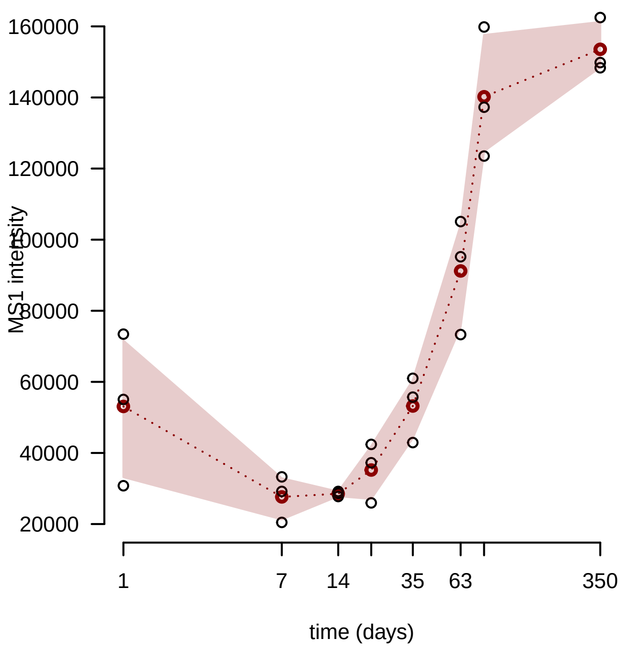

P86048
GN=Rpl10l
ribosomal protein L10−like

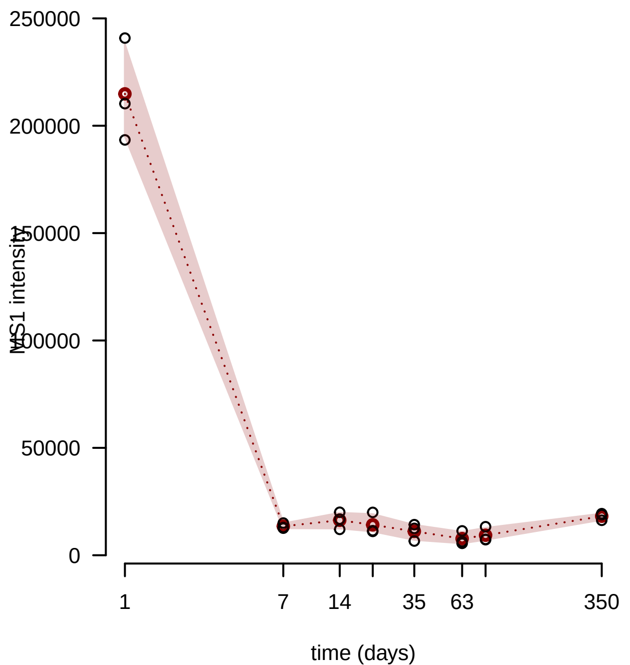

P97287
GN=Mcl1
myeloid cell leukemia sequence 1

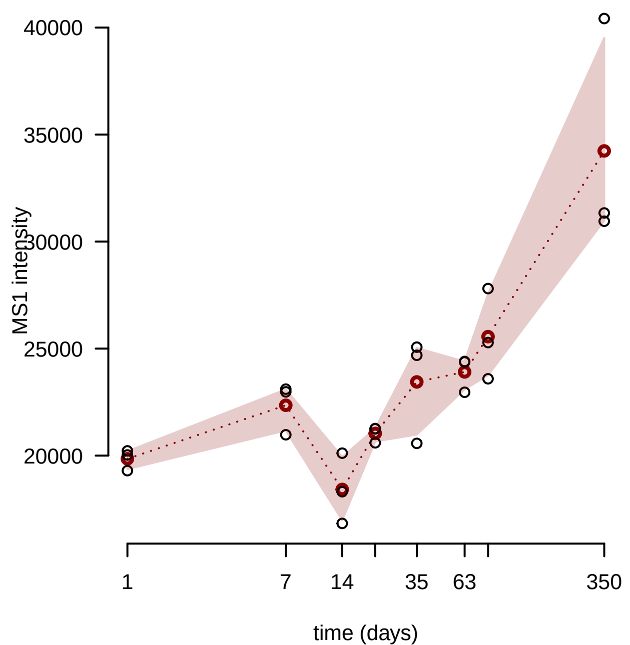

P97290
GN=Serping1
serine (or cysteine) peptidase inhibitor ...

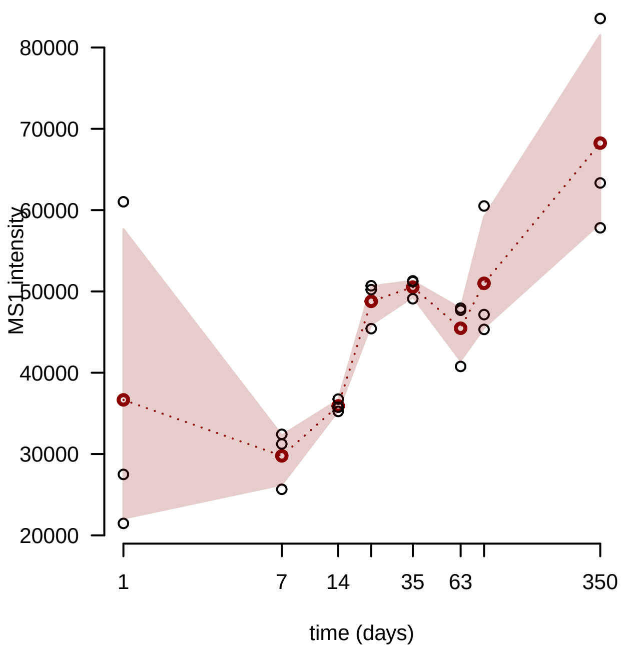

P97298
GN=Serpinf1
serine (or cysteine) peptidase inhibitor ...

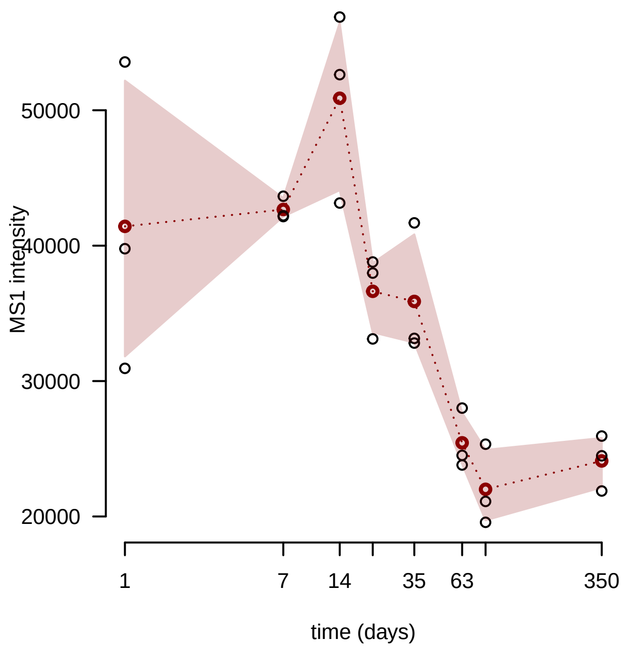

P97300
GN=Nptn
neuroplastin

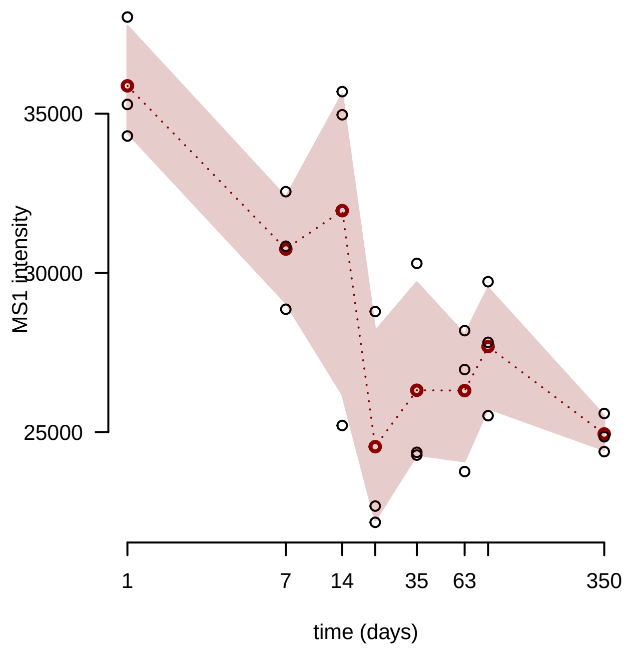

P97304
GN=Polr1d
polymerase (RNA) I polypeptide D

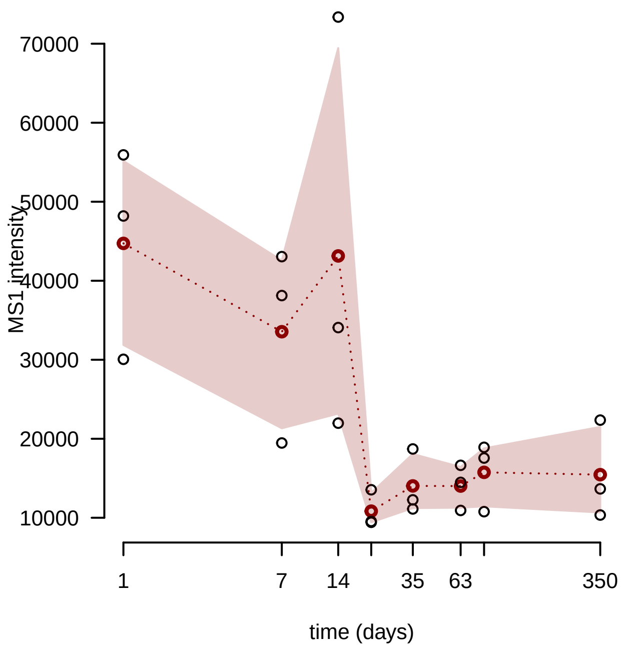

P97305
GN=Nfatc3
nuclear factor of activated T cells, cyt ...

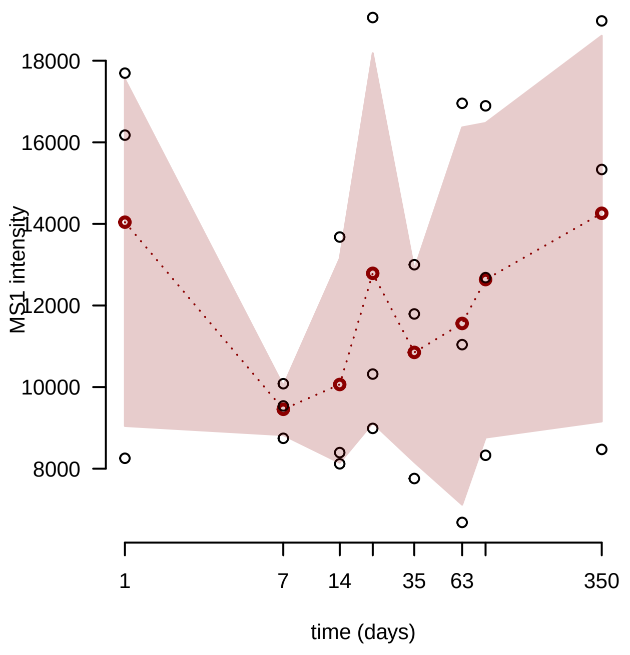

P97310
GN=Mcm2
minichromosome maintenance complex compo ...

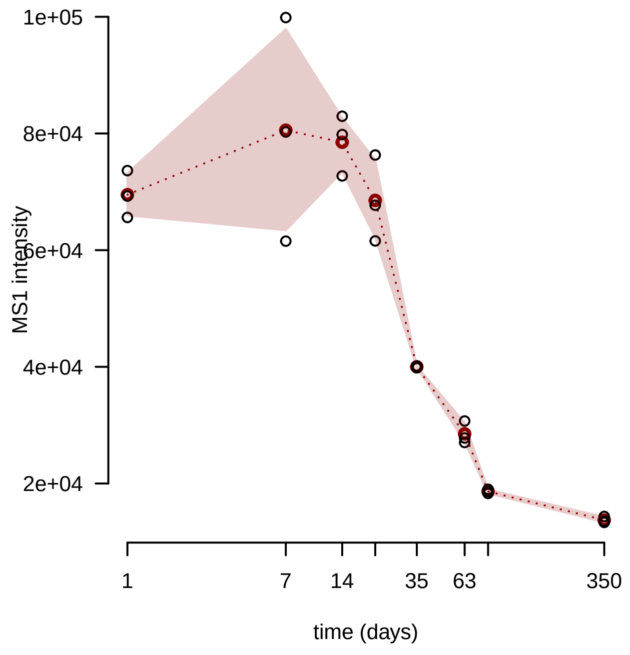

P97311
GN=Mcm6
minichromosome maintenance complex compo ...

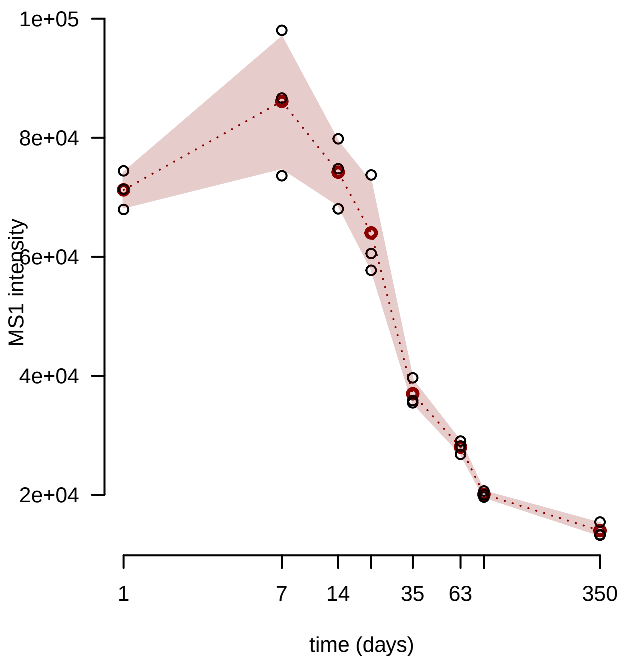

P97313
GN=Prkdc
protein kinase, DNA activated, catalytic ...

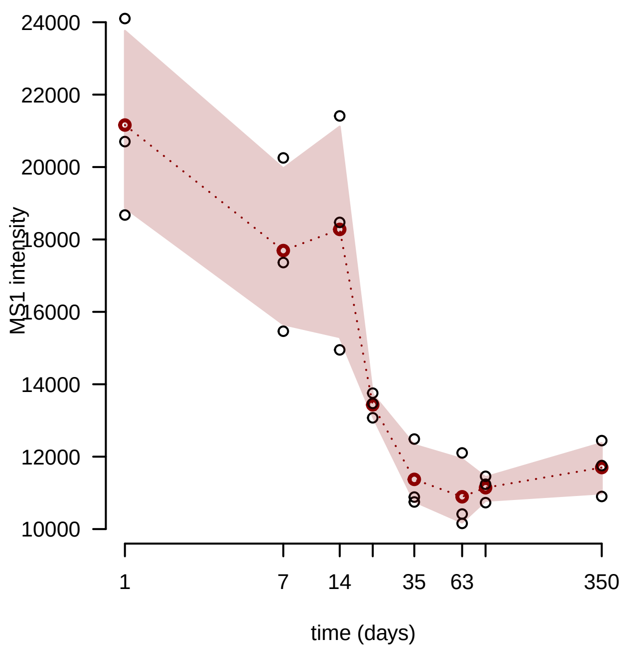

P97314
GN=Csrp2
cysteine and glycine−rich protein 2

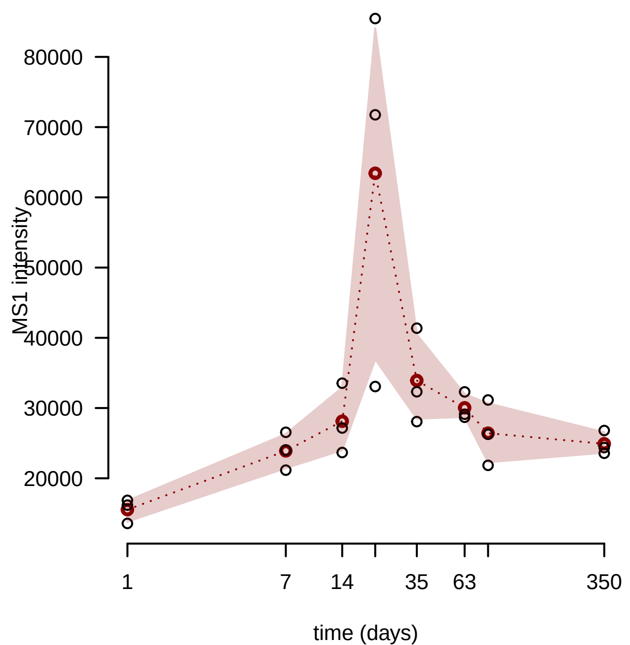

P97315
GN=Csrp1
cysteine and glycine−rich protein 1

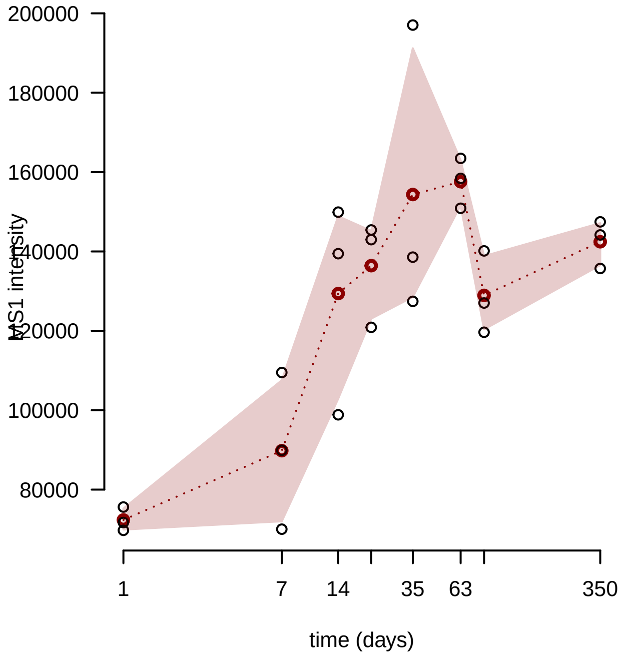

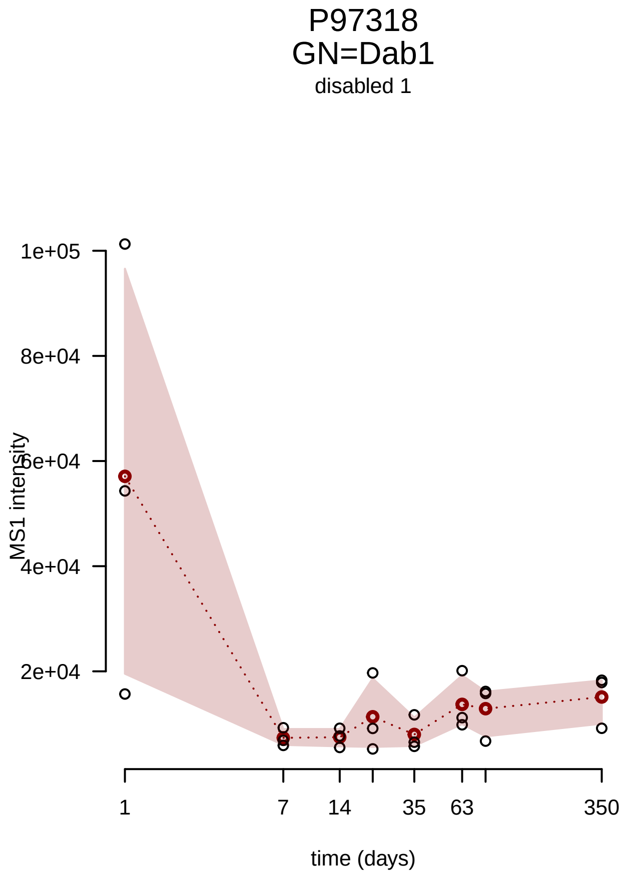
P97318
GN=Dab1
disabled 1
MS1 intensity
1e+05
8e+04
6e+04
4e+04
2e+04
1
7
14
35
63
350
time (days)

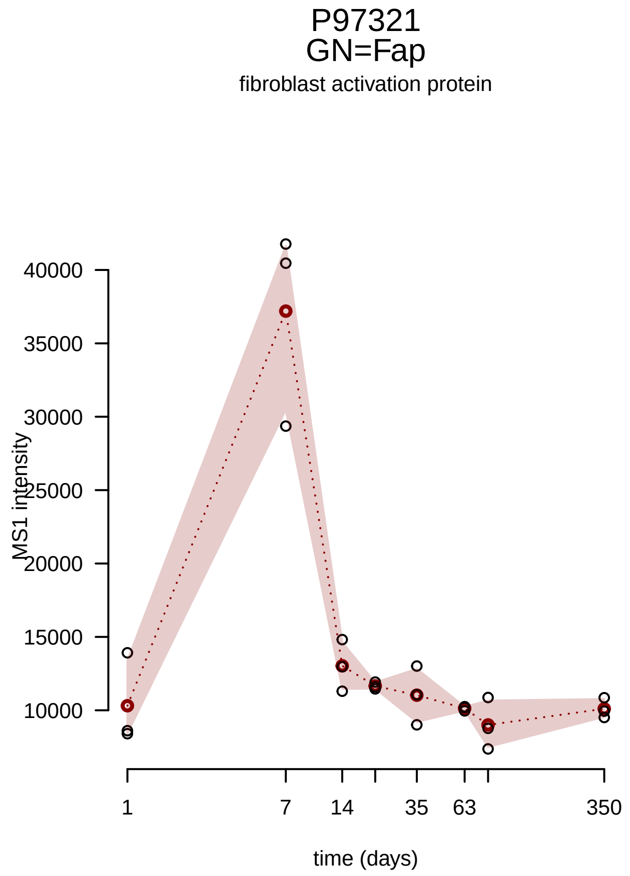
P97321
GN=Fap
fibroblast activation protein
MS1 intensity
40000
35000
30000
25000
20000
15000
10000
1
7
14
35
63
350
time (days)

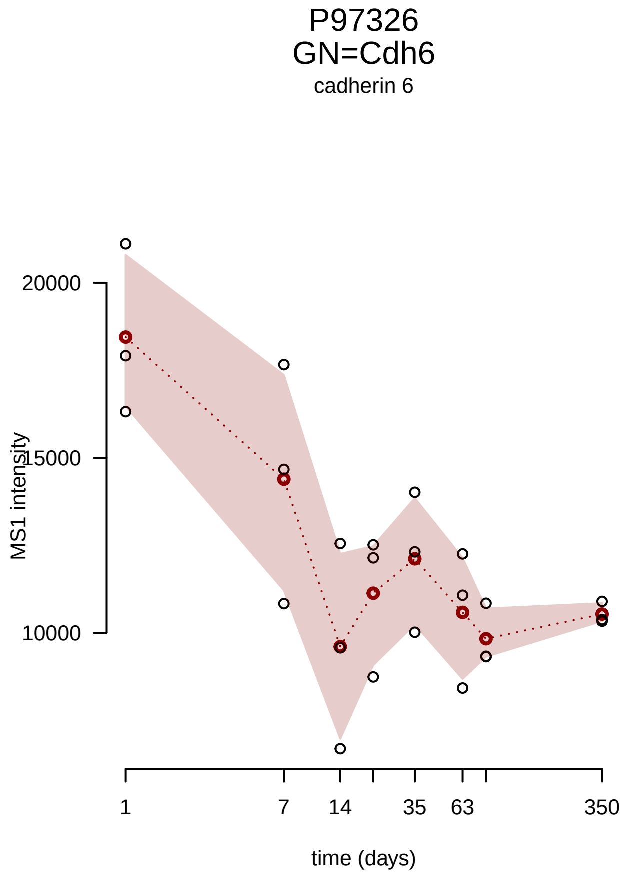
P97326
GN=Cdh6
cadherin 6
MS1 intensity
20000
15000
10000
1
7
14
35
63
350
time (days)

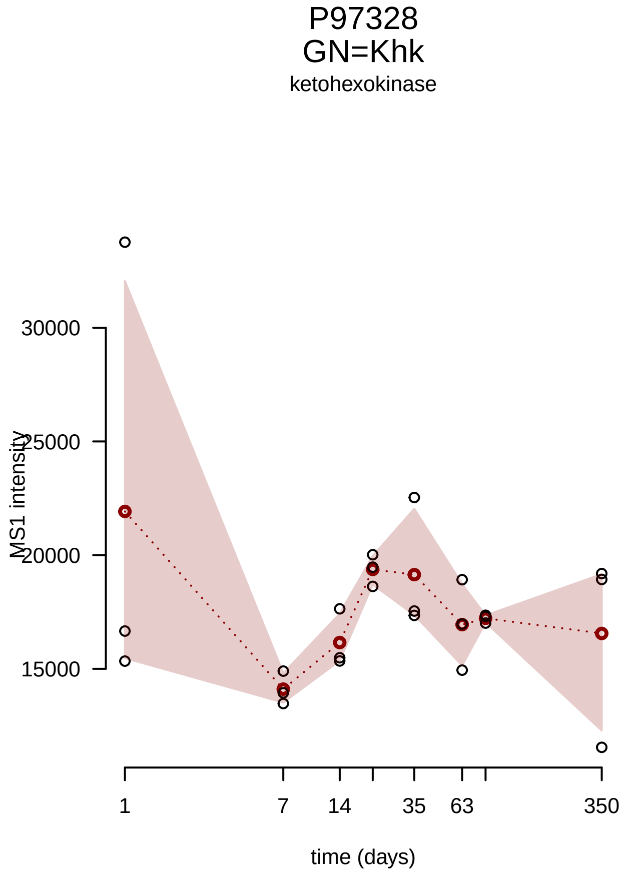
P97328
GN=Khk
ketohexokinase
MS1 intensity
30000
25000
20000
15000
1
7
14
35
63
350
time (days)

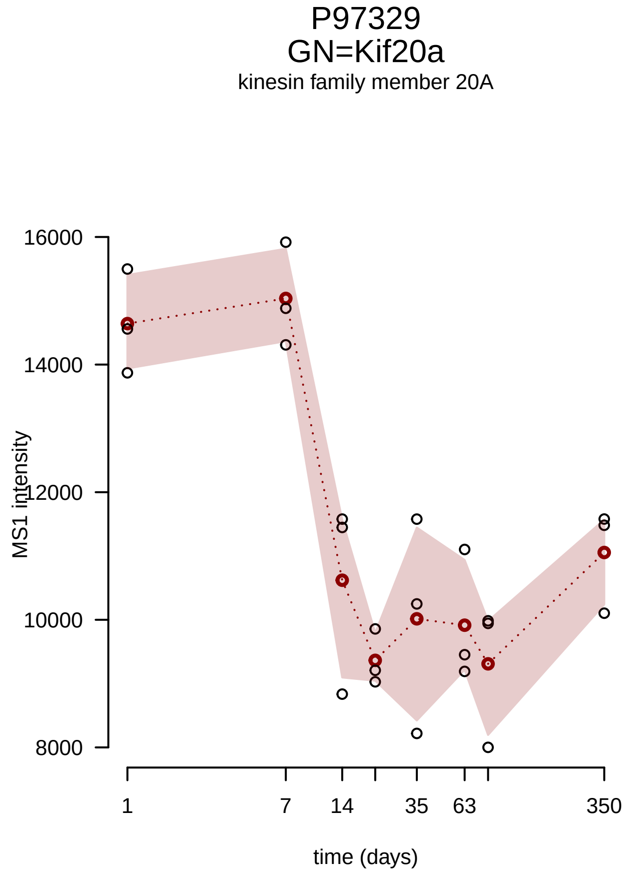
P97329
GN=Kif20a
kinesin family member 20A
MS1 intensity
16000
14000
12000
10000
8000
1
7
14
35
63
350
time (days)

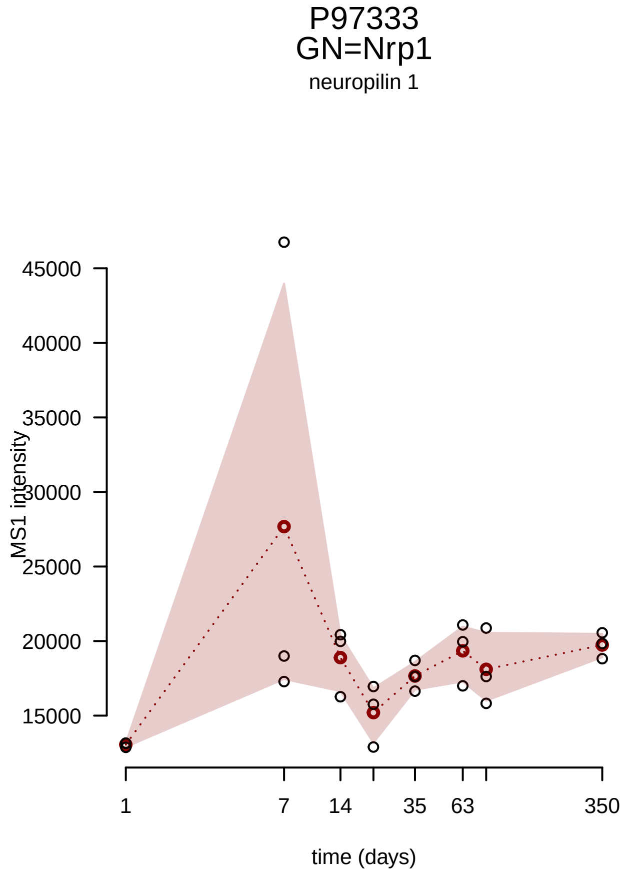
P97333
GN=Nrp1
neuropilin 1
MS1 intensity
45000
40000
35000
30000
25000
20000
15000
1
7
14
35
63
350
time (days)

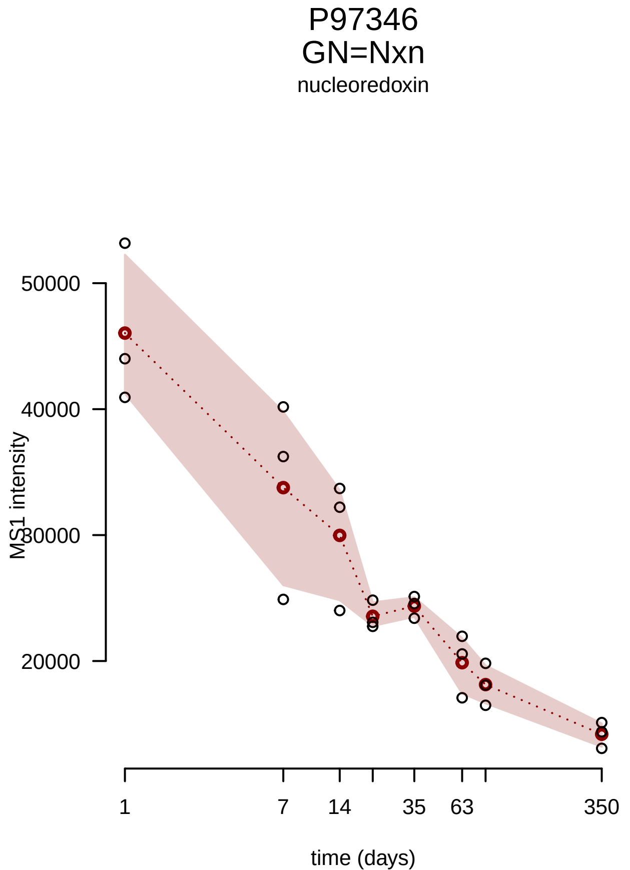
P97346
GN=Nxn
nucleoredoxin
MS1 intensity
50000
40000
30000
20000
1
7
14
35
63
350
time (days)

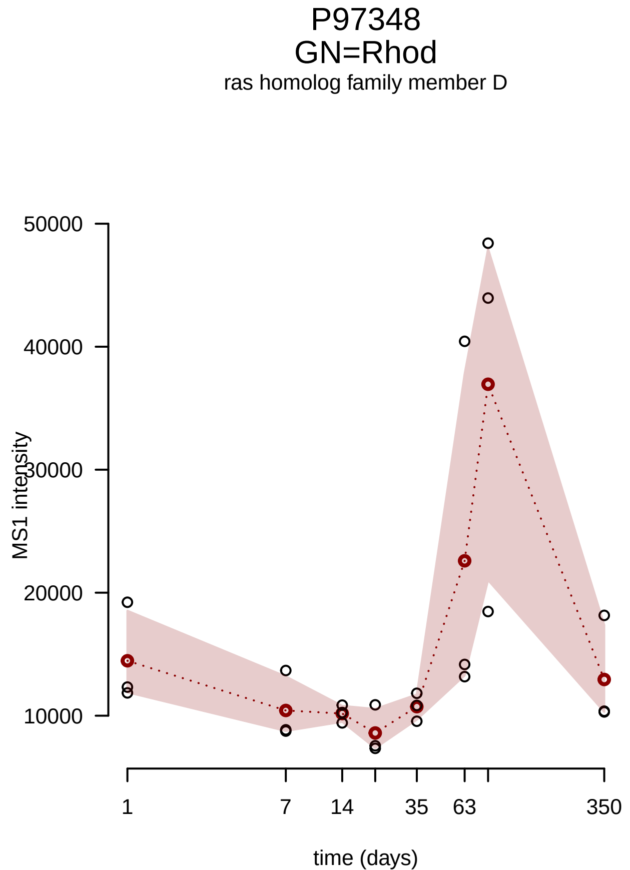
P97348
GN=Rhod
ras homolog family member D
MS1 intensity
50000
40000
30000
20000
10000
1
7
14
35
63
350
time (days)

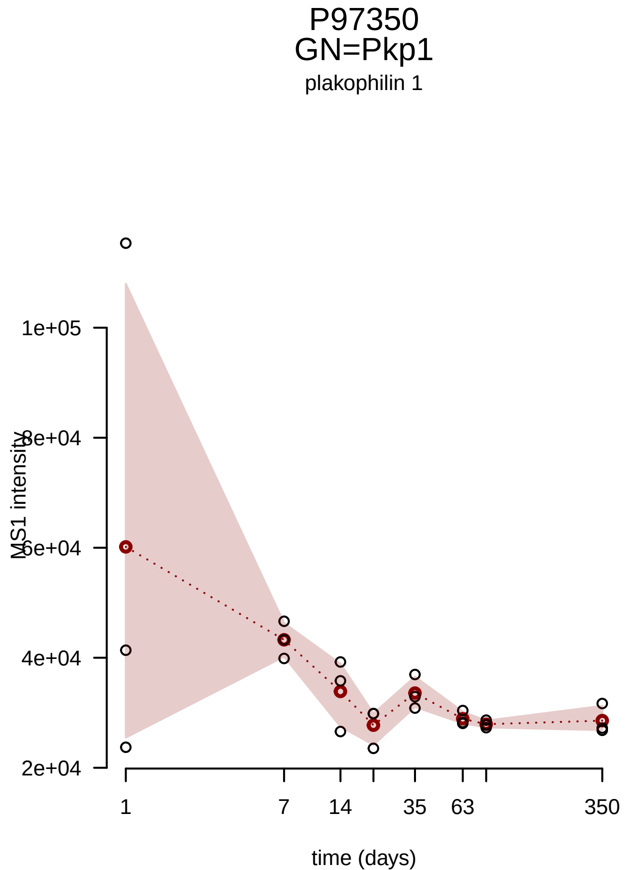
P97350
GN=Pkp1
plakophilin 1
MS1 intensity
1e+05
8e+04
6e+04
4e+04
2e+04
1
7
14
35
63
350
time (days)

P97351
GN=Rps3a

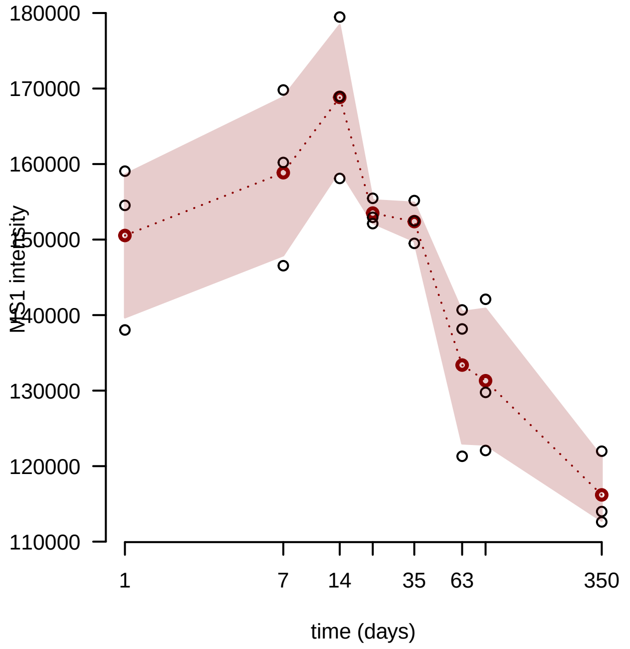

P97352
GN=S100a13
S100 calcium binding protein A13

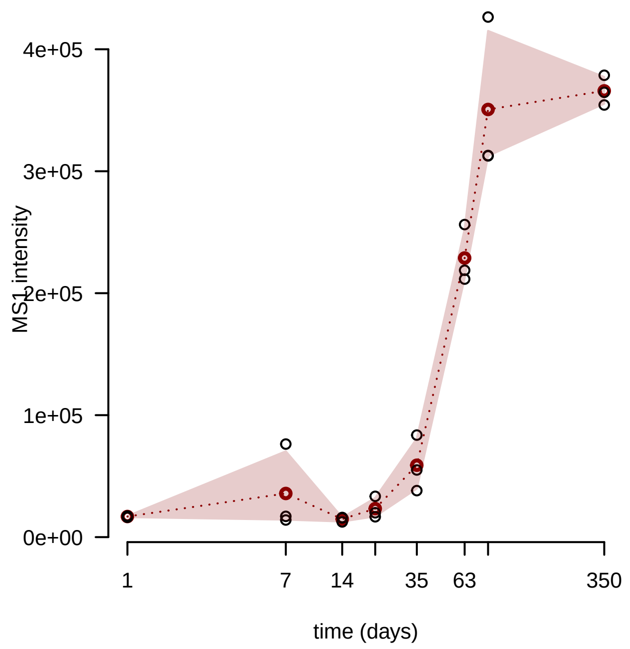

P97355
GN=Sms
spermine synthase

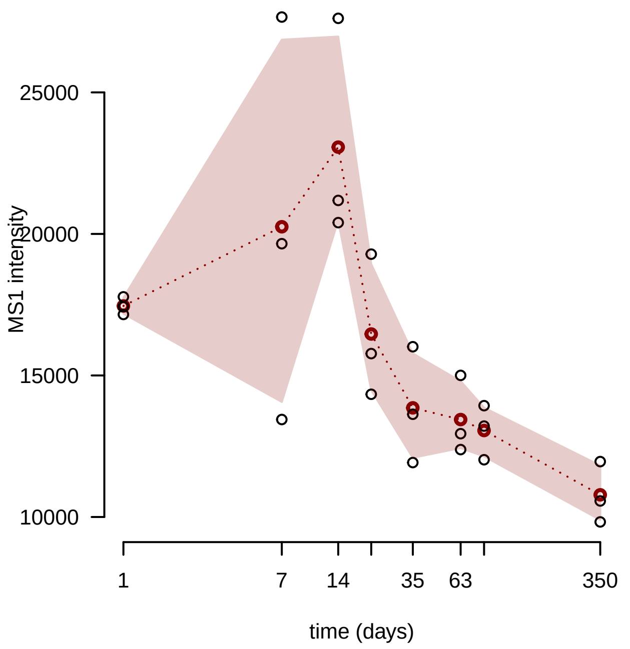

P97360
GN=Etv6
ets variant 6

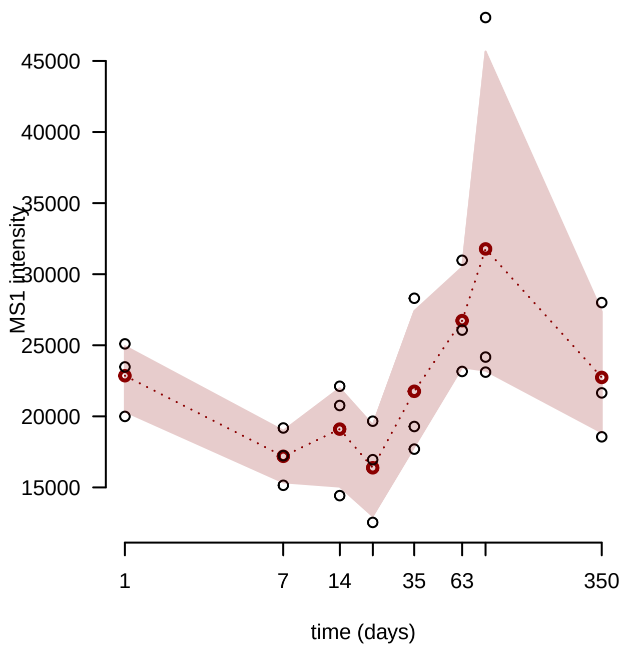

P97363
GN=Sptlc2
serine palmitoyltransferase, long chain ...

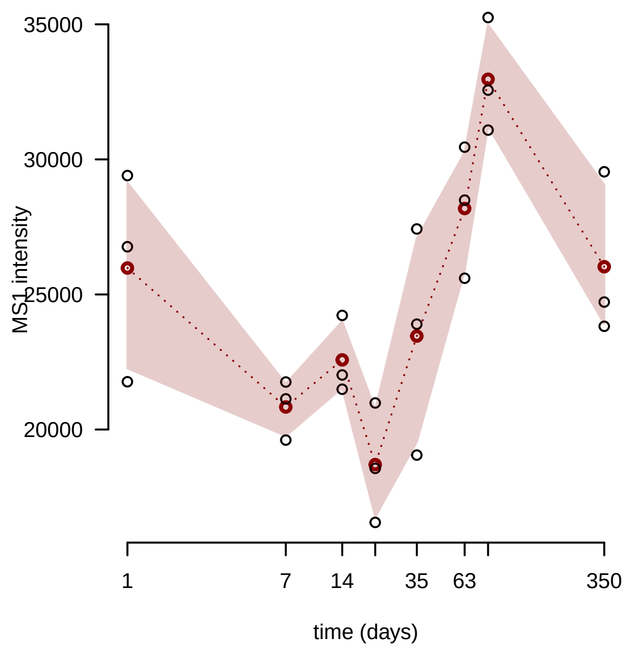

P97364
GN=Sephs2
selenophosphate synthetase 2

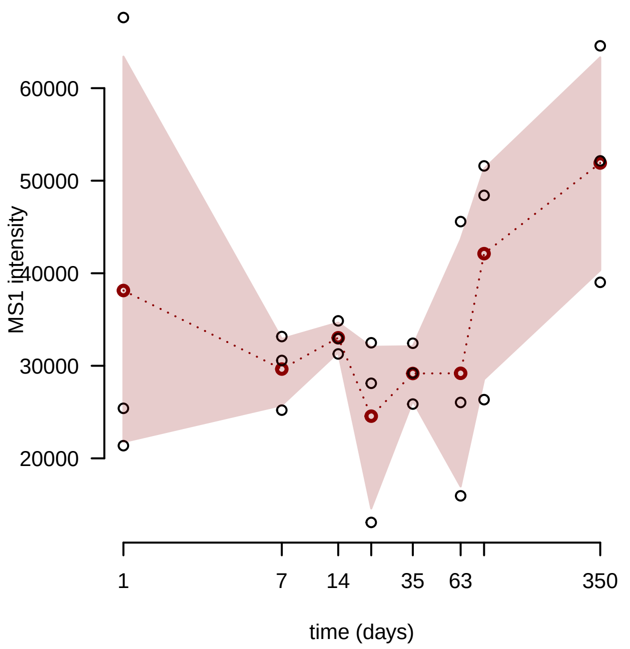

P97366
GN=Evi5
ecotropic viral integration site 5

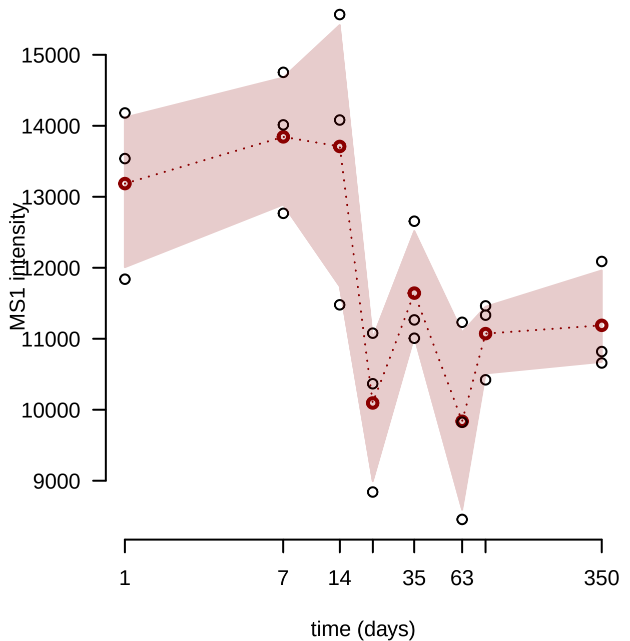

P97370
GN=Atp1b3
ATPase, Na+/K+ transporting, beta 3 poly ...

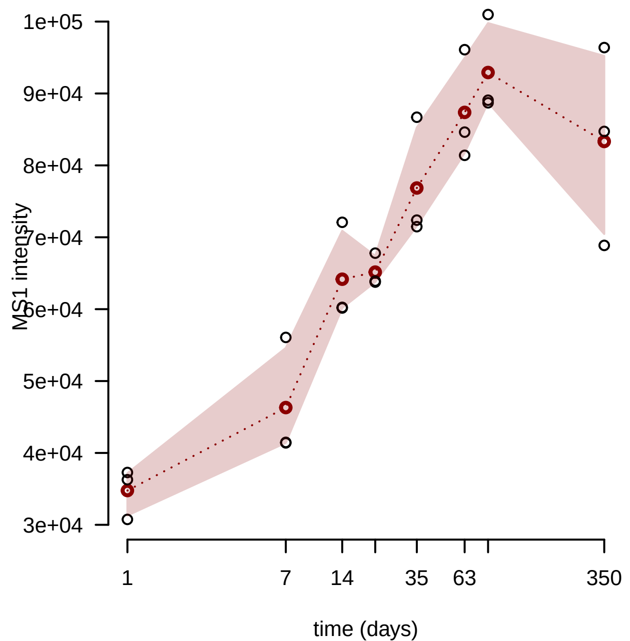

P97371
GN=Psme1
proteasome (prosome, macropain) activato ...

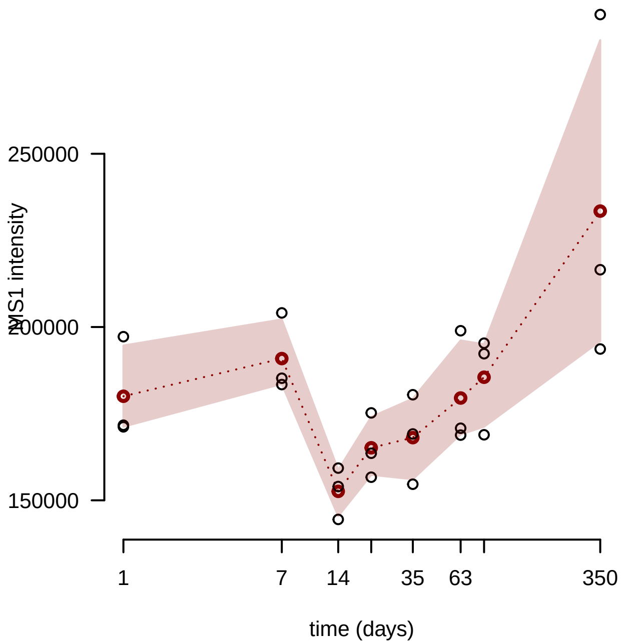

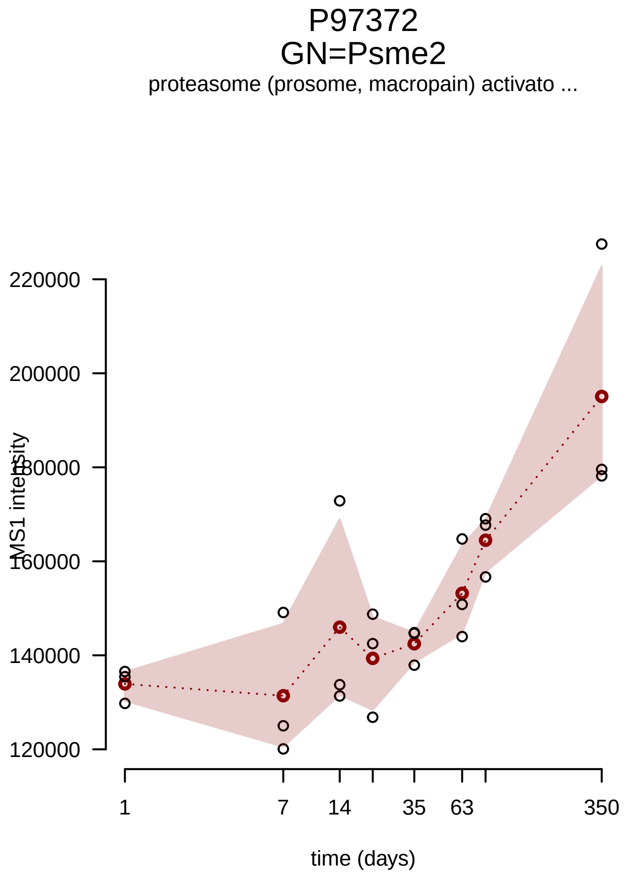
P97372
GN=Psme2
proteasome (prosome, macropain) activato ...
MS1 intensity
120000
140000
160000
180000
200000
220000
1
7
14
35
63
350
time (days)
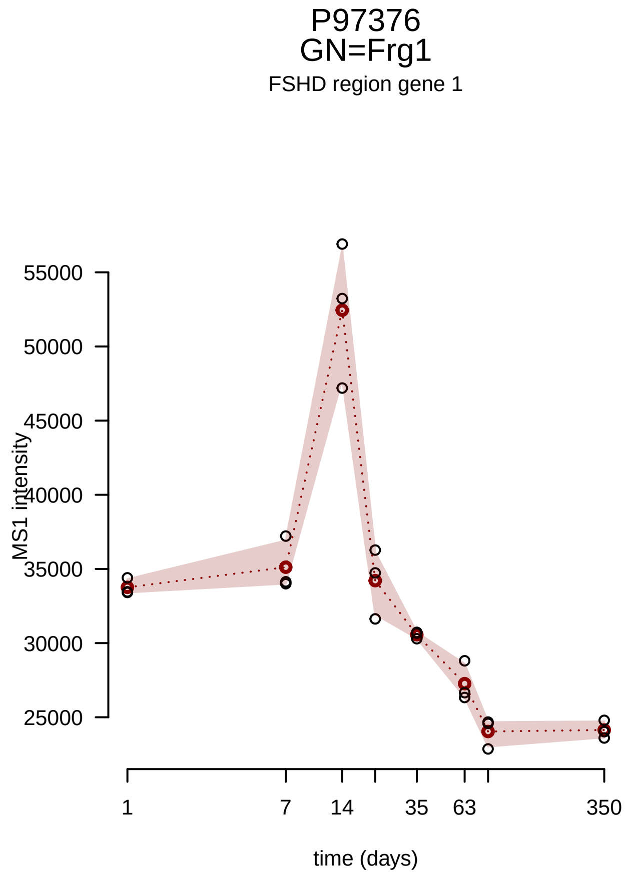
P97376
GN=Frg1
FSHD region gene 1
MS1 intensity
25000
30000
35000
40000
45000
50000
55000
1
7
14
35
63
350
time (days)
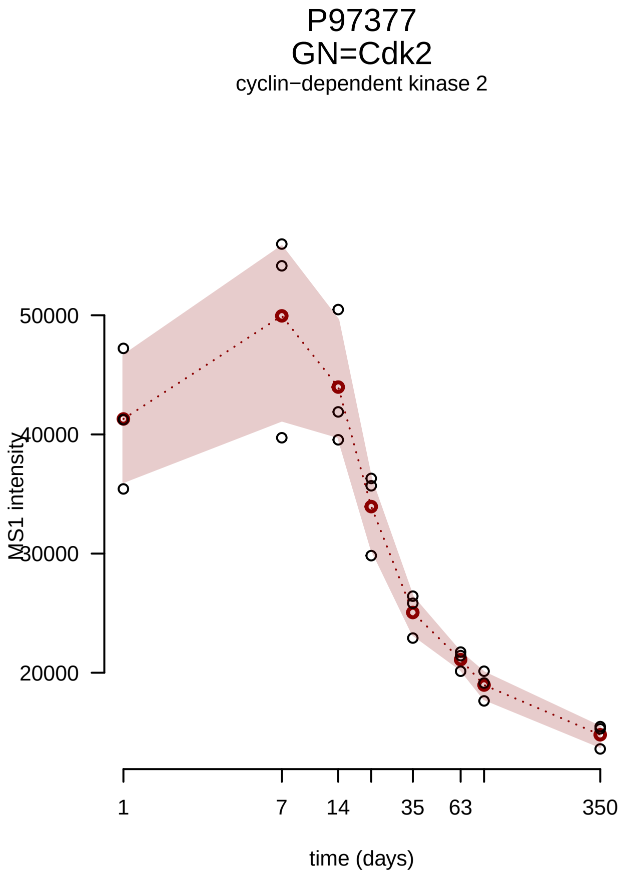
P97377
GN=Cdk2
cyclin−dependent kinase 2
MS1 intensity
20000
30000
40000
50000
1
7
14
35
63
350
time (days)
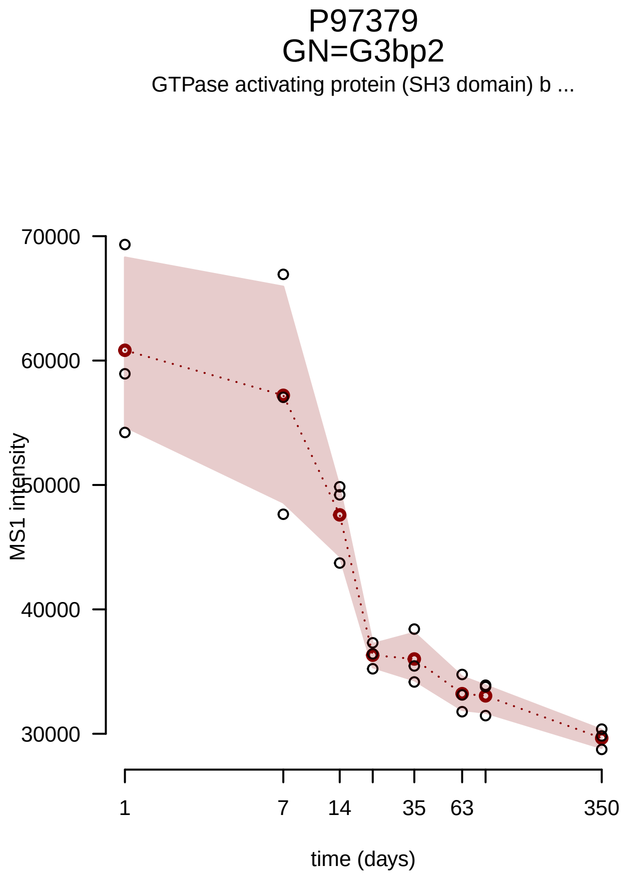
P97379
GN=G3bp2
GTPase activating protein (SH3 domain) b ...
MS1 intensity
30000
40000
50000
60000
70000
1
7
14
35
63
350
time (days)
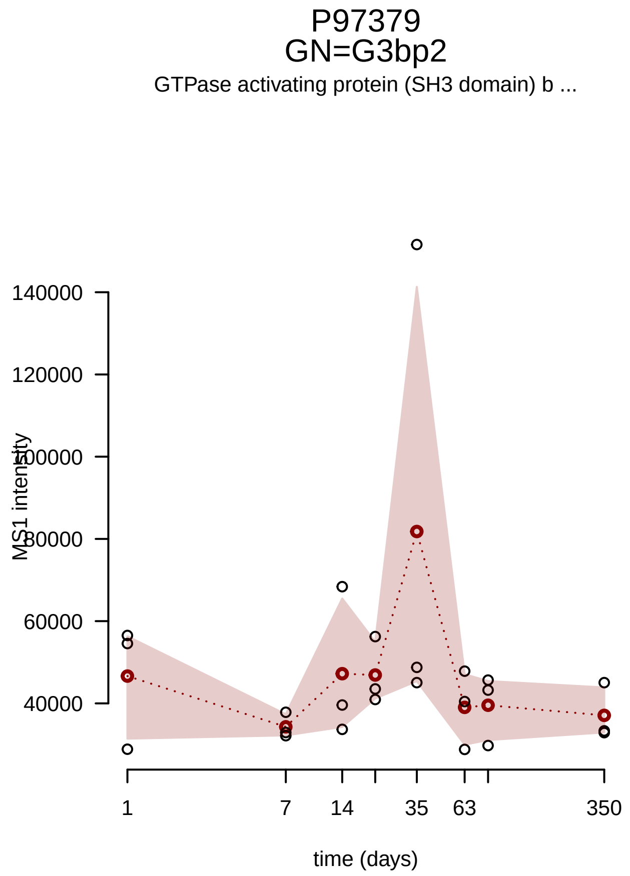
P97379
GN=G3bp2
GTPase activating protein (SH3 domain) b ...
MS1 intensity
40000
60000
80000
100000
120000
140000
1
7
14
35
63
350
time (days)
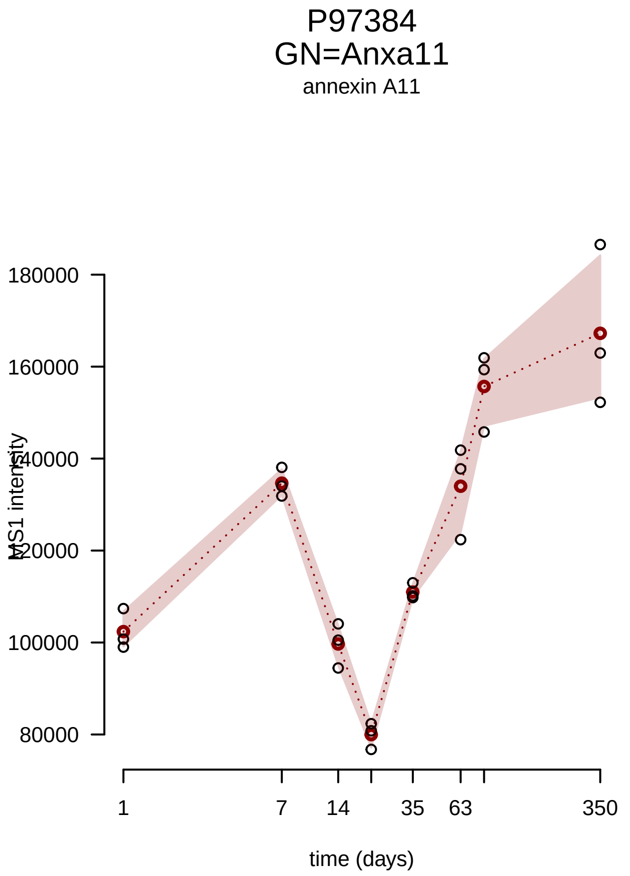
P97384
GN=Anxa11
annexin A11
MS1 intensity
80000
100000
120000
140000
160000
180000
1
7
14
35
63
350
time (days)
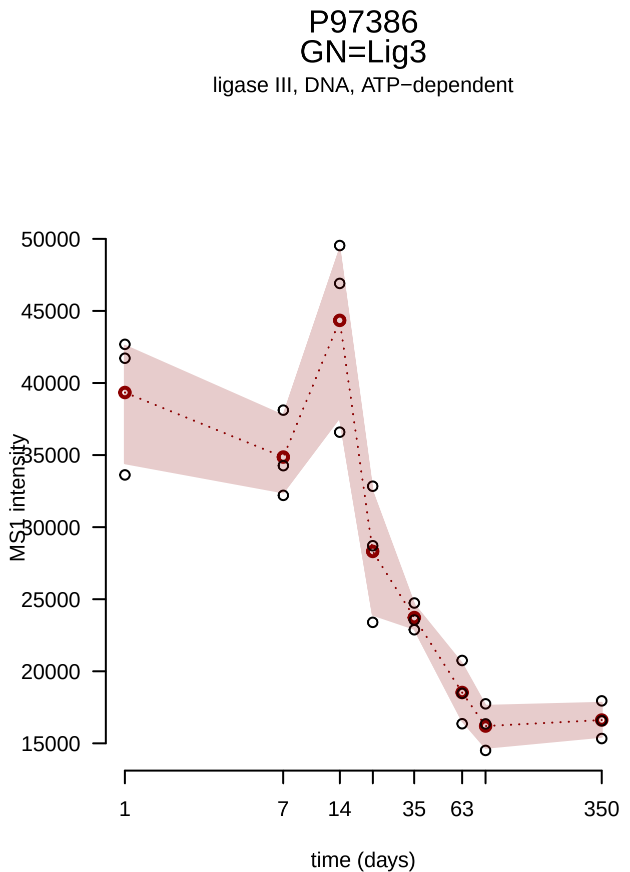
P97386
GN=Lig3
ligase III, DNA, ATP−dependent
MS1 intensity
15000
20000
25000
30000
35000
40000
45000
50000
1
7
14
35
63
350
time (days)
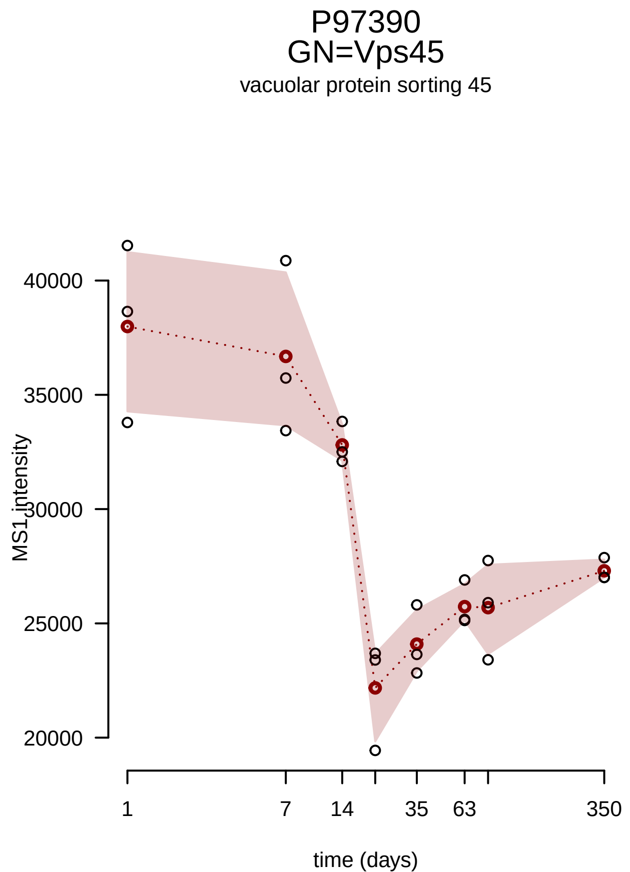
P97390
GN=Vps45
vacuolar protein sorting 45
MS1 intensity
20000
25000
30000
35000
40000
1
7
14
35
63
350
time (days)
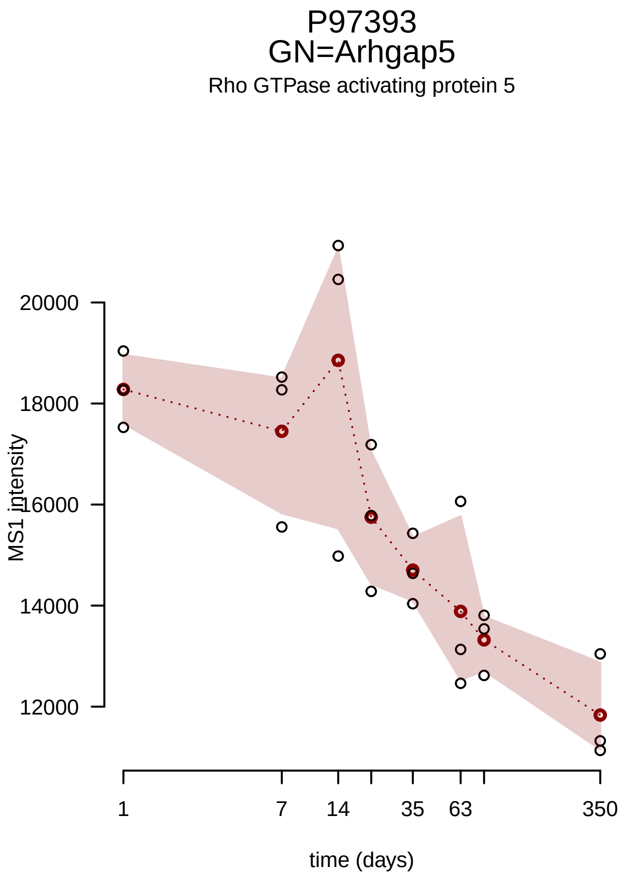
P97393
GN=Arhgap5
Rho GTPase activating protein 5
MS1 intensity
12000
14000
16000
18000
20000
1
7
14
35
63
350
time (days)

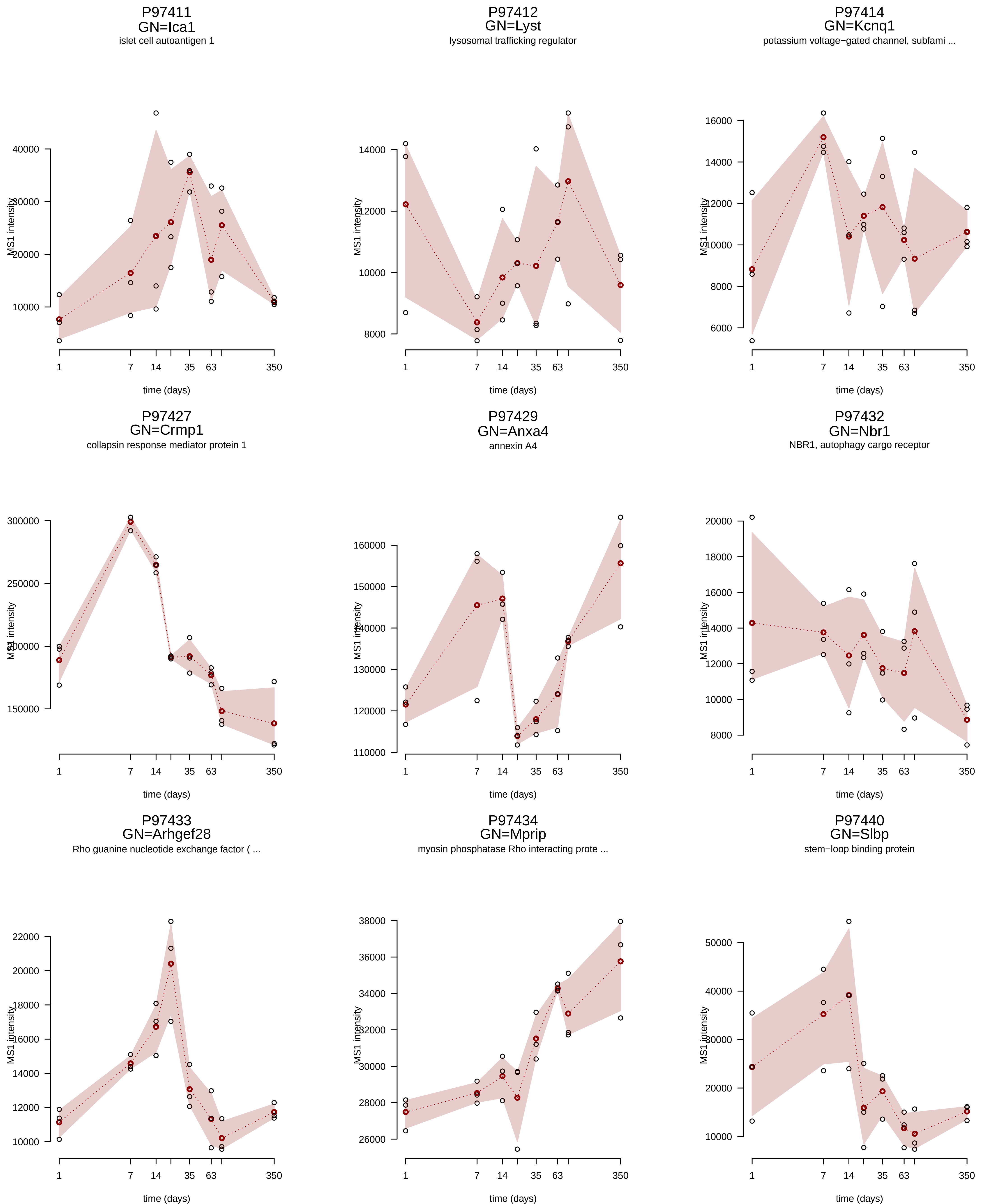
P97411
GN=Ica1
islet cell autoantigen 1
MS1 intensity
10000 20000 30000 40000
1 7 14 35 63 350
time (days)
P97412
GN=Lyst
lysosomal trafficking regulator
MS1 intensity
8000 10000 12000 14000
1 7 14 35 63 350
time (days)
P97414
GN=Kcnq1
potassium voltage−gated channel, subfami ...
MS1 intensity
6000 8000 10000 12000 14000 16000
1 7 14 35 63 350
time (days)
P97427
GN=Crmp1
collapsin response mediator protein 1
MS1 intensity
150000 200000 250000 300000
1 7 14 35 63 350
time (days)
P97429
GN=Anxa4
annexin A4
MS1 intensity
110000 120000 130000 140000 150000 160000
1 7 14 35 63 350
time (days)
P97432
GN=Nbr1
NBR1, autophagy cargo receptor
MS1 intensity
8000 10000 12000 14000 16000 18000 20000
1 7 14 35 63 350
time (days)
P97433
GN=Arhgef28
Rho guanine nucleotide exchange factor (...
MS1 intensity
10000 12000 14000 16000 18000 20000 22000
1 7 14 35 63 350
time (days)
P97434
GN=Mprip
myosin phosphatase Rho interacting prote ...
MS1 intensity
26000 28000 30000 32000 34000 36000 38000
1 7 14 35 63 350
time (days)
P97440
GN=Slbp
stem−loop binding protein
MS1 intensity
10000 20000 30000 40000 50000
1 7 14 35 63 350
time (days)

P97441
GN=Slc30a3
solute carrier family 30 (zinc transport ...

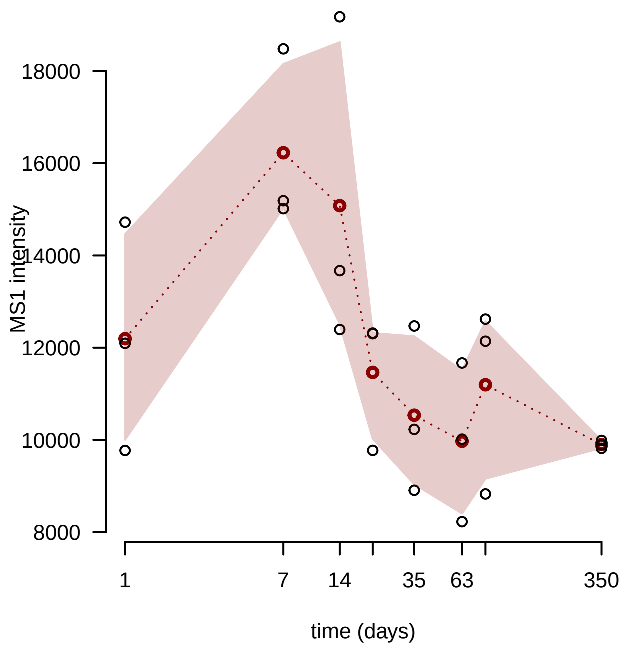

P97447
GN=Fhl1
four and a half LIM domains 1

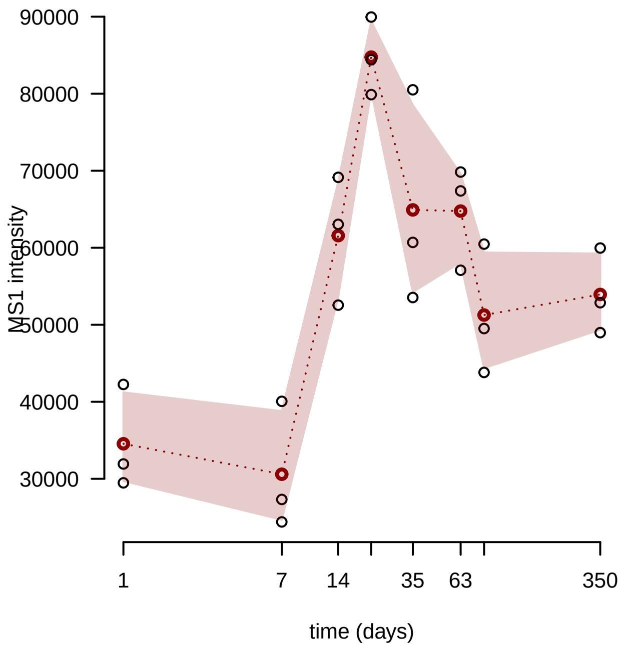

P97449
GN=Anpep
alanyl (membrane) aminopeptidase

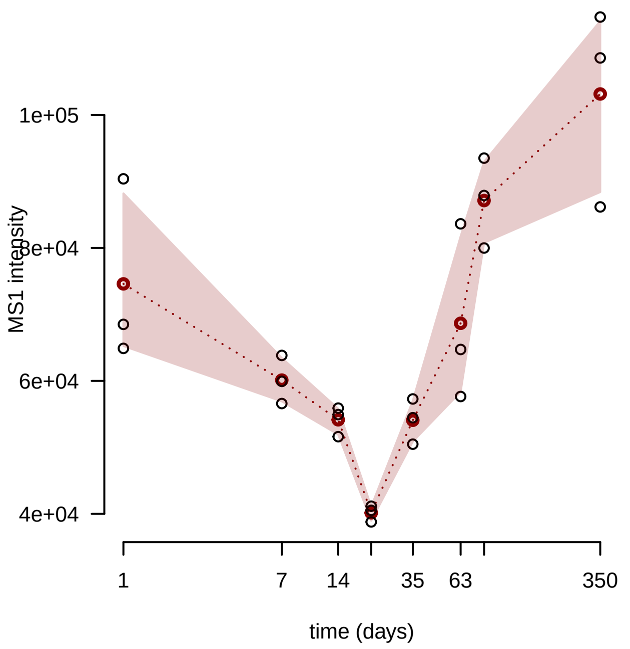

P97450
GN=Atp5pf

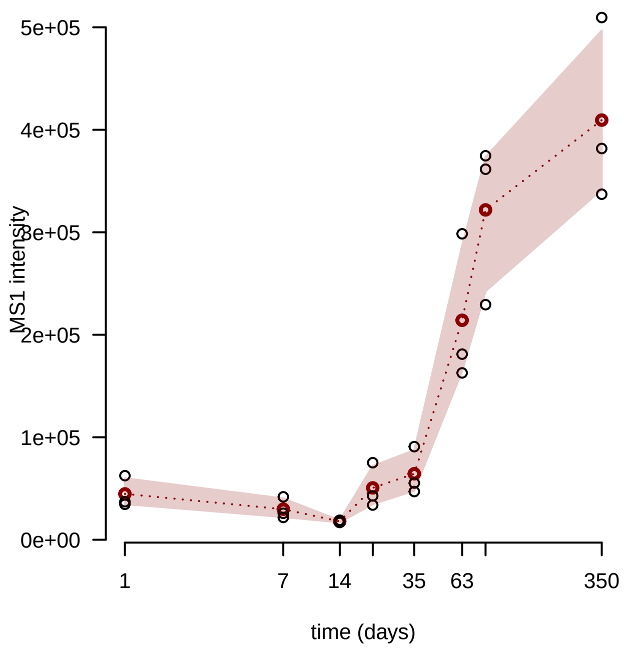

P97452
GN=Bop1
block of proliferation 1

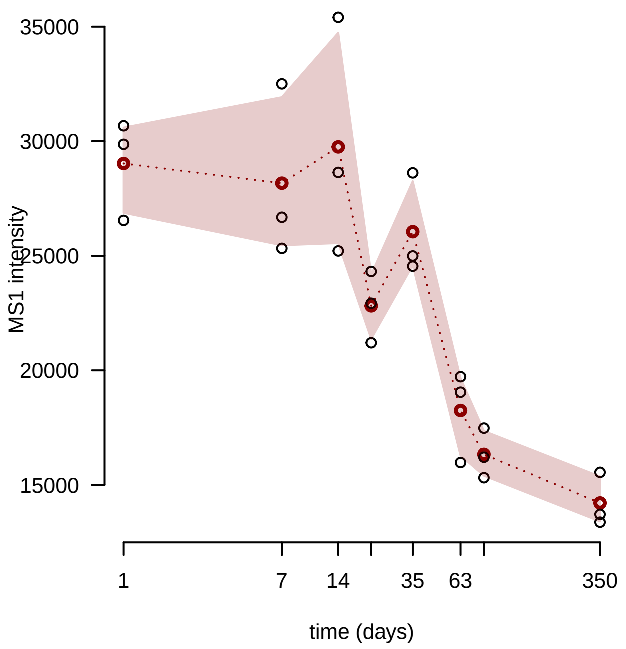

P97454
GN=Smad5
SMAD family member 5

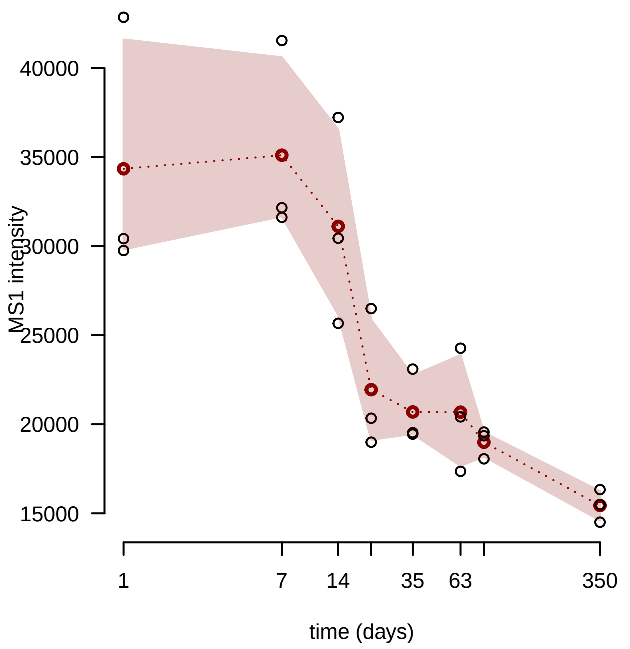

P97457
GN=Mylpf
myosin light chain, phosphorylatable, fa ...

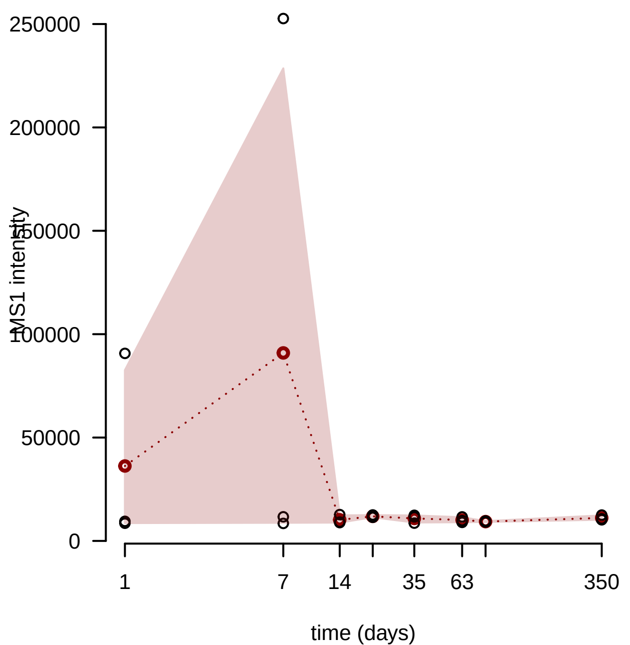

P97461
GN=Rps5
ribosomal protein S5

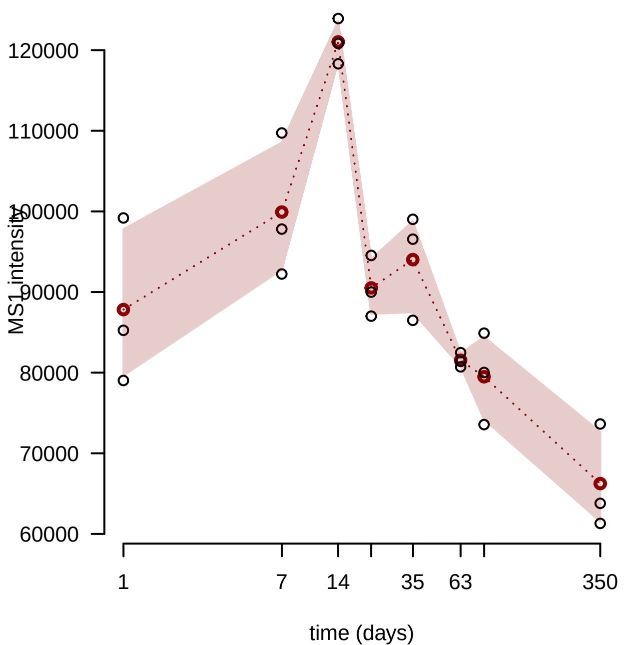

P97464
GN=Ext1
exostosin glycosyltransferase 1

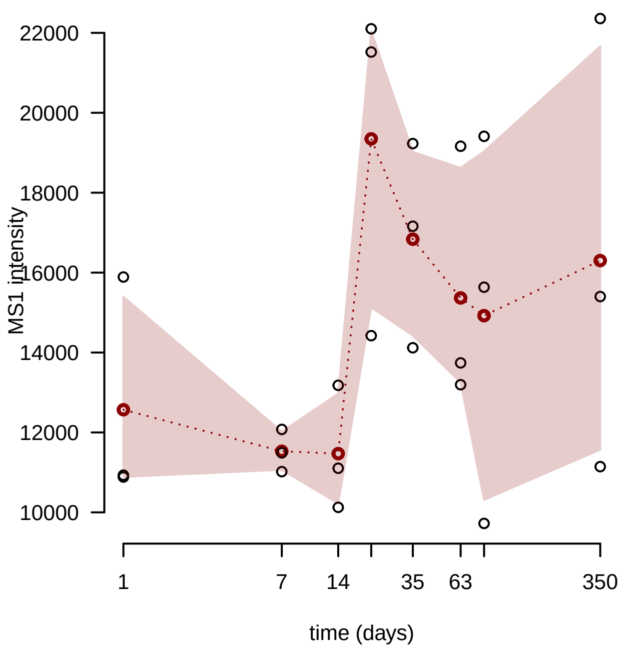

P97467
GN=Pam
peptidylglycine alpha−amidating monooxyg ...

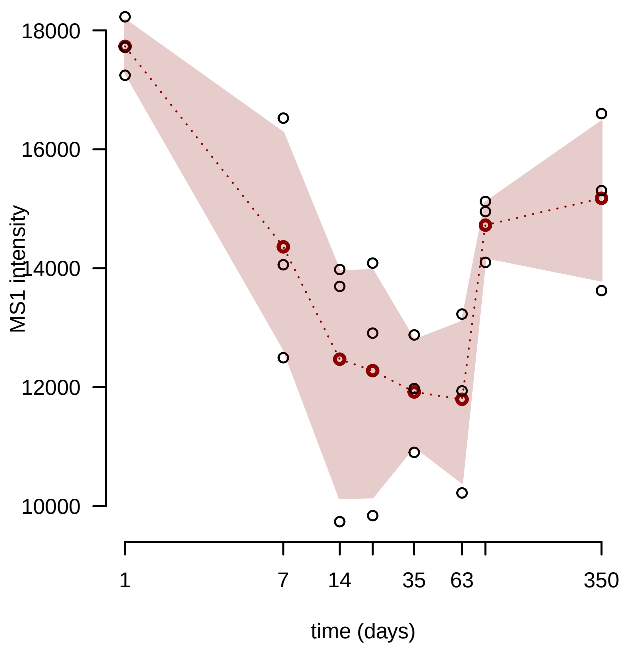

P97470
GN=Ppp4c
protein phosphatase 4, catalytic subunit

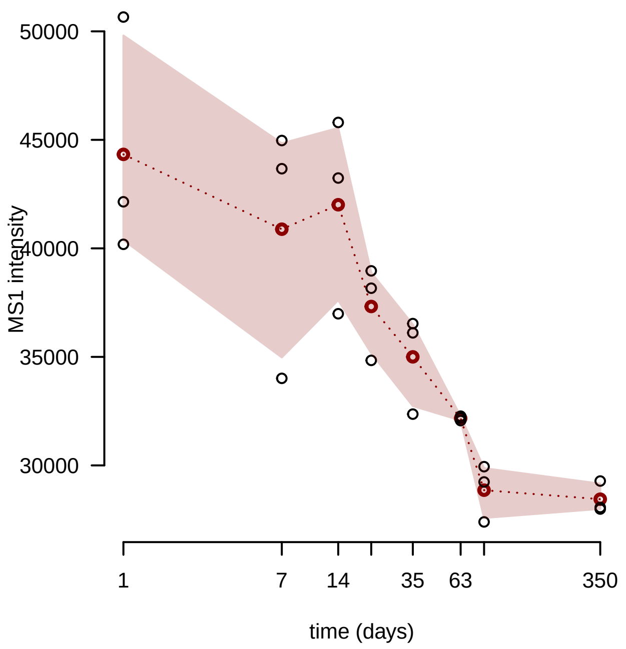

P97471
GN=Smad4
SMAD family member 4

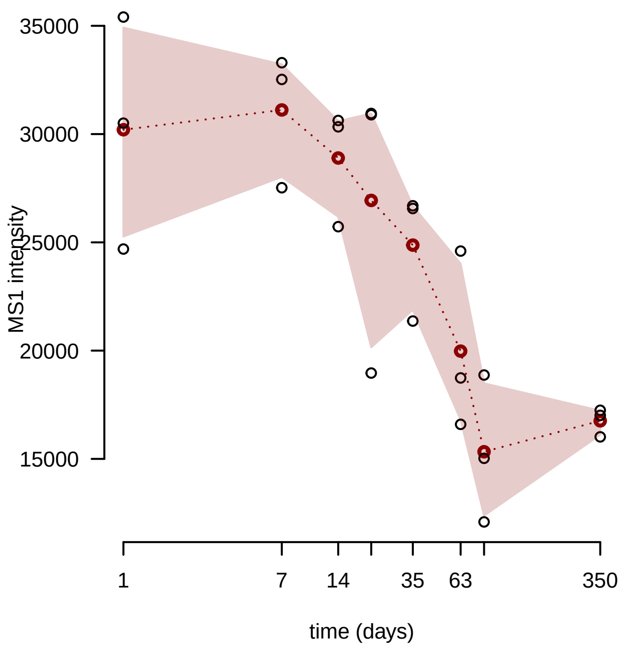

P97473
GN=Tarbp2
TARBP2, RISC loading complex RNA binding ...

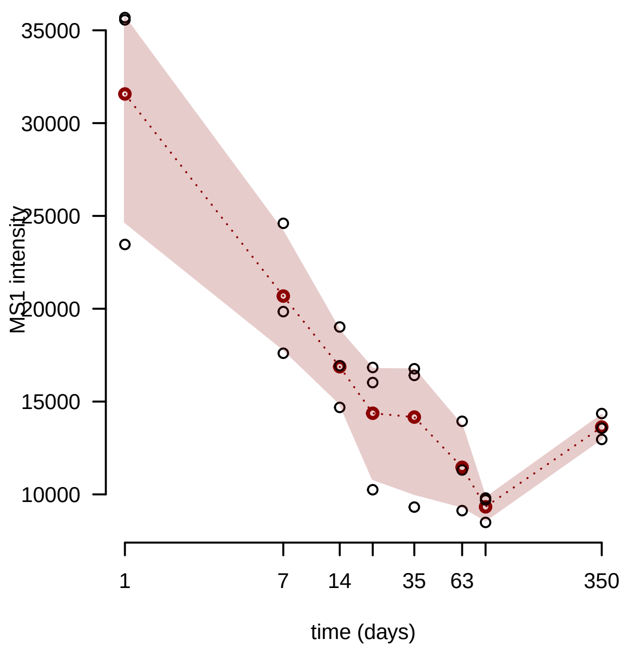

P97477
GN=Aurka
aurora kinase A

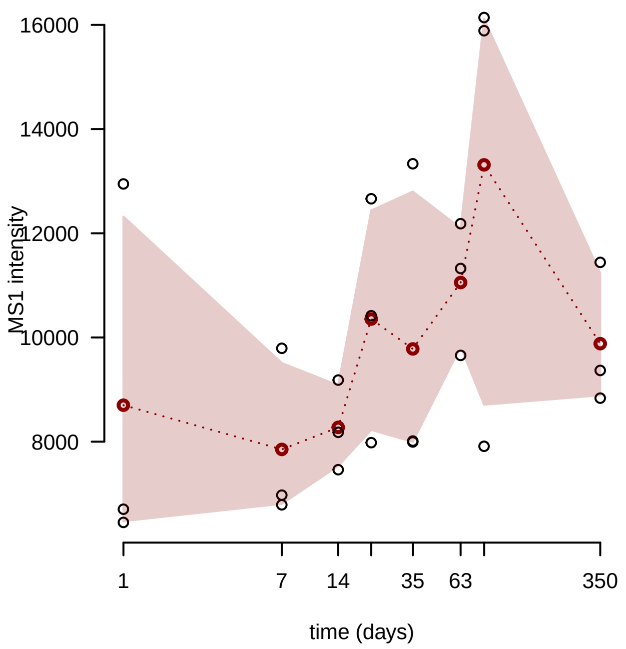

P97478
GN=Coq7
demethyl−Q 7

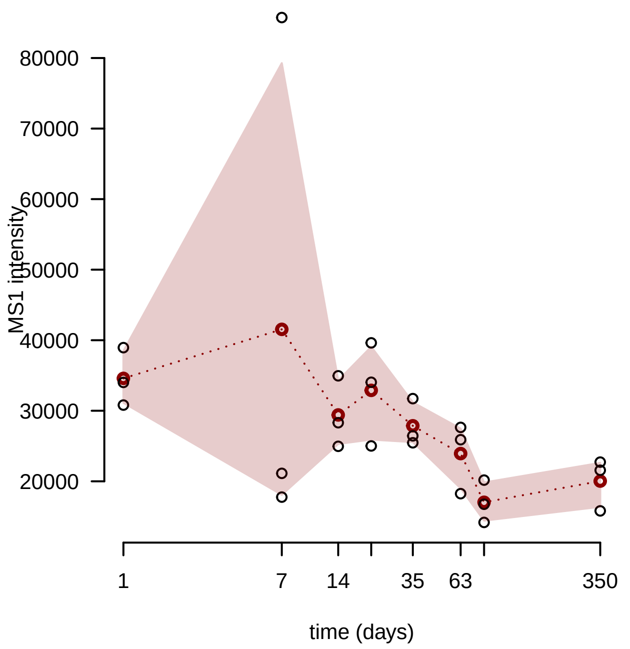

P97479
GN=Myo7a
myosin VIIA

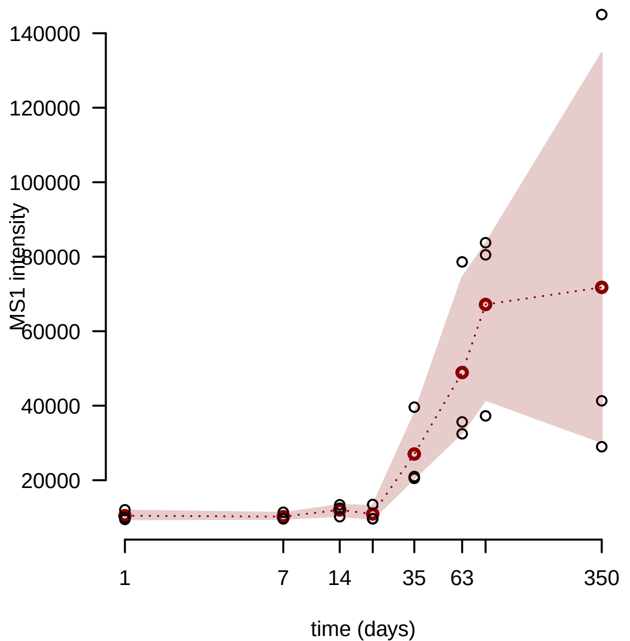

P97480
GN=Eya3
EYA transcriptional coactivator and phos ...

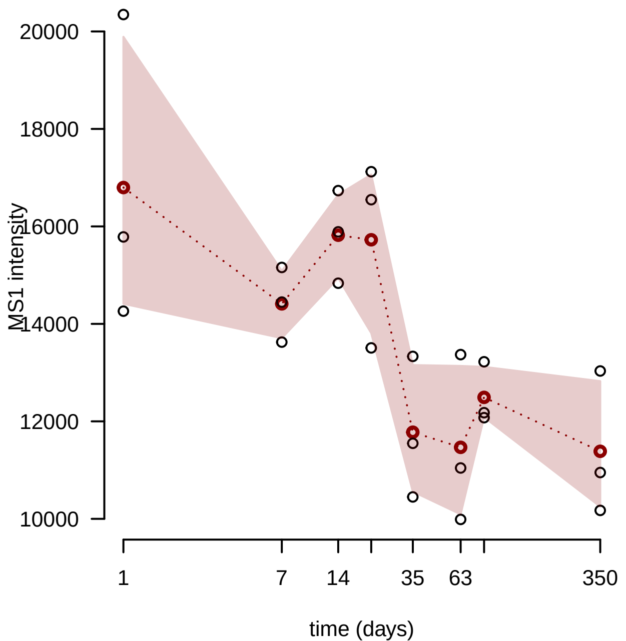

P97493
GN=Txn2
thioredoxin 2

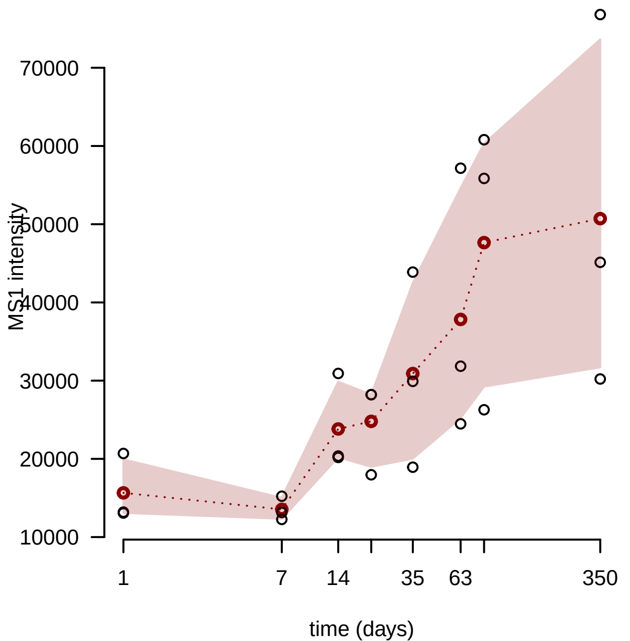

P97494
GN=Gclc
glutamate−cysteine ligase, catalytic sub ...

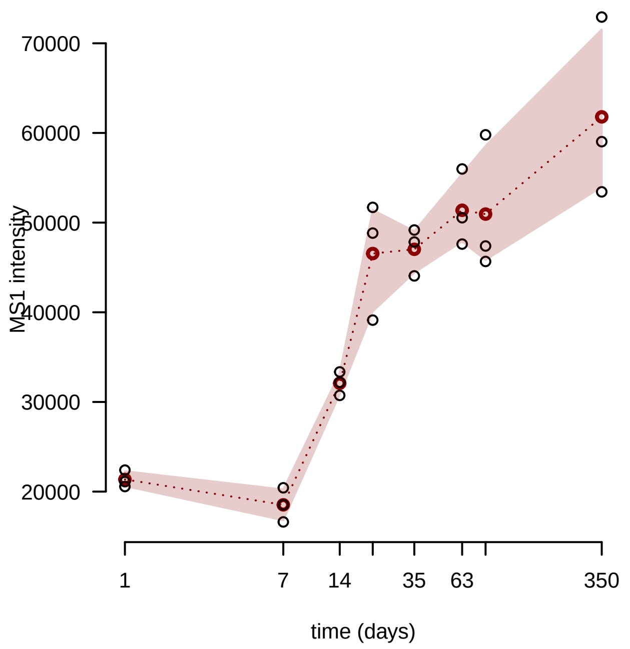

P97496
GN=Smarcc1
SWI/SNF related, matrix associated, acti ...

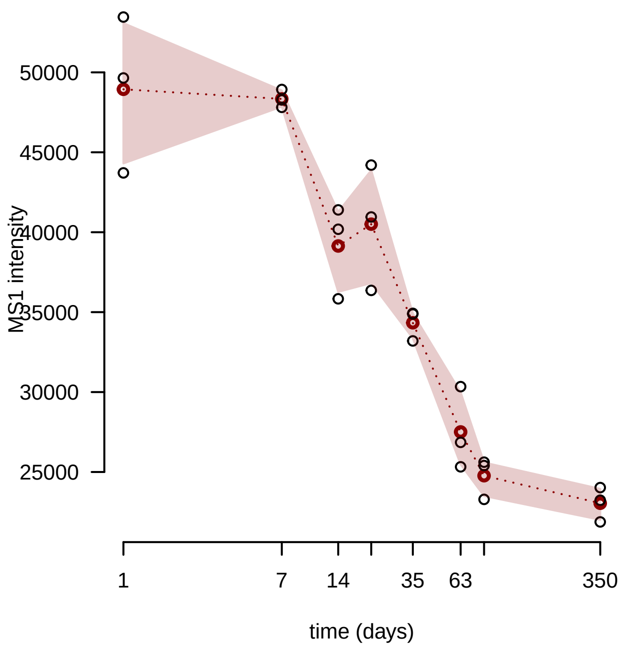

P97499
GN=Tep1
telomerase associated protein 1

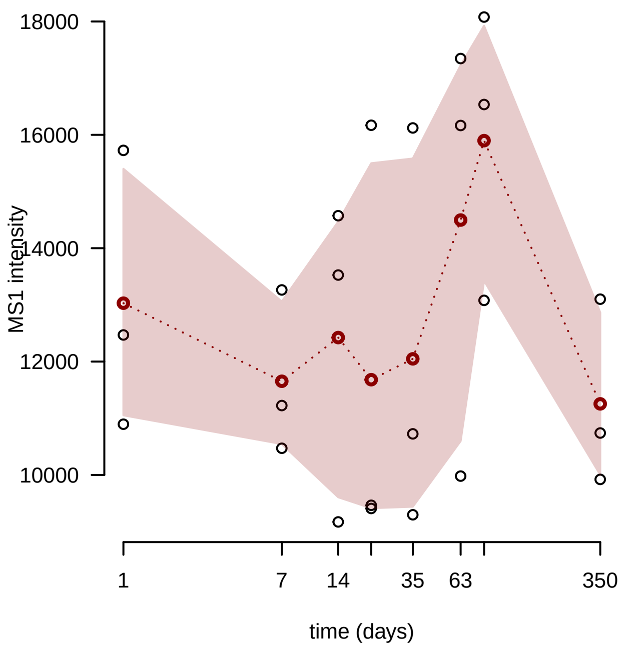

P97742
GN=Cpt1a
carnitine palmitoyltransferase 1a, liver

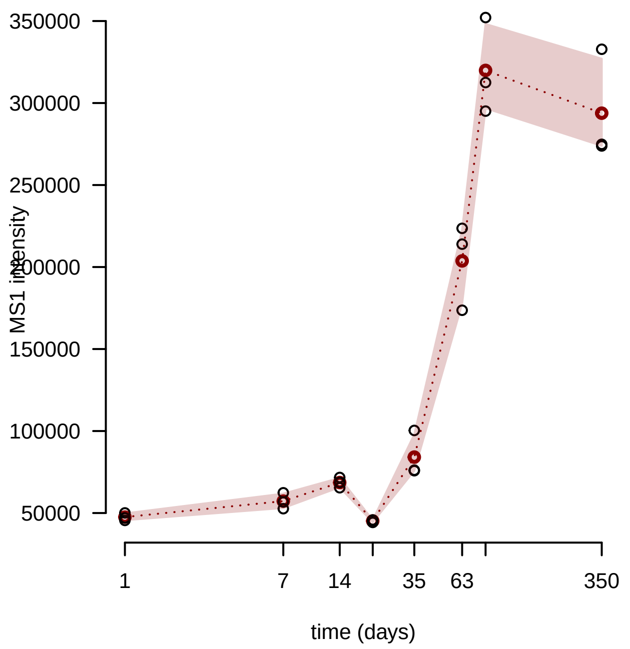

P97760
GN=Polr2c
polymerase (RNA) II (DNA directed) polyp ...

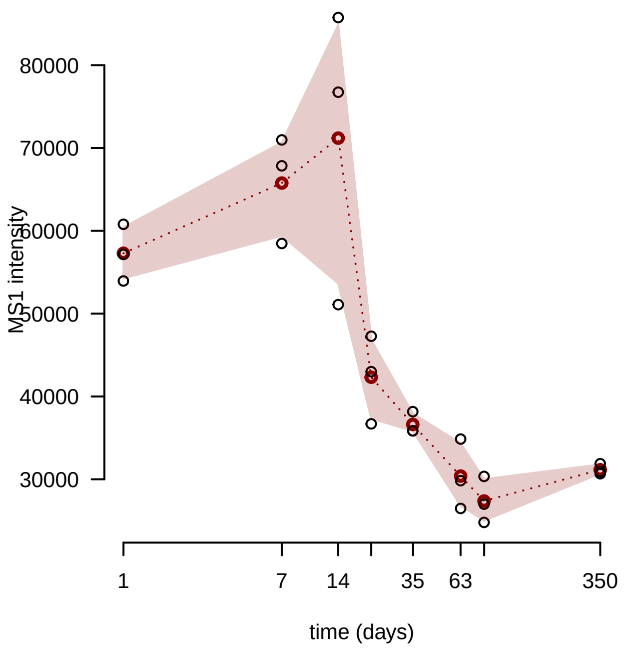

P97762
GN=rp9

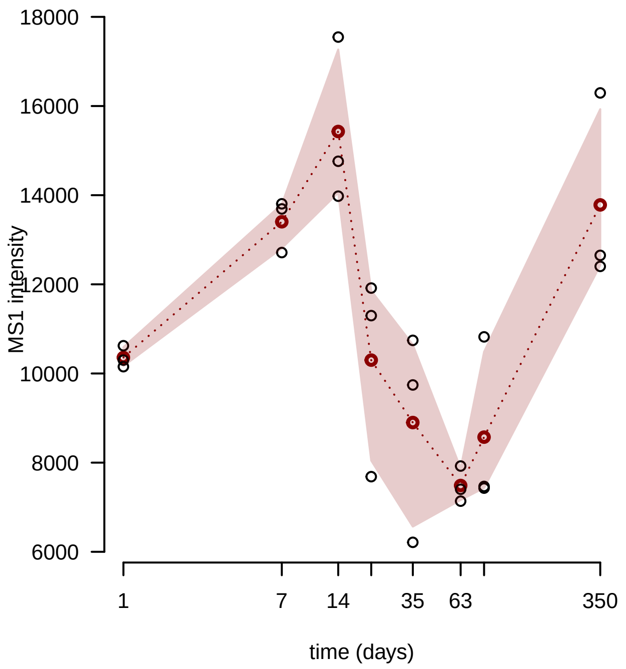

P97765
GN=Wbp2
WW domain binding protein 2

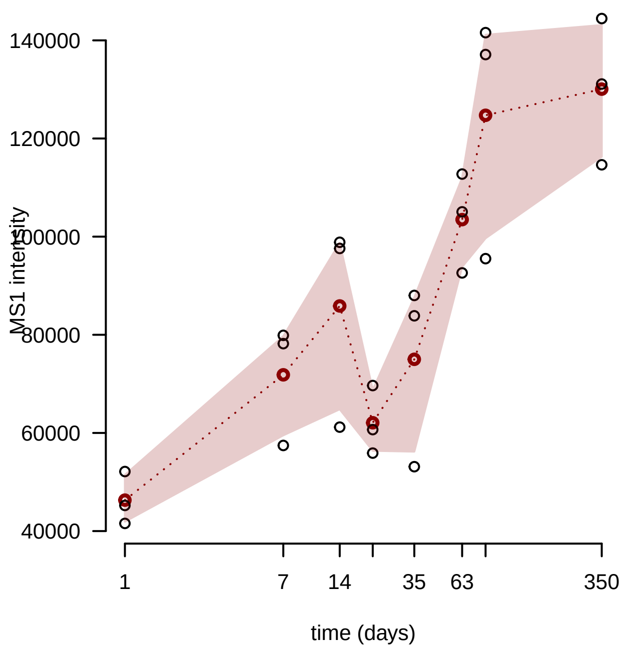

P97770
GN=Thumpd3
THUMP domain containing 3

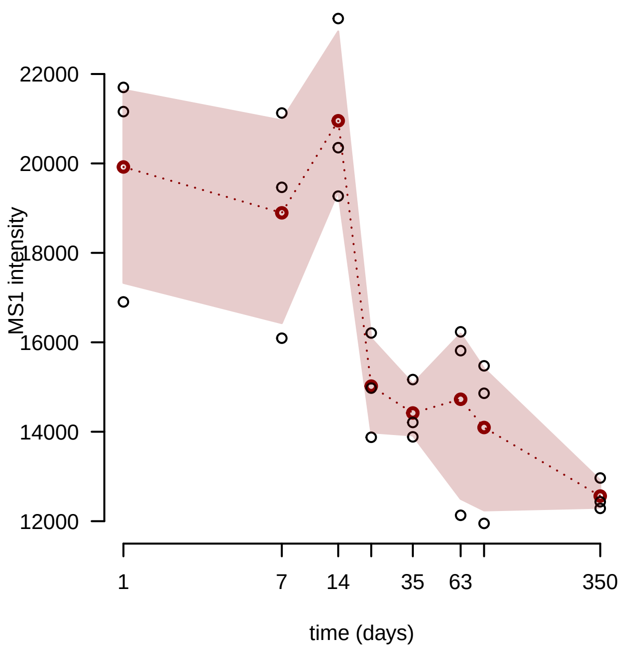

P97772
GN=Grm1
glutamate receptor, metabotropic 1

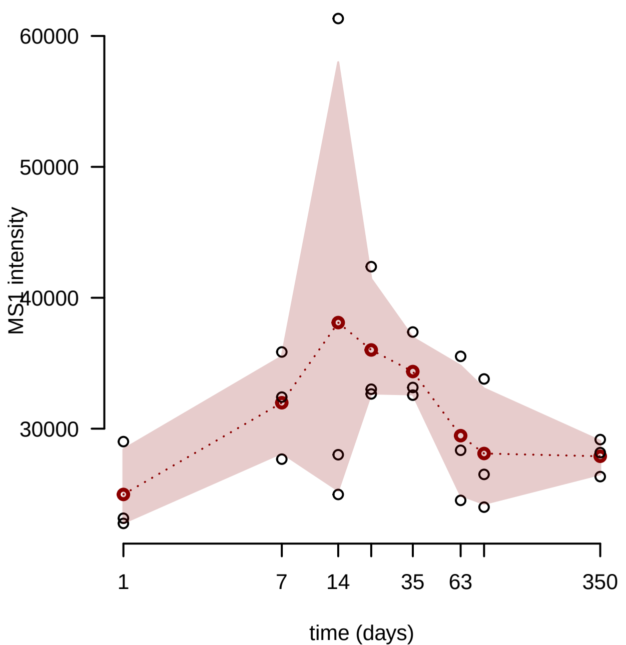

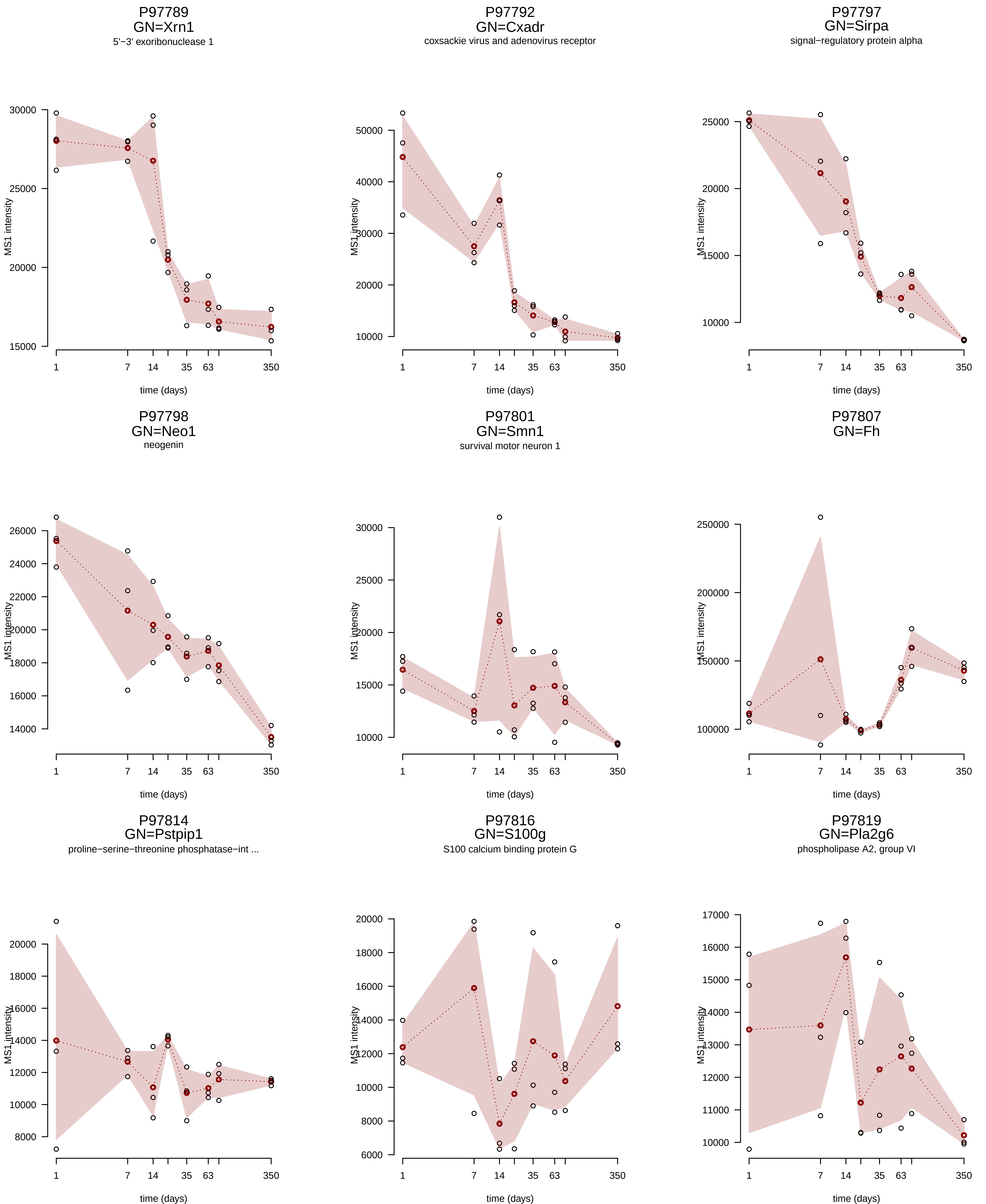
P97789
GN=Xrn1
5'−3' exoribonuclease 1
MS1 intensity
30000
25000
20000
15000
time (days)
1
7
14
35
63
350
P97792
GN=Cxadr
coxsackie virus and adenovirus receptor
MS1 intensity
50000
40000
30000
20000
10000
time (days)
P97797
GN=Sirpa
signal−regulatory protein alpha
MS1 intensity
25000
20000
15000
10000
time (days)
P97798
GN=Neo1
neogenin
MS1 intensity
26000
24000
22000
20000
18000
16000
14000
time (days)
P97801
GN=Smn1
survival motor neuron 1
MS1 intensity
30000
25000
20000
15000
10000
time (days)
P97807
GN=Fh
MS1 intensity
250000
200000
150000
100000
time (days)
P97814
GN=Pstpip1
proline−serine−threonine phosphatase−int ...
MS1 intensity
20000
18000
16000
14000
12000
10000
8000
time (days)
P97816
GN=S100g
S100 calcium binding protein G
MS1 intensity
20000
18000
16000
14000
12000
10000
8000
6000
time (days)
P97819
GN=Pla2g6
phospholipase A2, group VI
MS1 intensity
17000
16000
15000
14000
13000
12000
11000
10000
time (days)

P97820
GN=Map4k4
mitogen−activated protein kinase kinase ...

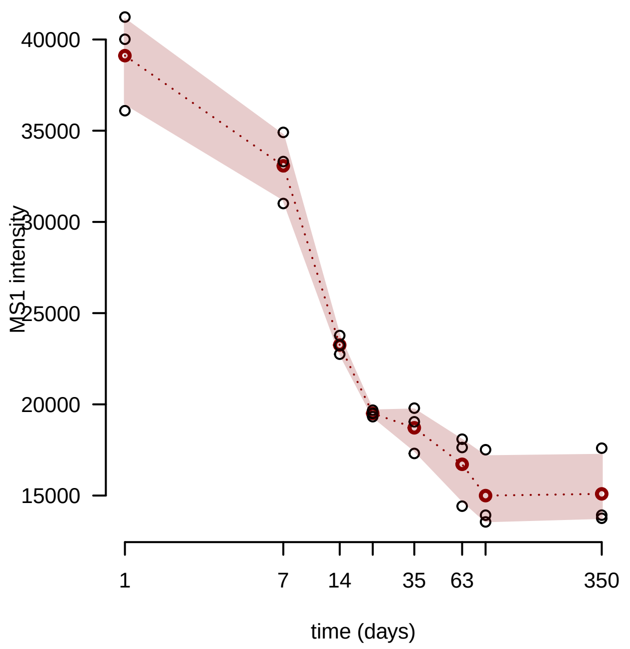

P97821
GN=Ctsc
cathepsin C

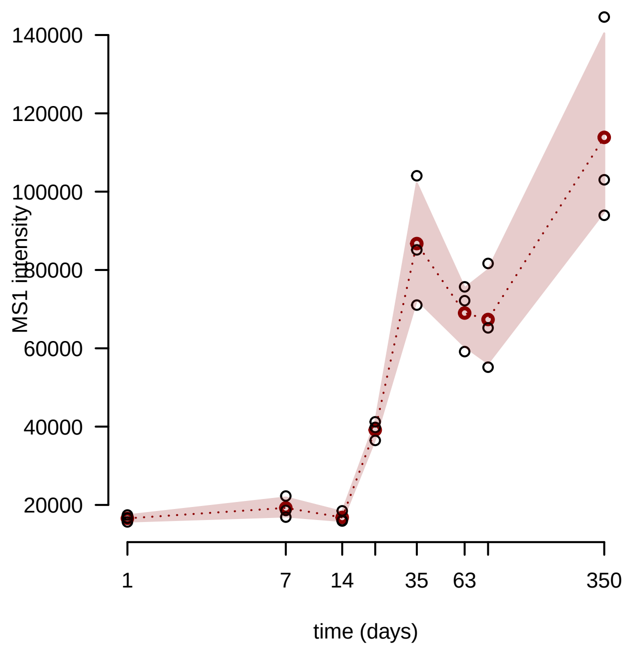

P97822
GN=Anp32e
acidic (leucine−rich) nuclear phosphopro ...

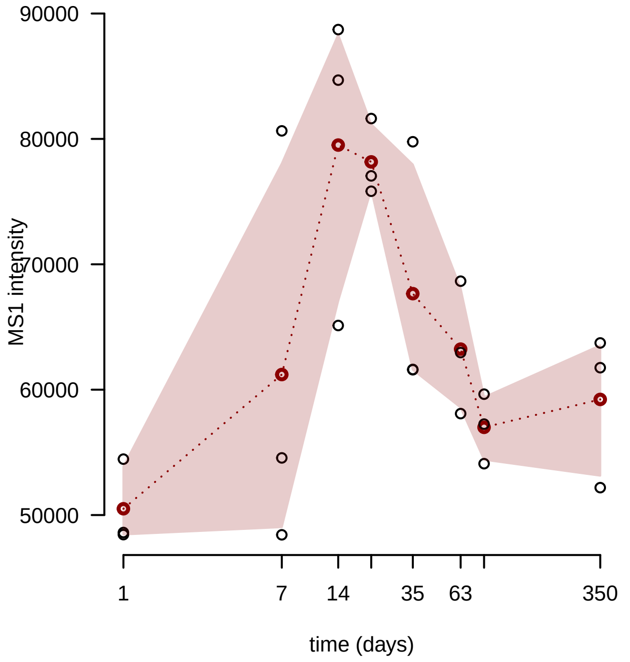

P97823
GN=Lypla1
lysophospholipase 1

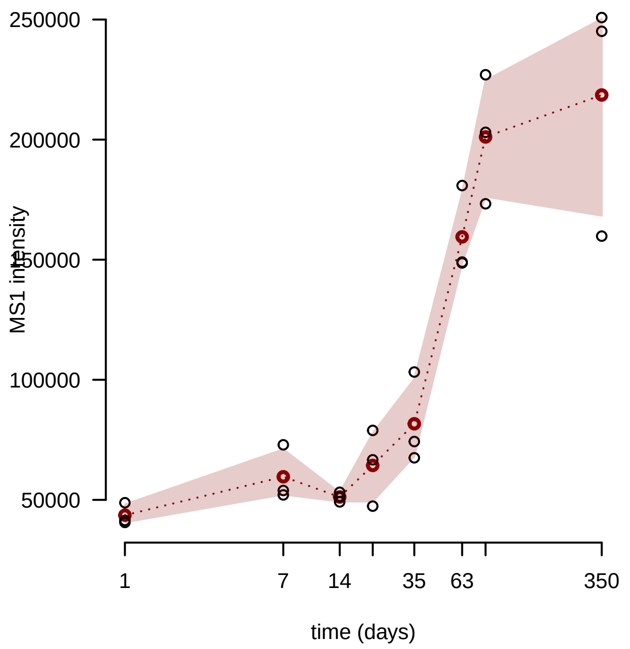

P97825
GN=Jpt1
Jupiter microtubule associated homolog 1

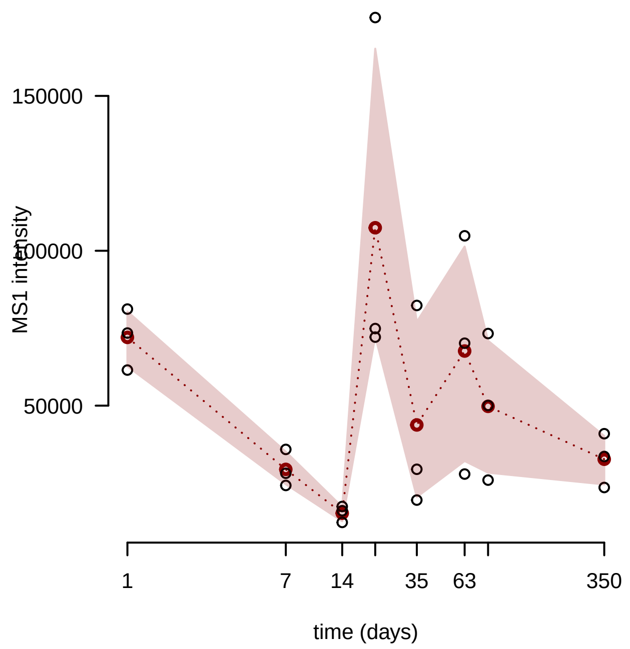

P97855
GN=G3bp1
GTPase activating protein (SH3 domain) b ...

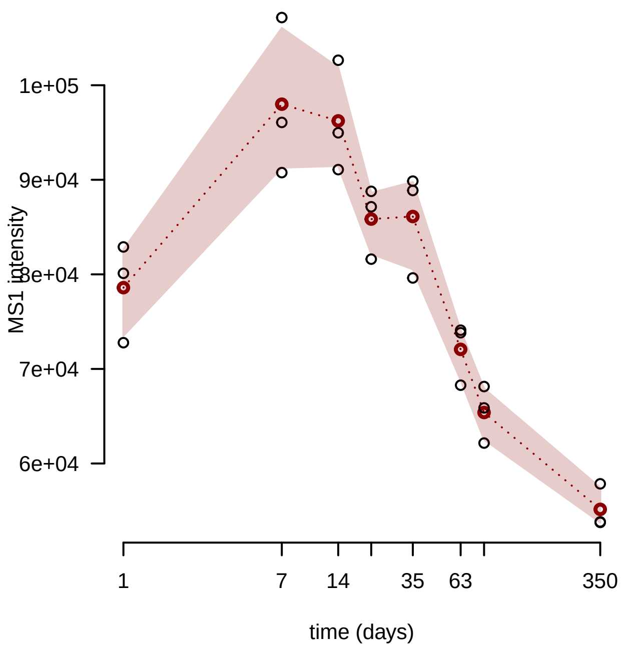

P97858
GN=Slc35b1
solute carrier family 35, member B1

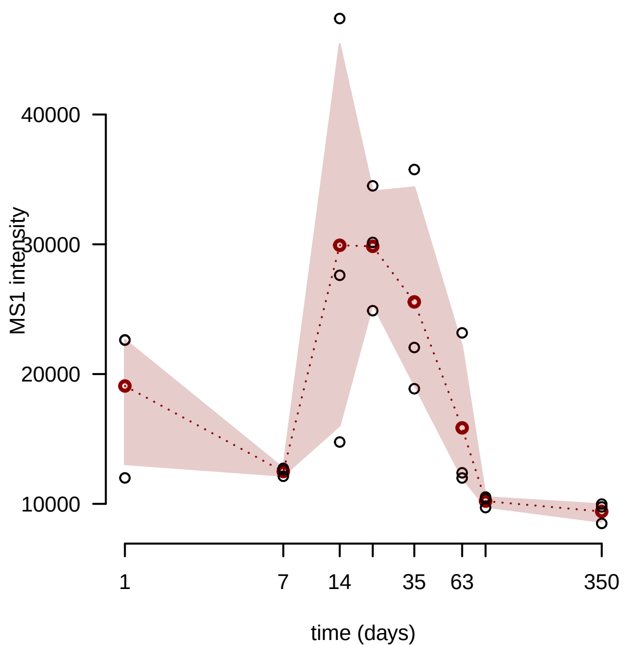

P97863
GN=Nfib
nuclear factor I/B

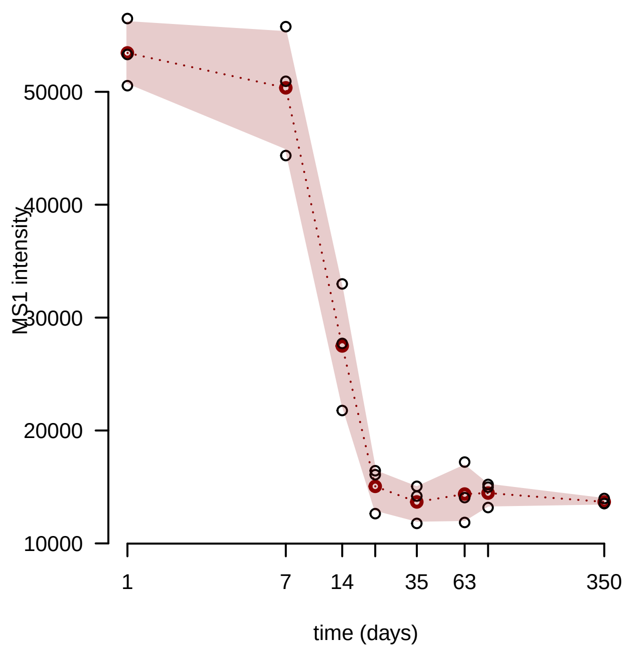

P97864
GN=Casp7
caspase 7

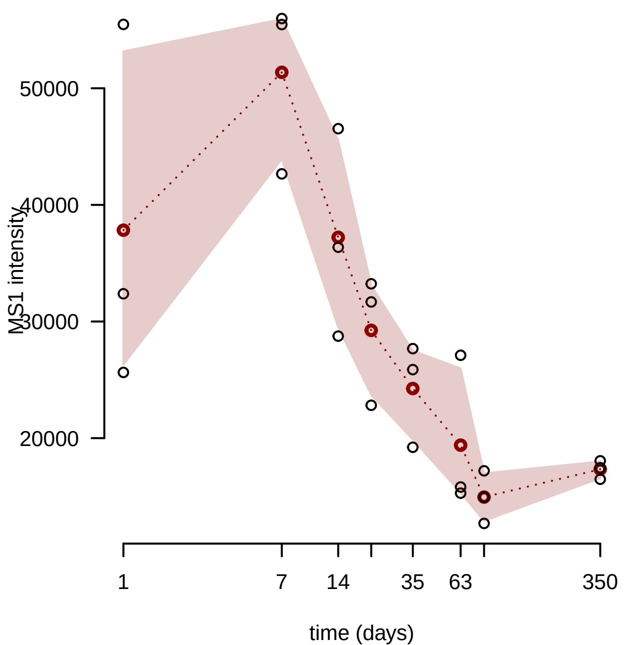

P97865
GN=Pex7
peroxisomal biogenesis factor 7

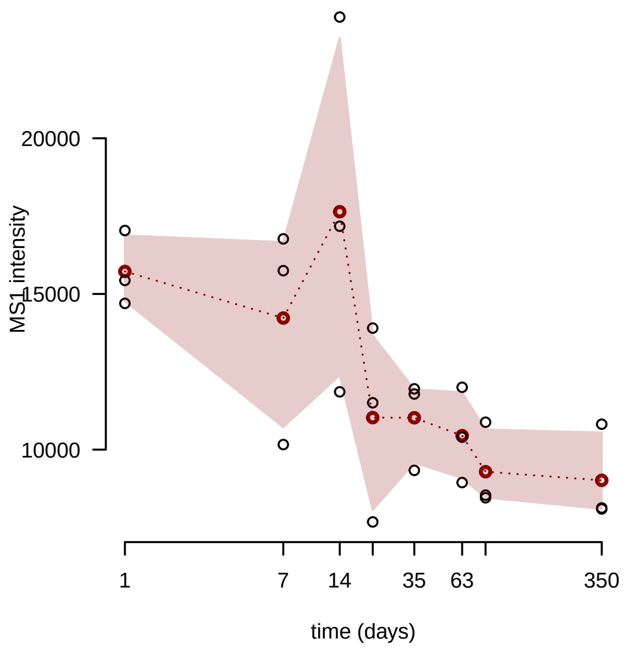

P97868
GN=Rbbp6
retinoblastoma binding protein 6, ubiqui ...

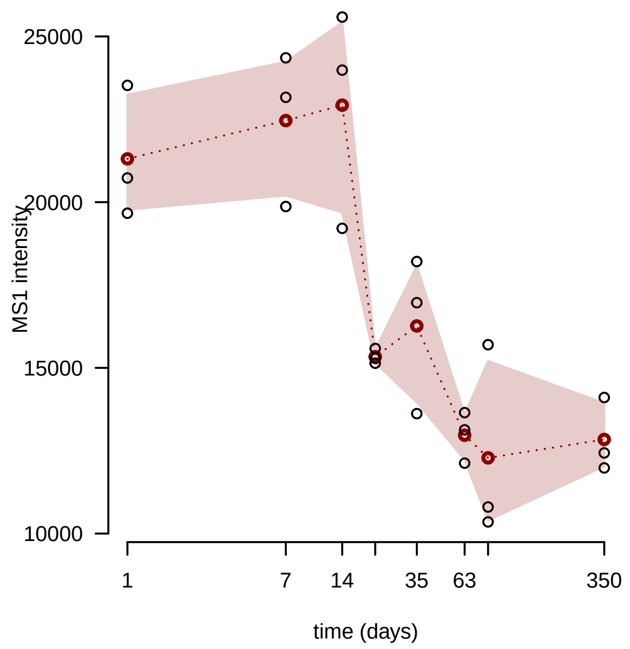

P97872
GN=Fmo5
flavin containing monooxygenase 5

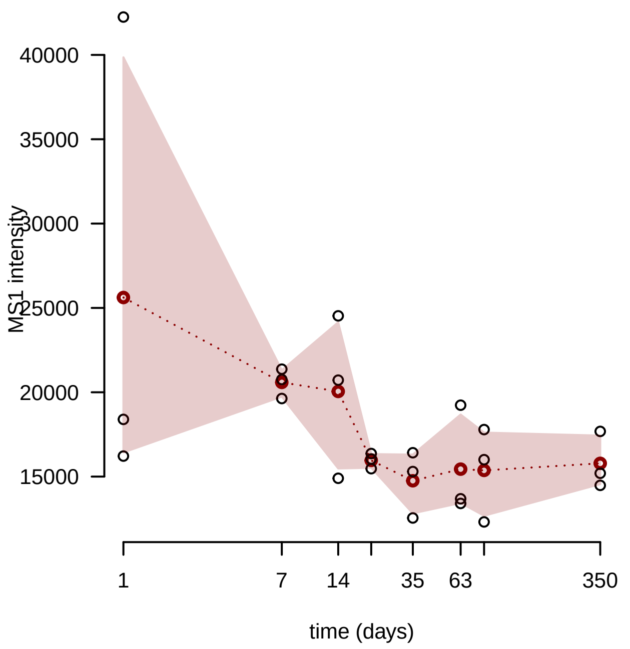

P97873
GN=Loxl1
lysyl oxidase−like 1

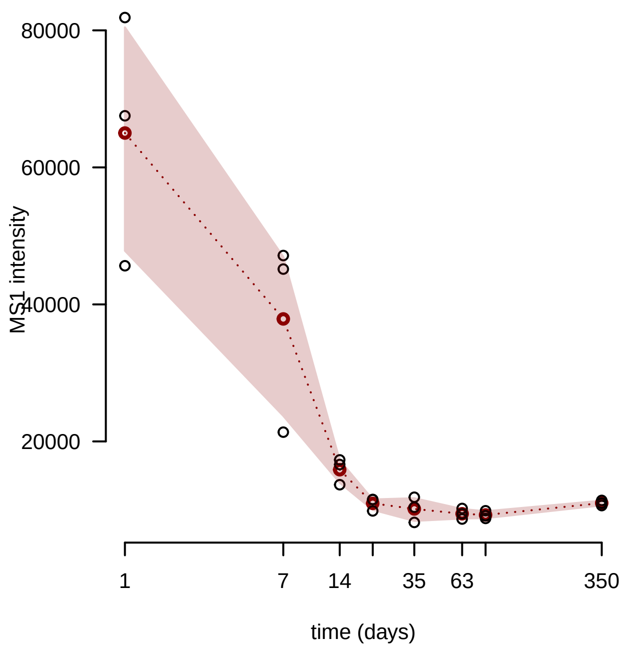

P97926
GN=Oxtr
oxytocin receptor

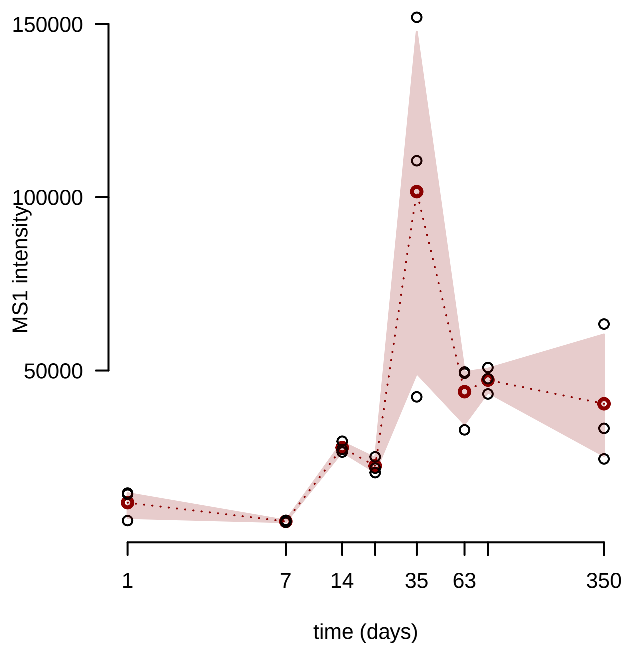

P97927
GN=Lama4
laminin, alpha 4

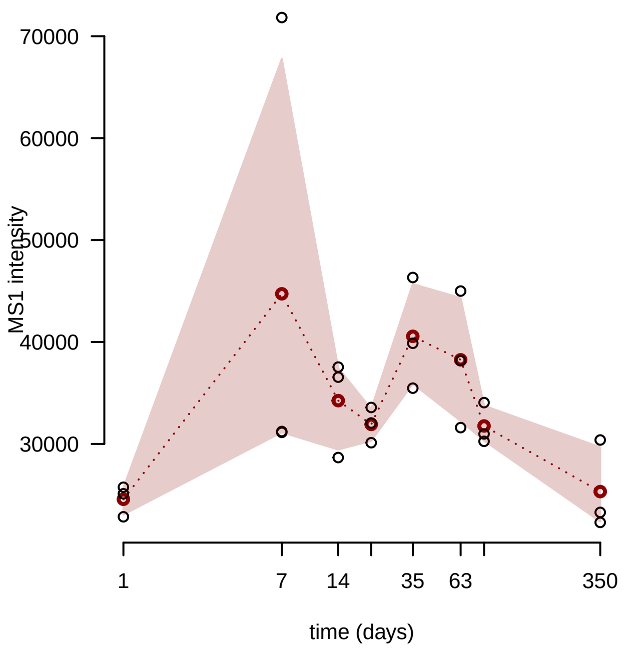

P97930
GN=Dtymk
deoxythymidylate kinase

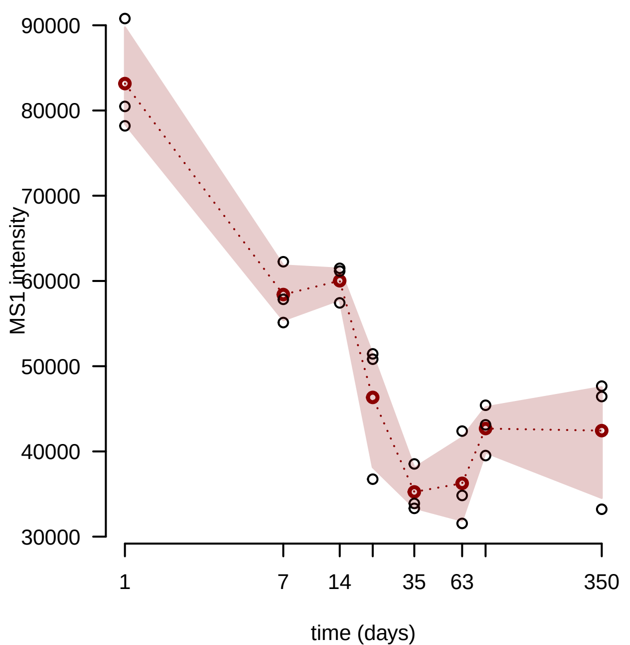

P97931
GN=Ung
uracil DNA glycosylase

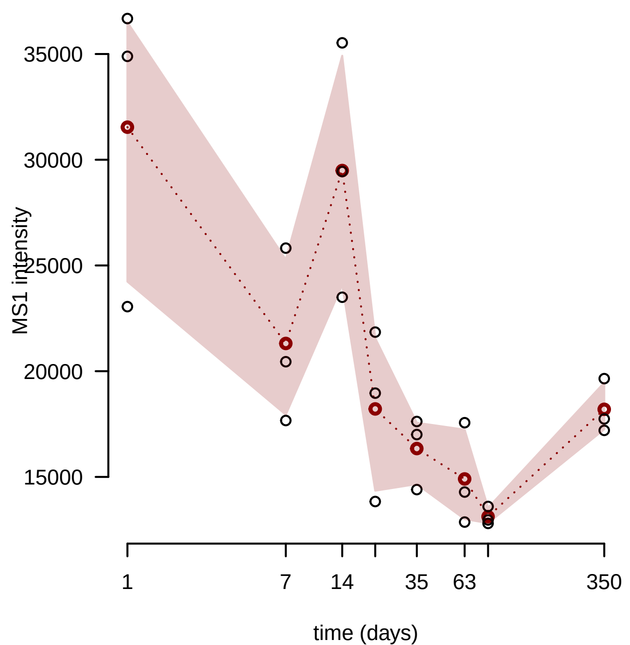

P97950
GN=Rab33a
RAB33A, member RAS oncogene family

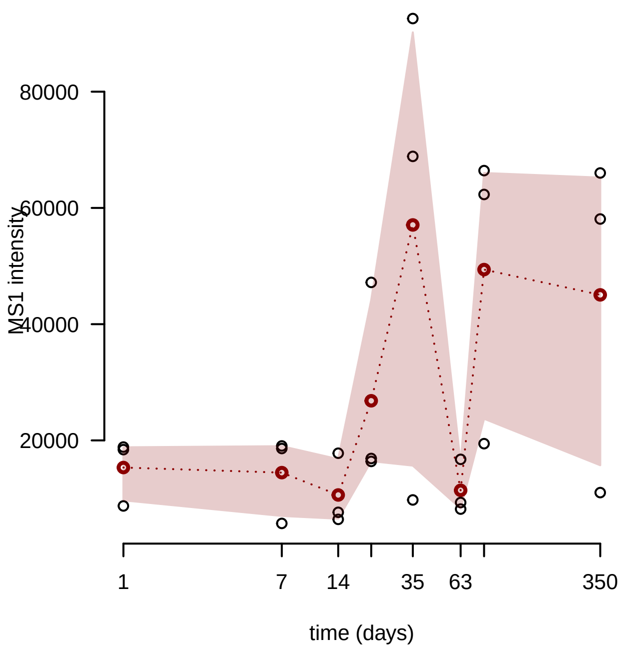

P98063
GN=Bmp1
bone morphogenetic protein 1

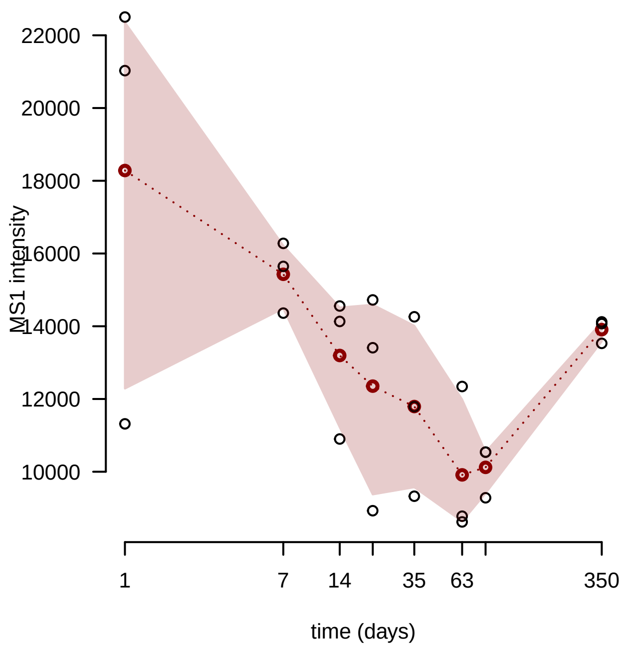

P98064
GN=Masp1
mannan−binding lectin serine peptidase 1

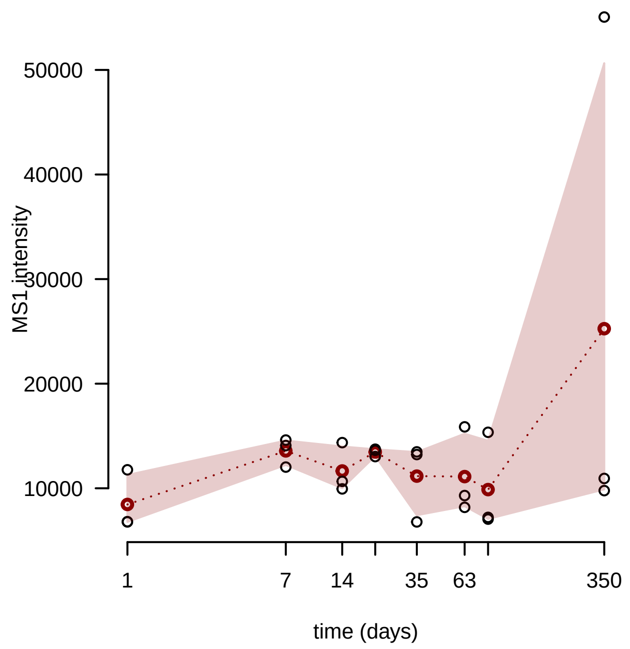

P98078
GN=Dab2
disabled 2, mitogen−responsive phosphopr ...

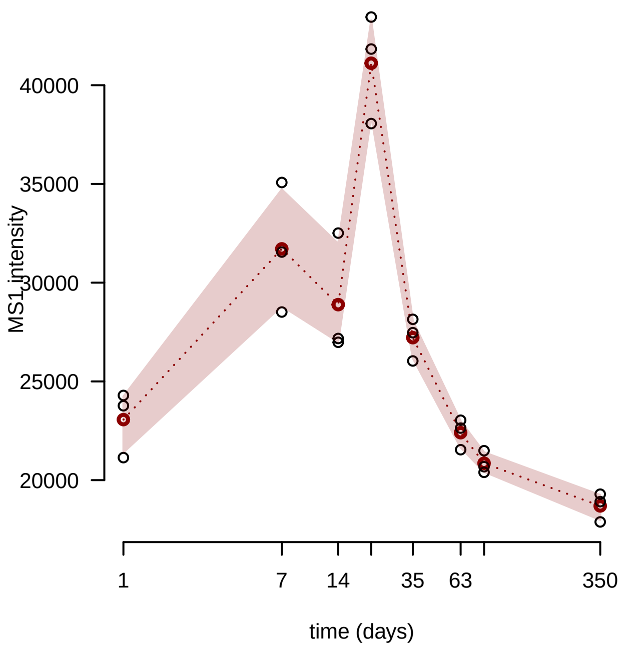

P98083
GN=Shc1
src homology 2 domain−containing transfo ...

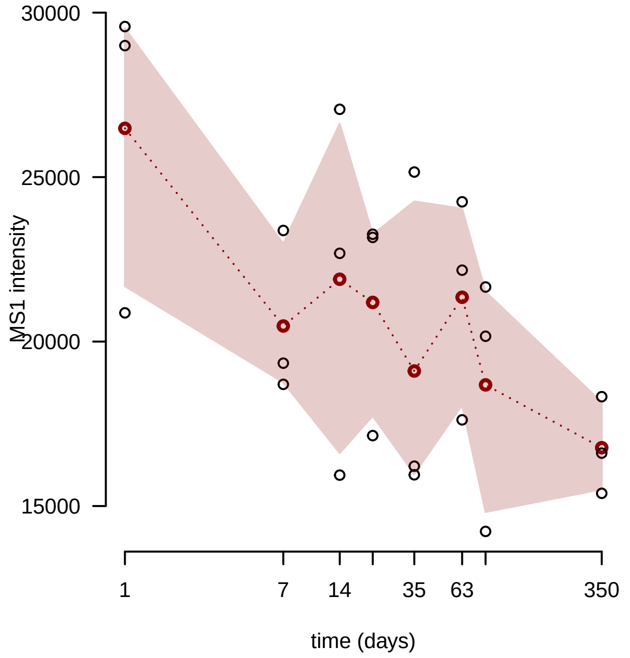

P98086
GN=C1qa
complement component 1, q subcomponent, ...

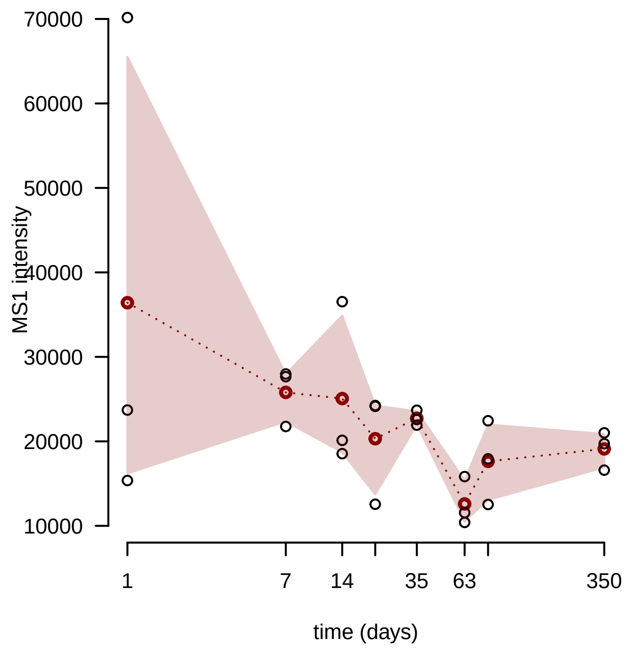

P98156
GN=Vldlr
very low density lipoprotein receptor

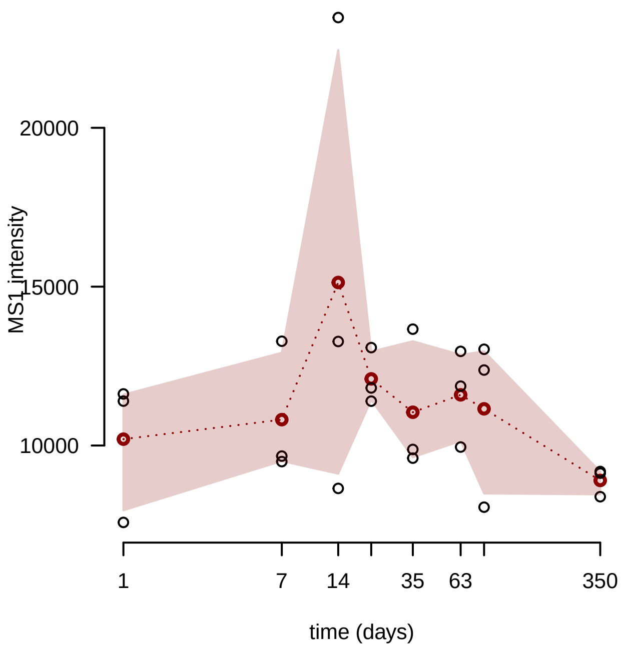

P98191
GN=Cds1
CDP−diacylglycerol synthase 1

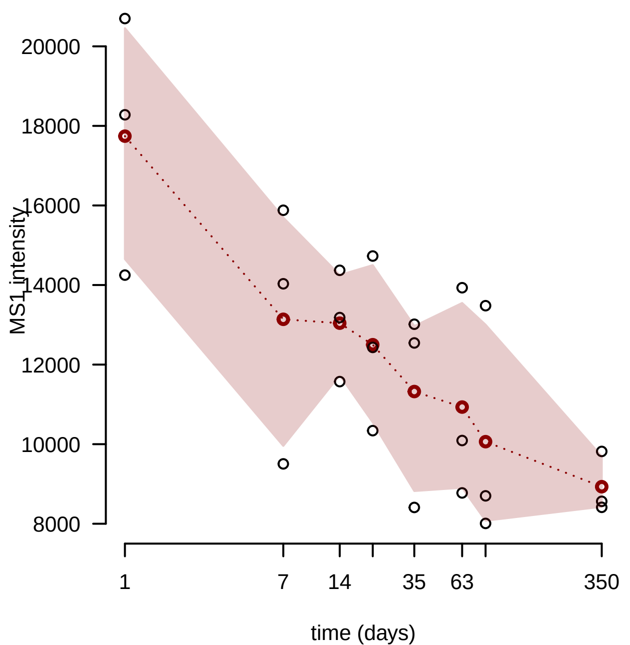

P98192
GN=Gnpat
glyceronephosphate O−acyltransferase

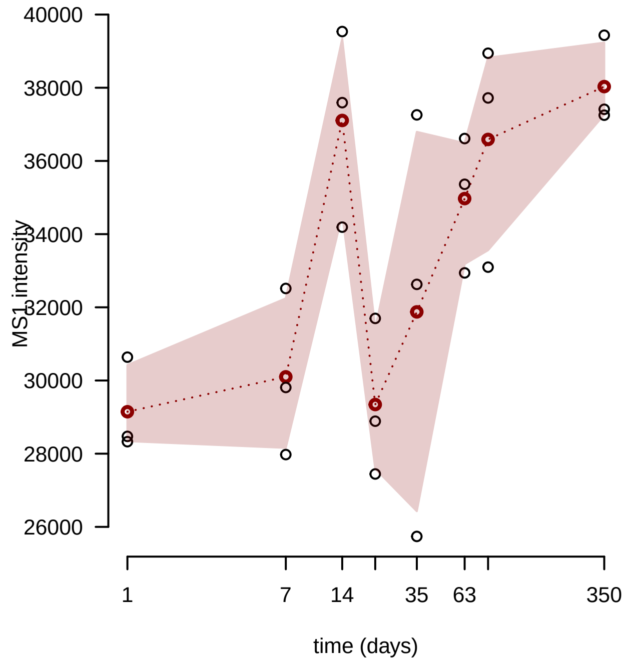

P98195
GN=Atp9b
ATPase, class II, type 9B

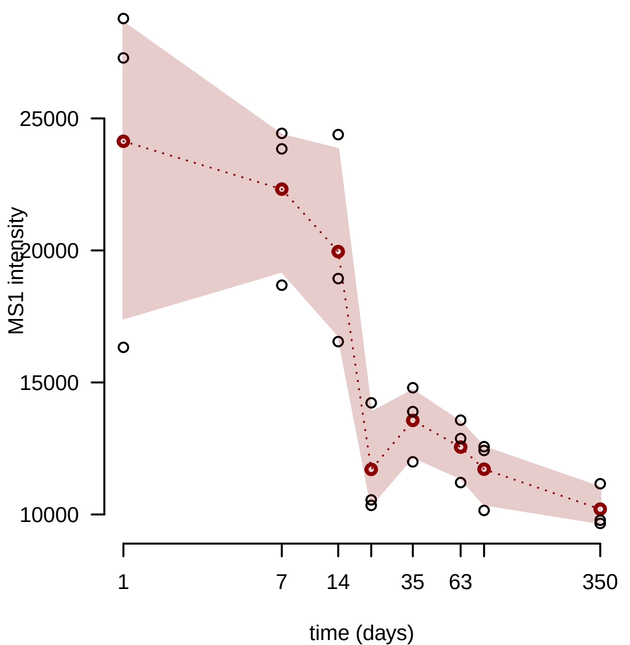

P98197
GN=Atp11a
ATPase, class VI, type 11A

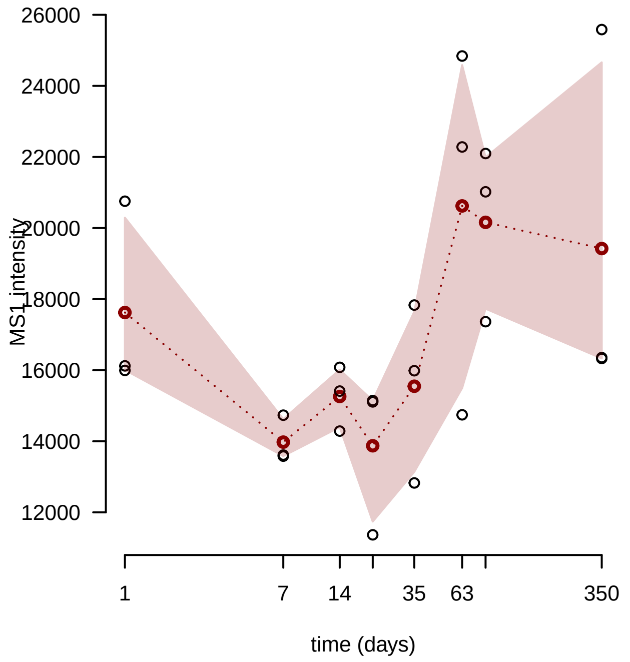

P98203
GN=Arvcf
armadillo repeat gene deleted in velocar ...

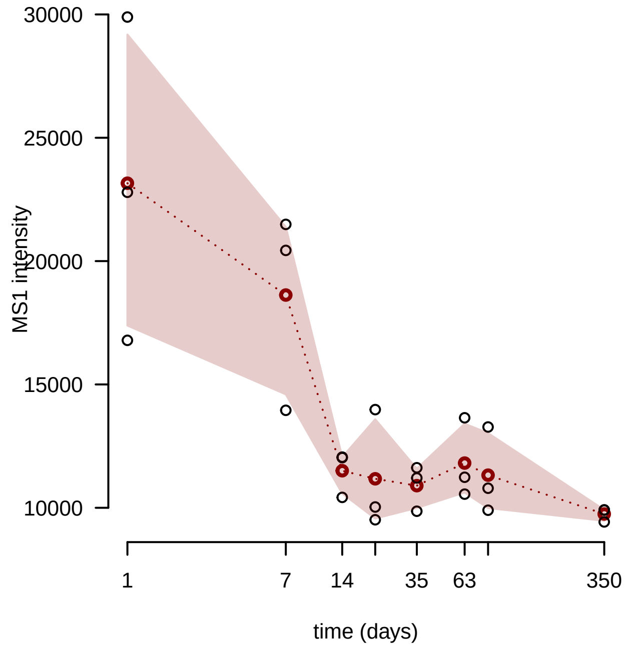

P99024
GN=Tubb5
tubulin, beta 5 class I

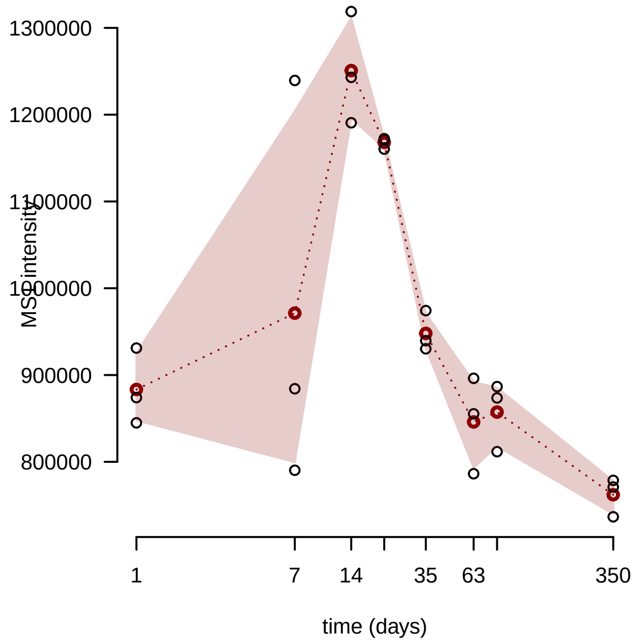

P99026
GN=Psmb4
proteasome (prosome, macropain) subunit, ...

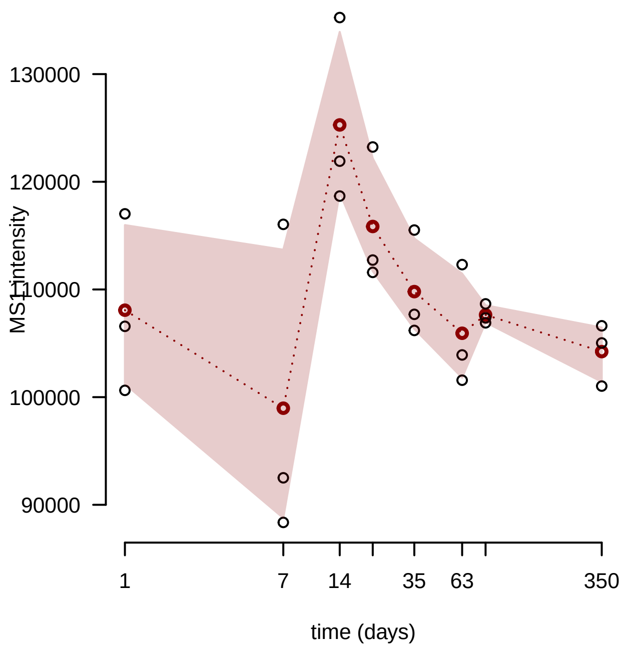

P99027
GN=Rplp2
ribosomal protein, large P2

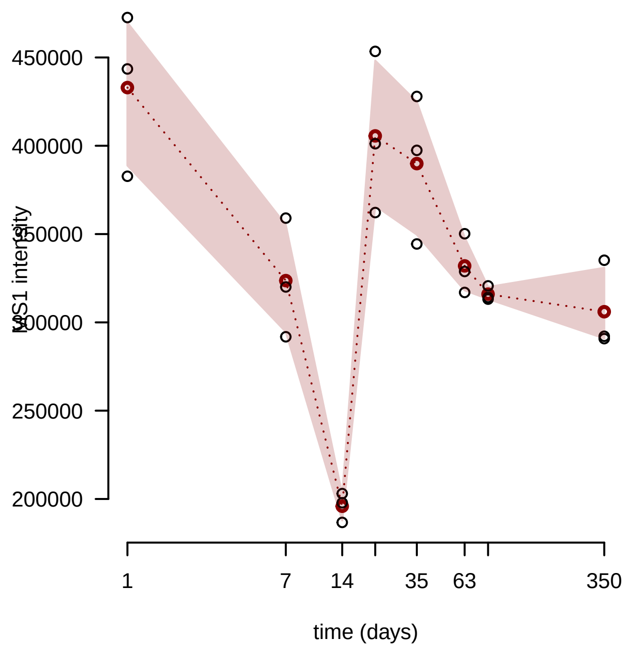

P99028
GN=Uqcrh
ubiquinol−cytochrome c reductase hinge p ...

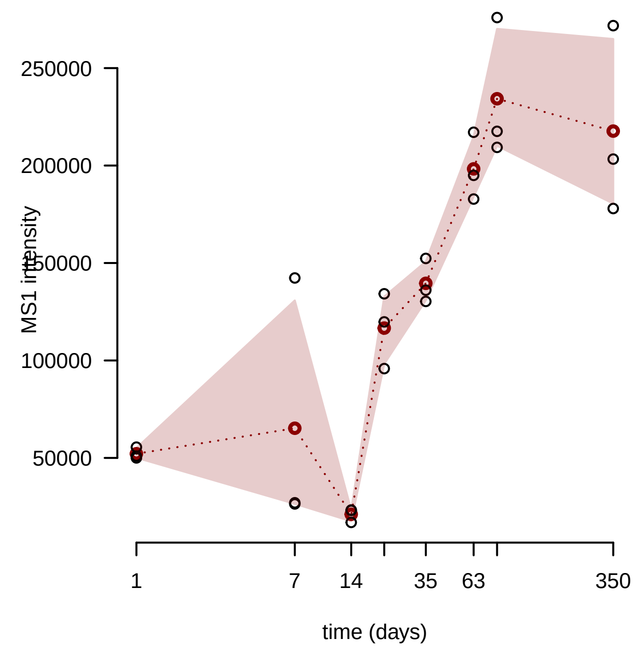

P99029
GN=Prdx5
peroxiredoxin 5

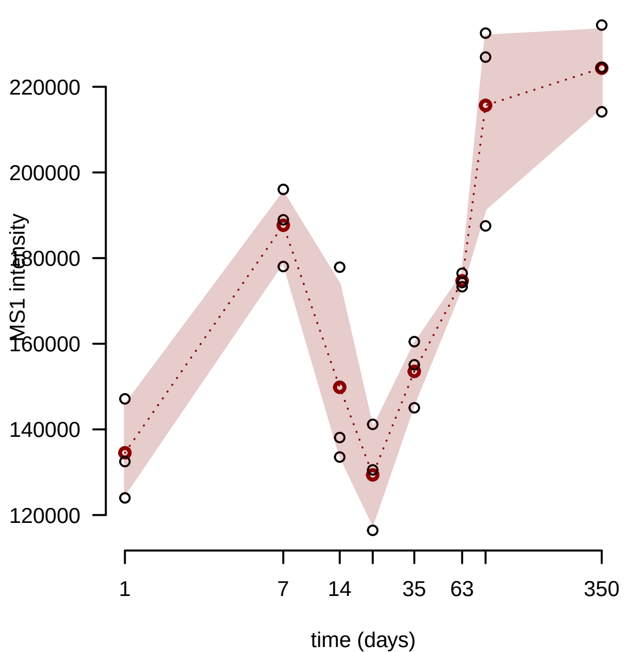

Q00262
GN=Stx2
syntaxin 2

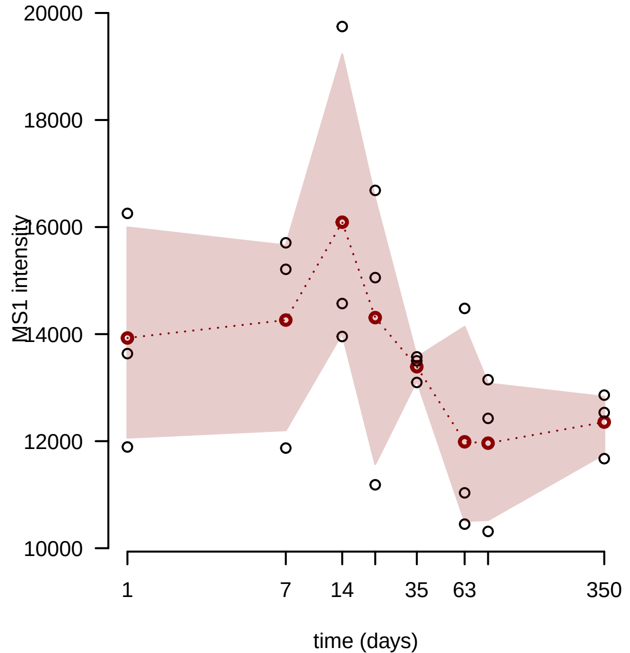

Q00420
GN=Gabpb1
GA repeat binding protein, beta 1

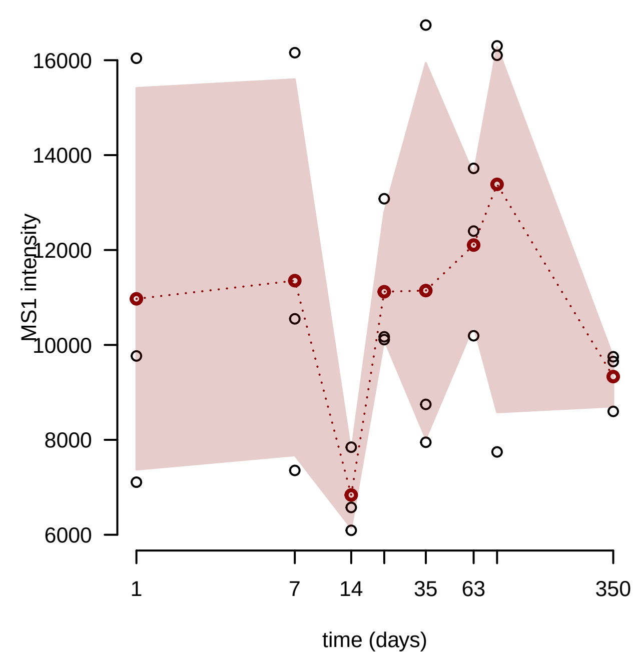

Q00422
GN=Gabpa
GA repeat binding protein, alpha

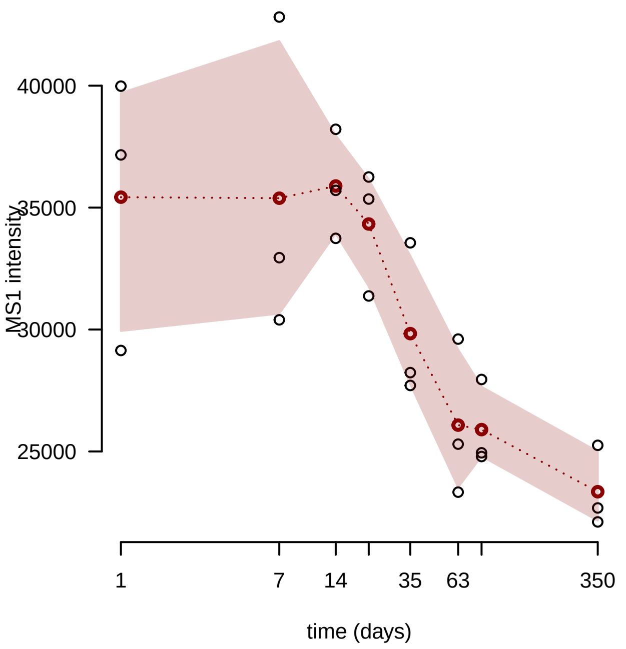

Q00493
GN=Cpe
carboxypeptidase E

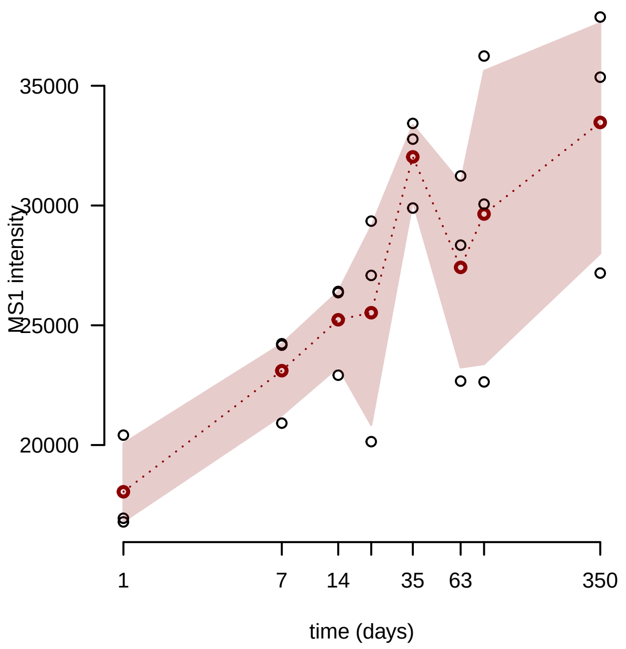

Q00519
GN=Xdh
xanthine dehydrogenase

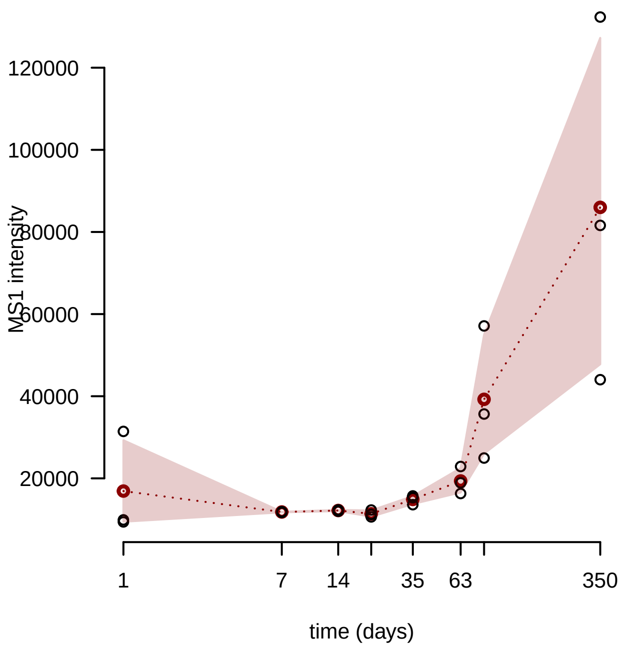

Q00547
GN=Hmmr
hyaluronan mediated motility receptor (R ...

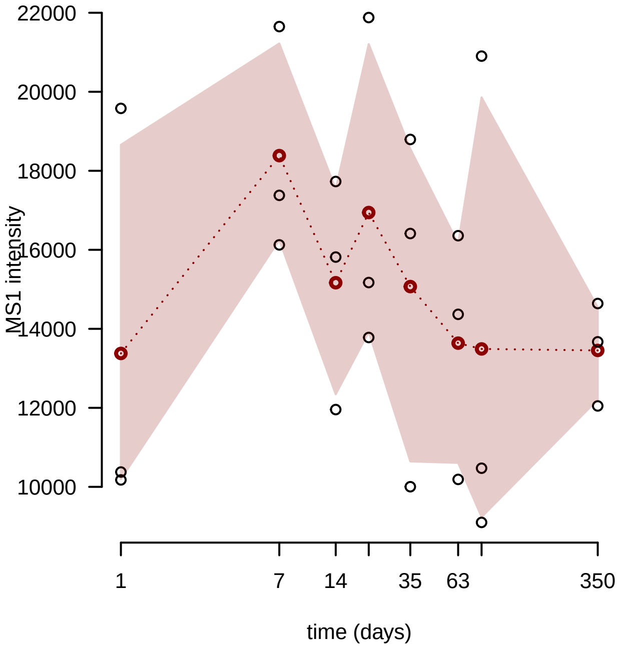

Q00558
GN=F8a1

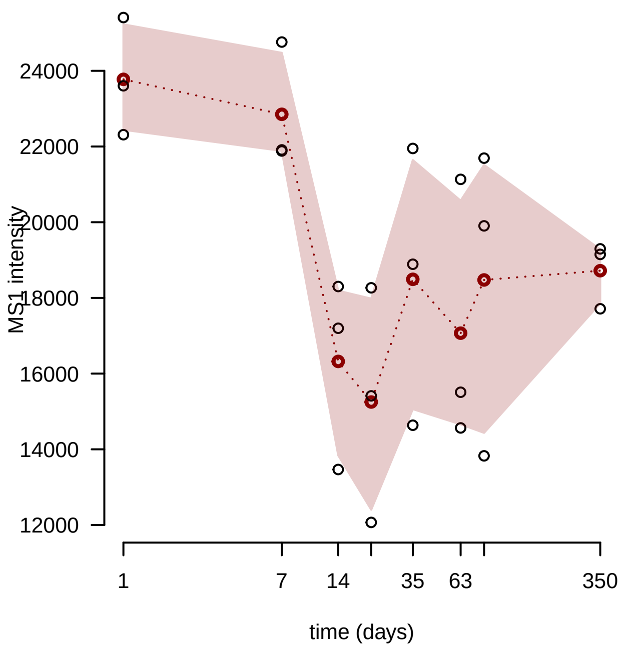

Q00560
GN=Il6st
interleukin 6 signal transducer

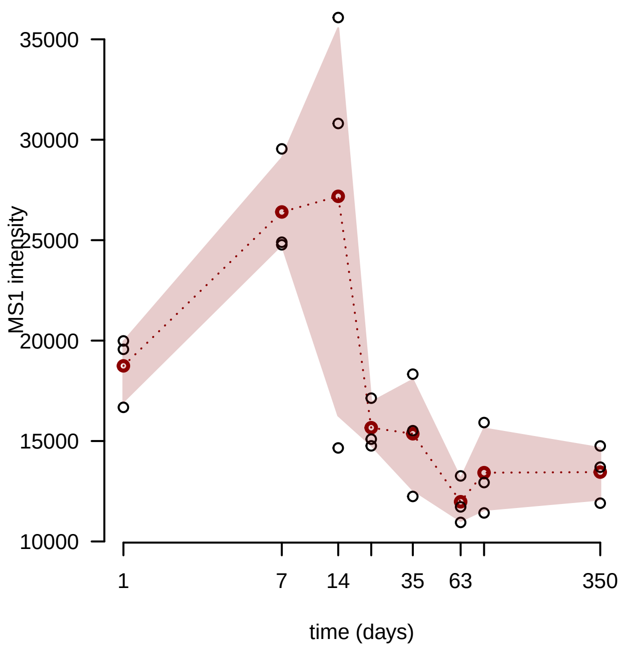

Q00612
GN=G6pdx
glucose−6−phosphate dehydrogenase X−link ...

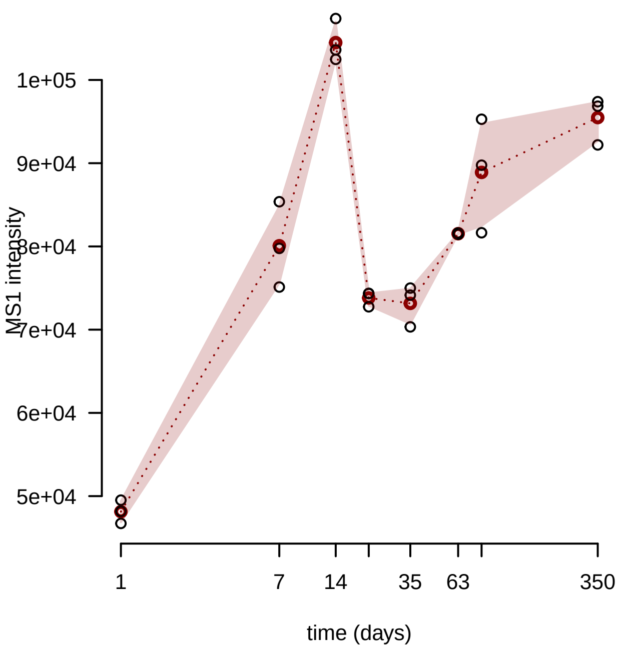

Q00623
GN=Apoa1
apolipoprotein A−I

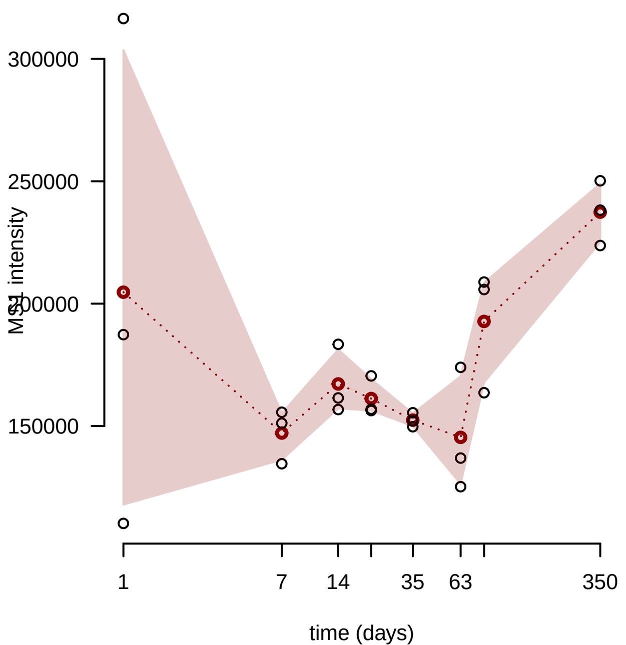

Q00651
GN=Itga4
integrin alpha 4

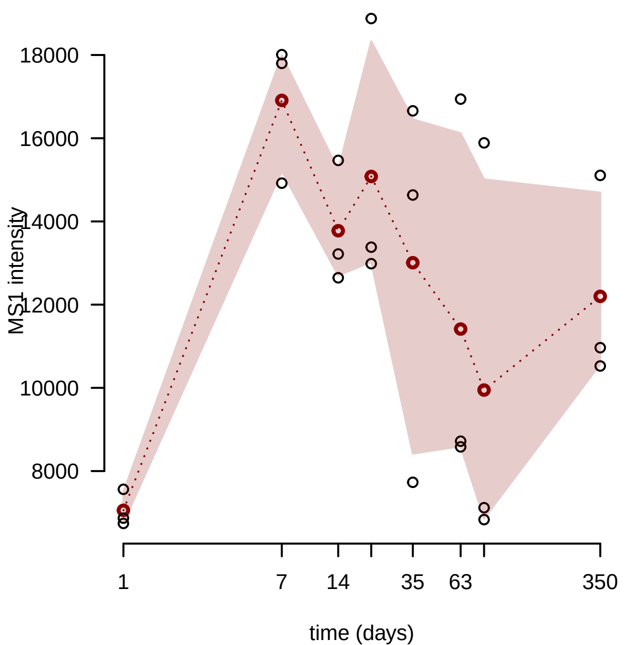

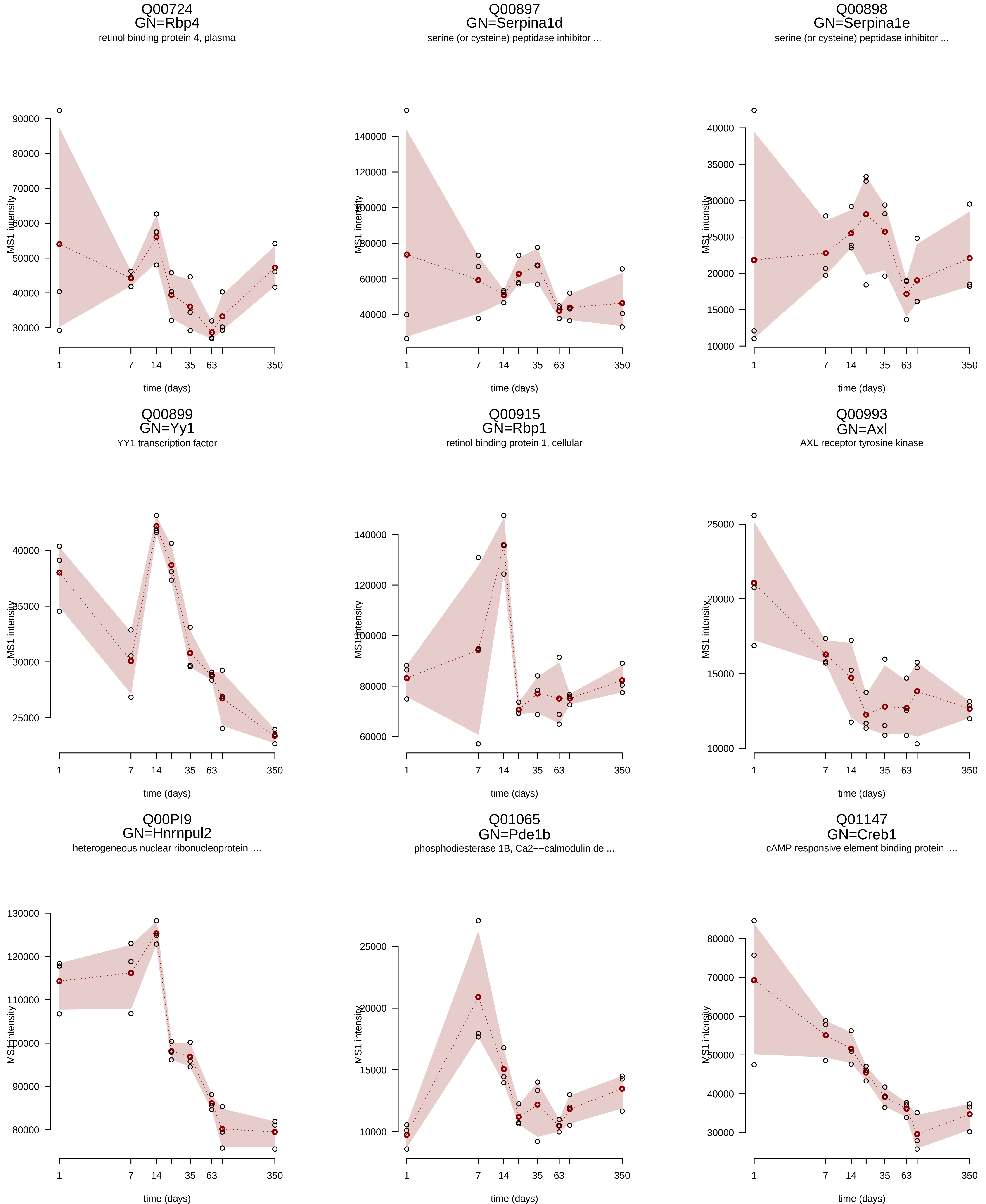

Q00724
GN=Rbp4
retinol binding protein 4, plasma
MS1 intensity
90000
80000
70000
60000
50000
40000
30000
1
7
14
35
63
350
time (days)
Q00897
GN=Serpina1d
serine (or cysteine) peptidase inhibitor ...
MS1 intensity
140000
120000
100000
80000
60000
40000
1
7
14
35
63
350
time (days)
Q00898
GN=Serpina1e
serine (or cysteine) peptidase inhibitor ...
MS1 intensity
40000
35000
30000
25000
20000
15000
10000
1
7
14
35
63
350
time (days)
Q00899
GN=Yy1
YY1 transcription factor
MS1 intensity
40000
35000
30000
25000
1
7
14
35
63
350
time (days)
Q00915
GN=Rbp1
retinol binding protein 1, cellular
MS1 intensity
140000
120000
100000
80000
60000
1
7
14
35
63
350
time (days)
Q00993
GN=Axl
AXL receptor tyrosine kinase
MS1 intensity
25000
20000
15000
10000
1
7
14
35
63
350
time (days)
Q00PI9
GN=Hnrnpul2
heterogeneous nuclear ribonucleoprotein ...
MS1 intensity
130000
120000
110000
100000
90000
80000
1
7
14
35
63
350
time (days)
Q01065
GN=Pde1b
phosphodiesterase 1B, Ca2+−calmodulin de ...
MS1 intensity
25000
20000
15000
10000
1
7
14
35
63
350
time (days)
Q01147
GN=Creb1
cAMP responsive element binding protein ...
MS1 intensity
80000
70000
60000
50000
40000
30000
1
7
14
35
63
350
time (days)

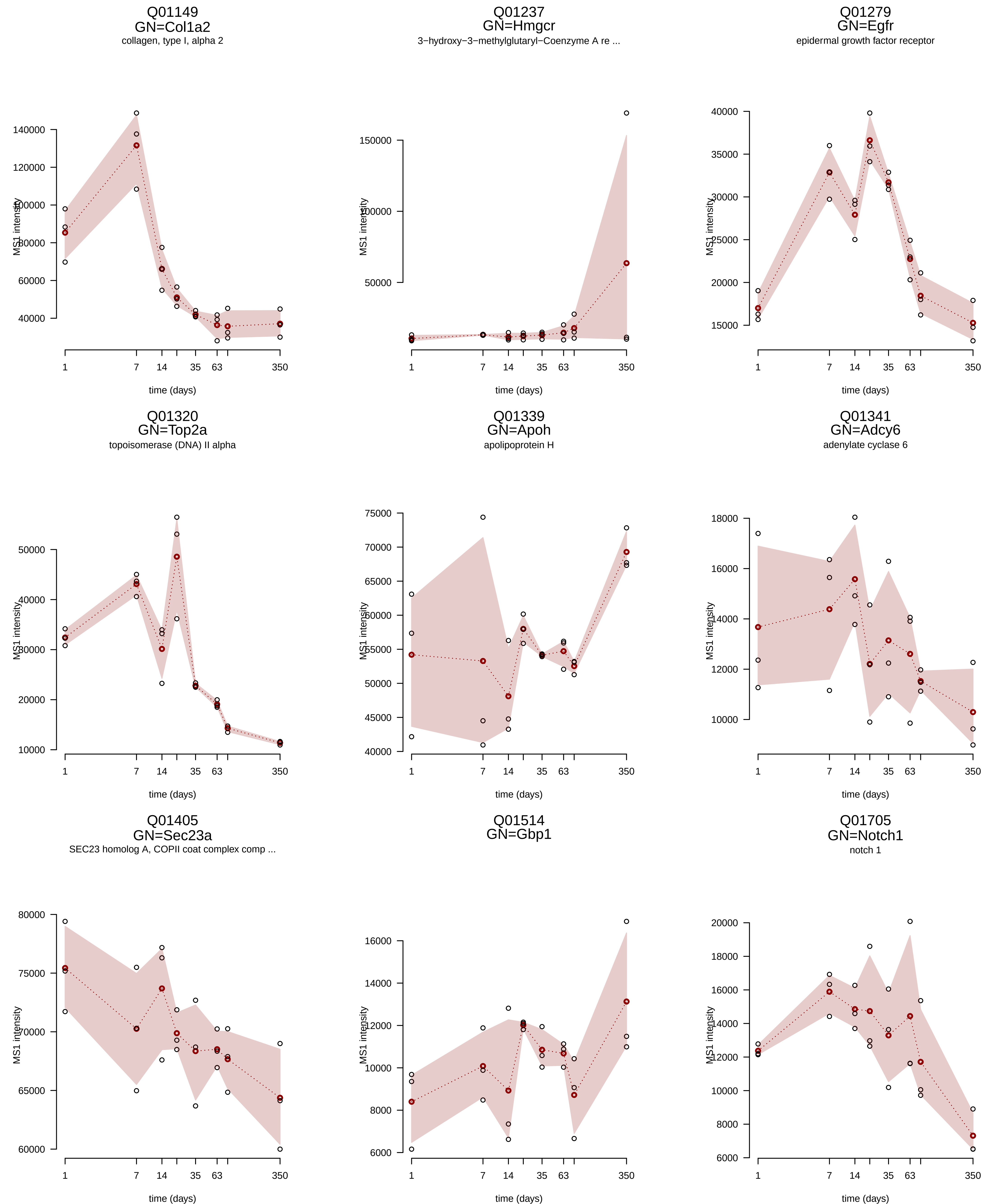
Q01149
GN=Col1a2
collagen, type I, alpha 2
MS1 intensity
time (days)
Q01237
GN=Hmgcr
3−hydroxy−3−methylglutaryl−Coenzyme A re ...
MS1 intensity
time (days)
Q01279
GN=Egfr
epidermal growth factor receptor
MS1 intensity
time (days)
Q01320
GN=Top2a
topoisomerase (DNA) II alpha
MS1 intensity
time (days)
Q01339
GN=Apoh
apolipoprotein H
MS1 intensity
time (days)
Q01341
GN=Adcy6
adenylate cyclase 6
MS1 intensity
time (days)
Q01405
GN=Sec23a
SEC23 homolog A, COPII coat complex comp ...
MS1 intensity
time (days)
Q01514
GN=Gbp1
MS1 intensity
time (days)
Q01705
GN=Notch1
notch 1
MS1 intensity
time (days)

Q01721
GN=Gas1
growth arrest specific 1

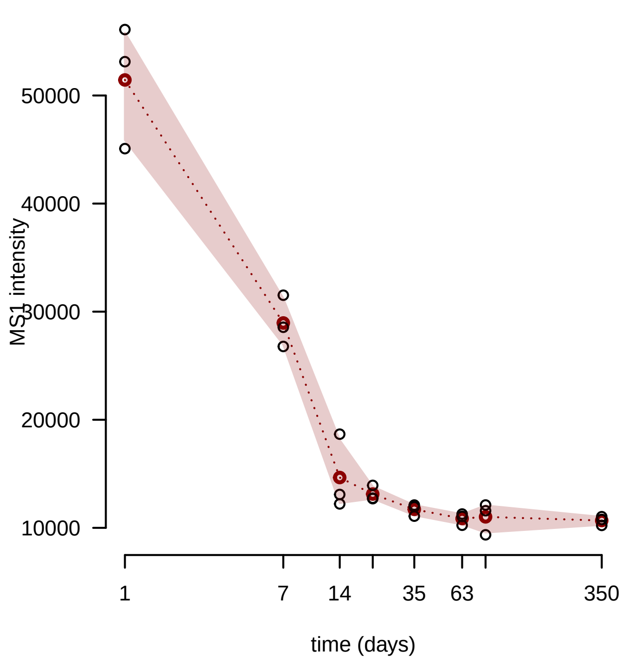

Q01730
GN=Rsu1
Ras suppressor protein 1

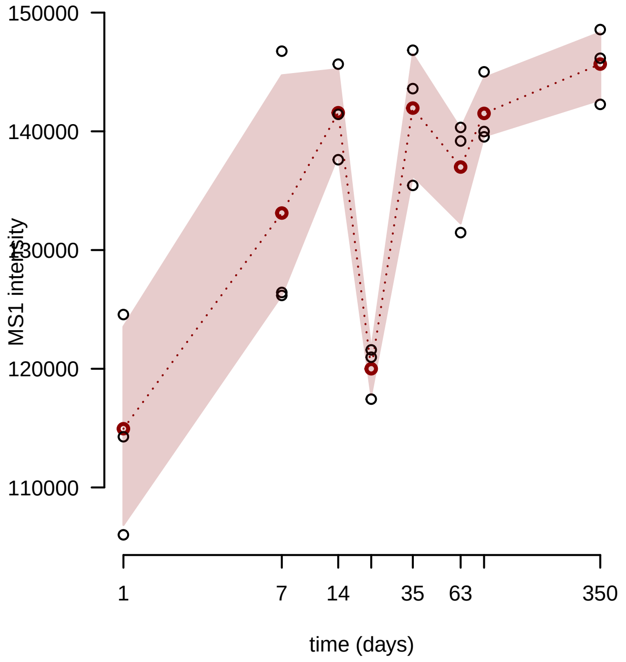

Q01768
GN=Nme2
NME/NM23 nucleoside diphosphate kinase 2

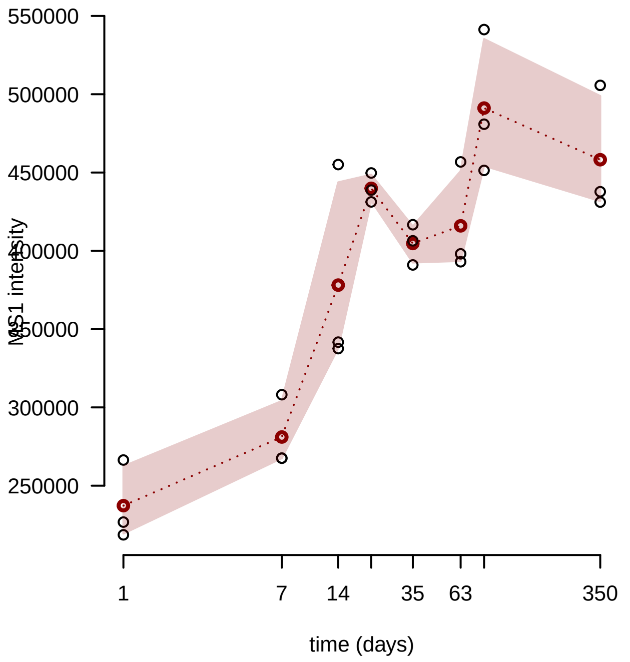

Q01853
GN=Vcp
valosin containing protein

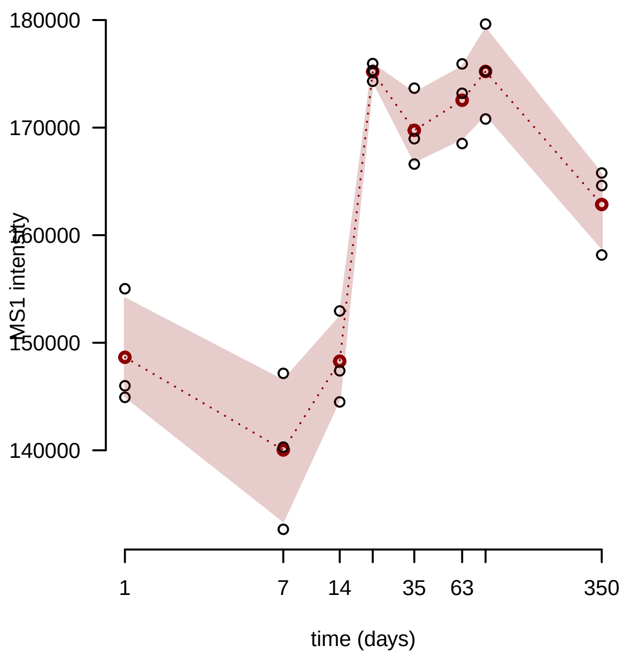

Q02013
GN=Aqp1
aquaporin 1

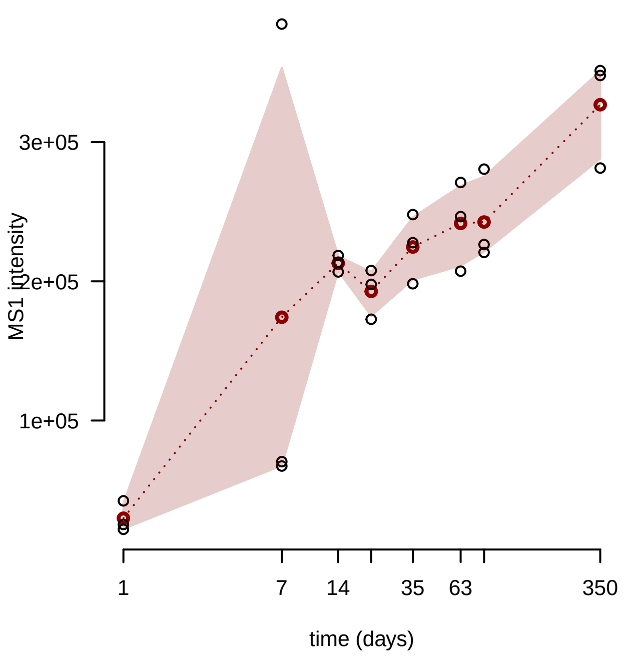

Q02053
GN=Uba1
ubiquitin−like modifier activating enzym ...

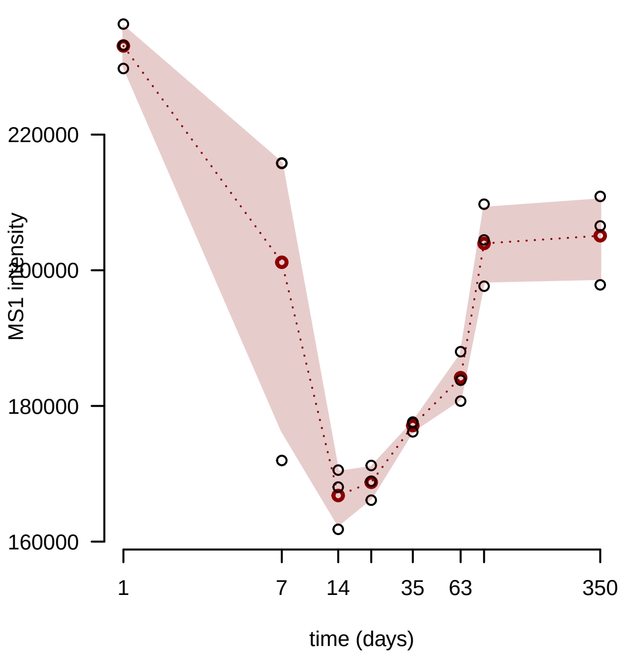

Q02105
GN=C1qc
complement component 1, q subcomponent, ...

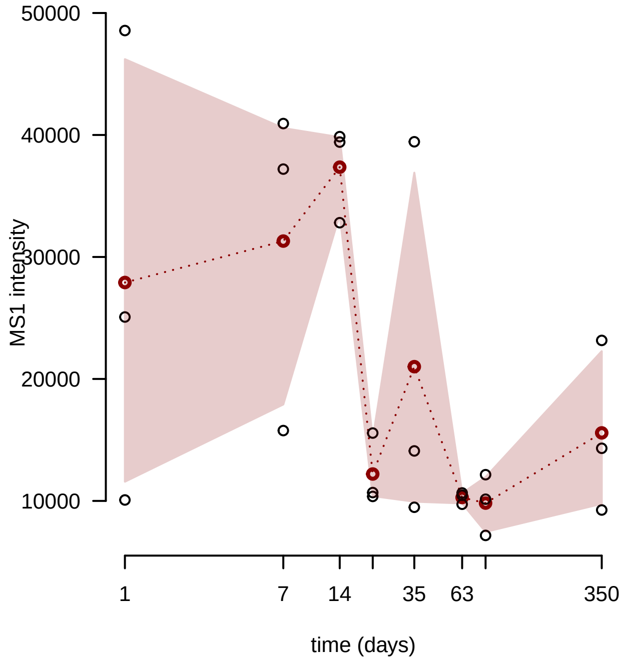

Q02242
GN=Pdcd1
programmed cell death 1

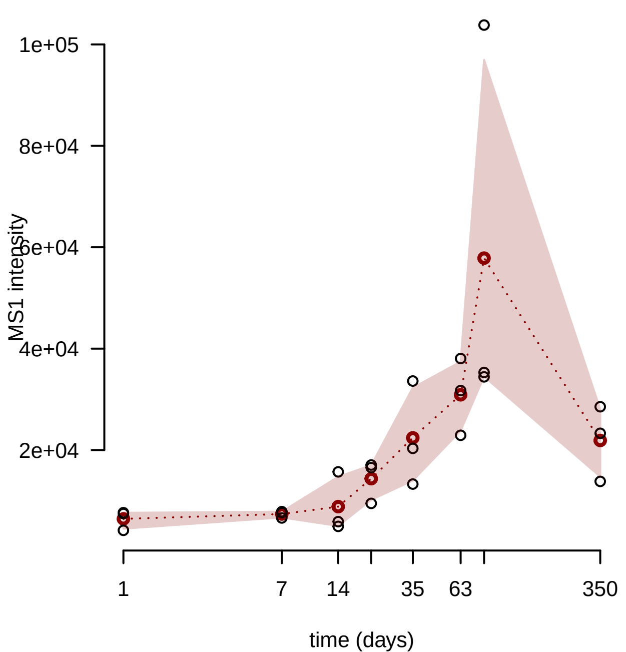

Q02248
GN=Ctnnb1
catenin (cadherin associated protein), b ...

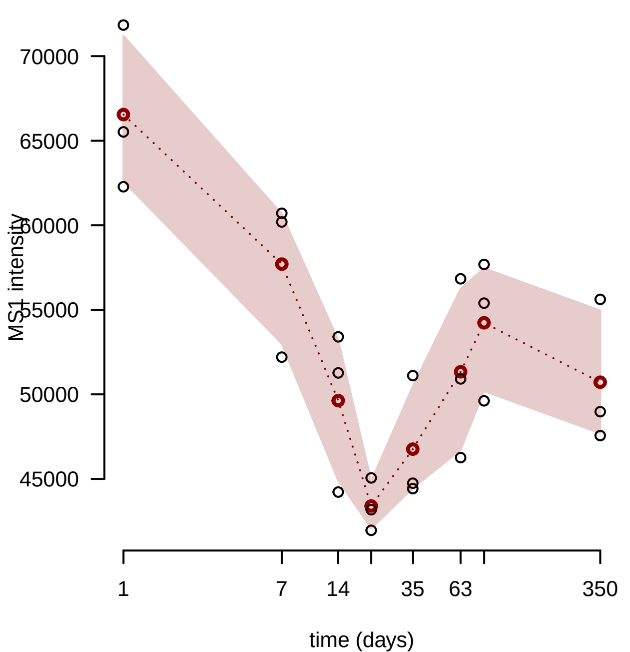

Q02257
GN=Jup
junction plakoglobin

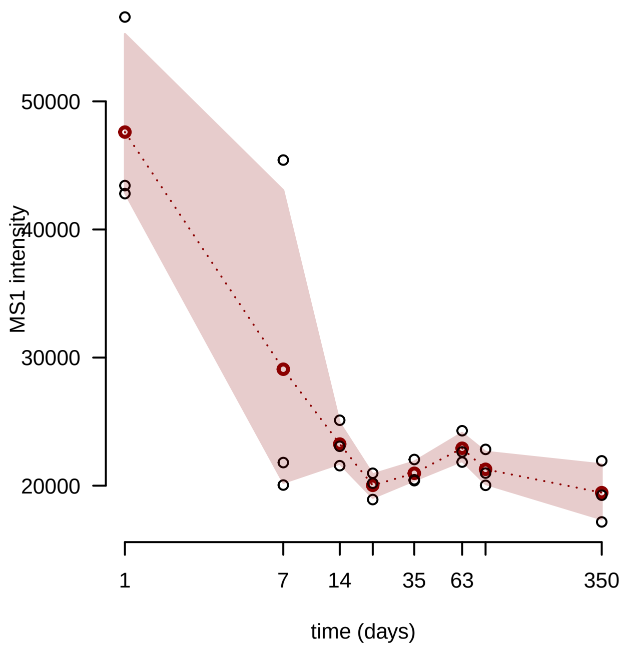

Q02357
GN=Ank1
ankyrin 1, erythroid

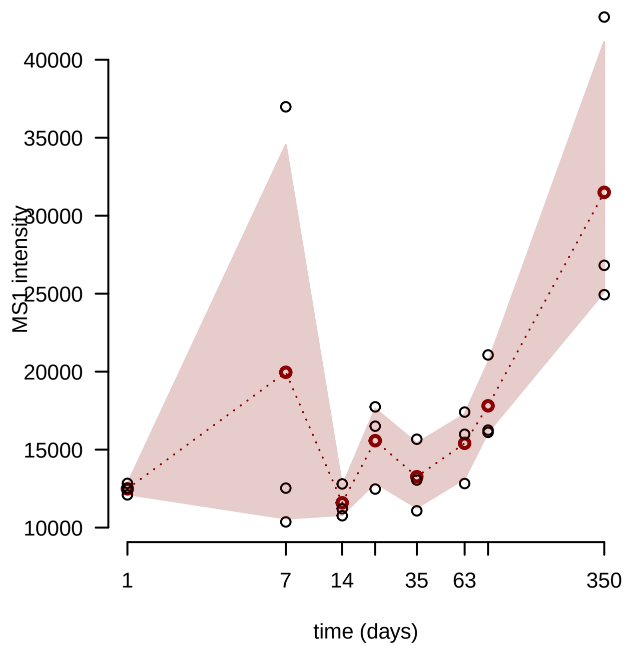

Q02384
GN=Sos2
SOS Ras/Rho guanine nucleotide exchange ...

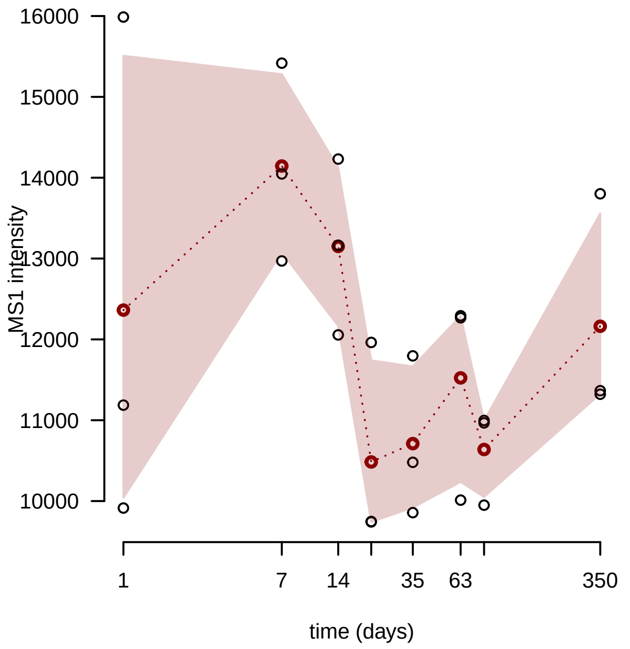

Q02395
GN=Mtf2
metal response element binding transcrip ...

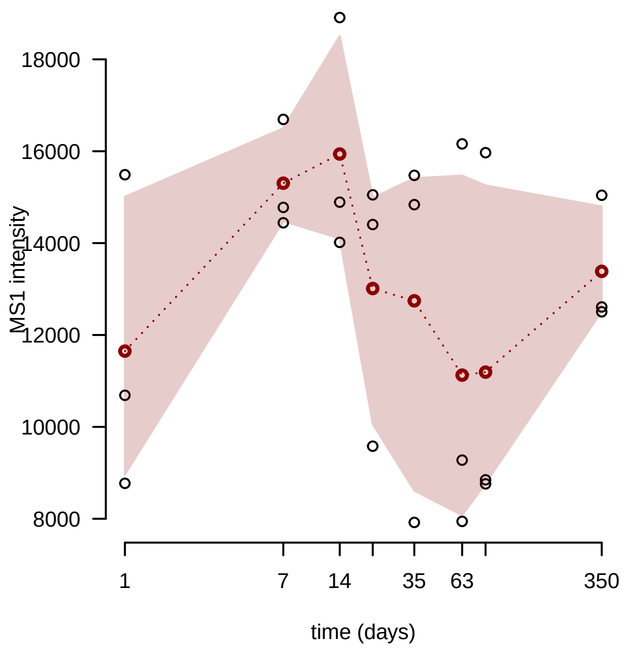

Q02526
GN=Zfp41
zinc finger protein 41

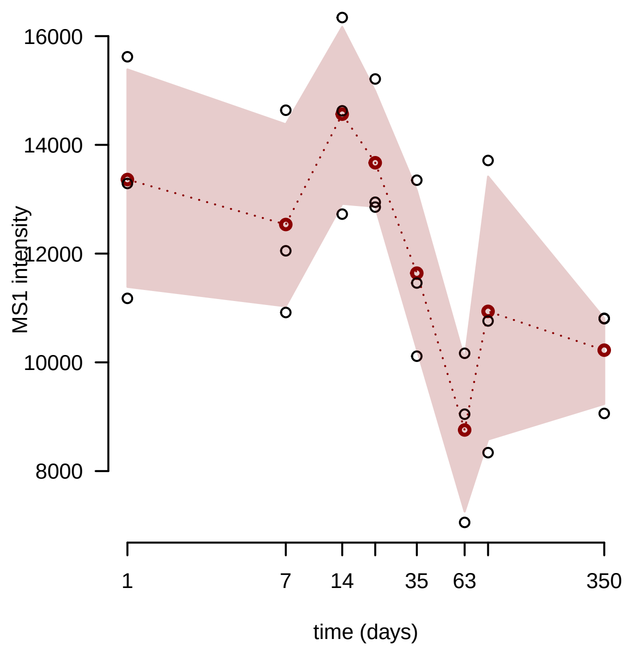

Q02614
GN=Sap30bp
SAP30 binding protein

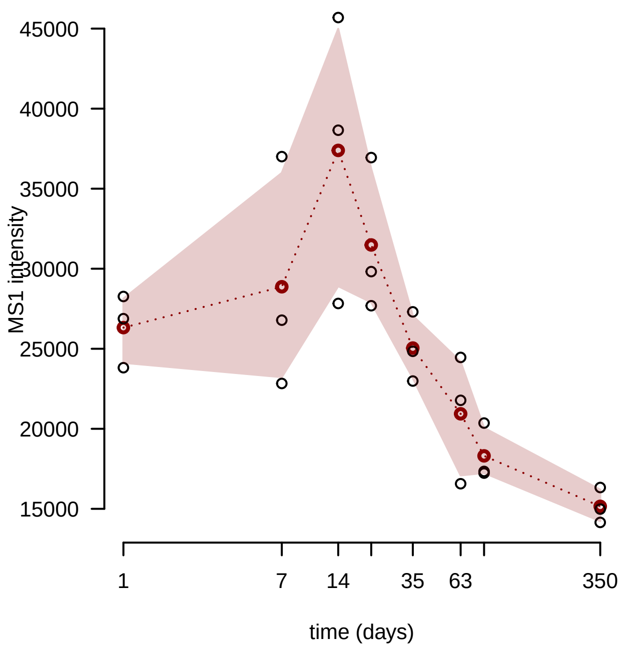

Q02780
GN=Nfia
nuclear factor I/A

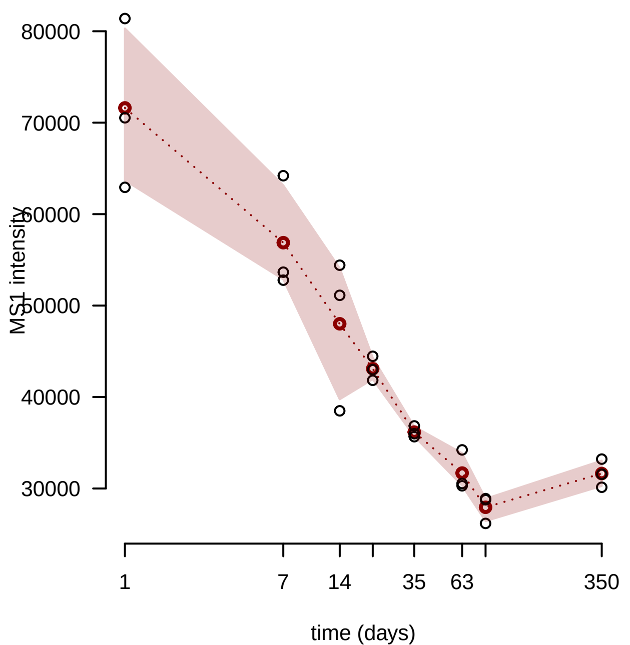

Q02788
GN=Col6a2
collagen, type VI, alpha 2

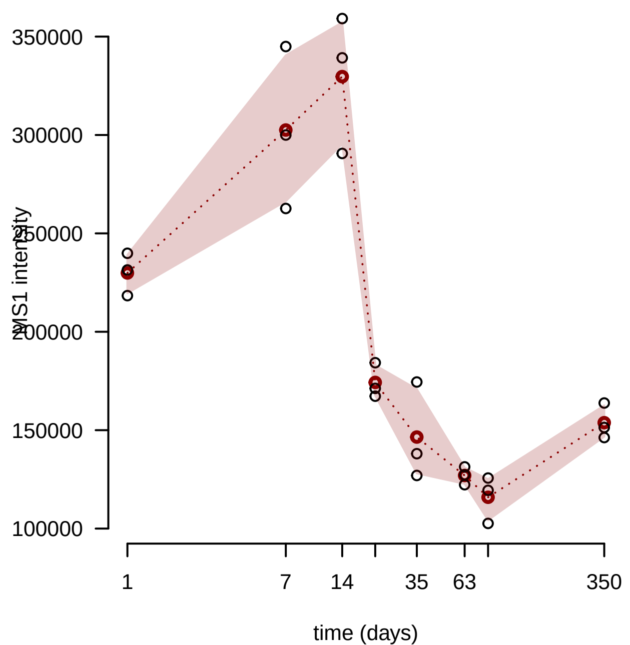

Q02819
GN=Nucb1
nucleobindin 1

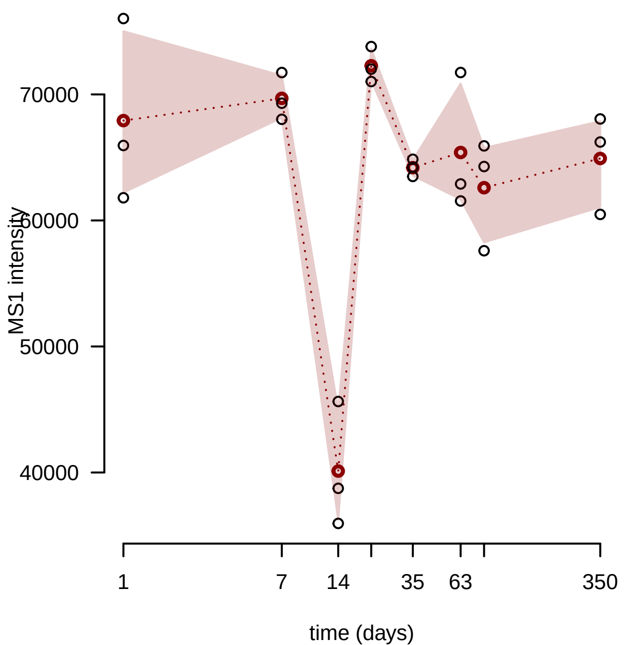

Q02844
GN=Tpsab1
tryptase alpha/beta 1

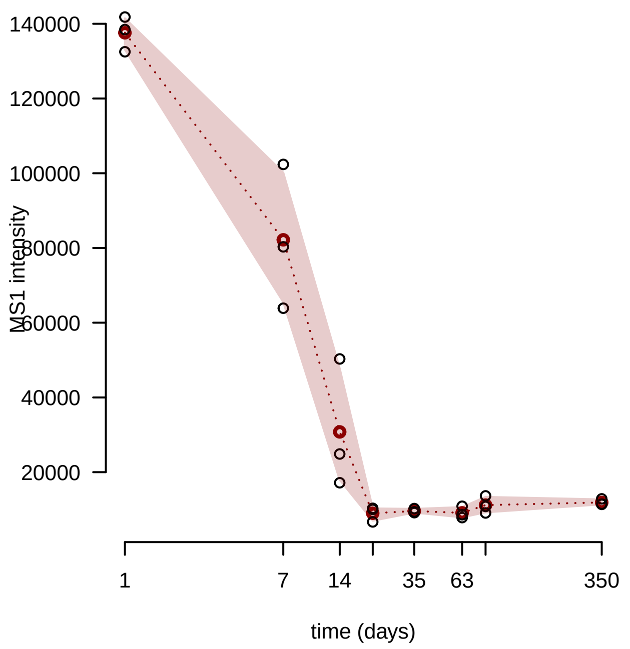

Q02853
GN=Mmp11
matrix metallopeptidase 11

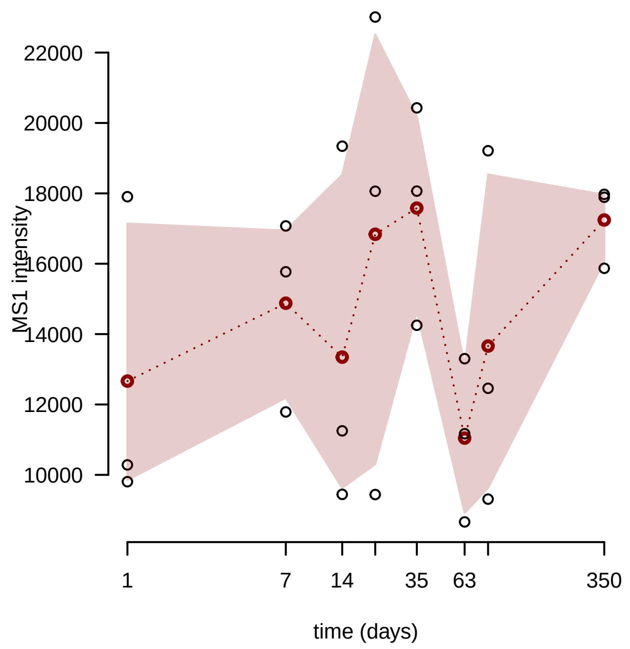

Q03137
GN=Epha4
Eph receptor A4

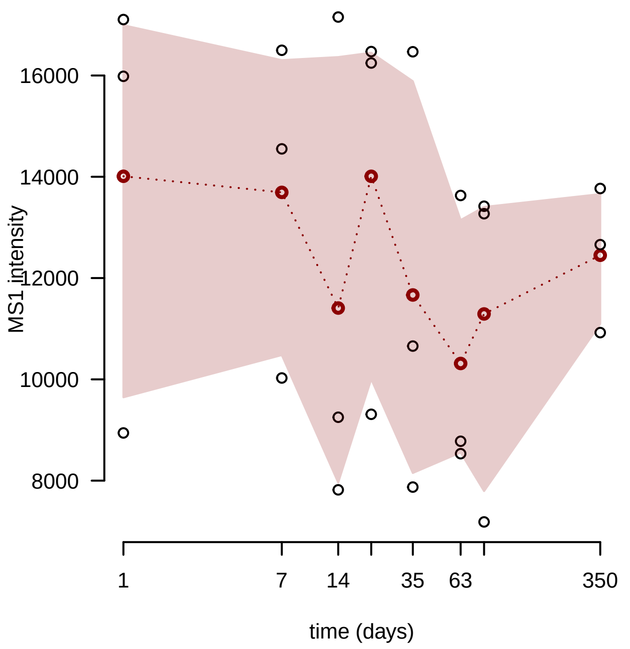

Q03141
GN=Mark3
MAP/microtubule affinity regulating kina ...

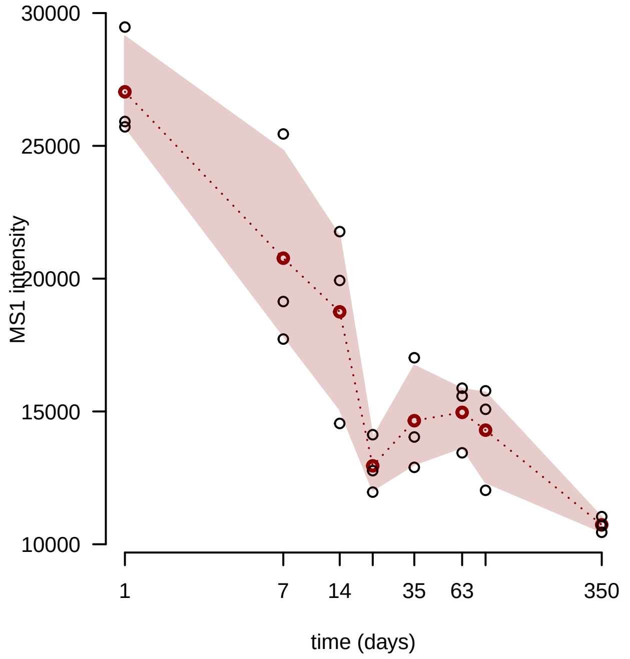

Q03145
GN=Epha2
Eph receptor A2

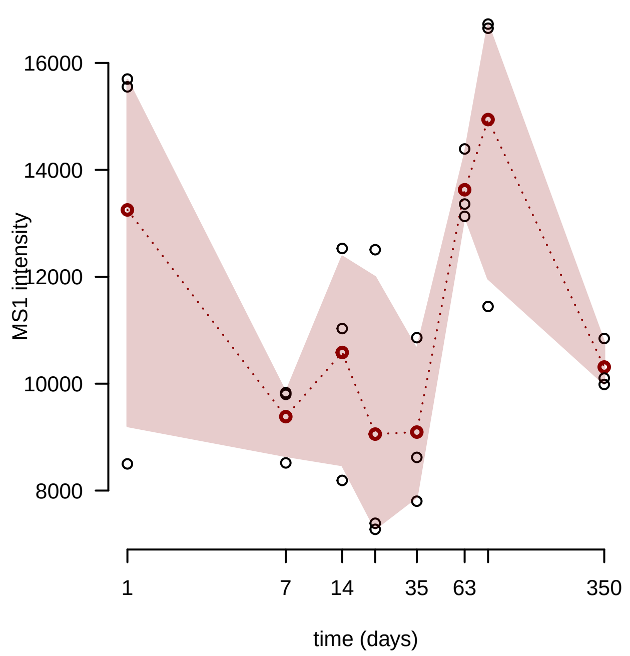

Q03147
GN=Cdk7
cyclin−dependent kinase 7

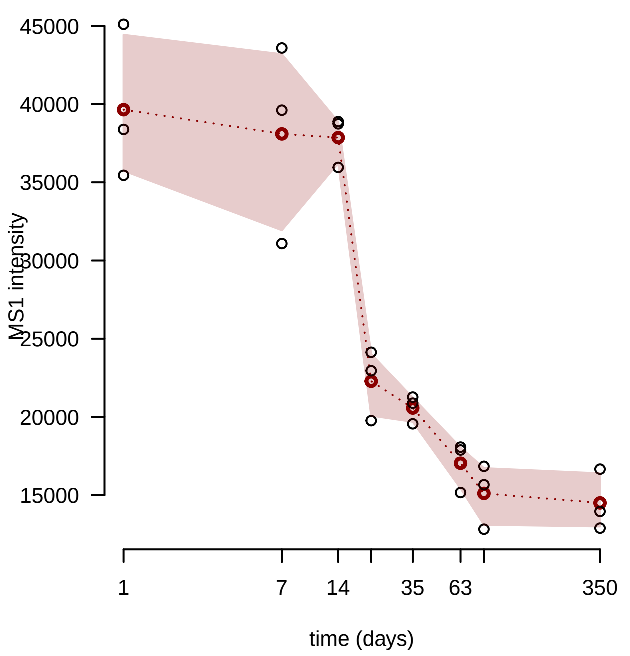

Q03173
GN=Enah
ENAH actin regulator

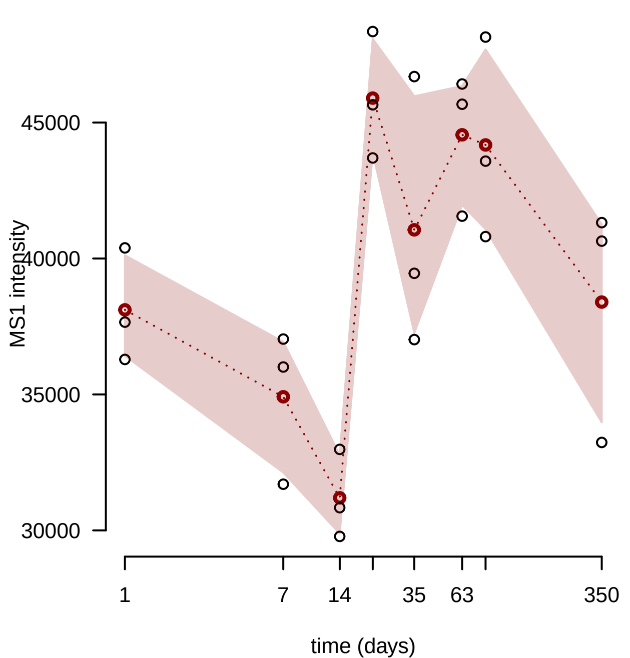

Q03249
GN=Galt
galactose−1−phosphate uridyl transferase

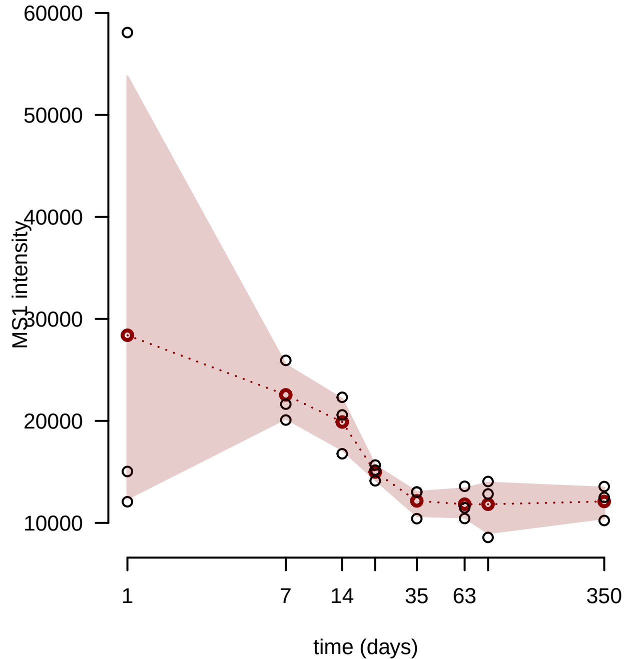

Q03265
GN=Atp5f1a

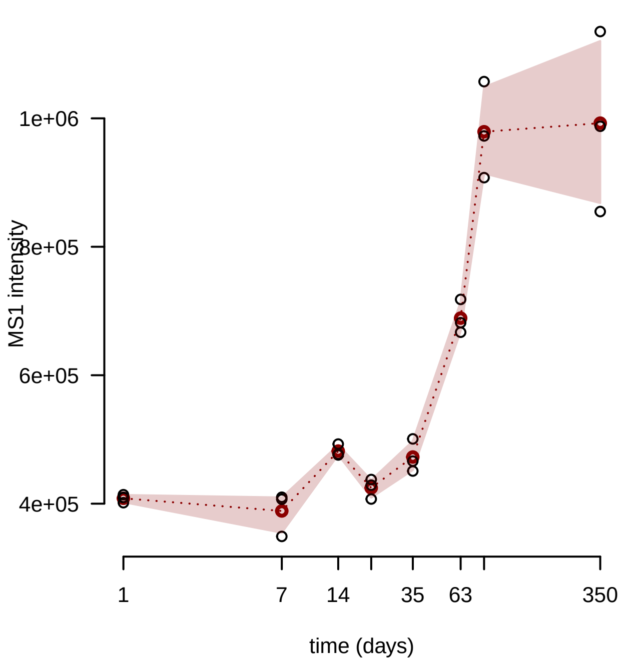

Q03347
GN=Runx1
runt related transcription factor 1

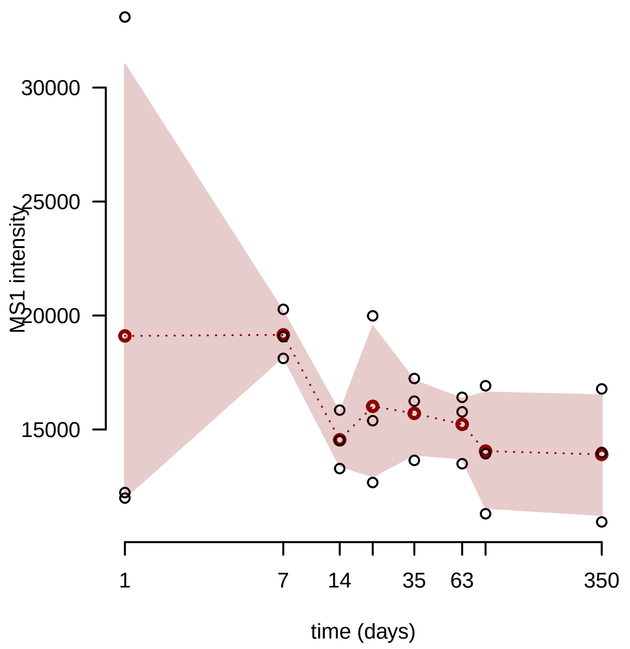

Q03350
GN=Thbs2
thrombospondin 2

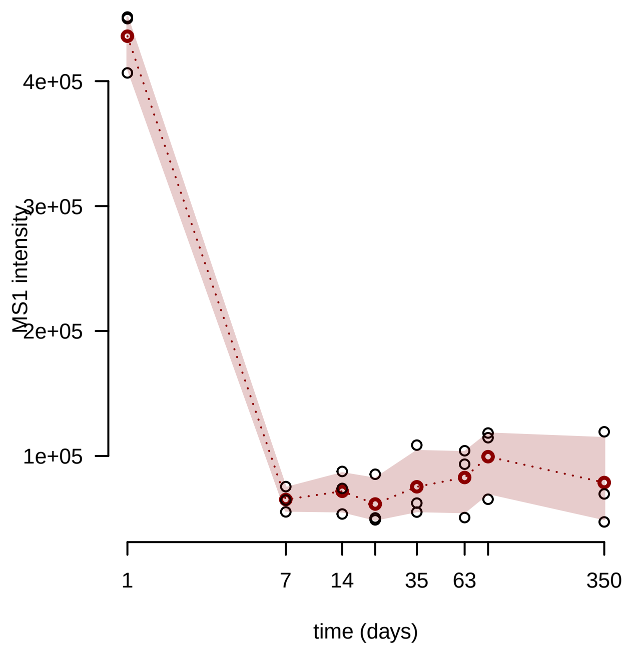

Q03517
GN=Scg2
secretogranin II

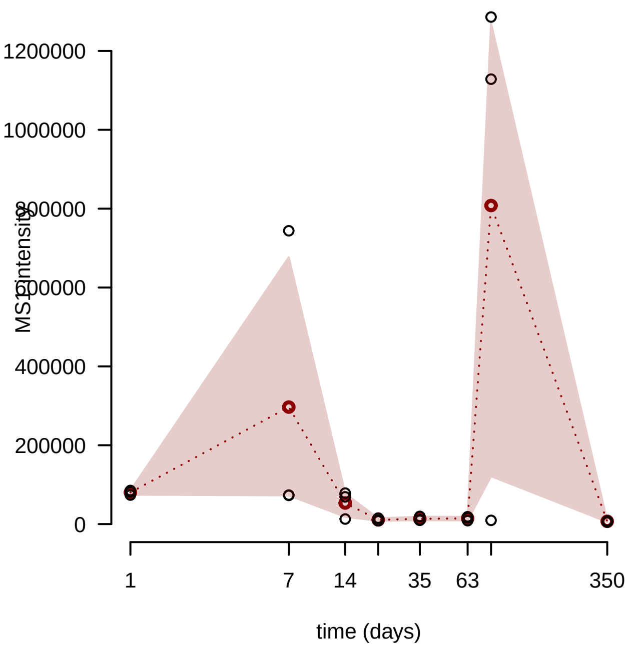

Q03734
GN=Serpina3m
serine (or cysteine) peptidase inhibitor ...

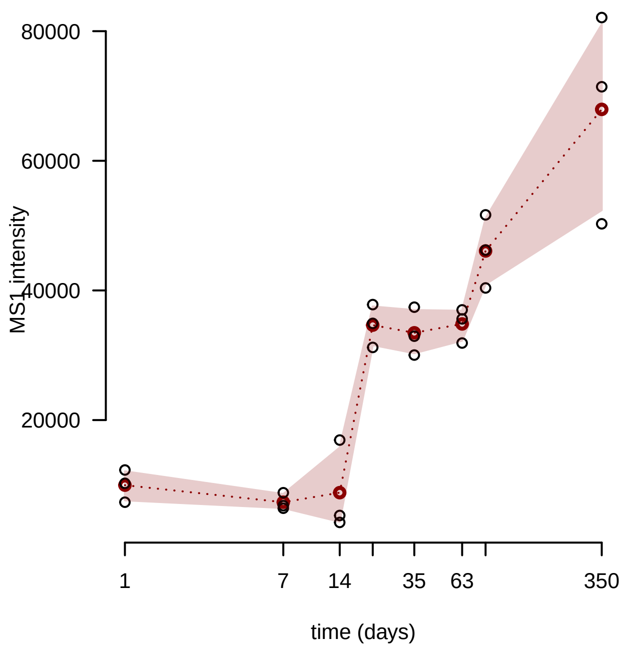

Q03958
GN=Pfdn6
prefoldin subunit 6

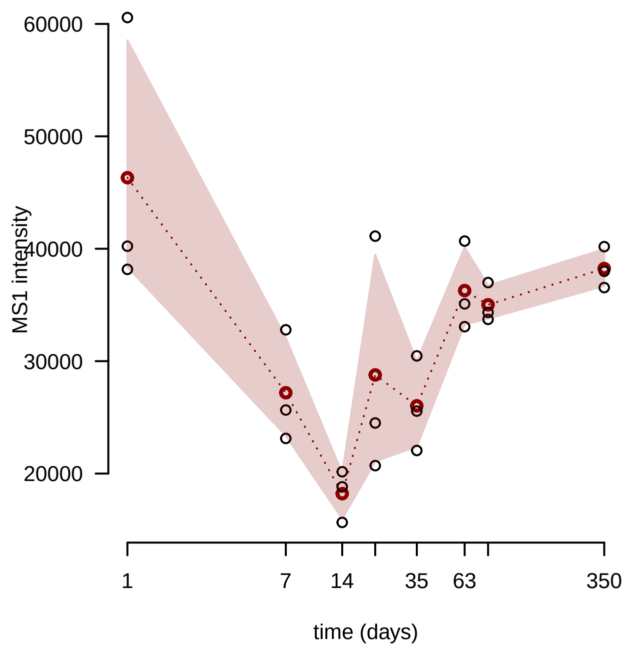

Q03963
GN=Eif2ak2
eukaryotic translation initiation factor ...

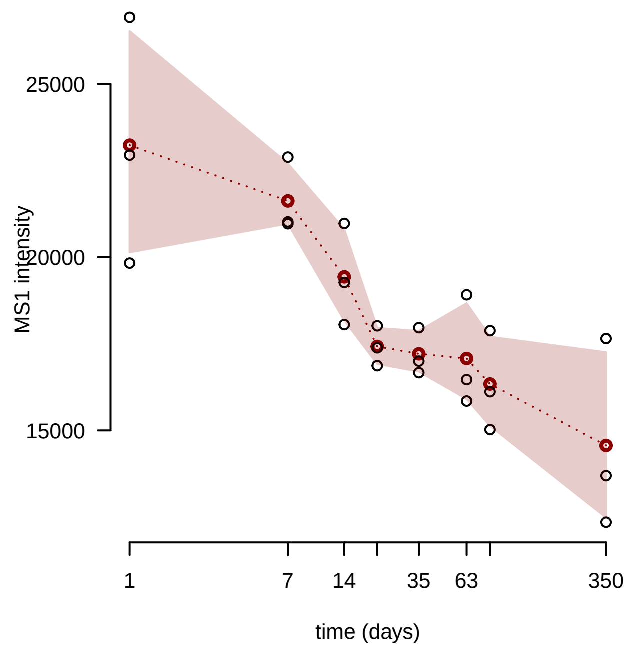

Q04207
GN=Rela
v−rel reticuloendotheliosis viral oncoge ...

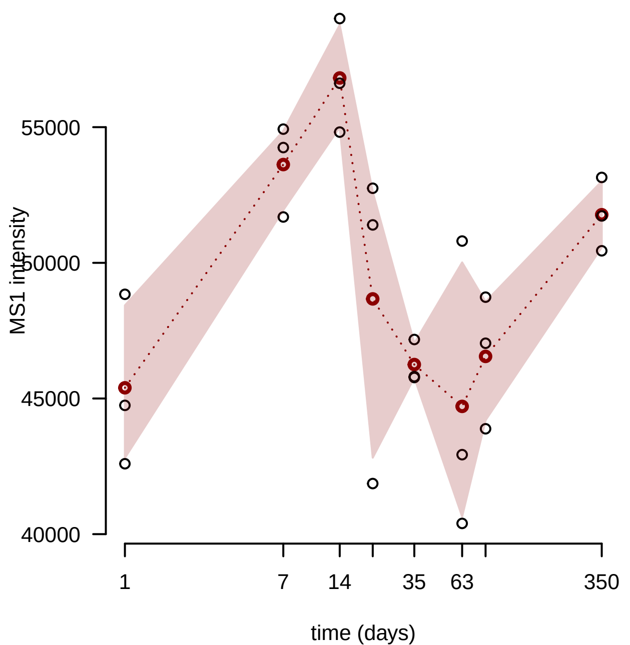

Q04447
GN=Ckb
creatine kinase, brain

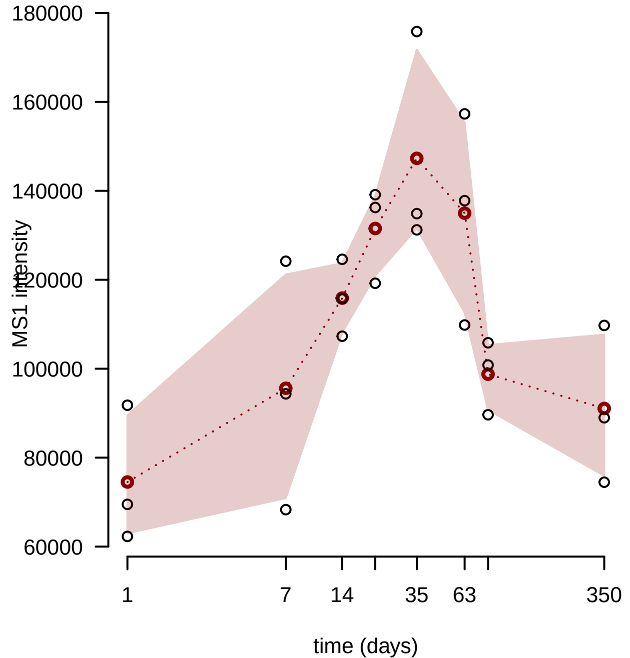

Q04519
GN=Smpd1
sphingomyelin phosphodiesterase 1, acid ...

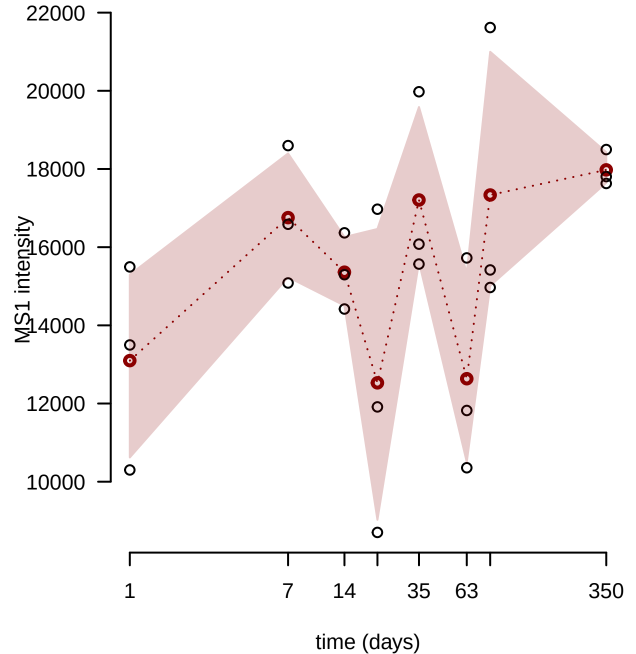

Q04592
GN=Pcsk5
proprotein convertase subtilisin/kexin t ...

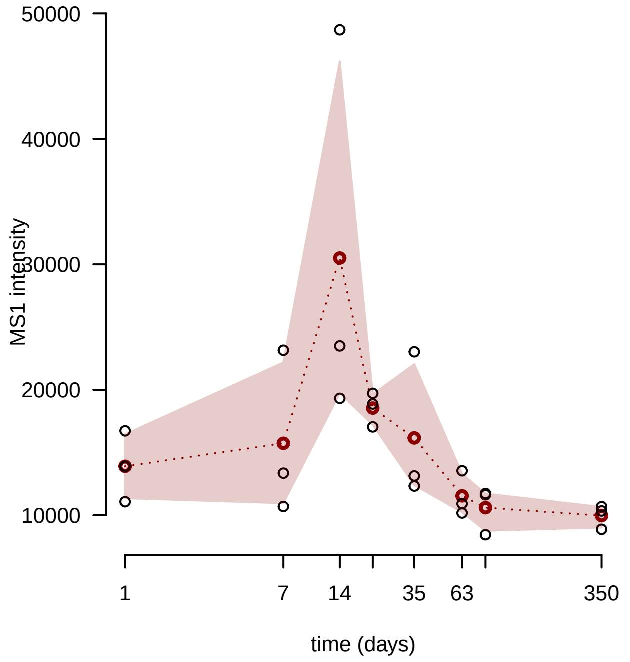

Q04690
GN=Nf1
neurofibromin 1

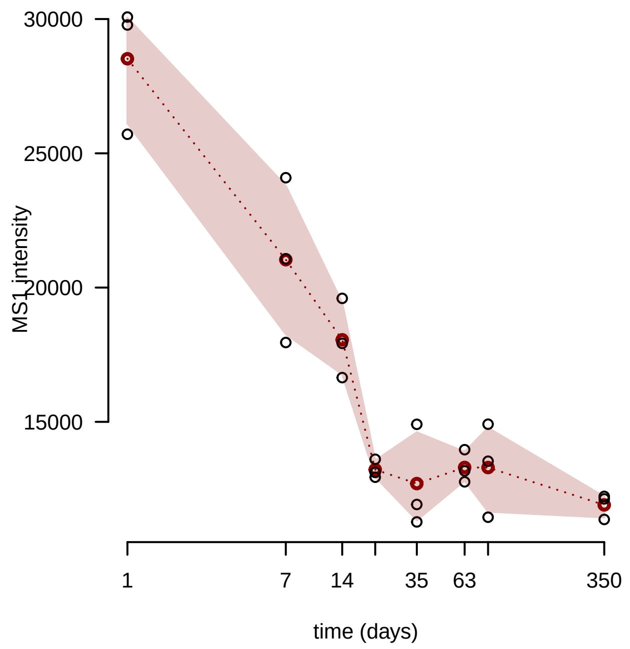

Q04692
GN=Smarcad1
SWI/SNF−related, matrix−associated actin ...

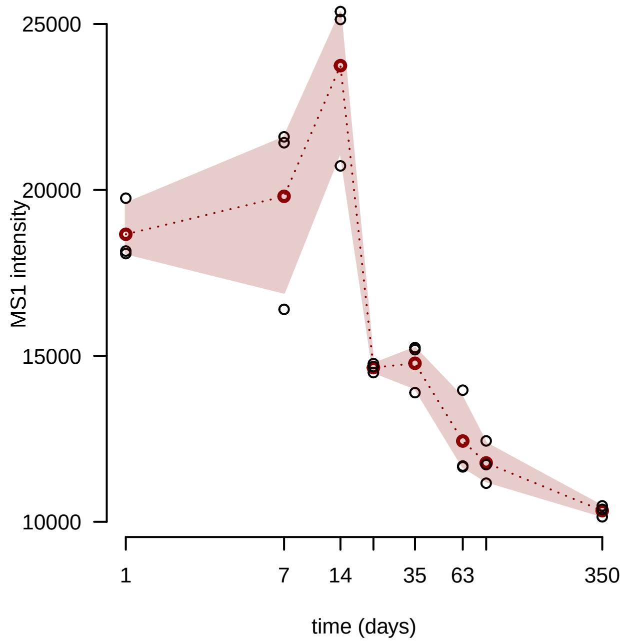

Q04735
GN=Cdk16
cyclin−dependent kinase 16

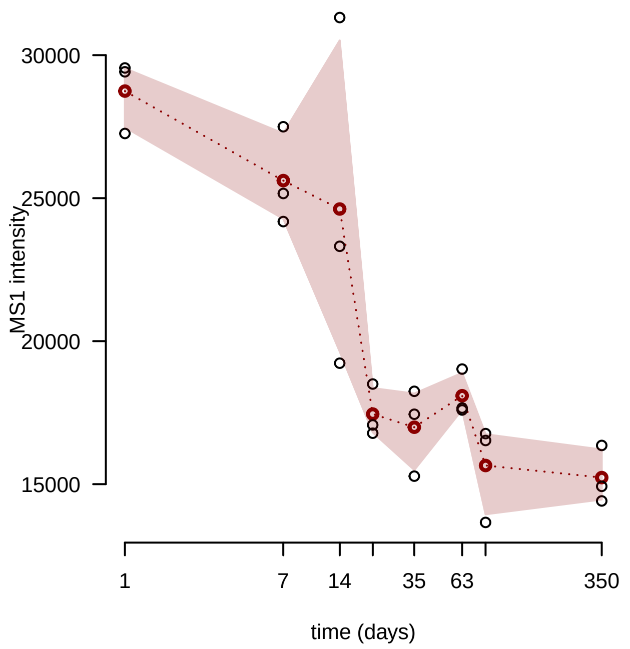

Q04736
GN=Yes1
YES proto−oncogene 1, Src family tyrosin ...

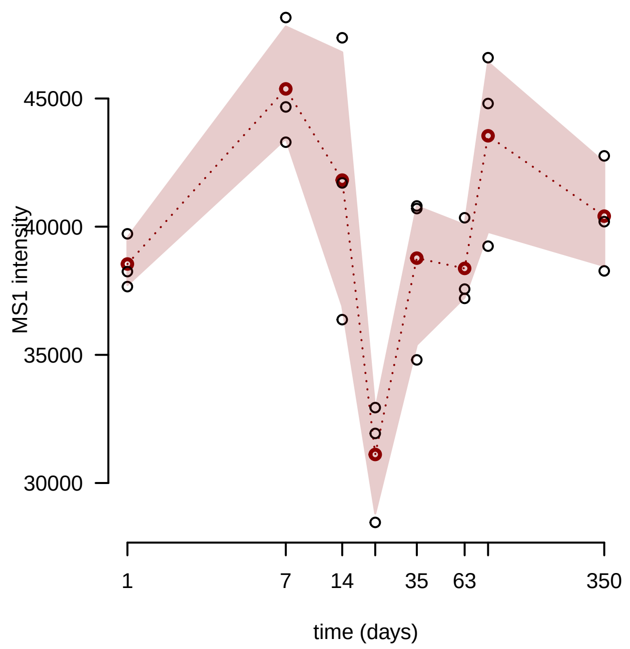

Q04744
GN=Emx2
empty spiracles homeobox 2

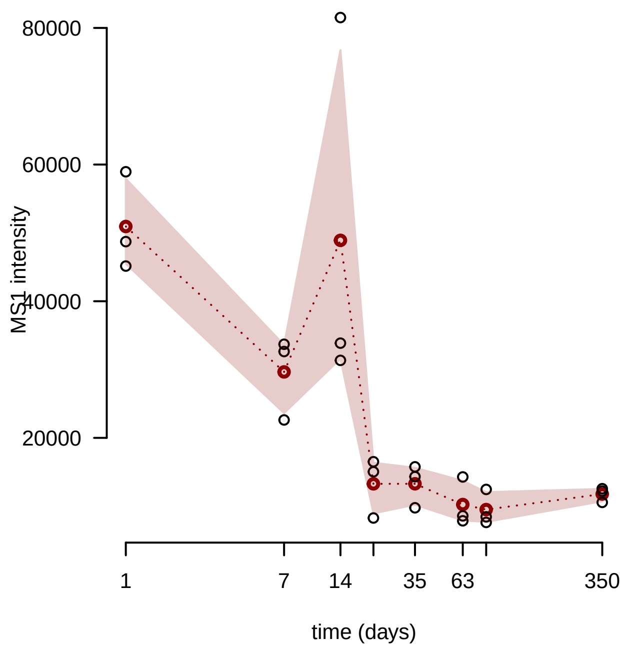

Q04750
GN=Top1
topoisomerase (DNA) I

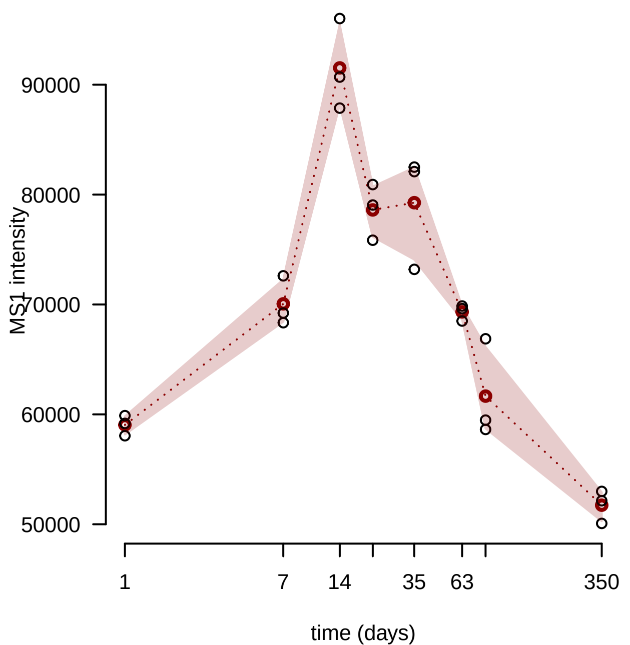

Q04841
GN=Mpg
N−methylpurine−DNA glycosylase

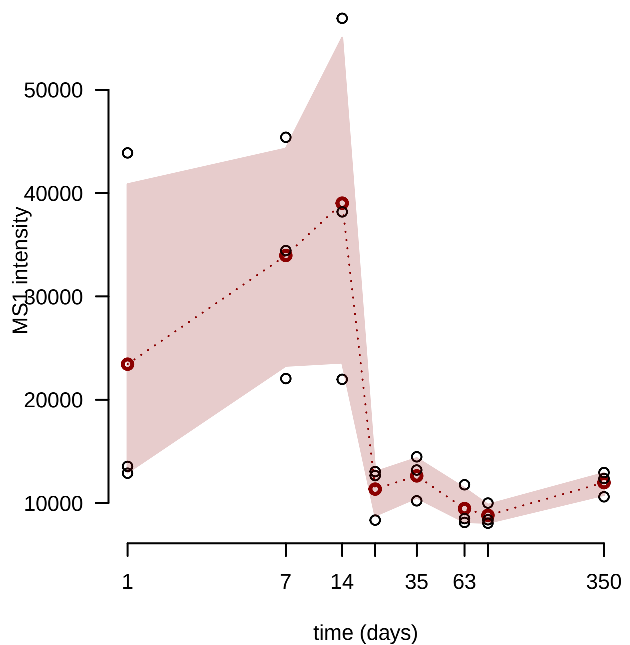

Q04857
GN=Col6a1
collagen, type VI, alpha 1

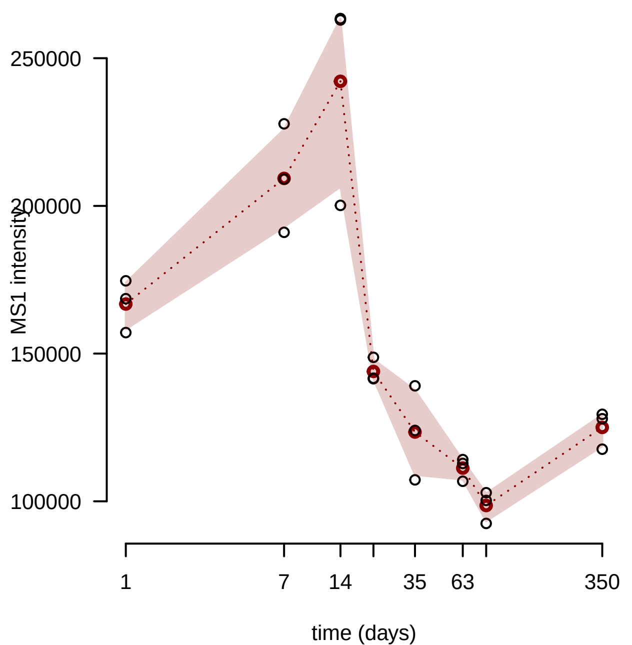

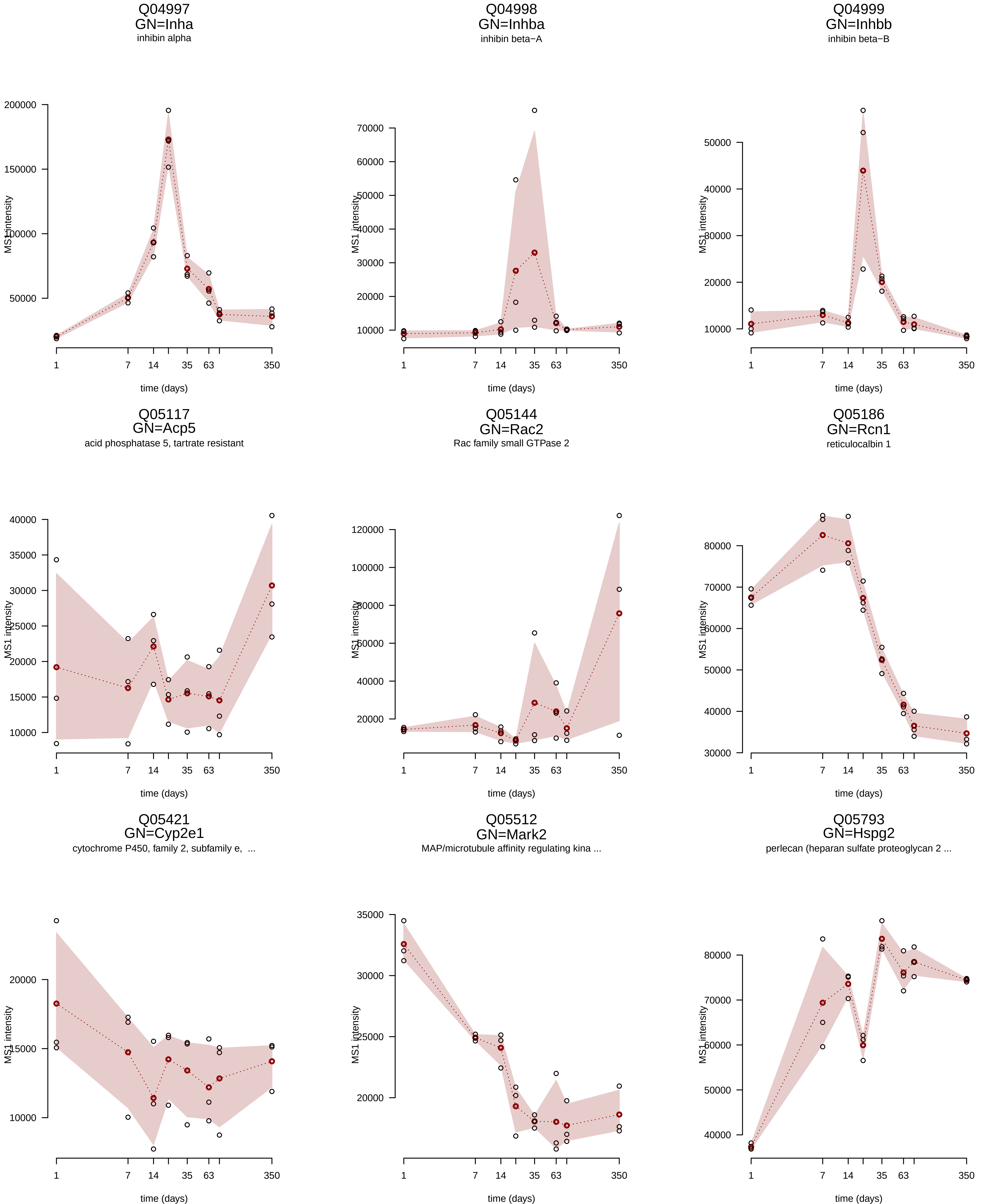
Q04997
GN=Inha
inhibin alpha
Q04998
GN=Inhba
inhibin beta−A
Q04999
GN=Inhbb
inhibin beta−B
Q05117
GN=Acp5
acid phosphatase 5, tartrate resistant
Q05144
GN=Rac2
Rac family small GTPase 2
Q05186
GN=Rcn1
reticulocalbin 1
Q05421
GN=Cyp2e1
cytochrome P450, family 2, subfamily e, ...
Q05512
GN=Mark2
MAP/microtubule affinity regulating kina ...
Q05793
GN=Hspg2
perlecan (heparan sulfate proteoglycan 2 ...
MS1 intensity
time (days)
1
7
14
35
63
350

Q05816
GN=Fabp5
fatty acid binding protein 5, epidermal

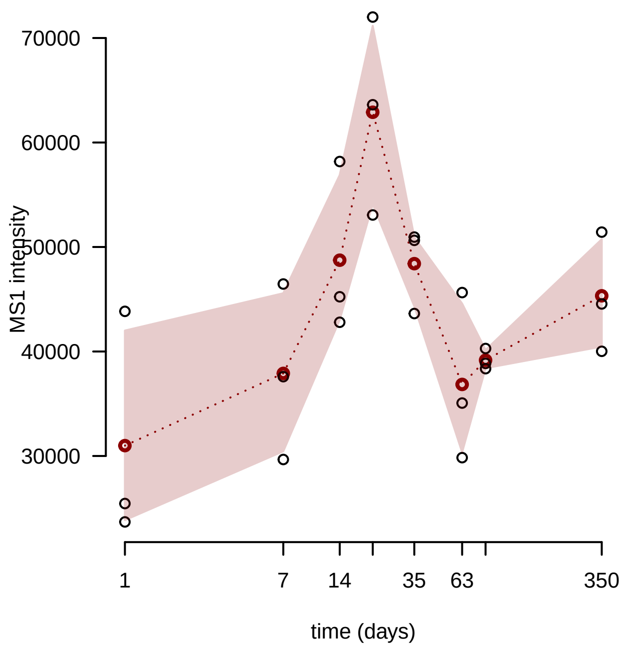

Q05860
GN=Fmn1
formin 1

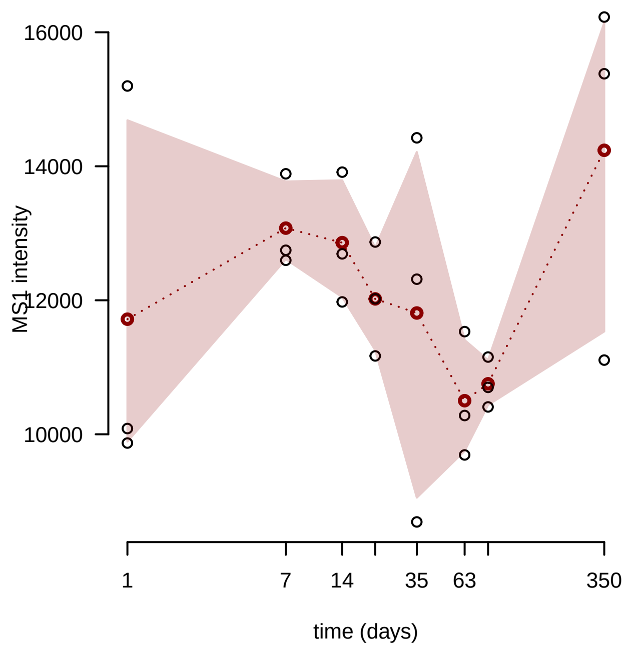

Q05895
GN=Thbs3
thrombospondin 3

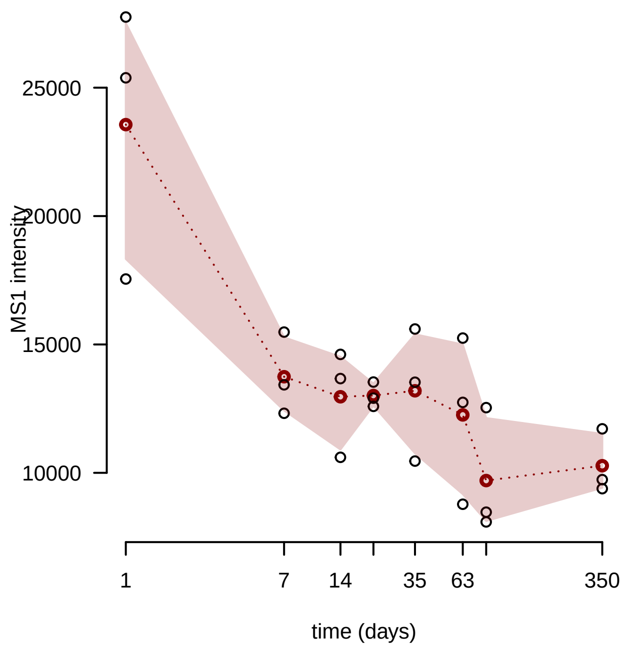

Q05909
GN=Ptprg
protein tyrosine phosphatase, receptor t ...

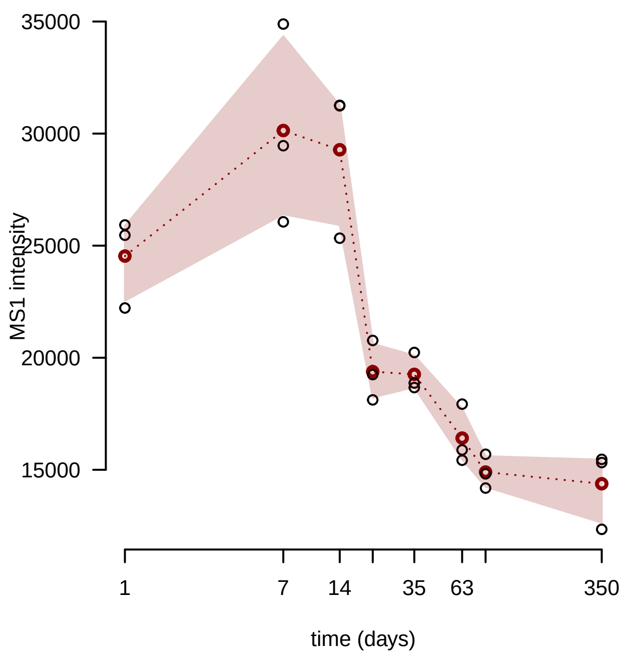

Q05915
GN=Gch1
GTP cyclohydrolase 1

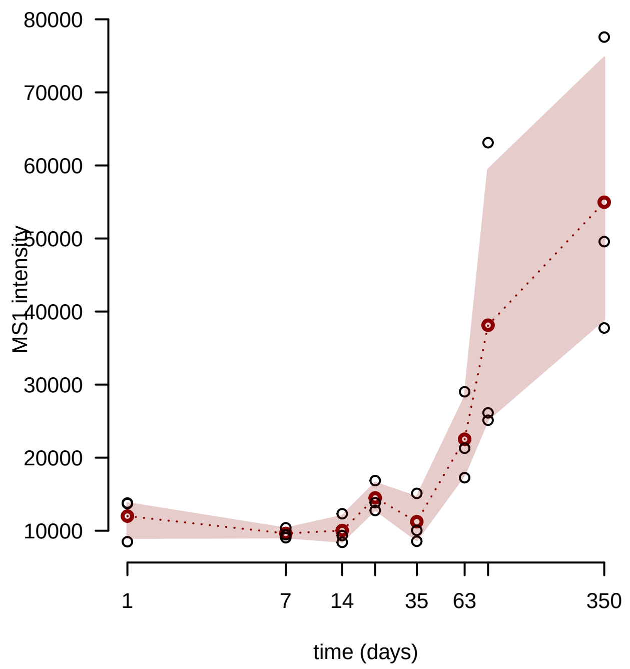

Q05920
GN=Pc

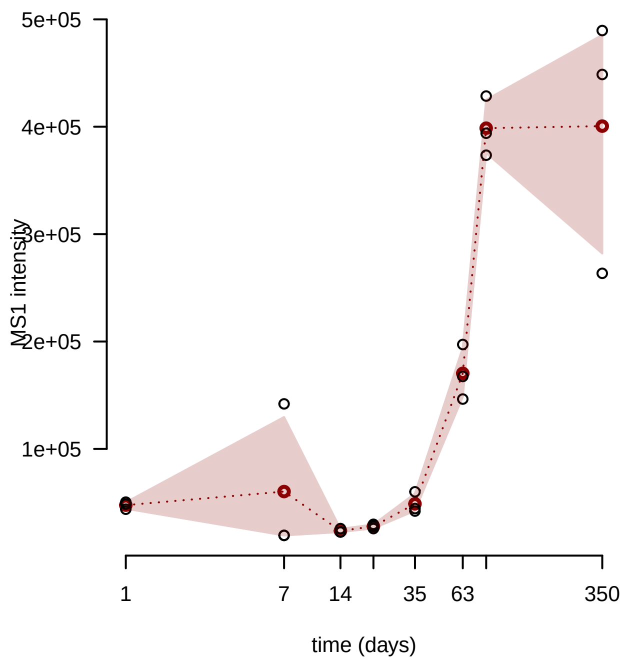

Q05921
GN=Rnasel
ribonuclease L (2', 5'−oligoisoadenylate ...

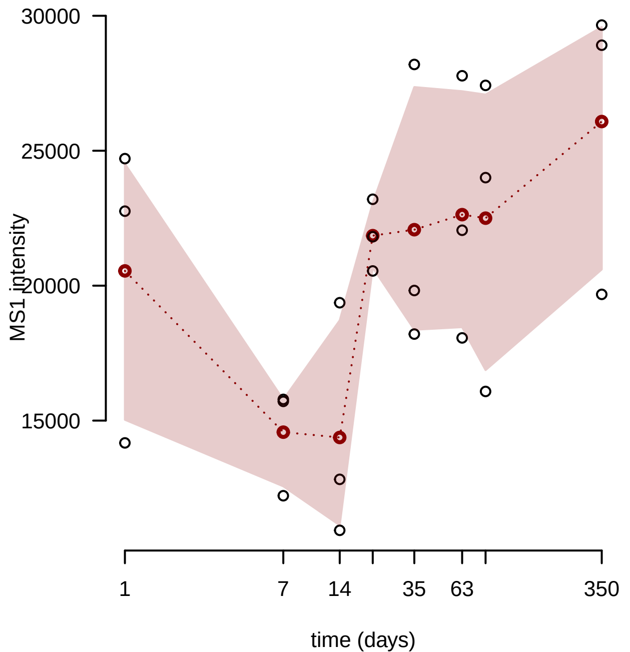

Q05A36
GN=Mex3c
mex3 RNA binding family member C

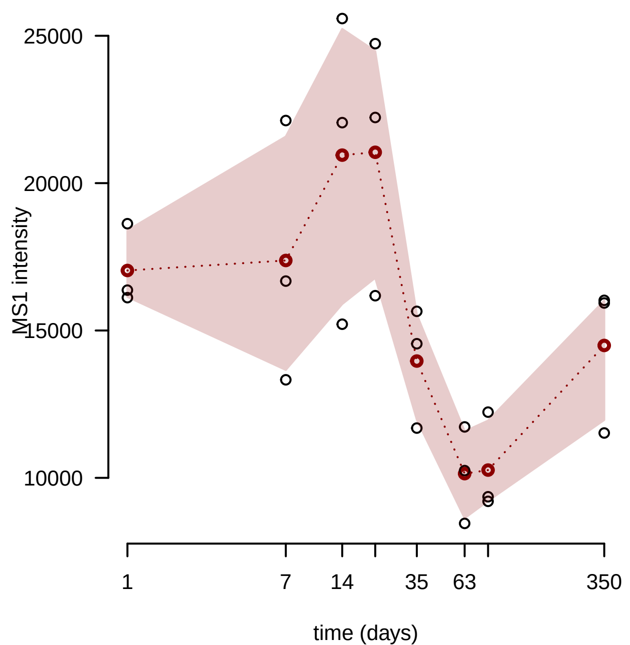

Q05A80
GN=Caprin2
caprin family member 2

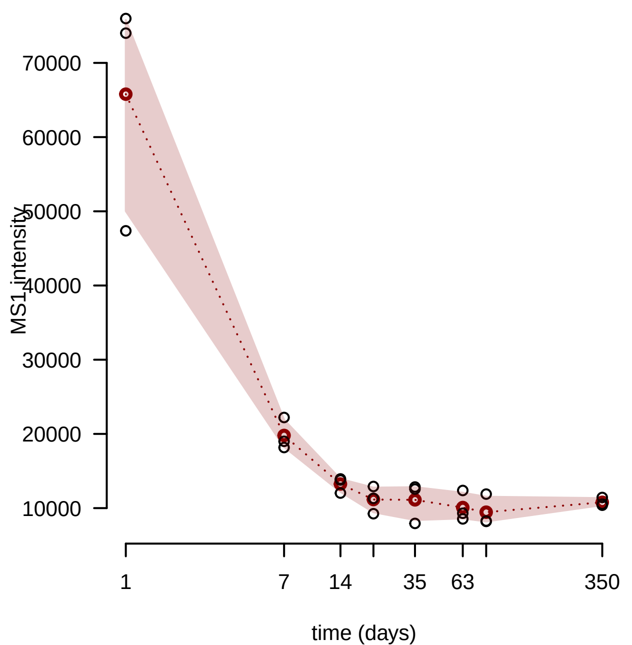

Q05AH6
GN=Spindoc
spindlin interactor and repressor of chr ...

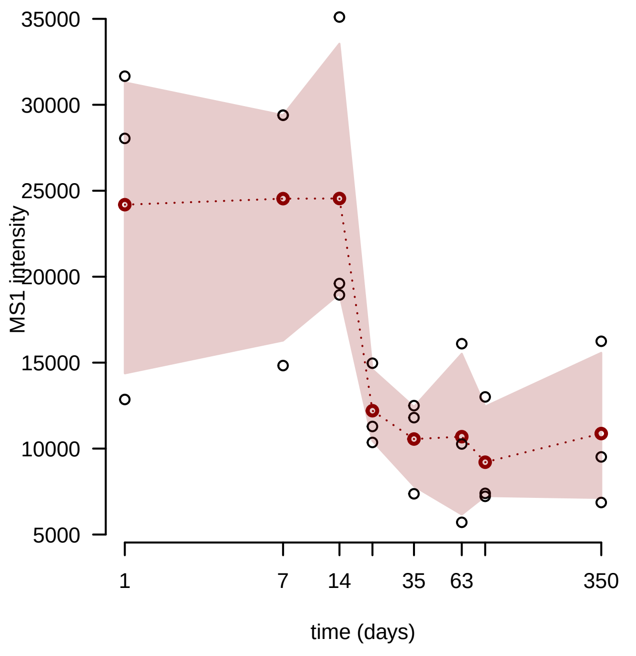

Q05BC3
GN=Eml1
echinoderm microtubule associated protei ...

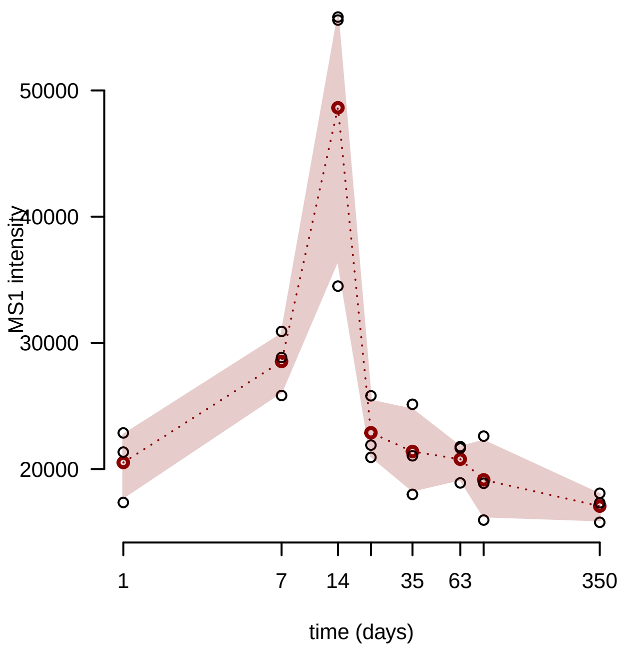

Q05CL8
GN=Larp7
La ribonucleoprotein 7, transcriptional ...

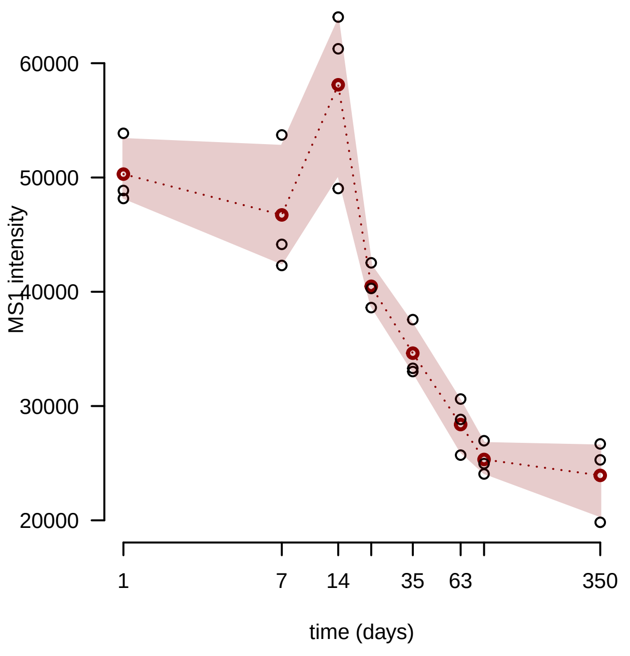

Q05D44
GN=Eif5b
eukaryotic translation initiation factor ...

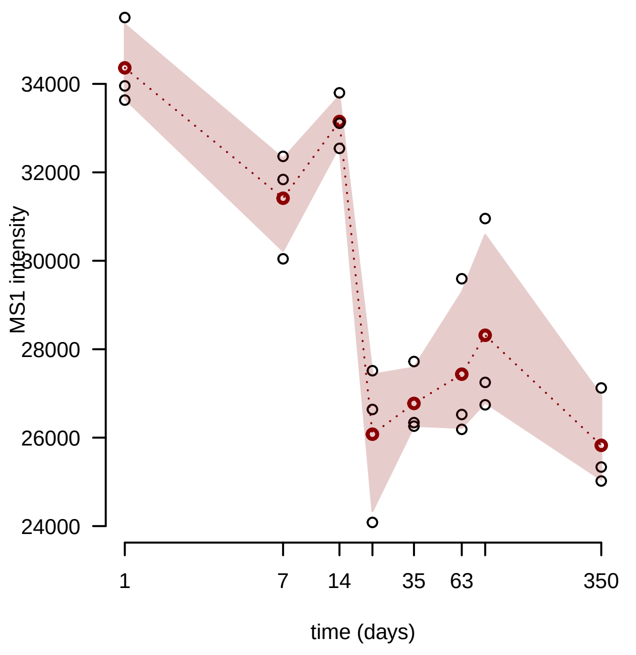

Q06138
GN=Cab39
calcium binding protein 39

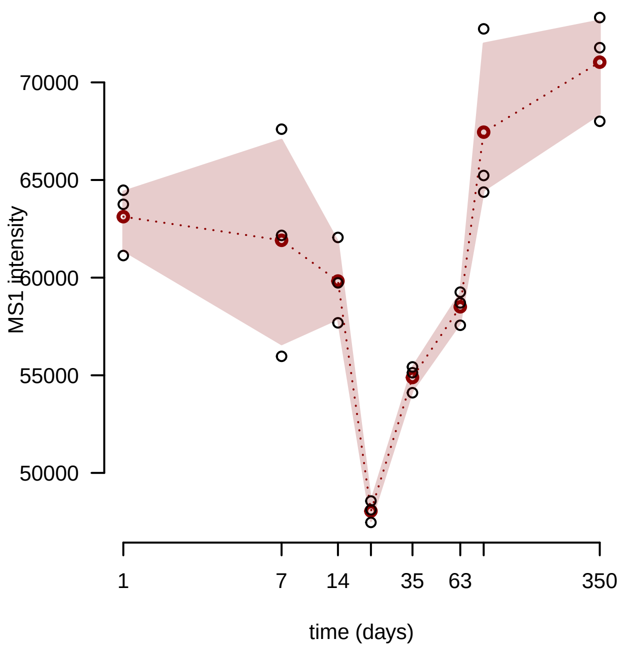

Q06180
GN=Ptpn2
protein tyrosine phosphatase, non−recept ...

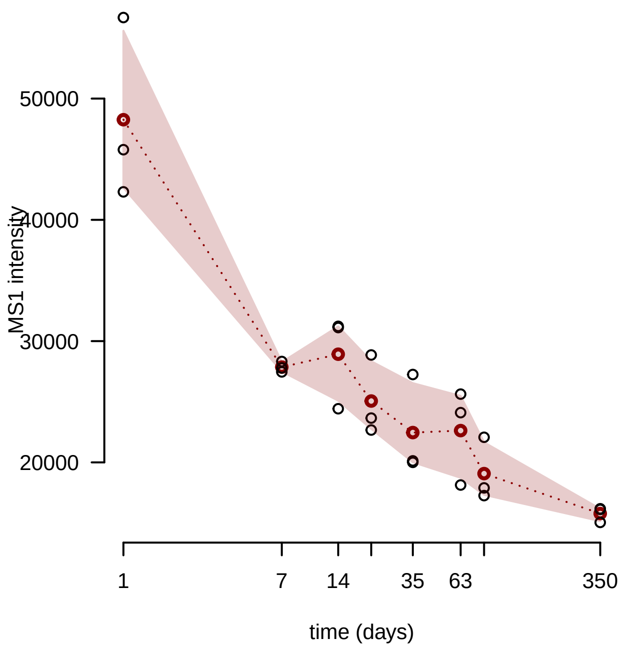

Q06185
GN=Atp5me

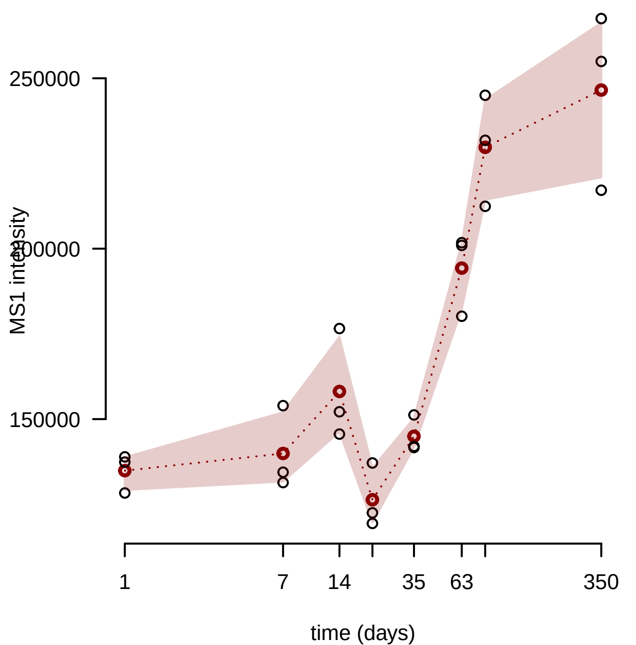

Q06335
GN=Aplp2
amyloid beta (A4) precursor−like protein ...

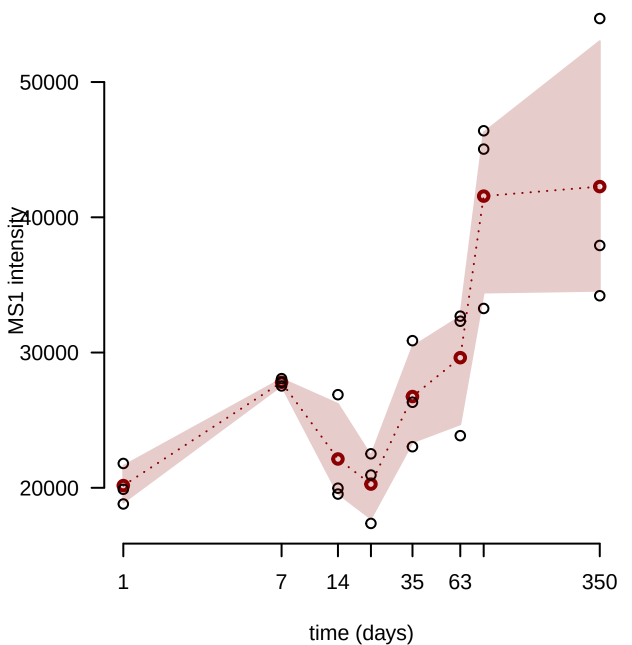

Q06348
GN=Prrx2
paired related homeobox 2

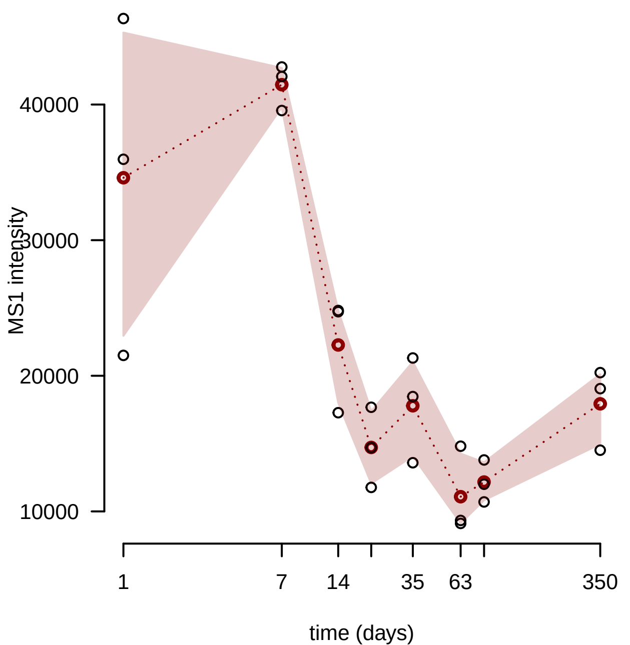

Q06770
GN=Serpina6
serine (or cysteine) peptidase inhibitor ...

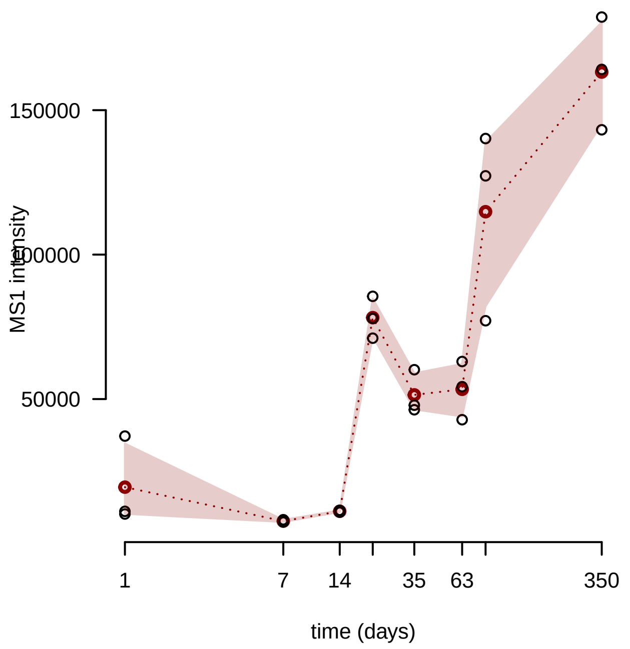

Q06831
GN=Sox4
SRY (sex determining region Y)−box 4

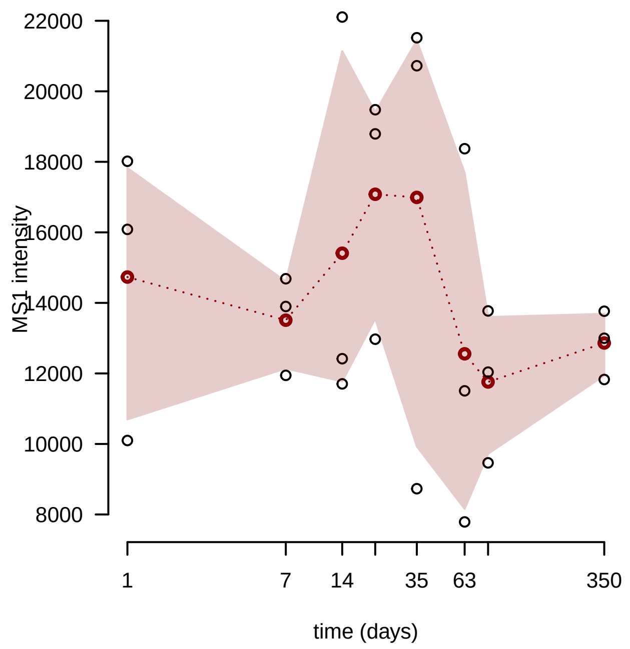

Q06890
GN=Clu
clusterin

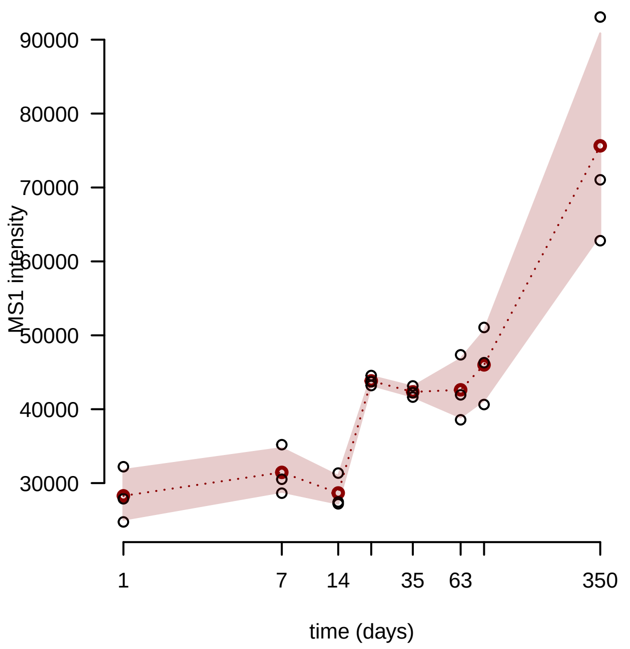

Q07076
GN=Anxa7
annexin A7

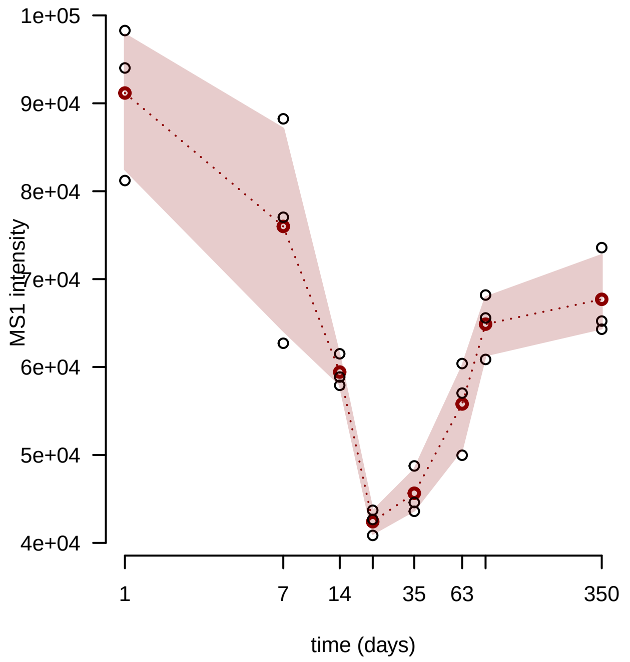

Q07079
GN=Igfbp5
insulin−like growth factor binding prote ...

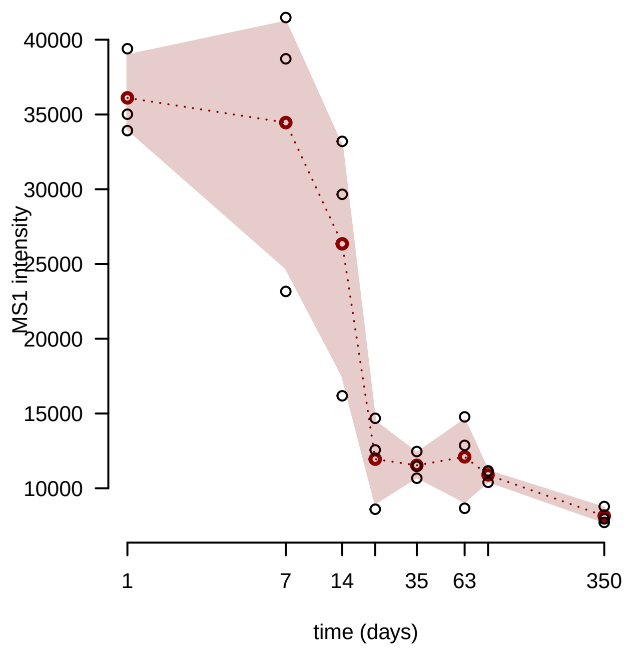

Q07105
GN=Gdf9
growth differentiation factor 9

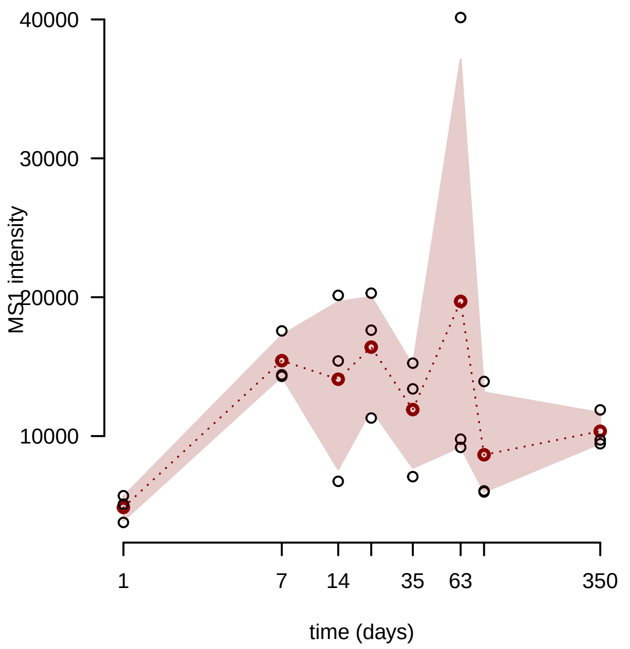

Q07113
GN=Igf2r
insulin−like growth factor 2 receptor

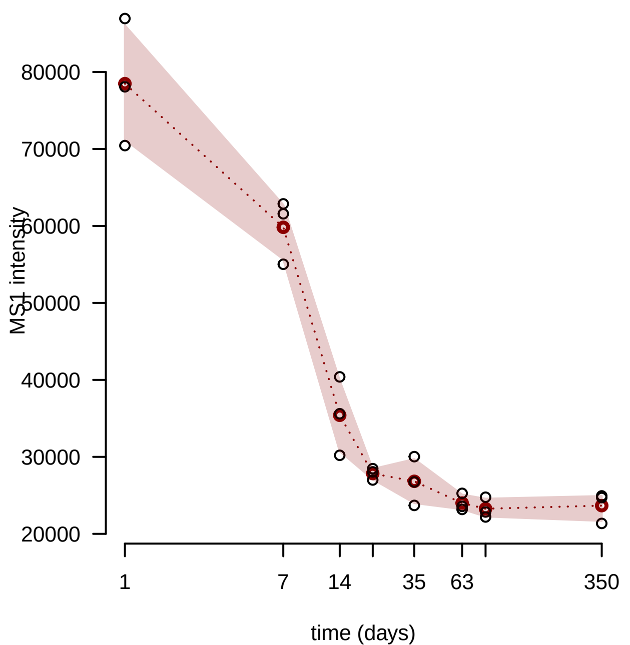

Q07133
GN=Hist1h1t

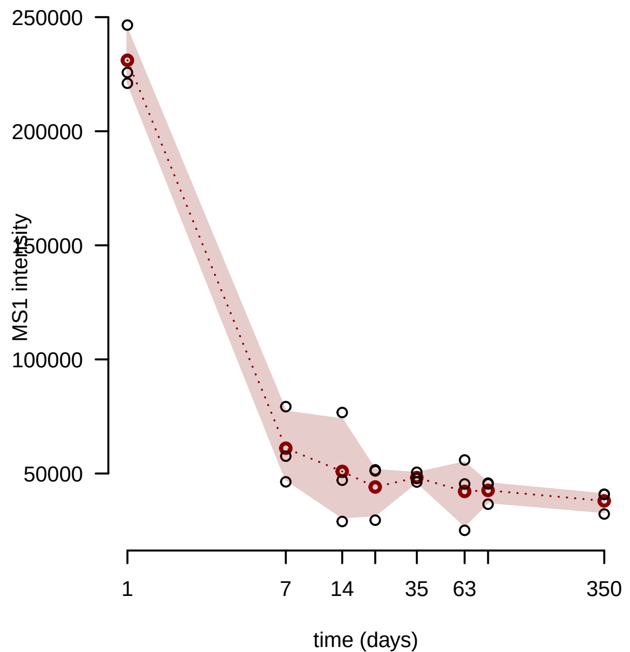

Q07139
GN=Ect2
ect2 oncogene

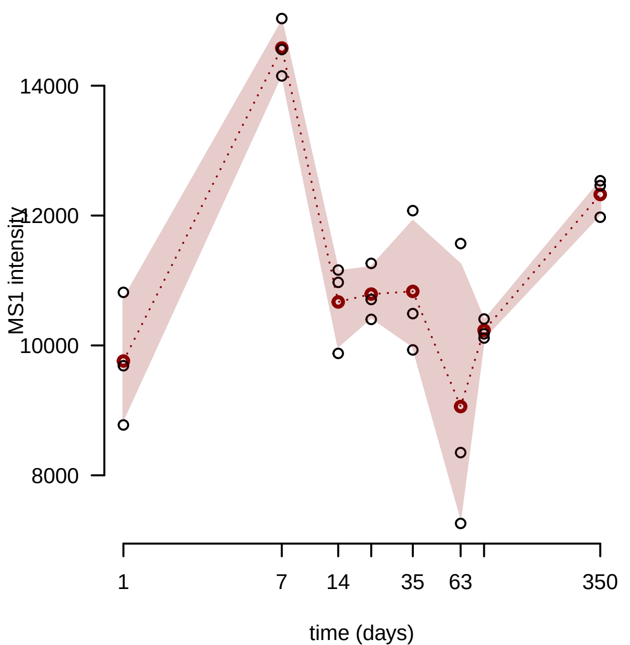

Q07231
GN=Zscan21
zinc finger and SCAN domain containing 2 ...

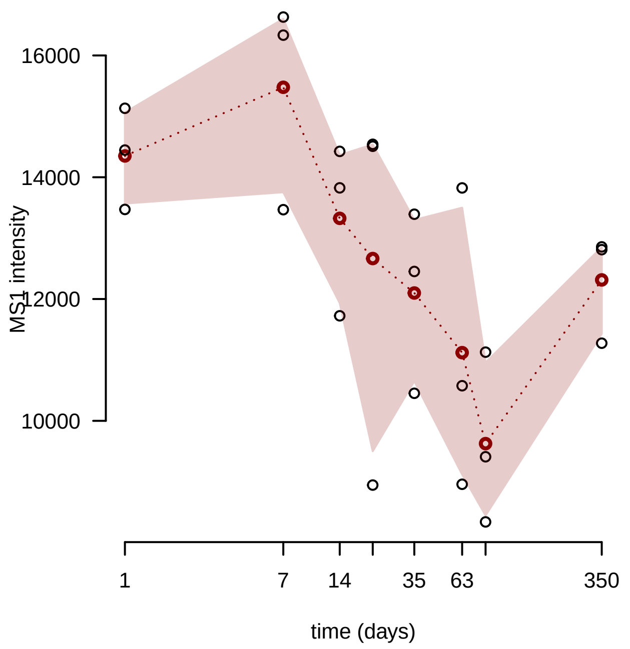

Q07235
GN=Serpine2
serine (or cysteine) peptidase inhibitor ...

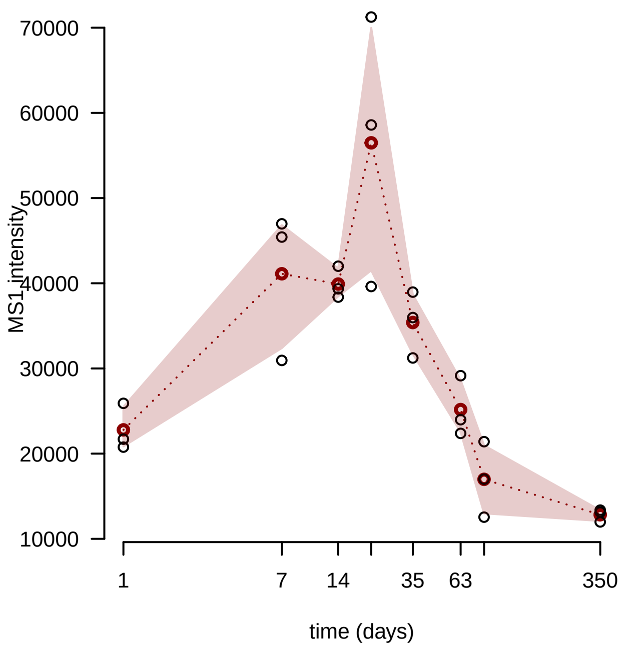

Q07417
GN=Acads
acyl−Coenzyme A dehydrogenase, short cha ...

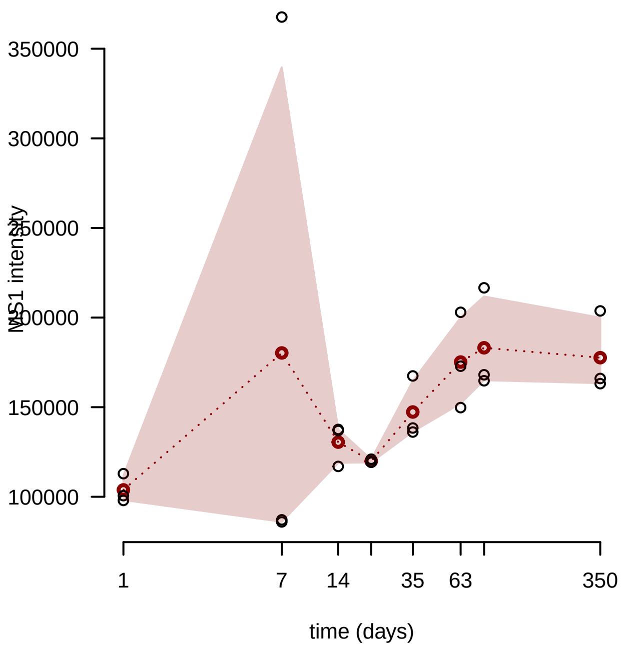

Q07456
GN=Ambp
alpha 1 microglobulin/bikunin precursor

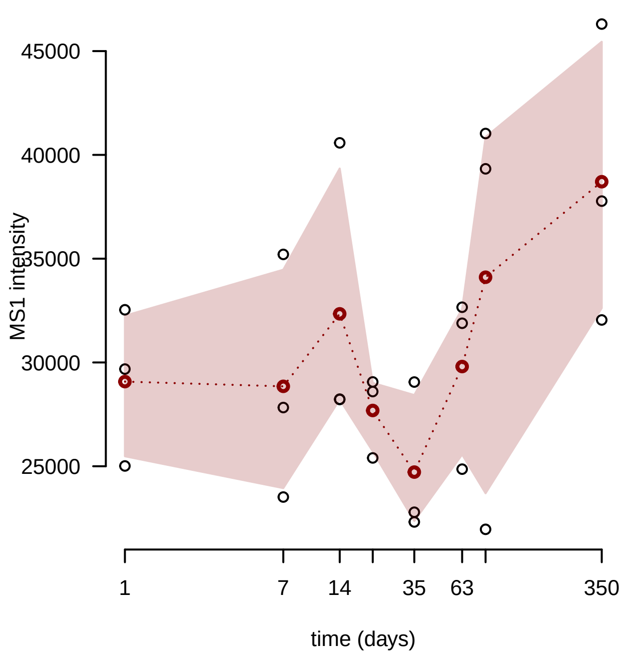

Q07643
GN=Col9a2
collagen, type IX, alpha 2

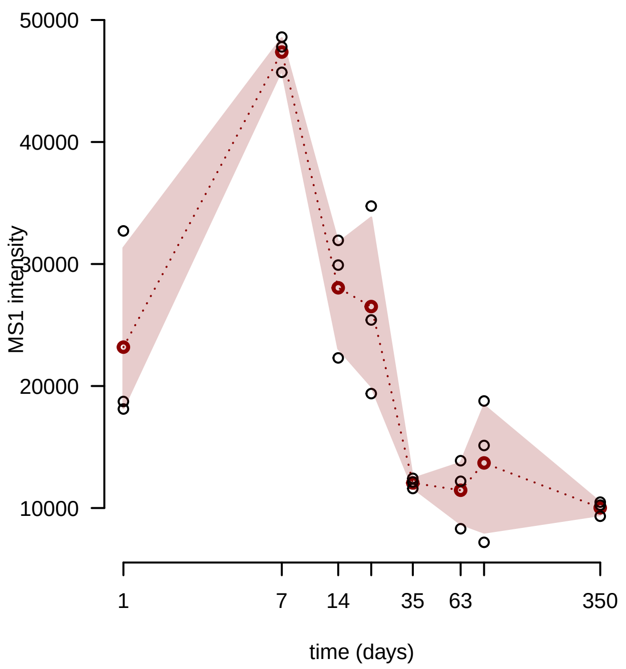

Q07646
GN=Mest
mesoderm specific transcript

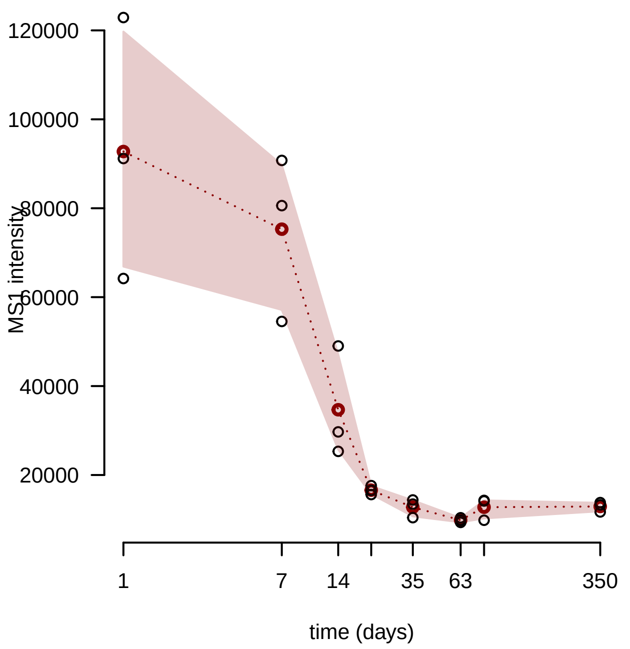

Q07797
GN=Lgals3bp
lectin, galactoside−binding, soluble, 3 ...

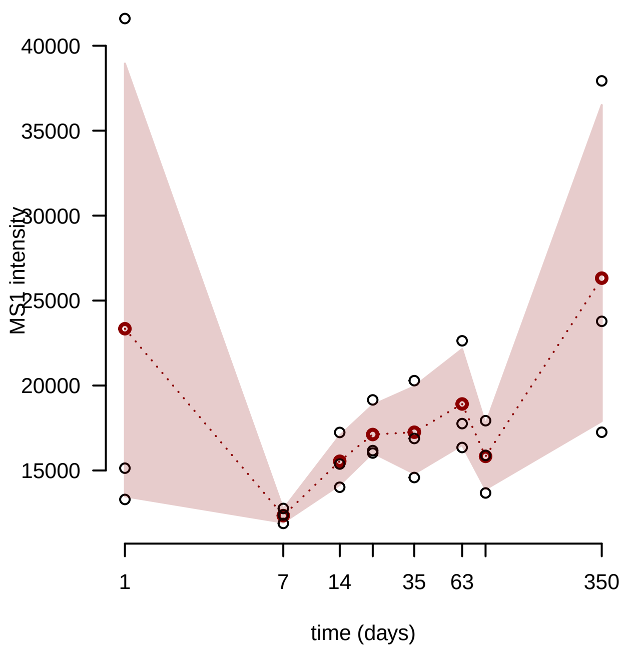

Q07802
GN=Ebf1
early B cell factor 1

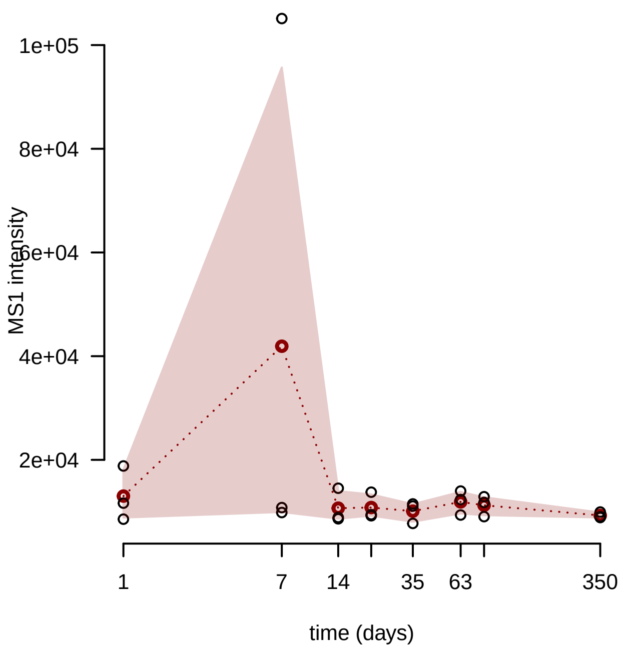

Q07813
GN=Bax
BCL2−associated X protein

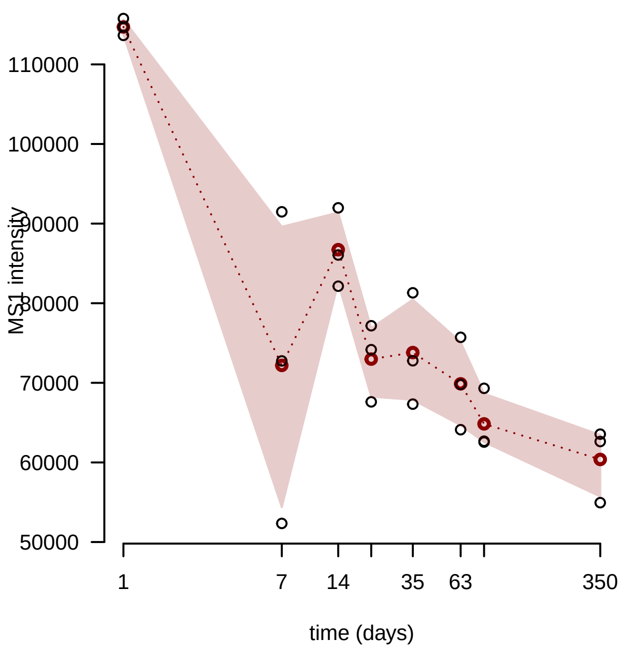

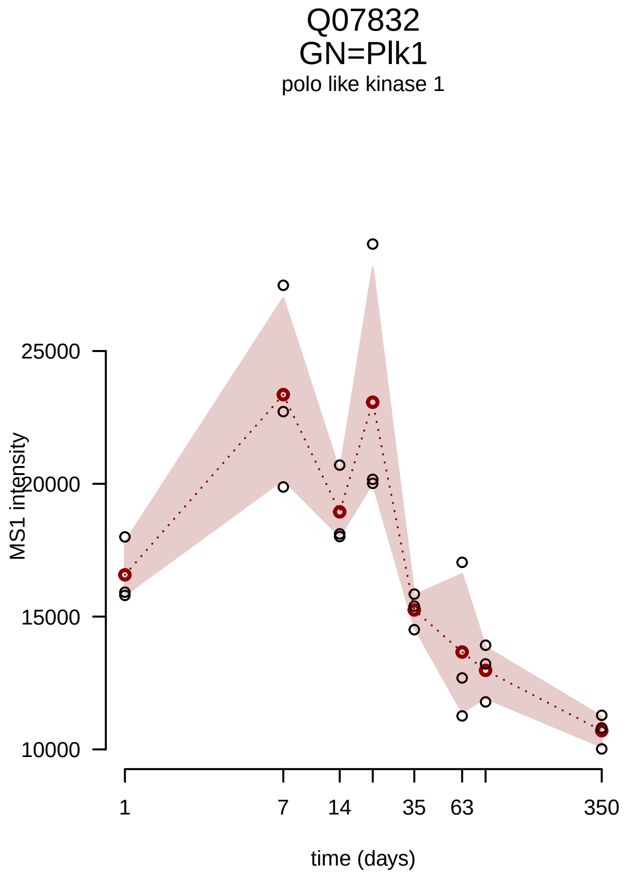
Q07832
GN=Plk1
polo like kinase 1
MS1 intensity
10000
15000
20000
25000
1
7
14
35
63
350
time (days)

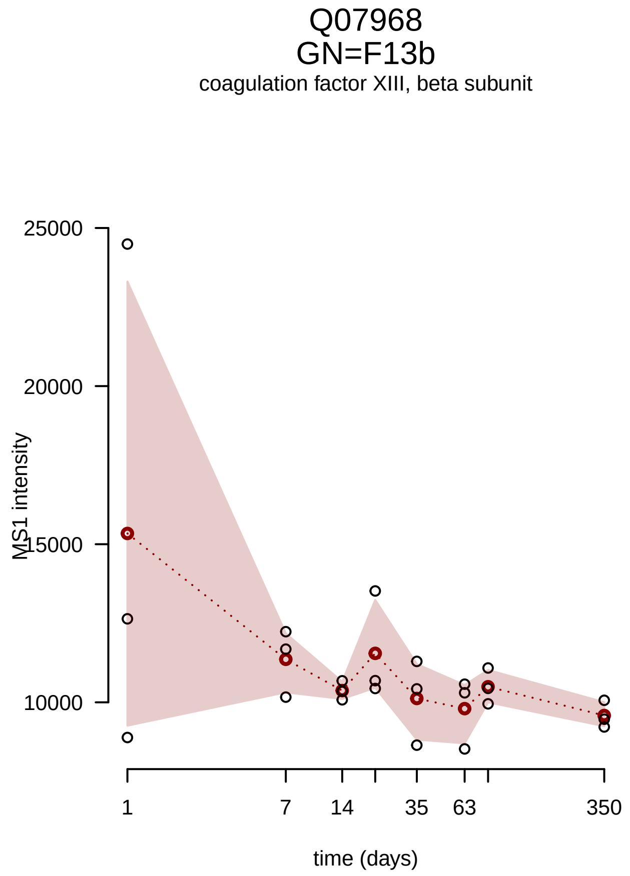
Q07968
GN=F13b
coagulation factor XIII, beta subunit
MS1 intensity
10000
15000
20000
25000
1
7
14
35
63
350
time (days)

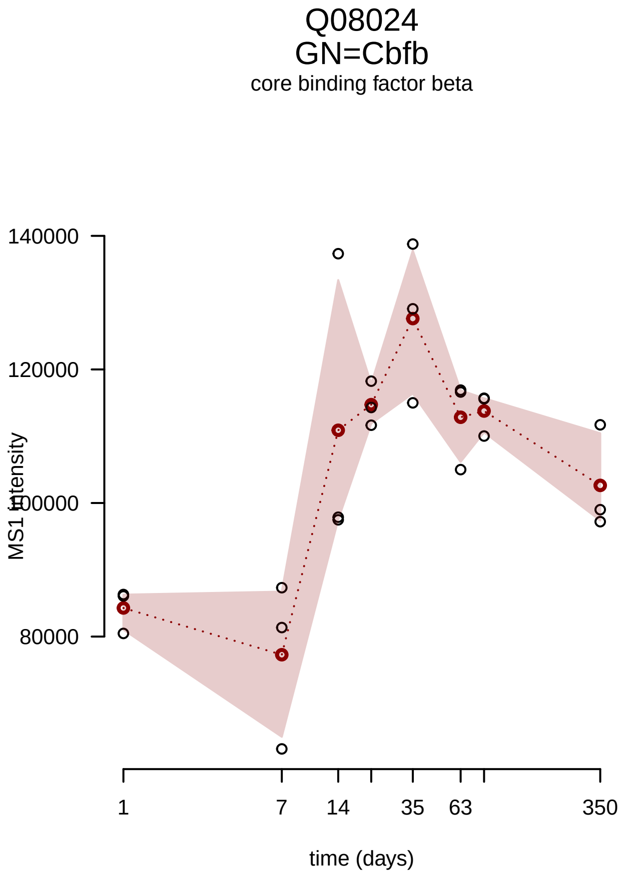
Q08024
GN=Cbfb
core binding factor beta
MS1 intensity
80000
100000
120000
140000
1
7
14
35
63
350
time (days)

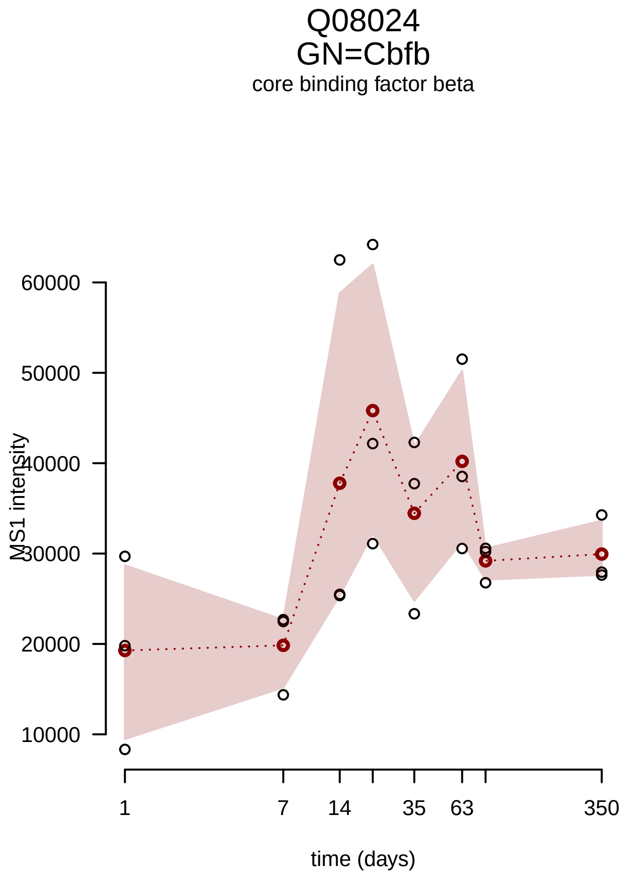
Q08024
GN=Cbfb
core binding factor beta
MS1 intensity
10000
20000
30000
40000
50000
60000
1
7
14
35
63
350
time (days)

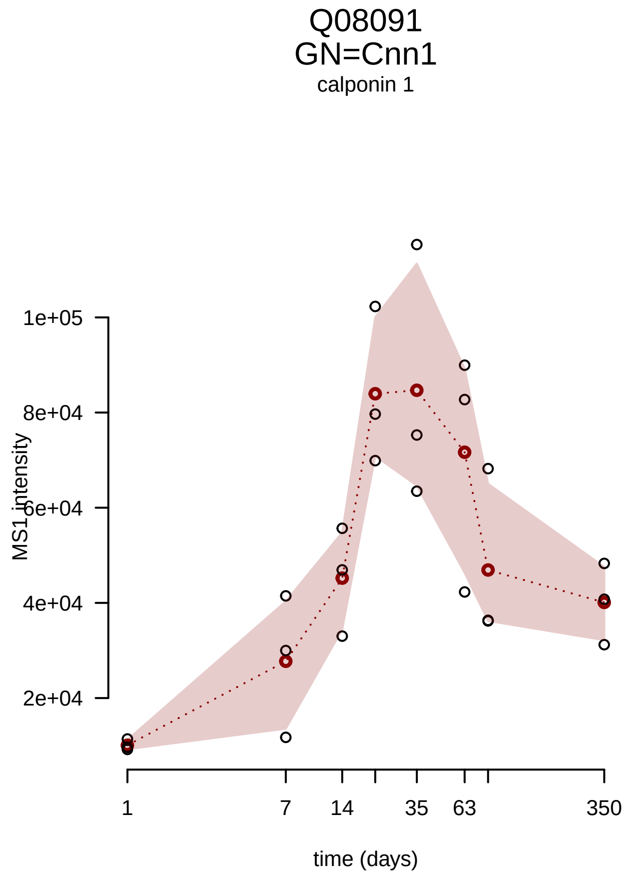
Q08091
GN=Cnn1
calponin 1
MS1 intensity
2e+04
4e+04
6e+04
8e+04
1e+05
1
7
14
35
63
350
time (days)

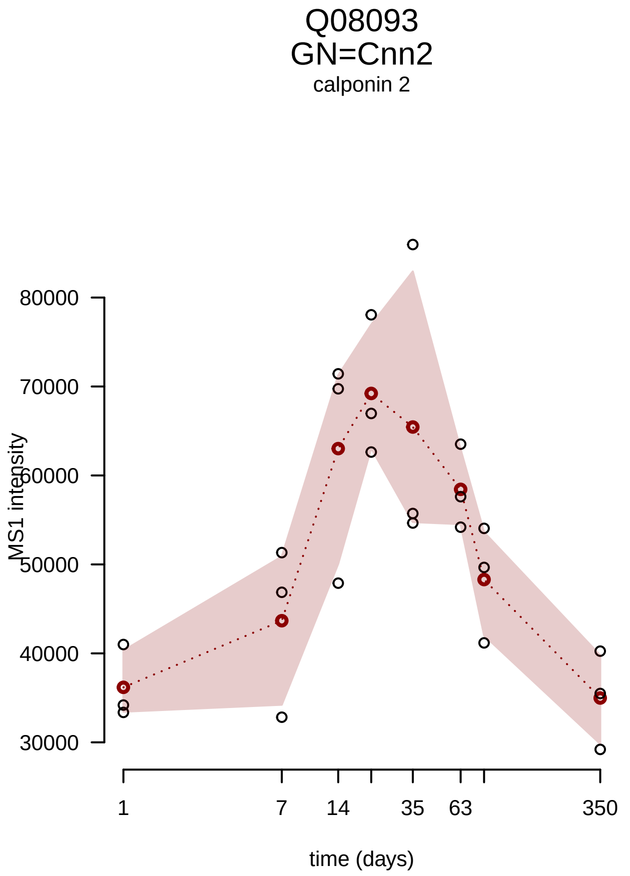
Q08093
GN=Cnn2
calponin 2
MS1 intensity
30000
40000
50000
60000
70000
80000
1
7
14
35
63
350
time (days)

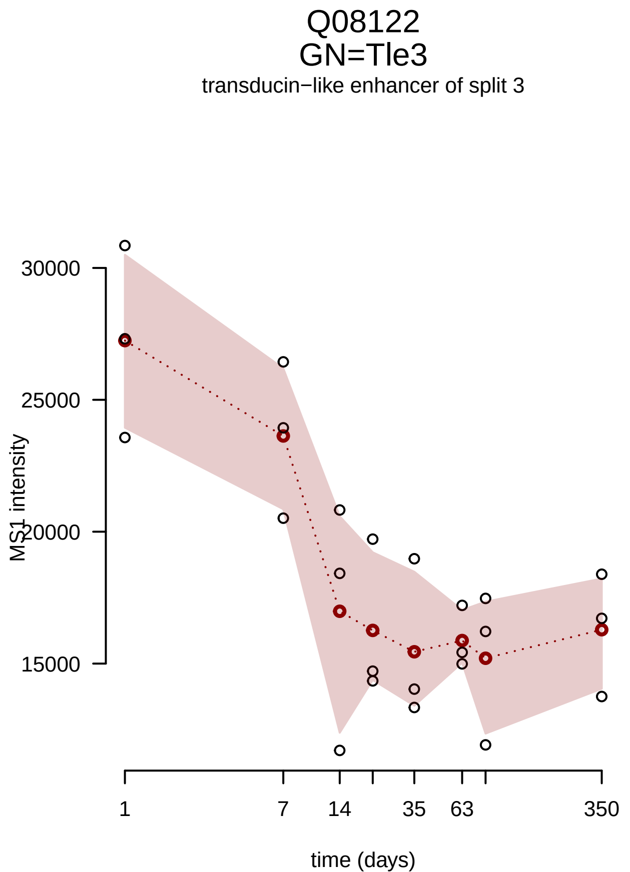
Q08122
GN=Tle3
transducin−like enhancer of split 3
MS1 intensity
15000
20000
25000
30000
1
7
14
35
63
350
time (days)

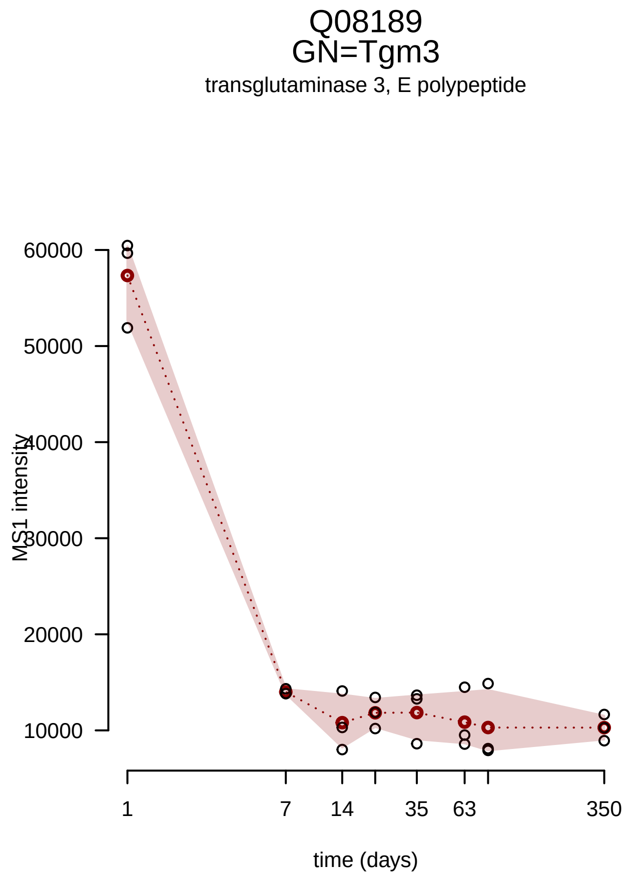
Q08189
GN=Tgm3
transglutaminase 3, E polypeptide
MS1 intensity
10000
20000
30000
40000
50000
60000
1
7
14
35
63
350
time (days)

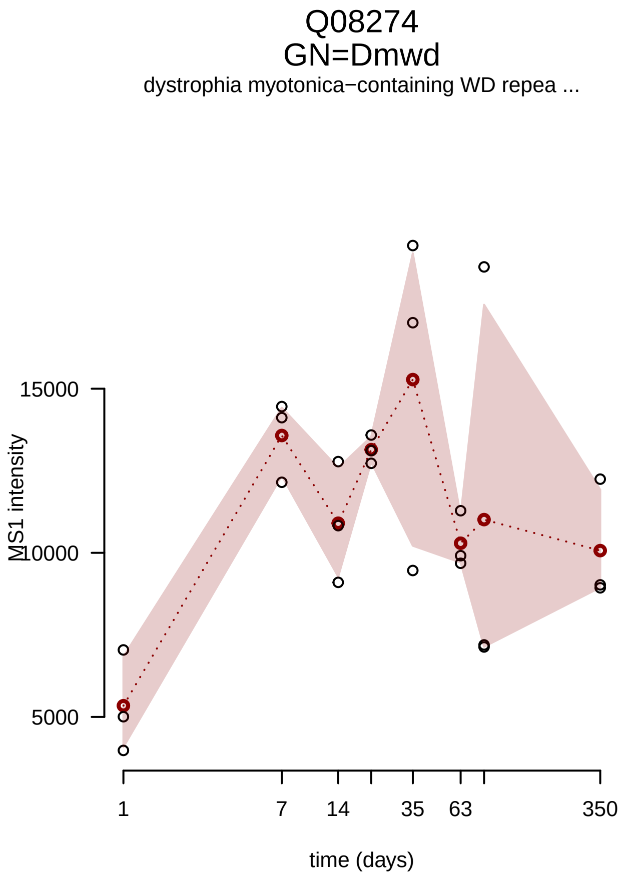
Q08274
GN=Dmwd
dystrophia myotonica−containing WD repea ...
MS1 intensity
5000
10000
15000
1
7
14
35
63
350
time (days)

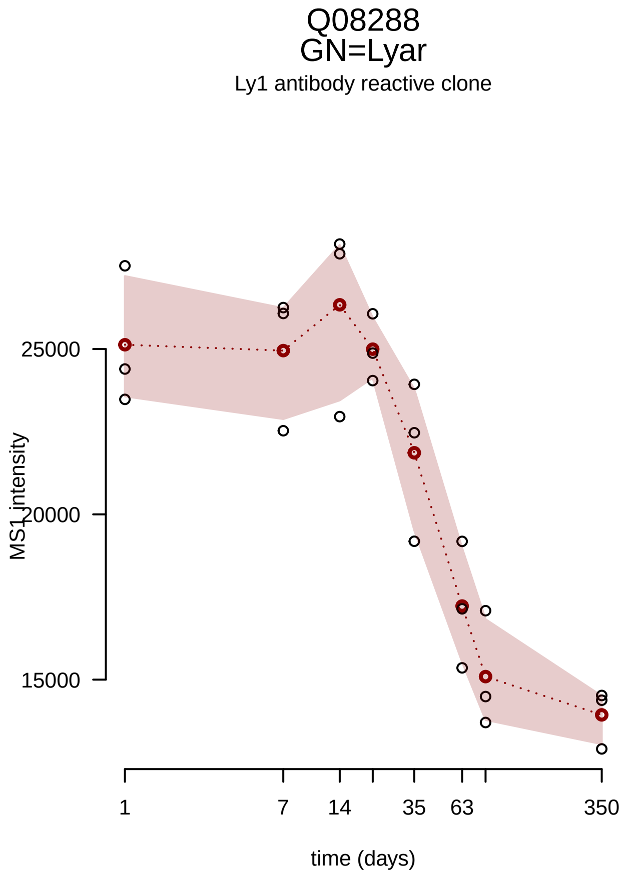
Q08288
GN=Lyar
Ly1 antibody reactive clone
MS1 intensity
15000
20000
25000
1
7
14
35
63
350
time (days)

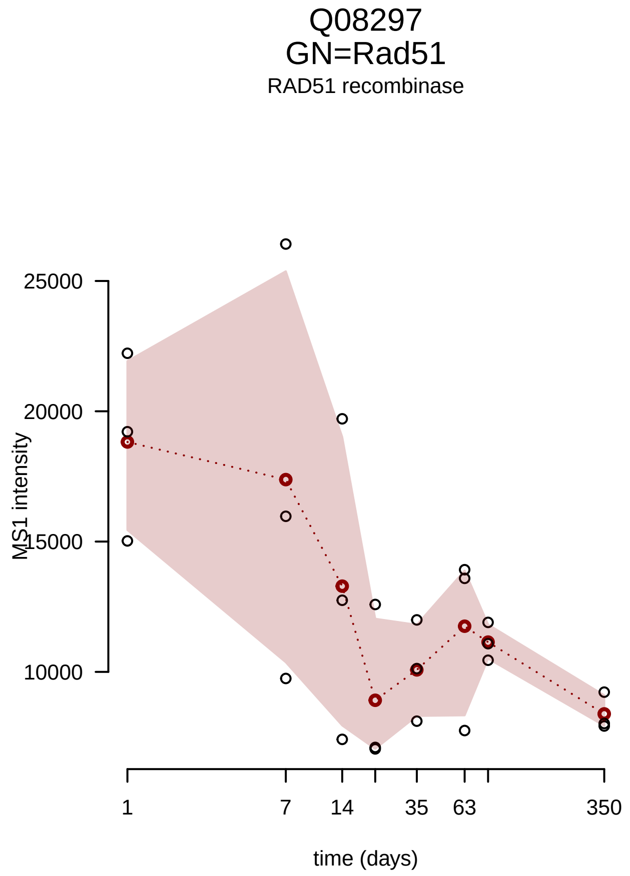
Q08297
GN=Rad51
RAD51 recombinase
MS1 intensity
10000
15000
20000
25000
1
7
14
35
63
350
time (days)

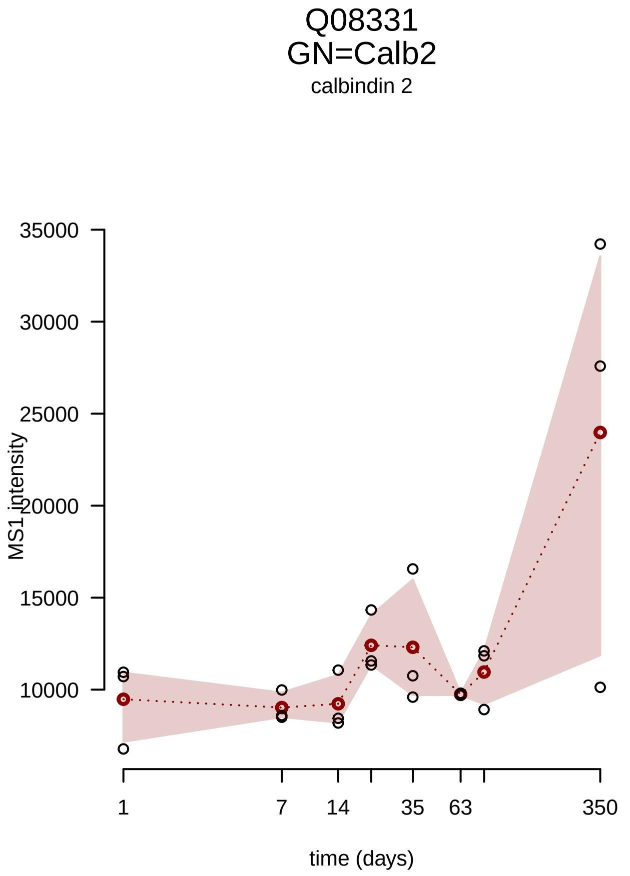
Q08331
GN=Calb2
calbindin 2
MS1 intensity
10000
15000
20000
25000
30000
35000
1
7
14
35
63
350
time (days)

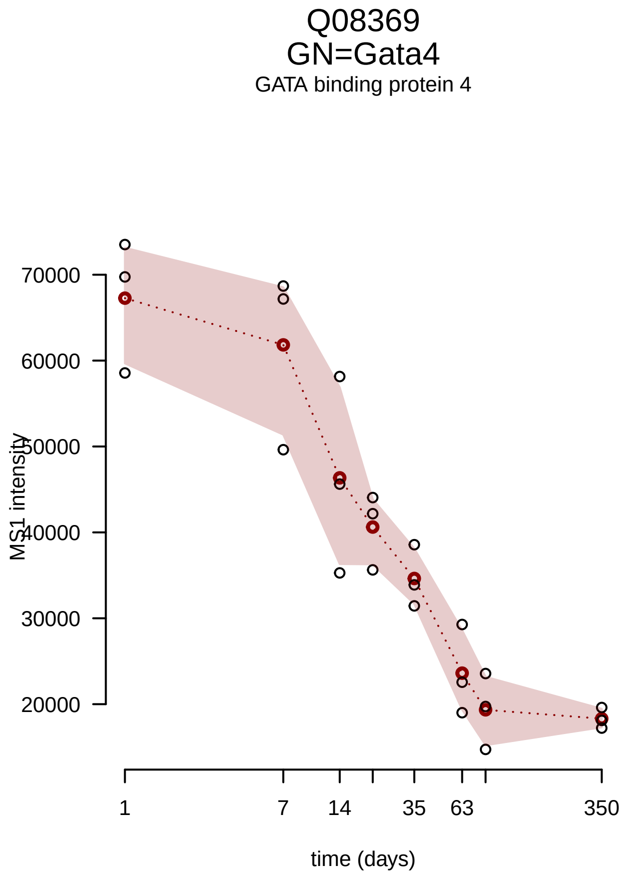
Q08369
GN=Gata4
GATA binding protein 4
MS1 intensity
20000
30000
40000
50000
60000
70000
1
7
14
35
63
350
time (days)

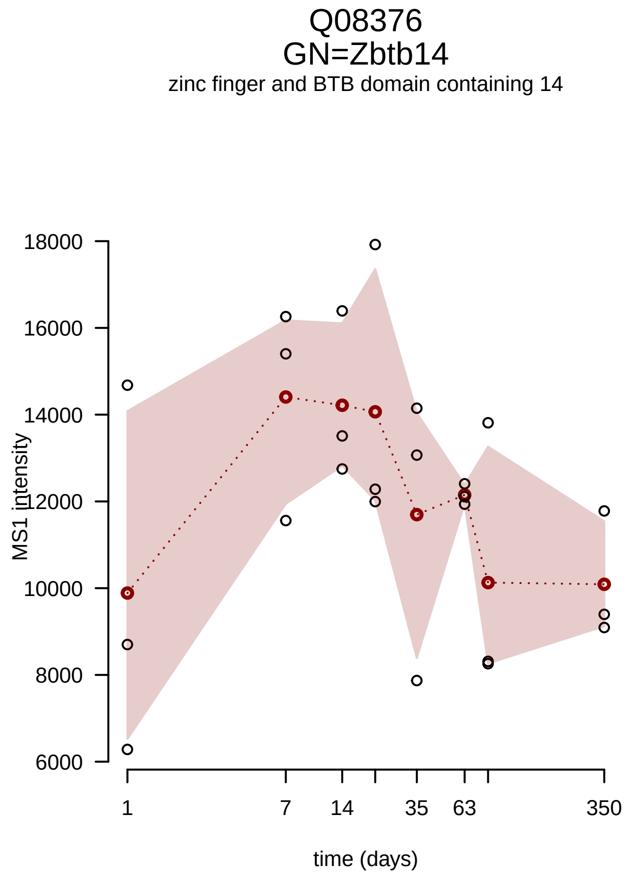
Q08376
GN=Zbtb14
zinc finger and BTB domain containing 14
MS1 intensity
6000
8000
10000
12000
14000
16000
18000
1
7
14
35
63
350
time (days)

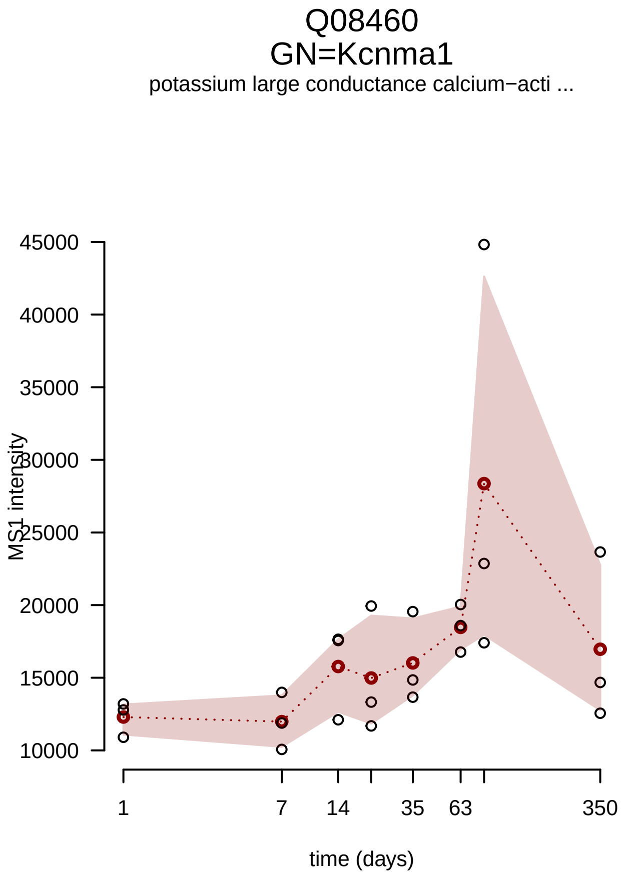
Q08460
GN=Kcnma1
potassium large conductance calcium−acti ...
MS1 intensity
10000
15000
20000
25000
30000
35000
40000
45000
1
7
14
35
63
350
time (days)

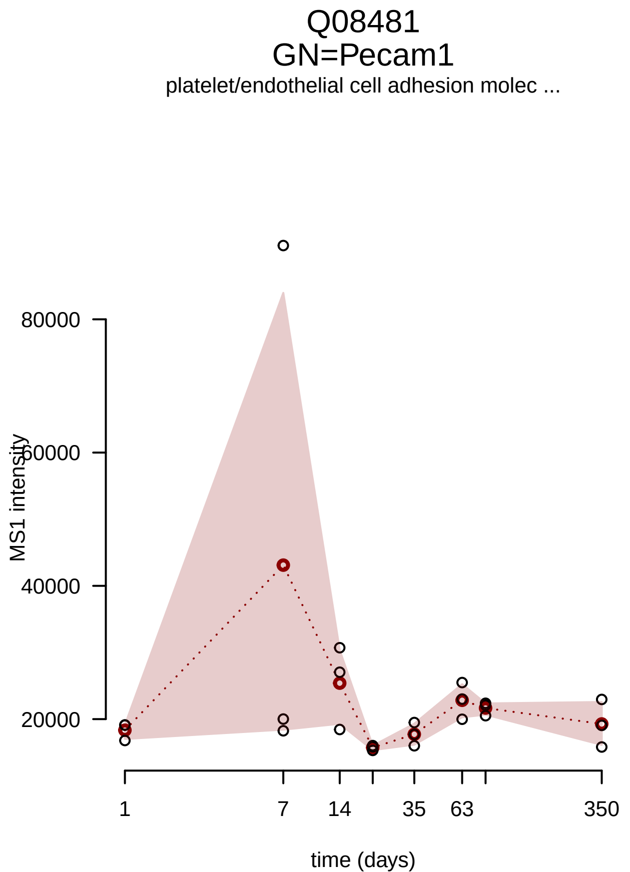
Q08481
GN=Pecam1
platelet/endothelial cell adhesion molec ...
MS1 intensity
20000
40000
60000
80000
1
7
14
35
63
350
time (days)

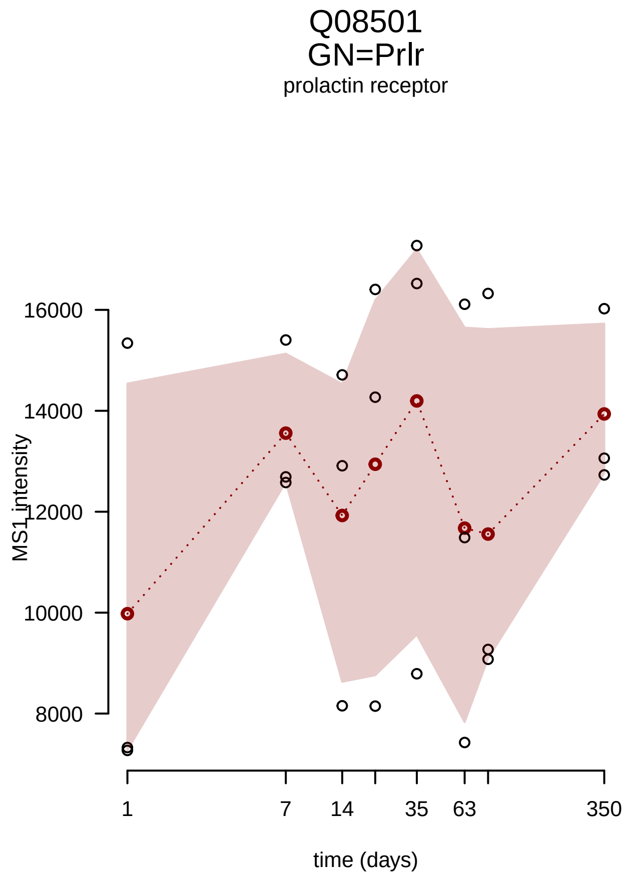
Q08501
GN=Prlr
prolactin receptor
MS1 intensity
8000
10000
12000
14000
16000
1
7
14
35
63
350
time (days)

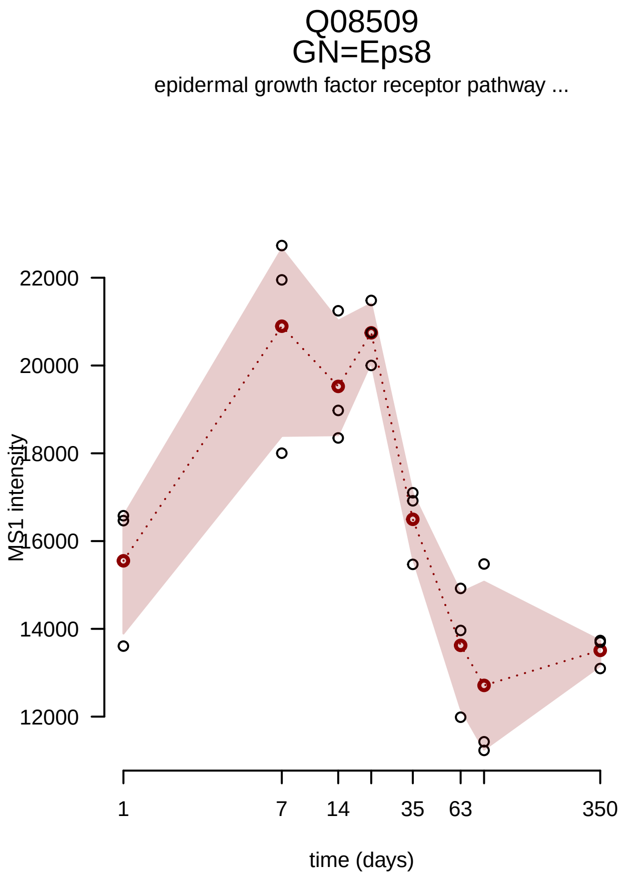
Q08509
GN=Eps8
epidermal growth factor receptor pathway ...
MS1 intensity
12000
14000
16000
18000
20000
22000
1
7
14
35
63
350
time (days)

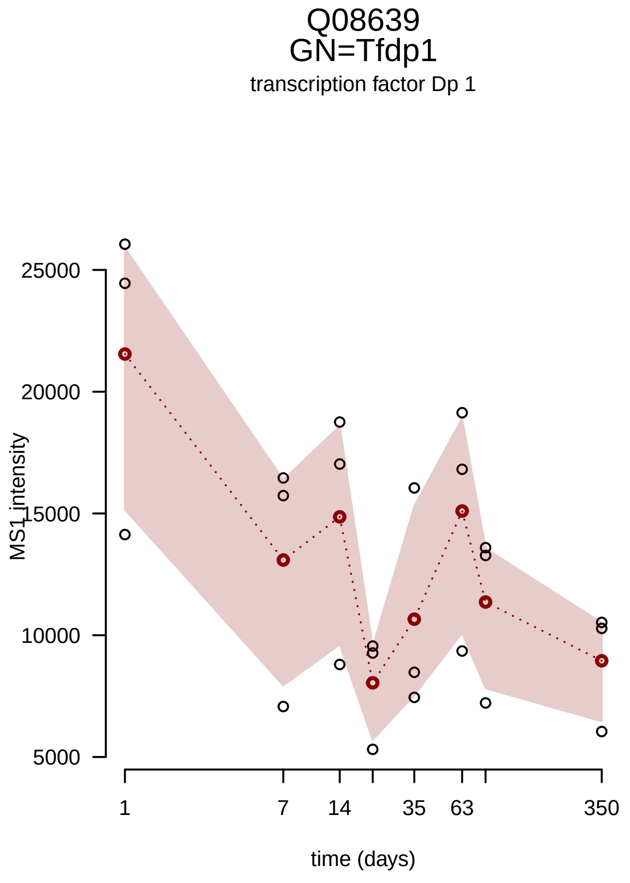
Q08639
GN=Tfdp1
transcription factor Dp 1
MS1 intensity
25000
20000
15000
10000
5000
1
7
14
35
63
350
time (days)

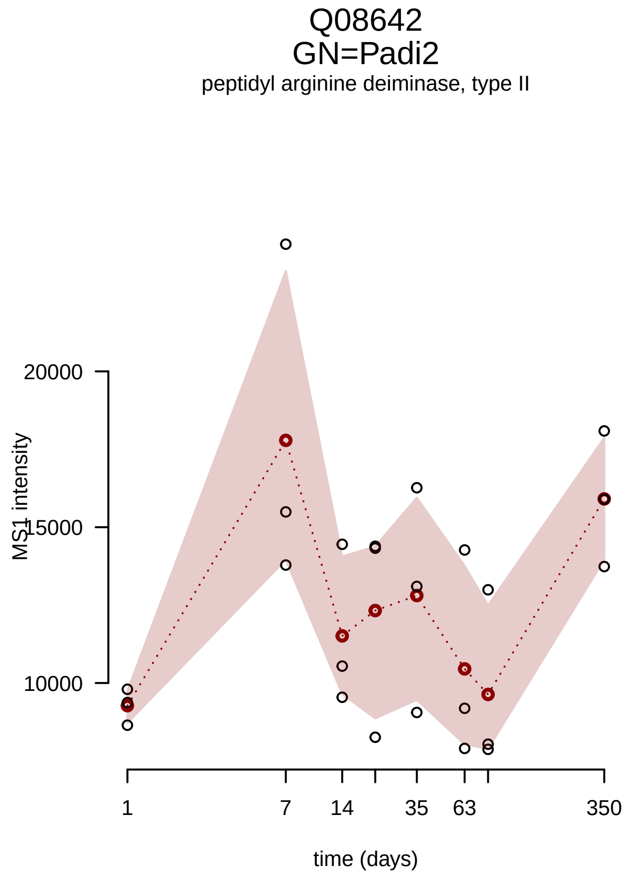
Q08642
GN=Padi2
peptidyl arginine deiminase, type II
MS1 intensity
20000
15000
10000
1
7
14
35
63
350
time (days)

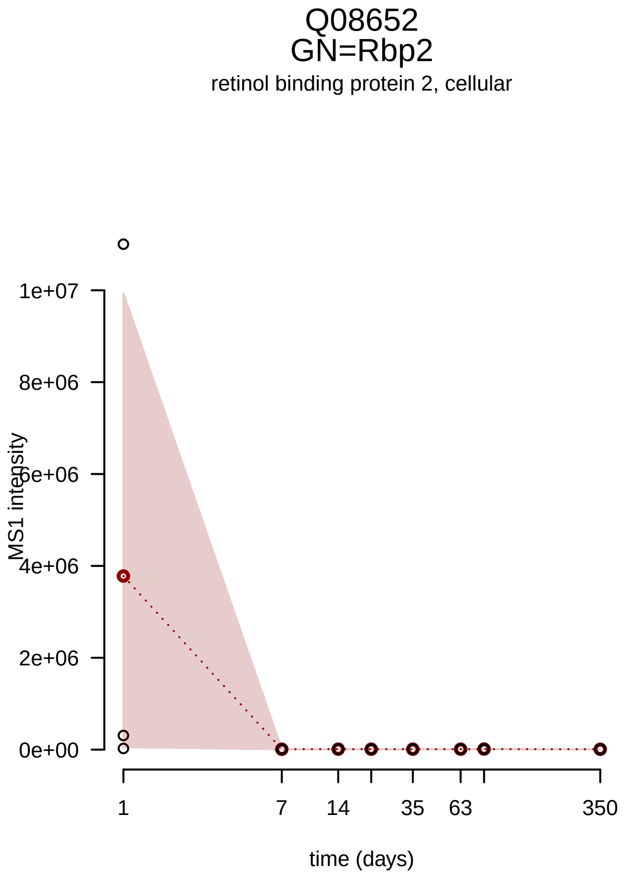
Q08652
GN=Rbp2
retinol binding protein 2, cellular
MS1 intensity
1e+07
8e+06
6e+06
4e+06
2e+06
0e+00
1
7
14
35
63
350
time (days)

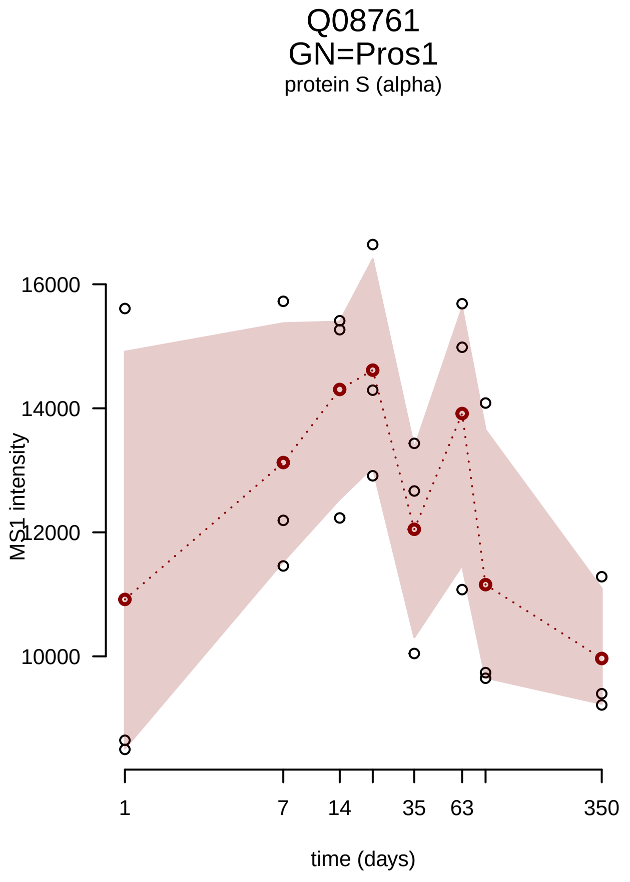
Q08761
GN=Pros1
protein S (alpha)
MS1 intensity
16000
14000
12000
10000
1
7
14
35
63
350
time (days)

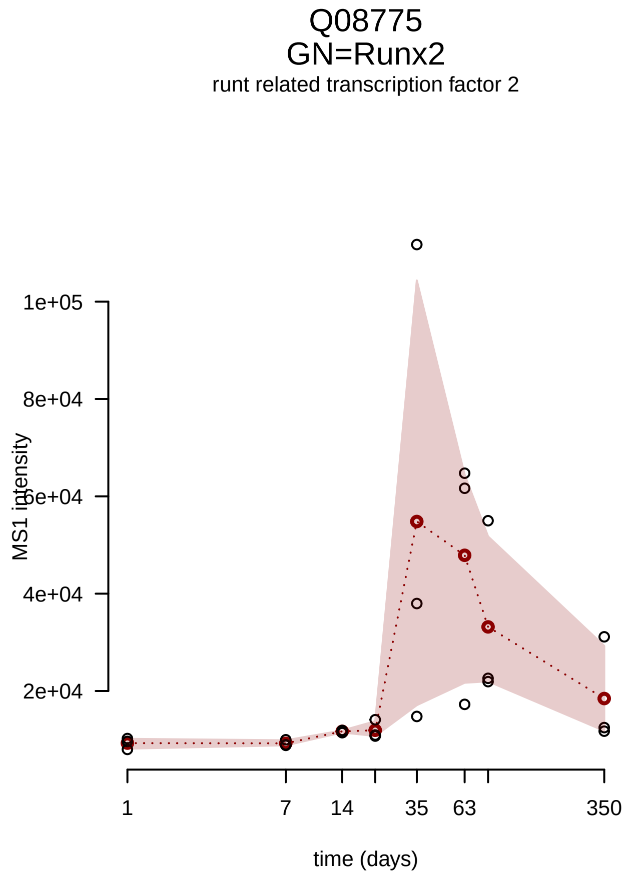
Q08775
GN=Runx2
runt related transcription factor 2
MS1 intensity
1e+05
8e+04
6e+04
4e+04
2e+04
1
7
14
35
63
350
time (days)

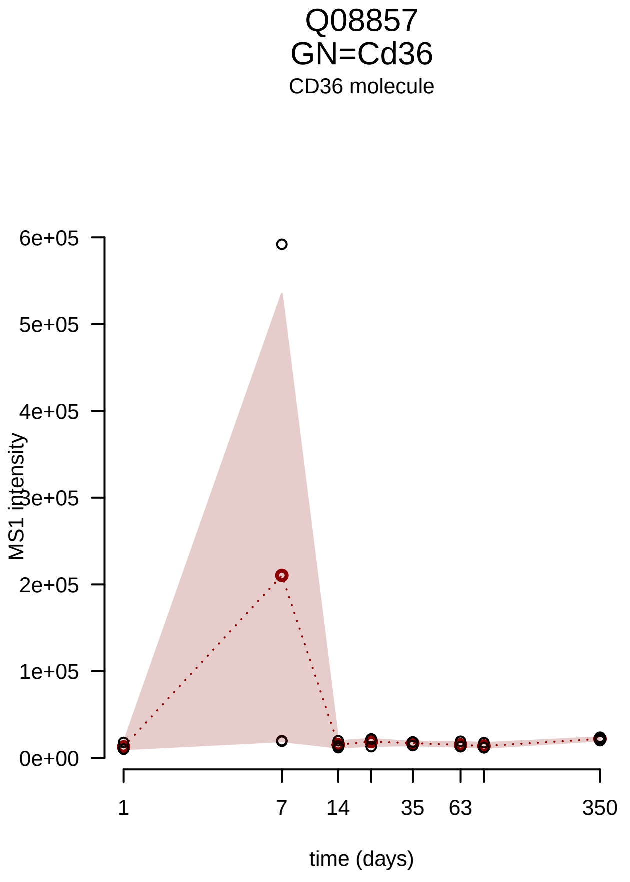
Q08857
GN=Cd36
CD36 molecule
MS1 intensity
6e+05
5e+05
4e+05
3e+05
2e+05
1e+05
0e+00
1
7
14
35
63
350
time (days)

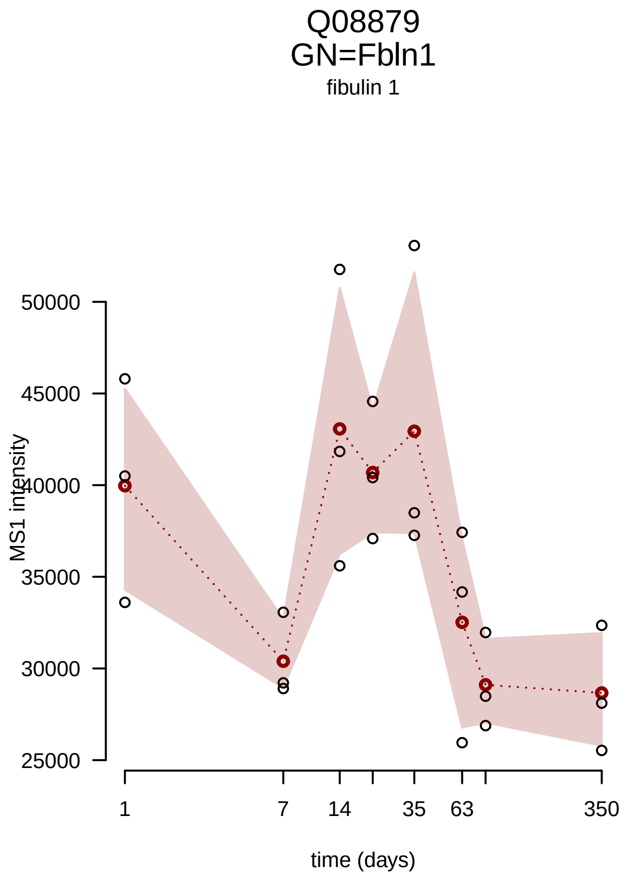
Q08879
GN=Fbln1
fibulin 1
MS1 intensity
50000
45000
40000
35000
30000
25000
1
7
14
35
63
350
time (days)

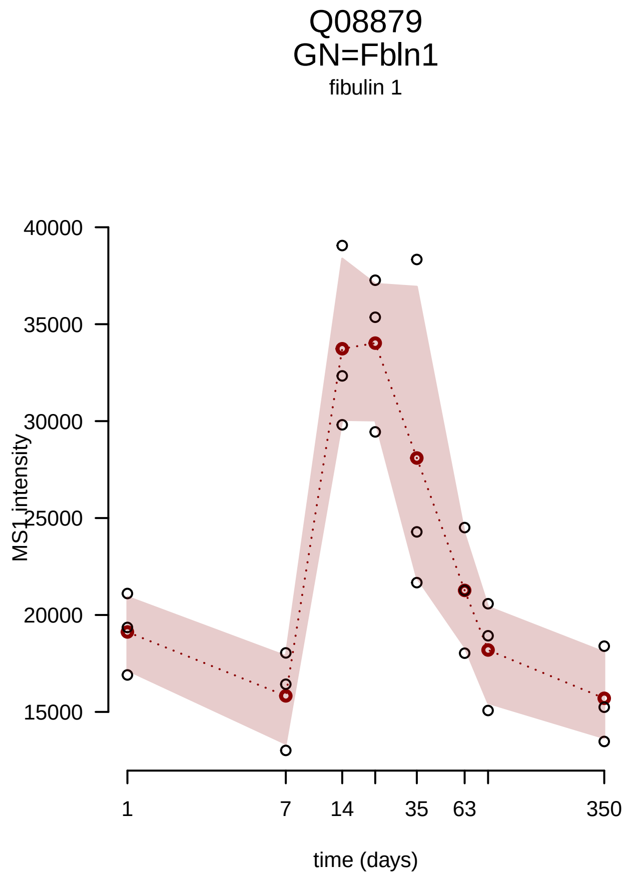
Q08879
GN=Fbln1
fibulin 1
MS1 intensity
40000
35000
30000
25000
20000
15000
1
7
14
35
63
350
time (days)

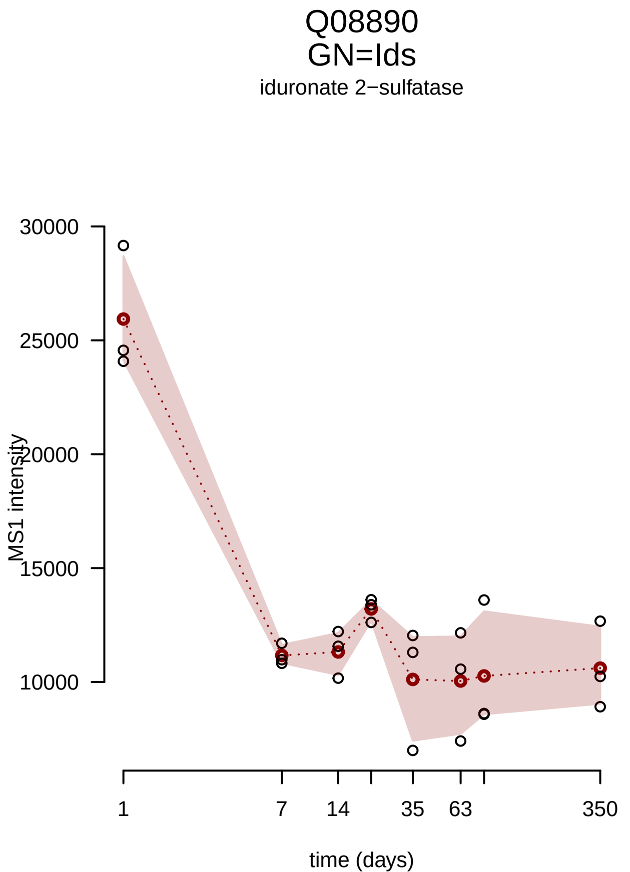
Q08890
GN=Ids
iduronate 2−sulfatase
MS1 intensity
30000
25000
20000
15000
10000
1
7
14
35
63
350
time (days)

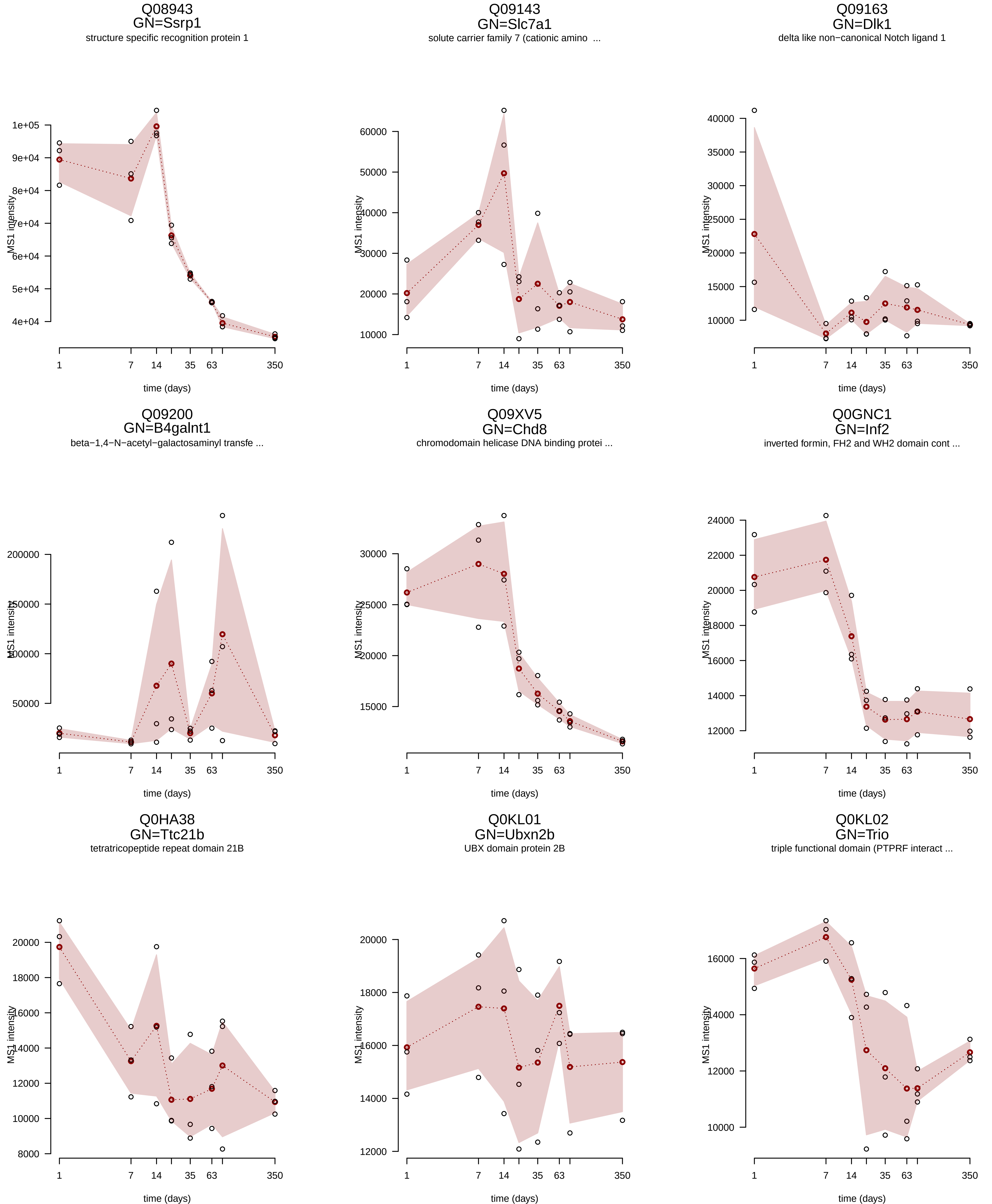
Q08943
GN=Ssrp1
structure specific recognition protein 1
1e+05
9e+04
8e+04
7e+04
6e+04
5e+04
4e+04
MS1 intensity
1
7
14
35
63
350
time (days)
Q09143
GN=Slc7a1
solute carrier family 7 (cationic amino ...
60000
50000
40000
30000
20000
10000
MS1 intensity
time (days)
Q09163
GN=Dlk1
delta like non−canonical Notch ligand 1
40000
35000
30000
25000
20000
15000
10000
MS1 intensity
time (days)
Q09200
GN=B4galnt1
beta−1,4−N−acetyl−galactosaminyl transfe ...
200000
150000
100000
50000
MS1 intensity
time (days)
Q09XV5
GN=Chd8
chromodomain helicase DNA binding protei ...
30000
25000
20000
15000
MS1 intensity
time (days)
Q0GNC1
GN=Inf2
inverted formin, FH2 and WH2 domain cont ...
24000
22000
20000
18000
16000
14000
12000
MS1 intensity
time (days)
Q0HA38
GN=Ttc21b
tetratricopeptide repeat domain 21B
20000
18000
16000
14000
12000
10000
8000
MS1 intensity
time (days)
Q0KL01
GN=Ubxn2b
UBX domain protein 2B
20000
18000
16000
14000
12000
MS1 intensity
time (days)
Q0KL02
GN=Trio
triple functional domain (PTPRF interact ...
16000
14000
12000
10000
MS1 intensity
time (days)

Q0P5W1
GN=Vps8
VPS8 CORVET complex subunit

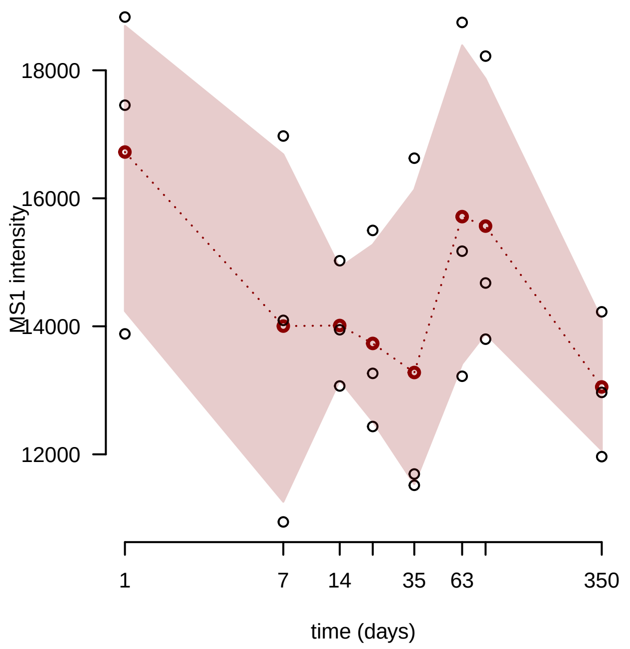

Q0P678
GN=Zc3h18
zinc finger CCCH−type containing 18

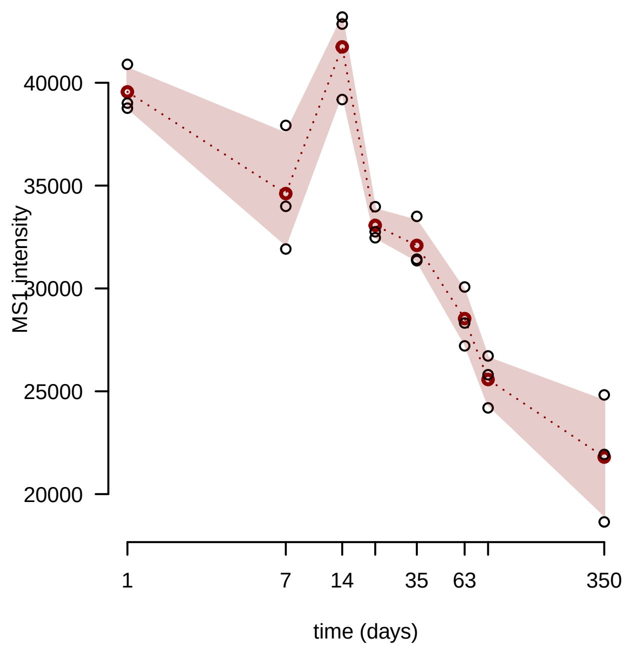

Q0PHV7
GN=Dact3
dishevelled−binding antagonist of beta−c ...

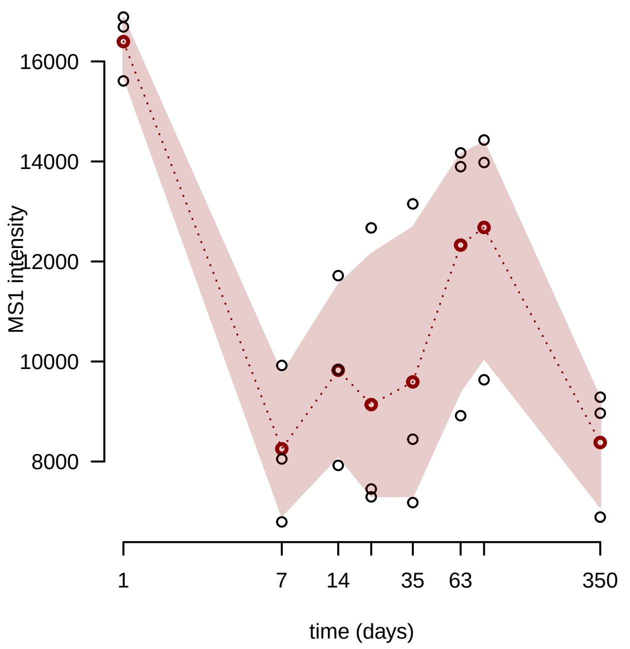

Q0VBF8
GN=Stum
mechanosensory transduction mediator

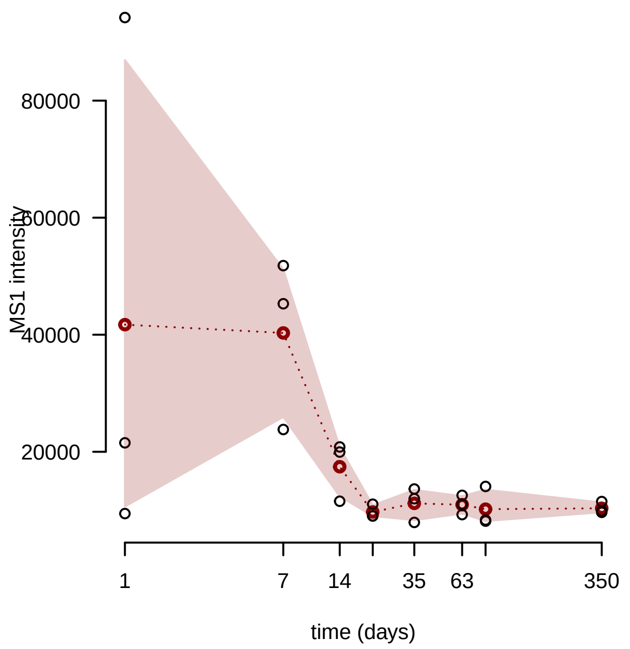

Q0VBK2
GN=Krt80
keratin 80

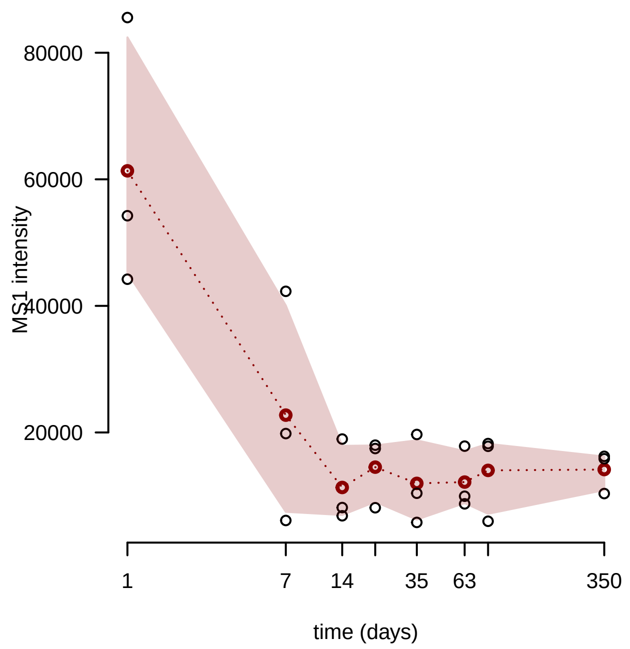

Q0VBL3
GN=Rbm15
RNA binding motif protein 15

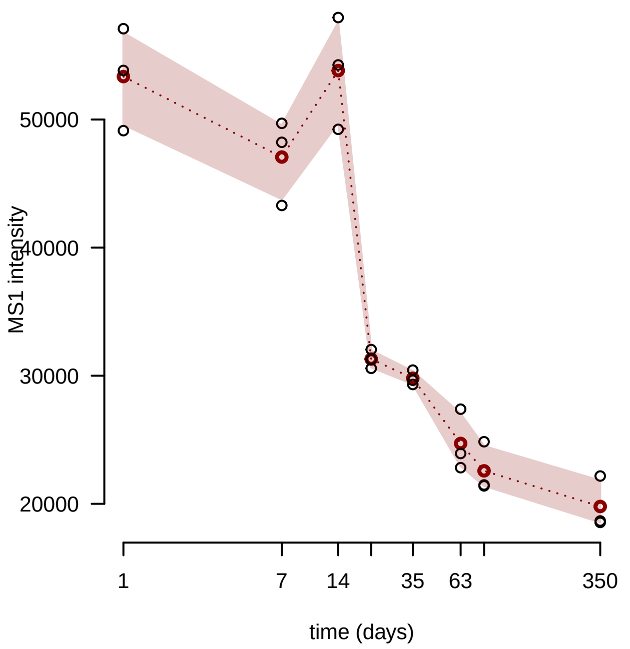

Q0VEJ0
GN=Cep76
centrosomal protein 76

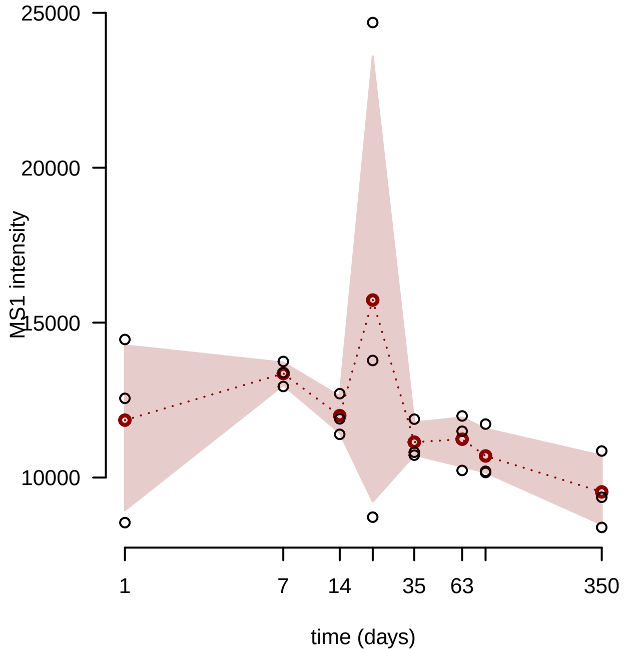

Q0VG62
GN=Rbis
ribosomal biogenesis factor

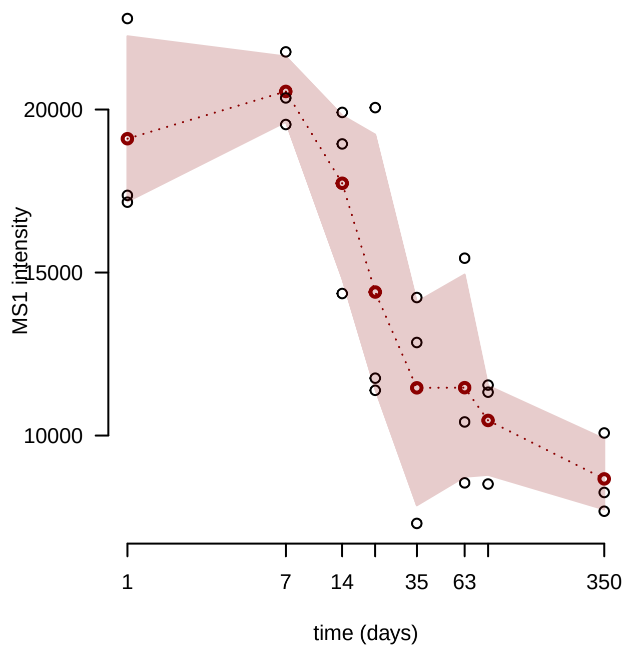

Q0VGB7
GN=Ppp4r2
protein phosphatase 4, regulatory subuni ...

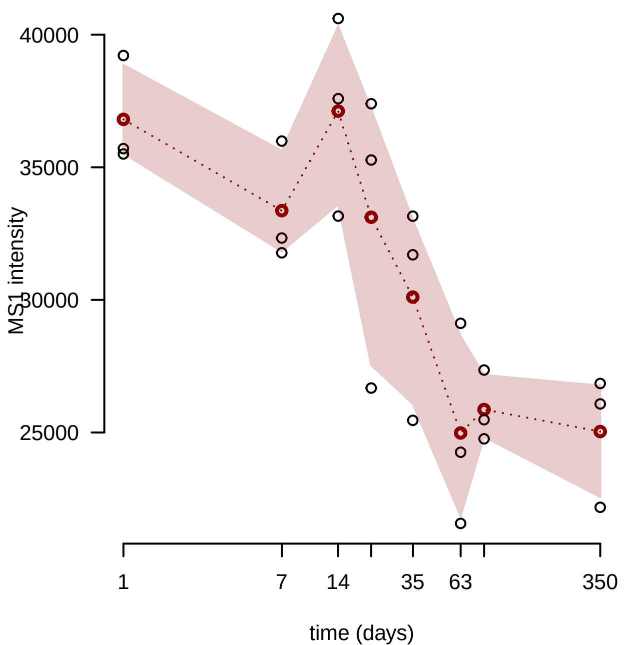

Q0VGY8
GN=Tanc1
tetratricopeptide repeat, ankyrin repeat ...

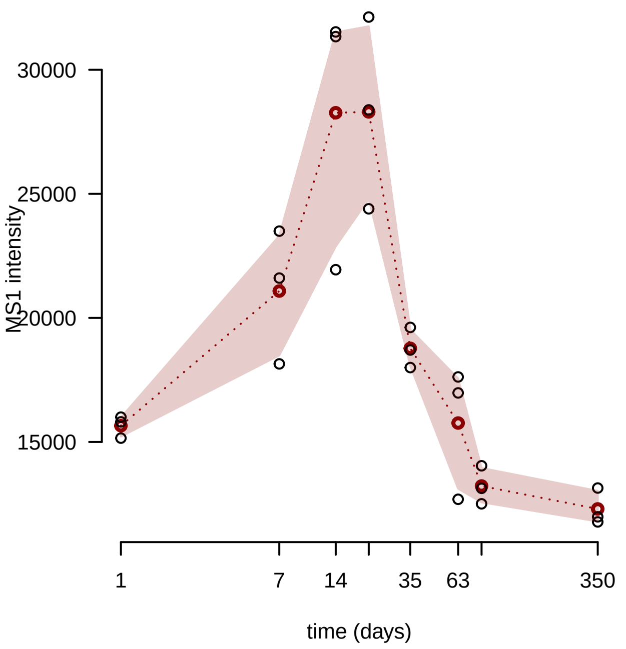

Q11011
GN=Npepps
aminopeptidase puromycin sensitive

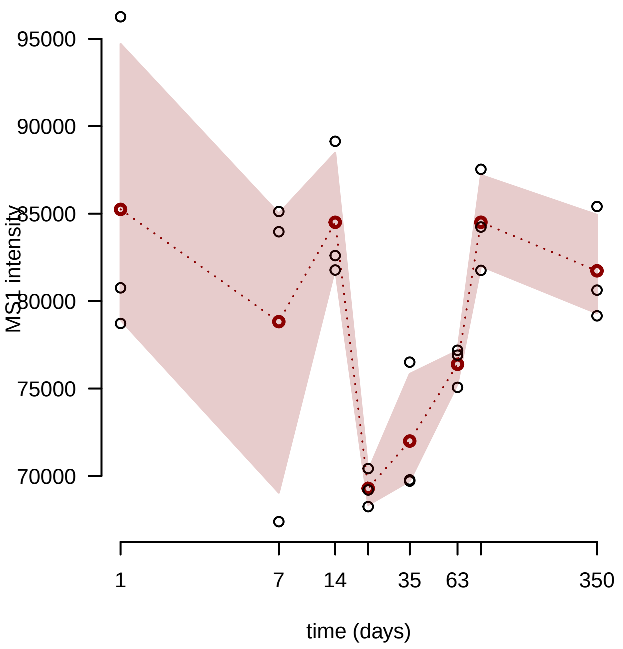

Q11136
GN=Pepd
peptidase D

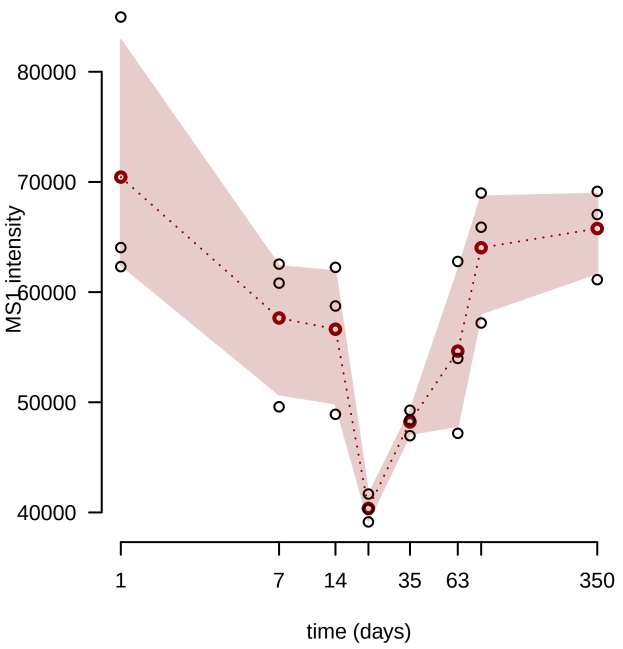

Q148V7
GN=Relch
RAB11 binding and LisH domain, coiled−co ...

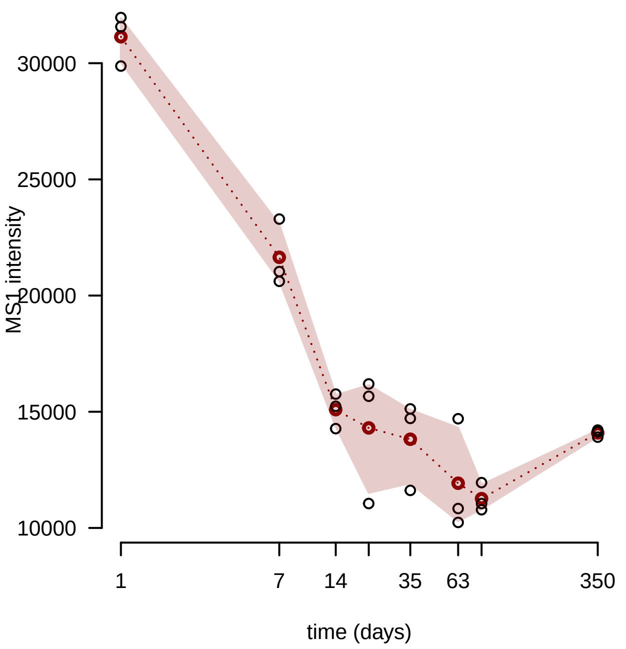

Q149C2
GN=Traf3ip1
TRAF3 interacting protein 1

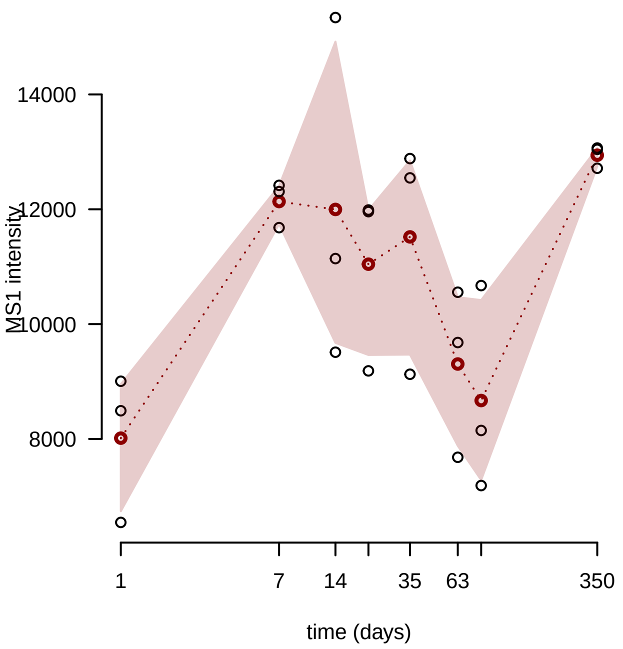

Q149F1
GN=Rpusd2
RNA pseudouridylate synthase domain cont ...

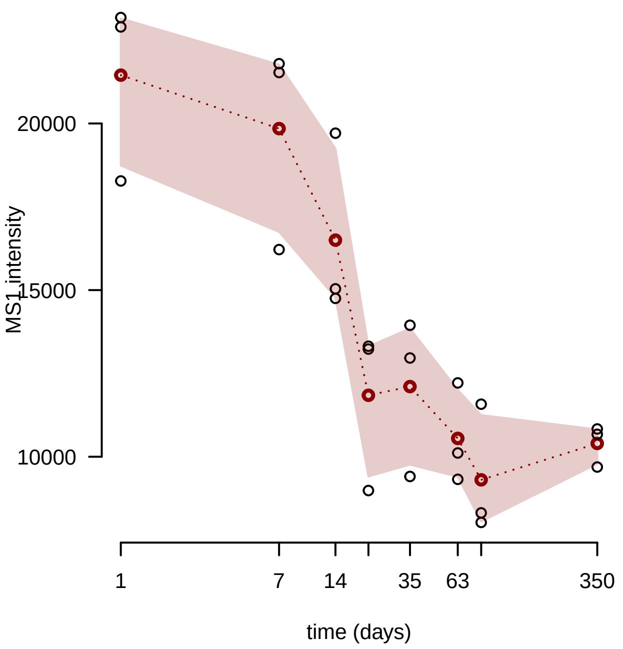

Q149F3
GN=Gspt2
G1 to S phase transition 2

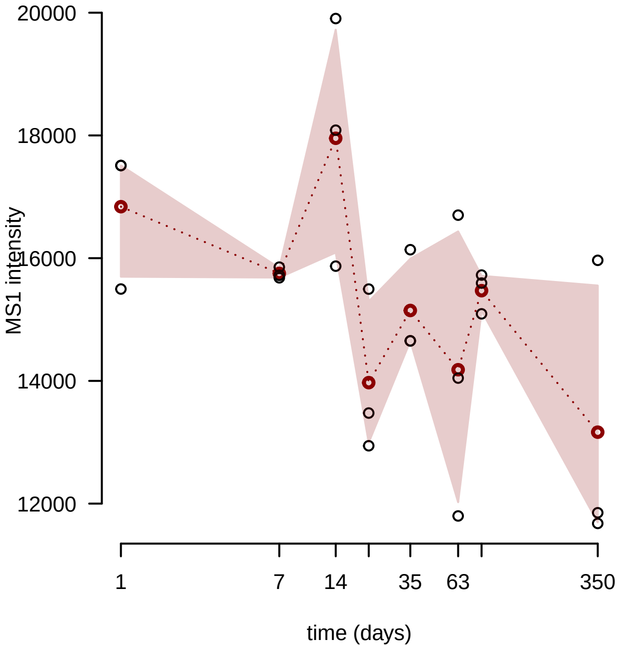

Q149L6
GN=Dnajb14
DnaJ heat shock protein family (Hsp40) m ...

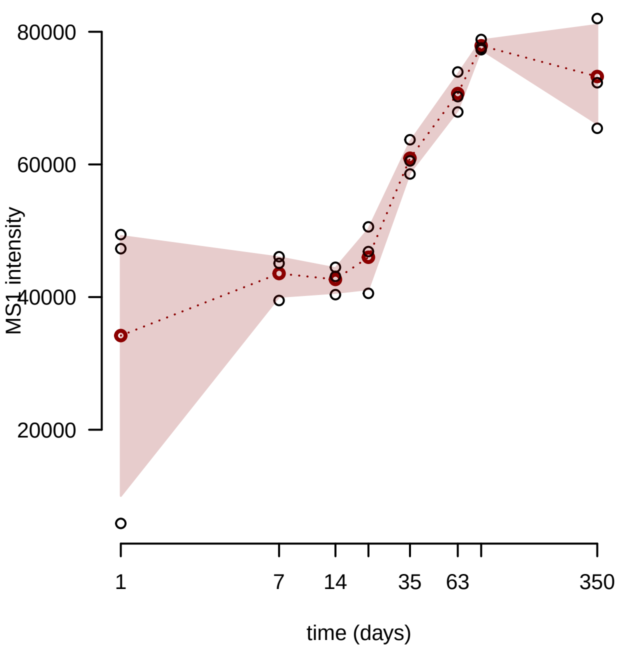

Q14AI0
GN=DSCC1

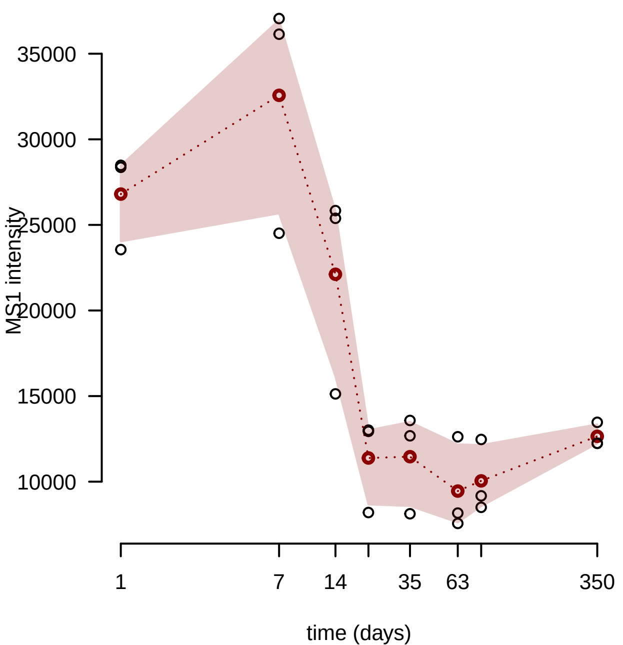

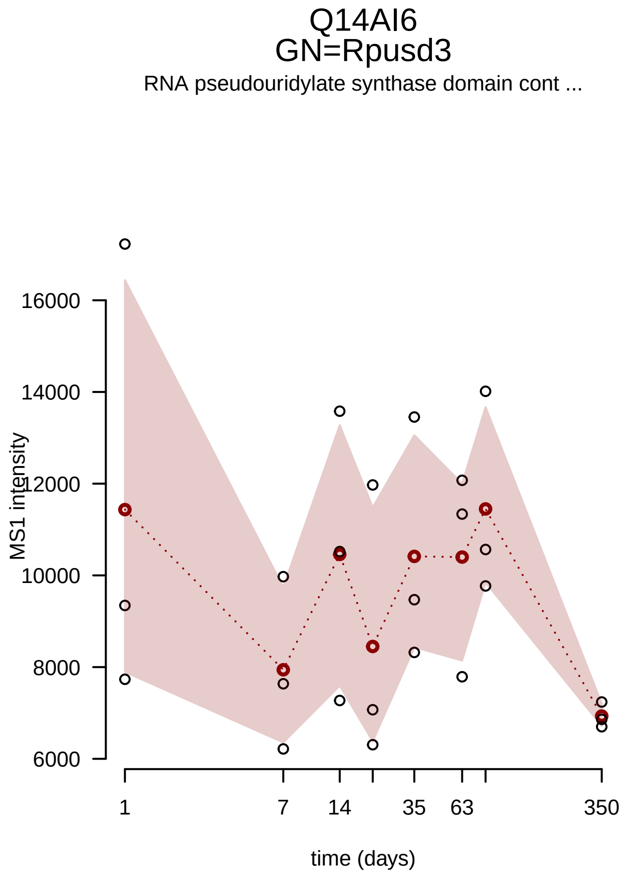
Q14AI6
GN=Rpusd3
RNA pseudouridylate synthase domain cont ...
MS1 intensity
6000
8000
10000
12000
14000
16000
1
7
14
35
63
350
time (days)

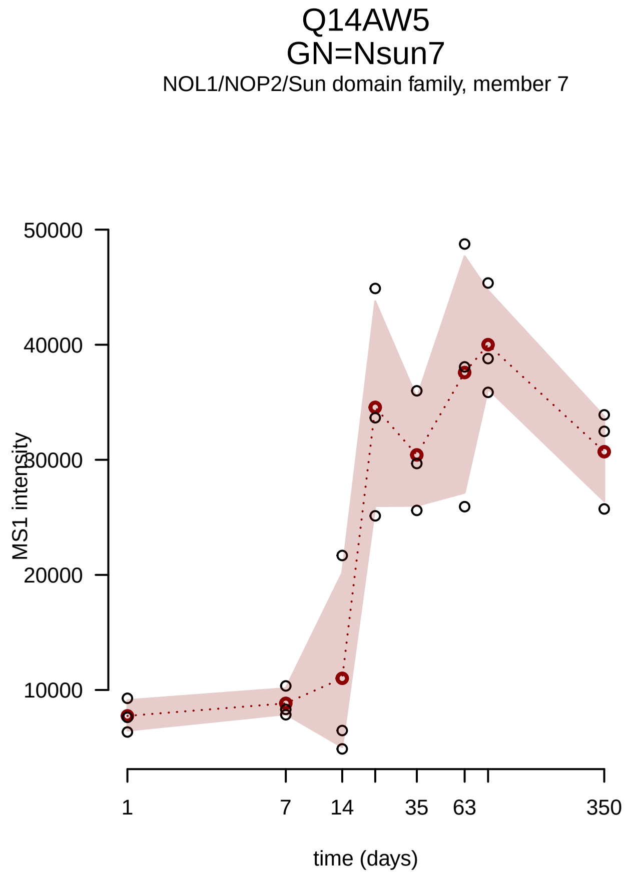
Q14AW5
GN=Nsun7
NOL1/NOP2/Sun domain family, member 7
MS1 intensity
10000
20000
30000
40000
50000
1
7
14
35
63
350
time (days)

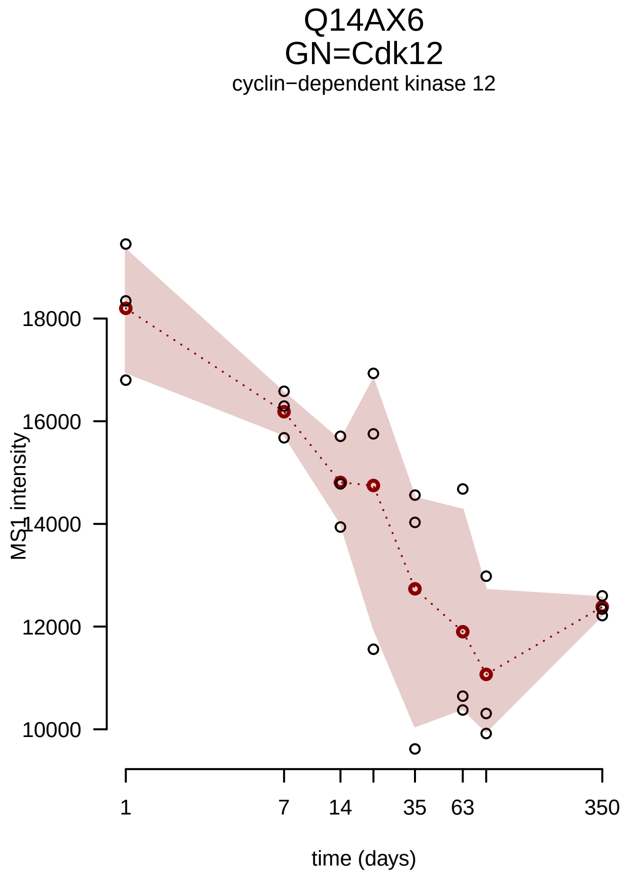
Q14AX6
GN=Cdk12
cyclin−dependent kinase 12
MS1 intensity
10000
12000
14000
16000
18000
1
7
14
35
63
350
time (days)

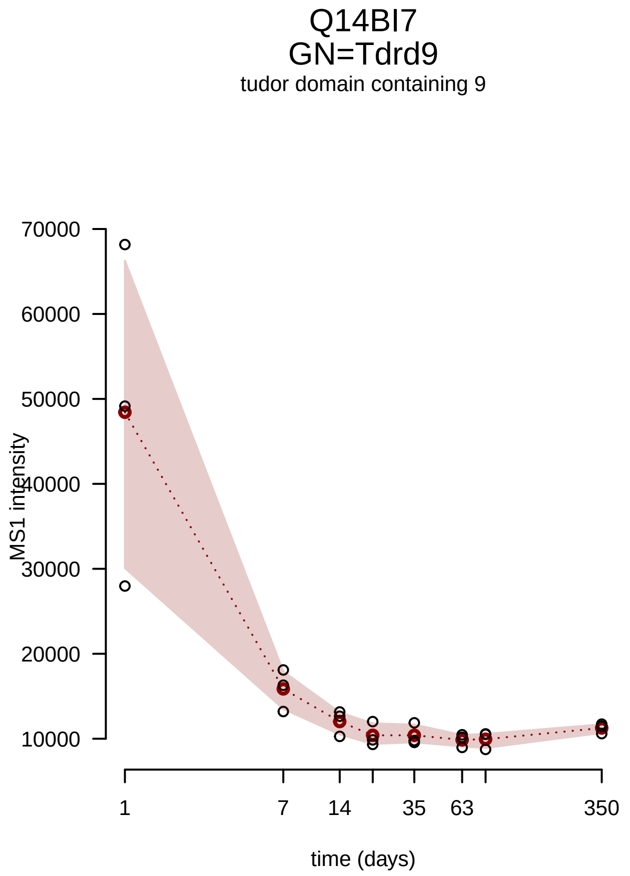
Q14BI7
GN=Tdrd9
tudor domain containing 9
MS1 intensity
10000
20000
30000
40000
50000
60000
70000
1
7
14
35
63
350
time (days)

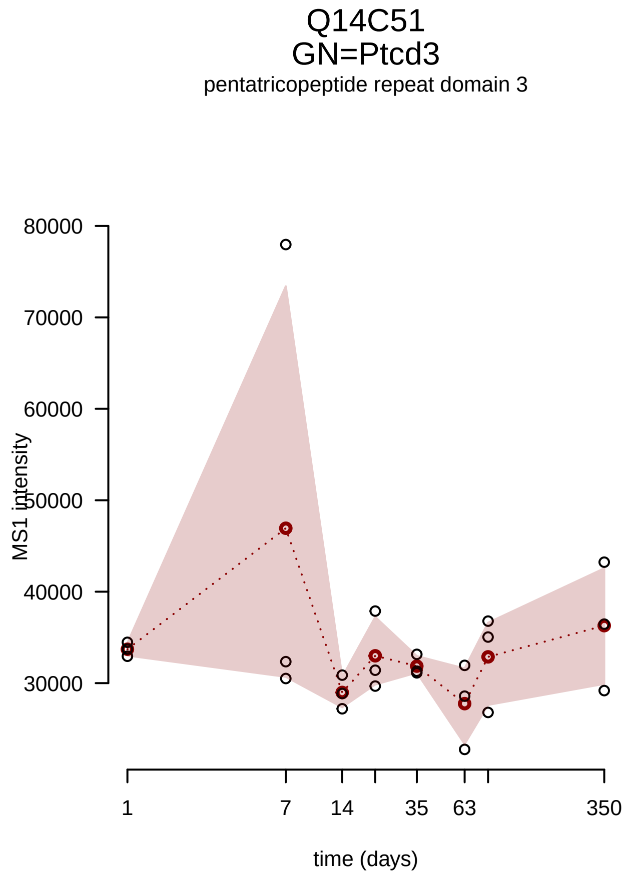
Q14C51
GN=Ptcd3
pentatricopeptide repeat domain 3
MS1 intensity
30000
40000
50000
60000
70000
80000
1
7
14
35
63
350
time (days)

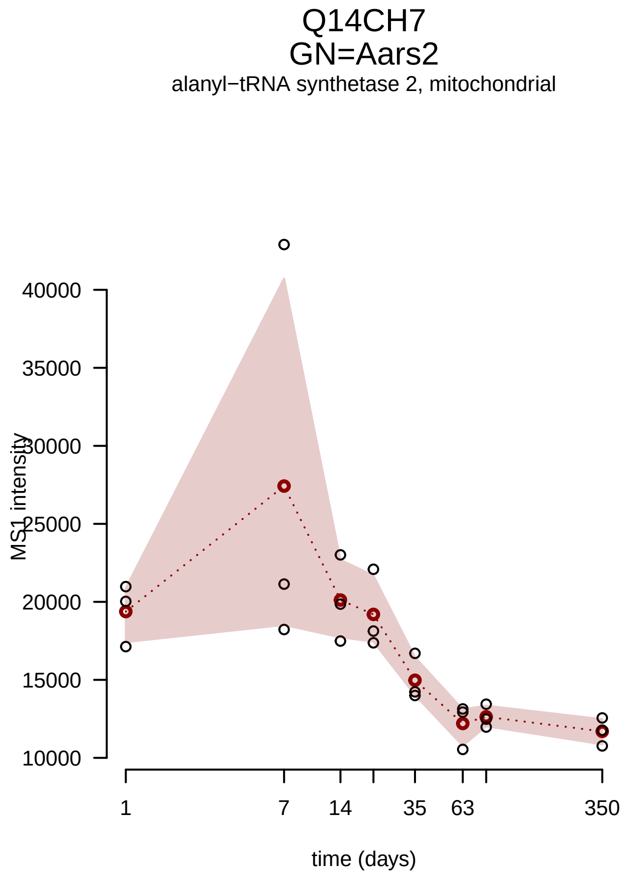
Q14CH7
GN=Aars2
alanyl−tRNA synthetase 2, mitochondrial
MS1 intensity
10000
15000
20000
25000
30000
35000
40000
1
7
14
35
63
350
time (days)

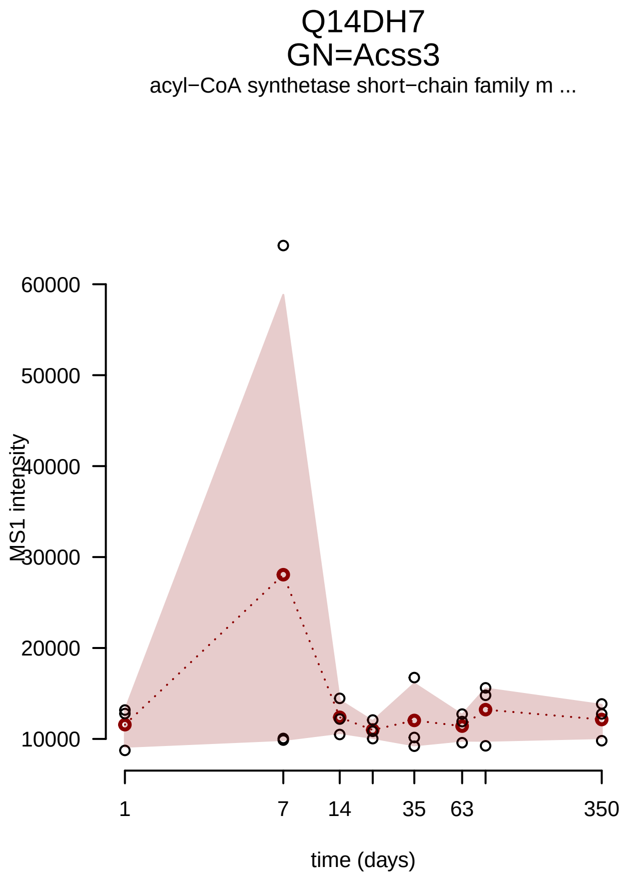
Q14DH7
GN=Acss3
acyl−CoA synthetase short−chain family m ...
MS1 intensity
10000
20000
30000
40000
50000
60000
1
7
14
35
63
350
time (days)

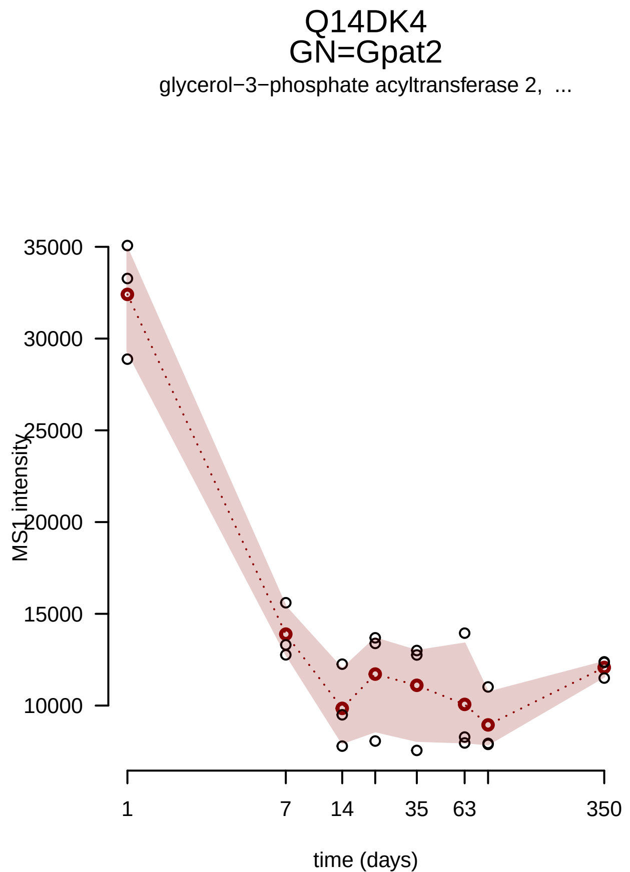
Q14DK4
GN=Gpat2
glycerol−3−phosphate acyltransferase 2, ...
MS1 intensity
10000
15000
20000
25000
30000
35000
1
7
14
35
63
350
time (days)

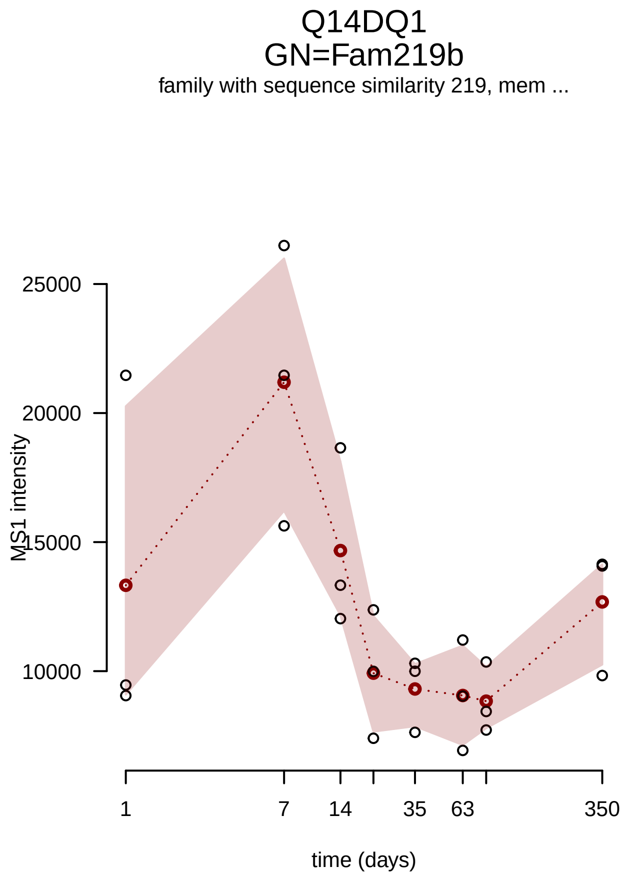
Q14DQ1
GN=Fam219b
family with sequence similarity 219, mem ...
MS1 intensity
10000
15000
20000
25000
1
7
14
35
63
350
time (days)

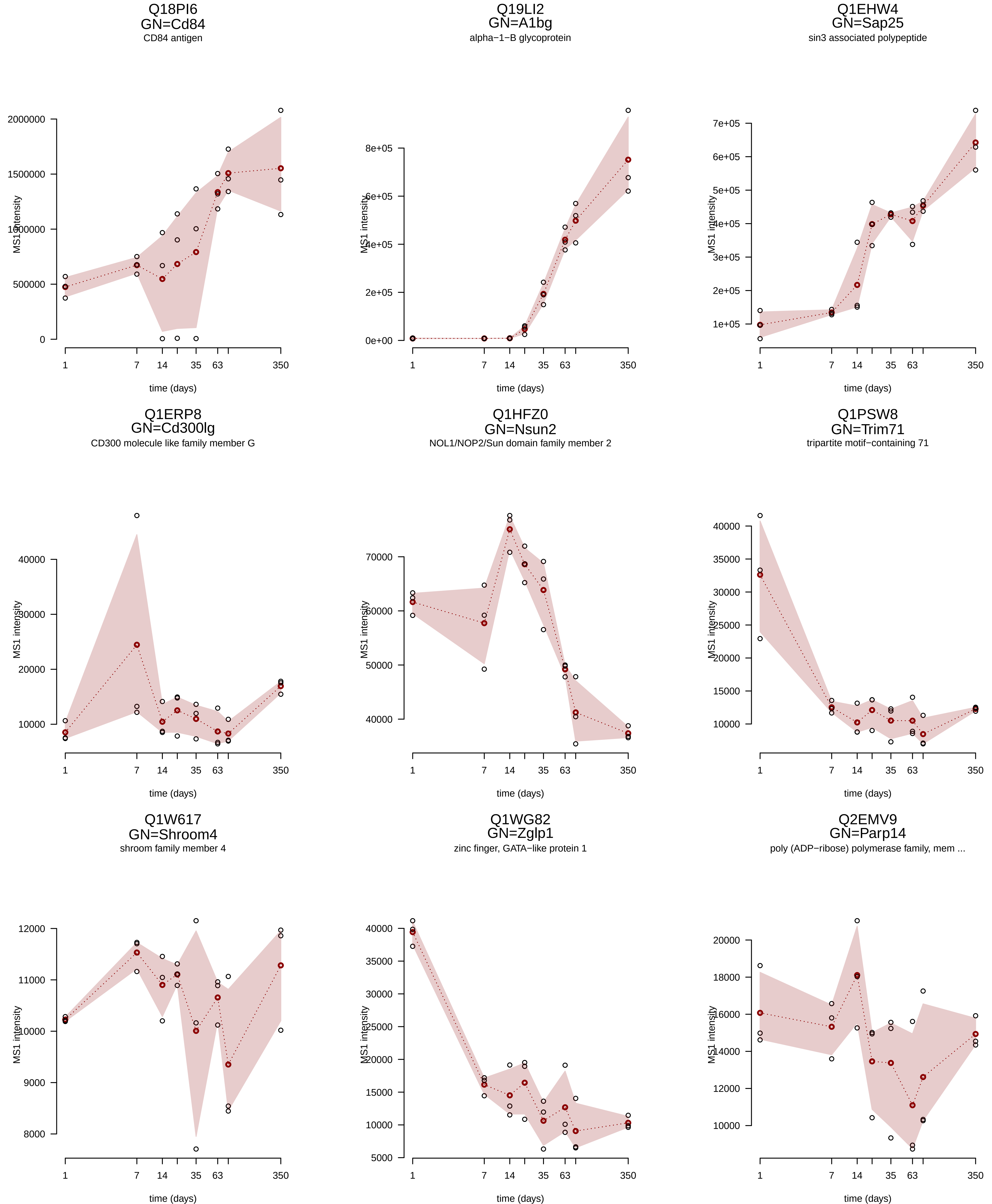
Q18PI6
GN=Cd84
CD84 antigen
MS1 intensity
0
500000
1000000
1500000
2000000
1 7 14 35 63 350
time (days)
Q19LI2
GN=A1bg
alpha−1−B glycoprotein
MS1 intensity
0e+00
2e+05
4e+05
6e+05
8e+05
1 7 14 35 63 350
time (days)
Q1EHW4
GN=Sap25
sin3 associated polypeptide
MS1 intensity
1e+05
2e+05
3e+05
4e+05
5e+05
6e+05
7e+05
1 7 14 35 63 350
time (days)
Q1ERP8
GN=Cd300lg
CD300 molecule like family member G
MS1 intensity
10000
20000
30000
40000
1 7 14 35 63 350
time (days)
Q1HFZ0
GN=Nsun2
NOL1/NOP2/Sun domain family member 2
MS1 intensity
40000
50000
60000
70000
1 7 14 35 63 350
time (days)
Q1PSW8
GN=Trim71
tripartite motif−containing 71
MS1 intensity
10000
15000
20000
25000
30000
35000
40000
1 7 14 35 63 350
time (days)
Q1W617
GN=Shroom4
shroom family member 4
MS1 intensity
8000
9000
10000
11000
12000
1 7 14 35 63 350
time (days)
Q1WG82
GN=Zglp1
zinc finger, GATA−like protein 1
MS1 intensity
5000
10000
15000
20000
25000
30000
35000
40000
1 7 14 35 63 350
time (days)
Q2EMV9
GN=Parp14
poly (ADP−ribose) polymerase family, mem ...
MS1 intensity
10000
12000
14000
16000
18000
20000
1 7 14 35 63 350
time (days)

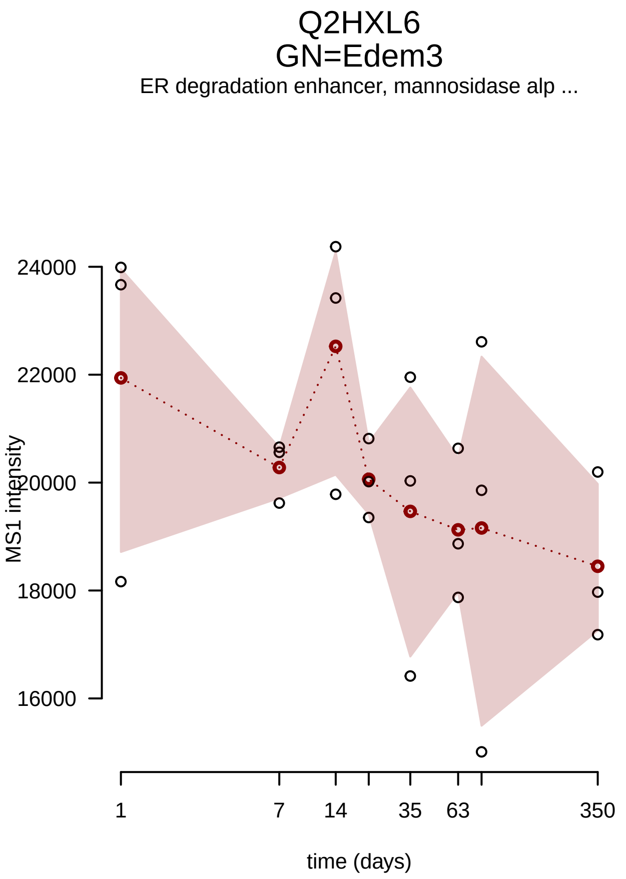
Q2HXL6
GN=Edem3
ER degradation enhancer, mannosidase alp ...
MS1 intensity
24000
22000
20000
18000
16000
1
7
14
35
63
350
time (days)
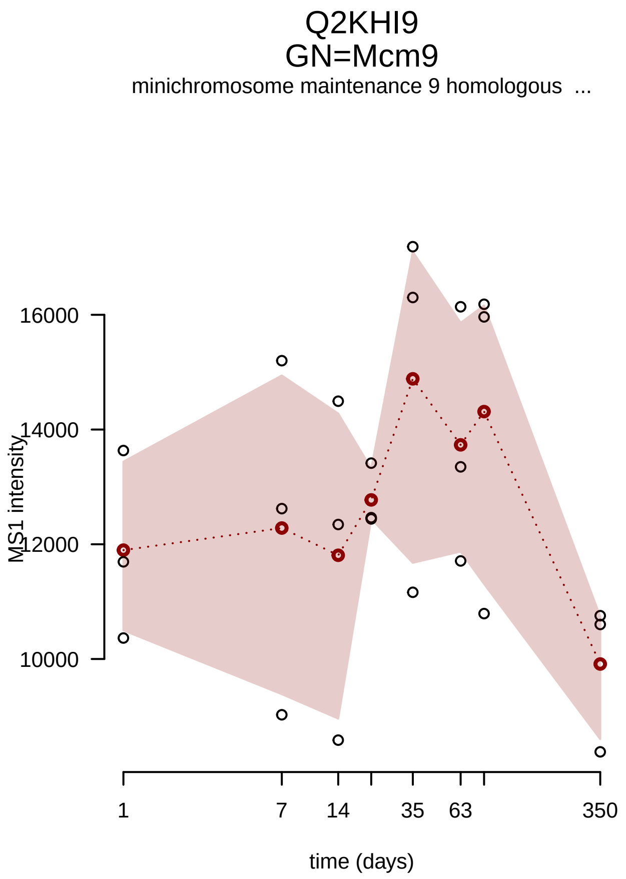
Q2KHI9
GN=Mcm9
minichromosome maintenance 9 homologous ...
MS1 intensity
16000
14000
12000
10000
1
7
14
35
63
350
time (days)
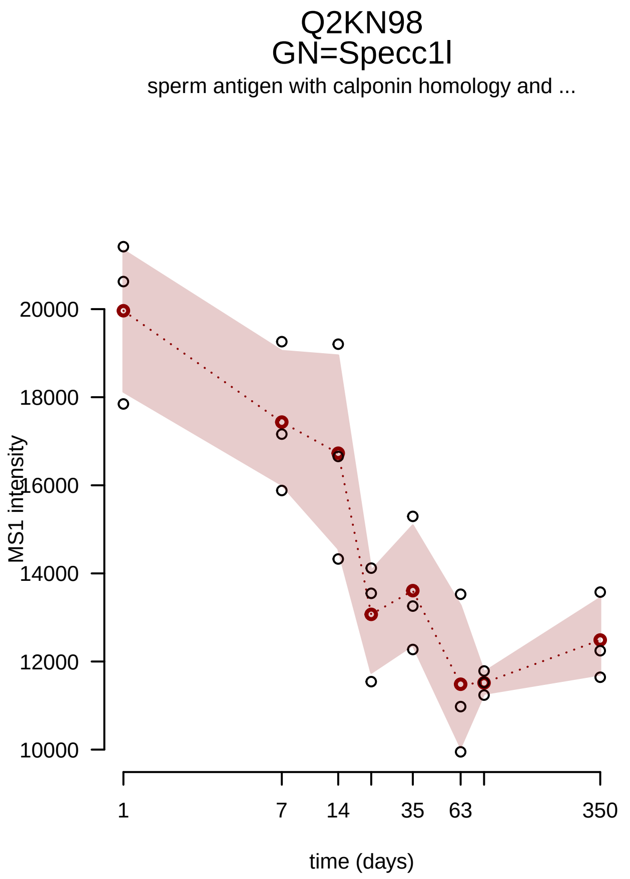
Q2KN98
GN=Specc1l
sperm antigen with calponin homology and ...
MS1 intensity
20000
18000
16000
14000
12000
10000
1
7
14
35
63
350
time (days)
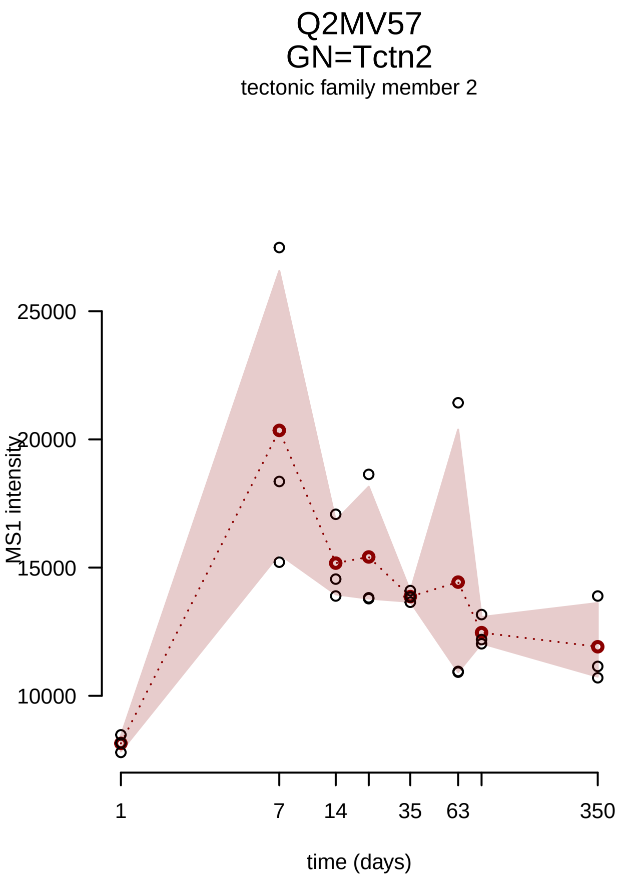
Q2MV57
GN=Tctn2
tectonic family member 2
MS1 intensity
25000
20000
15000
10000
1
7
14
35
63
350
time (days)
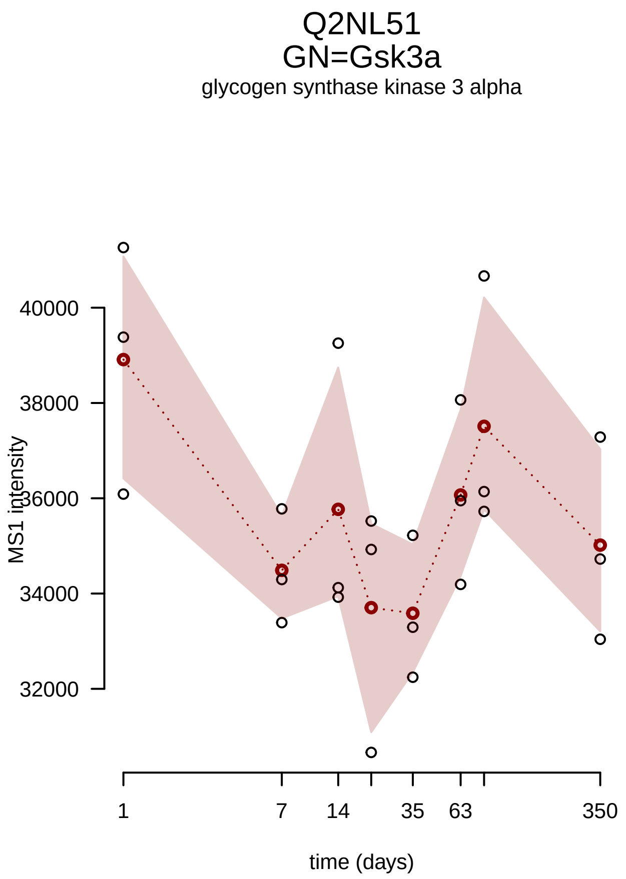
Q2NL51
GN=Gsk3a
glycogen synthase kinase 3 alpha
MS1 intensity
40000
38000
36000
34000
32000
1
7
14
35
63
350
time (days)
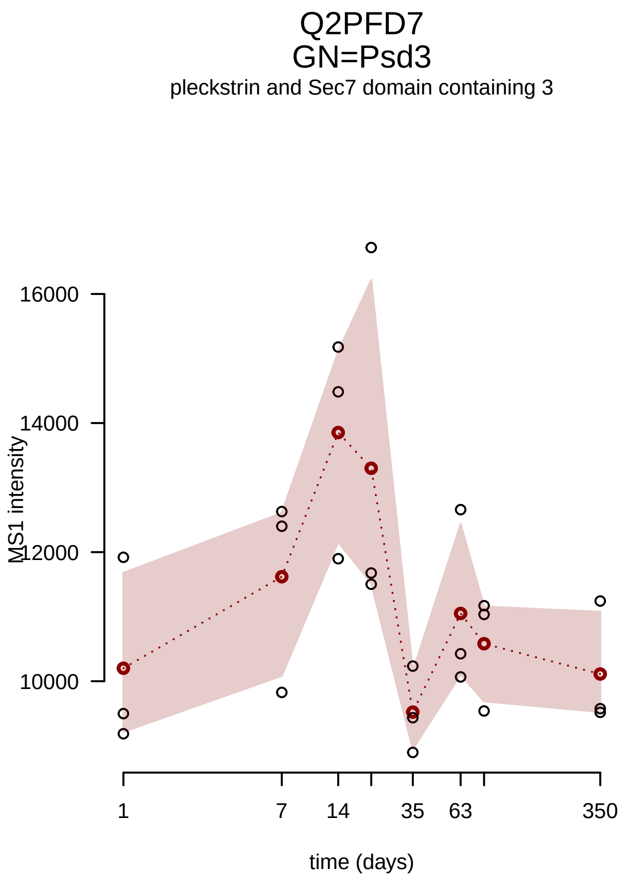
Q2PFD7
GN=Psd3
pleckstrin and Sec7 domain containing 3
MS1 intensity
16000
14000
12000
10000
1
7
14
35
63
350
time (days)
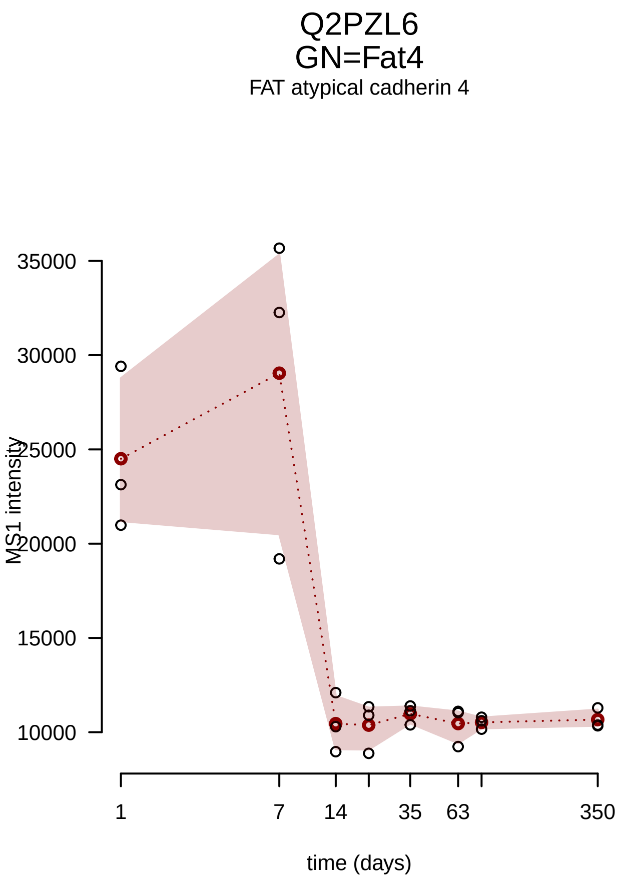
Q2PZL6
GN=Fat4
FAT atypical cadherin 4
MS1 intensity
35000
30000
25000
20000
15000
10000
1
7
14
35
63
350
time (days)
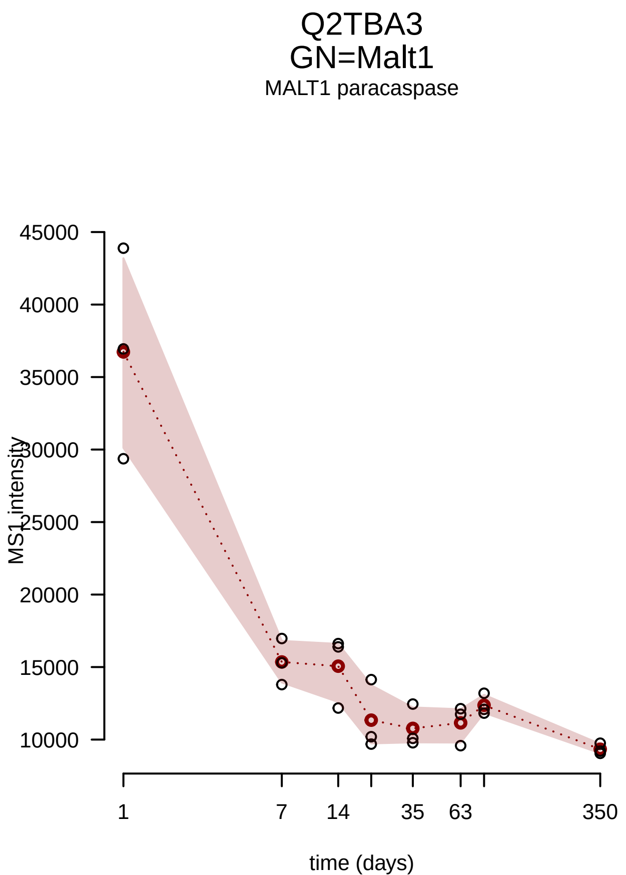
Q2TBA3
GN=Malt1
MALT1 paracaspase
MS1 intensity
45000
40000
35000
30000
25000
20000
15000
10000
1
7
14
35
63
350
time (days)
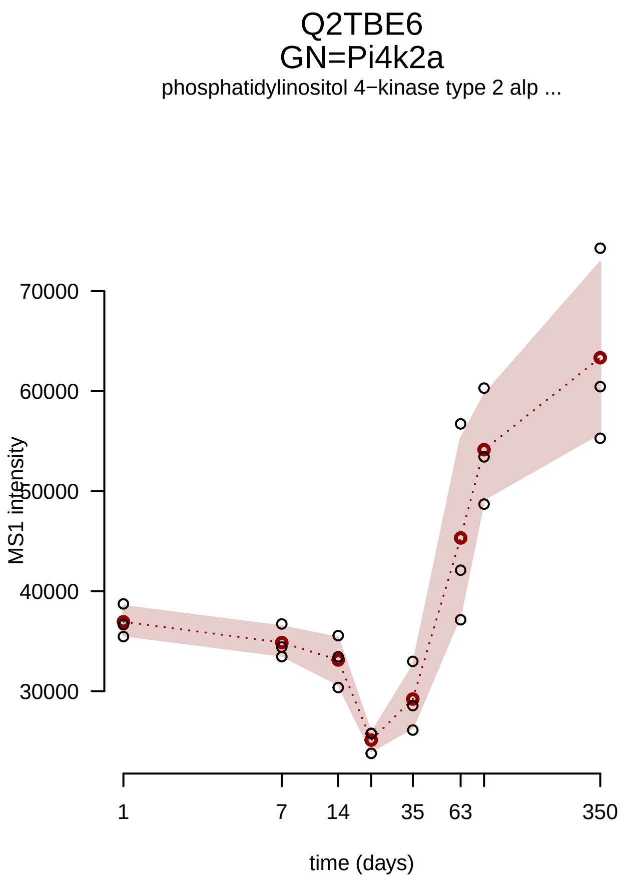
Q2TBE6
GN=Pi4k2a
phosphatidylinositol 4−kinase type 2 alp ...
MS1 intensity
70000
60000
50000
40000
30000
1
7
14
35
63
350
time (days)

Q2TL60
GN=Znf667

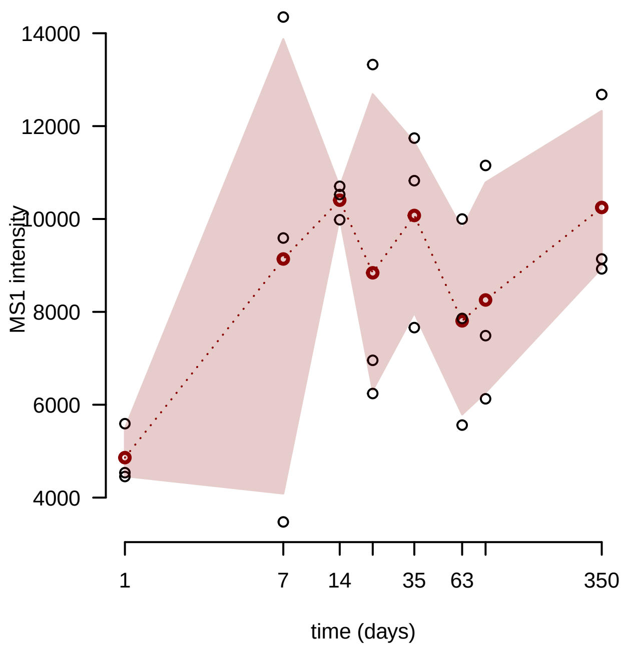

Q2TPA8
GN=Hsdl2
hydroxysteroid dehydrogenase like 2

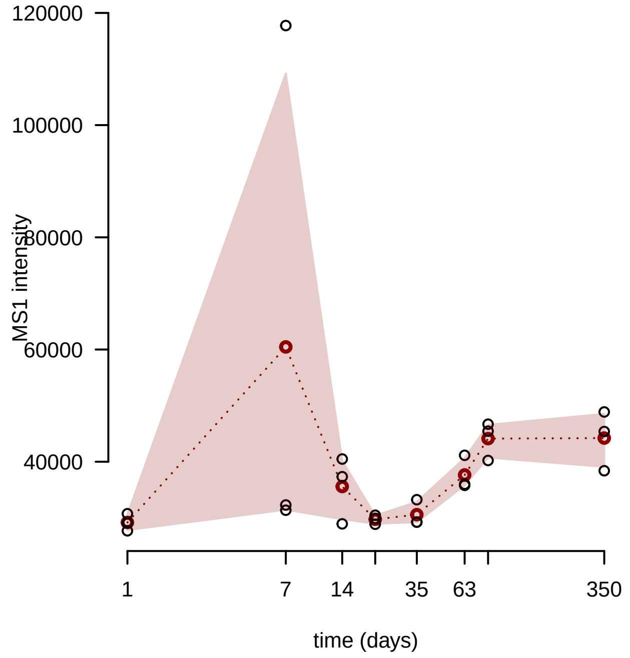

Q2VIS4
GN=Flg2
filaggrin family member 2

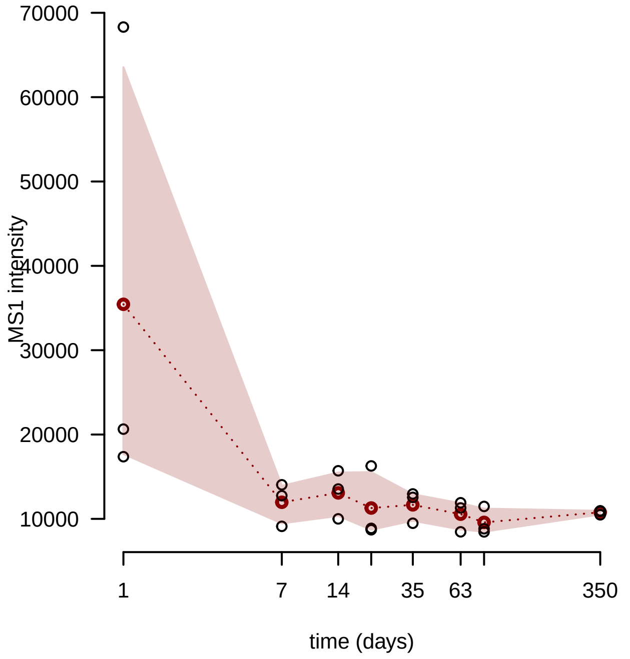

Q2VPQ9
GN=Meaf6
MYST/Esa1−associated factor 6

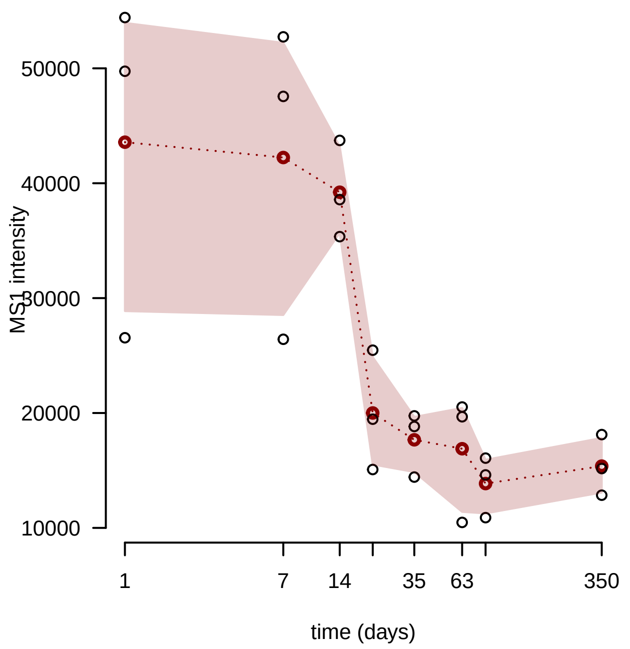

Q2VPU4
GN=Mlxip
MLX interacting protein

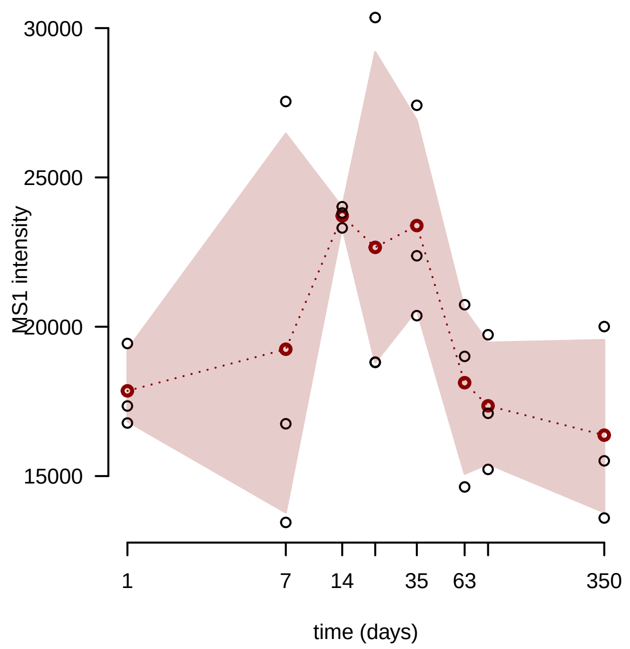

Q2YDW2
GN=Msto1
misato 1, mitochondrial distribution and ...

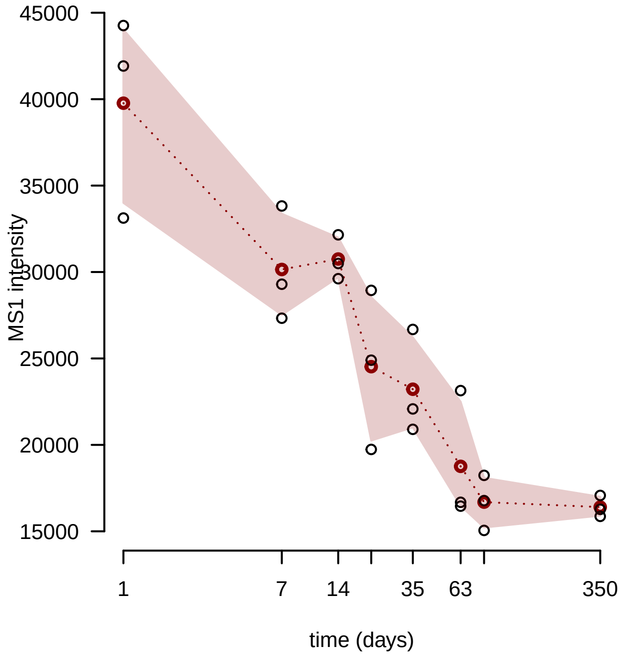

Q31125
GN=Slc39a7
solute carrier family 39 (zinc transport ...

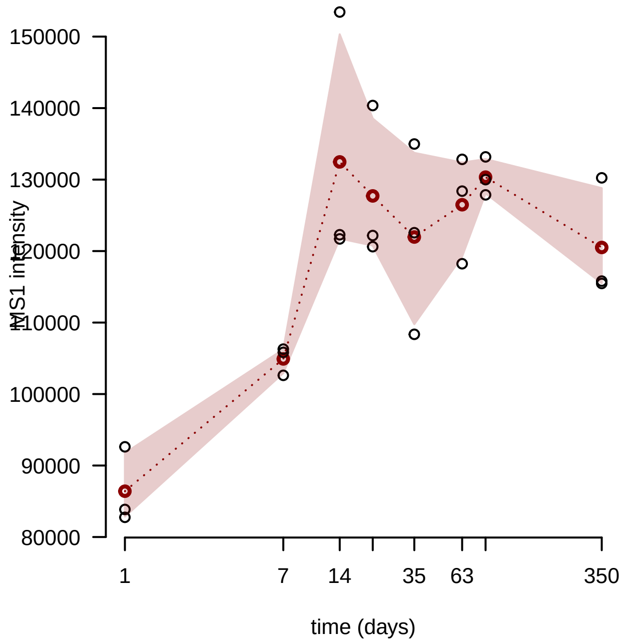

Q32NY4
GN=Cnnm3
cyclin M3

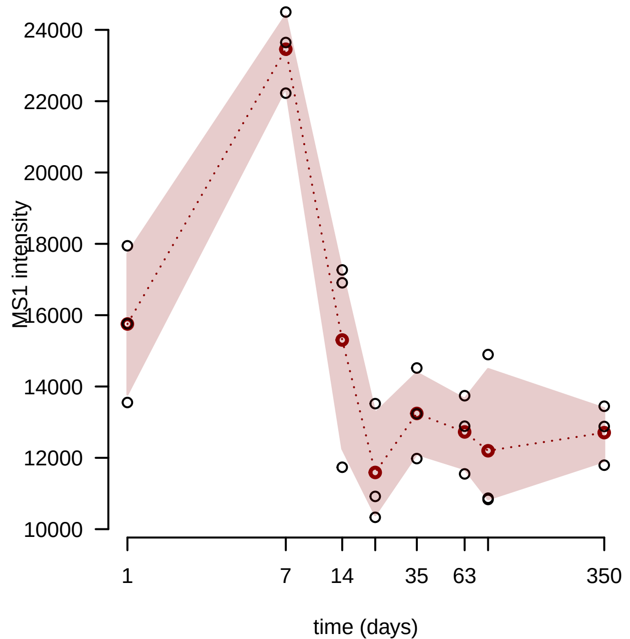

Q32Q92
GN=Acot6
acyl−CoA thioesterase 6

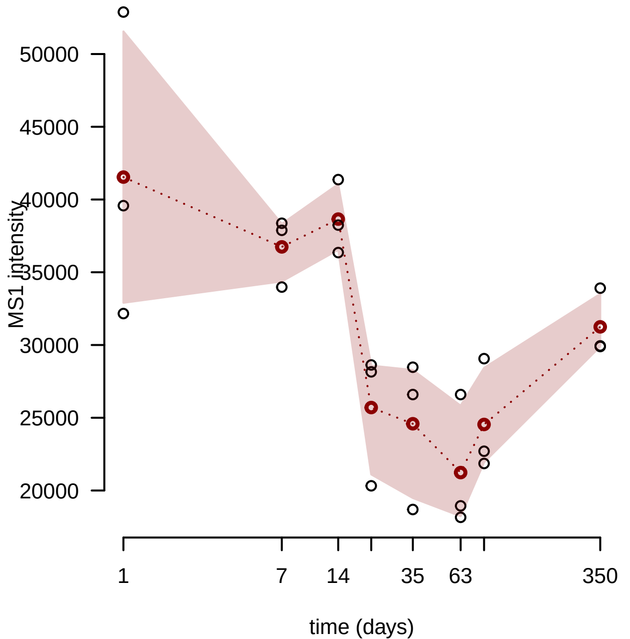

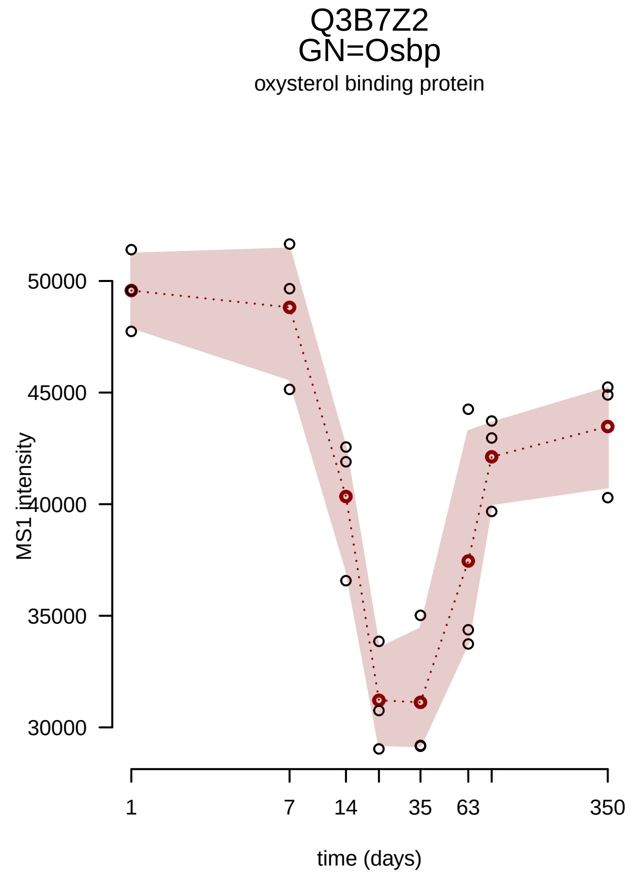
Q3B7Z2
GN=Osbp
oxysterol binding protein
MS1 intensity
50000
45000
40000
35000
30000
1
7
14
35
63
350
time (days)

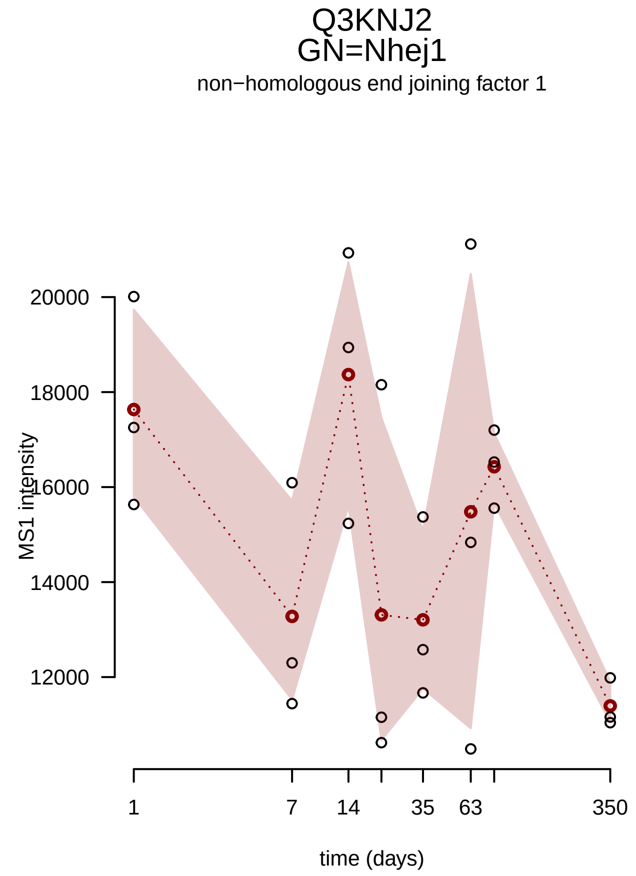
Q3KNJ2
GN=Nhej1
non−homologous end joining factor 1
MS1 intensity
20000
18000
16000
14000
12000
1
7
14
35
63
350
time (days)

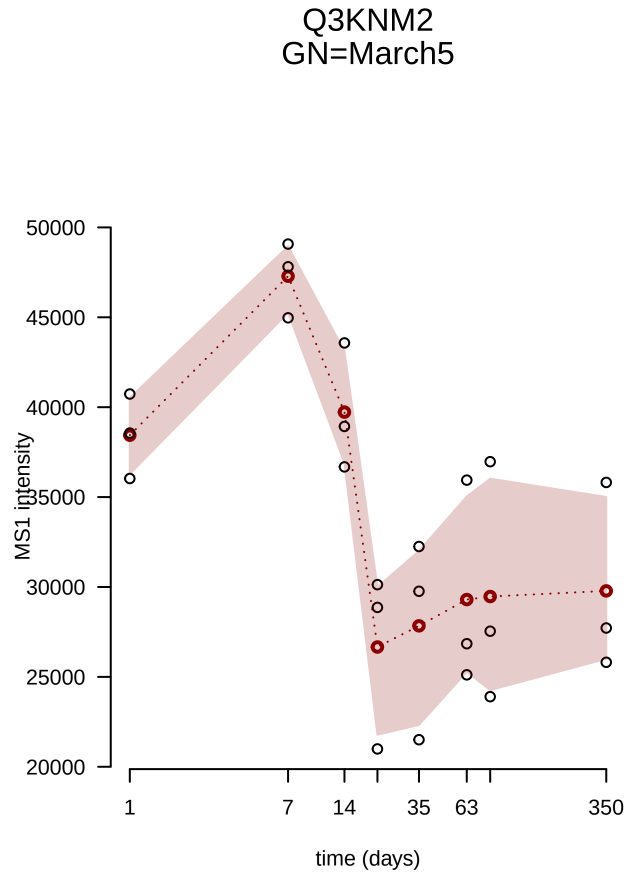
Q3KNM2
GN=March5
MS1 intensity
50000
45000
40000
35000
30000
25000
20000
1
7
14
35
63
350
time (days)

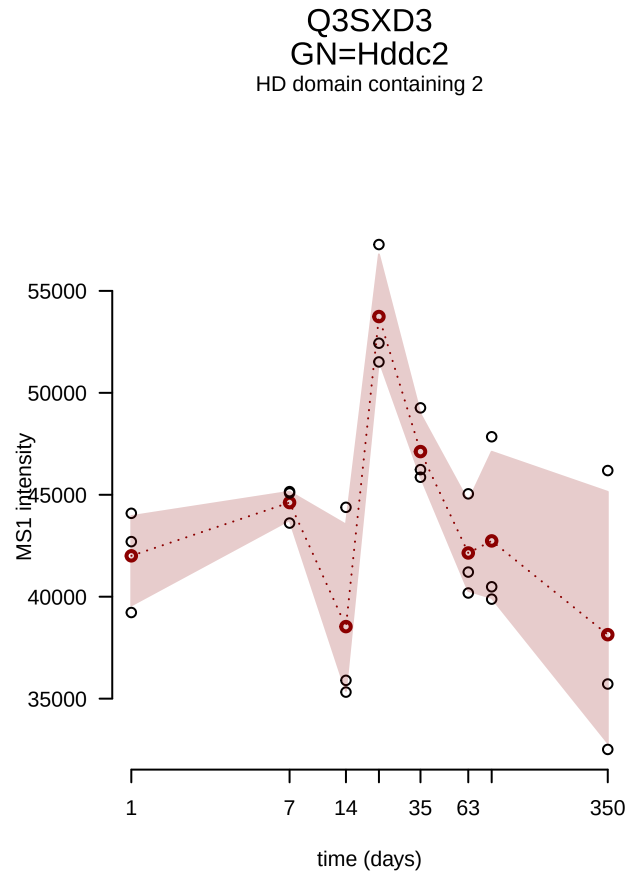
Q3SXD3
GN=Hddc2
HD domain containing 2
MS1 intensity
55000
50000
45000
40000
35000
1
7
14
35
63
350
time (days)

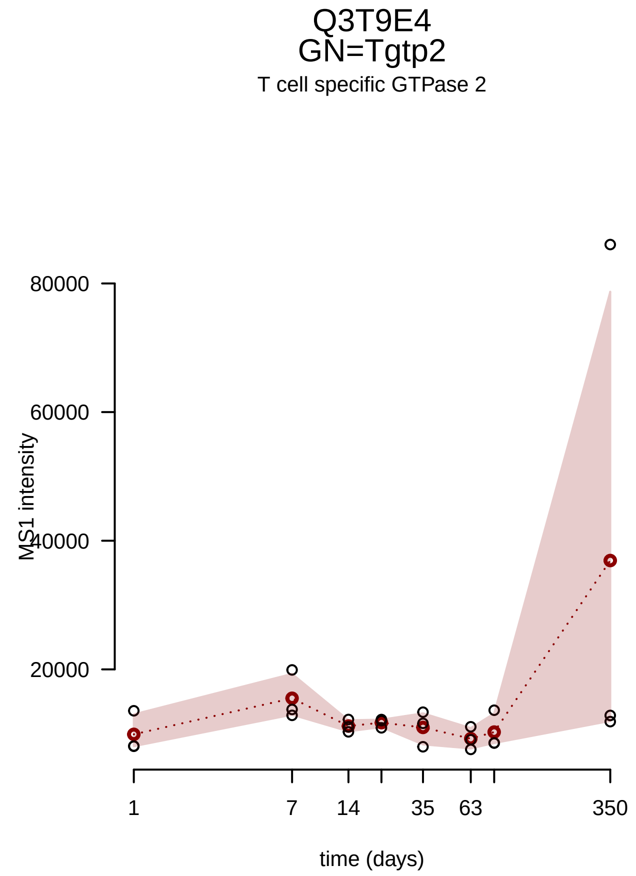
Q3T9E4
GN=Tgtp2
T cell specific GTPase 2
MS1 intensity
80000
60000
40000
20000
1
7
14
35
63
350
time (days)

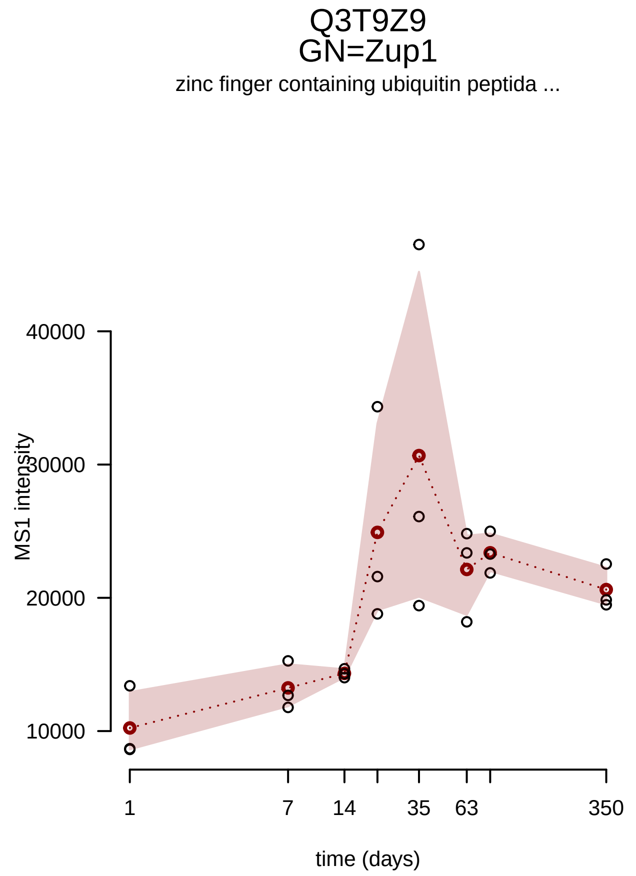
Q3T9Z9
GN=Zup1
zinc finger containing ubiquitin peptida ...
MS1 intensity
40000
30000
20000
10000
1
7
14
35
63
350
time (days)

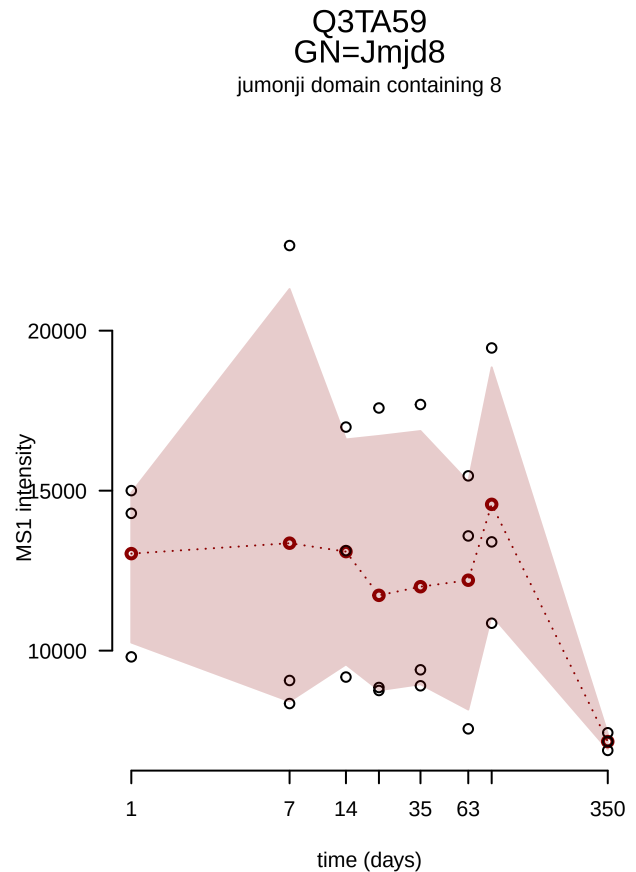
Q3TA59
GN=Jmjd8
jumonji domain containing 8
MS1 intensity
20000
15000
10000
1
7
14
35
63
350
time (days)

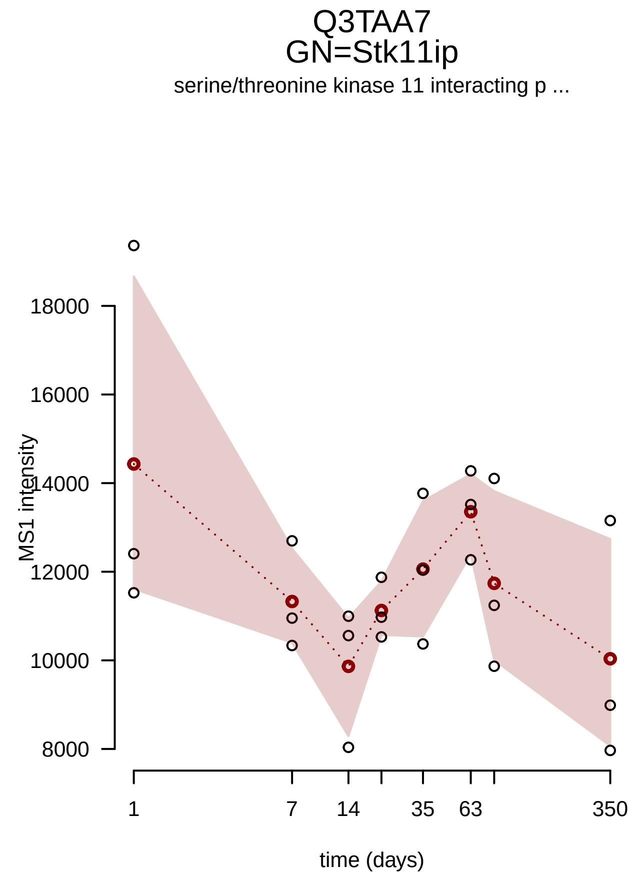
Q3TAA7
GN=Stk11ip
serine/threonine kinase 11 interacting p ...
MS1 intensity
18000
16000
14000
12000
10000
8000
1
7
14
35
63
350
time (days)

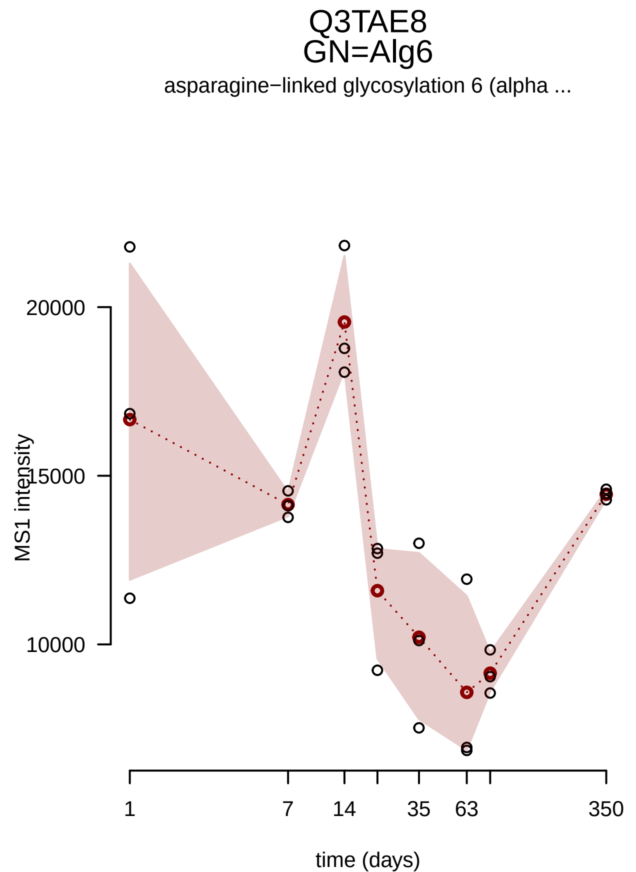
Q3TAE8
GN=Alg6
asparagine−linked glycosylation 6 (alpha ...
MS1 intensity
20000
15000
10000
1
7
14
35
63
350
time (days)

Q3TAP4
GN=Ap5b1
adaptor−related protein complex 5, beta ...

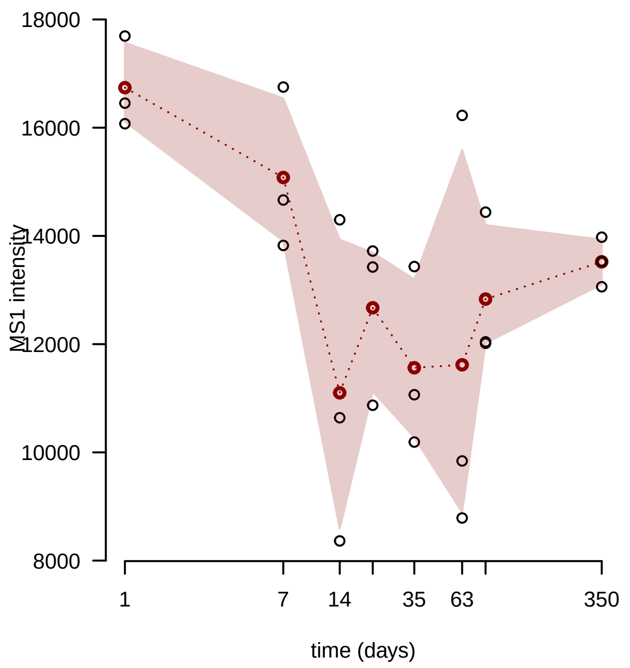

Q3TAS6
GN=Emc10
ER membrane protein complex subunit 10

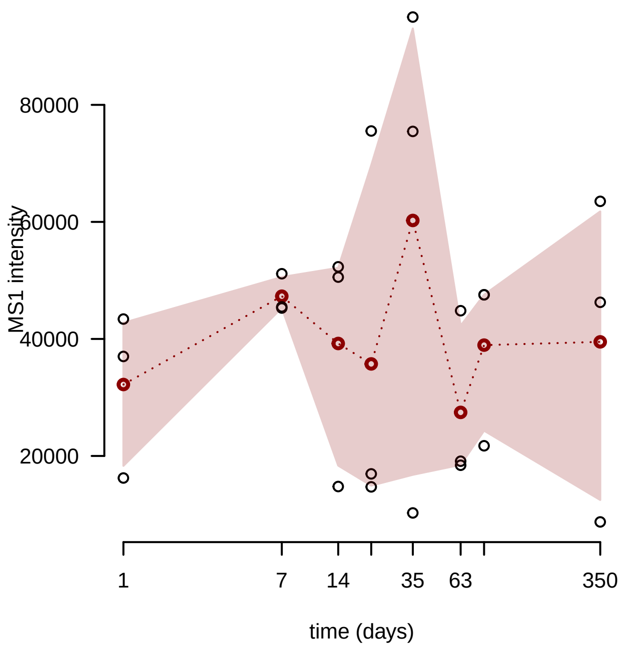

Q3TB48
GN=Tmem104
transmembrane protein 104

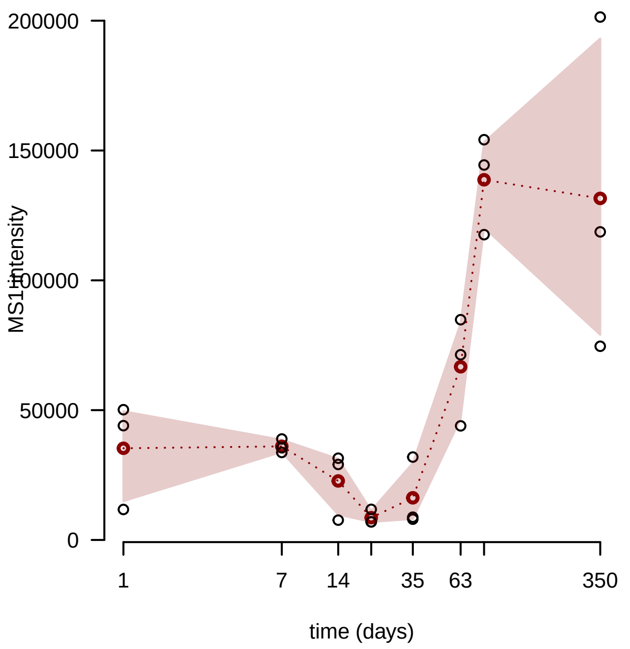

Q3TB82
GN=Plekhf1
pleckstrin homology domain containing, f ...

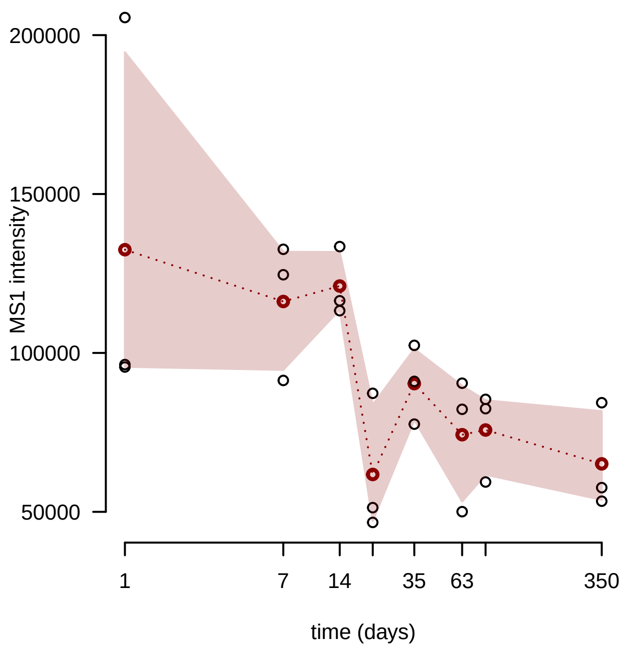

Q3TBD2
GN=Arhgap45
Rho GTPase activating protein 45

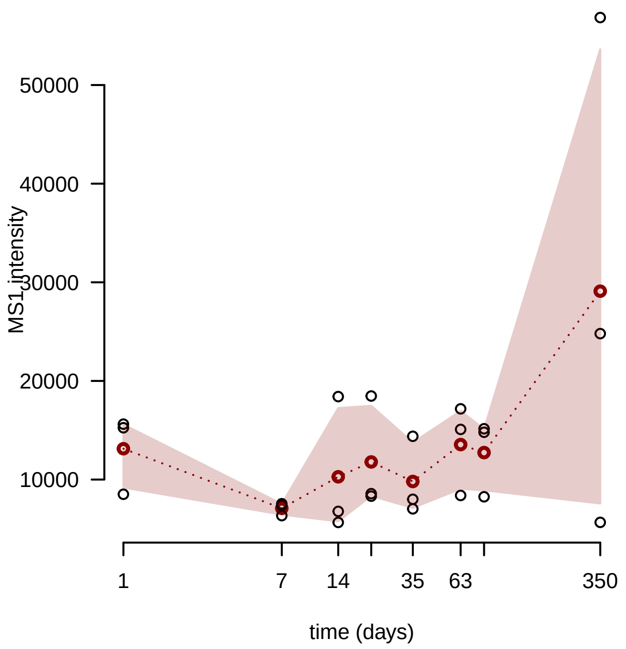

Q3TBT3
GN=Tmem173

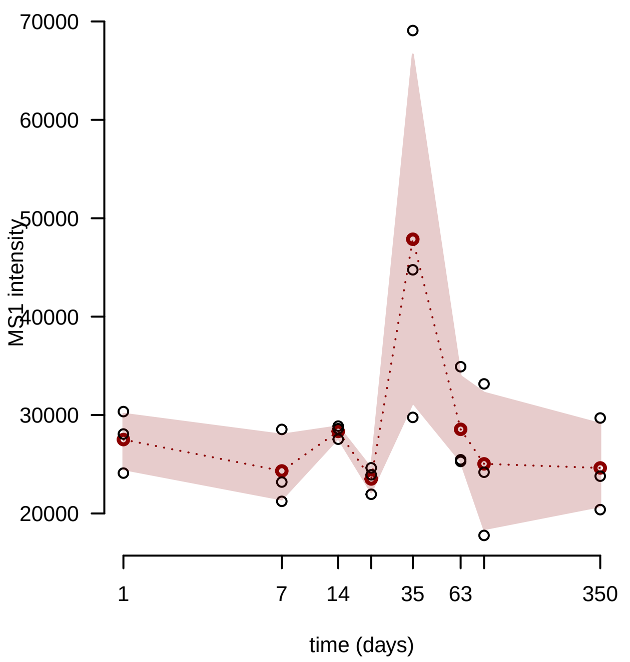

Q3TBW2
GN=Mrpl10
mitochondrial ribosomal protein L10

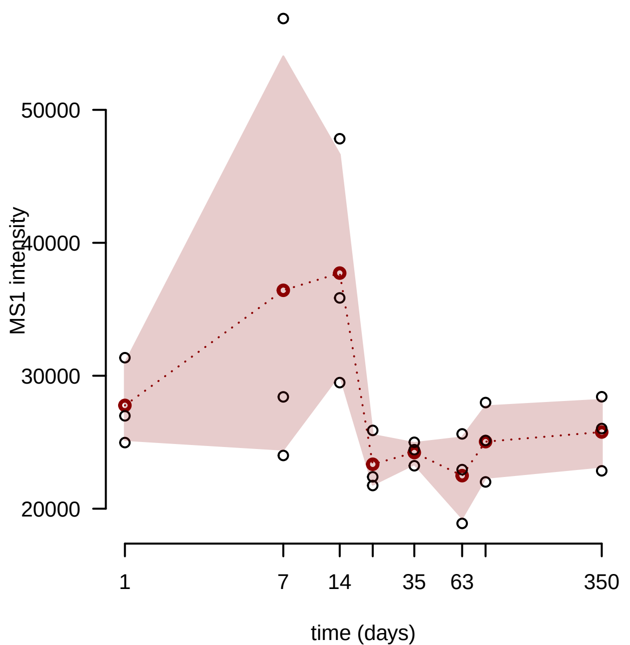

Q3TC33
GN=Ccdc127
coiled−coil domain containing 127

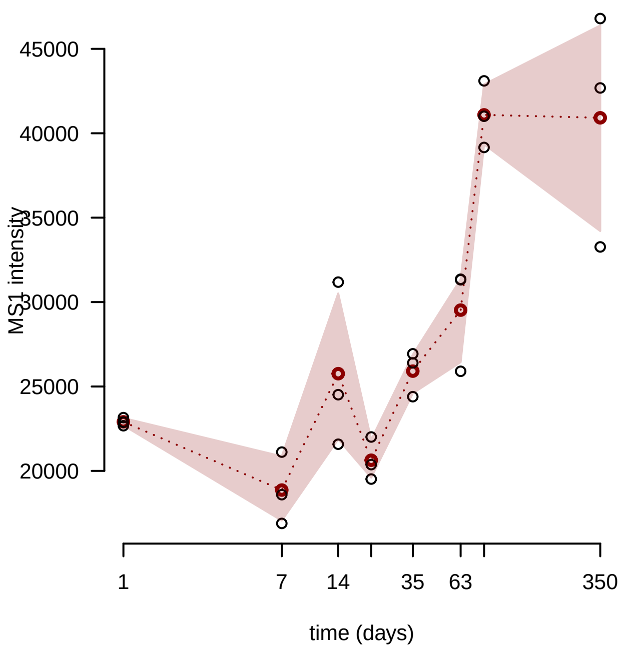

Q3TC46
GN=Patl1
protein associated with topoisomerase II ...

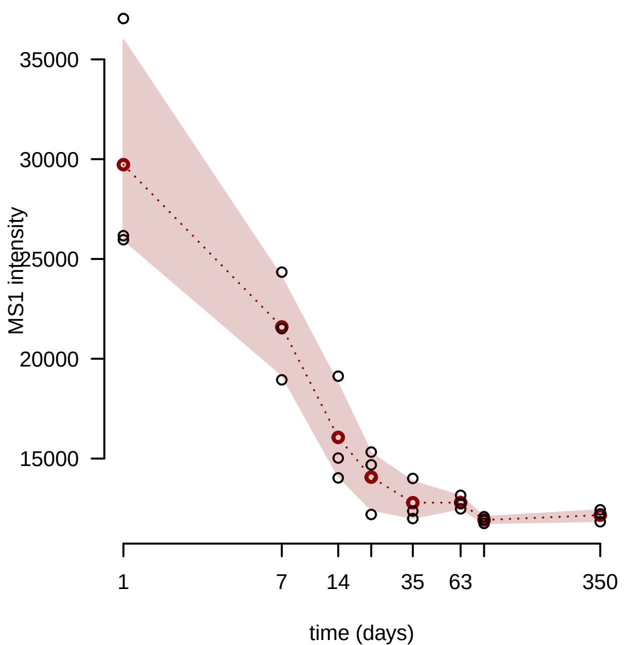

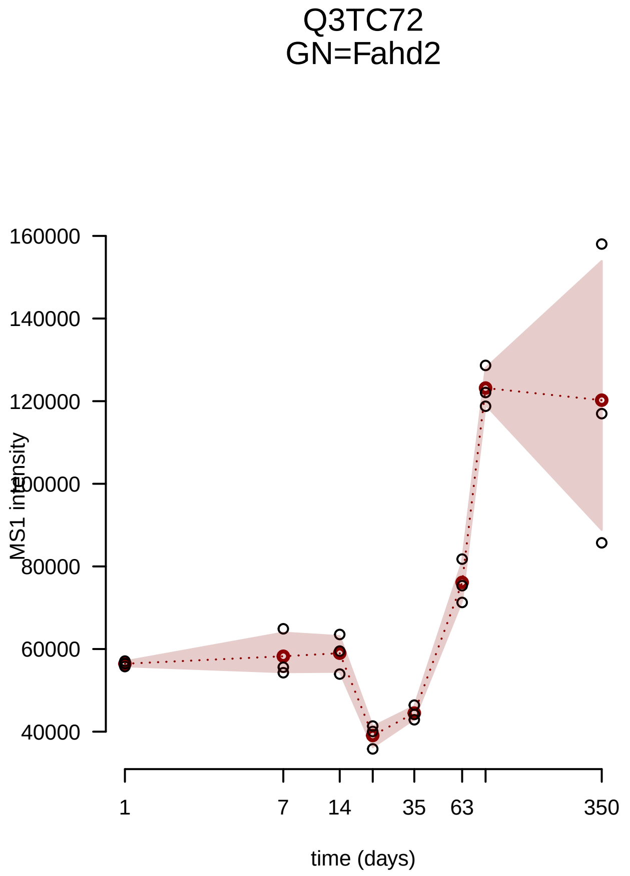
Q3TC72
GN=Fahd2
MS1 intensity
160000
140000
120000
100000
80000
60000
40000
1
7
14
35
63
350
time (days)

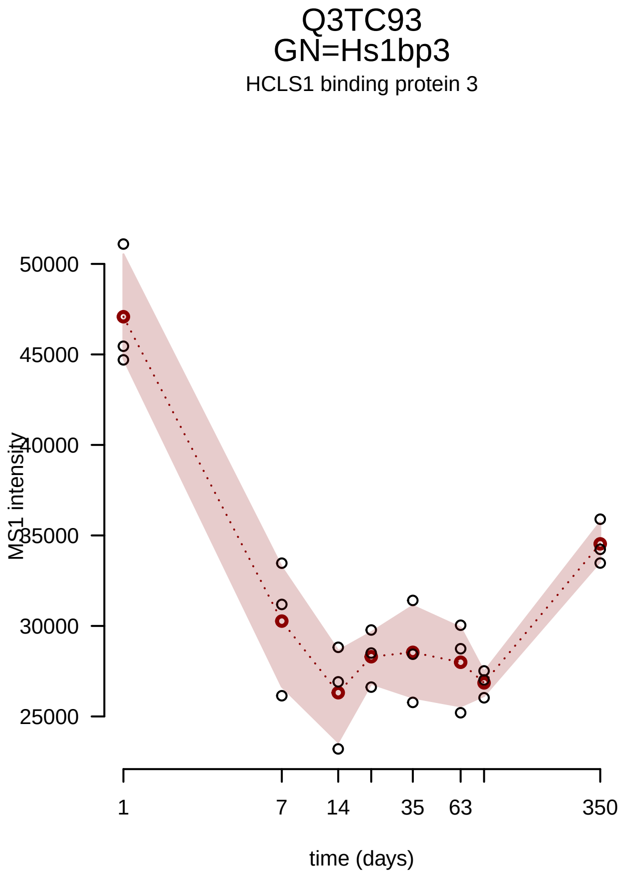
Q3TC93
GN=Hs1bp3
HCLS1 binding protein 3
MS1 intensity
50000
45000
40000
35000
30000
25000
1
7
14
35
63
350
time (days)

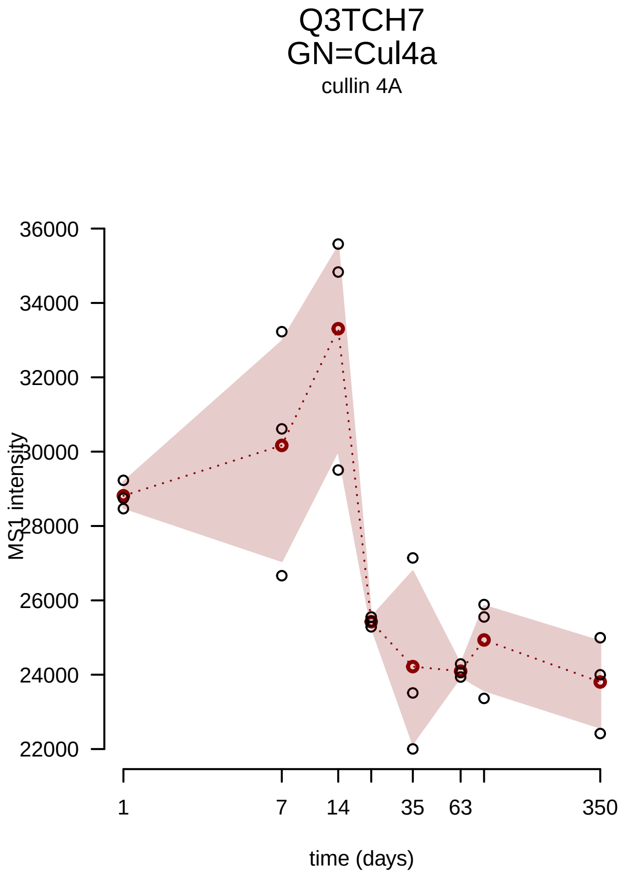
Q3TCH7
GN=Cul4a
cullin 4A
MS1 intensity
36000
34000
32000
30000
28000
26000
24000
22000
1
7
14
35
63
350
time (days)

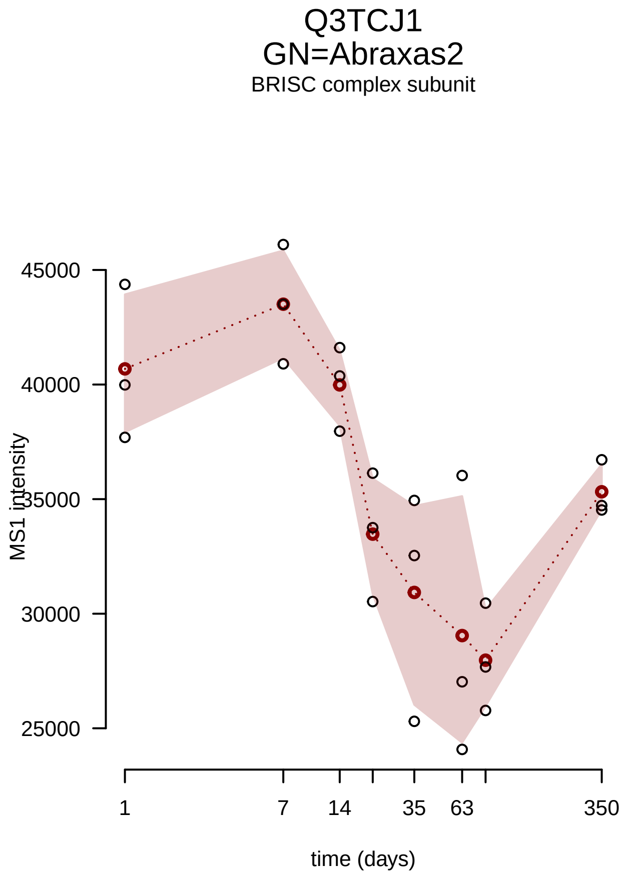
Q3TCJ1
GN=Abraxas2
BRISC complex subunit
MS1 intensity
45000
40000
35000
30000
25000
1
7
14
35
63
350
time (days)

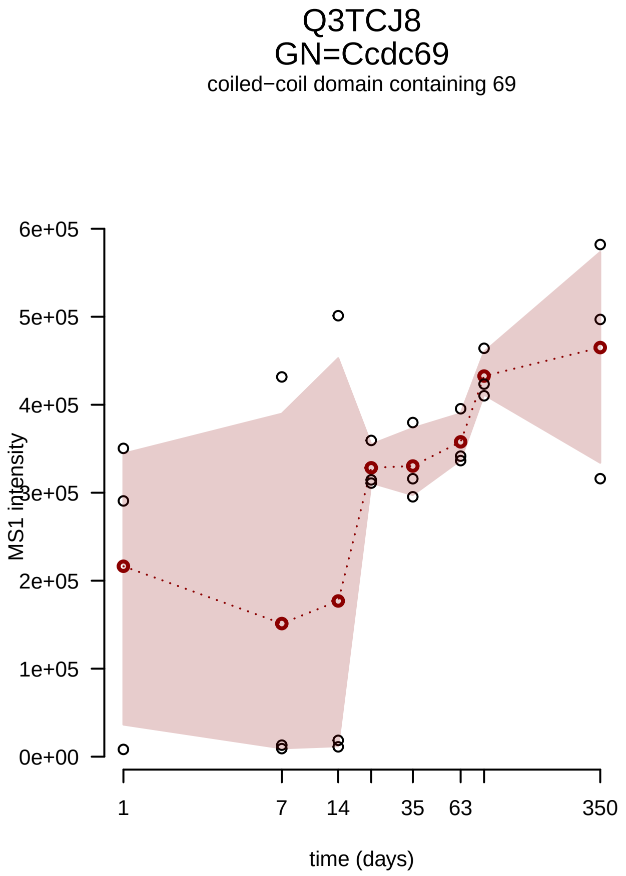
Q3TCJ8
GN=Ccdc69
coiled−coil domain containing 69
MS1 intensity
6e+05
5e+05
4e+05
3e+05
2e+05
1e+05
0e+00
1
7
14
35
63
350
time (days)

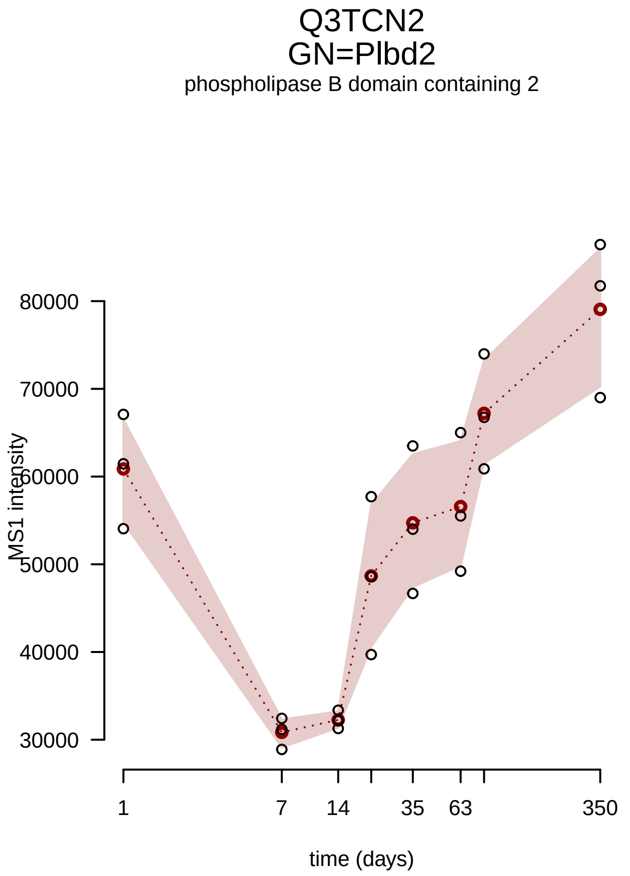
Q3TCN2
GN=Plbd2
phospholipase B domain containing 2
MS1 intensity
80000
70000
60000
50000
40000
30000
1
7
14
35
63
350
time (days)

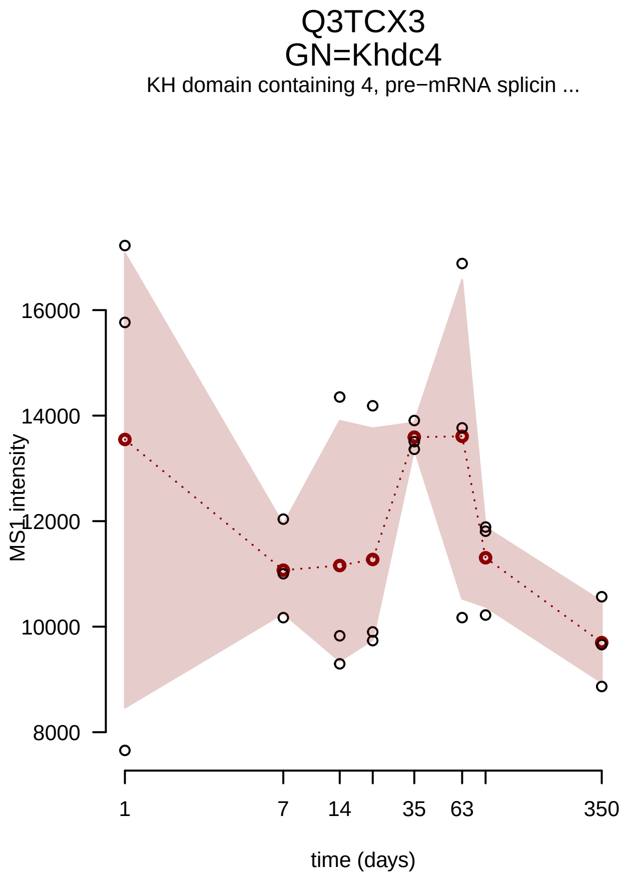
Q3TCX3
GN=Khdc4
KH domain containing 4, pre−mRNA splicin ...
MS1 intensity
16000
14000
12000
10000
8000
1
7
14
35
63
350
time (days)

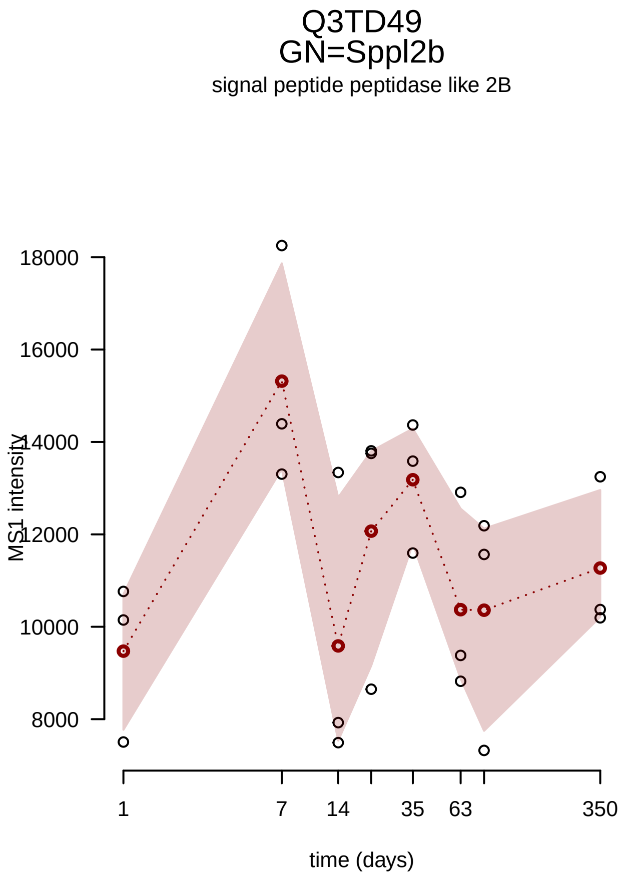
Q3TD49
GN=Sppl2b
signal peptide peptidase like 2B
MS1 intensity
18000
16000
14000
12000
10000
8000
1
7
14
35
63
350
time (days)

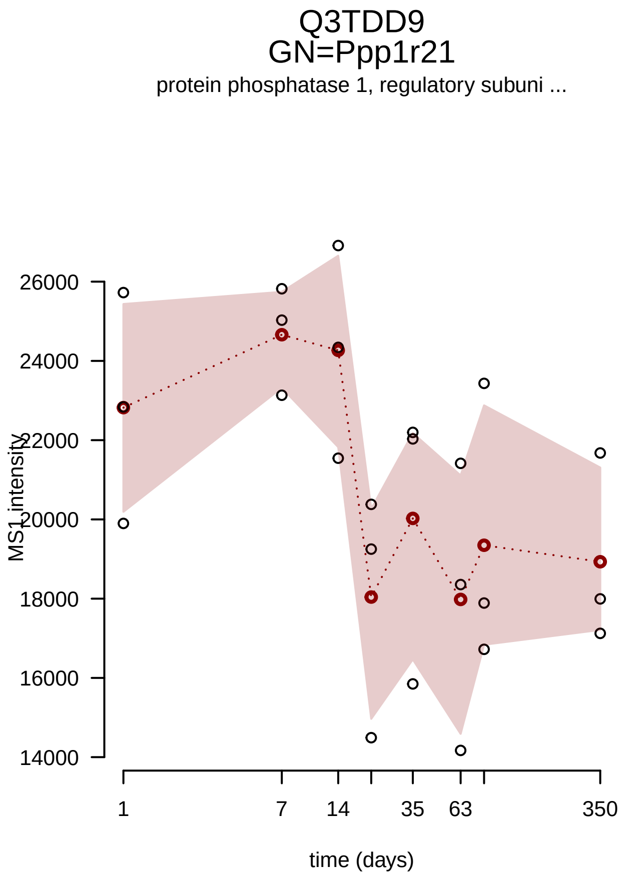
Q3TDD9
GN=Ppp1r21
protein phosphatase 1, regulatory subuni ...
MS1 intensity
26000
24000
22000
20000
18000
16000
14000
1
7
14
35
63
350
time (days)

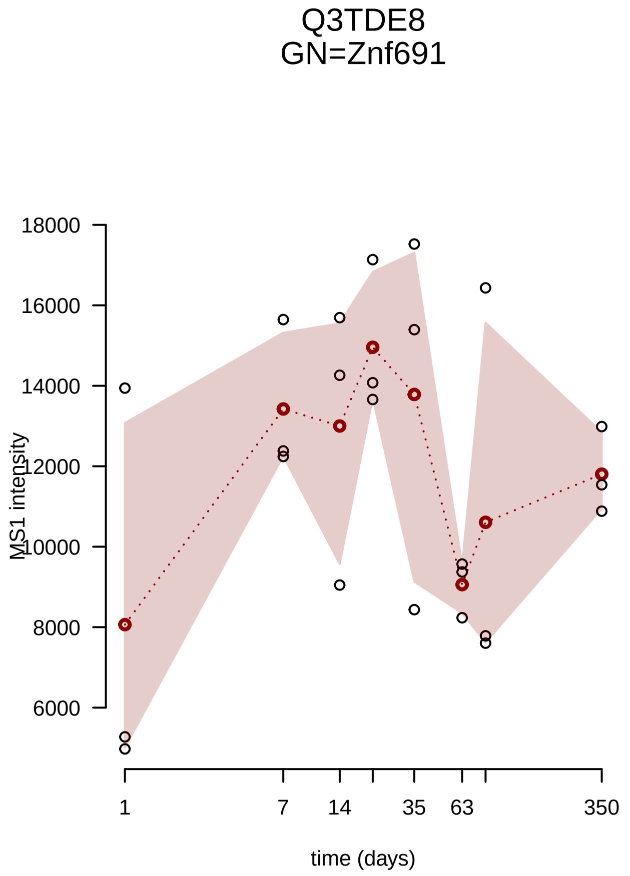
Q3TDE8
GN=Znf691
MS1 intensity
6000
8000
10000
12000
14000
16000
18000
time (days)
1
7
14
35
63
350

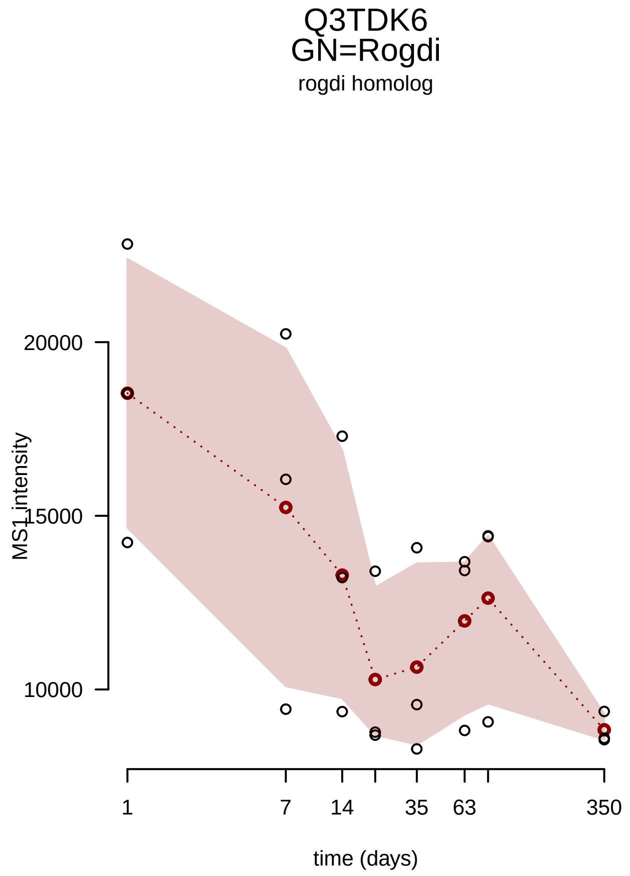
Q3TDK6
GN=Rogdi
rogdi homolog
MS1 intensity
10000
15000
20000
time (days)
1
7
14
35
63
350

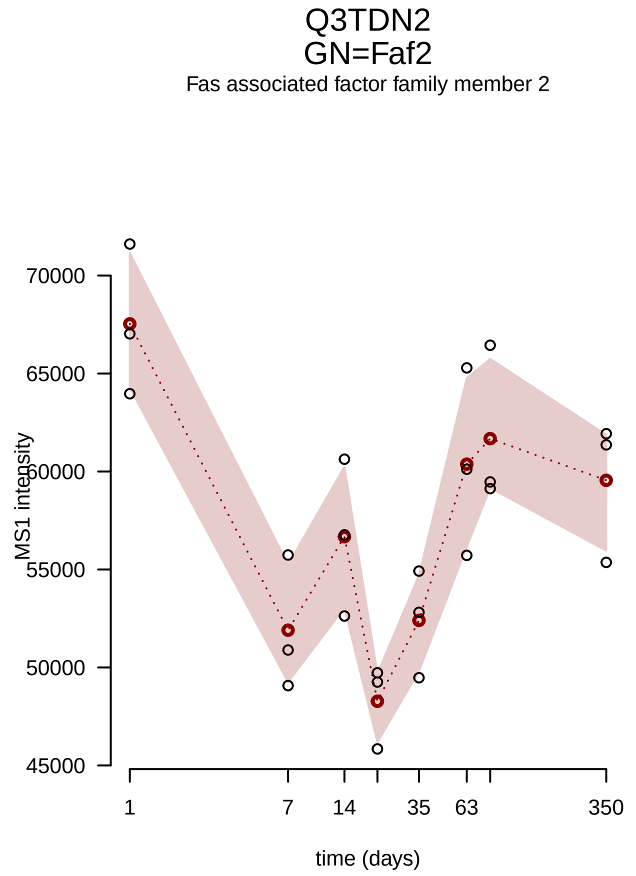
Q3TDN2
GN=Faf2
Fas associated factor family member 2
MS1 intensity
45000
50000
55000
60000
65000
70000
time (days)
1
7
14
35
63
350

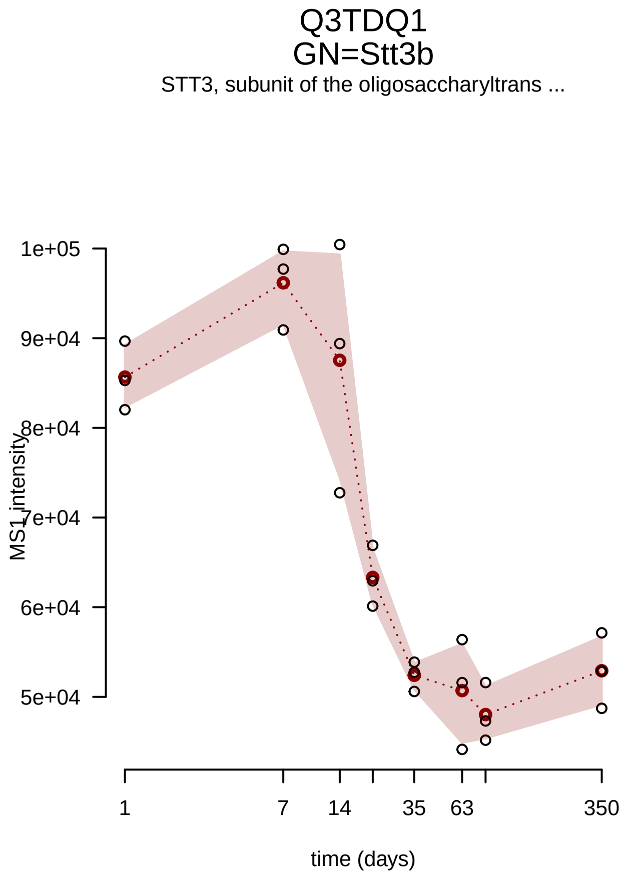
Q3TDQ1
GN=Stt3b
STT3, subunit of the oligosaccharyltrans ...
MS1 intensity
5e+04
6e+04
7e+04
8e+04
9e+04
1e+05
time (days)
1
7
14
35
63
350

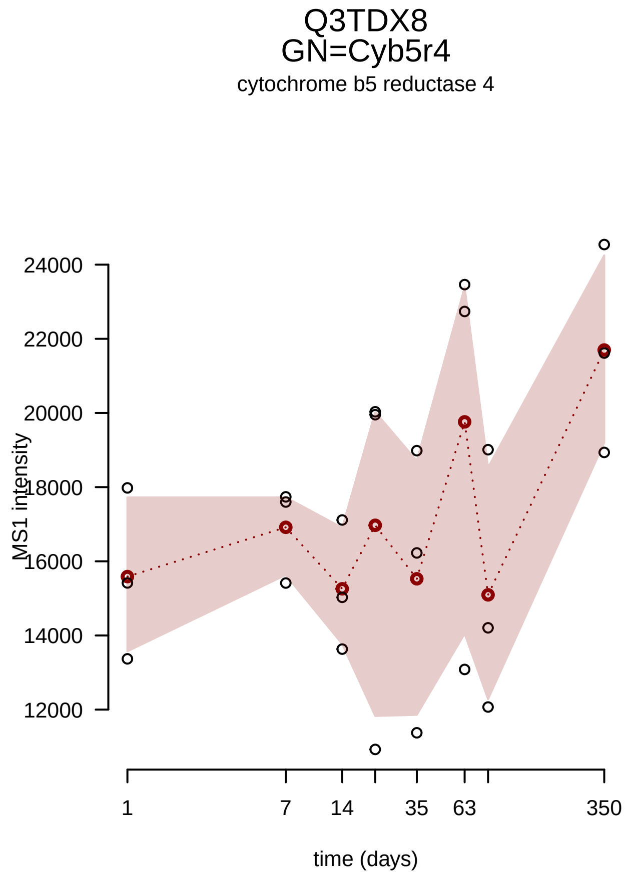
Q3TDX8
GN=Cyb5r4
cytochrome b5 reductase 4
MS1 intensity
12000
14000
16000
18000
20000
22000
24000
time (days)
1
7
14
35
63
350

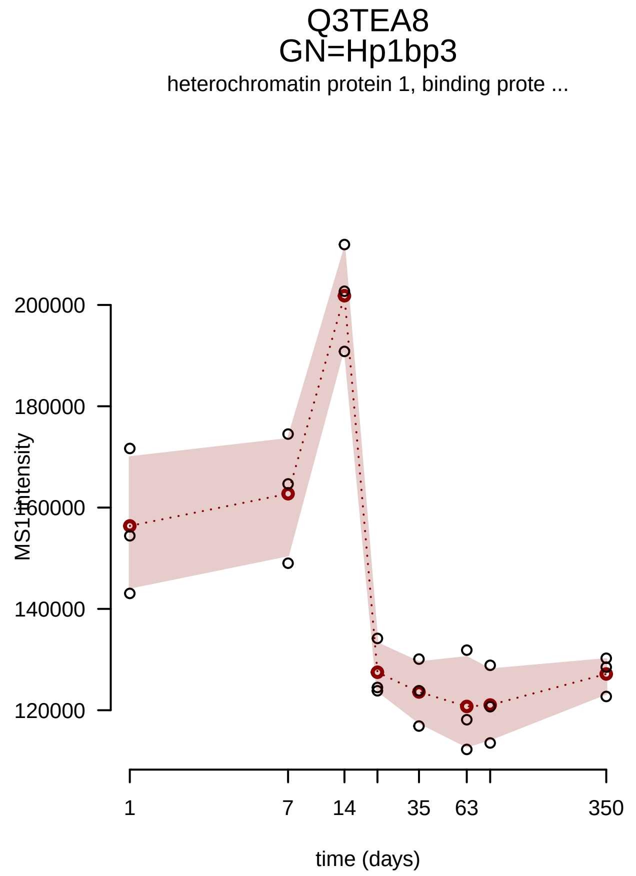
Q3TEA8
GN=Hp1bp3
heterochromatin protein 1, binding prote ...
MS1 intensity
120000
140000
160000
180000
200000
time (days)
1
7
14
35
63
350

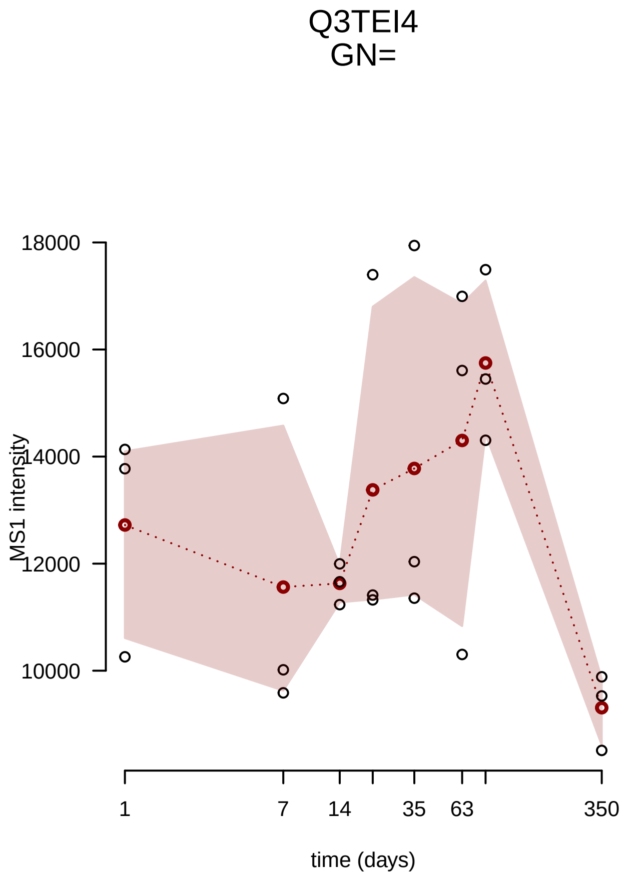
Q3TEI4
GN=
MS1 intensity
10000
12000
14000
16000
18000
time (days)
1
7
14
35
63
350

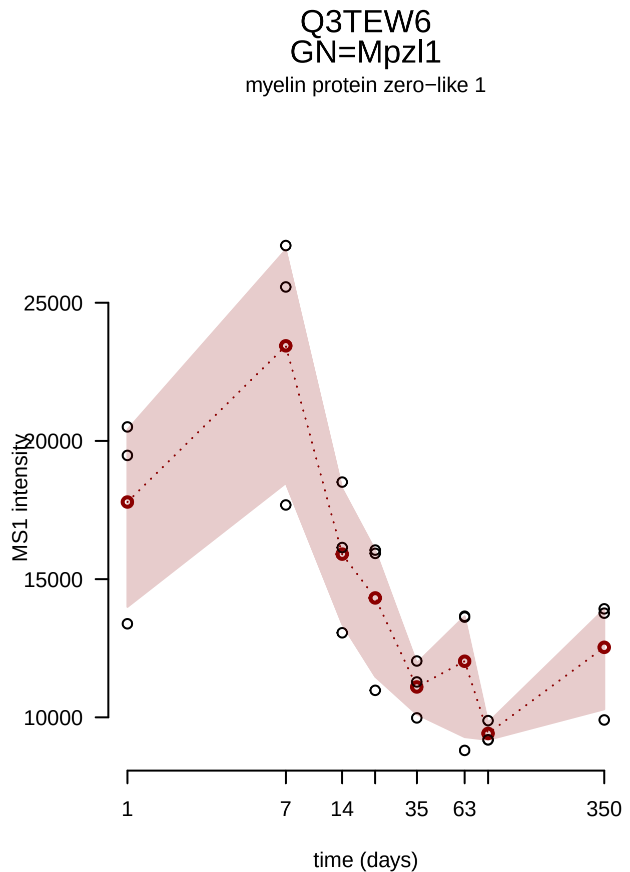
Q3TEW6
GN=Mpzl1
myelin protein zero−like 1
MS1 intensity
10000
15000
20000
25000
time (days)
1
7
14
35
63
350

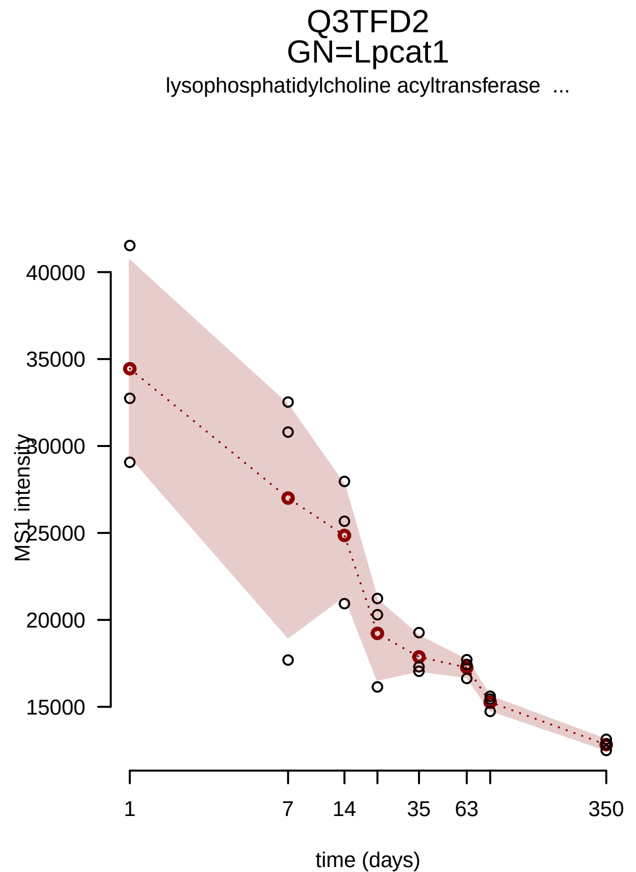
Q3TFD2
GN=Lpcat1
lysophosphatidylcholine acyltransferase ...
MS1 intensity
15000
20000
25000
30000
35000
40000
time (days)
1
7
14
35
63
350

Q3TFQ1
GN=Spryd7
SPRY domain containing 7

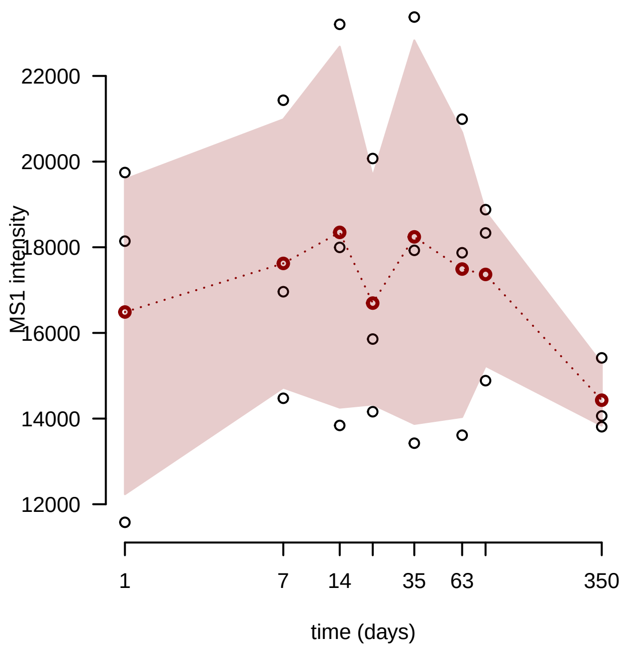

Q3TGF2
GN=Fam107b
family with sequence similarity 107, mem ...

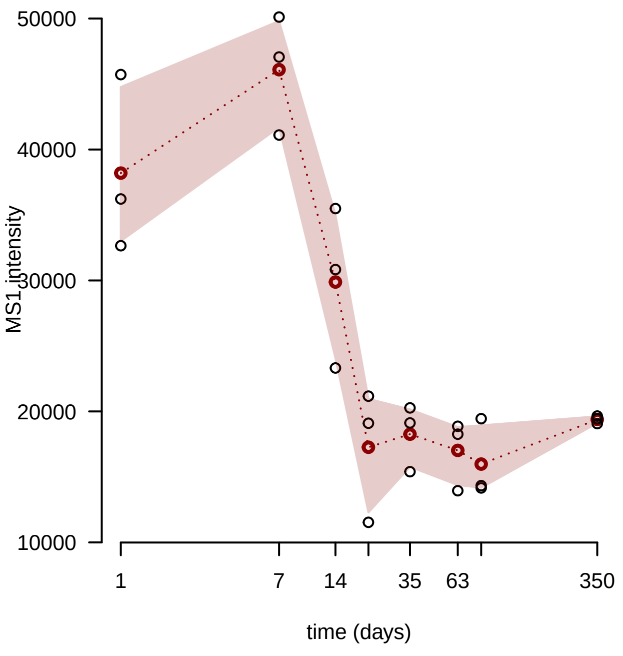

Q3THE2
GN=Myl12b
myosin, light chain 12B, regulatory

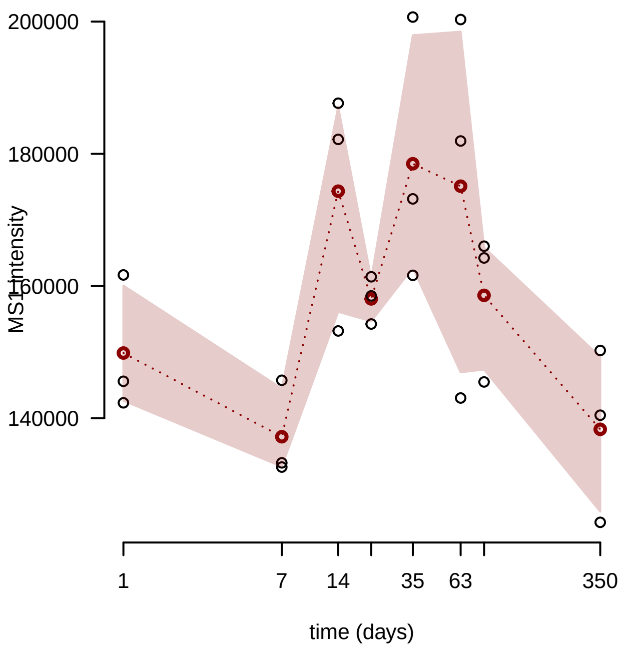

Q3THF9
GN=Coq10b
coenzyme Q10B

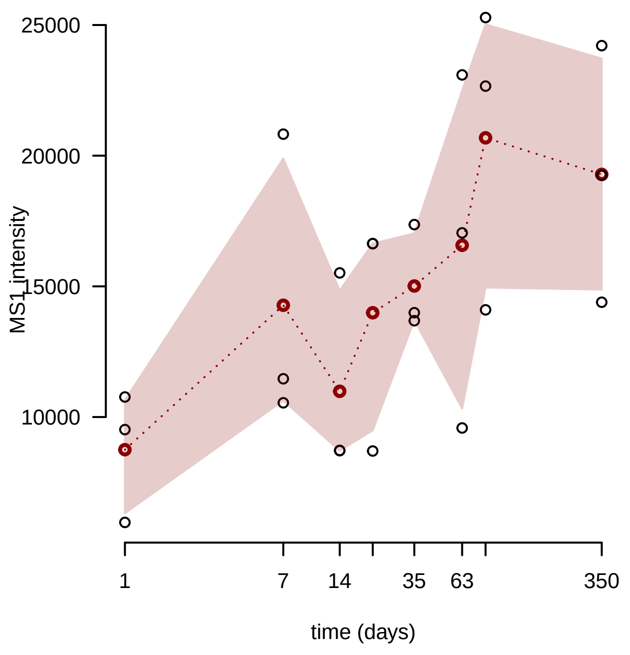

Q3THG9
GN=Aarsd1
alanyl−tRNA synthetase domain containing ...

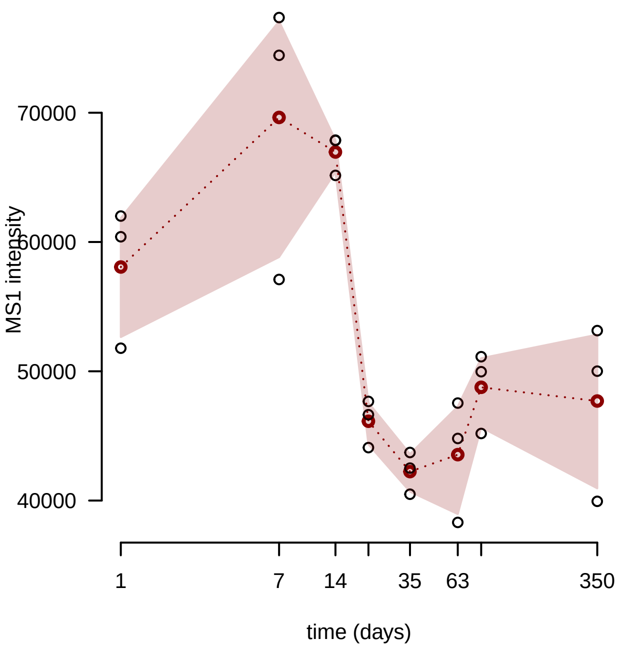

Q3THJ3
GN=Eif1ad
eukaryotic translation initiation factor ...

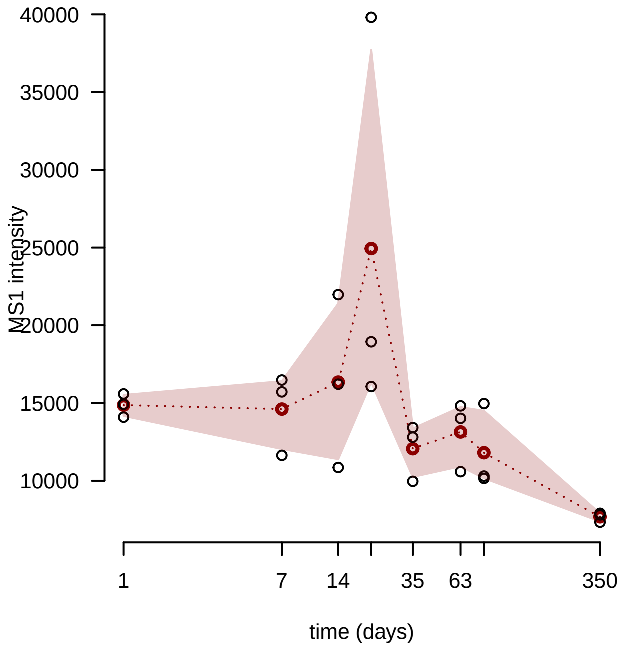

Q3THK3
GN=Gtf2f1
general transcription factor IIF, polype ...

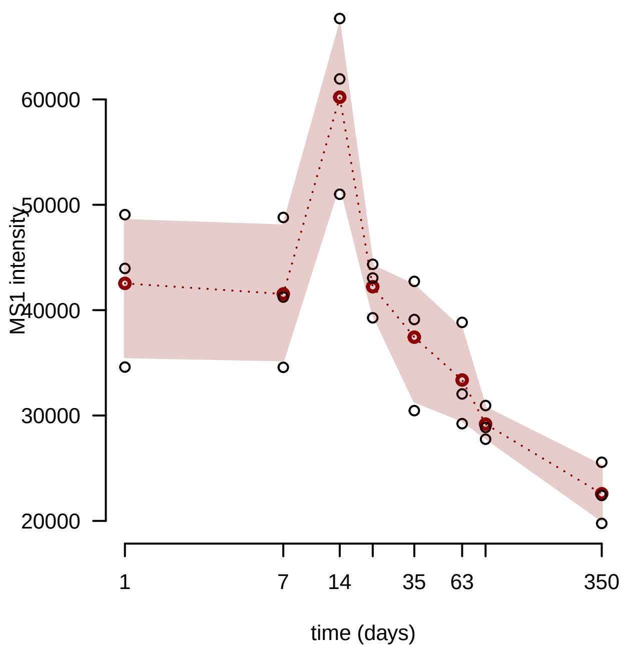

Q3THK7
GN=Gmps
guanine monophosphate synthetase

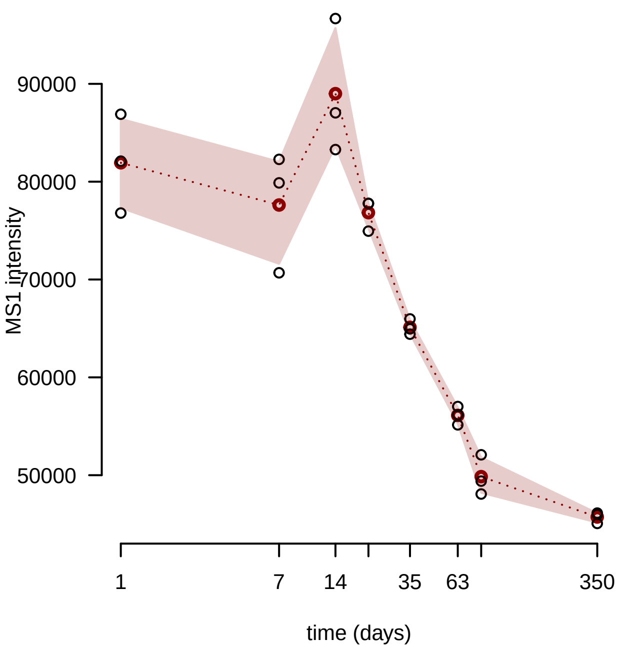

Q3THS6
GN=Mat2a
methionine adenosyltransferase II, alpha

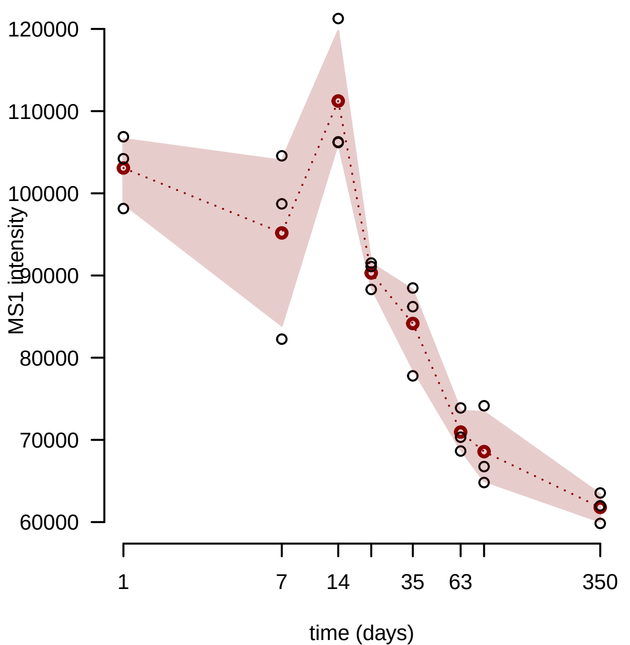

Q3TIR1
GN=Trappc13
trafficking protein particle complex 13

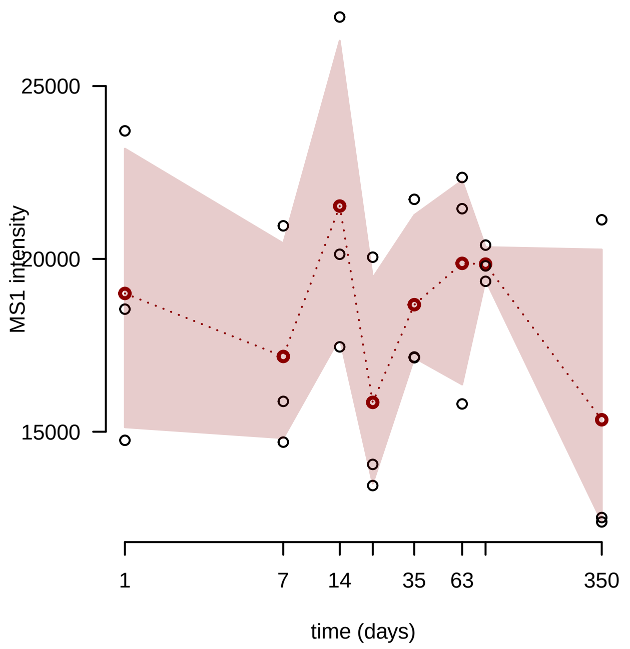

Q3TIR3
GN=Ric8a
RIC8 guanine nucleotide exchange factor ...

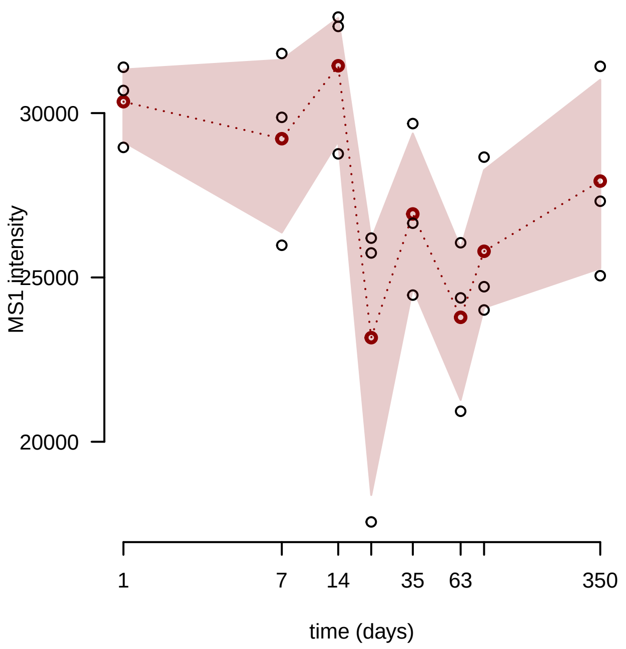

Q3TIT8
GN=Slc37a3
solute carrier family 37 (glycerol−3−pho ...

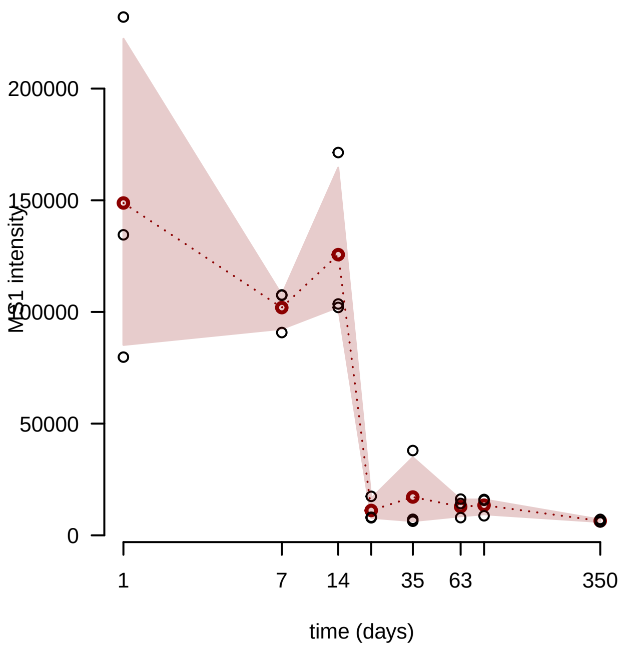

Q3TIU4
GN=Pde12
phosphodiesterase 12

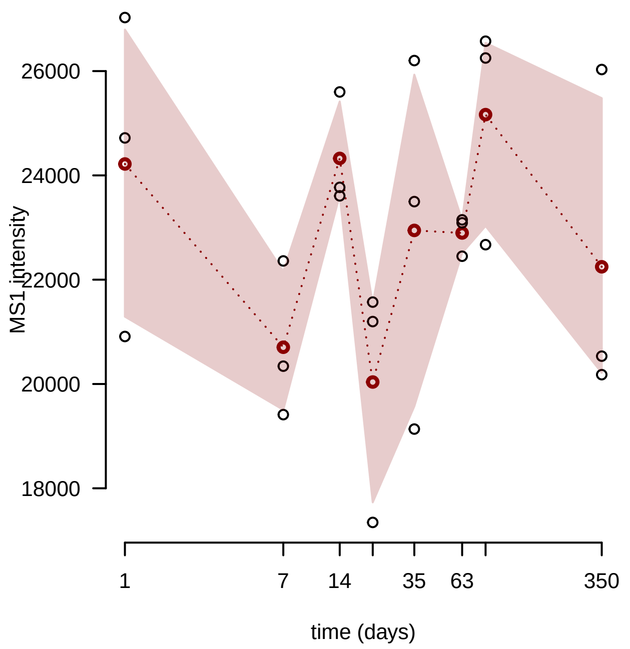

Q3TIV5
GN=Zc3h15
zinc finger CCCH−type containing 15

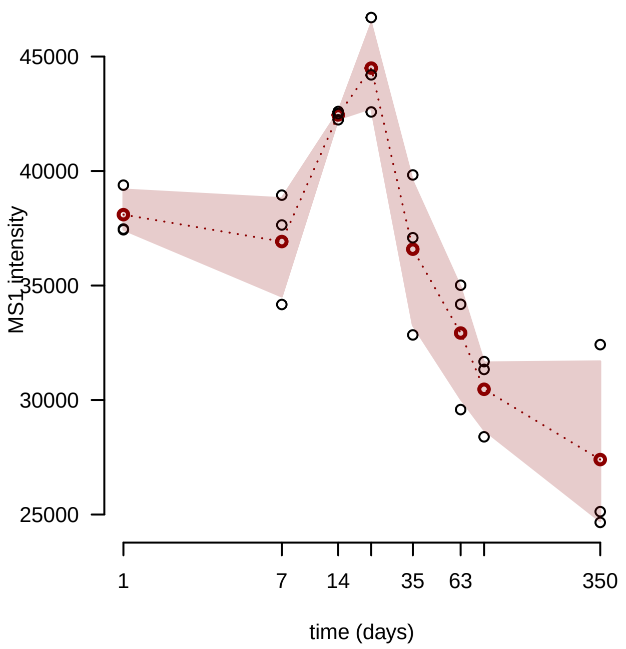

Q3TIX9
GN=Usp39
ubiquitin specific peptidase 39

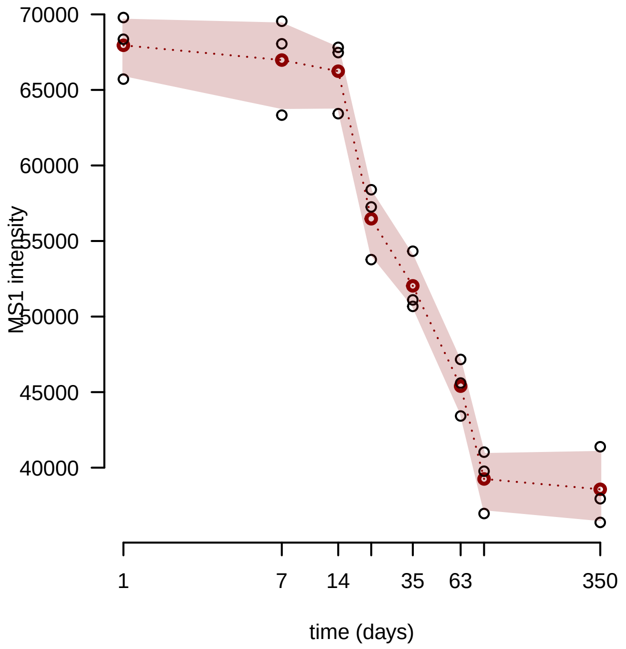

Q3TJD7
GN=Pdlim7
PDZ and LIM domain 7

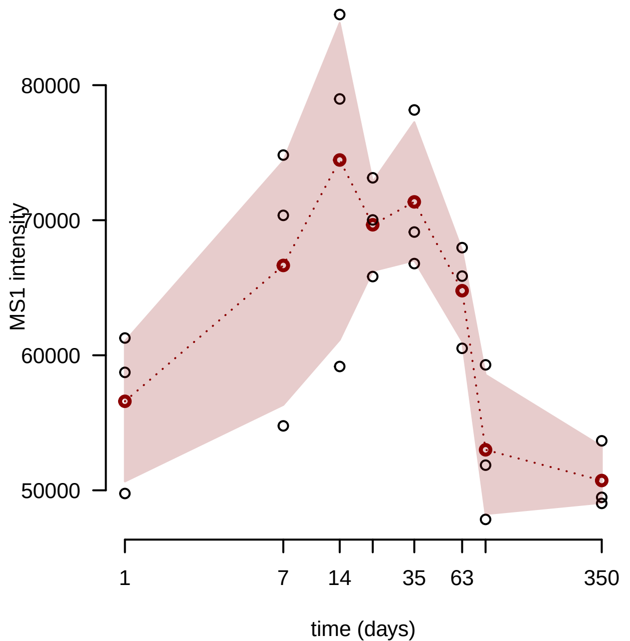

Q3TJM4
GN=Cenpt
centromere protein T

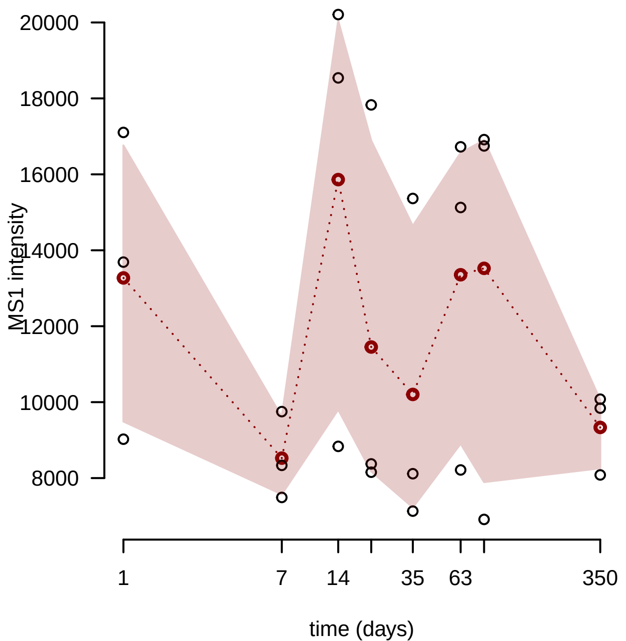

Q3TJZ6
GN=Fam98a
family with sequence similarity 98, memb ...

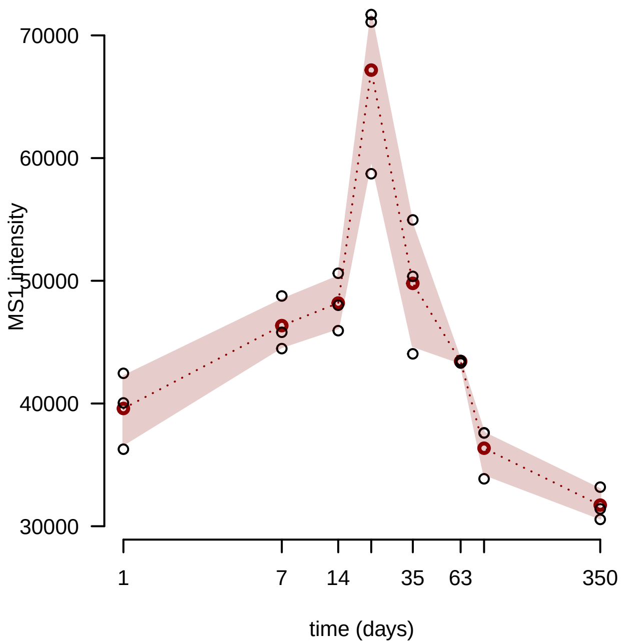

Q3TKR3
GN=Nlrp4c
NLR family, pyrin domain containing 4C

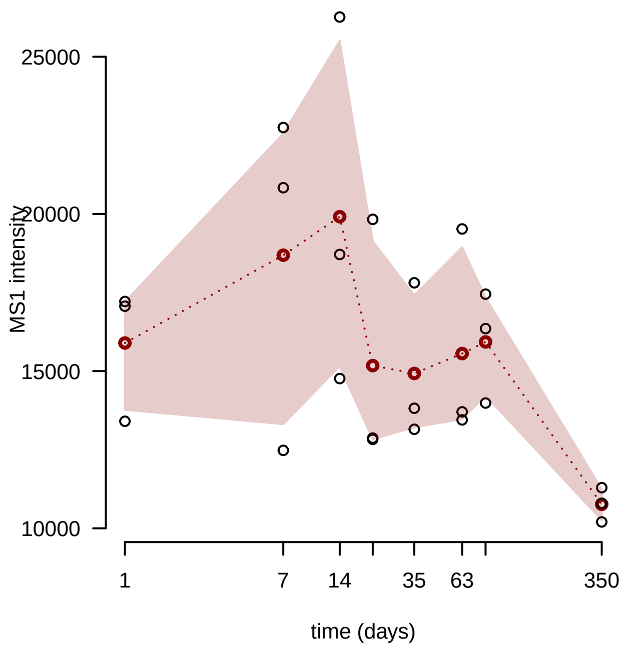

Q3TKT4
GN=Smarca4
SWI/SNF related, matrix associated, acti ...

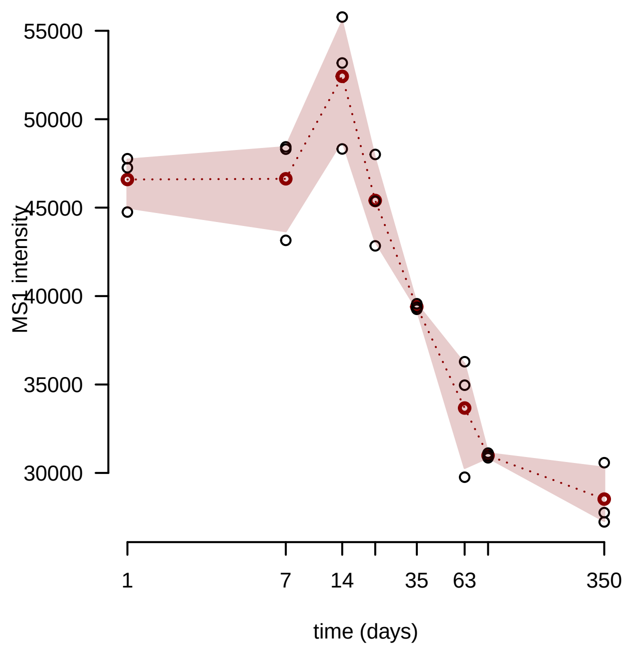

Q3TKY6
GN=Cwc27
CWC27 spliceosome−associated protein

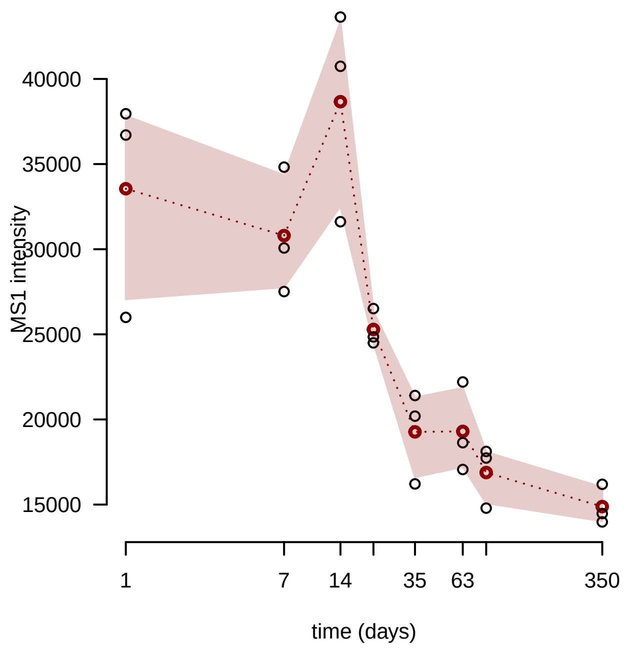

Q3TL26
GN=Tfb2m
transcription factor B2, mitochondrial

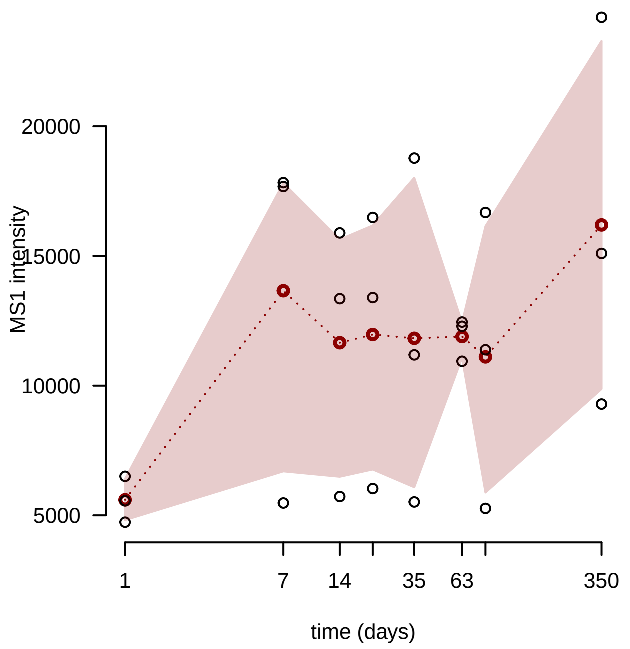

Q3TL44
GN=Nlrx1
NLR family member X1

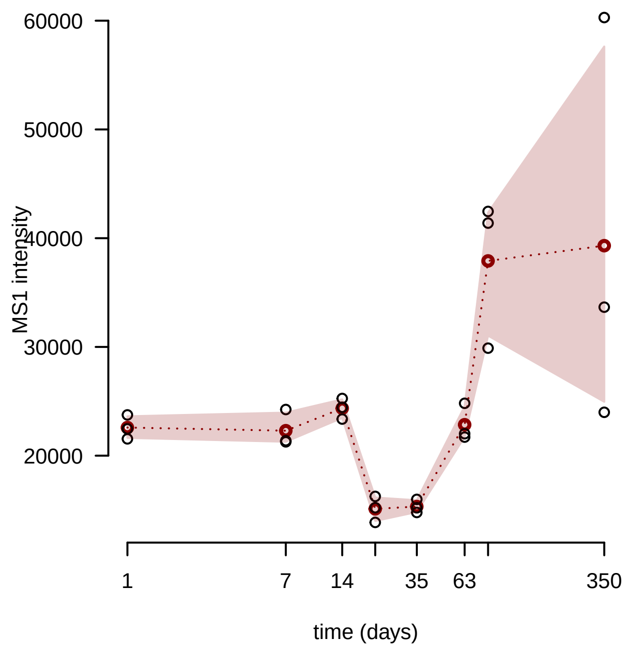

Q3TLD5
GN=Uri1
URI1, prefoldin−like chaperone

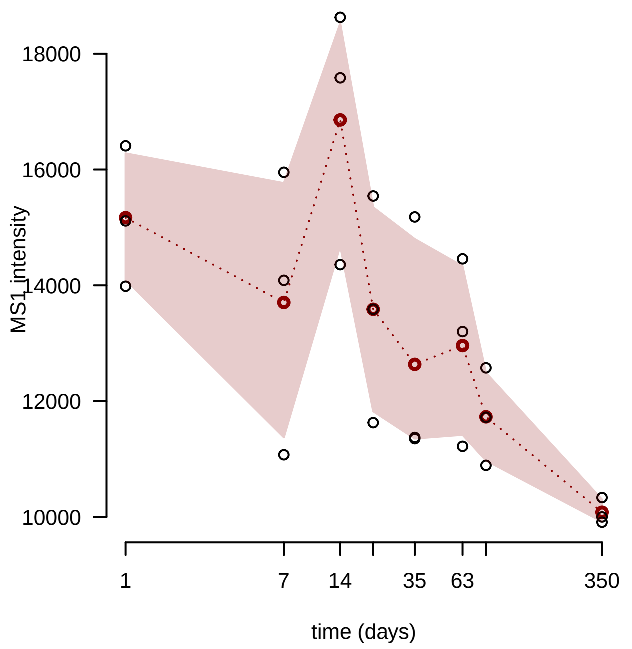

Q3TLH4
GN=Prrc2c
proline−rich coiled−coil 2C

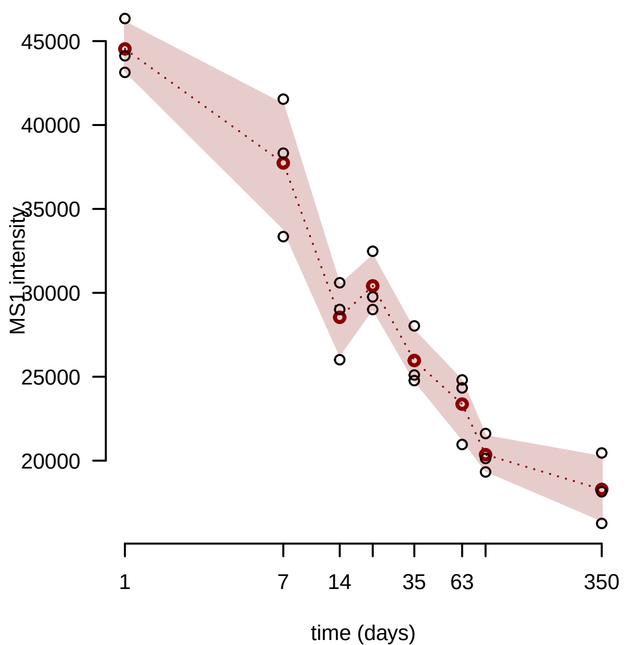

Q3TLI0
GN=Trappc10
trafficking protein particle complex 10

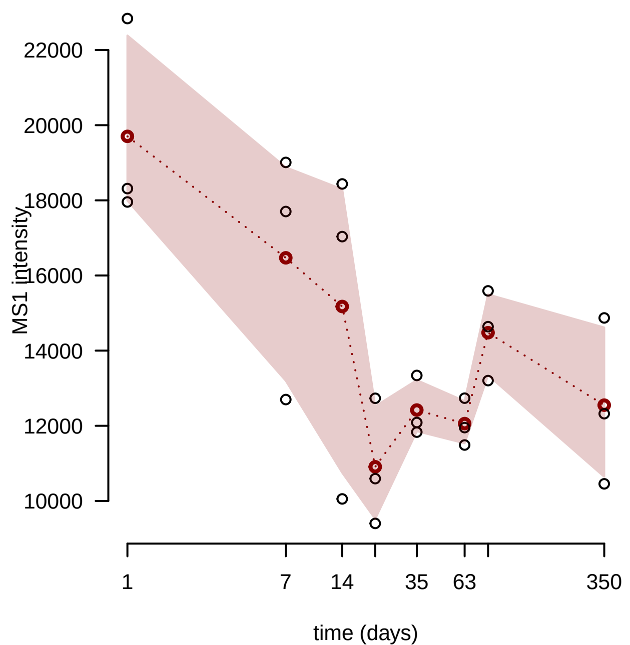

Q3TLP5
GN=Echdc2
enoyl Coenzyme A hydratase domain contai ...

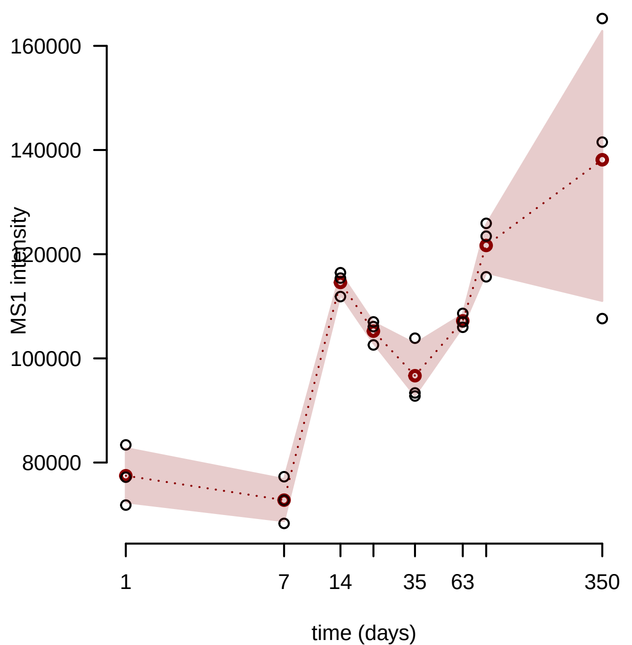

Q3TLS3
GN=Gdpgp1
GDP−D−glucose phosphorylase 1

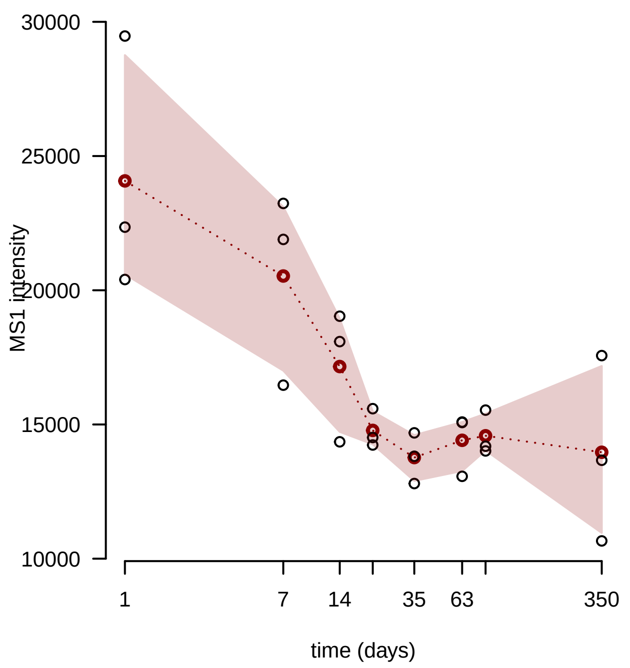

Q3TMH2
GN=Scrn3
secernin 3

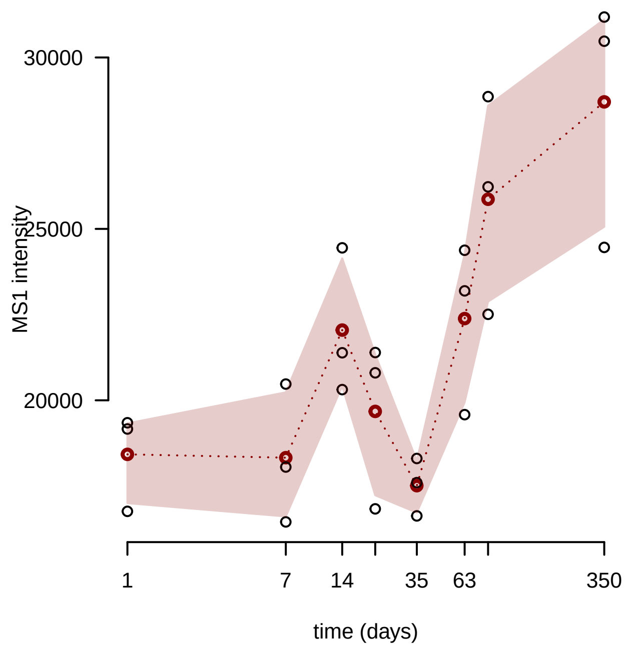

Q3TMW1
GN=Ccdc102a
coiled−coil domain containing 102A

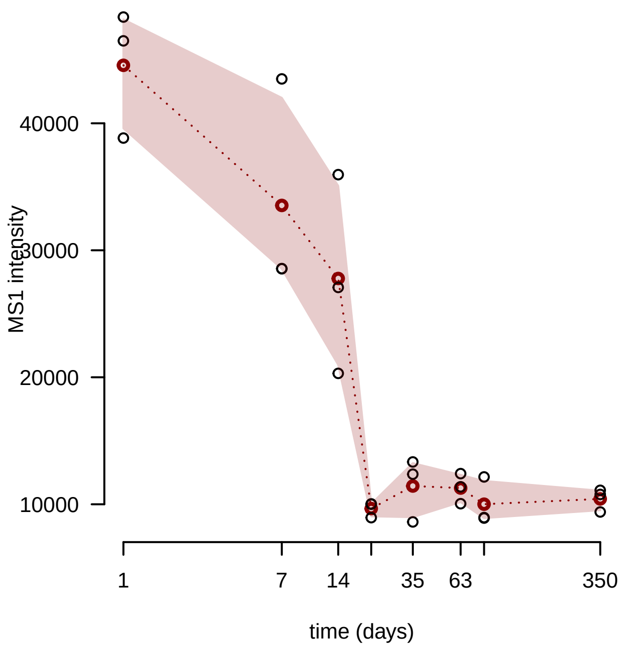

Q3TMX7
GN=Qsox2
quiescin Q6 sulfhydryl oxidase 2

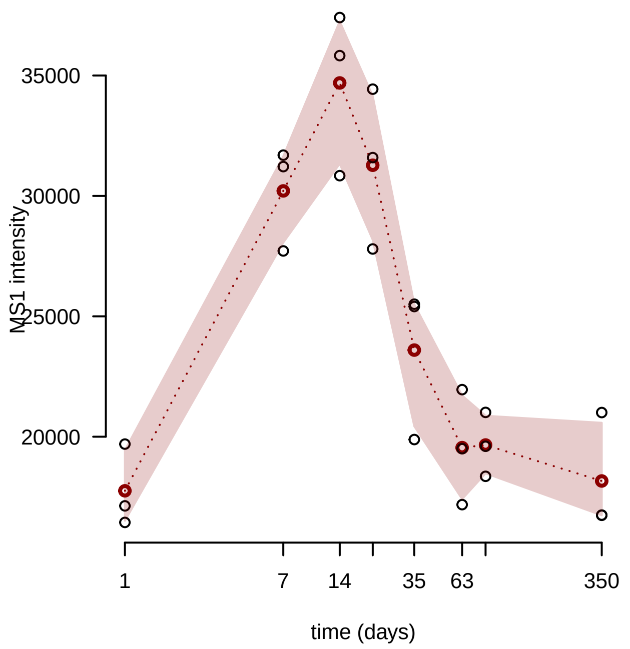

Q3TNA1
GN=Xylb
xylulokinase homolog (H. influenzae)

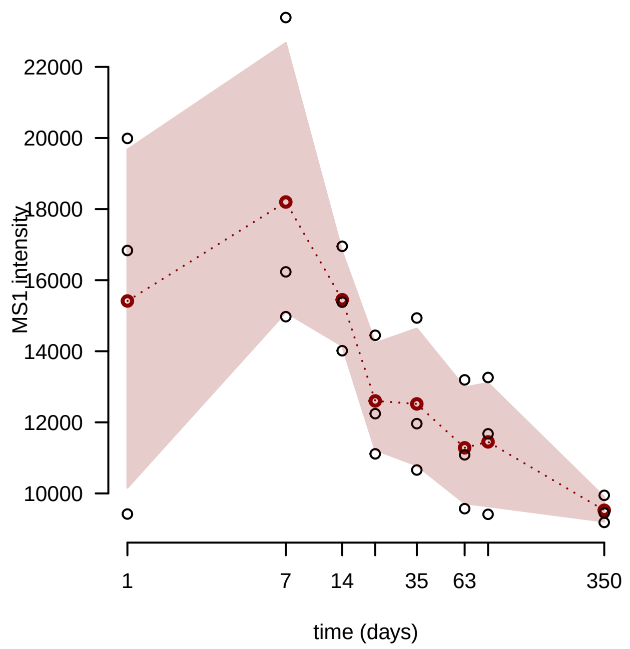

Q3TNH5
GN=Fam172a
family with sequence similarity 172, mem ...

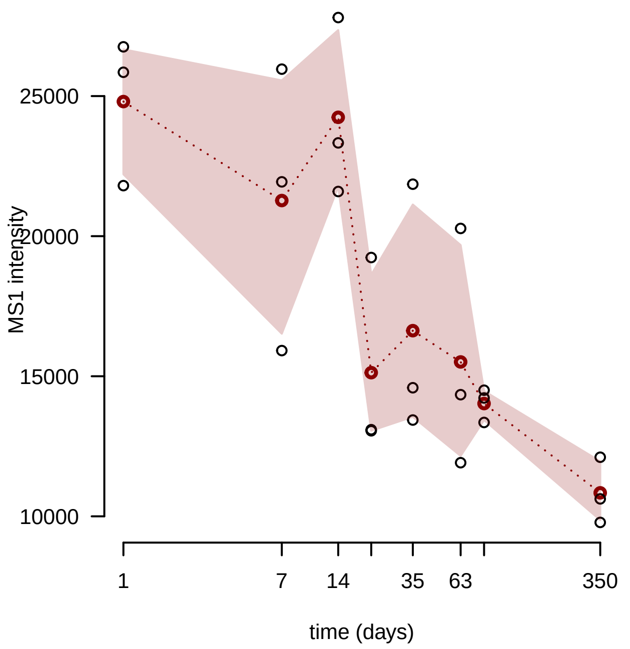

Q3TNL8
GN=Itprip
inositol 1,4,5−triphosphate receptor int ...

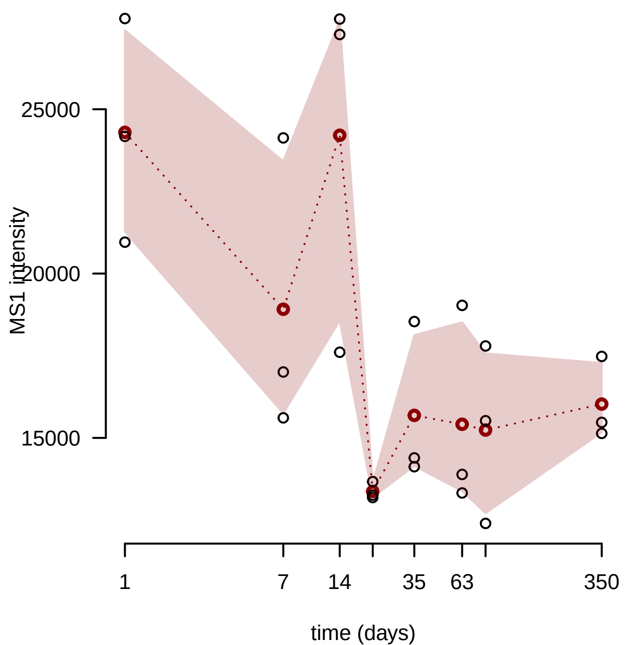

Q3TP92
GN=Ctdnep1
CTD nuclear envelope phosphatase 1

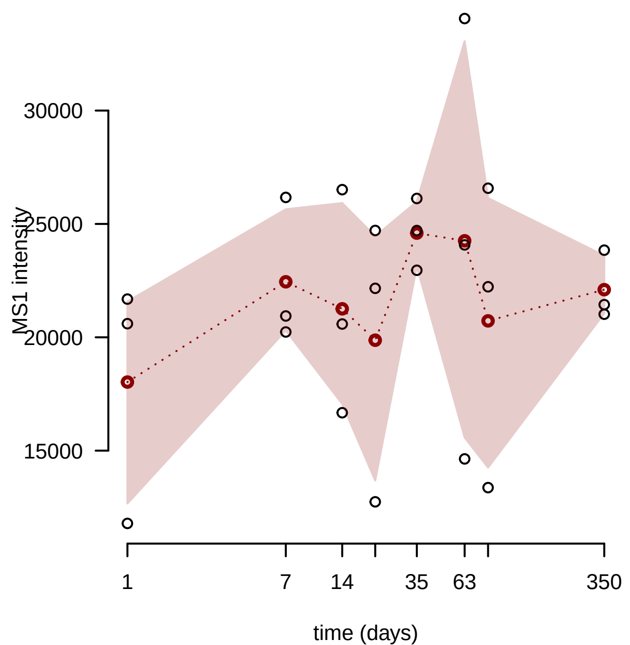

Q3TPE9
GN=Ankmy2
ankyrin repeat and MYND domain containin ...

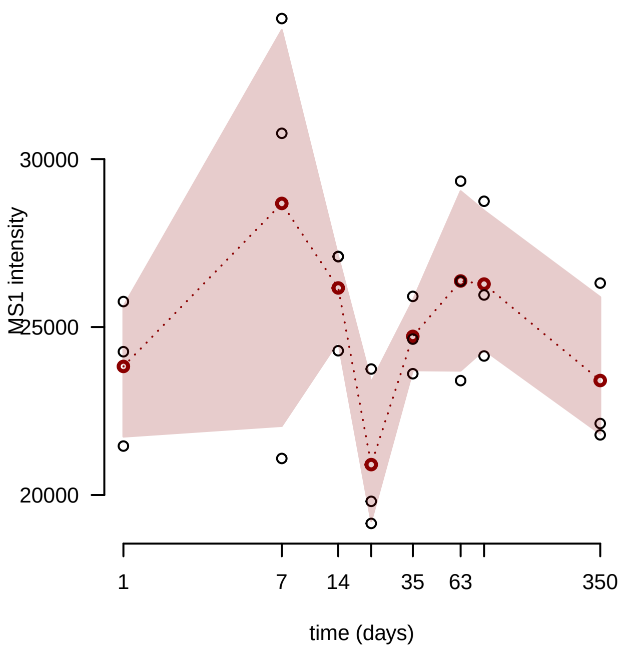

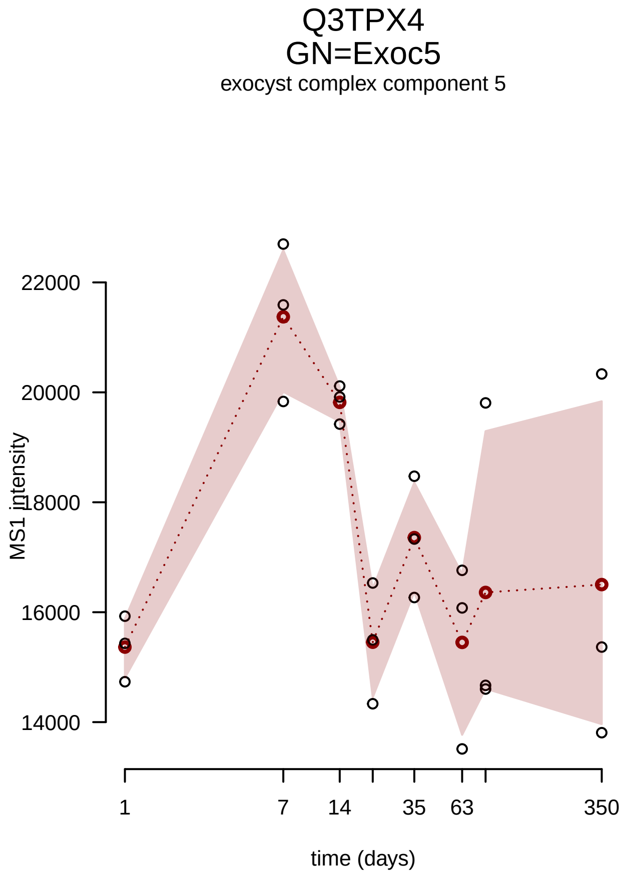
Q3TPX4
GN=Exoc5
exocyst complex component 5
MS1 intensity
22000
20000
18000
16000
14000
1
7
14
35
63
350
time (days)

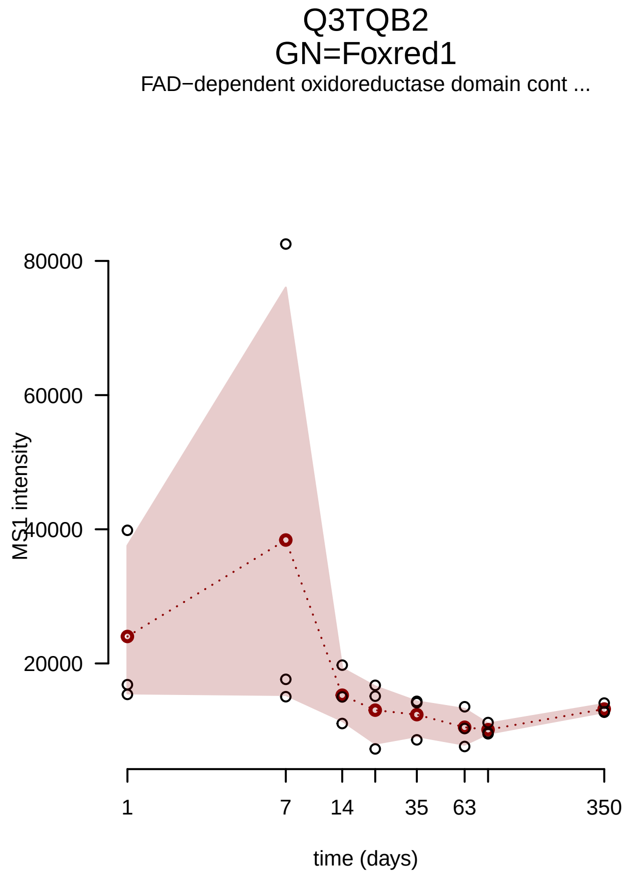
Q3TQB2
GN=Foxred1
FAD−dependent oxidoreductase domain cont ...
MS1 intensity
80000
60000
40000
20000
1
7
14
35
63
350
time (days)

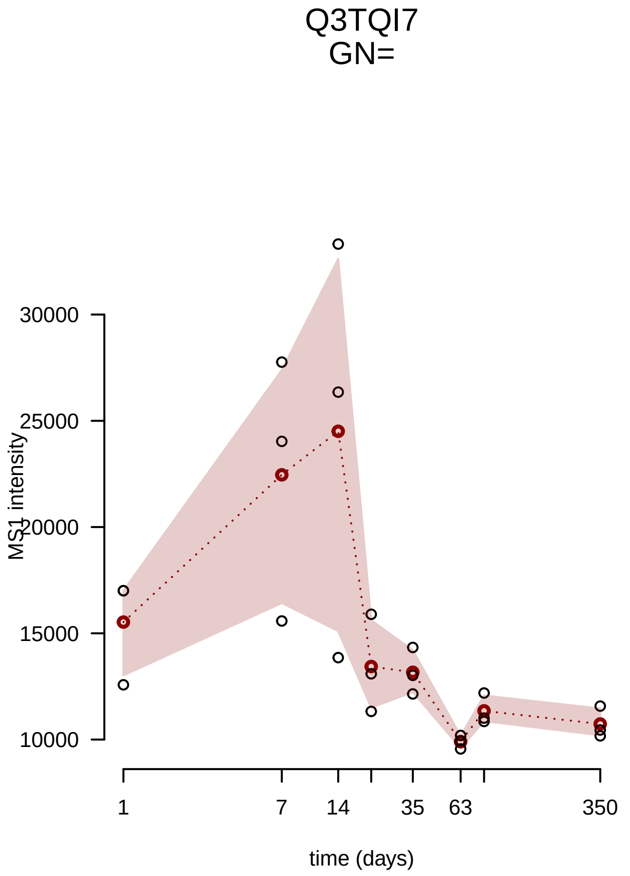
Q3TQI7
GN=
MS1 intensity
30000
25000
20000
15000
10000
1
7
14
35
63
350
time (days)

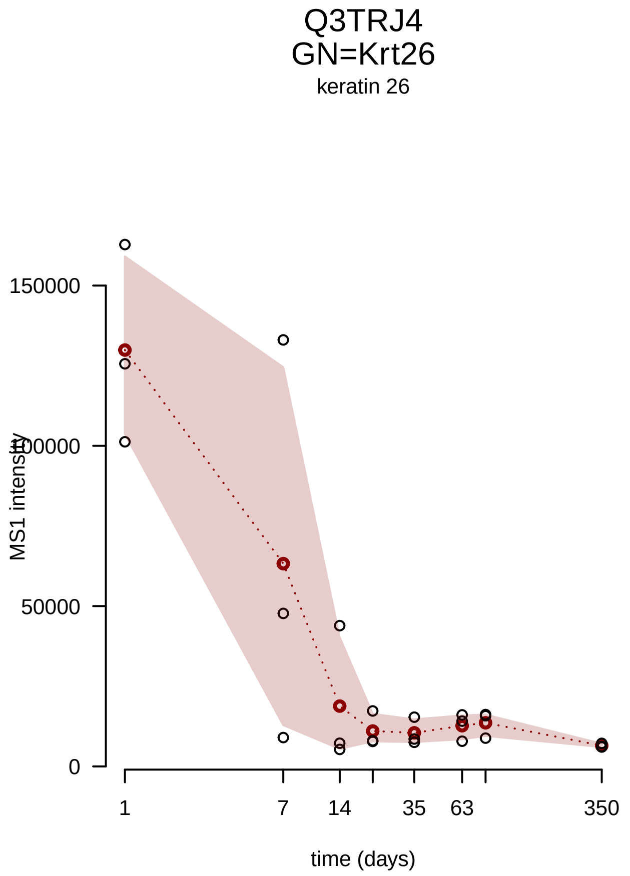
Q3TRJ4
GN=Krt26
keratin 26
MS1 intensity
150000
100000
50000
0
1
7
14
35
63
350
time (days)

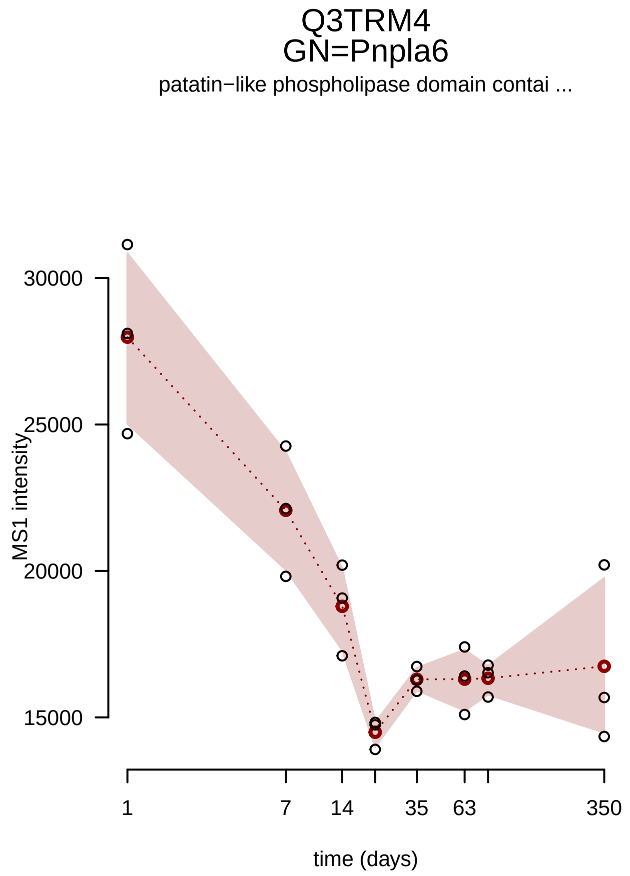
Q3TRM4
GN=Pnpla6
patatin−like phospholipase domain contai ...
MS1 intensity
30000
25000
20000
15000
1
7
14
35
63
350
time (days)

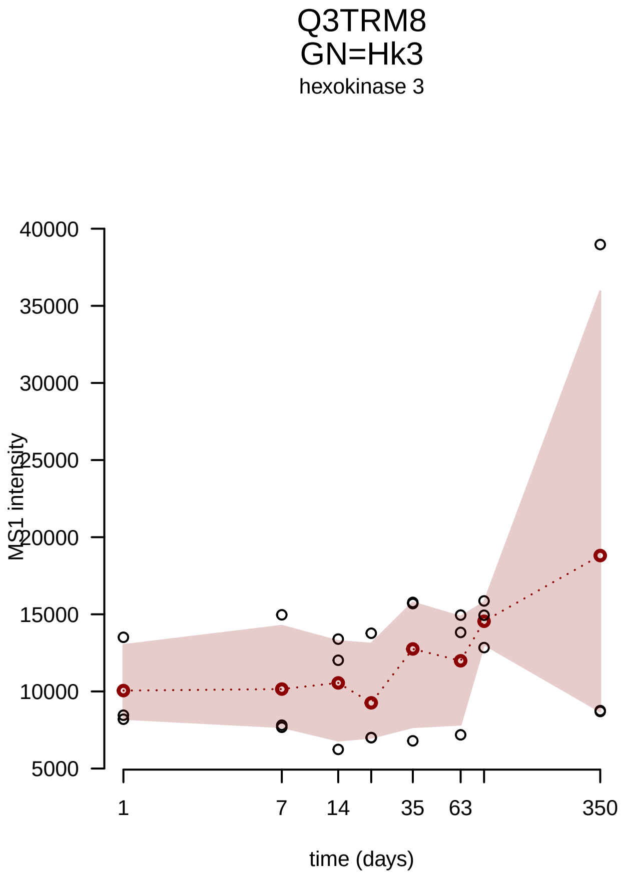
Q3TRM8
GN=Hk3
hexokinase 3
MS1 intensity
40000
35000
30000
25000
20000
15000
10000
5000
1
7
14
35
63
350
time (days)

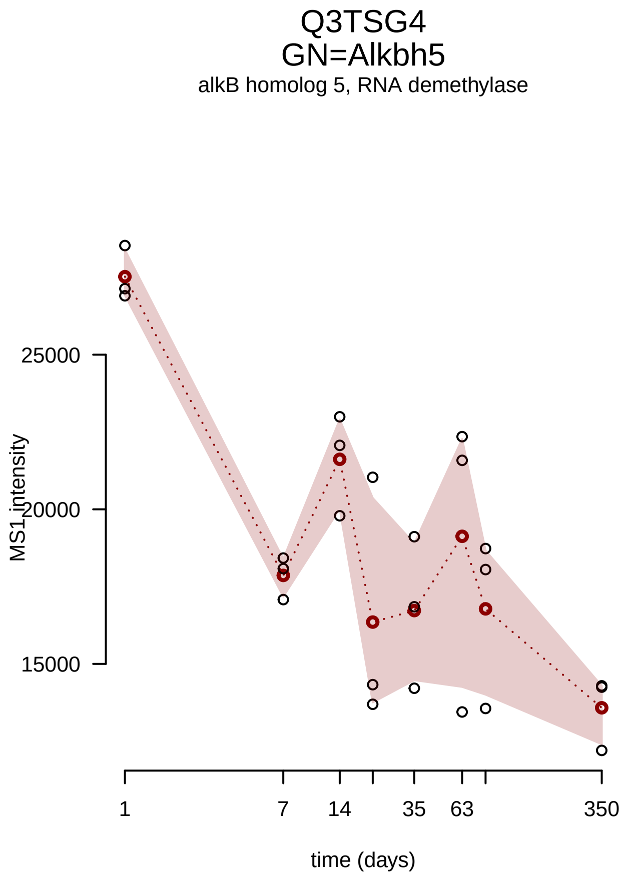
Q3TSG4
GN=Alkbh5
alkB homolog 5, RNA demethylase
MS1 intensity
25000
20000
15000
1
7
14
35
63
350
time (days)

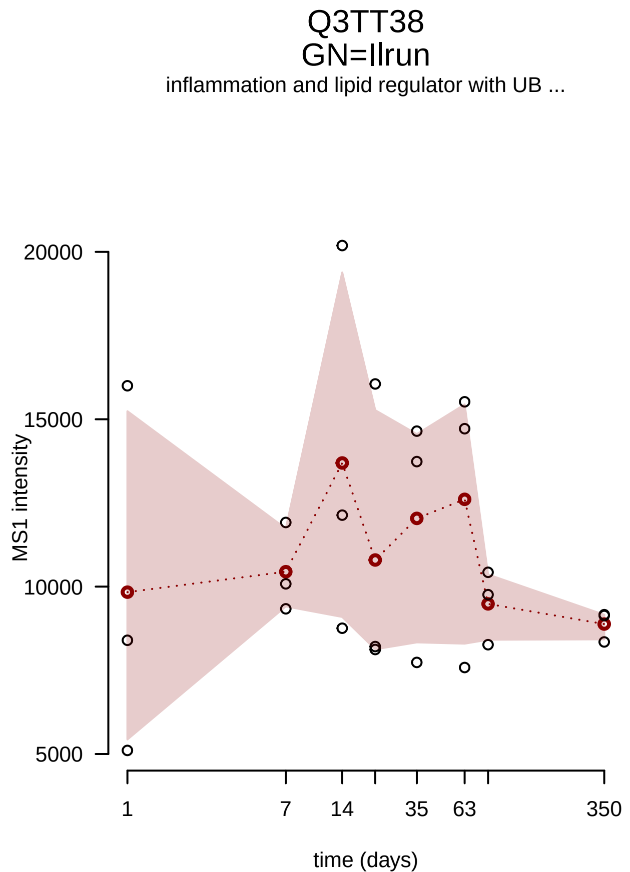
Q3TT38
GN=Ilrun
inflammation and lipid regulator with UB ...
MS1 intensity
20000
15000
10000
5000
1
7
14
35
63
350
time (days)

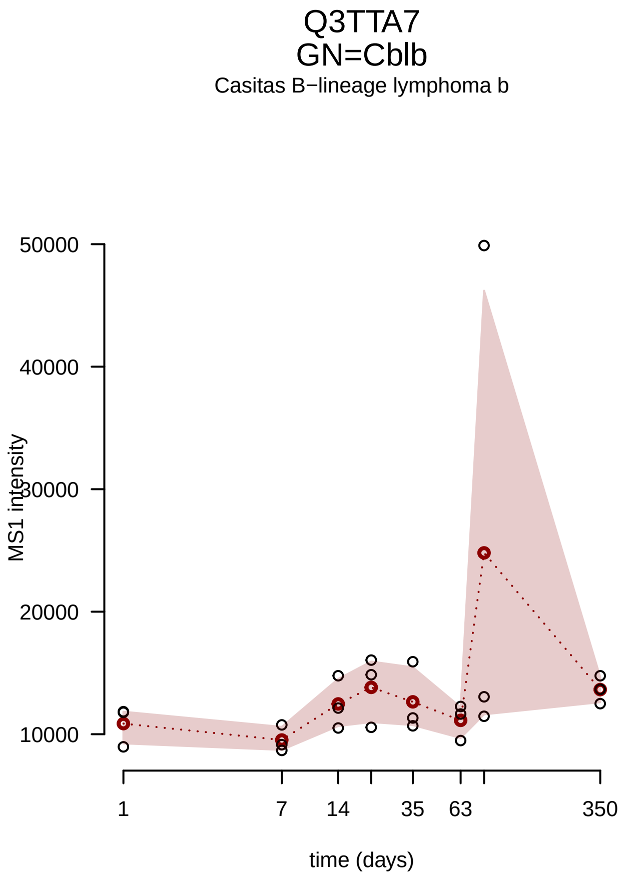
Q3TTA7
GN=Cblb
Casitas B−lineage lymphoma b
MS1 intensity
50000
40000
30000
20000
10000
1
7
14
35
63
350
time (days)

Q3TTY5
GN=Krt2
keratin 2

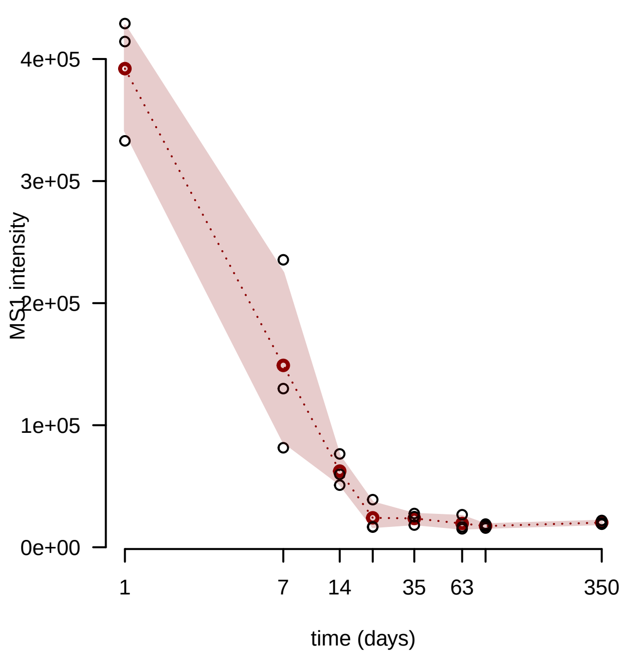

Q3TUA9
GN=Pomk
protein−O−mannose kinase

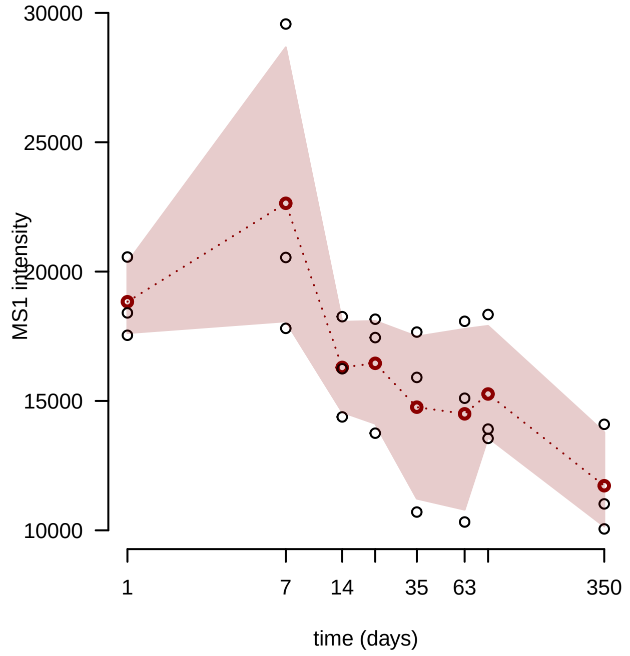

Q3TUD9
GN=Tmem18
transmembrane protein 18

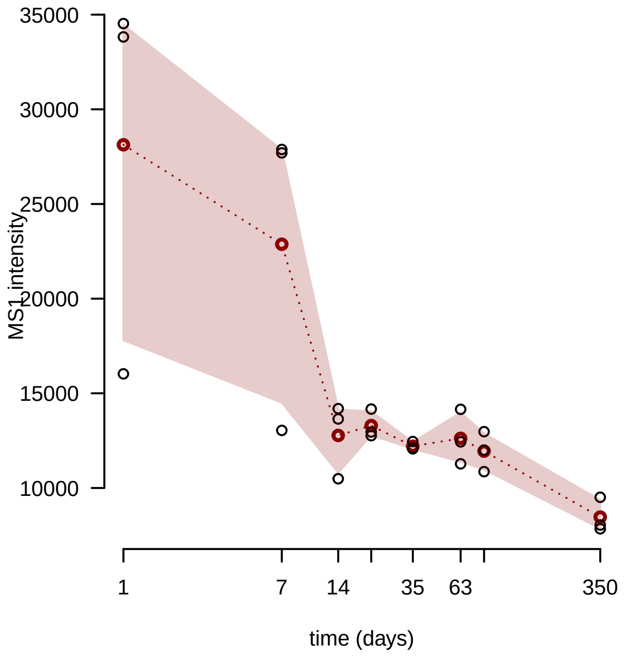

Q3TUF7
GN=Yeats2
YEATS domain containing 2

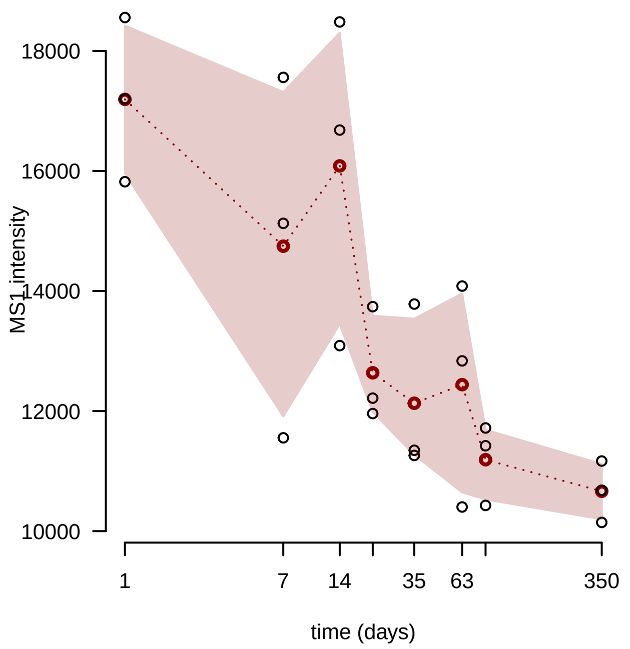

Q3TUH1
GN=Tamm41
TAM41 mitochondrial translocator assembl ...

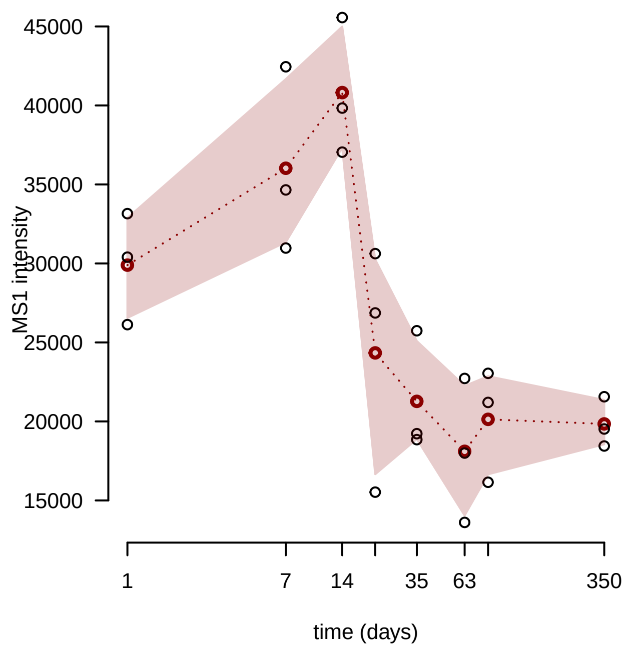

Q3TUU5
GN=Tex30
testis expressed 30

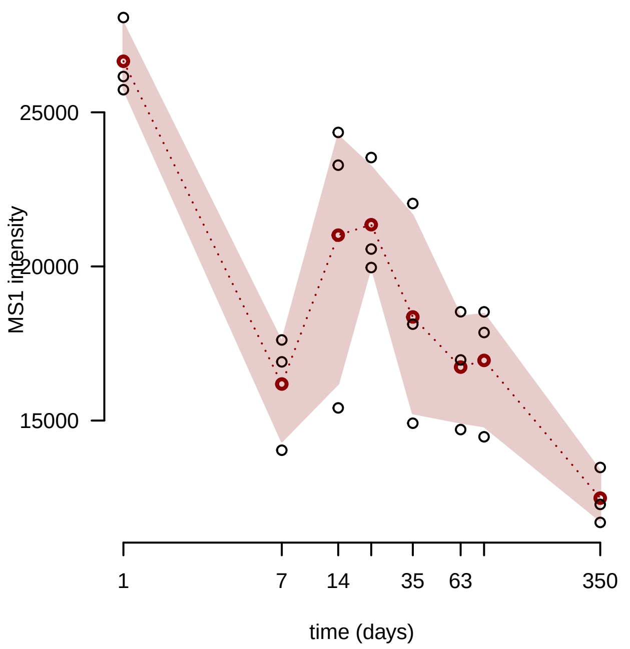

Q3TV65
GN=Mpnd
MPN domain containing

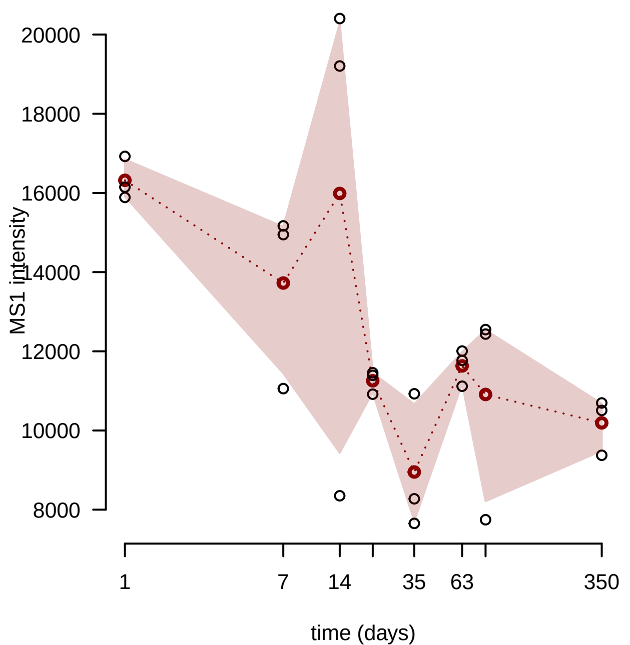

Q3TVI4
GN=Hexim2
hexamethylene bis−acetamide inducible 2

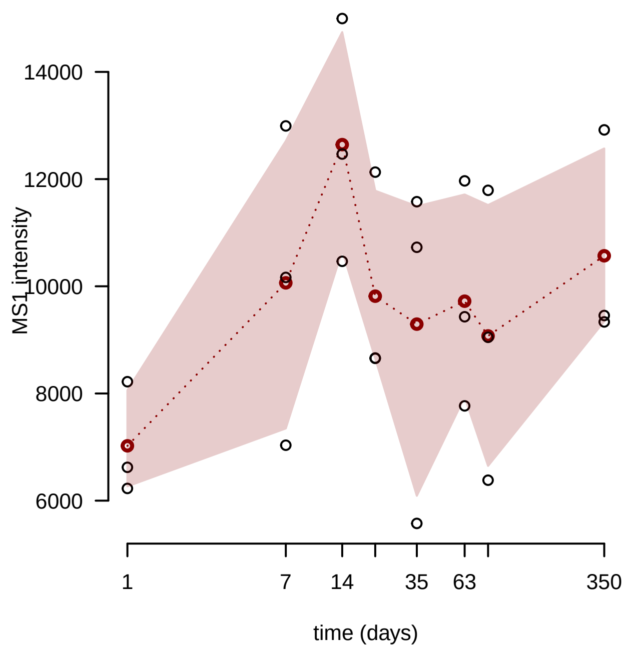

Q3TVI8
GN=Pbxip1
pre B cell leukemia transcription factor ...

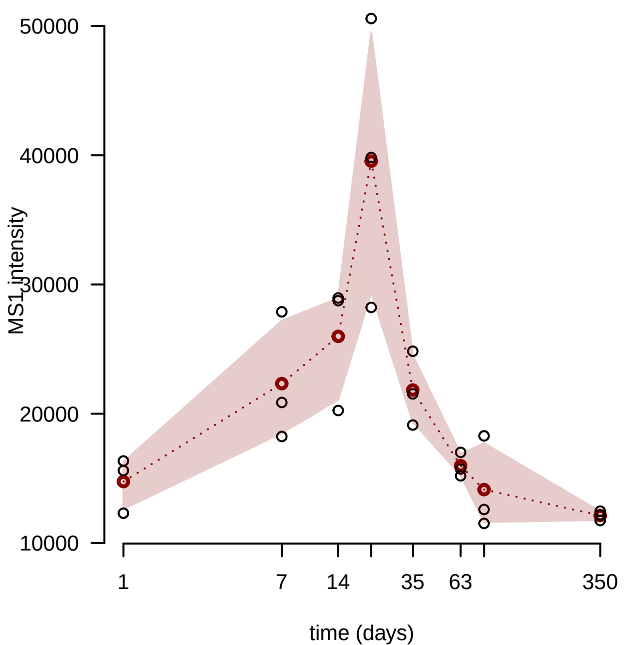

Q3TW96
GN=Uap1l1
UDP−N−acteylglucosamine pyrophosphorylas ...

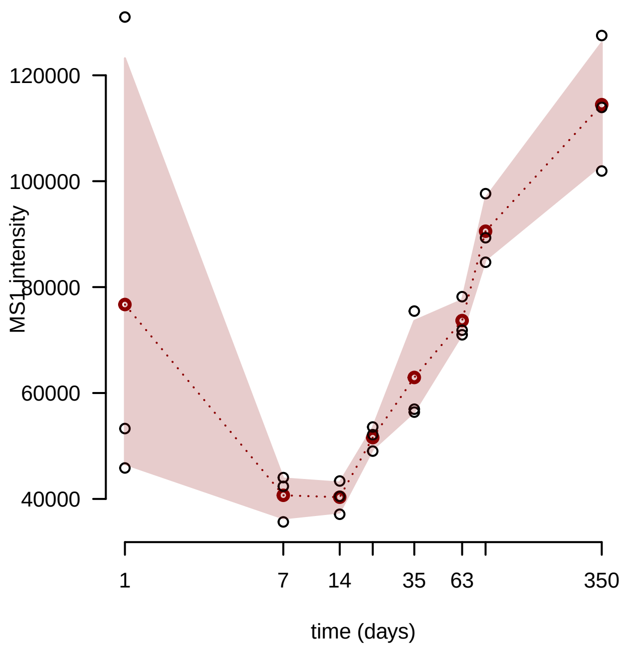

Q3TWF6
GN=Wdr70
WD repeat domain 70

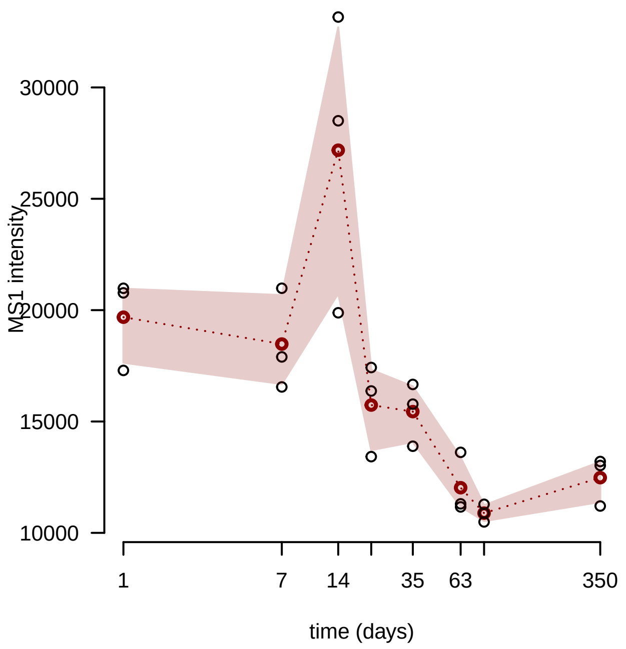

Q3TWI9
GN=Tmem63b
transmembrane protein 63b

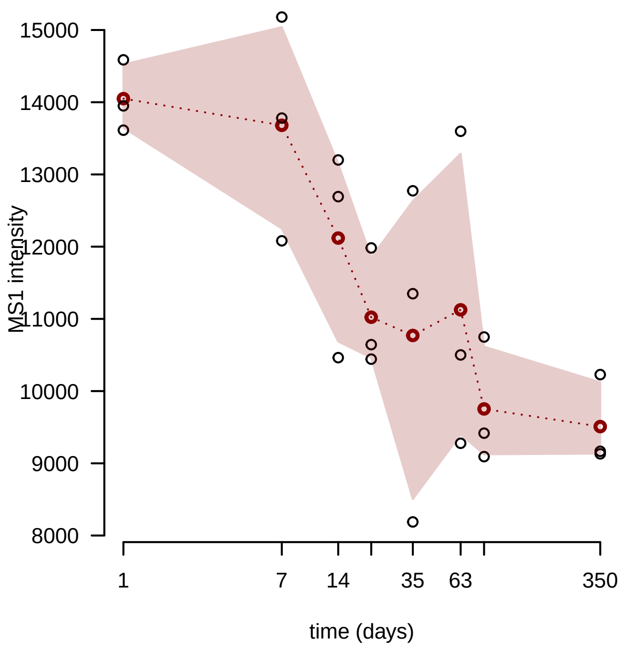

Q3TWL2
GN=Pip4p1
phosphatidylinositol−4,5−bisphosphate 4− ...

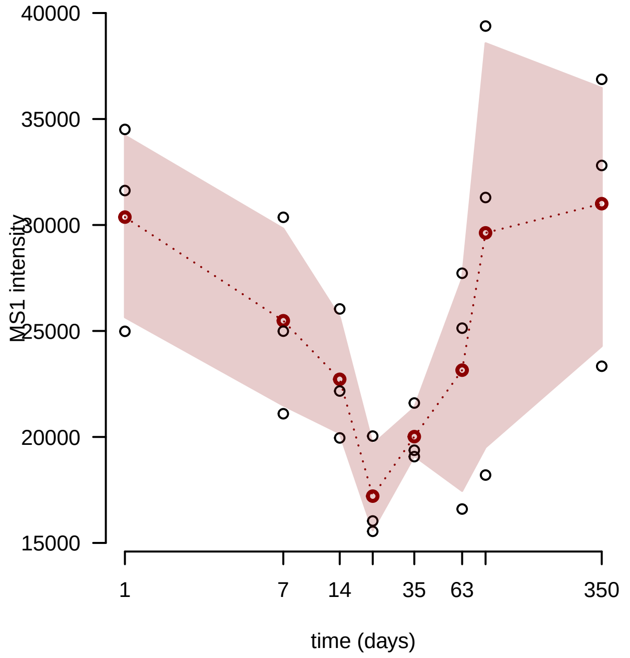

Q3TWN3
GN=Cnnm2
cyclin M2

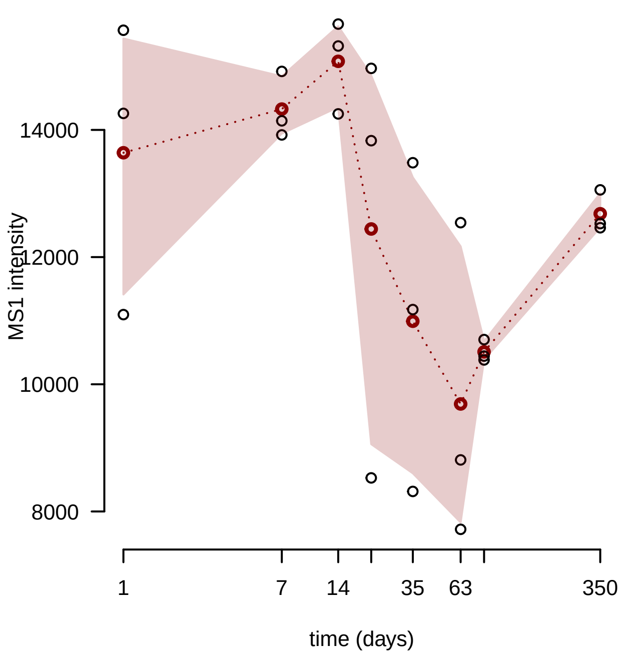

Q3TWW8
GN=Srsf6
serine and arginine−rich splicing factor ...

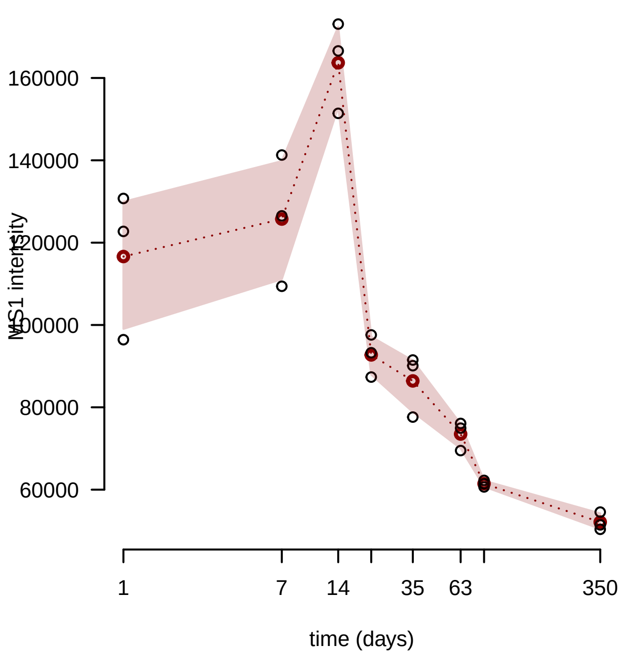

Q3TX08
GN=Trmt1
tRNA methyltransferase 1

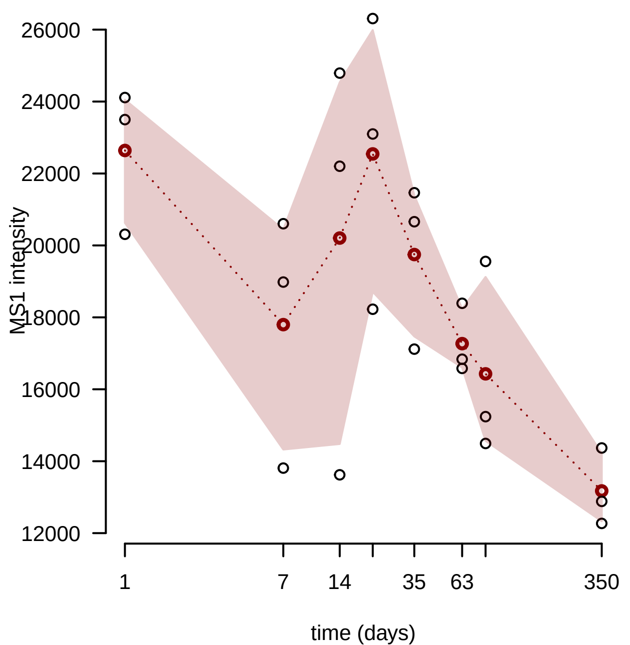

Q3TXS7
GN=Psmd1
proteasome (prosome, macropain) 26S subu ...

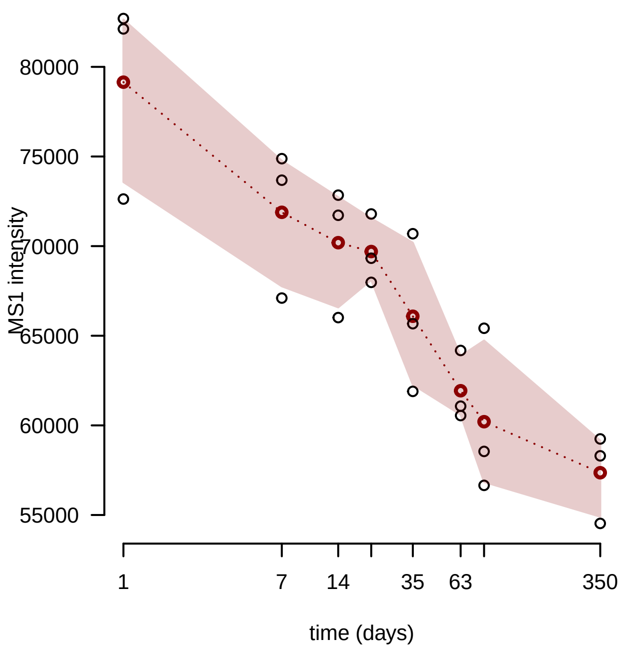

Q3TXT3
GN=Inip
INTS3 and NABP interacting protein

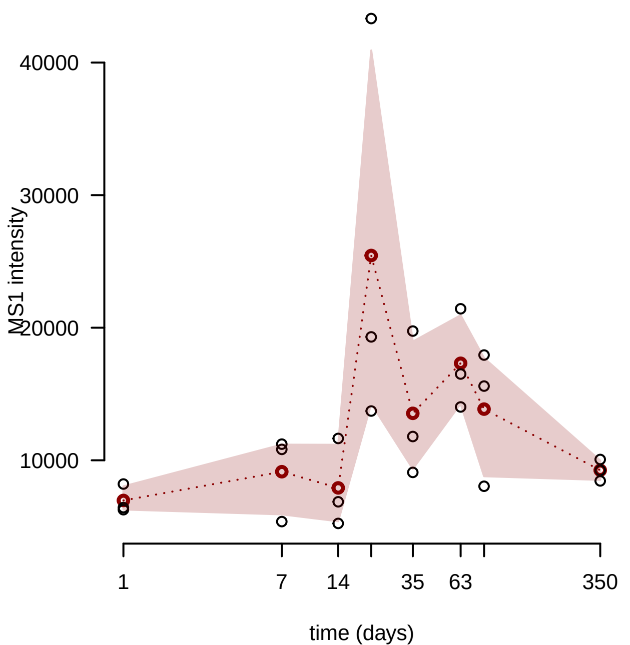

Q3TXU5
GN=Dhps
deoxyhypusine synthase

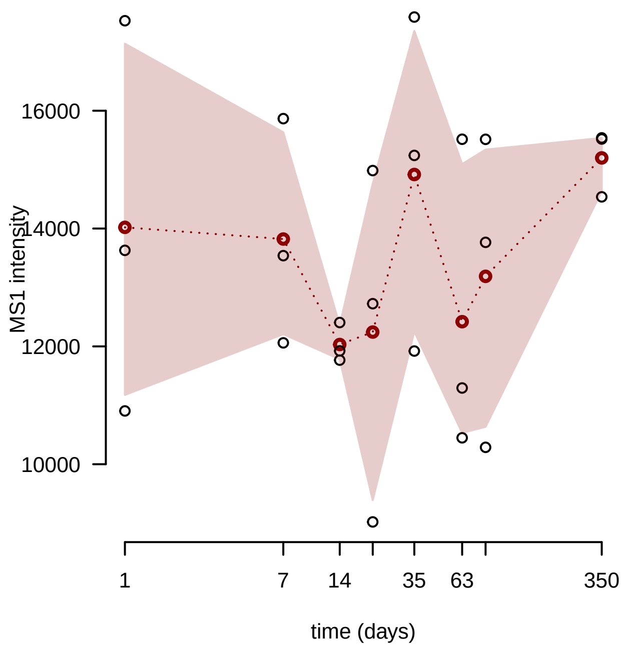

Q3TXX3
GN=Zfyve27
zinc finger, FYVE domain containing 27

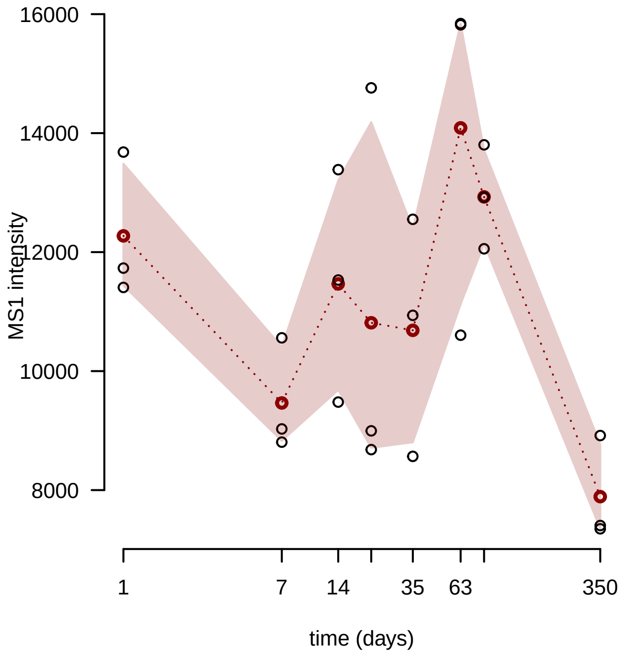

Q3TYA6
GN=Mphosph8
M−phase phosphoprotein 8

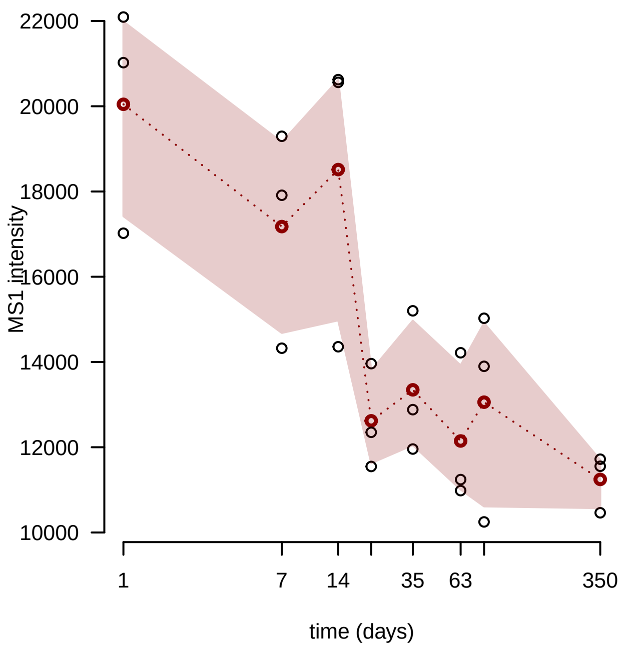

Q3TYD4
GN=Arsg
arylsulfatase G

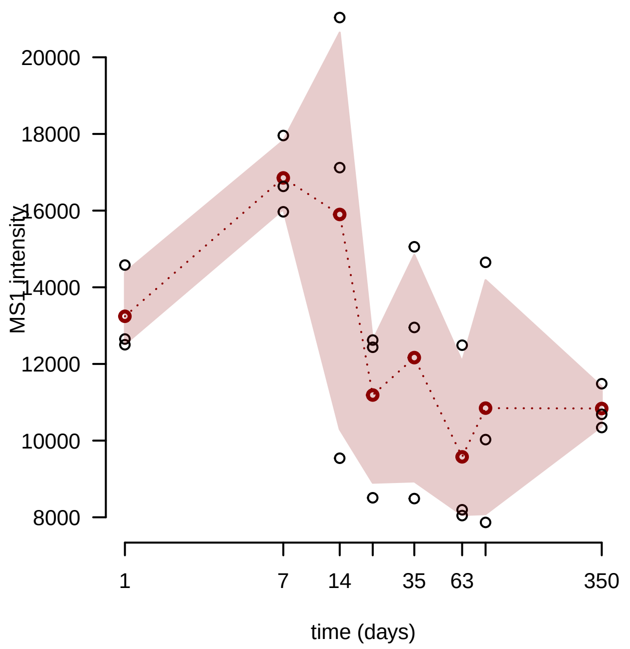

Q3TYD6
GN=Lmtk2
lemur tyrosine kinase 2

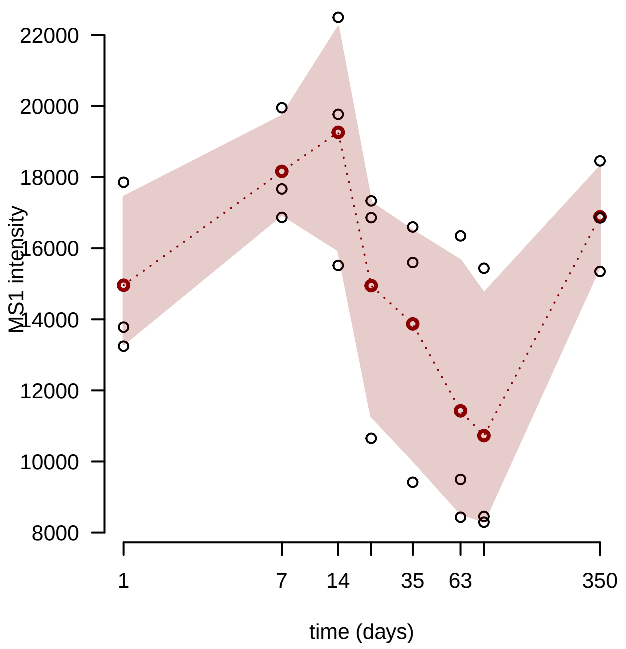

Q3TYS2
GN=Cybc1
cytochrome b 245 chaperone 1

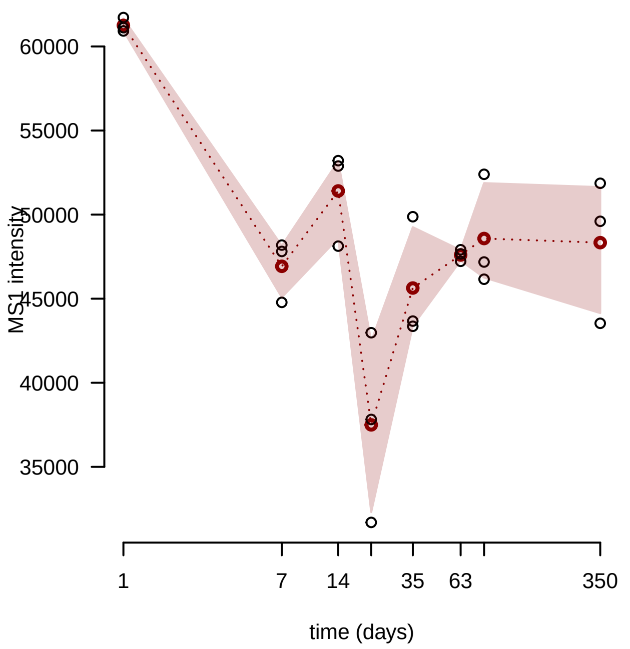

Q3TYX3
GN=Smyd5
SET and MYND domain containing 5

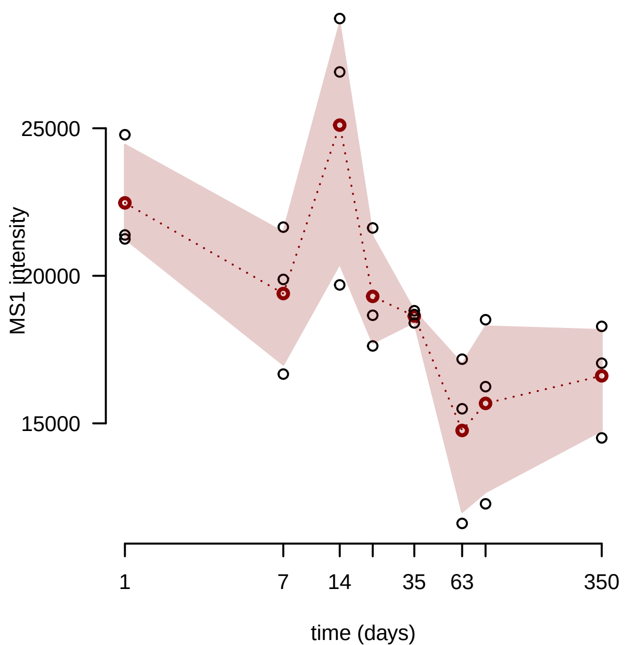

Q3TZM9
GN=Alg11
asparagine−linked glycosylation 11 (alph ...

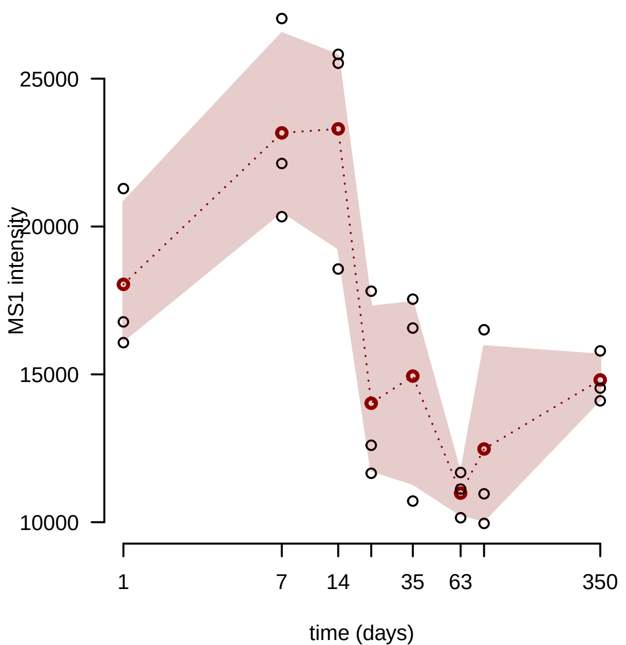

Q3TZX3
GN=Slc25a33
solute carrier family 25, member 33

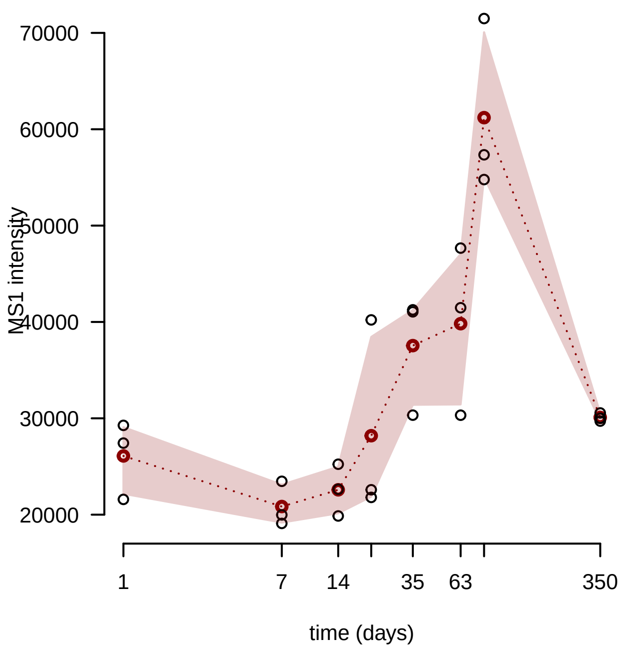

Q3TZX8
GN=Nol9
nucleolar protein 9

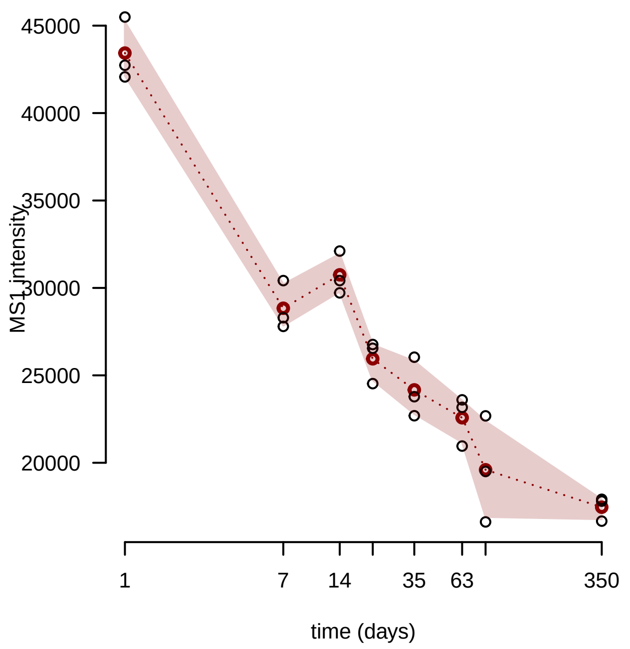

Q3TZZ7
GN=Esyt2
extended synaptotagmin−like protein 2

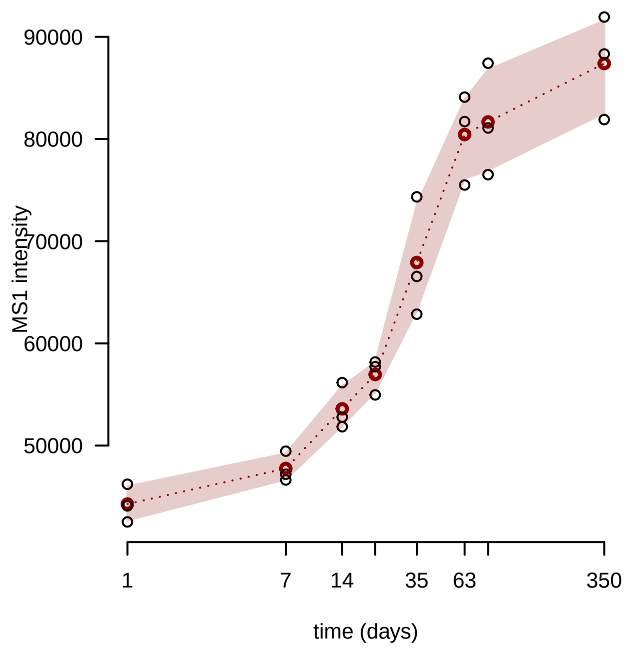

Q3U0B3
GN=Dhrs11
dehydrogenase/reductase (SDR family) mem ...

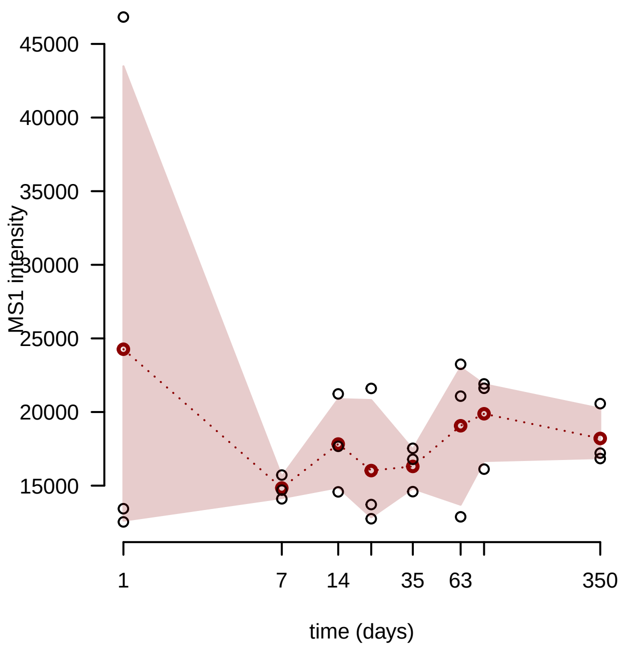

Q3U0D9
GN=Hace1
HECT domain and ankyrin repeat containin ...

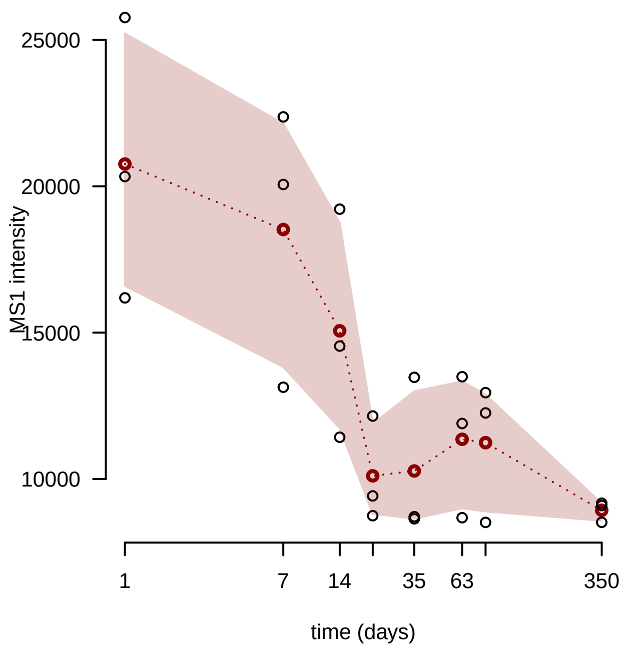

Q3U0J8
GN=Tbc1d2b
TBC1 domain family, member 2B

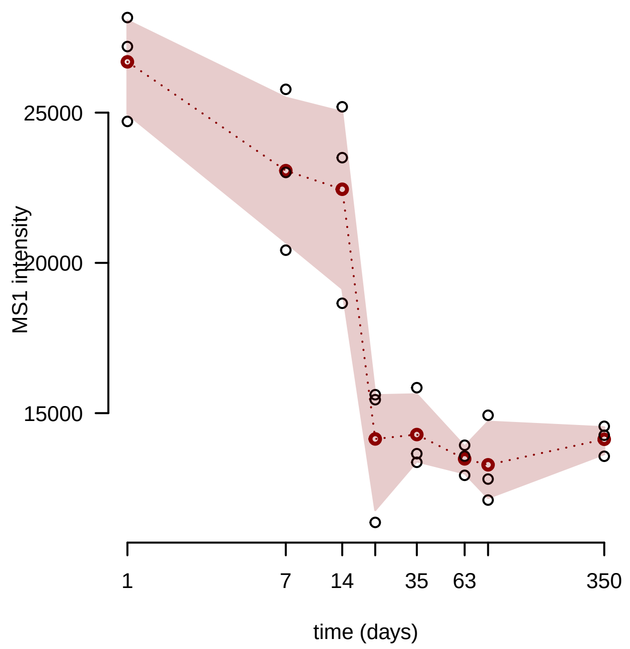

Q3U0K8
GN=Ogfod1
2−oxoglutarate and iron−dependent oxygen ...

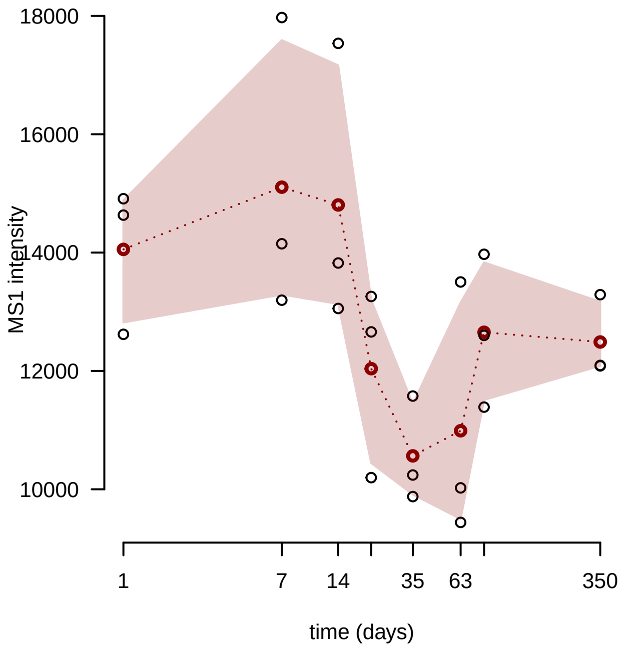

Q3U0M1
GN=Trappc9
trafficking protein particle complex 9

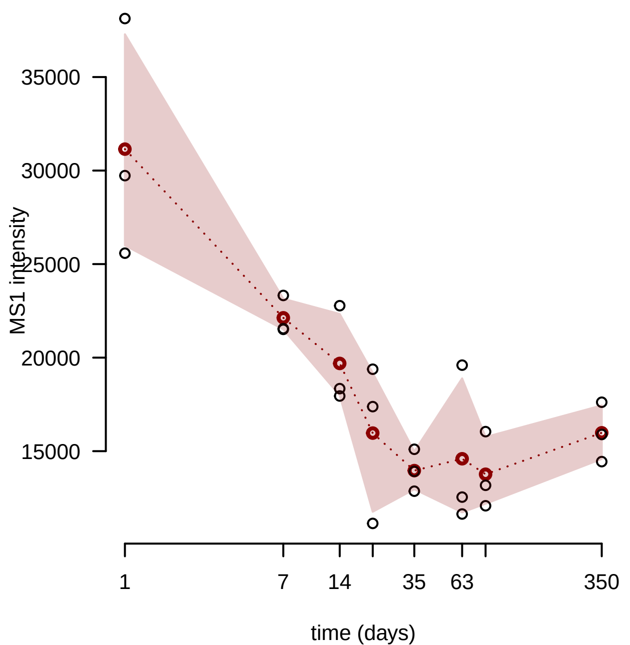

Q3U0P5
GN=

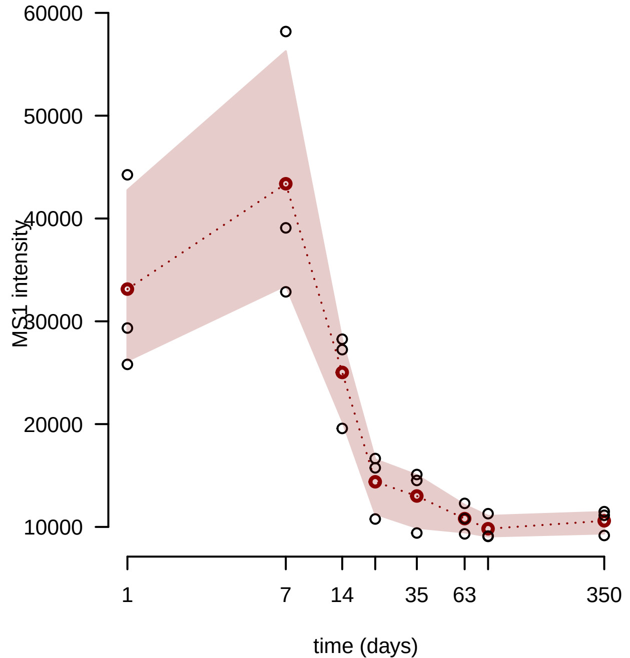

Q3U0S6
GN=Rasip1
Ras interacting protein 1

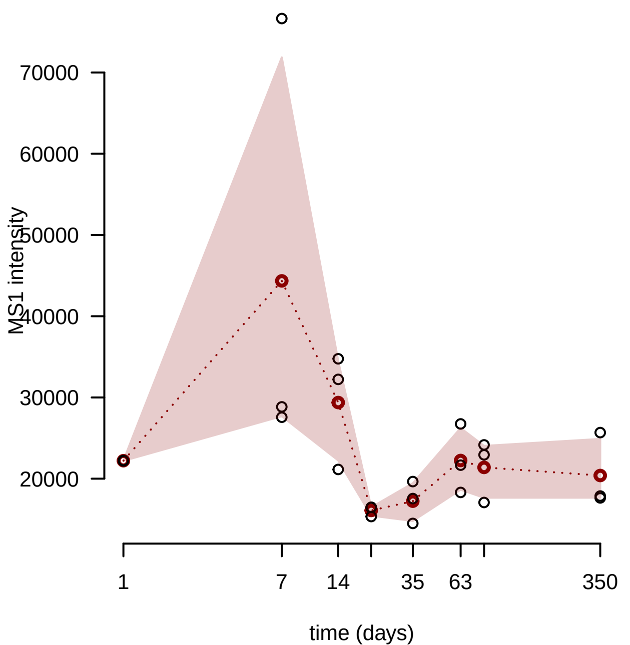

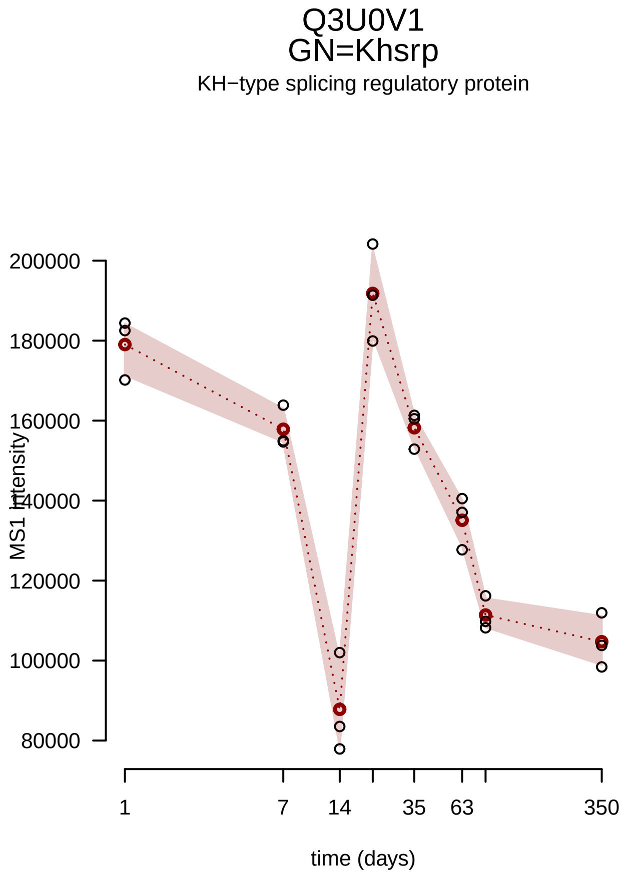
Q3U0V1
GN=Khsrp
KH−type splicing regulatory protein
200000
180000
160000
140000
120000
100000
80000
MS1 intensity
1
7
14
35
63
350
time (days)

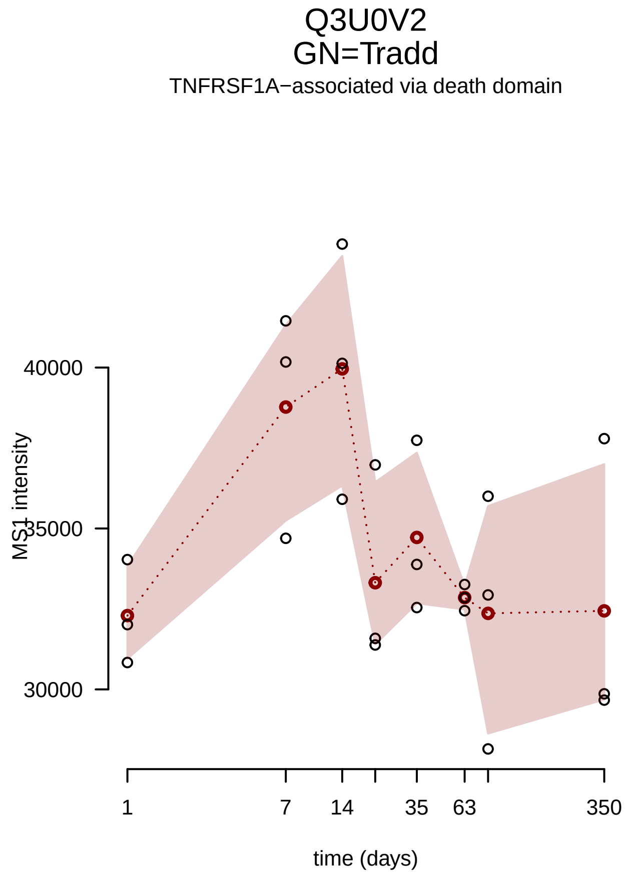
Q3U0V2
GN=Tradd
TNFRSF1A−associated via death domain
40000
35000
30000
MS1 intensity
1
7
14
35
63
350
time (days)

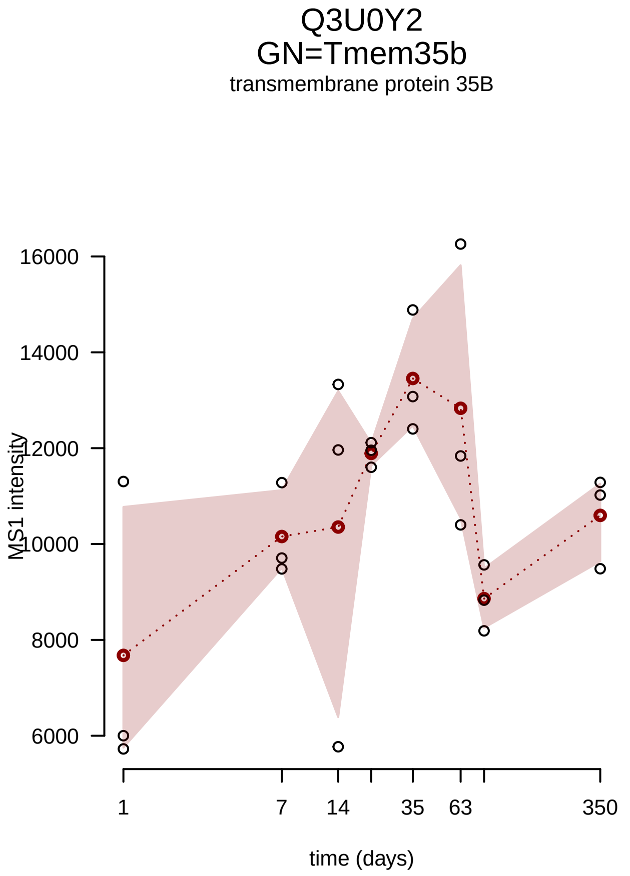
Q3U0Y2
GN=Tmem35b
transmembrane protein 35B
16000
14000
12000
10000
8000
6000
MS1 intensity
1
7
14
35
63
350
time (days)

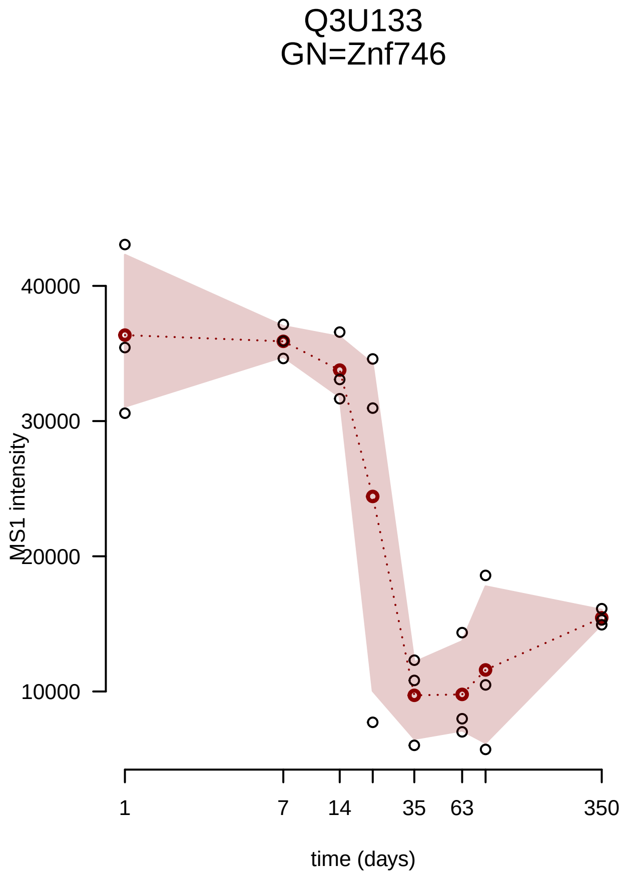
Q3U133
GN=Znf746
40000
30000
20000
10000
MS1 intensity
1
7
14
35
63
350
time (days)

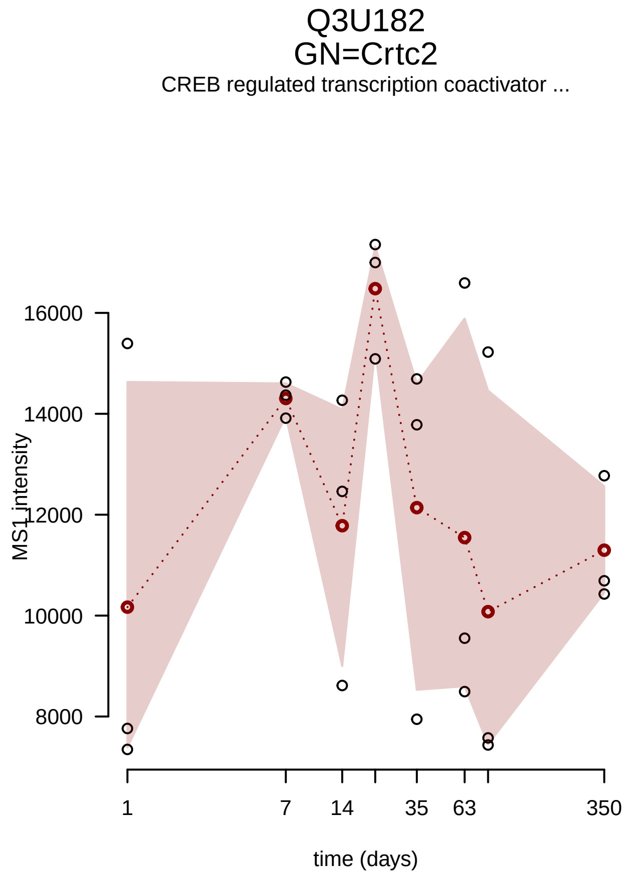
Q3U182
GN=Crtc2
CREB regulated transcription coactivator ...
16000
14000
12000
10000
8000
MS1 intensity
1
7
14
35
63
350
time (days)

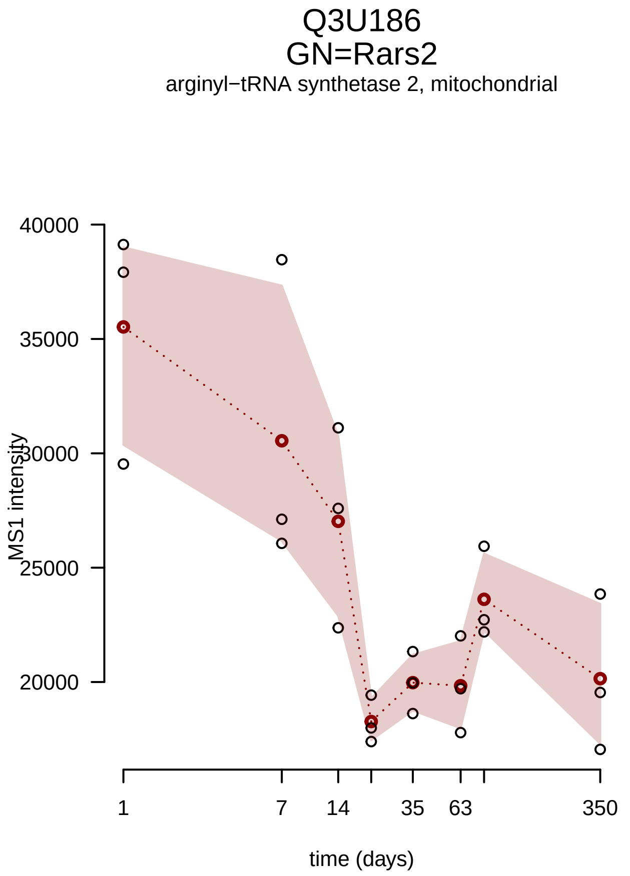
Q3U186
GN=Rars2
arginyl−tRNA synthetase 2, mitochondrial
40000
35000
30000
25000
20000
MS1 intensity
1
7
14
35
63
350
time (days)

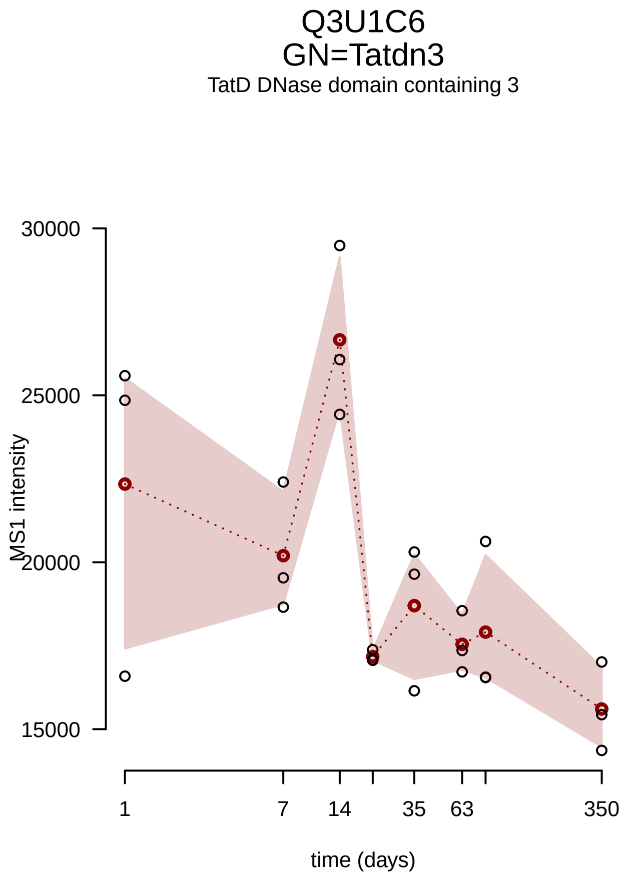
Q3U1C6
GN=Tatdn3
TatD DNase domain containing 3
30000
25000
20000
15000
MS1 intensity
1
7
14
35
63
350
time (days)

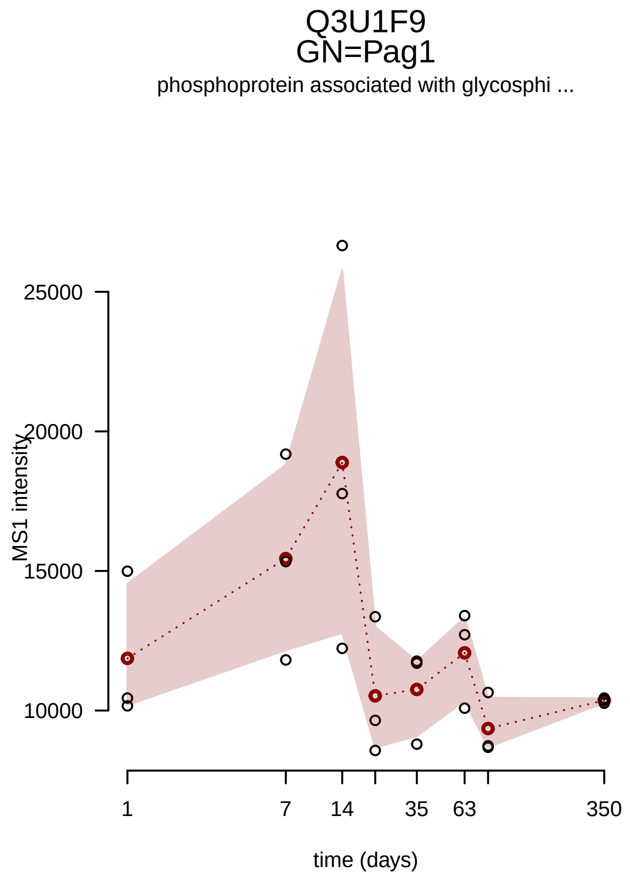
Q3U1F9
GN=Pag1
phosphoprotein associated with glycosphi ...
25000
20000
15000
10000
MS1 intensity
1
7
14
35
63
350
time (days)

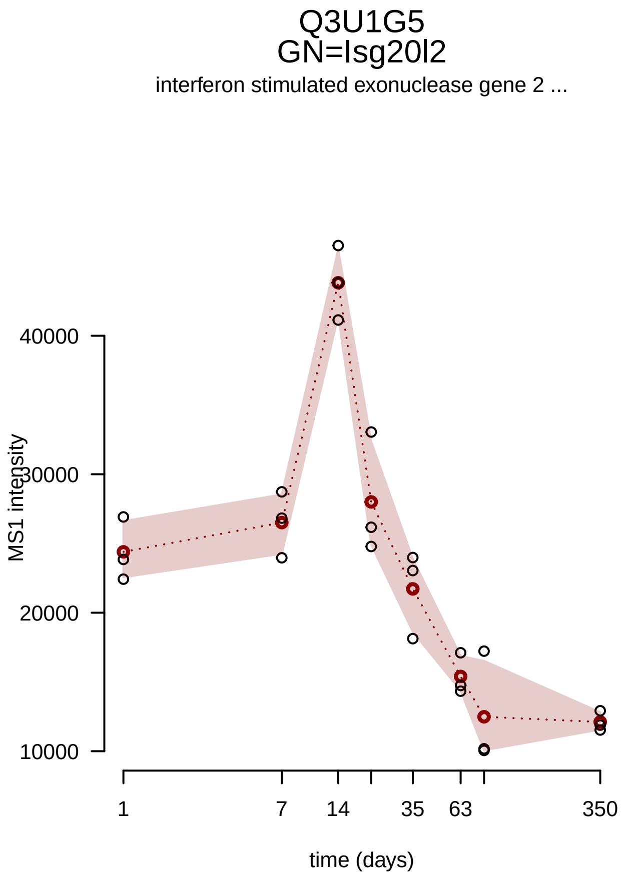
Q3U1G5
GN=Isg20l2
interferon stimulated exonuclease gene 2 ...
40000
30000
20000
10000
MS1 intensity
1
7
14
35
63
350
time (days)

Q3U1J0
GN=Slc38a5
solute carrier family 38, member 5

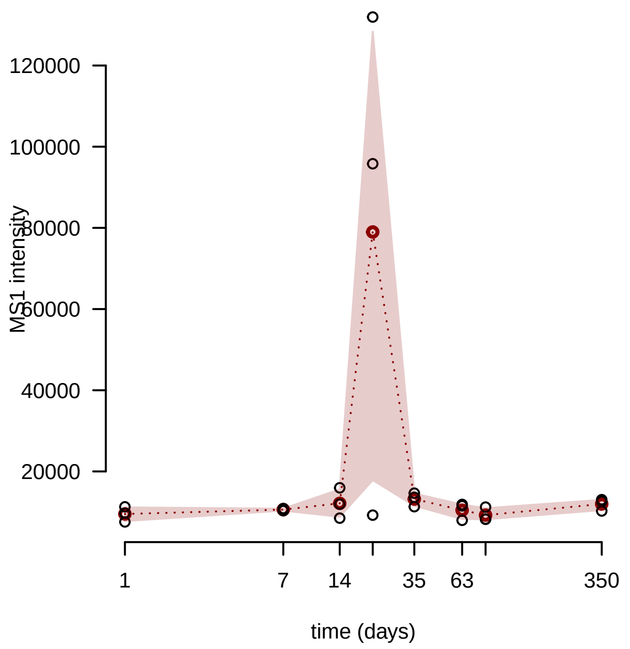

Q3U1J4
GN=Ddb1
damage specific DNA binding protein 1

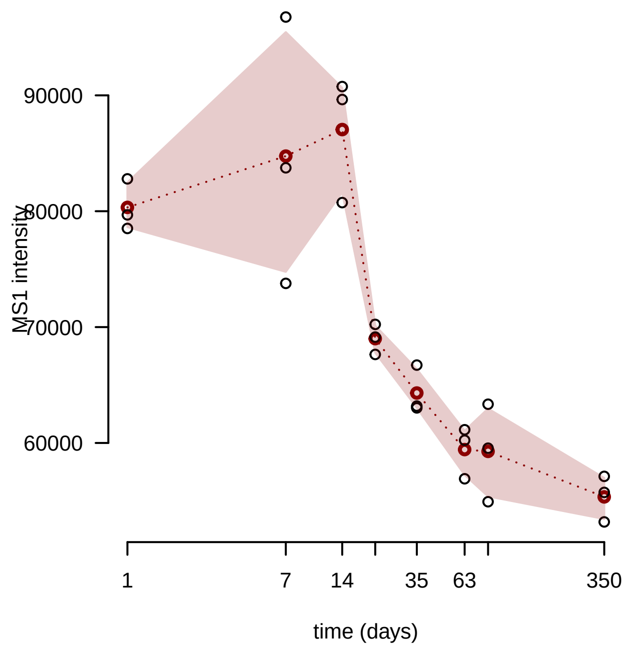

Q3U1N2
GN=Srebf2
sterol regulatory element binding factor ...

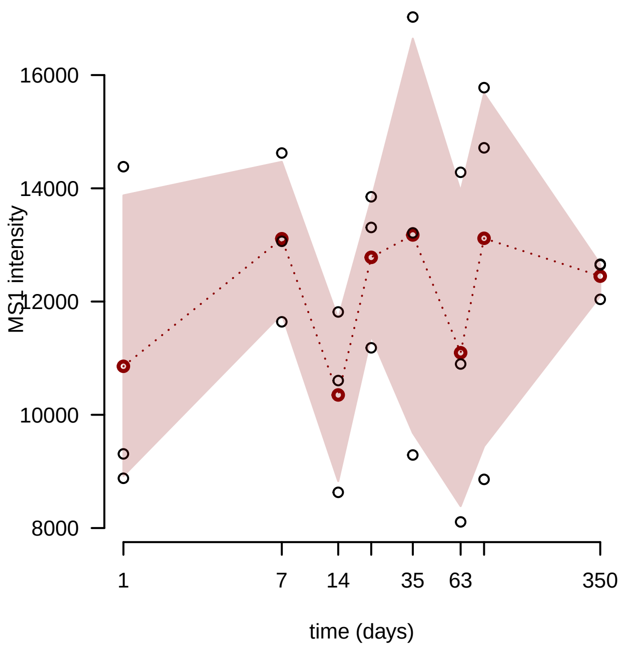

Q3U1T3
GN=Brms1l
breast cancer metastasis−suppressor 1−li ...

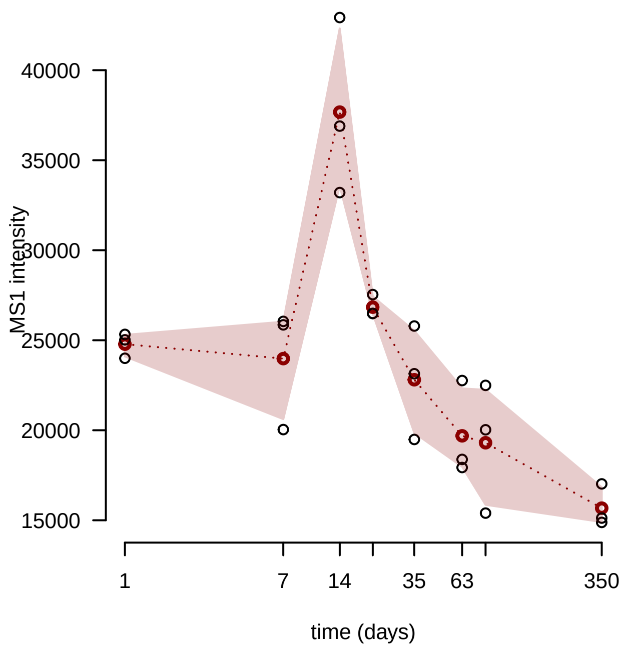

Q3U1T9
GN=Dennd1b
DENN/MADD domain containing 1B

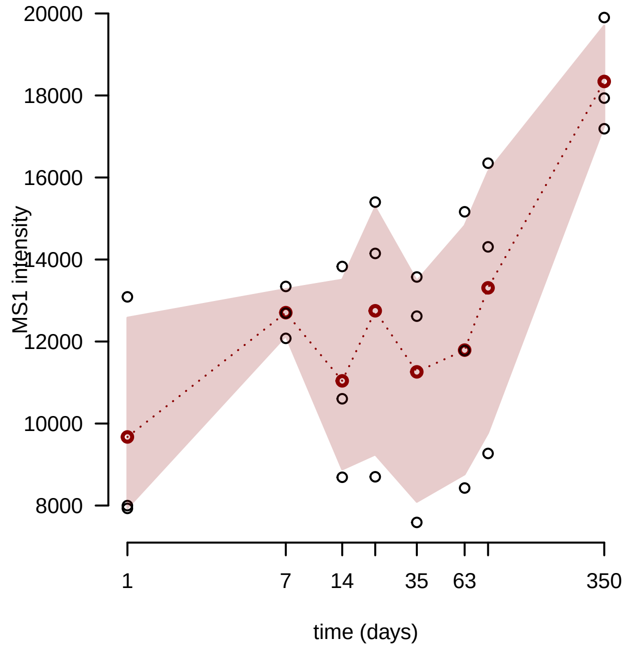

Q3U1V6
GN=Uevld
UEV and lactate/malate dehyrogenase doma ...

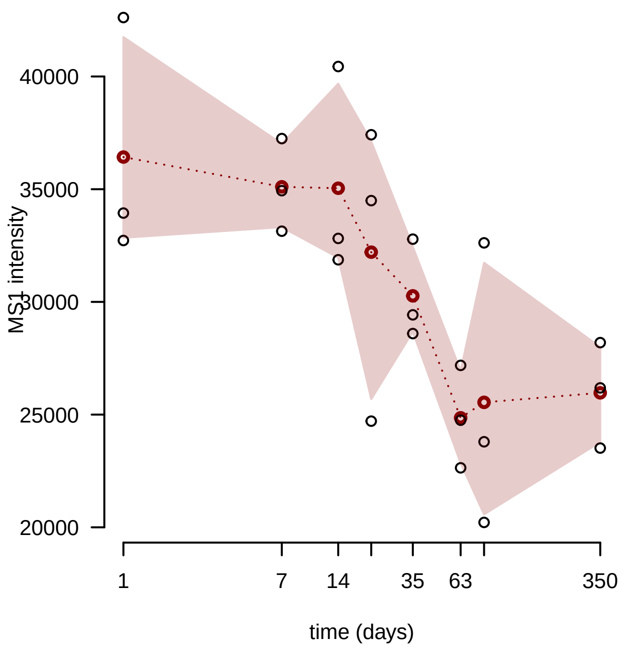

Q3U213
GN=Serac1
serine active site containing 1

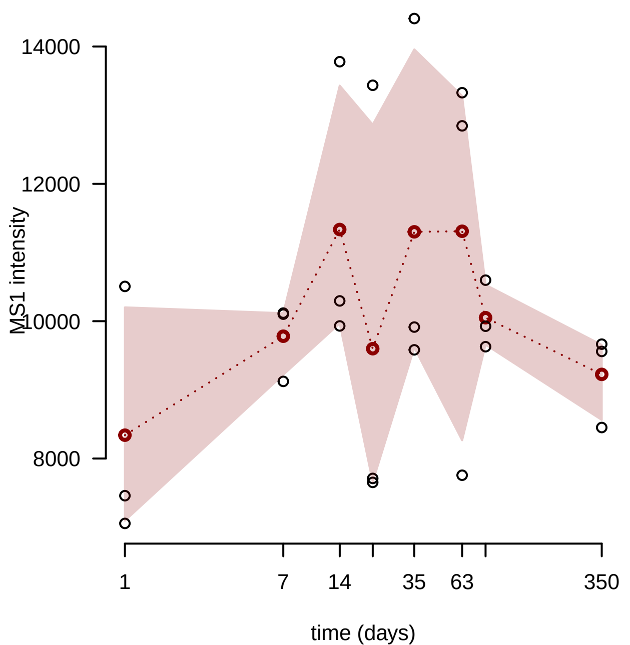

Q3U276
GN=Sdhaf1
succinate dehydrogenase complex assembly ...

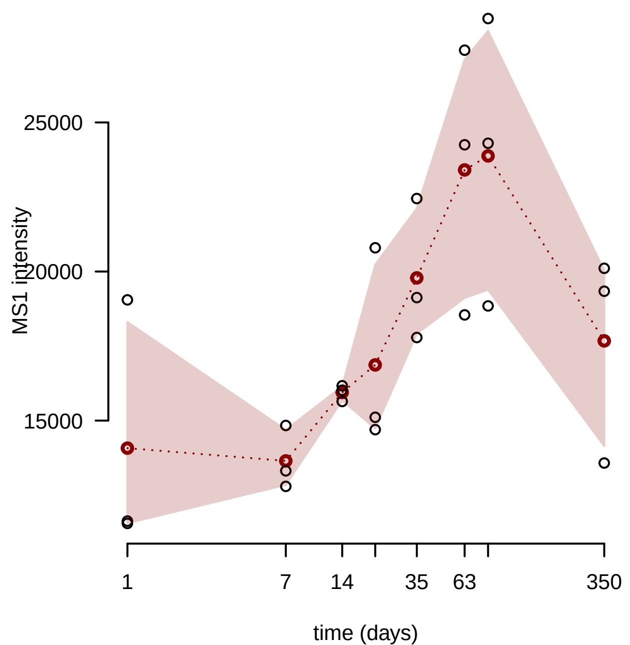

Q3U284
GN=Tmem231
transmembrane protein 231

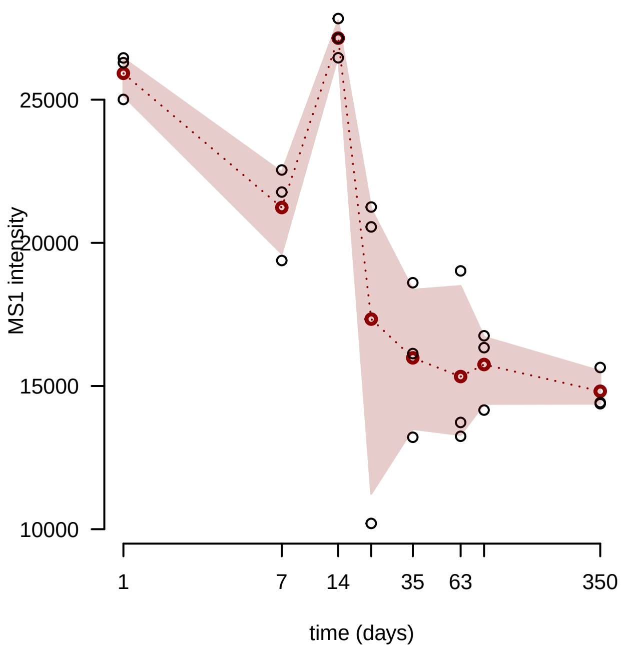

Q3U2A8
GN=Vars2
valyl−tRNA synthetase 2, mitochondrial

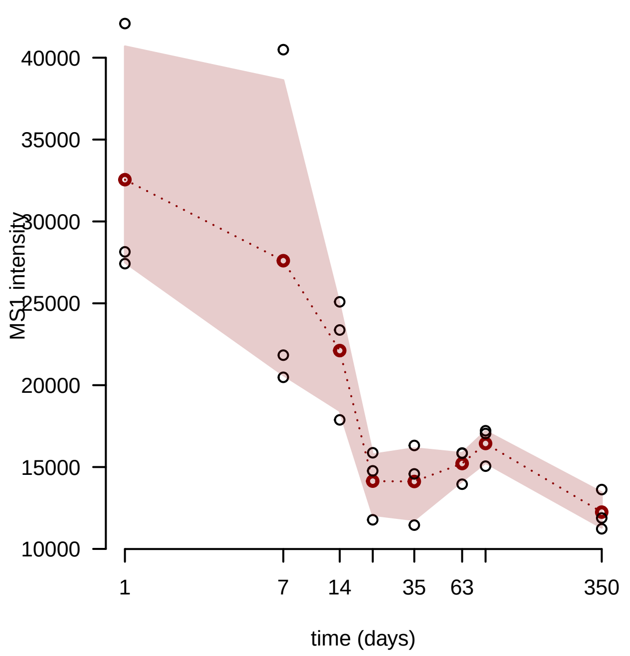

Q3U2E2
GN=Zmym5
zinc finger, MYM−type 5

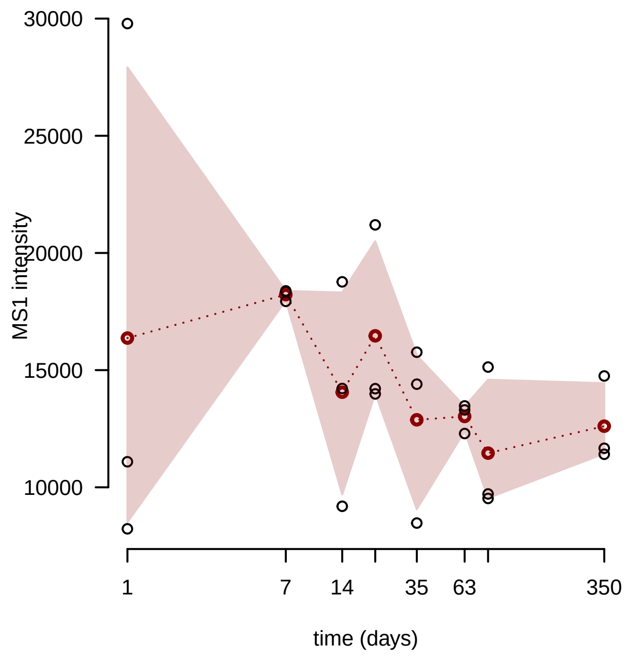

Q3U2I3
GN=Fam160a2

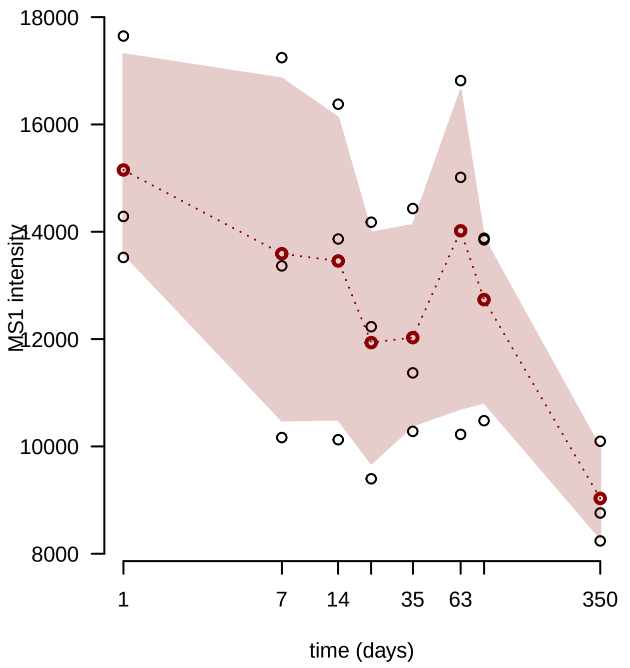

Q3U2J5
GN=Camkmt
calmodulin−lysine N−methyltransferase

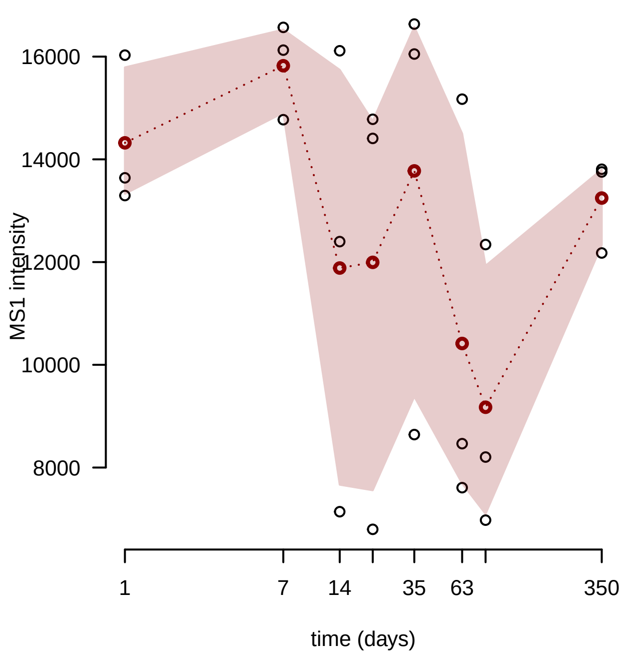

Q3U2K0
GN=Fam193b
family with sequence similarity 193, mem ...

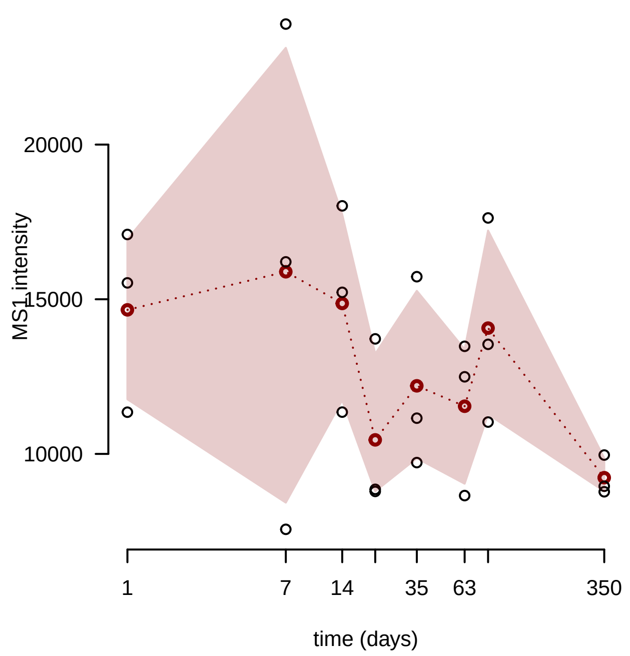

Q3U2P1
GN=Sec24a
Sec24 related gene family, member A (S. ...

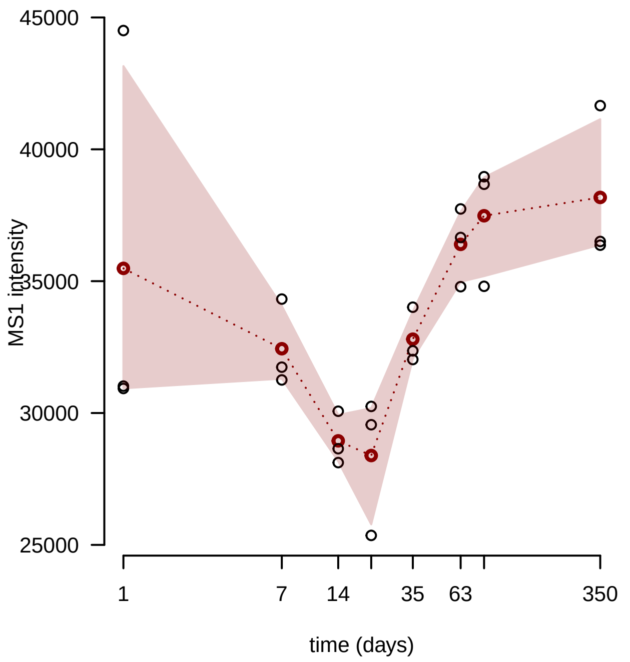

Q3U2V3
GN=Nudt18
nudix (nucleoside diphosphate linked moi ...

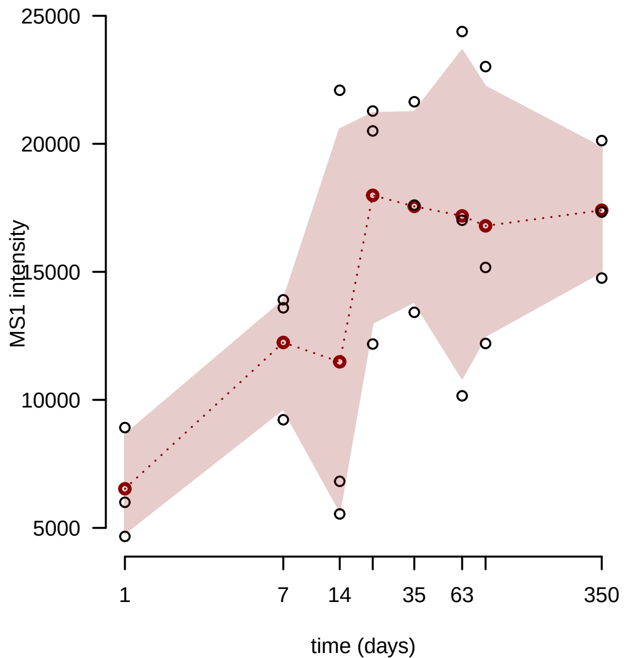

Q3U308
GN=Ctu2
cytosolic thiouridylase subunit 2

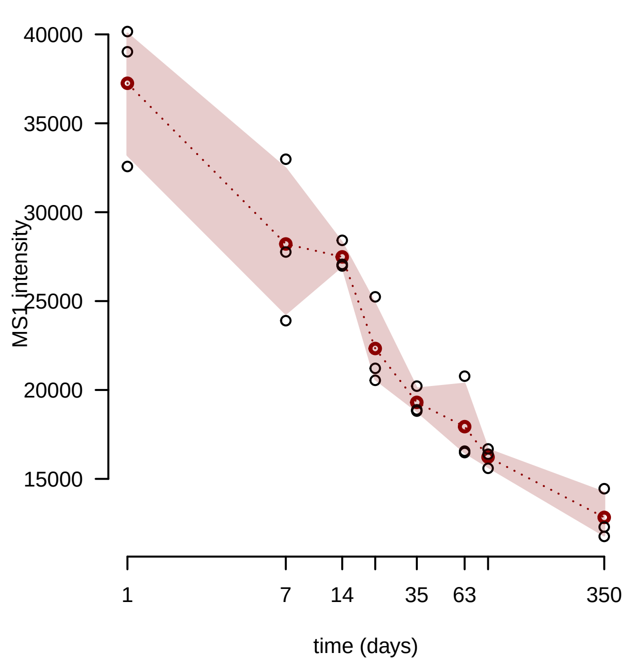

Q3U319
GN=Rnf40
ring finger protein 40

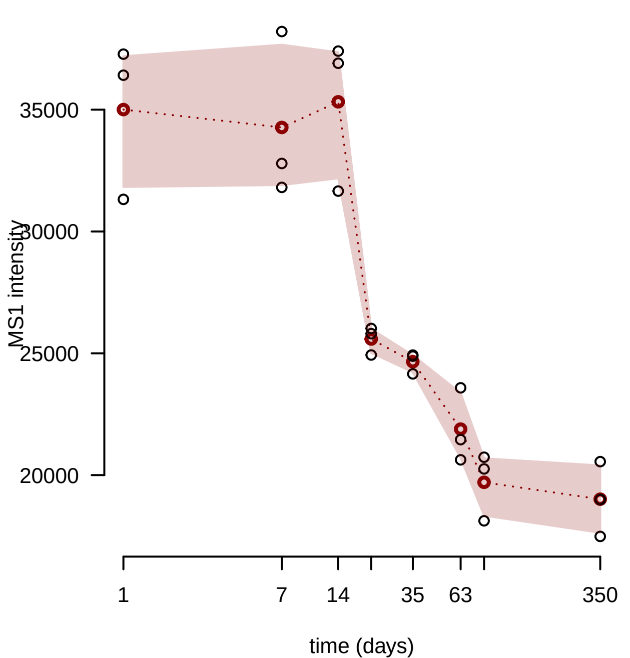

Q3U3C9
GN=Gse1
genetic suppressor element 1, coiled−coi ...

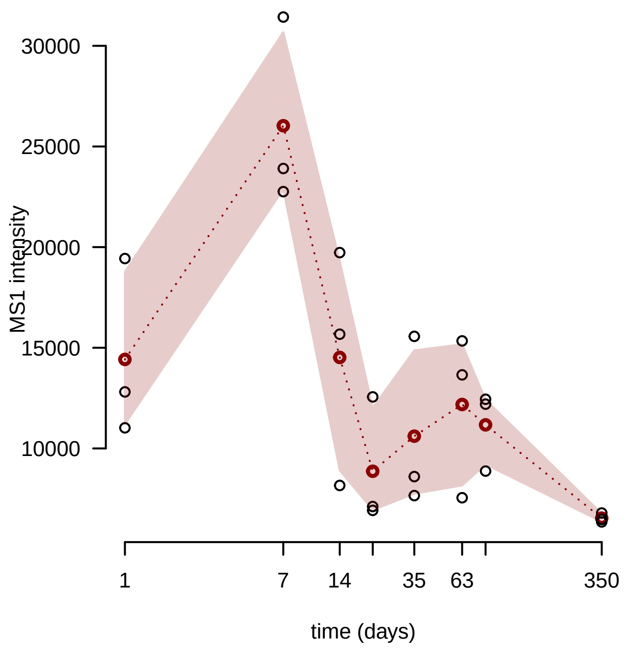

Q3U3E2
GN=Fam117b
family with sequence similarity 117, mem ...

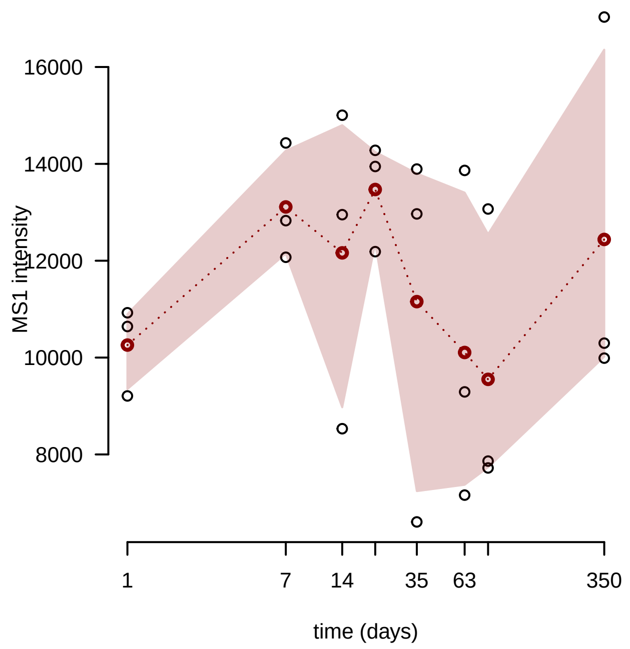

Q3U3I9
GN=Znf865

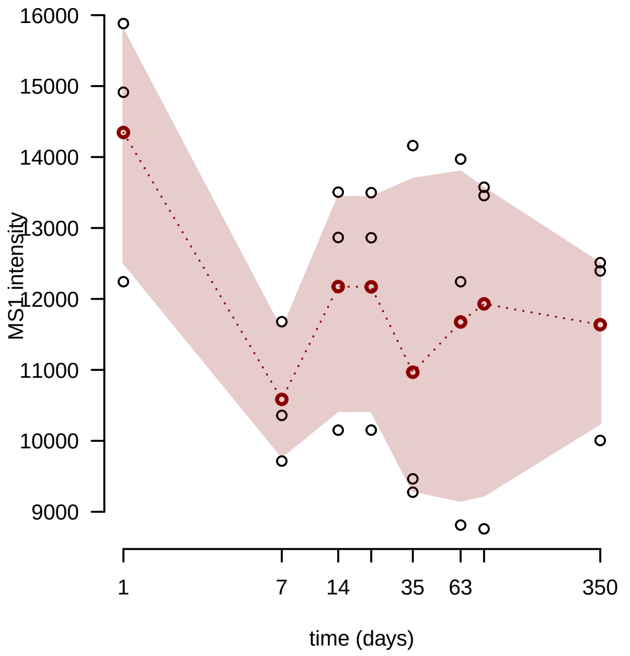

Q3U3Q1
GN=Ulk3
unc−51−like kinase 3

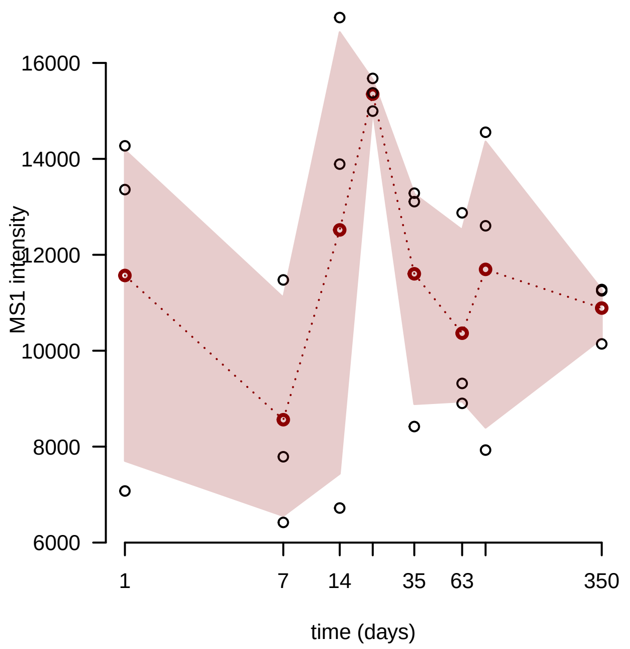

Q3U3R4
GN=Lmf1
lipase maturation factor 1

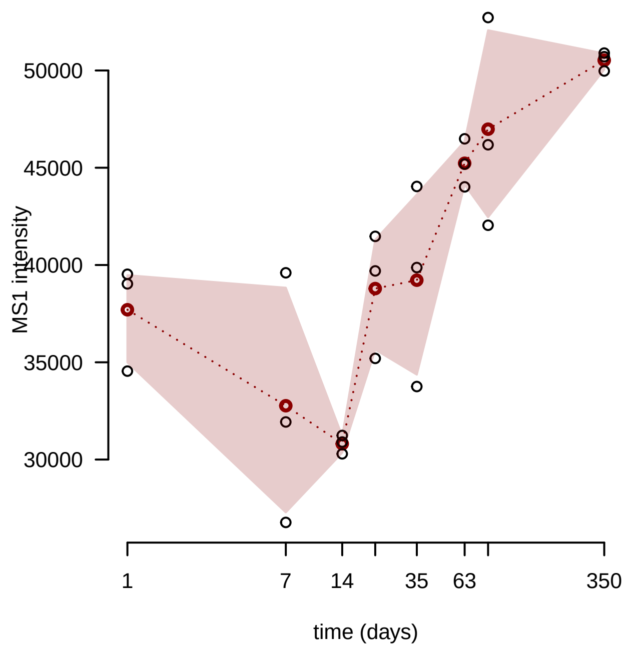

Q3U3S3
GN=Cenpl
centromere protein L

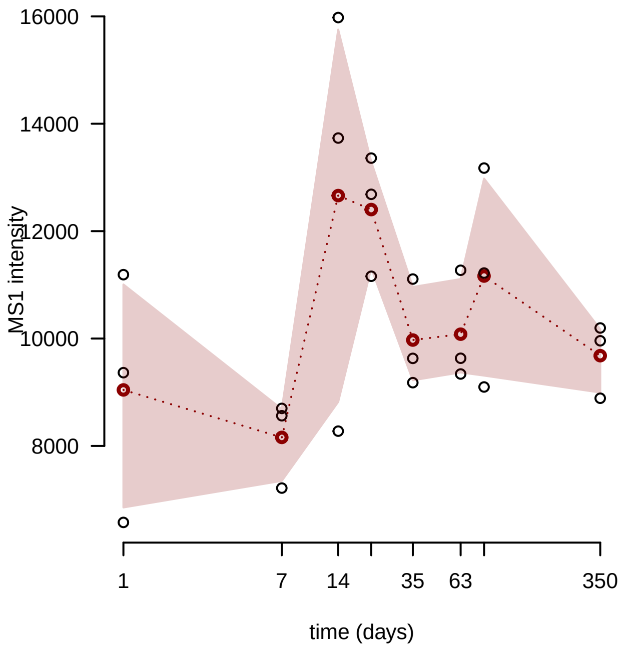

Q3U3T8
GN=Wdr62
WD repeat domain 62

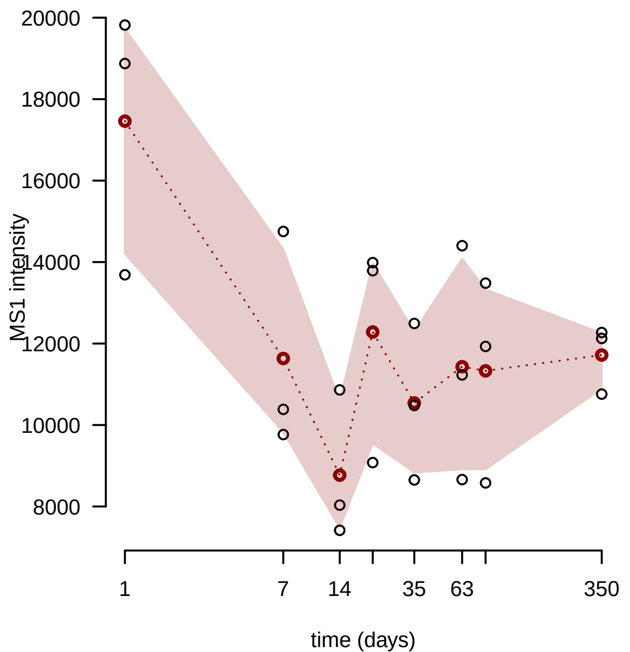

Q3U3W5
GN=Prmt9
protein arginine methyltransferase 9

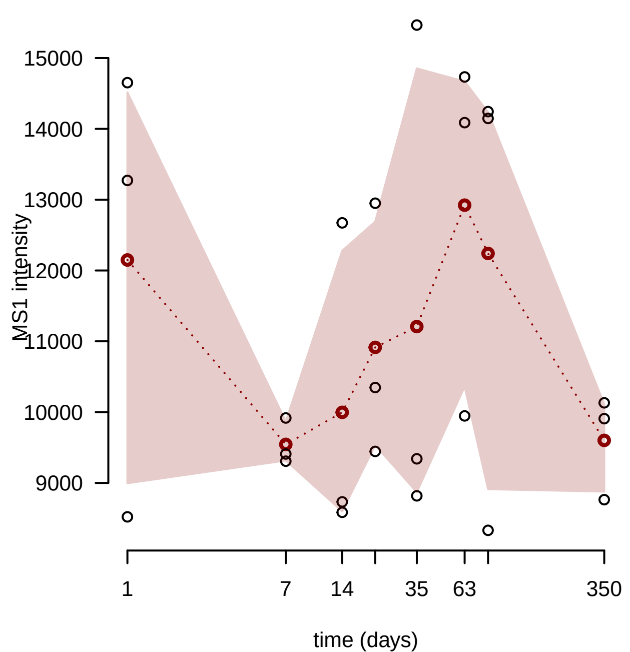

Q3U487
GN=Hectd3
HECT domain E3 ubiquitin protein ligase ...

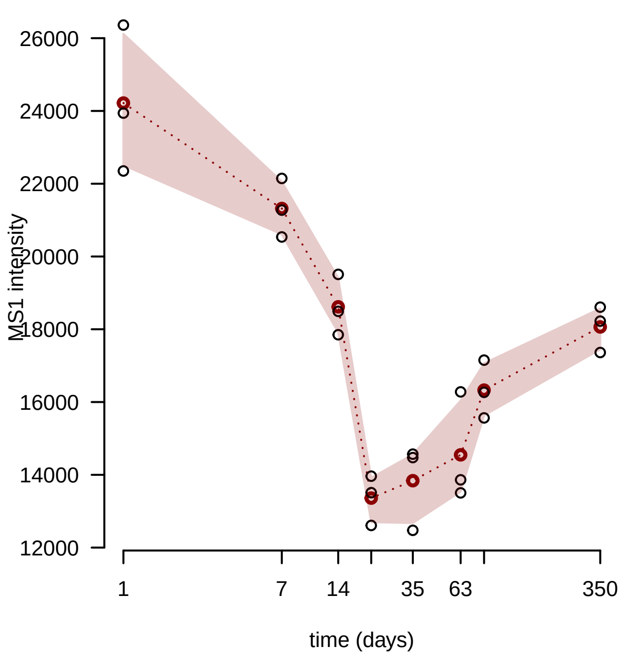

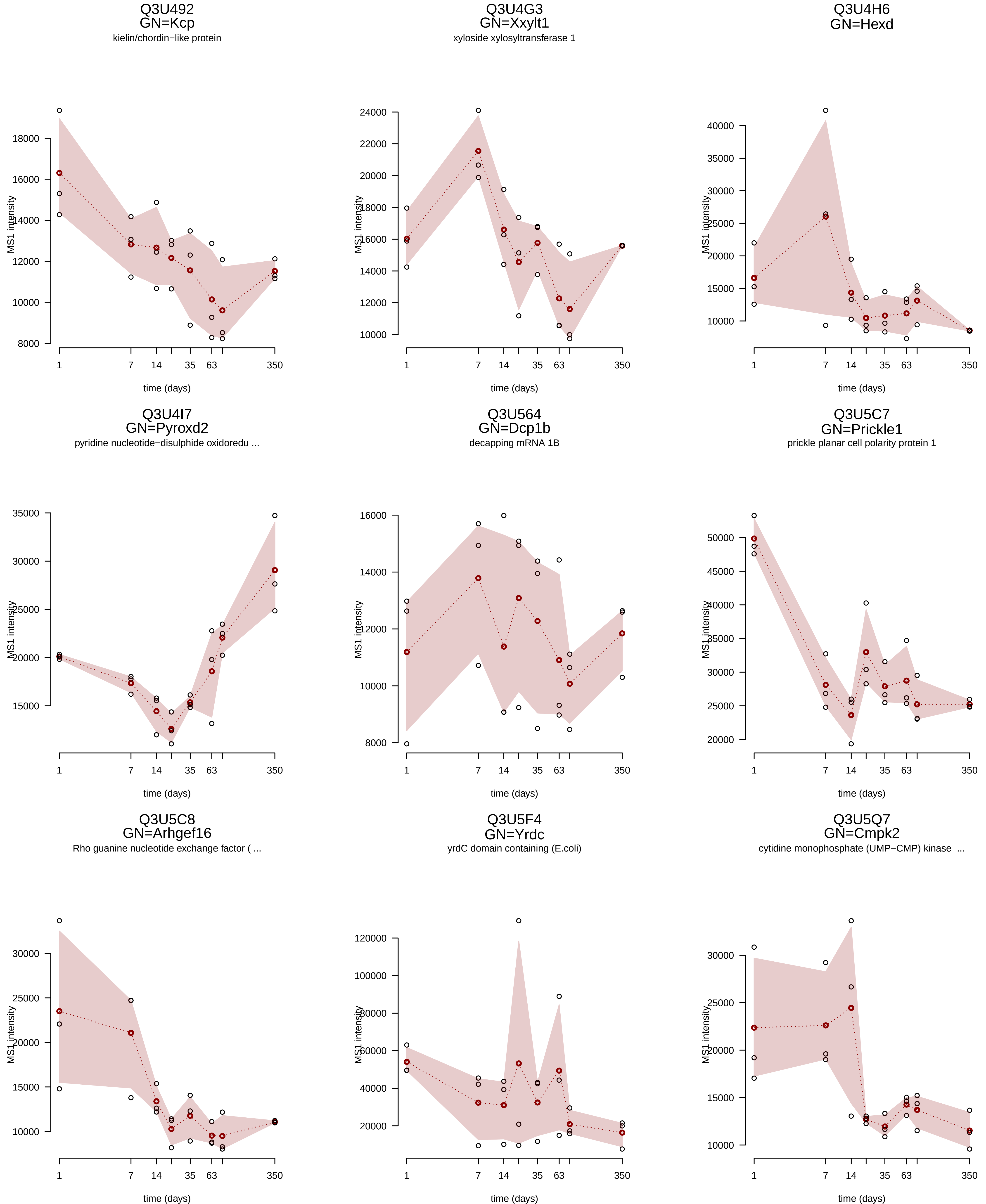
Q3U492
GN=Kcp
kielin/chordin−like protein
MS1 intensity
time (days)
Q3U4G3
GN=Xxylt1
xyloside xylosyltransferase 1
MS1 intensity
time (days)
Q3U4H6
GN=Hexd
MS1 intensity
time (days)
Q3U4I7
GN=Pyroxd2
pyridine nucleotide−disulphide oxidoredu ...
MS1 intensity
time (days)
Q3U564
GN=Dcp1b
decapping mRNA 1B
MS1 intensity
time (days)
Q3U5C7
GN=Prickle1
prickle planar cell polarity protein 1
MS1 intensity
time (days)
Q3U5C8
GN=Arhgef16
Rho guanine nucleotide exchange factor (...
MS1 intensity
time (days)
Q3U5F4
GN=Yrdc
yrdC domain containing (E.coli)
MS1 intensity
time (days)
Q3U5Q7
GN=Cmpk2
cytidine monophosphate (UMP−CMP) kinase ...
MS1 intensity
time (days)
1
7
14
35
63
350

Q3U6N9
GN=

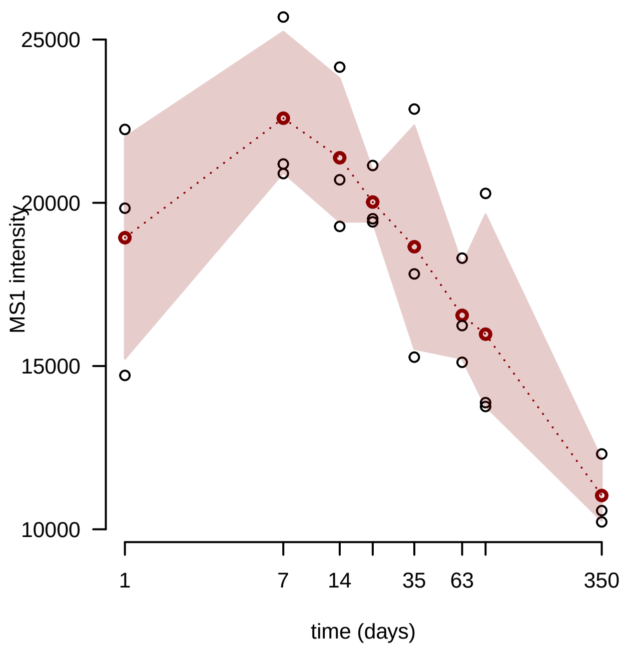

Q3U6U5
GN=Gtpbp6
GTP binding protein 6 (putative)

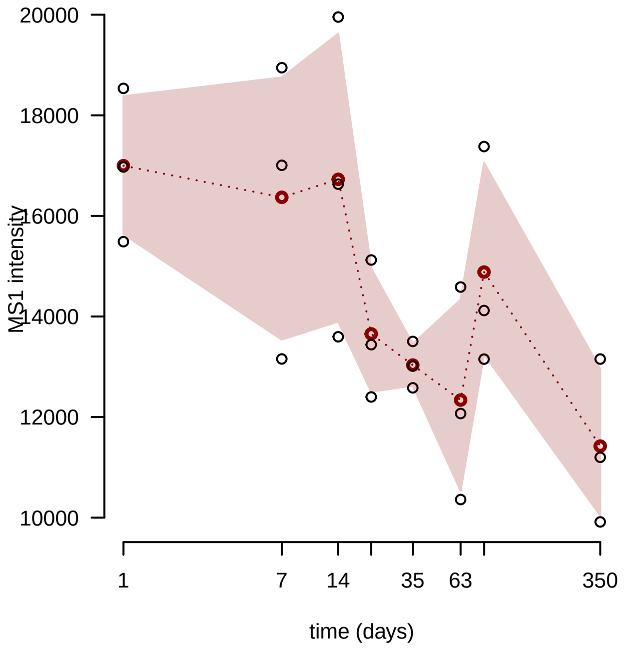

Q3U7R1
GN=Esyt1
extended synaptotagmin−like protein 1

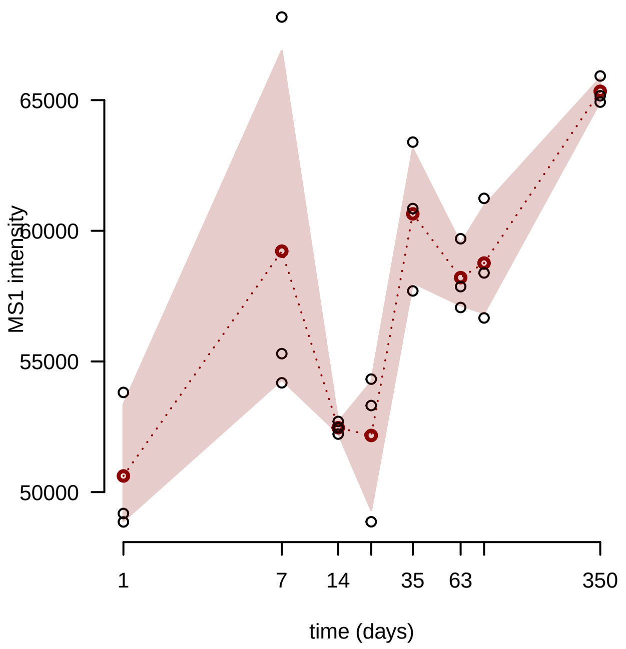

Q3U7U3
GN=Fbxo7
F−box protein 7

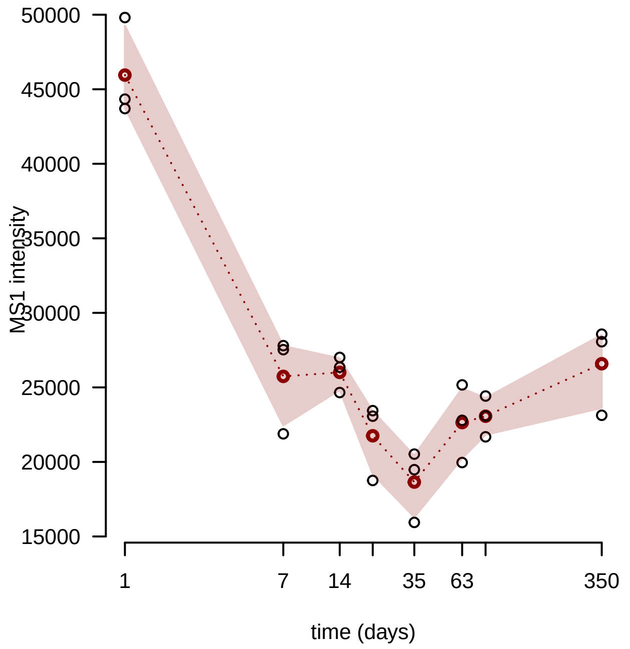

Q3U821
GN=Wdr75
WD repeat domain 75

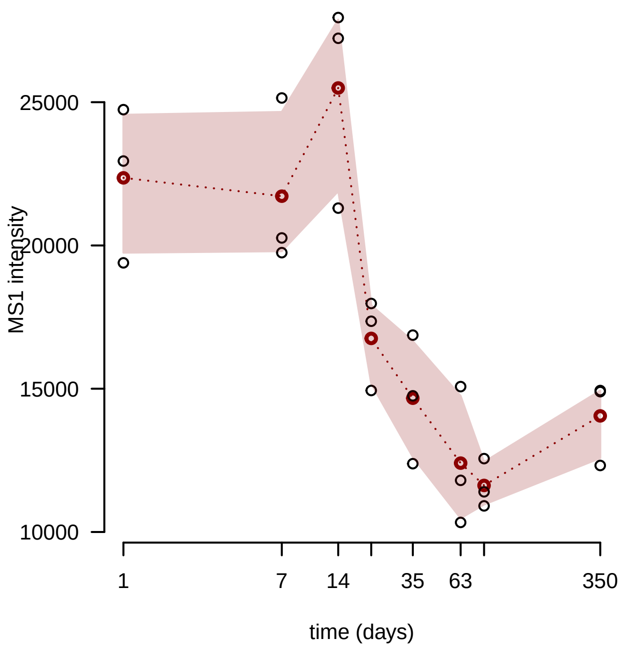

Q3U827
GN=Rnf180
ring finger protein 180

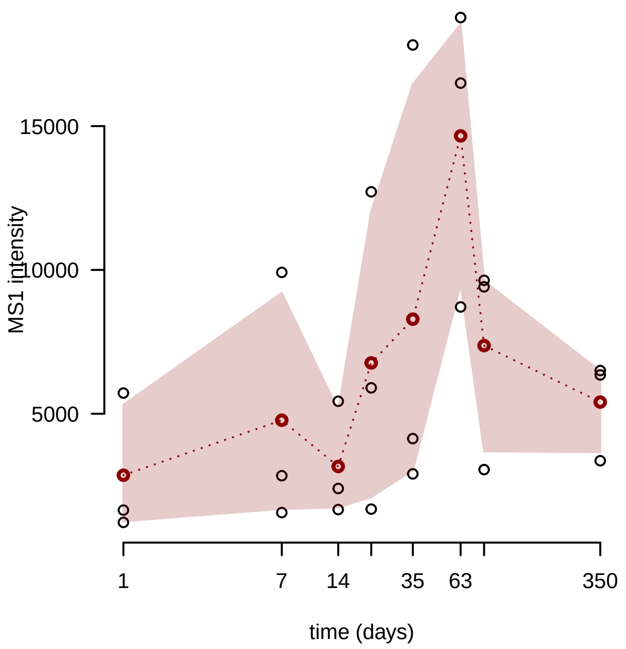

Q3U829
GN=Ap5z1
adaptor−related protein complex 5, zeta ...

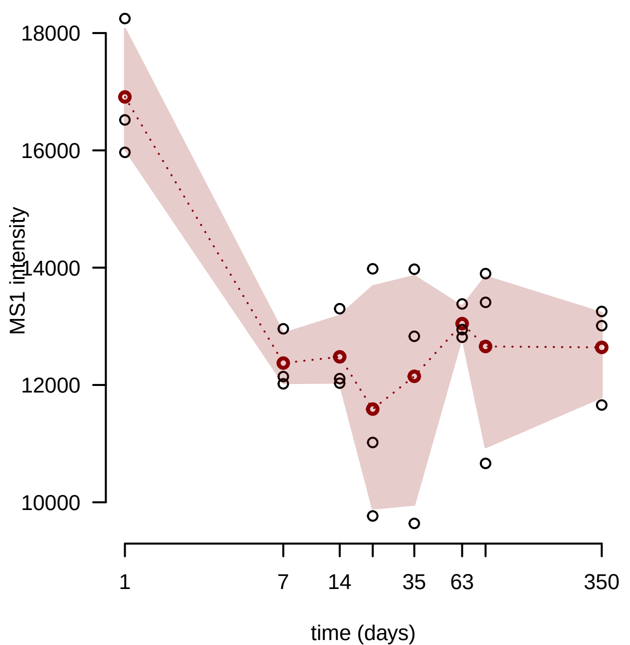

Q3U898
GN=Cops9
COP9 signalosome subunit 9

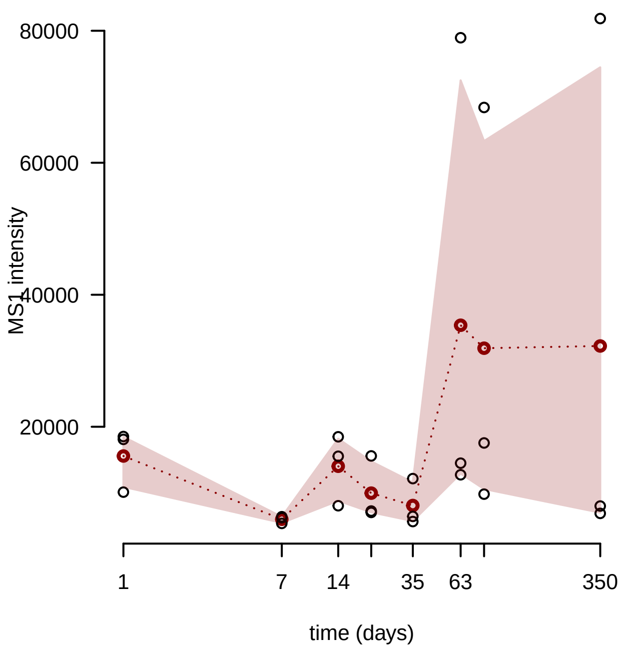

Q3U962
GN=Col5a2
collagen, type V, alpha 2

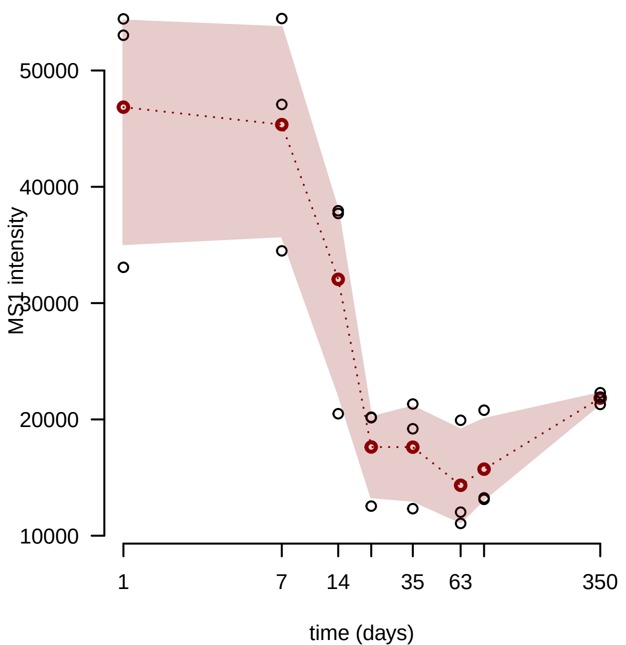

Q3U9G9
GN=Lbr
lamin B receptor

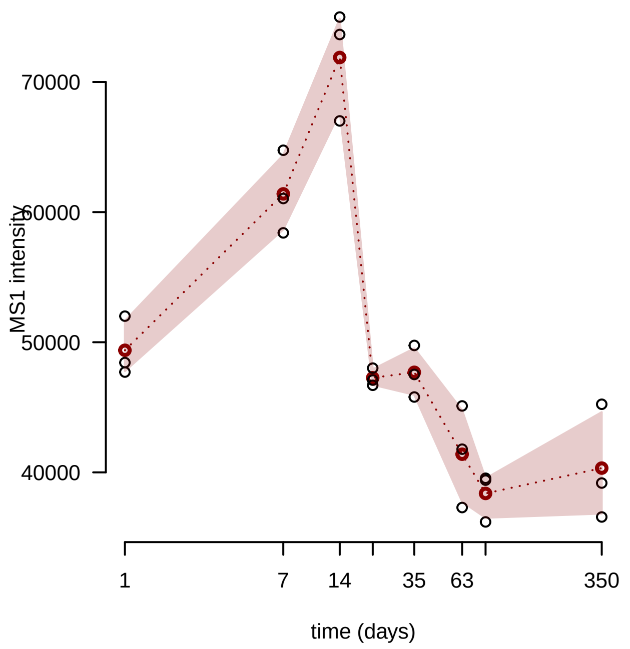

Q3UA06
GN=Trip13
thyroid hormone receptor interactor 13

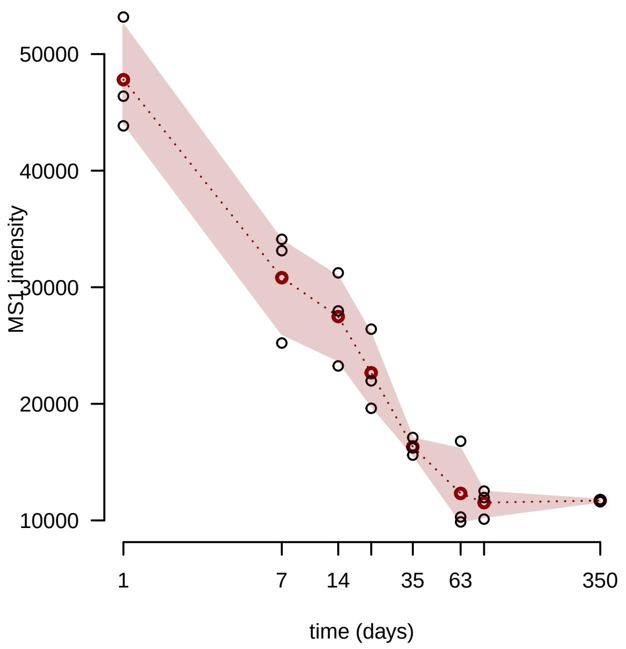

Q3UA16
GN=Spc25
SPC25, NDC80 kinetochore complex compone ...

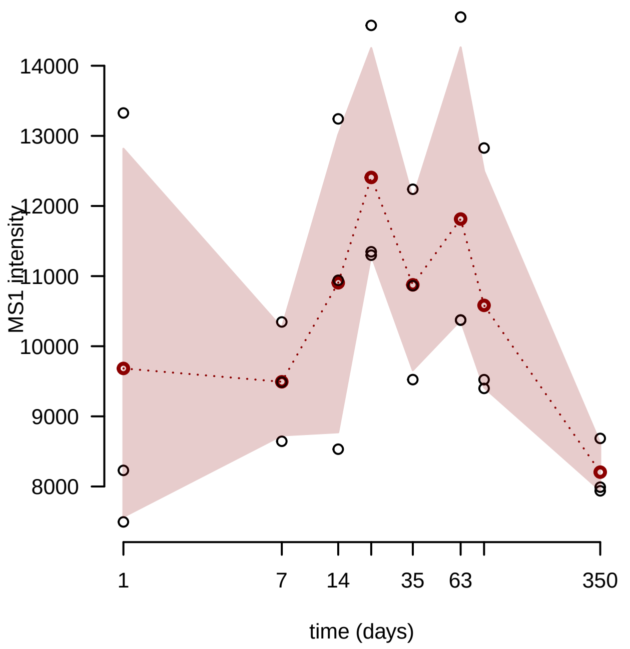

Q3UA37
GN=Qrich1
glutamine−rich 1

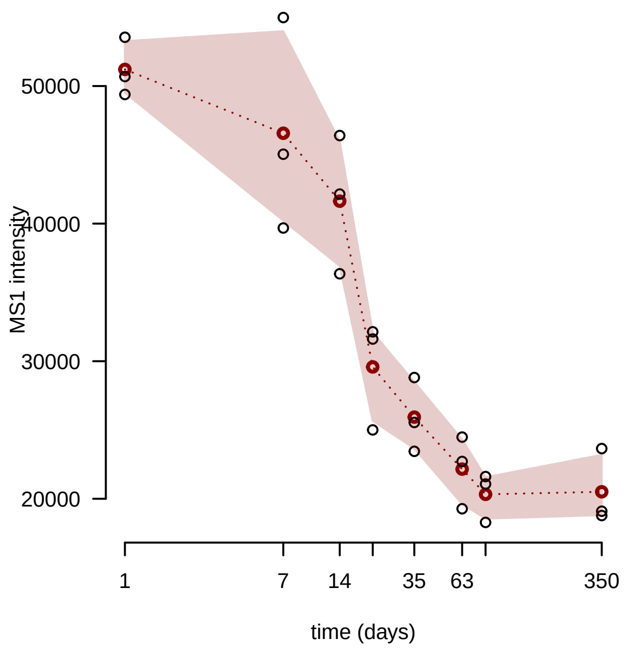

Q3UBG2
GN=Pid1
phosphotyrosine interaction domain conta ...

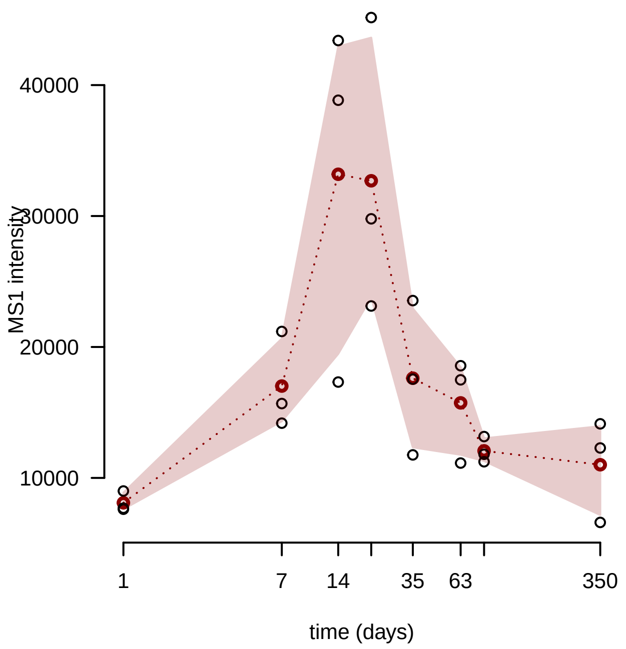

Q3UBX0
GN=Tmem109
transmembrane protein 109

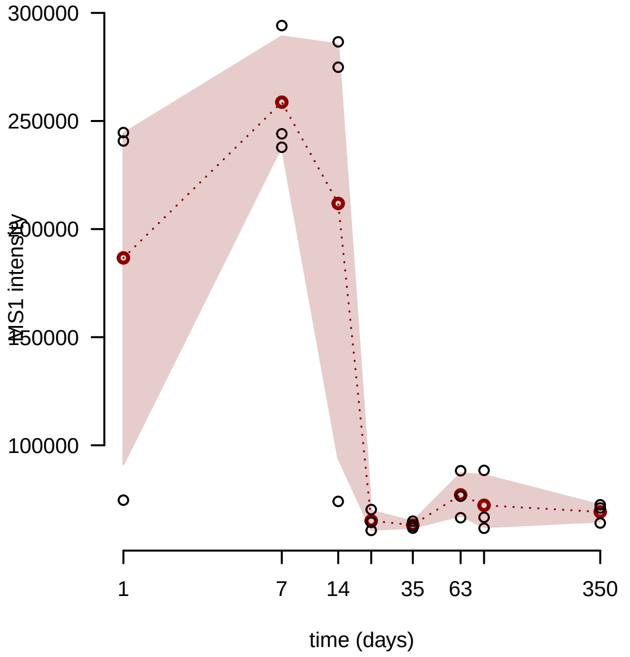

Q3UBZ5
GN=Mif4gd
MIF4G domain containing

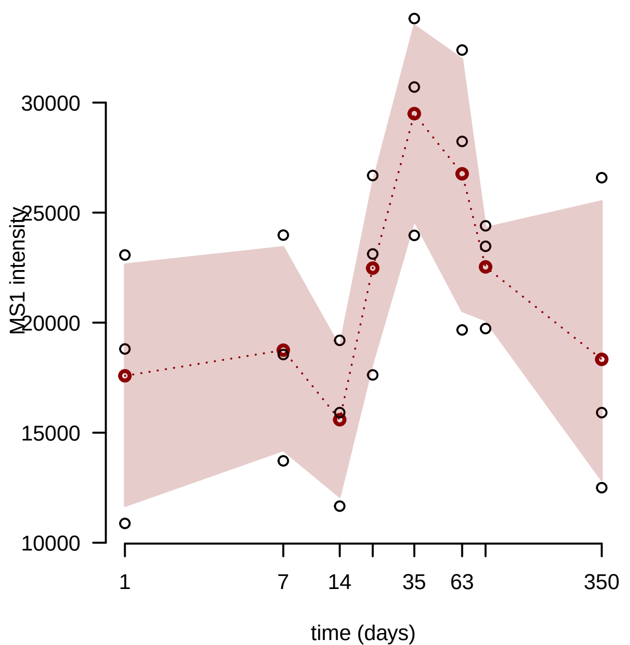

Q3UC65
GN=Rsrp1
arginine/serine rich protein 1

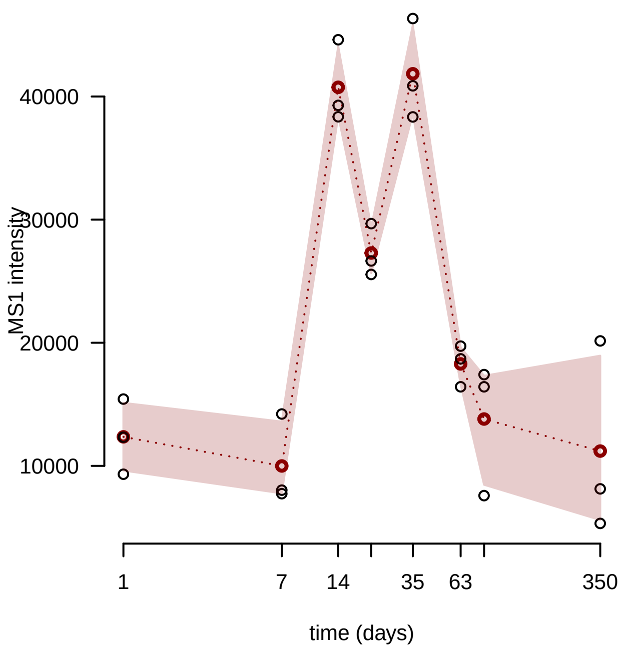

Q3UCQ1
GN=Foxk2
forkhead box K2

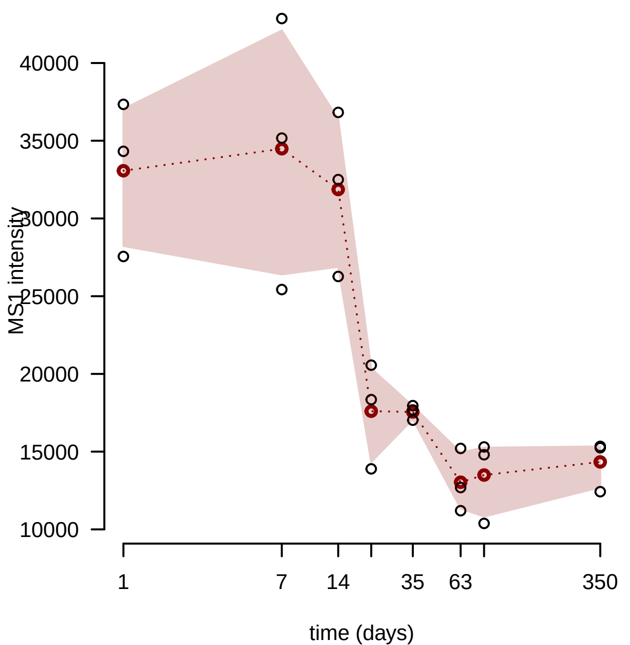

Q3UCV8
GN=Otulin
OTU deubiquitinase with linear linkage s ...

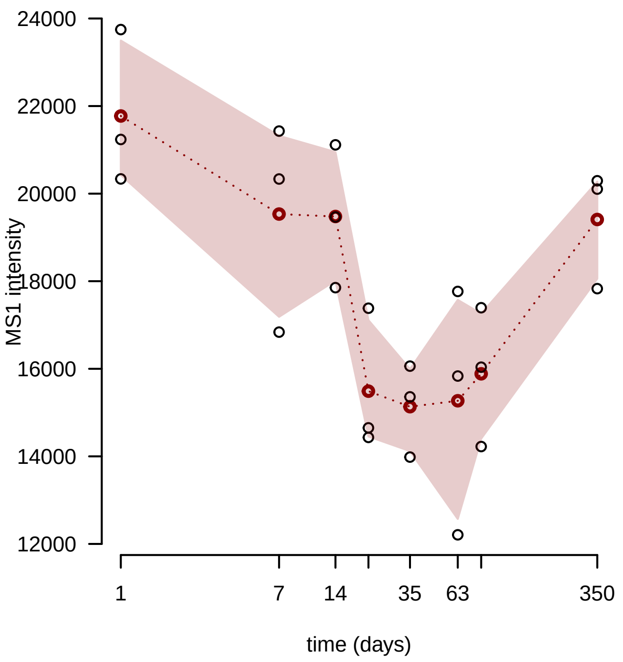

Q3UD01
GN=Atxn7l3b
ataxin 7−like 3B

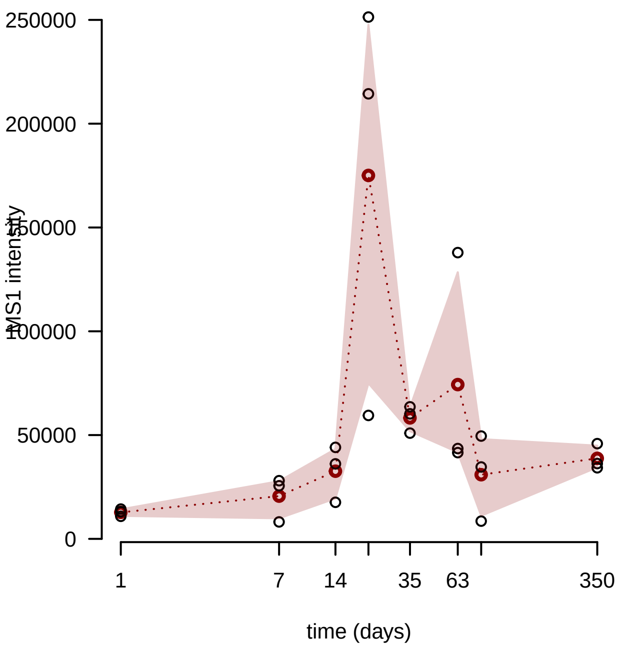

Q3UDE2
GN=Ttll12
tubulin tyrosine ligase−like family, mem ...

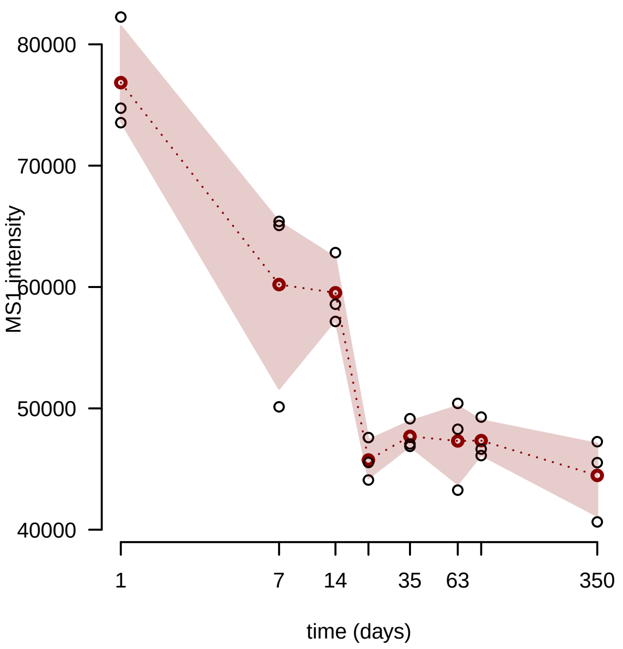

Q3UDK1
GN=Trafd1
TRAF type zinc finger domain containing ...

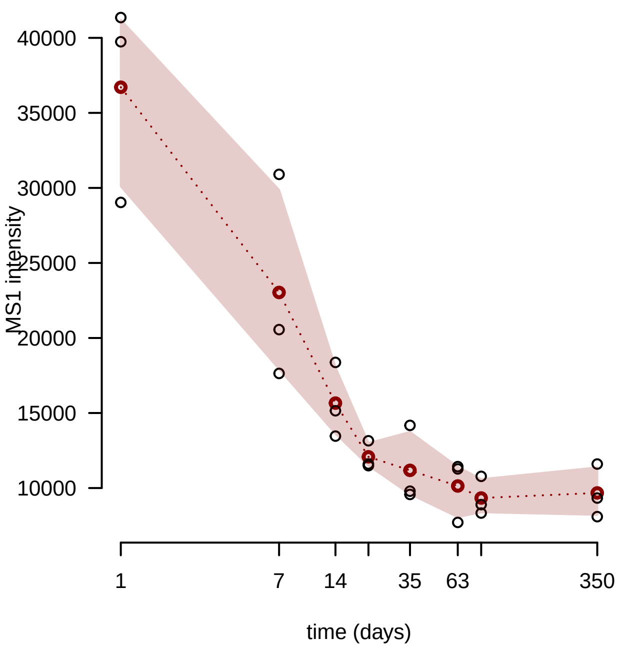

Q3UDP0
GN=Wdr41
WD repeat domain 41

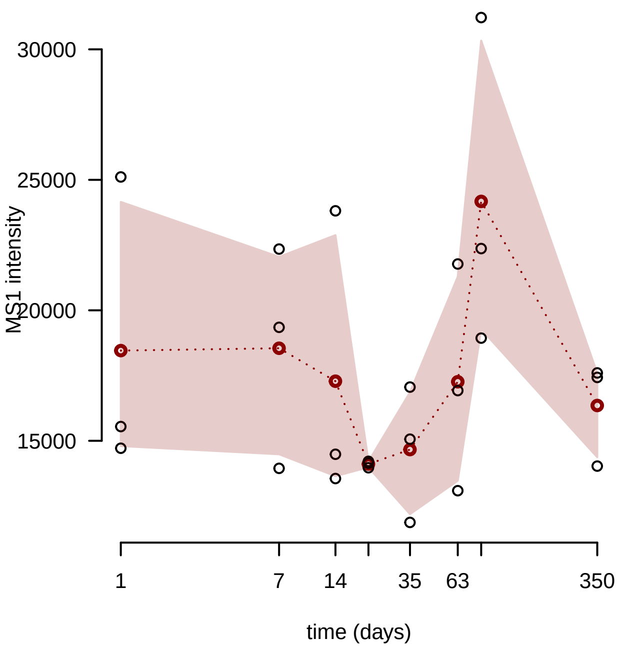

Q3UDR8
GN=Yipf3
Yip1 domain family, member 3

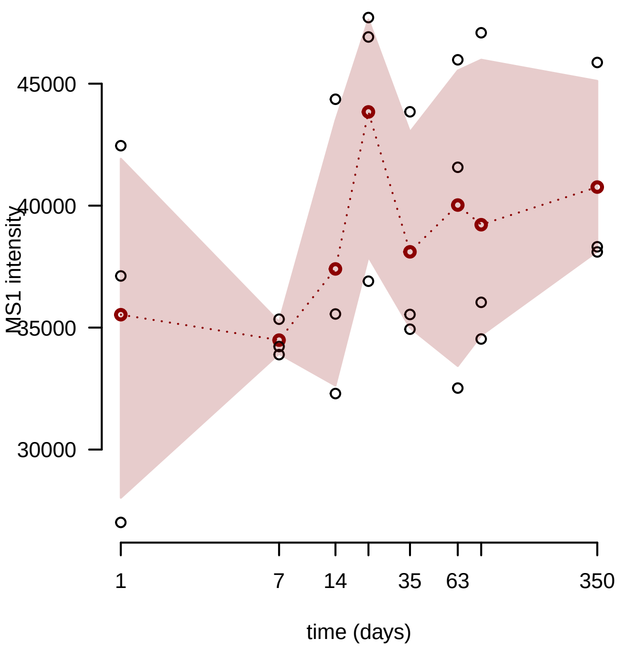

Q3UDW8
GN=Hgsnat
heparan−alpha−glucosaminide N−acetyltran ...

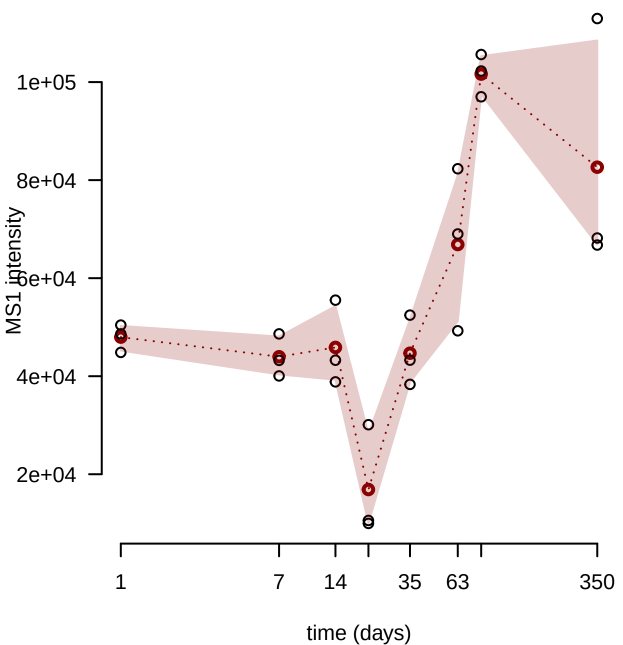

Q3UE17
GN=Mex3d
mex3 RNA binding family member D

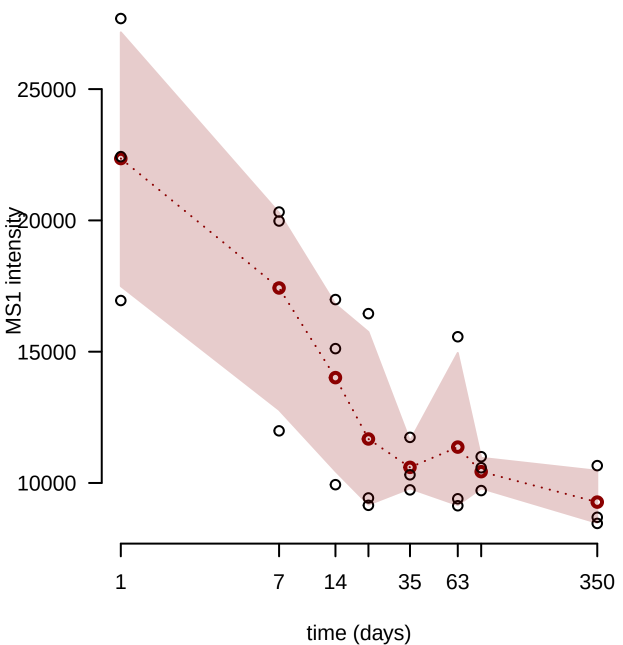

Q3UE31
GN=

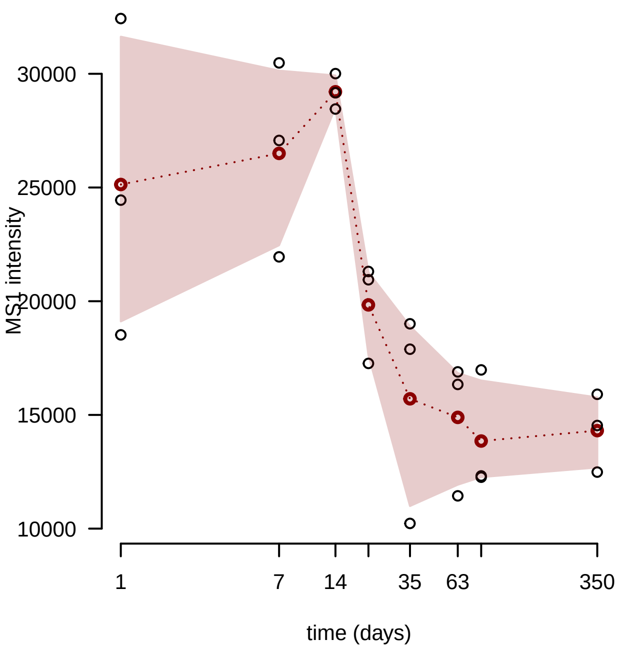

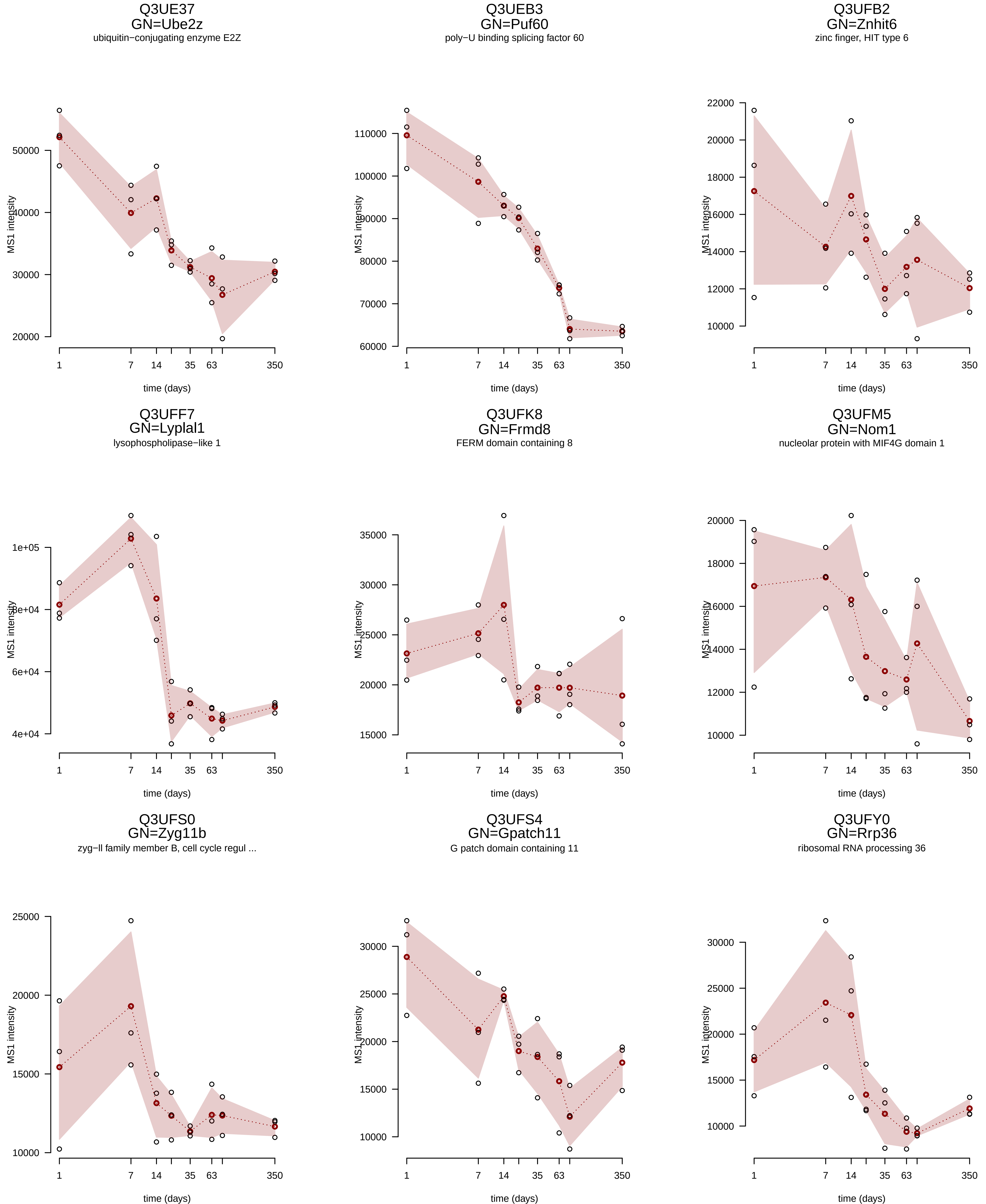
Q3UE37
GN=Ube2z
ubiquitin−conjugating enzyme E2Z
Q3UEB3
GN=Puf60
poly−U binding splicing factor 60
Q3UFB2
GN=Znhit6
zinc finger, HIT type 6
Q3UFF7
GN=Lyplal1
lysophospholipase−like 1
Q3UFK8
GN=Frmd8
FERM domain containing 8
Q3UFM5
GN=Nom1
nucleolar protein with MIF4G domain 1
Q3UFS0
GN=Zyg11b
zyg−ll family member B, cell cycle regul ...
Q3UFS4
GN=Gpatch11
G patch domain containing 11
Q3UFY0
GN=Rrp36
ribosomal RNA processing 36
MS1 intensity
time (days)
1
7
14
35
63
350

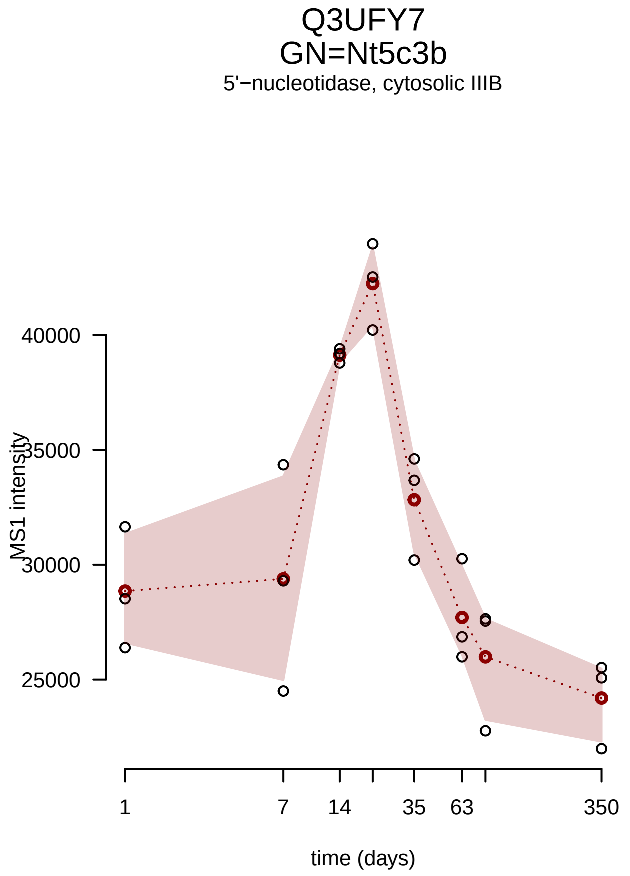
Q3UFY7
GN=Nt5c3b
5'−nucleotidase, cytosolic IIIB
MS1 intensity
time (days)
25000
30000
35000
40000
1
7
14
35
63
350

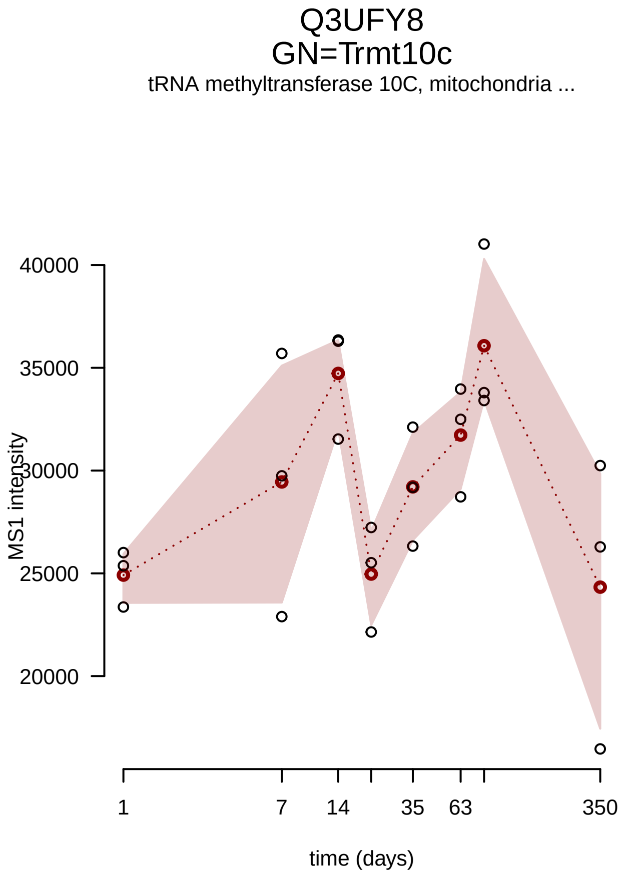
Q3UFY8
GN=Trmt10c
tRNA methyltransferase 10C, mitochondria ...
MS1 intensity
time (days)
20000
25000
30000
35000
40000
1
7
14
35
63
350

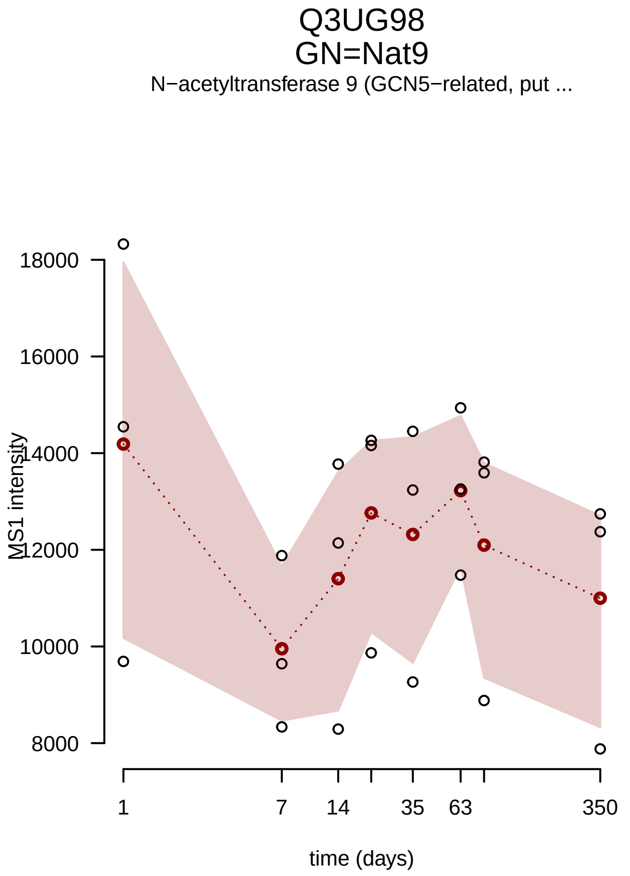
Q3UG98
GN=Nat9
N−acetyltransferase 9 (GCN5−related, put ...
MS1 intensity
time (days)
8000
10000
12000
14000
16000
18000
1
7
14
35
63
350

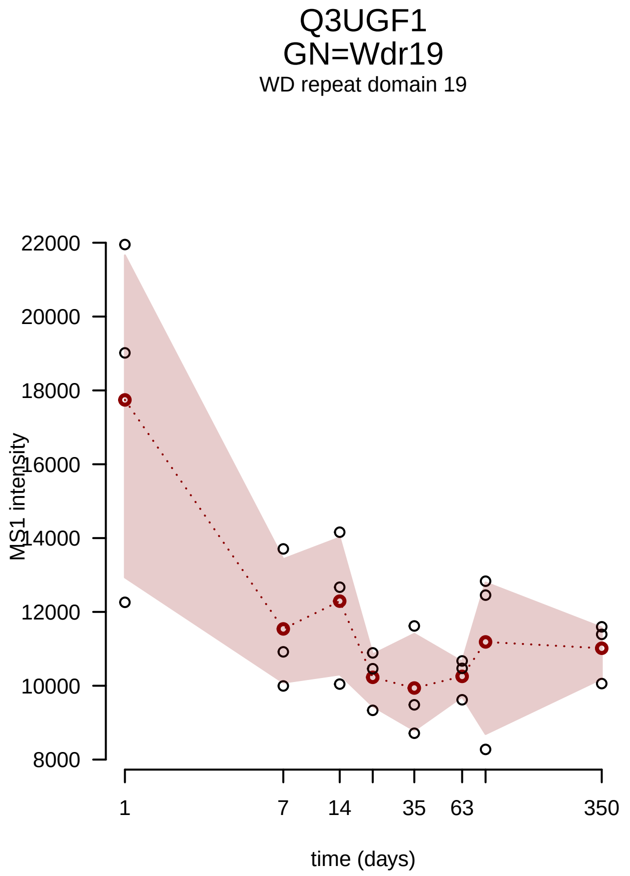
Q3UGF1
GN=Wdr19
WD repeat domain 19
MS1 intensity
time (days)
8000
10000
12000
14000
16000
18000
20000
22000
1
7
14
35
63
350

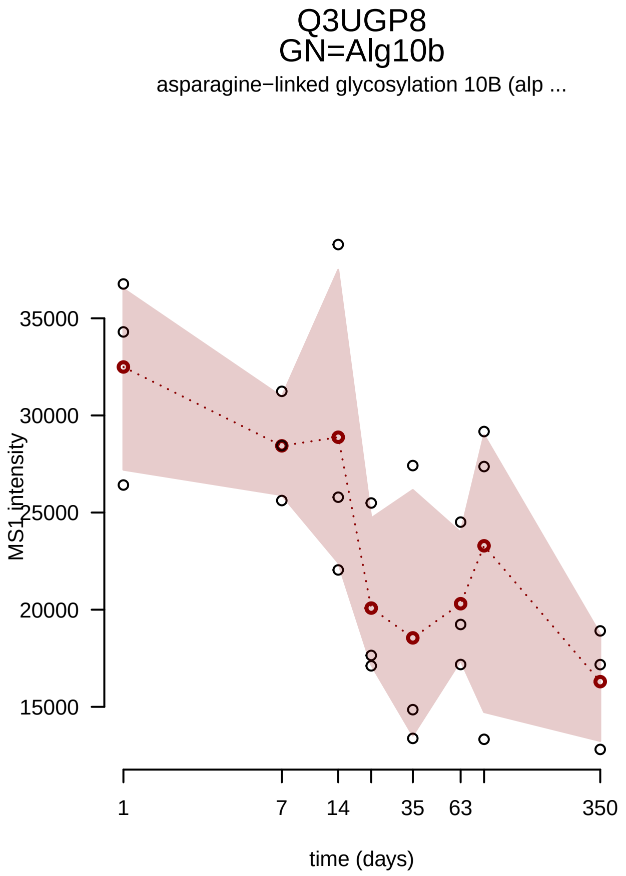
Q3UGP8
GN=Alg10b
asparagine−linked glycosylation 10B (alp ...
MS1 intensity
time (days)
15000
20000
25000
30000
35000
1
7
14
35
63
350

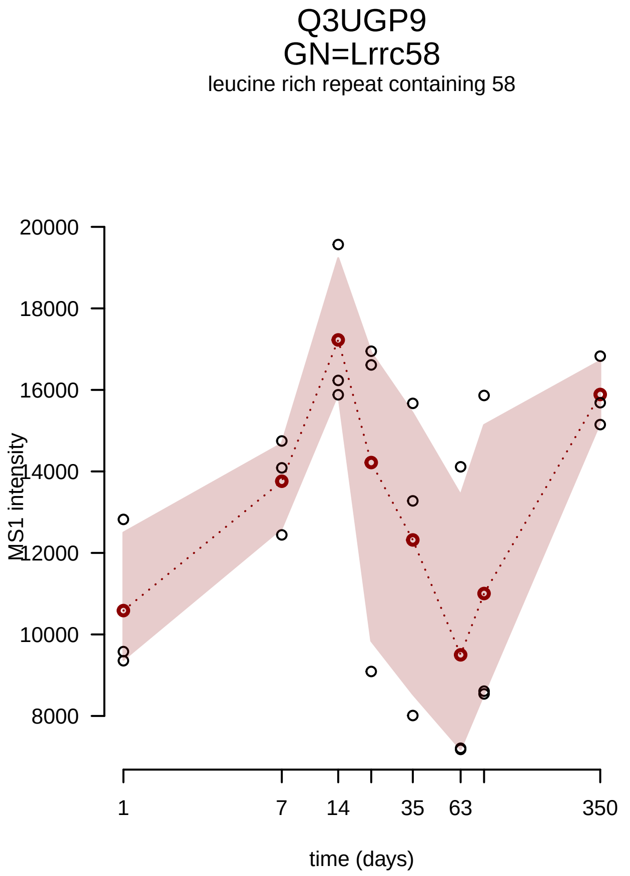
Q3UGP9
GN=Lrrc58
leucine rich repeat containing 58
MS1 intensity
time (days)
8000
10000
12000
14000
16000
18000
20000
1
7
14
35
63
350

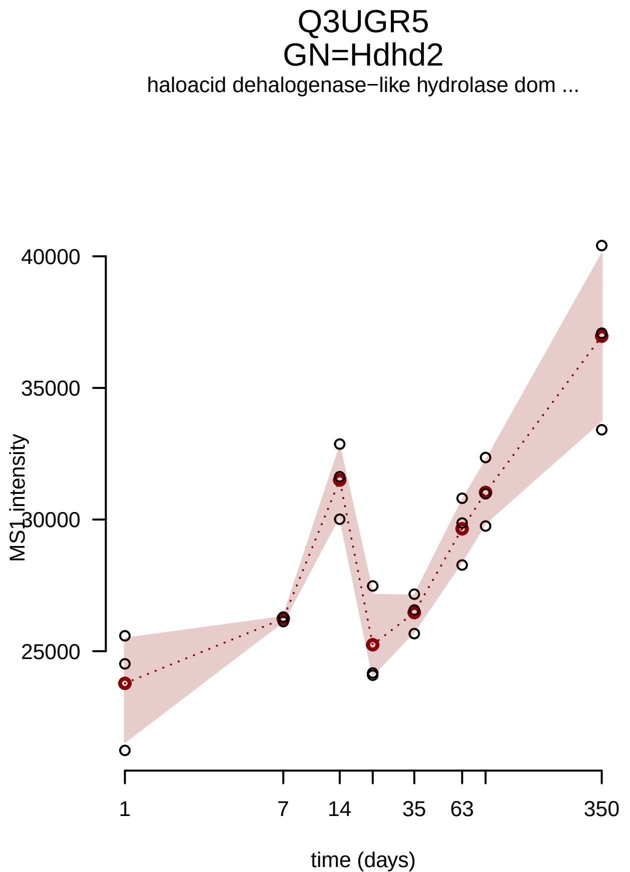
Q3UGR5
GN=Hdhd2
haloacid dehalogenase−like hydrolase dom ...
MS1 intensity
time (days)
25000
30000
35000
40000
1
7
14
35
63
350

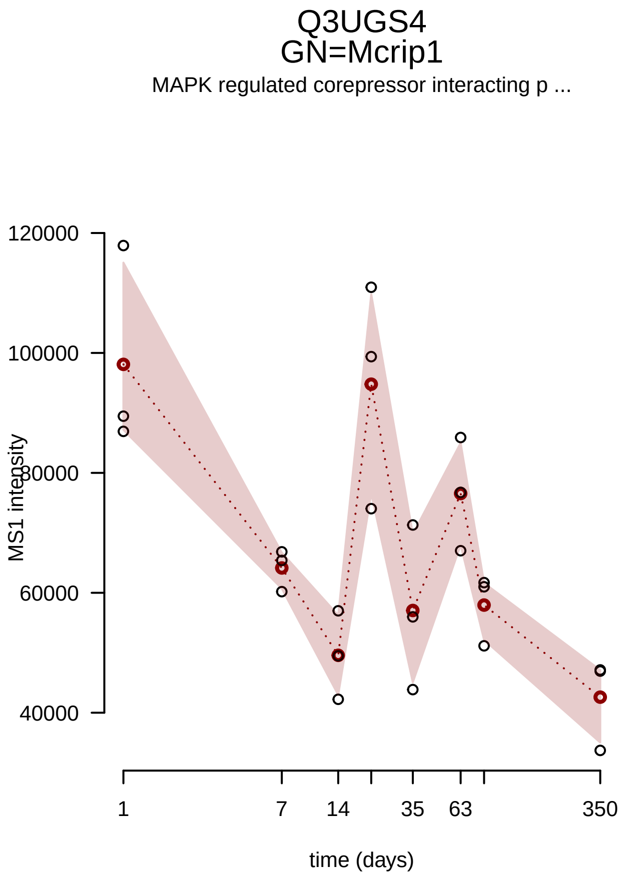
Q3UGS4
GN=Mcrip1
MAPK regulated corepressor interacting p ...
MS1 intensity
time (days)
40000
60000
80000
100000
120000
1
7
14
35
63
350

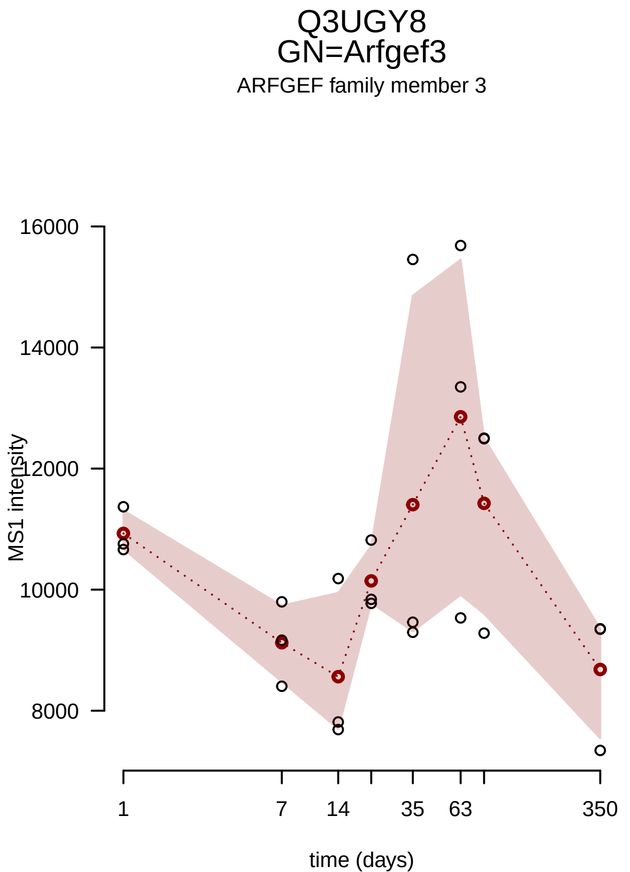
Q3UGY8
GN=Arfgef3
ARFGEF family member 3
MS1 intensity
time (days)
8000
10000
12000
14000
16000
1
7
14
35
63
350

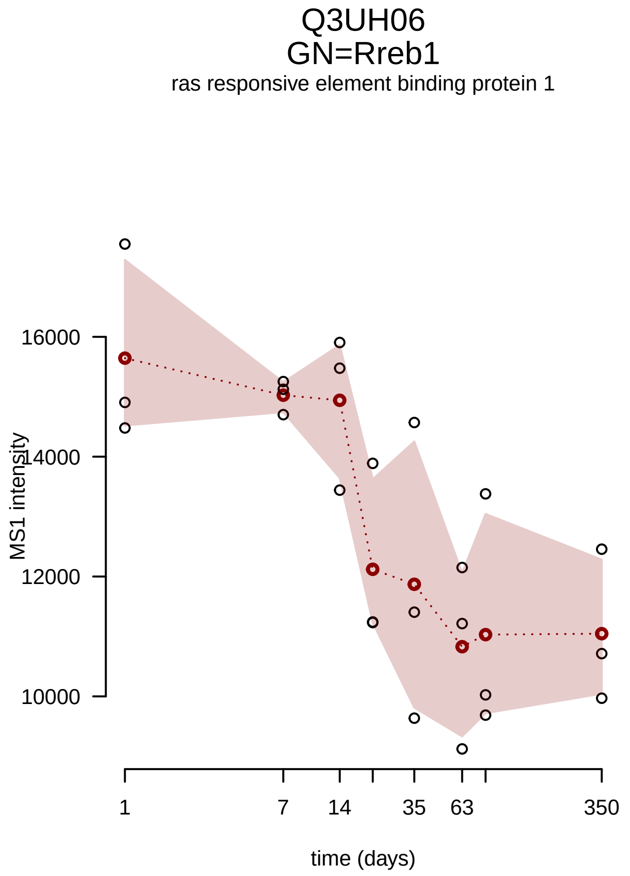
Q3UH06
GN=Rreb1
ras responsive element binding protein 1
MS1 intensity
10000
12000
14000
16000
1
7
14
35
63
350
time (days)

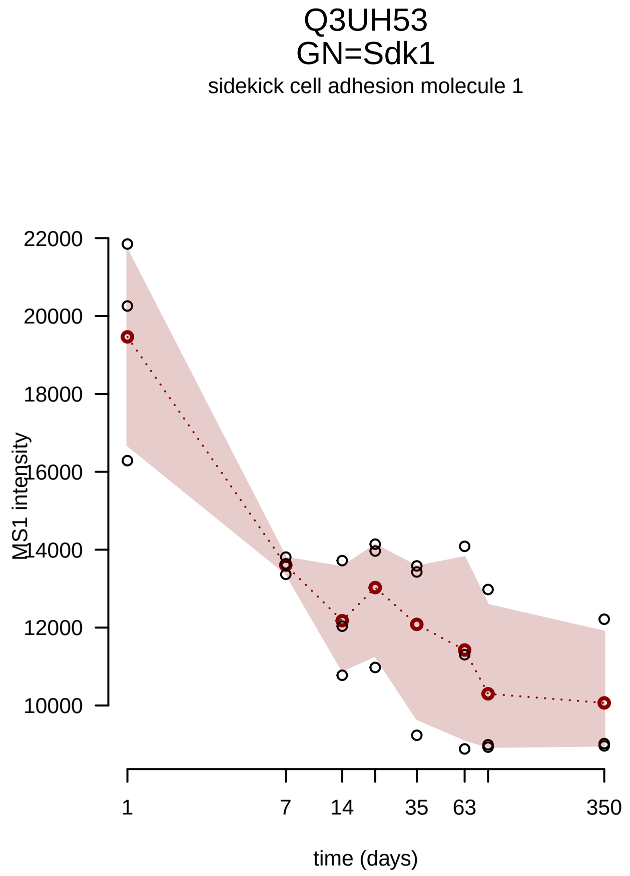
Q3UH53
GN=Sdk1
sidekick cell adhesion molecule 1
MS1 intensity
10000
12000
14000
16000
18000
20000
22000
1
7
14
35
63
350
time (days)

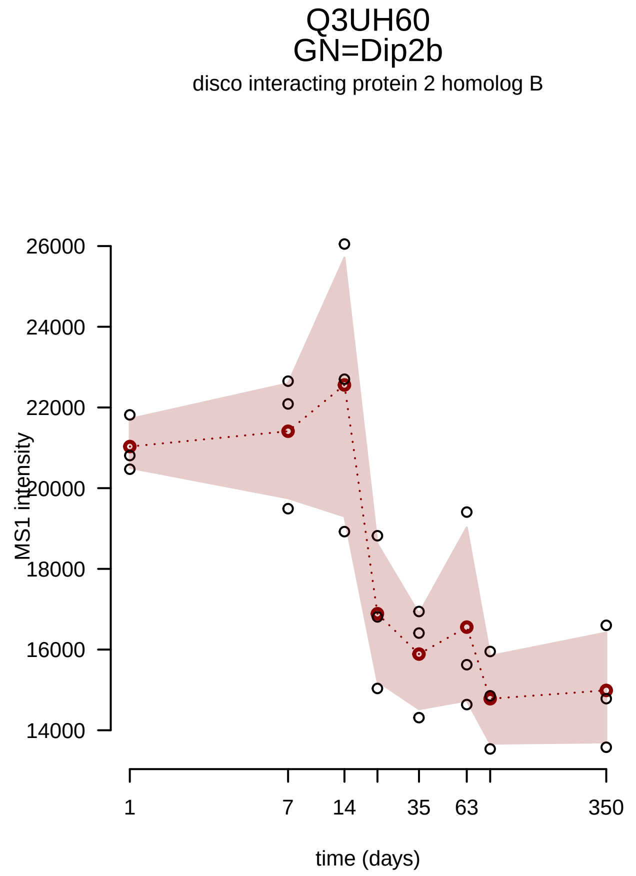
Q3UH60
GN=Dip2b
disco interacting protein 2 homolog B
MS1 intensity
14000
16000
18000
20000
22000
24000
26000
1
7
14
35
63
350
time (days)

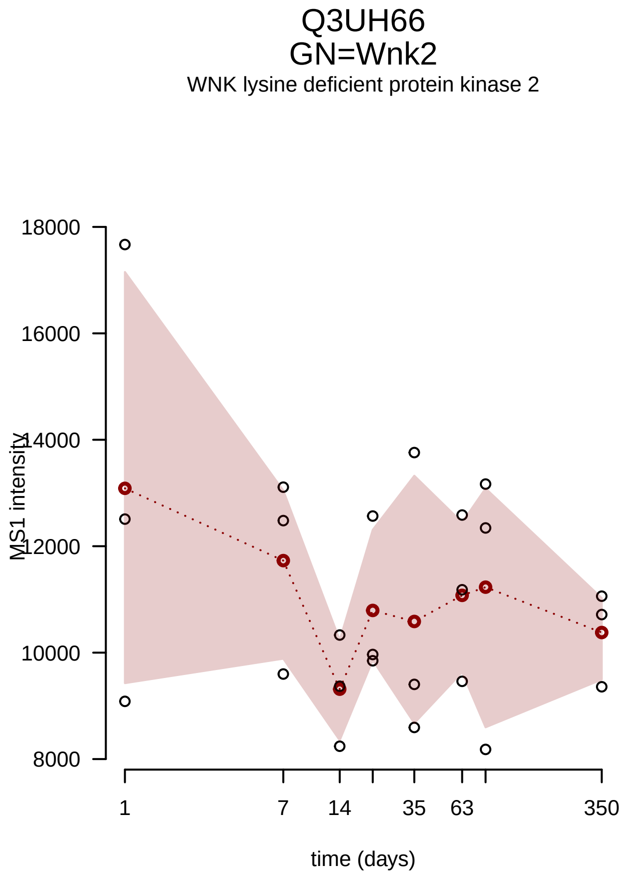
Q3UH66
GN=Wnk2
WNK lysine deficient protein kinase 2
MS1 intensity
8000
10000
12000
14000
16000
18000
1
7
14
35
63
350
time (days)

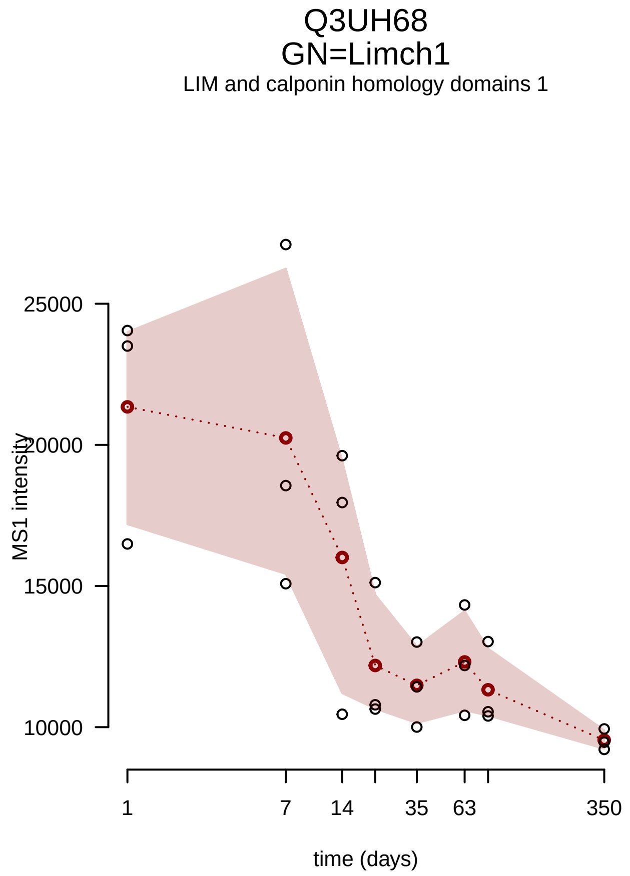
Q3UH68
GN=Limch1
LIM and calponin homology domains 1
MS1 intensity
10000
15000
20000
25000
1
7
14
35
63
350
time (days)

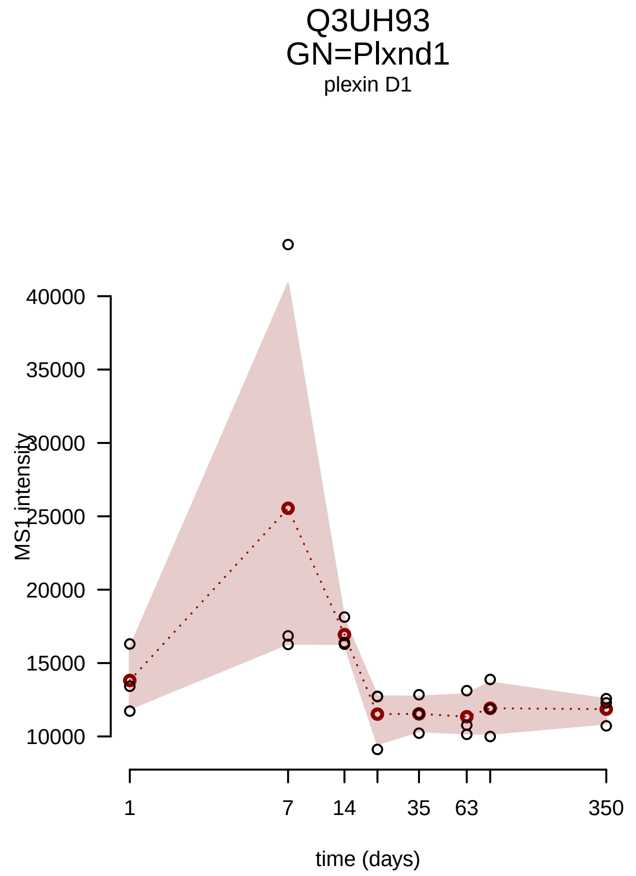
Q3UH93
GN=Plxnd1
plexin D1
MS1 intensity
10000
15000
20000
25000
30000
35000
40000
1
7
14
35
63
350
time (days)

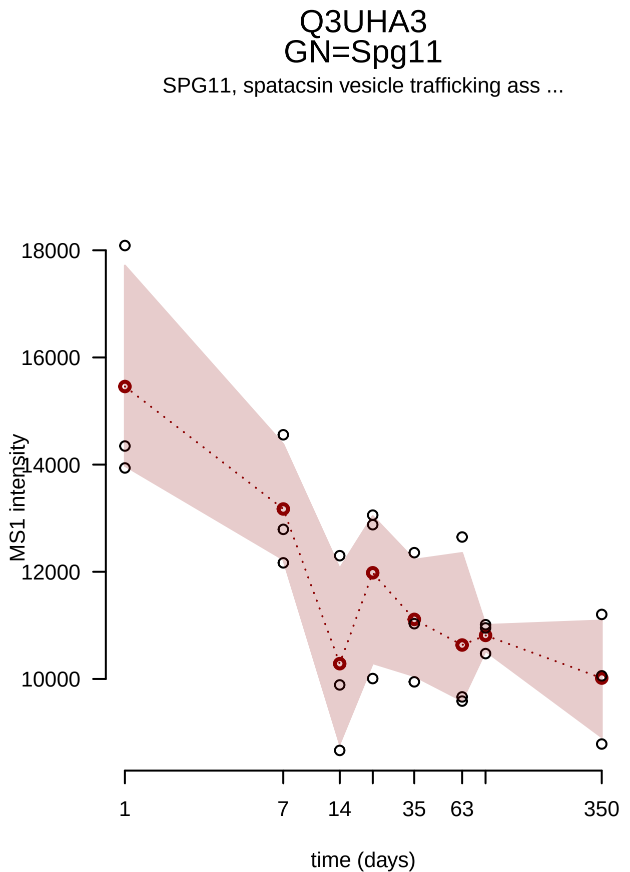
Q3UHA3
GN=Spg11
SPG11, spatacsin vesicle trafficking ass ...
MS1 intensity
10000
12000
14000
16000
18000
1
7
14
35
63
350
time (days)

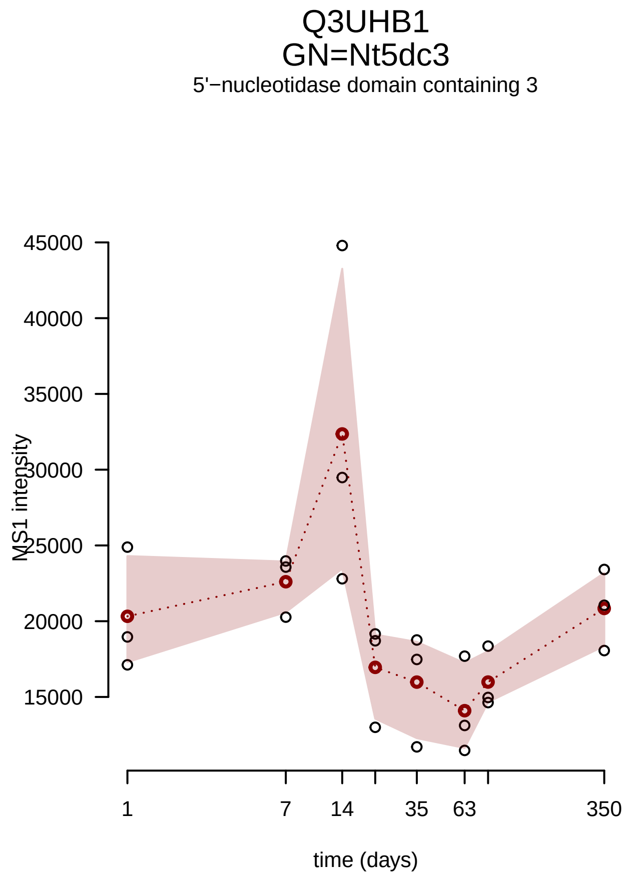
Q3UHB1
GN=Nt5dc3
5'−nucleotidase domain containing 3
MS1 intensity
15000
20000
25000
30000
35000
40000
45000
1
7
14
35
63
350
time (days)

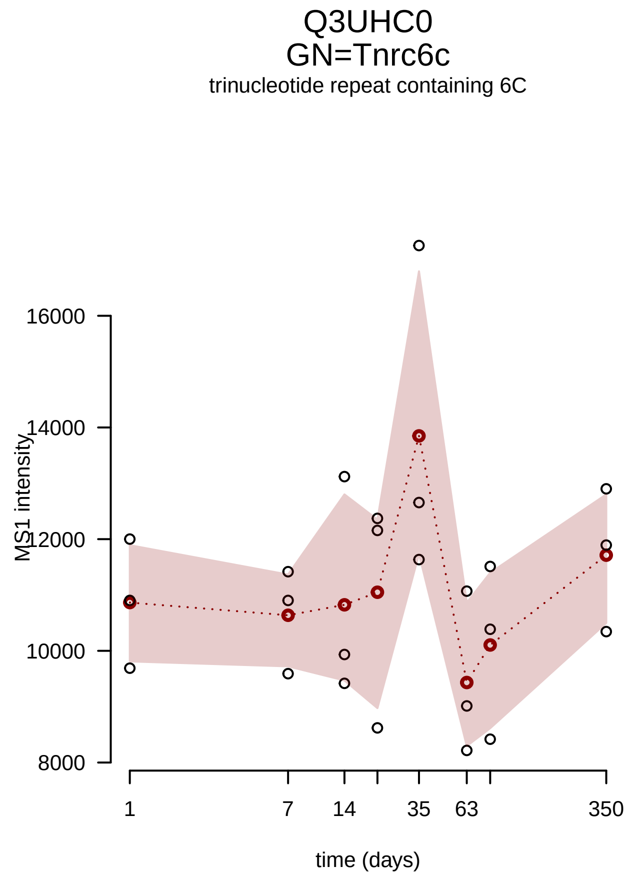
Q3UHC0
GN=Tnrc6c
trinucleotide repeat containing 6C
MS1 intensity
8000
10000
12000
14000
16000
1
7
14
35
63
350
time (days)

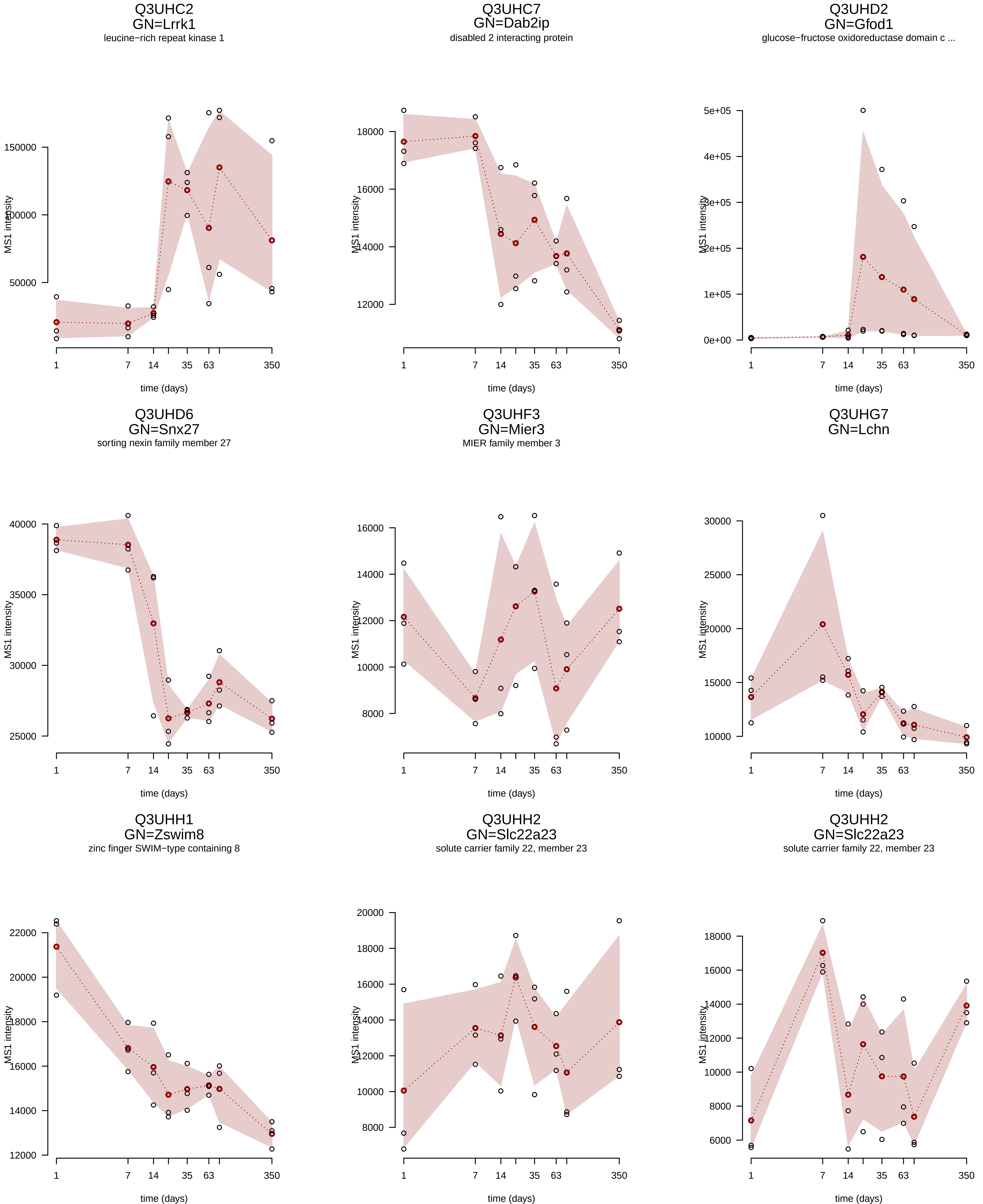
Q3UHC2
GN=Lrrk1
leucine−rich repeat kinase 1
MS1 intensity
150000
100000
50000
1 7 14 35 63 350
time (days)
Q3UHC7
GN=Dab2ip
disabled 2 interacting protein
MS1 intensity
18000
16000
14000
12000
1 7 14 35 63 350
time (days)
Q3UHD2
GN=Gfod1
glucose−fructose oxidoreductase domain c ...
MS1 intensity
5e+05
4e+05
3e+05
2e+05
1e+05
0e+00
1 7 14 35 63 350
time (days)
Q3UHD6
GN=Snx27
sorting nexin family member 27
MS1 intensity
40000
35000
30000
25000
1 7 14 35 63 350
time (days)
Q3UHF3
GN=Mier3
MIER family member 3
MS1 intensity
16000
14000
12000
10000
8000
1 7 14 35 63 350
time (days)
Q3UHG7
GN=Lchn
MS1 intensity
30000
25000
20000
15000
10000
1 7 14 35 63 350
time (days)
Q3UHH1
GN=Zswim8
zinc finger SWIM−type containing 8
MS1 intensity
22000
20000
18000
16000
14000
12000
1 7 14 35 63 350
time (days)
Q3UHH2
GN=Slc22a23
solute carrier family 22, member 23
MS1 intensity
20000
18000
16000
14000
12000
10000
8000
1 7 14 35 63 350
time (days)
Q3UHH2
GN=Slc22a23
solute carrier family 22, member 23
MS1 intensity
18000
16000
14000
12000
10000
8000
6000
1 7 14 35 63 350
time (days)

Q3UHH8
GN=Gxylt1
glucoside xylosyltransferase 1

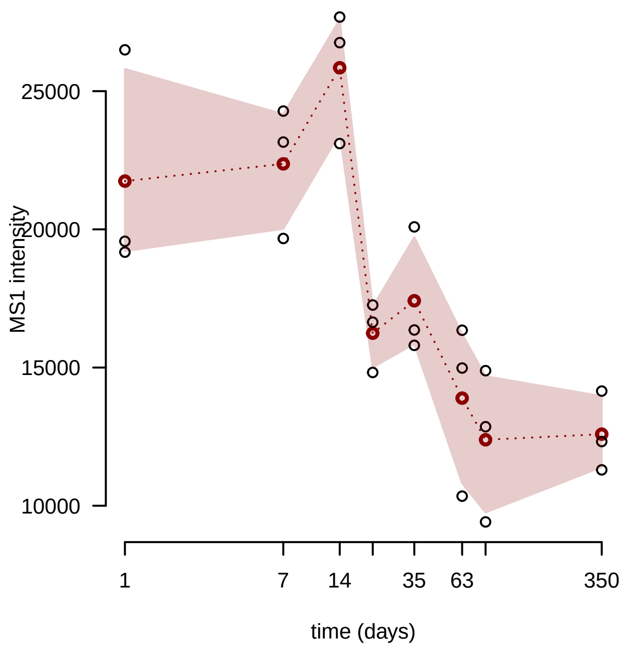

Q3UHI4
GN=Tmed8
transmembrane p24 trafficking protein 8

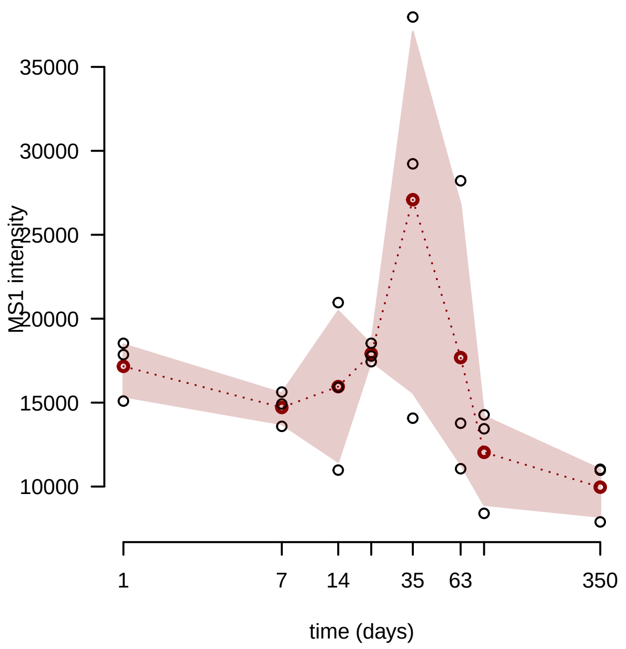

Q3UHJ0
GN=Aak1
AP2 associated kinase 1

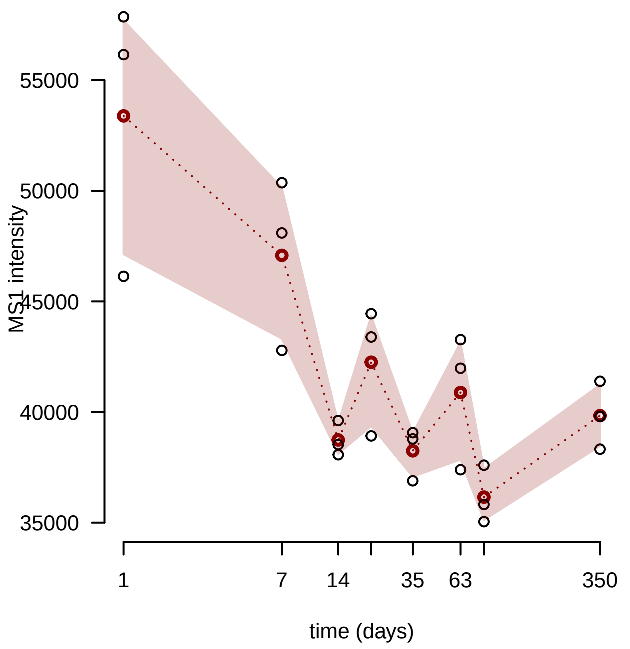

Q3UHK1
GN=Slc2a13
solute carrier family 2 (facilitated glu ...

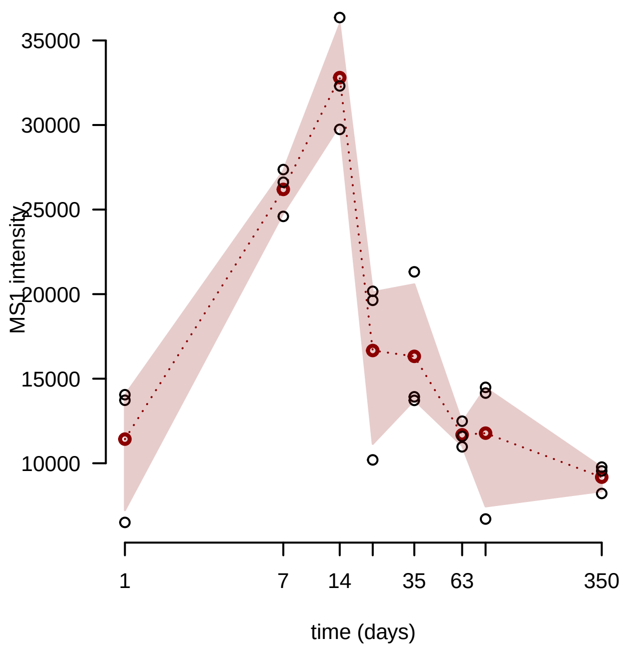

Q3UHK3
GN=Greb1
gene regulated by estrogen in breast can ...

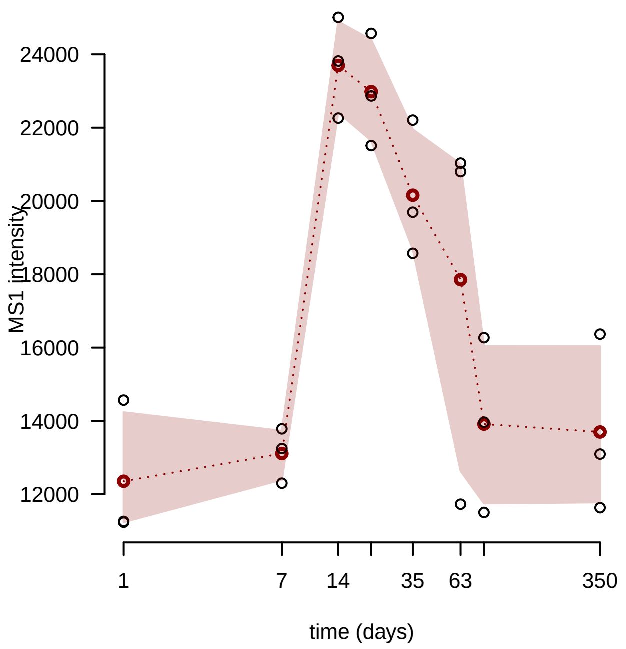

Q3UHK6
GN=Tenm4
teneurin transmembrane protein 4

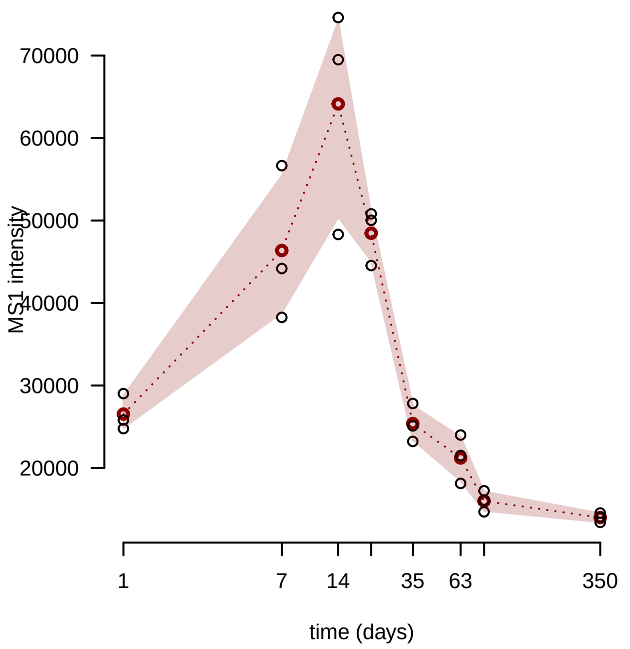

Q3UHK8
GN=Tnrc6a
trinucleotide repeat containing 6a

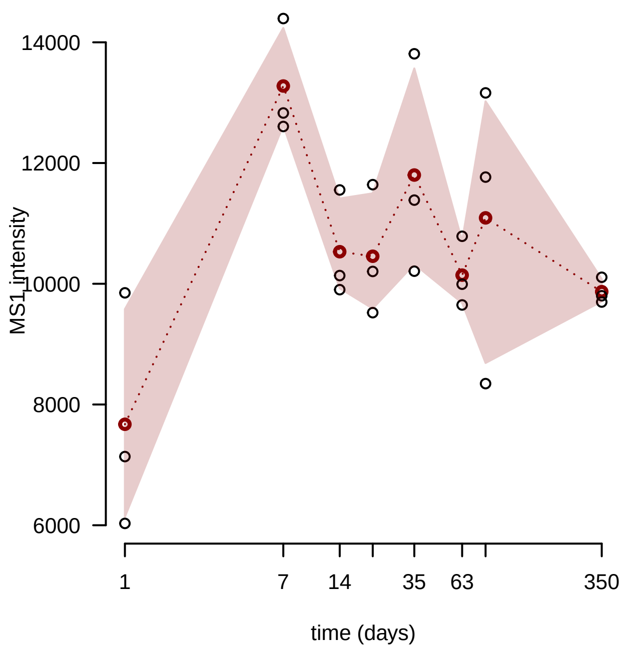

Q3UHN9
GN=Ndst1
N−deacetylase/N−sulfotransferase (hepara ...

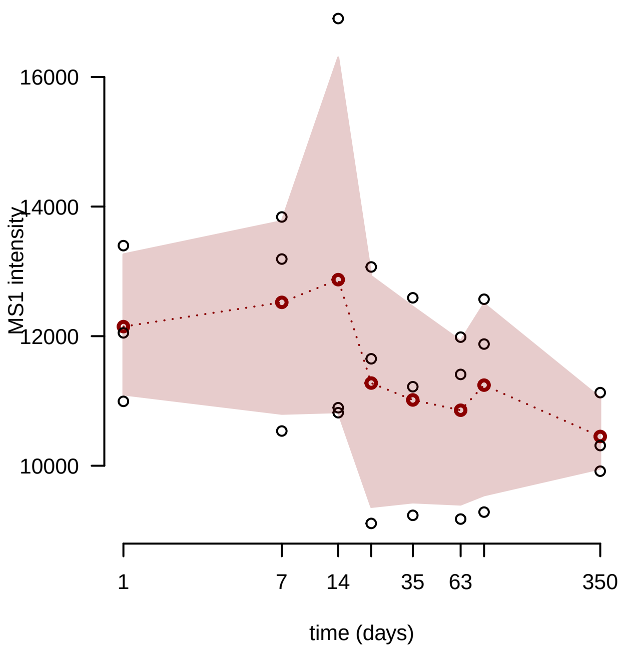

Q3UHQ6
GN=Dop1b
DOP1 leucine zipper like protein B

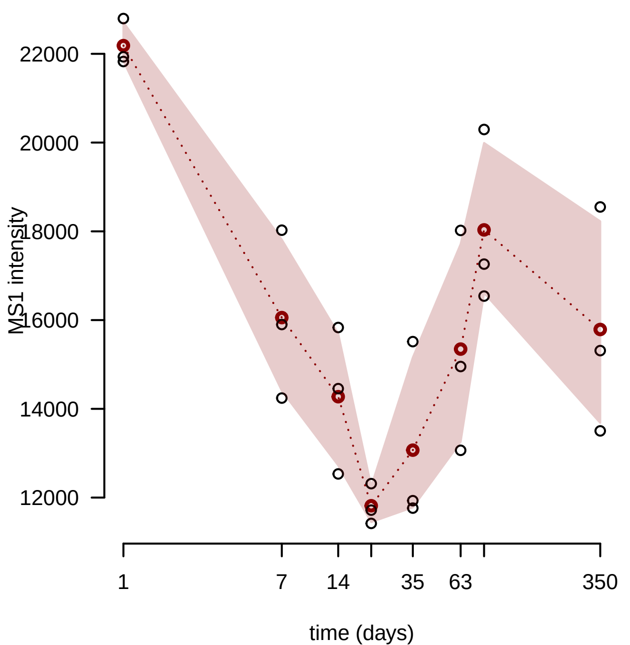

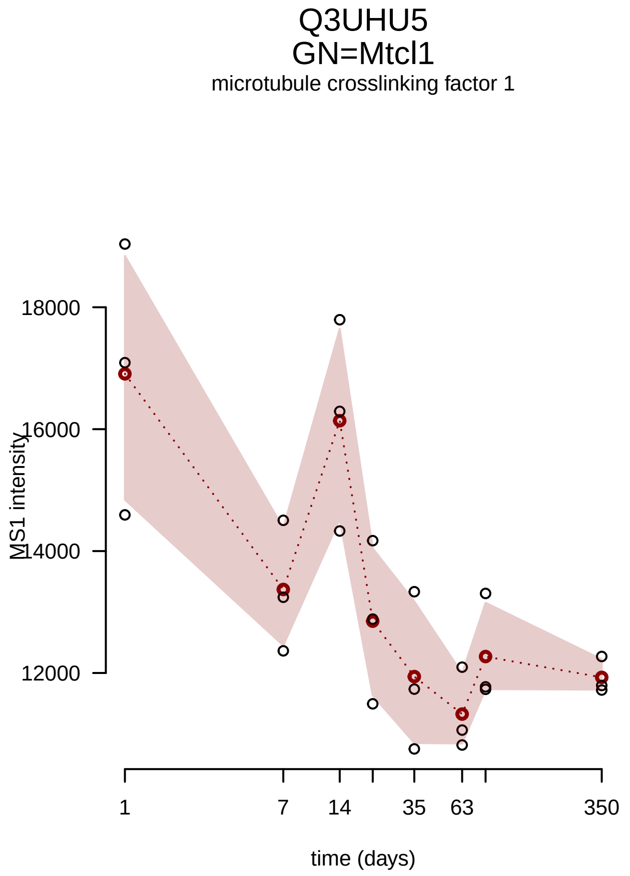
Q3UHU5
GN=Mtcl1
microtubule crosslinking factor 1
MS1 intensity
18000
16000
14000
12000
1
7
14
35
63
350
time (days)

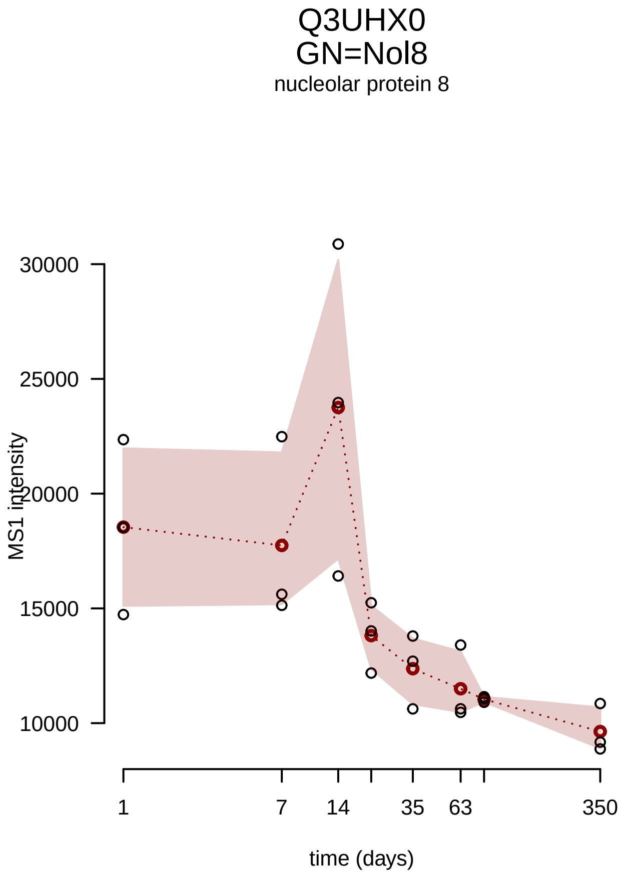
Q3UHX0
GN=Nol8
nucleolar protein 8
MS1 intensity
30000
25000
20000
15000
10000
1
7
14
35
63
350
time (days)

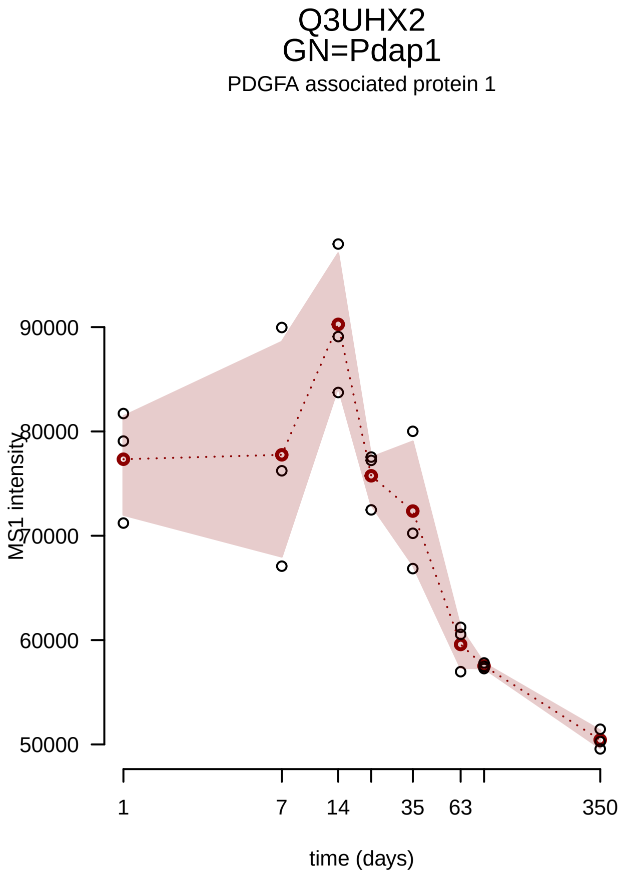
Q3UHX2
GN=Pdap1
PDGFA associated protein 1
MS1 intensity
90000
80000
70000
60000
50000
1
7
14
35
63
350
time (days)

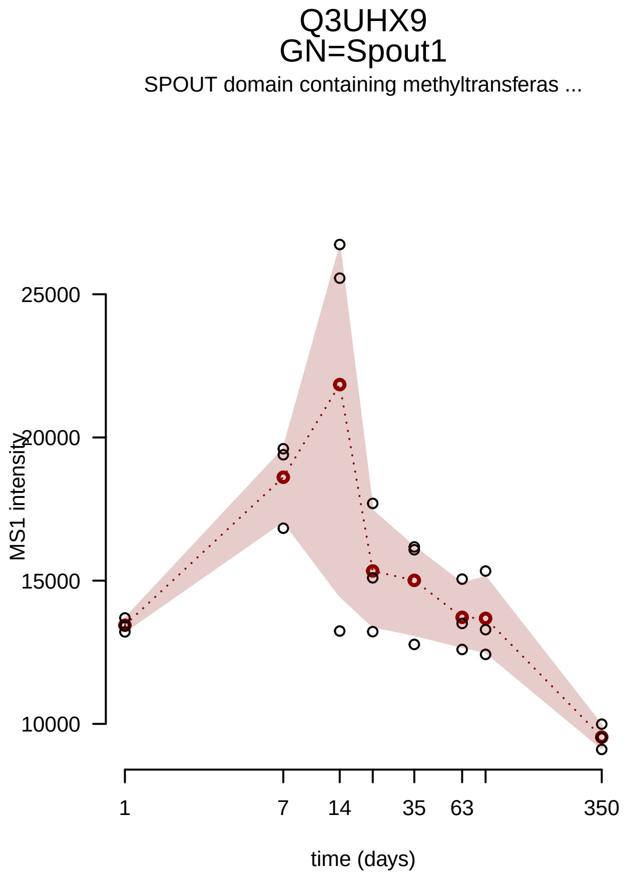
Q3UHX9
GN=Spout1
SPOUT domain containing methyltransferas ...
MS1 intensity
25000
20000
15000
10000
1
7
14
35
63
350
time (days)

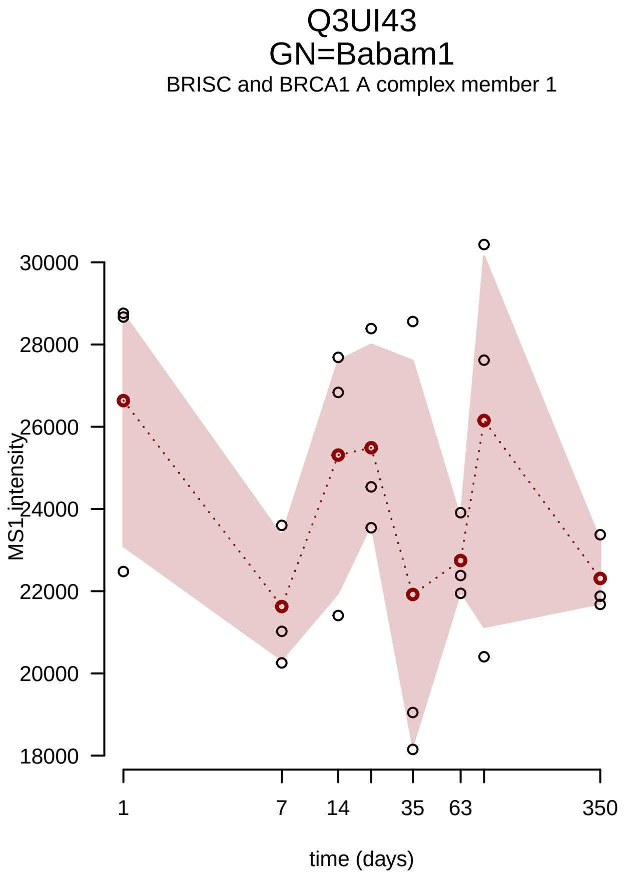
Q3UI43
GN=Babam1
BRISC and BRCA1 A complex member 1
MS1 intensity
30000
28000
26000
24000
22000
20000
18000
1
7
14
35
63
350
time (days)

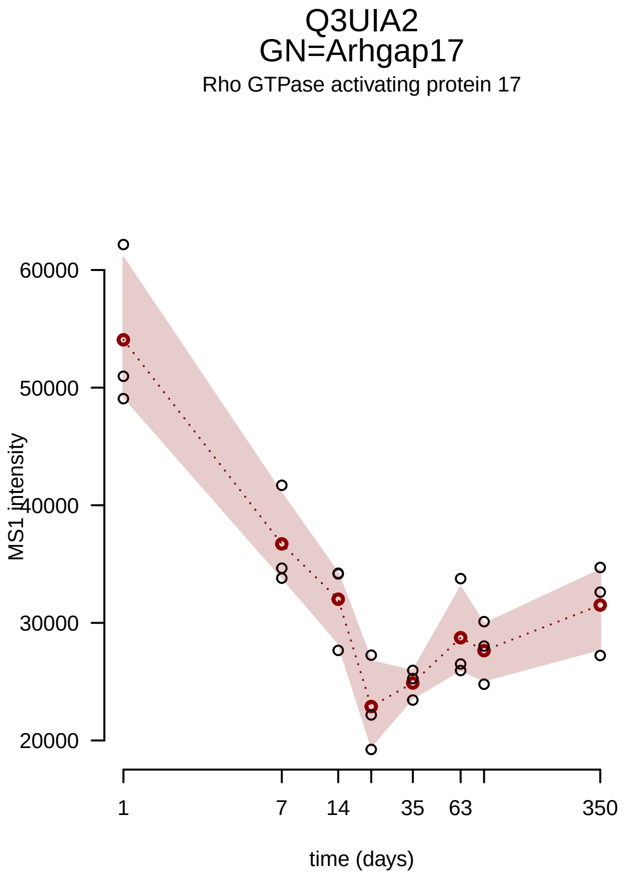
Q3UIA2
GN=Arhgap17
Rho GTPase activating protein 17
MS1 intensity
60000
50000
40000
30000
20000
1
7
14
35
63
350
time (days)

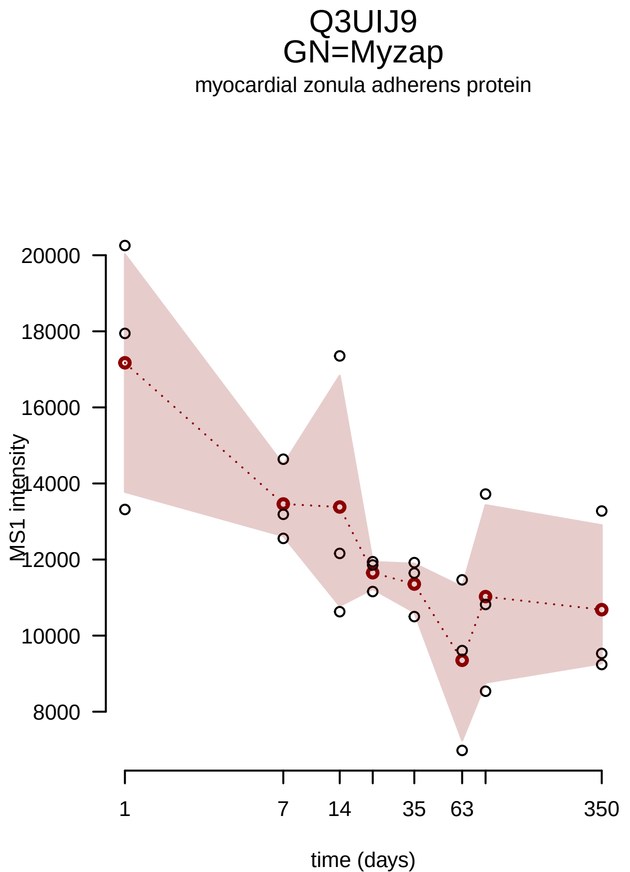
Q3UIJ9
GN=Myzap
myocardial zonula adherens protein
MS1 intensity
20000
18000
16000
14000
12000
10000
8000
1
7
14
35
63
350
time (days)

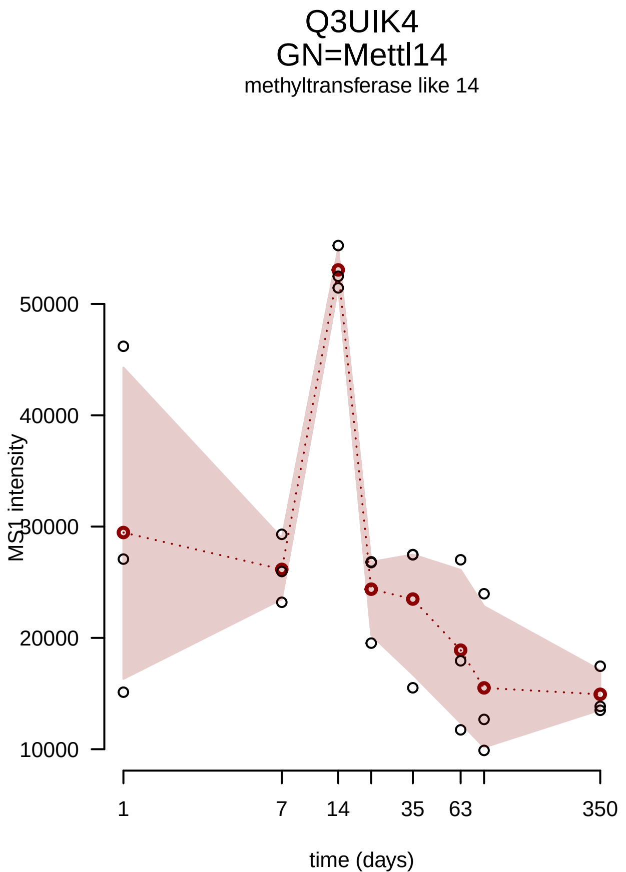
Q3UIK4
GN=Mettl14
methyltransferase like 14
MS1 intensity
50000
40000
30000
20000
10000
1
7
14
35
63
350
time (days)

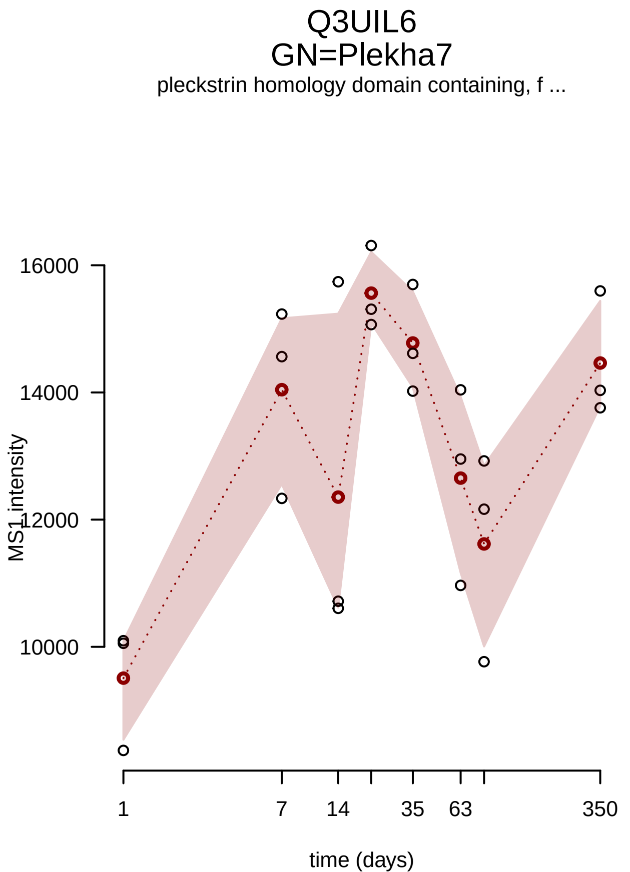
Q3UIL6
GN=Plekha7
pleckstrin homology domain containing, f ...
MS1 intensity
16000
14000
12000
10000
1
7
14
35
63
350
time (days)

Q3UIR3
GN=Dtx3l
deltex 3−like, E3 ubiquitin ligase

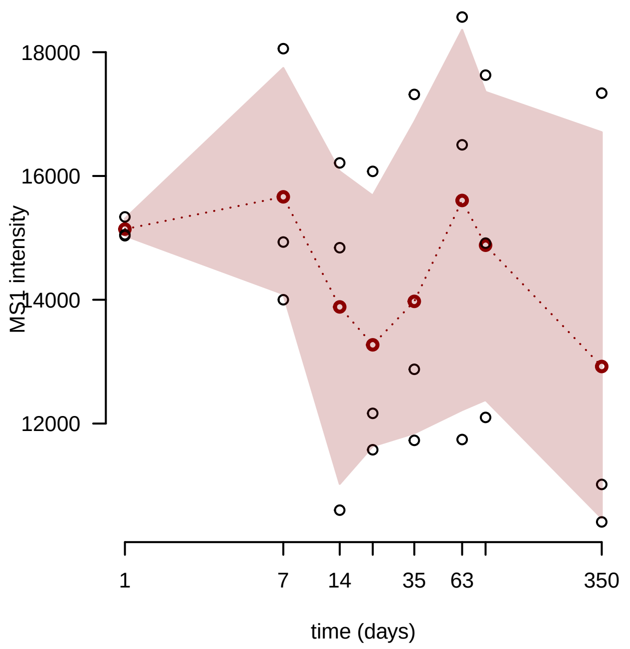

Q3UIU2
GN=Ndufb6
NADH:ubiquinone oxidoreductase subunit B ...

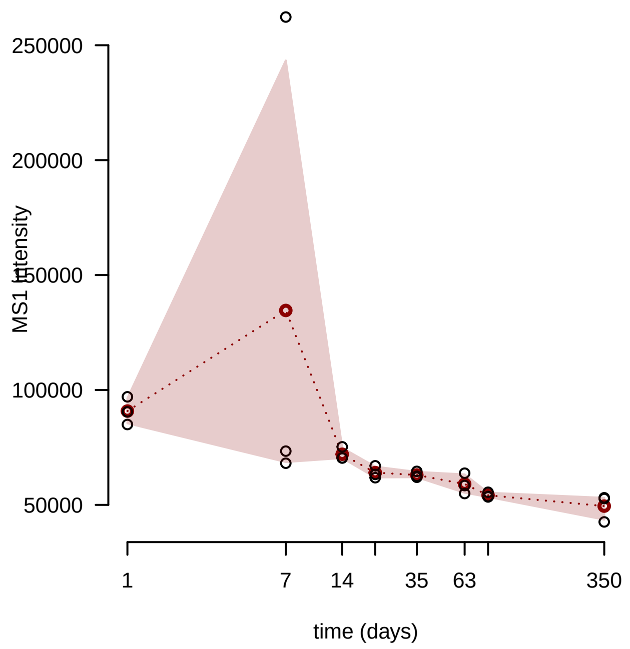

Q3UIW5
GN=Rnf10
ring finger protein 10

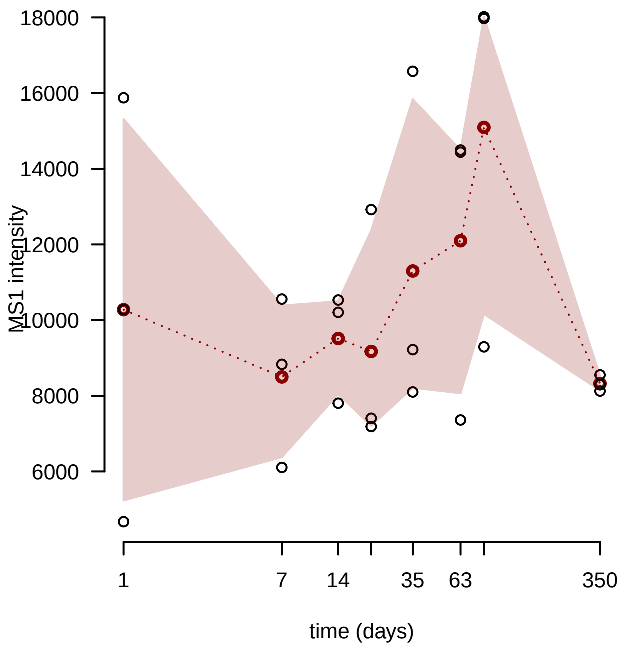

Q3UJ81
GN=Cnep1r1
CTD nuclear envelope phosphatase 1 regul ...

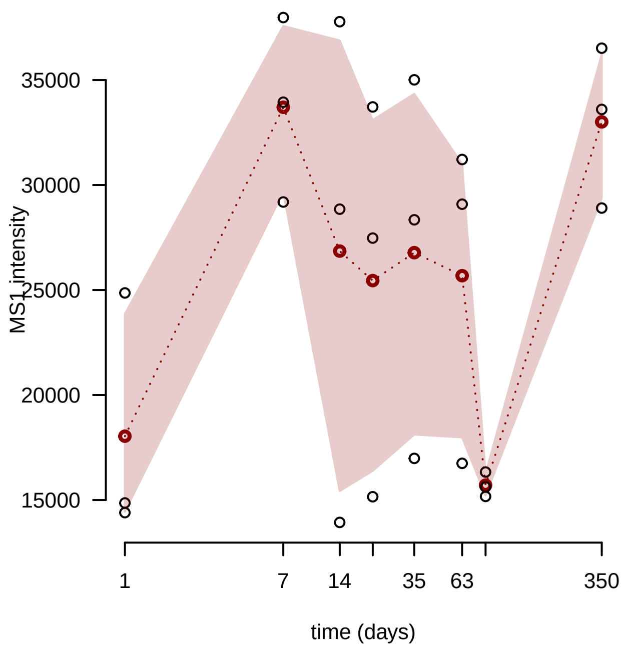

Q3UJB9
GN=Edc4
enhancer of mRNA decapping 4

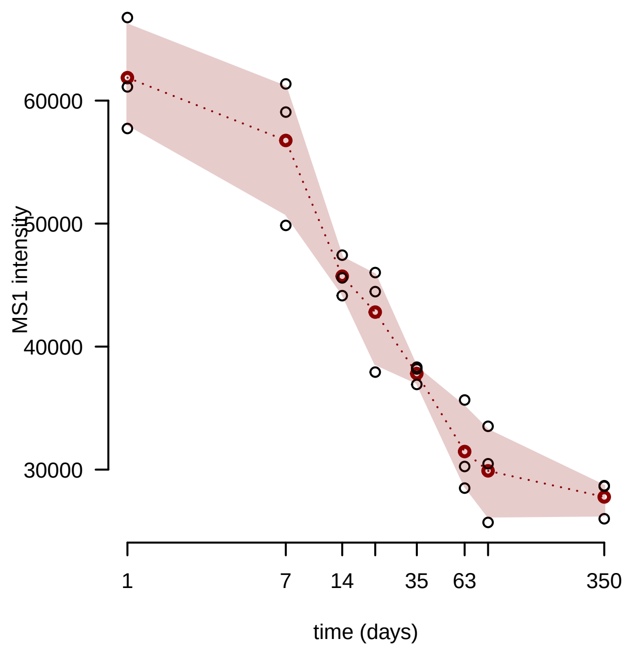

Q3UJD6
GN=Usp19
ubiquitin specific peptidase 19

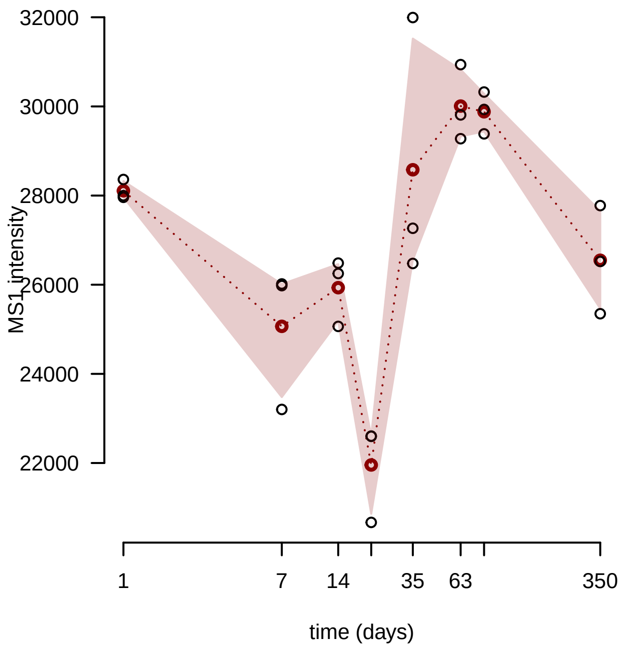

Q3UJK4
GN=Gtpbp2
GTP binding protein 2

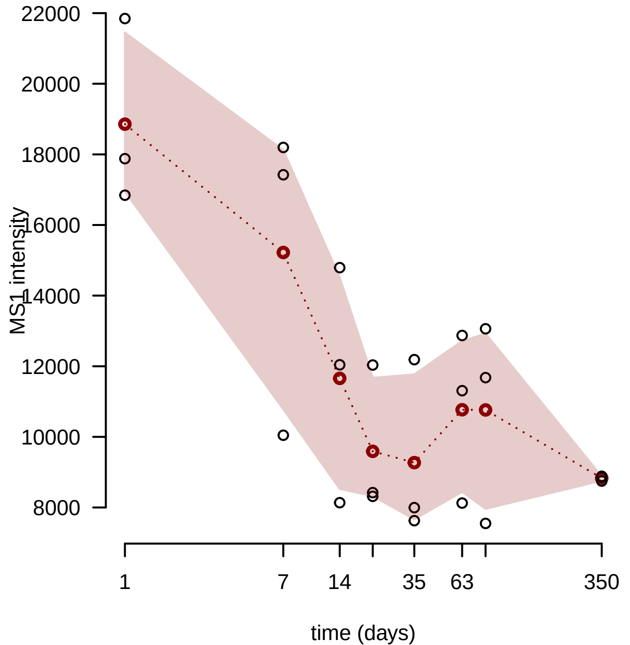

Q3UJP5
GN=

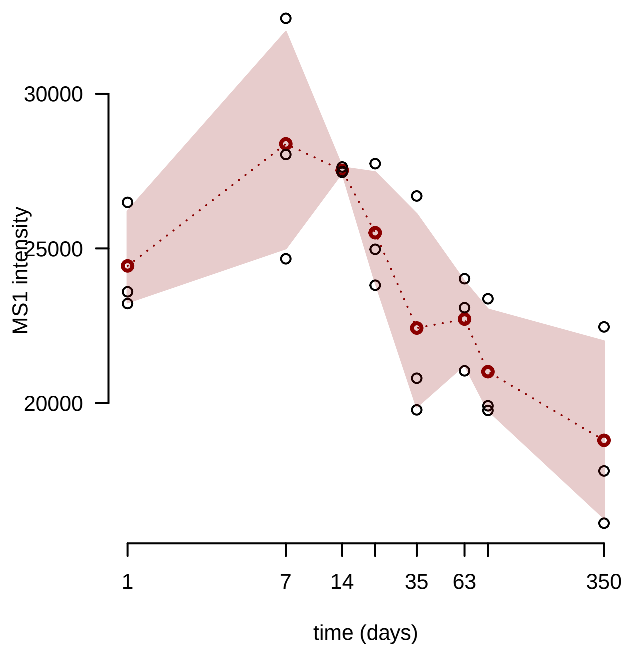

Q3UJU9
GN=Rmdn3
regulator of microtubule dynamics 3

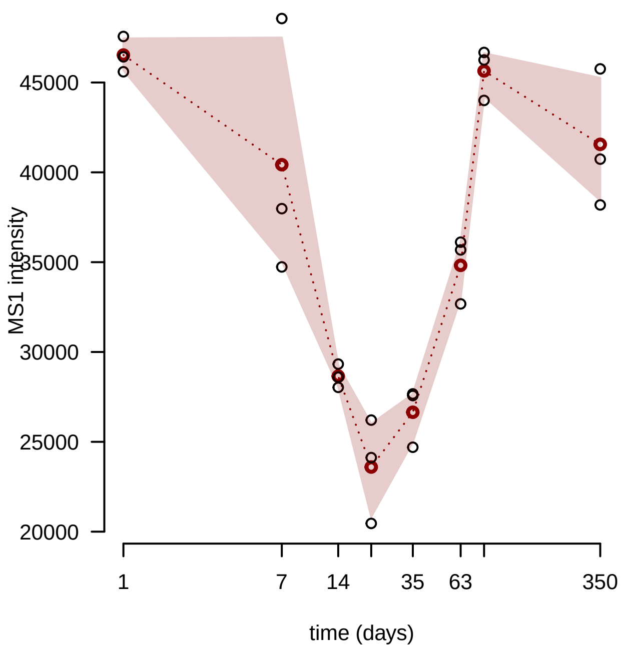

Q3UJV1
GN=Ccdc61
coiled−coil domain containing 61

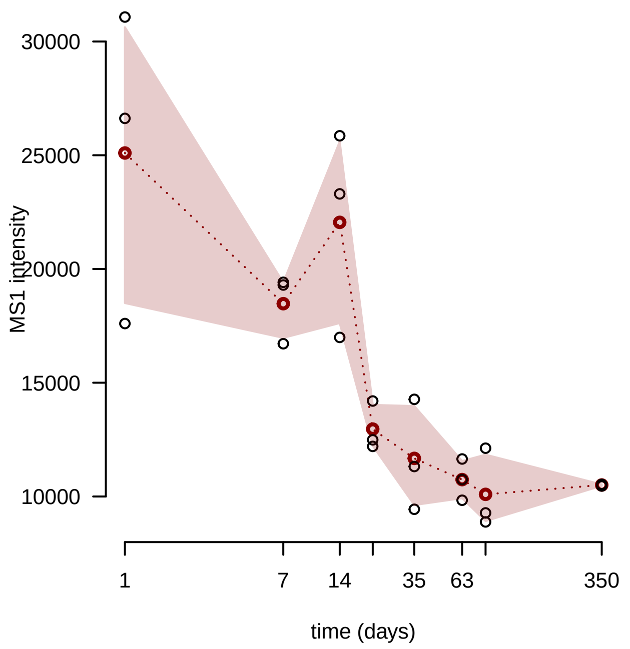

Q3UK10
GN=B9d2
B9 protein domain 2

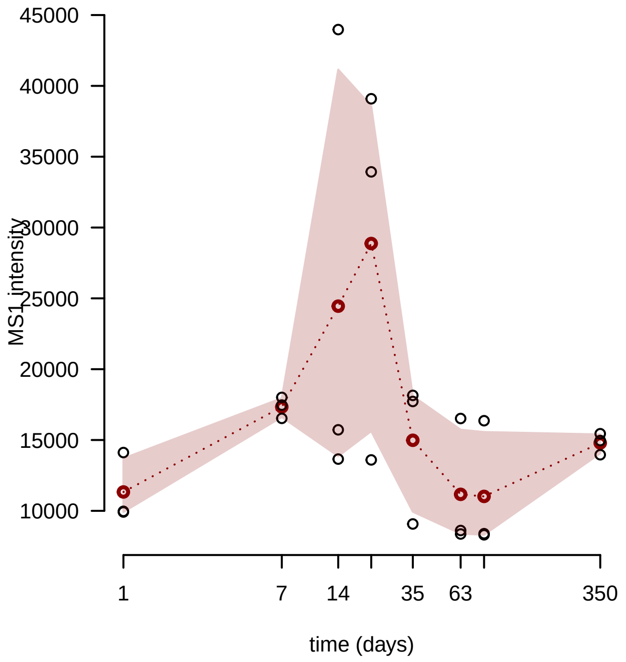

Q3UKC1
GN=Tax1bp1
Tax1 (human T cell leukemia virus type I ...

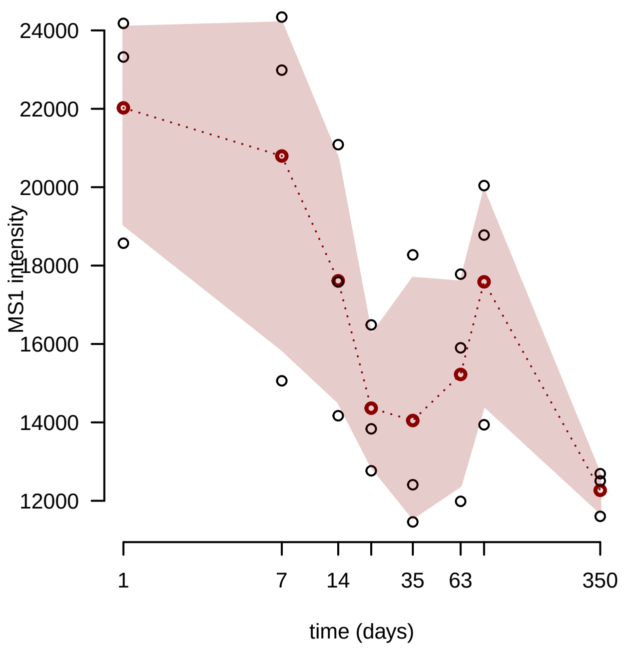

Q3UKJ7
GN=Smu1
smu−1 suppressor of mec−8 and unc−52 hom ...

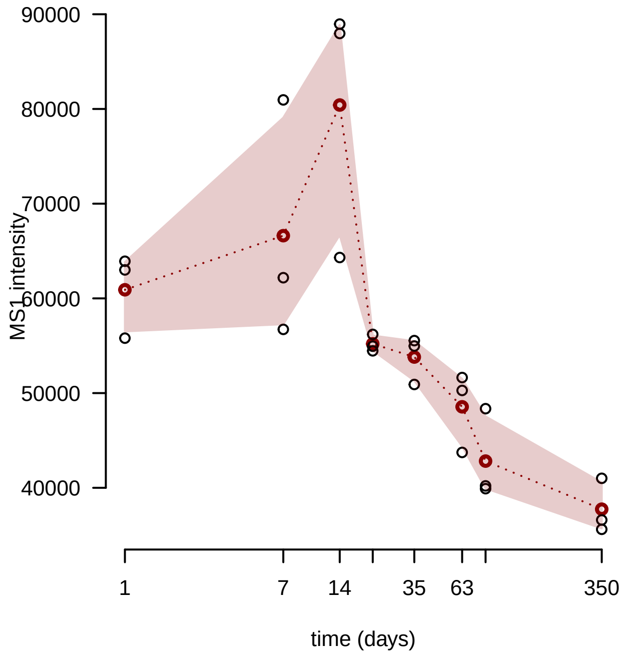

Q3UL36
GN=Arglu1
arginine and glutamate rich 1

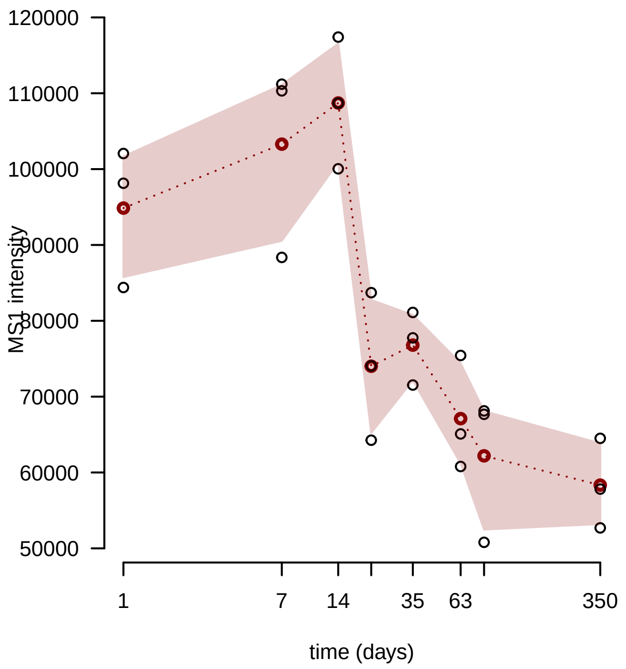

Q3ULA2
GN=Btrc
beta−transducin repeat containing protei ...

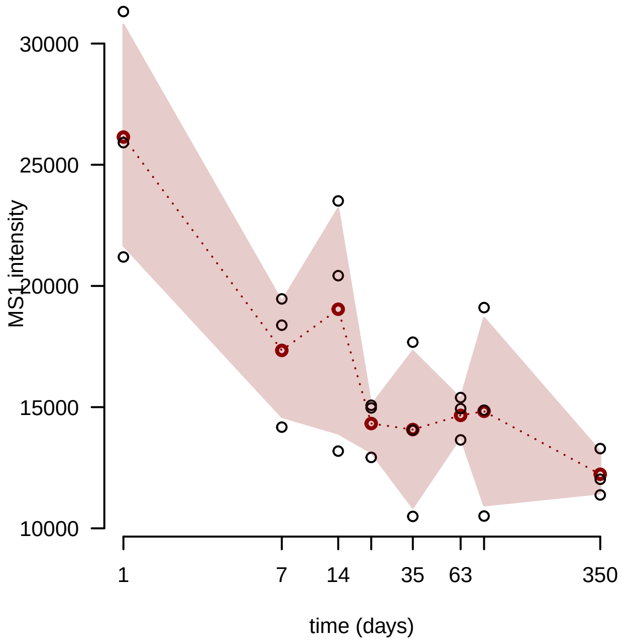

Q3ULD5
GN=Mccc2
methylcrotonoyl−Coenzyme A carboxylase 2 ...

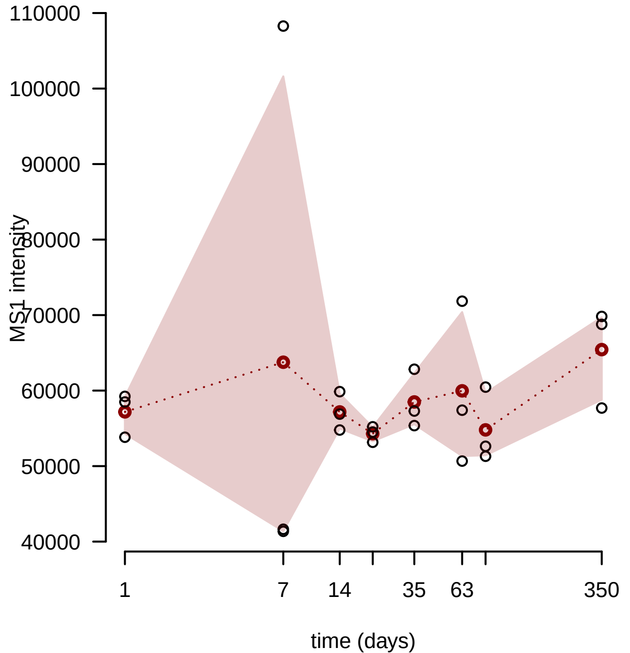

Q3ULF4
GN=Spg7
SPG7, paraplegin matrix AAA peptidase su ...

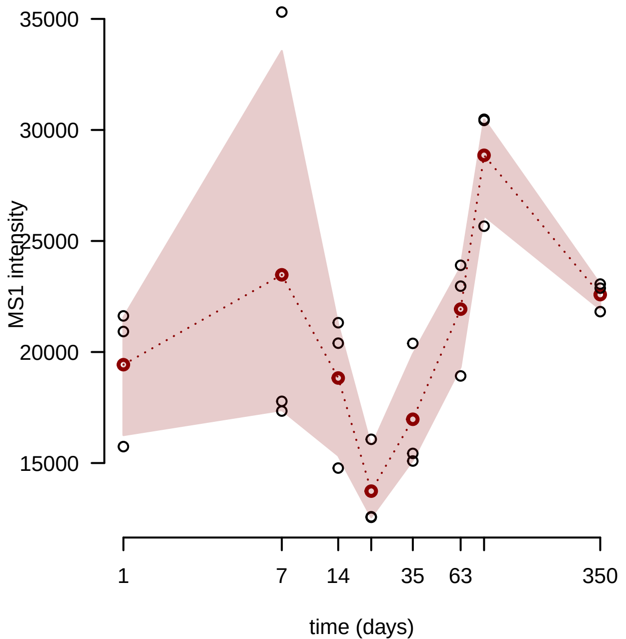

Q3ULJ0
GN=Gpd1l
glycerol−3−phosphate dehydrogenase 1−lik ...

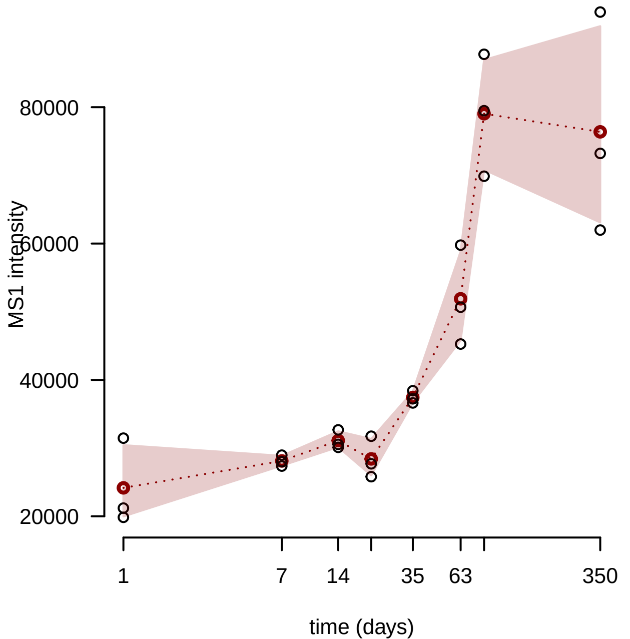

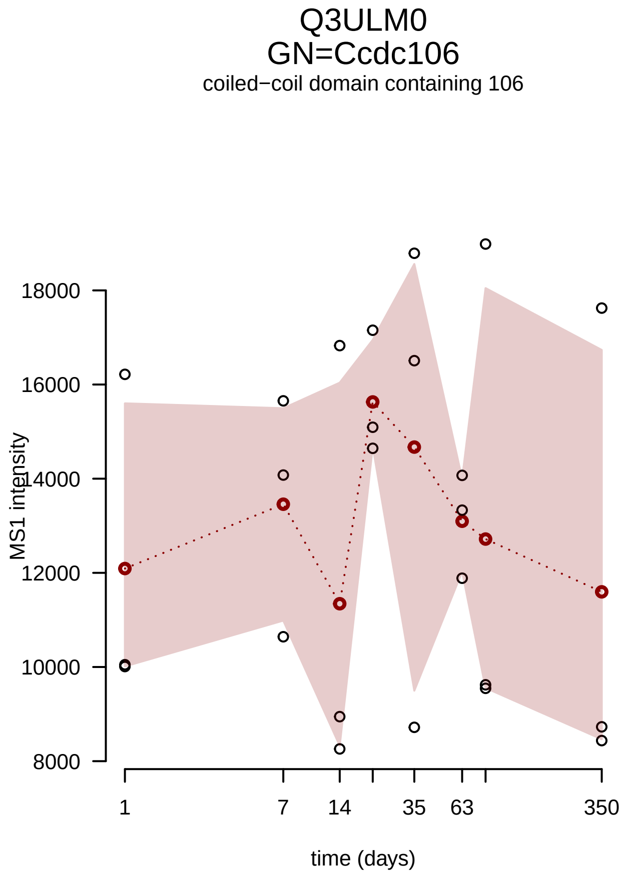
Q3ULM0
GN=Ccdc106
coiled−coil domain containing 106
MS1 intensity
8000
10000
12000
14000
16000
18000
1
7
14
35
63
350
time (days)

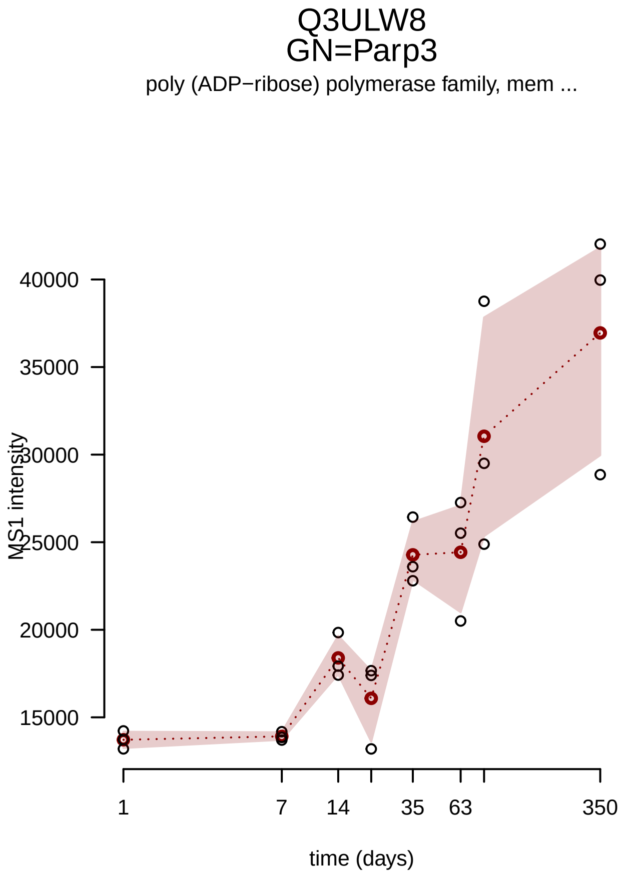
Q3ULW8
GN=Parp3
poly (ADP−ribose) polymerase family, mem ...
MS1 intensity
15000
20000
25000
30000
35000
40000
1
7
14
35
63
350
time (days)

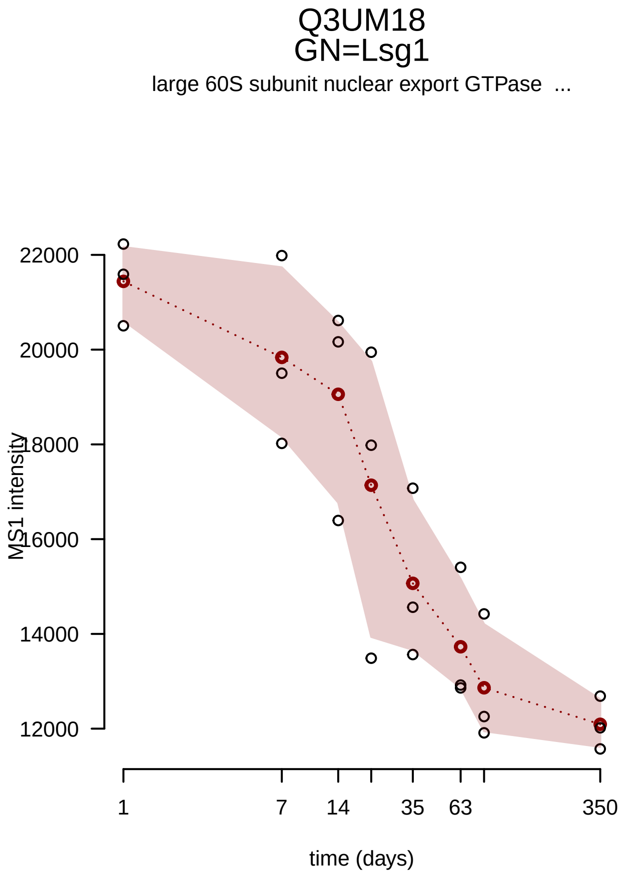
Q3UM18
GN=Lsg1
large 60S subunit nuclear export GTPase ...
MS1 intensity
12000
14000
16000
18000
20000
22000
1
7
14
35
63
350
time (days)

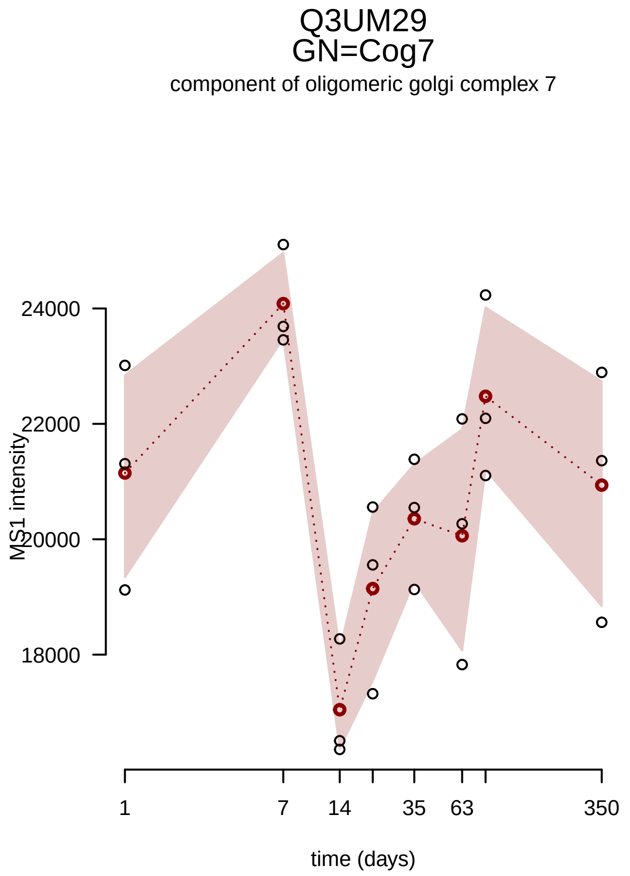
Q3UM29
GN=Cog7
component of oligomeric golgi complex 7
MS1 intensity
18000
20000
22000
24000
1
7
14
35
63
350
time (days)

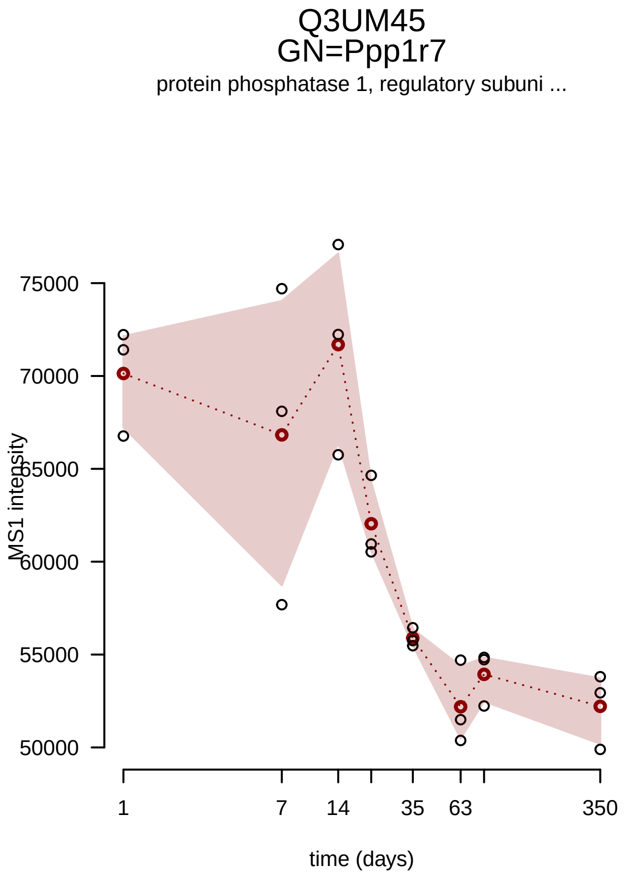
Q3UM45
GN=Ppp1r7
protein phosphatase 1, regulatory subuni ...
MS1 intensity
50000
55000
60000
65000
70000
75000
1
7
14
35
63
350
time (days)

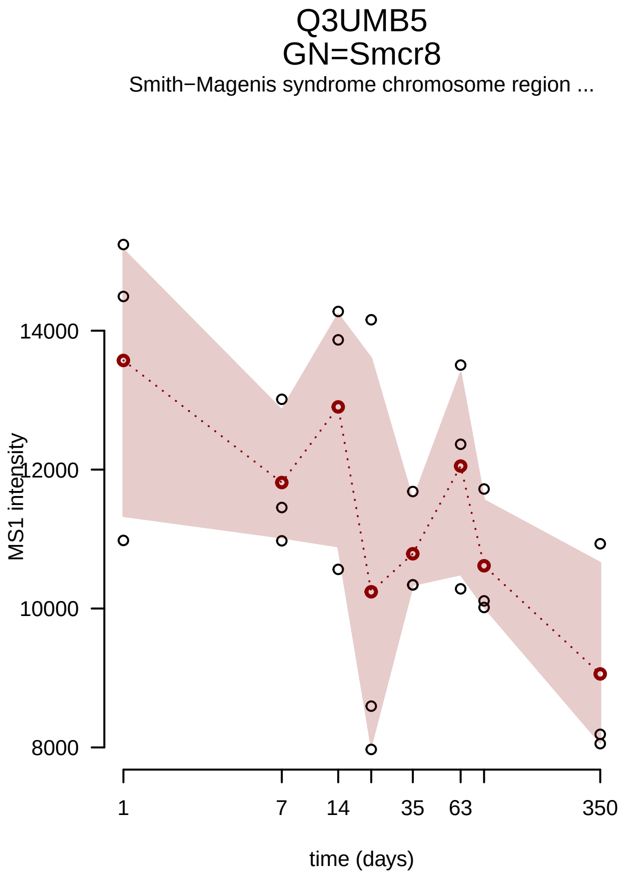
Q3UMB5
GN=Smcr8
Smith−Magenis syndrome chromosome region ...
MS1 intensity
8000
10000
12000
14000
1
7
14
35
63
350
time (days)

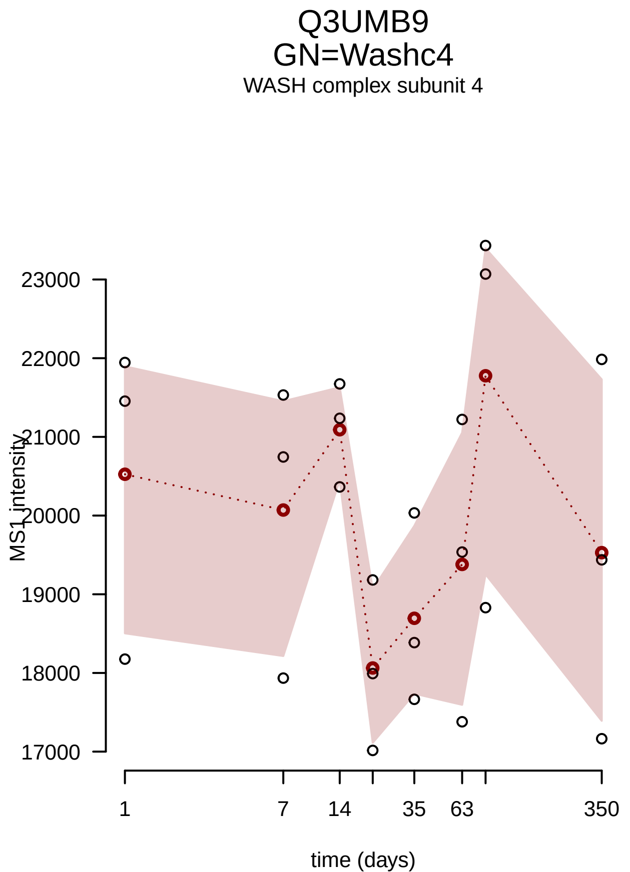
Q3UMB9
GN=Washc4
WASH complex subunit 4
MS1 intensity
17000
18000
19000
20000
21000
22000
23000
1
7
14
35
63
350
time (days)

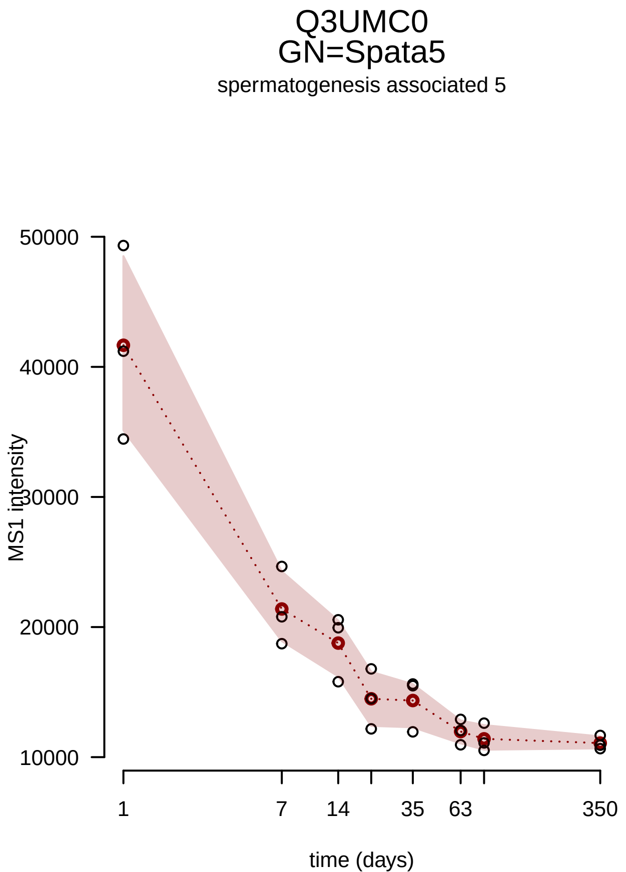
Q3UMC0
GN=Spata5
spermatogenesis associated 5
MS1 intensity
10000
20000
30000
40000
50000
1
7
14
35
63
350
time (days)

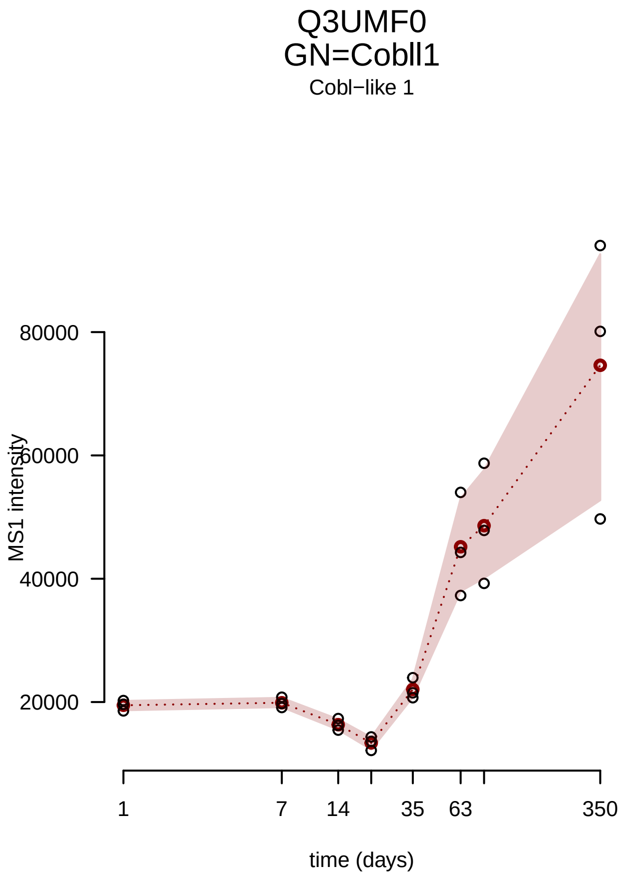
Q3UMF0
GN=Cobll1
Cobl−like 1
MS1 intensity
20000
40000
60000
80000
1
7
14
35
63
350
time (days)

Q3UMF0
GN=Cobll1
Cobl−like 1

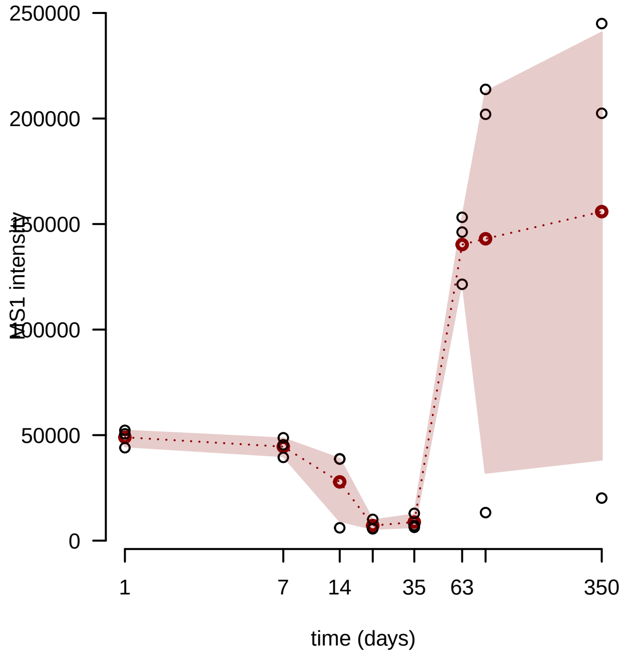

Q3UMG5
GN=Lrch2
leucine−rich repeats and calponin homolo ...

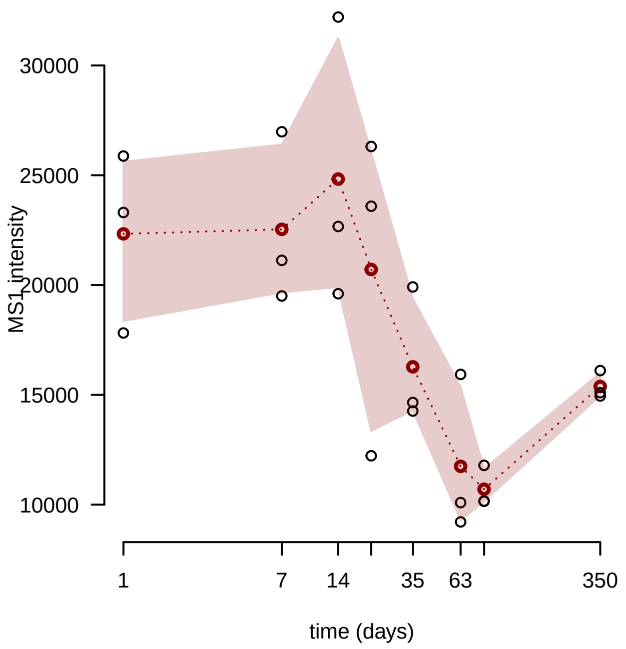

Q3UMM4
GN=Cdk10
cyclin−dependent kinase 10

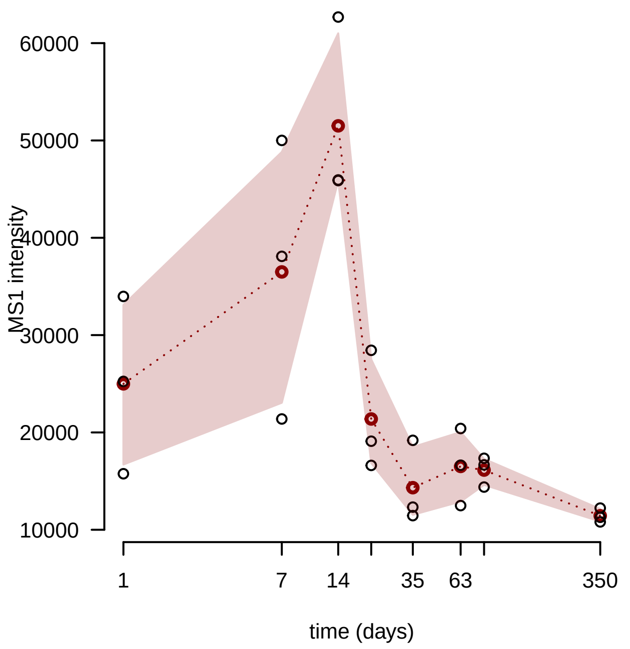

Q3UMR0
GN=Ankrd27
ankyrin repeat domain 27 (VPS9 domain)

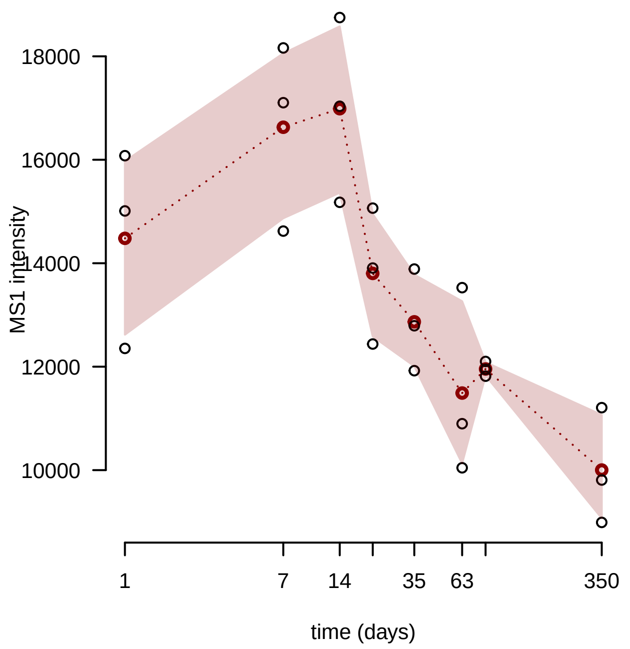

Q3UMR5
GN=Mcu
mitochondrial calcium uniporter

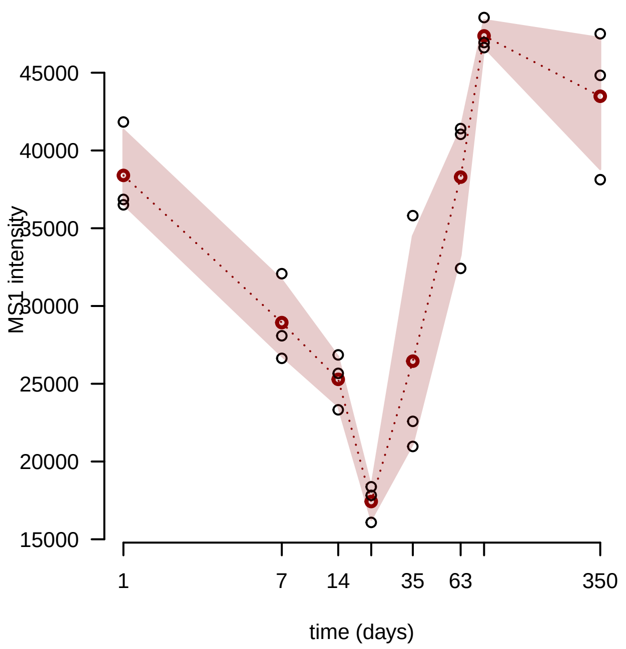

Q3UMT1
GN=Ppp1r12c
protein phosphatase 1, regulatory subuni ...

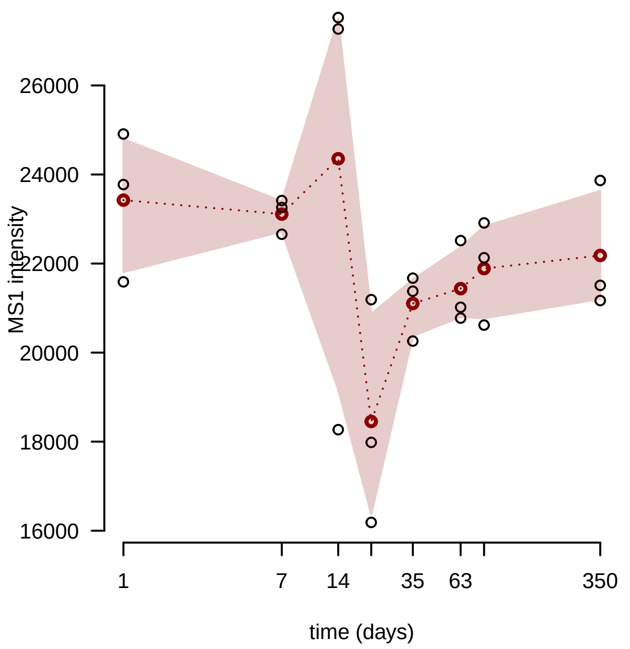

Q3UMU9
GN=Hdgfl2
HDGF like 2

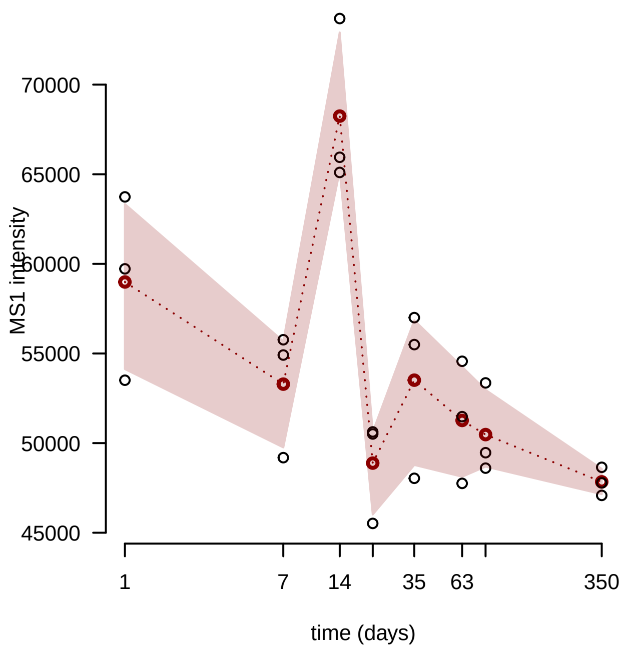

Q3UMW7
GN=Mapkapk3
mitogen−activated protein kinase−activat ...

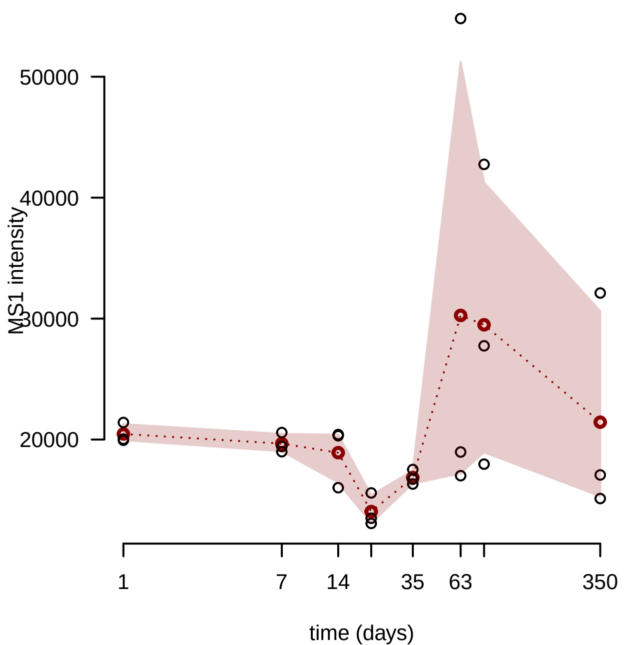

Q3UMW8
GN=Cln5
ceroid−lipofuscinosis, neuronal 5

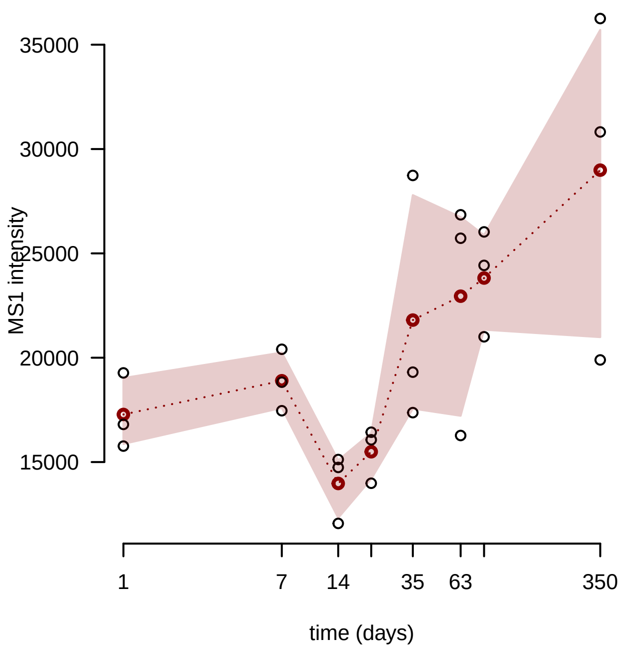

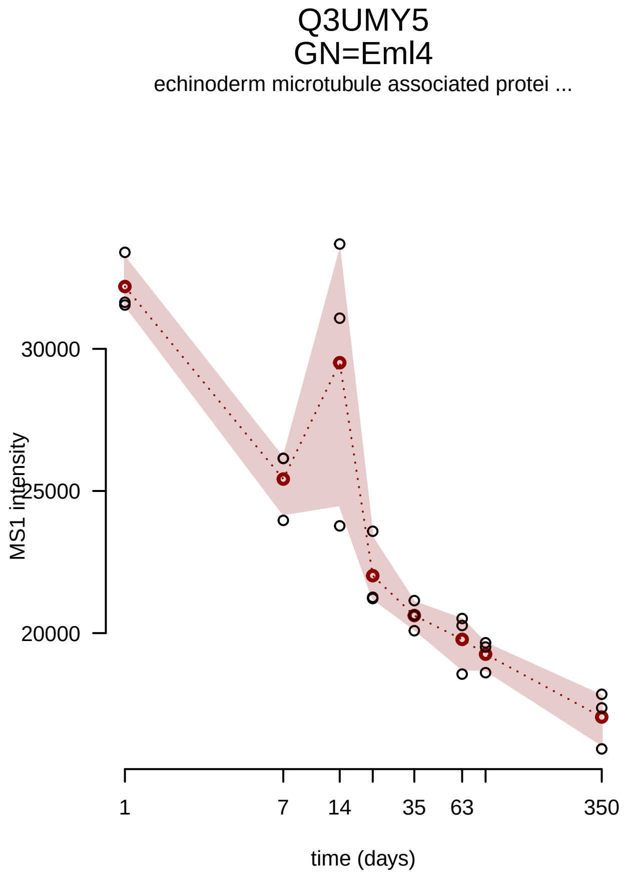
Q3UMY5
GN=Eml4
echinoderm microtubule associated protei ...
MS1 intensity
30000
25000
20000
1
7
14
35
63
350
time (days)

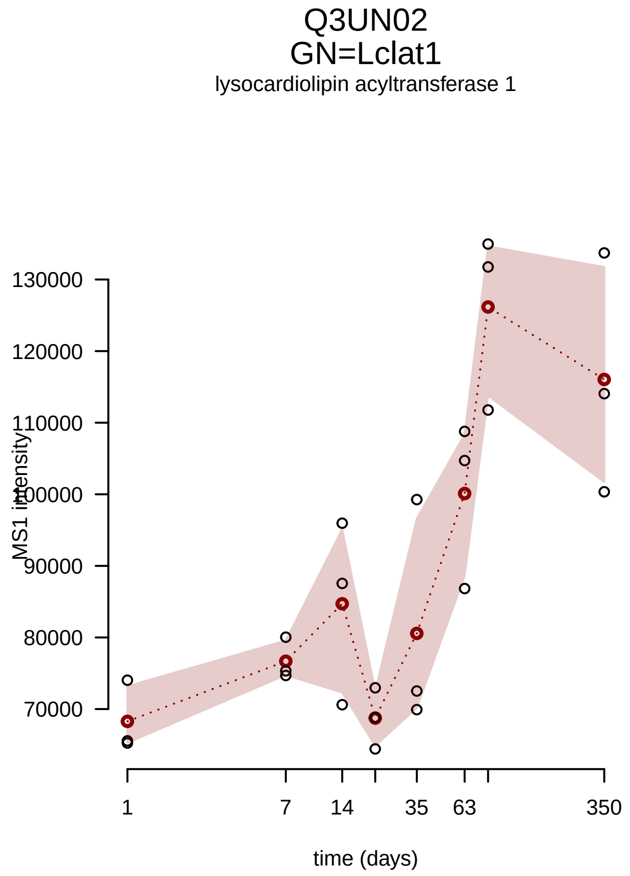
Q3UN02
GN=Lclat1
lysocardiolipin acyltransferase 1
MS1 intensity
130000
120000
110000
100000
90000
80000
70000
1
7
14
35
63
350
time (days)

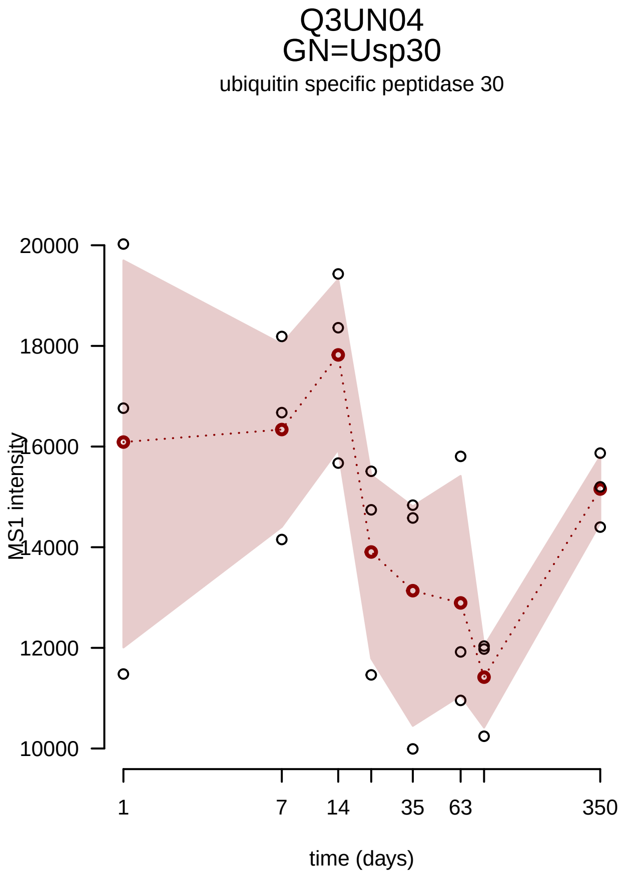
Q3UN04
GN=Usp30
ubiquitin specific peptidase 30
MS1 intensity
20000
18000
16000
14000
12000
10000
1
7
14
35
63
350
time (days)

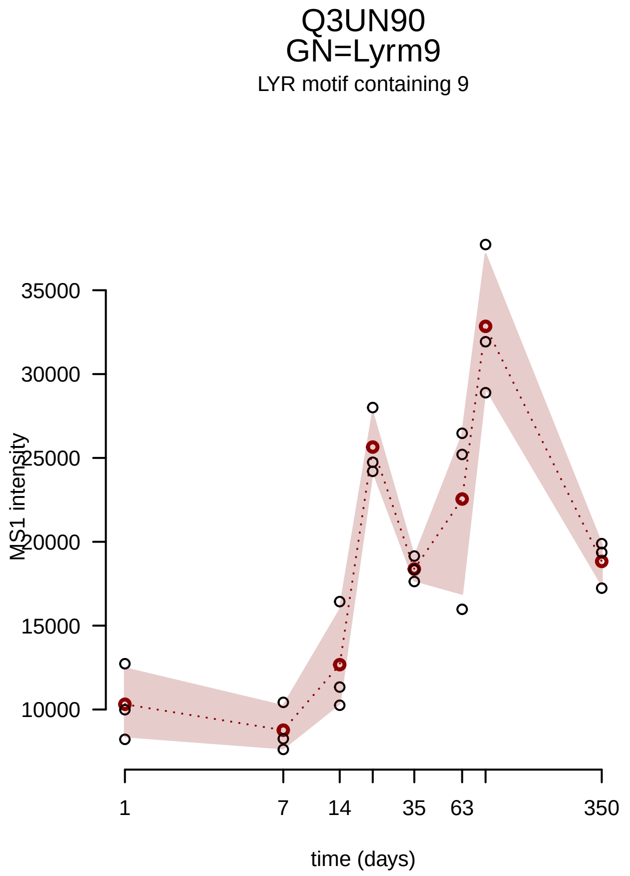
Q3UN90
GN=Lyrm9
LYR motif containing 9
MS1 intensity
35000
30000
25000
20000
15000
10000
1
7
14
35
63
350
time (days)

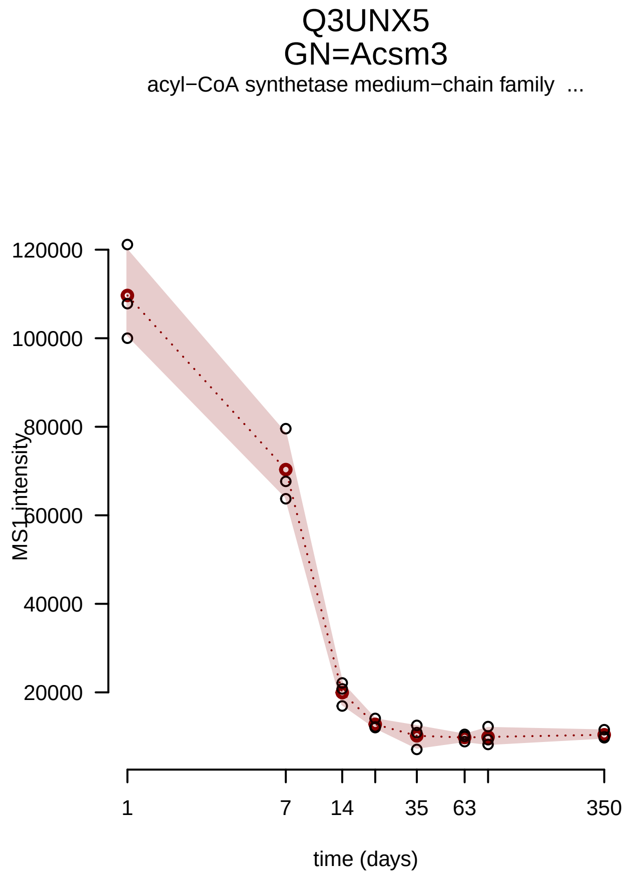
Q3UNX5
GN=Acsm3
acyl−CoA synthetase medium−chain family ...
MS1 intensity
120000
100000
80000
60000
40000
20000
1
7
14
35
63
350
time (days)

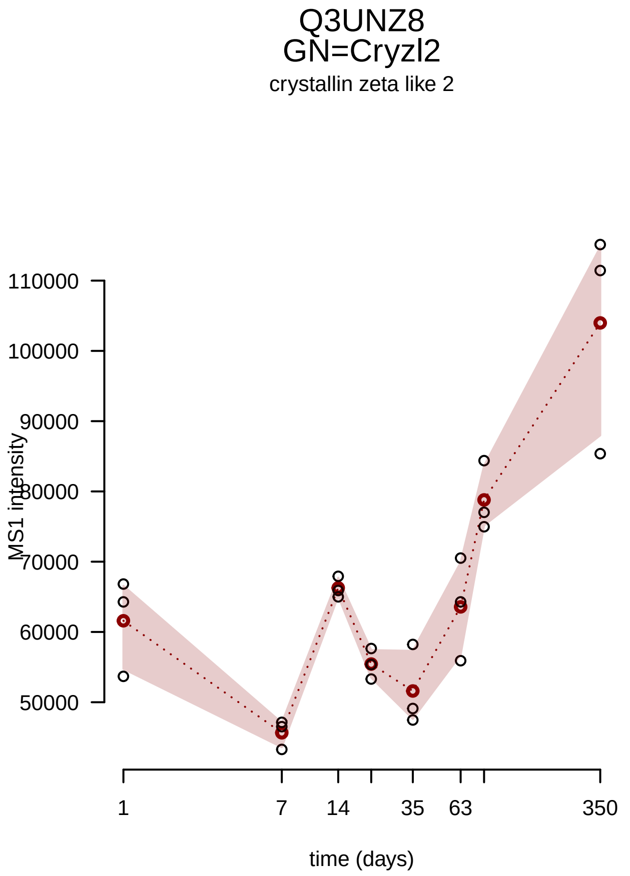
Q3UNZ8
GN=Cryzl2
crystallin zeta like 2
MS1 intensity
110000
100000
90000
80000
70000
60000
50000
1
7
14
35
63
350
time (days)

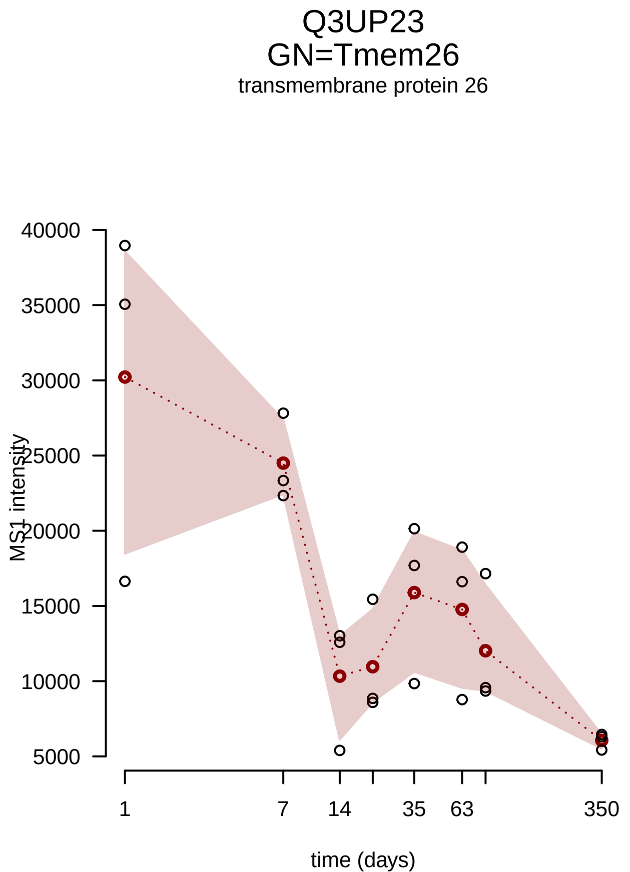
Q3UP23
GN=Tmem26
transmembrane protein 26
MS1 intensity
40000
35000
30000
25000
20000
15000
10000
5000
1
7
14
35
63
350
time (days)

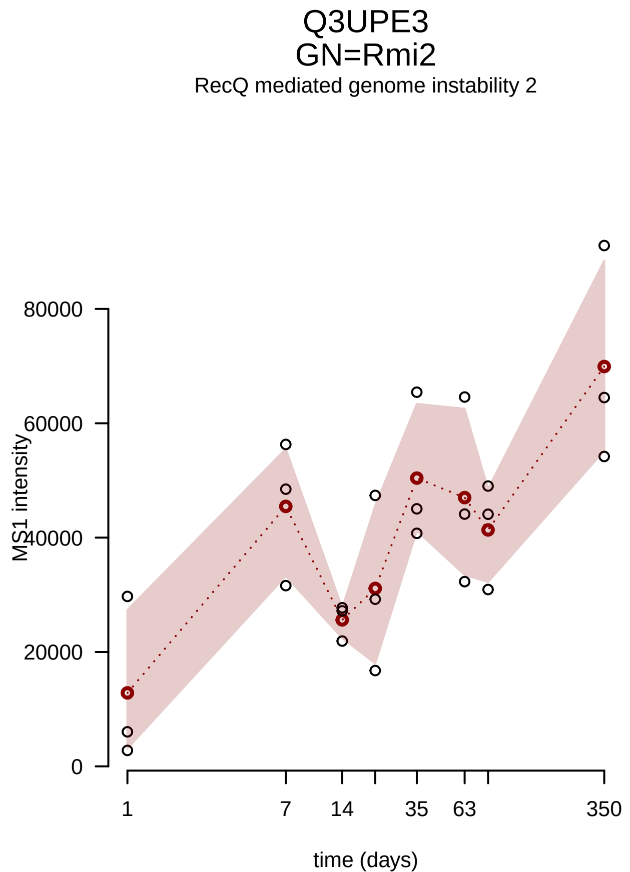
Q3UPE3
GN=Rmi2
RecQ mediated genome instability 2
MS1 intensity
80000
60000
40000
20000
0
1
7
14
35
63
350
time (days)

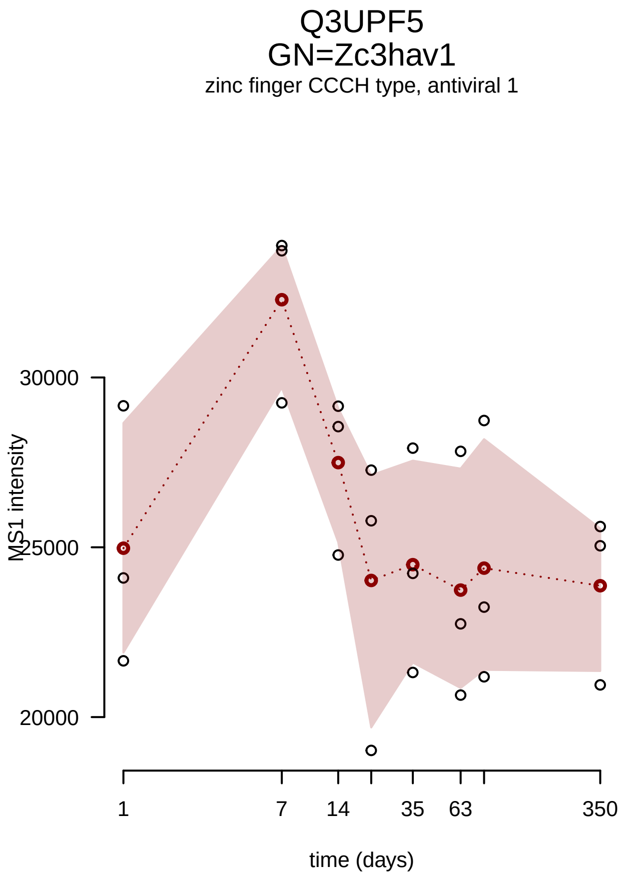
Q3UPF5
GN=Zc3hav1
zinc finger CCCH type, antiviral 1
MS1 intensity
30000
25000
20000
1
7
14
35
63
350
time (days)

Q3UPH1
GN=Prrc1
proline−rich coiled−coil 1

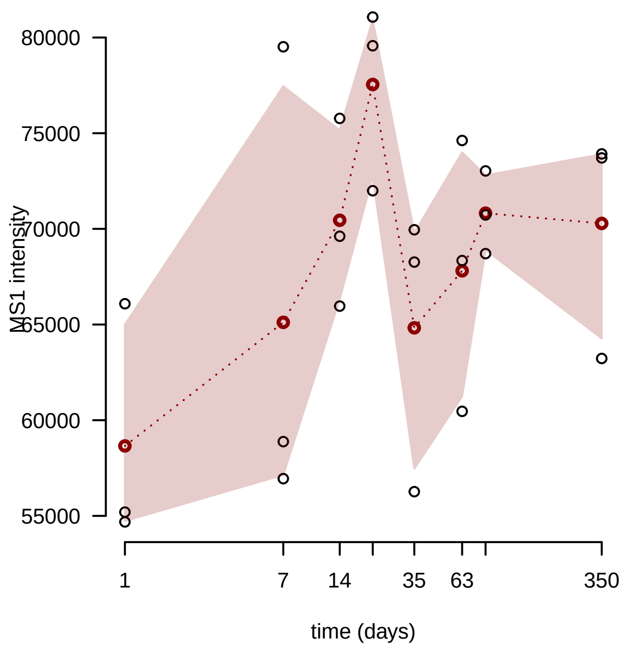

Q3UPH7
GN=Arhgef40
Rho guanine nucleotide exchange factor (...

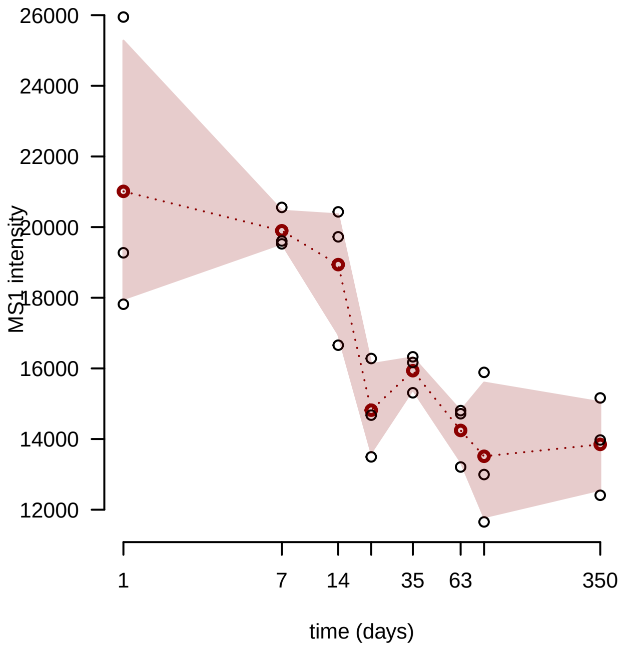

Q3UPL0
GN=Sec31a
Sec31 homolog A (S. cerevisiae)

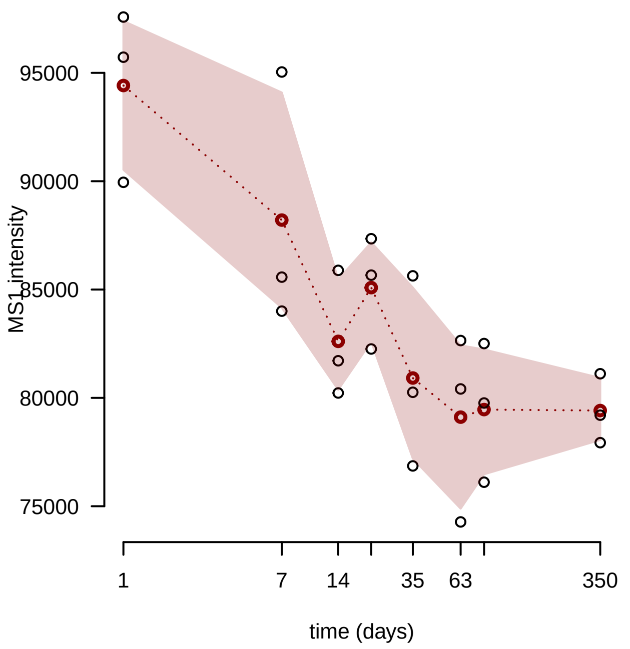

Q3UPP8
GN=Cep63
centrosomal protein 63

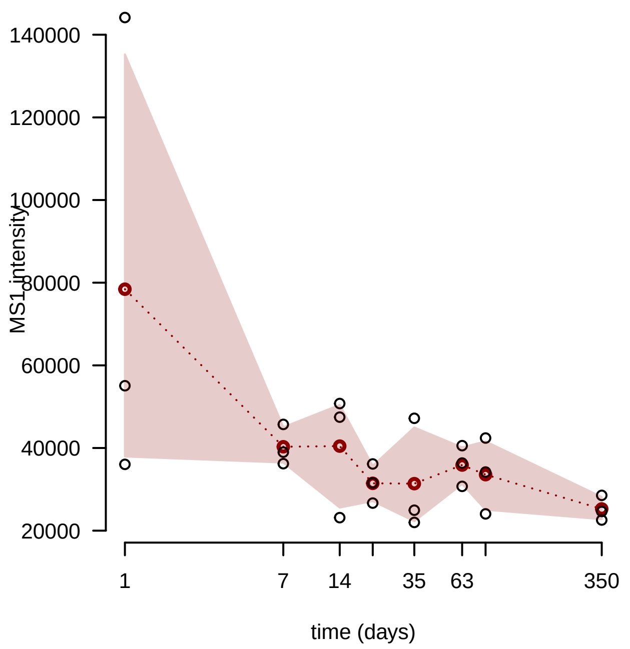

Q3UPR9
GN=Sbspon
somatomedin B and thrombospondin, type 1 ...

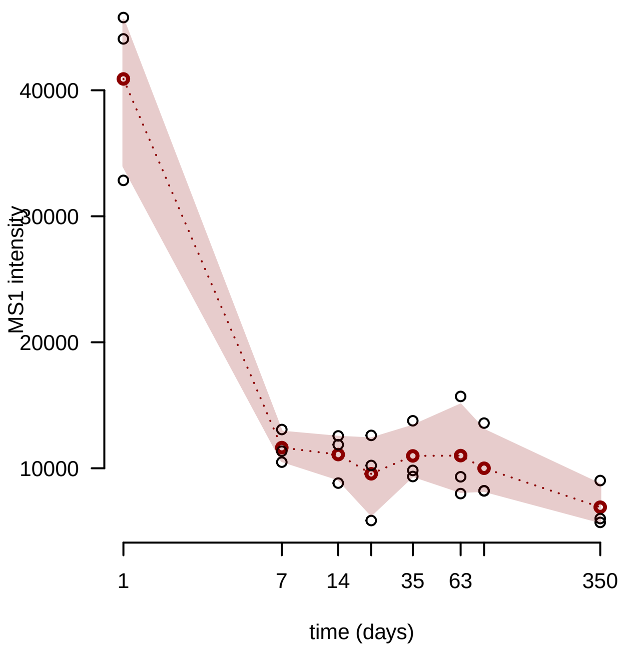

Q3UPY5
GN=Glb1l2
galactosidase, beta 1−like 2

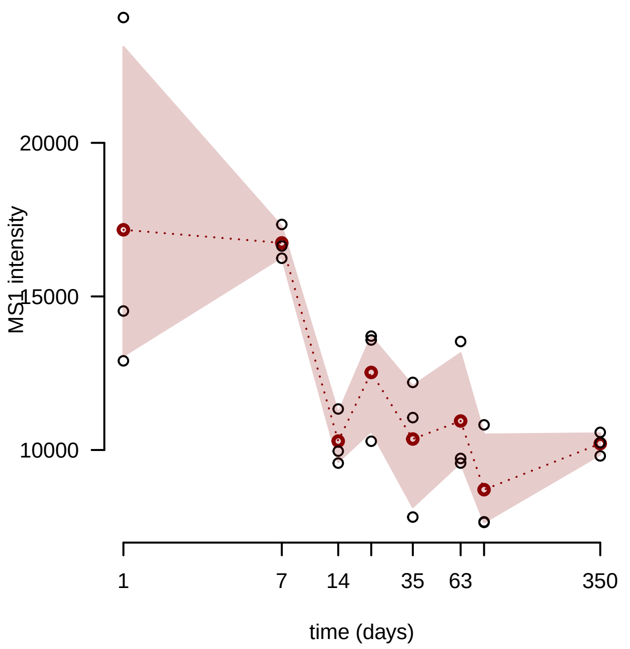

Q3UQ28
GN=Pxdn
peroxidasin

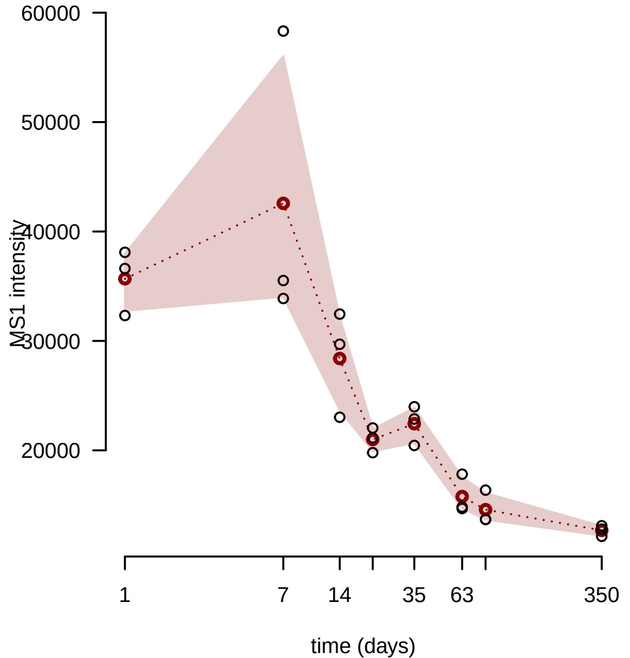

Q3UQ44
GN=Iqgap2
IQ motif containing GTPase activating pr ...

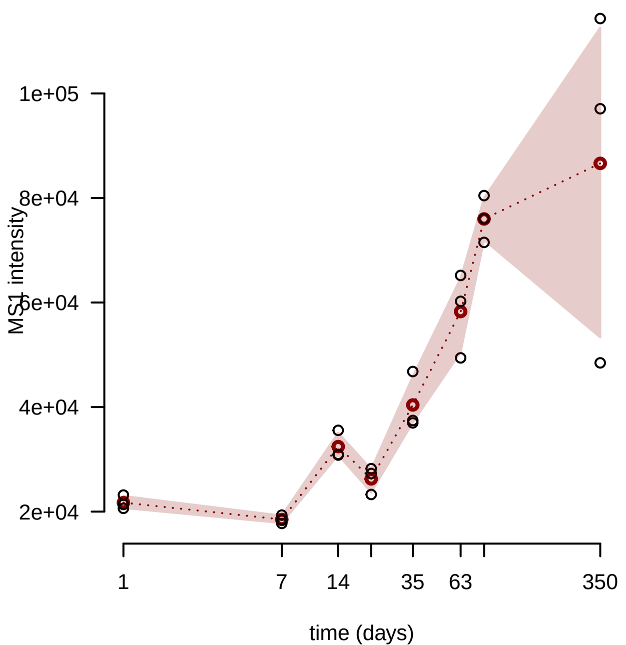

Q3UQ84
GN=Tars2
threonyl−tRNA synthetase 2, mitochondria ...

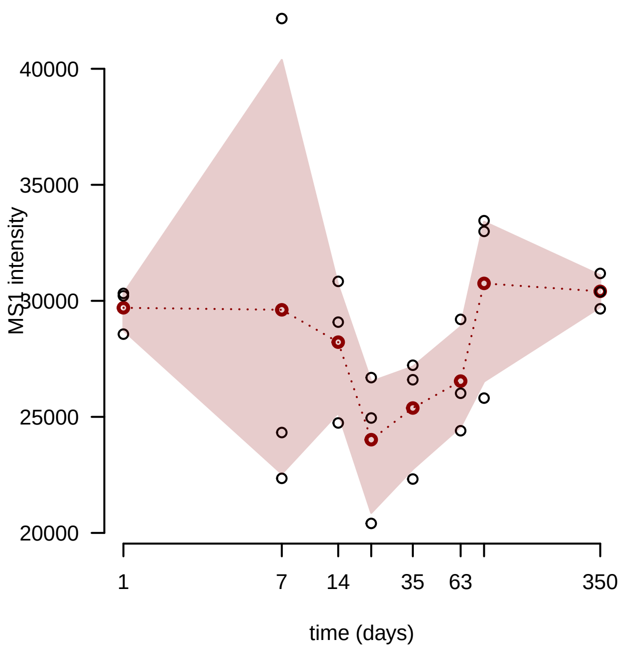

Q3UQA7
GN=Selenoh
selenoprotein H

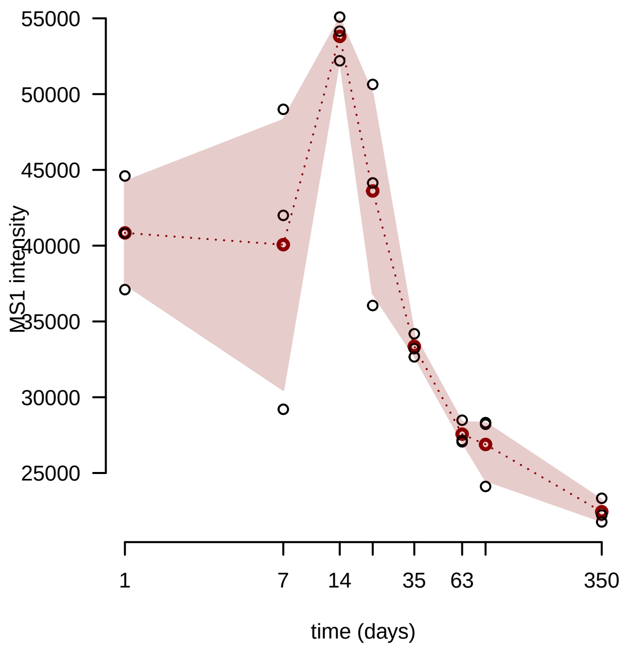

Q3UQN2
GN=Fcho2
FCH domain only 2

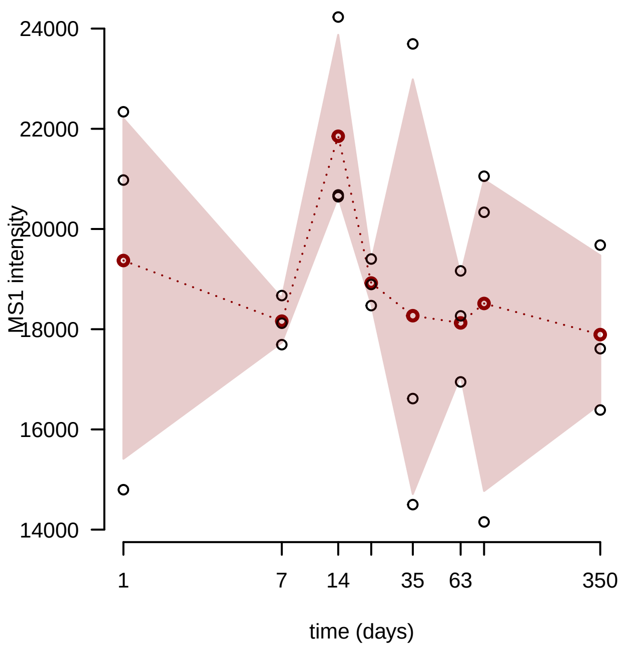

Q3UQU0
GN=Brd9
bromodomain containing 9

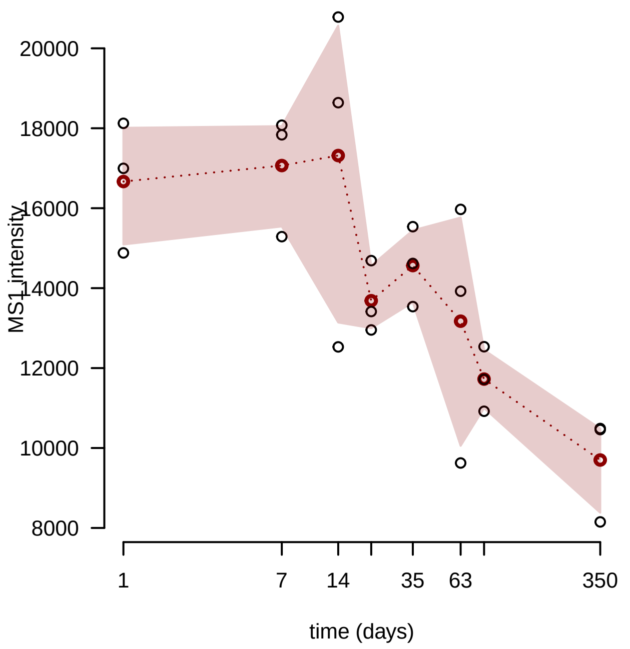

Q3UQV5
GN=Kbtbd8
kelch repeat and BTB (POZ) domain contai ...

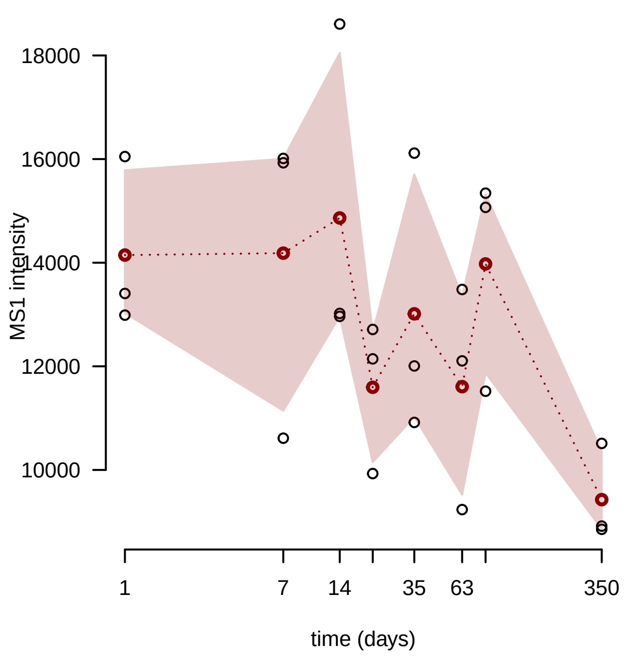

Q3UR70
GN=Tgfbrap1
transforming growth factor, beta recepto ...

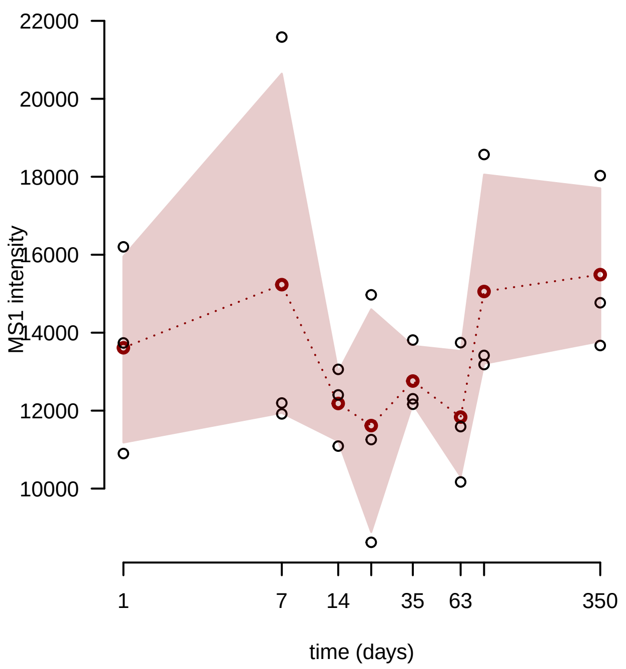

Q3UR85
GN=Myrf
myelin regulatory factor

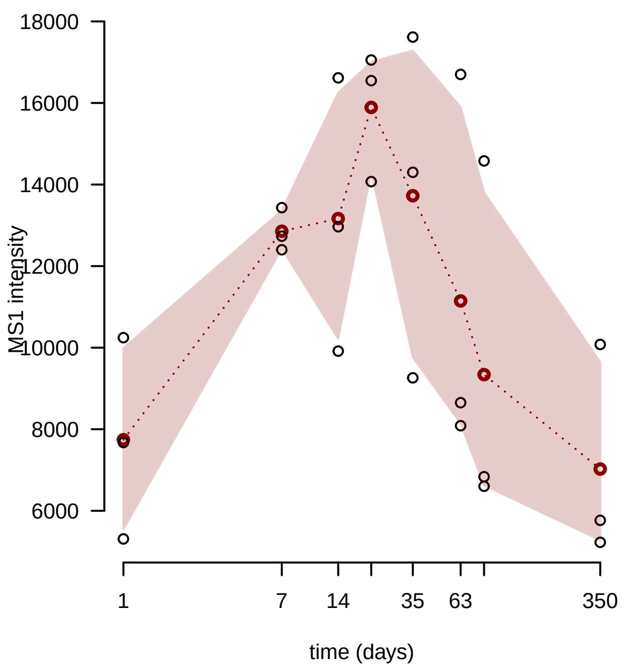

Q3UR97
GN=Snx21
sorting nexin family member 21

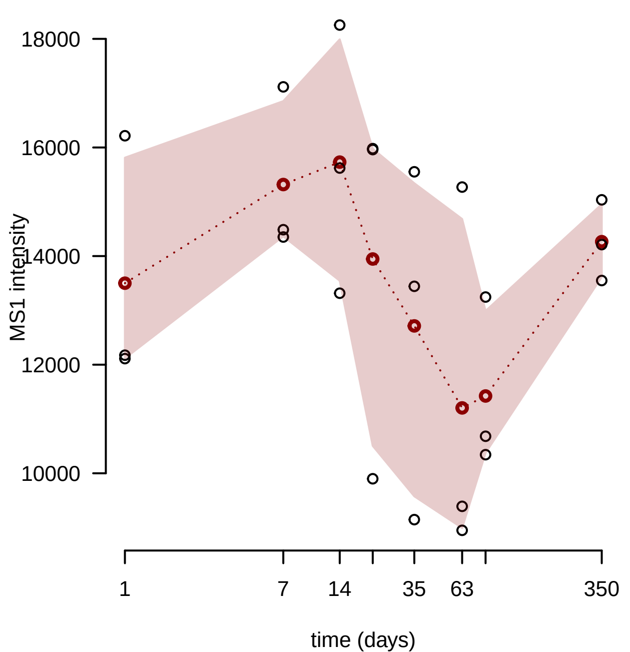

Q3URD3
GN=Slmap
sarcolemma associated protein

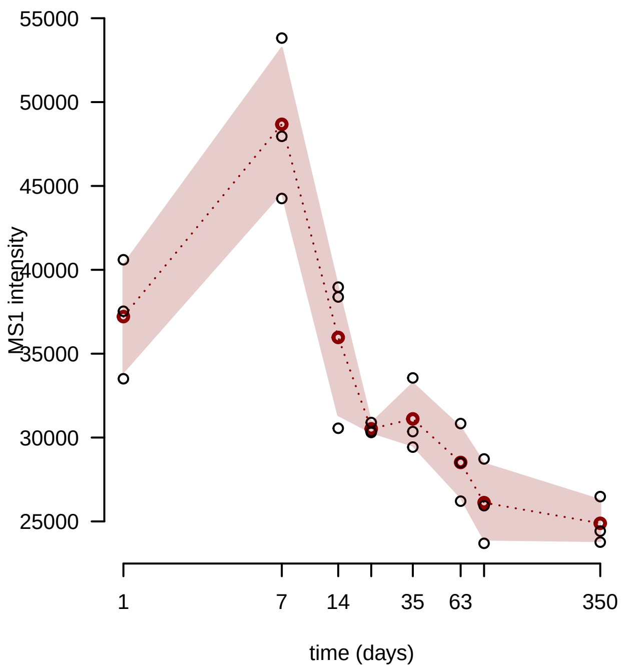

Q3URD3
GN=Slmap
sarcolemma associated protein

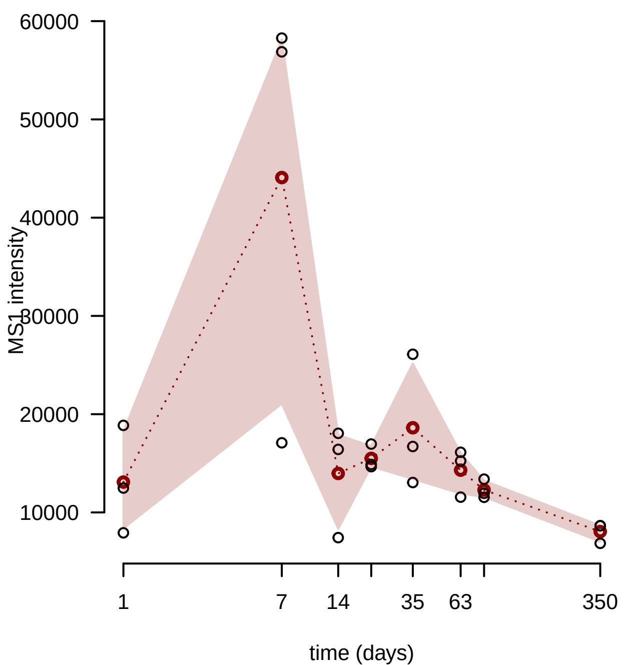

Q3URE1
GN=Acsf3
acyl−CoA synthetase family member 3

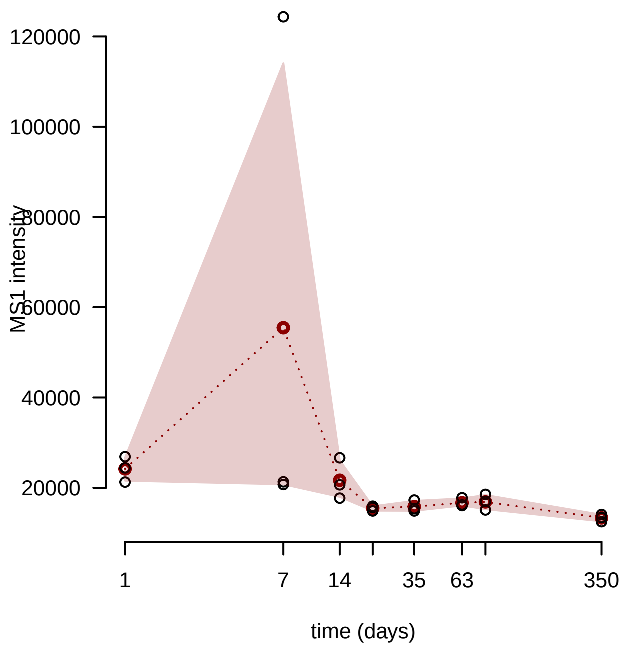

Q3URQ0
GN=Tex10
testis expressed gene 10

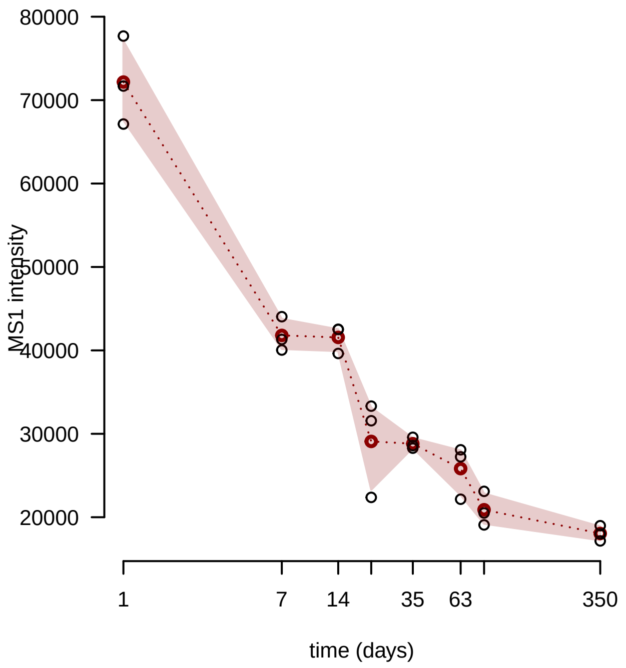

Q3URQ7
GN=Mthfsd
methenyltetrahydrofolate synthetase doma ...

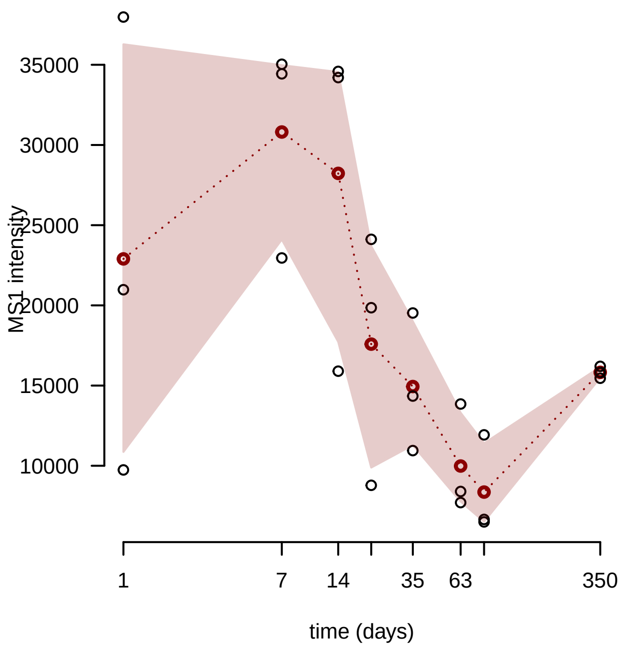

Q3URS9
GN=Ccdc51
coiled−coil domain containing 51

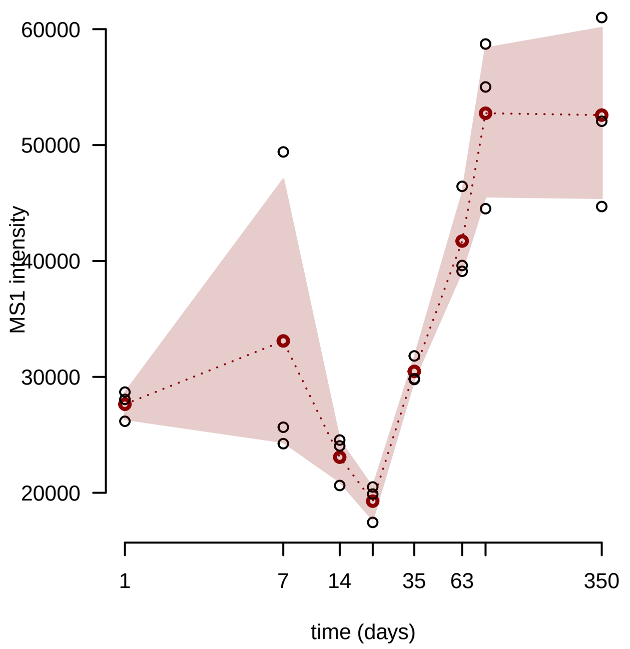

Q3URU2
GN=Peg3
paternally expressed 3

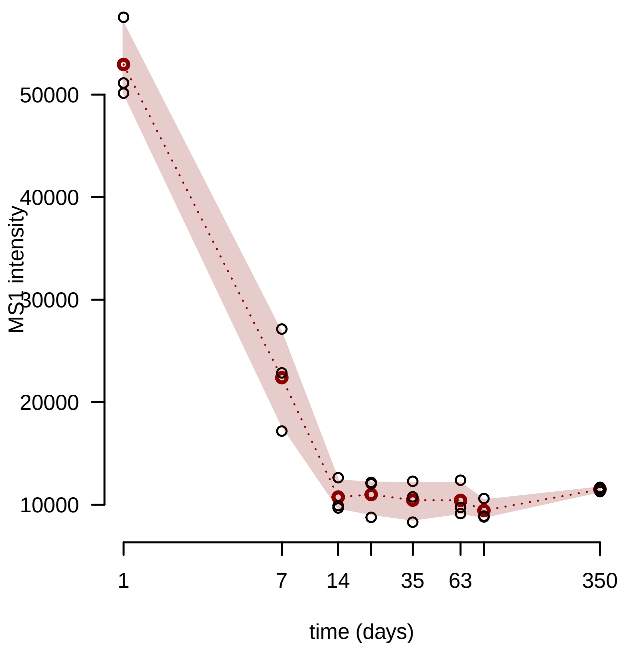

Q3US17
GN=Znf48

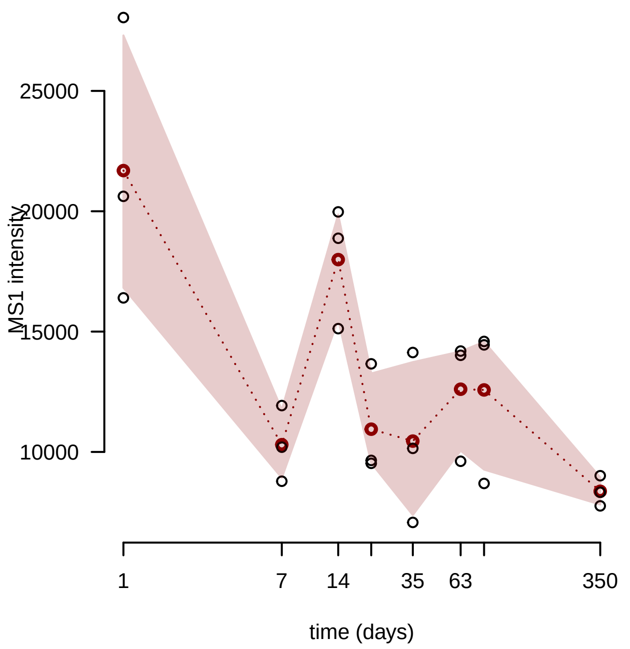

Q3US41
GN=Esrp1
epithelial splicing regulatory protein 1

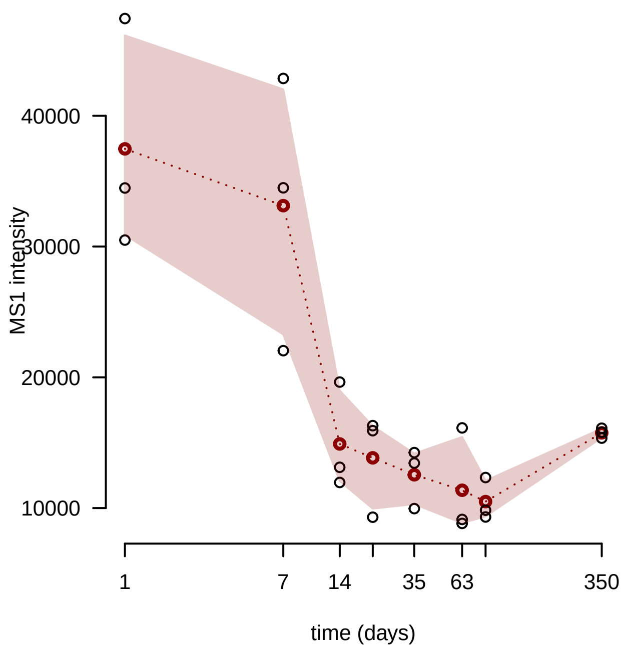

Q3USB7
GN=Plcl1
phospholipase C−like 1

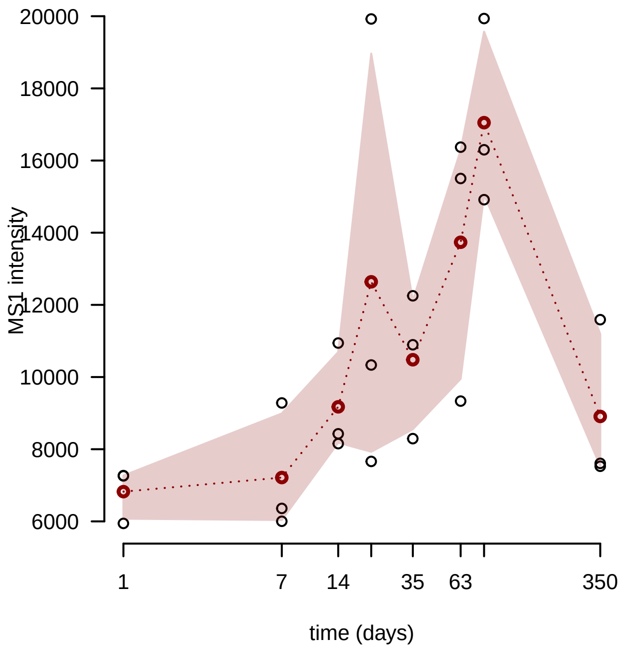

Q3USH5
GN=Sfswap
splicing factor SWAP

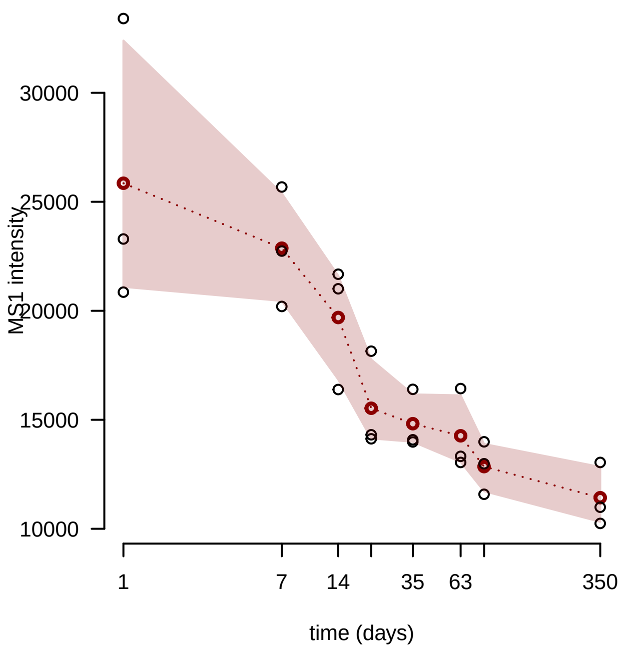

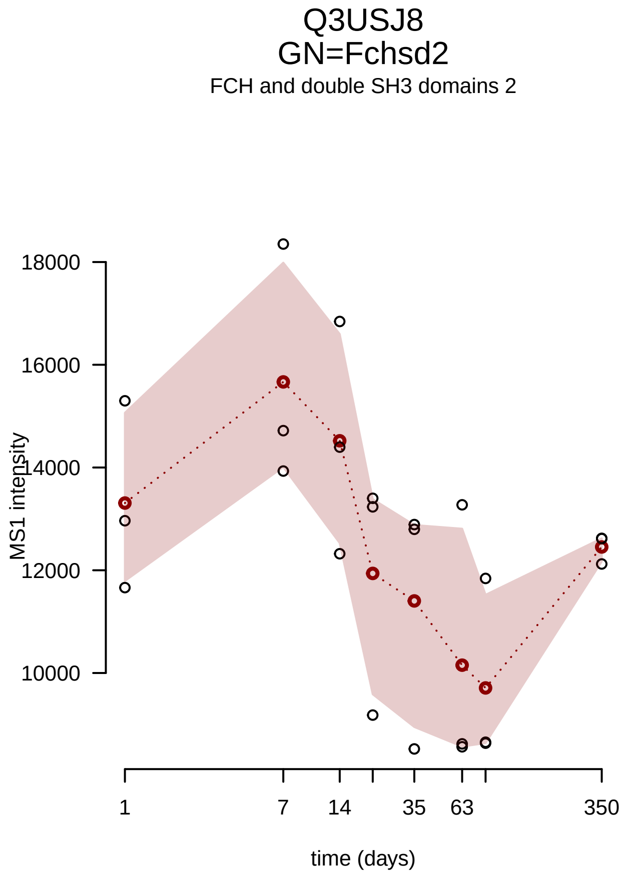
Q3USJ8
GN=Fchsd2
FCH and double SH3 domains 2
18000
16000
14000
12000
10000
MS1 intensity
1
7
14
35
63
350
time (days)

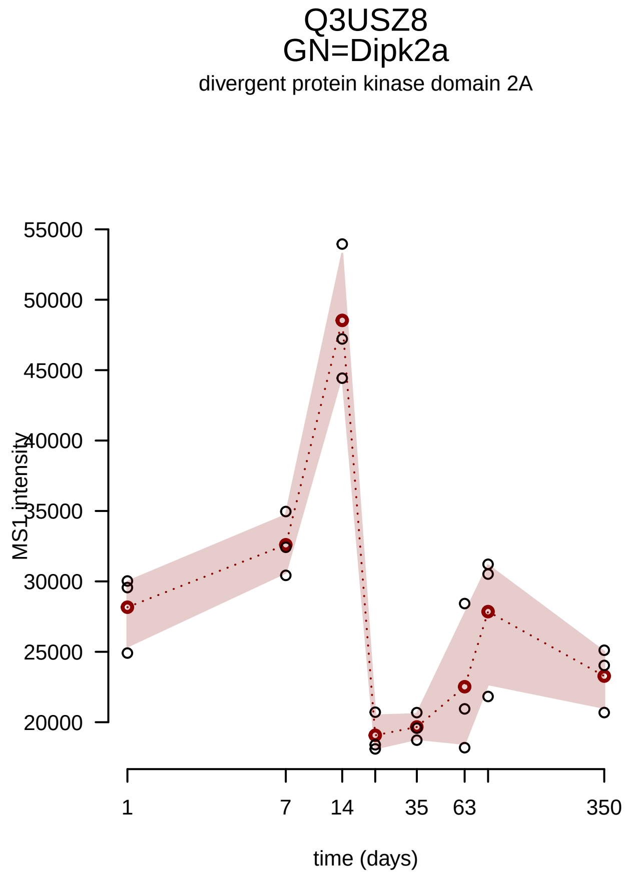
Q3USZ8
GN=Dipk2a
divergent protein kinase domain 2A
55000
50000
45000
40000
35000
30000
25000
20000
MS1 intensity
1
7
14
35
63
350
time (days)

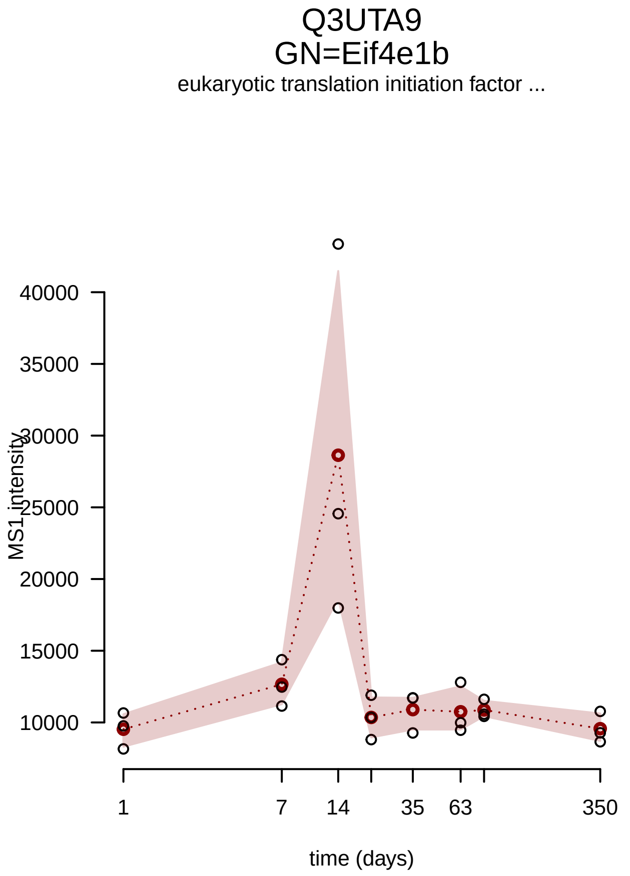
Q3UTA9
GN=Eif4e1b
eukaryotic translation initiation factor ...
40000
35000
30000
25000
20000
15000
10000
MS1 intensity
1
7
14
35
63
350
time (days)

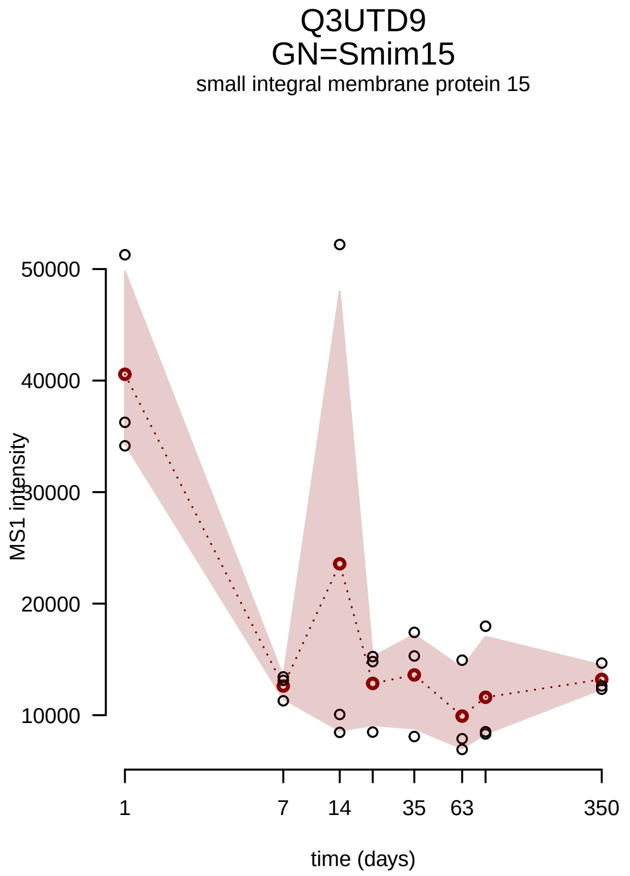
Q3UTD9
GN=Smim15
small integral membrane protein 15
50000
40000
30000
20000
10000
MS1 intensity
1
7
14
35
63
350
time (days)

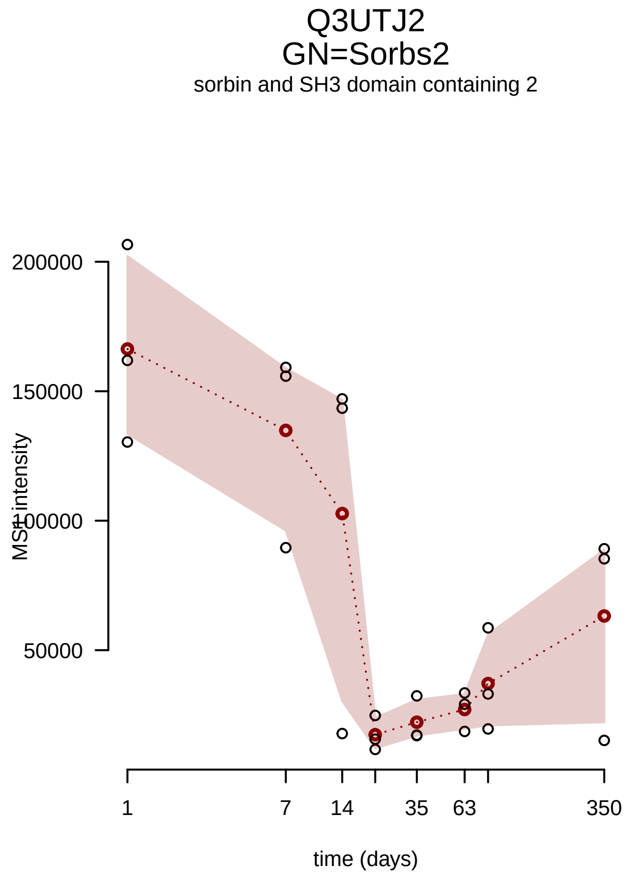
Q3UTJ2
GN=Sorbs2
sorbin and SH3 domain containing 2
200000
150000
100000
50000
MS1 intensity
1
7
14
35
63
350
time (days)

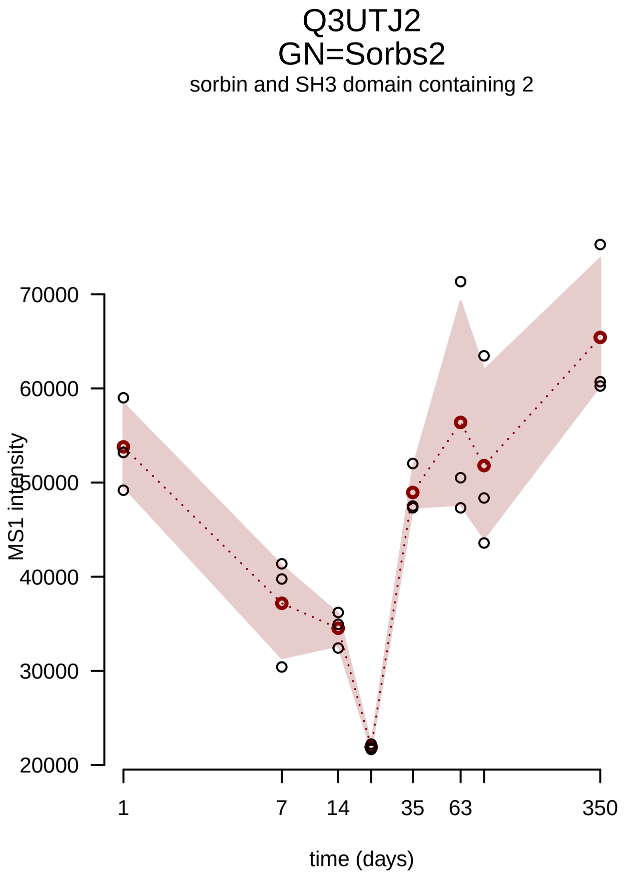
Q3UTJ2
GN=Sorbs2
sorbin and SH3 domain containing 2
70000
60000
50000
40000
30000
20000
MS1 intensity
1
7
14
35
63
350
time (days)

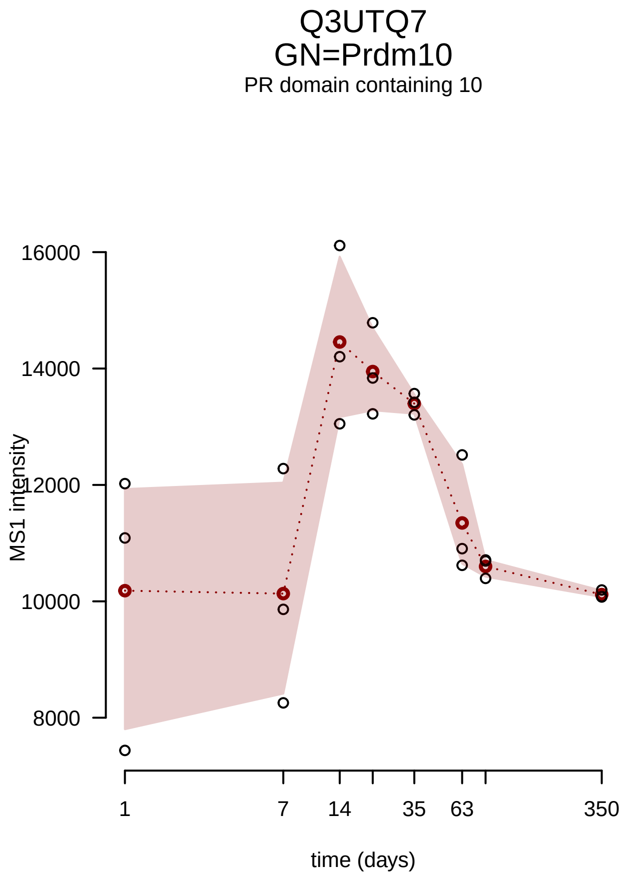
Q3UTQ7
GN=Prdm10
PR domain containing 10
16000
14000
12000
10000
8000
MS1 intensity
1
7
14
35
63
350
time (days)

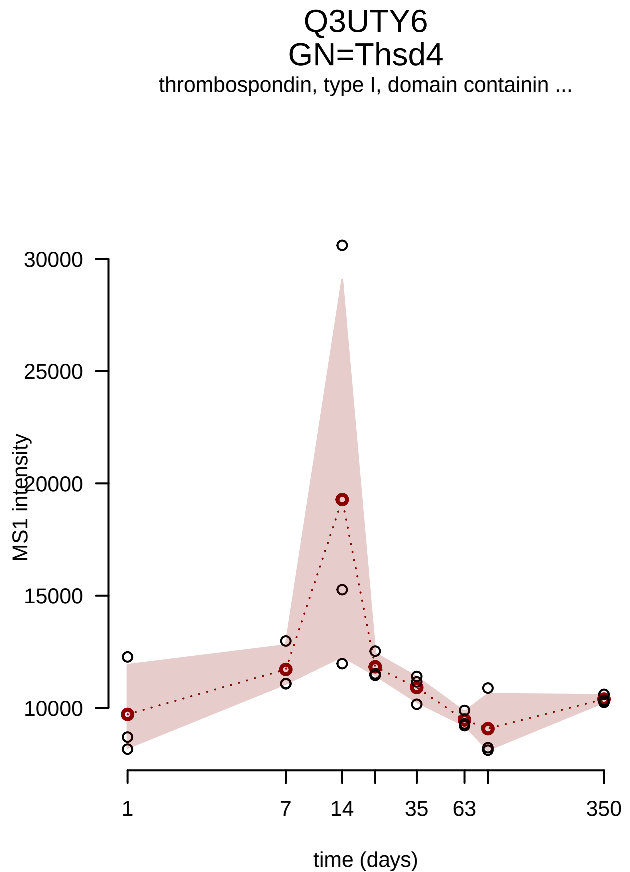
Q3UTY6
GN=Thsd4
thrombospondin, type I, domain containin ...
30000
25000
20000
15000
10000
MS1 intensity
1
7
14
35
63
350
time (days)

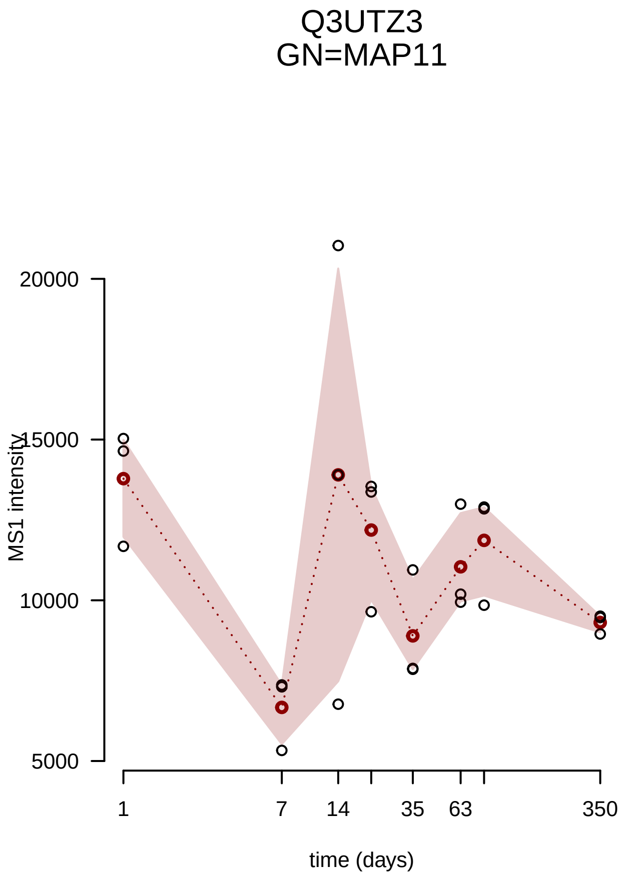
Q3UTZ3
GN=MAP11
20000
15000
10000
5000
MS1 intensity
1
7
14
35
63
350
time (days)

Q3UU96
GN=Cdc42bpa
CDC42 binding protein kinase alpha

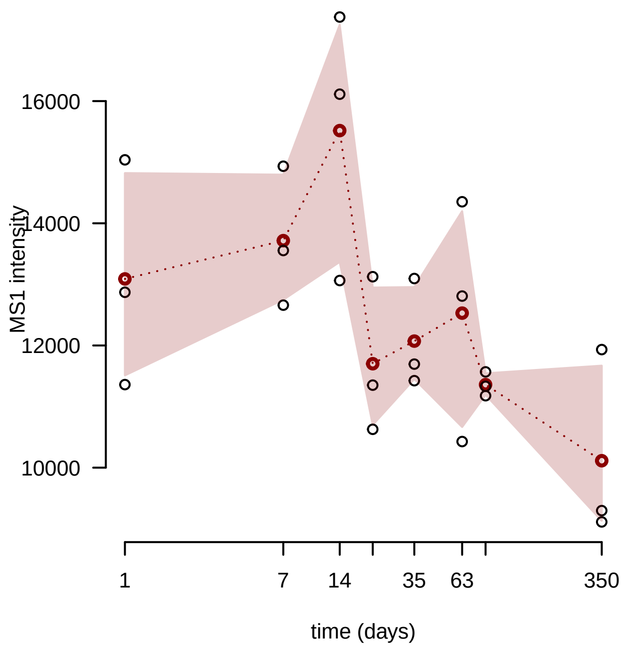

Q3UUG6
GN=Tbc1d24
TBC1 domain family, member 24

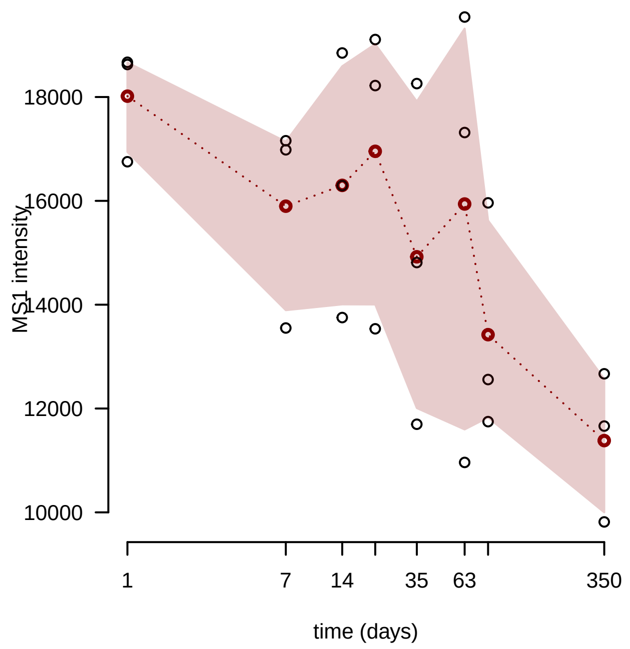

Q3UUI3
GN=Them4
thioesterase superfamily member 4

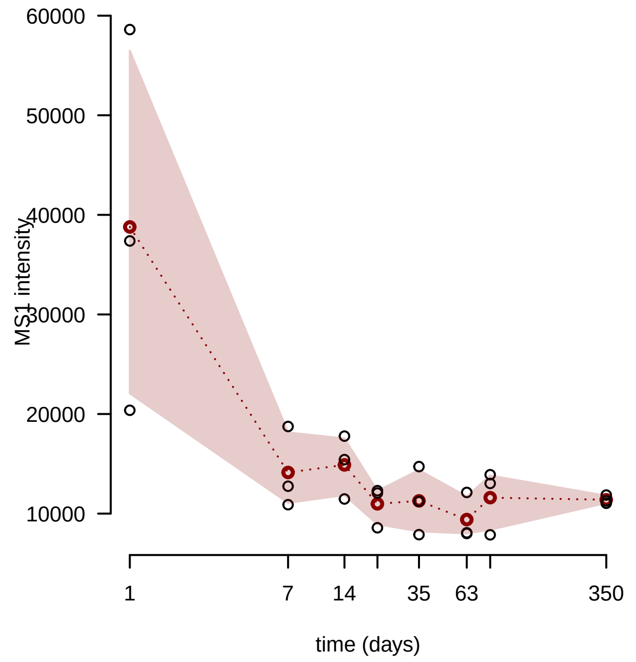

Q3UUJ4
GN=Strada
STE20−related kinase adaptor alpha

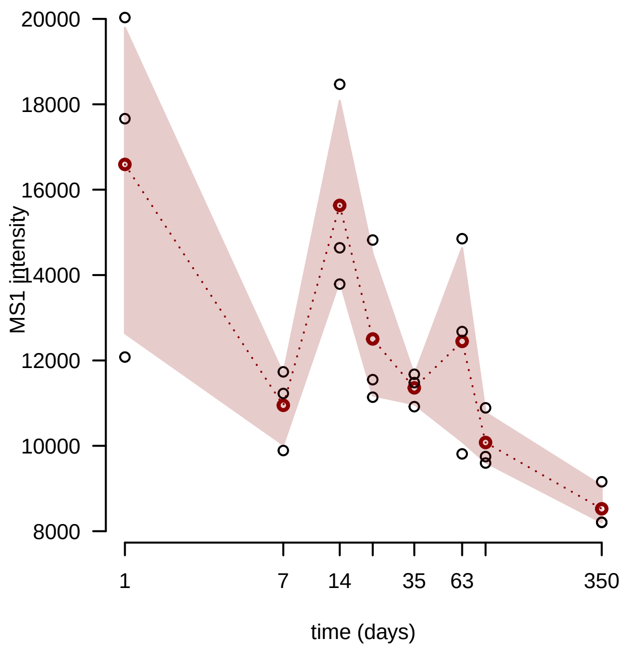

Q3UUQ7
GN=Pgap1
post−GPI attachment to proteins 1

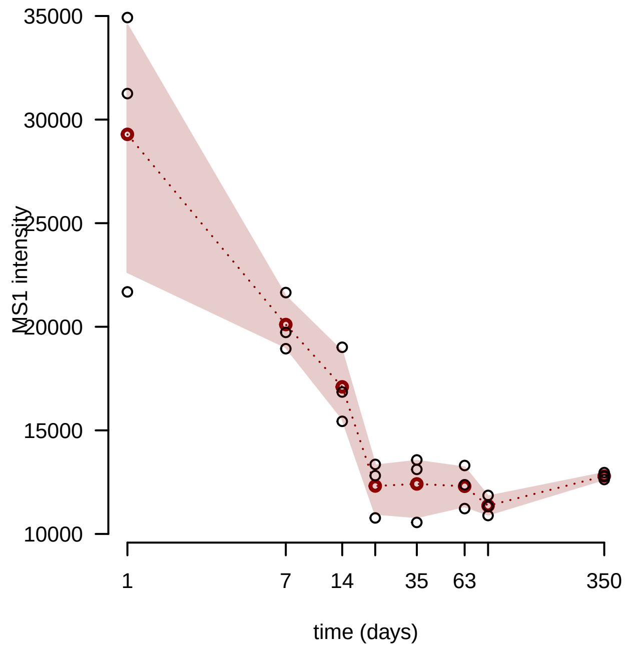

Q3UV17
GN=Krt76
keratin 76

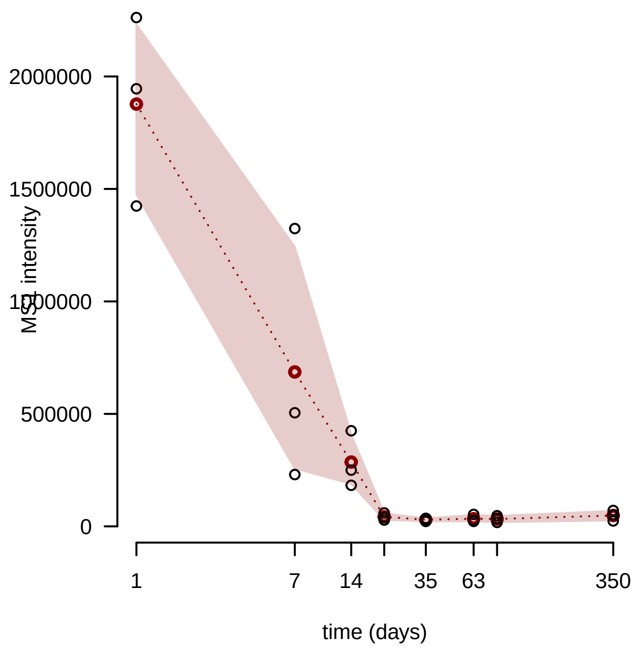

Q3UV70
GN=Pdp1
pyruvate dehydrogenase phosphatase catal ...

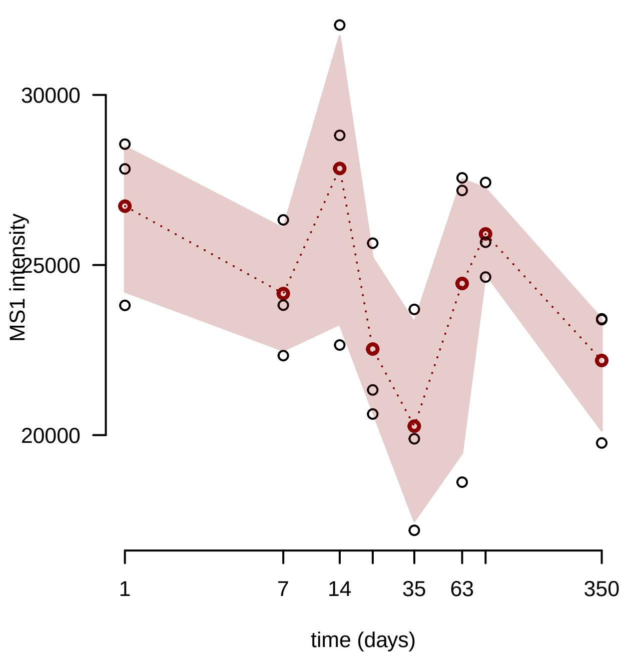

Q3UVG3
GN=Fam91a1
family with sequence similarity 91, memb ...

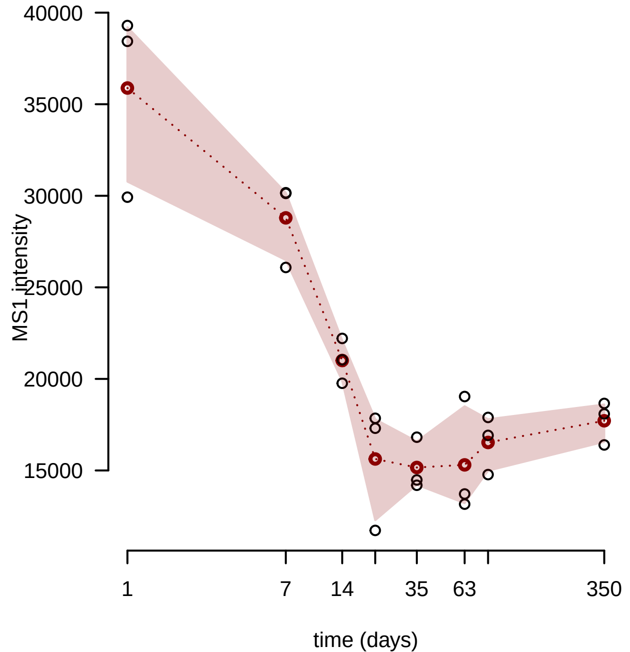

Q3UVK0
GN=Ermp1
endoplasmic reticulum metallopeptidase 1

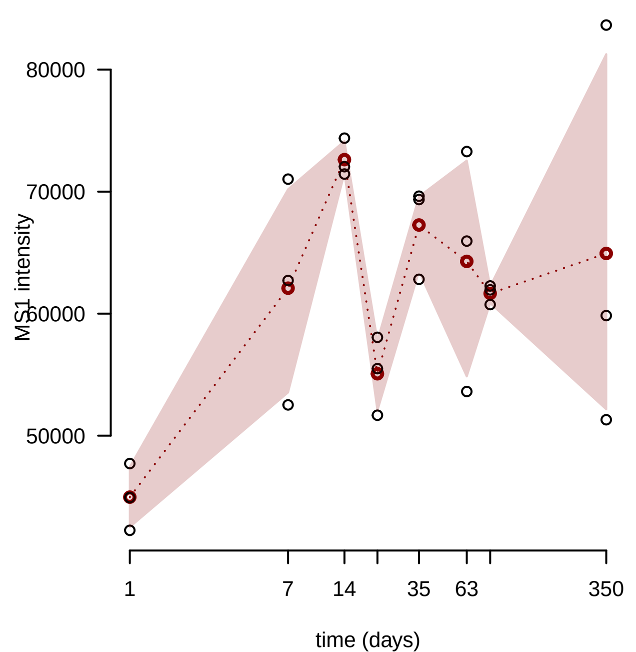

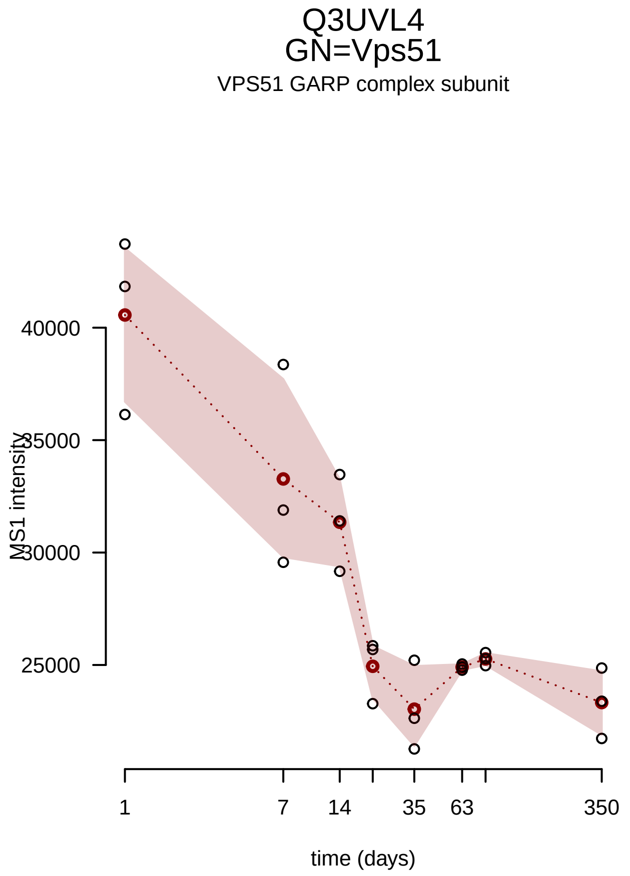
Q3UVL4
GN=Vps51
VPS51 GARP complex subunit
MS1 intensity
25000
30000
35000
40000
1
7
14
35
63
350
time (days)

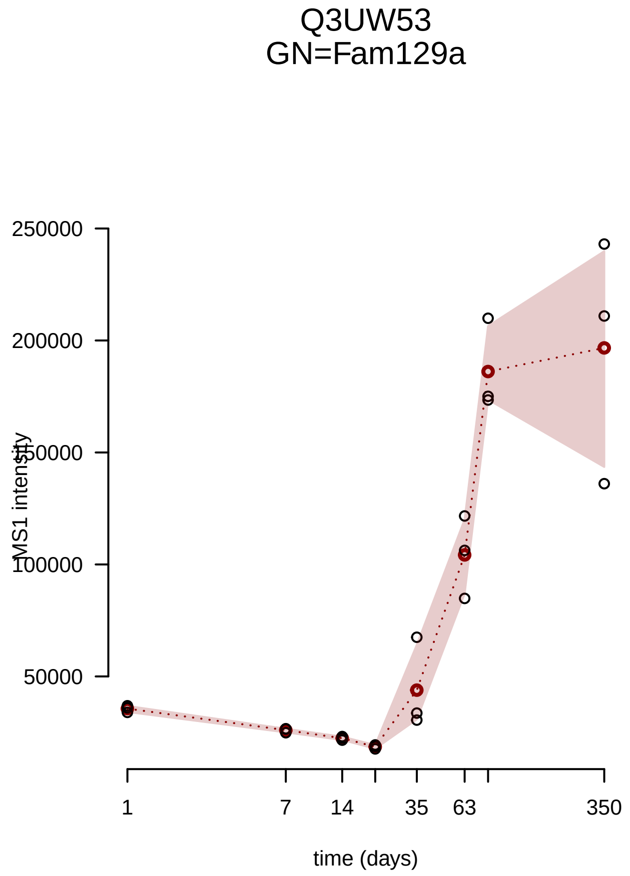
Q3UW53
GN=Fam129a
MS1 intensity
50000
100000
150000
200000
250000
1
7
14
35
63
350
time (days)

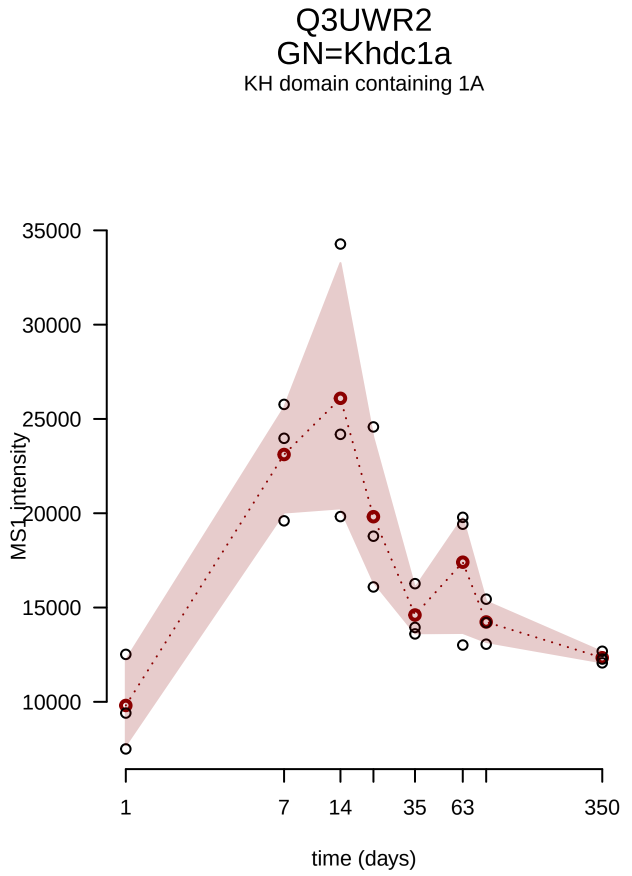
Q3UWR2
GN=Khdc1a
KH domain containing 1A
MS1 intensity
10000
15000
20000
25000
30000
35000
1
7
14
35
63
350
time (days)

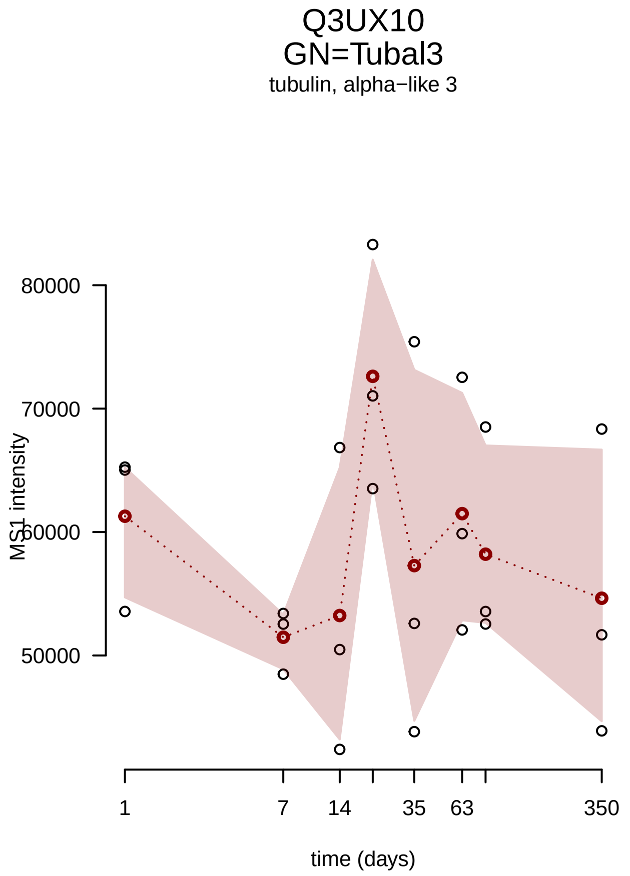
Q3UX10
GN=Tubal3
tubulin, alpha−like 3
MS1 intensity
50000
60000
70000
80000
1
7
14
35
63
350
time (days)

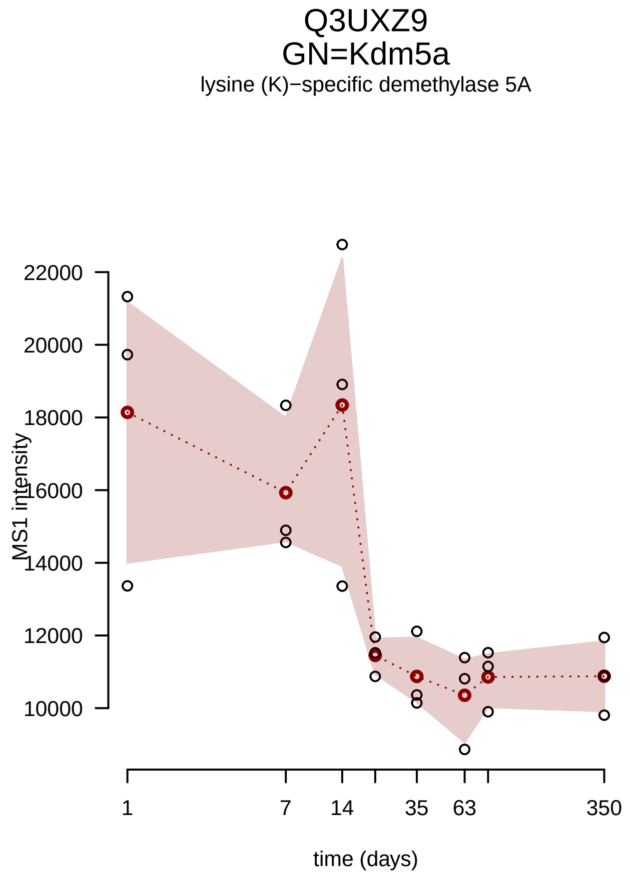
Q3UXZ9
GN=Kdm5a
lysine (K)−specific demethylase 5A
MS1 intensity
10000
12000
14000
16000
18000
20000
22000
1
7
14
35
63
350
time (days)

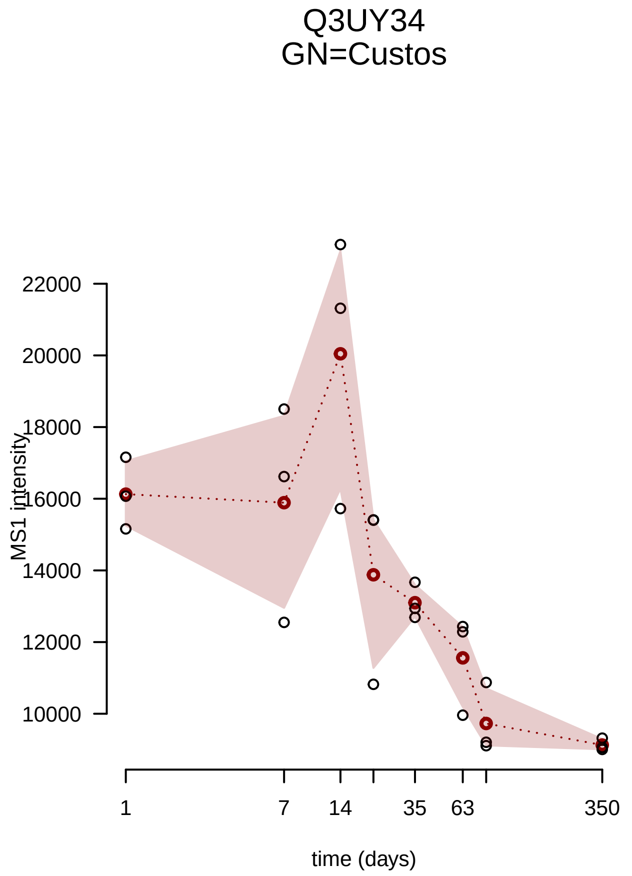
Q3UY34
GN=Custos
MS1 intensity
10000
12000
14000
16000
18000
20000
22000
1
7
14
35
63
350
time (days)

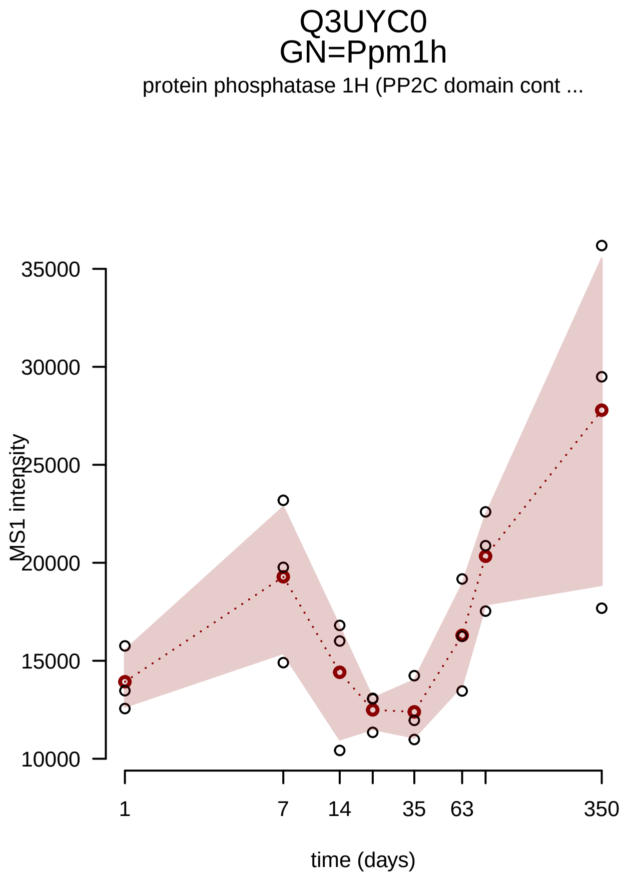
Q3UYC0
GN=Ppm1h
protein phosphatase 1H (PP2C domain cont ...
MS1 intensity
10000
15000
20000
25000
30000
35000
1
7
14
35
63
350
time (days)

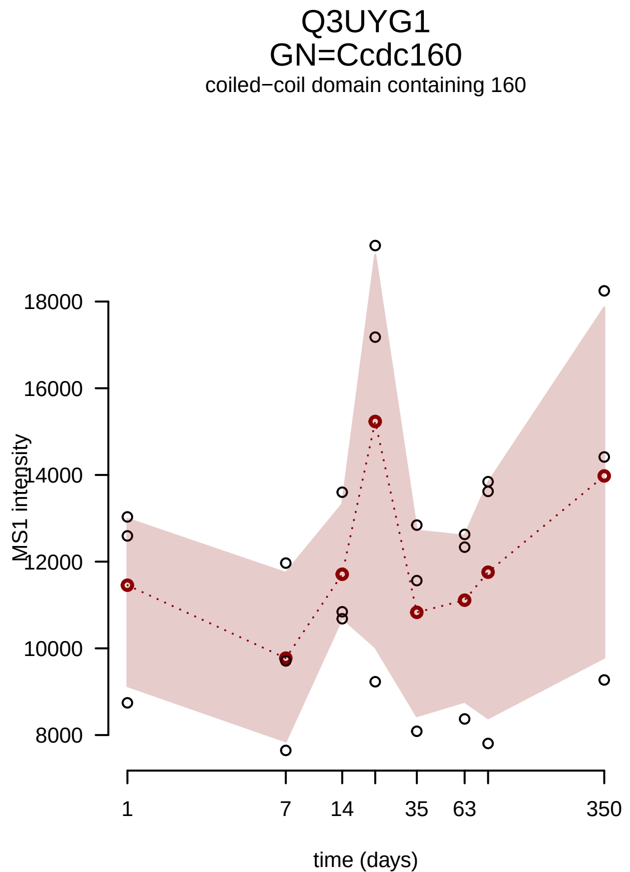
Q3UYG1
GN=Ccdc160
coiled−coil domain containing 160
MS1 intensity
8000
10000
12000
14000
16000
18000
1
7
14
35
63
350
time (days)

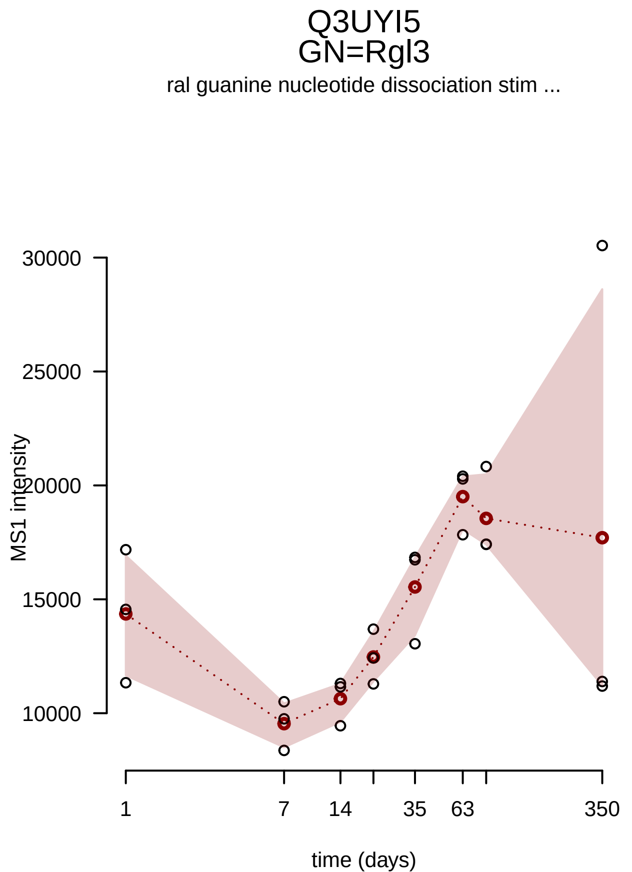
Q3UYI5
GN=Rgl3
ral guanine nucleotide dissociation stim ...
MS1 intensity
10000
15000
20000
25000
30000
1
7
14
35
63
350
time (days)

Q3UYV9
GN=Ncbp1
nuclear cap binding protein subunit 1

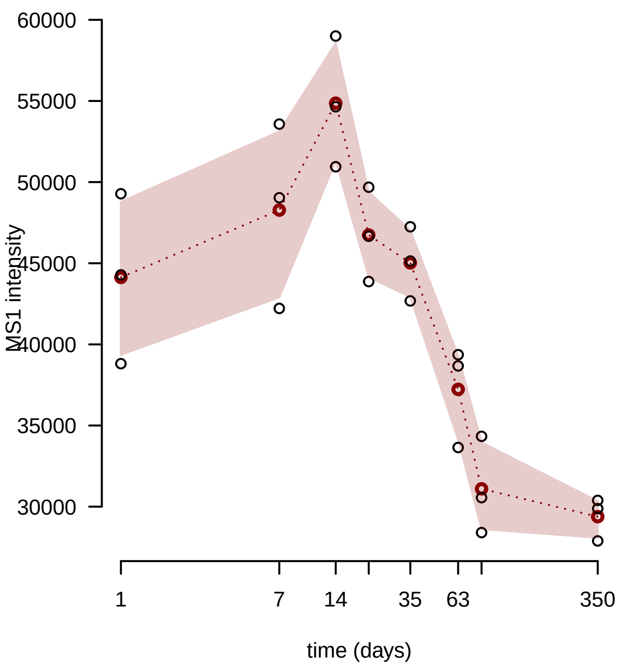

Q3UZ01
GN=Rnpc3
RNA−binding region (RNP1, RRM) containin ...

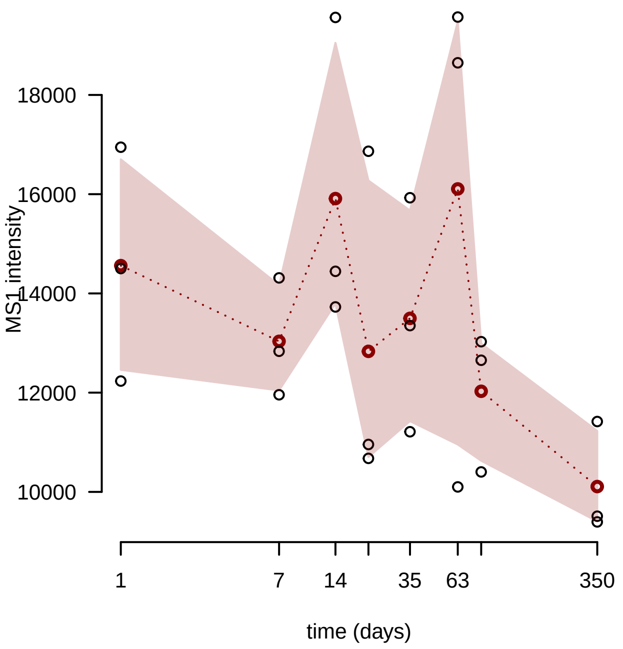

Q3UZ09
GN=C1rl
complement component 1, r subcomponent−l ...

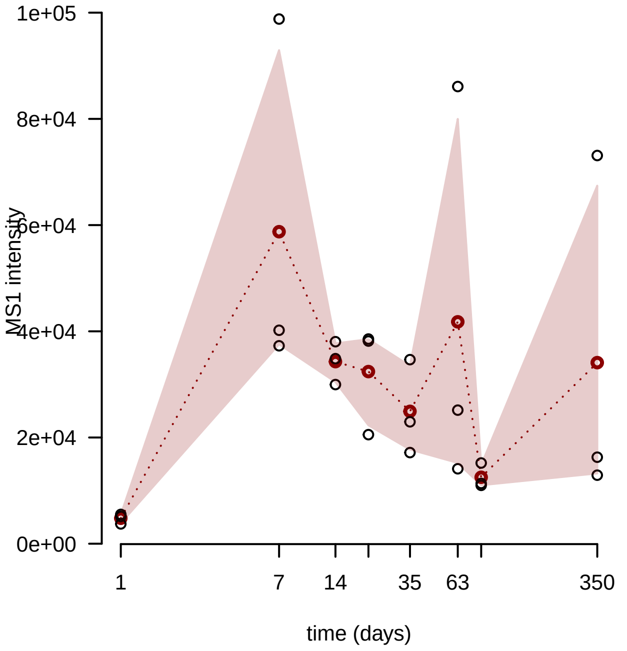

Q3UZ18
GN=Ice2
interactor of little elongation complex ...

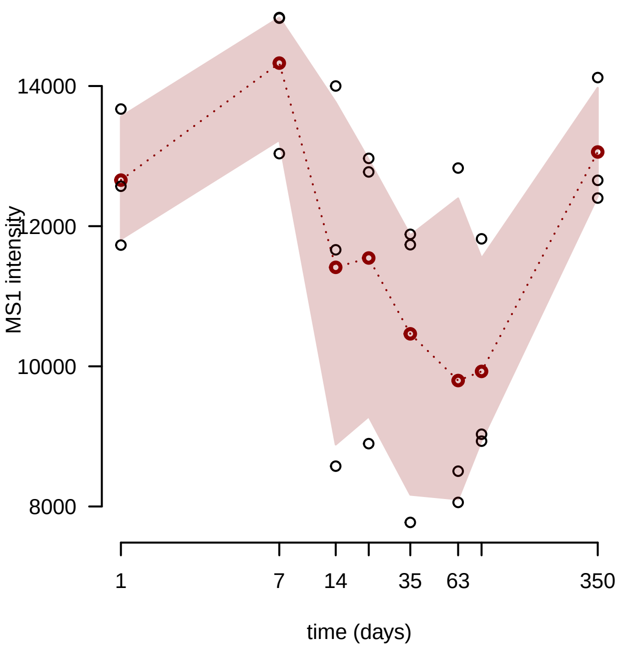

Q3UZ39
GN=Lrrfip1
leucine rich repeat (in FLII) interactin ...

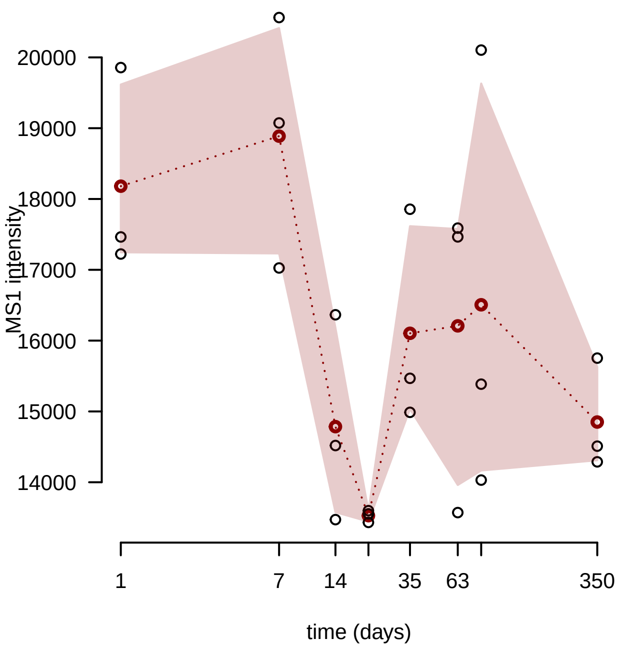

Q3UZA1
GN=Rcsd1
RCSD domain containing 1

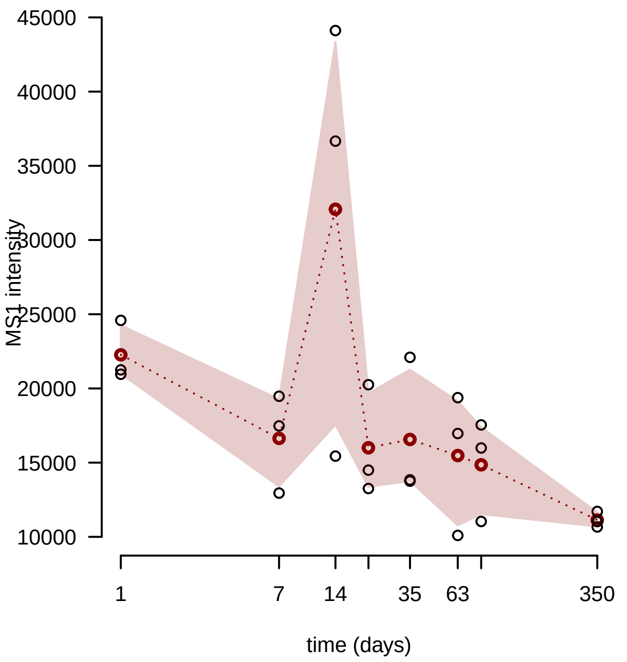

Q3UZB0
GN=Armcx5
armadillo repeat containing, X−linked 5

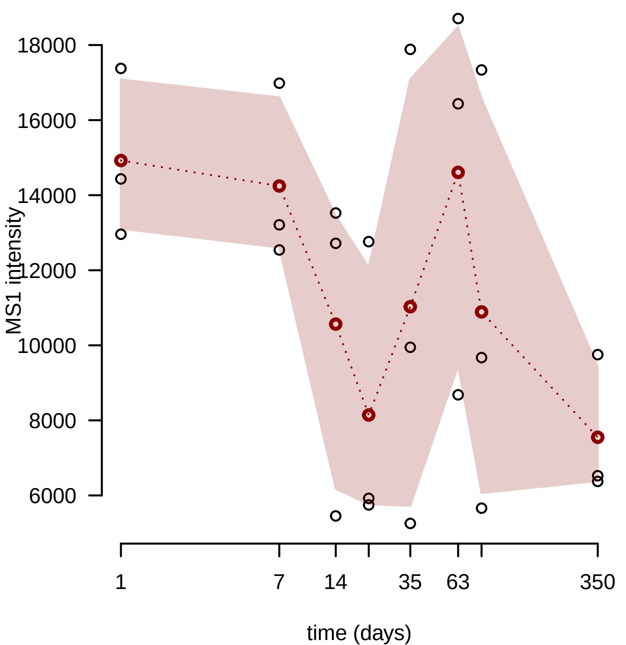

Q3UZP0
GN=Marveld2
MARVEL (membrane−associating) domain con ...

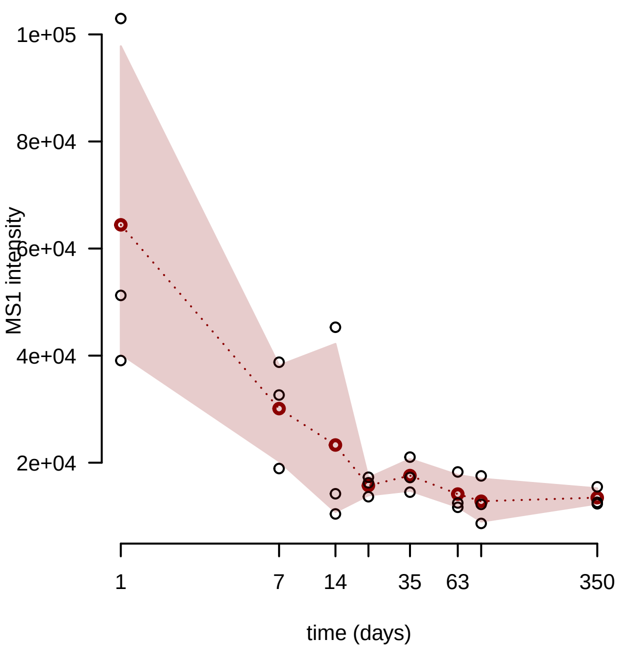

Q3UZW7
GN=Eef2kmt
eukaryotic elongation factor 2 lysine me ...

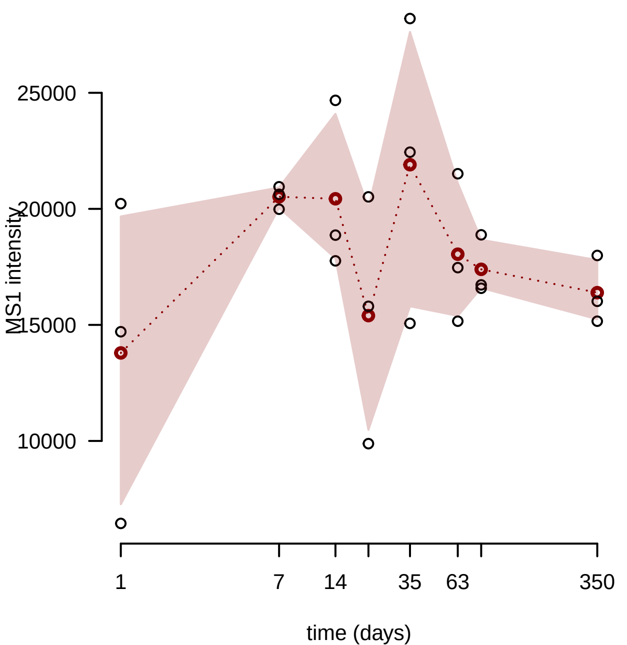

Q3UZZ6
GN=Sult1d1
sulfotransferase family 1D, member 1

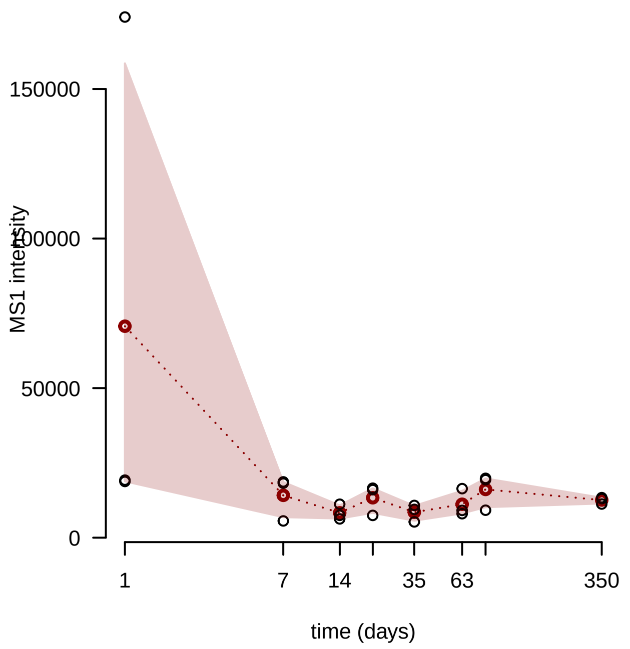

Q3V009
GN=Tmed1
transmembrane p24 trafficking protein 1

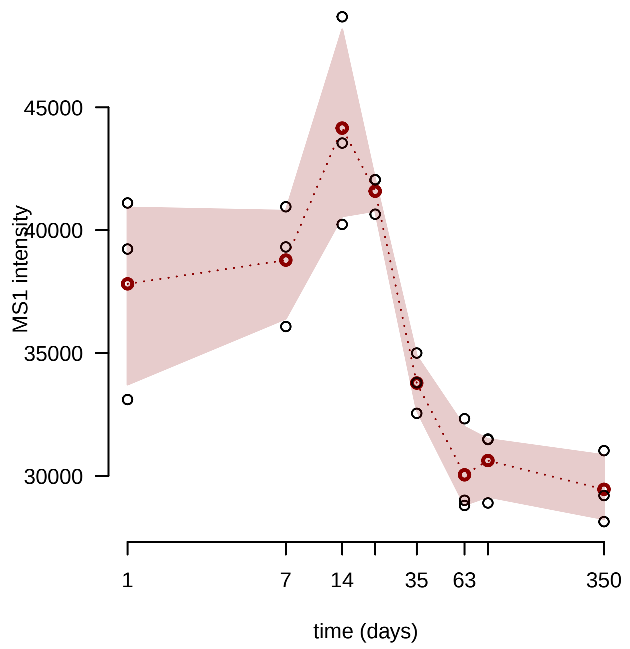

Q3V080
GN=Znf583

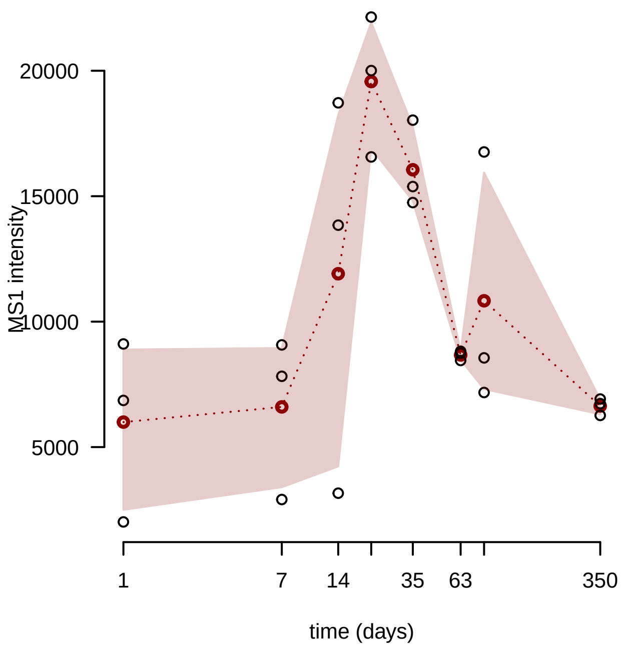

Q3V0C1
GN=Zmat1
zinc finger, matrin type 1

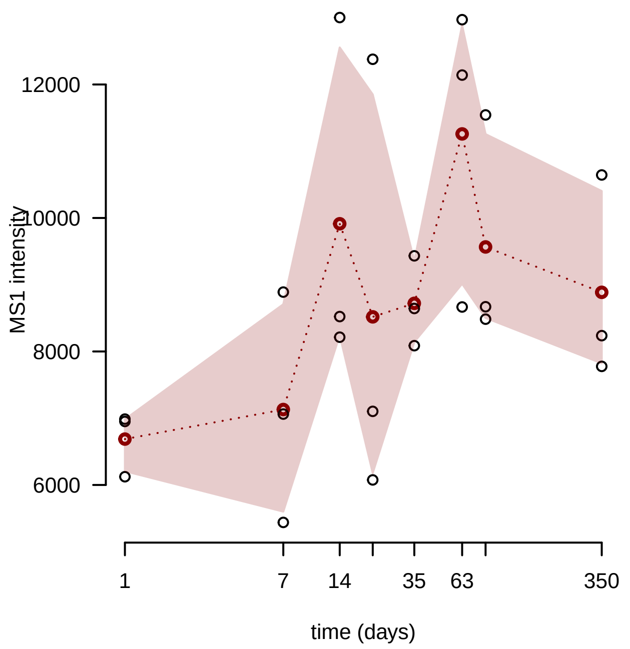

Q3V0C5
GN=Usp48
ubiquitin specific peptidase 48

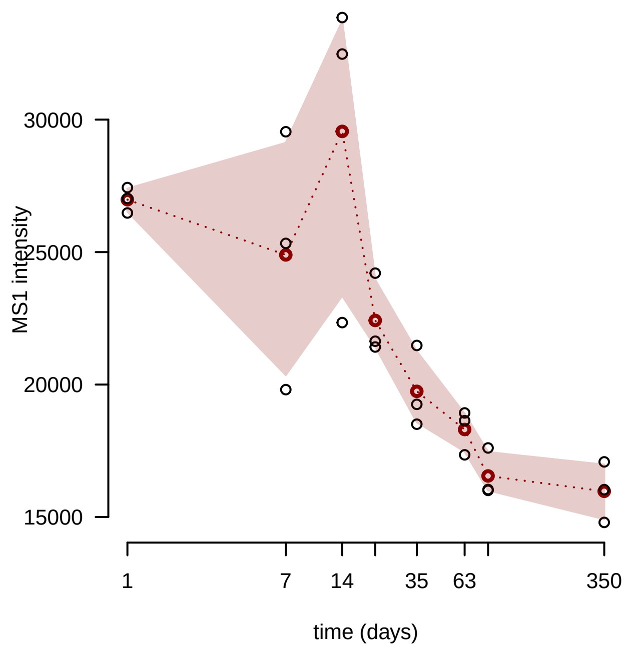

Q3V0J1
GN=Tmem237
transmembrane protein 237

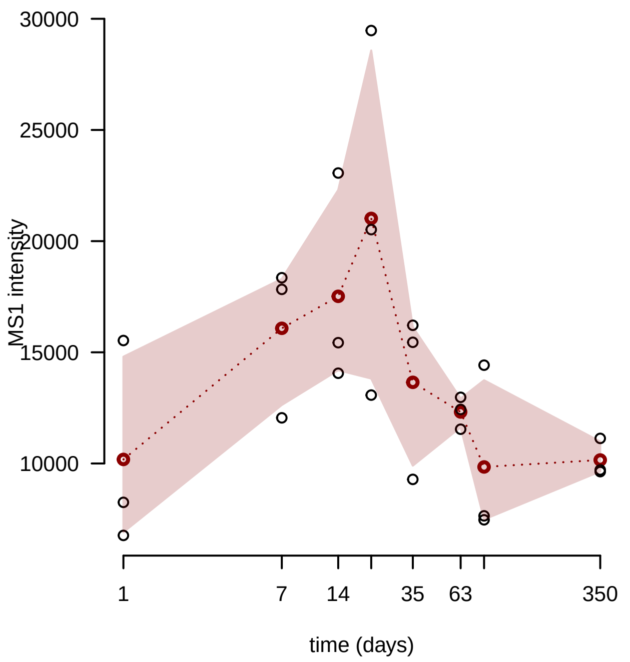

Q3V0K9
GN=Pls1
plastin 1 (I−isoform)

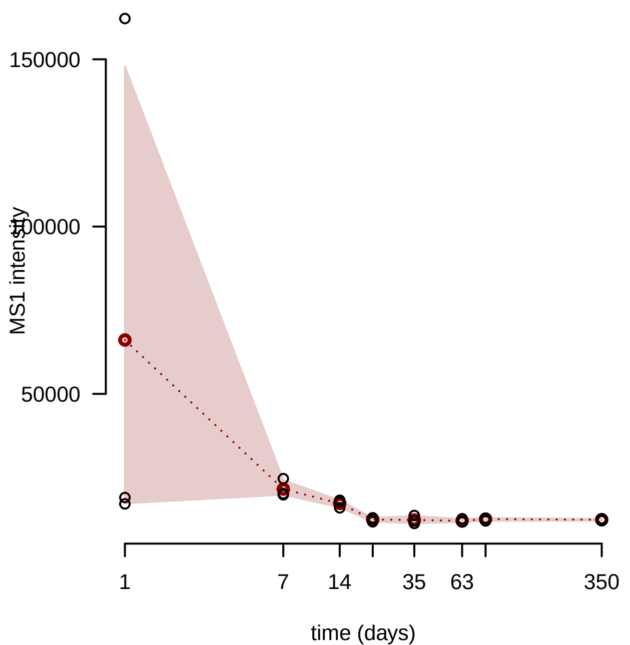

Q3V132
GN=Slc25a31
solute carrier family 25 (mitochondrial ...

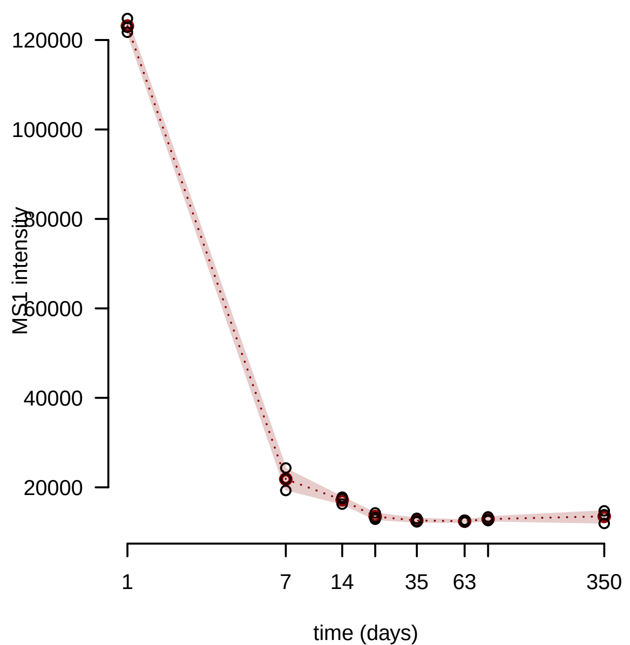

Q3V1H1
GN=Ckap2
cytoskeleton associated protein 2

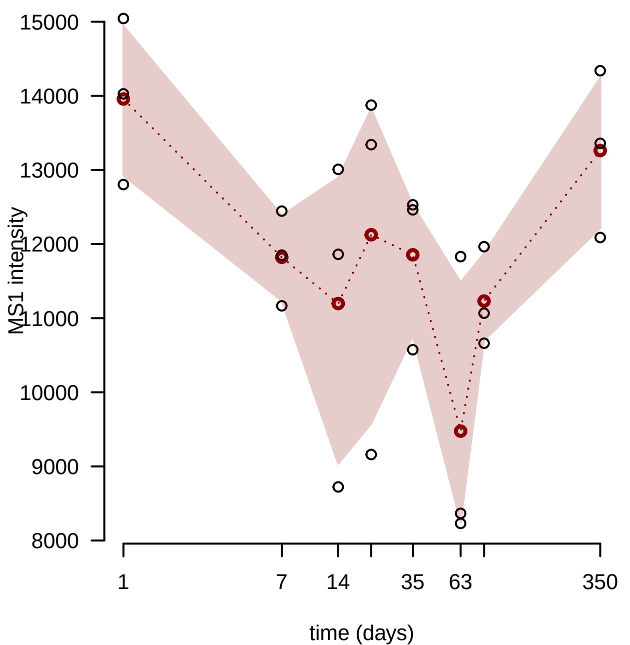

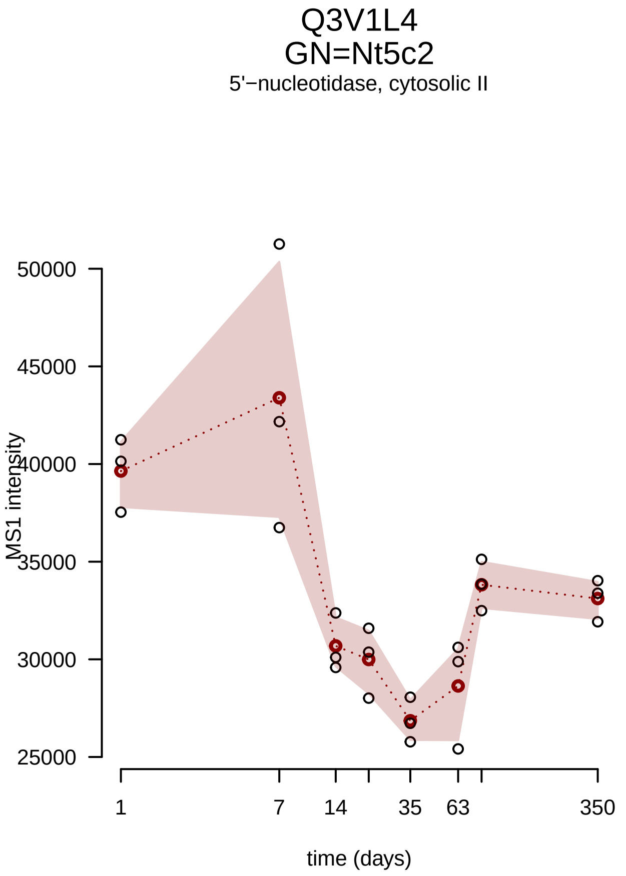
Q3V1L4
GN=Nt5c2
5'−nucleotidase, cytosolic II
MS1 intensity
50000
45000
40000
35000
30000
25000
1
7
14
35
63
350
time (days)

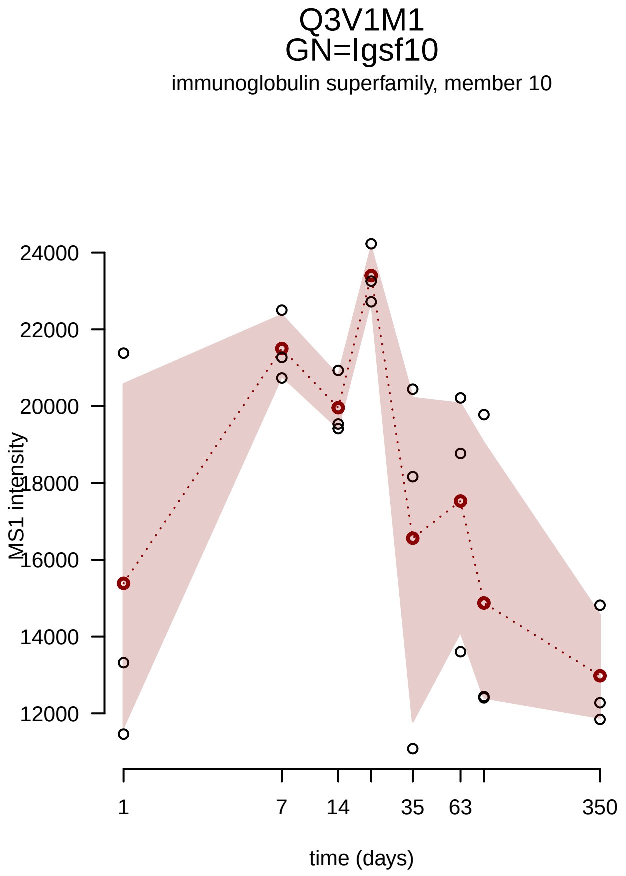
Q3V1M1
GN=Igsf10
immunoglobulin superfamily, member 10
MS1 intensity
24000
22000
20000
18000
16000
14000
12000
1
7
14
35
63
350
time (days)

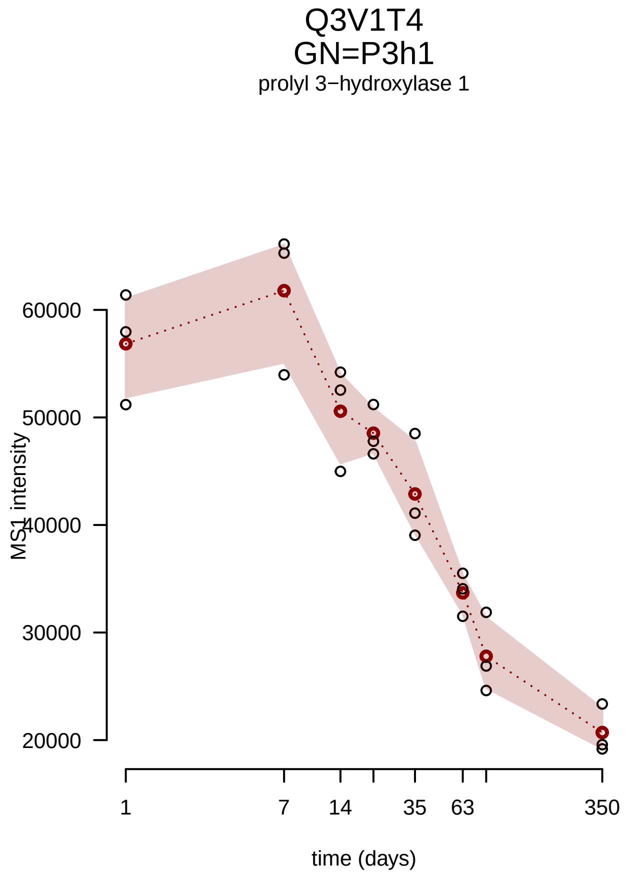
Q3V1T4
GN=P3h1
prolyl 3−hydroxylase 1
MS1 intensity
60000
50000
40000
30000
20000
1
7
14
35
63
350
time (days)

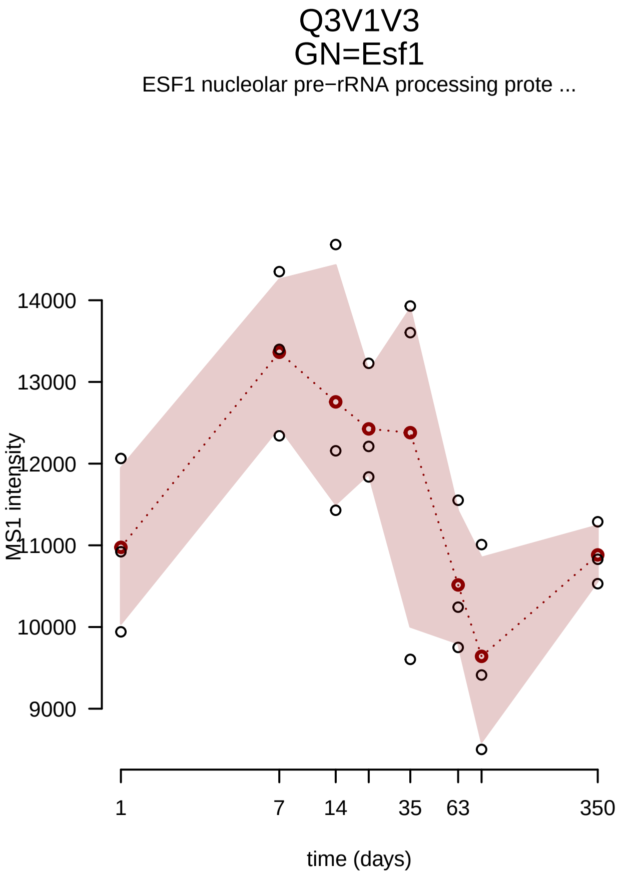
Q3V1V3
GN=Esf1
ESF1 nucleolar pre−rRNA processing prote ...
MS1 intensity
14000
13000
12000
11000
10000
9000
1
7
14
35
63
350
time (days)

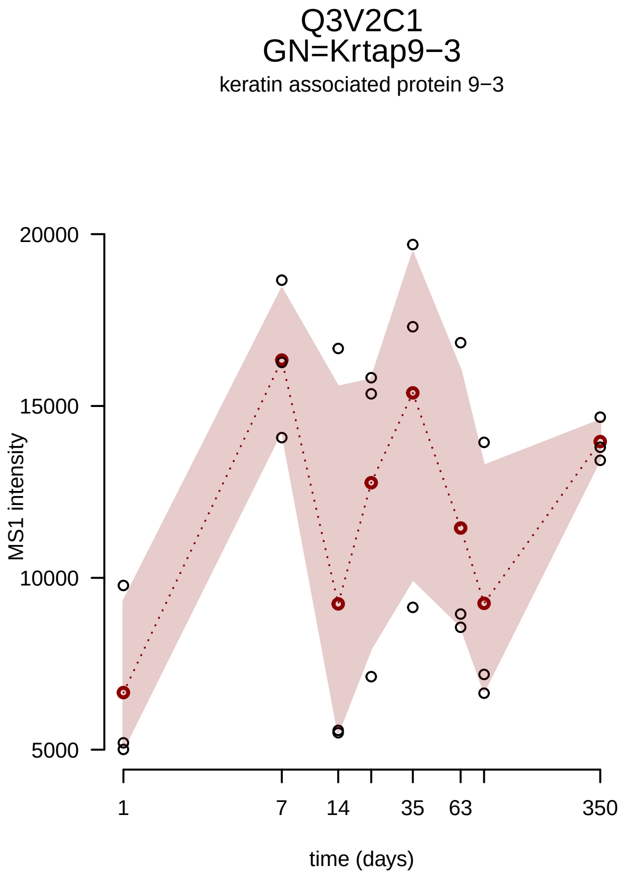
Q3V2C1
GN=Krtap9−3
keratin associated protein 9−3
MS1 intensity
20000
15000
10000
5000
1
7
14
35
63
350
time (days)

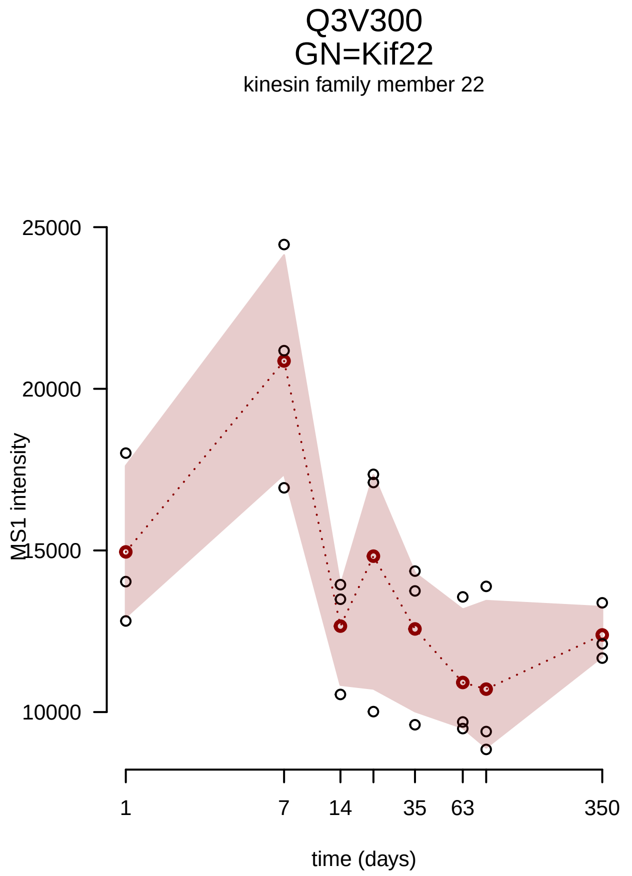
Q3V300
GN=Kif22
kinesin family member 22
MS1 intensity
25000
20000
15000
10000
1
7
14
35
63
350
time (days)

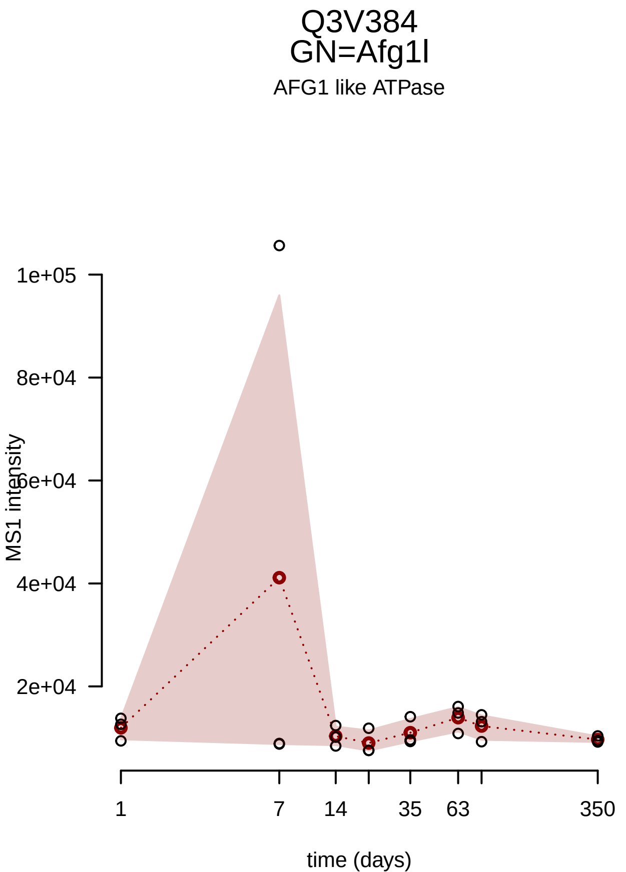
Q3V384
GN=Afg1l
AFG1 like ATPase
MS1 intensity
1e+05
8e+04
6e+04
4e+04
2e+04
1
7
14
35
63
350
time (days)

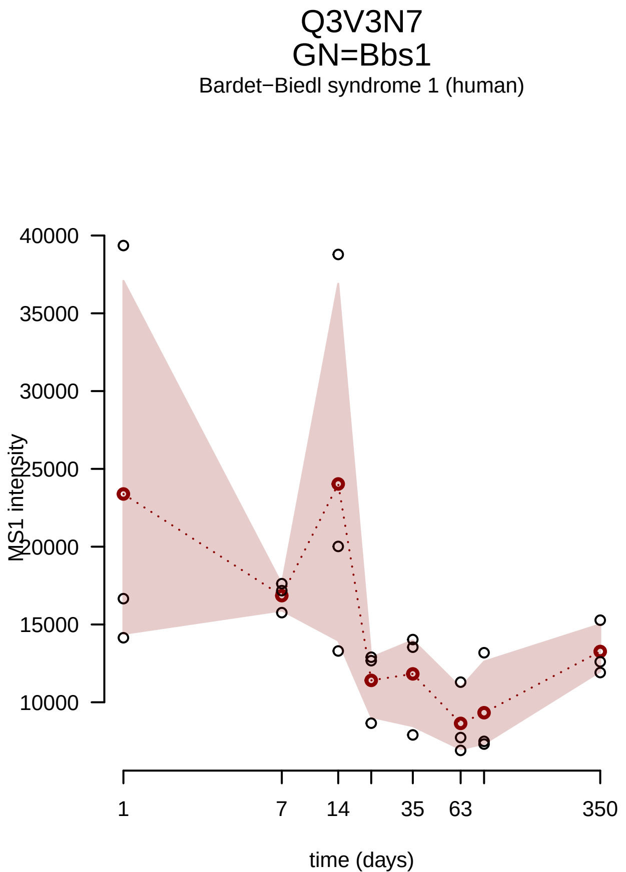
Q3V3N7
GN=Bbs1
Bardet−Biedl syndrome 1 (human)
MS1 intensity
40000
35000
30000
25000
20000
15000
10000
1
7
14
35
63
350
time (days)

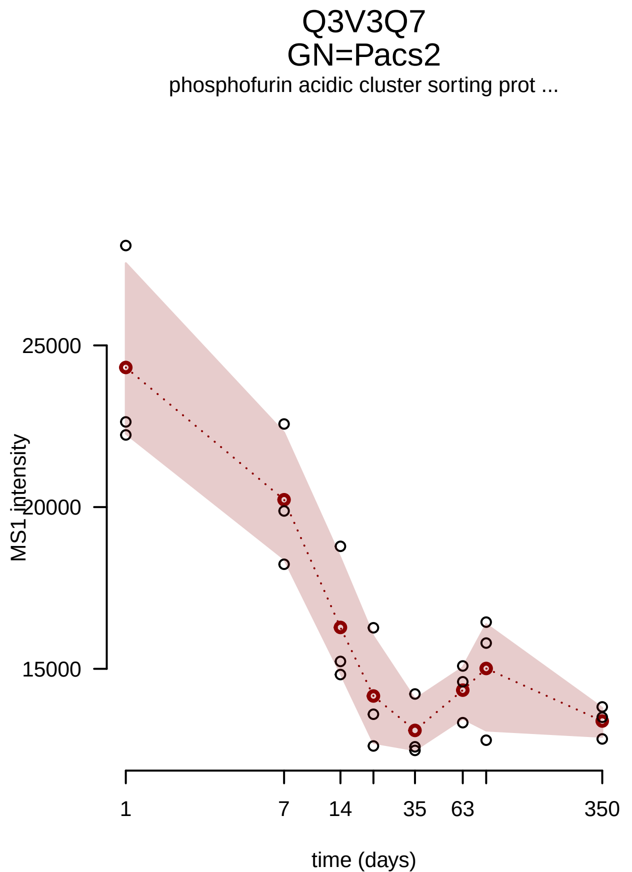
Q3V3Q7
GN=Pacs2
phosphofurin acidic cluster sorting prot ...
MS1 intensity
25000
20000
15000
1
7
14
35
63
350
time (days)

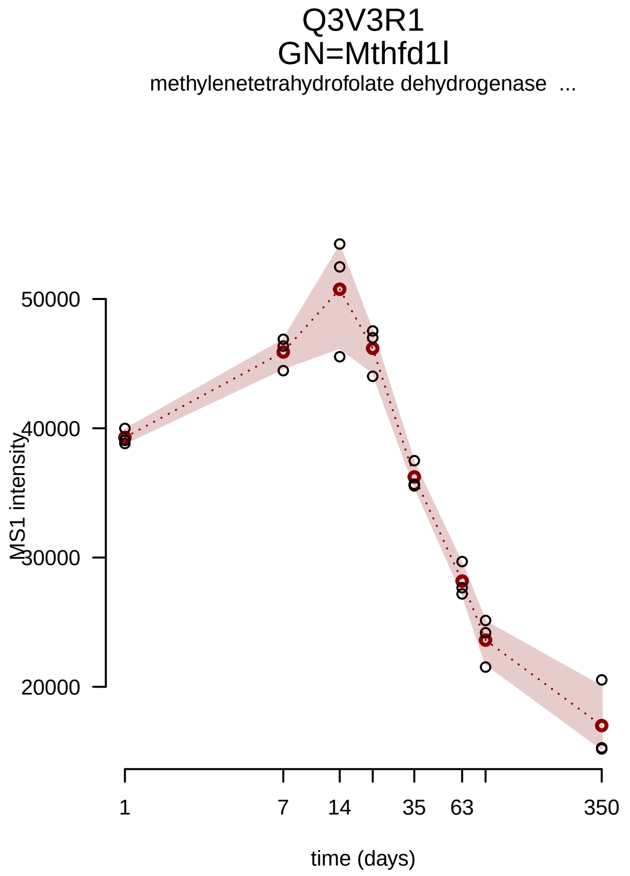
Q3V3R1
GN=Mthfd1l
methylenetetrahydrofolate dehydrogenase ...
50000
40000
30000
20000
MS1 intensity
1
7
14
35
63
350
time (days)

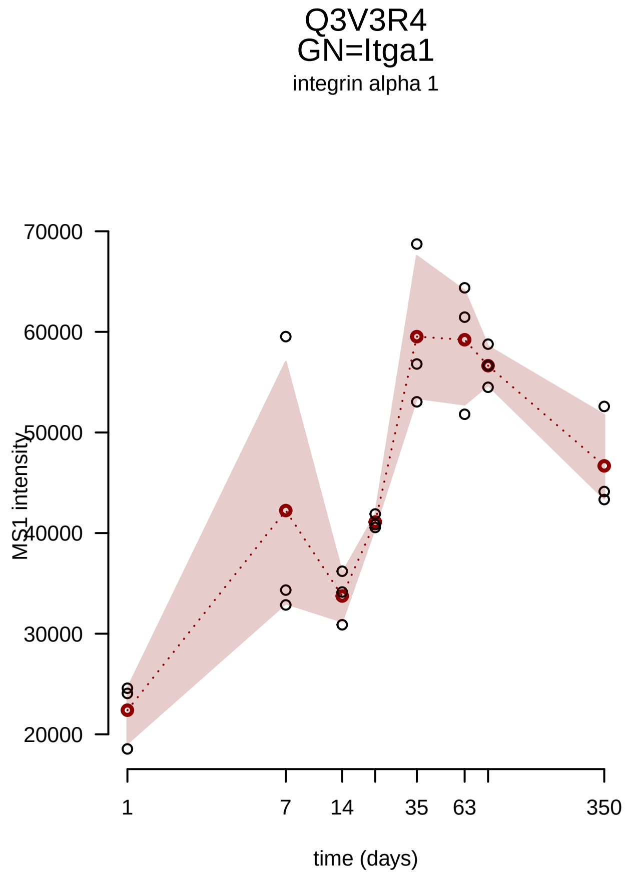
Q3V3R4
GN=Itga1
integrin alpha 1
70000
60000
50000
40000
30000
20000
MS1 intensity
1
7
14
35
63
350
time (days)

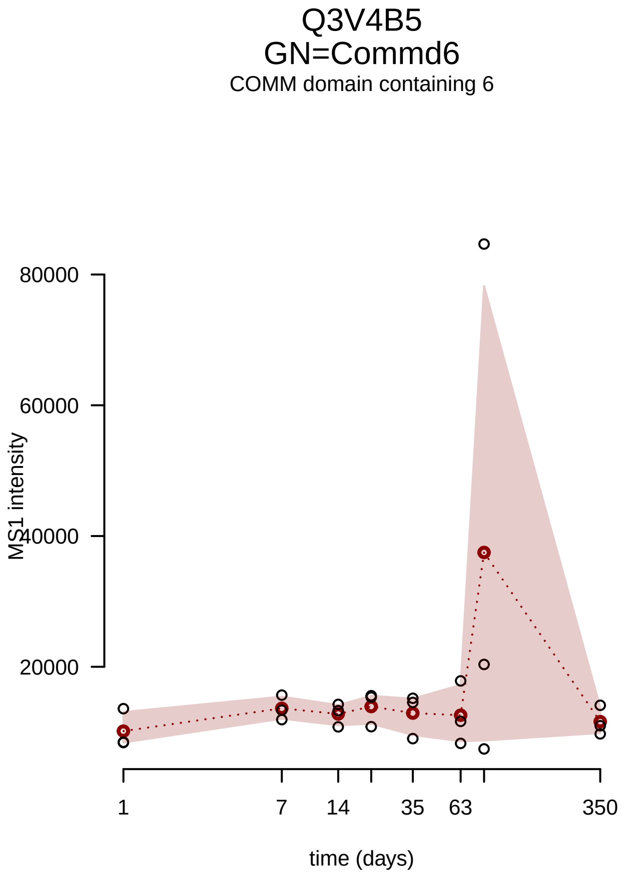
Q3V4B5
GN=Commd6
COMM domain containing 6
80000
60000
40000
20000
MS1 intensity
1
7
14
35
63
350
time (days)

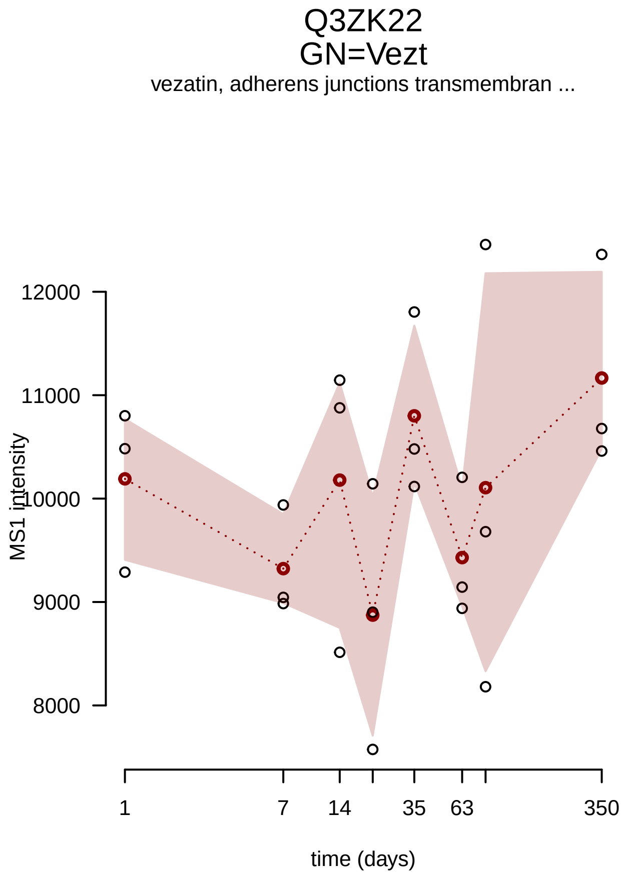
Q3ZK22
GN=Vezt
vezatin, adherens junctions transmembran ...
12000
11000
10000
9000
8000
MS1 intensity
1
7
14
35
63
350
time (days)

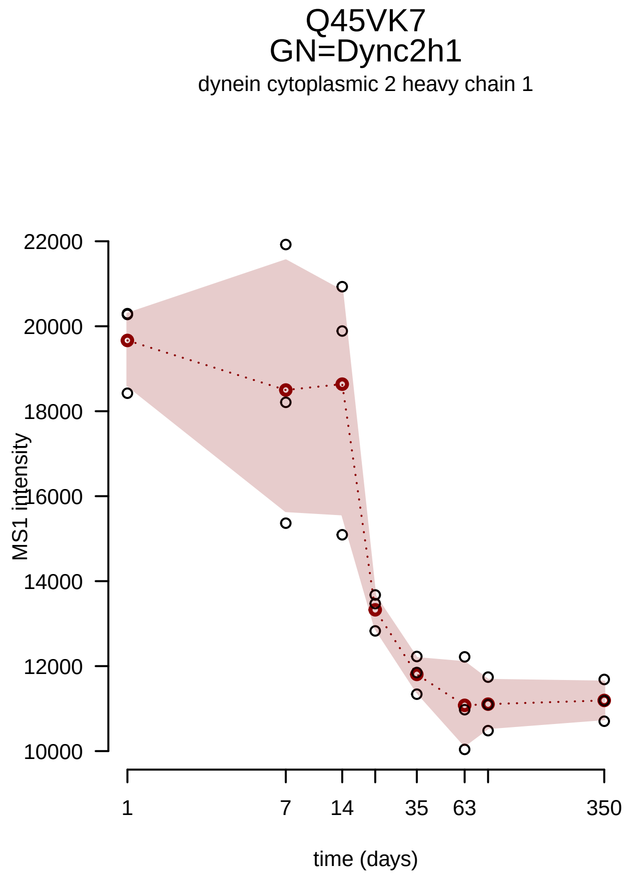
Q45VK7
GN=Dync2h1
dynein cytoplasmic 2 heavy chain 1
22000
20000
18000
16000
14000
12000
10000
MS1 intensity
1
7
14
35
63
350
time (days)

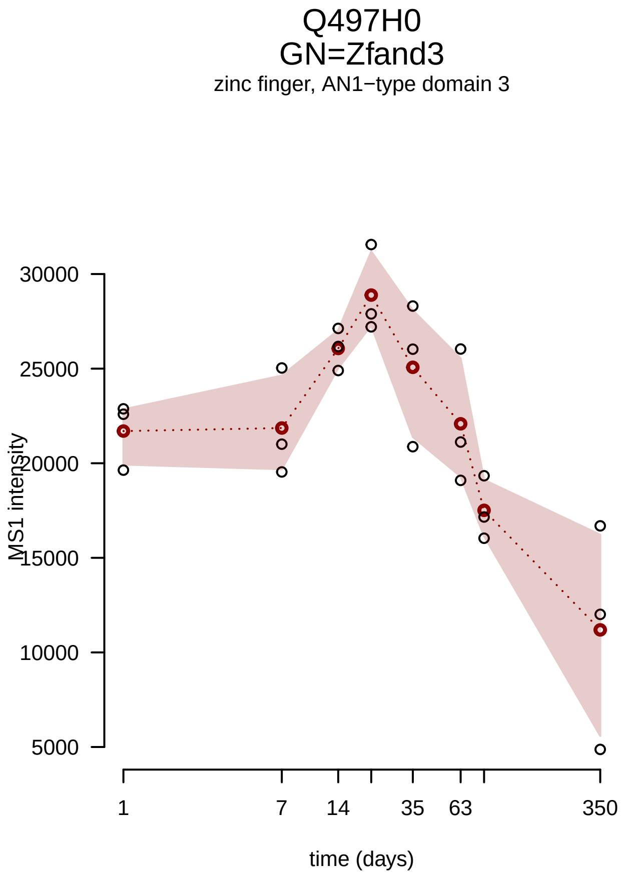
Q497H0
GN=Zfand3
zinc finger, AN1−type domain 3
30000
25000
20000
15000
10000
5000
MS1 intensity
1
7
14
35
63
350
time (days)

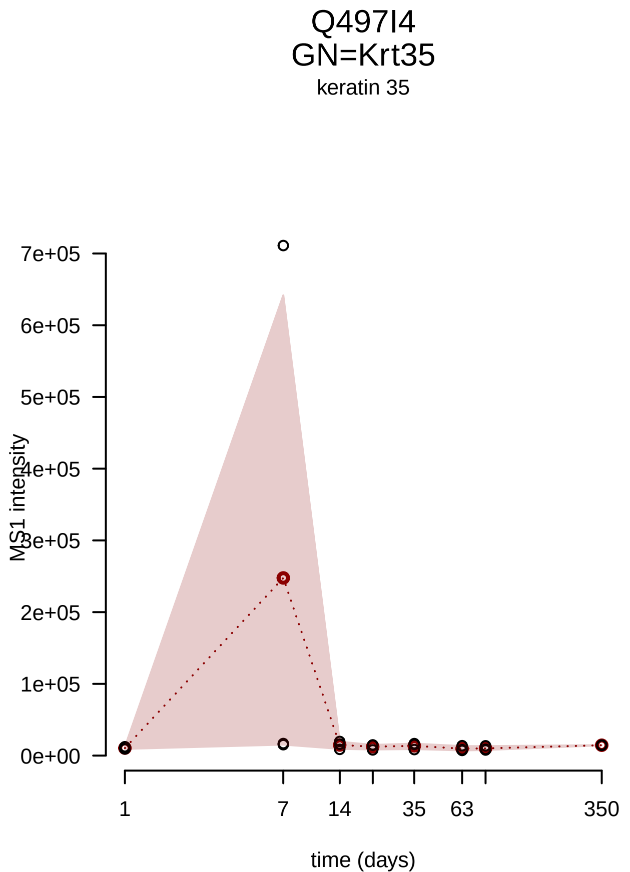
Q497I4
GN=Krt35
keratin 35
7e+05
6e+05
5e+05
4e+05
3e+05
2e+05
1e+05
0e+00
MS1 intensity
1
7
14
35
63
350
time (days)

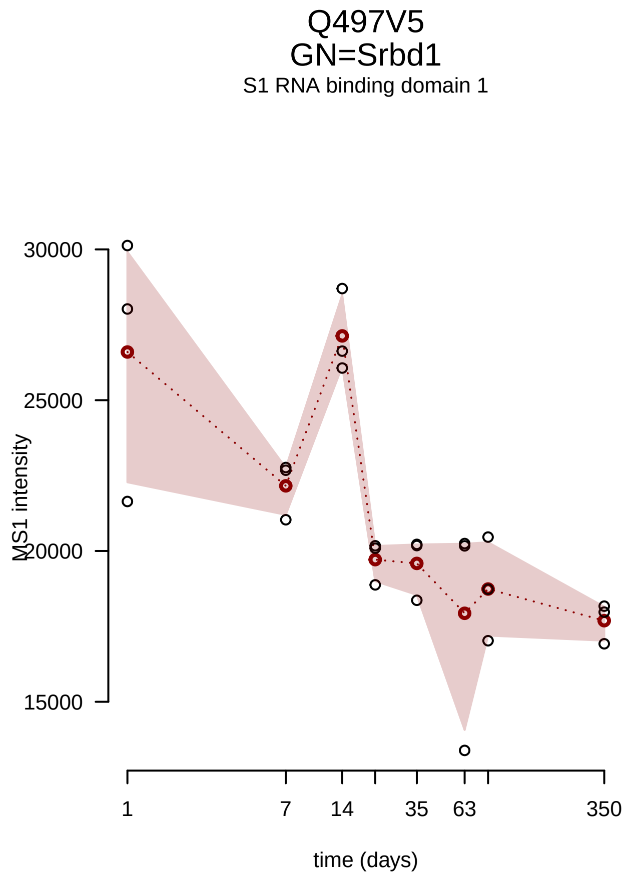
Q497V5
GN=Srbd1
S1 RNA binding domain 1
30000
25000
20000
15000
MS1 intensity
1
7
14
35
63
350
time (days)

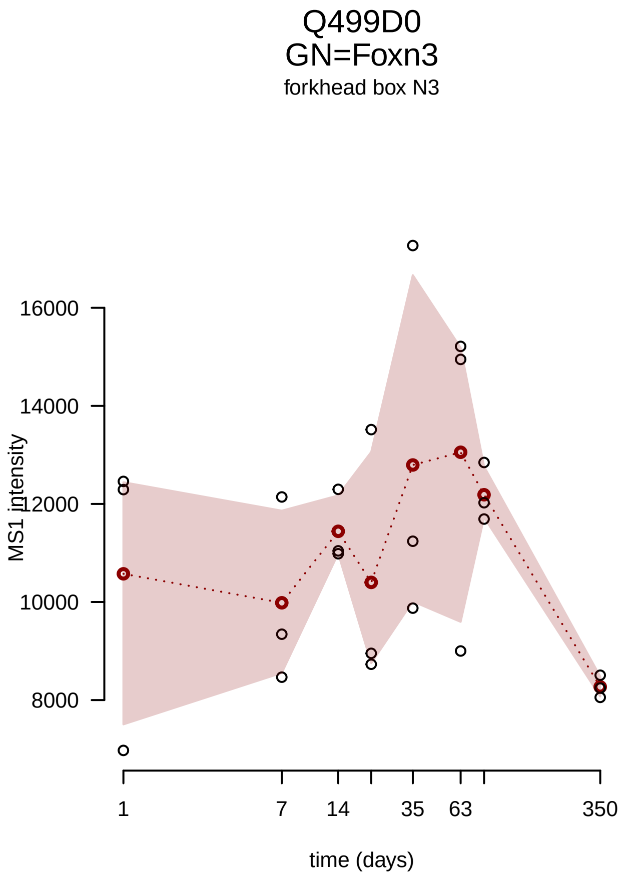
Q499D0
GN=Foxn3
forkhead box N3
16000
14000
12000
10000
8000
MS1 intensity
1
7
14
35
63
350
time (days)

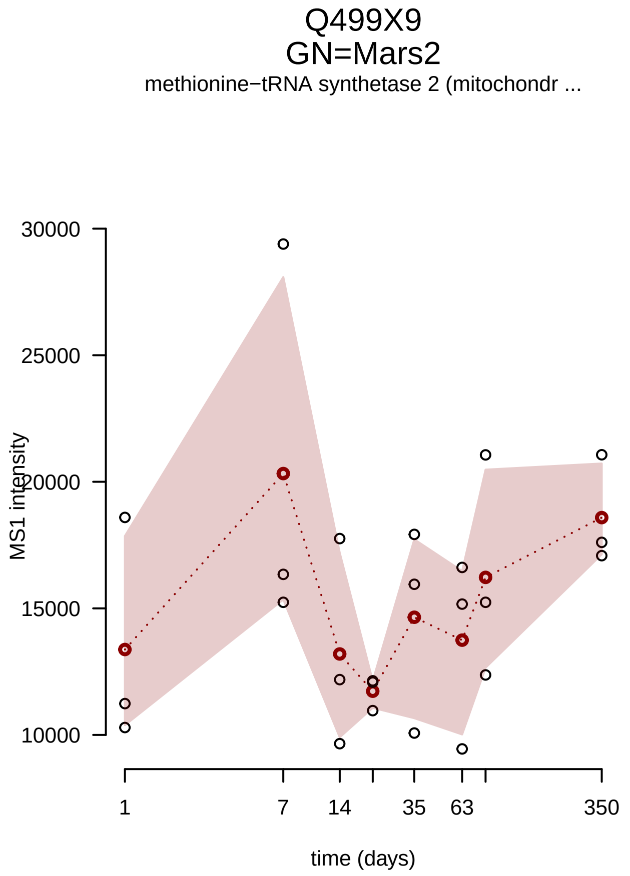
Q499X9
GN=Mars2
methionine−tRNA synthetase 2 (mitochondr ...
MS1 intensity
10000 15000 20000 25000 30000
1 7 14 35 63 350
time (days)

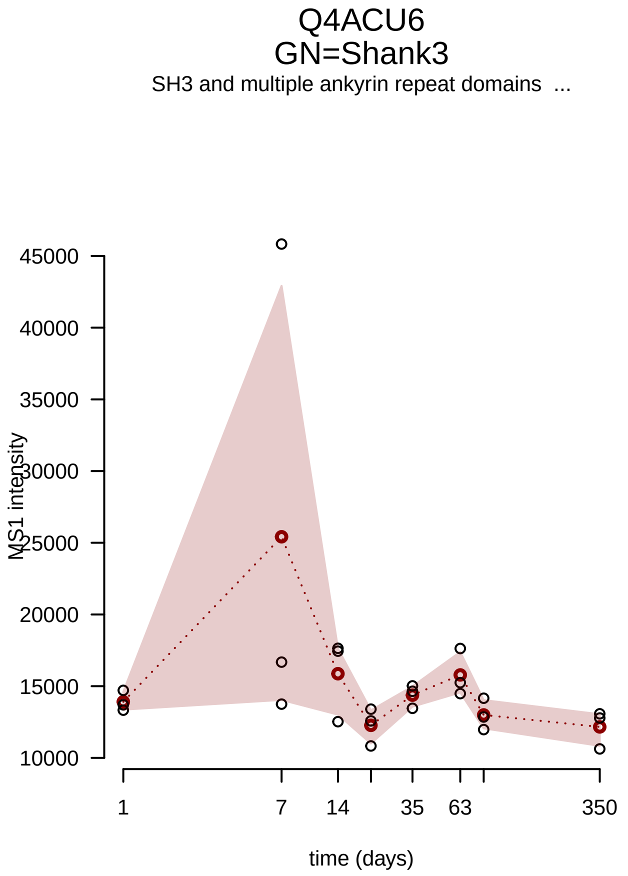
Q4ACU6
GN=Shank3
SH3 and multiple ankyrin repeat domains ...
MS1 intensity
10000 15000 20000 25000 30000 35000 40000 45000
1 7 14 35 63 350
time (days)

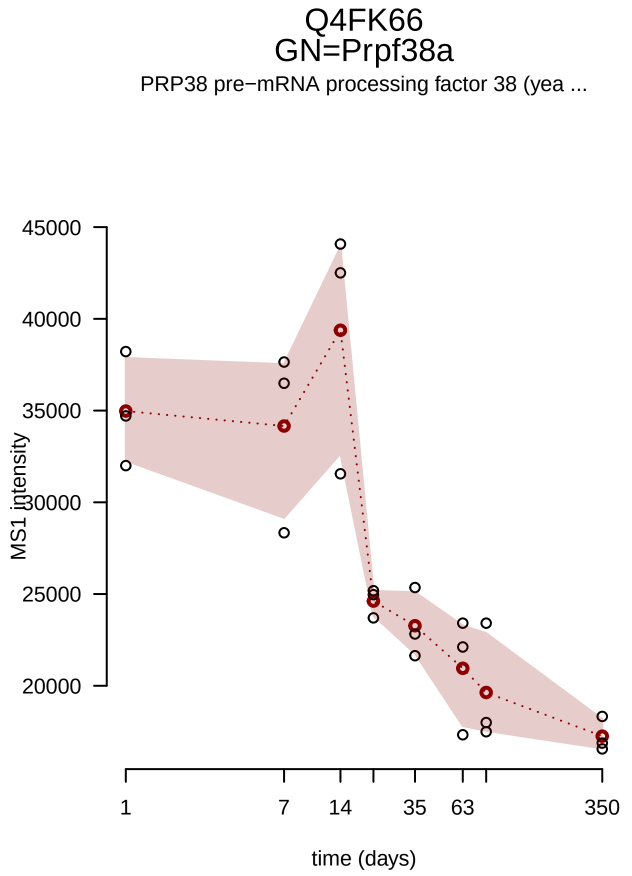
Q4FK66
GN=Prpf38a
PRP38 pre−mRNA processing factor 38 (yea ...
MS1 intensity
20000 25000 30000 35000 40000 45000
1 7 14 35 63 350
time (days)

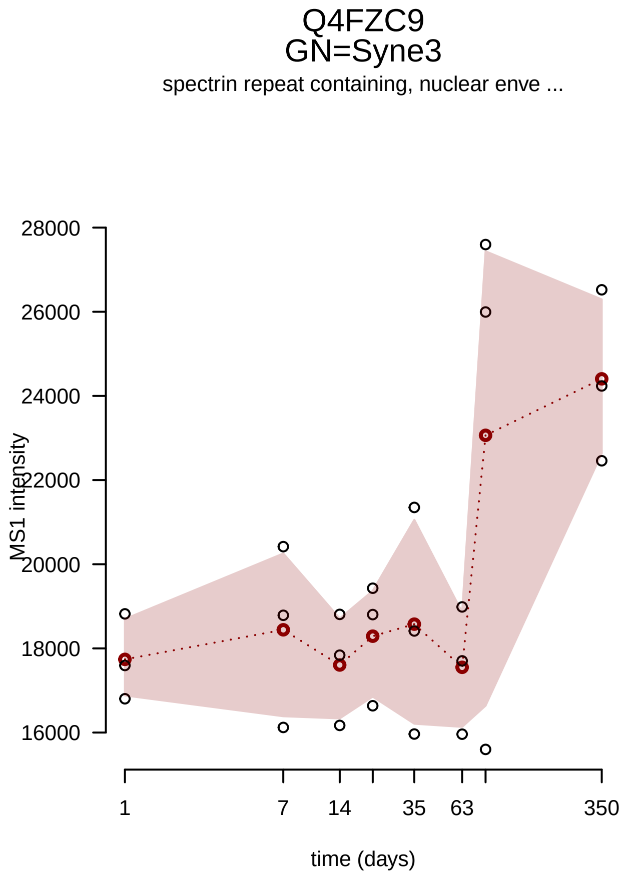
Q4FZC9
GN=Syne3
spectrin repeat containing, nuclear enve ...
MS1 intensity
16000 18000 20000 22000 24000 26000 28000
1 7 14 35 63 350
time (days)

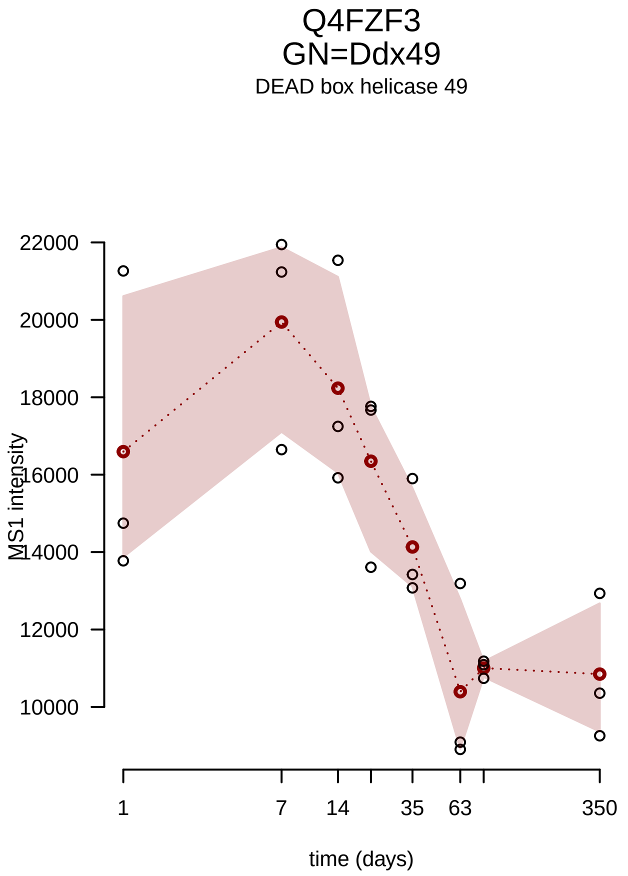
Q4FZF3
GN=Ddx49
DEAD box helicase 49
MS1 intensity
10000 12000 14000 16000 18000 20000 22000
1 7 14 35 63 350
time (days)

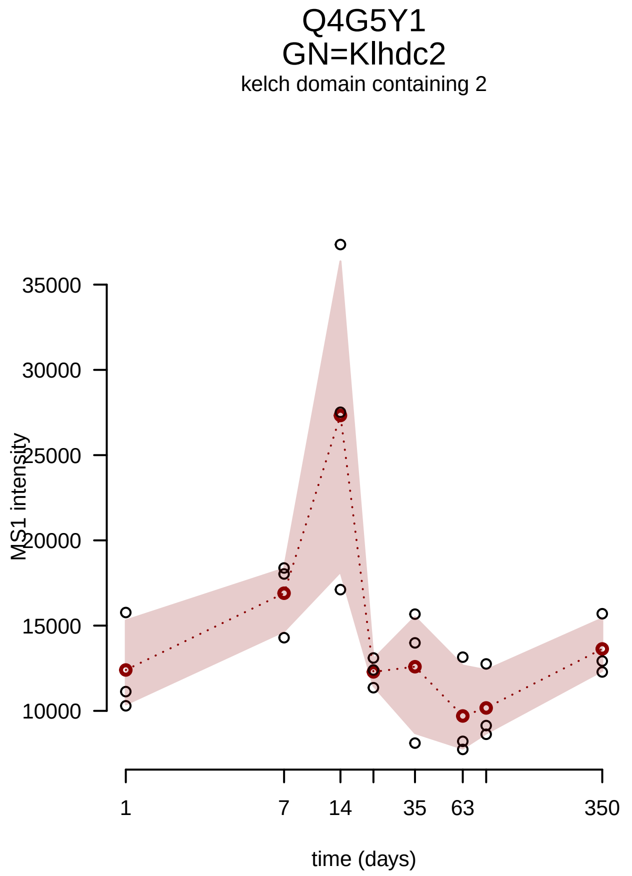
Q4G5Y1
GN=Klhdc2
kelch domain containing 2
MS1 intensity
10000 15000 20000 25000 30000 35000
1 7 14 35 63 350
time (days)

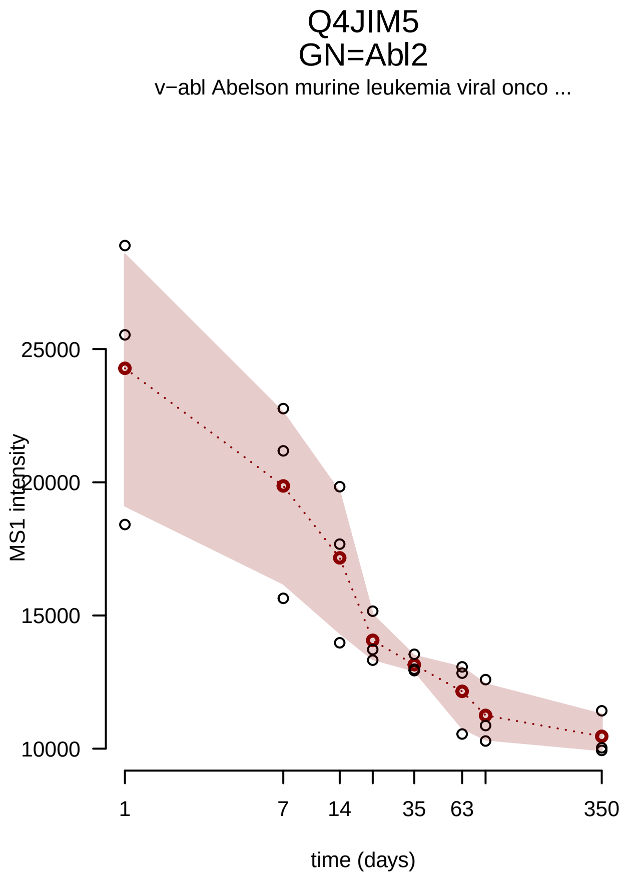
Q4JIM5
GN=Abl2
v−abl Abelson murine leukemia viral onco ...
MS1 intensity
10000 15000 20000 25000
1 7 14 35 63 350
time (days)

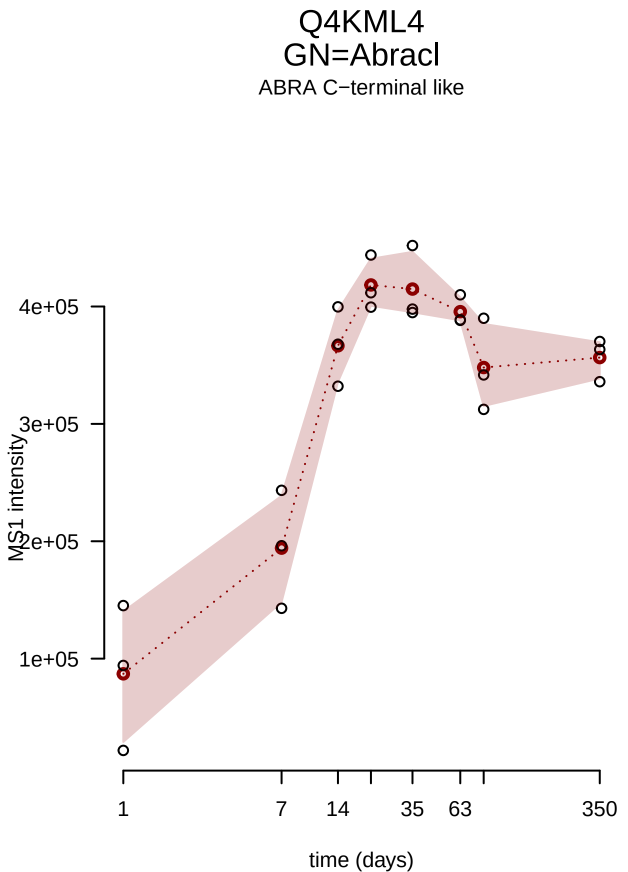
Q4KML4
GN=Abracl
ABRA C−terminal like
MS1 intensity
1e+05 2e+05 3e+05 4e+05
1 7 14 35 63 350
time (days)

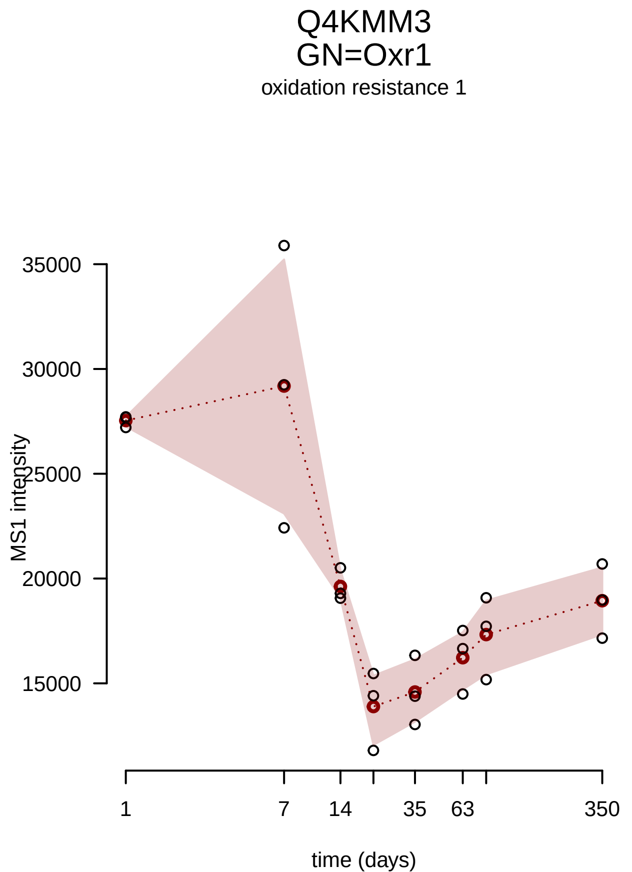
Q4KMM3
GN=Oxr1
oxidation resistance 1
MS1 intensity
15000 20000 25000 30000 35000
1 7 14 35 63 350
time (days)

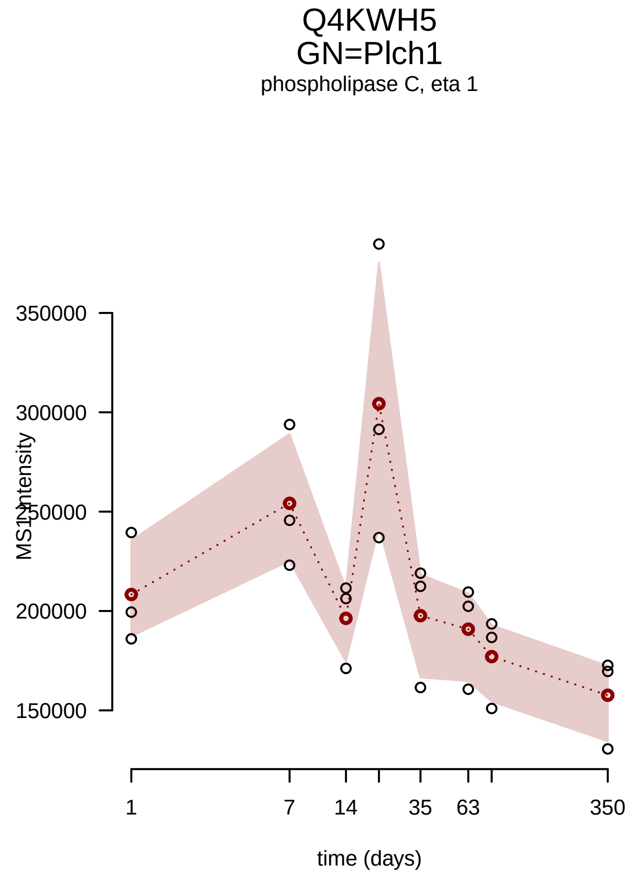
Q4KWH5
GN=Plch1
phospholipase C, eta 1
MS1 intensity
150000
200000
250000
300000
350000
1
7
14
35
63
350
time (days)

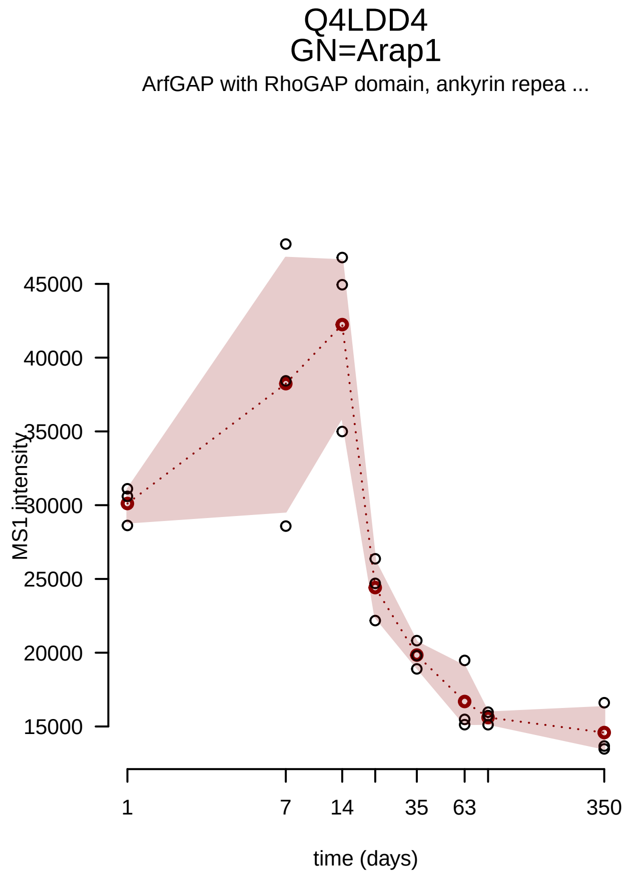
Q4LDD4
GN=Arap1
ArfGAP with RhoGAP domain, ankyrin repea ...
MS1 intensity
15000
20000
25000
30000
35000
40000
45000
1
7
14
35
63
350
time (days)

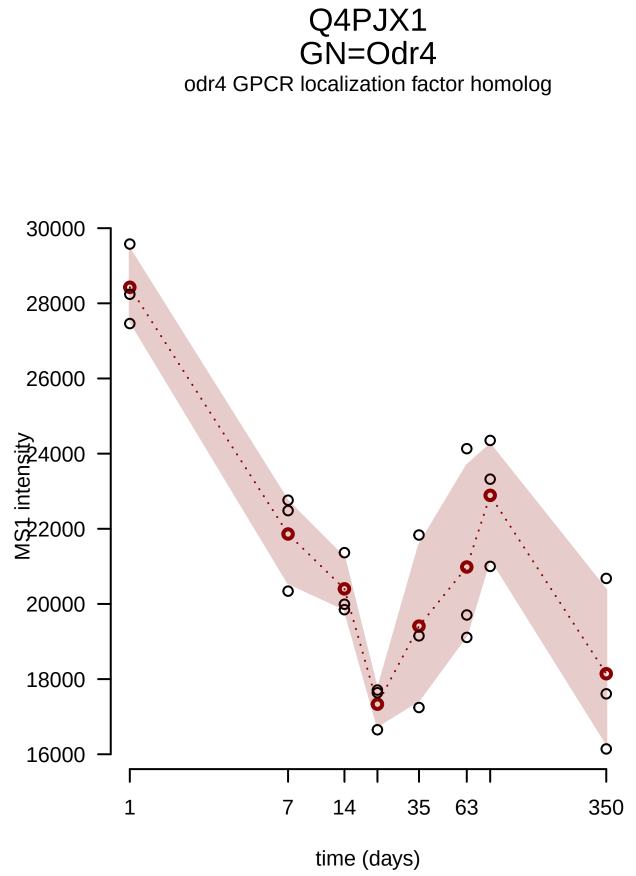
Q4PJX1
GN=Odr4
odr4 GPCR localization factor homolog
MS1 intensity
16000
18000
20000
22000
24000
26000
28000
30000
1
7
14
35
63
350
time (days)

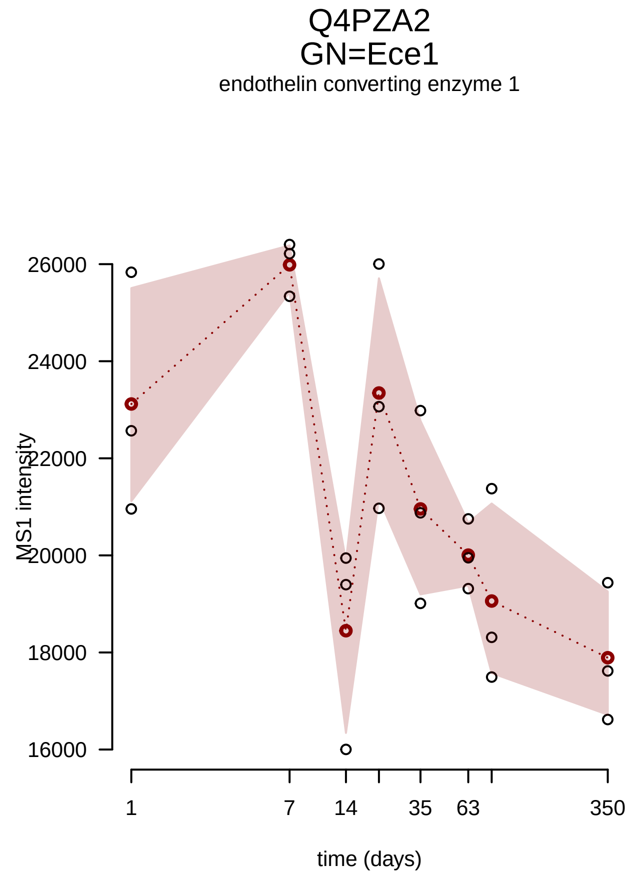
Q4PZA2
GN=Ece1
endothelin converting enzyme 1
MS1 intensity
16000
18000
20000
22000
24000
26000
1
7
14
35
63
350
time (days)

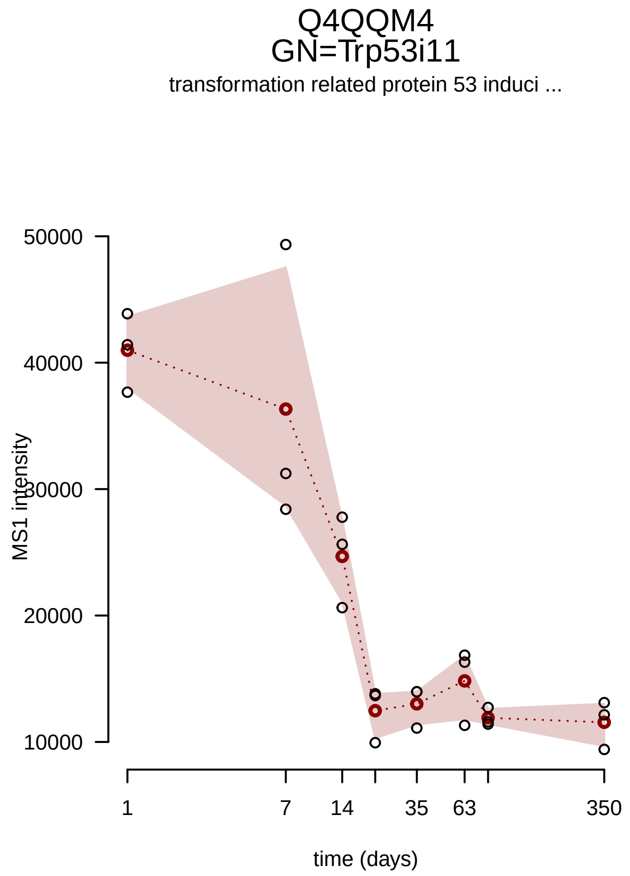
Q4QQM4
GN=Trp53i11
transformation related protein 53 induci ...
MS1 intensity
10000
20000
30000
40000
50000
1
7
14
35
63
350
time (days)

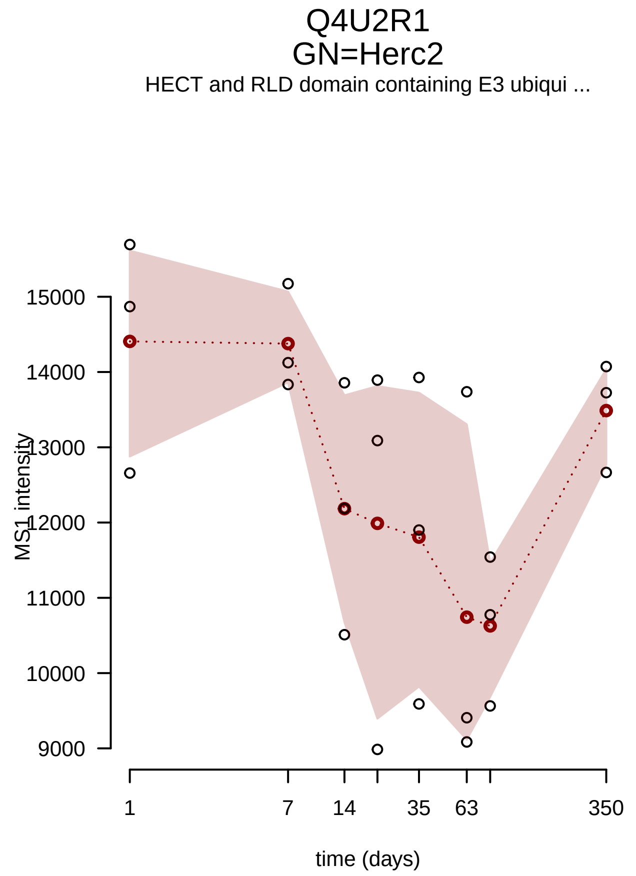
Q4U2R1
GN=Herc2
HECT and RLD domain containing E3 ubiqui ...
MS1 intensity
9000
10000
11000
12000
13000
14000
15000
1
7
14
35
63
350
time (days)

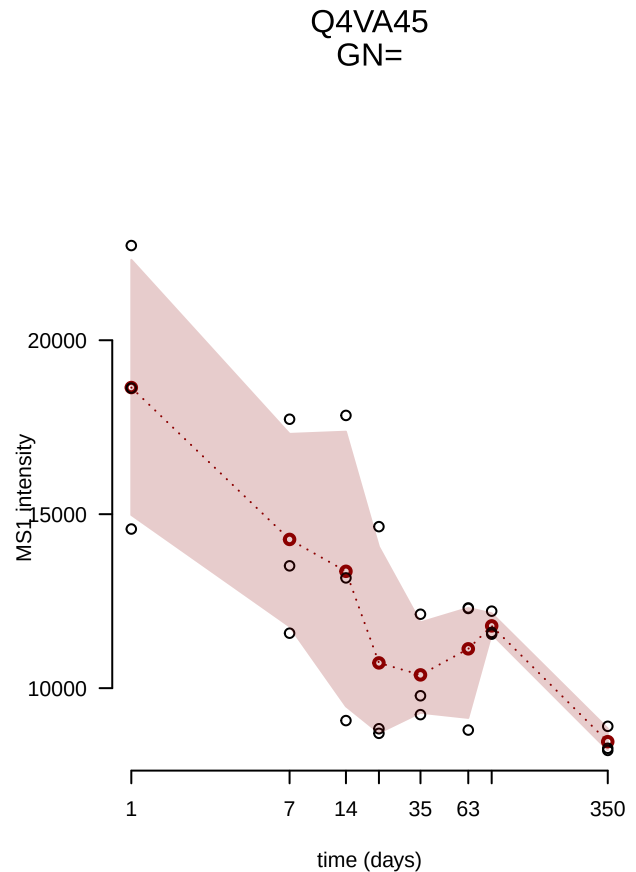
Q4VA45
GN=
MS1 intensity
10000
15000
20000
1
7
14
35
63
350
time (days)

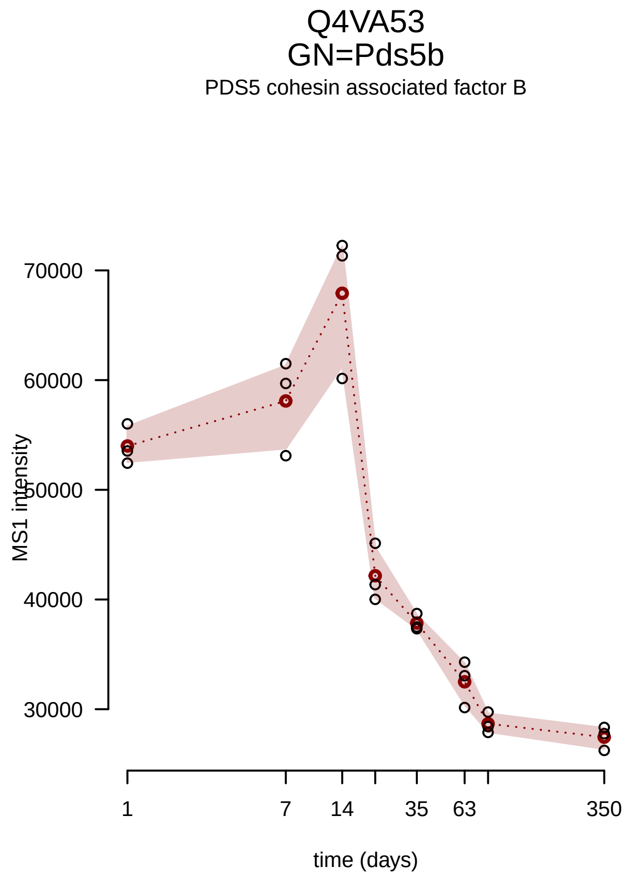
Q4VA53
GN=Pds5b
PDS5 cohesin associated factor B
MS1 intensity
30000
40000
50000
60000
70000
1
7
14
35
63
350
time (days)

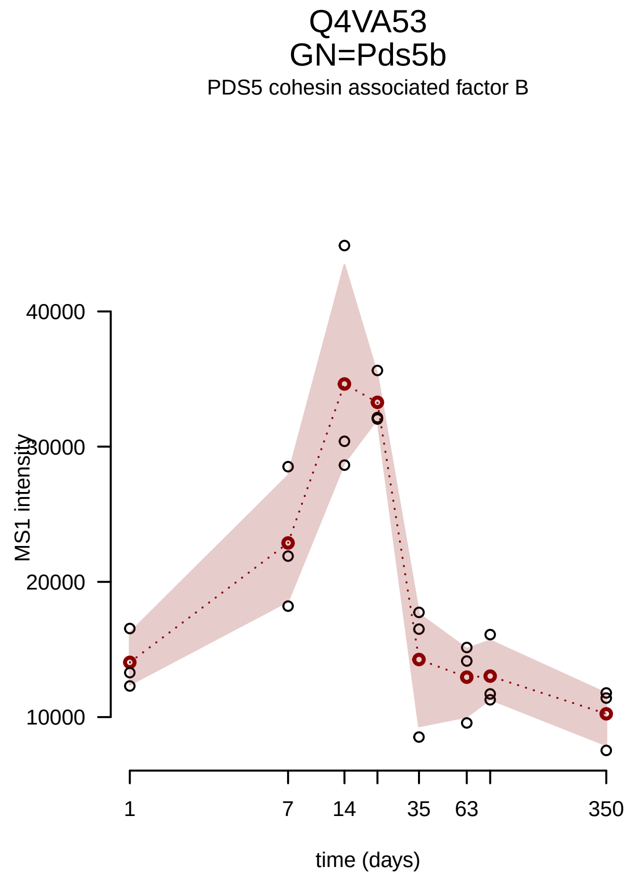
Q4VA53
GN=Pds5b
PDS5 cohesin associated factor B
MS1 intensity
10000
20000
30000
40000
1
7
14
35
63
350
time (days)

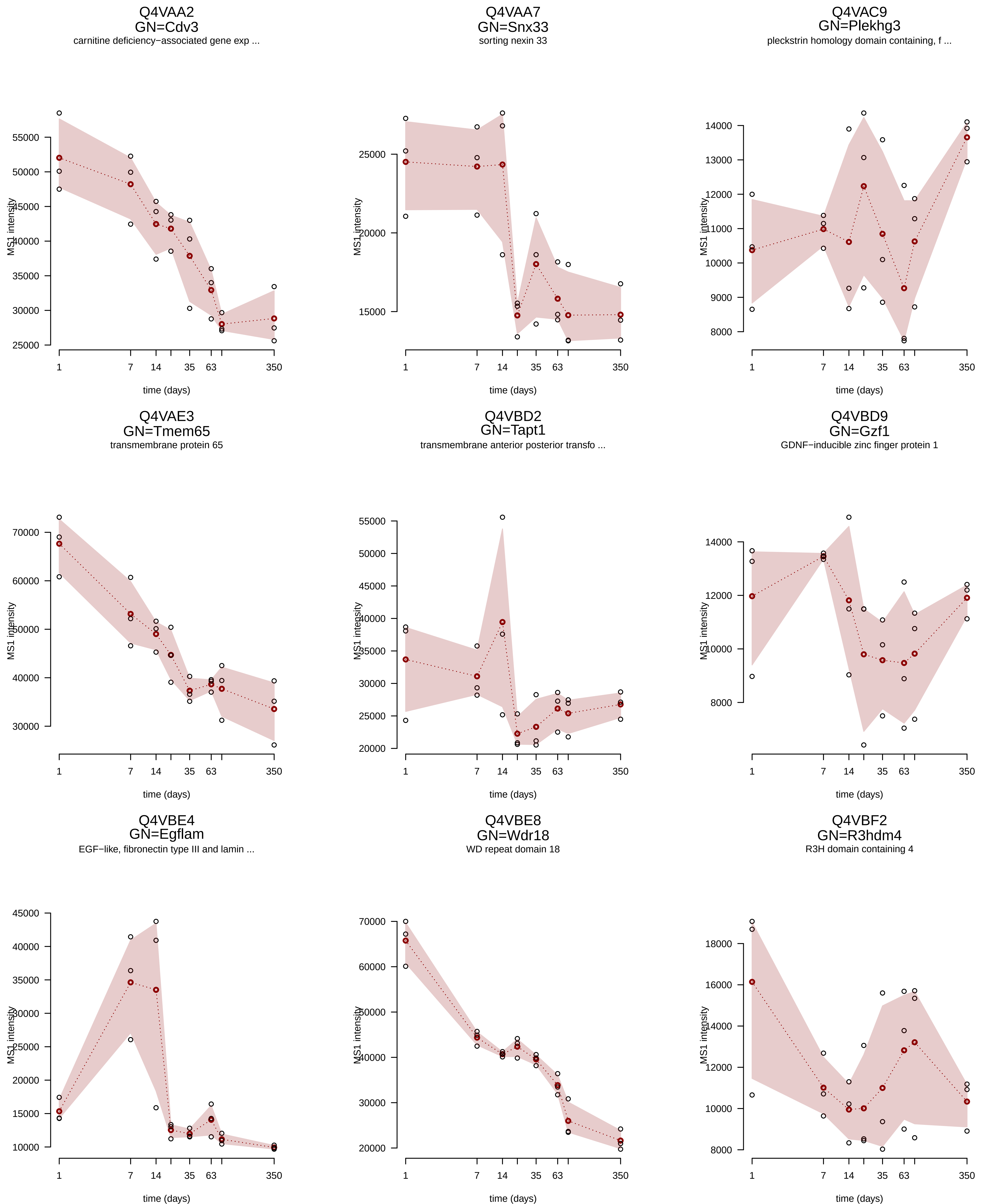
Q4VAA2
GN=Cdv3
carnitine deficiency−associated gene exp ...
MS1 intensity
25000 30000 35000 40000 45000 50000 55000
1 7 14 35 63 350
time (days)
Q4VAA7
GN=Snx33
sorting nexin 33
MS1 intensity
15000 20000 25000
1 7 14 35 63 350
time (days)
Q4VAC9
GN=Plekhg3
pleckstrin homology domain containing, f ...
MS1 intensity
8000 9000 10000 11000 12000 13000 14000
1 7 14 35 63 350
time (days)
Q4VAE3
GN=Tmem65
transmembrane protein 65
MS1 intensity
30000 40000 50000 60000 70000
1 7 14 35 63 350
time (days)
Q4VBD2
GN=Tapt1
transmembrane anterior posterior transfo ...
MS1 intensity
20000 25000 30000 35000 40000 45000 50000 55000
1 7 14 35 63 350
time (days)
Q4VBD9
GN=Gzf1
GDNF−inducible zinc finger protein 1
MS1 intensity
8000 10000 12000 14000
1 7 14 35 63 350
time (days)
Q4VBE4
GN=Egflam
EGF−like, fibronectin type III and lamin ...
MS1 intensity
10000 15000 20000 25000 30000 35000 40000 45000
1 7 14 35 63 350
time (days)
Q4VBE8
GN=Wdr18
WD repeat domain 18
MS1 intensity
20000 30000 40000 50000 60000 70000
1 7 14 35 63 350
time (days)
Q4VBF2
GN=R3hdm4
R3H domain containing 4
MS1 intensity
8000 10000 12000 14000 16000 18000
1 7 14 35 63 350
time (days)

Q4VC33
GN=Maea
macrophage erythroblast attacher

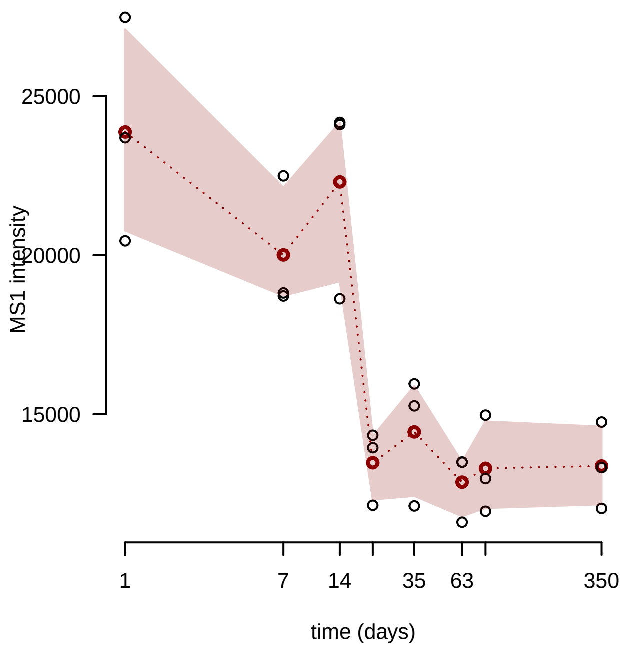

Q4VGL6
GN=Rc3h1
RING CCCH (C3H) domains 1

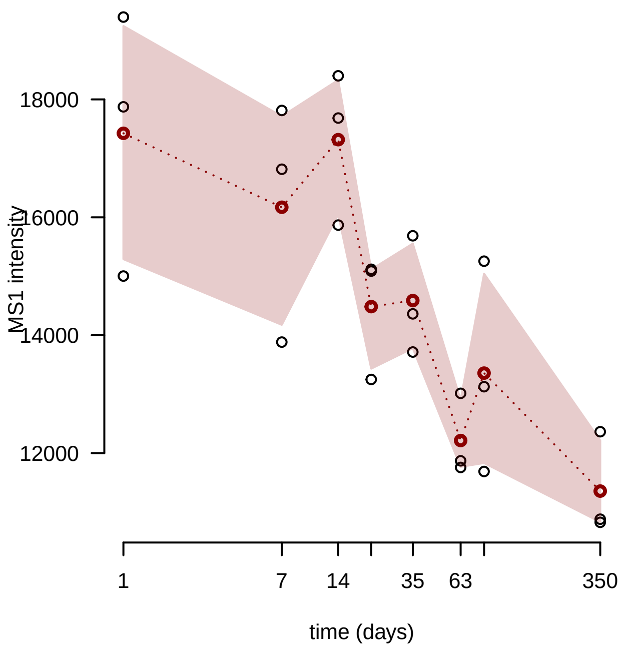

Q501J2
GN=Fam173a

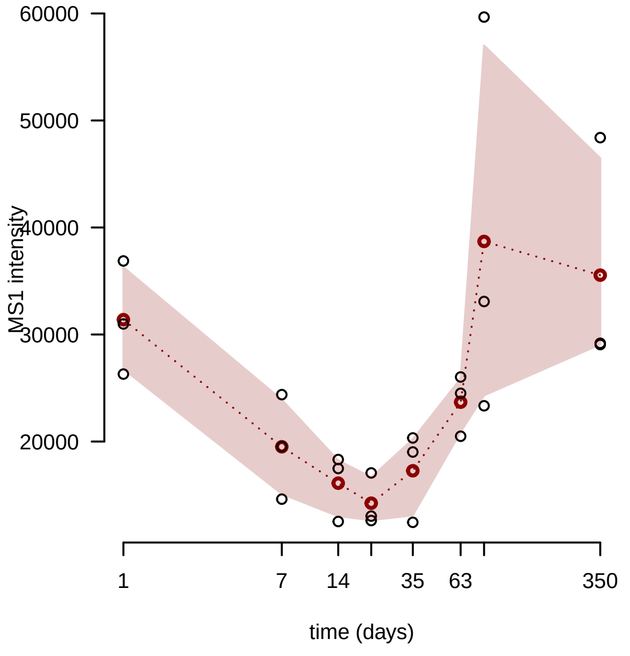

Q501J6
GN=Ddx17
DEAD box helicase 17

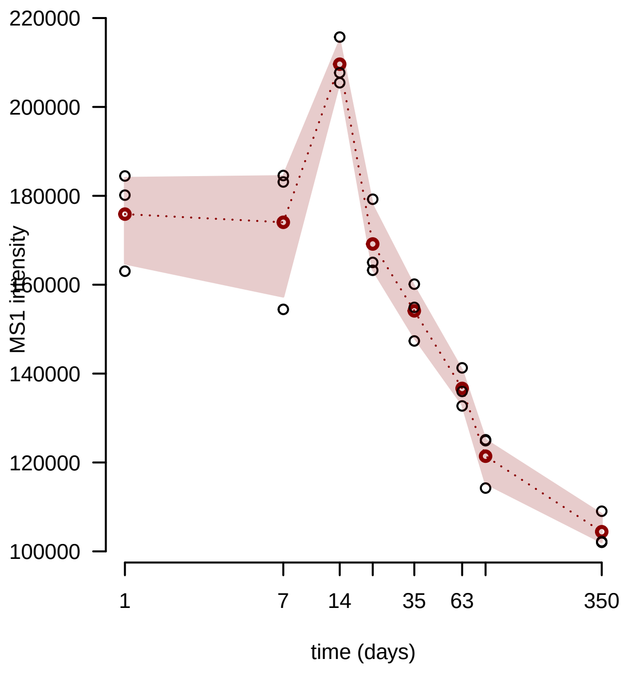

Q501J7
GN=Phactr4
phosphatase and actin regulator 4

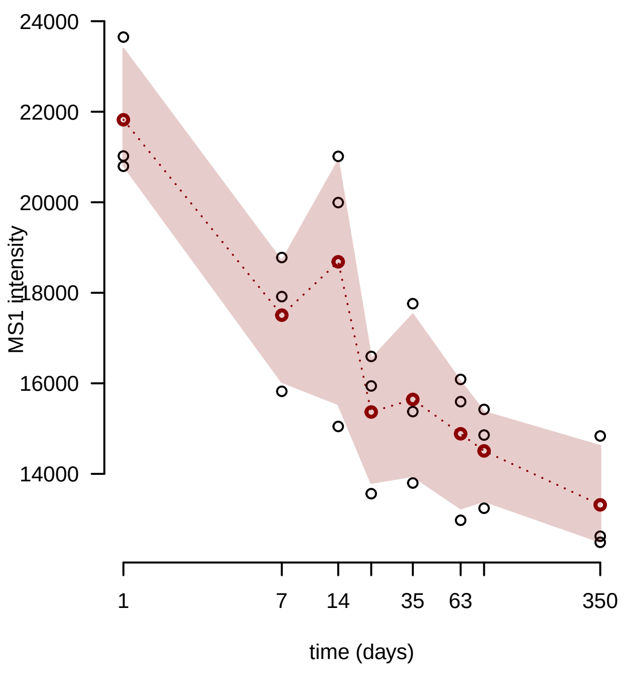

Q504N0
GN=Cpa2
carboxypeptidase A2, pancreatic

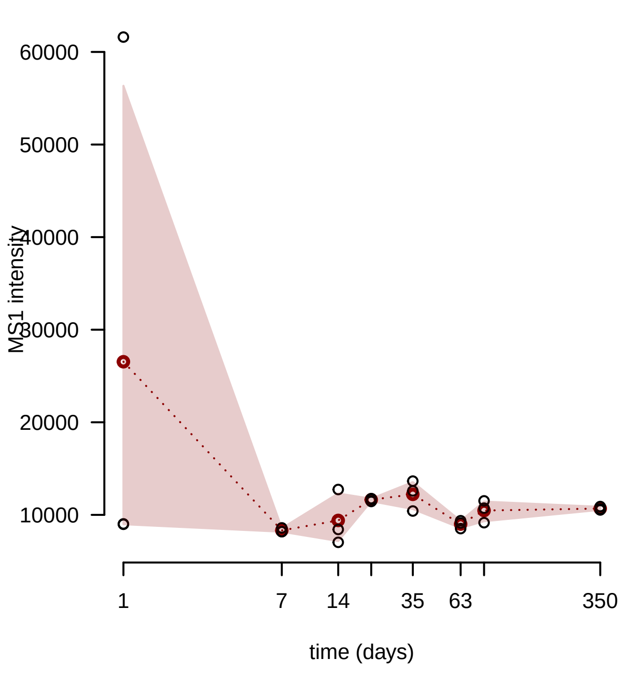

Q504N7
GN=

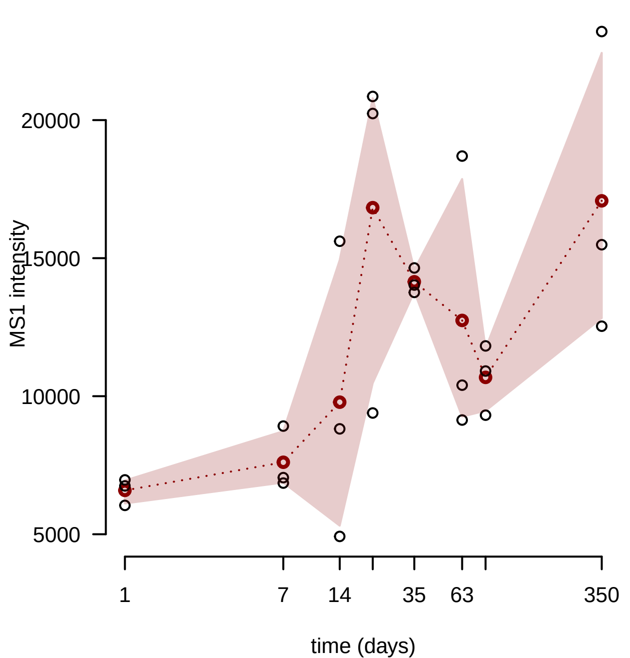

Q505B7
GN=Zbtb8os
zinc finger and BTB domain containing 8 ...

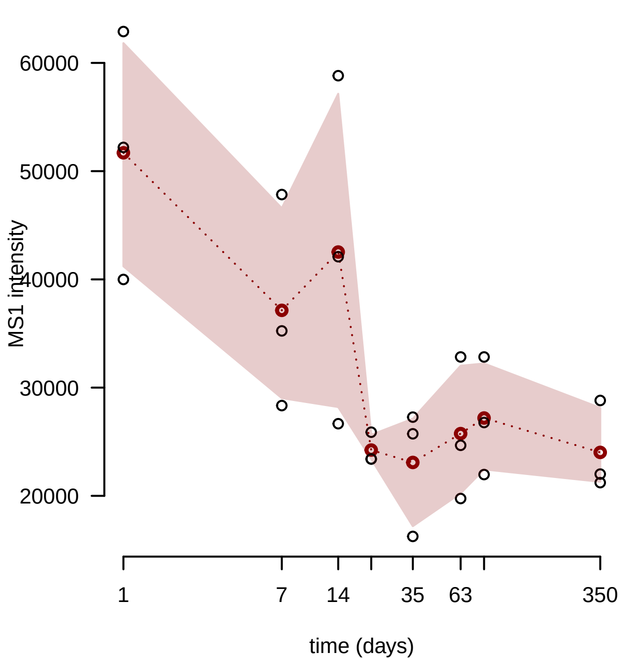

Q505B8
GN=Syce2
synaptonemal complex central element pro ...

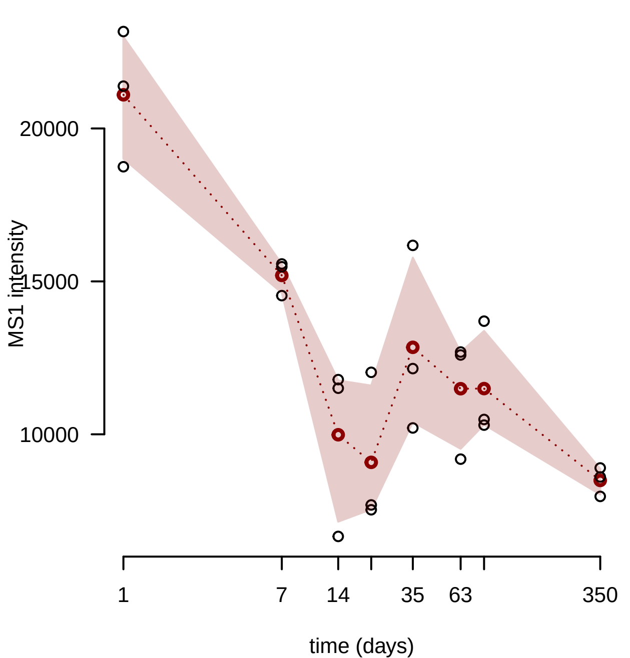

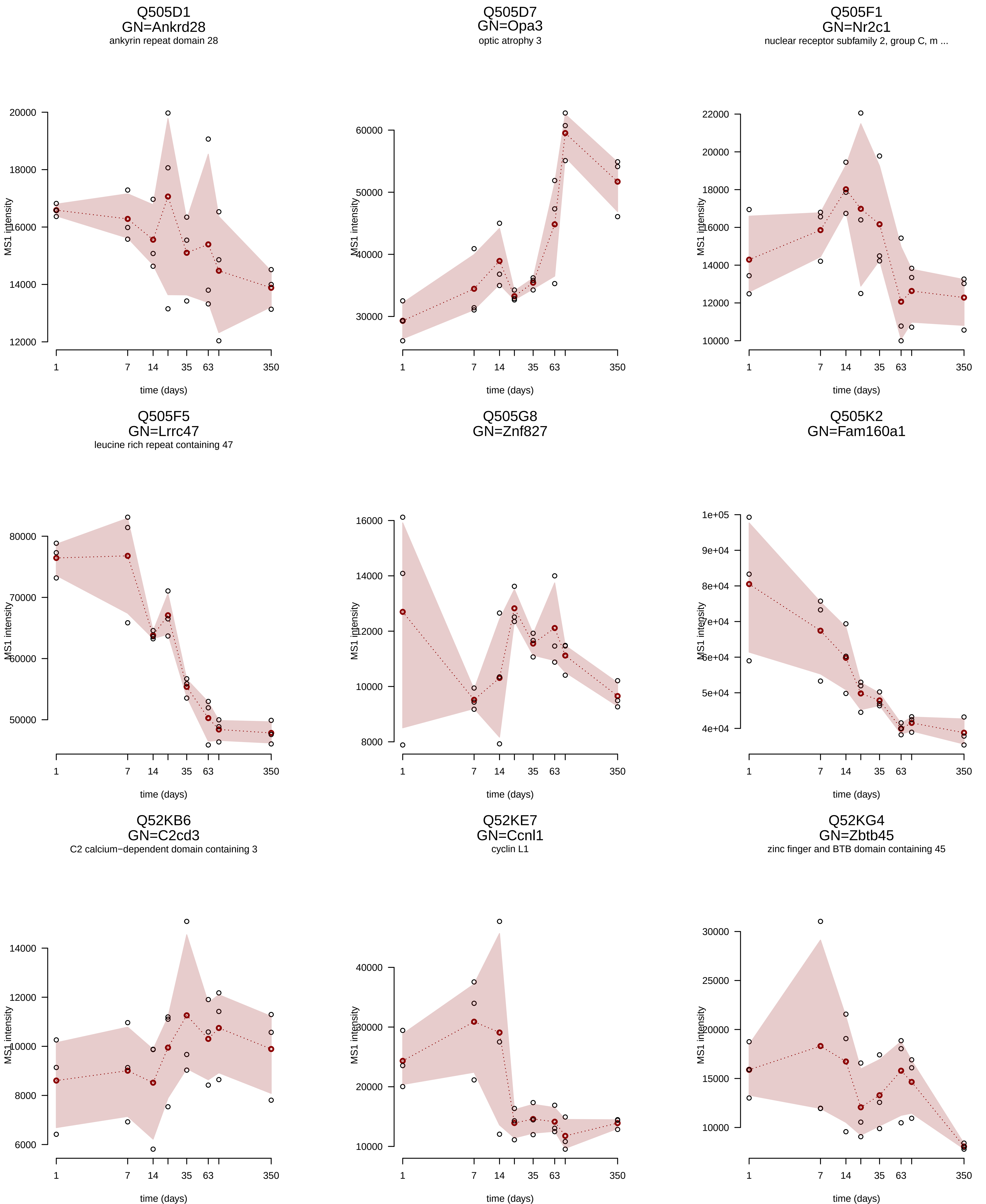
Q505D1
GN=Ankrd28
ankyrin repeat domain 28
MS1 intensity
12000 14000 16000 18000 20000
1 7 14 35 63 350
time (days)
Q505D7
GN=Opa3
optic atrophy 3
MS1 intensity
30000 40000 50000 60000
1 7 14 35 63 350
time (days)
Q505F1
GN=Nr2c1
nuclear receptor subfamily 2, group C, m ...
MS1 intensity
10000 12000 14000 16000 18000 20000 22000
1 7 14 35 63 350
time (days)
Q505F5
GN=Lrrc47
leucine rich repeat containing 47
MS1 intensity
50000 60000 70000 80000
1 7 14 35 63 350
time (days)
Q505G8
GN=Znf827
MS1 intensity
8000 10000 12000 14000 16000
1 7 14 35 63 350
time (days)
Q505K2
GN=Fam160a1
MS1 intensity
4e+04 5e+04 6e+04 7e+04 8e+04 9e+04 1e+05
1 7 14 35 63 350
time (days)
Q52KB6
GN=C2cd3
C2 calcium−dependent domain containing 3
MS1 intensity
6000 8000 10000 12000 14000
1 7 14 35 63 350
time (days)
Q52KE7
GN=Ccnl1
cyclin L1
MS1 intensity
10000 20000 30000 40000
1 7 14 35 63 350
time (days)
Q52KG4
GN=Zbtb45
zinc finger and BTB domain containing 45
MS1 intensity
10000 15000 20000 25000 30000
1 7 14 35 63 350
time (days)

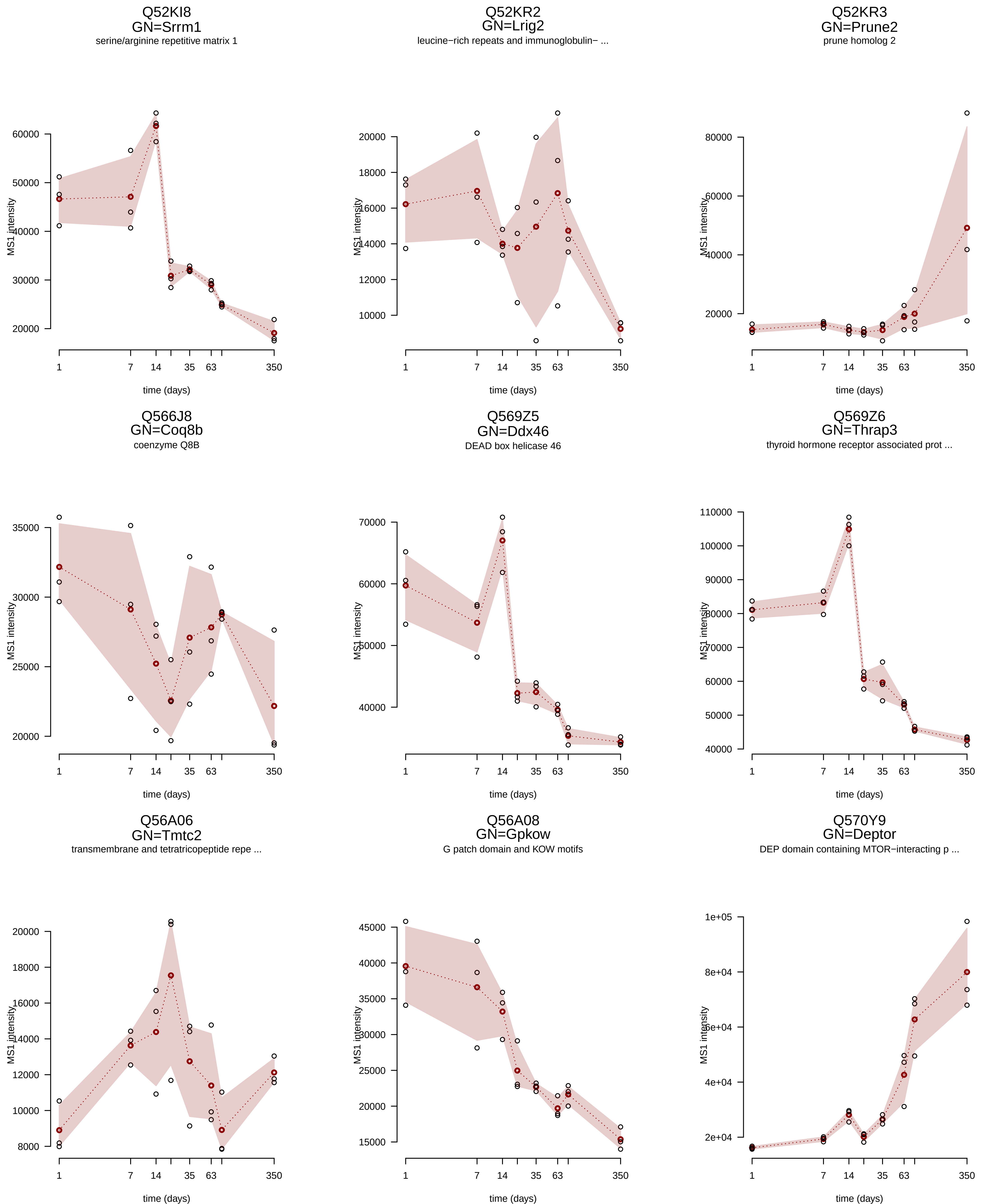
Q52KI8
GN=Srrm1
serine/arginine repetitive matrix 1
MS1 intensity
20000 30000 40000 50000 60000
1 7 14 35 63 350
time (days)
Q52KR2
GN=Lrig2
leucine−rich repeats and immunoglobulin− ...
MS1 intensity
10000 12000 14000 16000 18000 20000
1 7 14 35 63 350
time (days)
Q52KR3
GN=Prune2
prune homolog 2
MS1 intensity
20000 40000 60000 80000
1 7 14 35 63 350
time (days)
Q566J8
GN=Coq8b
coenzyme Q8B
MS1 intensity
20000 25000 30000 35000
1 7 14 35 63 350
time (days)
Q569Z5
GN=Ddx46
DEAD box helicase 46
MS1 intensity
40000 50000 60000 70000
1 7 14 35 63 350
time (days)
Q569Z6
GN=Thrap3
thyroid hormone receptor associated prot ...
MS1 intensity
40000 50000 60000 70000 80000 90000 100000 110000
1 7 14 35 63 350
time (days)
Q56A06
GN=Tmtc2
transmembrane and tetratricopeptide repe ...
MS1 intensity
8000 10000 12000 14000 16000 18000 20000
1 7 14 35 63 350
time (days)
Q56A08
GN=Gpkow
G patch domain and KOW motifs
MS1 intensity
15000 20000 25000 30000 35000 40000 45000
1 7 14 35 63 350
time (days)
Q570Y9
GN=Deptor
DEP domain containing MTOR−interacting p ...
MS1 intensity
2e+04 4e+04 6e+04 8e+04 1e+05
1 7 14 35 63 350
time (days)

Q571E4
GN=Galns
galactosamine (N−acetyl)−6−sulfate sulfa ...

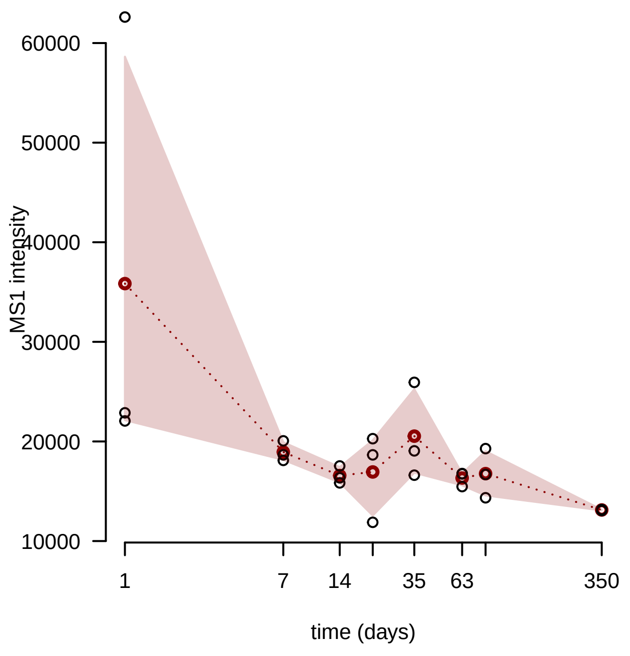

Q571G4
GN=Lin54
lin−54 DREAM MuvB core complex component

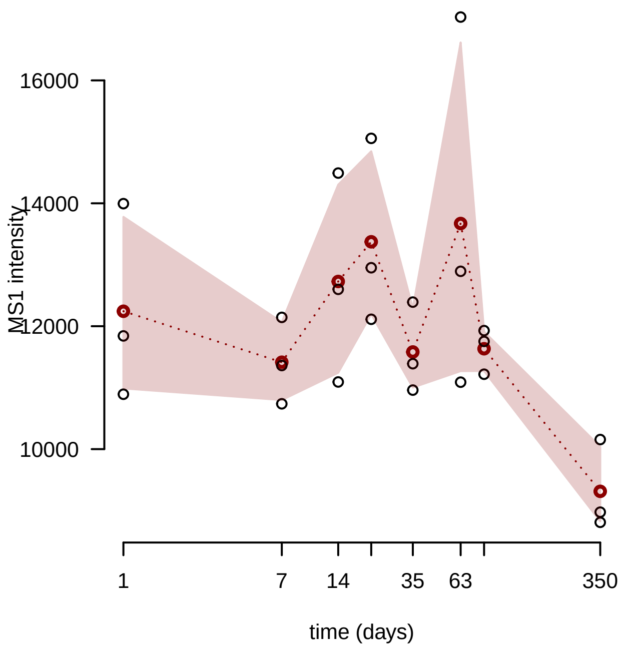

Q571H0
GN=Urb1
URB1 ribosome biogenesis 1 homolog (S. c ...

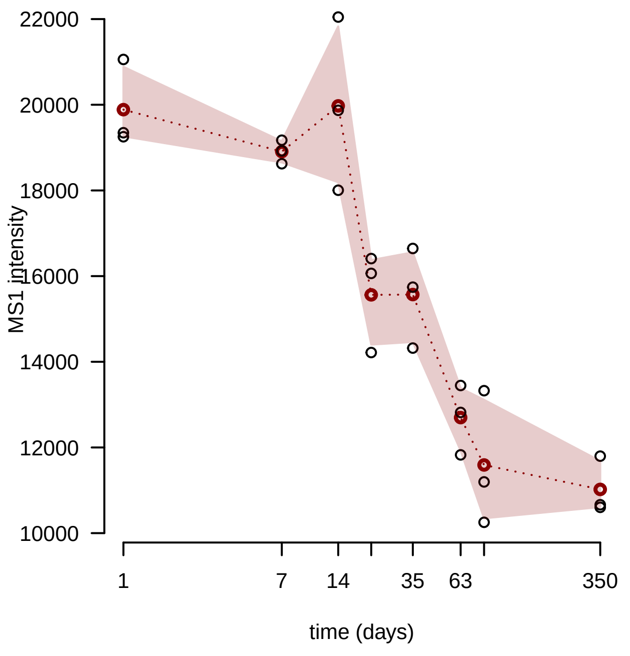

Q571I9
GN=Aldh16a1
aldehyde dehydrogenase 16 family, member ...

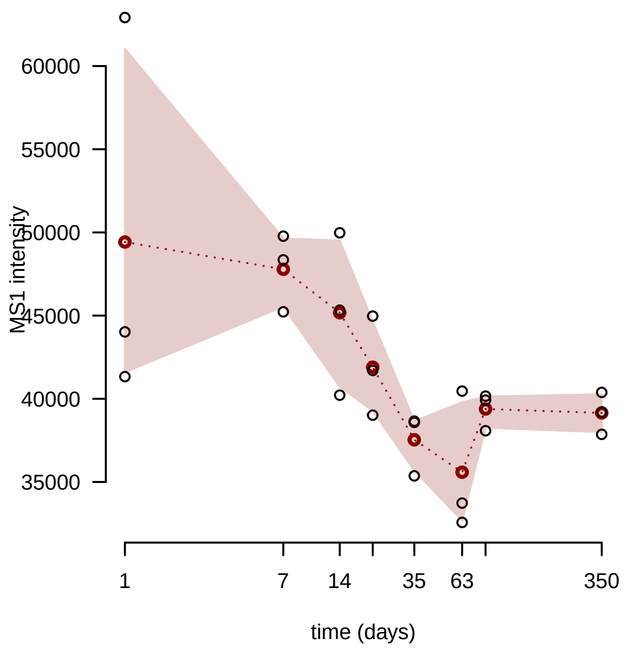

Q571J5
GN=Znf354c

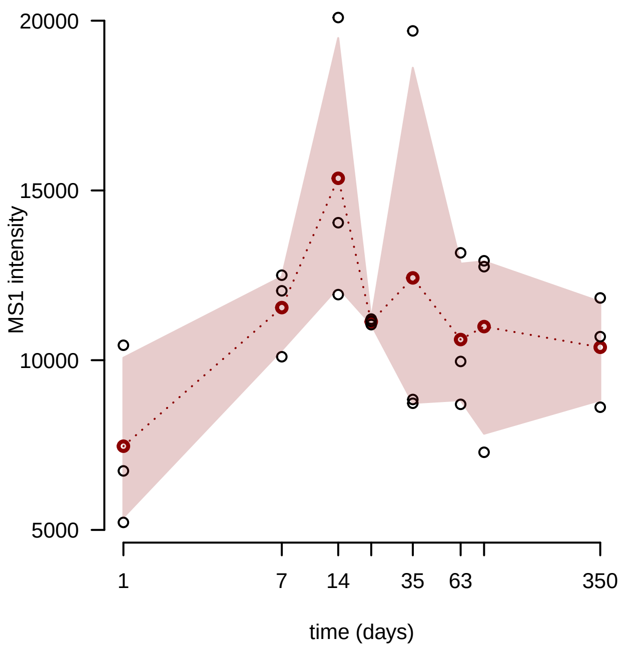

Q571K4
GN=Tab3
TGF−beta activated kinase 1/MAP3K7 bindi ...

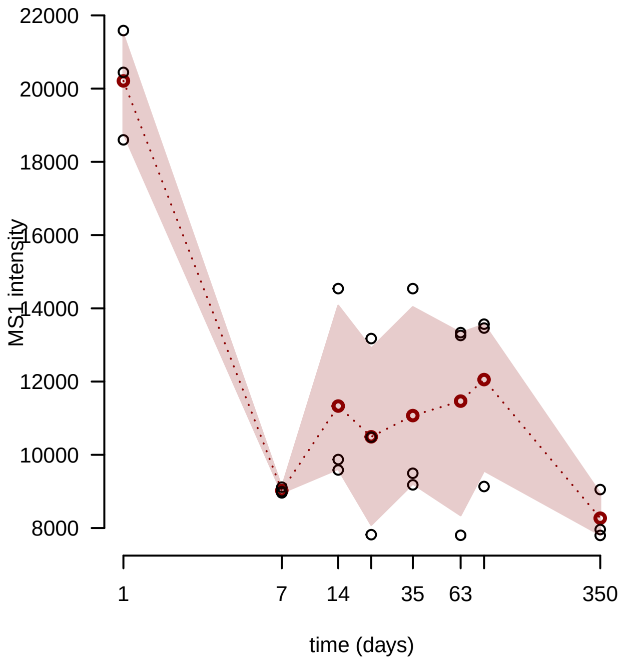

Q58A65
GN=Spag9
sperm associated antigen 9

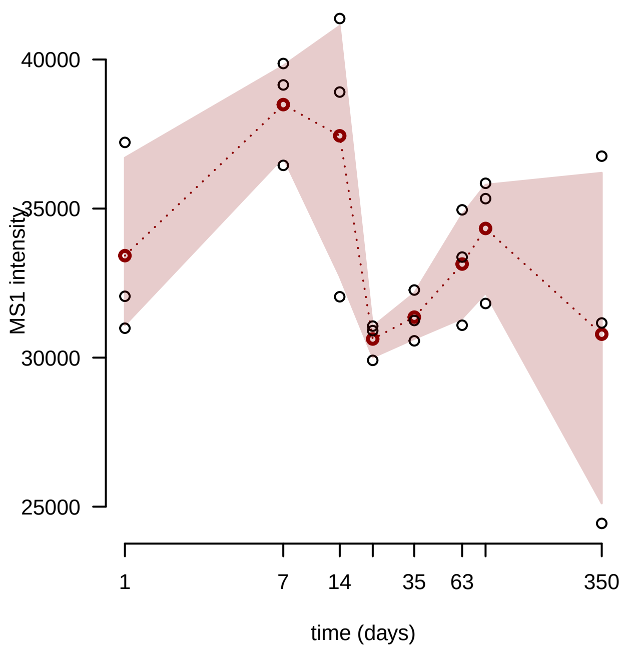

Q59J78
GN=Ndufaf2
NADH:ubiquinone oxidoreductase complex a ...

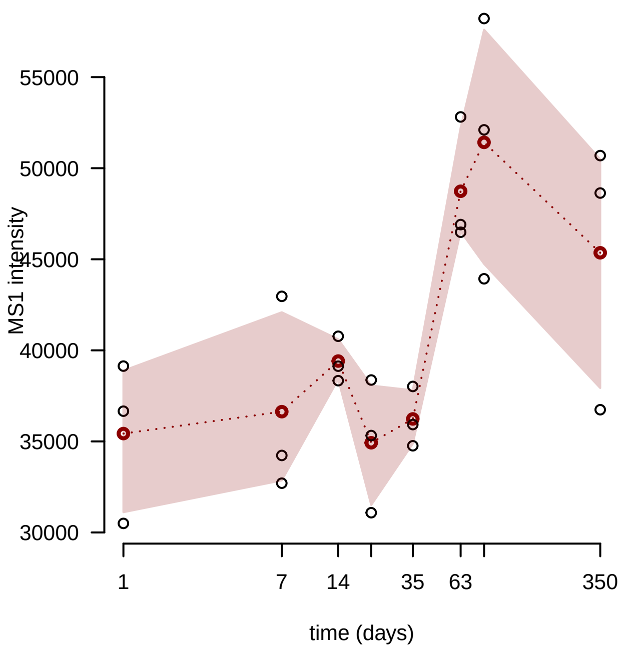

Q5BKQ4
GN=Pnliprp1
pancreatic lipase related protein 1

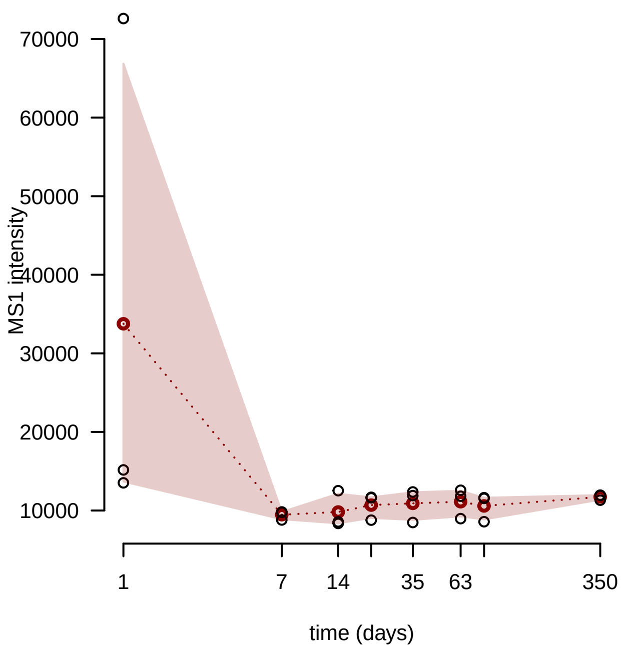

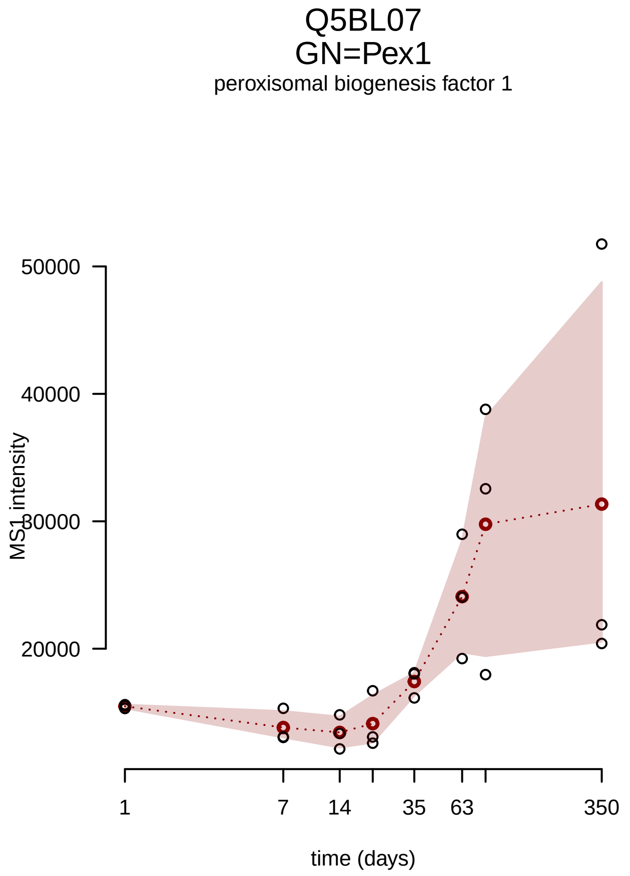
Q5BL07
GN=Pex1
peroxisomal biogenesis factor 1
MS1 intensity
50000
40000
30000
20000
1
7
14
35
63
350
time (days)

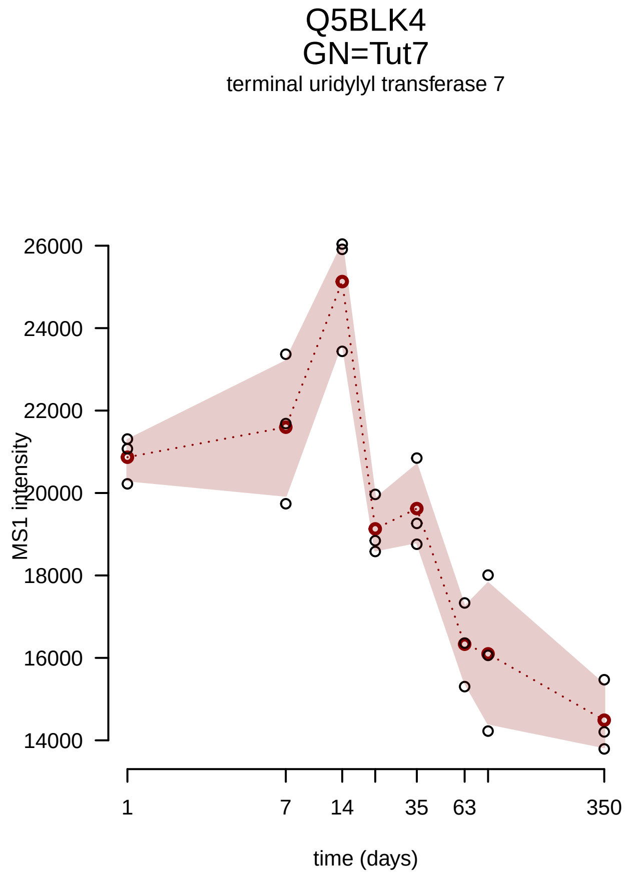
Q5BLK4
GN=Tut7
terminal uridylyl transferase 7
MS1 intensity
26000
24000
22000
20000
18000
16000
14000
1
7
14
35
63
350
time (days)

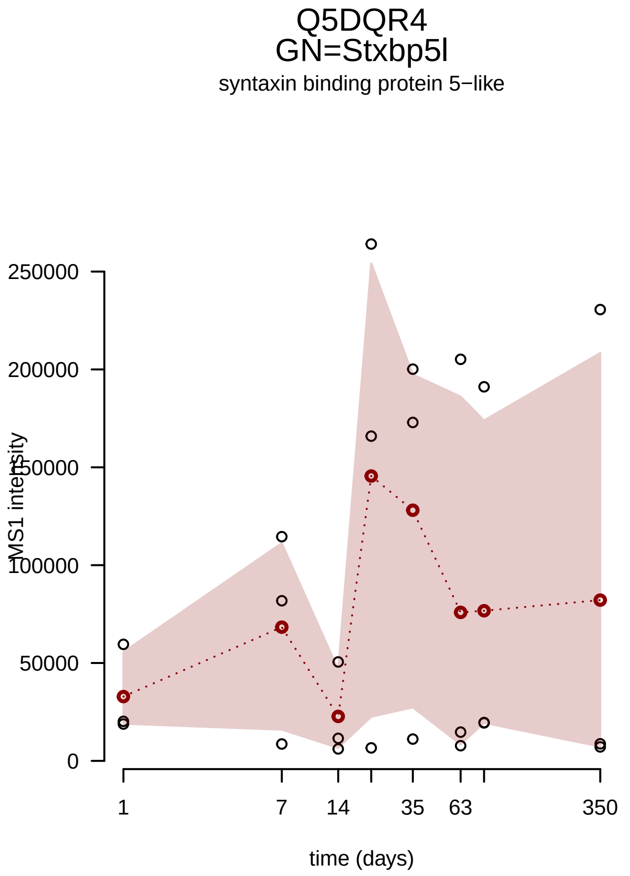
Q5DQR4
GN=Stxbp5l
syntaxin binding protein 5−like
MS1 intensity
250000
200000
150000
100000
50000
0
1
7
14
35
63
350
time (days)

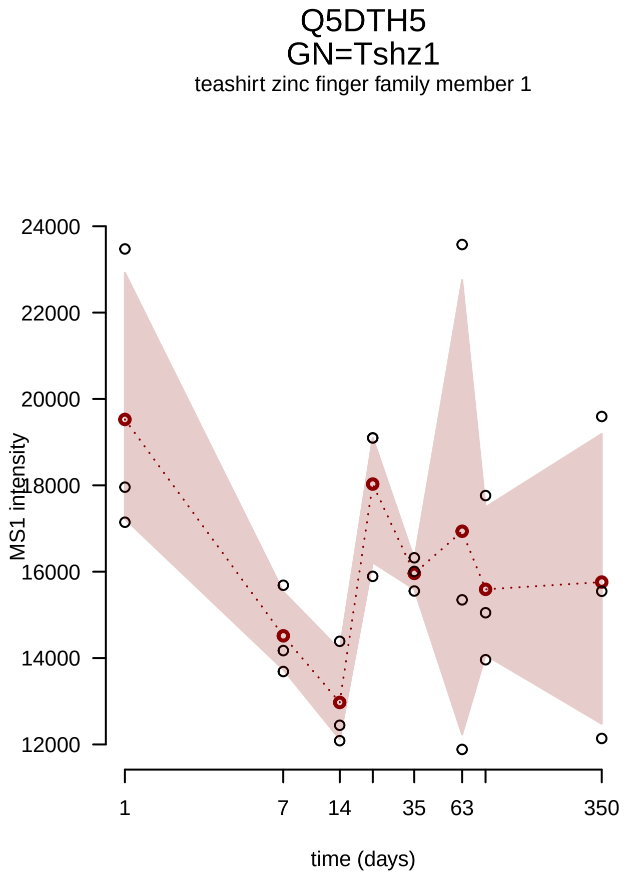
Q5DTH5
GN=Tshz1
teashirt zinc finger family member 1
MS1 intensity
24000
22000
20000
18000
16000
14000
12000
1
7
14
35
63
350
time (days)

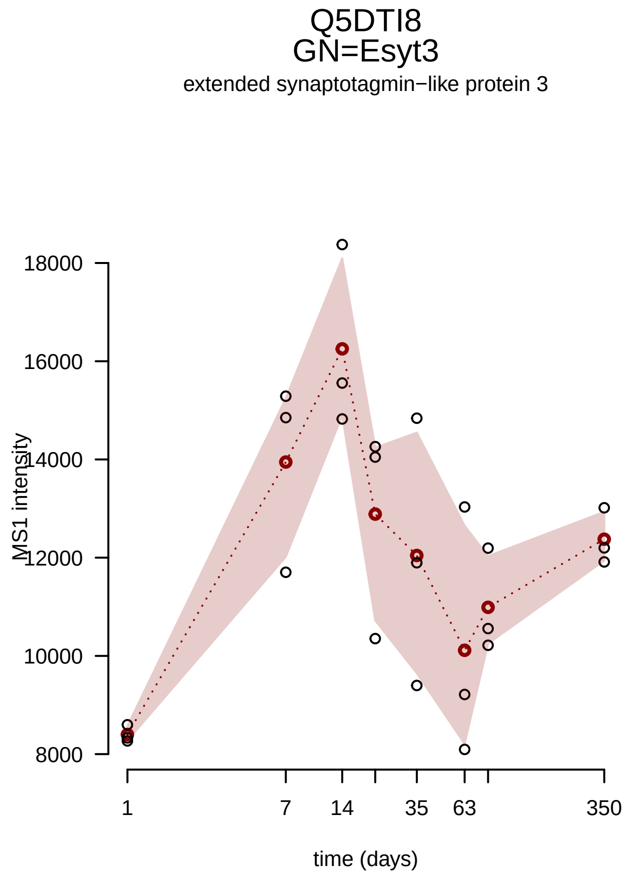
Q5DTI8
GN=Esyt3
extended synaptotagmin−like protein 3
MS1 intensity
18000
16000
14000
12000
10000
8000
1
7
14
35
63
350
time (days)

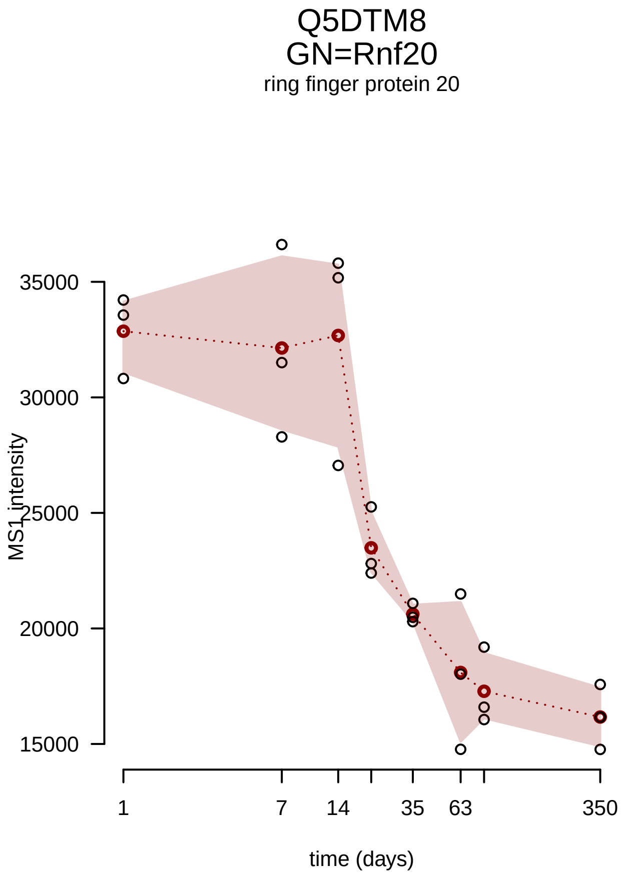
Q5DTM8
GN=Rnf20
ring finger protein 20
MS1 intensity
35000
30000
25000
20000
15000
1
7
14
35
63
350
time (days)

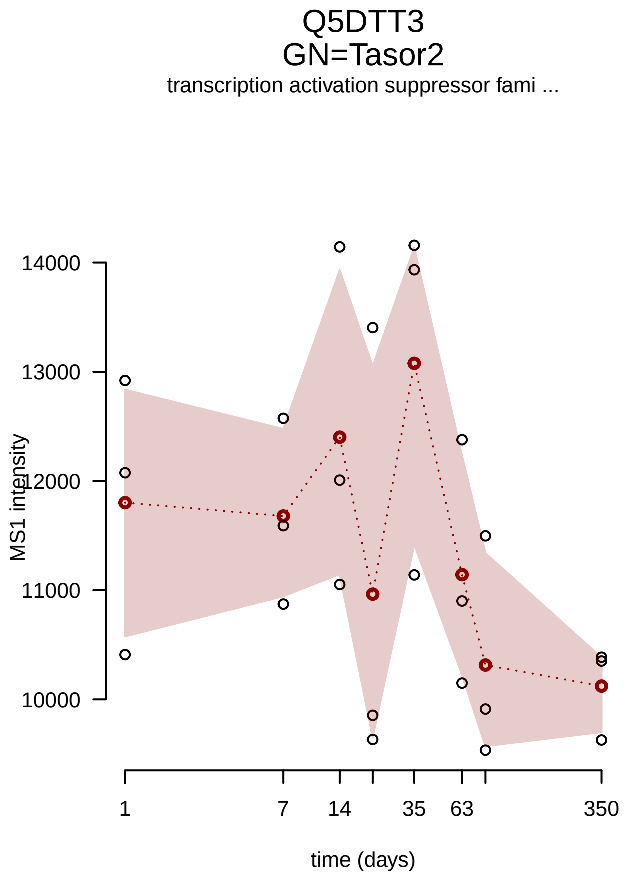
Q5DTT3
GN=Tasor2
transcription activation suppressor fami ...
MS1 intensity
14000
13000
12000
11000
10000
1
7
14
35
63
350
time (days)

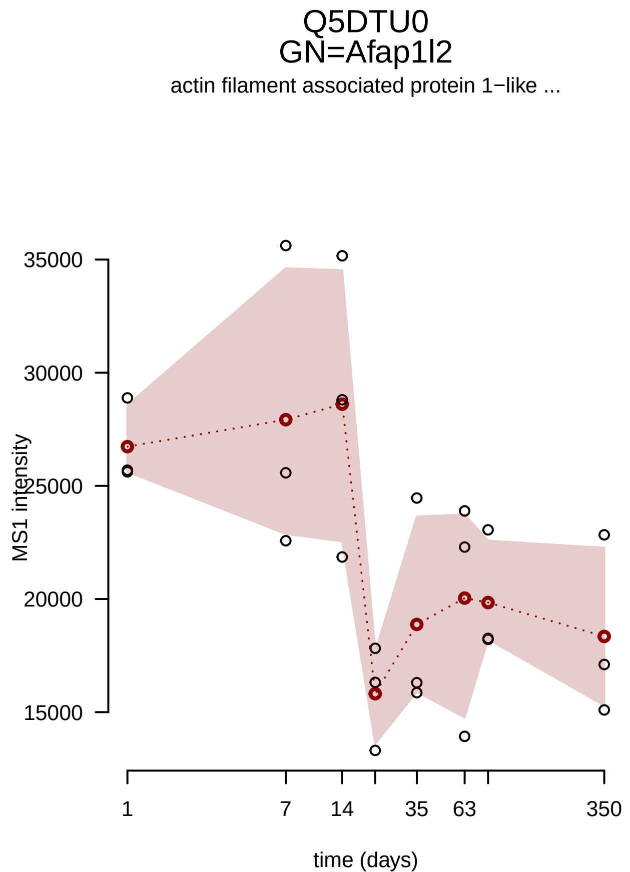
Q5DTU0
GN=Afap1l2
actin filament associated protein 1−like ...
MS1 intensity
35000
30000
25000
20000
15000
1
7
14
35
63
350
time (days)

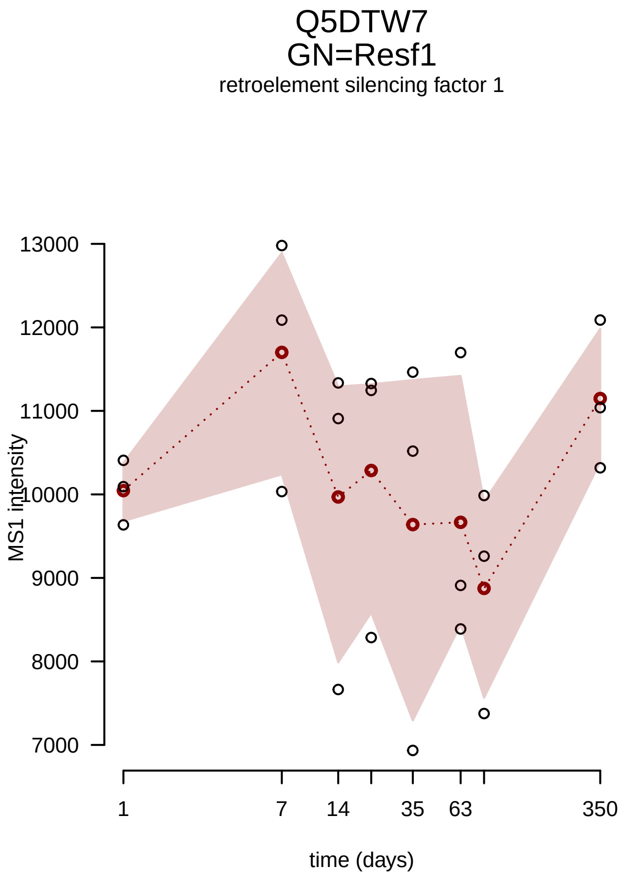
Q5DTW7
GN=Resf1
retroelement silencing factor 1
MS1 intensity
13000
12000
11000
10000
9000
8000
7000
1
7
14
35
63
350
time (days)

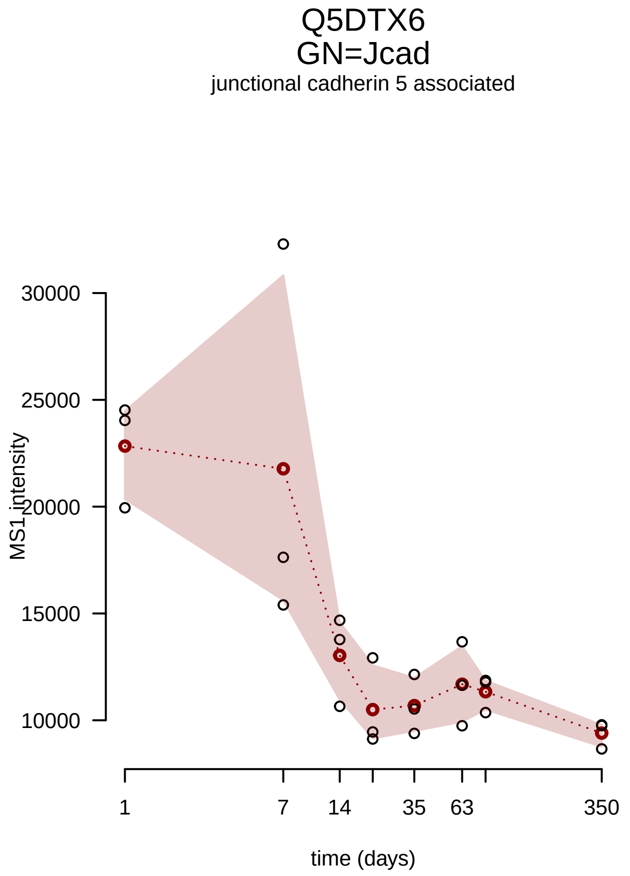
Q5DTX6
GN=Jcad
junctional cadherin 5 associated
MS1 intensity
30000
25000
20000
15000
10000
1
7
14
35
63
350
time (days)

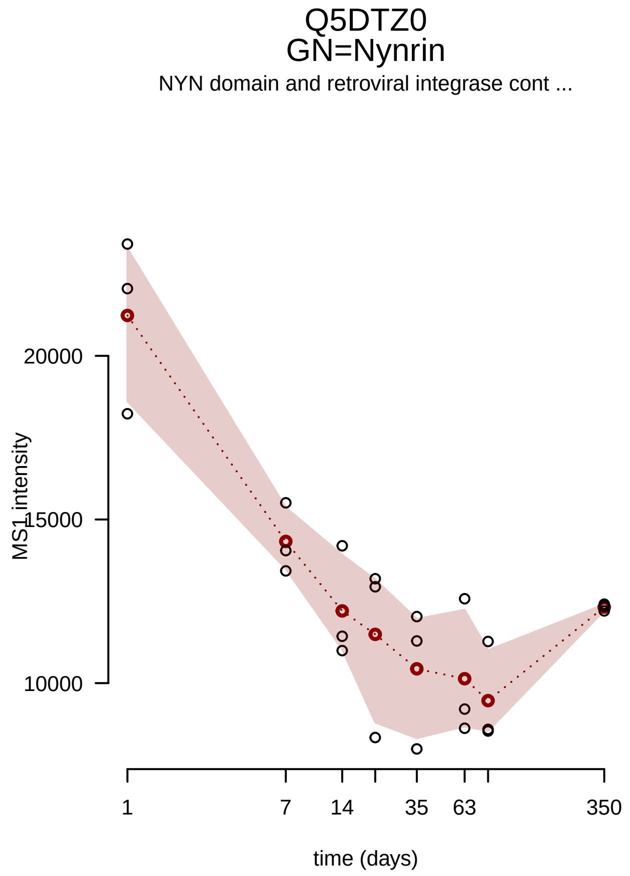
Q5DTZ0
GN=Nynrin
NYN domain and retroviral integrase cont ...
MS1 intensity
20000
15000
10000
1
7
14
35
63
350
time (days)

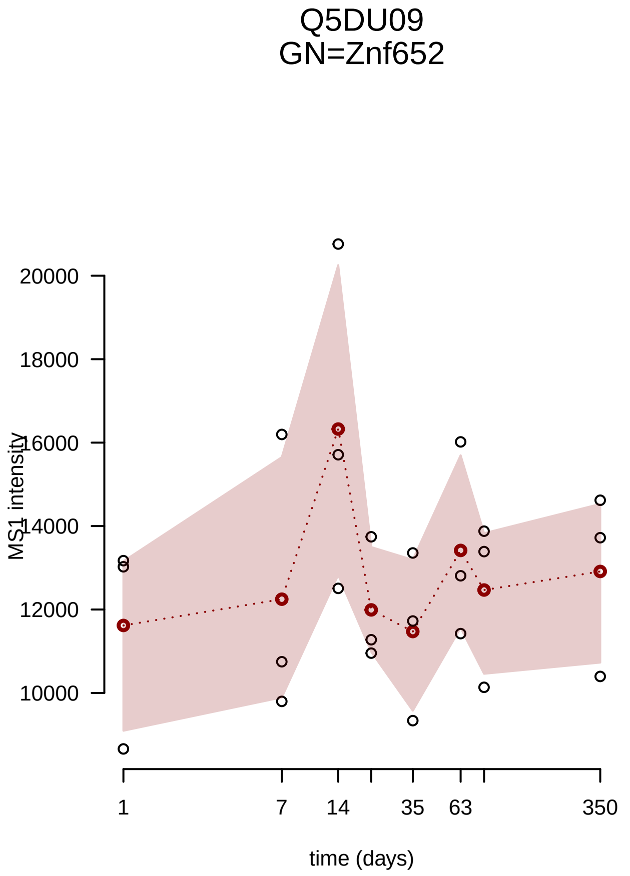
Q5DU09
GN=Znf652
MS1 intensity
20000
18000
16000
14000
12000
10000
1
7
14
35
63
350
time (days)

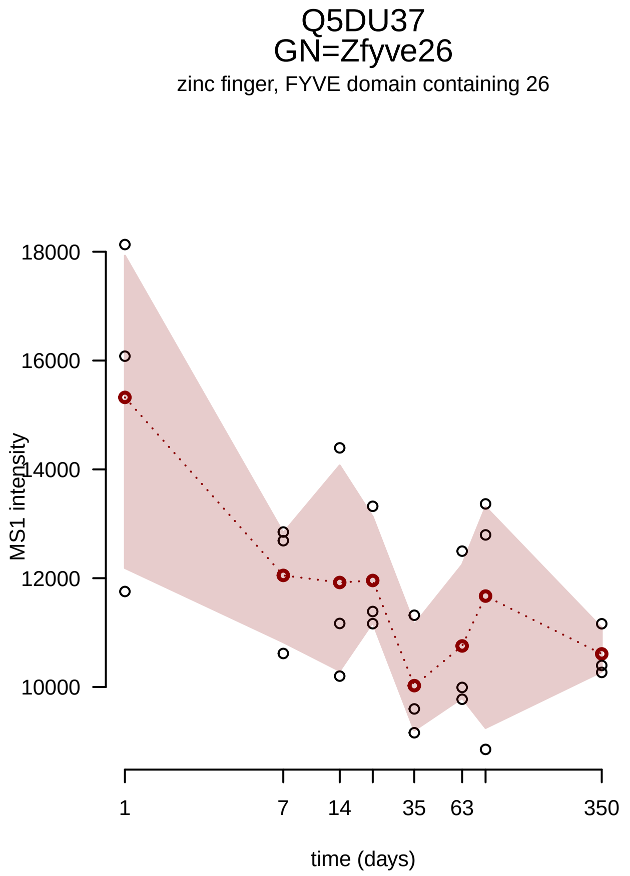
Q5DU37
GN=Zfyve26
zinc finger, FYVE domain containing 26
MS1 intensity
18000
16000
14000
12000
10000
1
7
14
35
63
350
time (days)

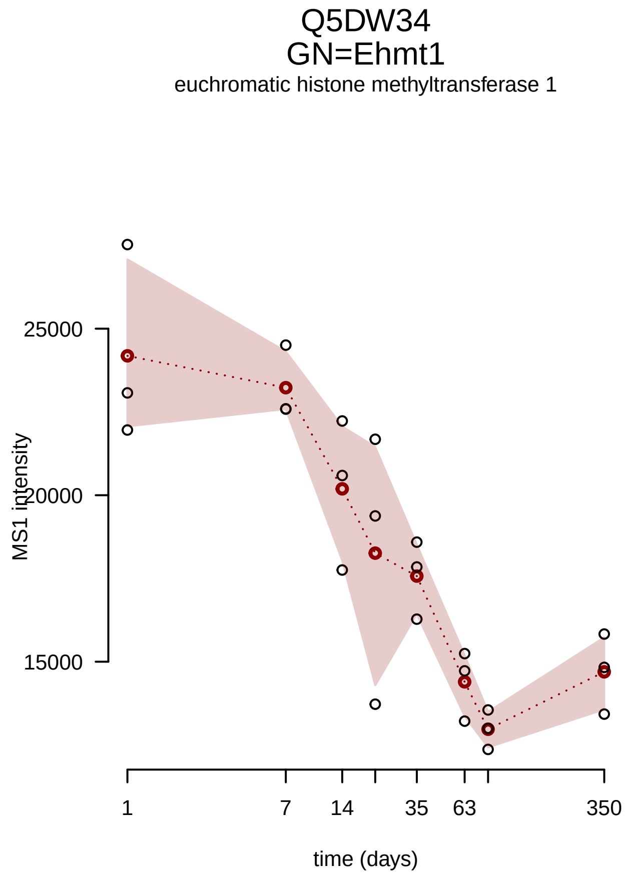
Q5DW34
GN=Ehmt1
euchromatic histone methyltransferase 1
MS1 intensity
25000
20000
15000
1
7
14
35
63
350
time (days)

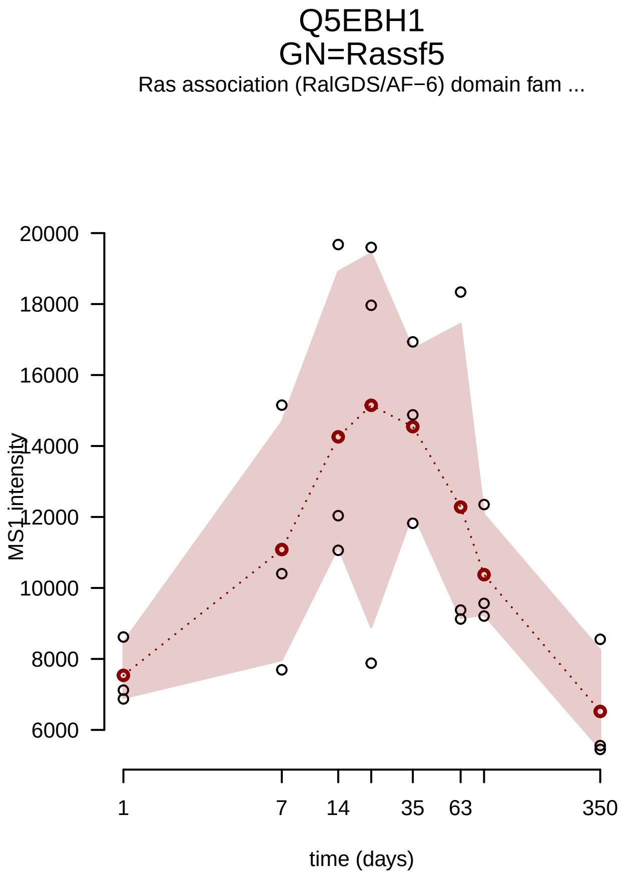
Q5EBH1
GN=Rassf5
Ras association (RalGDS/AF−6) domain fam ...
MS1 intensity
20000
18000
16000
14000
12000
10000
8000
6000
1
7
14
35
63
350
time (days)

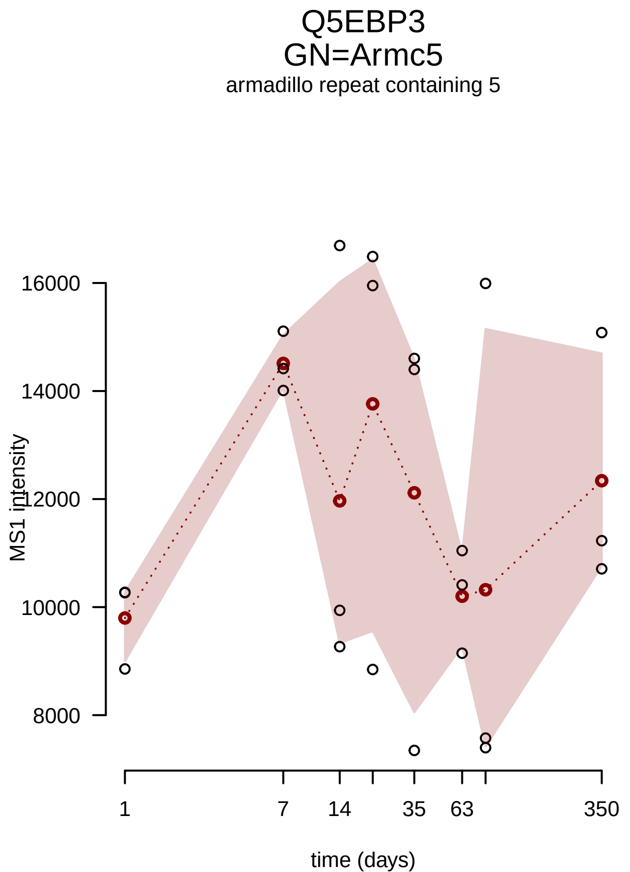
Q5EBP3
GN=Armc5
armadillo repeat containing 5
MS1 intensity
16000
14000
12000
10000
8000
1
7
14
35
63
350
time (days)

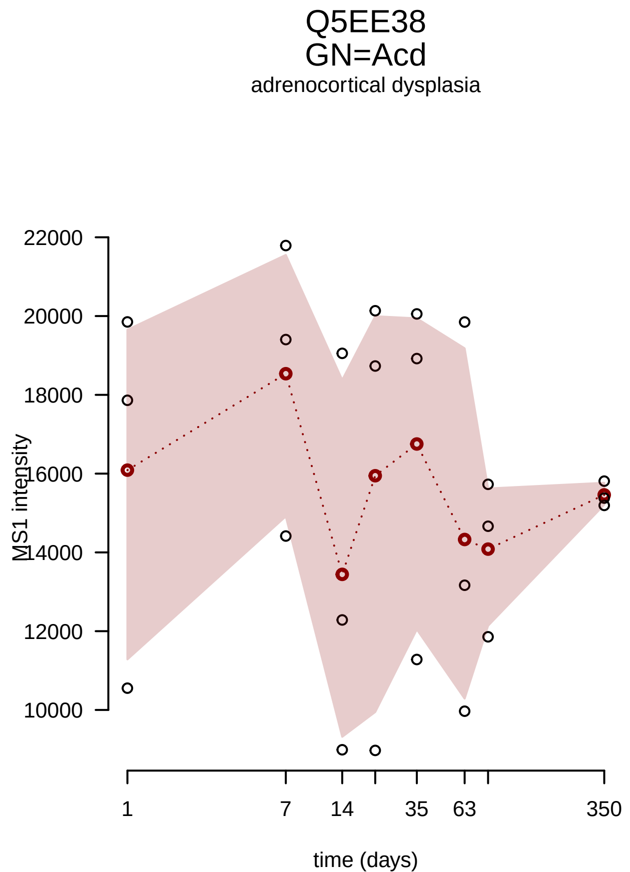
Q5EE38
GN=Acd
adrenocortical dysplasia
MS1 intensity
22000
20000
18000
16000
14000
12000
10000
1
7
14
35
63
350
time (days)

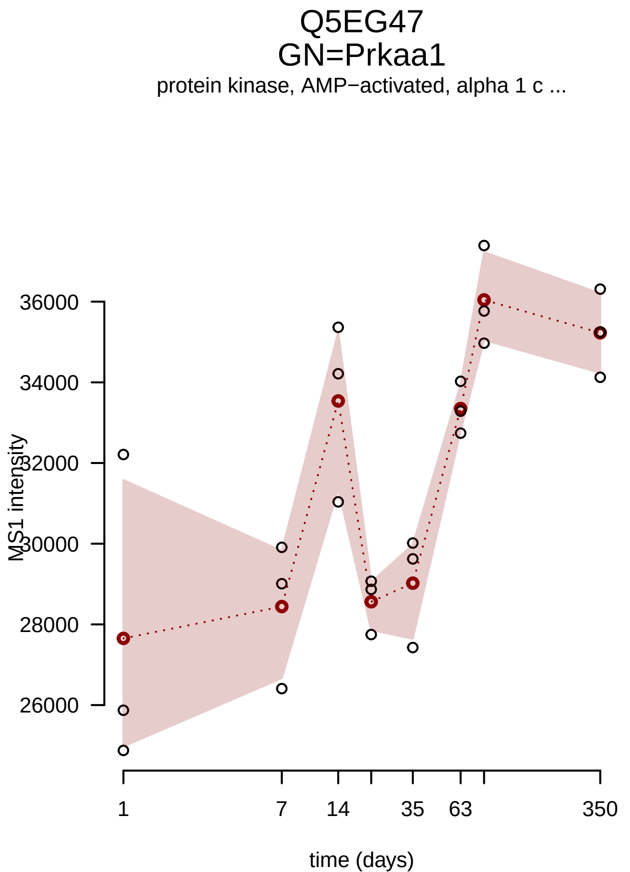
Q5EG47
GN=Prkaa1
protein kinase, AMP−activated, alpha 1 c ...
MS1 intensity
36000
34000
32000
30000
28000
26000
1
7
14
35
63
350
time (days)

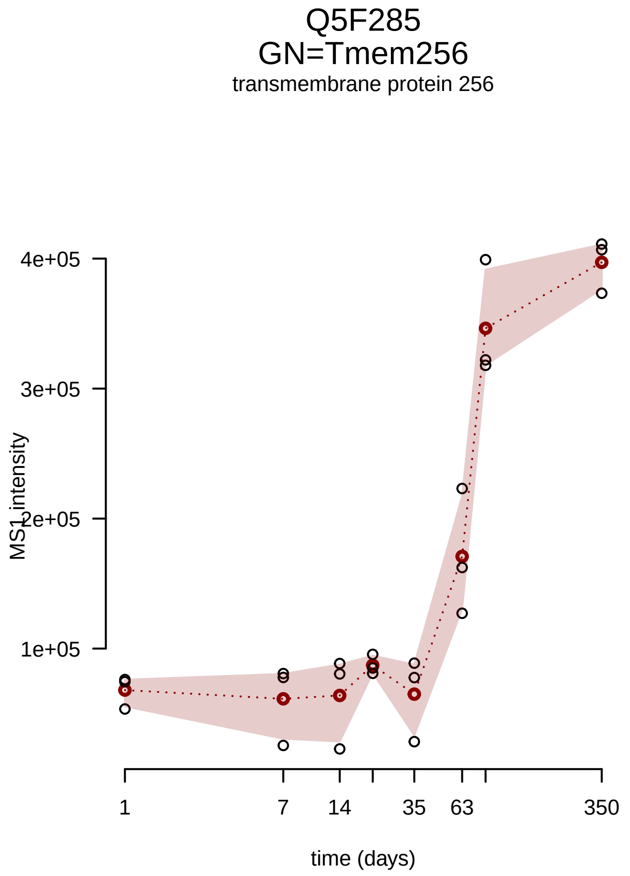
Q5F285
GN=Tmem256
transmembrane protein 256
MS1 intensity
4e+05
3e+05
2e+05
1e+05
time (days)
1
7
14
35
63
350

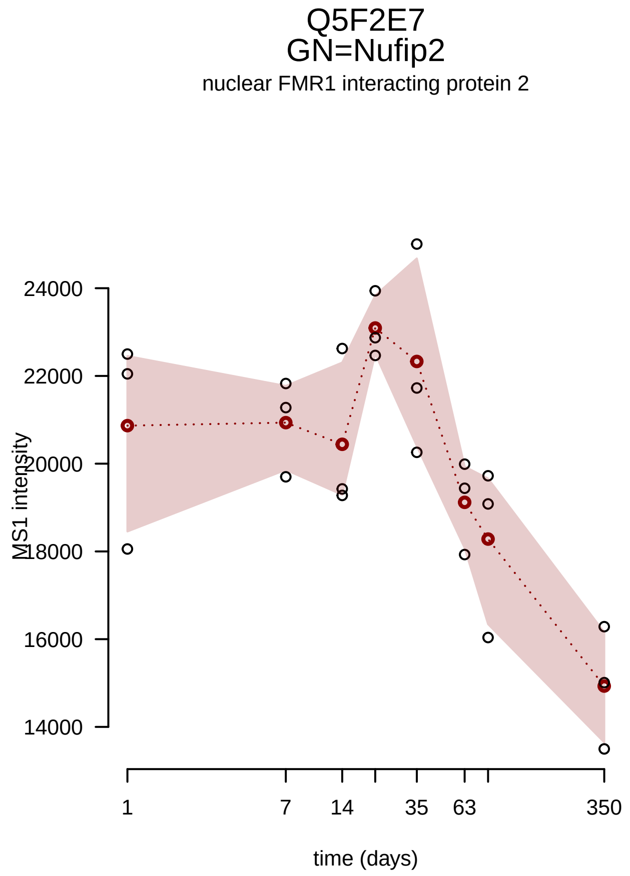
Q5F2E7
GN=Nufip2
nuclear FMR1 interacting protein 2
MS1 intensity
24000
22000
20000
18000
16000
14000
time (days)
1
7
14
35
63
350

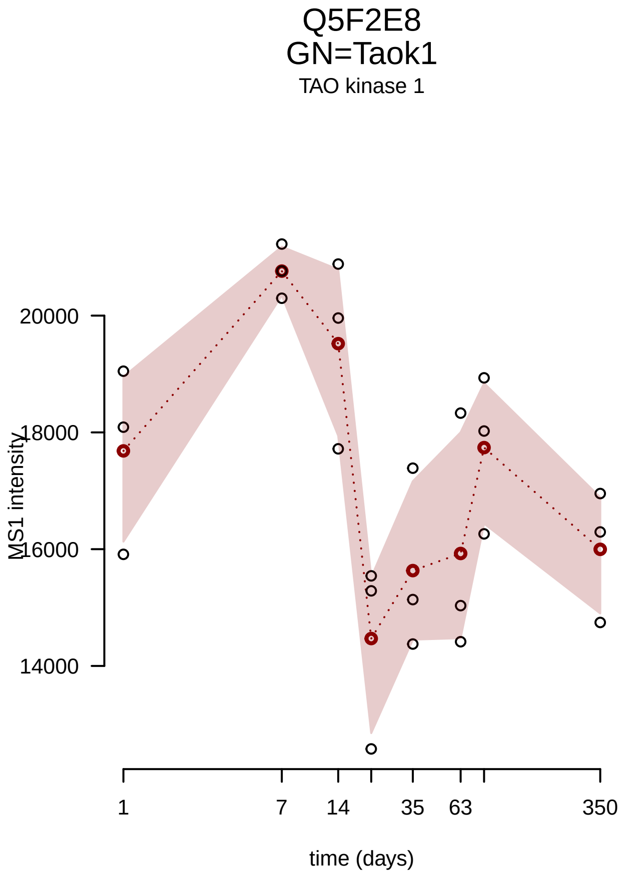
Q5F2E8
GN=Taok1
TAO kinase 1
MS1 intensity
20000
18000
16000
14000
time (days)
1
7
14
35
63
350

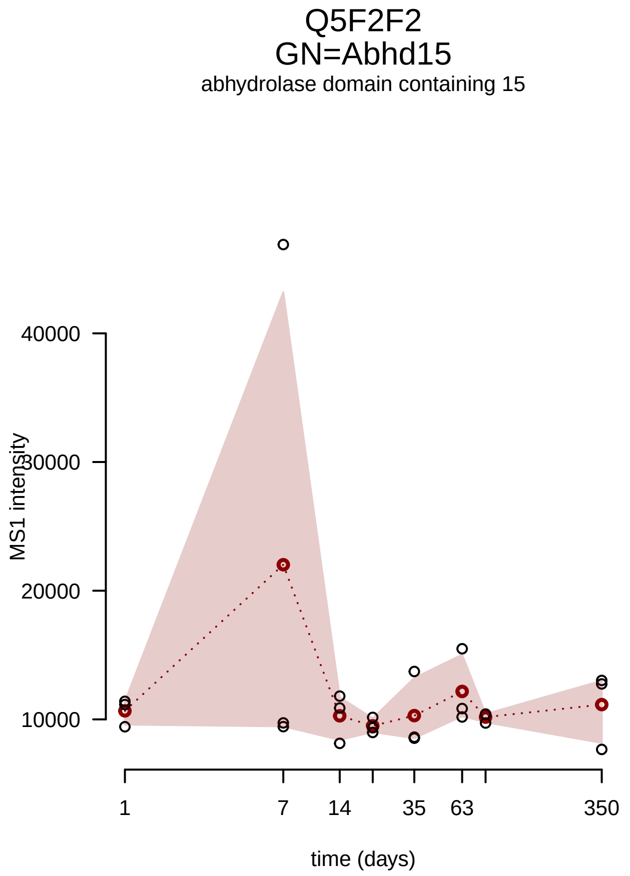
Q5F2F2
GN=Abhd15
abhydrolase domain containing 15
MS1 intensity
40000
30000
20000
10000
time (days)
1
7
14
35
63
350

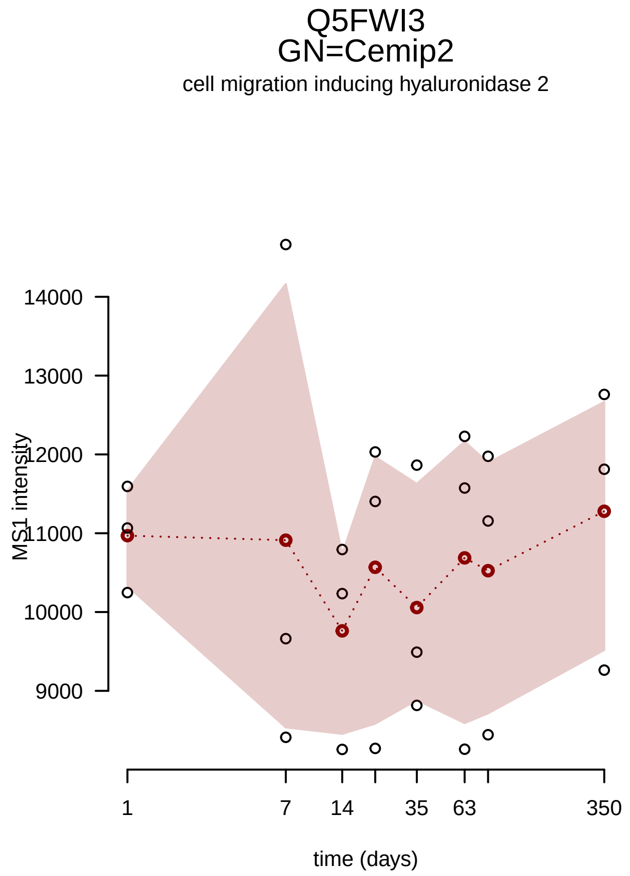
Q5FWI3
GN=Cemip2
cell migration inducing hyaluronidase 2
MS1 intensity
14000
13000
12000
11000
10000
9000
time (days)
1
7
14
35
63
350

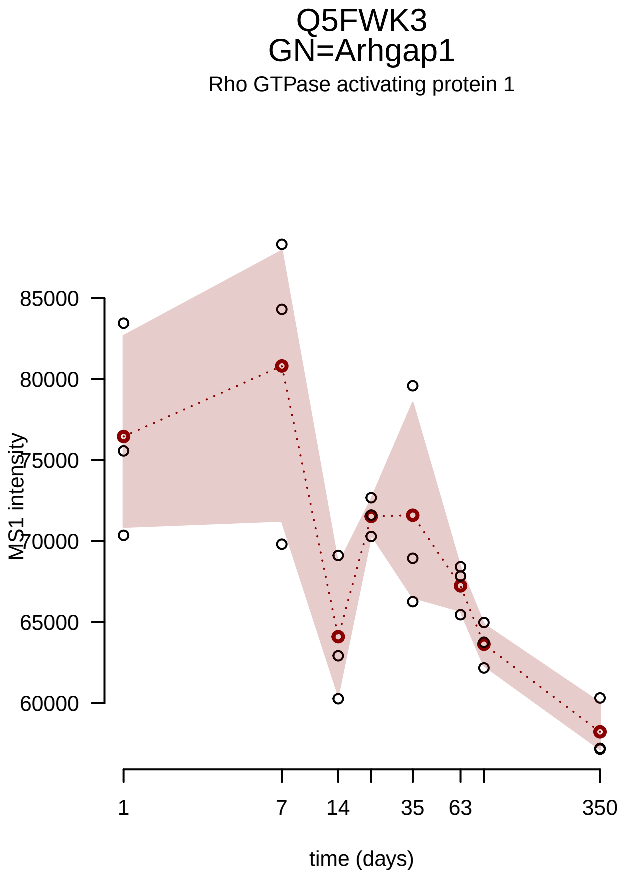
Q5FWK3
GN=Arhgap1
Rho GTPase activating protein 1
MS1 intensity
85000
80000
75000
70000
65000
60000
time (days)
1
7
14
35
63
350

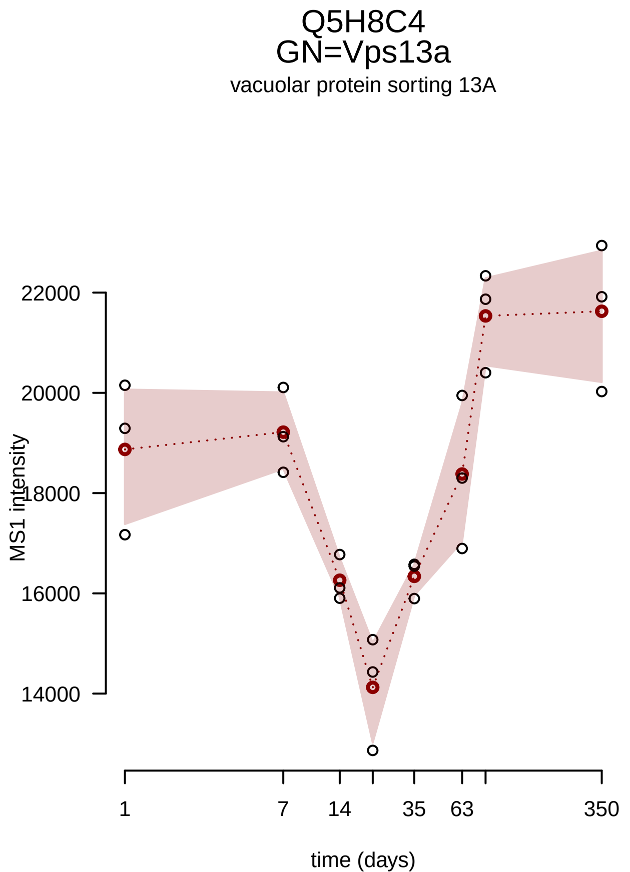
Q5H8C4
GN=Vps13a
vacuolar protein sorting 13A
MS1 intensity
22000
20000
18000
16000
14000
time (days)
1
7
14
35
63
350

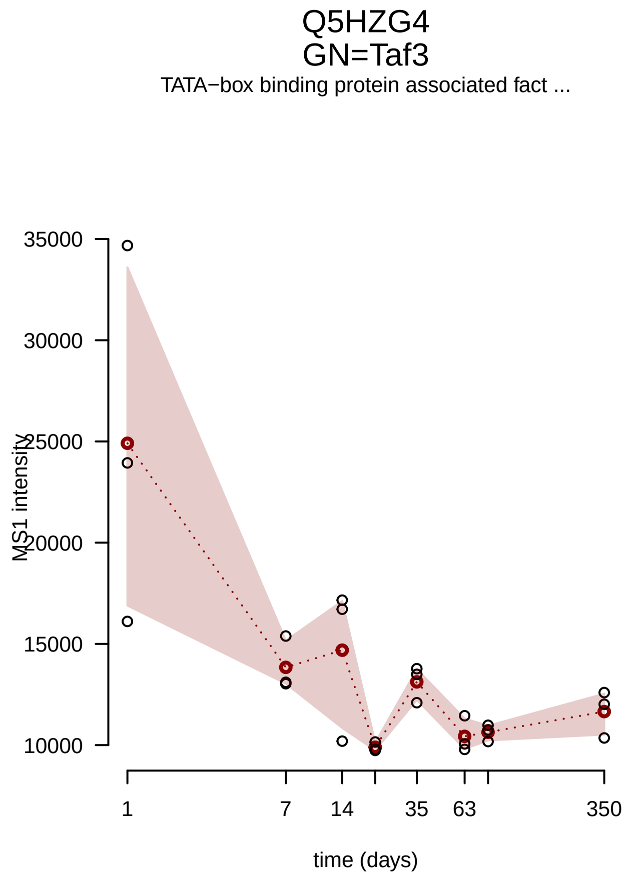
Q5HZG4
GN=Taf3
TATA−box binding protein associated fact ...
MS1 intensity
35000
30000
25000
20000
15000
10000
time (days)
1
7
14
35
63
350

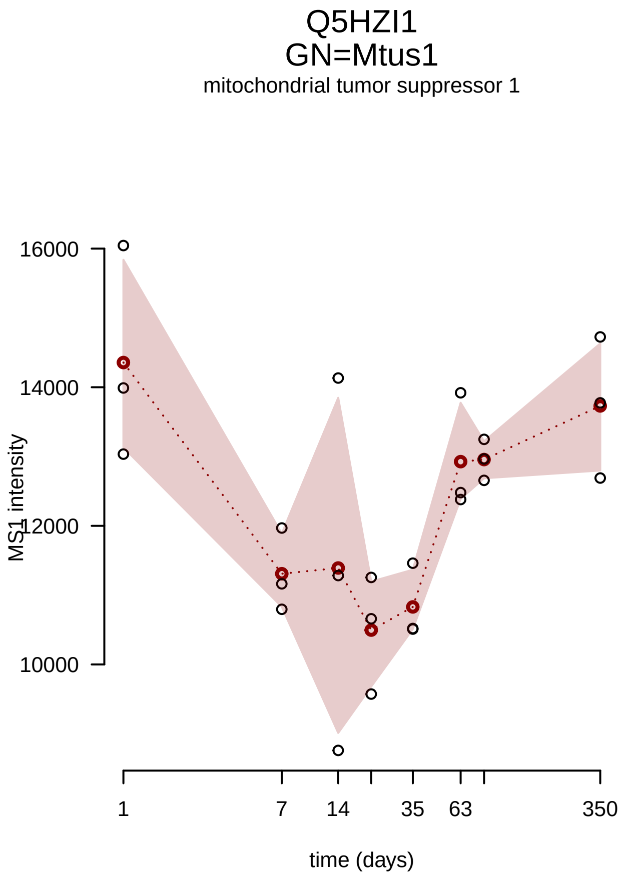
Q5HZI1
GN=Mtus1
mitochondrial tumor suppressor 1
MS1 intensity
16000
14000
12000
10000
time (days)
1
7
14
35
63
350

Q5HZI9
GN=Slc25a51
solute carrier family 25, member 51

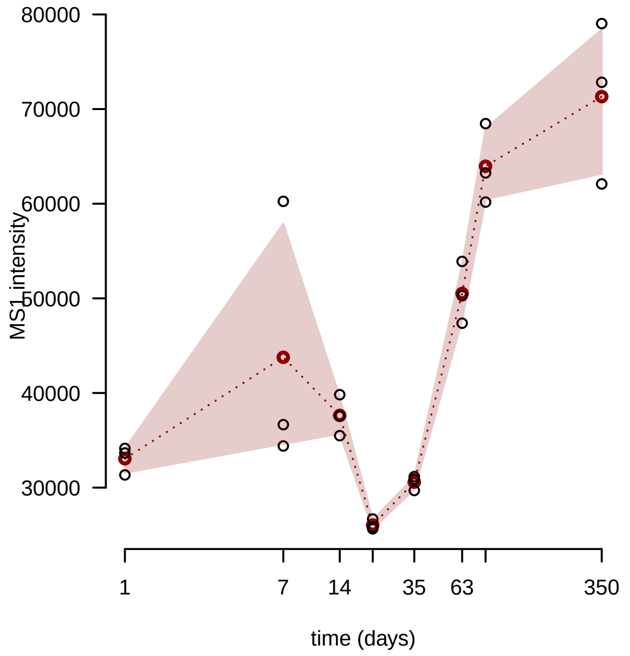

Q5HZJ0
GN=Drosha
drosha, ribonuclease type III

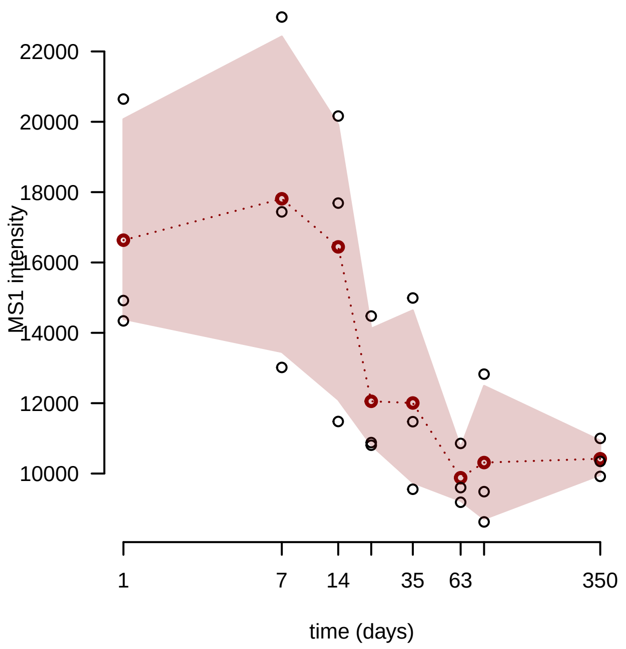

Q5I012
GN=Slc38a10
solute carrier family 38, member 10

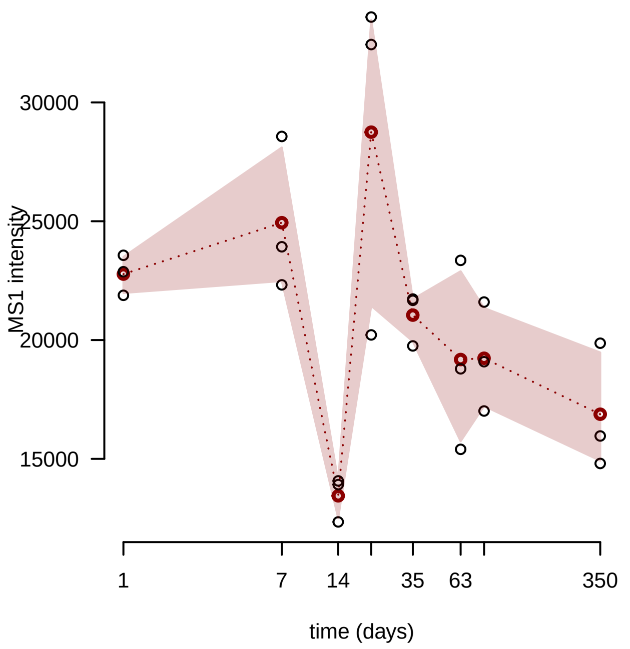

Q5I043
GN=Usp28
ubiquitin specific peptidase 28

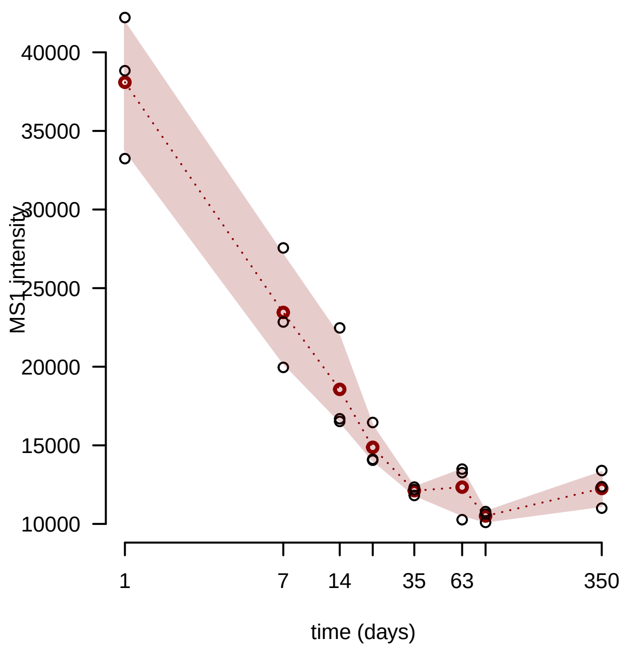

Q5I2A0
GN=Serpina3g
serine (or cysteine) peptidase inhibitor ...

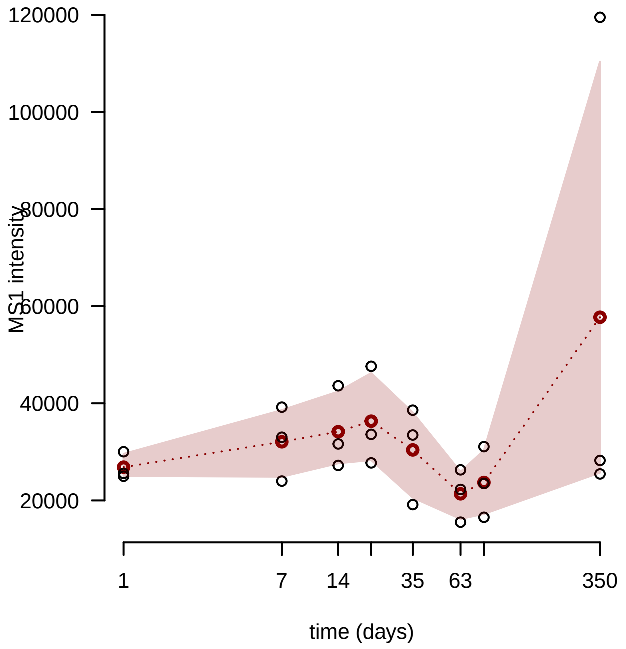

Q5IRJ6
GN=Slc30a9
solute carrier family 30 (zinc transport ...

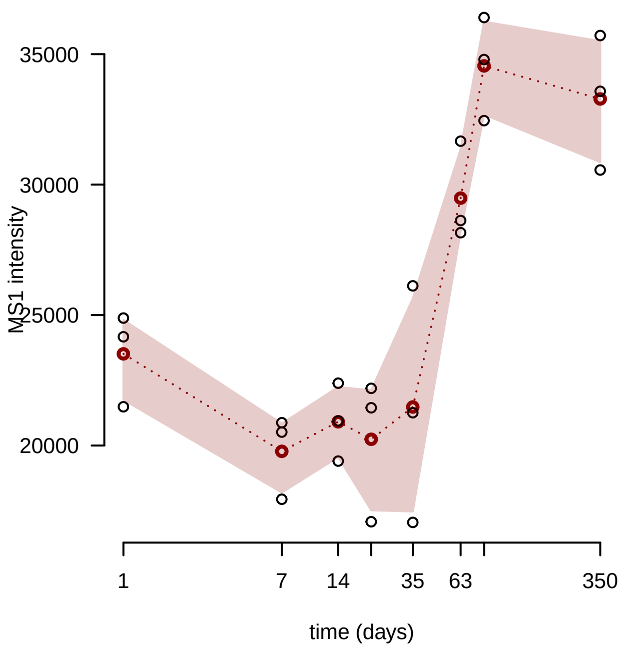

Q5KU39
GN=Vps41
VPS41 HOPS complex subunit

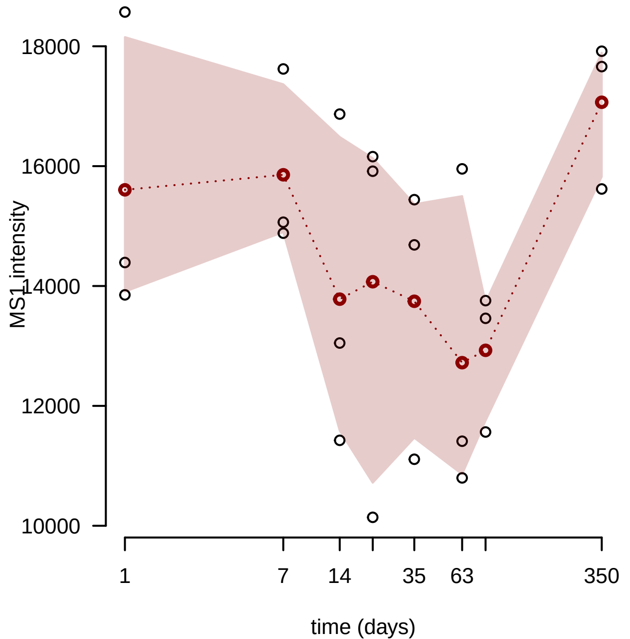

Q5M8N0
GN=Cnrip1
cannabinoid receptor interacting protein ...

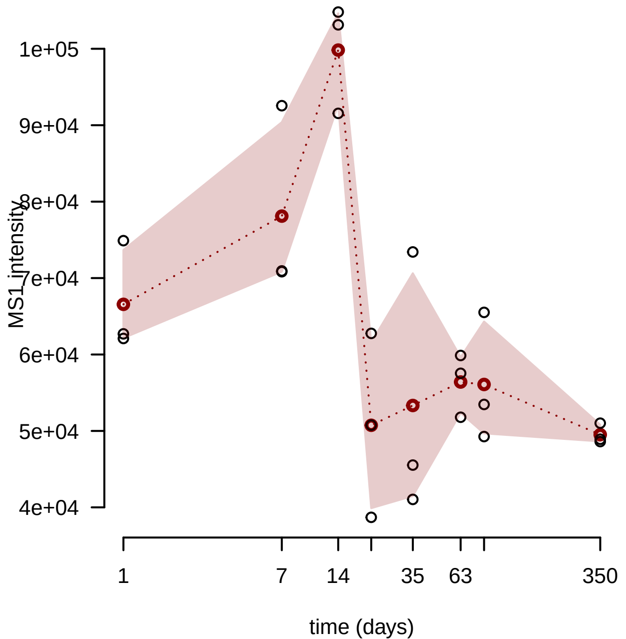

Q5M8N4
GN=Sdr39u1
short chain dehydrogenase/reductase fami ...

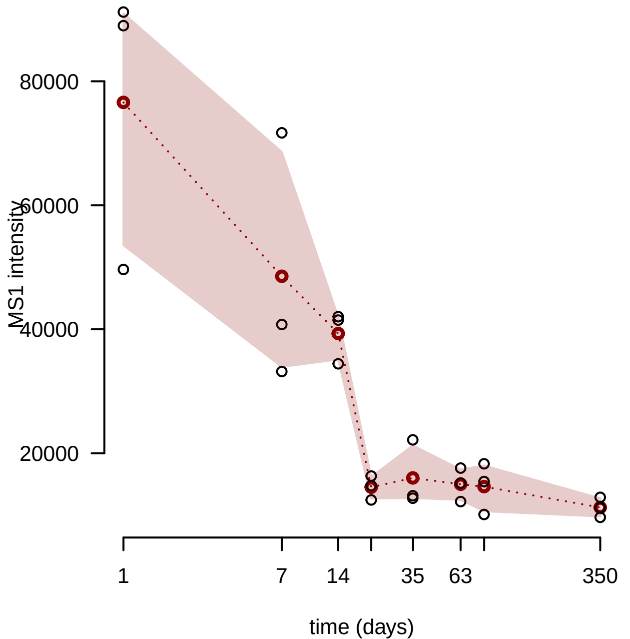

Q5M956
GN=Kctd1
potassium channel tetramerisation domain ...

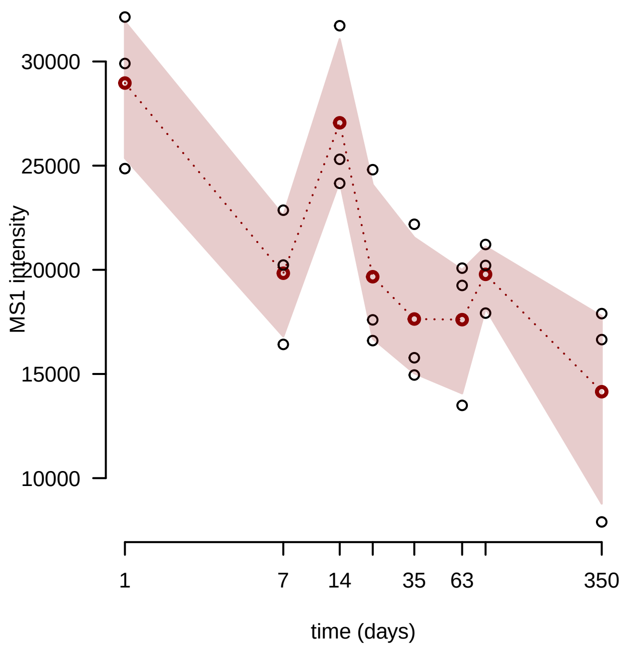

Q5NBU8
GN=Xaf1
XIAP associated factor 1

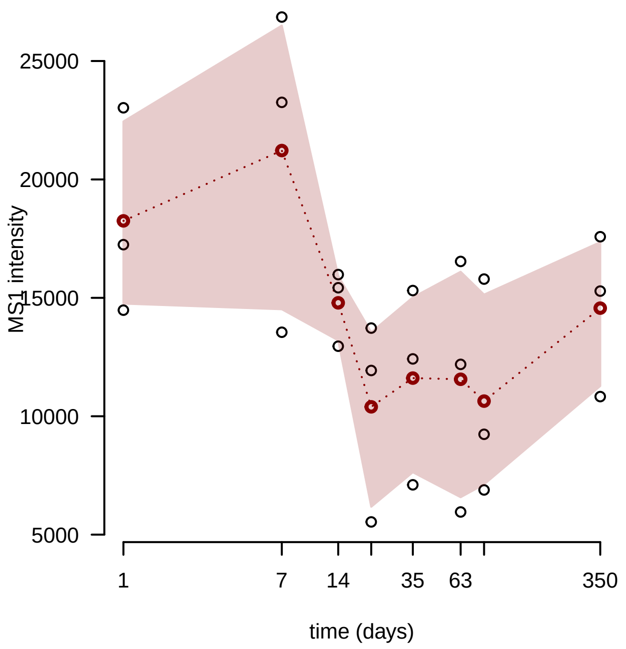

Q5NC05
GN=Ttf2
transcription termination factor, RNA po ...

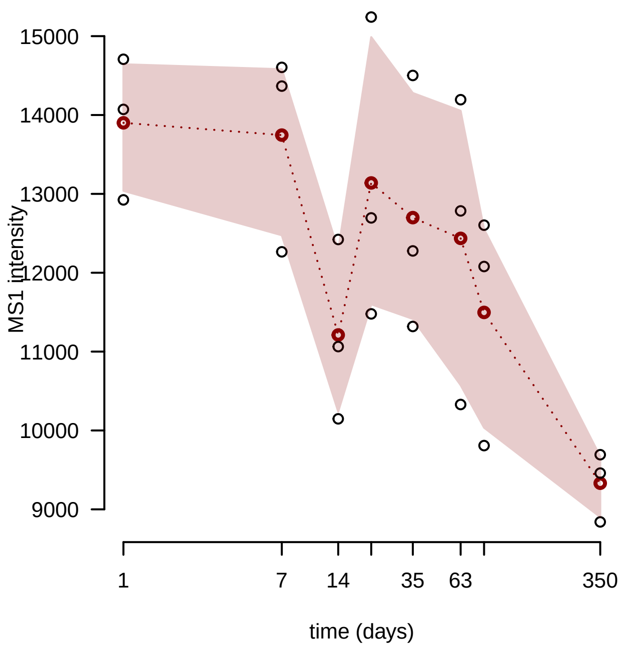

Q5NCC3
GN=Trim41
tripartite motif−containing 41

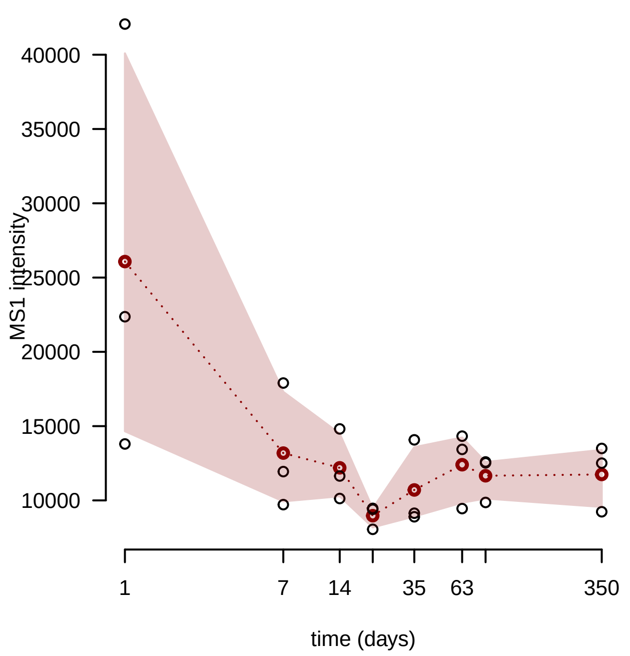

Q5NCE8
GN=Mrs2
MRS2 magnesium transporter

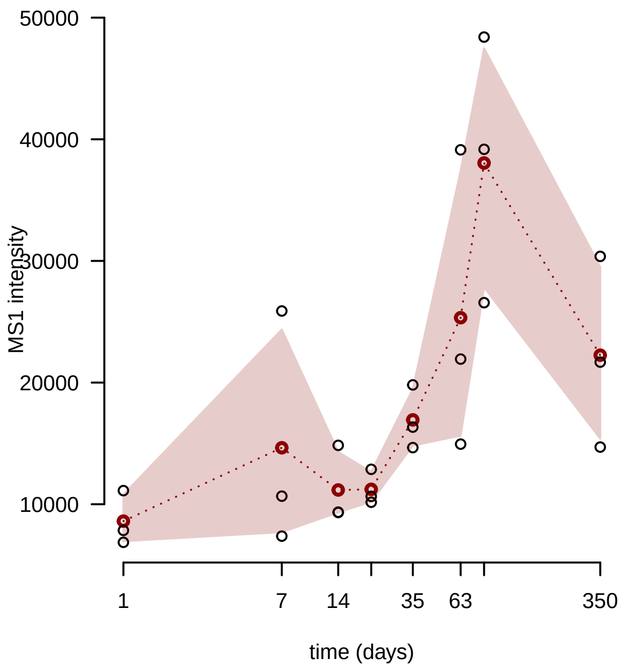

Q5NCF2
GN=Trappc1
trafficking protein particle complex 1

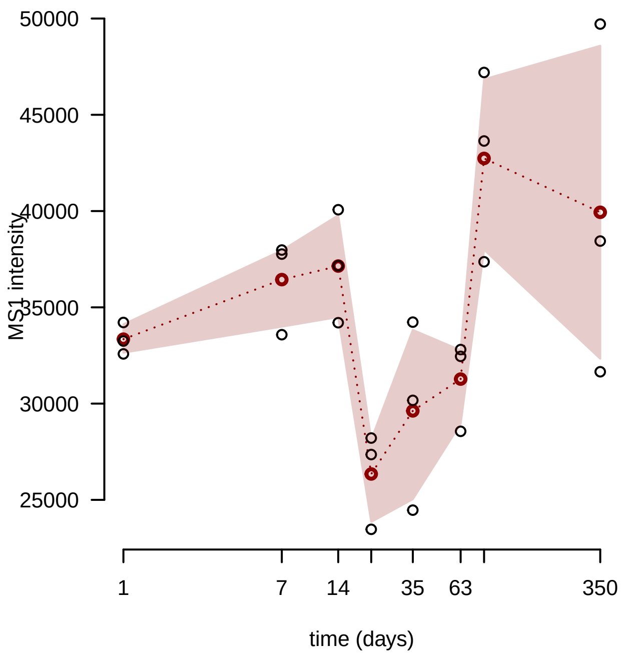

Q5NCI0
GN=Urgcp
upregulator of cell proliferation

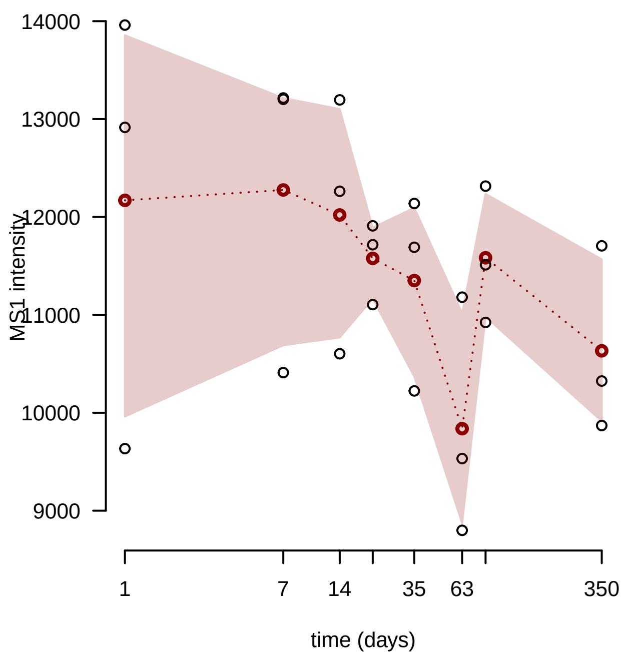

Q5NCQ5
GN=Dph1
diphthamide biosynthesis 1

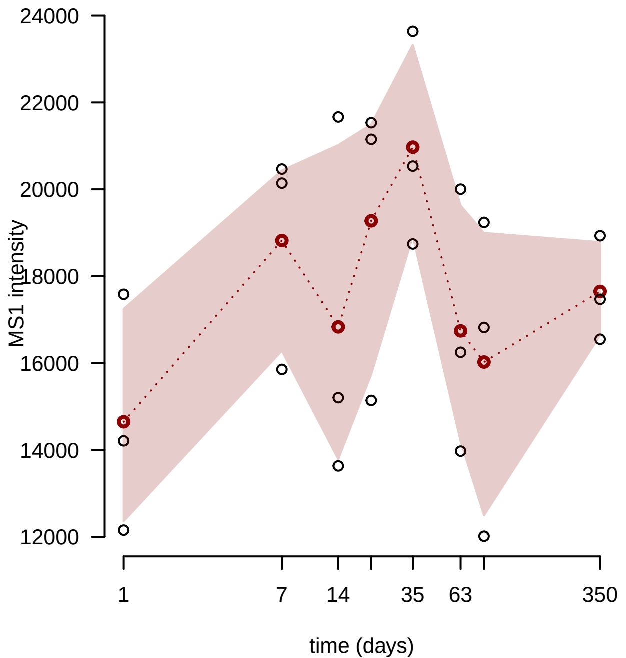

Q5NCR9
GN=Nsrp1
nuclear speckle regulatory protein 1

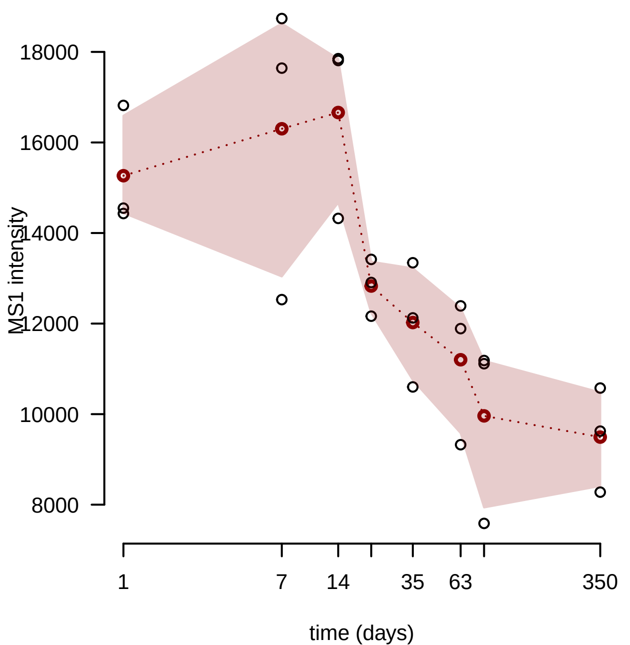

Q5NCS9
GN=Mief2
mitochondrial elongation factor 2

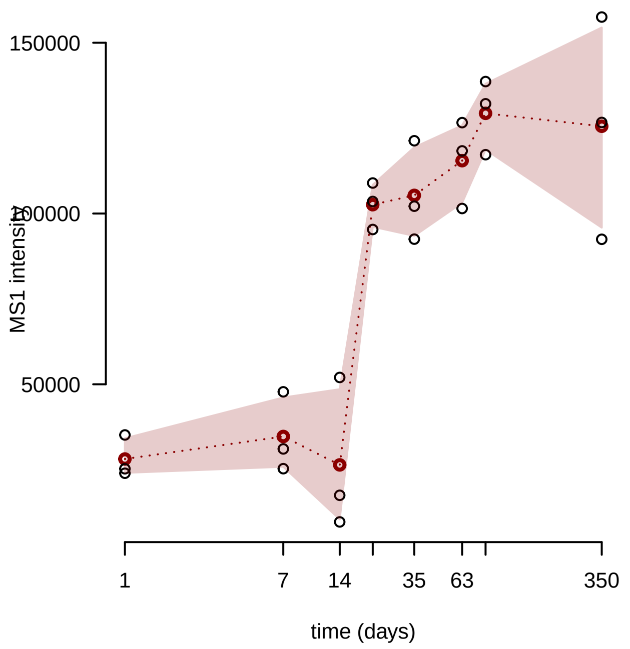

Q5NCX5
GN=Neurl4
neuralized E3 ubiquitin protein ligase 4

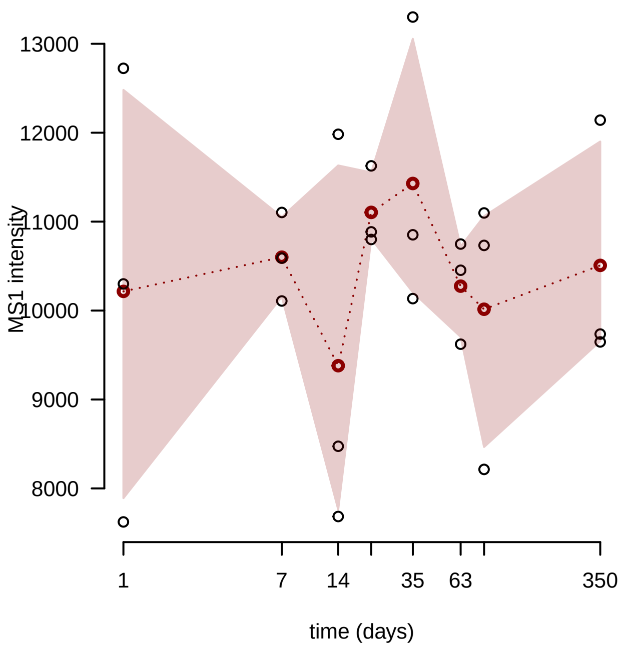

Q5ND34
GN=Wdr81
WD repeat domain 81

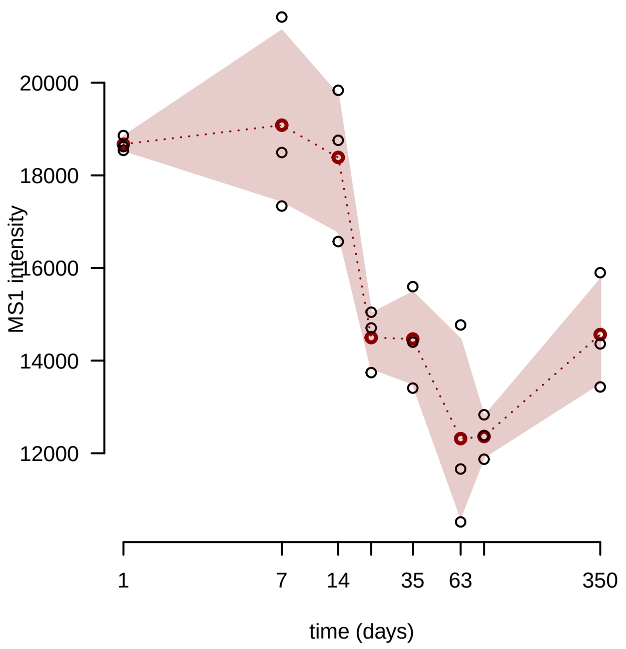

Q5ND52
GN=Mrm3
mitochondrial rRNA methyltransferase 3

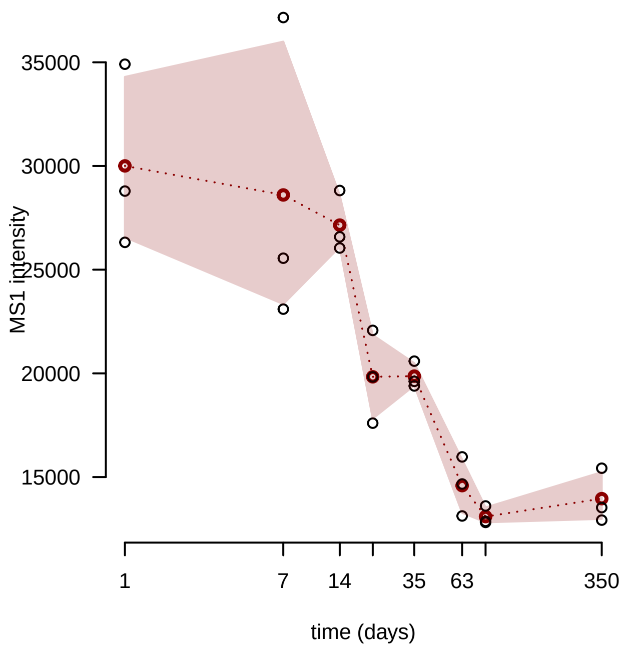

Q5ND56
GN=Fam57a

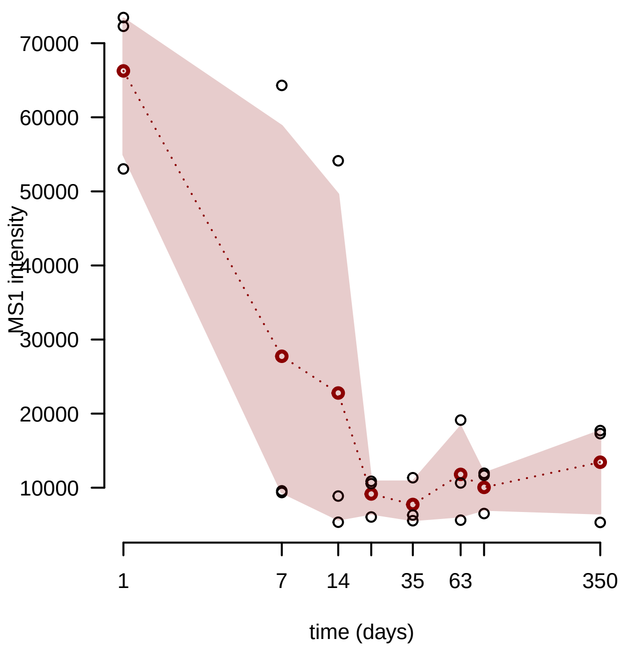

Q5PR68
GN=Cep112
centrosomal protein 112

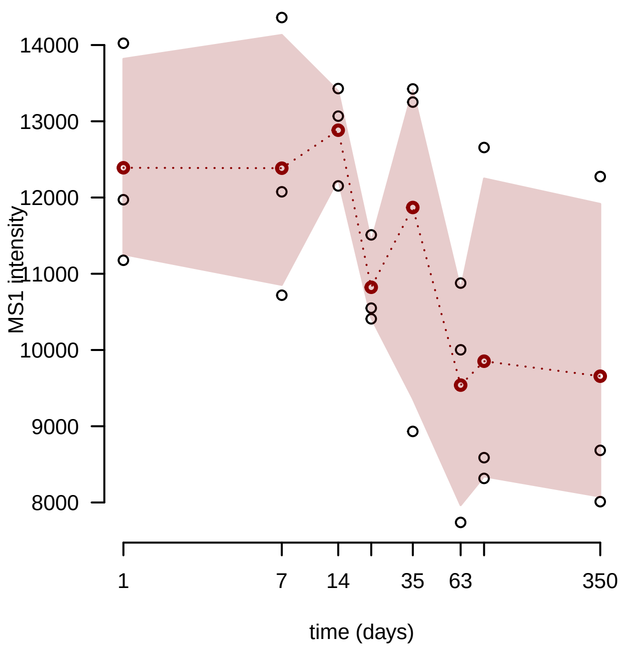

Q5PRE5
GN=Proser1
proline and serine rich 1

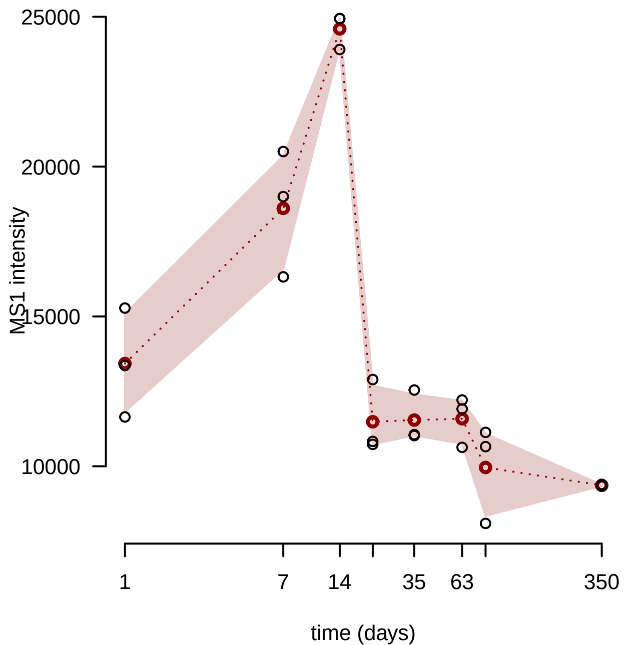

Q5PRF0
GN=Heatr5a
HEAT repeat containing 5A

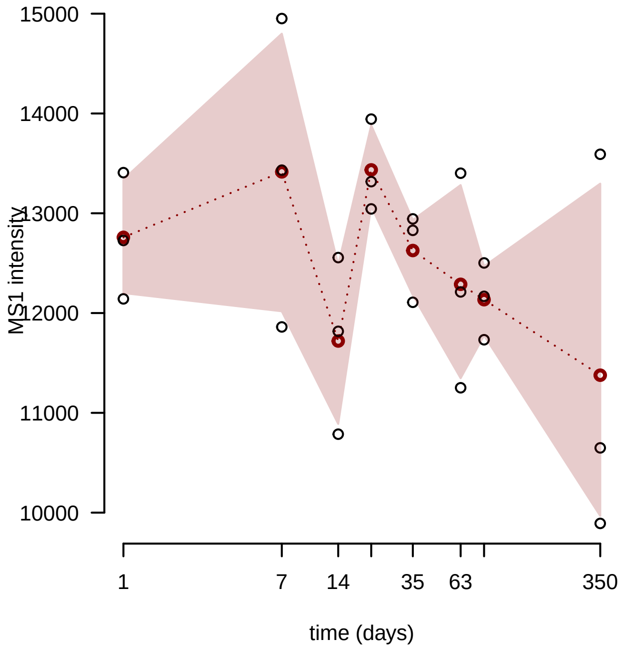

Q5PSV9
GN=Mdc1
mediator of DNA damage checkpoint 1

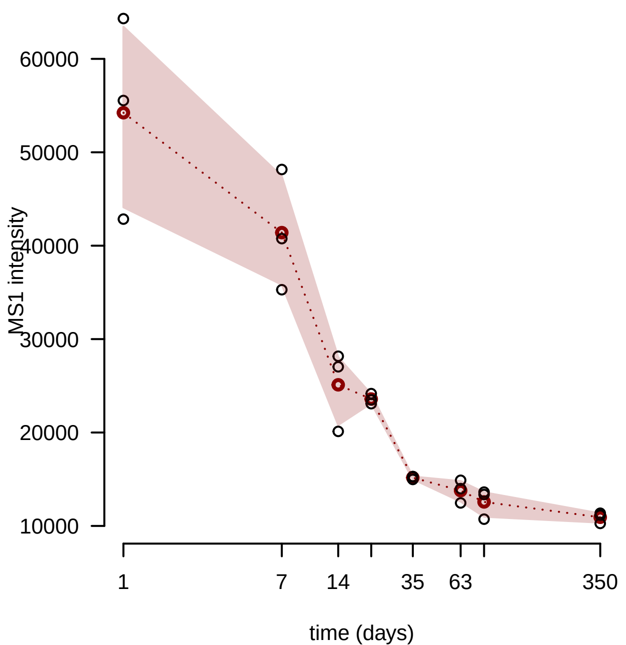

Q5PT53
GN=Slc10a7
solute carrier family 10 (sodium/bile ac ...

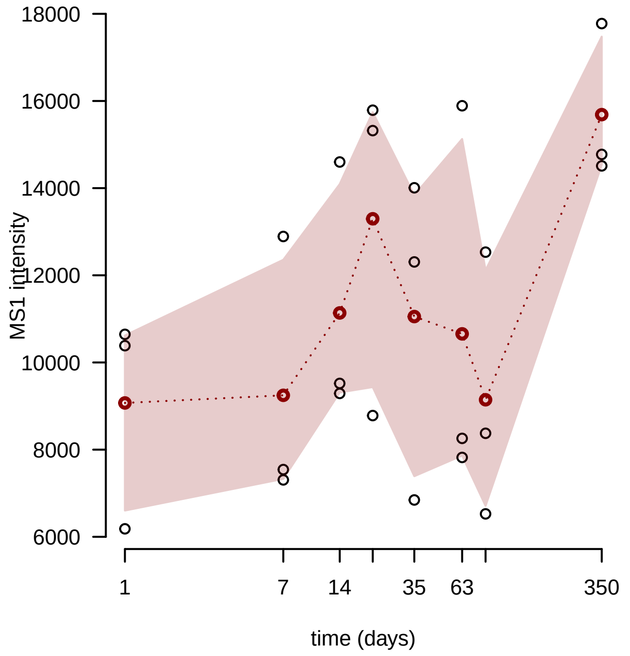

Q5QNQ6
GN=Osbp2
oxysterol binding protein 2

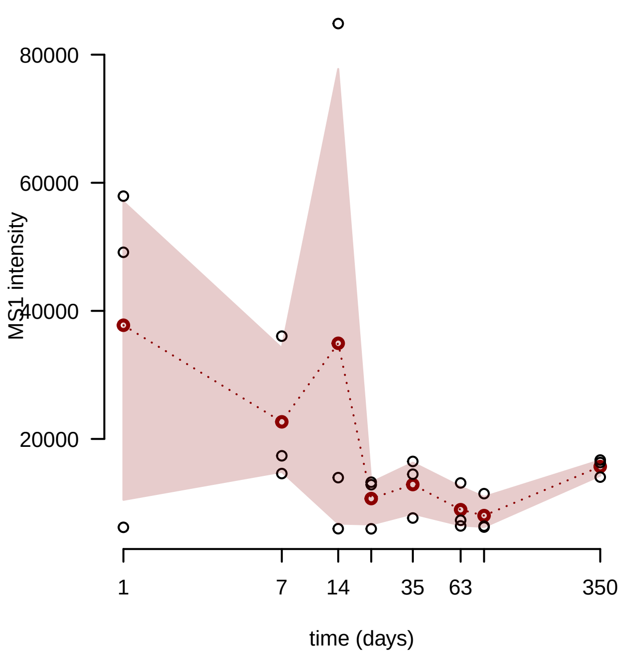

Q5RJ54
GN=Zscan26
zinc finger and SCAN domain containing 2 ...

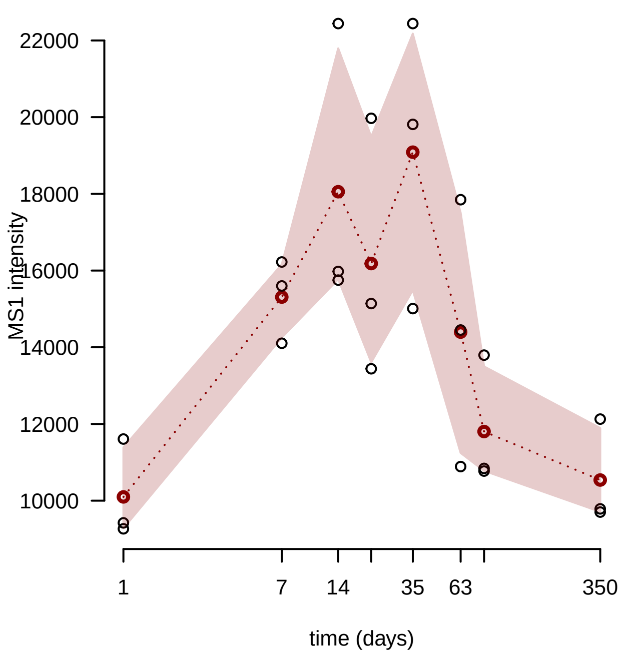

Q5RJG1
GN=Nol10
nucleolar protein 10

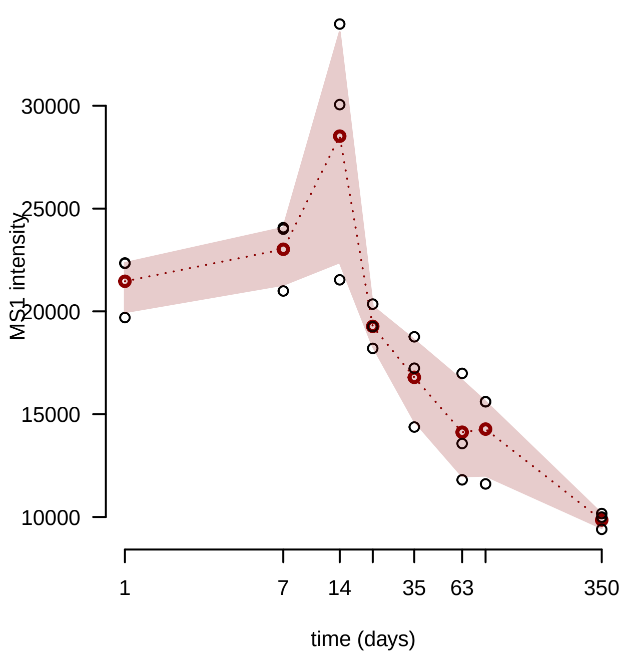

Q5RJG7
GN=Crppa
CDP−L−ribitol pyrophosphorylase A

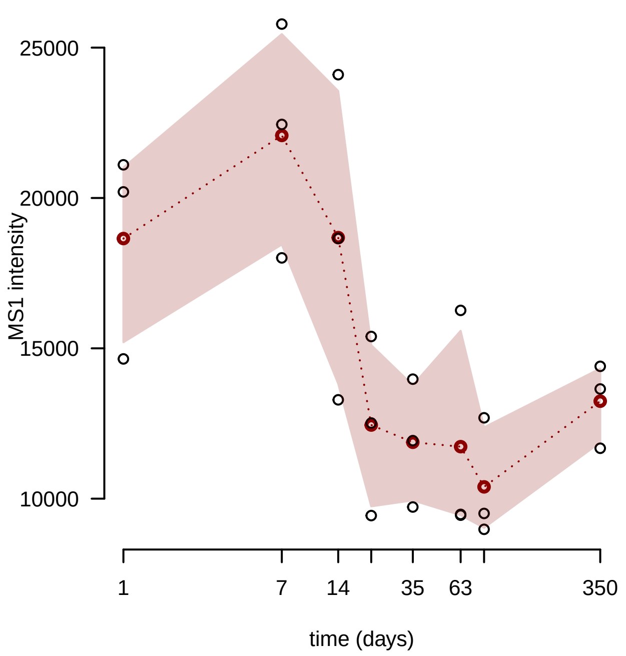

Q5RJH2
GN=Mctp2
multiple C2 domains, transmembrane 2

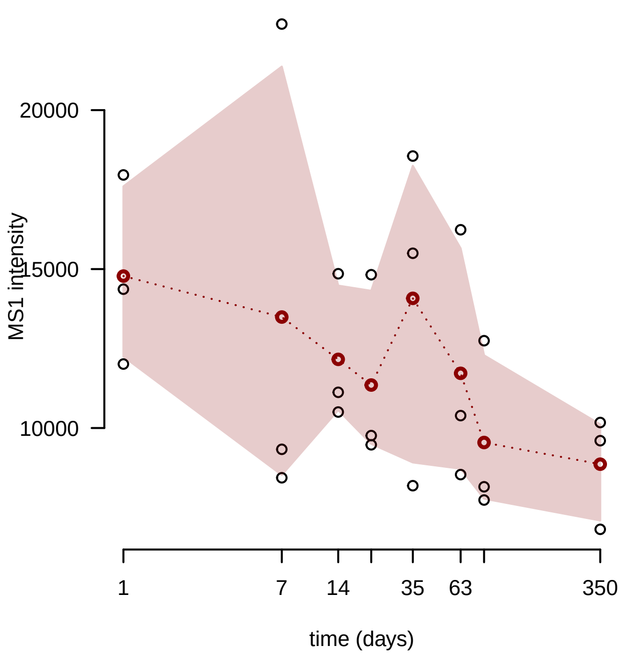

Q5RJH6
GN=Smg7
SMG7 nonsense mediated mRNA decay factor

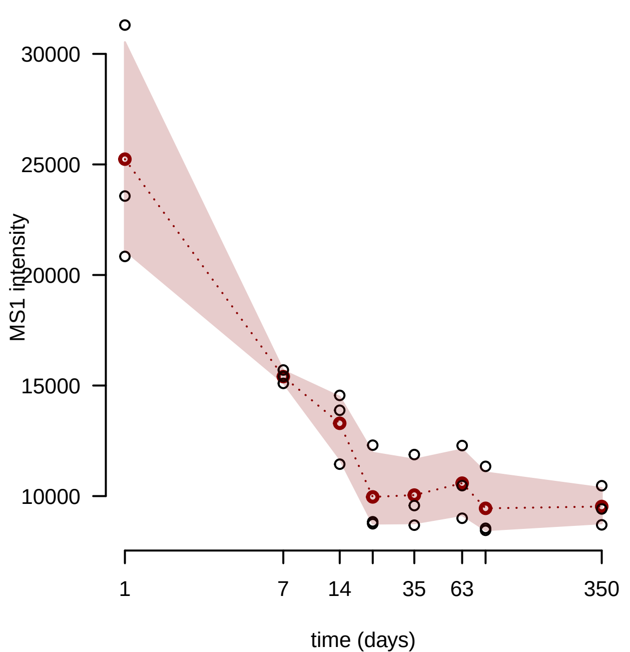

Q5RKS2
GN=Smim7
small integral membrane protein 7

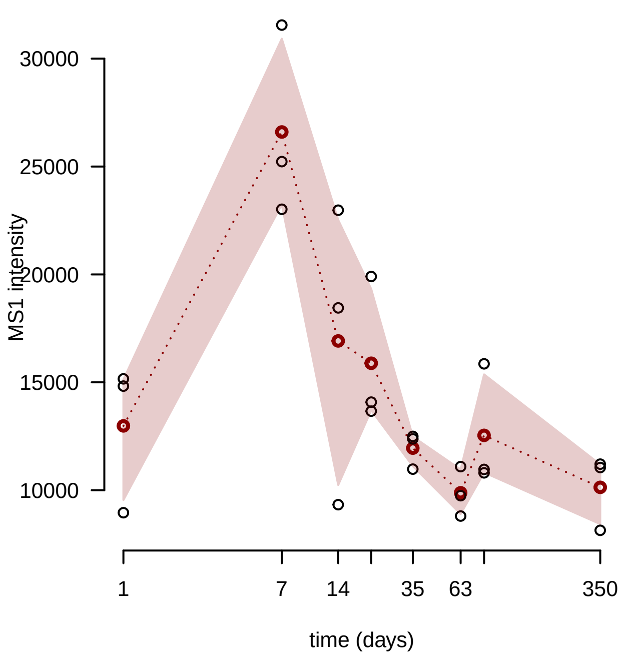

Q5RKZ7
GN=Mocs1
molybdenum cofactor synthesis 1

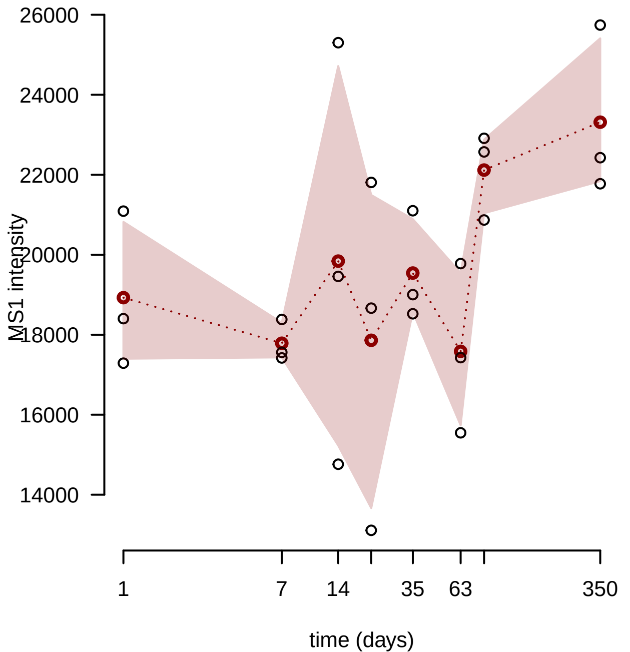

Q5RL51
GN=Gstcd
glutathione S−transferase, C−terminal do ...

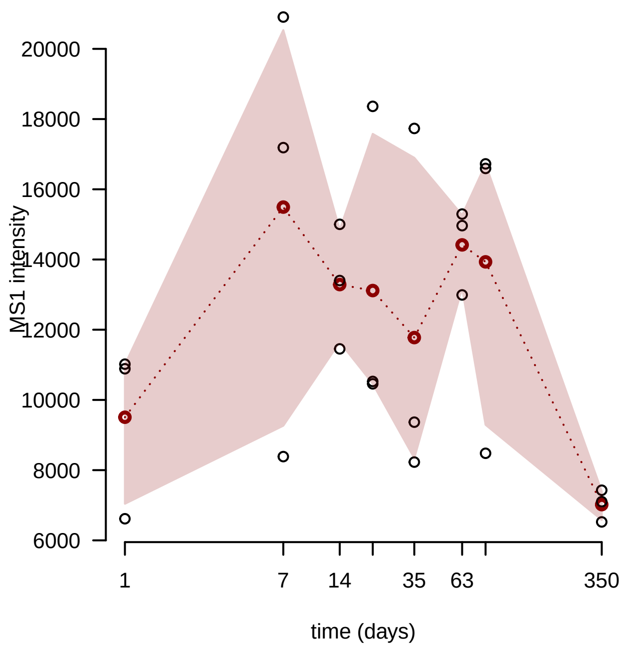

Q5RL79
GN=Krtcap2
keratinocyte associated protein 2

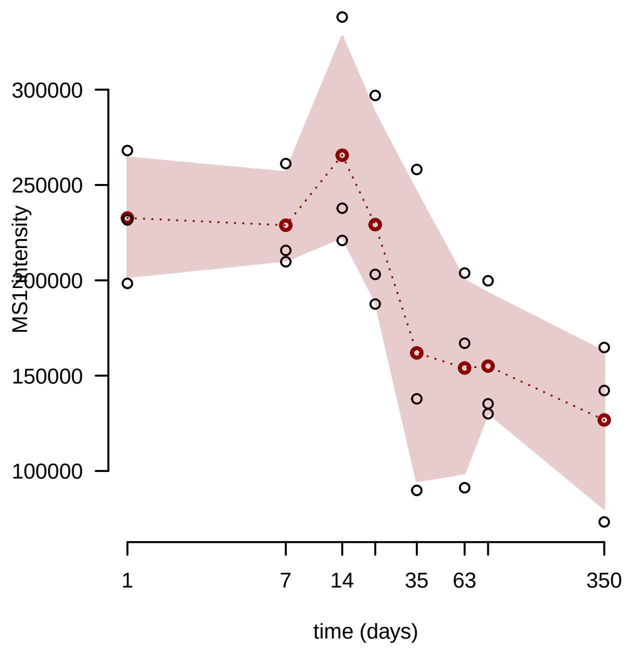

Q5S006
GN=Lrrk2
leucine−rich repeat kinase 2

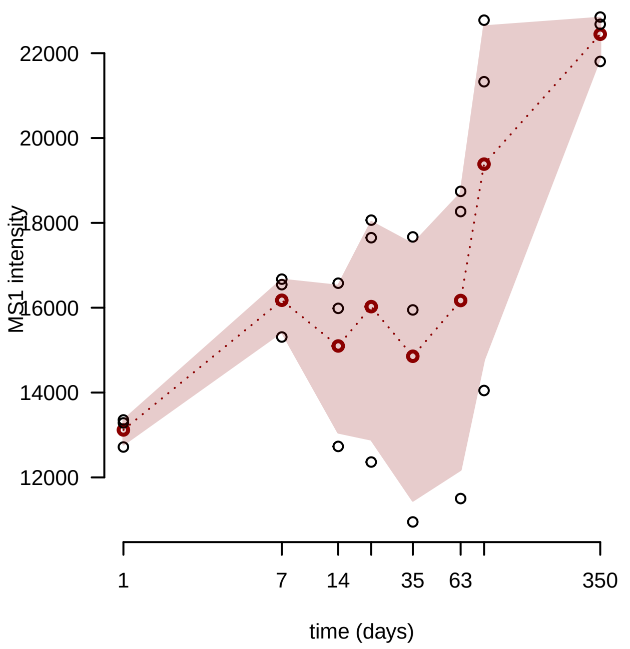

Q5SF07
GN=Igf2bp2
insulin−like growth factor 2 mRNA bindin ...

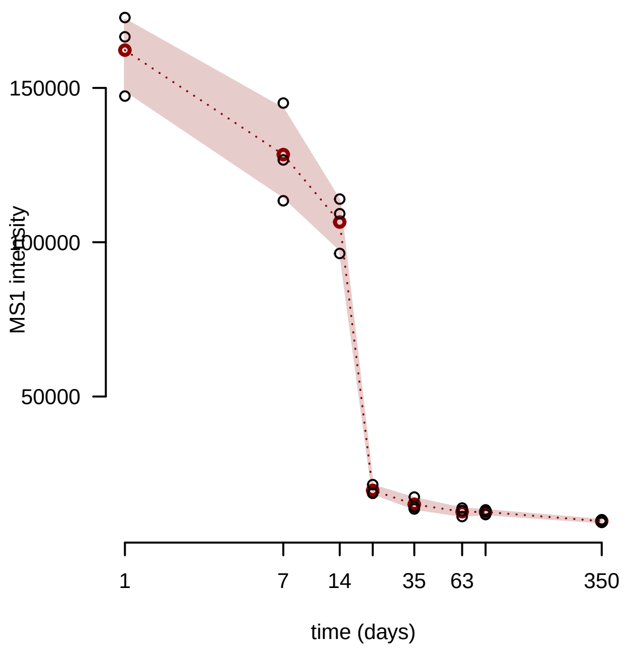

Q5SFM8
GN=Rbm27
RNA binding motif protein 27

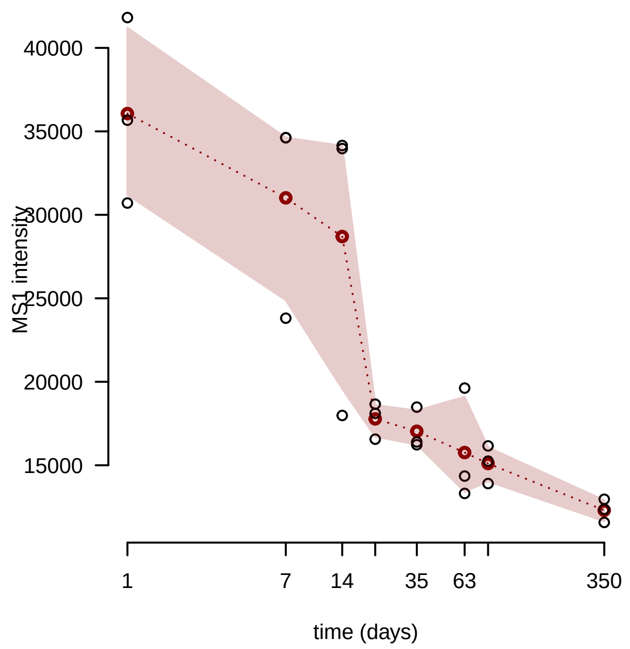

Q5SNZ0
GN=Ccdc88a
coiled coil domain containing 88A

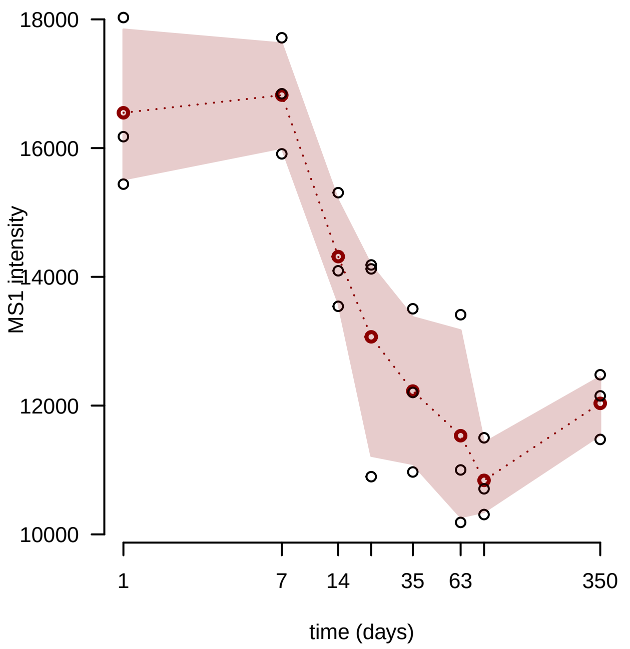

Q5SPL2
GN=Phf12
PHD finger protein 12

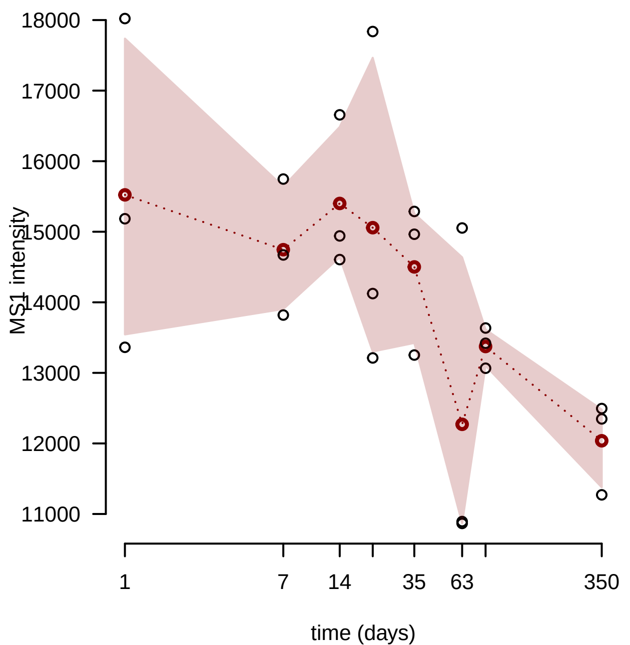

Q5SQF8
GN=Sap30l
SAP30−like

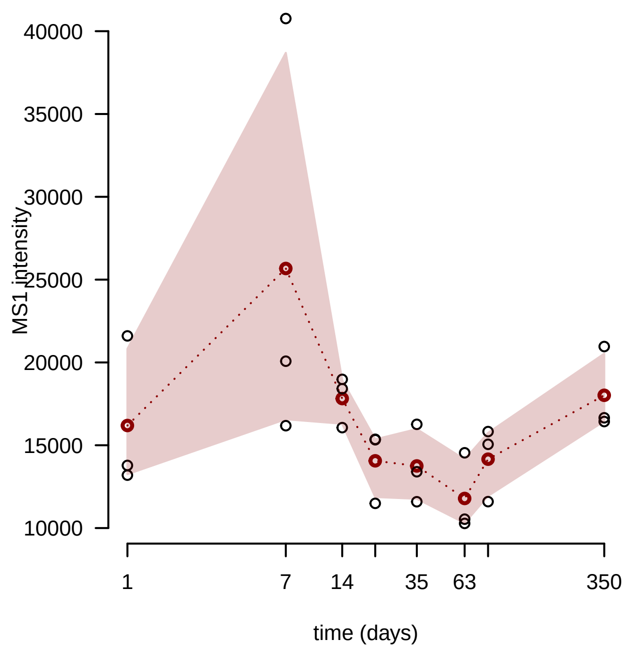

Q5SQX6
GN=Cyfip2
cytoplasmic FMR1 interacting protein 2

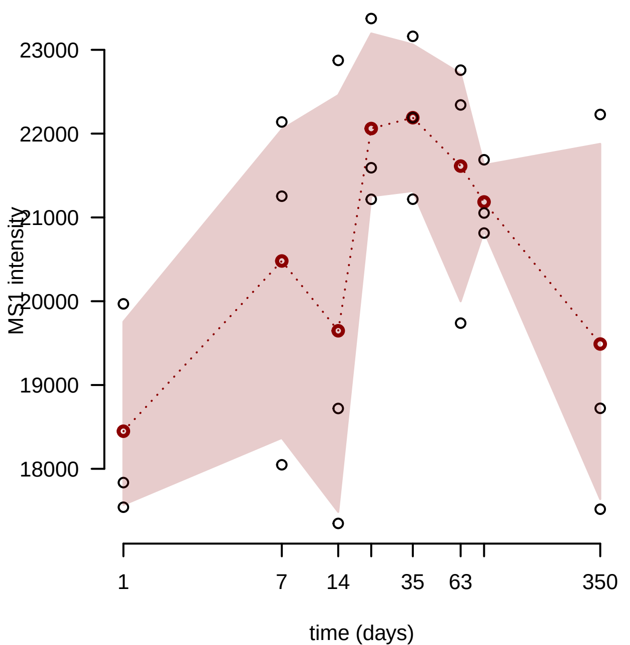

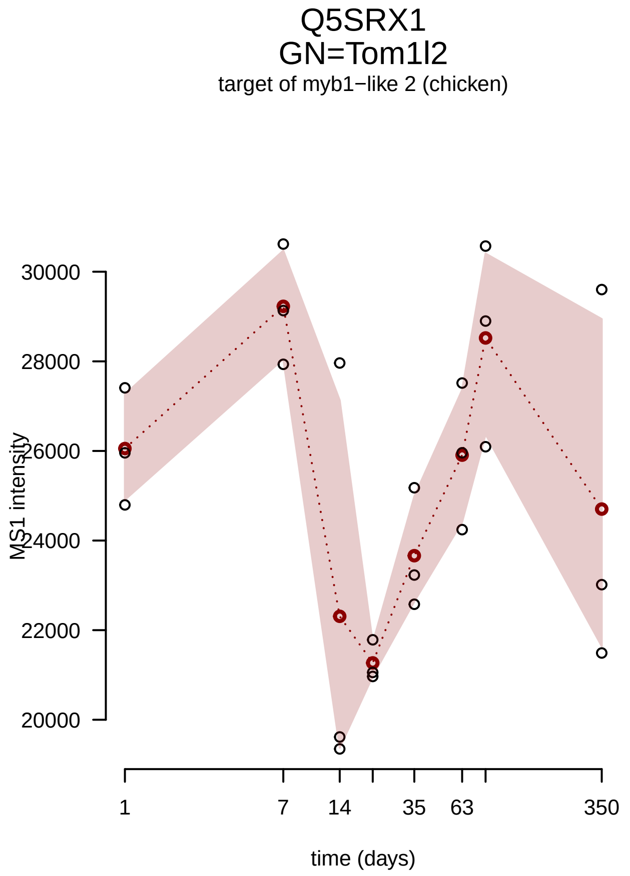
Q5SRX1
GN=Tom1l2
target of myb1−like 2 (chicken)
MS1 intensity
20000
22000
24000
26000
28000
30000
1
7
14
35
63
350
time (days)

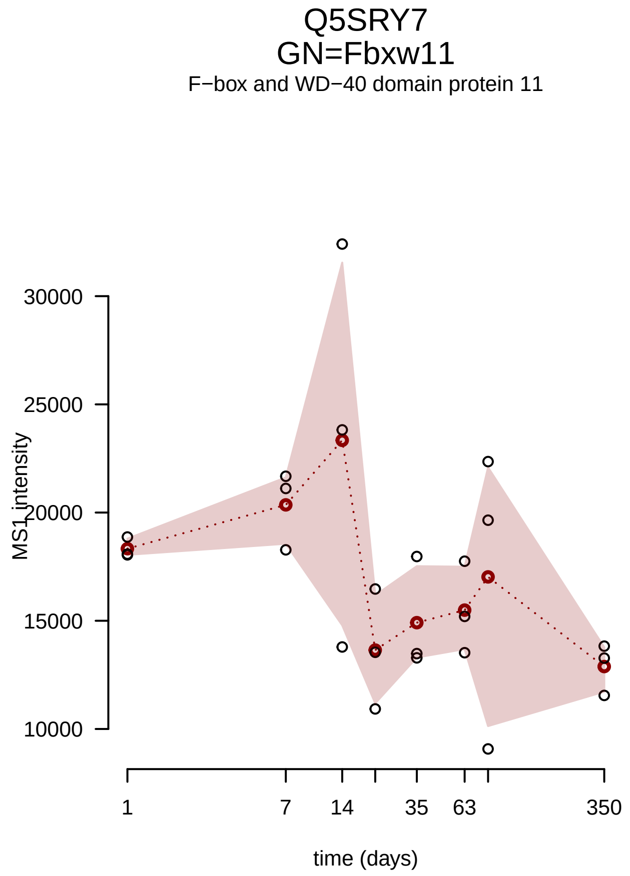
Q5SRY7
GN=Fbxw11
F−box and WD−40 domain protein 11
MS1 intensity
10000
15000
20000
25000
30000
1
7
14
35
63
350
time (days)

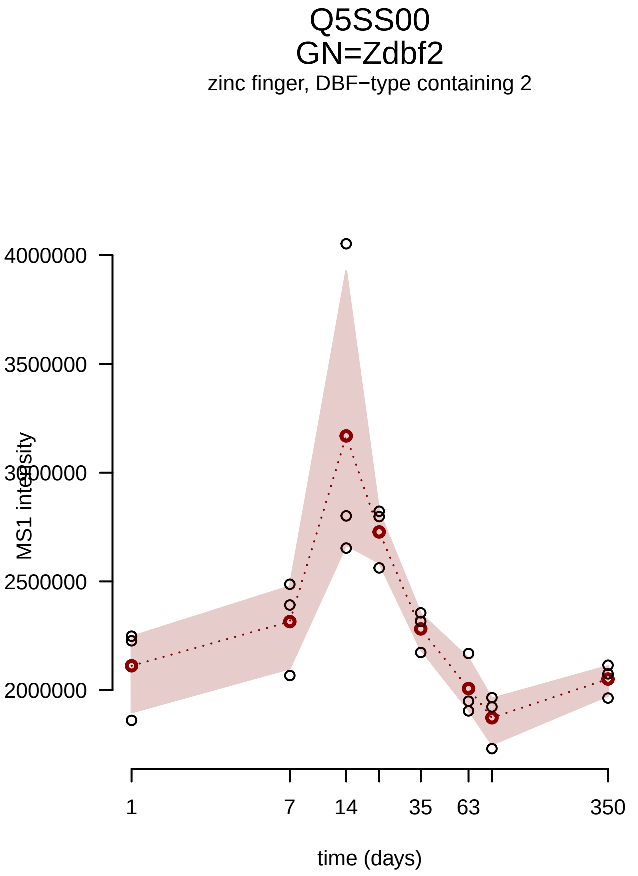
Q5SS00
GN=Zdbf2
zinc finger, DBF−type containing 2
MS1 intensity
2000000
2500000
3000000
3500000
4000000
1
7
14
35
63
350
time (days)

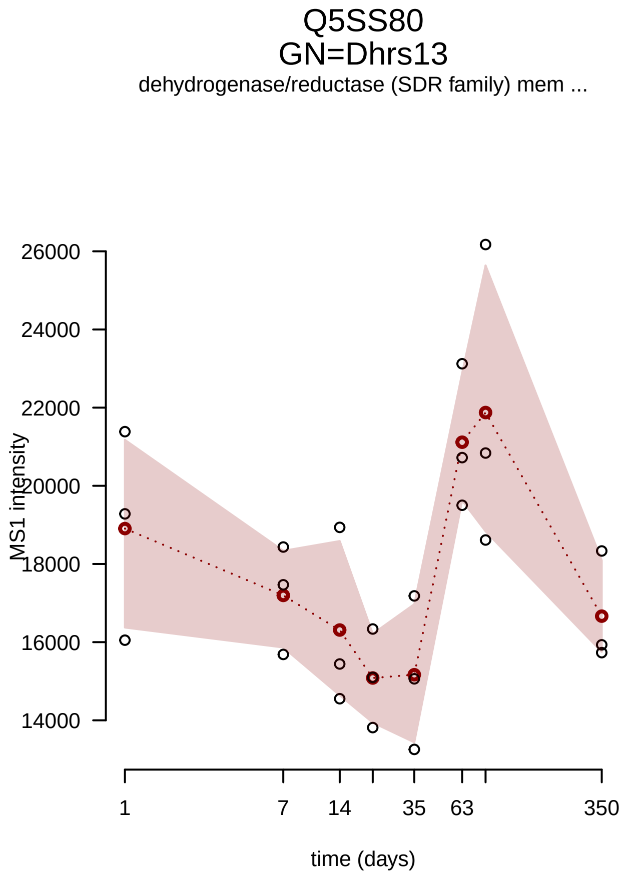
Q5SS80
GN=Dhrs13
dehydrogenase/reductase (SDR family) mem ...
MS1 intensity
14000
16000
18000
20000
22000
24000
26000
1
7
14
35
63
350
time (days)

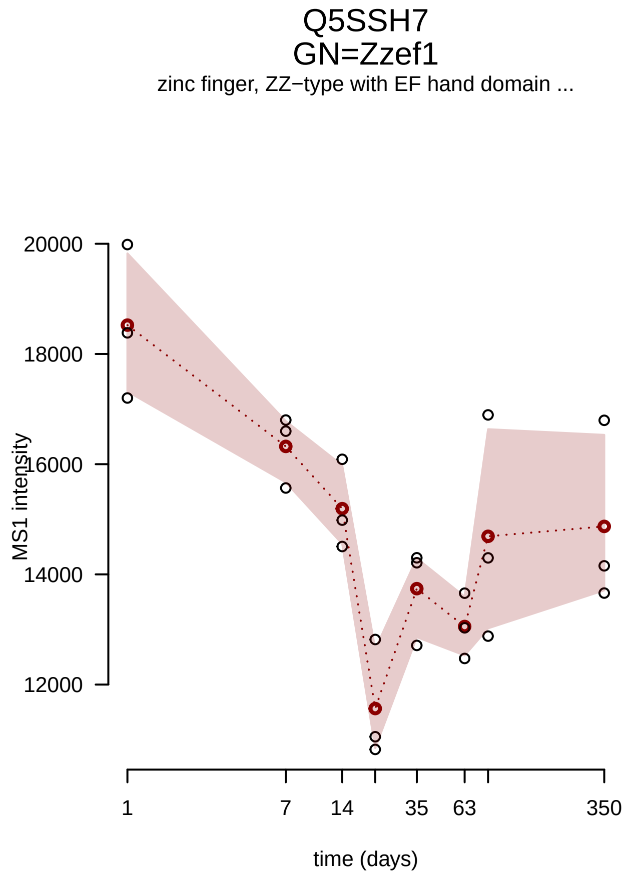
Q5SSH7
GN=Zzef1
zinc finger, ZZ−type with EF hand domain ...
MS1 intensity
12000
14000
16000
18000
20000
1
7
14
35
63
350
time (days)

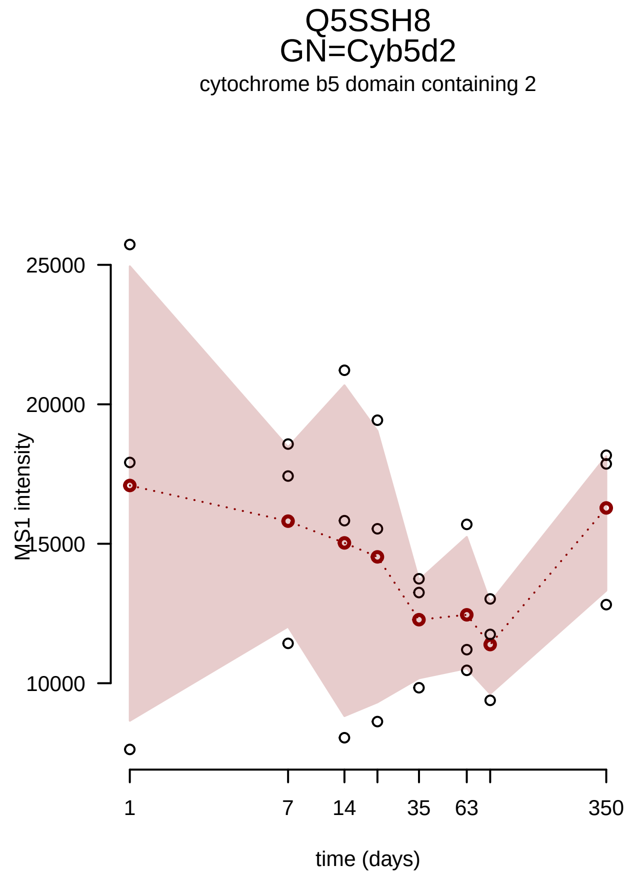
Q5SSH8
GN=Cyb5d2
cytochrome b5 domain containing 2
MS1 intensity
10000
15000
20000
25000
1
7
14
35
63
350
time (days)

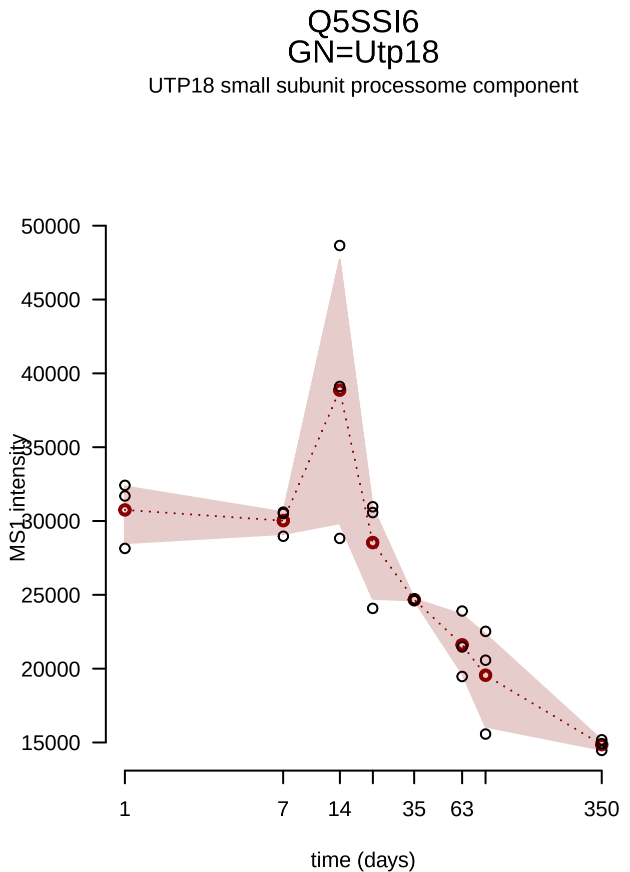
Q5SSI6
GN=Utp18
UTP18 small subunit processome component
MS1 intensity
15000
20000
25000
30000
35000
40000
45000
50000
1
7
14
35
63
350
time (days)

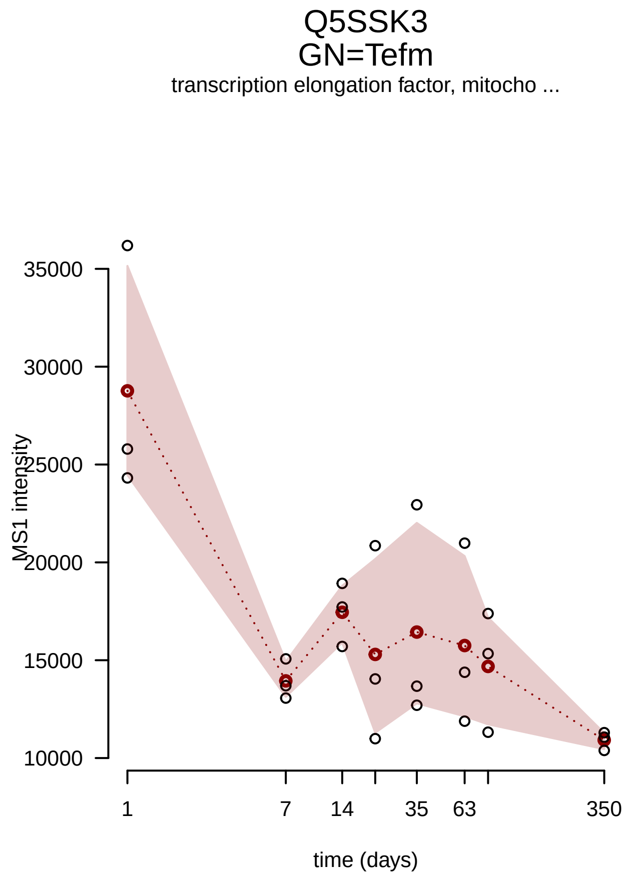
Q5SSK3
GN=Tefm
transcription elongation factor, mitocho ...
MS1 intensity
10000
15000
20000
25000
30000
35000
1
7
14
35
63
350
time (days)

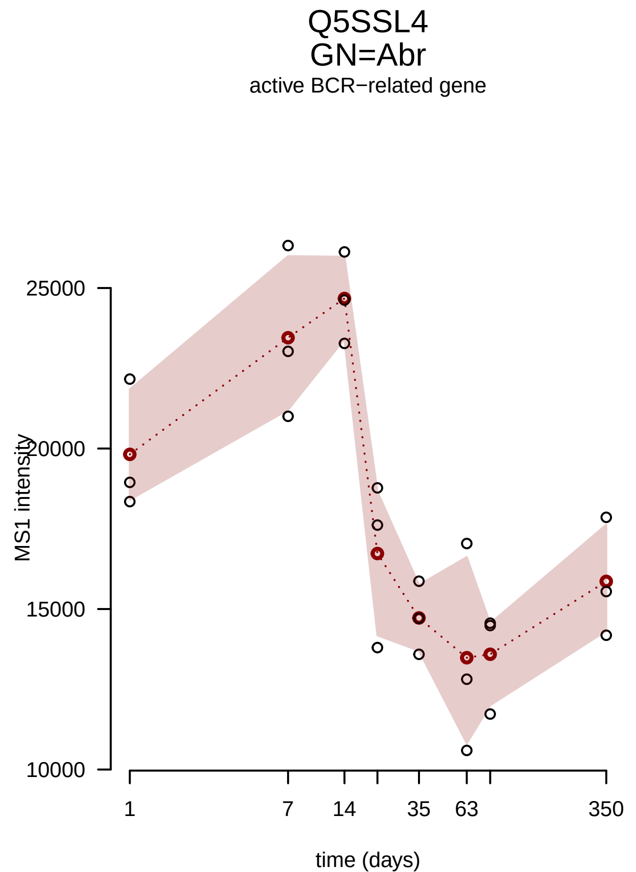
Q5SSL4
GN=Abr
active BCR−related gene
MS1 intensity
10000
15000
20000
25000
1
7
14
35
63
350
time (days)

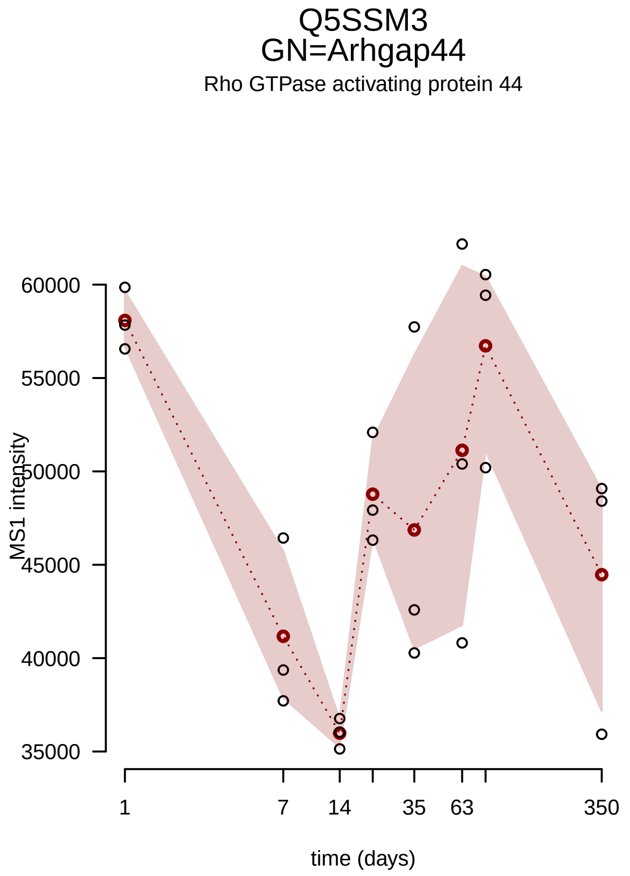
Q5SSM3
GN=Arhgap44
Rho GTPase activating protein 44
MS1 intensity
60000
55000
50000
45000
40000
35000
1
7
14
35
63
350
time (days)

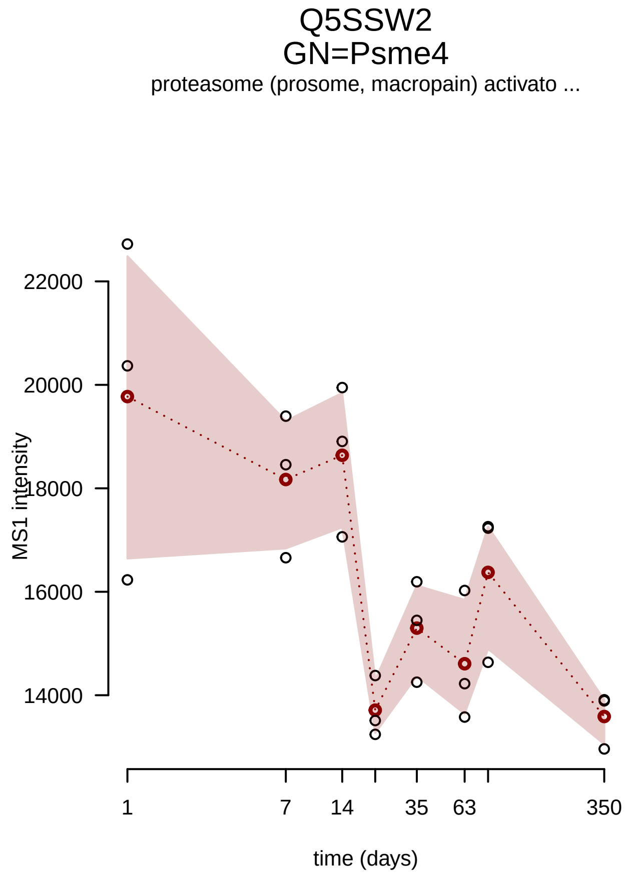
Q5SSW2
GN=Psme4
proteasome (prosome, macropain) activato ...
MS1 intensity
22000
20000
18000
16000
14000
1
7
14
35
63
350
time (days)

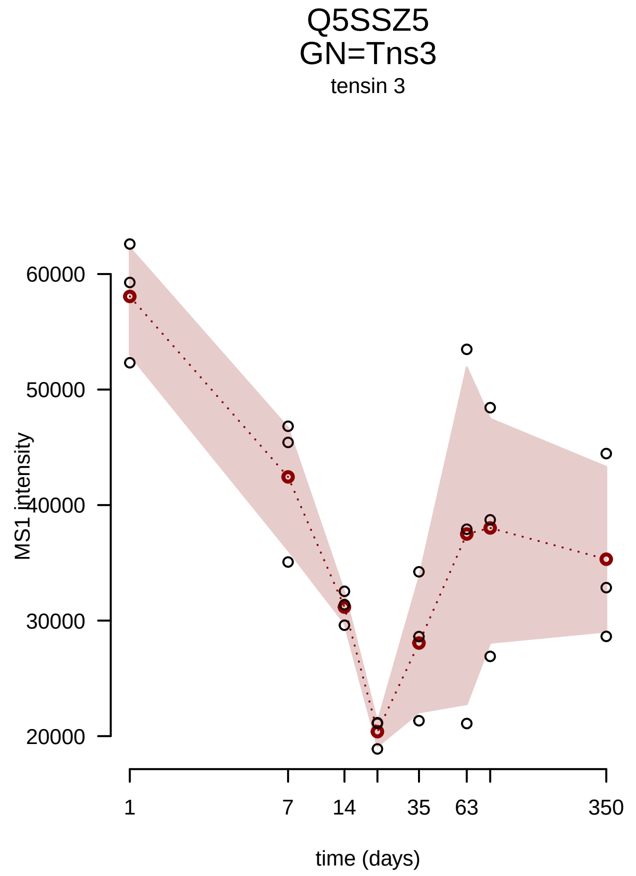
Q5SSZ5
GN=Tns3
tensin 3
MS1 intensity
60000
50000
40000
30000
20000
1
7
14
35
63
350
time (days)

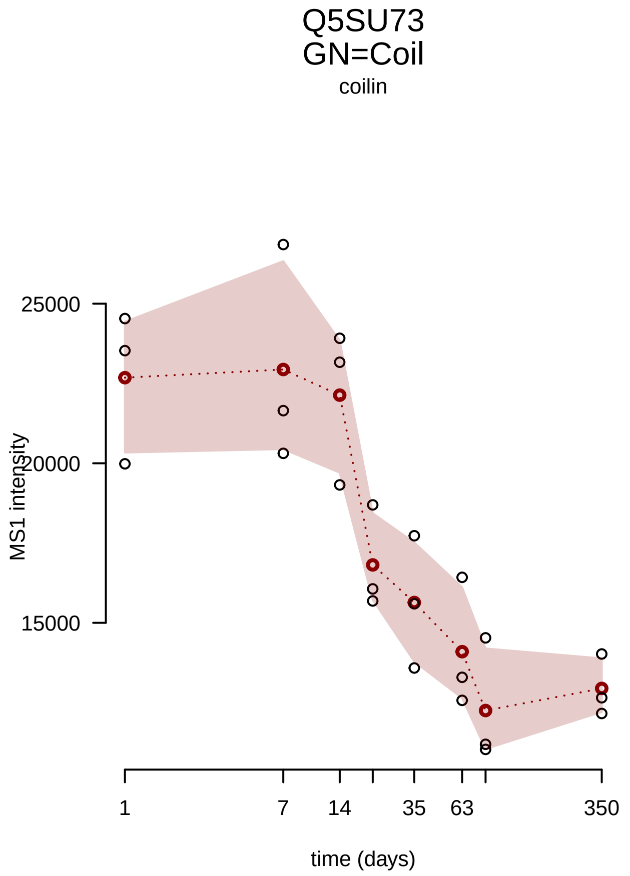
Q5SU73
GN=Coil
coilin
MS1 intensity
25000
20000
15000
1
7
14
35
63
350
time (days)

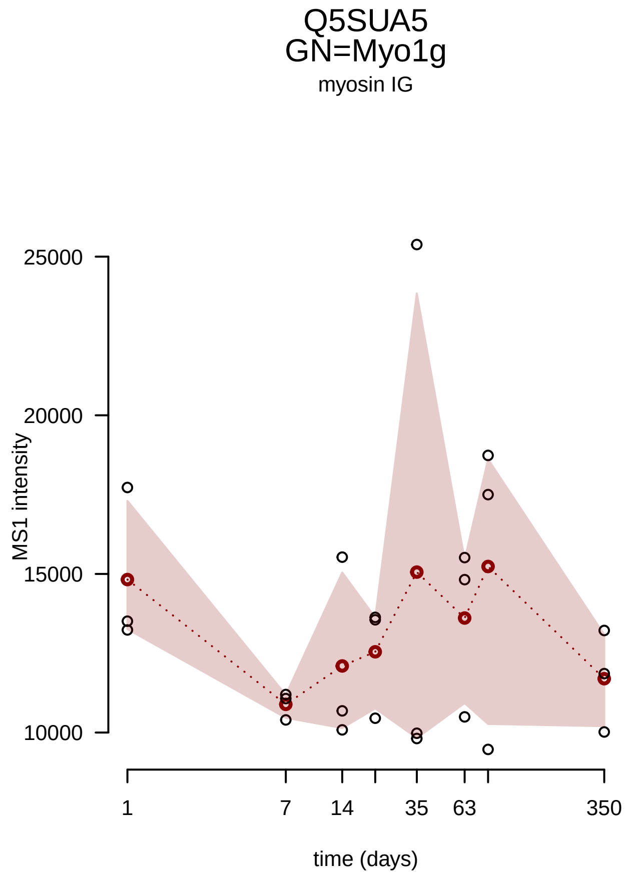
Q5SUA5
GN=Myo1g
myosin IG
MS1 intensity
25000
20000
15000
10000
1
7
14
35
63
350
time (days)

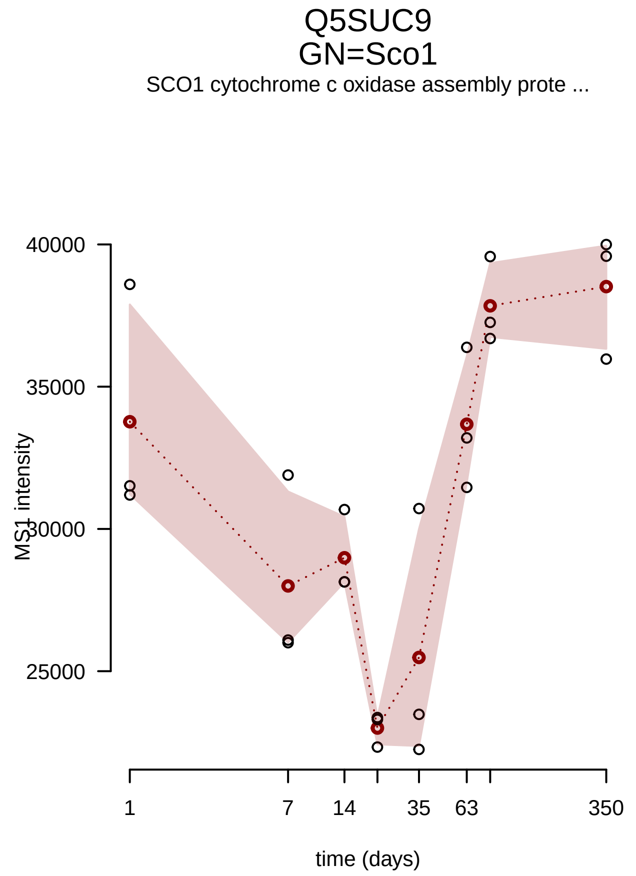
Q5SUC9
GN=Sco1
SCO1 cytochrome c oxidase assembly prote ...
MS1 intensity
40000
35000
30000
25000
1
7
14
35
63
350
time (days)

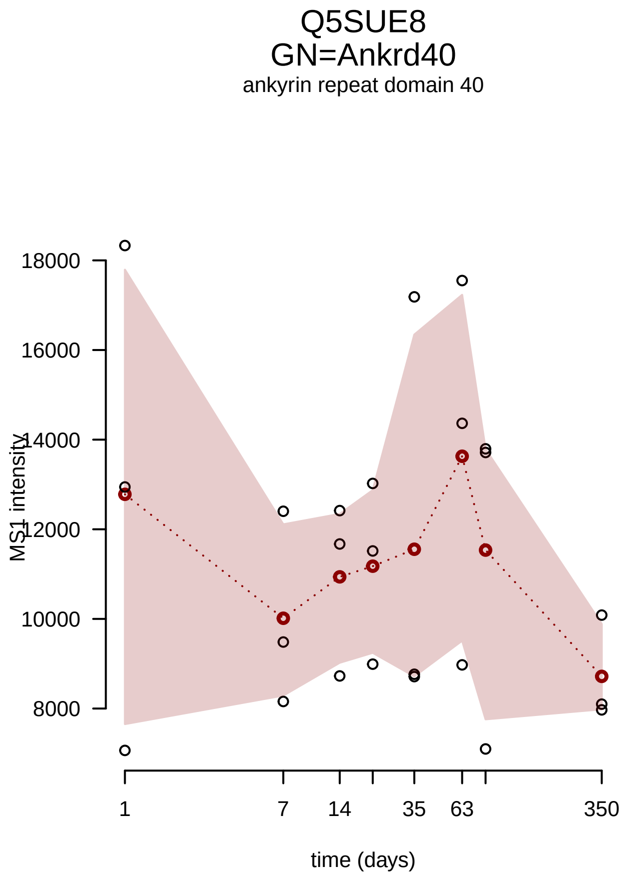
Q5SUE8
GN=Ankrd40
ankyrin repeat domain 40
MS1 intensity
18000
16000
14000
12000
10000
8000
1
7
14
35
63
350
time (days)

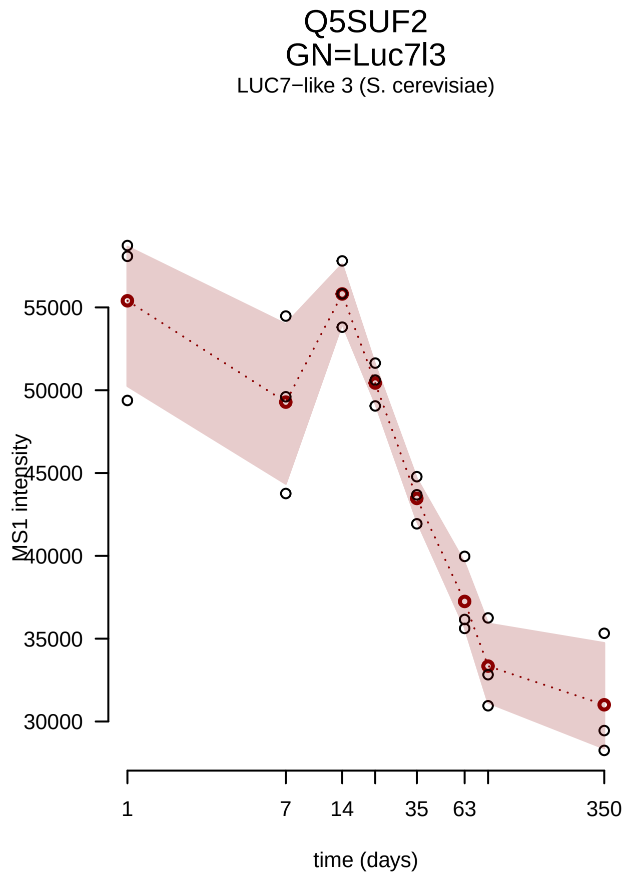
Q5SUF2
GN=Luc7l3
LUC7−like 3 (S. cerevisiae)
MS1 intensity
55000
50000
45000
40000
35000
30000
1
7
14
35
63
350
time (days)

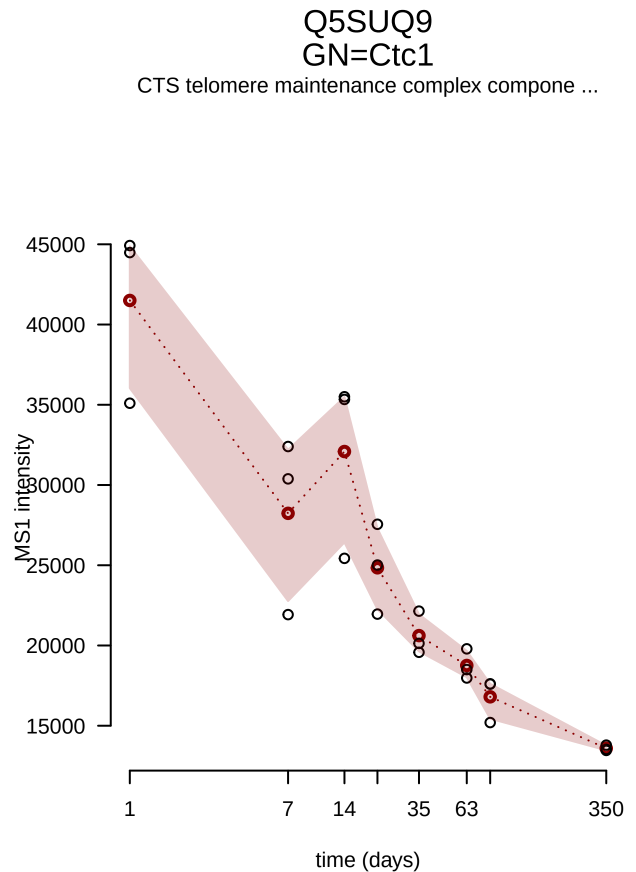
Q5SUQ9
GN=Ctc1
CTS telomere maintenance complex compone ...
MS1 intensity
45000
40000
35000
30000
25000
20000
15000
1
7
14
35
63
350
time (days)

Q5SUQ9
GN=Ctc1
CTS telomere maintenance complex compone ...

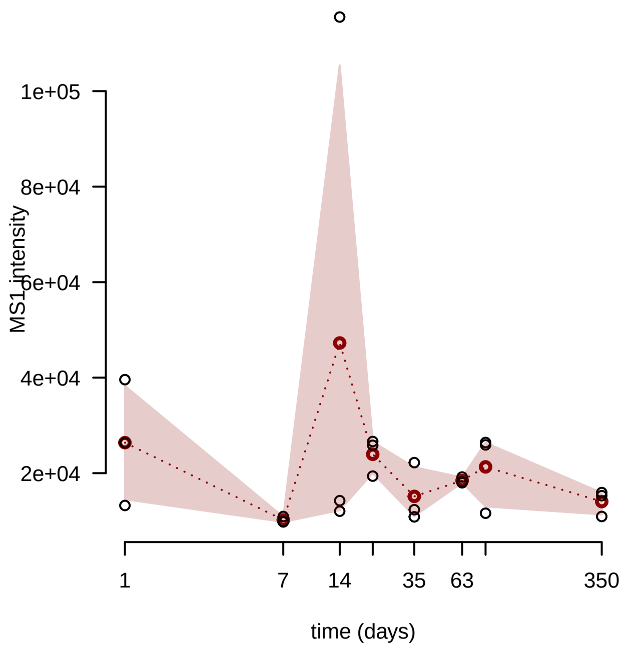

Q5SUR0
GN=Pfas
phosphoribosylformylglycinamidine syntha ...

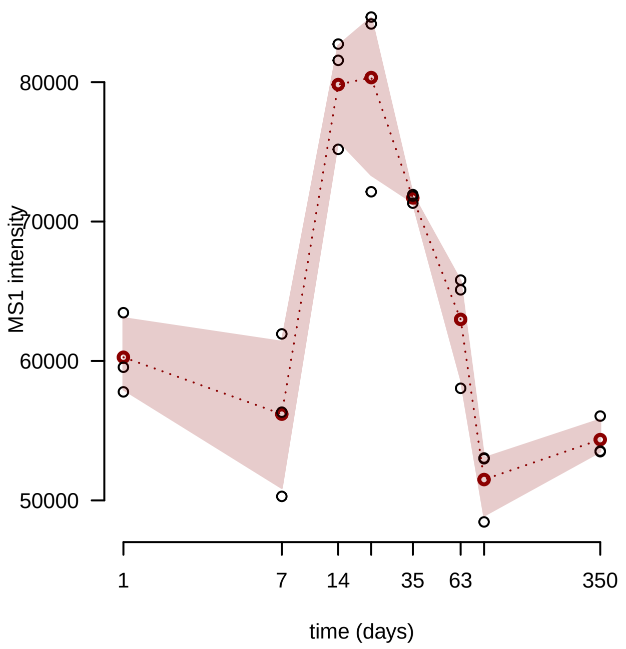

Q5SV80
GN=Myo19
myosin XIX

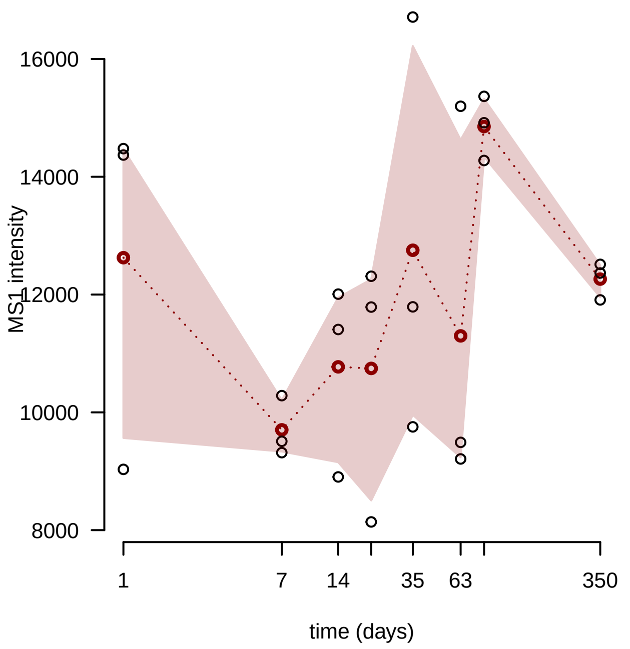

Q5SV85
GN=Synrg
synergin, gamma

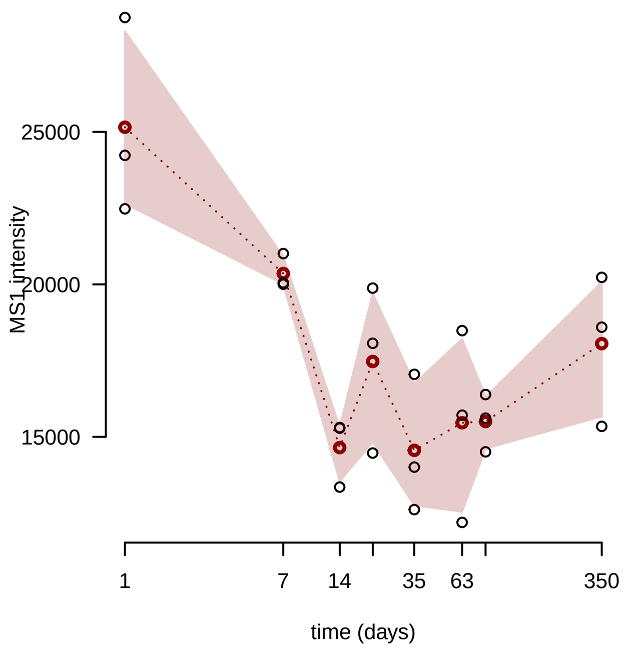

Q5SVD0
GN=Rflnb
refilin B

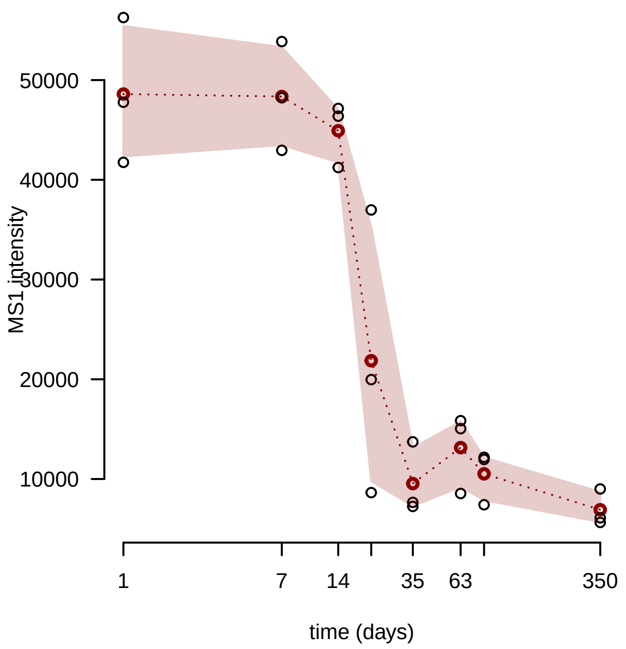

Q5SVQ0
GN=Kat7
K(lysine) acetyltransferase 7

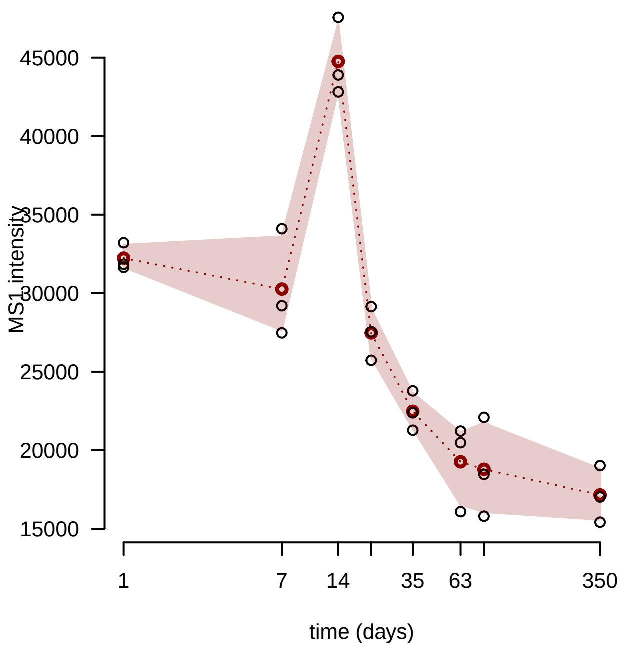

Q5SVR0
GN=Tbc1d9b
TBC1 domain family, member 9B

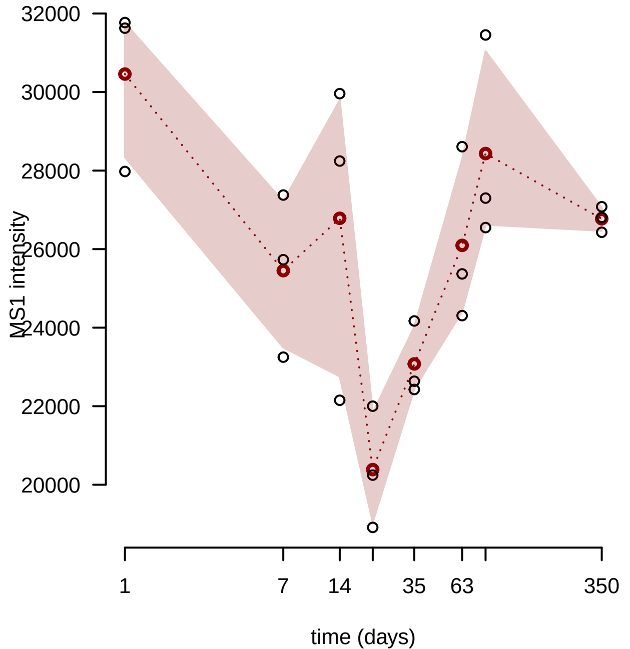

Q5SW19
GN=Cluh
clustered mitochondria (cluA/CLU1) homol ...

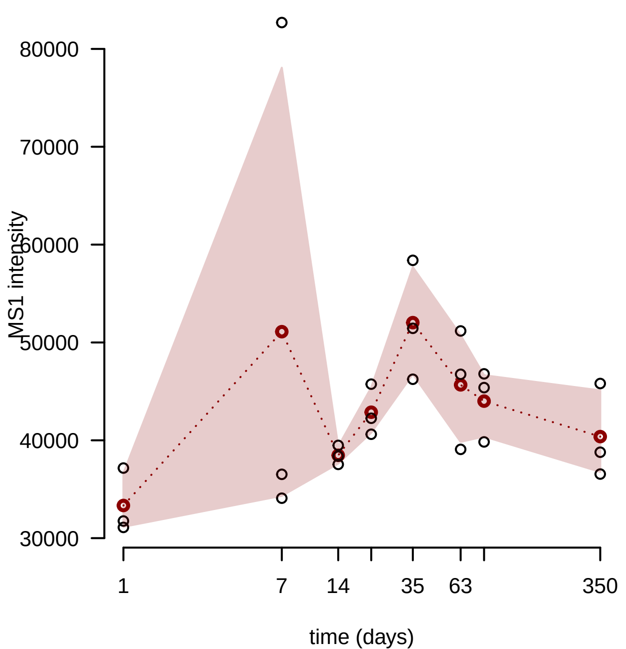

Q5SWD9
GN=Tsr1
TSR1 20S rRNA accumulation

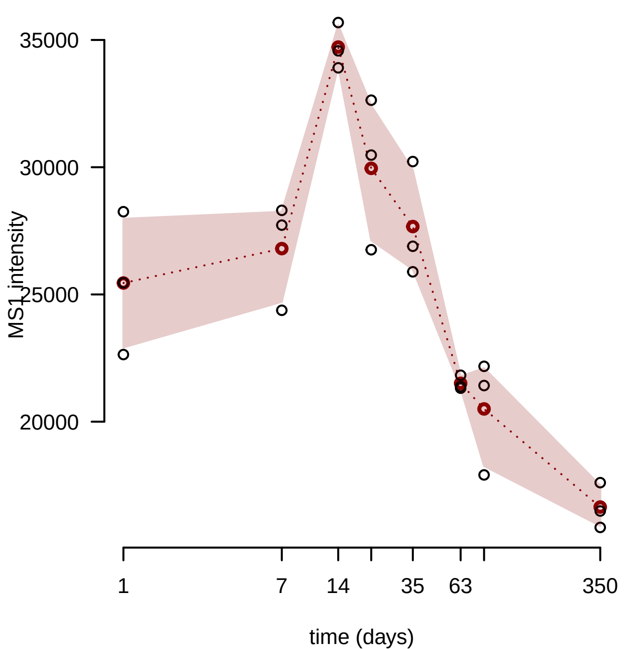

Q5SWK7
GN=Rnf145
ring finger protein 145

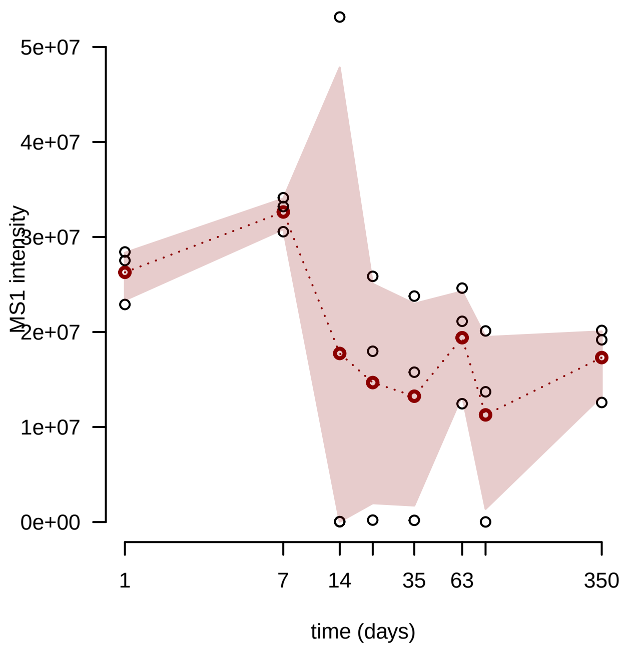

Q5SWP3
GN=Nacad
NAC alpha domain containing

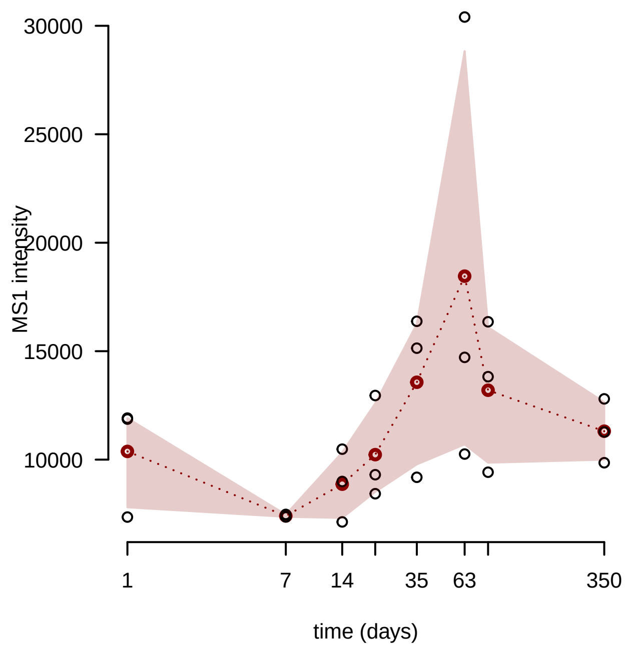

Q5SWT3
GN=Slc25a35
solute carrier family 25, member 35

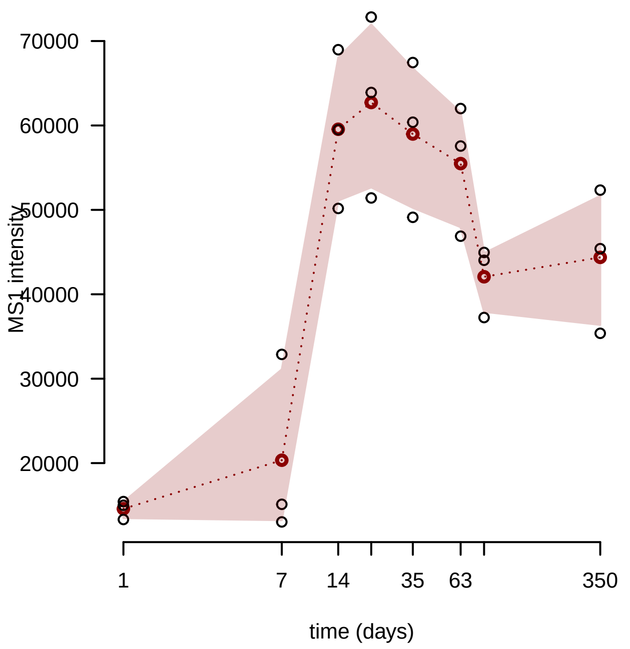

Q5SWU9
GN=Acaca
acetyl−Coenzyme A carboxylase alpha

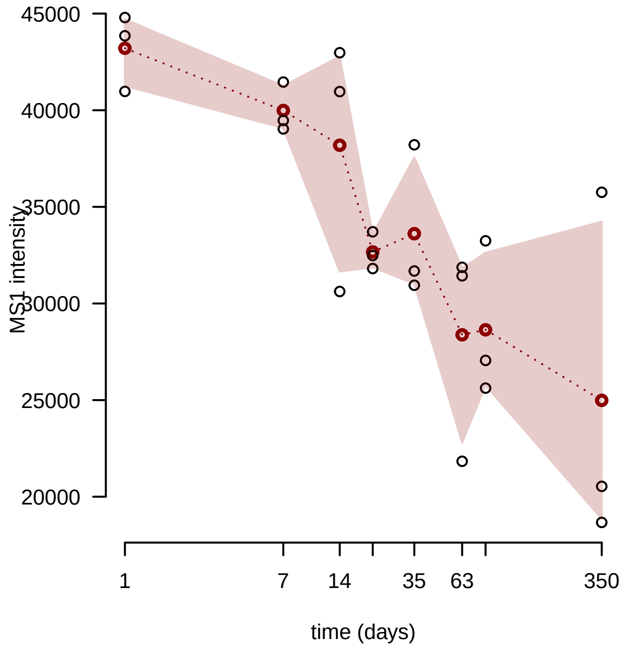

Q5SWW4
GN=Med13
mediator complex subunit 13

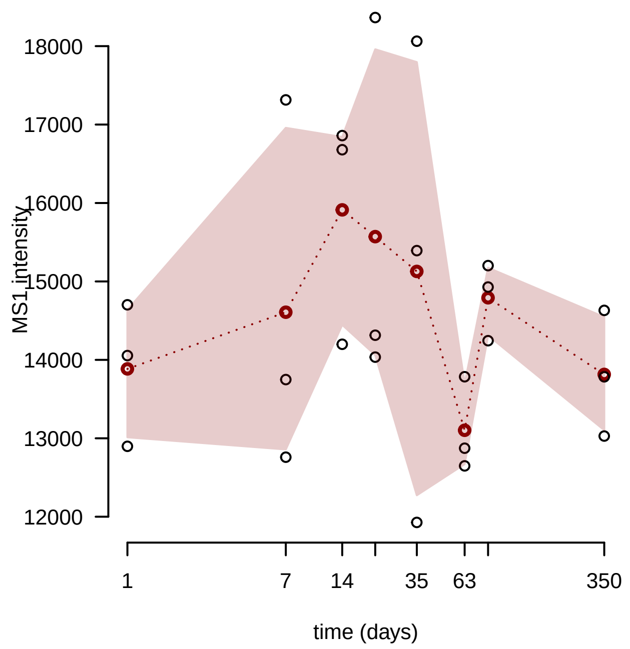

Q5SWY7
GN=Fam83g
family with sequence similarity 83, memb ...

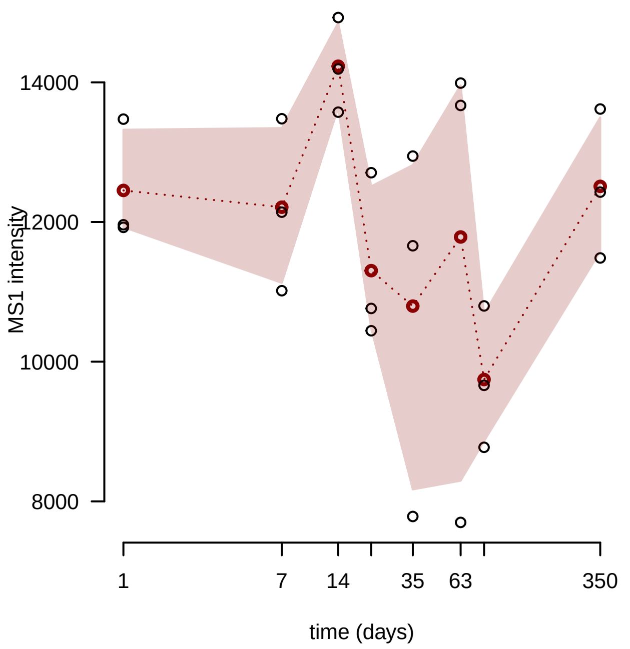

Q5SX19
GN=Pigl
phosphatidylinositol glycan anchor biosy ...

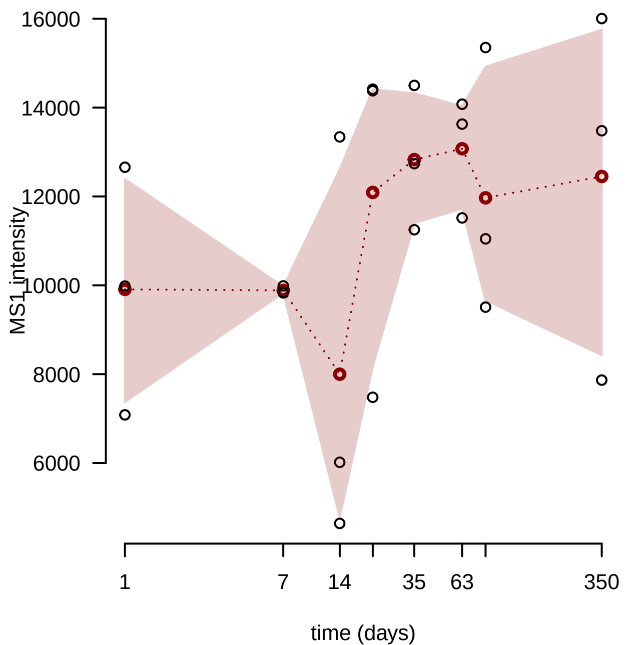

Q5SX39
GN=Myh4
myosin, heavy polypeptide 4, skeletal mu ...

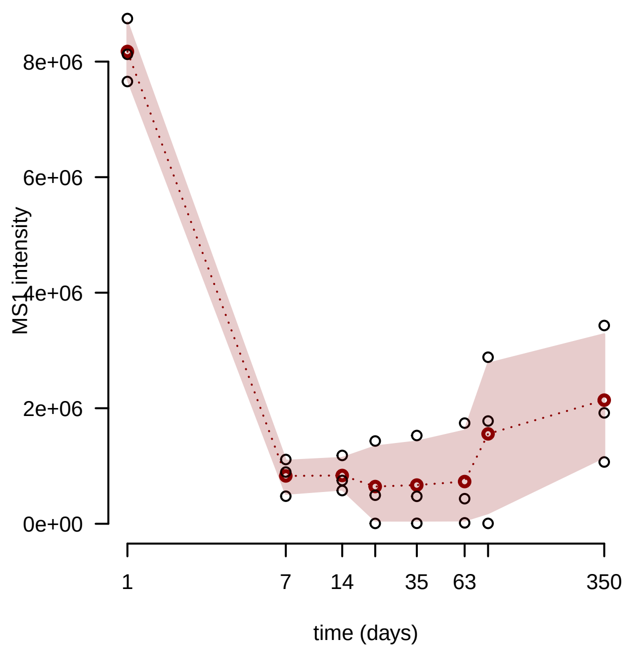

Q5SX40
GN=Myh1
myosin, heavy polypeptide 1, skeletal mu ...

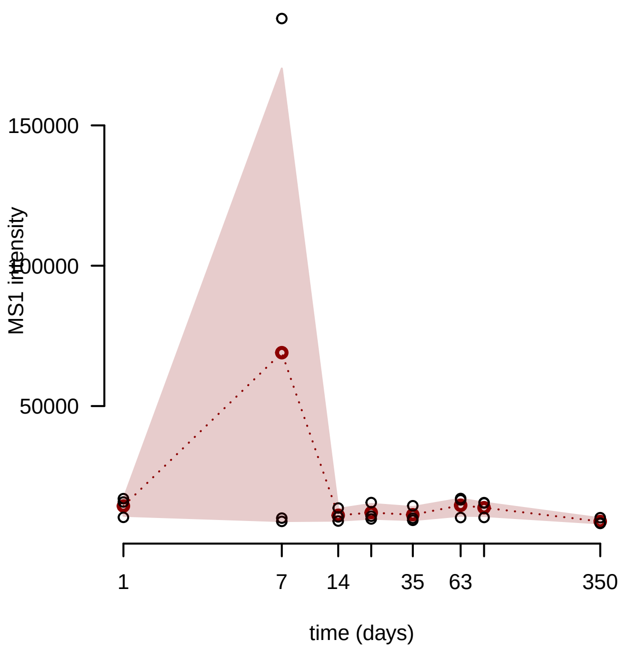

Q5SXY1
GN=Specc1
sperm antigen with calponin homology and ...

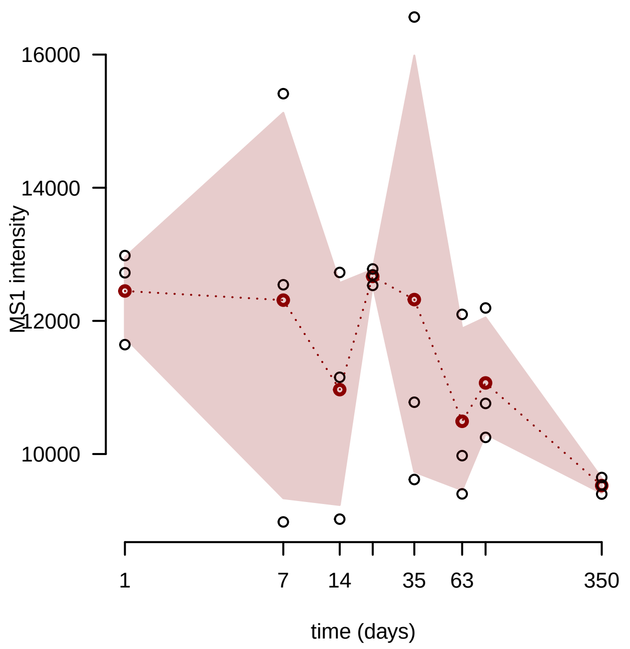

Q5SYD0
GN=Myo1d
myosin ID

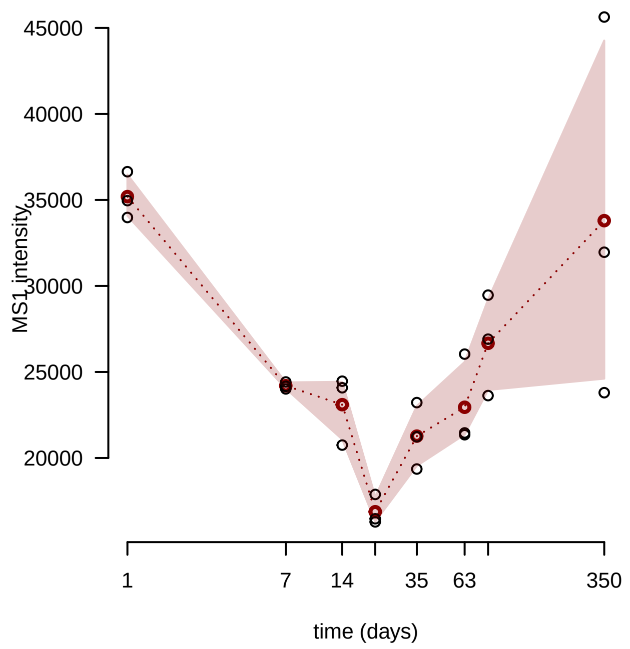

Q5SYH2
GN=Tmem199
transmembrane protein 199

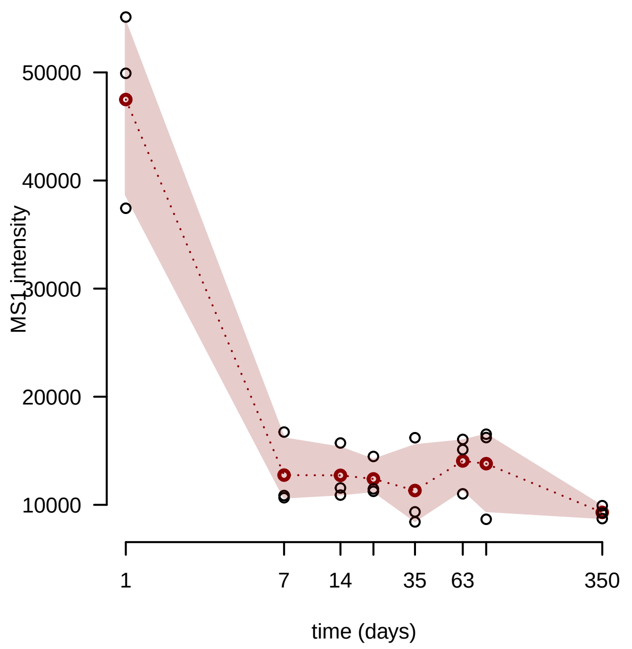

Q5SYL3
GN=Kiaa0100

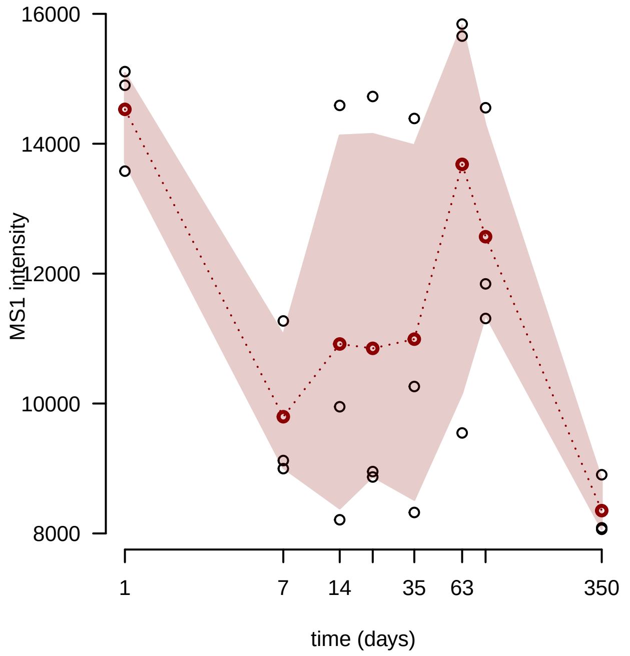

Q5TIS6
GN=Noto
notochord homeobox

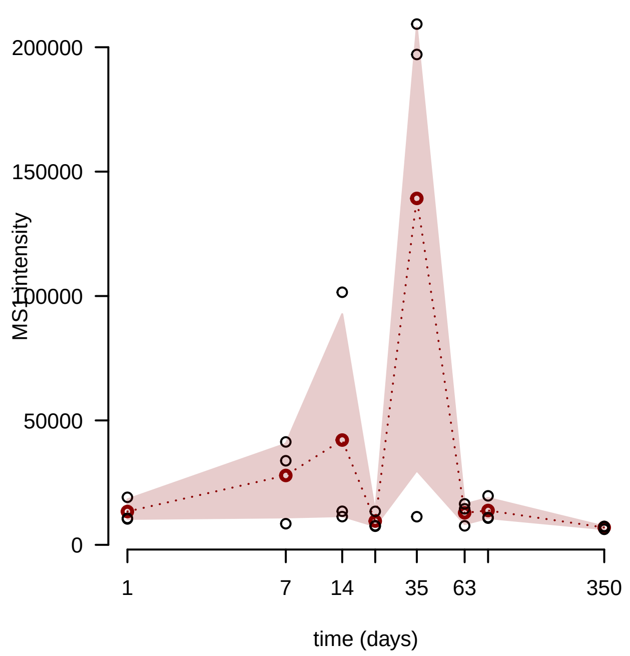

Q5U3K5
GN=Rabl6
RAB, member RAS oncogene family−like 6

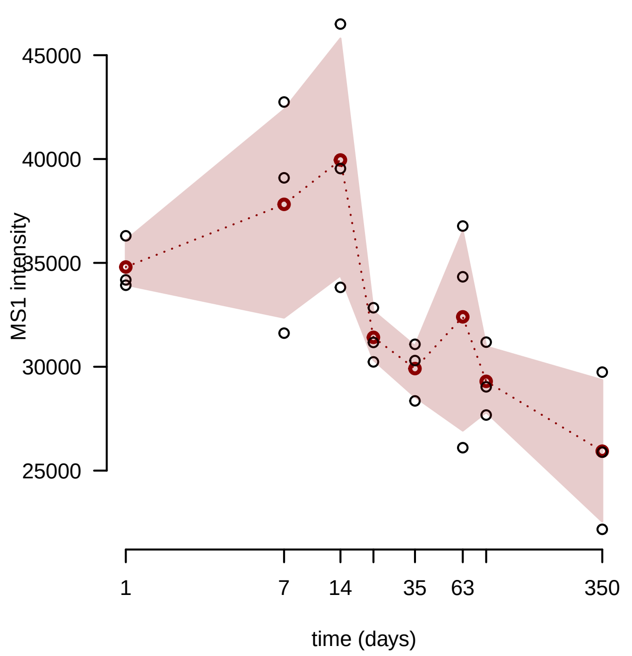

Q5U419
GN=Mfsd3
major facilitator superfamily domain con ...

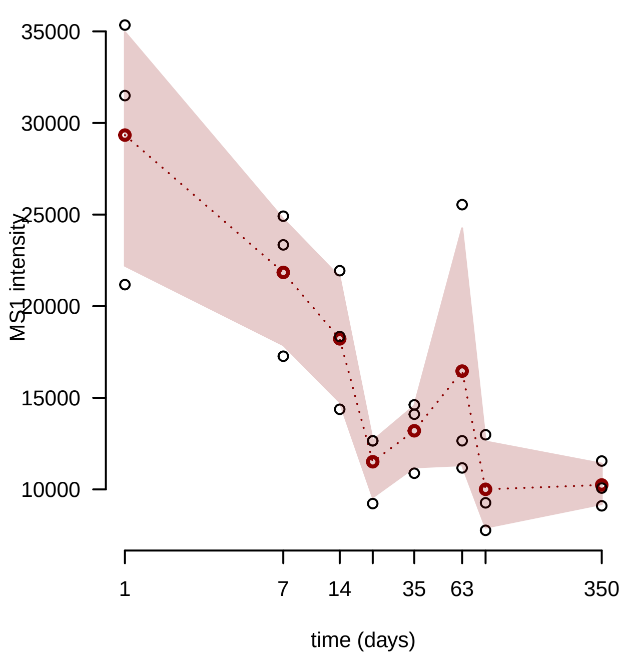

Q5U430
GN=Ubr3
ubiquitin protein ligase E3 component n− ...

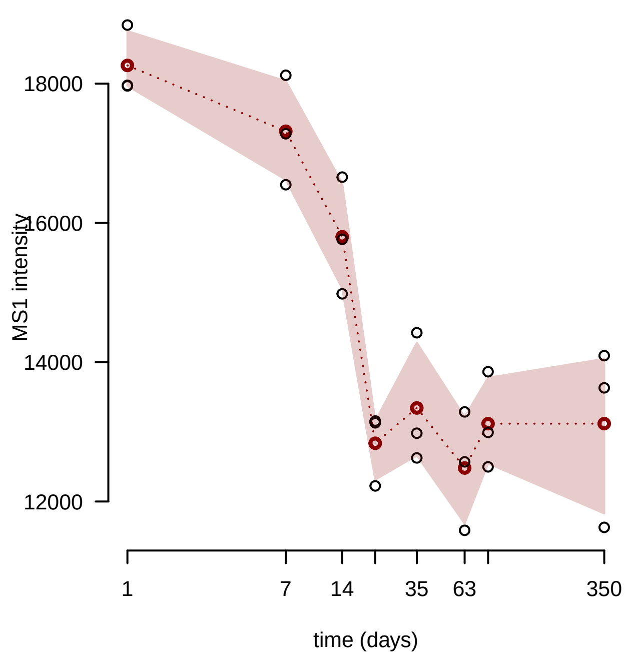

Q5U458
GN=Dnajc11
DnaJ heat shock protein family (Hsp40) m ...

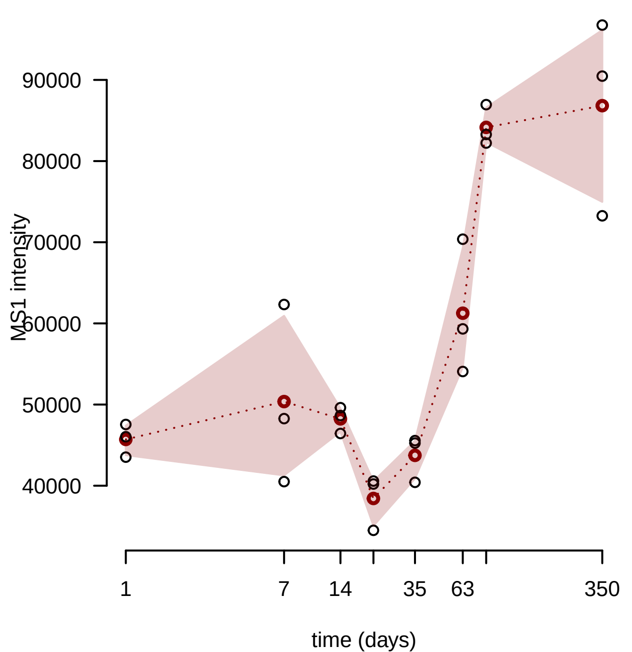

Q5U4C1
GN=Gprasp1
G protein−coupled receptor associated so ...

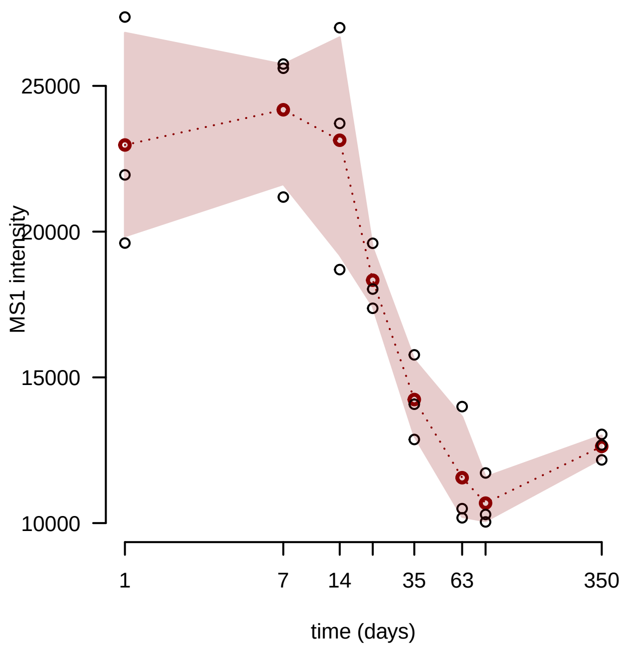

Q5U4C3
GN=Scaf1
SR−related CTD−associated factor 1

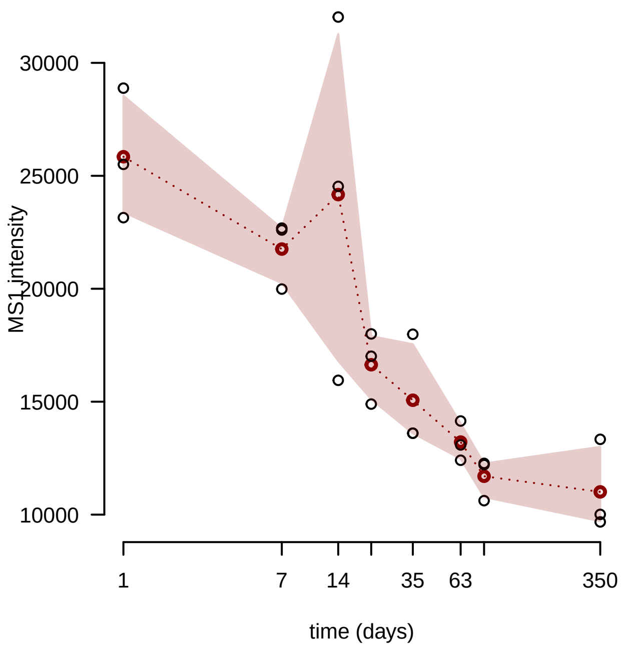

Q5U4D9
GN=Thoc6
THO complex 6

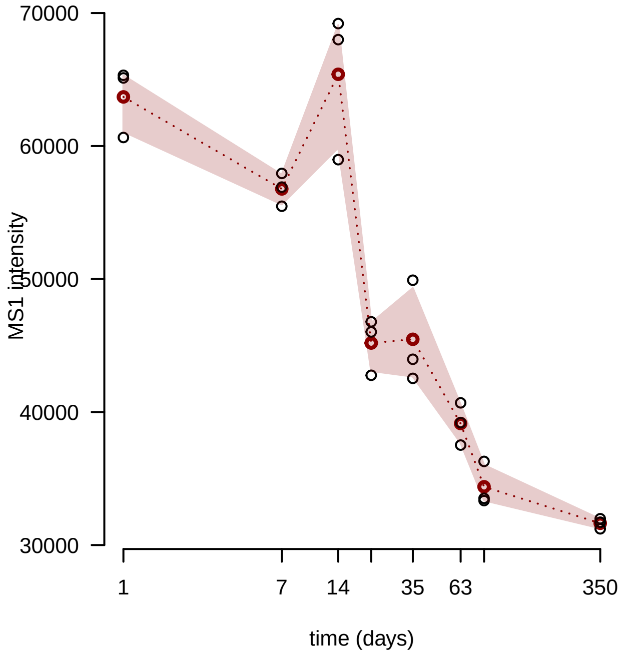

Q5U4E2
GN=Repin1
replication initiator 1

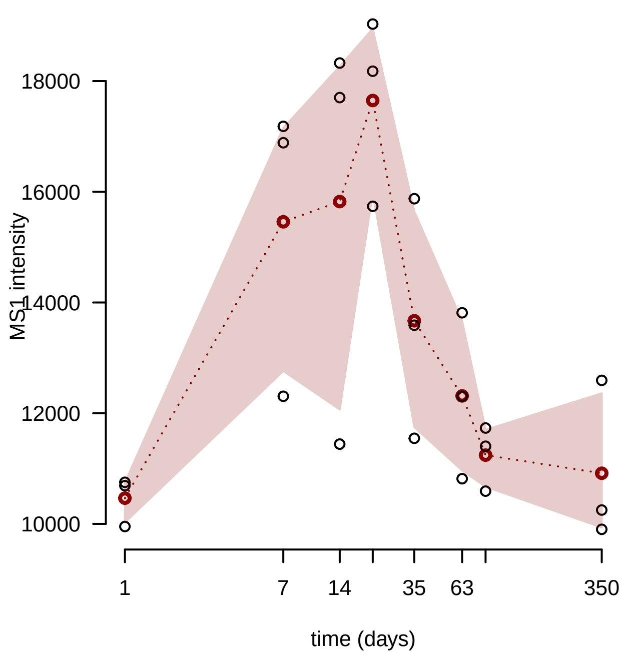

Q5U4F6
GN=Wdr34

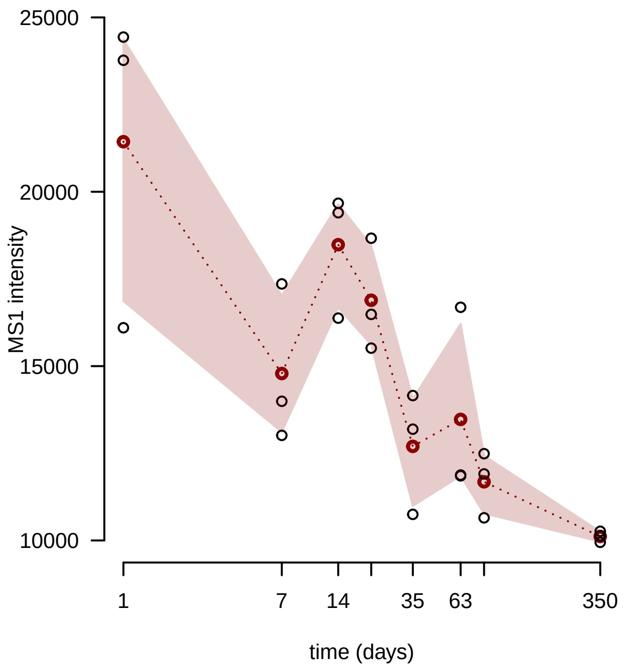

Q5U5M8
GN=Bloc1s3
biogenesis of lysosomal organelles compl ...

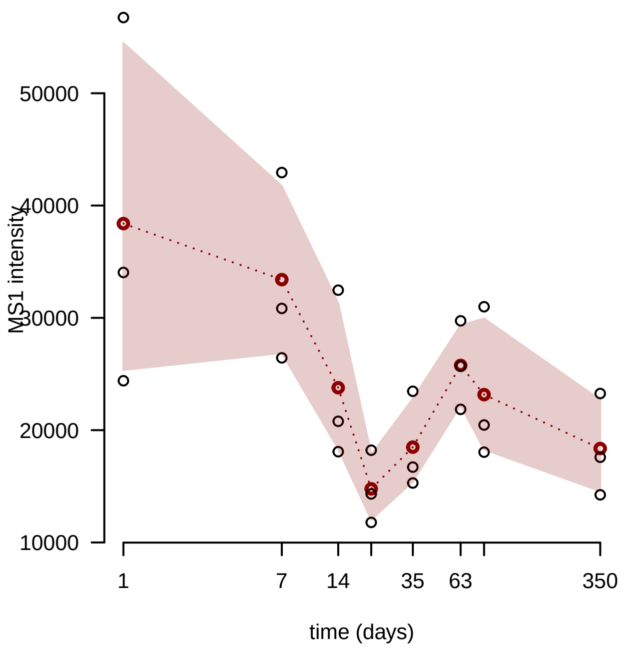

Q5U5Q9
GN=Uimc1
ubiquitin interaction motif containing 1

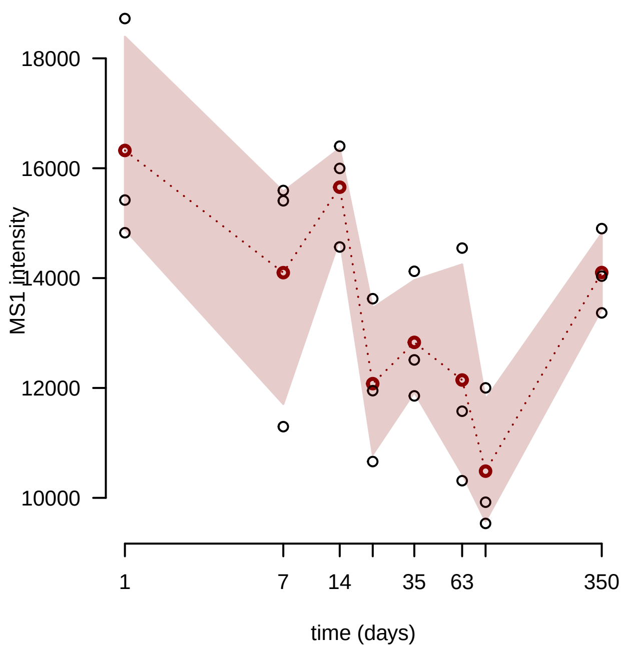

Q5U5V2
GN=Hykk
hydroxylysine kinase 1

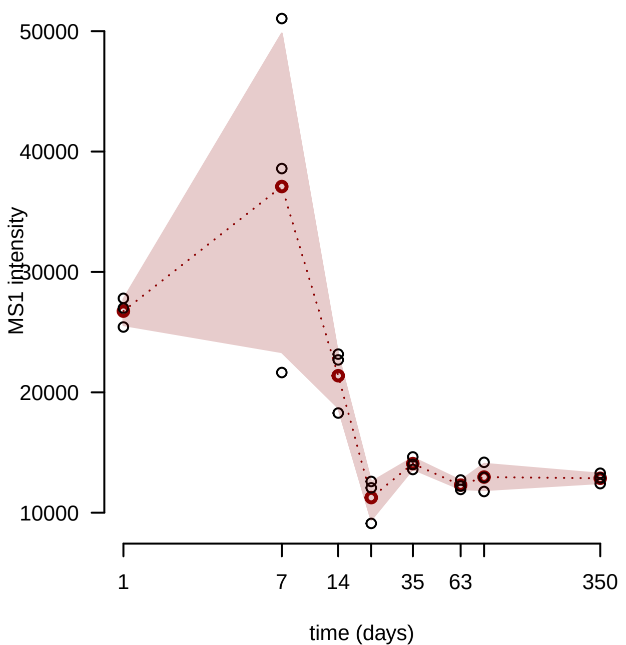

Q5U680
GN=Slc25a26
solute carrier family 25 (mitochondrial ...

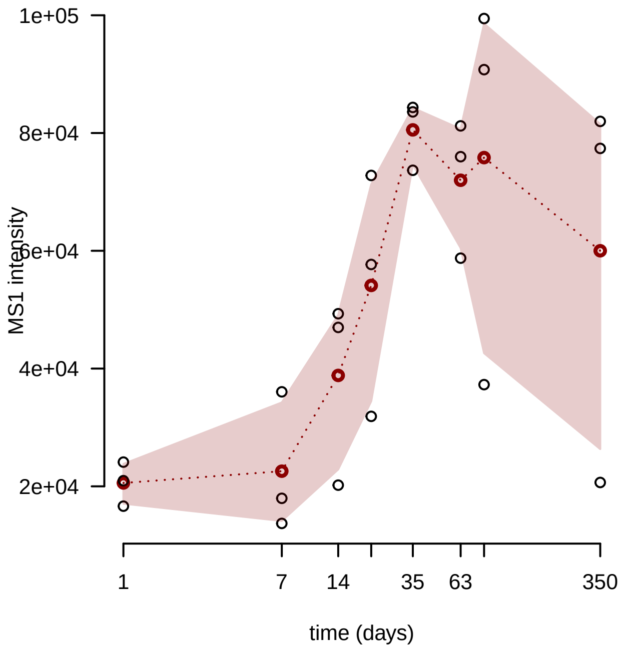

Q5UAK0
GN=Mier1
MEIR1 treanscription regulator

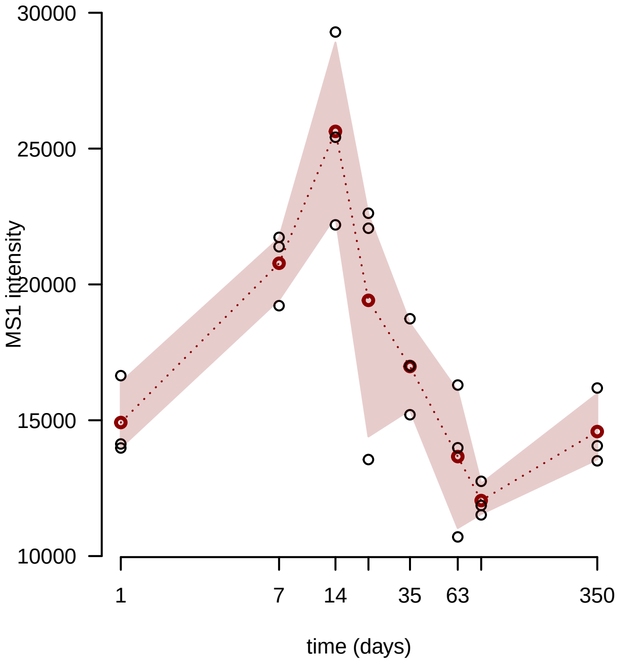

Q5VCS6
GN=Tdrd5
tudor domain containing 5

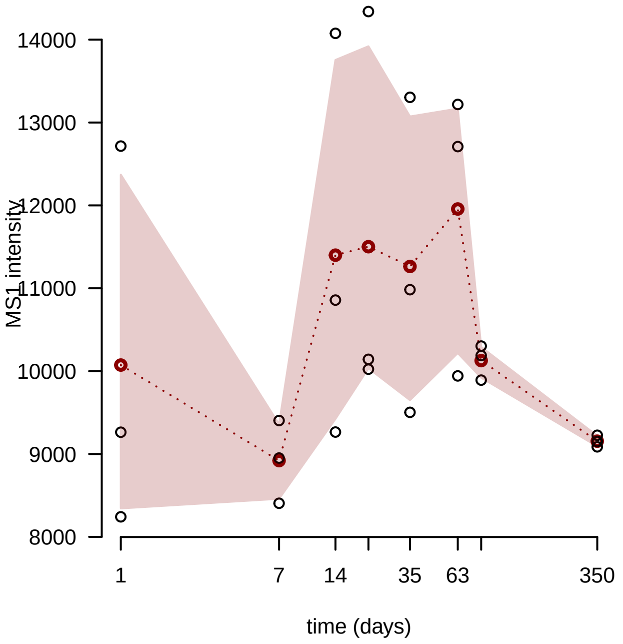

Q5XF89
GN=Atp13a3
ATPase type 13A3

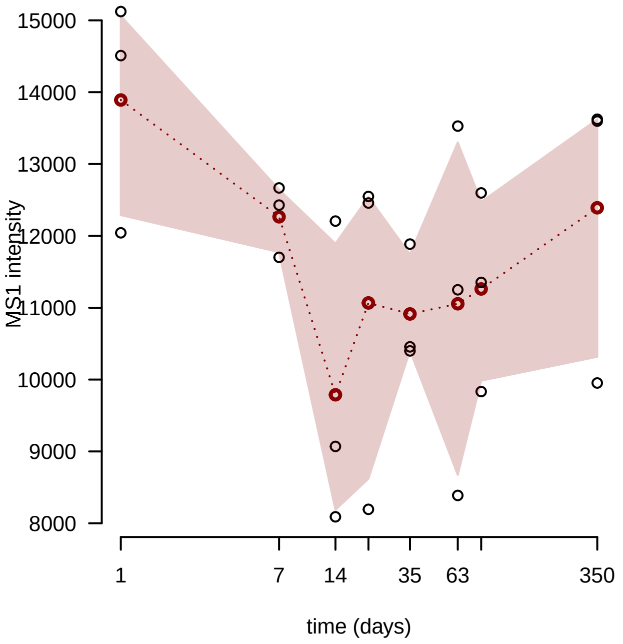

Q5XFZ0
GN=

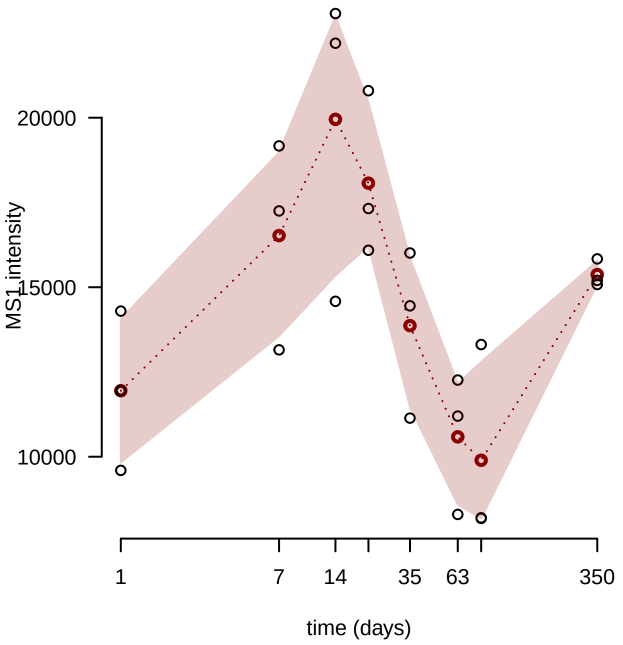

Q5XG69
GN=Fam169a
family with sequence similarity 169, mem ...

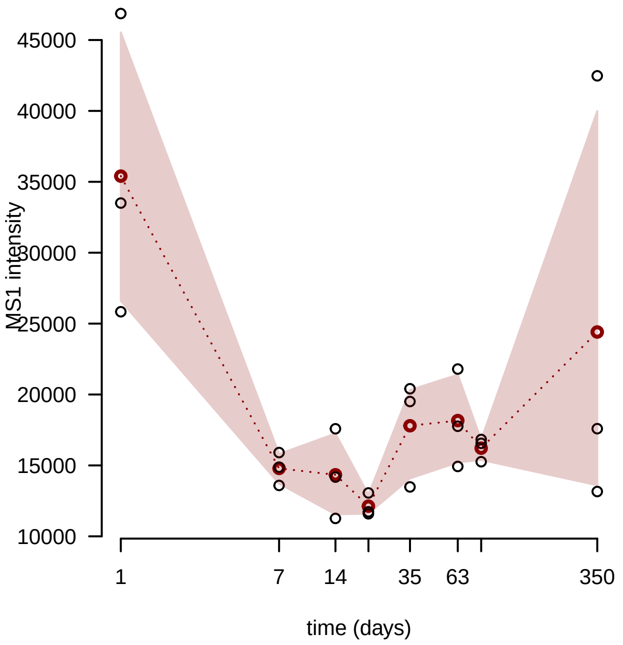

Q5XG71
GN=Utp20
UTP20 small subunit processome component

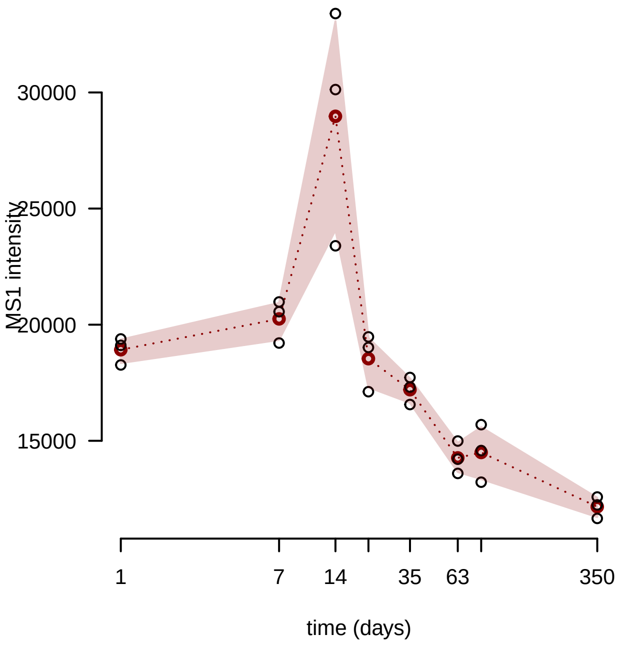

Q5XG73
GN=Acbd5
acyl−Coenzyme A binding domain containin ...

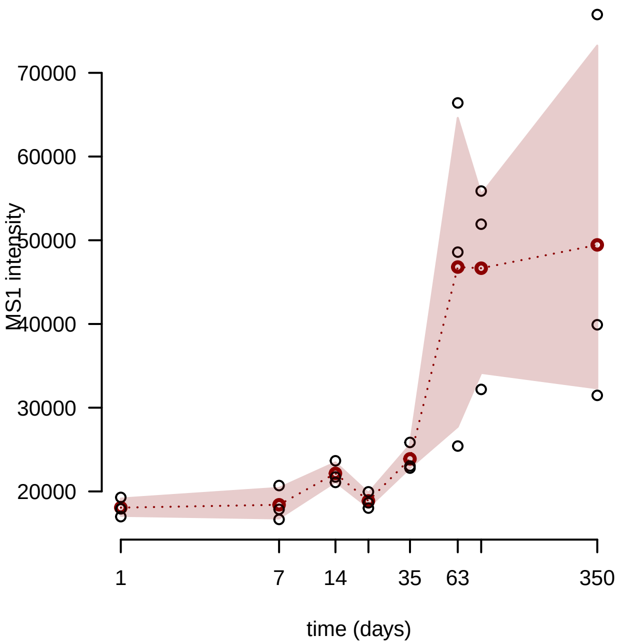

Q5XJE5
GN=Leo1
Leo1, Paf1/RNA polymerase II complex com ...

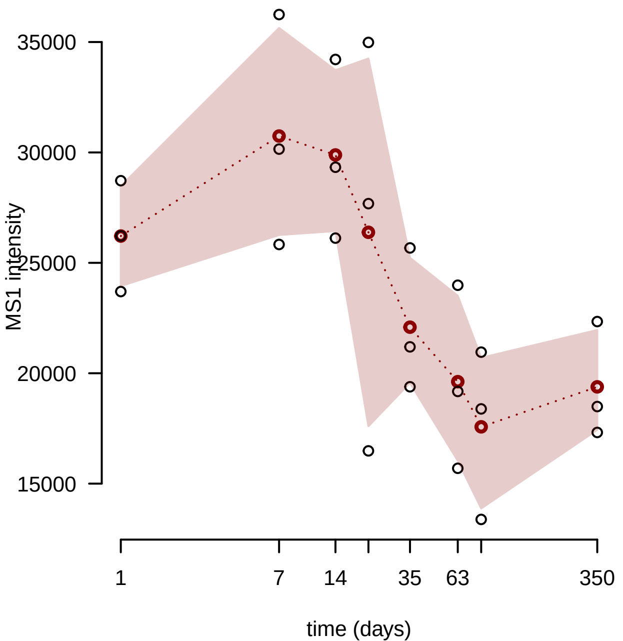

Q5XJY4
GN=Parl
presenilin associated, rhomboid−like

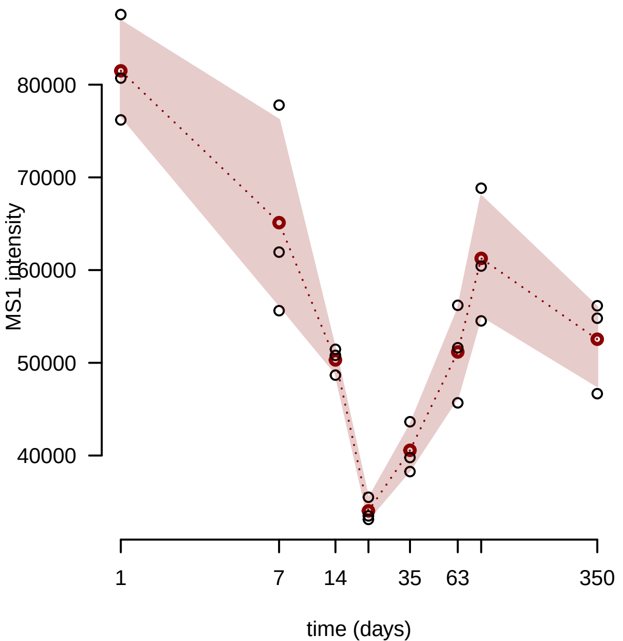

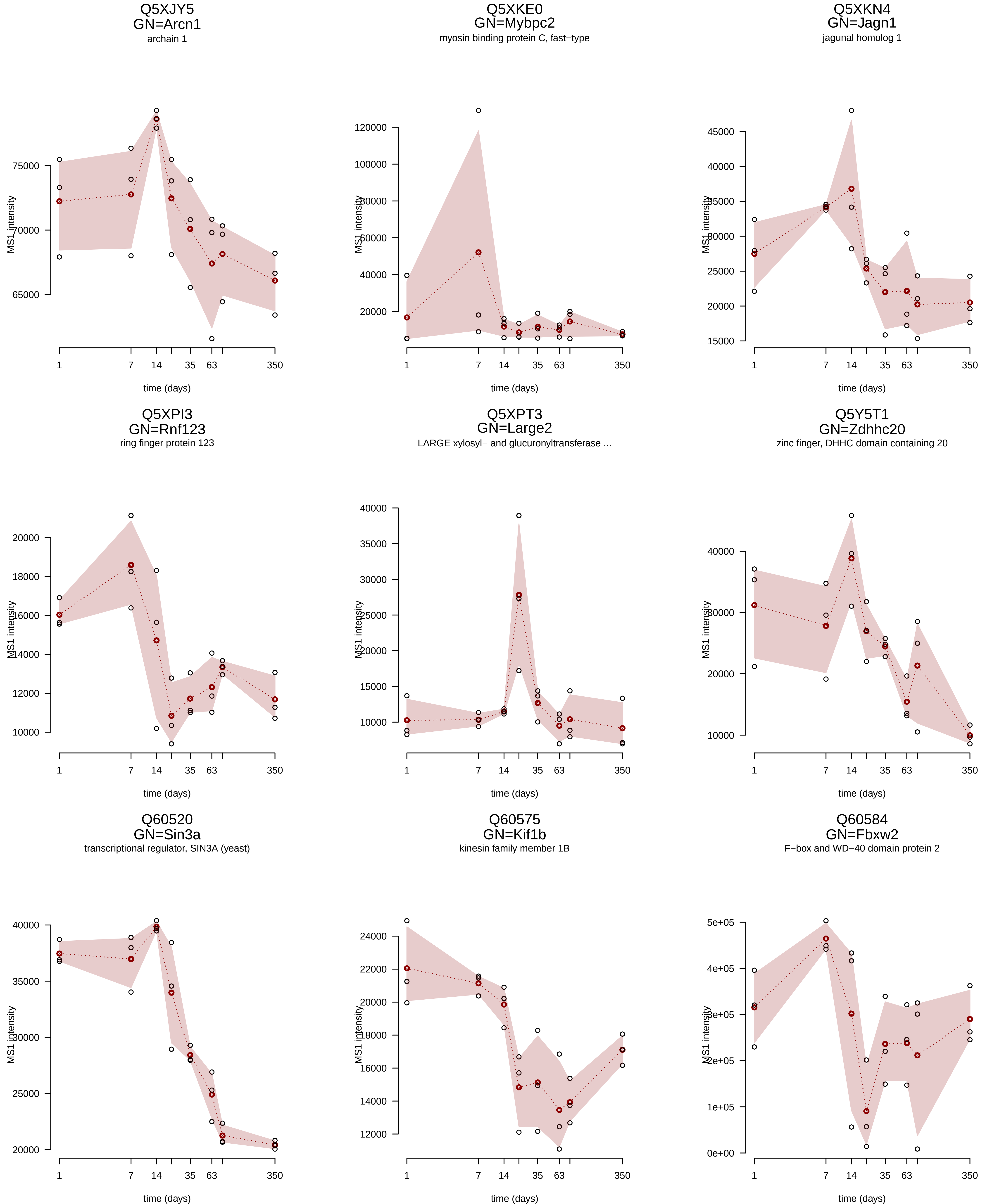
Q5XJY5
GN=Arcn1
archain 1
MS1 intensity
65000
70000
75000
1
7
14
35
63
350
time (days)
Q5XKE0
GN=Mybpc2
myosin binding protein C, fast−type
MS1 intensity
20000
40000
60000
80000
100000
120000
1
7
14
35
63
350
time (days)
Q5XKN4
GN=Jagn1
jagunal homolog 1
MS1 intensity
15000
20000
25000
30000
35000
40000
45000
1
7
14
35
63
350
time (days)
Q5XPI3
GN=Rnf123
ring finger protein 123
MS1 intensity
10000
12000
14000
16000
18000
20000
1
7
14
35
63
350
time (days)
Q5XPT3
GN=Large2
LARGE xylosyl− and glucuronyltransferase ...
MS1 intensity
10000
15000
20000
25000
30000
35000
40000
1
7
14
35
63
350
time (days)
Q5Y5T1
GN=Zdhhc20
zinc finger, DHHC domain containing 20
MS1 intensity
10000
20000
30000
40000
1
7
14
35
63
350
time (days)
Q60520
GN=Sin3a
transcriptional regulator, SIN3A (yeast)
MS1 intensity
20000
25000
30000
35000
40000
1
7
14
35
63
350
time (days)
Q60575
GN=Kif1b
kinesin family member 1B
MS1 intensity
12000
14000
16000
18000
20000
22000
24000
1
7
14
35
63
350
time (days)
Q60584
GN=Fbxw2
F−box and WD−40 domain protein 2
MS1 intensity
0e+00
1e+05
2e+05
3e+05
4e+05
5e+05
1
7
14
35
63
350
time (days)

Q60590
GN=Orm1
orosomucoid 1

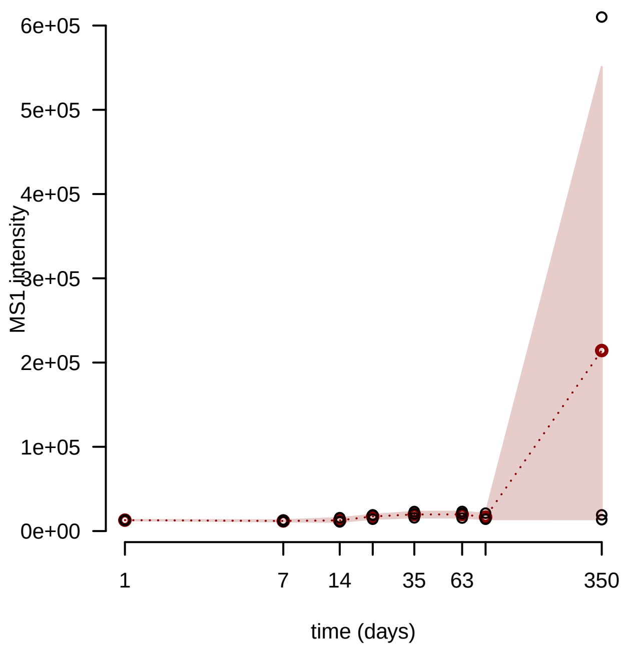

Q60592
GN=Mast2
microtubule associated serine/threonine ...

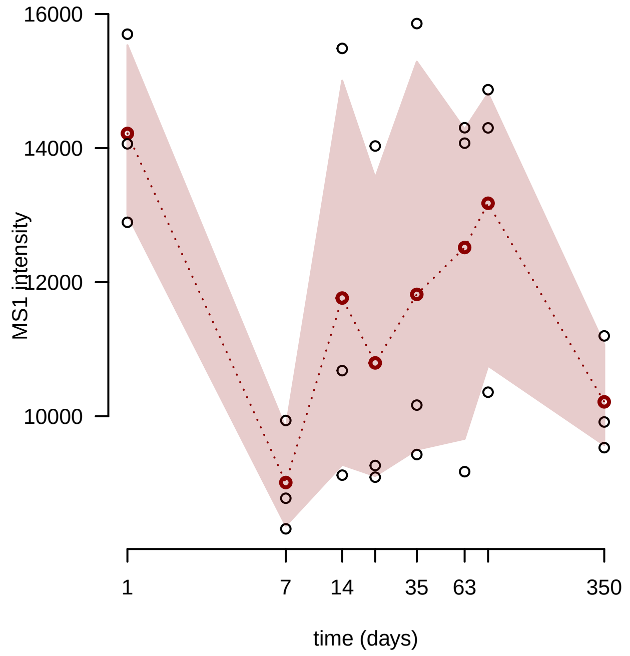

Q60595
GN=Xlr3a
X−linked lymphocyte−regulated 3A

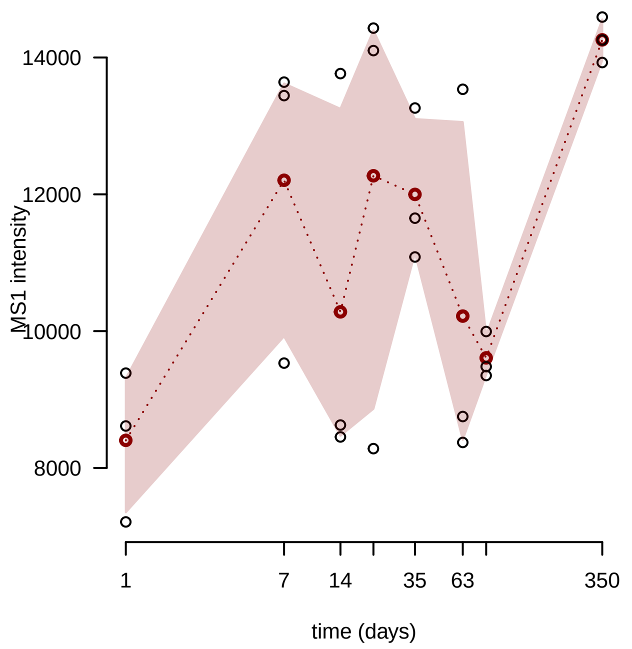

Q60596
GN=Xrcc1
X−ray repair complementing defective rep ...

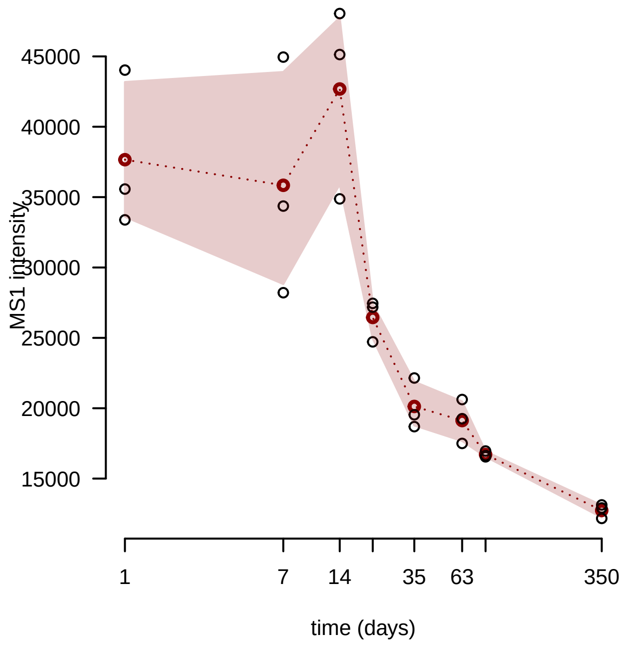

Q60597
GN=Ogdh
oxoglutarate (alpha−ketoglutarate) dehyd ...

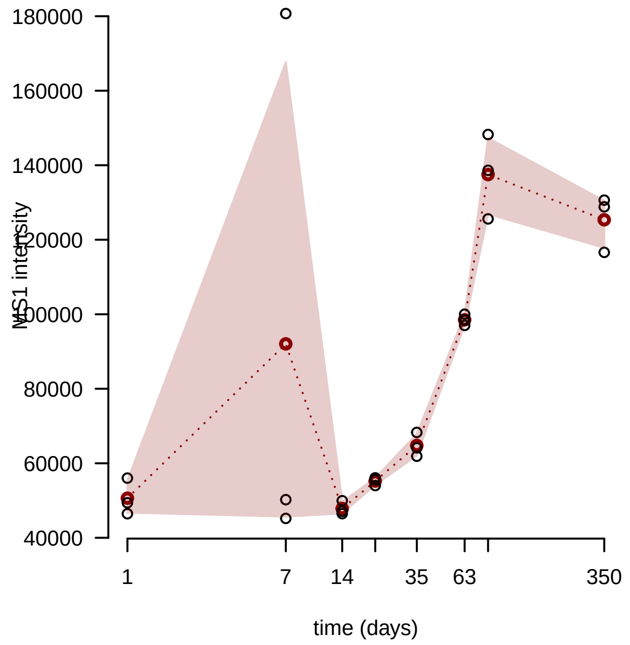

Q60598
GN=Cttn
cortactin

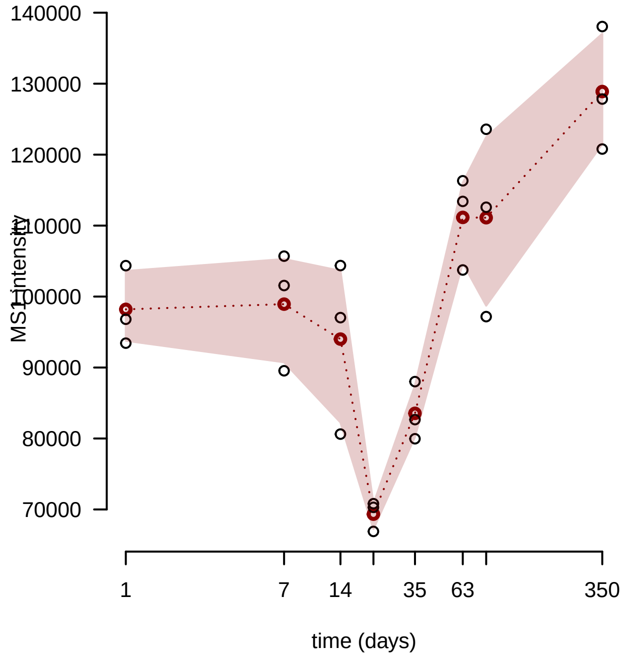

Q60604
GN=Scin
scinderin

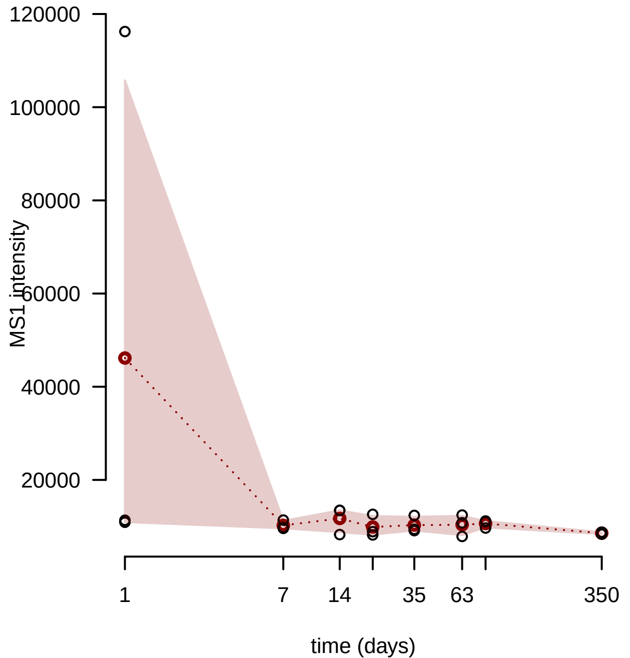

Q60605
GN=Myl6
myosin, light polypeptide 6, alkali, smo ...

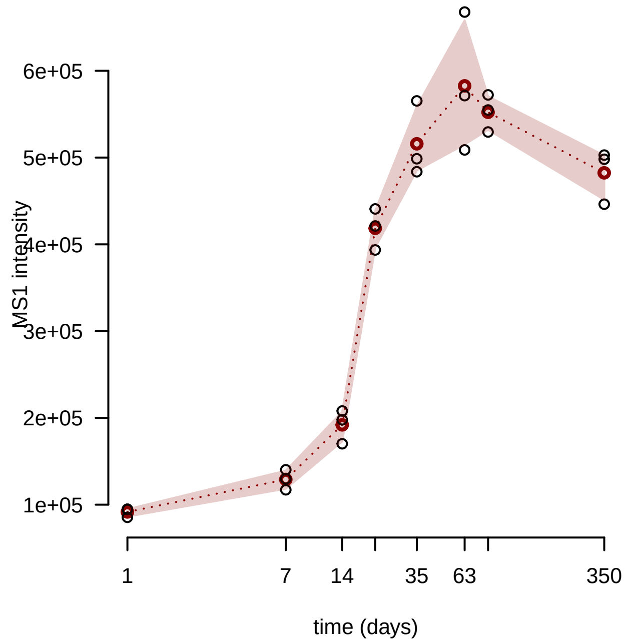

Q60631
GN=Grb2
growth factor receptor bound protein 2

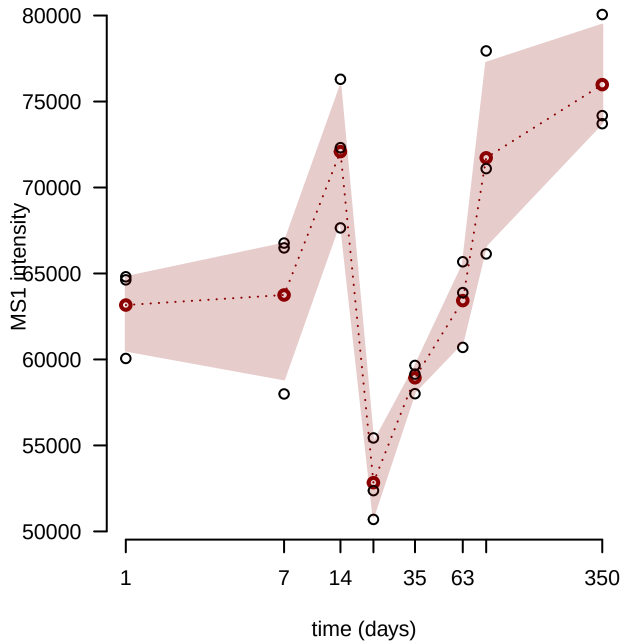

Q60634
GN=Flot2
flotillin 2

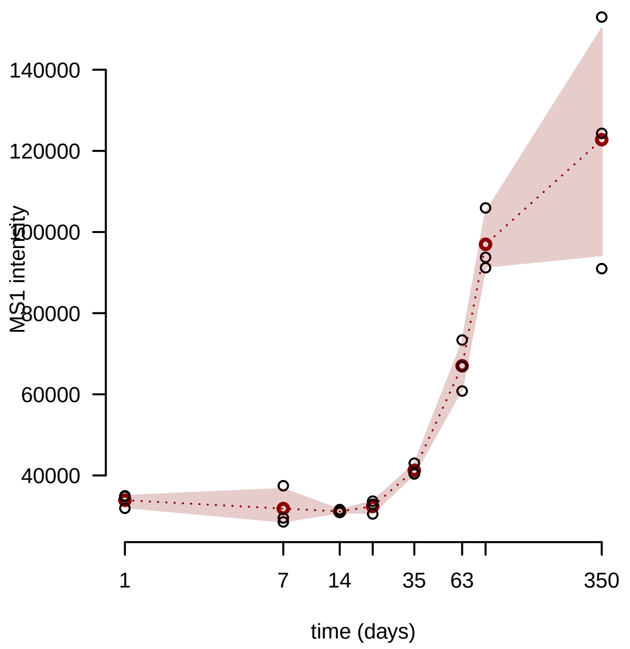

Q60648
GN=Gm2a
GM2 ganglioside activator protein

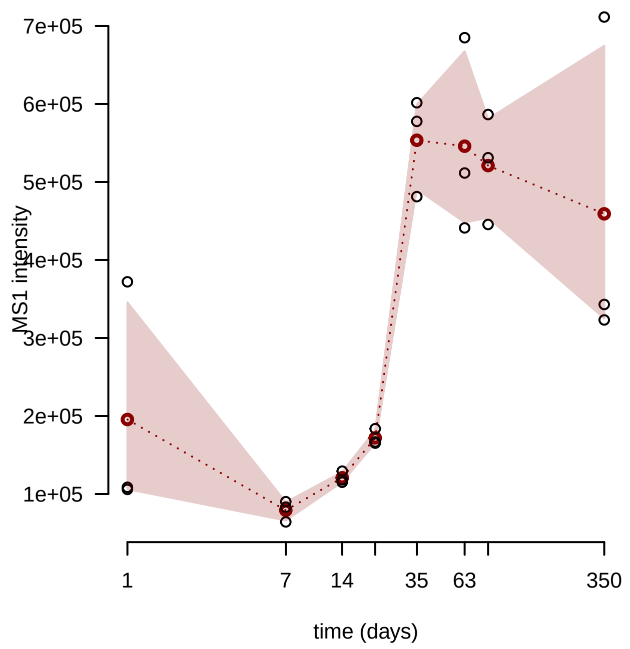

Q60649
GN=Clpb
ClpB caseinolytic peptidase B

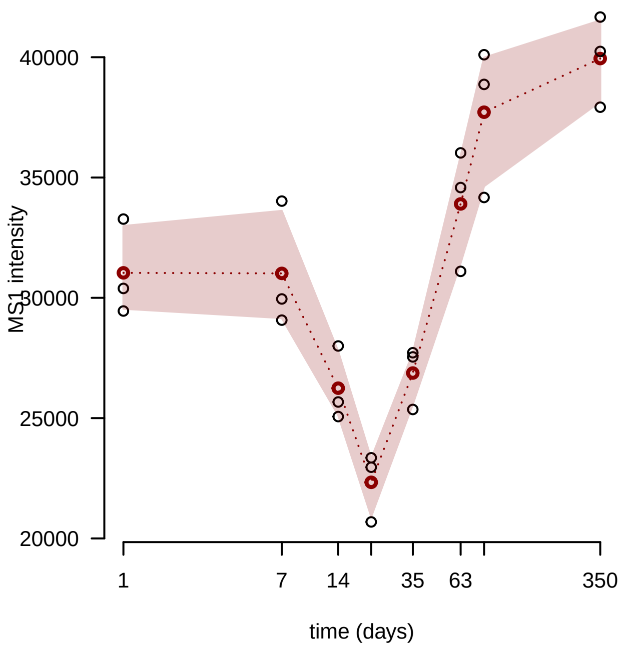

Q60668
GN=Hnrnpd
heterogeneous nuclear ribonucleoprotein ...

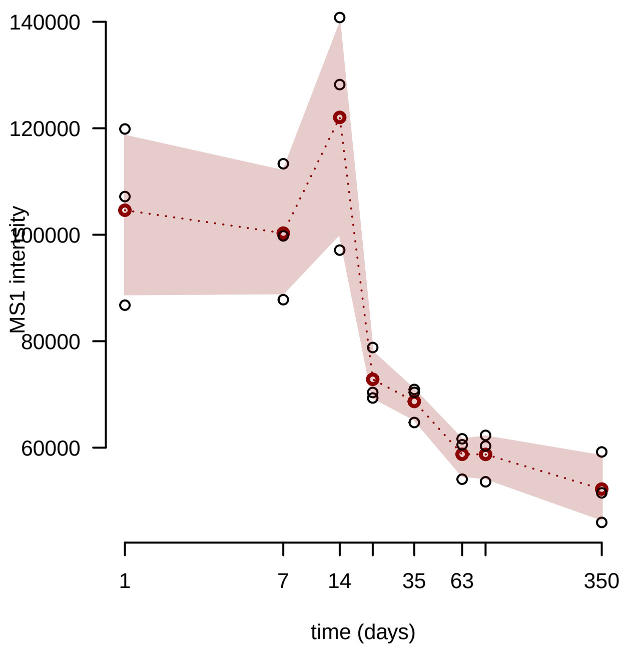

Q60674
GN=Nr1d2
nuclear receptor subfamily 1, group D, m ...

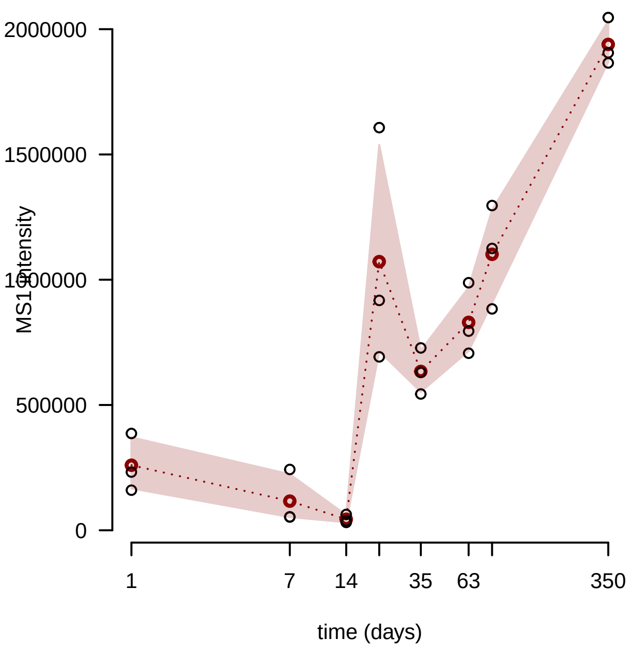

Q60675
GN=Lama2
laminin, alpha 2

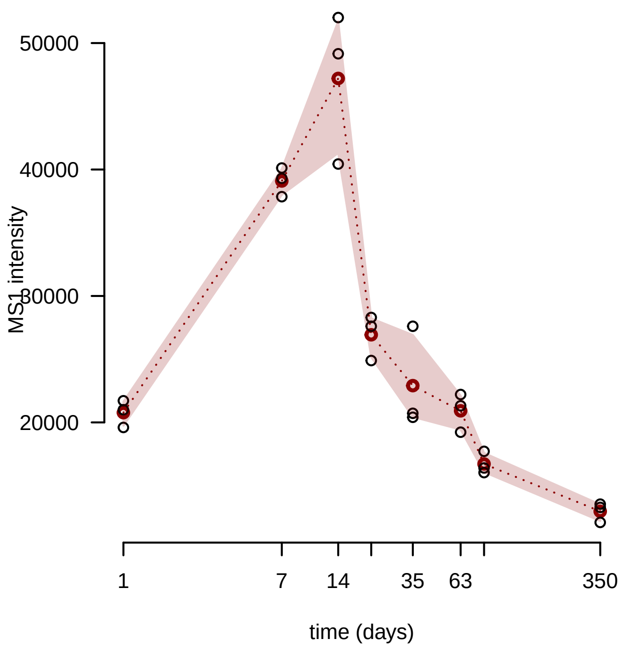

Q60676
GN=Ppp5c
protein phosphatase 5, catalytic subunit

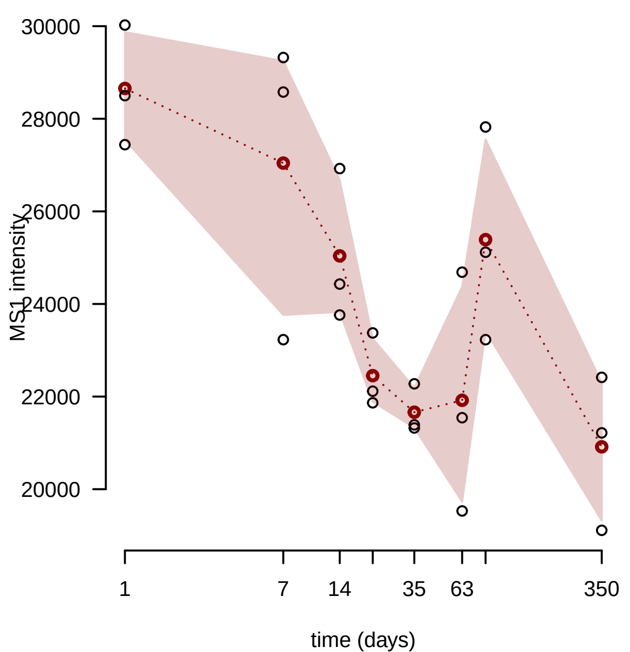

Q60680
GN=Chuk
conserved helix−loop−helix ubiquitous ki ...

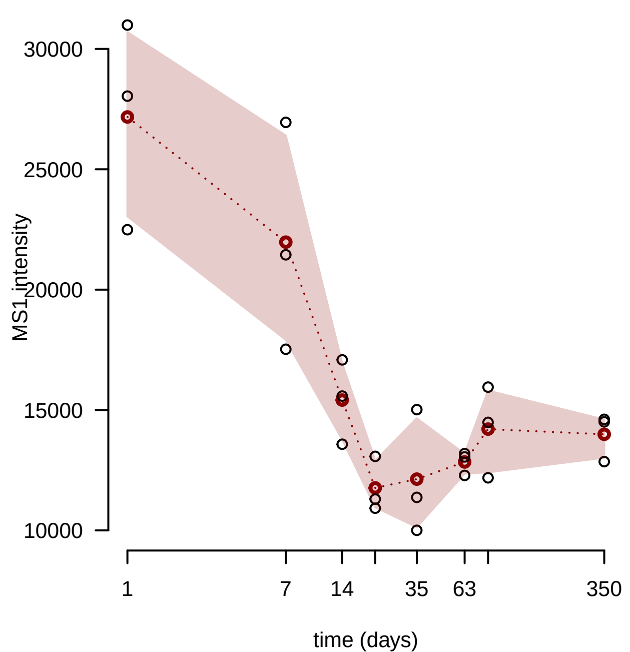

Q60692
GN=Psmb6
proteasome (prosome, macropain) subunit, ...

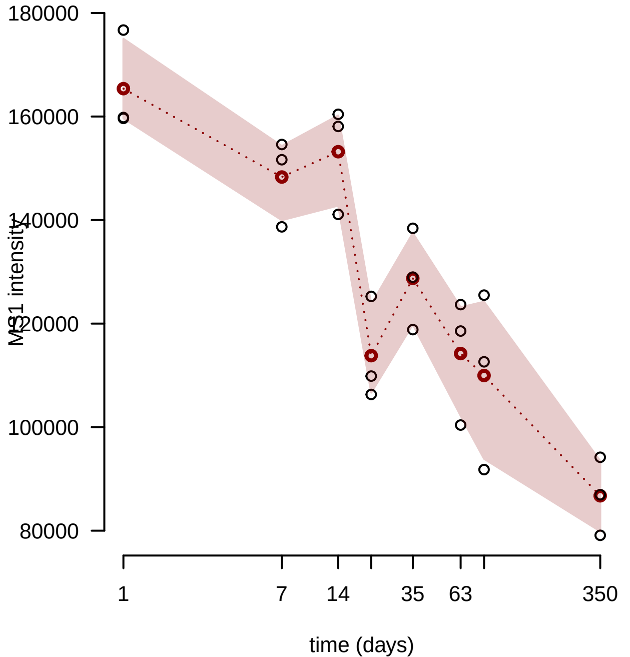

Q60695
GN=Rgl1
ral guanine nucleotide dissociation stim ...

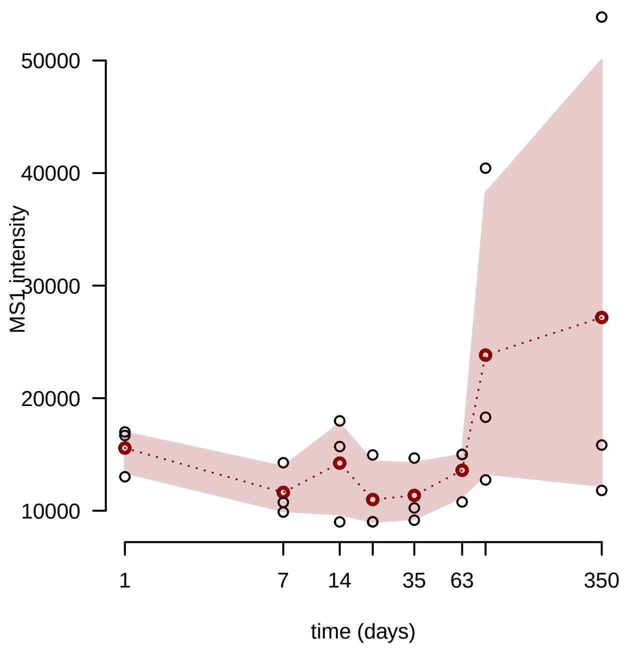

Q60698
GN=Ski
ski sarcoma viral oncogene homolog (avia ...

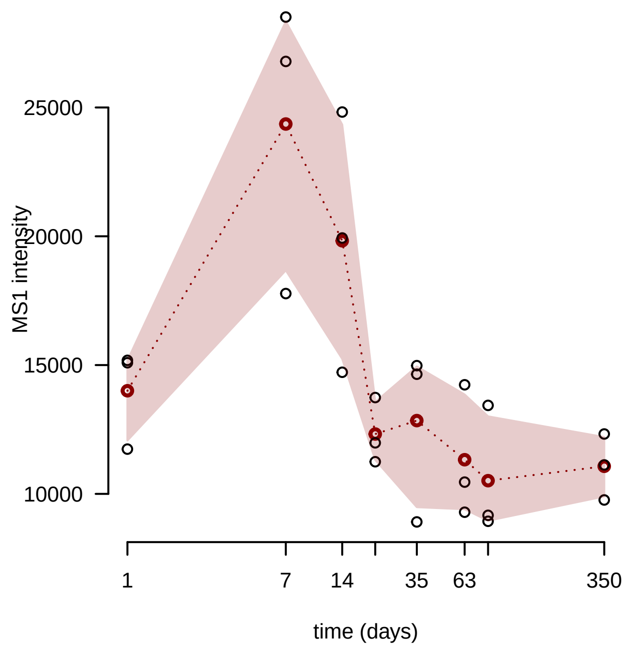

Q60707
GN=Tbx2
T−box 2

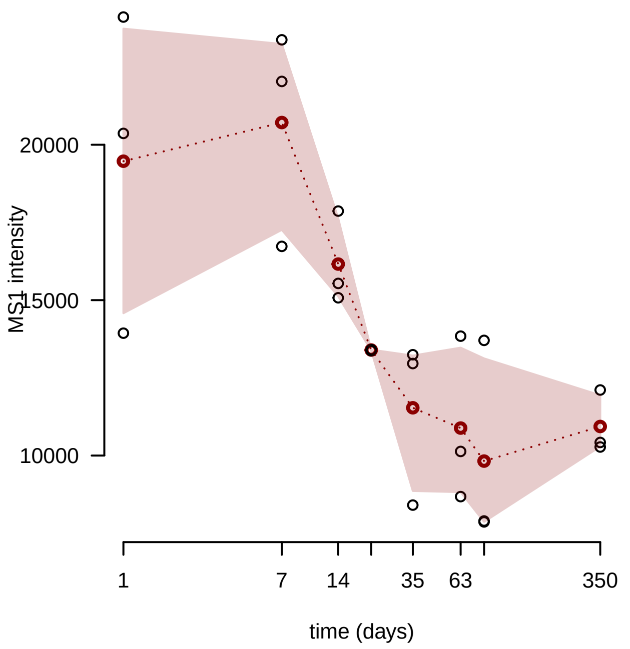

Q60710
GN=Samhd1
SAM domain and HD domain, 1

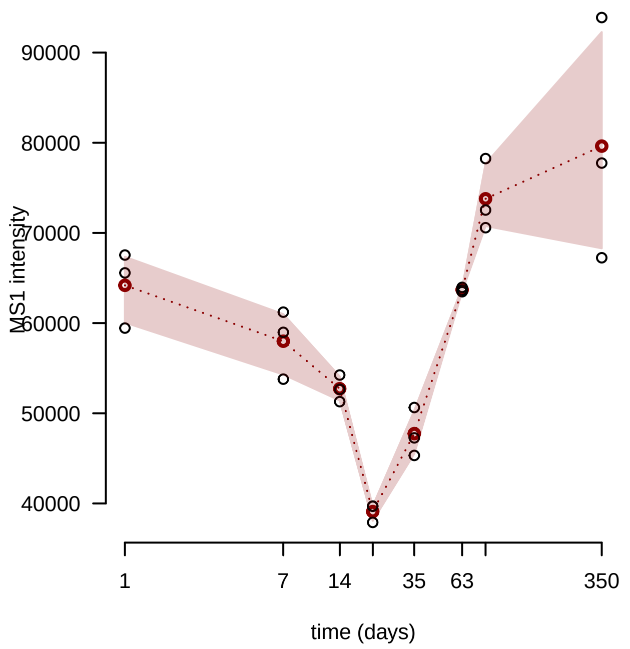

Q60714
GN=Slc27a1
solute carrier family 27 (fatty acid tra ...

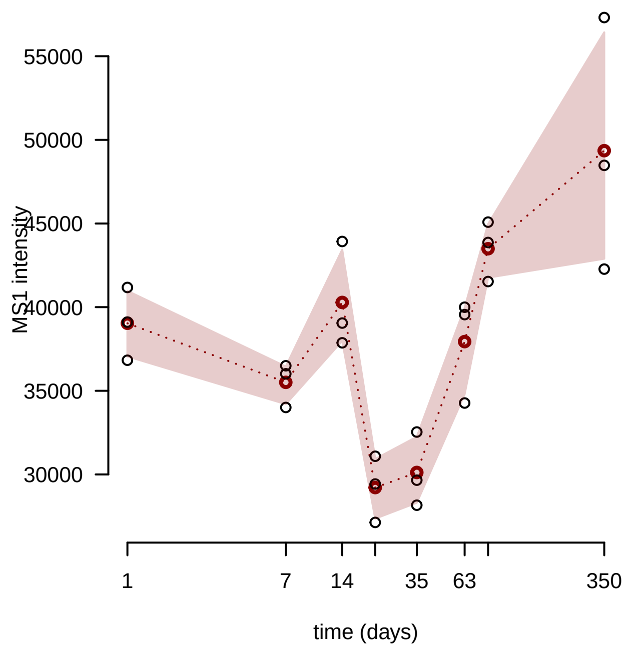

Q60715
GN=P4ha1
procollagen−proline, 2−oxoglutarate 4−di ...

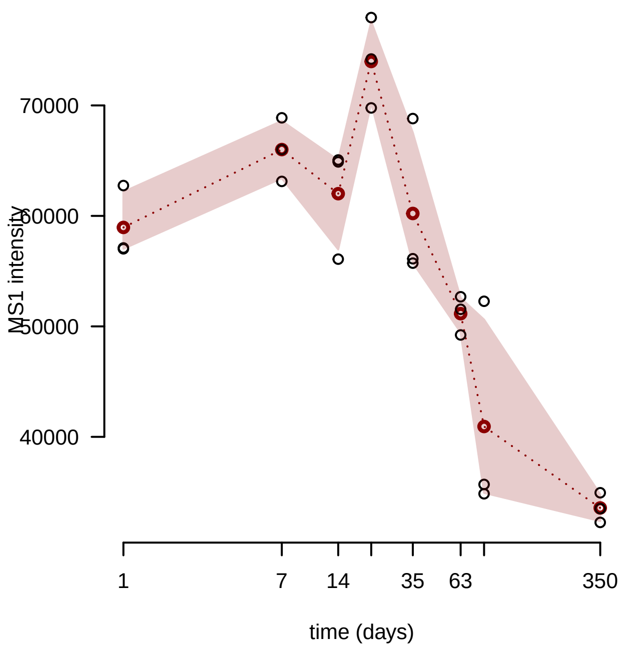

Q60715
GN=P4ha1
procollagen−proline, 2−oxoglutarate 4−di ...

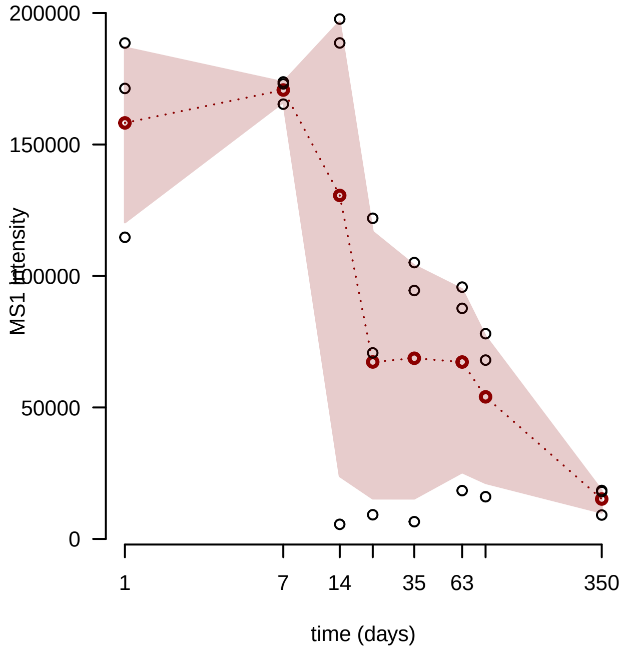

Q60716
GN=P4ha2
procollagen−proline, 2−oxoglutarate 4−di ...

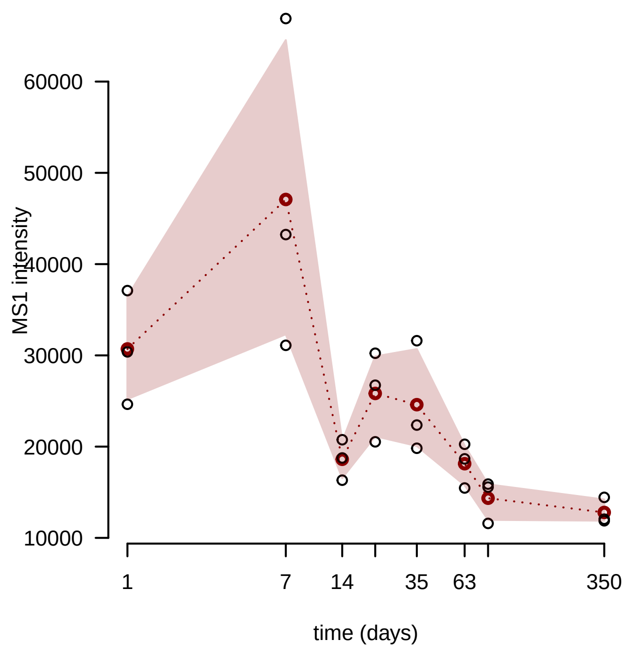

Q60722
GN=Tcf4
transcription factor 4

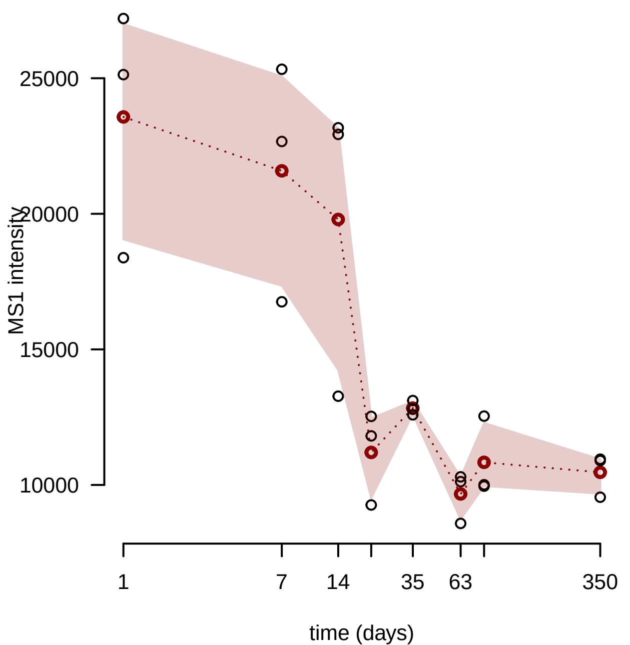

Q60722
GN=Tcf4
transcription factor 4

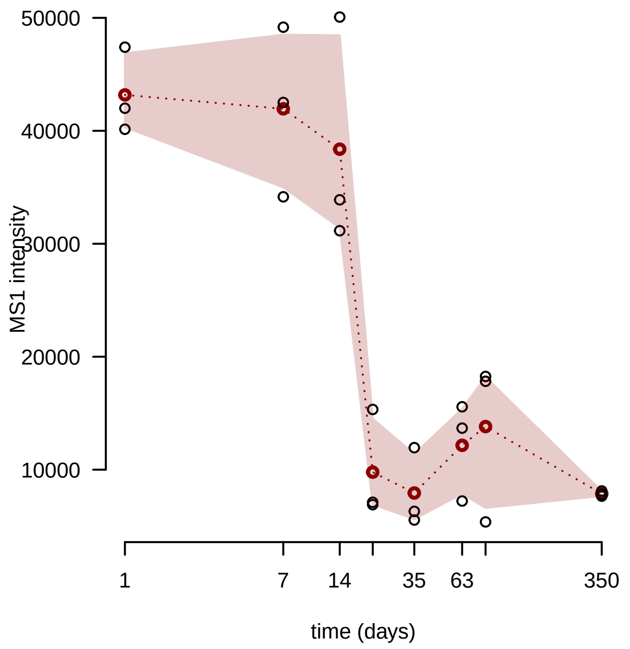

Q60737
GN=Csnk2a1
casein kinase 2, alpha 1 polypeptide

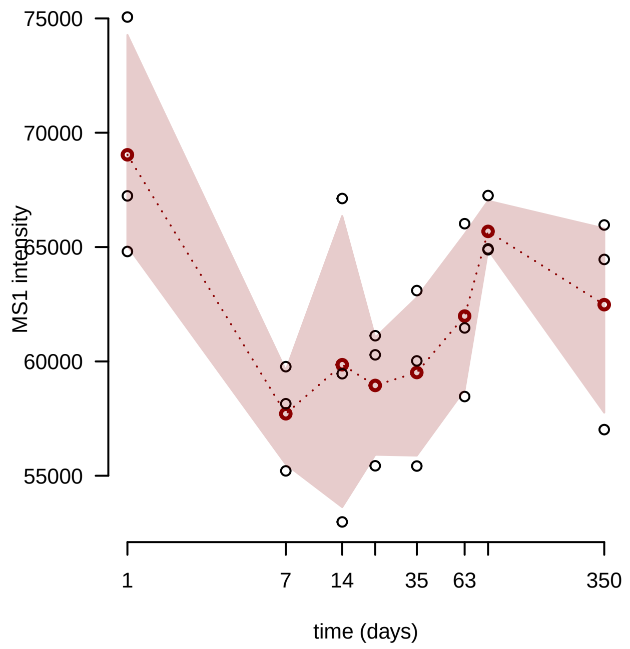

Q60738
GN=Slc30a1
solute carrier family 30 (zinc transport ...

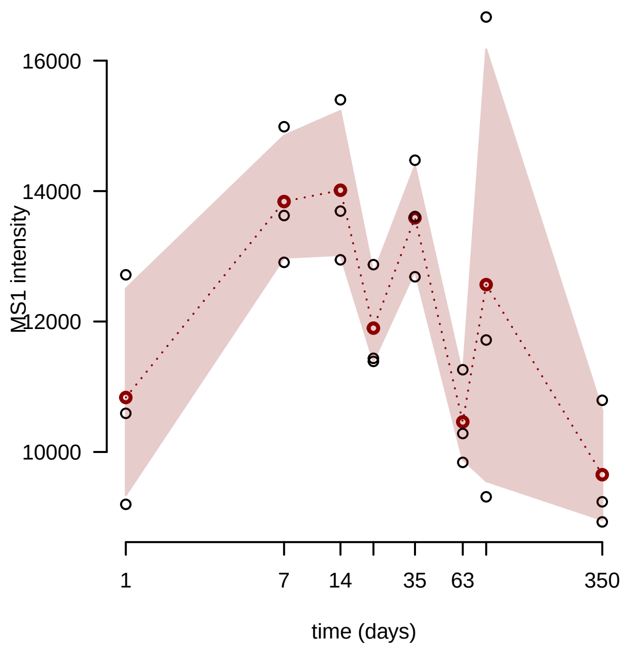

Q60739
GN=Bag1
BCL2−associated athanogene 1

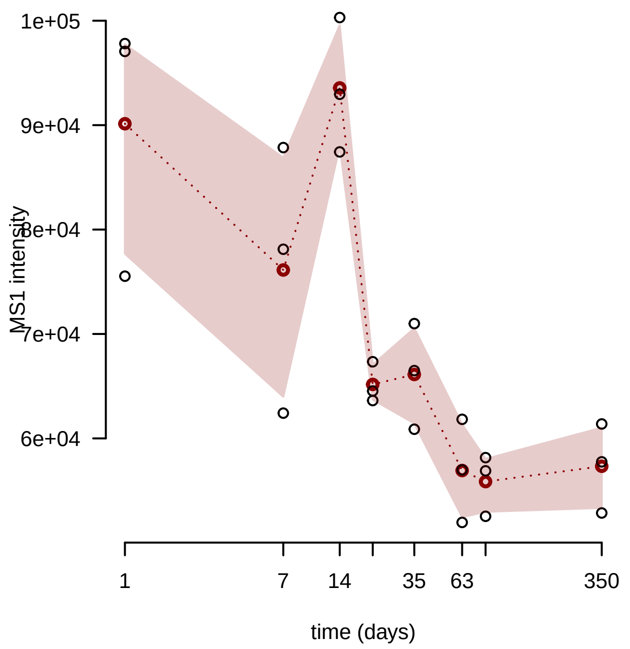

Q60749
GN=Khdrbs1
KH domain containing, RNA binding, signa ...

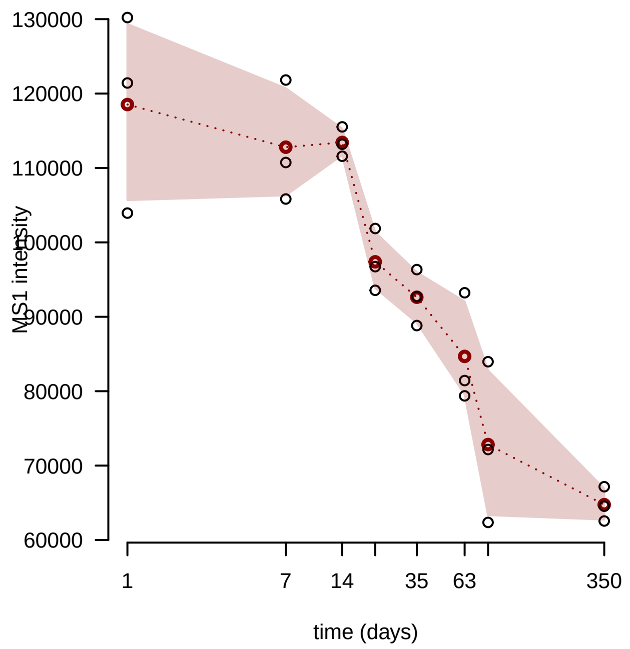

Q60751
GN=Igf1r
insulin−like growth factor I receptor

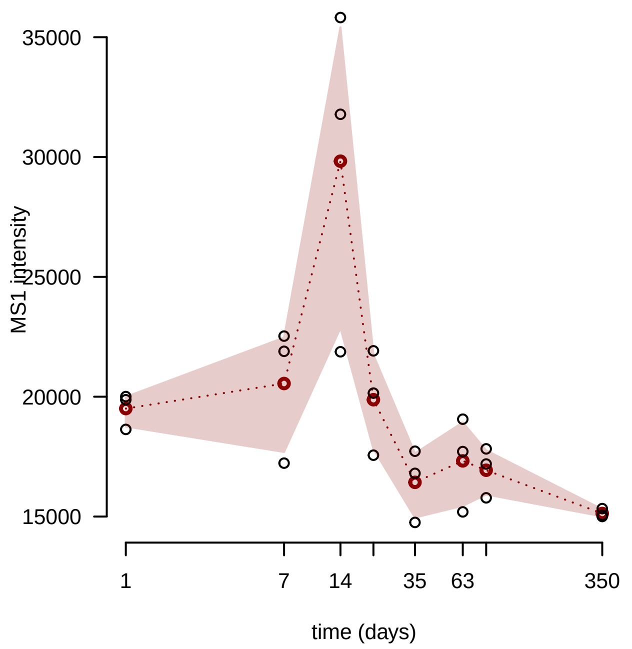

Q60759
GN=Gcdh
glutaryl−Coenzyme A dehydrogenase

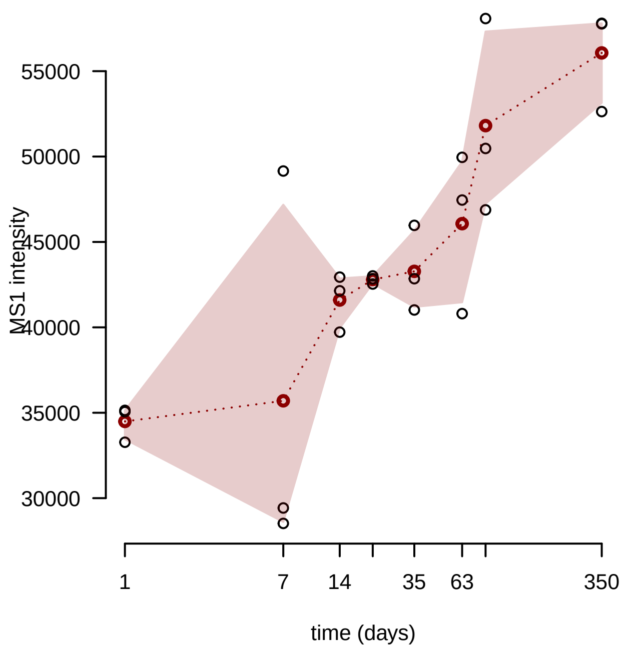

Q60760
GN=Grb10
growth factor receptor bound protein 10

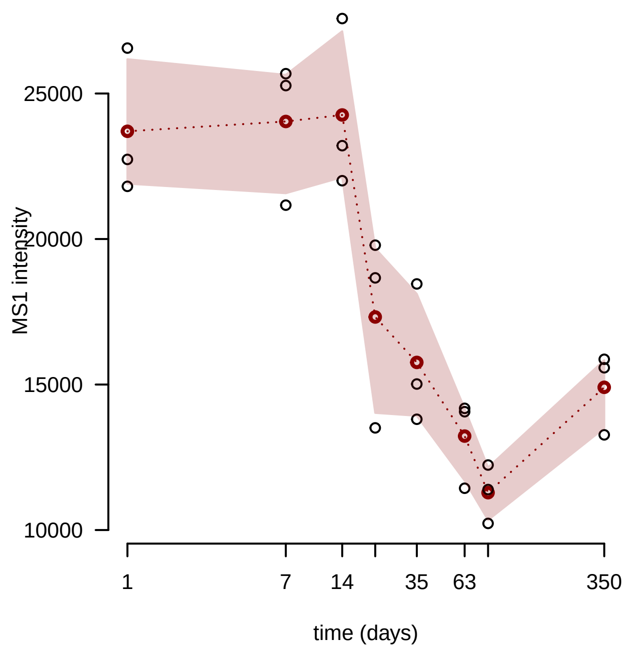

Q60766
GN=Irgm1
immunity−related GTPase family M member ...

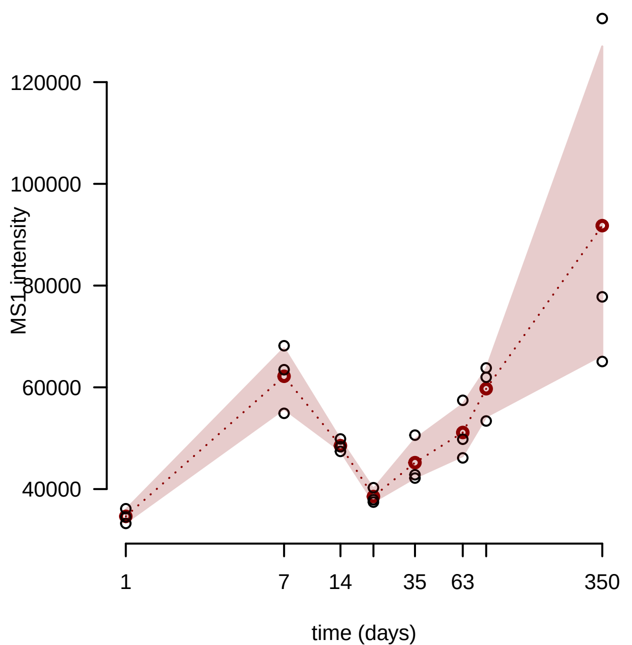

Q60767
GN=Ly75
lymphocyte antigen 75

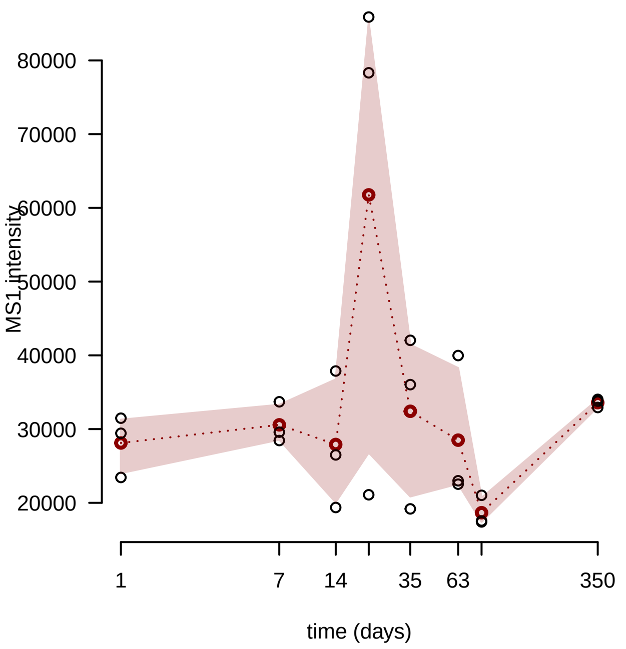

Q60770
GN=Stxbp3
syntaxin binding protein 3

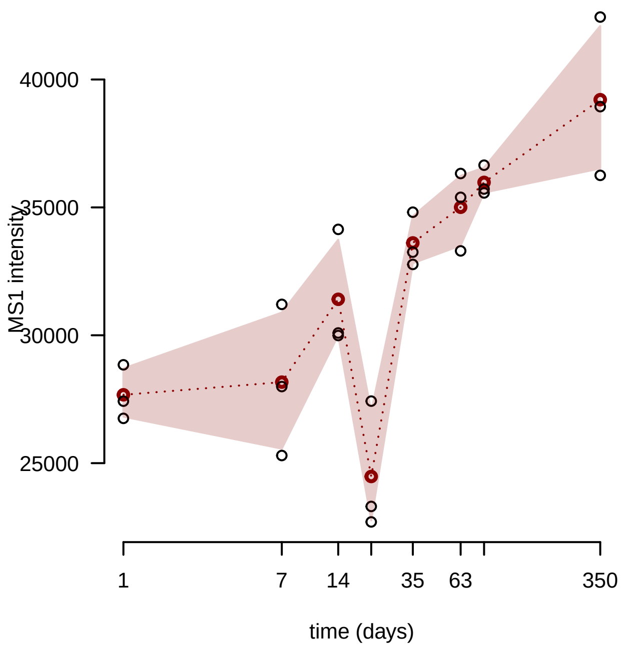

Q60772
GN=Cdkn2c
cyclin dependent kinase inhibitor 2C

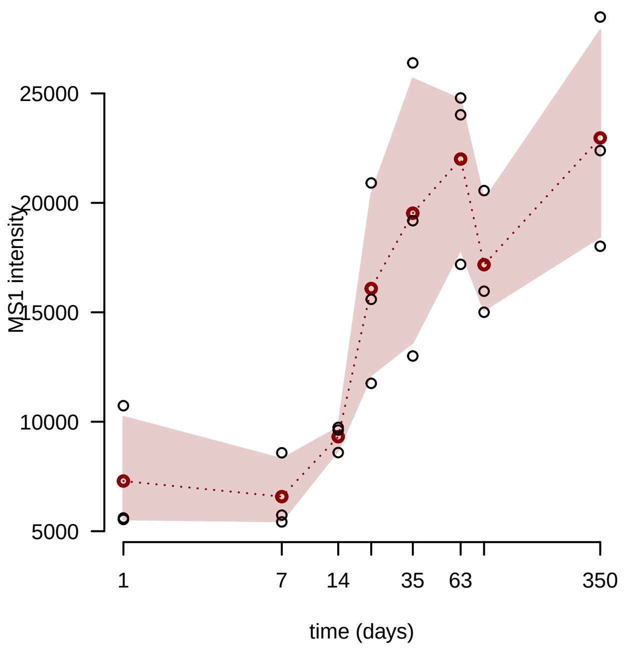

Q60775
GN=Elf1
E74 like ETS transcription factor 1

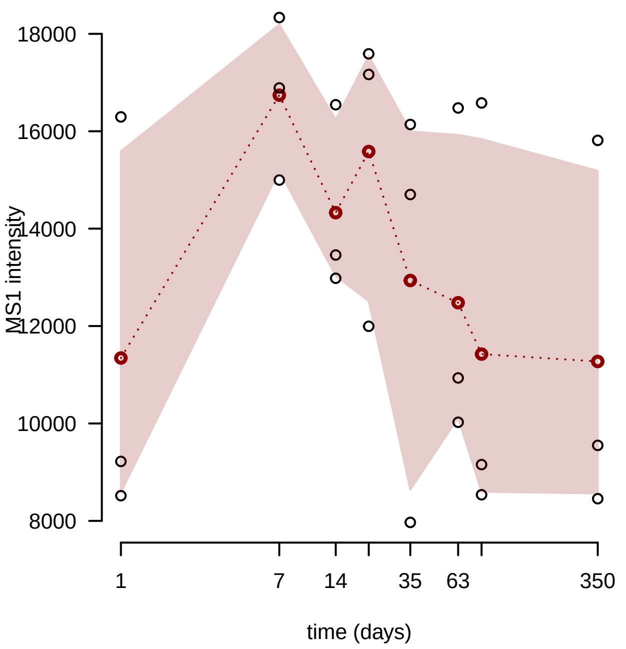

Q60778
GN=Nfkbib
nuclear factor of kappa light polypeptid ...

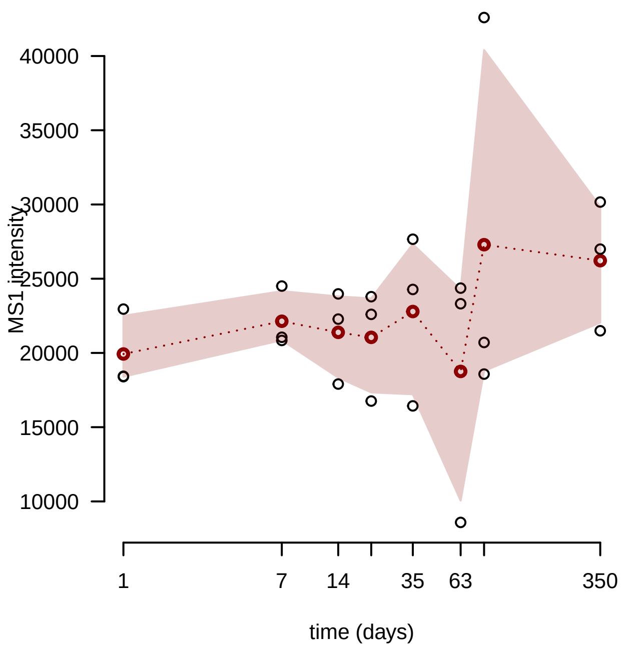

Q60780
GN=Gas7
growth arrest specific 7

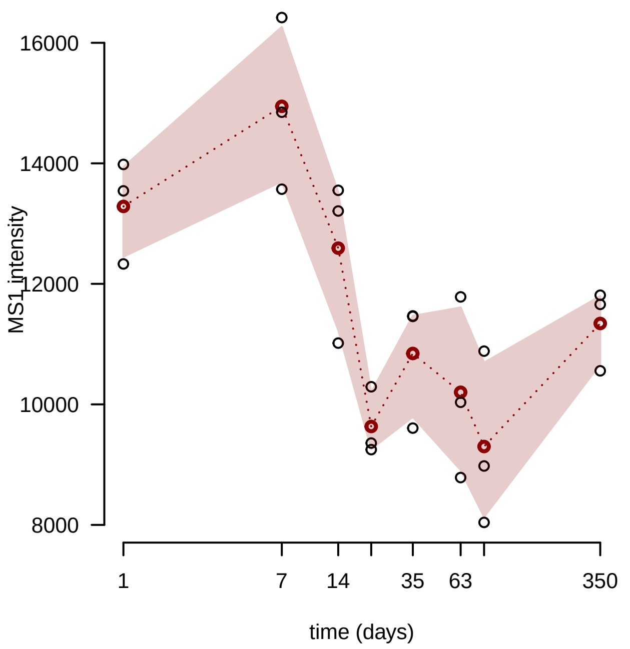

Q60787
GN=Lcp2
lymphocyte cytosolic protein 2

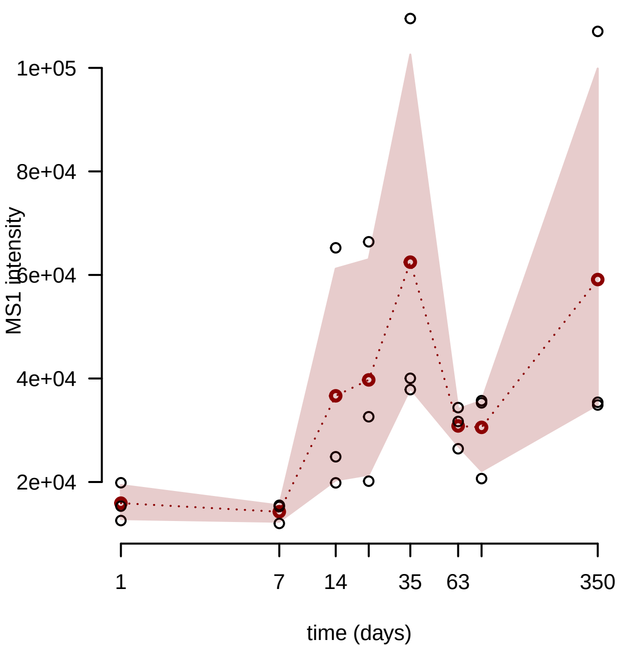

Q60790
GN=Rasa3
RAS p21 protein activator 3

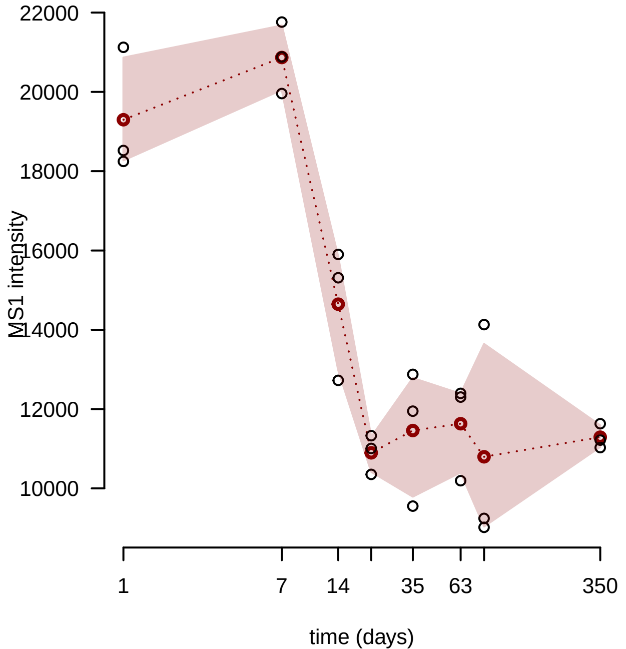

Q60803
GN=Traf3
TNF receptor−associated factor 3

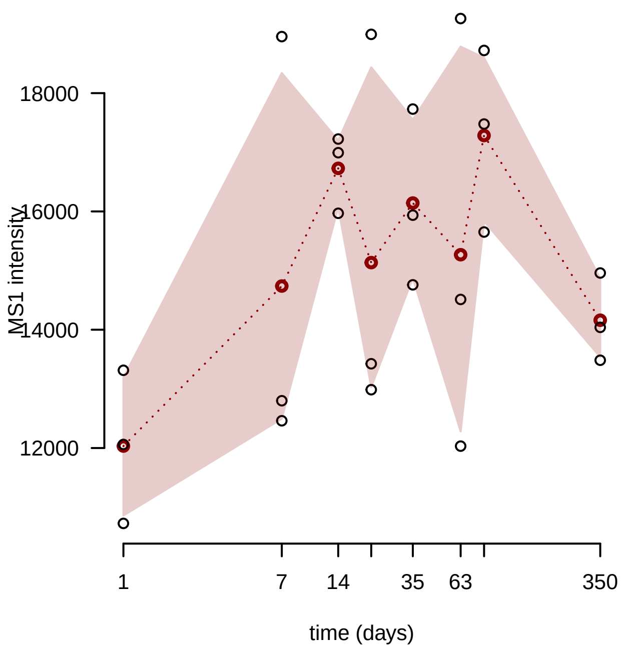

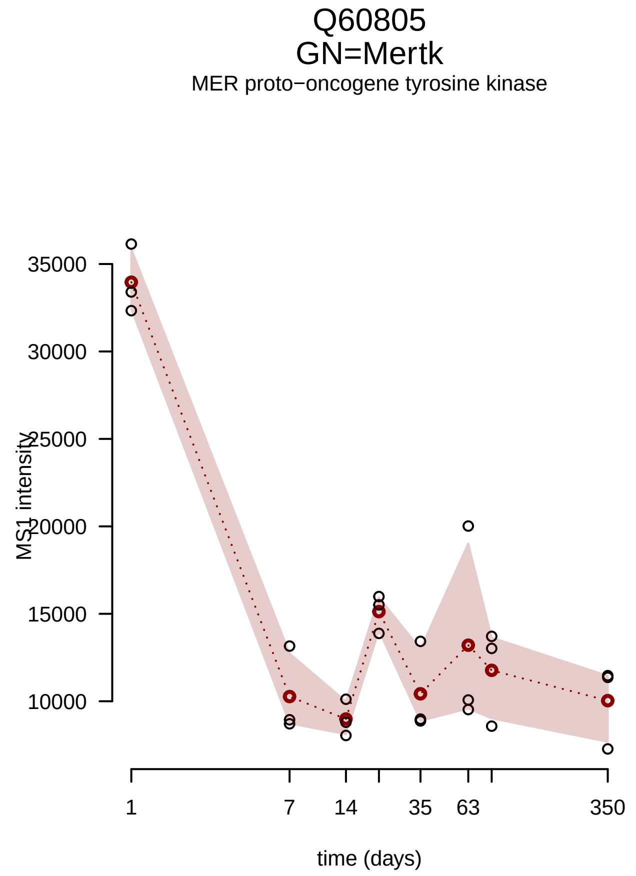
Q60805
GN=Mertk
MER proto−oncogene tyrosine kinase
MS1 intensity
10000
15000
20000
25000
30000
35000
1
7
14
35
63
350
time (days)
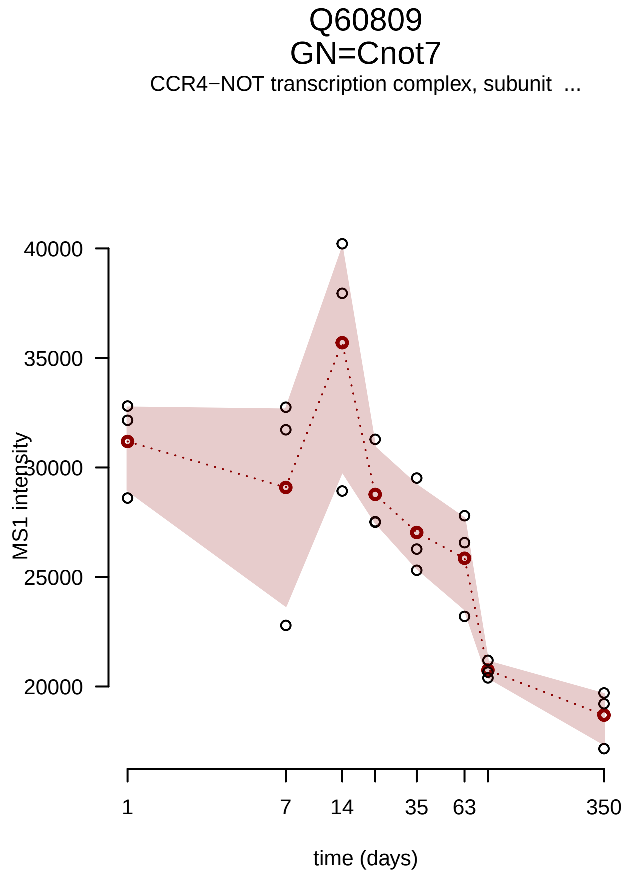
Q60809
GN=Cnot7
CCR4−NOT transcription complex, subunit ...
MS1 intensity
20000
25000
30000
35000
40000
1
7
14
35
63
350
time (days)
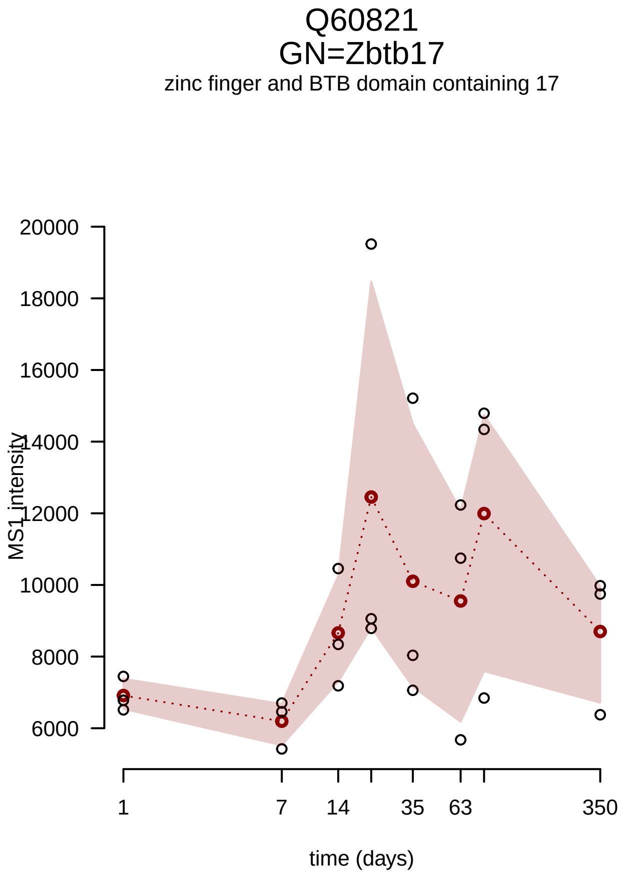
Q60821
GN=Zbtb17
zinc finger and BTB domain containing 17
MS1 intensity
6000
8000
10000
12000
14000
16000
18000
20000
1
7
14
35
63
350
time (days)
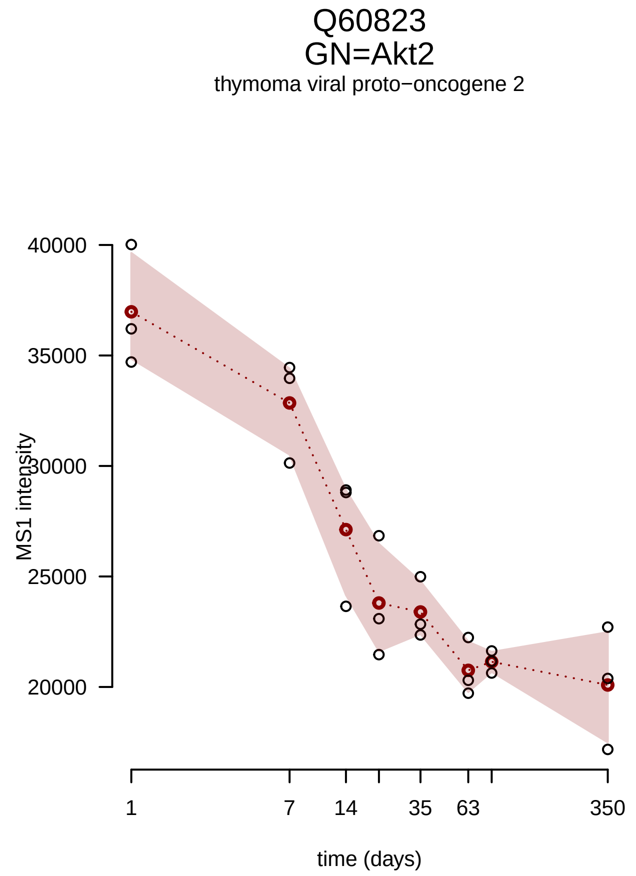
Q60823
GN=Akt2
thymoma viral proto−oncogene 2
MS1 intensity
20000
25000
30000
35000
40000
1
7
14
35
63
350
time (days)
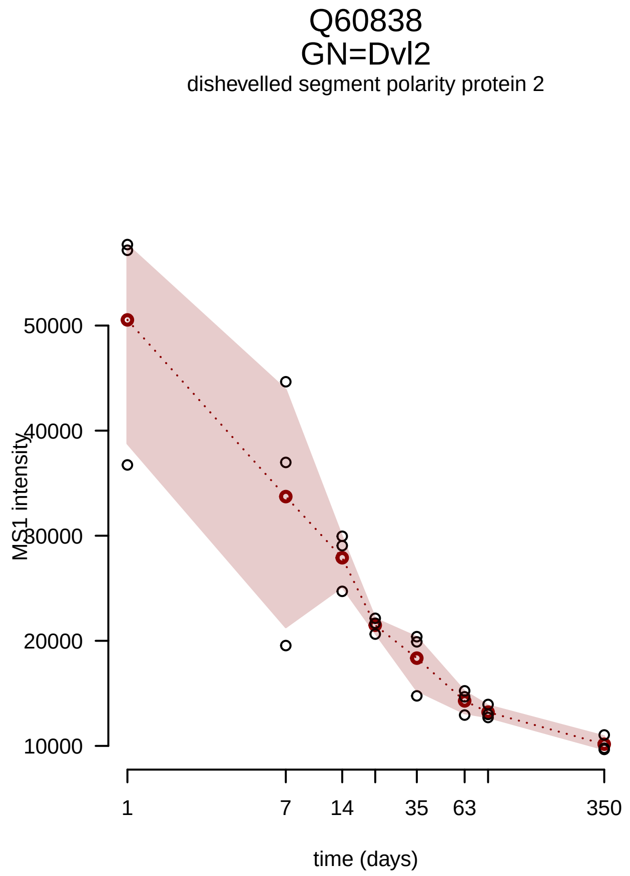
Q60838
GN=Dvl2
dishevelled segment polarity protein 2
MS1 intensity
10000
20000
30000
40000
50000
1
7
14
35
63
350
time (days)
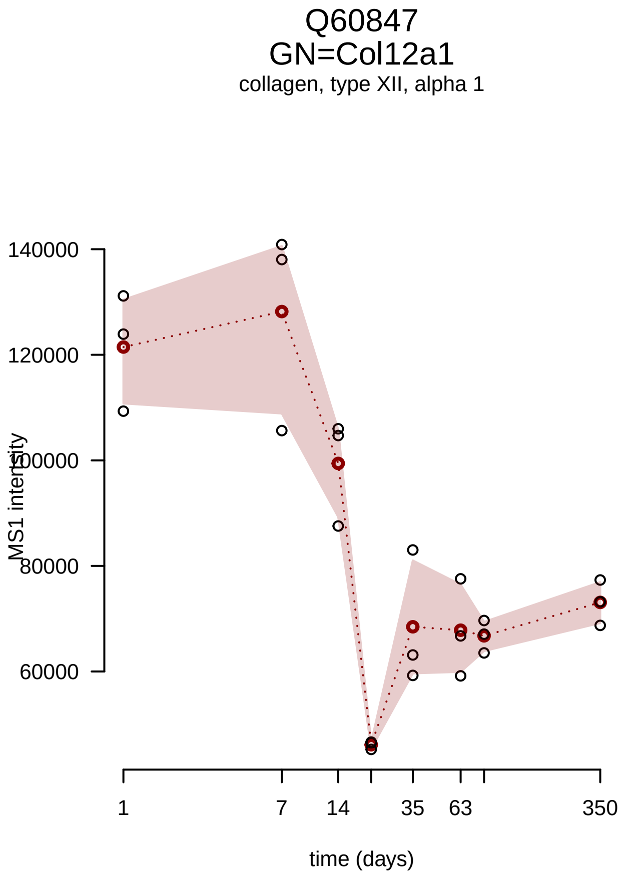
Q60847
GN=Col12a1
collagen, type XII, alpha 1
MS1 intensity
60000
80000
100000
120000
140000
1
7
14
35
63
350
time (days)
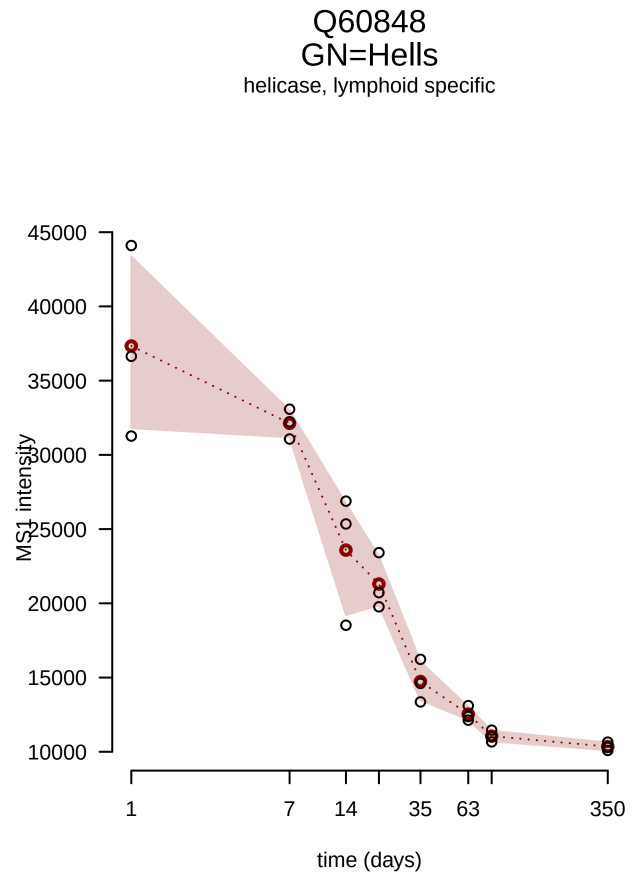
Q60848
GN=Hells
helicase, lymphoid specific
MS1 intensity
10000
15000
20000
25000
30000
35000
40000
45000
1
7
14
35
63
350
time (days)
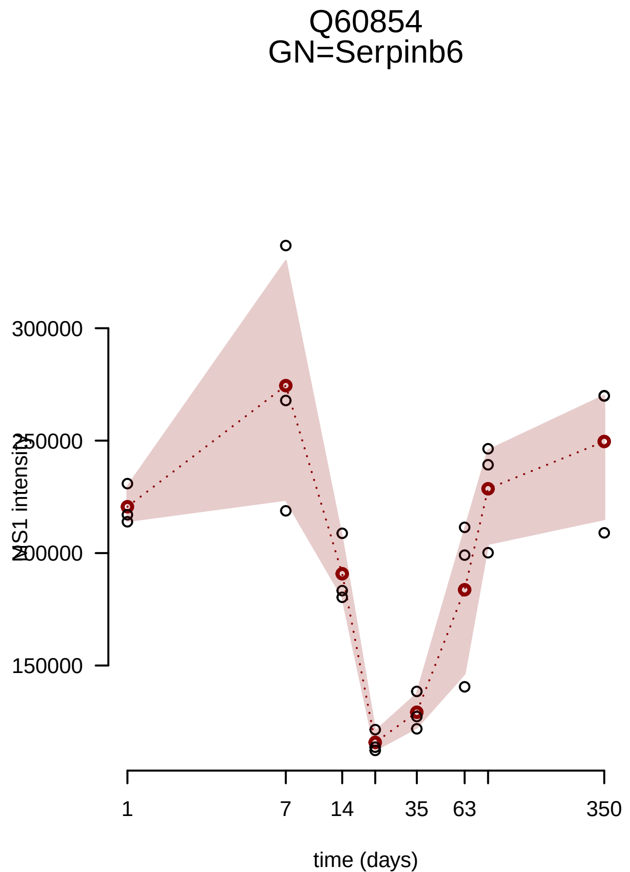
Q60854
GN=Serpinb6
MS1 intensity
150000
200000
250000
300000
1
7
14
35
63
350
time (days)
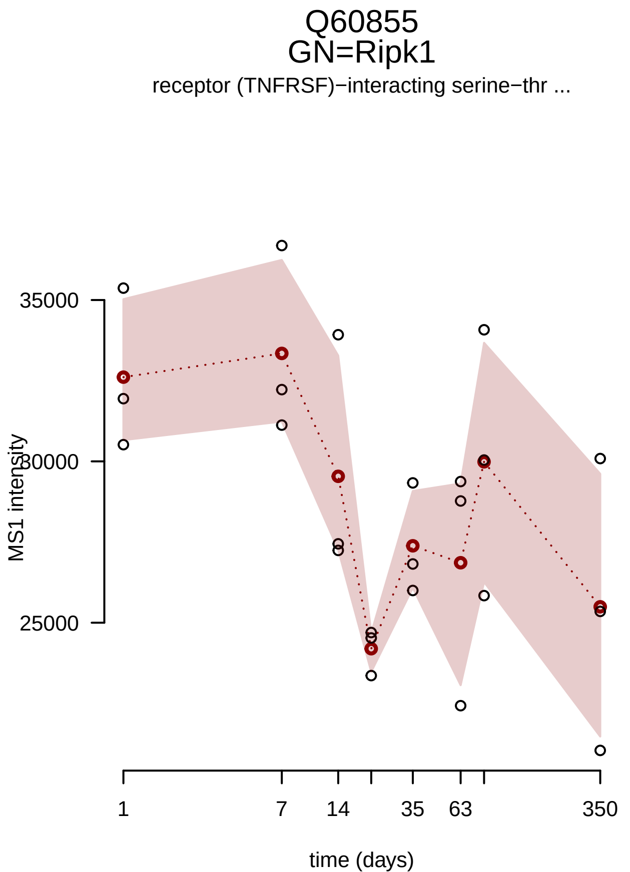
Q60855
GN=Ripk1
receptor (TNFRSF)−interacting serine−thr ...
MS1 intensity
25000
30000
35000
1
7
14
35
63
350
time (days)

Q60860
GN=Ltc4s
leukotriene C4 synthase

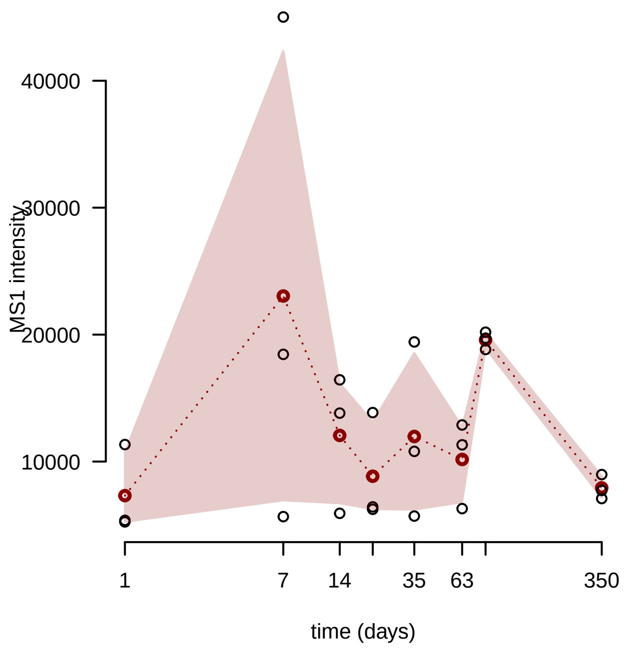

Q60862
GN=Orc2
origin recognition complex, subunit 2

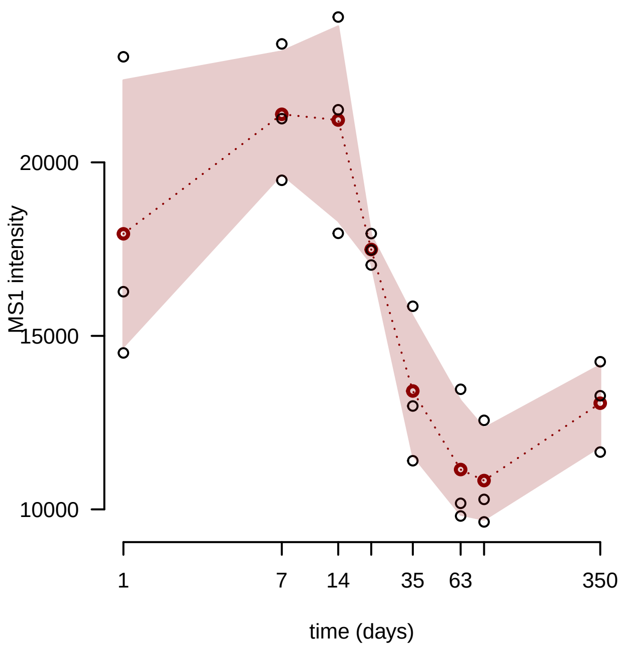

Q60864
GN=Stip1
stress−induced phosphoprotein 1

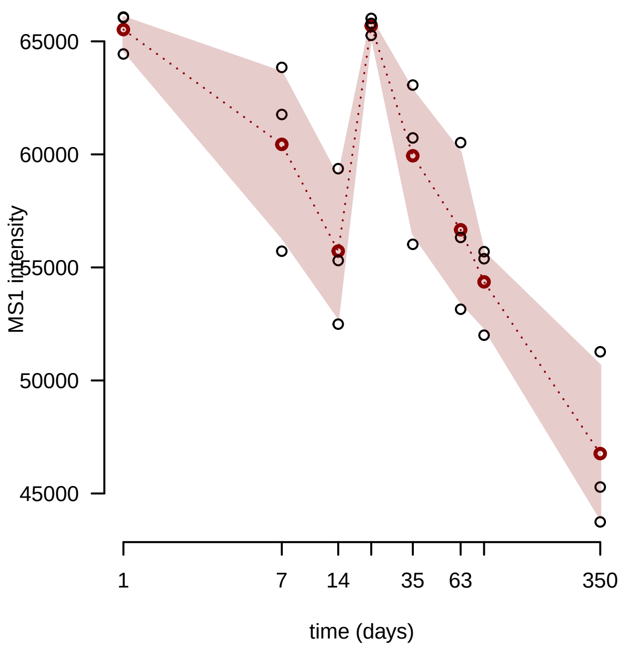

Q60865
GN=Caprin1
cell cycle associated protein 1

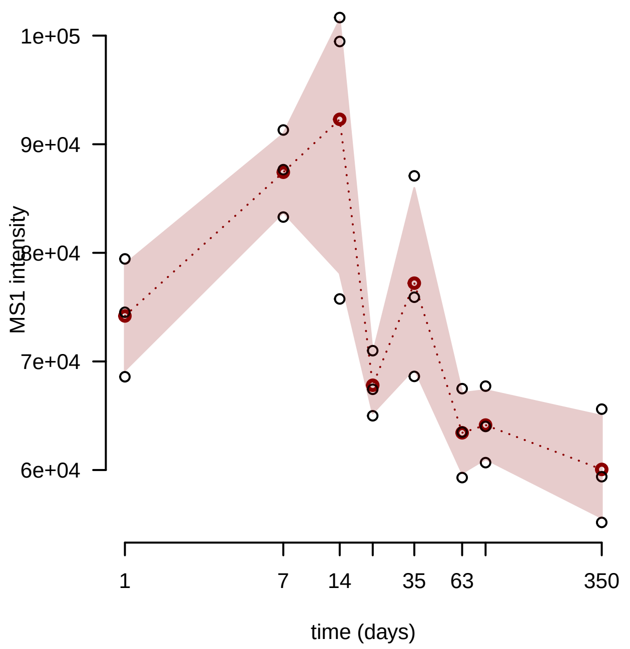

Q60866
GN=Pter
phosphotriesterase related

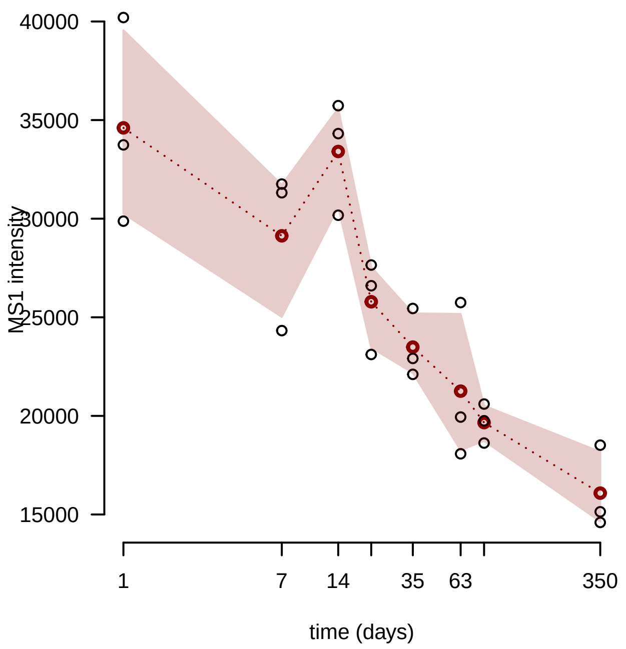

Q60870
GN=Reep5
receptor accessory protein 5

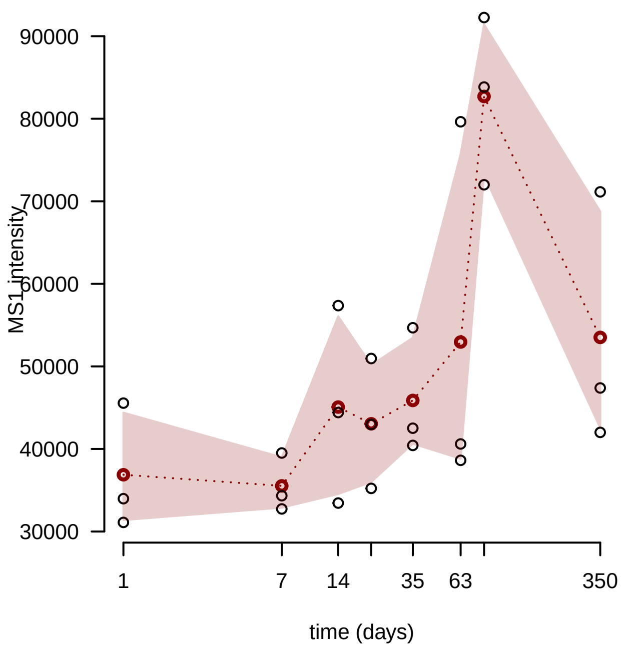

Q60872
GN=Eif1a
eukaryotic translation initiation factor ...

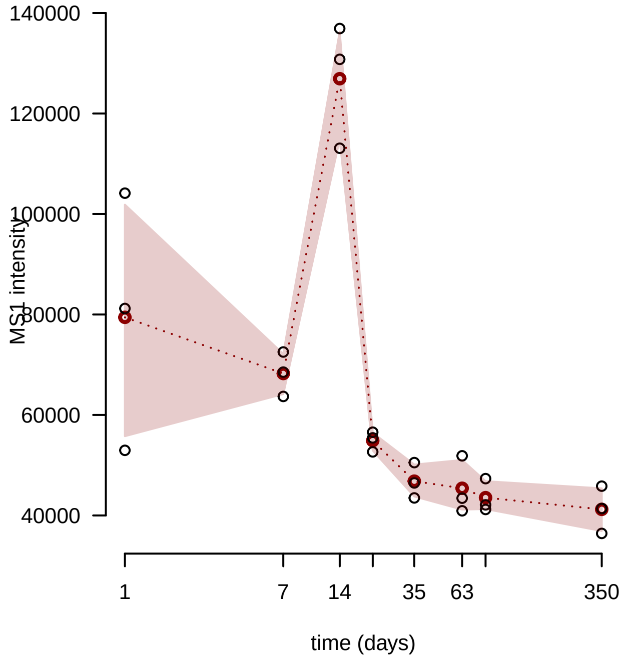

Q60875
GN=Arhgef2
rho/rac guanine nucleotide exchange fact ...

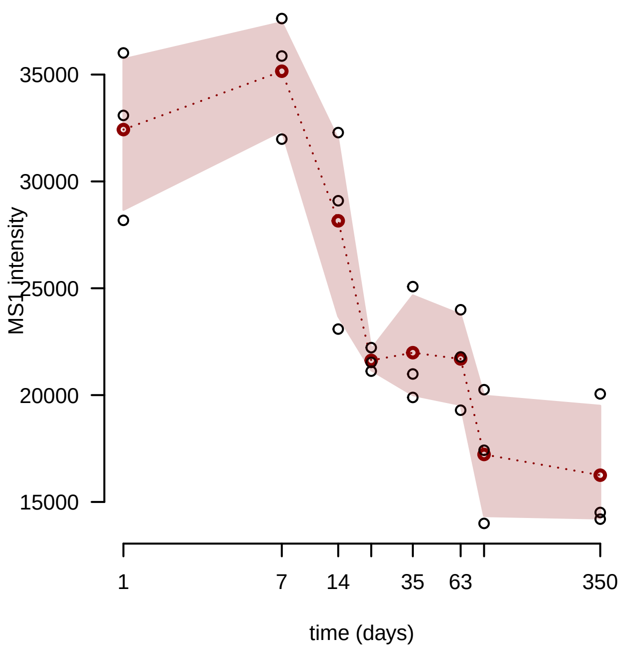

Q60899
GN=Elavl2
ELAV like RNA binding protein 1

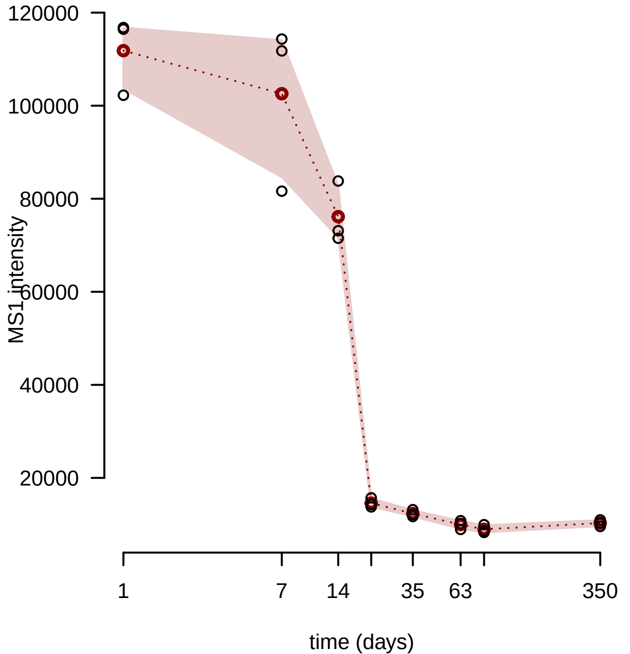

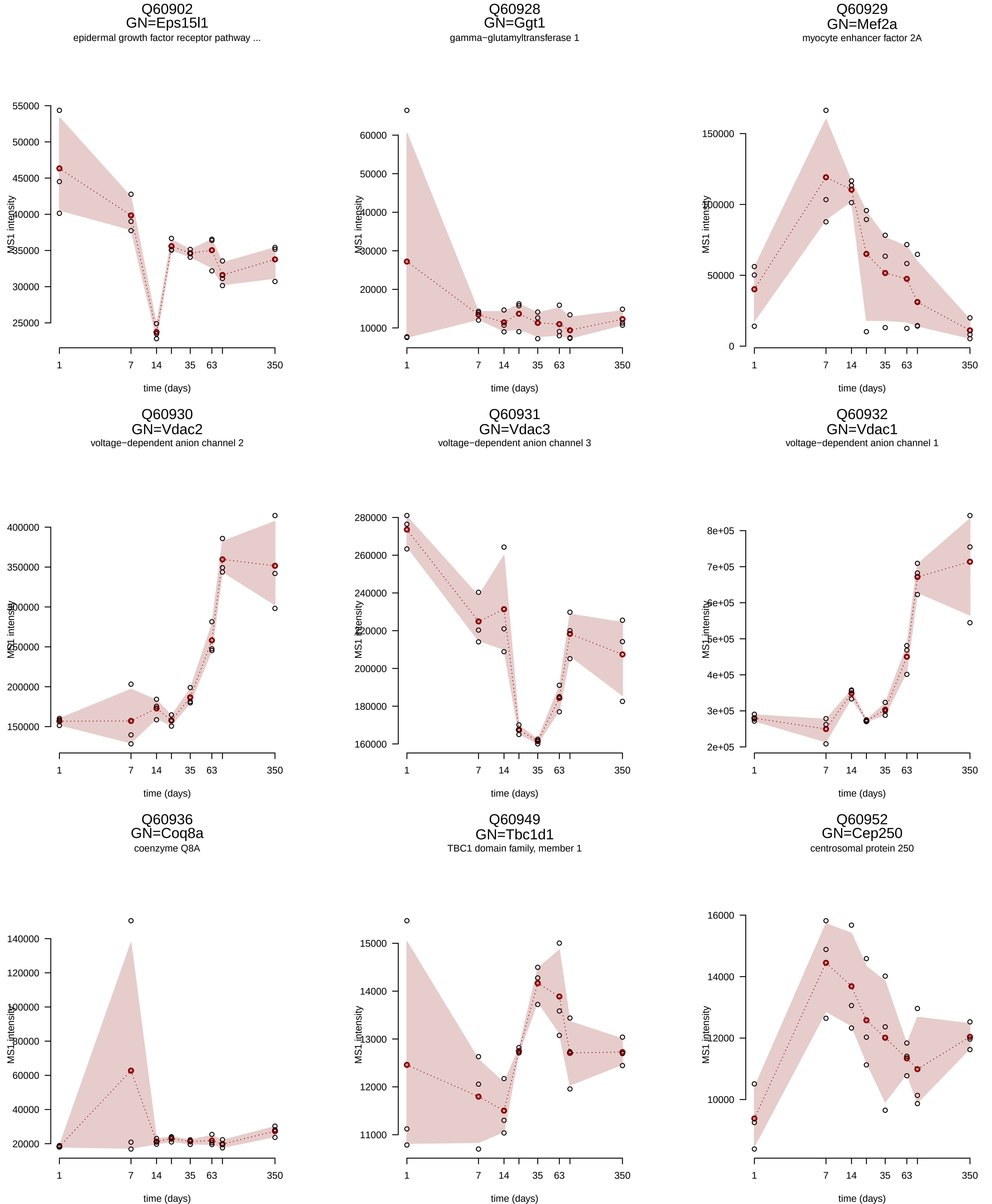
Q60902
GN=Eps15l1
epidermal growth factor receptor pathway ...
55000
50000
45000
40000
35000
30000
25000
MS1 intensity
1
7
14
35
63
350
time (days)
Q60928
GN=Ggt1
gamma−glutamyltransferase 1
60000
50000
40000
30000
20000
10000
MS1 intensity
1
7
14
35
63
350
time (days)
Q60929
GN=Mef2a
myocyte enhancer factor 2A
150000
100000
50000
0
MS1 intensity
1
7
14
35
63
350
time (days)
Q60930
GN=Vdac2
voltage−dependent anion channel 2
400000
350000
300000
250000
200000
150000
MS1 intensity
1
7
14
35
63
350
time (days)
Q60931
GN=Vdac3
voltage−dependent anion channel 3
280000
260000
240000
220000
200000
180000
160000
MS1 intensity
1
7
14
35
63
350
time (days)
Q60932
GN=Vdac1
voltage−dependent anion channel 1
8e+05
7e+05
6e+05
5e+05
4e+05
3e+05
2e+05
MS1 intensity
1
7
14
35
63
350
time (days)
Q60936
GN=Coq8a
coenzyme Q8A
140000
120000
100000
80000
60000
40000
20000
MS1 intensity
1
7
14
35
63
350
time (days)
Q60949
GN=Tbc1d1
TBC1 domain family, member 1
15000
14000
13000
12000
11000
MS1 intensity
1
7
14
35
63
350
time (days)
Q60952
GN=Cep250
centrosomal protein 250
16000
14000
12000
10000
MS1 intensity
1
7
14
35
63
350
time (days)

Q60953
GN=Pml
promyelocytic leukemia

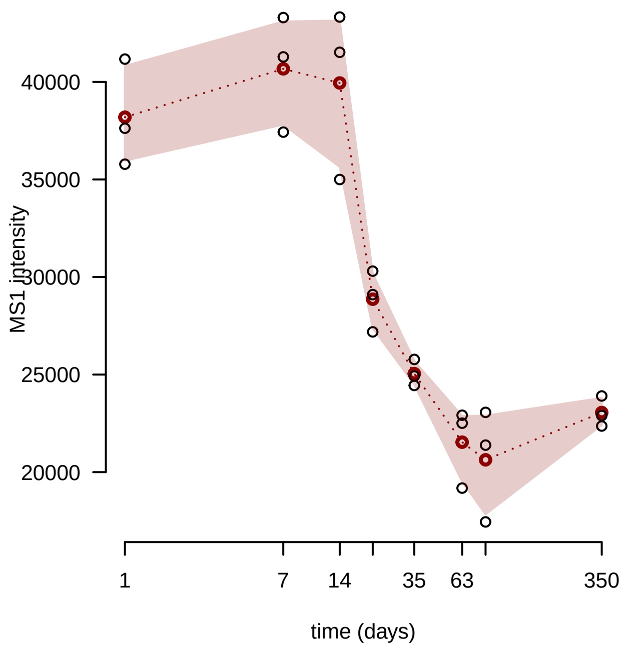

Q60954
GN=Meis1
Meis homeobox 1

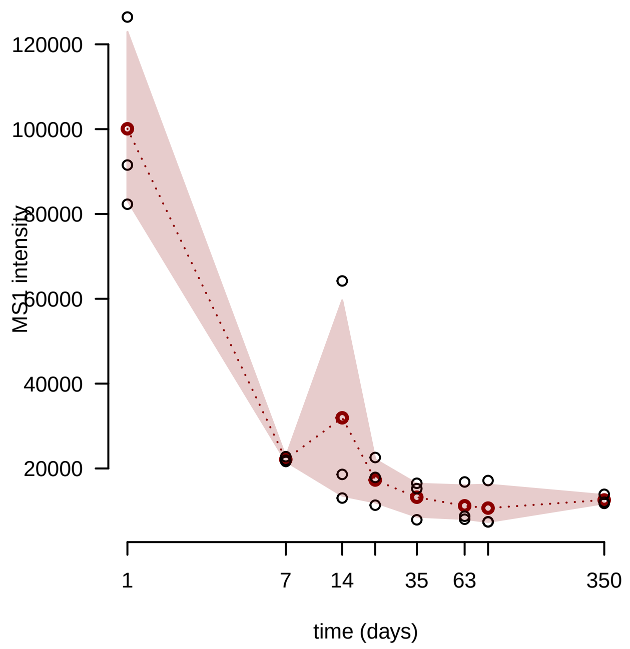

Q60960
GN=Kpna1
karyopherin (importin) alpha 1

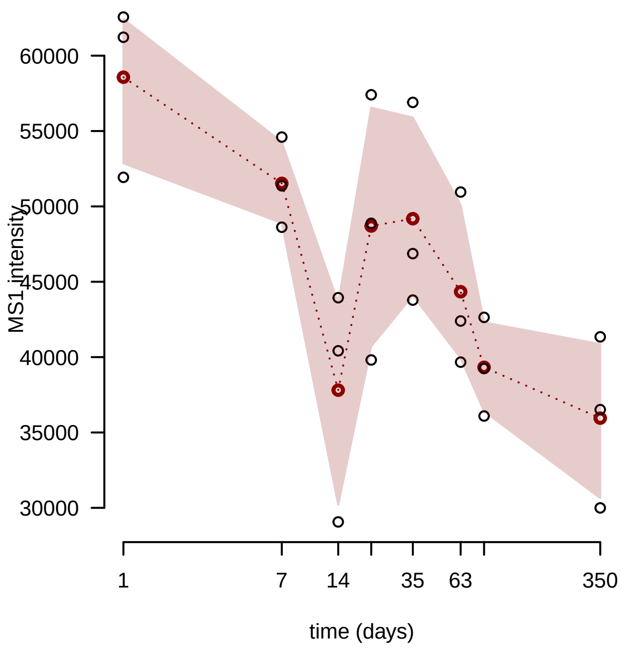

Q60961
GN=Laptm4a
lysosomal−associated protein transmembra ...

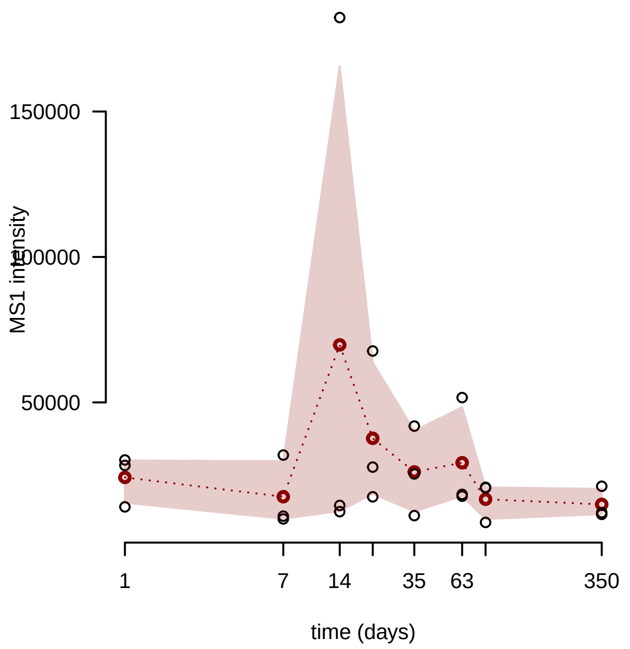

Q60963
GN=Pla2g7
phospholipase A2, group VII (platelet−ac ...

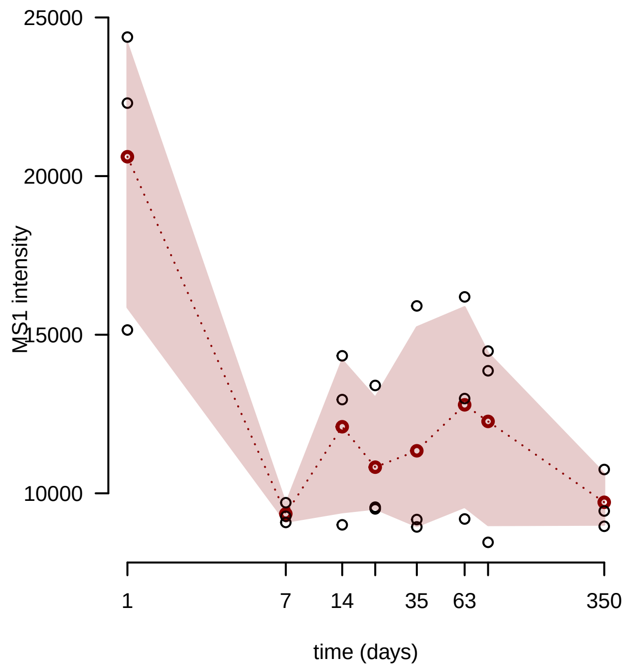

Q60967
GN=Papss1
3'−phosphoadenosine 5'−phosphosulfate sy ...

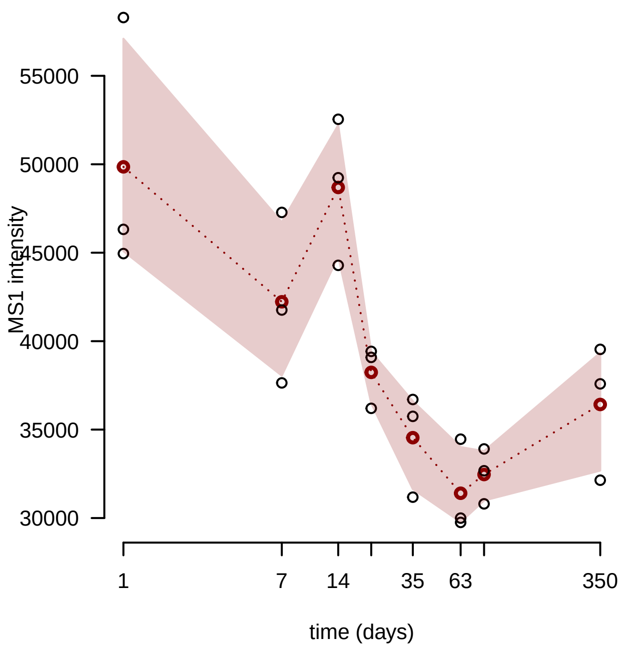

Q60969
GN=Styx
serine/threonine/tyrosine interaction pr ...

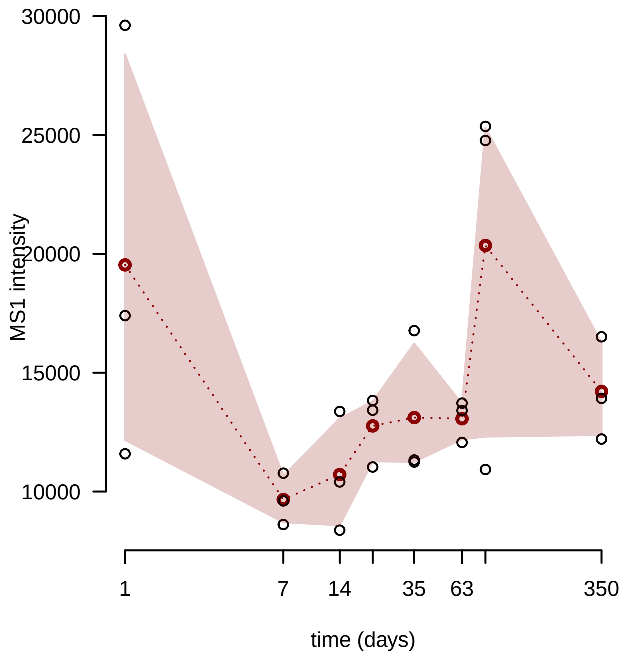

Q60972
GN=Rbbp4
retinoblastoma binding protein 4, chroma ...

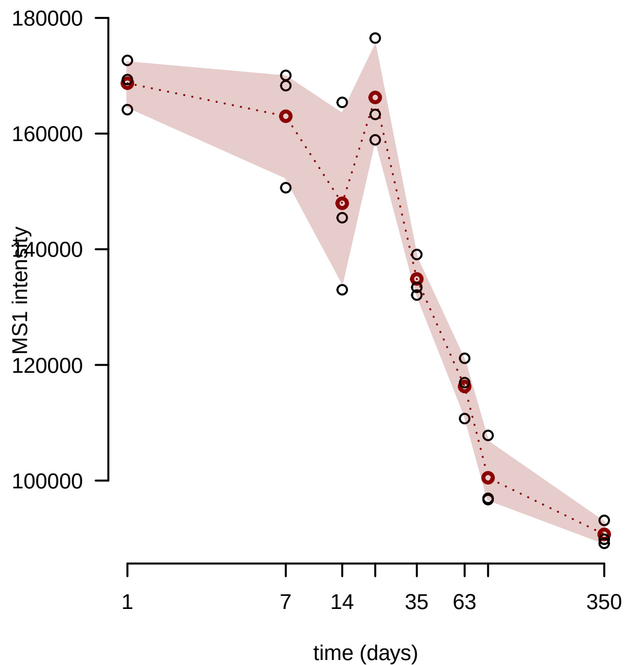

Q60973
GN=Rbbp7
retinoblastoma binding protein 7, chroma ...

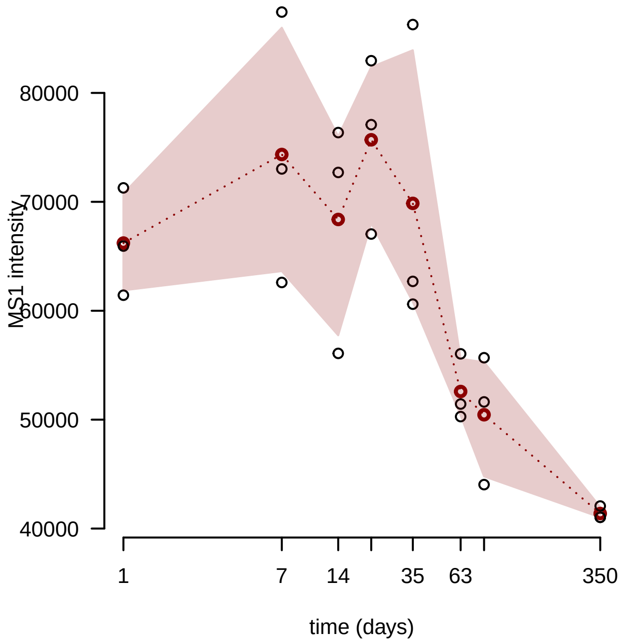

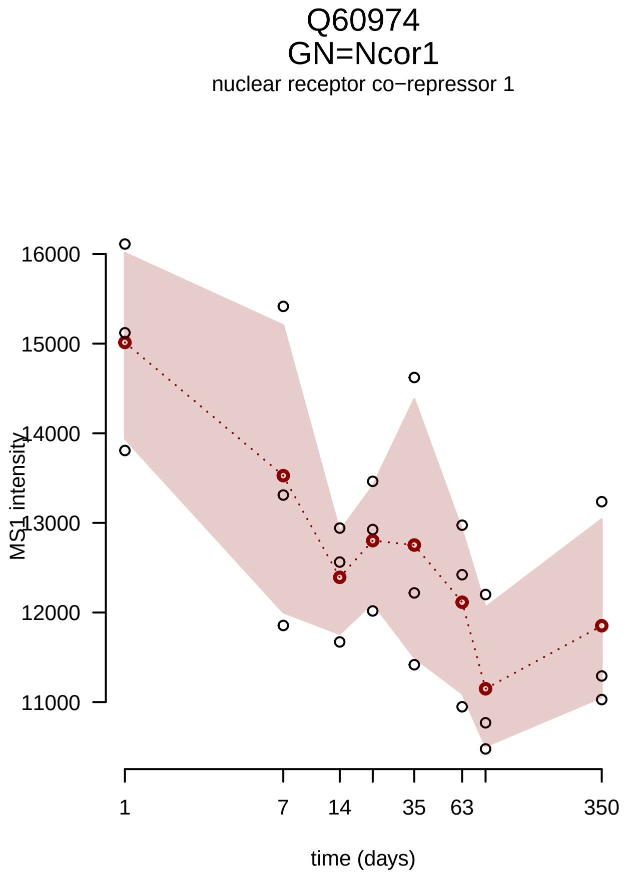
Q60974
GN=Ncor1
nuclear receptor co−repressor 1
MS1 intensity
16000
15000
14000
13000
12000
11000
1
7
14
35
63
350
time (days)

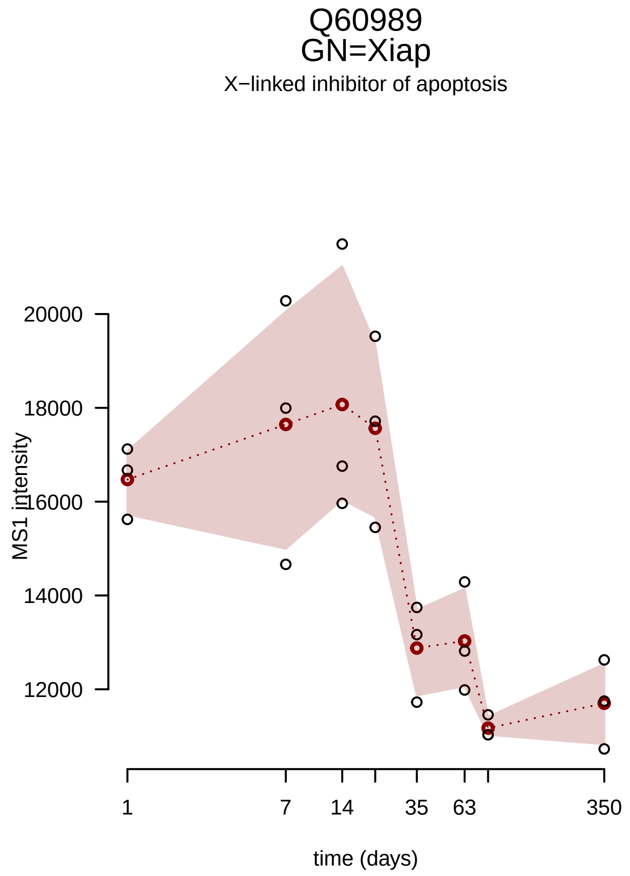
Q60989
GN=Xiap
X−linked inhibitor of apoptosis
MS1 intensity
20000
18000
16000
14000
12000
1
7
14
35
63
350
time (days)

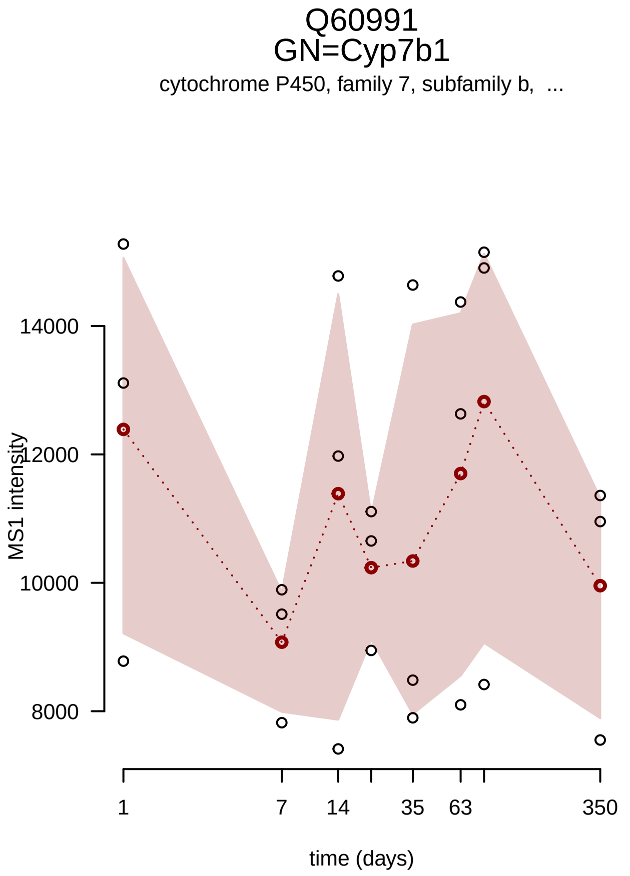
Q60991
GN=Cyp7b1
cytochrome P450, family 7, subfamily b, ...
MS1 intensity
14000
12000
10000
8000
1
7
14
35
63
350
time (days)

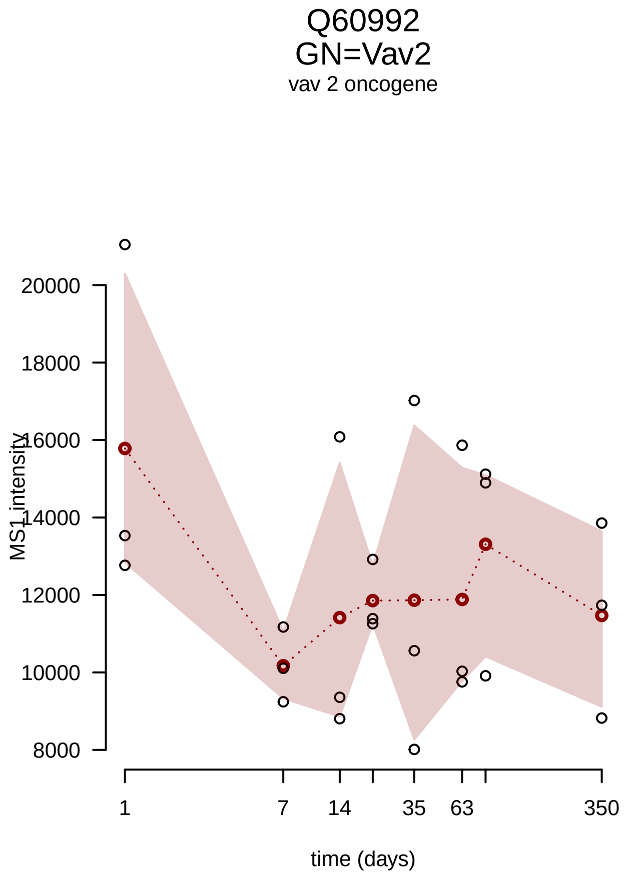
Q60992
GN=Vav2
vav 2 oncogene
MS1 intensity
20000
18000
16000
14000
12000
10000
8000
1
7
14
35
63
350
time (days)

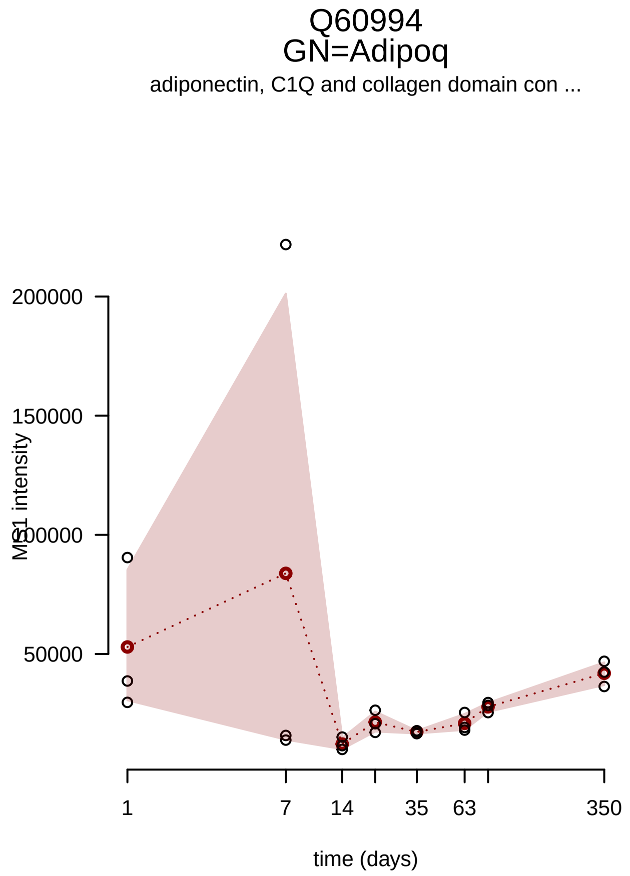
Q60994
GN=Adipoq
adiponectin, C1Q and collagen domain con ...
MS1 intensity
200000
150000
100000
50000
1
7
14
35
63
350
time (days)

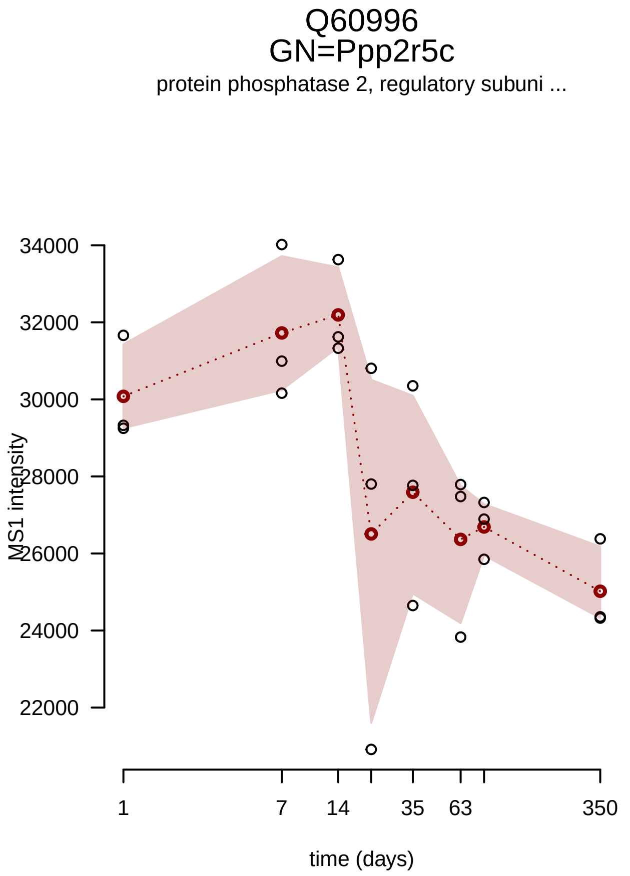
Q60996
GN=Ppp2r5c
protein phosphatase 2, regulatory subuni ...
MS1 intensity
34000
32000
30000
28000
26000
24000
22000
1
7
14
35
63
350
time (days)

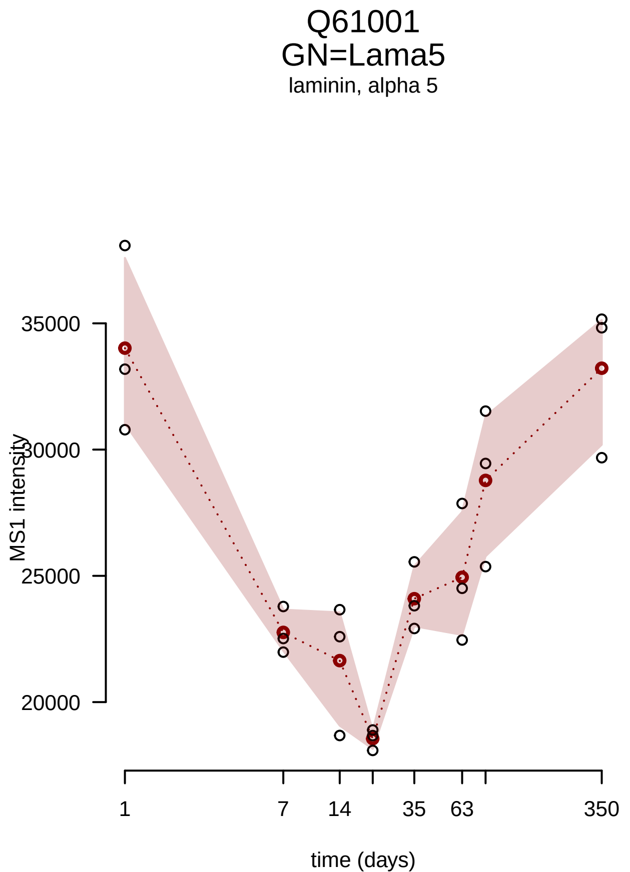
Q61001
GN=Lama5
laminin, alpha 5
MS1 intensity
35000
30000
25000
20000
1
7
14
35
63
350
time (days)

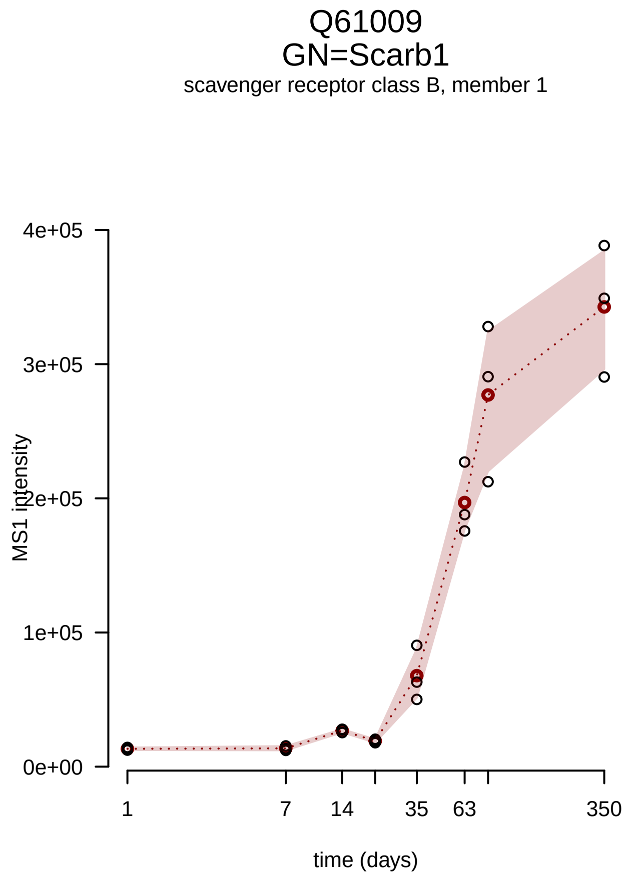
Q61009
GN=Scarb1
scavenger receptor class B, member 1
MS1 intensity
4e+05
3e+05
2e+05
1e+05
0e+00
1
7
14
35
63
350
time (days)

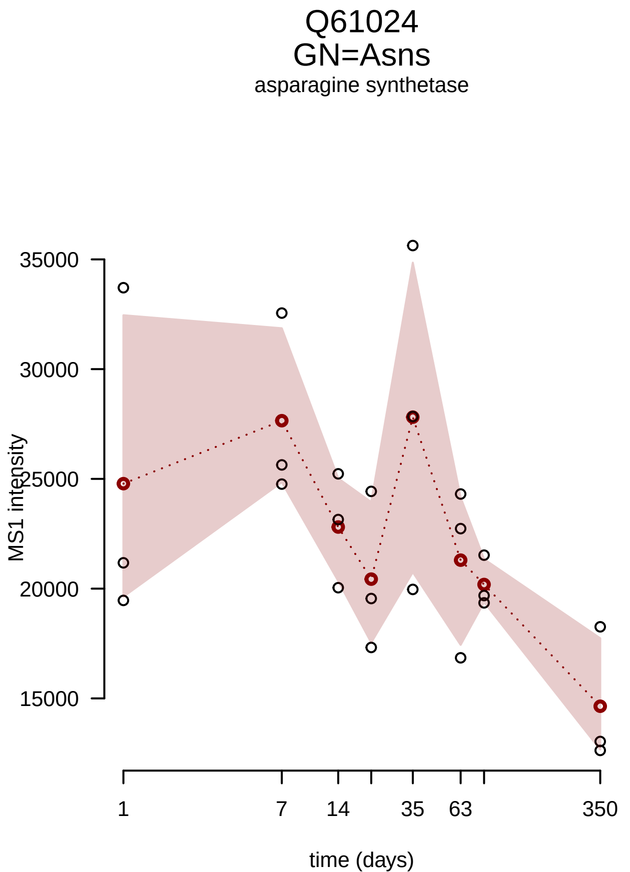
Q61024
GN=Asns
asparagine synthetase
MS1 intensity
35000
30000
25000
20000
15000
1
7
14
35
63
350
time (days)

Q61026
GN=Ncoa2
nuclear receptor coactivator 2

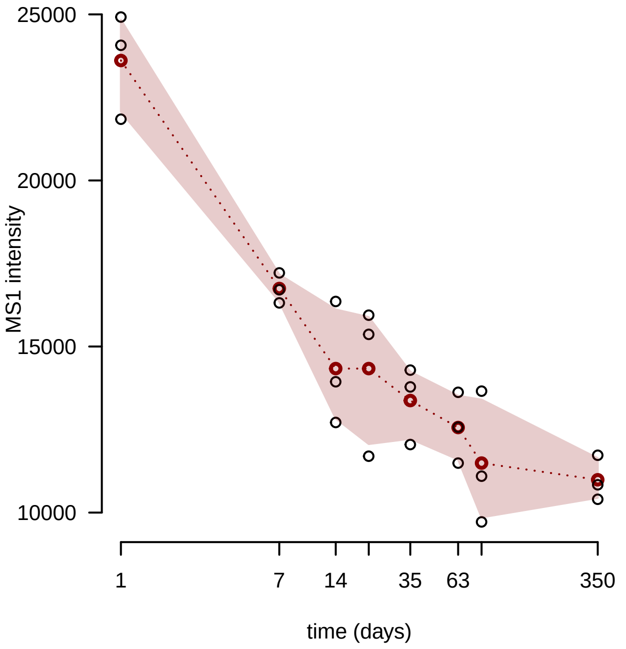

Q61029
GN=Tmpo
thymopoietin

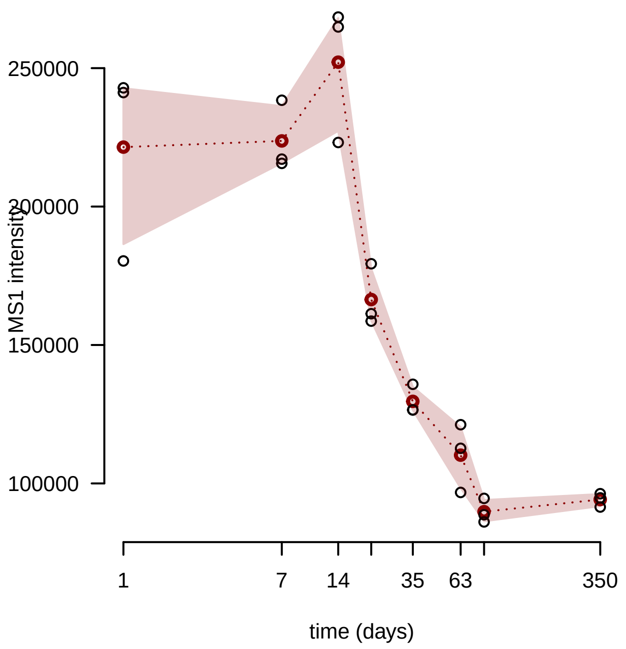

Q61029
GN=Tmpo
thymopoietin

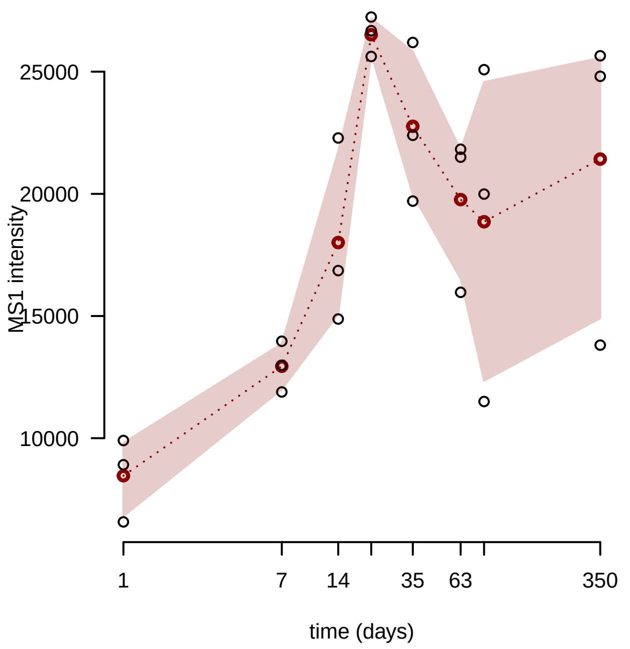

Q61033
GN=Tmpo

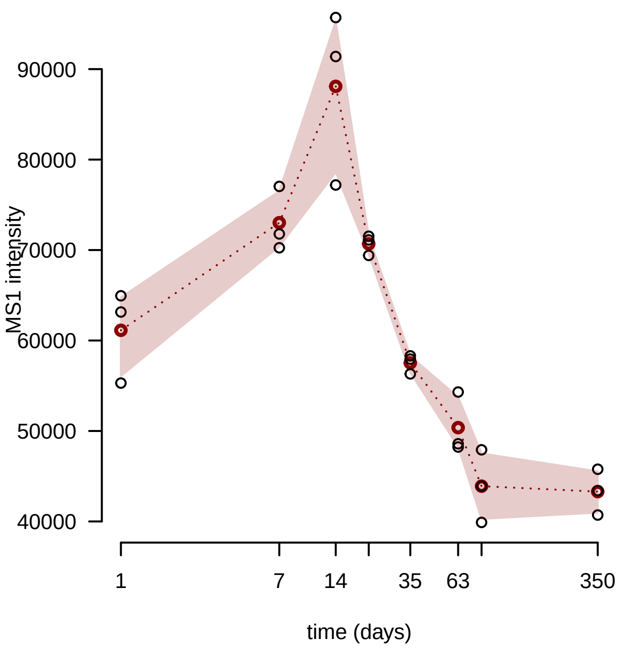

Q61035
GN=Hars
histidyl−tRNA synthetase

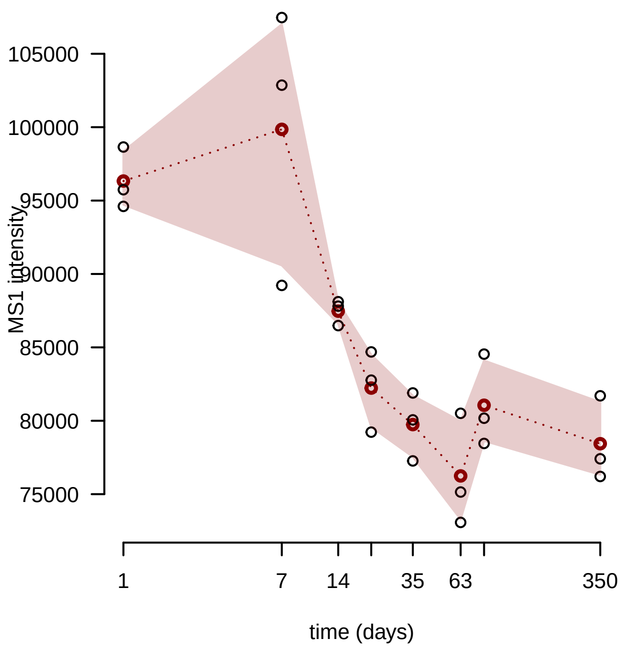

Q61036
GN=Pak3
p21 (RAC1) activated kinase 3

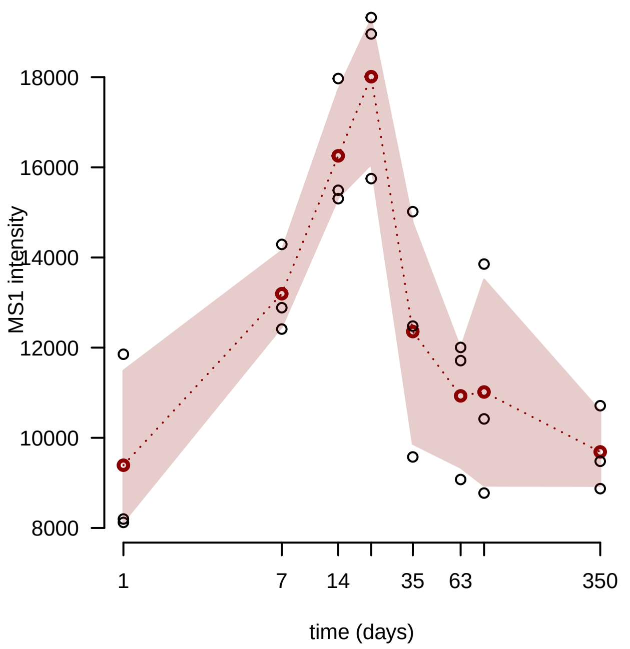

Q61037
GN=Tsc2
TSC complex subunit 2

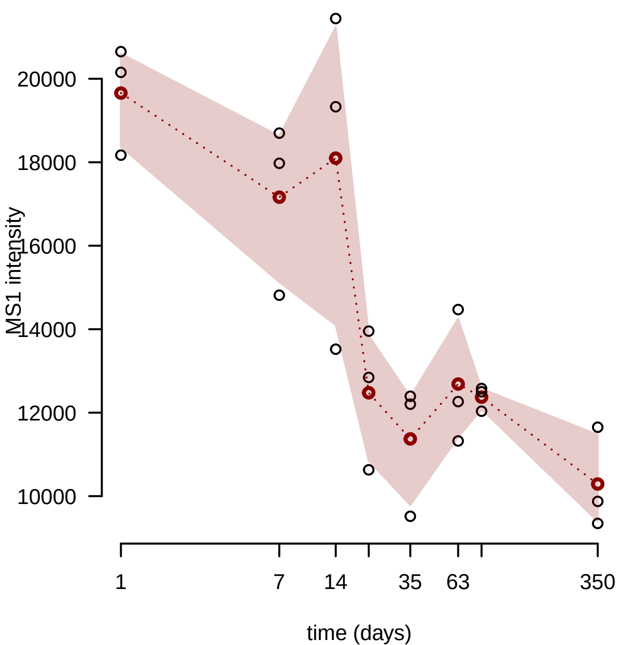

Q61048
GN=Wbp4
WW domain binding protein 4

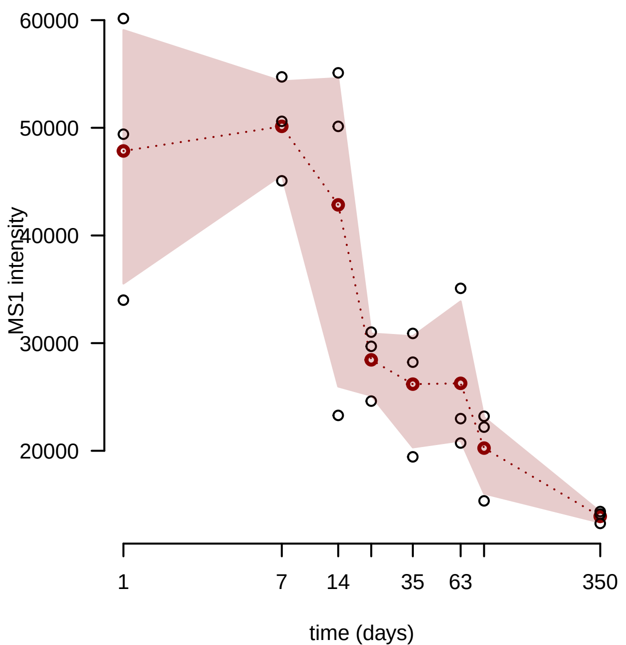

Q61062
GN=Dvl3
dishevelled segment polarity protein 3

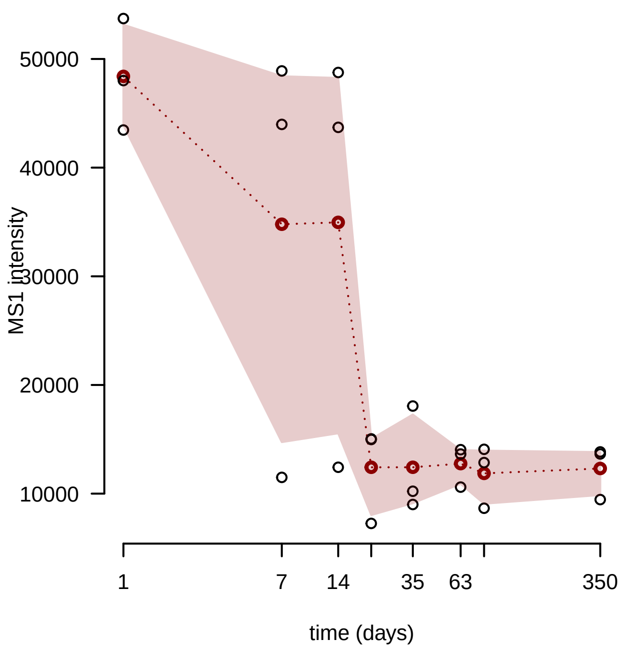

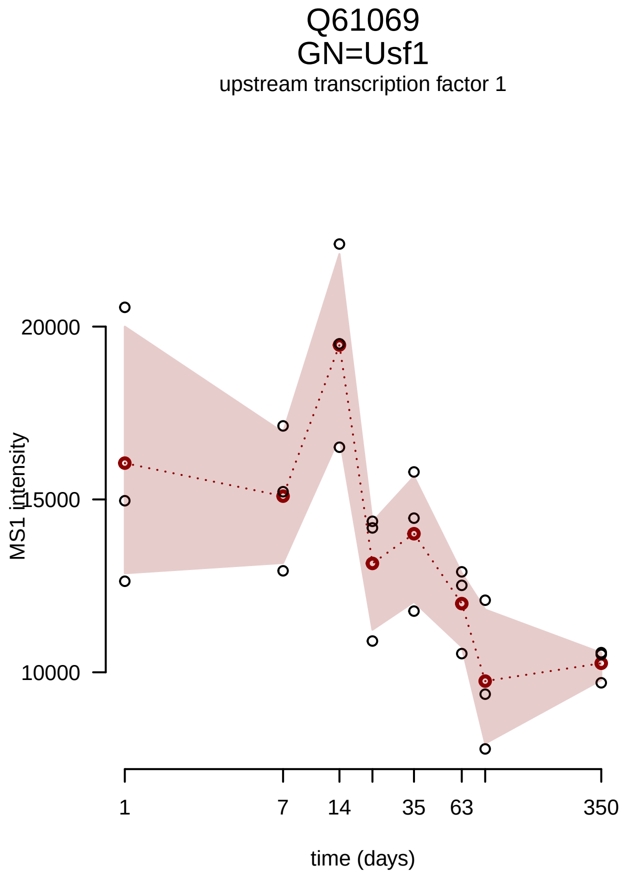
Q61069
GN=Usf1
upstream transcription factor 1
20000
15000
10000
MS1 intensity
1
7
14
35
63
350
time (days)

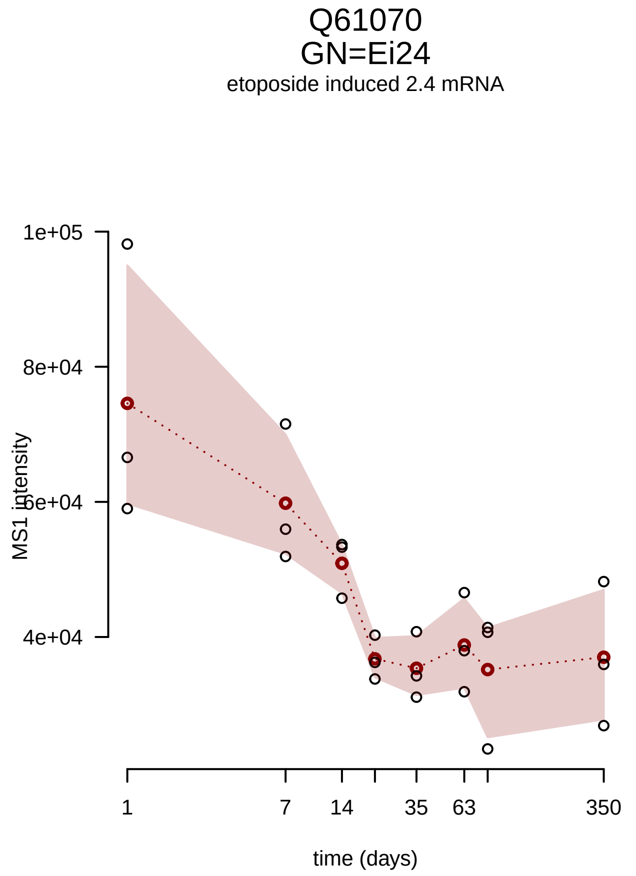
Q61070
GN=Ei24
etoposide induced 2.4 mRNA
1e+05
8e+04
6e+04
4e+04
MS1 intensity
1
7
14
35
63
350
time (days)

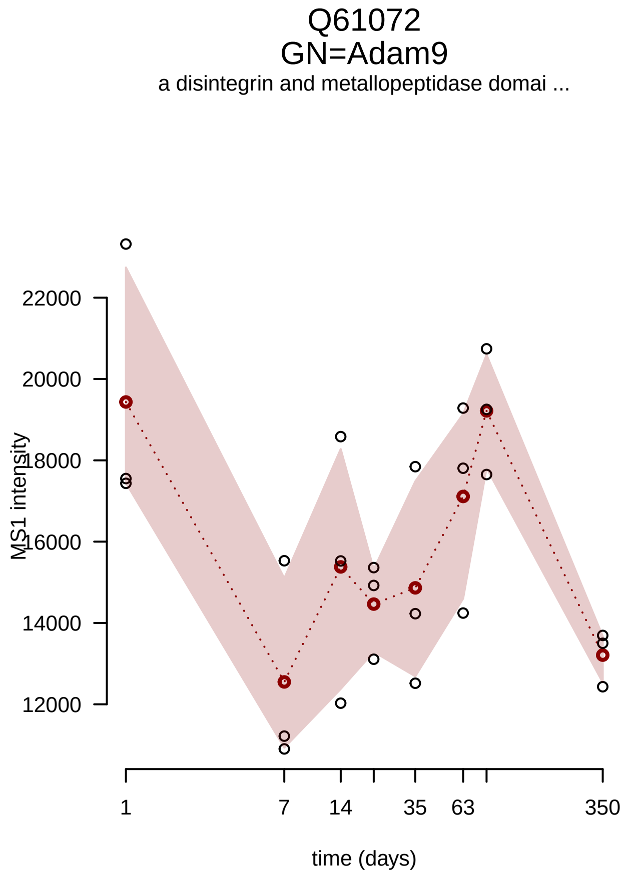
Q61072
GN=Adam9
a disintegrin and metallopeptidase domai ...
22000
20000
18000
16000
14000
12000
MS1 intensity
1
7
14
35
63
350
time (days)

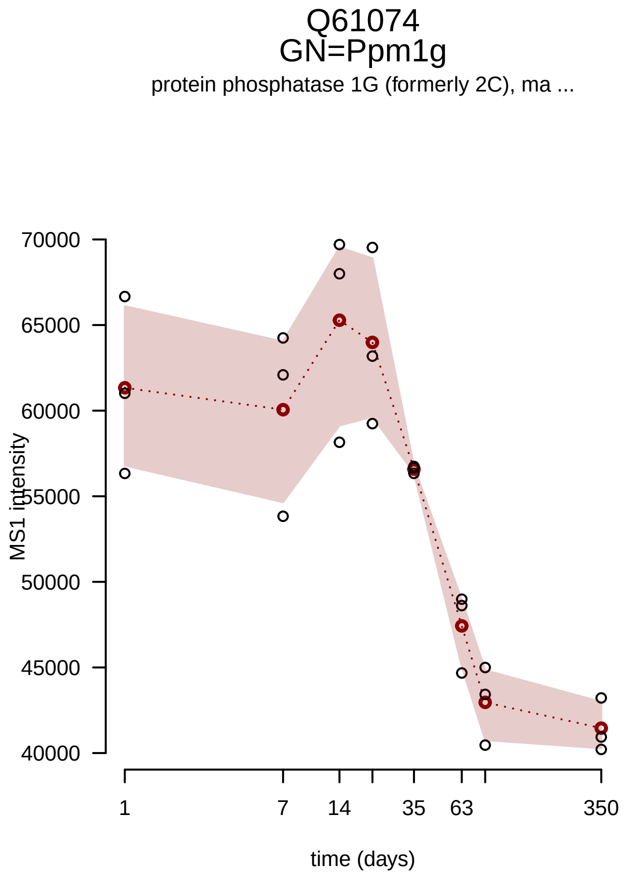
Q61074
GN=Ppm1g
protein phosphatase 1G (formerly 2C), ma ...
70000
65000
60000
55000
50000
45000
40000
MS1 intensity
1
7
14
35
63
350
time (days)

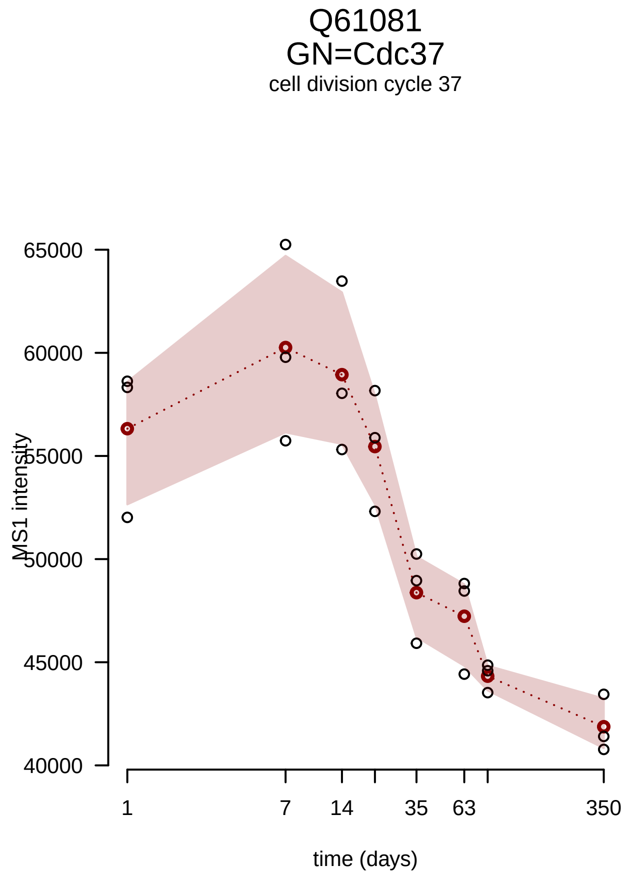
Q61081
GN=Cdc37
cell division cycle 37
65000
60000
55000
50000
45000
40000
MS1 intensity
1
7
14
35
63
350
time (days)

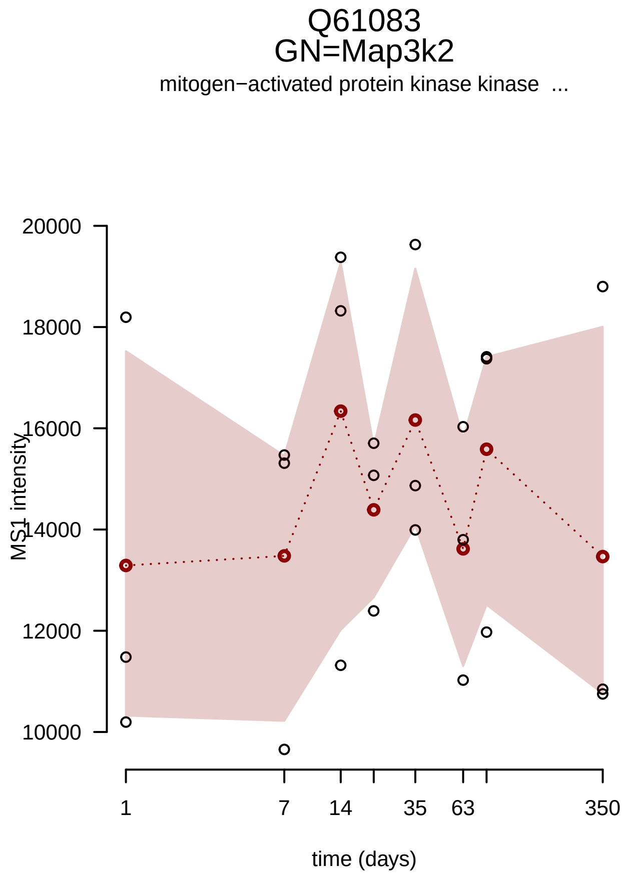
Q61083
GN=Map3k2
mitogen−activated protein kinase kinase ...
20000
18000
16000
14000
12000
10000
MS1 intensity
1
7
14
35
63
350
time (days)

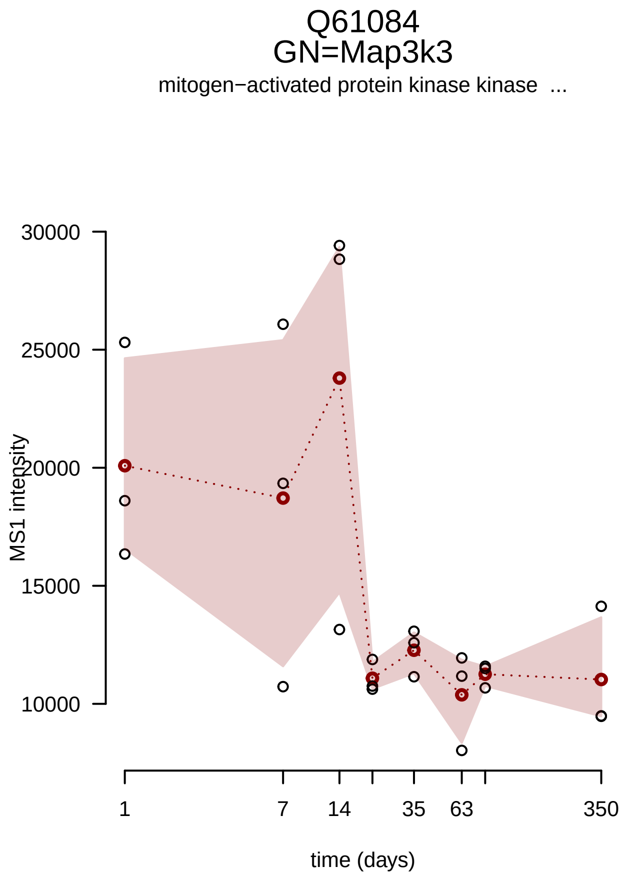
Q61084
GN=Map3k3
mitogen−activated protein kinase kinase ...
30000
25000
20000
15000
10000
MS1 intensity
1
7
14
35
63
350
time (days)

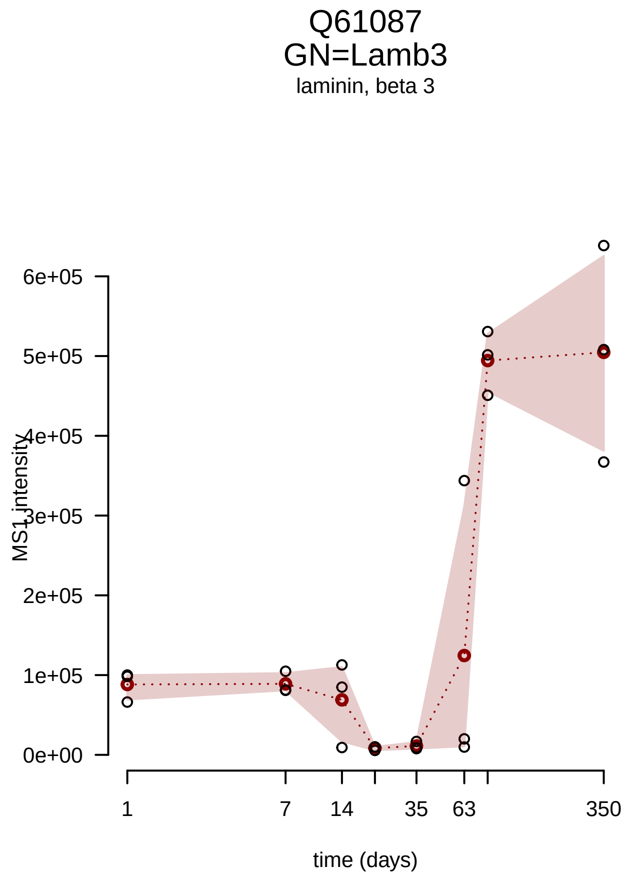
Q61087
GN=Lamb3
laminin, beta 3
6e+05
5e+05
4e+05
3e+05
2e+05
1e+05
0e+00
MS1 intensity
1
7
14
35
63
350
time (days)

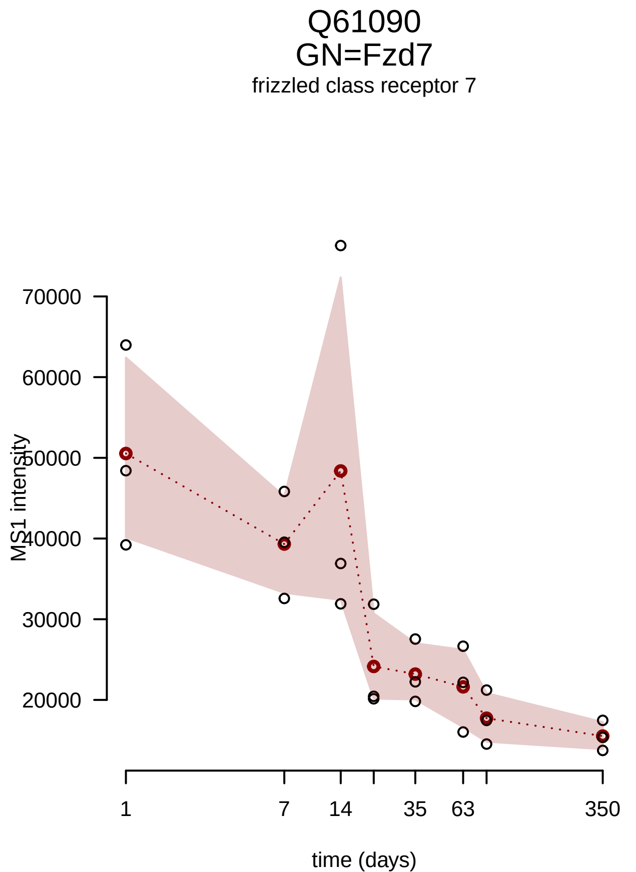
Q61090
GN=Fzd7
frizzled class receptor 7
70000
60000
50000
40000
30000
20000
MS1 intensity
1
7
14
35
63
350
time (days)

Q61097
GN=Ksr1
kinase suppressor of ras 1

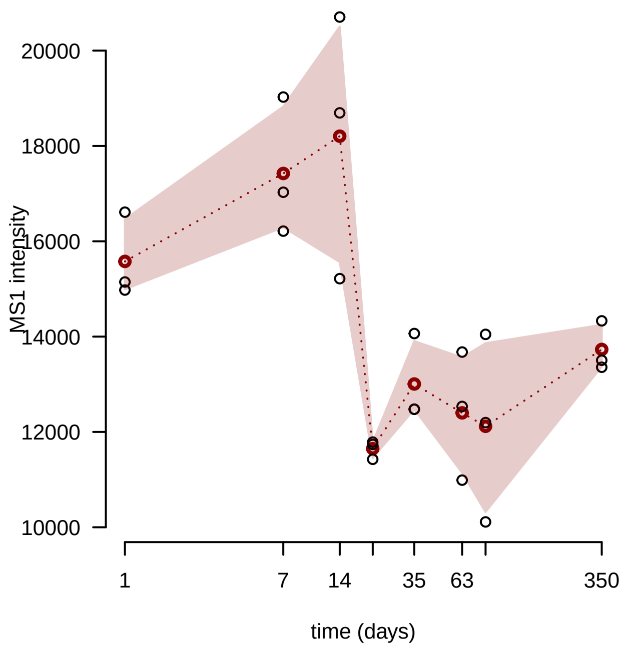

Q61102
GN=Abcb7
ATP−binding cassette, sub−family B (MDR/ ...

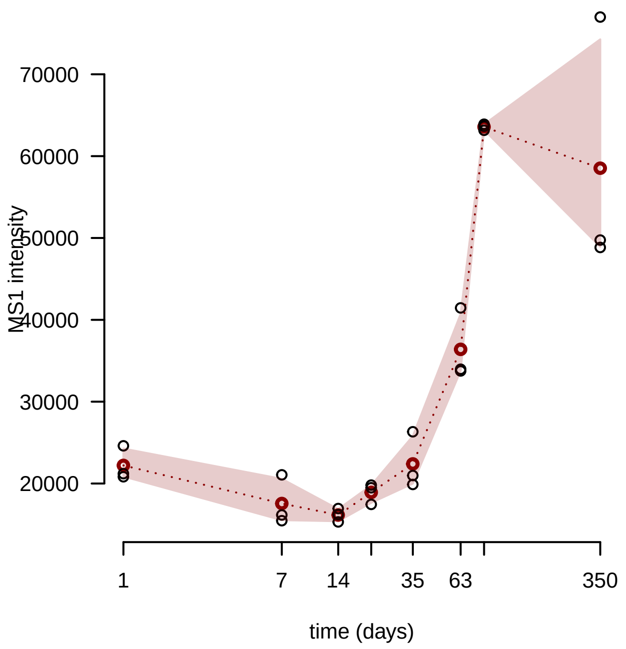

Q61103
GN=Dpf2
double PHD fingers 2

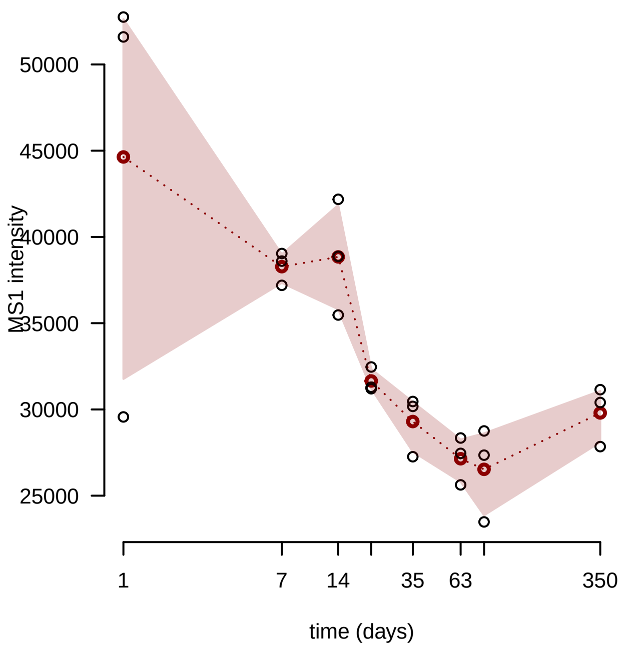

Q61107
GN=Gbp4

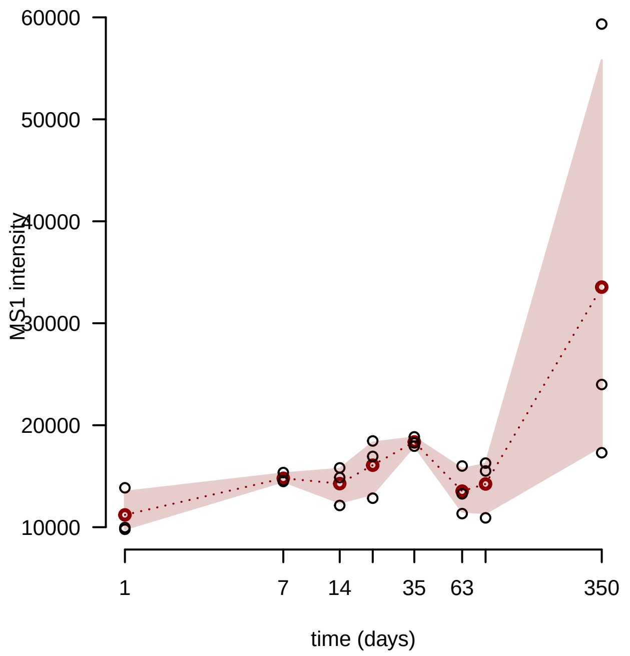

Q61112
GN=Sdf4
stromal cell derived factor 4

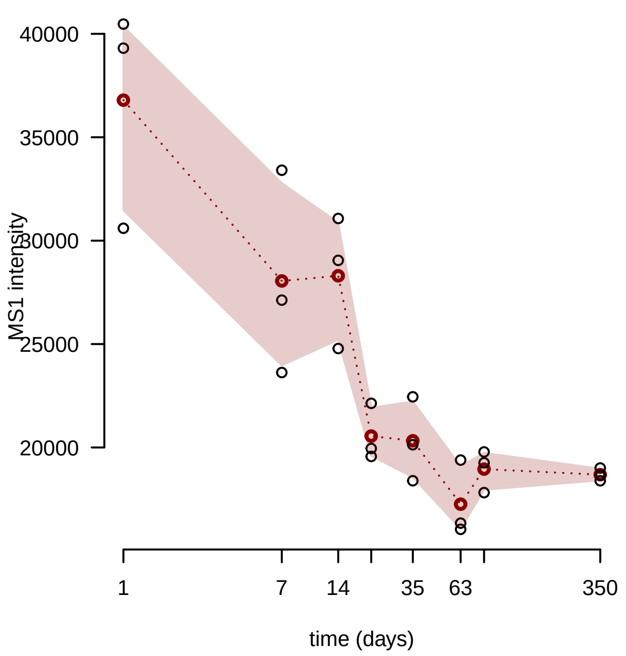

Q61122
GN=Nab1
Ngfi−A binding protein 1

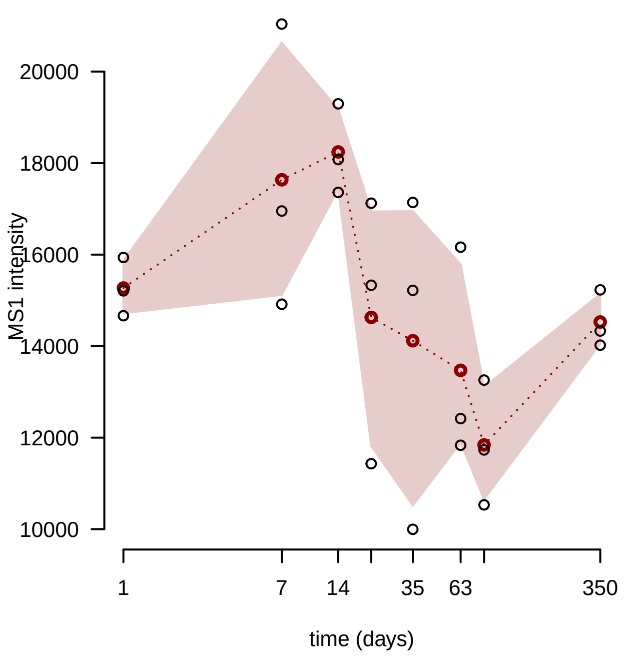

Q61124
GN=Cln3
ceroid lipofuscinosis, neuronal 3, juven ...

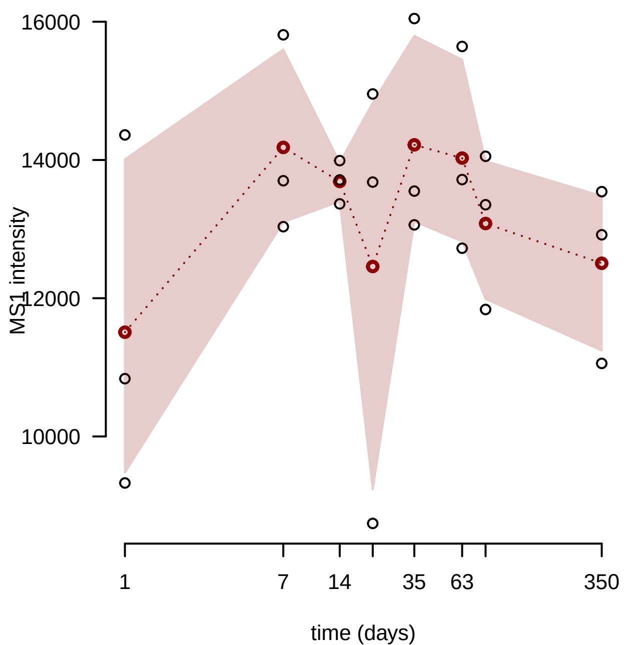

Q61127
GN=Nab2
Ngfi−A binding protein 2

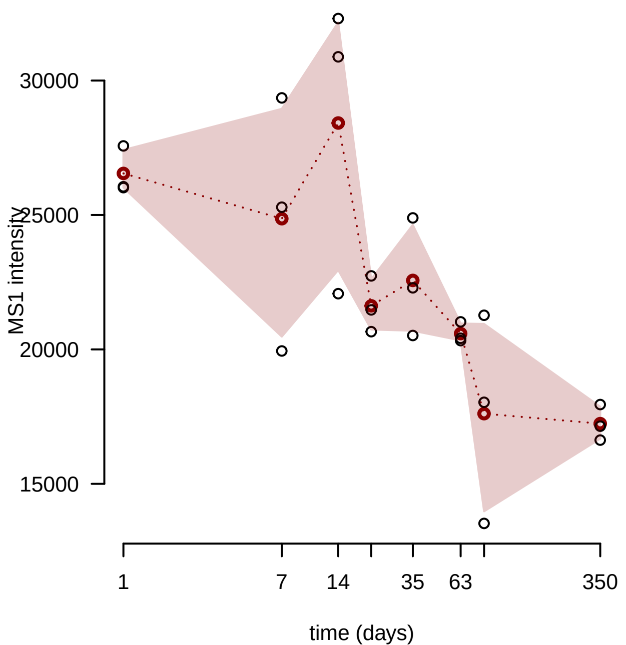

Q61129
GN=Cfi
complement component factor i

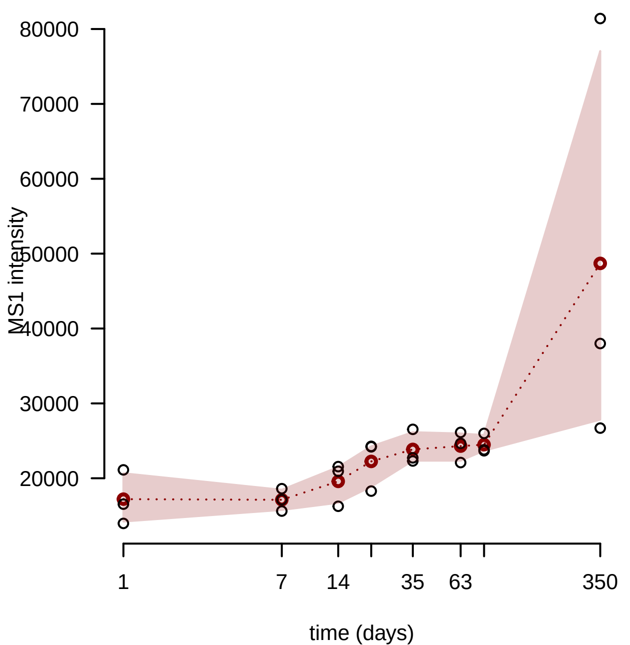

Q61133
GN=Gstt2
glutathione S−transferase, theta 2

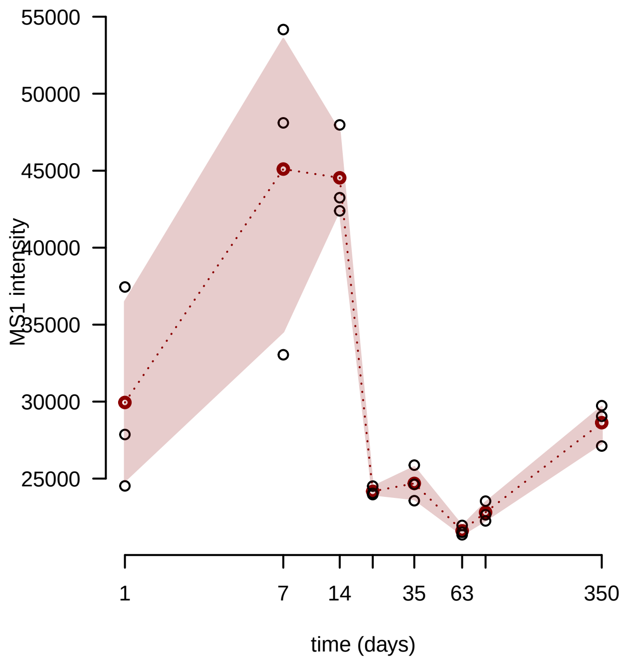

Q61136
GN=Prpf4b
pre−mRNA processing factor 4B

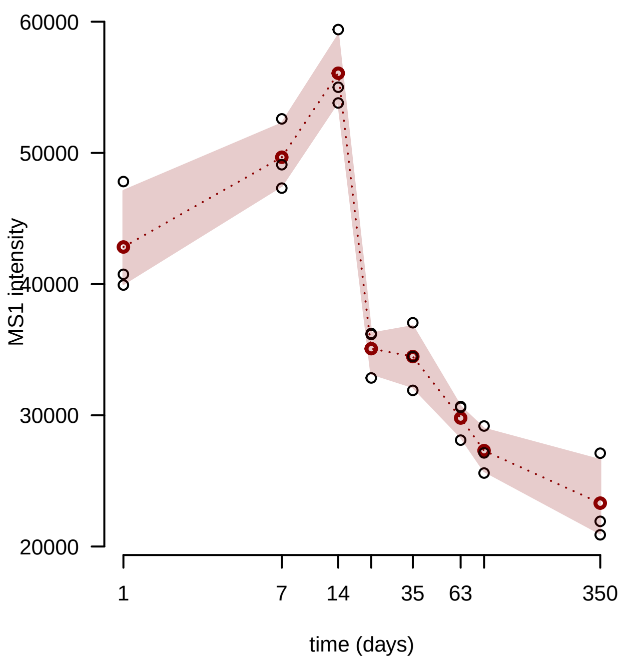

Q61140
GN=Bcar1
breast cancer anti−estrogen resistance 1

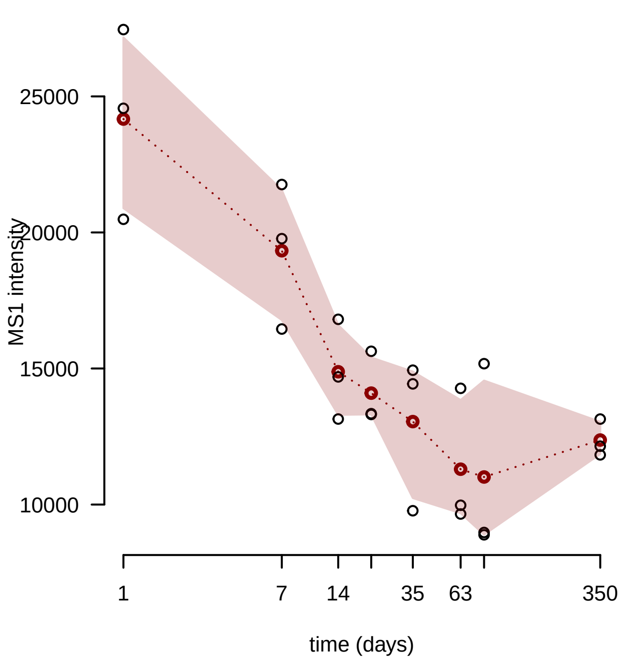

Q61142
GN=Spin1
spindlin 1

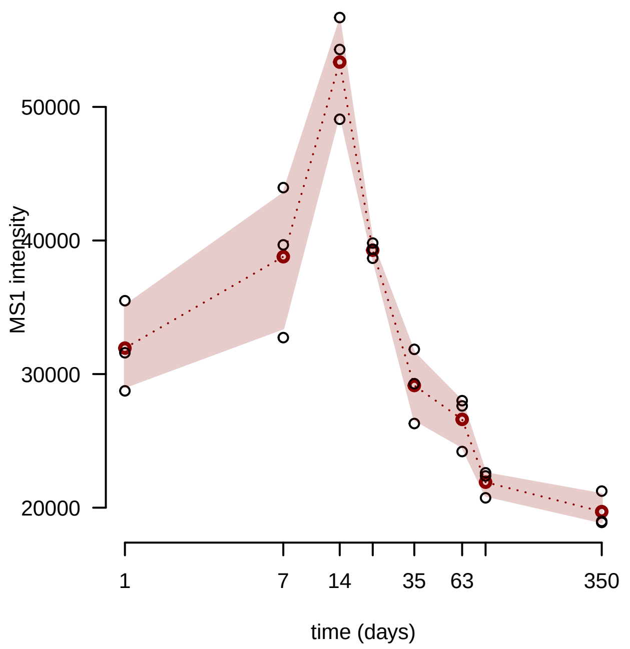

Q61144
GN=Psen2
presenilin 2

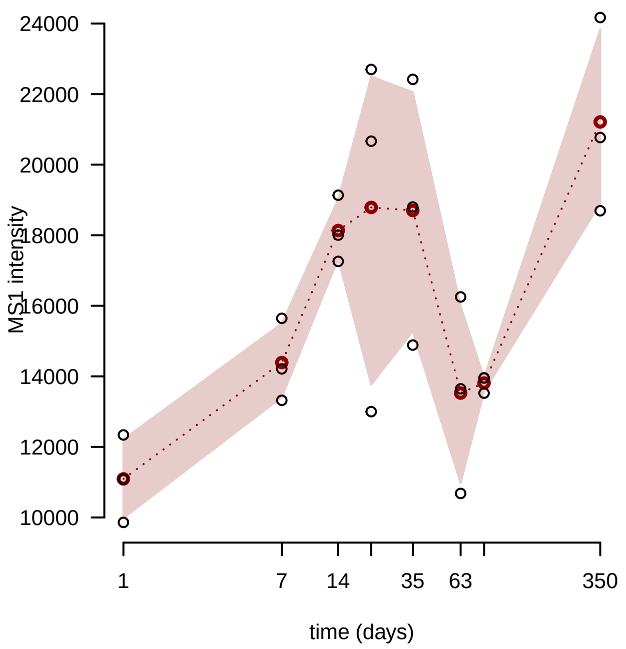

Q61147
GN=Cp
ceruloplasmin

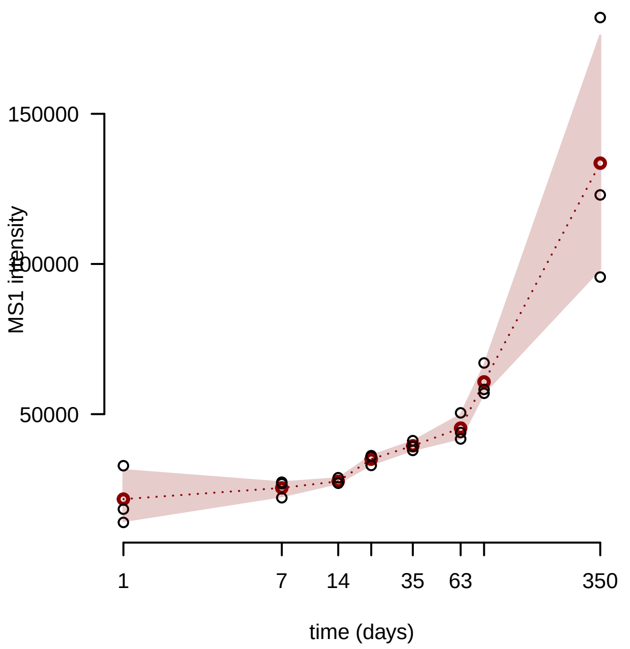

Q61151
GN=Ppp2r5e
protein phosphatase 2, regulatory subuni ...

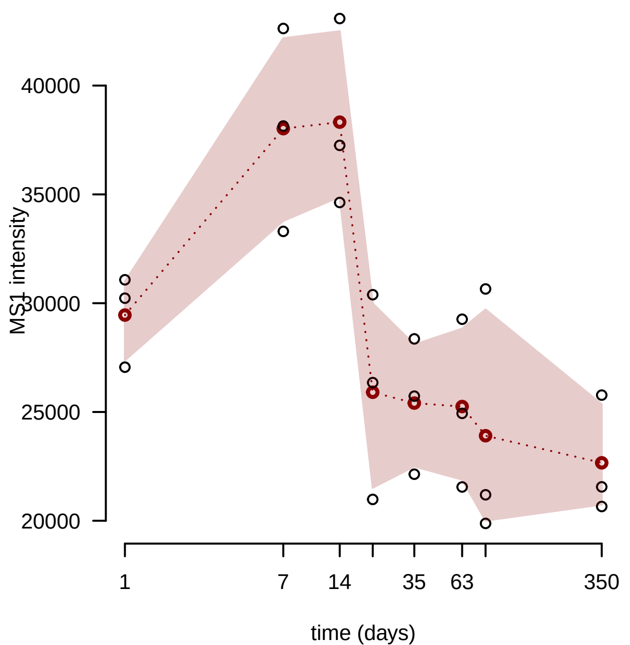

Q61152
GN=Ptpn18
protein tyrosine phosphatase, non−recept ...

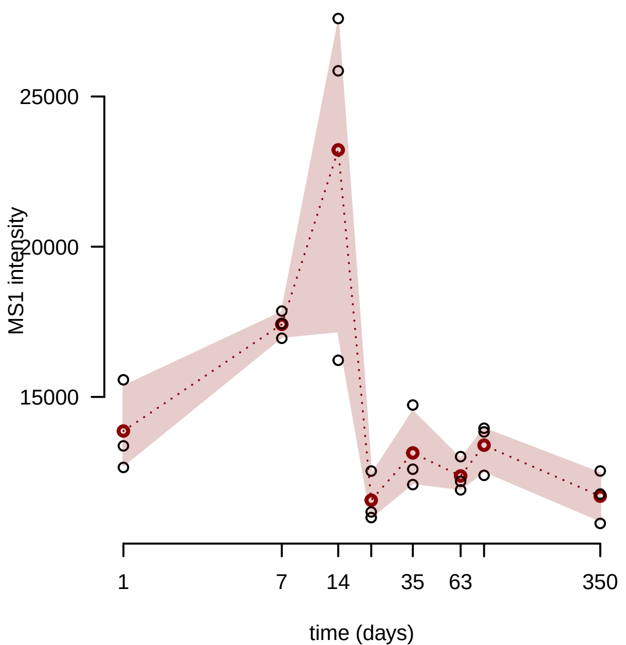

Q61160
GN=Fadd
Fas (TNFRSF6)−associated via death domai ...

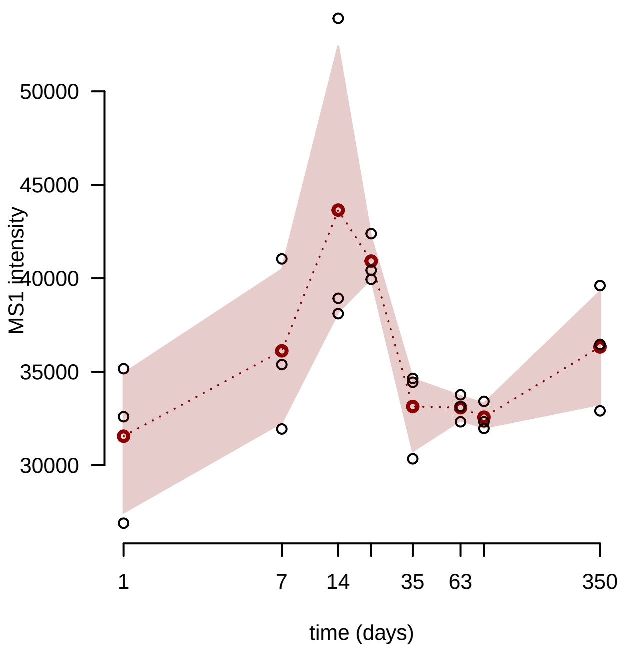

Q61161
GN=Map4k2
mitogen−activated protein kinase kinase ...

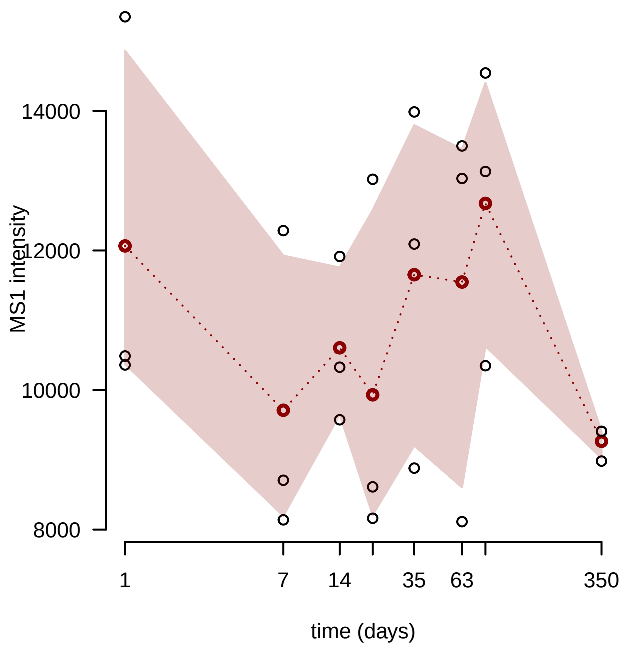

Q61164
GN=Ctcf
CCCTC−binding factor

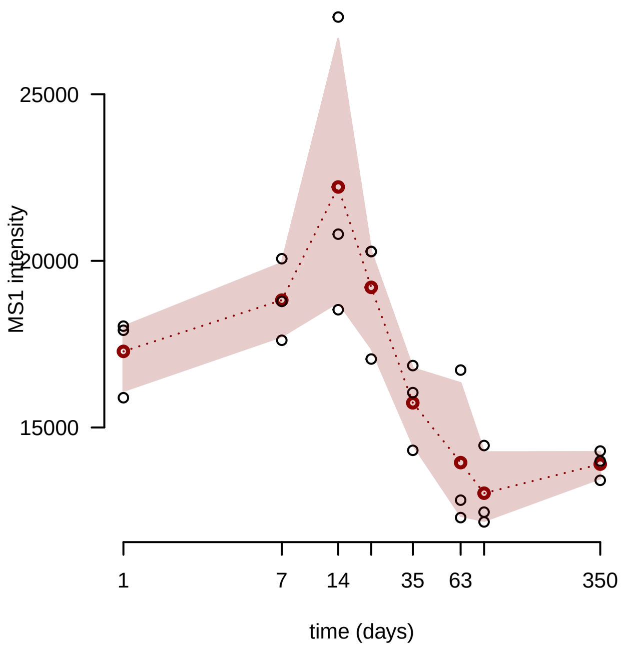

Q61165
GN=Slc9a1
solute carrier family 9 (sodium/hydrogen ...

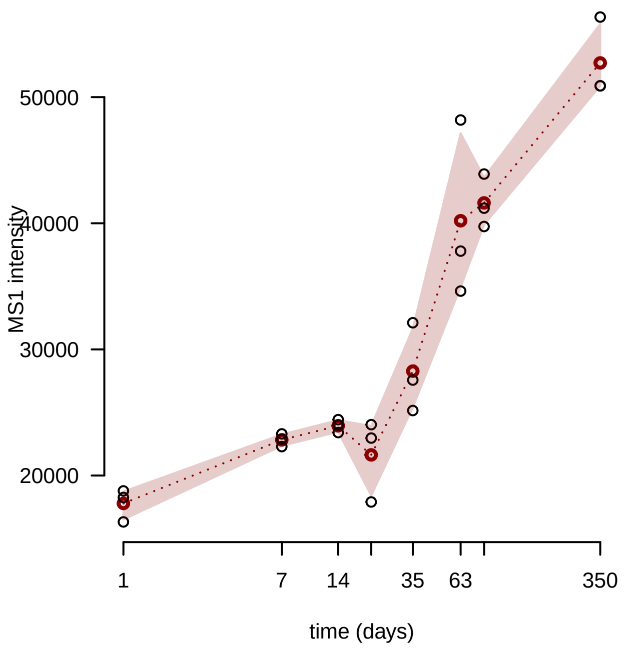

Q61166
GN=Mapre1
microtubule−associated protein, RP/EB fa ...

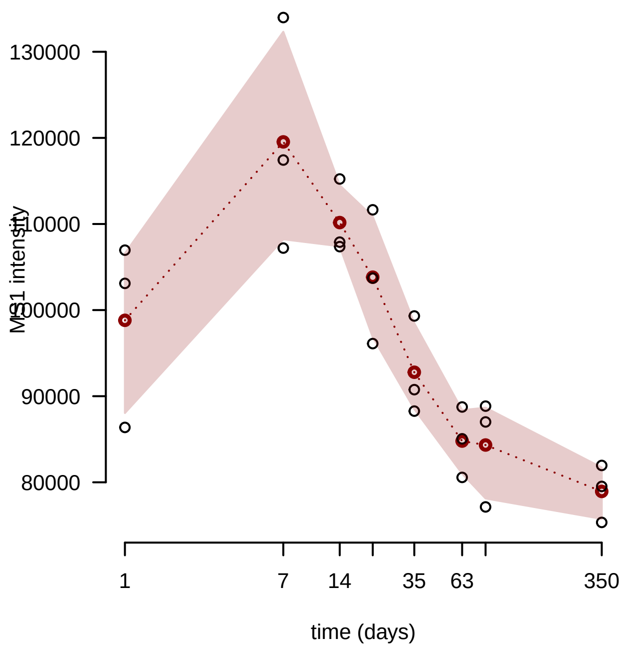

Q61169
GN=Gata6
GATA binding protein 6

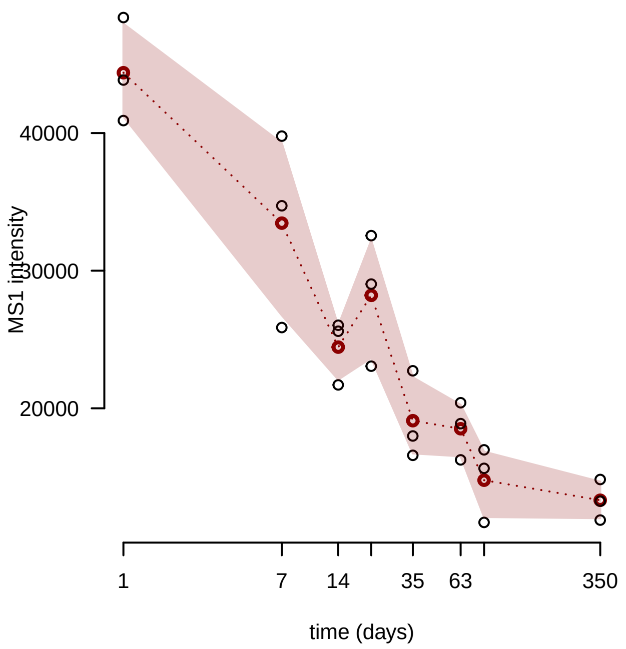

Q61171
GN=Prdx2
peroxiredoxin 2

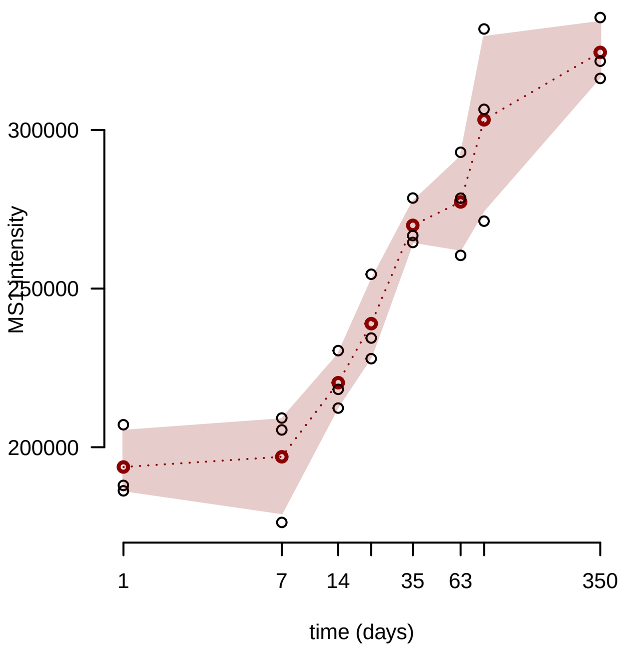

Q61176
GN=Arg1
arginase, liver

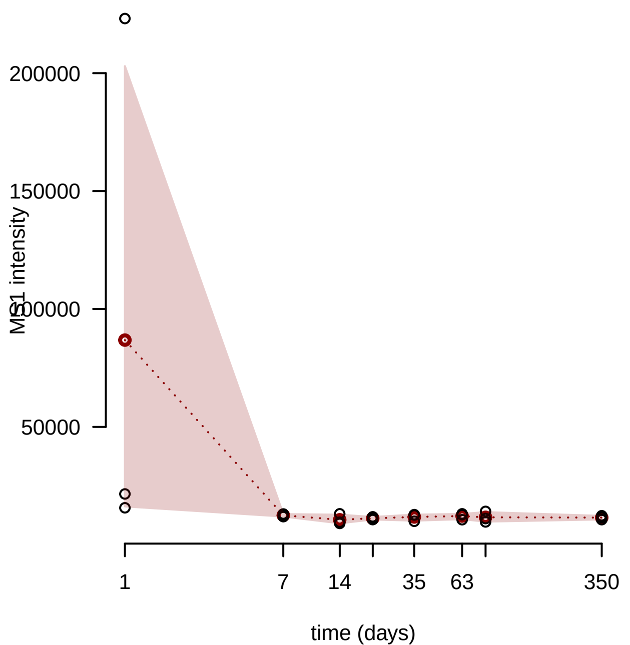

Q61179
GN=Irf9
interferon regulatory factor 9

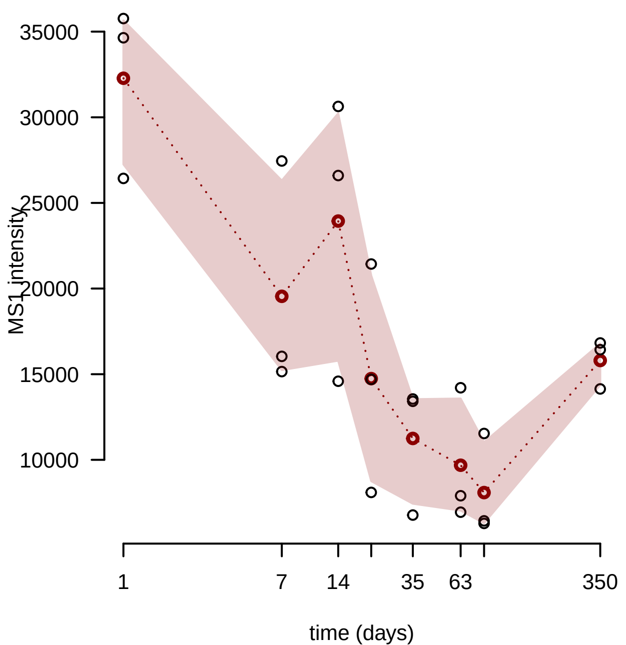

Q61183
GN=Papola
poly (A) polymerase alpha

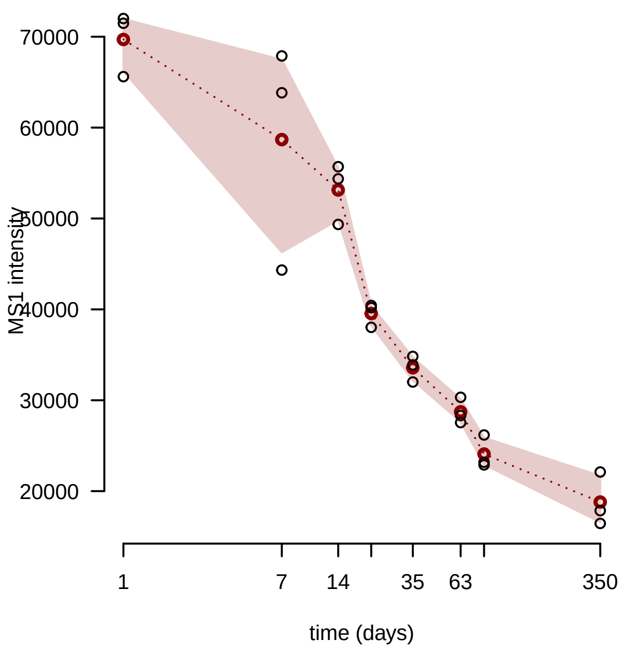

Q61187
GN=Tsg101
tumor susceptibility gene 101

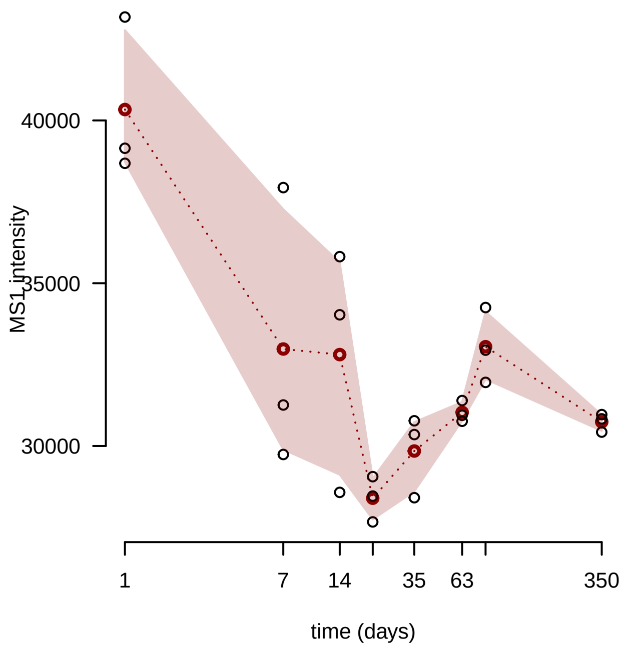

Q61188
GN=Ezh2
enhancer of zeste 2 polycomb repressive ...

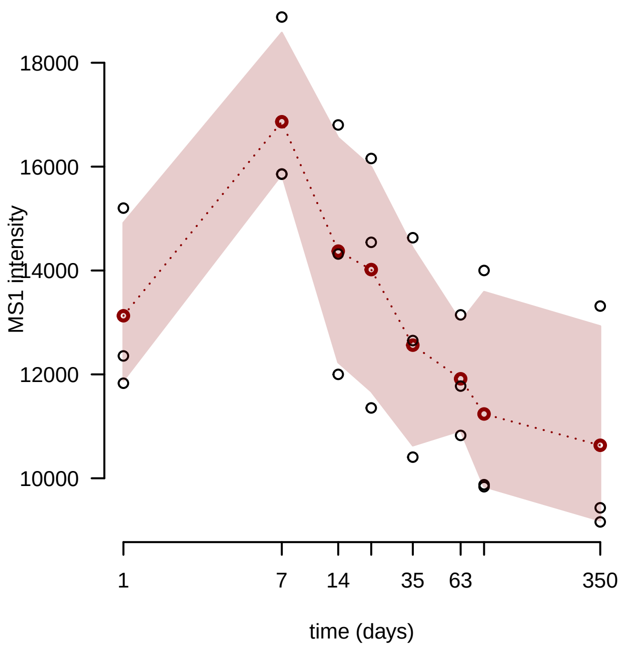

Q61189
GN=Clns1a
chloride channel, nucleotide−sensitive, ...

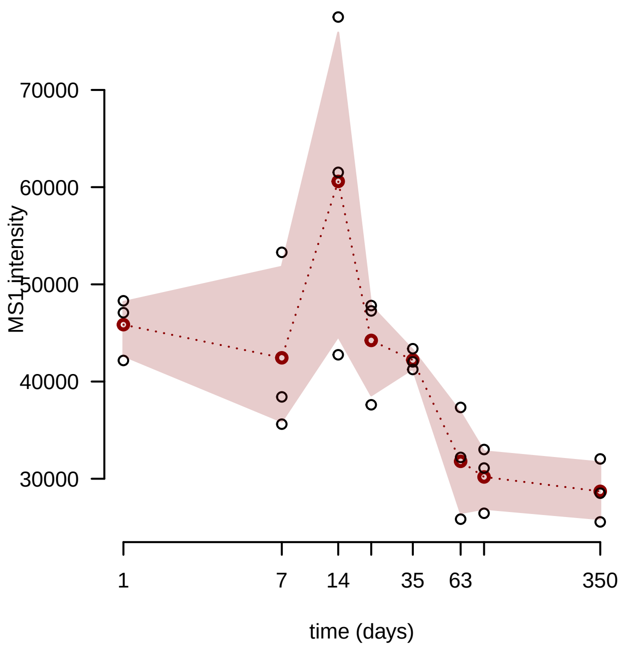

Q61190
GN=Il10rb
interleukin 10 receptor, beta

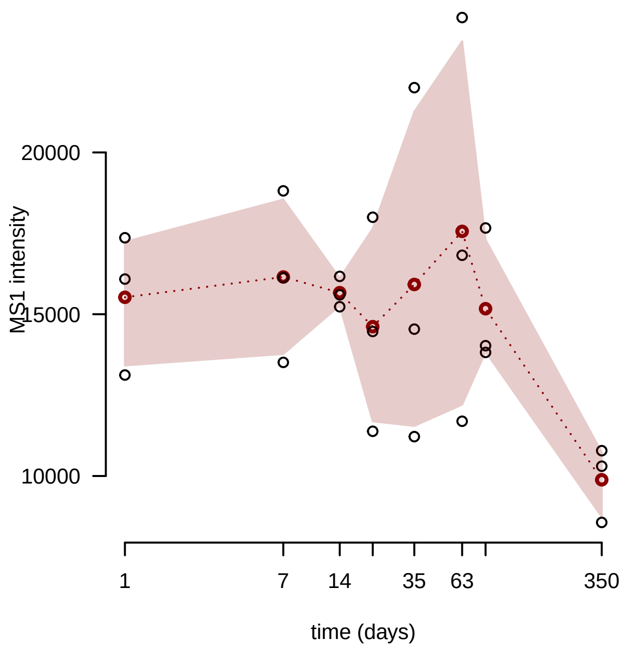

Q61191
GN=Hcfc1
host cell factor C1

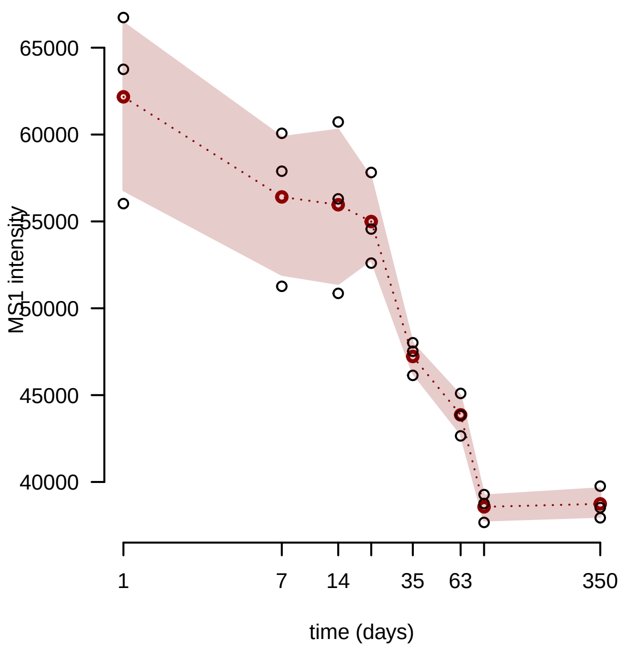

Q61193
GN=Rgl2
ral guanine nucleotide dissociation stim ...

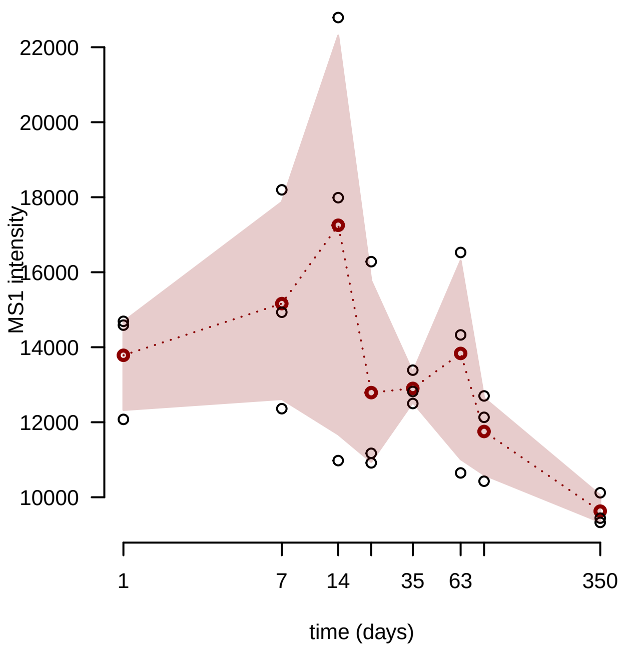

Q61194
GN=Pik3c2a
phosphatidylinositol−4−phosphate 3−kinas ...

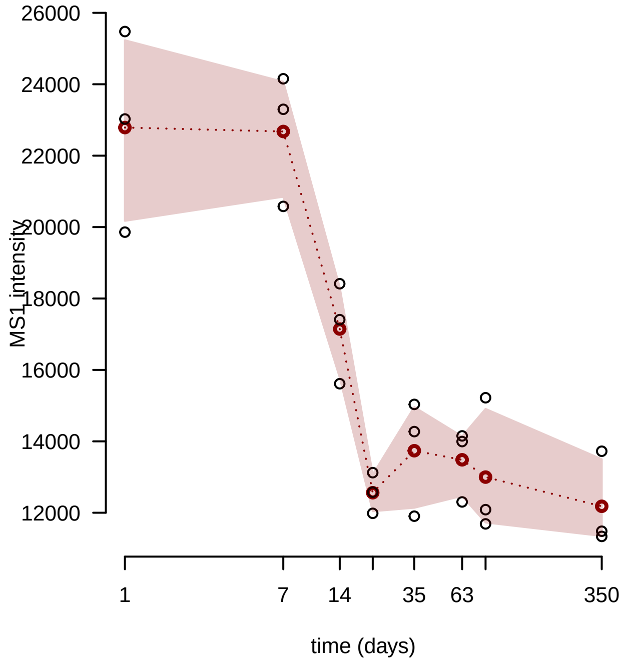

Q61205
GN=Pafah1b3
platelet−activating factor acetylhydrola ...

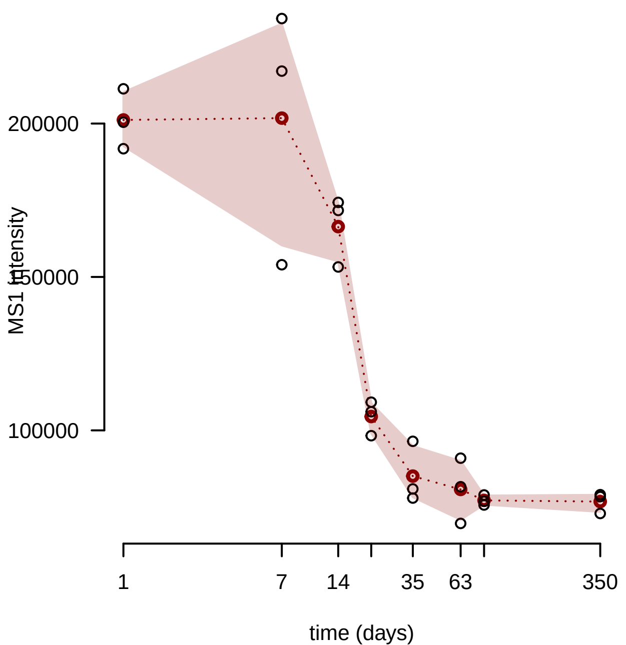

Q61206
GN=Pafah1b2
platelet−activating factor acetylhydrola ...

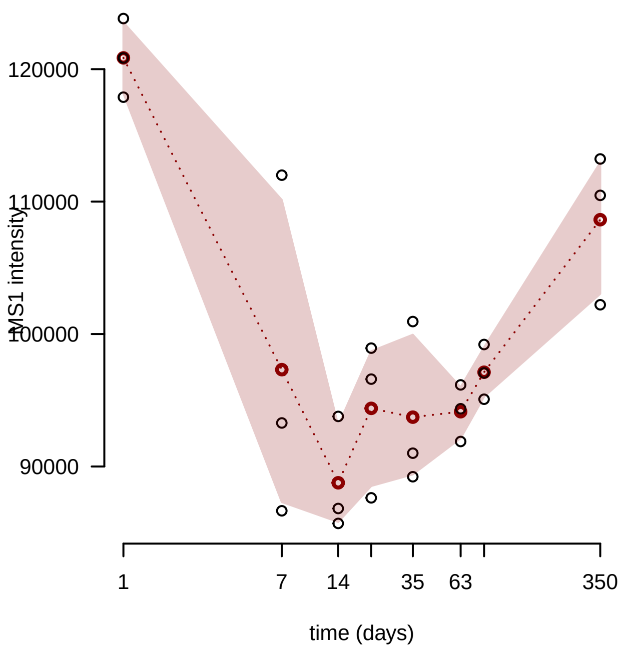

Q61207
GN=Psap
prosaposin

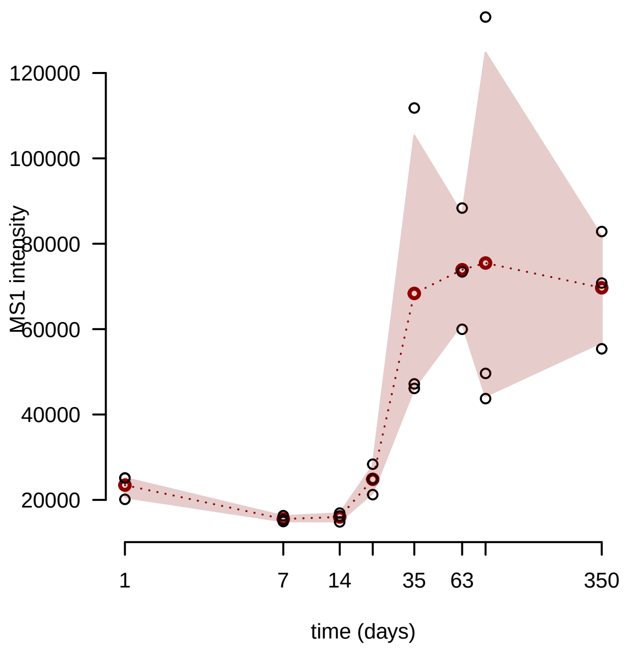

Q61210
GN=Arhgef1
Rho guanine nucleotide exchange factor (...

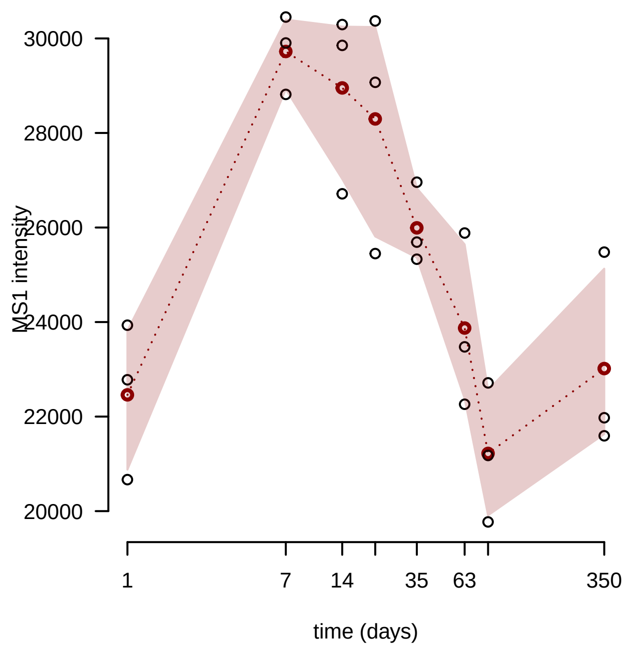

Q61211
GN=Eif2d
eukaryotic translation initiation factor ...

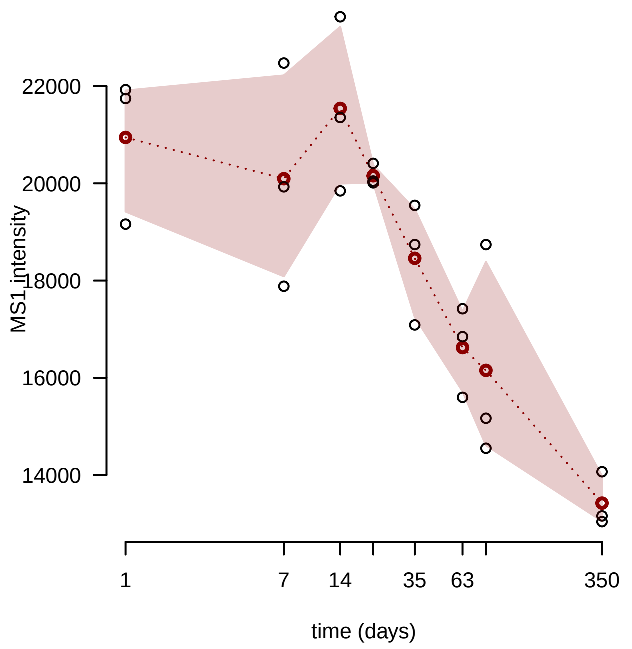

Q61214
GN=Dyrk1a
dual−specificity tyrosine−(Y)−phosphoryl ...

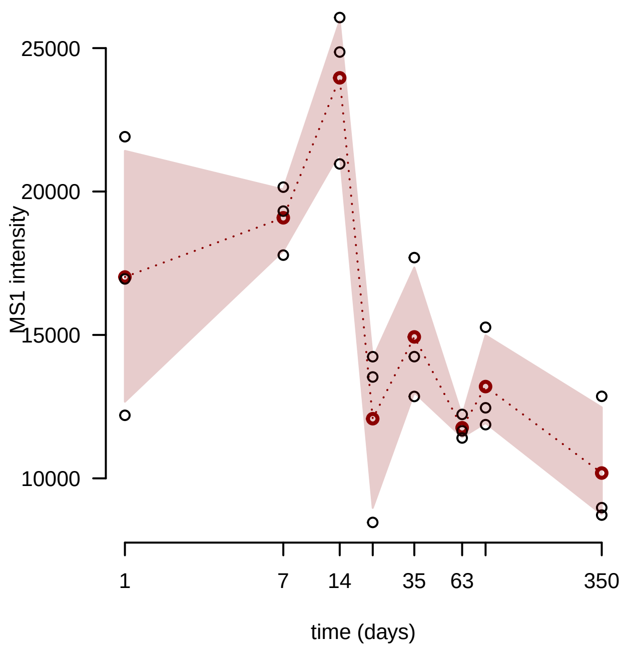

Q61216
GN=Mre11

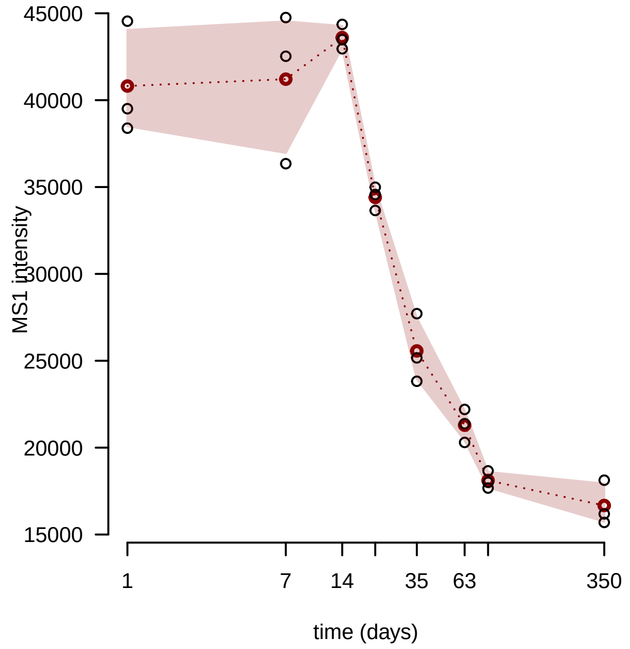

Q61220
GN=Nell2
NEL−like 2

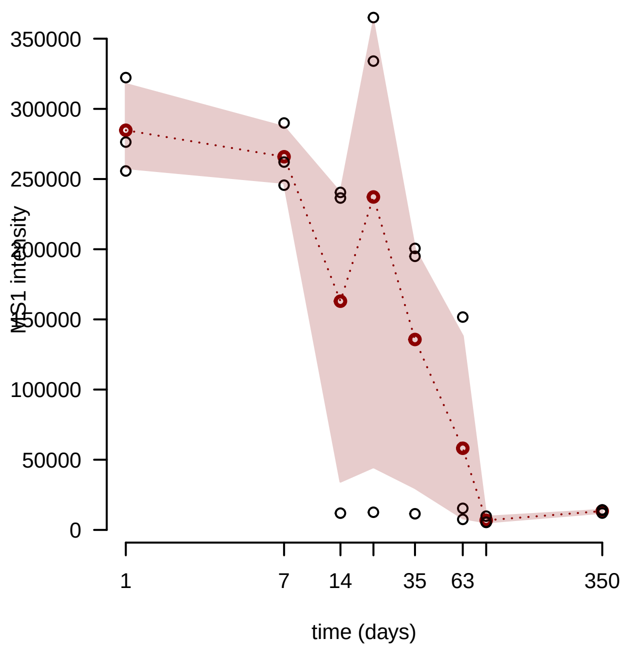

Q61233
GN=Lcp1
lymphocyte cytosolic protein 1

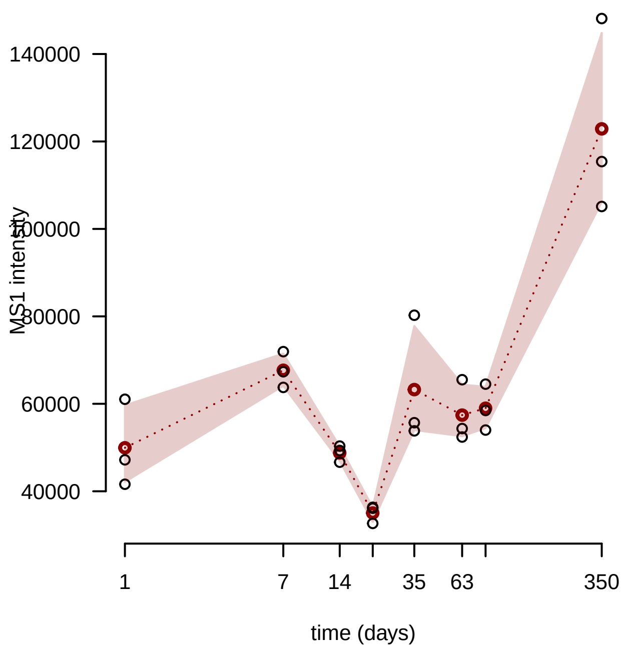

Q61234
GN=Snta1
syntrophin, acidic 1

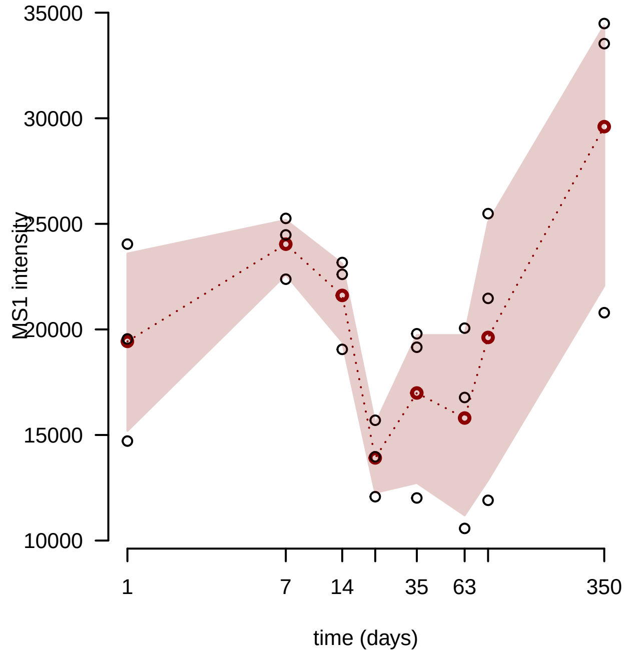

Q61235
GN=Sntb2
syntrophin, basic 2

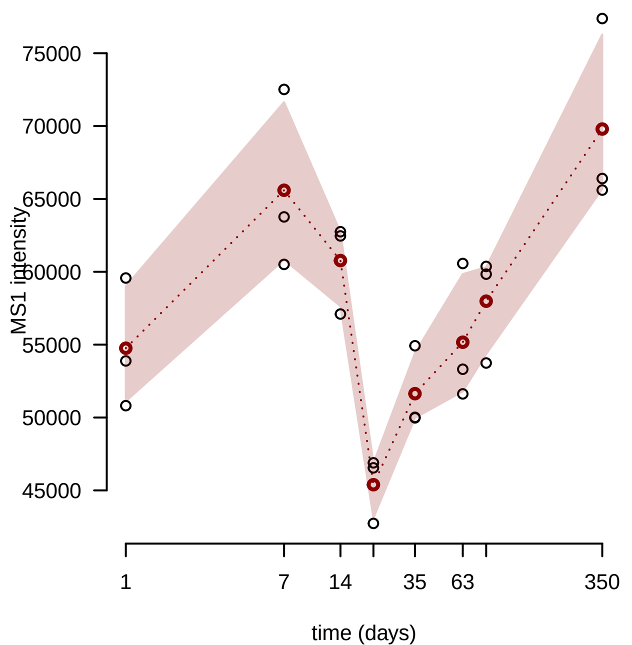

Q61239
GN=Fnta
farnesyltransferase, CAAX box, alpha

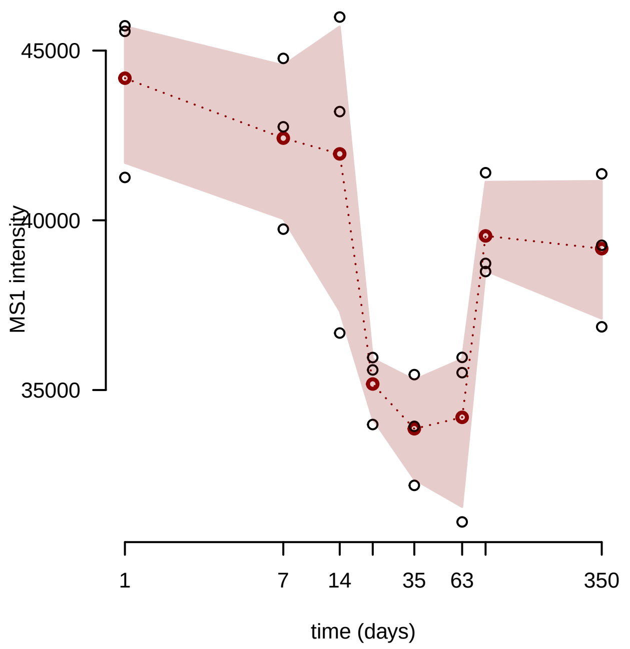

Q61245
GN=Col11a1
collagen, type XI, alpha 1

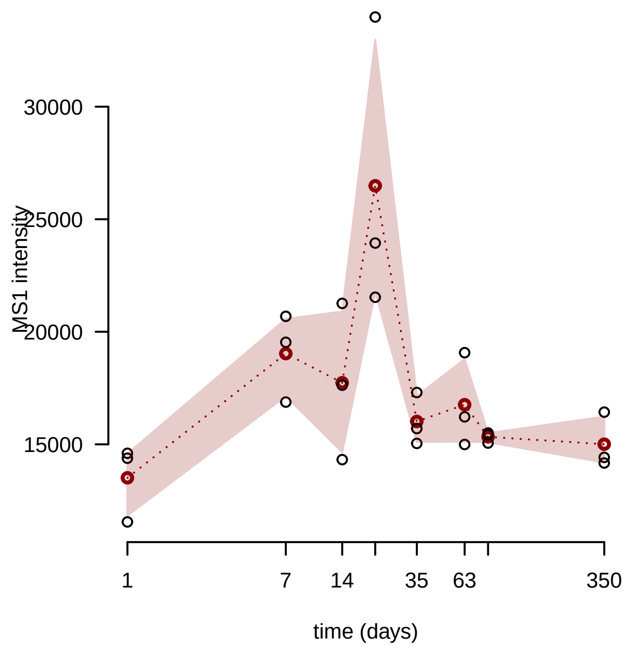

Q61247
GN=Serpinf2
serine (or cysteine) peptidase inhibitor ...

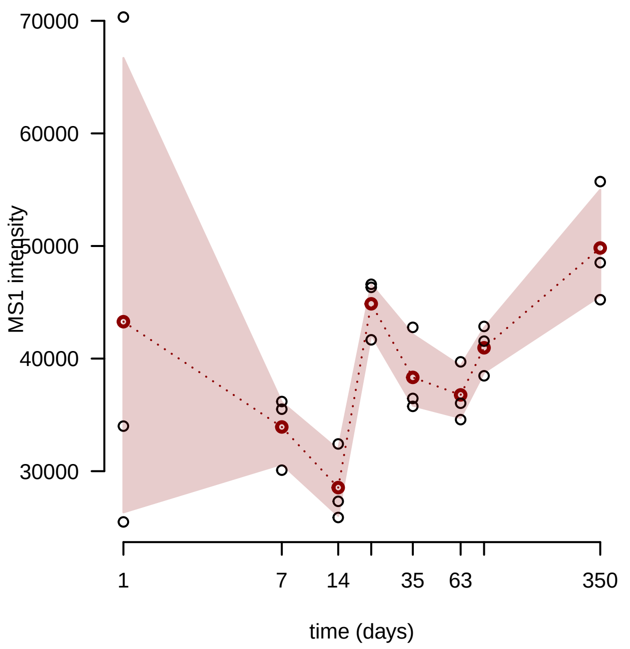

Q61249
GN=Igbp1
immunoglobulin (CD79A) binding protein 1

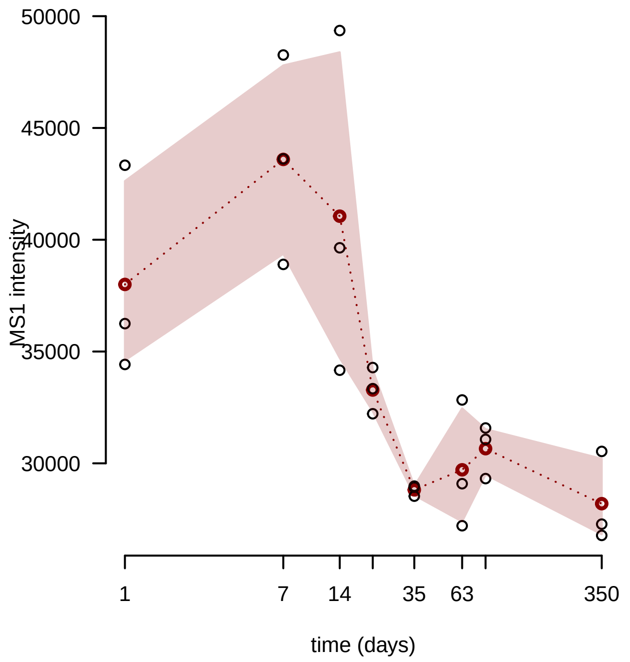

Q61263
GN=Soat1
sterol O−acyltransferase 1

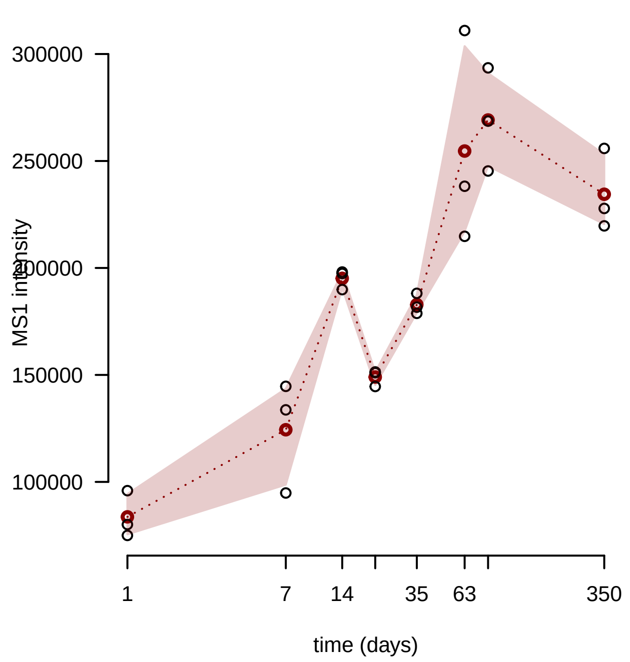

Q61268
GN=Apoc4
apolipoprotein C−IV

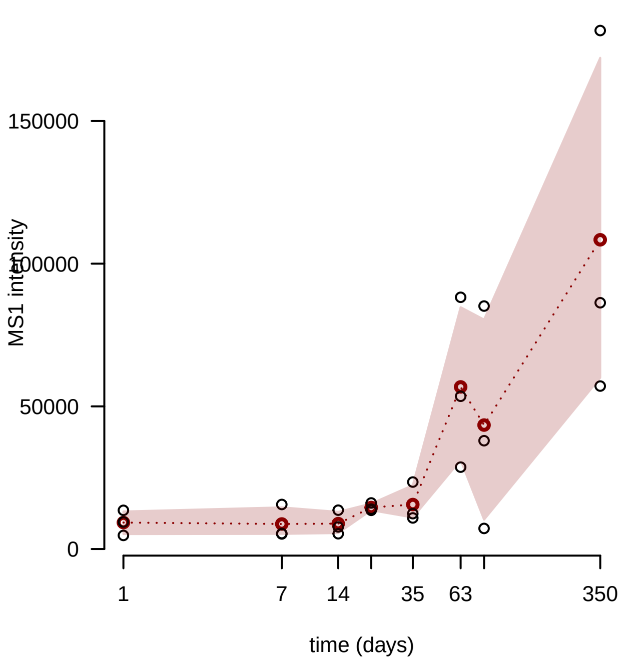

Q61285
GN=Abcd2
ATP−binding cassette, sub−family D (ALD) ...

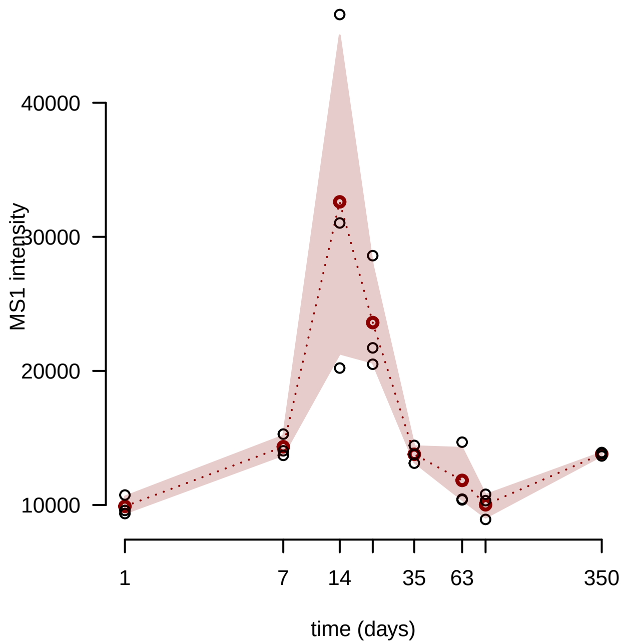

Q61286
GN=Tcf12
transcription factor 12

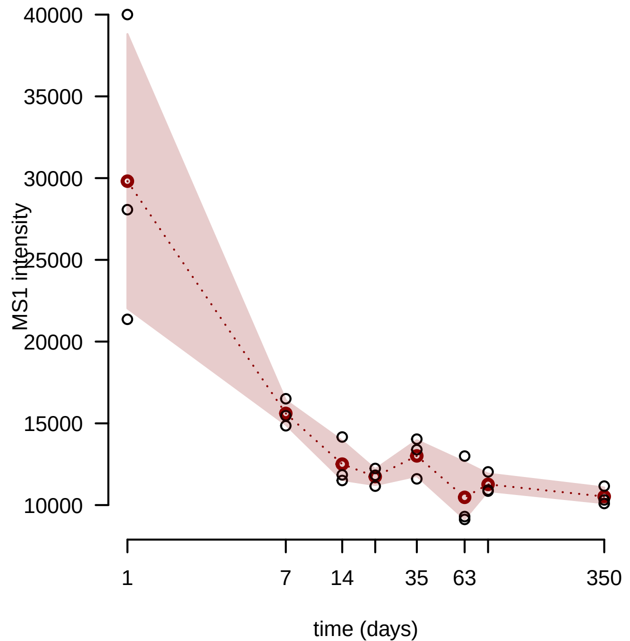

Q61286
GN=Tcf12
transcription factor 12

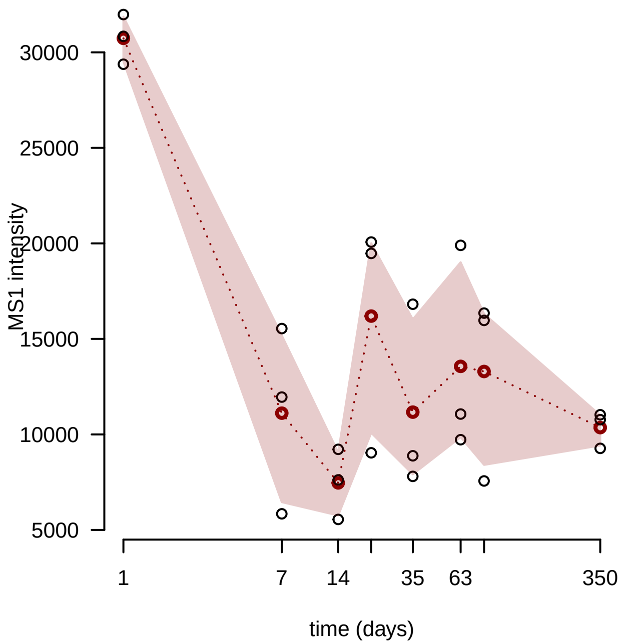

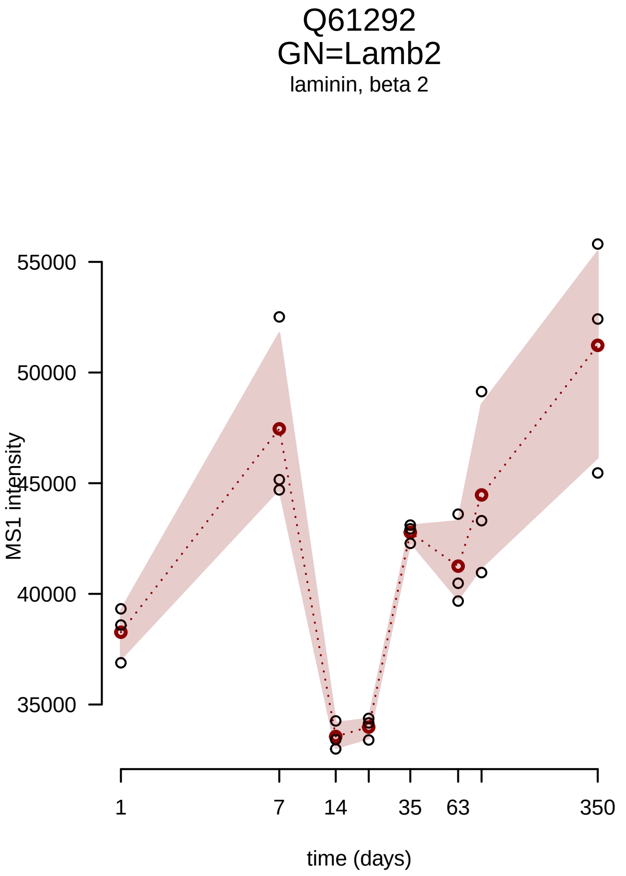
Q61292
GN=Lamb2
laminin, beta 2
MS1 intensity
35000
40000
45000
50000
55000
1
7
14
35
63
350
time (days)

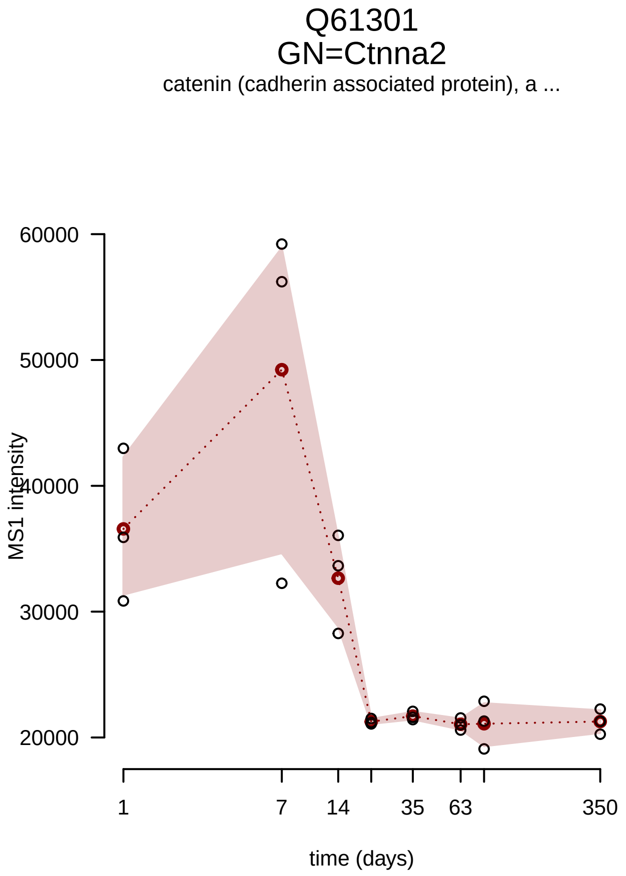
Q61301
GN=Ctnna2
catenin (cadherin associated protein), a ...
MS1 intensity
20000
30000
40000
50000
60000
1
7
14
35
63
350
time (days)

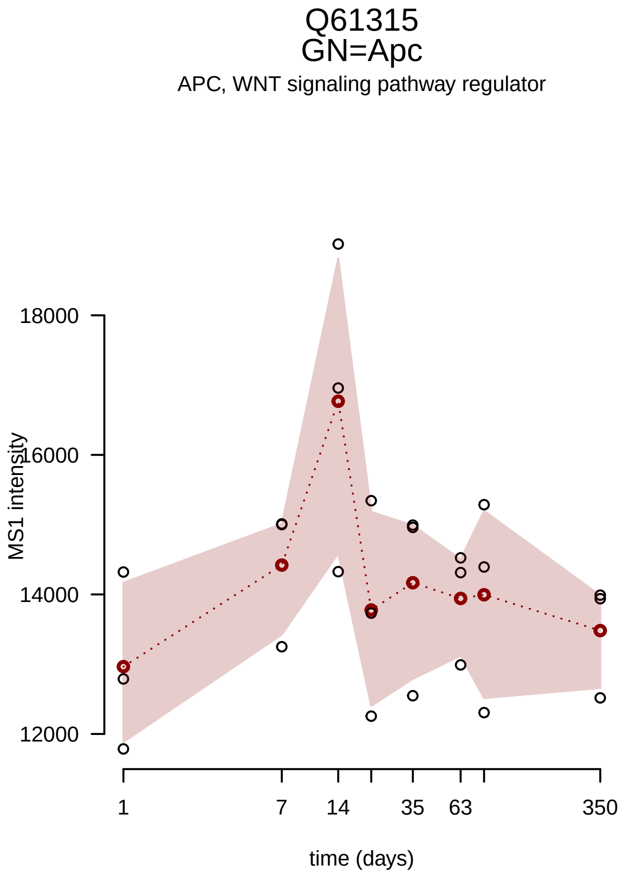
Q61315
GN=Apc
APC, WNT signaling pathway regulator
MS1 intensity
12000
14000
16000
18000
1
7
14
35
63
350
time (days)

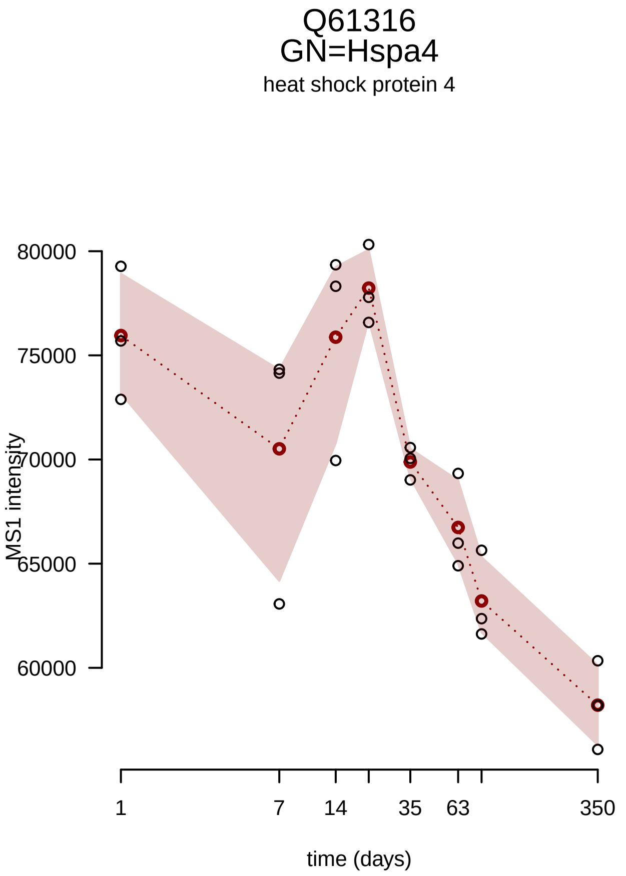
Q61316
GN=Hspa4
heat shock protein 4
MS1 intensity
60000
65000
70000
75000
80000
1
7
14
35
63
350
time (days)

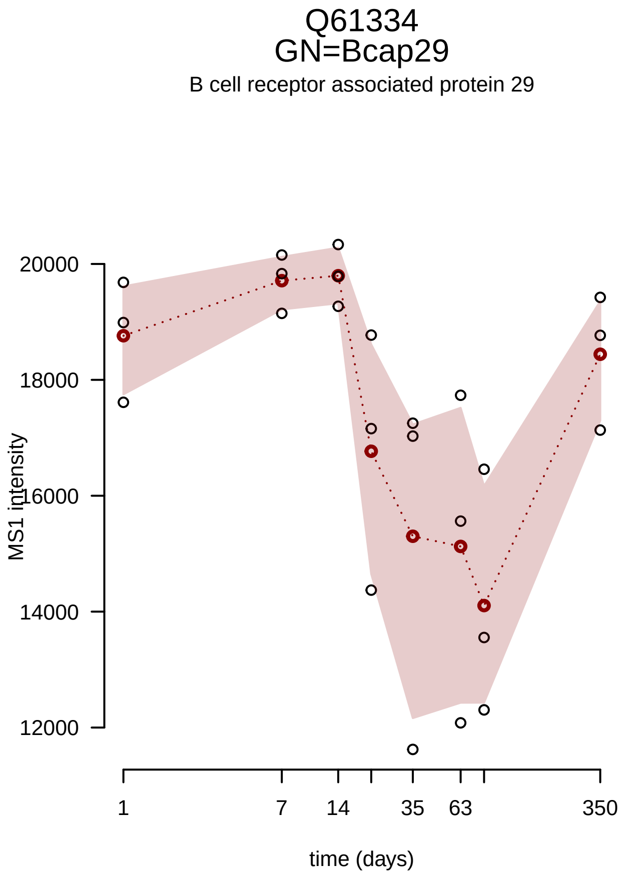
Q61334
GN=Bcap29
B cell receptor associated protein 29
MS1 intensity
12000
14000
16000
18000
20000
1
7
14
35
63
350
time (days)

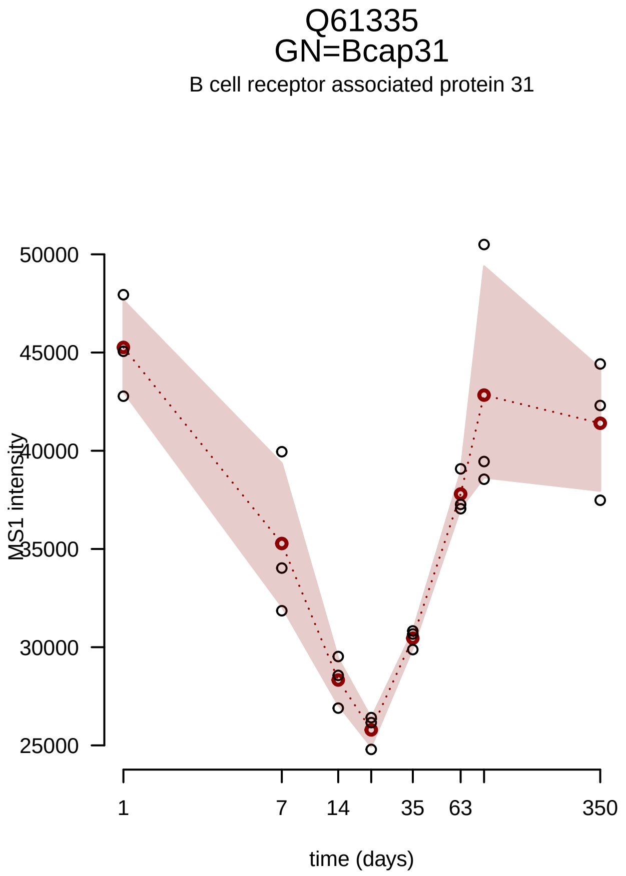
Q61335
GN=Bcap31
B cell receptor associated protein 31
MS1 intensity
25000
30000
35000
40000
45000
50000
1
7
14
35
63
350
time (days)

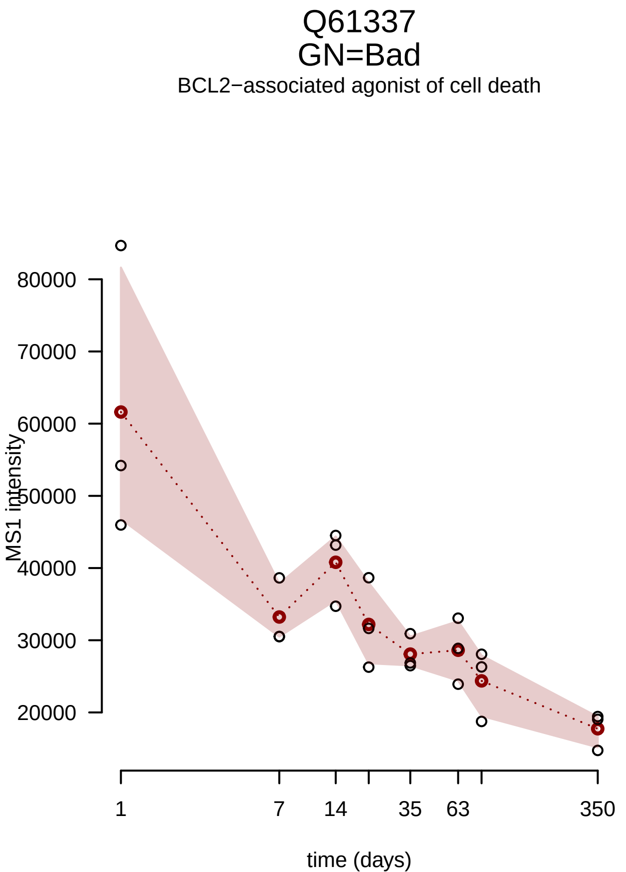
Q61337
GN=Bad
BCL2−associated agonist of cell death
MS1 intensity
20000
30000
40000
50000
60000
70000
80000
1
7
14
35
63
350
time (days)

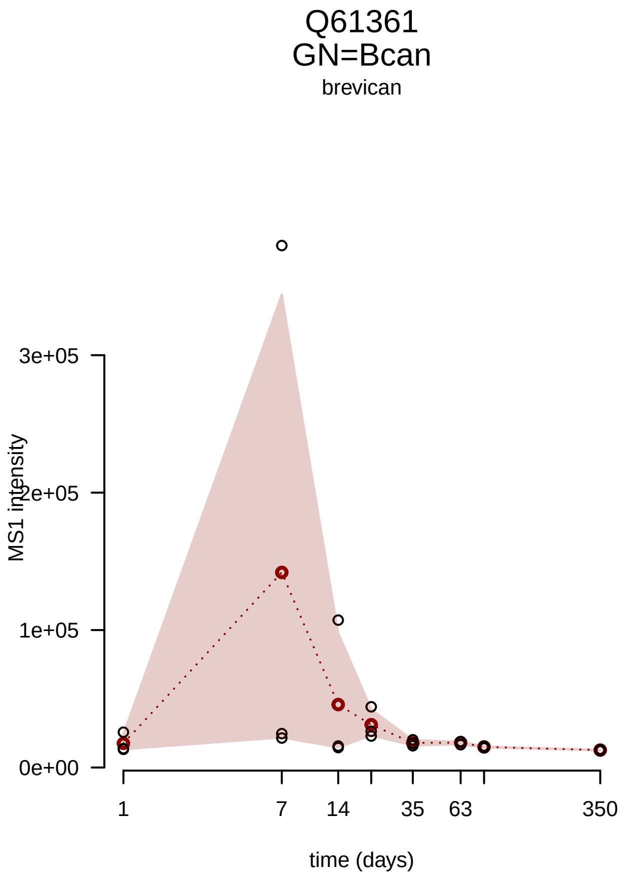
Q61361
GN=Bcan
brevican
MS1 intensity
0e+00
1e+05
2e+05
3e+05
1
7
14
35
63
350
time (days)

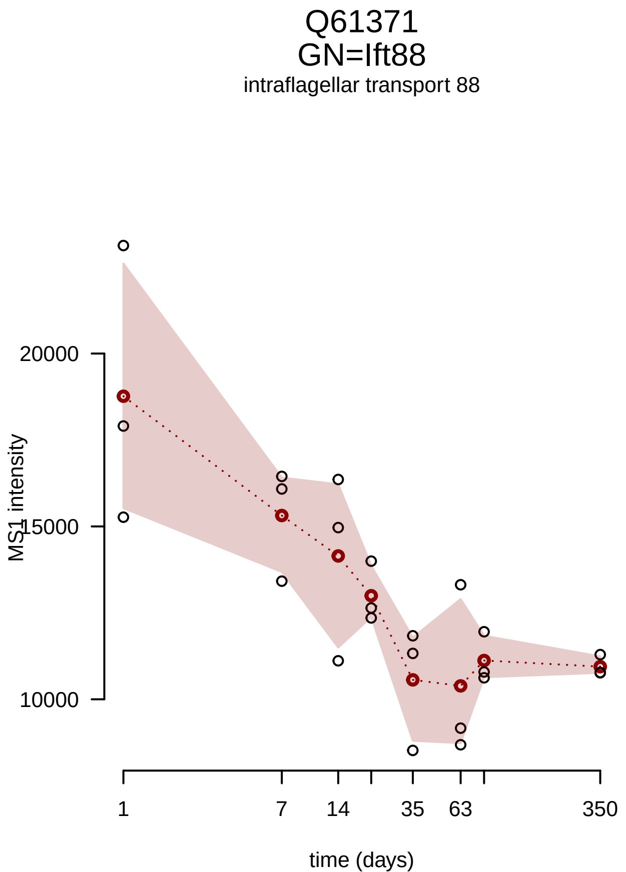
Q61371
GN=Ift88
intraflagellar transport 88
MS1 intensity
10000
15000
20000
1
7
14
35
63
350
time (days)

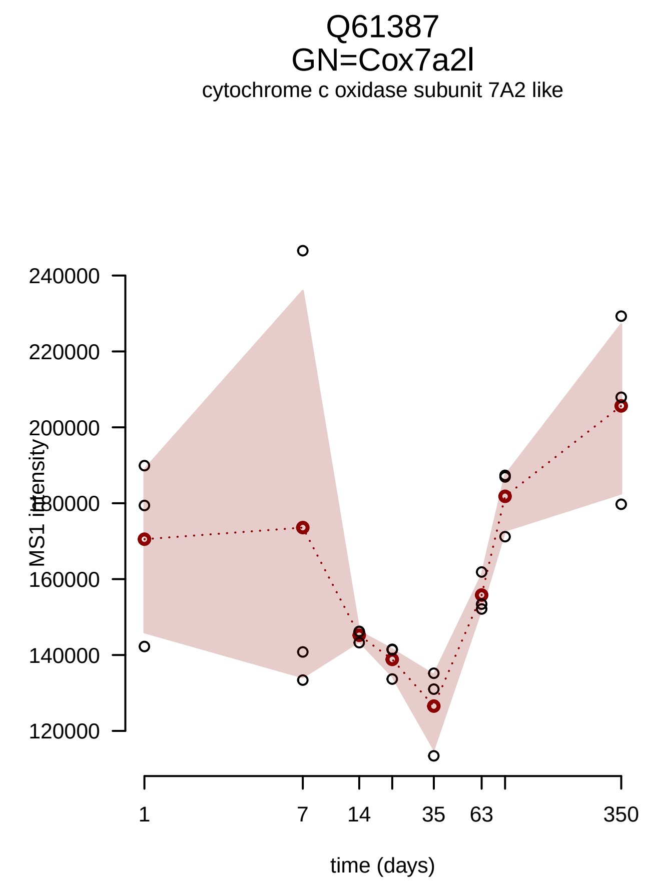
Q61387
GN=Cox7a2l
cytochrome c oxidase subunit 7A2 like
MS1 intensity
120000
140000
160000
180000
200000
220000
240000
1
7
14
35
63
350
time (days)
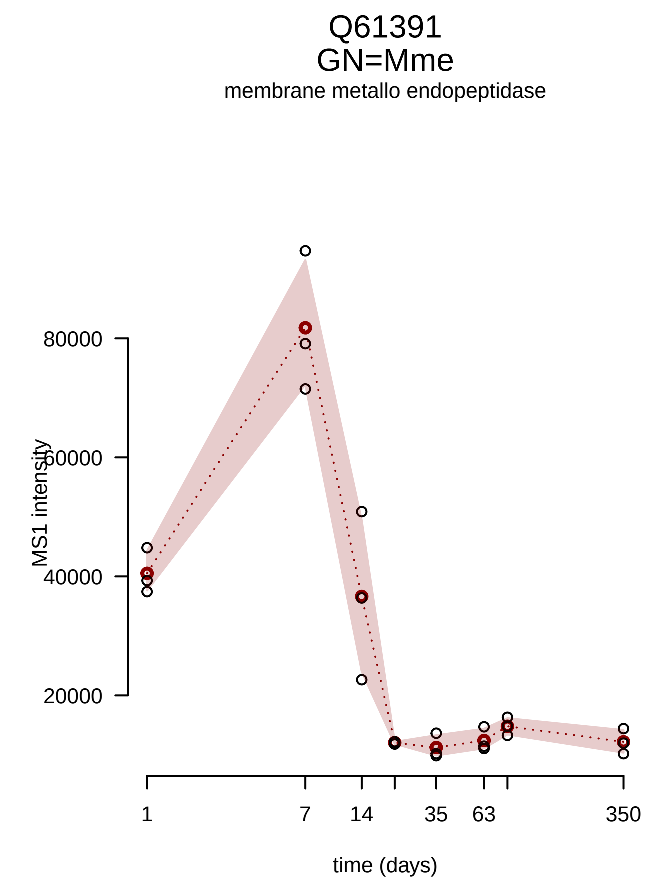
Q61391
GN=Mme
membrane metallo endopeptidase
MS1 intensity
20000
40000
60000
80000
1
7
14
35
63
350
time (days)
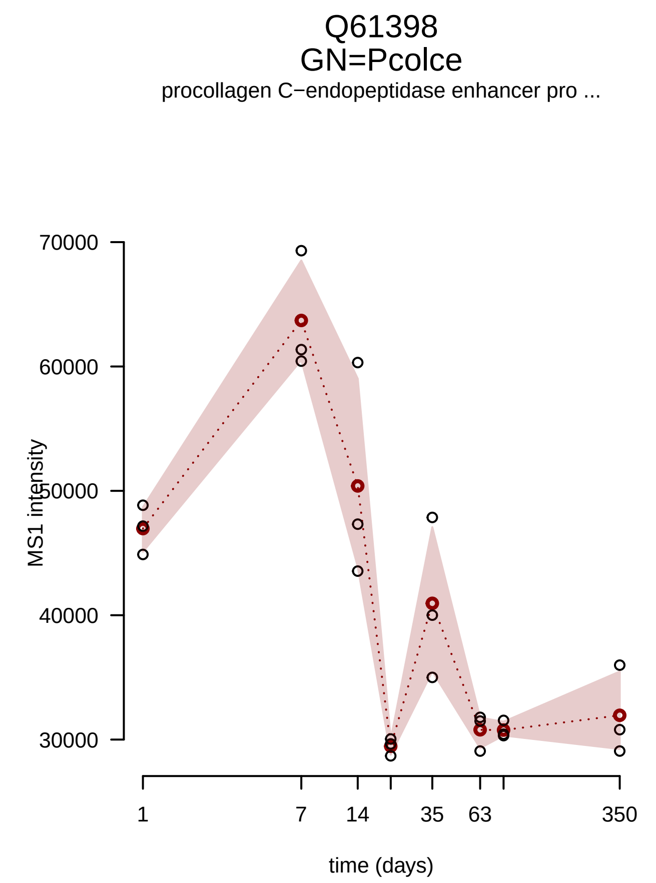
Q61398
GN=Pcolce
procollagen C−endopeptidase enhancer pro ...
MS1 intensity
30000
40000
50000
60000
70000
1
7
14
35
63
350
time (days)
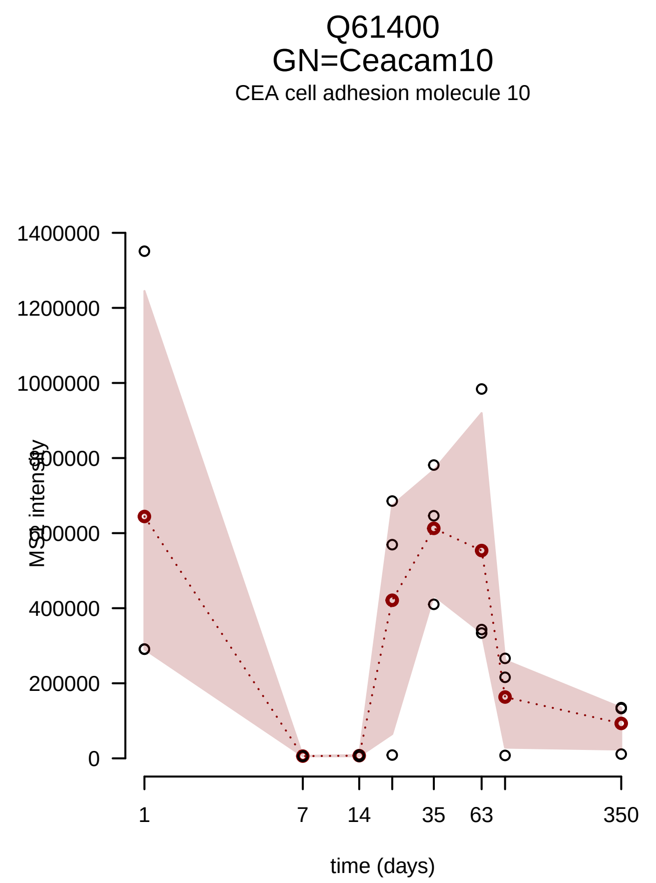
Q61400
GN=Ceacam10
CEA cell adhesion molecule 10
MS1 intensity
0
200000
400000
600000
800000
1000000
1200000
1400000
1
7
14
35
63
350
time (days)
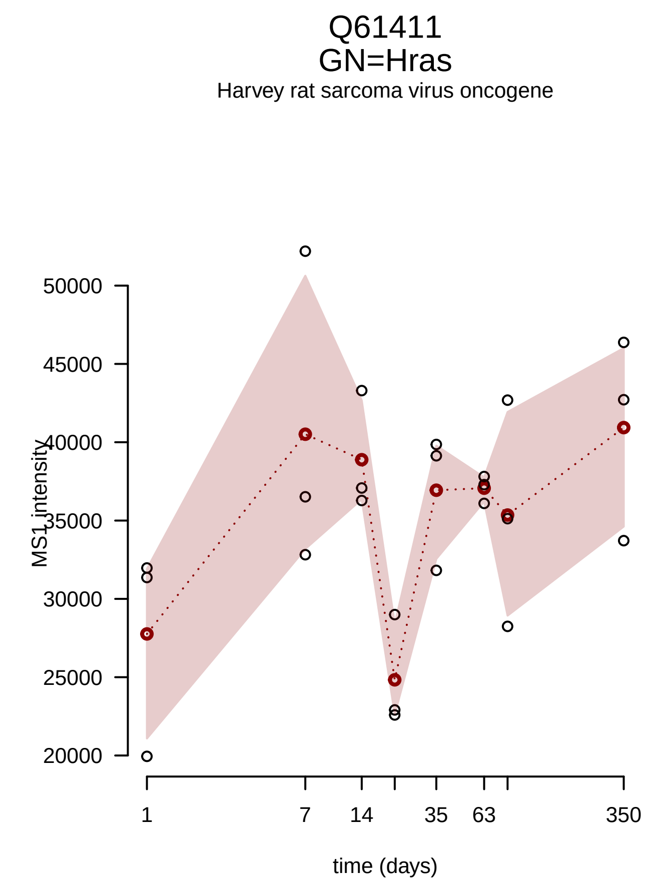
Q61411
GN=Hras
Harvey rat sarcoma virus oncogene
MS1 intensity
20000
25000
30000
35000
40000
45000
50000
1
7
14
35
63
350
time (days)
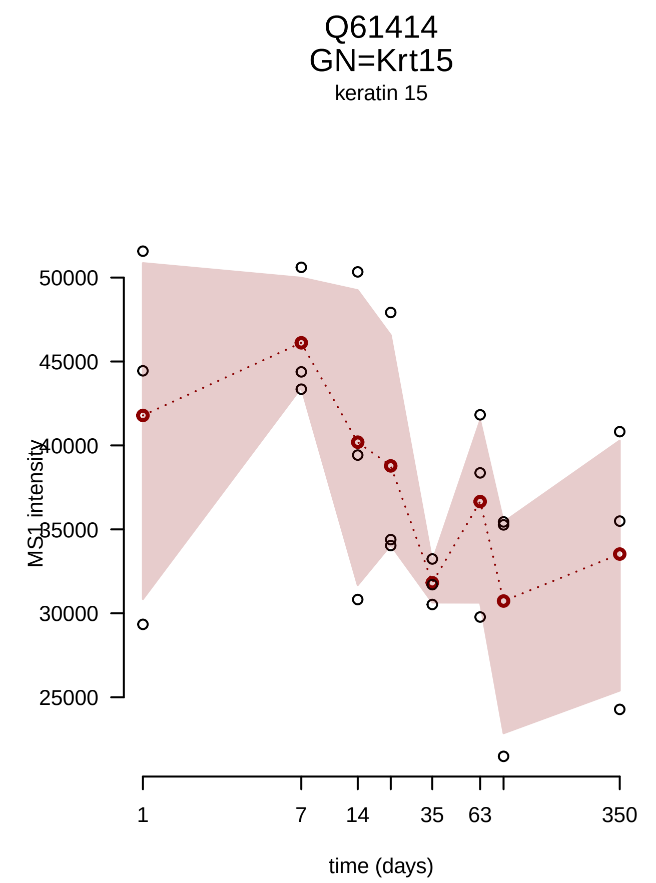
Q61414
GN=Krt15
keratin 15
MS1 intensity
25000
30000
35000
40000
45000
50000
1
7
14
35
63
350
time (days)
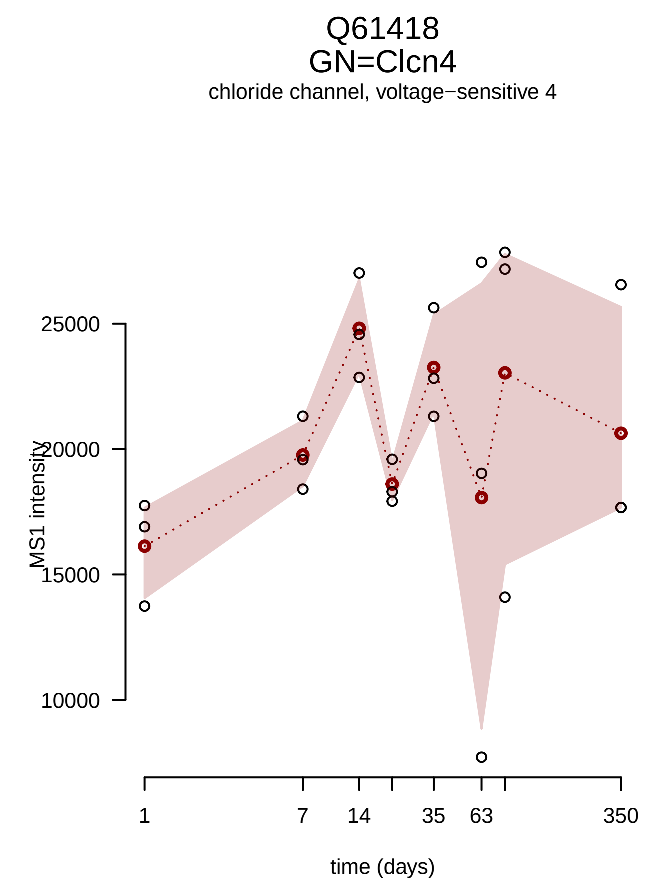
Q61418
GN=Clcn4
chloride channel, voltage−sensitive 4
MS1 intensity
10000
15000
20000
25000
1
7
14
35
63
350
time (days)
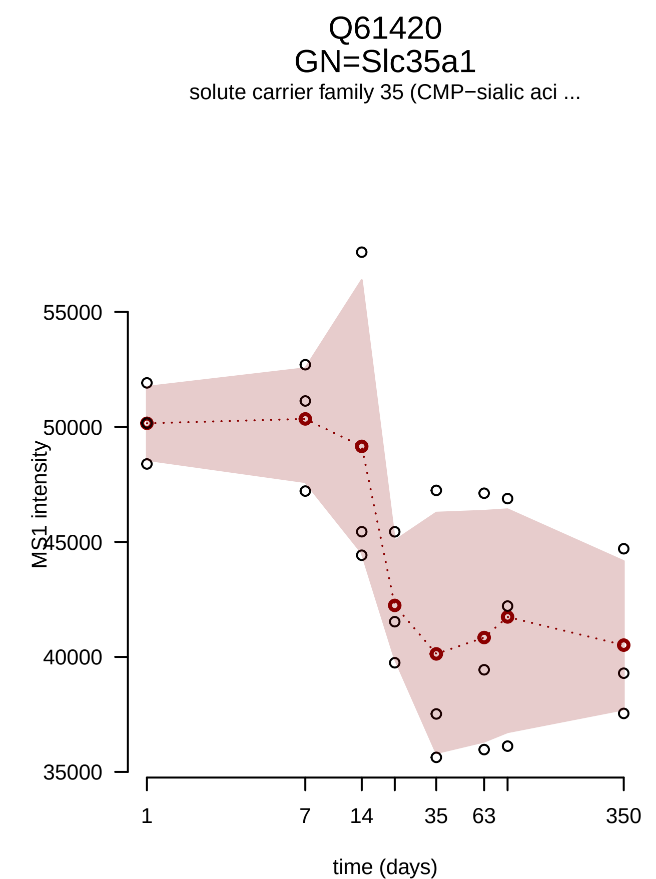
Q61420
GN=Slc35a1
solute carrier family 35 (CMP−sialic aci ...
MS1 intensity
35000
40000
45000
50000
55000
1
7
14
35
63
350
time (days)
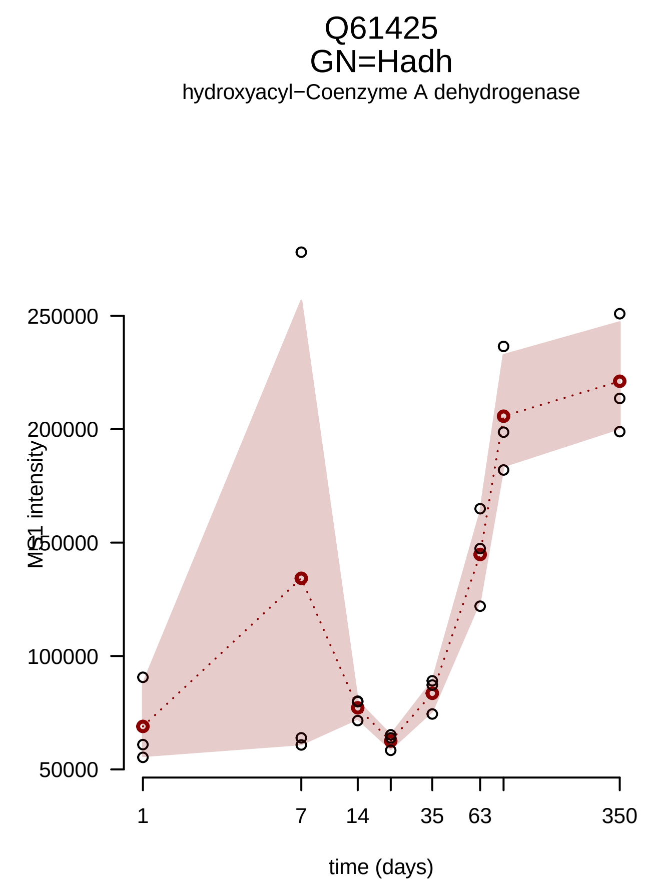
Q61425
GN=Hadh
hydroxyacyl−Coenzyme A dehydrogenase
MS1 intensity
50000
100000
150000
200000
250000
1
7
14
35
63
350
time (days)

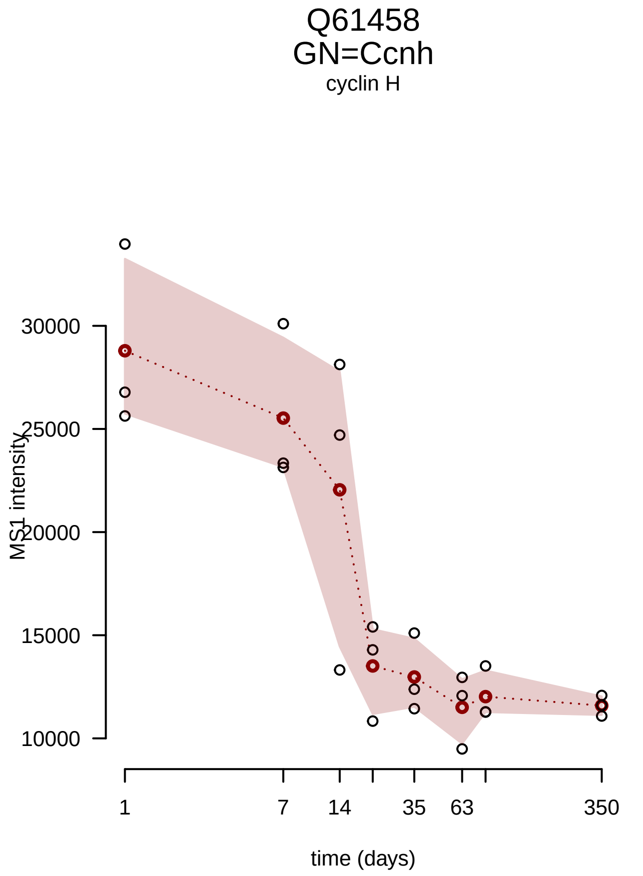
Q61458
GN=Ccnh
cyclin H
30000
25000
20000
15000
10000
MS1 intensity
1
7
14
35
63
350
time (days)

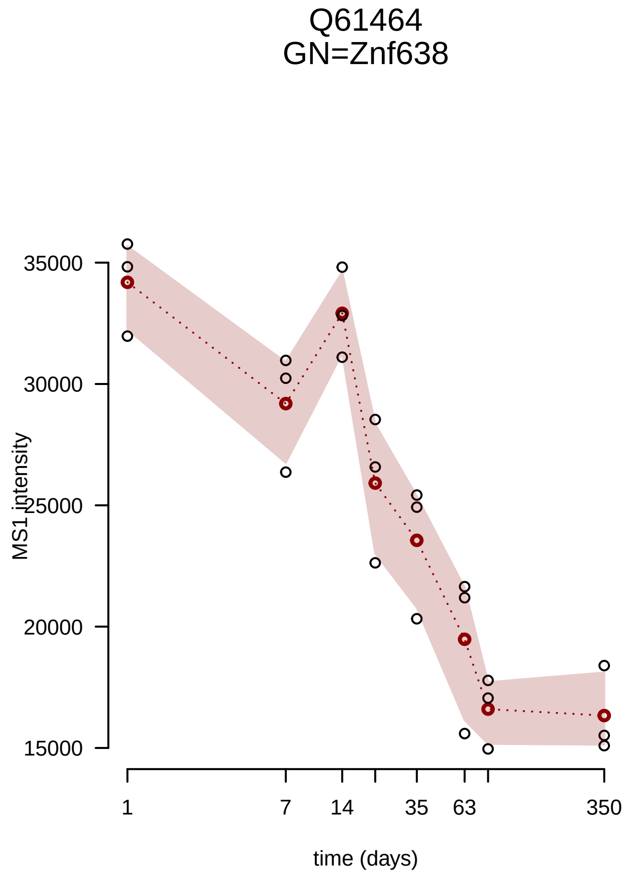
Q61464
GN=Znf638
35000
30000
25000
20000
15000
MS1 intensity
1
7
14
35
63
350
time (days)

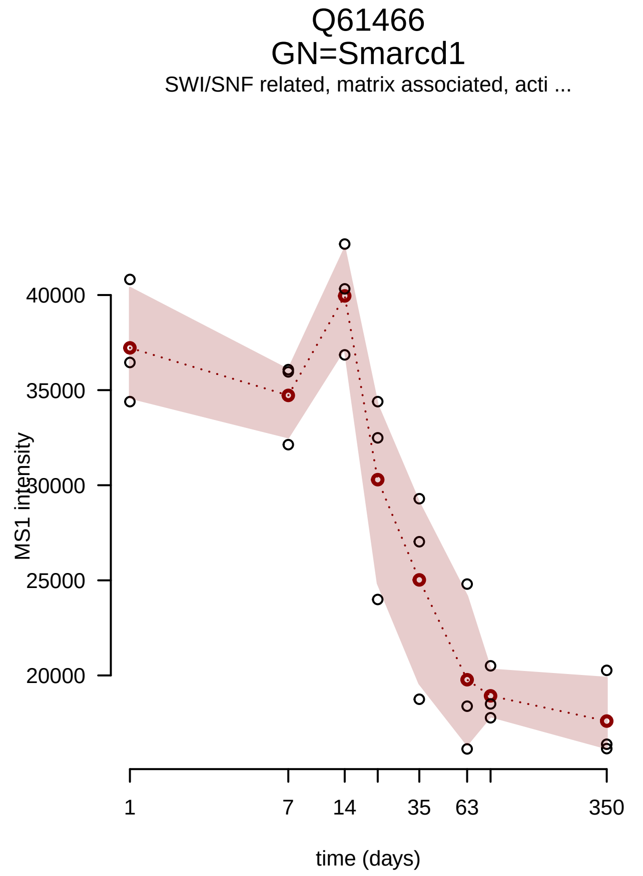
Q61466
GN=Smarcd1
SWI/SNF related, matrix associated, acti ...
40000
35000
30000
25000
20000
MS1 intensity
1
7
14
35
63
350
time (days)

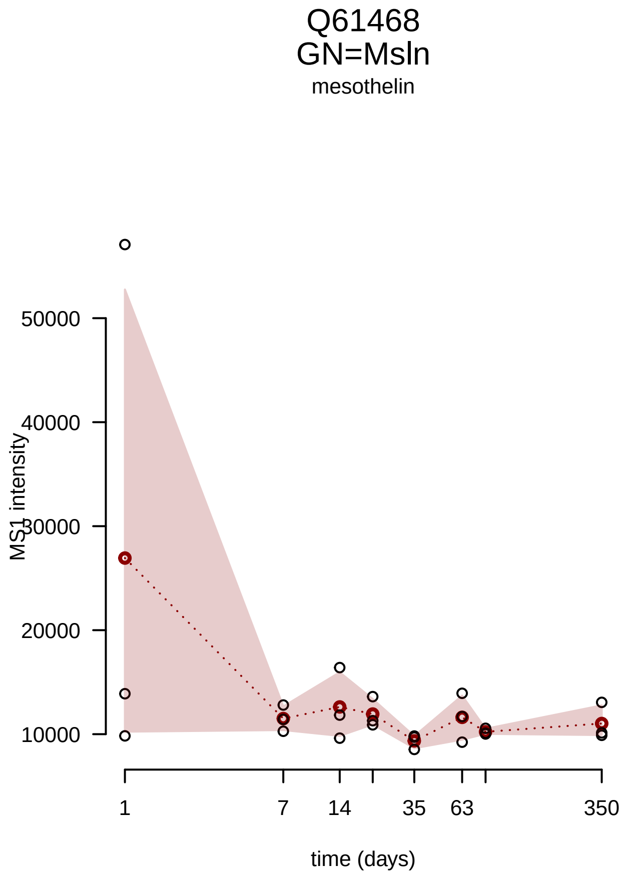
Q61468
GN=Msln
mesothelin
50000
40000
30000
20000
10000
MS1 intensity
1
7
14
35
63
350
time (days)

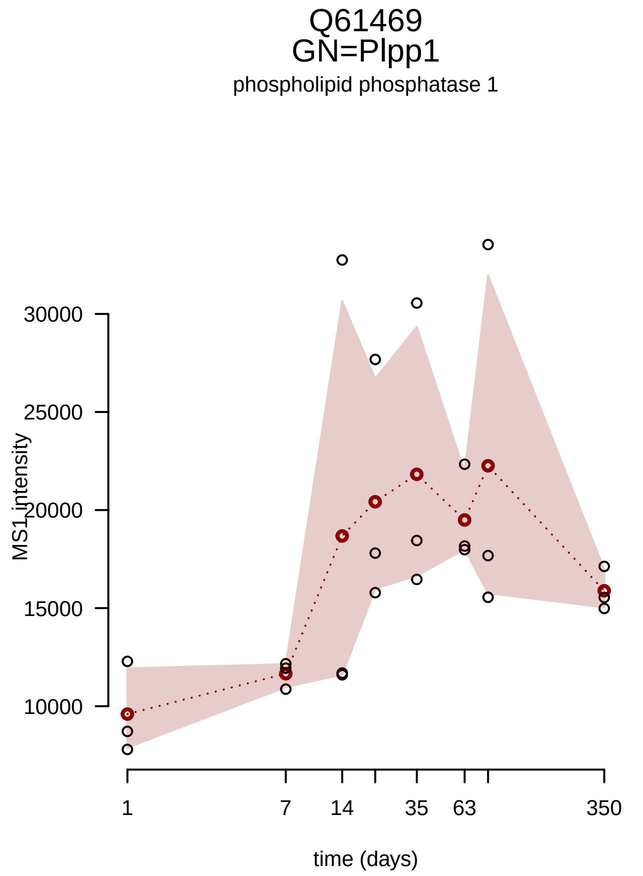
Q61469
GN=Plpp1
phospholipid phosphatase 1
30000
25000
20000
15000
10000
MS1 intensity
1
7
14
35
63
350
time (days)

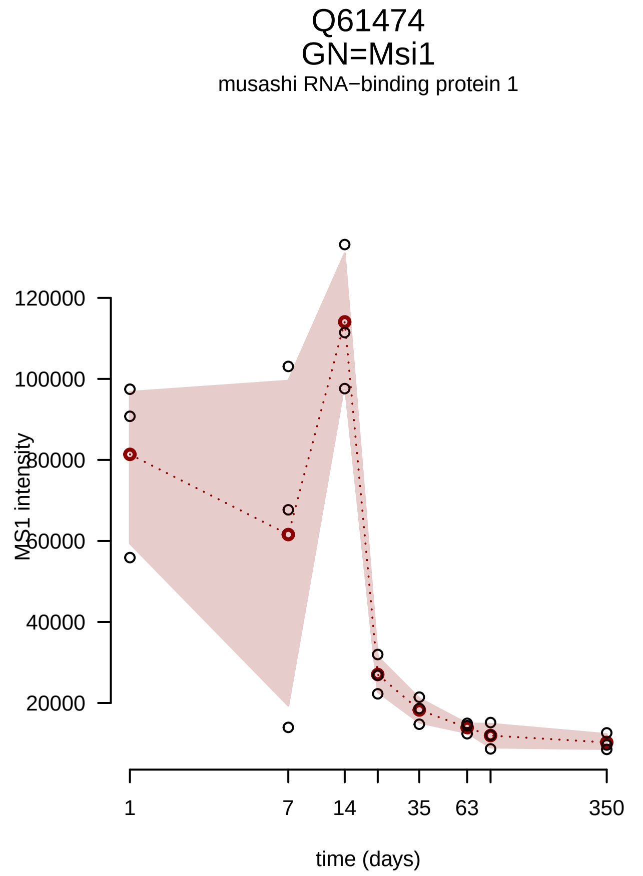
Q61474
GN=Msi1
musashi RNA−binding protein 1
120000
100000
80000
60000
40000
20000
MS1 intensity
1
7
14
35
63
350
time (days)

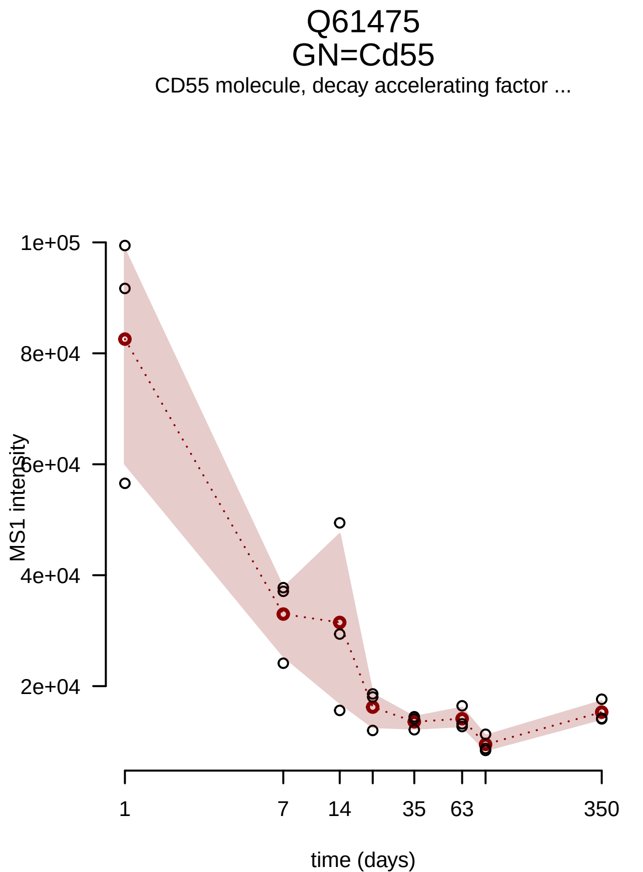
Q61475
GN=Cd55
CD55 molecule, decay accelerating factor ...
1e+05
8e+04
6e+04
4e+04
2e+04
MS1 intensity
1
7
14
35
63
350
time (days)

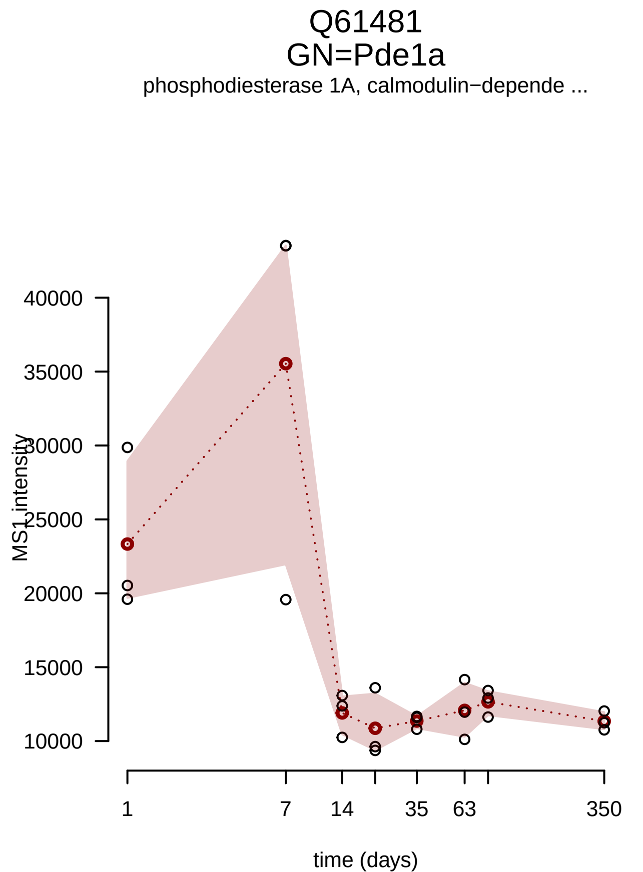
Q61481
GN=Pde1a
phosphodiesterase 1A, calmodulin−depende ...
40000
35000
30000
25000
20000
15000
10000
MS1 intensity
1
7
14
35
63
350
time (days)

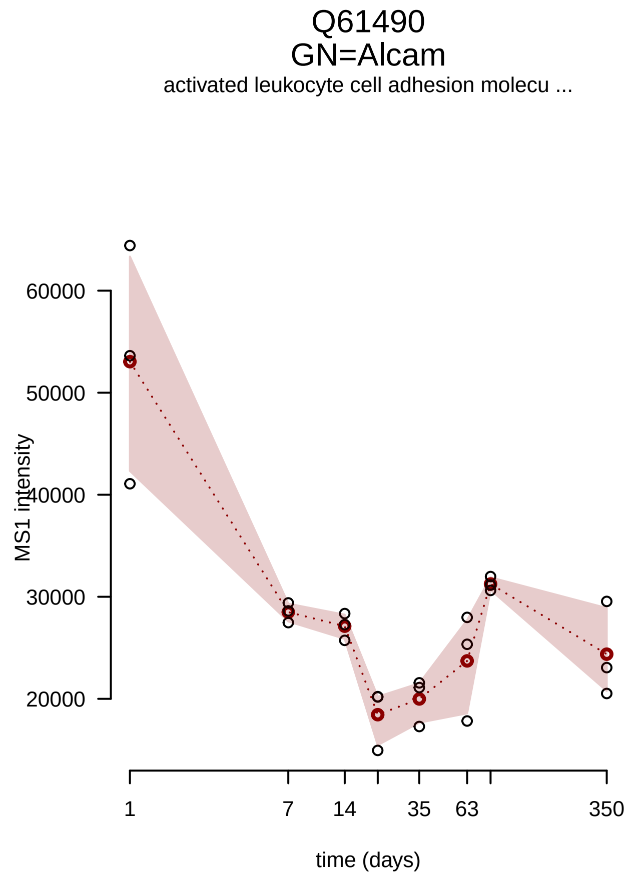
Q61490
GN=Alcam
activated leukocyte cell adhesion molecu ...
60000
50000
40000
30000
20000
MS1 intensity
1
7
14
35
63
350
time (days)

Q61493
GN=Rev3l
REV3 like, DNA directed polymerase zeta ...

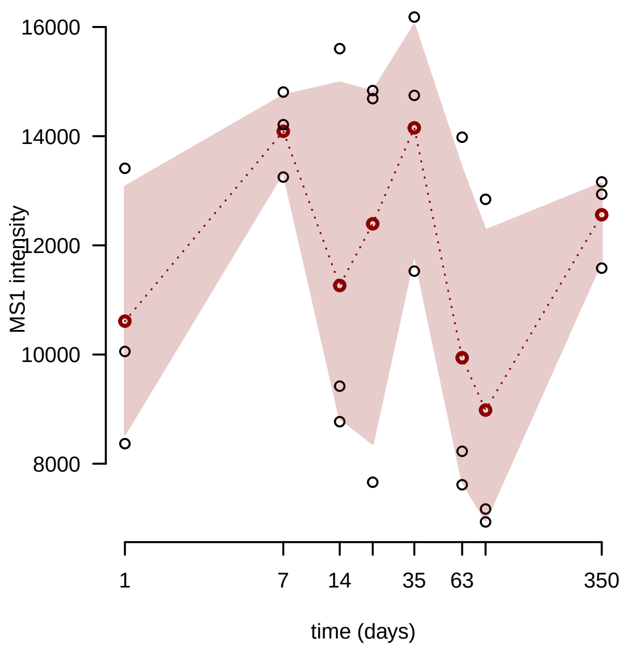

Q61495
GN=Dsg1a
desmoglein 1 alpha

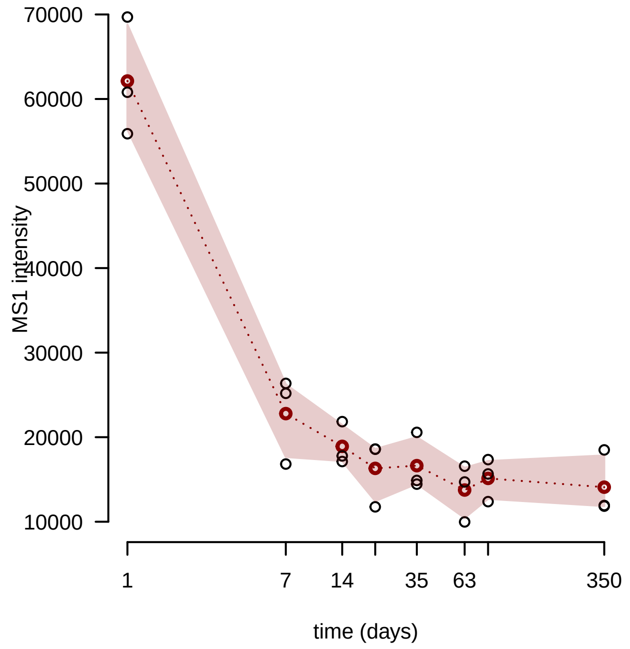

Q61496
GN=Ddx4
DEAD box helicase 4

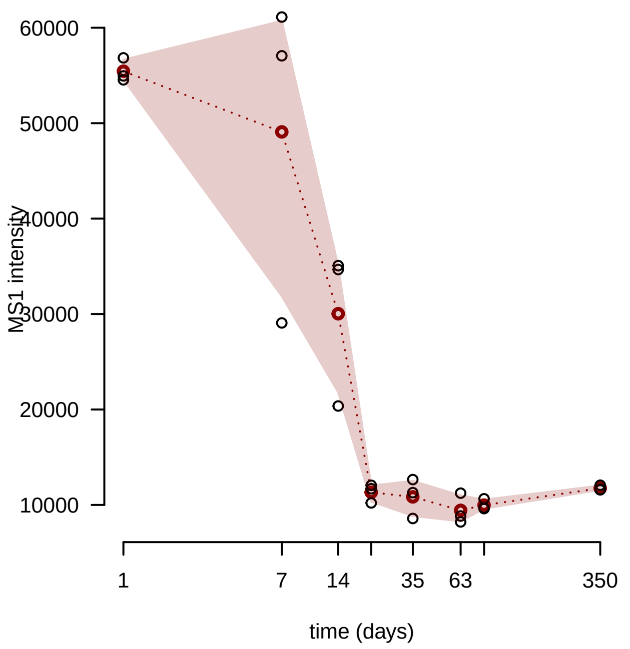

Q61500
GN=Itm2a
integral membrane protein 2A

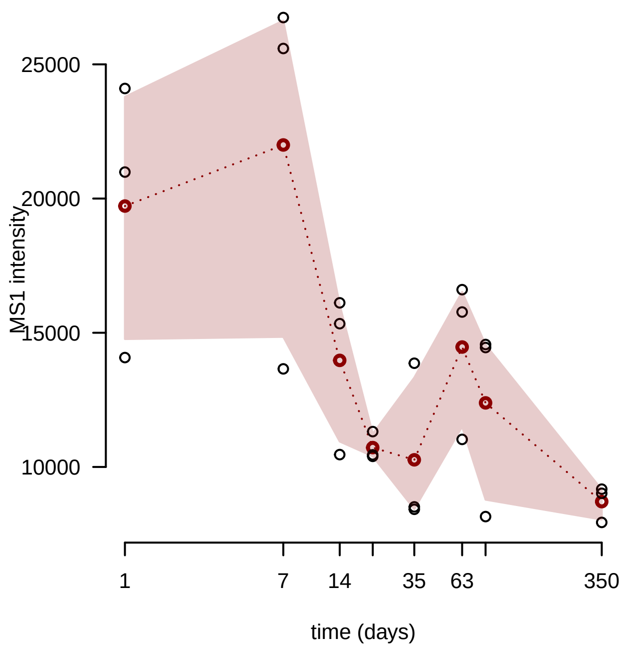

Q61503
GN=Nt5e
5' nucleotidase, ecto

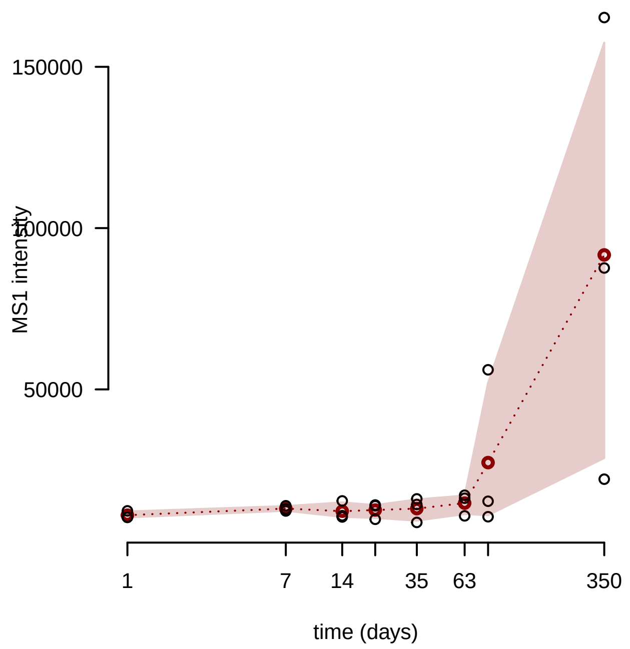

Q61508
GN=Ecm1
extracellular matrix protein 1

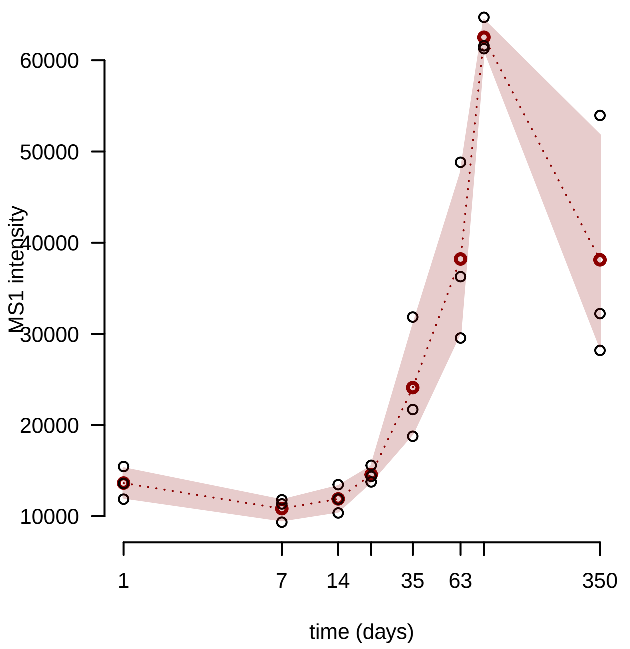

Q61510
GN=Trim25
tripartite motif−containing 25

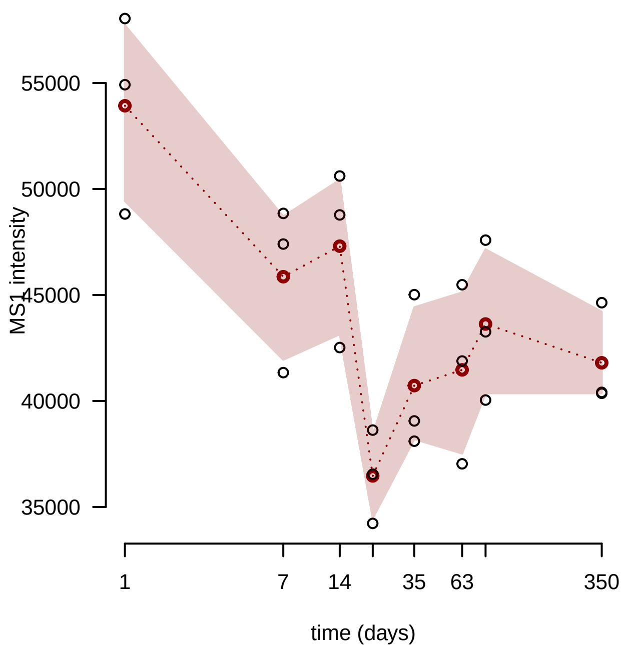

Q61542
GN=Stard3
START domain containing 3

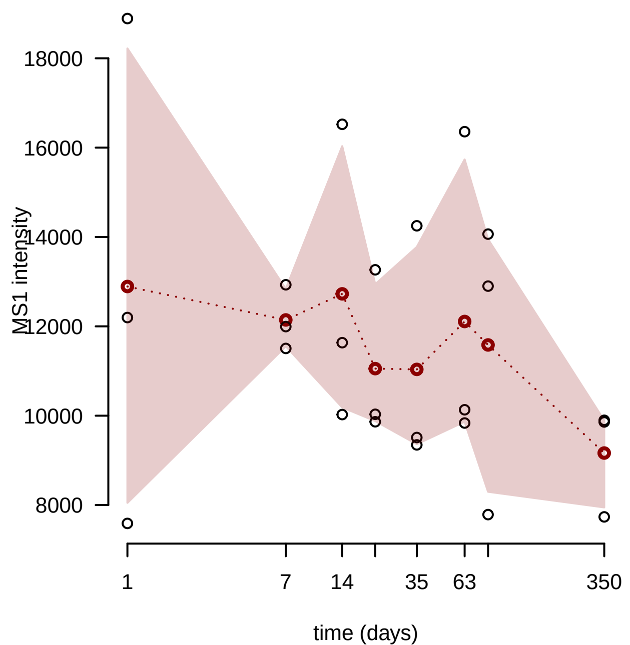

Q61543
GN=Glg1
golgi apparatus protein 1

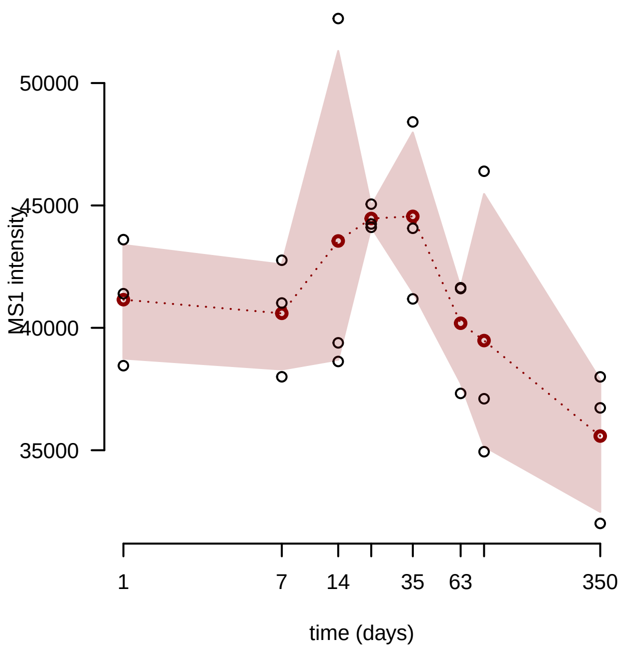

Q61545
GN=Ewsr1
Ewing sarcoma breakpoint region 1

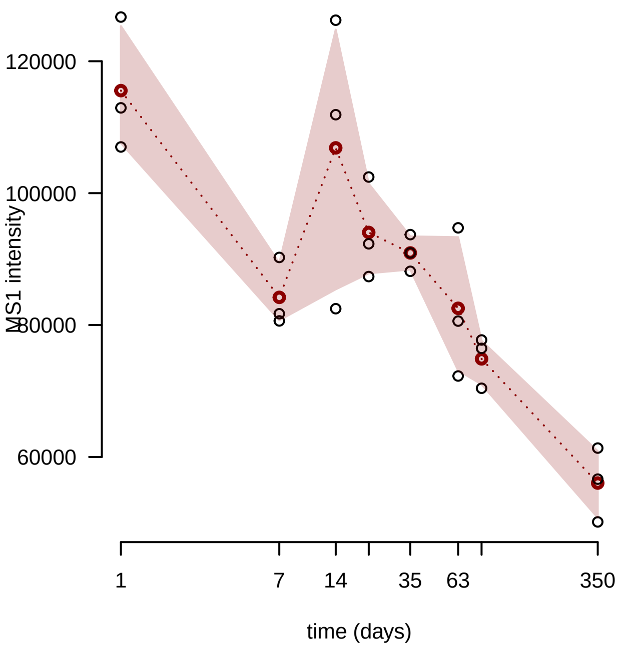

Q61548
GN=Snap91
synaptosomal−associated protein 91

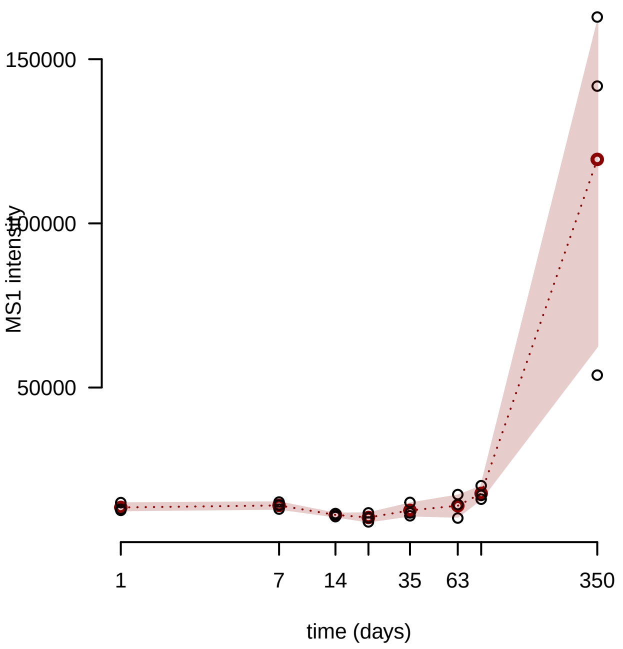

Q61550
GN=Rad21
RAD21 cohesin complex component

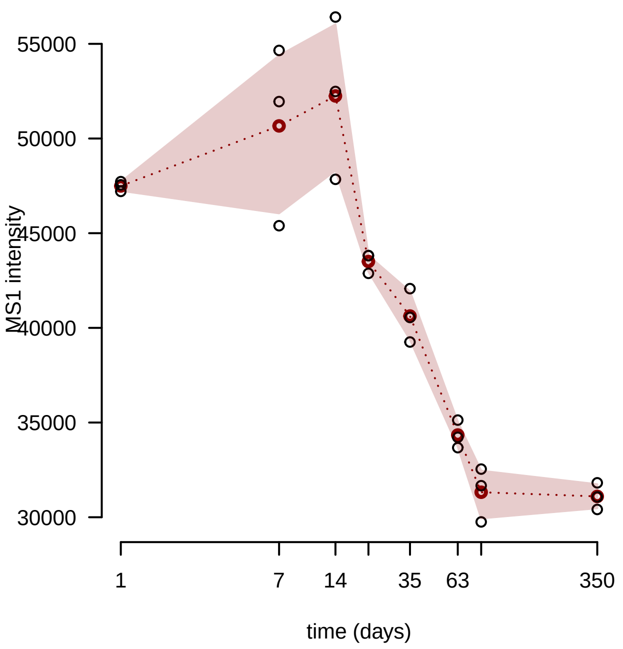

Q61553
GN=Fscn1
fascin actin−bundling protein 1

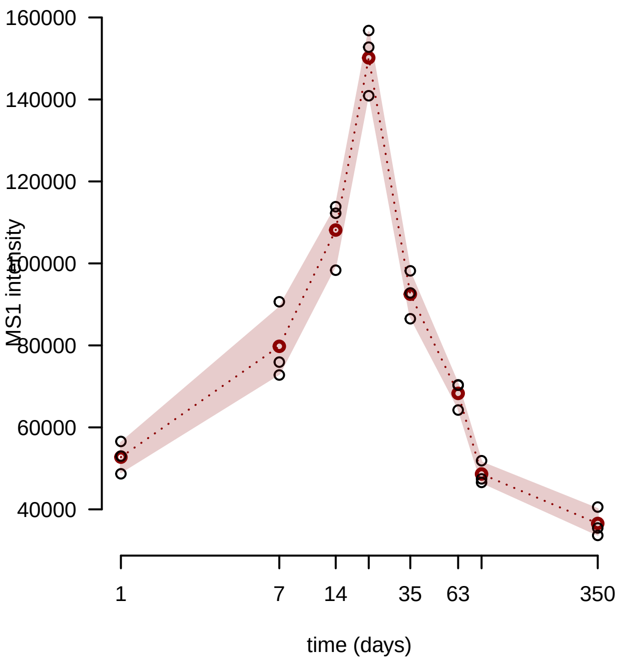

Q61554
GN=Fbn1
fibrillin 1

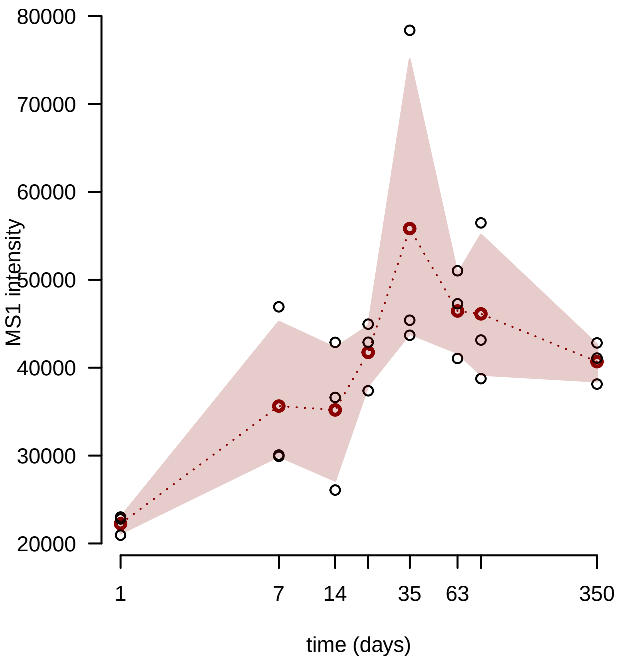

Q61555
GN=Fbn2
fibrillin 2

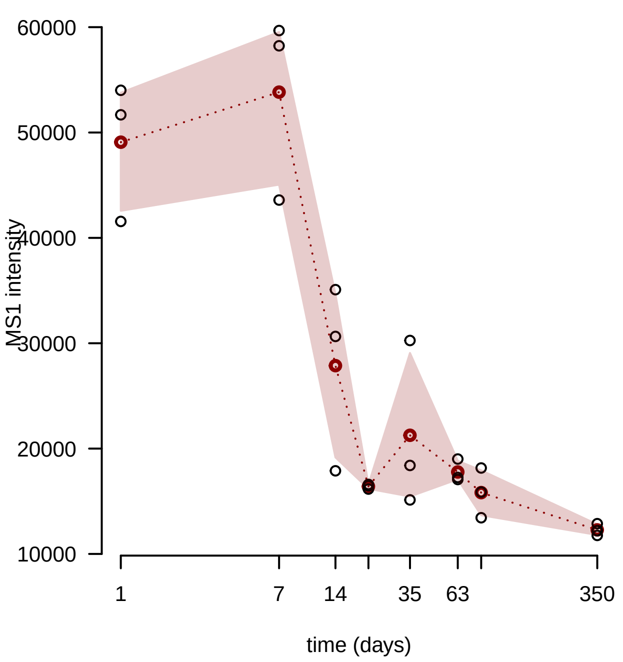

Q61559
GN=Fcgrt
Fc fragment of IgG receptor and transpor ...

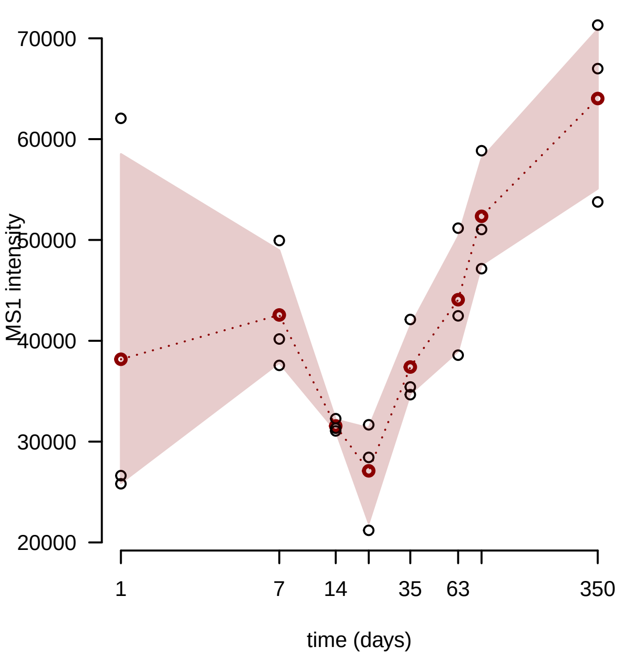

Q61576
GN=Fkbp10
FK506 binding protein 10

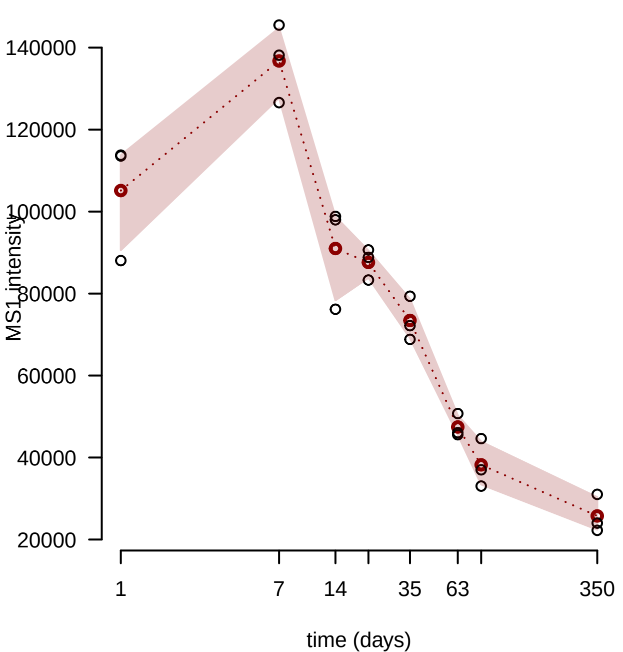

Q61578
GN=Fdxr
ferredoxin reductase

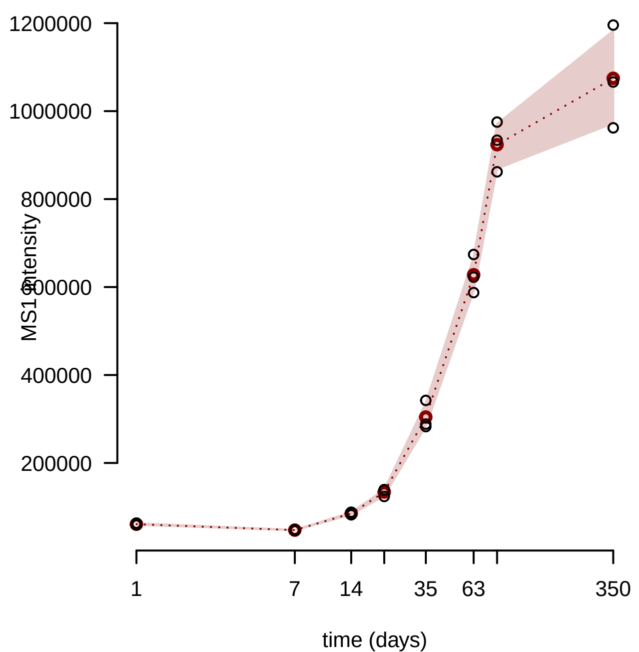

Q61581
GN=Igfbp7
insulin−like growth factor binding prote ...

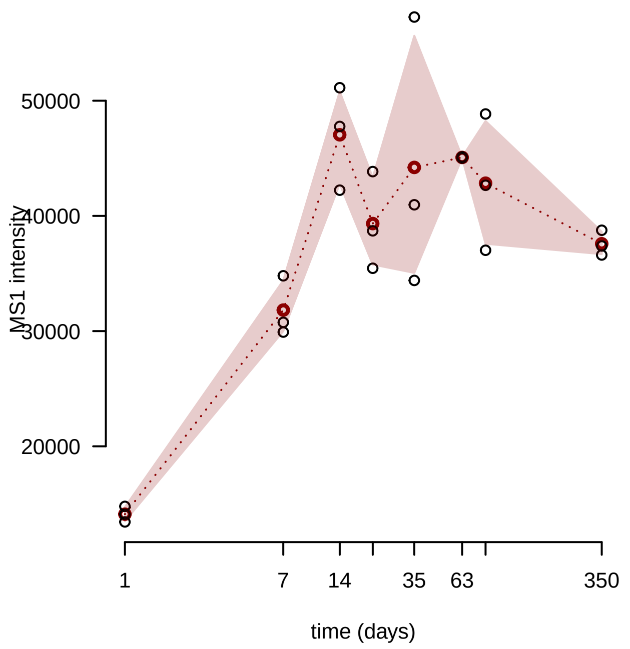

Q61584
GN=Fxr1
FMR1 autosomal homolog 1

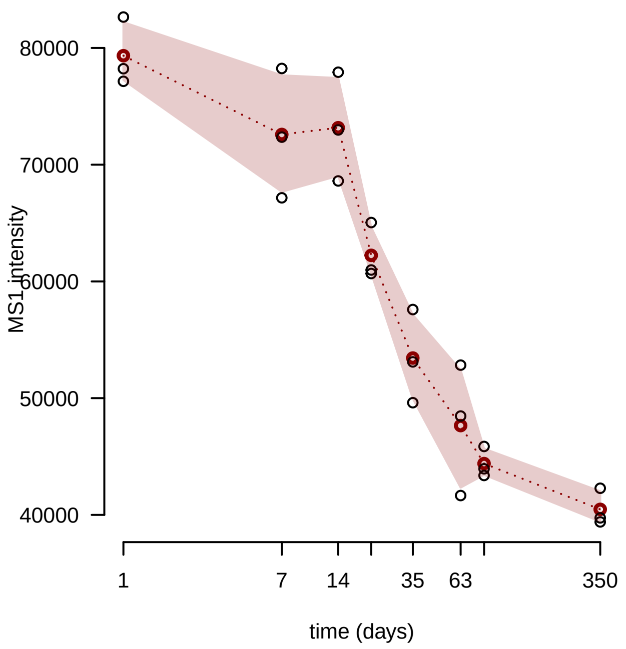

Q61586
GN=Gpam
glycerol−3−phosphate acyltransferase, mi ...

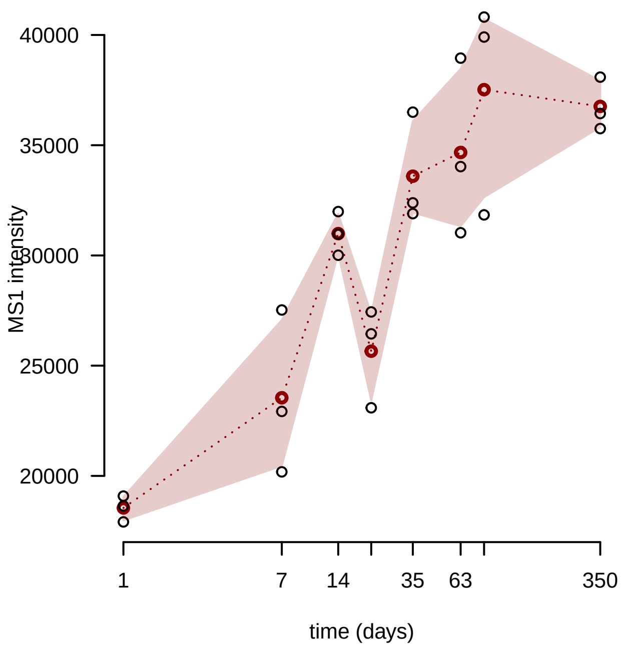

Q61592
GN=Gas6
growth arrest specific 6

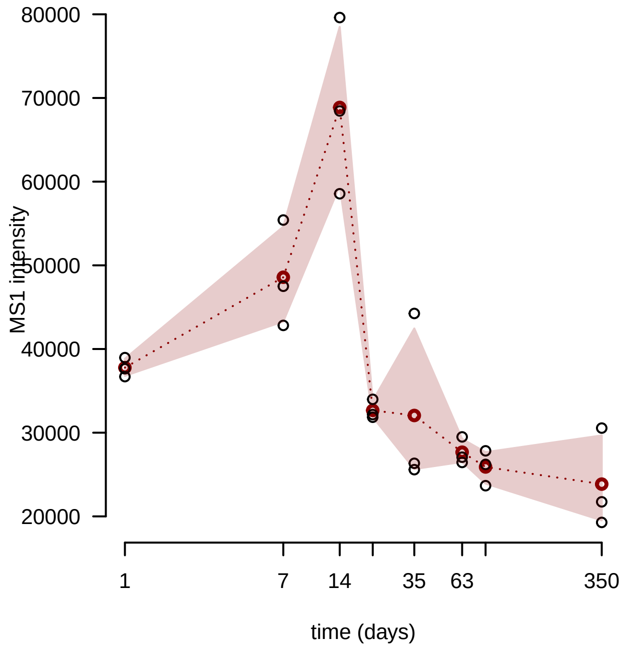

Q61595
GN=Ktn1
kinectin 1

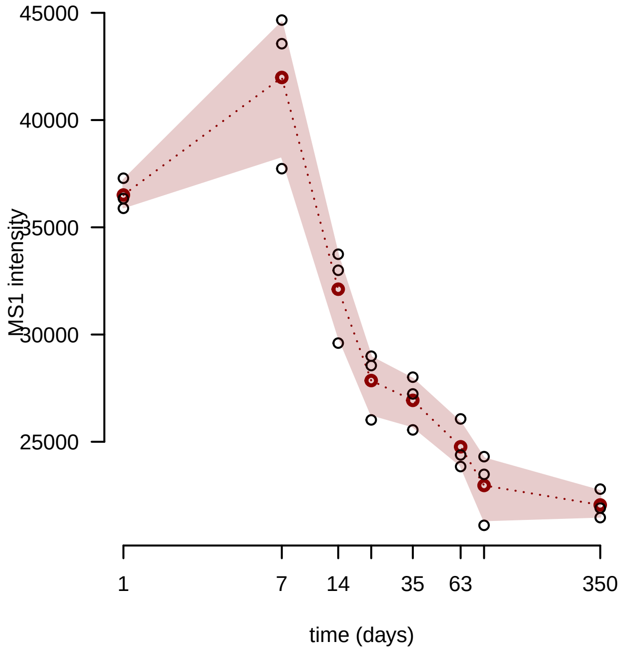

Q61595
GN=Ktn1
kinectin 1

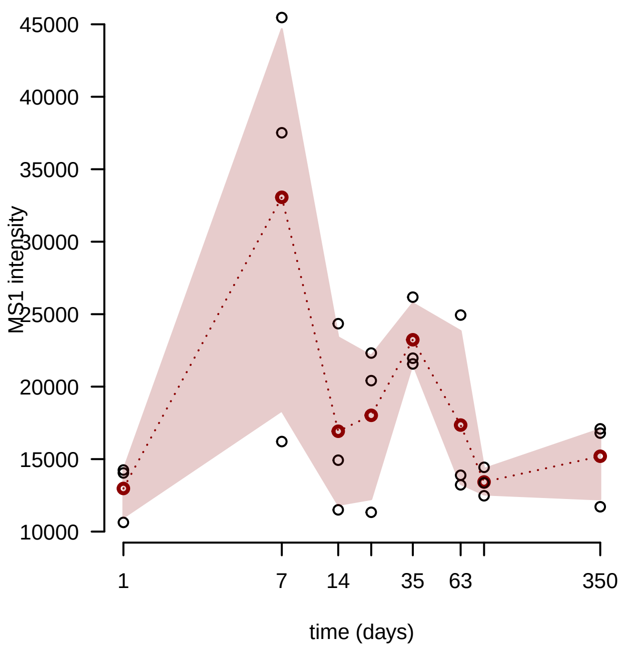

Q61598
GN=Gdi2
guanosine diphosphate (GDP) dissociation ...

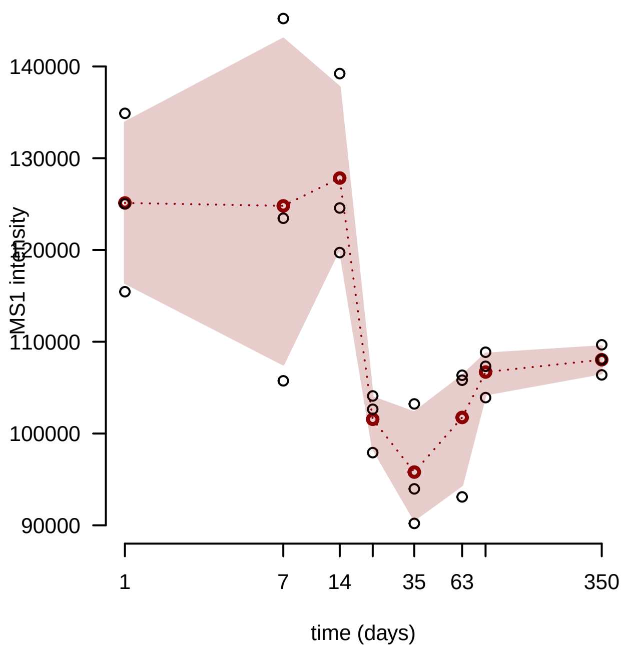

Q61599
GN=Arhgdib
Rho, GDP dissociation inhibitor (GDI) be ...

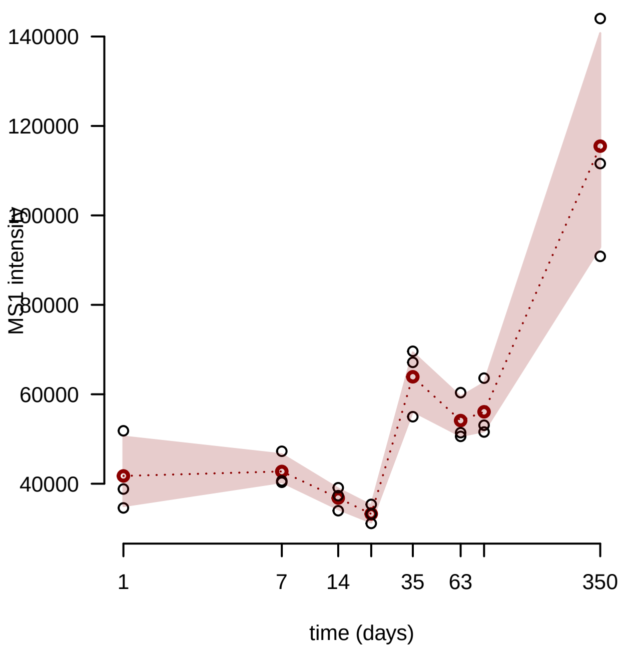

Q61602
GN=Gli3
GLI−Kruppel family member GLI3

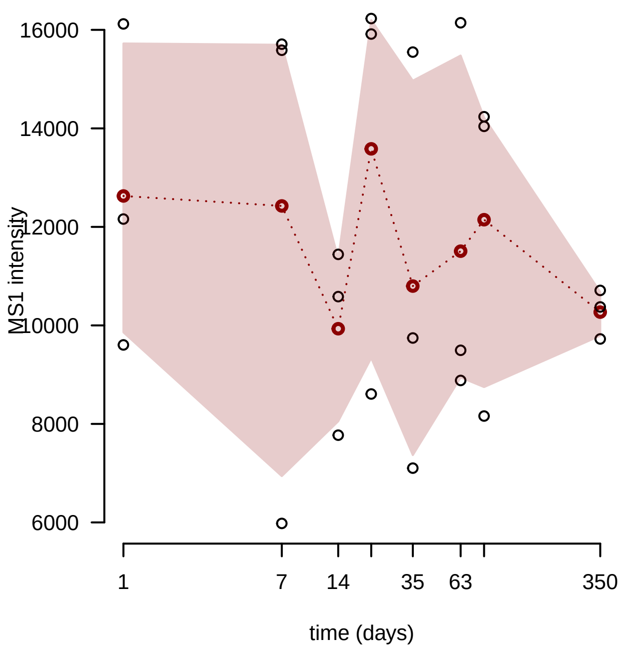

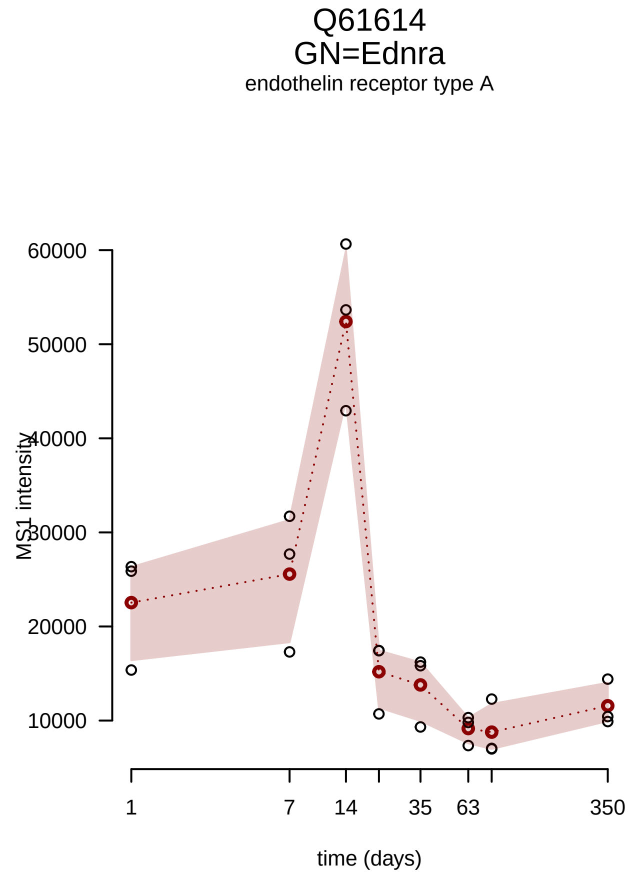
Q61614
GN=Ednra
endothelin receptor type A
MS1 intensity
60000
50000
40000
30000
20000
10000
1
7
14
35
63
350
time (days)

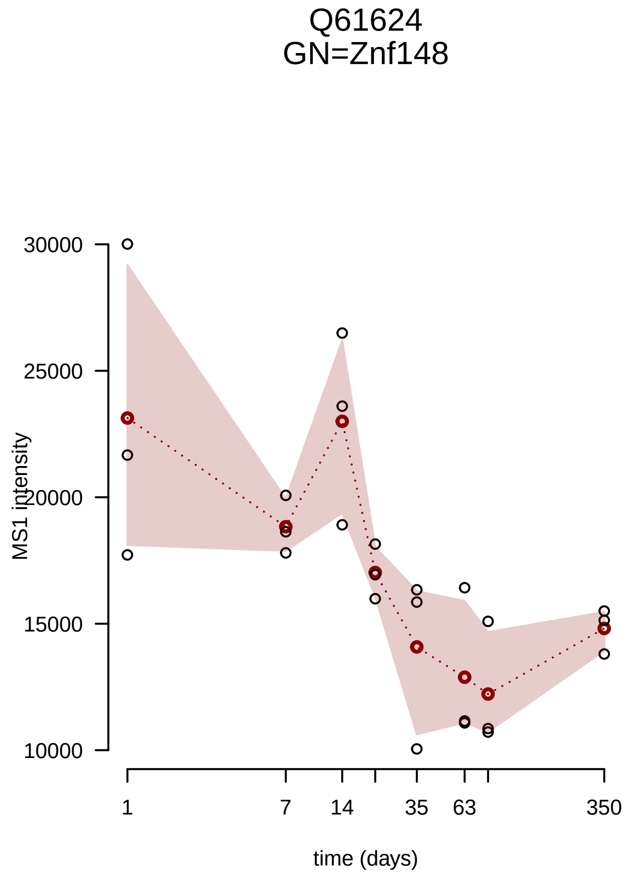
Q61624
GN=Znf148
MS1 intensity
30000
25000
20000
15000
10000
1
7
14
35
63
350
time (days)

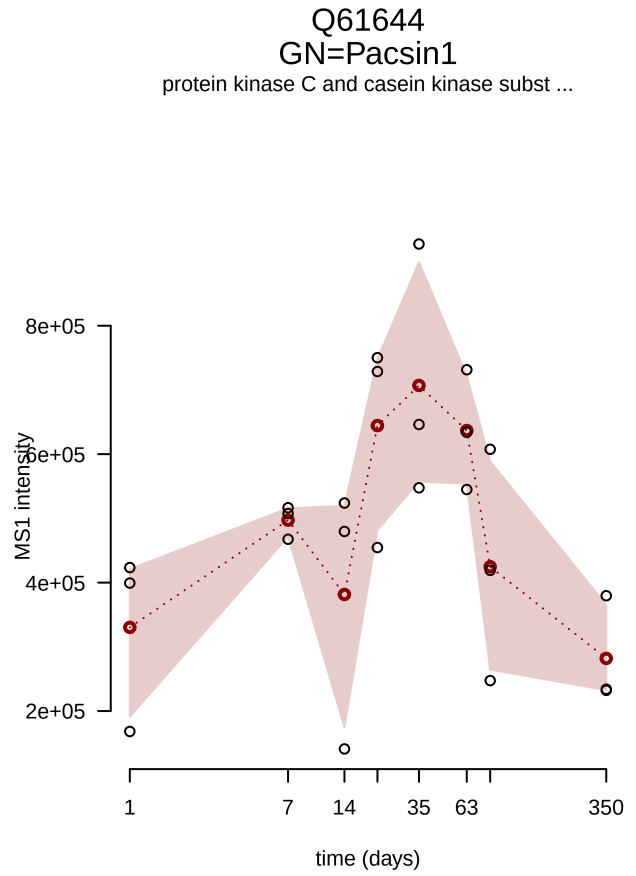
Q61644
GN=Pacsin1
protein kinase C and casein kinase subst ...
MS1 intensity
8e+05
6e+05
4e+05
2e+05
1
7
14
35
63
350
time (days)

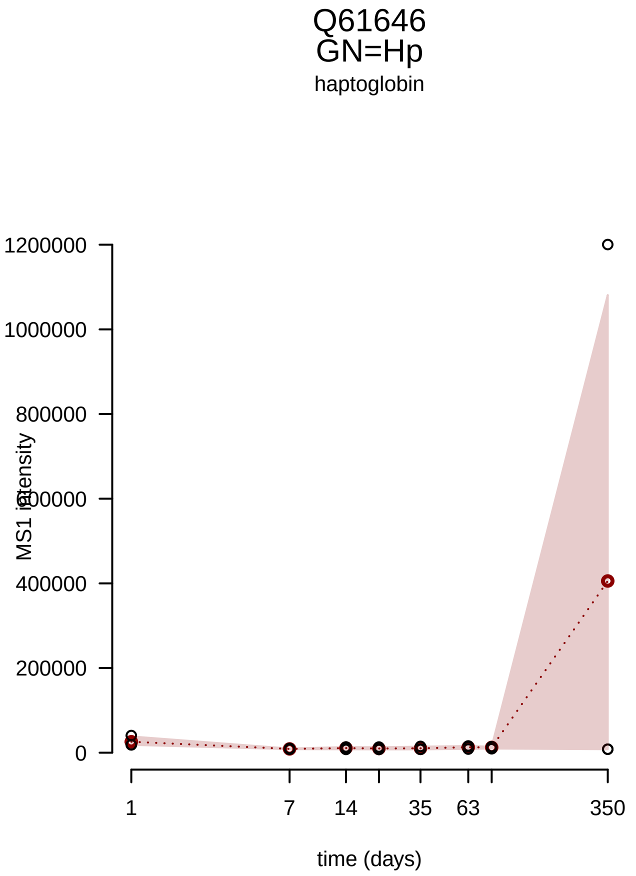
Q61646
GN=Hp
haptoglobin
MS1 intensity
1200000
1000000
800000
600000
400000
200000
0
1
7
14
35
63
350
time (days)

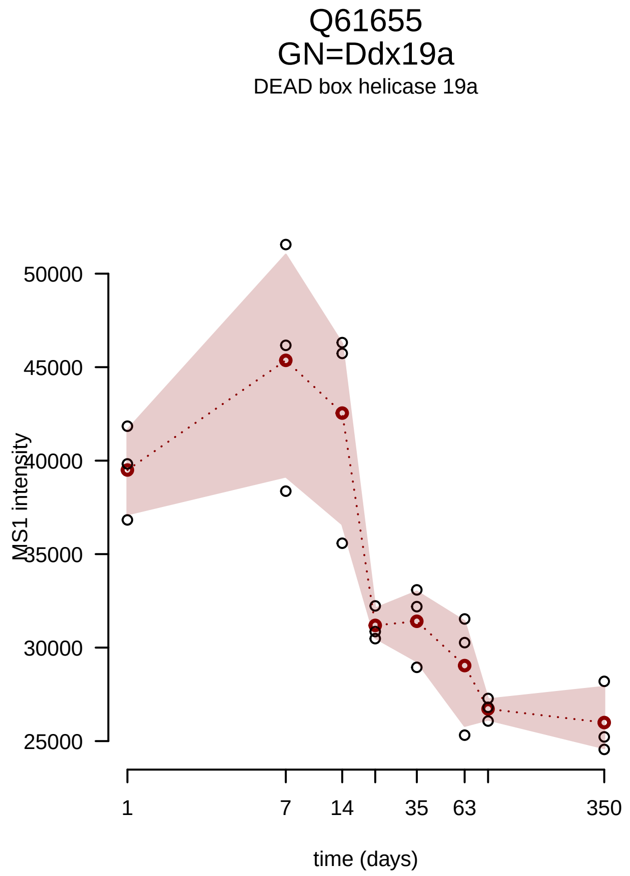
Q61655
GN=Ddx19a
DEAD box helicase 19a
MS1 intensity
50000
45000
40000
35000
30000
25000
1
7
14
35
63
350
time (days)

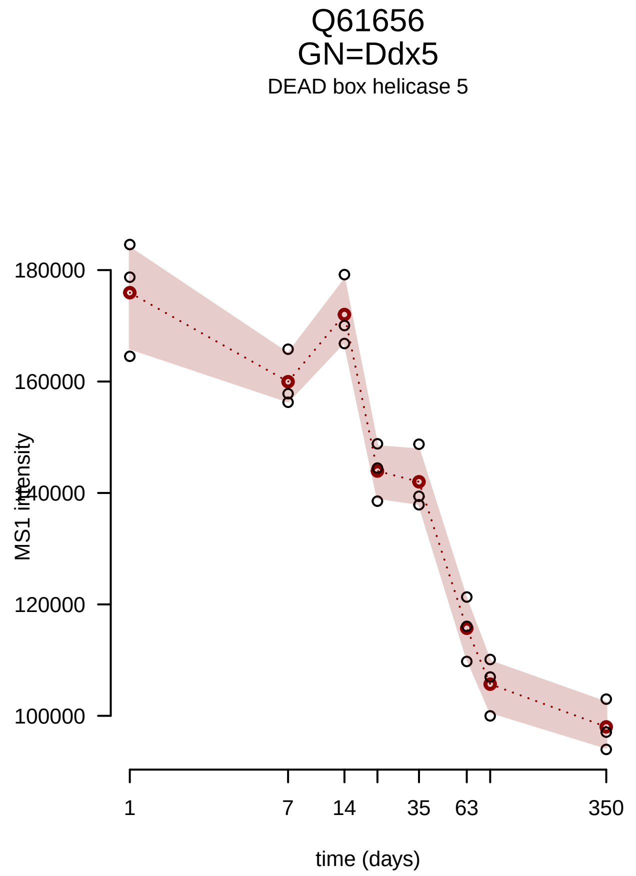
Q61656
GN=Ddx5
DEAD box helicase 5
MS1 intensity
180000
160000
140000
120000
100000
1
7
14
35
63
350
time (days)

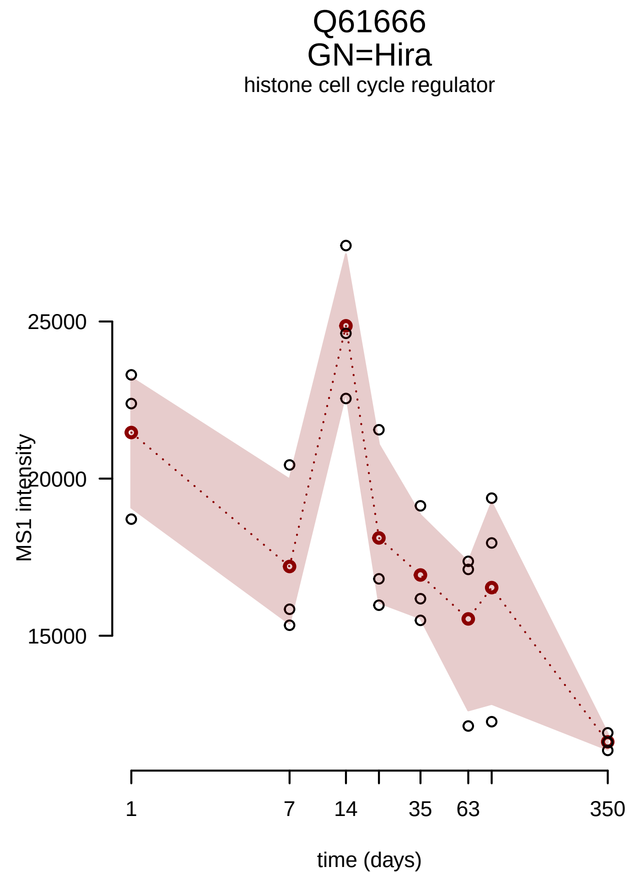
Q61666
GN=Hira
histone cell cycle regulator
MS1 intensity
25000
20000
15000
1
7
14
35
63
350
time (days)

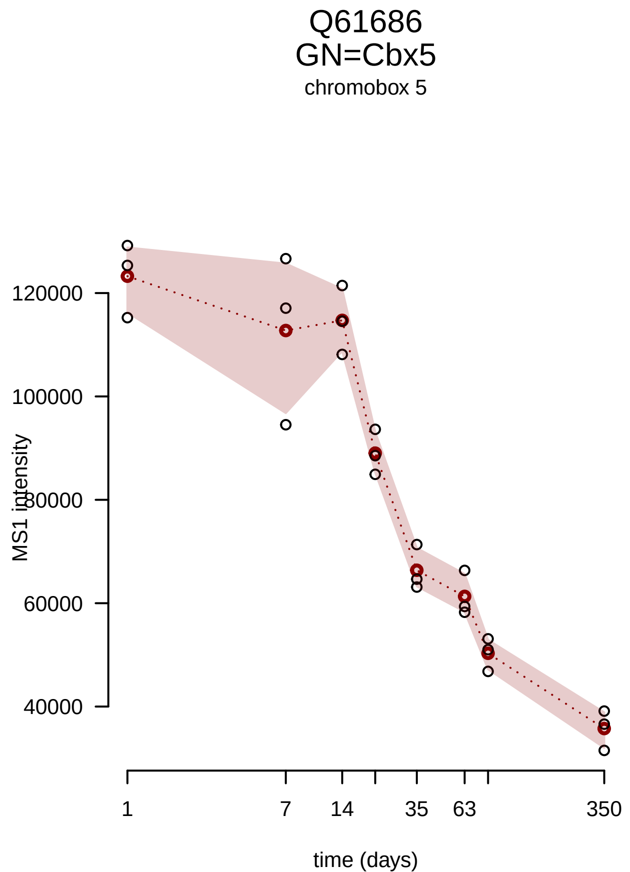
Q61686
GN=Cbx5
chromobox 5
MS1 intensity
120000
100000
80000
60000
40000
1
7
14
35
63
350
time (days)

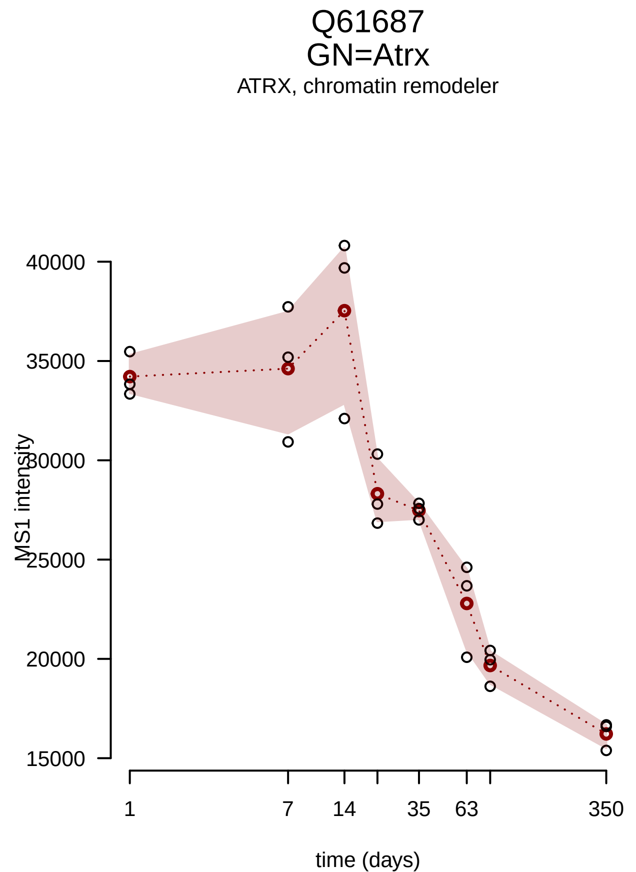
Q61687
GN=Atrx
ATRX, chromatin remodeler
MS1 intensity
40000
35000
30000
25000
20000
15000
1
7
14
35
63
350
time (days)

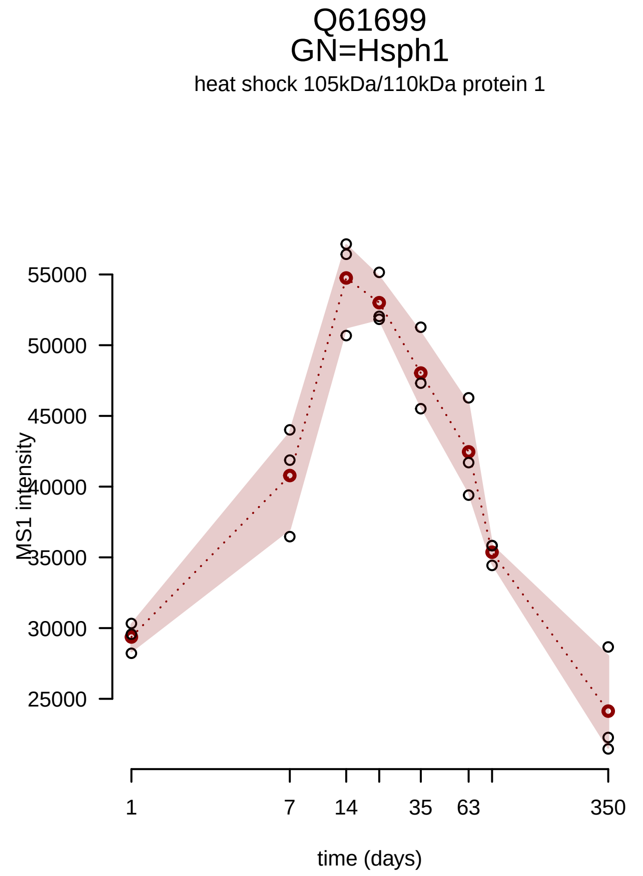
Q61699
GN=Hsph1
heat shock 105kDa/110kDa protein 1
MS1 intensity
25000 30000 35000 40000 45000 50000 55000
1 7 14 35 63 350
time (days)

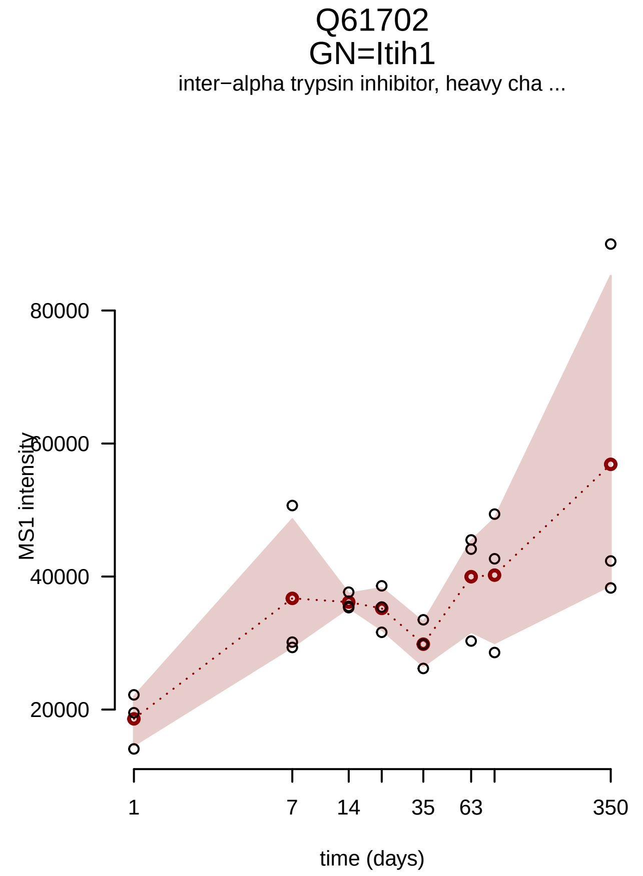
Q61702
GN=Itih1
inter−alpha trypsin inhibitor, heavy cha ...
MS1 intensity
20000 40000 60000 80000
1 7 14 35 63 350
time (days)

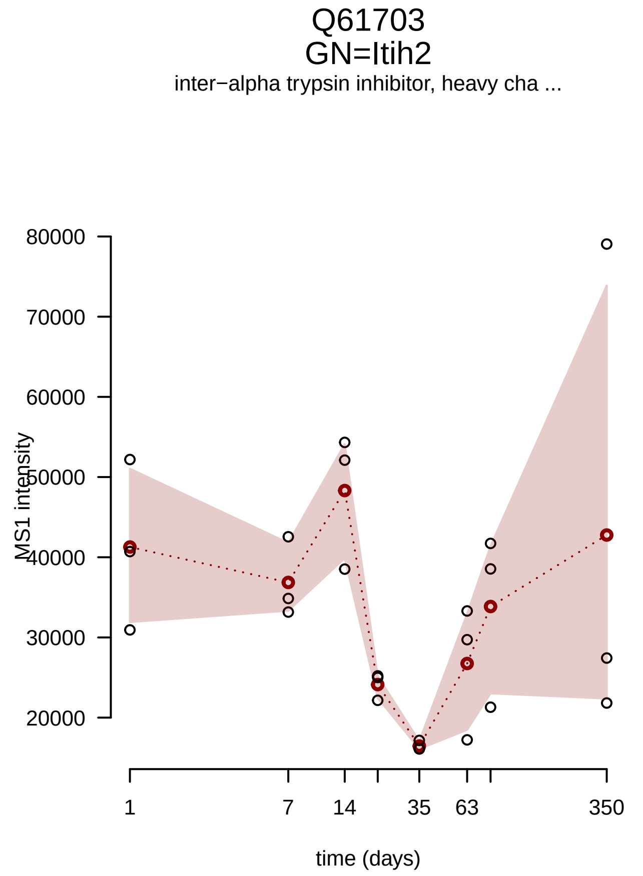
Q61703
GN=Itih2
inter−alpha trypsin inhibitor, heavy cha ...
MS1 intensity
20000 30000 40000 50000 60000 70000 80000
1 7 14 35 63 350
time (days)

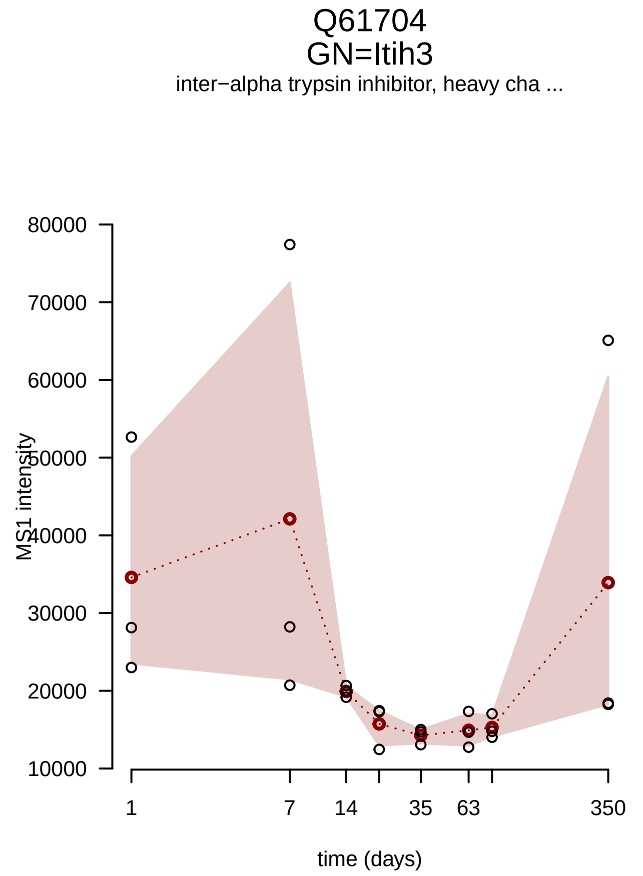
Q61704
GN=Itih3
inter−alpha trypsin inhibitor, heavy cha ...
MS1 intensity
10000 20000 30000 40000 50000 60000 70000 80000
1 7 14 35 63 350
time (days)

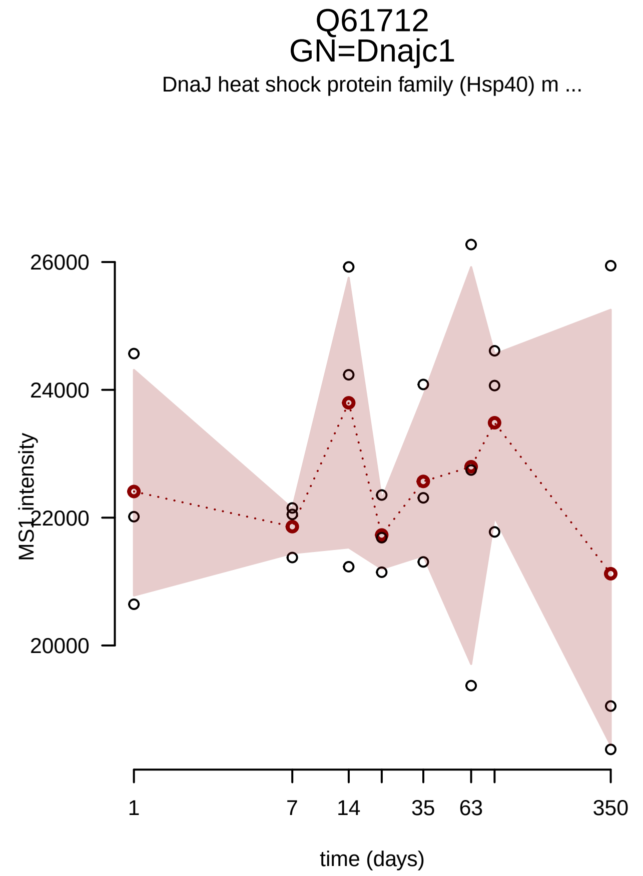
Q61712
GN=Dnajc1
DnaJ heat shock protein family (Hsp40) m ...
MS1 intensity
20000 22000 24000 26000
1 7 14 35 63 350
time (days)

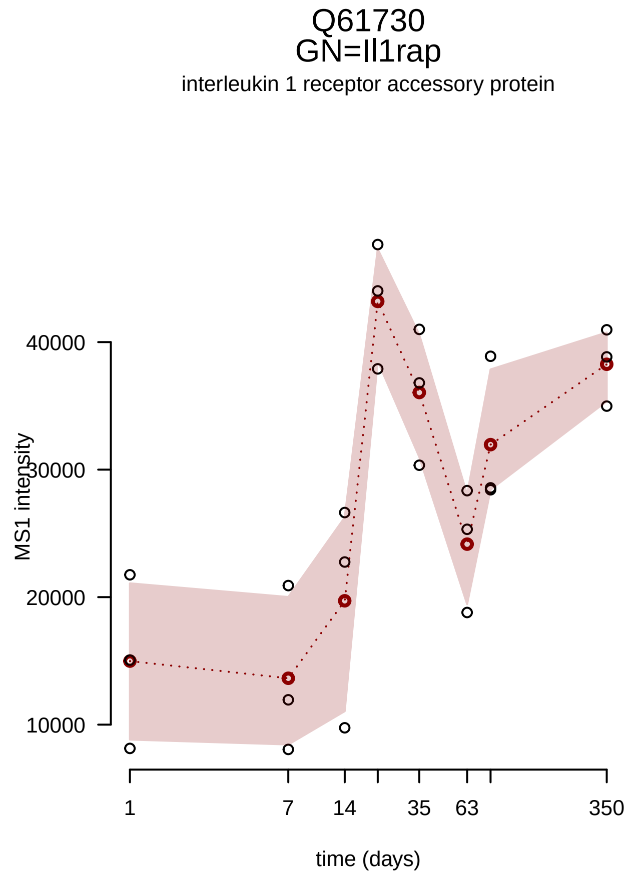
Q61730
GN=Il1rap
interleukin 1 receptor accessory protein
MS1 intensity
10000 20000 30000 40000
1 7 14 35 63 350
time (days)

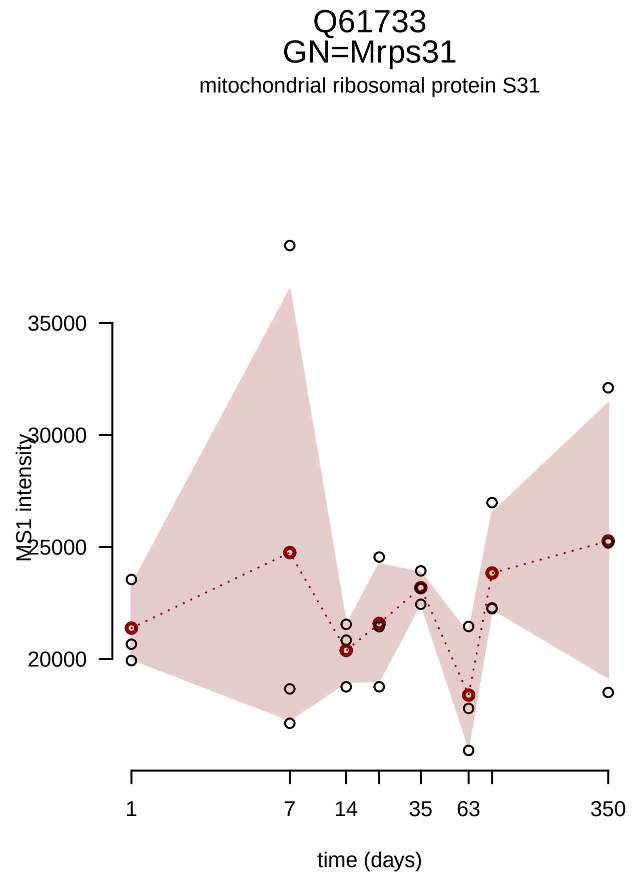
Q61733
GN=Mrps31
mitochondrial ribosomal protein S31
MS1 intensity
20000 25000 30000 35000
1 7 14 35 63 350
time (days)

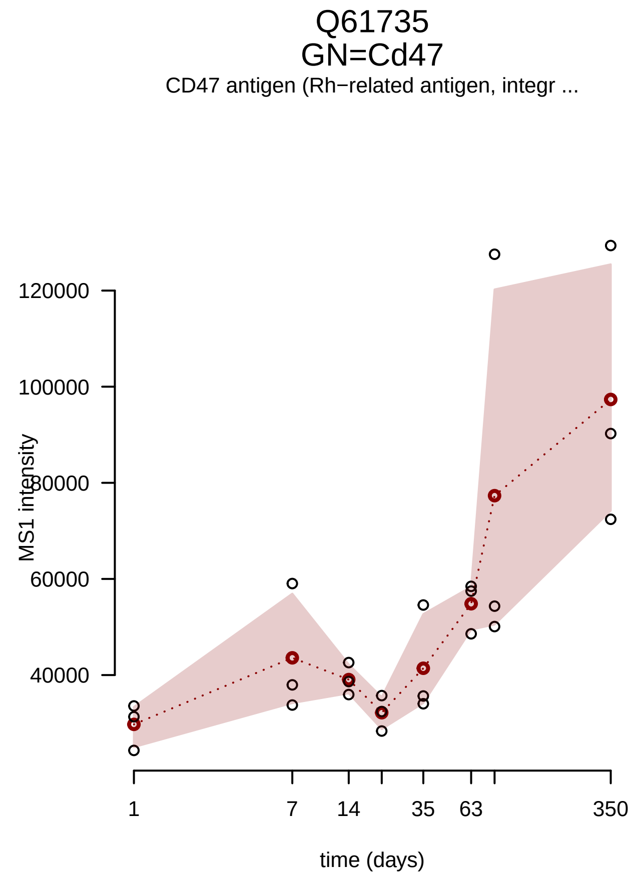
Q61735
GN=Cd47
CD47 antigen (Rh−related antigen, integr ...
MS1 intensity
40000 60000 80000 100000 120000
1 7 14 35 63 350
time (days)

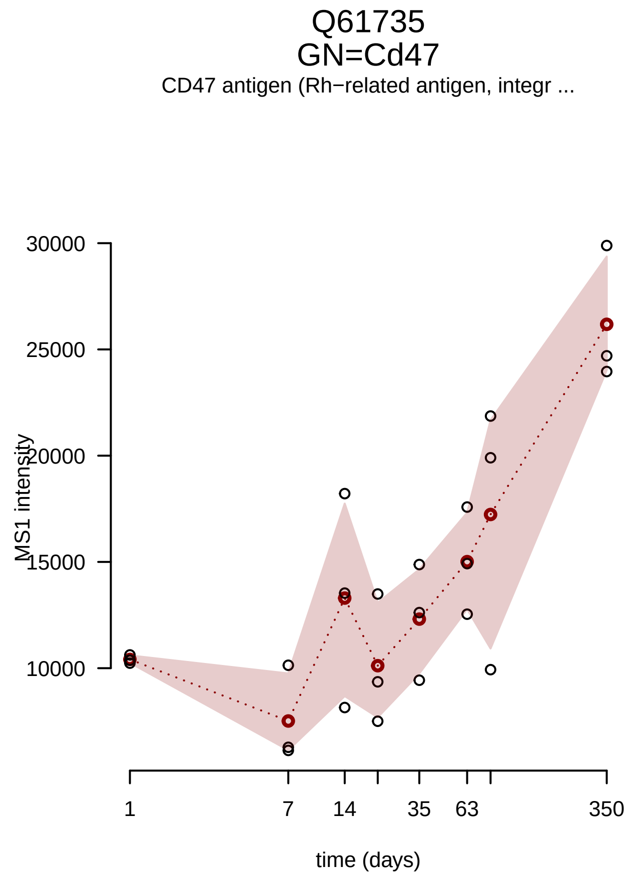
Q61735
GN=Cd47
CD47 antigen (Rh−related antigen, integr ...
MS1 intensity
10000 15000 20000 25000 30000
1 7 14 35 63 350
time (days)

Q61738
GN=Itga7
integrin alpha 7

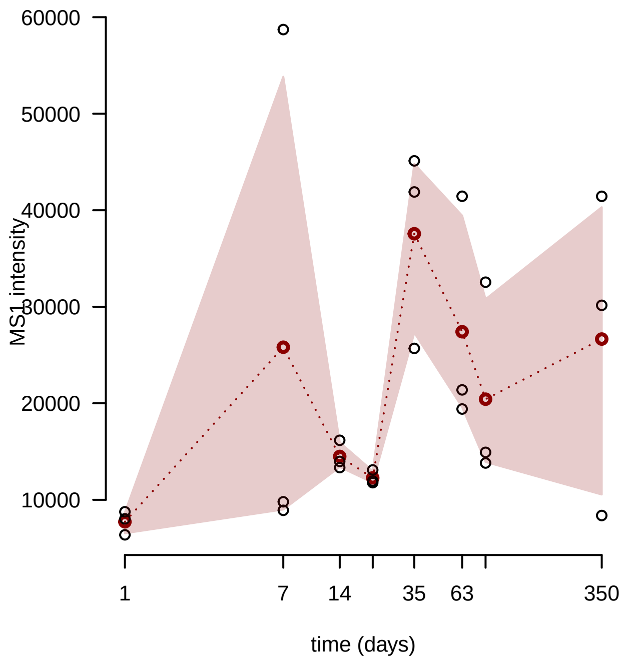

Q61739
GN=Itga6
integrin alpha 6

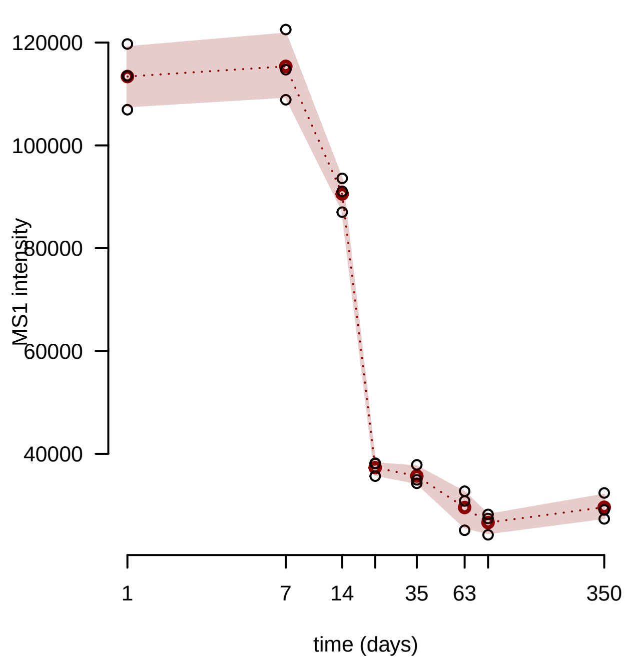

Q61739
GN=Itga6
integrin alpha 6

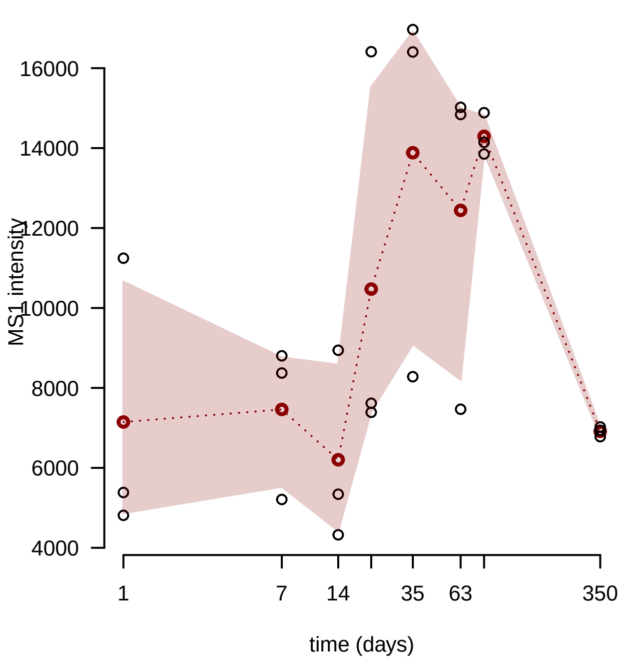

Q61749
GN=Eif2b4
eukaryotic translation initiation factor ...

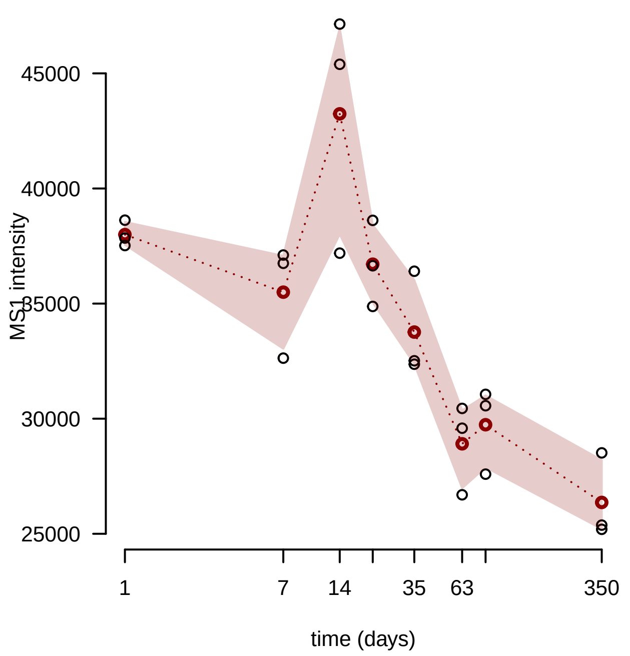

Q61753
GN=Phgdh
3−phosphoglycerate dehydrogenase

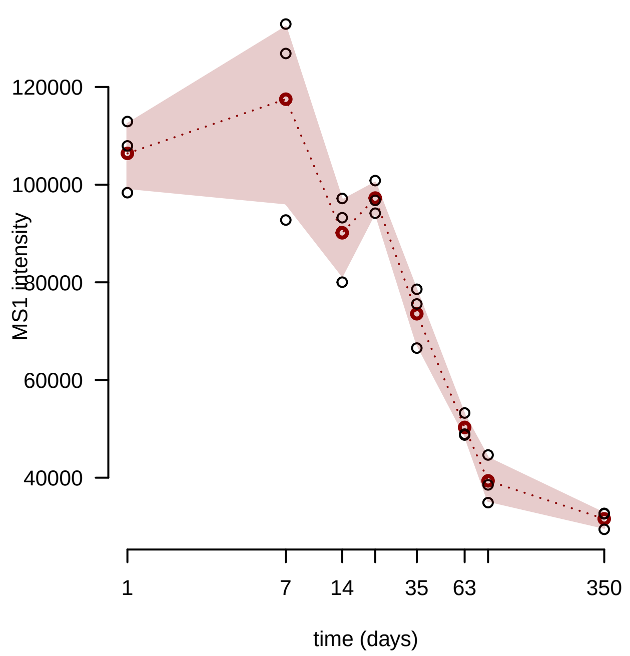

Q61765
GN=Krt31
keratin 31

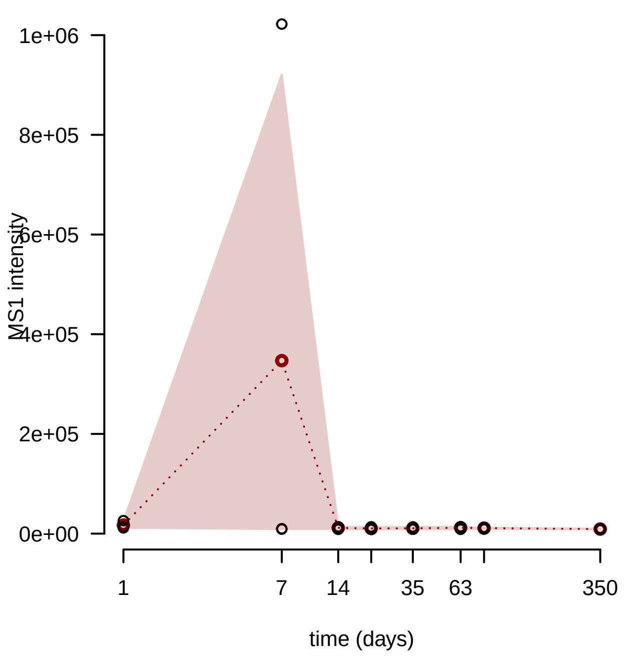

Q61768
GN=Kif5b
kinesin family member 5B

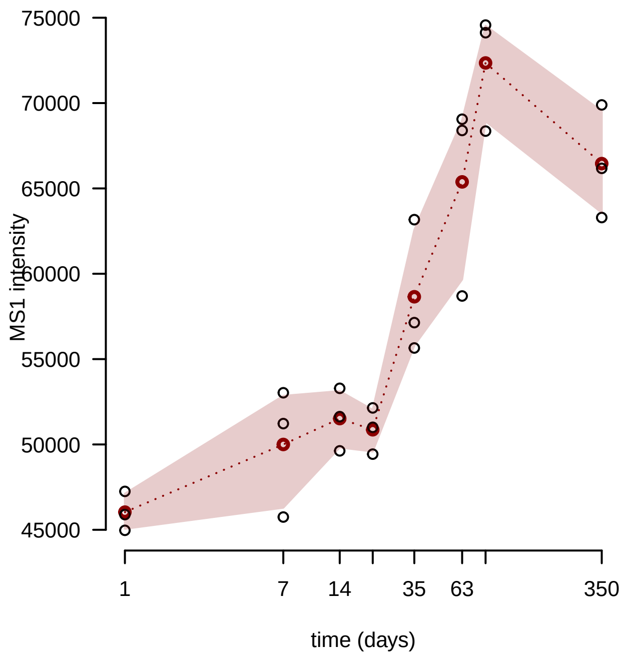

Q61771
GN=Kif3b
kinesin family member 3B

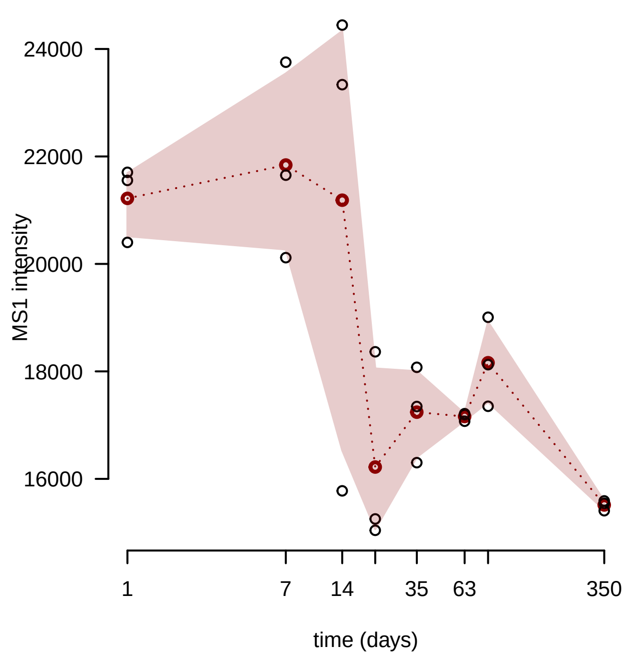

Q61781
GN=Krt14
keratin 14

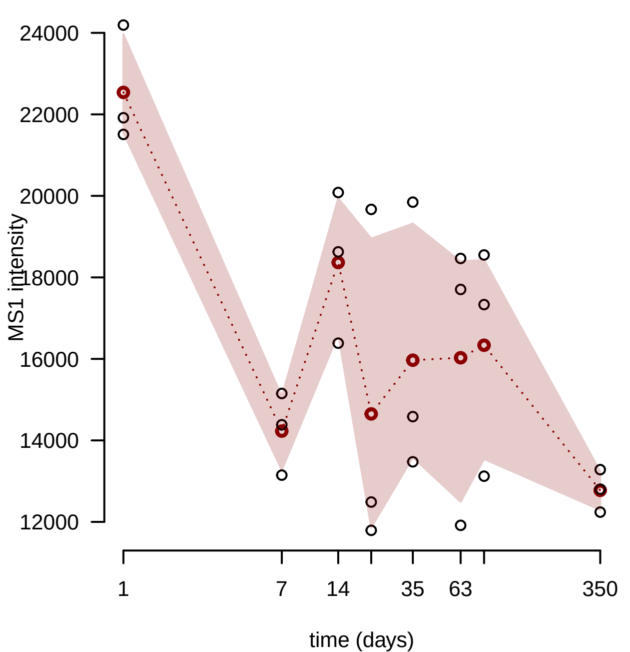

Q61789
GN=Lama3
laminin, alpha 3

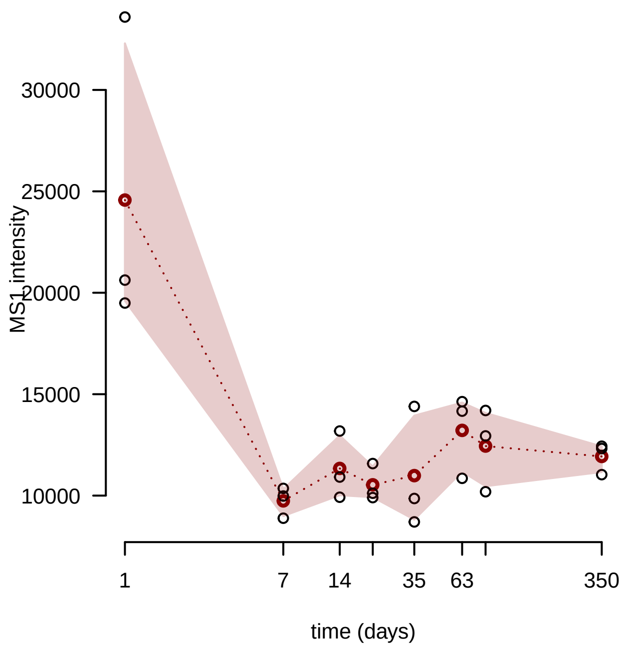

Q61792
GN=Lasp1
LIM and SH3 protein 1

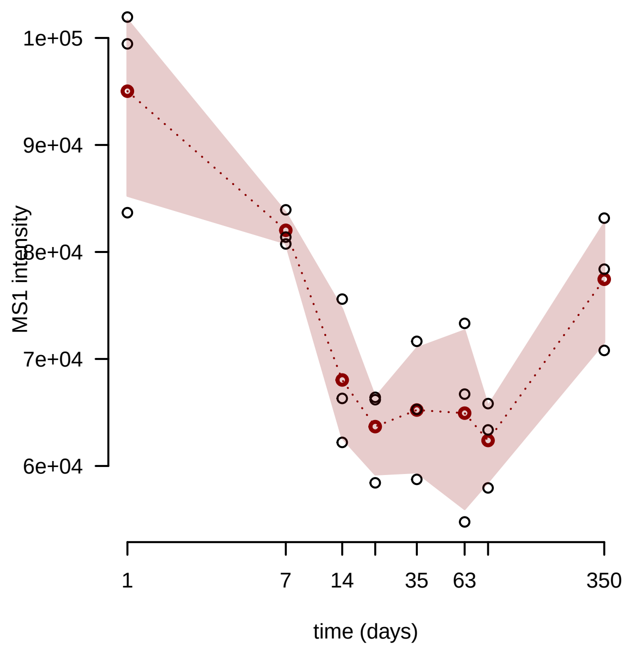

Q61805
GN=Lbp
lipopolysaccharide binding protein

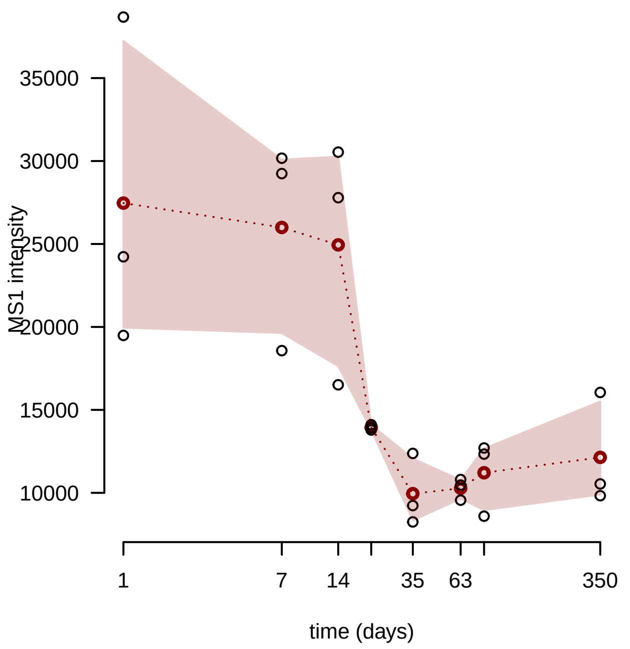

Q61809
GN=Lrrn1
leucine rich repeat protein 1, neuronal

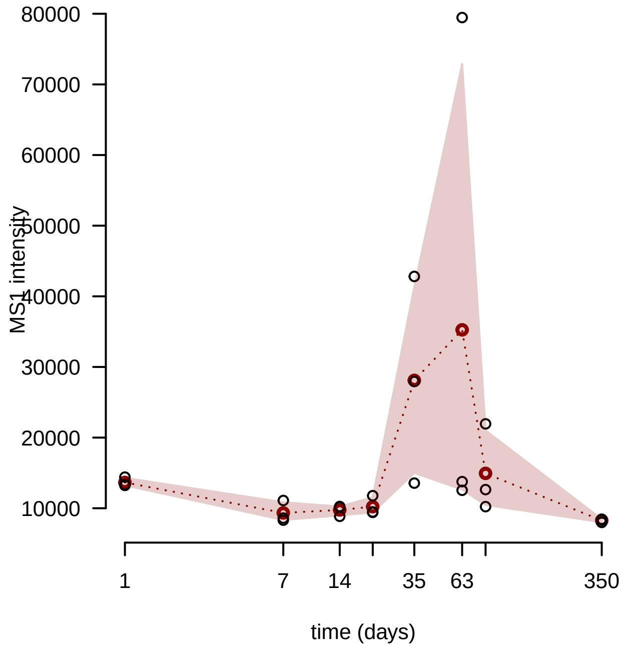

Q61810
GN=Ltbp3
latent transforming growth factor beta b ...

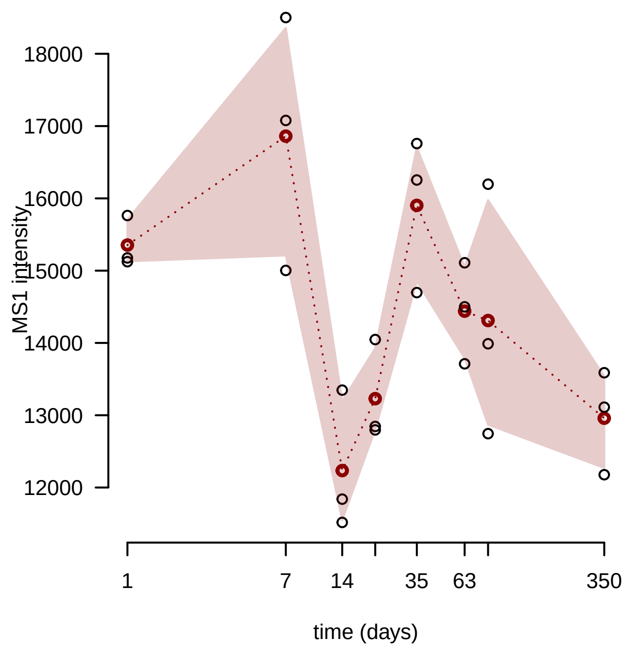

Q61818
GN=Rai1
retinoic acid induced 1

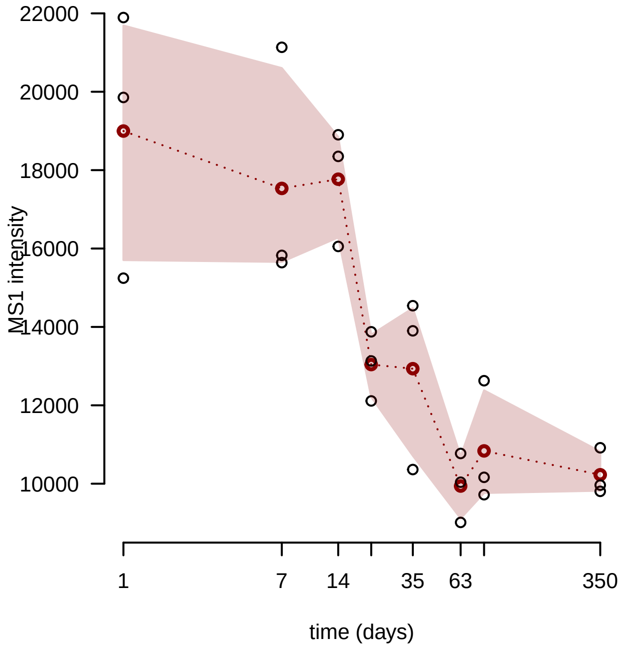

Q61823
GN=Pdcd4
programmed cell death 4

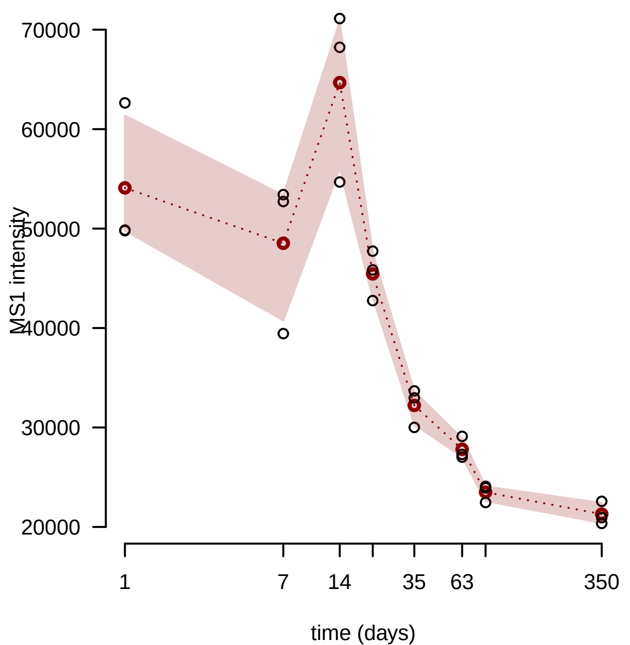

Q61824
GN=Adam12
a disintegrin and metallopeptidase domai ...

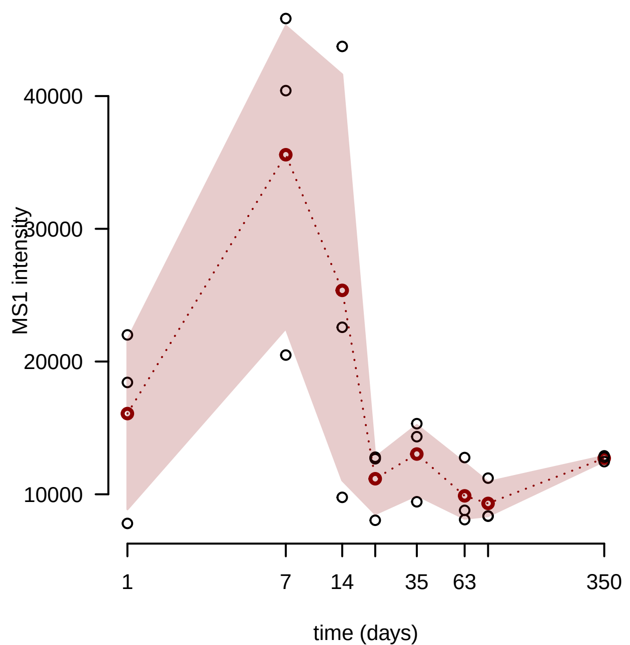

Q61830
GN=Mrc1
mannose receptor, C type 1

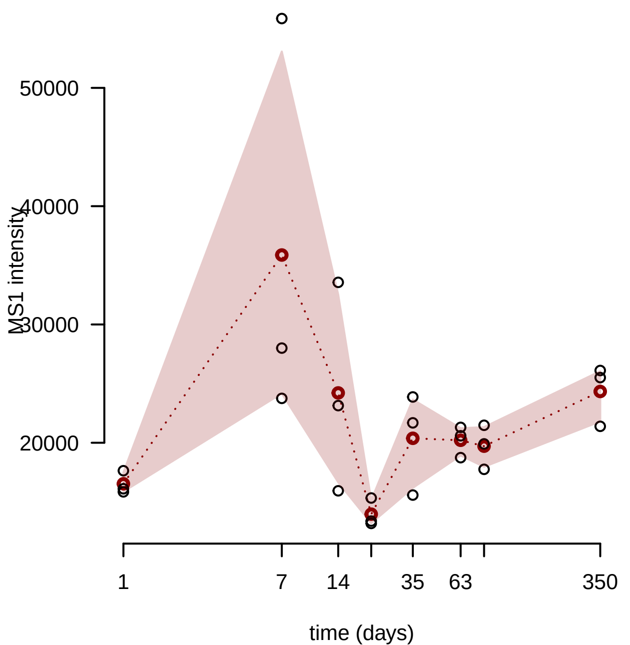

Q61838
GN=Pzp
PZP, alpha−2−macroglobulin like

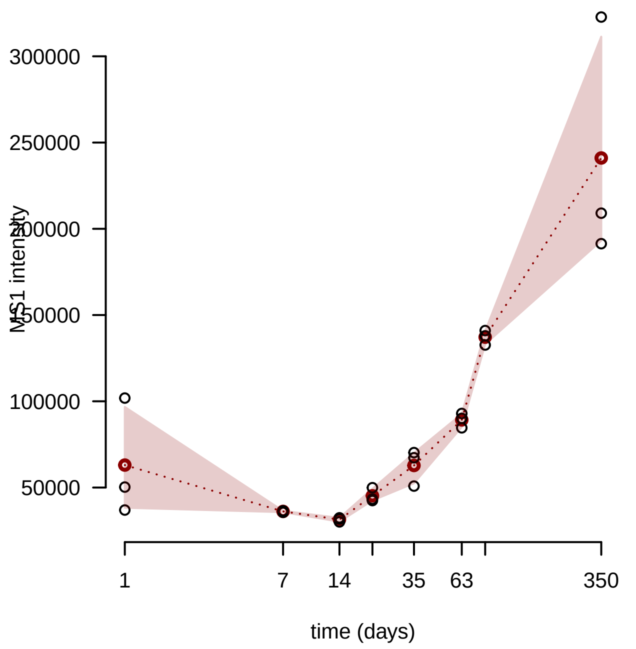

Q61846
GN=Melk
maternal embryonic leucine zipper kinase

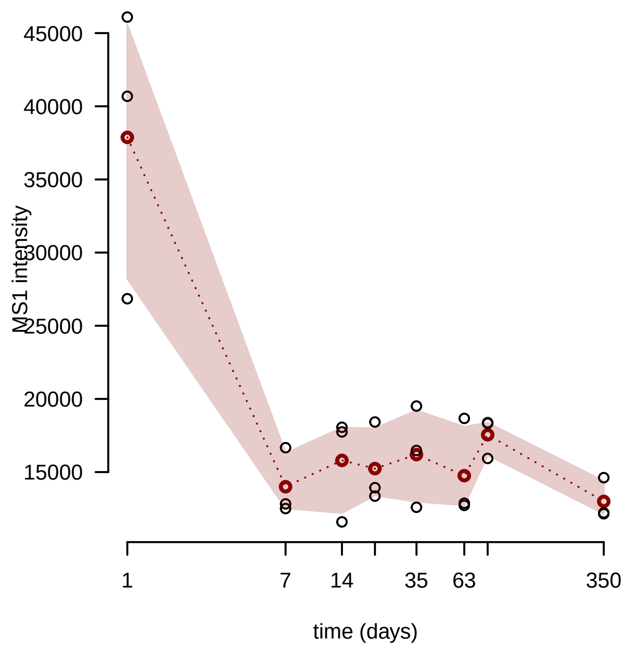

Q61878
GN=Prg2
proteoglycan 2, bone marrow

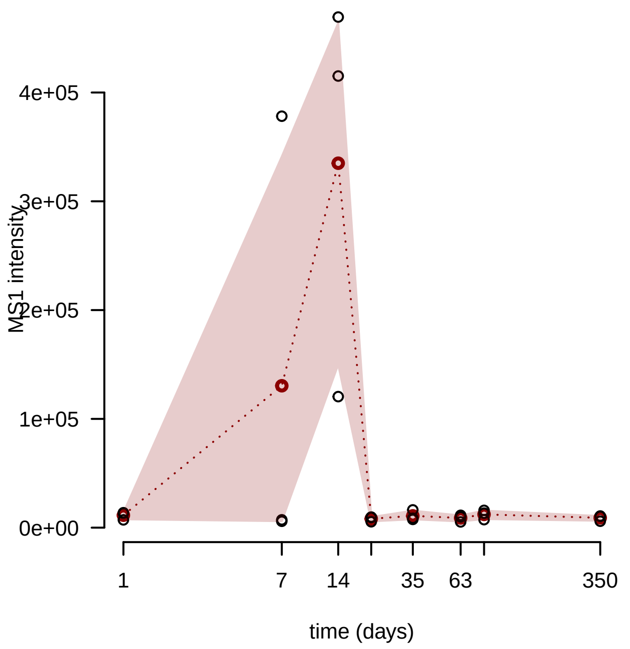

Q61879
GN=Myh10
myosin, heavy polypeptide 10, non−muscle

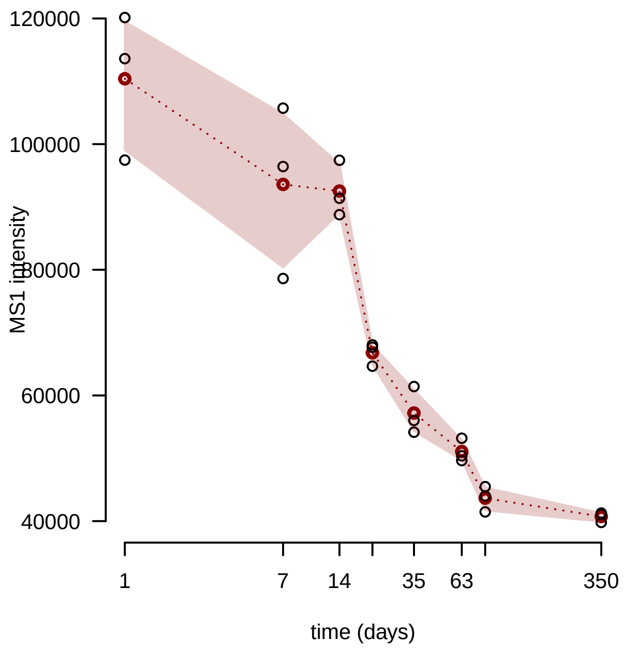

Q61881
GN=Mcm7
minichromosome maintenance complex compo ...

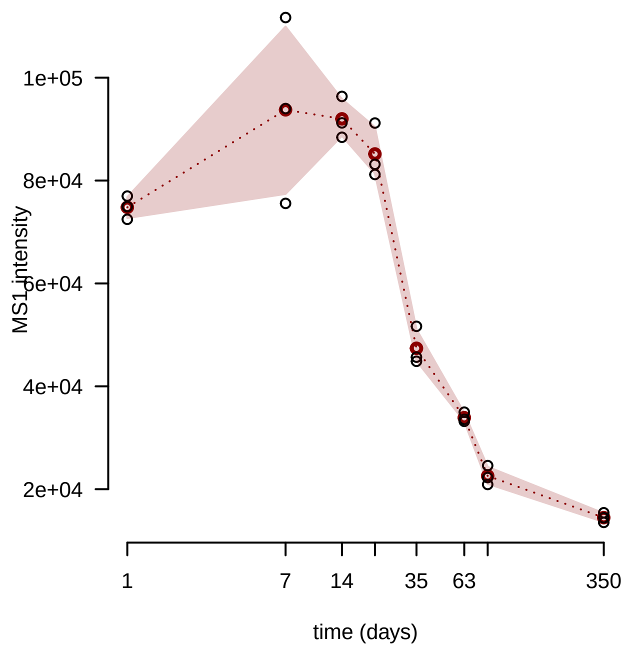

Q61884
GN=Mns1
meiosis−specific nuclear structural prot ...

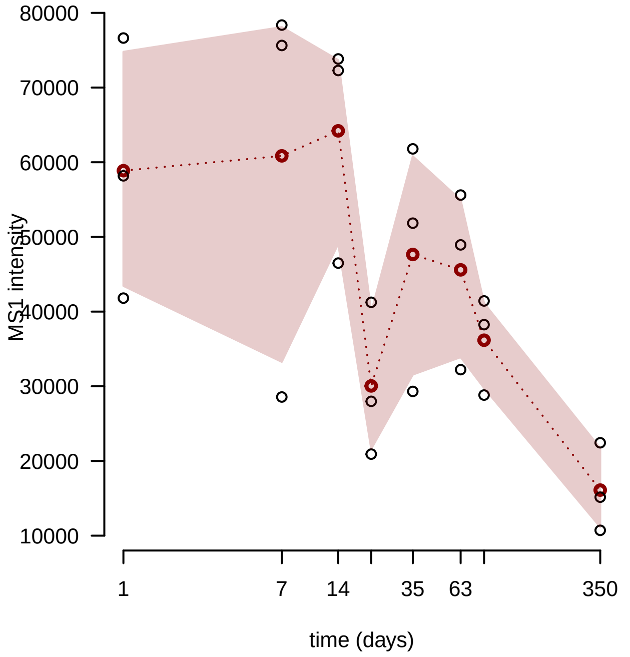

Q61897
GN=Krt33b
keratin 33B

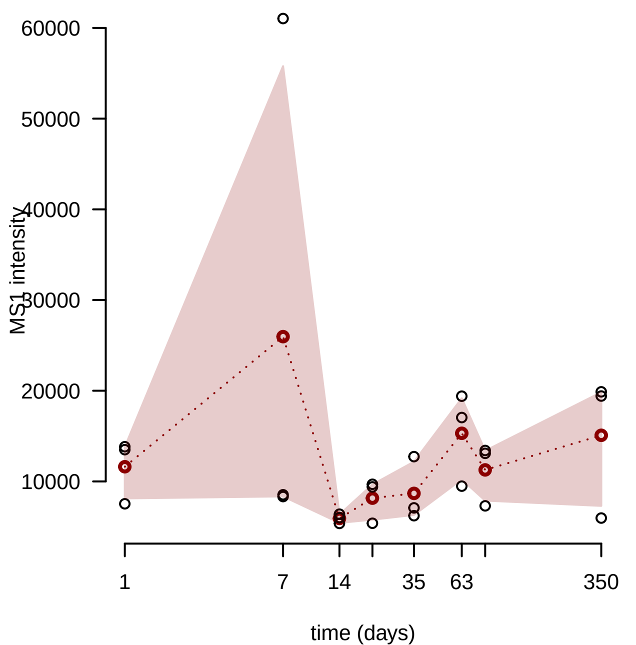

Q61937
GN=Npm1
nucleophosmin 1

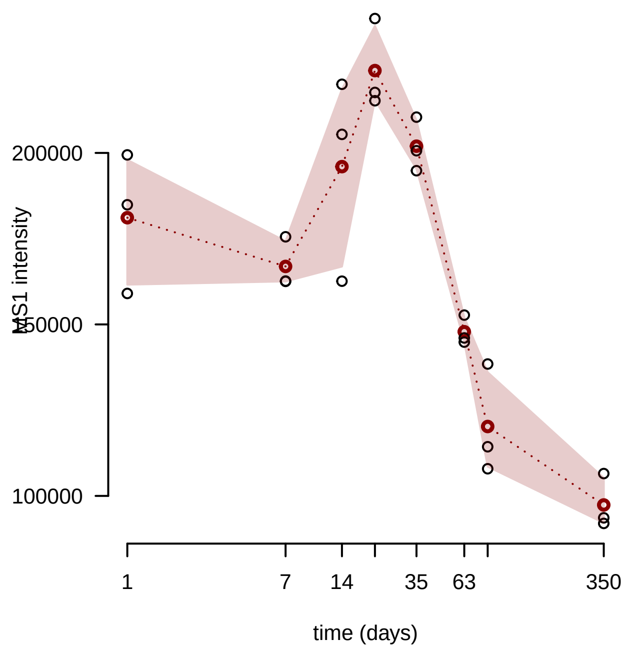

Q61941
GN=Nnt
nicotinamide nucleotide transhydrogenase

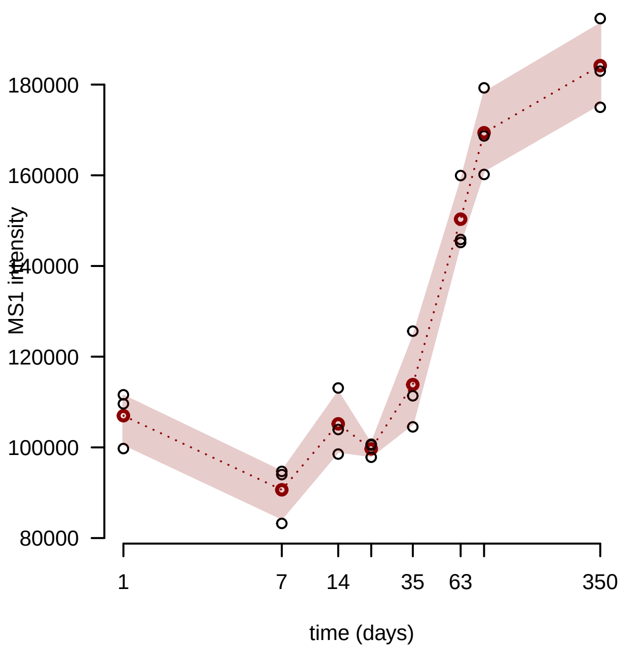

Q61967
GN=Zfp90
zinc finger protein 90

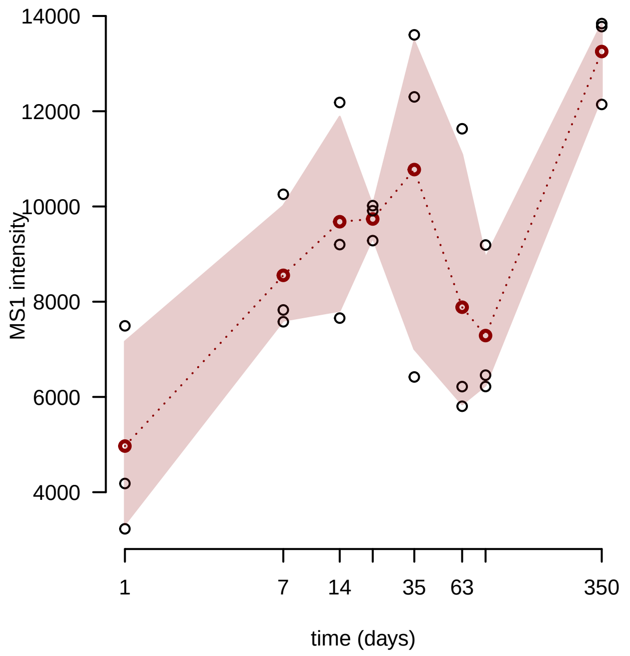

Q61979
GN=Nnat
neuronatin

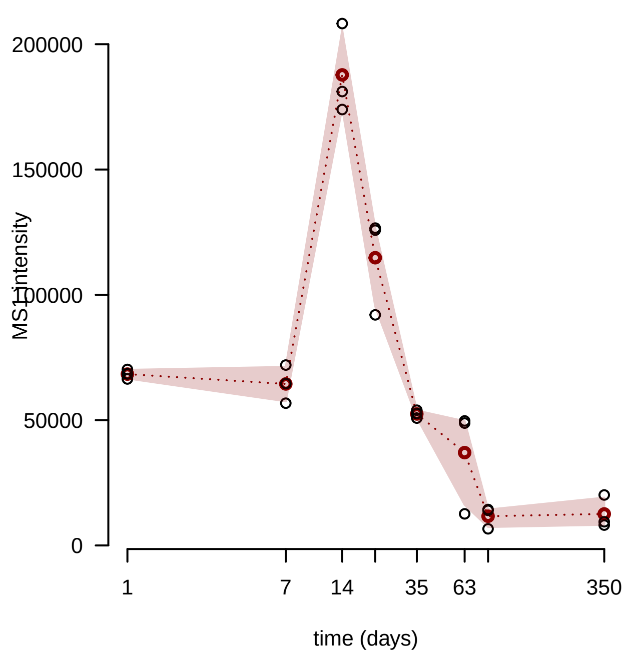

Q61990
GN=Pcbp2
poly(rC) binding protein 2

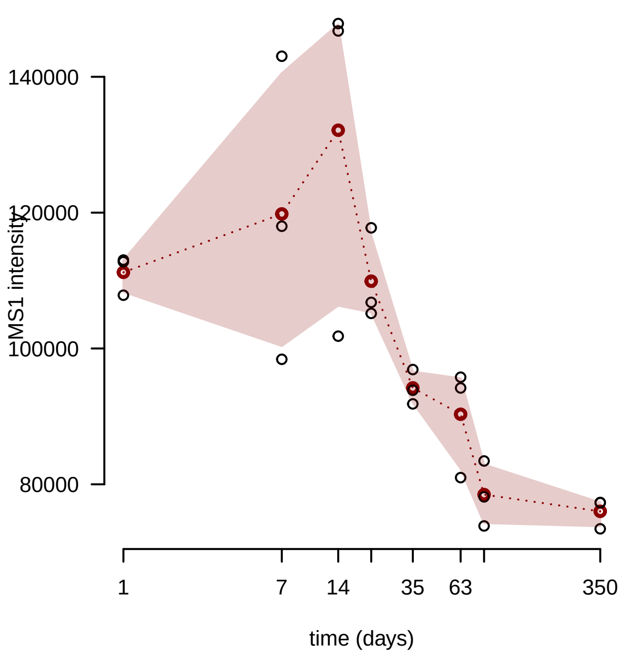

Q61990
GN=Pcbp2
poly(rC) binding protein 2

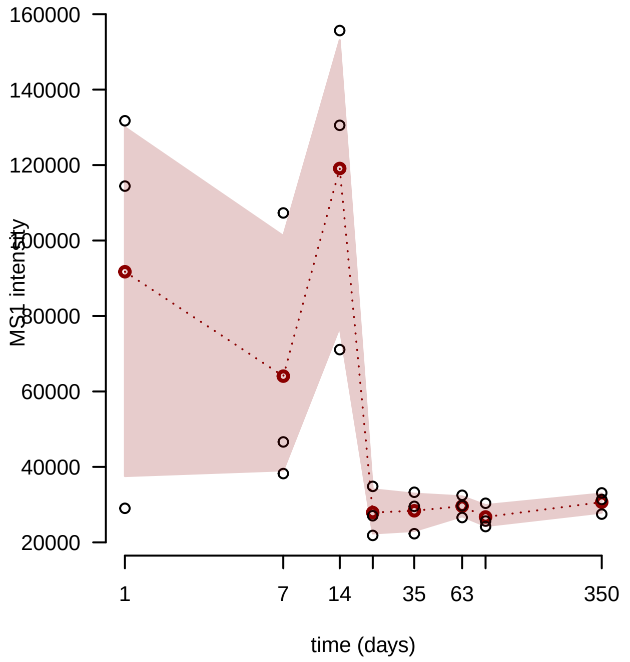

Q62000
GN=Ogn
osteoglycin

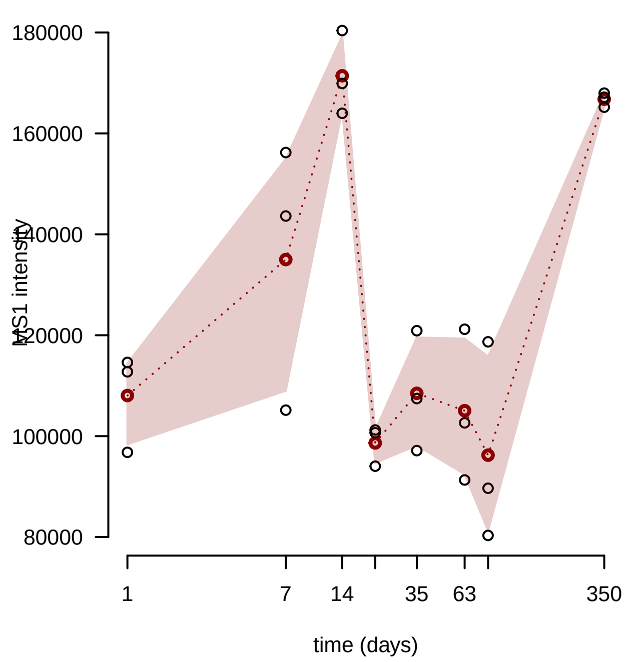

Q62005
GN=Zp1
zona pellucida glycoprotein 1

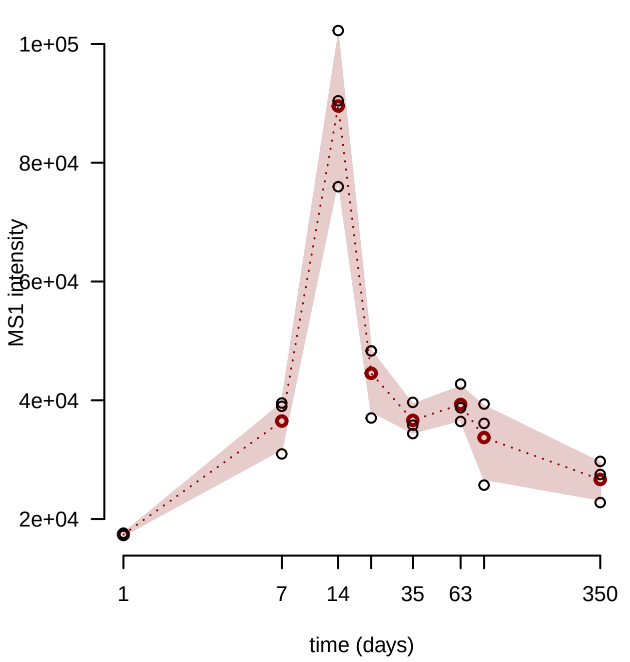

Q62009
GN=Postn
periostin, osteoblast specific factor

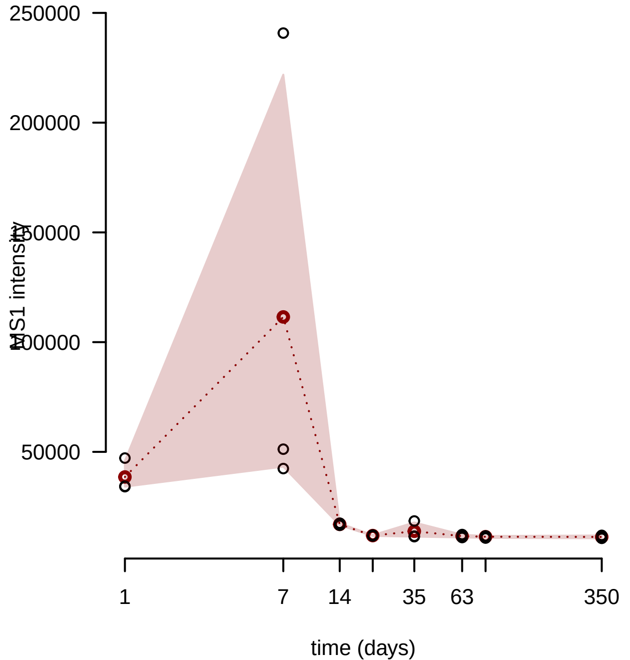

Q62009
GN=Postn
periostin, osteoblast specific factor

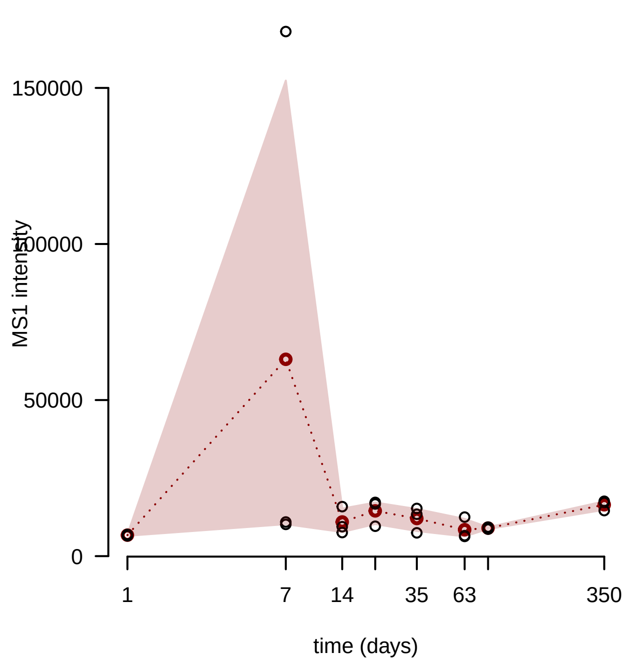

Q62018
GN=Ctr9
CTR9 homolog, Paf1/RNA polymerase II com ...

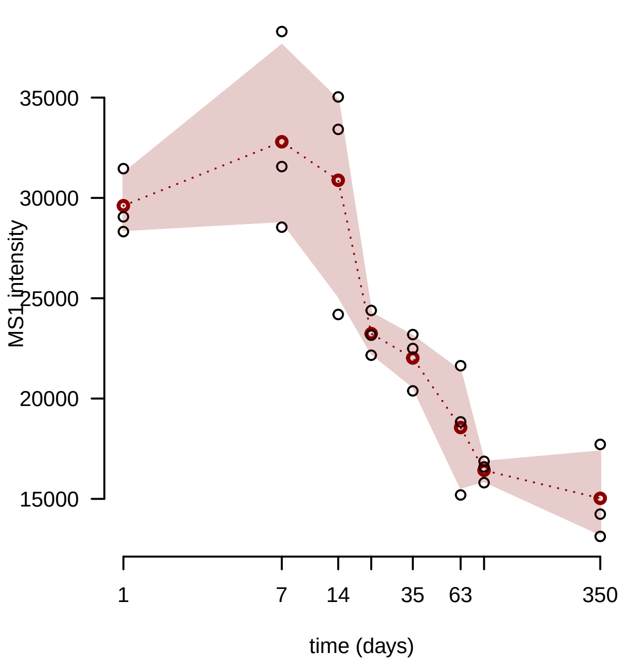

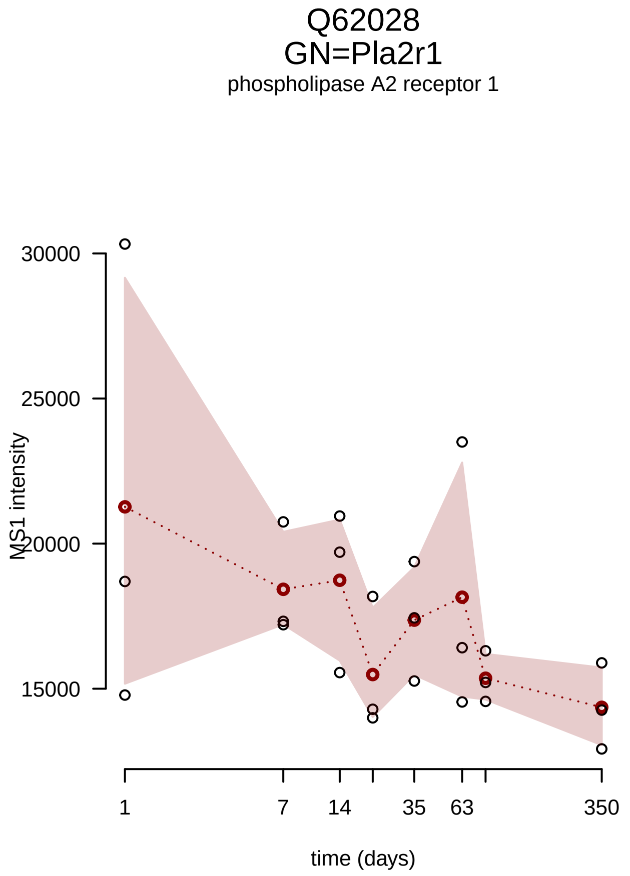
Q62028
GN=Pla2r1
phospholipase A2 receptor 1
MS1 intensity
30000
25000
20000
15000
1
7
14
35
63
350
time (days)

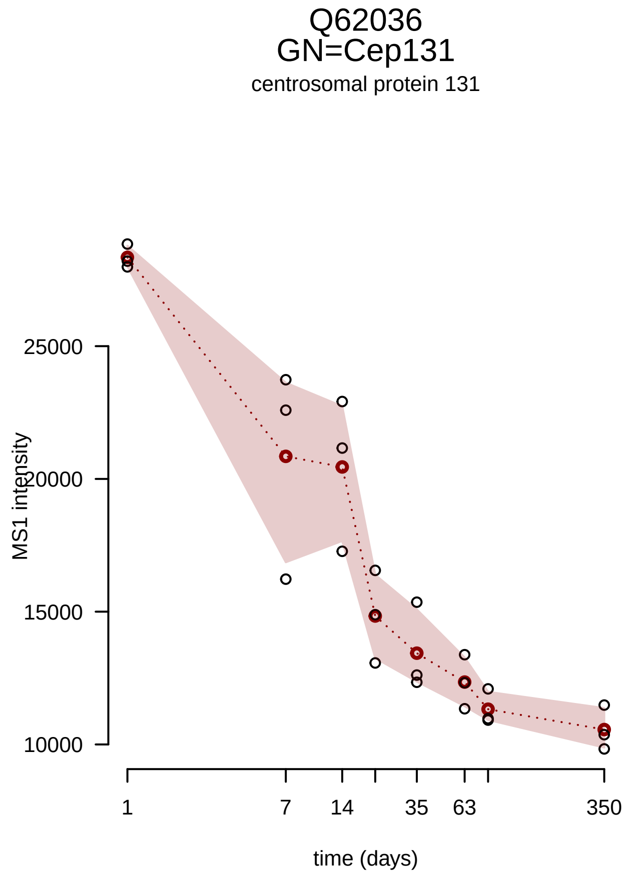
Q62036
GN=Cep131
centrosomal protein 131
MS1 intensity
25000
20000
15000
10000
1
7
14
35
63
350
time (days)

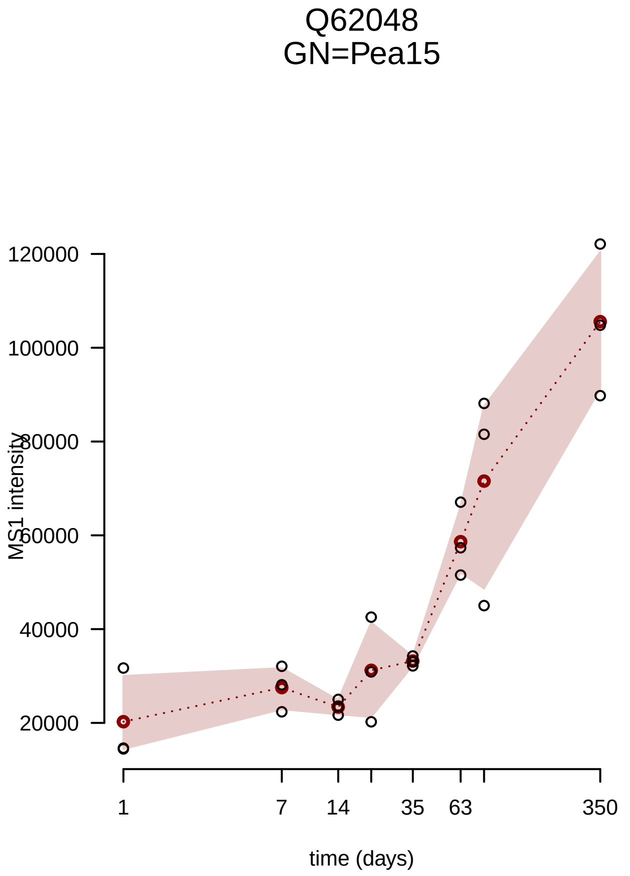
Q62048
GN=Pea15
MS1 intensity
120000
100000
80000
60000
40000
20000
1
7
14
35
63
350
time (days)

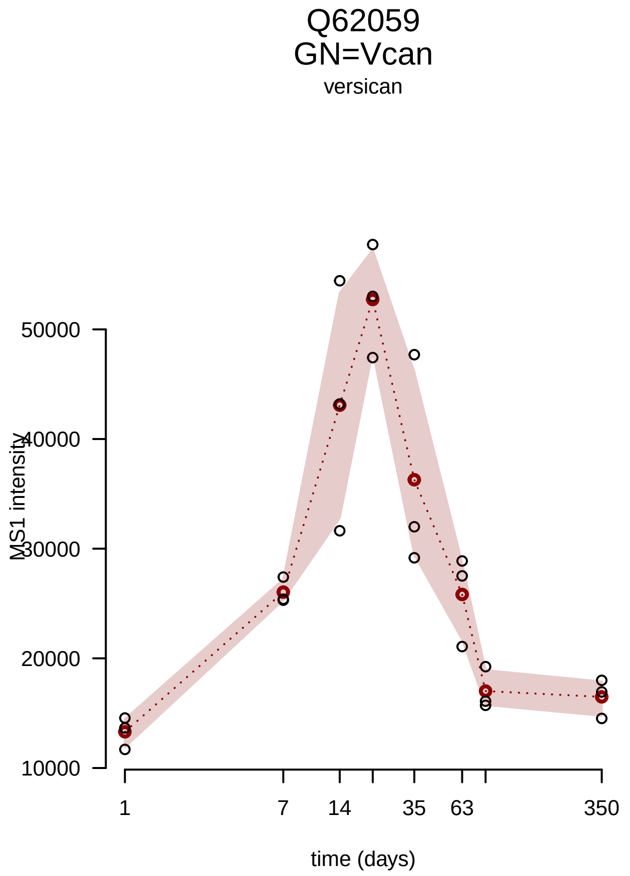
Q62059
GN=Vcan
versican
MS1 intensity
50000
40000
30000
20000
10000
1
7
14
35
63
350
time (days)

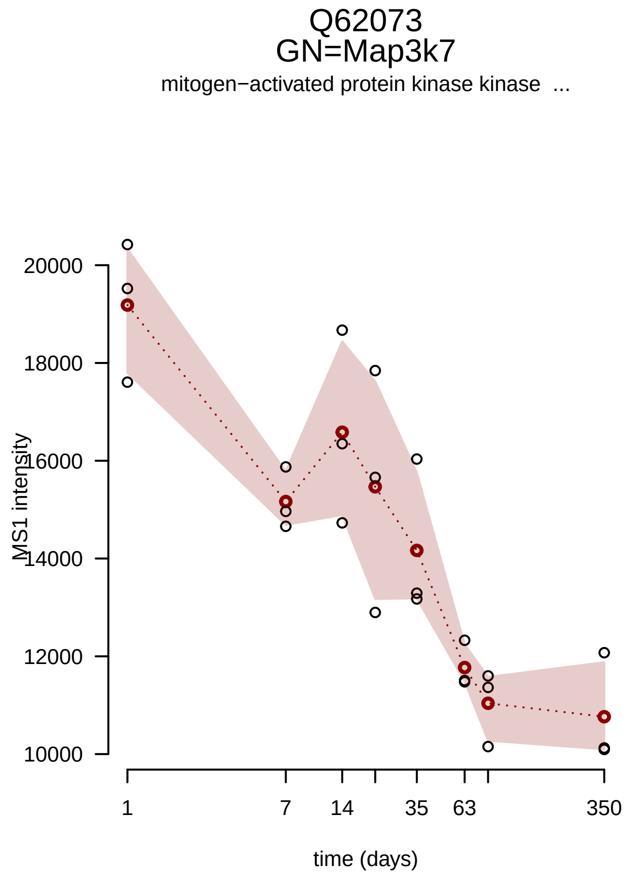
Q62073
GN=Map3k7
mitogen−activated protein kinase kinase ...
MS1 intensity
20000
18000
16000
14000
12000
10000
1
7
14
35
63
350
time (days)

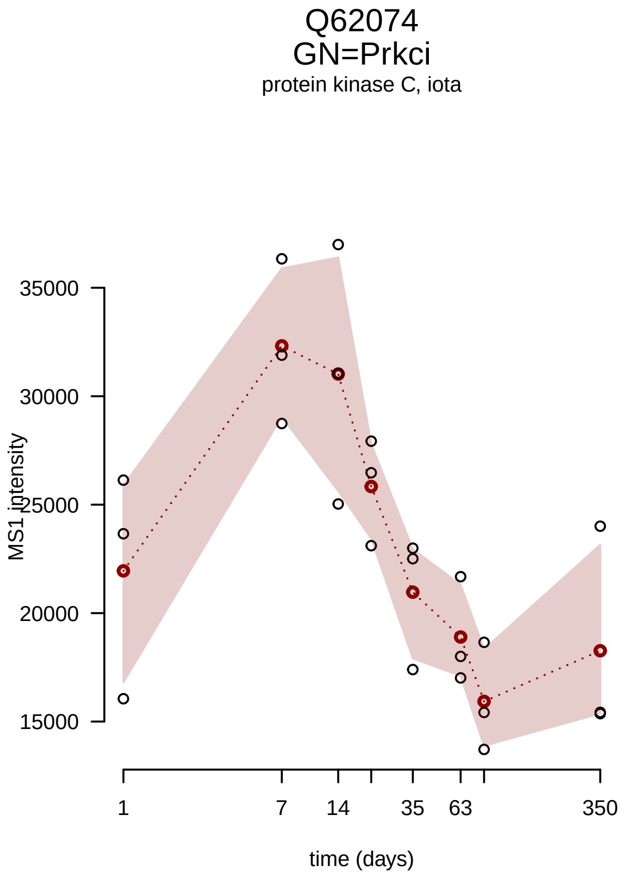
Q62074
GN=Prkci
protein kinase C, iota
MS1 intensity
35000
30000
25000
20000
15000
1
7
14
35
63
350
time (days)

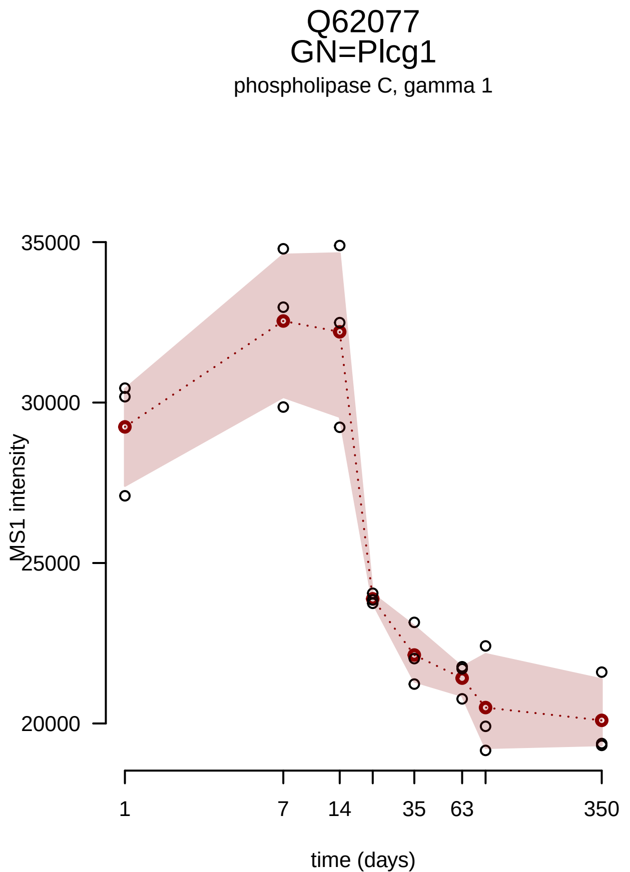
Q62077
GN=Plcg1
phospholipase C, gamma 1
MS1 intensity
35000
30000
25000
20000
1
7
14
35
63
350
time (days)

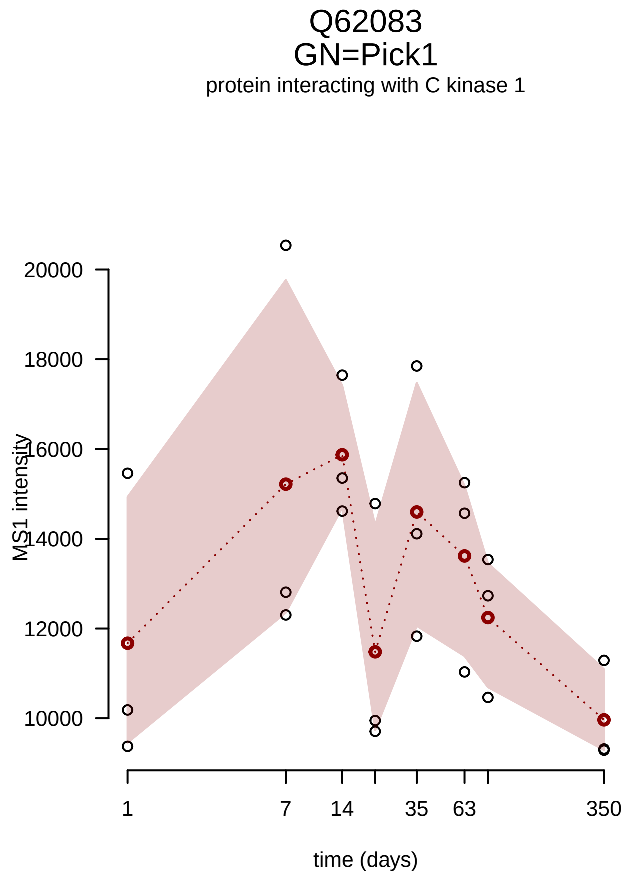
Q62083
GN=Pick1
protein interacting with C kinase 1
MS1 intensity
20000
18000
16000
14000
12000
10000
1
7
14
35
63
350
time (days)

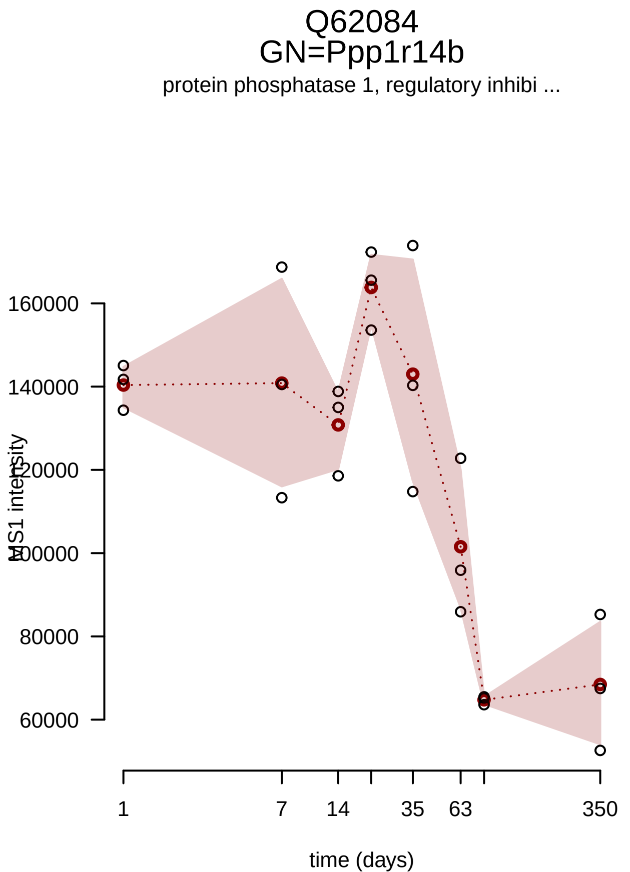
Q62084
GN=Ppp1r14b
protein phosphatase 1, regulatory inhibi ...
MS1 intensity
160000
140000
120000
100000
80000
60000
1
7
14
35
63
350
time (days)

Q62086
GN=Pon2
paraoxonase 2

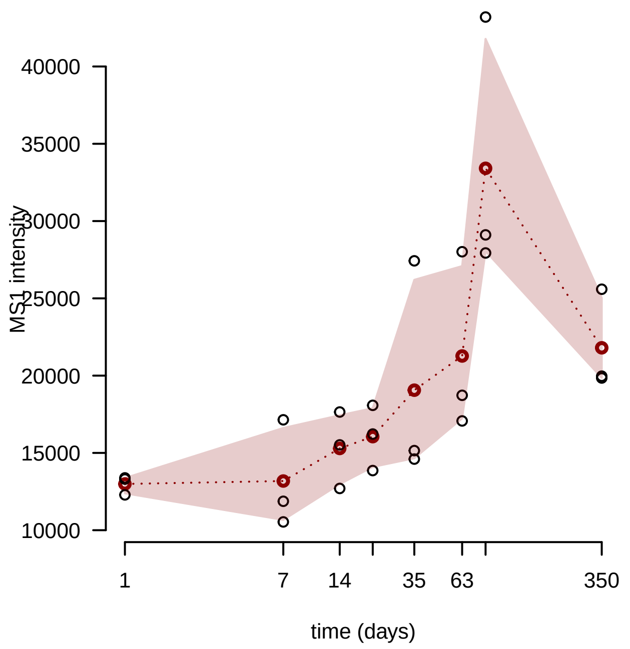

Q62087
GN=Pon3
paraoxonase 3

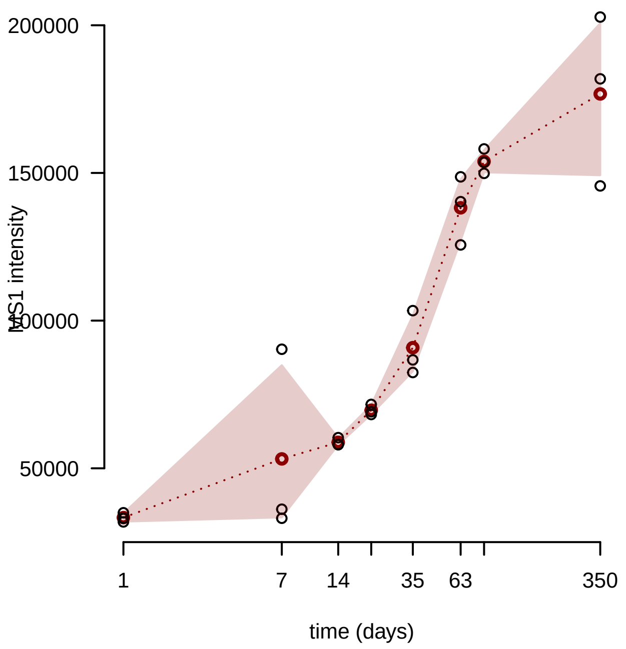

Q62093
GN=Srsf2
serine and arginine−rich splicing factor ...

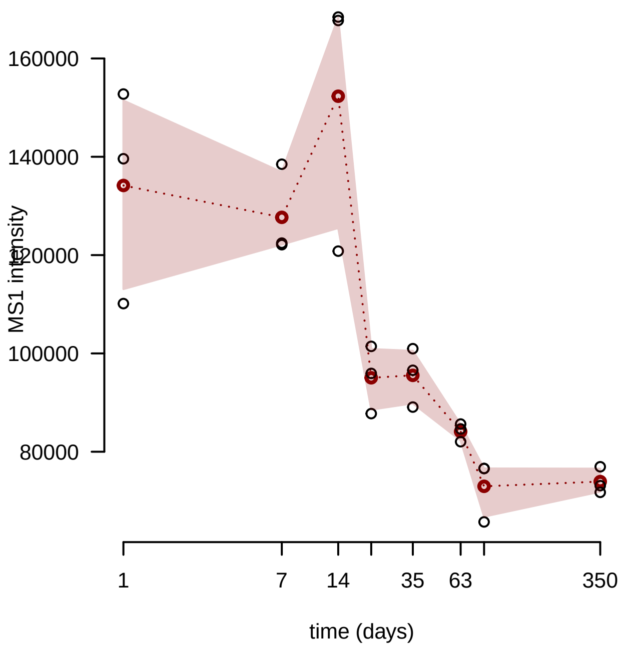

Q62101
GN=Prkd1
protein kinase D1

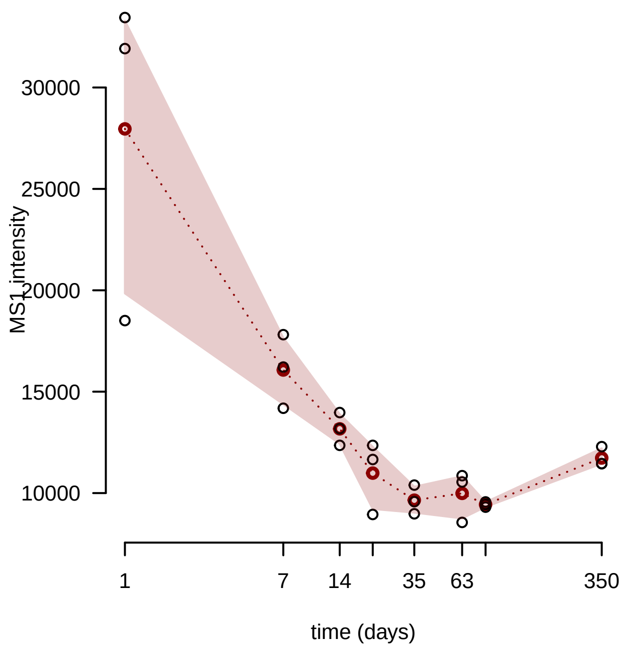

Q62120
GN=Jak2
Janus kinase 2

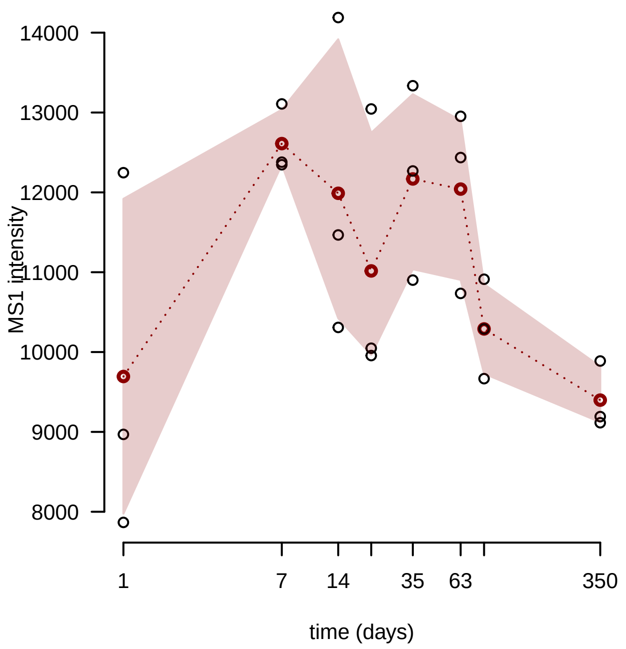

Q62130
GN=Ptpn14
protein tyrosine phosphatase, non−recept ...

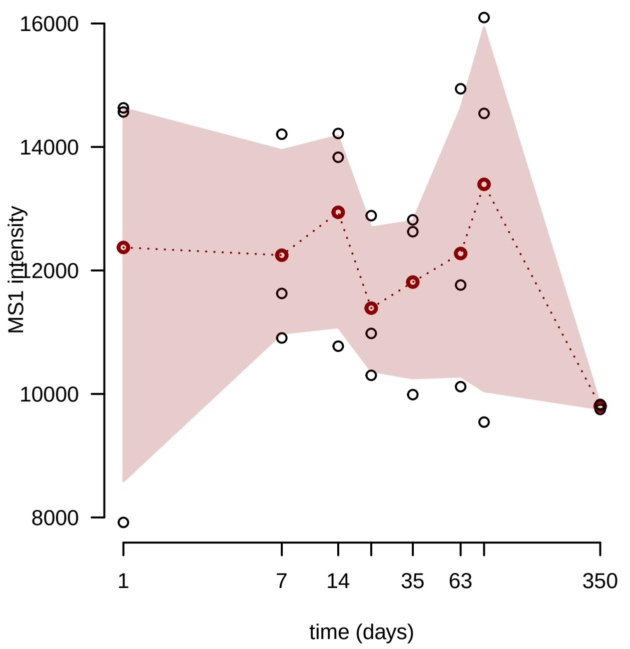

Q62136
GN=Ptpn21
protein tyrosine phosphatase, non−recept ...

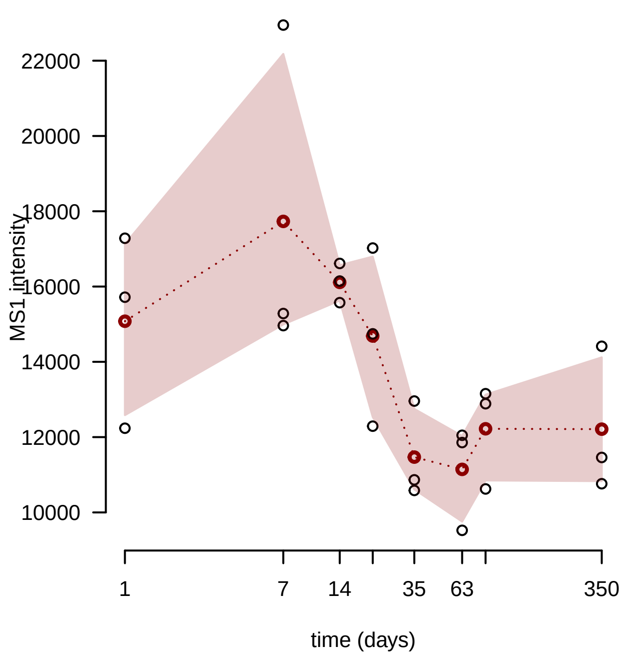

Q62141
GN=Sin3b
transcriptional regulator, SIN3B (yeast)

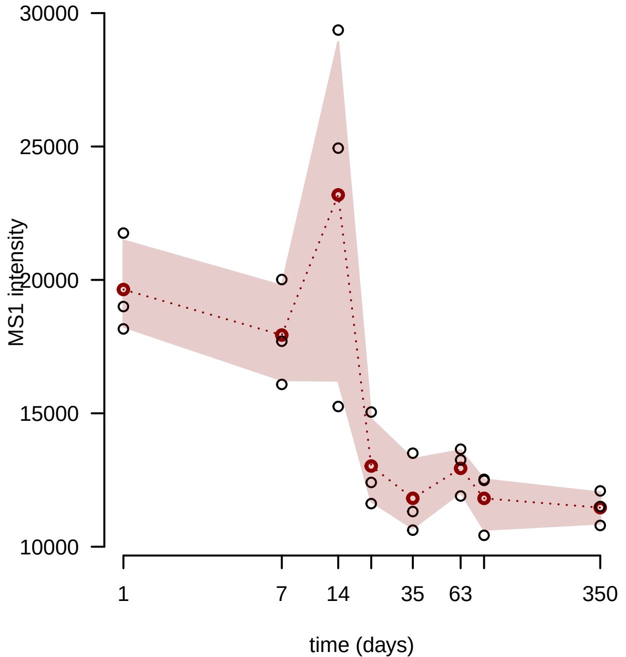

Q62147
GN=Sspn
sarcospan

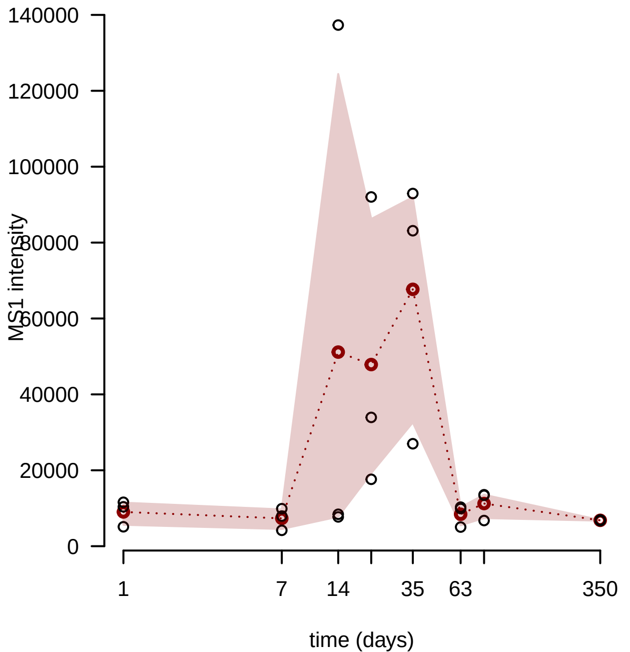

Q62148
GN=Aldh1a2
aldehyde dehydrogenase family 1, subfami ...

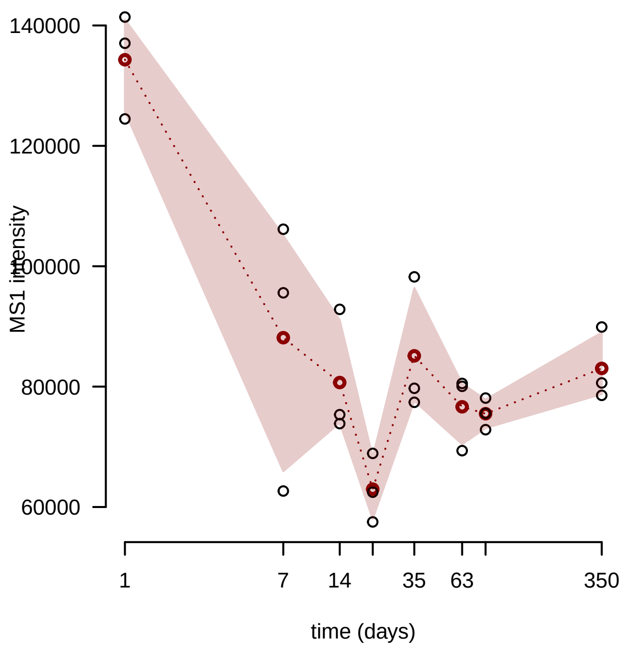

Q62158
GN=Trim27
tripartite motif−containing 27

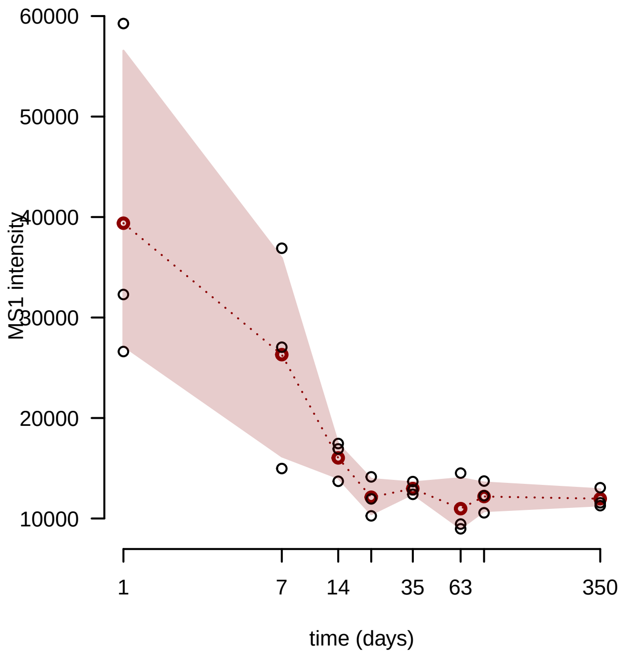

Q62159
GN=Rhoc
ras homolog family member C

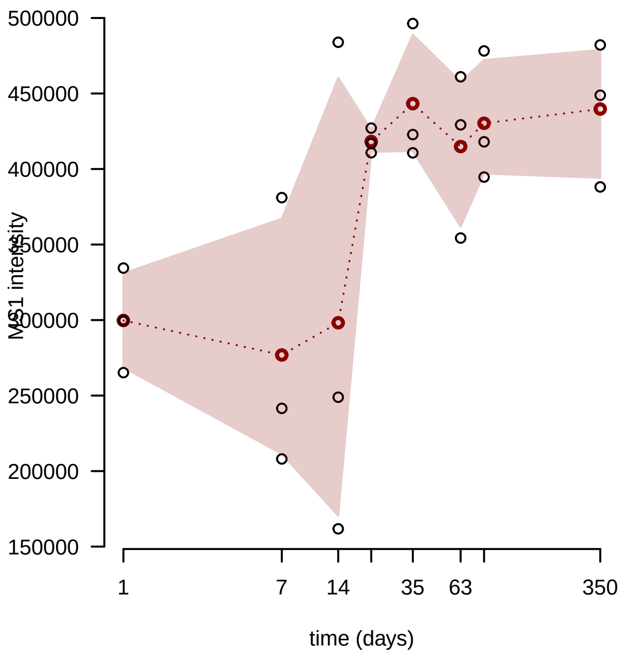

Q62165
GN=Dag1
dystroglycan 1

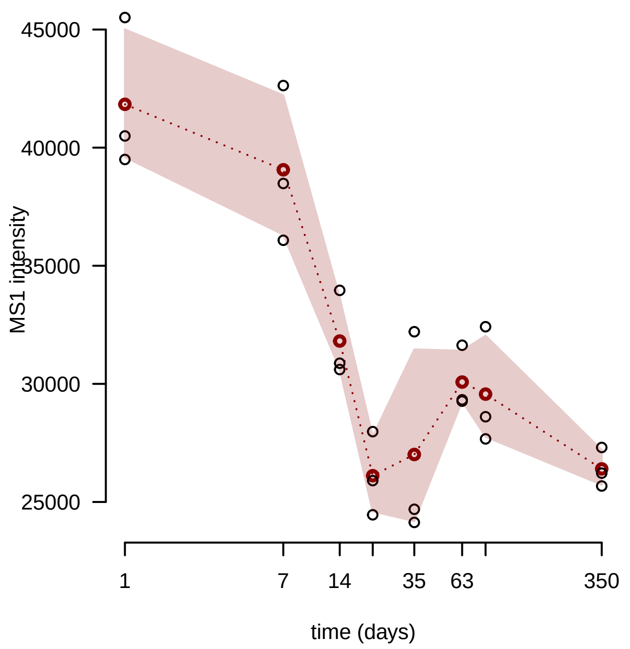

Q62167
GN=Ddx3x
DEAD box helicase 3, X−linked

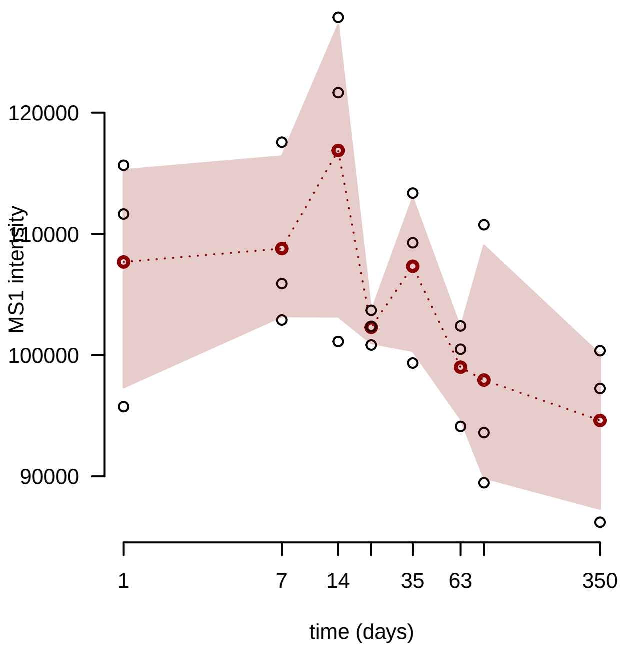

Q62168
GN=Krt32
keratin 32

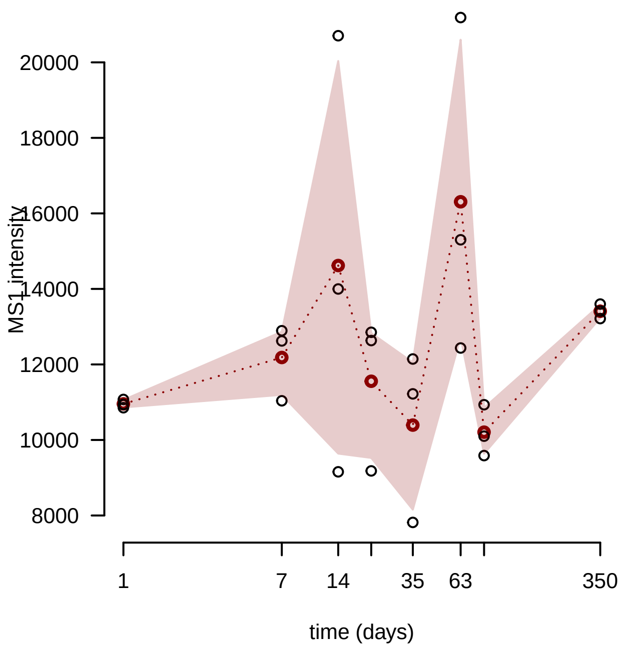

Q62172
GN=Ralbp1
ralA binding protein 1

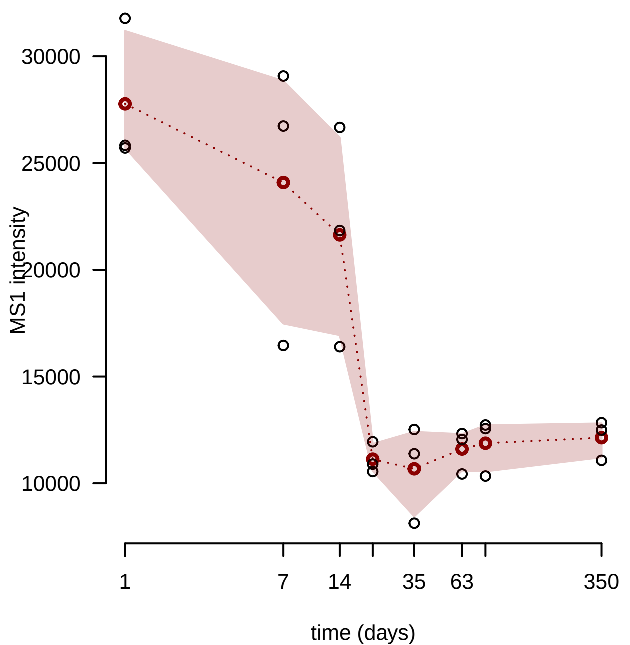

Q62179
GN=Sema4b
sema domain, immunoglobulin domain (Ig), ...

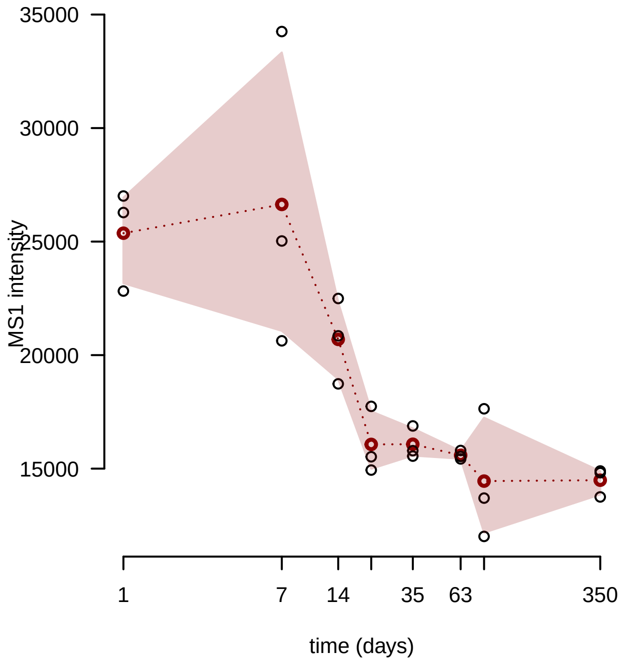

Q62181
GN=Sema3c
sema domain, immunoglobulin domain (Ig), ...

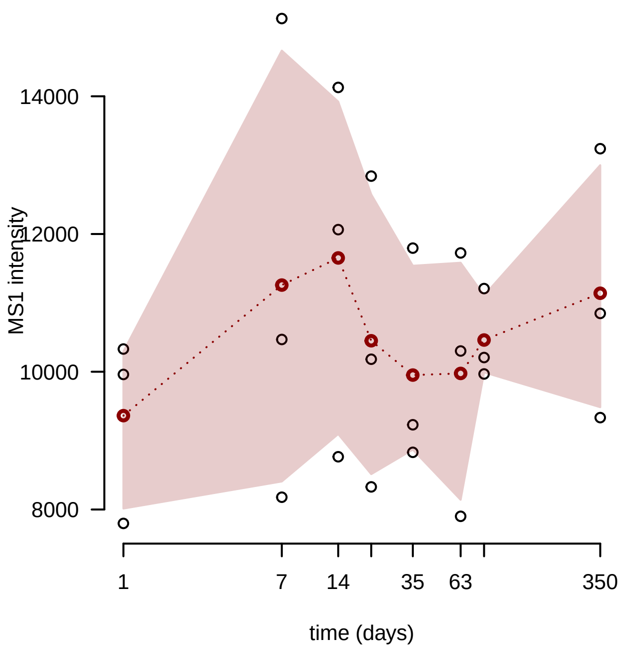

Q62186
GN=Ssr4
signal sequence receptor, delta

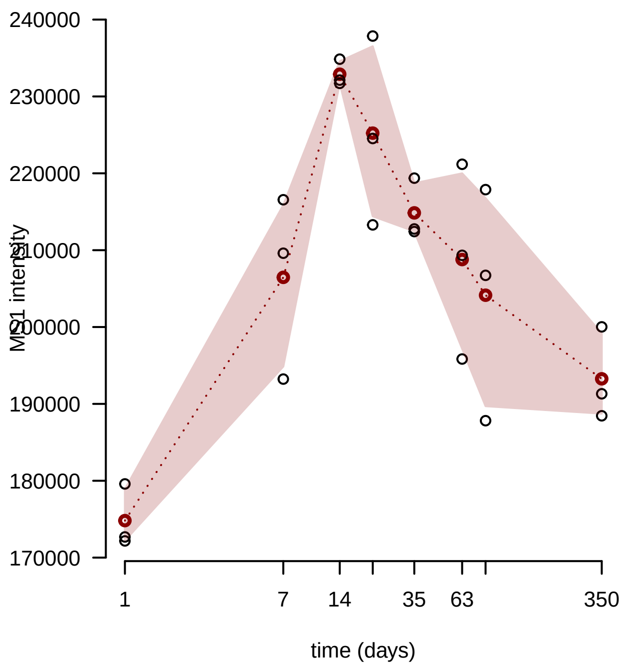

Q62187
GN=Ttf1
transcription termination factor, RNA po ...

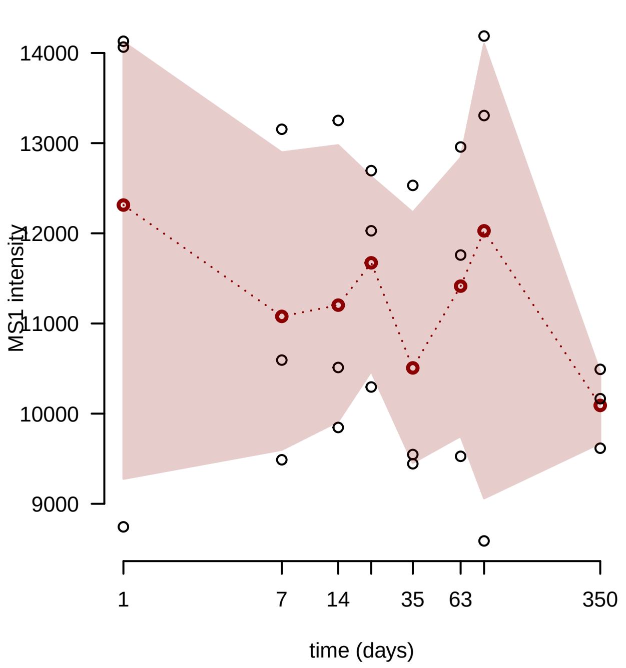

Q62188
GN=Dpysl3
dihydropyrimidinase−like 3

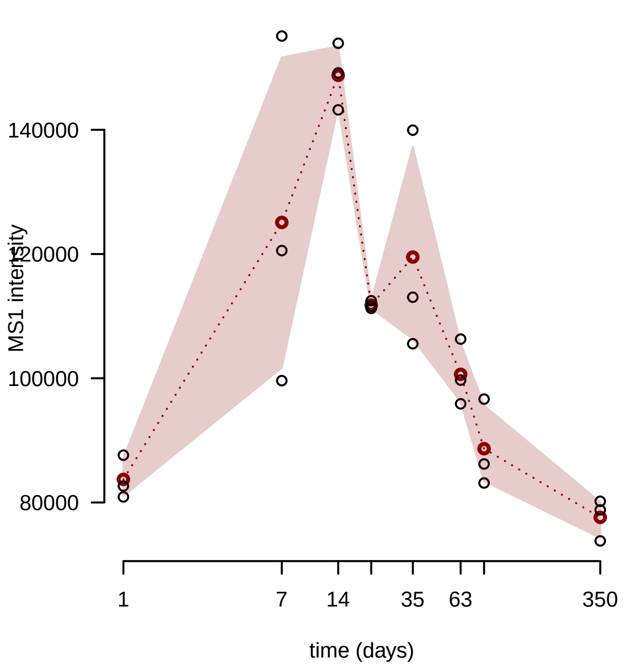

Q62189
GN=Snrpa
small nuclear ribonucleoprotein polypept ...

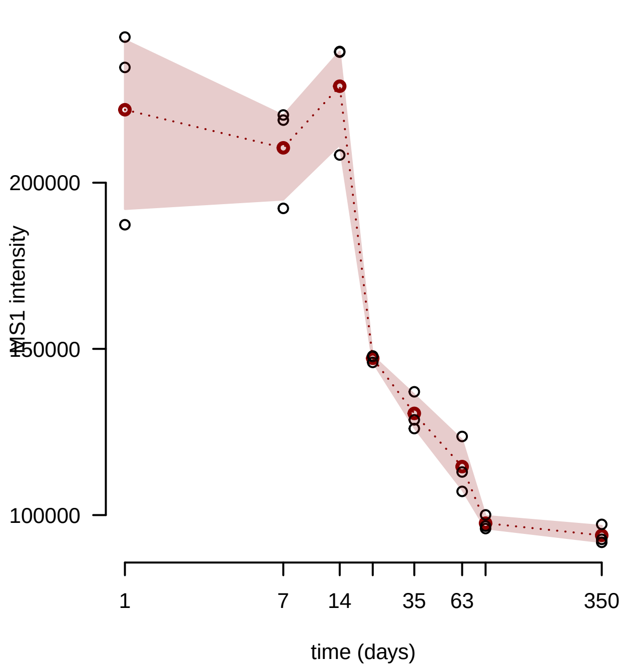

Q62190
GN=Mst1r
macrophage stimulating 1 receptor (c−met ...

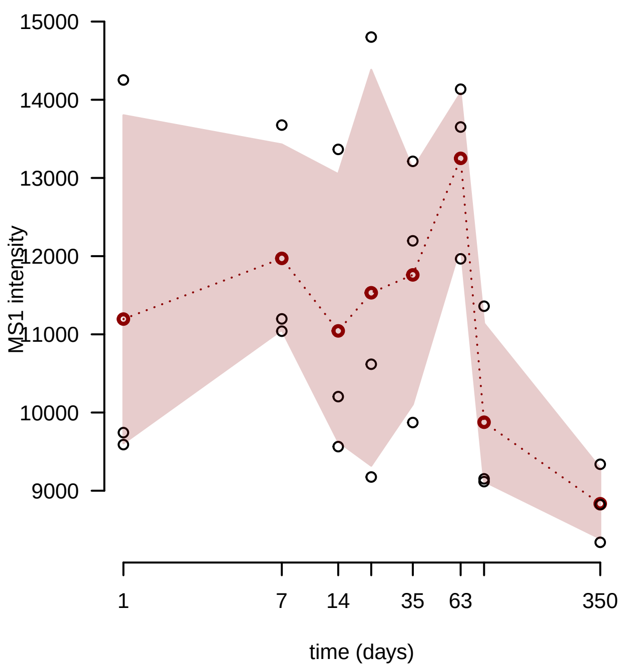

Q62191
GN=Trim21
tripartite motif−containing 21

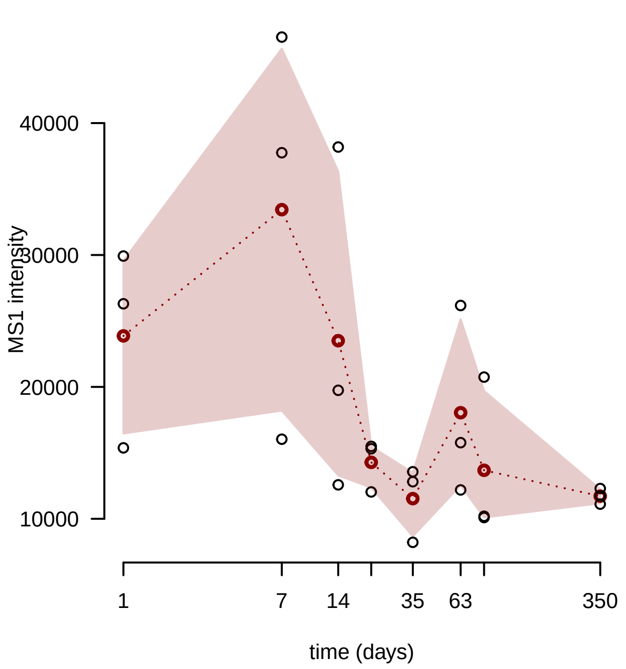

Q62193
GN=Rpa2
replication protein A2

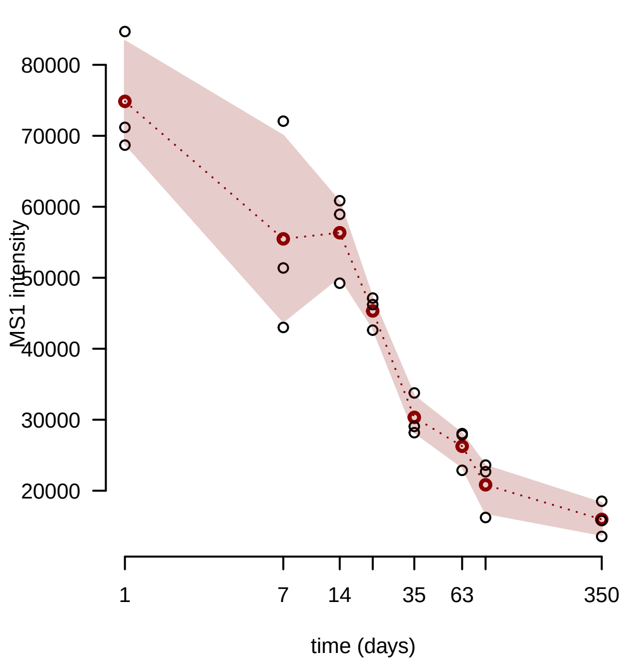

Q62203
GN=Sf3a2
splicing factor 3a, subunit 2

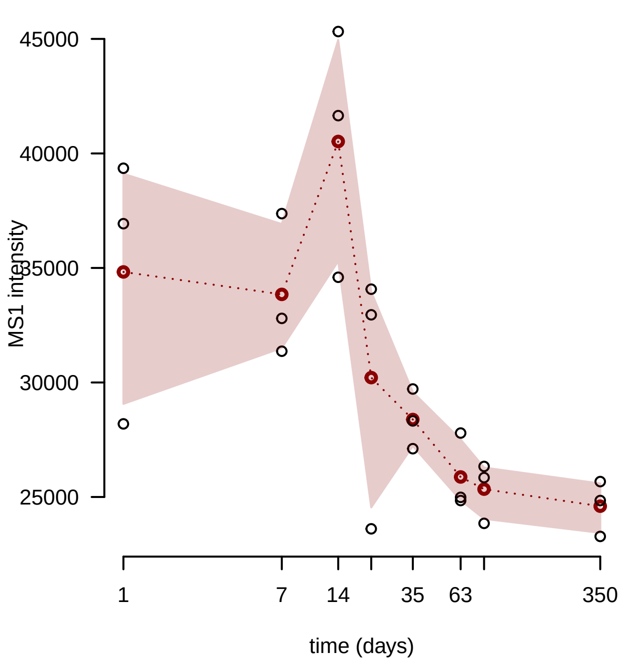

Q62210
GN=Birc2
baculoviral IAP repeat−containing 2

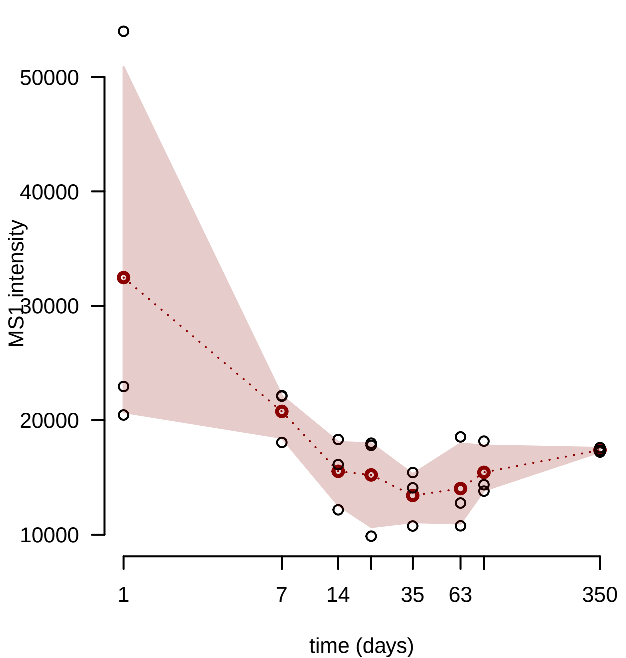

Q62217
GN=Sema5a
sema domain, seven thrombospondin repeat ...

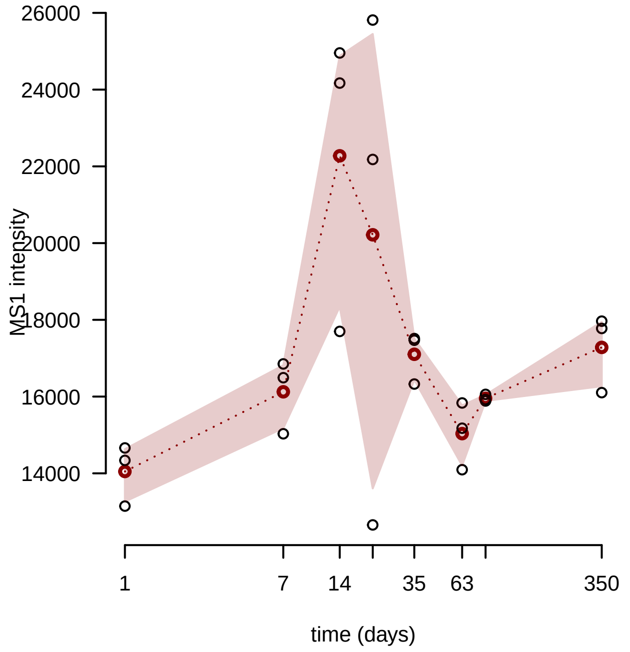

Q62219
GN=Tgfb1i1
transforming growth factor beta 1 induce ...

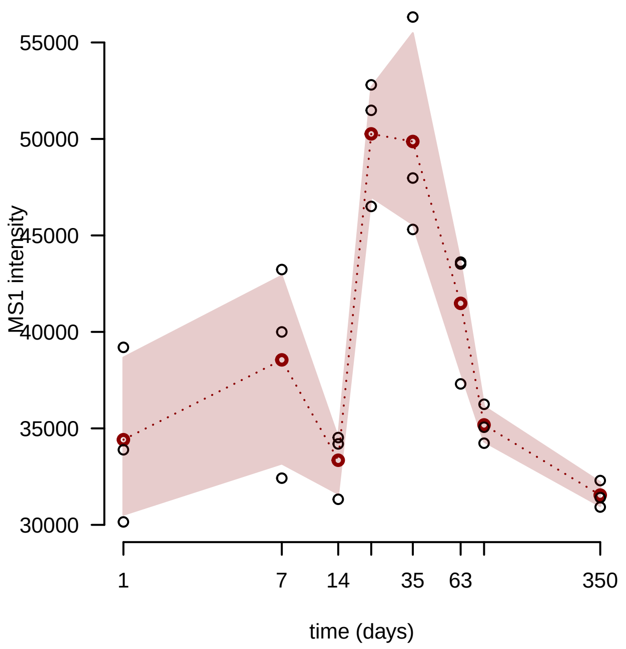

Q62230
GN=Siglec1
sialic acid binding Ig−like lectin 1, si ...

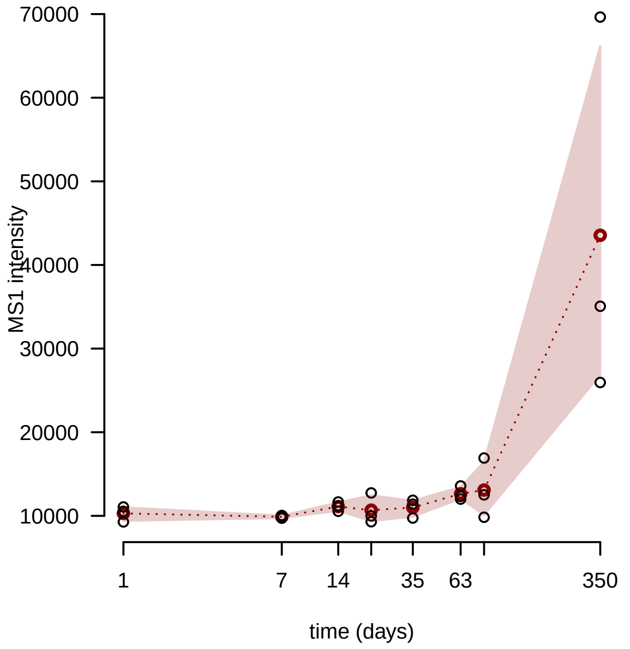

Q62234
GN=Myom1
myomesin 1

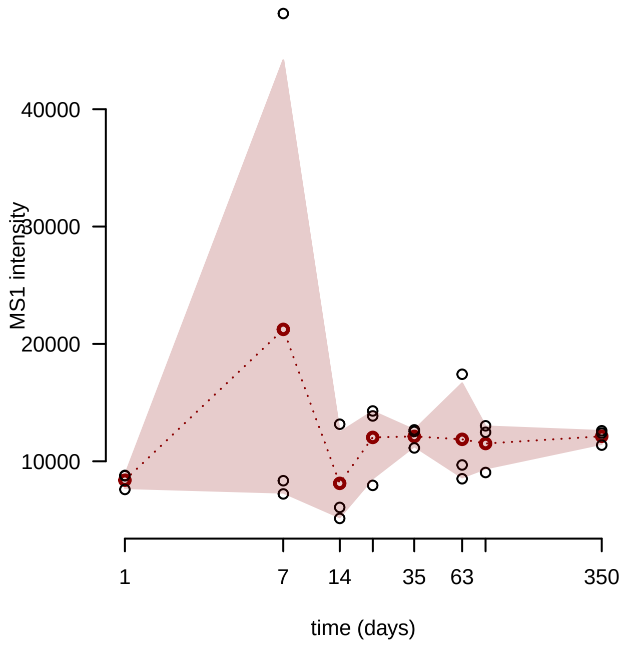

Q62241
GN=Snrpc
U1 small nuclear ribonucleoprotein C

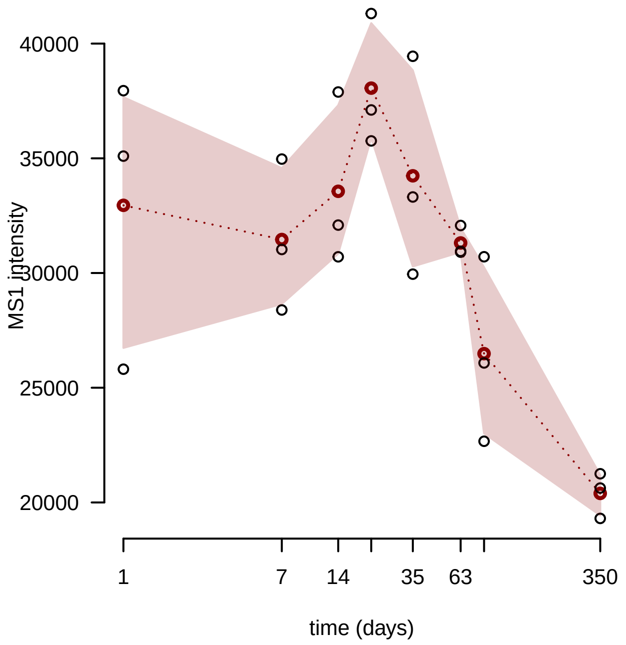

Q62245
GN=Sos1
SOS Ras/Rac guanine nucleotide exchange ...

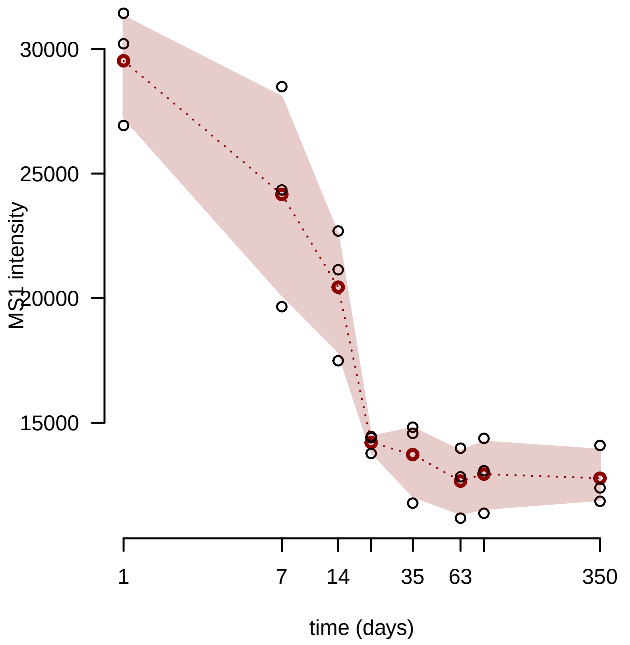

Q62252
GN=Spa17
sperm autoantigenic protein 17

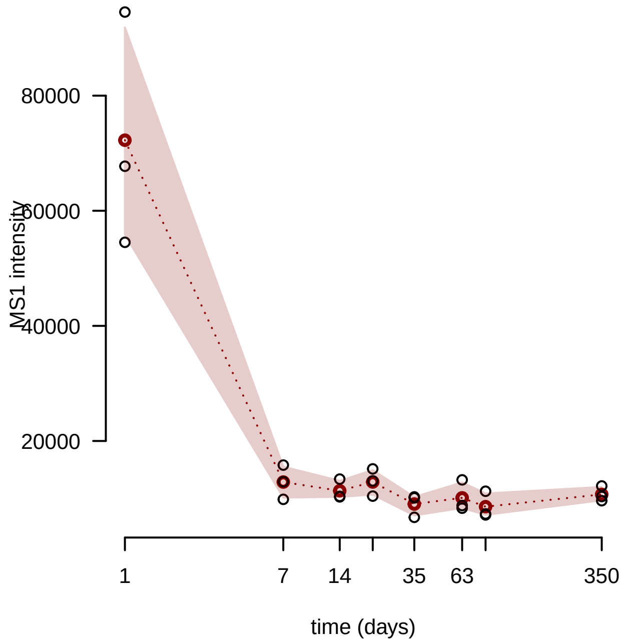

Q62261
GN=Sptbn1
spectrin beta, non−erythrocytic 1

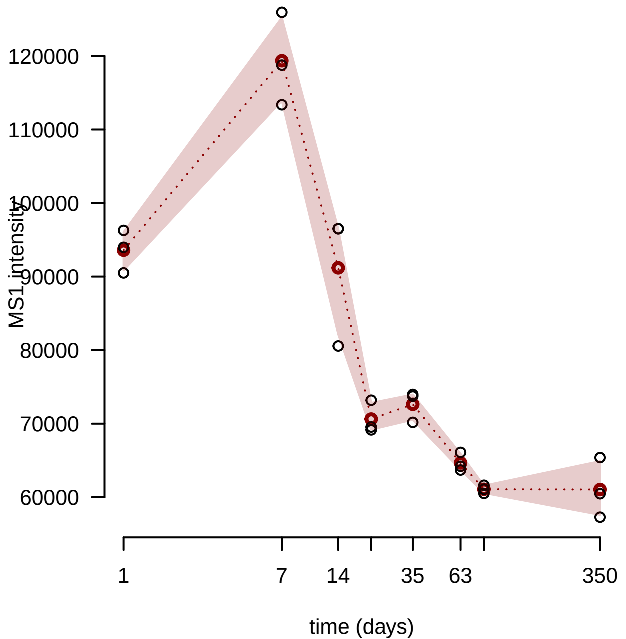

Q62266
GN=Sprr1a
small proline−rich protein 1A

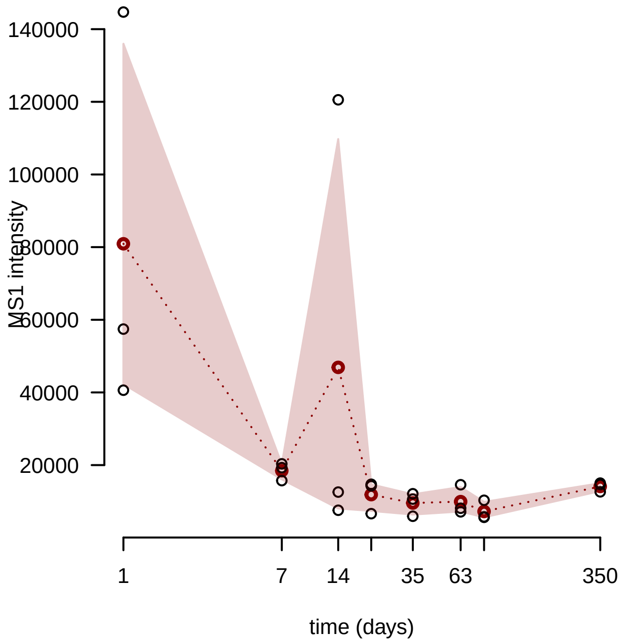

Q62267
GN=Sprr1b
small proline−rich protein 1B

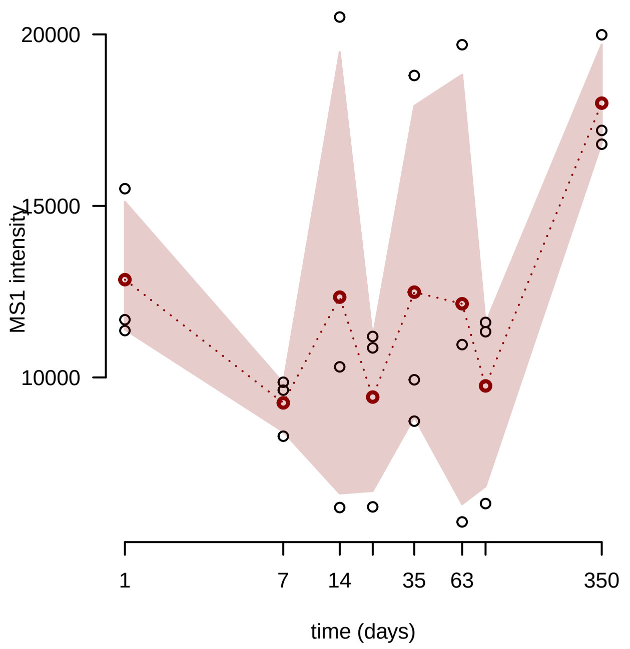

Q62273
GN=Slc26a2
solute carrier family 26 (sulfate transp ...

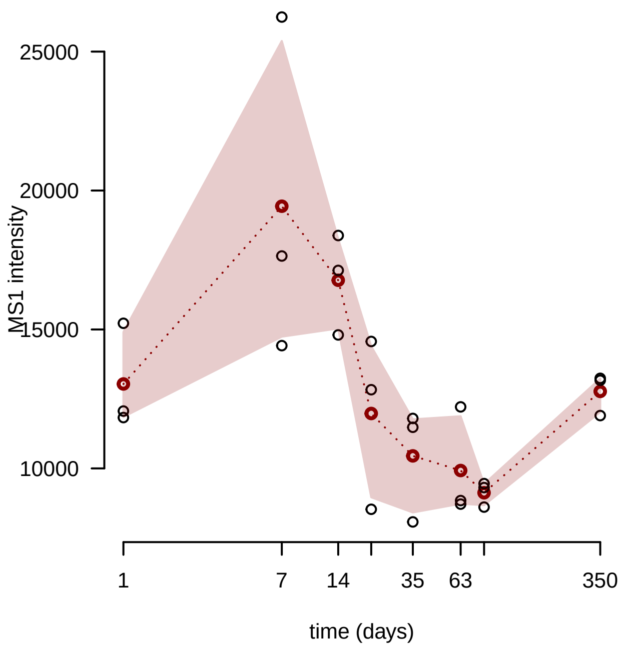

Q62276
GN=Med22
mediator complex subunit 22

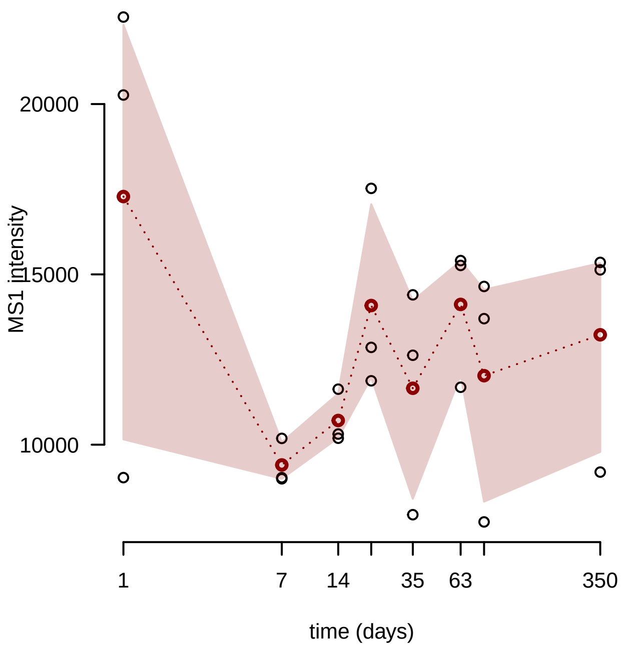

Q62283
GN=Tspan7
tetraspanin 7

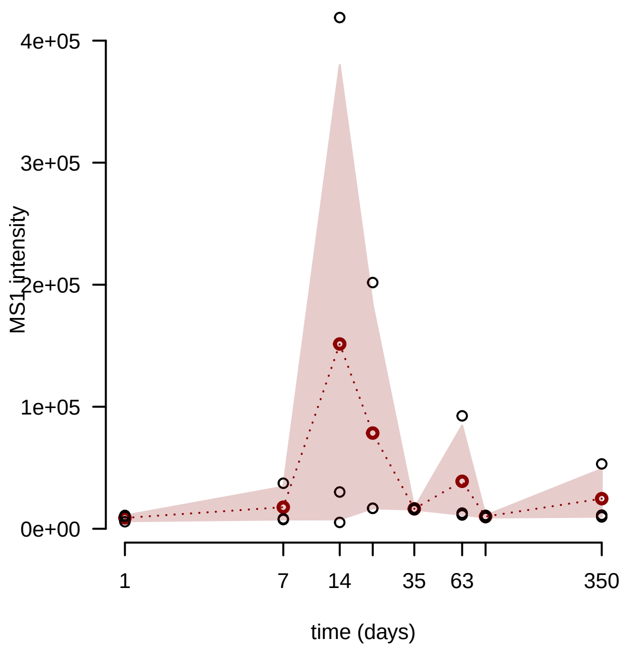

Q62302
GN=Tex261
testis expressed gene 261

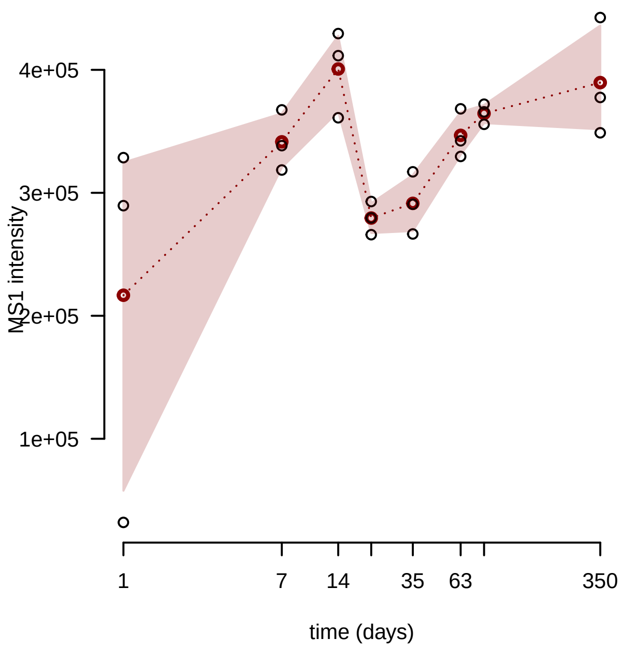

Q62311
GN=Taf6
TATA−box binding protein associated fact ...

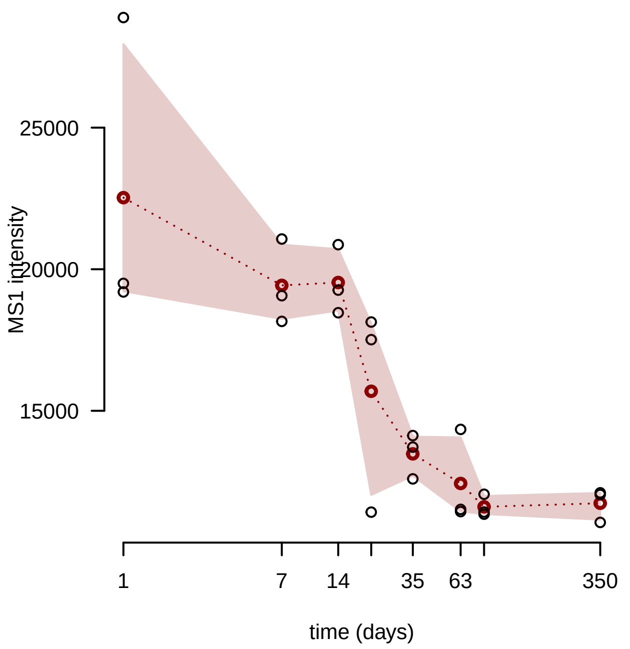

Q62312
GN=Tgfbr2
transforming growth factor, beta recepto ...

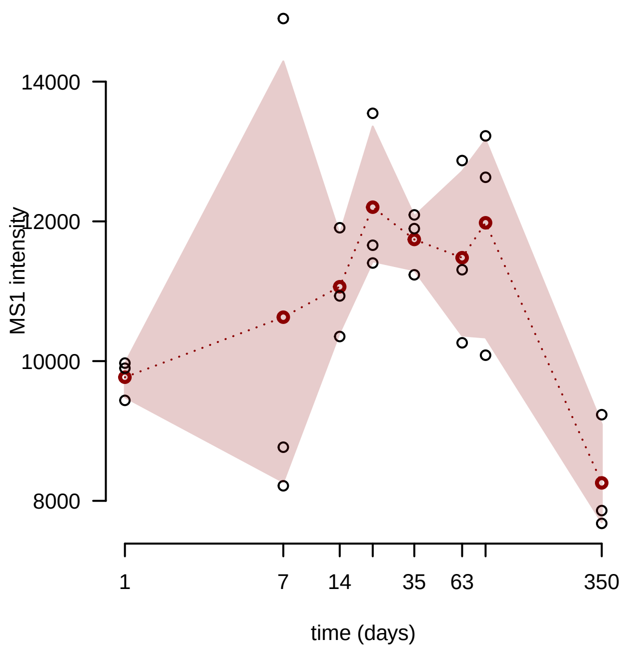

Q62313
GN=Tgoln1
trans−golgi network protein

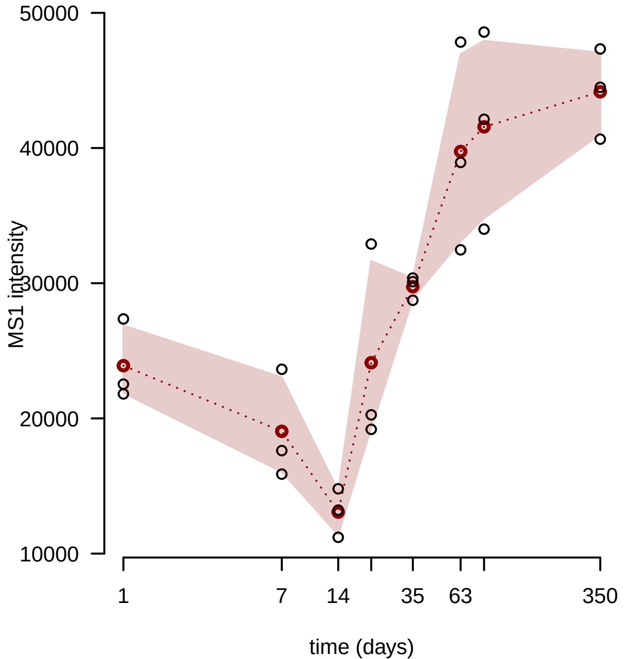

Q62315
GN=Jarid2
jumonji, AT rich interactive domain 2

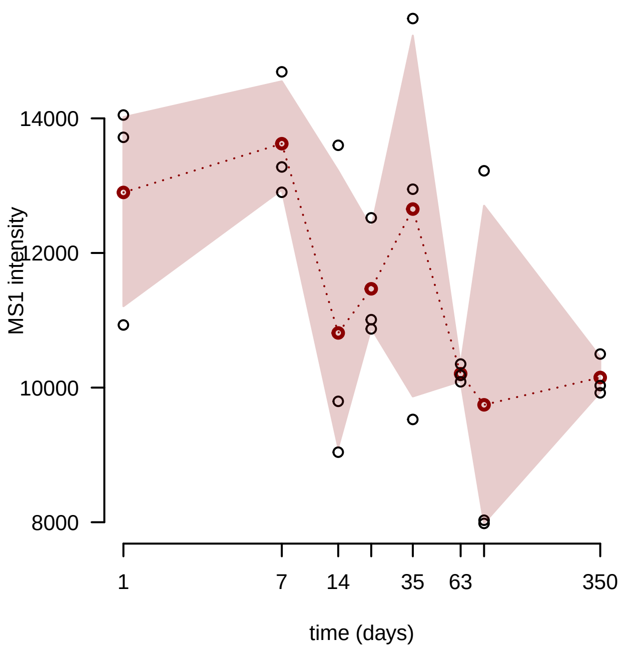

Q62318
GN=Trim28
tripartite motif−containing 28

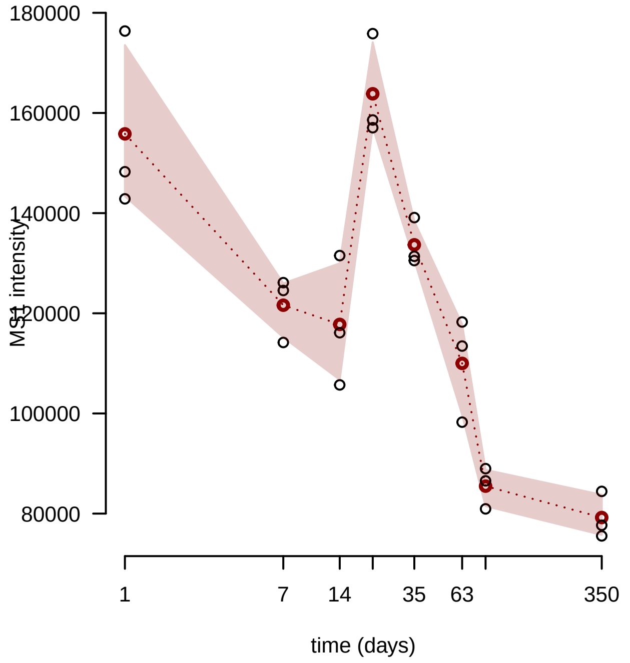

Q62348
GN=Tsn
translin

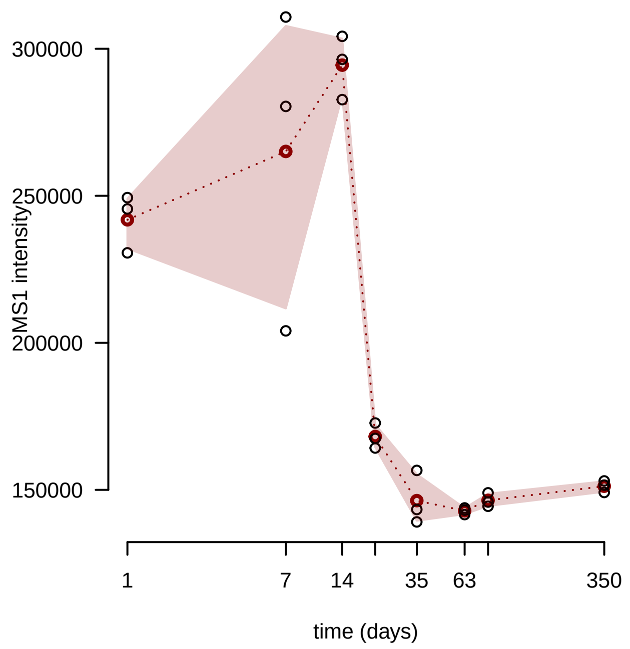

Q62351
GN=Tfrc
transferrin receptor

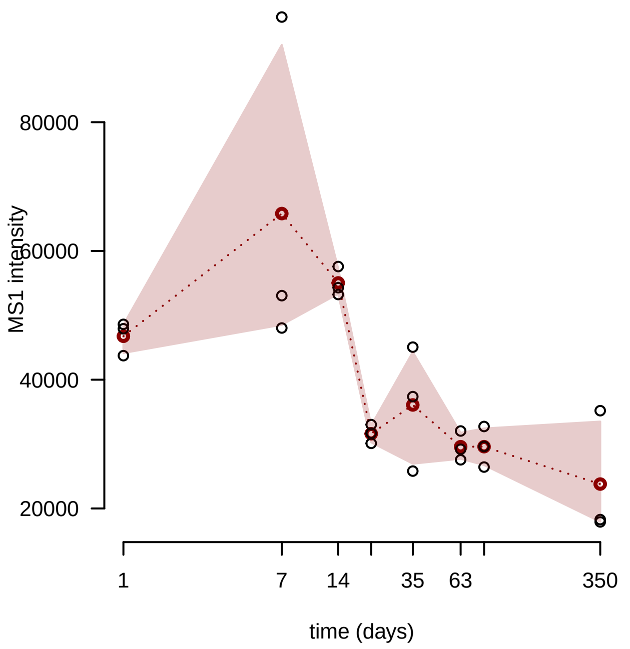

Q62356
GN=Fstl1
follistatin−like 1

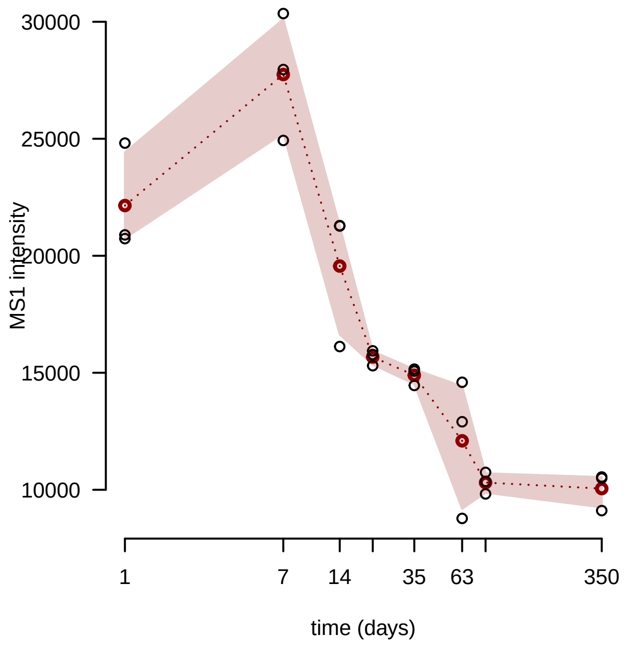

Q62371
GN=Ddr2
discoidin domain receptor family, member ...

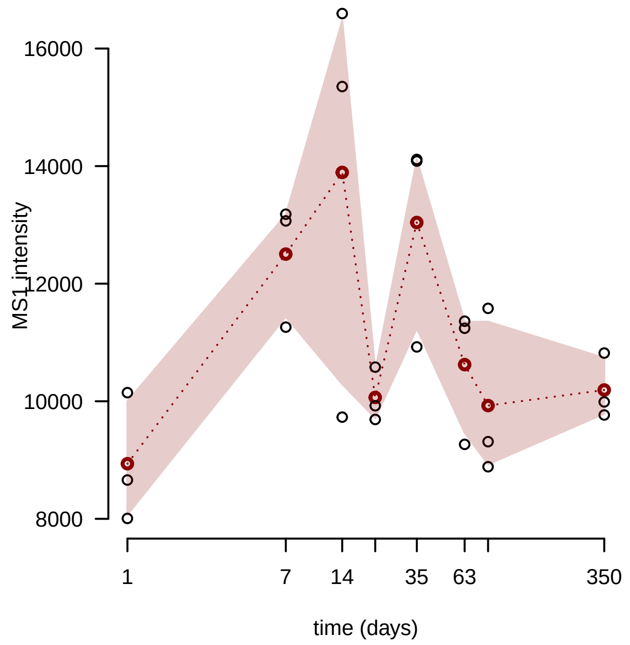

Q62376
GN=Snrnp70
small nuclear ribonucleoprotein 70 (U1)

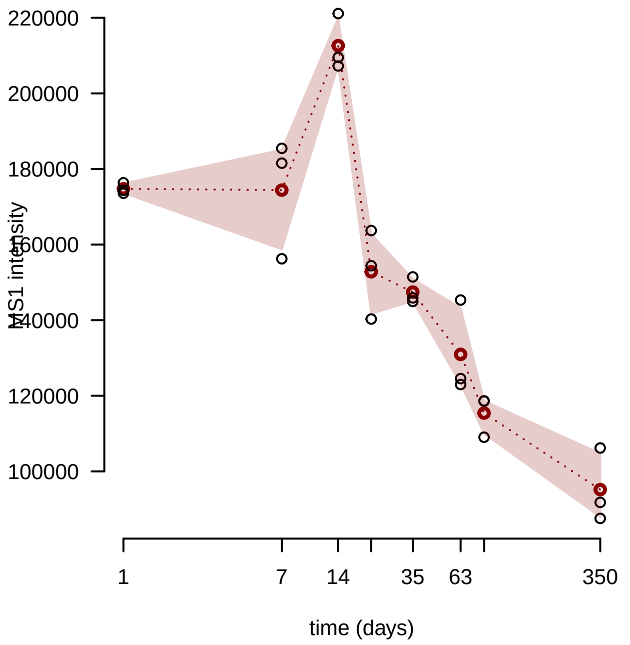

Q62377
GN=Zrsr2
zinc finger (CCCH type), RNA binding mot ...

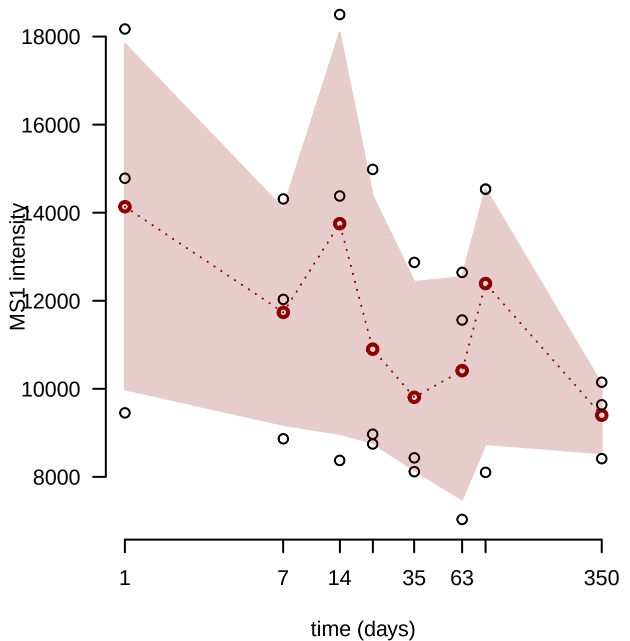

Q62383
GN=Supt6h

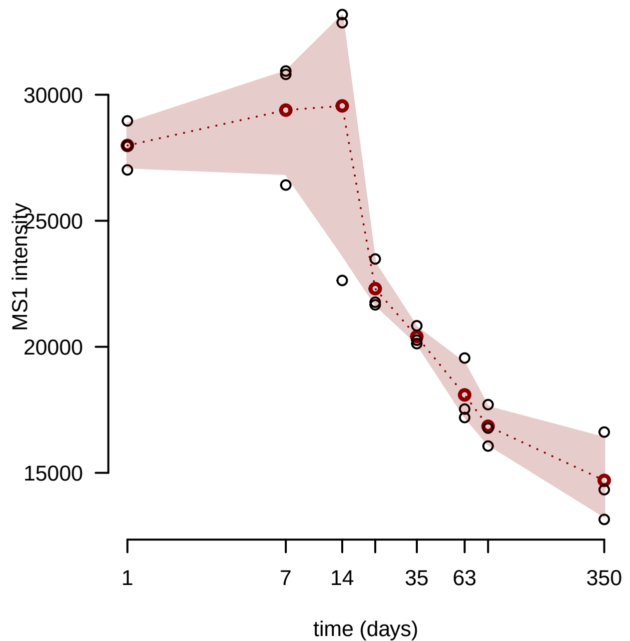

Q62384
GN=Zpr1
ZPR1 zinc finger

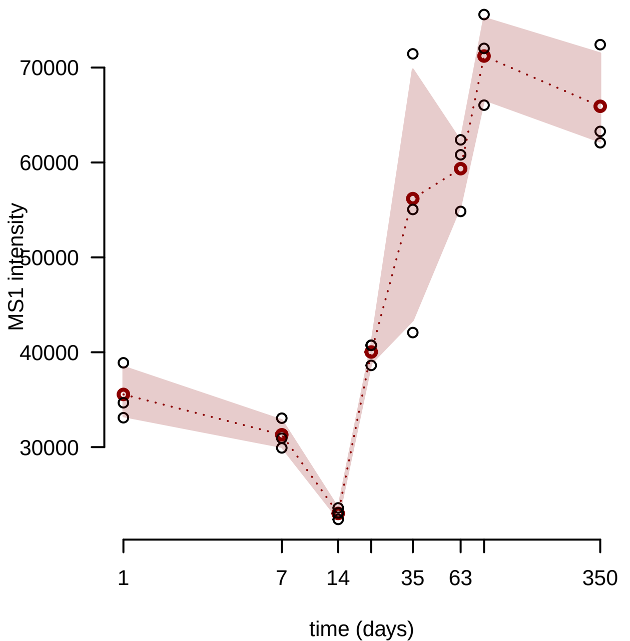

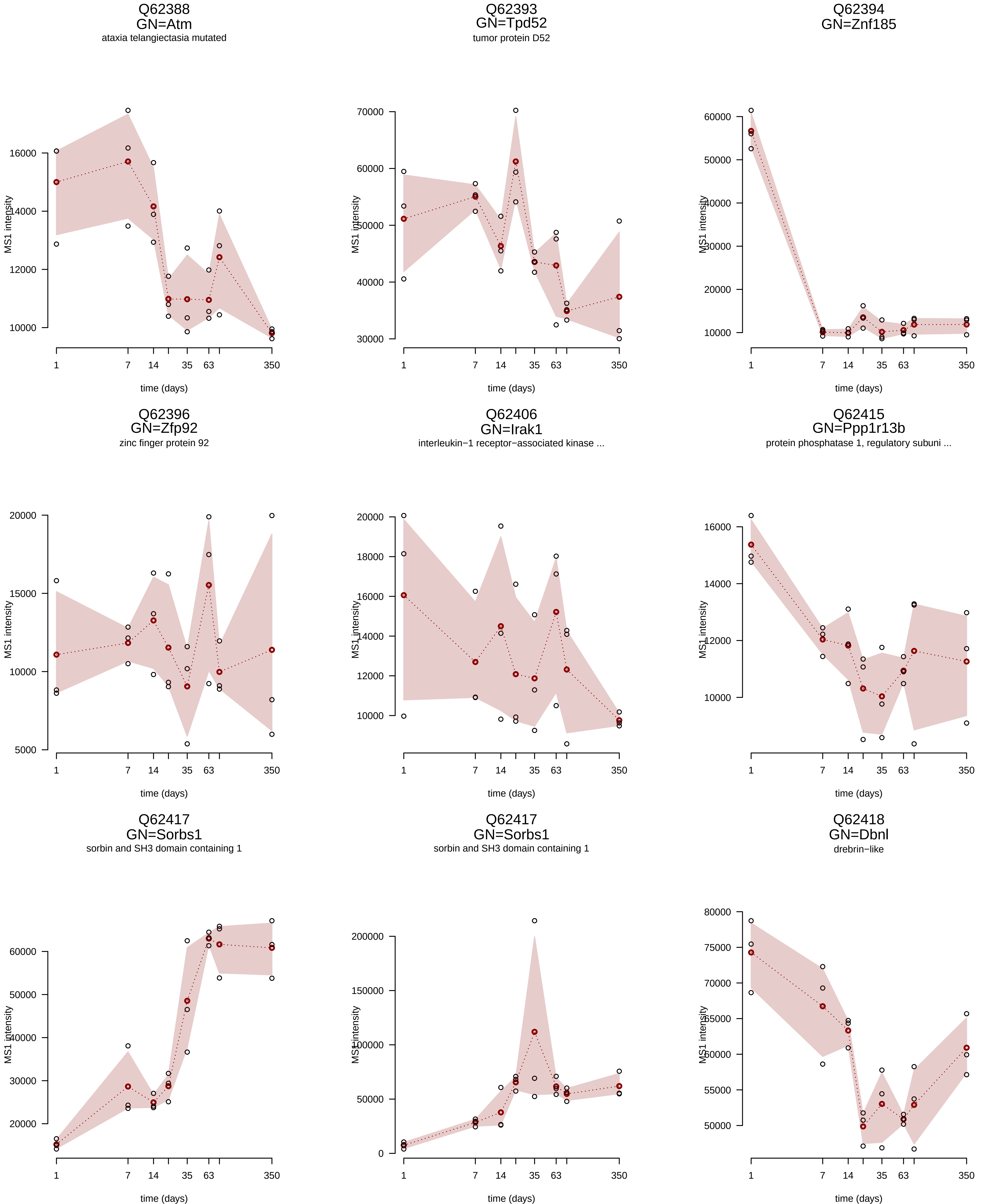
Q62388
GN=Atm
ataxia telangiectasia mutated
MS1 intensity
10000
12000
14000
16000
1
7
14
35
63
350
time (days)
Q62393
GN=Tpd52
tumor protein D52
MS1 intensity
30000
40000
50000
60000
70000
1
7
14
35
63
350
time (days)
Q62394
GN=Znf185
MS1 intensity
10000
20000
30000
40000
50000
60000
1
7
14
35
63
350
time (days)
Q62396
GN=Zfp92
zinc finger protein 92
MS1 intensity
5000
10000
15000
20000
1
7
14
35
63
350
time (days)
Q62406
GN=Irak1
interleukin−1 receptor−associated kinase ...
MS1 intensity
10000
12000
14000
16000
18000
20000
1
7
14
35
63
350
time (days)
Q62415
GN=Ppp1r13b
protein phosphatase 1, regulatory subuni ...
MS1 intensity
10000
12000
14000
16000
1
7
14
35
63
350
time (days)
Q62417
GN=Sorbs1
sorbin and SH3 domain containing 1
MS1 intensity
20000
30000
40000
50000
60000
1
7
14
35
63
350
time (days)
Q62417
GN=Sorbs1
sorbin and SH3 domain containing 1
MS1 intensity
0
50000
100000
150000
200000
1
7
14
35
63
350
time (days)
Q62418
GN=Dbnl
drebrin−like
MS1 intensity
50000
55000
60000
65000
70000
75000
80000
1
7
14
35
63
350
time (days)

Q62419
GN=Sh3gl1
SH3−domain GRB2−like 1

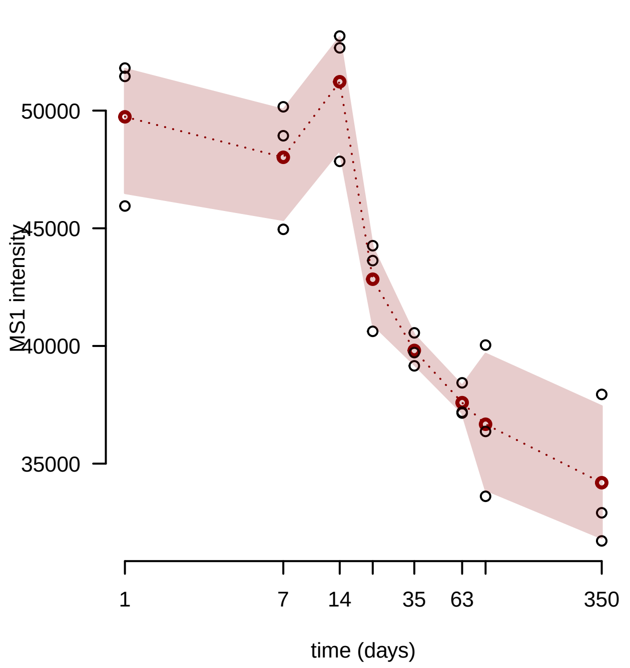

Q62422
GN=Ostf1
osteoclast stimulating factor 1

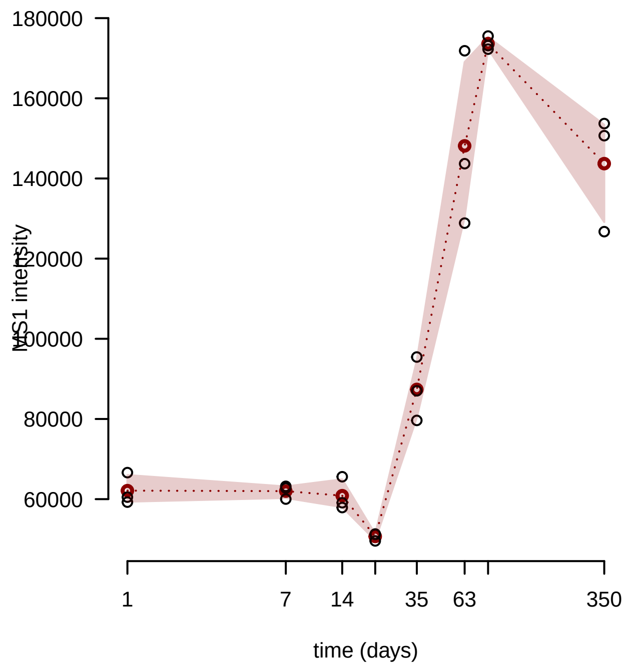

Q62425
GN=Ndufa4
Ndufa4, mitochondrial complex associated

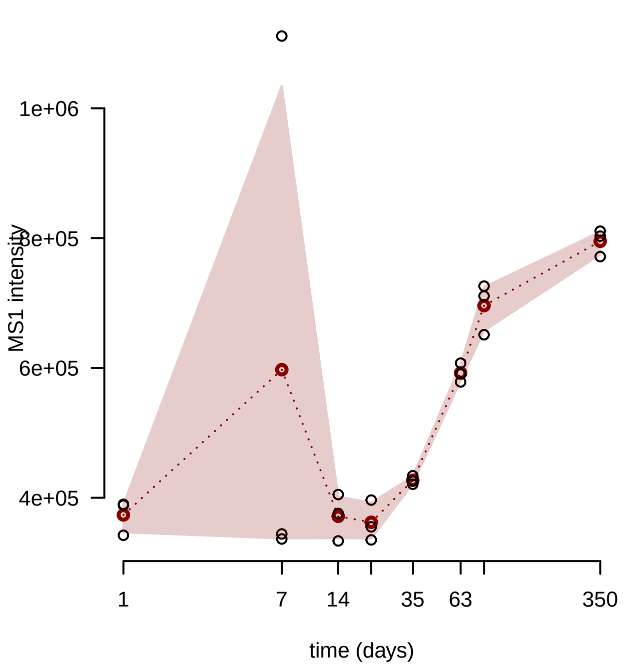

Q62426
GN=Cstb
cystatin B

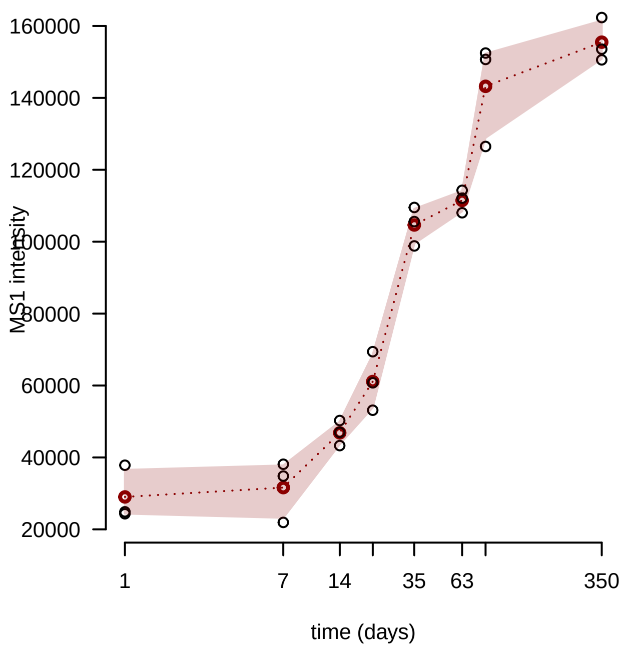

Q62432
GN=Smad2
SMAD family member 2

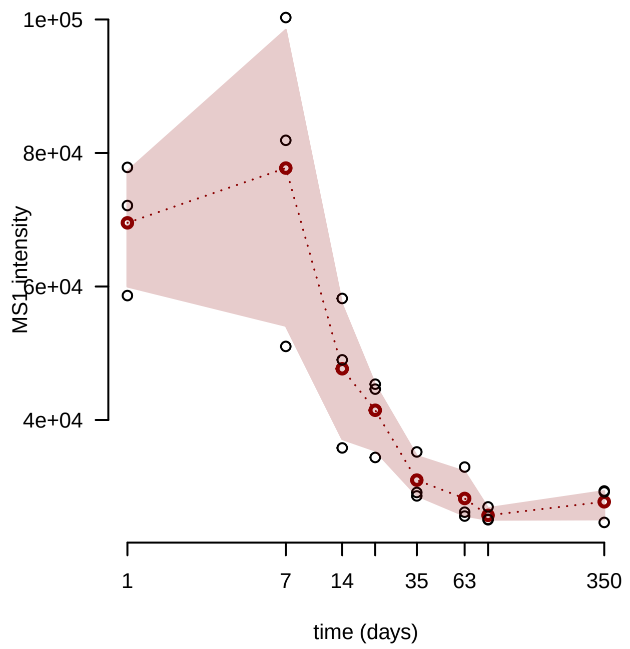

Q62433
GN=Ndrg1
N−myc downstream regulated gene 1

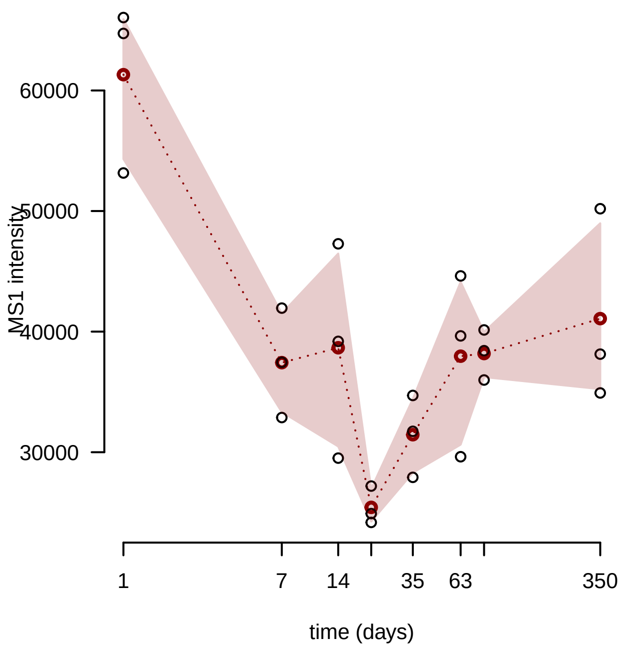

Q62440
GN=Tle1
transducin−like enhancer of split 1

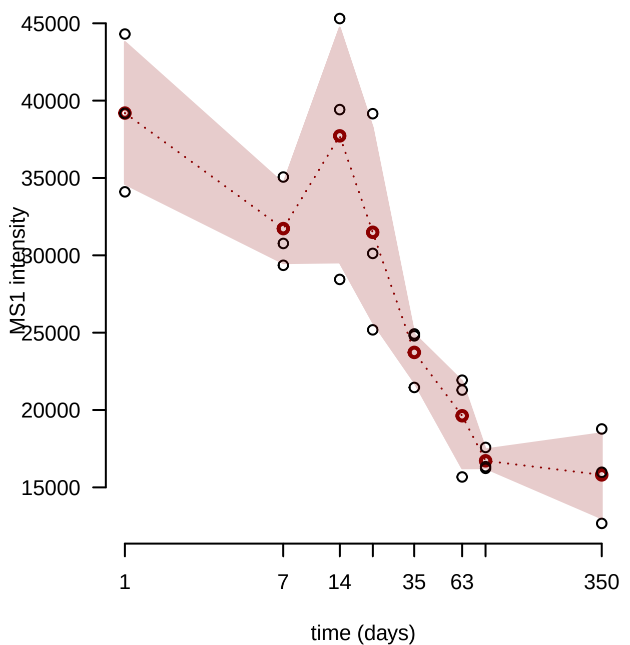

Q62441
GN=Tle4
transducin−like enhancer of split 4

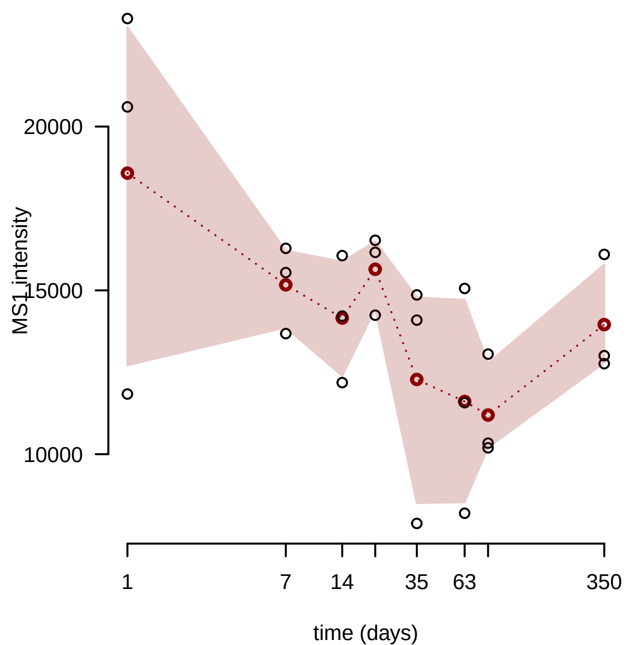

Q62446
GN=Fkbp3
FK506 binding protein 3

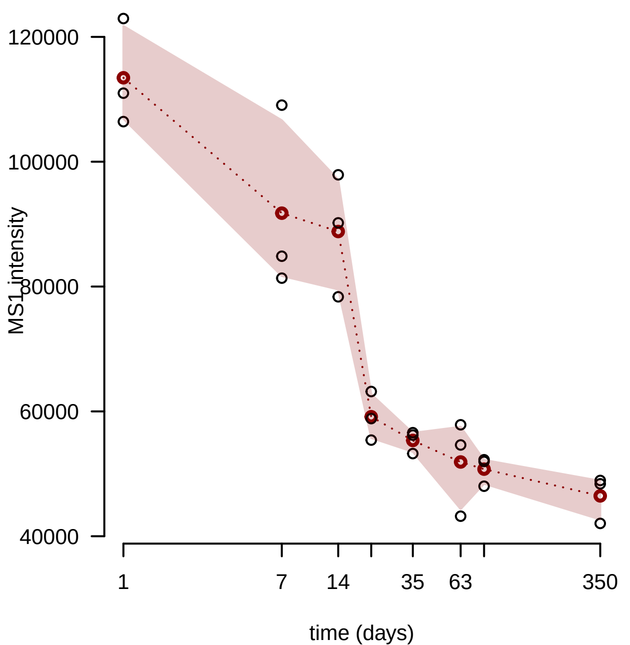

Q62447
GN=Ccnc
cyclin C

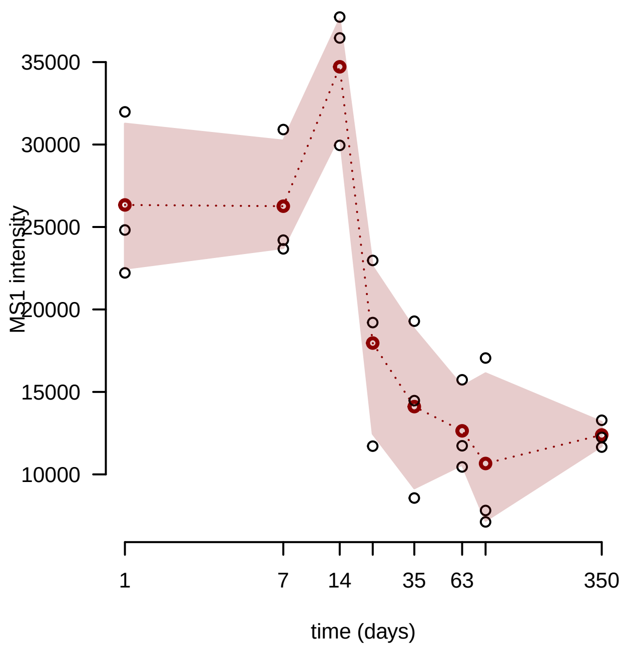

Q62448
GN=Eif4g2
eukaryotic translation initiation factor ...

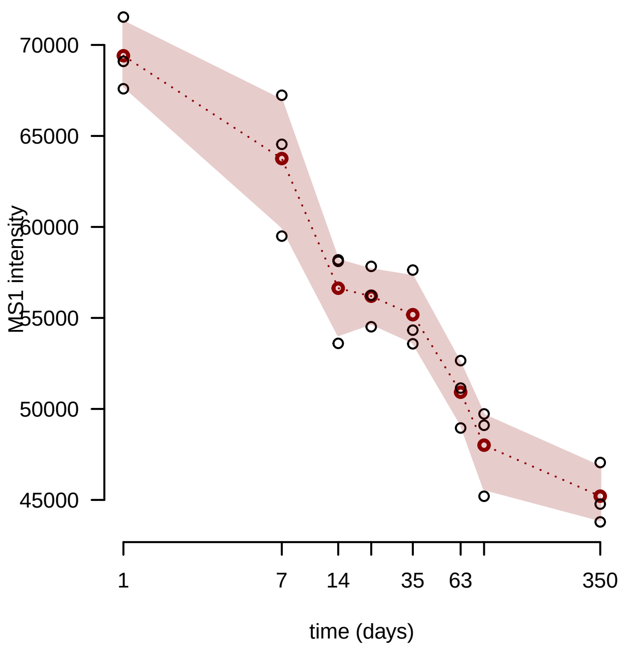

Q62465
GN=Vat1
vesicle amine transport 1

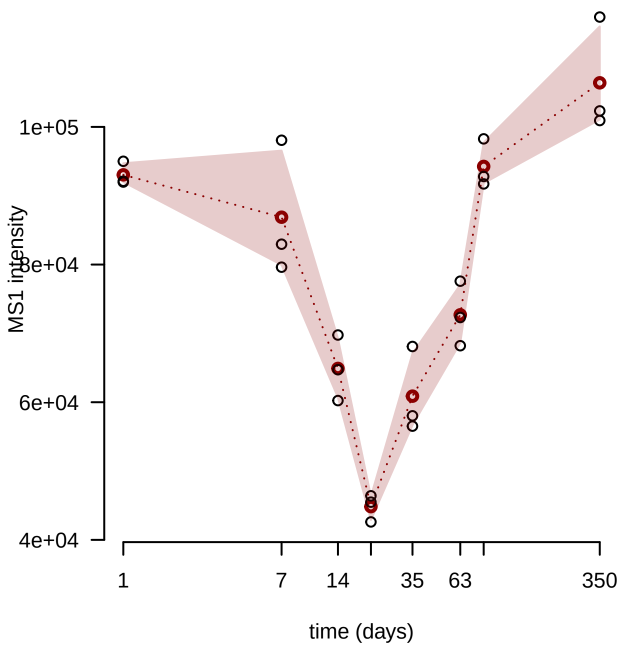

Q62468
GN=Vil1
villin 1

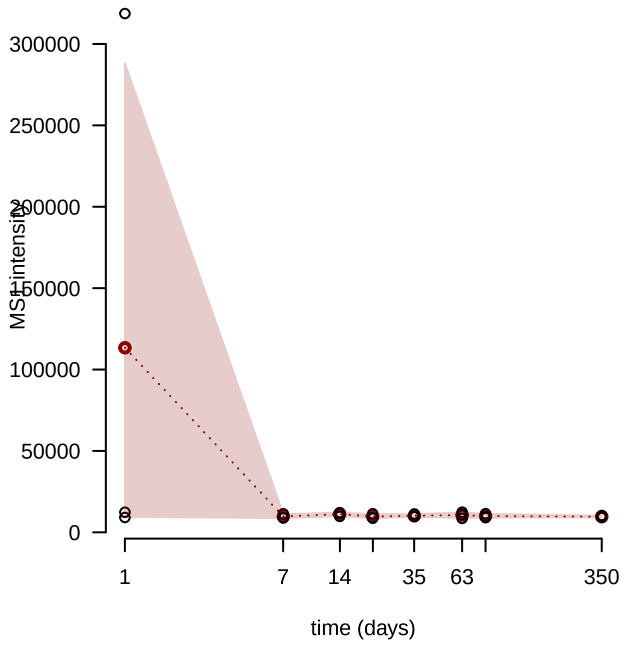

Q62469
GN=Itga2
integrin alpha 2

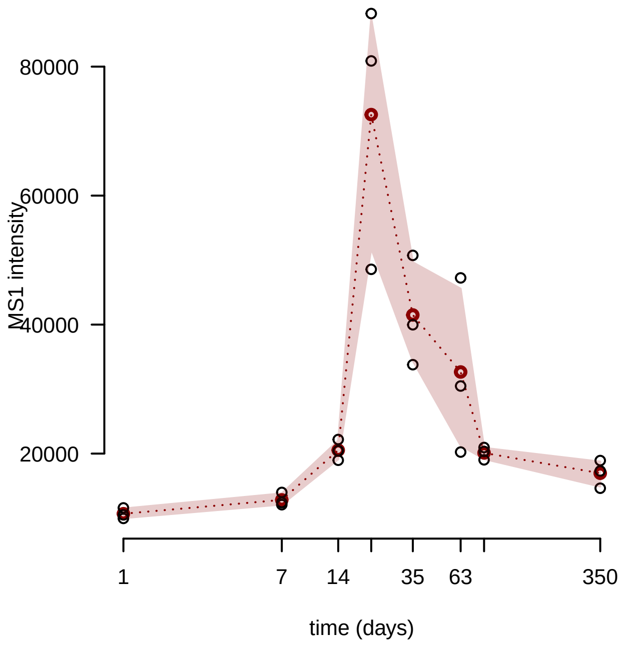

Q62470
GN=Itga3
integrin alpha 3

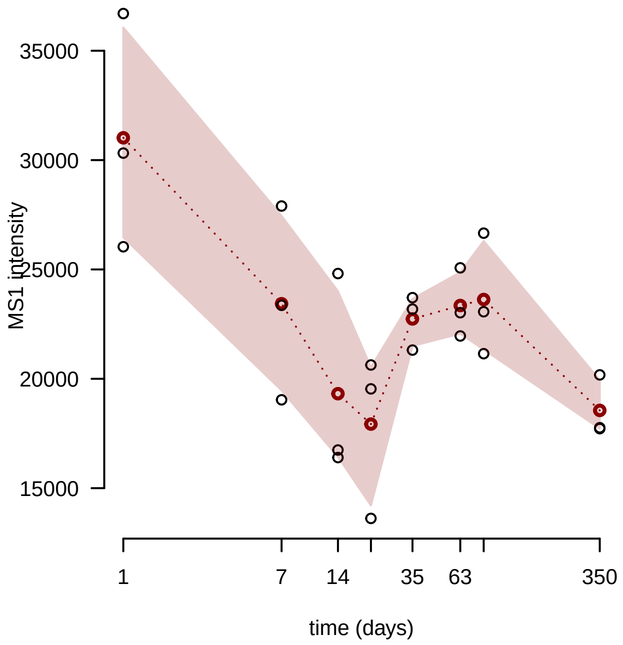

Q62481
GN=Vps72
vacuolar protein sorting 72

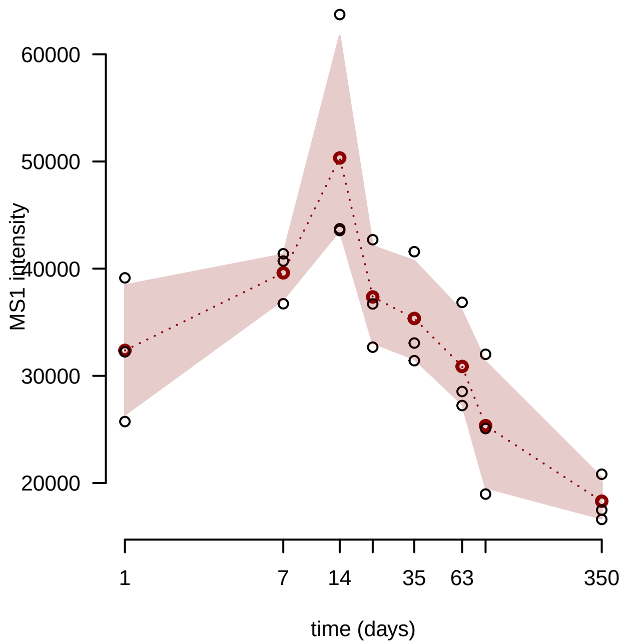

Q62504
GN=Spen
spen family transcription repressor

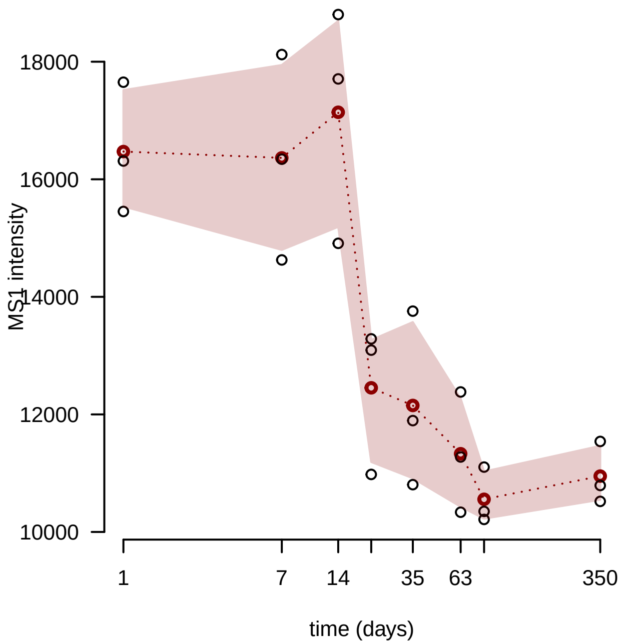

Q62507
GN=Coch
cochlin

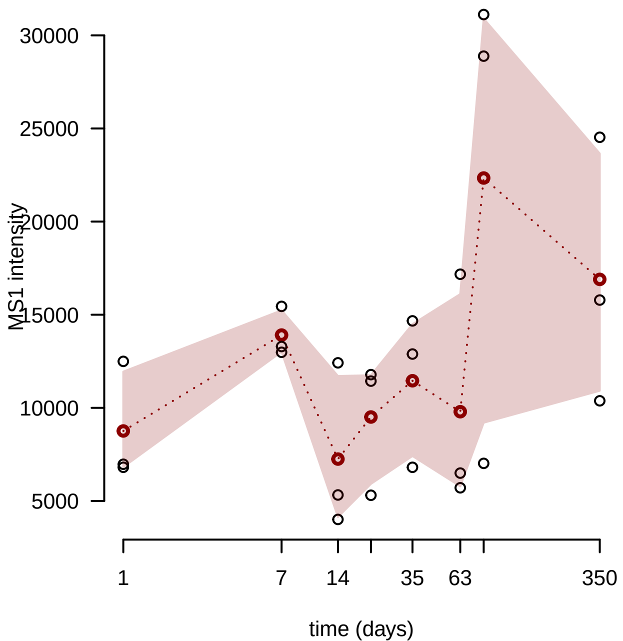

Q62511
GN=Zfp91
zinc finger protein 91

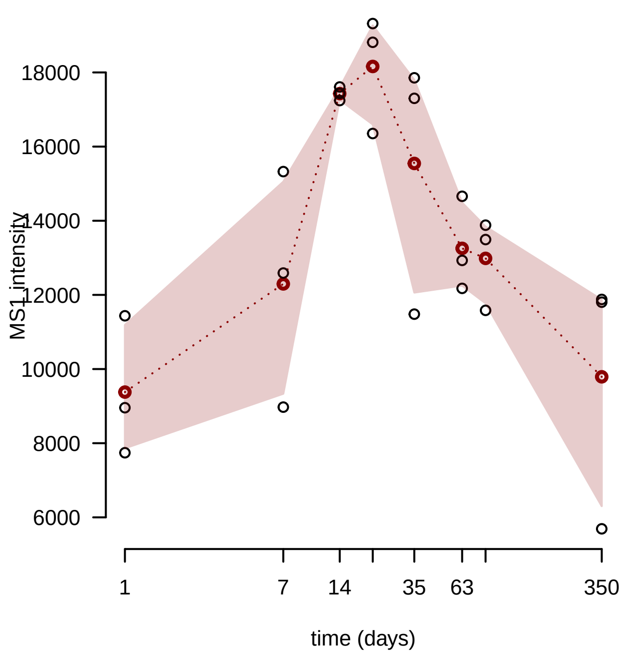

Q62513
GN=Znf260

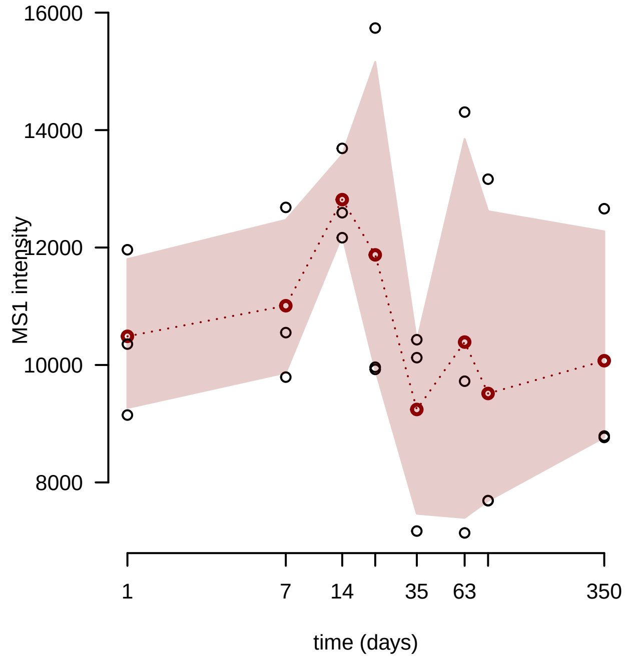

Q62523
GN=Zyx
zyxin

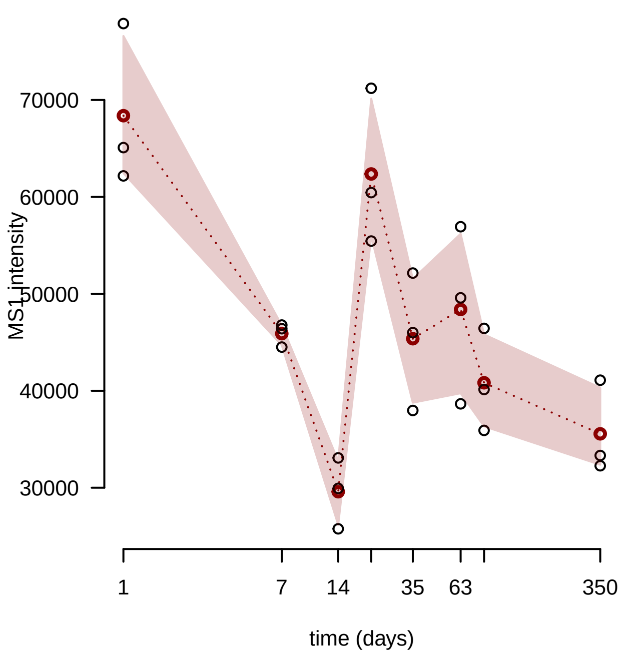

Q62559
GN=Ift52
intraflagellar transport 52

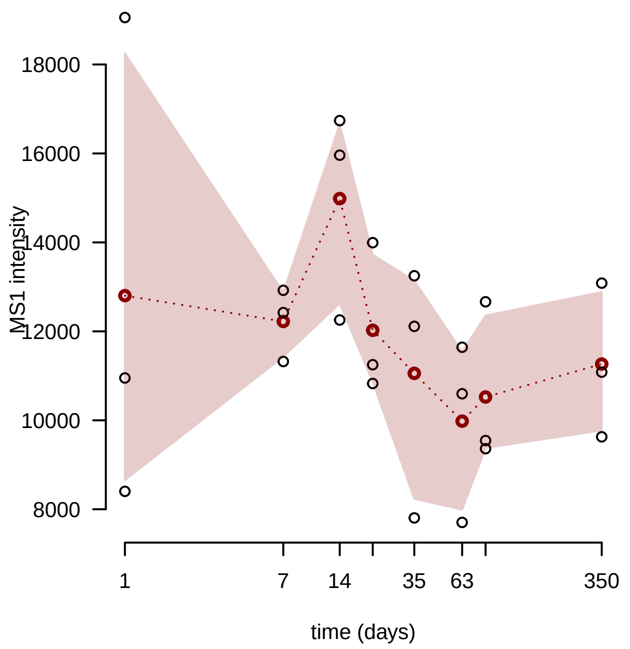

Q63739
GN=Ptp4a1
protein tyrosine phosphatase 4a1

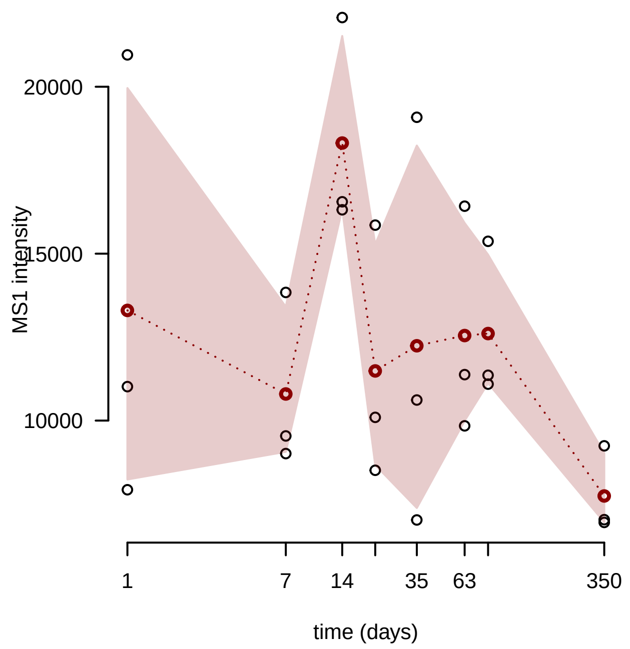

Q63810
GN=Ppp3r1
protein phosphatase 3, regulatory subuni ...

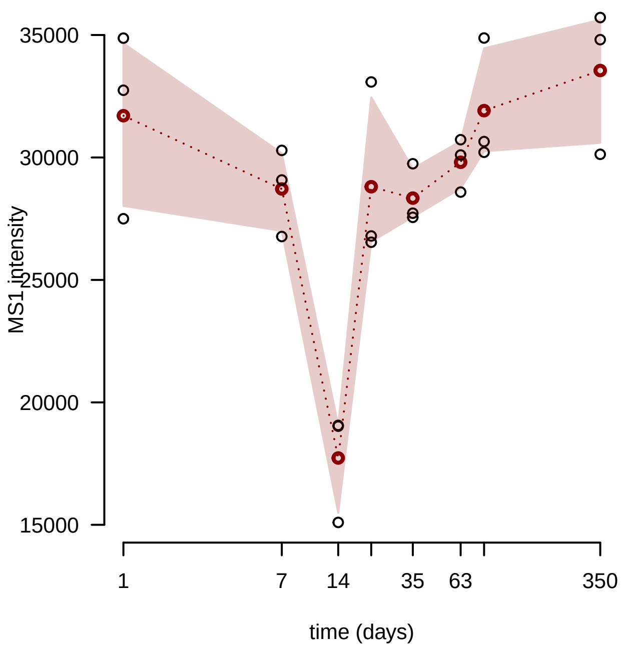

Q63829
GN=Commd3
COMM domain containing 3

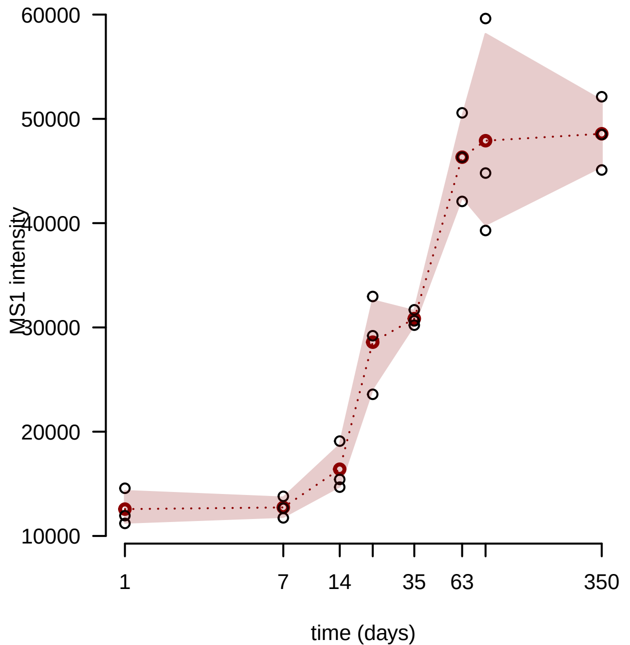

Q63844
GN=Mapk3
mitogen−activated protein kinase 3

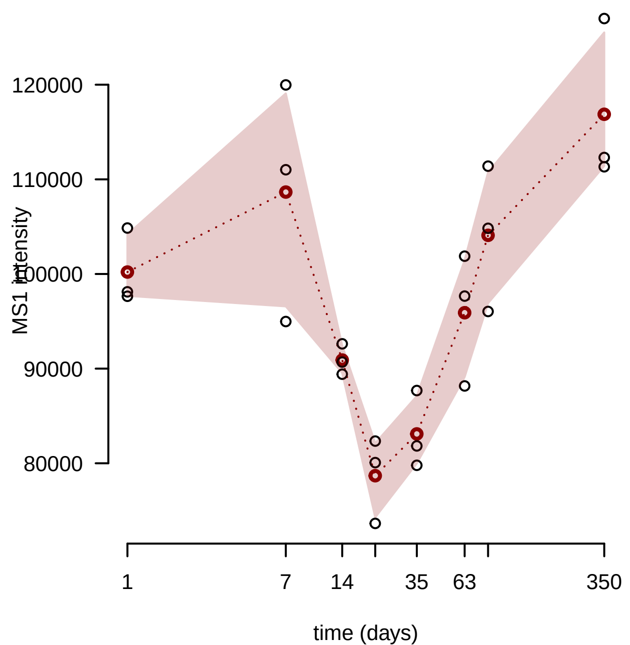

Q63850
GN=Nup62
nucleoporin 62

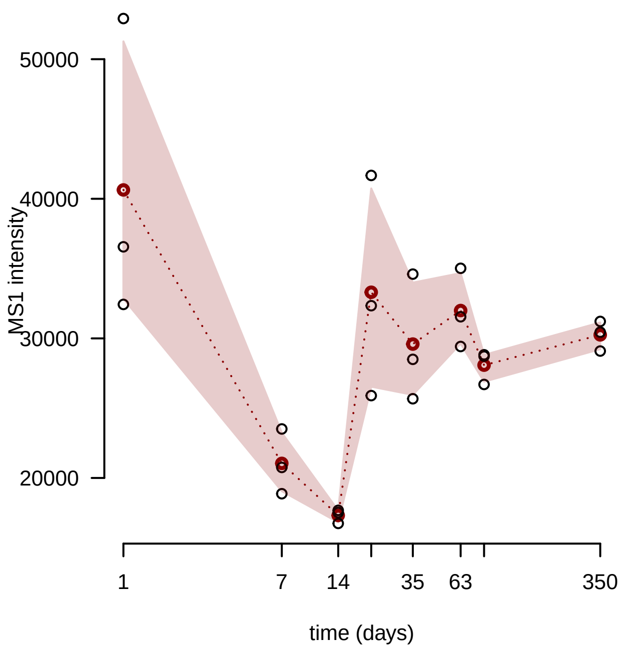

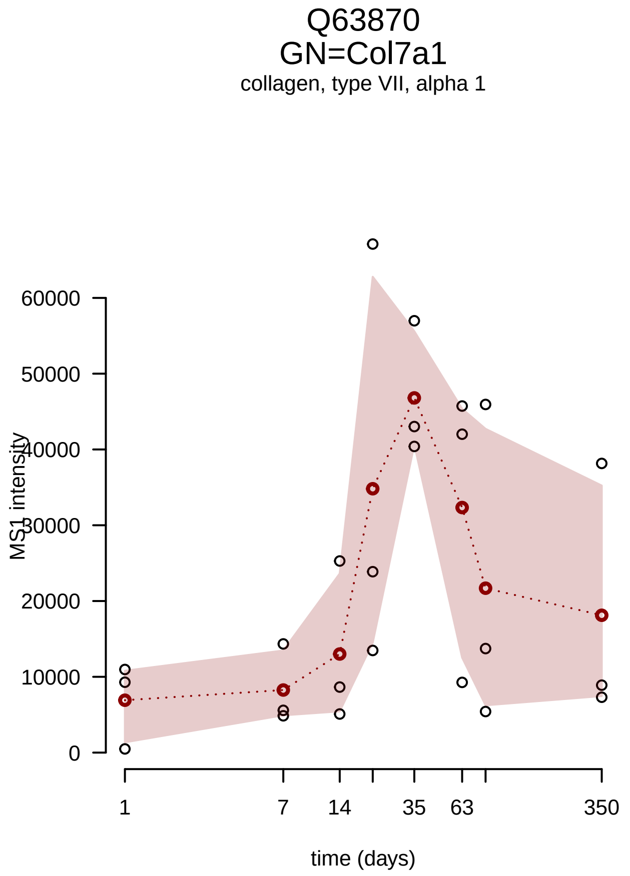
Q63870
GN=Col7a1
collagen, type VII, alpha 1
MS1 intensity
0
10000
20000
30000
40000
50000
60000
1
7
14
35
63
350
time (days)

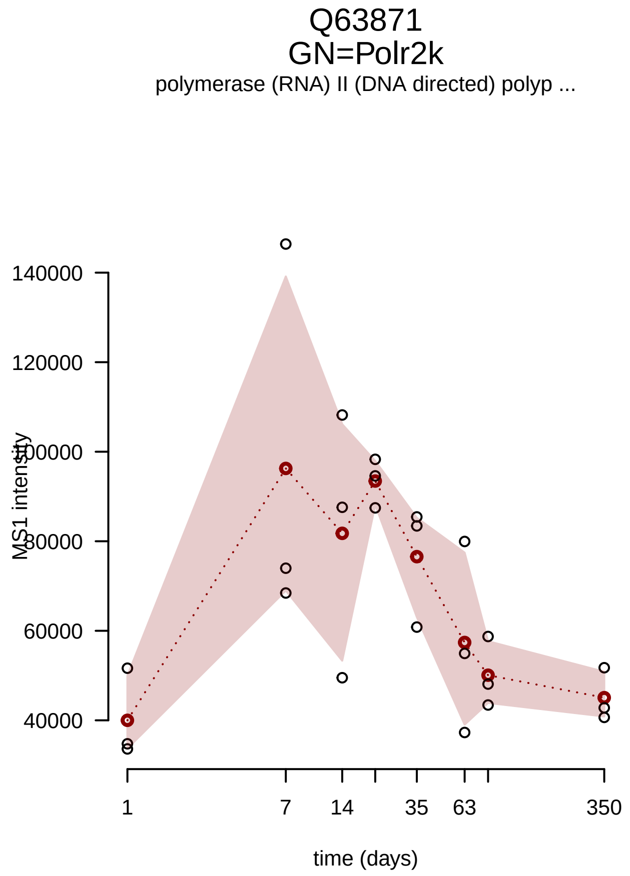
Q63871
GN=Polr2k
polymerase (RNA) II (DNA directed) polyp ...
MS1 intensity
40000
60000
80000
100000
120000
140000
1
7
14
35
63
350
time (days)

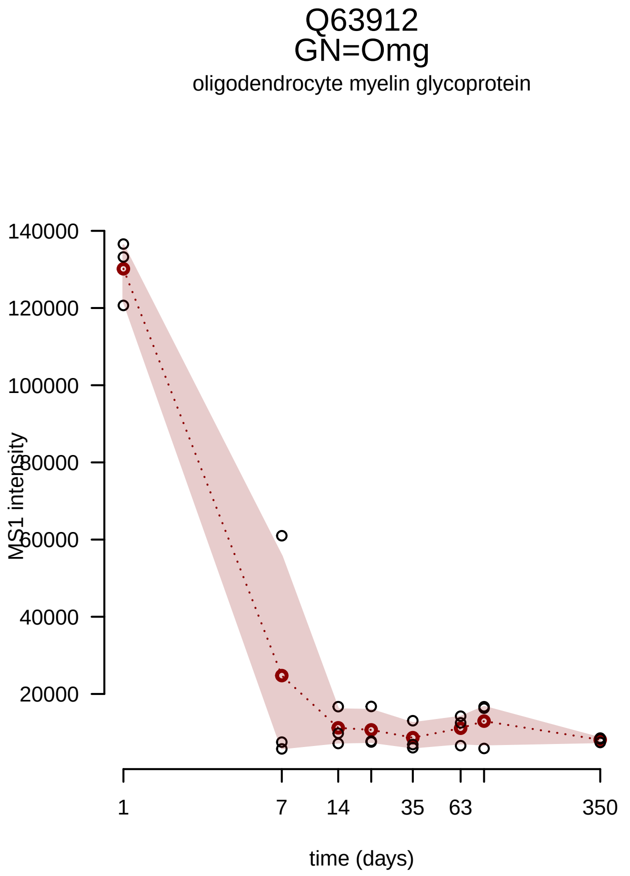
Q63912
GN=Omg
oligodendrocyte myelin glycoprotein
MS1 intensity
20000
40000
60000
80000
100000
120000
140000
1
7
14
35
63
350
time (days)

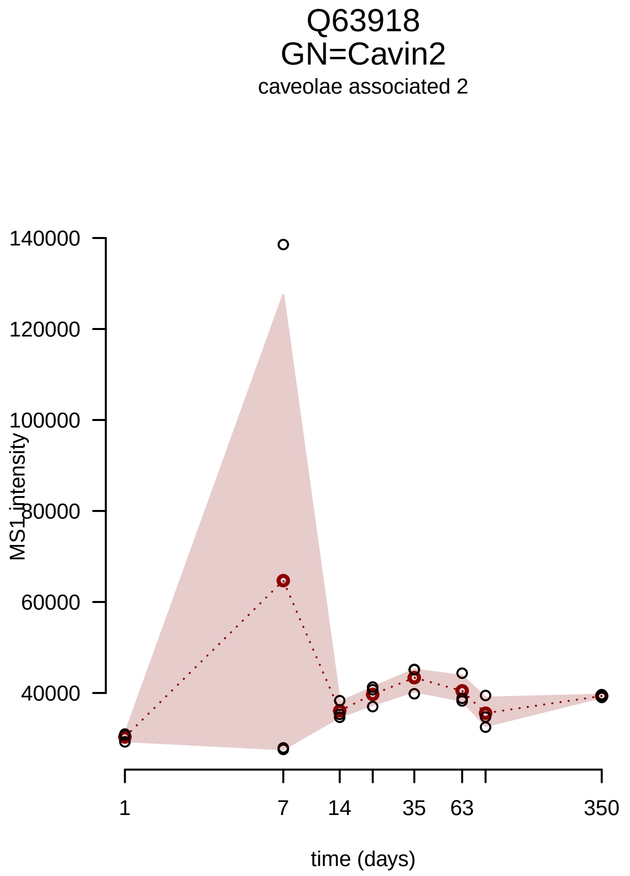
Q63918
GN=Cavin2
caveolae associated 2
MS1 intensity
40000
60000
80000
100000
120000
140000
1
7
14
35
63
350
time (days)

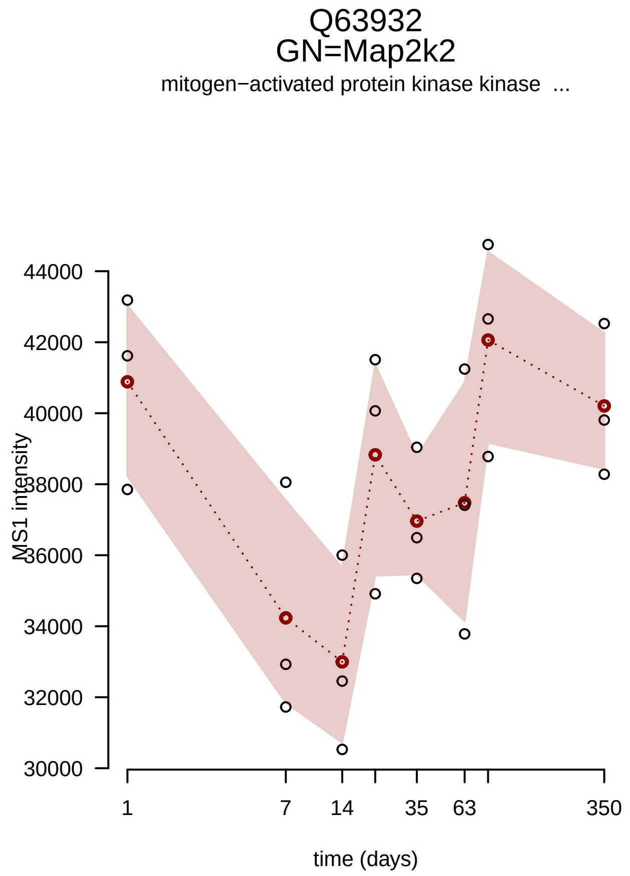
Q63932
GN=Map2k2
mitogen−activated protein kinase kinase ...
MS1 intensity
30000
32000
34000
36000
38000
40000
42000
44000
1
7
14
35
63
350
time (days)

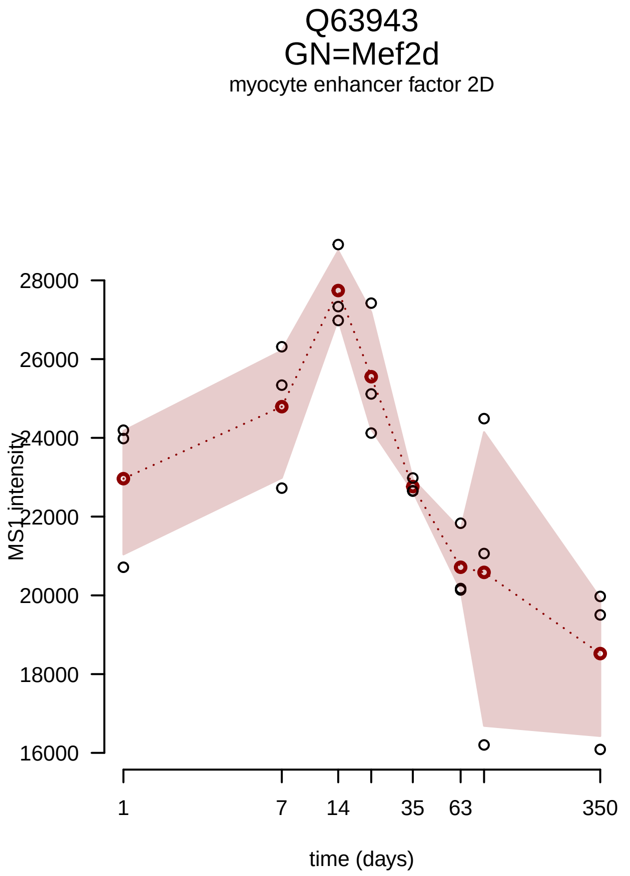
Q63943
GN=Mef2d
myocyte enhancer factor 2D
MS1 intensity
16000
18000
20000
22000
24000
26000
28000
1
7
14
35
63
350
time (days)

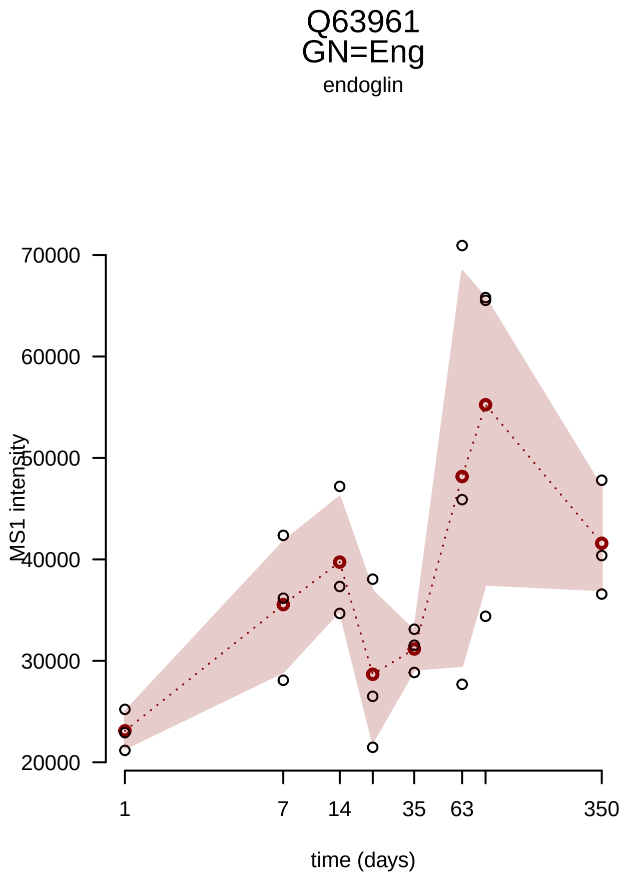
Q63961
GN=Eng
endoglin
MS1 intensity
20000
30000
40000
50000
60000
70000
1
7
14
35
63
350
time (days)

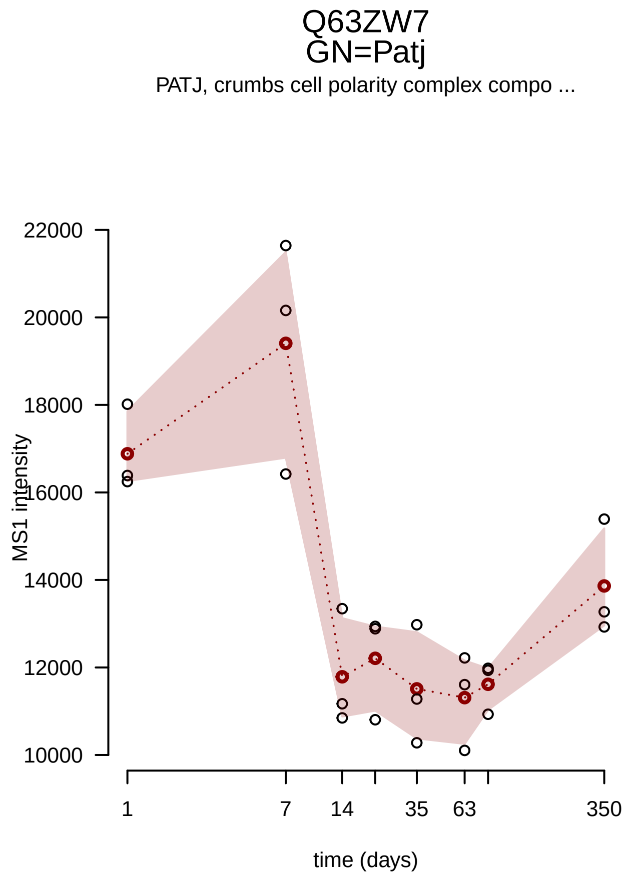
Q63ZW7
GN=Patj
PATJ, crumbs cell polarity complex compo ...
MS1 intensity
10000
12000
14000
16000
18000
20000
22000
1
7
14
35
63
350
time (days)

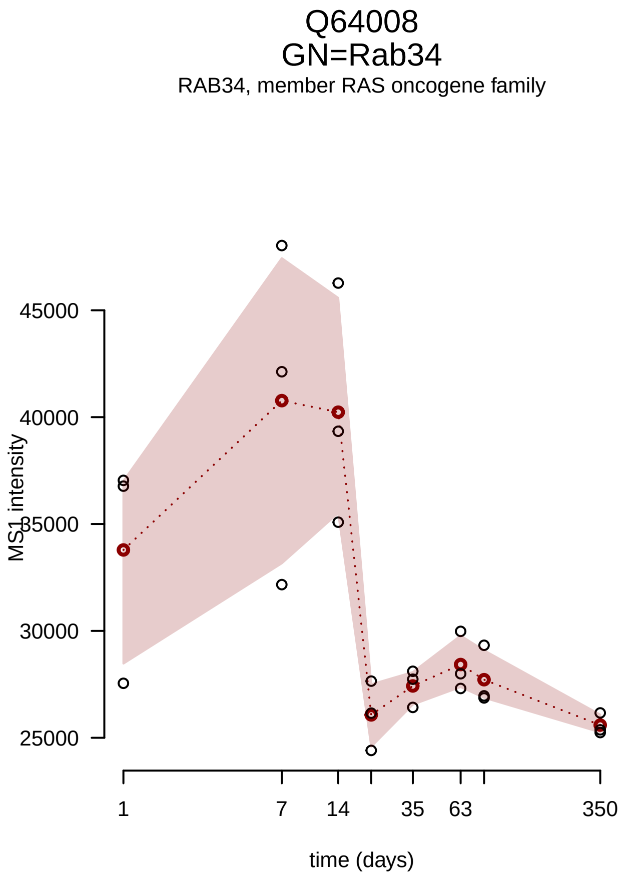
Q64008
GN=Rab34
RAB34, member RAS oncogene family
MS1 intensity
25000
30000
35000
40000
45000
1
7
14
35
63
350
time (days)

Q64010
GN=Crk
v−crk avian sarcoma virus CT10 oncogene ...

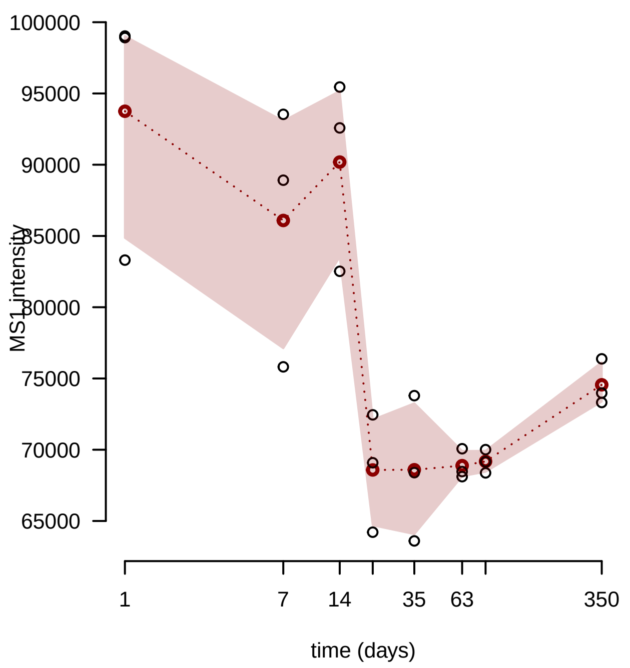

Q64012
GN=Raly
hnRNP−associated with lethal yellow

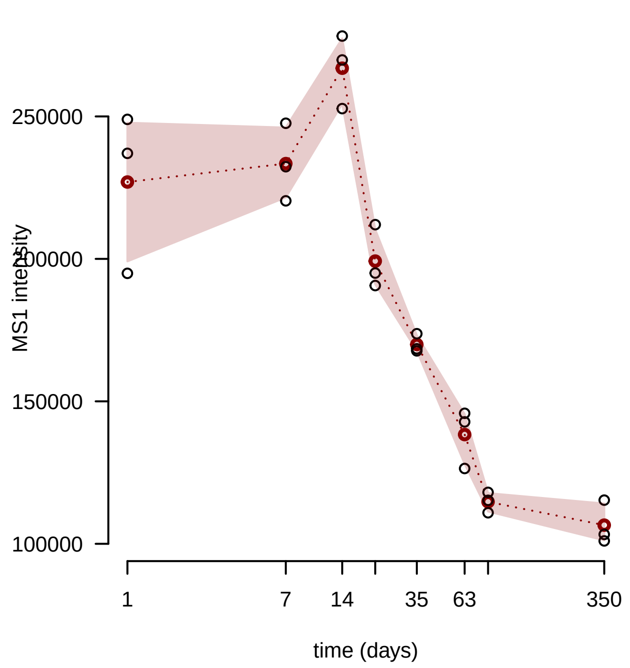

Q64028
GN=Phc1
polyhomeotic 1

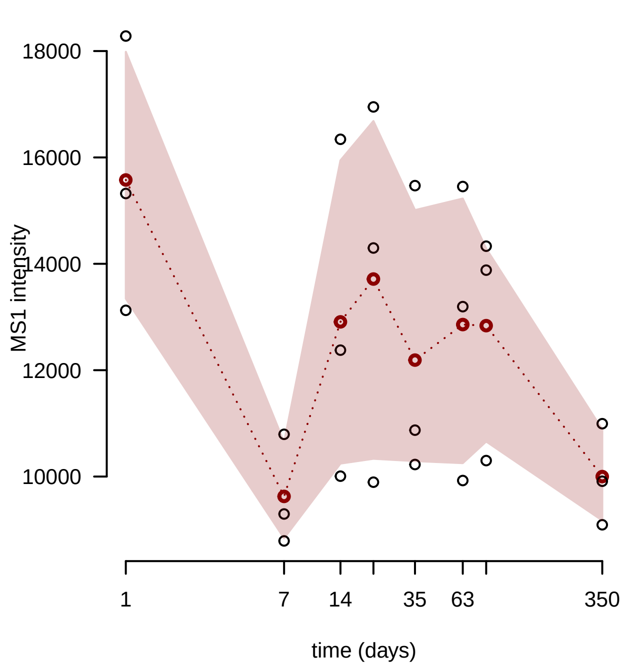

Q64092
GN=Tfe3
transcription factor E3

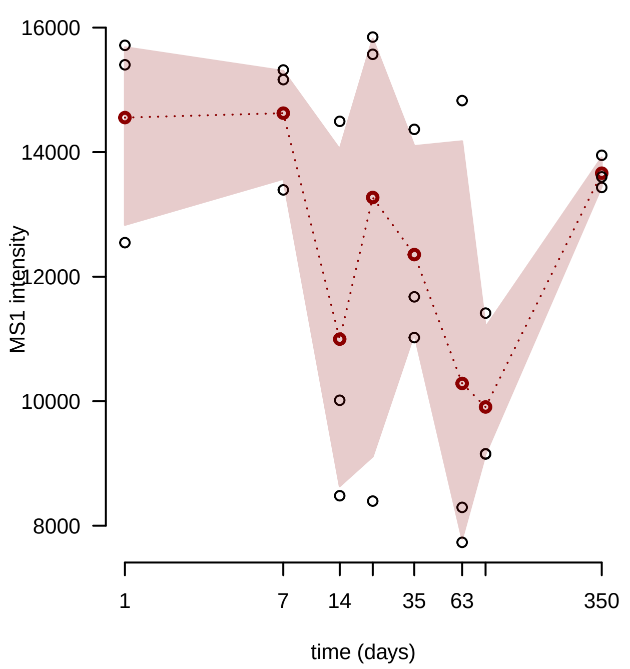

Q64096
GN=Mcf2l
mcf.2 transforming sequence−like

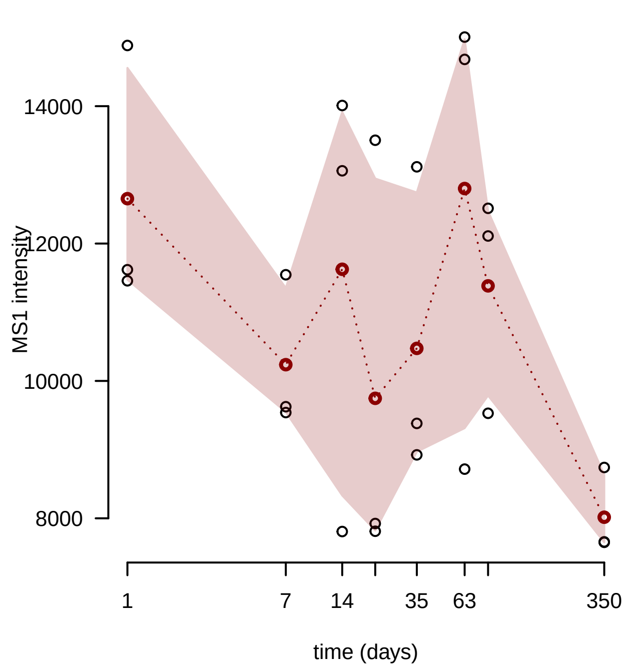

Q640L3
GN=Ccpg1
cell cycle progression 1

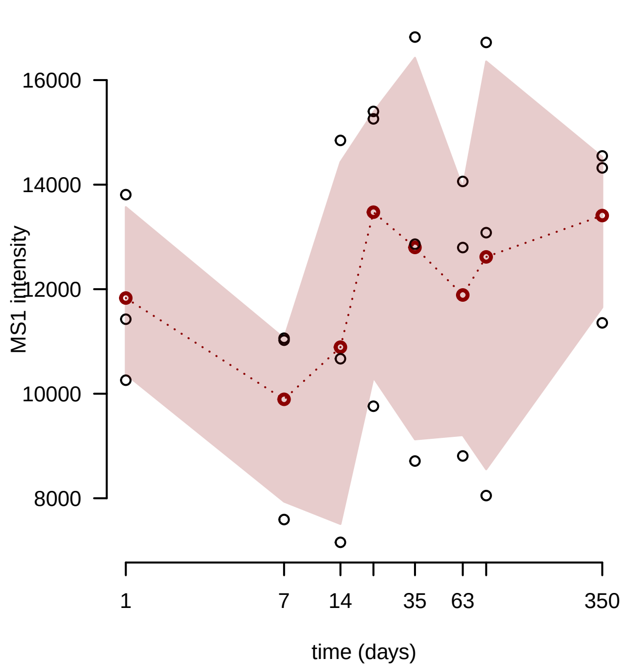

Q640M1
GN=Utp14a
UTP14A small subunit processome componen ...

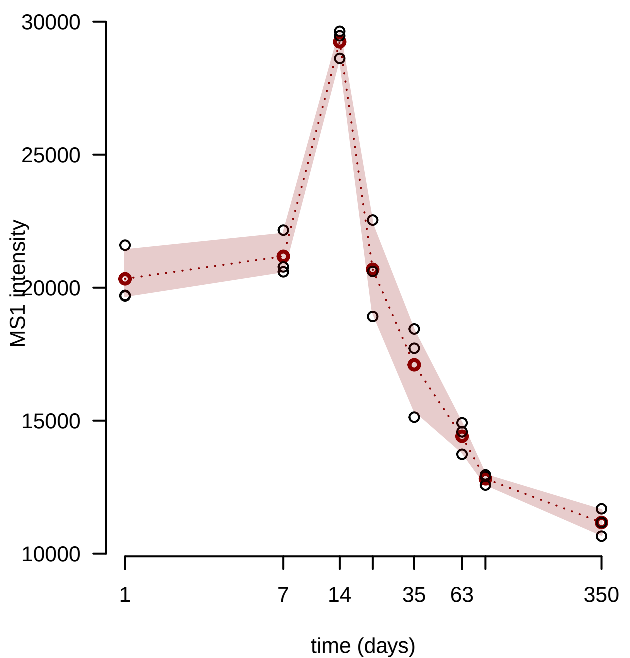

Q640N1
GN=Aebp1
AE binding protein 1

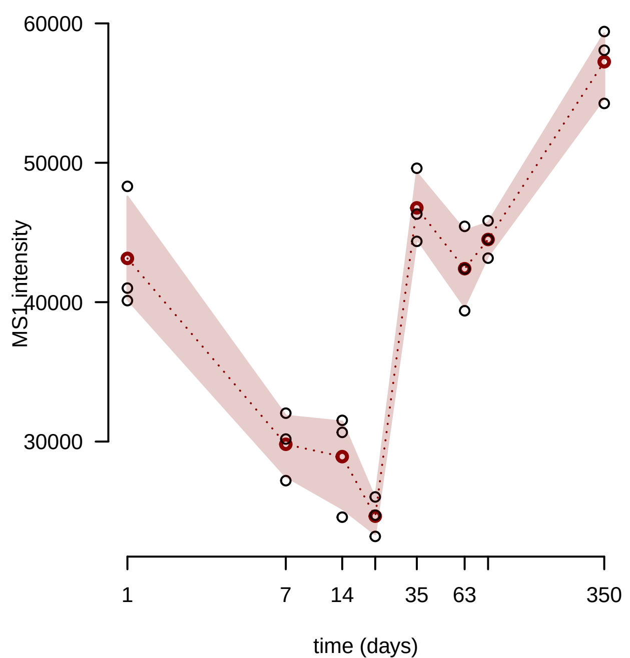

Q640P2
GN=Angptl1
angiopoietin−like 1

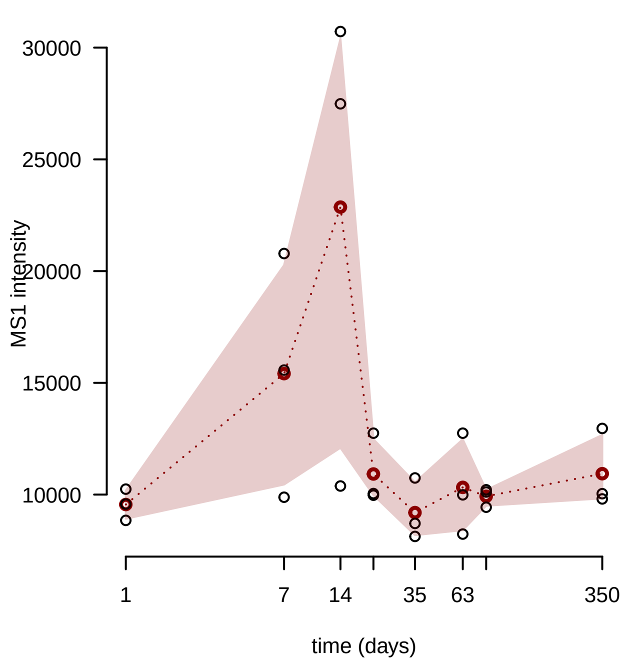

Q640Q5
GN=Pan3
PAN3 poly(A) specific ribonuclease subun ...

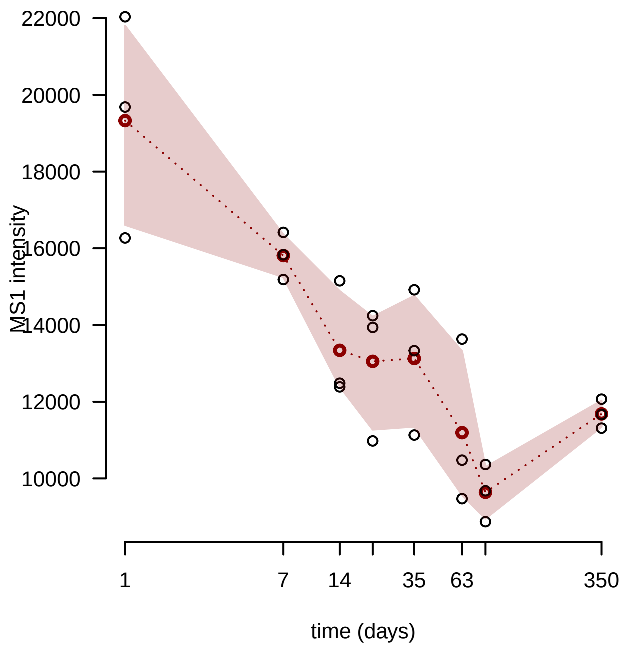

Q64105
GN=Spr
sepiapterin reductase

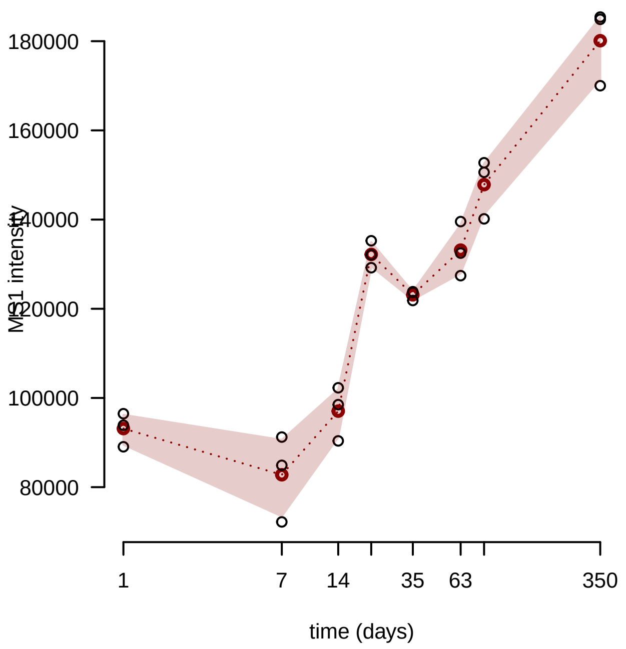

Q64127
GN=Trim24
tripartite motif−containing 24

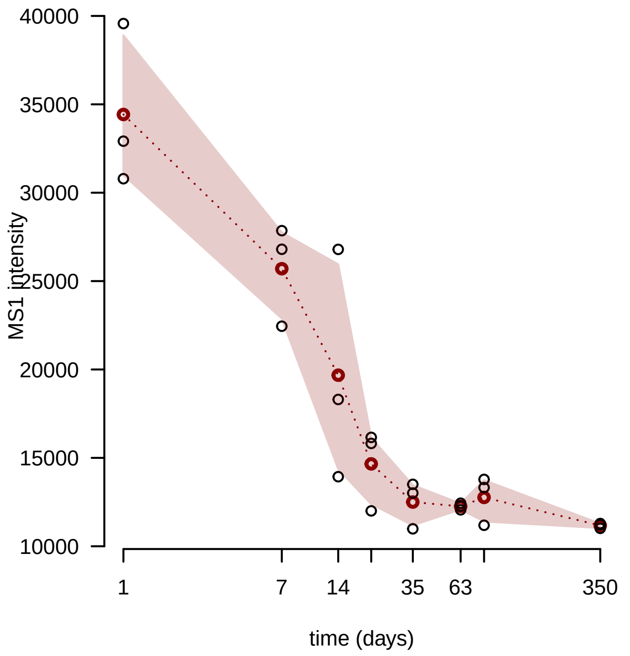

Q64133
GN=Maoa
monoamine oxidase A

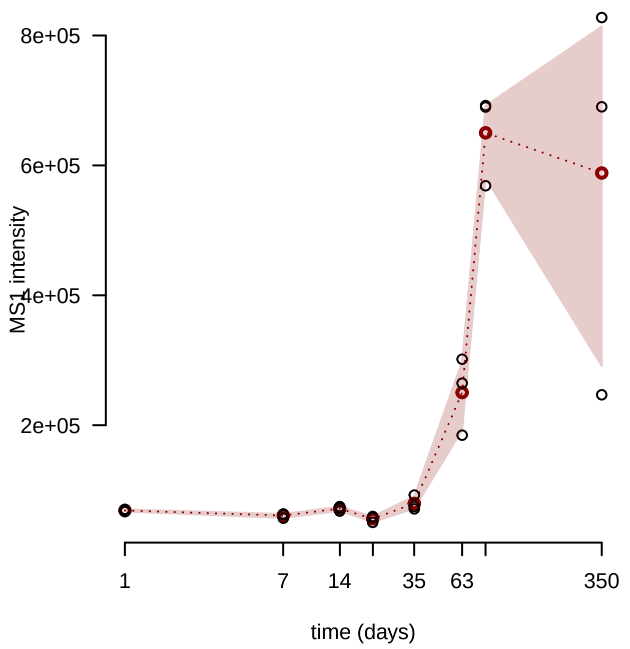

Q64151
GN=Sema4c
sema domain, immunoglobulin domain (Ig), ...

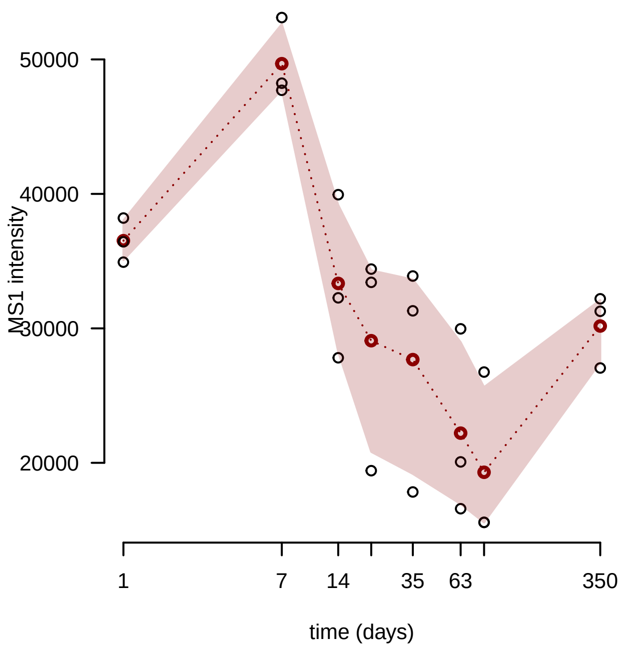

Q64152
GN=Btf3
basic transcription factor 3

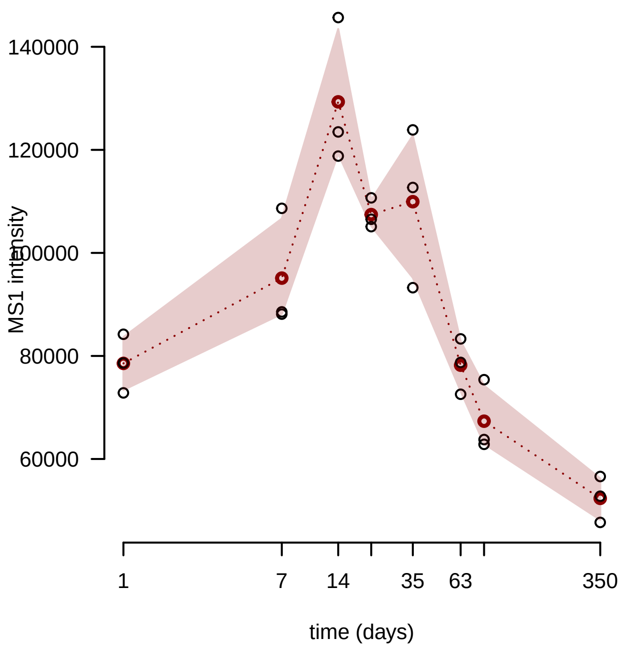

Q64152
GN=Btf3
basic transcription factor 3

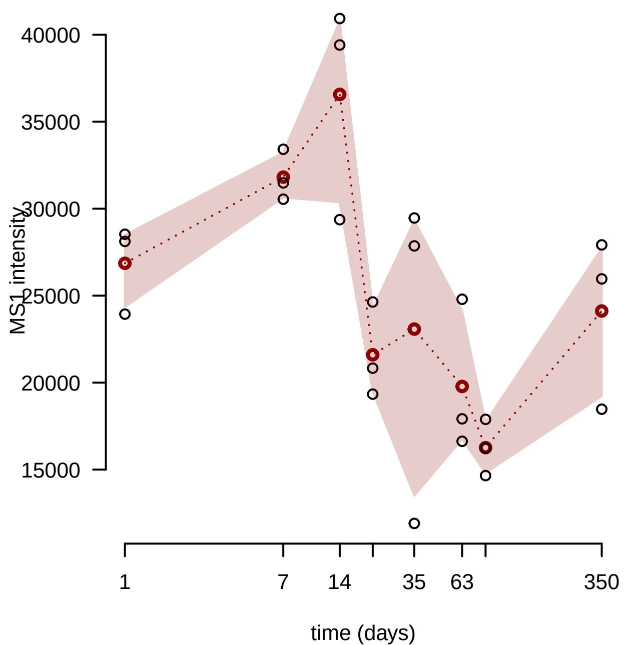

Q64163
GN=Tfdp2
transcription factor Dp 2

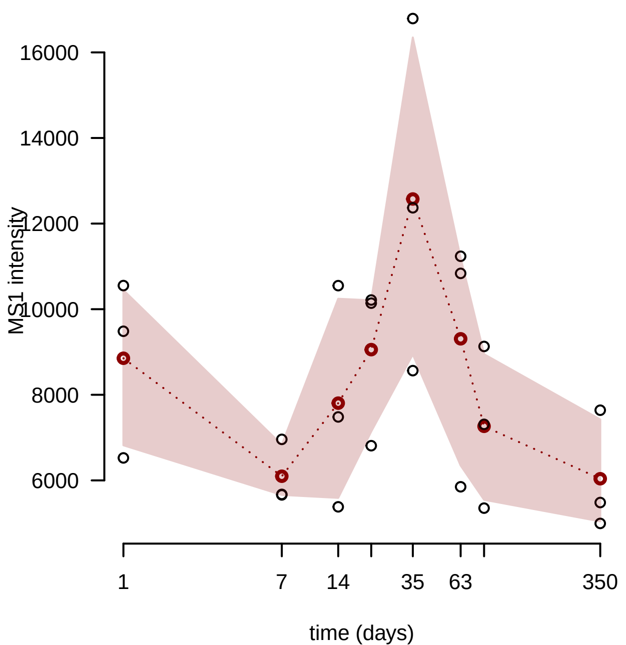

Q64191
GN=Aga
aspartylglucosaminidase

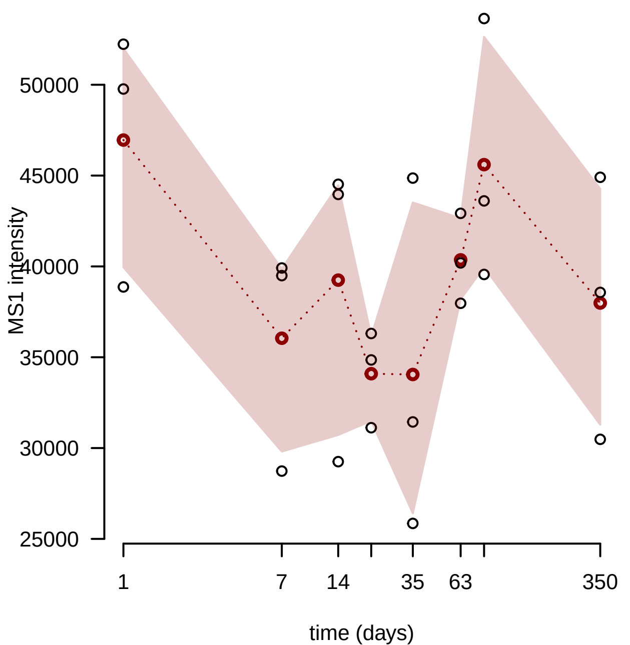

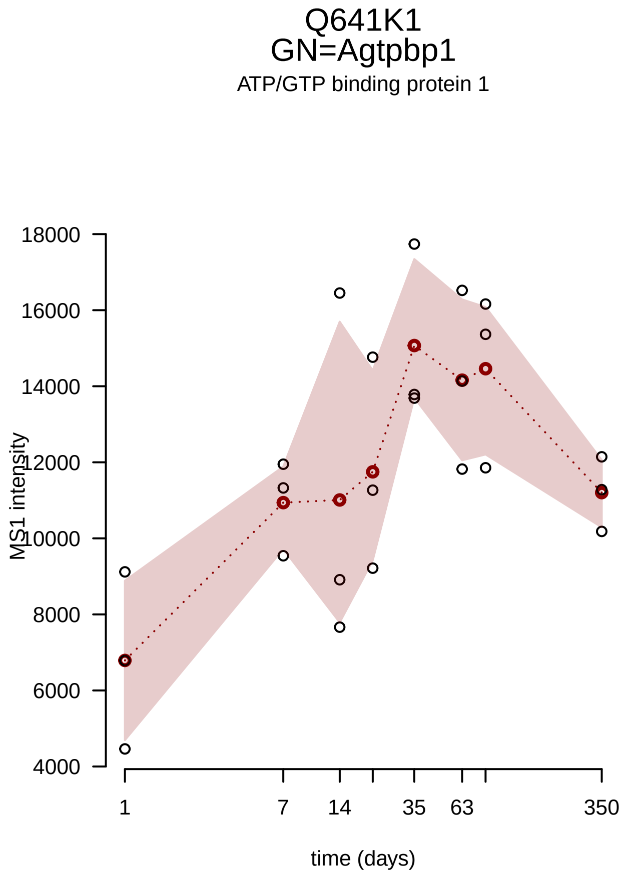
Q641K1
GN=Agtpbp1
ATP/GTP binding protein 1
MS1 intensity
4000
6000
8000
10000
12000
14000
16000
18000
1
7
14
35
63
350
time (days)

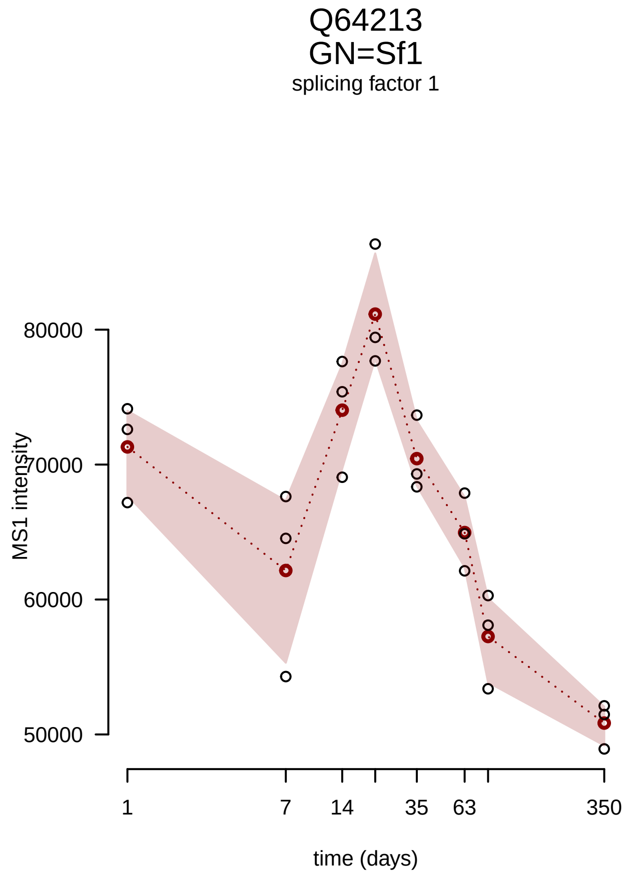
Q64213
GN=Sf1
splicing factor 1
MS1 intensity
50000
60000
70000
80000
1
7
14
35
63
350
time (days)

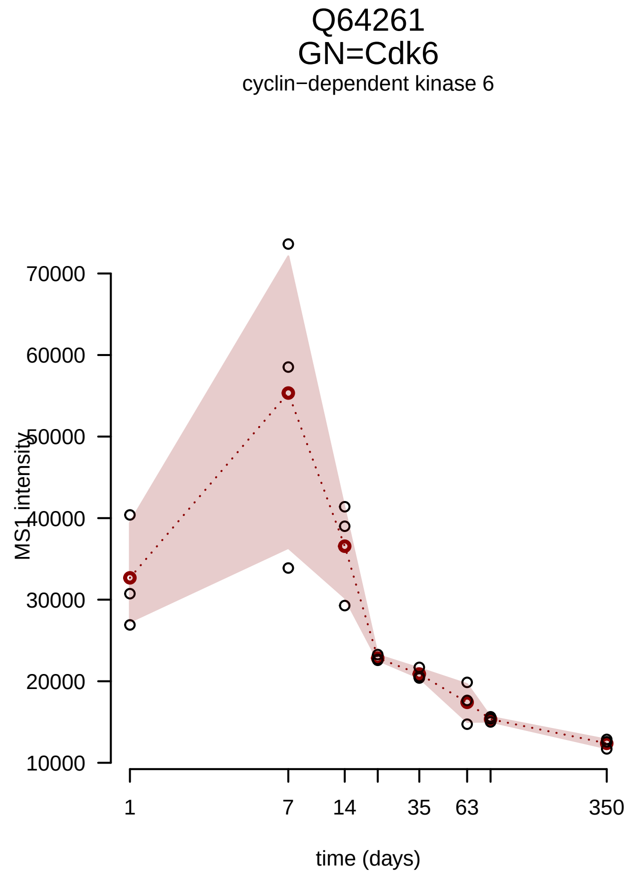
Q64261
GN=Cdk6
cyclin−dependent kinase 6
MS1 intensity
10000
20000
30000
40000
50000
60000
70000
1
7
14
35
63
350
time (days)

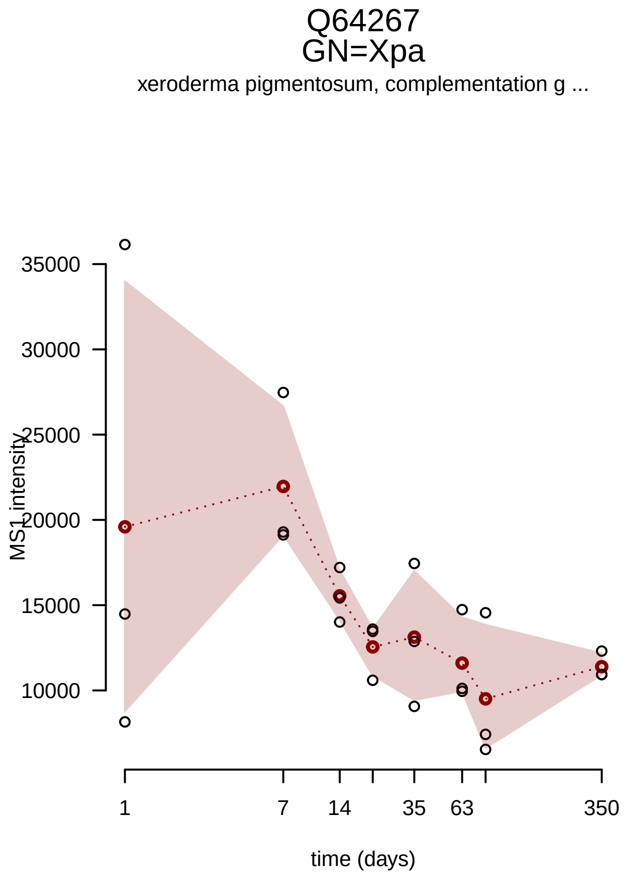
Q64267
GN=Xpa
xeroderma pigmentosum, complementation g ...
MS1 intensity
10000
15000
20000
25000
30000
35000
1
7
14
35
63
350
time (days)

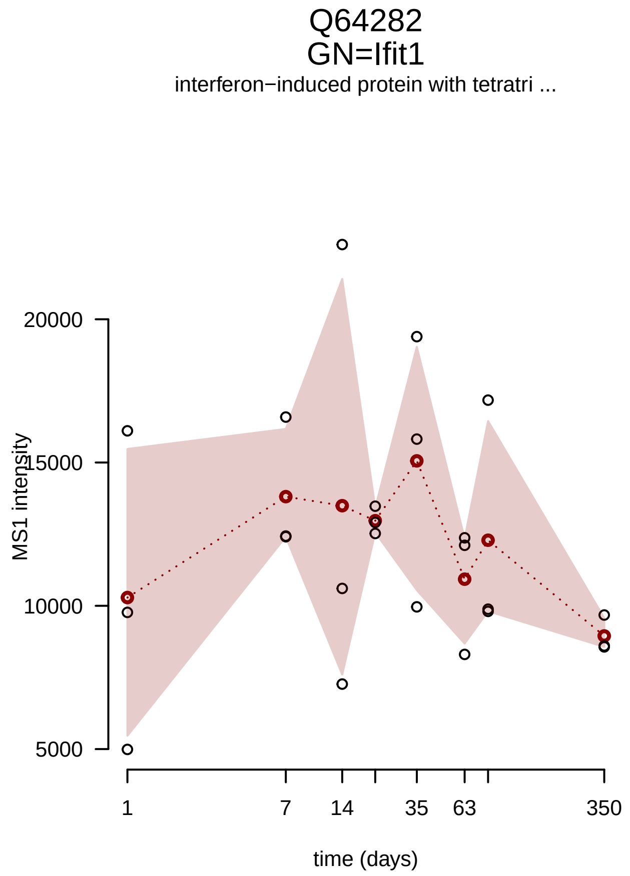
Q64282
GN=Ifit1
interferon−induced protein with tetratri ...
MS1 intensity
5000
10000
15000
20000
1
7
14
35
63
350
time (days)

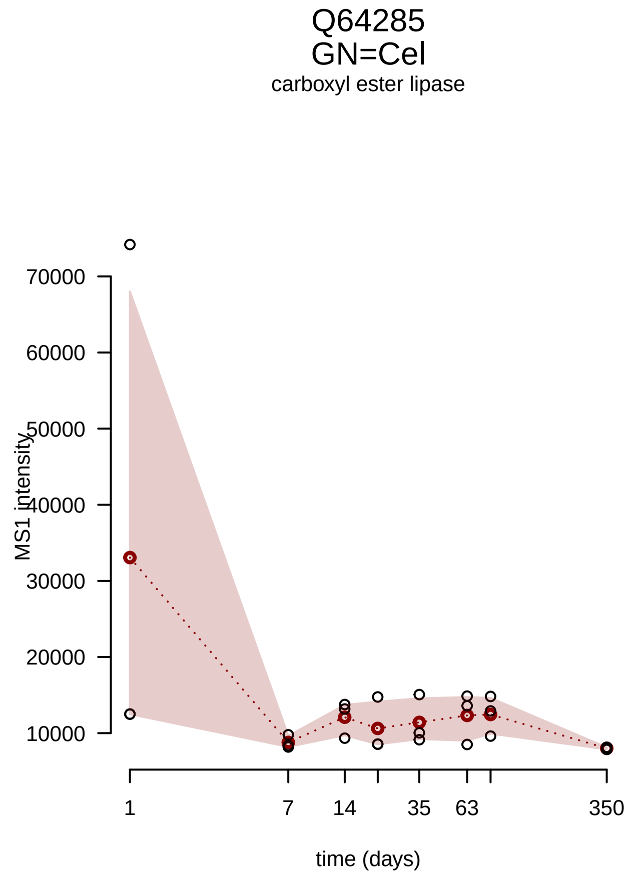
Q64285
GN=Cel
carboxyl ester lipase
MS1 intensity
10000
20000
30000
40000
50000
60000
70000
1
7
14
35
63
350
time (days)

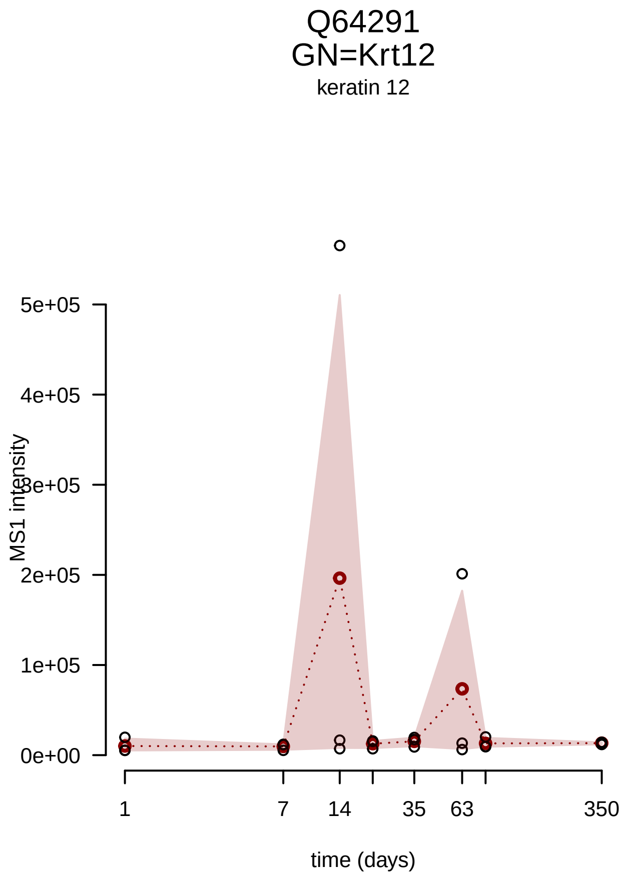
Q64291
GN=Krt12
keratin 12
MS1 intensity
0e+00
1e+05
2e+05
3e+05
4e+05
5e+05
1
7
14
35
63
350
time (days)

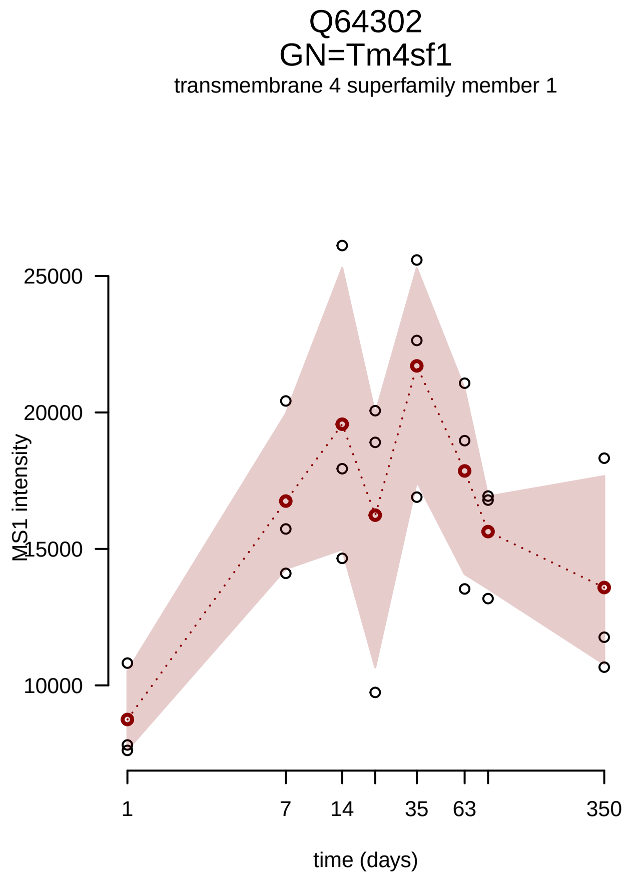
Q64302
GN=Tm4sf1
transmembrane 4 superfamily member 1
MS1 intensity
10000
15000
20000
25000
1
7
14
35
63
350
time (days)

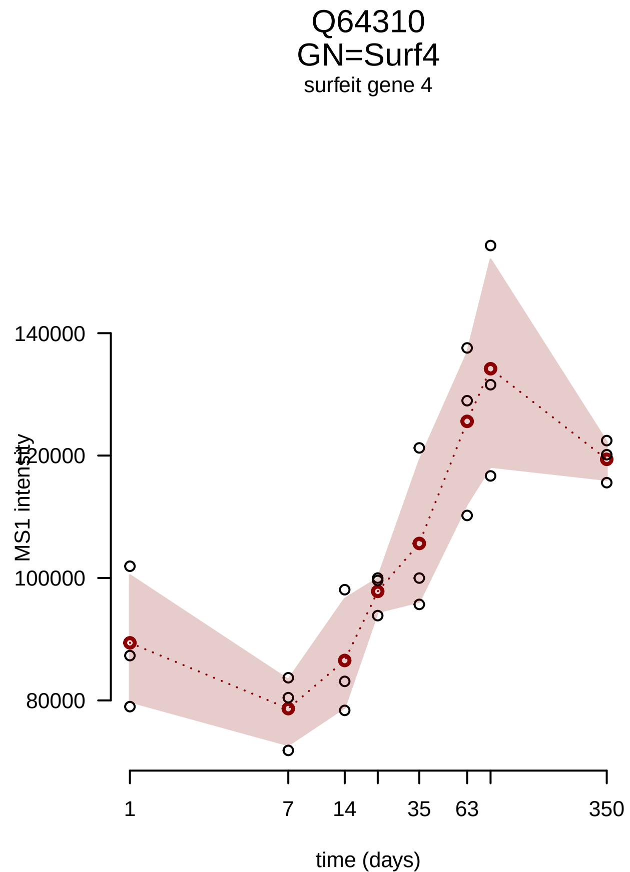
Q64310
GN=Surf4
surfeit gene 4
MS1 intensity
80000
100000
120000
140000
1
7
14
35
63
350
time (days)

Q64311
GN=Ntan1
N−terminal Asn amidase

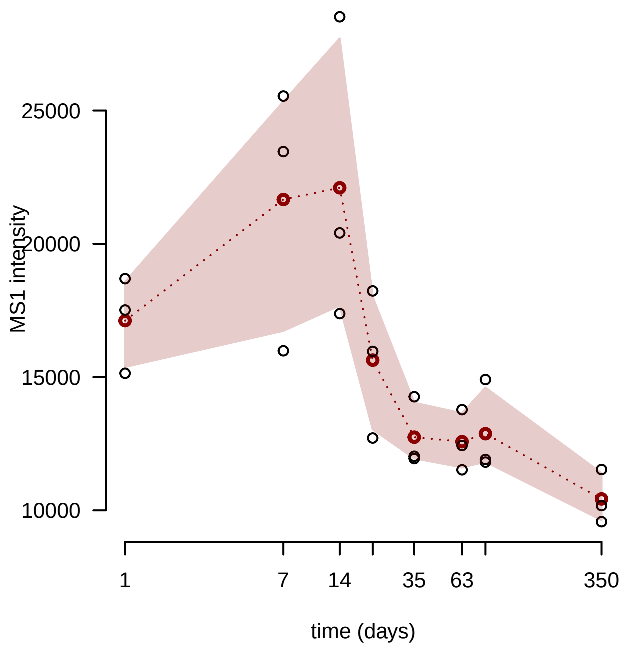

Q64314
GN=Cd34
CD34 antigen

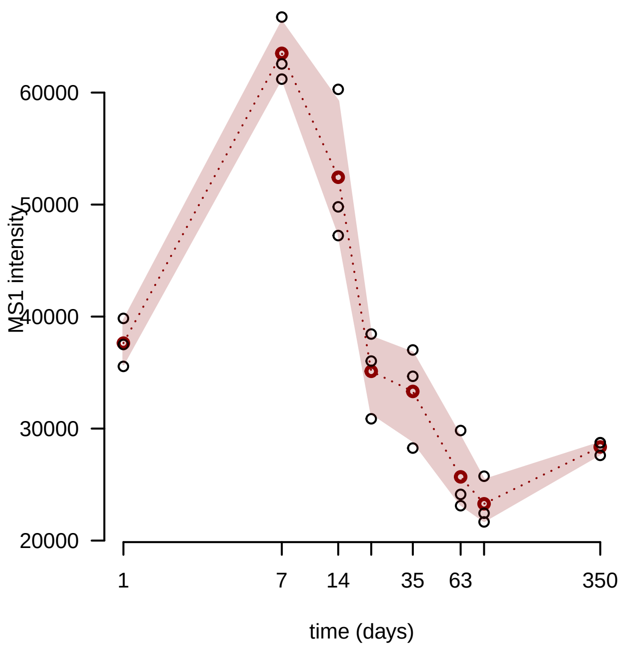

Q64318
GN=Zeb1
zinc finger E−box binding homeobox 1

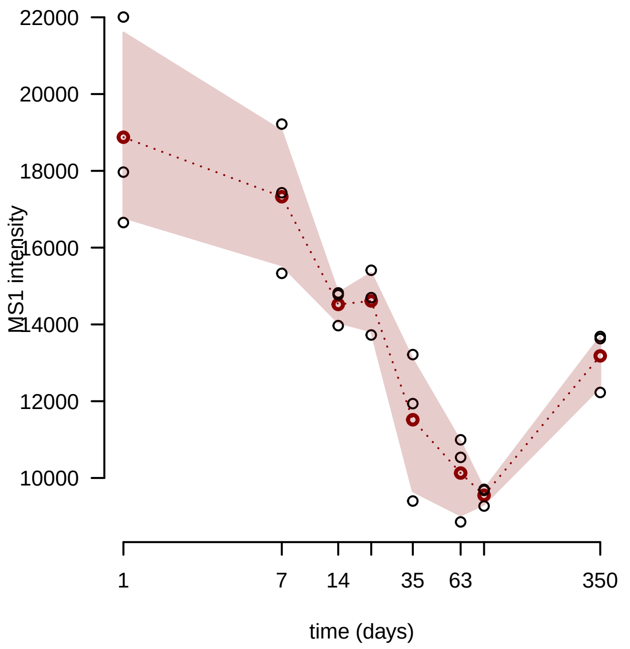

Q64324
GN=Stxbp2
syntaxin binding protein 2

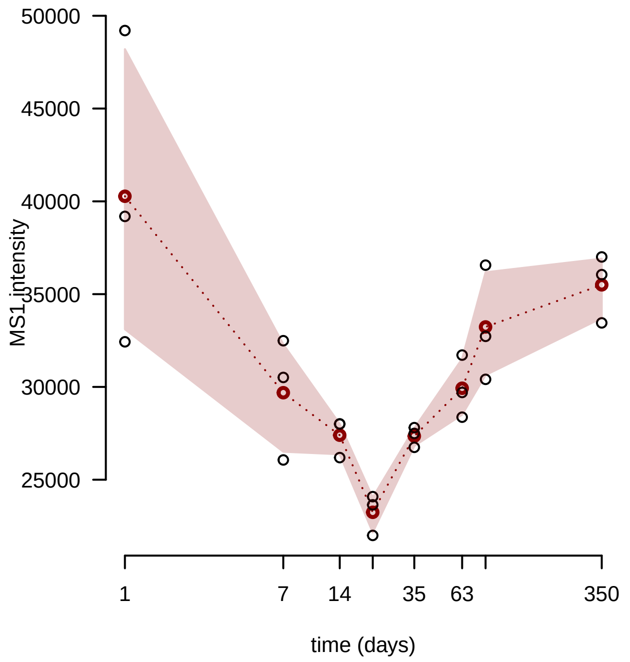

Q64327
GN=Mea1
male enhanced antigen 1

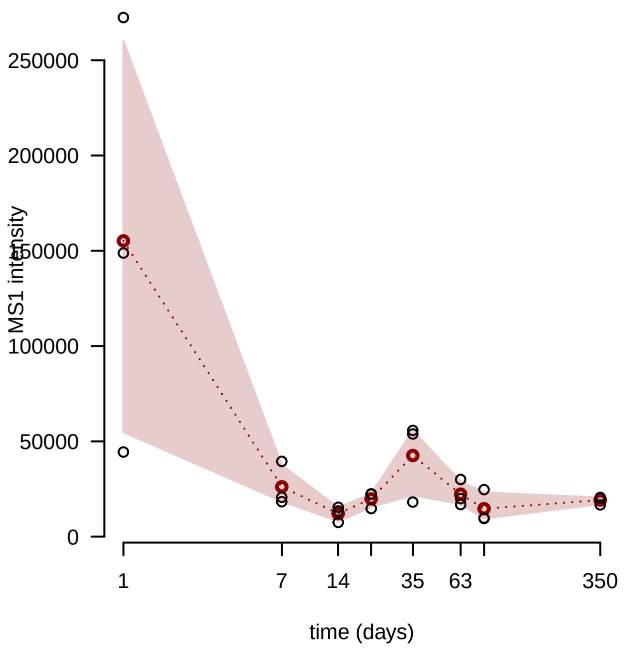

Q64331
GN=Myo6
myosin VI

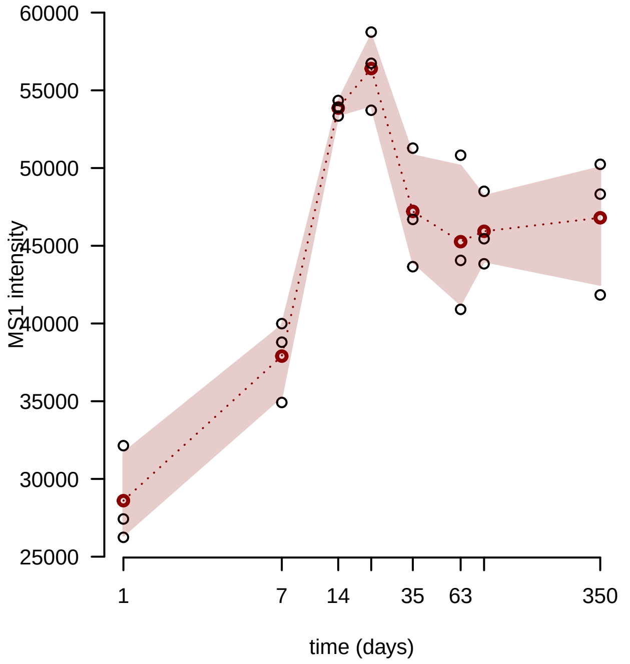

Q64337
GN=Sqstm1
sequestosome 1

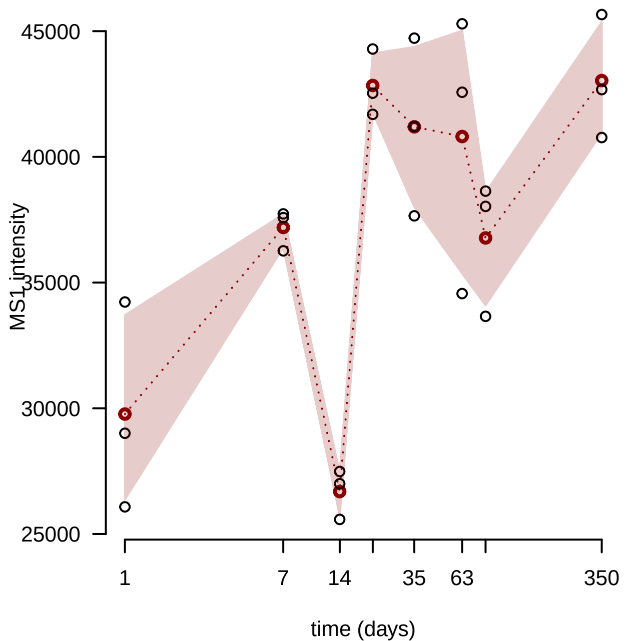

Q64337
GN=Sqstm1
sequestosome 1

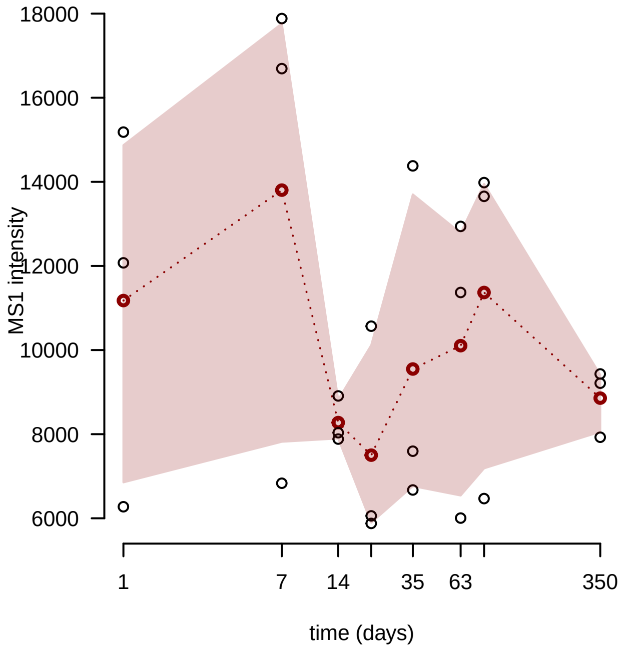

Q64339
GN=Isg15
ISG15 ubiquitin−like modifier

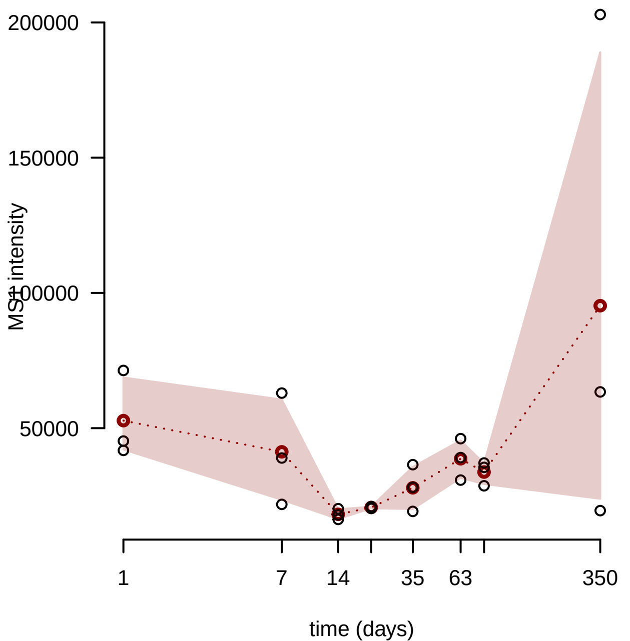

Q64355
GN=Efs
embryonal Fyn−associated substrate

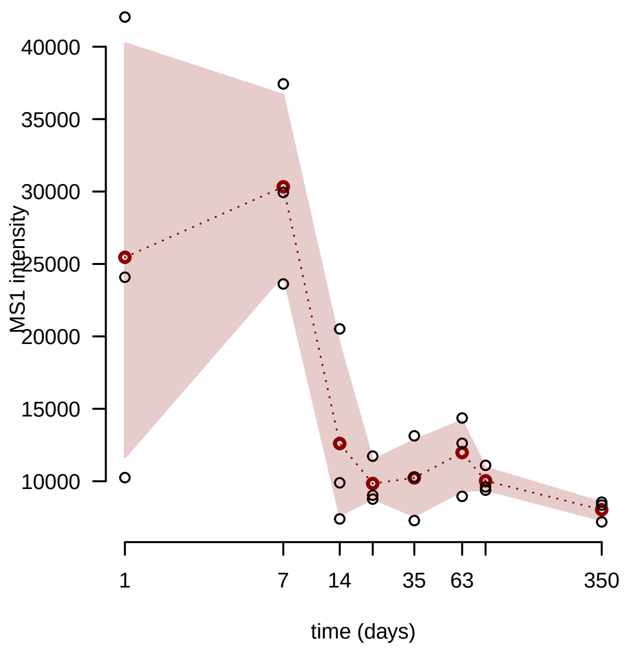

Q64362
GN=Aktip
AKT interacting protein

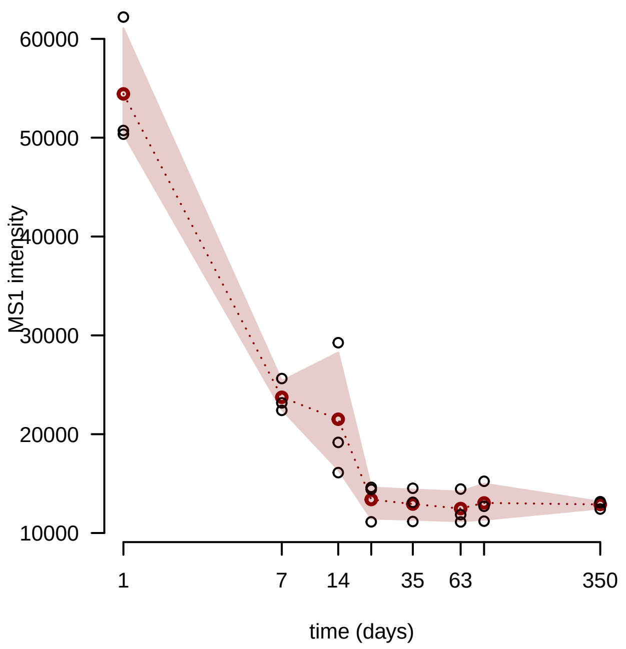

Q64368
GN=Dazl
deleted in azoospermia−like

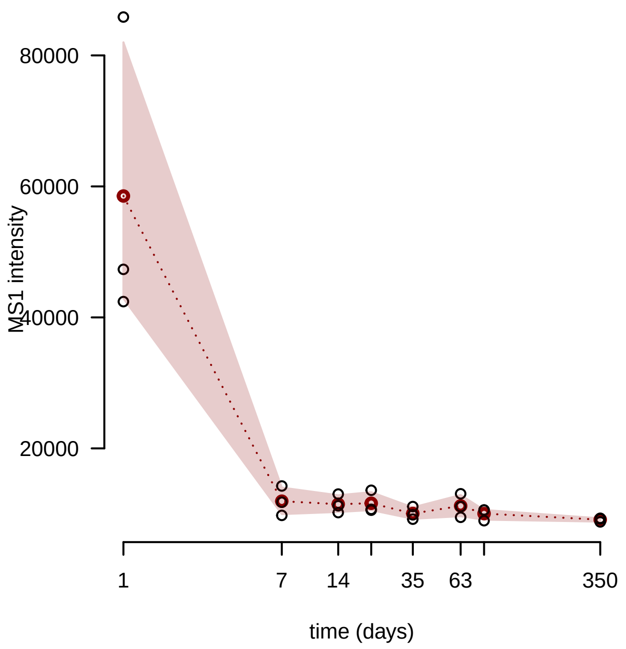

Q64373
GN=Bcl2l1
BCL2−like 1

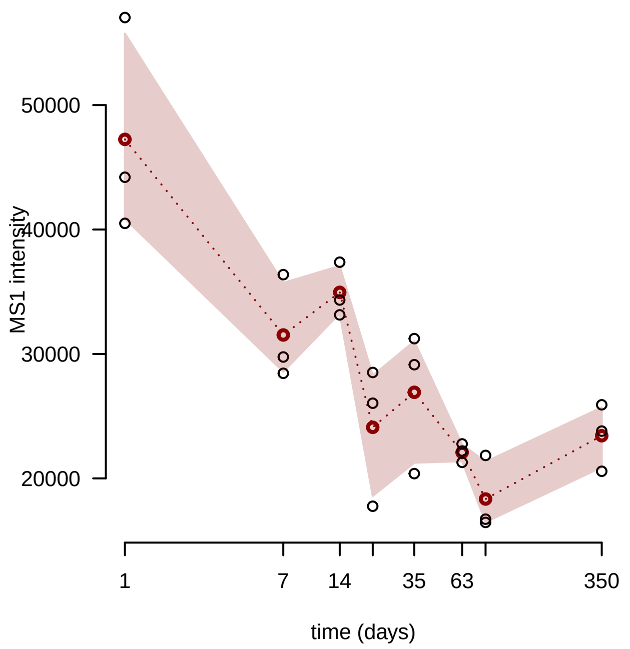

Q64374
GN=Rgn
regucalcin

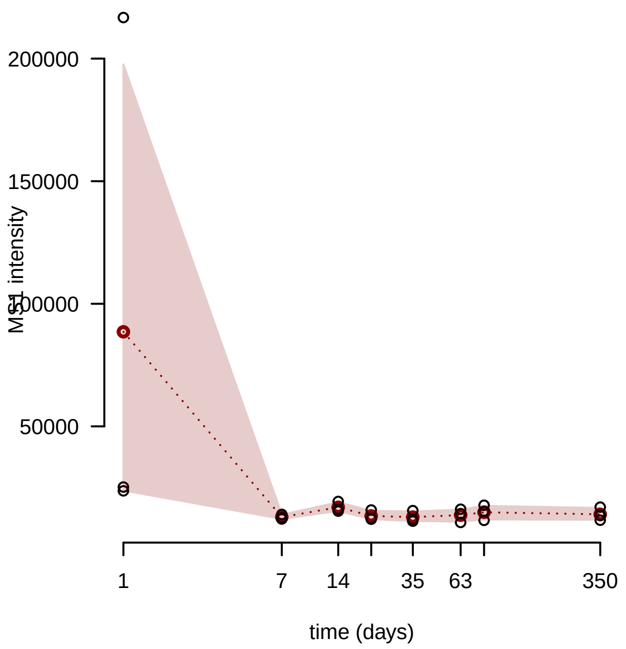

Q64378
GN=Fkbp5
FK506 binding protein 5

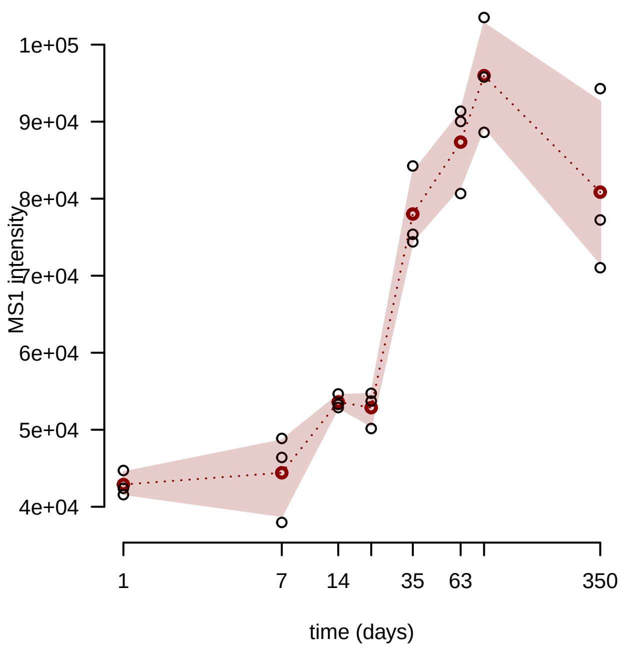

Q64385
GN=Il11ra1
interleukin 11 receptor, alpha chain 1

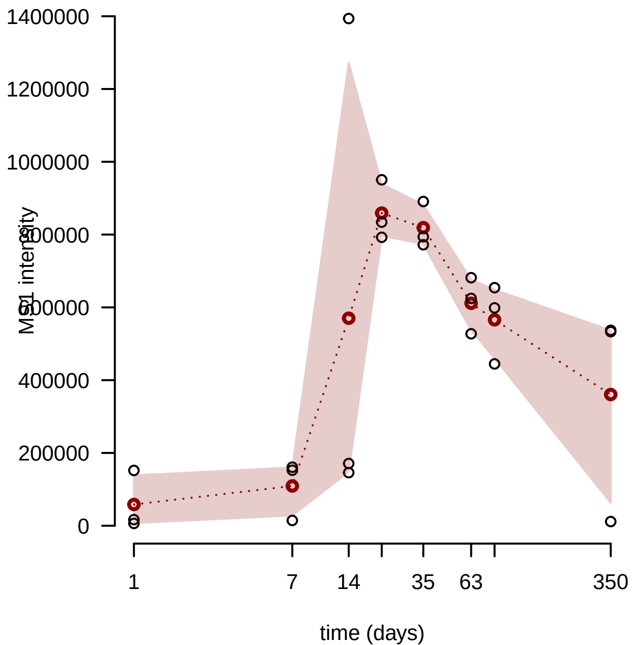

Q64429
GN=Cyp1b1
cytochrome P450, family 1, subfamily b, ...

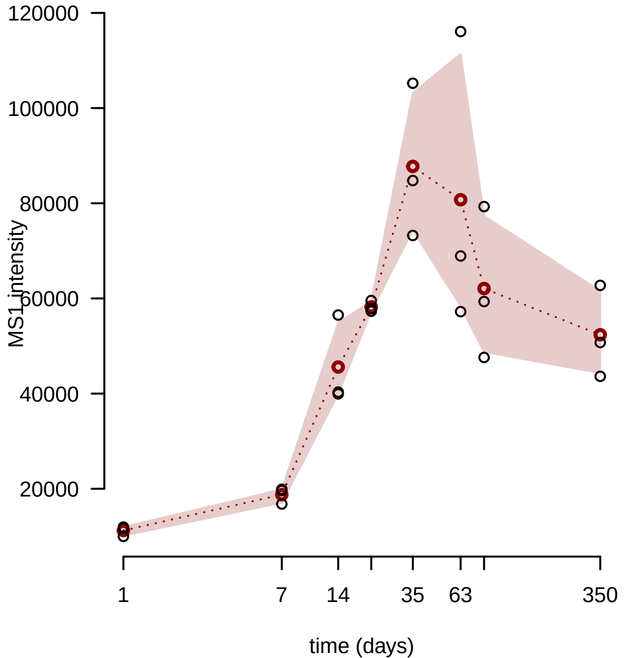

Q64430
GN=Atp7a
ATPase, Cu++ transporting, alpha polypep ...

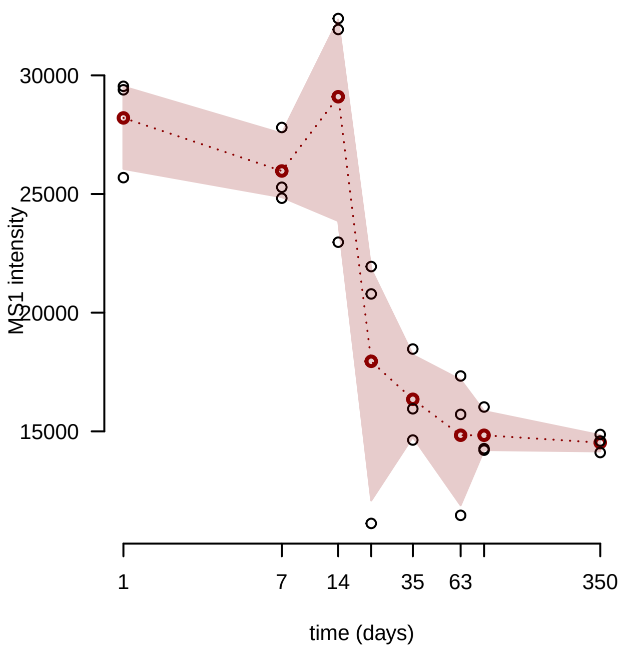

Q64433
GN=Hspe1
heat shock protein 1 (chaperonin 10)

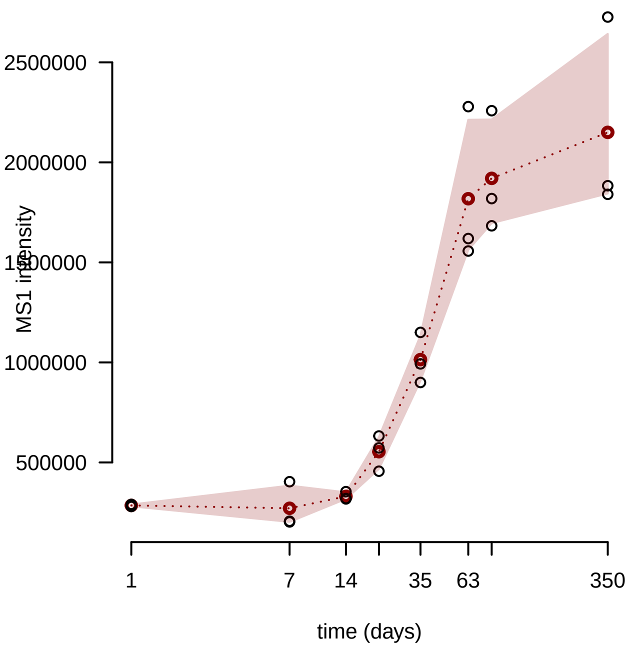

Q64435
GN=Ugt1a6

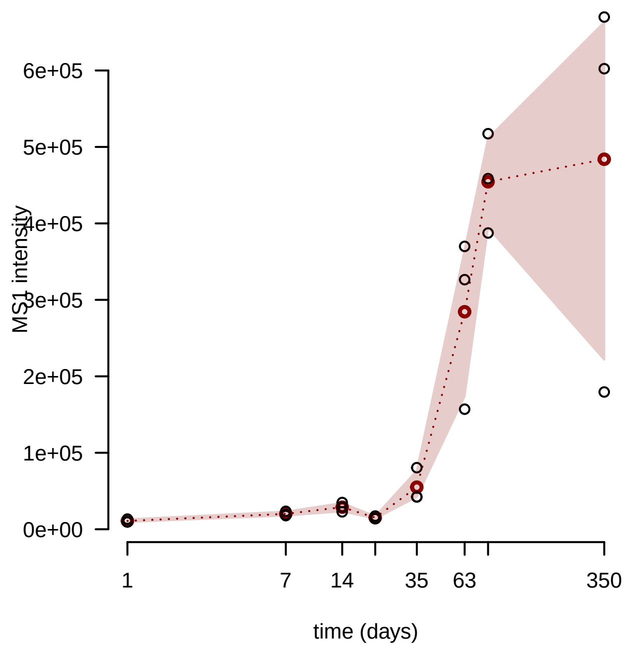

Q64437
GN=Adh7
alcohol dehydrogenase 7 (class IV), mu o ...

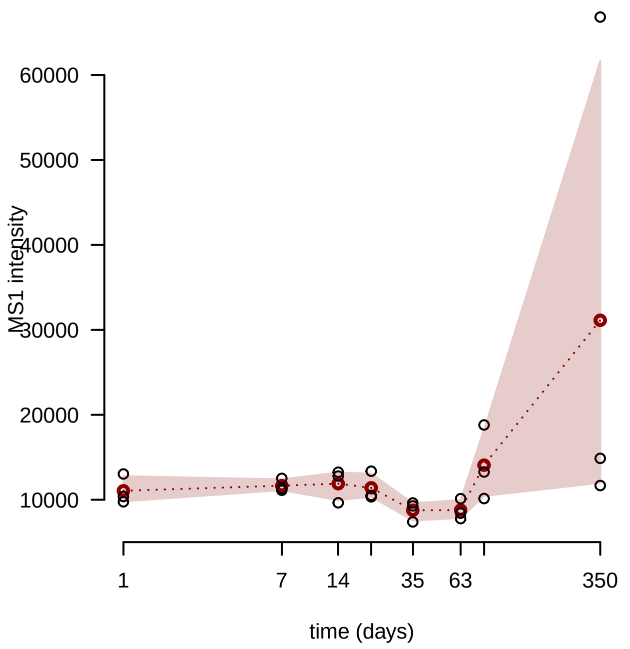

Q64442
GN=Sord
sorbitol dehydrogenase

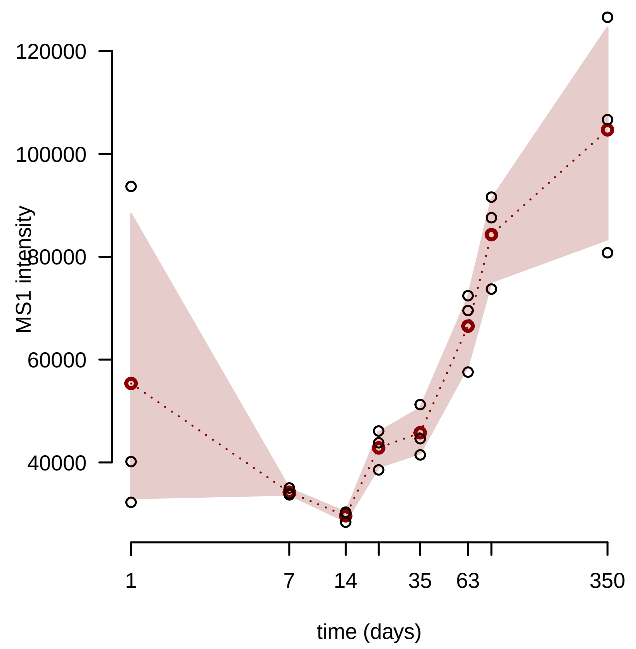

Q64444
GN=Ca4

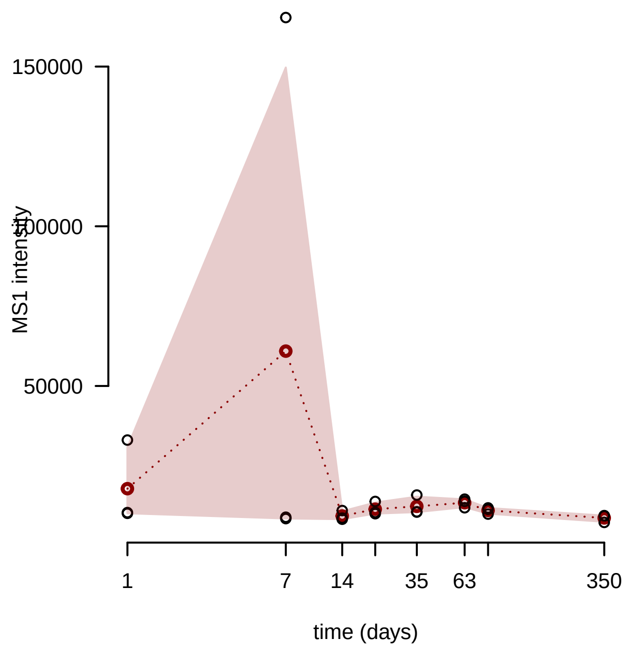

Q64449
GN=Mrc2
mannose receptor, C type 2

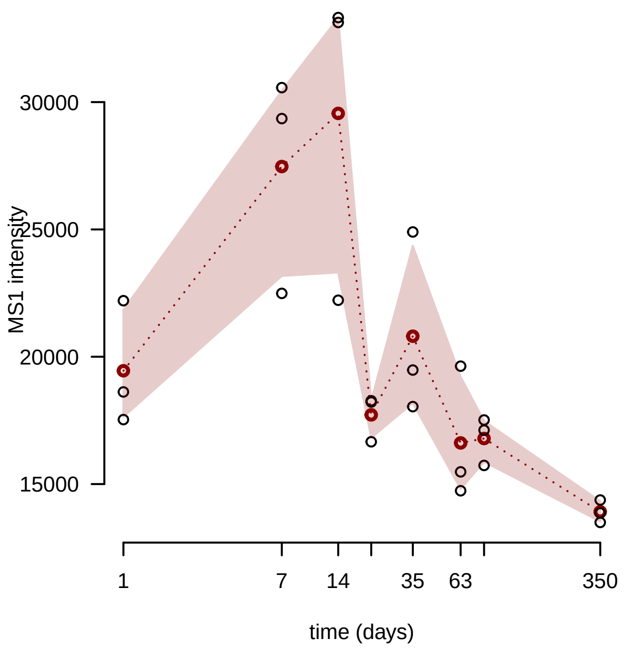

Q64455
GN=Ptprj
protein tyrosine phosphatase, receptor t ...

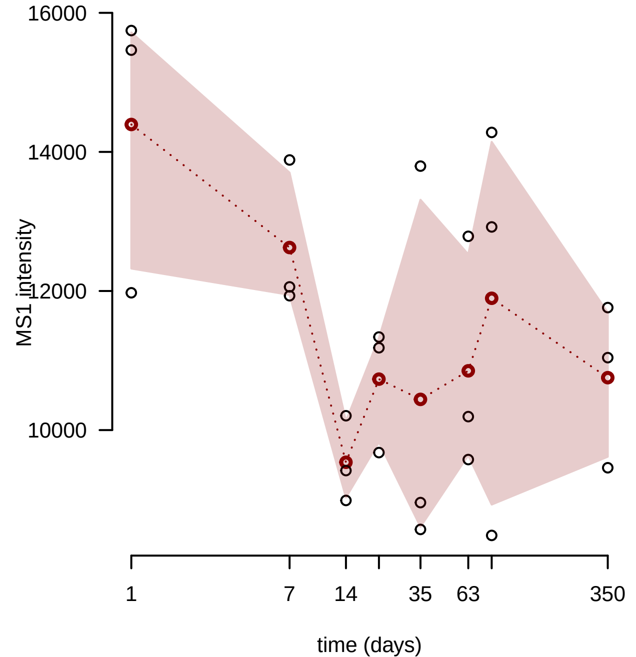

Q64462
GN=Cyp4b1
cytochrome P450, family 4, subfamily b, ...

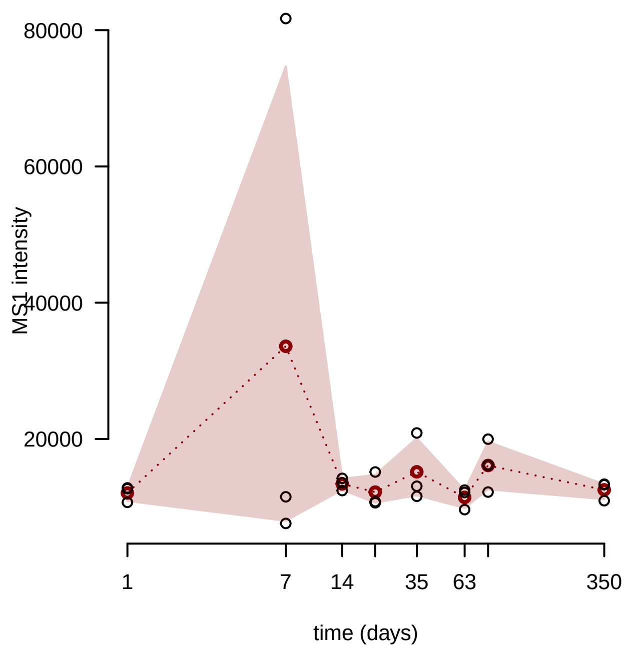

Q64471
GN=Gstt1
glutathione S−transferase, theta 1

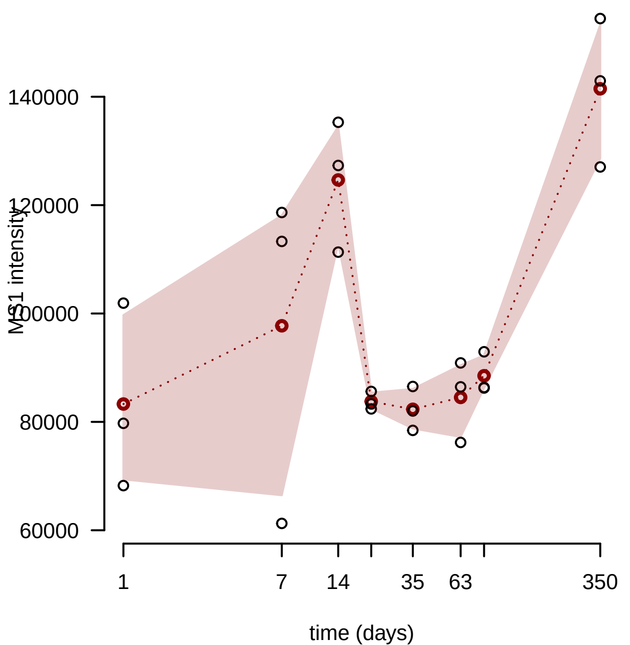

Q64478
GN=Hist1h2bh

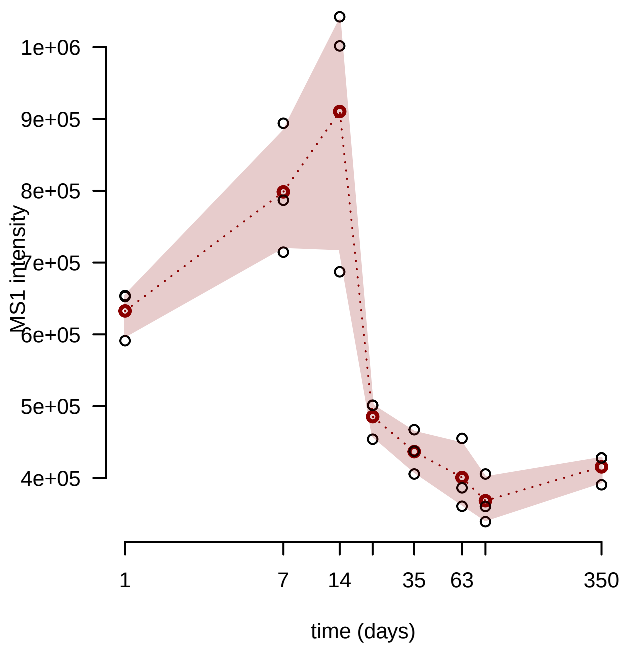

Q64487
GN=Ptprd
protein tyrosine phosphatase, receptor t ...

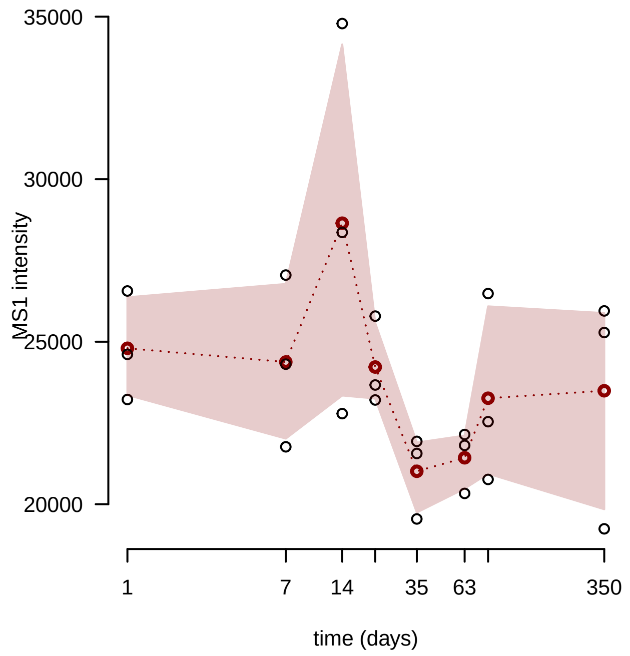

Q64511
GN=Top2b
topoisomerase (DNA) II beta

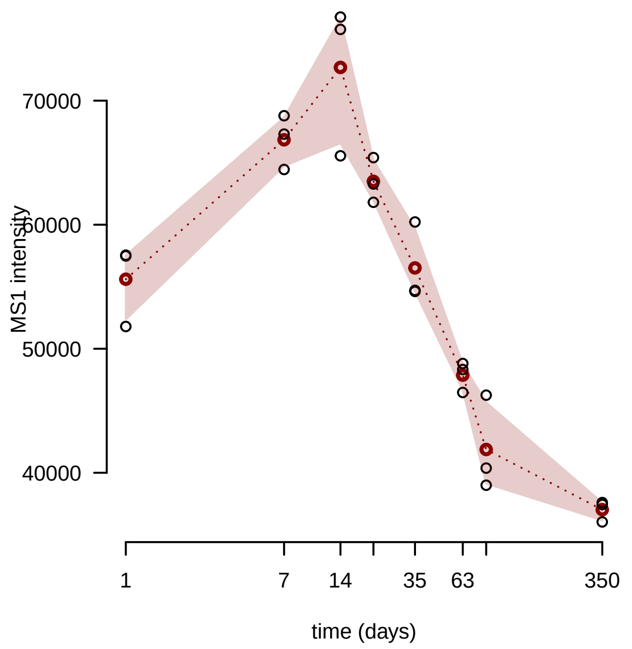

Q64512
GN=Ptpn13
protein tyrosine phosphatase, non−recept ...

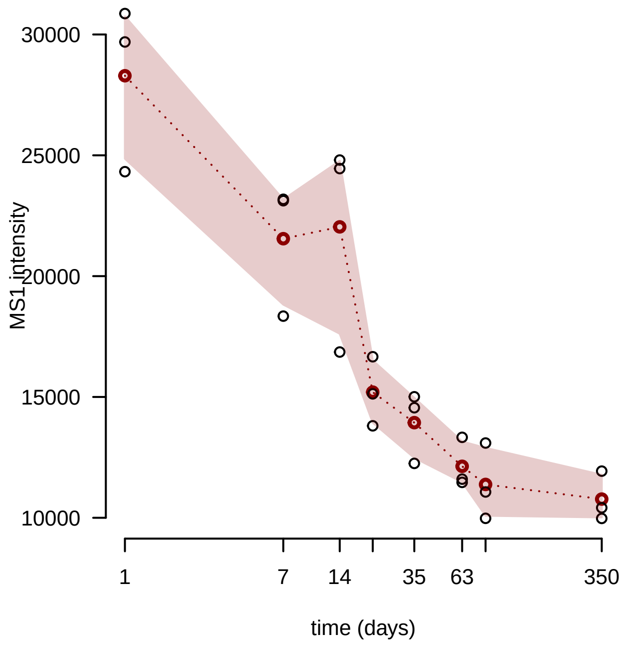

Q64514
GN=Tpp2
tripeptidyl peptidase II

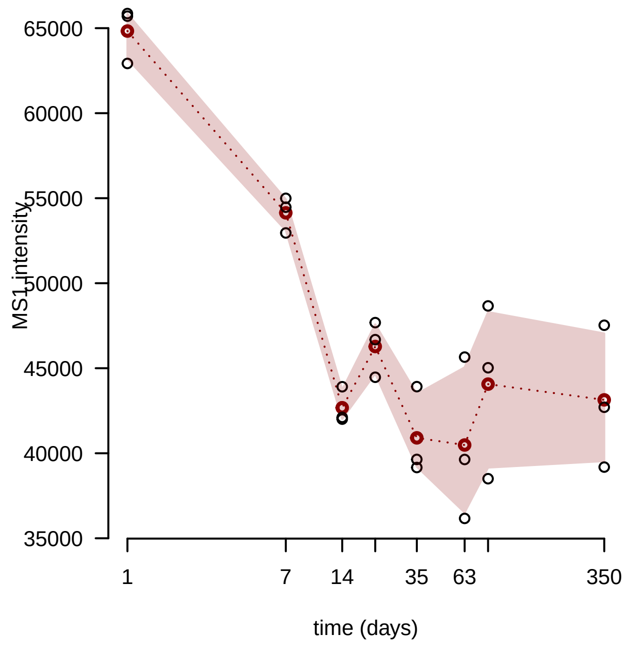

Q64514
GN=Tpp2
tripeptidyl peptidase II

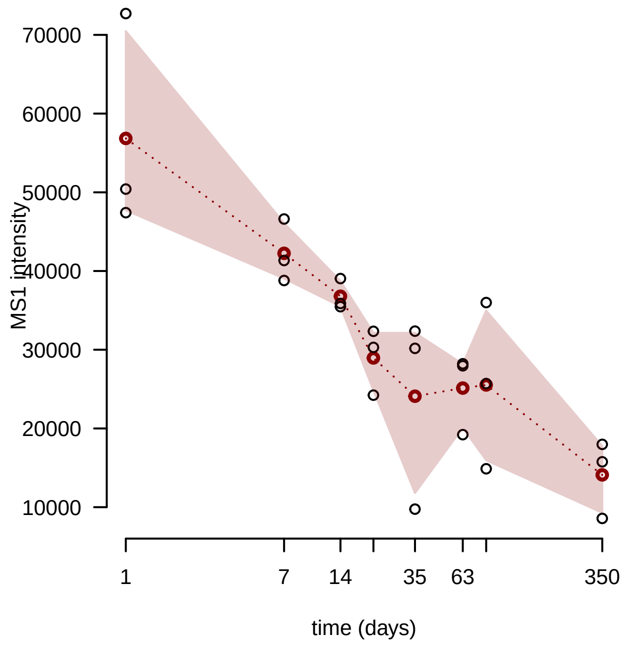

Q64516
GN=Gk
glycerol kinase

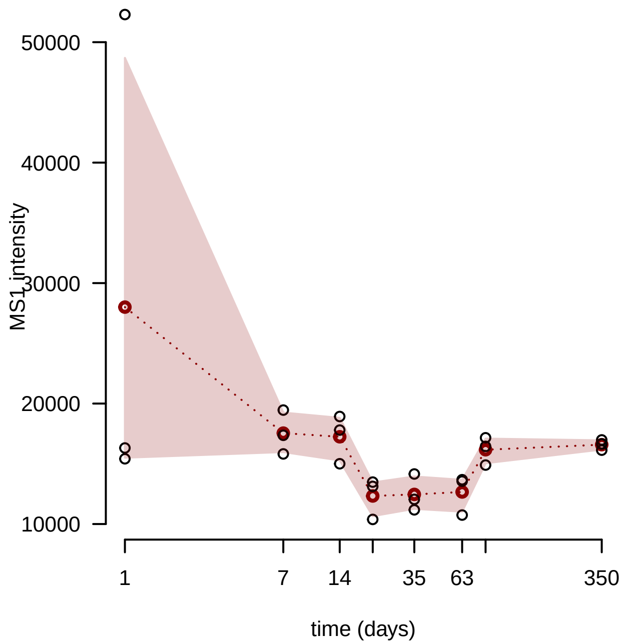

Q64518
GN=Atp2a3
ATPase, Ca++ transporting, ubiquitous

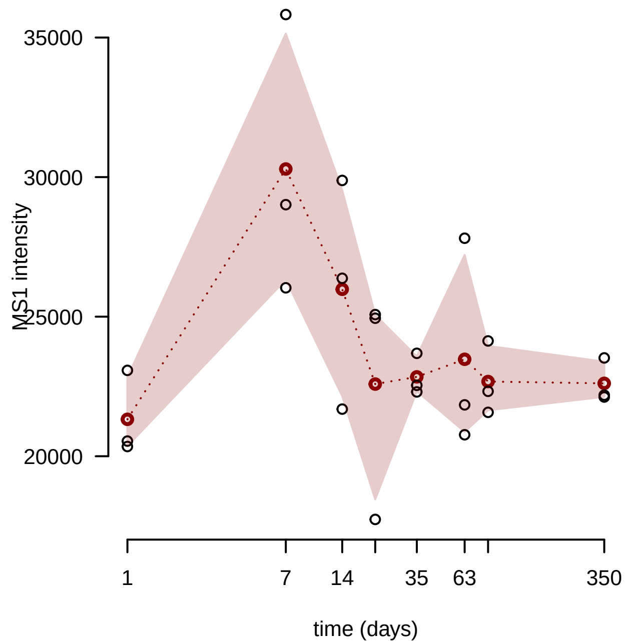

Q64519
GN=Sdc3
syndecan 3

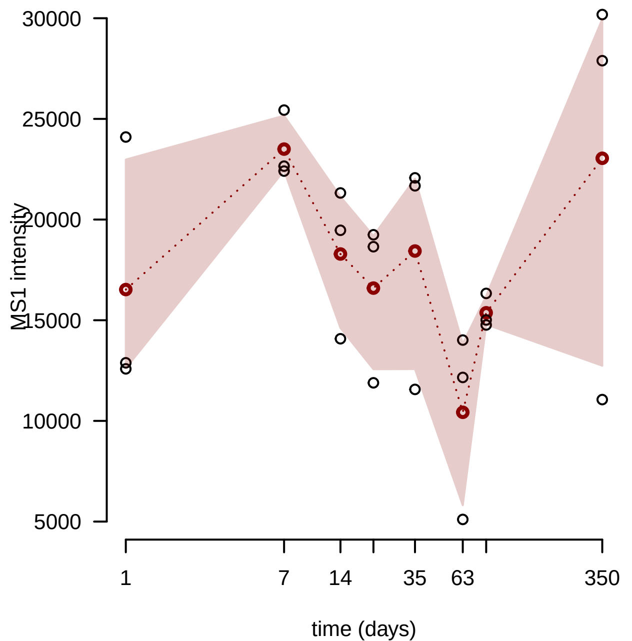

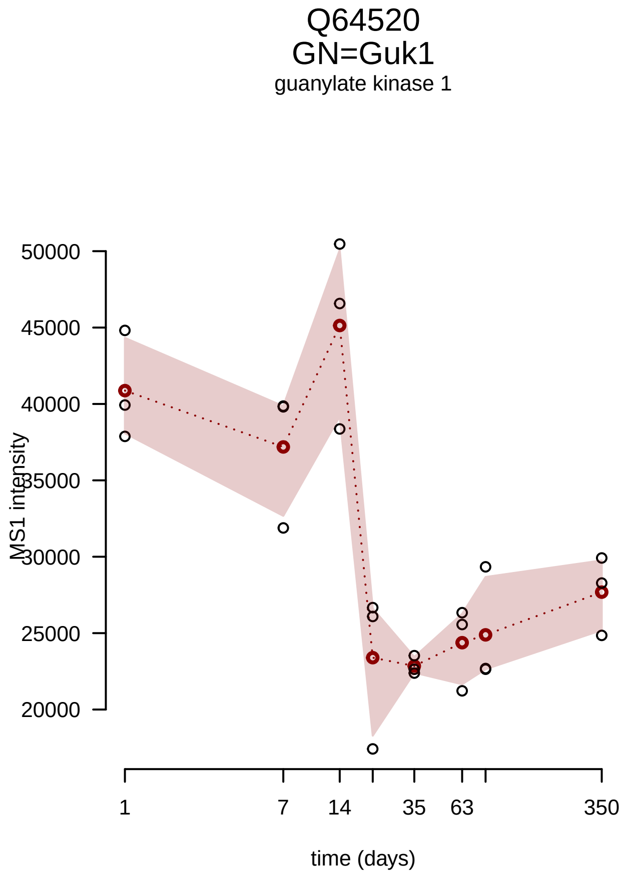
Q64520
GN=Guk1
guanylate kinase 1
MS1 intensity
50000
45000
40000
35000
30000
25000
20000
1
7
14
35
63
350
time (days)

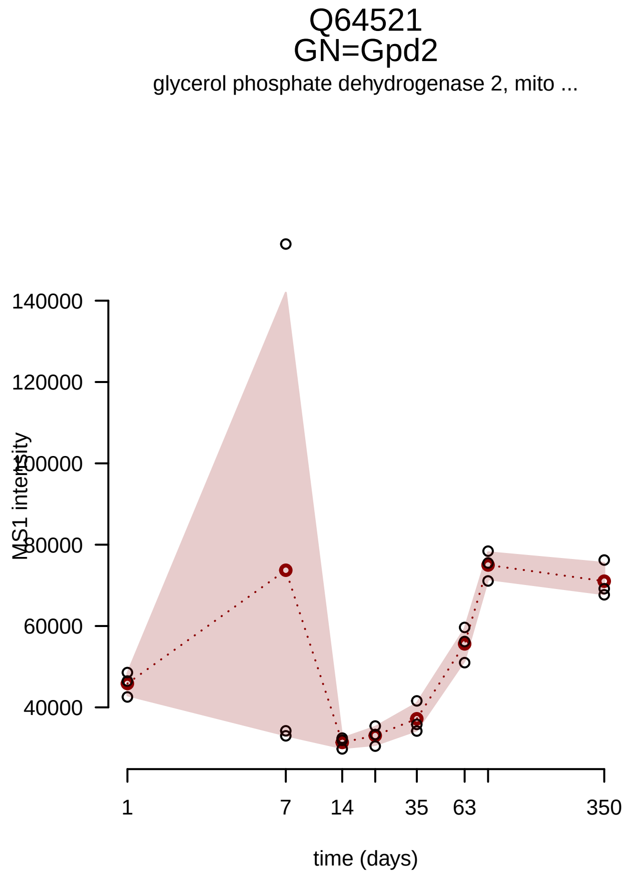
Q64521
GN=Gpd2
glycerol phosphate dehydrogenase 2, mito ...
MS1 intensity
140000
120000
100000
80000
60000
40000
1
7
14
35
63
350
time (days)

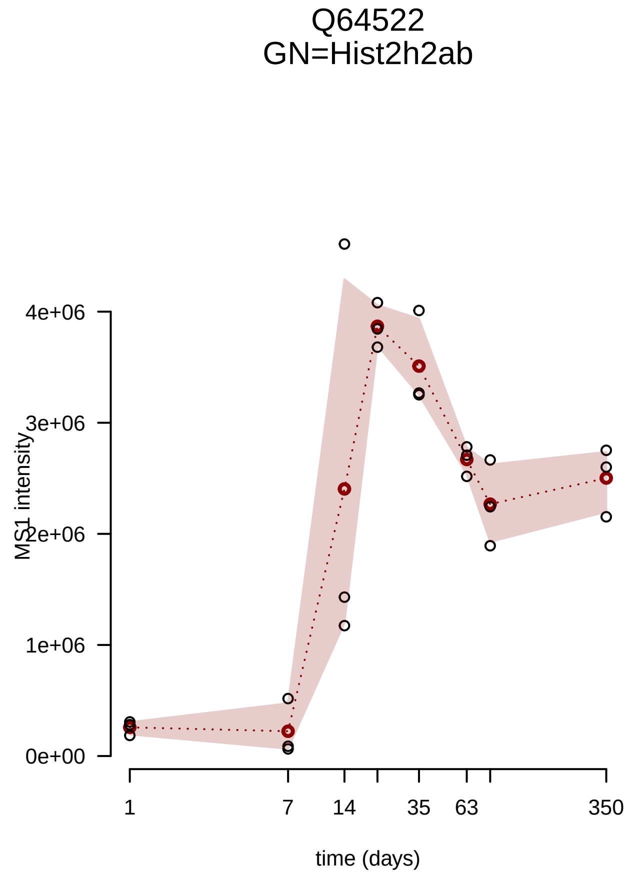
Q64522
GN=Hist2h2ab
MS1 intensity
4e+06
3e+06
2e+06
1e+06
0e+00
1
7
14
35
63
350
time (days)

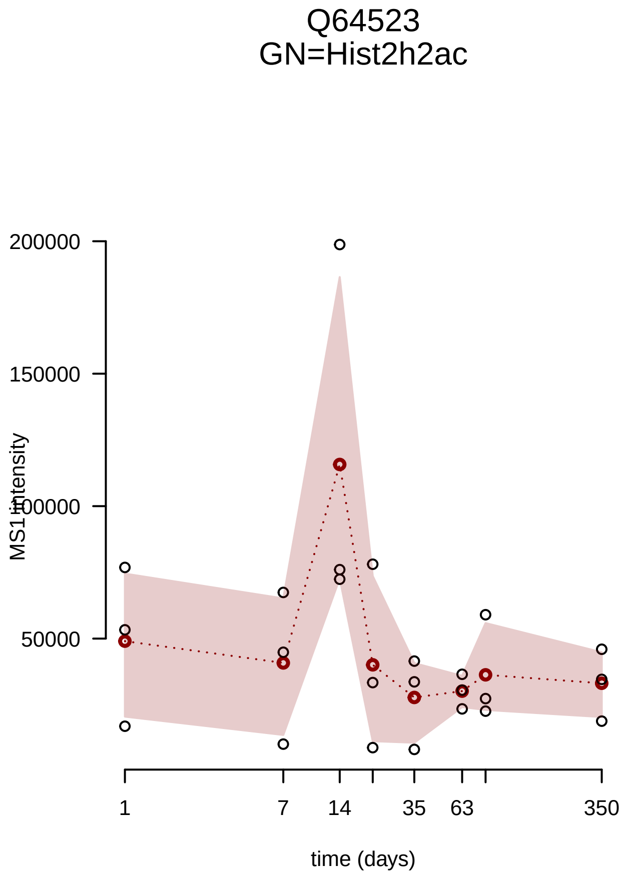
Q64523
GN=Hist2h2ac
MS1 intensity
200000
150000
100000
50000
1
7
14
35
63
350
time (days)

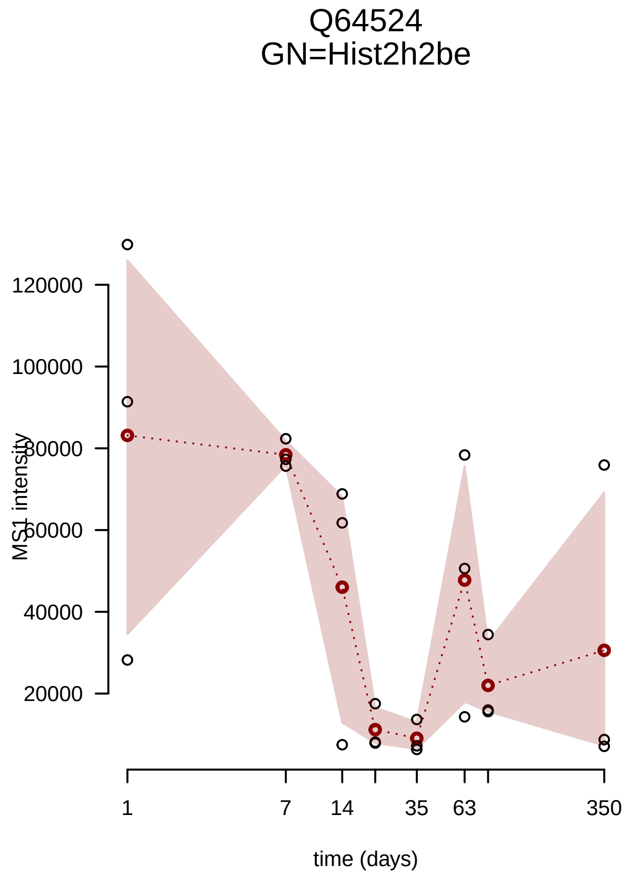
Q64524
GN=Hist2h2be
MS1 intensity
120000
100000
80000
60000
40000
20000
1
7
14
35
63
350
time (days)

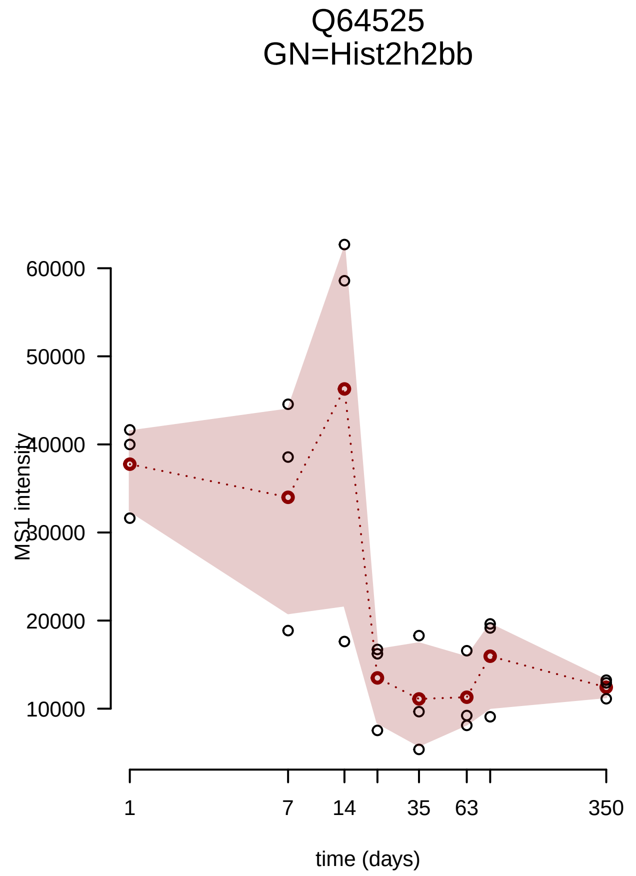
Q64525
GN=Hist2h2bb
MS1 intensity
60000
50000
40000
30000
20000
10000
1
7
14
35
63
350
time (days)

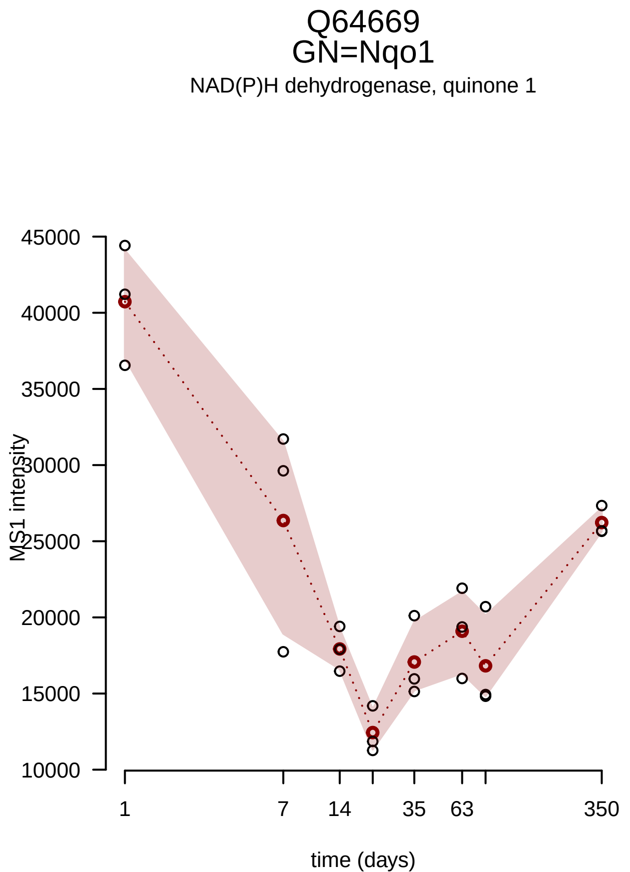
Q64669
GN=Nqo1
NAD(P)H dehydrogenase, quinone 1
MS1 intensity
45000
40000
35000
30000
25000
20000
15000
10000
1
7
14
35
63
350
time (days)

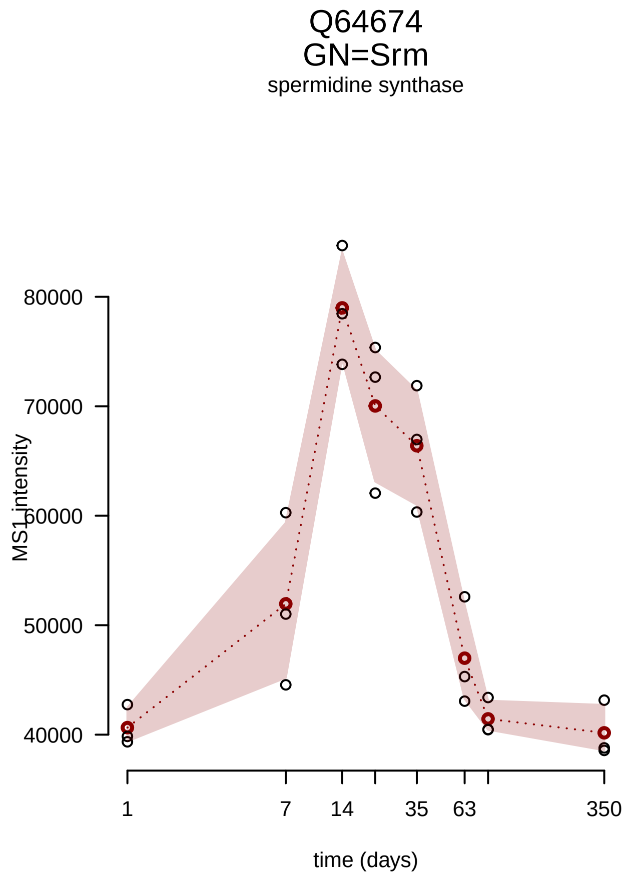
Q64674
GN=Srm
spermidine synthase
MS1 intensity
80000
70000
60000
50000
40000
1
7
14
35
63
350
time (days)

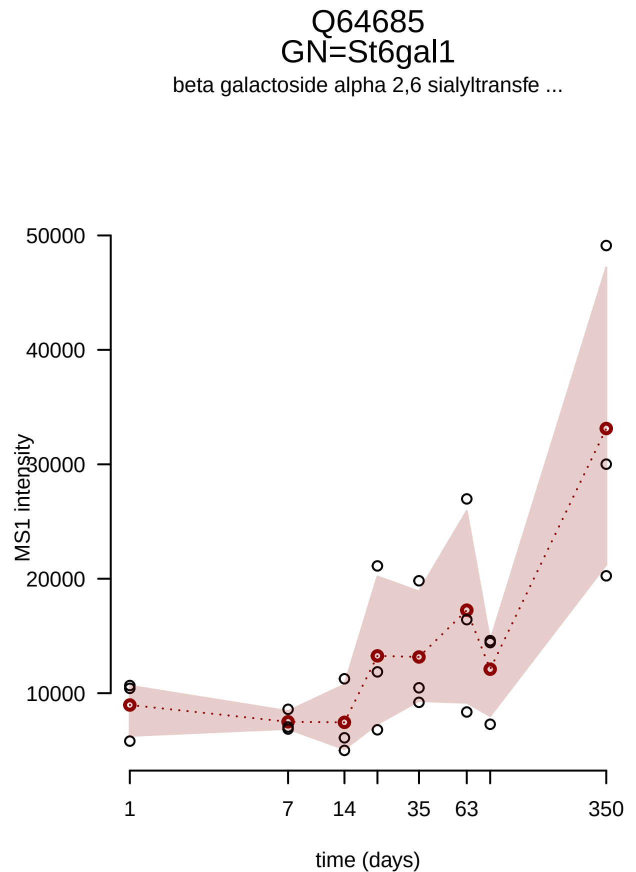
Q64685
GN=St6gal1
beta galactoside alpha 2,6 sialyltransfe ...
MS1 intensity
50000
40000
30000
20000
10000
1
7
14
35
63
350
time (days)

Q64687
GN=St8sia1
ST8 alpha−N−acetyl−neuraminide alpha−2,8 ...

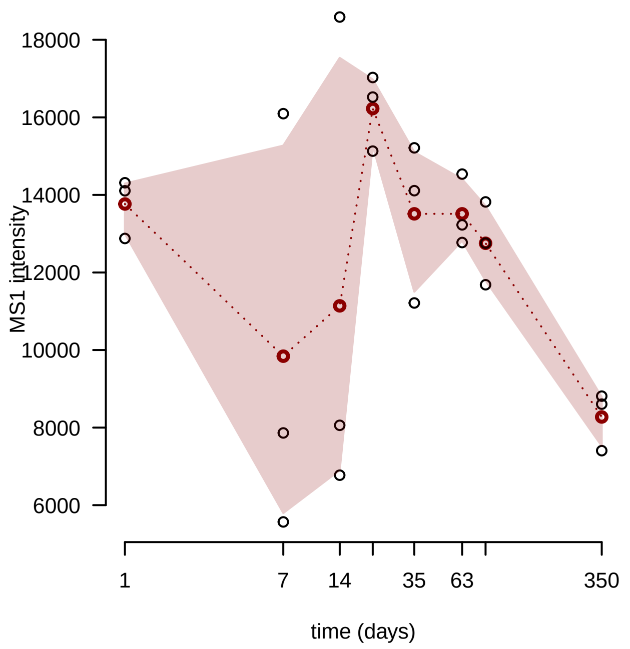

Q64695
GN=Procr
protein C receptor, endothelial

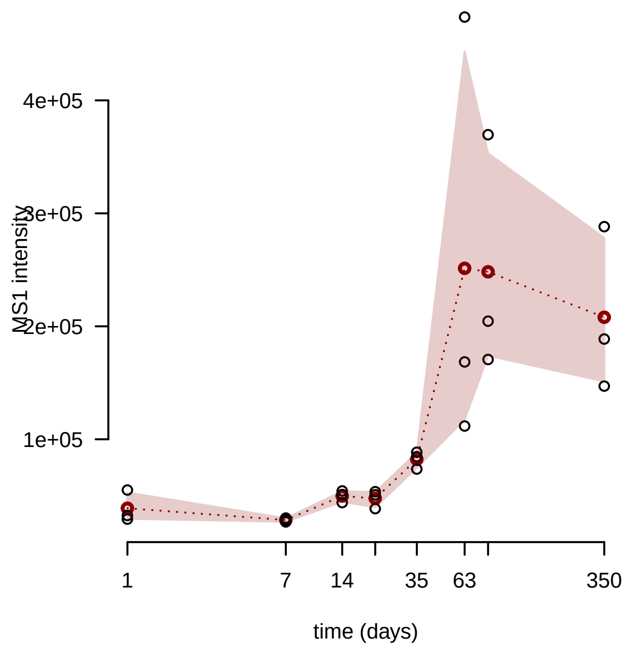

Q64700
GN=Rbl2
RB transcriptional corepressor like 2

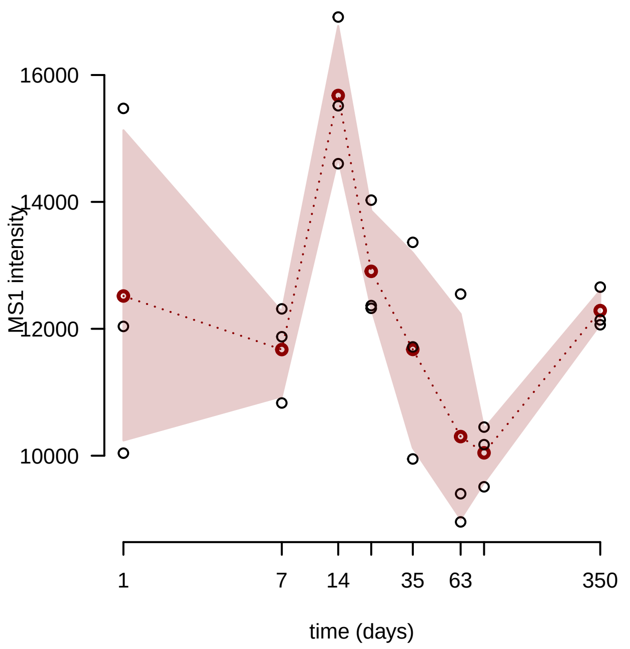

Q64704
GN=Stx3
syntaxin 3

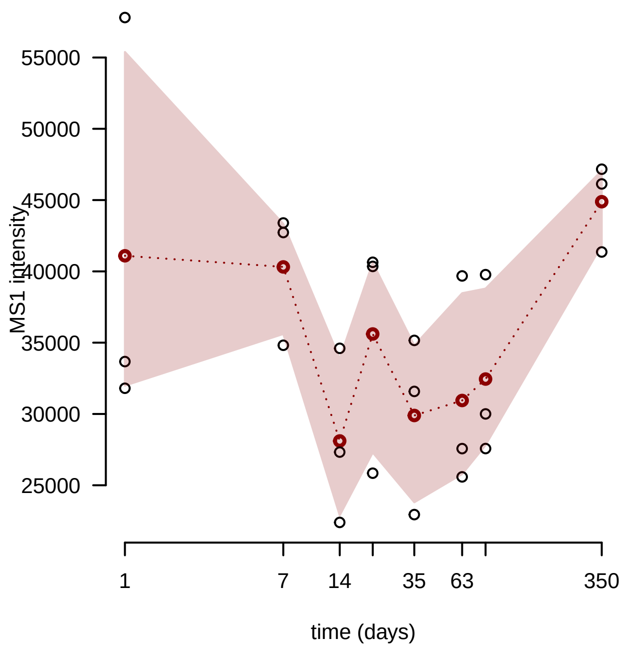

Q64705
GN=Usf2
upstream transcription factor 2

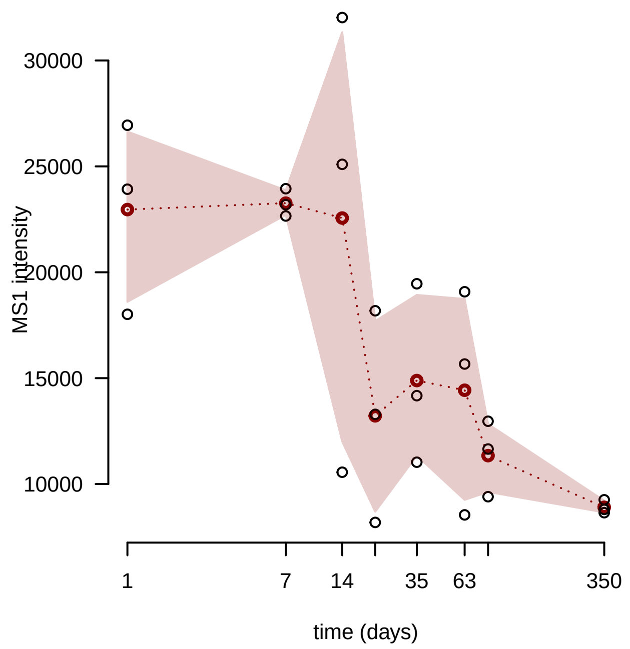

Q64707
GN=Zrsr1
zinc finger (CCCH type), RNA binding mot ...

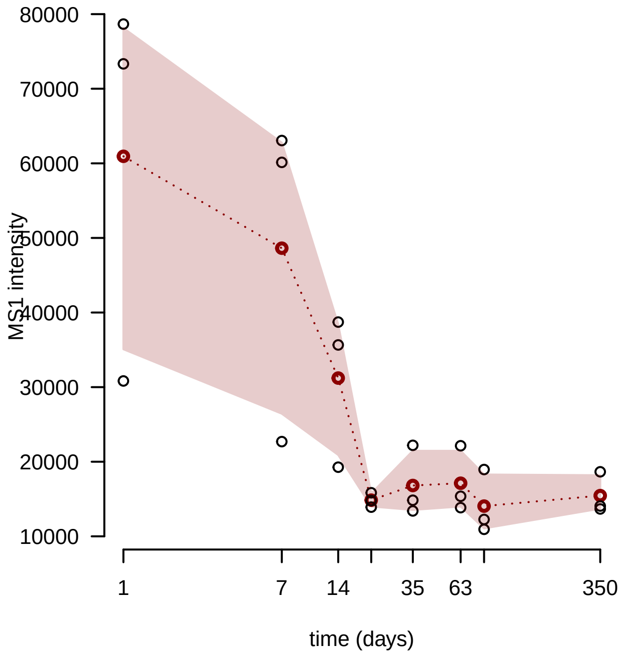

Q64726
GN=Azgp1
alpha−2−glycoprotein 1, zinc

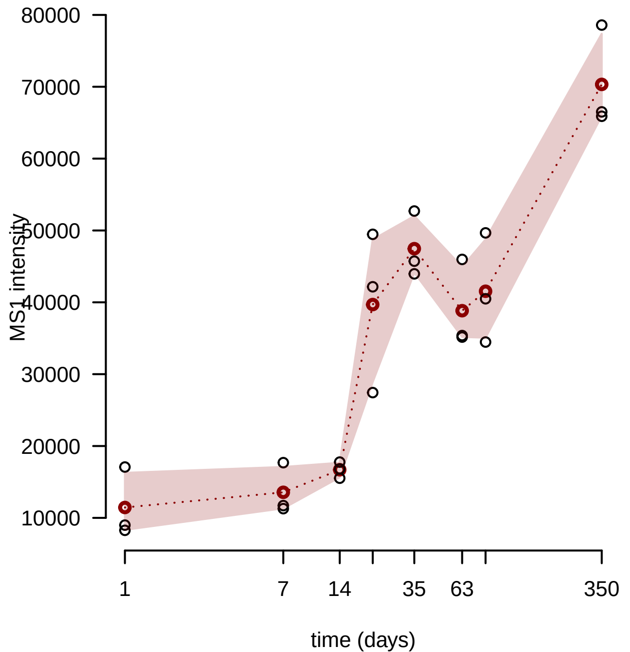

Q64727
GN=Vcl
vinculin

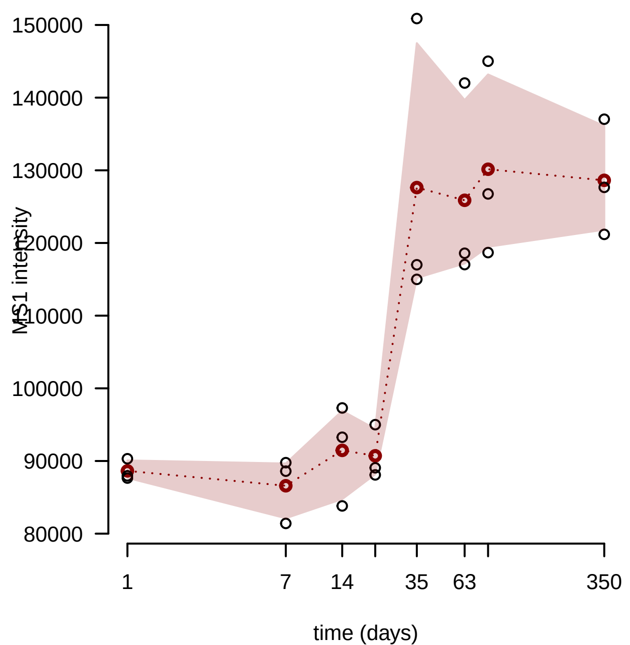

Q64729
GN=Tgfbr1
transforming growth factor, beta recepto ...

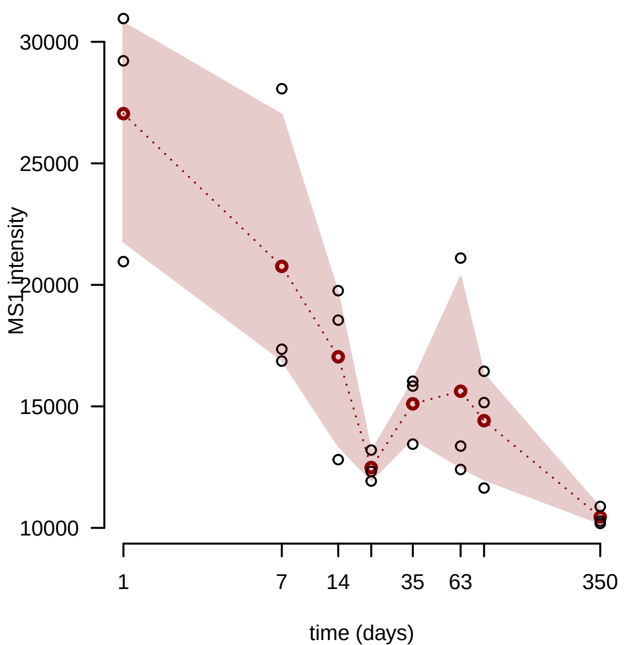

Q64735
GN=Cr1l
complement component (3b/4b) receptor 1− ...

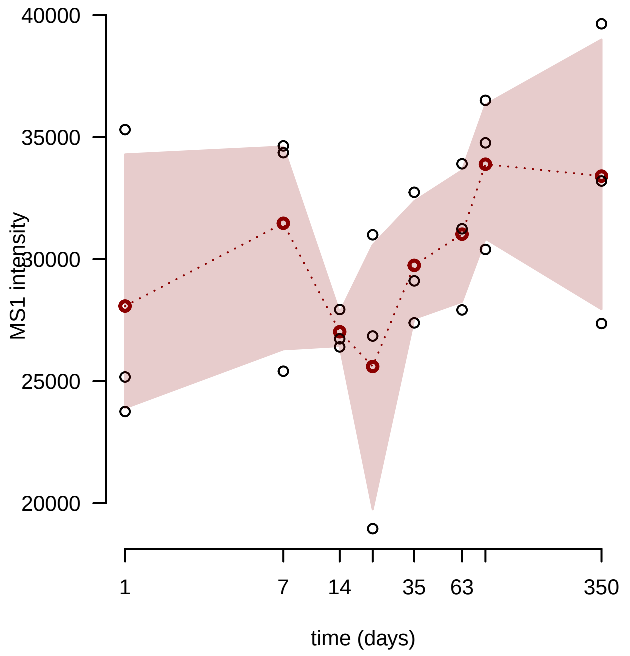

Q64737
GN=Gart
phosphoribosylglycinamide formyltransfer ...

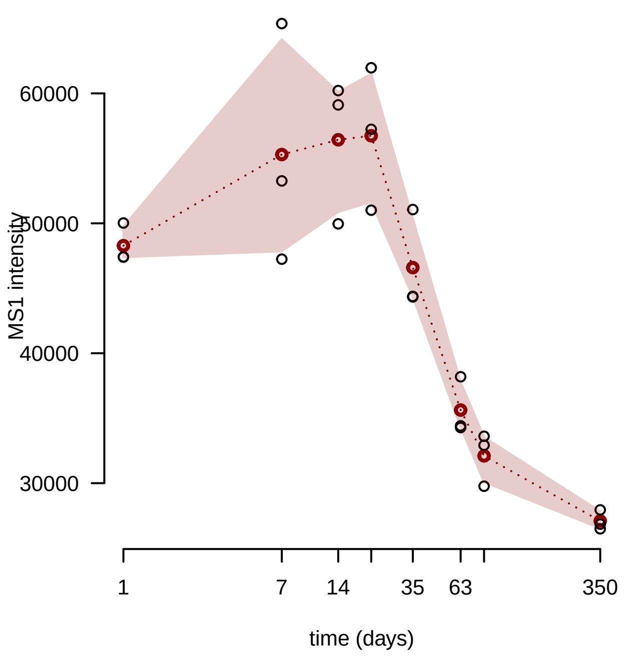

Q64FW2
GN=Retsat
retinol saturase (all trans retinol 13,1 ...

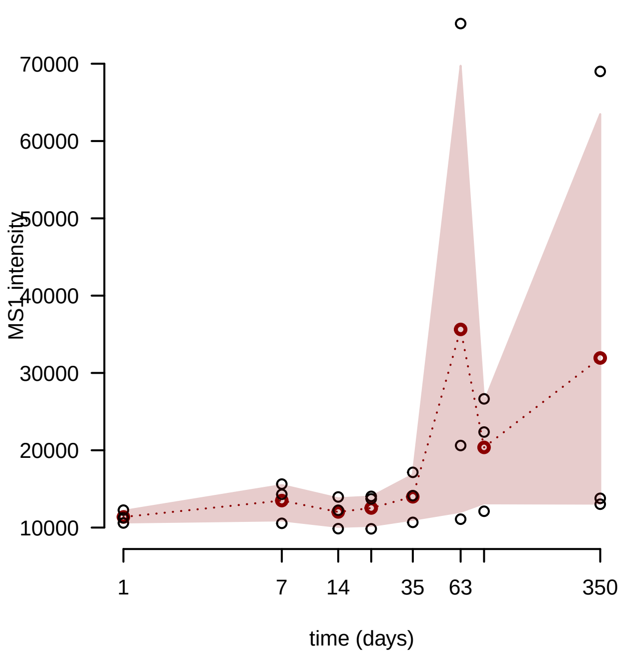

Q64GA5
GN=Pla2g4c
phospholipase A2, group IVC (cytosolic, ...

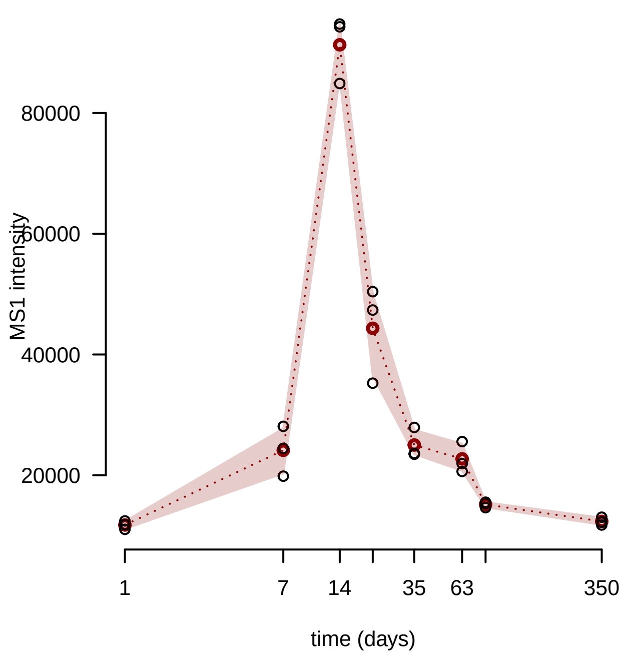

Q65Z40
GN=Wapl
WAPL cohesin release factor

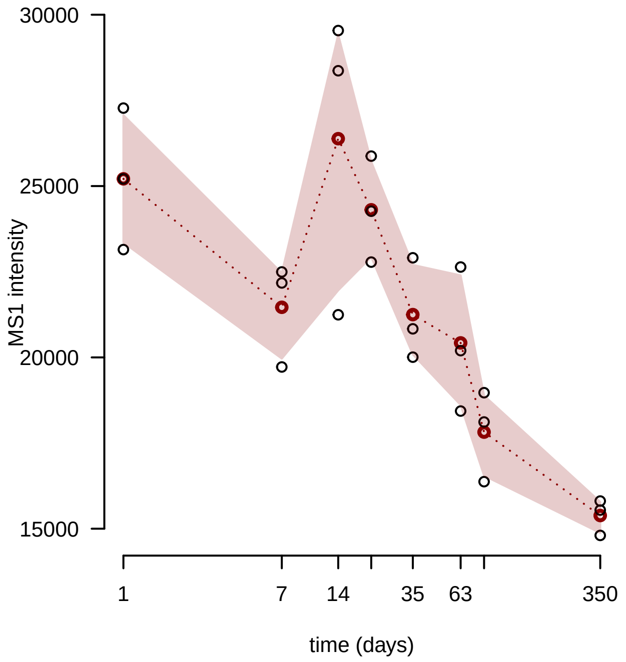

Q66GT5
GN=Ptpmt1
protein tyrosine phosphatase, mitochondr ...

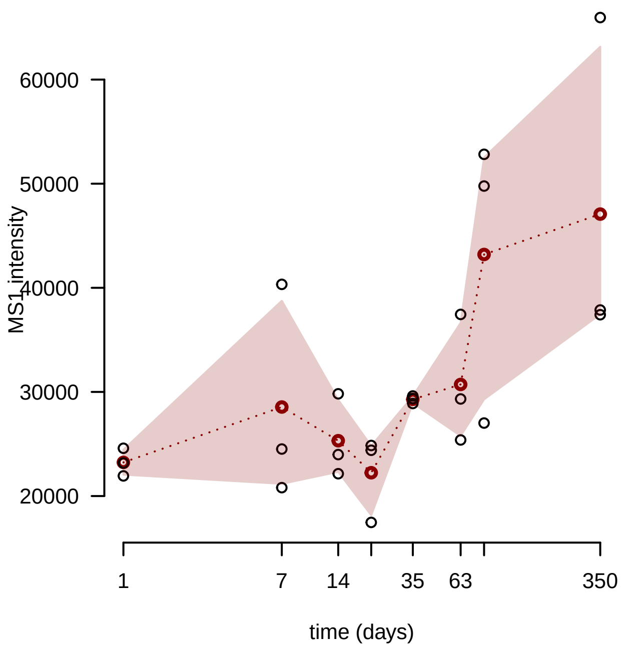

Q66JQ7
GN=Knl1
kinetochore scaffold 1

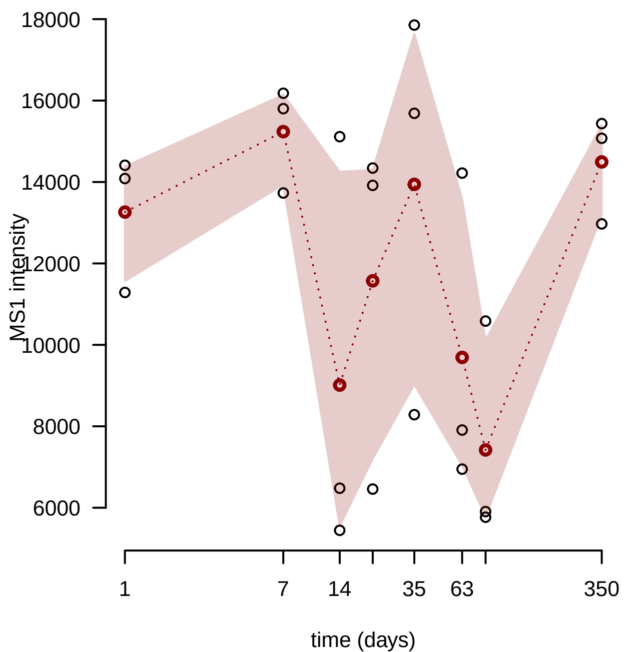

Q66JS6
GN=Eif3j2
eukaryotic translation initiation factor ...

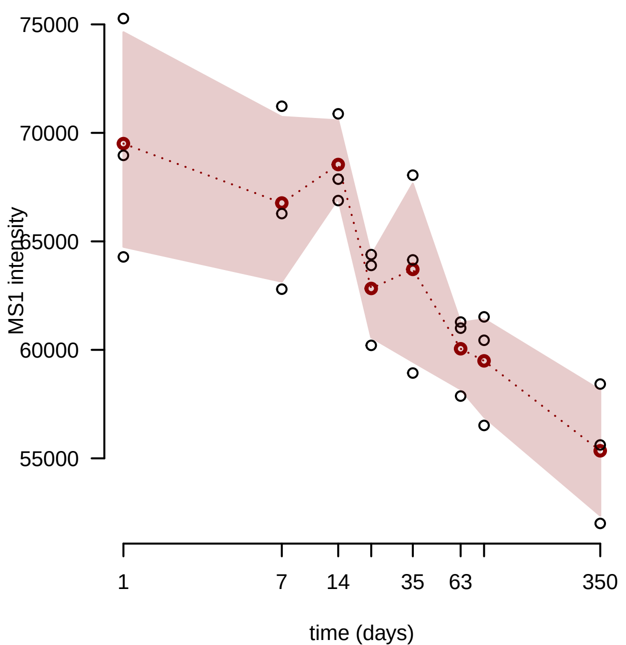

Q66JV4
GN=Rbm12b2
RNA binding motif protein 12 B2

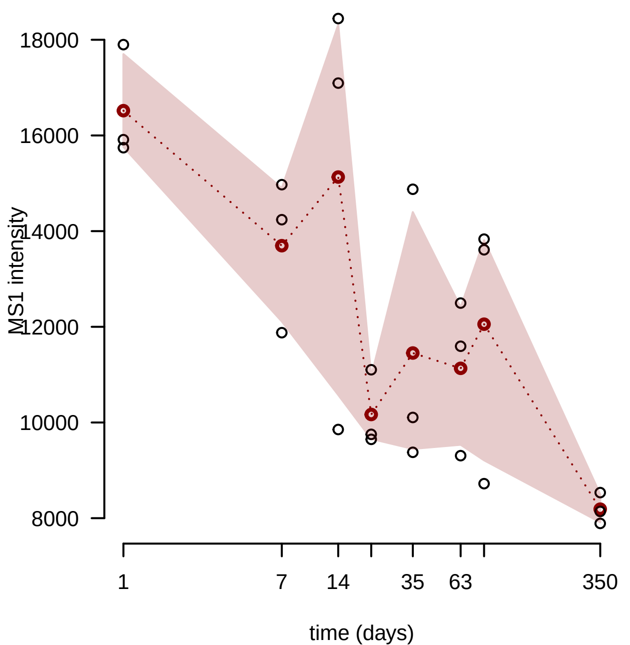

Q66JX5
GN=Fgfr1op

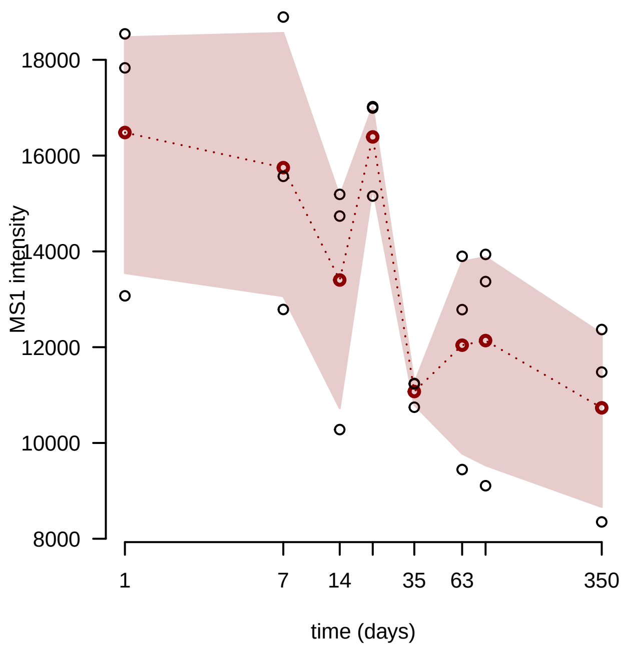

Q66T02
GN=Plekhg5
pleckstrin homology domain containing, f ...

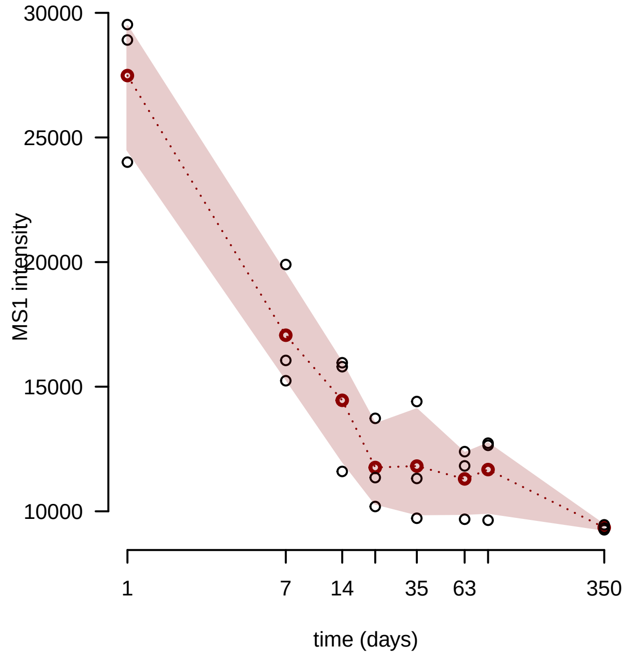

Q66X03
GN=Nlrp9a
NLR family, pyrin domain containing 9A

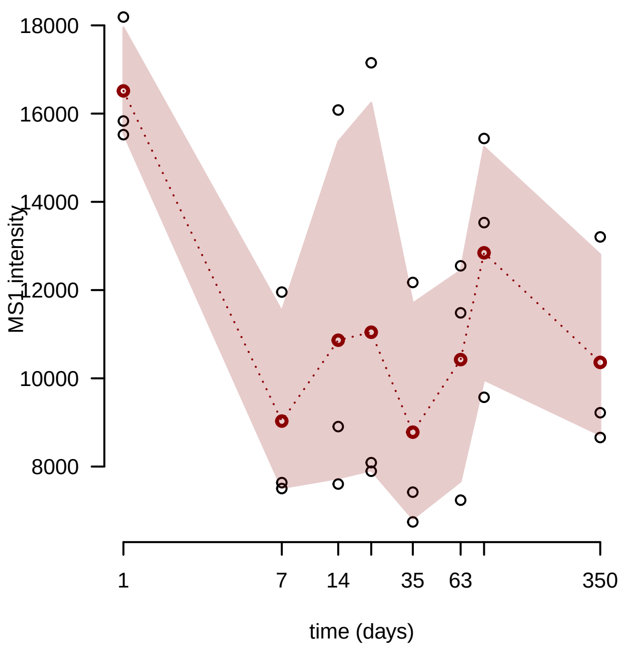

Q66X05
GN=Nlrp4f
NLR family, pyrin domain containing 4F

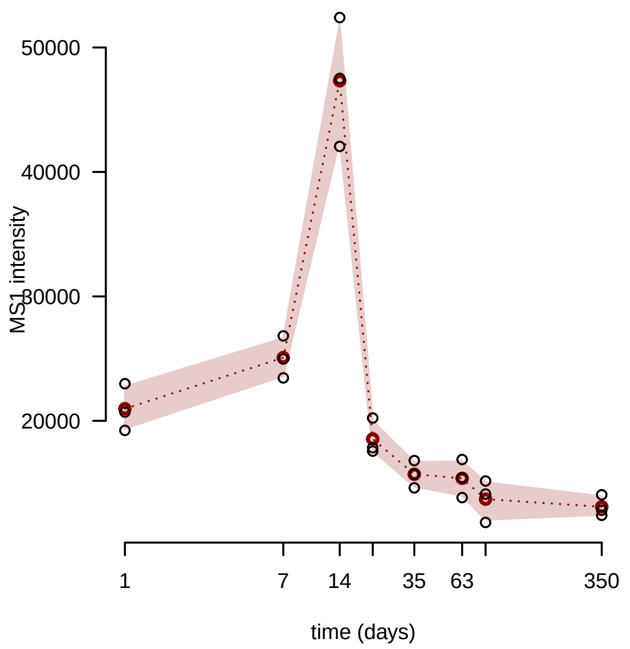

Q66X19
GN=Nlrp4e
NLR family, pyrin domain containing 4E

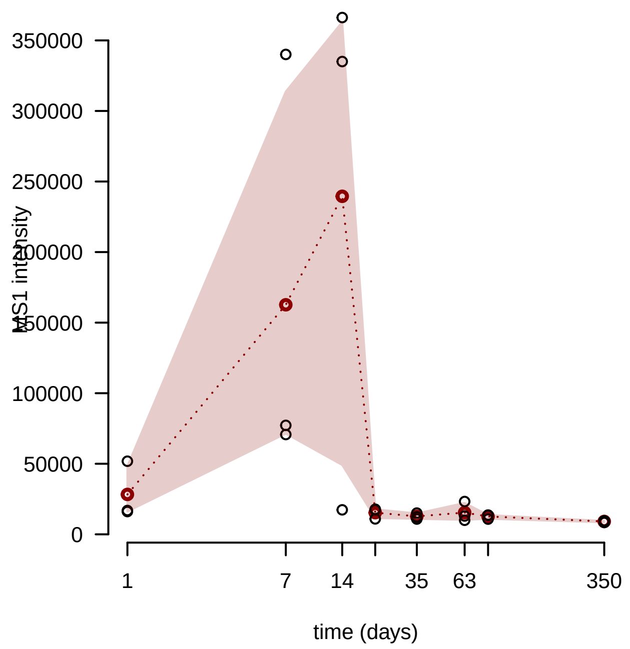

Q66X22
GN=Nlrp9b
NLR family, pyrin domain containing 9B

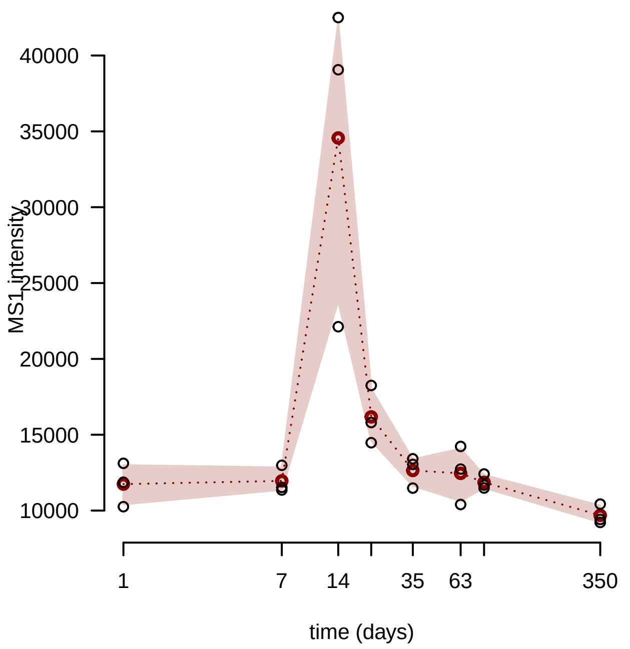

Q673H1
GN=Tusc1
tumor suppressor candidate 1

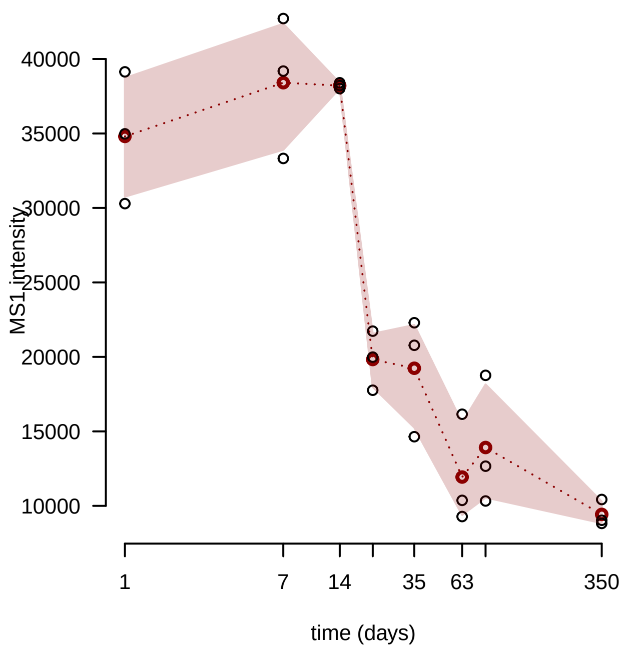

Q67FY2
GN=Bcl9l
B cell CLL/lymphoma 9−like

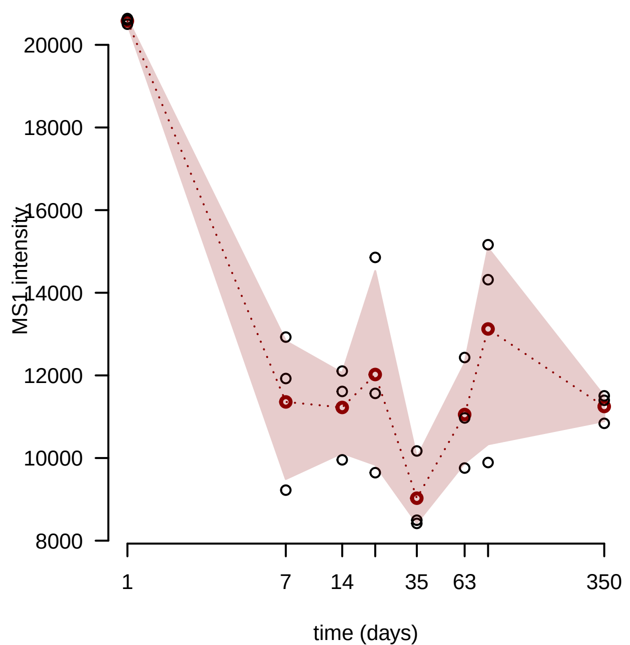

Q684R7
GN=Frem1
Fras1 related extracellular matrix prote ...

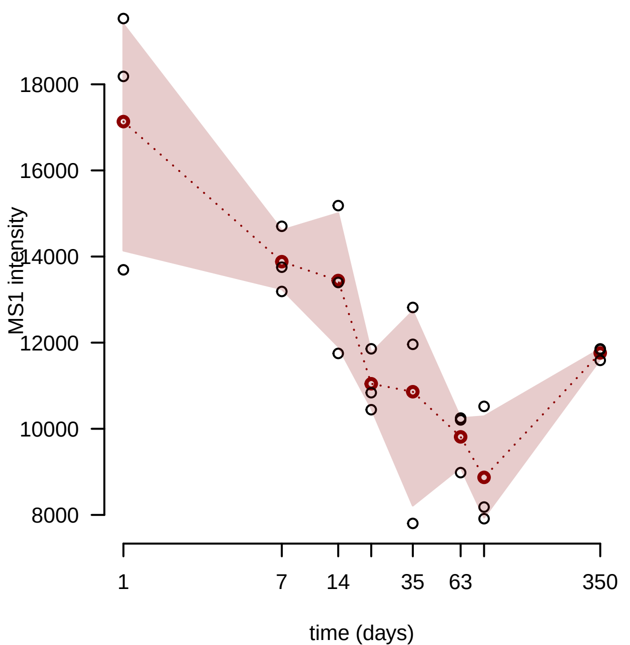

Q689Z5
GN=Sbno1
strawberry notch 1

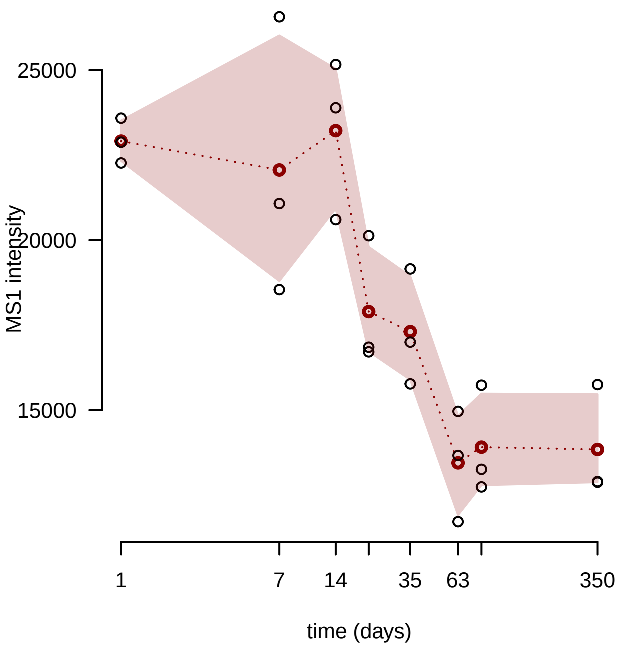

Q68ED3
GN=Tent4b
terminal nucleotidyltransferase 4B

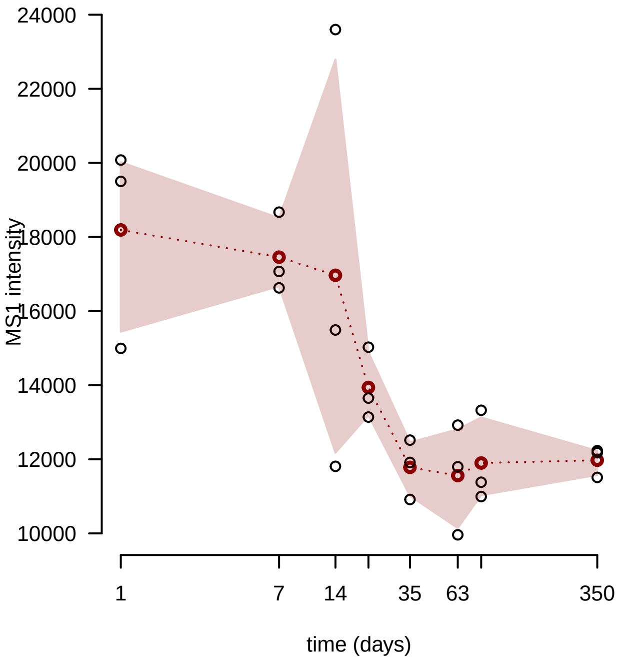

Q68ED7
GN=Crtc1
CREB regulated transcription coactivator ...

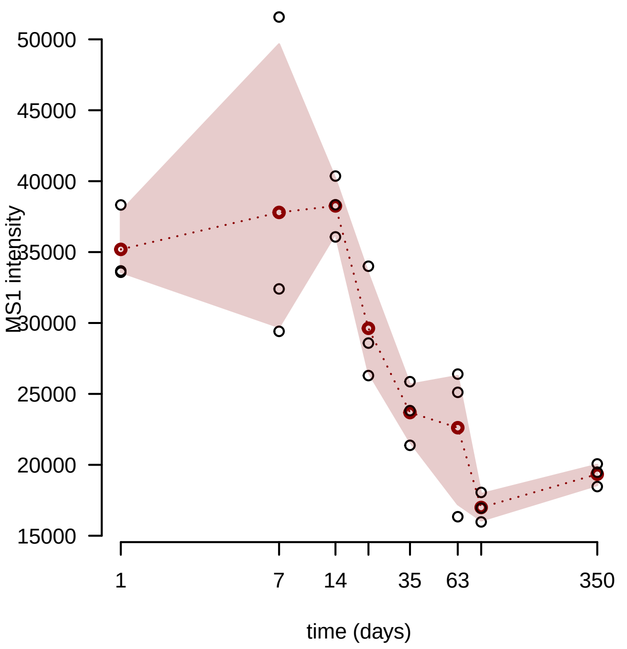

Q68EF0
GN=Rab3ip
RAB3A interacting protein

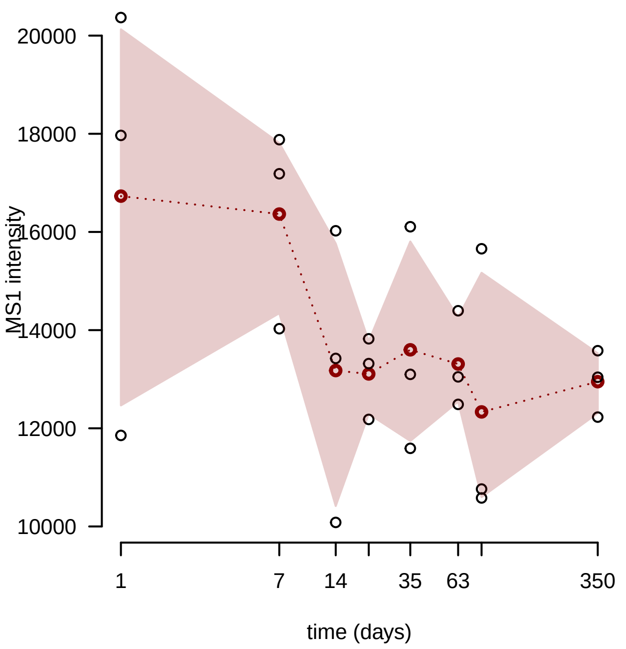

Q68FD5
GN=Cltc
clathrin, heavy polypeptide (Hc)

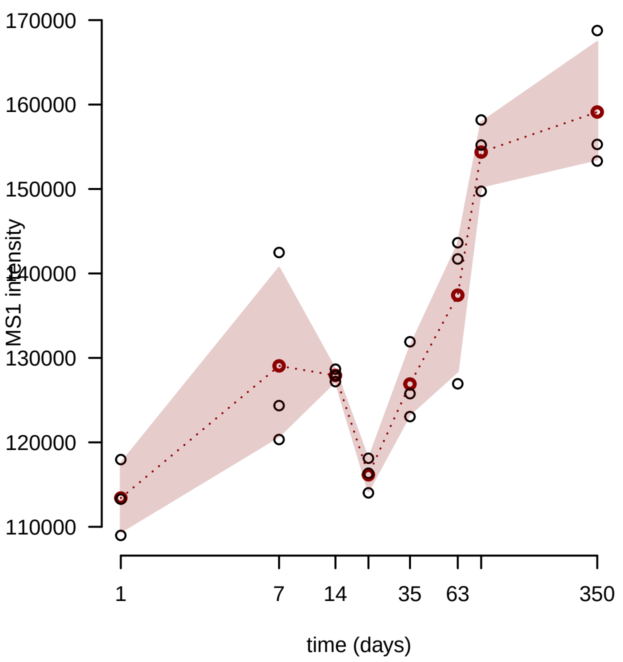

Q68FD7
GN=Fnip1
folliculin interacting protein 1

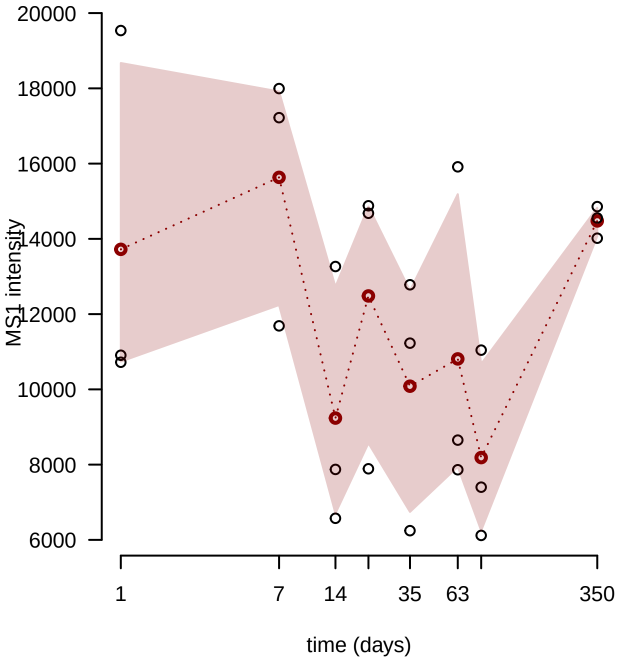

Q68FE2
GN=Atg9a
autophagy related 9A

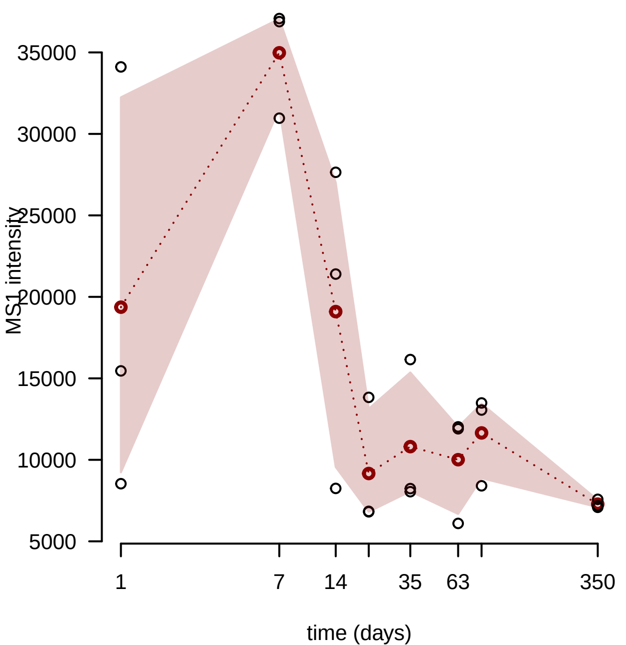

Q68FE6
GN=Ripor1
RHO family interacting cell polarization ...

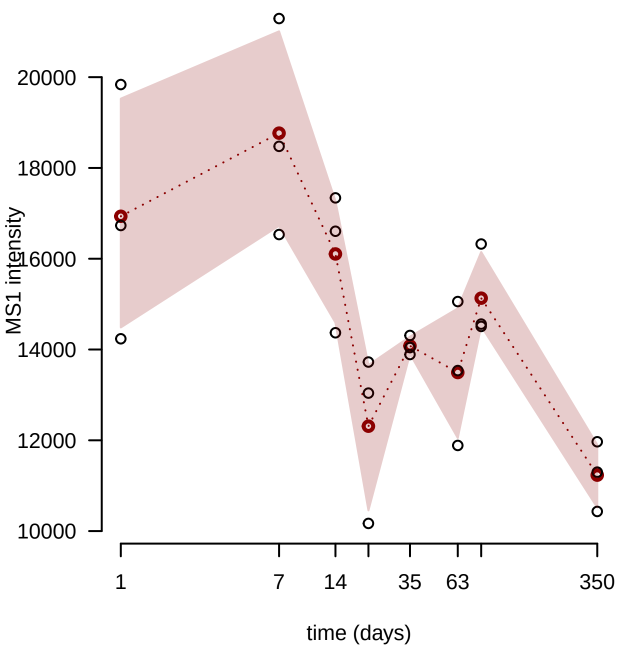

Q68FE8
GN=Znf280d

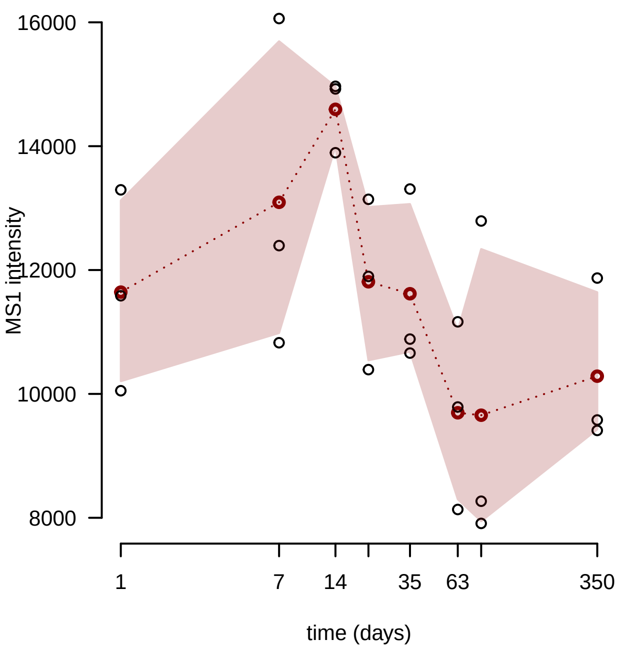

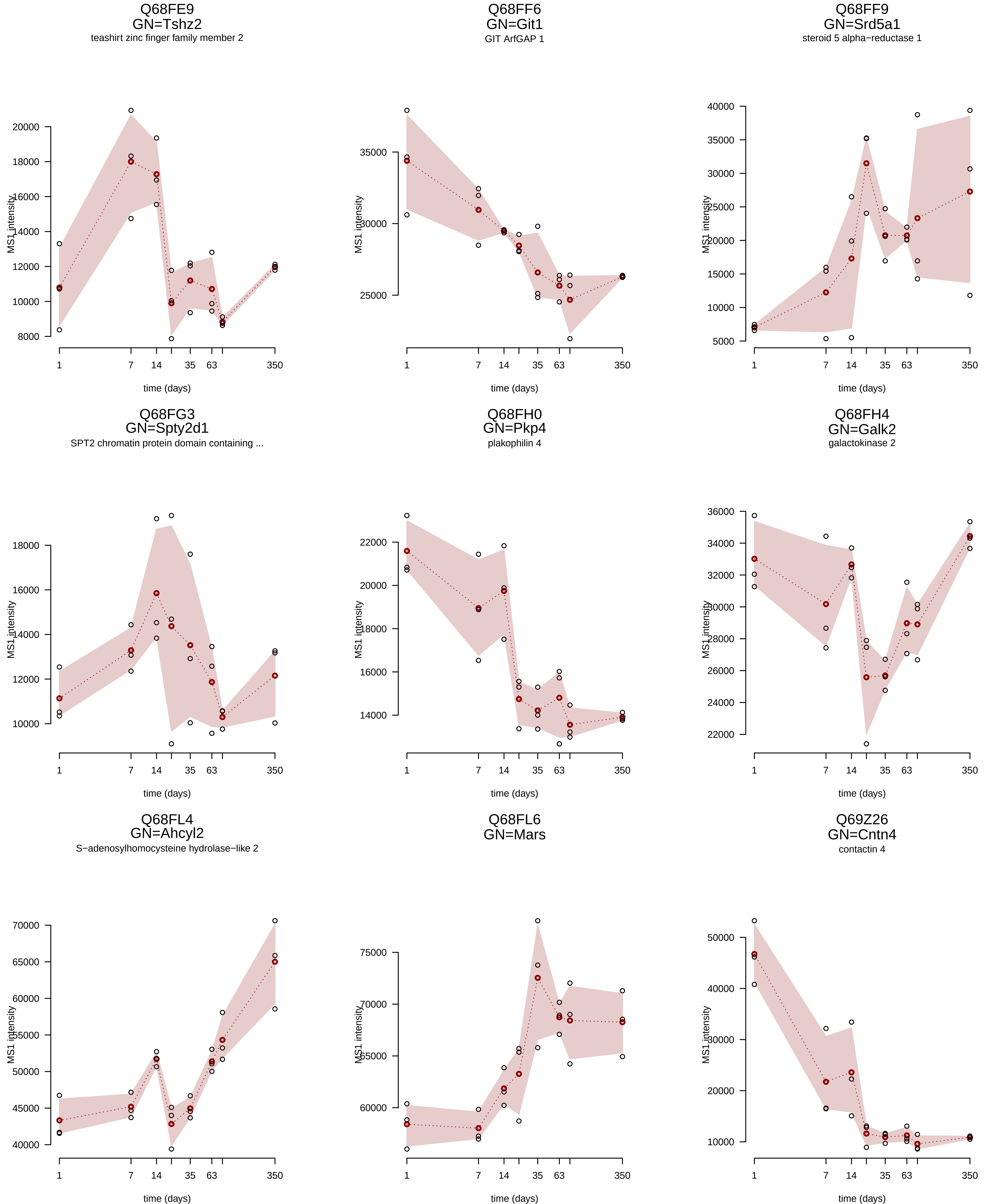

Q68FE9
GN=Tshz2
teashirt zinc finger family member 2
Q68FF6
GN=Git1
GIT ArfGAP 1
Q68FF9
GN=Srd5a1
steroid 5 alpha−reductase 1
Q68FG3
GN=Spty2d1
SPT2 chromatin protein domain containing ...
Q68FH0
GN=Pkp4
plakophilin 4
Q68FH4
GN=Galk2
galactokinase 2
Q68FL4
GN=Ahcyl2
S−adenosylhomocysteine hydrolase−like 2
Q68FL6
GN=Mars
Q69Z26
GN=Cntn4
contactin 4
MS1 intensity
time (days)
1
7
14
35
63
350

Q69Z36
GN=Mex3b
mex3 RNA binding family member B

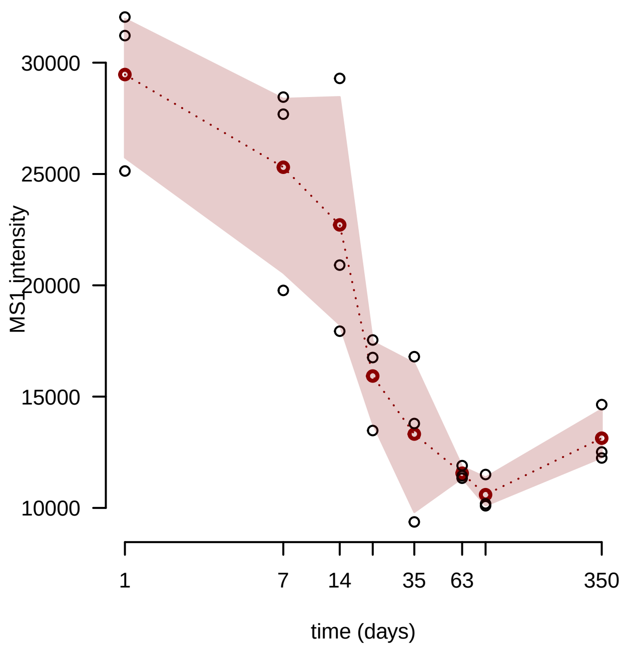

Q69Z37
GN=Samd9l
sterile alpha motif domain containing 9− ...

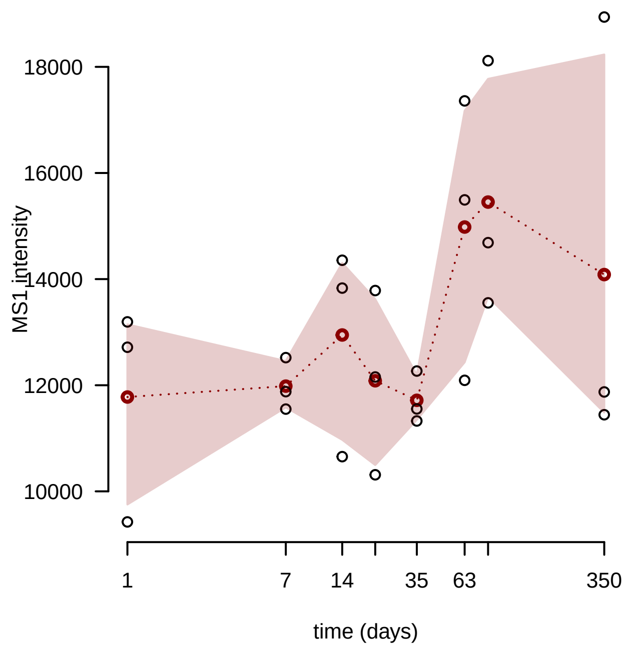

Q69Z38
GN=Peak1
pseudopodium−enriched atypical kinase 1

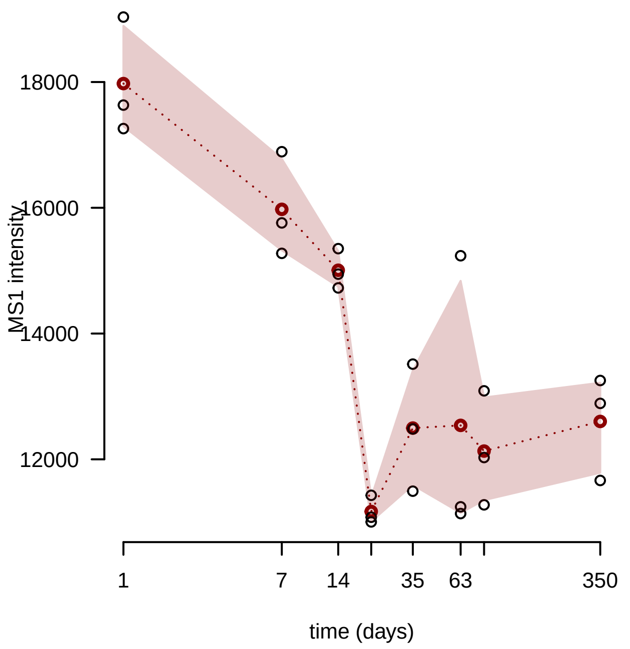

Q69Z61
GN=Pwwp2a
PWWP domain containing 2A

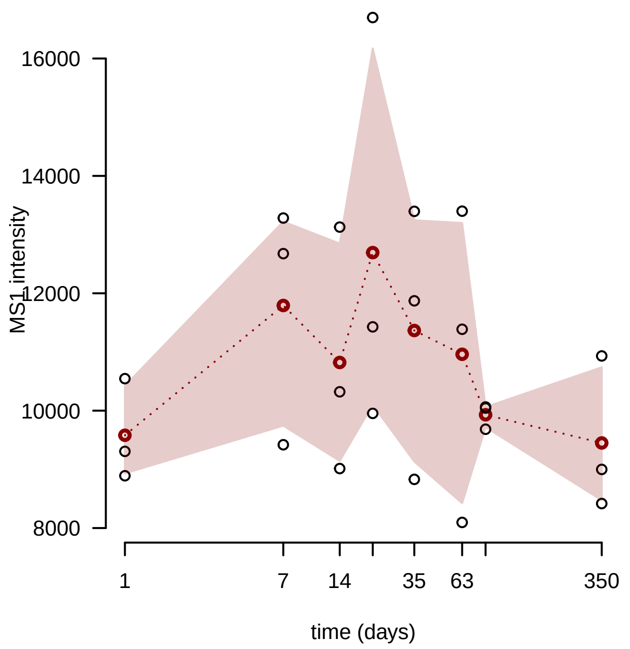

Q69Z99
GN=Znf512

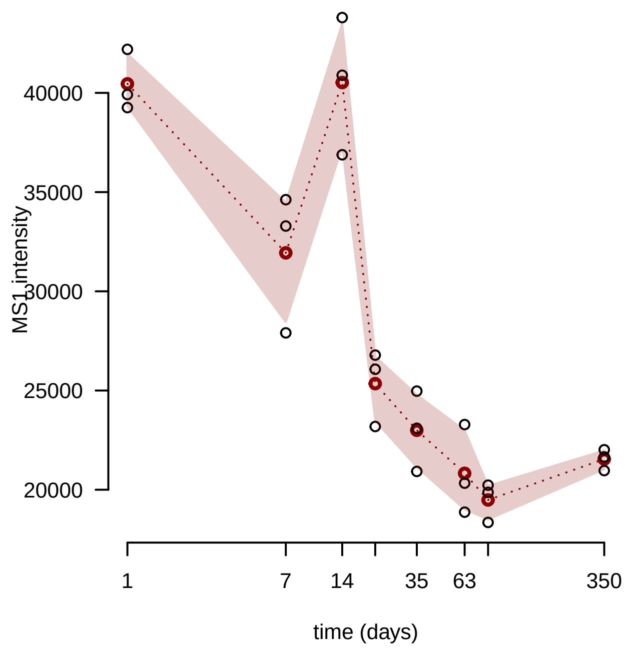

Q69ZA1
GN=Cdk13
cyclin−dependent kinase 13

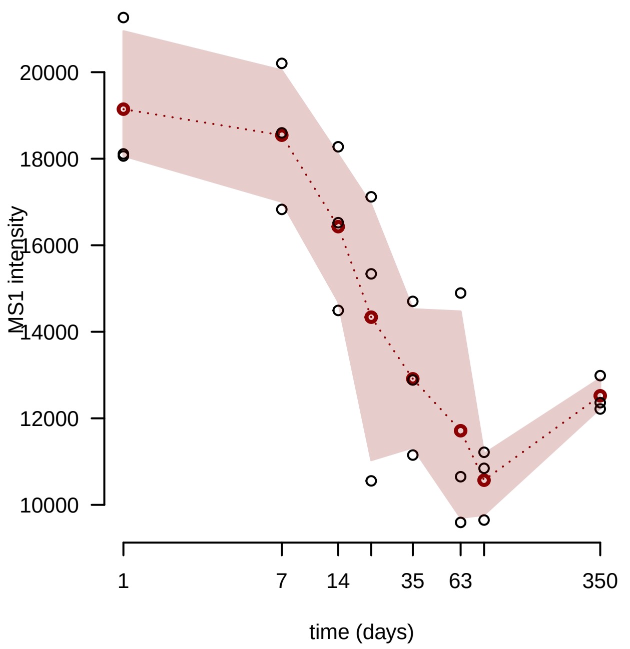

Q69ZB0
GN=Lrrcc1
leucine rich repeat and coiled−coil doma ...

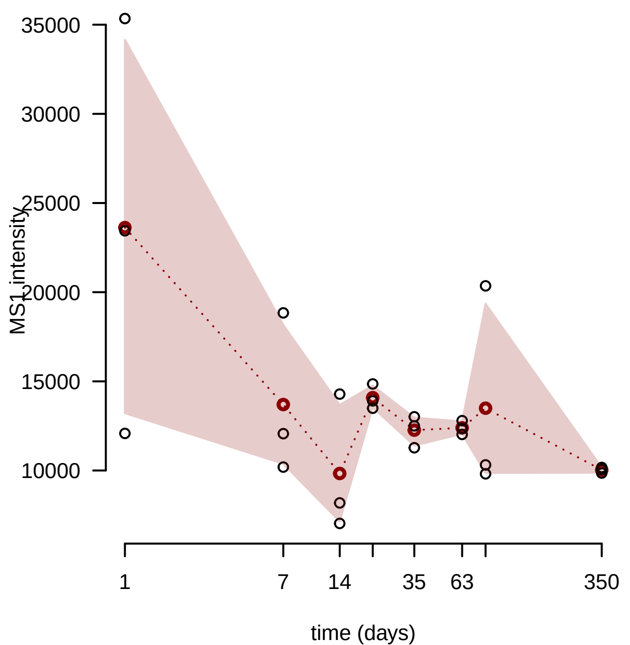

Q69ZB3
GN=Tspyl5
testis−specific protein, Y−encoded−like ...

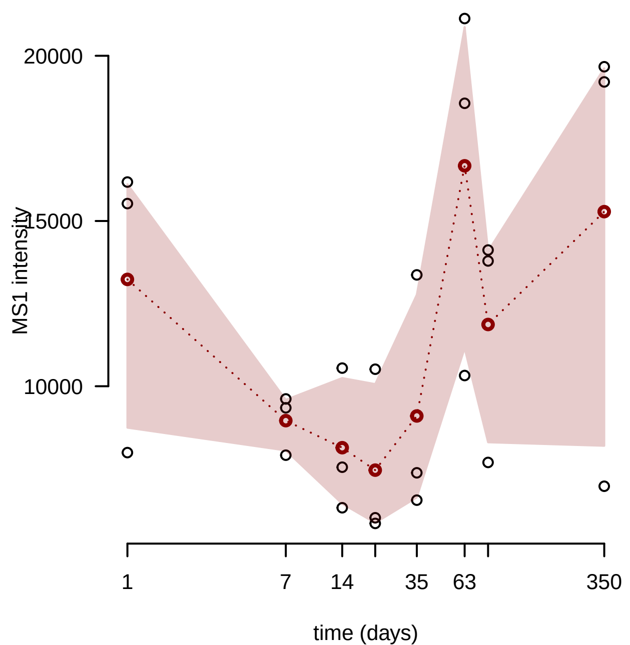

Q69ZC8
GN=Gpalpp1
GPALPP motifs containing 1

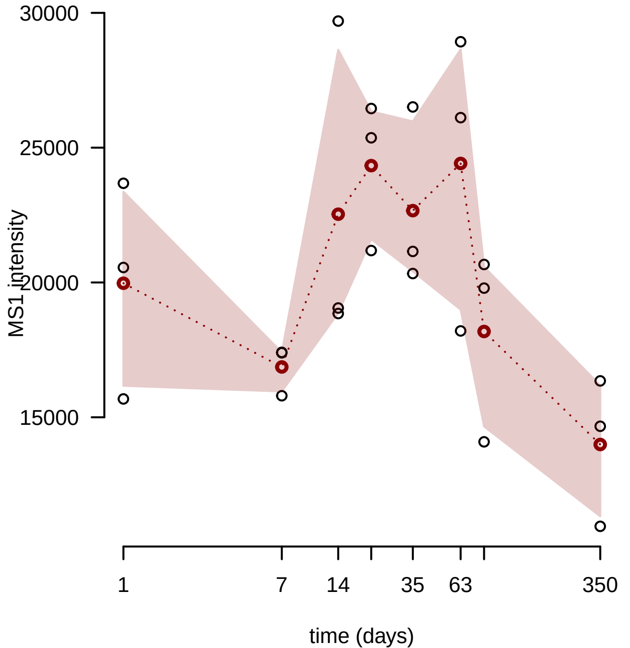

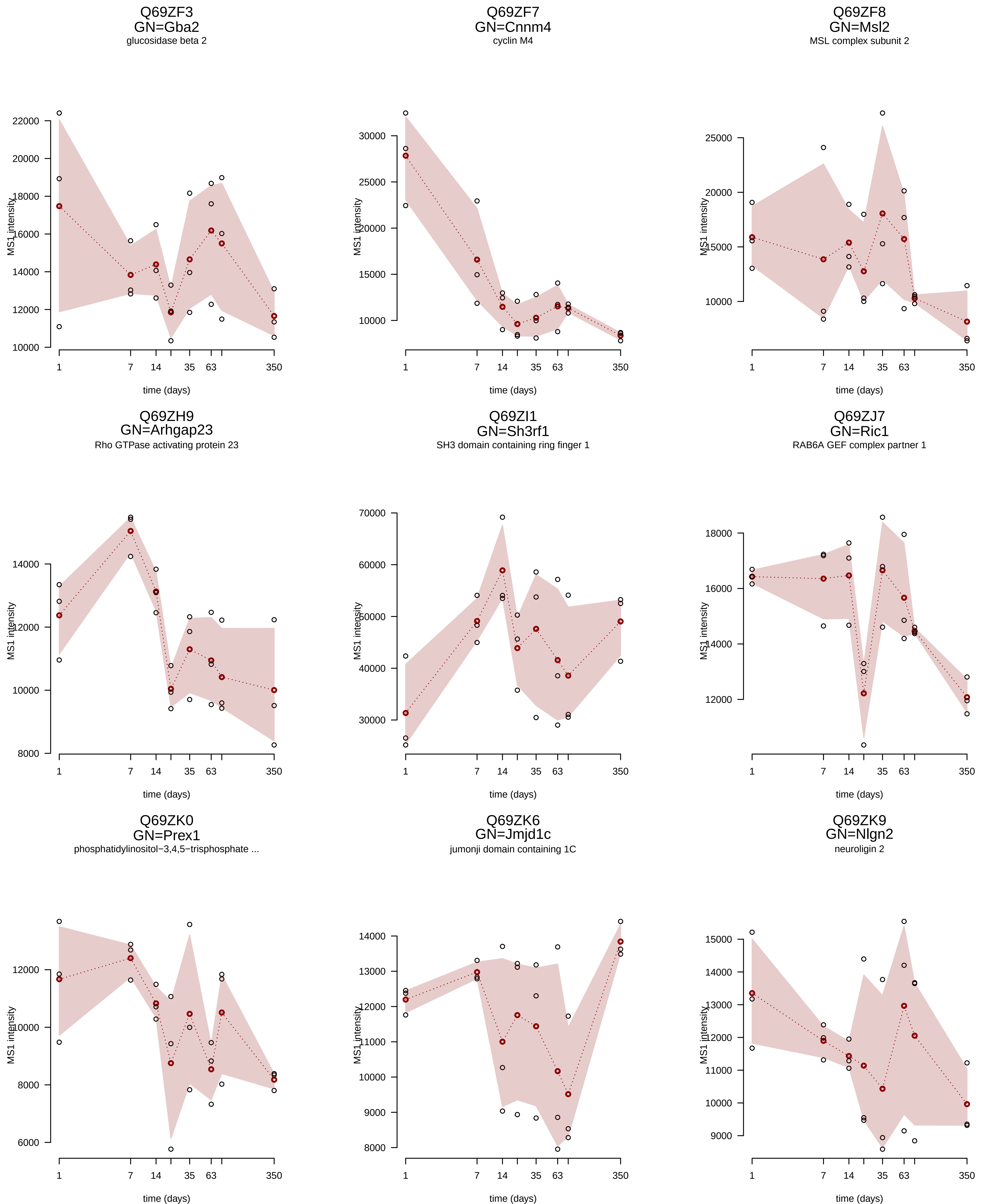
Q69ZF3
GN=Gba2
glucosidase beta 2
Q69ZF7
GN=Cnnm4
cyclin M4
Q69ZF8
GN=Msl2
MSL complex subunit 2
Q69ZH9
GN=Arhgap23
Rho GTPase activating protein 23
Q69ZI1
GN=Sh3rf1
SH3 domain containing ring finger 1
Q69ZJ7
GN=Ric1
RAB6A GEF complex partner 1
Q69ZK0
GN=Prex1
phosphatidylinositol−3,4,5−trisphosphate ...
Q69ZK6
GN=Jmjd1c
jumonji domain containing 1C
Q69ZK9
GN=Nlgn2
neuroligin 2
MS1 intensity
time (days)
1
7
14
35
63
350

Q69ZL1
GN=Fgd6
FYVE, RhoGEF and PH domain containing 6

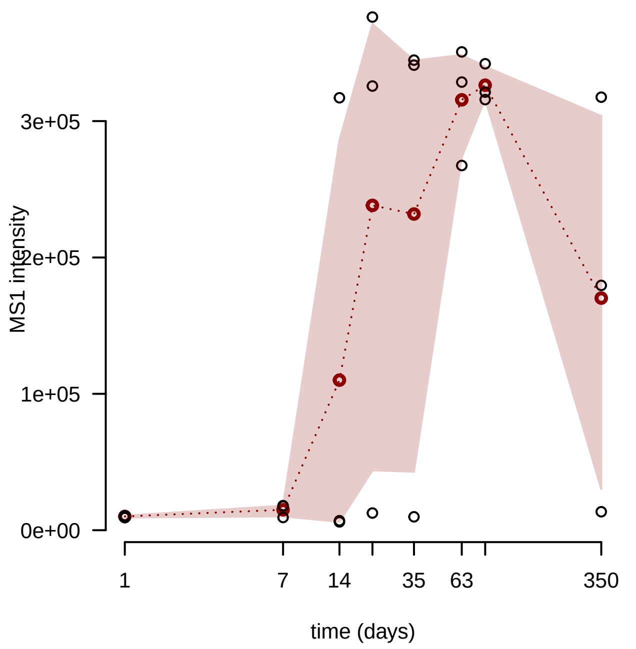

Q69ZM6
GN=Stk36
serine/threonine kinase 36

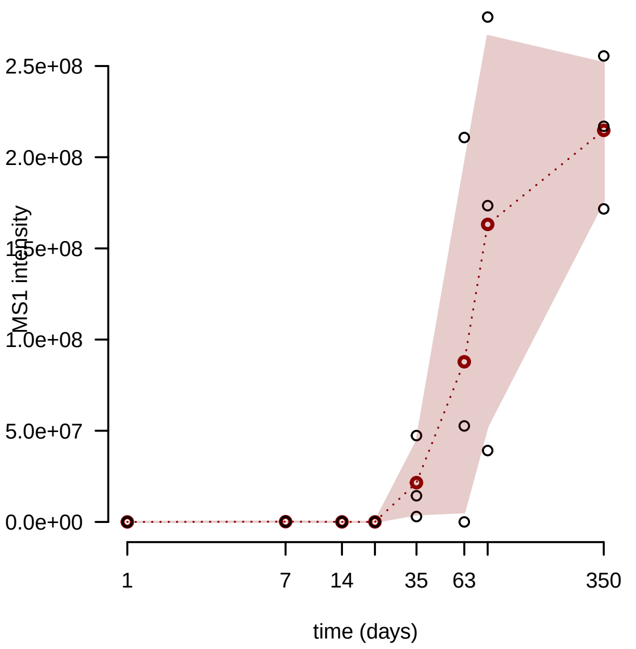

Q69ZN7
GN=Myof
myoferlin

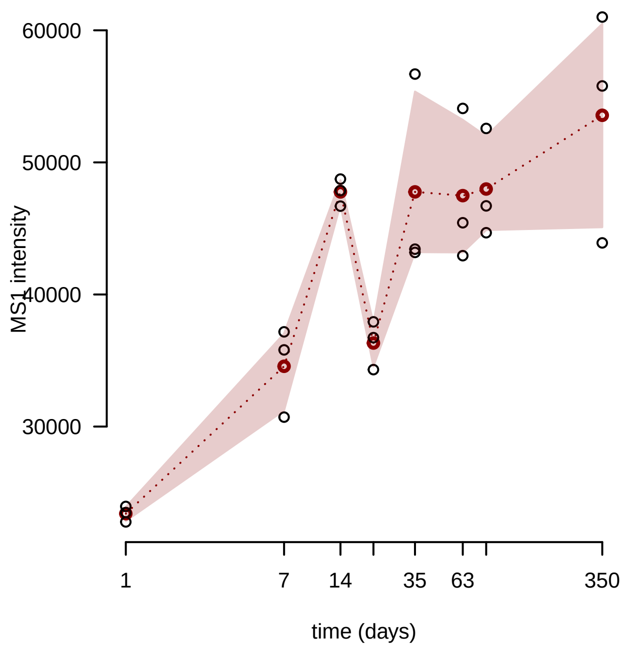

Q69ZP3
GN=Pnkd
paroxysmal nonkinesiogenic dyskinesia

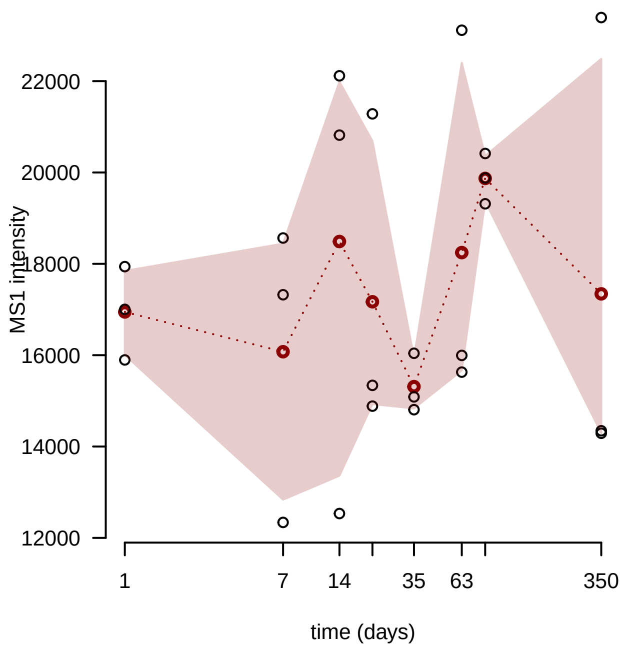

Q69ZP3
GN=Pnkd
paroxysmal nonkinesiogenic dyskinesia

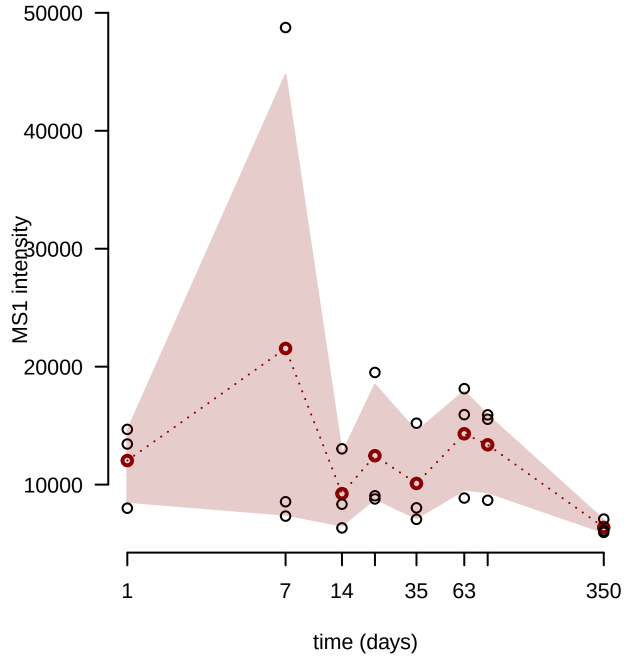

Q69ZQ1
GN=Myorg
myogenesis regulating glycosidase (putat ...

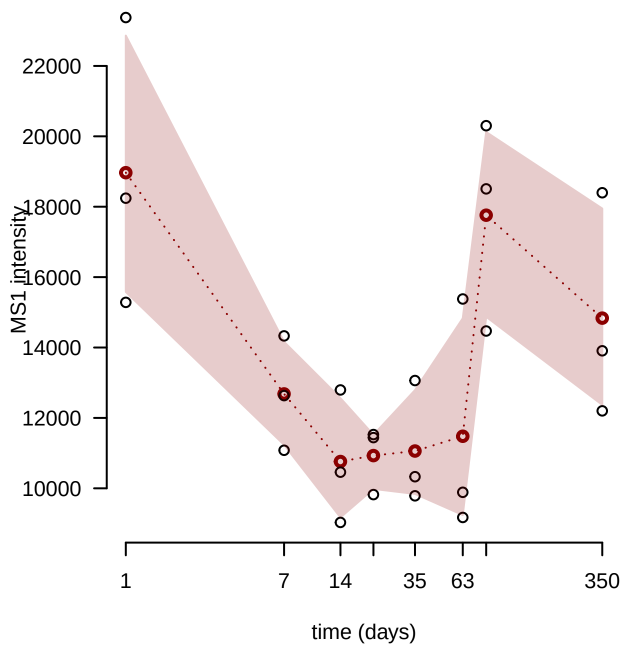

Q69ZQ2
GN=Isy1
ISY1 splicing factor homolog

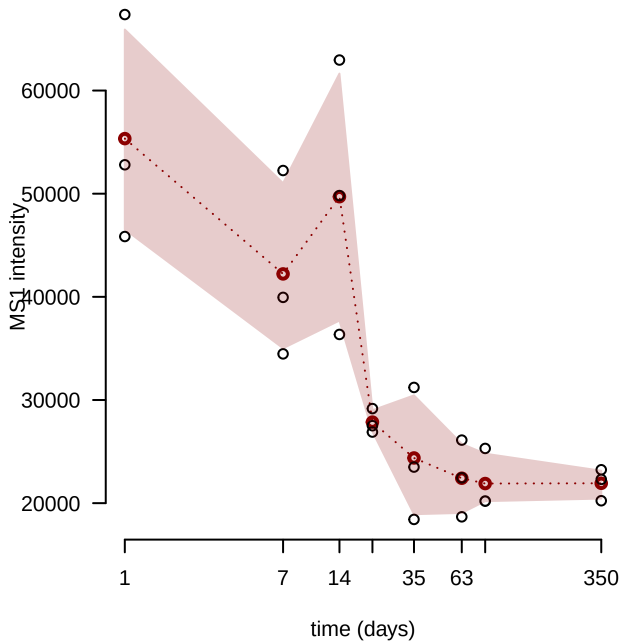

Q69ZR2
GN=Hectd1
HECT domain E3 ubiquitin protein ligase ...

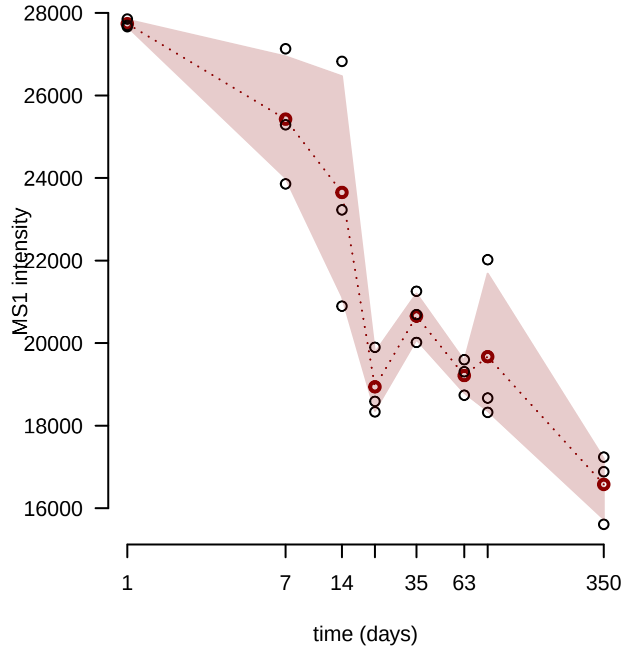

Q69ZR9
GN=Tasor
transcription activation suppressor

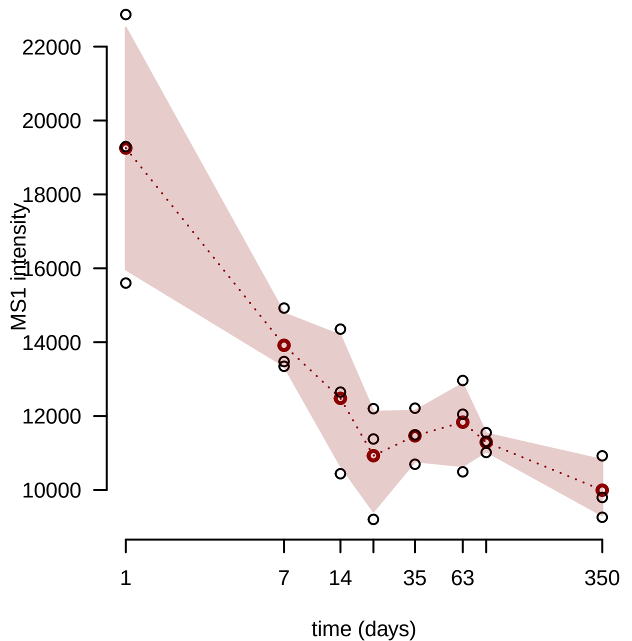

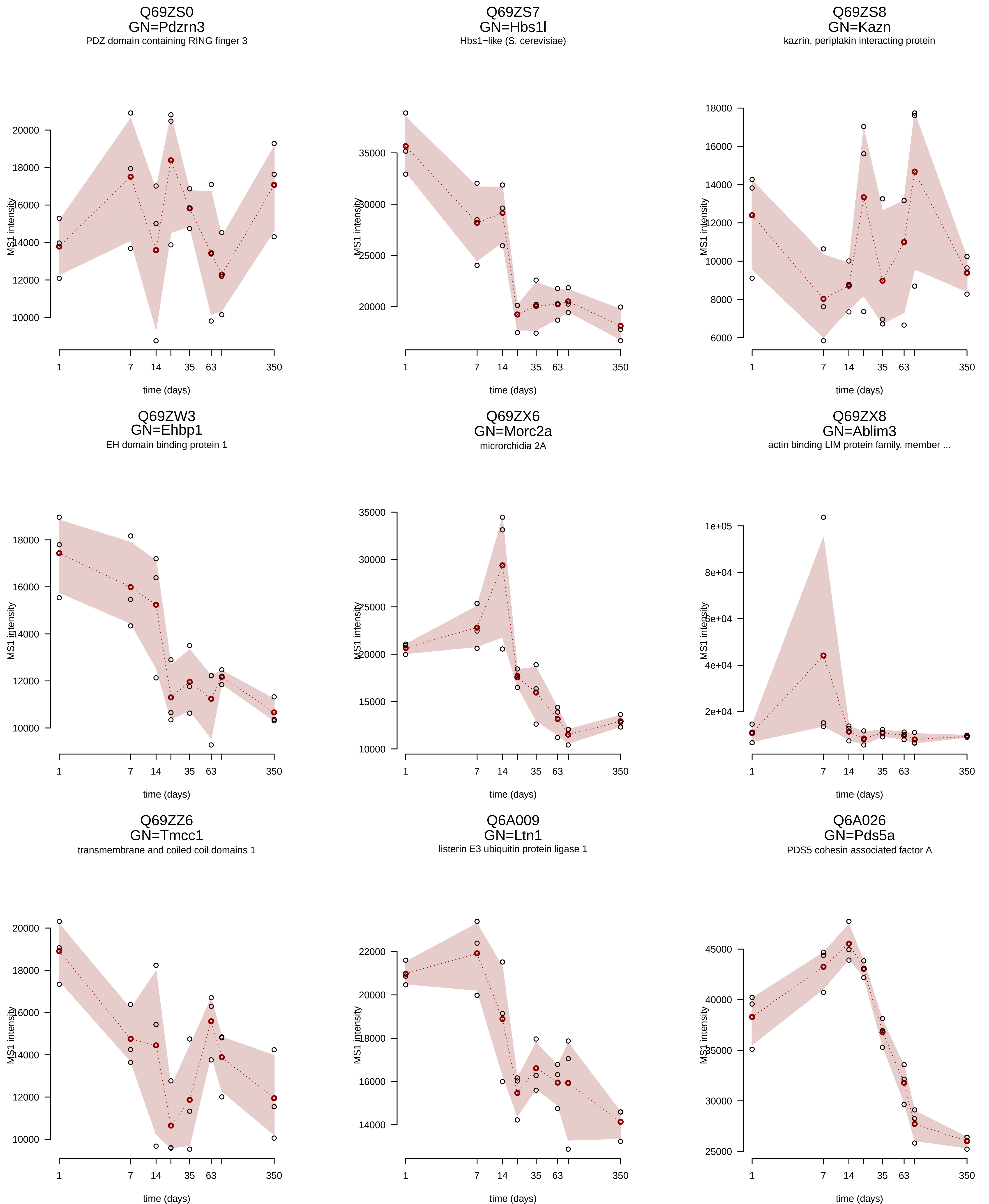
Q69ZS0
GN=Pdzrn3
PDZ domain containing RING finger 3
Q69ZS7
GN=Hbs1l
Hbs1−like (S. cerevisiae)
Q69ZS8
GN=Kazn
kazrin, periplakin interacting protein
Q69ZW3
GN=Ehbp1
EH domain binding protein 1
Q69ZX6
GN=Morc2a
microrchidia 2A
Q69ZX8
GN=Ablim3
actin binding LIM protein family, member ...
Q69ZZ6
GN=Tmcc1
transmembrane and coiled coil domains 1
Q6A009
GN=Ltn1
listerin E3 ubiquitin protein ligase 1
Q6A026
GN=Pds5a
PDS5 cohesin associated factor A
MS1 intensity
time (days)
1
7
14
35
63
350

Q6A028
GN=Swap70
SWA−70 protein

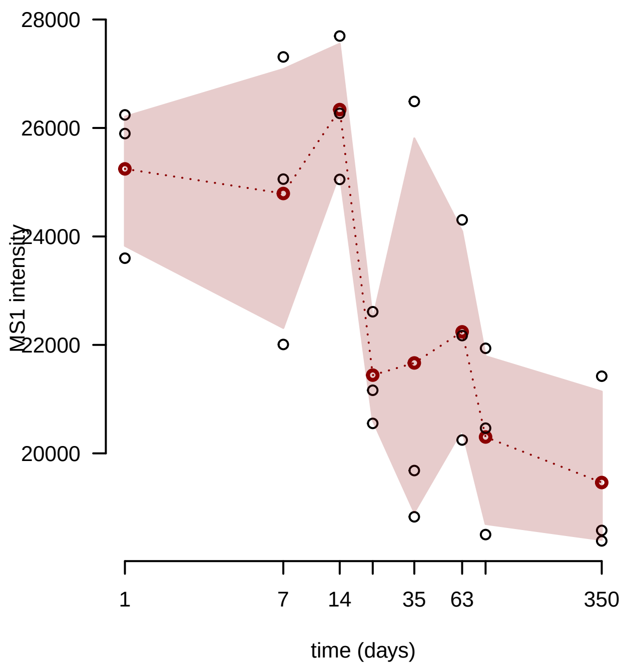

Q6A037
GN=N4bp1
NEDD4 binding protein 1

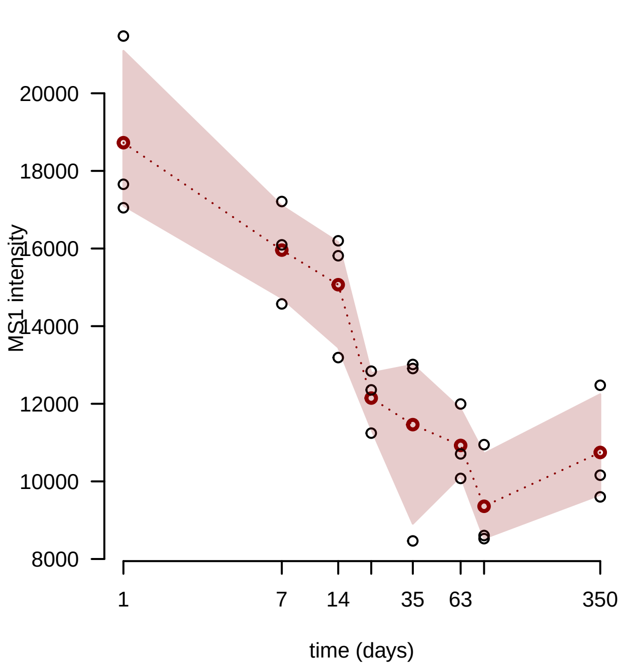

Q6A039
GN=Tbc1d12
TBC1D12: TBC1 domain family, member 12

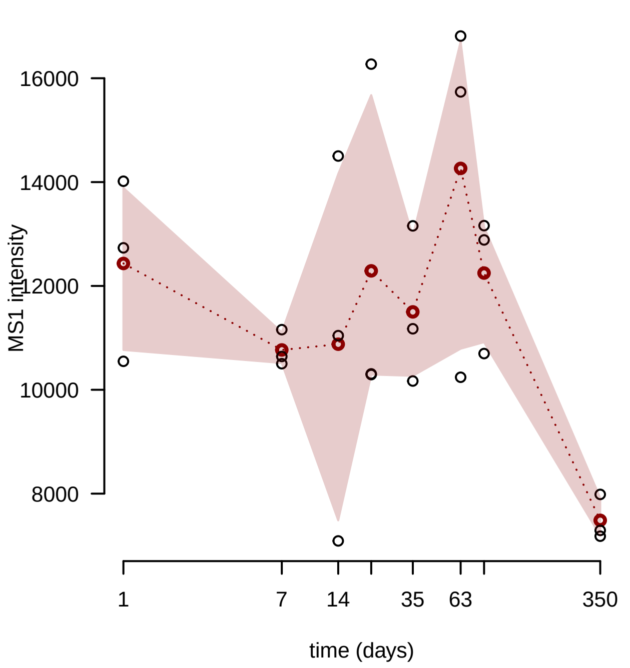

Q6A058
GN=Armcx2
armadillo repeat containing, X−linked 2

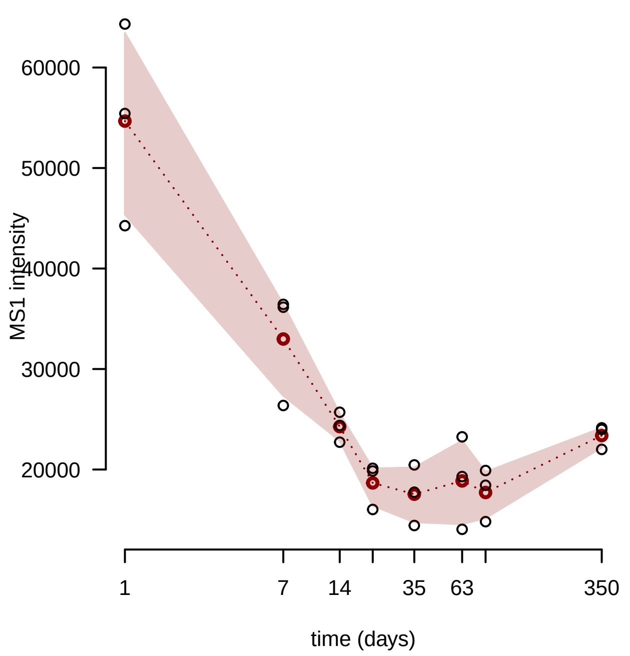

Q6A065
GN=Cep170
centrosomal protein 170

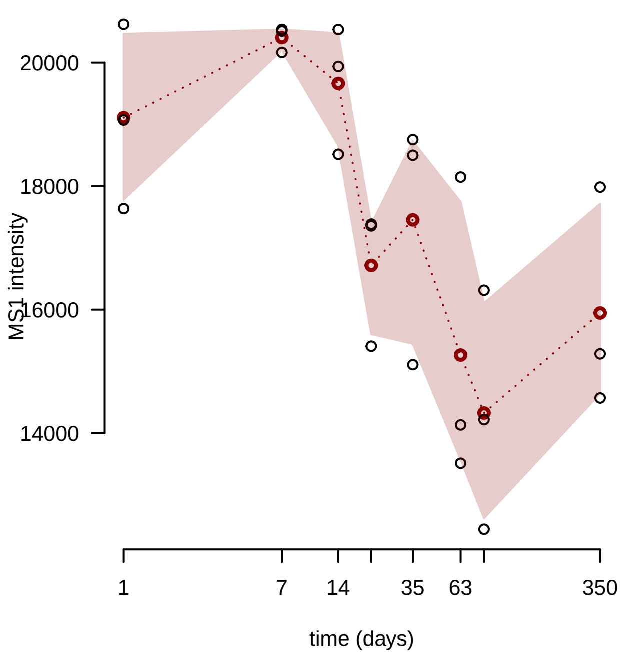

Q6A068
GN=Cdc5l
cell division cycle 5−like (S. pombe)

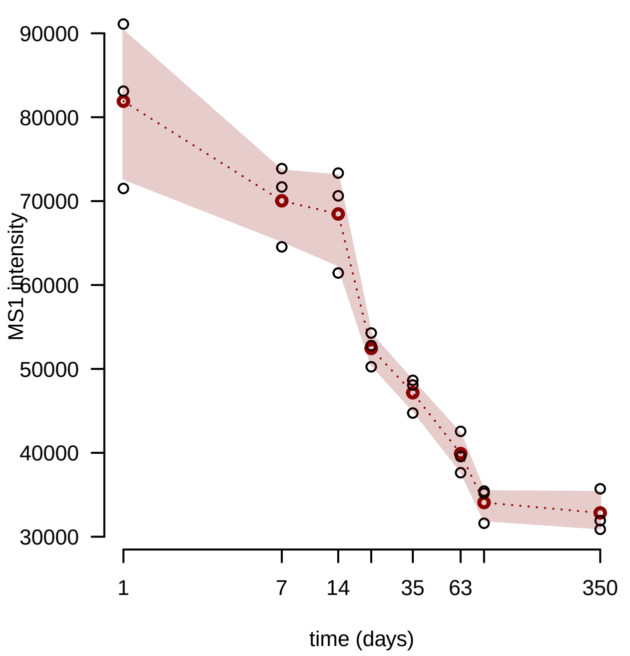

Q6A078
GN=Cep290
centrosomal protein 290

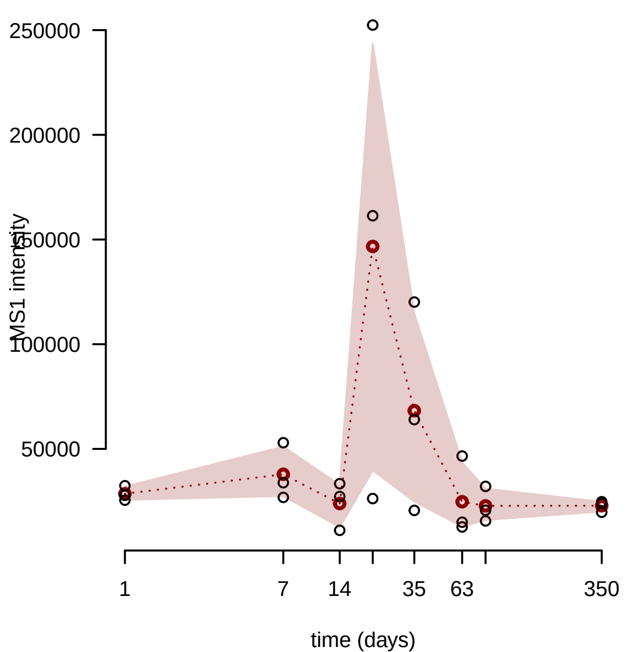

Q6A085
GN=Znf629

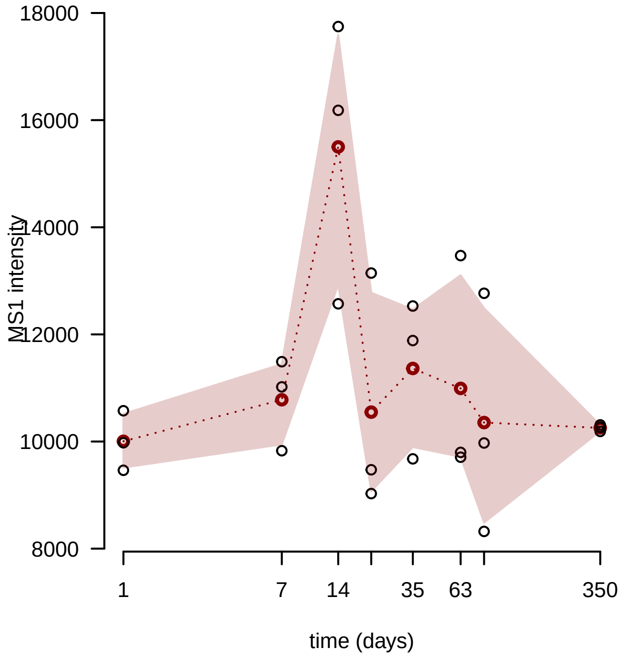

Q6A098
GN=Secisbp2l
SECIS binding protein 2−like

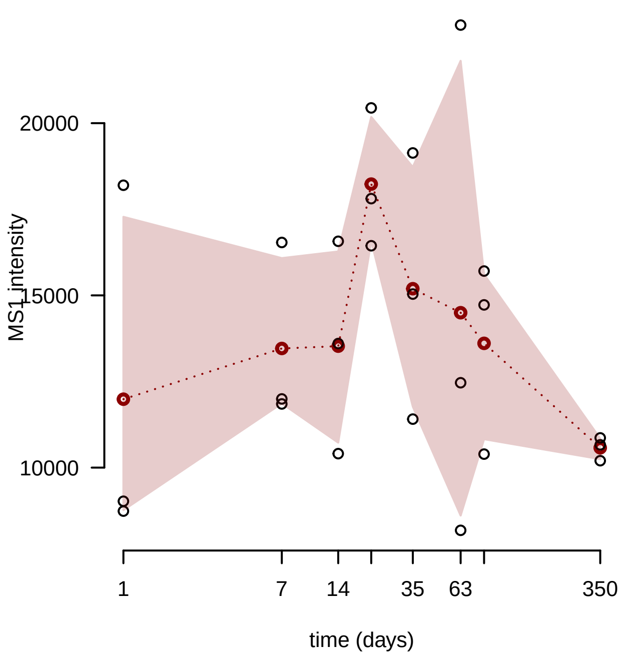

Q6A0A2
GN=Larp4b
La ribonucleoprotein 4B

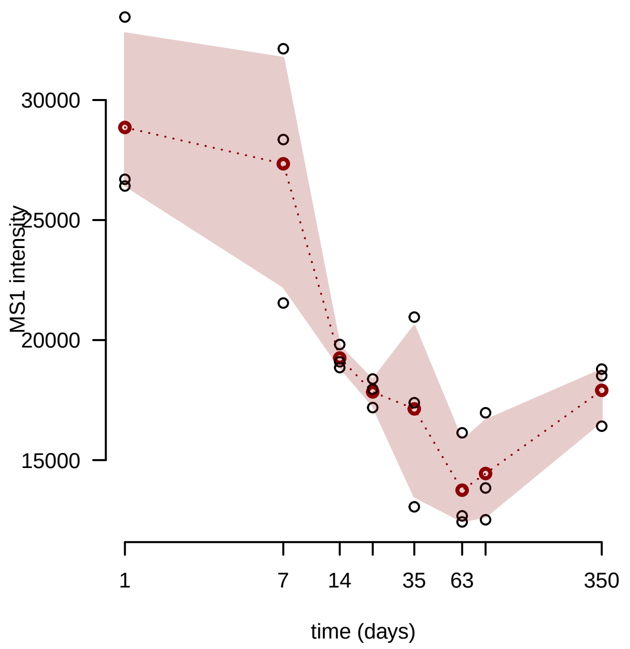

Q6A0A9
GN=FAM120A

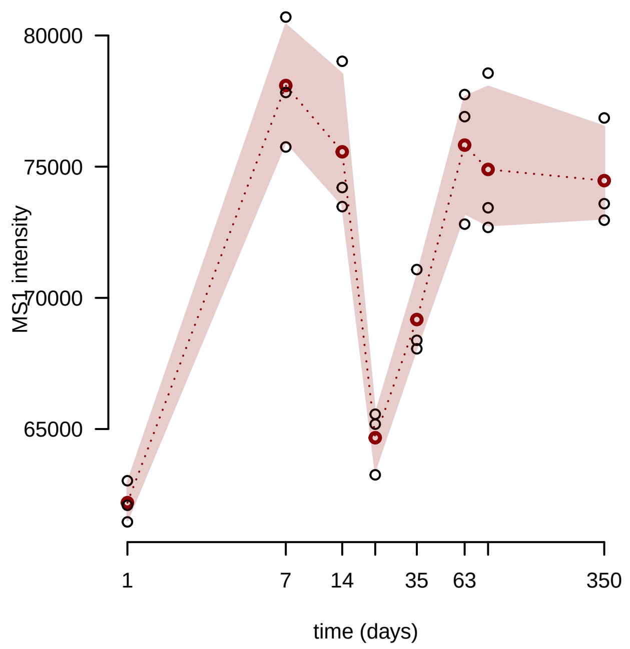

Q6A0D4
GN=Rftn1
raftlin lipid raft linker 1

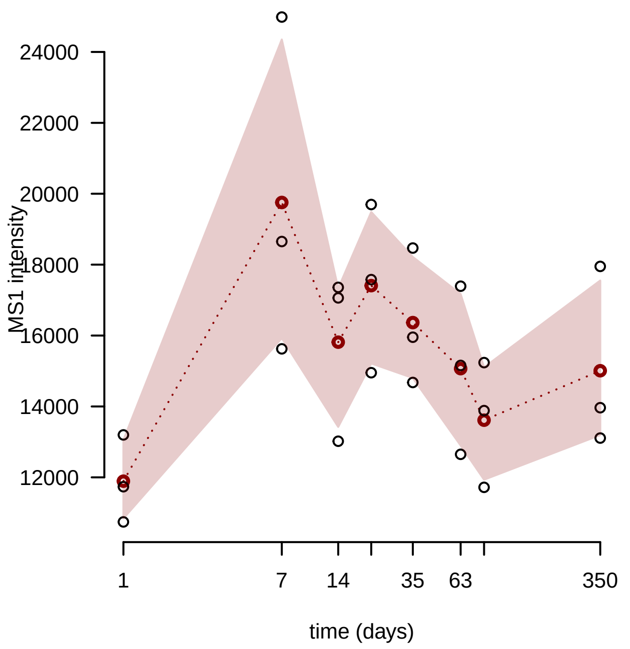

Q6A4J8
GN=Usp7
ubiquitin specific peptidase 7

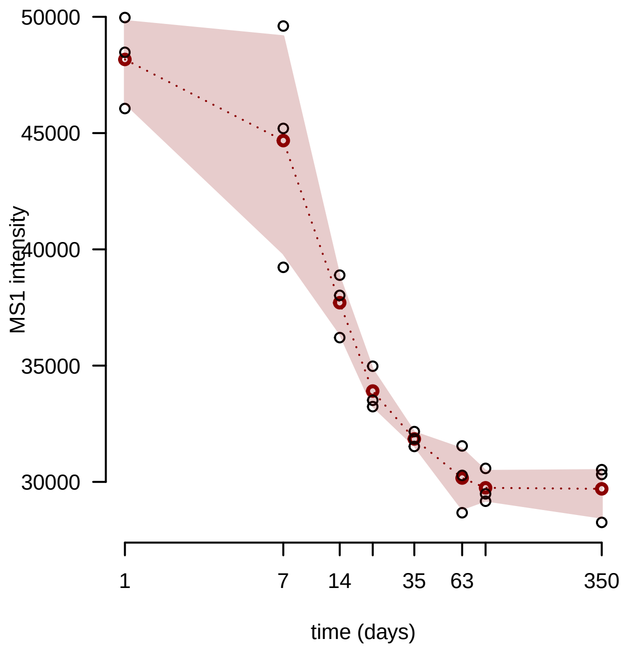

Q6A4J8
GN=Usp7
ubiquitin specific peptidase 7

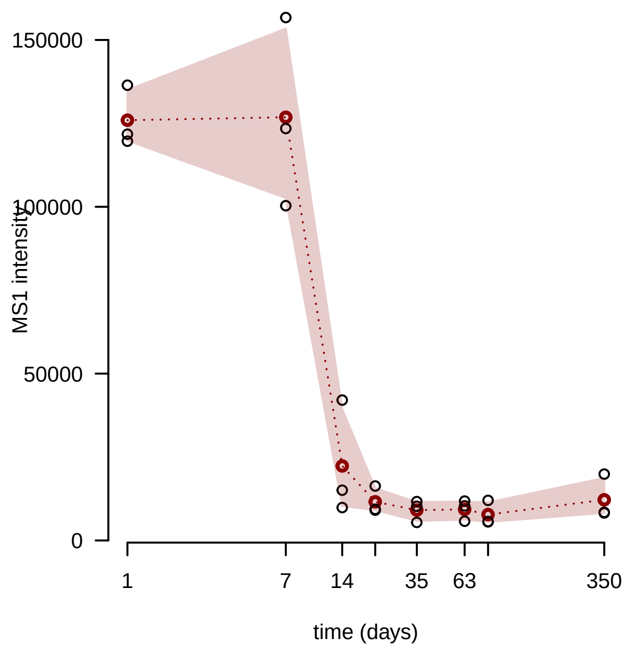

Q6AW69
GN=Cgnl1
cingulin−like 1

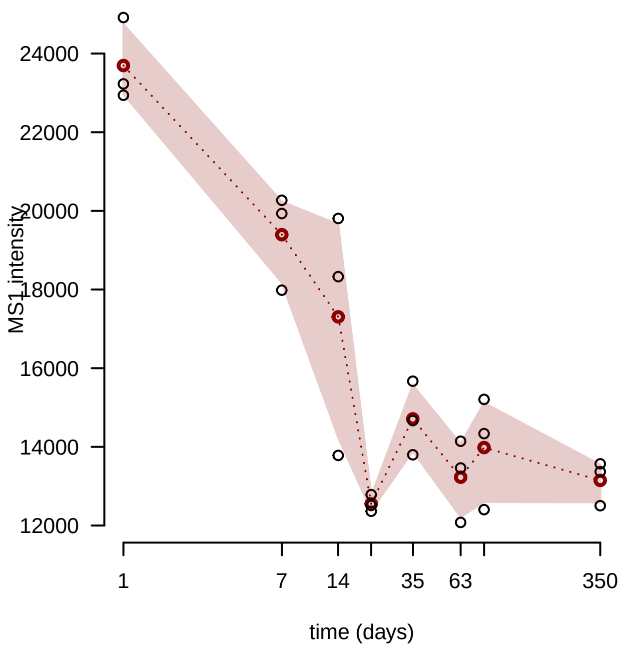

Q6AXC6
GN=Ddx11
DEAD/H box helicase 11

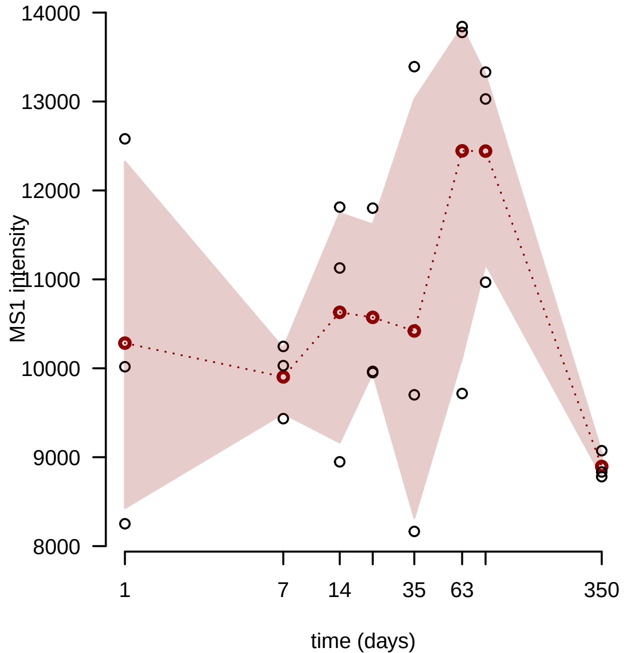

Q6B966
GN=Nlrp14
NLR family, pyrin domain containing 14

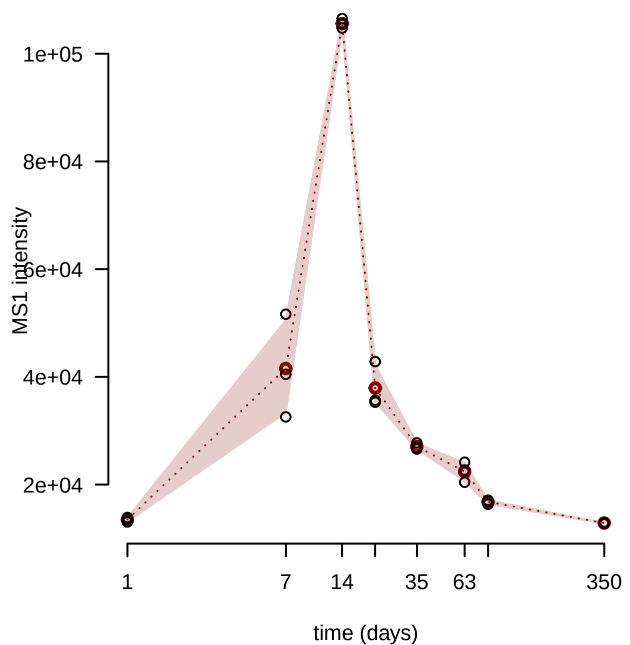

Q6DFV1
GN=Ncapg2
non−SMC condensin II complex, subunit G2

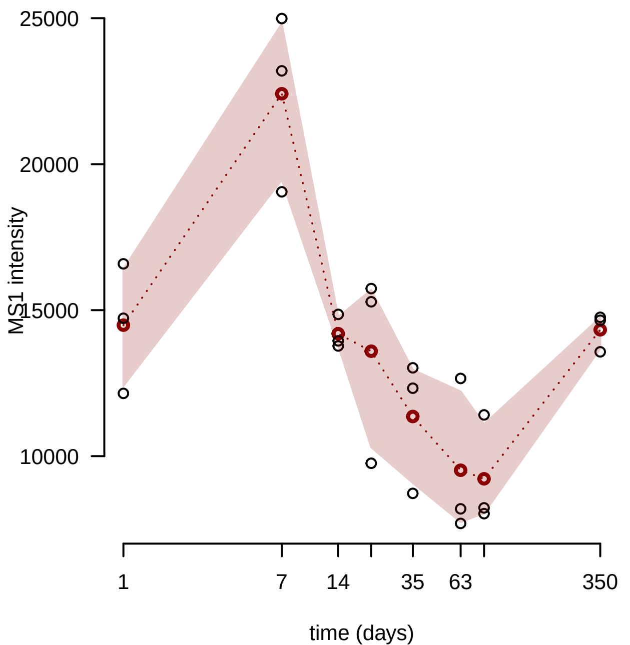

Q6DFV3
GN=Arhgap21
Rho GTPase activating protein 21

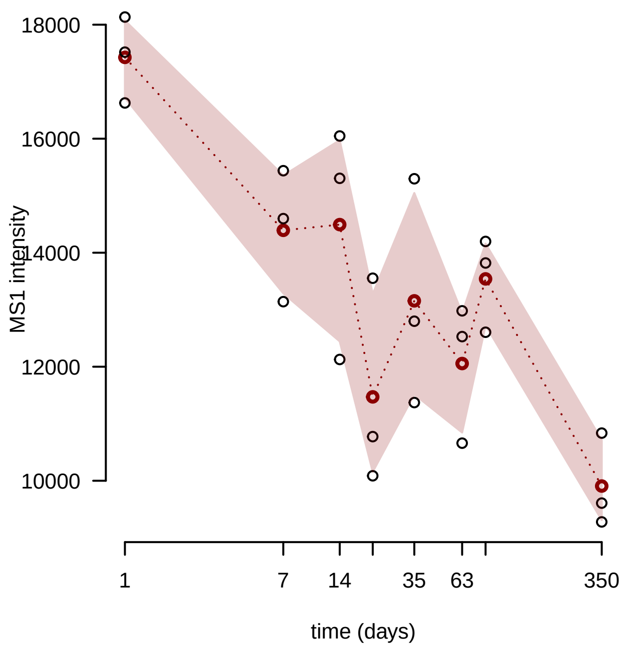

Q6DFV5
GN=Helz
helicase with zinc finger domain

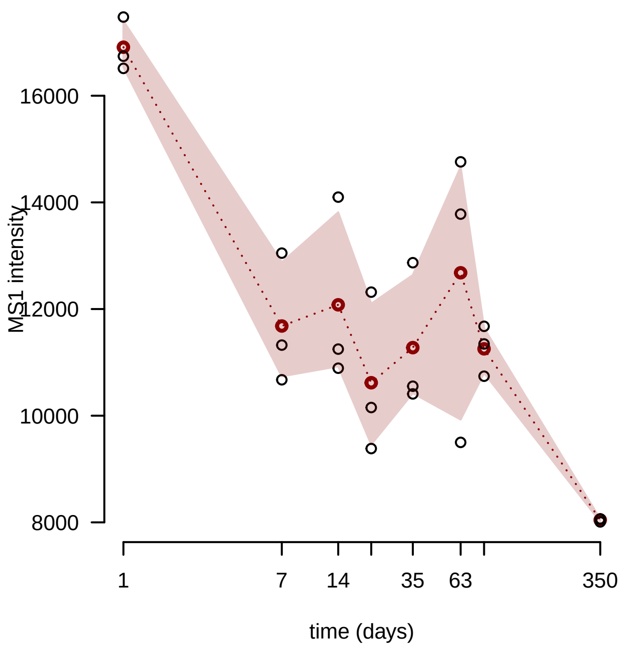

Q6DFV7
GN=Ncoa7
nuclear receptor coactivator 7

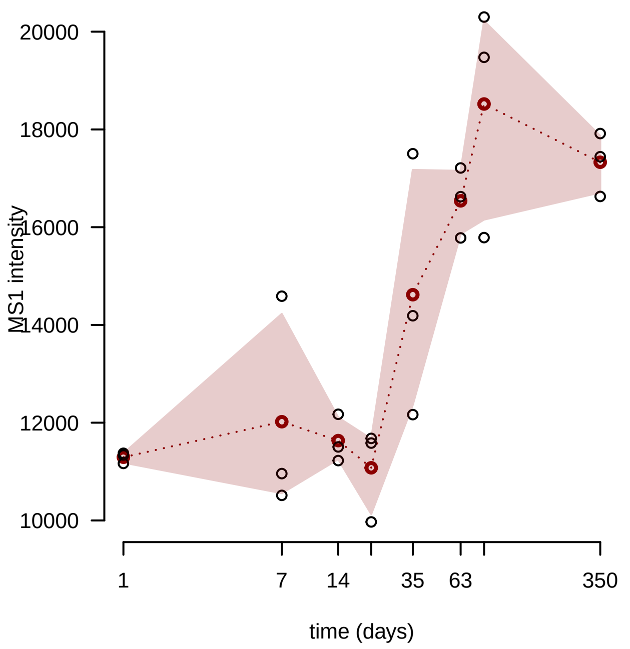

Q6DFW4
GN=Nop58
NOP58 ribonucleoprotein

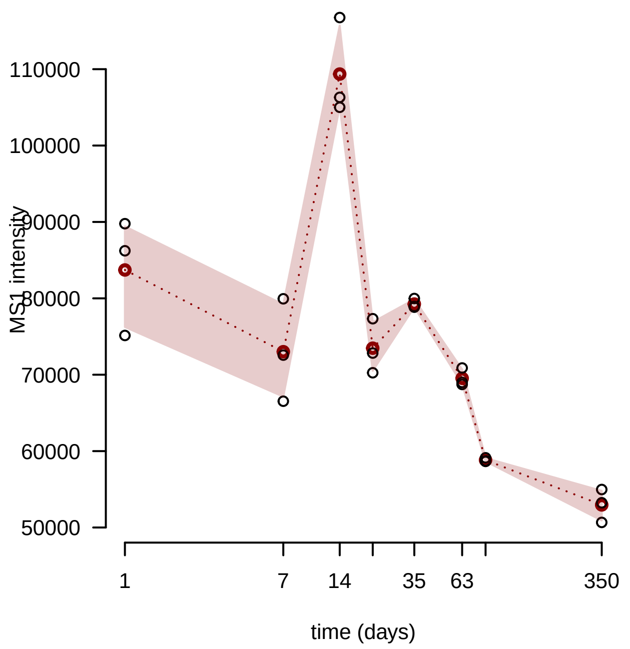

Q6DFW5
GN=

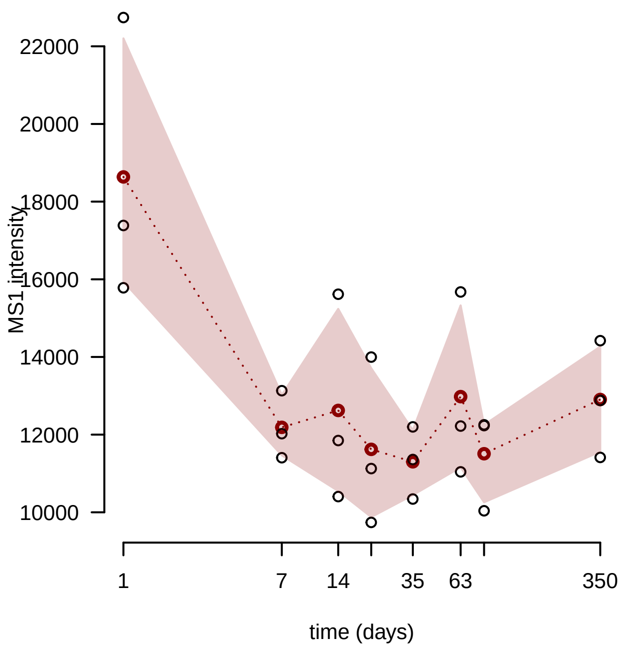

Q6DFX2
GN=Antxr2
anthrax toxin receptor 2

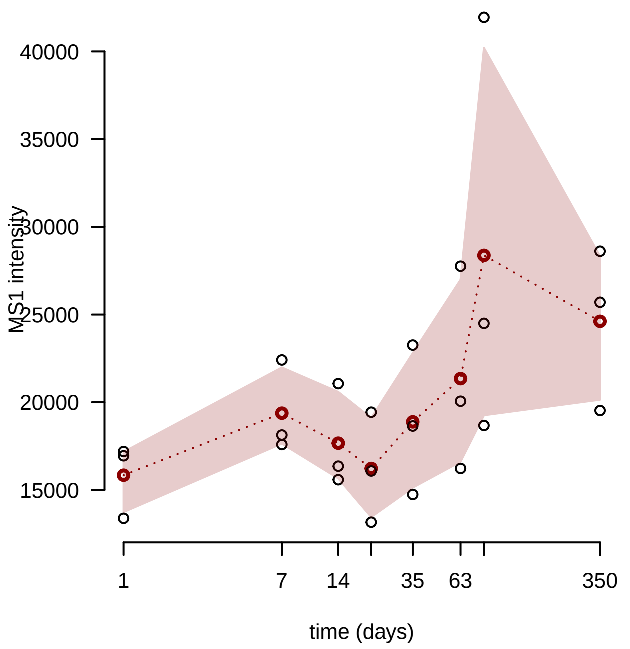

Q6DI86
GN=Fastkd1
FAST kinase domains 1

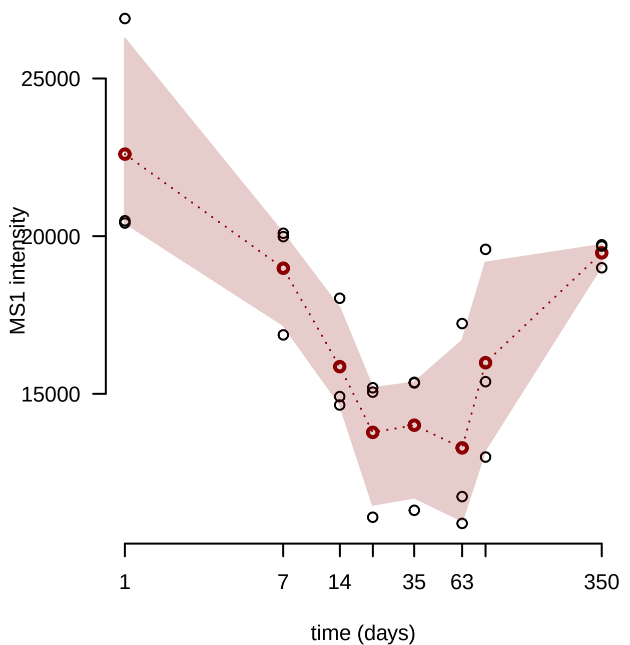

Q6DI92
GN=Zswim9
zinc finger SWIM−type containing 9

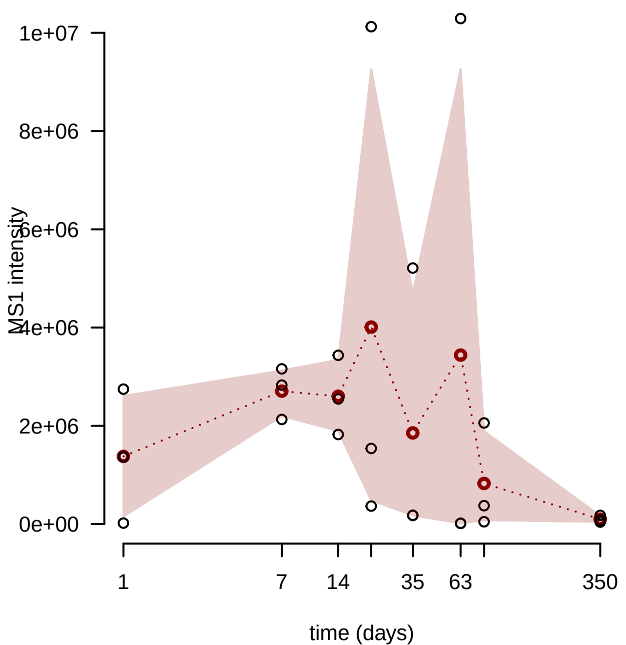

Q6DIA2
GN=Exoc3l4
exocyst complex component 3−like 4

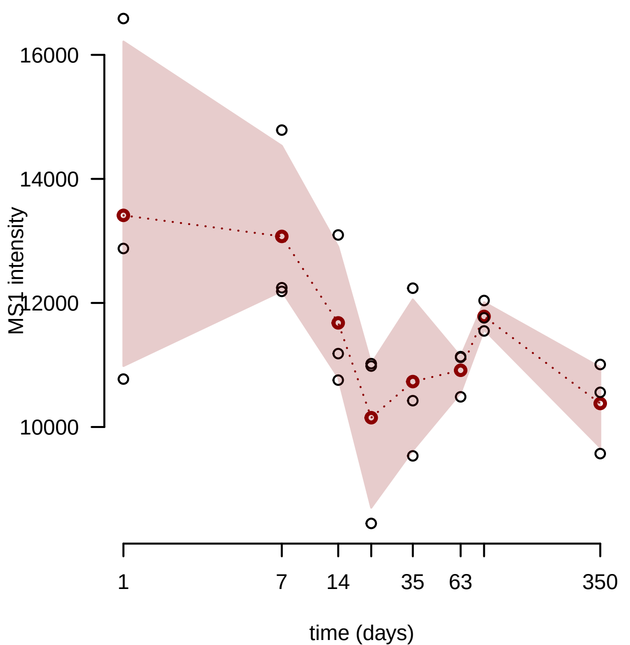

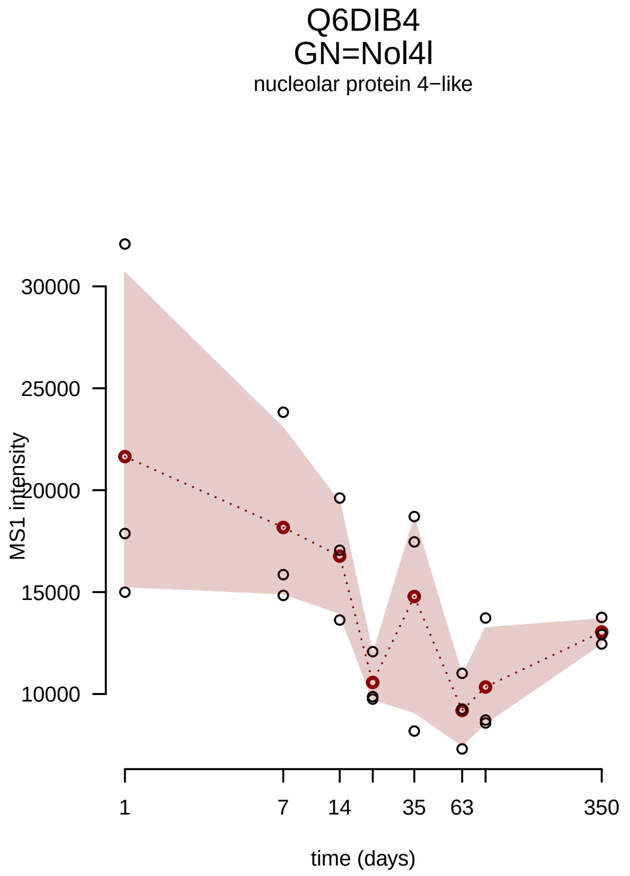
Q6DIB4
GN=Nol4l
nucleolar protein 4−like
MS1 intensity
30000
25000
20000
15000
10000
1
7
14
35
63
350
time (days)

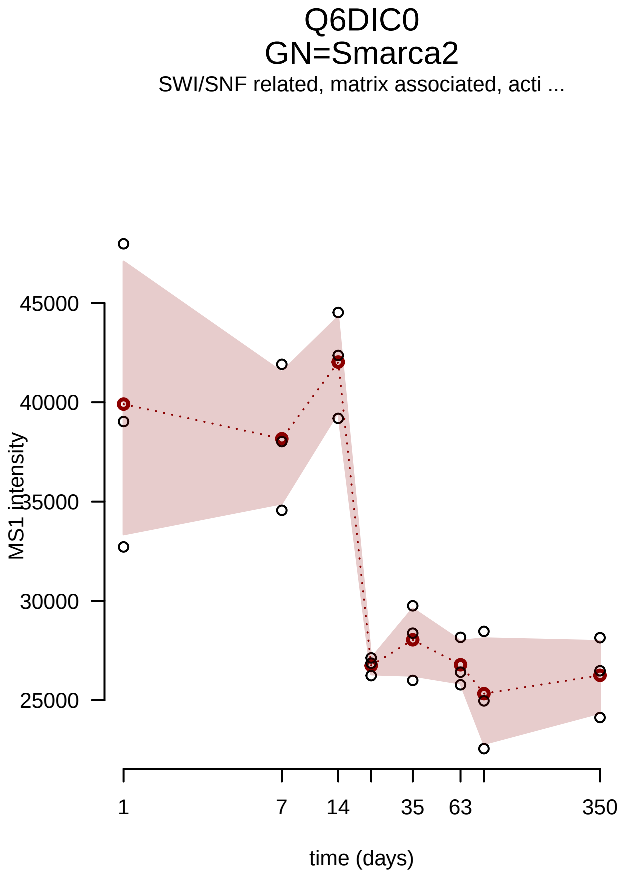
Q6DIC0
GN=Smarca2
SWI/SNF related, matrix associated, acti ...
MS1 intensity
45000
40000
35000
30000
25000
1
7
14
35
63
350
time (days)

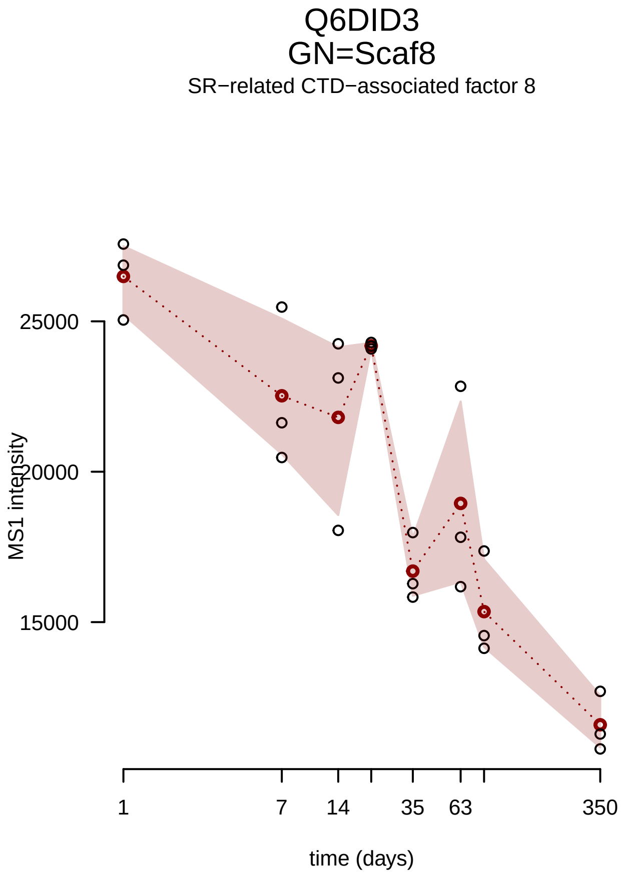
Q6DID3
GN=Scaf8
SR−related CTD−associated factor 8
MS1 intensity
25000
20000
15000
1
7
14
35
63
350
time (days)

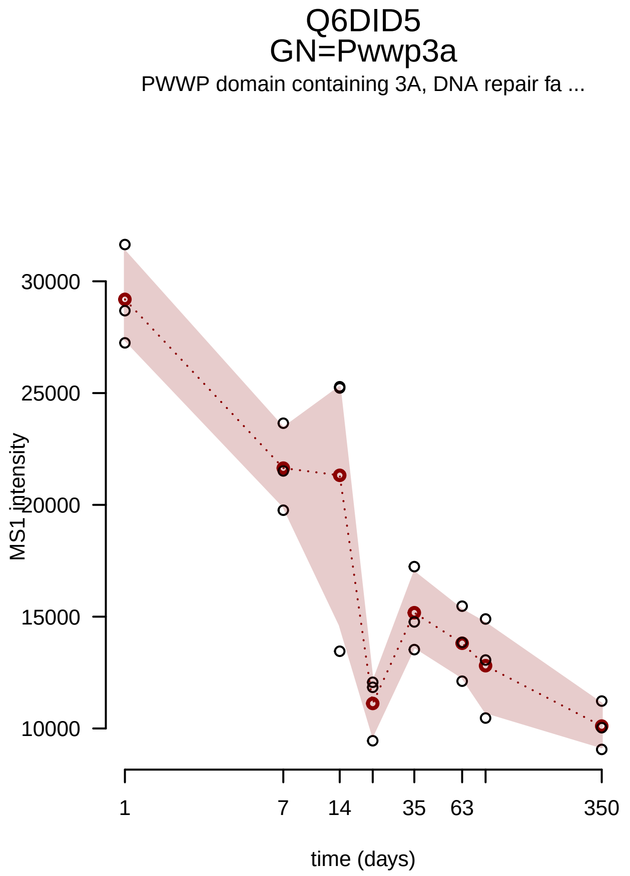
Q6DID5
GN=Pwwp3a
PWWP domain containing 3A, DNA repair fa ...
MS1 intensity
30000
25000
20000
15000
10000
1
7
14
35
63
350
time (days)

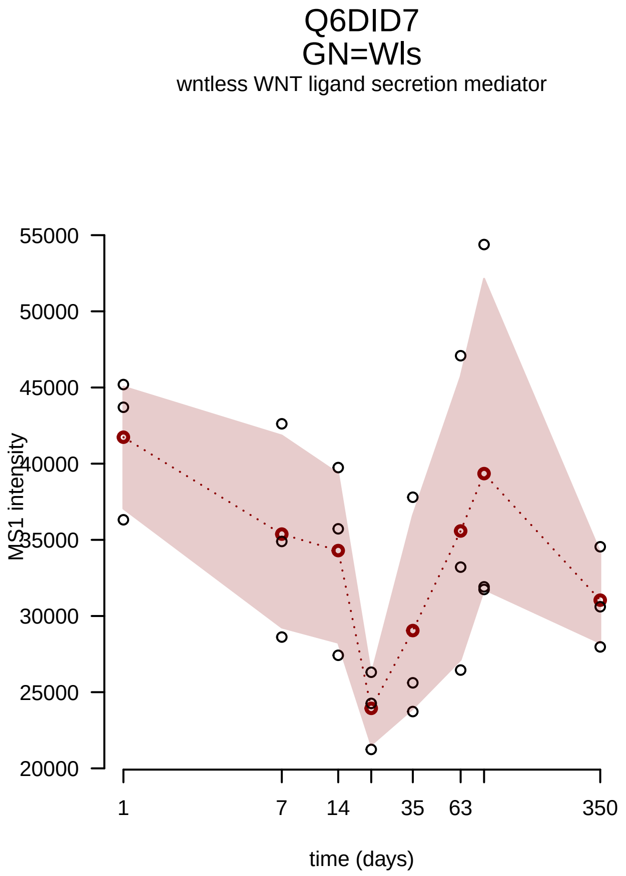
Q6DID7
GN=Wls
wntless WNT ligand secretion mediator
MS1 intensity
55000
50000
45000
40000
35000
30000
25000
20000
1
7
14
35
63
350
time (days)

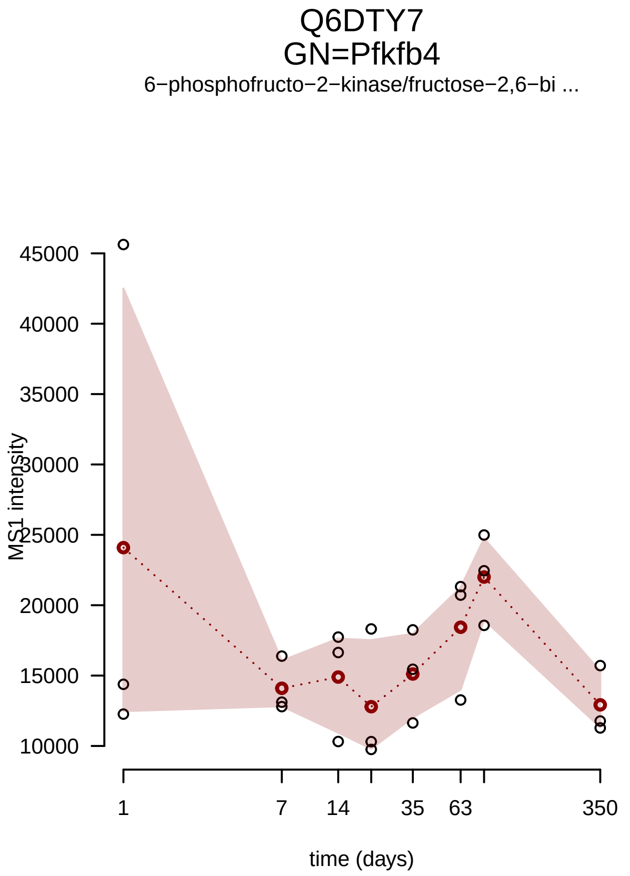
Q6DTY7
GN=Pfkfb4
6−phosphofructo−2−kinase/fructose−2,6−bi ...
MS1 intensity
45000
40000
35000
30000
25000
20000
15000
10000
1
7
14
35
63
350
time (days)

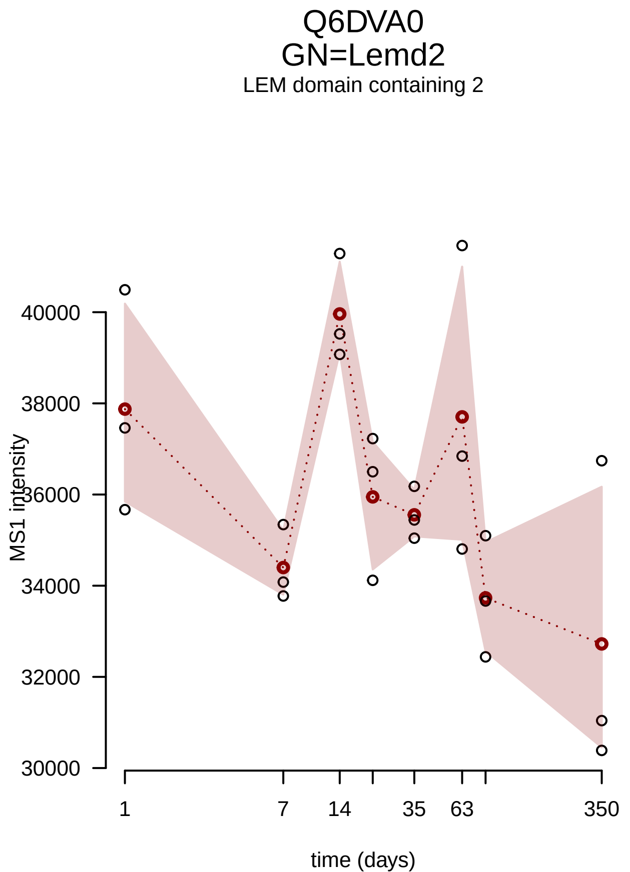
Q6DVA0
GN=Lemd2
LEM domain containing 2
MS1 intensity
40000
38000
36000
34000
32000
30000
1
7
14
35
63
350
time (days)

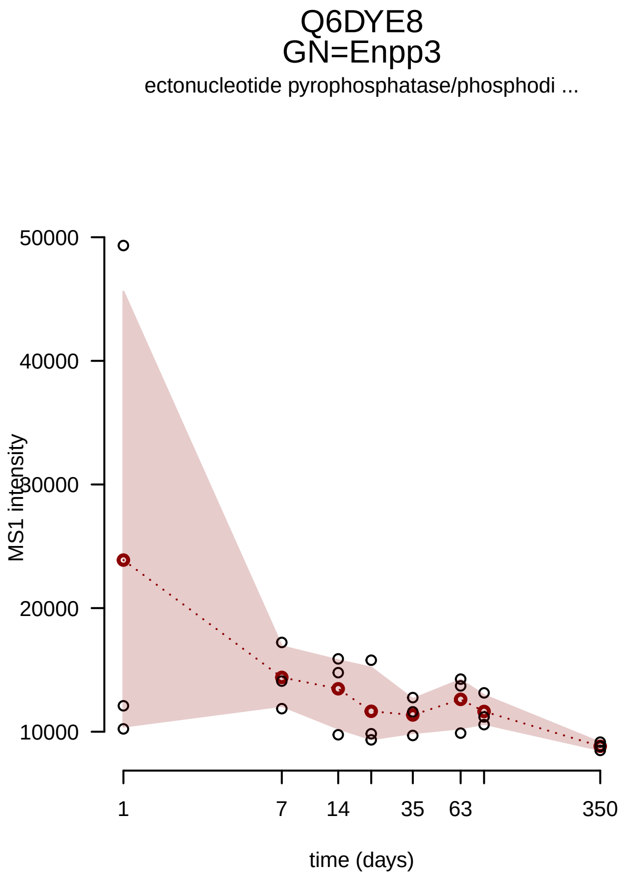
Q6DYE8
GN=Enpp3
ectonucleotide pyrophosphatase/phosphodi ...
MS1 intensity
50000
40000
30000
20000
10000
1
7
14
35
63
350
time (days)

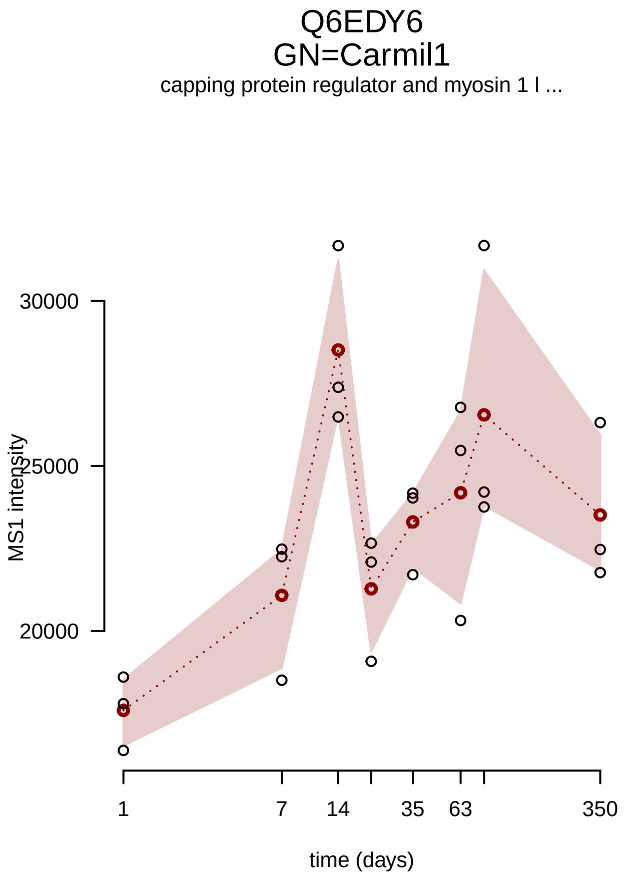
Q6EDY6
GN=Carmil1
capping protein regulator and myosin 1 l ...
MS1 intensity
30000
25000
20000
1
7
14
35
63
350
time (days)

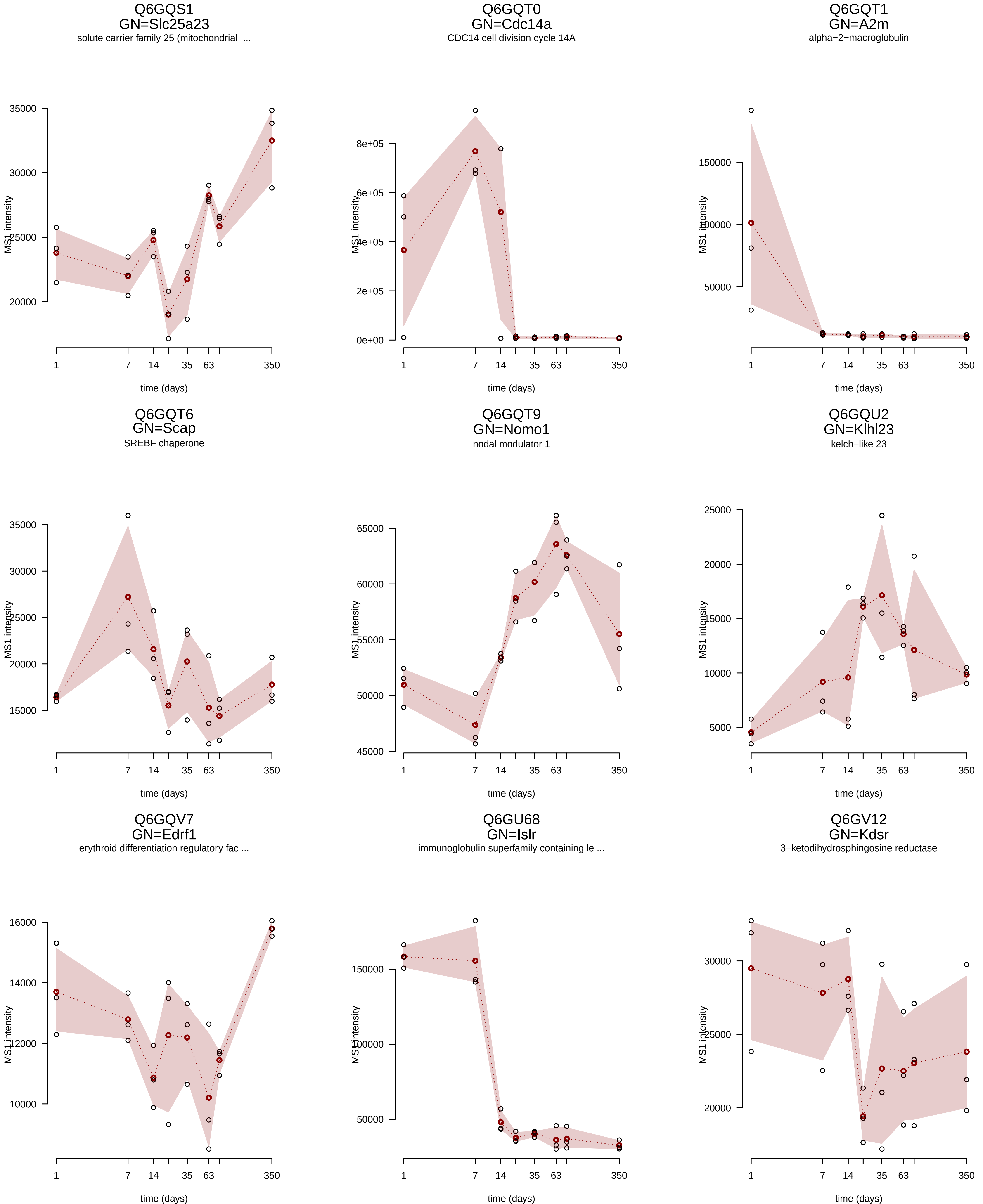
Q6GQS1
GN=Slc25a23
solute carrier family 25 (mitochondrial ...
Q6GQT0
GN=Cdc14a
CDC14 cell division cycle 14A
Q6GQT1
GN=A2m
alpha−2−macroglobulin
Q6GQT6
GN=Scap
SREBF chaperone
Q6GQT9
GN=Nomo1
nodal modulator 1
Q6GQU2
GN=Klhl23
kelch−like 23
Q6GQV7
GN=Edrf1
erythroid differentiation regulatory fac ...
Q6GU68
GN=Islr
immunoglobulin superfamily containing le ...
Q6GV12
GN=Kdsr
3−ketodihydrosphingosine reductase
MS1 intensity
time (days)
1
7
14
35
63
350

Q6GYP7
GN=Ralgapa1
Ral GTPase activating protein, alpha sub ...

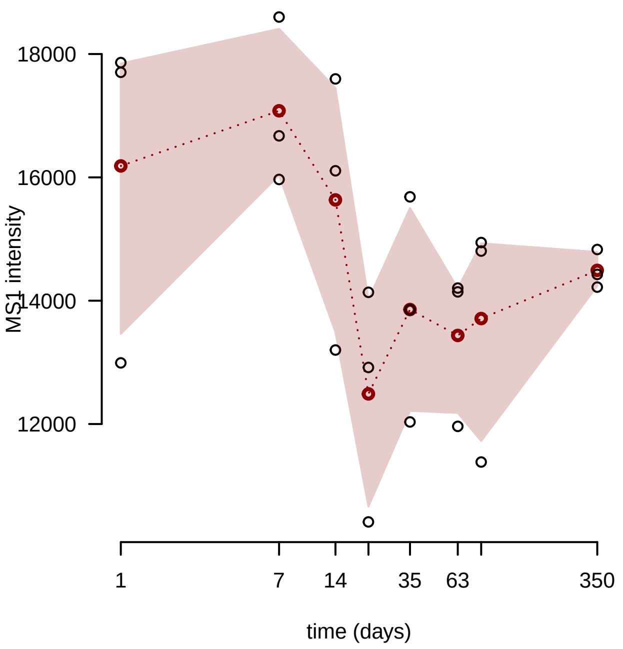

Q6HA09
GN=Astl
astacin−like metalloendopeptidase (M12 f ...

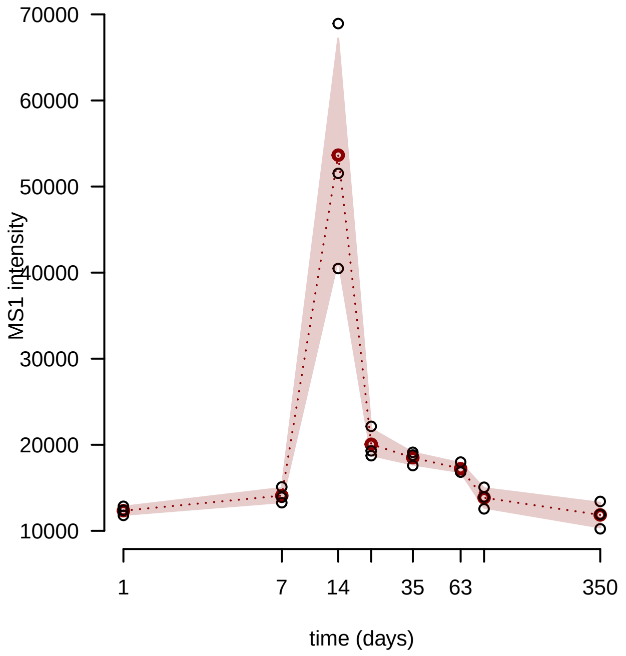

Q6I6G8
GN=Hecw2
HECT, C2 and WW domain containing E3 ubi ...

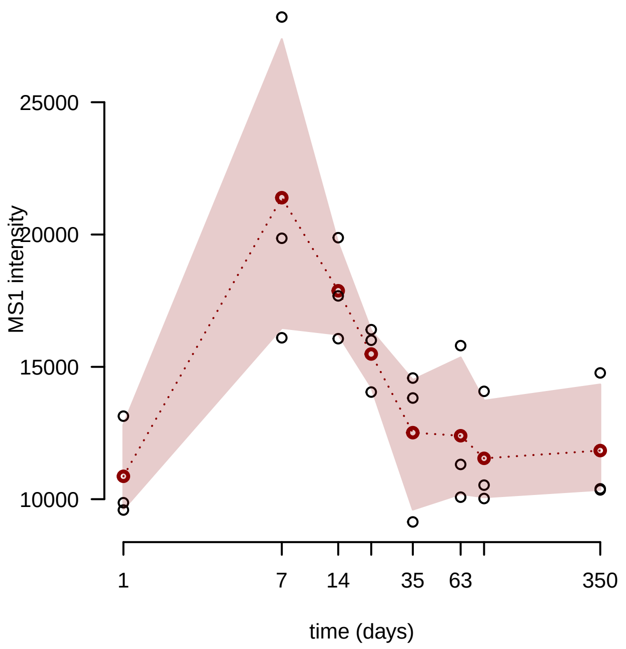

Q6IE82
GN=Jade3
jade family PHD finger 3

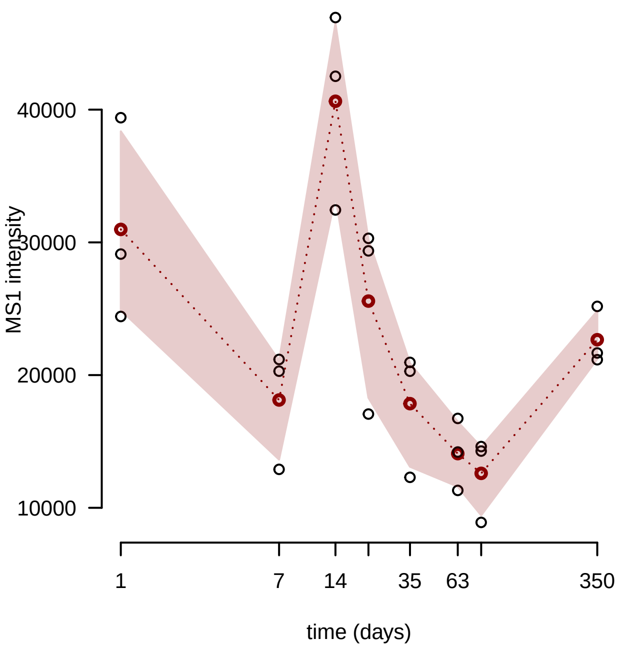

Q6IFX2
GN=Krt42
keratin 42

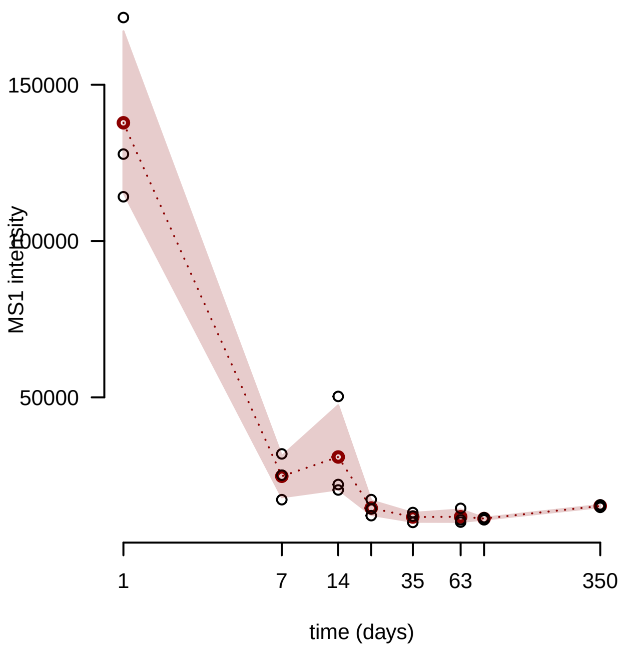

Q6IFX4
GN=Krt39
keratin 39

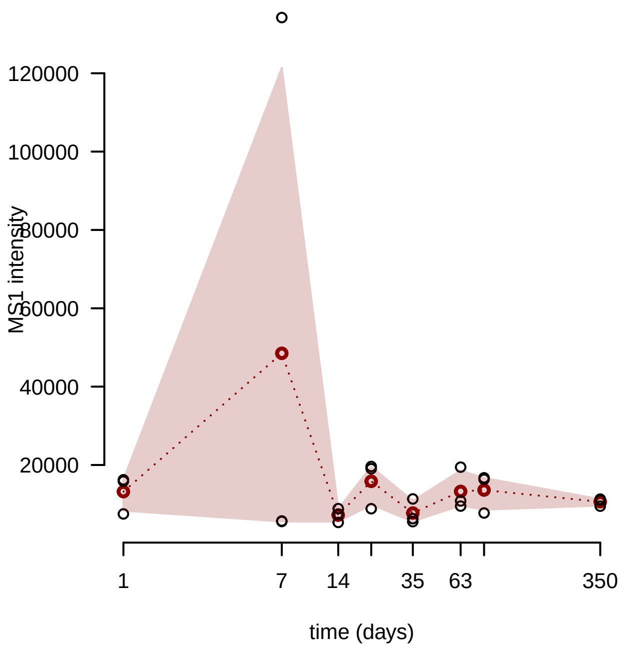

Q6IFZ6
GN=Krt77
keratin 77

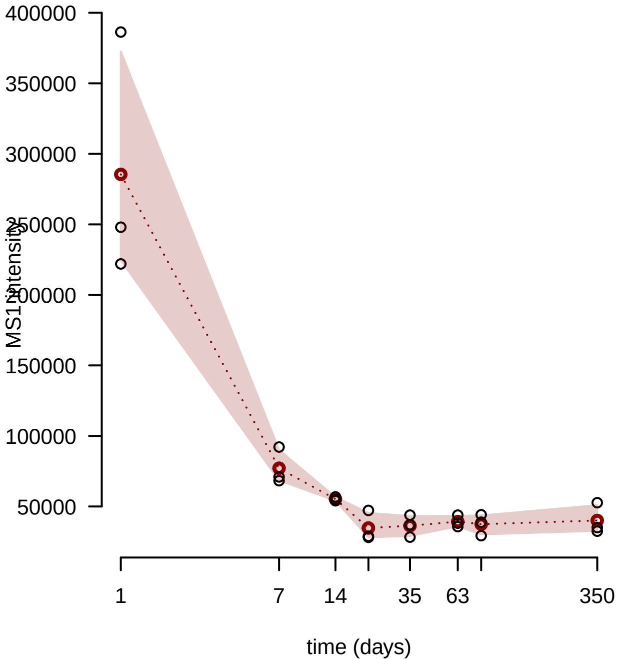

Q6IMB1
GN=Rasl11a
RAS−like, family 11, member A

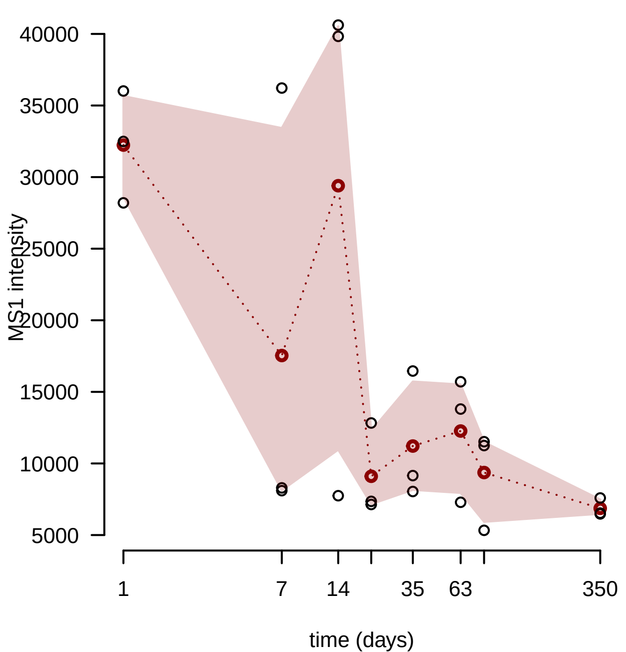

Q6IME9
GN=Krt72
keratin 72

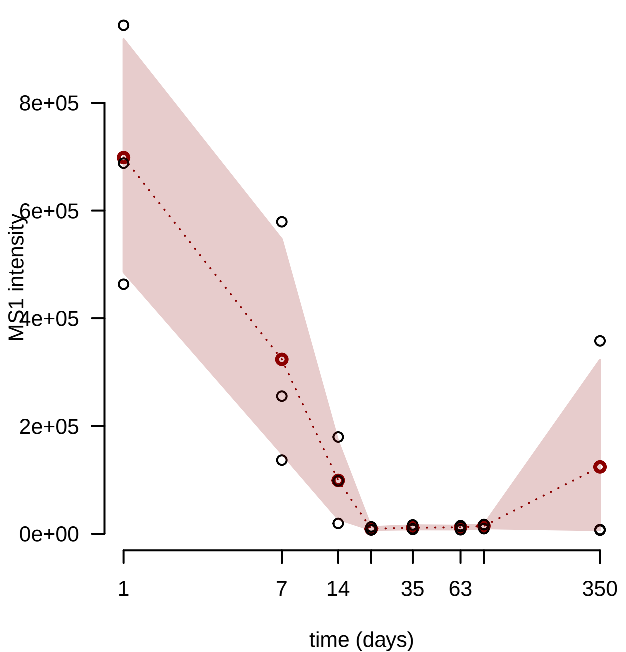

Q6IMF0
GN=Krt87
keratin 87

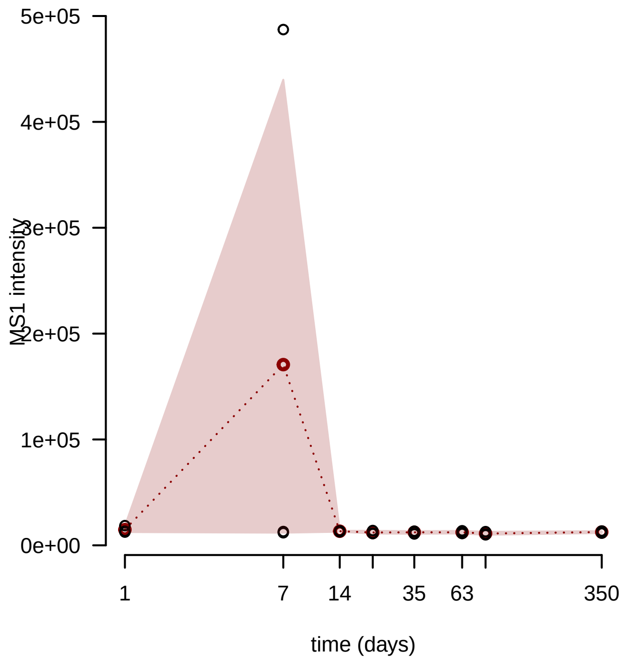

Q6IQX7
GN=Chpf
chondroitin polymerizing factor

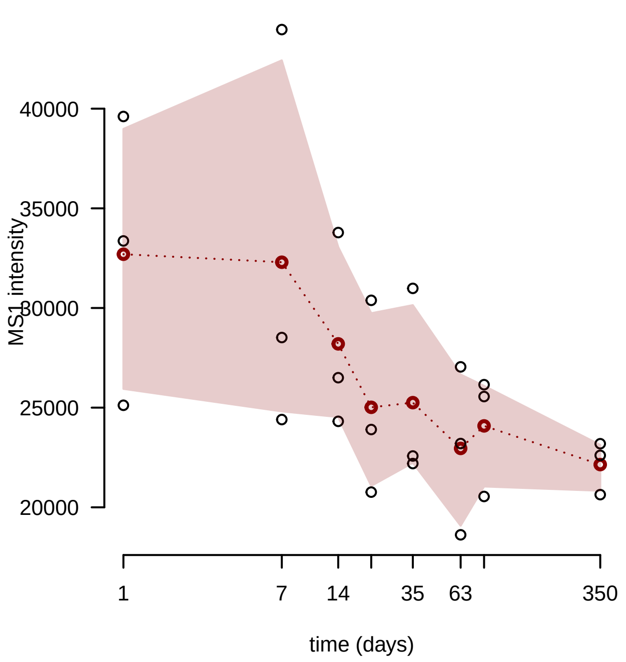

Q6IQX8
GN=Znf219

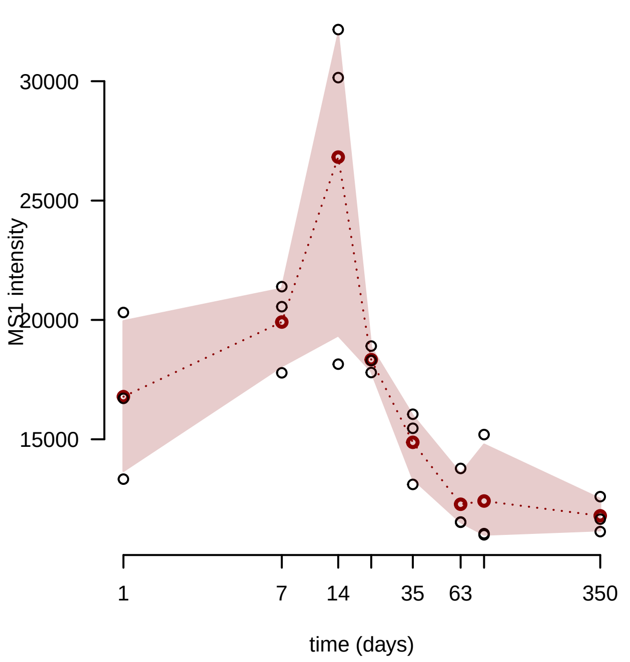

Q6IQY5
GN=Cep70
centrosomal protein 70

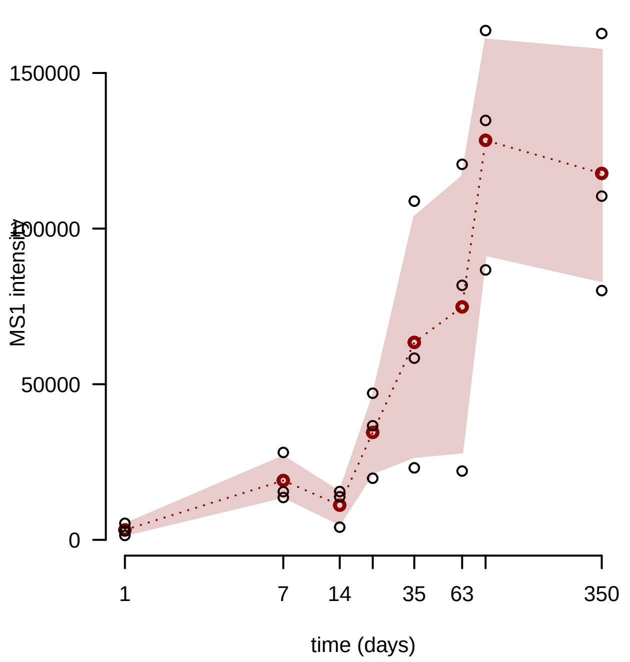

Q6IR34
GN=Gpsm1
G−protein signalling modulator 1 (AGS3−l ...

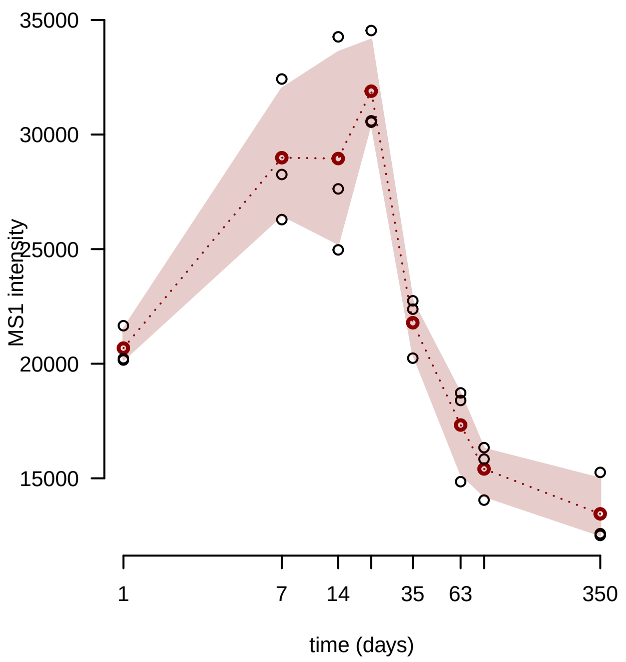

Q6IR41
GN=C1qtnf6
C1q and tumor necrosis factor related pr ...

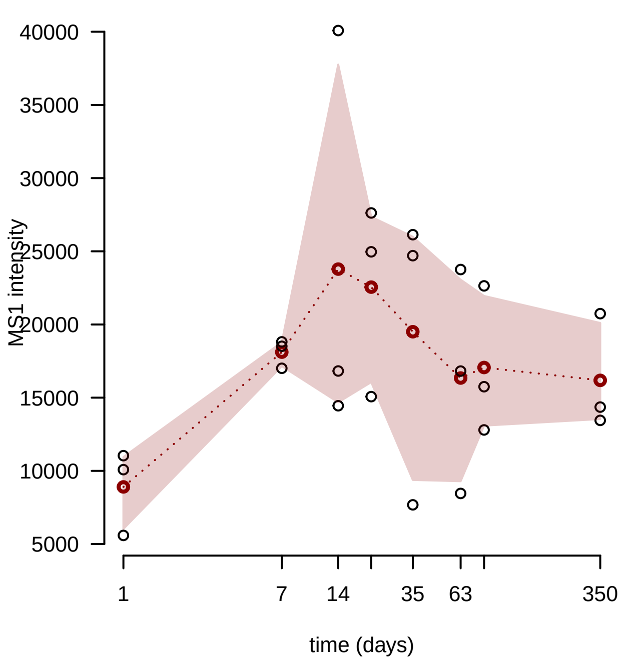

Q6IRT3
GN=Parpbp
PARP1 binding protein

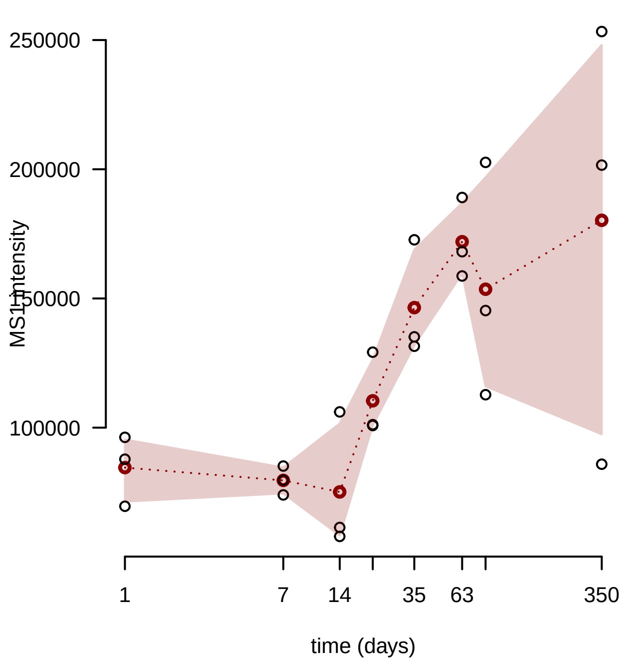

Q6IRU2
GN=Tpm4
tropomyosin 4

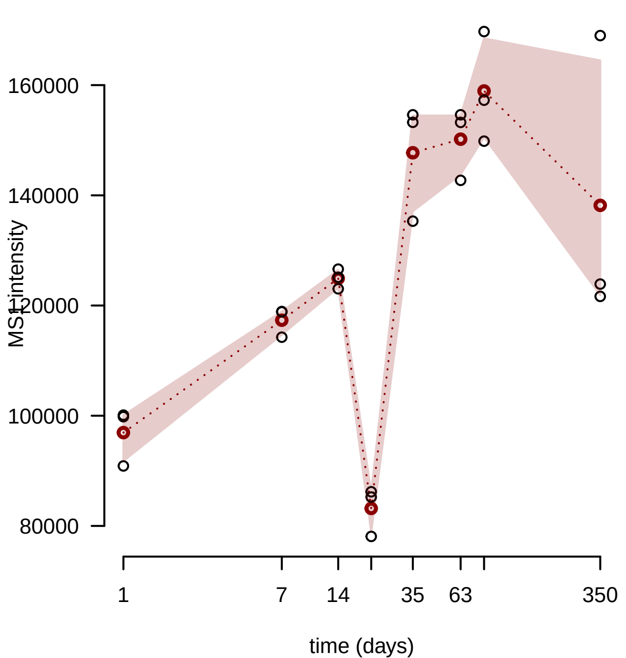

Q6IRU5
GN=Cltb
clathrin, light polypeptide (Lcb)

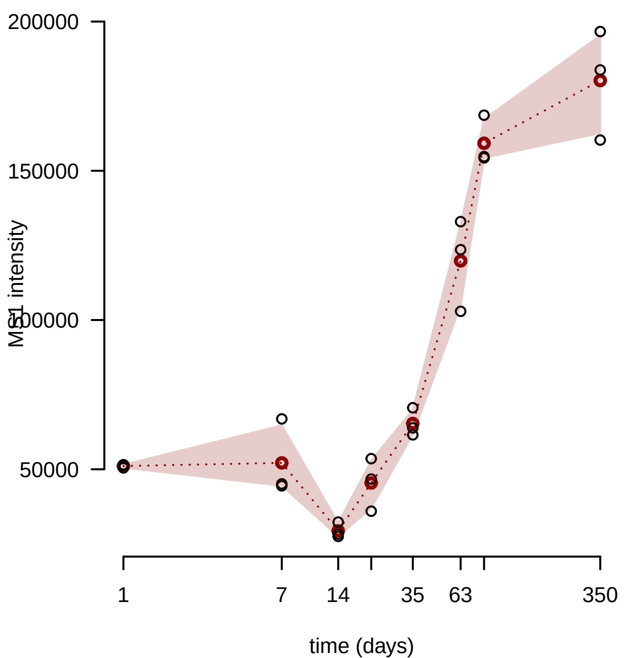

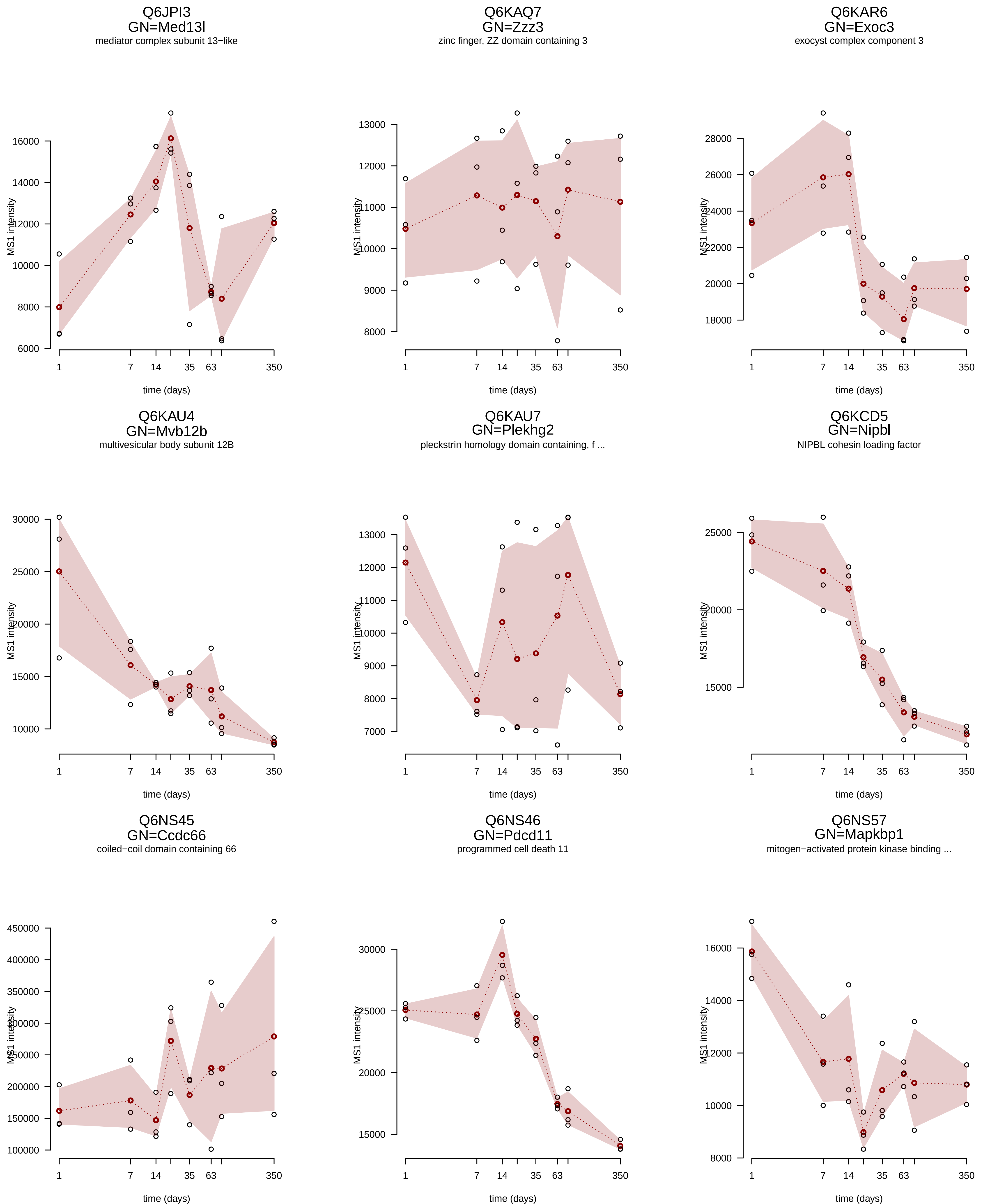
Q6JPI3
GN=Med13l
mediator complex subunit 13−like
Q6KAQ7
GN=Zzz3
zinc finger, ZZ domain containing 3
Q6KAR6
GN=Exoc3
exocyst complex component 3
Q6KAU4
GN=Mvb12b
multivesicular body subunit 12B
Q6KAU7
GN=Plekhg2
pleckstrin homology domain containing, f ...
Q6KCD5
GN=Nipbl
NIPBL cohesin loading factor
Q6NS45
GN=Ccdc66
coiled−coil domain containing 66
Q6NS46
GN=Pdcd11
programmed cell death 11
Q6NS57
GN=Mapkbp1
mitogen−activated protein kinase binding ...
MS1 intensity
time (days)
1
7
14
35
63
350

Q6NS59
GN=Fam135a
family with sequence similarity 135, mem ...

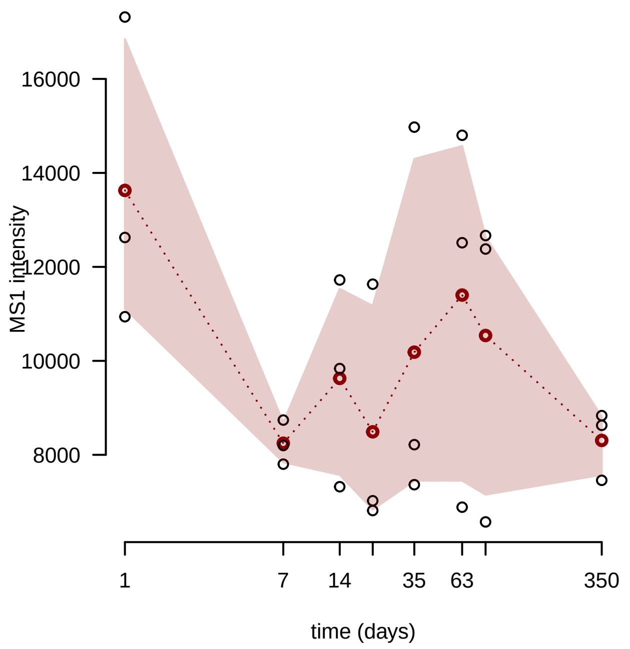

Q6NS82
GN=Retreg2
reticulophagy regulator family member 2

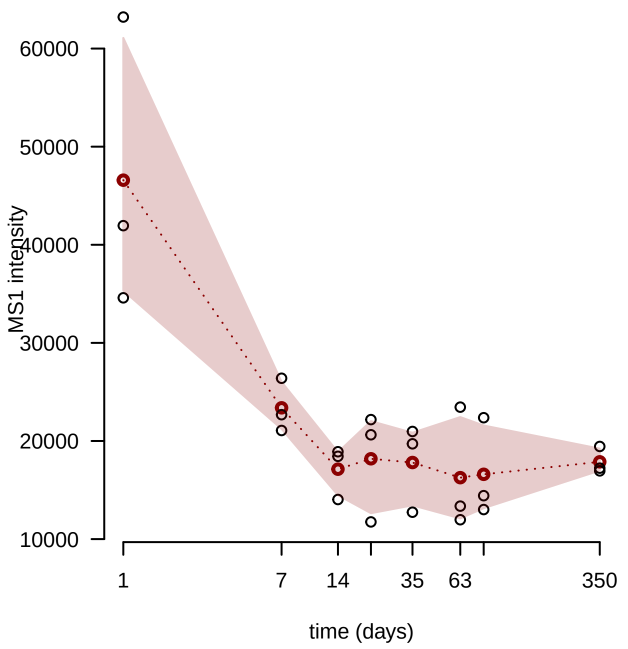

Q6NSQ7
GN=Ltv1
LTV1 ribosome biogenesis factor

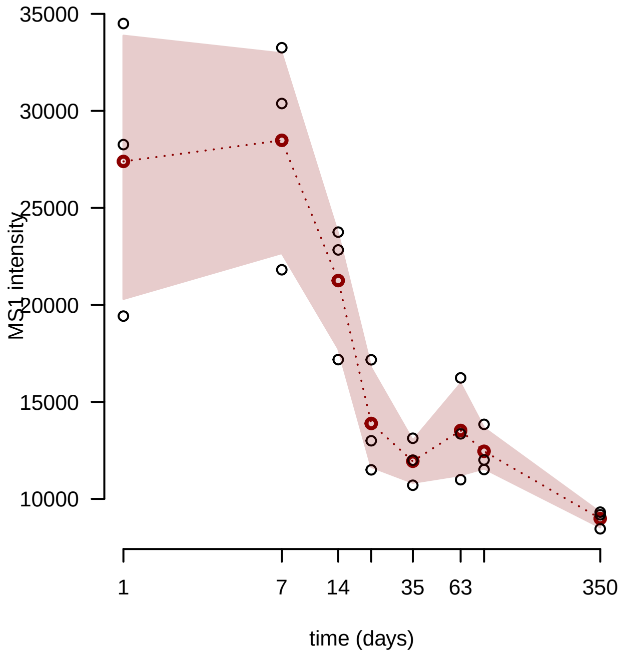

Q6NSQ9
GN=G6pc3
glucose 6 phosphatase, catalytic, 3

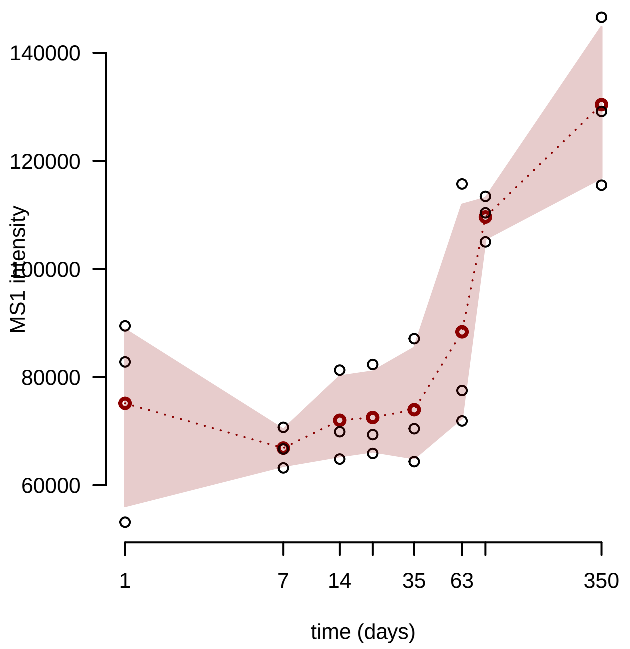

Q6NSR8
GN=Npepl1
aminopeptidase−like 1

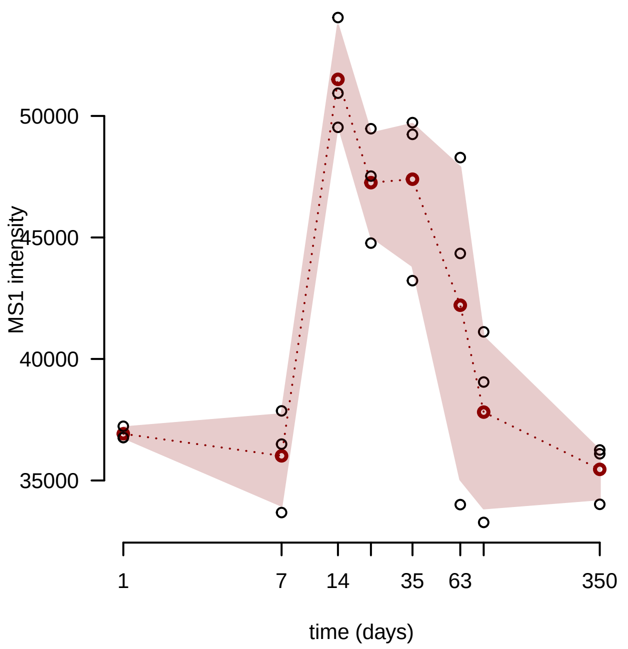

Q6NSU3
GN=Glt8d1
glycosyltransferase 8 domain containing ...

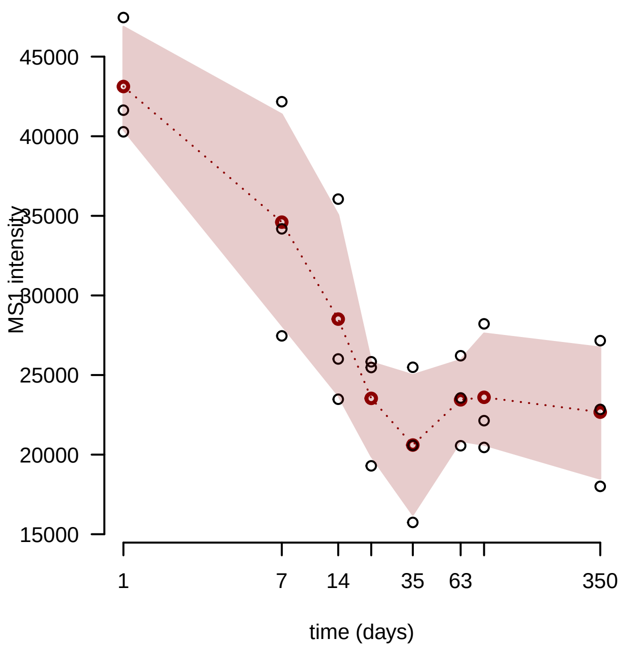

Q6NT99
GN=Dusp23
dual specificity phosphatase 23

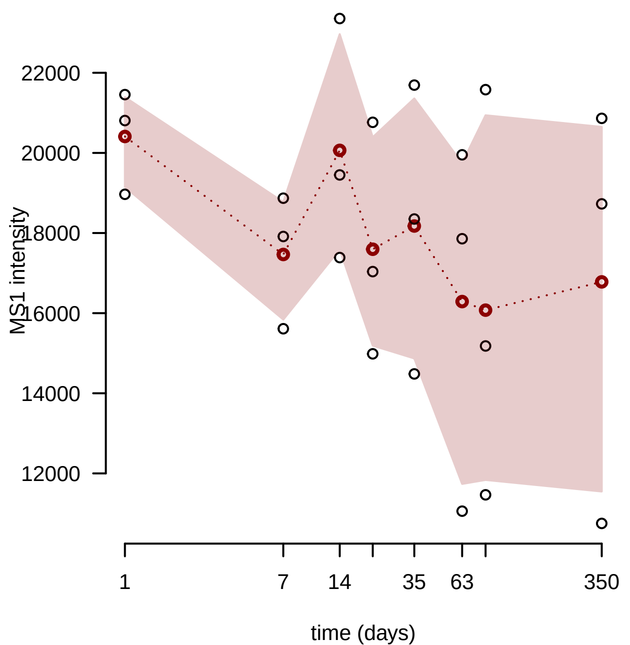

Q6NV66
GN=Zfp646
zinc finger protein 646

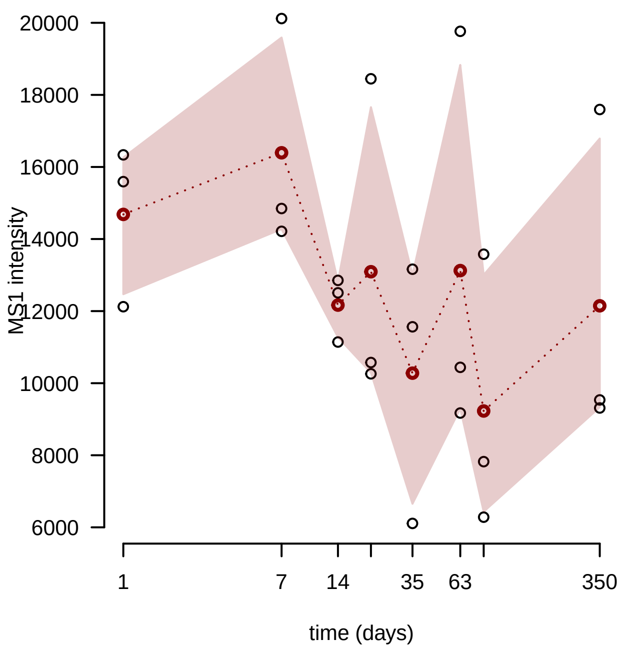

Q6NV83
GN=U2surp
U2 snRNP−associated SURP domain containi ...

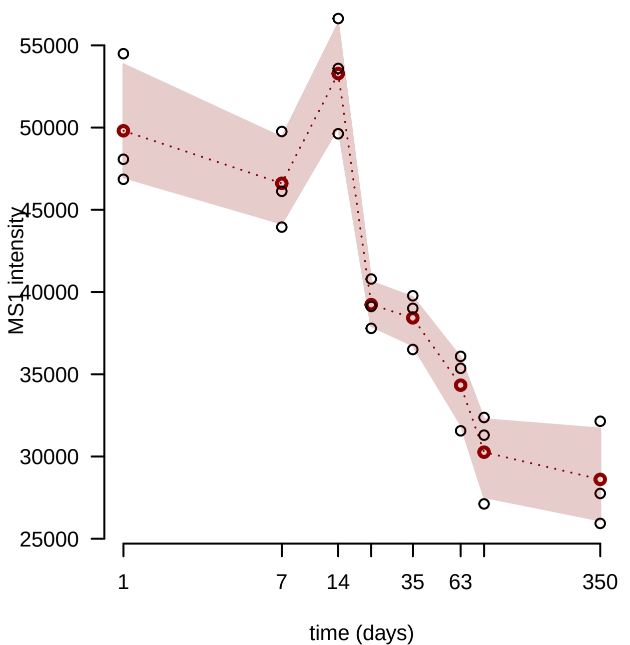

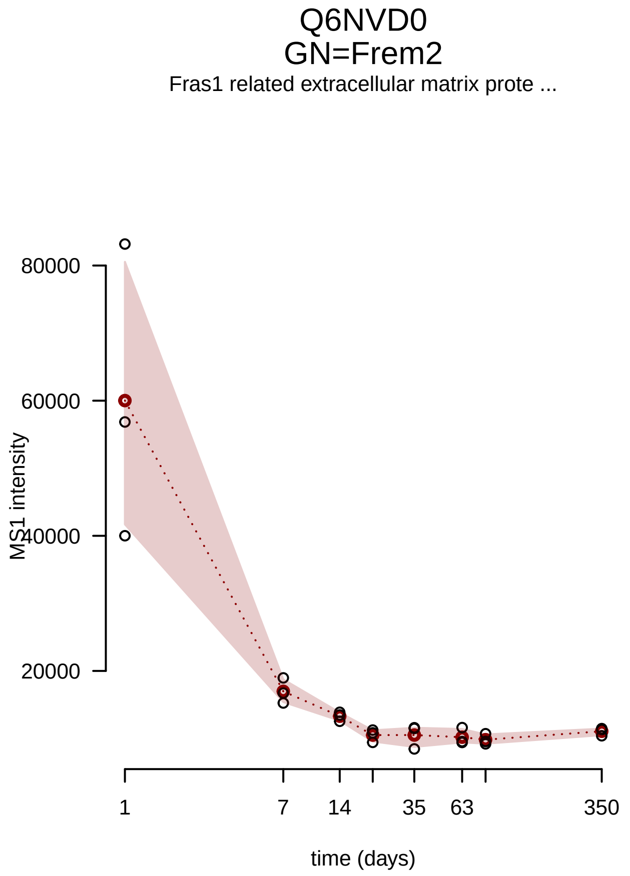
Q6NVD0
GN=Frem2
Fras1 related extracellular matrix prote ...
MS1 intensity
80000
60000
40000
20000
1
7
14
35
63
350
time (days)

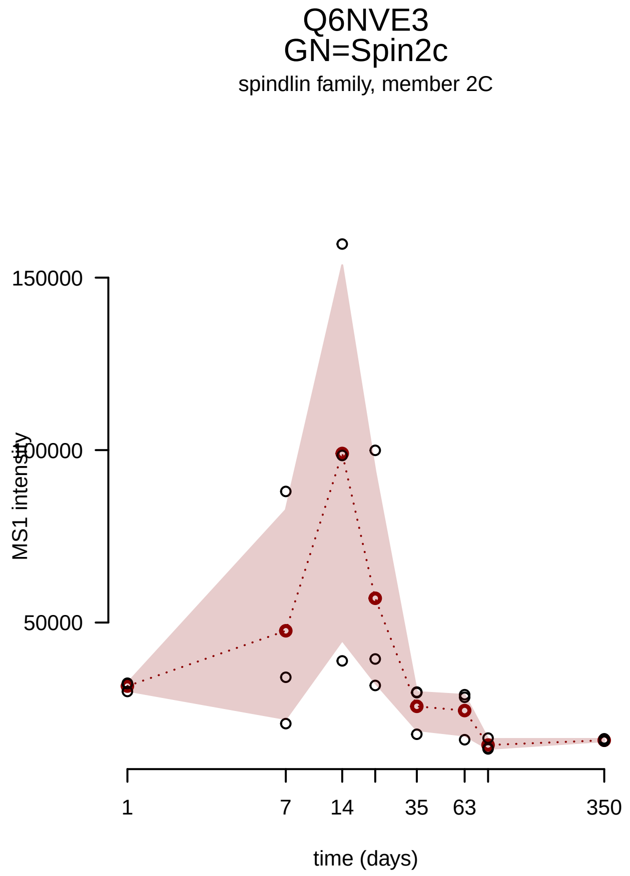
Q6NVE3
GN=Spin2c
spindlin family, member 2C
MS1 intensity
150000
100000
50000
1
7
14
35
63
350
time (days)

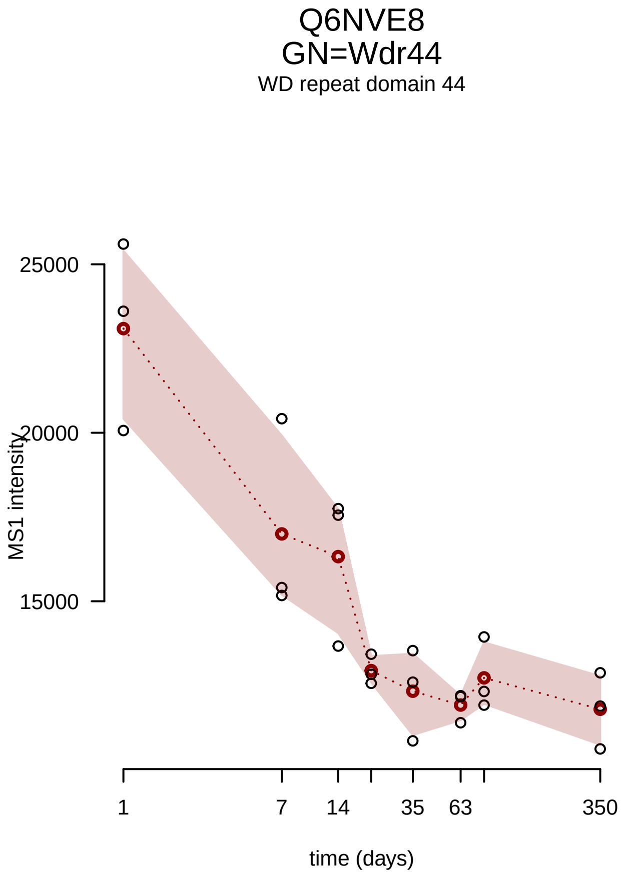
Q6NVE8
GN=Wdr44
WD repeat domain 44
MS1 intensity
25000
20000
15000
1
7
14
35
63
350
time (days)

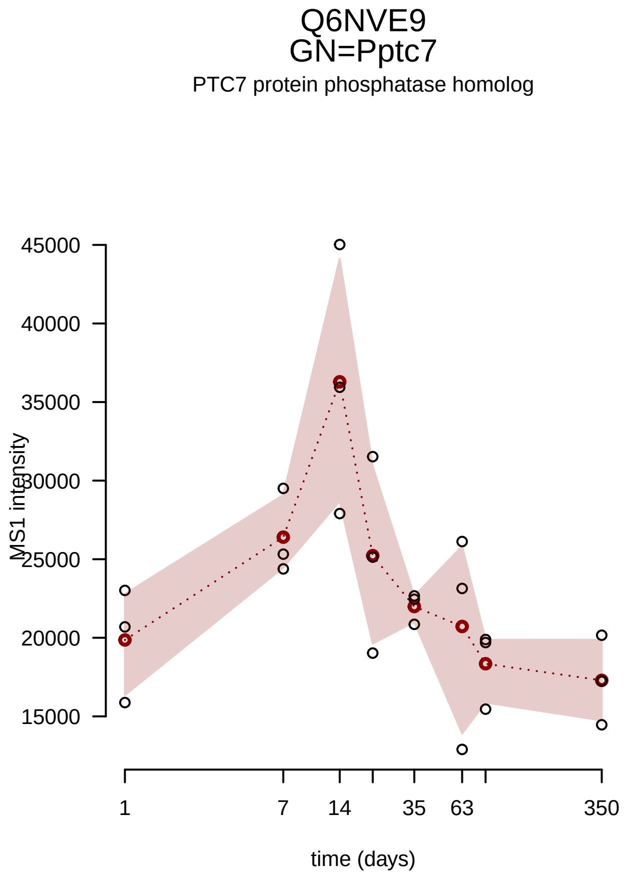
Q6NVE9
GN=Pptc7
PTC7 protein phosphatase homolog
MS1 intensity
45000
40000
35000
30000
25000
20000
15000
1
7
14
35
63
350
time (days)

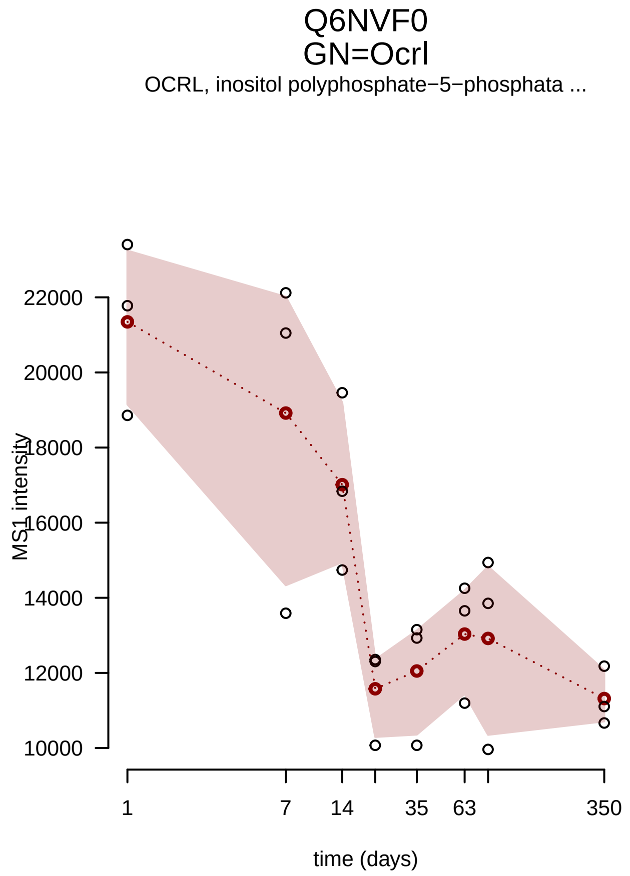
Q6NVF0
GN=Ocrl
OCRL, inositol polyphosphate−5−phosphata ...
MS1 intensity
22000
20000
18000
16000
14000
12000
10000
1
7
14
35
63
350
time (days)

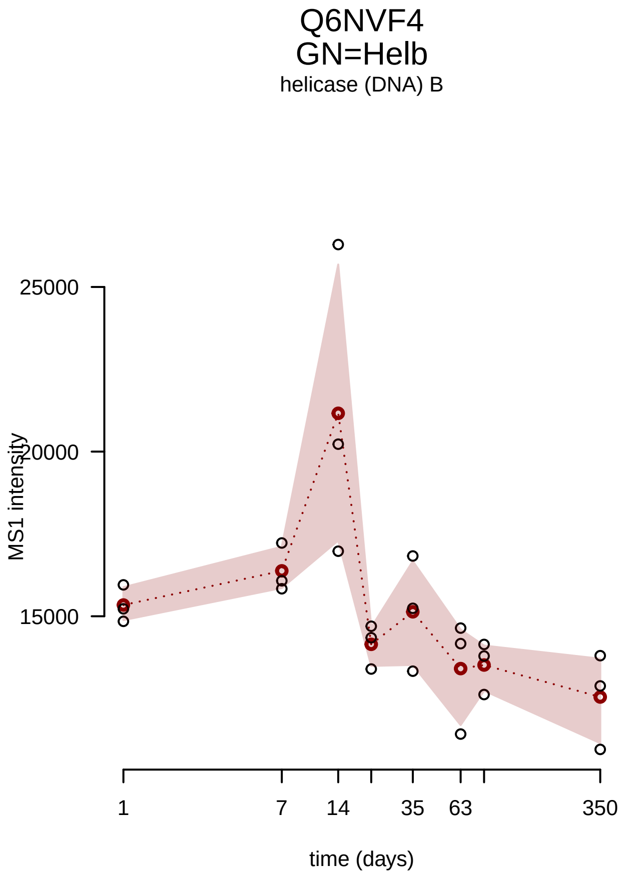
Q6NVF4
GN=Helb
helicase (DNA) B
MS1 intensity
25000
20000
15000
1
7
14
35
63
350
time (days)

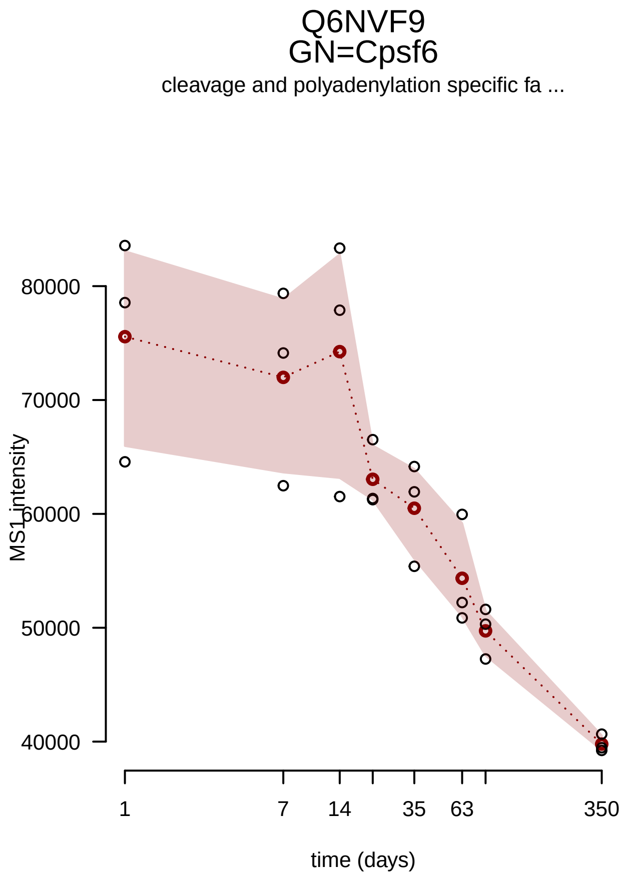
Q6NVF9
GN=Cpsf6
cleavage and polyadenylation specific fa ...
MS1 intensity
80000
70000
60000
50000
40000
1
7
14
35
63
350
time (days)

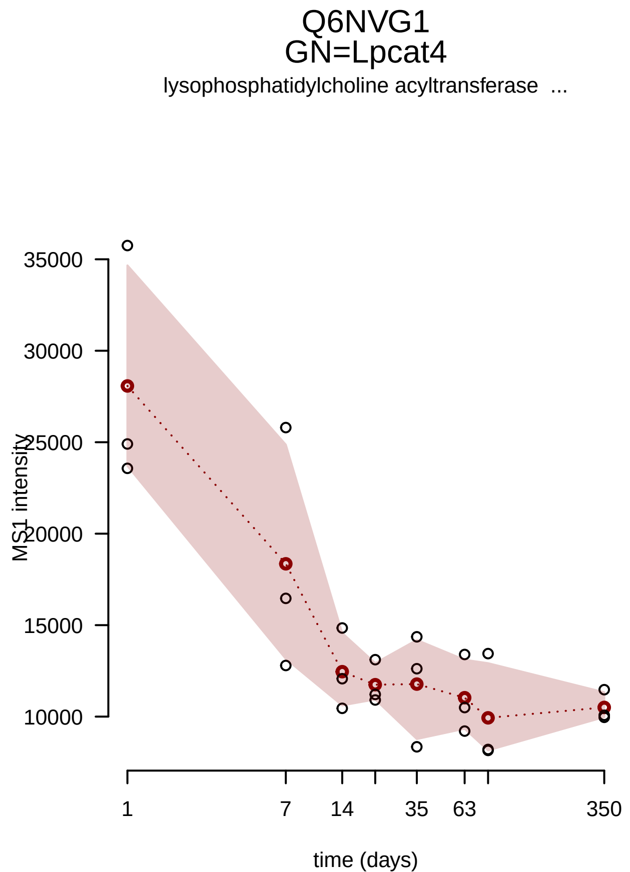
Q6NVG1
GN=Lpcat4
lysophosphatidylcholine acyltransferase ...
MS1 intensity
35000
30000
25000
20000
15000
10000
1
7
14
35
63
350
time (days)

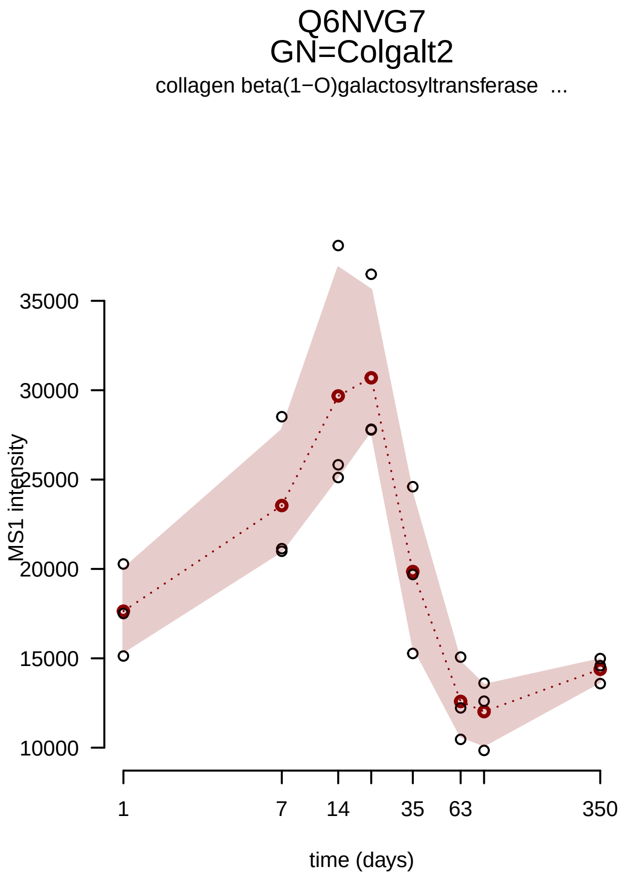
Q6NVG7
GN=Colgalt2
collagen beta(1−O)galactosyltransferase ...
MS1 intensity
35000
30000
25000
20000
15000
10000
1
7
14
35
63
350
time (days)

Q6NWV3
GN=Ift122
intraflagellar transport 122

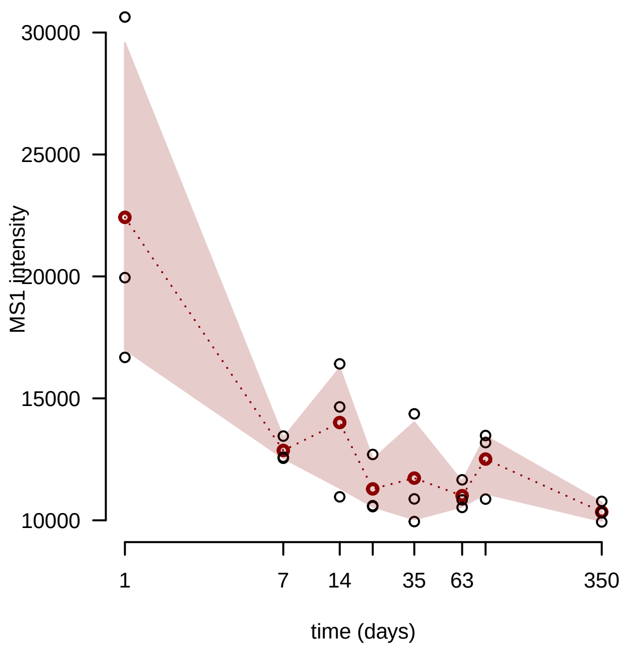

Q6NWW9
GN=Fndc3b
fibronectin type III domain containing 3 ...

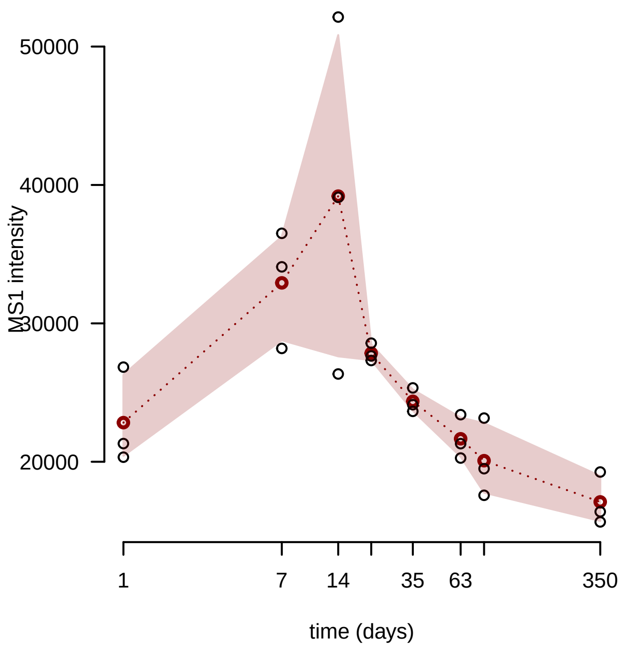

Q6NXH2
GN=Manea
mannosidase, endo−alpha

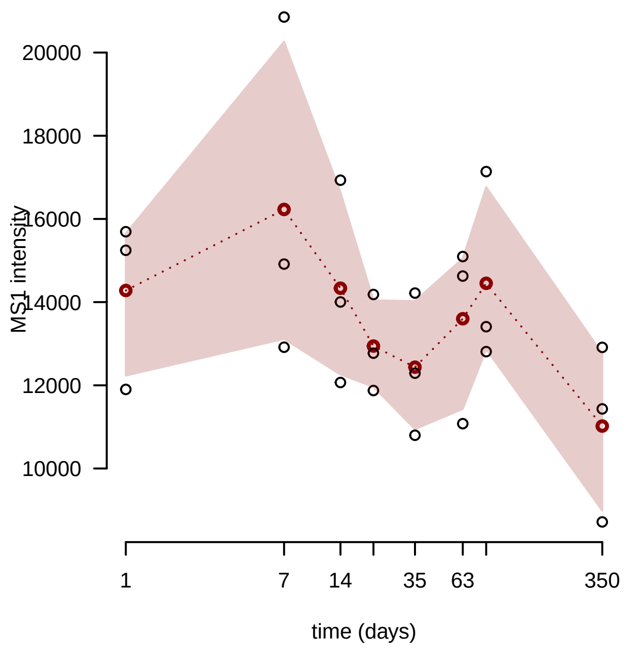

Q6NXH3
GN=Gpbp1
GC−rich promoter binding protein 1

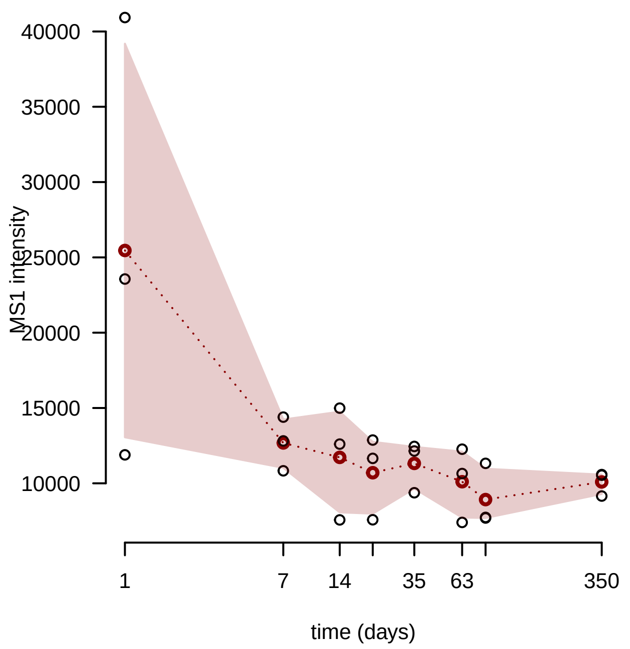

Q6NXH9
GN=Krt73
keratin 73

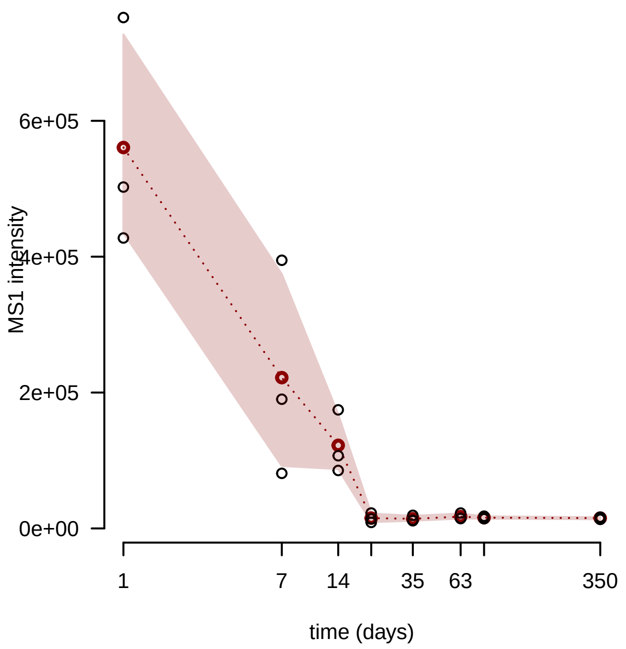

Q6NXI6
GN=Rprd2
regulation of nuclear pre−mRNA domain co ...

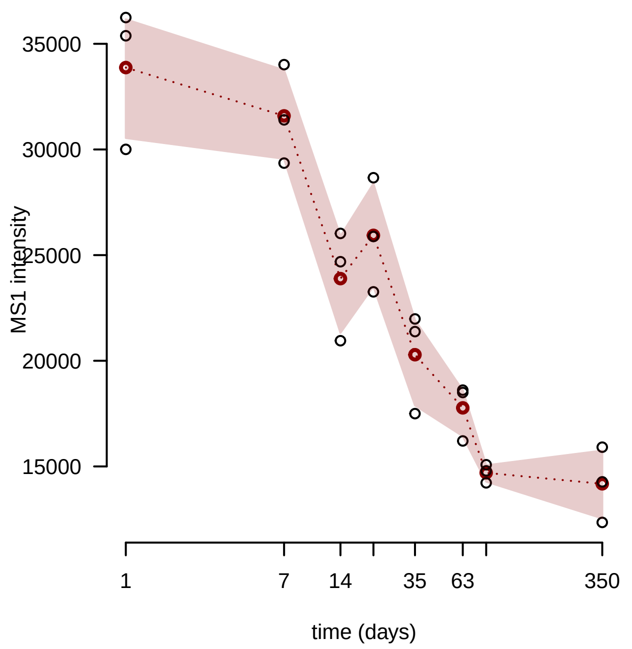

Q6NXJ0
GN=Wwc2
WW, C2 and coiled−coil domain containing ...

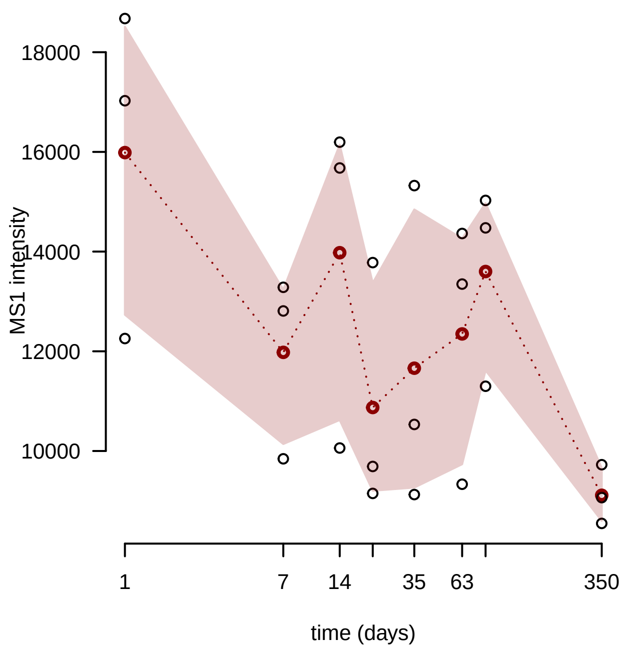

Q6NXK2
GN=Znf532

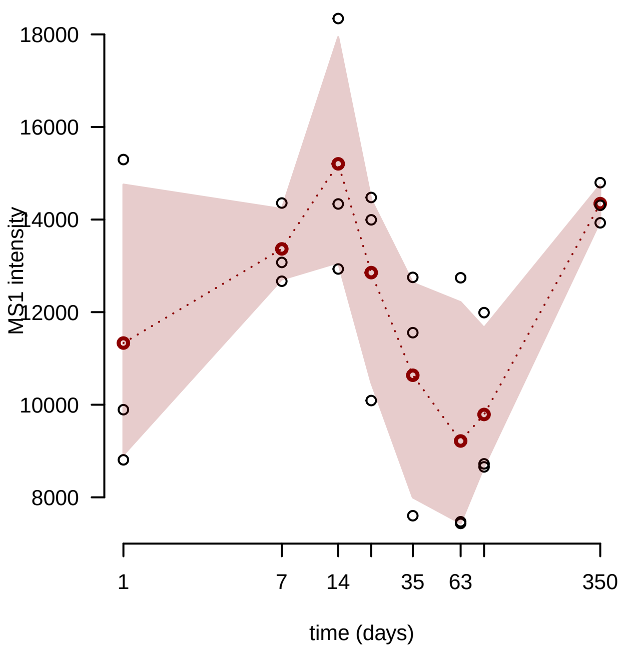

Q6NXL6
GN=Senp5
SUMO/sentrin specific peptidase 5

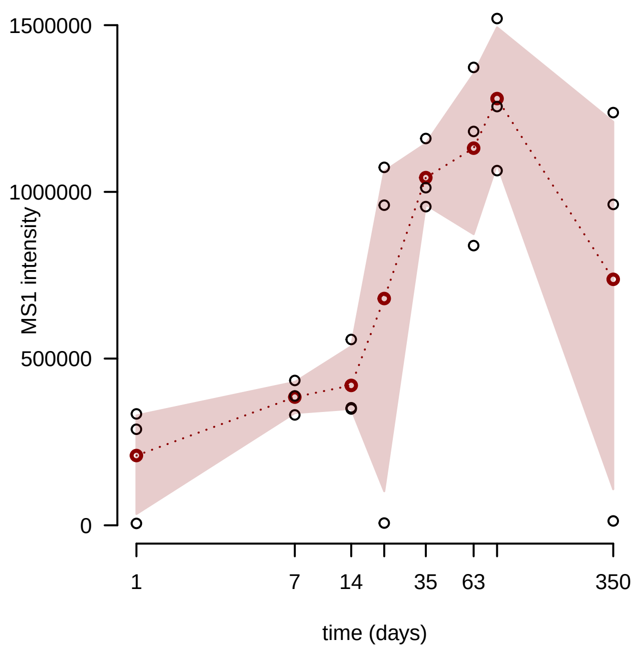

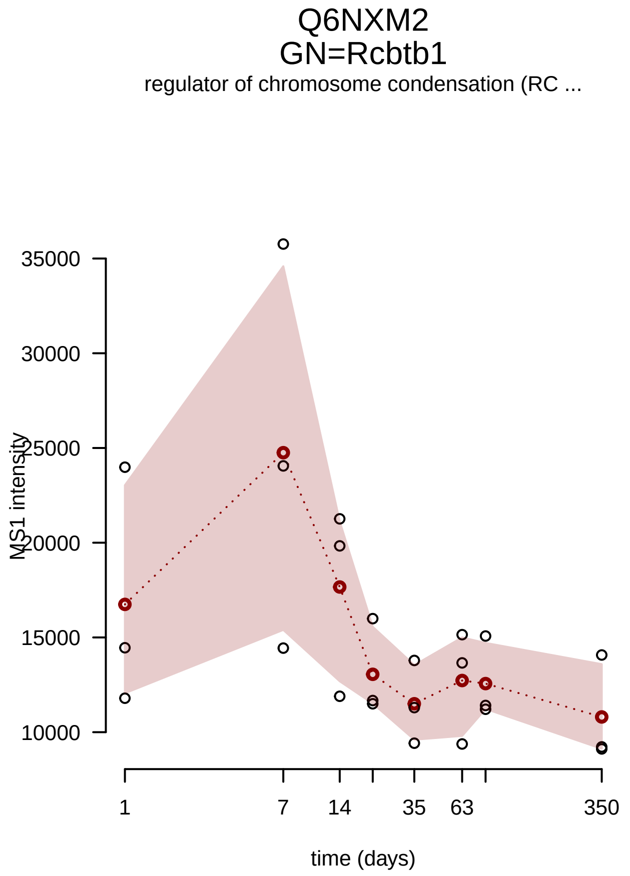
Q6NXM2
GN=Rcbtb1
regulator of chromosome condensation (RC ...
MS1 intensity
35000
30000
25000
20000
15000
10000
1
7
14
35
63
350
time (days)
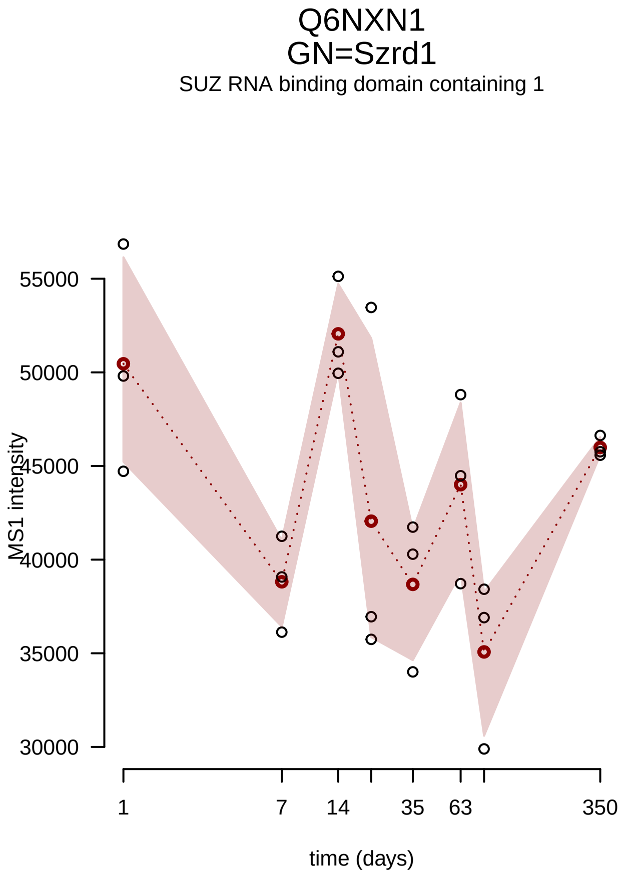
Q6NXN1
GN=Szrd1
SUZ RNA binding domain containing 1
MS1 intensity
55000
50000
45000
40000
35000
30000
1
7
14
35
63
350
time (days)
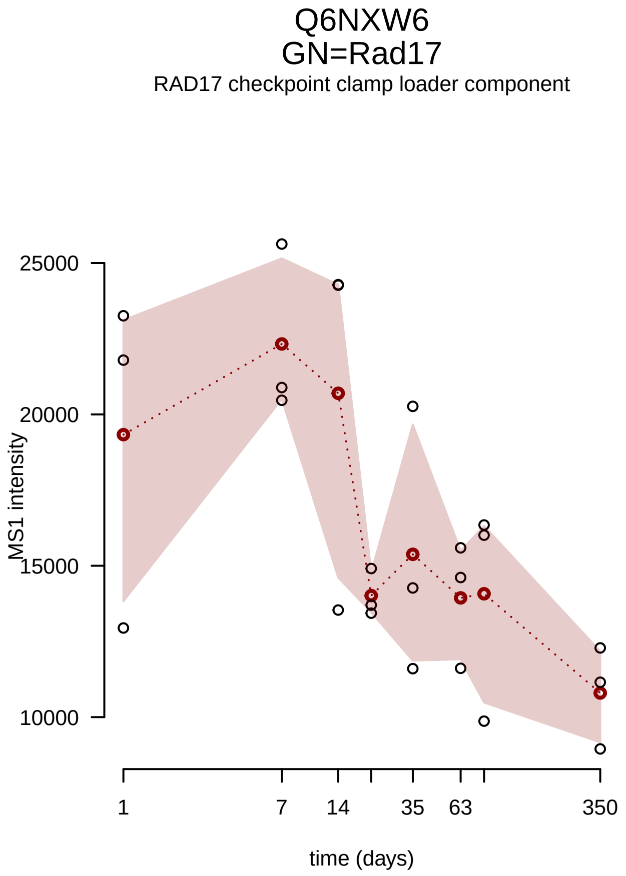
Q6NXW6
GN=Rad17
RAD17 checkpoint clamp loader component
MS1 intensity
25000
20000
15000
10000
1
7
14
35
63
350
time (days)
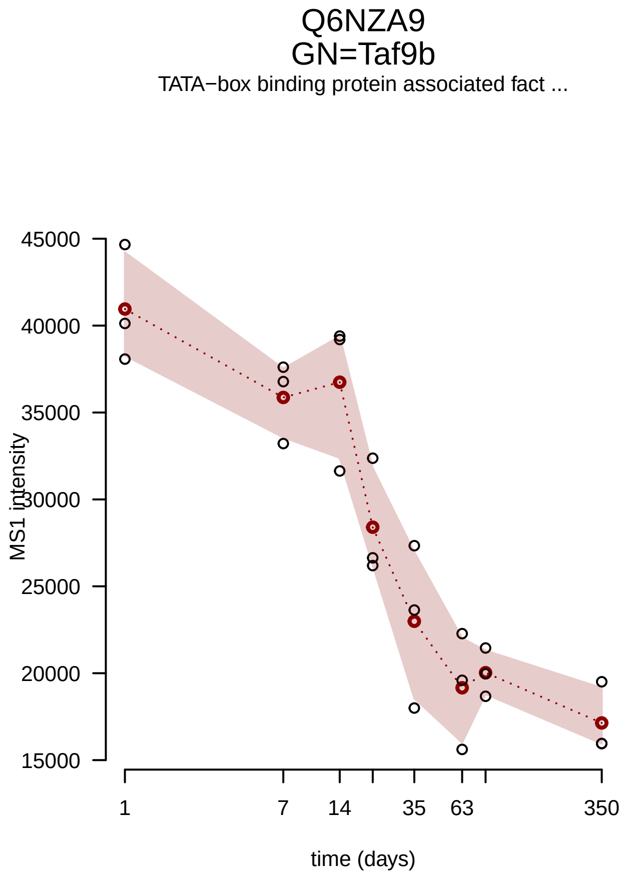
Q6NZA9
GN=Taf9b
TATA−box binding protein associated fact ...
MS1 intensity
45000
40000
35000
30000
25000
20000
15000
1
7
14
35
63
350
time (days)
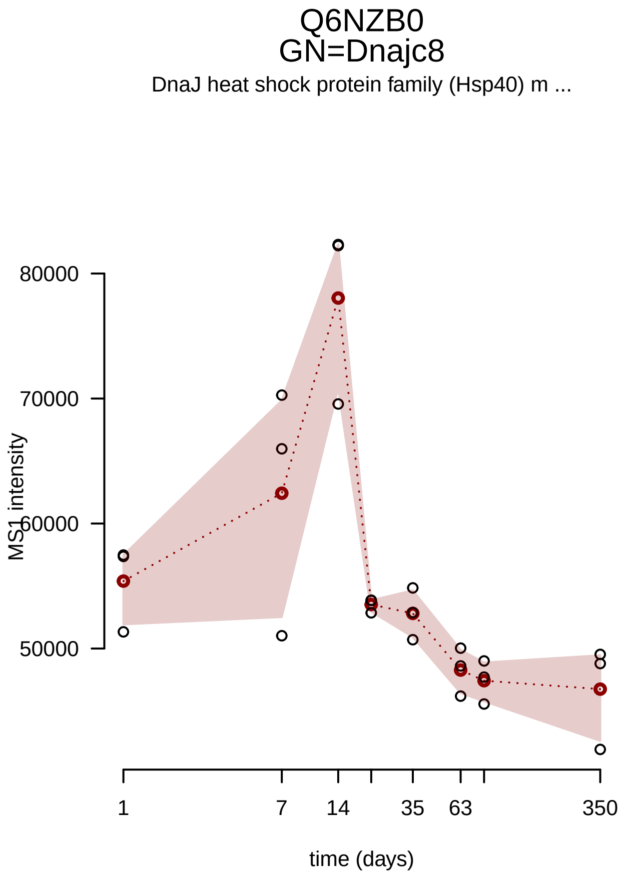
Q6NZB0
GN=Dnajc8
DnaJ heat shock protein family (Hsp40) m ...
MS1 intensity
80000
70000
60000
50000
1
7
14
35
63
350
time (days)
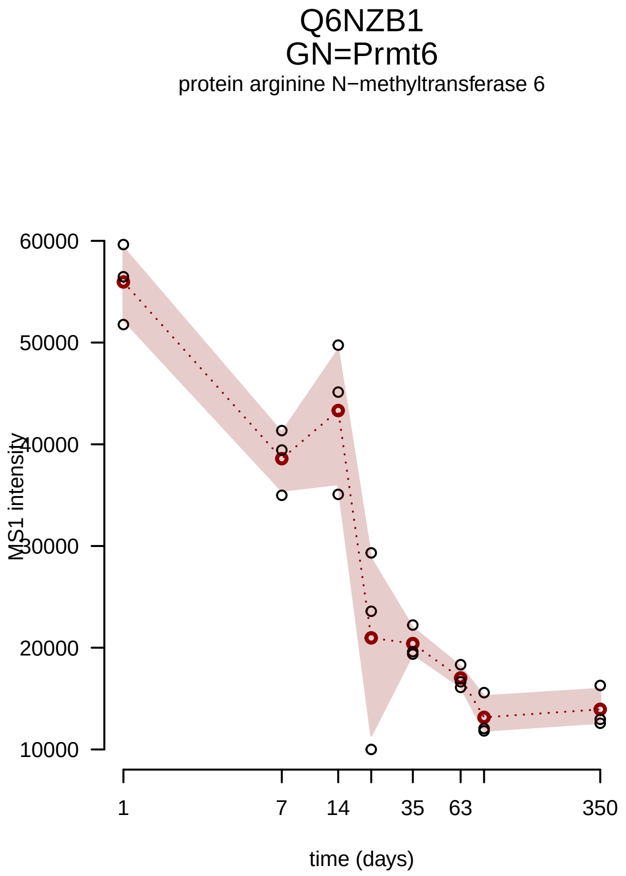
Q6NZB1
GN=Prmt6
protein arginine N−methyltransferase 6
MS1 intensity
60000
50000
40000
30000
20000
10000
1
7
14
35
63
350
time (days)
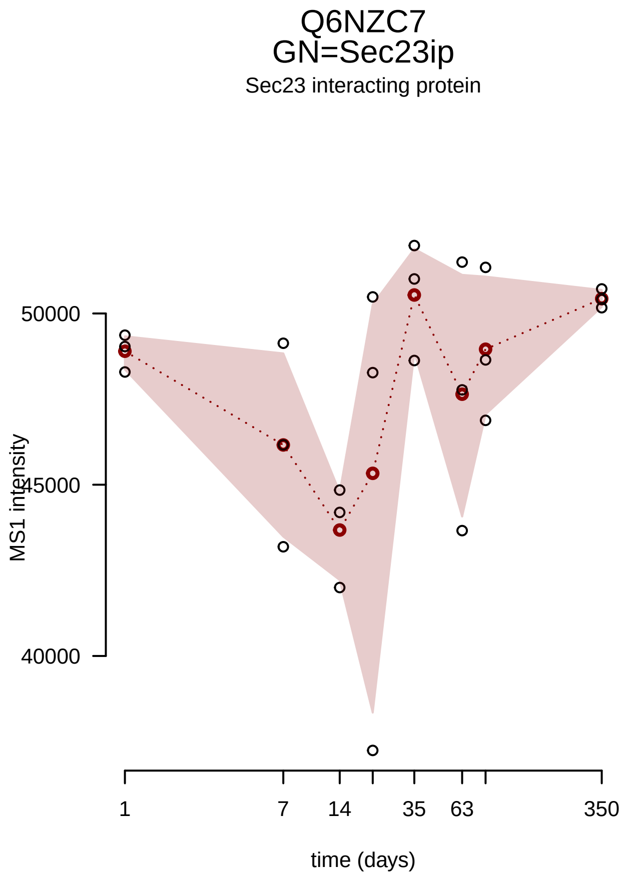
Q6NZC7
GN=Sec23ip
Sec23 interacting protein
MS1 intensity
50000
45000
40000
1
7
14
35
63
350
time (days)
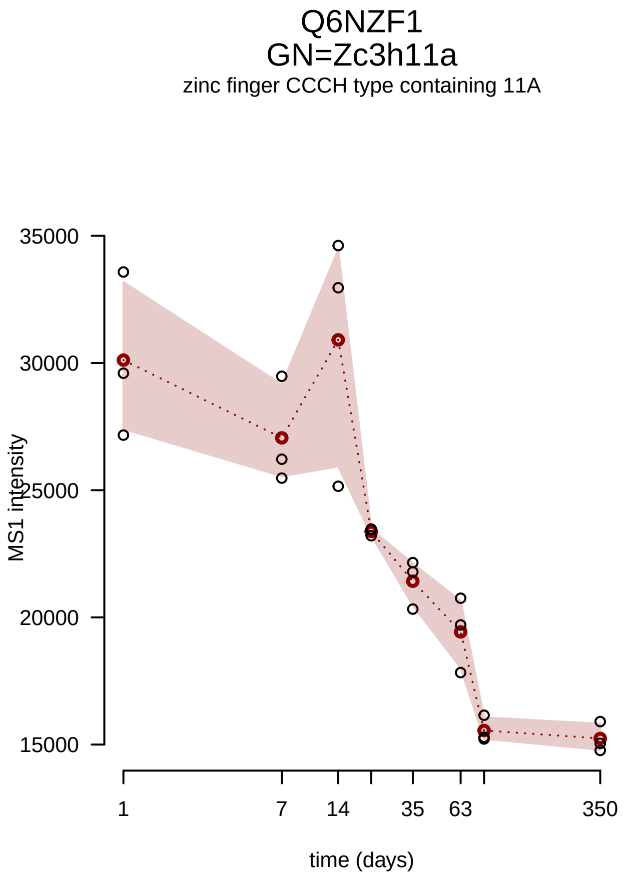
Q6NZF1
GN=Zc3h11a
zinc finger CCCH type containing 11A
MS1 intensity
35000
30000
25000
20000
15000
1
7
14
35
63
350
time (days)
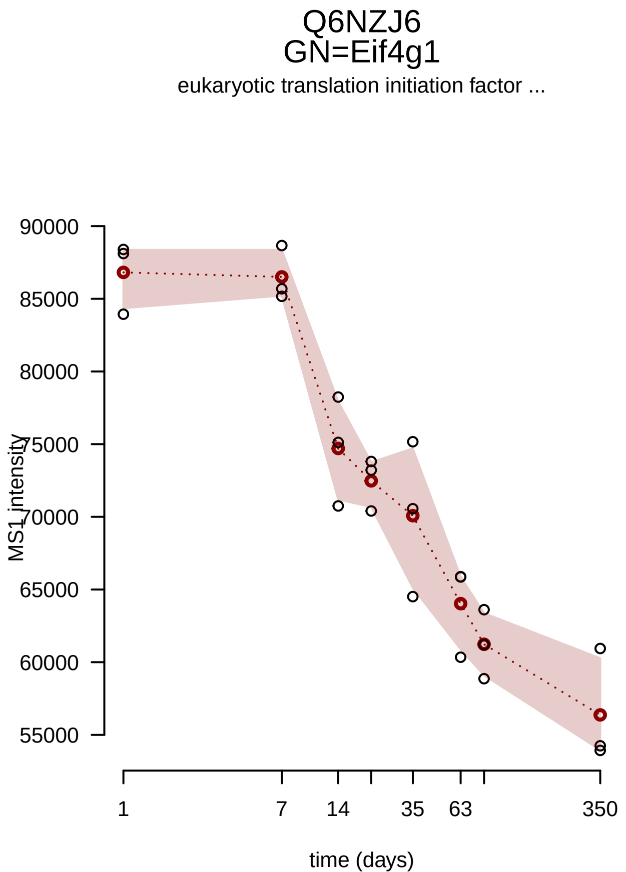
Q6NZJ6
GN=Eif4g1
eukaryotic translation initiation factor ...
MS1 intensity
90000
85000
80000
75000
70000
65000
60000
55000
1
7
14
35
63
350
time (days)

Q6NZK8
GN=Ptpdc1
protein tyrosine phosphatase domain cont ...

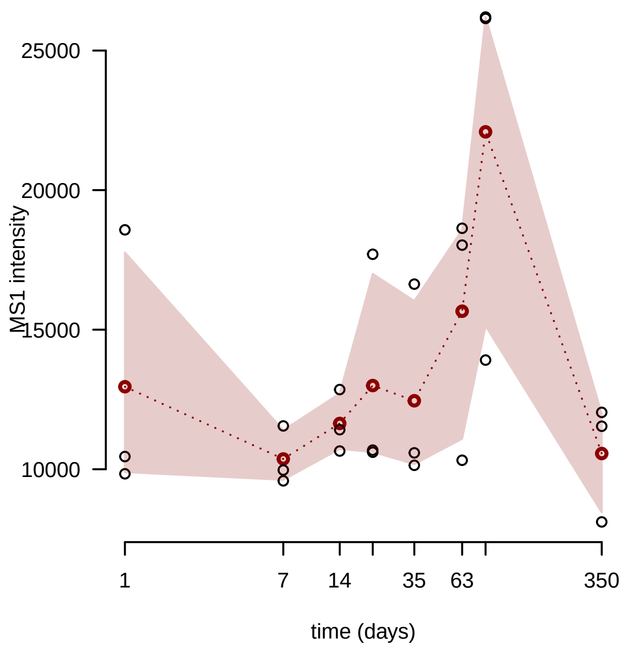

Q6NZL6
GN=Tonsl
tonsoku−like, DNA repair protein

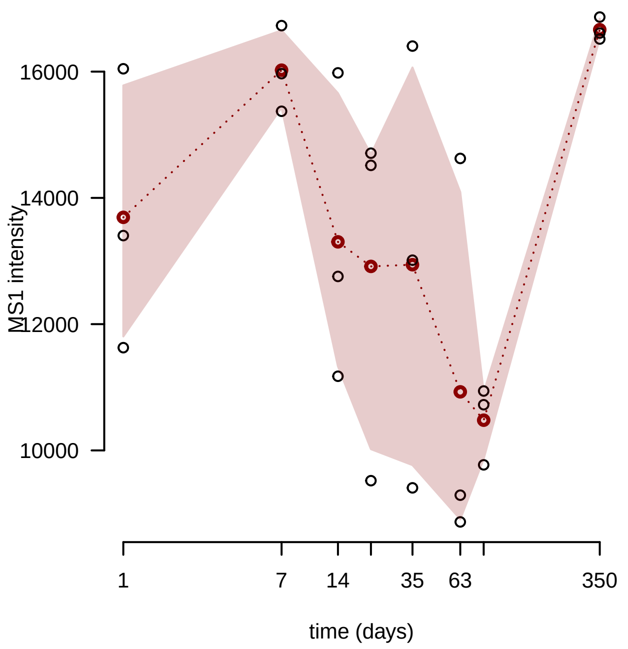

Q6NZL8
GN=Scube1
signal peptide, CUB domain, EGF−like 1

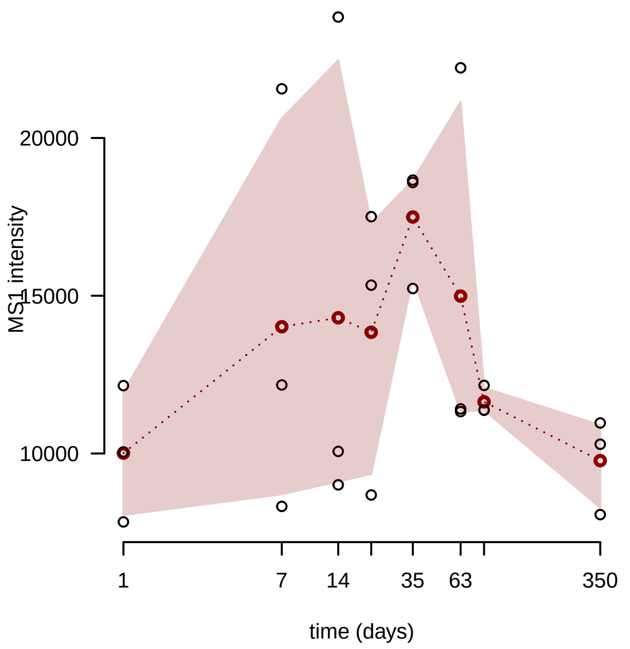

Q6NZM9
GN=Hdac4
histone deacetylase 4

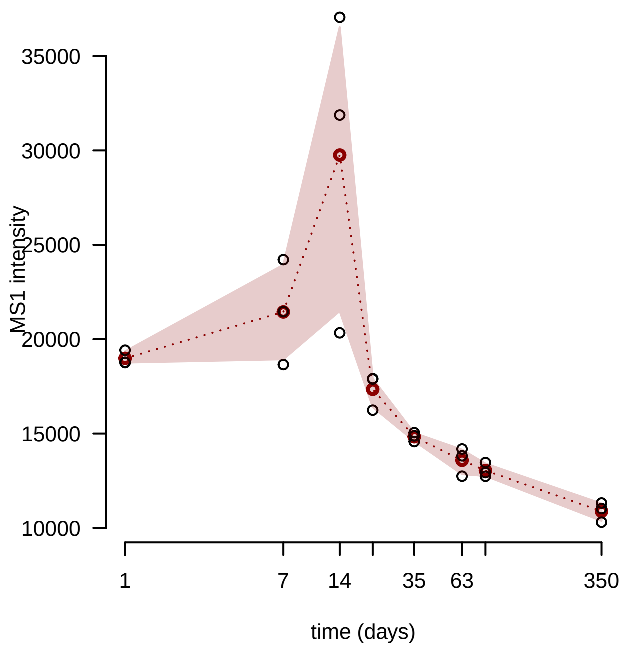

Q6NZM9
GN=Hdac4
histone deacetylase 4

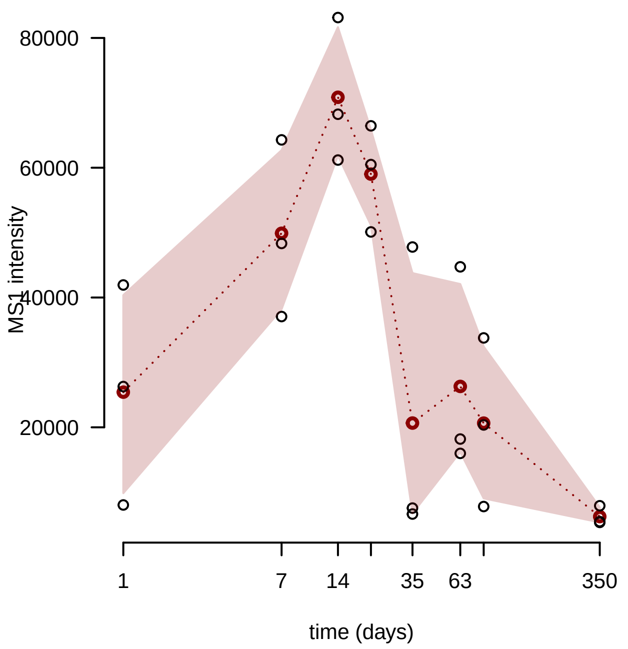

Q6NZN0
GN=Rbm26
RNA binding motif protein 26

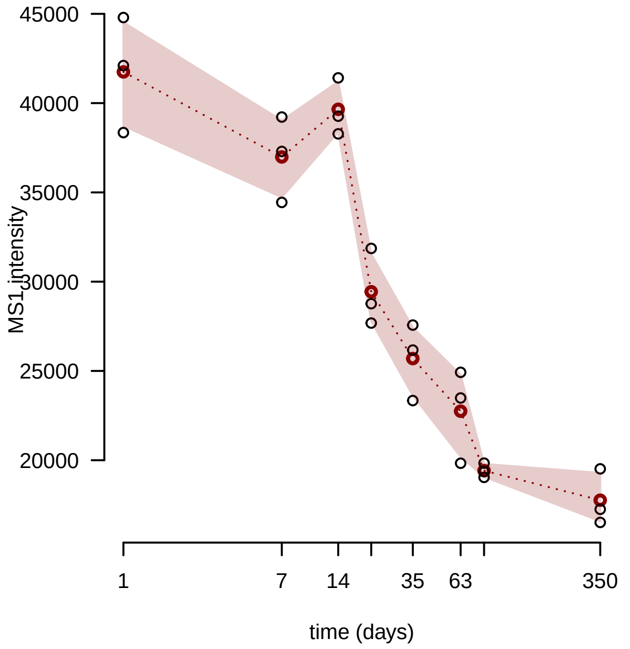

Q6NZP1
GN=Zranb3
zinc finger, RAN−binding domain containi ...

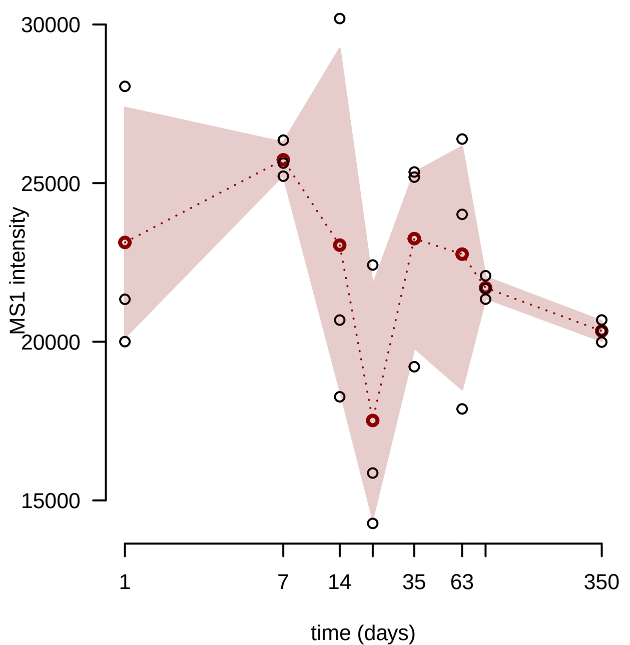

Q6NZQ2
GN=Ddx31
DEAD/H box helicase 31

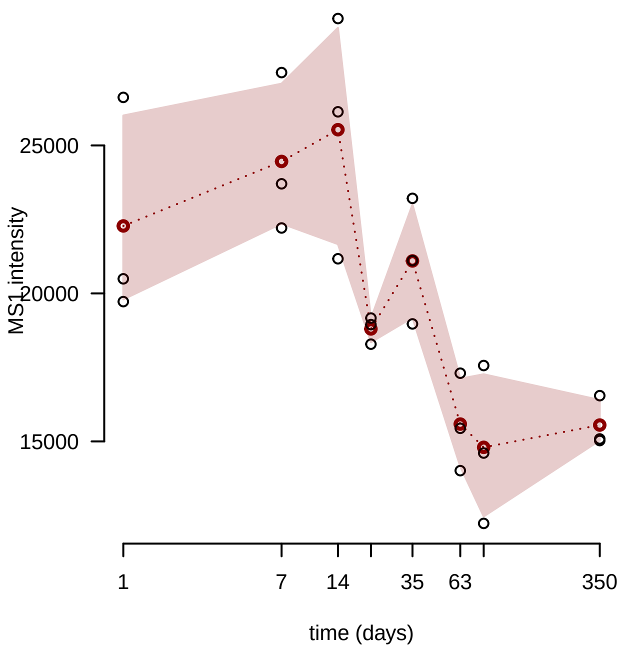

Q6NZQ4
GN=Paxip1
PAX interacting (with transcription−acti ...

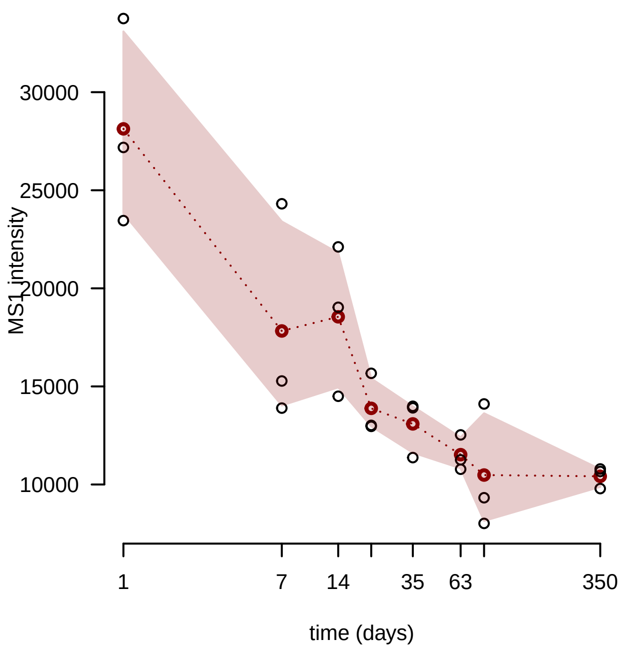

Q6NZQ6
GN=Znf740

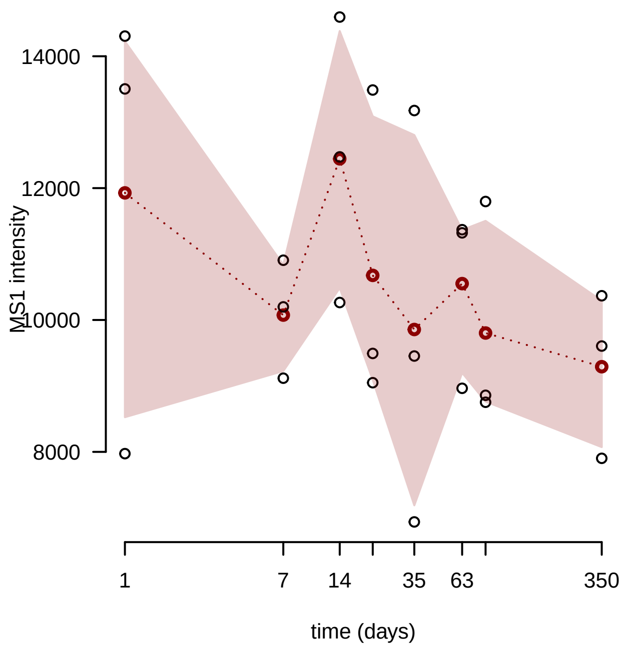

Q6NZR2
GN=Msantd2
Myb/SANT−like DNA−binding domain contain ...

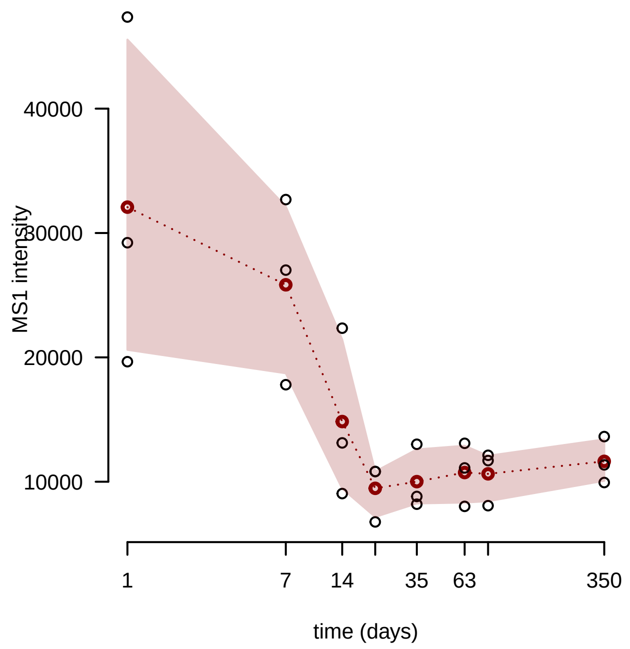

Q6P069
GN=Sri
sorcin

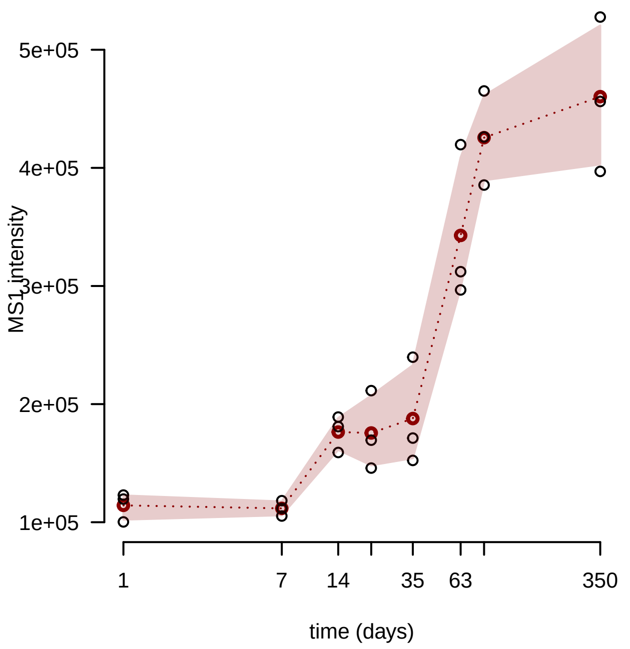

Q6P073
GN=Ube2j2
ubiquitin−conjugating enzyme E2J 2

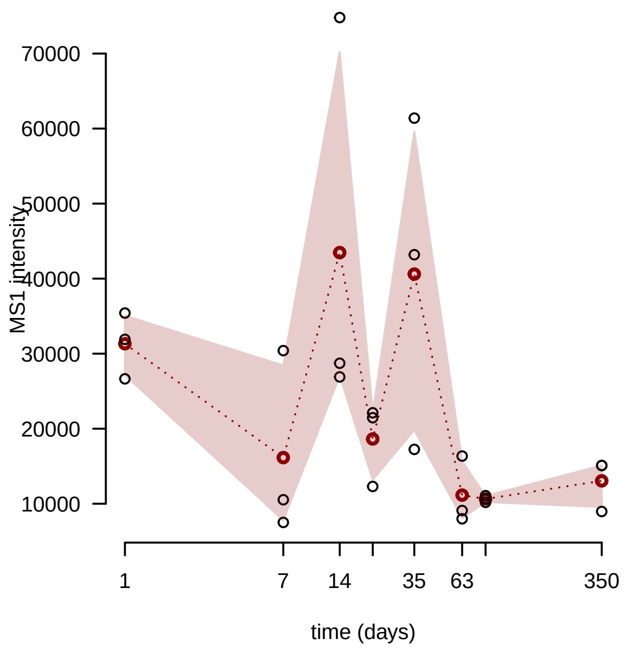

Q6P0X2
GN=Znf511

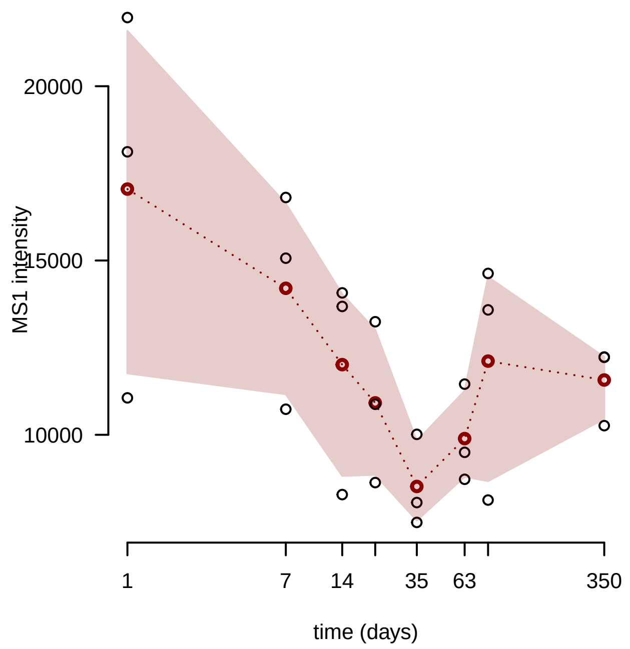

Q6P1B1
GN=Xpnpep1
X−prolyl aminopeptidase (aminopeptidase ...

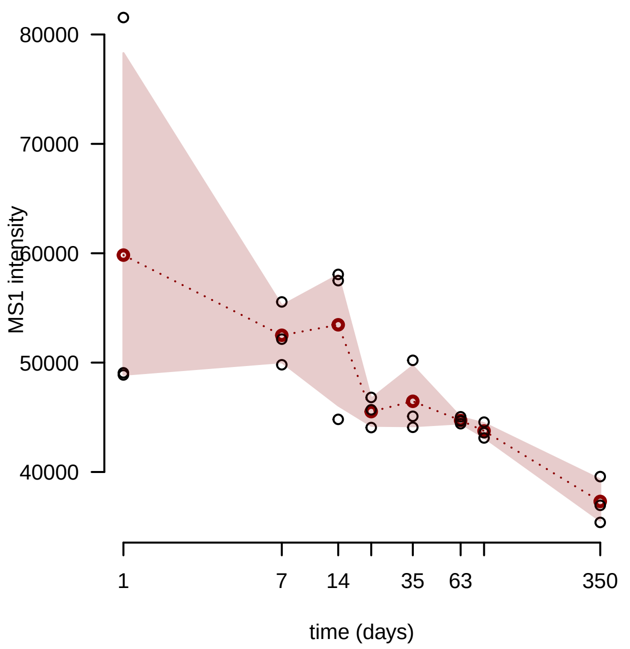

Q6P1D7
GN=Slx4
SLX4 structure−specific endonuclease sub ...

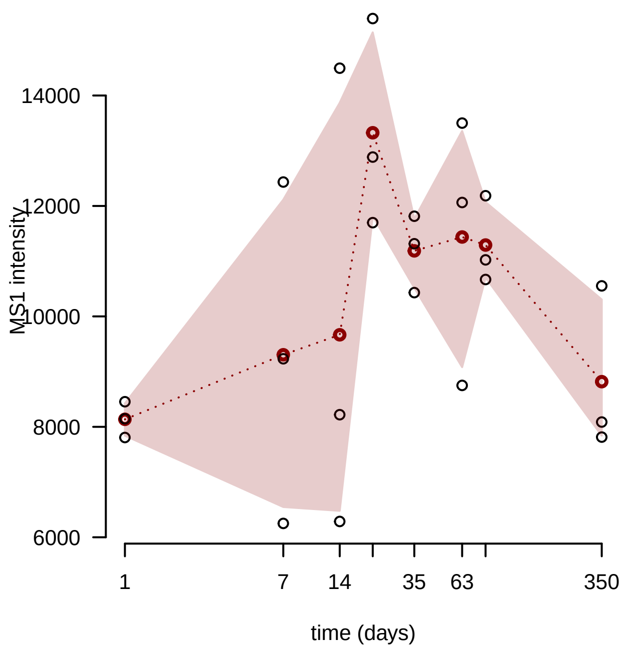

Q6P1E1
GN=Zmiz1
zinc finger, MIZ−type containing 1

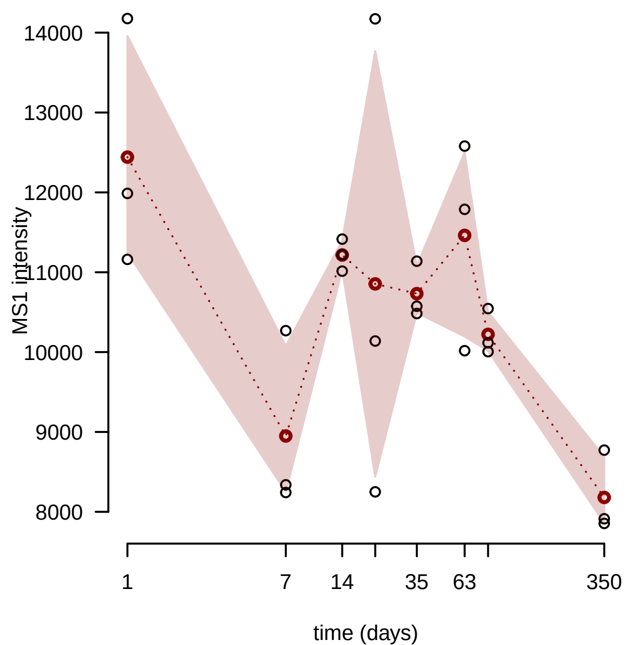

Q6P1F6
GN=Ppp2r2a
protein phosphatase 2, regulatory subuni ...

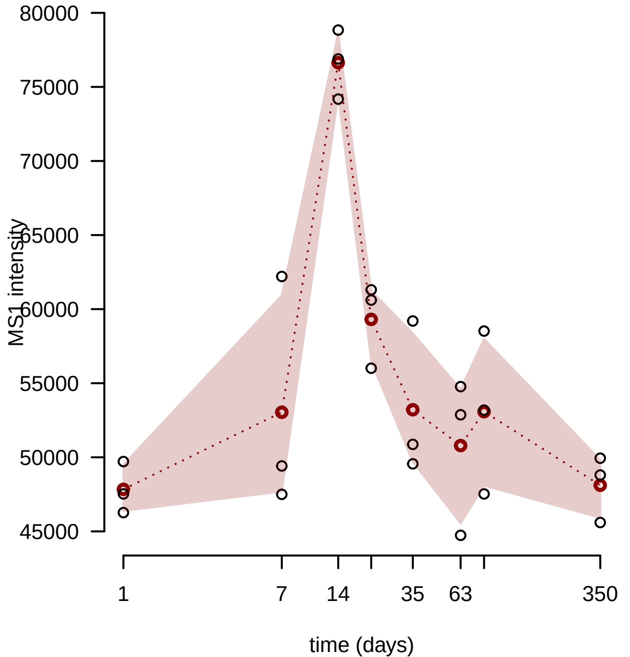

Q6P1G0
GN=Heatr6
HEAT repeat containing 6

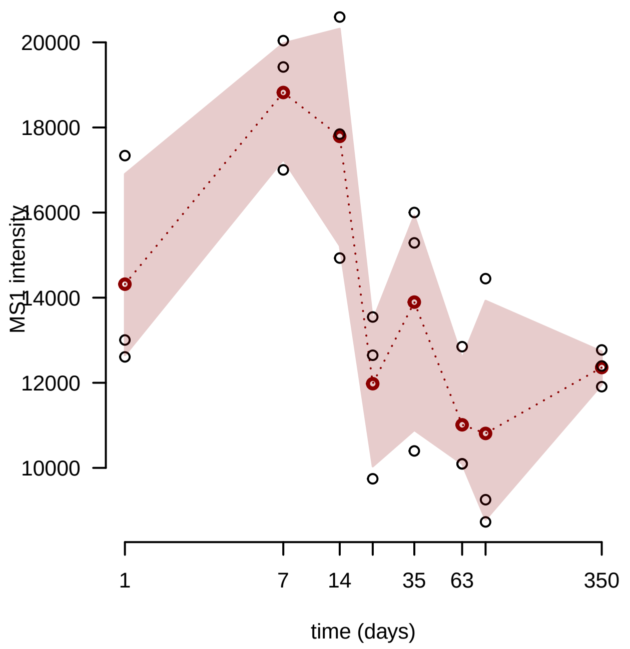

Q6P1G2
GN=Kdm2b
lysine (K)−specific demethylase 2B

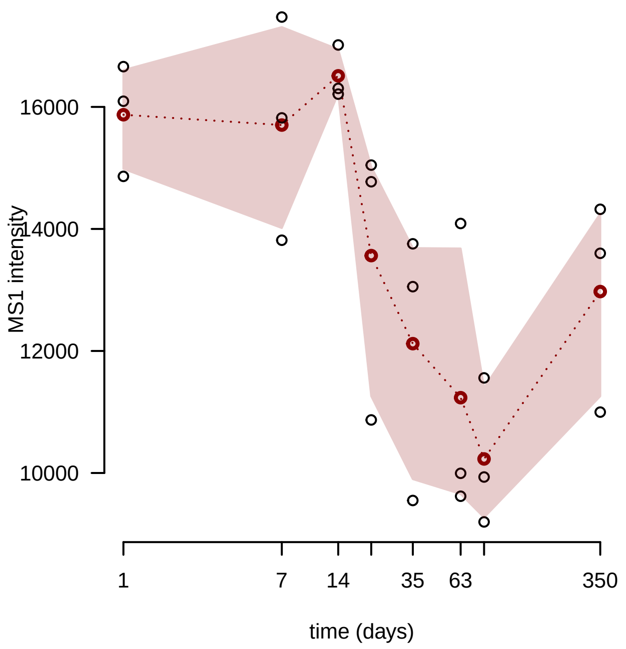

Q6P1H1
GN=Cyb561a3
cytochrome b561 family, member A3

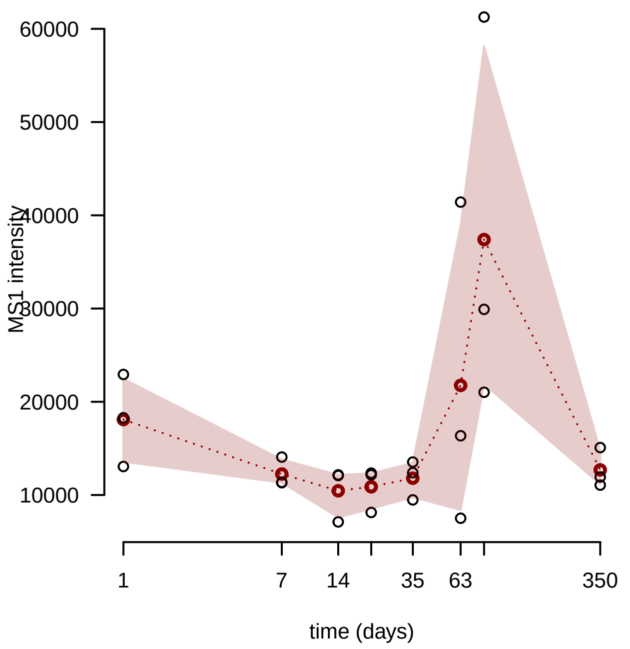

Q6P1H6
GN=Ankle2
ankyrin repeat and LEM domain containing ...

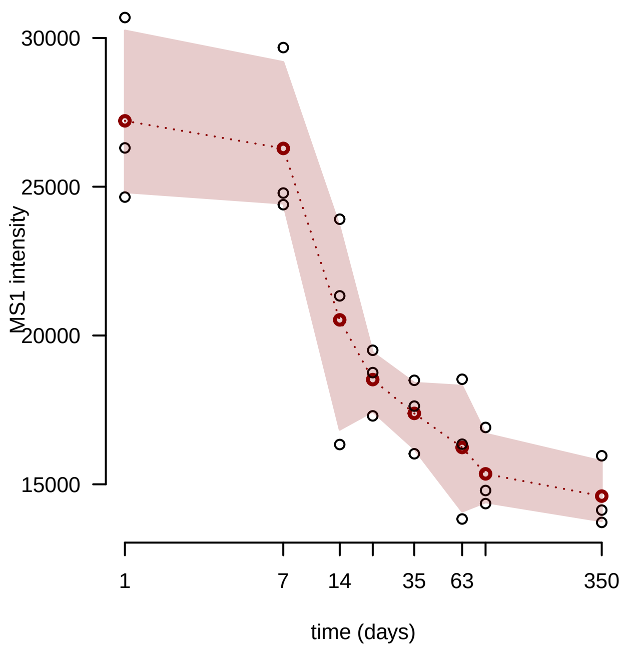

Q6P1I3
GN=

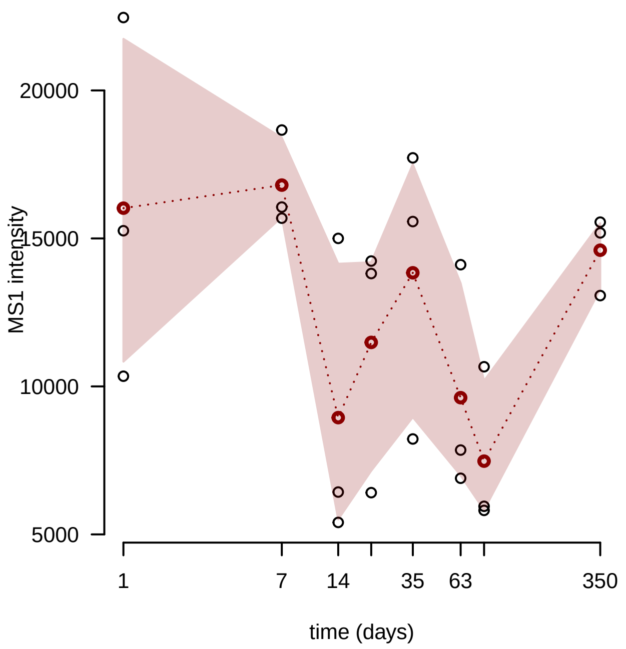

Q6P1Y1
GN=Rtl3
retrotransposon Gag like 3

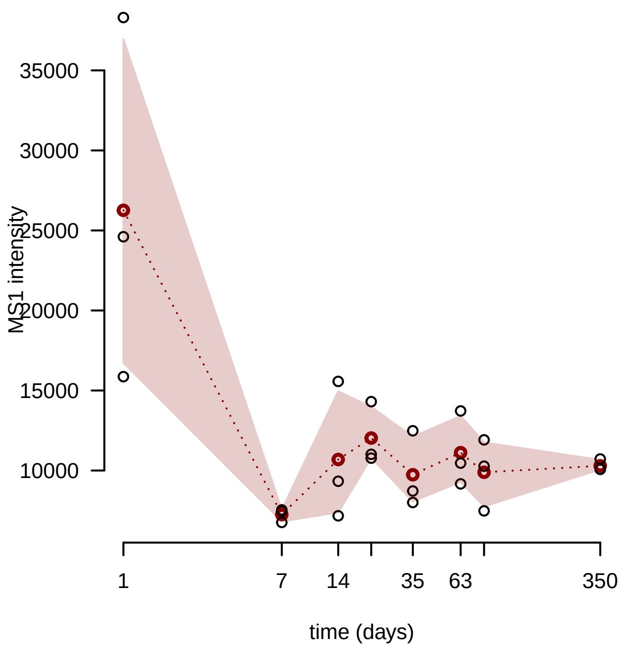

Q6P2B1
GN=Tnpo3
transportin 3

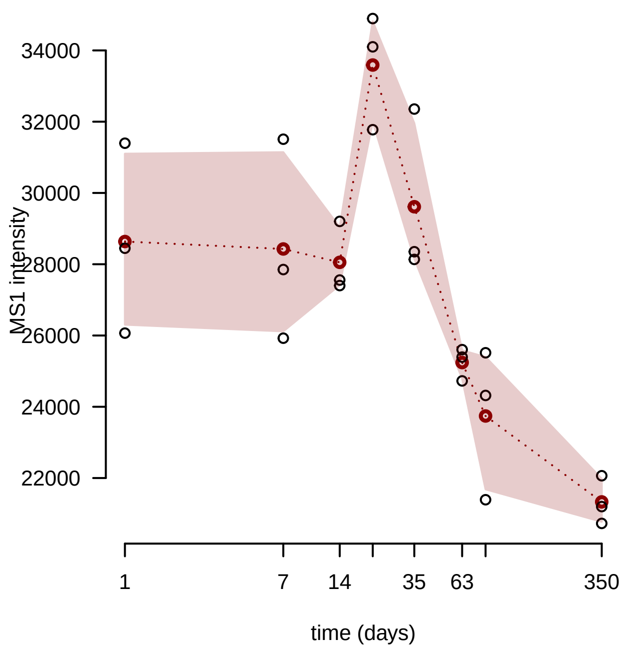

Q6P2K6
GN=Ppp4r3a
protein phosphatase 4 regulatory subunit ...

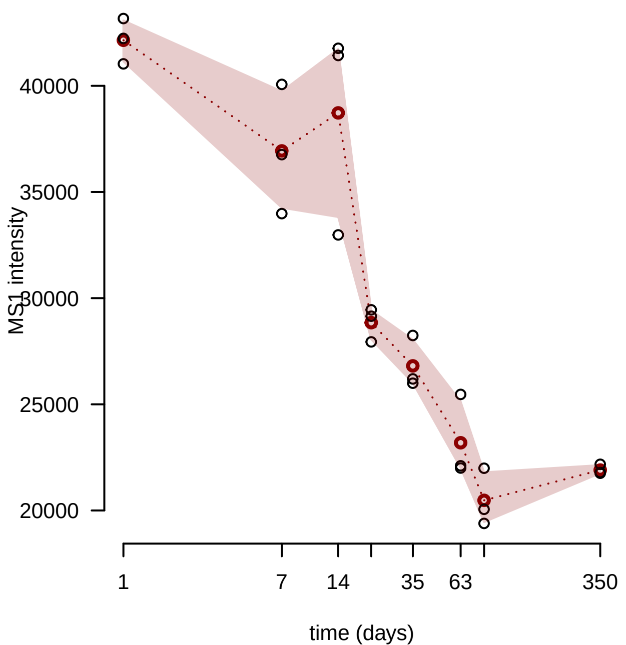

Q6P2L6
GN=Nsd3
nuclear receptor binding SET domain prot ...

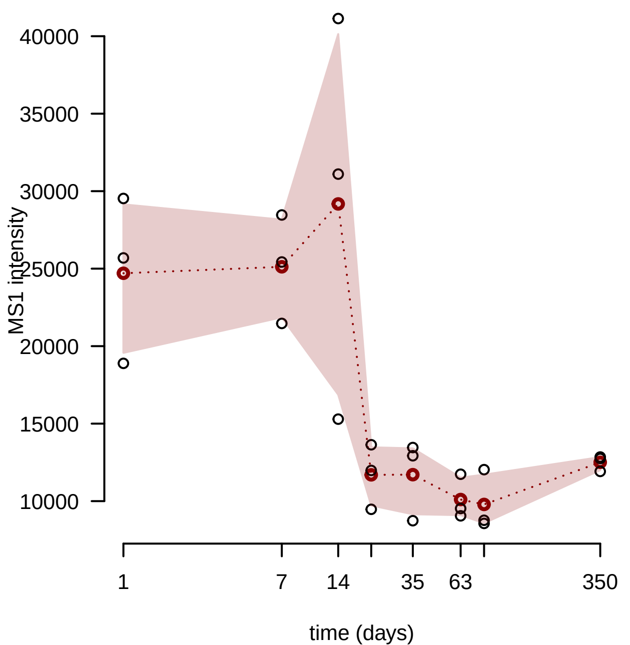

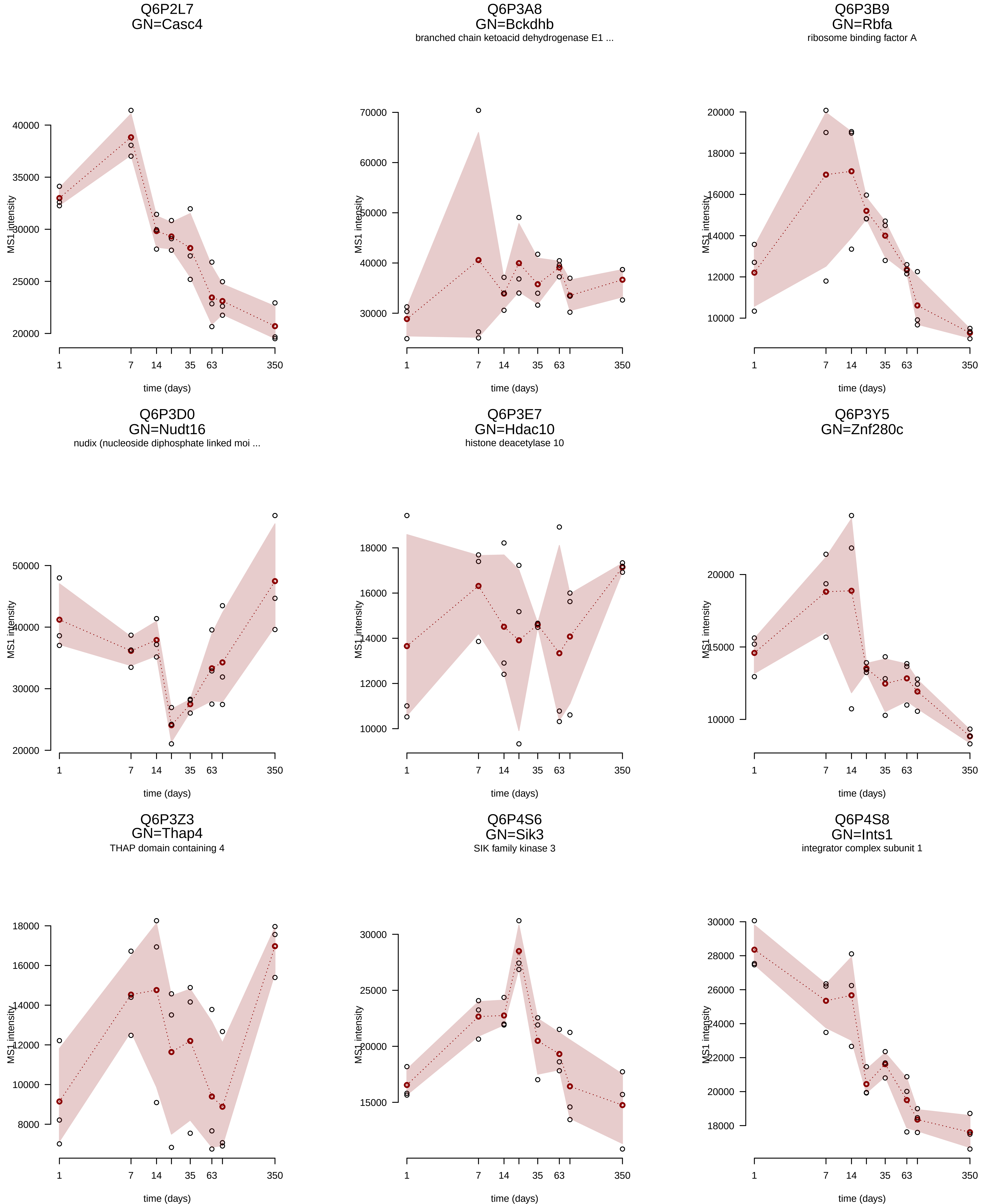
Q6P2L7
GN=Casc4
Q6P3A8
GN=Bckdhb
branched chain ketoacid dehydrogenase E1 ...
Q6P3B9
GN=Rbfa
ribosome binding factor A
Q6P3D0
GN=Nudt16
nudix (nucleoside diphosphate linked moi ...
Q6P3E7
GN=Hdac10
histone deacetylase 10
Q6P3Y5
GN=Znf280c
Q6P3Z3
GN=Thap4
THAP domain containing 4
Q6P4S6
GN=Sik3
SIK family kinase 3
Q6P4S8
GN=Ints1
integrator complex subunit 1
MS1 intensity
time (days)
1
7
14
35
63
350

Q6P4T0
GN=Atg2a
autophagy related 2A

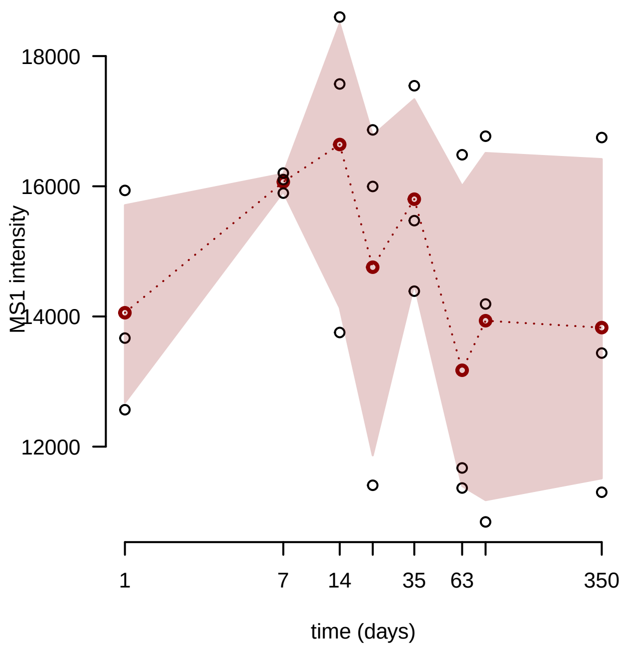

Q6P4T2
GN=Snrnp200
small nuclear ribonucleoprotein 200 (U5)

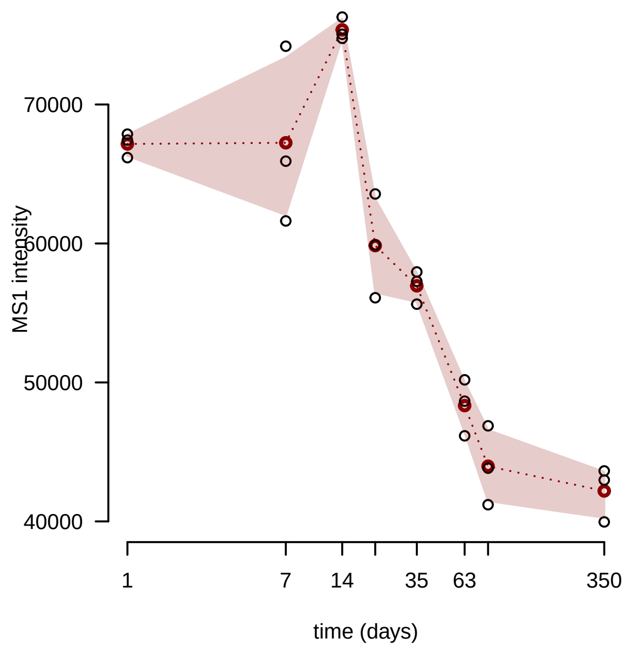

Q6P542
GN=Abcf1
ATP−binding cassette, sub−family F (GCN2 ...

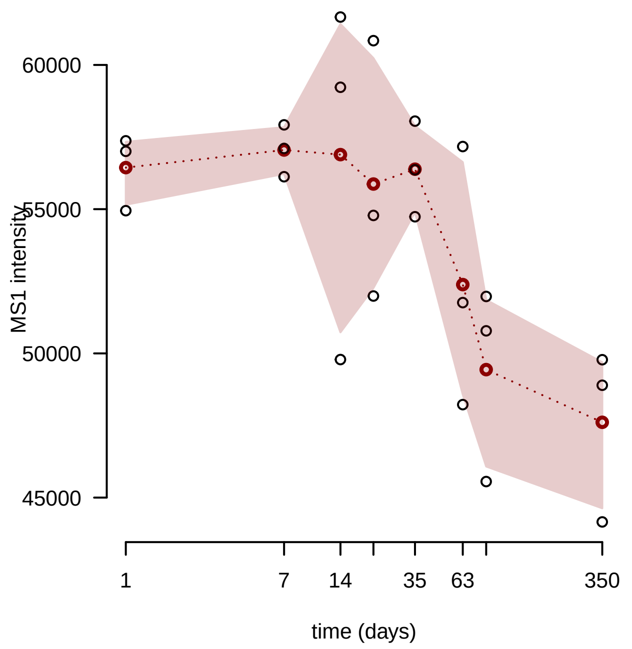

Q6P549
GN=Inppl1
inositol polyphosphate phosphatase−like ...

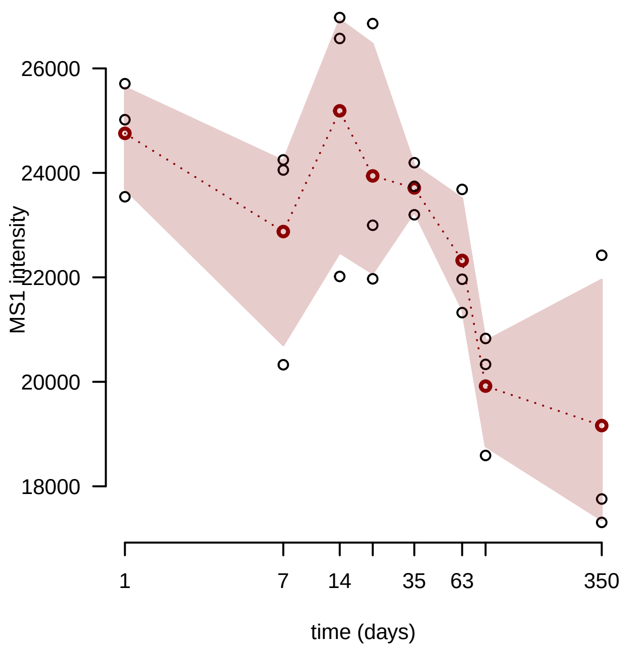

Q6P5B0
GN=Rrp12
ribosomal RNA processing 12 homolog

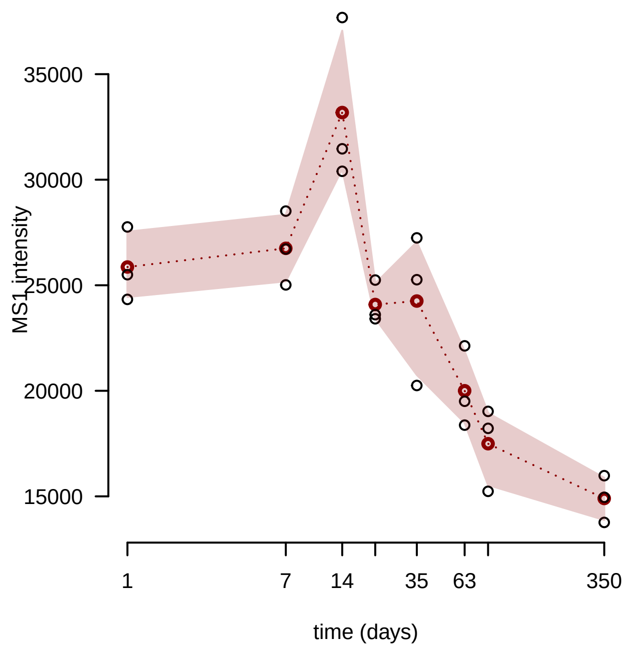

Q6P5C5
GN=Smug1
single−strand selective monofunctional u ...

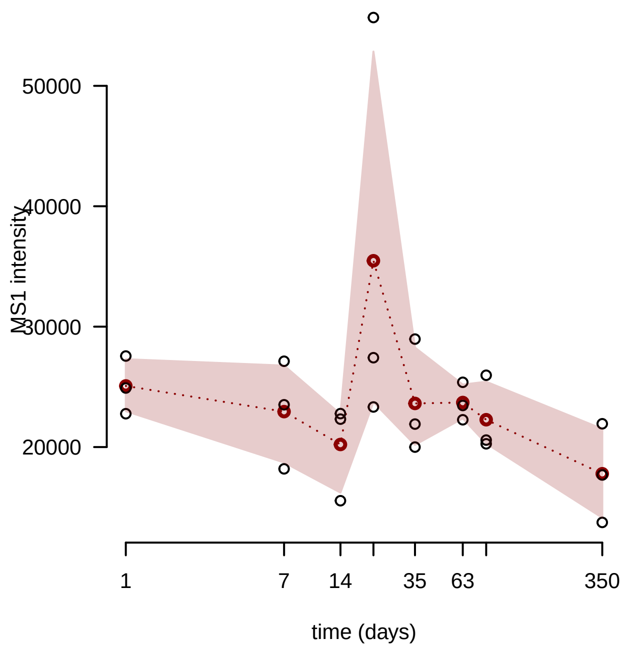

Q6P5C7
GN=Znf728

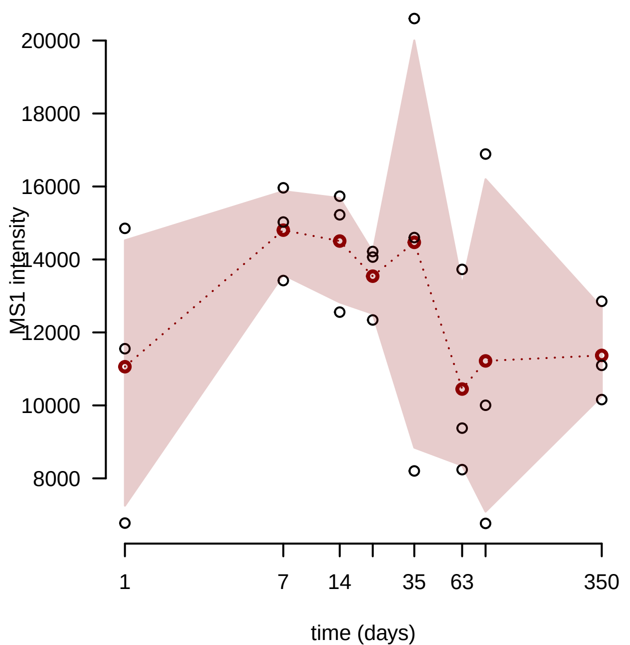

Q6P5D3
GN=Dhx57
DEAH (Asp−Glu−Ala−Asp/His) box polypepti ...

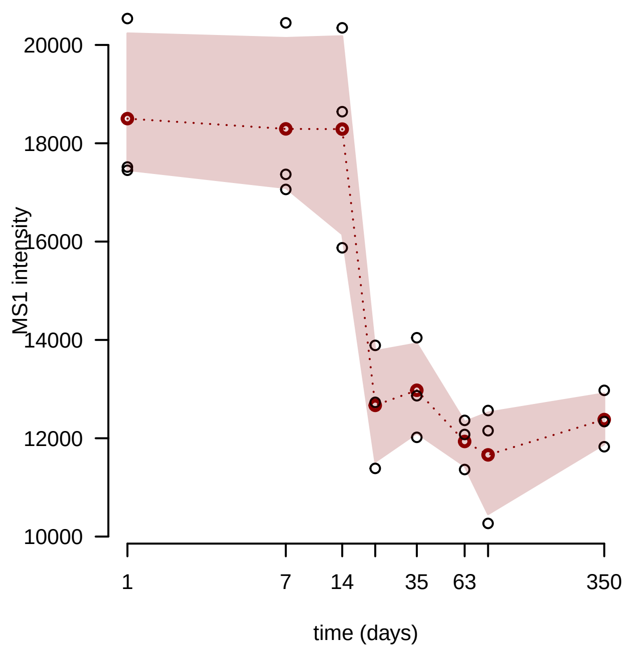

Q6P5D4
GN=Cep135
centrosomal protein 135

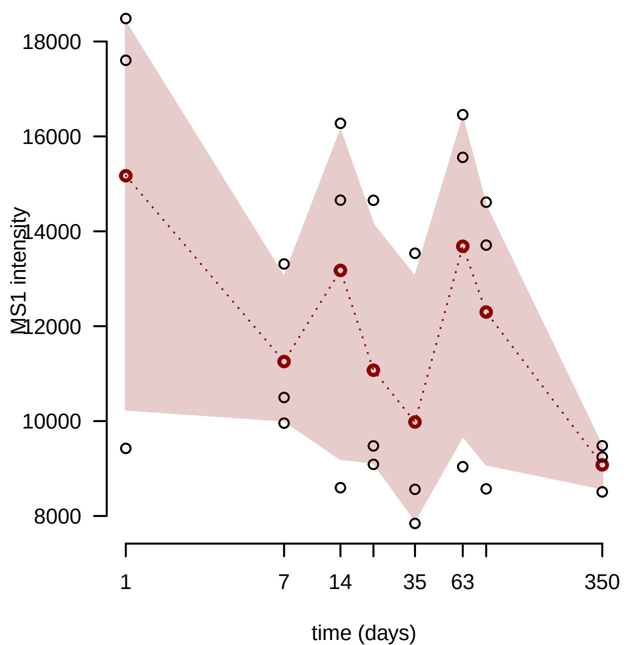

Q6P5D8
GN=Smchd1
SMC hinge domain containing 1

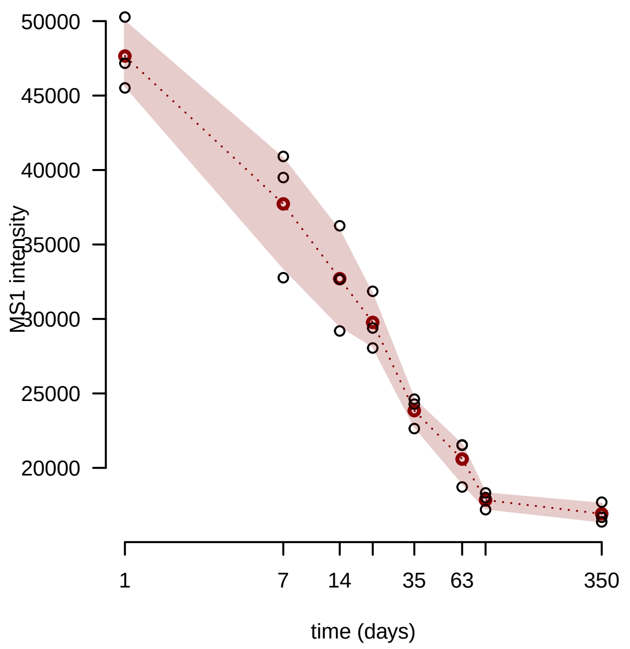

Q6P5E4
GN=Uggt1
UDP−glucose glycoprotein glucosyltransfe ...

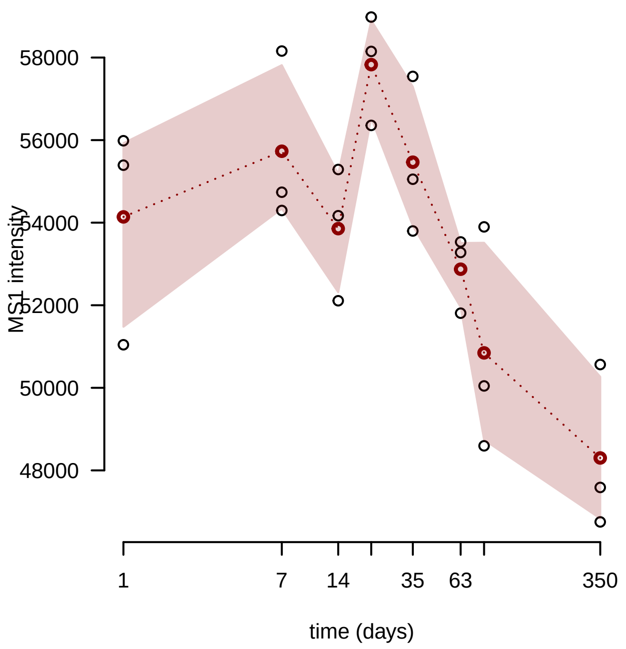

Q6P5E6
GN=Gga2
golgi associated, gamma adaptin ear cont ...

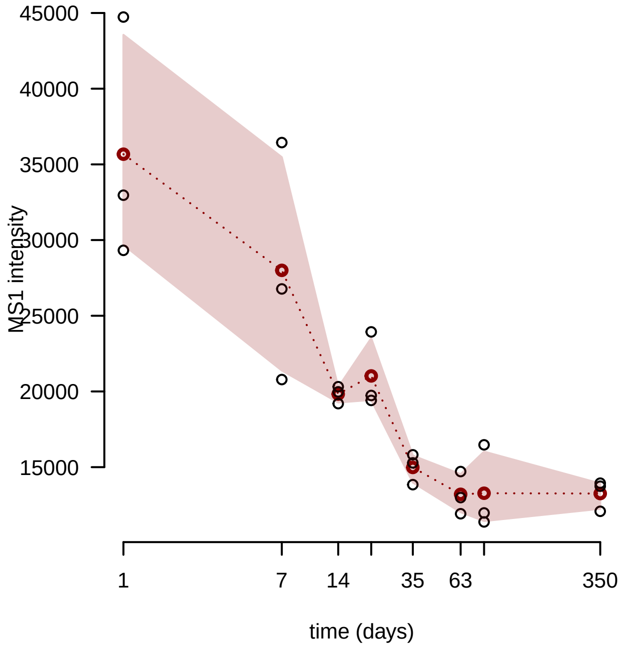

Q6P5E8
GN=Dgkq
diacylglycerol kinase, theta

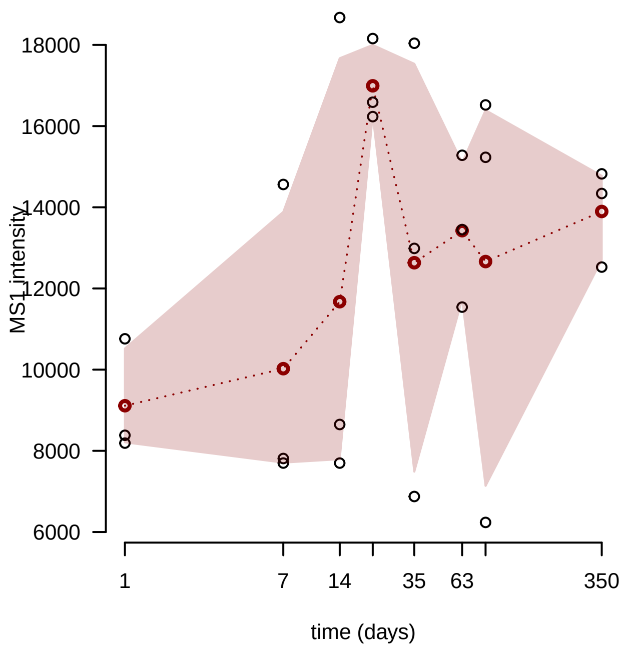

Q6P5F6
GN=Slc39a10
solute carrier family 39 (zinc transport ...

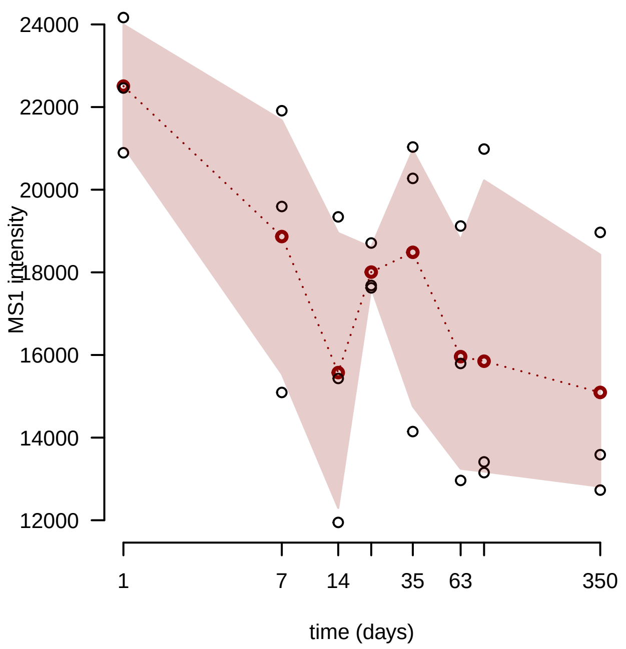

Q6P5F7
GN=Ttyh3
tweety family member 3

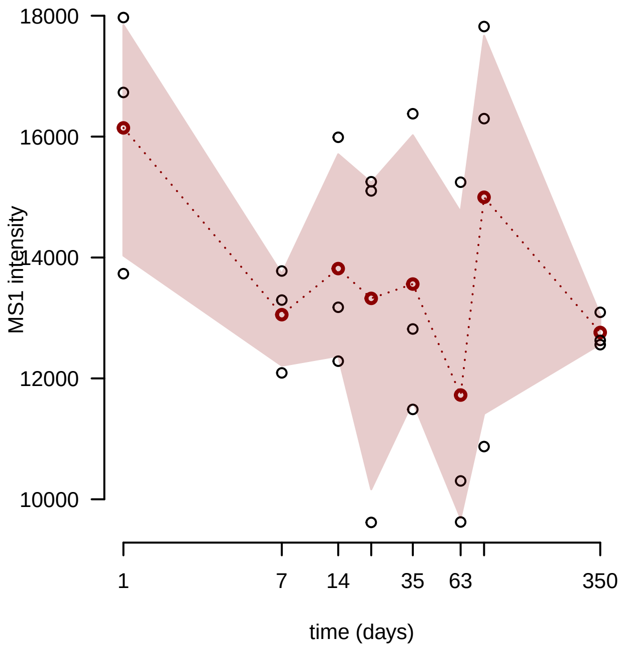

Q6P5F9
GN=Xpo1
exportin 1

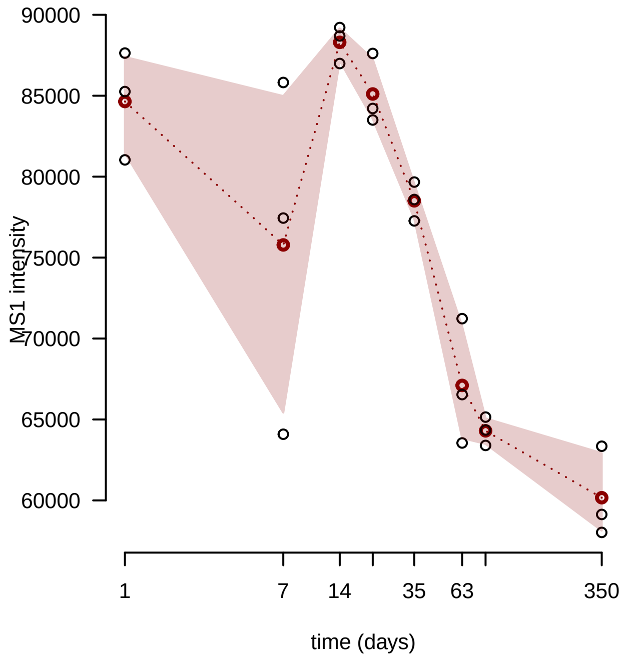

Q6P5G3
GN=Mbtd1
mbt domain containing 1

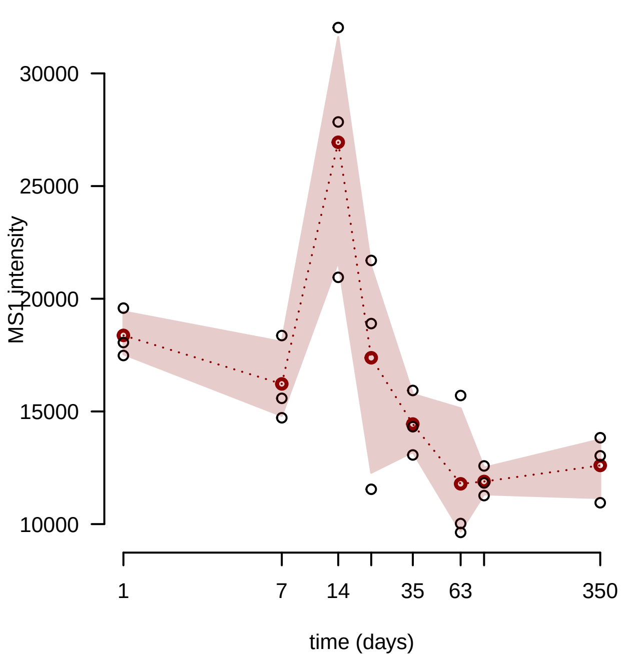

Q6P5G6
GN=Ubxn7
UBX domain protein 7

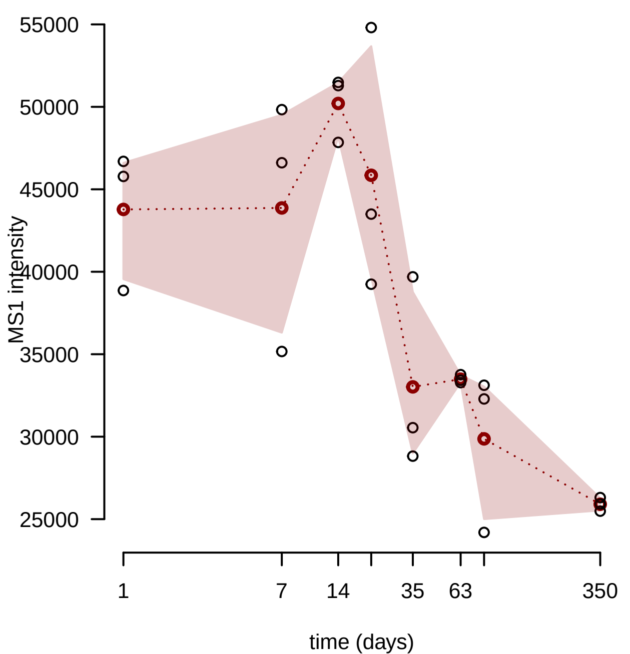

Q6P5H2
GN=Nes
nestin

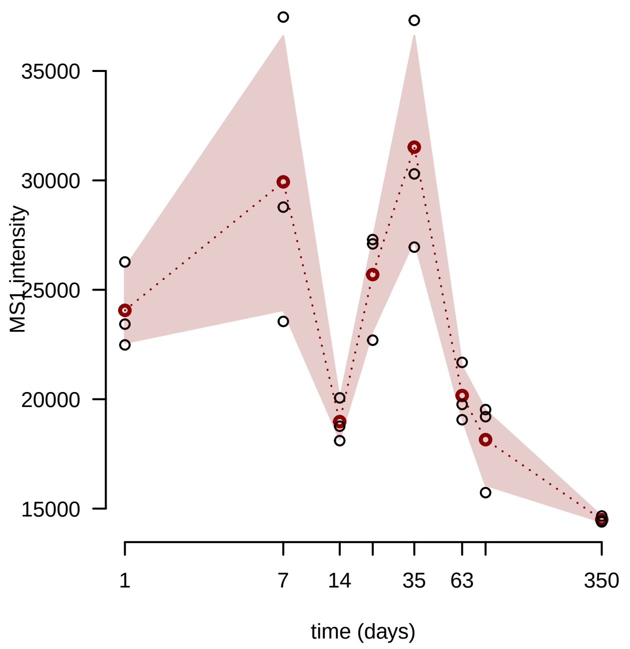

Q6P5H6
GN=Frmd5
FERM domain containing 5

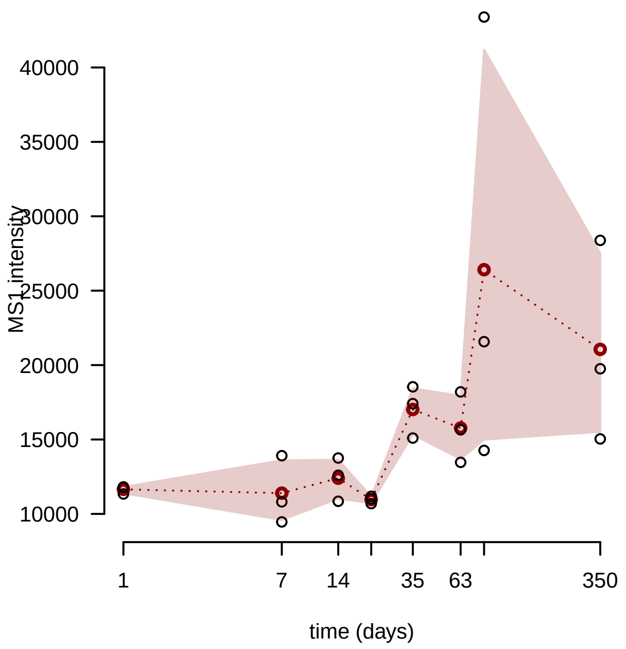

Q6P5H6
GN=Frmd5
FERM domain containing 5

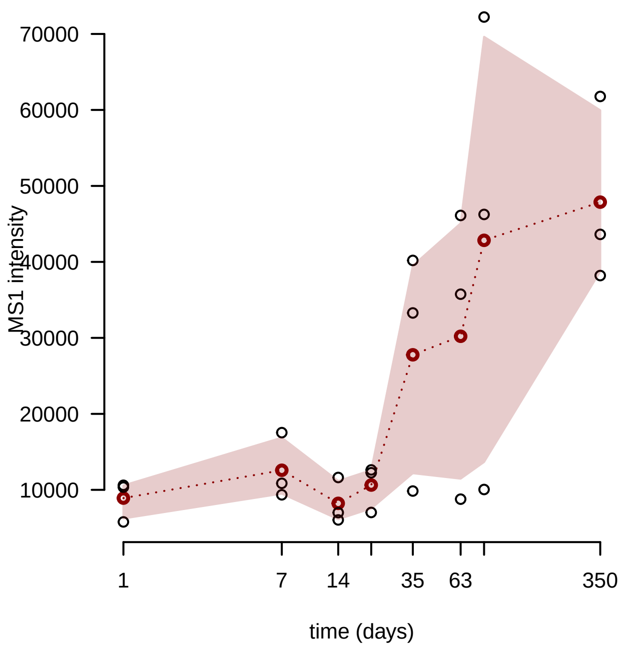

Q6P6I6
GN=Polr2m
polymerase (RNA) II (DNA directed) polyp ...

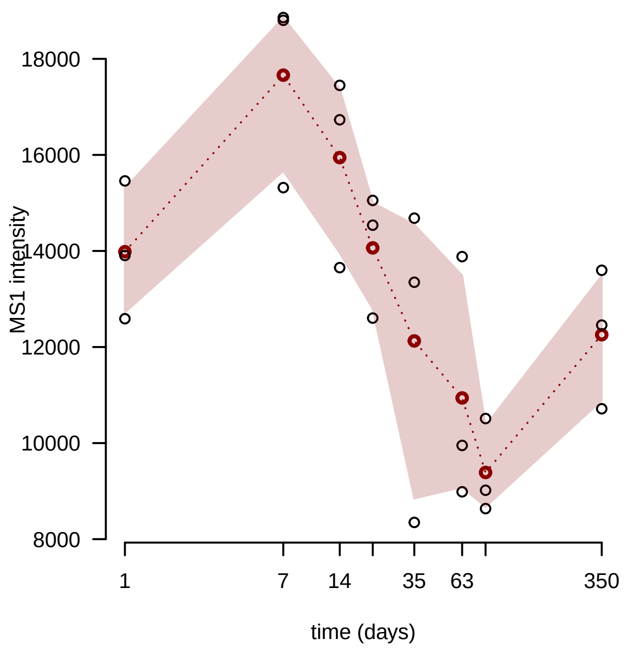

Q6P6J9
GN=Txndc15
thioredoxin domain containing 15

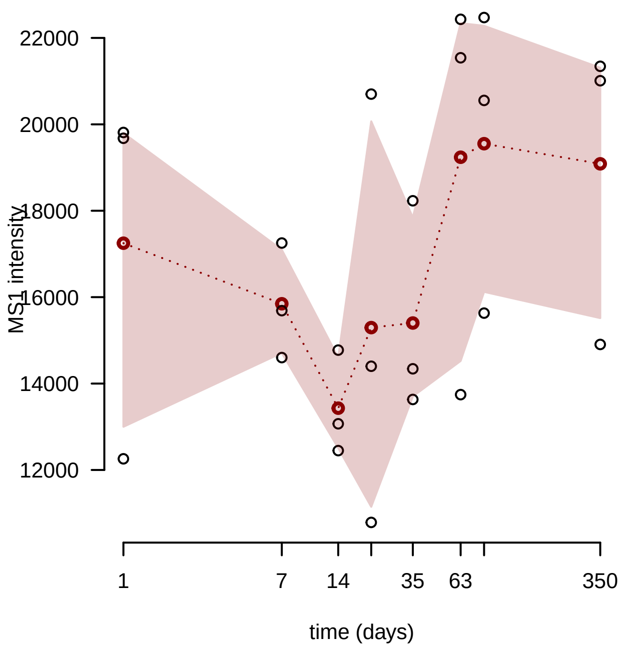

Q6P6L0
GN=Filip1l
filamin A interacting protein 1−like

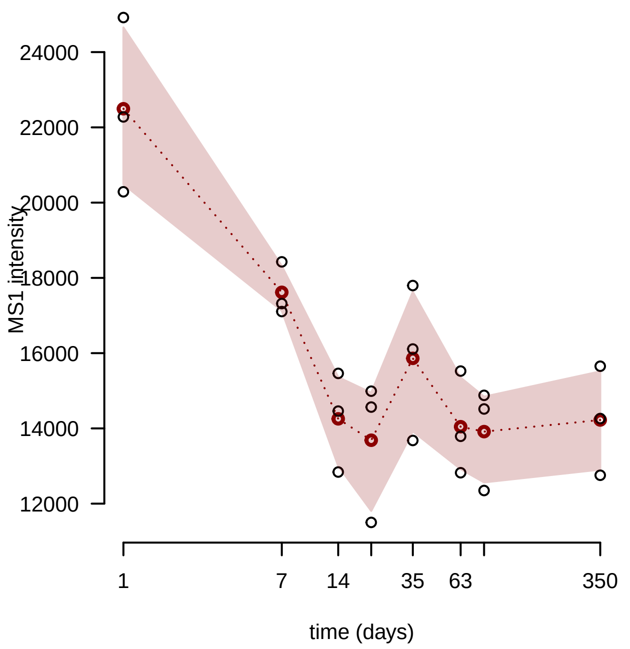

Q6P6M7
GN=Sepsecs
Sep (O−phosphoserine) tRNA:Sec (selenocy ...

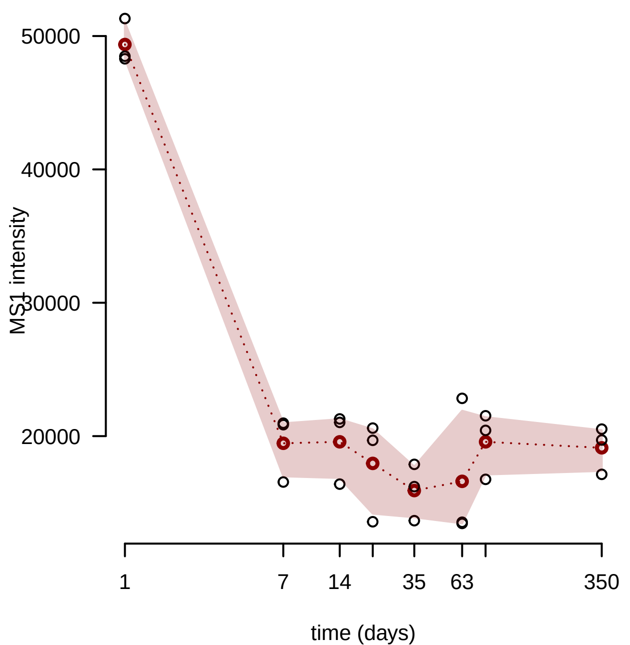

Q6P7W0
GN=Senp6
SUMO/sentrin specific peptidase 6

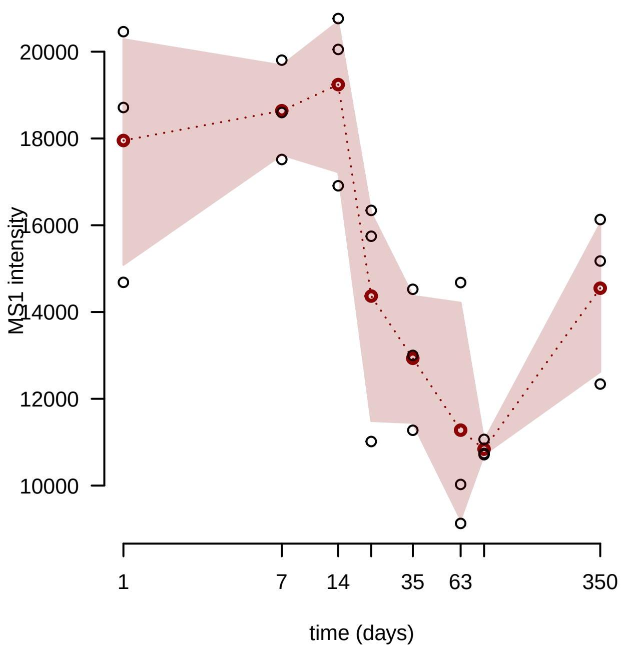

Q6P7W5
GN=Tsen2
tRNA splicing endonuclease subunit 2

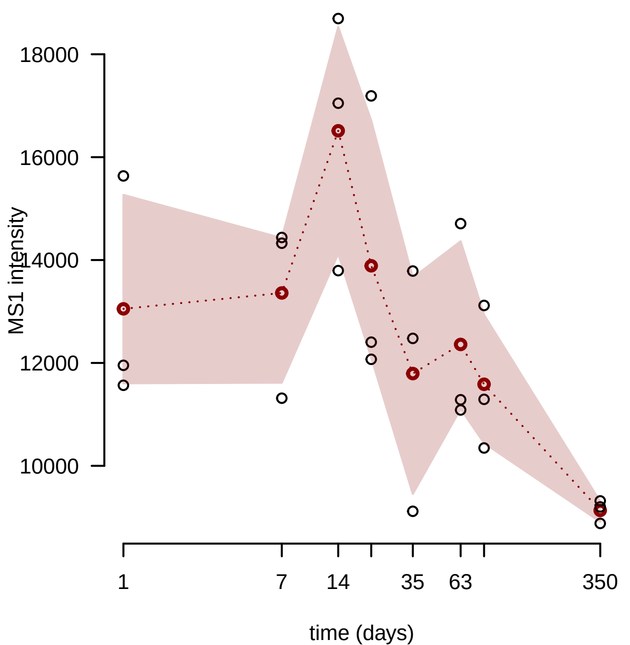

Q6P8H8
GN=Alg8
asparagine−linked glycosylation 8 (alpha ...

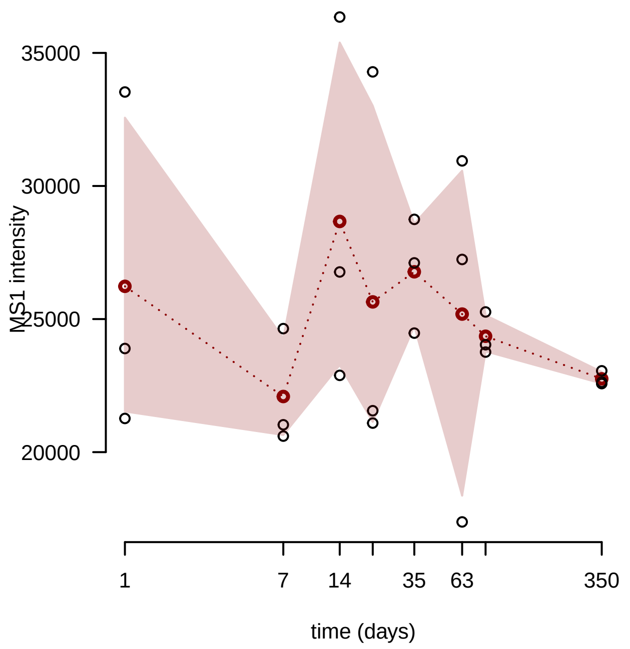

Q6P8I4
GN=Pcnp
PEST proteolytic signal containing nucle ...

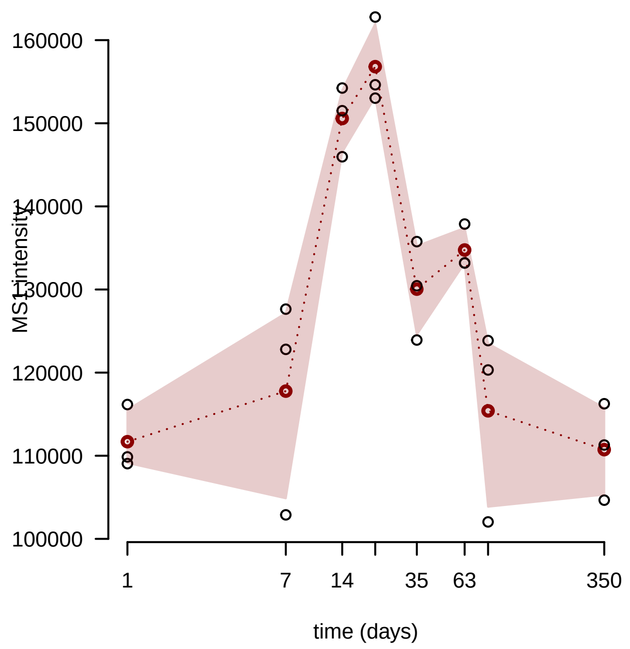

Q6P8I6
GN=Cox11
cytochrome c oxidase assembly protein 11 ...

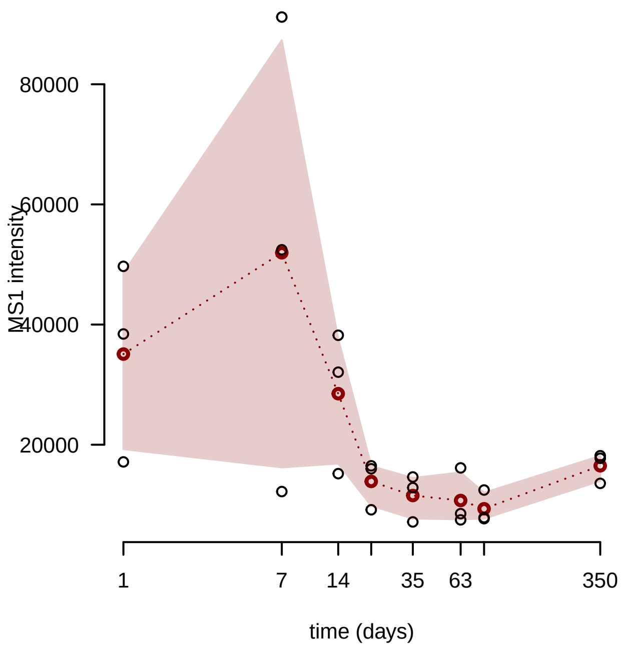

Q6P8J2
GN=Sat2
spermidine/spermine N1−acetyl transferas ...

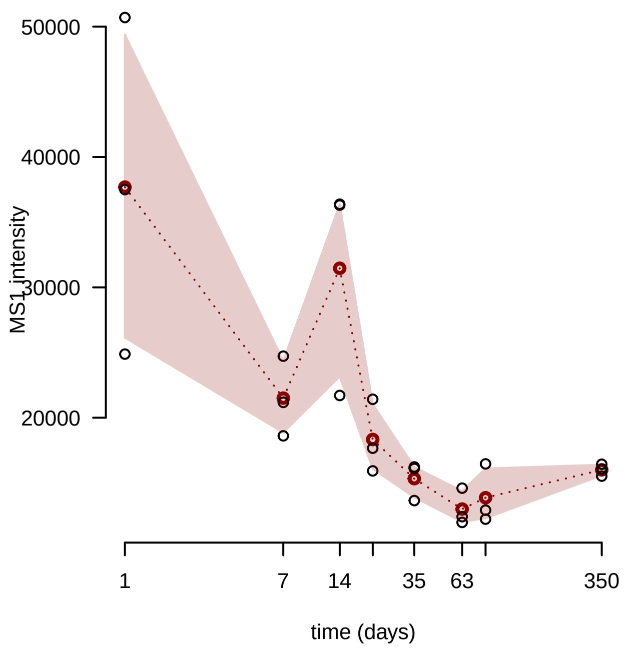

Q6P8M1
GN=Tatdn1
TatD DNase domain containing 1

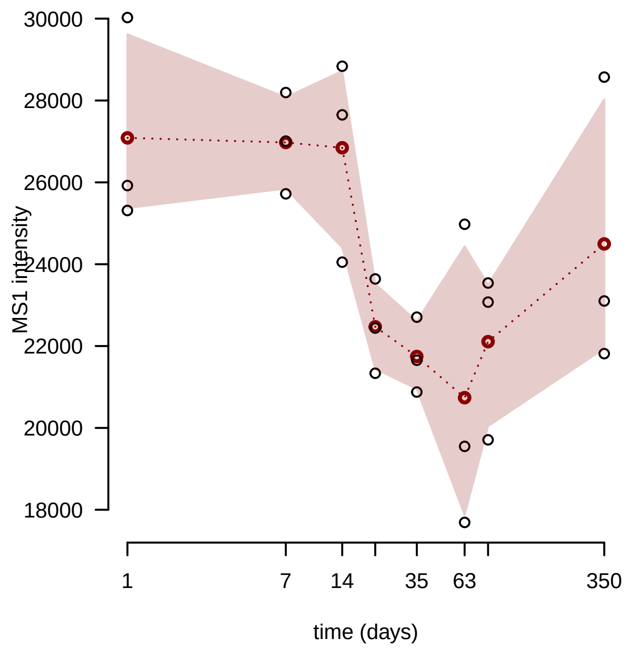

Q6P8X1
GN=Snx6
sorting nexin 6

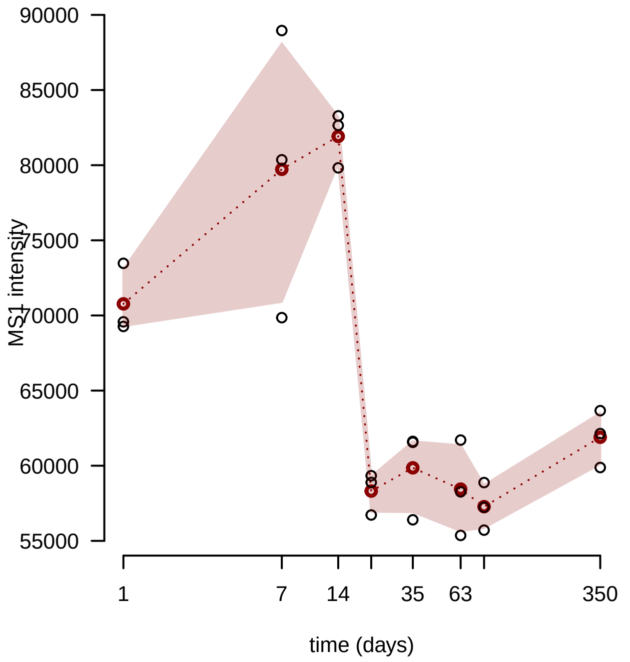

Q6P9J5
GN=Kank4
KN motif and ankyrin repeat domains 4

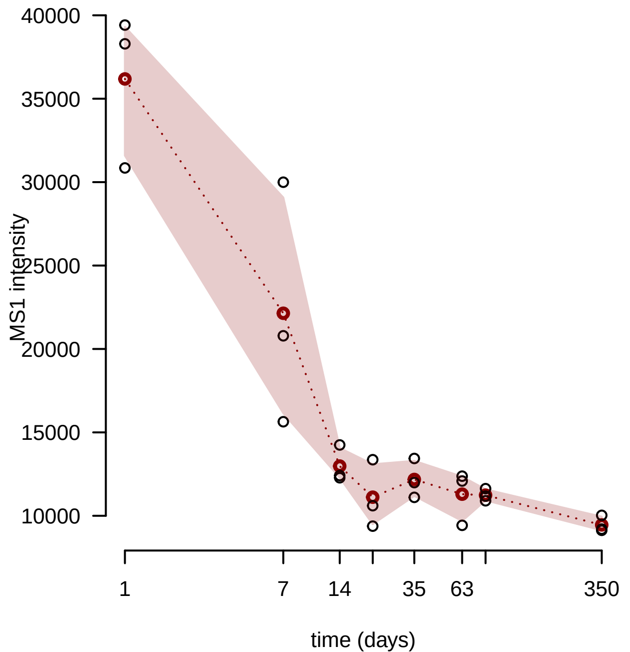

Q6P9J9
GN=Ano6
anoctamin 6

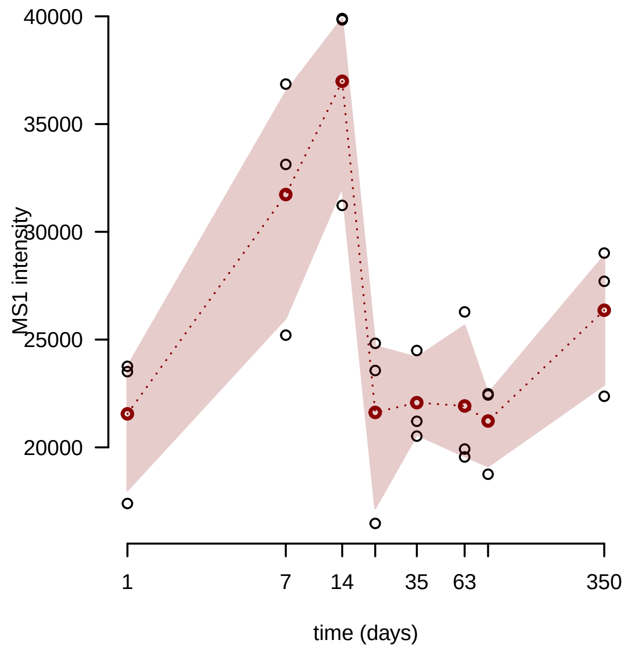

Q6P9K8
GN=Caskin1
CASK interacting protein 1

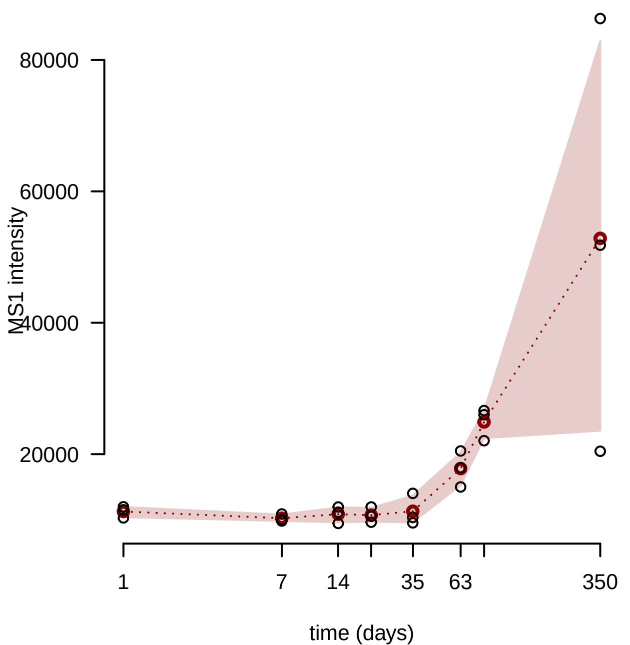

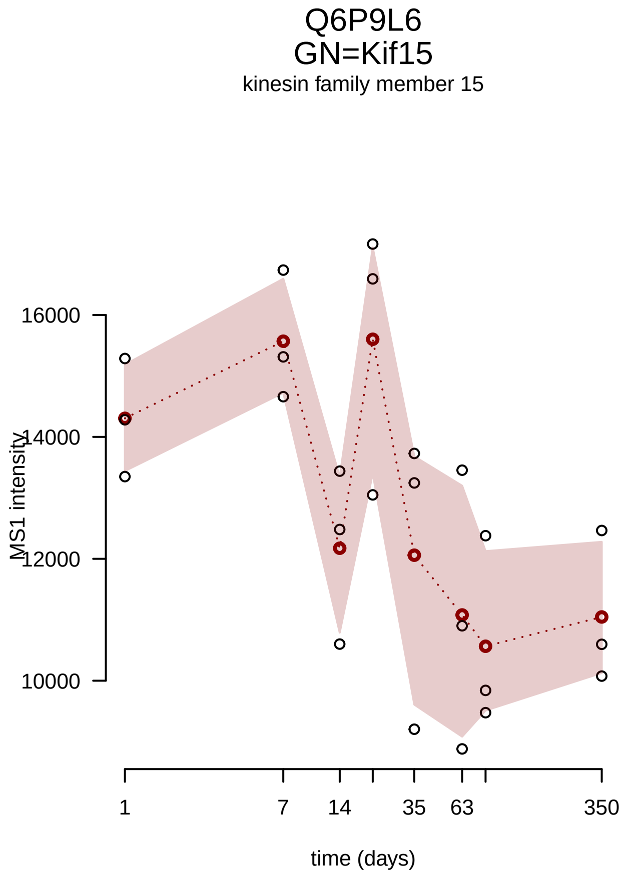
Q6P9L6
GN=Kif15
kinesin family member 15
MS1 intensity
10000
12000
14000
16000
1
7
14
35
63
350
time (days)
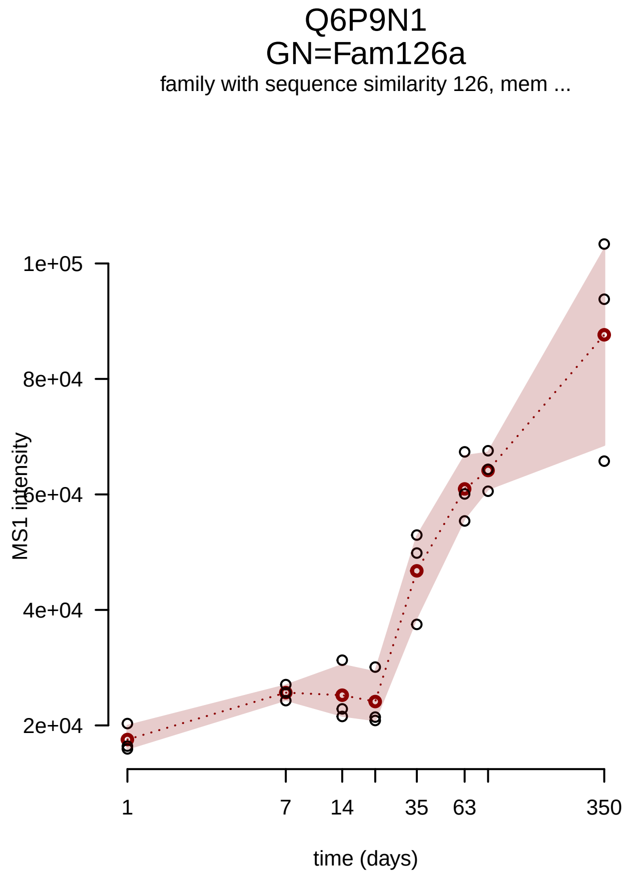
Q6P9N1
GN=Fam126a
family with sequence similarity 126, mem ...
MS1 intensity
2e+04
4e+04
6e+04
8e+04
1e+05
1
7
14
35
63
350
time (days)
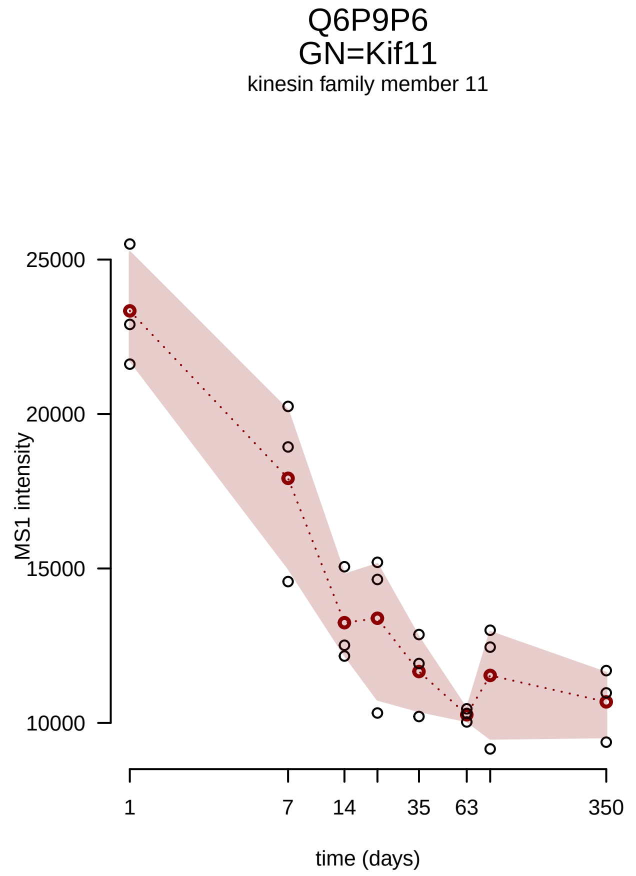
Q6P9P6
GN=Kif11
kinesin family member 11
MS1 intensity
10000
15000
20000
25000
1
7
14
35
63
350
time (days)
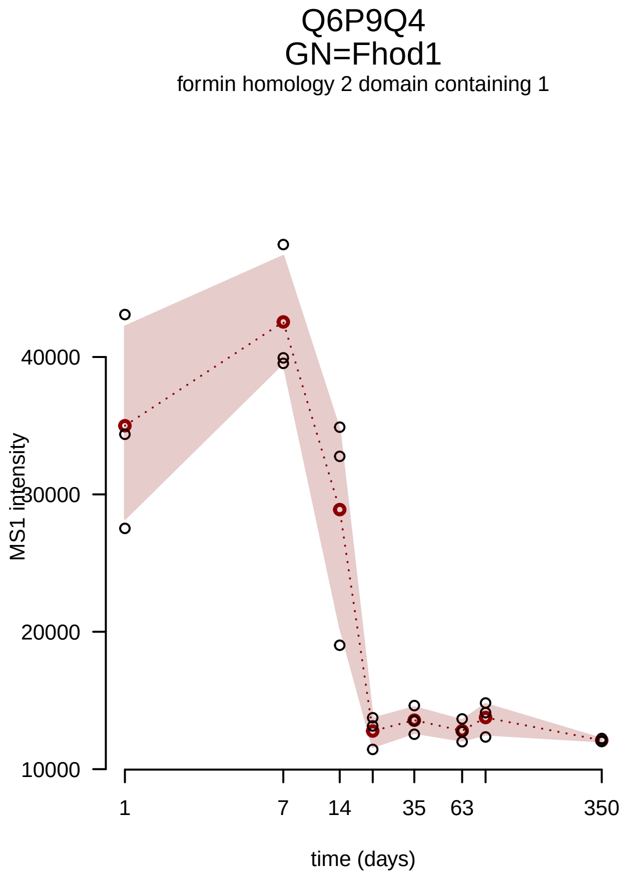
Q6P9Q4
GN=Fhod1
formin homology 2 domain containing 1
MS1 intensity
10000
20000
30000
40000
1
7
14
35
63
350
time (days)
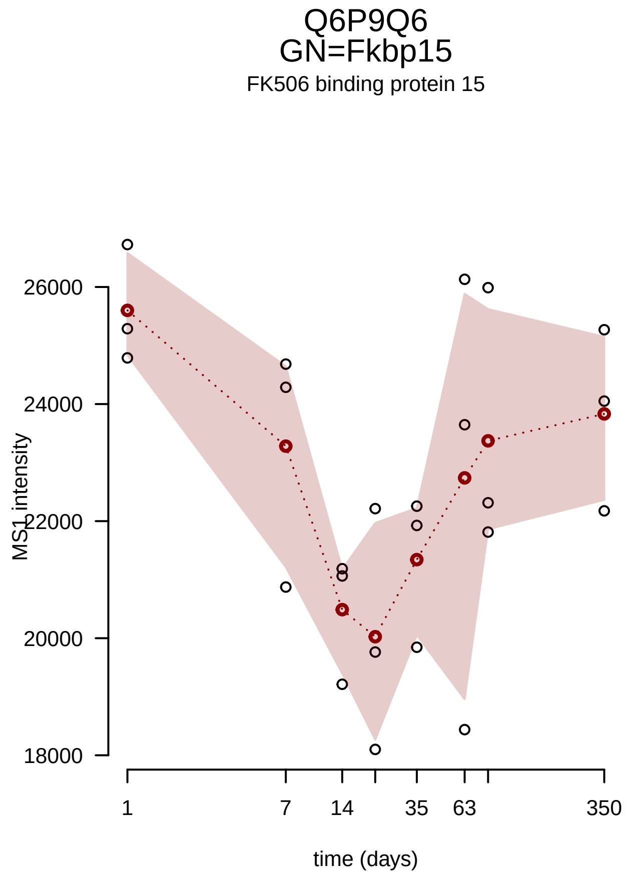
Q6P9Q6
GN=Fkbp15
FK506 binding protein 15
MS1 intensity
18000
20000
22000
24000
26000
1
7
14
35
63
350
time (days)
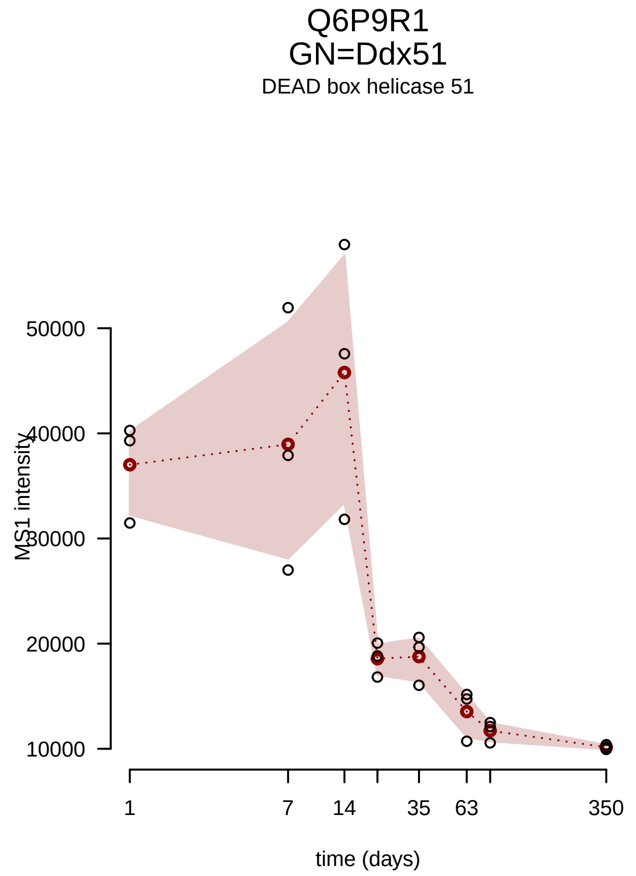
Q6P9R1
GN=Ddx51
DEAD box helicase 51
MS1 intensity
10000
20000
30000
40000
50000
1
7
14
35
63
350
time (days)
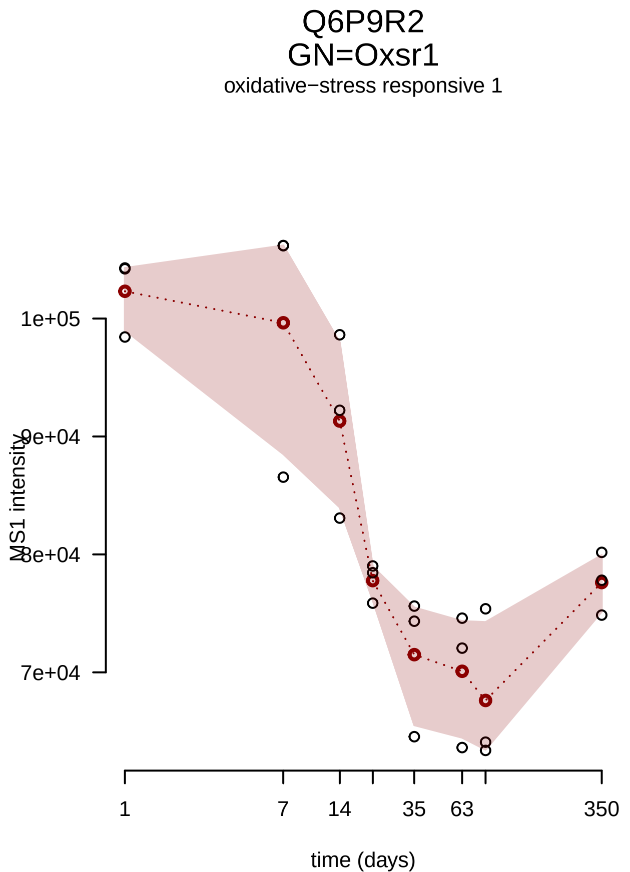
Q6P9R2
GN=Oxsr1
oxidative−stress responsive 1
MS1 intensity
7e+04
8e+04
9e+04
1e+05
1
7
14
35
63
350
time (days)
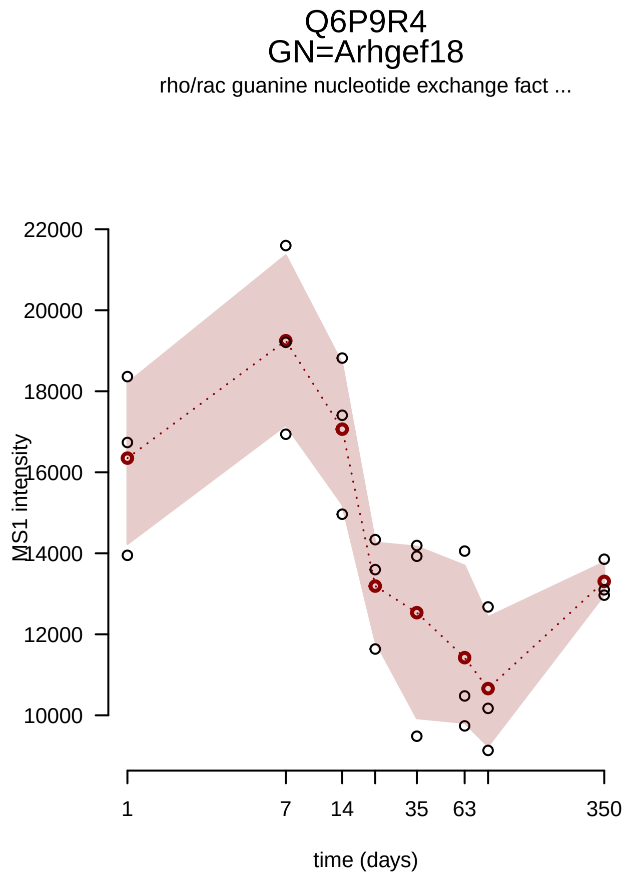
Q6P9R4
GN=Arhgef18
rho/rac guanine nucleotide exchange fact ...
MS1 intensity
10000
12000
14000
16000
18000
20000
22000
1
7
14
35
63
350
time (days)
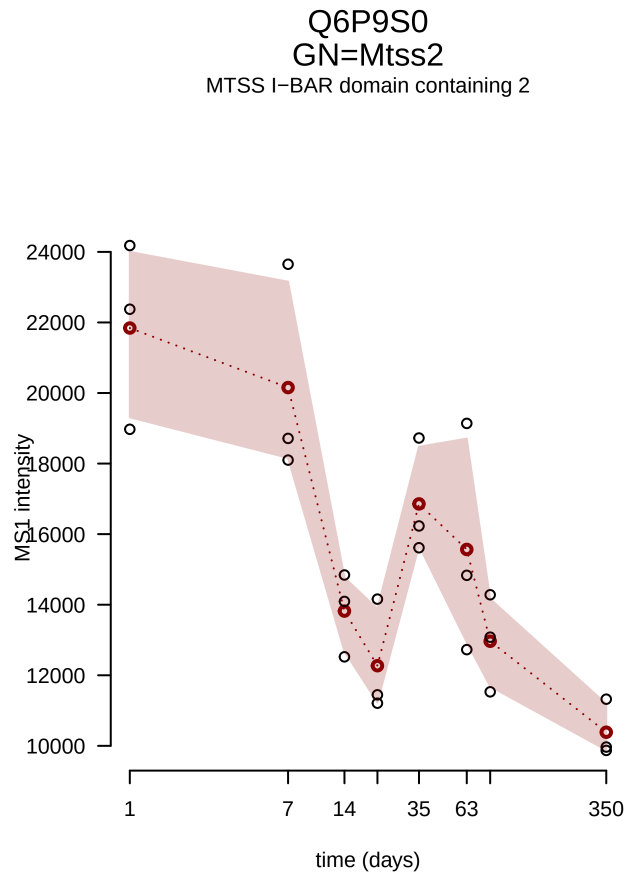
Q6P9S0
GN=Mtss2
MTSS I−BAR domain containing 2
MS1 intensity
10000
12000
14000
16000
18000
20000
22000
24000
1
7
14
35
63
350
time (days)

Q6P9Z1
GN=Smarcd3
SWI/SNF related, matrix associated, acti ...

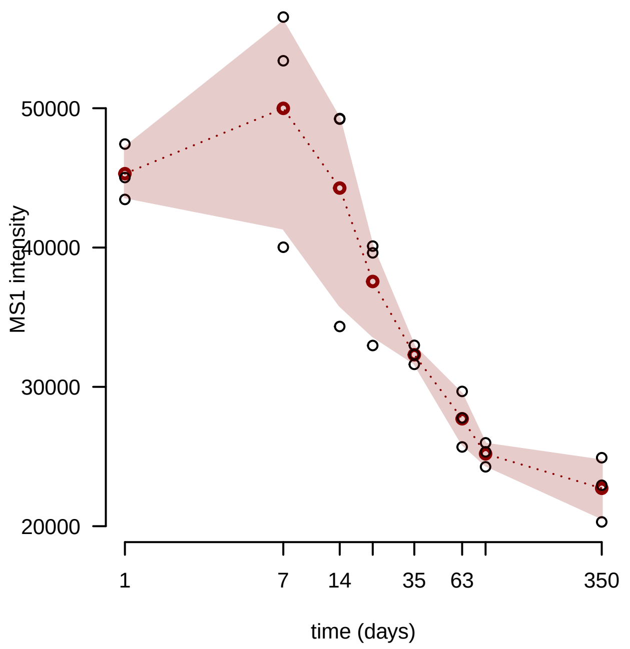

Q6PA06
GN=Atl2
atlastin GTPase 2

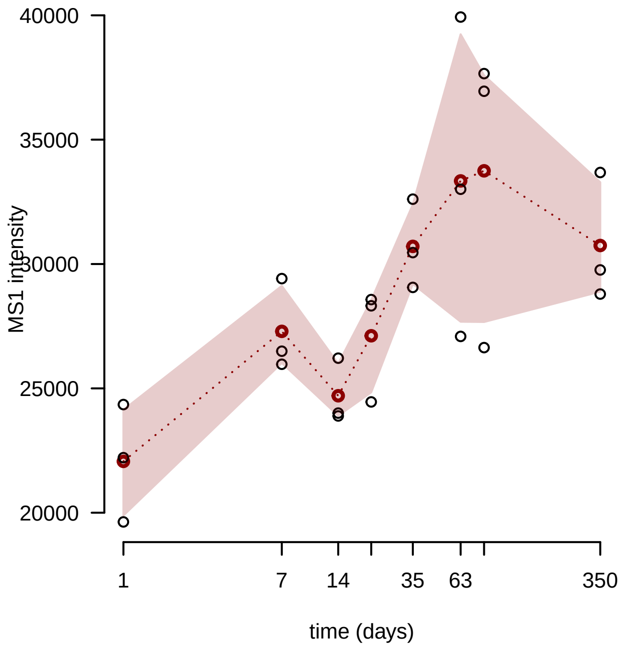

Q6PAC3
GN=Dcaf13
DDB1 and CUL4 associated factor 13

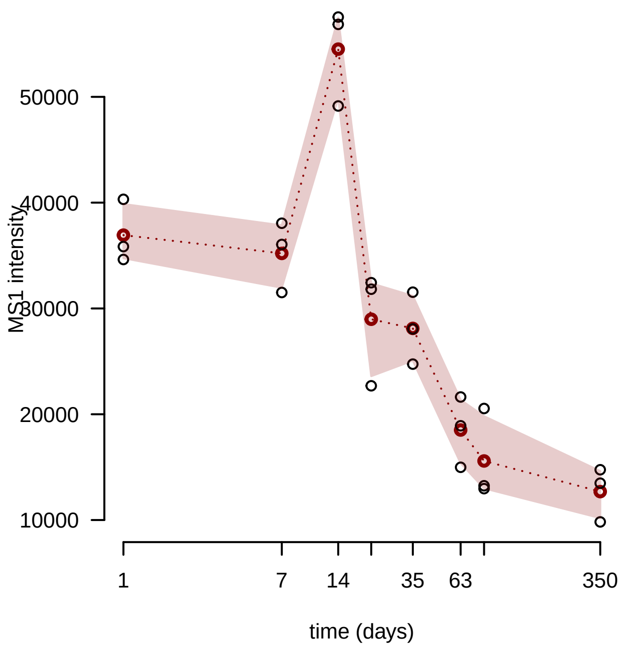

Q6PAJ1
GN=Bcr
BCR activator of RhoGEF and GTPase

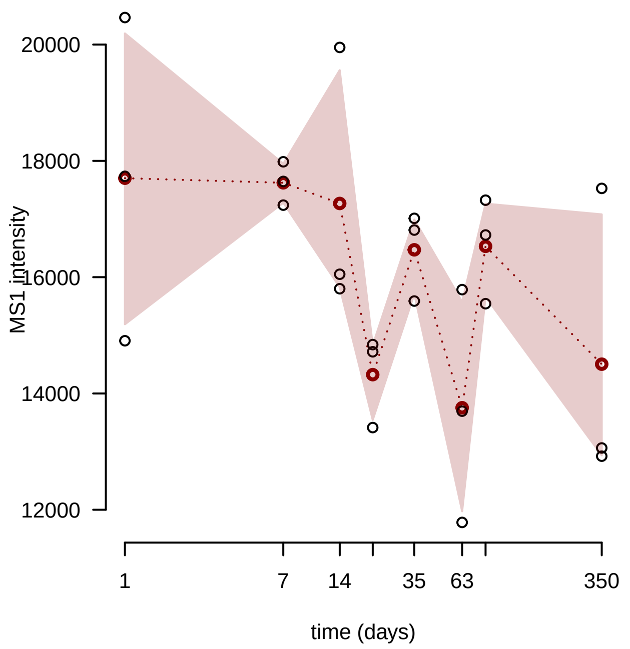

Q6PAL0
GN=Bend3
BEN domain containing 3

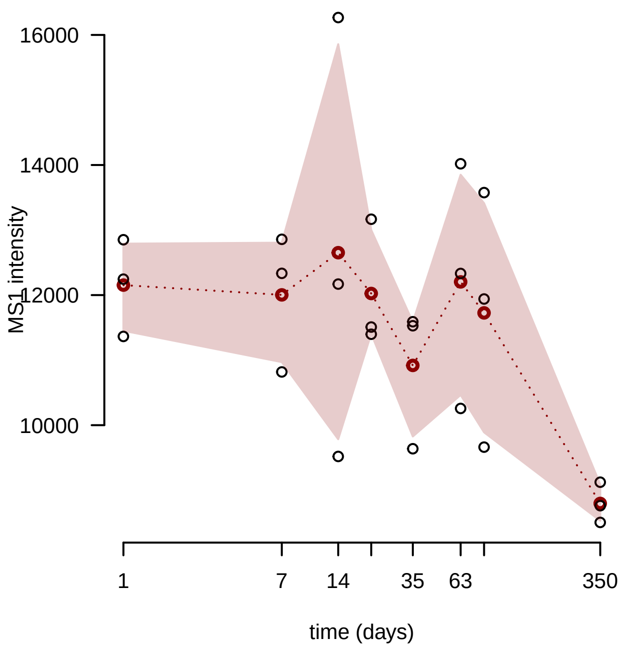

Q6PAL7
GN=Ahdc1
AT hook, DNA binding motif, containing 1

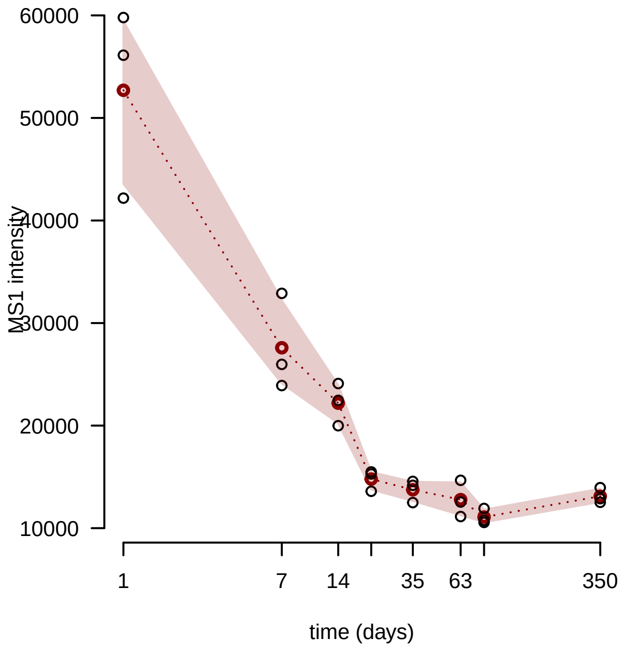

Q6PAL8
GN=Dennd5a
DENN/MADD domain containing 5A

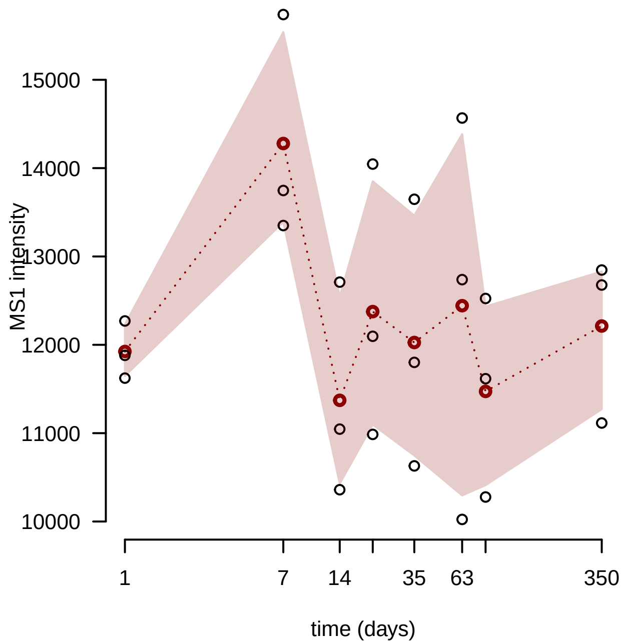

Q6PAM0
GN=Prkab2
protein kinase, AMP−activated, beta 2 no ...

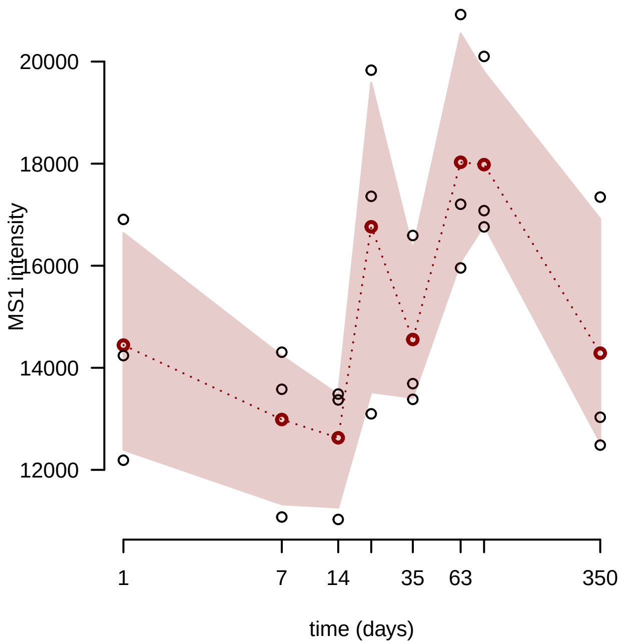

Q6PAM1
GN=Txlna
taxilin alpha

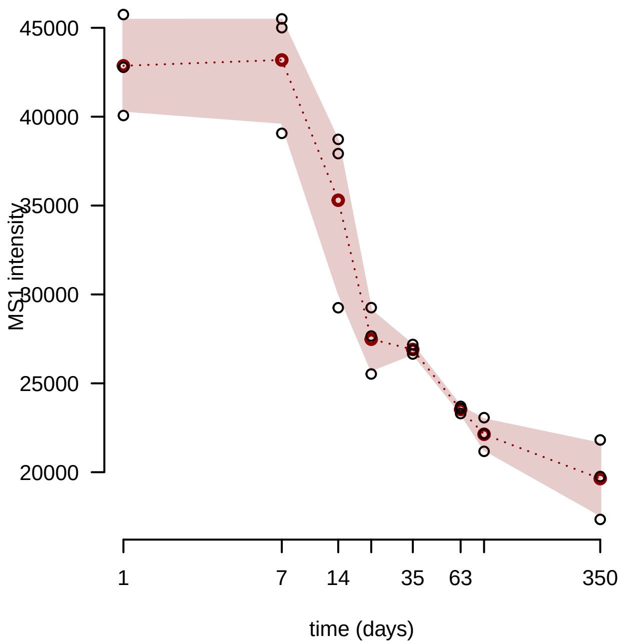

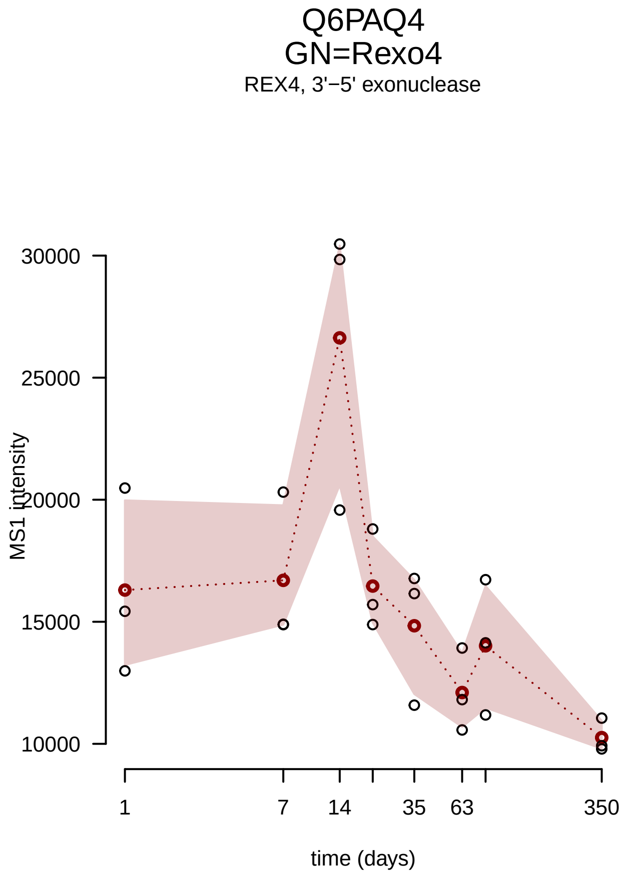
Q6PAQ4
GN=Rexo4
REX4, 3'−5' exonuclease
MS1 intensity
30000
25000
20000
15000
10000
1
7
14
35
63
350
time (days)

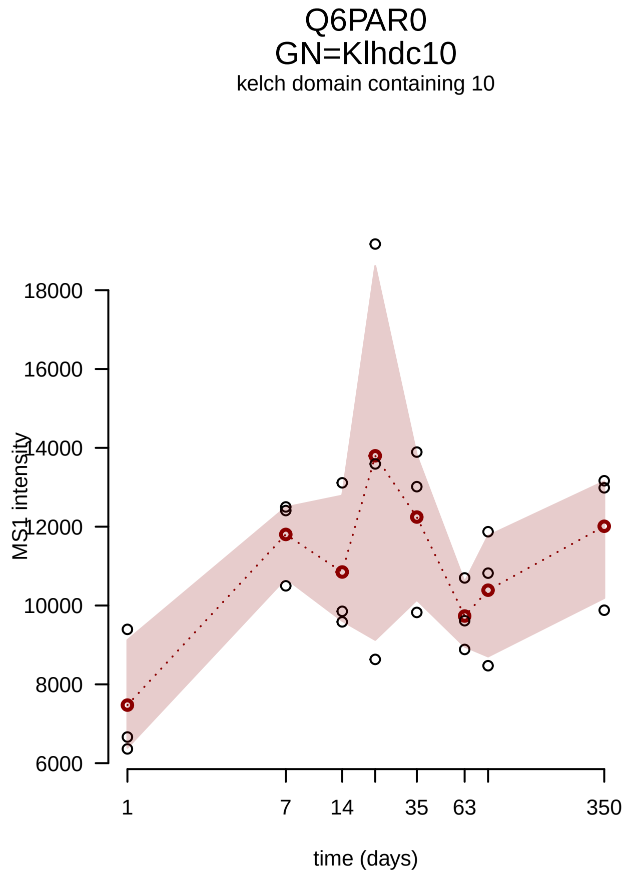
Q6PAR0
GN=Klhdc10
kelch domain containing 10
MS1 intensity
18000
16000
14000
12000
10000
8000
6000
1
7
14
35
63
350
time (days)

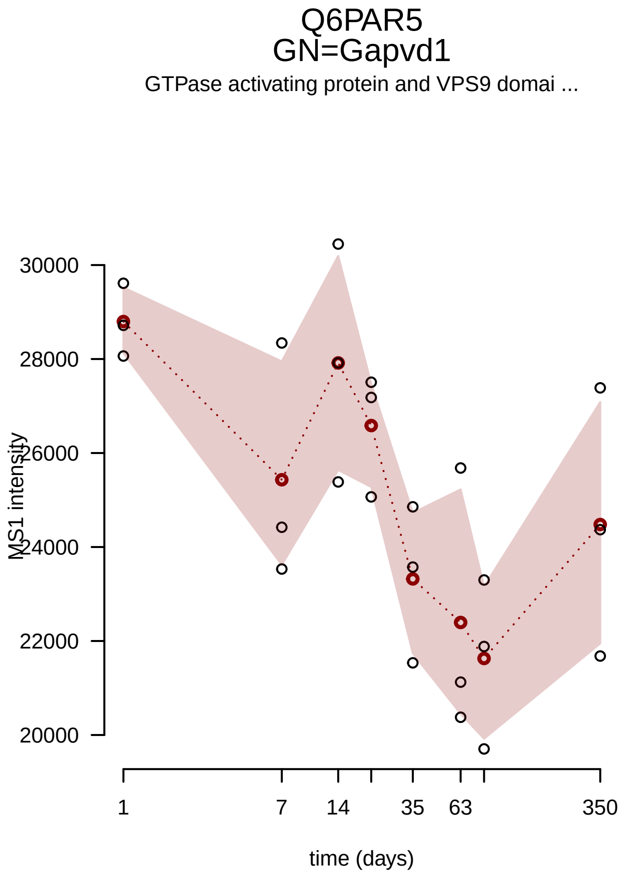
Q6PAR5
GN=Gapvd1
GTPase activating protein and VPS9 domai ...
MS1 intensity
30000
28000
26000
24000
22000
20000
1
7
14
35
63
350
time (days)

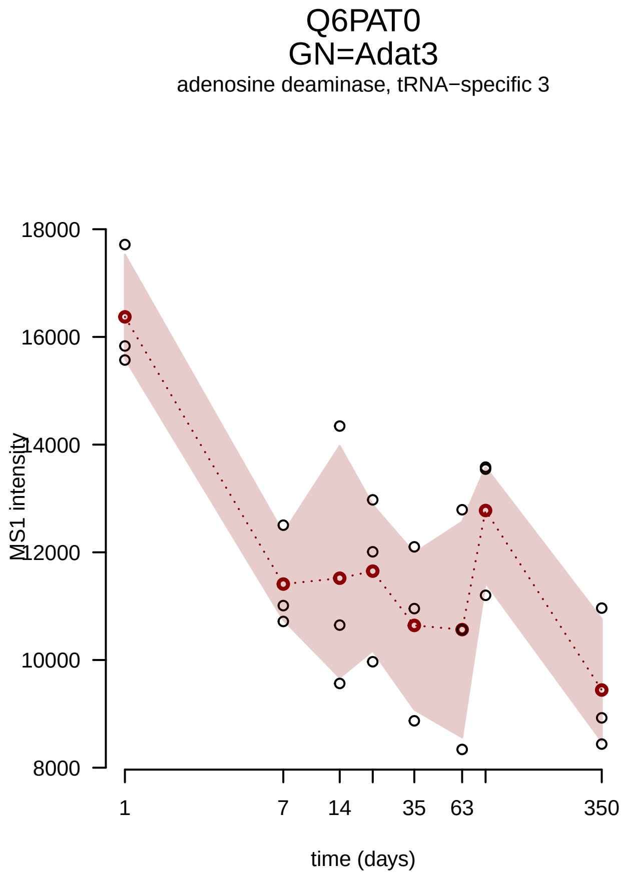
Q6PAT0
GN=Adat3
adenosine deaminase, tRNA−specific 3
MS1 intensity
18000
16000
14000
12000
10000
8000
1
7
14
35
63
350
time (days)

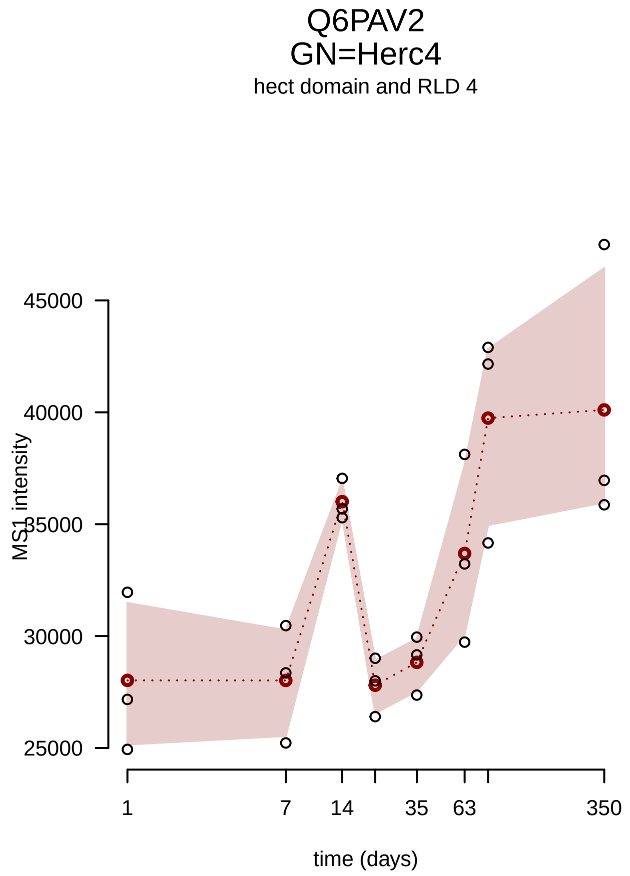
Q6PAV2
GN=Herc4
hect domain and RLD 4
MS1 intensity
45000
40000
35000
30000
25000
1
7
14
35
63
350
time (days)

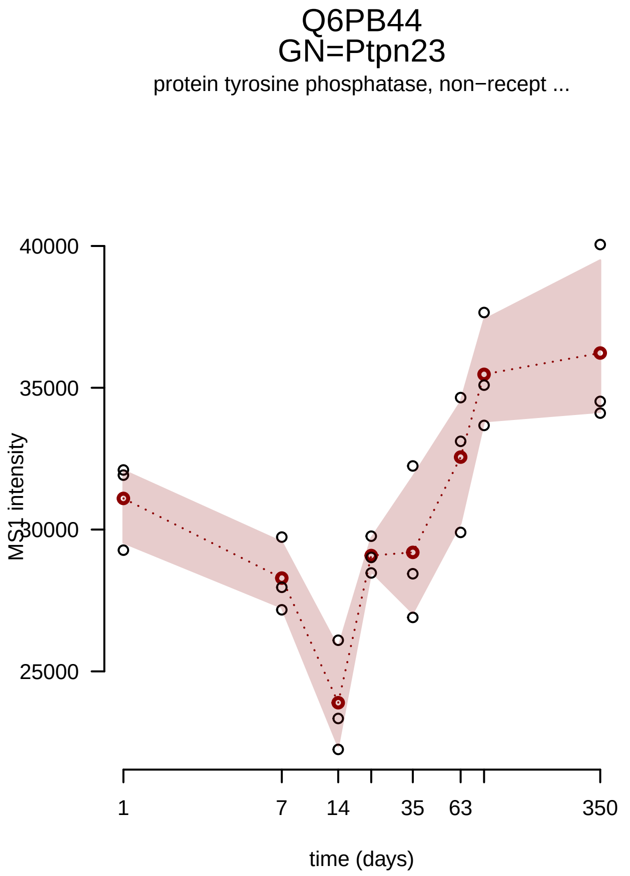
Q6PB44
GN=Ptpn23
protein tyrosine phosphatase, non−recept ...
MS1 intensity
40000
35000
30000
25000
1
7
14
35
63
350
time (days)

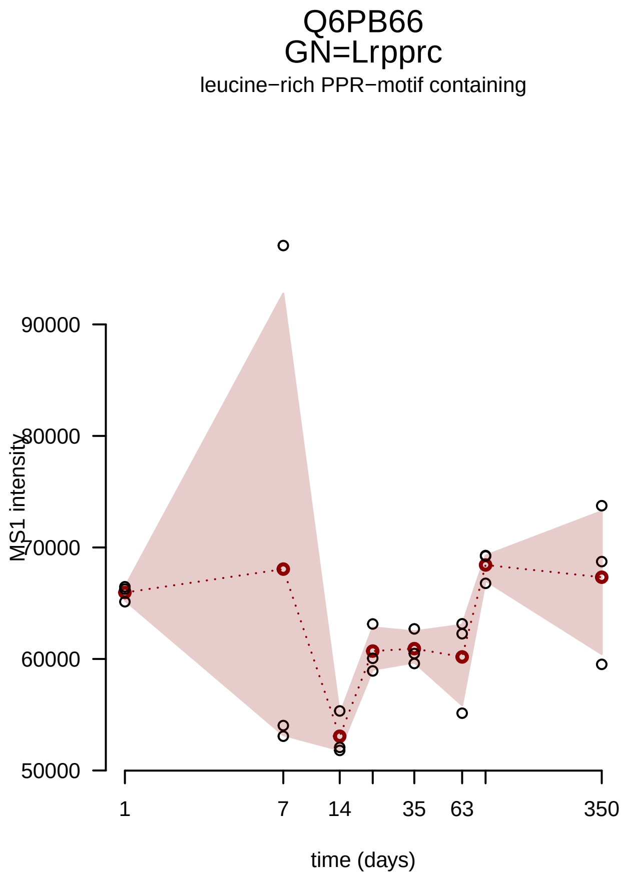
Q6PB66
GN=Lrpprc
leucine−rich PPR−motif containing
MS1 intensity
90000
80000
70000
60000
50000
1
7
14
35
63
350
time (days)

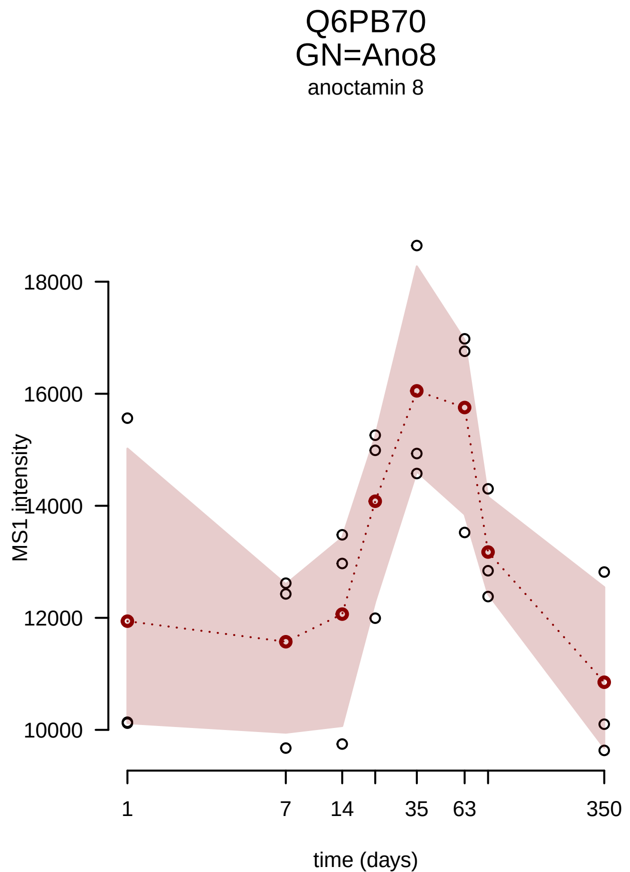
Q6PB70
GN=Ano8
anoctamin 8
MS1 intensity
18000
16000
14000
12000
10000
1
7
14
35
63
350
time (days)

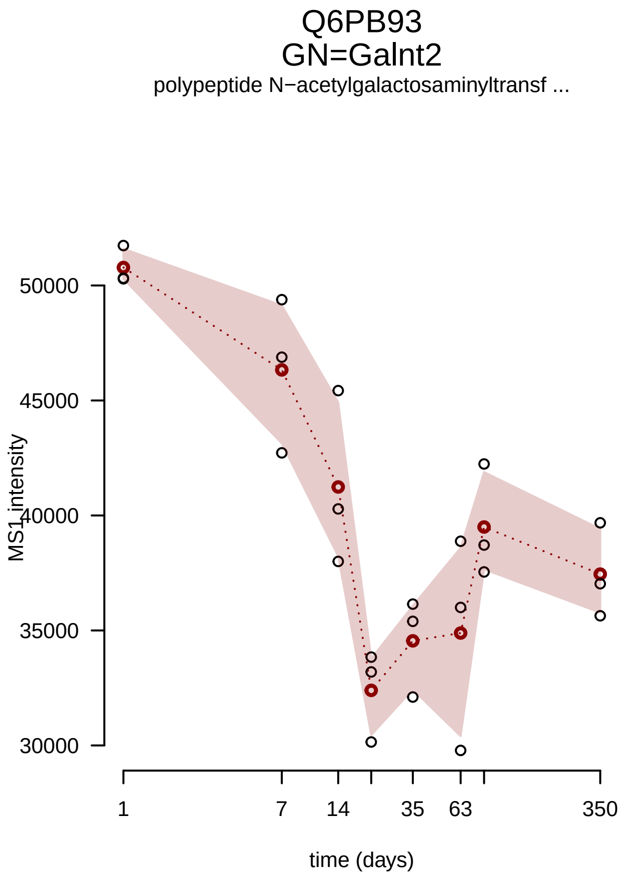
Q6PB93
GN=Galnt2
polypeptide N−acetylgalactosaminyltransf ...
MS1 intensity
50000
45000
40000
35000
30000
1
7
14
35
63
350
time (days)

Q6PB97
GN=Fbxl19
F−box and leucine−rich repeat protein 19

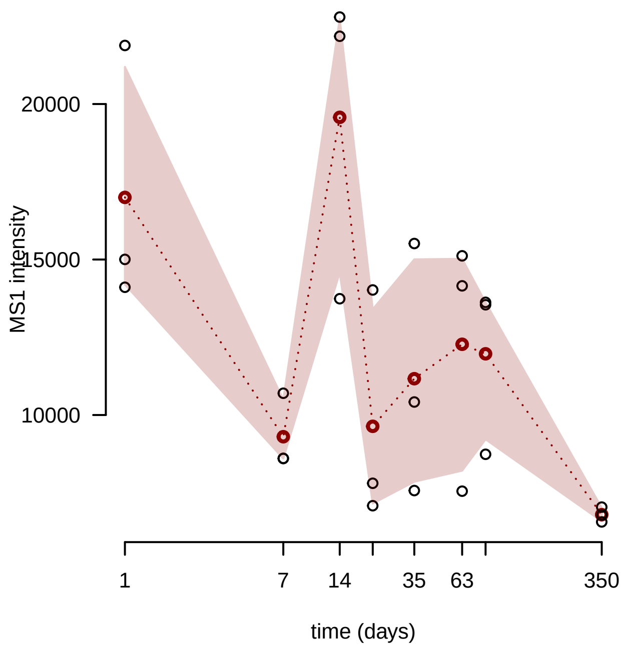

Q6PCL9
GN=Papolg
poly(A) polymerase gamma

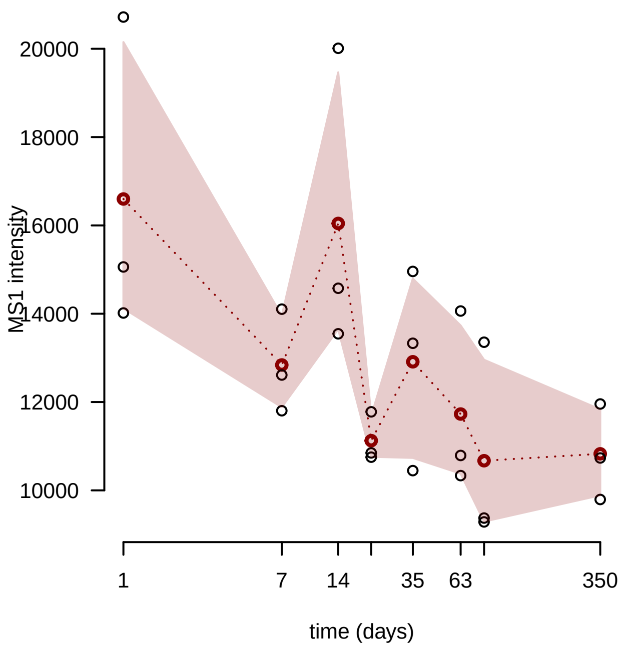

Q6PCM1
GN=Kdm3a
lysine (K)−specific demethylase 3A

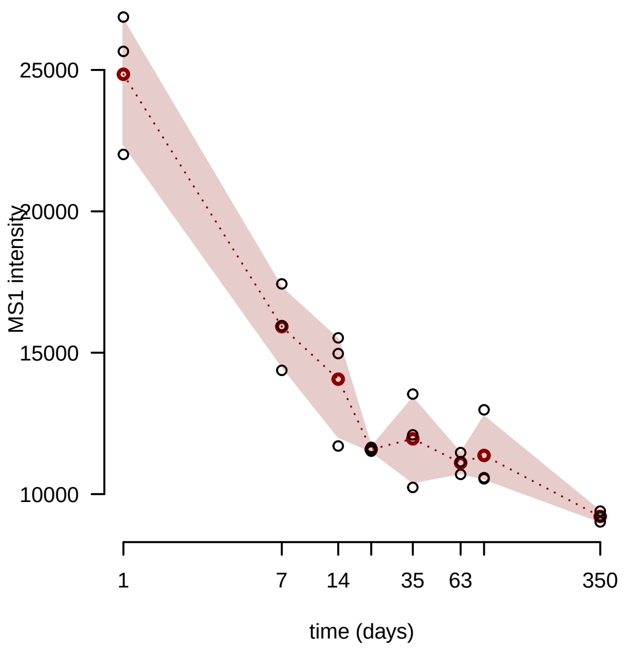

Q6PCM2
GN=Ints6
integrator complex subunit 6

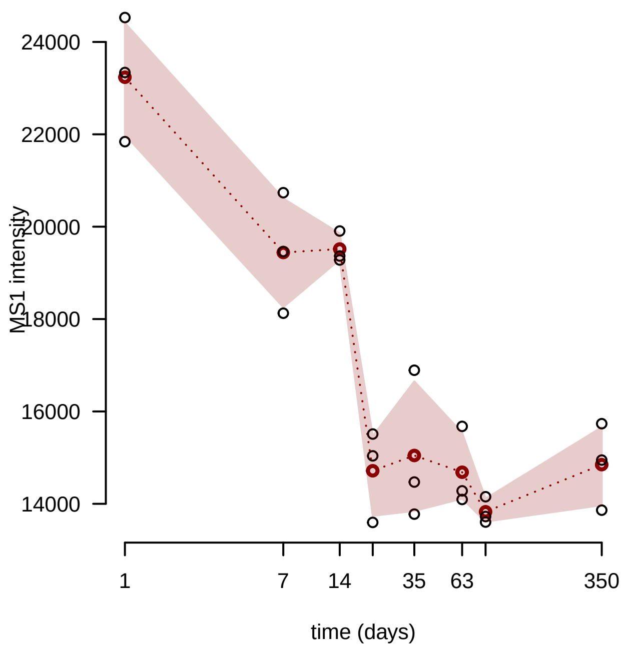

Q6PCN7
GN=Hltf
helicase−like transcription factor

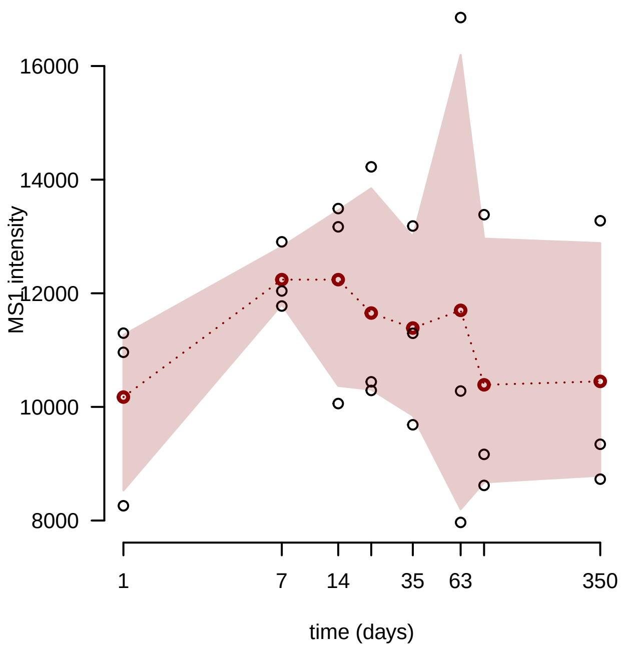

Q6PCP5
GN=Mff
mitochondrial fission factor

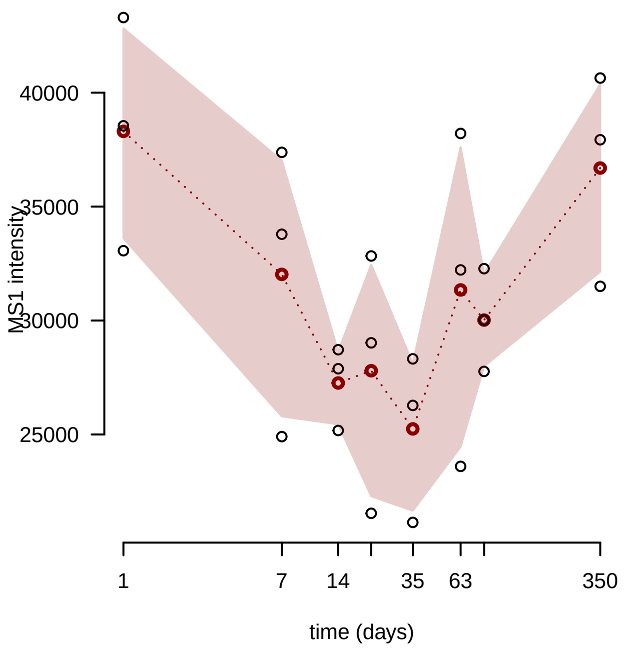

Q6PCP5
GN=Mff
mitochondrial fission factor

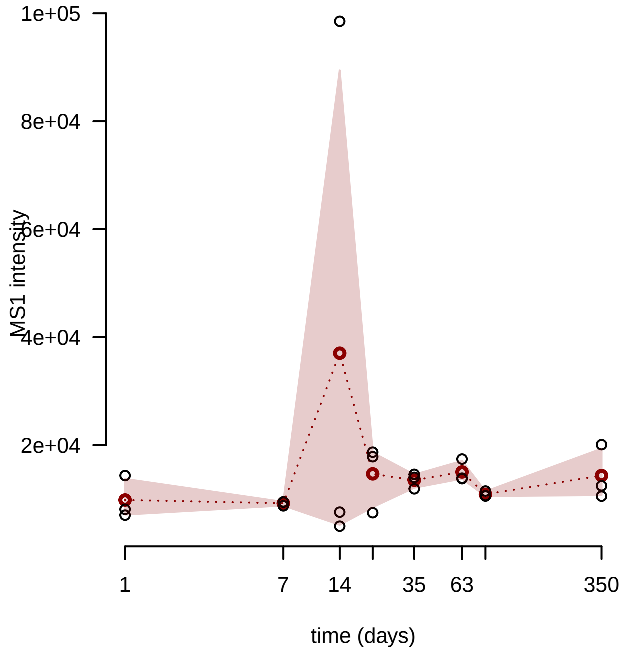

Q6PCQ0
GN=Iqce
IQ motif containing E

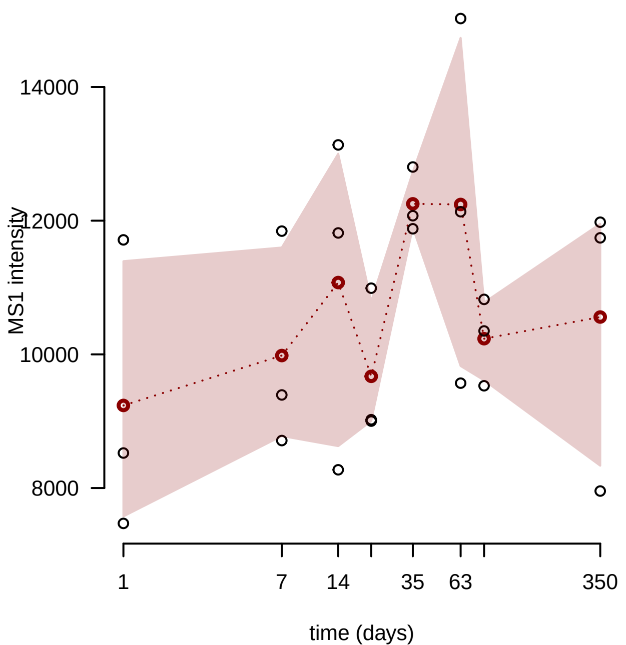

Q6PCX9
GN=Trim37
tripartite motif−containing 37

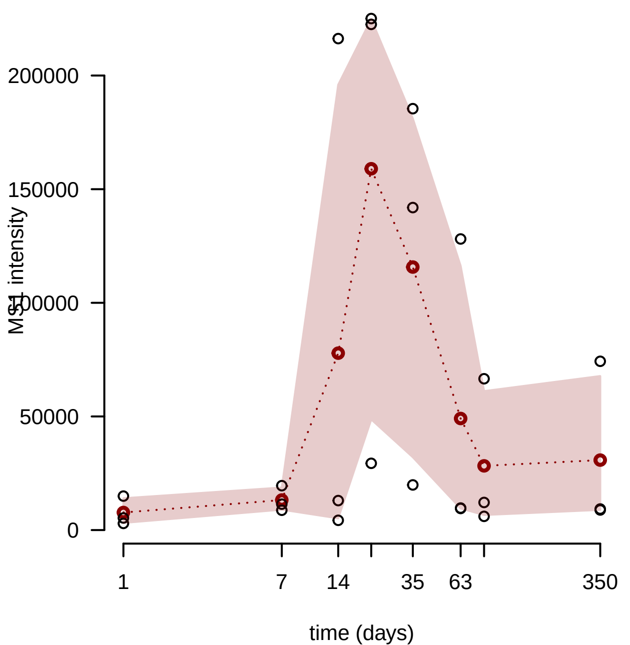

Q6PCZ4
GN=Magee1
MAGE family member E1

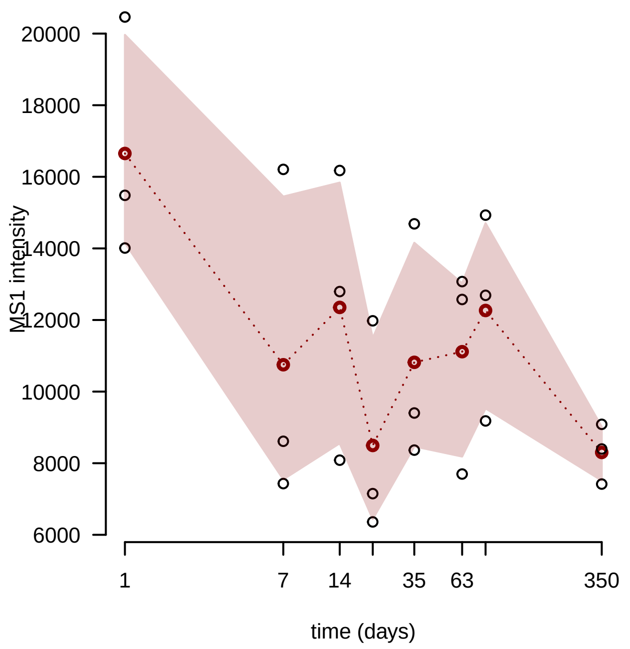

Q6PD03
GN=Ppp2r5a
protein phosphatase 2, regulatory subuni ...

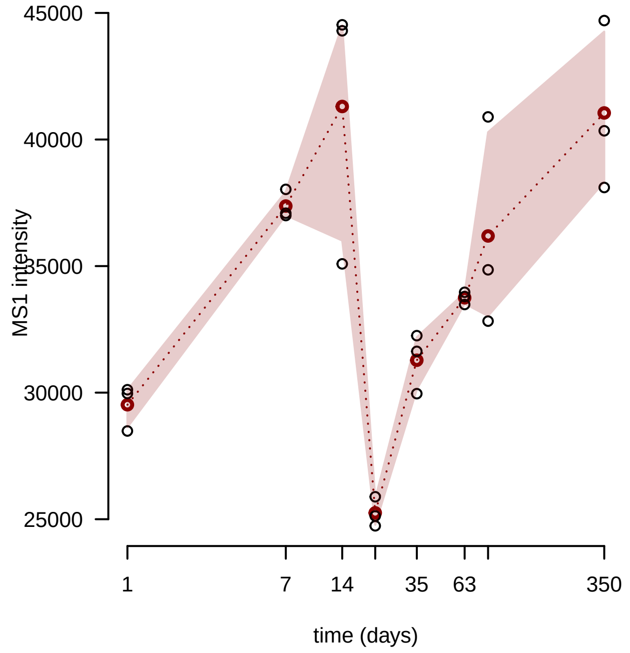

Q6PD10
GN=Ip6k1
inositol hexaphosphate kinase 1

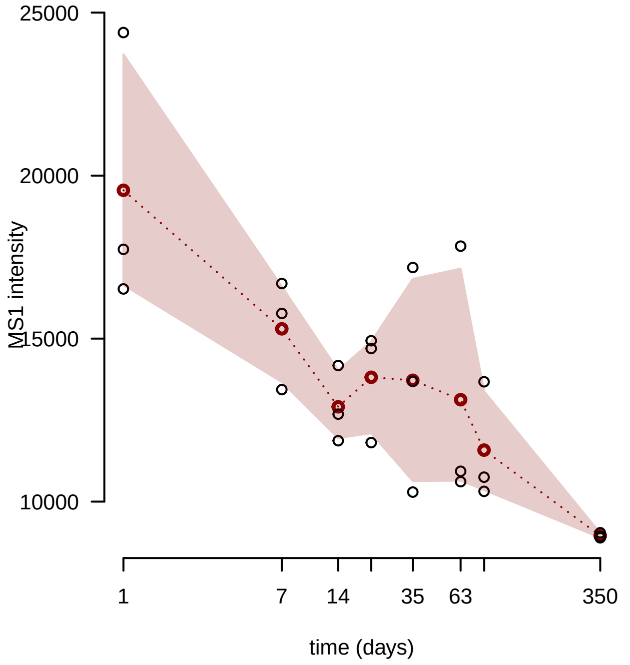

Q6PD19
GN=Armh3
armadillo−like helical domain containing ...

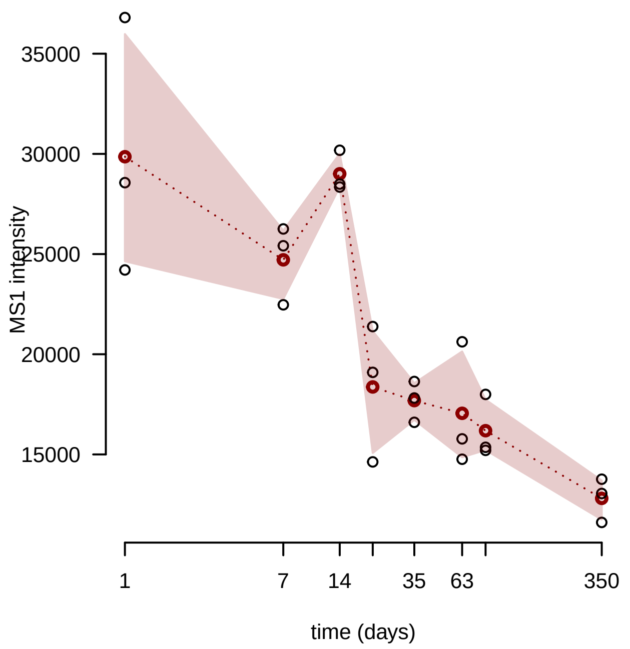

Q6PD26
GN=Pigs
phosphatidylinositol glycan anchor biosy ...

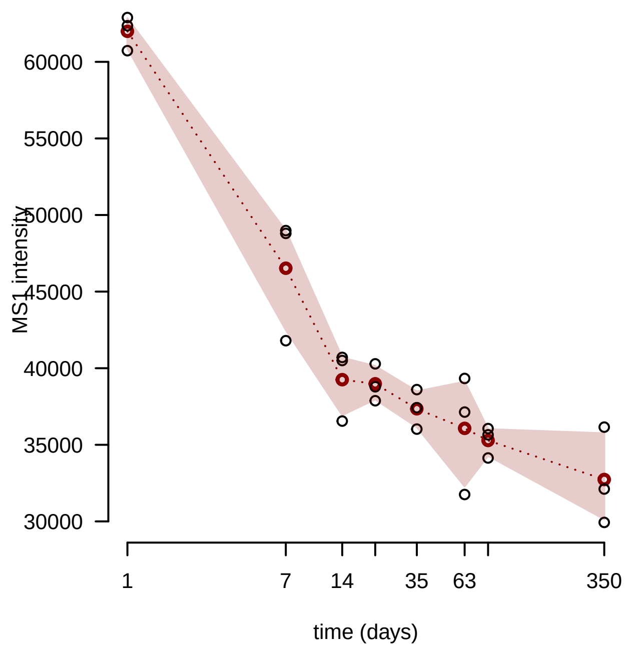

Q6PD28
GN=Ppp2r5b
protein phosphatase 2, regulatory subuni ...

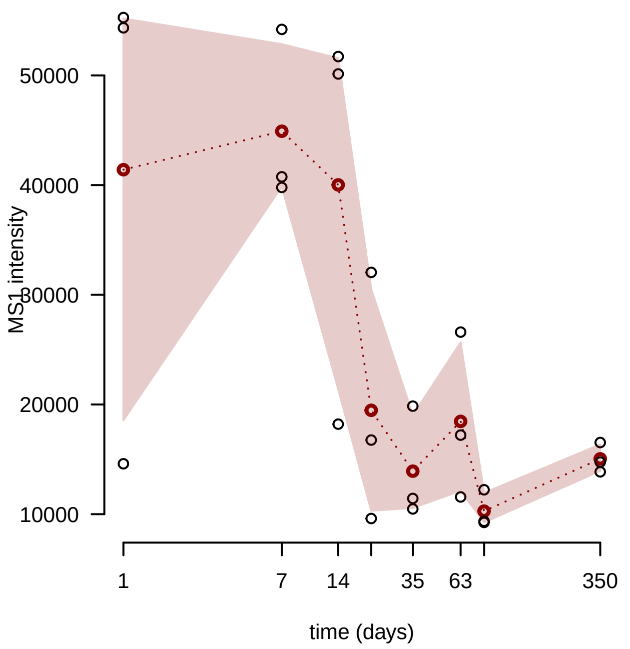

Q6PDG5
GN=Smarcc2
SWI/SNF related, matrix associated, acti ...

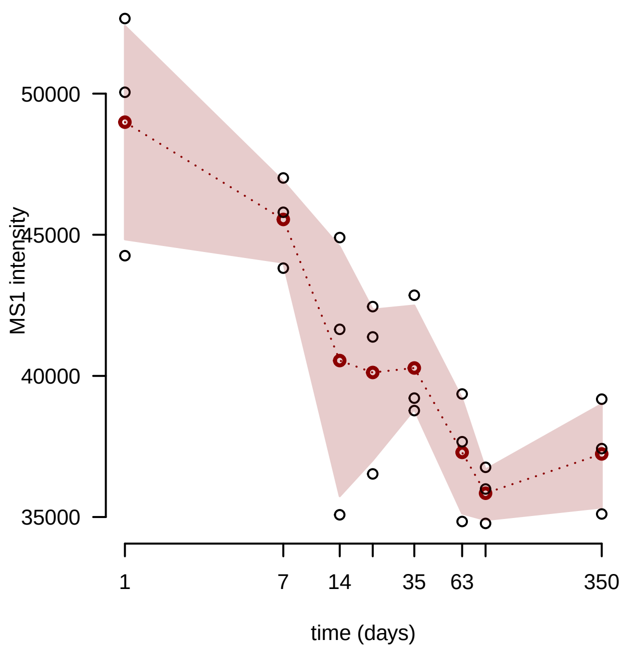

Q6PDG8
GN=Mon1a
MON1 homolog A, secretory traffciking as ...

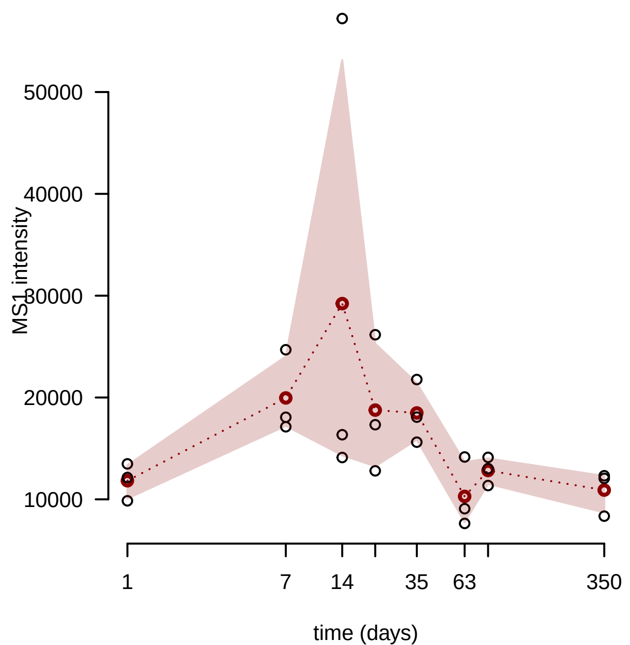

Q6PDH0
GN=Phldb1
pleckstrin homology like domain, family ...

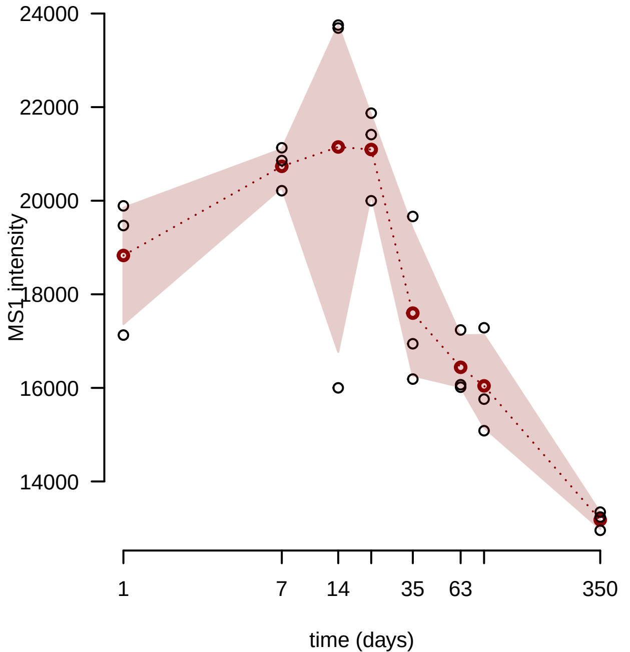

Q6PDI5
GN=Ecpas
Ecm29 proteasome adaptor and scaffold

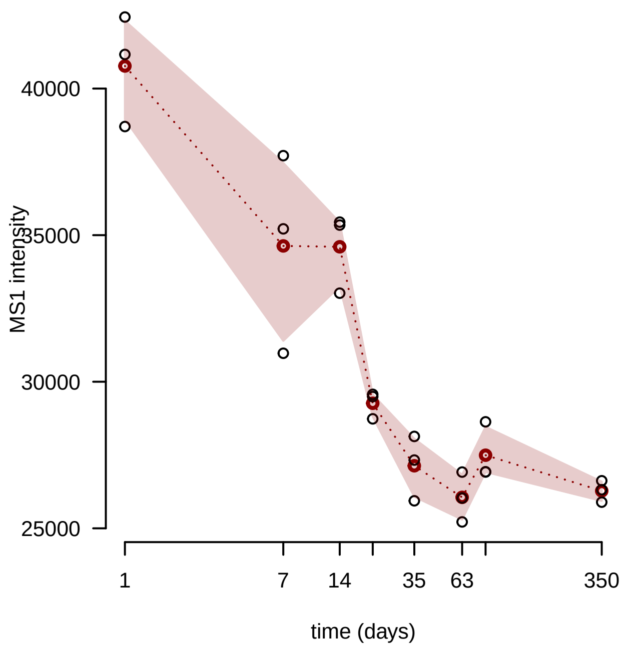

Q6PDI6
GN=Mindy2
MINDY lysine 48 deubiquitinase 2

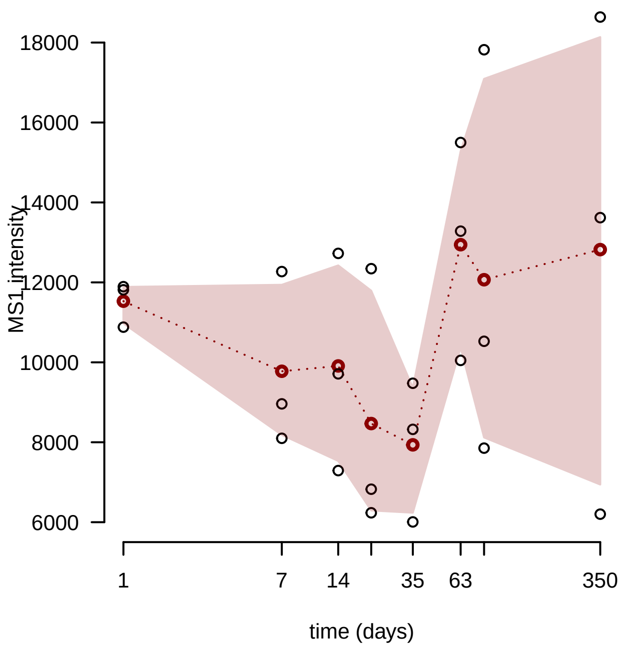

Q6PDJ1
GN=Cachd1
cache domain containing 1

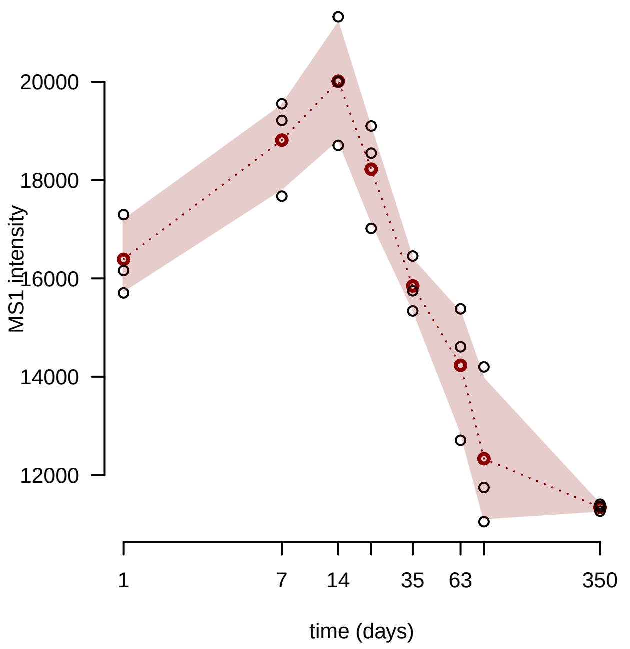

Q6PDJ6
GN=Fbxo42
F−box protein 42

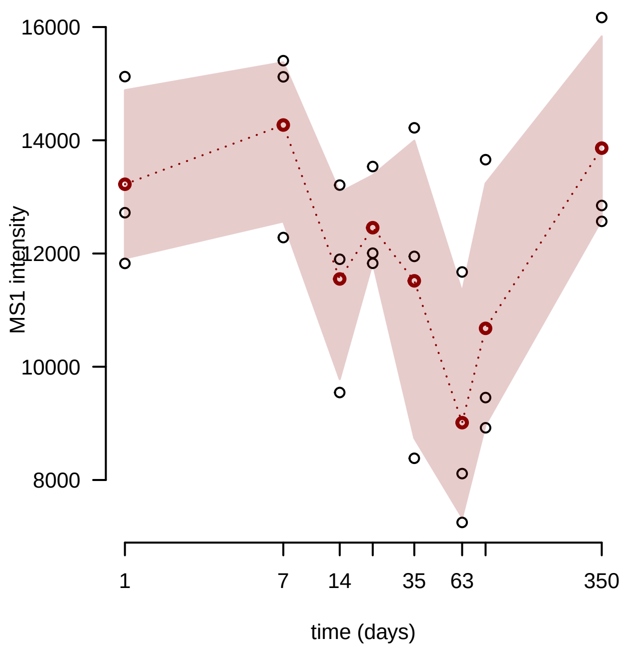

Q6PDK2
GN=Kmt2d
lysine (K)−specific methyltransferase 2D

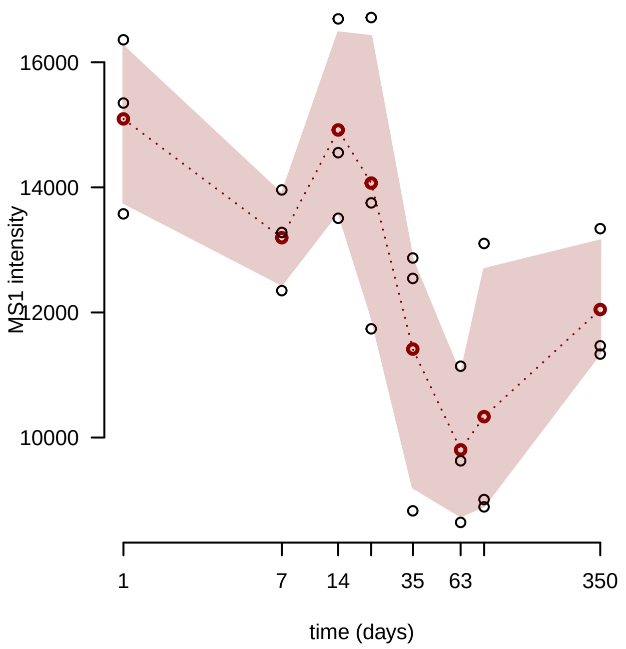

Q6PDL0
GN=Dync1li2
dynein, cytoplasmic 1 light intermediate ...

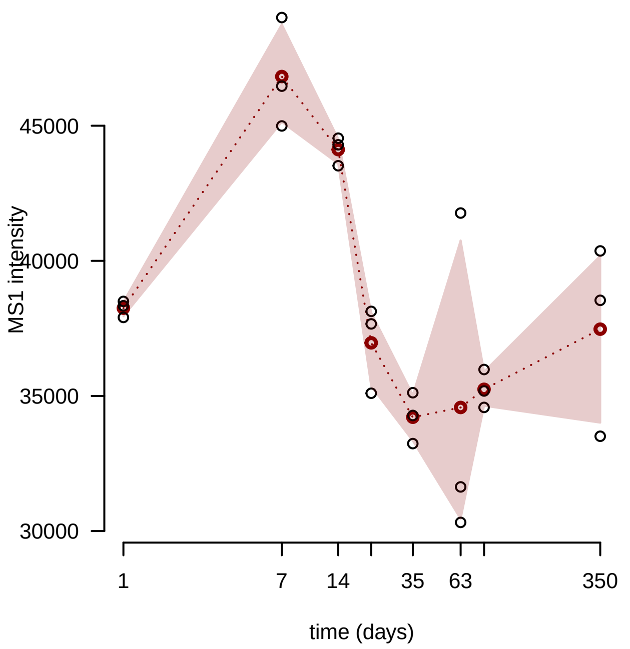

Q6PDM1
GN=Msl1
male specific lethal 1

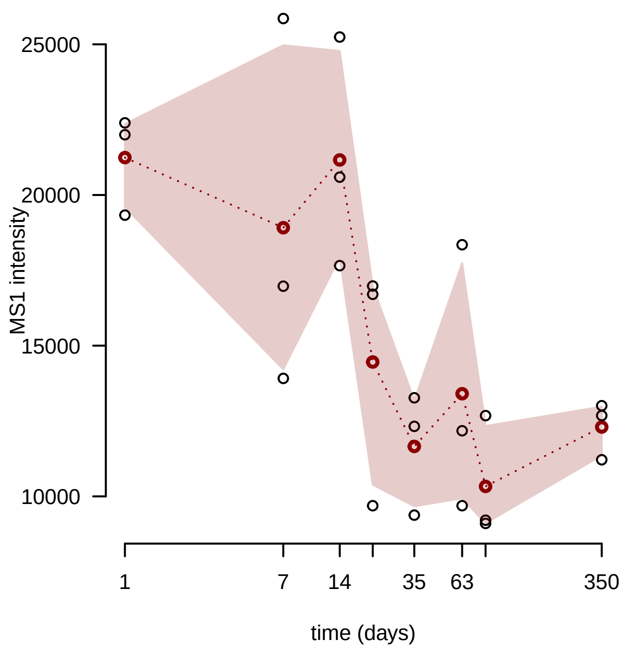

Q6PDM2
GN=Srsf1
serine and arginine−rich splicing factor ...

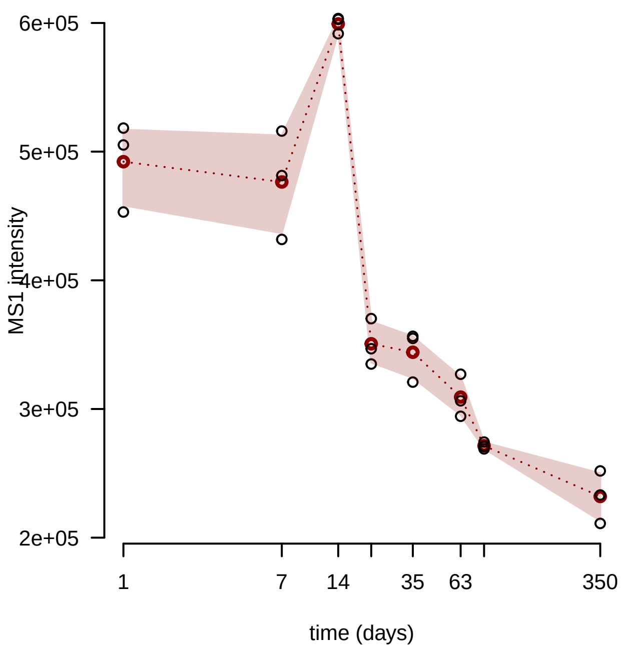

Q6PDN3
GN=Mylk
myosin, light polypeptide kinase

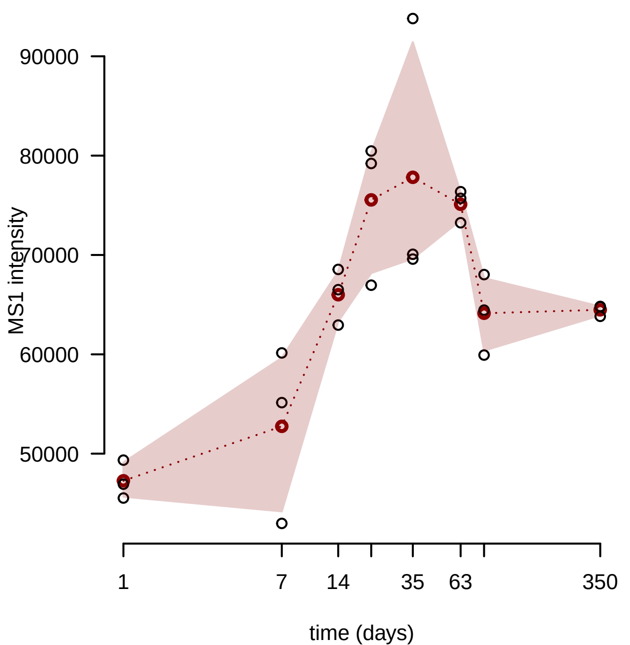

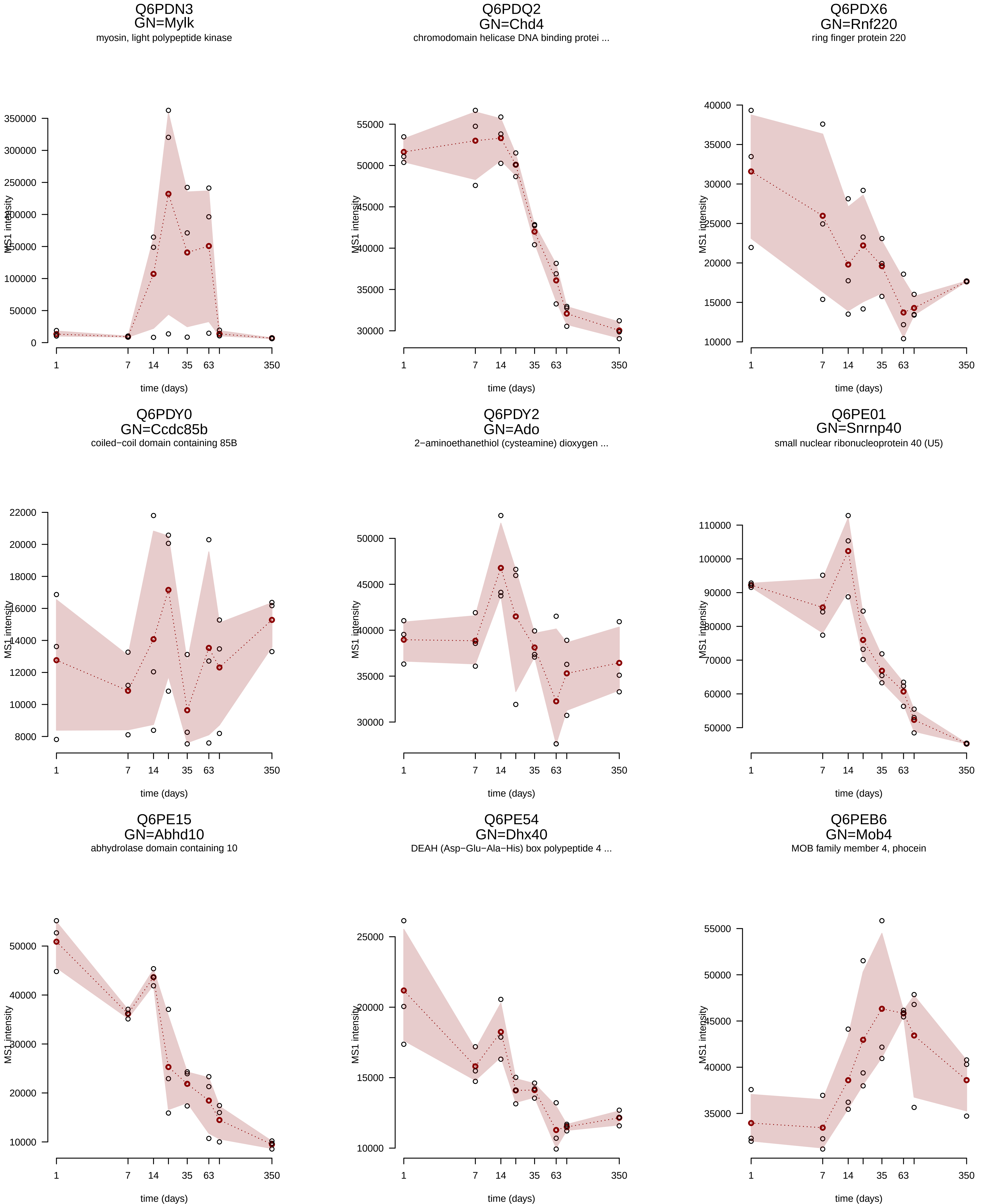
Q6PDN3
GN=Mylk
myosin, light polypeptide kinase
MS1 intensity
350000
300000
250000
200000
150000
100000
50000
0
1
7
14
35
63
350
time (days)
Q6PDQ2
GN=Chd4
chromodomain helicase DNA binding protei ...
MS1 intensity
55000
50000
45000
40000
35000
30000
1
7
14
35
63
350
time (days)
Q6PDX6
GN=Rnf220
ring finger protein 220
MS1 intensity
40000
35000
30000
25000
20000
15000
10000
1
7
14
35
63
350
time (days)
Q6PDY0
GN=Ccdc85b
coiled−coil domain containing 85B
MS1 intensity
22000
20000
18000
16000
14000
12000
10000
8000
1
7
14
35
63
350
time (days)
Q6PDY2
GN=Ado
2−aminoethanethiol (cysteamine) dioxygen ...
MS1 intensity
50000
45000
40000
35000
30000
1
7
14
35
63
350
time (days)
Q6PE01
GN=Snrnp40
small nuclear ribonucleoprotein 40 (U5)
MS1 intensity
110000
100000
90000
80000
70000
60000
50000
1
7
14
35
63
350
time (days)
Q6PE15
GN=Abhd10
abhydrolase domain containing 10
MS1 intensity
50000
40000
30000
20000
10000
1
7
14
35
63
350
time (days)
Q6PE54
GN=Dhx40
DEAH (Asp−Glu−Ala−His) box polypeptide 4 ...
MS1 intensity
25000
20000
15000
10000
1
7
14
35
63
350
time (days)
Q6PEB6
GN=Mob4
MOB family member 4, phocein
MS1 intensity
55000
50000
45000
40000
35000
1
7
14
35
63
350
time (days)

Q6PEE2
GN=Ctif
CBP80/20−dependent translation initiatio ...

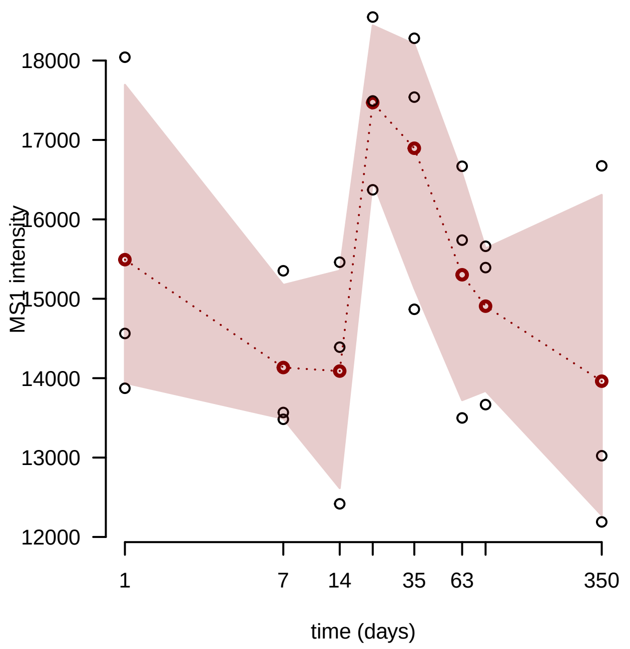

Q6PEE3
GN=Rrm2b
ribonucleotide reductase M2 B (TP53 indu ...

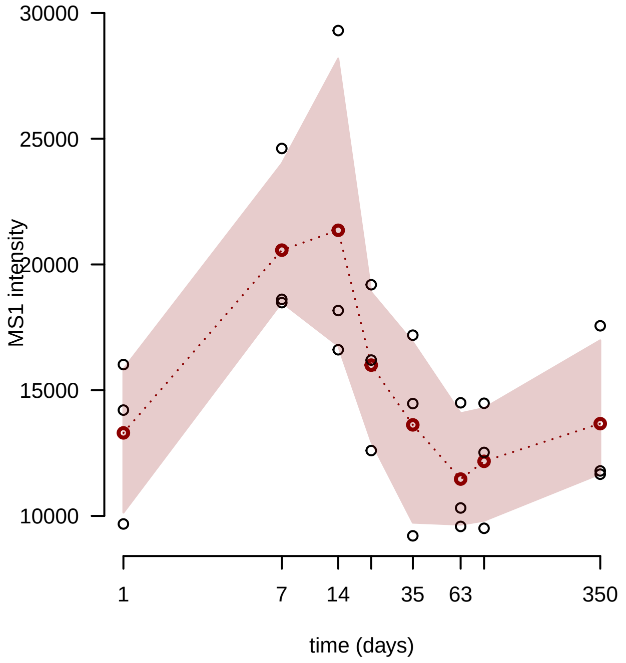

Q6PEM6
GN=Gramd2b

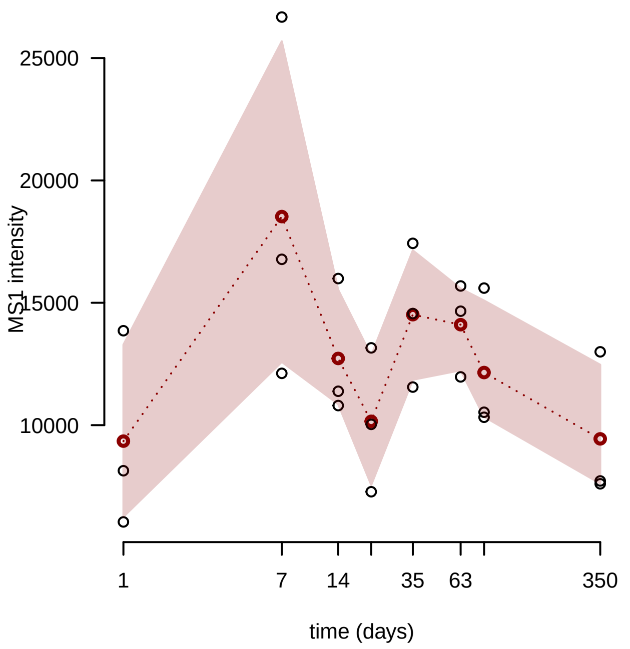

Q6PER3
GN=Mapre3
microtubule−associated protein, RP/EB fa ...

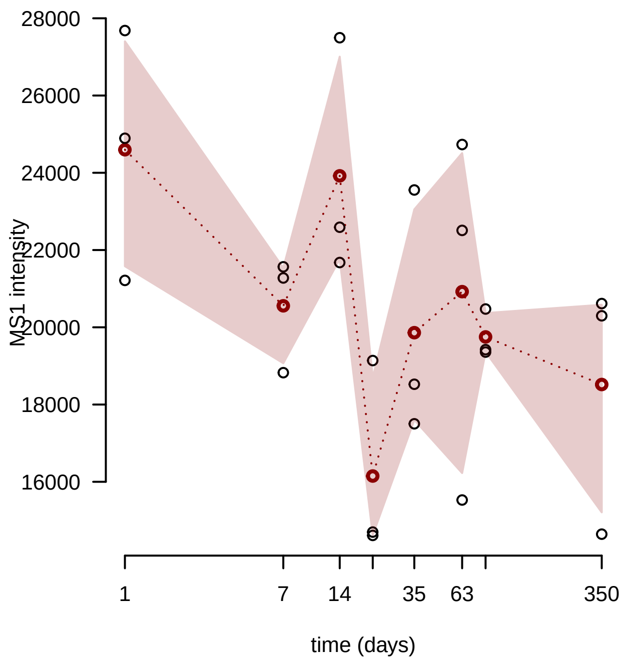

Q6PEV3
GN=Wipf2
WAS/WASL interacting protein family, mem ...

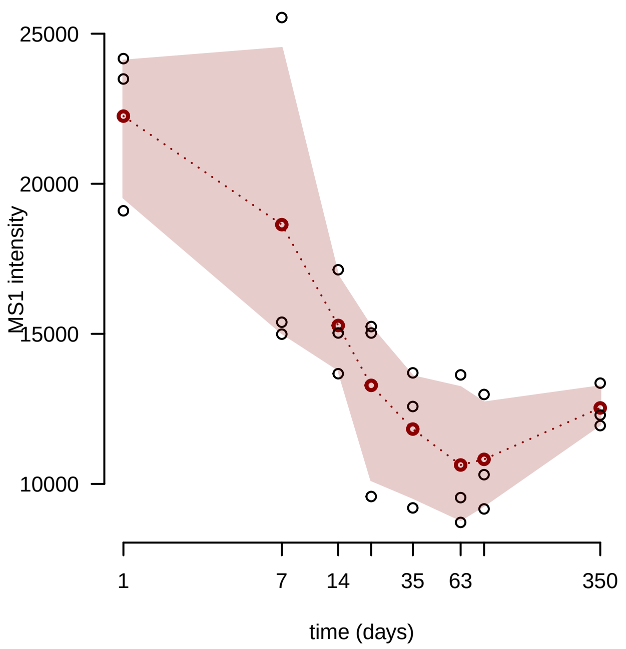

Q6PF93
GN=Pik3c3
phosphatidylinositol 3−kinase catalytic ...

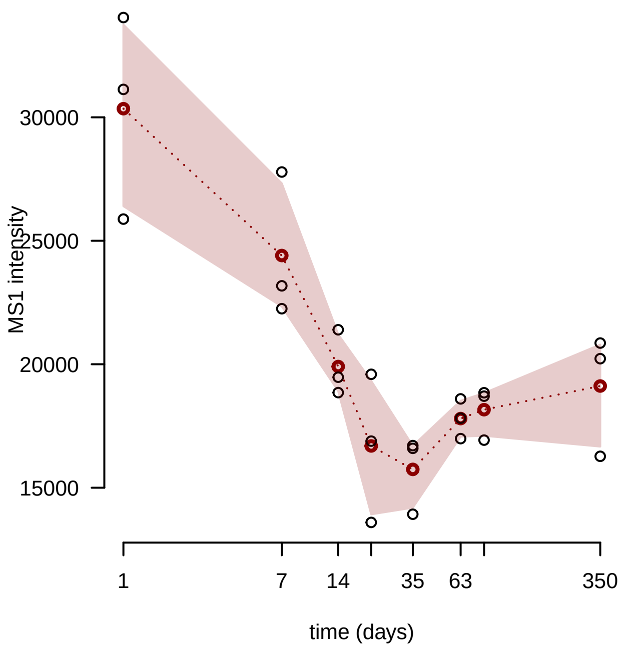

Q6PFD6
GN=Kif18b
kinesin family member 18B

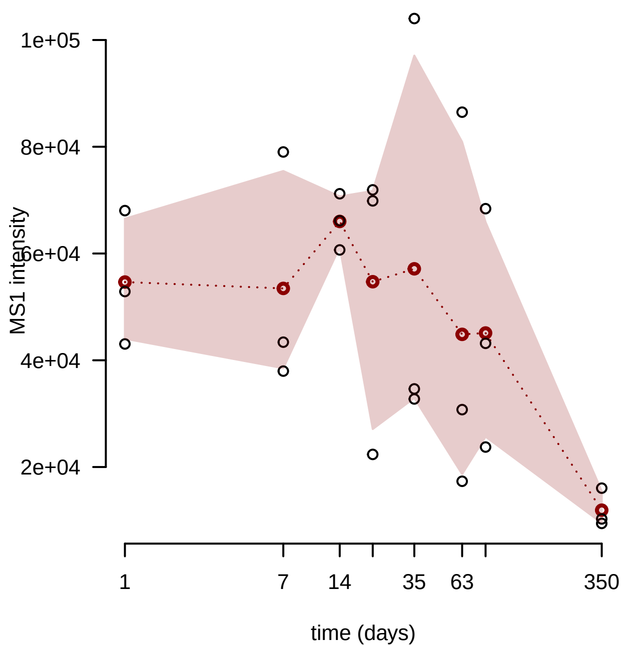

Q6PFD9
GN=Nup98
nucleoporin 98

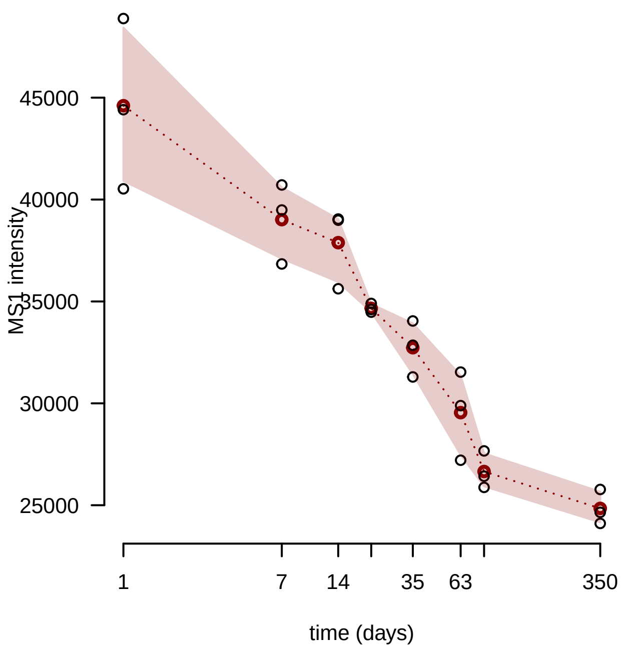

Q6PFE7
GN=Tmeff1
transmembrane protein with EGF−like and ...

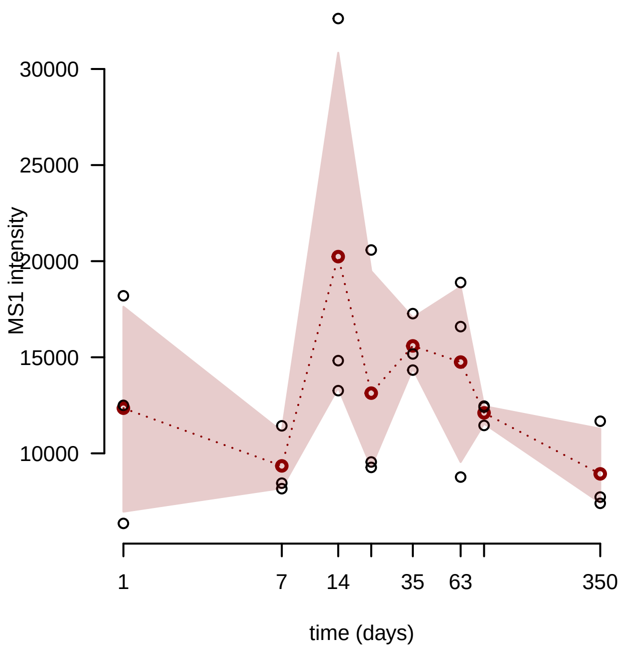

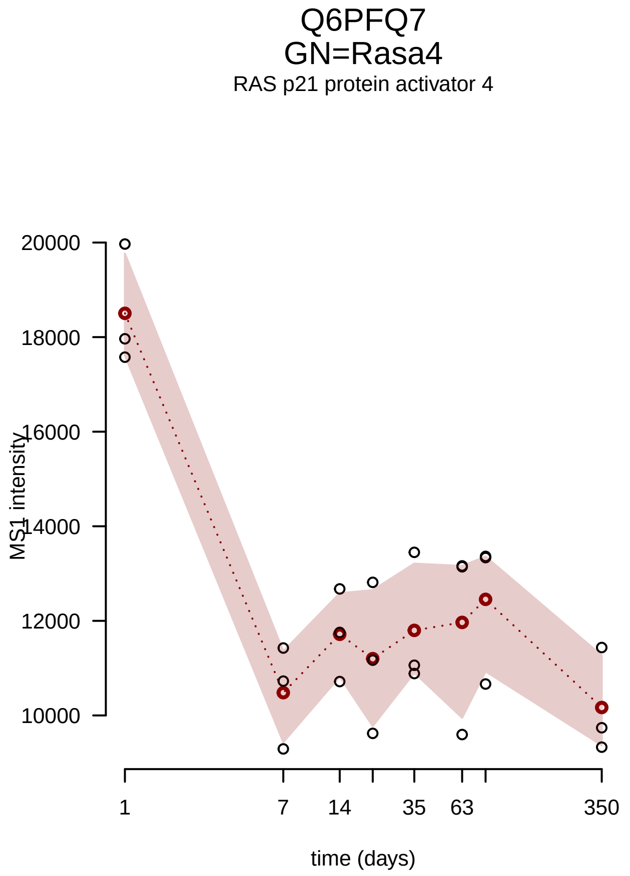
Q6PFQ7
GN=Rasa4
RAS p21 protein activator 4
MS1 intensity
10000 12000 14000 16000 18000 20000
1 7 14 35 63 350
time (days)
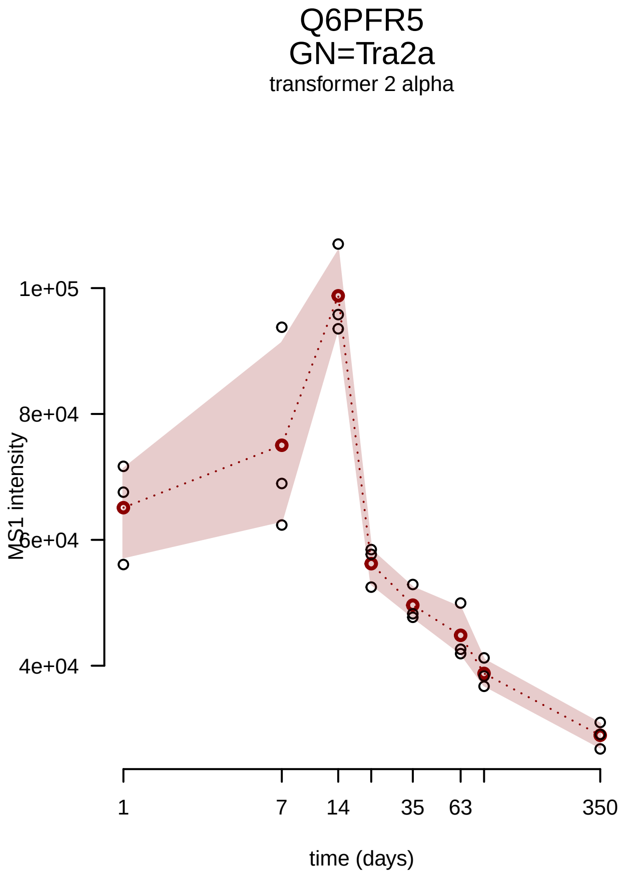
Q6PFR5
GN=Tra2a
transformer 2 alpha
MS1 intensity
4e+04 6e+04 8e+04 1e+05
1 7 14 35 63 350
time (days)
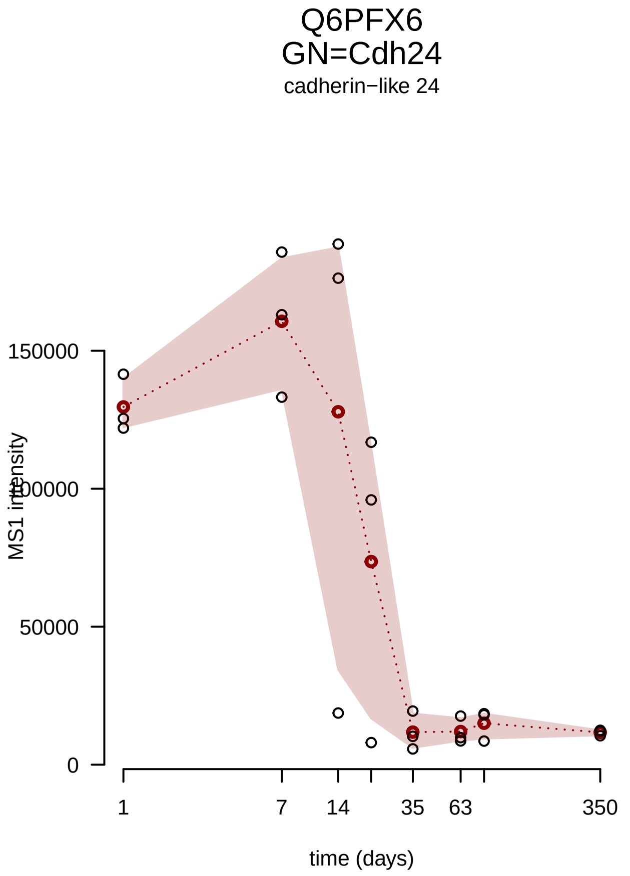
Q6PFX6
GN=Cdh24
cadherin−like 24
MS1 intensity
0 50000 100000 150000
1 7 14 35 63 350
time (days)
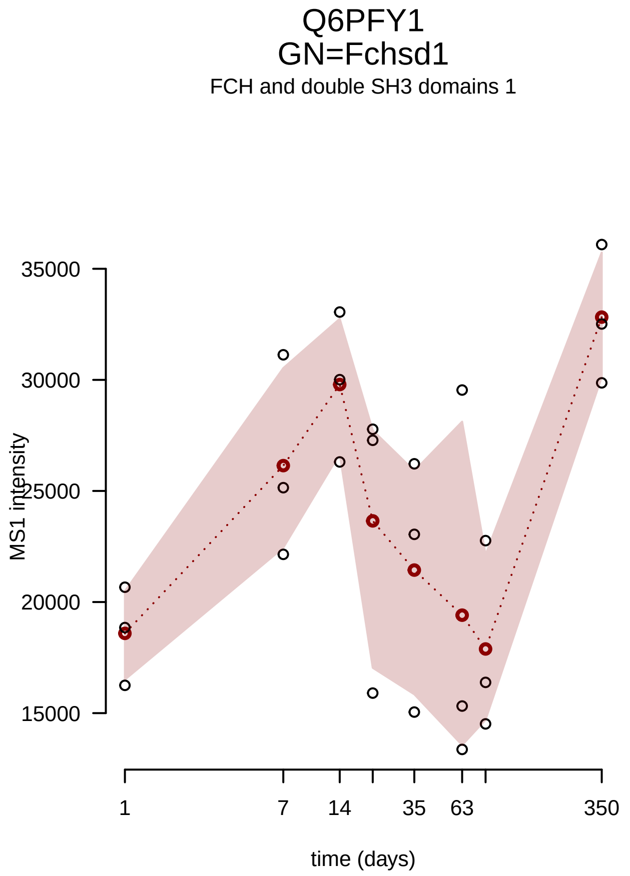
Q6PFY1
GN=Fchsd1
FCH and double SH3 domains 1
MS1 intensity
15000 20000 25000 30000 35000
1 7 14 35 63 350
time (days)
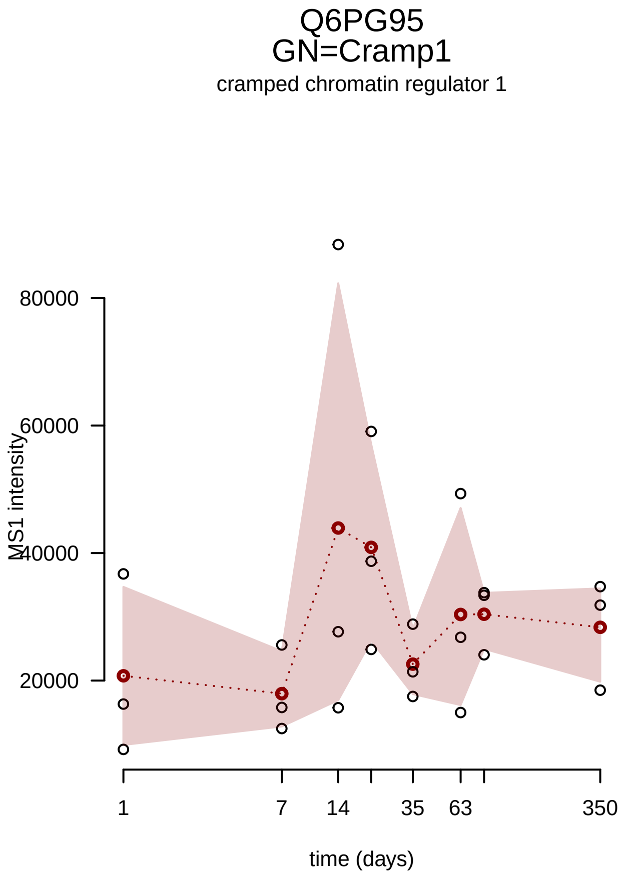
Q6PG95
GN=Cramp1
cramped chromatin regulator 1
MS1 intensity
20000 40000 60000 80000
1 7 14 35 63 350
time (days)
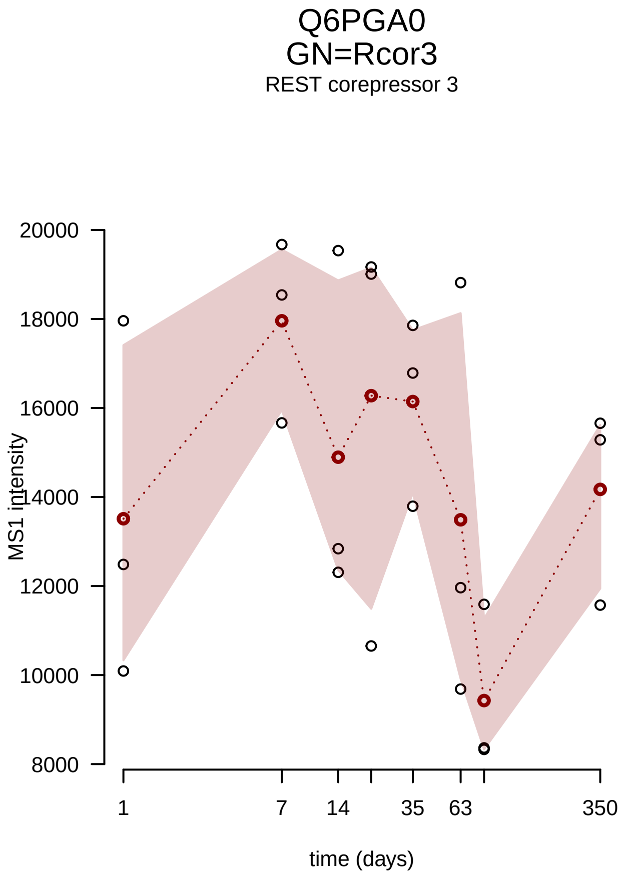
Q6PGA0
GN=Rcor3
REST corepressor 3
MS1 intensity
8000 10000 12000 14000 16000 18000 20000
1 7 14 35 63 350
time (days)
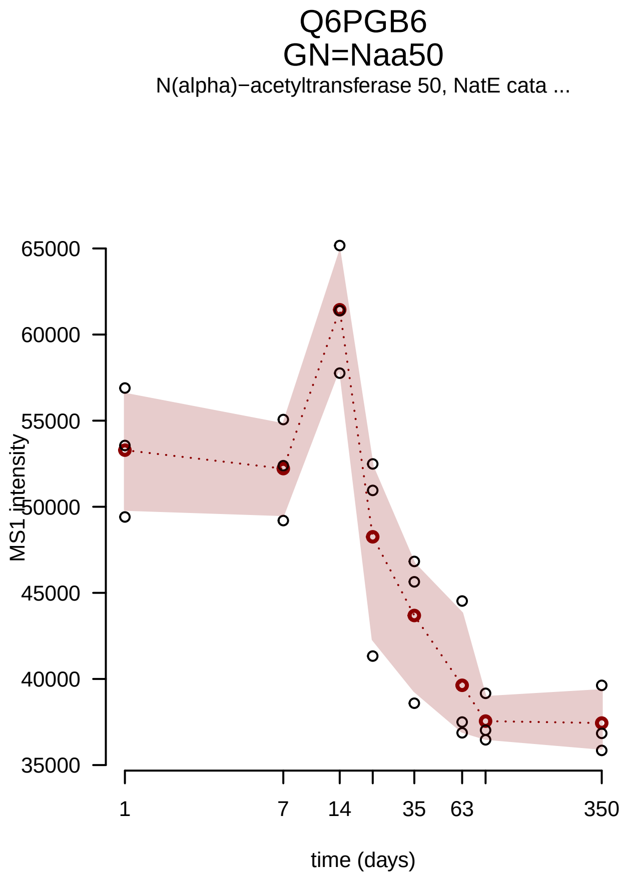
Q6PGB6
GN=Naa50
N(alpha)−acetyltransferase 50, NatE cata ...
MS1 intensity
35000 40000 45000 50000 55000 60000 65000
1 7 14 35 63 350
time (days)
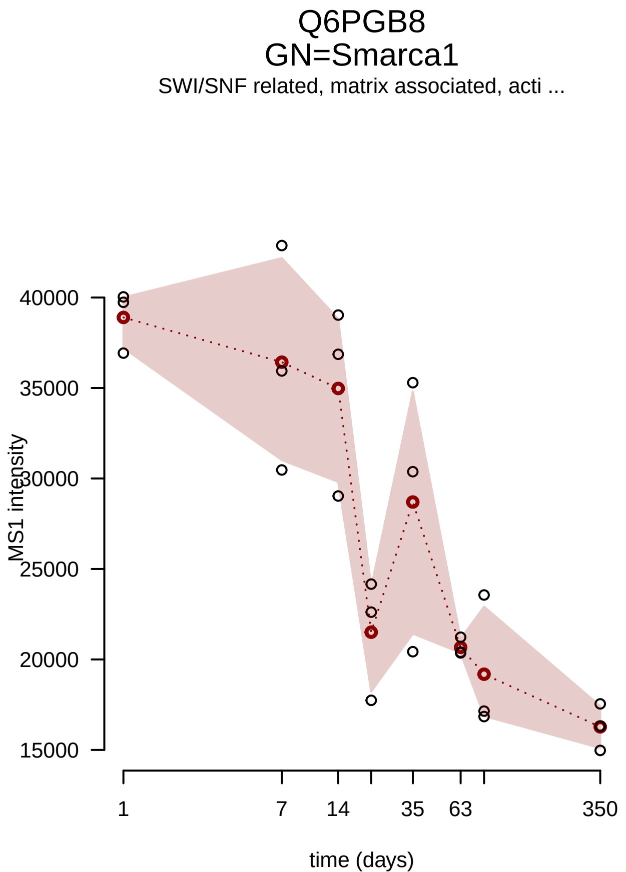
Q6PGB8
GN=Smarca1
SWI/SNF related, matrix associated, acti ...
MS1 intensity
15000 20000 25000 30000 35000 40000
1 7 14 35 63 350
time (days)
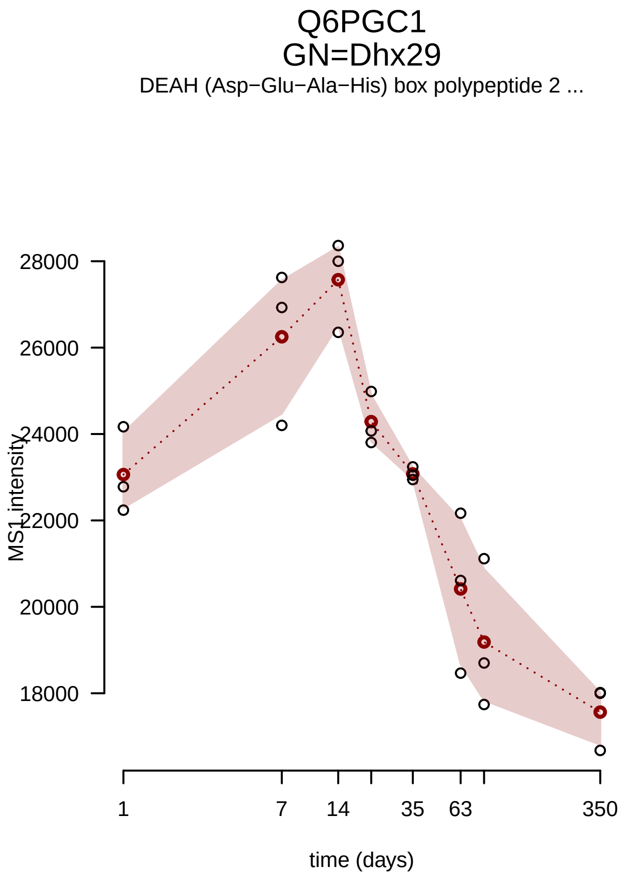
Q6PGC1
GN=Dhx29
DEAH (Asp−Glu−Ala−His) box polypeptide 2 ...
MS1 intensity
18000 20000 22000 24000 26000 28000
1 7 14 35 63 350
time (days)

Q6PGF3
GN=Med16
mediator complex subunit 16

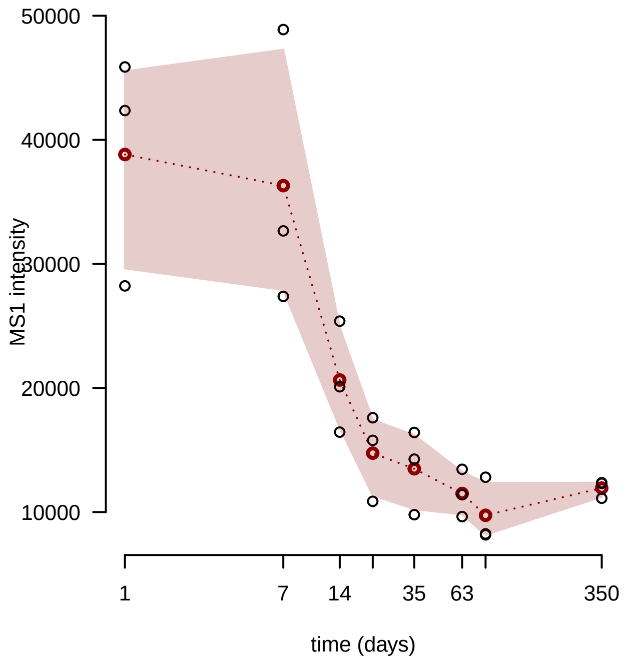

Q6PGF7
GN=Exoc8
exocyst complex component 8

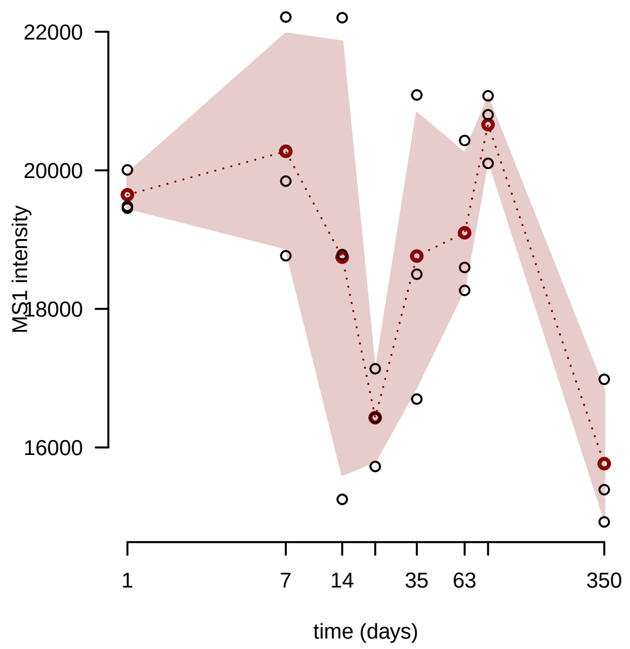

Q6PGG2
GN=Gmip
Gem−interacting protein

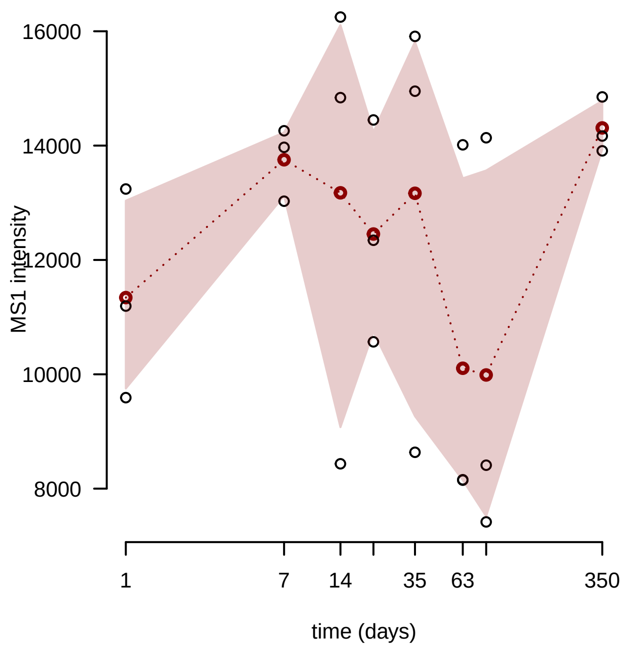

Q6PGG6
GN=Gnl3l
guanine nucleotide binding protein−like ...

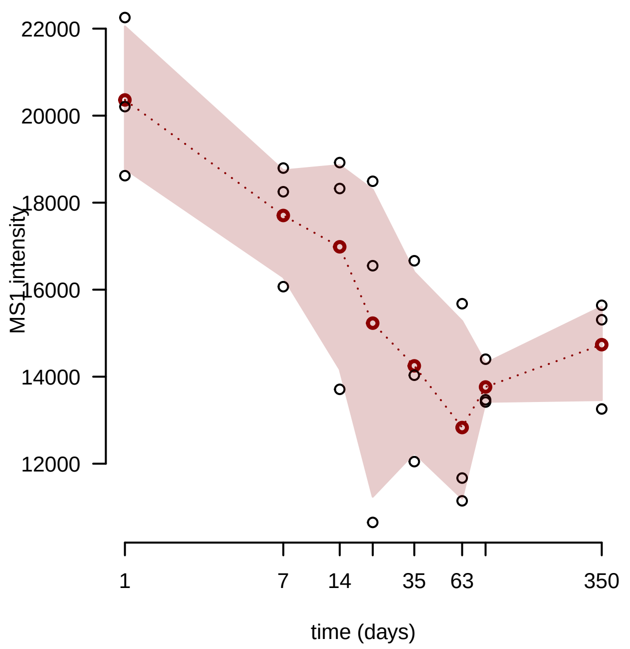

Q6PGH0
GN=Ubtd2
ubiquitin domain containing 2

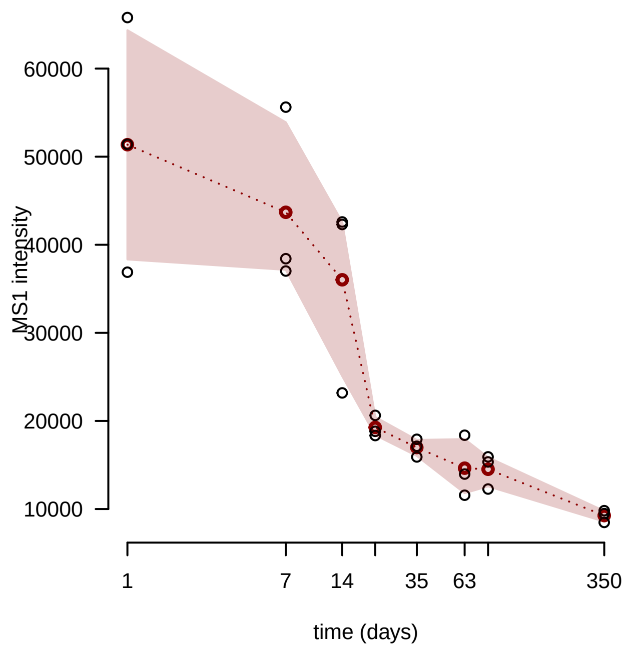

Q6PGH1
GN=Bud31
BUD31 homolog

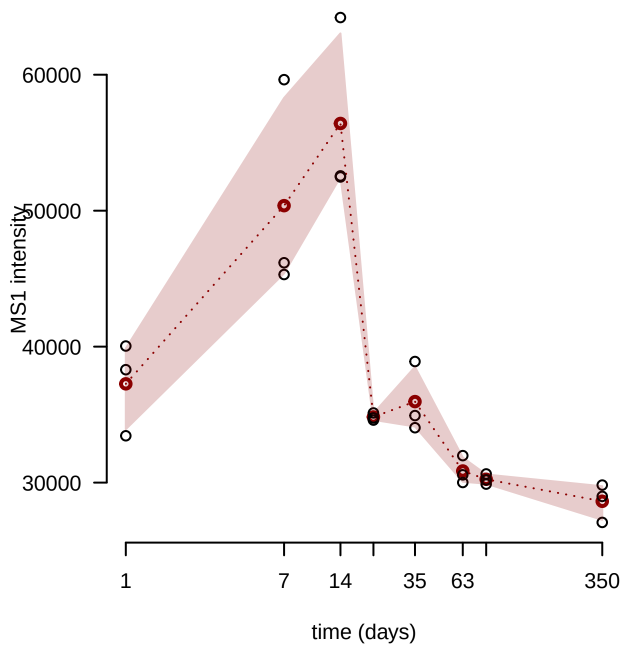

Q6PGH2
GN=Jpt2
Jupiter microtubule associated homolog 2

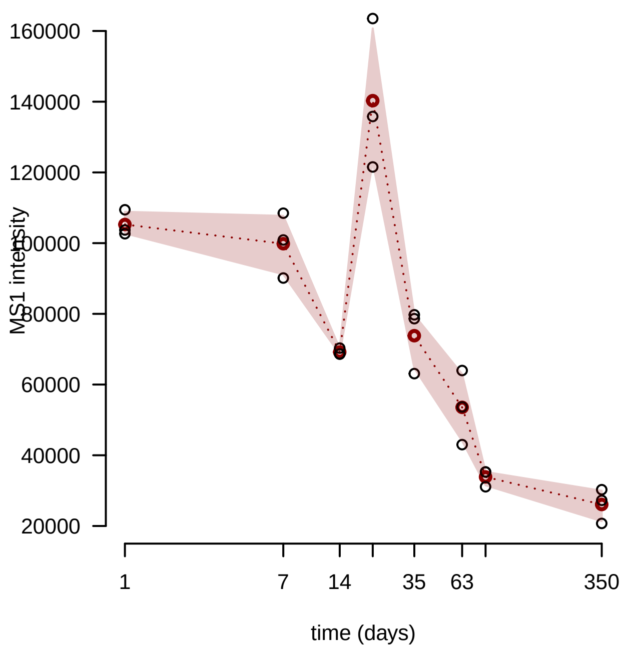

Q6PGL7
GN=Washc2
WASH complex subunit 2

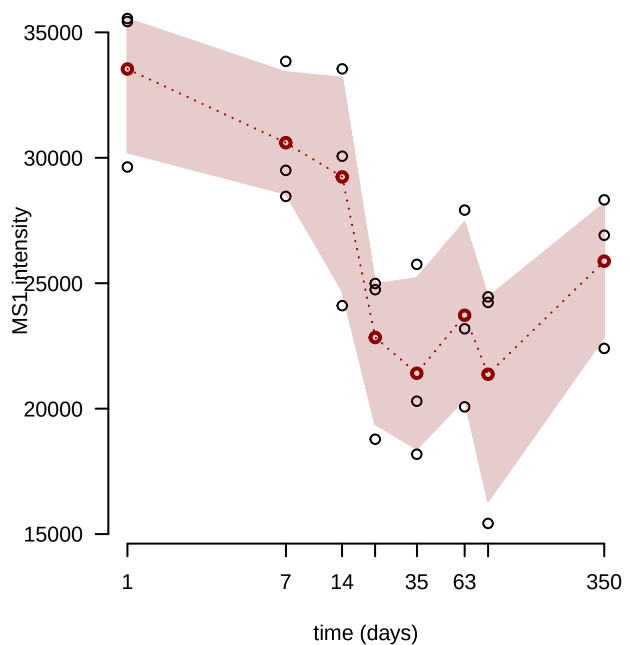

Q6PGN3
GN=Dclk2
doublecortin−like kinase 2

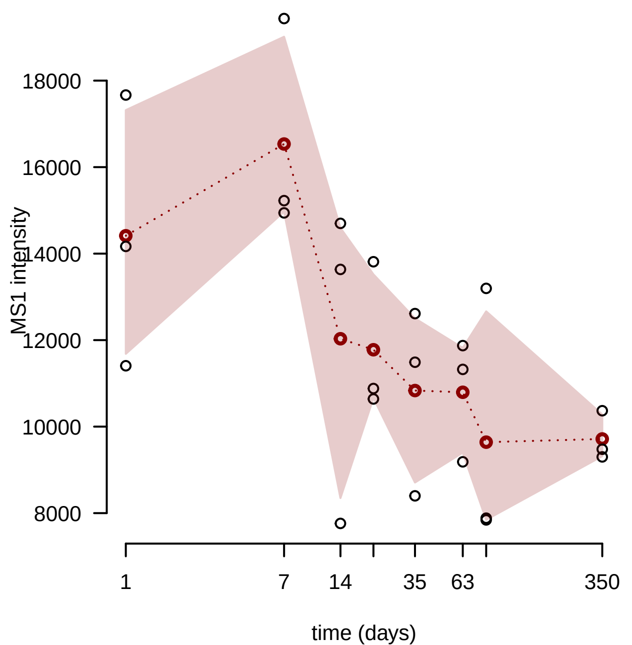

Q6PHN7
GN=Tmem164
transmembrane protein 164

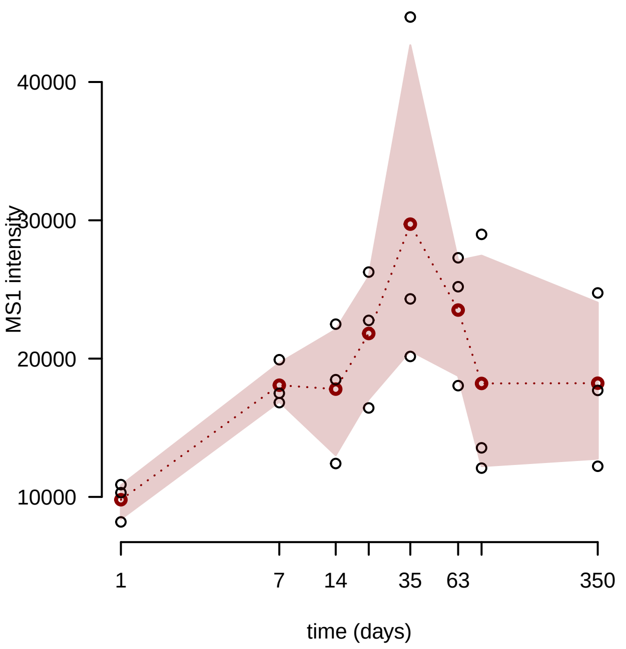

Q6PHN9
GN=Rab35
RAB35, member RAS oncogene family

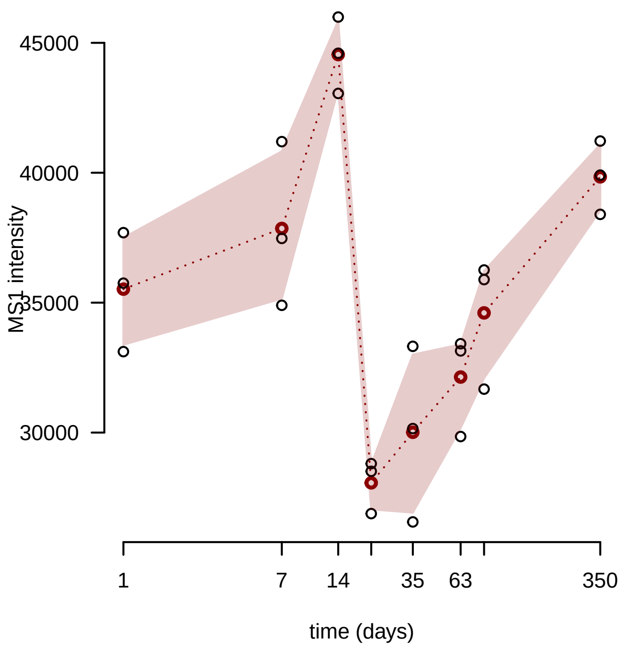

Q6PHQ8
GN=Naa35
N(alpha)−acetyltransferase 35, NatC auxi ...

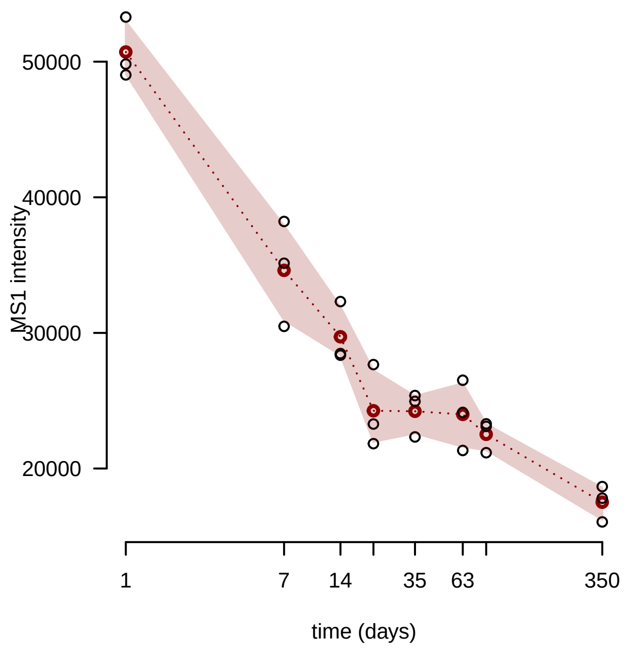

Q6PHS6
GN=Snx13
sorting nexin 13

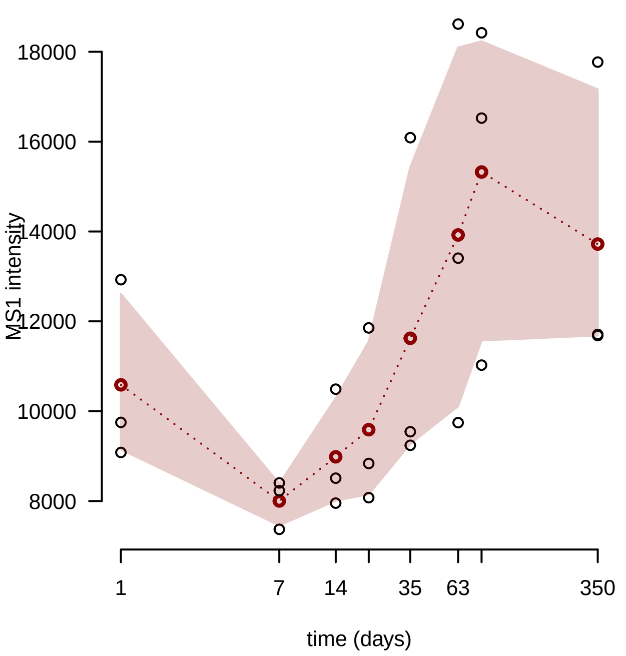

Q6PHS9
GN=Cacna2d2
calcium channel, voltage−dependent, alph ...

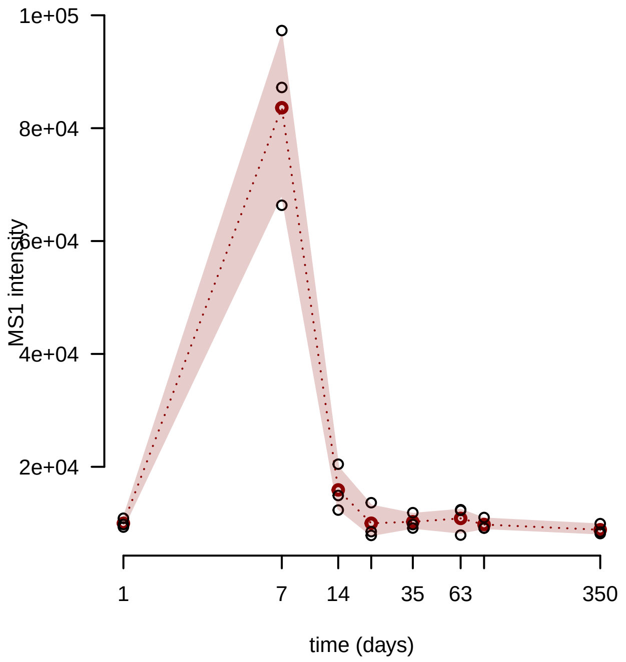

Q6PHU5
GN=Sort1
sortilin 1

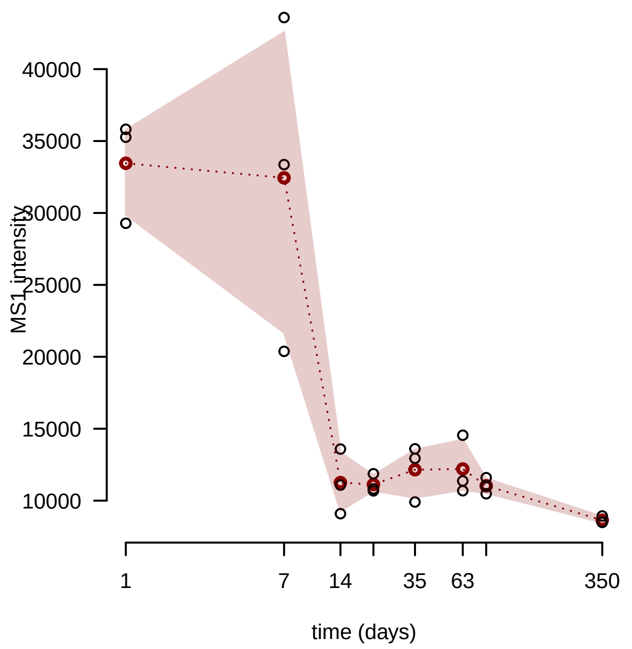

Q6PHZ2
GN=Camk2d
calcium/calmodulin−dependent protein kin ...

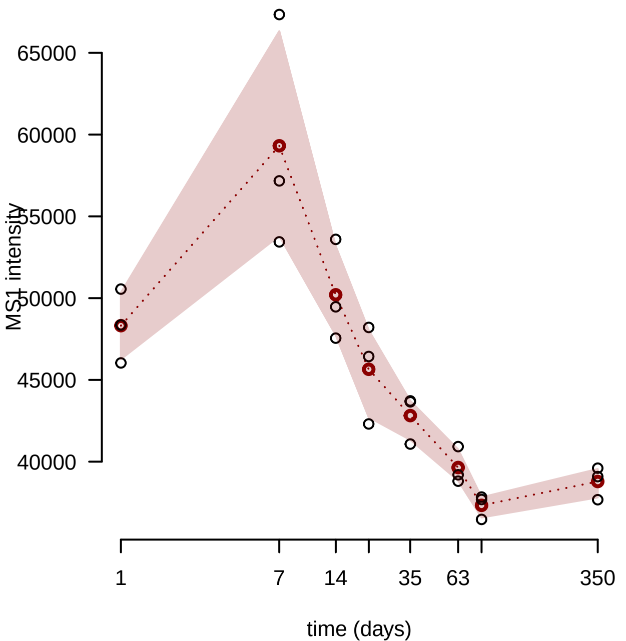

Q6PHZ5
GN=Rbm15b
RNA binding motif protein 15B

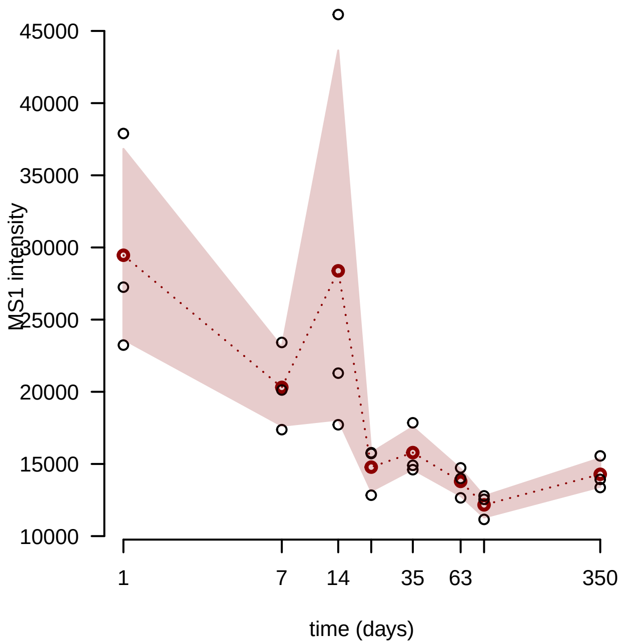

Q6PIC6
GN=Atp1a3
ATPase, Na+/K+ transporting, alpha 3 pol ...

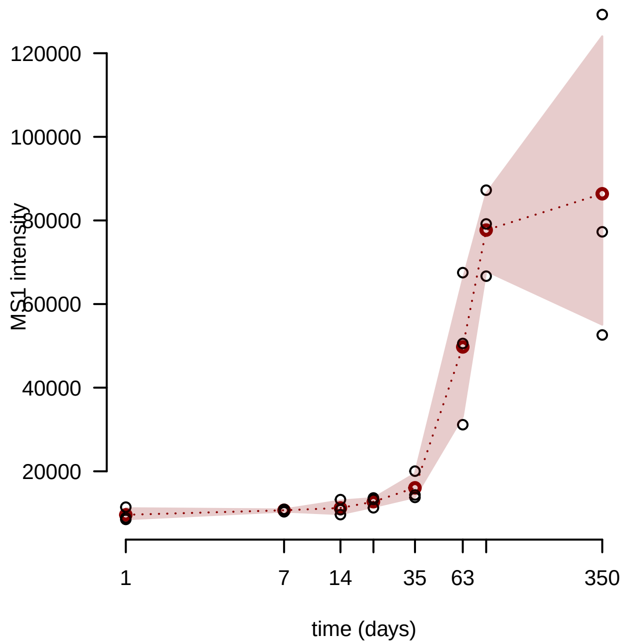

Q6PIE5
GN=Atp1a2
ATPase, Na+/K+ transporting, alpha 2 pol ...

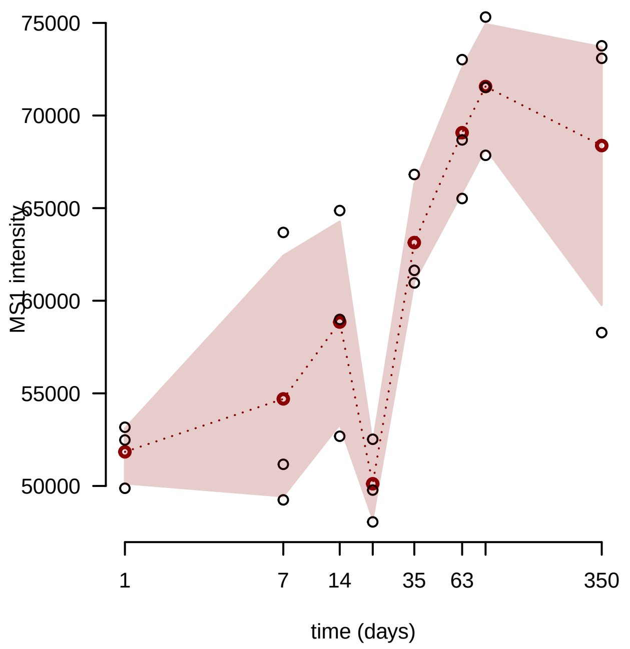

Q6PIJ4
GN=Nfrkb
nuclear factor related to kappa B bindin ...

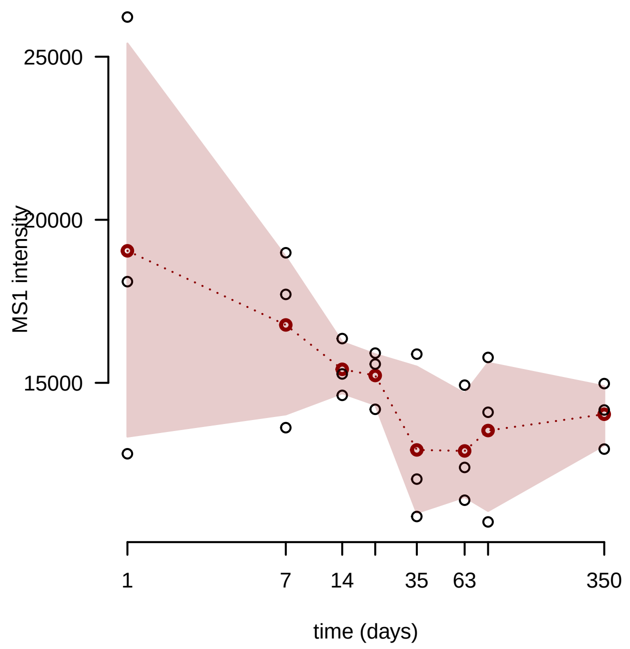

Q6PIP5
GN=Nudcd1
NudC domain containing 1

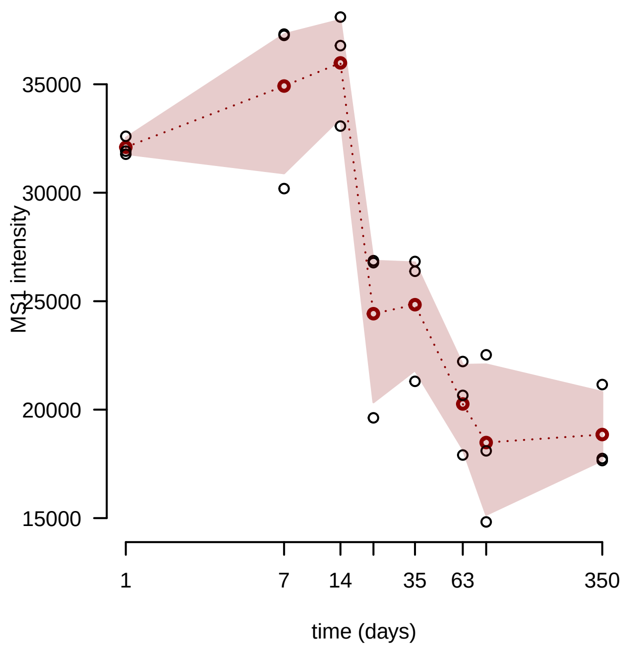

Q6PIU9
GN=

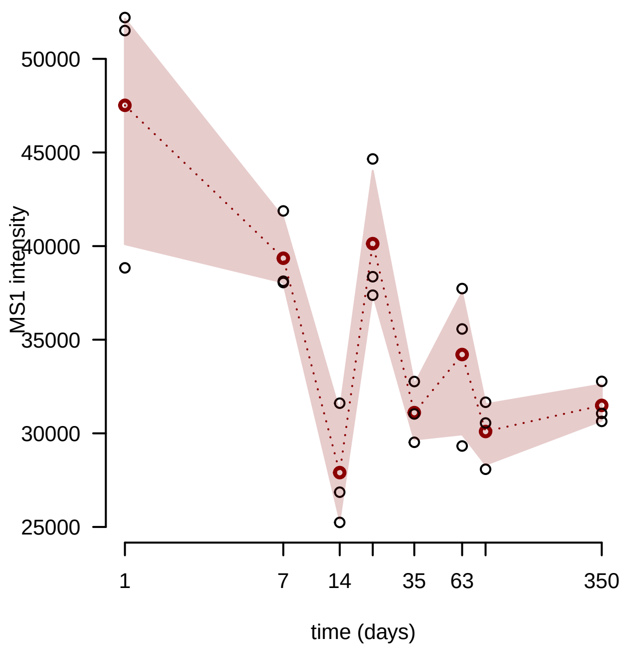

Q6PIX5
GN=Rhbdf1
rhomboid 5 homolog 1

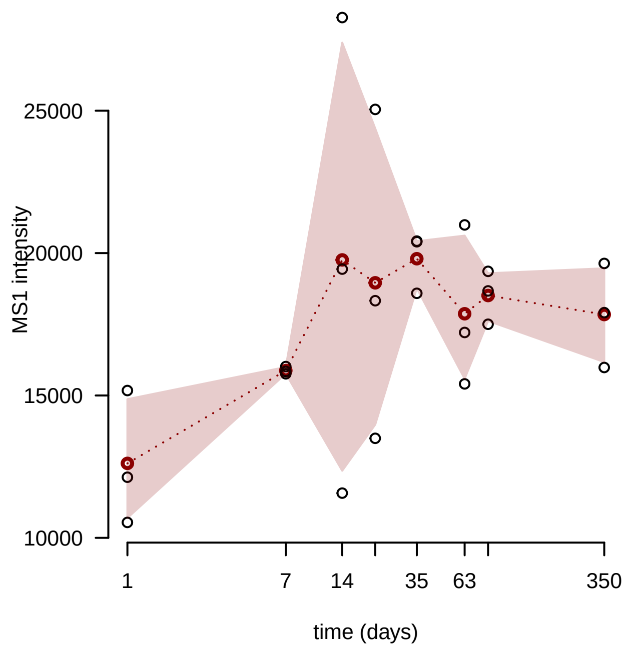

Q6PIX9
GN=D11Wsu47e
DNA segment, Chr 11, Wayne State Univers ...

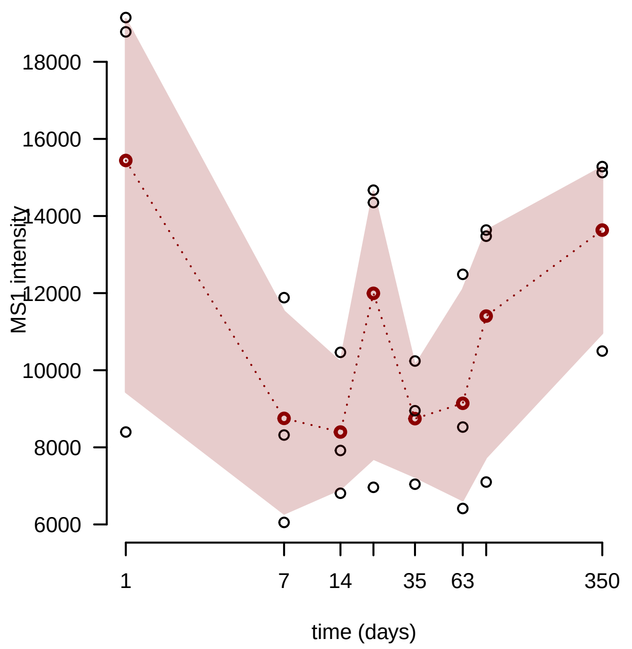

Q6PJN8
GN=Dalrd3
DALR anticodon binding domain containing ...

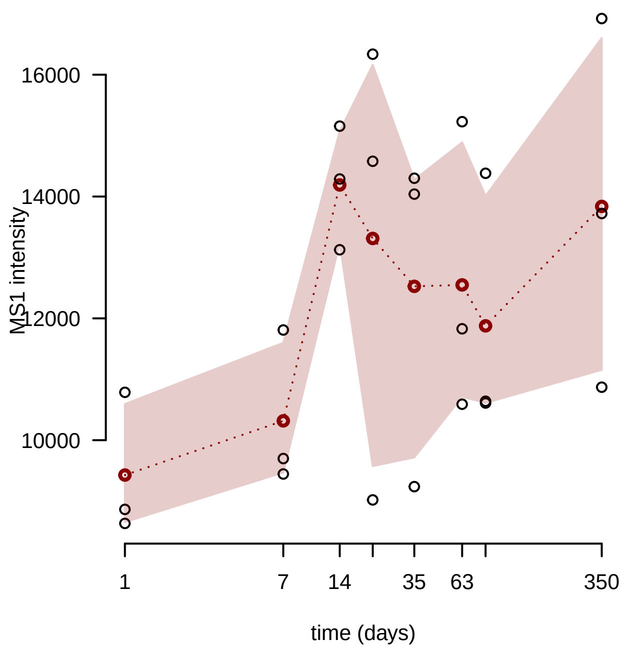

Q6PNC0
GN=Dmxl1
Dmx−like 1

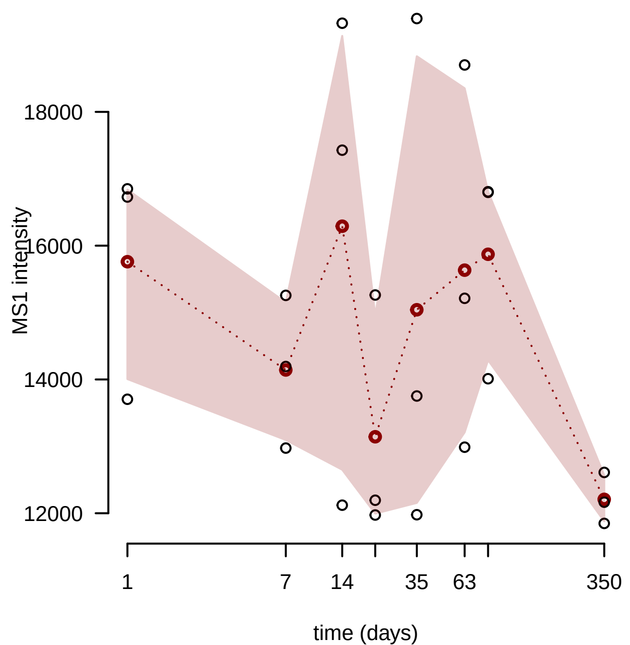

Q6PR54
GN=Rif1
replication timing regulatory factor 1

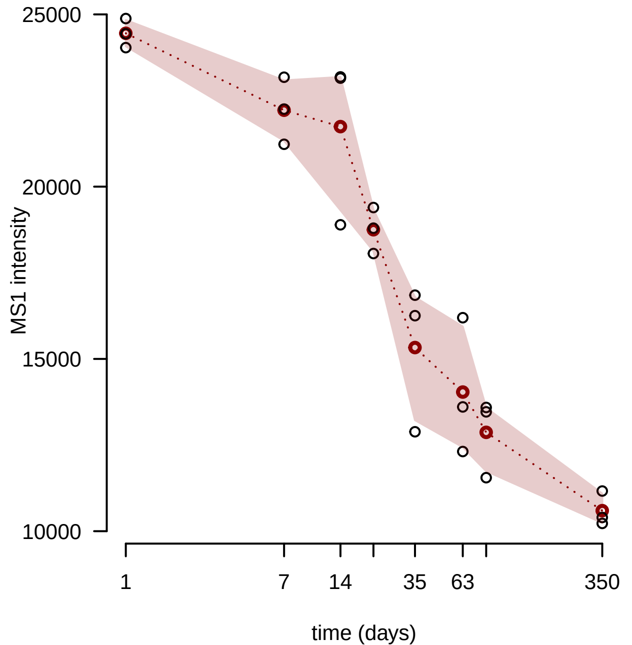

Q6Q477
GN=Atp2b4
ATPase, Ca++ transporting, plasma membra ...

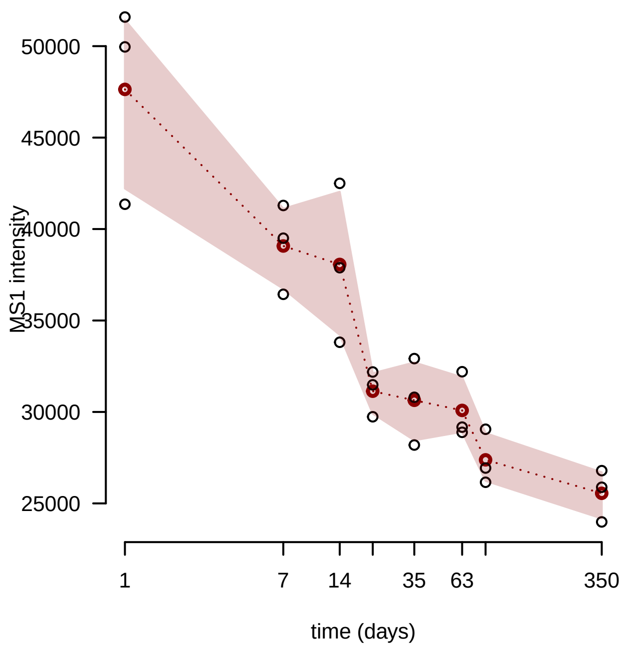

Q6Q783
GN=Kmt5c
lysine methyltransferase 5C

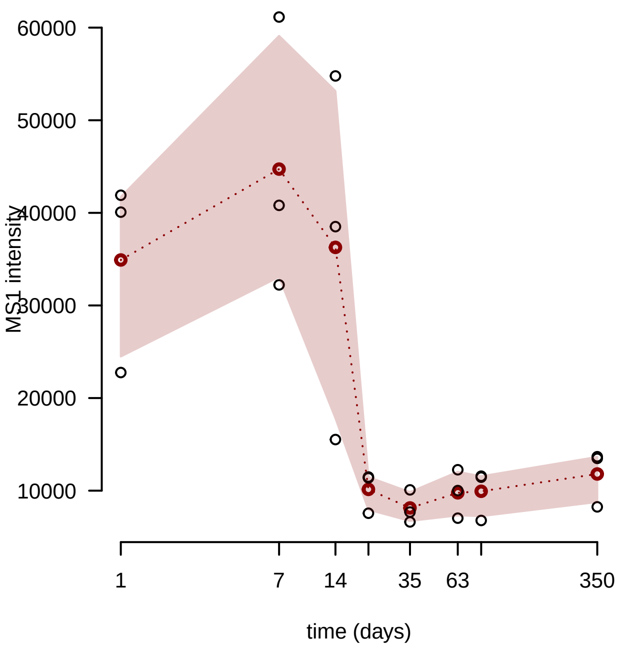

Q6Q899
GN=Ddx58
DEAD/H box helicase 58

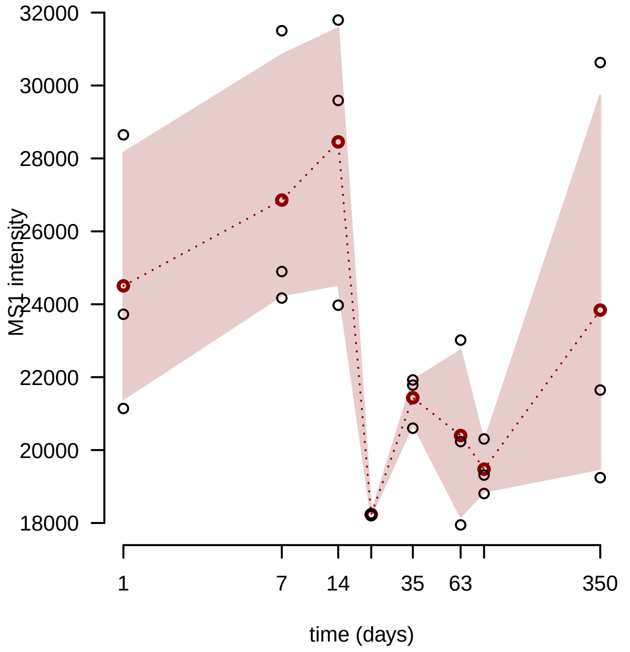

Q6QD59
GN=Bnip1
BCL2/adenovirus E1B interacting protein ...

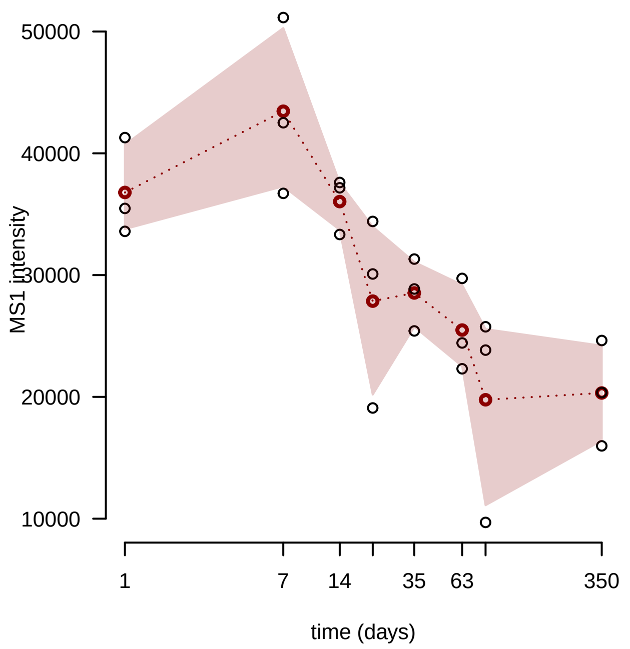

Q6QI06
GN=Rictor
RPTOR independent companion of MTOR, com ...

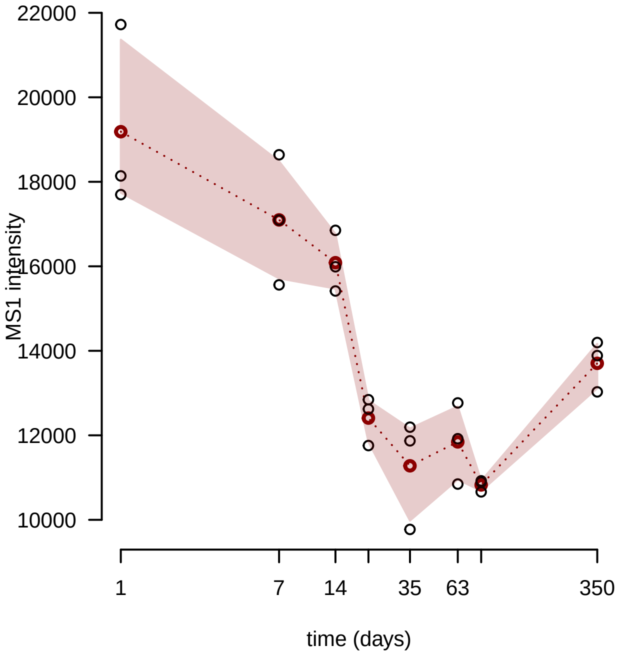

Q6R2P8
GN=Neil2
nei like 2 (E. coli)

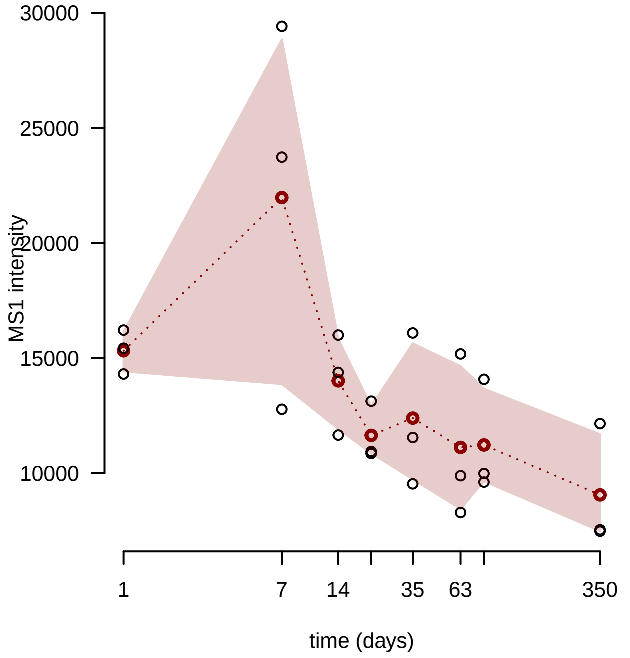

Q6R891
GN=Ppp1r9b
protein phosphatase 1, regulatory subuni ...

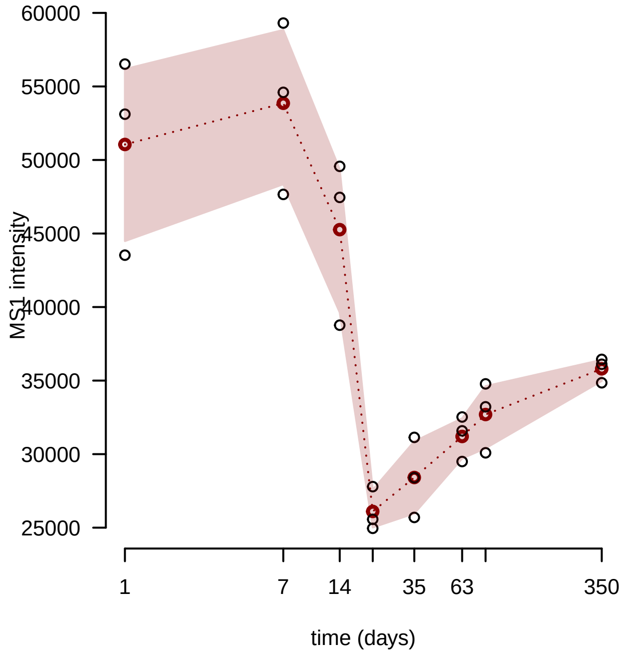

Q6RHR9
GN=Magi1
membrane associated guanylate kinase, WW ...

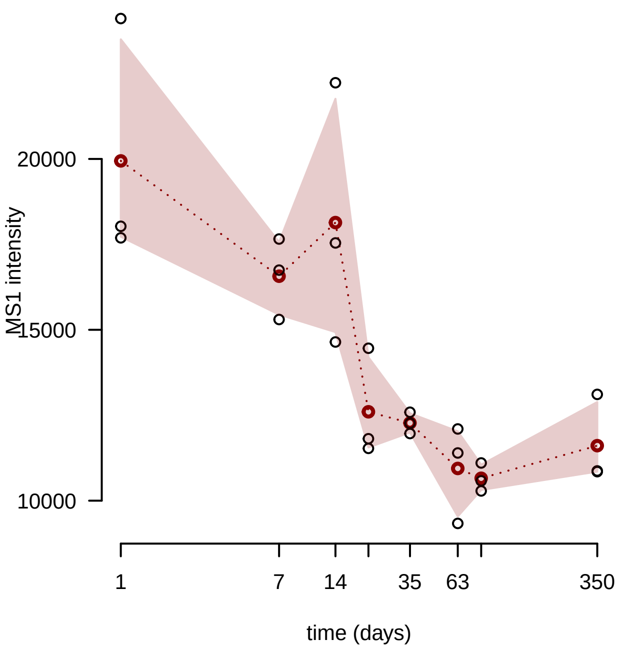

Q6RI63
GN=Fam120b
family with sequence similarity 120, mem ...

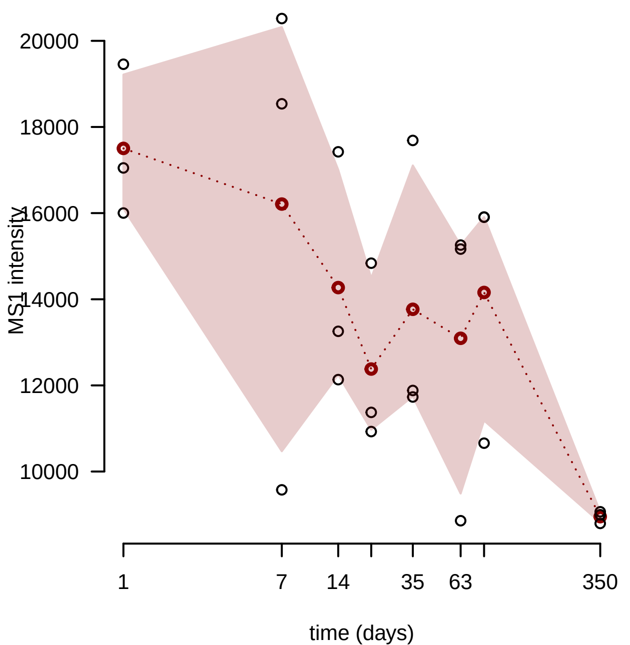

Q6RT24
GN=Cenpe
centromere protein E

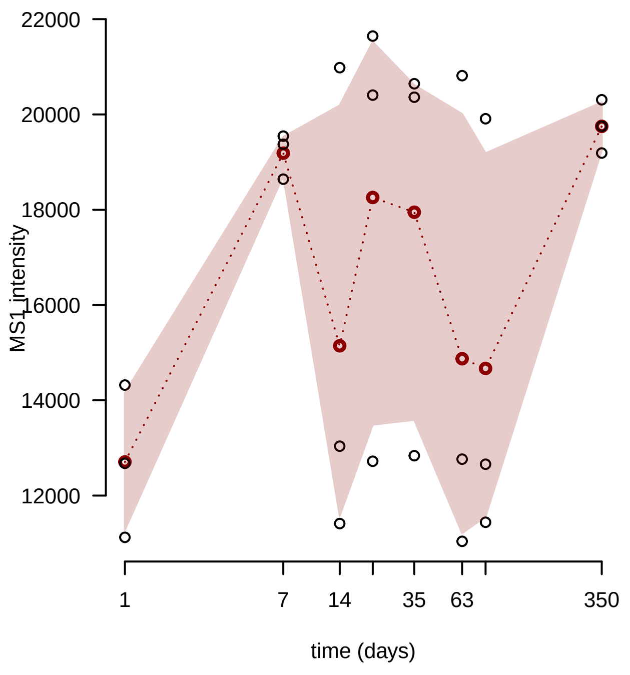

Q6RUT7
GN=Ccsmst1

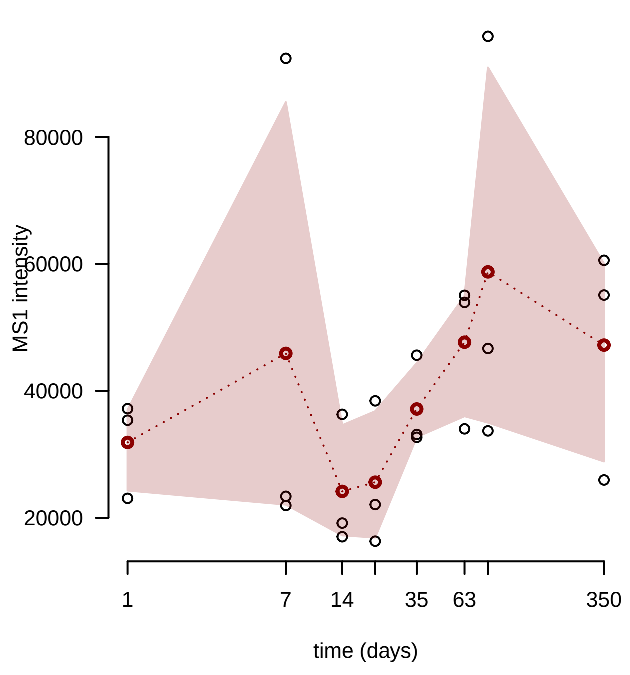

Q6S5J6
GN=Krit1
KRIT1, ankyrin repeat containing

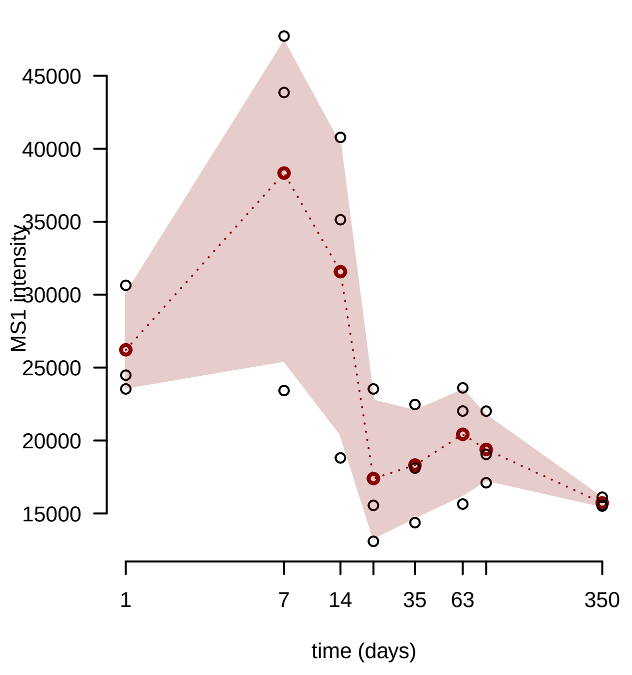

Q6SKR2
GN=N6amt1
N−6 adenine−specific DNA methyltransfera ...

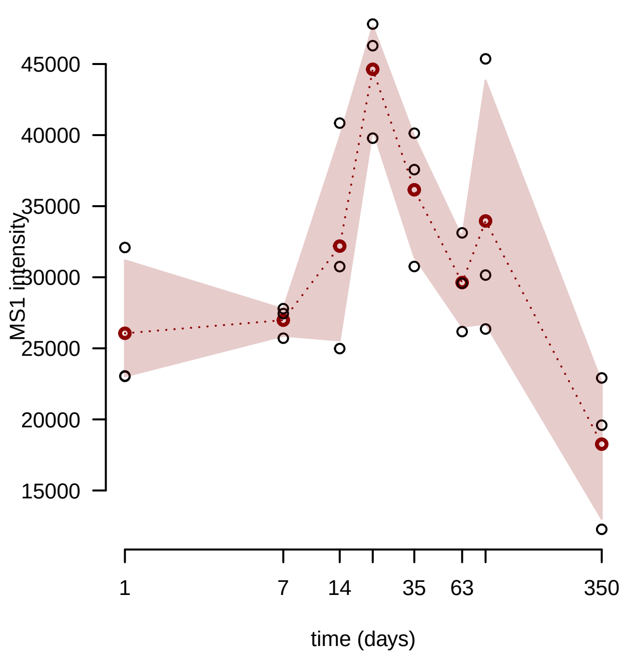

Q6TCG8
GN=Paqr3
progestin and adipoQ receptor family mem ...

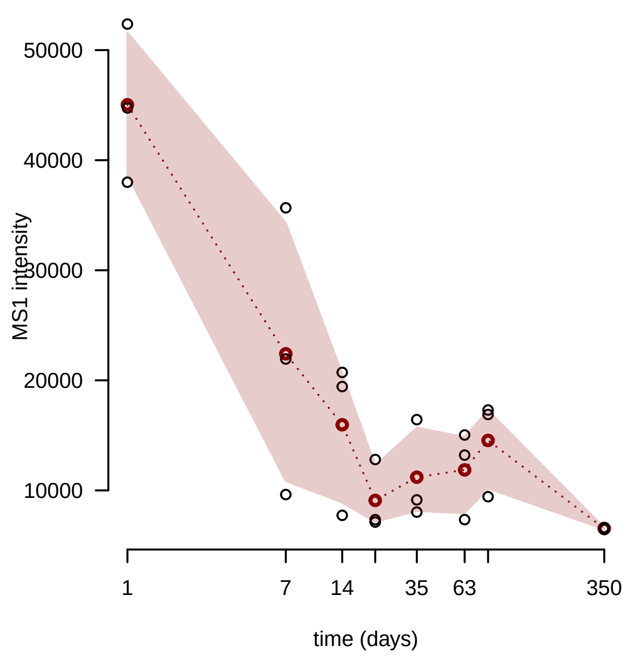

Q6TEK5
GN=Vkorc1l1
vitamin K epoxide reductase complex, sub ...

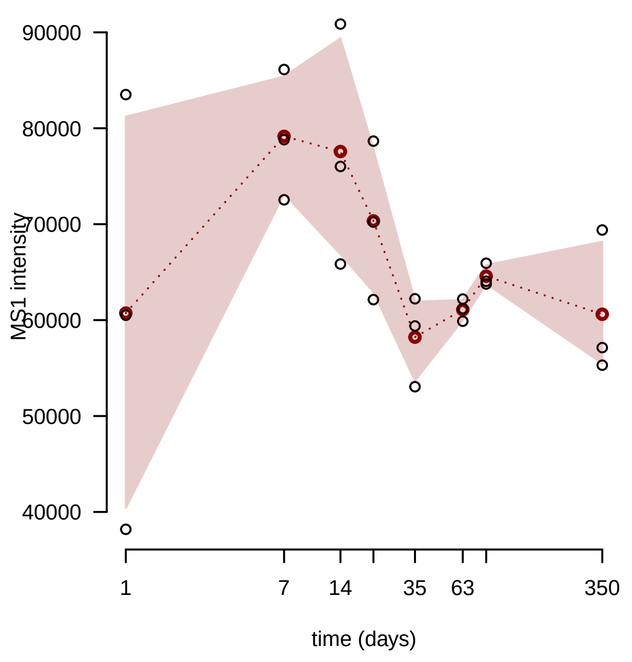

Q6TXD4
GN=Dnmbp
dynamin binding protein

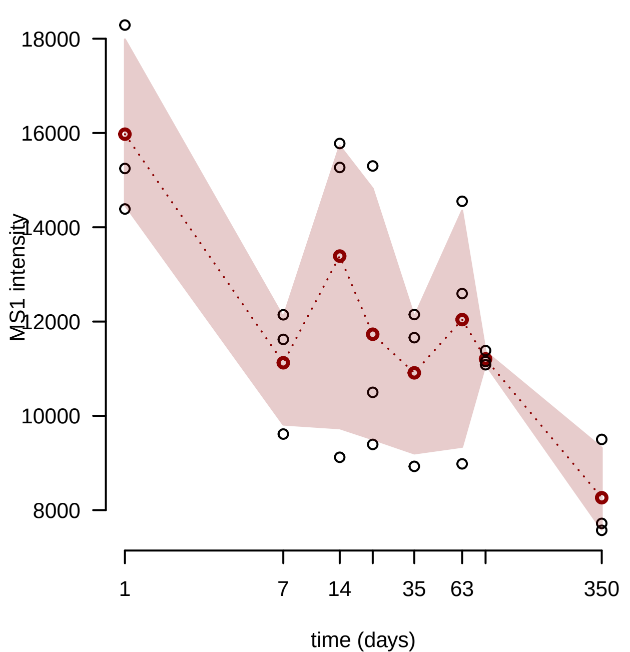

Q6TYB5
GN=Fez2
fasciculation and elongation protein zet ...

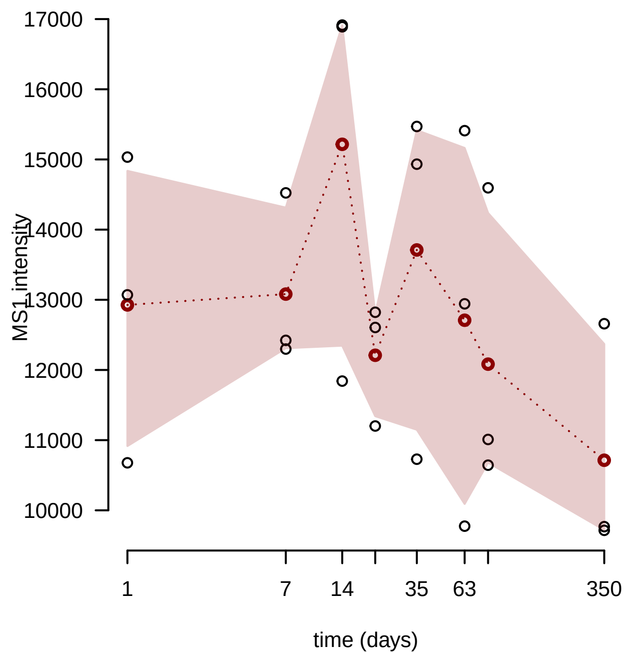

Q6URW6
GN=Myh14
myosin, heavy polypeptide 14

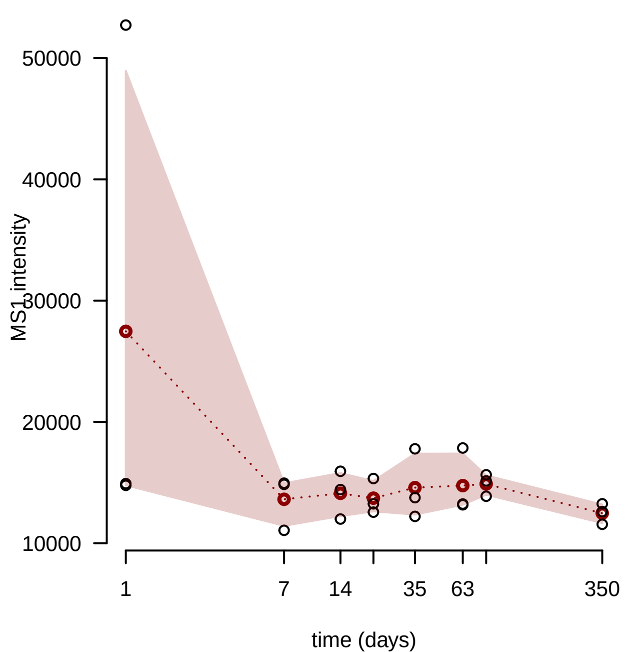

Q6V4S5
GN=Sdk2
sidekick cell adhesion molecule 2

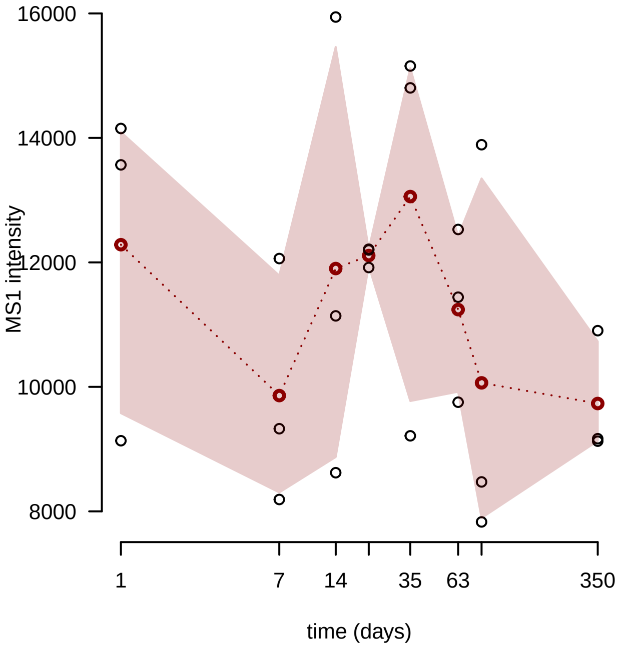

Q6VGS5
GN=Ccdc88c
coiled−coil domain containing 88C

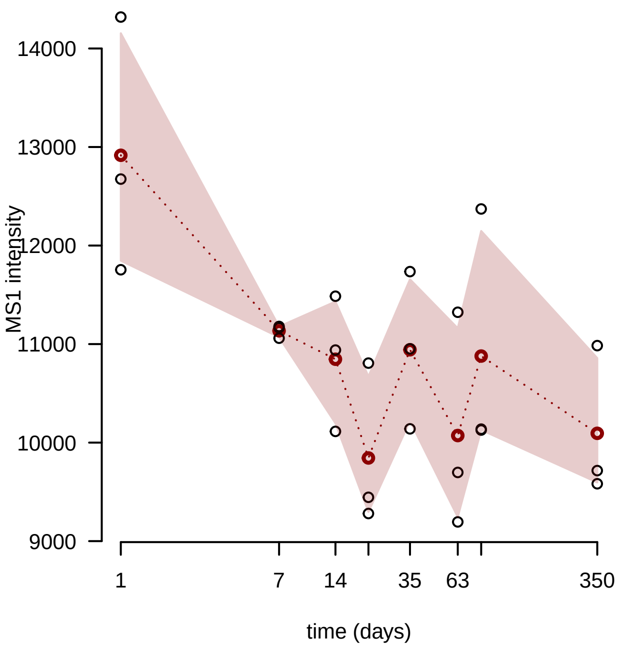

Q6VH22
GN=Ift172
intraflagellar transport 172

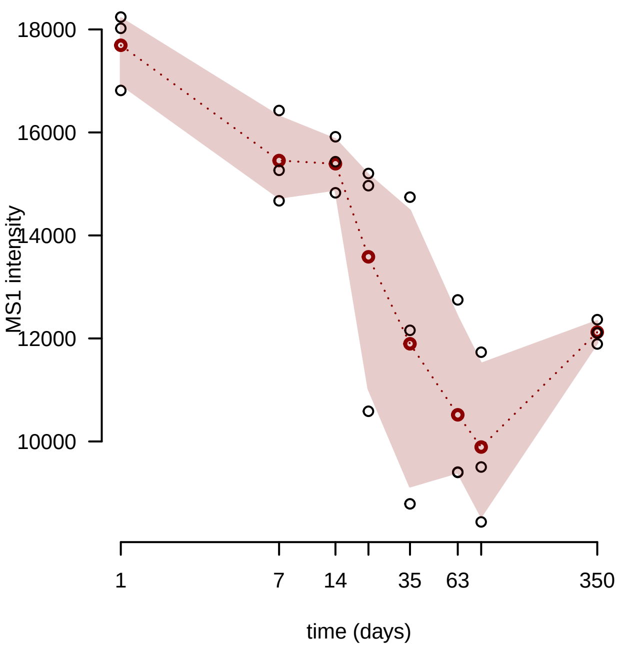

Q6VN19
GN=Ranbp10
RAN binding protein 10

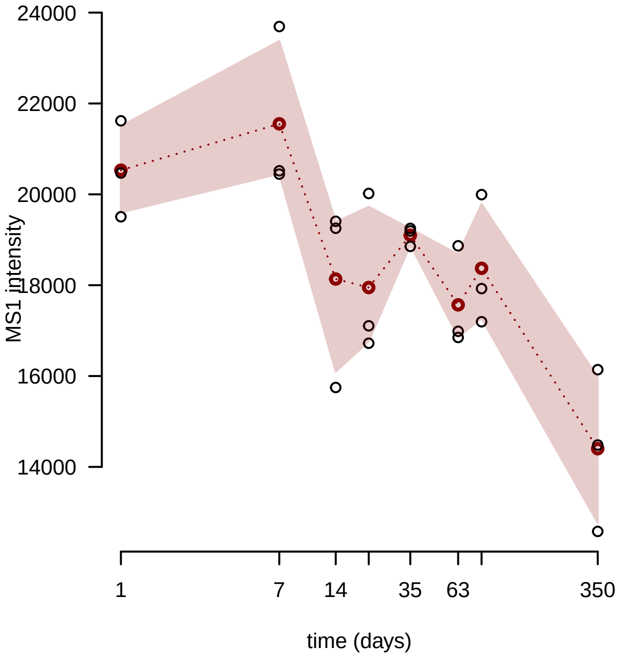

Q6VNB8
GN=Wdfy3
WD repeat and FYVE domain containing 3

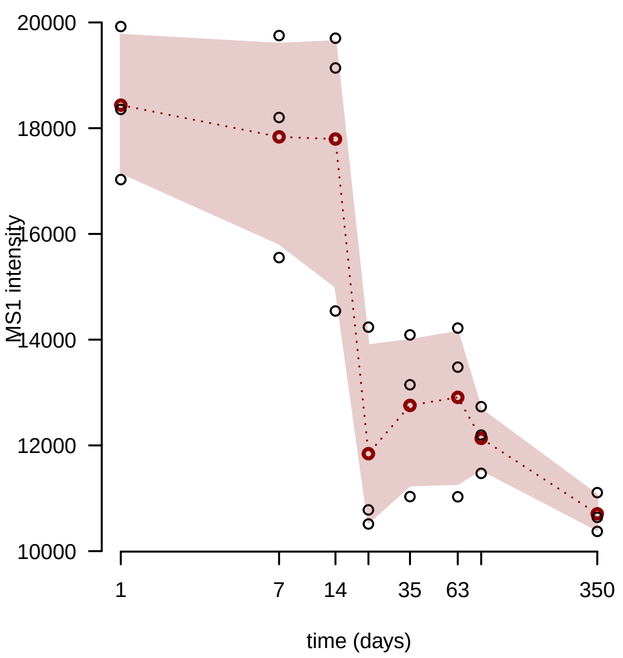

Q6VNS1
GN=Ntrk3
neurotrophic tyrosine kinase, receptor, ...

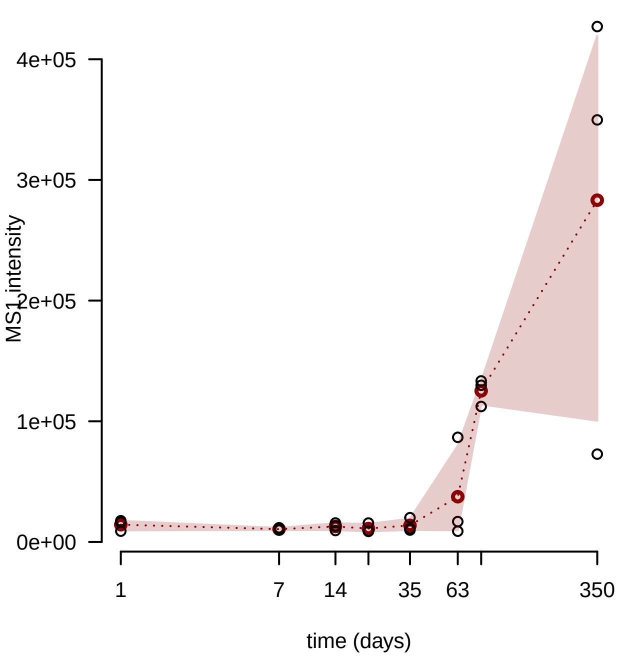

Q6VVW5
GN=Npr2
natriuretic peptide receptor 2

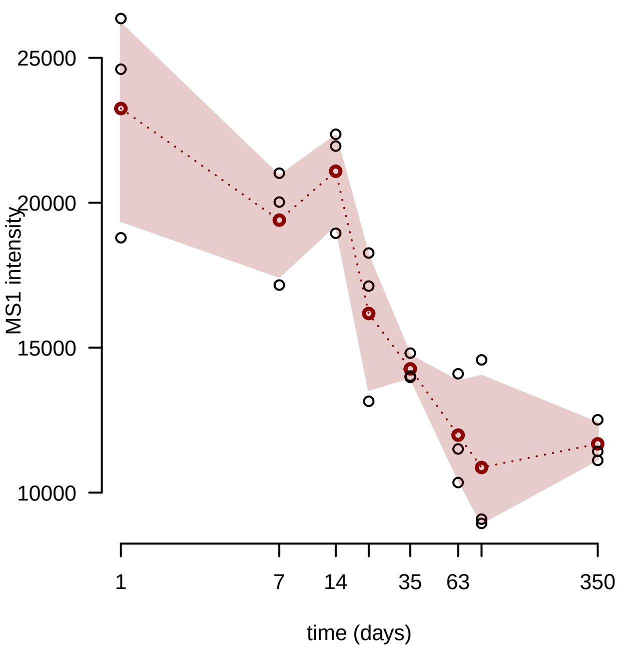

Q6W8Q3
GN=Pcp4l1
Purkinje cell protein 4−like 1

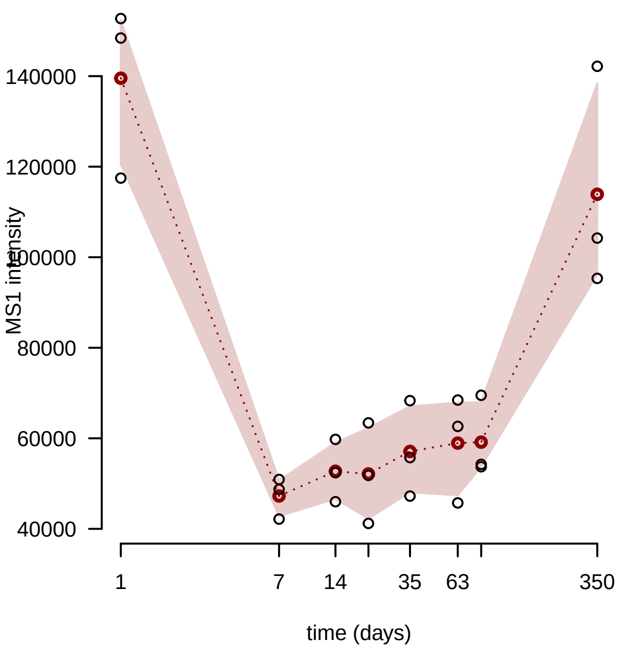

Q6WKZ8
GN=Ubr2
ubiquitin protein ligase E3 component n− ...

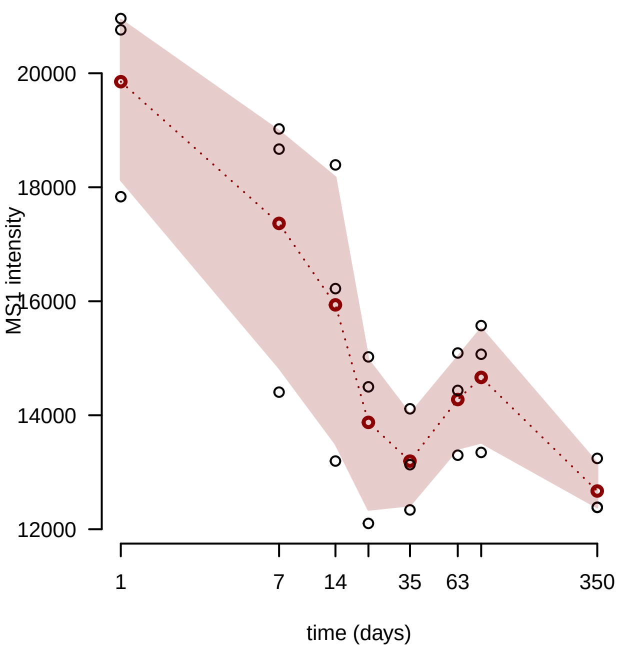

Q6WQJ1
GN=Dagla
diacylglycerol lipase, alpha

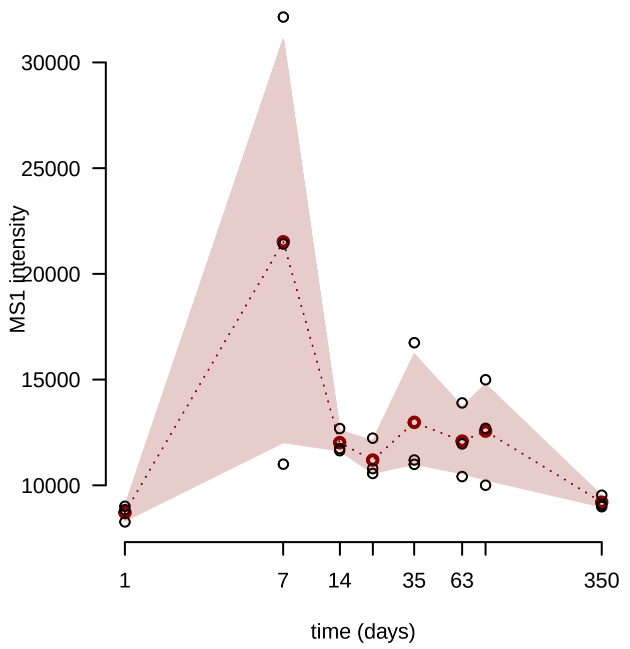

Q6WVG3
GN=Kctd12
potassium channel tetramerisation domain ...

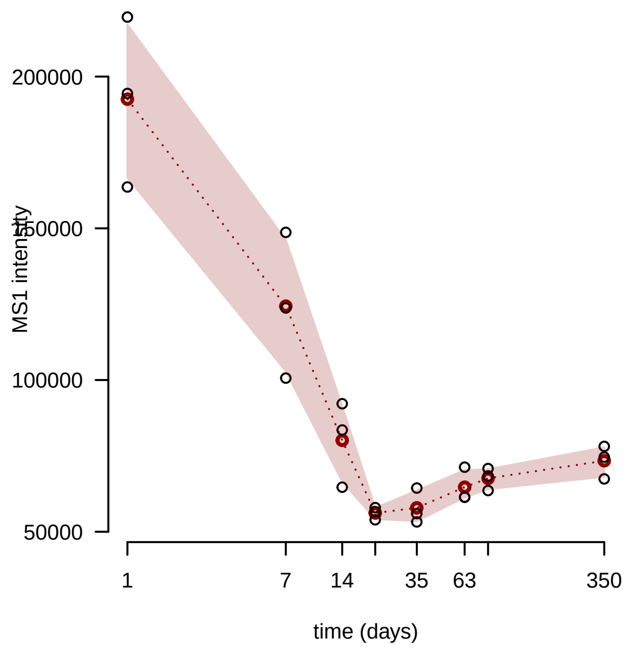

Q6X893
GN=Slc44a1
solute carrier family 44, member 1

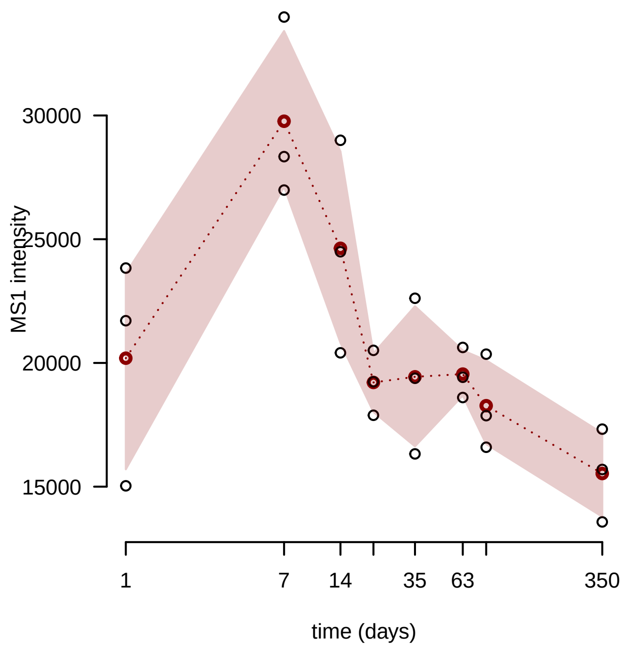

Q6XBJ3
GN=Onecut2
one cut domain, family member 2

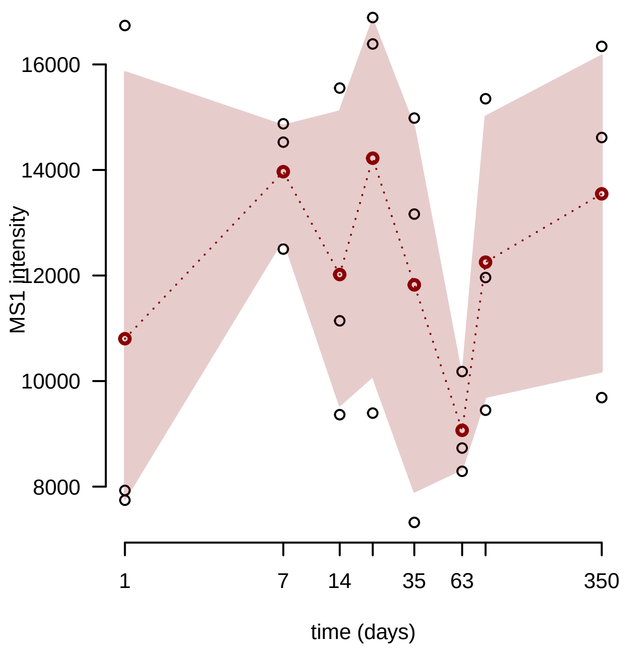

Q6XP49
GN=Glis3
GLIS family zinc finger 3

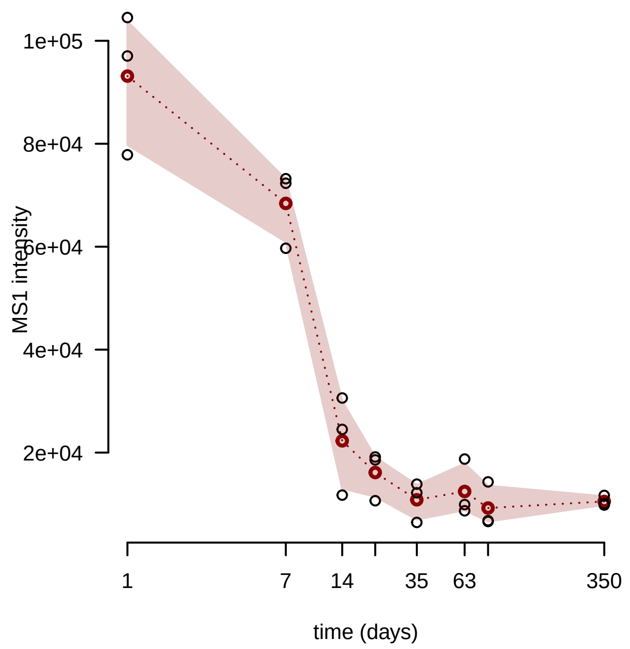

Q6XUX1
GN=Dstyk
dual serine/threonine and tyrosine prote ...

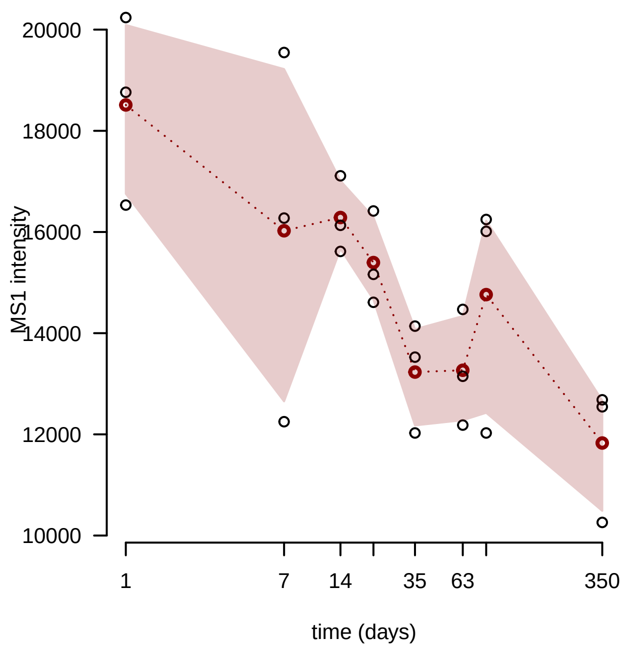

Q6Y5D8
GN=Arhgap10
Rho GTPase activating protein 10

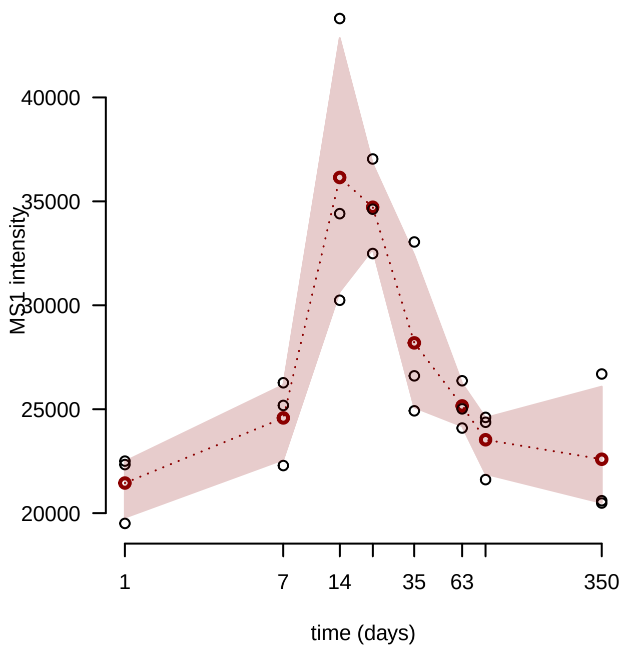

Q6Y685
GN=Tacc1
transforming, acidic coiled−coil contain ...

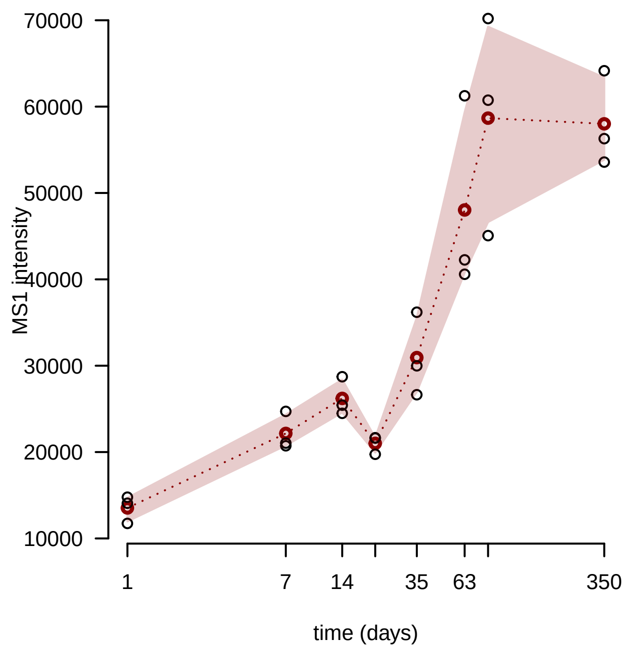

Q6Y7W8
GN=Gigyf2
GRB10 interacting GYF protein 2

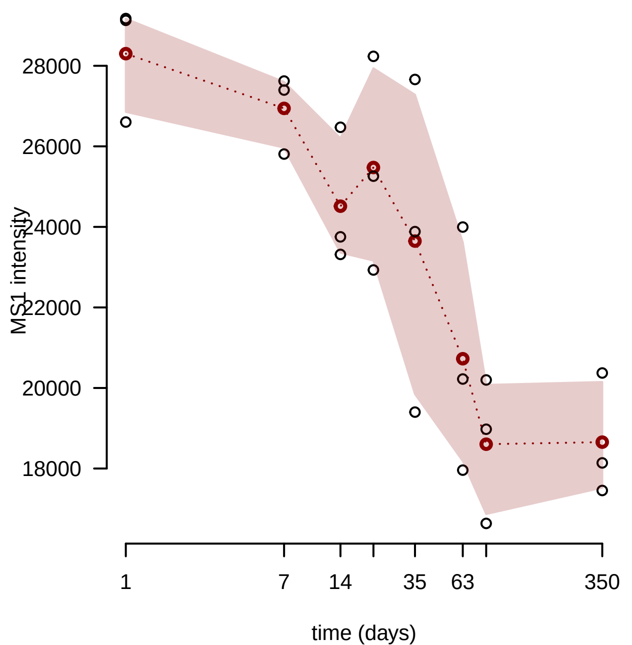

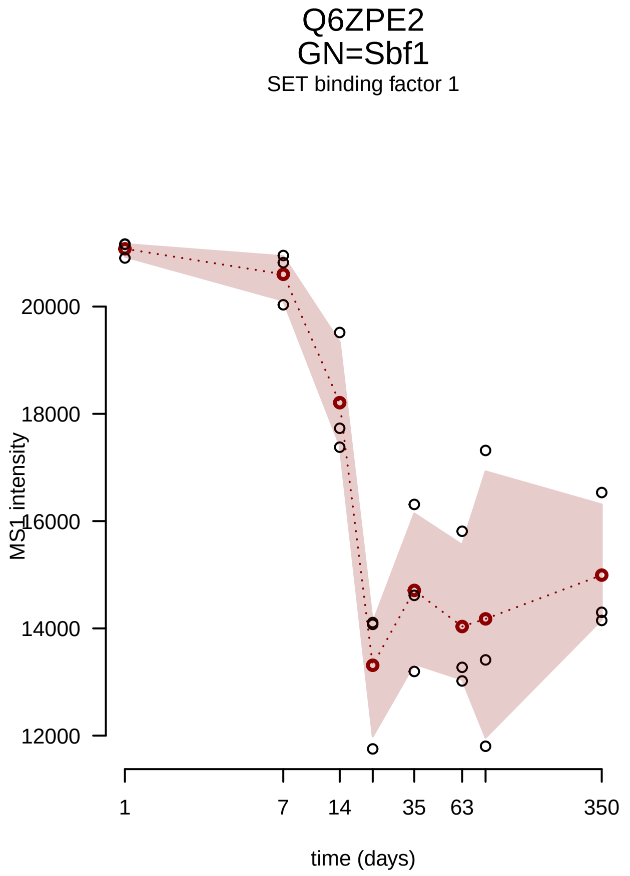
Q6ZPE2
GN=Sbf1
SET binding factor 1
MS1 intensity
12000
14000
16000
18000
20000
1
7
14
35
63
350
time (days)

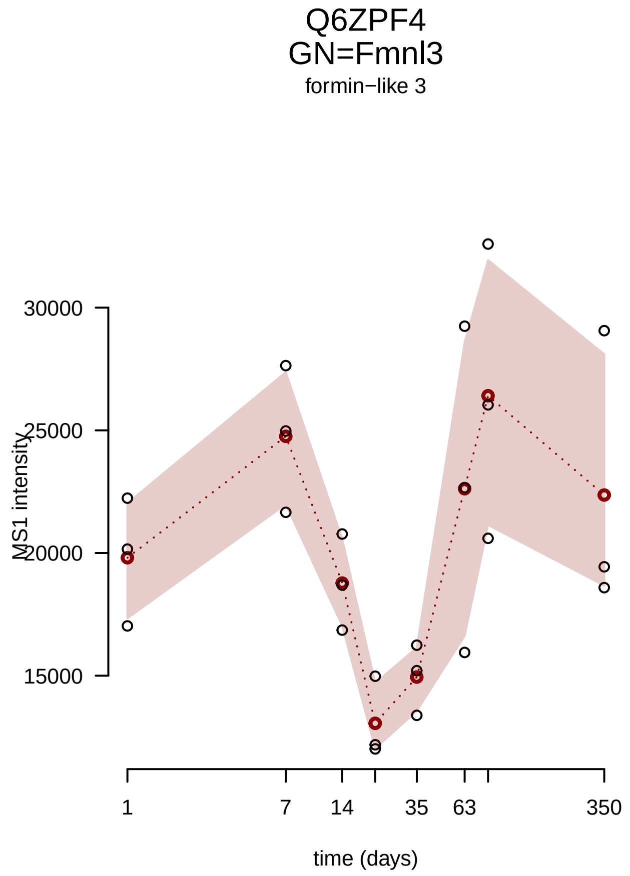
Q6ZPF4
GN=Fmnl3
formin−like 3
MS1 intensity
15000
20000
25000
30000
1
7
14
35
63
350
time (days)

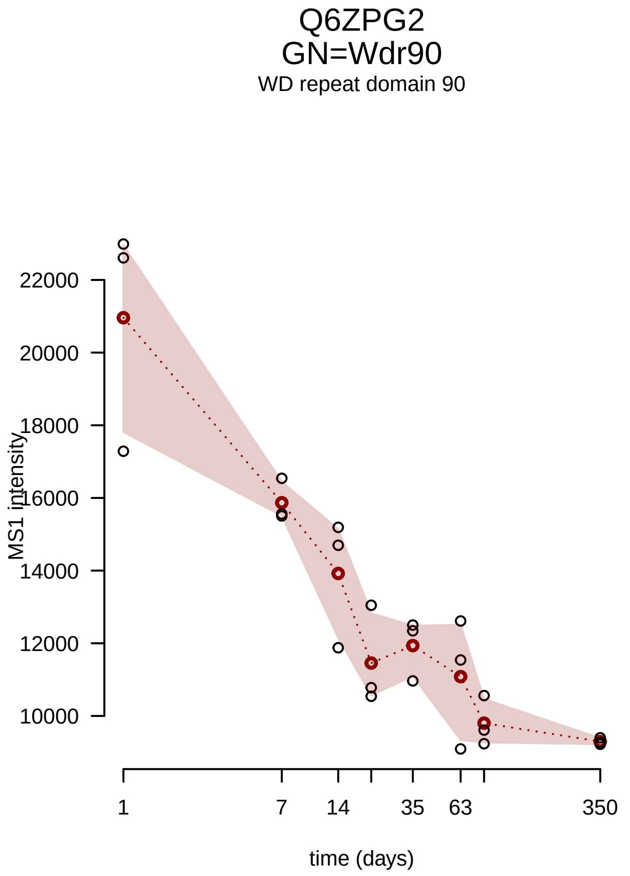
Q6ZPG2
GN=Wdr90
WD repeat domain 90
MS1 intensity
10000
12000
14000
16000
18000
20000
22000
1
7
14
35
63
350
time (days)

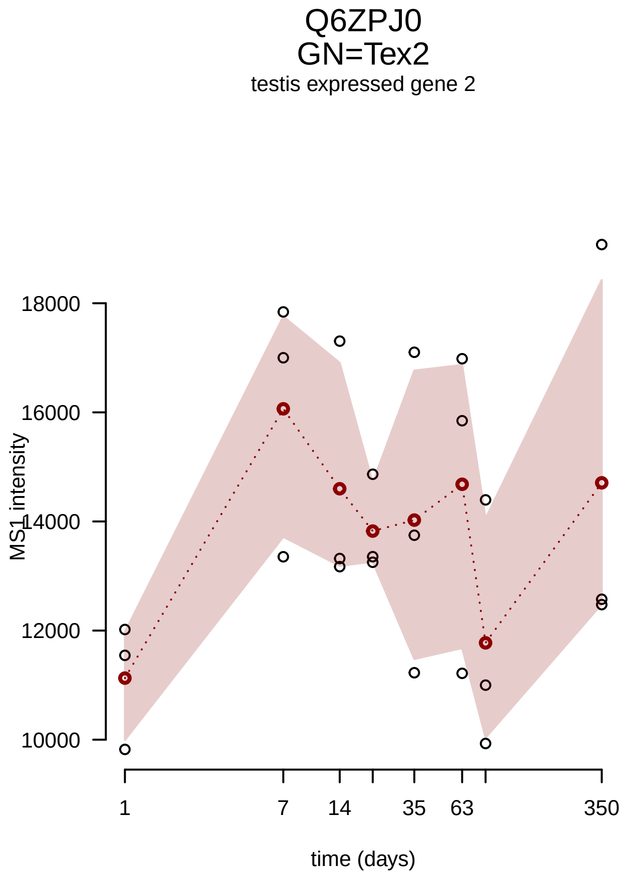
Q6ZPJ0
GN=Tex2
testis expressed gene 2
MS1 intensity
10000
12000
14000
16000
18000
1
7
14
35
63
350
time (days)

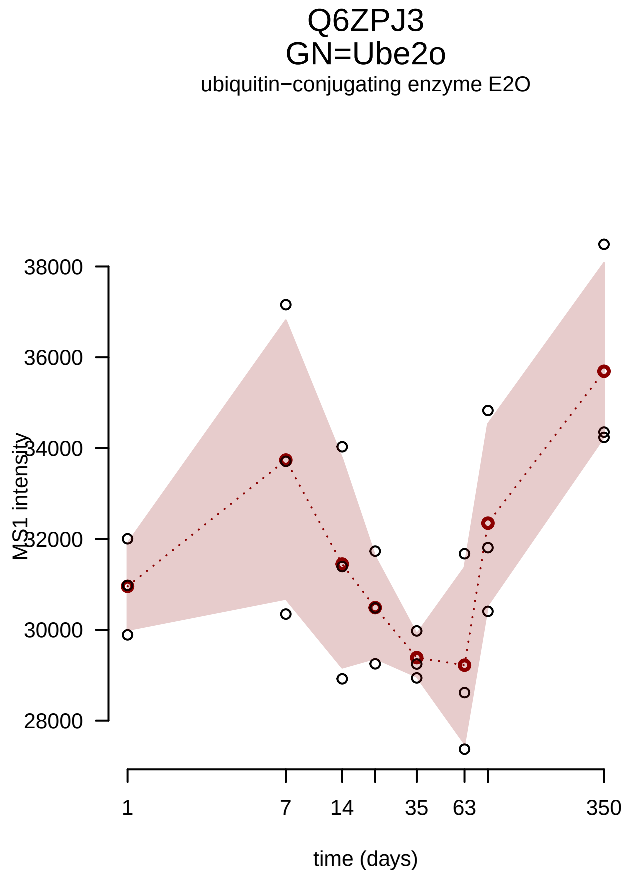
Q6ZPJ3
GN=Ube2o
ubiquitin−conjugating enzyme E2O
MS1 intensity
28000
30000
32000
34000
36000
38000
1
7
14
35
63
350
time (days)

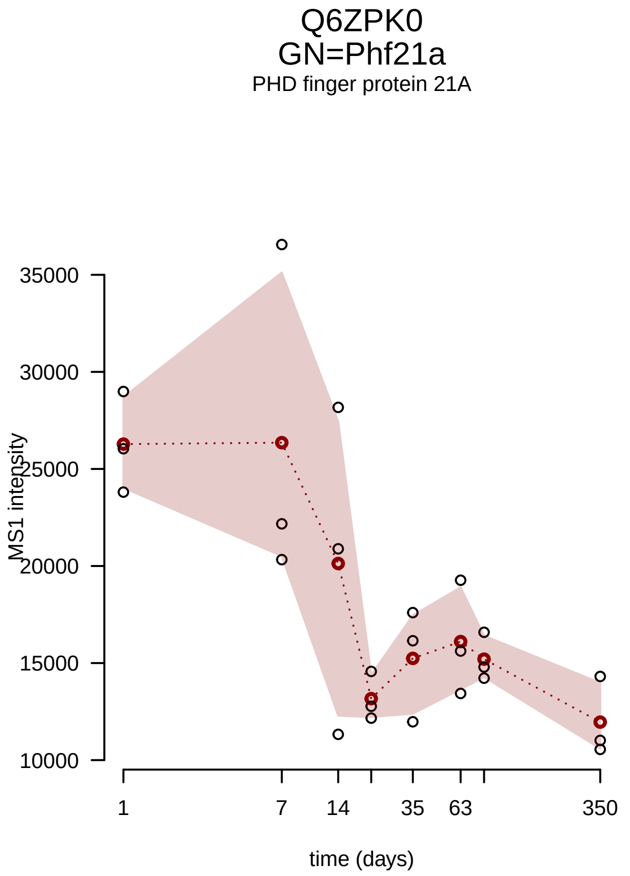
Q6ZPK0
GN=Phf21a
PHD finger protein 21A
MS1 intensity
10000
15000
20000
25000
30000
35000
1
7
14
35
63
350
time (days)

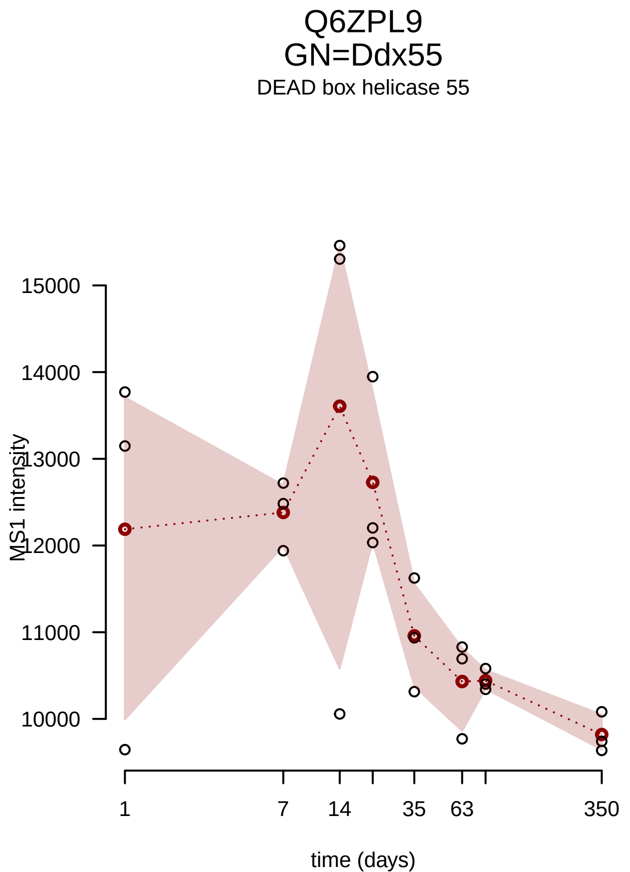
Q6ZPL9
GN=Ddx55
DEAD box helicase 55
MS1 intensity
10000
11000
12000
13000
14000
15000
1
7
14
35
63
350
time (days)

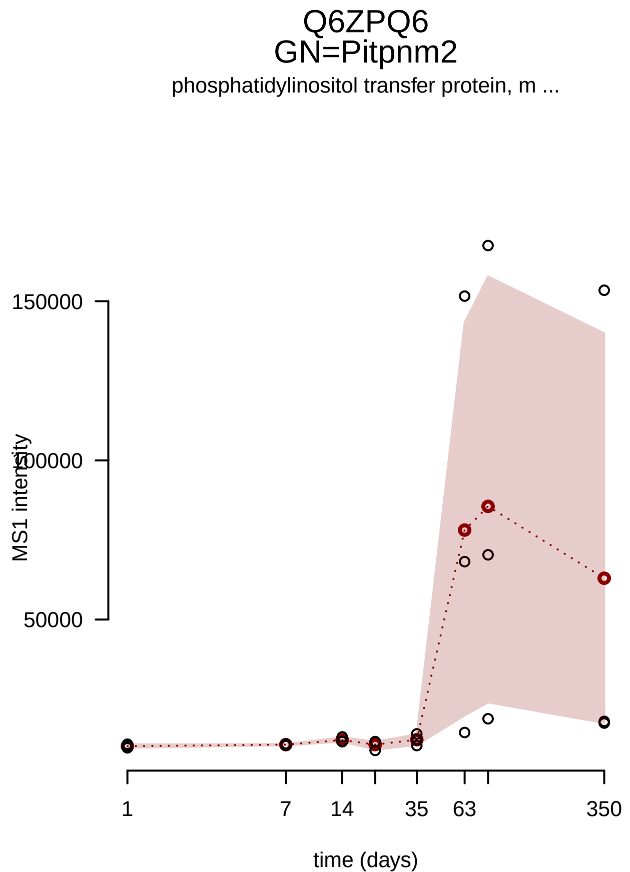
Q6ZPQ6
GN=Pitpnm2
phosphatidylinositol transfer protein, m ...
MS1 intensity
50000
100000
150000
1
7
14
35
63
350
time (days)

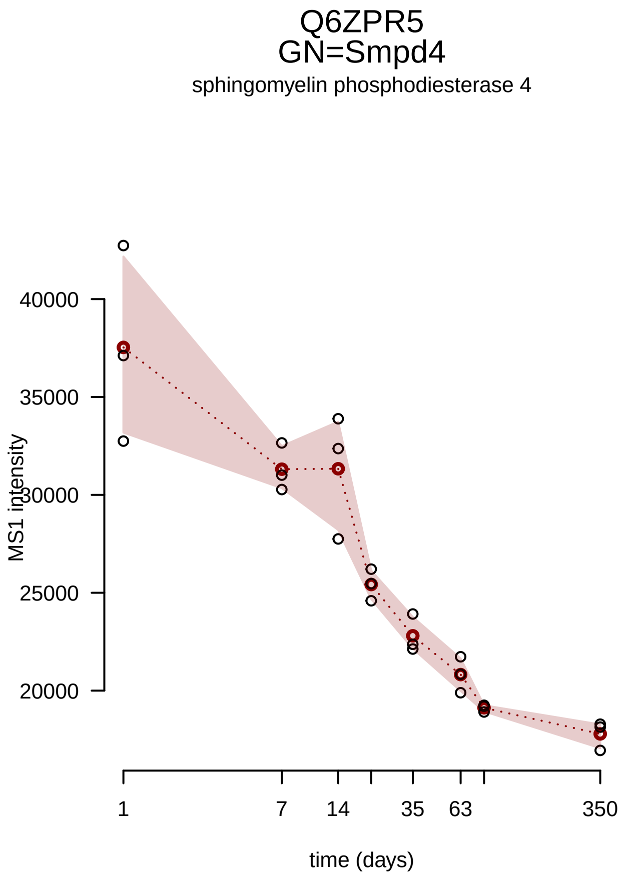
Q6ZPR5
GN=Smpd4
sphingomyelin phosphodiesterase 4
MS1 intensity
20000
25000
30000
35000
40000
1
7
14
35
63
350
time (days)

Q6ZPR6
GN=Ibtk
inhibitor of Bruton agammaglobulinemia t ...

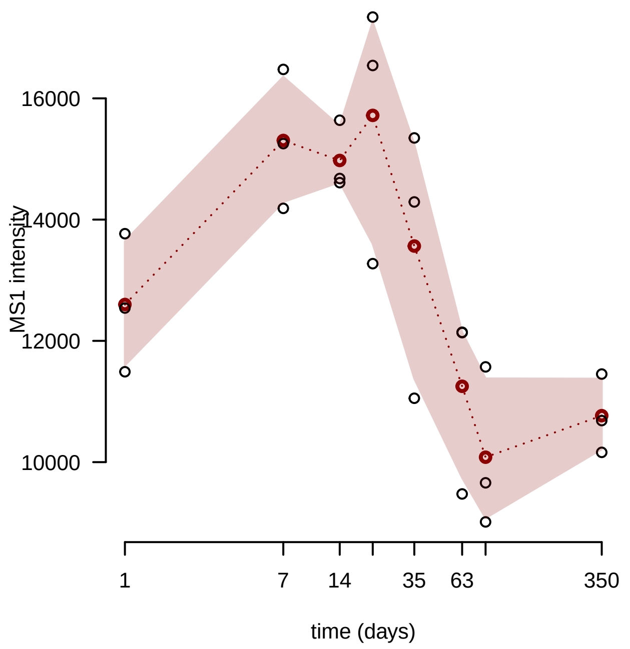

Q6ZPS6
GN=Ankib1
ankyrin repeat and IBR domain containing ...

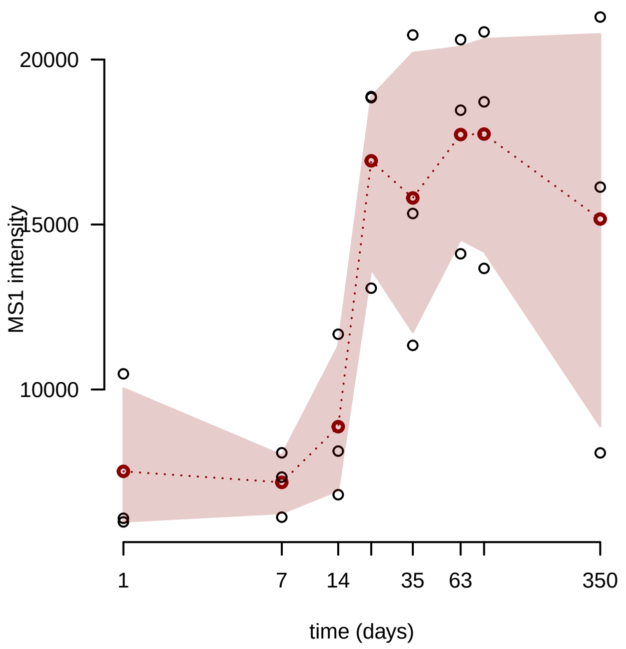

Q6ZPT1
GN=Klhl9
kelch−like 9

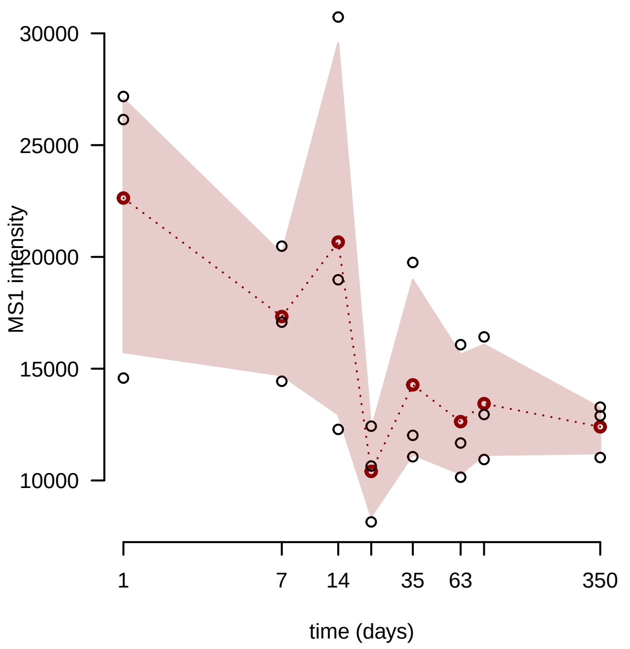

Q6ZPU9
GN=Kif1bp

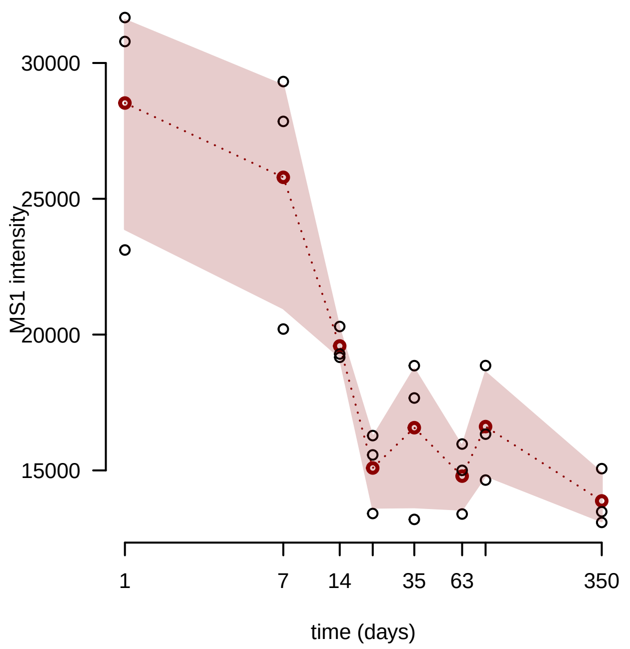

Q6ZPV2
GN=Ino80
INO80 complex subunit

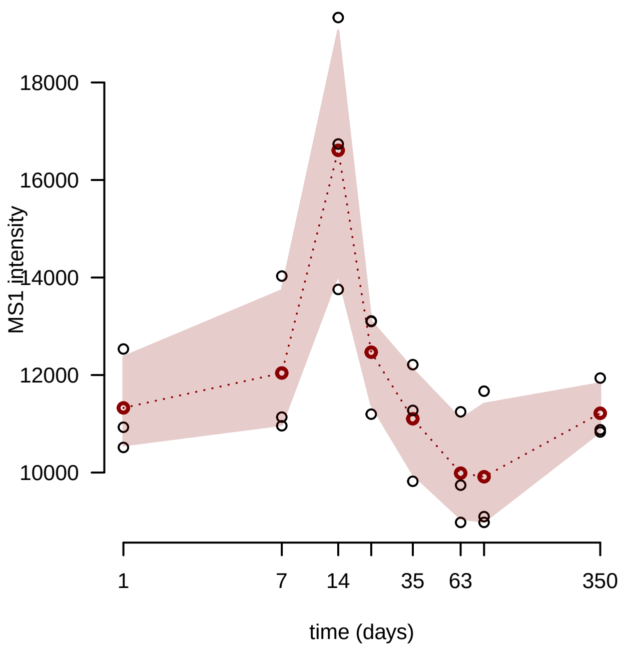

Q6ZPY2
GN=Smg5
SMG5 nonsense mediated mRNA decay factor

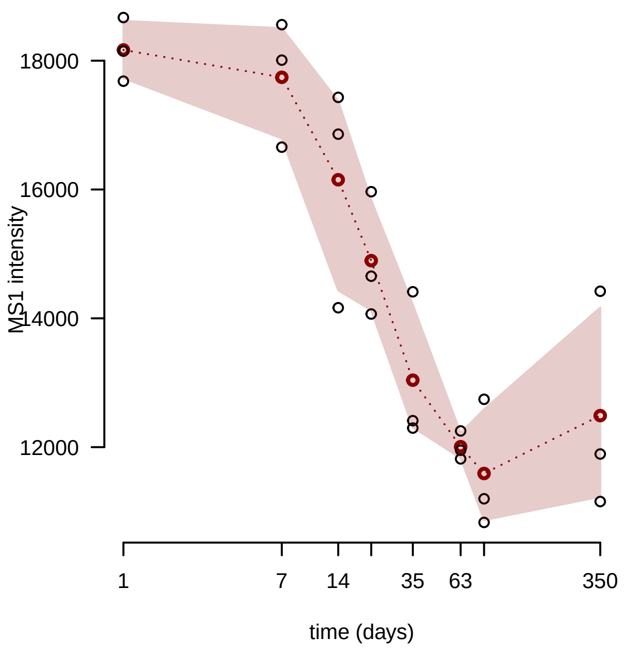

Q6ZPY7
GN=Kdm3b
KDM3B lysine (K)−specific demethylase 3B

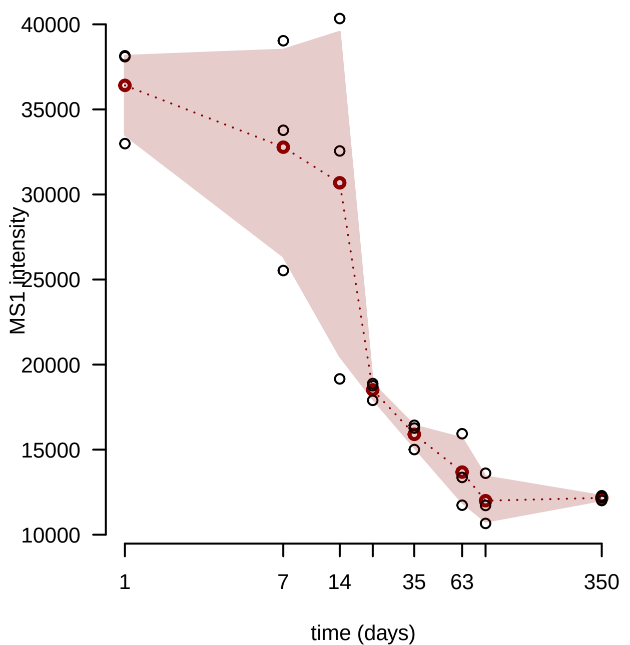

Q6ZPZ3
GN=Zc3h4
zinc finger CCCH−type containing 4

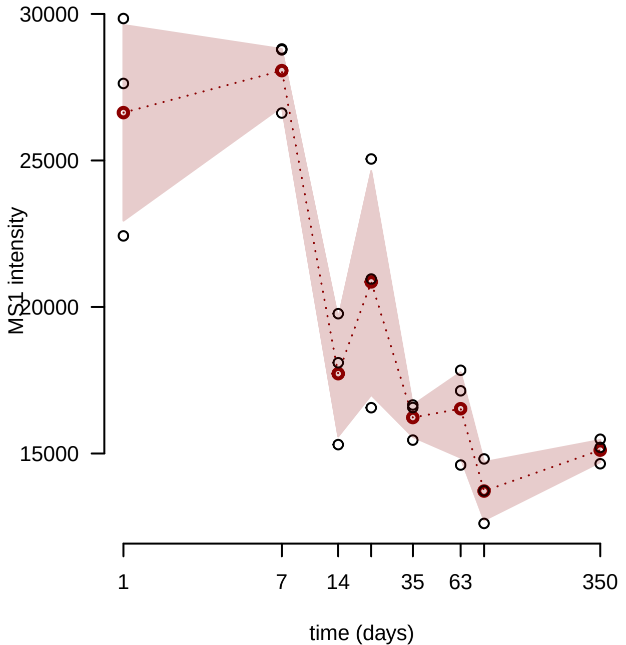

Q6ZQ03
GN=Fnbp4
formin binding protein 4

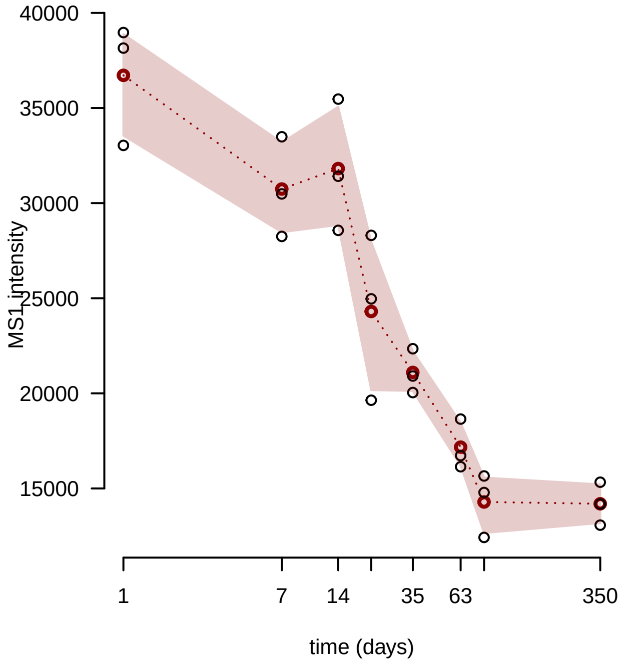

Q6ZQ08
GN=Cnot1
CCR4−NOT transcription complex, subunit ...

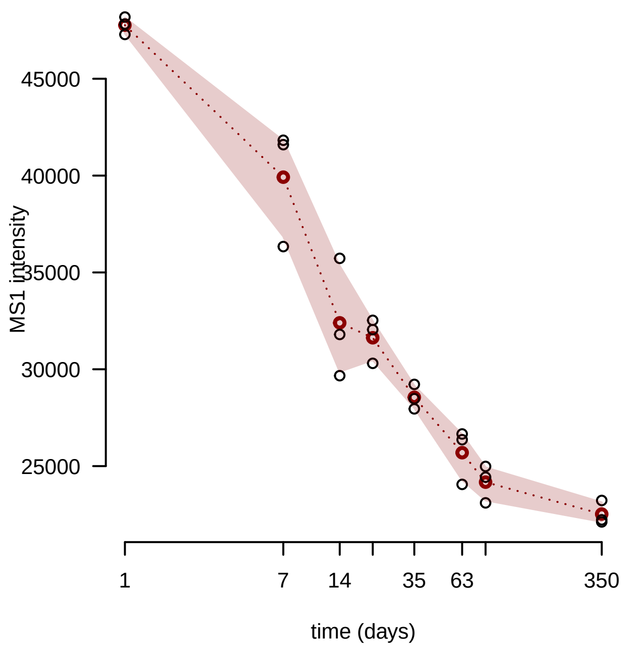

Q6ZQ11
GN=Chsy1
chondroitin sulfate synthase 1

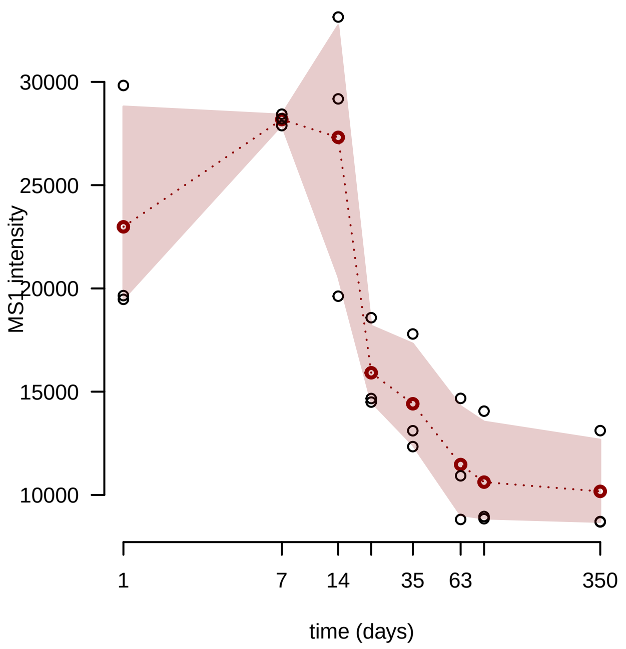

Q6ZQ29
GN=Taok2
TAO kinase 2

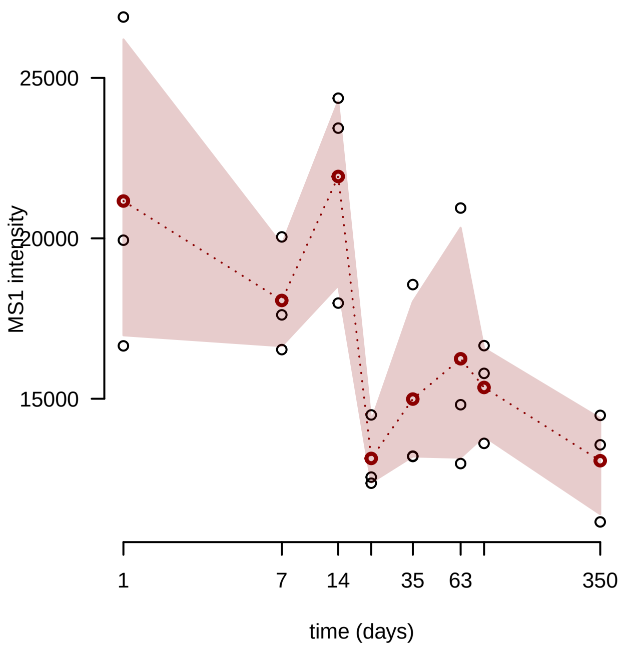

Q6ZQ29
GN=Taok2
TAO kinase 2

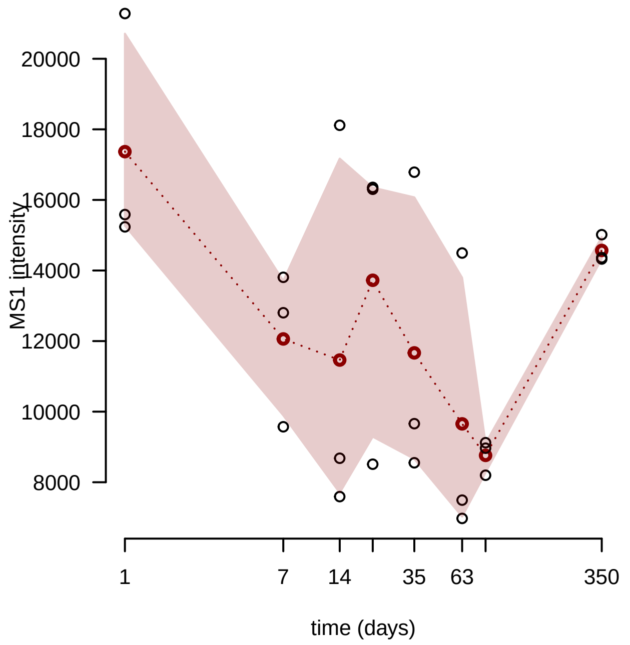

Q6ZQ38
GN=Cand1
cullin associated and neddylation disass ...

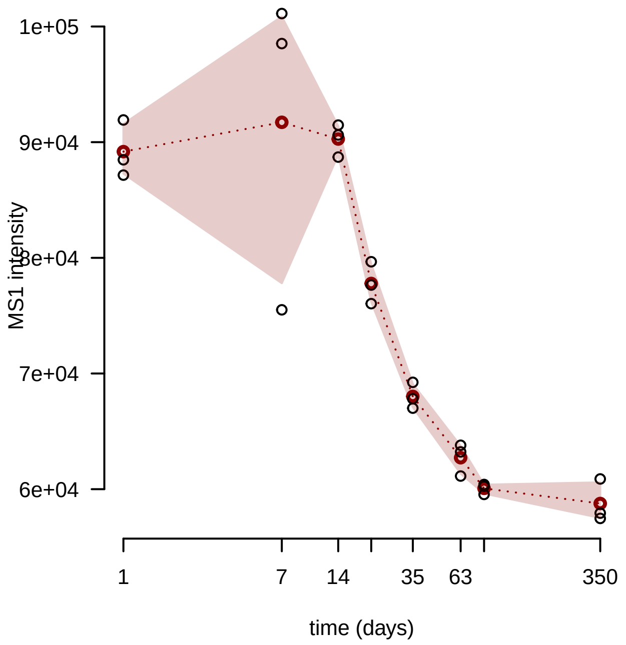

Q6ZQ58
GN=Larp1
La ribonucleoprotein 1, translational re ...

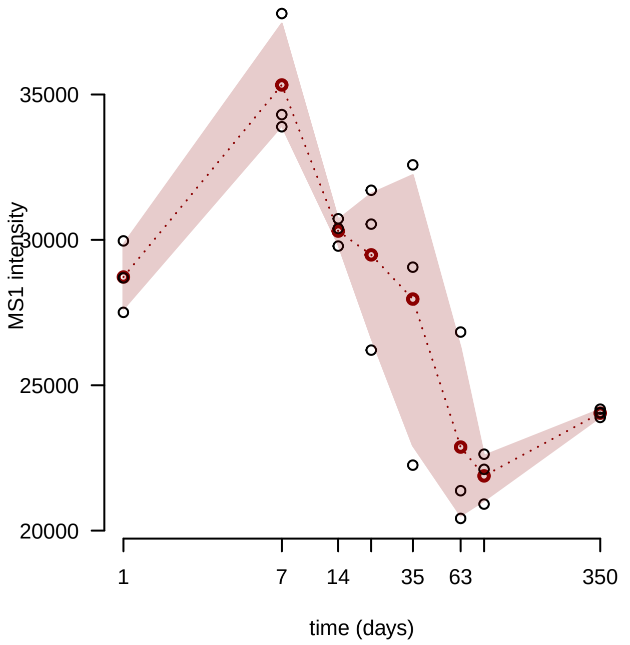

Q6ZQ73
GN=Cand2
cullin−associated and neddylation−dissoc ...

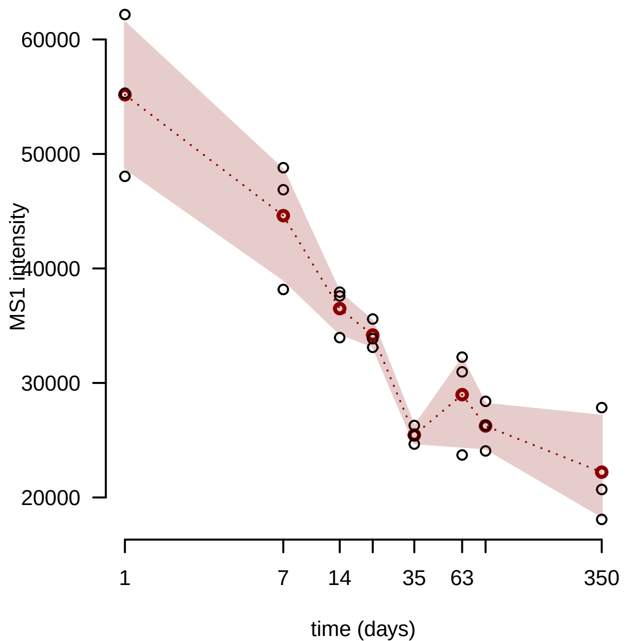

Q6ZQ82
GN=Arhgap26
Rho GTPase activating protein 26

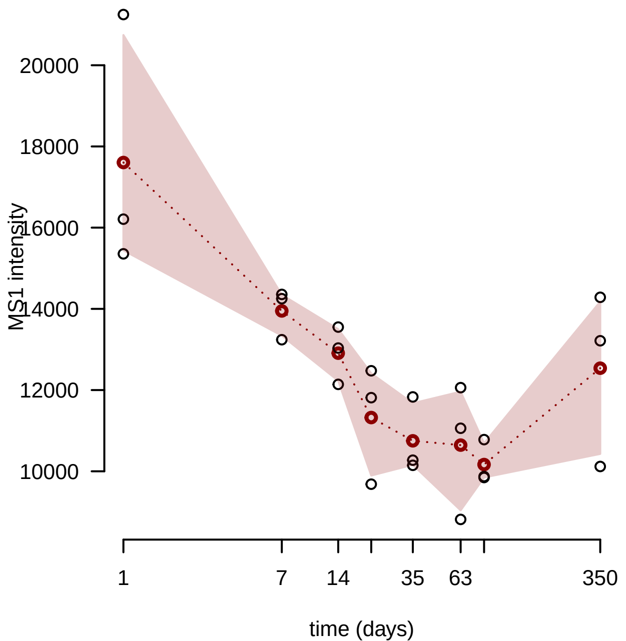

Q6ZQ88
GN=Kdm1a
lysine (K)−specific demethylase 1A

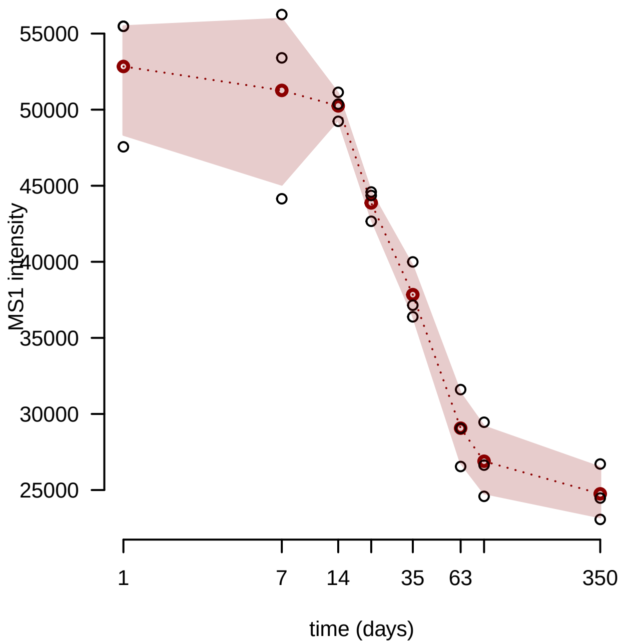

Q6ZQ89
GN=March6

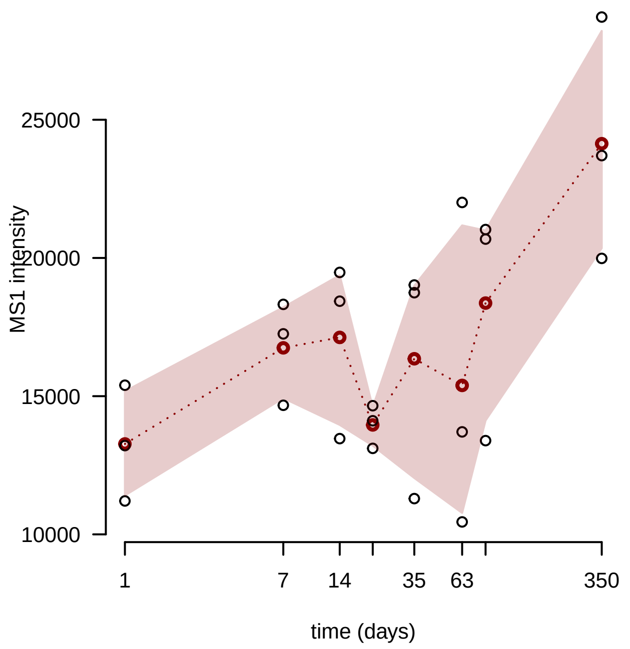

Q6ZQ93
GN=Usp34
ubiquitin specific peptidase 34

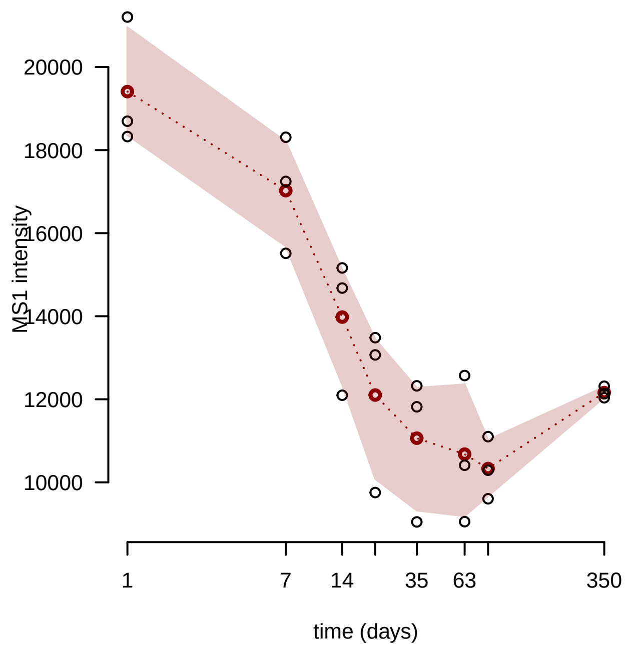

Q6ZQA6
GN=Igsf3
immunoglobulin superfamily, member 3

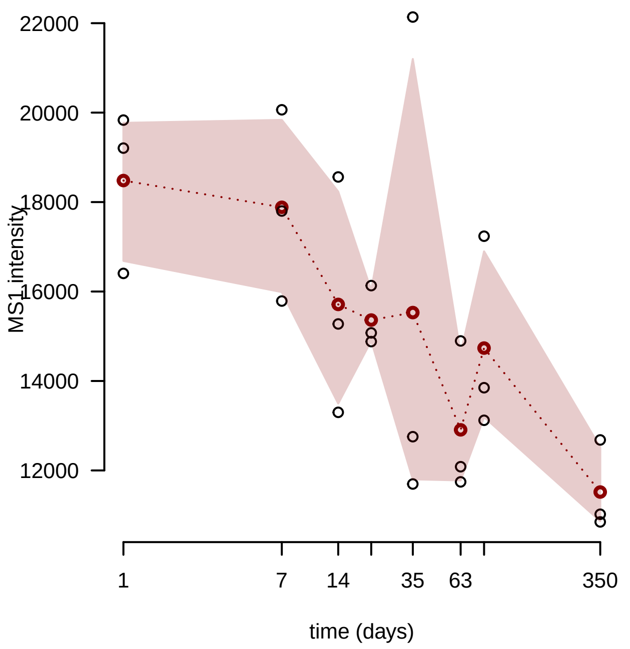

Q6ZQB6
GN=Ppip5k2
diphosphoinositol pentakisphosphate kina ...

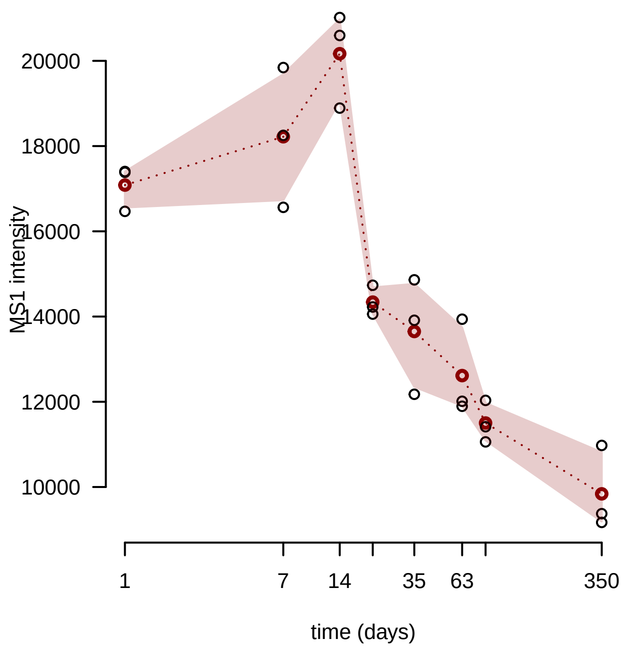

Q6ZQE4
GN=Nemp1
nuclear envelope integral membrane prote ...

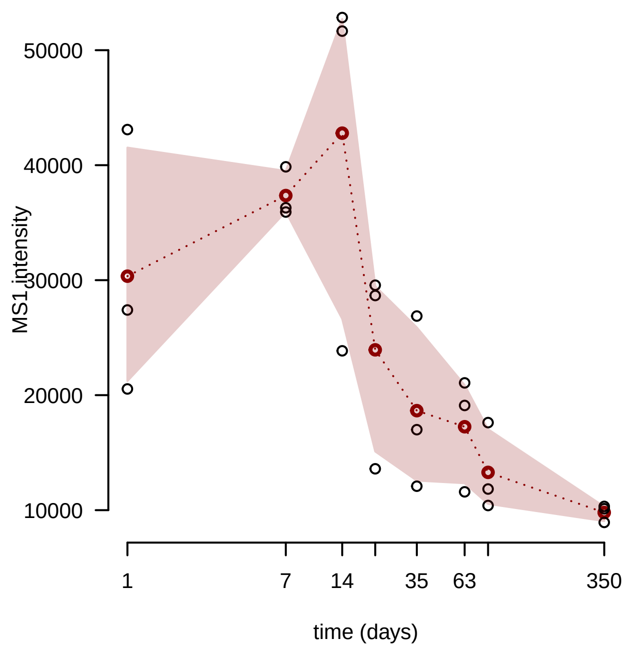

Q6ZQF0
GN=Topbp1
topoisomerase (DNA) II binding protein 1

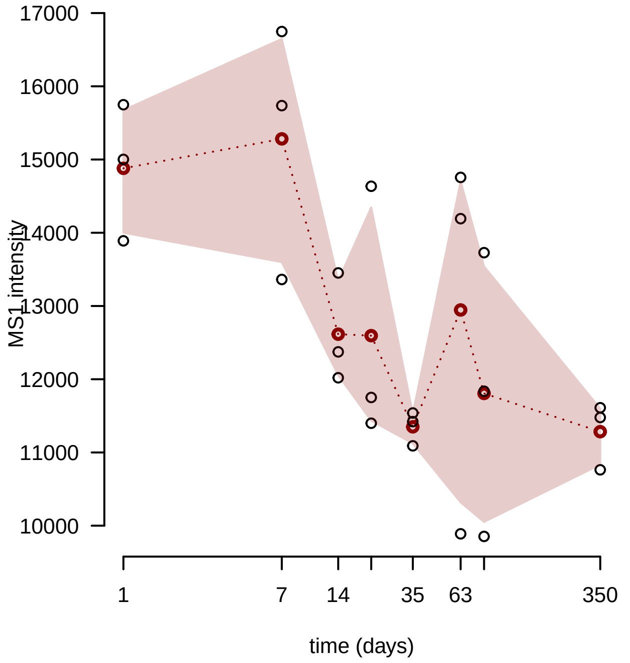

Q6ZQH8
GN=Nup188
nucleoporin 188

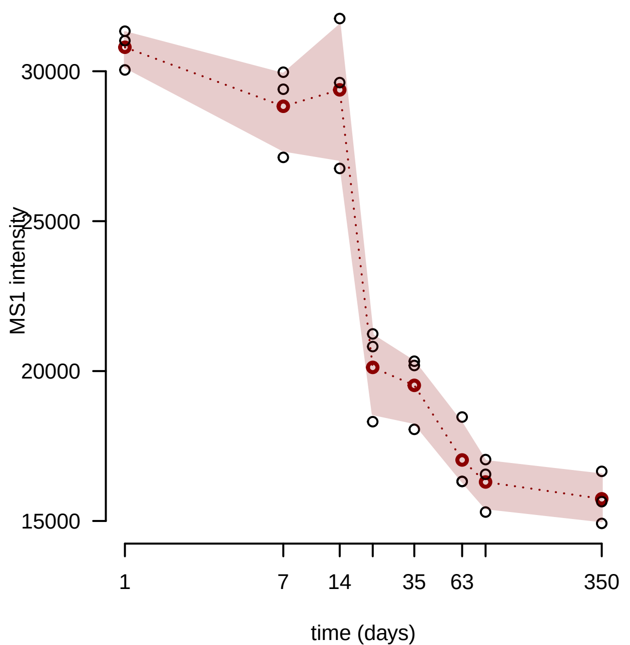

Q6ZQI3
GN=Mlec
malectin

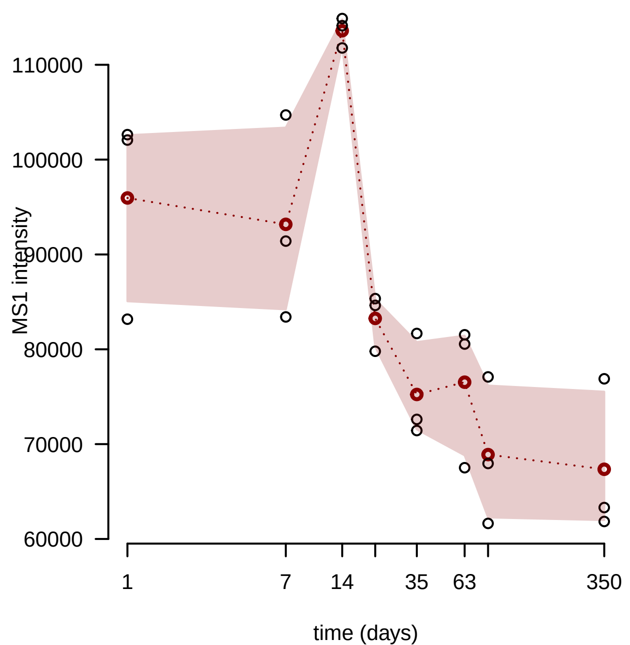

Q6ZQK0
GN=Ncapd3
non−SMC condensin II complex, subunit D3

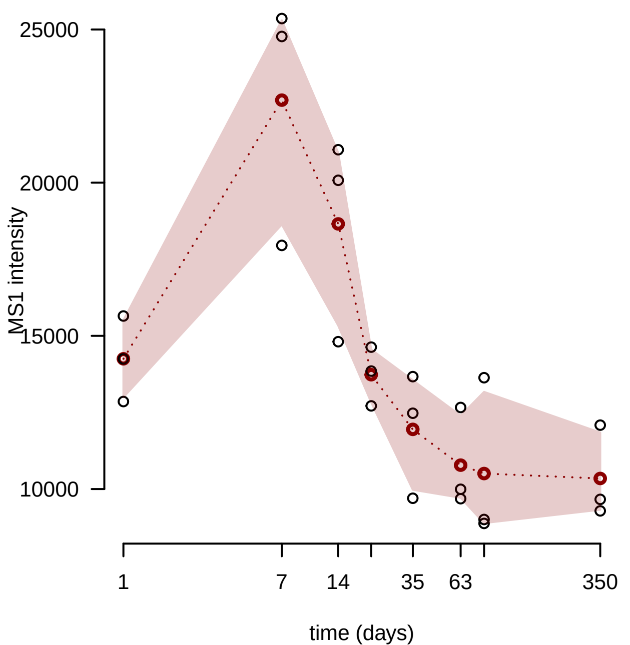

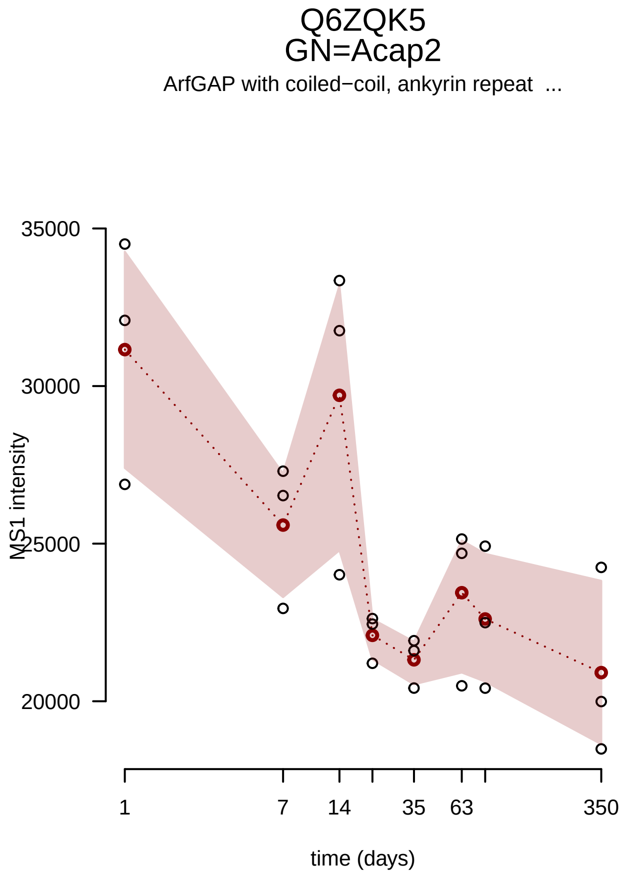
Q6ZQK5
GN=Acap2
ArfGAP with coiled−coil, ankyrin repeat ...
MS1 intensity
35000
30000
25000
20000
1
7
14
35
63
350
time (days)
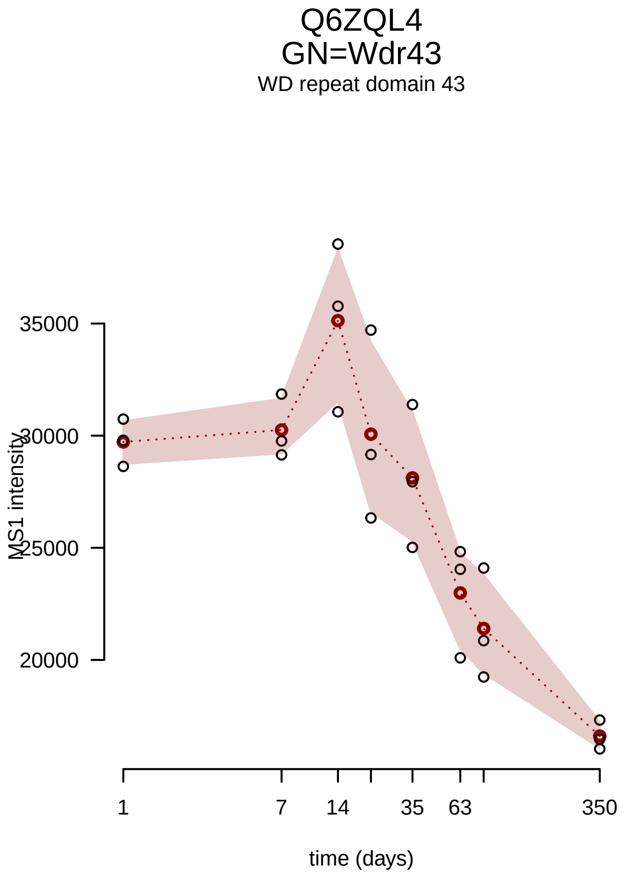
Q6ZQL4
GN=Wdr43
WD repeat domain 43
MS1 intensity
35000
30000
25000
20000
1
7
14
35
63
350
time (days)
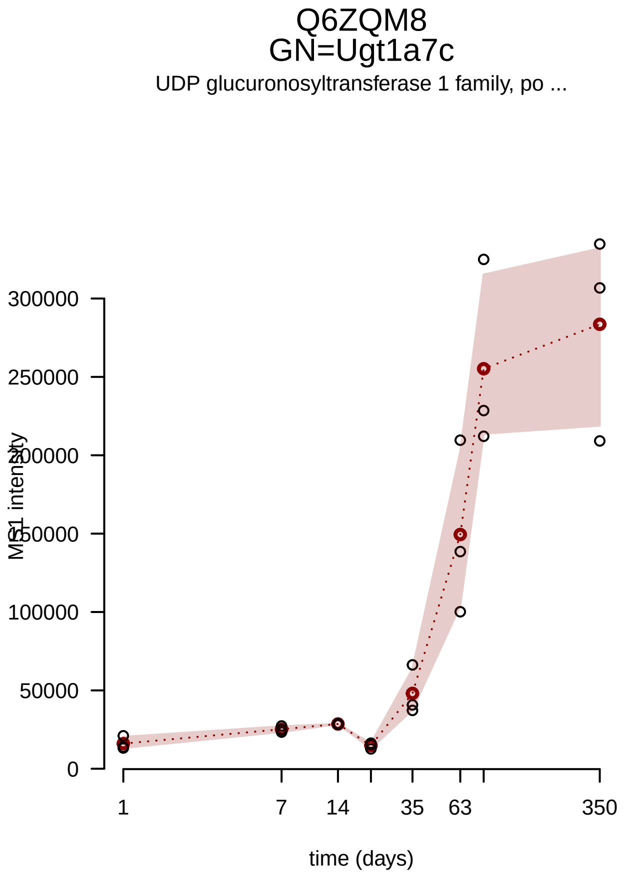
Q6ZQM8
GN=Ugt1a7c
UDP glucuronosyltransferase 1 family, po ...
MS1 intensity
300000
250000
200000
150000
100000
50000
0
1
7
14
35
63
350
time (days)
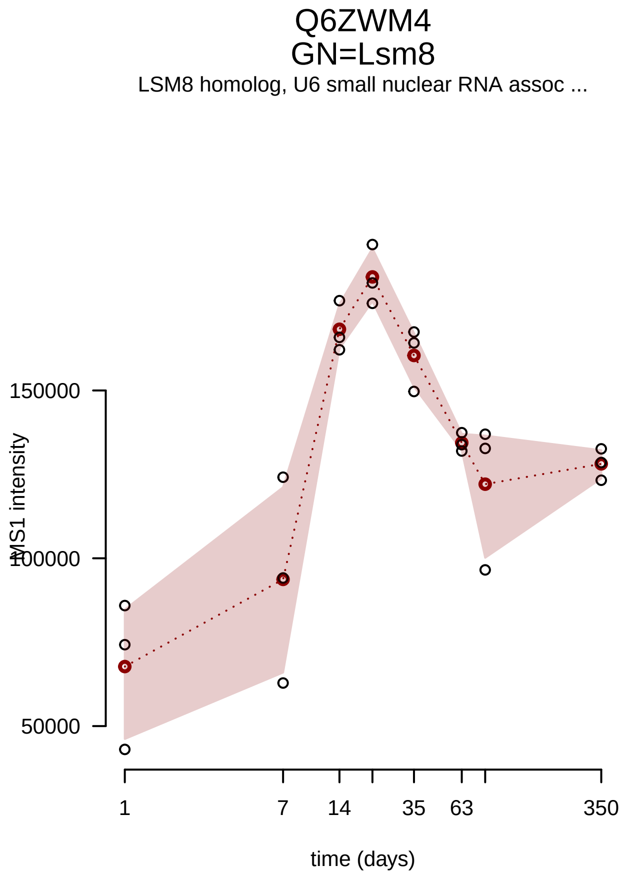
Q6ZWM4
GN=Lsm8
LSM8 homolog, U6 small nuclear RNA assoc ...
MS1 intensity
150000
100000
50000
1
7
14
35
63
350
time (days)
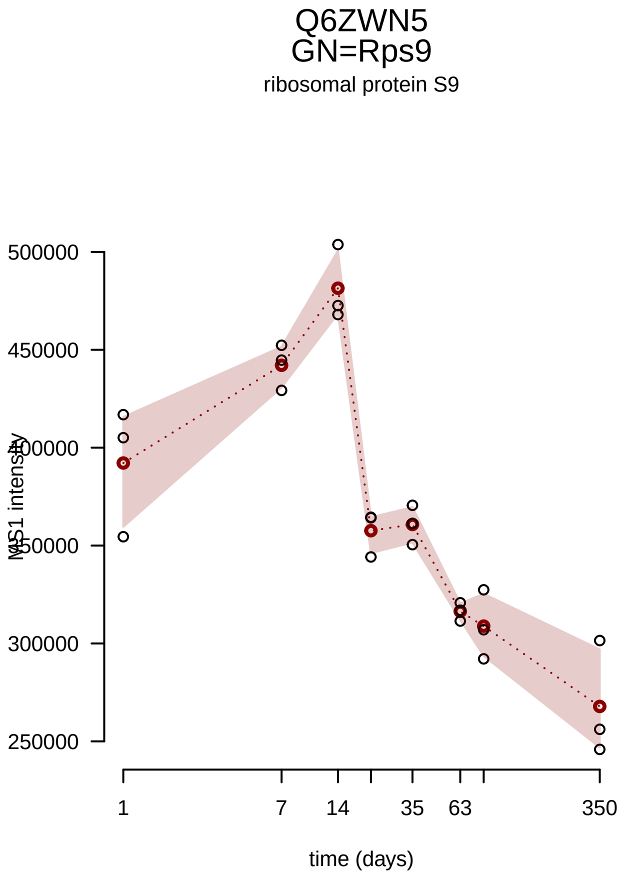
Q6ZWN5
GN=Rps9
ribosomal protein S9
MS1 intensity
500000
450000
400000
350000
300000
250000
1
7
14
35
63
350
time (days)
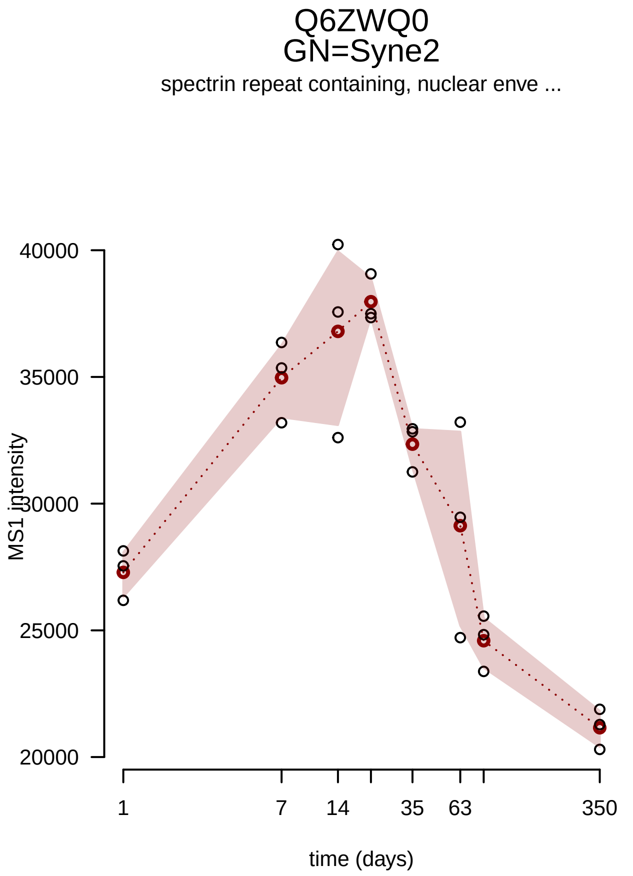
Q6ZWQ0
GN=Syne2
spectrin repeat containing, nuclear enve ...
MS1 intensity
40000
35000
30000
25000
20000
1
7
14
35
63
350
time (days)
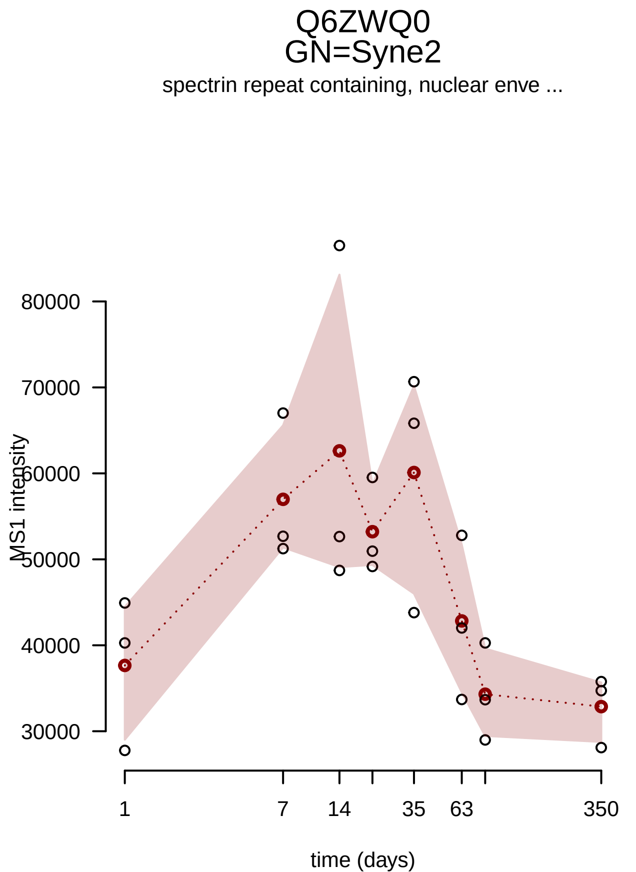
Q6ZWQ0
GN=Syne2
spectrin repeat containing, nuclear enve ...
MS1 intensity
80000
70000
60000
50000
40000
30000
1
7
14
35
63
350
time (days)
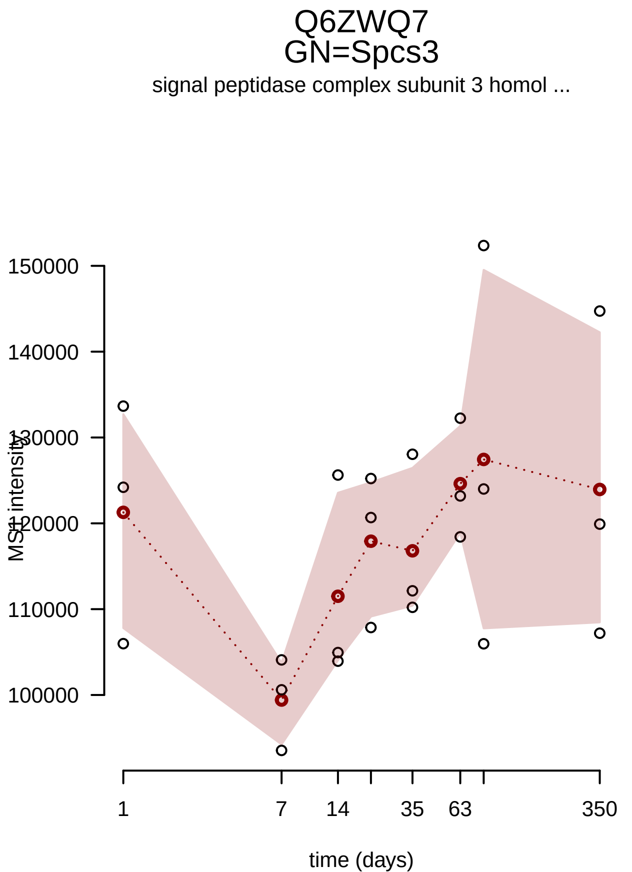
Q6ZWQ7
GN=Spcs3
signal peptidase complex subunit 3 homol ...
MS1 intensity
150000
140000
130000
120000
110000
100000
1
7
14
35
63
350
time (days)
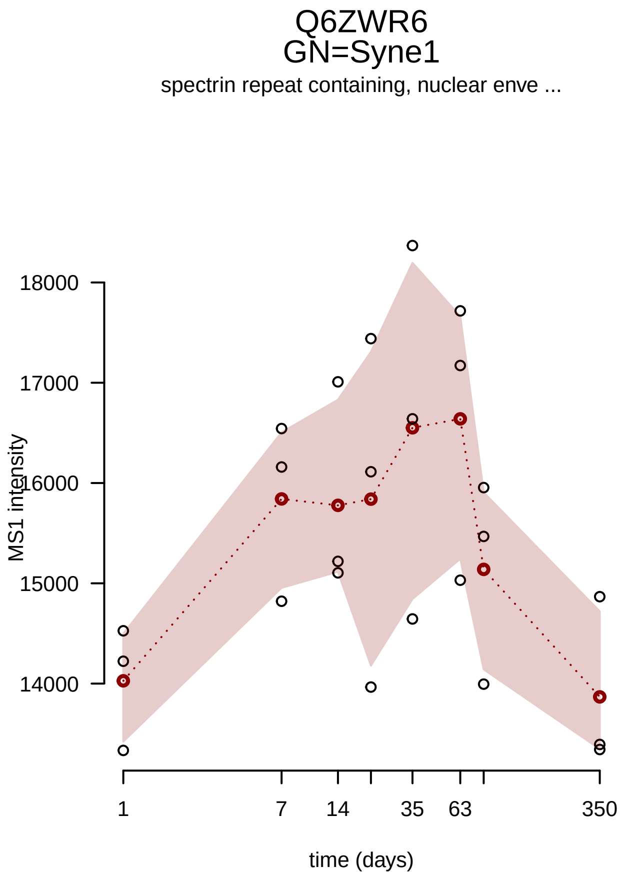
Q6ZWR6
GN=Syne1
spectrin repeat containing, nuclear enve ...
MS1 intensity
18000
17000
16000
15000
14000
1
7
14
35
63
350
time (days)

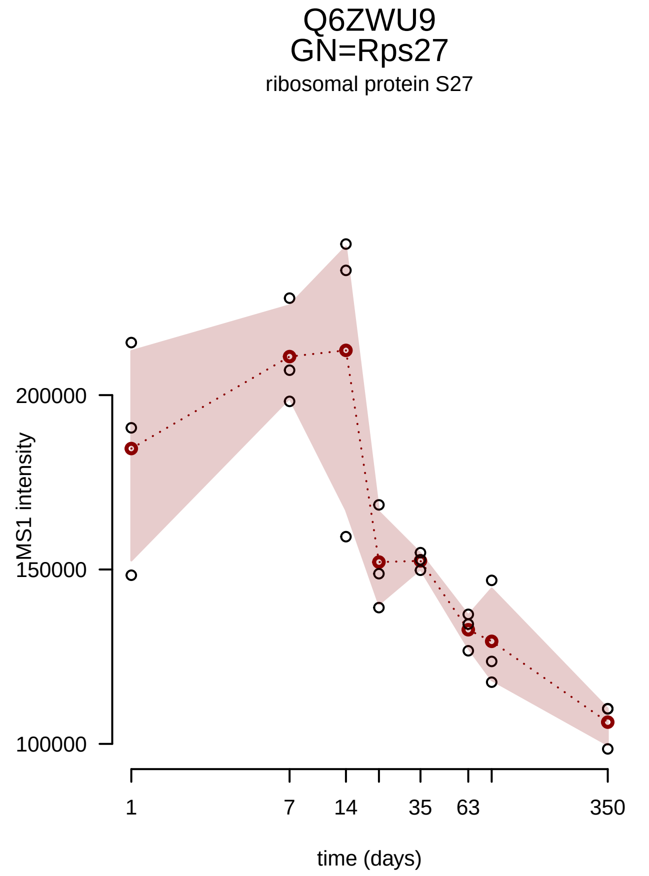
Q6ZWU9
GN=Rps27
ribosomal protein S27
MS1 intensity
100000
150000
200000
1
7
14
35
63
350
time (days)

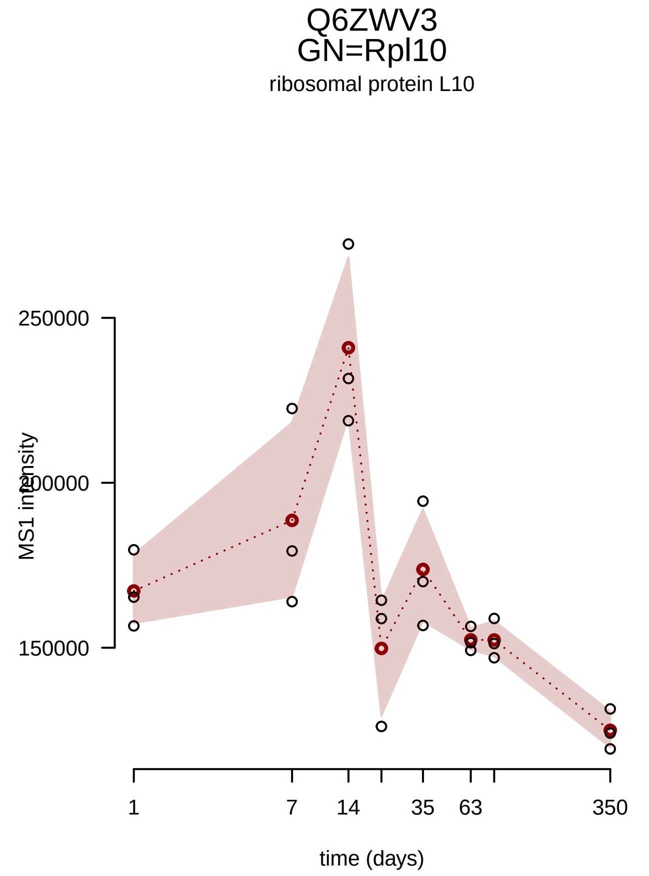
Q6ZWV3
GN=Rpl10
ribosomal protein L10
MS1 intensity
150000
200000
250000
1
7
14
35
63
350
time (days)

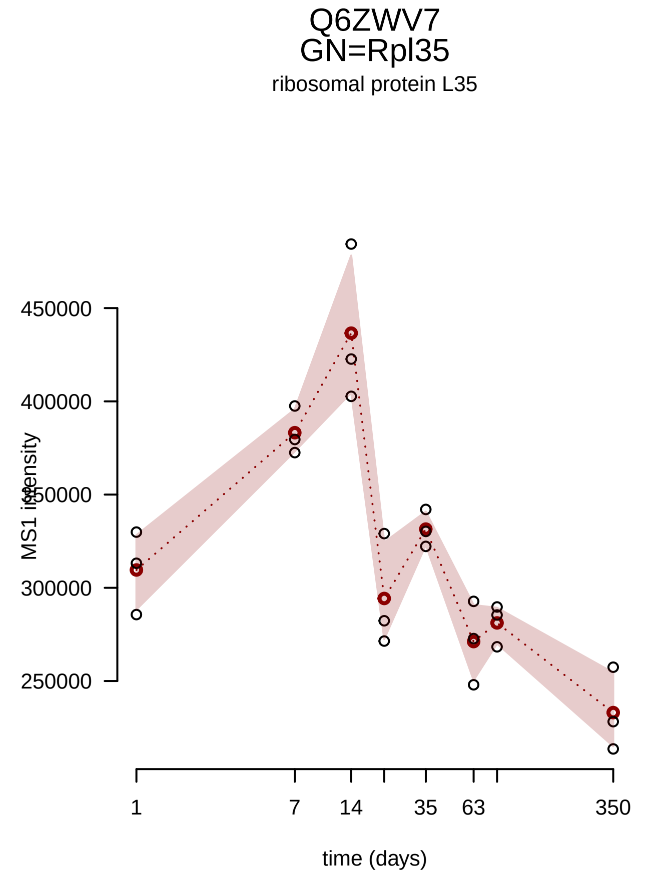
Q6ZWV7
GN=Rpl35
ribosomal protein L35
MS1 intensity
250000
300000
350000
400000
450000
1
7
14
35
63
350
time (days)

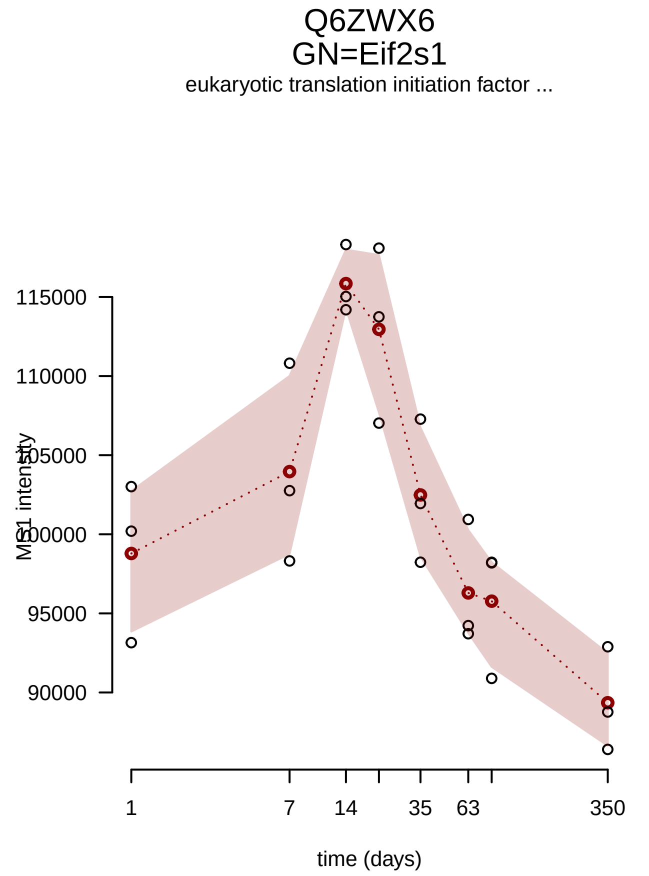
Q6ZWX6
GN=Eif2s1
eukaryotic translation initiation factor ...
MS1 intensity
90000
95000
100000
105000
110000
115000
1
7
14
35
63
350
time (days)

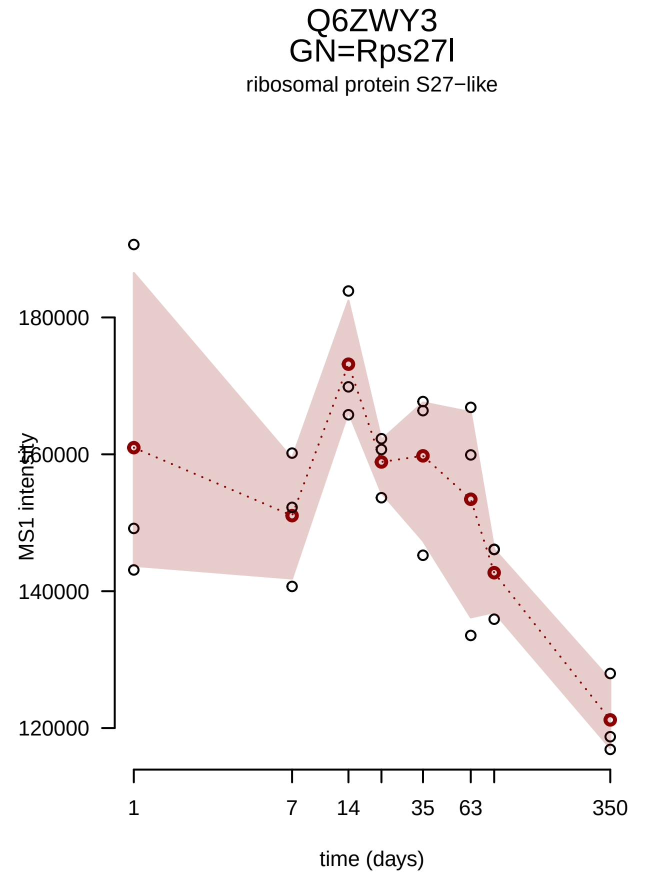
Q6ZWY3
GN=Rps27l
ribosomal protein S27−like
MS1 intensity
120000
140000
160000
180000
1
7
14
35
63
350
time (days)

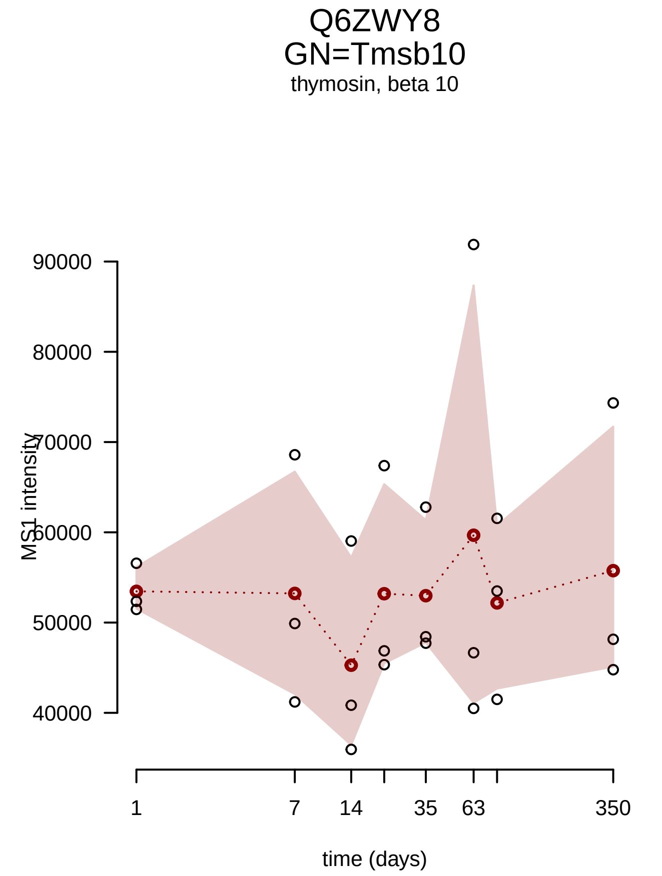
Q6ZWY8
GN=Tmsb10
thymosin, beta 10
MS1 intensity
40000
50000
60000
70000
80000
90000
1
7
14
35
63
350
time (days)

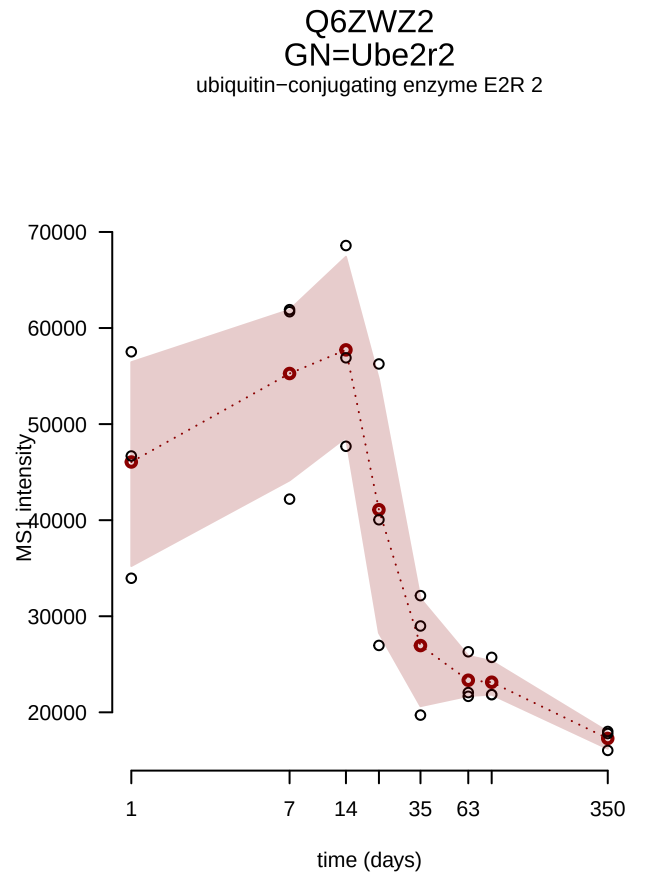
Q6ZWZ2
GN=Ube2r2
ubiquitin−conjugating enzyme E2R 2
MS1 intensity
20000
30000
40000
50000
60000
70000
1
7
14
35
63
350
time (days)

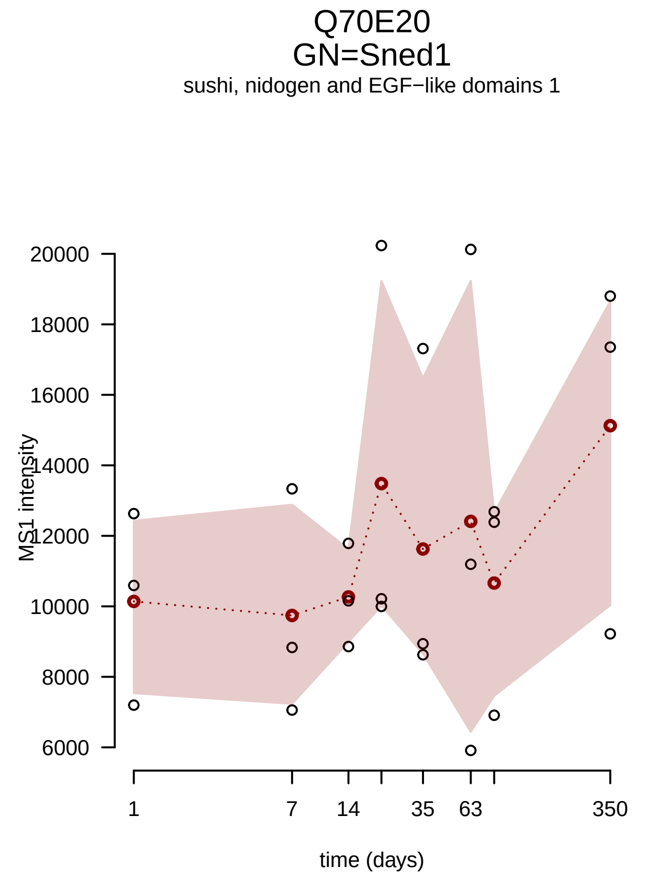
Q70E20
GN=Sned1
sushi, nidogen and EGF−like domains 1
MS1 intensity
6000
8000
10000
12000
14000
16000
18000
20000
1
7
14
35
63
350
time (days)

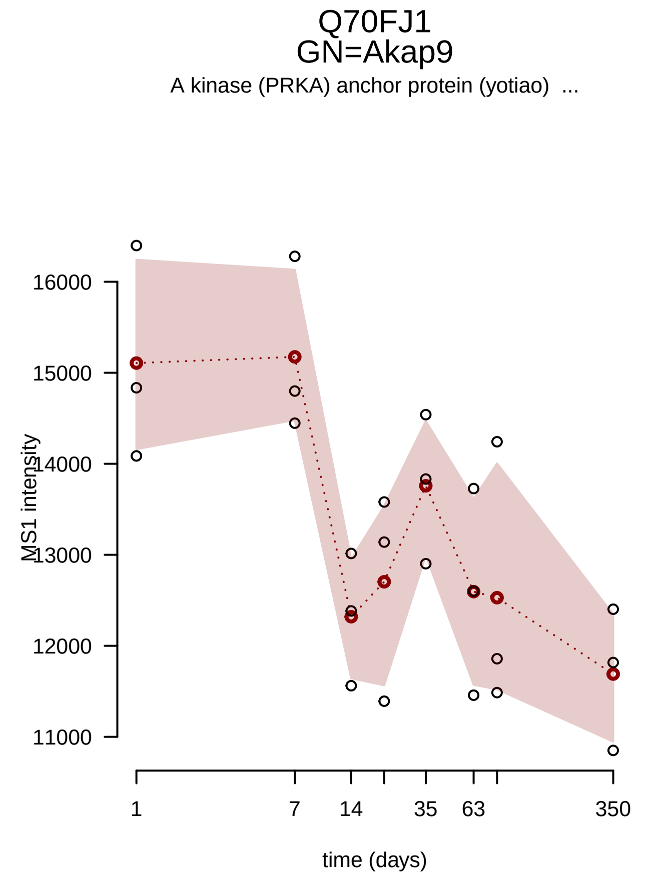
Q70FJ1
GN=Akap9
A kinase (PRKA) anchor protein (yotiao) ...
MS1 intensity
11000
12000
13000
14000
15000
16000
1
7
14
35
63
350
time (days)

Q70IV5
GN=Synm
synemin, intermediate filament protein

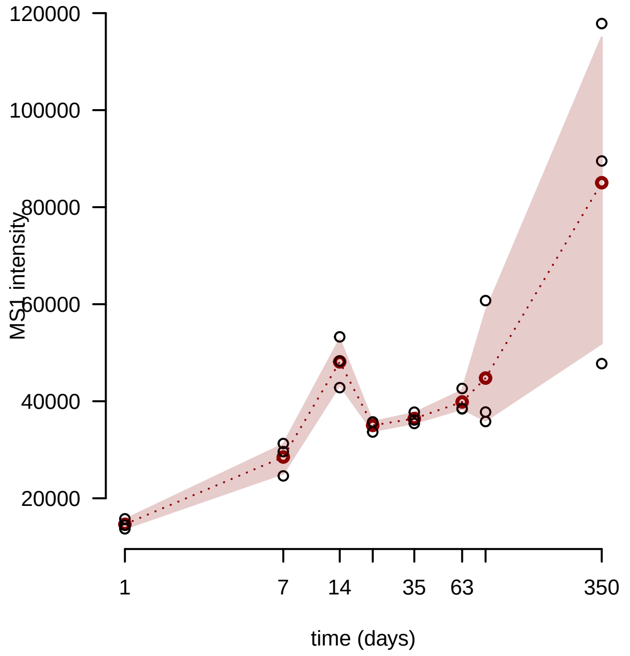

Q70IV5
GN=Synm
synemin, intermediate filament protein

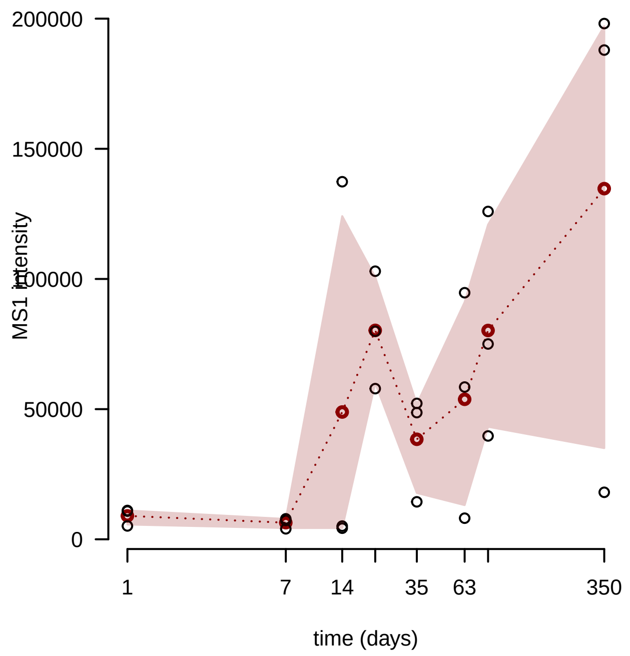

Q711T7
GN=Nadsyn1
NAD synthetase 1

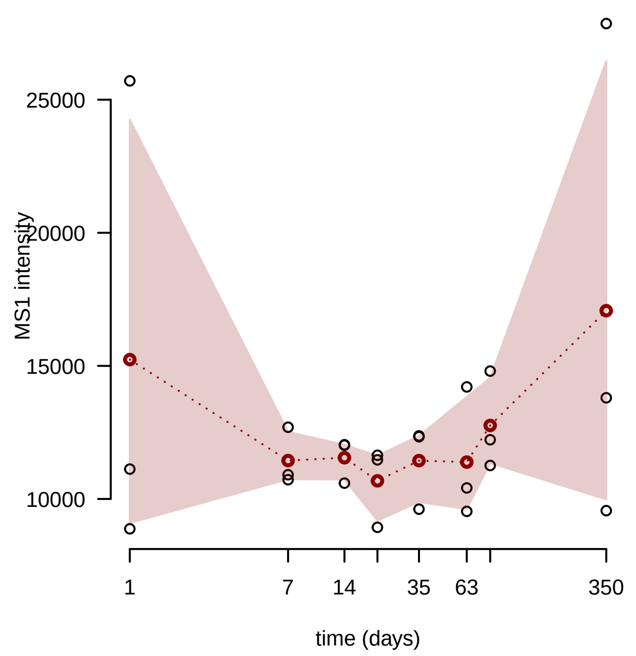

Q71B07
GN=Dpy19l3
dpy−19−like 3 (C. elegans)

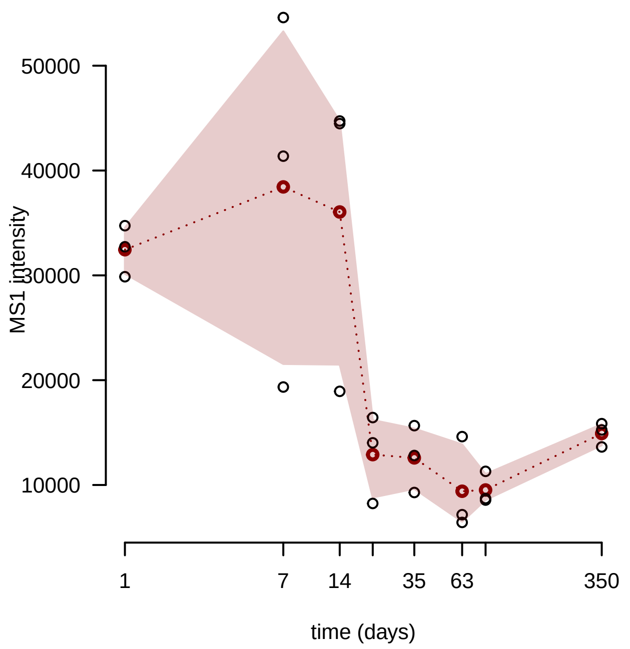

Q71FD5
GN=Znrf2
zinc and ring finger 2

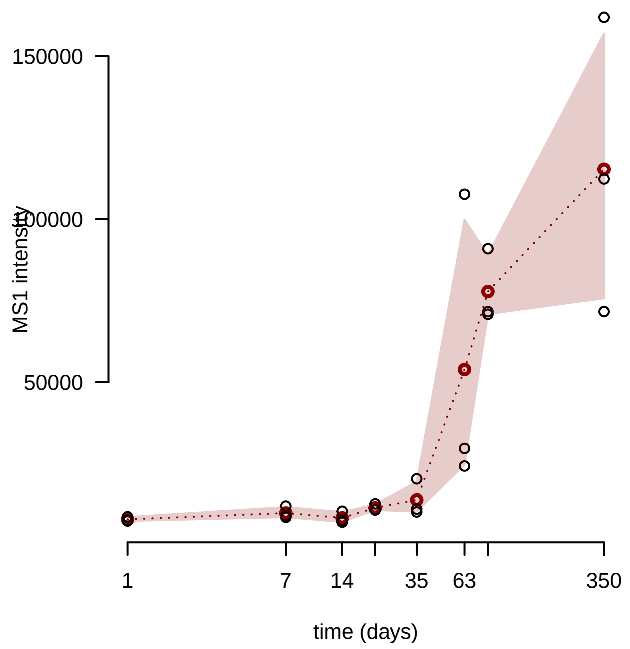

Q71FD7
GN=Fblim1
filamin binding LIM protein 1

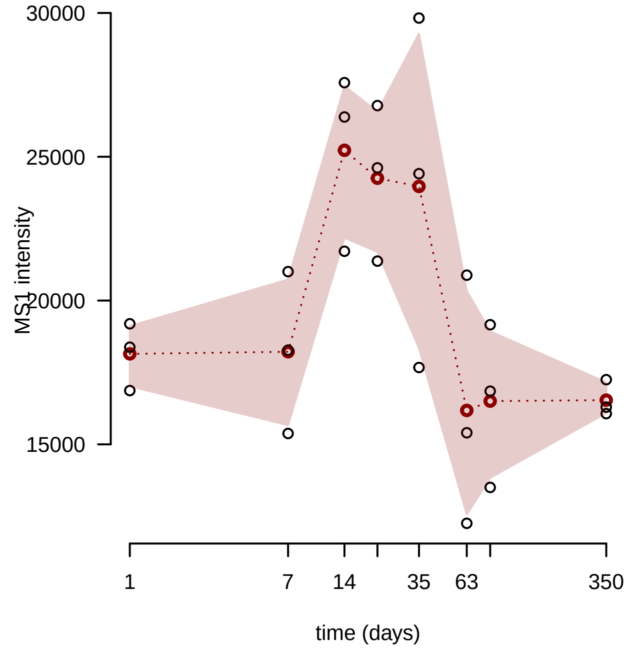

Q71KT5
GN=Tm7sf2
transmembrane 7 superfamily member 2

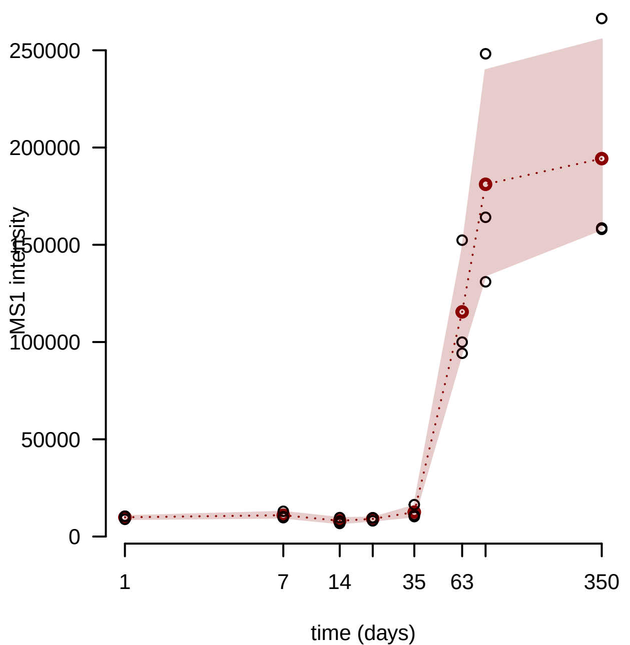

Q71LX4
GN=Tln2
talin 2

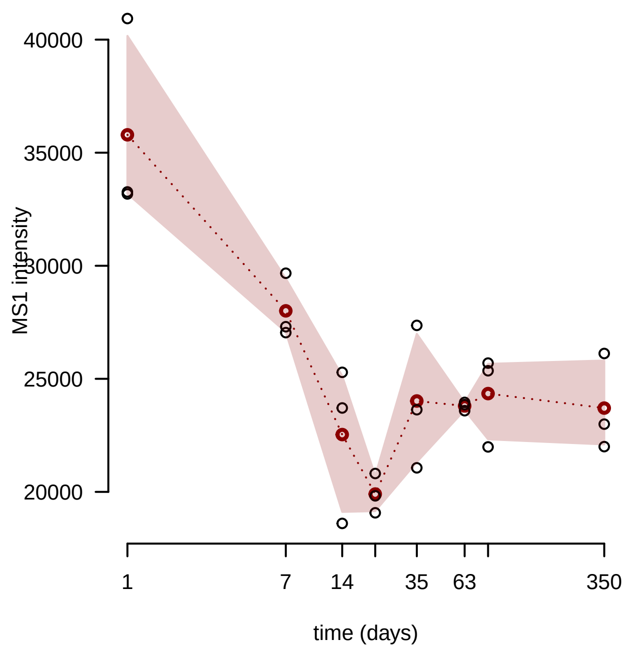

Q71RI9
GN=Kyat3
kynurenine aminotransferase 3

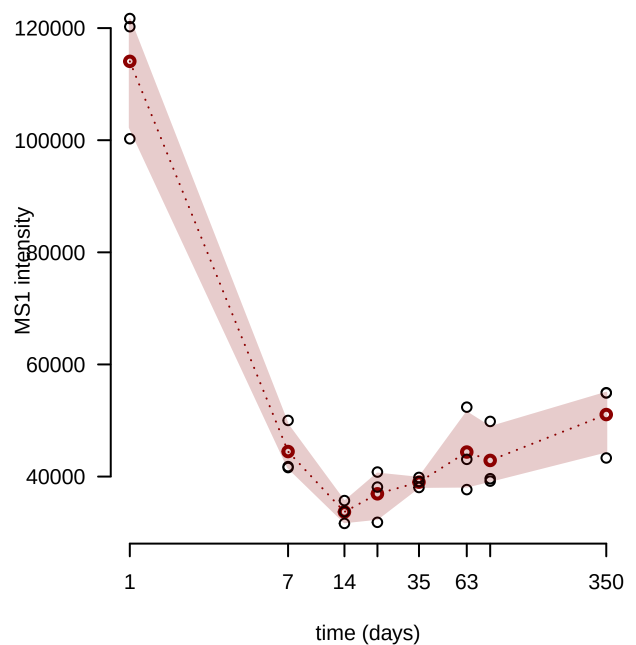

Q75N62
GN=Gimap8
GTPase, IMAP family member 8

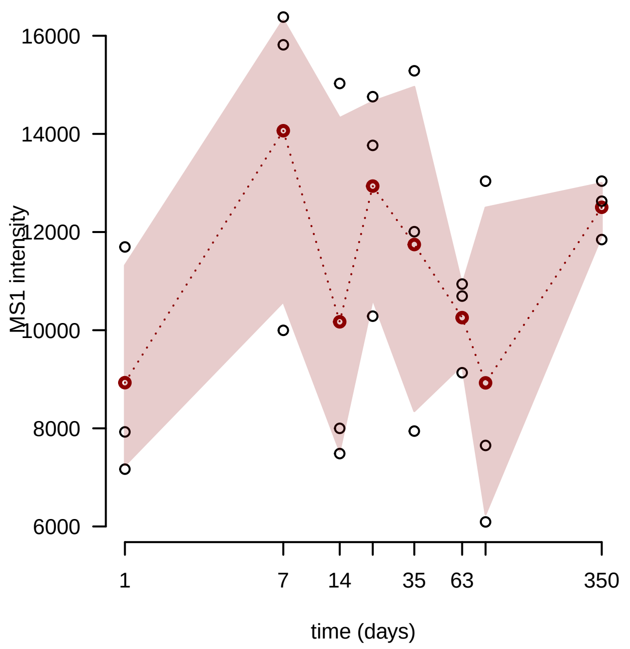

Q76I79
GN=Ssh1
slingshot protein phosphatase 1

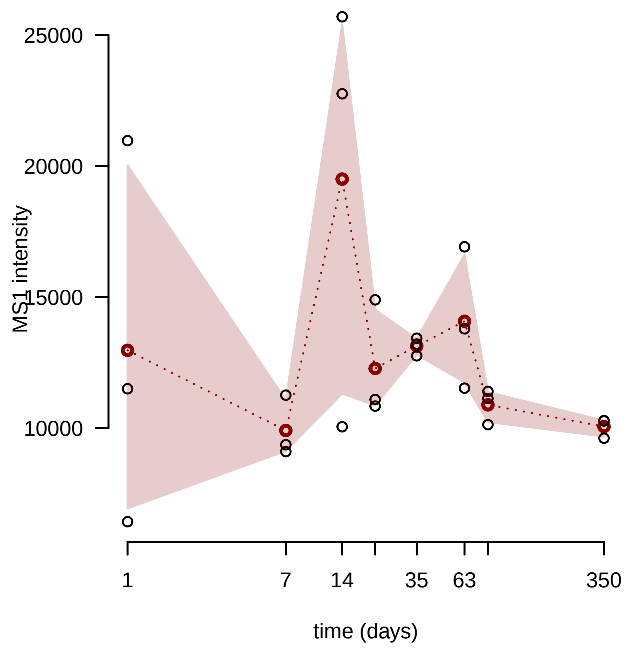

Q76KF0
GN=Sema6d
sema domain, transmembrane domain (TM), ...

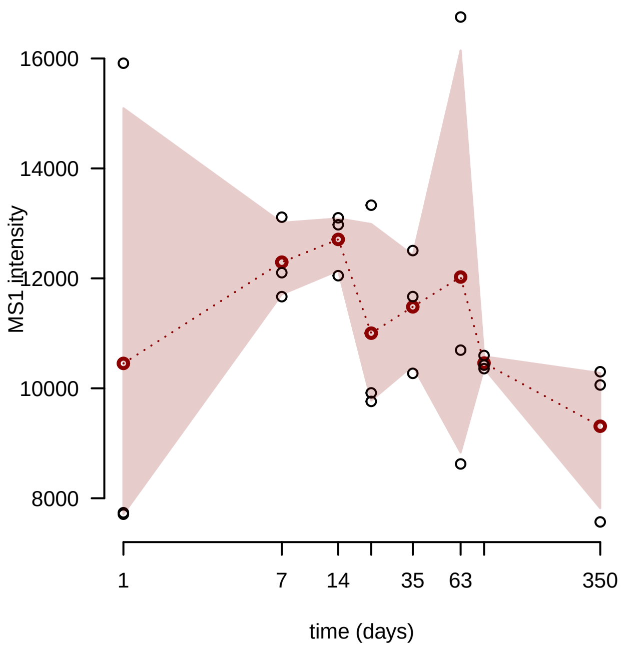

Q76KJ5
GN=Cd3eap
CD3E antigen, epsilon polypeptide associ ...

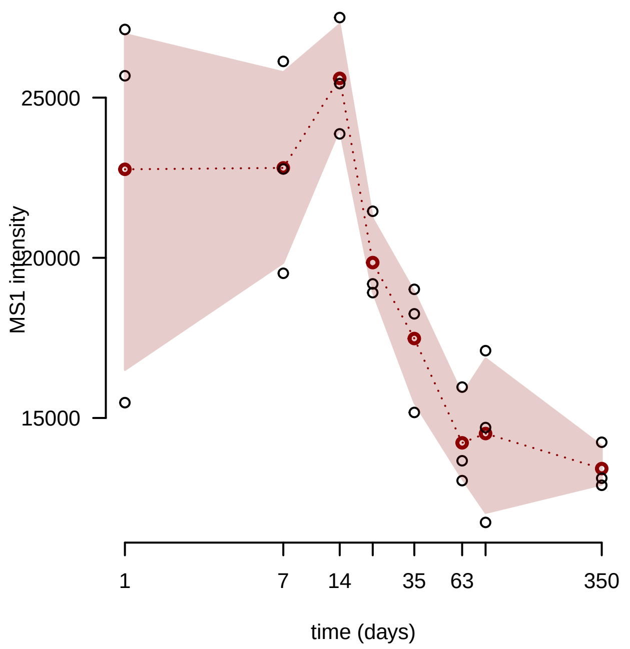

Q76LS9
GN=Mindy1
MINDY lysine 48 deubiquitinase 1

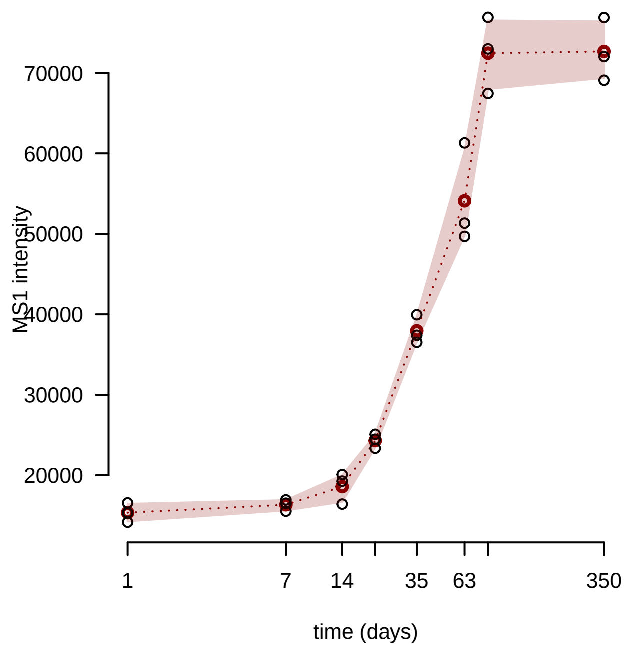

Q76MZ3
GN=Ppp2r1a
protein phosphatase 2, regulatory subuni ...

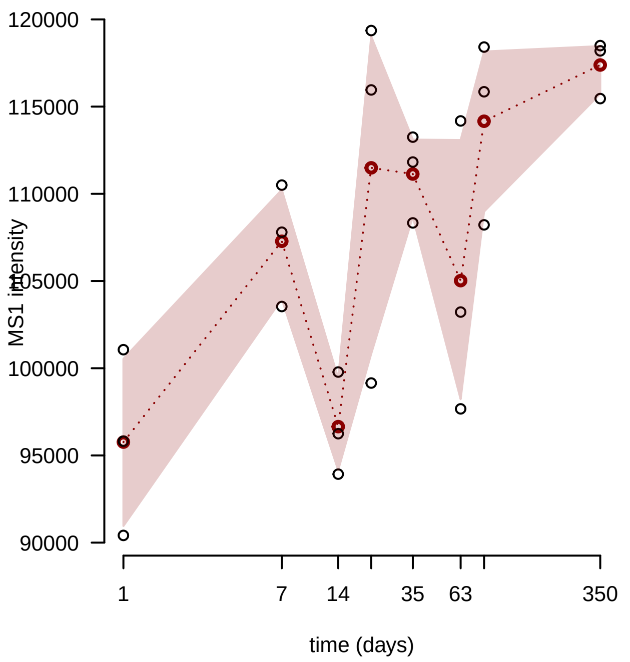

Q76N33
GN=Stambpl1
STAM binding protein like 1

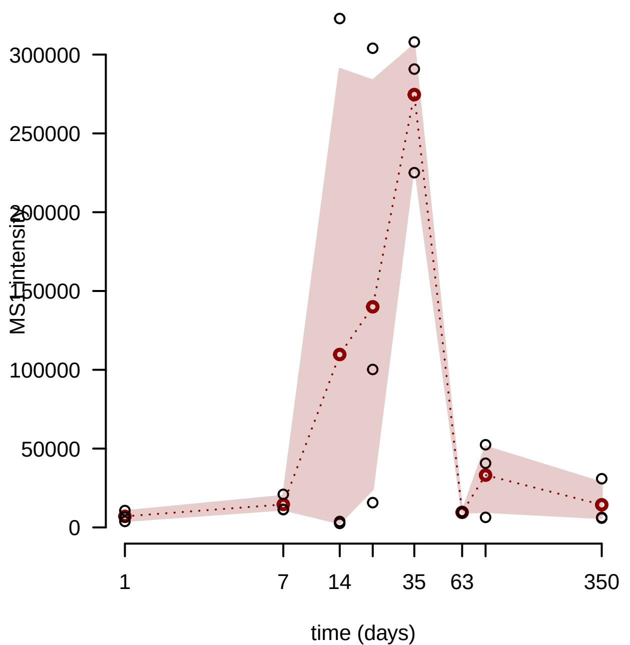

Q78HU3
GN=Mvb12a
multivesicular body subunit 12A

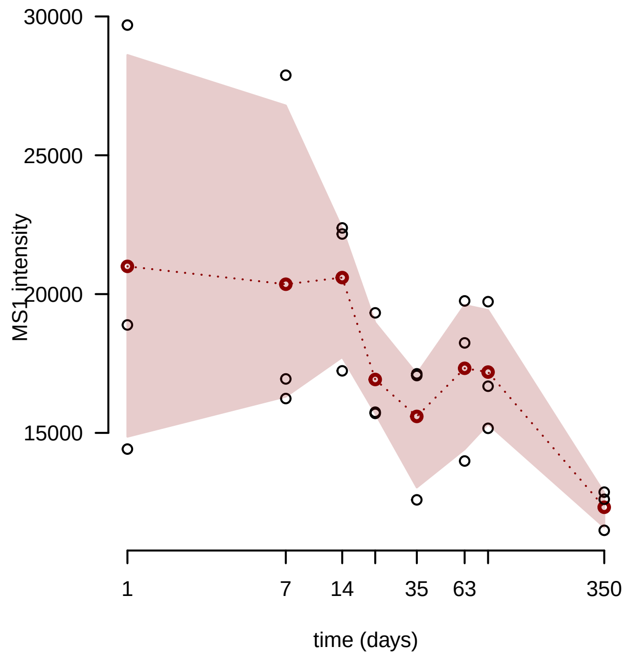

Q78HU7
GN=Gypc
glycophorin C

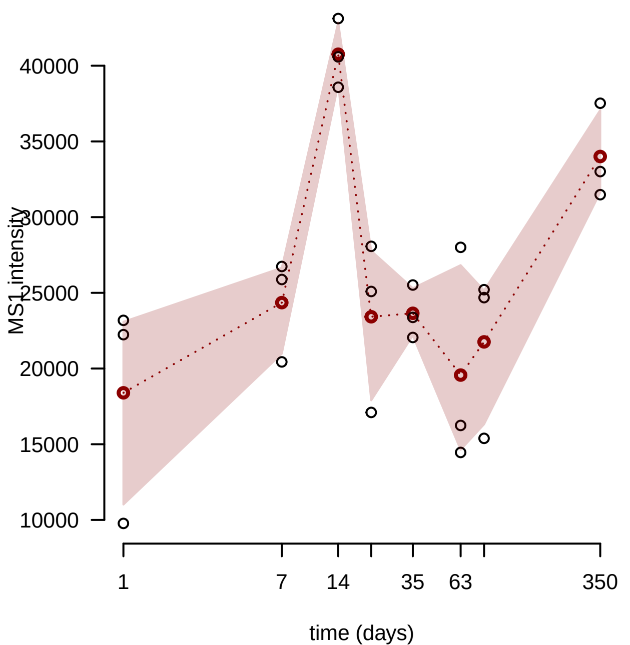

Q78IK2
GN=Atp5md
ATP synthase membrane subunit DAPIT

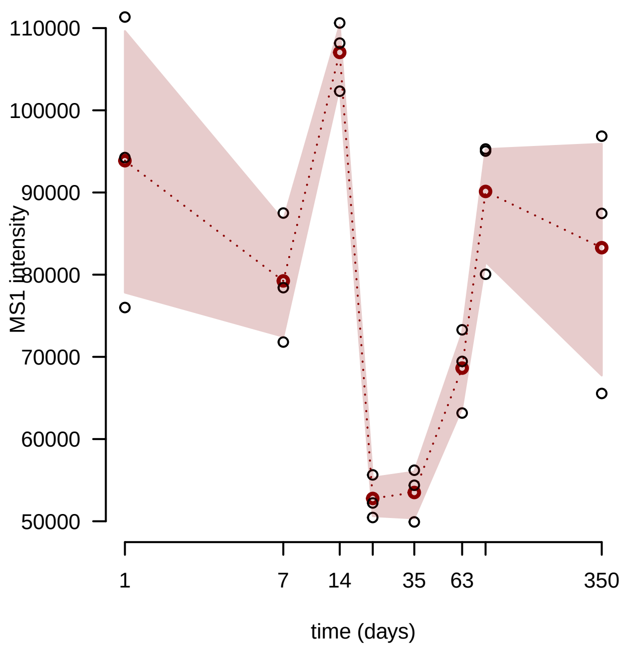

Q78IK4
GN=Apool
apolipoprotein O−like

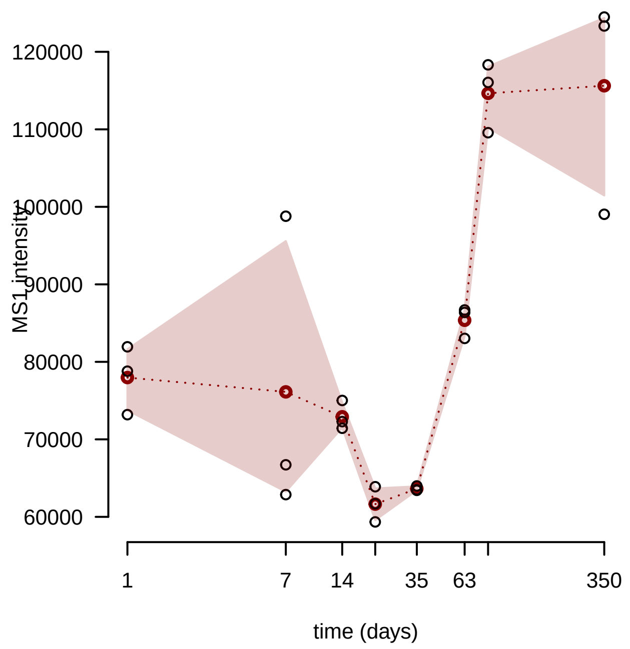

Q78IS1
GN=Tmed3
transmembrane p24 trafficking protein 3

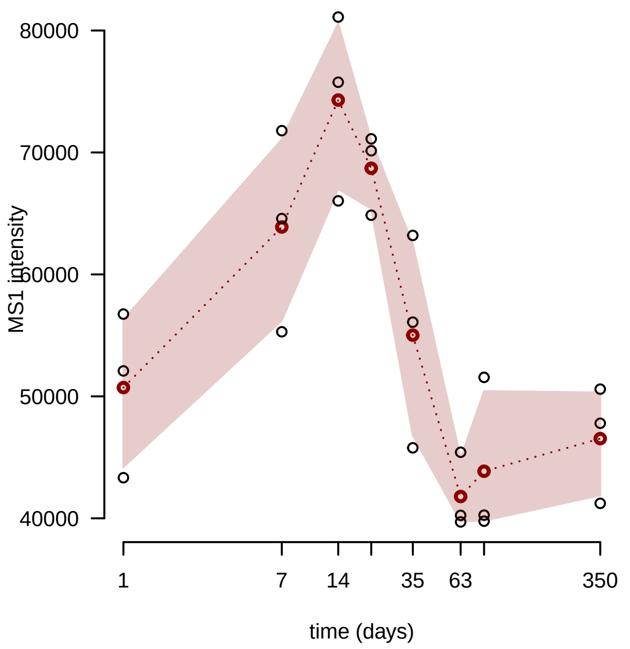

Q78J03
GN=Msrb2
methionine sulfoxide reductase B2

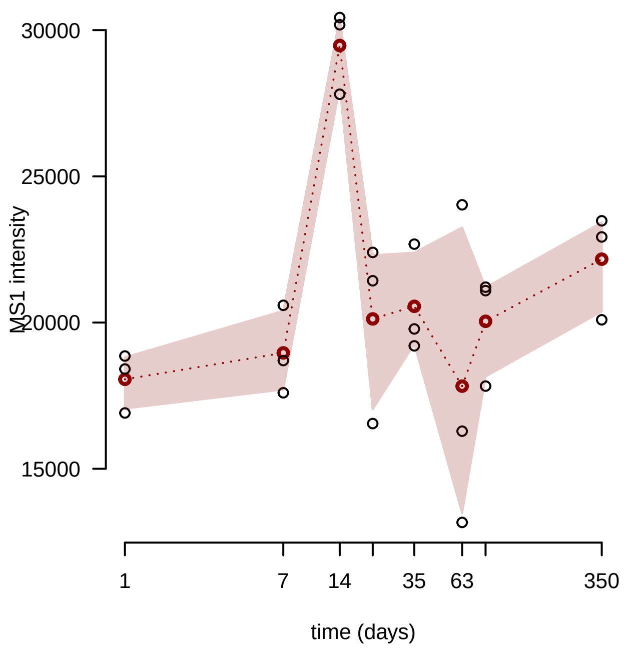

Q78JE5
GN=Fbxo22
F−box protein 22

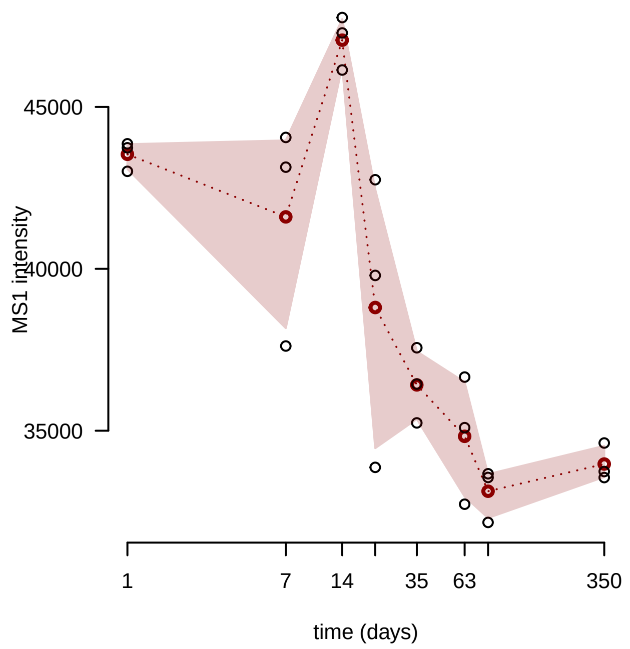

Q78JT3
GN=Haao
3−hydroxyanthranilate 3,4−dioxygenase

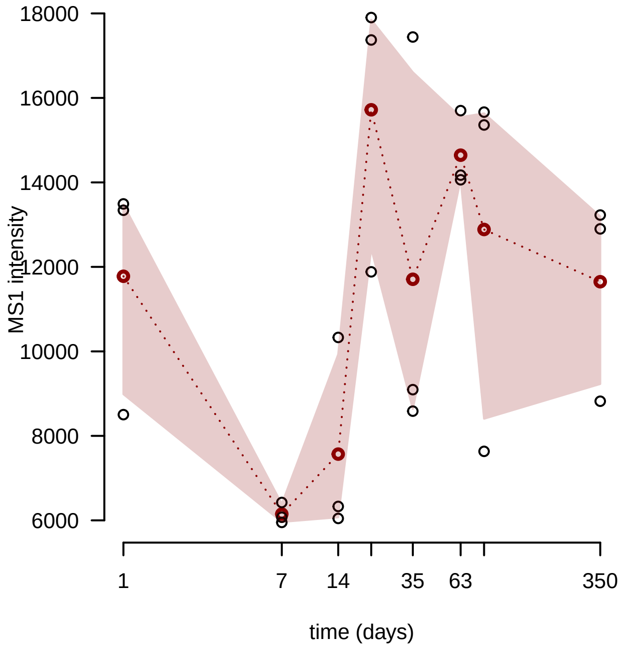

Q78JW9
GN=Ubfd1
ubiquitin family domain containing 1

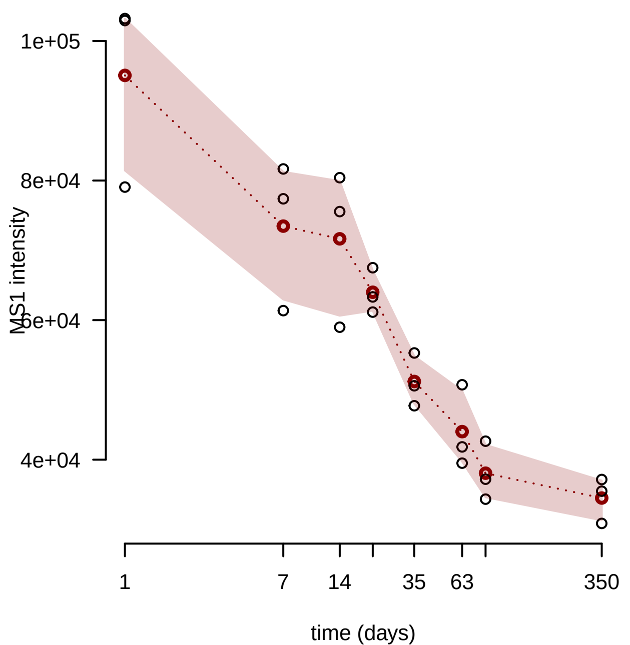

Q78PG9
GN=Ccdc25
coiled−coil domain containing 25

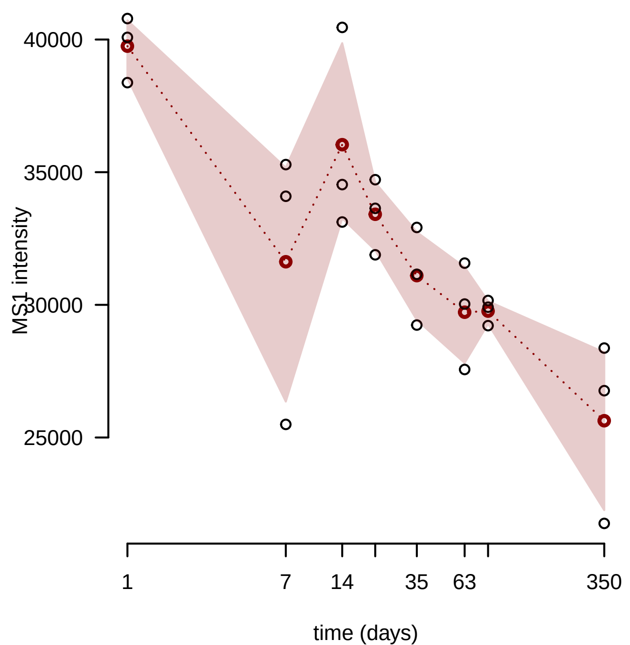

Q78PY7
GN=Snd1
staphylococcal nuclease and tudor domain ...

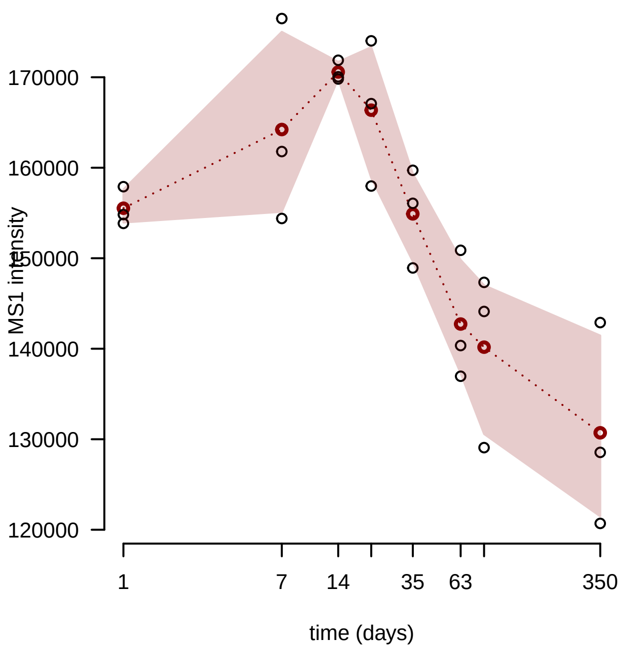

Q78RX3
GN=Smim12
small integral membrane protein 12

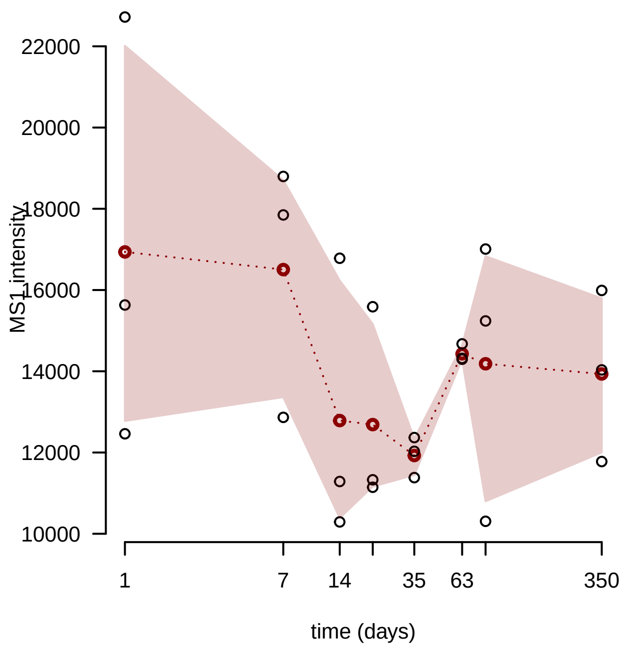

Q78T54
GN=Vma21
VMA21 vacuolar H+−ATPase homolog (S. cer ...

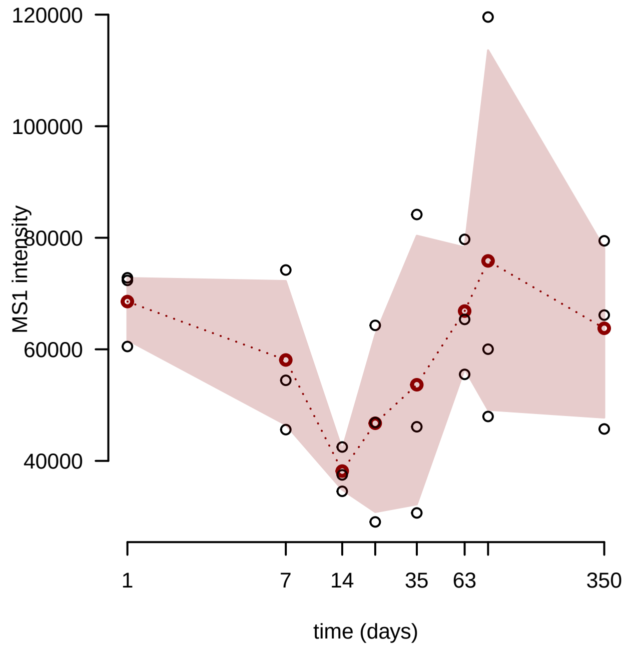

Q78WZ7
GN=Twistnb

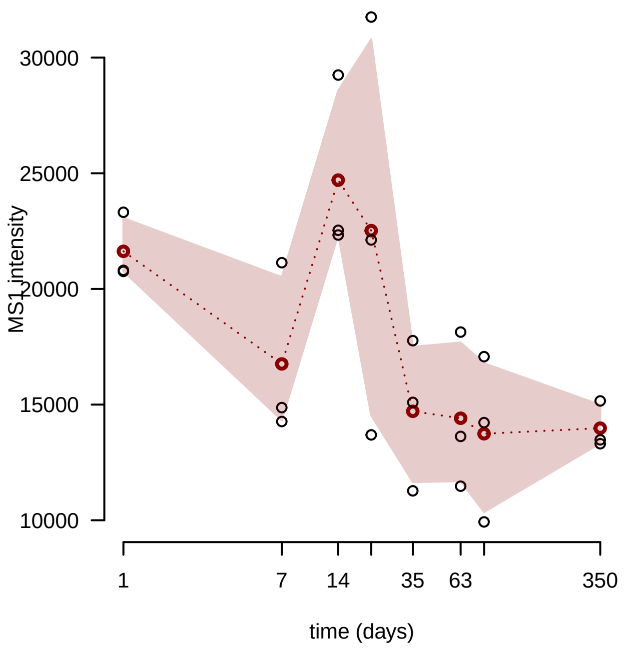

Q78XF5
GN=Ostc
oligosaccharyltransferase complex subuni ...

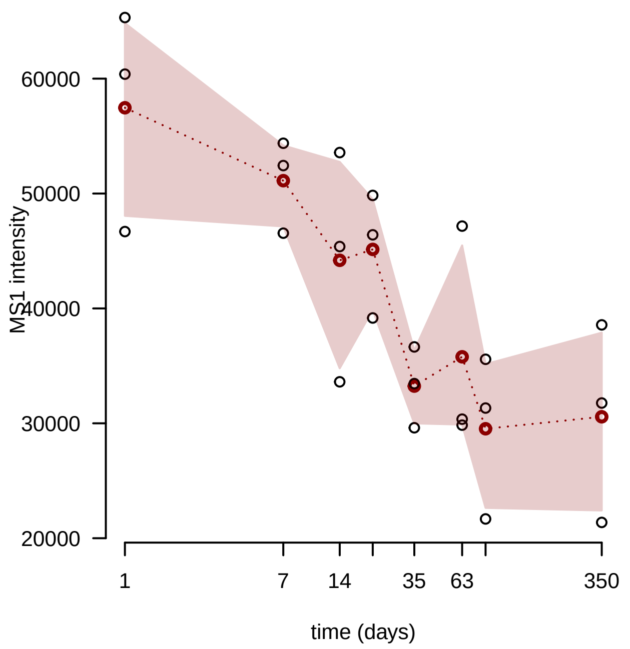

Q78YY6
GN=Dnajc15
DnaJ heat shock protein family (Hsp40) m ...

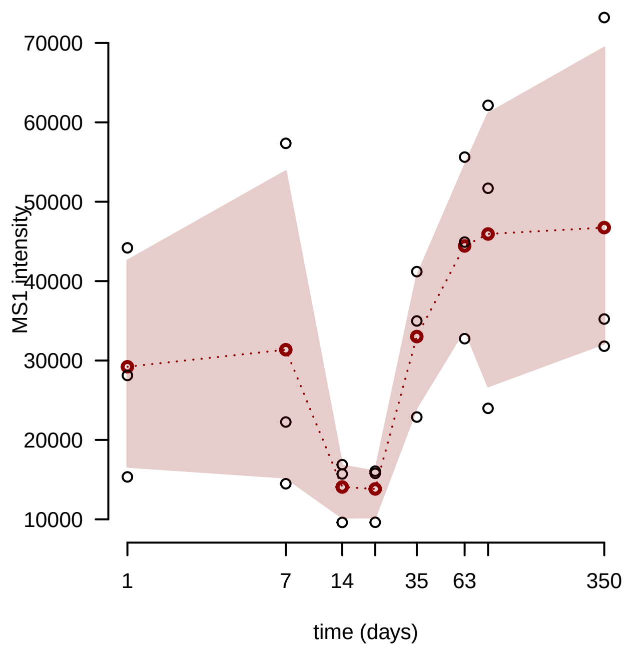

Q78YZ6
GN=Scoc
short coiled−coil protein

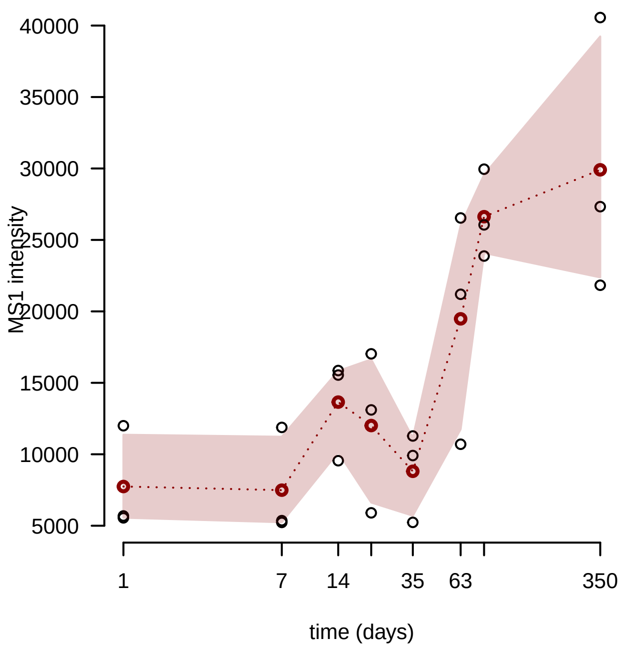

Q78ZA7
GN=Nap1l4
nucleosome assembly protein 1−like 4

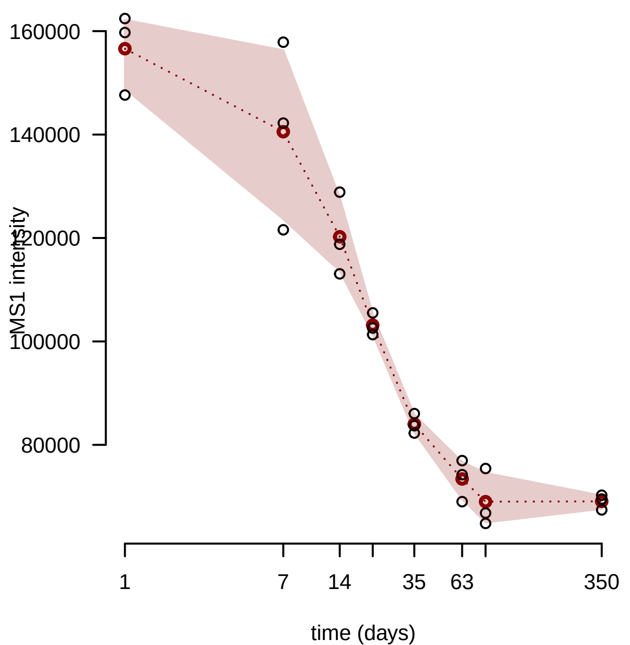

Q791T5
GN=Mtch1
mitochondrial carrier 1

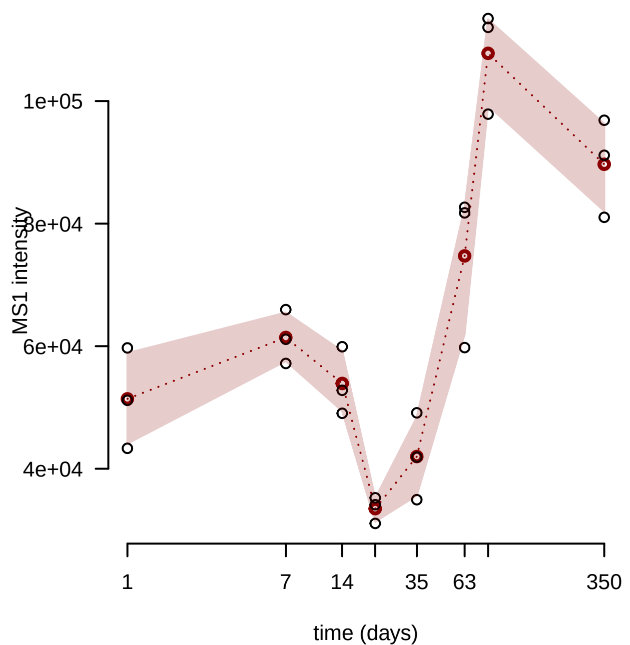

Q791V5
GN=Mtch2
mitochondrial carrier 2

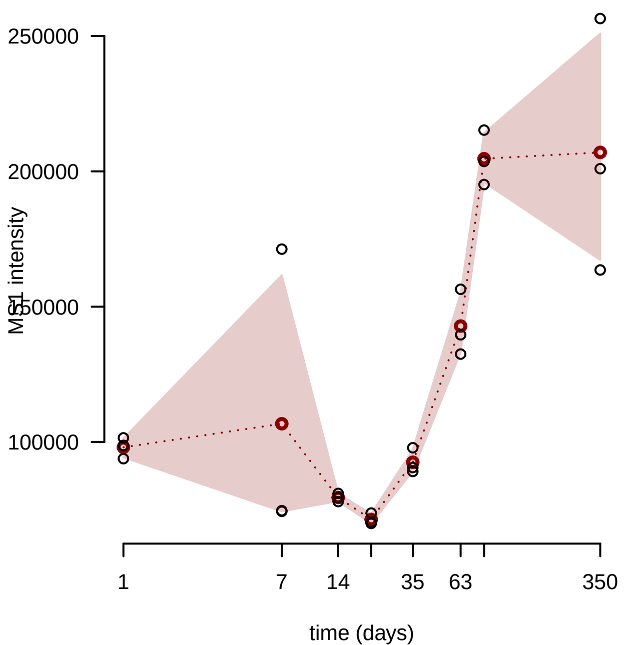

Q793I8
GN=Tifa
TRAF−interacting protein with forkhead−a ...

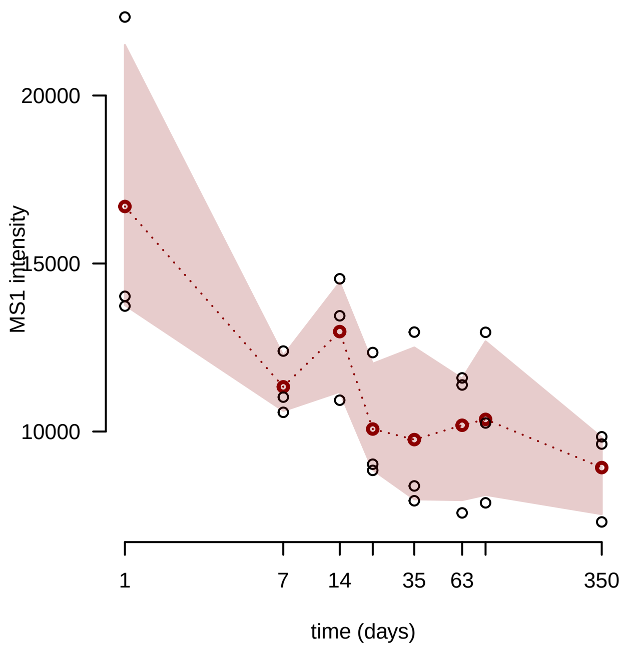

Q7JJ13
GN=Brd2
bromodomain containing 2

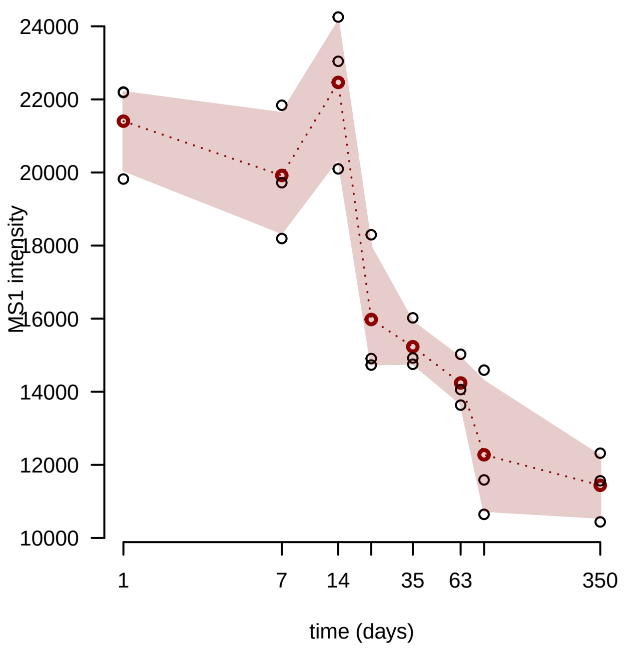

Q7M6U3
GN=Tex14
testis expressed gene 14

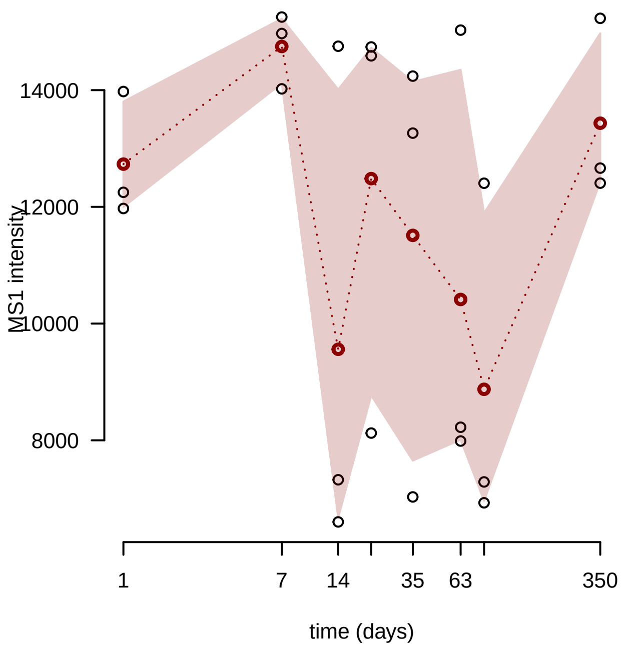

Q7M6Y3
GN=Picalm
phosphatidylinositol binding clathrin as ...

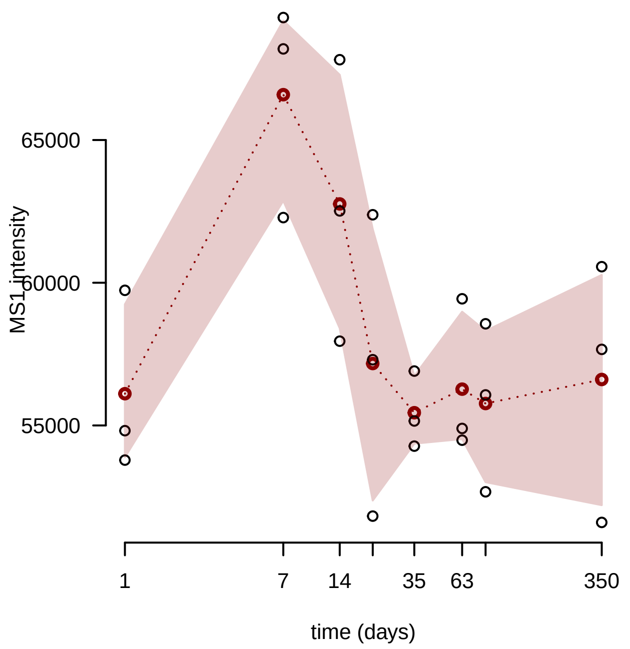

Q7M753
GN=

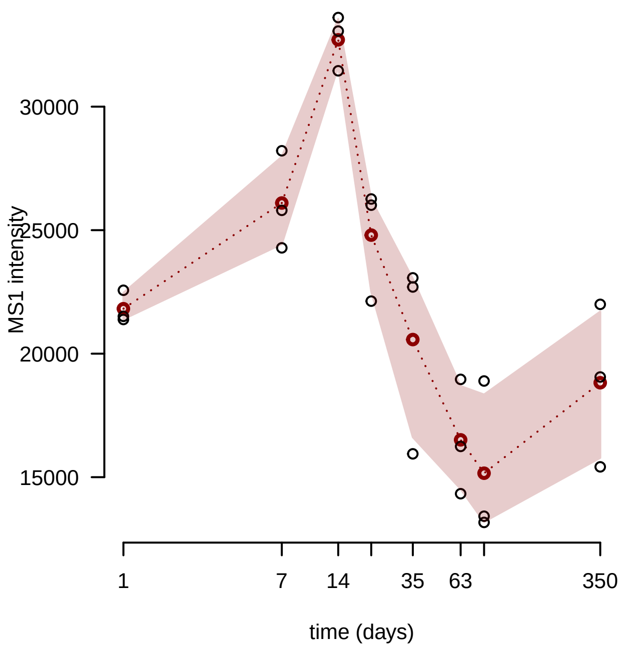

Q7SIG6
GN=Asap2
ArfGAP with SH3 domain, ankyrin repeat a ...

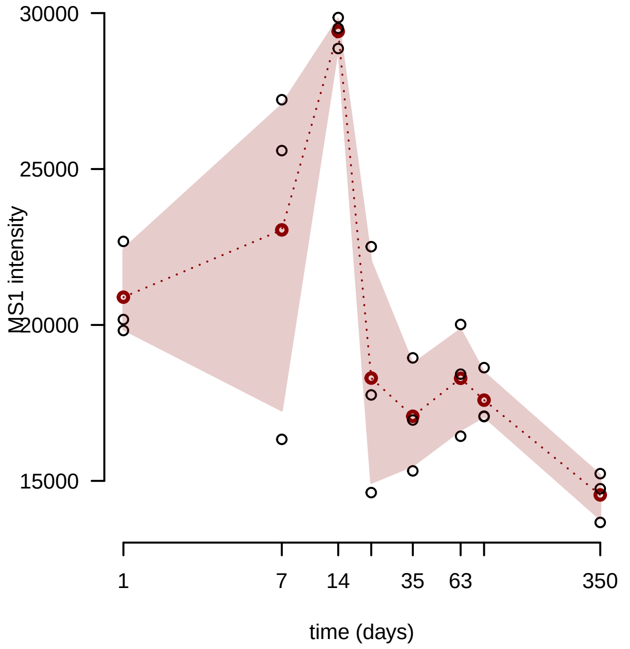

Q7TMA2
GN=Znf503

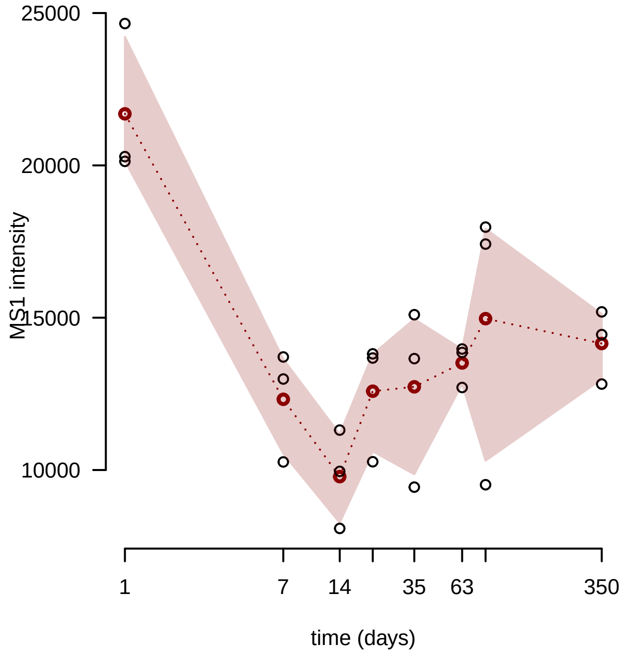

Q7TMB8
GN=Cyfip1
cytoplasmic FMR1 interacting protein 1

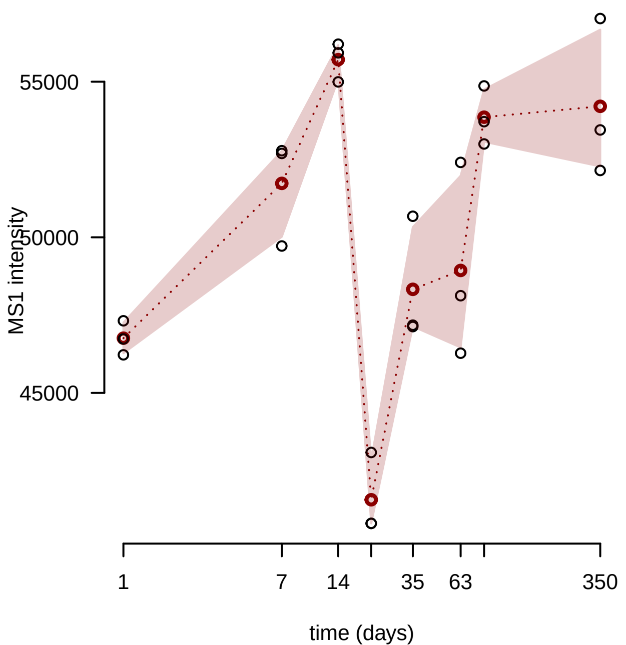

Q7TMC8
GN=Fcsk
fucose kinase

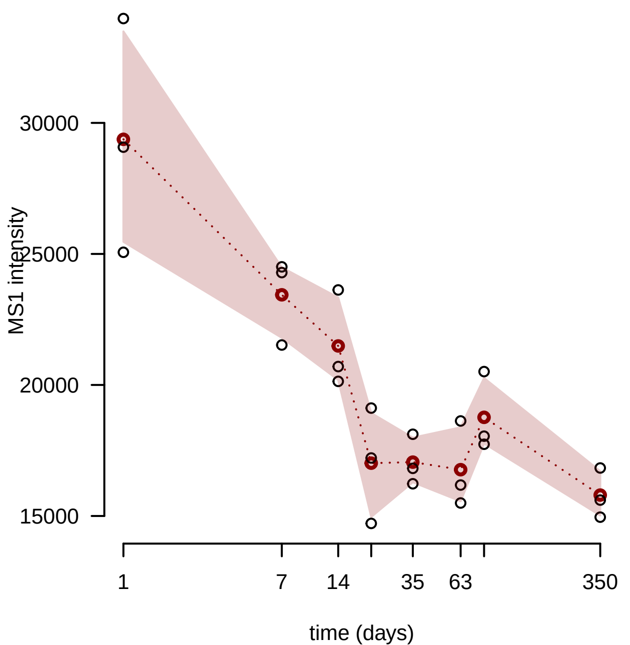

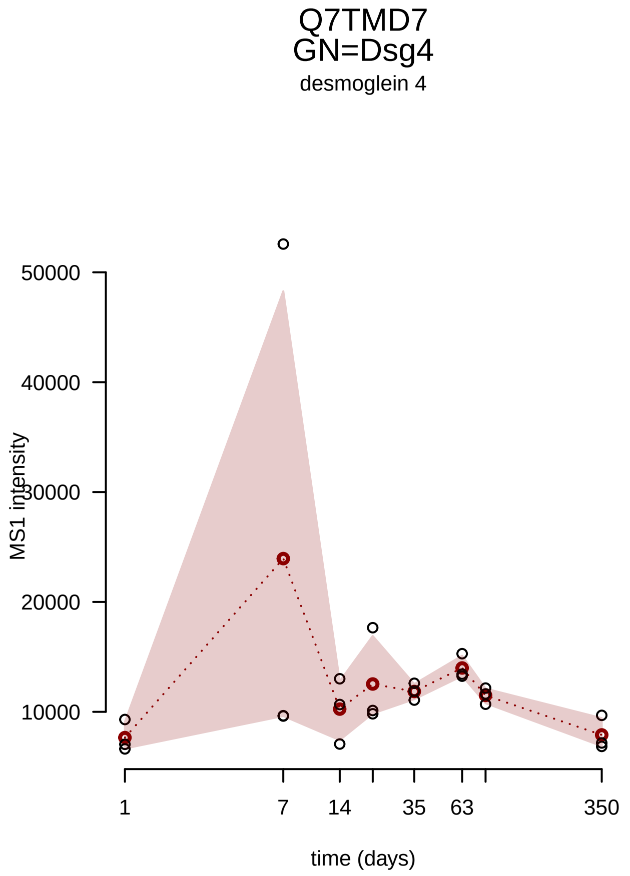
Q7TMD7
GN=Dsg4
desmoglein 4
MS1 intensity
10000
20000
30000
40000
50000
1
7
14
35
63
350
time (days)

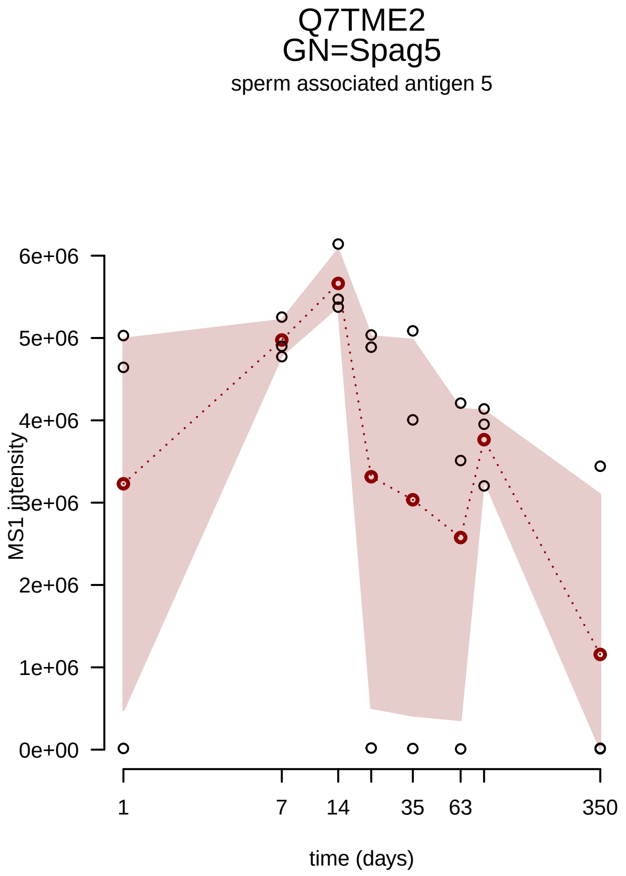
Q7TME2
GN=Spag5
sperm associated antigen 5
MS1 intensity
0e+00
1e+06
2e+06
3e+06
4e+06
5e+06
6e+06
1
7
14
35
63
350
time (days)

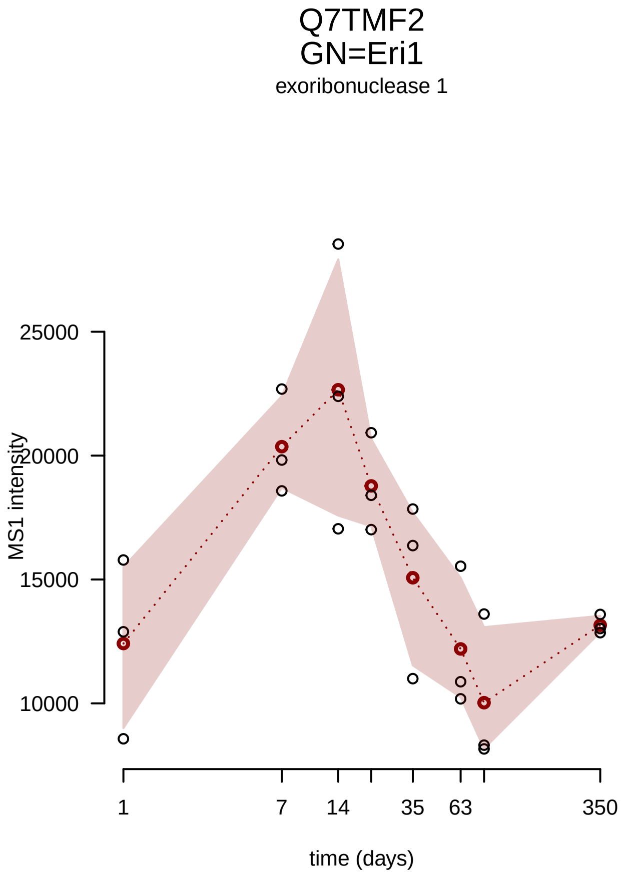
Q7TMF2
GN=Eri1
exoribonuclease 1
MS1 intensity
10000
15000
20000
25000
1
7
14
35
63
350
time (days)

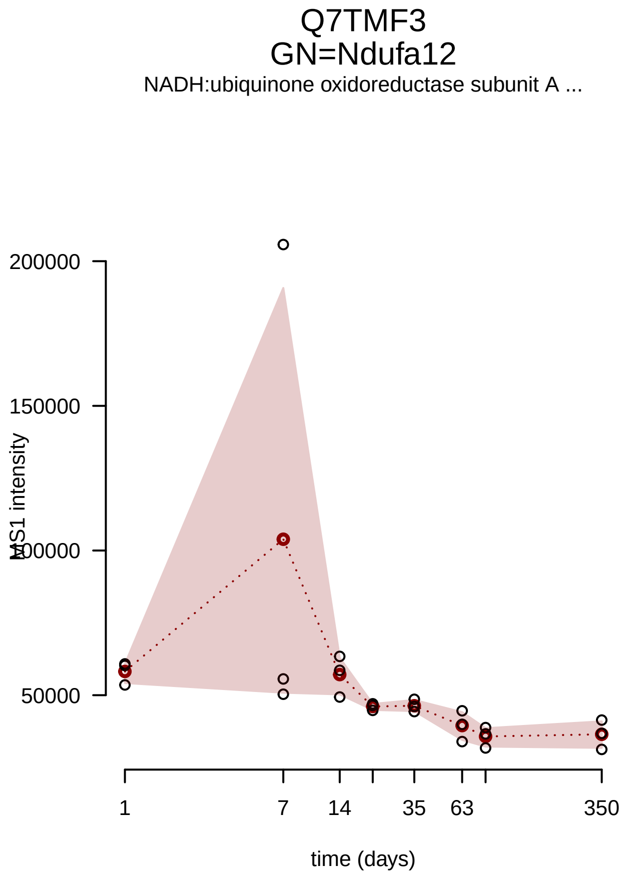
Q7TMF3
GN=Ndufa12
NADH:ubiquinone oxidoreductase subunit A ...
MS1 intensity
50000
100000
150000
200000
1
7
14
35
63
350
time (days)

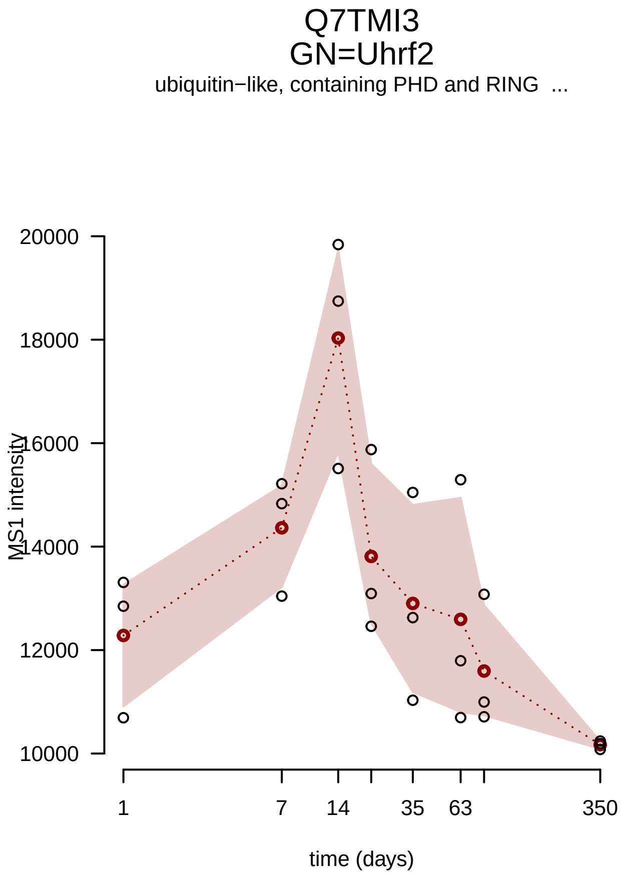
Q7TMI3
GN=Uhrf2
ubiquitin−like, containing PHD and RING ...
MS1 intensity
10000
12000
14000
16000
18000
20000
1
7
14
35
63
350
time (days)

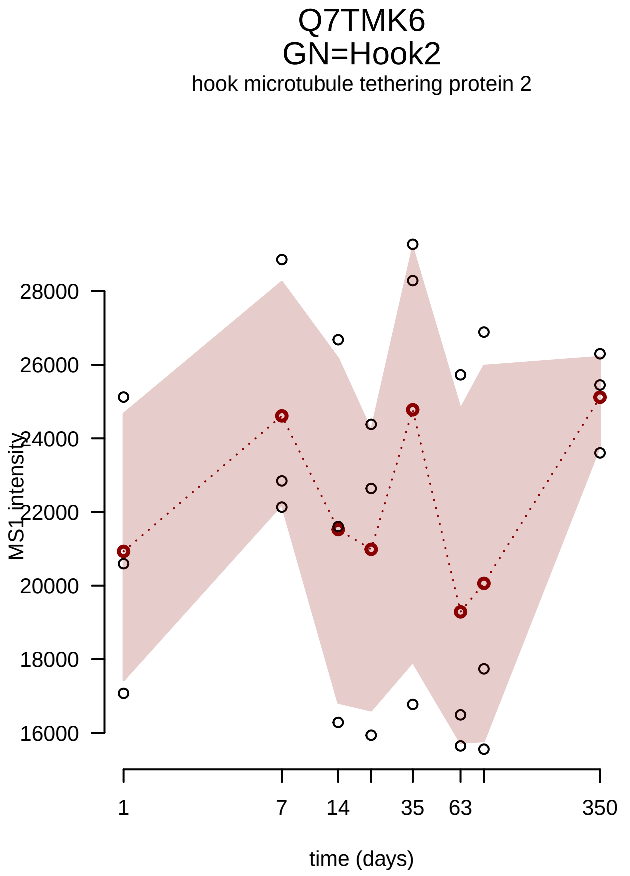
Q7TMK6
GN=Hook2
hook microtubule tethering protein 2
MS1 intensity
16000
18000
20000
22000
24000
26000
28000
1
7
14
35
63
350
time (days)

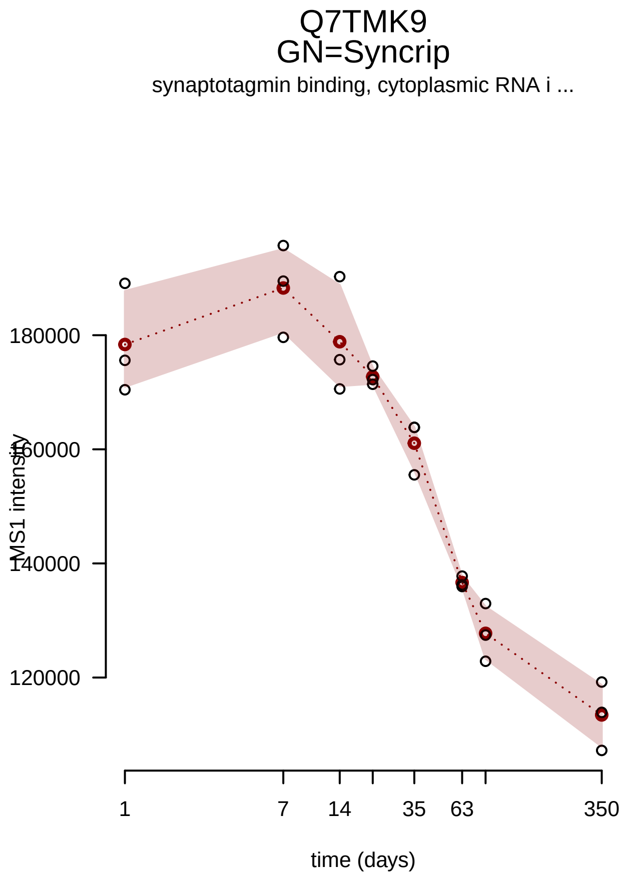
Q7TMK9
GN=Syncrip
synaptotagmin binding, cytoplasmic RNA i ...
MS1 intensity
120000
140000
160000
180000
1
7
14
35
63
350
time (days)

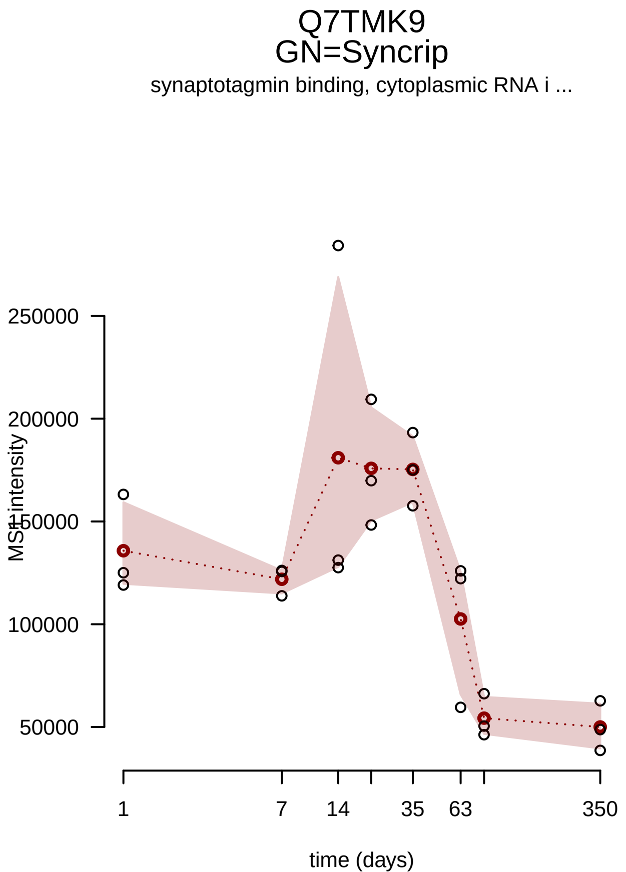
Q7TMK9
GN=Syncrip
synaptotagmin binding, cytoplasmic RNA i ...
MS1 intensity
50000
100000
150000
200000
250000
1
7
14
35
63
350
time (days)

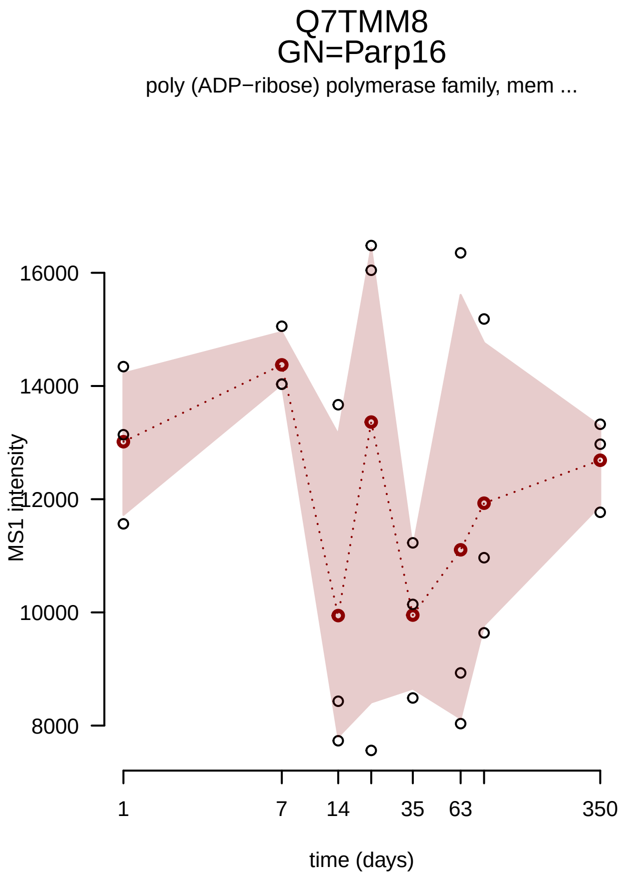
Q7TMM8
GN=Parp16
poly (ADP−ribose) polymerase family, mem ...
MS1 intensity
8000
10000
12000
14000
16000
1
7
14
35
63
350
time (days)

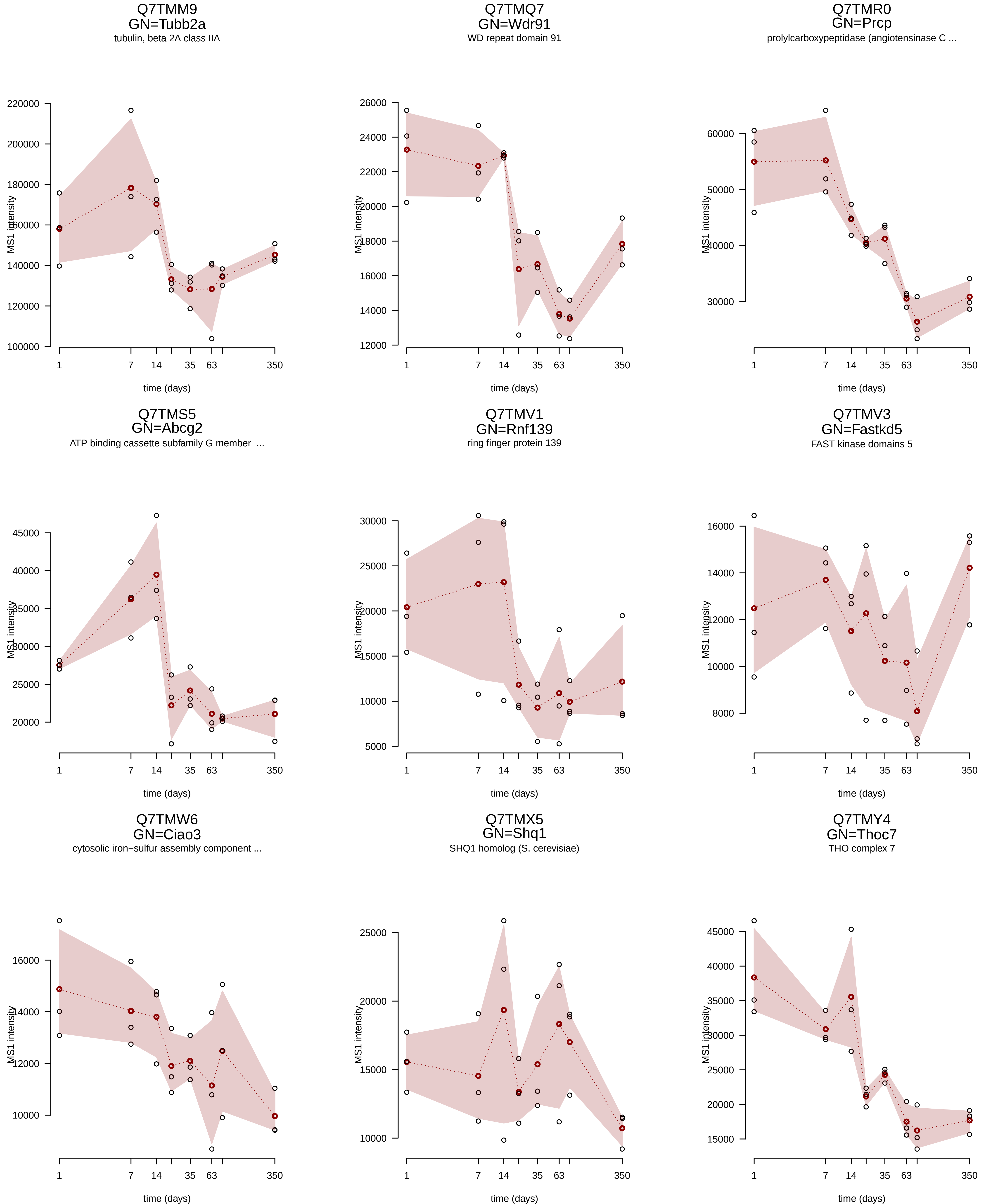

Q7TMM9
GN=Tubb2a
tubulin, beta 2A class IIA
MS1 intensity
220000
200000
180000
160000
140000
120000
100000
1
7
14
35
63
350
time (days)
Q7TMQ7
GN=Wdr91
WD repeat domain 91
MS1 intensity
26000
24000
22000
20000
18000
16000
14000
12000
time (days)
Q7TMR0
GN=Prcp
prolylcarboxypeptidase (angiotensinase C ...
MS1 intensity
60000
50000
40000
30000
time (days)
Q7TMS5
GN=Abcg2
ATP binding cassette subfamily G member ...
MS1 intensity
45000
40000
35000
30000
25000
20000
time (days)
Q7TMV1
GN=Rnf139
ring finger protein 139
MS1 intensity
30000
25000
20000
15000
10000
5000
time (days)
Q7TMV3
GN=Fastkd5
FAST kinase domains 5
MS1 intensity
16000
14000
12000
10000
8000
time (days)
Q7TMW6
GN=Ciao3
cytosolic iron−sulfur assembly component ...
MS1 intensity
16000
14000
12000
10000
time (days)
Q7TMX5
GN=Shq1
SHQ1 homolog (S. cerevisiae)
MS1 intensity
25000
20000
15000
10000
time (days)
Q7TMY4
GN=Thoc7
THO complex 7
MS1 intensity
45000
40000
35000
30000
25000
20000
15000
time (days)

Q7TMY7
GN=Ipo8
importin 8

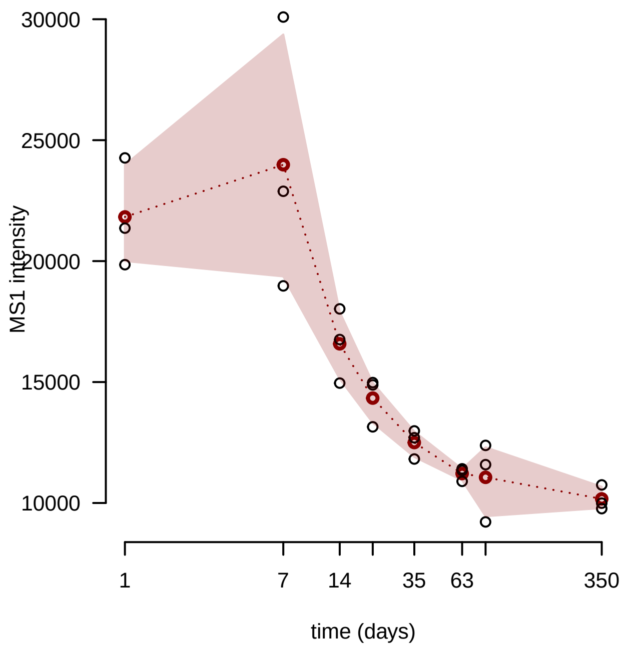

Q7TMY8
GN=Huwe1
HECT, UBA and WWE domain containing 1

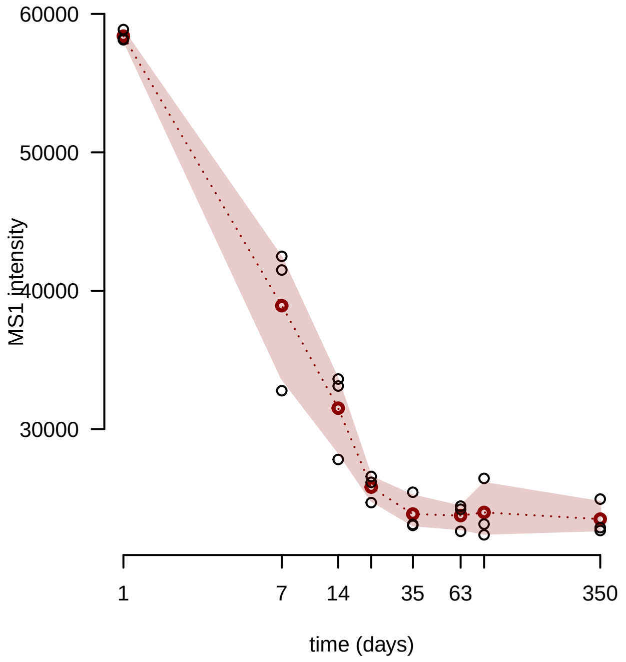

Q7TN02
GN=Med26
mediator complex subunit 26

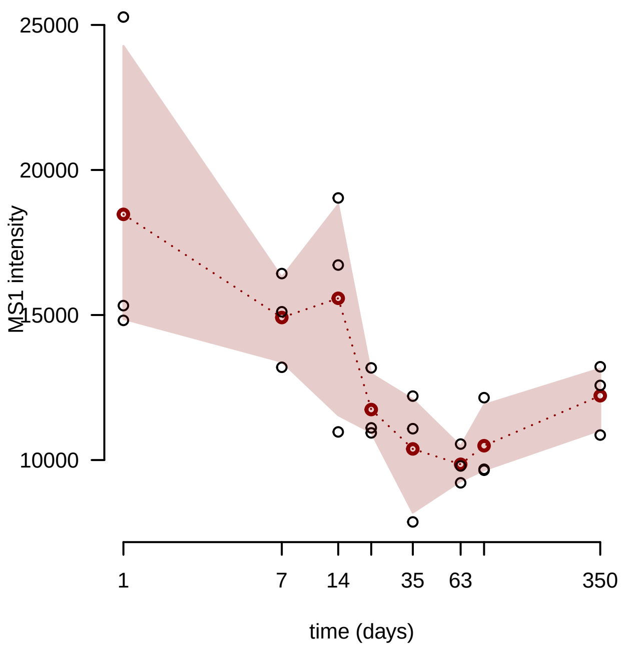

Q7TN22
GN=Txndc16
thioredoxin domain containing 16

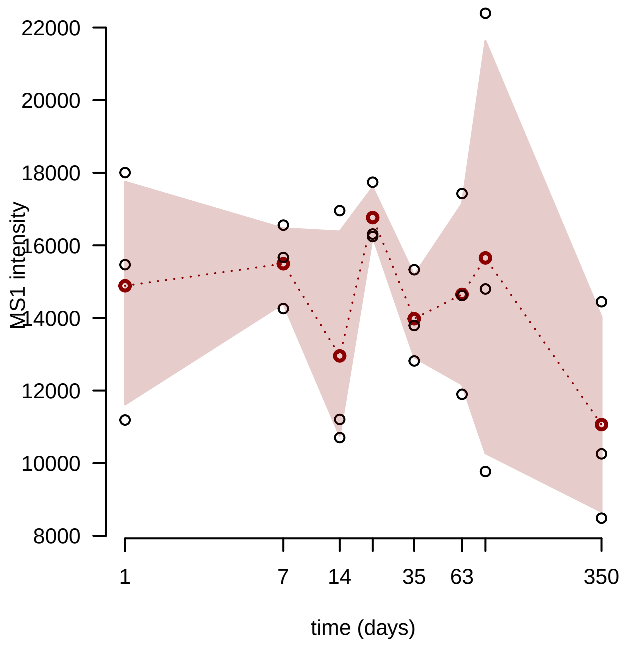

Q7TN29
GN=Smap2
small ArfGAP 2

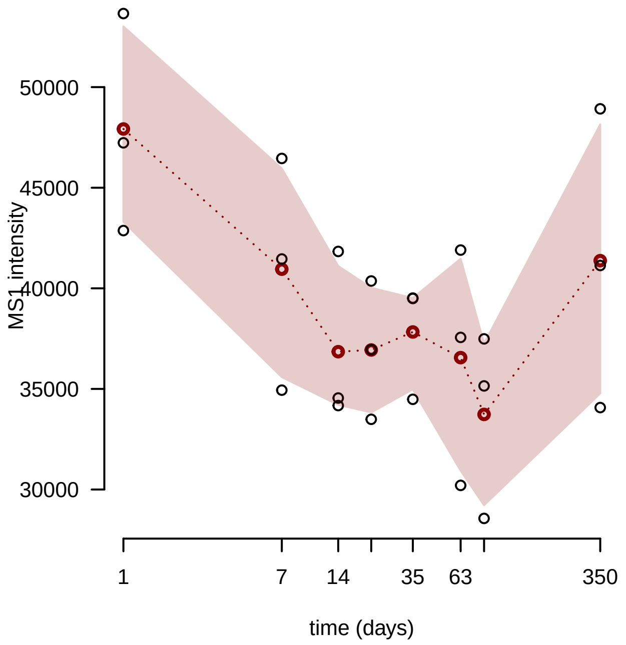

Q7TN31
GN=Aggf1
angiogenic factor with G patch and FHA d ...

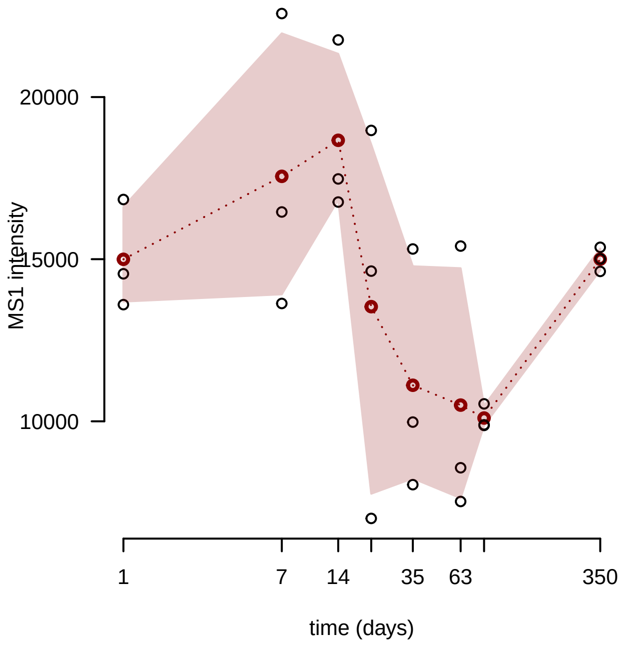

Q7TN37
GN=Trpm4
transient receptor potential cation chan ...

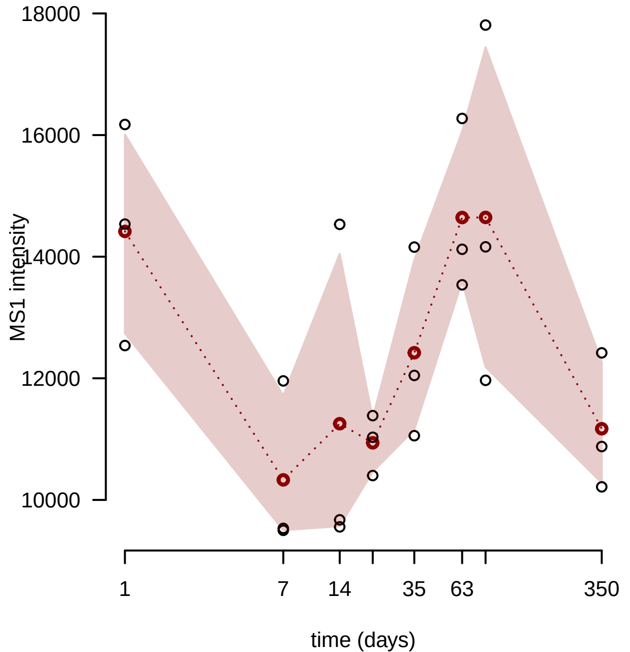

Q7TN73
GN=Casd1
CAS1 domain containing 1

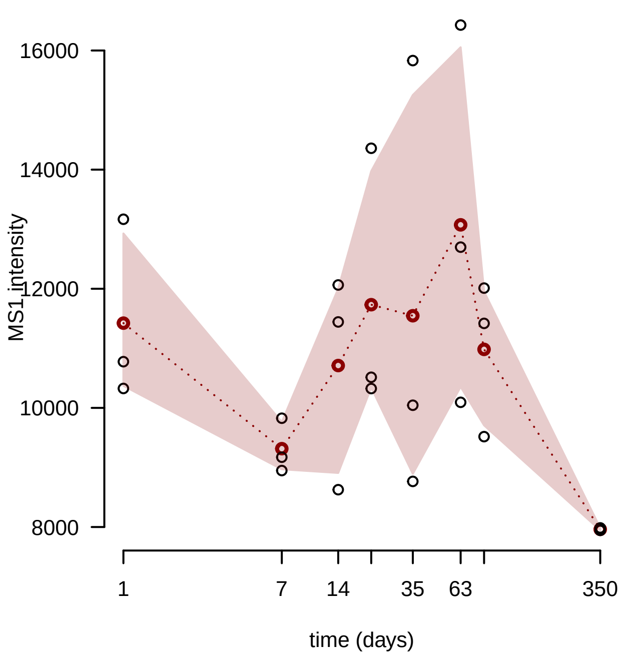

Q7TN75
GN=Peg10
paternally expressed 10

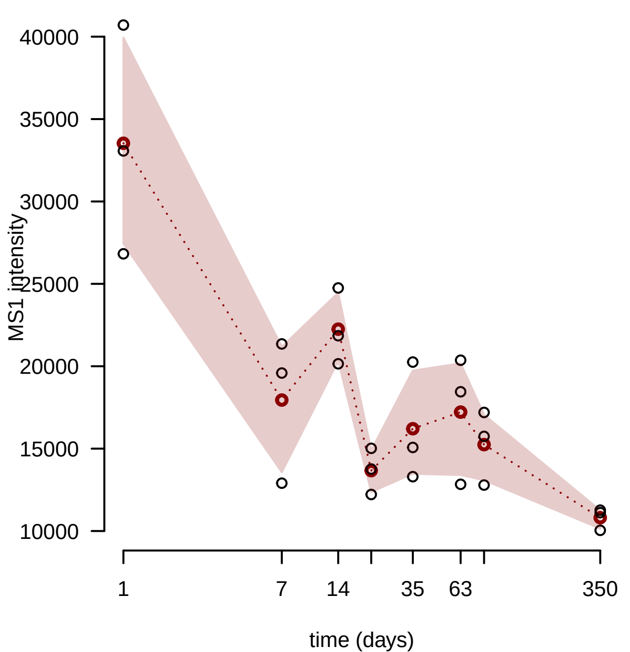

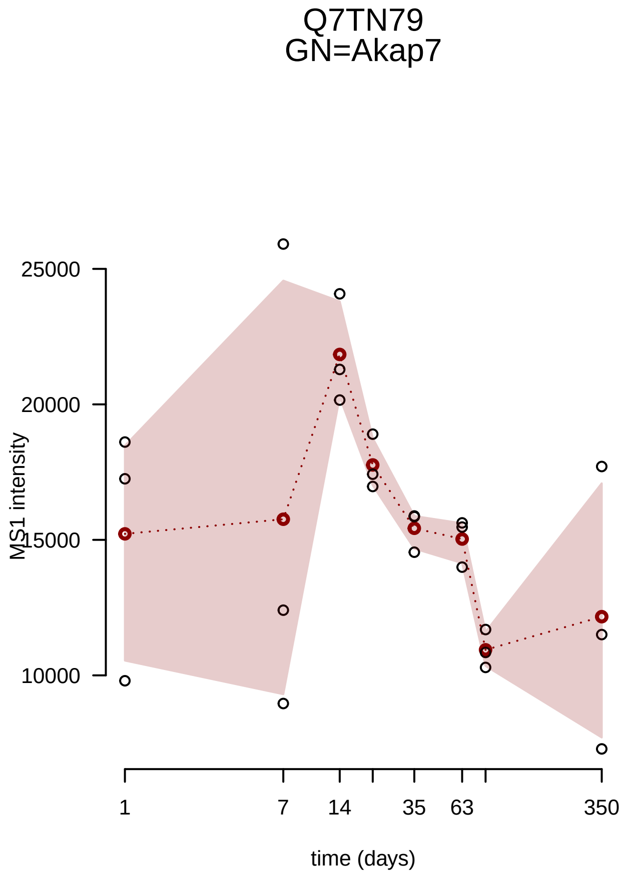
Q7TN79
GN=Akap7
25000
20000
15000
10000
MS1 intensity
1
7
14
35
63
350
time (days)

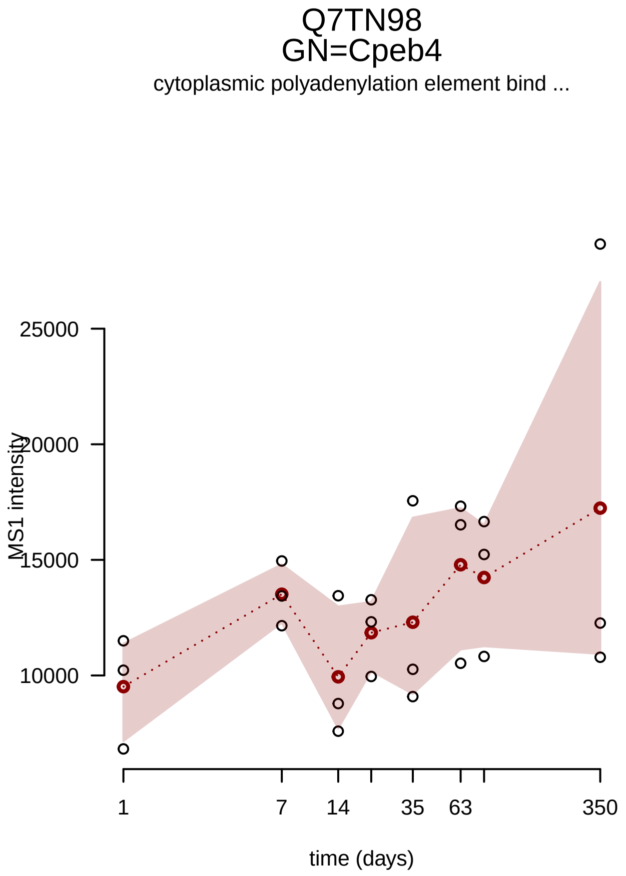
Q7TN98
GN=Cpeb4
cytoplasmic polyadenylation element bind ...
25000
20000
15000
10000
MS1 intensity
1
7
14
35
63
350
time (days)

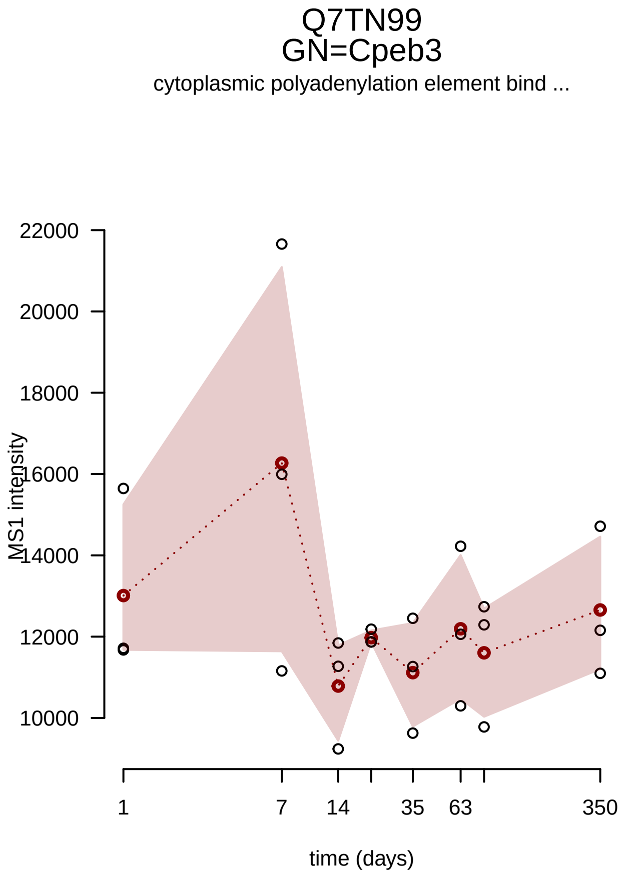
Q7TN99
GN=Cpeb3
cytoplasmic polyadenylation element bind ...
22000
20000
18000
16000
14000
12000
10000
MS1 intensity
1
7
14
35
63
350
time (days)

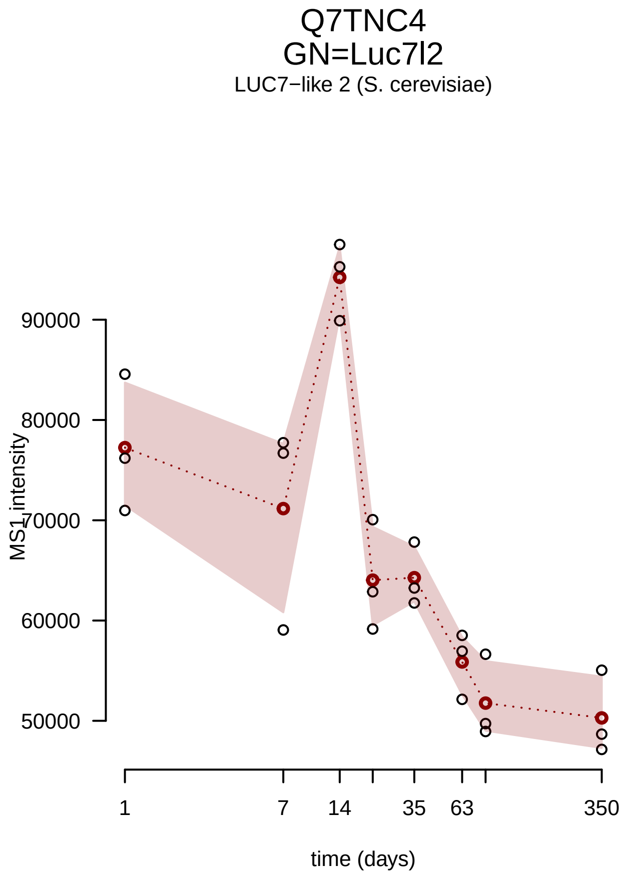
Q7TNC4
GN=Luc7l2
LUC7−like 2 (S. cerevisiae)
90000
80000
70000
60000
50000
MS1 intensity
1
7
14
35
63
350
time (days)

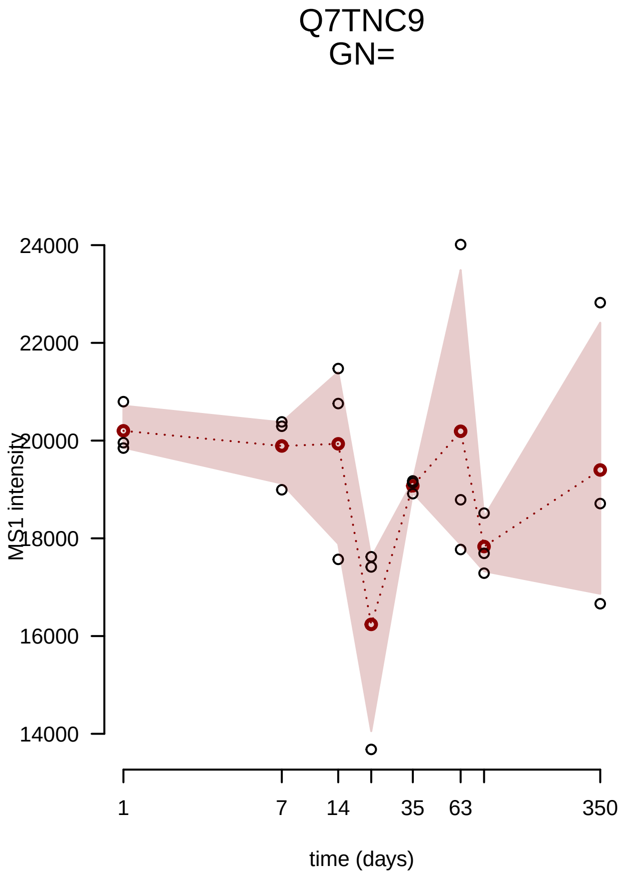
Q7TNC9
GN=
24000
22000
20000
18000
16000
14000
MS1 intensity
1
7
14
35
63
350
time (days)

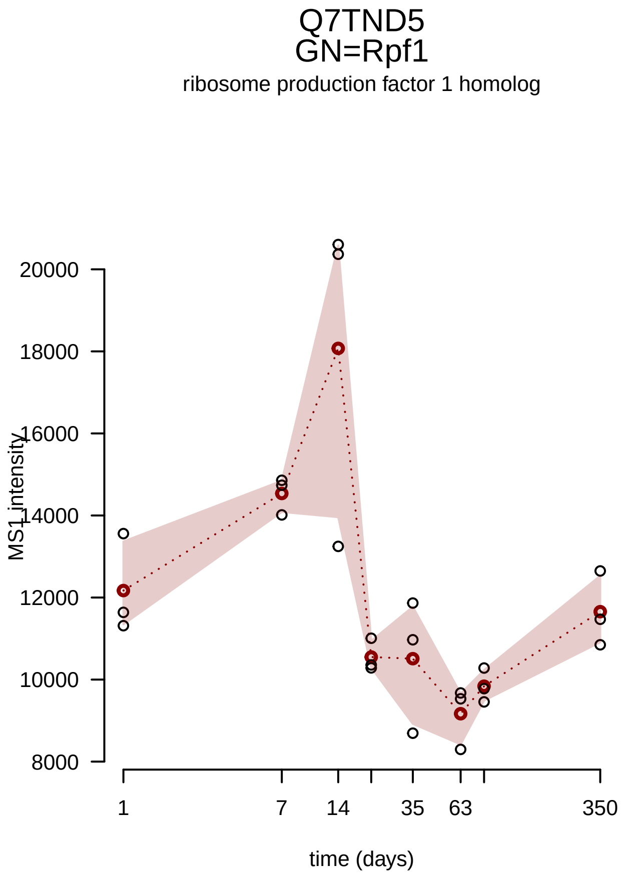
Q7TND5
GN=Rpf1
ribosome production factor 1 homolog
20000
18000
16000
14000
12000
10000
8000
MS1 intensity
1
7
14
35
63
350
time (days)

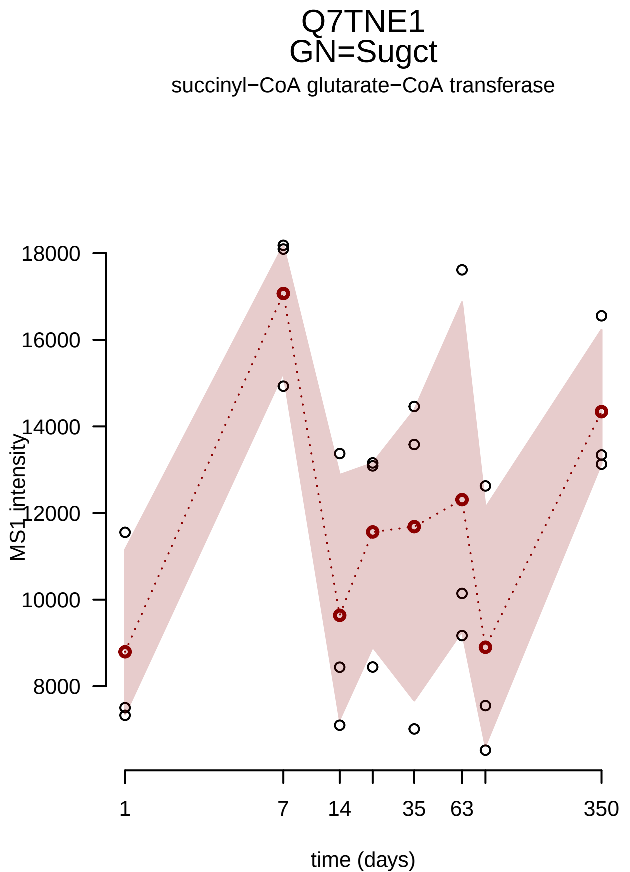
Q7TNE1
GN=Sugct
succinyl−CoA glutarate−CoA transferase
18000
16000
14000
12000
10000
8000
MS1 intensity
1
7
14
35
63
350
time (days)

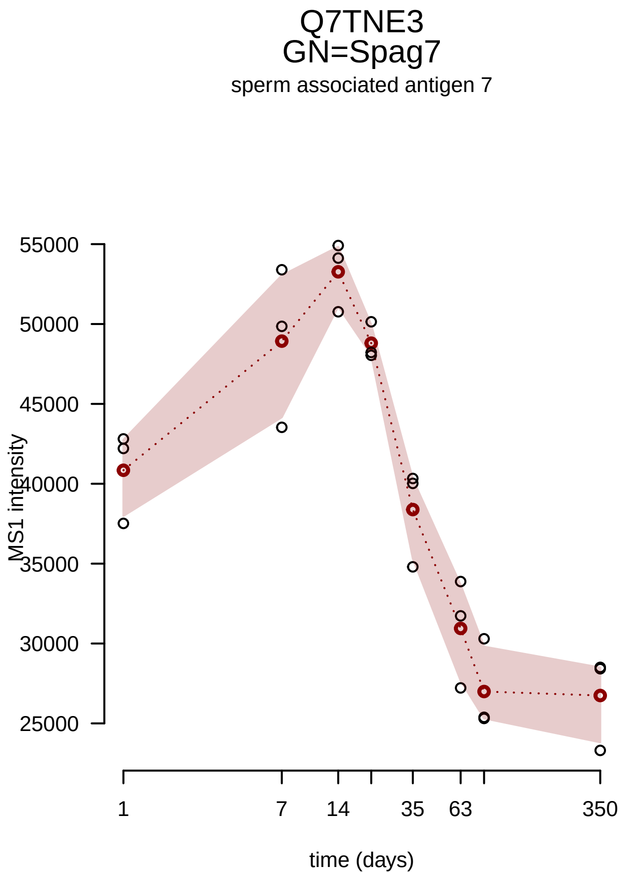
Q7TNE3
GN=Spag7
sperm associated antigen 7
55000
50000
45000
40000
35000
30000
25000
MS1 intensity
1
7
14
35
63
350
time (days)

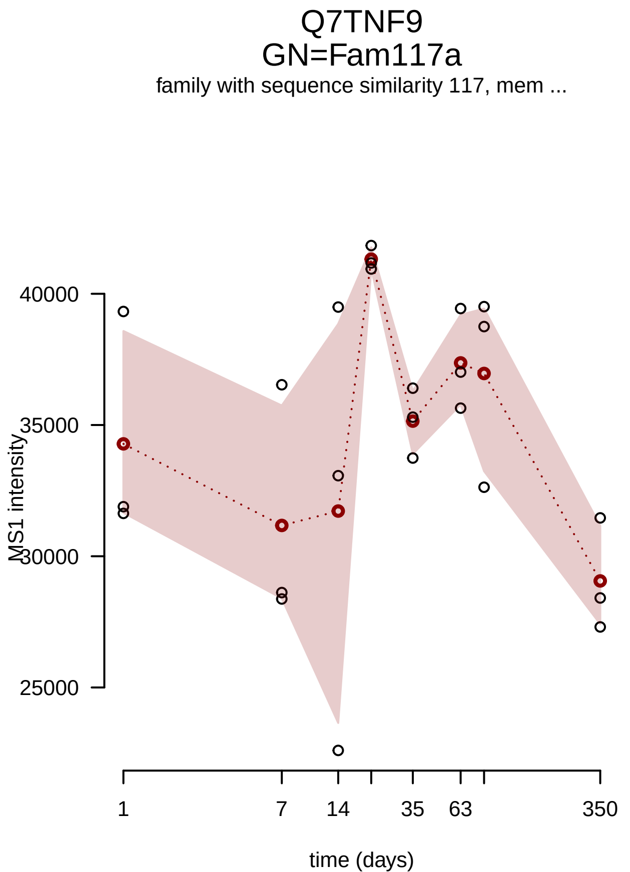
Q7TNF9
GN=Fam117a
family with sequence similarity 117, mem ...
40000
35000
30000
25000
MS1 intensity
1
7
14
35
63
350
time (days)

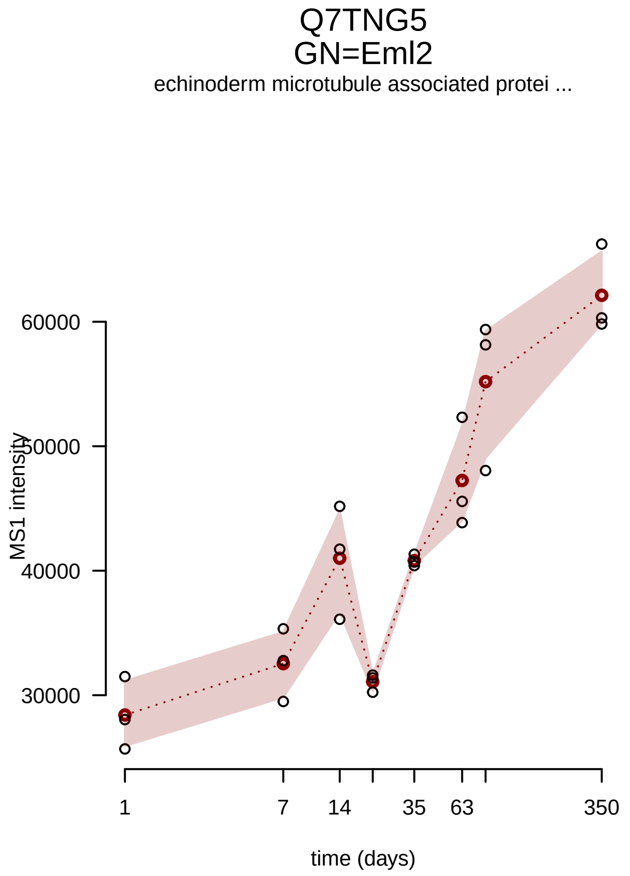
Q7TNG5
GN=Eml2
echinoderm microtubule associated protei ...
MS1 intensity
60000
50000
40000
30000
1
7
14
35
63
350
time (days)

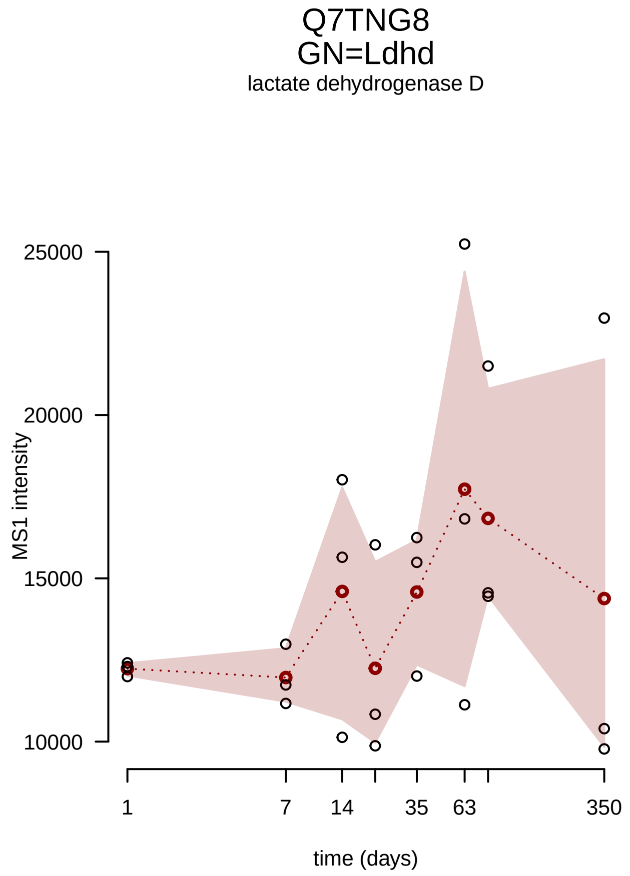
Q7TNG8
GN=Ldhd
lactate dehydrogenase D
MS1 intensity
25000
20000
15000
10000
1
7
14
35
63
350
time (days)

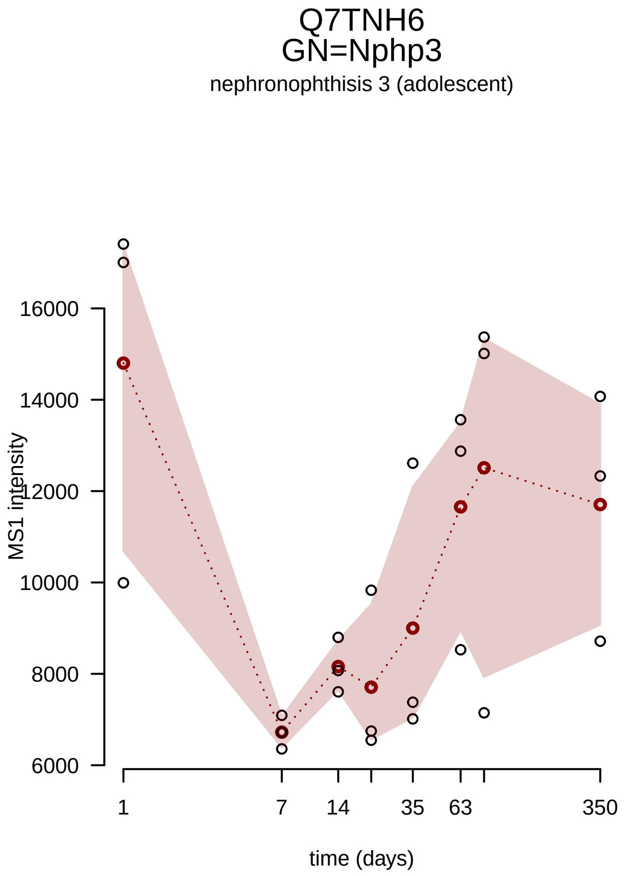
Q7TNH6
GN=Nphp3
nephronophthisis 3 (adolescent)
MS1 intensity
16000
14000
12000
10000
8000
6000
1
7
14
35
63
350
time (days)

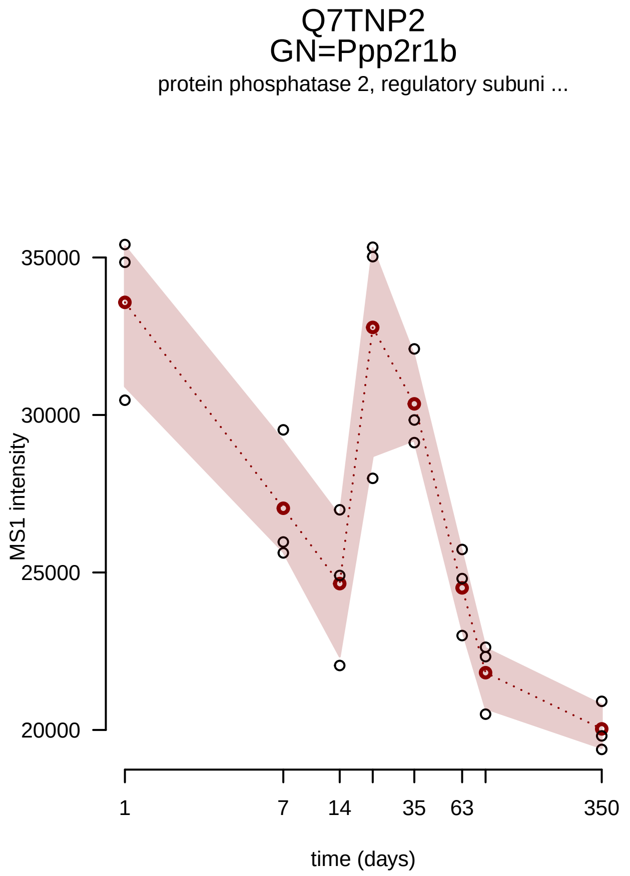
Q7TNP2
GN=Ppp2r1b
protein phosphatase 2, regulatory subuni ...
MS1 intensity
35000
30000
25000
20000
1
7
14
35
63
350
time (days)

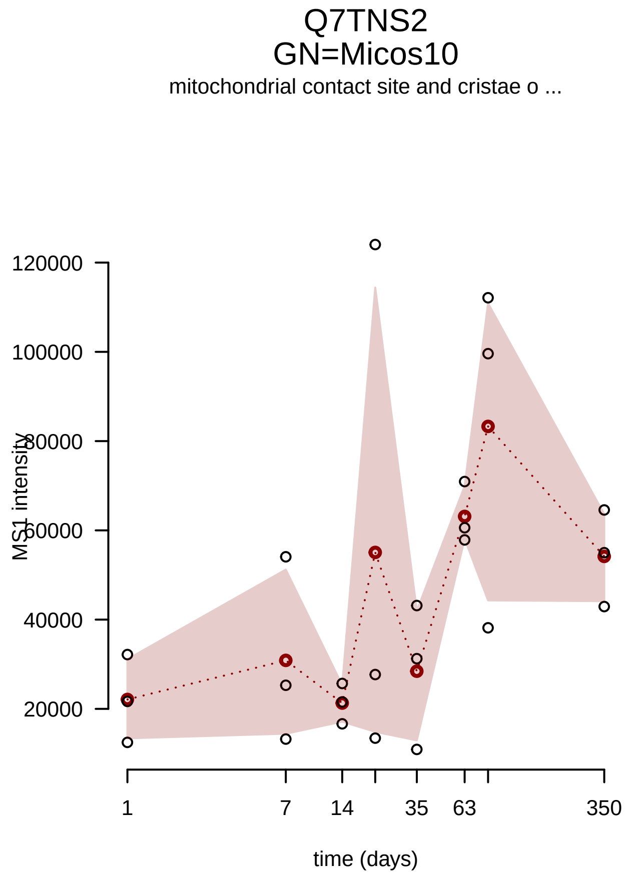
Q7TNS2
GN=Micos10
mitochondrial contact site and cristae o ...
MS1 intensity
120000
100000
80000
60000
40000
20000
1
7
14
35
63
350
time (days)

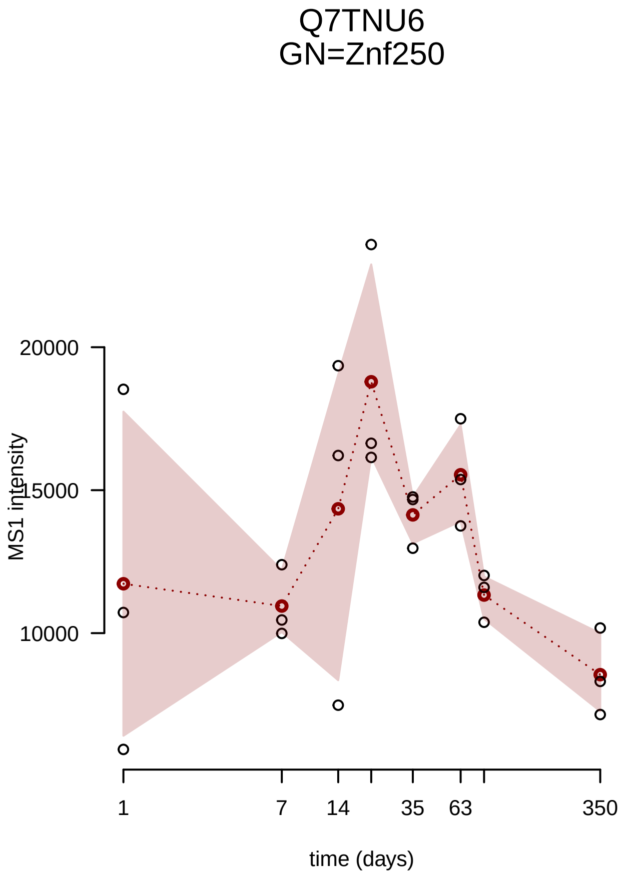
Q7TNU6
GN=Znf250
MS1 intensity
20000
15000
10000
1
7
14
35
63
350
time (days)

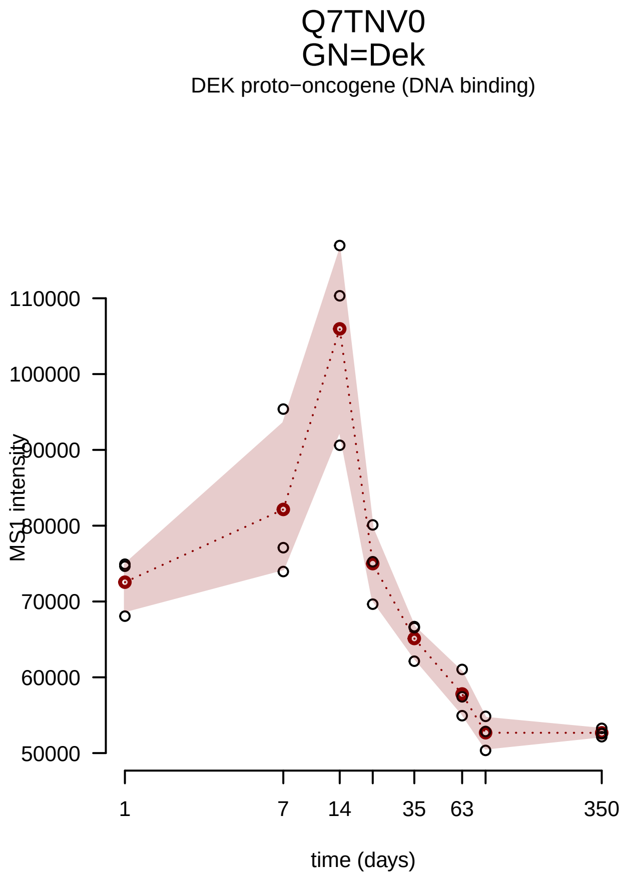
Q7TNV0
GN=Dek
DEK proto−oncogene (DNA binding)
MS1 intensity
110000
100000
90000
80000
70000
60000
50000
1
7
14
35
63
350
time (days)

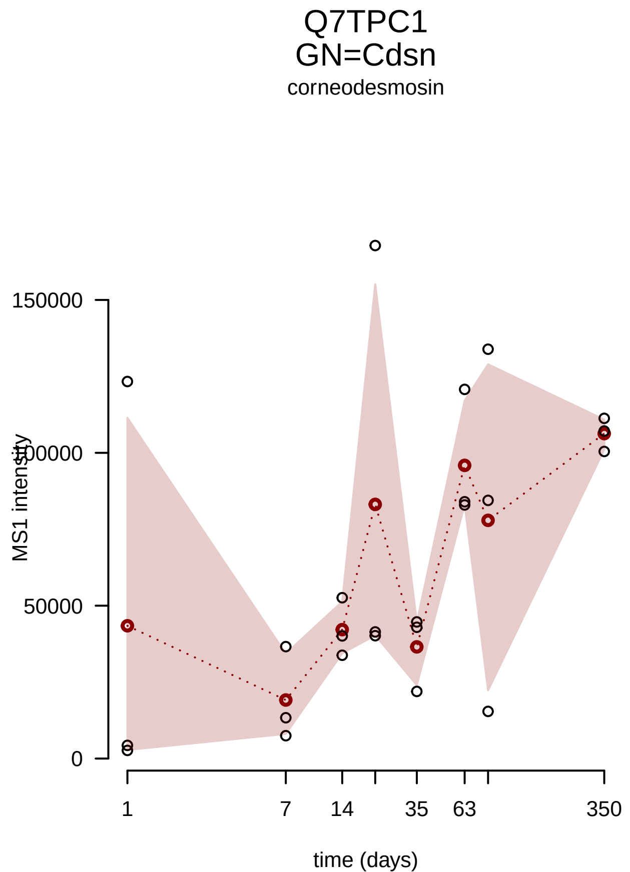
Q7TPC1
GN=Cdsn
corneodesmosin
MS1 intensity
150000
100000
50000
0
1
7
14
35
63
350
time (days)

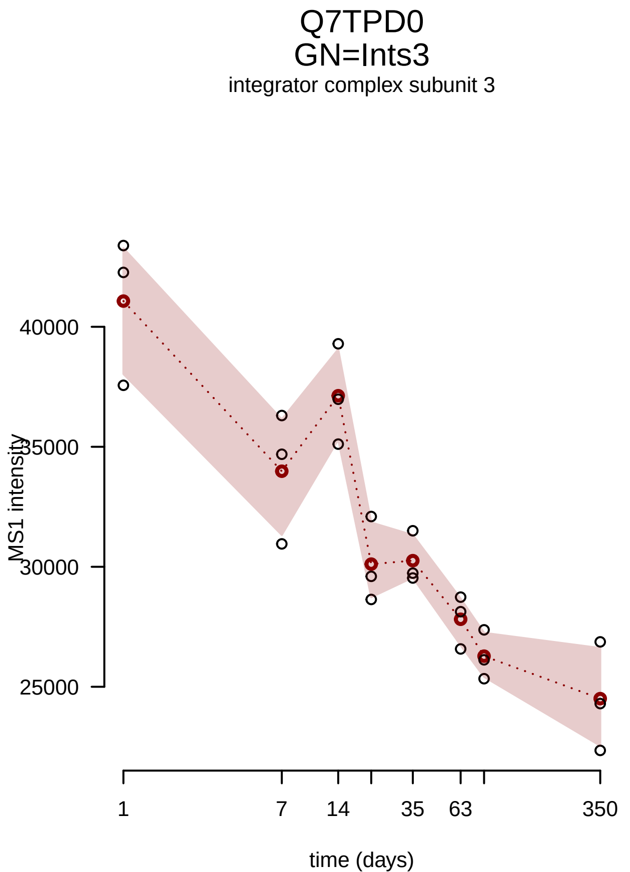
Q7TPD0
GN=Ints3
integrator complex subunit 3
MS1 intensity
40000
35000
30000
25000
1
7
14
35
63
350
time (days)

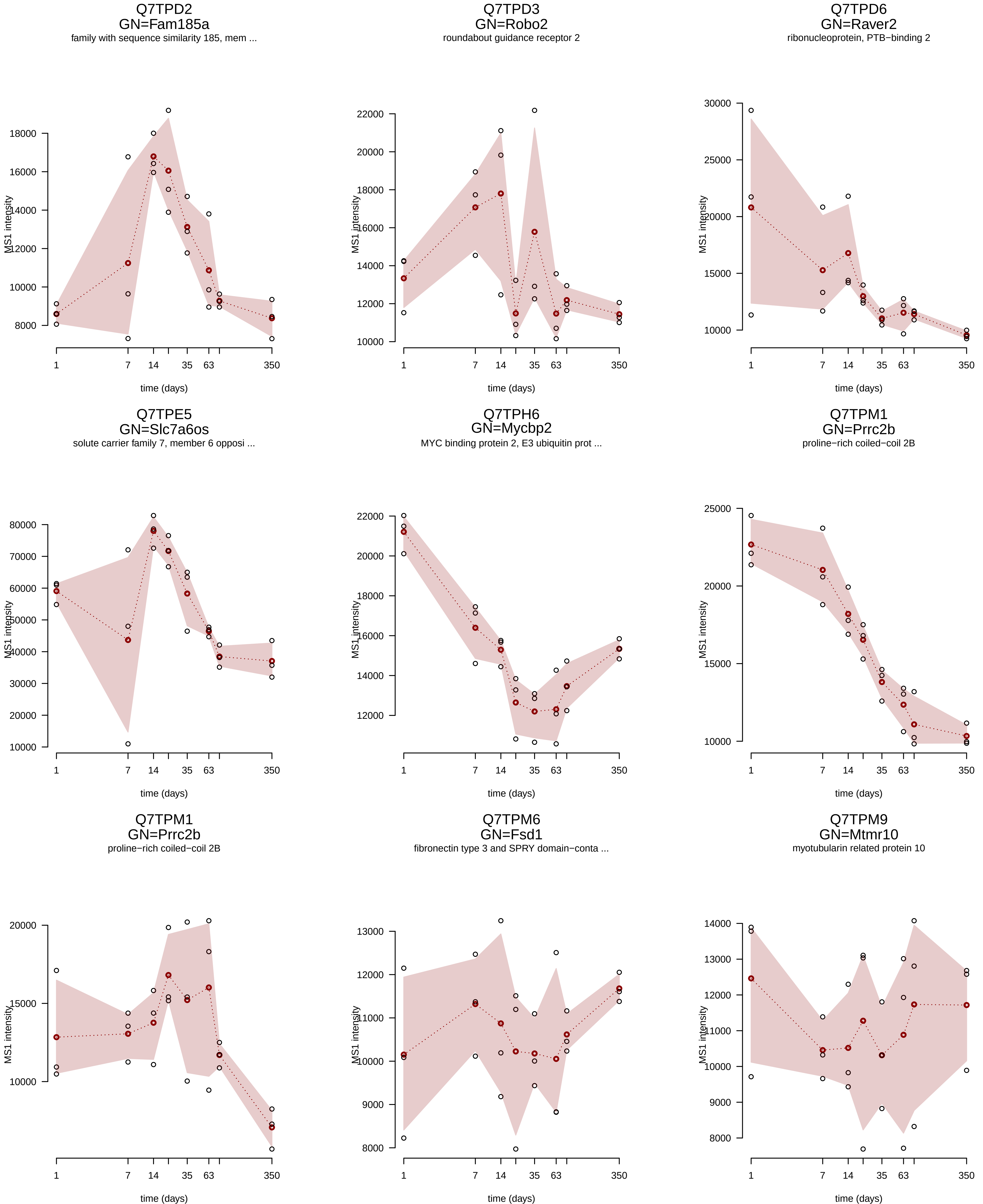
Q7TPD2
GN=Fam185a
family with sequence similarity 185, mem ...
MS1 intensity
8000
10000
12000
14000
16000
18000
1
7
14
35
63
350
time (days)
Q7TPD3
GN=Robo2
roundabout guidance receptor 2
MS1 intensity
10000
12000
14000
16000
18000
20000
22000
1
7
14
35
63
350
time (days)
Q7TPD6
GN=Raver2
ribonucleoprotein, PTB−binding 2
MS1 intensity
10000
15000
20000
25000
30000
1
7
14
35
63
350
time (days)
Q7TPE5
GN=Slc7a6os
solute carrier family 7, member 6 opposi ...
MS1 intensity
10000
20000
30000
40000
50000
60000
70000
80000
1
7
14
35
63
350
time (days)
Q7TPH6
GN=Mycbp2
MYC binding protein 2, E3 ubiquitin prot ...
MS1 intensity
12000
14000
16000
18000
20000
22000
1
7
14
35
63
350
time (days)
Q7TPM1
GN=Prrc2b
proline−rich coiled−coil 2B
MS1 intensity
10000
15000
20000
25000
1
7
14
35
63
350
time (days)
Q7TPM1
GN=Prrc2b
proline−rich coiled−coil 2B
MS1 intensity
10000
15000
20000
1
7
14
35
63
350
time (days)
Q7TPM6
GN=Fsd1
fibronectin type 3 and SPRY domain−conta ...
MS1 intensity
8000
9000
10000
11000
12000
13000
1
7
14
35
63
350
time (days)
Q7TPM9
GN=Mtmr10
myotubularin related protein 10
MS1 intensity
8000
9000
10000
11000
12000
13000
14000
1
7
14
35
63
350
time (days)

Q7TPN9
GN=Prr14
proline rich 14

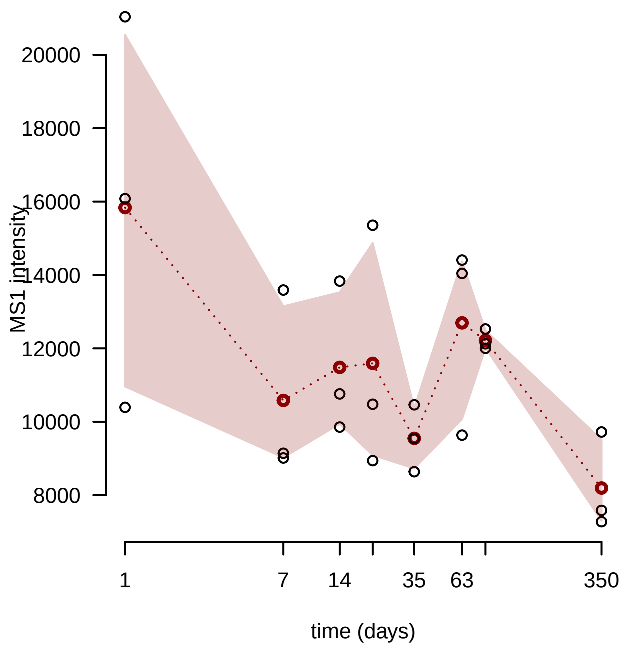

Q7TPQ3
GN=Shprh
SNF2 histone linker PHD RING helicase

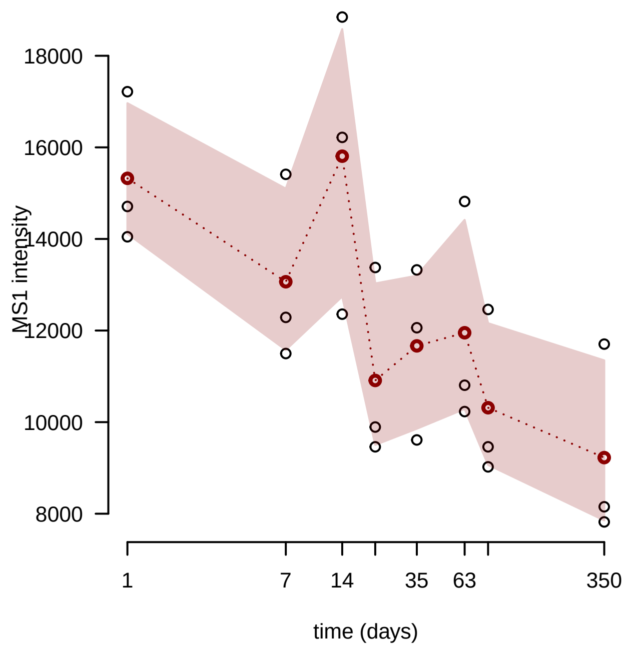

Q7TPR4
GN=Actn1
actinin, alpha 1

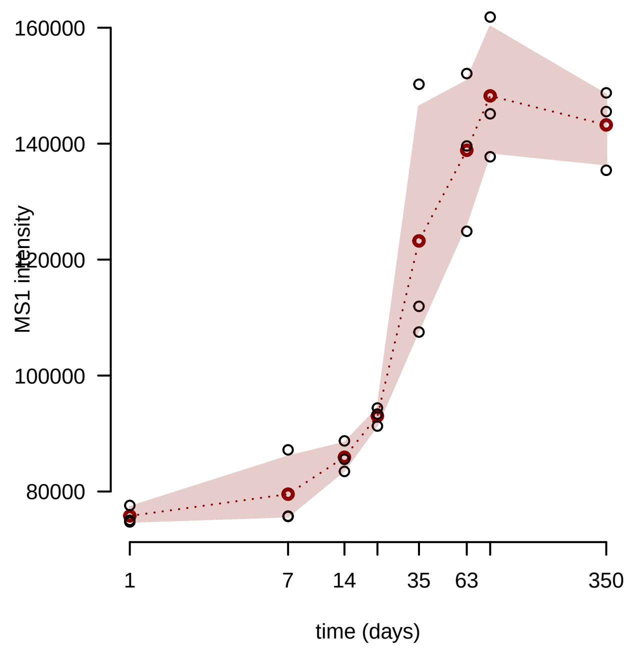

Q7TPS5
GN=C2cd5
C2 calcium−dependent domain containing 5

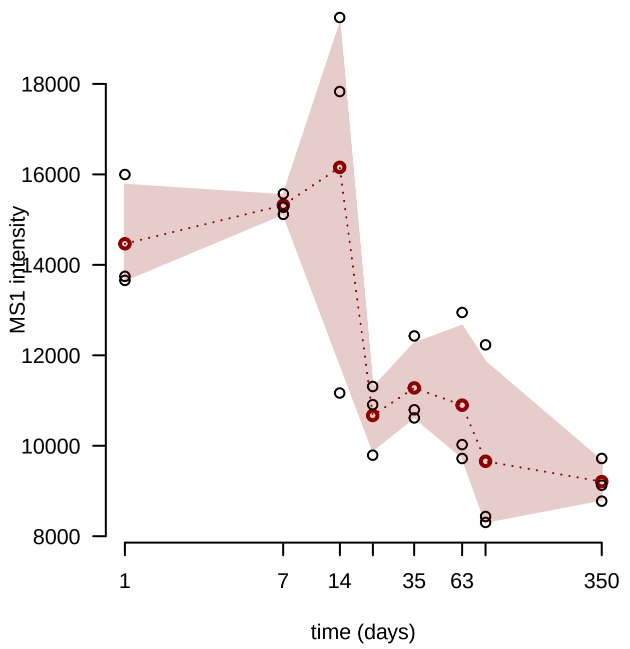

Q7TPV2
GN=Dzip3
DAZ interacting protein 3, zinc finger

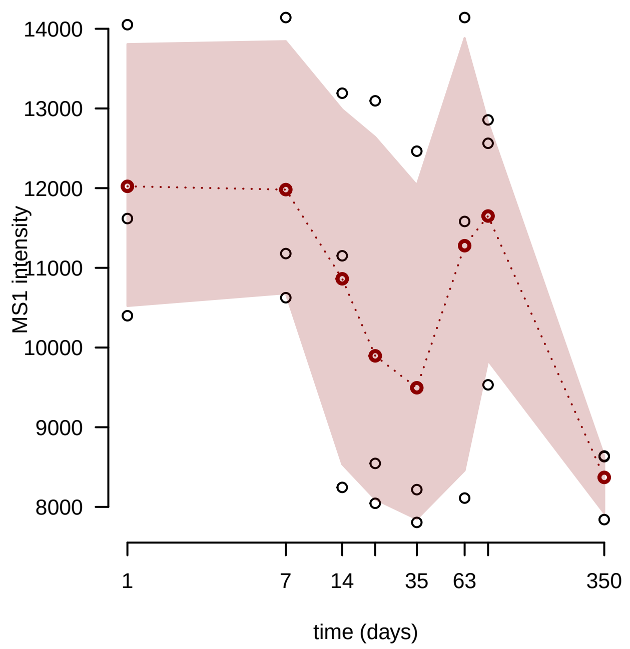

Q7TPV4
GN=Mybbp1a
MYB binding protein (P160) 1a

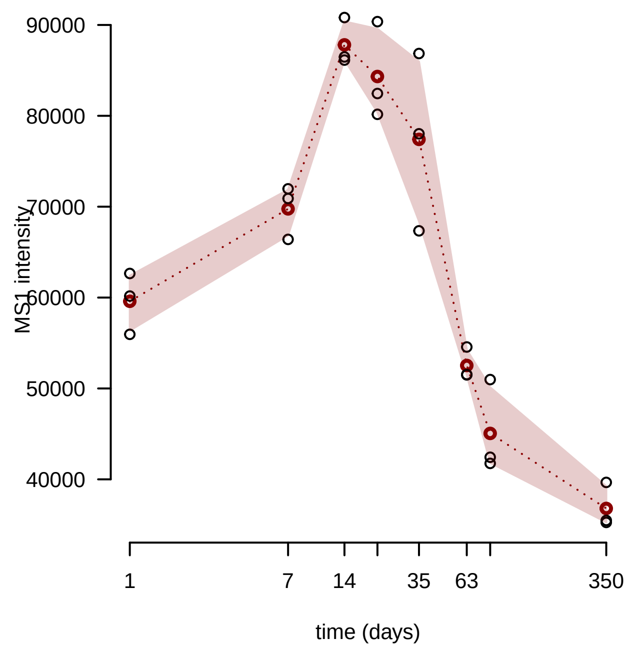

Q7TPW1
GN=Nexn
nexilin

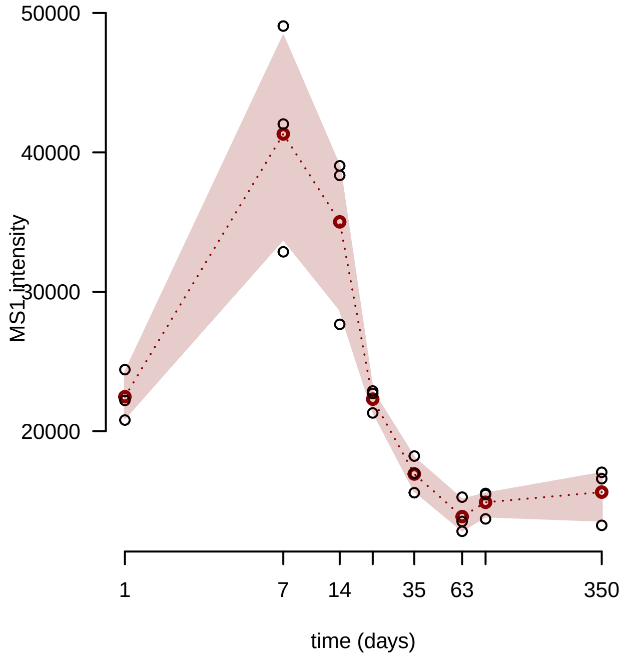

Q7TPZ8
GN=Cpa1
carboxypeptidase A1, pancreatic

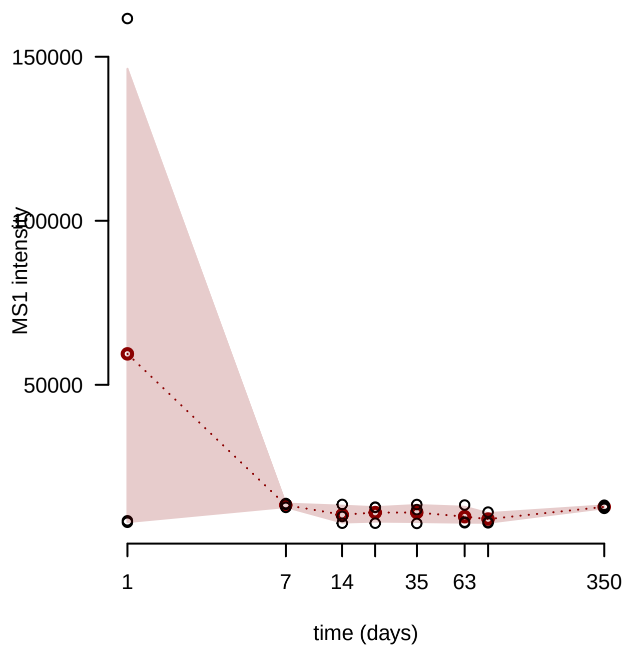

Q7TQ95
GN=Lnpk
lunapark, ER junction formation factor

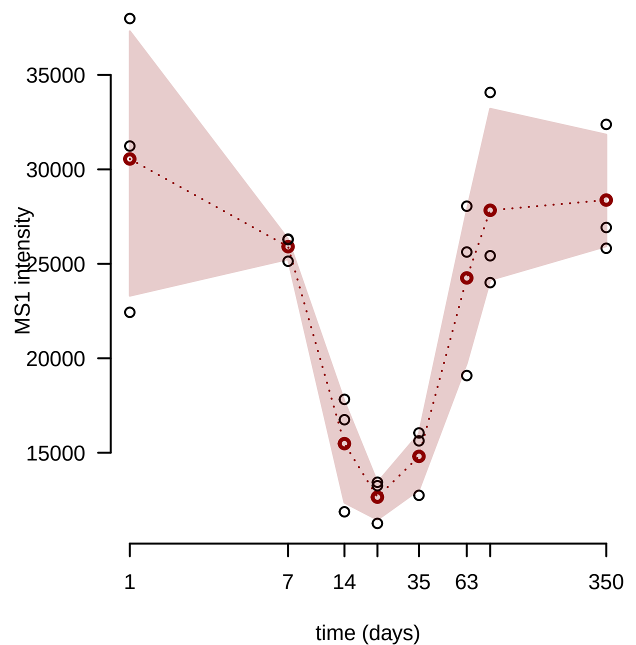

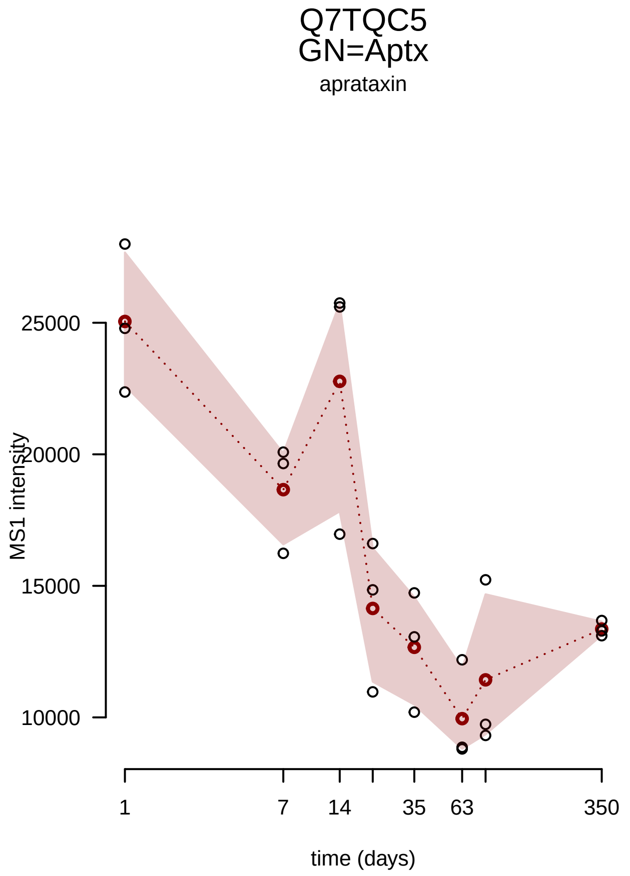
Q7TQC5
GN=Aptx
aprataxin
MS1 intensity
25000
20000
15000
10000
1
7
14
35
63
350
time (days)

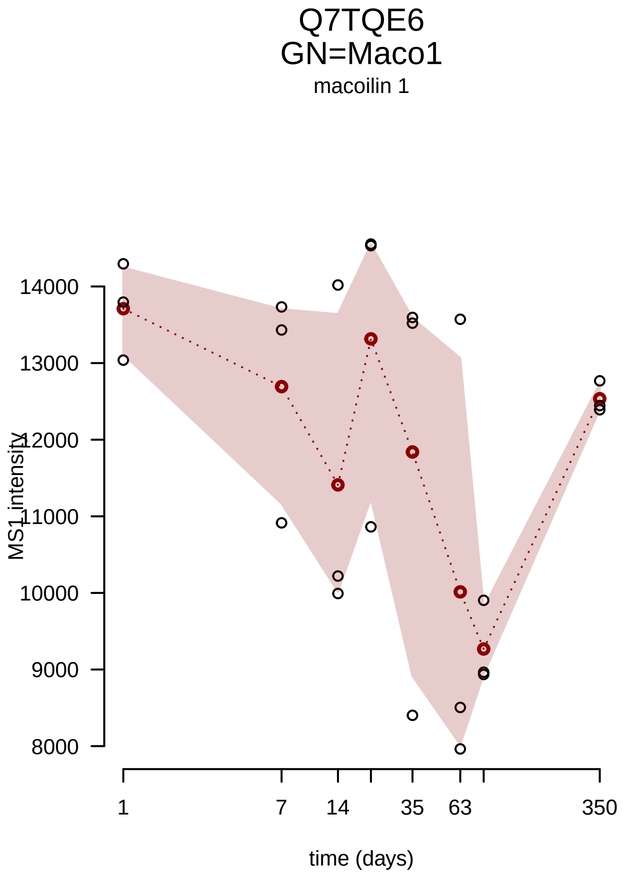
Q7TQE6
GN=Maco1
macoilin 1
MS1 intensity
14000
13000
12000
11000
10000
9000
8000
1
7
14
35
63
350
time (days)

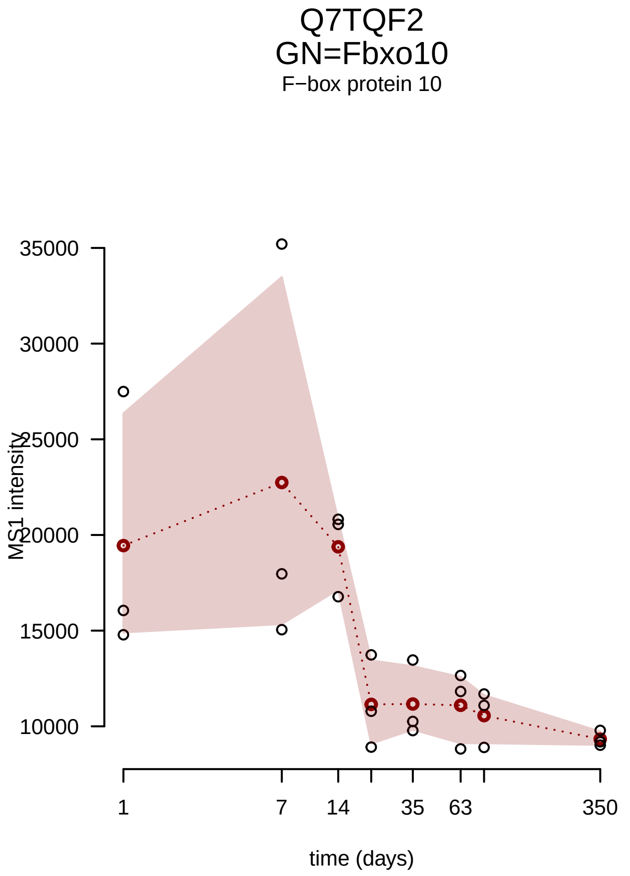
Q7TQF2
GN=Fbxo10
F−box protein 10
MS1 intensity
35000
30000
25000
20000
15000
10000
1
7
14
35
63
350
time (days)

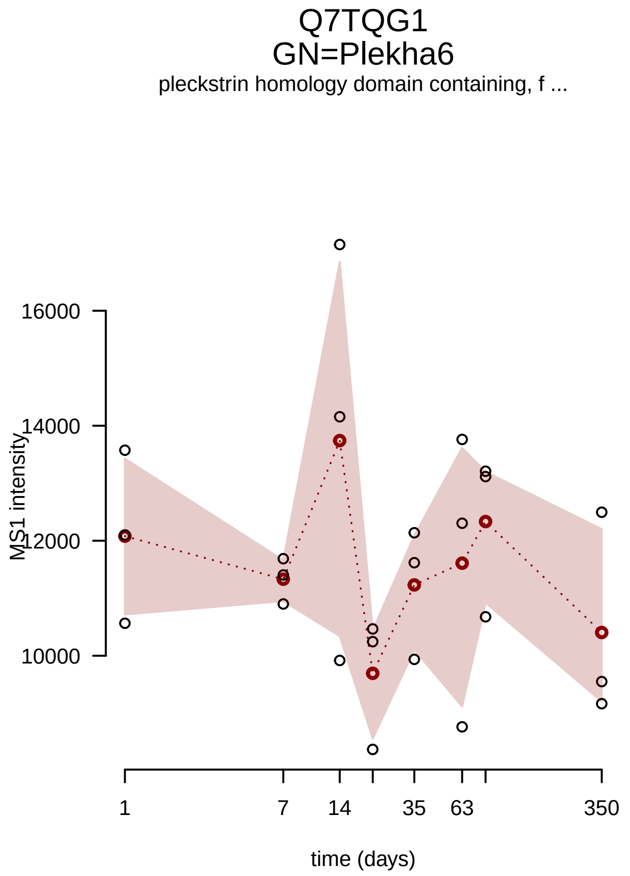
Q7TQG1
GN=Plekha6
pleckstrin homology domain containing, f ...
MS1 intensity
16000
14000
12000
10000
1
7
14
35
63
350
time (days)

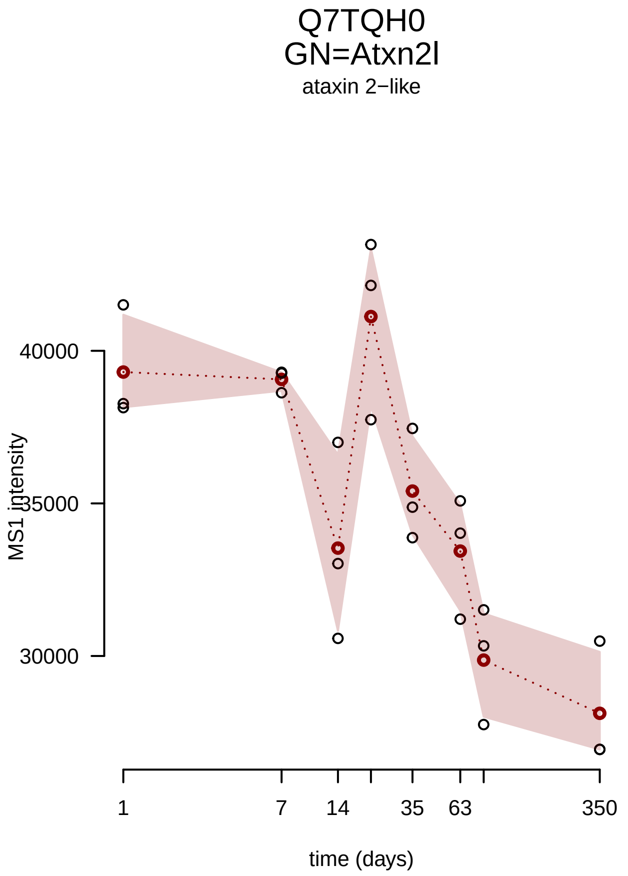
Q7TQH0
GN=Atxn2l
ataxin 2−like
MS1 intensity
40000
35000
30000
1
7
14
35
63
350
time (days)

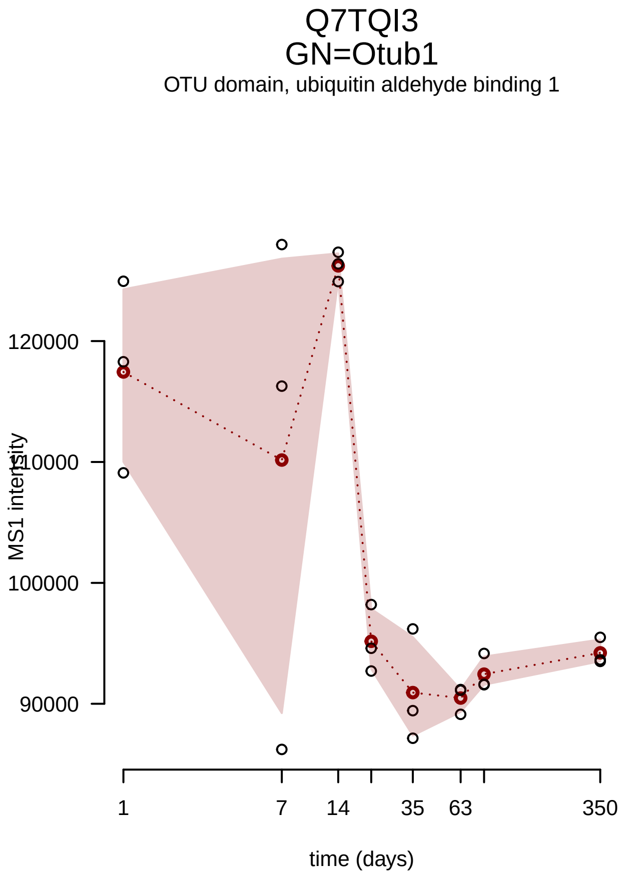
Q7TQI3
GN=Otub1
OTU domain, ubiquitin aldehyde binding 1
MS1 intensity
120000
110000
100000
90000
1
7
14
35
63
350
time (days)

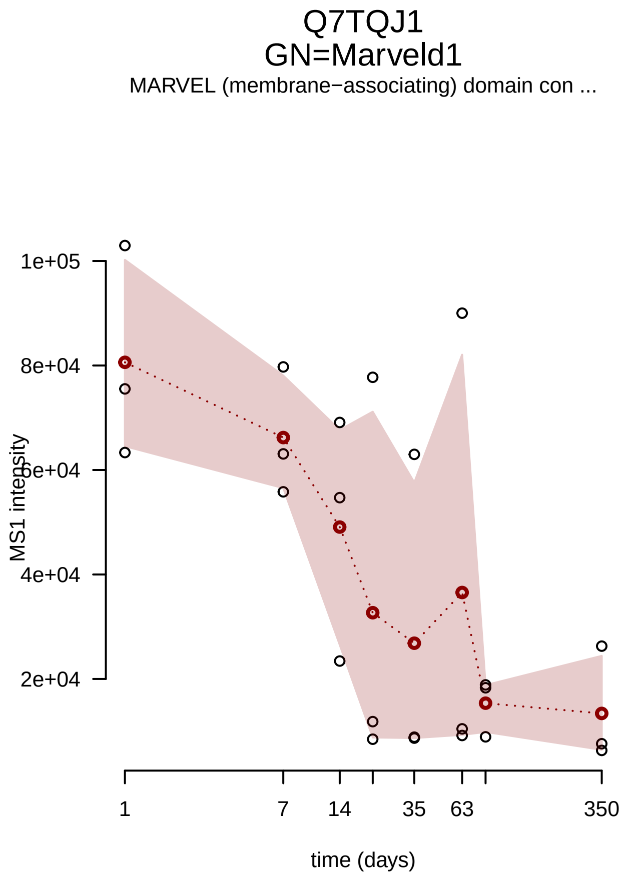
Q7TQJ1
GN=Marveld1
MARVEL (membrane−associating) domain con ...
MS1 intensity
1e+05
8e+04
6e+04
4e+04
2e+04
1
7
14
35
63
350
time (days)

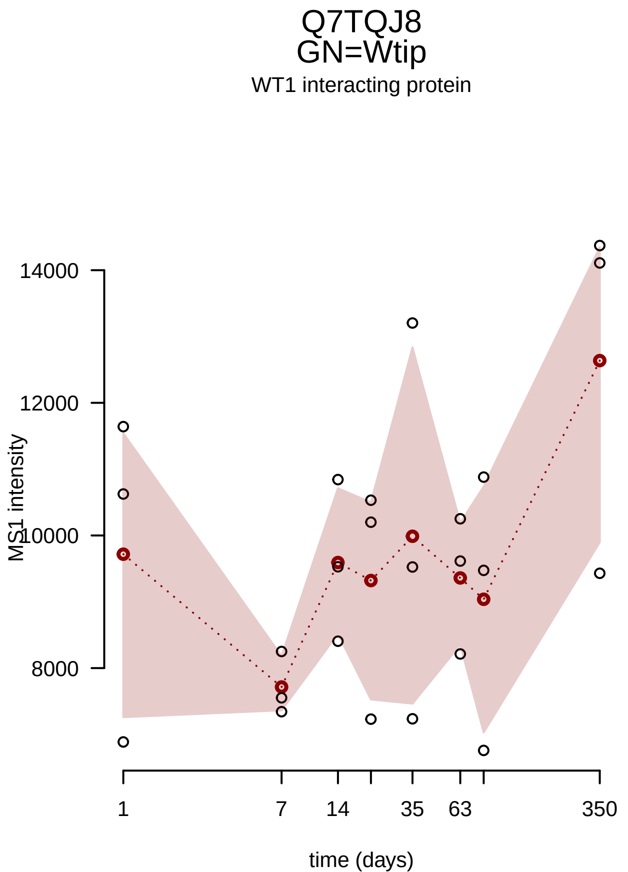
Q7TQJ8
GN=Wtip
WT1 interacting protein
MS1 intensity
14000
12000
10000
8000
1
7
14
35
63
350
time (days)

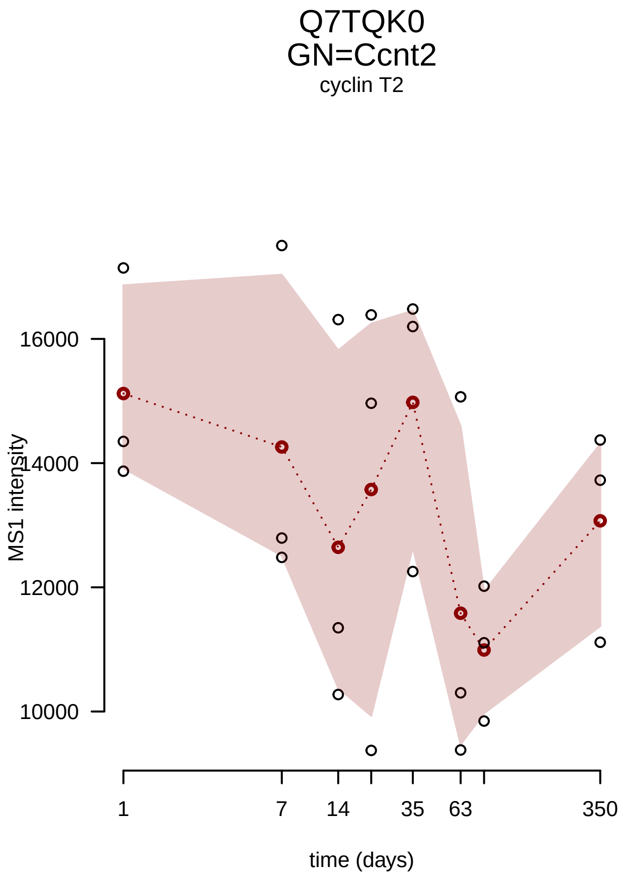
Q7TQK0
GN=Ccnt2
cyclin T2
MS1 intensity
16000
14000
12000
10000
1
7
14
35
63
350
time (days)

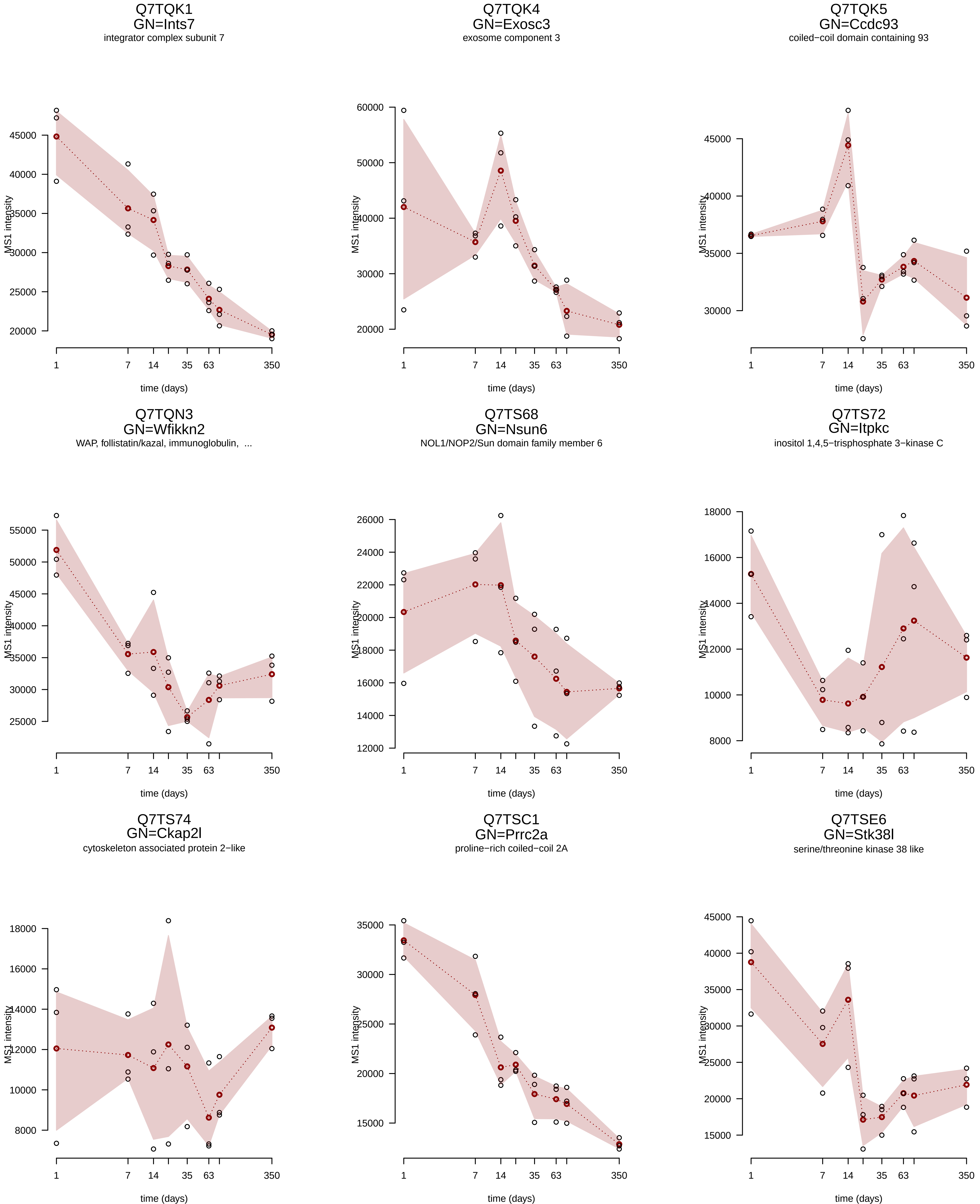
Q7TQK1
GN=Ints7
integrator complex subunit 7
Q7TQK4
GN=Exosc3
exosome component 3
Q7TQK5
GN=Ccdc93
coiled−coil domain containing 93
Q7TQN3
GN=Wfikkn2
WAP, follistatin/kazal, immunoglobulin, ...
Q7TS68
GN=Nsun6
NOL1/NOP2/Sun domain family member 6
Q7TS72
GN=Itpkc
inositol 1,4,5−trisphosphate 3−kinase C
Q7TS74
GN=Ckap2l
cytoskeleton associated protein 2−like
Q7TSC1
GN=Prrc2a
proline−rich coiled−coil 2A
Q7TSE6
GN=Stk38l
serine/threonine kinase 38 like
MS1 intensity
time (days)
1
7
14
35
63
350

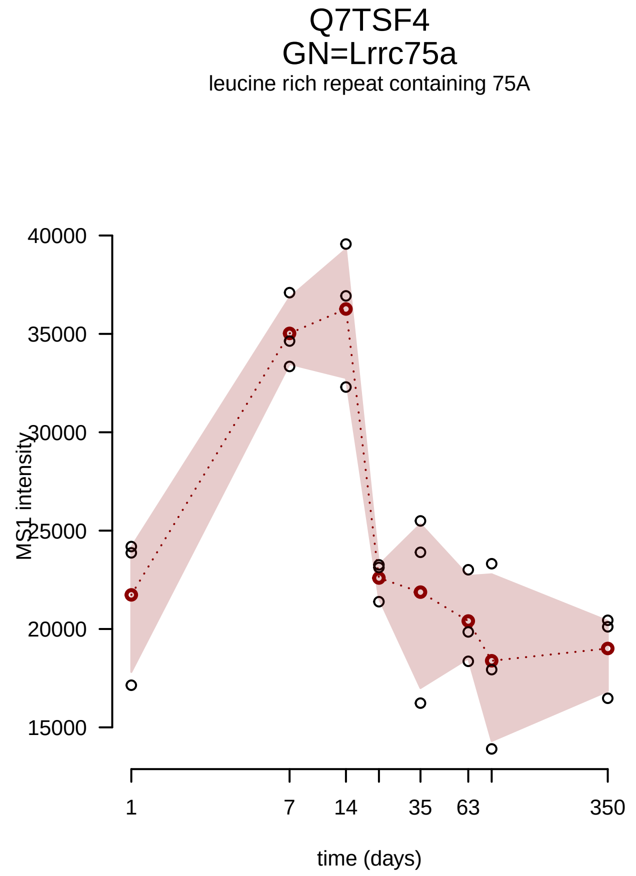
Q7TSF4
GN=Lrrc75a
leucine rich repeat containing 75A
MS1 intensity
40000
35000
30000
25000
20000
15000
1
7
14
35
63
350
time (days)
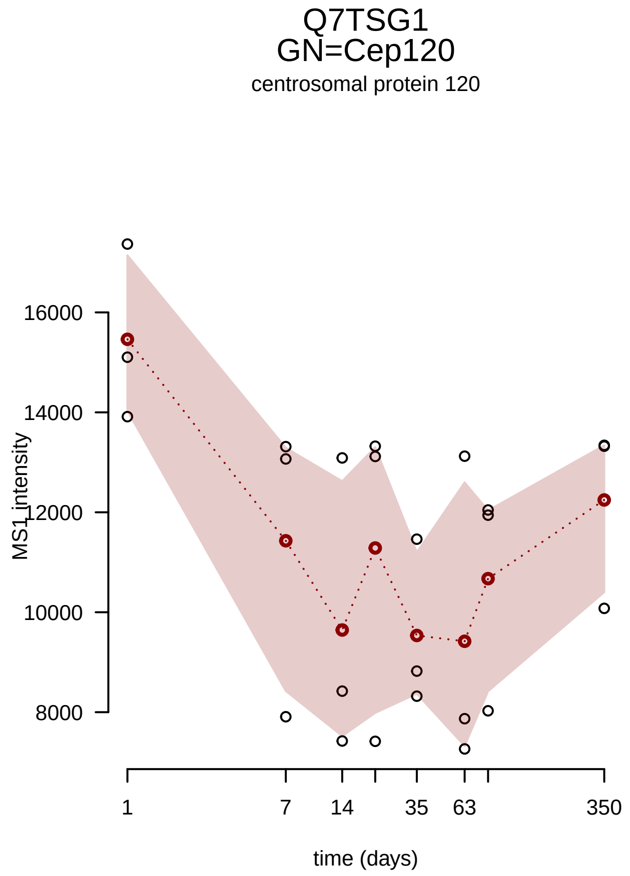
Q7TSG1
GN=Cep120
centrosomal protein 120
MS1 intensity
16000
14000
12000
10000
8000
1
7
14
35
63
350
time (days)
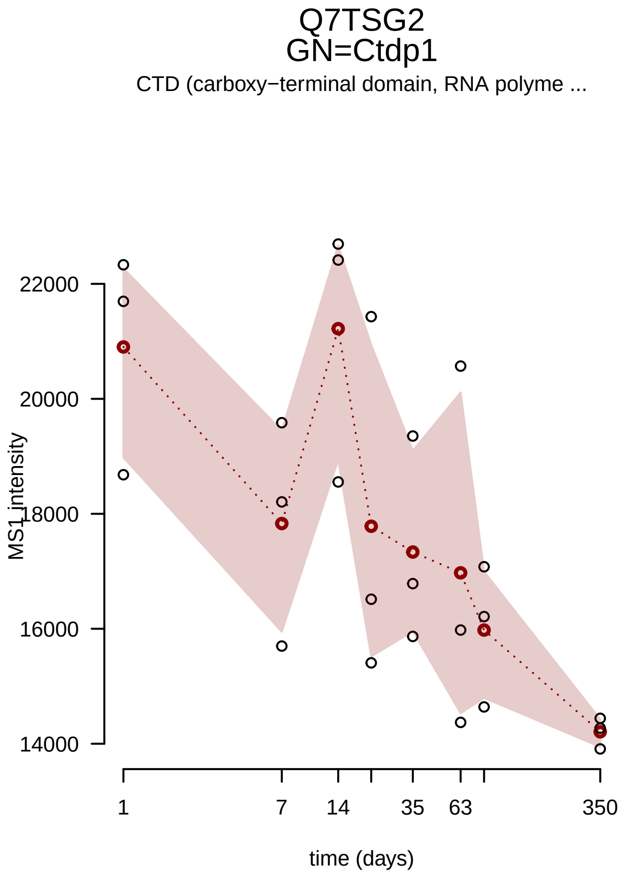
Q7TSG2
GN=Ctdp1
CTD (carboxy−terminal domain, RNA polyme ...
MS1 intensity
22000
20000
18000
16000
14000
1
7
14
35
63
350
time (days)
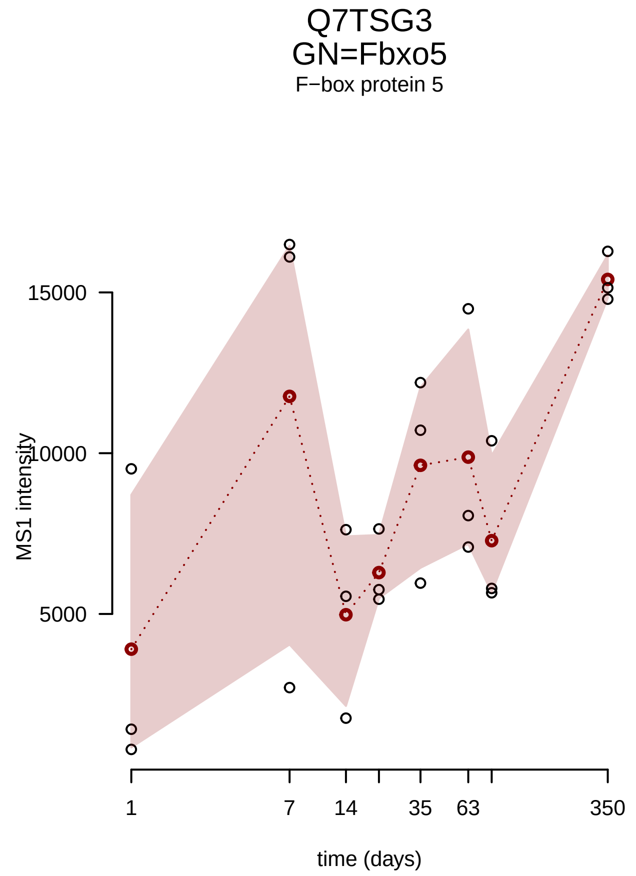
Q7TSG3
GN=Fbxo5
F−box protein 5
MS1 intensity
15000
10000
5000
1
7
14
35
63
350
time (days)
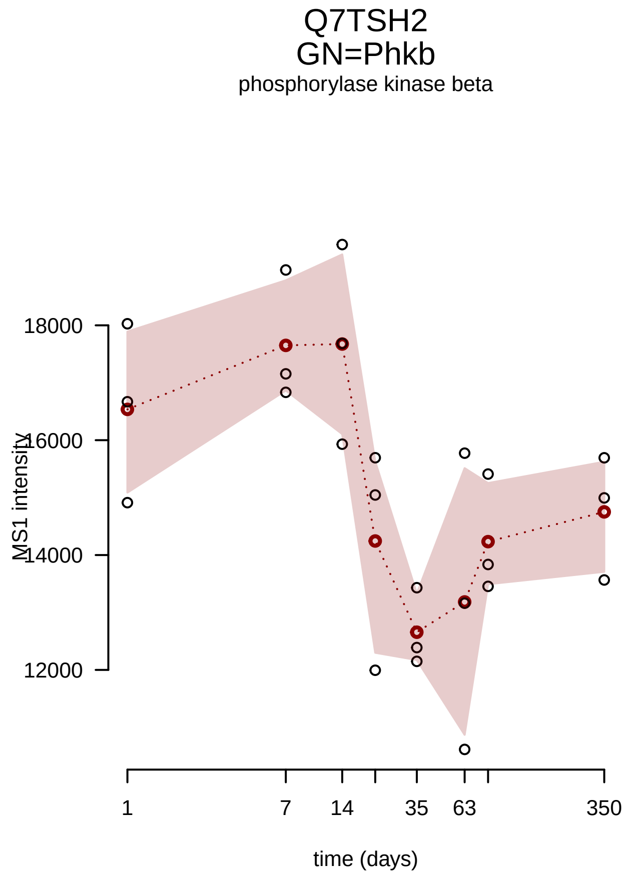
Q7TSH2
GN=Phkb
phosphorylase kinase beta
MS1 intensity
18000
16000
14000
12000
1
7
14
35
63
350
time (days)
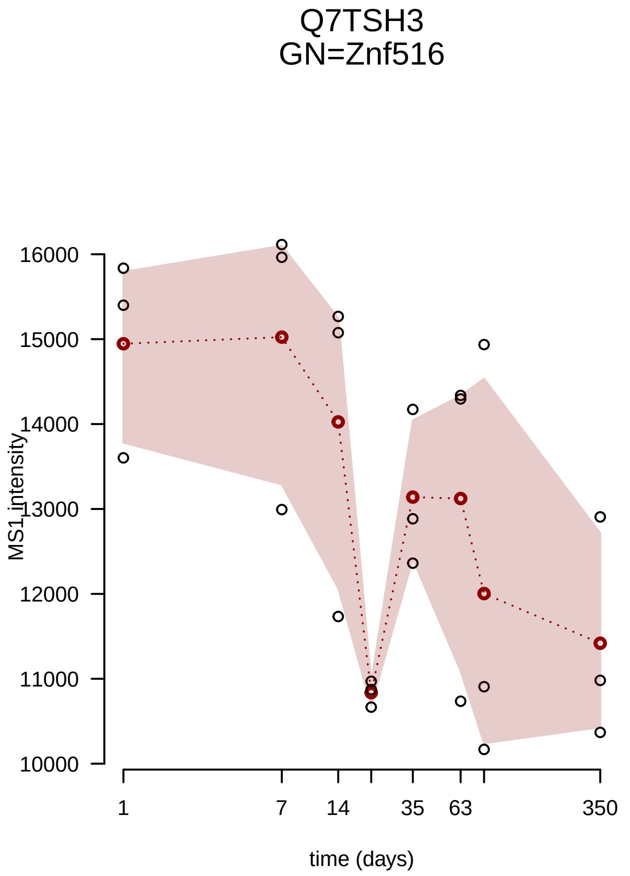
Q7TSH3
GN=Znf516
MS1 intensity
16000
15000
14000
13000
12000
11000
10000
1
7
14
35
63
350
time (days)
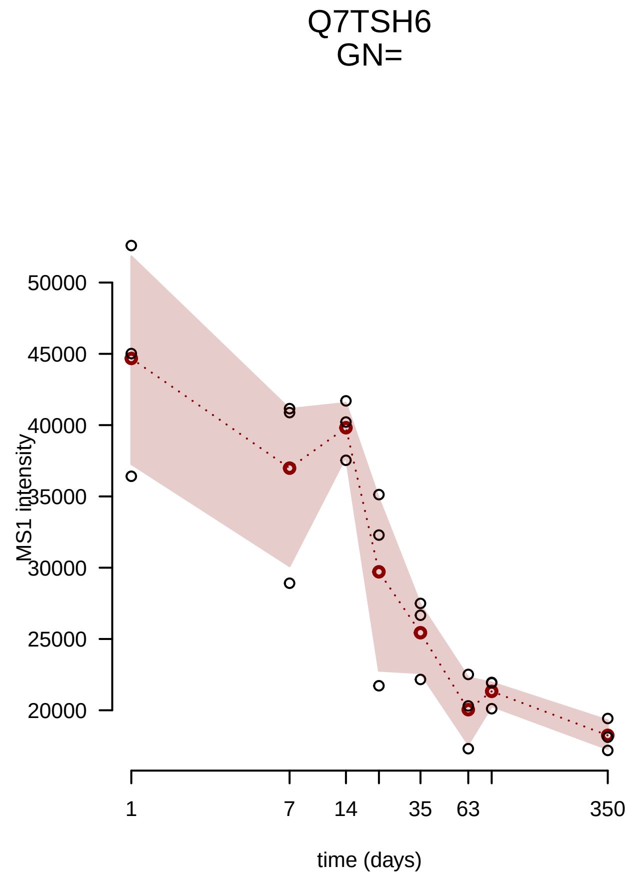
Q7TSH6
GN=
MS1 intensity
50000
45000
40000
35000
30000
25000
20000
1
7
14
35
63
350
time (days)
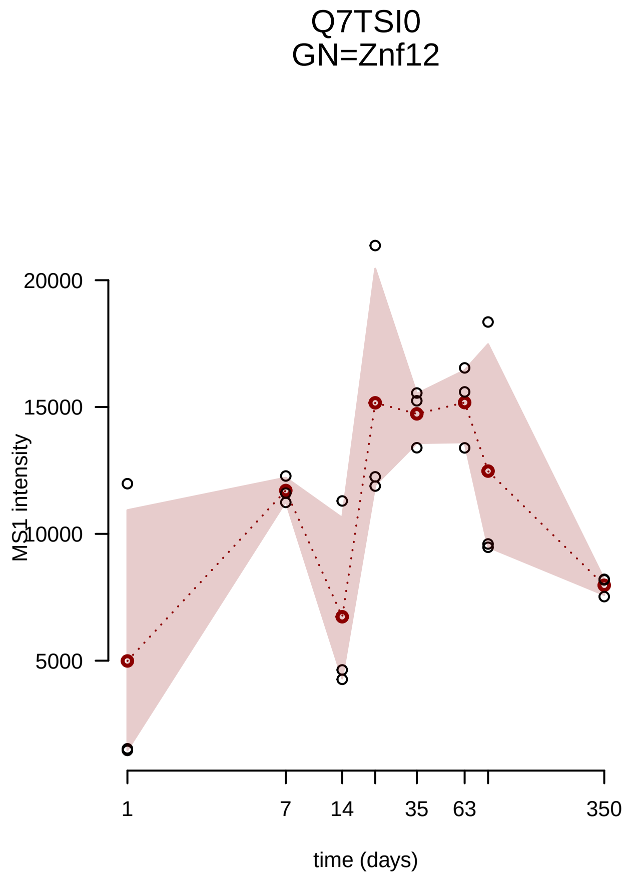
Q7TSI0
GN=Znf12
MS1 intensity
20000
15000
10000
5000
1
7
14
35
63
350
time (days)
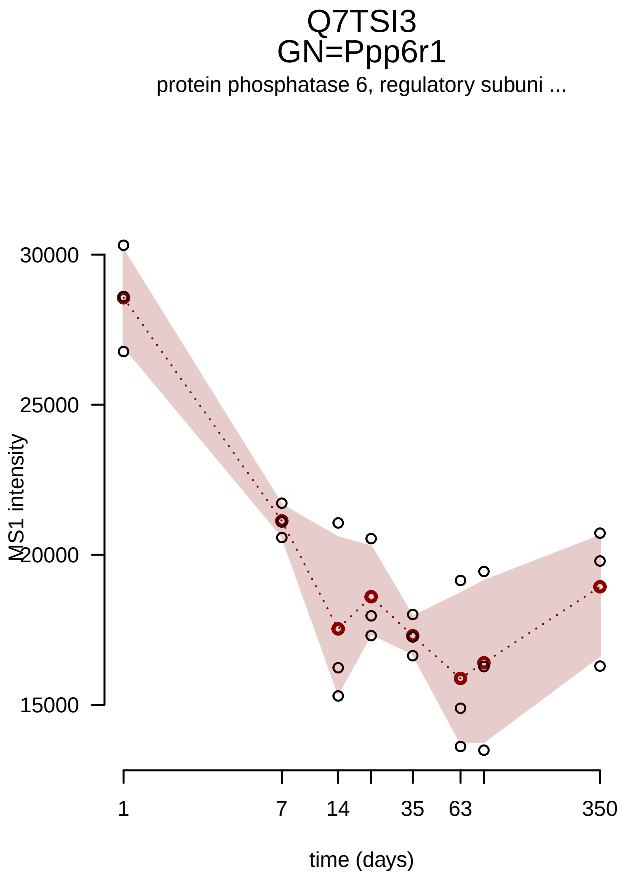
Q7TSI3
GN=Ppp6r1
protein phosphatase 6, regulatory subuni ...
MS1 intensity
30000
25000
20000
15000
1
7
14
35
63
350
time (days)

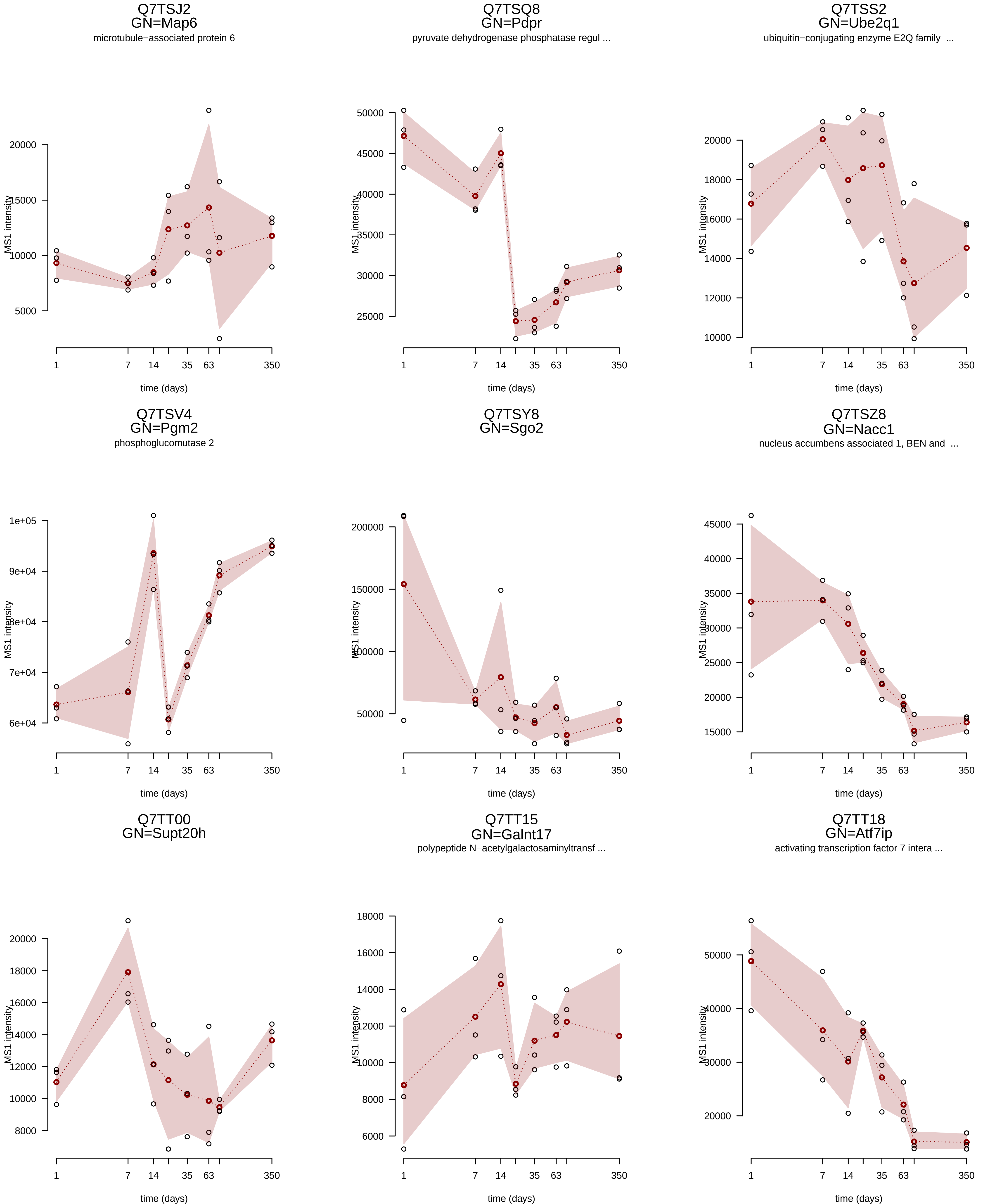
Q7TSJ2
GN=Map6
microtubule−associated protein 6
Q7TSQ8
GN=Pdpr
pyruvate dehydrogenase phosphatase regul ...
Q7TSS2
GN=Ube2q1
ubiquitin−conjugating enzyme E2Q family ...
Q7TSV4
GN=Pgm2
phosphoglucomutase 2
Q7TSY8
GN=Sgo2
Q7TSZ8
GN=Nacc1
nucleus accumbens associated 1, BEN and ...
Q7TT00
GN=Supt20h
Q7TT15
GN=Galnt17
polypeptide N−acetylgalactosaminyltransf ...
Q7TT18
GN=Atf7ip
activating transcription factor 7 intera ...
MS1 intensity
time (days)
1
7
14
35
63
350

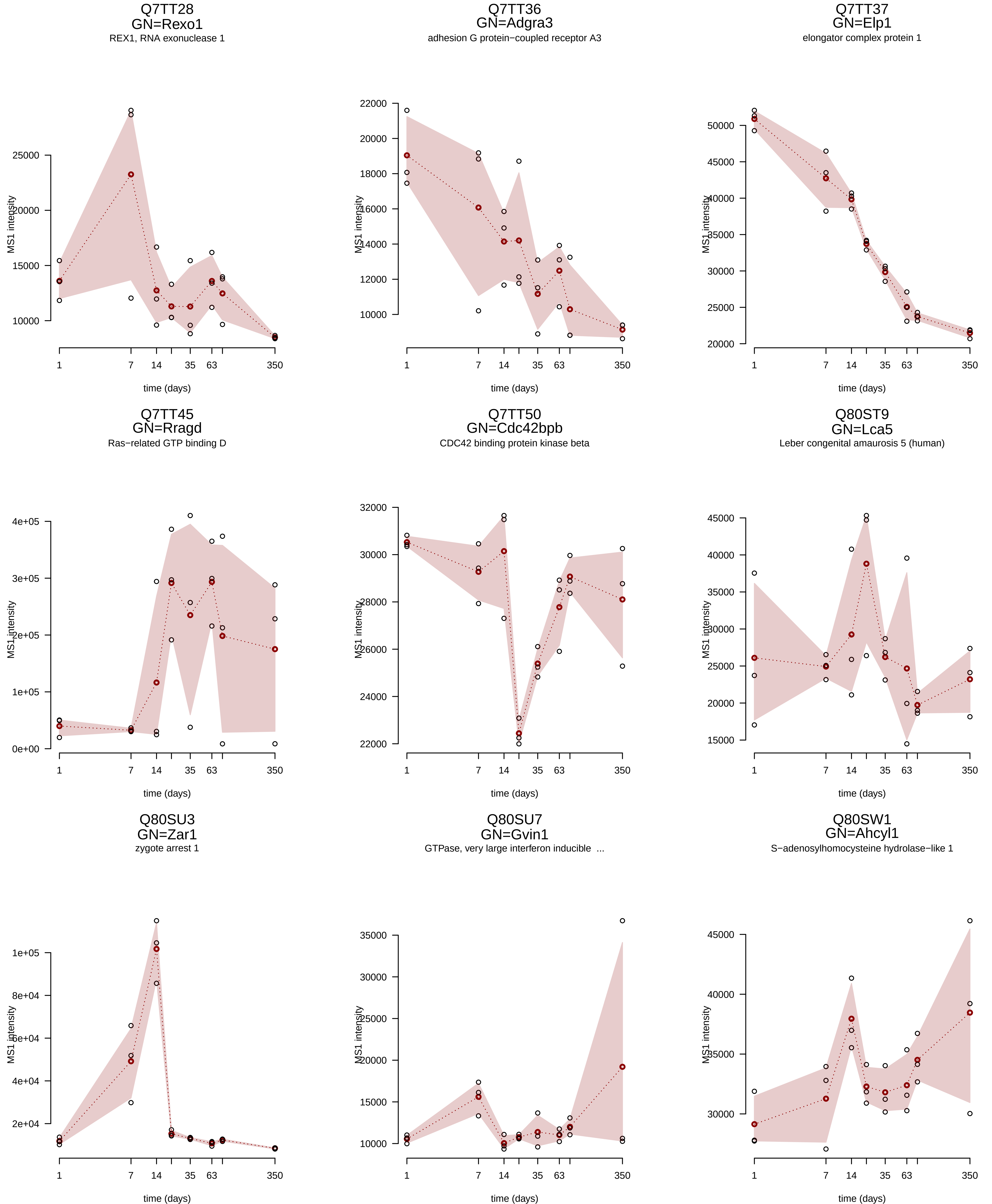
Q7TT28
GN=Rexo1
REX1, RNA exonuclease 1
Q7TT36
GN=Adgra3
adhesion G protein−coupled receptor A3
Q7TT37
GN=Elp1
elongator complex protein 1
Q7TT45
GN=Rragd
Ras−related GTP binding D
Q7TT50
GN=Cdc42bpb
CDC42 binding protein kinase beta
Q80ST9
GN=Lca5
Leber congenital amaurosis 5 (human)
Q80SU3
GN=Zar1
zygote arrest 1
Q80SU7
GN=Gvin1
GTPase, very large interferon inducible ...
Q80SW1
GN=Ahcyl1
S−adenosylhomocysteine hydrolase−like 1
MS1 intensity
time (days)
1
7
14
35
63
350

Q80SY4
GN=Mib1
MIB E3 ubiquitin protein ligase 1

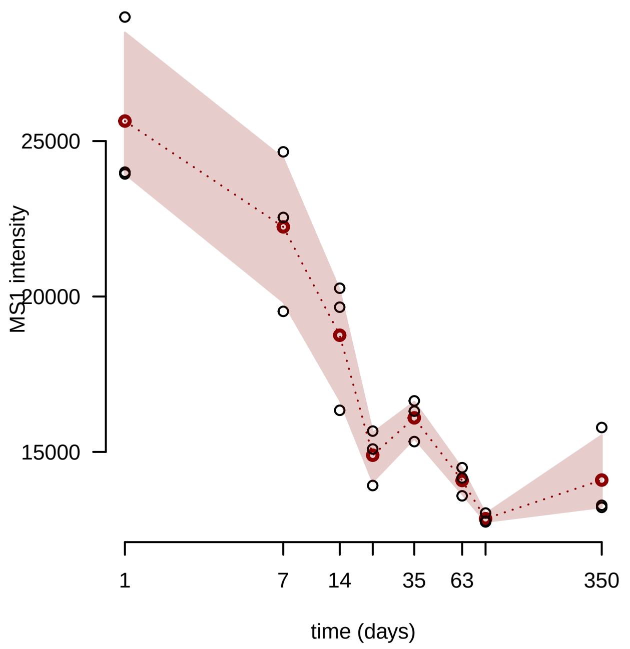

Q80SY5
GN=Prpf38b
PRP38 pre−mRNA processing factor 38 (yea ...

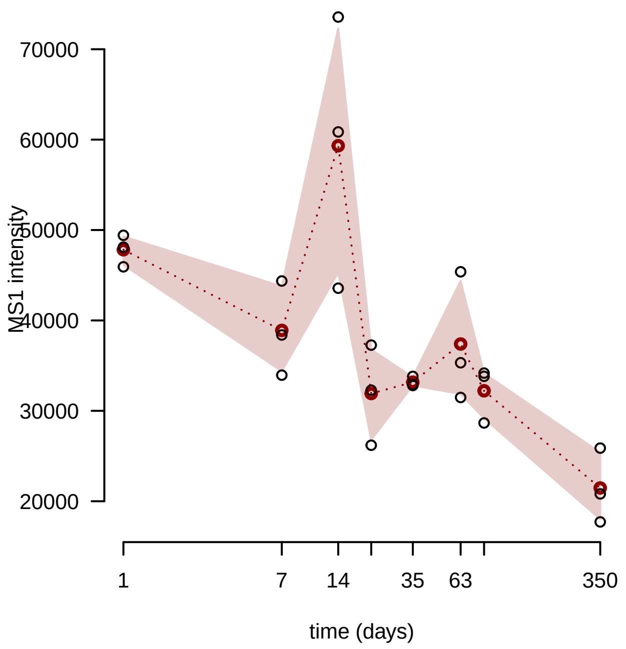

Q80SY6
GN=Adal
adenosine deaminase−like

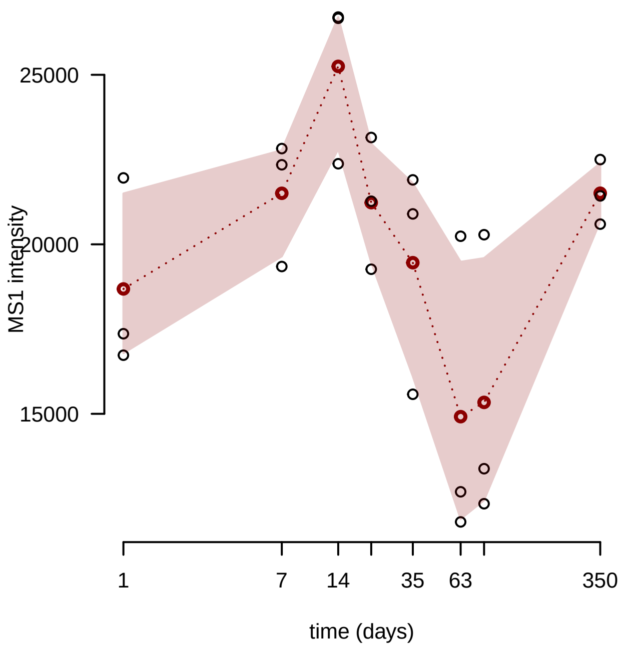

Q80SZ7
GN=Gng5
guanine nucleotide binding protein (G pr ...

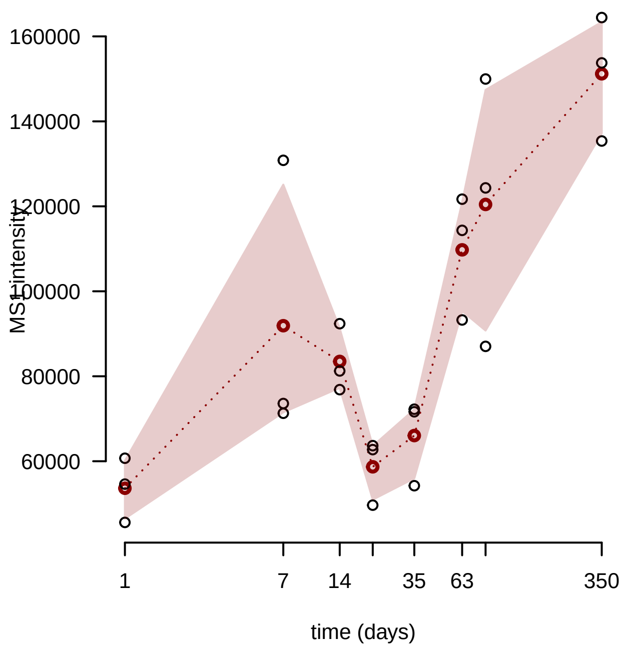

Q80T14
GN=Fras1
Fraser extracellular matrix complex subu ...

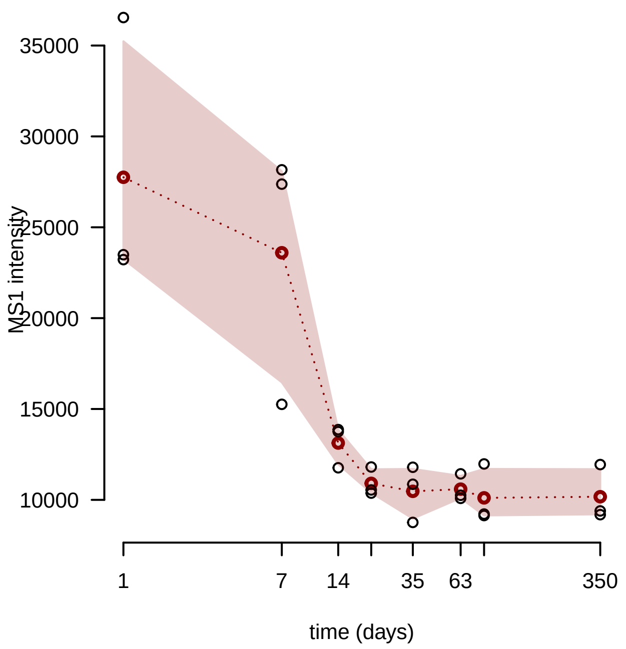

Q80T69
GN=Rsbn1
rosbin, round spermatid basic protein 1

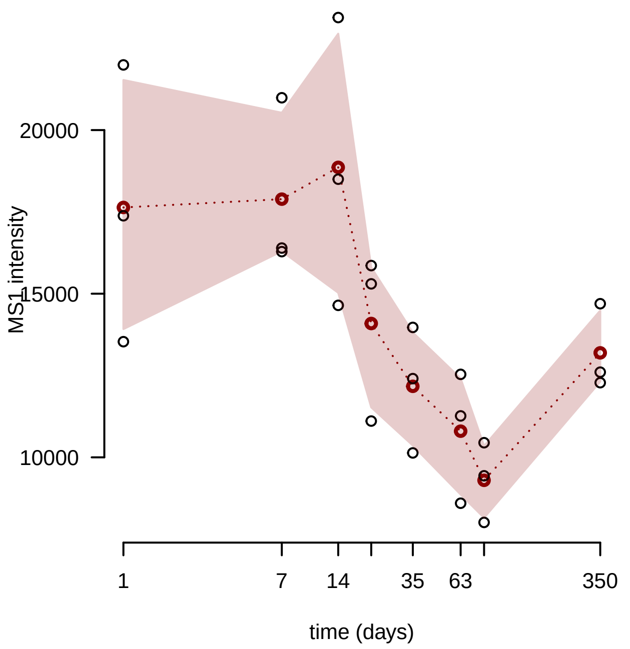

Q80T85
GN=Dcaf5
DDB1 and CUL4 associated factor 5

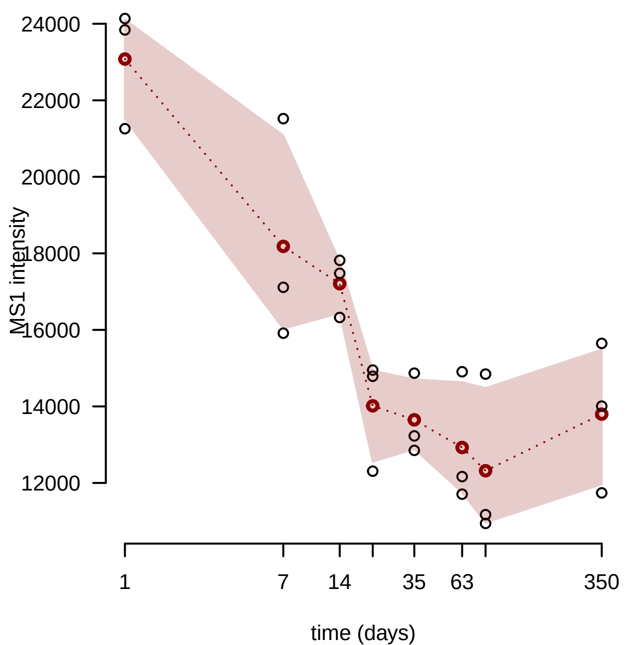

Q80TA1
GN=Selenoi
selenoprotein I

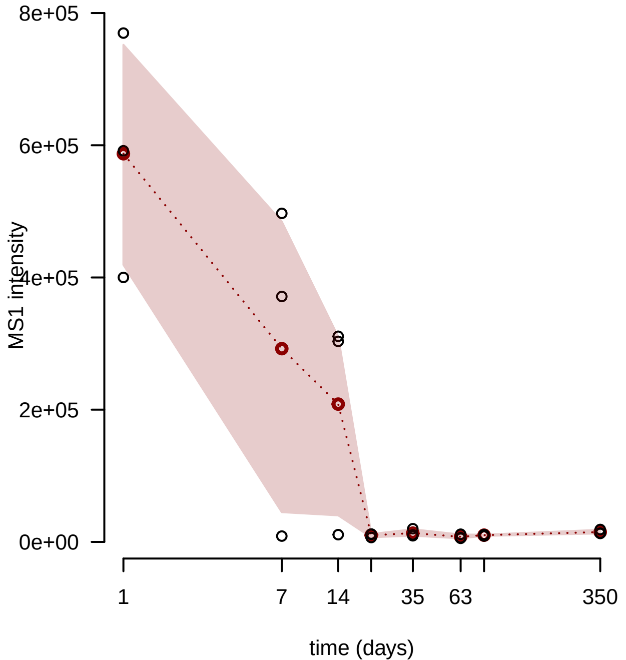

Q80TA6
GN=Mtmr12
myotubularin related protein 12

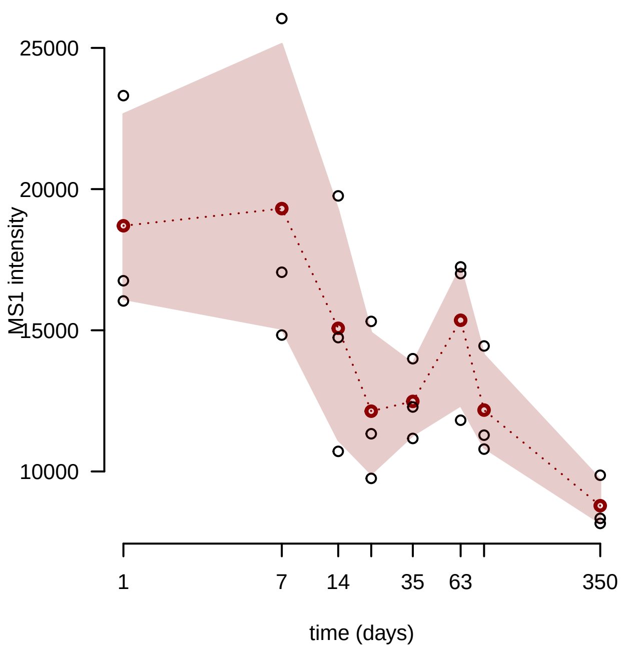

Q80TA9
GN=Epg5
ectopic P−granules autophagy protein 5 h ...

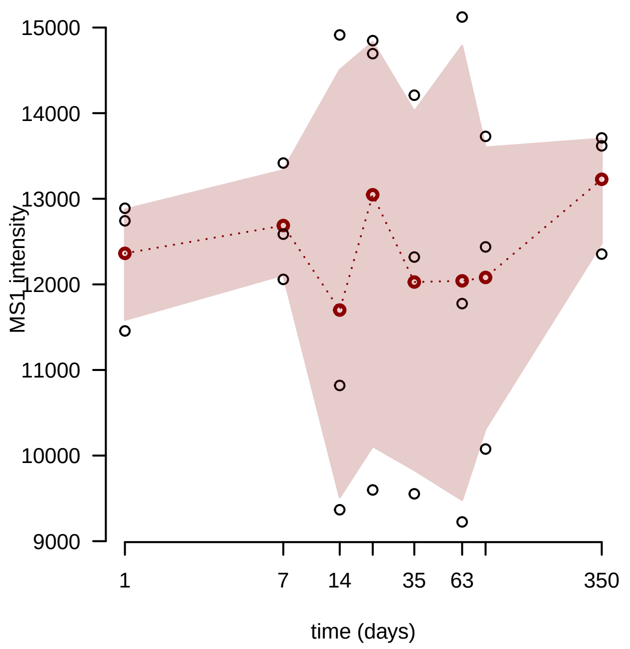

Q80TB8
GN=Vat1l
vesicle amine transport protein 1 like

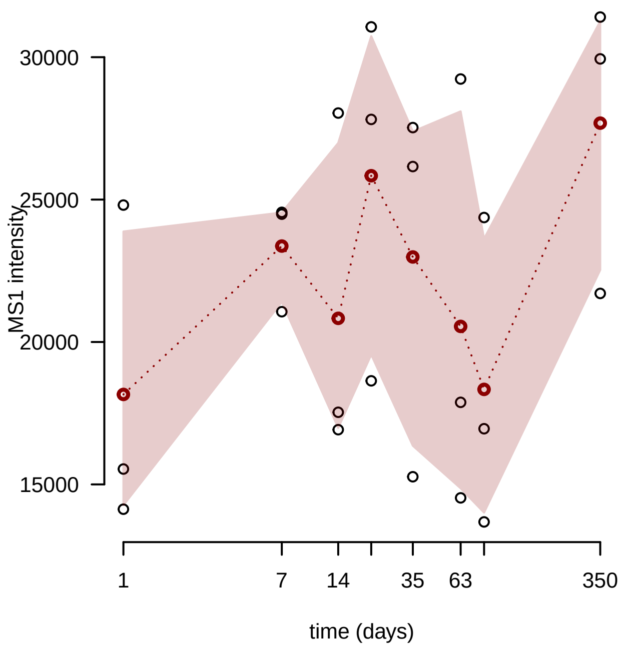

Q80TC5
GN=Pogk
pogo transposable element with KRAB doma ...

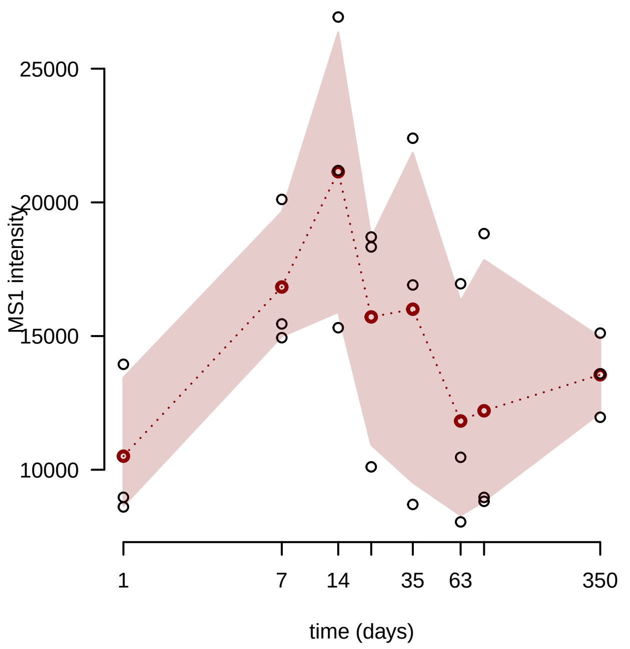

Q80TE0
GN=Rpap1
RNA polymerase II associated protein 1

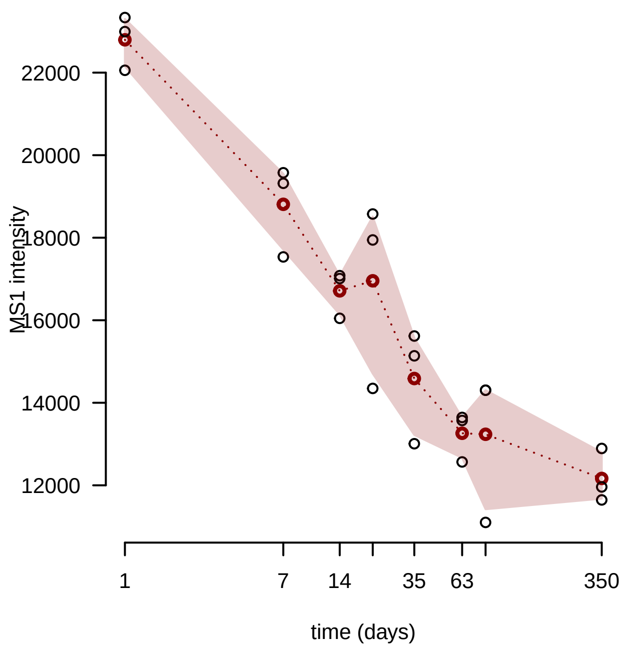

Q80TE4
GN=Sipa1l2
signal−induced proliferation−associated ...

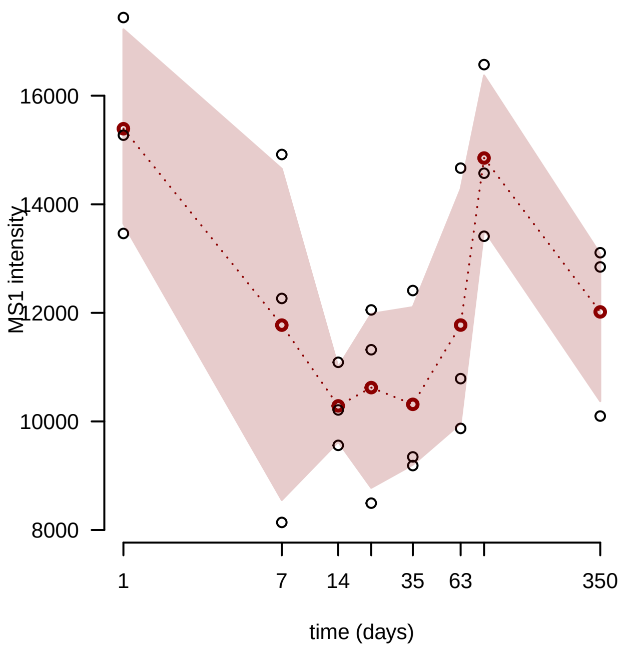

Q80TF4
GN=Klhl13
kelch−like 13

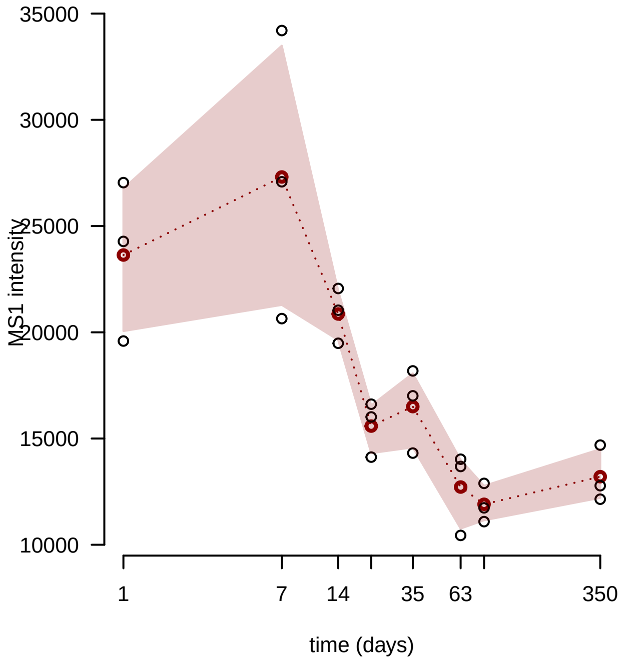

Q80TG1
GN=Kansl1
KAT8 regulatory NSL complex subunit 1

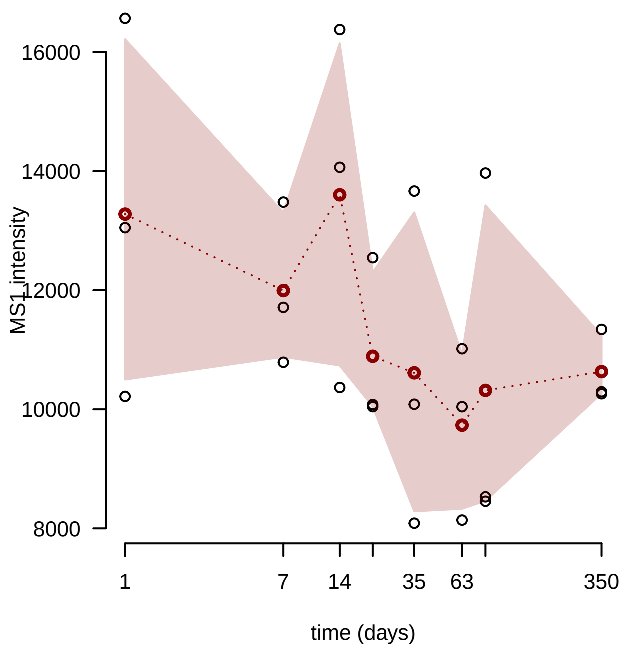

Q80TH2
GN=Erbin
Erbb2 interacting protein

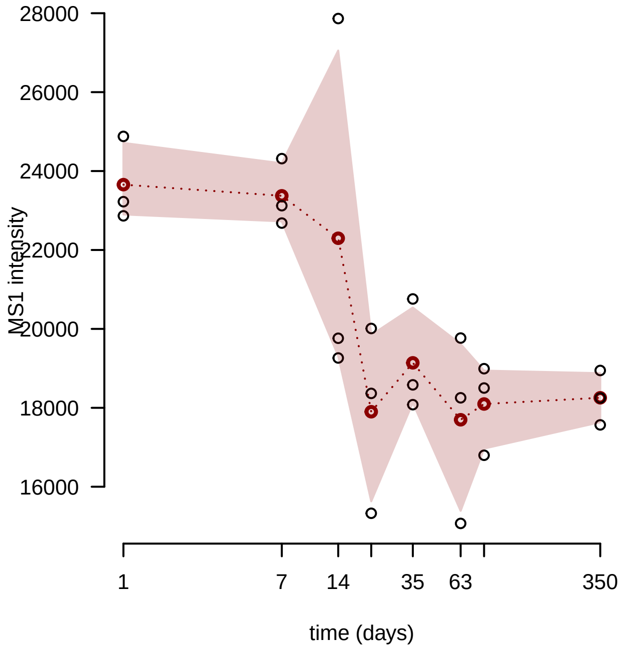

Q80TI0
GN=Gramd1b
GRAM domain containing 1B

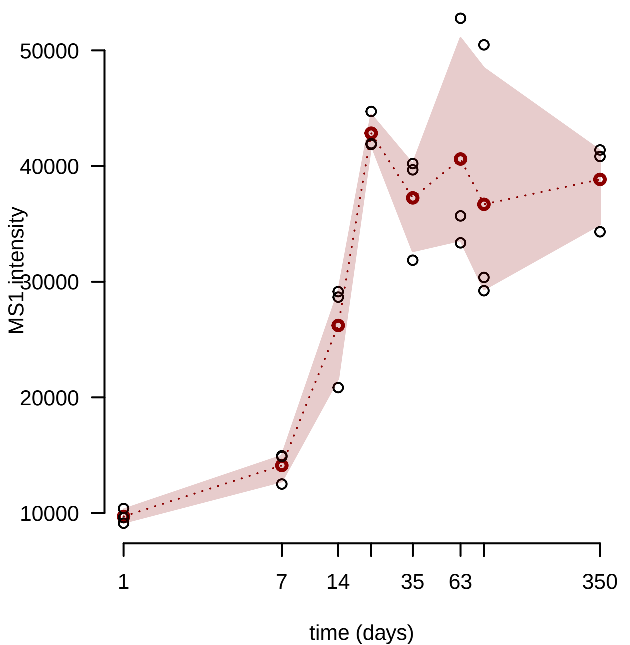

Q80TJ7
GN=Phf8
PHD finger protein 8

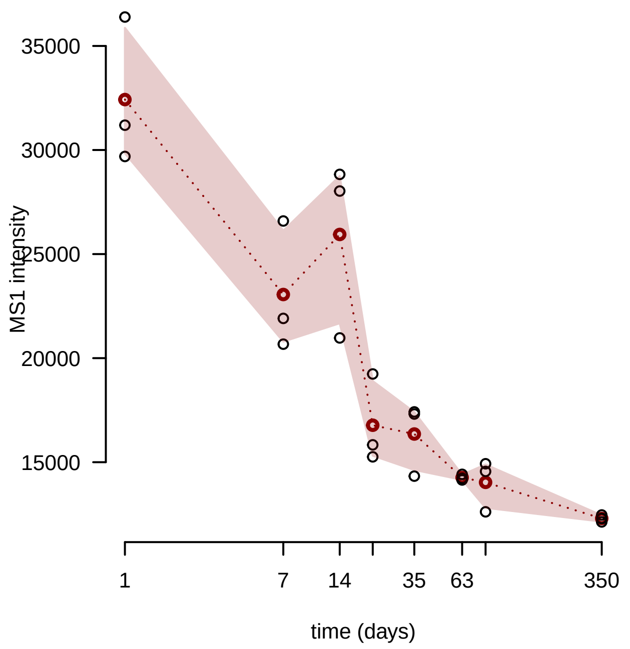

Q80TL0
GN=Ppm1e
protein phosphatase 1E (PP2C domain cont ...

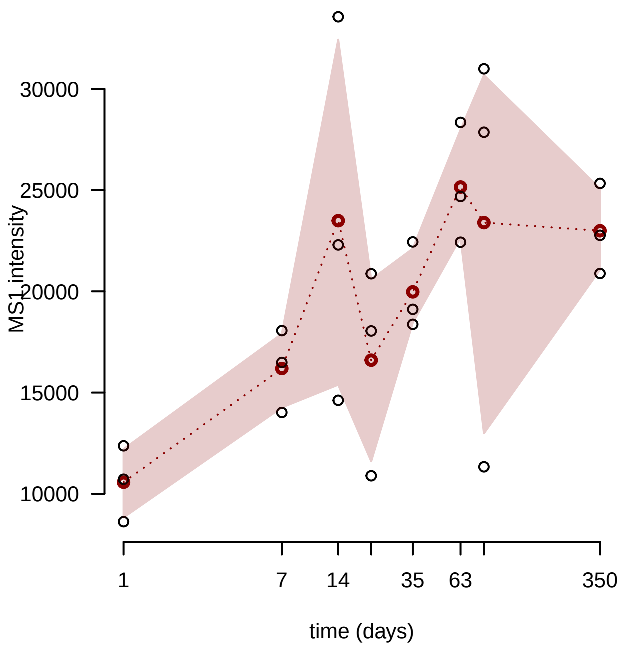

Q80TL7
GN=Mon2
MON2 homolog, regulator of endosome to G ...

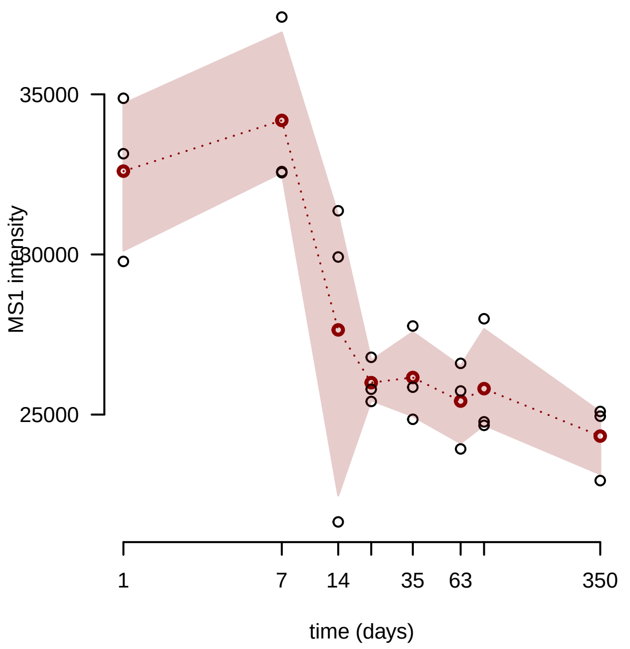

Q80TM6
GN=R3hdm2
R3H domain containing 2

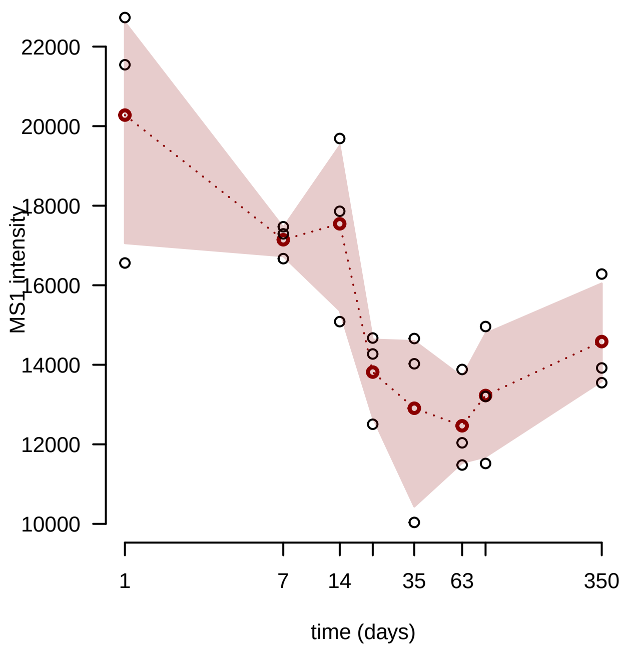

Q80TM9
GN=Nisch
nischarin

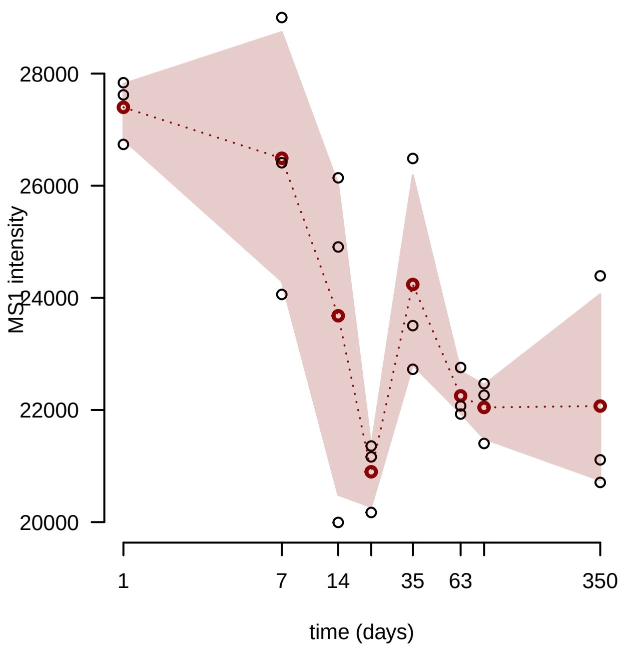

Q80TN4
GN=Dnajc16
DnaJ heat shock protein family (Hsp40) m ...

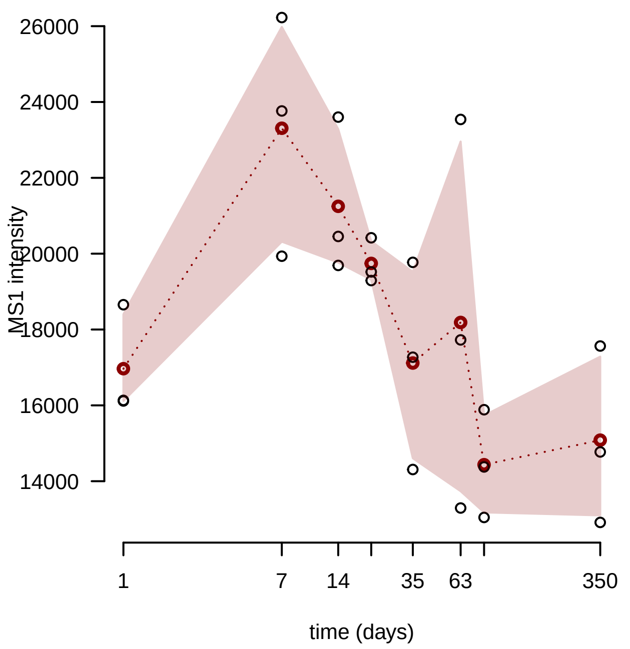

Q80TN5
GN=Zdhhc17
zinc finger, DHHC domain containing 17

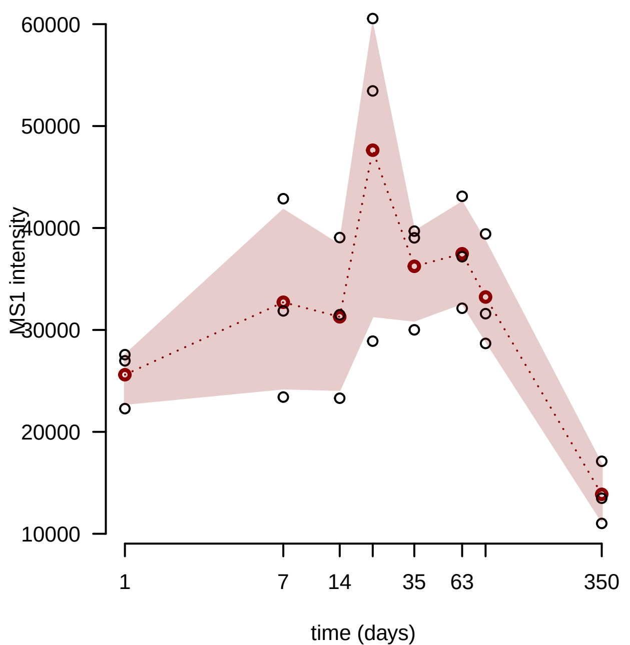

Q80TP3
GN=Ubr5
ubiquitin protein ligase E3 component n− ...

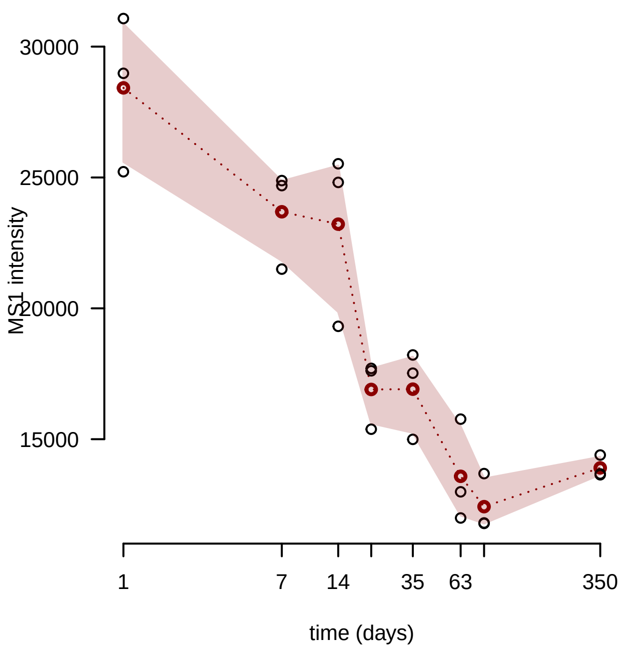

Q80TQ2
GN=Cyld
CYLD lysine 63 deubiquitinase

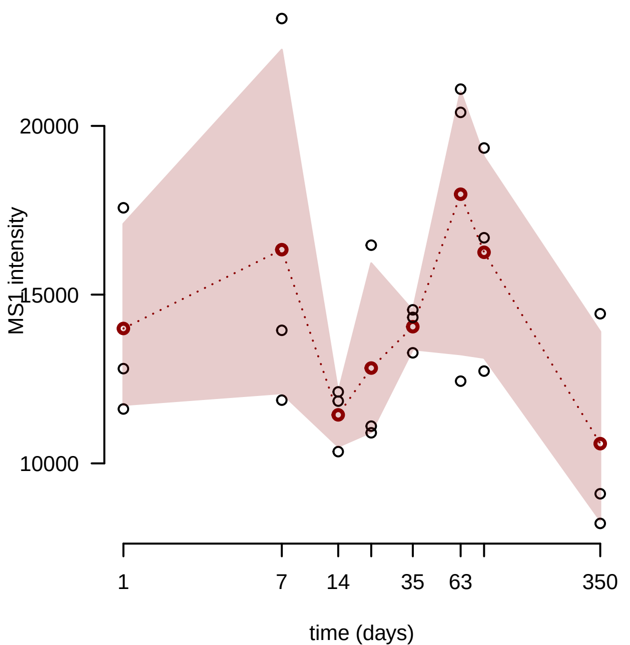

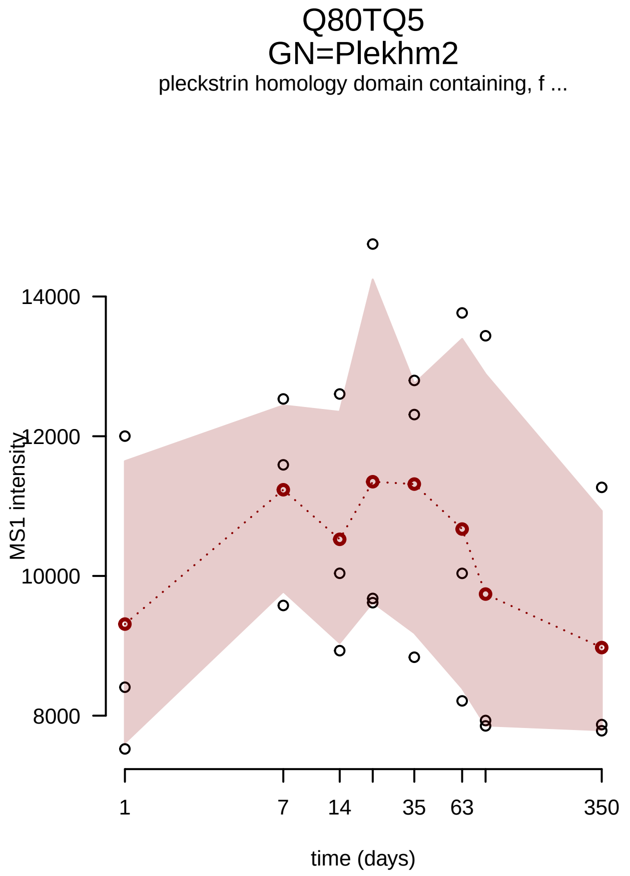
Q80TQ5
GN=Plekhm2
pleckstrin homology domain containing, f ...
MS1 intensity
8000 10000 12000 14000
1 7 14 35 63 350
time (days)

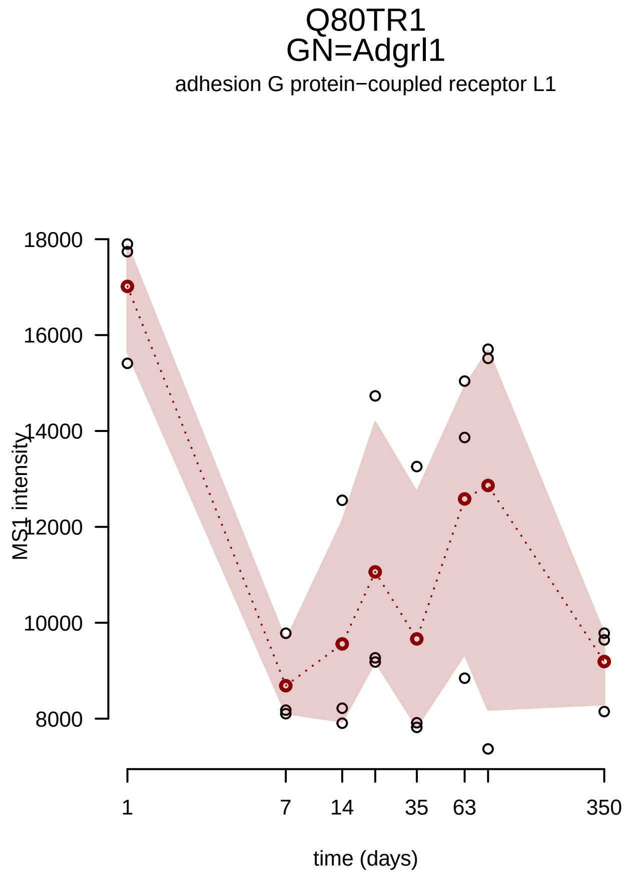
Q80TR1
GN=Adgrl1
adhesion G protein−coupled receptor L1
MS1 intensity
8000 10000 12000 14000 16000 18000
1 7 14 35 63 350
time (days)

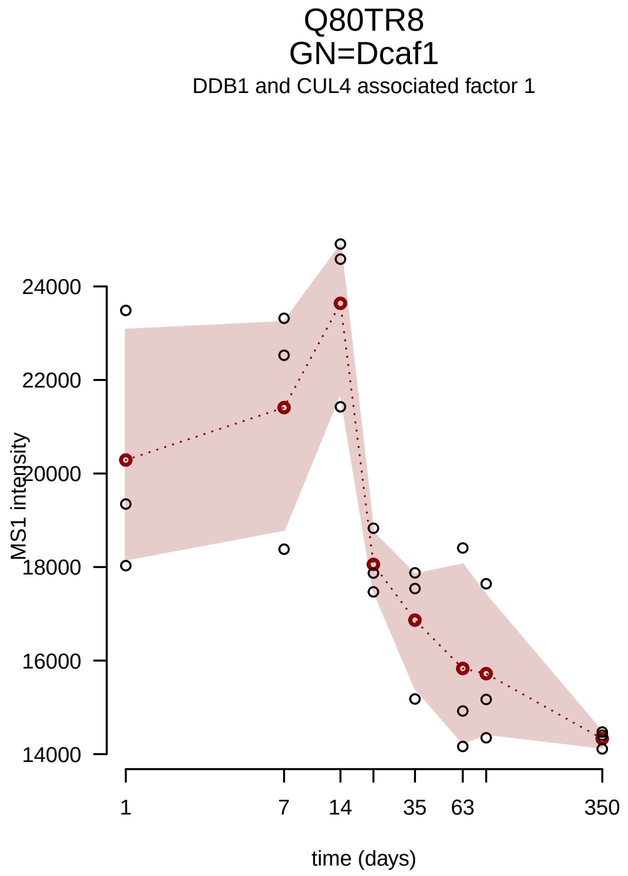
Q80TR8
GN=Dcaf1
DDB1 and CUL4 associated factor 1
MS1 intensity
14000 16000 18000 20000 22000 24000
1 7 14 35 63 350
time (days)

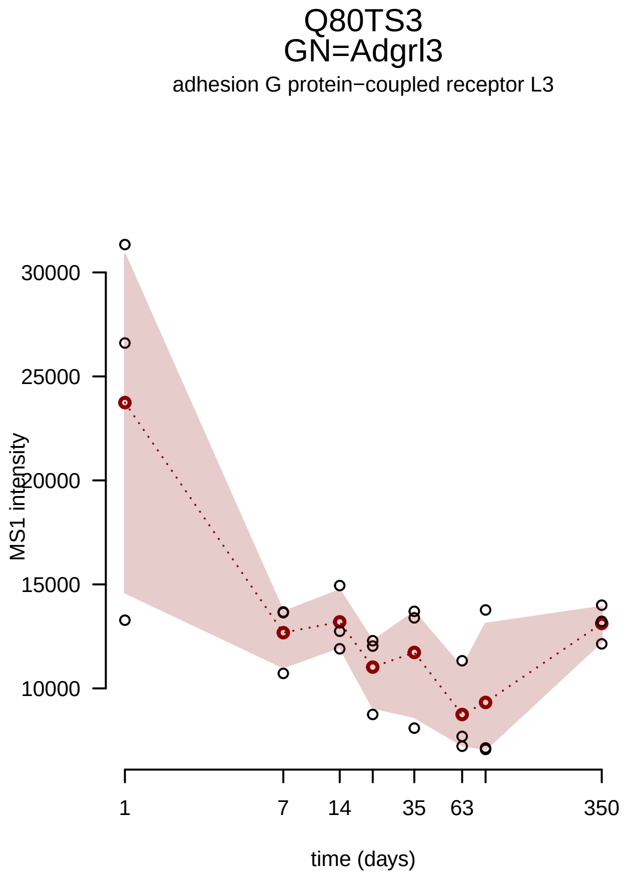
Q80TS3
GN=Adgrl3
adhesion G protein−coupled receptor L3
MS1 intensity
10000 15000 20000 25000 30000
1 7 14 35 63 350
time (days)

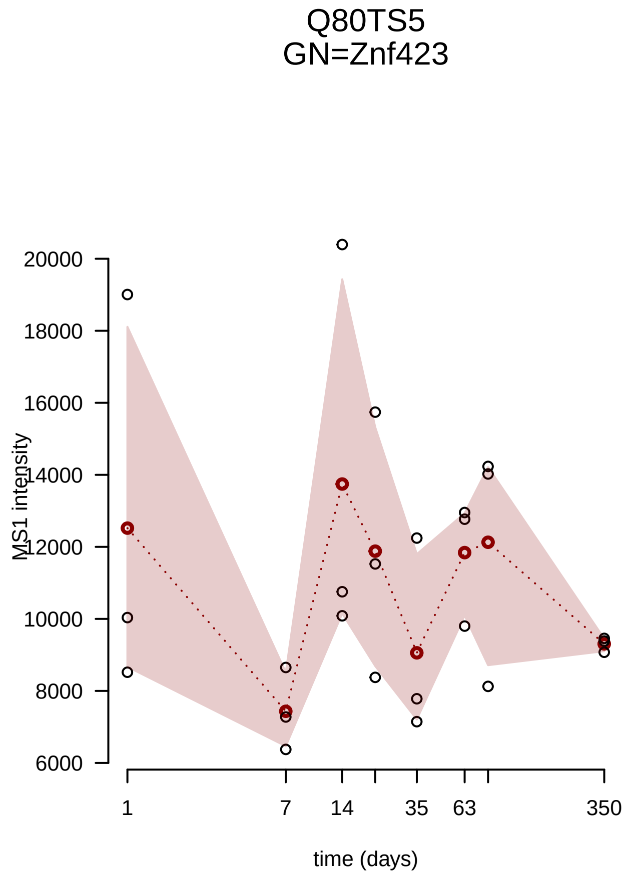
Q80TS5
GN=Znf423
MS1 intensity
6000 8000 10000 12000 14000 16000 18000 20000
1 7 14 35 63 350
time (days)

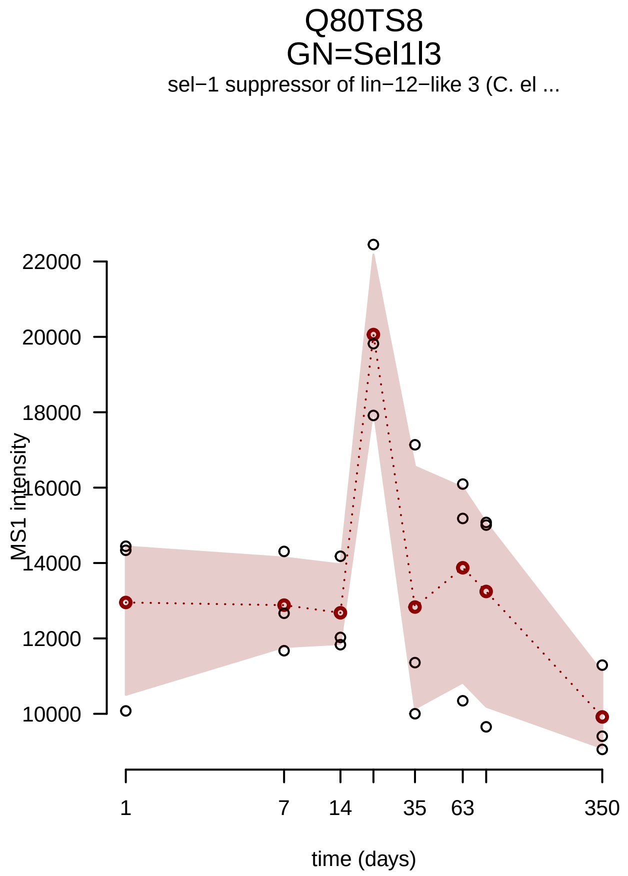
Q80TS8
GN=Sel1l3
sel−1 suppressor of lin−12−like 3 (C. el ...
MS1 intensity
10000 12000 14000 16000 18000 20000 22000
1 7 14 35 63 350
time (days)

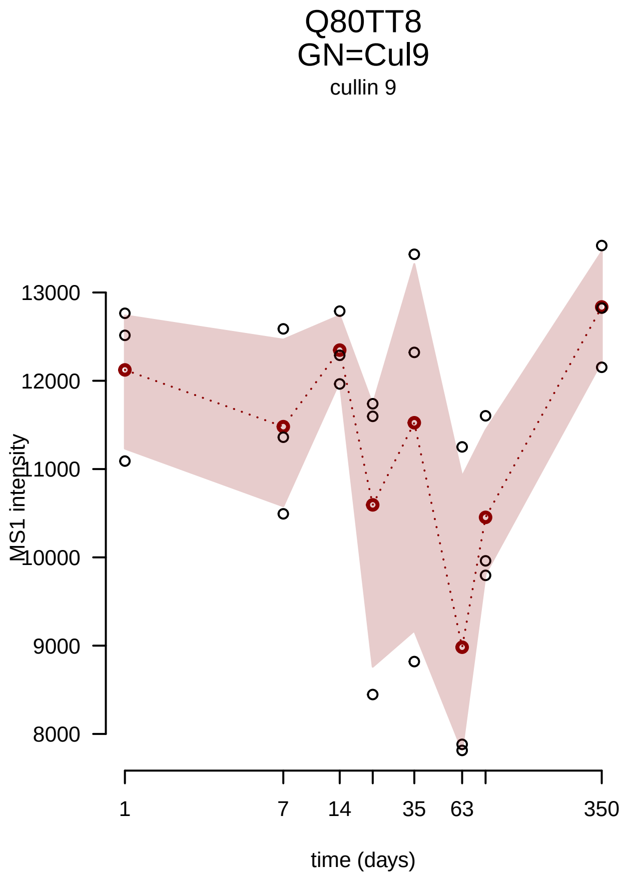
Q80TT8
GN=Cul9
cullin 9
MS1 intensity
8000 9000 10000 11000 12000 13000
1 7 14 35 63 350
time (days)

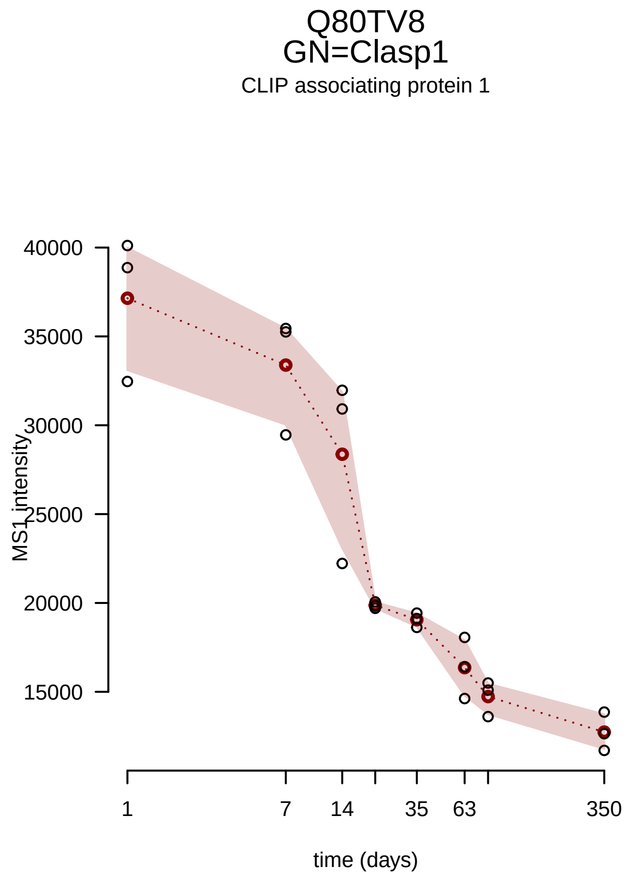
Q80TV8
GN=Clasp1
CLIP associating protein 1
MS1 intensity
15000 20000 25000 30000 35000 40000
1 7 14 35 63 350
time (days)

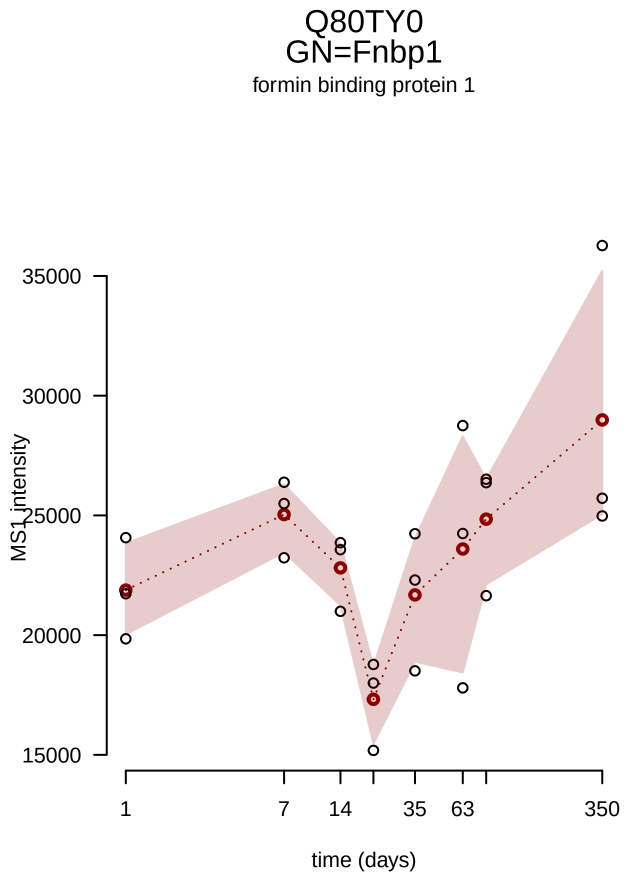
Q80TY0
GN=Fnbp1
formin binding protein 1
MS1 intensity
15000 20000 25000 30000 35000
1 7 14 35 63 350
time (days)

Q80TY5
GN=Vps13b
vacuolar protein sorting 13B

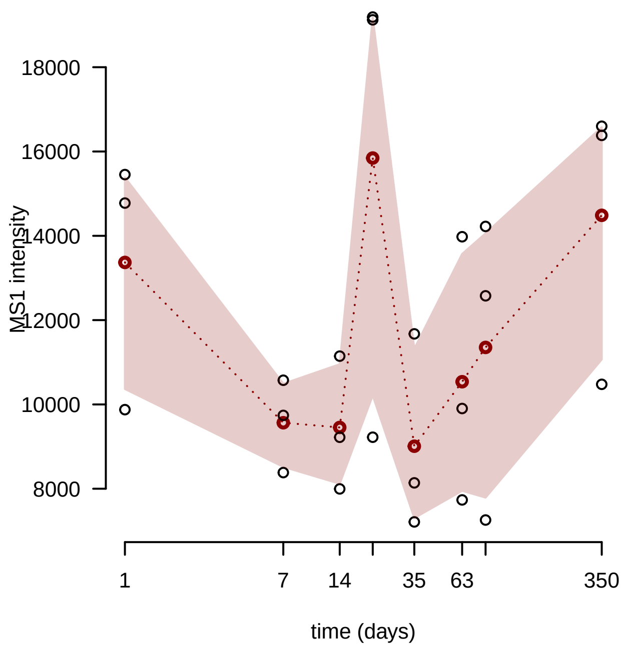

Q80TZ9
GN=Rere
arginine glutamic acid dipeptide (RE) re ...

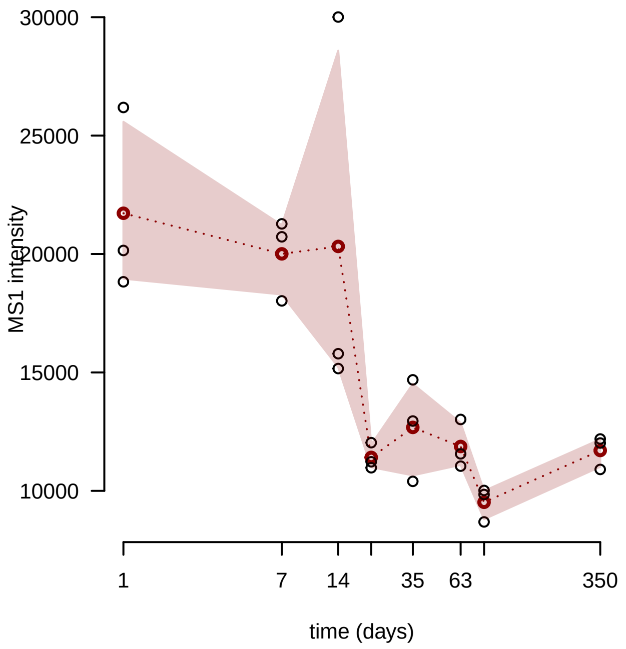

Q80U04
GN=Pja2
praja ring finger ubiquitin ligase 2

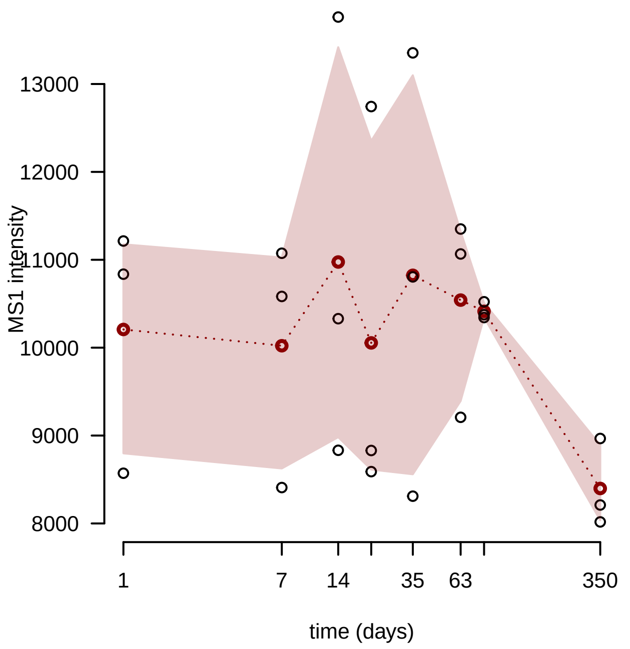

Q80U16
GN=Ripor2
RHO family interacting cell polarization ...

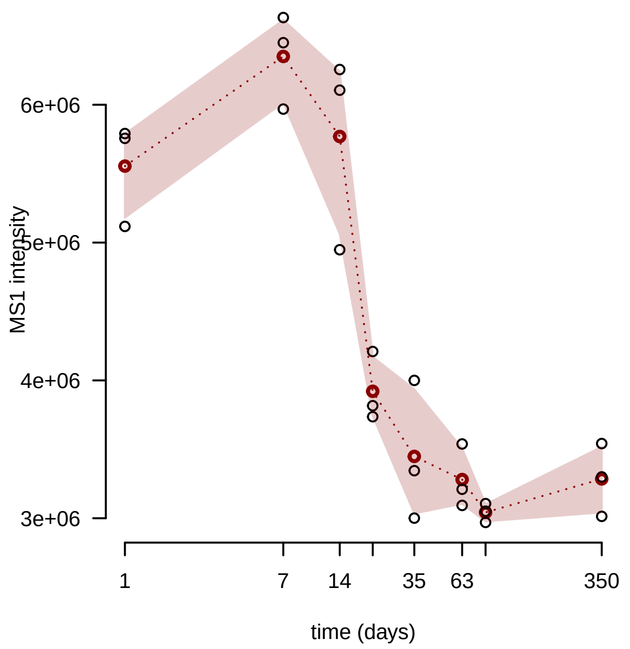

Q80U28
GN=Madd
MAP−kinase activating death domain

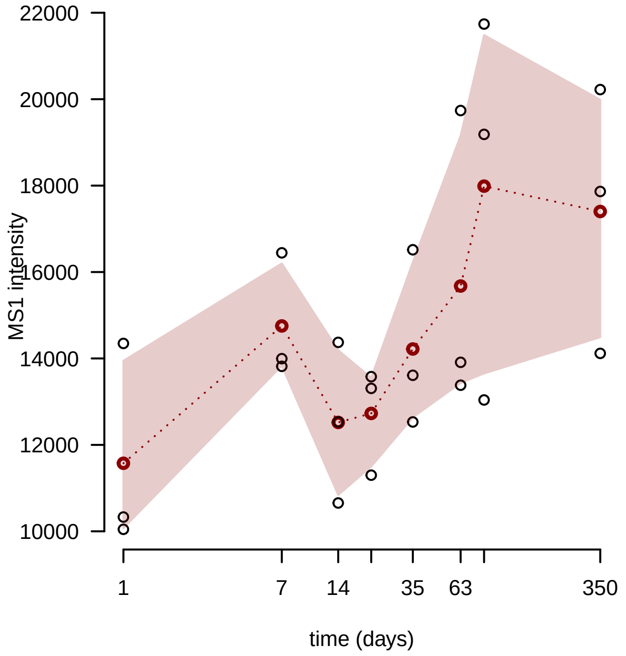

Q80U30
GN=Clec16a
C−type lectin domain family 16, member A

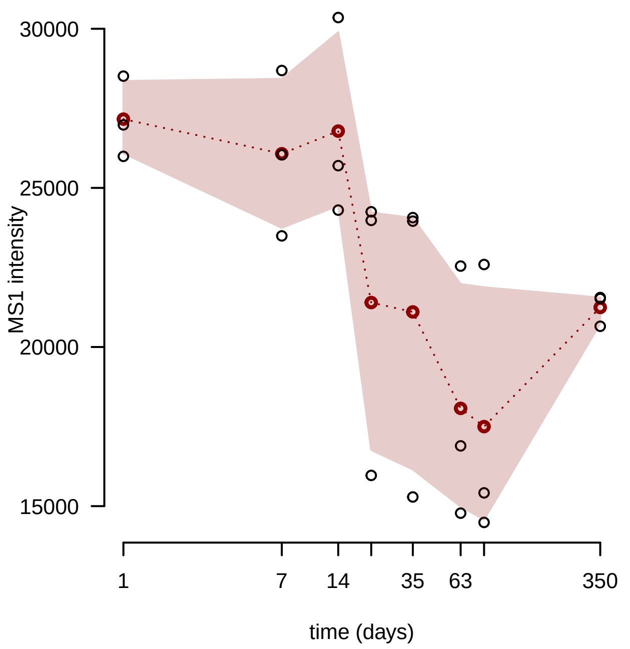

Q80U35
GN=Arhgef17
Rho guanine nucleotide exchange factor (...

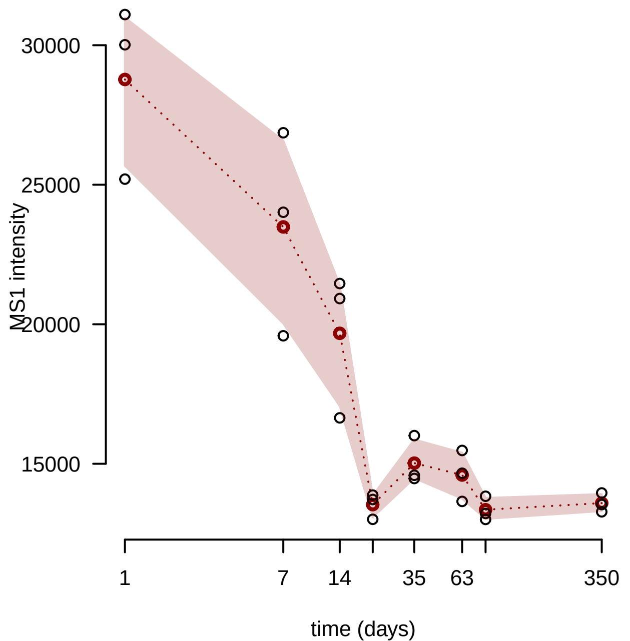

Q80U38
GN=Khnyn
KH and NYN domain containing

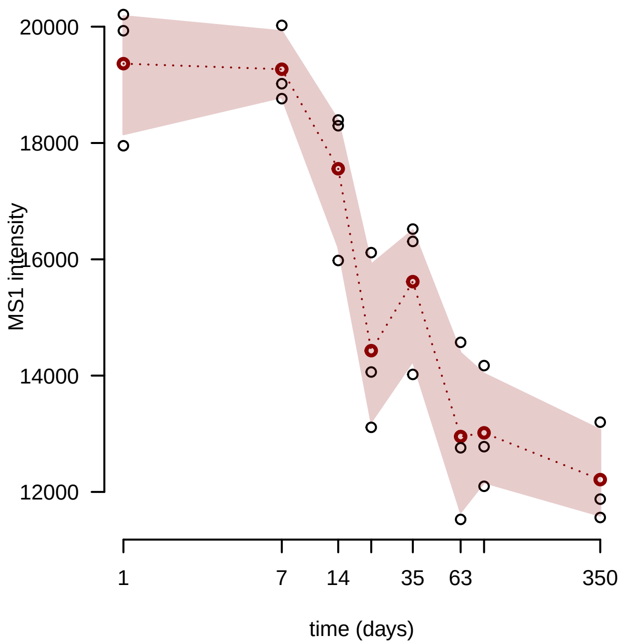

Q80U44
GN=Zfyve16
zinc finger, FYVE domain containing 16

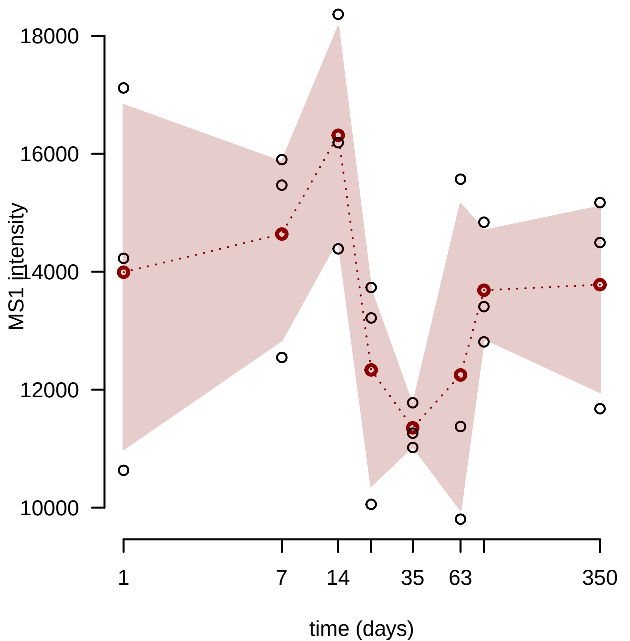

Q80U49
GN=Cep170b
centrosomal protein 170B

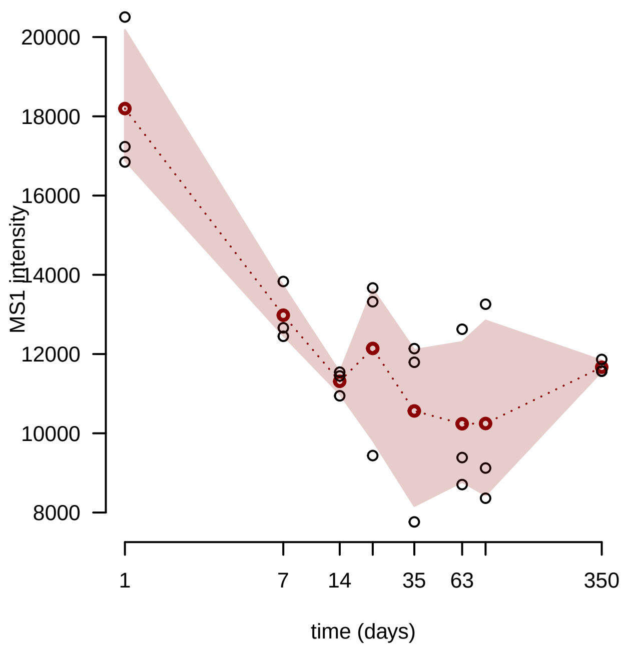

Q80U56
GN=Avl9
AVL9 cell migration associated

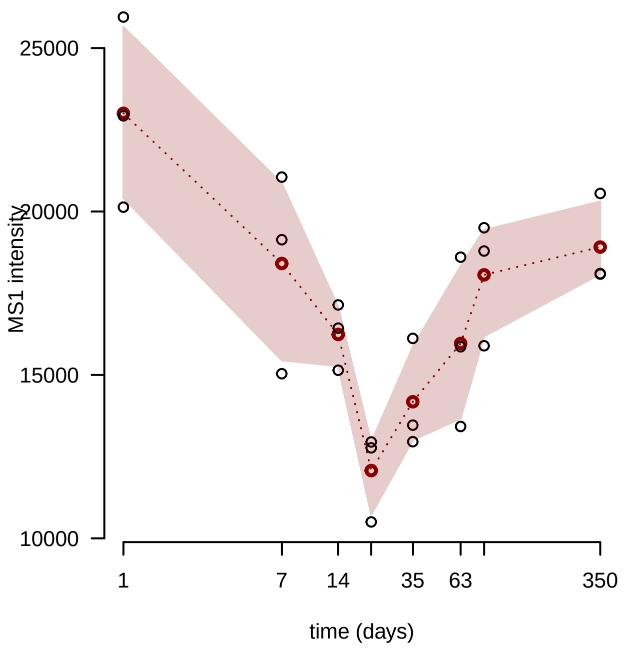

Q80U58
GN=Pum2
pumilio RNA−binding family member 2

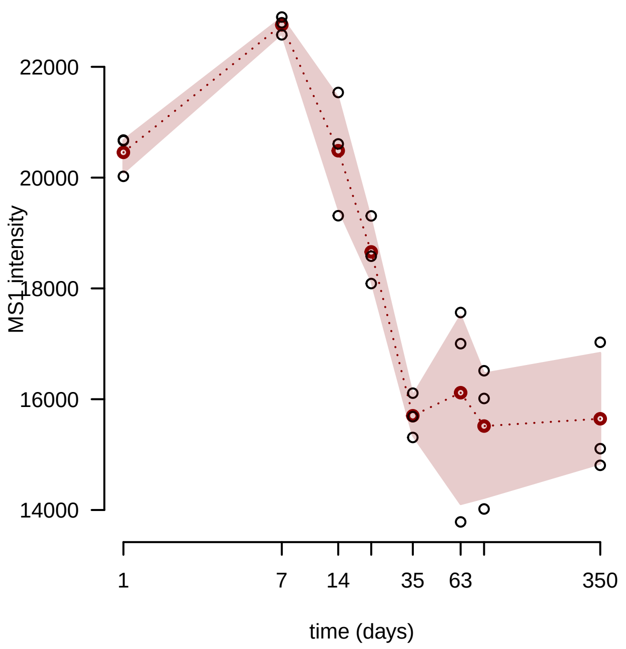

Q80U63
GN=Mfn2
mitofusin 2

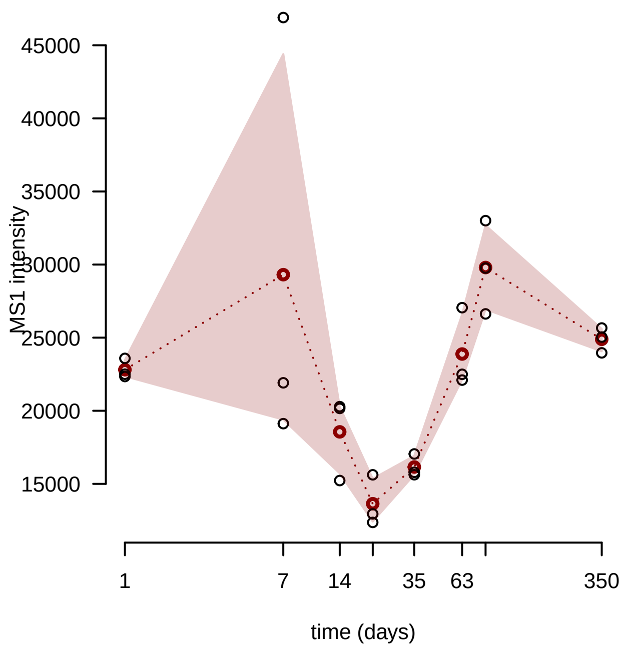

Q80U70
GN=Suz12
SUZ12 polycomb repressive complex 2 subu ...

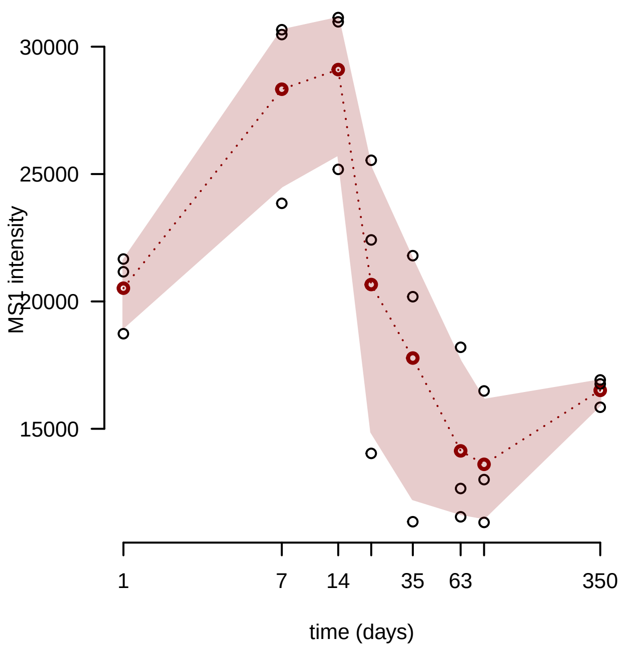

Q80U72
GN=Scrib
scribbled planar cell polarity

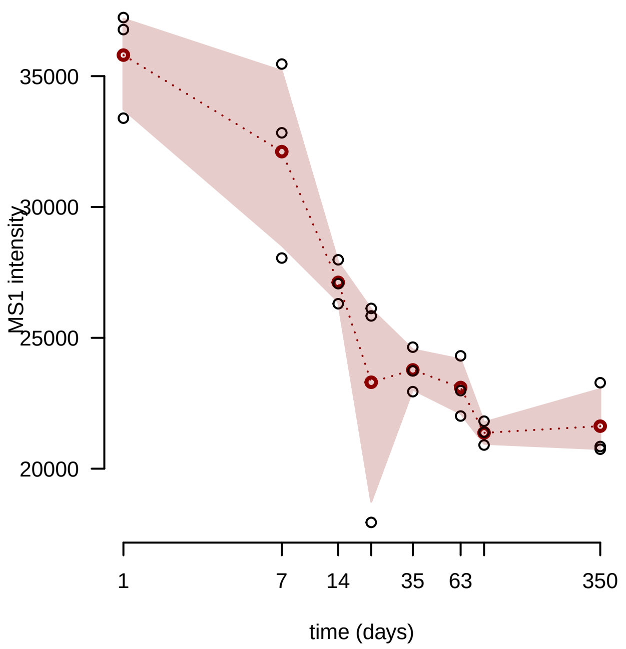

Q80U78
GN=Pum1
pumilio RNA−binding family member 1

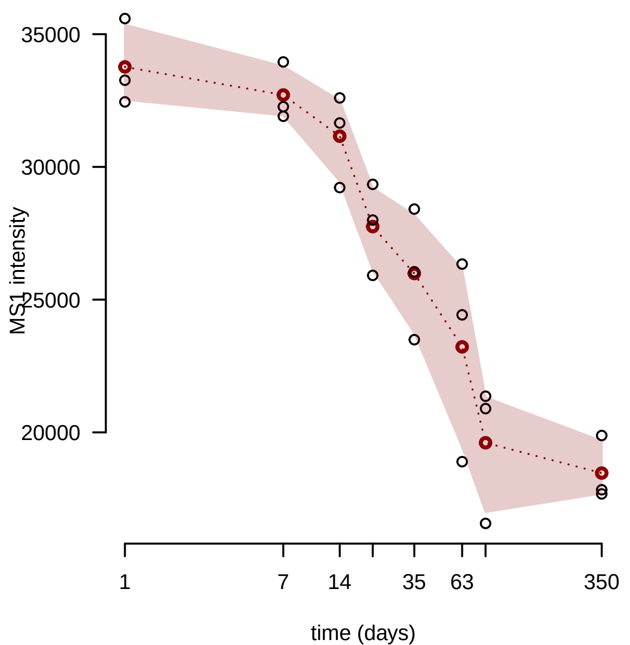

Q80U87
GN=Usp8
ubiquitin specific peptidase 8

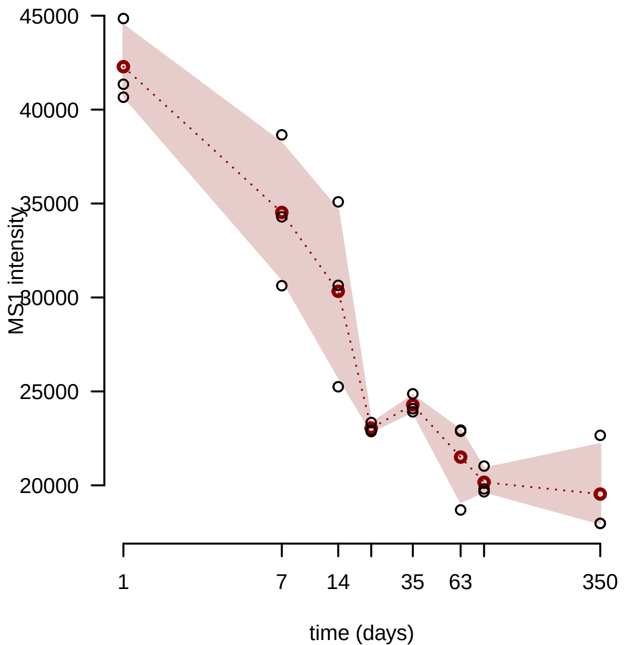

Q80U93
GN=Nup214
nucleoporin 214

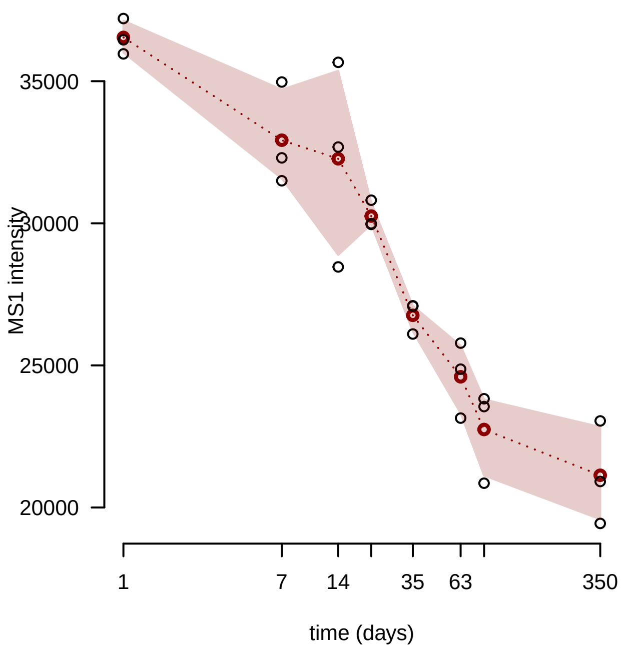

Q80U95
GN=Ube3c
ubiquitin protein ligase E3C

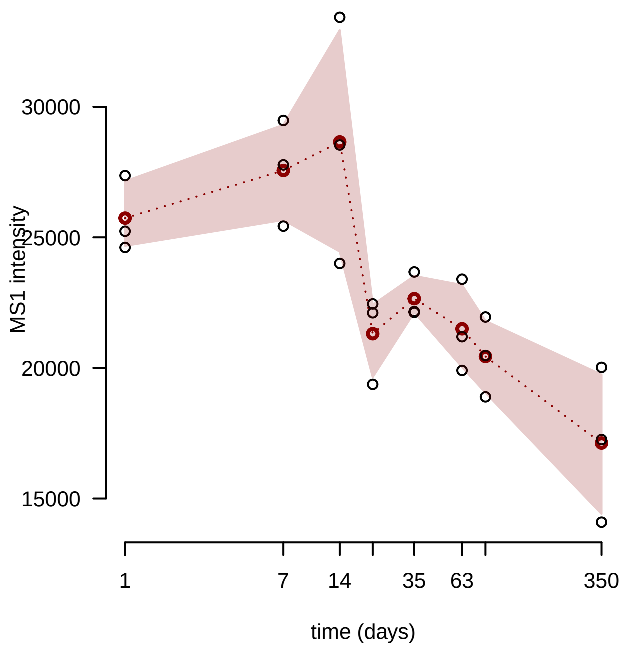

Q80UG1
GN=Fads6
fatty acid desaturase domain family, mem ...

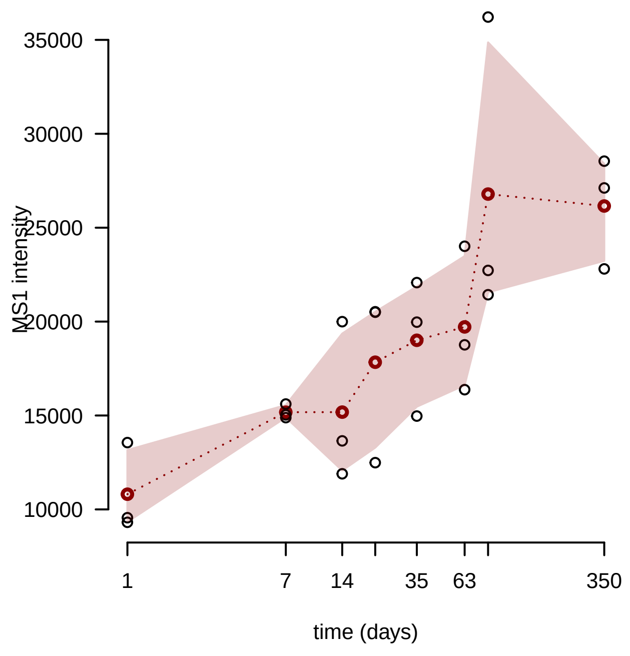

Q80UG2
GN=Plxna4
plexin A4

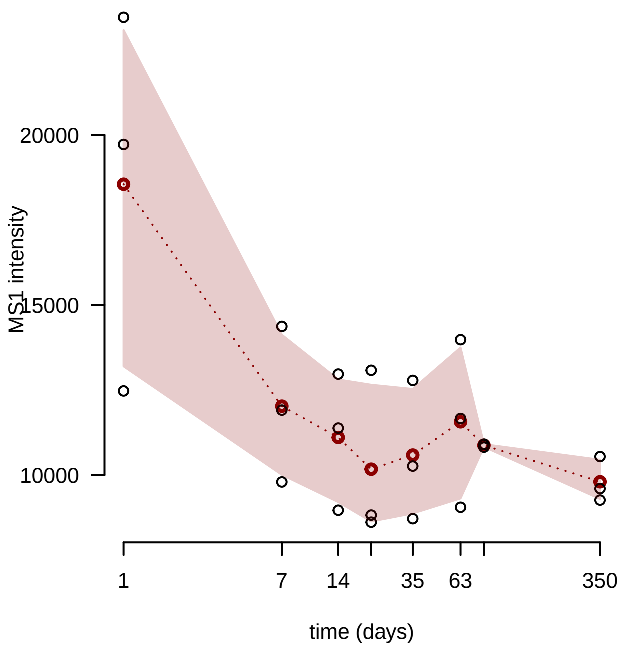

Q80UG5
GN=Sept9

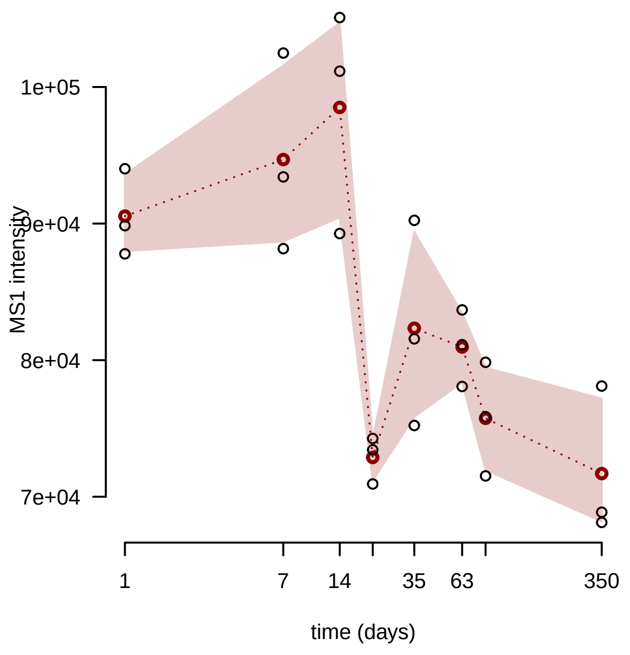

Q80UJ7
GN=Rab3gap1
RAB3 GTPase activating protein subunit 1

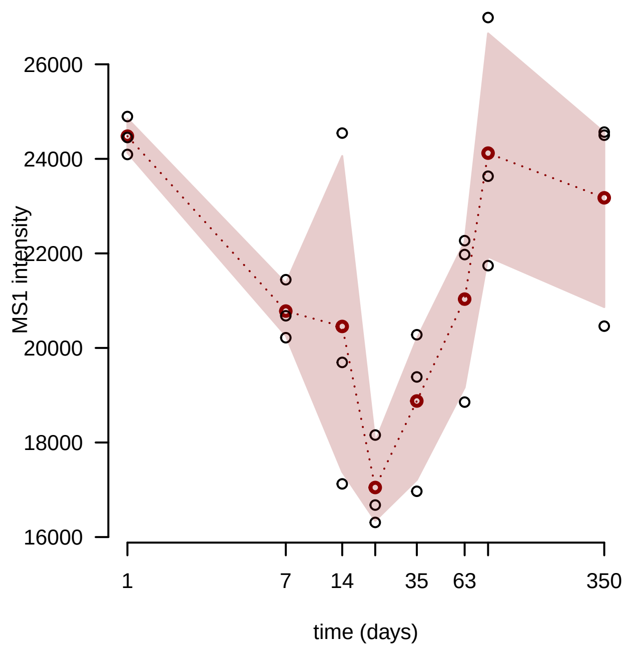

Q80UK0
GN=Sestd1
SEC14 and spectrin domains 1

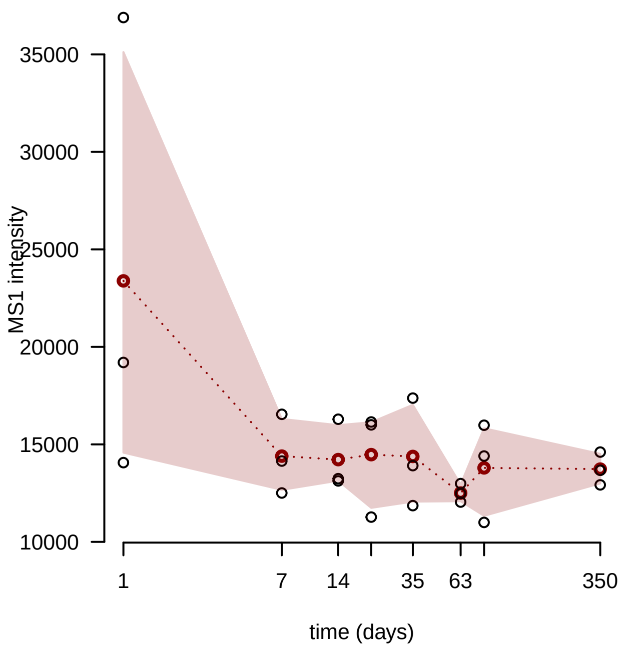

Q80UK8
GN=Ints2
integrator complex subunit 2

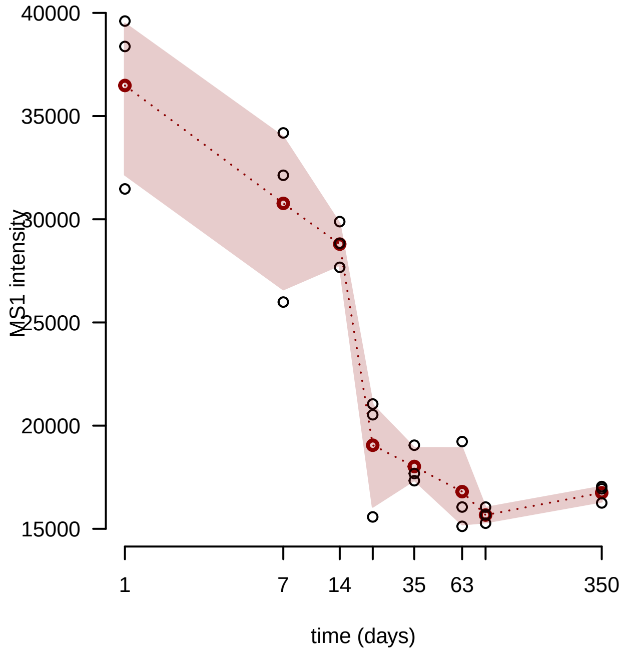

Q80UM3
GN=Naa15
N(alpha)−acetyltransferase 15, NatA auxi ...

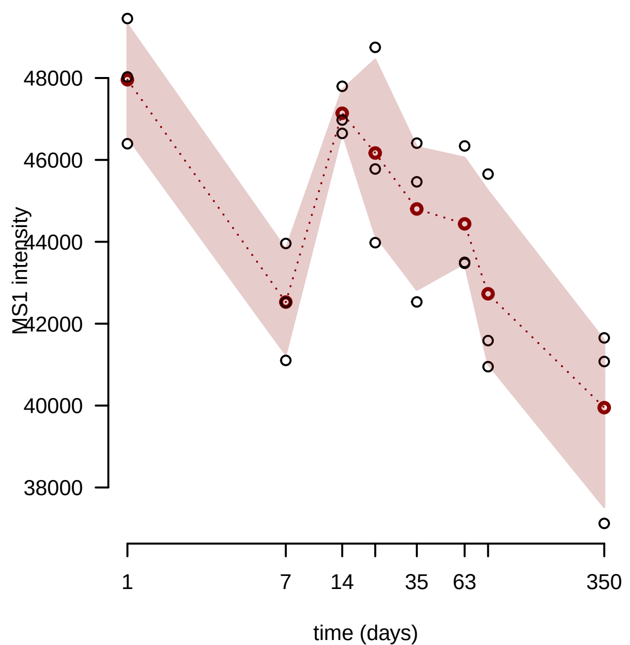

Q80UM7
GN=Mogs
mannosyl−oligosaccharide glucosidase

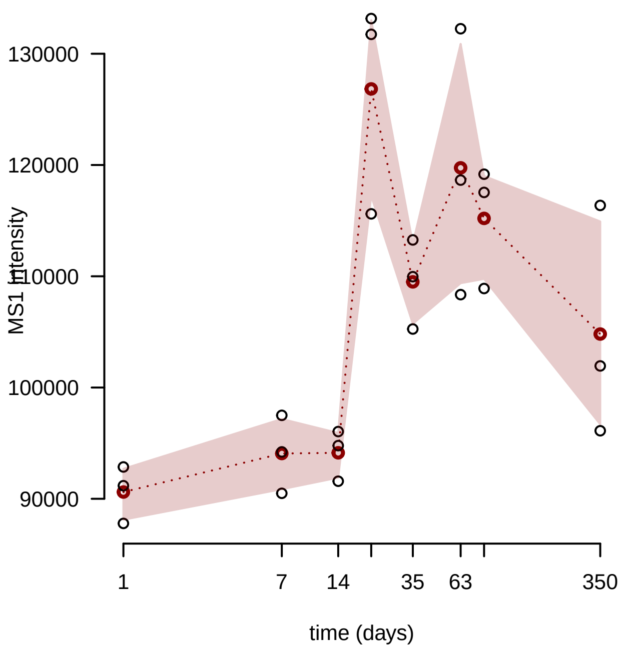

Q80UN9
GN=Trit1
tRNA isopentenyltransferase 1

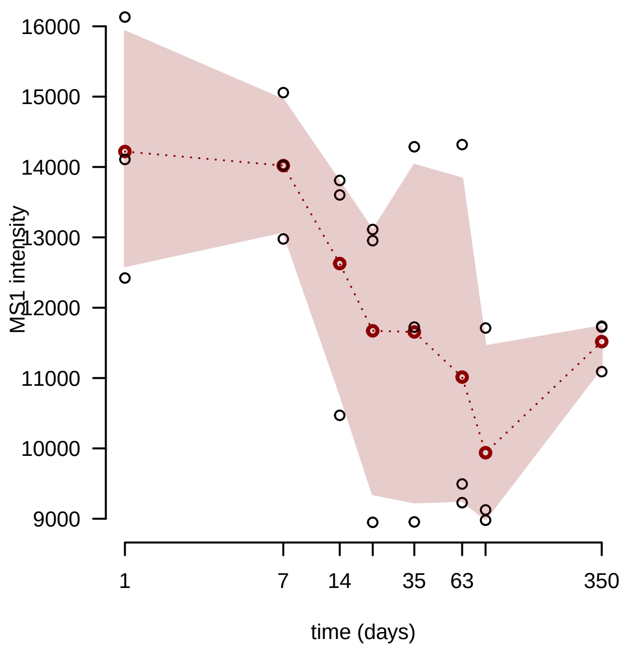

Q80UP3
GN=Dgkz
diacylglycerol kinase zeta

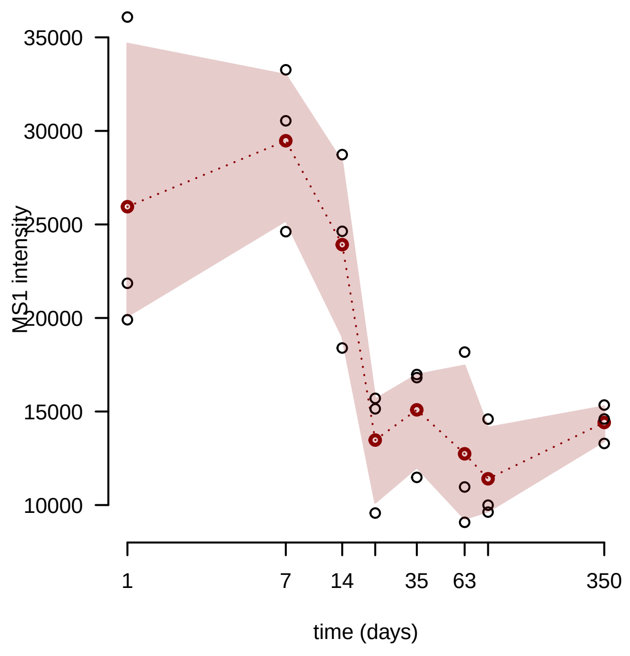

Q80UP5
GN=Ankrd13a
ankyrin repeat domain 13a

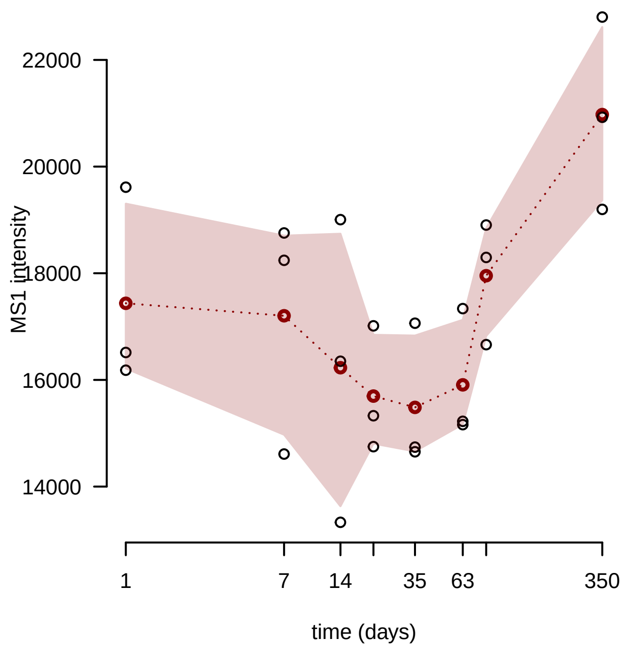

Q80UP8
GN=Slc20a2
solute carrier family 20, member 2

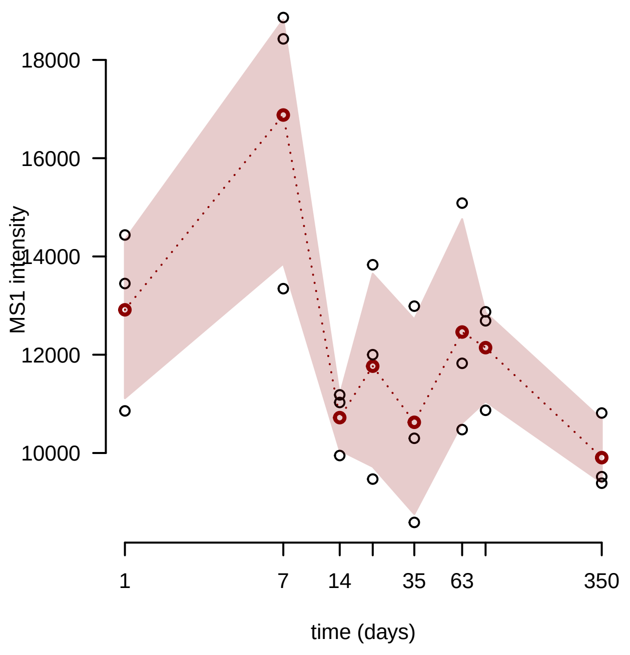

Q80US4
GN=Actr5
ARP5 actin−related protein 5

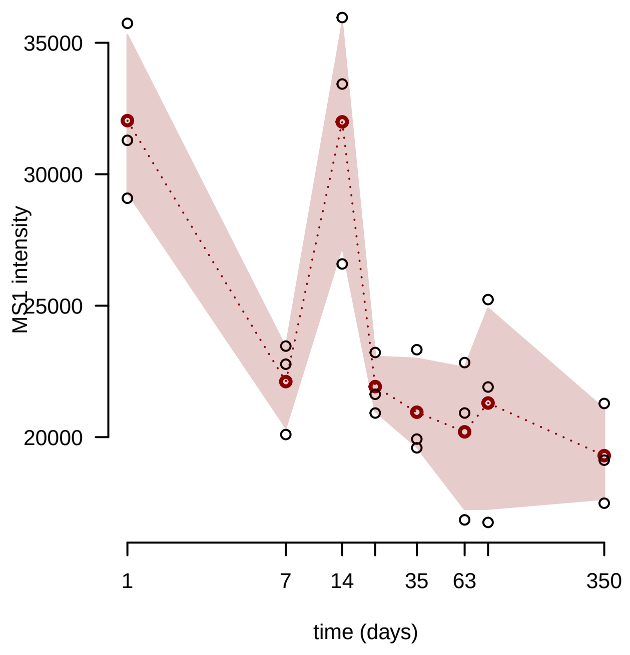

Q80UU1
GN=Ankzf1
ankyrin repeat and zinc finger domain co ...

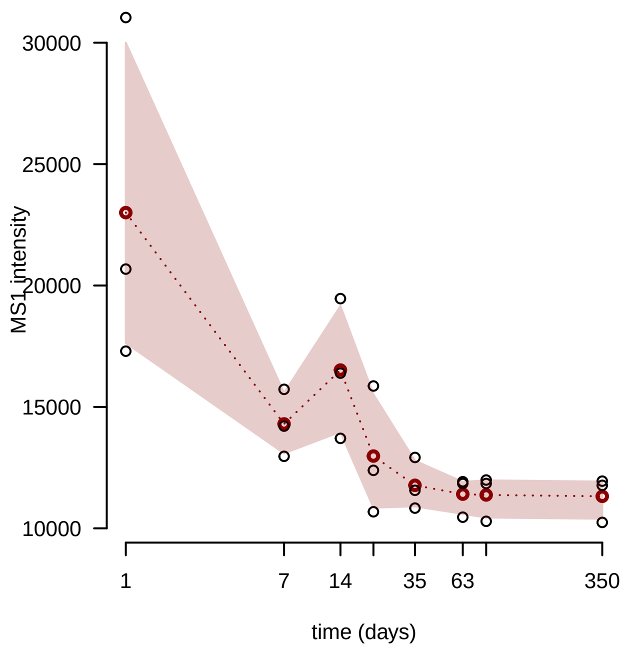

Q80UU2
GN=Rpp38
ribonuclease P/MRP 38 subunit

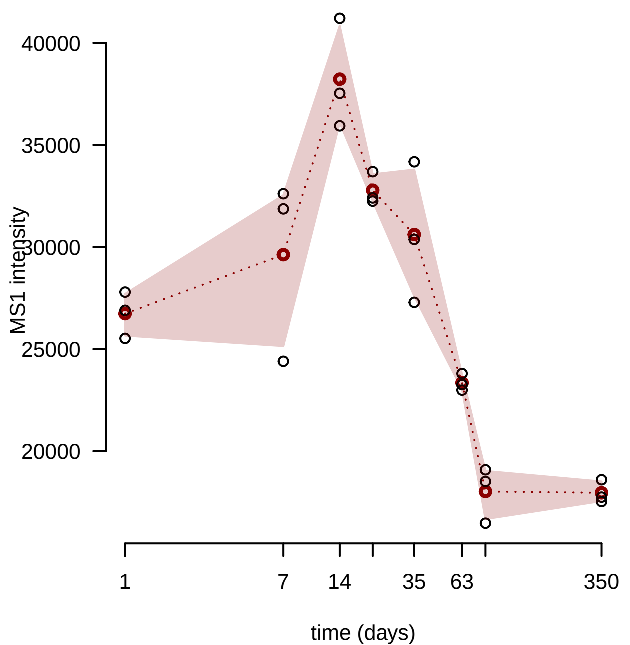

Q80UU9
GN=Pgrmc2
progesterone receptor membrane component ...

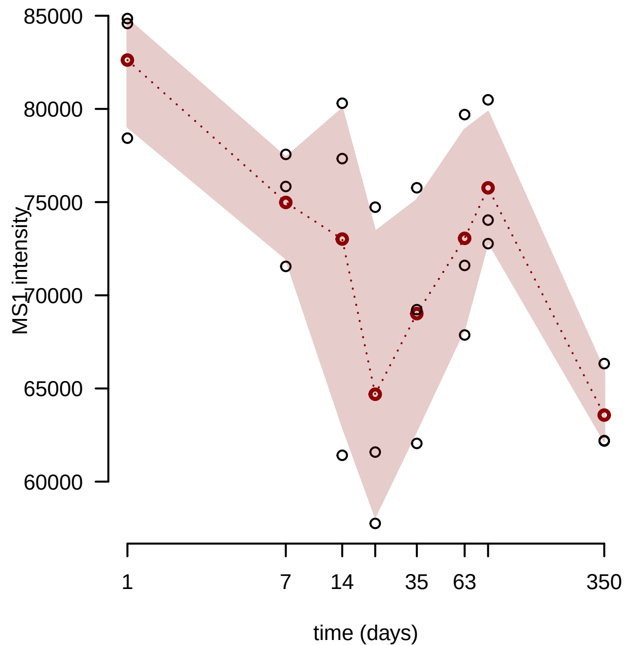

Q80UV9
GN=Taf1
TATA−box binding protein associated fact ...

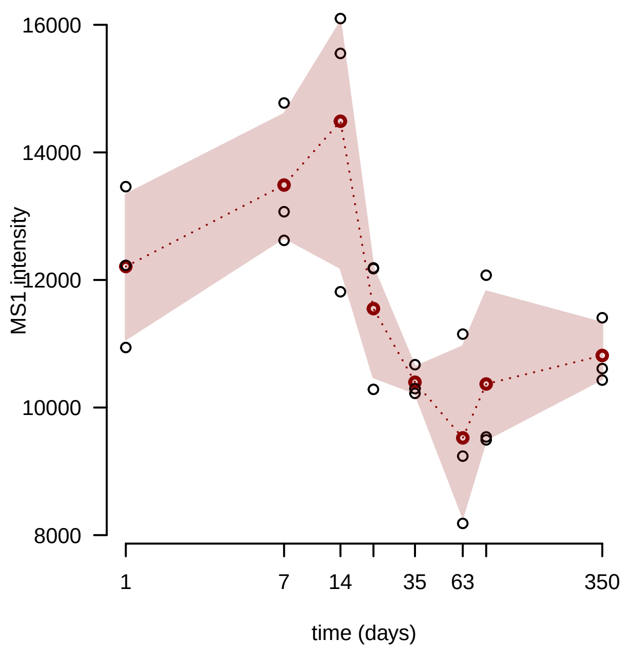

Q80UW0
GN=Hs6st2
heparan sulfate 6−O−sulfotransferase 2

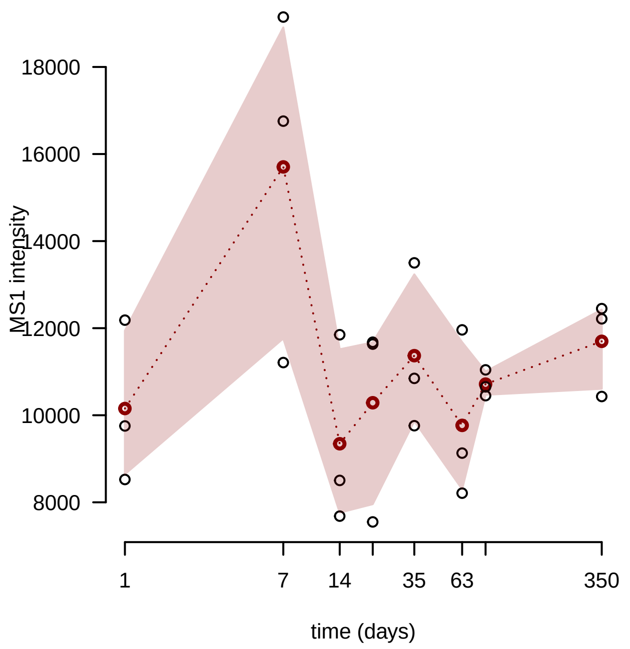

Q80UW5
GN=Cdc42bpg
CDC42 binding protein kinase gamma (DMPK ...

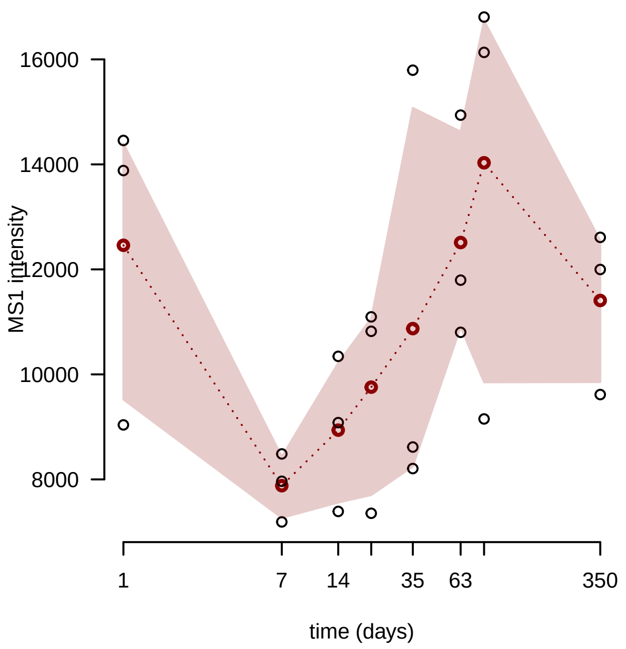

Q80UW8
GN=Polr2e
polymerase (RNA) II (DNA directed) polyp ...

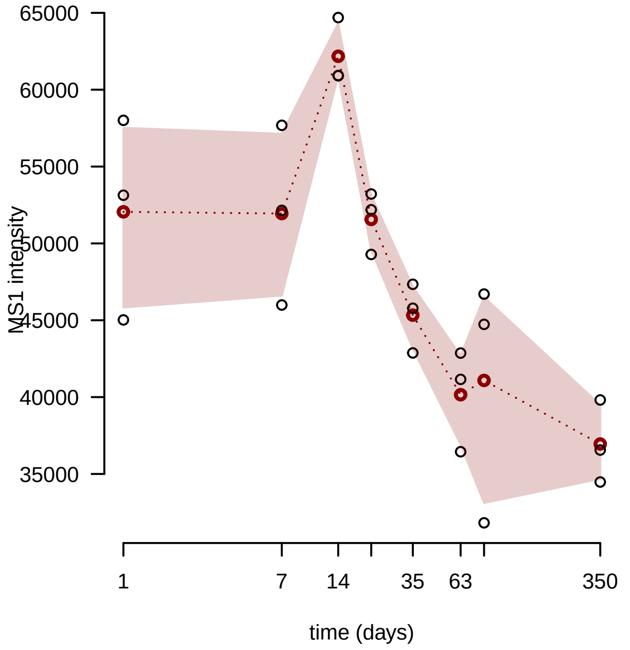

Q80UX8
GN=Abhd13
abhydrolase domain containing 13

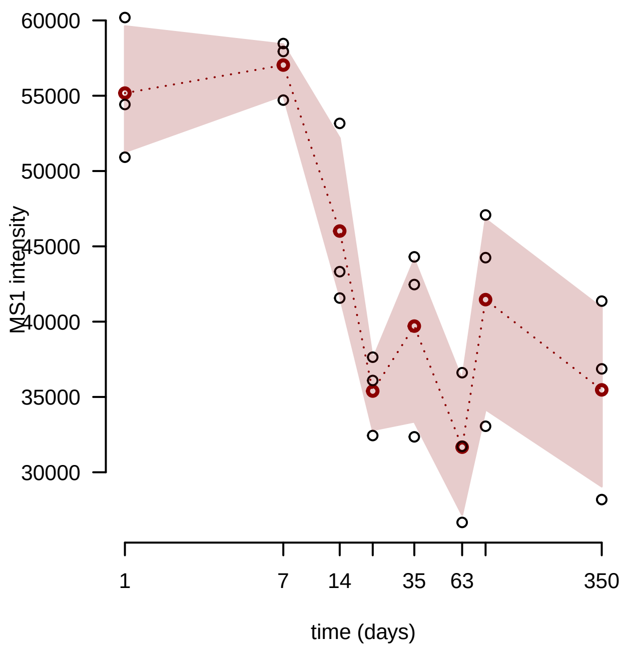

Q80UY1
GN=Carnmt1
carnosine N−methyltransferase 1

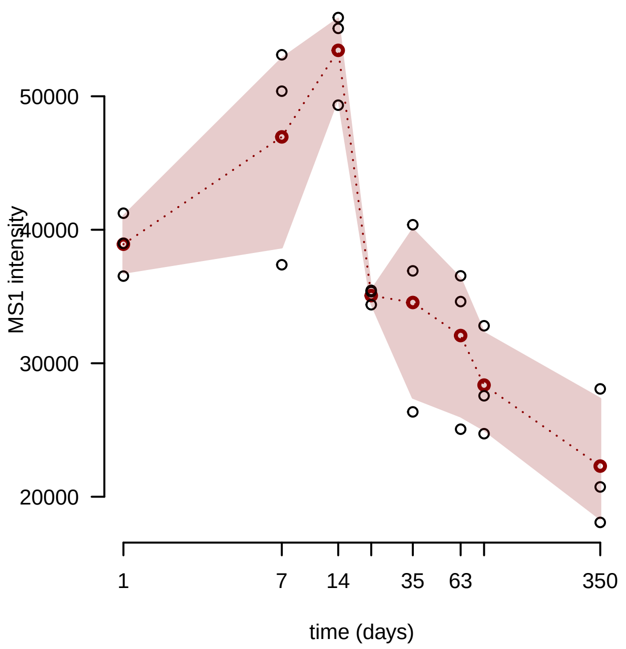

Q80UY2
GN=Kcmf1
potassium channel modulatory factor 1

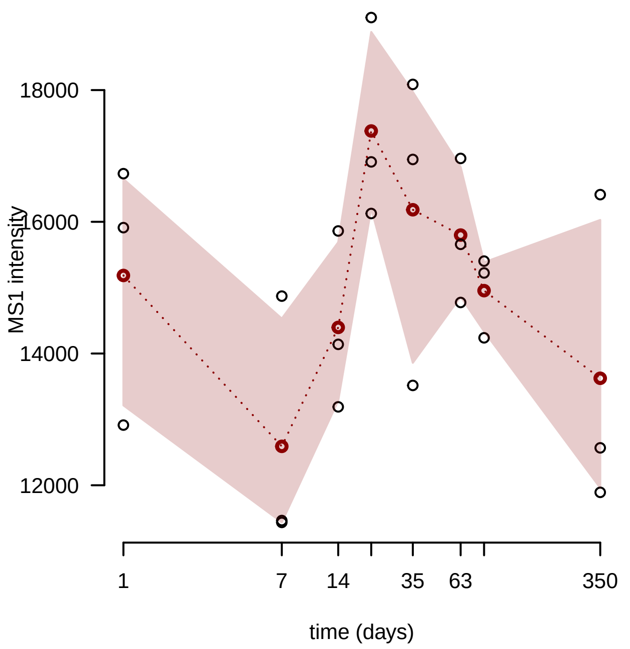

Q80UZ0
GN=Fgd5
FYVE, RhoGEF and PH domain containing 5

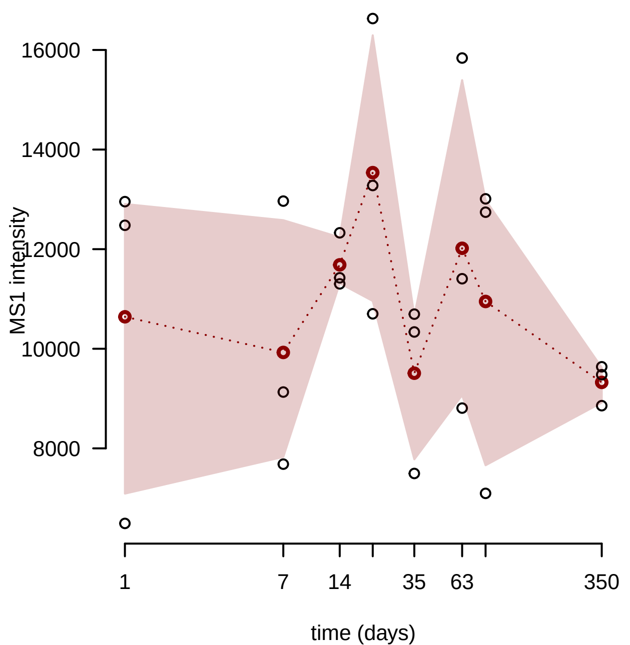

Q80UZ2
GN=Sdad1
SDA1 domain containing 1

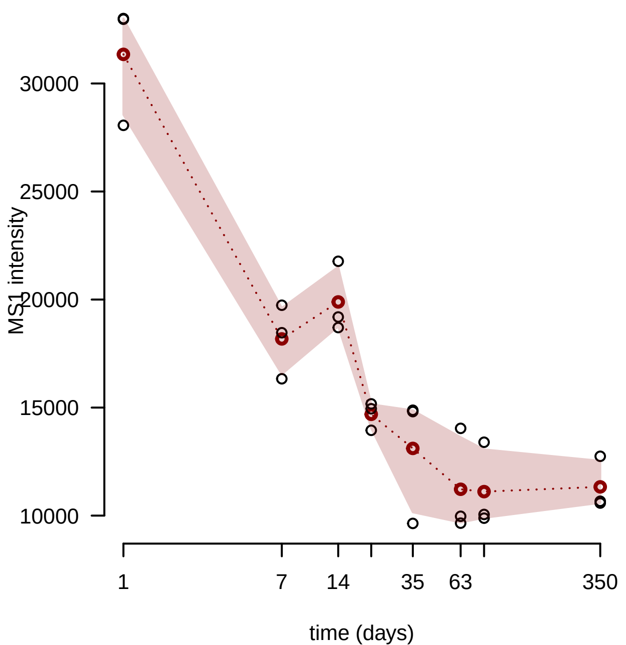

Q80V03
GN=Adck5
aarF domain containing kinase 5

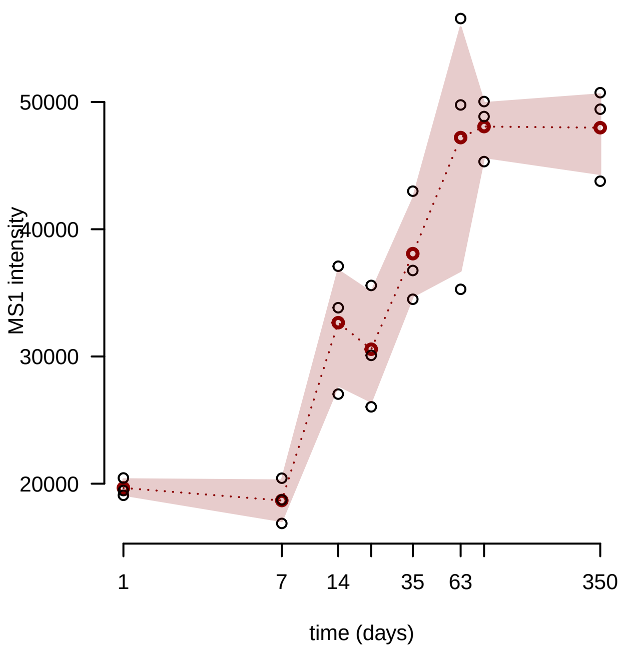

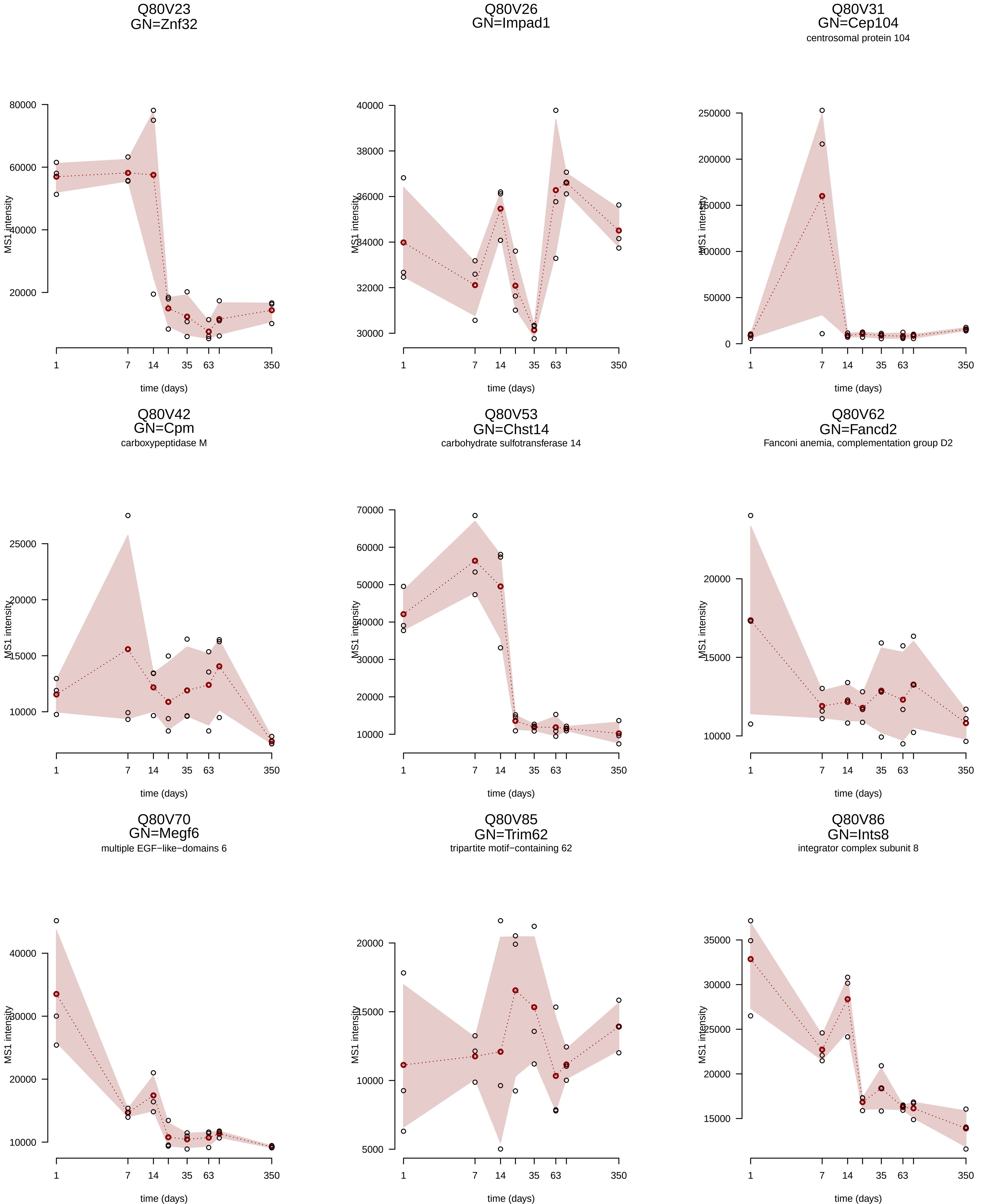
Q80V23
GN=Znf32
80000
60000
40000
20000
MS1 intensity
1
7
14
35
63
350
time (days)
Q80V26
GN=Impad1
40000
38000
36000
34000
32000
30000
MS1 intensity
1
7
14
35
63
350
time (days)
Q80V31
GN=Cep104
centrosomal protein 104
250000
200000
150000
100000
50000
0
MS1 intensity
1
7
14
35
63
350
time (days)
Q80V42
GN=Cpm
carboxypeptidase M
25000
20000
15000
10000
MS1 intensity
1
7
14
35
63
350
time (days)
Q80V53
GN=Chst14
carbohydrate sulfotransferase 14
70000
60000
50000
40000
30000
20000
10000
MS1 intensity
1
7
14
35
63
350
time (days)
Q80V62
GN=Fancd2
Fanconi anemia, complementation group D2
20000
15000
10000
MS1 intensity
1
7
14
35
63
350
time (days)
Q80V70
GN=Megf6
multiple EGF−like−domains 6
40000
30000
20000
10000
MS1 intensity
1
7
14
35
63
350
time (days)
Q80V85
GN=Trim62
tripartite motif−containing 62
20000
15000
10000
5000
MS1 intensity
1
7
14
35
63
350
time (days)
Q80V86
GN=Ints8
integrator complex subunit 8
35000
30000
25000
20000
15000
MS1 intensity
1
7
14
35
63
350
time (days)

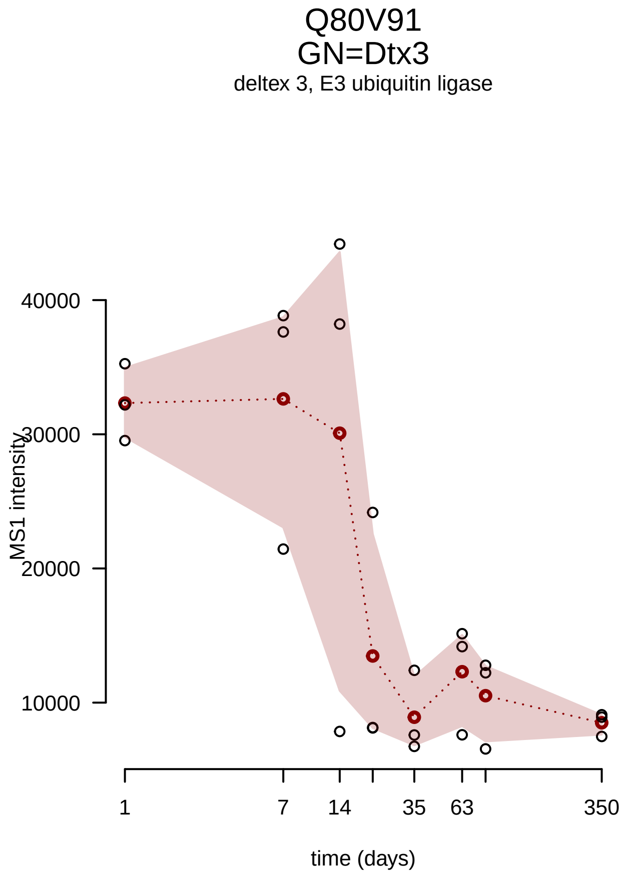
Q80V91
GN=Dtx3
deltex 3, E3 ubiquitin ligase
MS1 intensity
40000
30000
20000
10000
1
7
14
35
63
350
time (days)

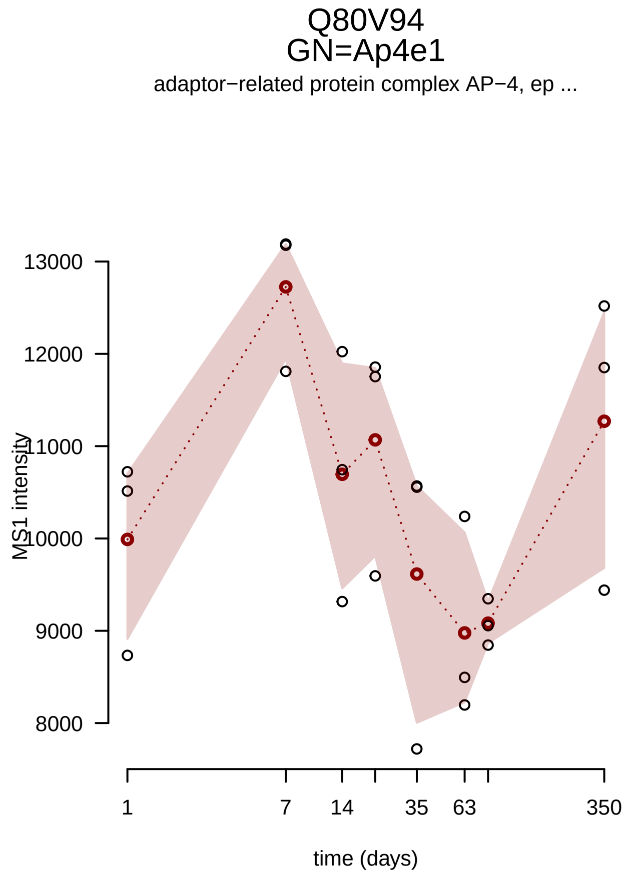
Q80V94
GN=Ap4e1
adaptor−related protein complex AP−4, ep ...
MS1 intensity
13000
12000
11000
10000
9000
8000
1
7
14
35
63
350
time (days)

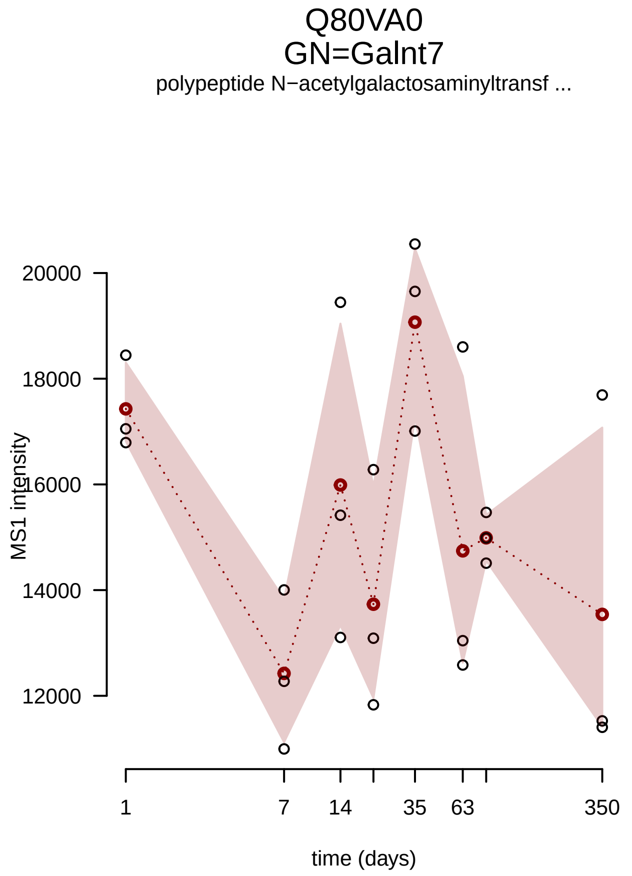
Q80VA0
GN=Galnt7
polypeptide N−acetylgalactosaminyltransf ...
MS1 intensity
20000
18000
16000
14000
12000
1
7
14
35
63
350
time (days)

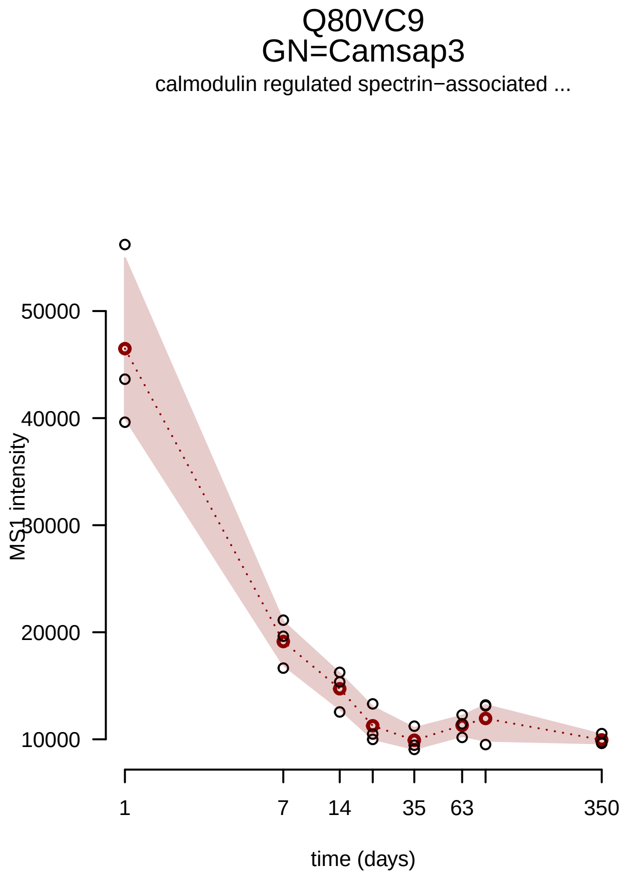
Q80VC9
GN=Camsap3
calmodulin regulated spectrin−associated ...
MS1 intensity
50000
40000
30000
20000
10000
1
7
14
35
63
350
time (days)

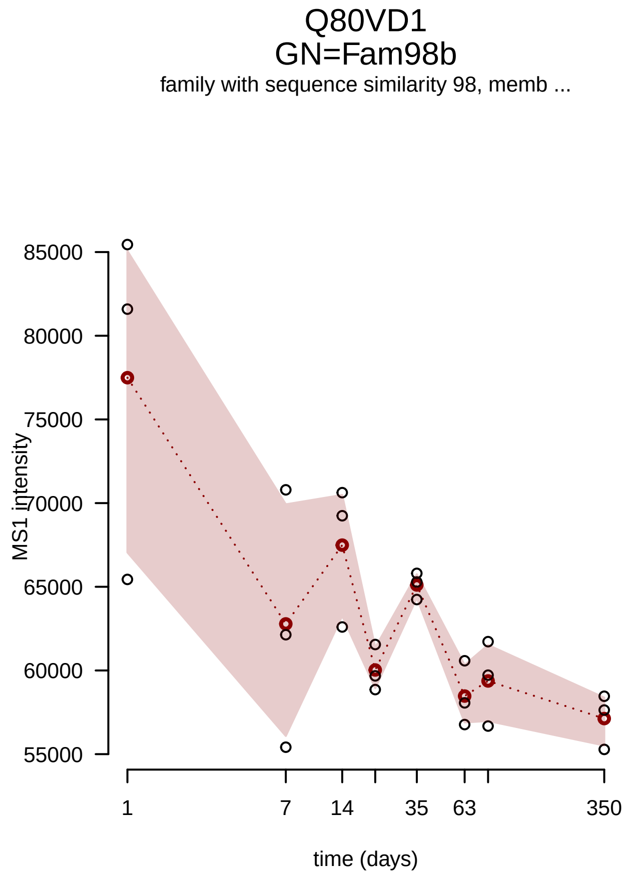
Q80VD1
GN=Fam98b
family with sequence similarity 98, memb ...
MS1 intensity
85000
80000
75000
70000
65000
60000
55000
1
7
14
35
63
350
time (days)

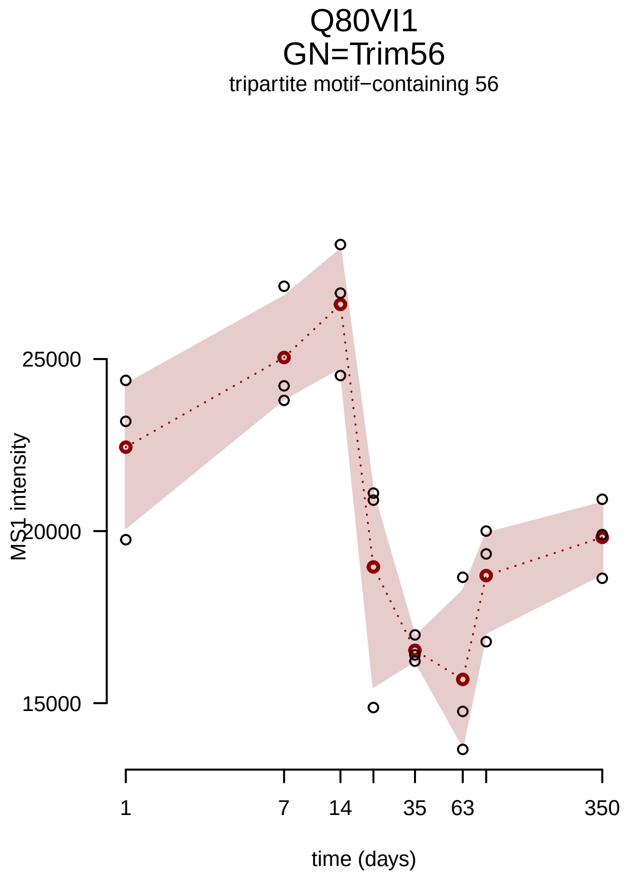
Q80VI1
GN=Trim56
tripartite motif−containing 56
MS1 intensity
25000
20000
15000
1
7
14
35
63
350
time (days)

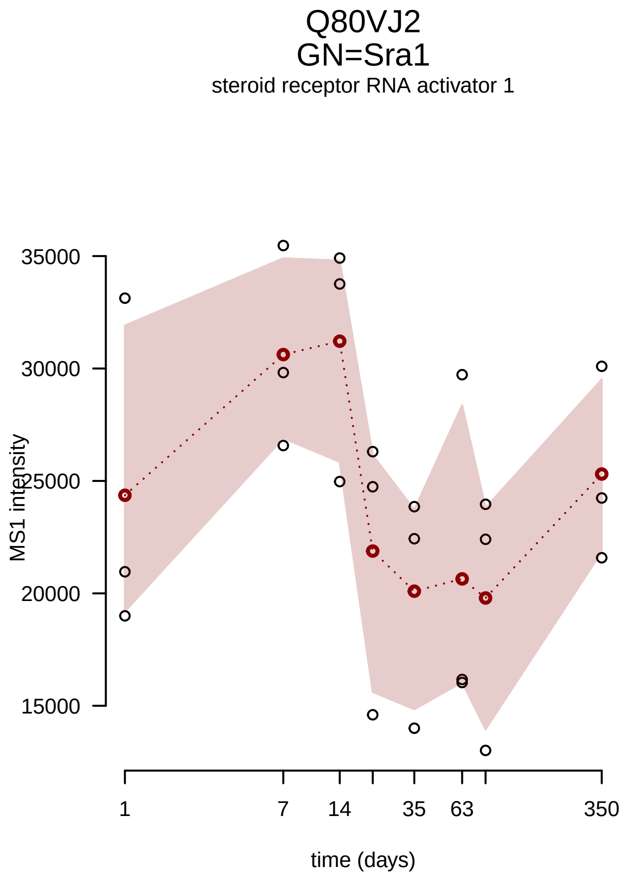
Q80VJ2
GN=Sra1
steroid receptor RNA activator 1
MS1 intensity
35000
30000
25000
20000
15000
1
7
14
35
63
350
time (days)

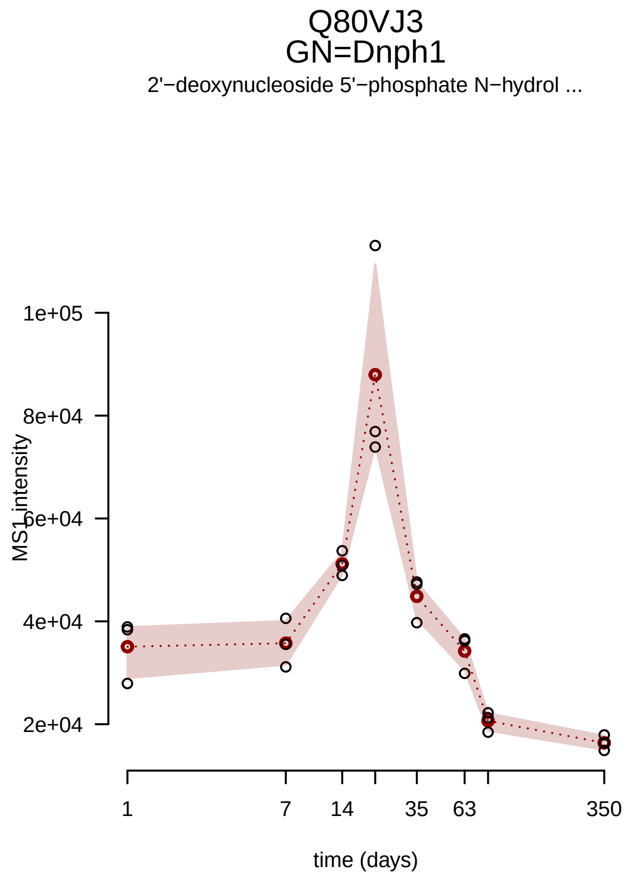
Q80VJ3
GN=Dnph1
2'−deoxynucleoside 5'−phosphate N−hydrol ...
MS1 intensity
1e+05
8e+04
6e+04
4e+04
2e+04
1
7
14
35
63
350
time (days)

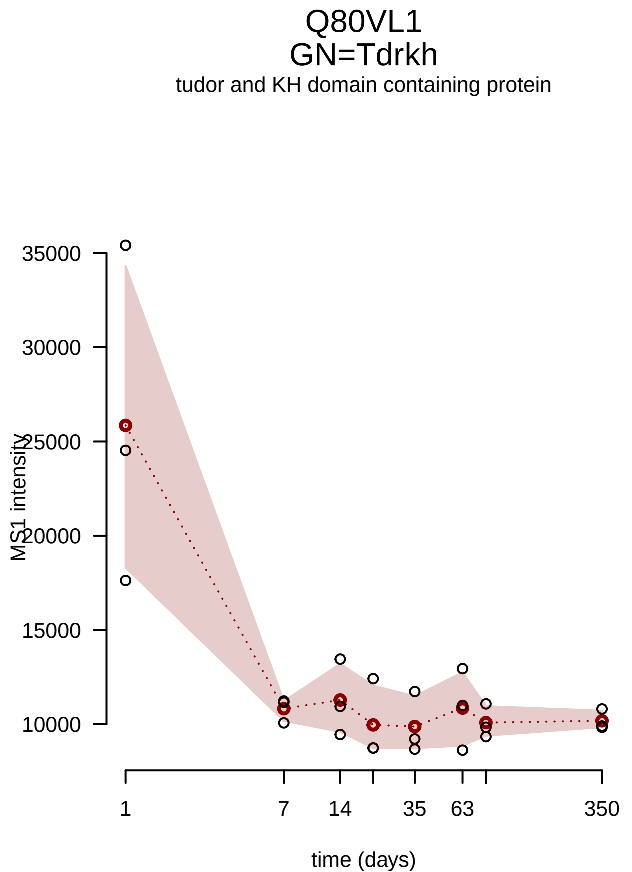
Q80VL1
GN=Tdrkh
tudor and KH domain containing protein
MS1 intensity
35000
30000
25000
20000
15000
10000
1
7
14
35
63
350
time (days)

Q80VL3
GN=Prickle3
prickle planar cell polarity protein 3

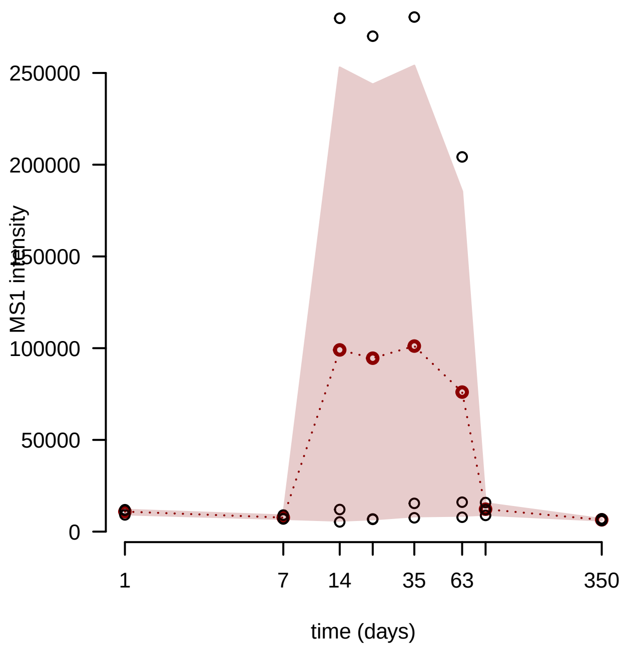

Q80VM4
GN=Znf579

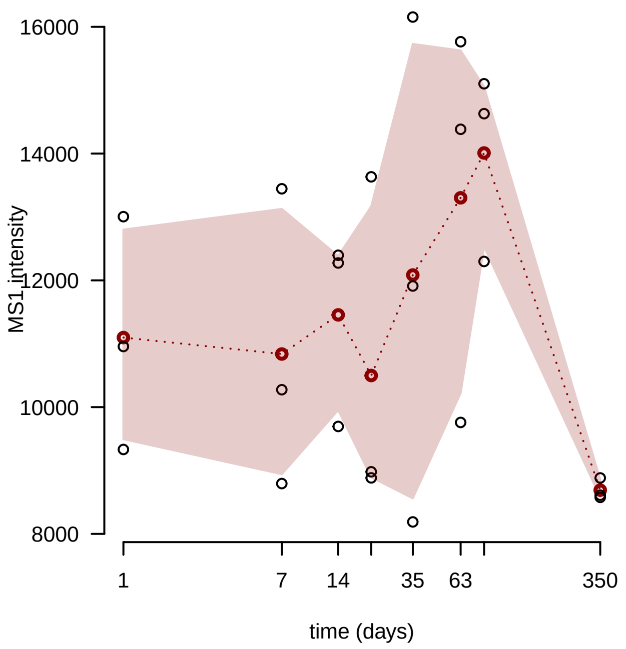

Q80VP0
GN=Tecpr1
tectonin beta−propeller repeat containin ...

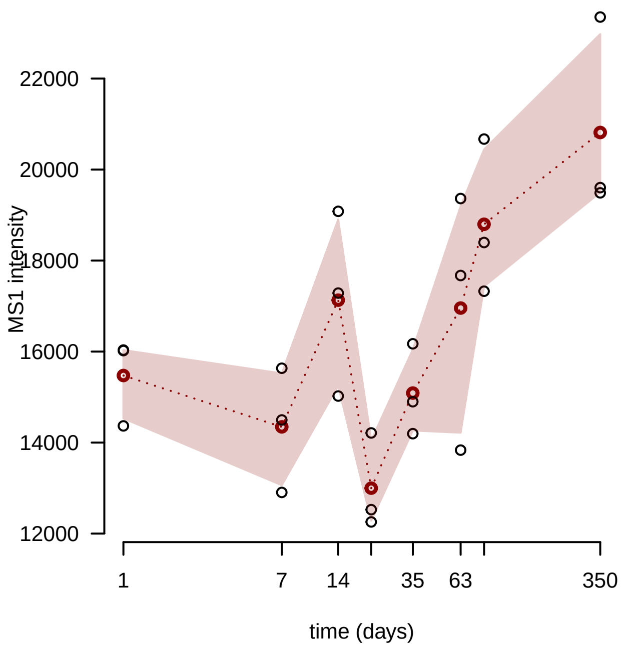

Q80VP1
GN=Epn1
epsin 1

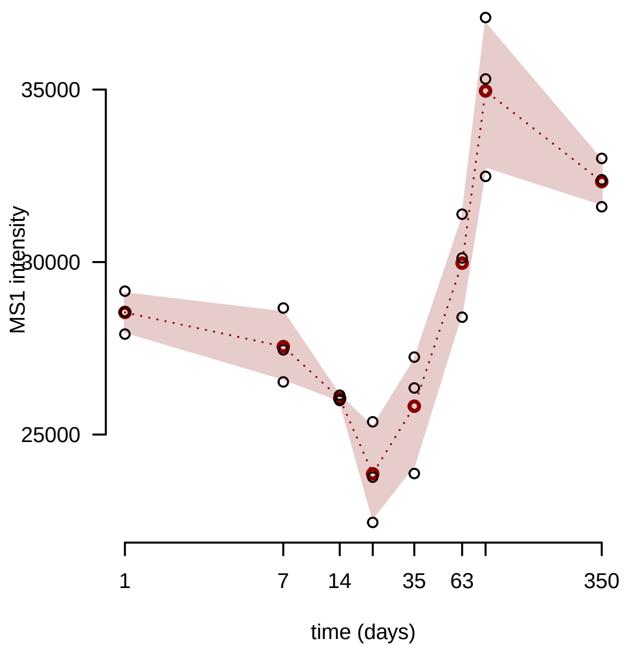

Q80VP2
GN=Spata7
spermatogenesis associated 7

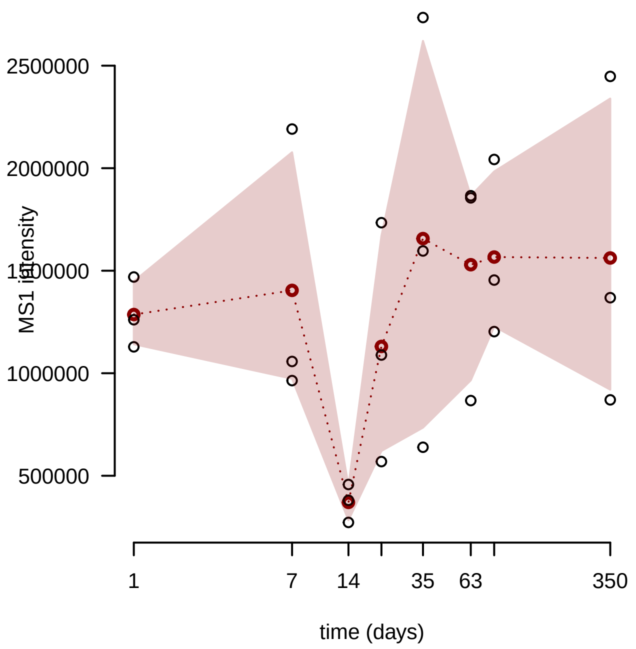

Q80VP5
GN=

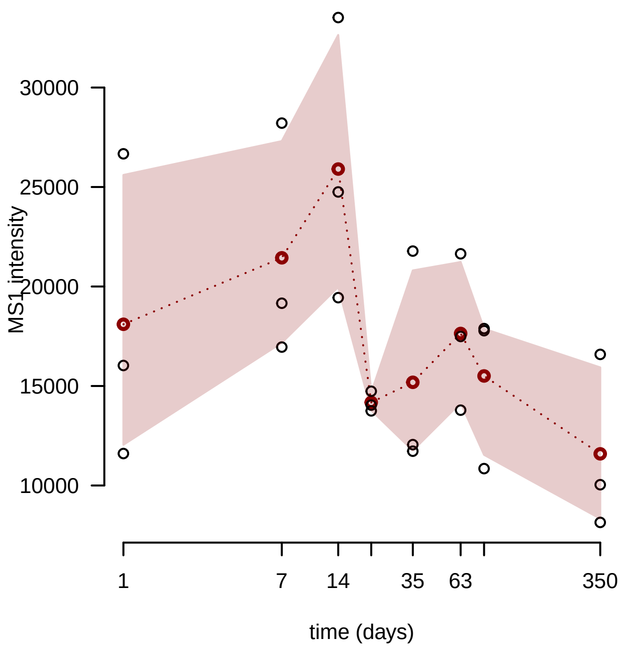

Q80VQ0
GN=Aldh3b1
aldehyde dehydrogenase 3 family, member ...

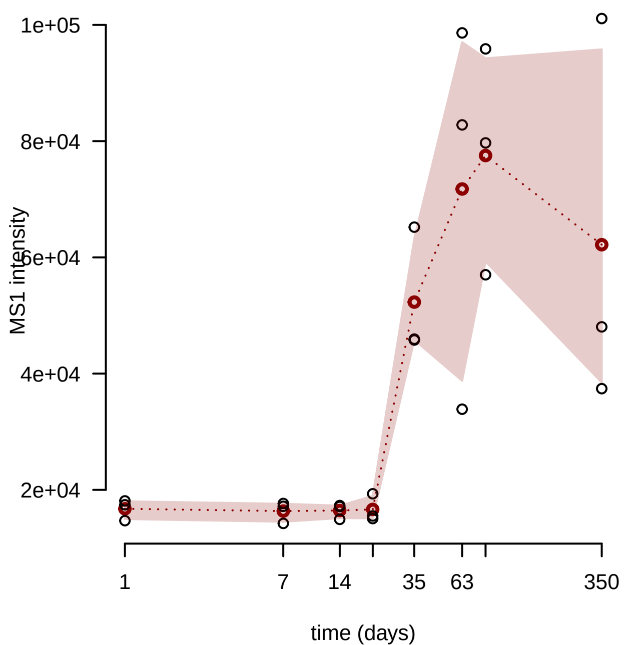

Q80VQ1
GN=Lrrc1
leucine rich repeat containing 1

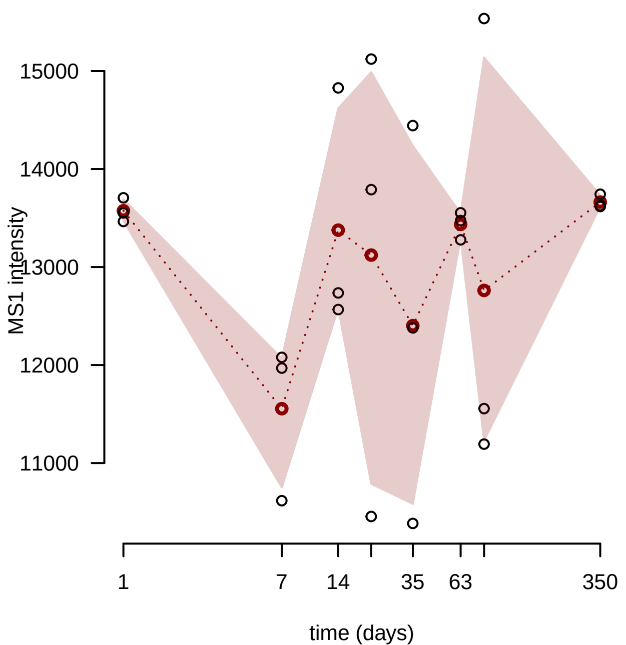

Q80VY9
GN=Dhx33
DEAH (Asp−Glu−Ala−His) box polypeptide 3 ...

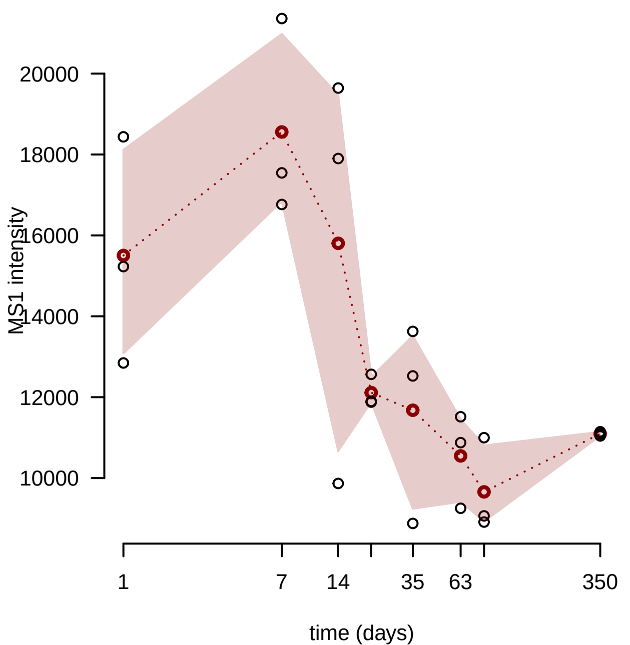

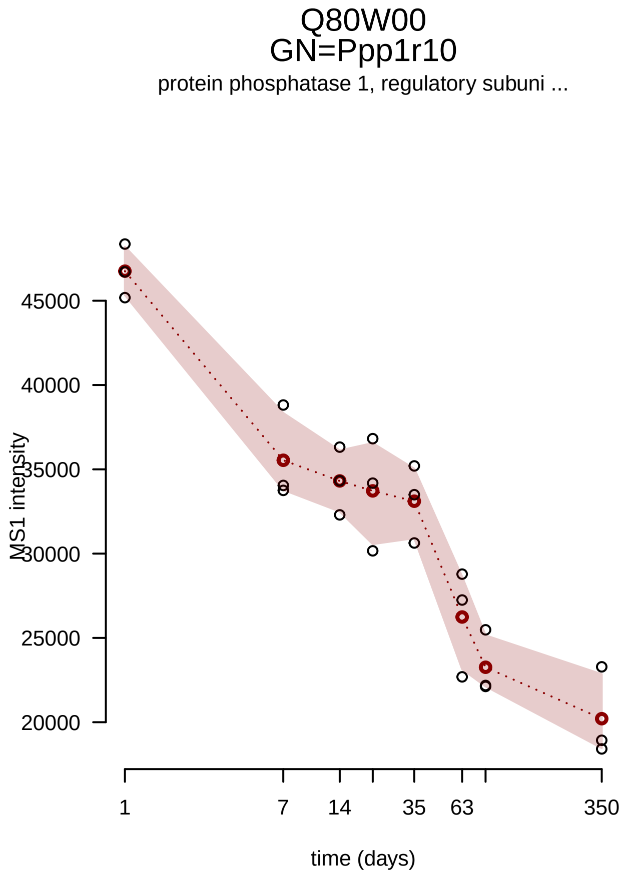
Q80W00
GN=Ppp1r10
protein phosphatase 1, regulatory subuni ...
MS1 intensity
20000
25000
30000
35000
40000
45000
1
7
14
35
63
350
time (days)

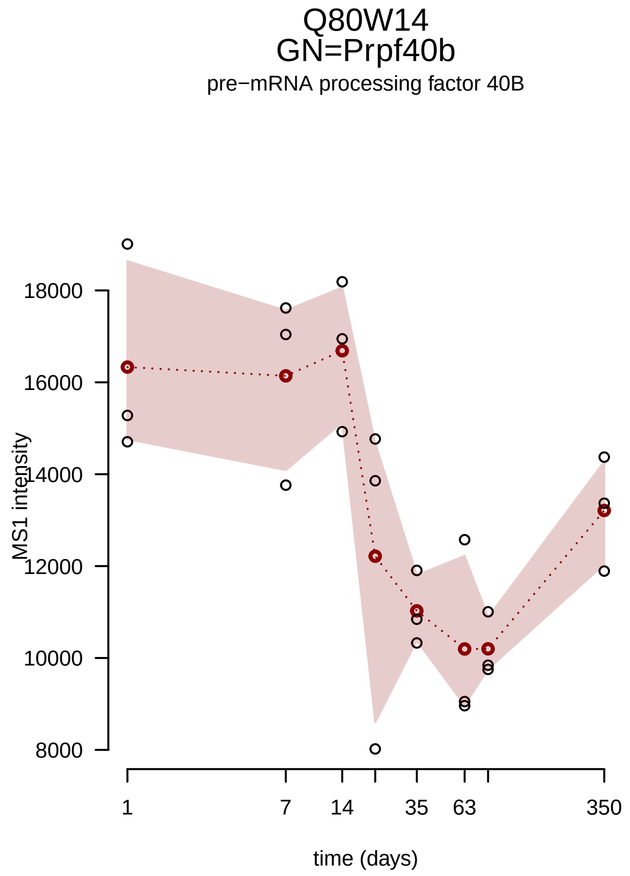
Q80W14
GN=Prpf40b
pre−mRNA processing factor 40B
MS1 intensity
8000
10000
12000
14000
16000
18000
1
7
14
35
63
350
time (days)

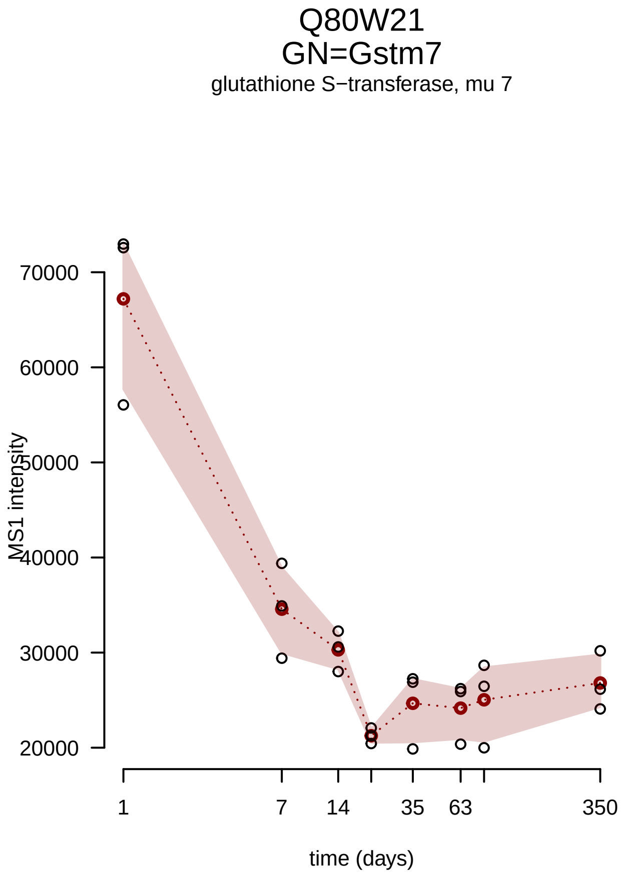
Q80W21
GN=Gstm7
glutathione S−transferase, mu 7
MS1 intensity
20000
30000
40000
50000
60000
70000
1
7
14
35
63
350
time (days)

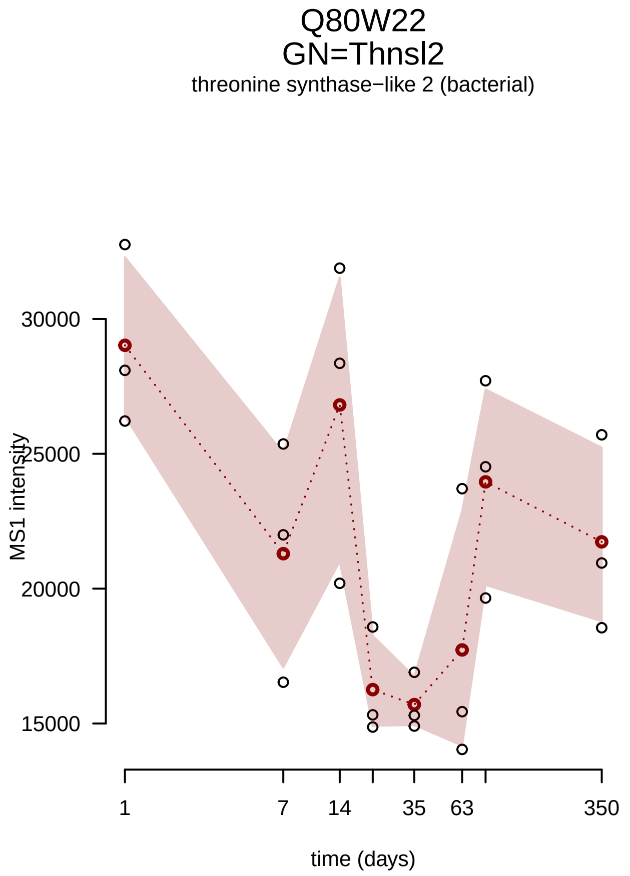
Q80W22
GN=Thnsl2
threonine synthase−like 2 (bacterial)
MS1 intensity
15000
20000
25000
30000
1
7
14
35
63
350
time (days)

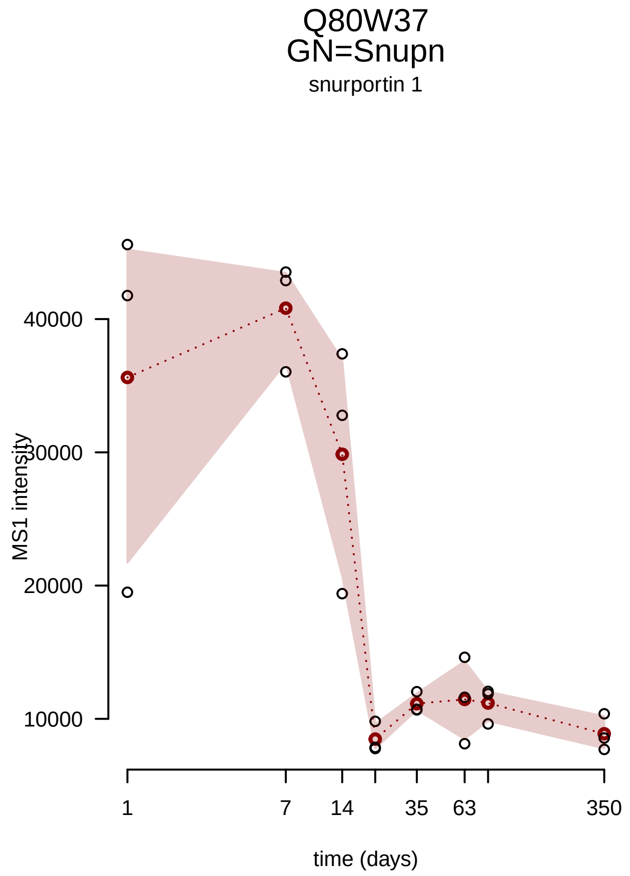
Q80W37
GN=Snupn
snurportin 1
MS1 intensity
10000
20000
30000
40000
1
7
14
35
63
350
time (days)

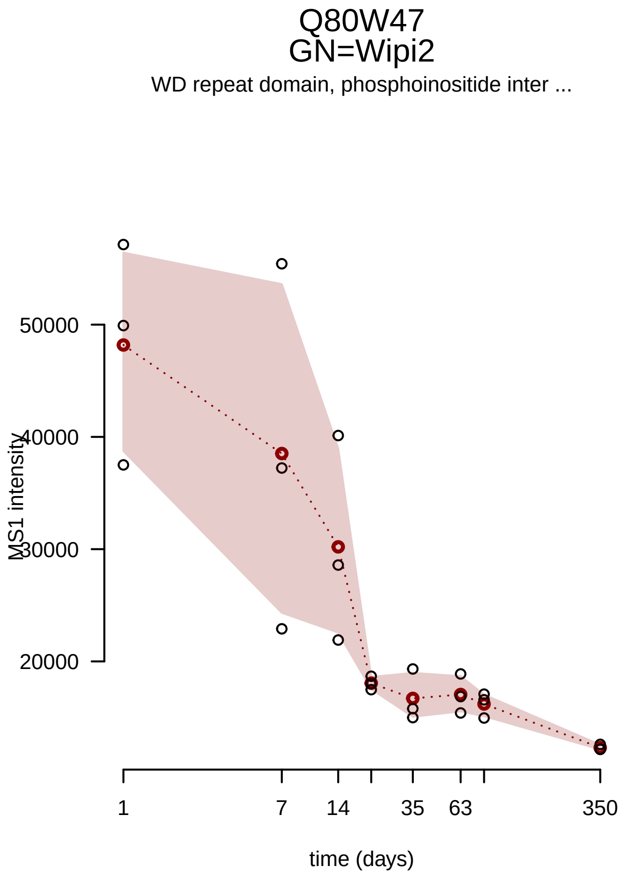
Q80W47
GN=Wipi2
WD repeat domain, phosphoinositide inter ...
MS1 intensity
20000
30000
40000
50000
1
7
14
35
63
350
time (days)

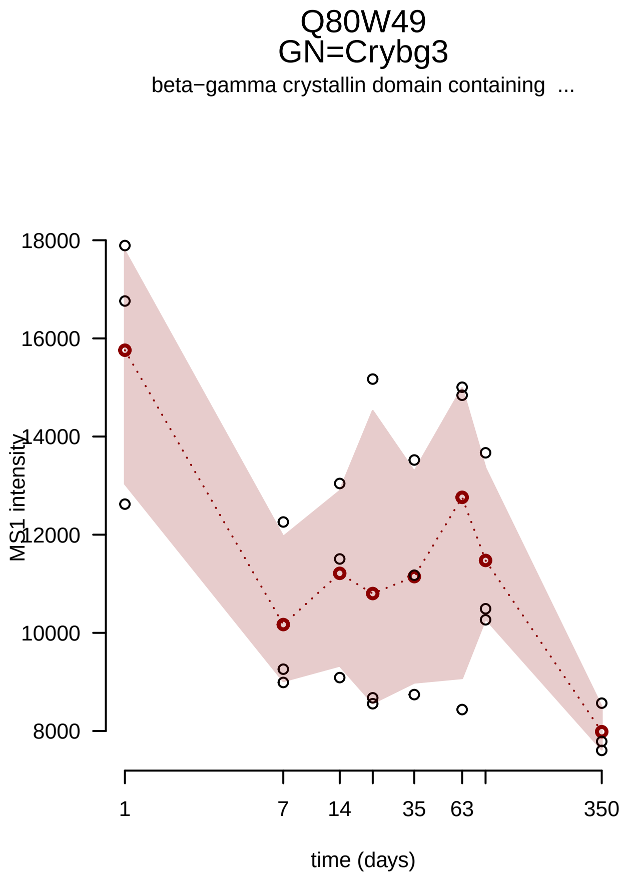
Q80W49
GN=Crybg3
beta−gamma crystallin domain containing ...
MS1 intensity
8000
10000
12000
14000
16000
18000
1
7
14
35
63
350
time (days)

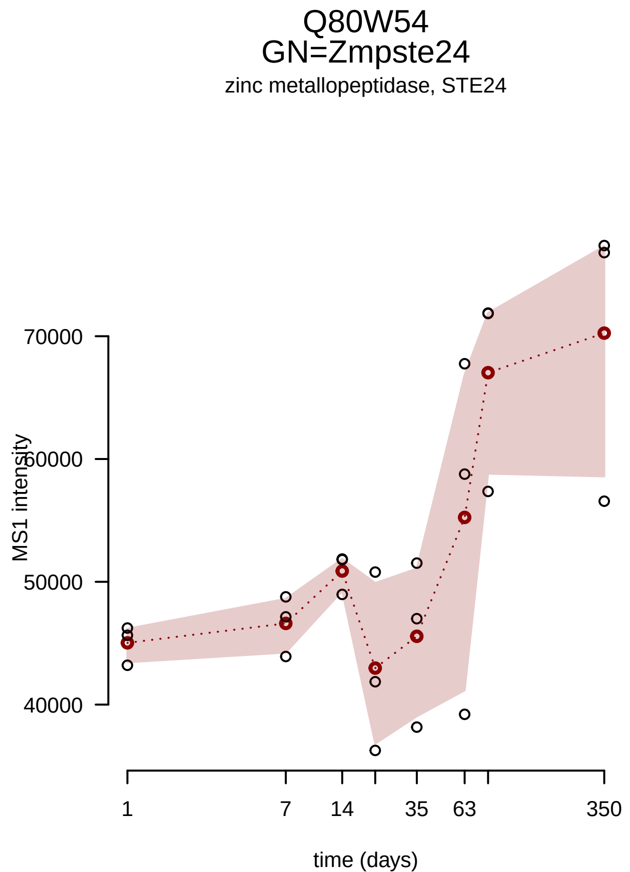
Q80W54
GN=Zmpste24
zinc metallopeptidase, STE24
MS1 intensity
40000
50000
60000
70000
1
7
14
35
63
350
time (days)

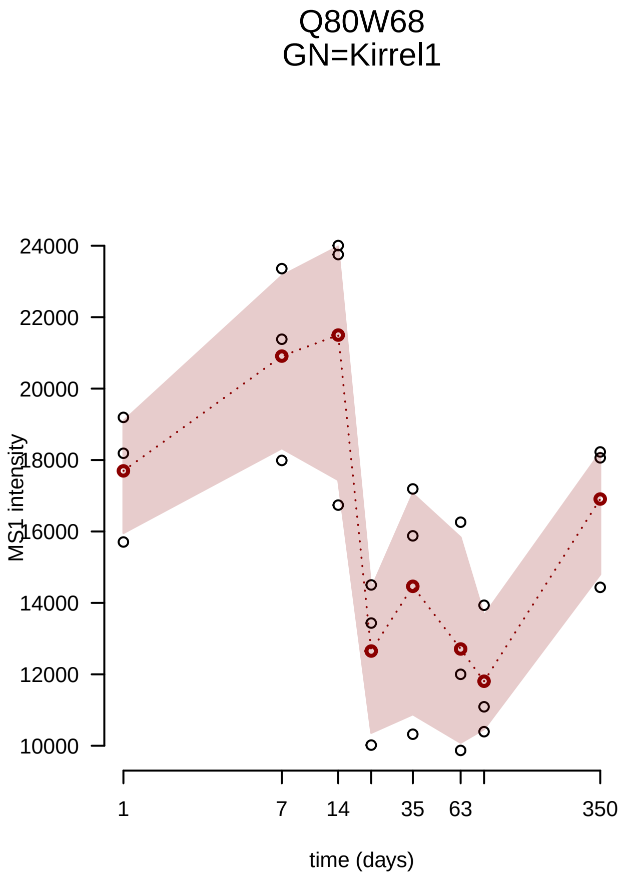
Q80W68
GN=Kirrel1
MS1 intensity
10000
12000
14000
16000
18000
20000
22000
24000
1
7
14
35
63
350
time (days)

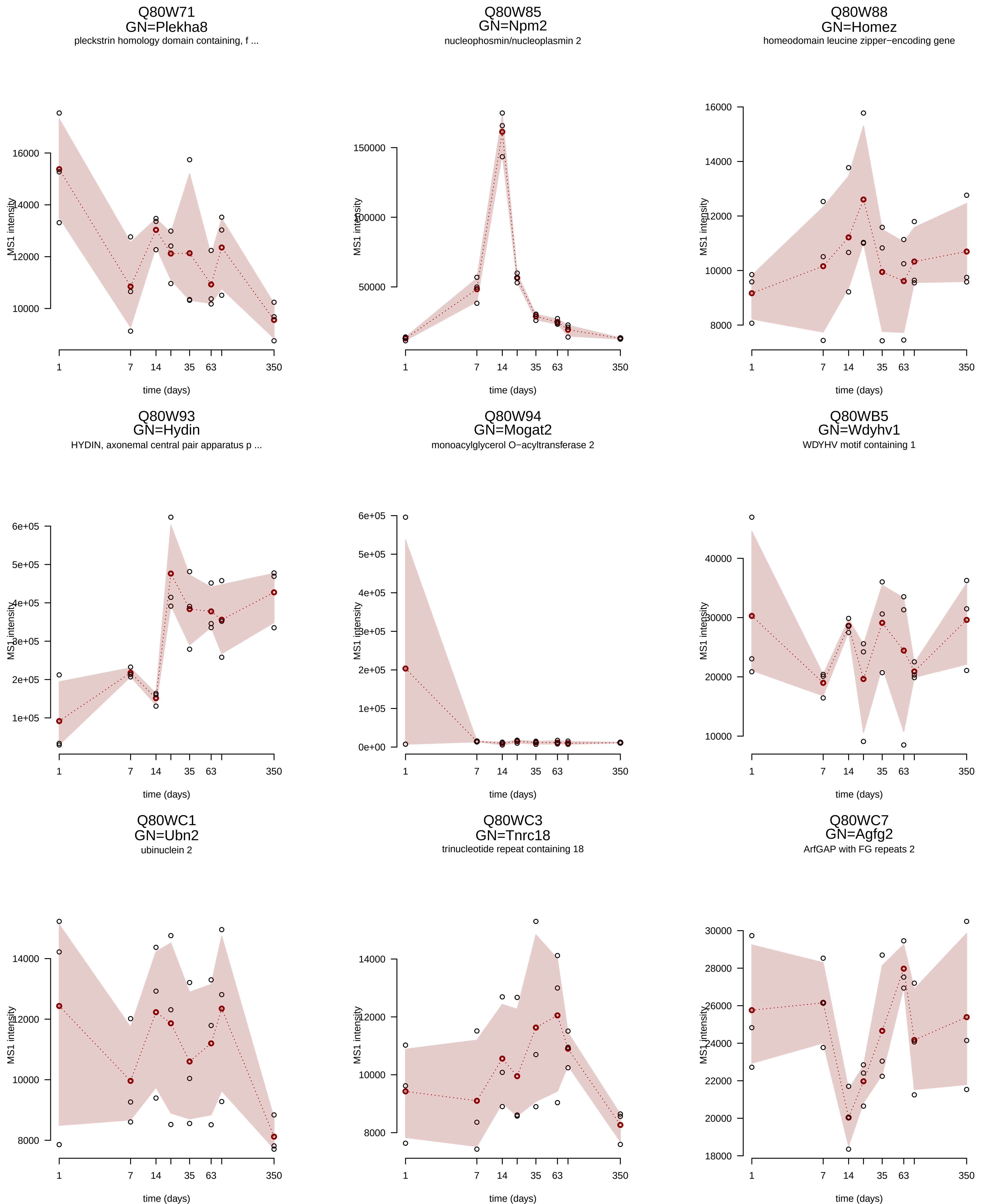
Q80W71
GN=Plekha8
pleckstrin homology domain containing, f ...
Q80W85
GN=Npm2
nucleophosmin/nucleoplasmin 2
Q80W88
GN=Homez
homeodomain leucine zipper−encoding gene
Q80W93
GN=Hydin
HYDIN, axonemal central pair apparatus p ...
Q80W94
GN=Mogat2
monoacylglycerol O−acyltransferase 2
Q80WB5
GN=Wdyhv1
WDYHV motif containing 1
Q80WC1
GN=Ubn2
ubinuclein 2
Q80WC3
GN=Tnrc18
trinucleotide repeat containing 18
Q80WC7
GN=Agfg2
ArfGAP with FG repeats 2
MS1 intensity
time (days)
1
7
14
35
63
350

Q80WG5
GN=Lrrc8a
leucine rich repeat containing 8A VRAC s ...

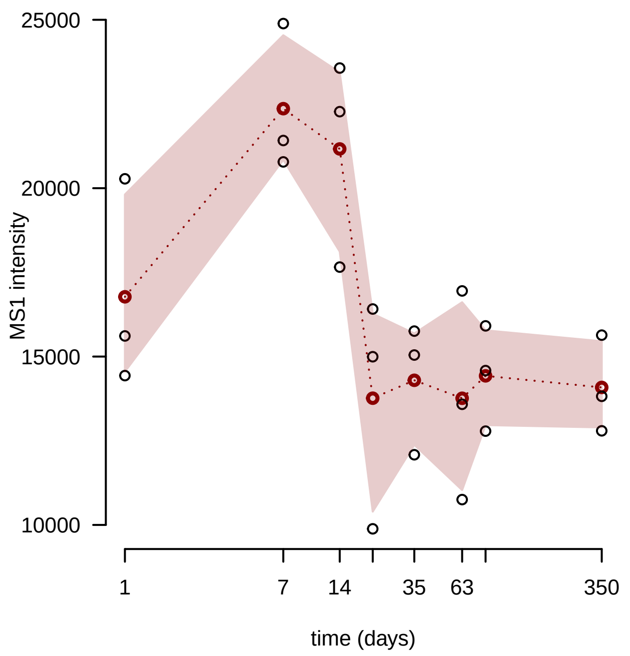

Q80WJ7
GN=Mtdh
metadherin

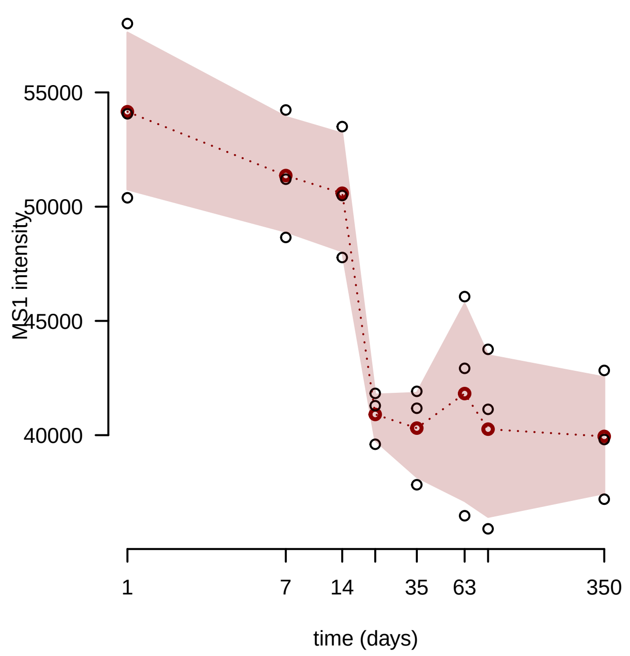

Q80WQ2
GN=Vac14
Vac14 homolog (S. cerevisiae)

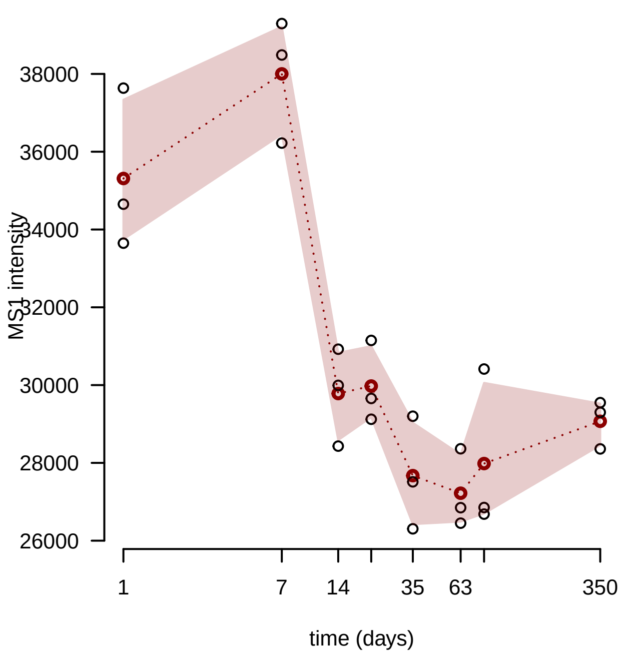

Q80WR1
GN=Tspan18
tetraspanin 18

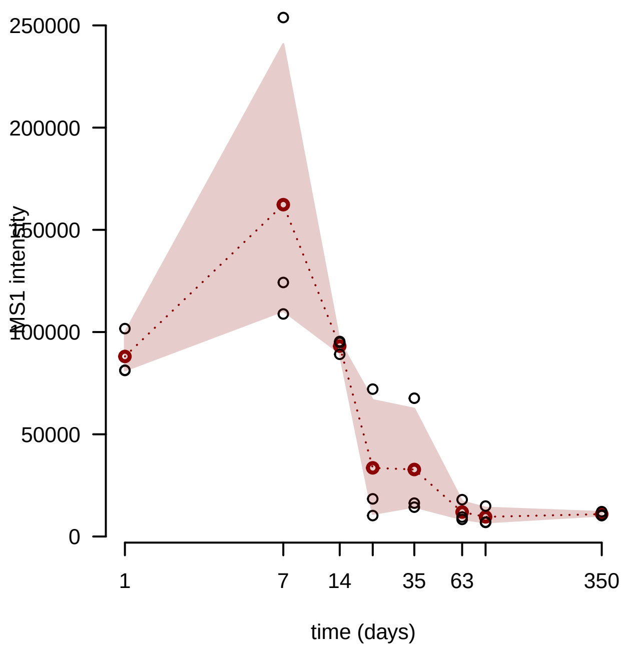

Q80WR5
GN=

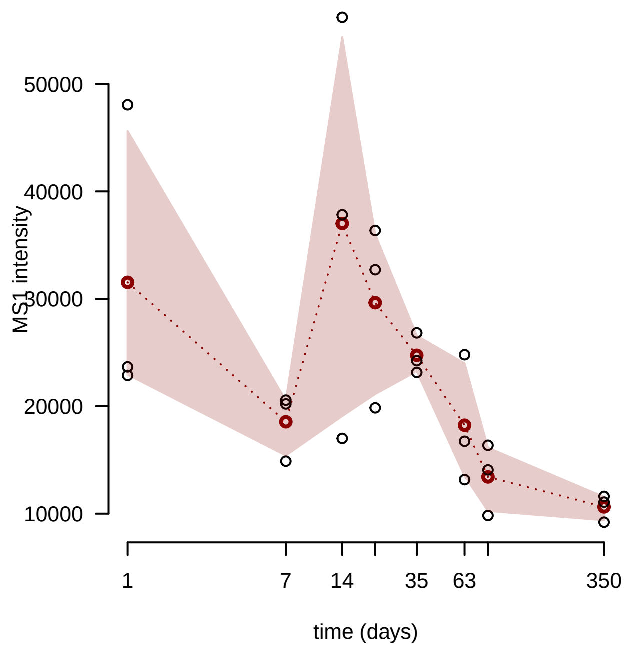

Q80WS1
GN=Rimklb
ribosomal modification protein rimK−like ...

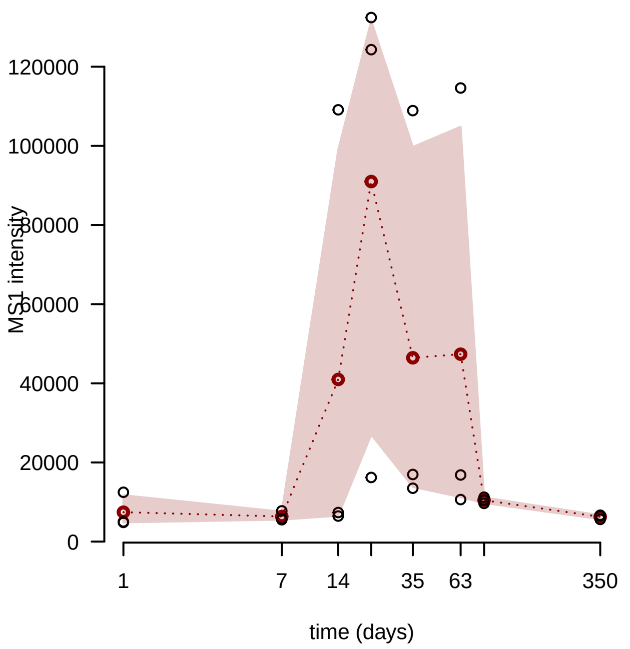

Q80WT5
GN=Aftph
aftiphilin

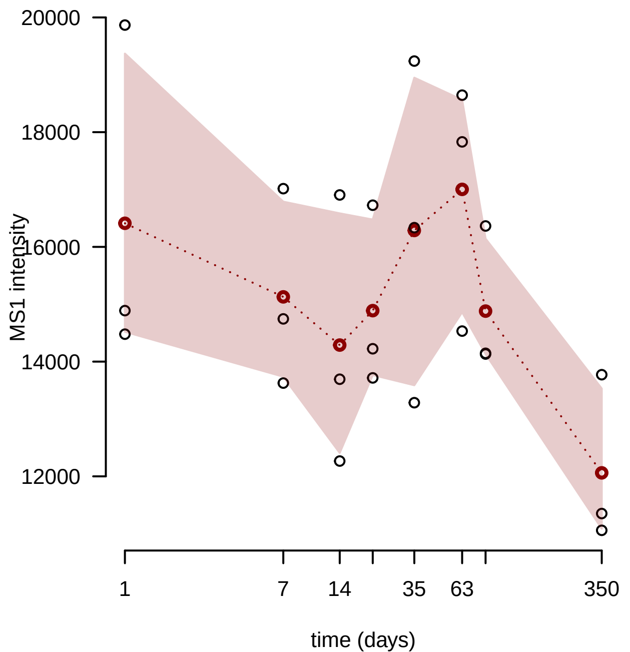

Q80WV3
GN=Chst2
carbohydrate sulfotransferase 2

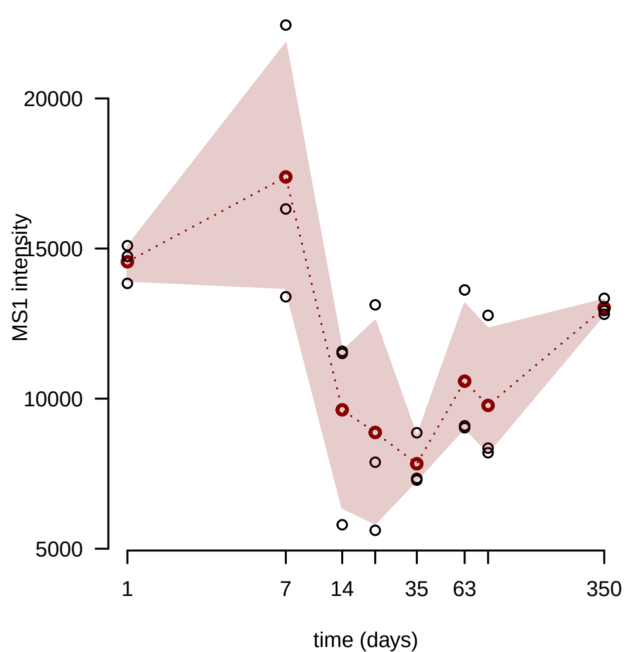

Q80WW9
GN=Ddrgk1
DDRGK domain containing 1

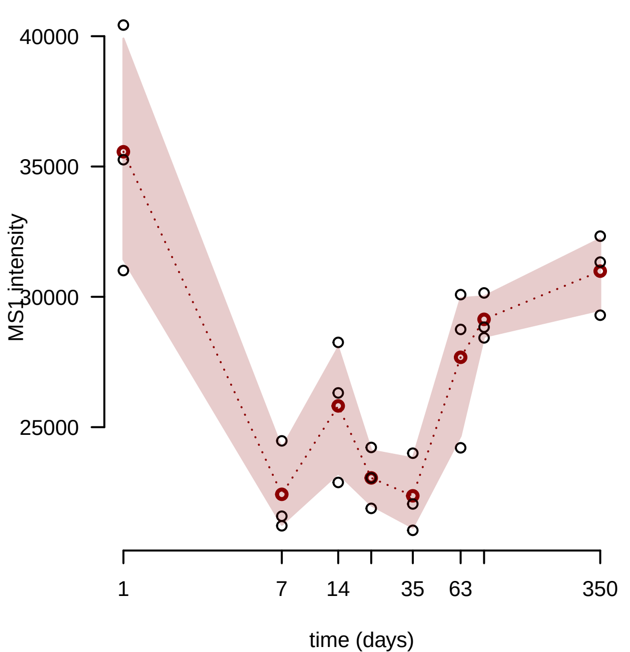

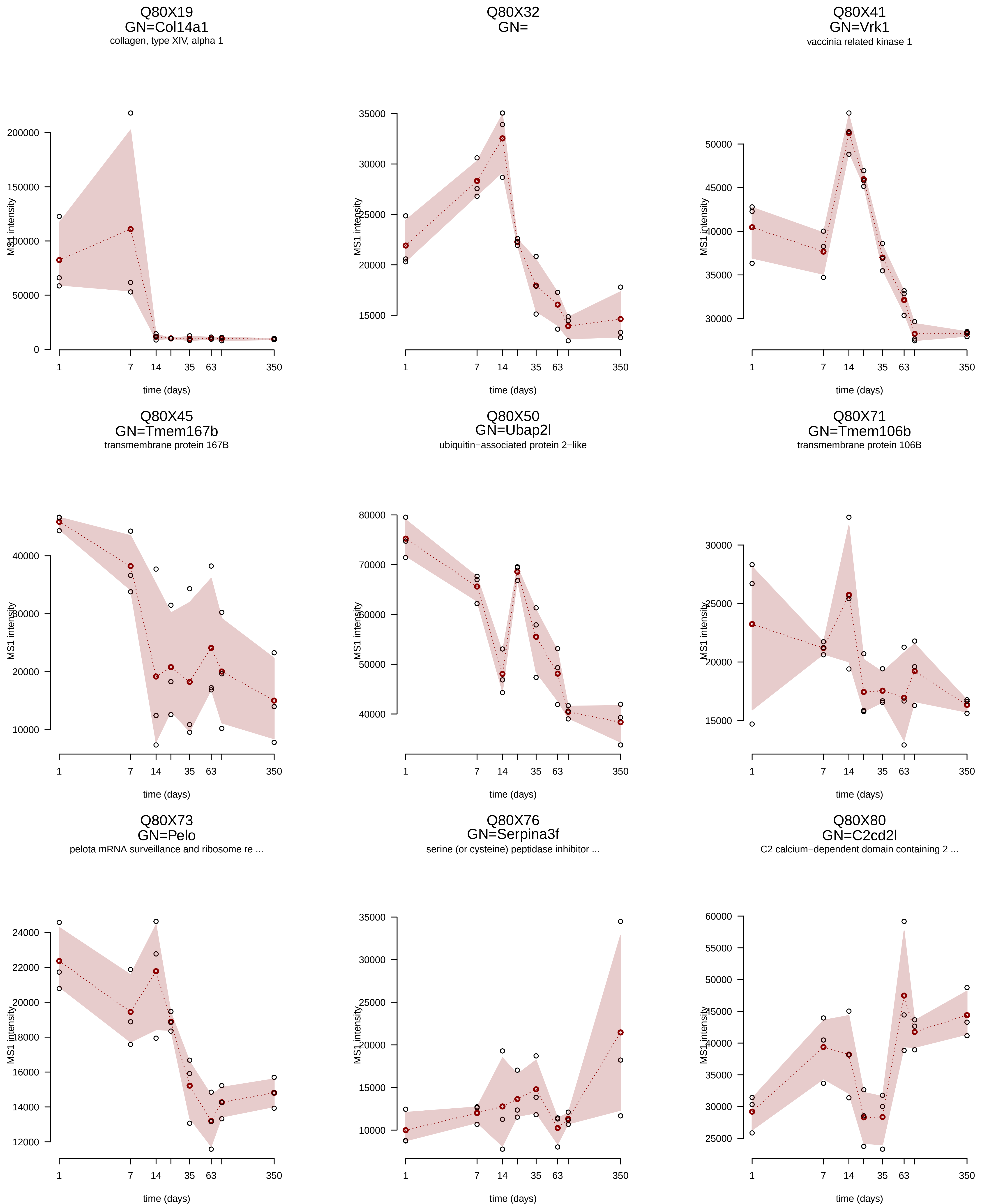
Q80X19
GN=Col14a1
collagen, type XIV, alpha 1
MS1 intensity
time (days)
Q80X32
GN=
MS1 intensity
time (days)
Q80X41
GN=Vrk1
vaccinia related kinase 1
MS1 intensity
time (days)
Q80X45
GN=Tmem167b
transmembrane protein 167B
MS1 intensity
time (days)
Q80X50
GN=Ubap2l
ubiquitin−associated protein 2−like
MS1 intensity
time (days)
Q80X71
GN=Tmem106b
transmembrane protein 106B
MS1 intensity
time (days)
Q80X73
GN=Pelo
pelota mRNA surveillance and ribosome re ...
MS1 intensity
time (days)
Q80X76
GN=Serpina3f
serine (or cysteine) peptidase inhibitor ...
MS1 intensity
time (days)
Q80X80
GN=C2cd2l
C2 calcium−dependent domain containing 2 ...
MS1 intensity
time (days)

Q80X82
GN=Sympk
symplekin

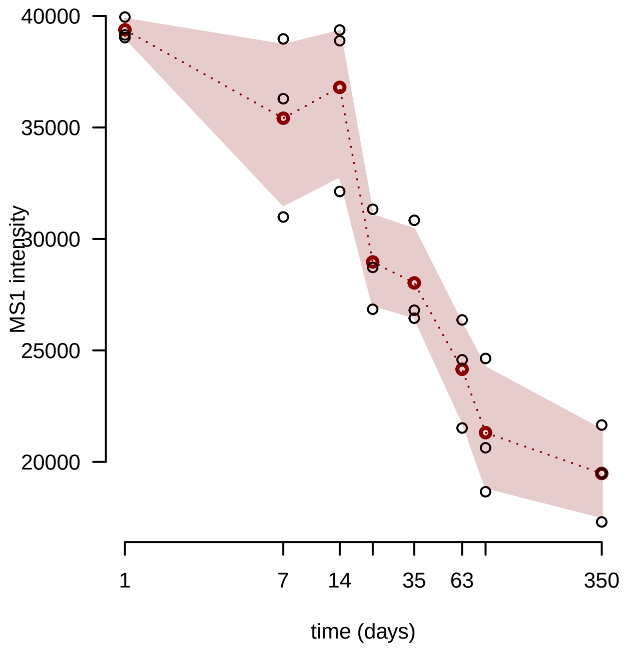

Q80X85
GN=Mrps7
mitchondrial ribosomal protein S7

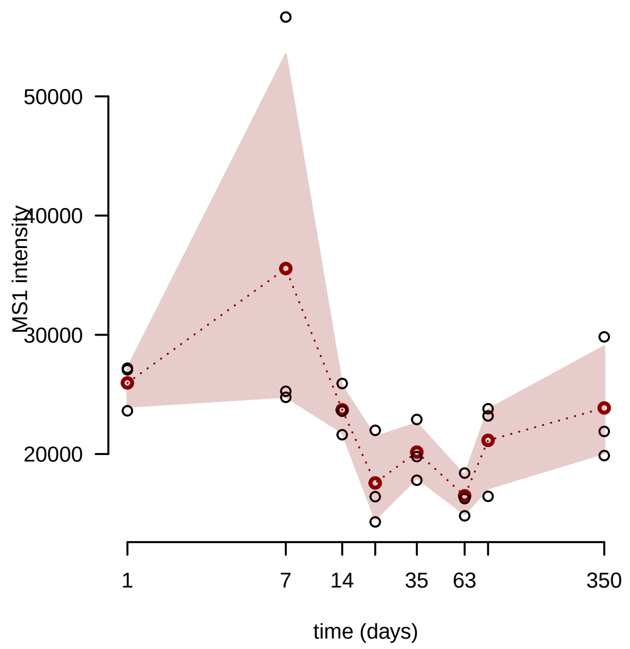

Q80X90
GN=Flnb
filamin, beta

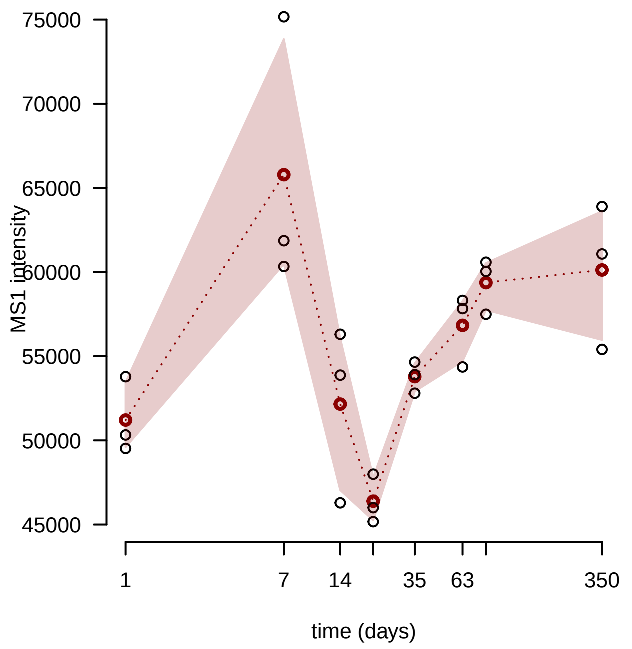

Q80X95
GN=Rraga
Ras−related GTP binding A

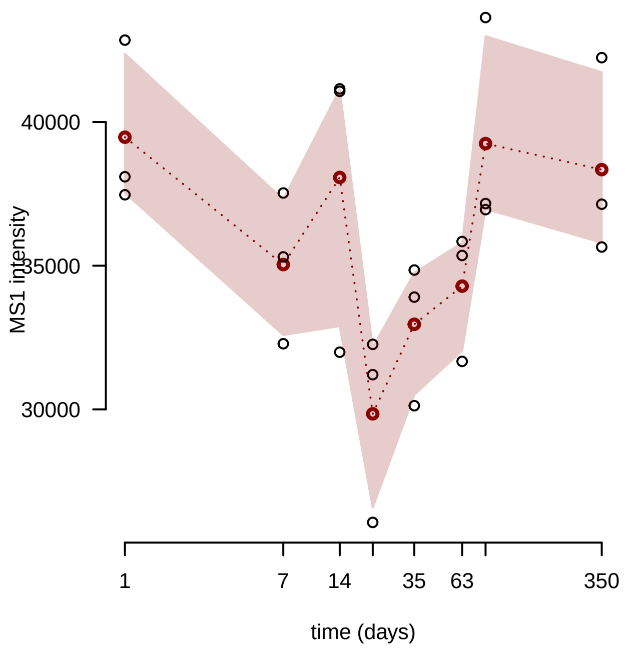

Q80XA6
GN=Reps2
RALBP1 associated Eps domain containing ...

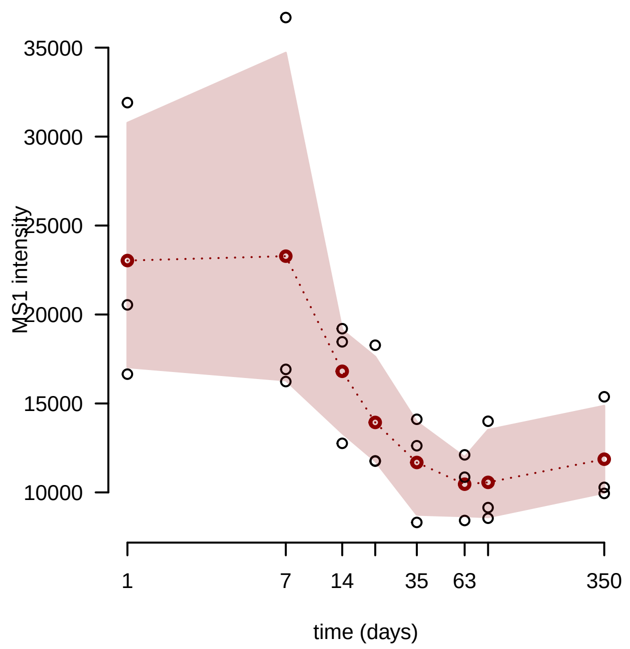

Q80XC2
GN=Trmt61a
tRNA methyltransferase 61A

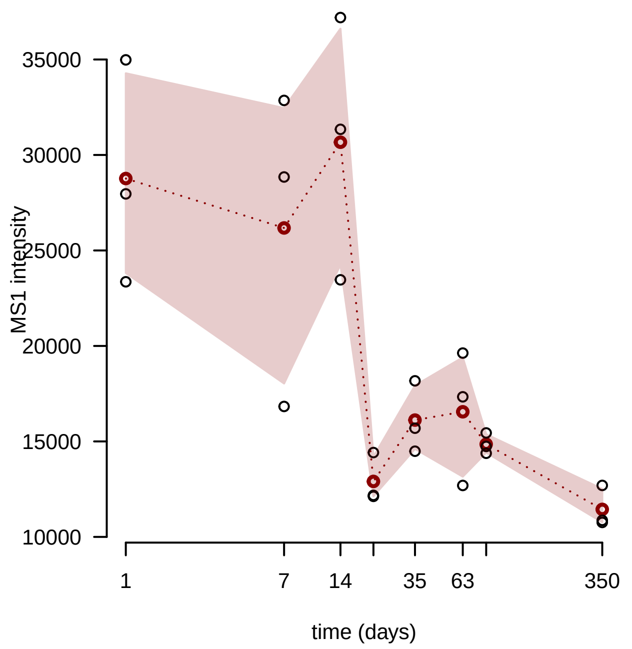

Q80XC6
GN=Nrde2
nrde−2 necessary for RNA interference, d ...

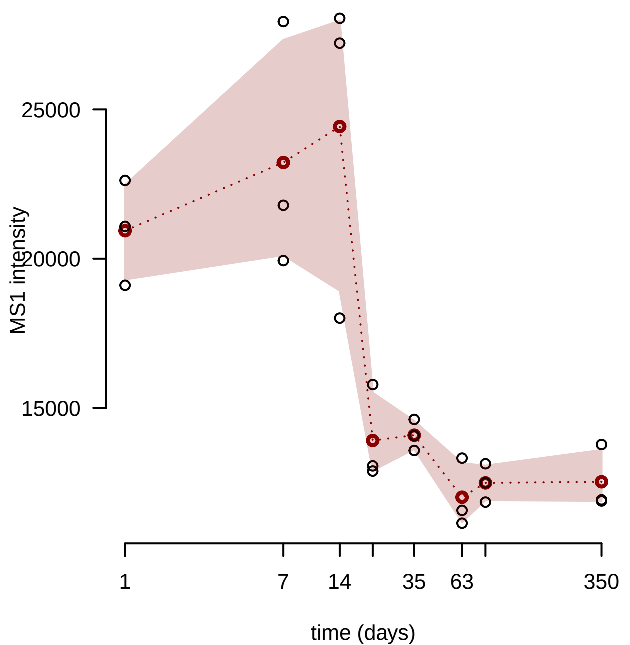

Q80XD1
GN=Chn2
chimerin 2

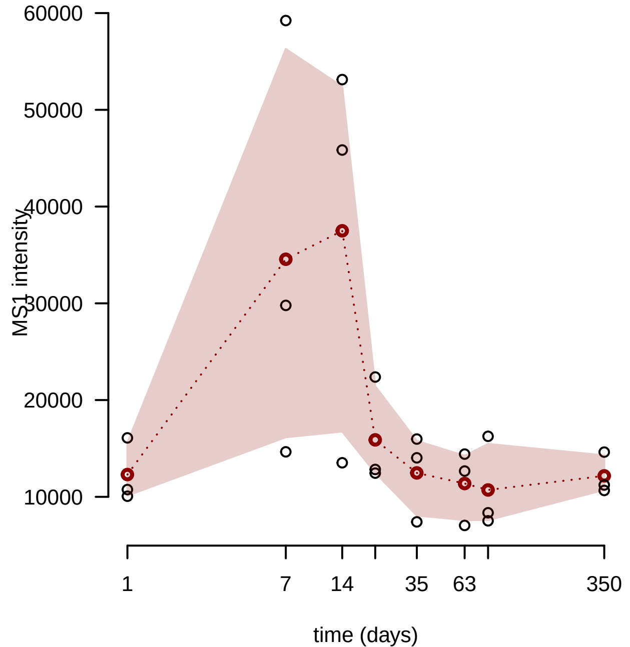

Q80XE1
GN=Ric8b
RIC8 guanine nucleotide exchange factor ...

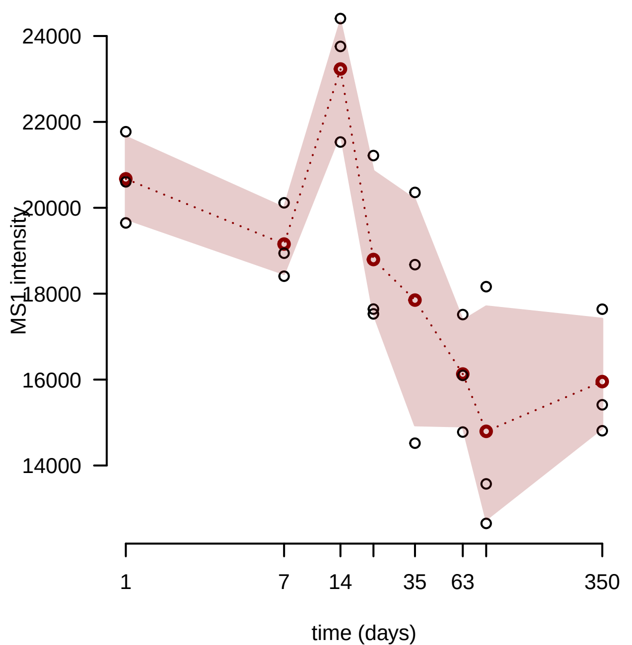

Q80XH1
GN=Kxd1
KxDL motif containing 1

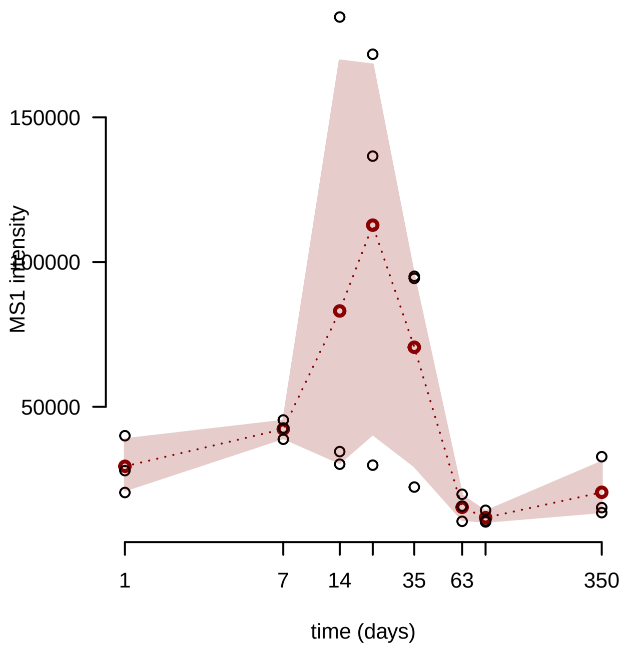

Q80XI3
GN=Eif4g3
eukaryotic translation initiation factor ...

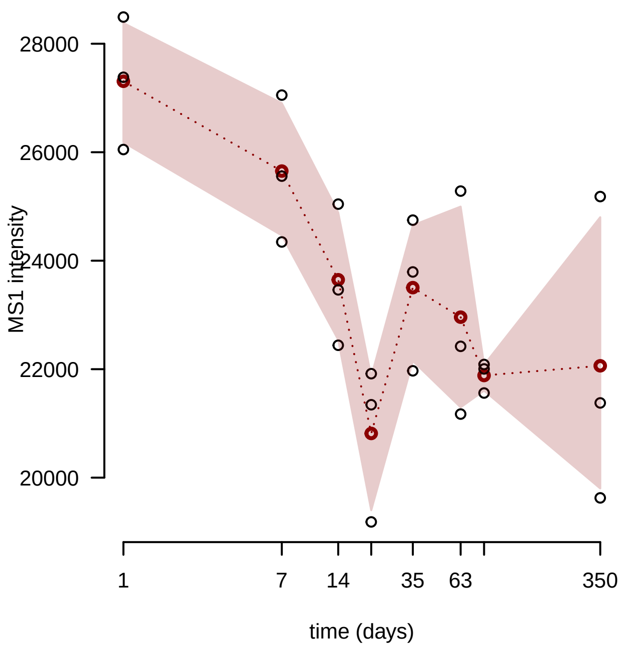

Q80XI3
GN=Eif4g3
eukaryotic translation initiation factor ...

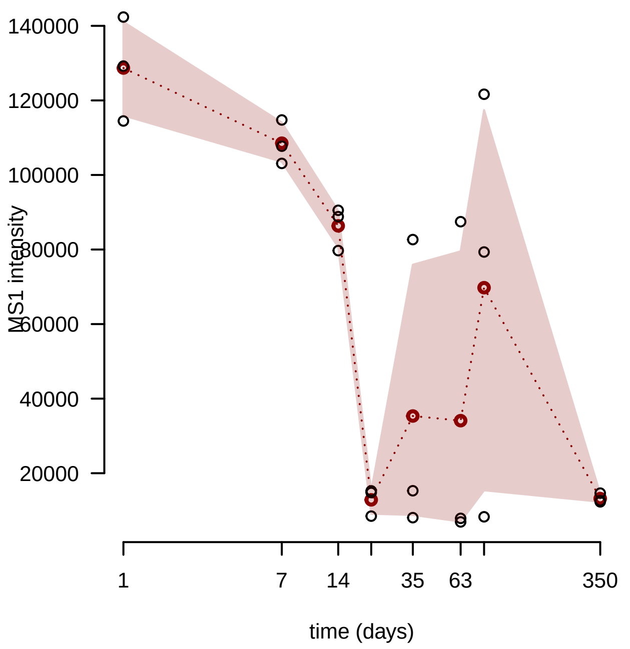

Q80XI4
GN=Pip4k2b
phosphatidylinositol−5−phosphate 4−kinas ...

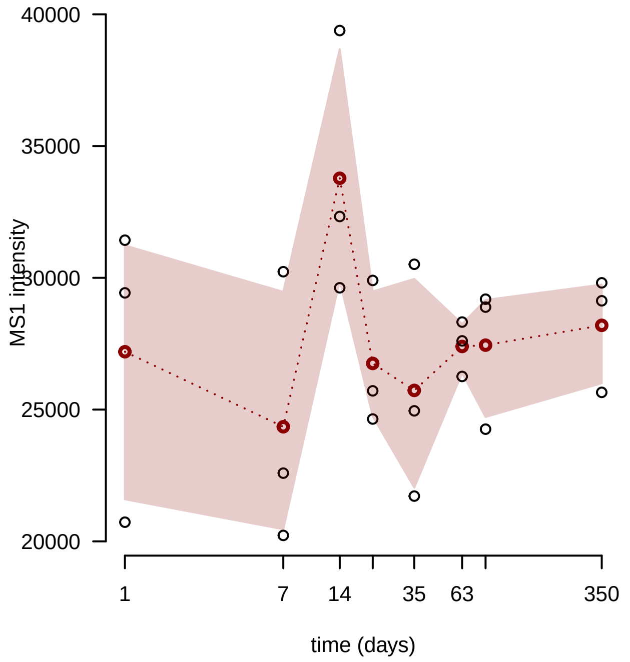

Q80XJ2
GN=Rnf168
ring finger protein 168

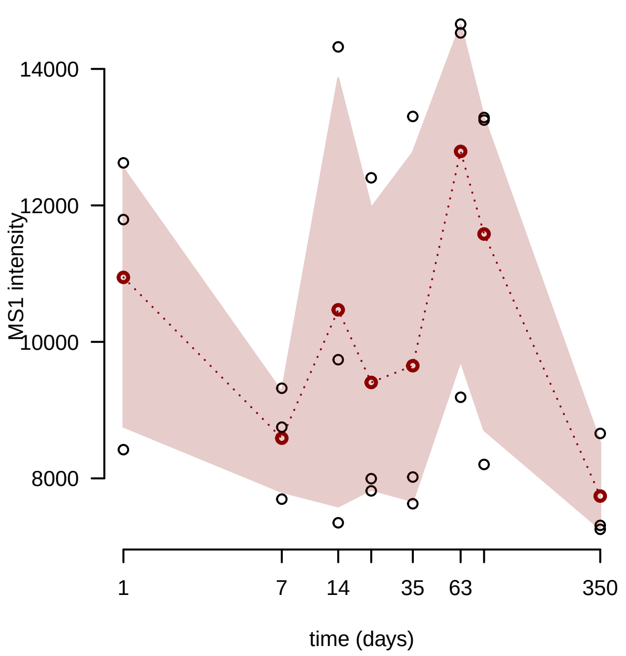

Q80XJ3
GN=Ttc28
tetratricopeptide repeat domain 28

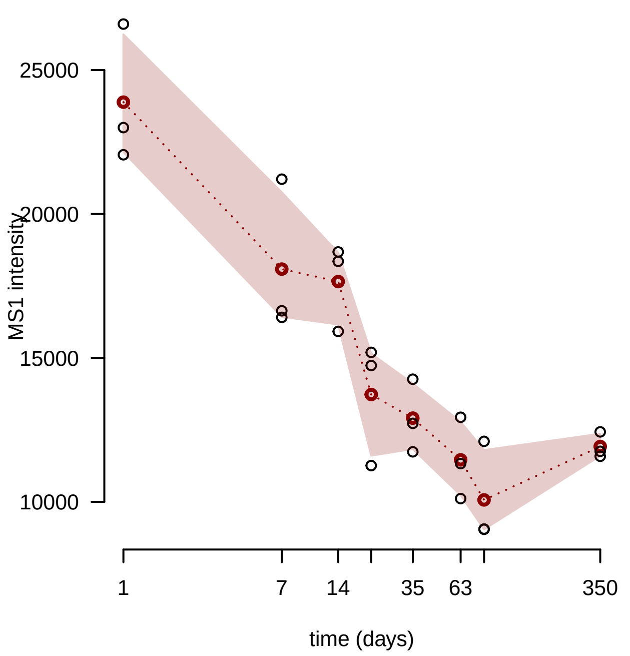

Q80XK6
GN=Atg2b
autophagy related 2B

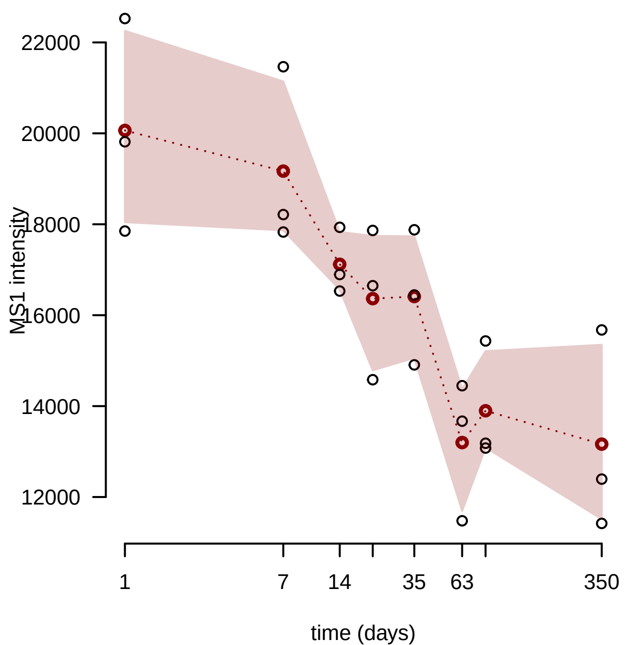

Q80XL6
GN=Acad11
acyl−Coenzyme A dehydrogenase family, me ...

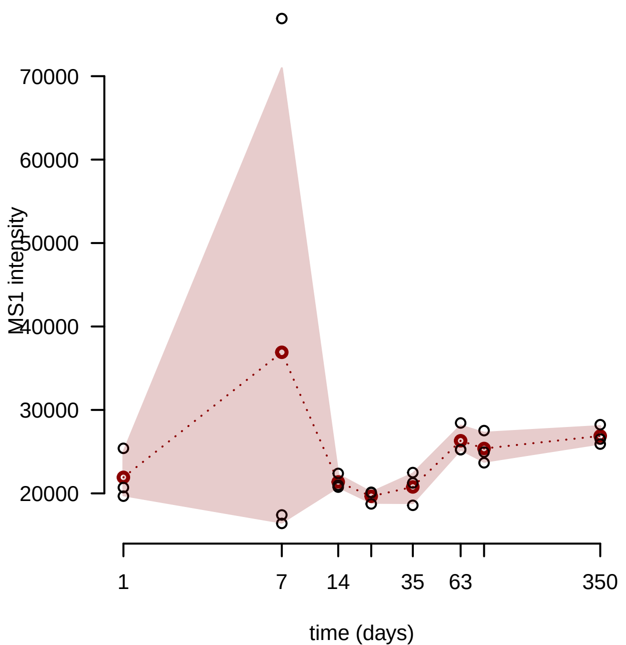

Q80XL7
GN=Mppe1
metallophosphoesterase 1

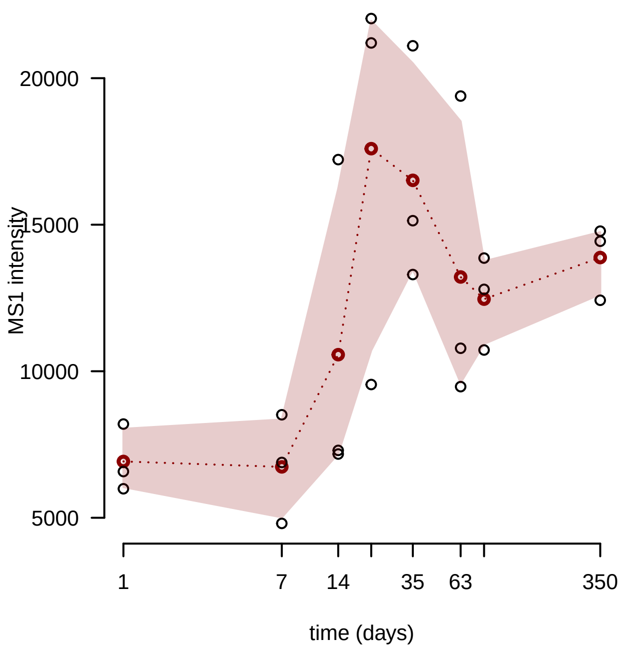

Q80XN0
GN=Bdh1
3−hydroxybutyrate dehydrogenase, type 1

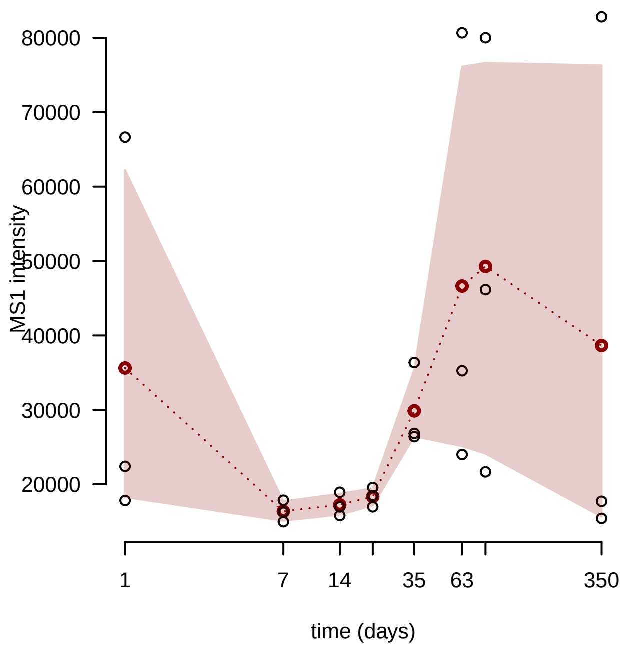

Q80XP8
GN=Fam76b
family with sequence similarity 76, memb ...

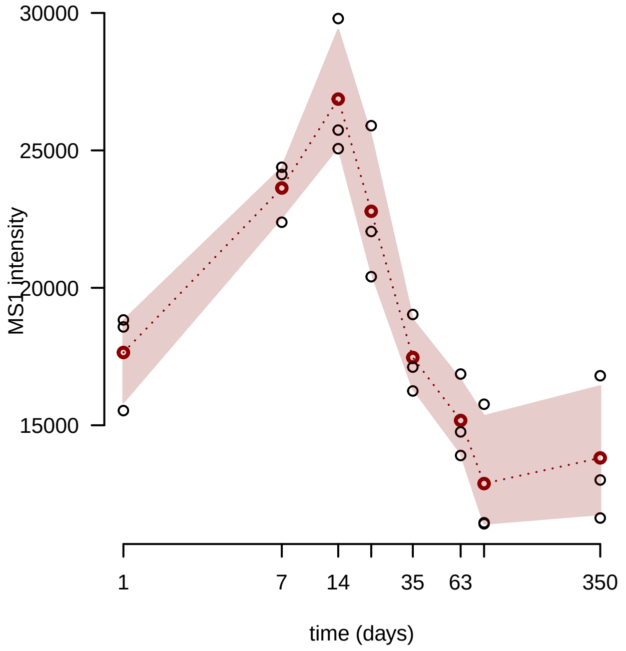

Q80XP9
GN=Wnk3
WNK lysine deficient protein kinase 3

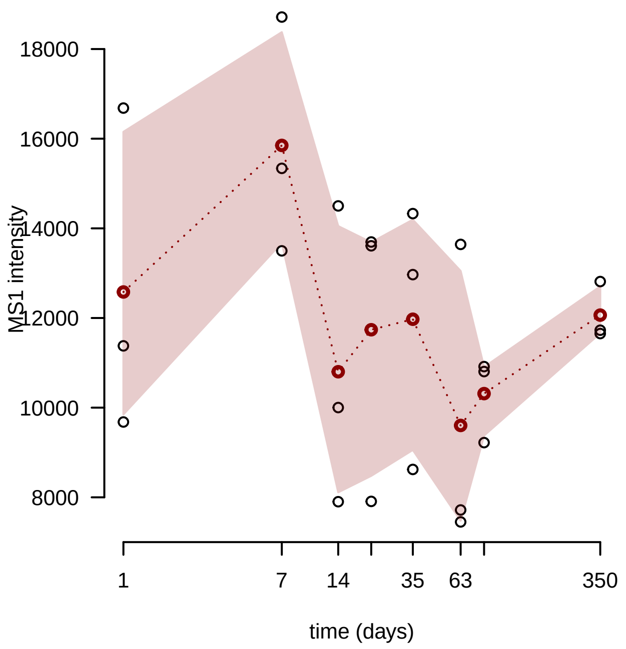

Q80XQ2
GN=Tbc1d5
TBC1 domain family, member 5

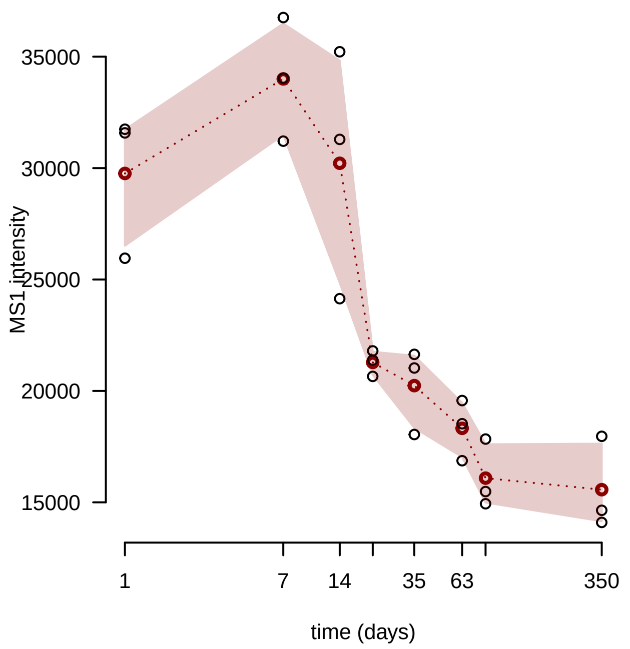

Q80XR2
GN=Atp2c1
ATPase, Ca++−sequestering

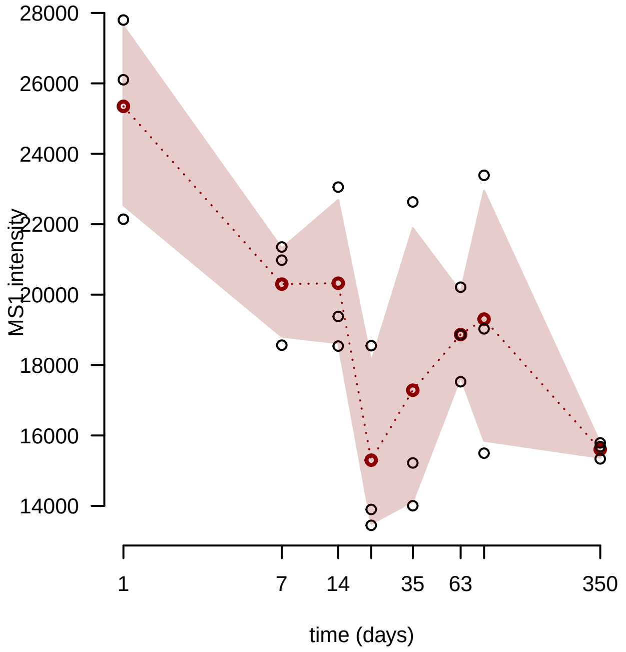

Q80XS6
GN=Samd4b
sterile alpha motif domain containing 4B

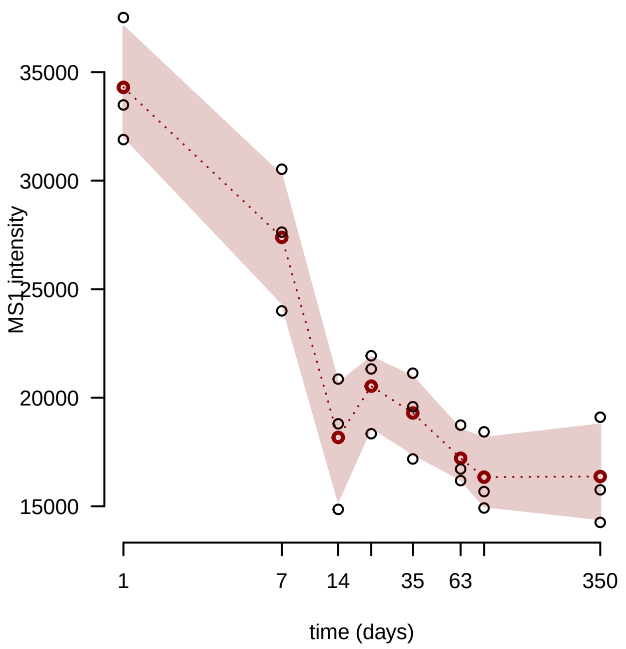

Q80XU3
GN=Nucks1
nuclear casein kinase and cyclin−depende ...

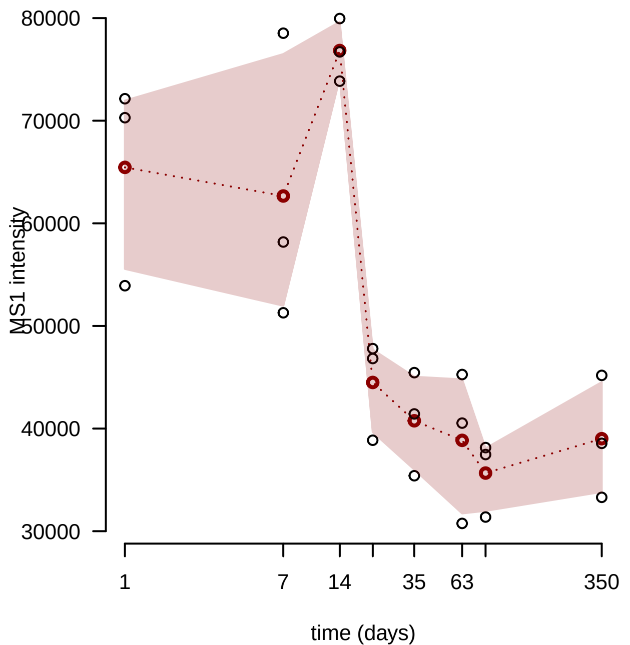

Q80Y14
GN=Glrx5
glutaredoxin 5

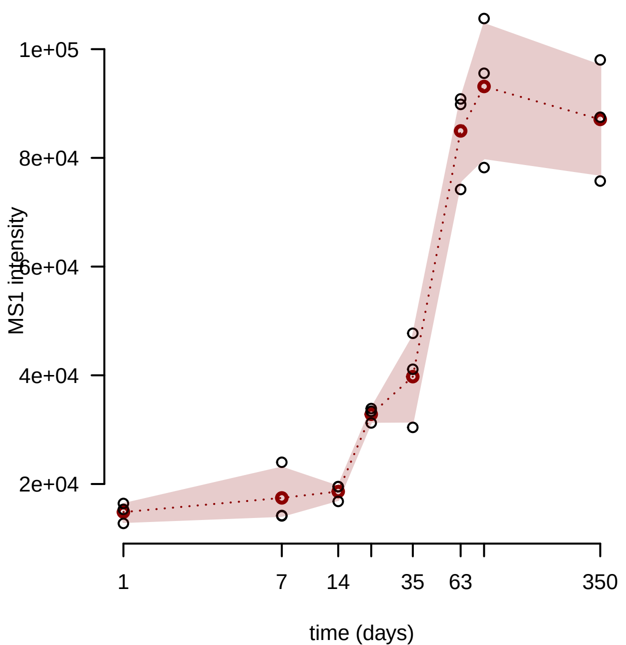

Q80Y17
GN=Llgl1
LLGL1 scribble cell polarity complex com ...

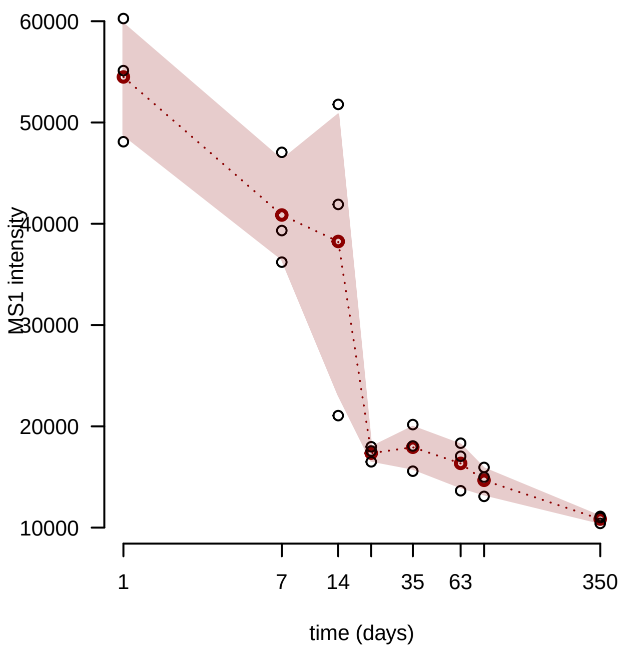

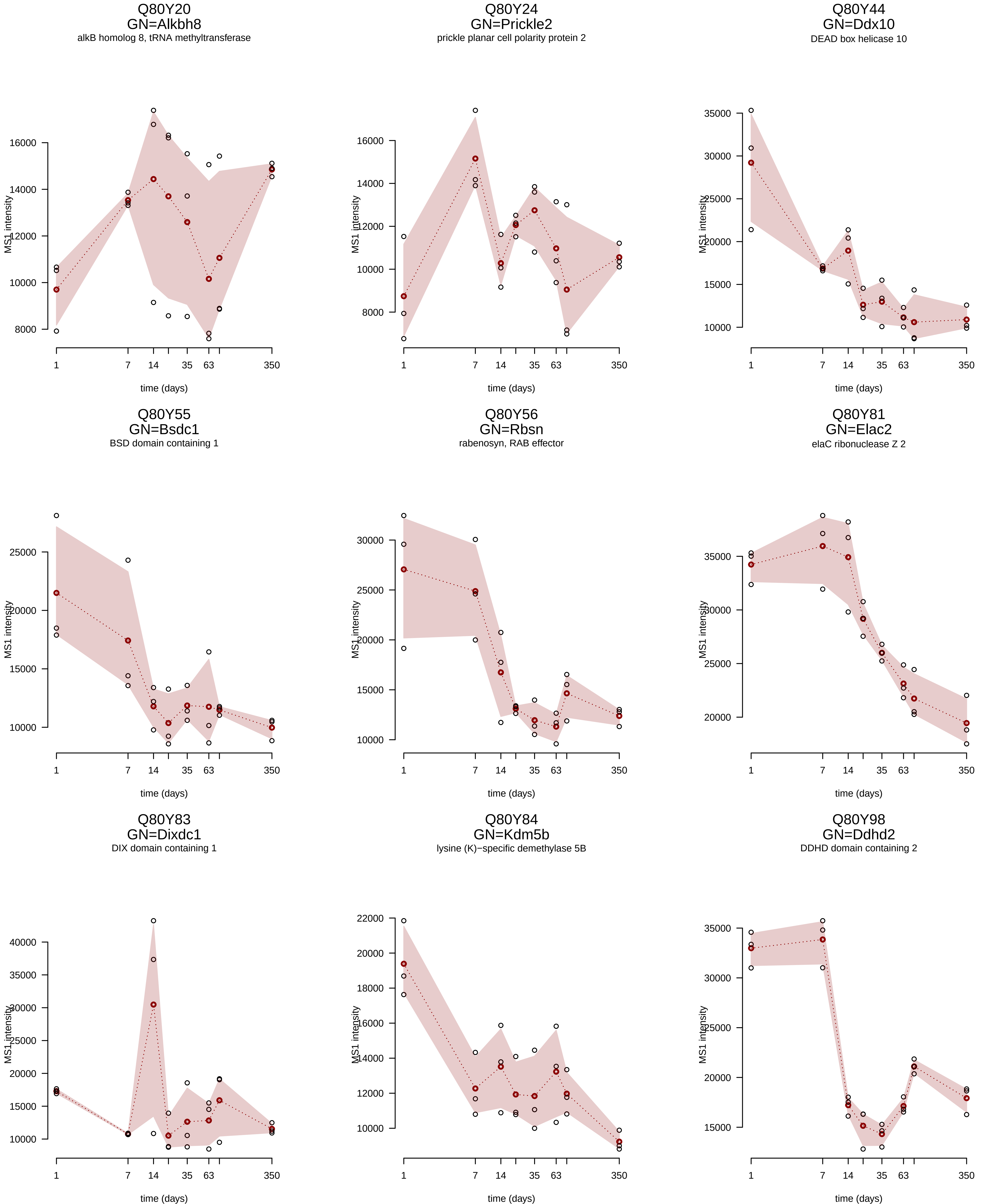
Q80Y20
GN=Alkbh8
alkB homolog 8, tRNA methyltransferase
MS1 intensity
8000
10000
12000
14000
16000
1
7
14
35
63
350
time (days)
Q80Y24
GN=Prickle2
prickle planar cell polarity protein 2
MS1 intensity
8000
10000
12000
14000
16000
time (days)
Q80Y44
GN=Ddx10
DEAD box helicase 10
MS1 intensity
10000
15000
20000
25000
30000
35000
time (days)
Q80Y55
GN=Bsdc1
BSD domain containing 1
MS1 intensity
10000
15000
20000
25000
time (days)
Q80Y56
GN=Rbsn
rabenosyn, RAB effector
MS1 intensity
10000
15000
20000
25000
30000
time (days)
Q80Y81
GN=Elac2
elaC ribonuclease Z 2
MS1 intensity
20000
25000
30000
35000
time (days)
Q80Y83
GN=Dixdc1
DIX domain containing 1
MS1 intensity
10000
15000
20000
25000
30000
35000
40000
time (days)
Q80Y84
GN=Kdm5b
lysine (K)−specific demethylase 5B
MS1 intensity
10000
12000
14000
16000
18000
20000
22000
time (days)
Q80Y98
GN=Ddhd2
DDHD domain containing 2
MS1 intensity
15000
20000
25000
30000
35000
time (days)

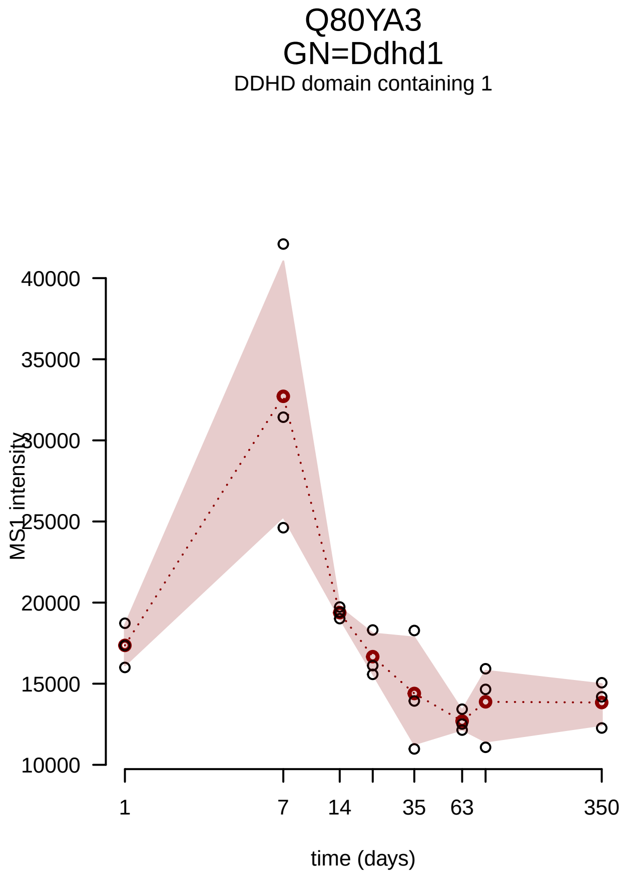
Q80YA3
GN=Ddhd1
DDHD domain containing 1
MS1 intensity
time (days)

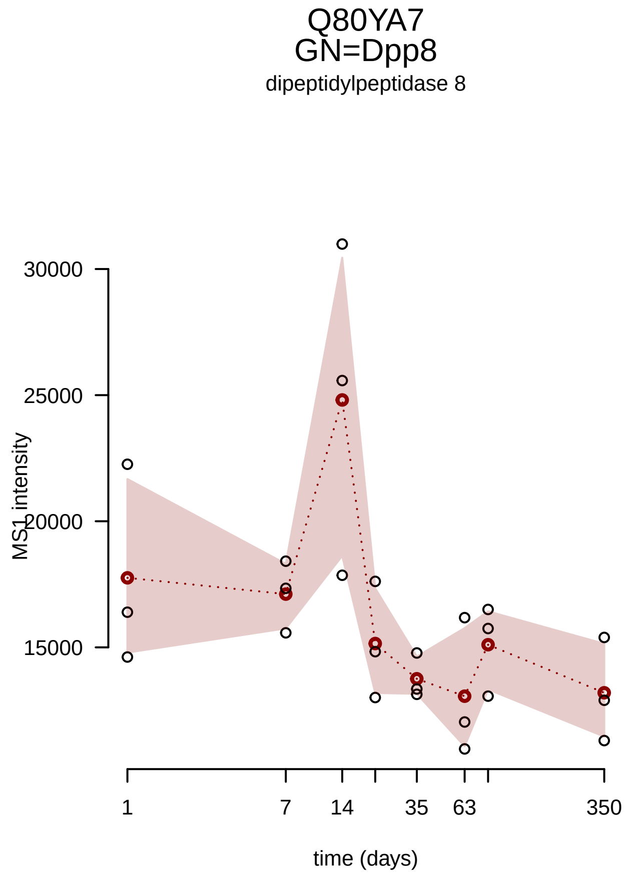
Q80YA7
GN=Dpp8
dipeptidylpeptidase 8
MS1 intensity
time (days)

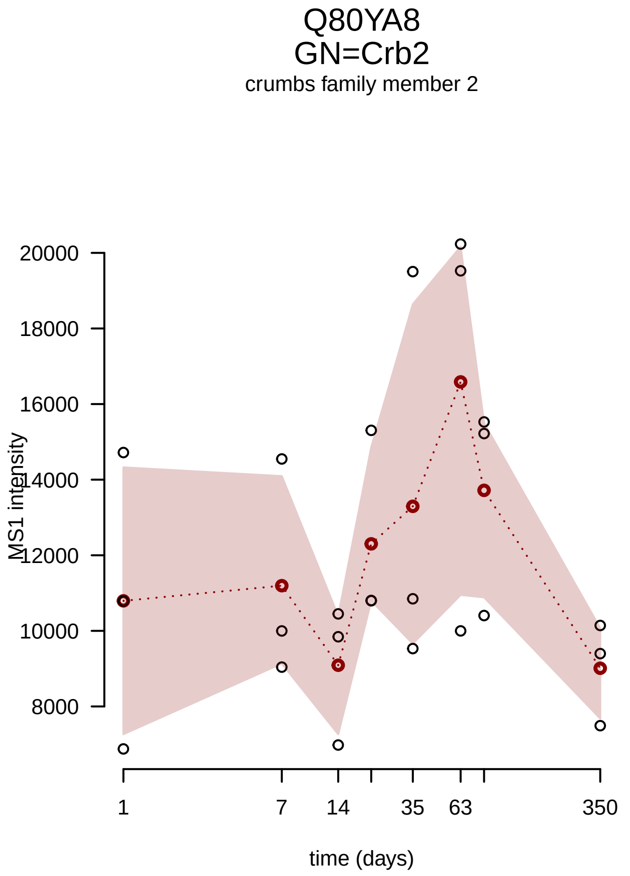
Q80YA8
GN=Crb2
crumbs family member 2
MS1 intensity
time (days)

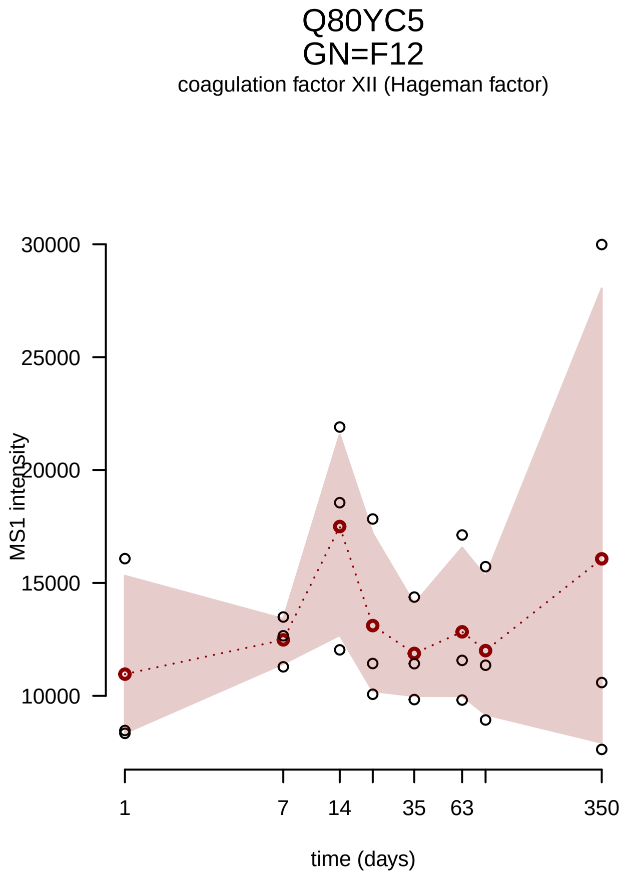
Q80YC5
GN=F12
coagulation factor XII (Hageman factor)
MS1 intensity
time (days)

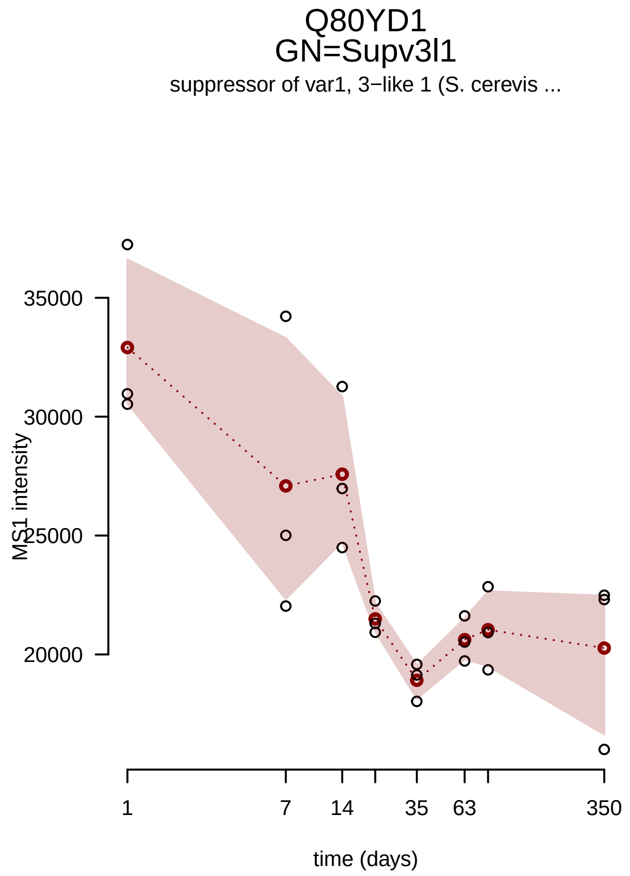
Q80YD1
GN=Supv3l1
suppressor of var1, 3−like 1 (S. cerevis ...
MS1 intensity
time (days)

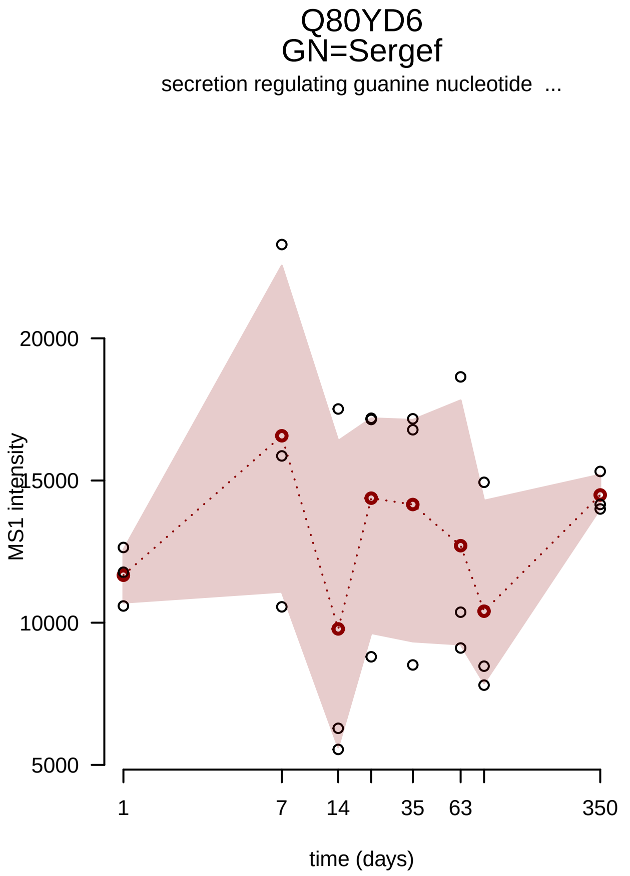
Q80YD6
GN=Sergef
secretion regulating guanine nucleotide ...
MS1 intensity
time (days)

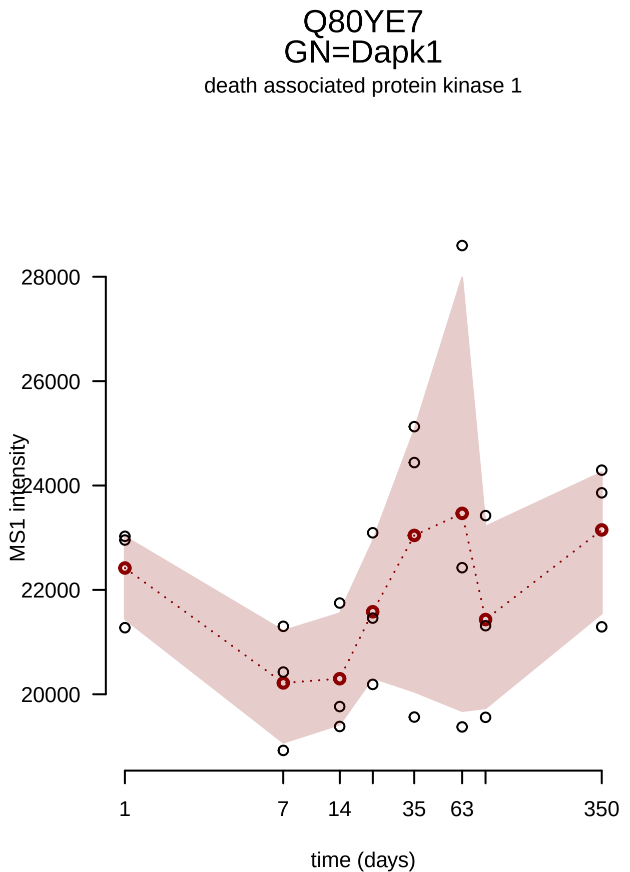
Q80YE7
GN=Dapk1
death associated protein kinase 1
MS1 intensity
time (days)

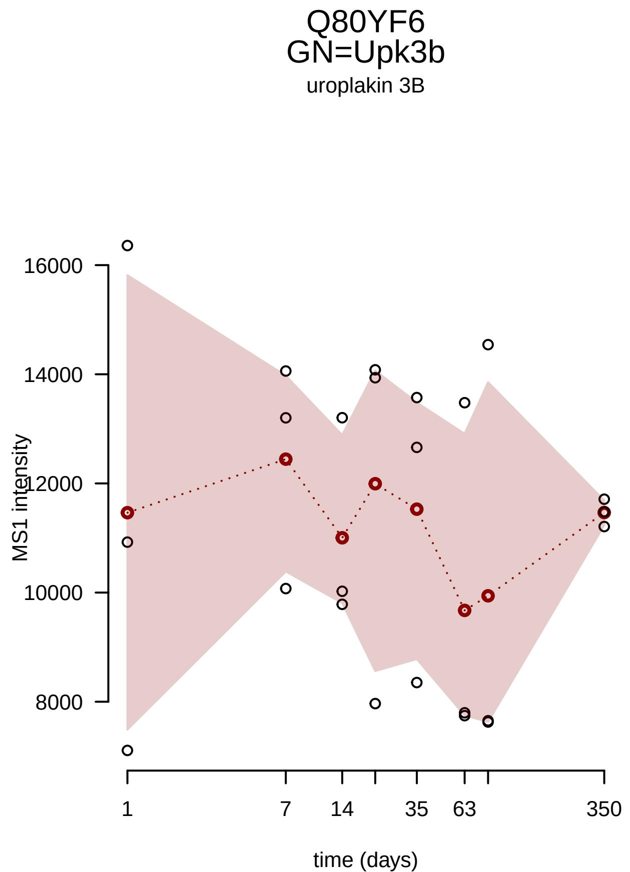
Q80YF6
GN=Upk3b
uroplakin 3B
MS1 intensity
time (days)

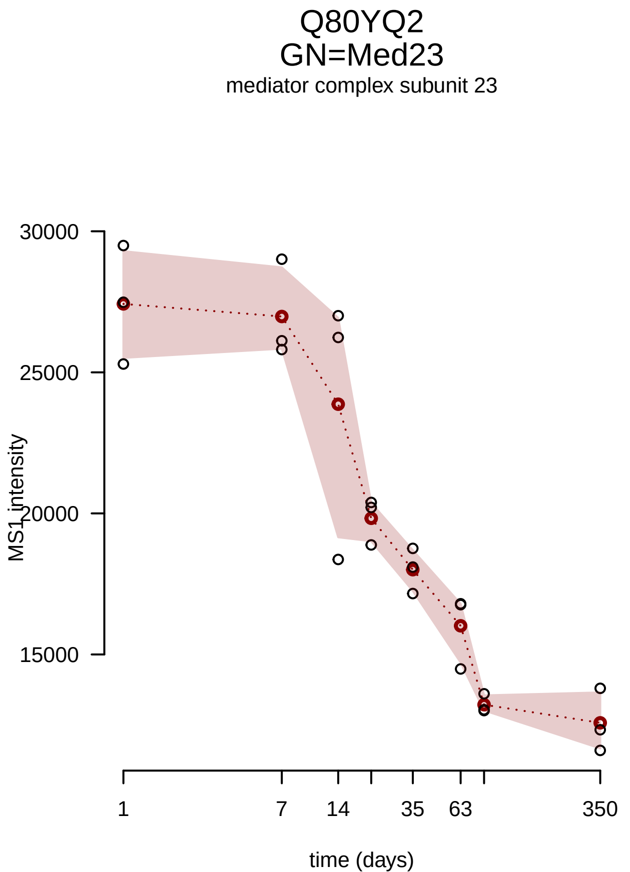
Q80YQ2
GN=Med23
mediator complex subunit 23
MS1 intensity
time (days)

Q80YQ8
GN=Rmnd5a
required for meiotic nuclear division 5 ...

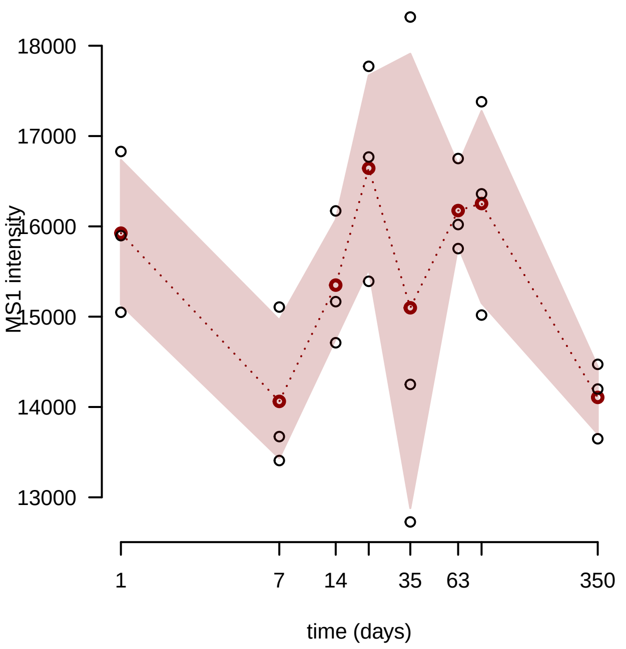

Q80YR4
GN=Znf598

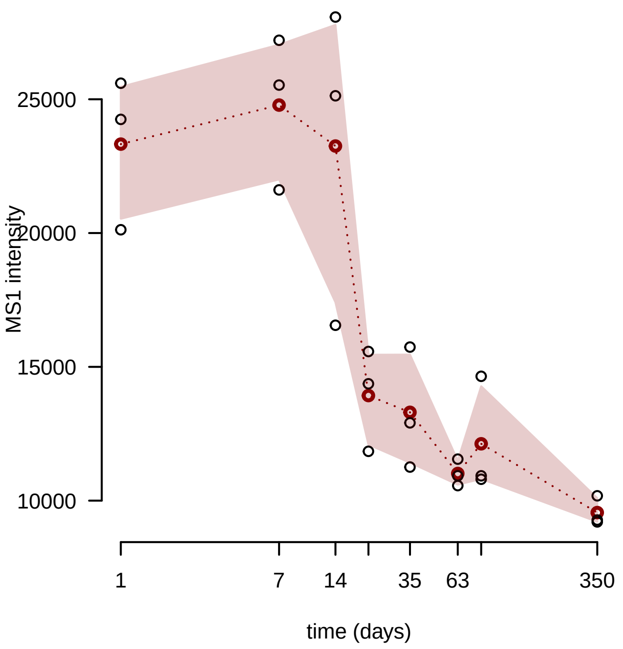

Q80YR5
GN=Safb2
scaffold attachment factor B2

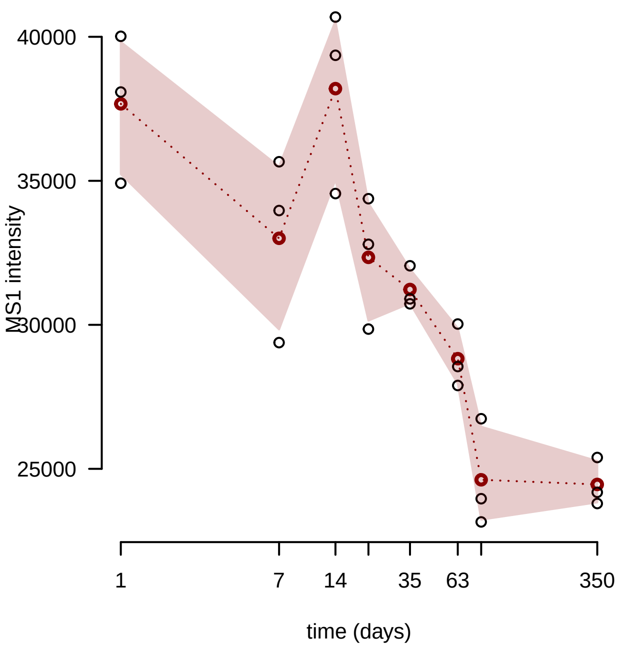

Q80YR9
GN=Rbm12b1
RNA binding motif protein 12 B1

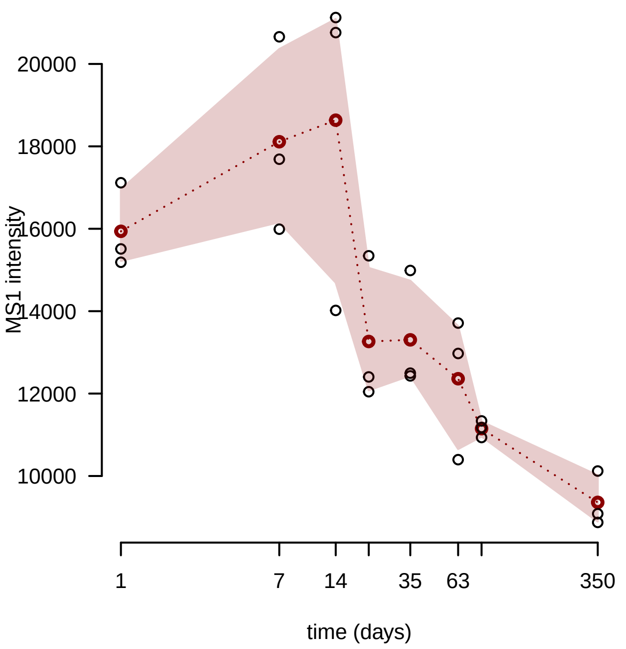

Q80YS6
GN=Afap1
actin filament associated protein 1

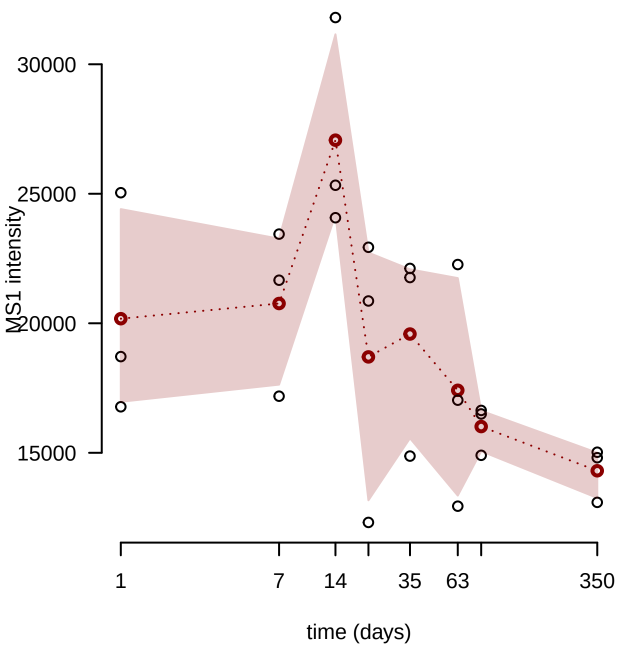

Q80YT7
GN=Pde4dip
phosphodiesterase 4D interacting protein ...

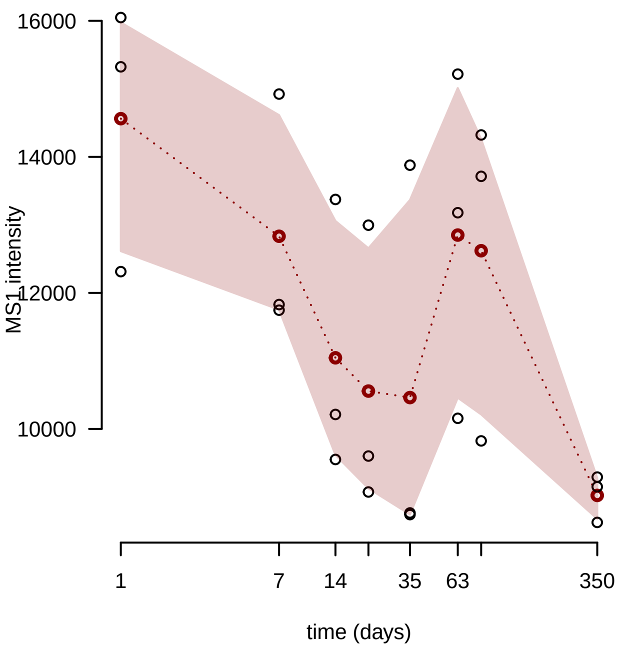

Q80YV2
GN=Zc3hc1
zinc finger, C3HC type 1

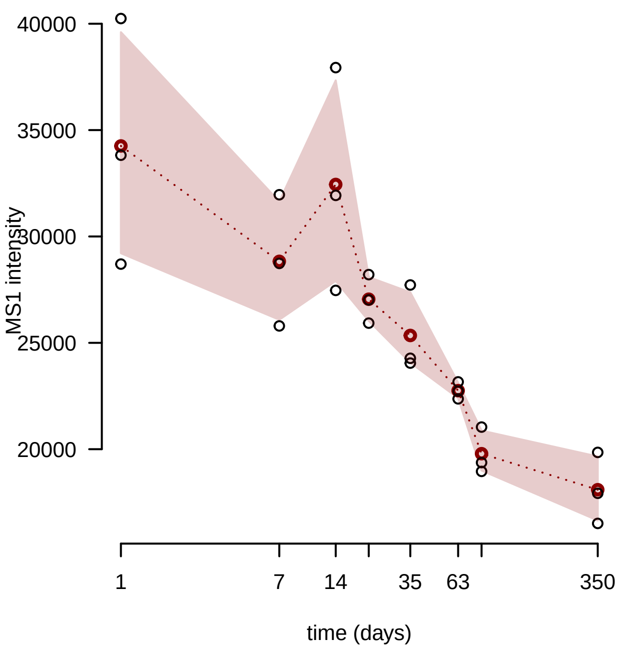

Q80YV3
GN=Trrap
transformation/transcription domain−asso ...

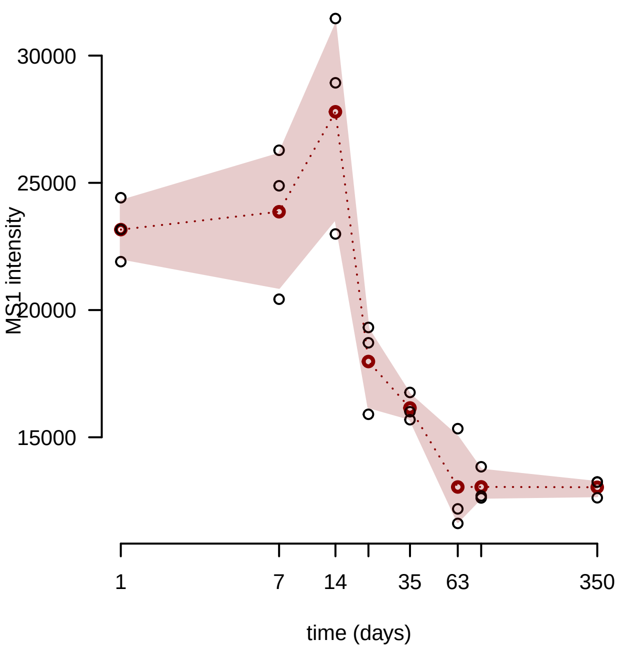

Q80YV4
GN=Pank4
pantothenate kinase 4

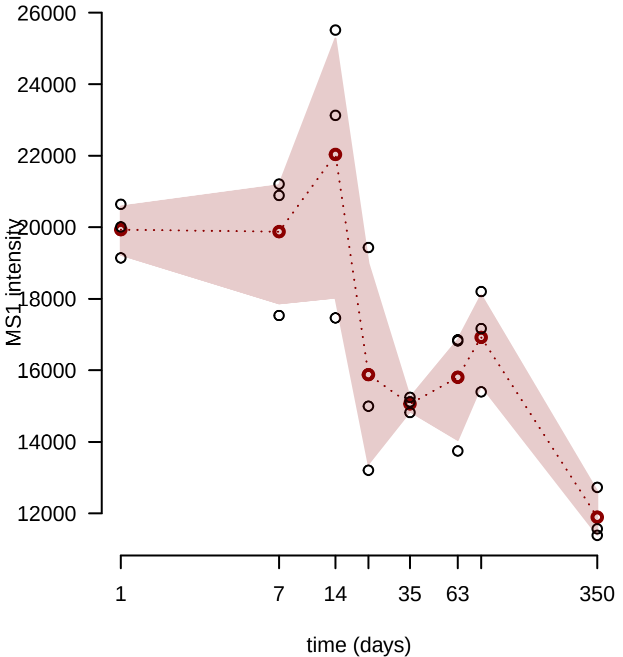

Q80YW0
GN=Cyth4
cytohesin 4

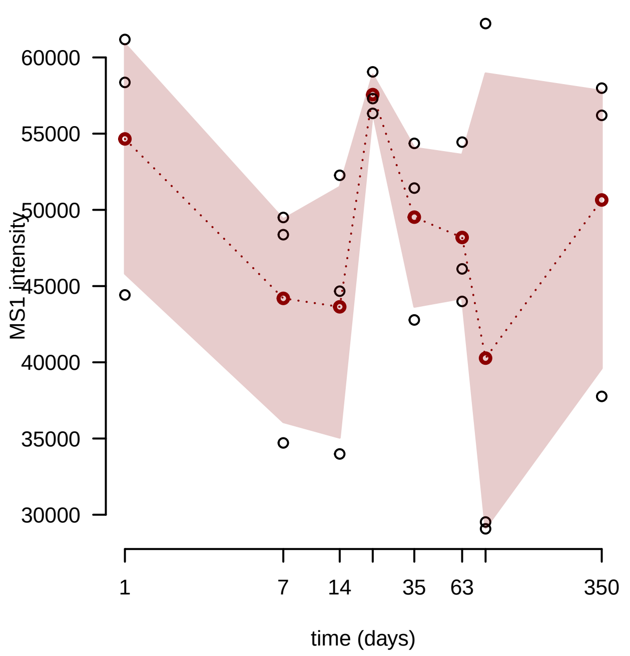

Q80YX1
GN=Tnc
tenascin C

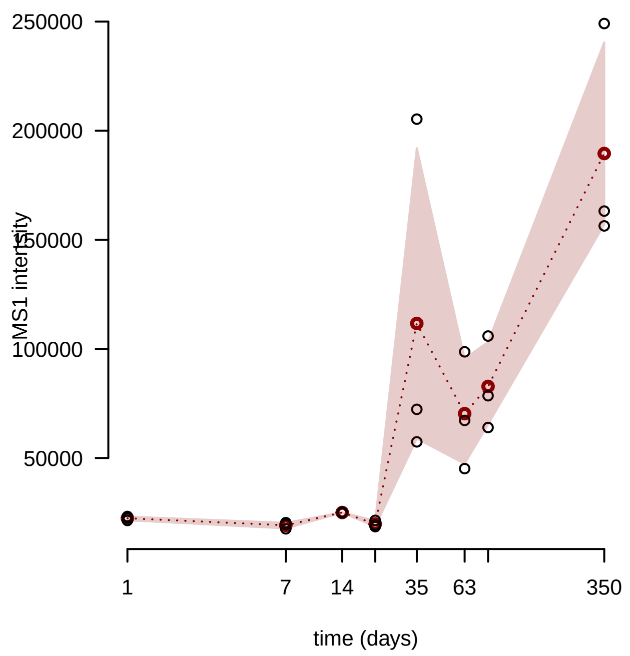

Q80YX1
GN=Tnc
tenascin C

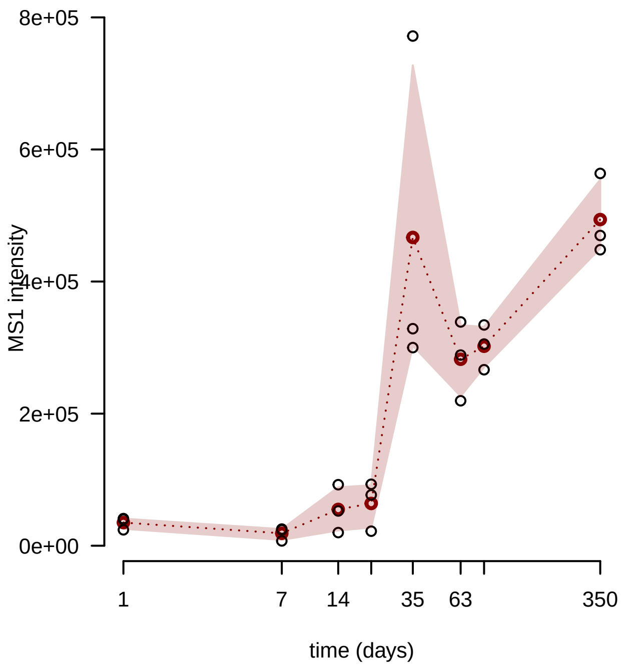

Q80YY7
GN=Znf618

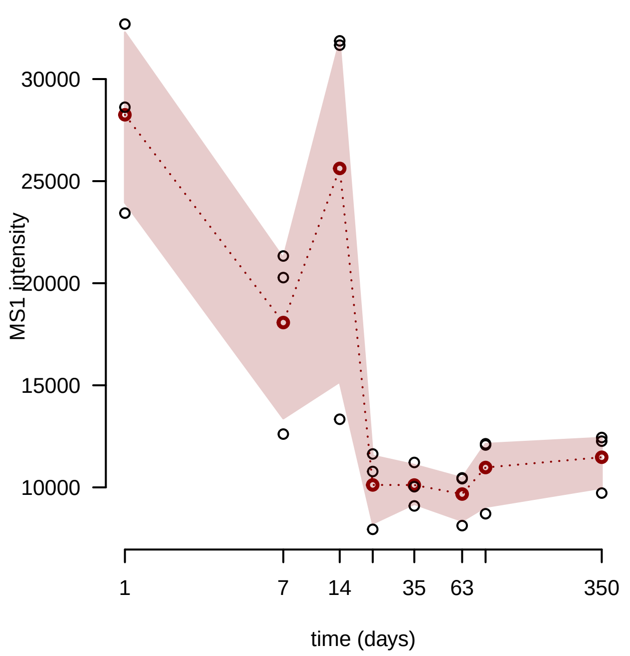

Q80Z25
GN=Ofd1
OFD1, centriole and centriolar satellite ...

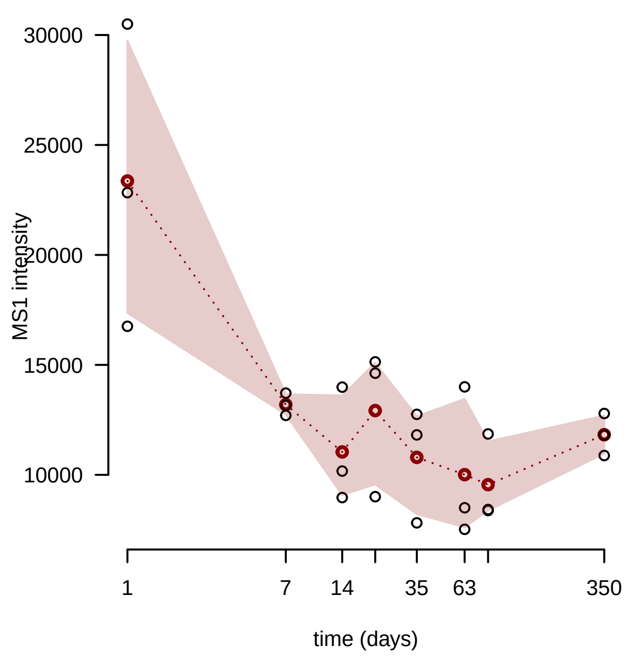

Q80Z96
GN=Vangl1
VANGL planar cell polarity 1

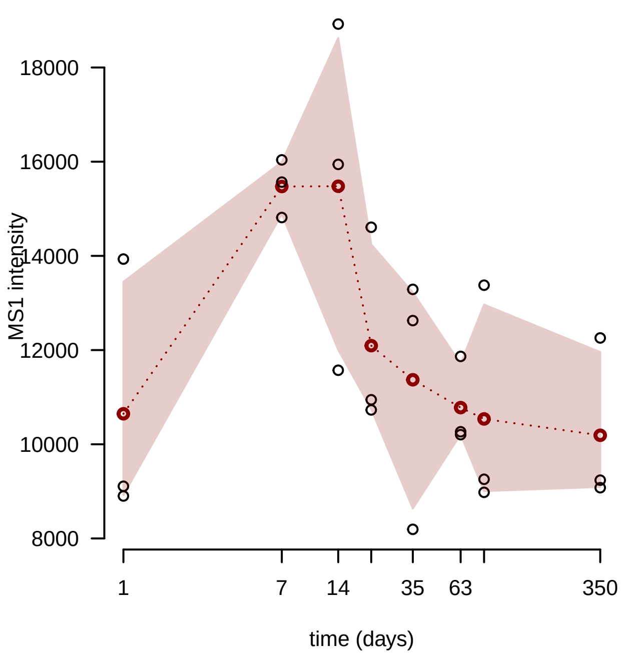

Q80ZA4
GN=Pkhd1l1
polycystic kidney and hepatic disease 1− ...

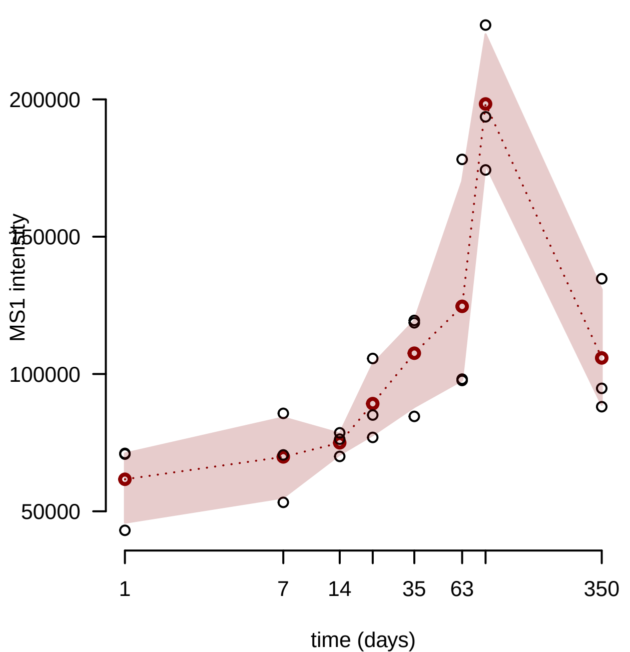

Q80ZA7
GN=Fmr1nb
Fmr1 neighbor

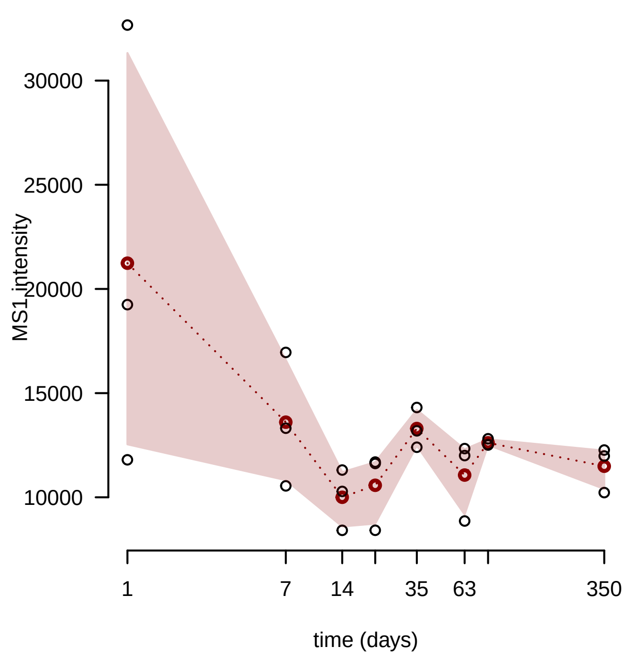

Q80ZI6
GN=Lrsam1
leucine rich repeat and sterile alpha mo ...

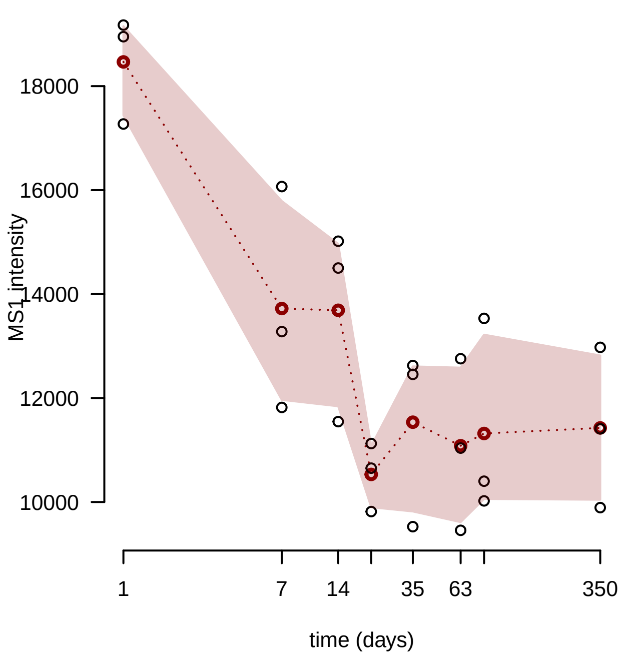

Q80ZJ1
GN=Rap2a
RAS related protein 2a

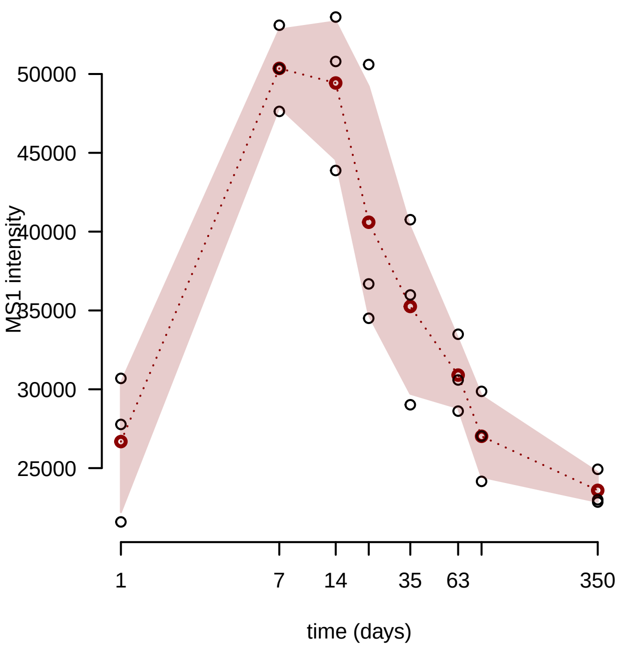

Q80ZJ6
GN=Zer1
zyg−11 related, cell cycle regulator

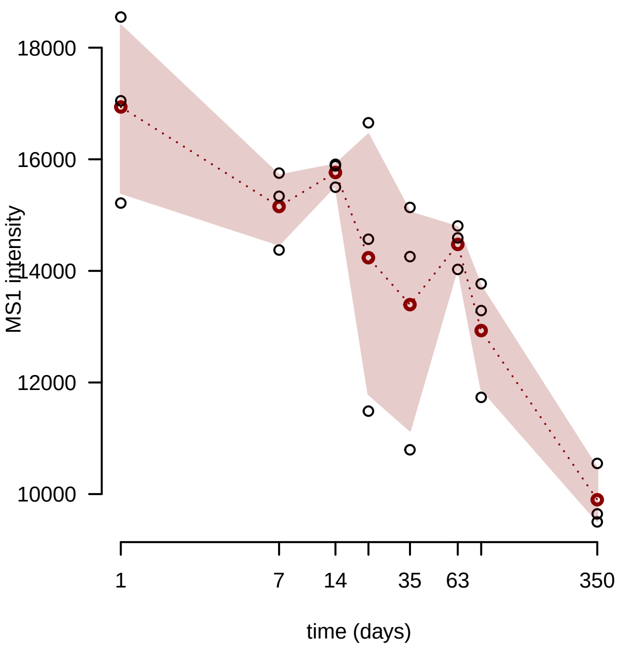

Q80ZK0
GN=Mrps10
mitochondrial ribosomal protein S10

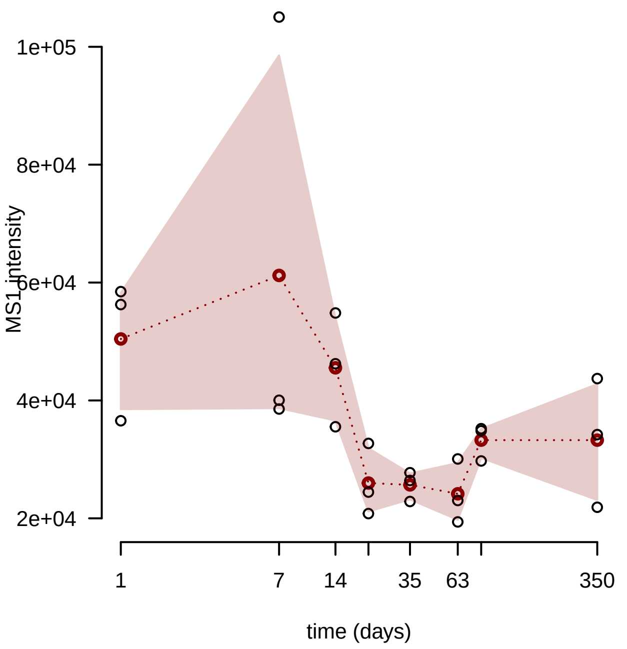

Q80ZK9
GN=Wdtc1
WD and tetratricopeptide repeats 1

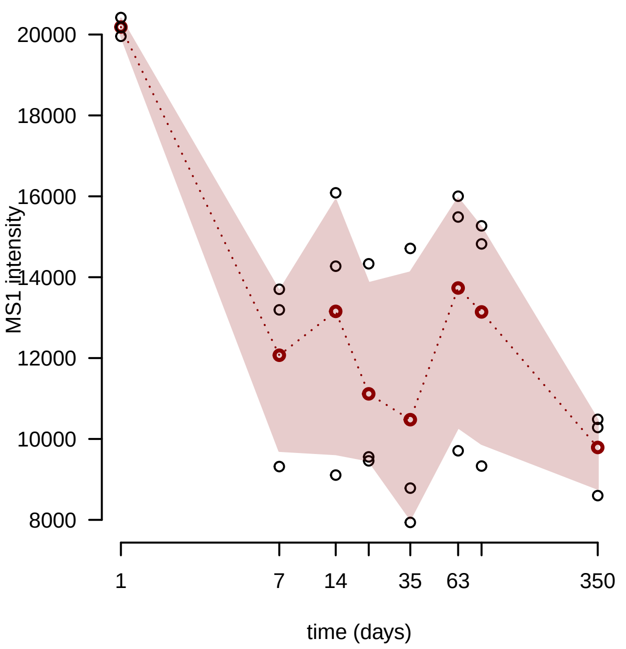

Q80ZM7
GN=Gtf2a2
general transcription factor II A, 2

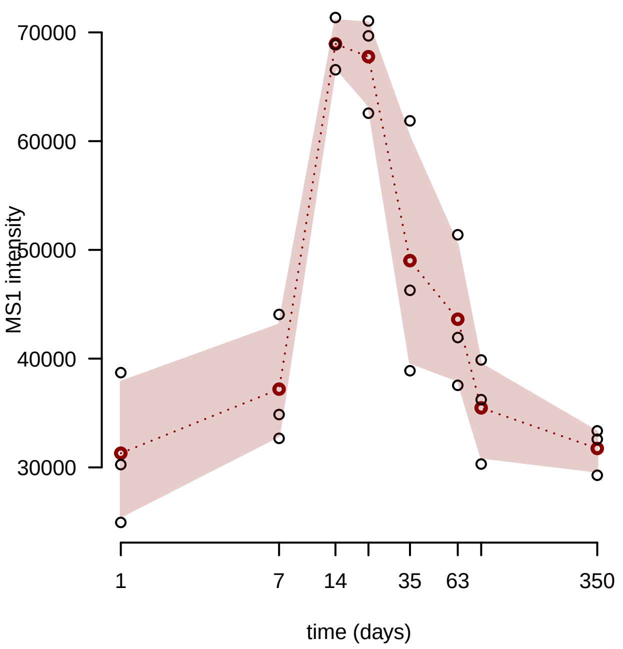

Q80ZM8
GN=Crls1
cardiolipin synthase 1

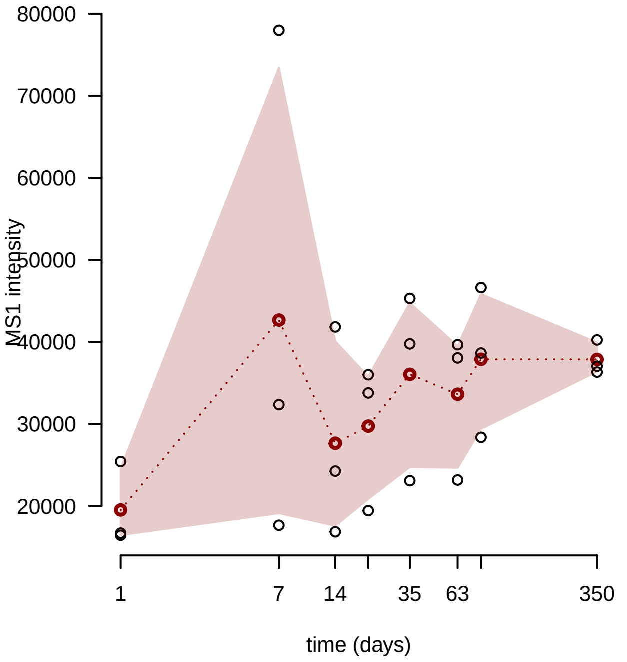

Q80ZN9
GN=Cox6b2
cytochrome c oxidase subunit 6B2

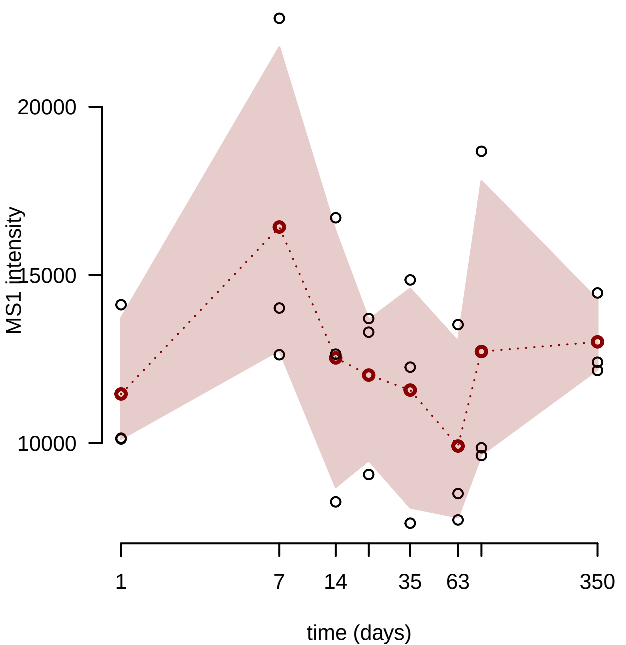

Q80ZQ9
GN=Abitram
actin binding transcription modulator

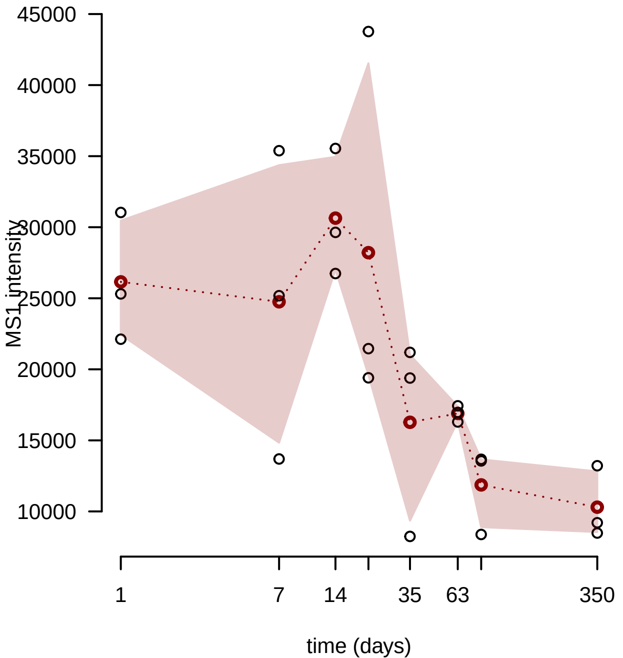

Q80ZS3
GN=Mrps26
mitochondrial ribosomal protein S26

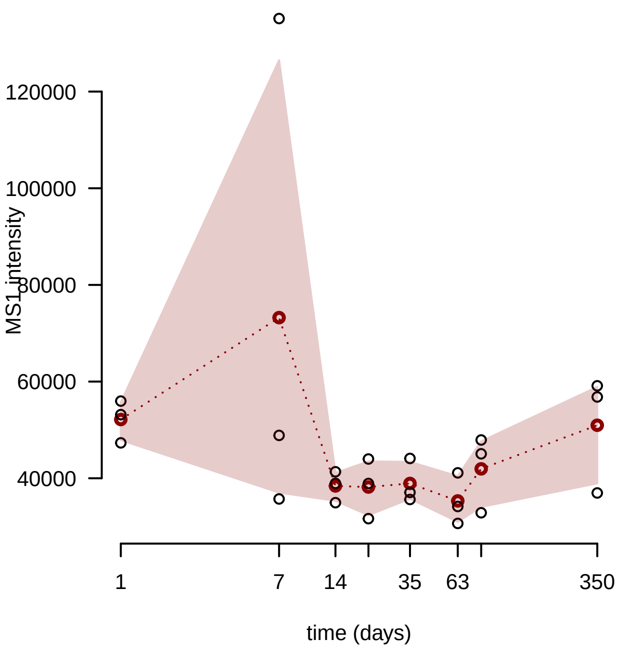

Q80ZU0
GN=Arl5a
ADP−ribosylation factor−like 5A

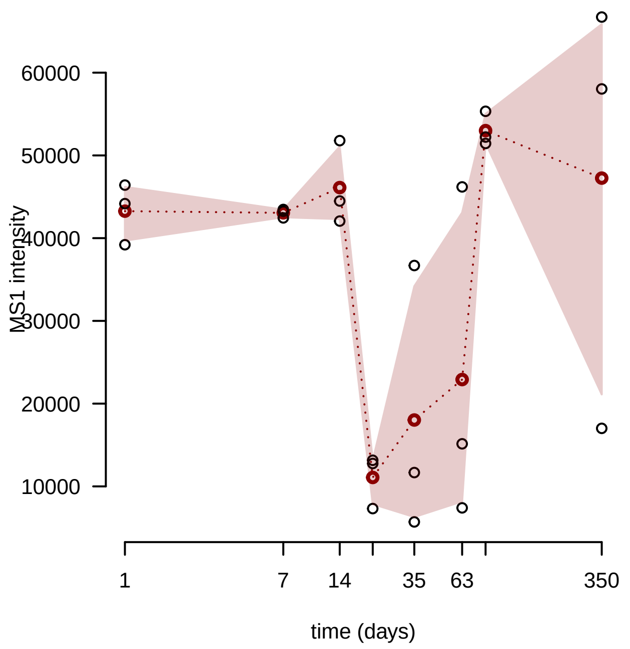

Q80ZV0
GN=Rnaseh2b
ribonuclease H2, subunit B

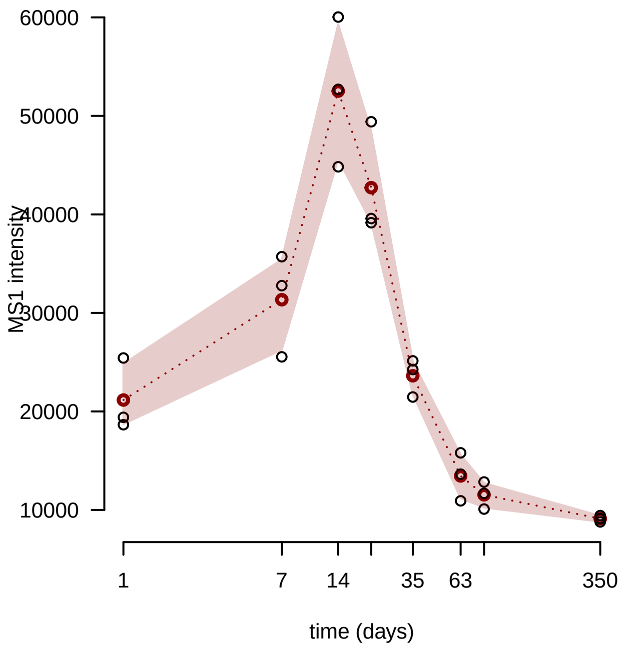

Q80ZW2
GN=Them6
thioesterase superfamily member 6

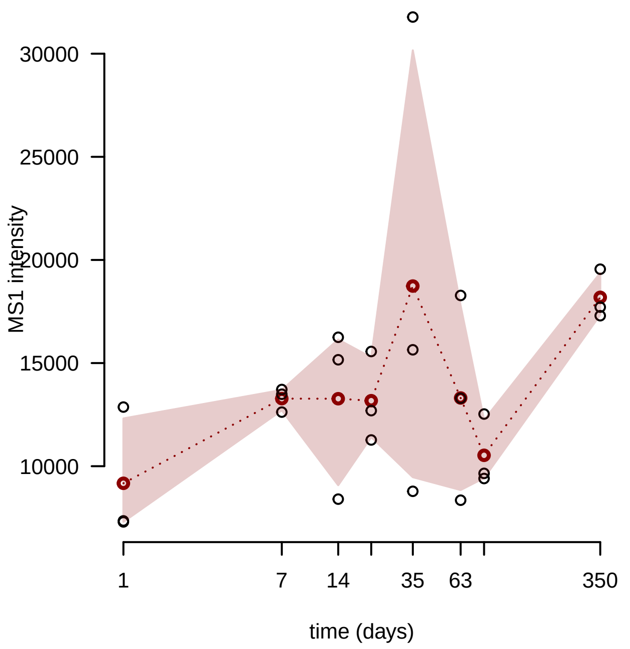

Q80ZX8
GN=Spag1
sperm associated antigen 1

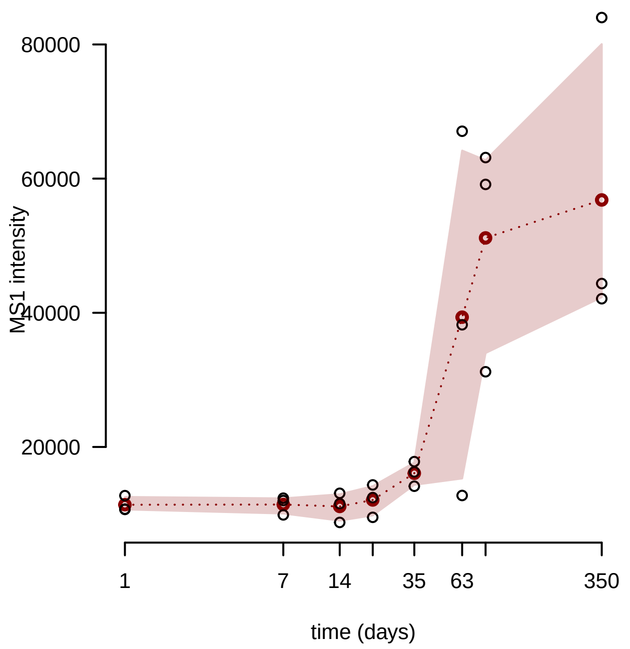

Q810A3
GN=Ttc9c
tetratricopeptide repeat domain 9C

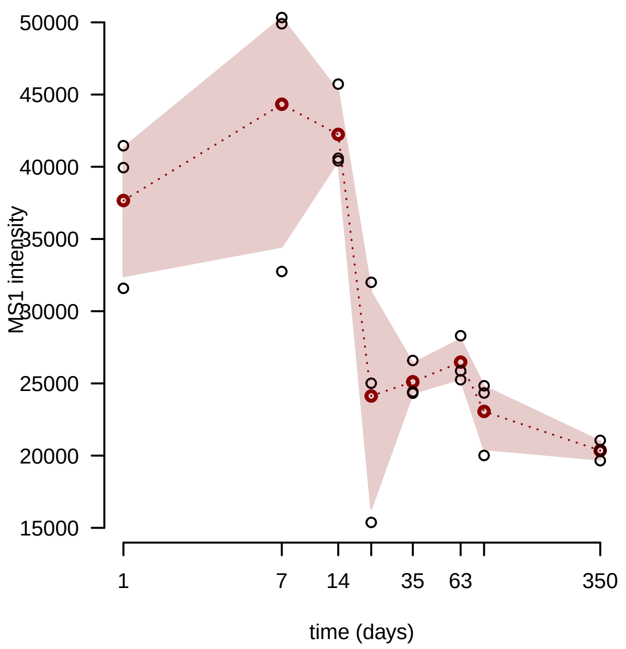

Q810A7
GN=Ddx42
DEAD box helicase 42

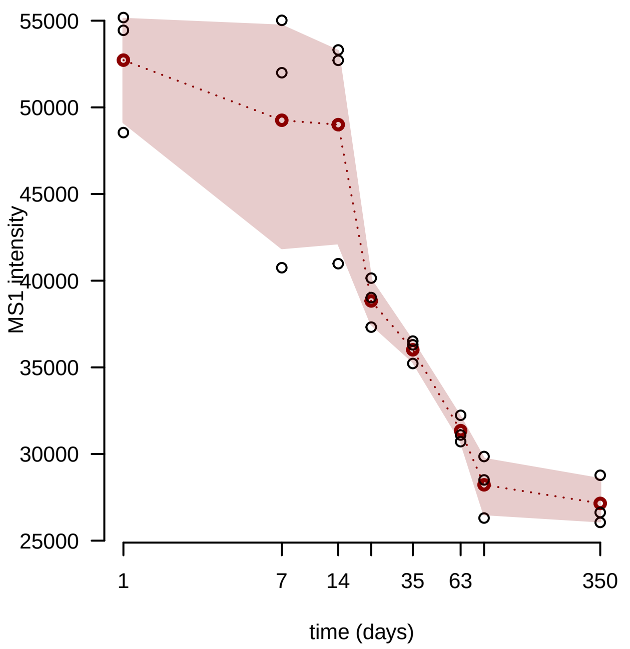

Q810B6
GN=Ankfy1
ankyrin repeat and FYVE domain containin ...

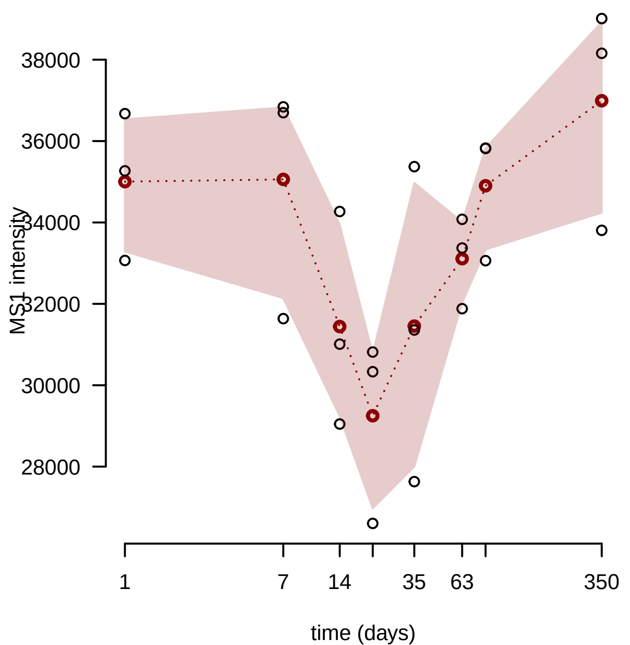

Q810D6
GN=Grwd1
glutamate−rich WD repeat containing 1

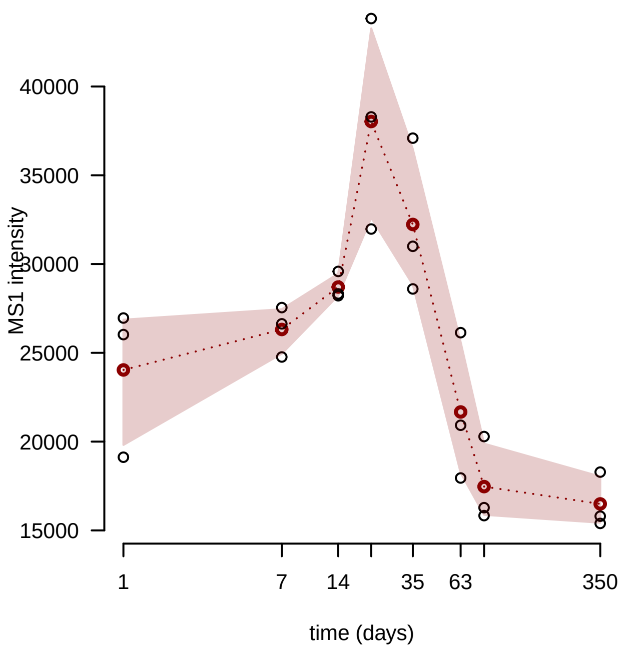

Q810J8
GN=Zfyve1
zinc finger, FYVE domain containing 1

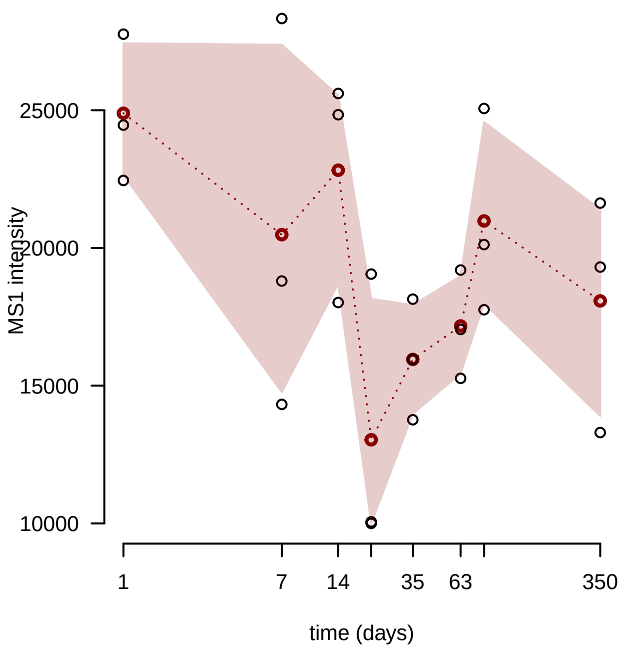

Q810K9
GN=Gxylt2
glucoside xylosyltransferase 2

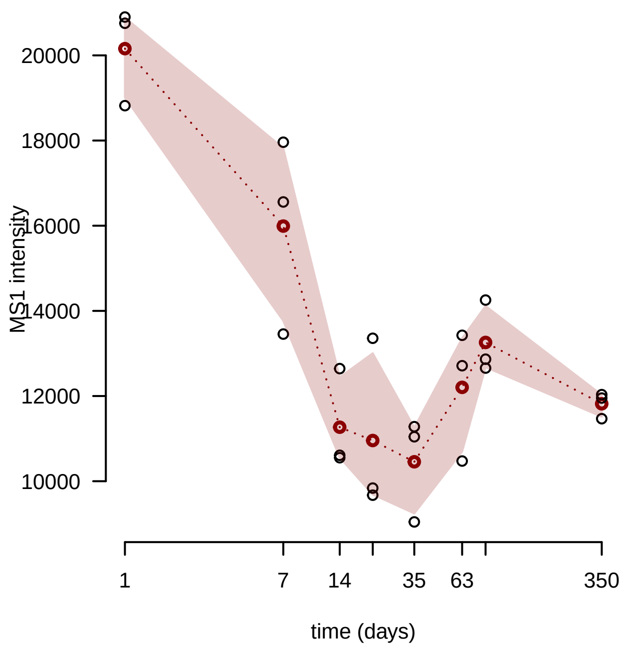

Q810L3
GN=Chfr
checkpoint with forkhead and ring finger ...

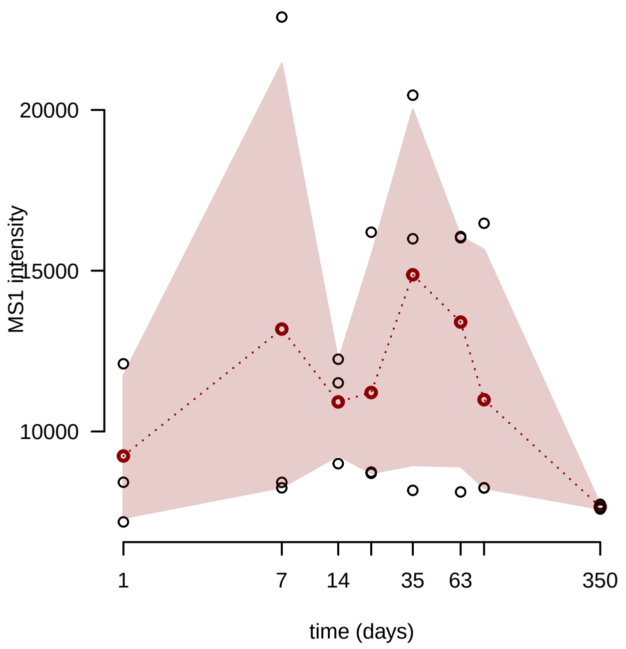

Q810L4
GN=Tmem39b
transmembrane protein 39b

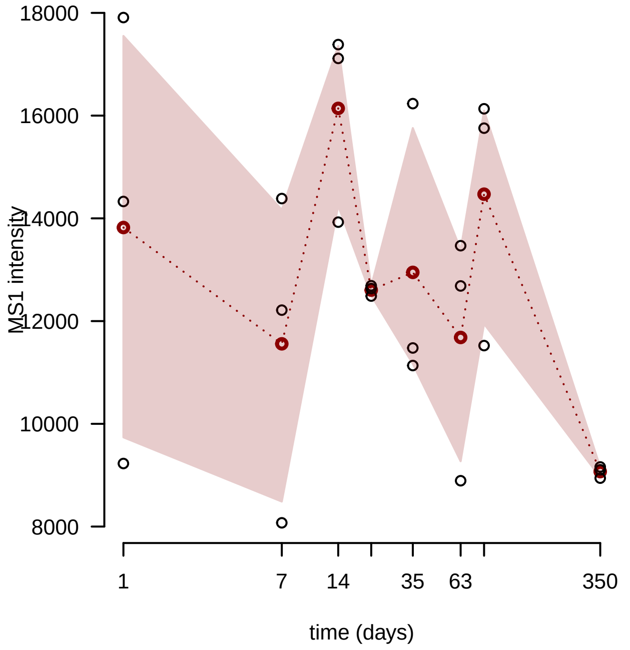

Q810U4
GN=Nrcam
neuronal cell adhesion molecule

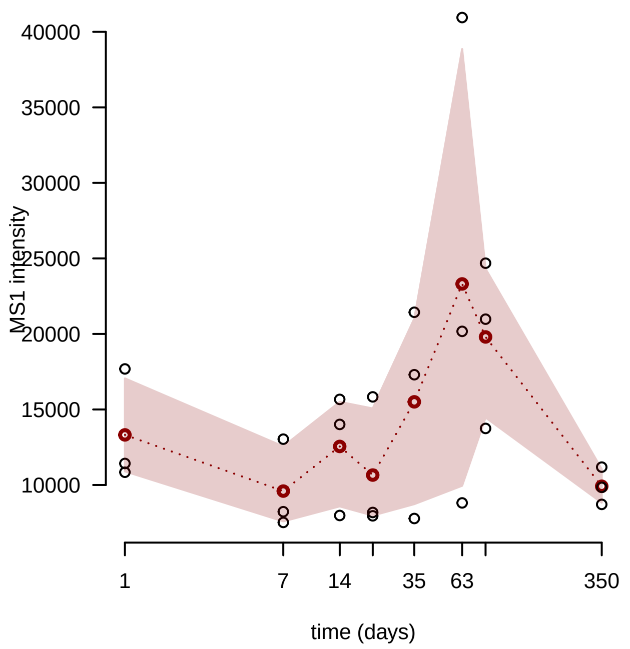

Q810U5
GN=Ccdc50
coiled−coil domain containing 50

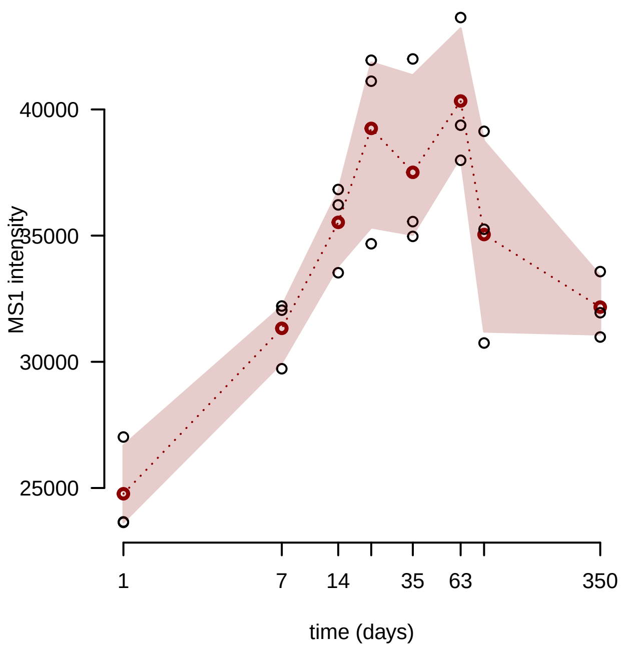

Q810V0
GN=Mphosph10
M−phase phosphoprotein 10 (U3 small nucl ...

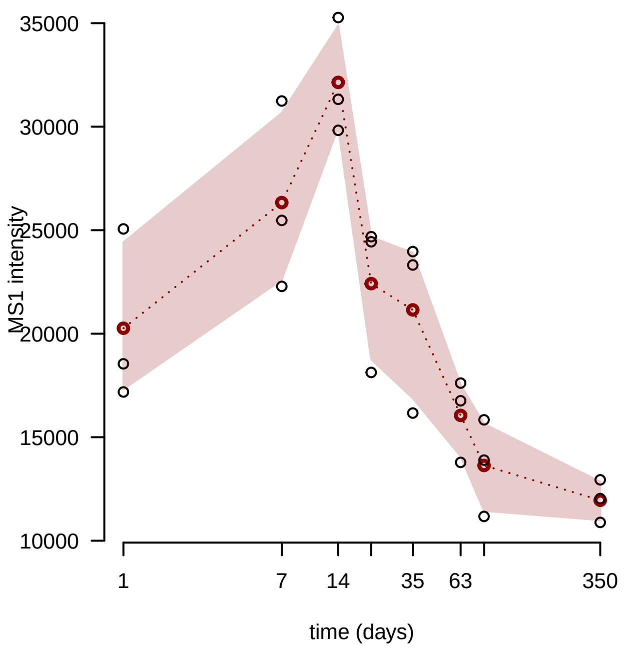

Q811B1
GN=Xylt1
xylosyltransferase 1

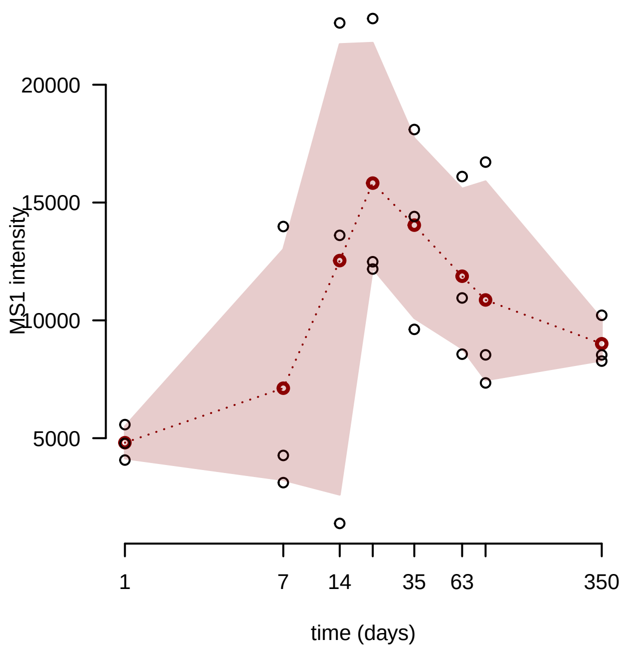

Q811B5
GN=Prr3
proline−rich polypeptide 3

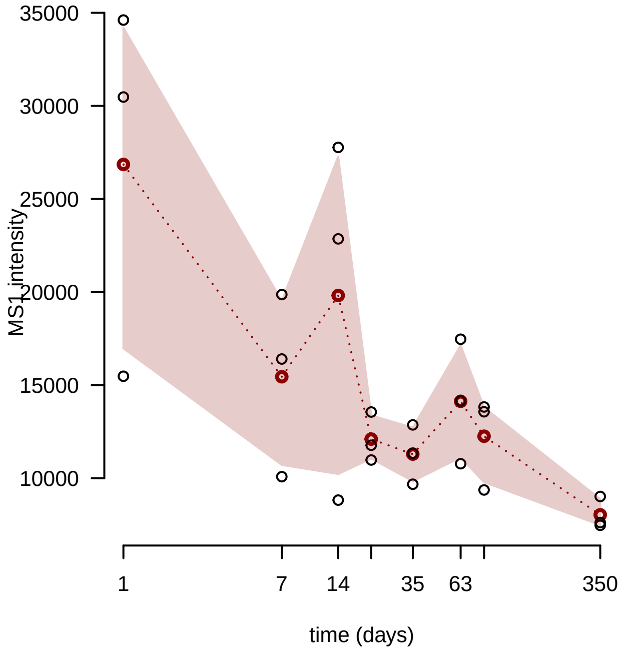

Q811C2
GN=Atg4c
autophagy related 4C, cysteine peptidase

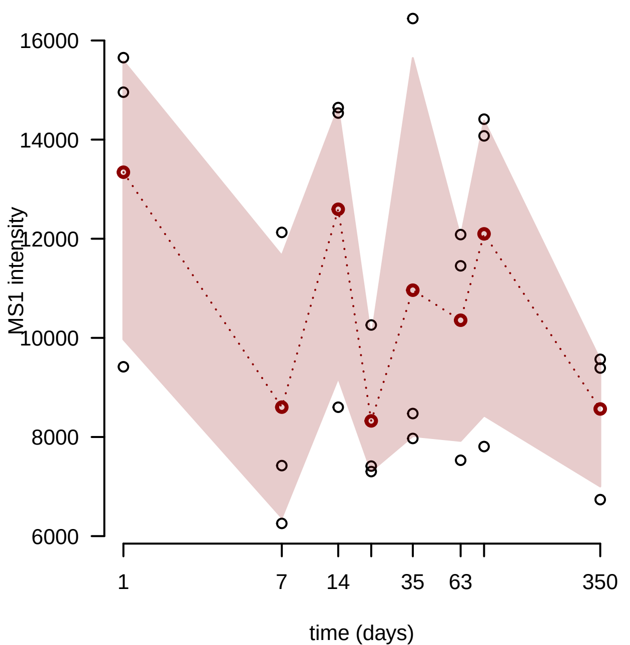

Q811D0
GN=Dlg1
discs large MAGUK scaffold protein 1

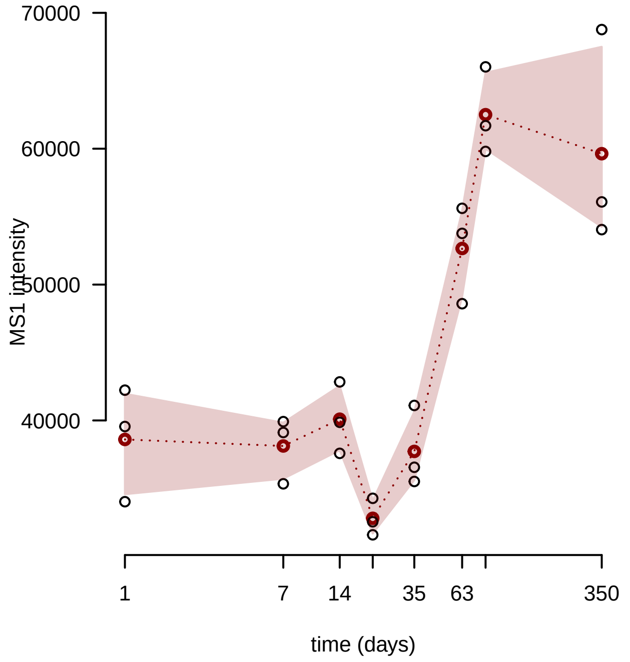

Q811D0
GN=Dlg1
discs large MAGUK scaffold protein 1

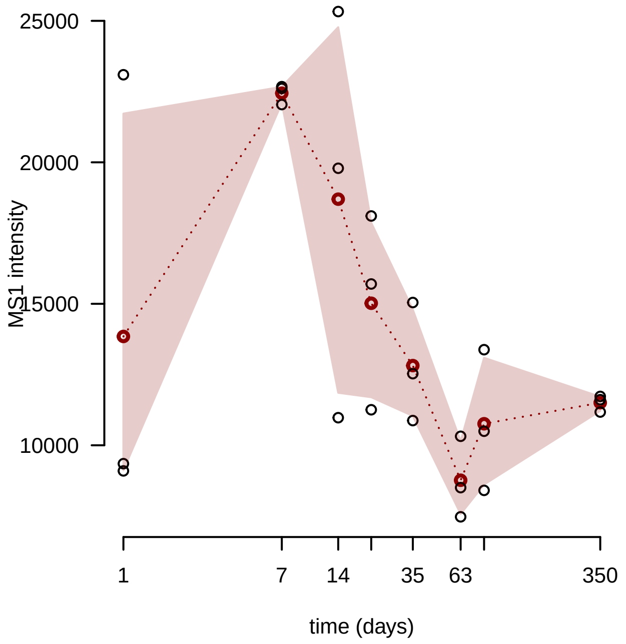

Q811G0
GN=Bbs9
Bardet−Biedl syndrome 9 (human)

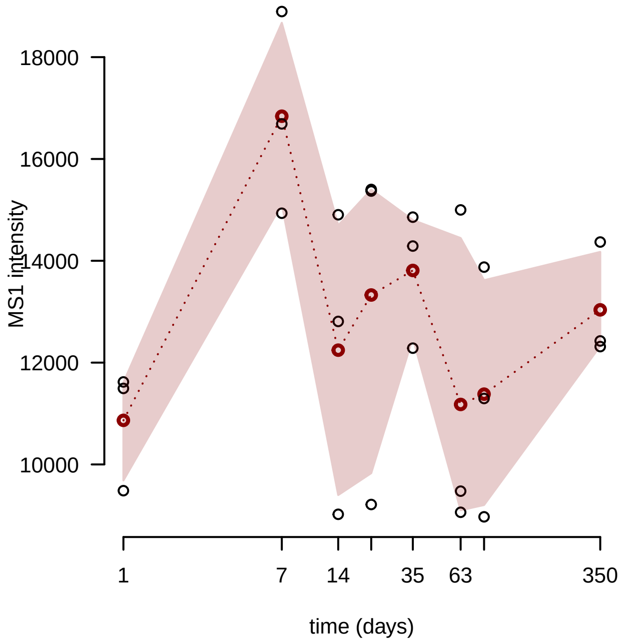

Q811I0
GN=Atpaf1
ATP synthase mitochondrial F1 complex as ...

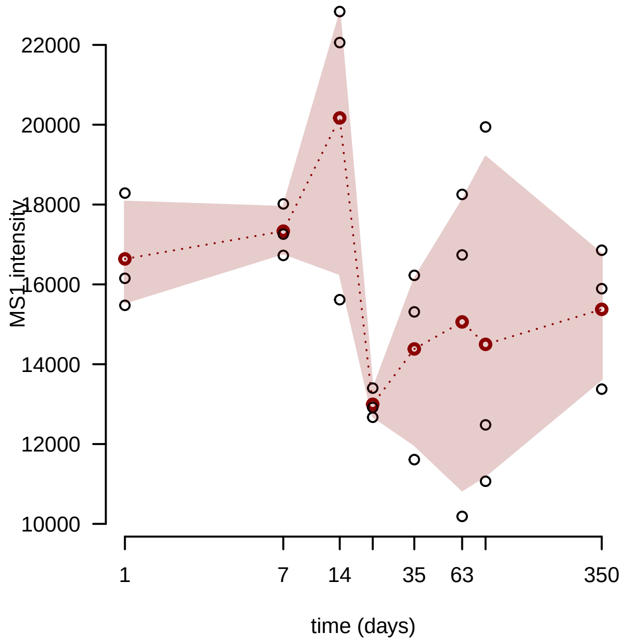

Q811J3
GN=Ireb2
iron responsive element binding protein ...

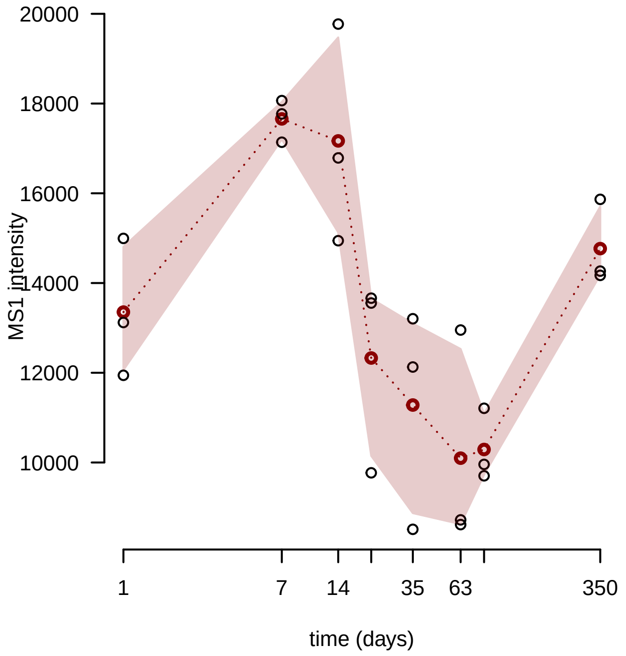

Q811L6
GN=Mast4
microtubule associated serine/threonine ...

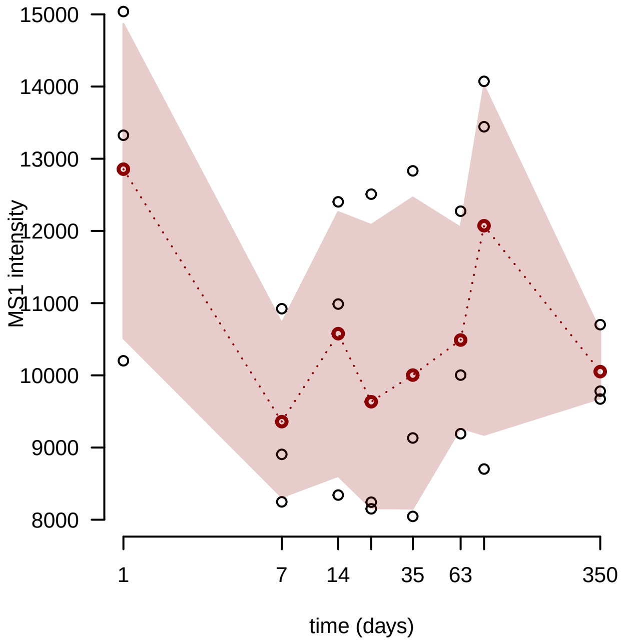

Q811P8
GN=Arhgap32
Rho GTPase activating protein 32

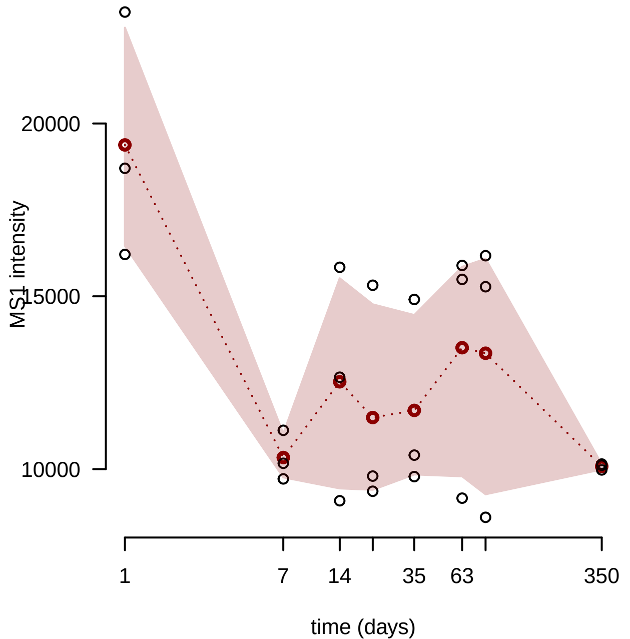

Q811S7
GN=Ubp1
upstream binding protein 1

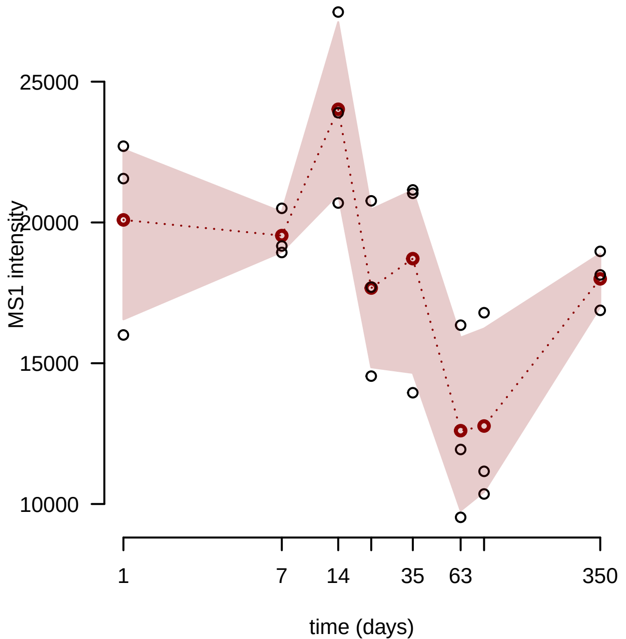

Q811U4
GN=Mfn1
mitofusin 1

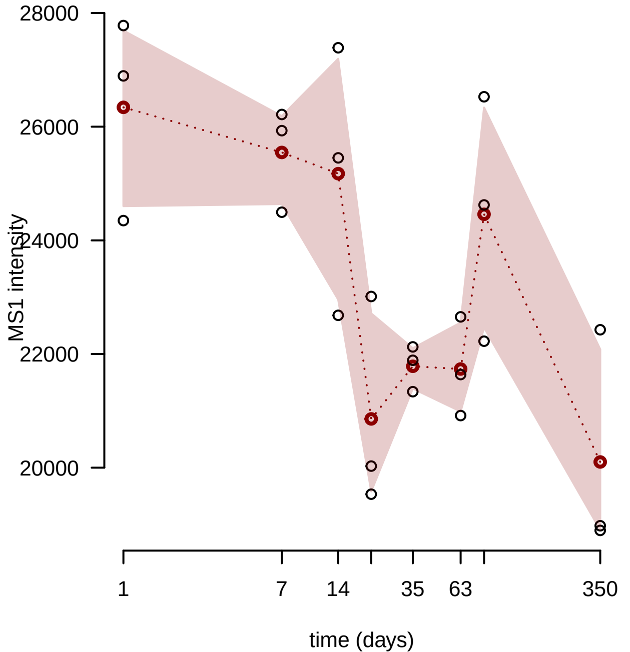

Q812A2
GN=Srgap3
SLIT−ROBO Rho GTPase activating protein ...

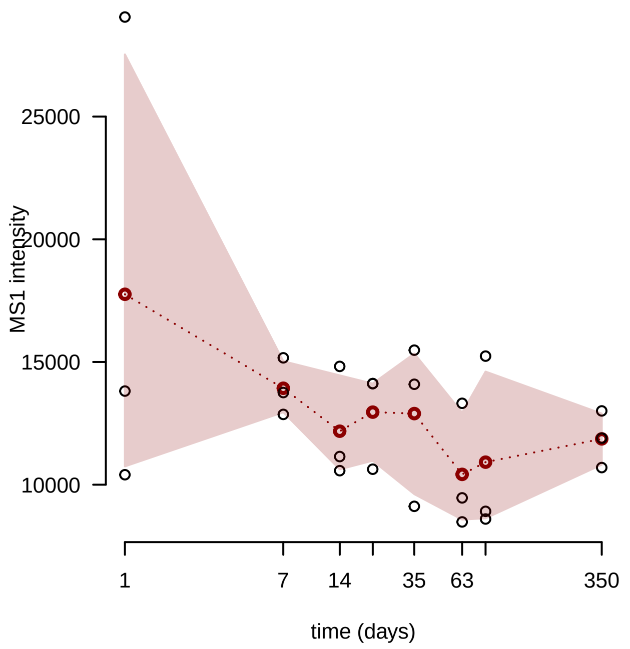

Q812G0
GN=Mgat4a
mannoside acetylglucosaminyltransferase ...

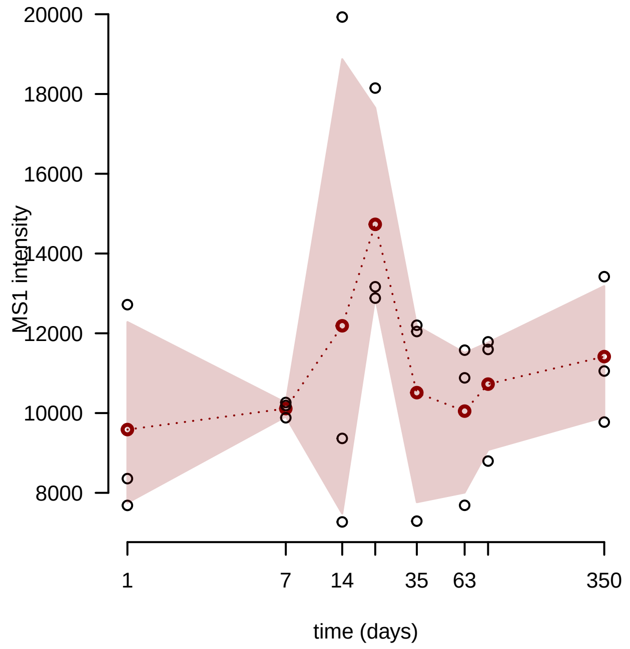

Q8BFP9
GN=Pdk1
pyruvate dehydrogenase kinase, isoenzyme ...

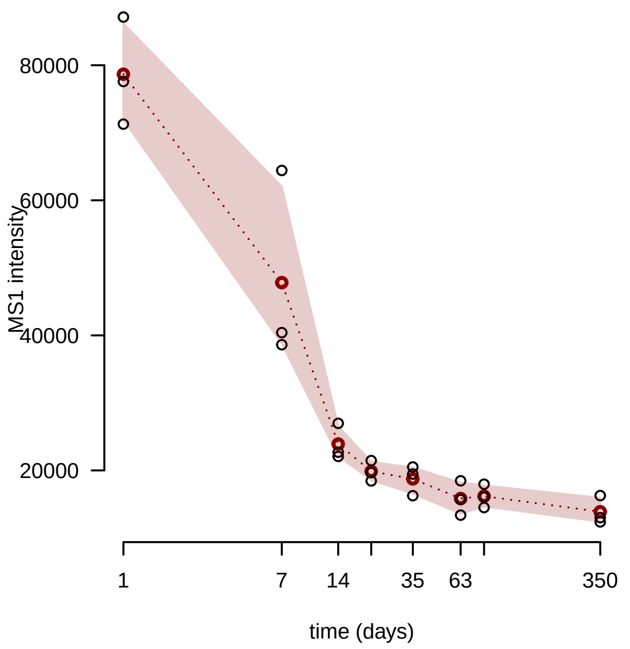

Q8BFQ4
GN=Wdr82
WD repeat domain containing 82

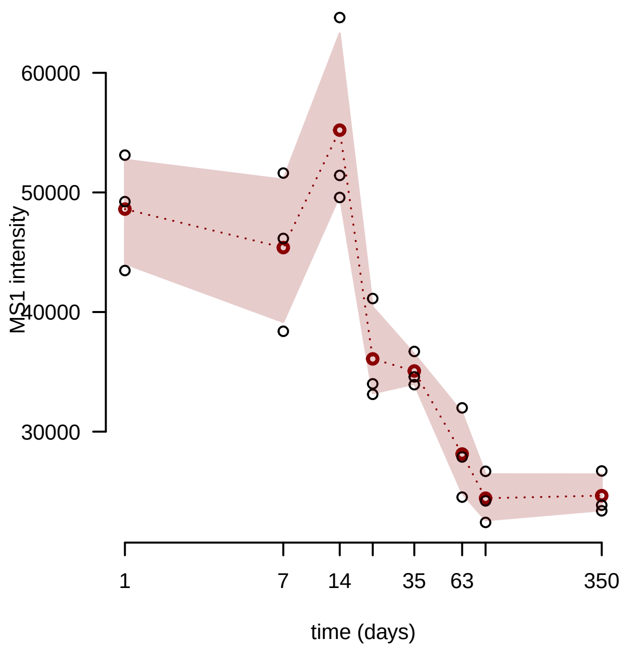

Q8BFQ6
GN=Slc49a4
solute carrier family 49 member 4

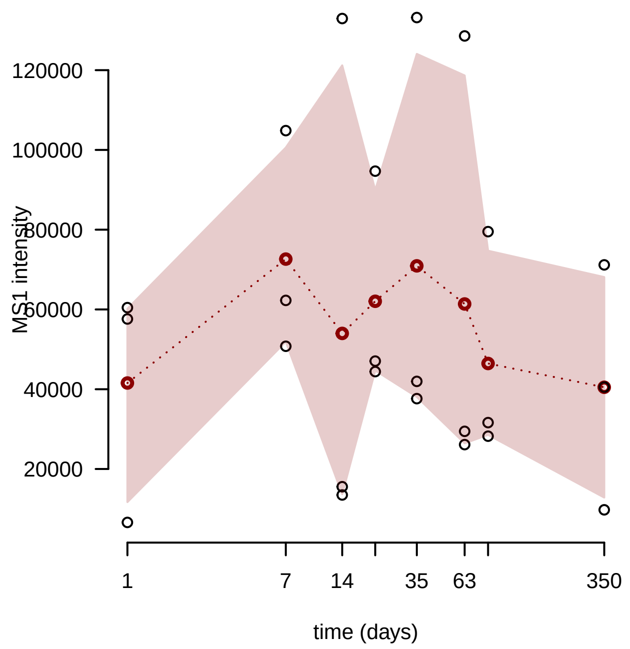

Q8BFQ8
GN=Gatd1
glutamine amidotransferase like class 1 ...

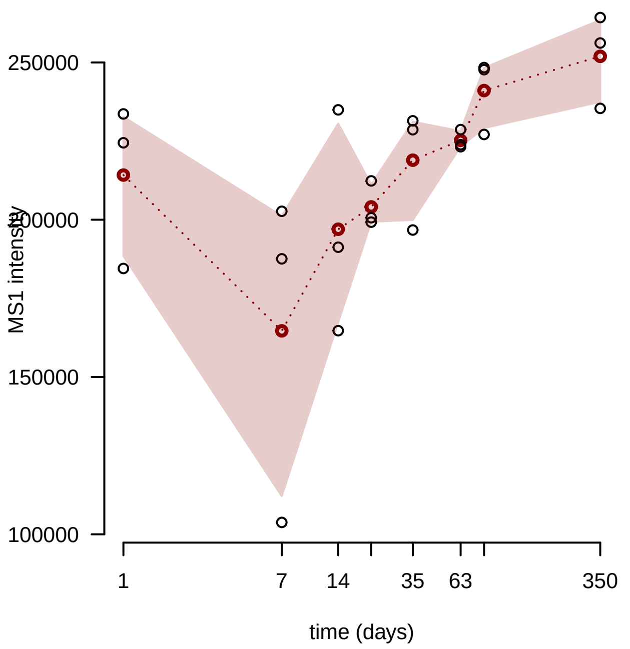

Q8BFQ9
GN=Klhl42
kelch−like 42

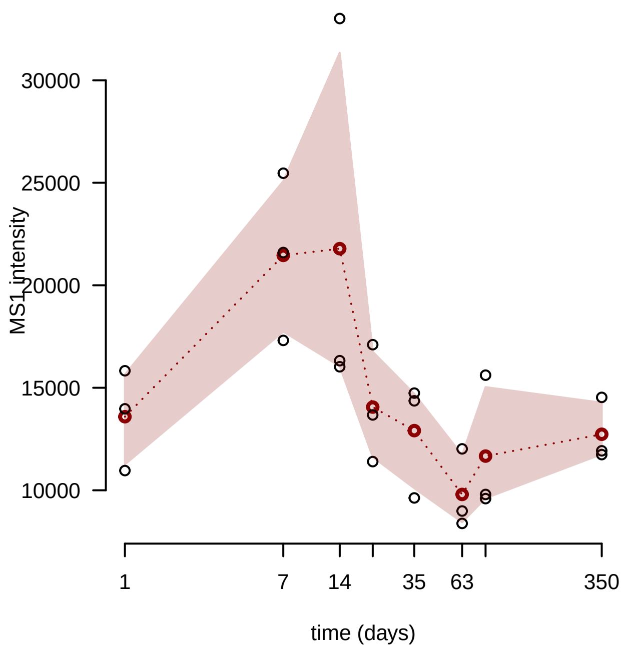

Q8BFR1
GN=Zc3hav1l
zinc finger CCCH−type, antiviral 1−like

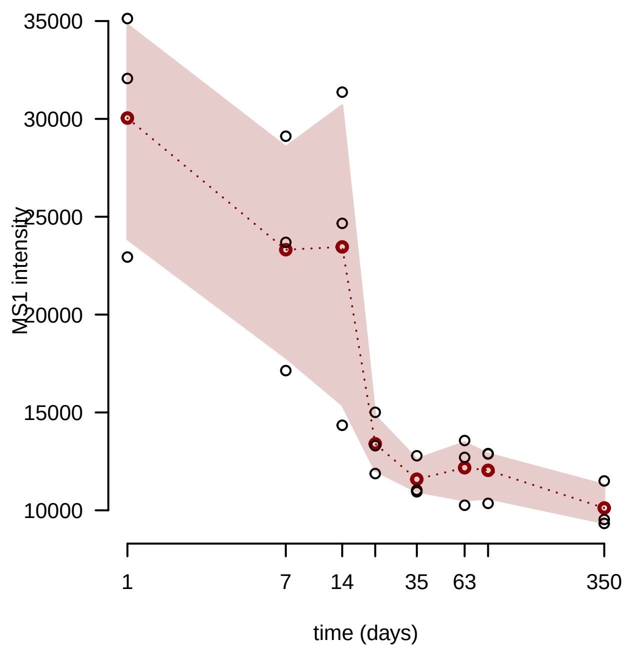

Q8BFR2
GN=Fstl5
follistatin−like 5

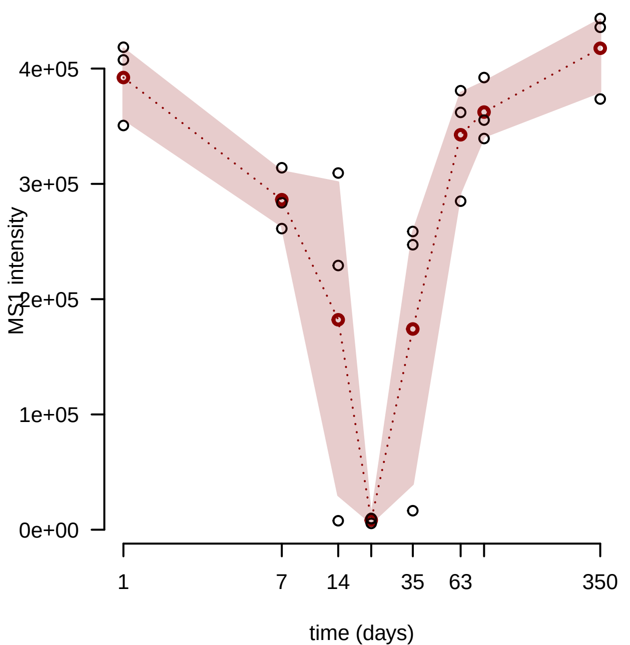

Q8BFR4
GN=Gns
glucosamine (N−acetyl)−6−sulfatase

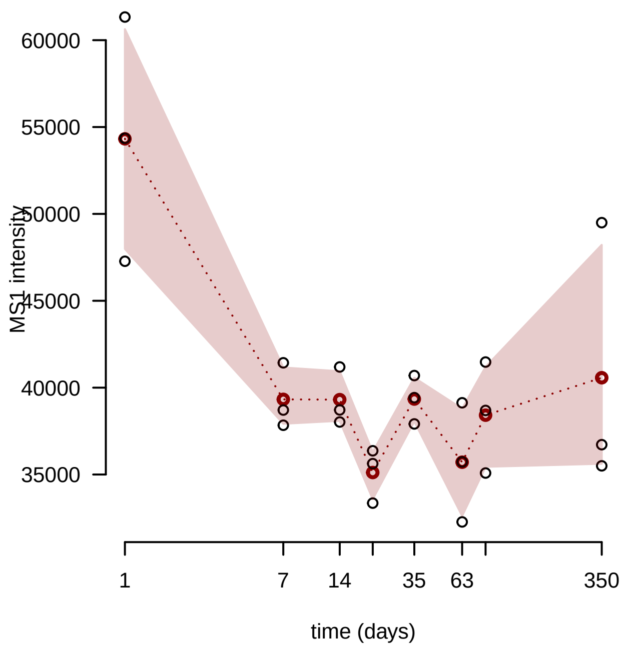

Q8BFR5
GN=Tufm
Tu translation elongation factor, mitoch ...

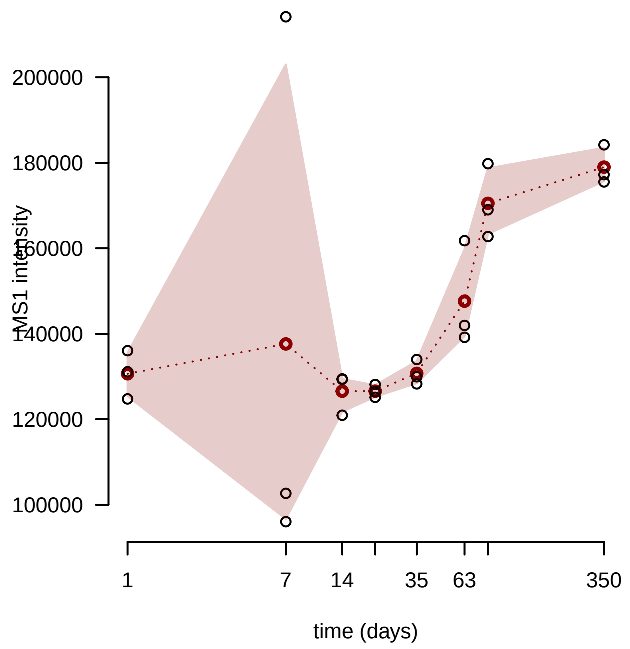

Q8BFR6
GN=Zfand1
zinc finger, AN1−type domain 1

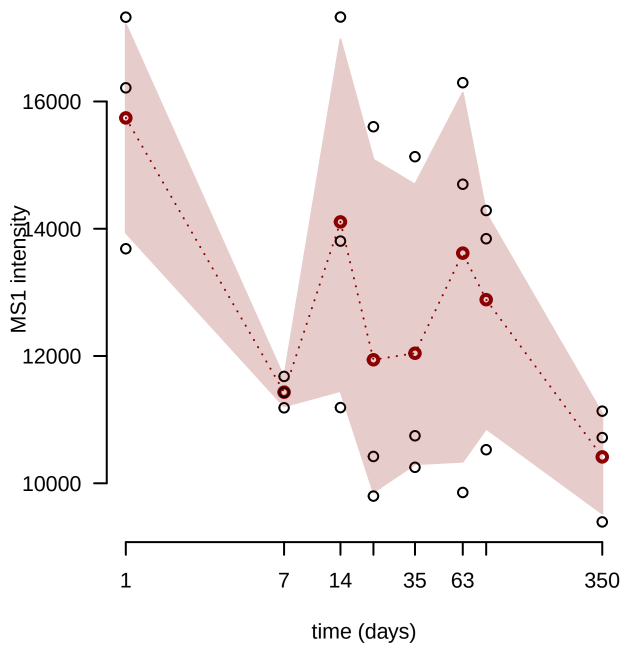

Q8BFS6
GN=Cpped1
calcineurin−like phosphoesterase domain ...

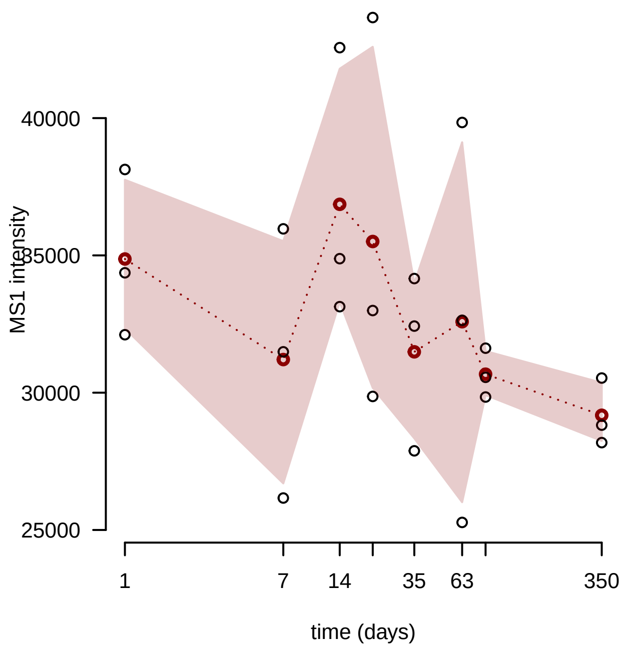

Q8BFS9
GN=Asnsd1
asparagine synthetase domain containing ...

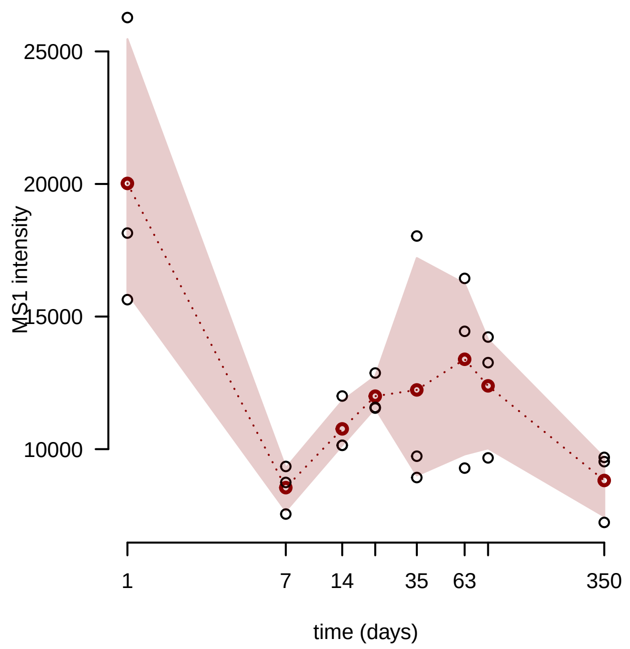

Q8BFT2
GN=Haus4
HAUS augmin−like complex, subunit 4

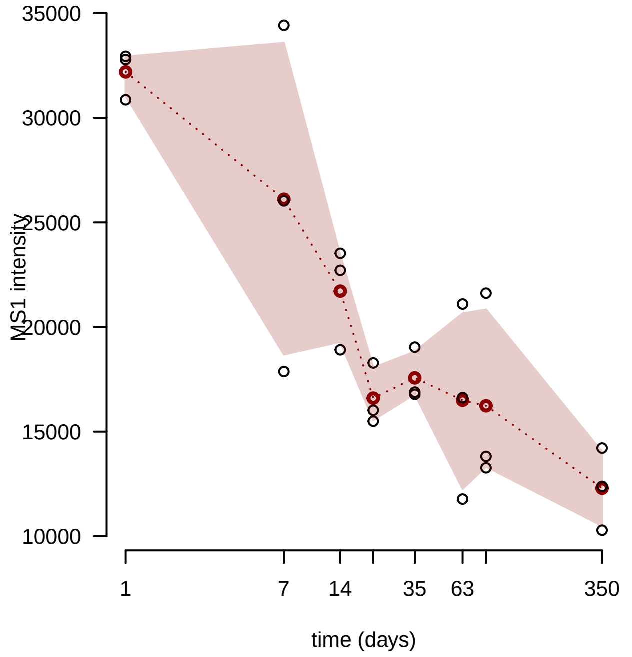

Q8BFU3
GN=Rnf214
ring finger protein 214

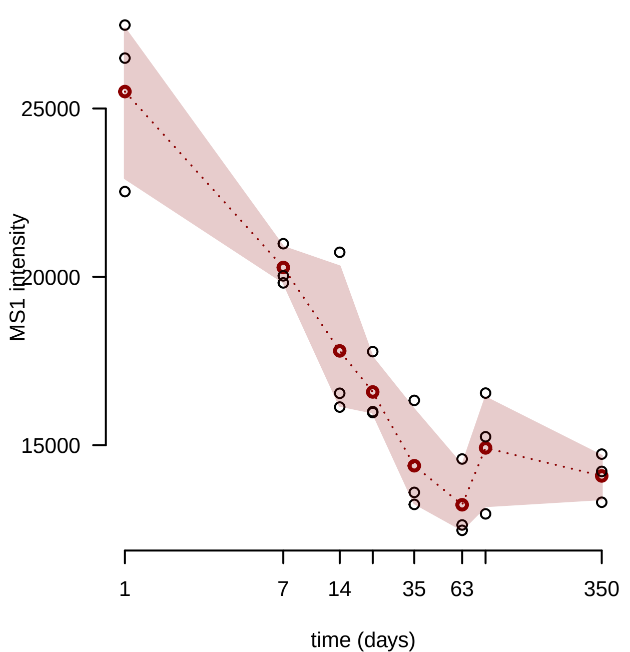

Q8BFU3
GN=Rnf214
ring finger protein 214

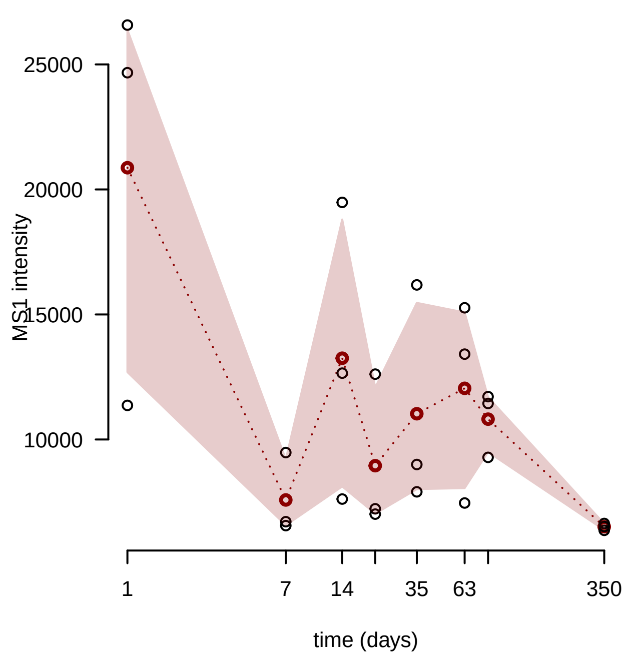

Q8BFV2
GN=Pcid2
PCI domain containing 2

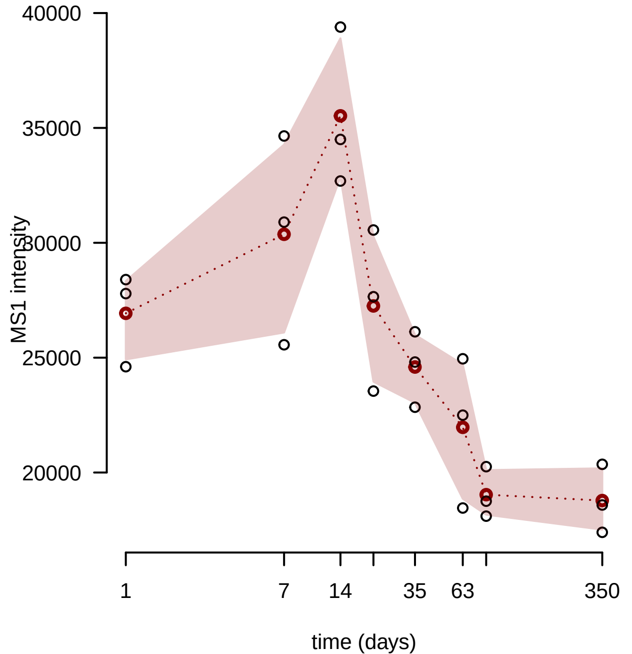

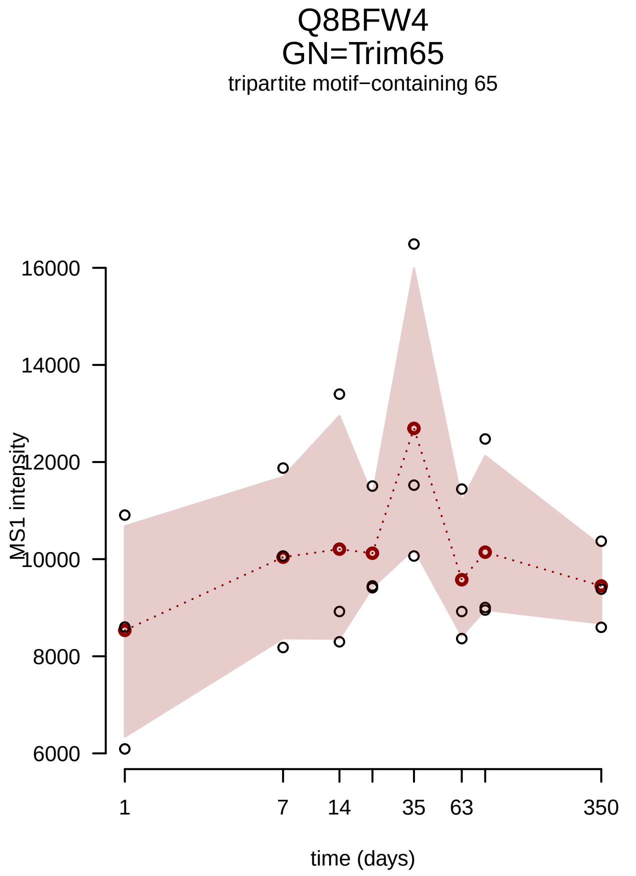
Q8BFW4
GN=Trim65
tripartite motif−containing 65
MS1 intensity
6000
8000
10000
12000
14000
16000
1
7
14
35
63
350
time (days)

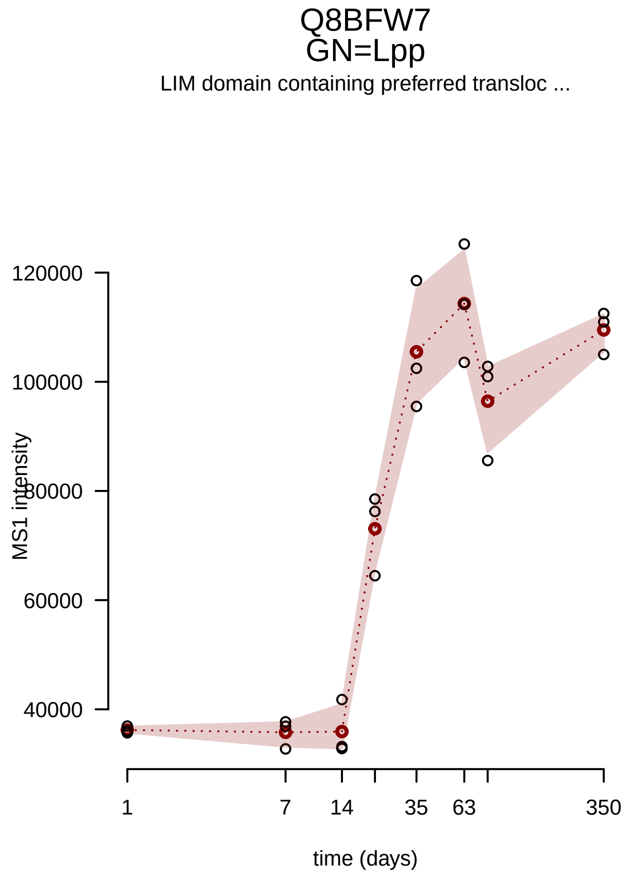
Q8BFW7
GN=Lpp
LIM domain containing preferred transloc ...
MS1 intensity
40000
60000
80000
100000
120000
1
7
14
35
63
350
time (days)

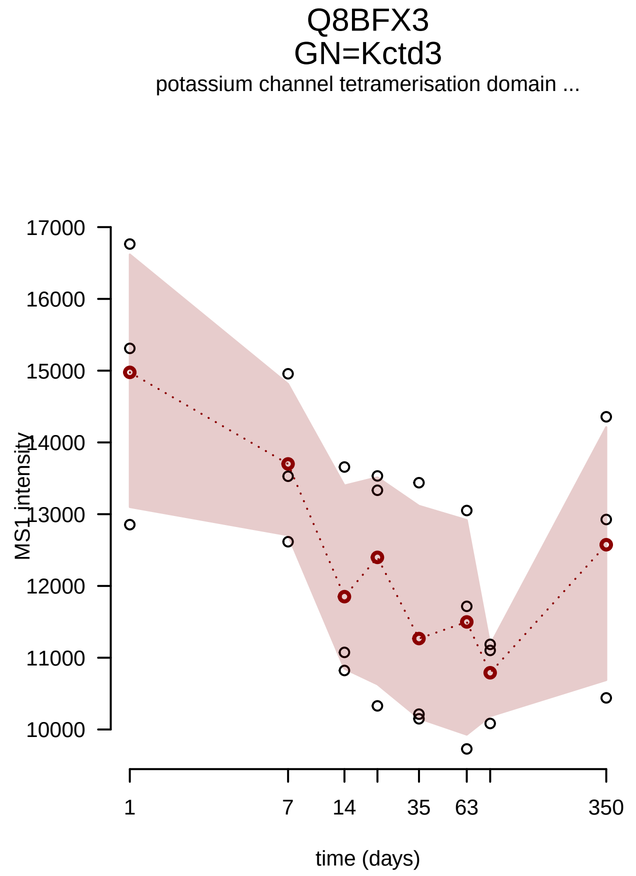
Q8BFX3
GN=Kctd3
potassium channel tetramerisation domain ...
MS1 intensity
10000
11000
12000
13000
14000
15000
16000
17000
1
7
14
35
63
350
time (days)

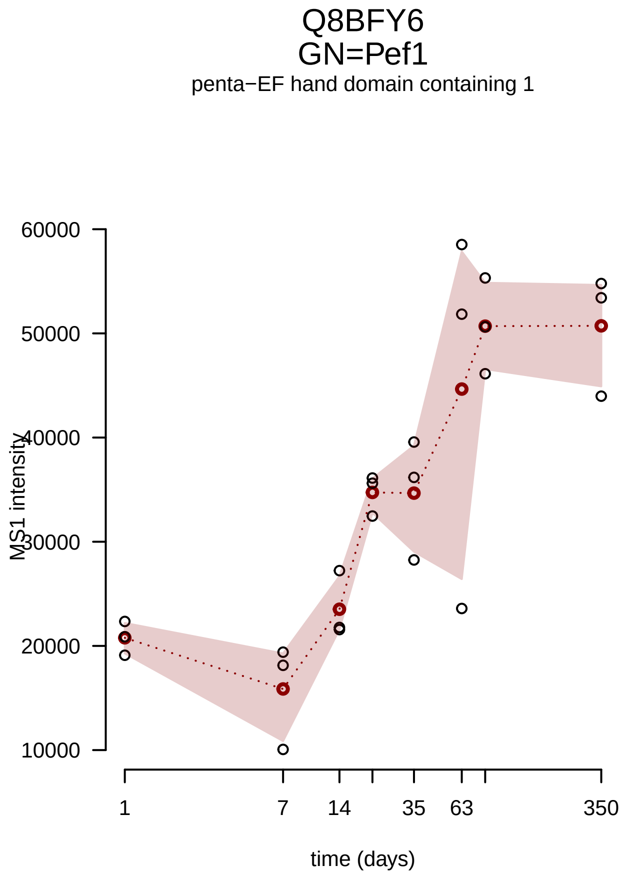
Q8BFY6
GN=Pef1
penta−EF hand domain containing 1
MS1 intensity
10000
20000
30000
40000
50000
60000
1
7
14
35
63
350
time (days)

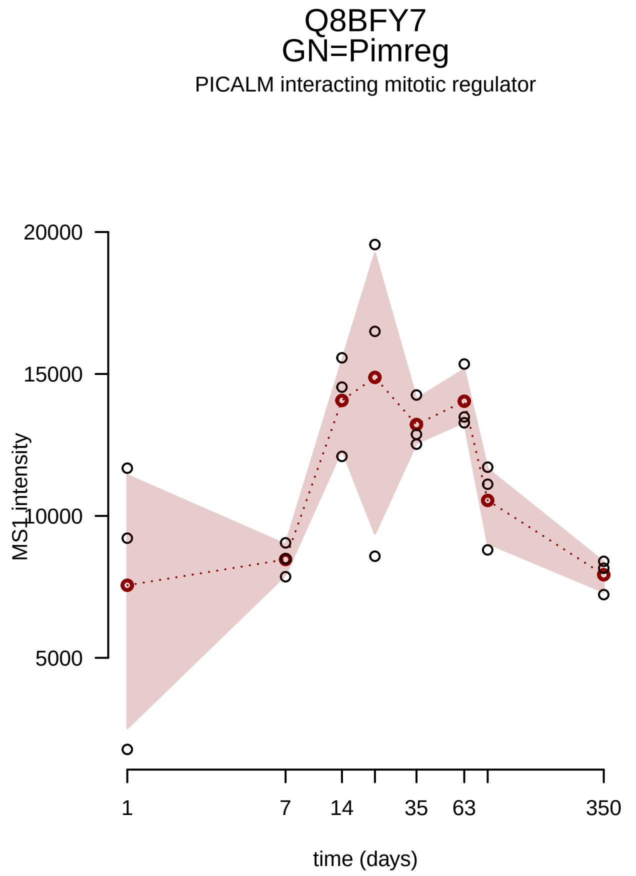
Q8BFY7
GN=Pimreg
PICALM interacting mitotic regulator
MS1 intensity
5000
10000
15000
20000
1
7
14
35
63
350
time (days)

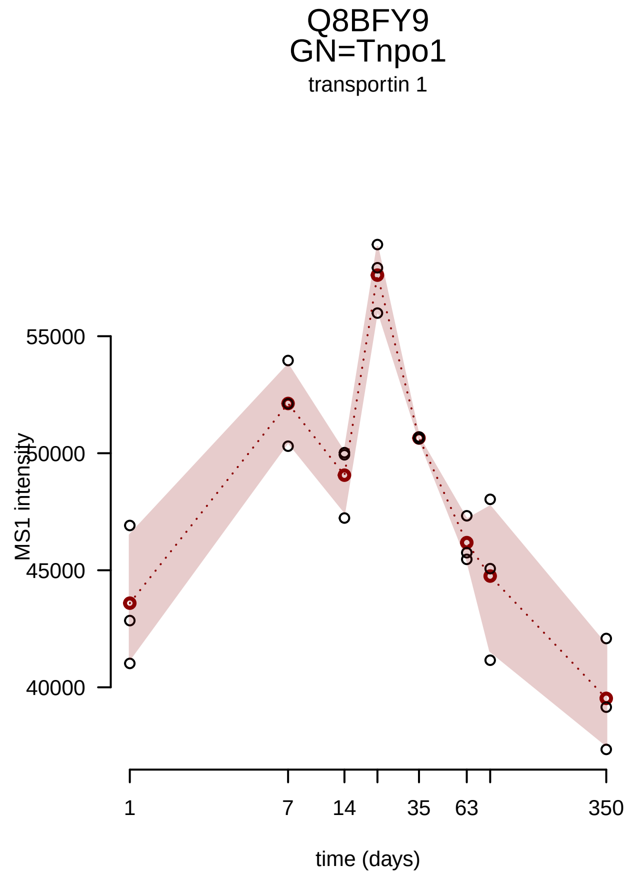
Q8BFY9
GN=Tnpo1
transportin 1
MS1 intensity
40000
45000
50000
55000
1
7
14
35
63
350
time (days)

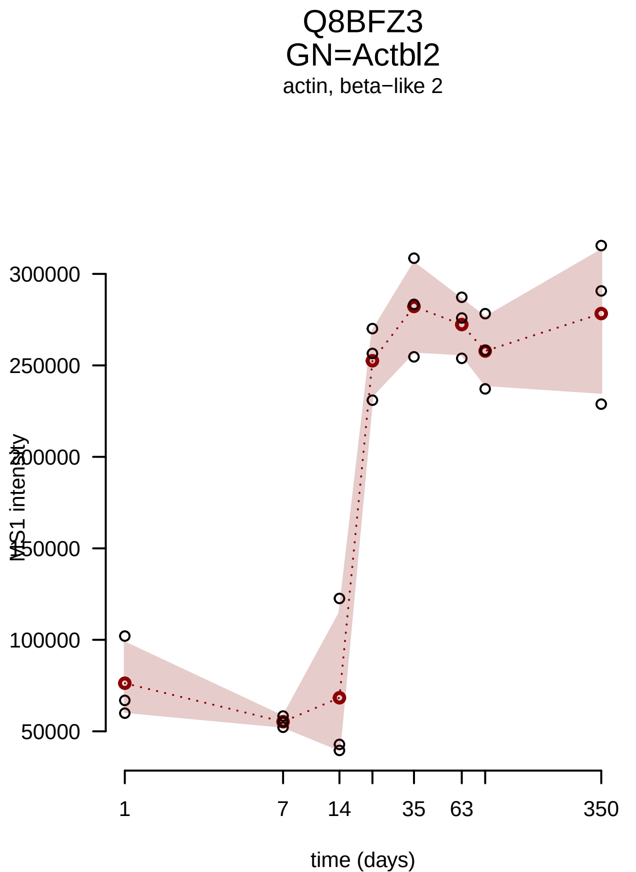
Q8BFZ3
GN=Actbl2
actin, beta−like 2
MS1 intensity
50000
100000
150000
200000
250000
300000
1
7
14
35
63
350
time (days)

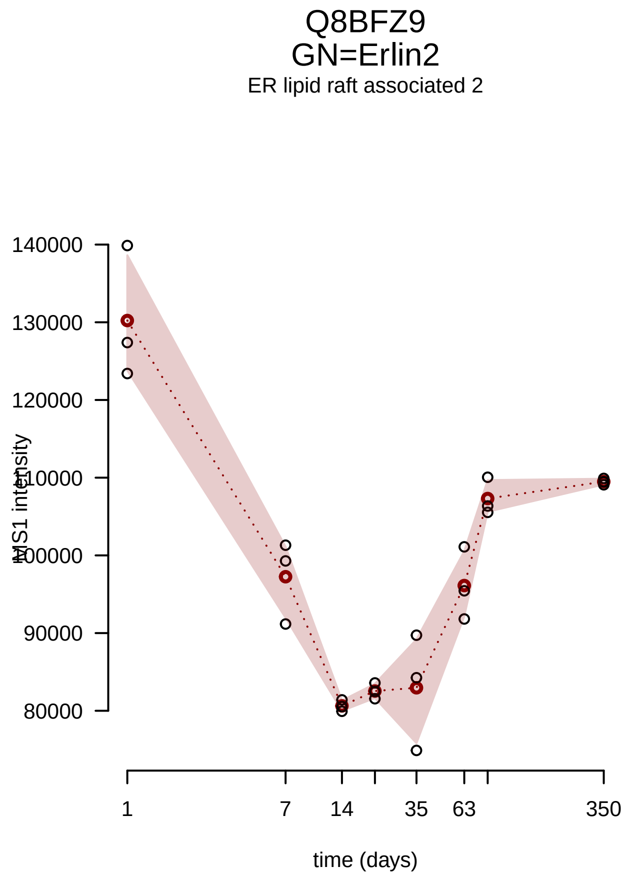
Q8BFZ9
GN=Erlin2
ER lipid raft associated 2
MS1 intensity
80000
90000
100000
110000
120000
130000
140000
1
7
14
35
63
350
time (days)

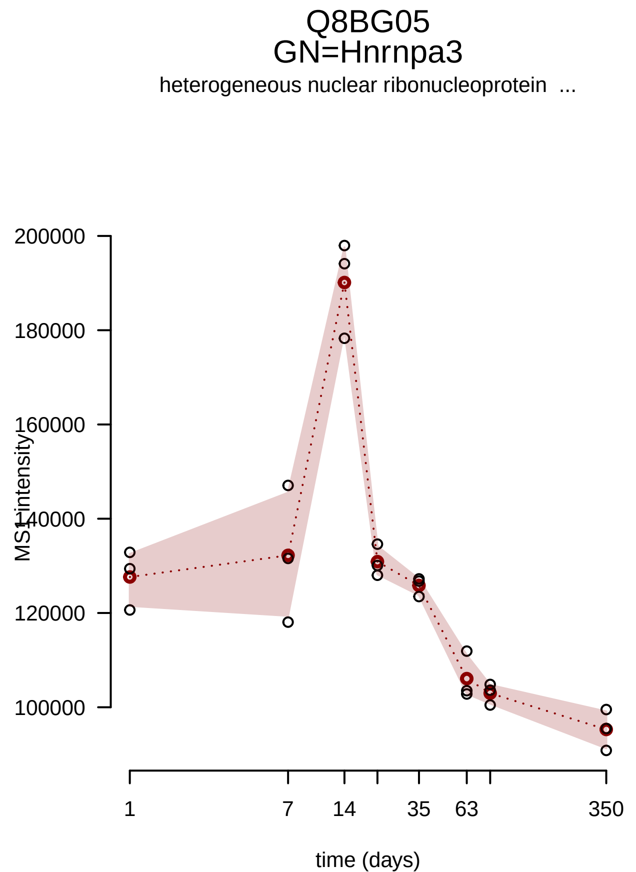
Q8BG05
GN=Hnrnpa3
heterogeneous nuclear ribonucleoprotein ...
MS1 intensity
100000
120000
140000
160000
180000
200000
1
7
14
35
63
350
time (days)

Q8BG05
GN=Hnrnpa3
heterogeneous nuclear ribonucleoprotein ...

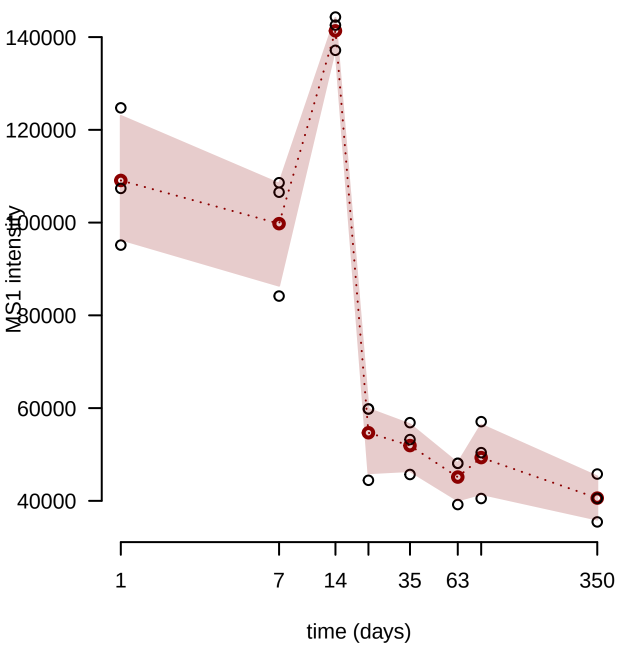

Q8BG07
GN=Pld4
phospholipase D family, member 4

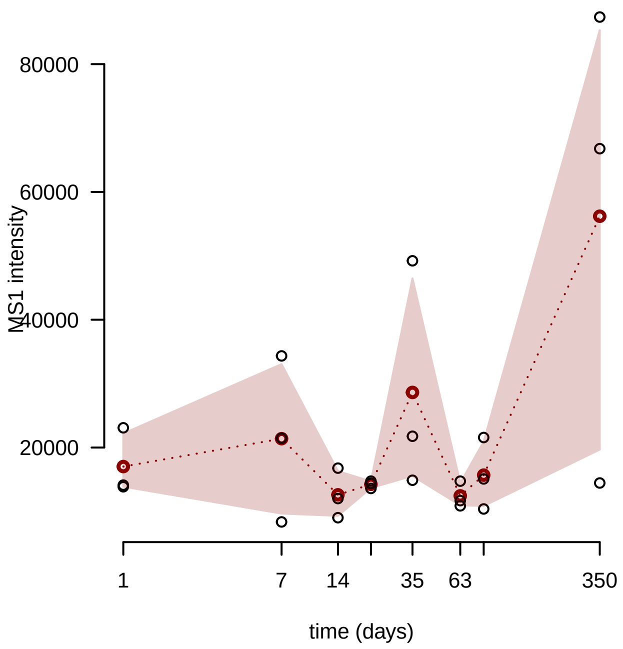

Q8BG15
GN=Ctdspl2
CTD (carboxy−terminal domain, RNA polyme ...

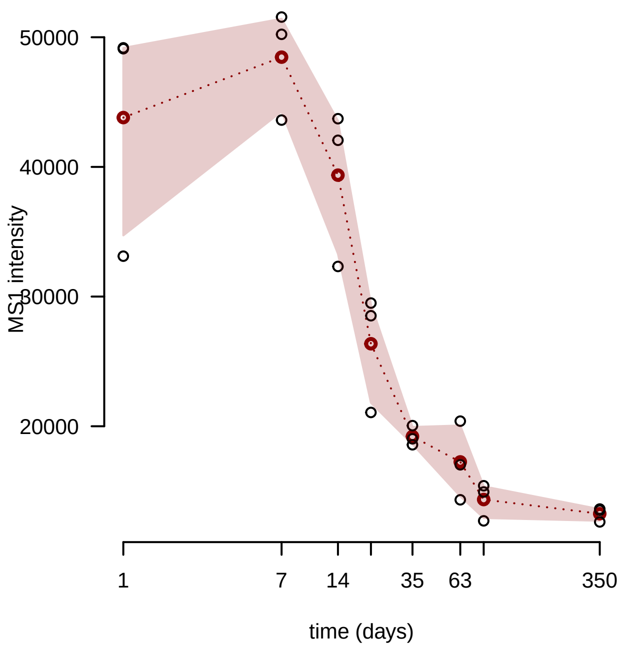

Q8BG17
GN=Nol12
nucleolar protein 12

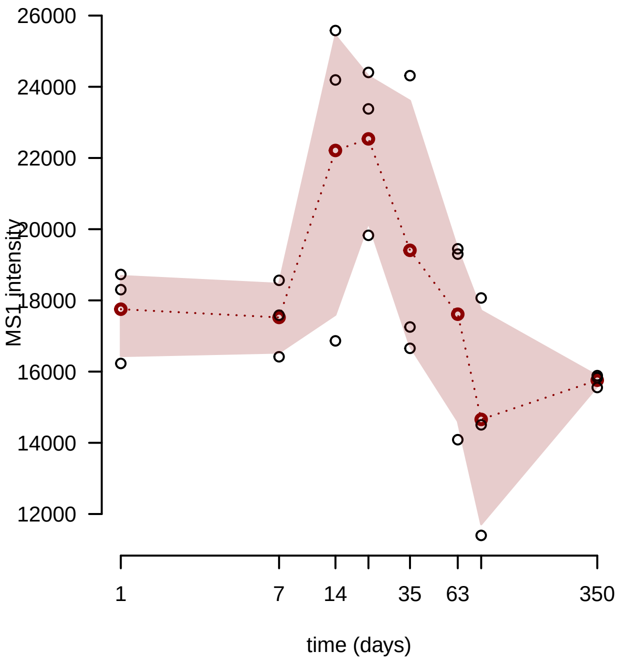

Q8BG19
GN=Tmtc4
transmembrane and tetratricopeptide repe ...

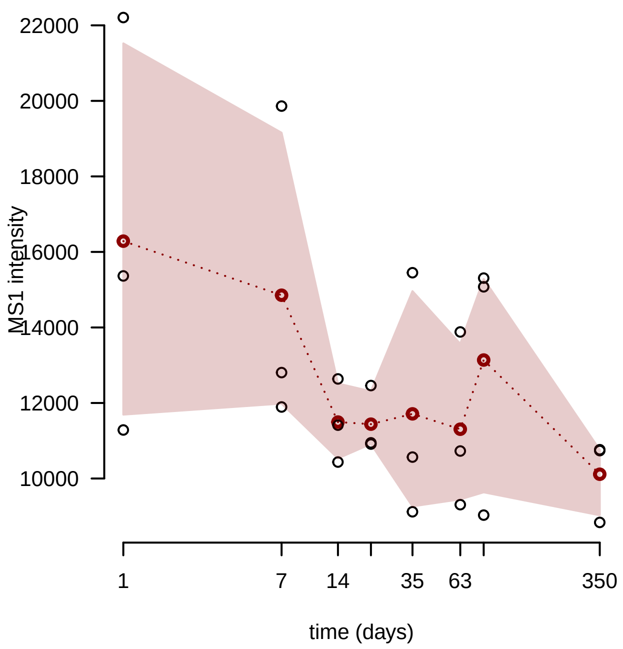

Q8BG21
GN=Cacfd1
calcium channel flower domain containing ...

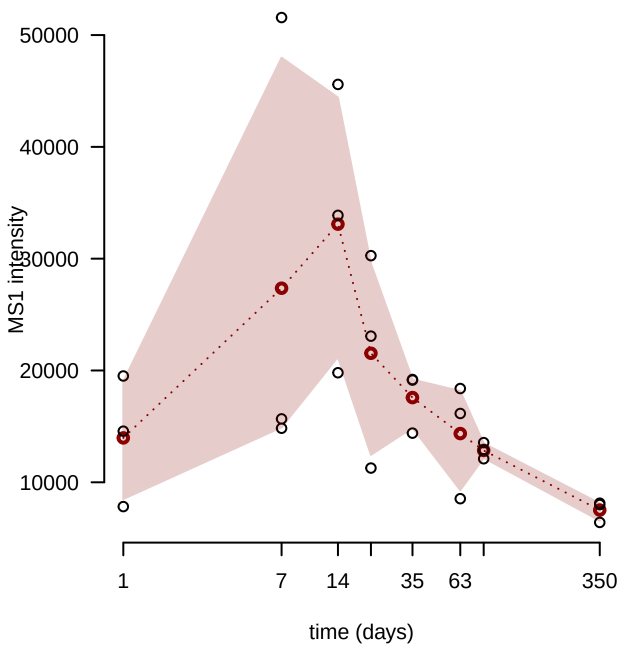

Q8BG30
GN=Nelfa
negative elongation factor complex membe ...

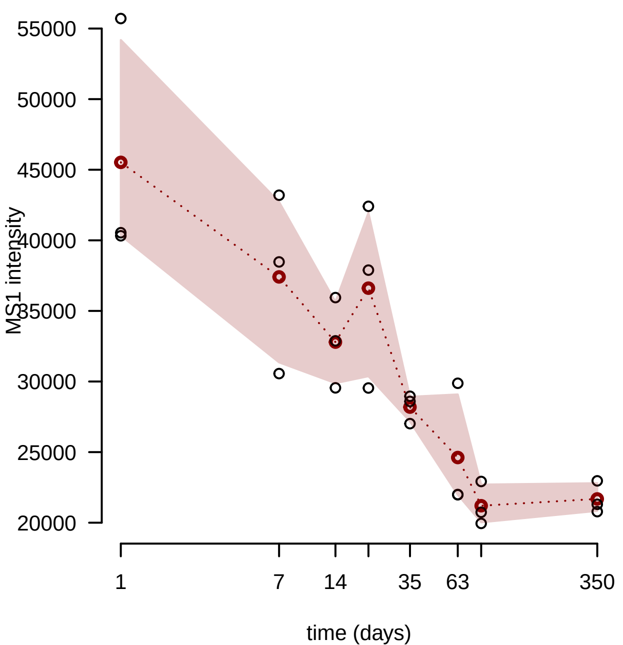

Q8BG32
GN=Psmd11
proteasome (prosome, macropain) 26S subu ...

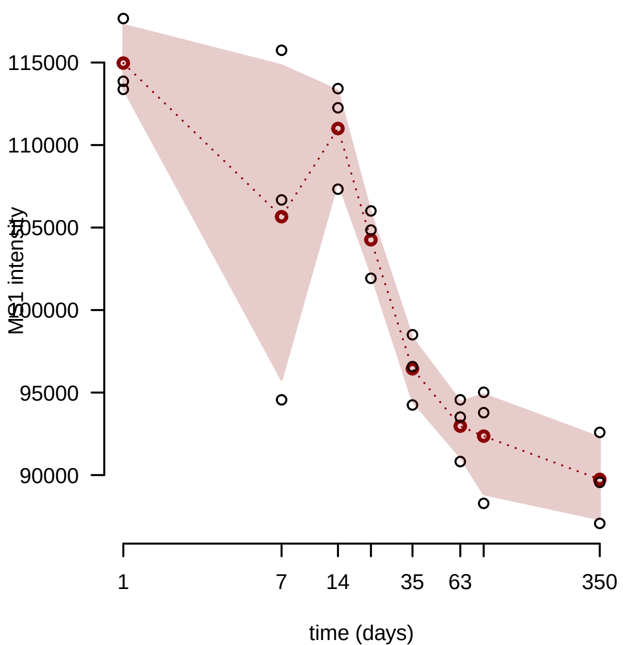

Q8BG40
GN=Katnb1
katanin p80 (WD40−containing) subunit B ...

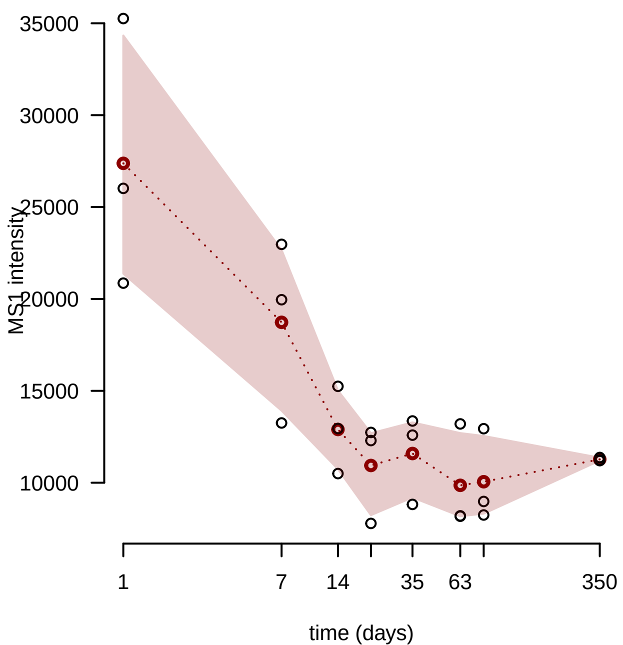

Q8BG48
GN=Stk17b
serine/threonine kinase 17b (apoptosis−i ...

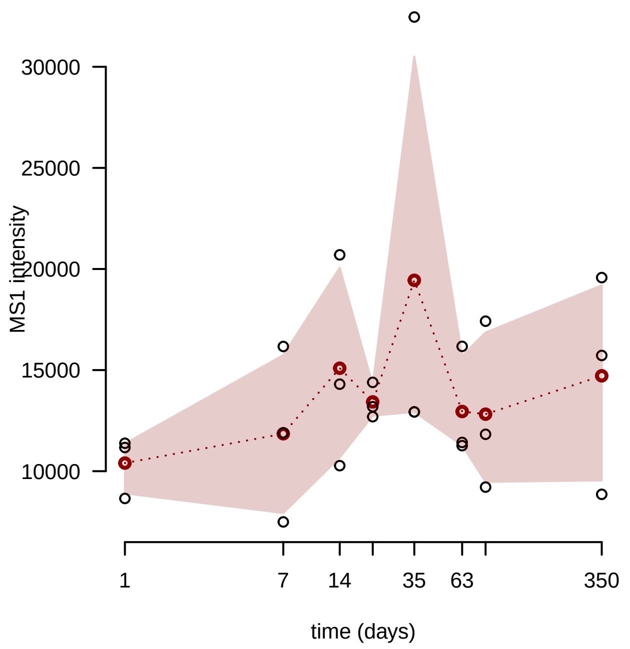

Q8BG51
GN=Rhot1
ras homolog family member T1

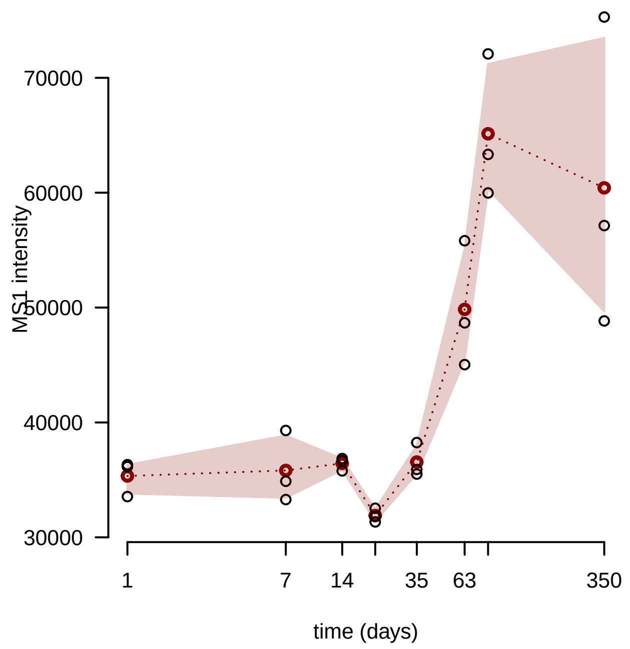

Q8BG60
GN=Txnip
thioredoxin interacting protein

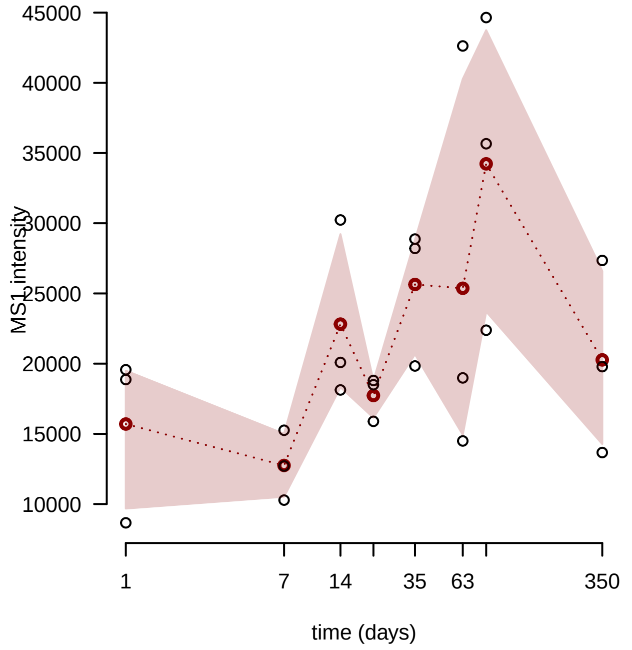

Q8BG67
GN=Efr3a
EFR3 homolog A

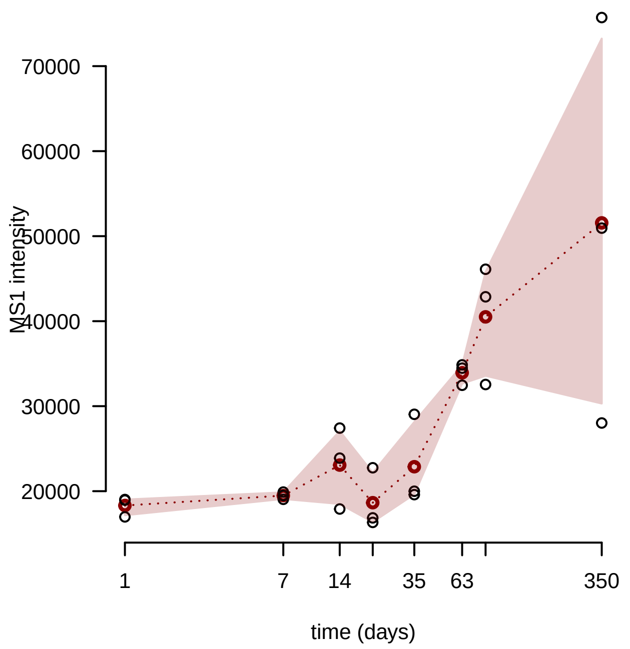

Q8BG73
GN=Sh3bgrl2
SH3 domain binding glutamic acid−rich pr ...

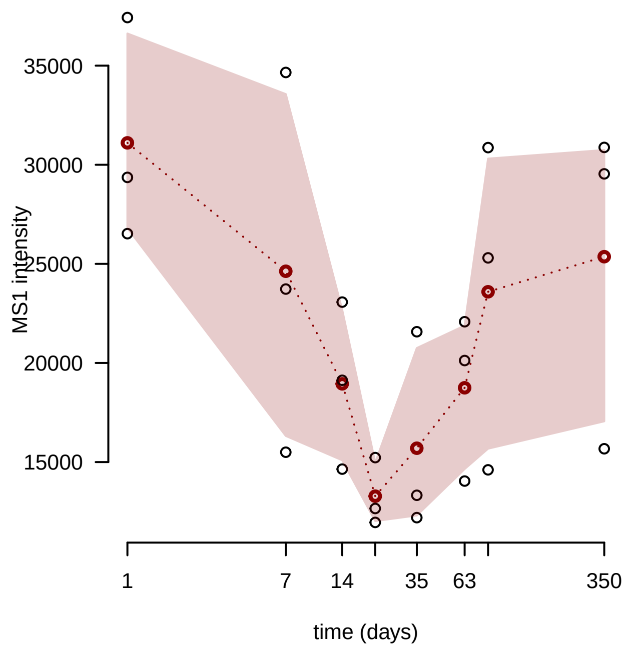

Q8BG79
GN=Cwf19l2
CWF19−like 2, cell cycle control (S. pom ...

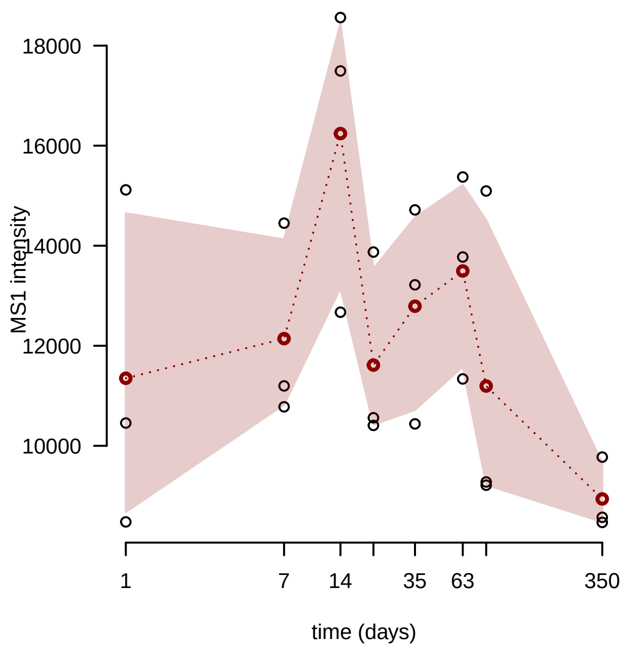

Q8BG81
GN=Poldip3
polymerase (DNA−directed), delta interac ...

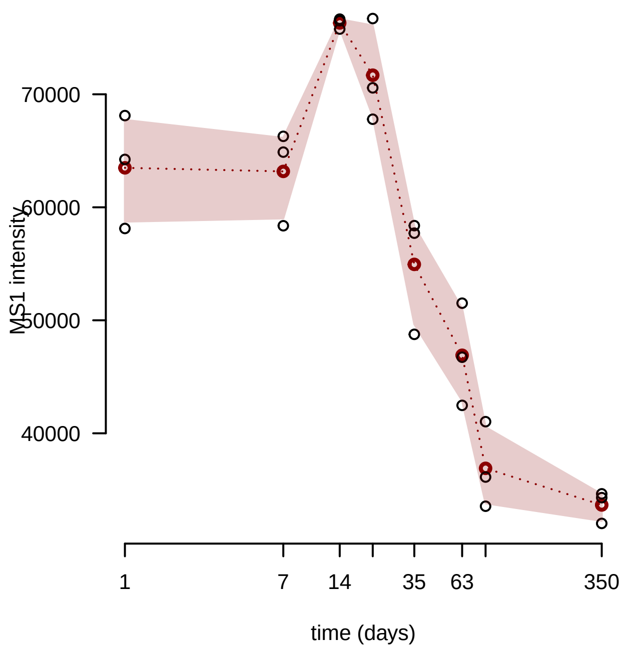

Q8BG87
GN=Tet3
tet methylcytosine dioxygenase 3

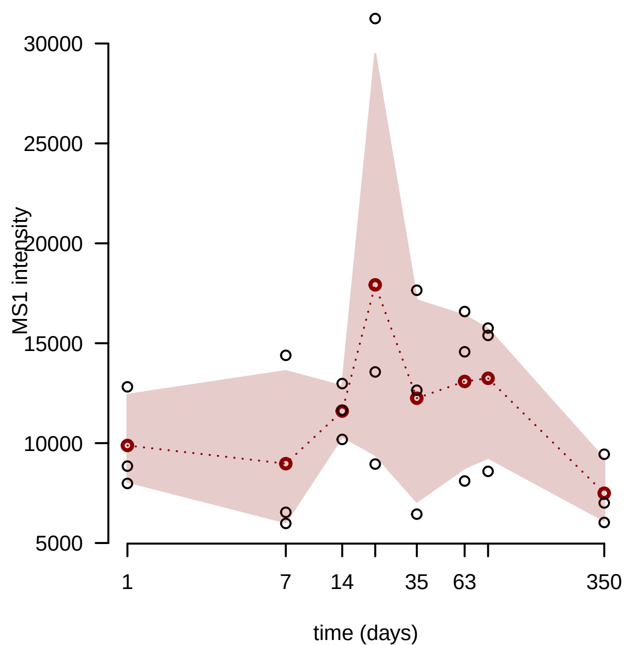

Q8BG94
GN=Commd7
COMM domain containing 7

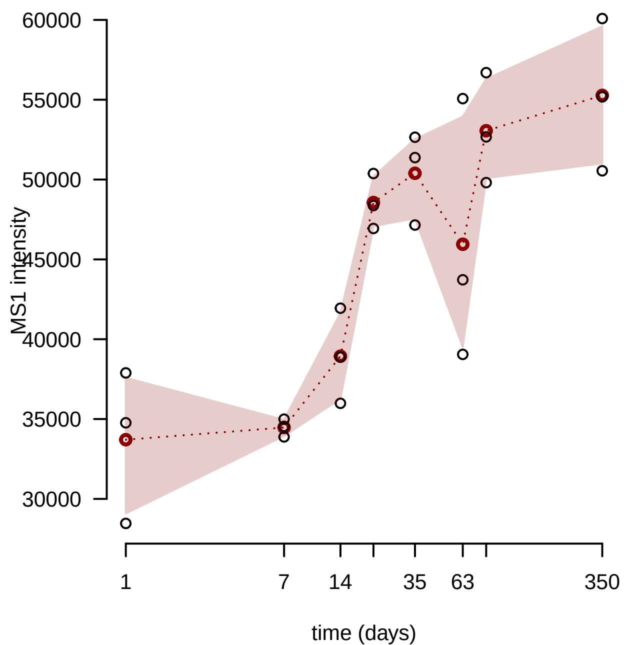

Q8BG95
GN=Ppp1r12b
protein phosphatase 1, regulatory subuni ...

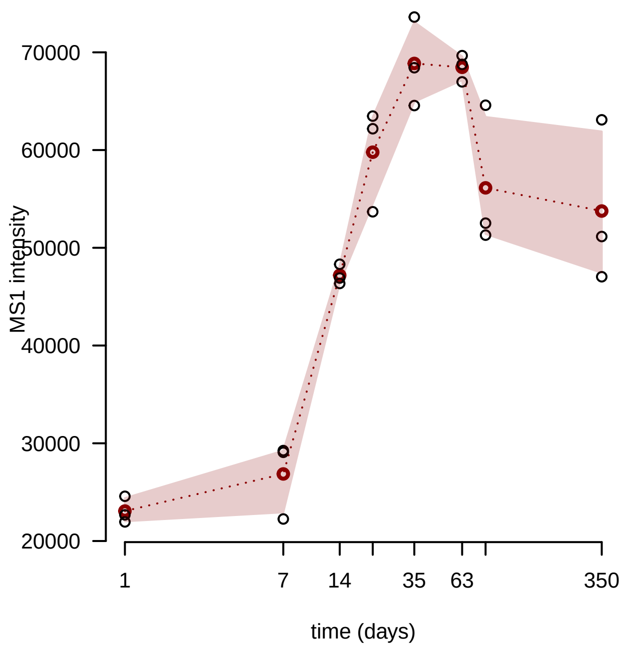

Q8BG99
GN=Pknox2
Pbx/knotted 1 homeobox 2

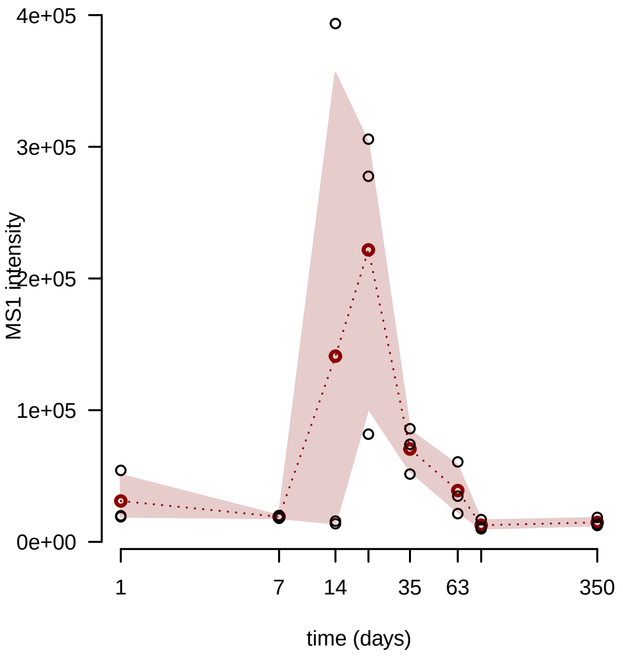

Q8BGA2
GN=Lhfpl2
lipoma HMGIC fusion partner−like 2

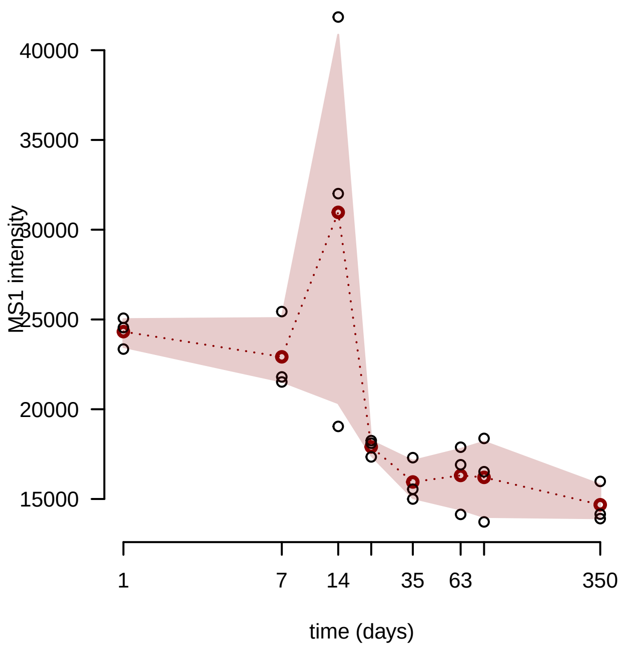

Q8BGA5
GN=Krr1
KRR1, small subunit (SSU) processome com ...

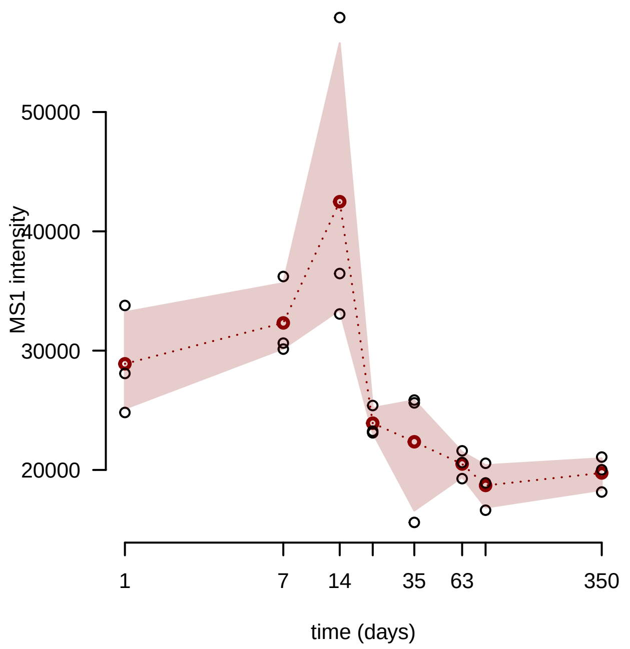

Q8BGA7
GN=

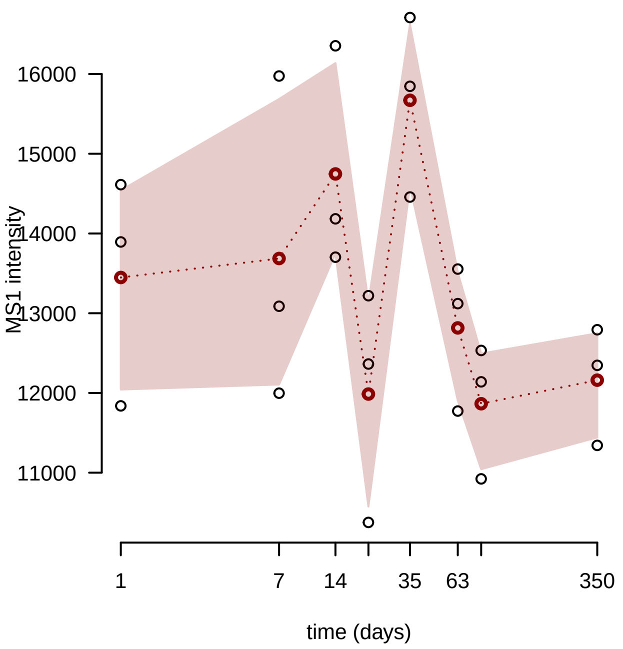

Q8BGA9
GN=Oxa1l
oxidase assembly 1−like

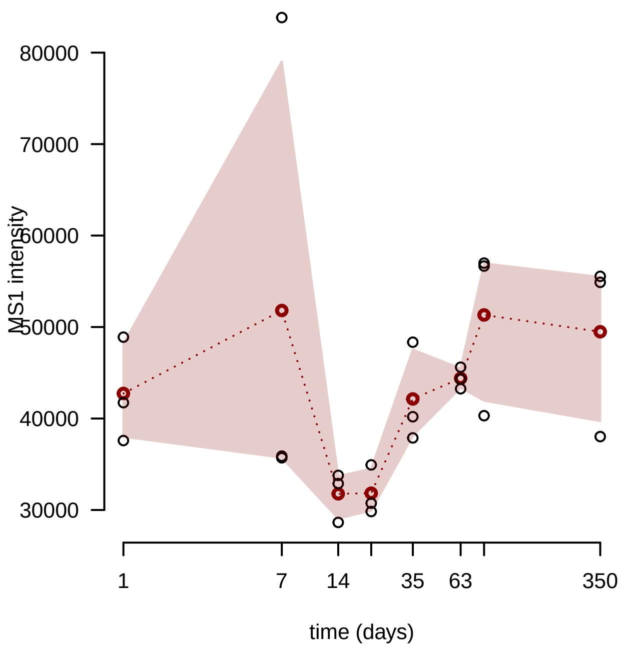

Q8BGB5
GN=Limd2
LIM domain containing 2

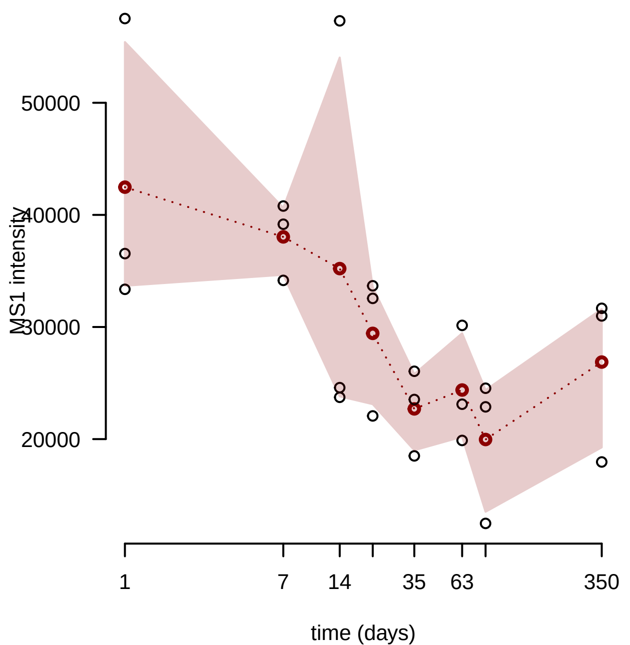

Q8BGB7
GN=Enoph1
enolase−phosphatase 1

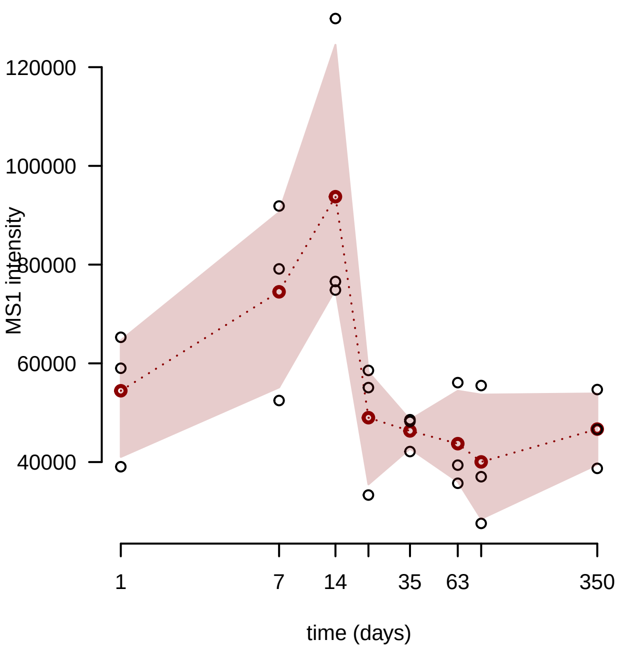

Q8BGB8
GN=Coq4
coenzyme Q4

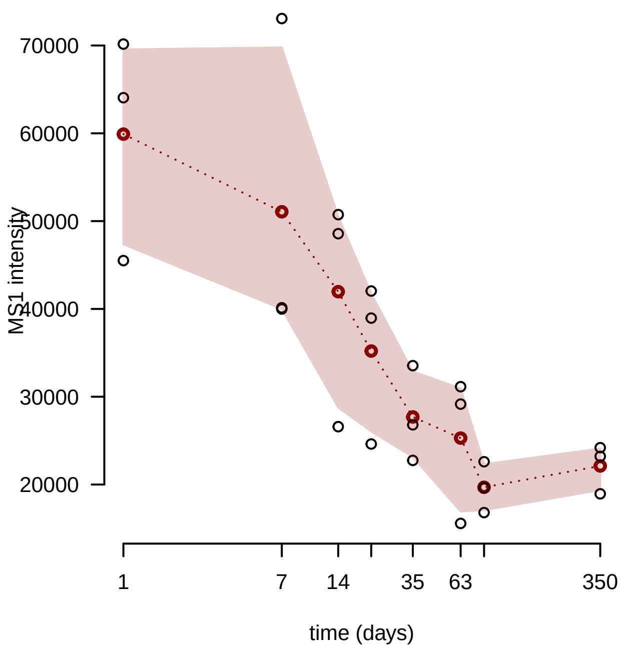

Q8BGC0
GN=Htatsf1
HIV TAT specific factor 1

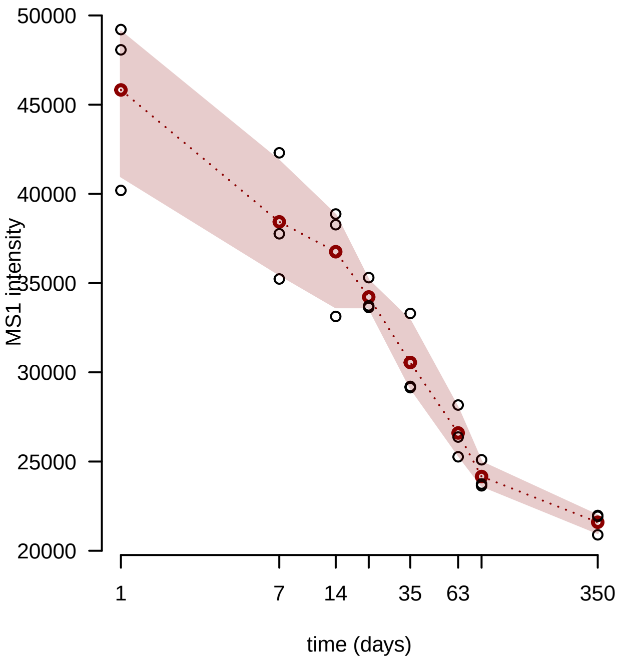

Q8BGC1
GN=

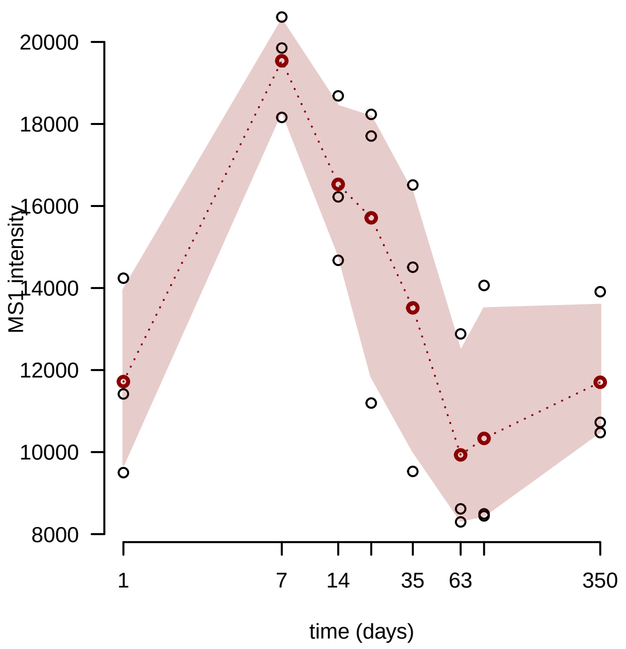

Q8BGC4
GN=Zadh2
zinc binding alcohol dehydrogenase, doma ...

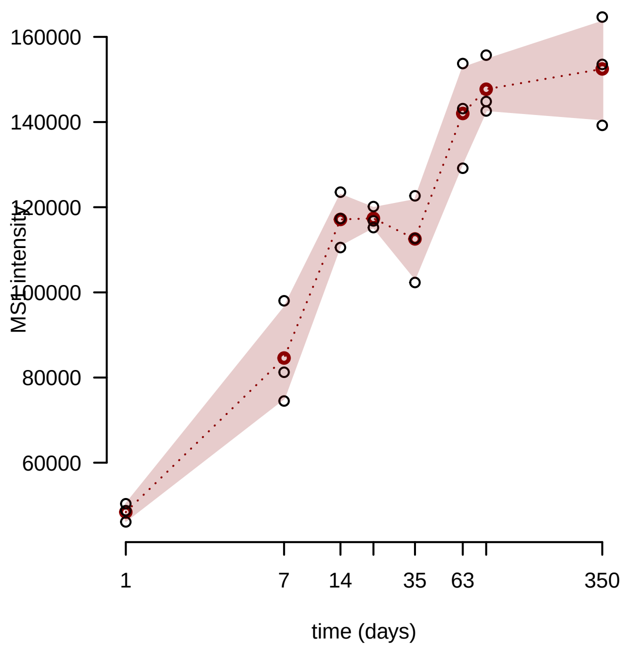

Q8BGD5
GN=Cpt1c
carnitine palmitoyltransferase 1c

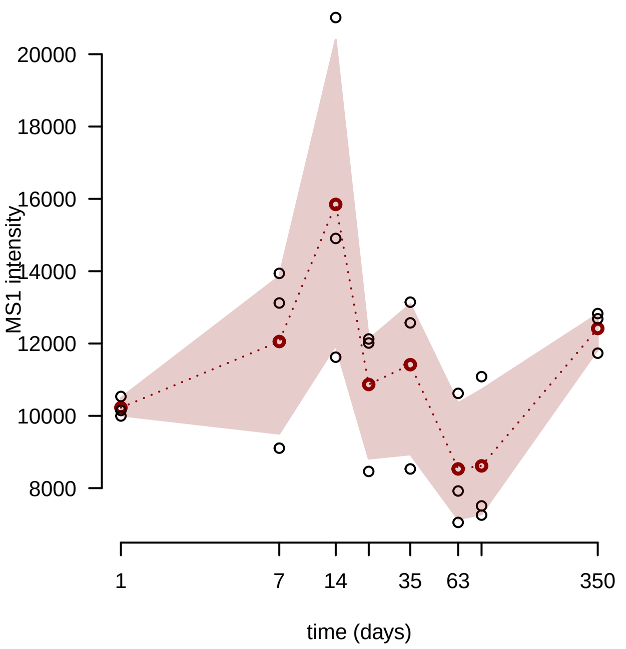

Q8BGD8
GN=Coa6
cytochrome c oxidase assembly factor 6

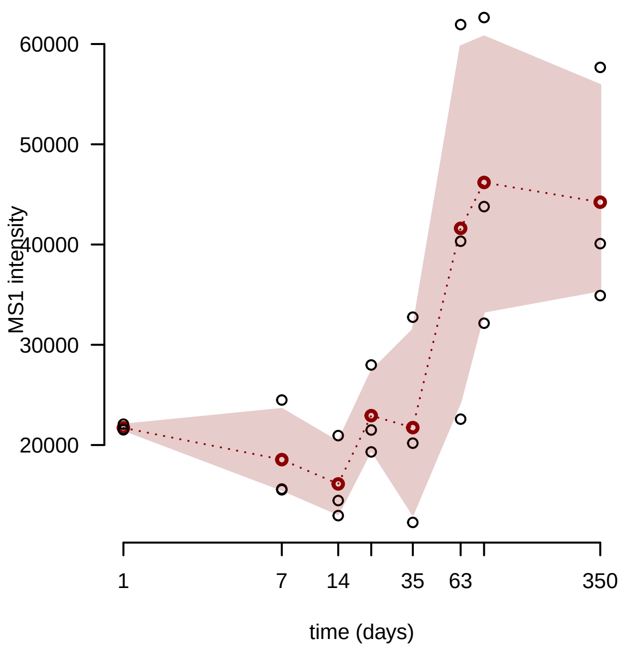

Q8BGD9
GN=Eif4b
eukaryotic translation initiation factor ...

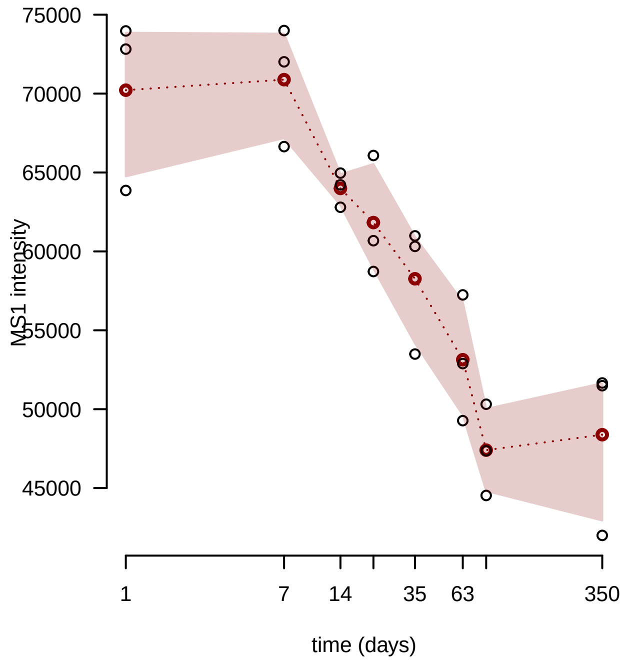

Q8BGE6
GN=Atg4b
autophagy related 4B, cysteine peptidase

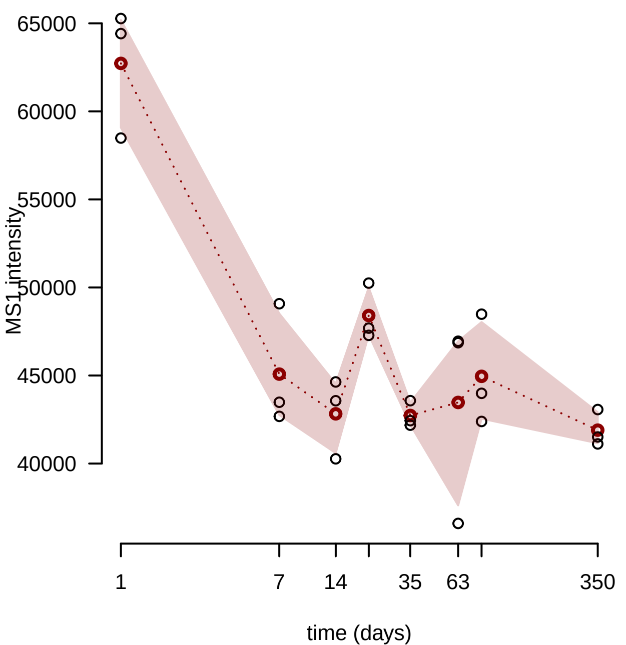

Q8BGE7
GN=Trim6
tripartite motif−containing 6

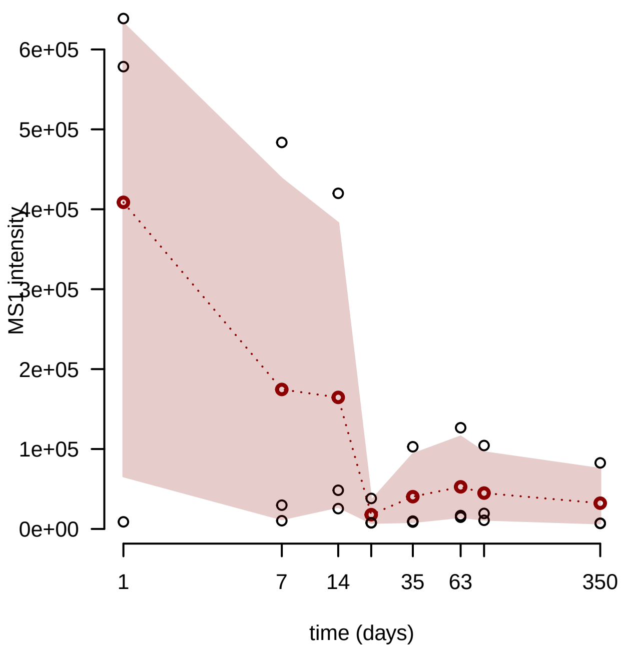

Q8BGF3
GN=Wdr92

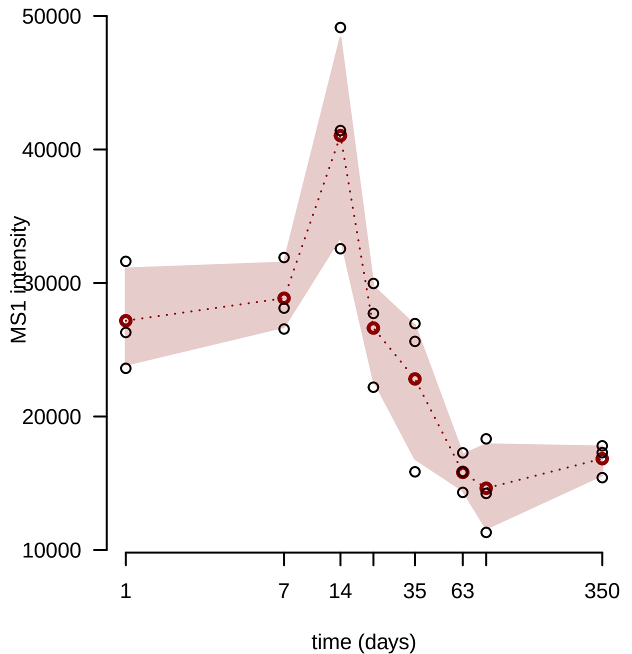

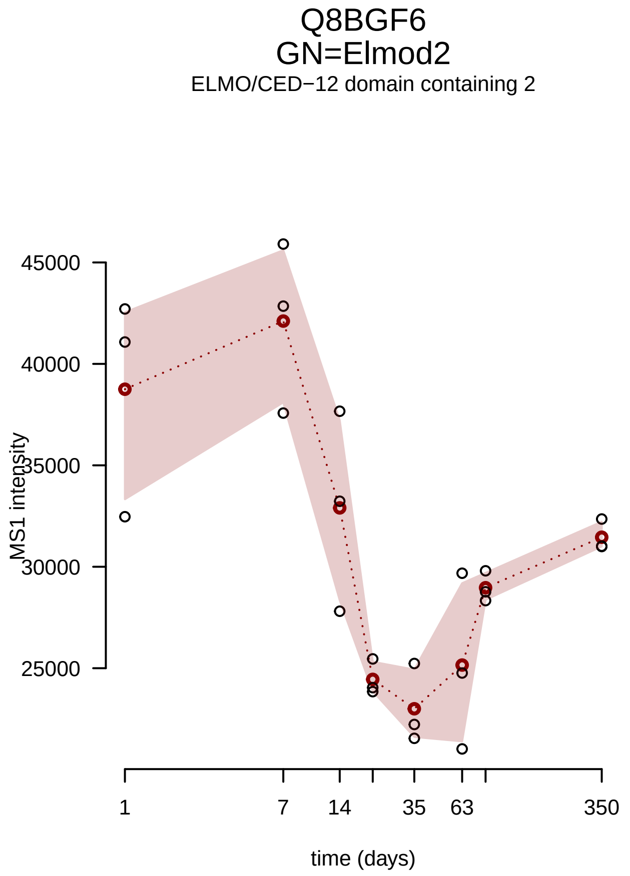
Q8BGF6
GN=Elmod2
ELMO/CED−12 domain containing 2
MS1 intensity
45000
40000
35000
30000
25000
1
7
14
35
63
350
time (days)

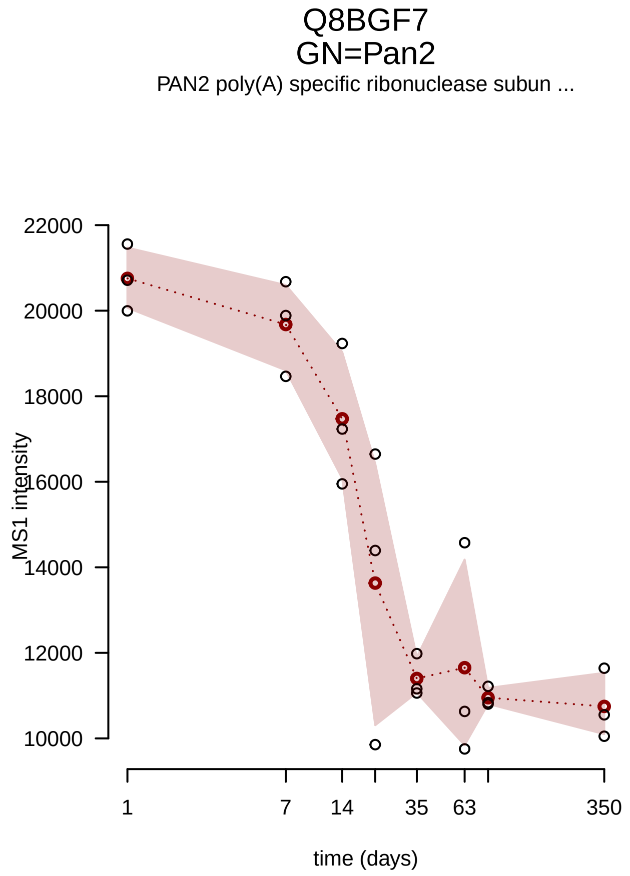
Q8BGF7
GN=Pan2
PAN2 poly(A) specific ribonuclease subun ...
MS1 intensity
22000
20000
18000
16000
14000
12000
10000
1
7
14
35
63
350
time (days)

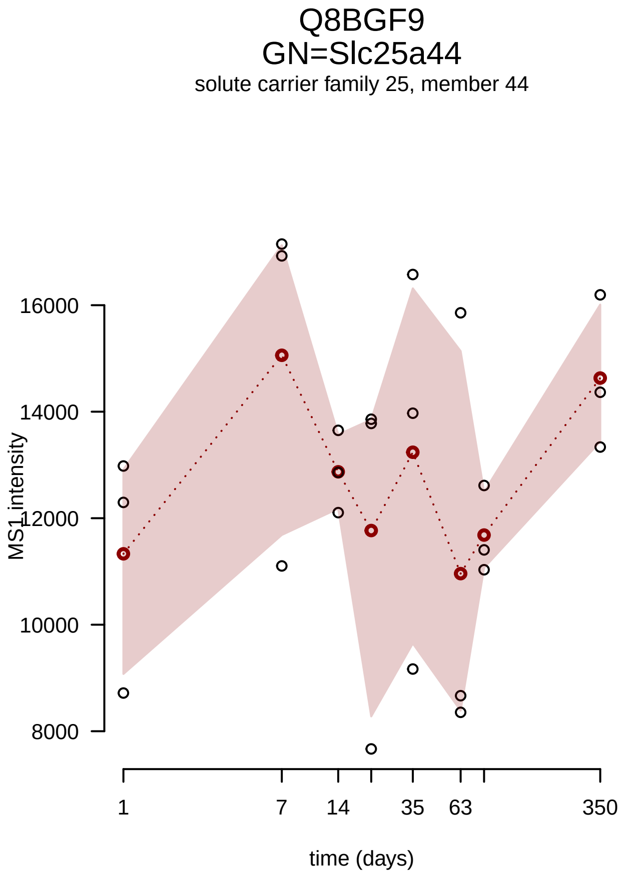
Q8BGF9
GN=Slc25a44
solute carrier family 25, member 44
MS1 intensity
16000
14000
12000
10000
8000
1
7
14
35
63
350
time (days)

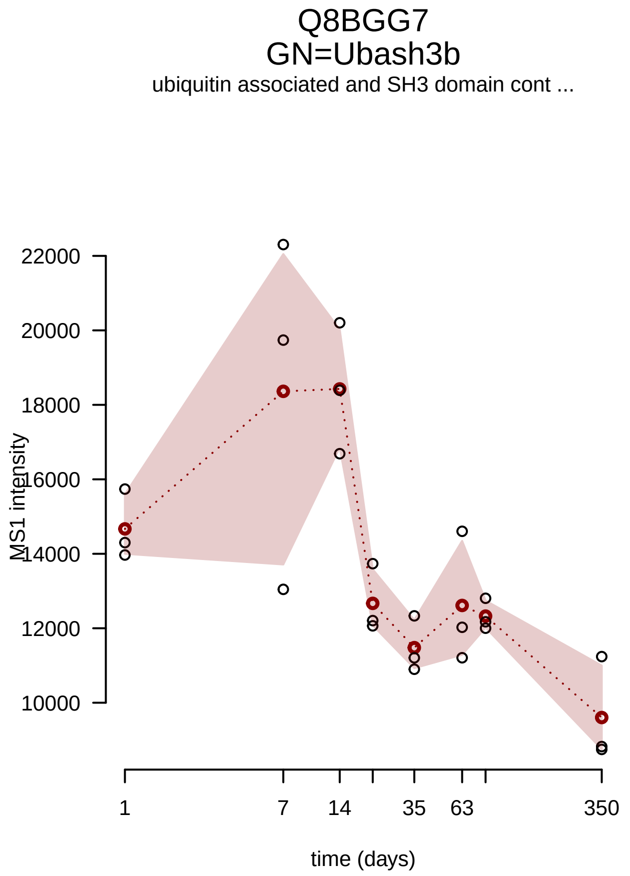
Q8BGG7
GN=Ubash3b
ubiquitin associated and SH3 domain cont ...
MS1 intensity
22000
20000
18000
16000
14000
12000
10000
1
7
14
35
63
350
time (days)

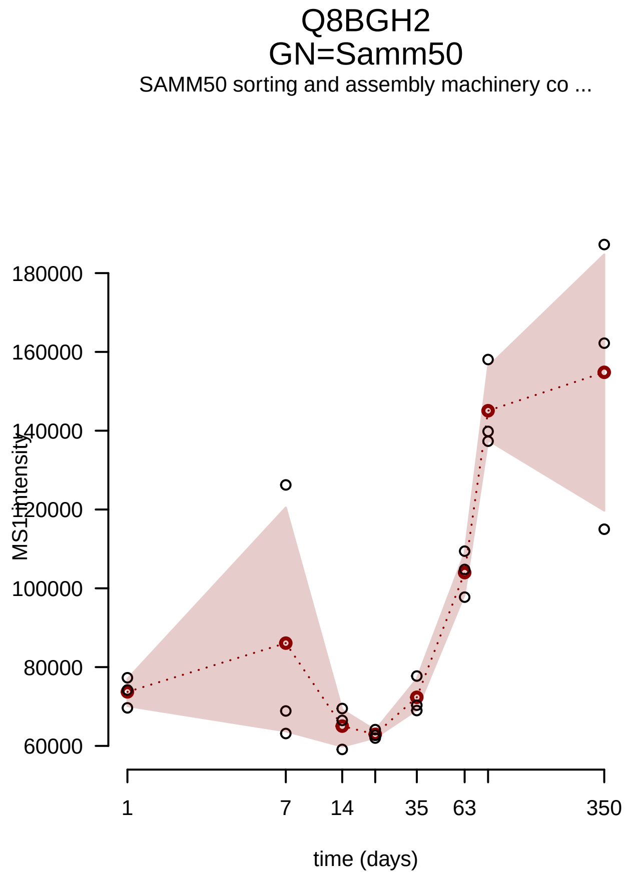
Q8BGH2
GN=Samm50
SAMM50 sorting and assembly machinery co ...
MS1 intensity
180000
160000
140000
120000
100000
80000
60000
1
7
14
35
63
350
time (days)

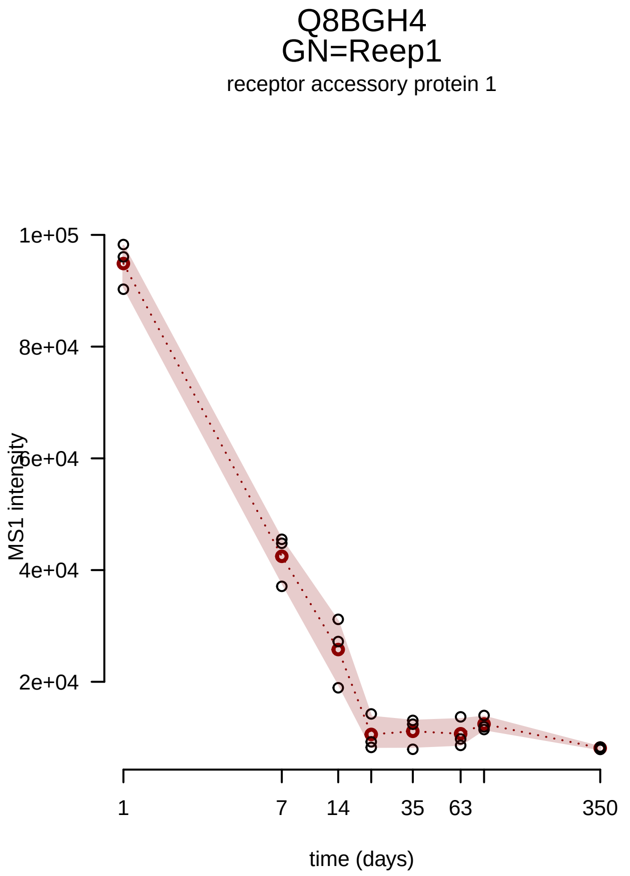
Q8BGH4
GN=Reep1
receptor accessory protein 1
MS1 intensity
1e+05
8e+04
6e+04
4e+04
2e+04
1
7
14
35
63
350
time (days)

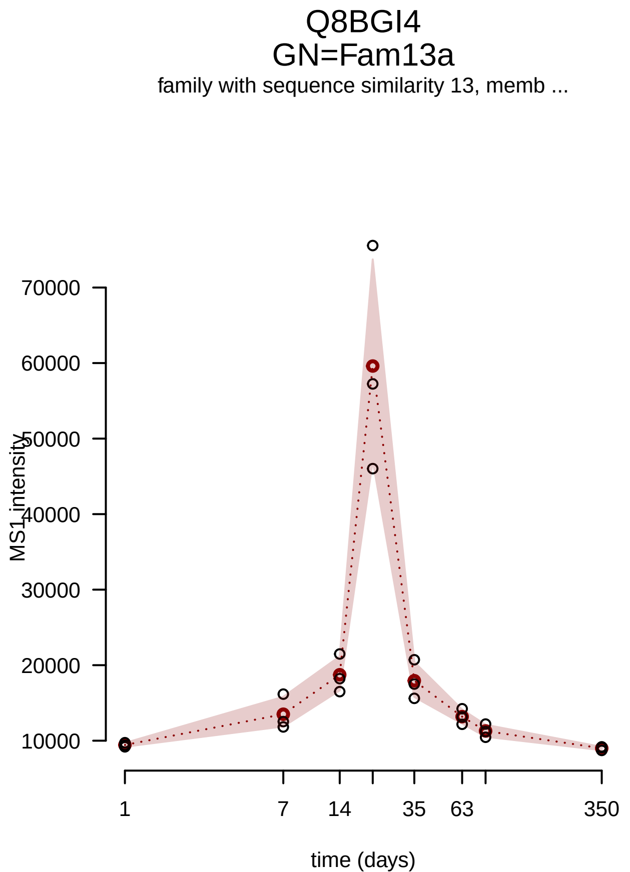
Q8BGI4
GN=Fam13a
family with sequence similarity 13, memb ...
MS1 intensity
70000
60000
50000
40000
30000
20000
10000
1
7
14
35
63
350
time (days)

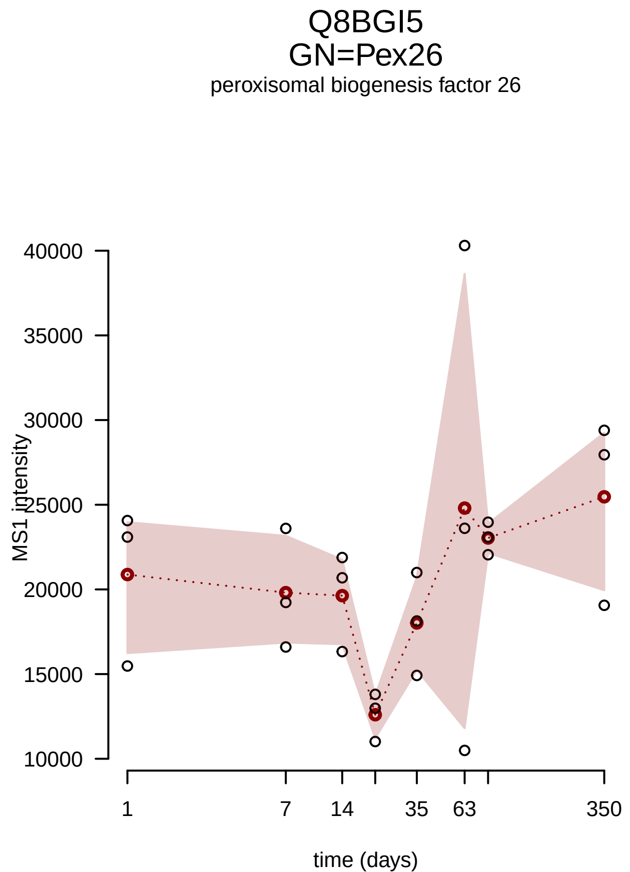
Q8BGI5
GN=Pex26
peroxisomal biogenesis factor 26
MS1 intensity
40000
35000
30000
25000
20000
15000
10000
1
7
14
35
63
350
time (days)

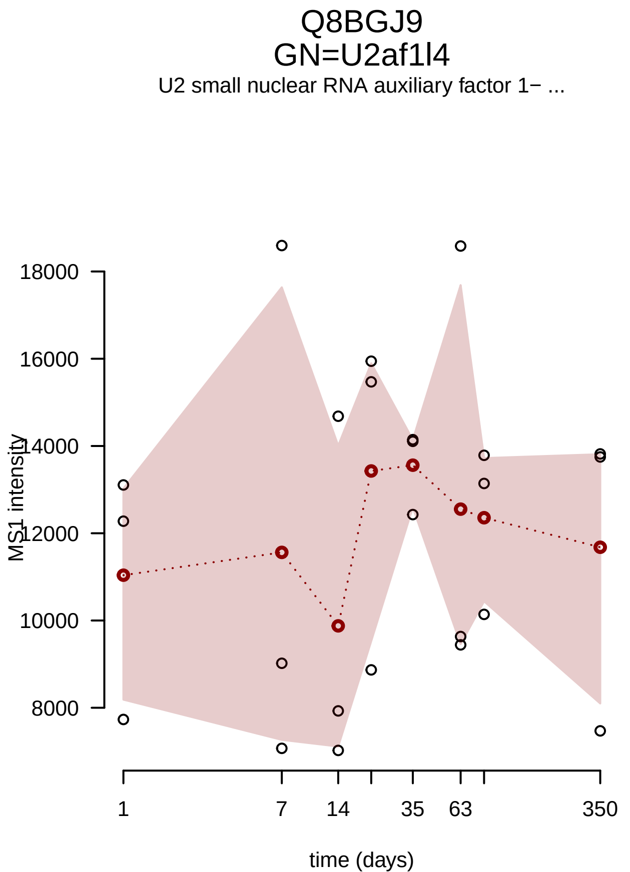
Q8BGJ9
GN=U2af1l4
U2 small nuclear RNA auxiliary factor 1− ...
MS1 intensity
18000
16000
14000
12000
10000
8000
1
7
14
35
63
350
time (days)

Q8BGK6
GN=Slc7a6
solute carrier family 7 (cationic amino ...

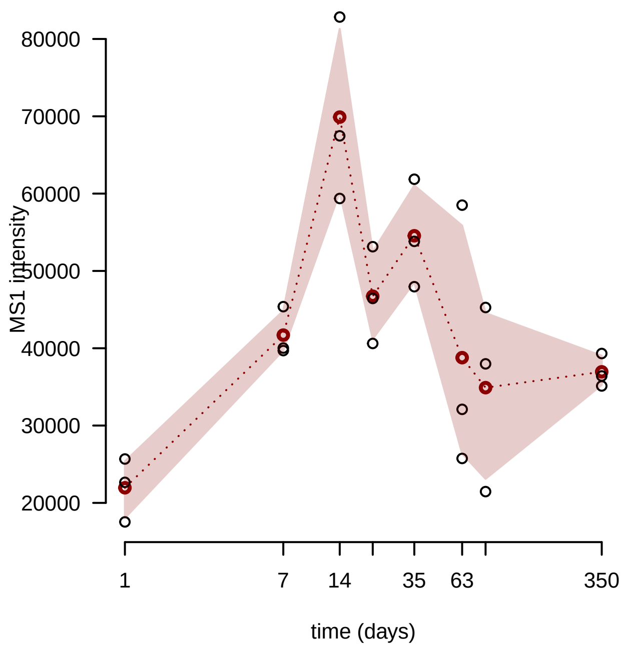

Q8BGN2
GN=D3Ertd751e
DNA segment, Chr 3, ERATO Doi 751, expre ...

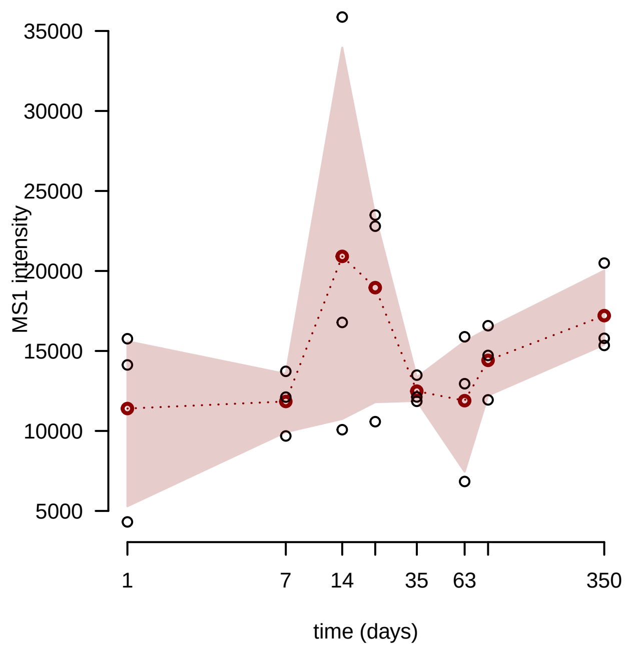

Q8BGN3
GN=Enpp6
ectonucleotide pyrophosphatase/phosphodi ...

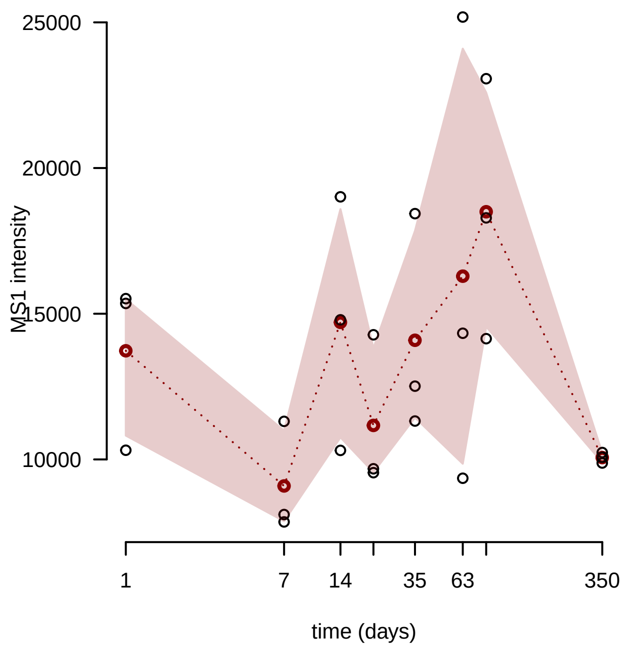

Q8BGP6
GN=Slc25a40
solute carrier family 25, member 40

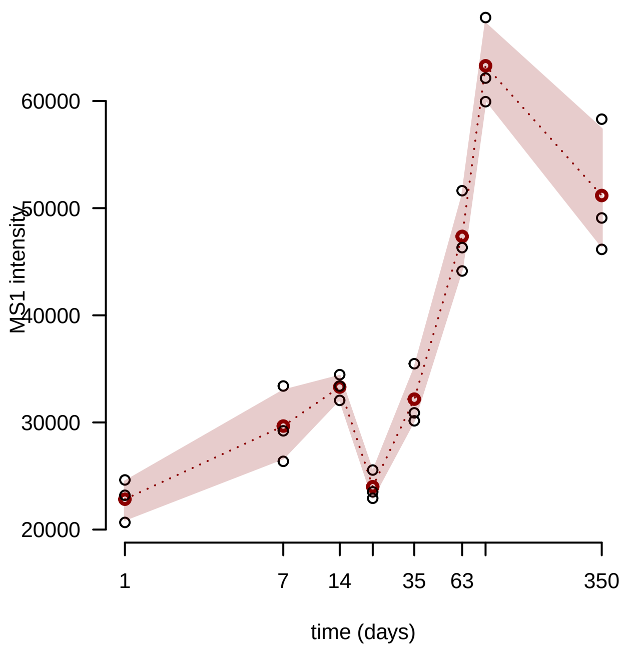

Q8BGQ1
GN=Vipas39
VPS33B interacting protein, apical−basol ...

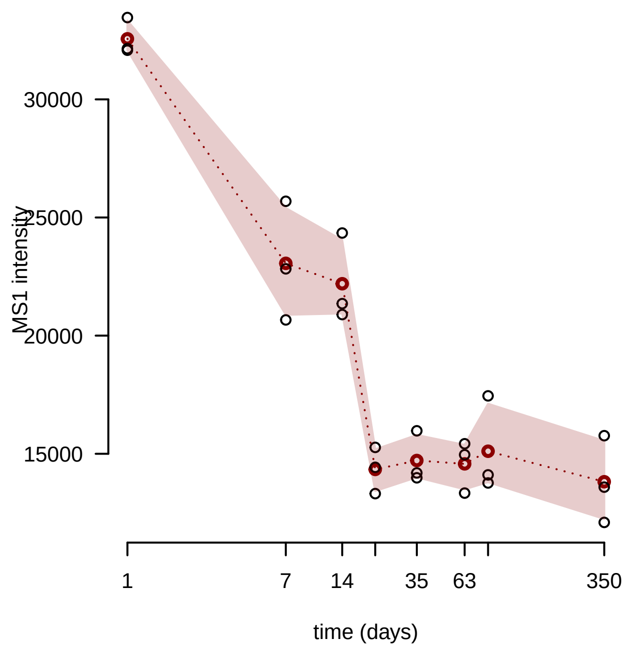

Q8BGQ4
GN=Pomt2
protein−O−mannosyltransferase 2

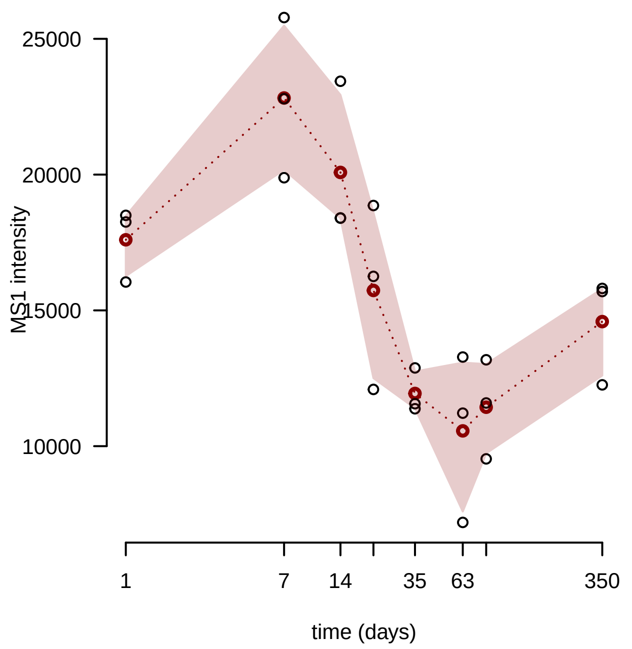

Q8BGQ6
GN=Efcab14
EF−hand calcium binding domain 14

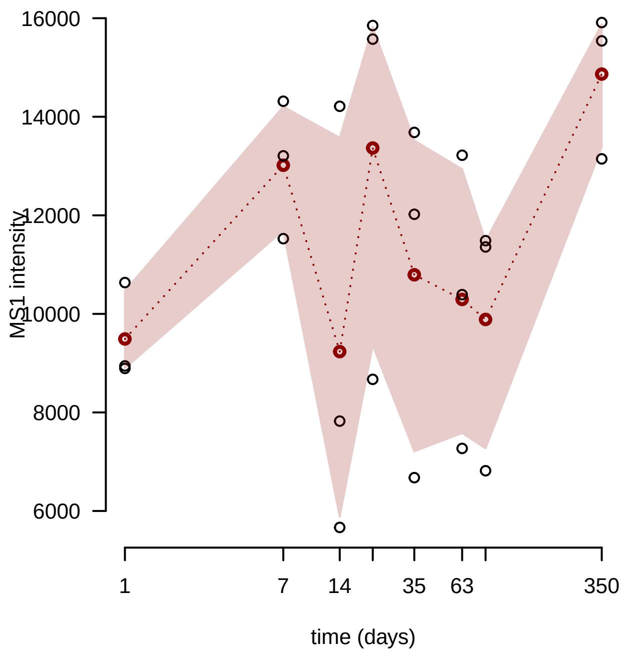

Q8BGQ7
GN=Aars
alanyl−tRNA synthetase

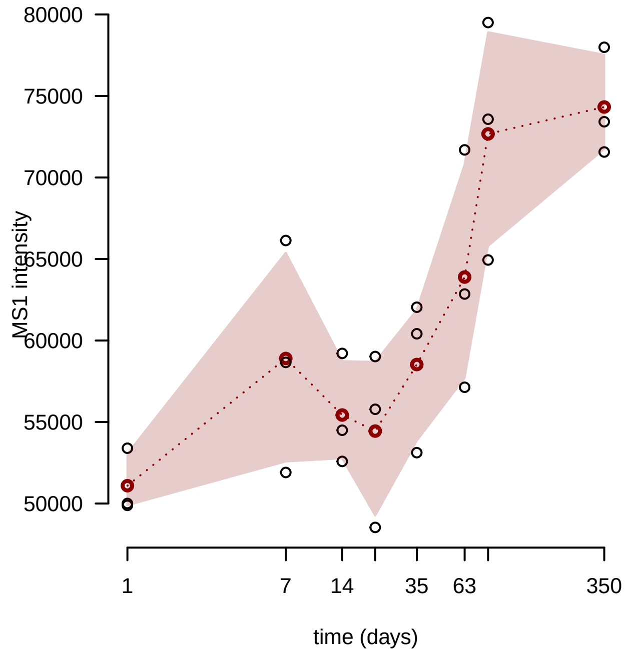

Q8BGR2
GN=Lrrc8d
leucine rich repeat containing 8D

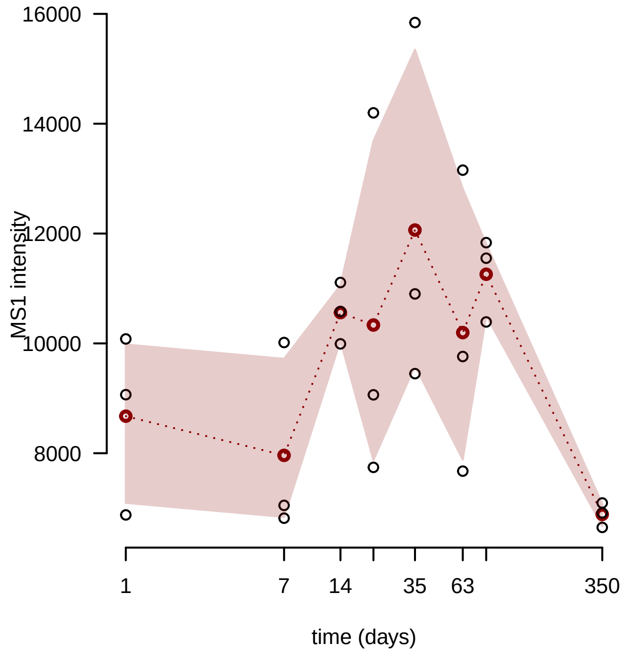

Q8BGR6
GN=Arl15
ADP−ribosylation factor−like 15

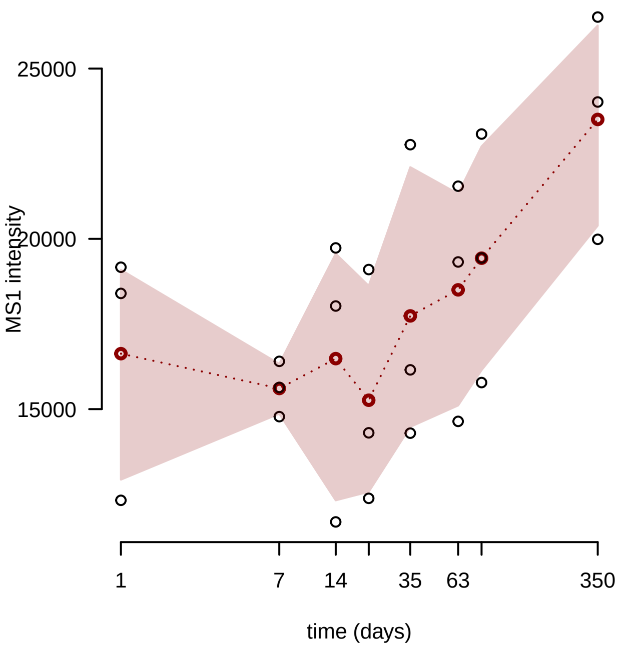

Q8BGR9
GN=Ublcp1
ubiquitin−like domain containing CTD pho ...

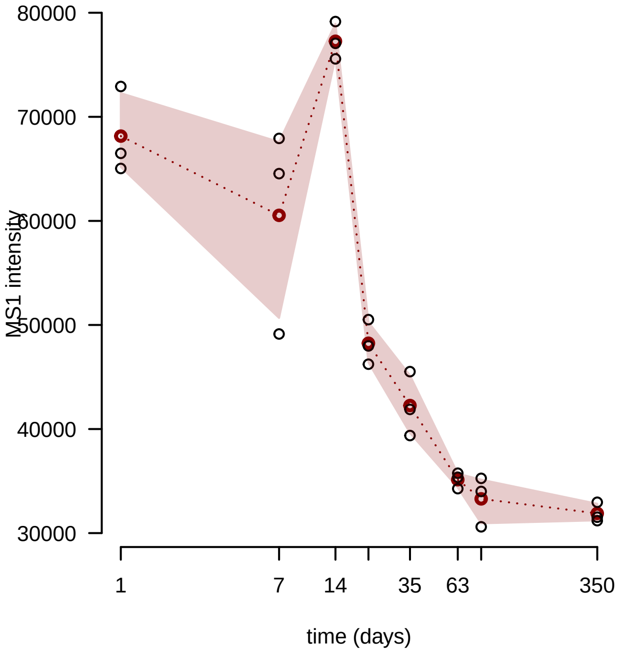

Q8BGS0
GN=Mak16
MAK16 homolog

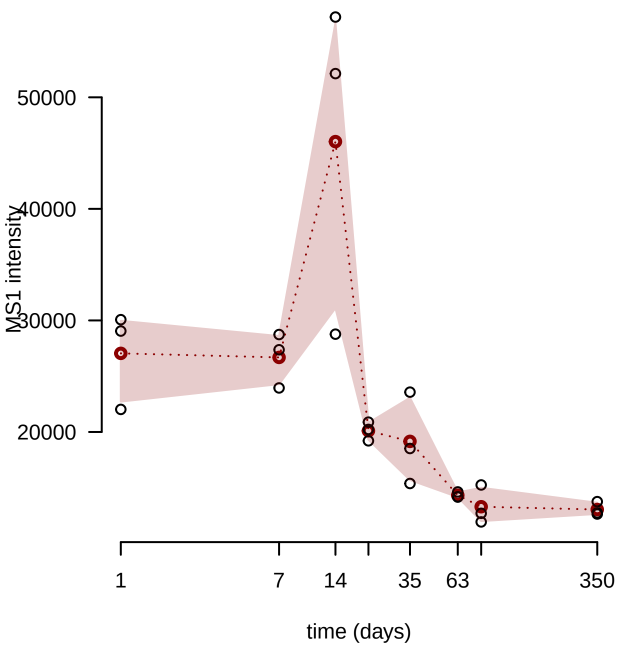

Q8BGS1
GN=Epb41l5
erythrocyte membrane protein band 4.1 li ...

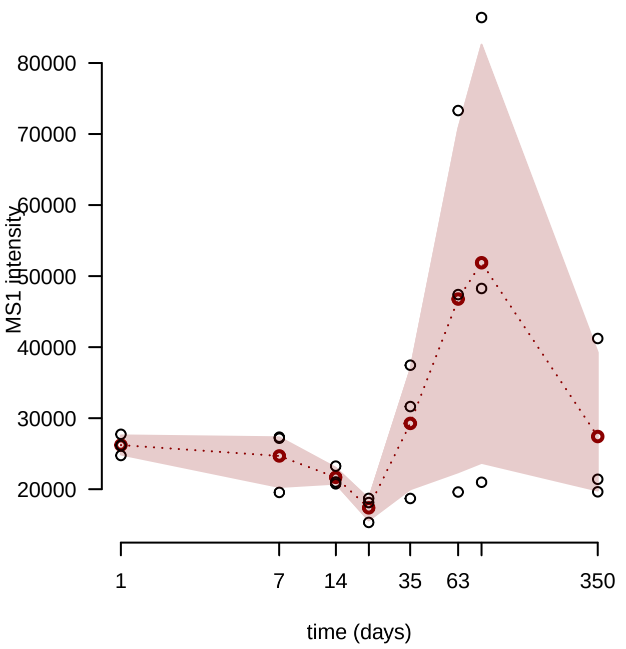

Q8BGS2
GN=Bola2
bolA−like 2 (E. coli)

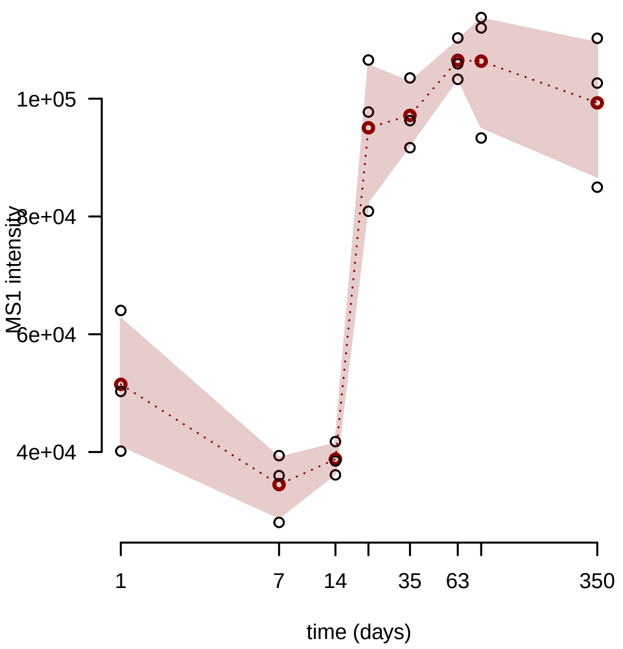

Q8BGS3
GN=Zkscan1
zinc finger with KRAB and SCAN domains 1

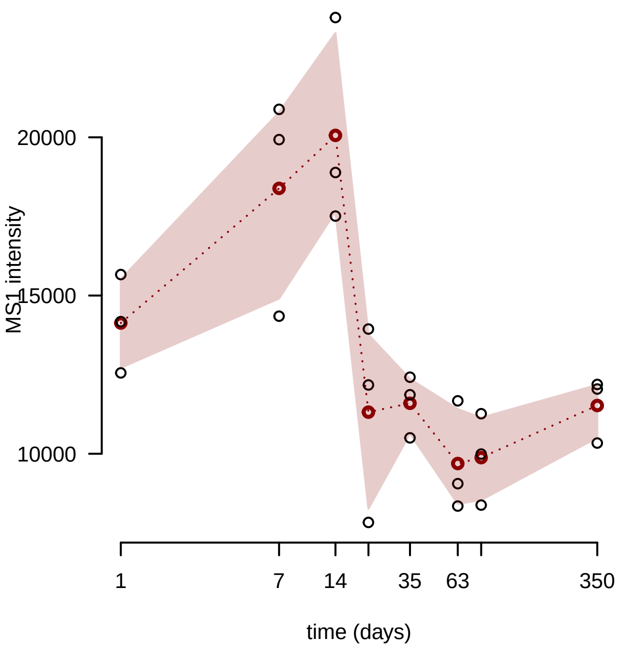

Q8BGS7
GN=Cept1
choline/ethanolaminephosphotransferase 1

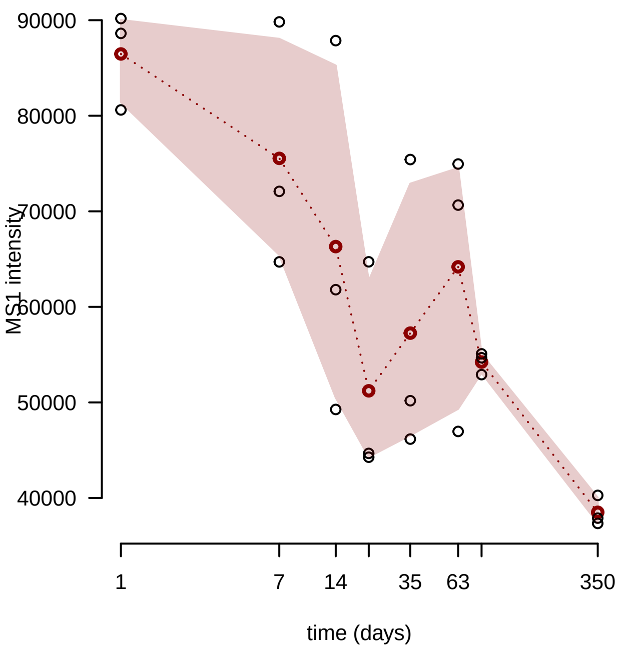

Q8BGT5
GN=Gpt2
glutamic pyruvate transaminase (alanine ...

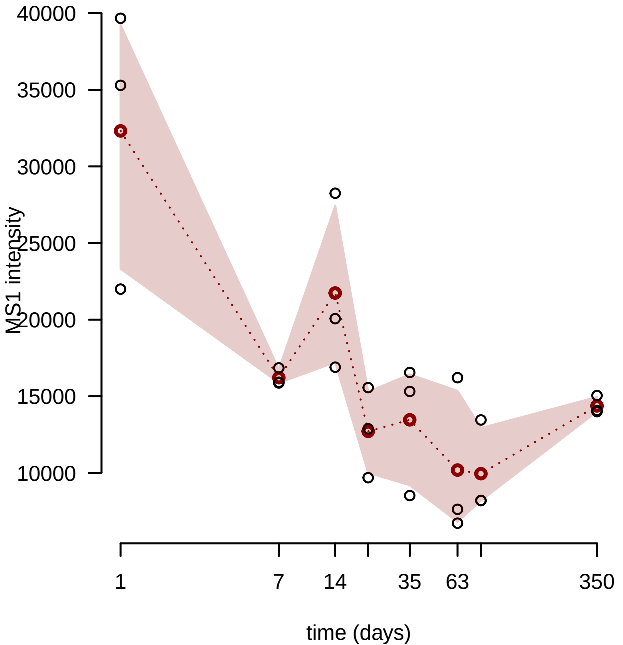

Q8BGT7
GN=Smndc1
survival motor neuron domain containing ...

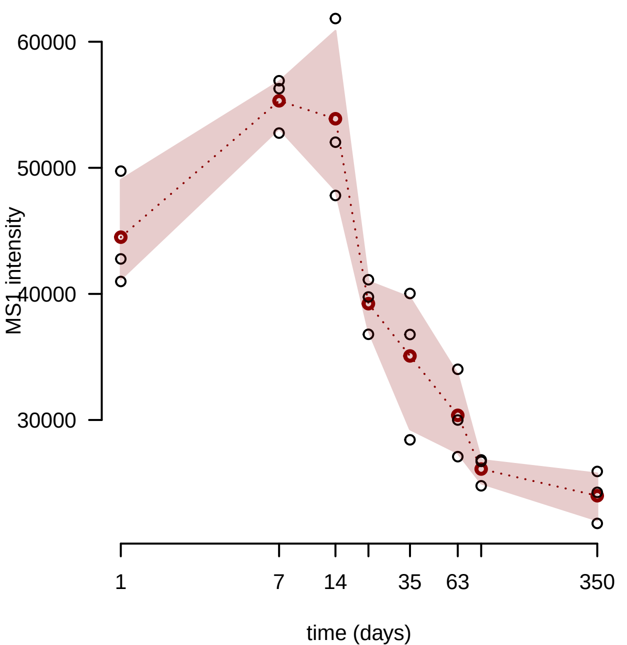

Q8BGT9
GN=Galnt12
polypeptide N−acetylgalactosaminyltransf ...

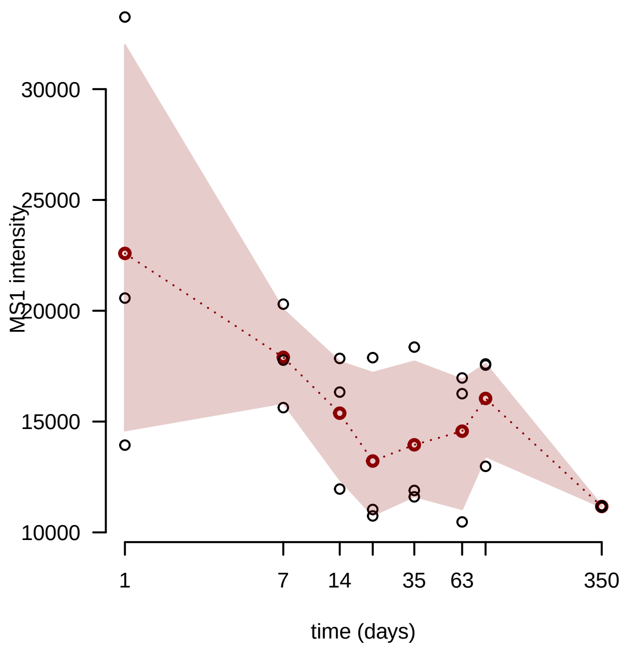

Q8BGU5
GN=Ccny
cyclin Y

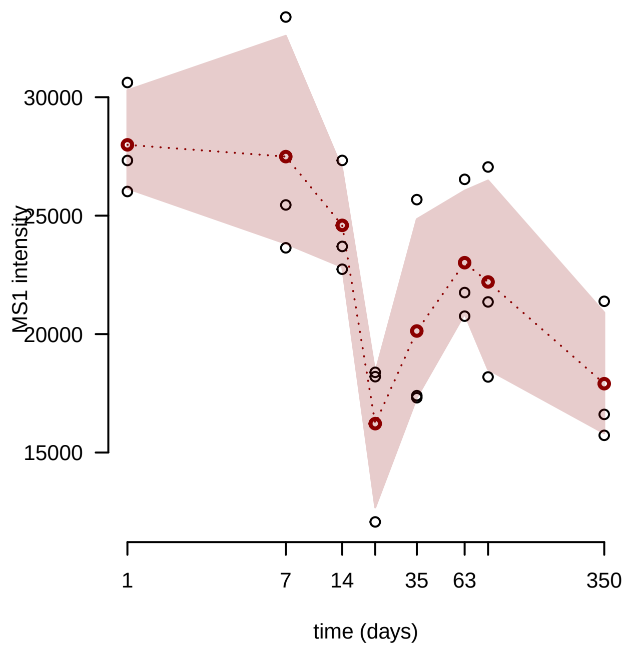

Q8BGV0
GN=Nars2
asparaginyl−tRNA synthetase 2 (mitochond ...

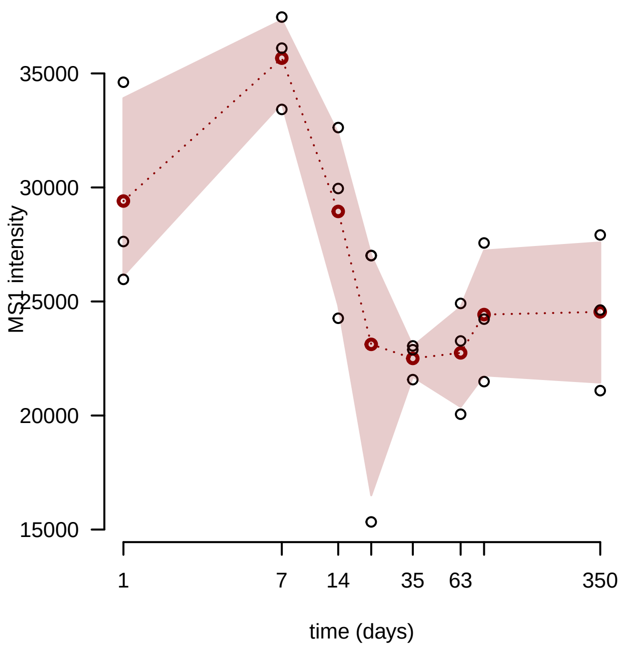

Q8BGV4
GN=Tti2
TELO2 interacting protein 2

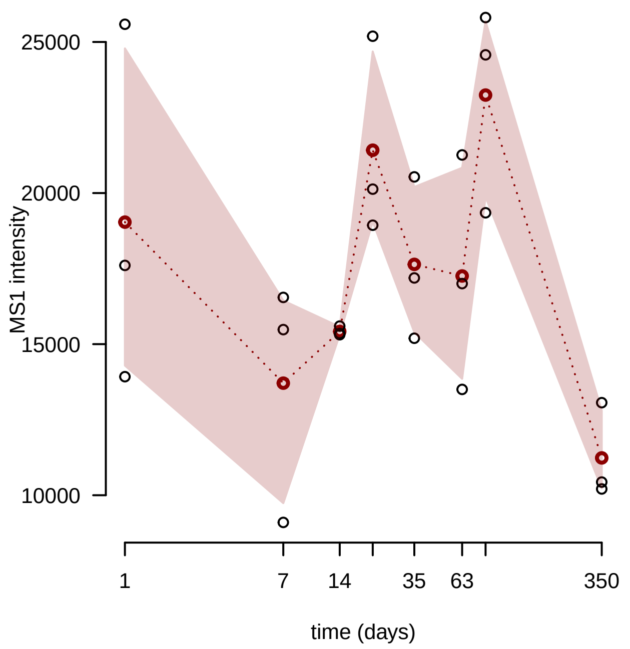

Q8BGV8
GN=Mief1
mitochondrial elongation factor 1

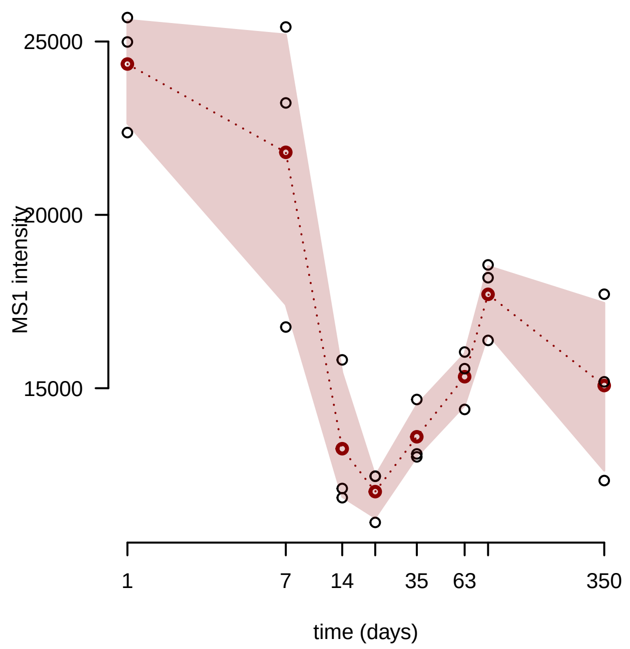

Q8BGW1
GN=Fto
fat mass and obesity associated

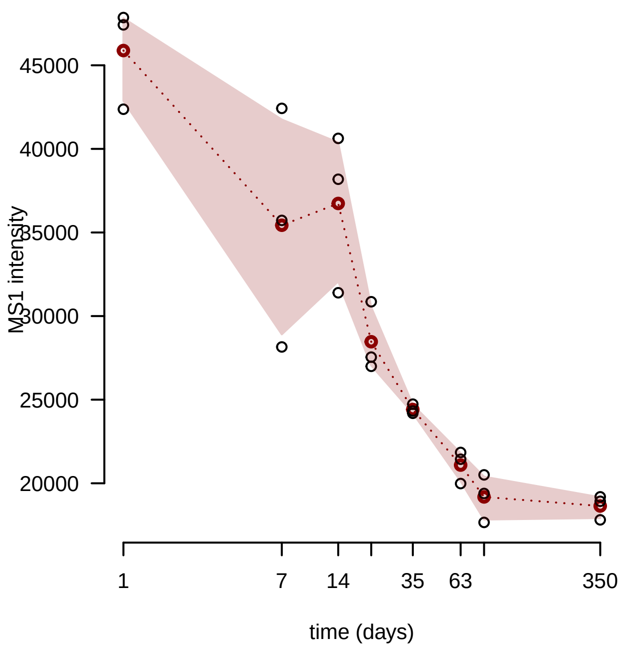

Q8BGX1
GN=Pced1b
PC−esterase domain containing 1B

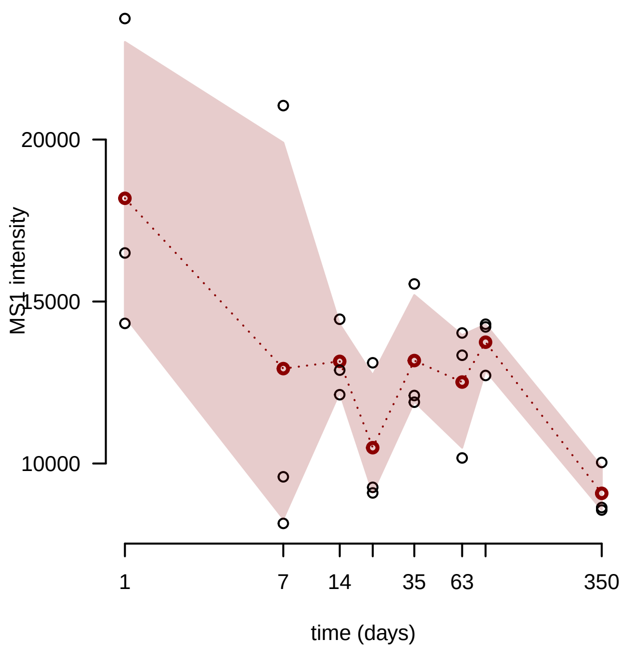

Q8BGX2
GN=Timm29
translocase of inner mitochondrial membr ...

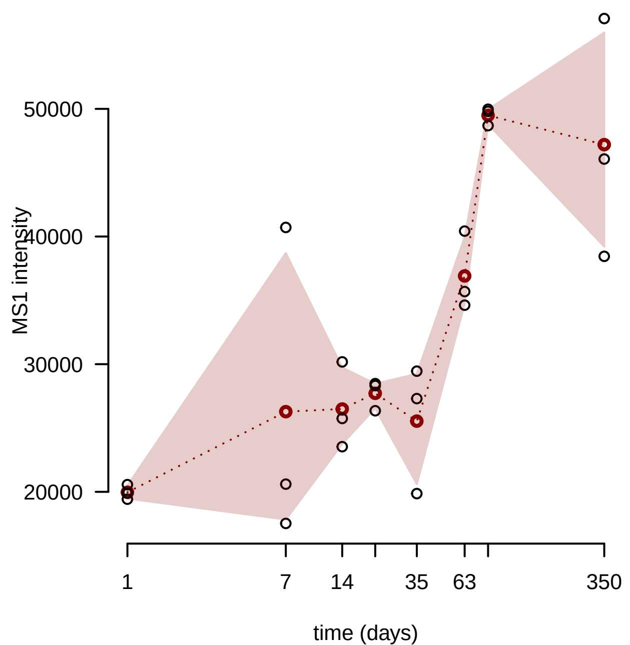

Q8BGX7
GN=Spidr
scaffolding protein involved in DNA repa ...

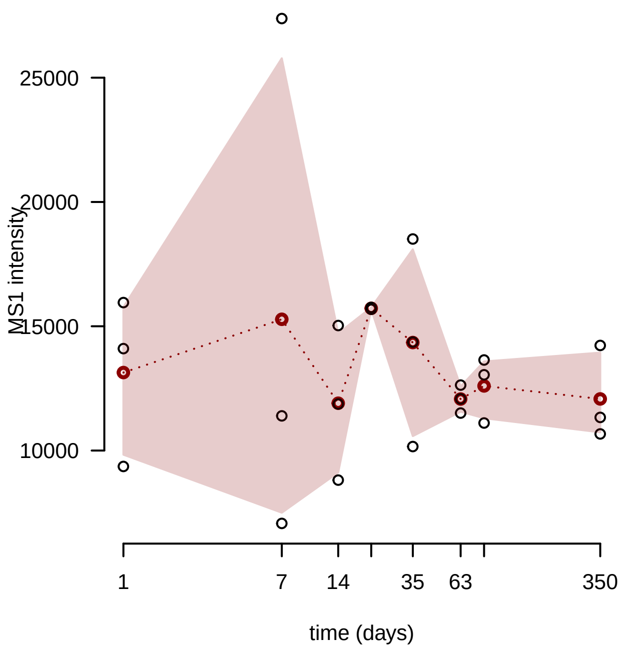

Q8BGY2
GN=Eif5a2
eukaryotic translation initiation factor ...

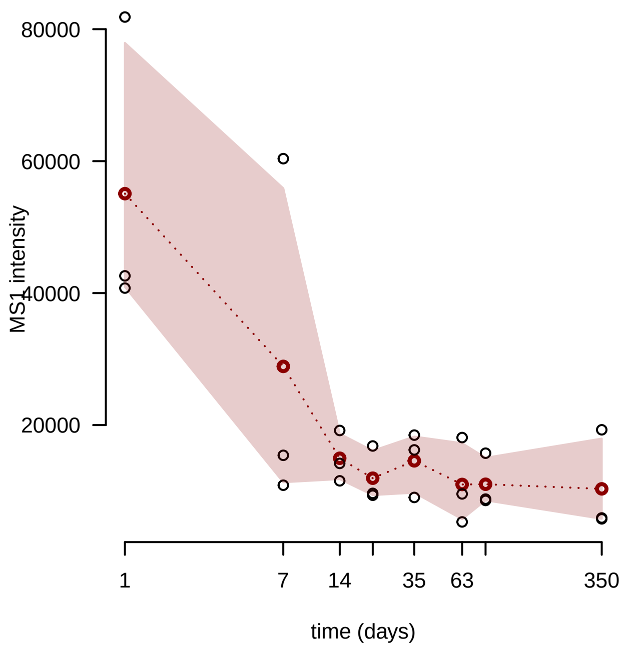

Q8BGY4
GN=Klhl26
kelch−like 26

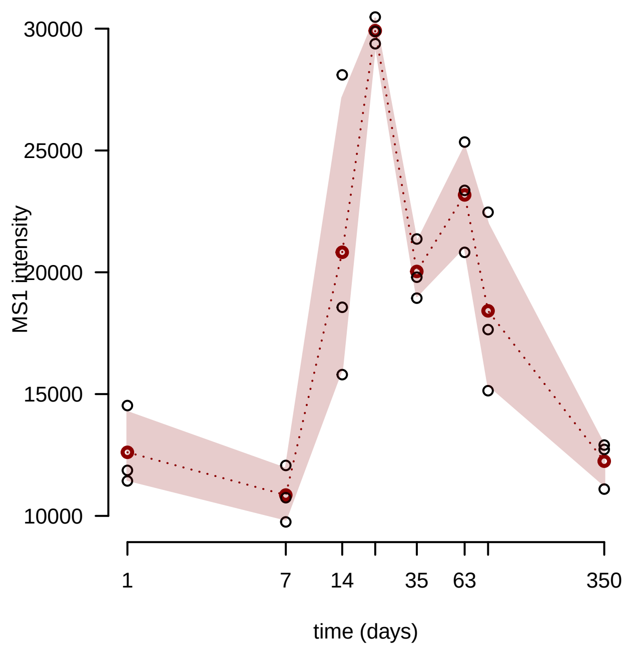

Q8BGY7
GN=Fam210a
family with sequence similarity 210, mem ...

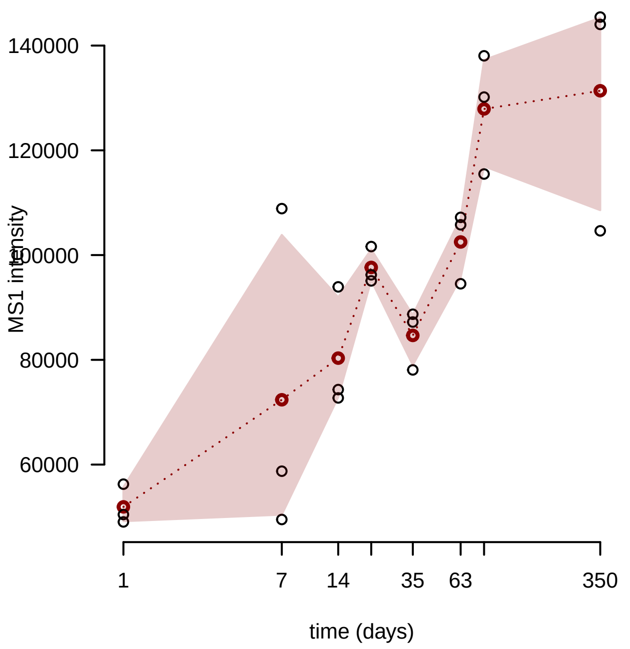

Q8BGZ4
GN=Cdc23
CDC23 cell division cycle 23

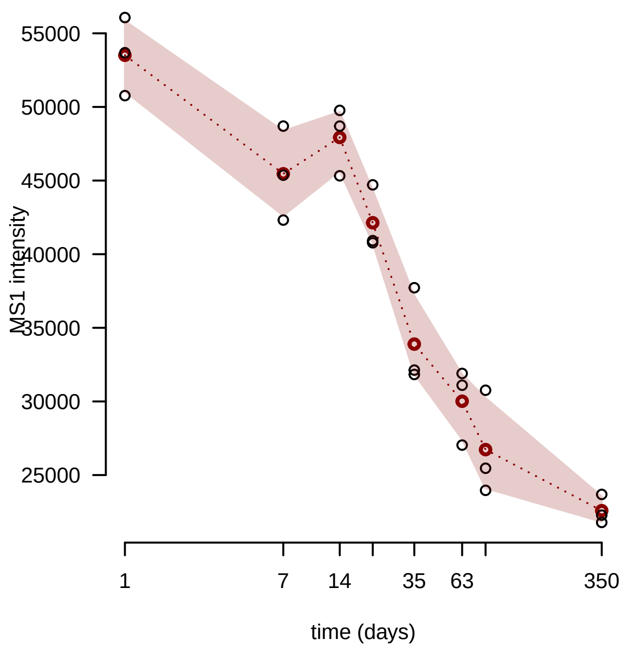

Q8BGZ7
GN=Krt75
keratin 75

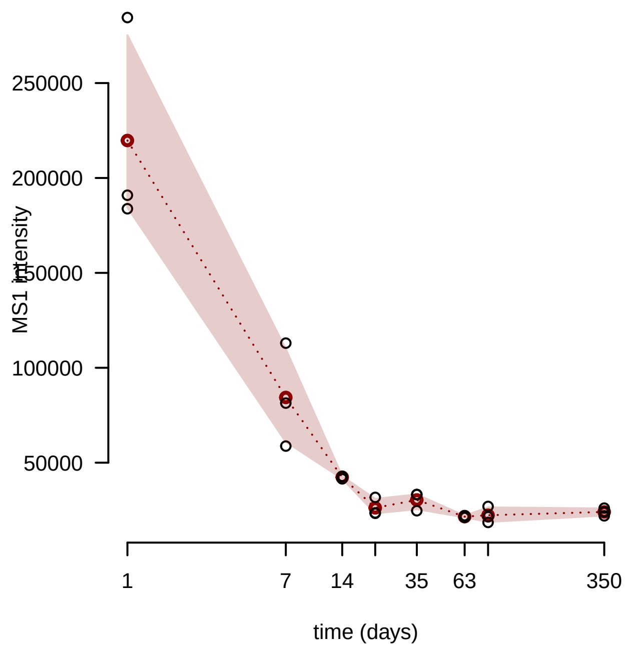

Q8BGZ8
GN=Zpld1
zona pellucida like domain containing 1

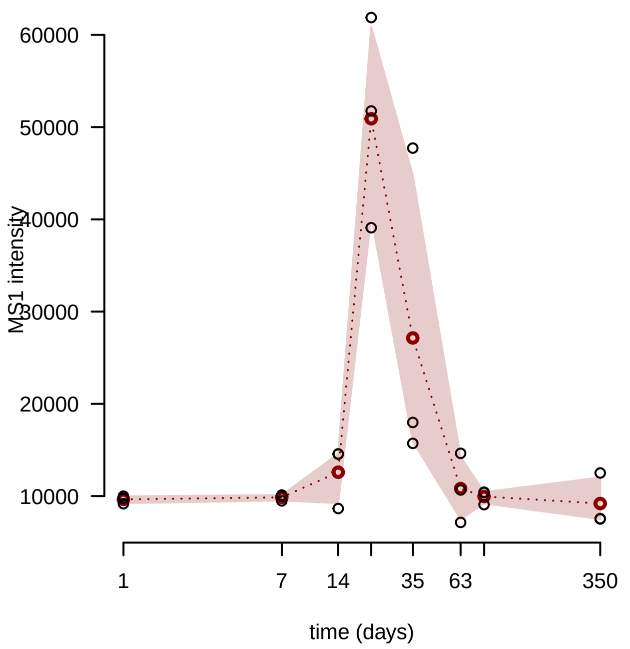

Q8BH00
GN=Aldh8a1
aldehyde dehydrogenase 8 family, member ...

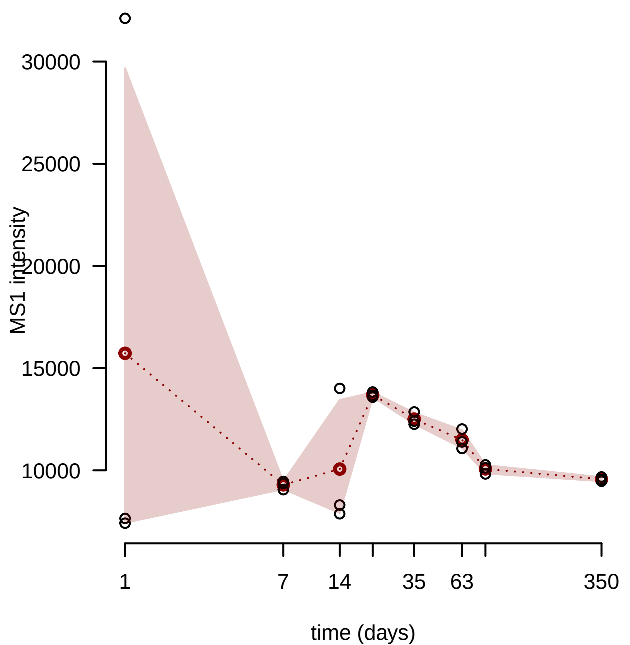

Q8BH02
GN=Tor4a
torsin family 4, member A

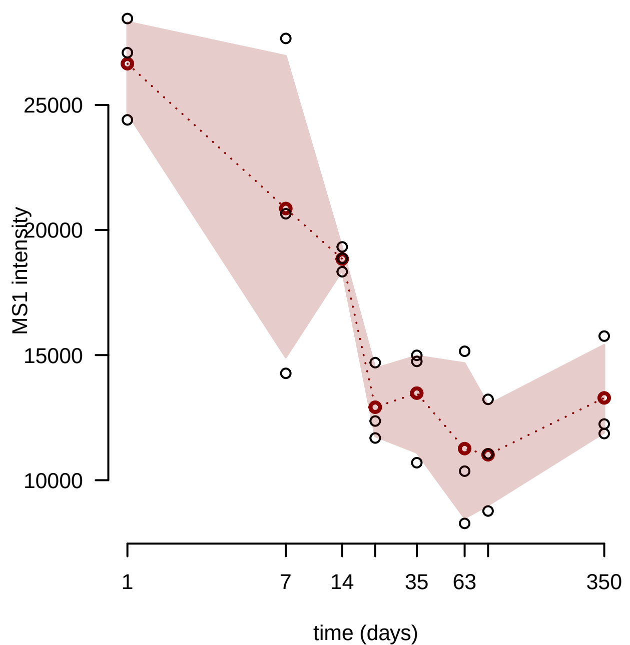

Q8BH04
GN=Pck2
phosphoenolpyruvate carboxykinase 2 (mit ...

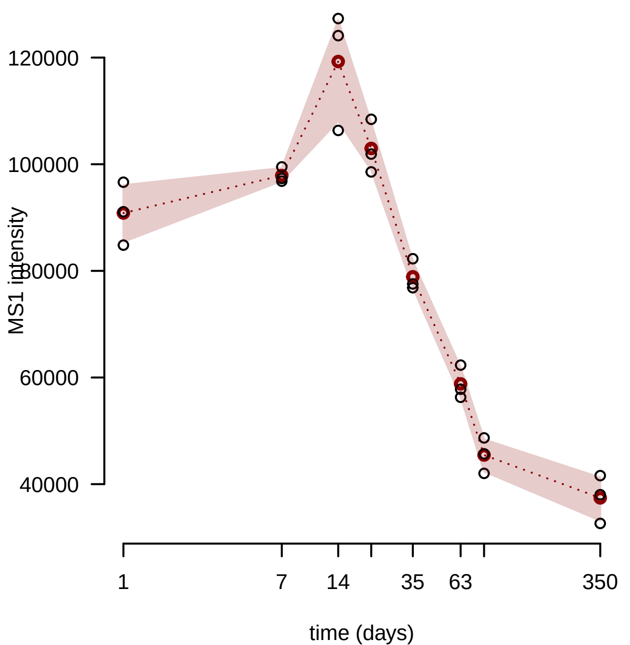

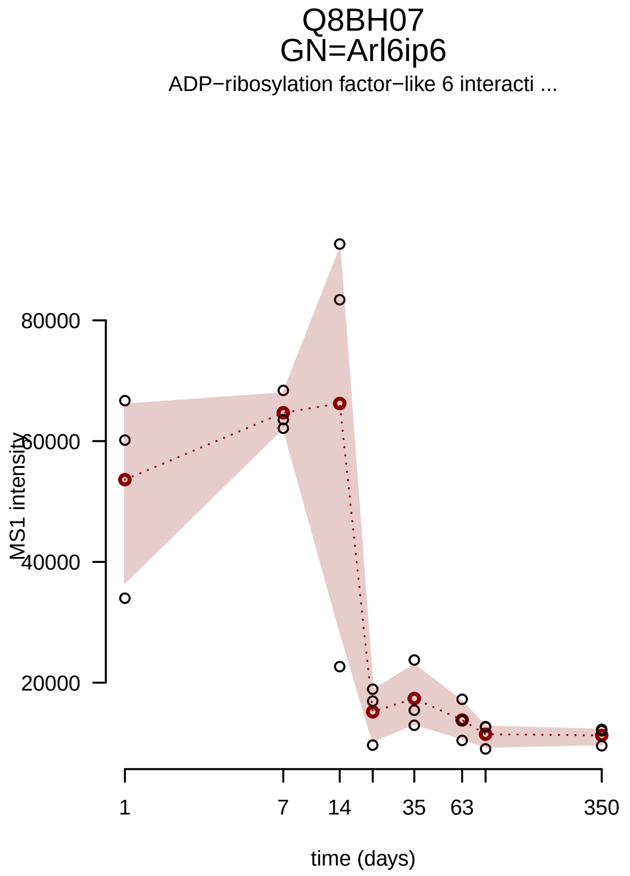
Q8BH07
GN=Arl6ip6
ADP−ribosylation factor−like 6 interacti ...
MS1 intensity
20000
40000
60000
80000
1
7
14
35
63
350
time (days)

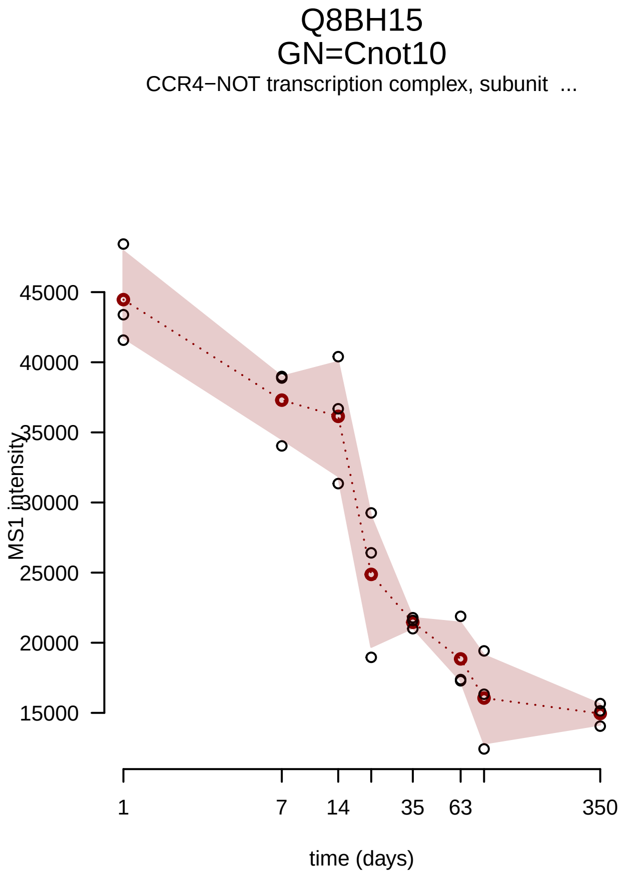
Q8BH15
GN=Cnot10
CCR4−NOT transcription complex, subunit ...
MS1 intensity
15000
20000
25000
30000
35000
40000
45000
1
7
14
35
63
350
time (days)

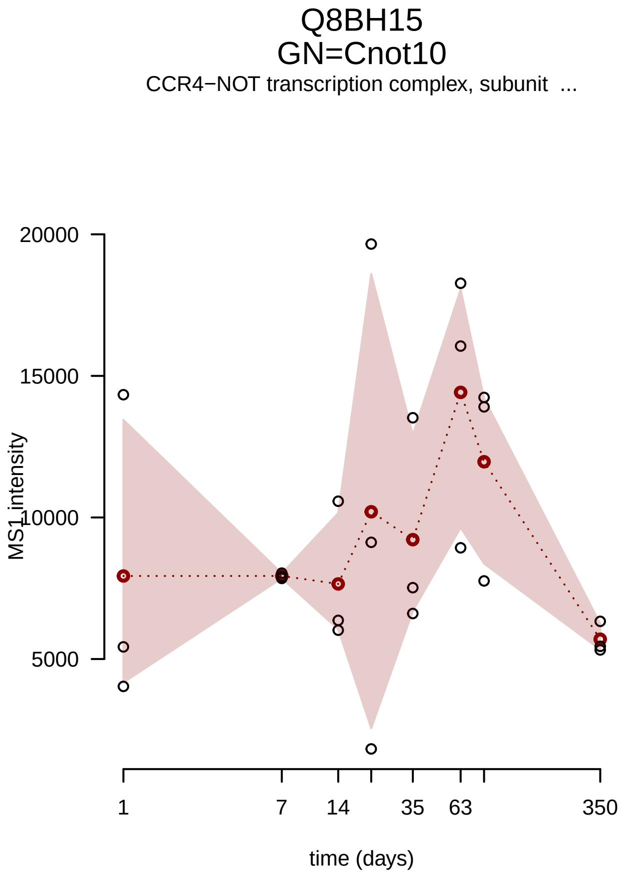
Q8BH15
GN=Cnot10
CCR4−NOT transcription complex, subunit ...
MS1 intensity
5000
10000
15000
20000
1
7
14
35
63
350
time (days)

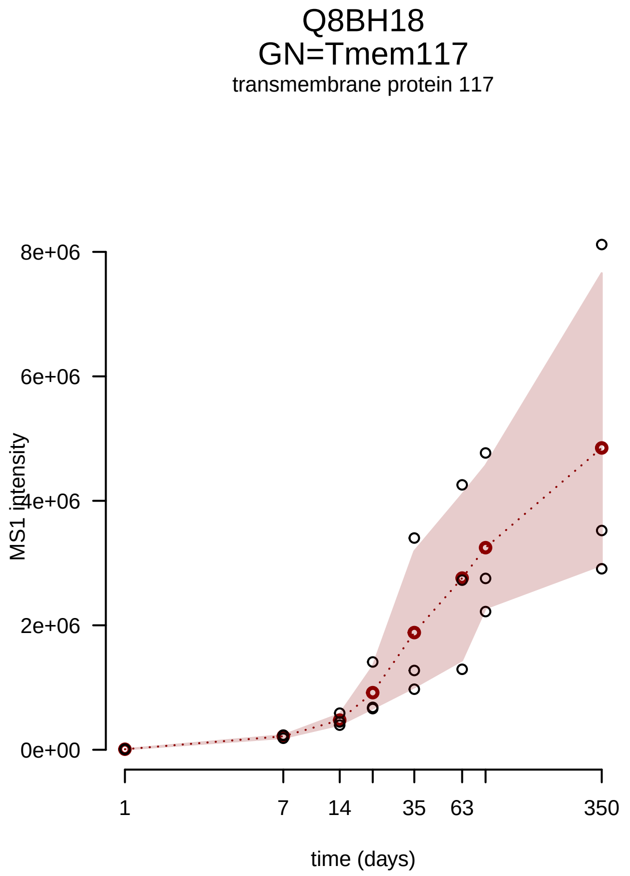
Q8BH18
GN=Tmem117
transmembrane protein 117
MS1 intensity
0e+00
2e+06
4e+06
6e+06
8e+06
1
7
14
35
63
350
time (days)

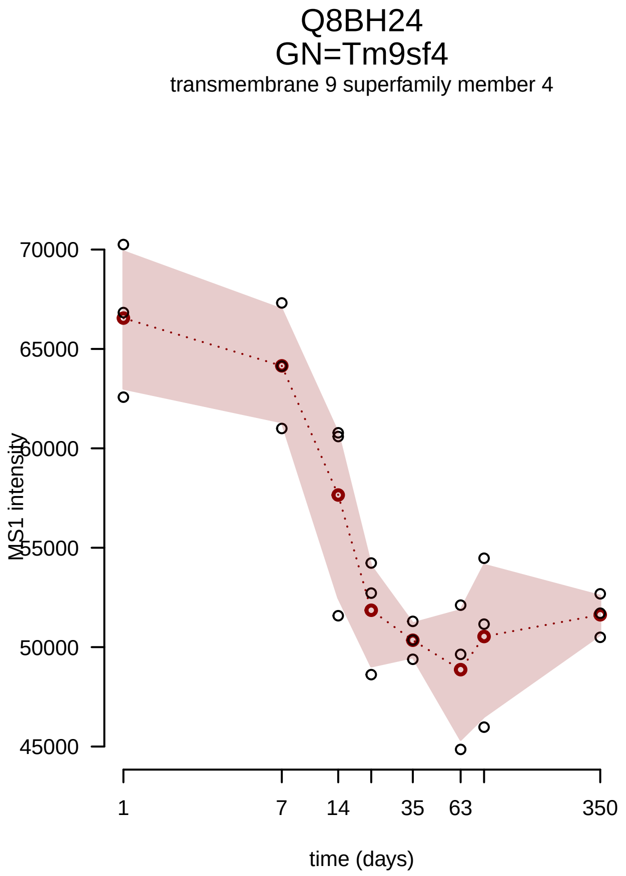
Q8BH24
GN=Tm9sf4
transmembrane 9 superfamily member 4
MS1 intensity
45000
50000
55000
60000
65000
70000
1
7
14
35
63
350
time (days)

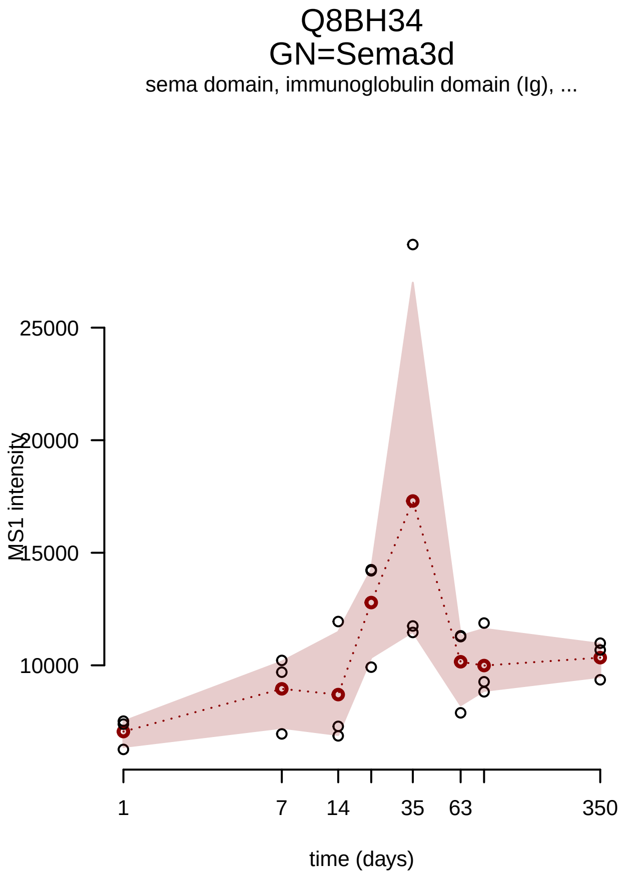
Q8BH34
GN=Sema3d
sema domain, immunoglobulin domain (Ig), ...
MS1 intensity
10000
15000
20000
25000
1
7
14
35
63
350
time (days)

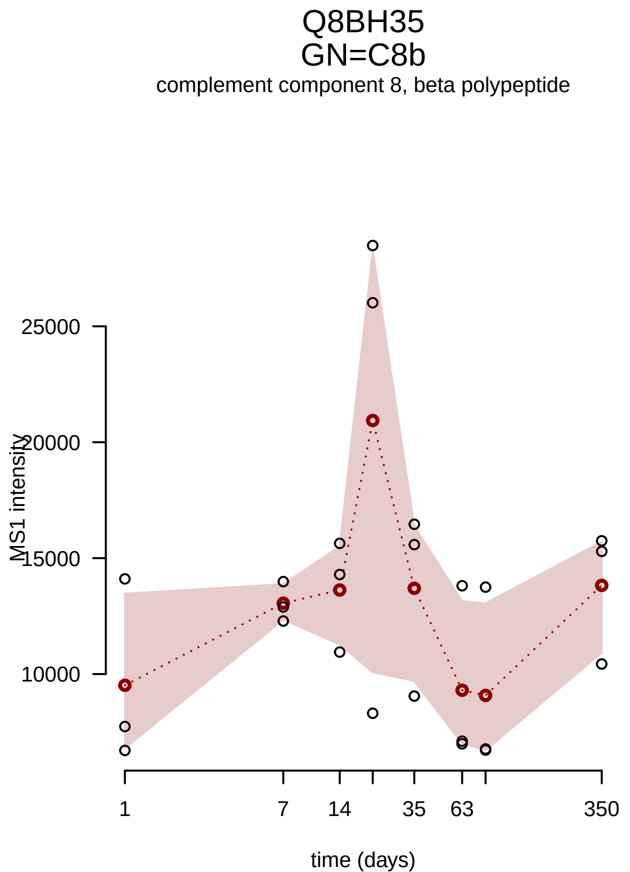
Q8BH35
GN=C8b
complement component 8, beta polypeptide
MS1 intensity
10000
15000
20000
25000
1
7
14
35
63
350
time (days)

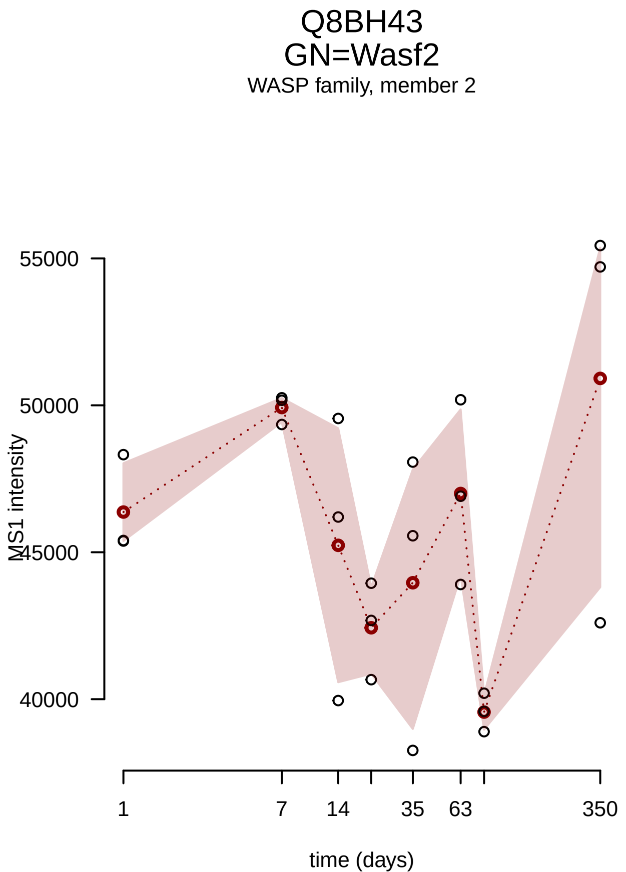
Q8BH43
GN=Wasf2
WASP family, member 2
MS1 intensity
40000
45000
50000
55000
1
7
14
35
63
350
time (days)

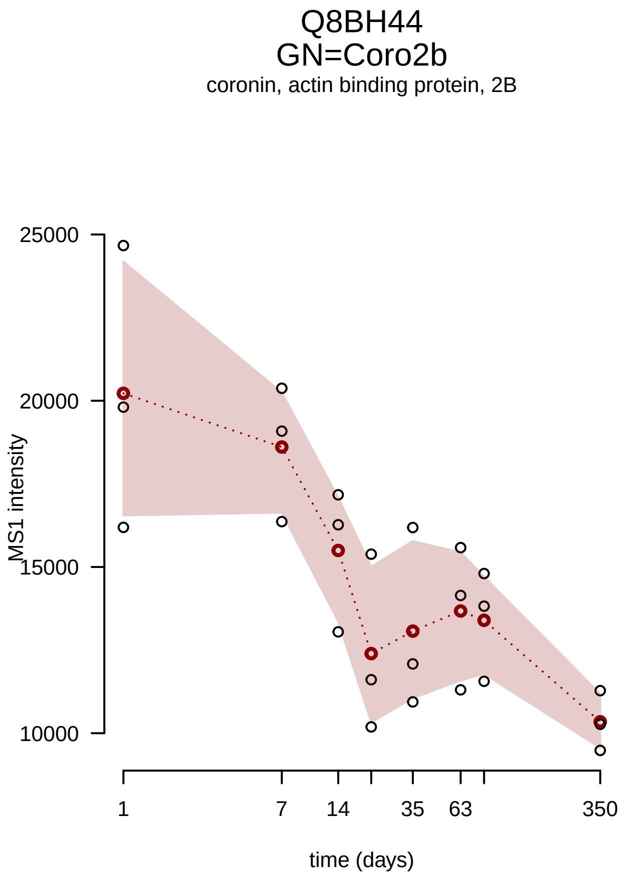
Q8BH44
GN=Coro2b
coronin, actin binding protein, 2B
MS1 intensity
10000
15000
20000
25000
1
7
14
35
63
350
time (days)

Q8BH47
GN=Sec22a
SEC22 homolog A, vesicle trafficking pro ...

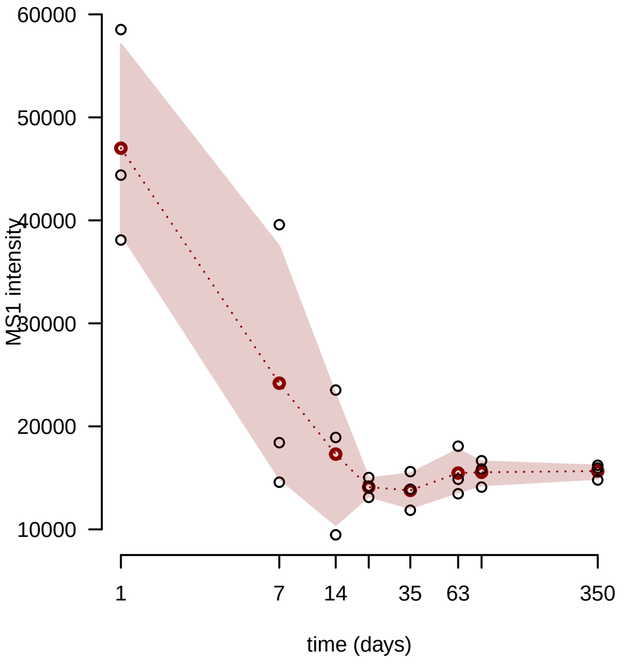

Q8BH48
GN=Ubap1
ubiquitin−associated protein 1

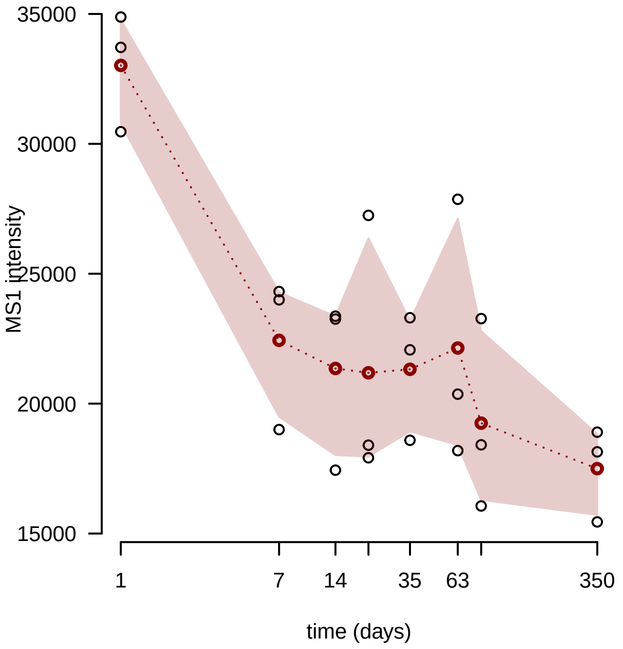

Q8BH50
GN=

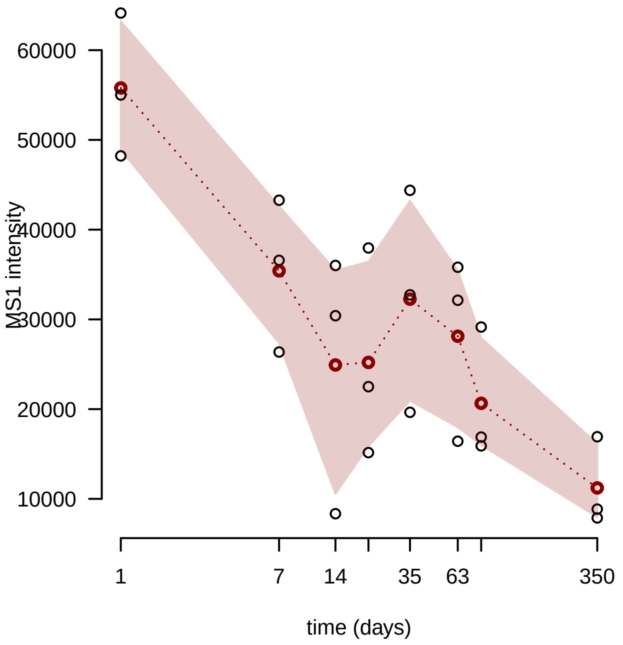

Q8BH51
GN=Cox14
cytochrome c oxidase assembly protein 14

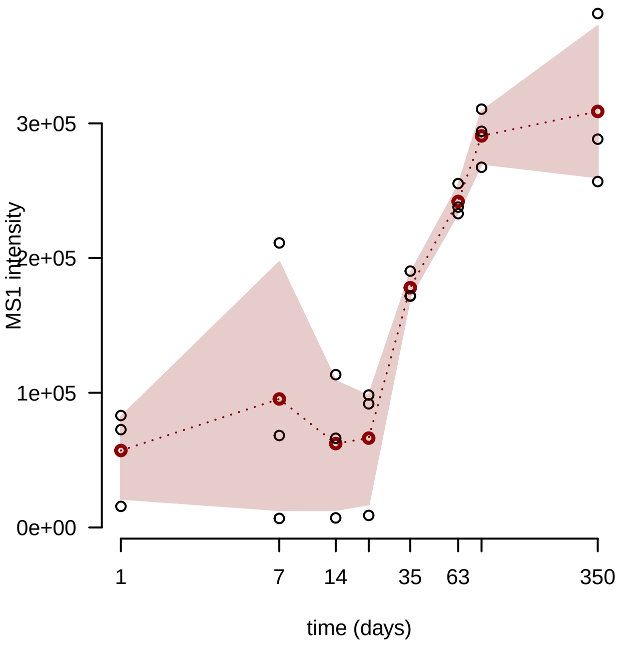

Q8BH55
GN=Thnsl1
threonine synthase−like 1 (bacterial)

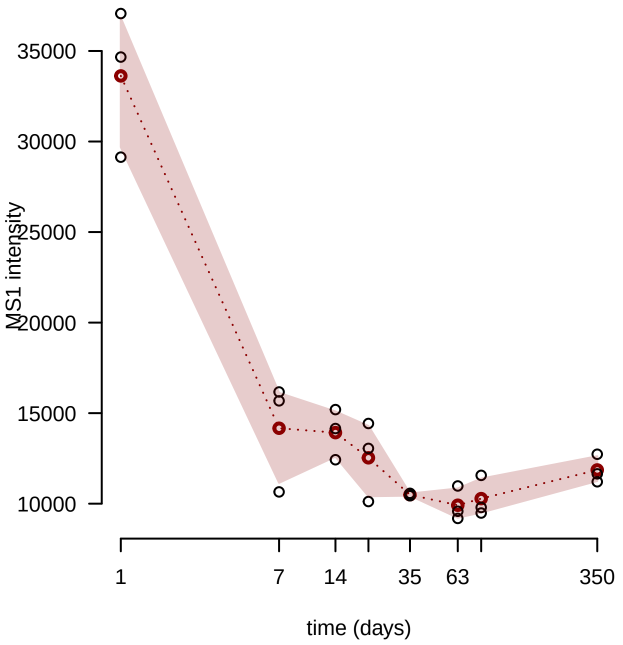

Q8BH57
GN=Wdr48
WD repeat domain 48

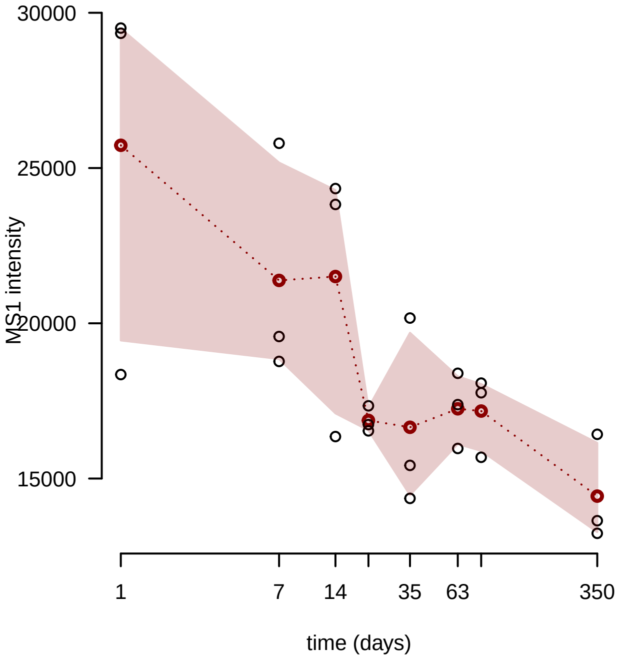

Q8BH58
GN=Tiprl
TIP41, TOR signalling pathway regulator− ...

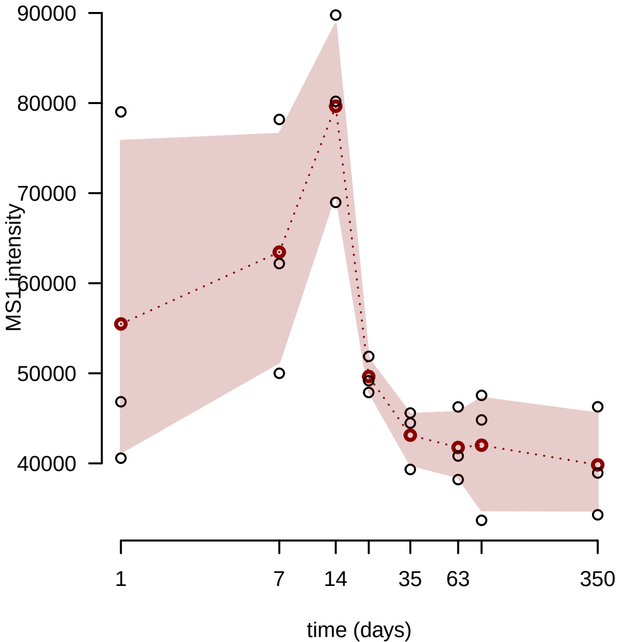

Q8BH59
GN=Slc25a12
solute carrier family 25 (mitochondrial ...

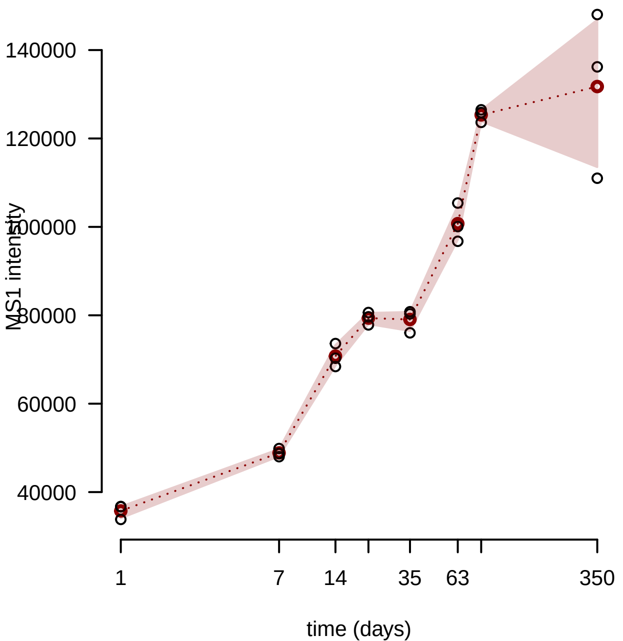

Q8BH60
GN=Gopc
golgi associated PDZ and coiled−coil mot ...

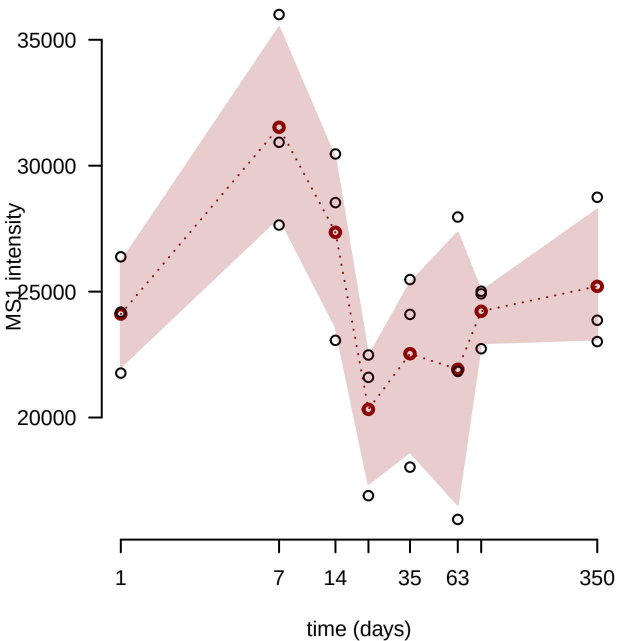

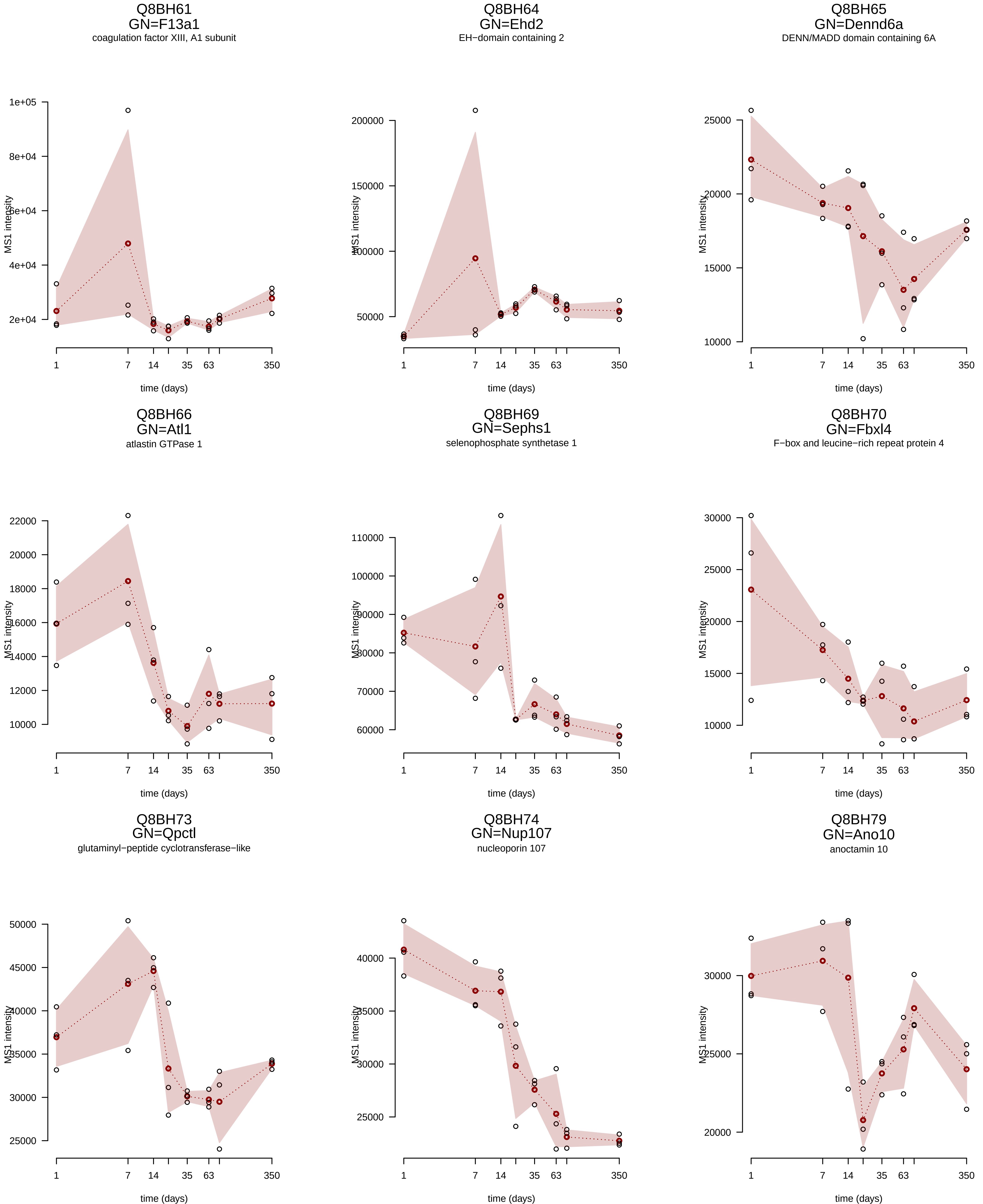

Q8BH61
GN=F13a1
coagulation factor XIII, A1 subunit
MS1 intensity
2e+04
4e+04
6e+04
8e+04
1e+05
time (days)
1
7
14
35
63
350
Q8BH64
GN=Ehd2
EH−domain containing 2
MS1 intensity
50000
100000
150000
200000
time (days)
Q8BH65
GN=Dennd6a
DENN/MADD domain containing 6A
MS1 intensity
10000
15000
20000
25000
time (days)
Q8BH66
GN=Atl1
atlastin GTPase 1
MS1 intensity
10000
12000
14000
16000
18000
20000
22000
time (days)
Q8BH69
GN=Sephs1
selenophosphate synthetase 1
MS1 intensity
60000
70000
80000
90000
100000
110000
time (days)
Q8BH70
GN=Fbxl4
F−box and leucine−rich repeat protein 4
MS1 intensity
10000
15000
20000
25000
30000
time (days)
Q8BH73
GN=Qpctl
glutaminyl−peptide cyclotransferase−like
MS1 intensity
25000
30000
35000
40000
45000
50000
time (days)
Q8BH74
GN=Nup107
nucleoporin 107
MS1 intensity
25000
30000
35000
40000
time (days)
Q8BH79
GN=Ano10
anoctamin 10
MS1 intensity
20000
25000
30000
time (days)

Q8BH82
GN=Napepld
N−acyl phosphatidylethanolamine phosphol ...

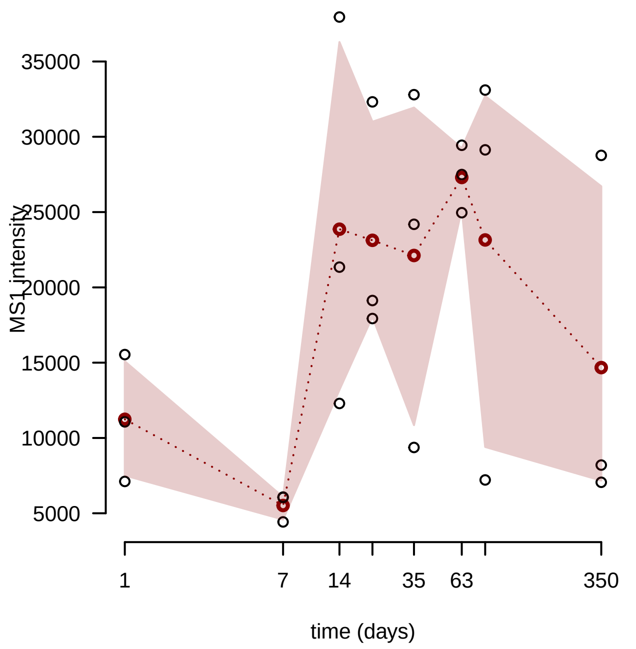

Q8BH86
GN=Dglucy
D−glutamate cyclase

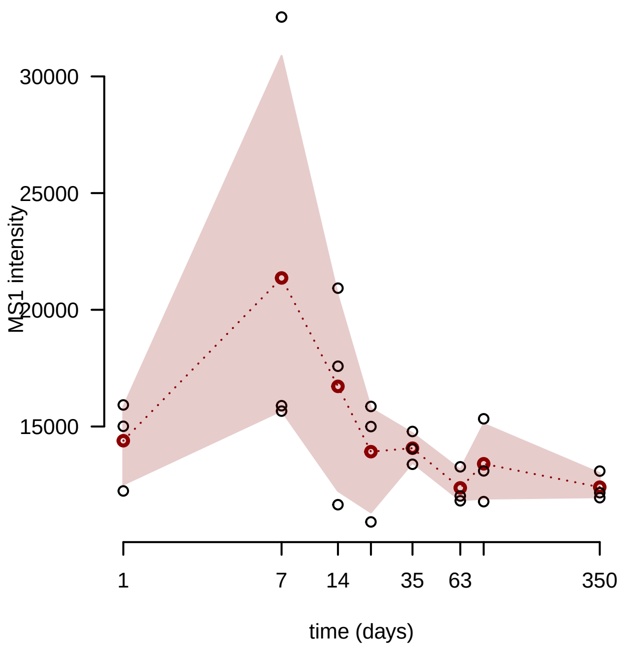

Q8BH95
GN=Echs1
enoyl Coenzyme A hydratase, short chain, ...

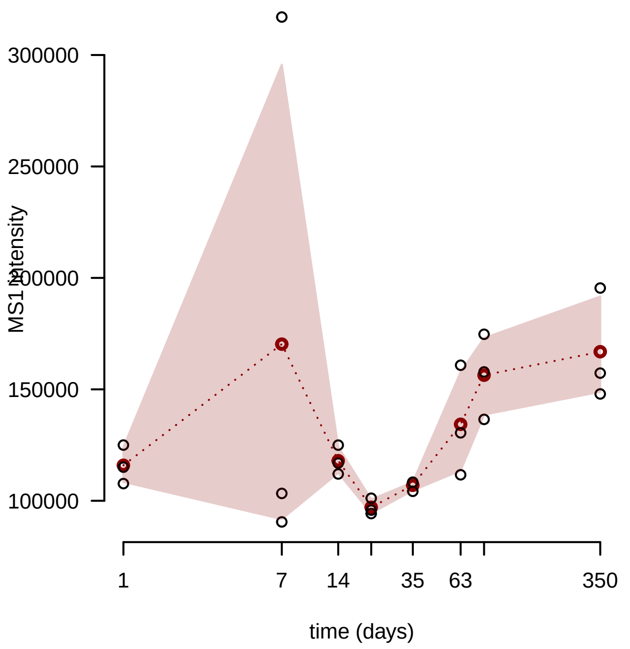

Q8BH97
GN=Rcn3
reticulocalbin 3, EF−hand calcium bindin ...

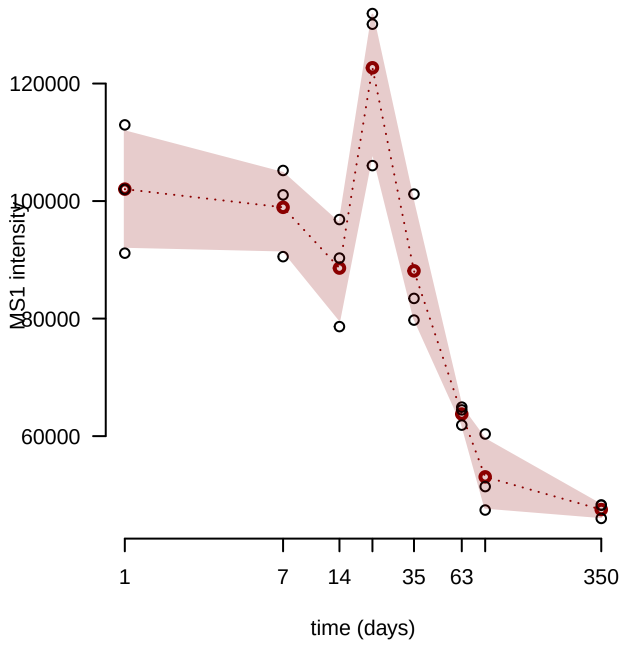

Q8BHA0
GN=Ino80c
INO80 complex subunit C

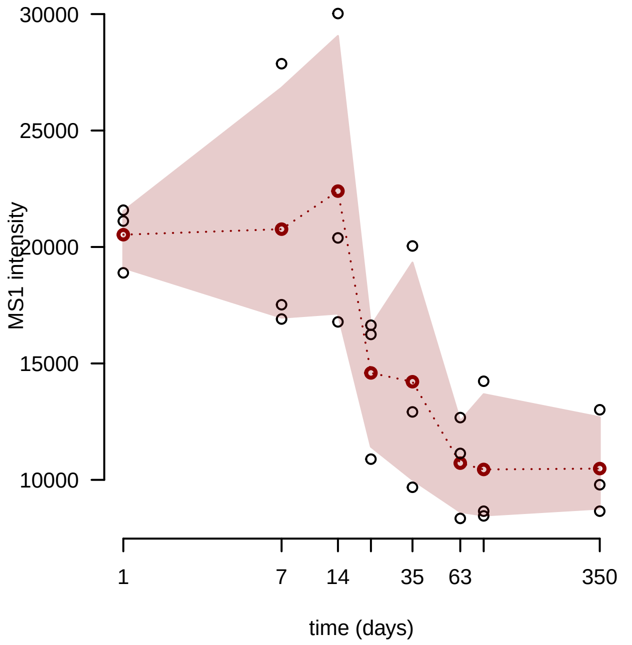

Q8BHA3
GN=Dtd2
D−tyrosyl−tRNA deacylase 2

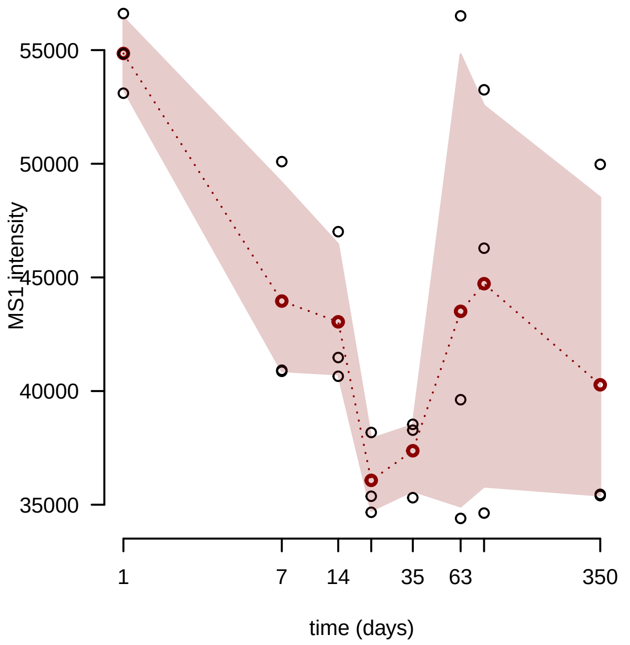

Q8BHB4
GN=Wdr3
WD repeat domain 3

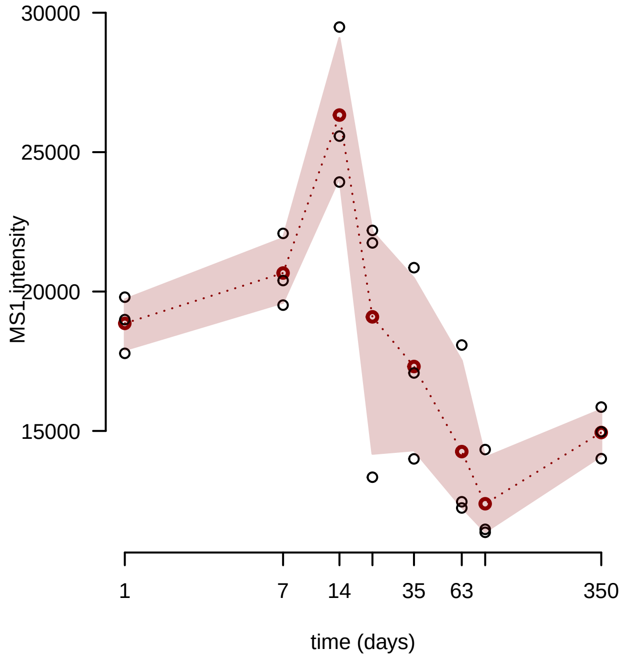

Q8BHB9
GN=Clic6
chloride intracellular channel 6

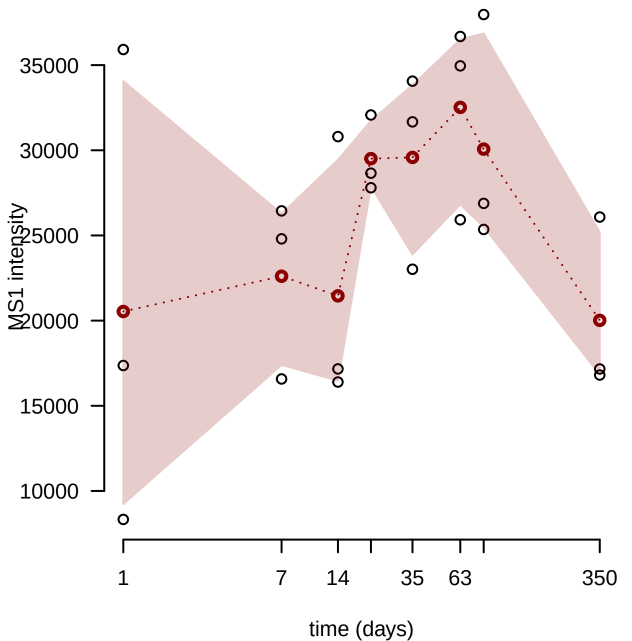

Q8BHC0
GN=Lyve1
lymphatic vessel endothelial hyaluronan ...

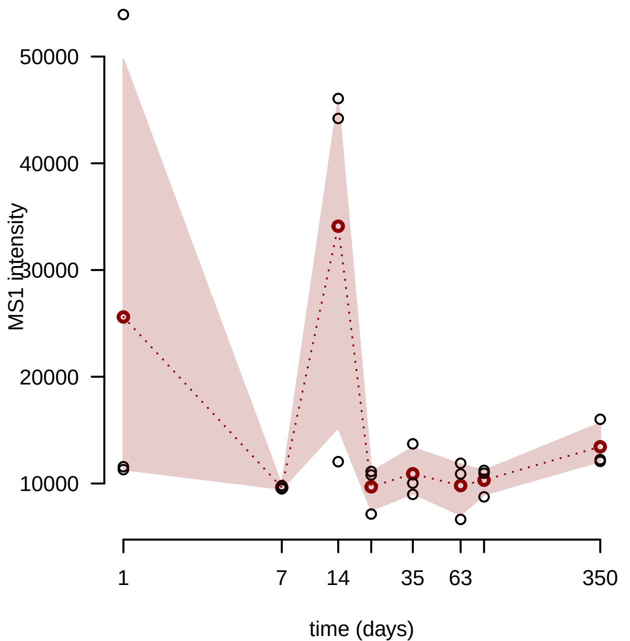

Q8BHC4
GN=Dcakd
dephospho−CoA kinase domain containing

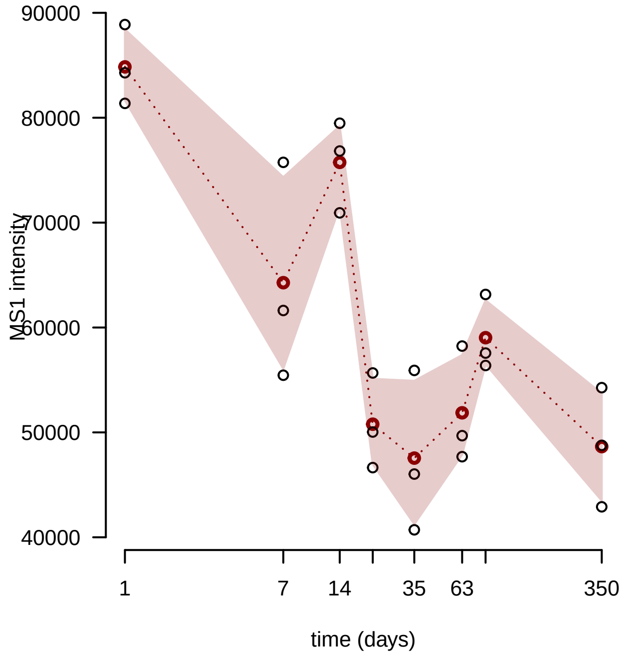

Q8BHC7
GN=Rhbdd1
rhomboid domain containing 1

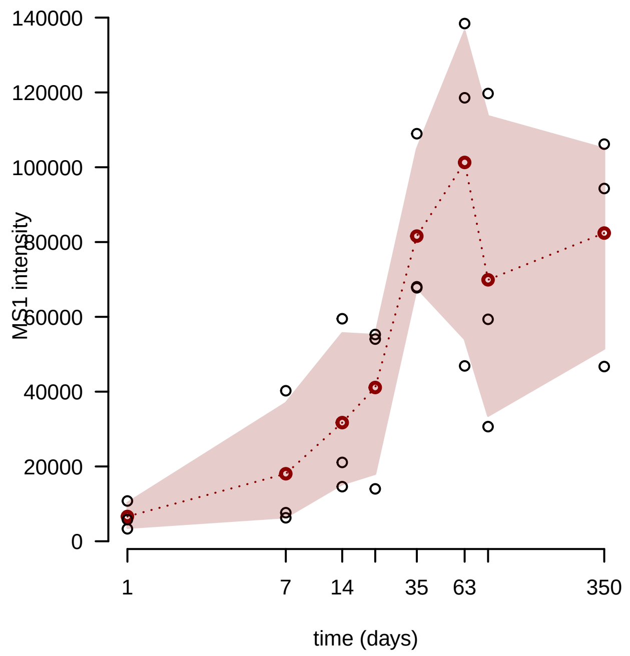

Q8BHC9
GN=Fut11
fucosyltransferase 11

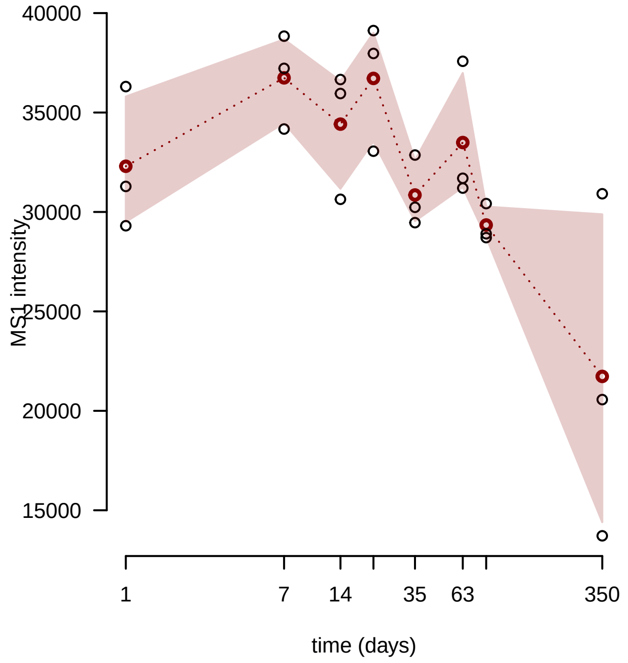

Q8BHD7
GN=Ptbp3
polypyrimidine tract binding protein 3

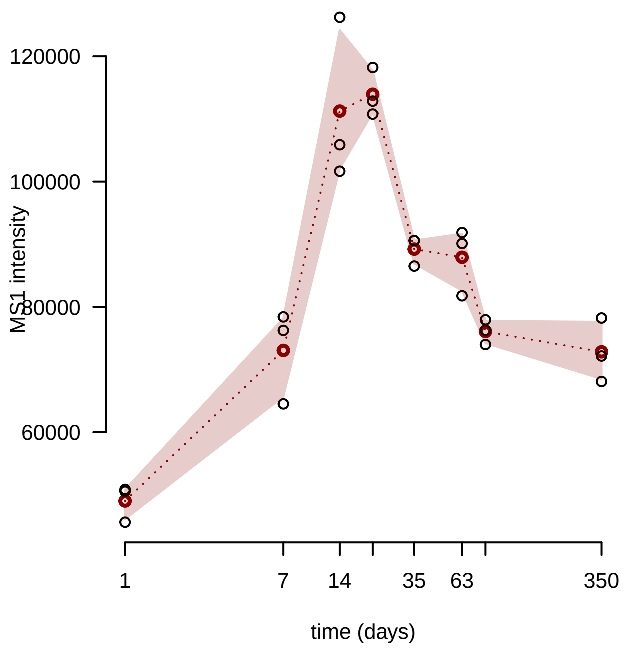

Q8BHD8
GN=Pcmtd2
protein−L−isoaspartate (D−aspartate) O−m ...

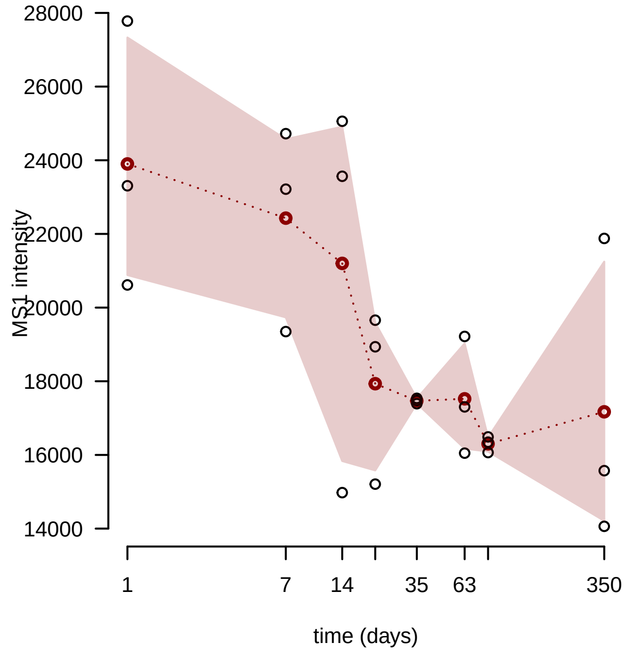

Q8BHE1
GN=Gemin8
gem nuclear organelle associated protein ...

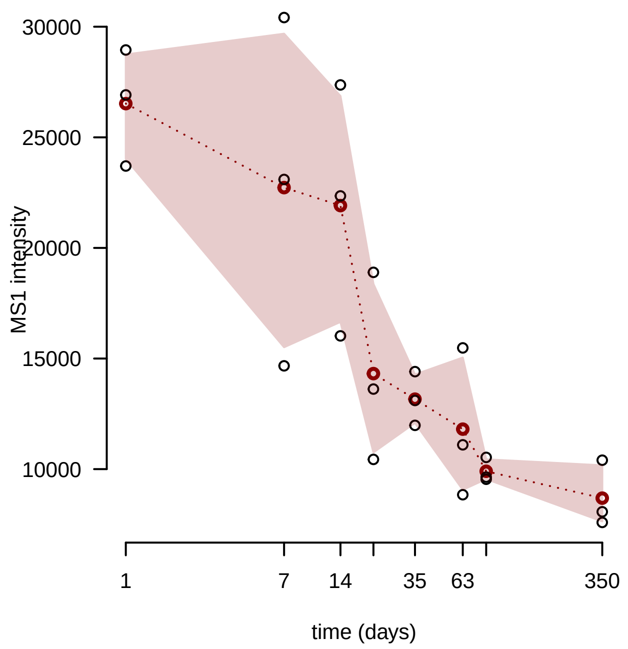

Q8BHE8
GN=Maip1
matrix AAA peptidase interacting protein ...

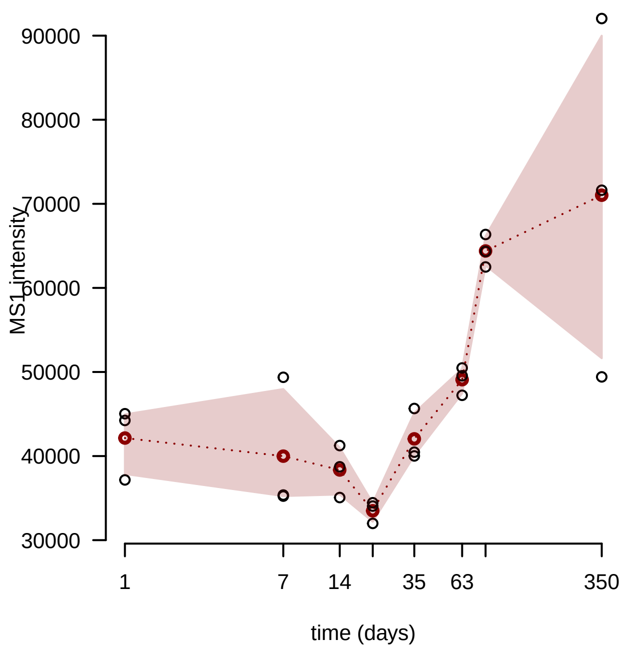

Q8BHF7
GN=Pgs1
phosphatidylglycerophosphate synthase 1

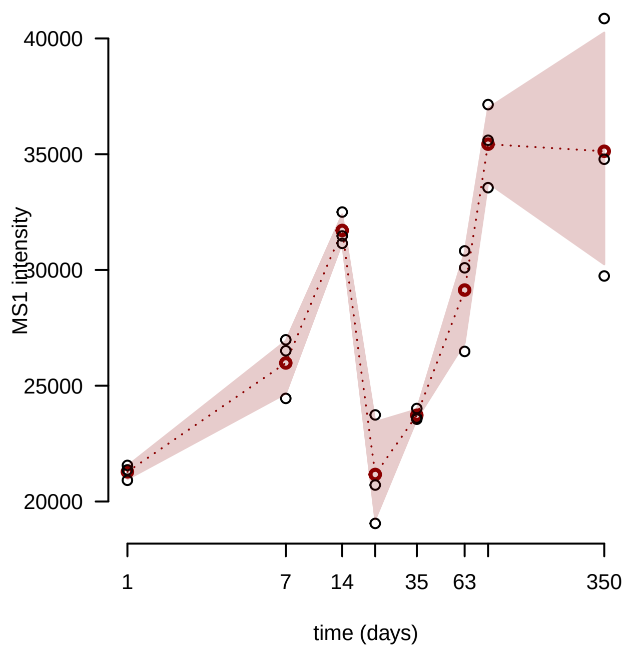

Q8BHG1
GN=Nrdc

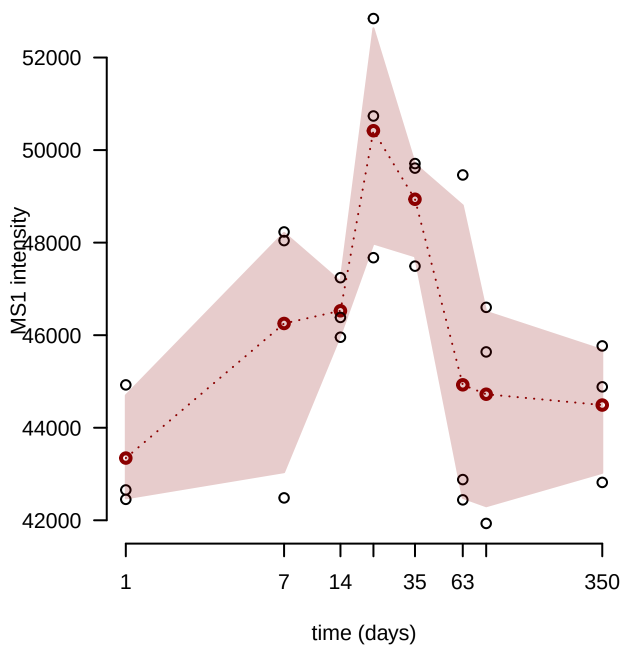

Q8BHG2
GN=Czib
CXXC motif containing zinc binding prote ...

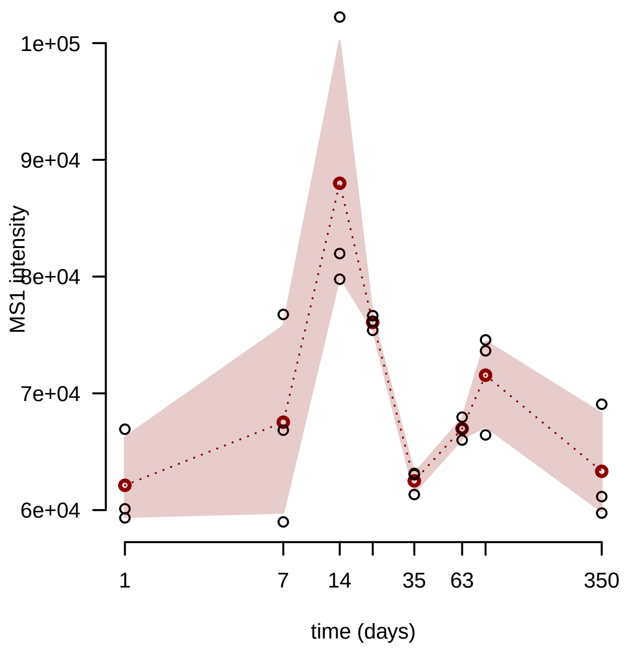

Q8BHG9
GN=Cggbp1
CGG triplet repeat binding protein 1

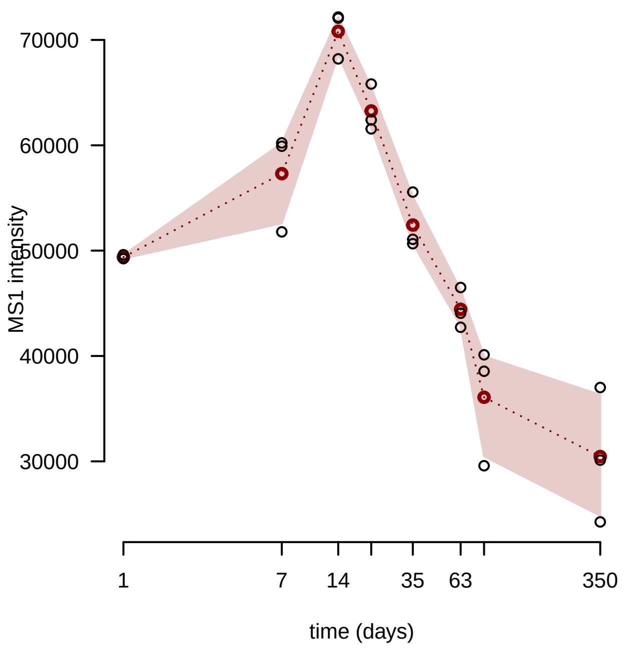

Q8BHI7
GN=Elovl5
ELOVL family member 5, elongation of lon ...

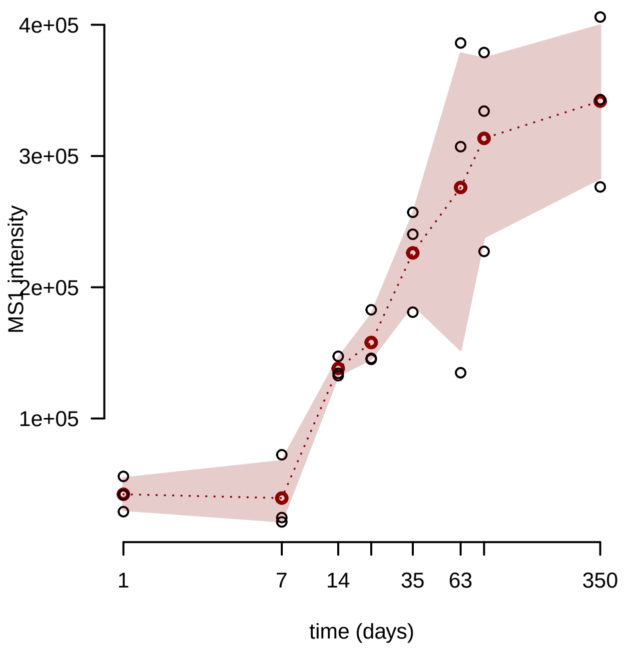

Q8BHJ5
GN=Tbl1xr1
transducin (beta)−like 1X−linked recepto ...

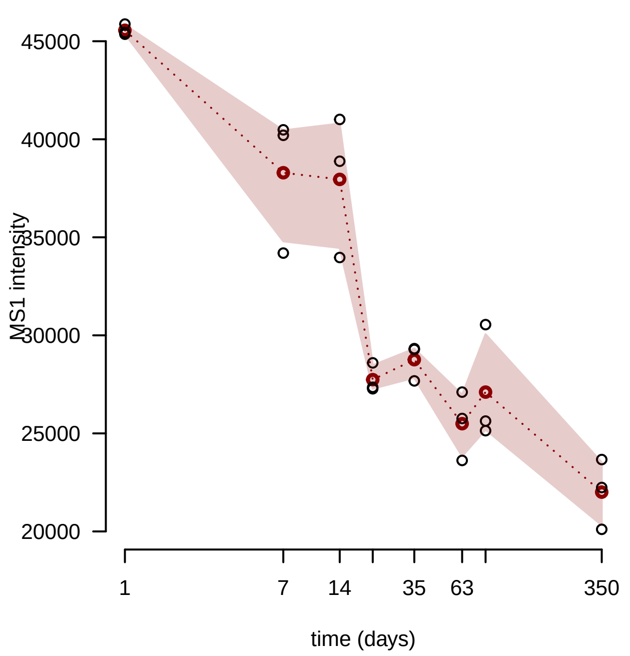

Q8BHJ6
GN=Serinc5
serine incorporator 5

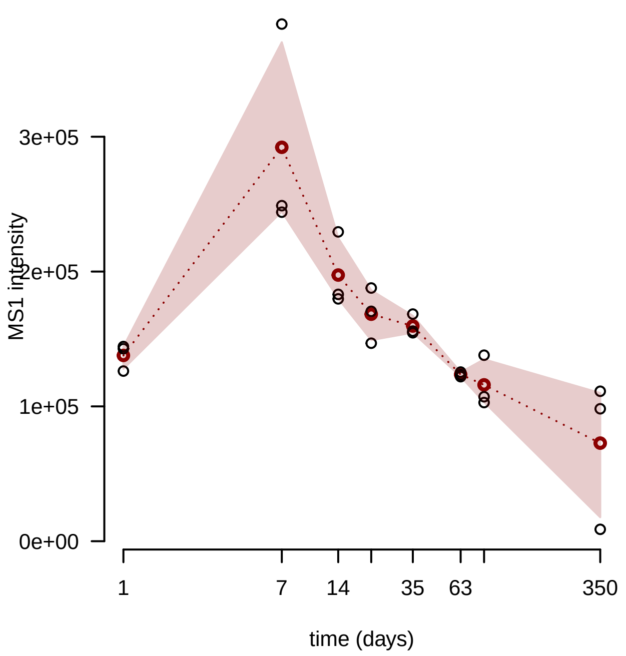

Q8BHJ9
GN=Slu7
SLU7 splicing factor homolog (S. cerevis ...

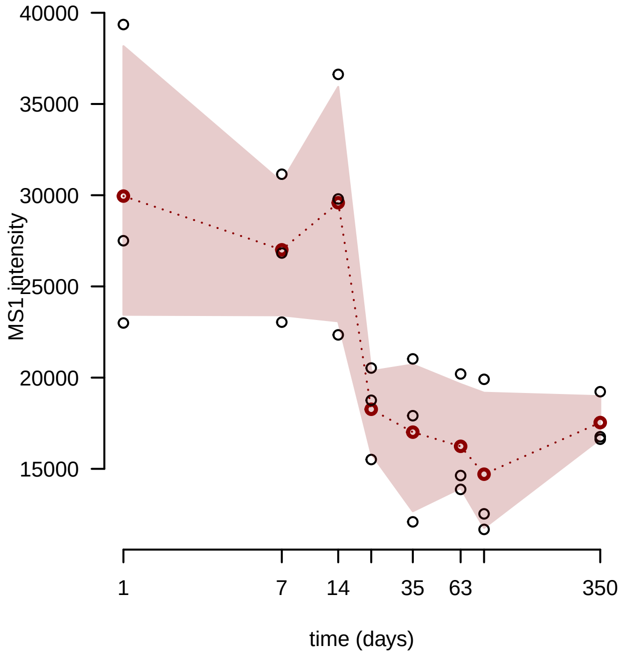

Q8BHK1
GN=Nipa1
non imprinted in Prader−Willi/Angelman s ...

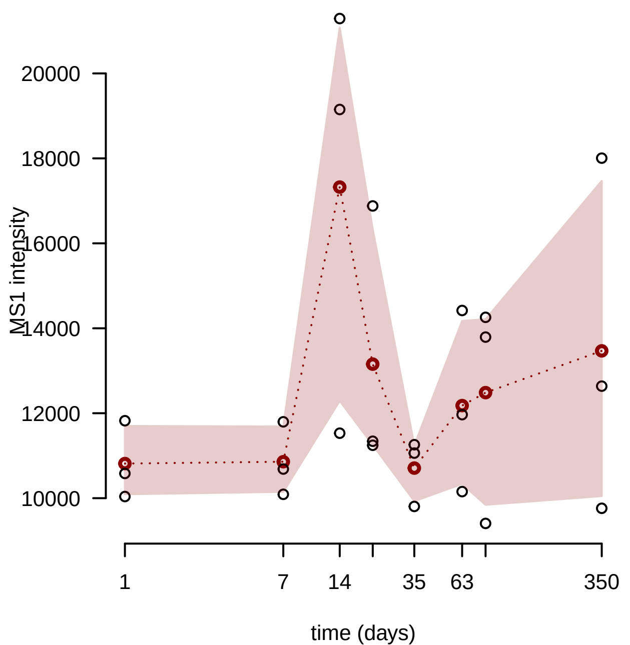

Q8BHK9
GN=Ercc6l
excision repair cross−complementing rode ...

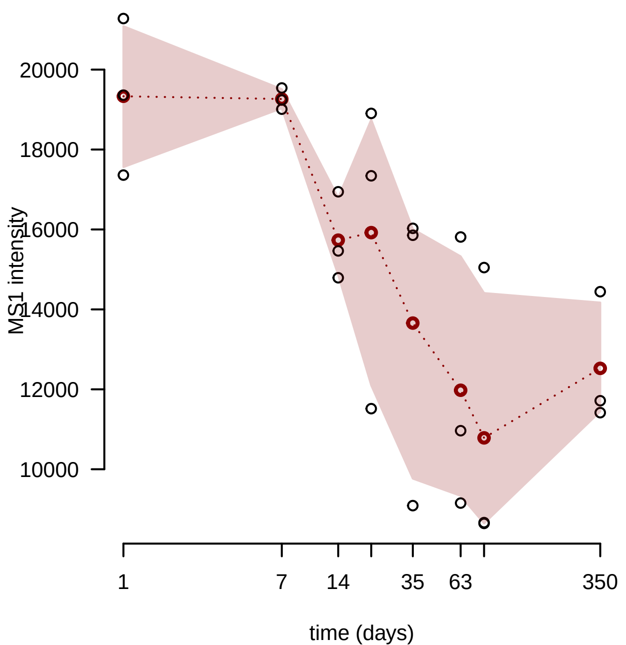

Q8BHL3
GN=Tbc1d10b
TBC1 domain family, member 10b

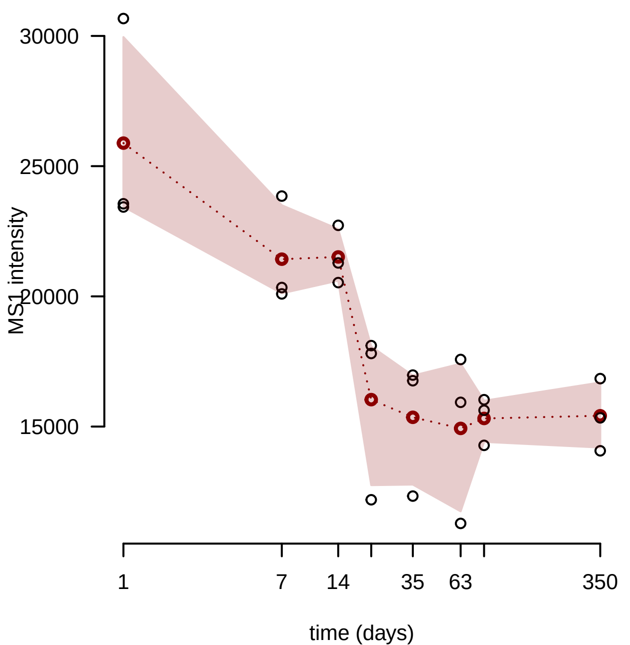

Q8BHL5
GN=Elmo2
engulfment and cell motility 2

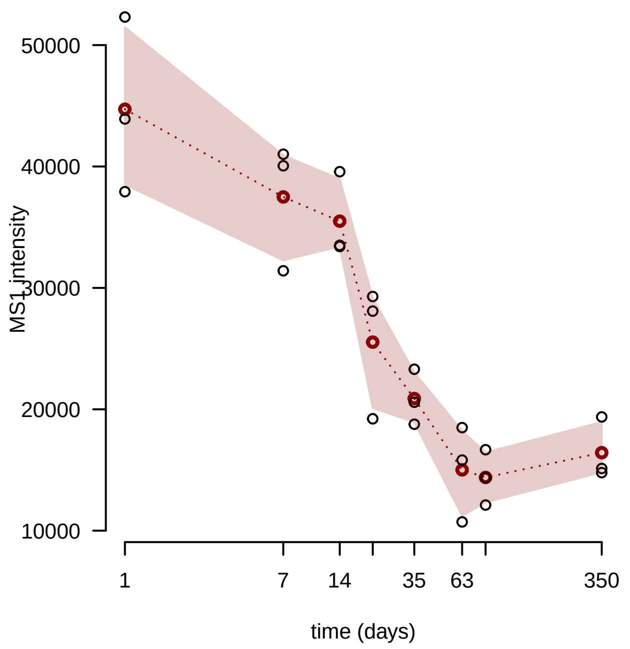

Q8BHL8
GN=Psmf1
proteasome (prosome, macropain) inhibito ...

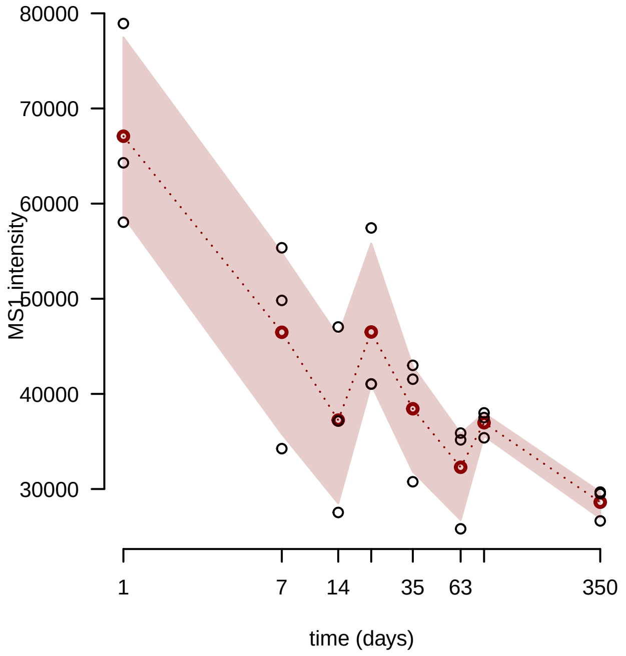

Q8BHN0
GN=Ppm1l
protein phosphatase 1 (formerly 2C)−like

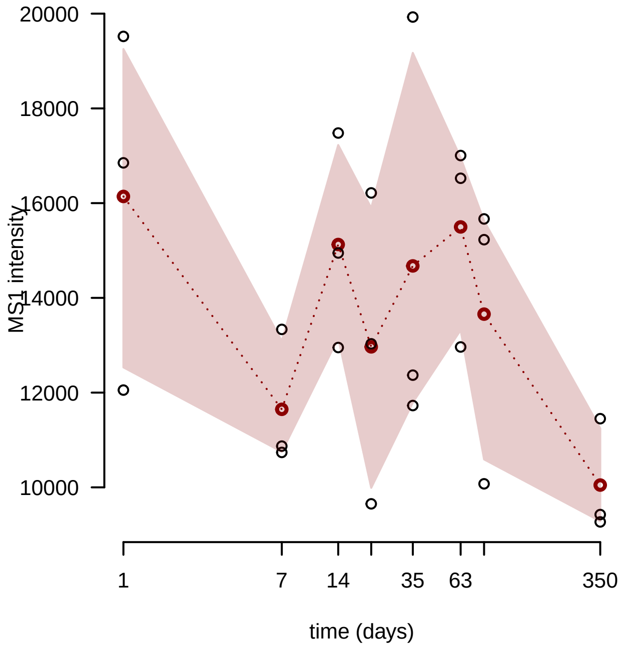

Q8BHN1
GN=Txlng
taxilin gamma

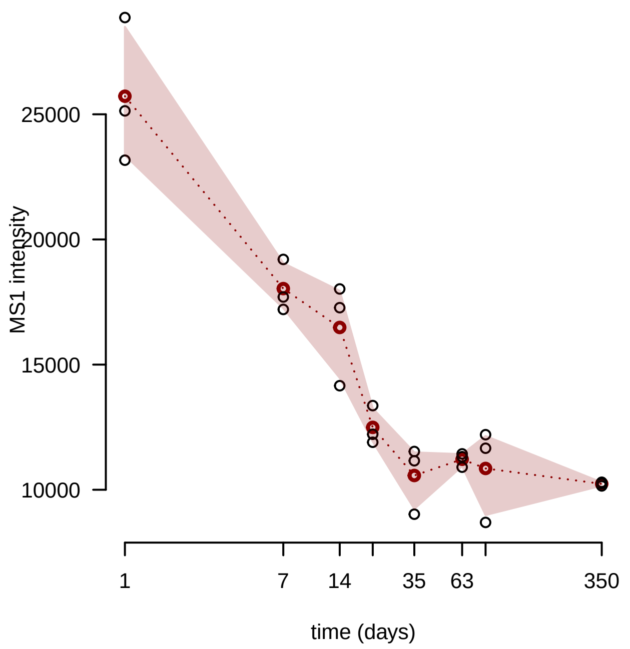

Q8BHN3
GN=Ganab
alpha glucosidase 2 alpha neutral subuni ...

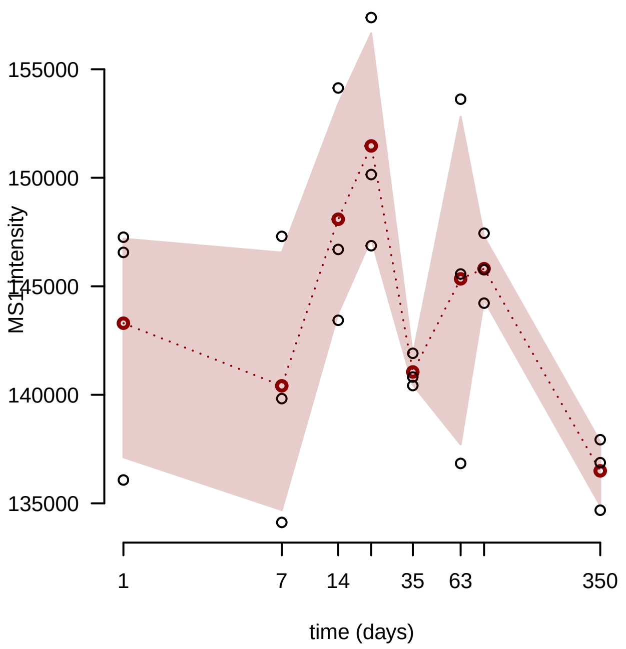

Q8BHN3
GN=Ganab
alpha glucosidase 2 alpha neutral subuni ...

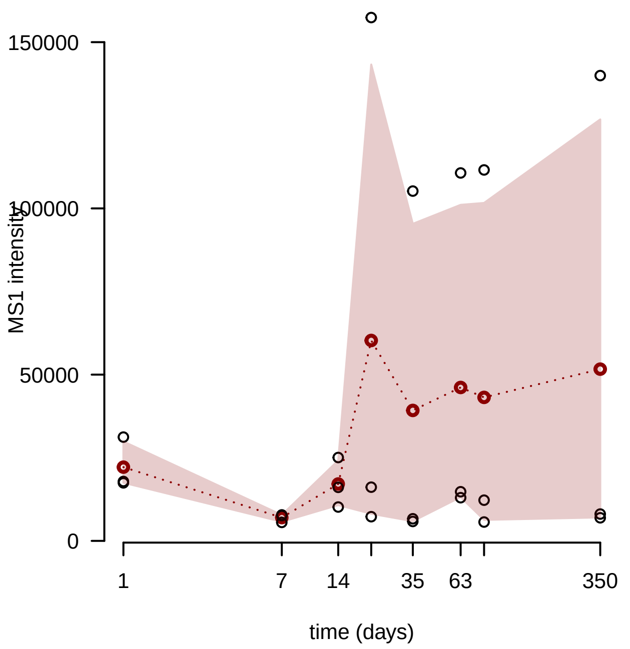

Q8BHN5
GN=Rbm45
RNA binding motif protein 45

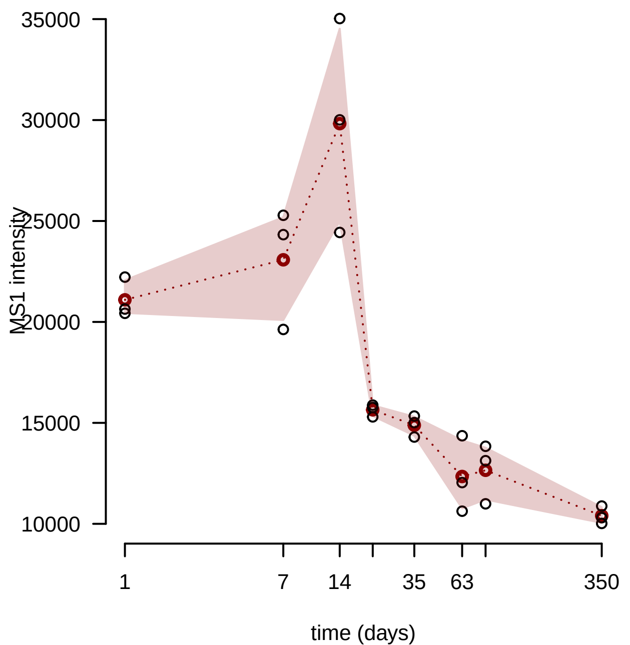

Q8BHN7
GN=

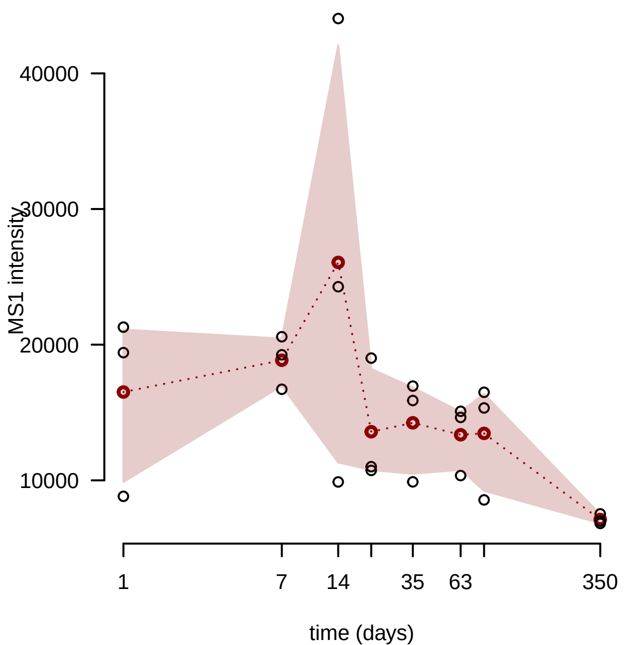

Q8BHS3
GN=Rbm22
RNA binding motif protein 22

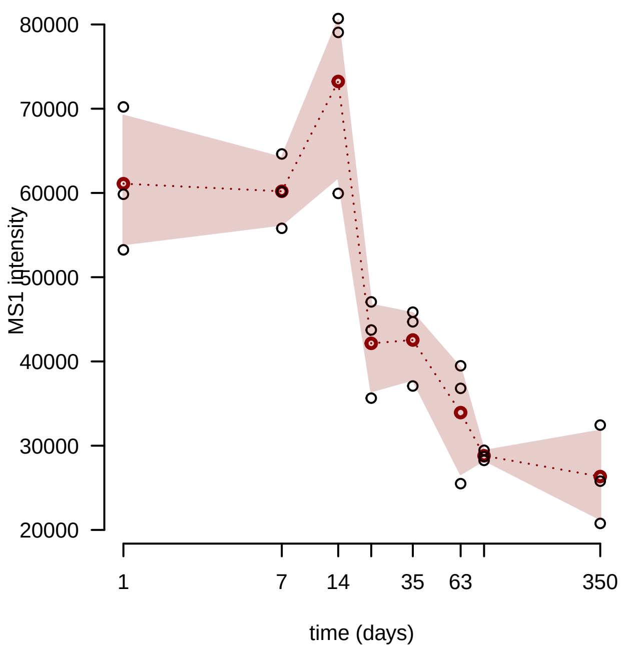

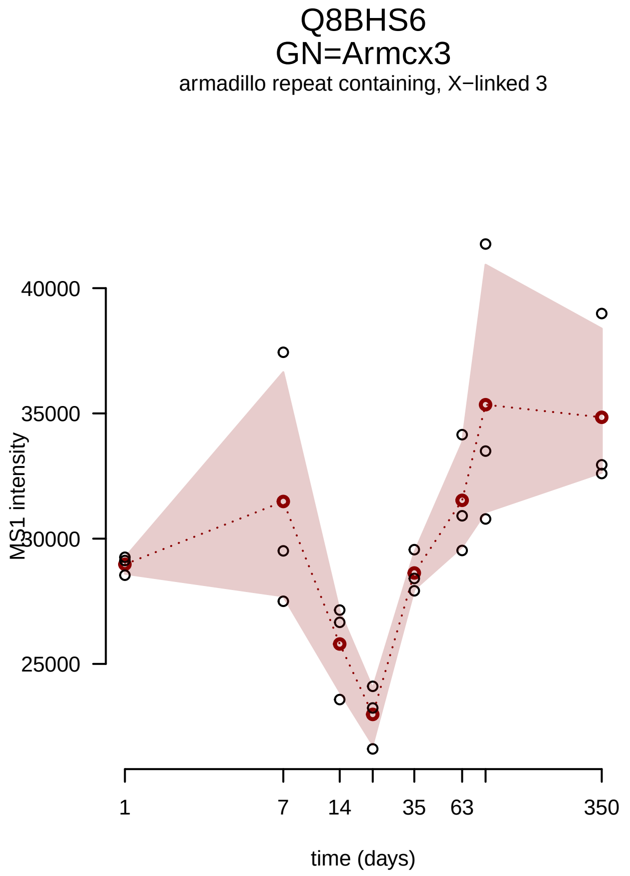
Q8BHS6
GN=Armcx3
armadillo repeat containing, X−linked 3
MS1 intensity
25000
30000
35000
40000
1
7
14
35
63
350
time (days)

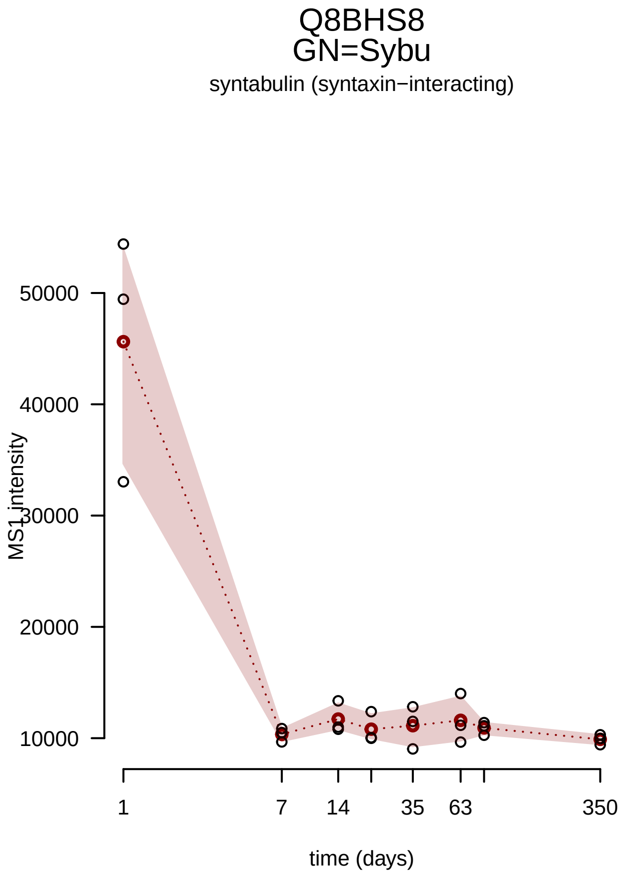
Q8BHS8
GN=Sybu
syntabulin (syntaxin−interacting)
MS1 intensity
10000
20000
30000
40000
50000
1
7
14
35
63
350
time (days)

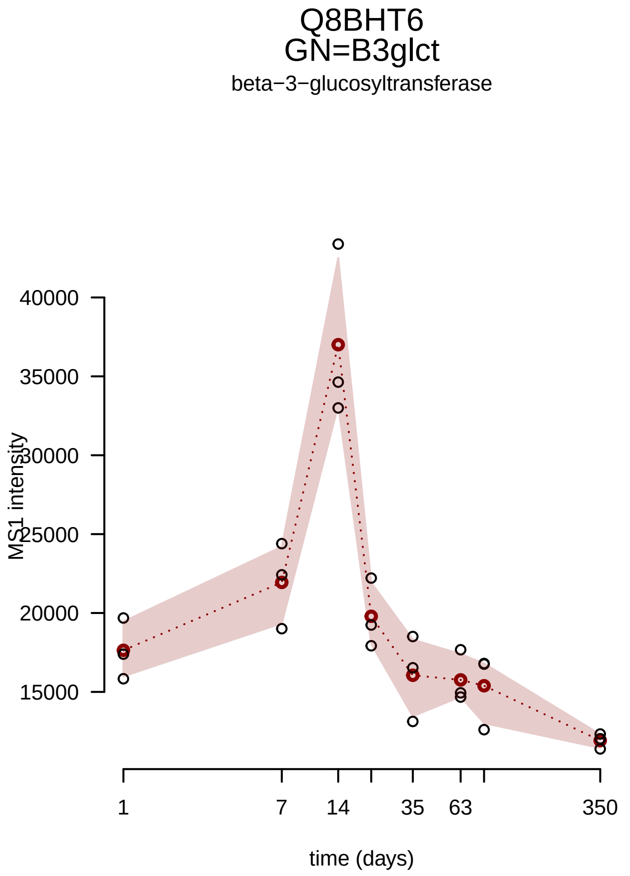
Q8BHT6
GN=B3glct
beta−3−glucosyltransferase
MS1 intensity
15000
20000
25000
30000
35000
40000
1
7
14
35
63
350
time (days)

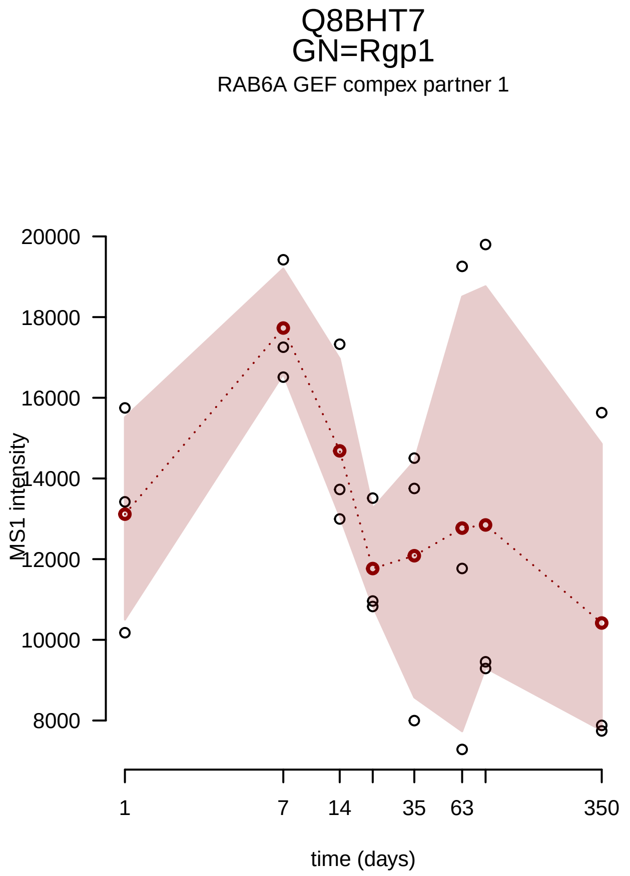
Q8BHT7
GN=Rgp1
RAB6A GEF compex partner 1
MS1 intensity
8000
10000
12000
14000
16000
18000
20000
1
7
14
35
63
350
time (days)

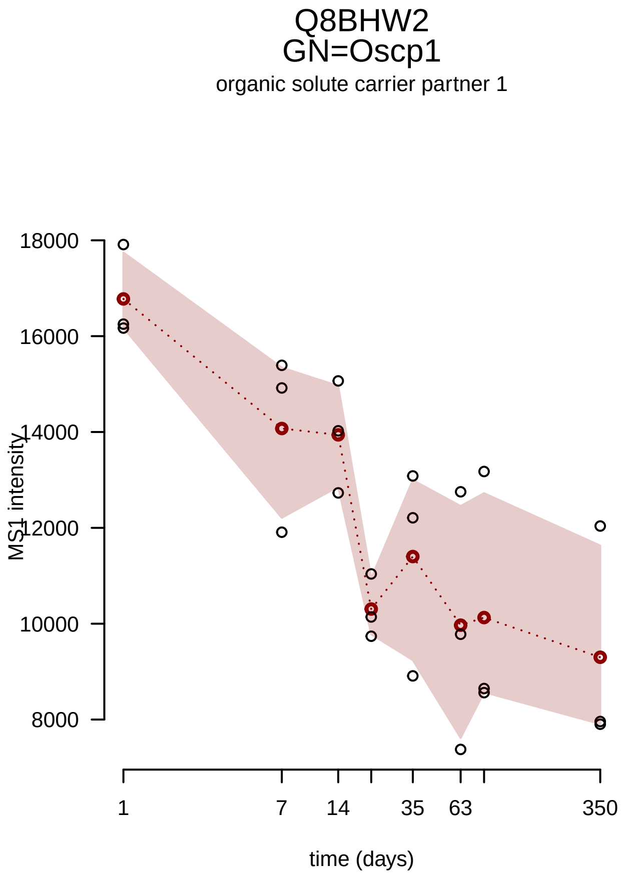
Q8BHW2
GN=Oscp1
organic solute carrier partner 1
MS1 intensity
8000
10000
12000
14000
16000
18000
1
7
14
35
63
350
time (days)

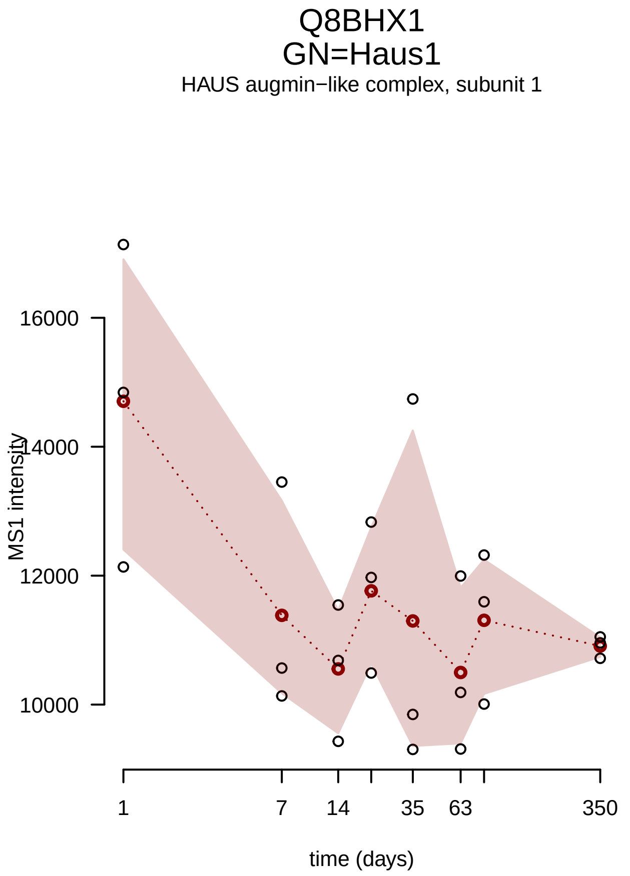
Q8BHX1
GN=Haus1
HAUS augmin−like complex, subunit 1
MS1 intensity
10000
12000
14000
16000
1
7
14
35
63
350
time (days)

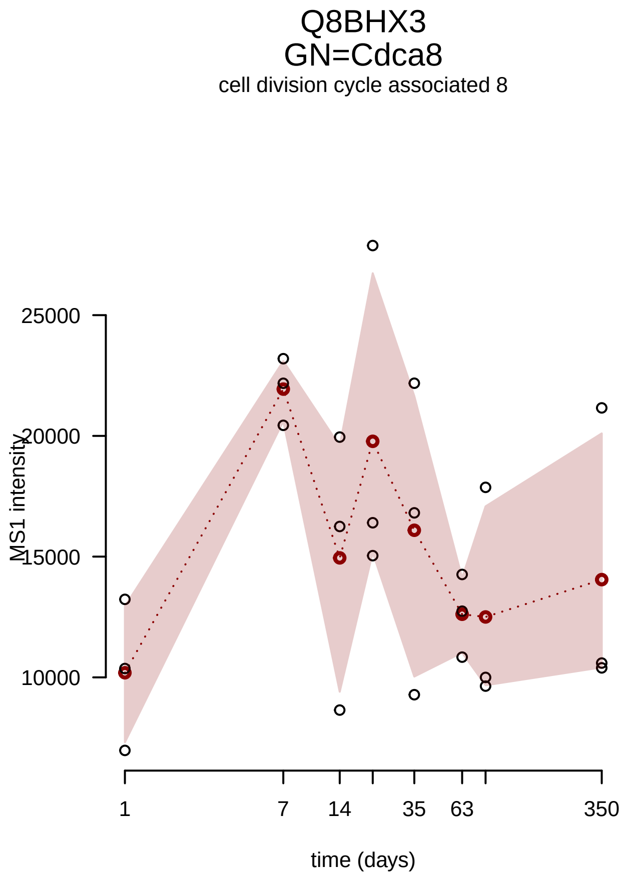
Q8BHX3
GN=Cdca8
cell division cycle associated 8
MS1 intensity
10000
15000
20000
25000
1
7
14
35
63
350
time (days)

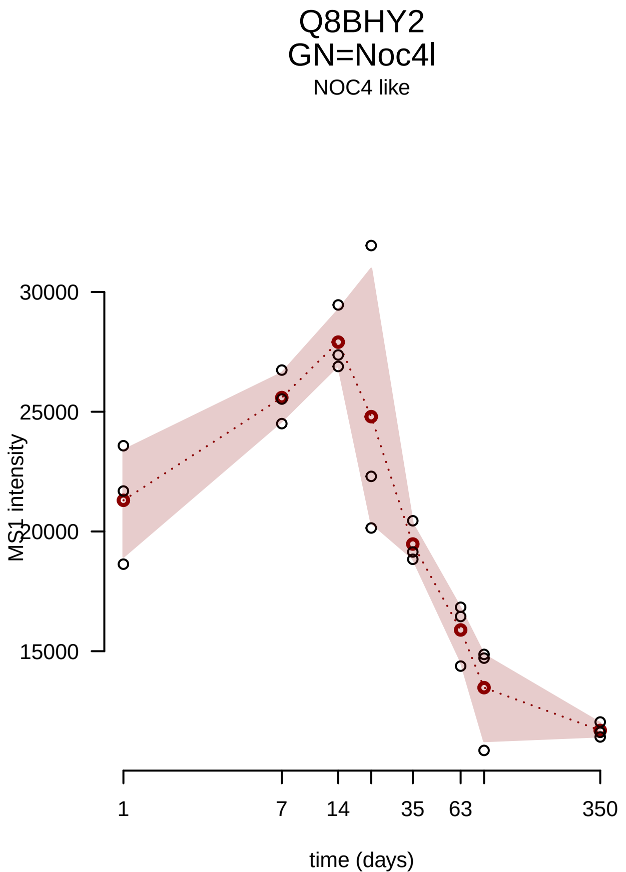
Q8BHY2
GN=Noc4l
NOC4 like
MS1 intensity
15000
20000
25000
30000
1
7
14
35
63
350
time (days)

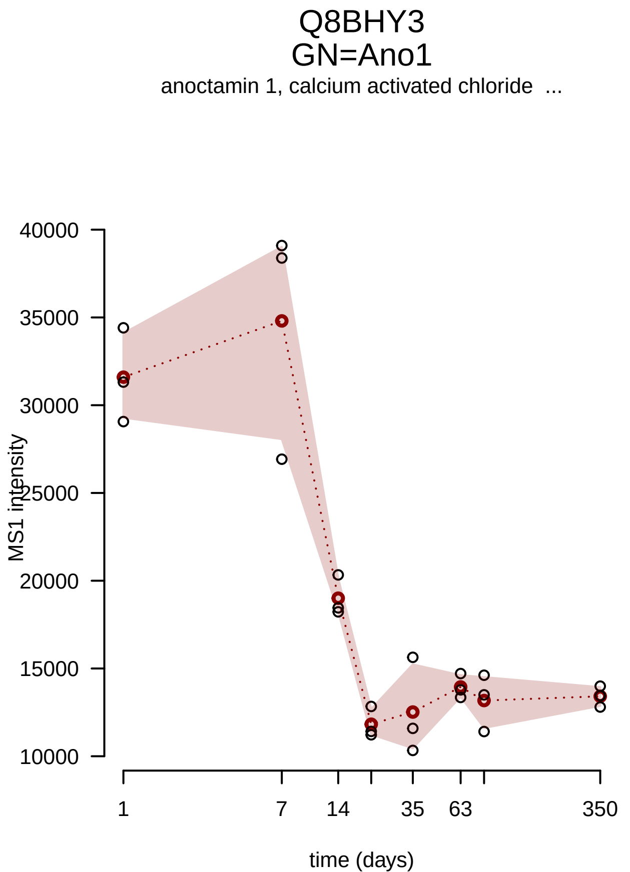
Q8BHY3
GN=Ano1
anoctamin 1, calcium activated chloride ...
MS1 intensity
10000
15000
20000
25000
30000
35000
40000
1
7
14
35
63
350
time (days)

Q8BHY8
GN=Snx14
sorting nexin 14

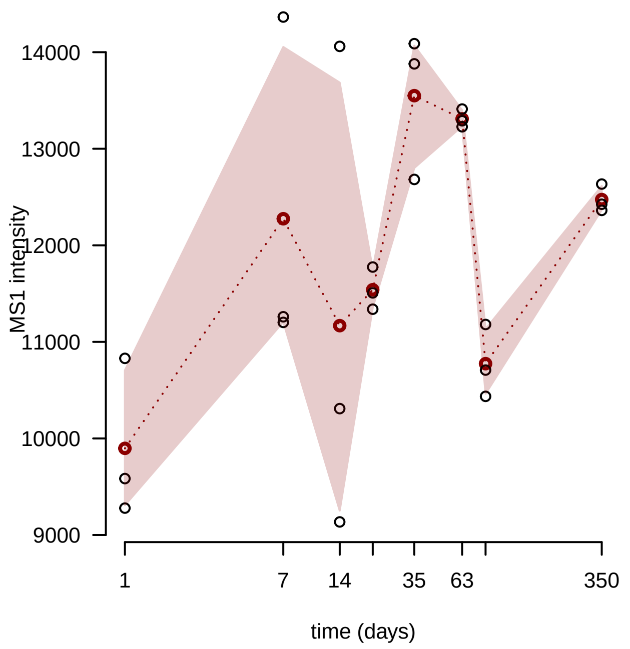

Q8BHZ0
GN=Fam49a

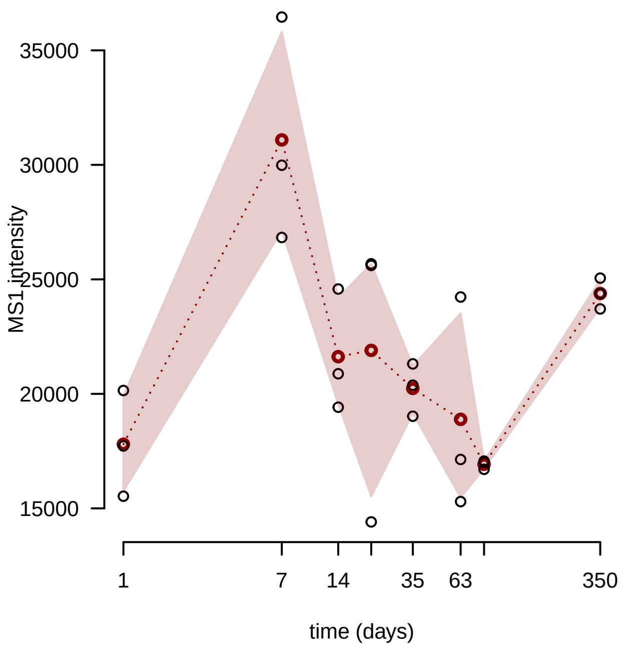

Q8BHZ4
GN=Znf592

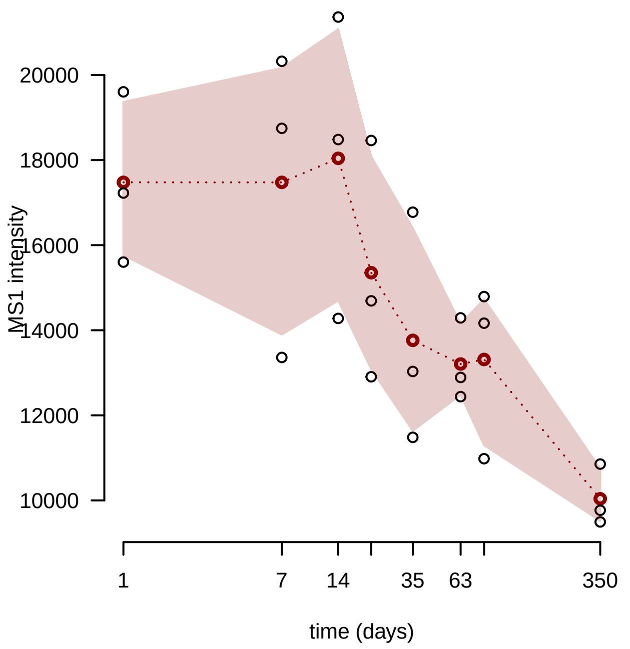

Q8BI06
GN=Cemip
cell migration inducing protein, hyaluro ...

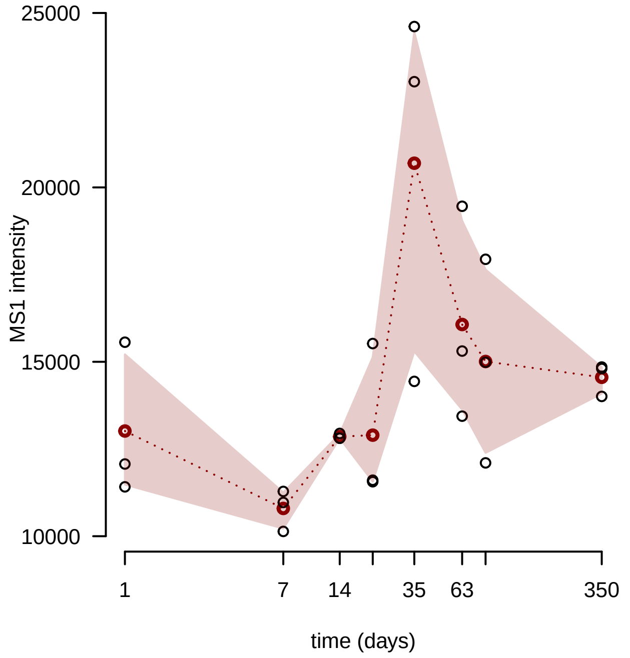

Q8BI08
GN=Mal2
mal, T cell differentiation protein 2

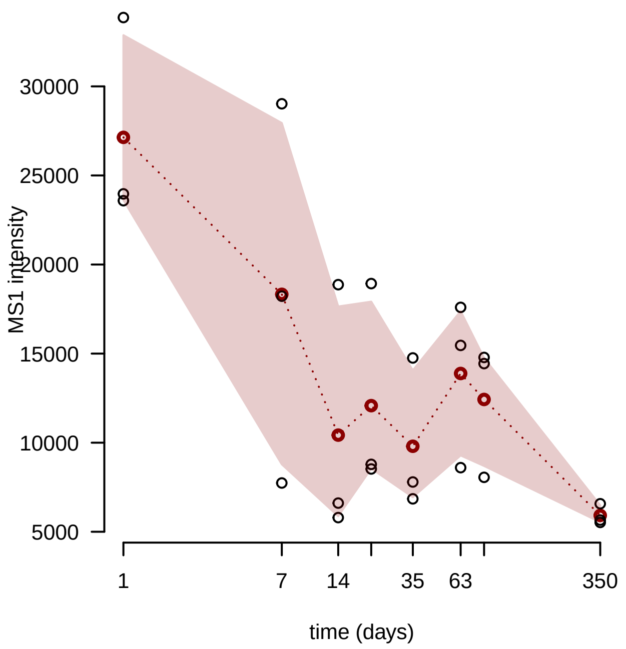

Q8BI72
GN=Cdkn2aip
CDKN2A interacting protein

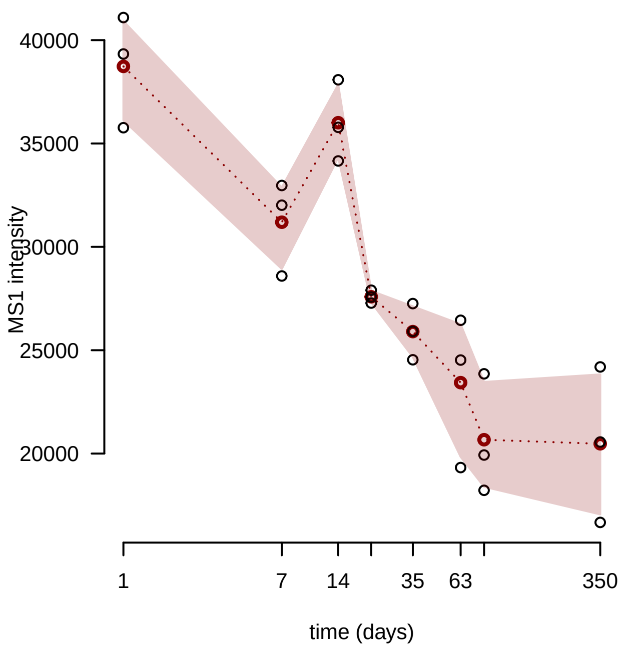

Q8BI84
GN=Mia3
MIA SH3 domain ER export factor 3

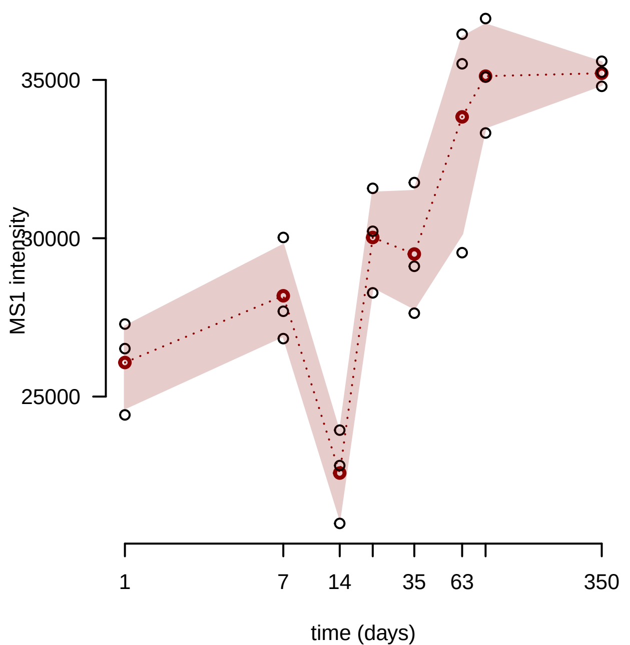

Q8BIA4
GN=Fbxw8
F−box and WD−40 domain protein 8

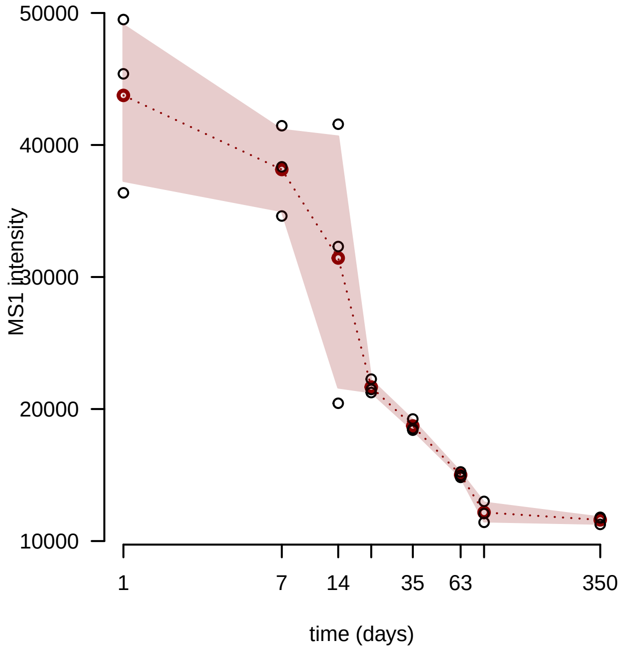

Q8BIE6
GN=Frmd4a
FERM domain containing 4A

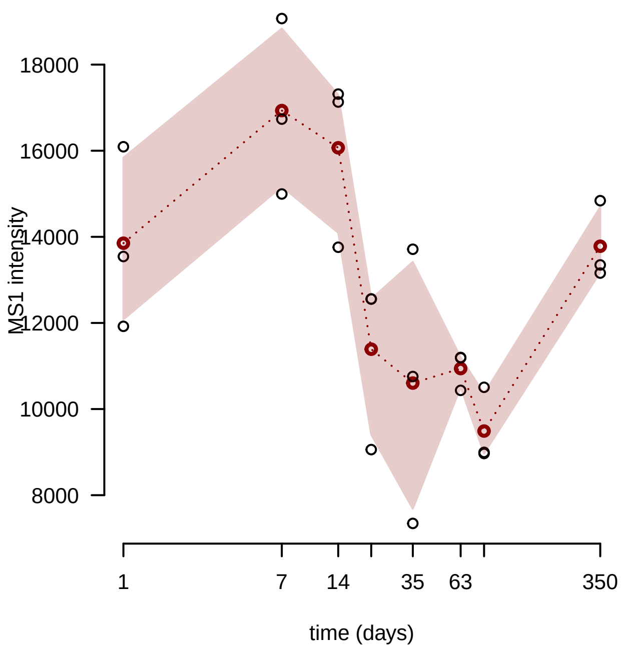

Q8BIF0
GN=Cd99l2
CD99 antigen−like 2

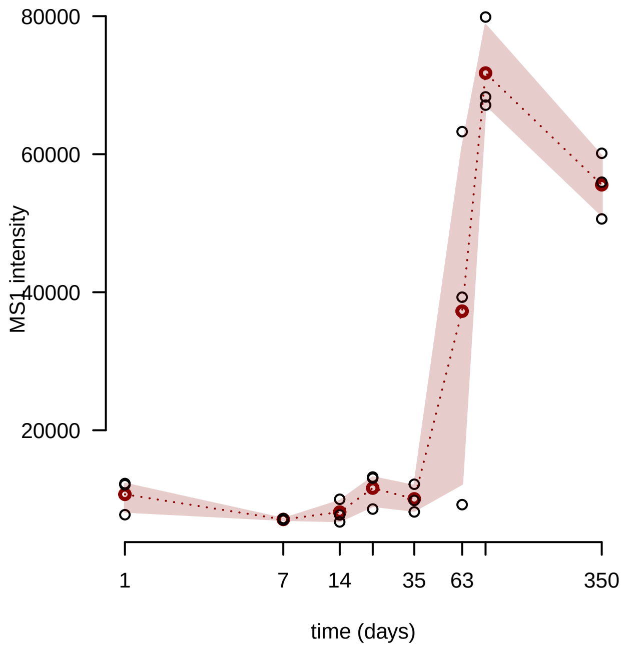

Q8BIF9
GN=Znf787

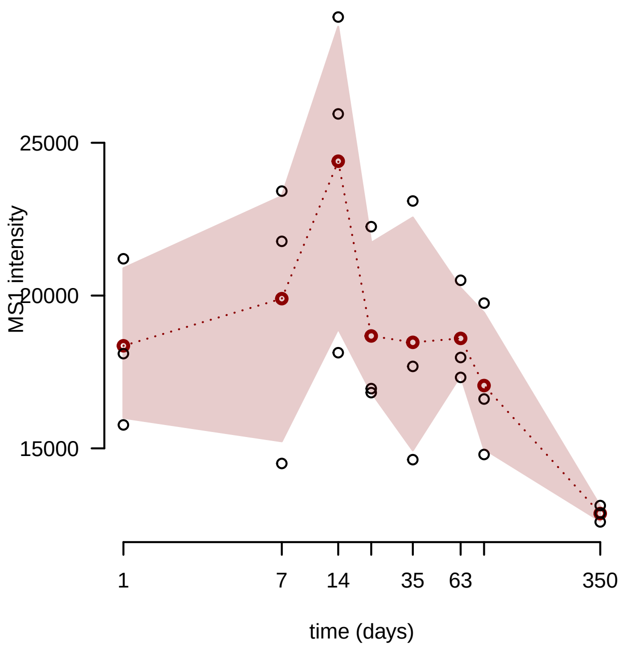

Q8BIG4
GN=Fbxo28
F−box protein 28

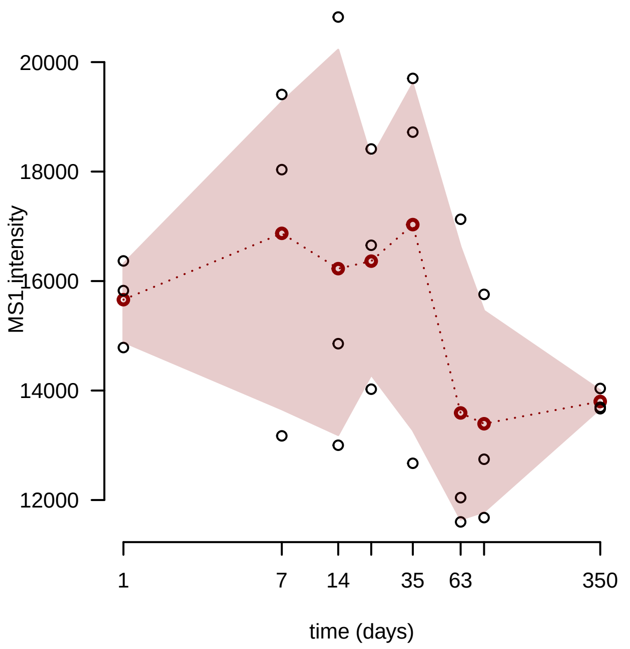

Q8BIG7
GN=Comtd1
catechol−O−methyltransferase domain cont ...

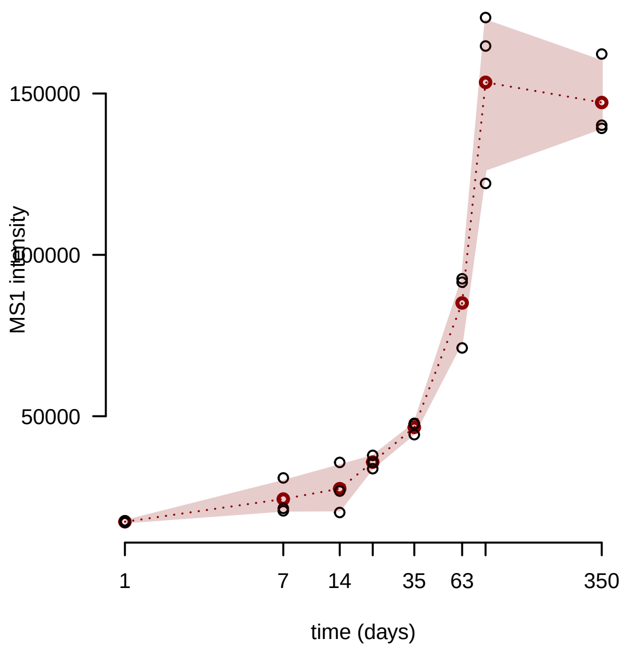

Q8BIH0
GN=Sap130
Sin3A associated protein

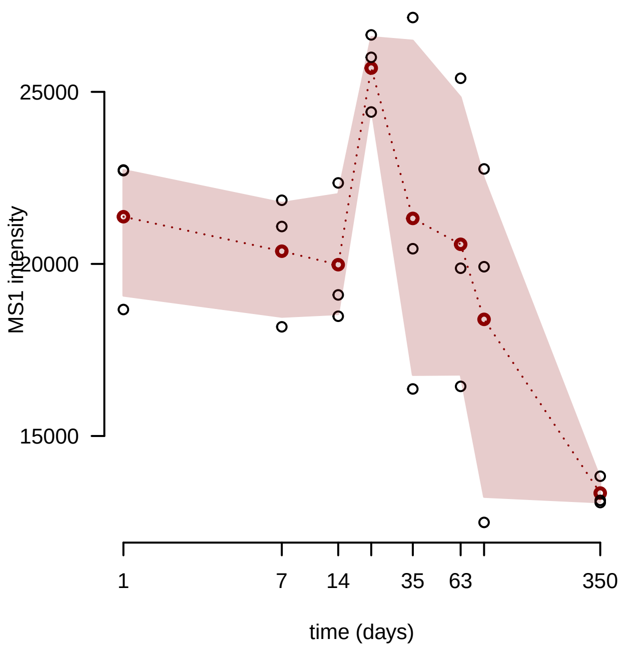

Q8BIJ6
GN=Iars2
isoleucine−tRNA synthetase 2, mitochondr ...

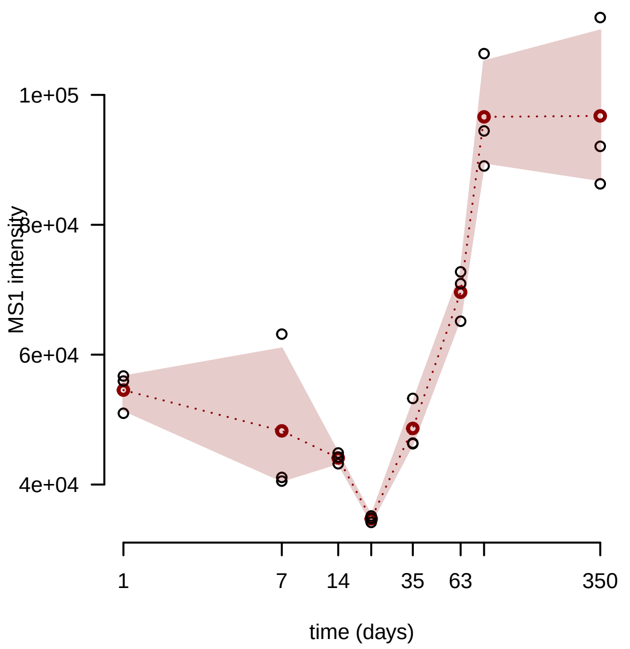

Q8BIJ7
GN=Rufy1
RUN and FYVE domain containing 1

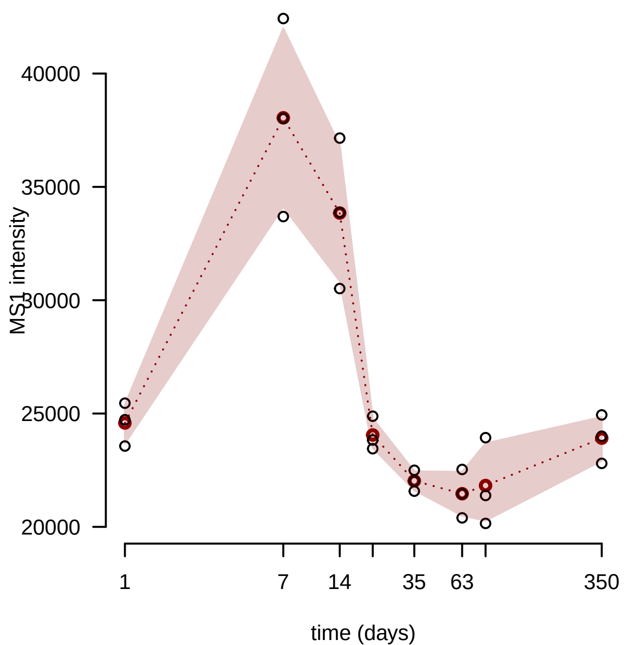

Q8BIK4
GN=Dock9
dedicator of cytokinesis 9

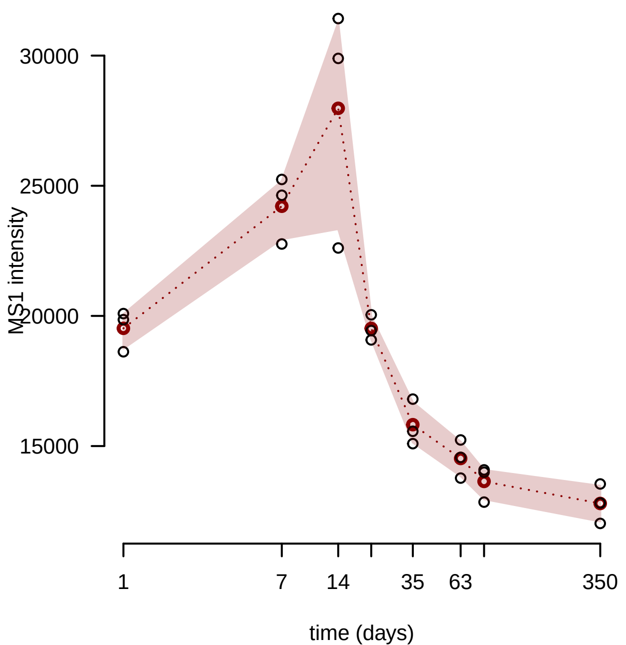

Q8BIL5
GN=Hook1
hook microtubule tethering protein 1

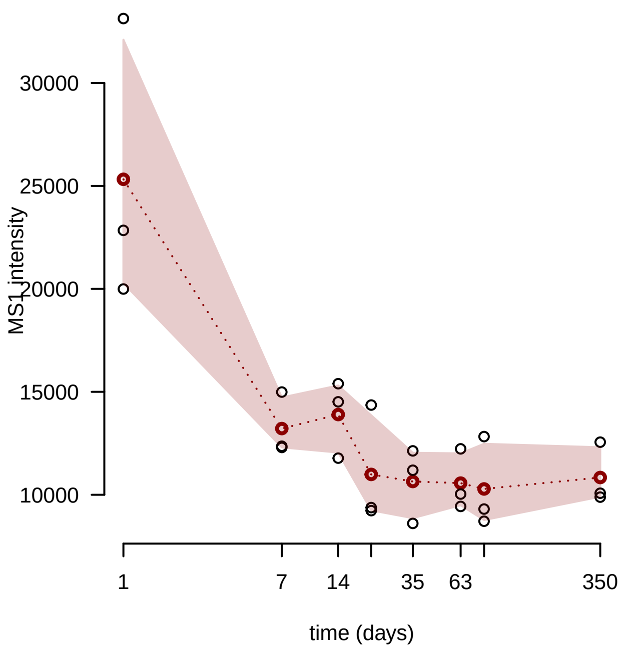

Q8BIP0
GN=Dars2
aspartyl−tRNA synthetase 2 (mitochondria ...

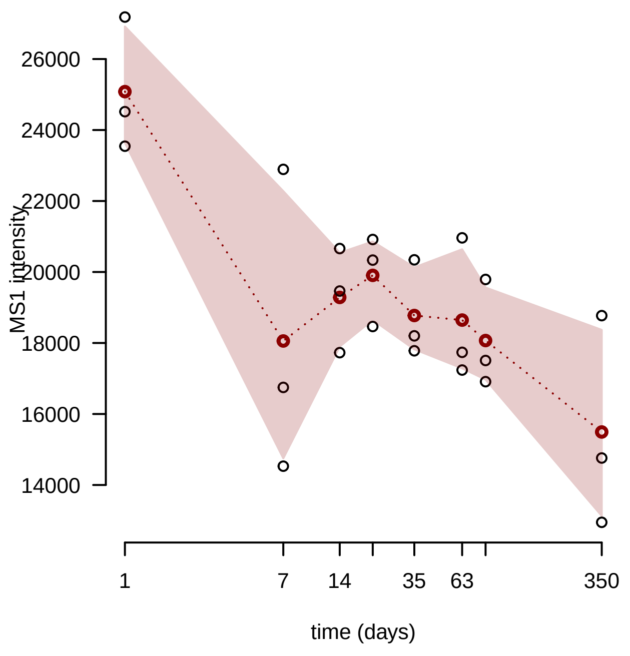

Q8BIQ5
GN=Cstf2
cleavage stimulation factor, 3' pre−RNA ...

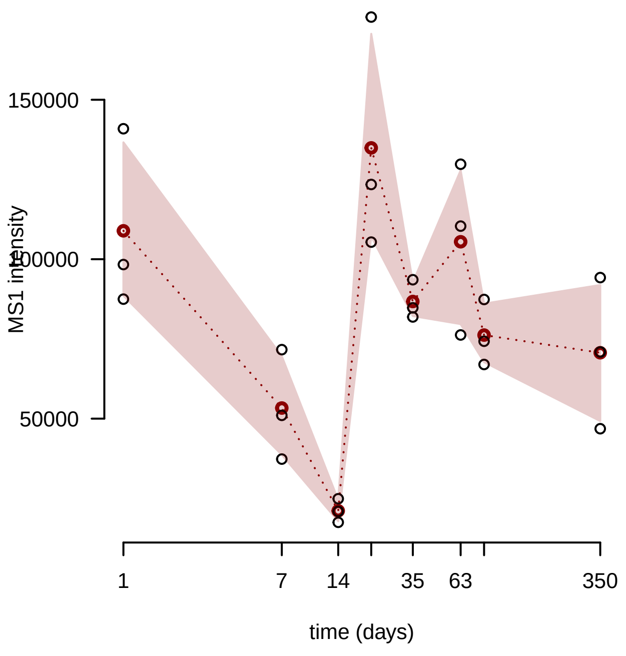

Q8BIS8
GN=Ccdc126
coiled−coil domain containing 126

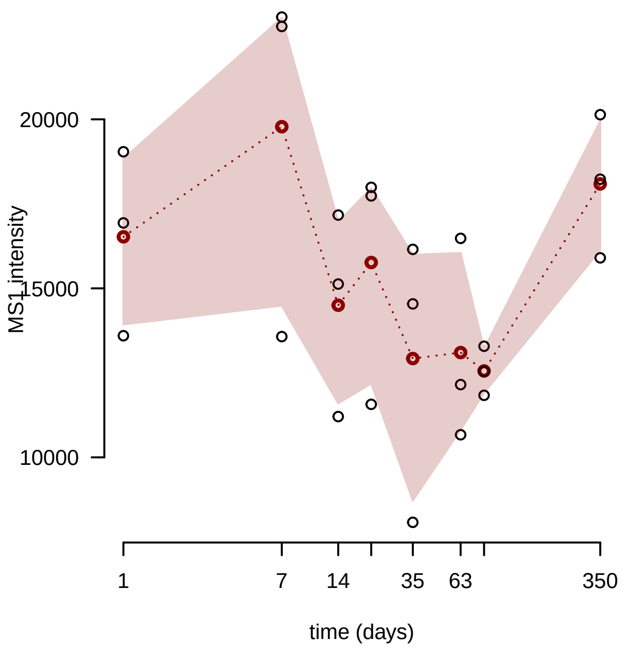

Q8BIV3
GN=Ranbp6
RAN binding protein 6

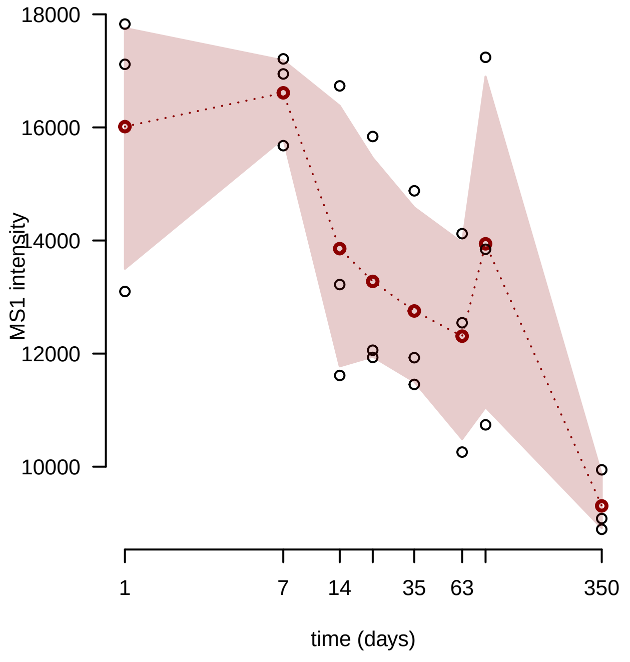

Q8BIW1
GN=Prune1
prune exopolyphosphatase

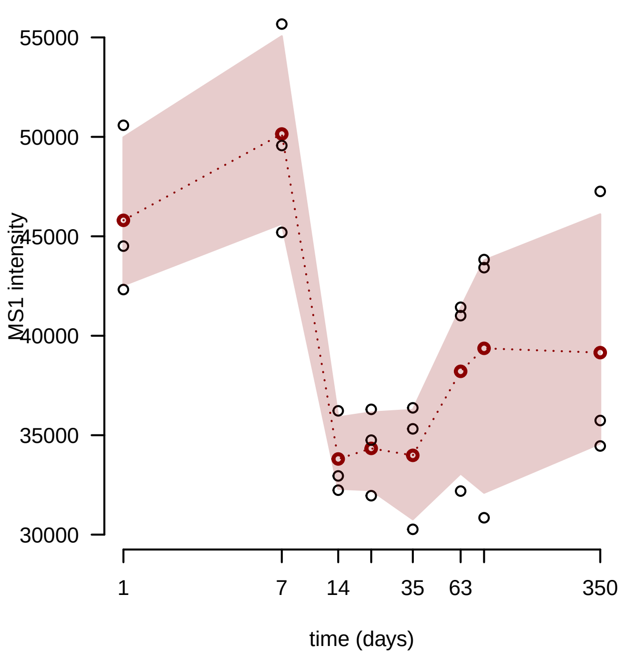

Q8BIW9
GN=Chtf18
CTF18, chromosome transmission fidelity ...

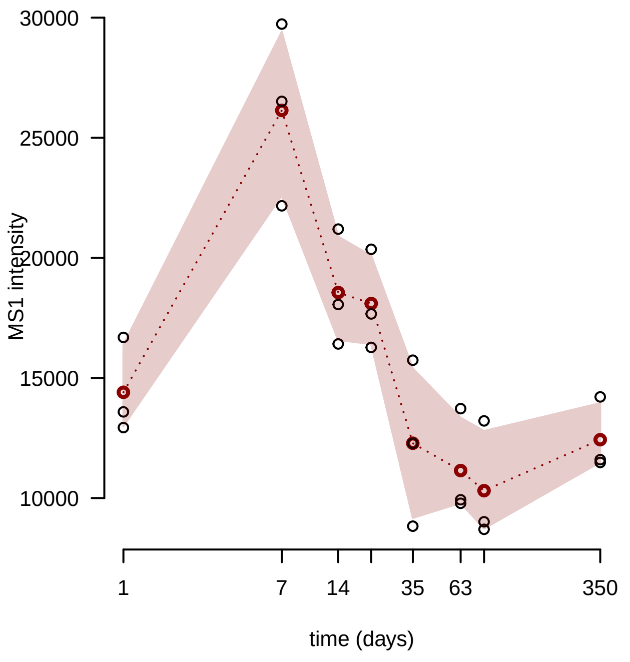

Q8BIX3
GN=Arl14ep
ADP−ribosylation factor−like 14 effector ...

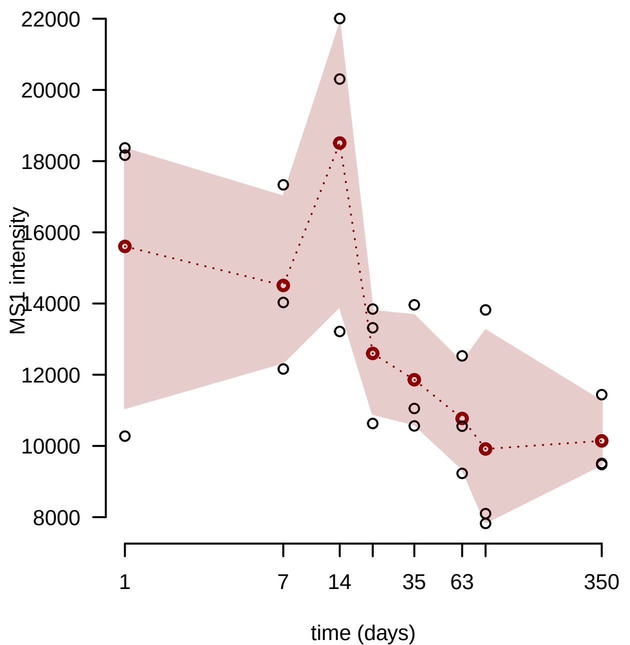

Q8BIZ6
GN=Snip1
Smad nuclear interacting protein 1

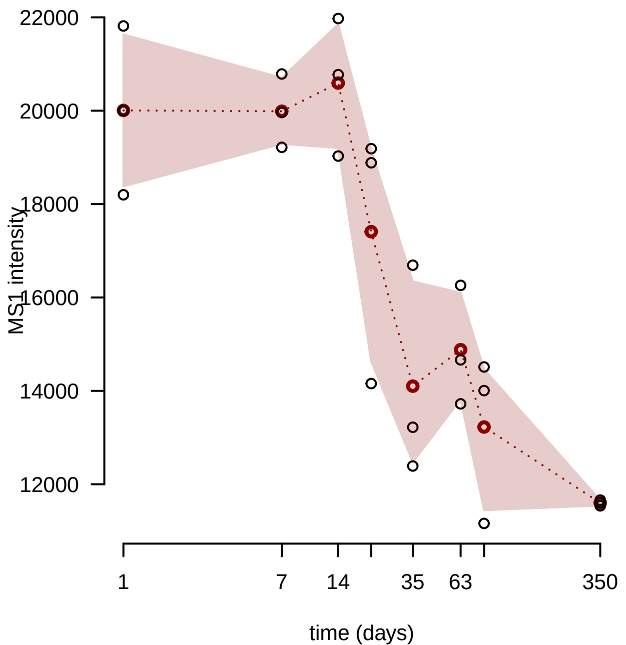

Q8BJ03
GN=Cox15
cytochrome c oxidase assembly protein 15

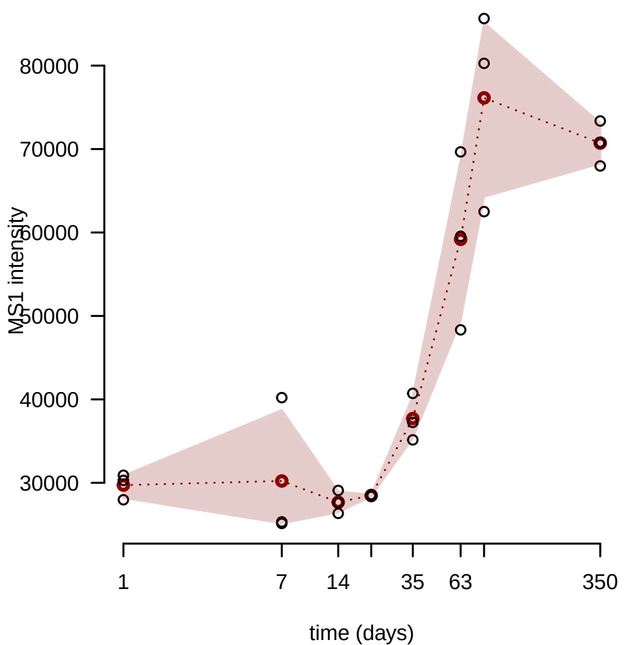

Q8BJ05
GN=Zc3h14
zinc finger CCCH type containing 14

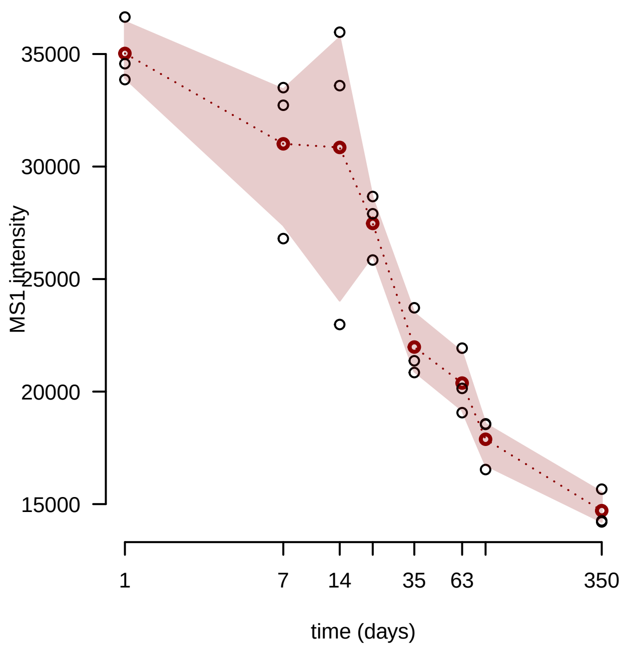

Q8BJ34
GN=Marf1
meiosis regulator and mRNA stability 1

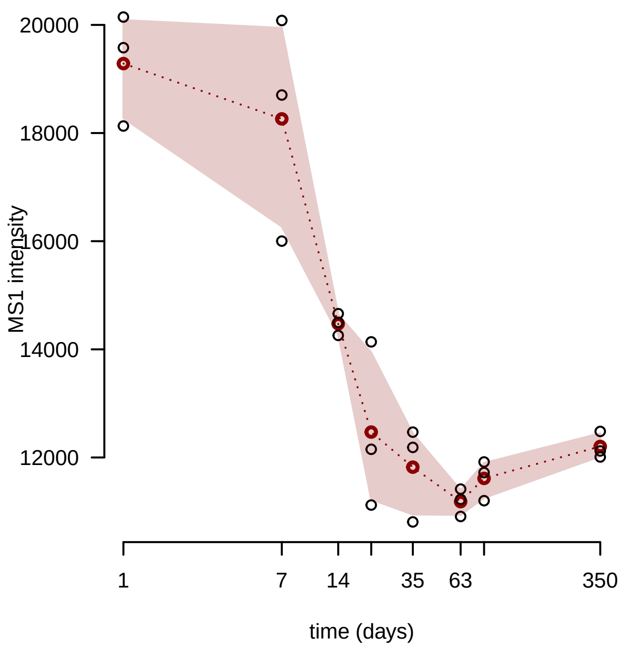

Q8BJ37
GN=Tdp1
tyrosyl−DNA phosphodiesterase 1

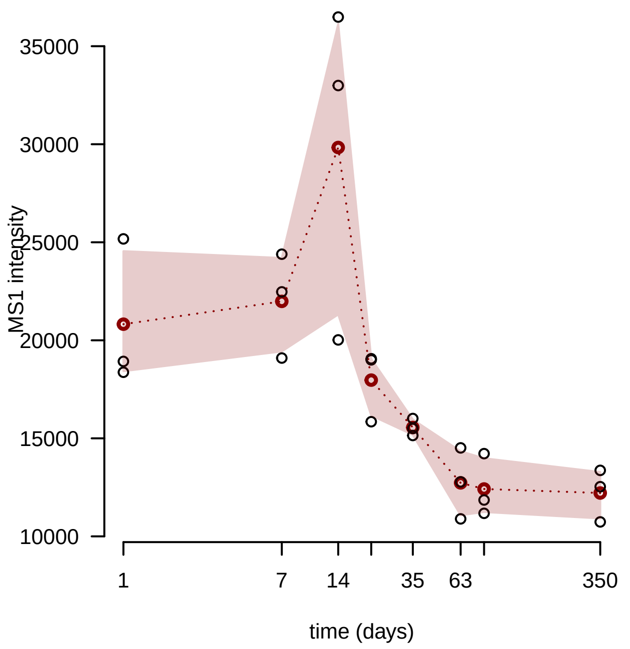

Q8BJ48
GN=Nagpa
N−acetylglucosamine−1−phosphodiester alp ...

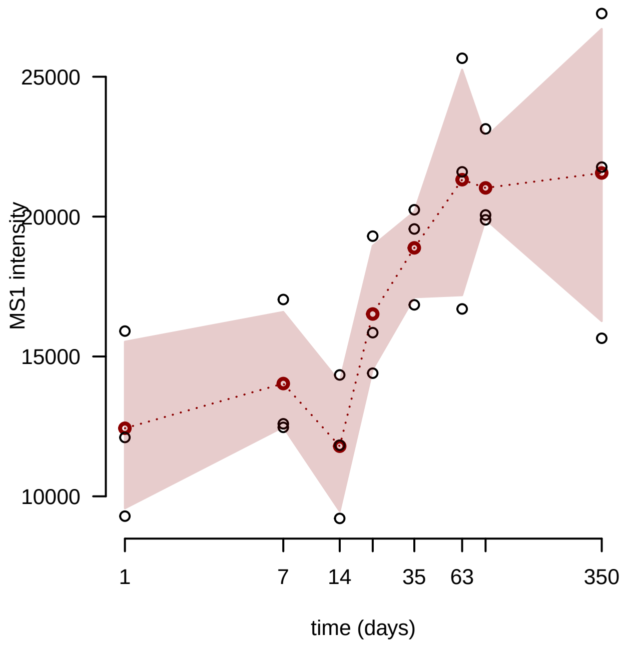

Q8BJ56
GN=Pnpla2
patatin−like phospholipase domain contai ...

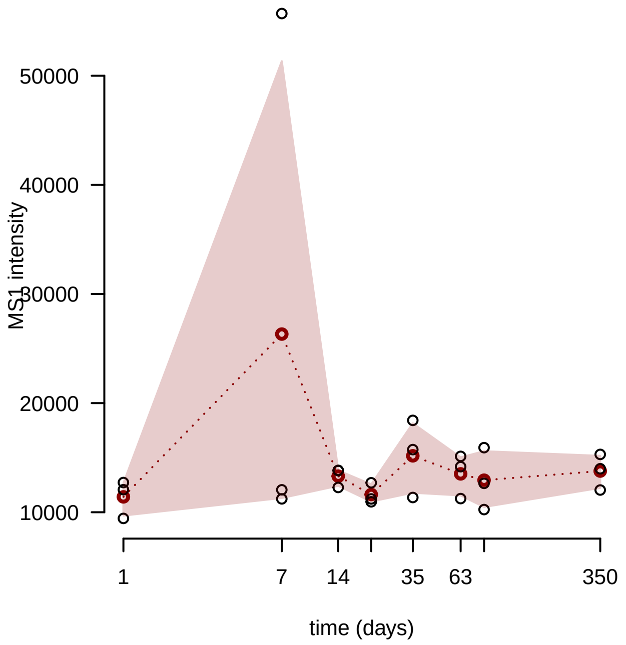

Q8BJ63
GN=Ap5m1
adaptor−related protein complex 5, mu 1 ...

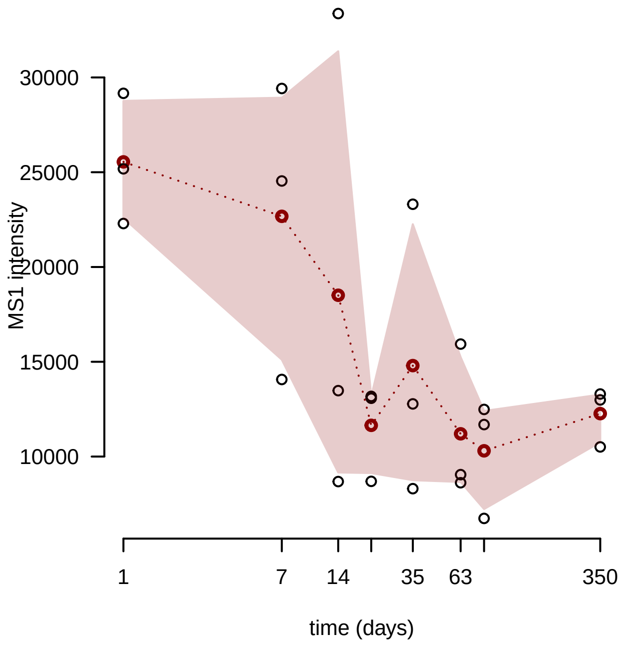

Q8BJ64
GN=Chdh
choline dehydrogenase

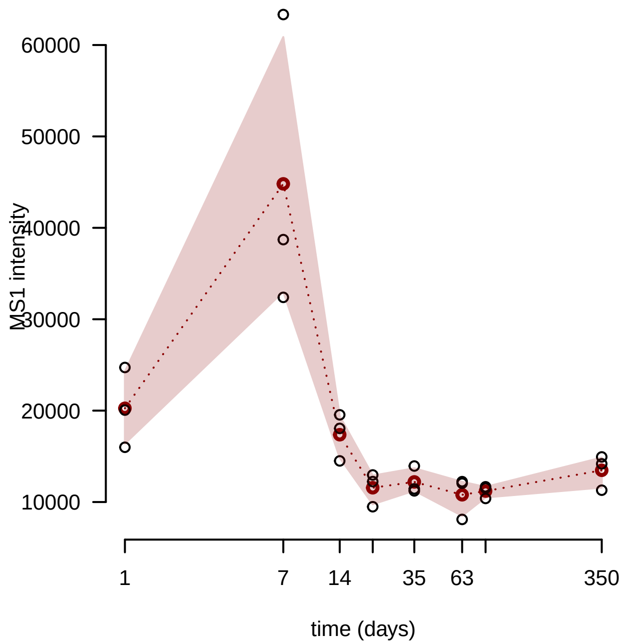

Q8BJ71
GN=Nup93
nucleoporin 93

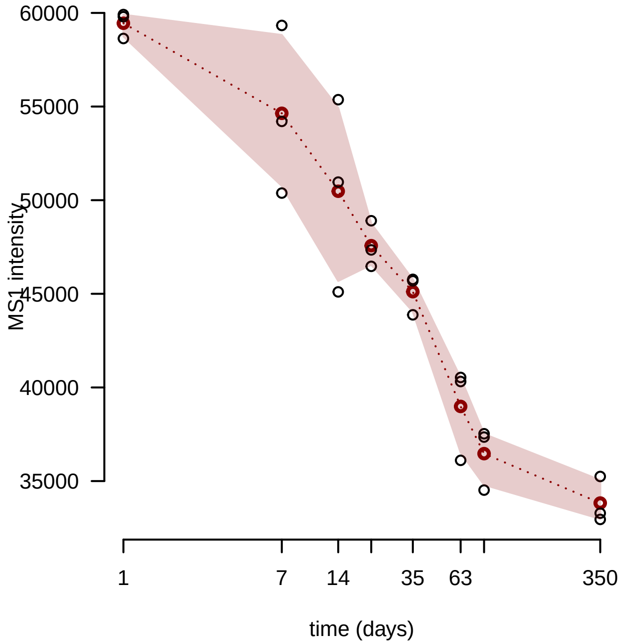

Q8BJ83
GN=Tm2d3
TM2 domain containing 3

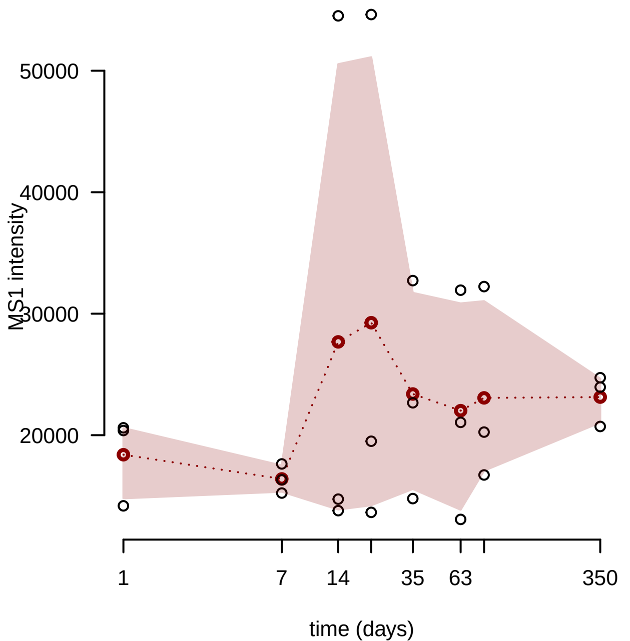

Q8BJ90
GN=Znf771

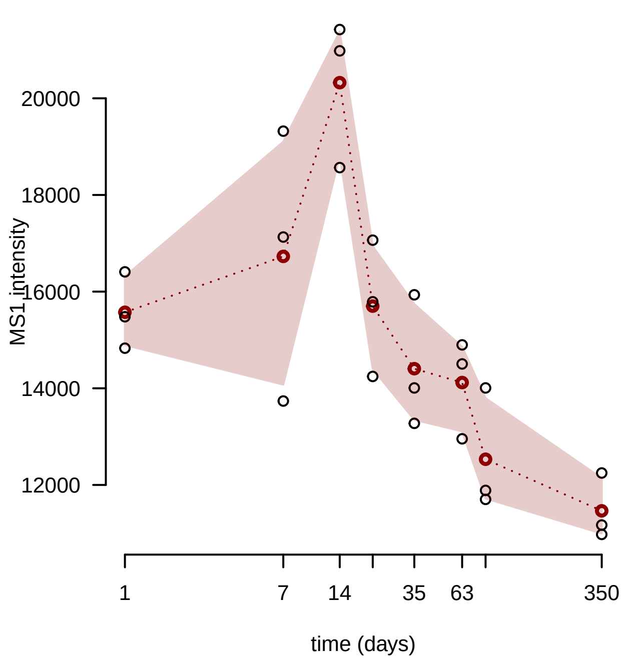

Q8BJA3
GN=Hmbox1
homeobox containing 1

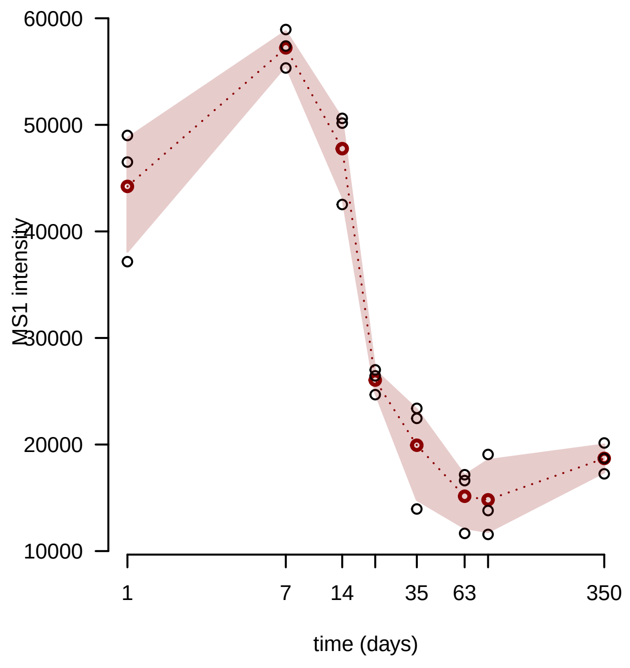

Q8BJD1
GN=Itih5
inter−alpha−trypsin inhibitor, heavy cha ...

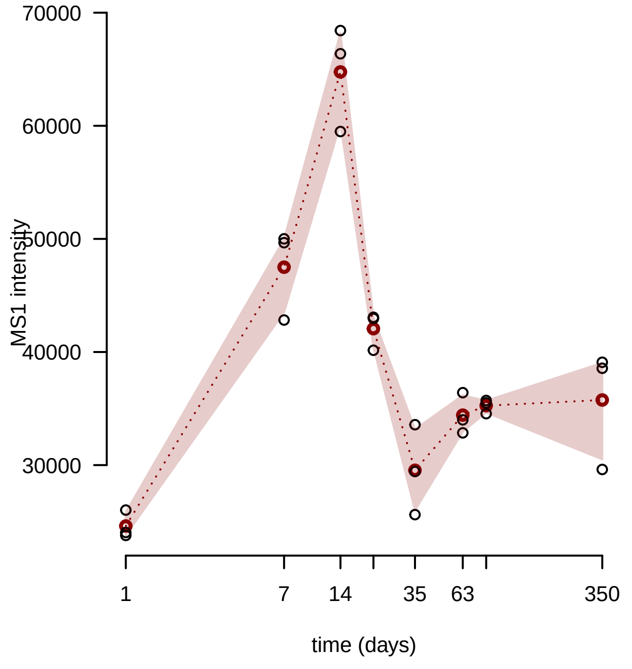

Q8BJE2
GN=Btnl9
butyrophilin−like 9

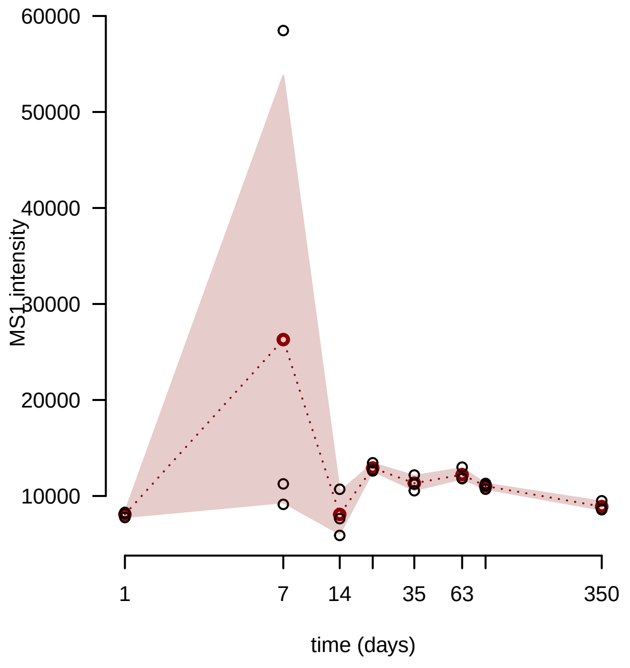

Q8BJF9
GN=Chmp2b
charged multivesicular body protein 2B

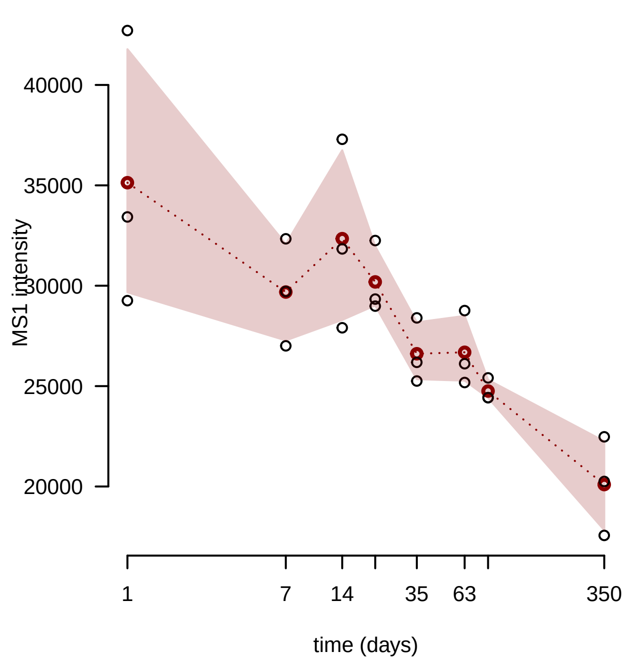

Q8BJG4
GN=Mob3c
MOB kinase activator 3C

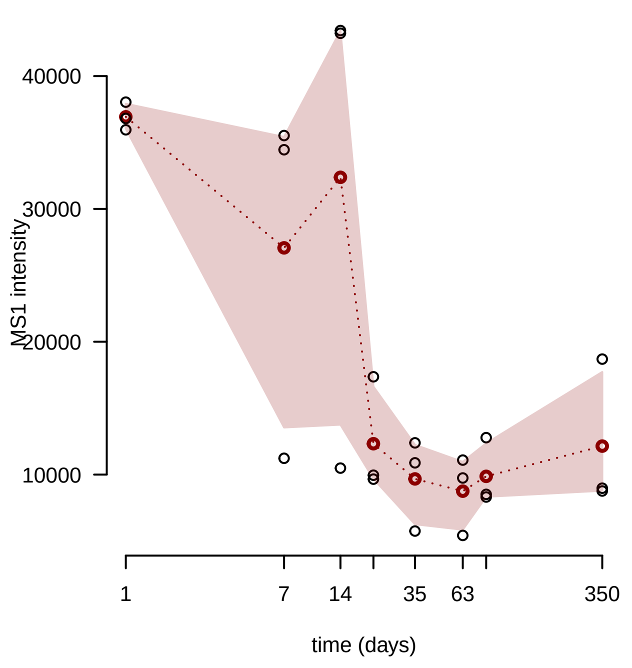

Q8BJH1
GN=Zc2hc1a
zinc finger, C2HC−type containing 1A

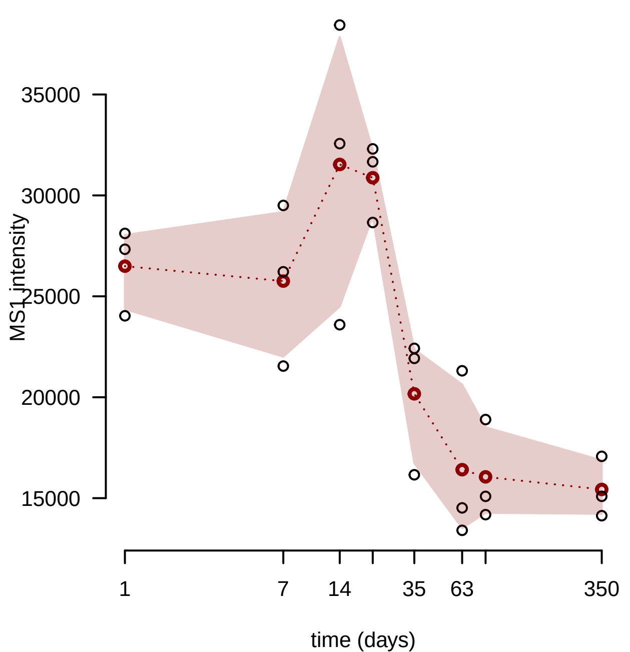

Q8BJK1
GN=Kctd7
potassium channel tetramerisation domain ...

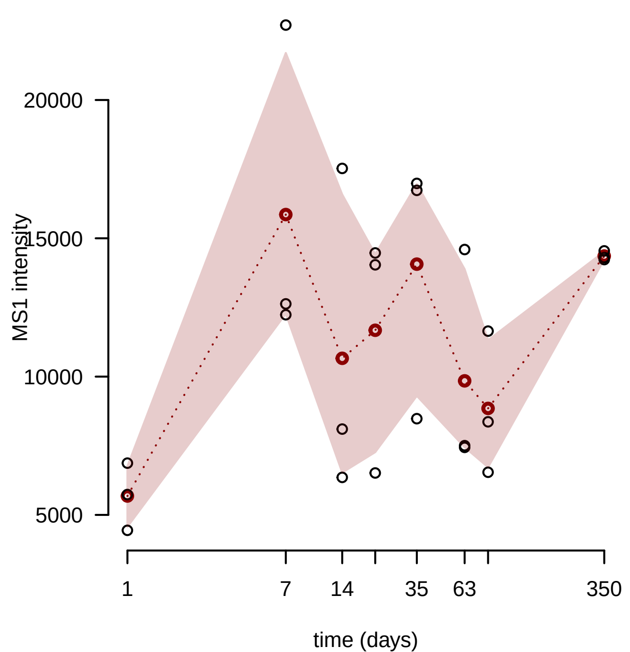

Q8BJL0
GN=Smarcal1
SWI/SNF related matrix associated, actin ...

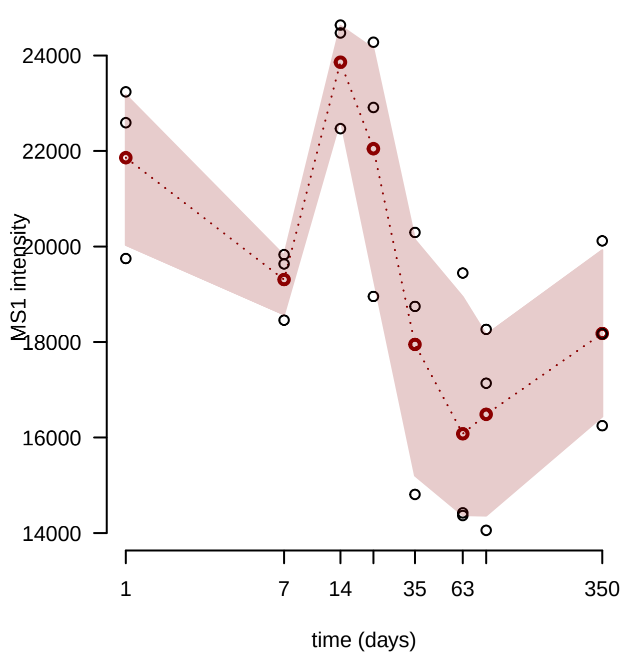

Q8BJL1
GN=Fbxo30
F−box protein 30

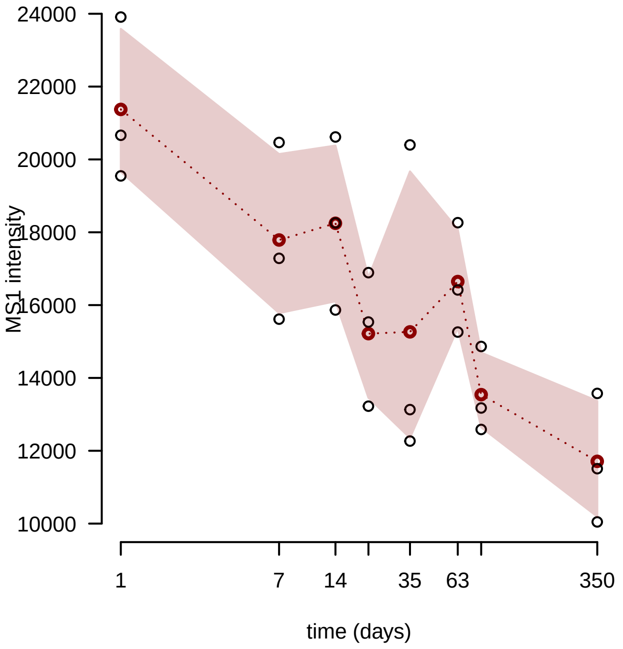

Q8BJM3
GN=R3hcc1l
R3H domain and coiled−coil containing 1 ...

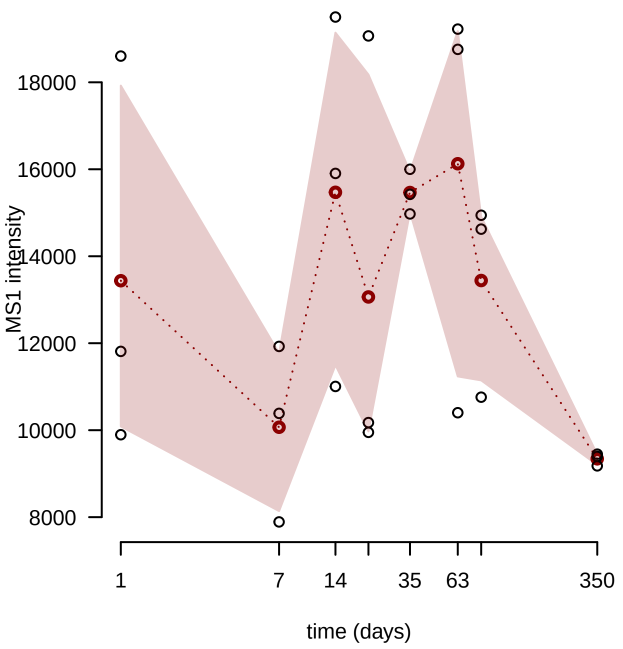

Q8BJM5
GN=Slc30a6
solute carrier family 30 (zinc transport ...

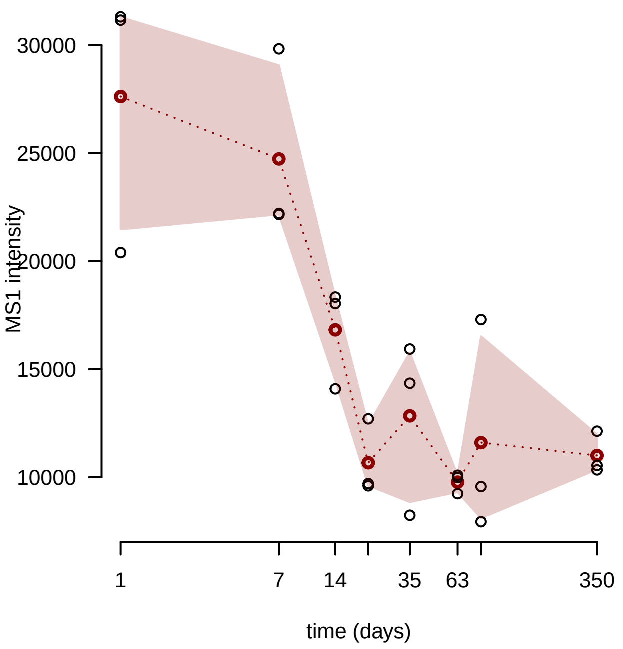

Q8BJM7
GN=Tyw1
tRNA−yW synthesizing protein 1 homolog (...

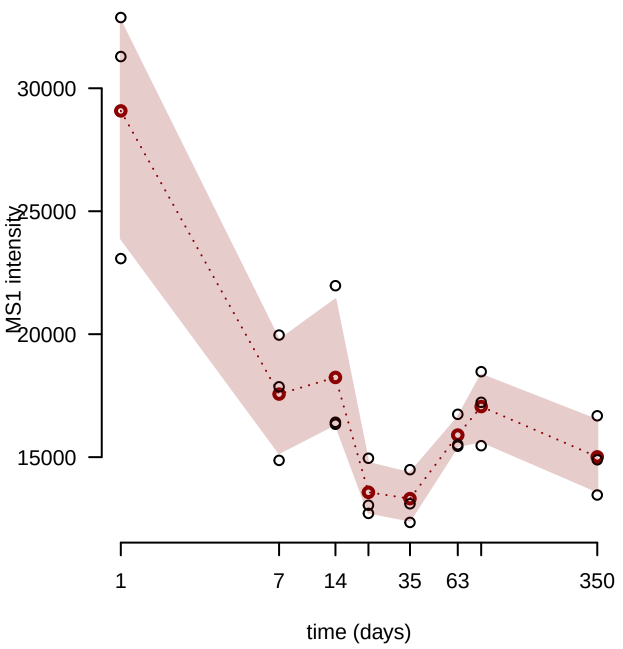

Q8BJQ2
GN=Usp1
ubiquitin specific peptidase 1

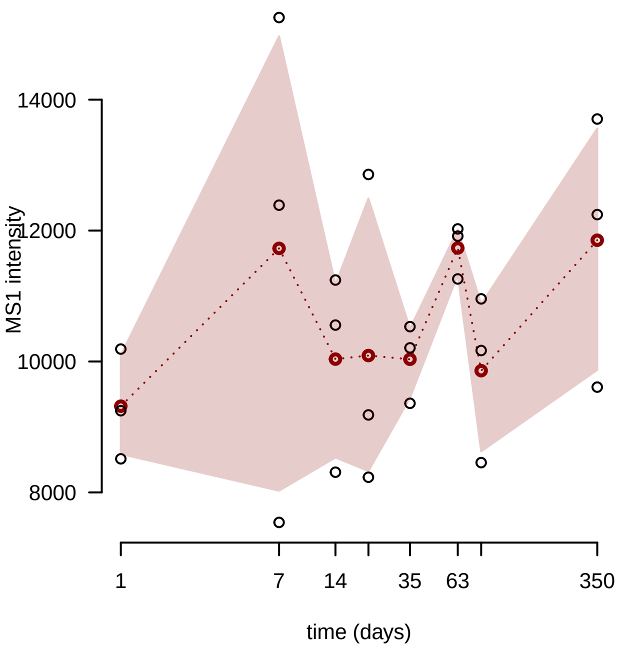

Q8BJQ9
GN=Csgalnact1
chondroitin sulfate N−acetylgalactosamin ...

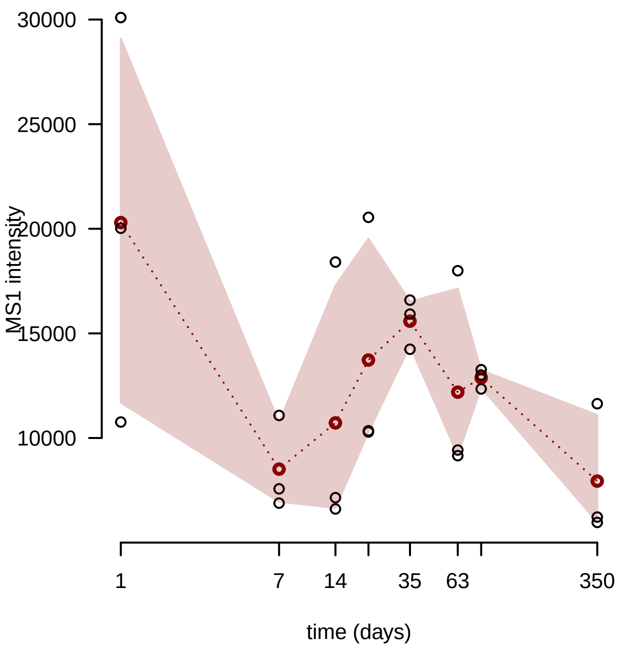

Q8BJS4
GN=Sun2
Sad1 and UNC84 domain containing 2

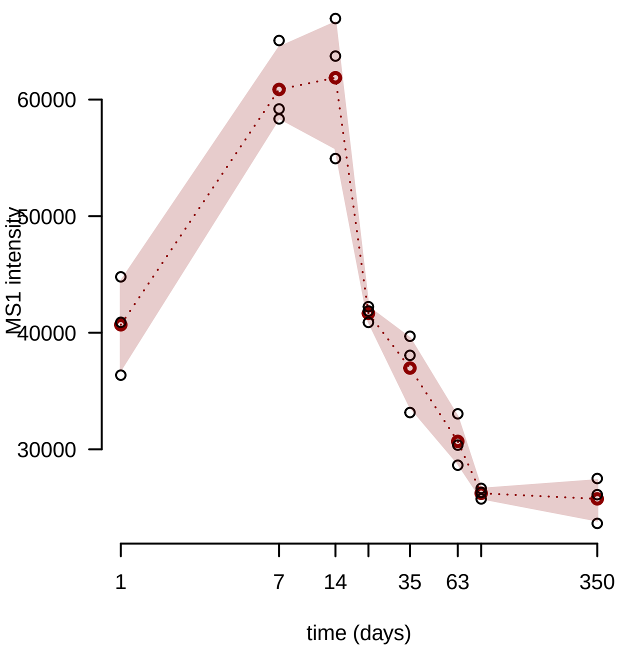

Q8BJT9
GN=Edem2
ER degradation enhancer, mannosidase alp ...

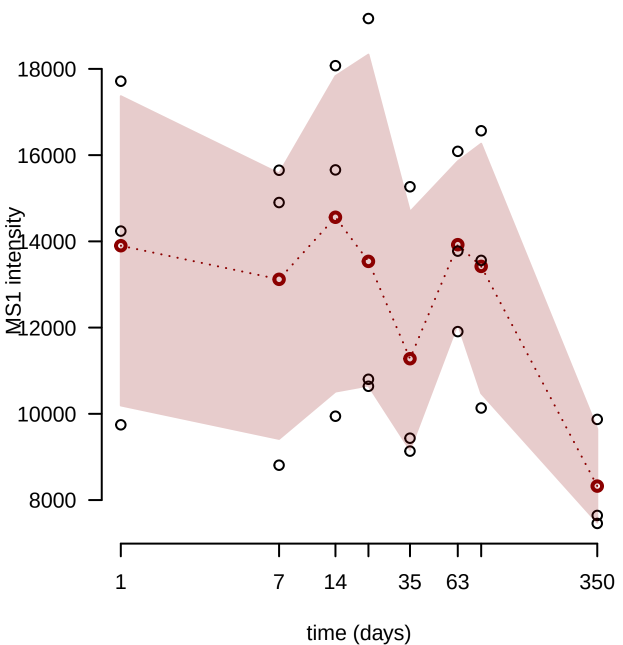

Q8BJU0
GN=Sgta
small glutamine−rich tetratricopeptide r ...

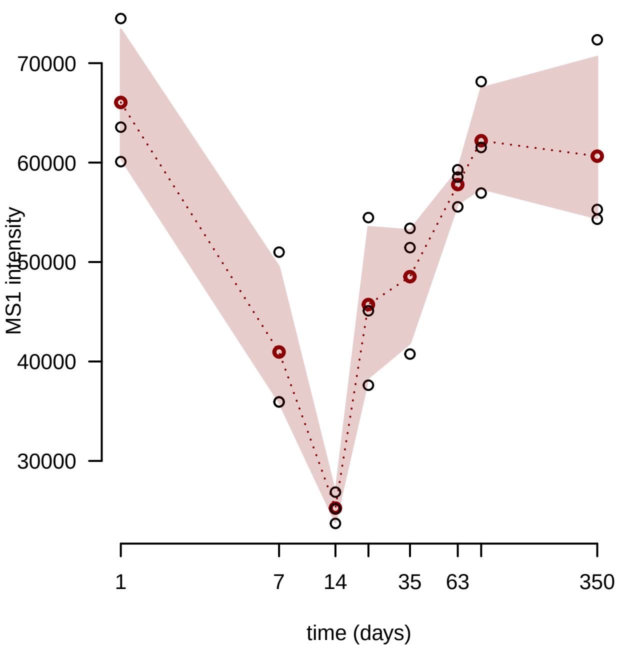

Q8BJU2
GN=Tspan9
tetraspanin 9

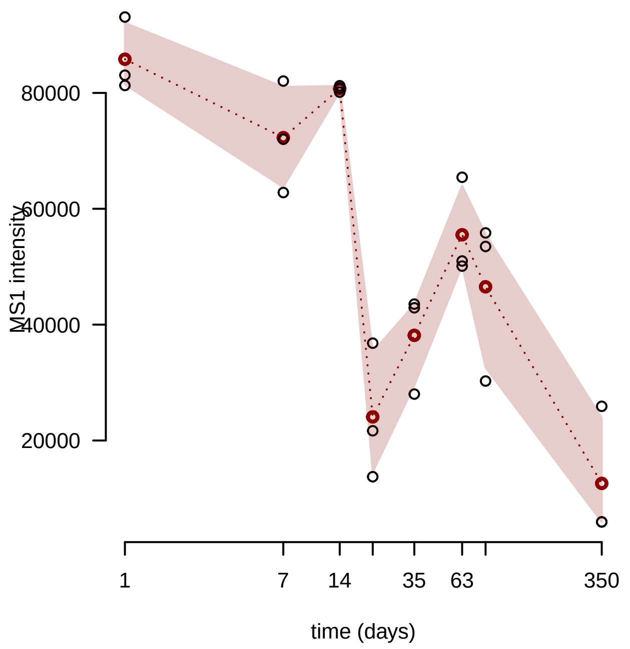

Q8BJU9
GN=Mtrf1l
mitochondrial translational release fact ...

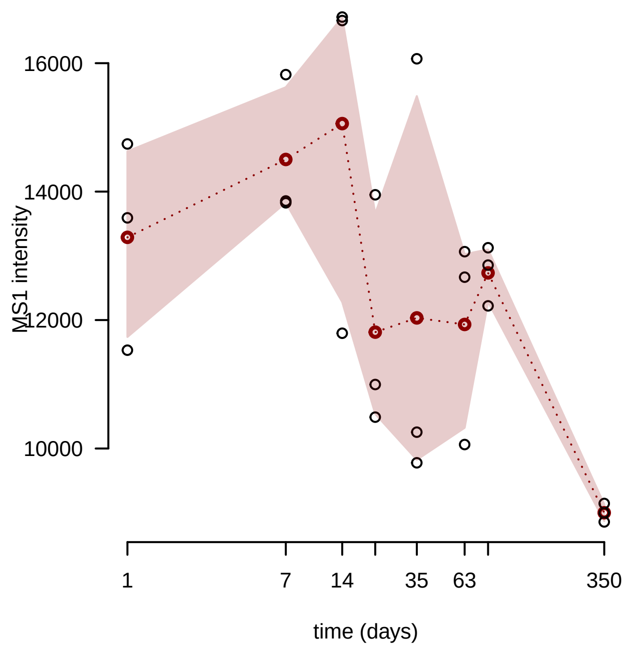

Q8BJW5
GN=Nol11
nucleolar protein 11

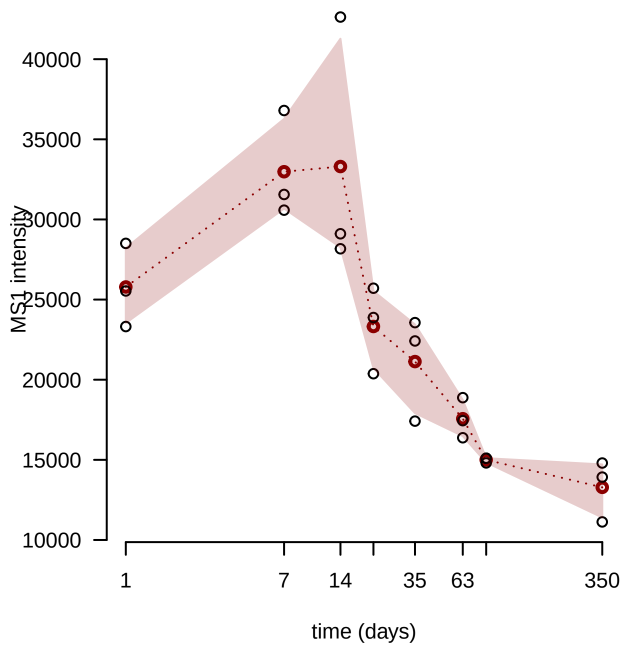

Q8BJW6
GN=Eif2a
eukaryotic translation initiation factor ...

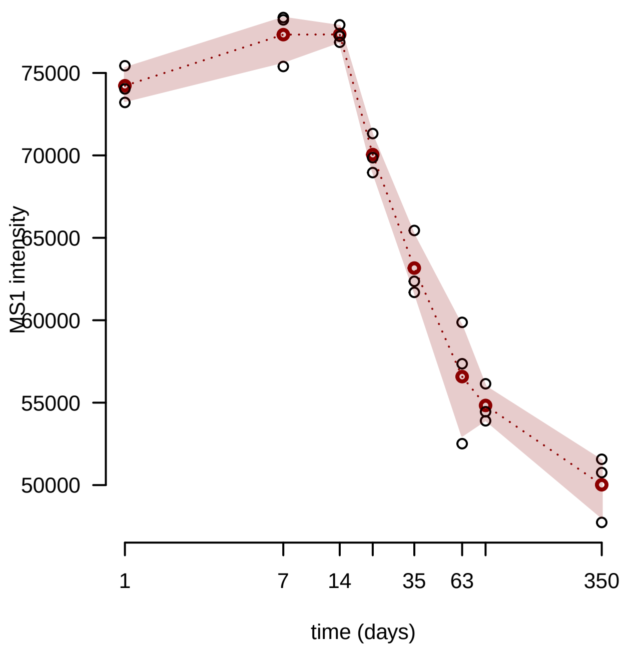

Q8BJY1
GN=Psmd5
proteasome (prosome, macropain) 26S subu ...

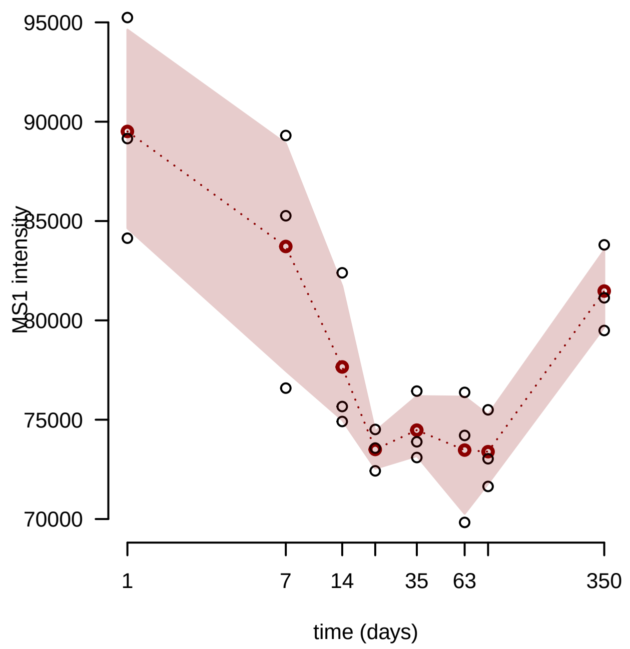

Q8BJZ3
GN=Tmbim1
transmembrane BAX inhibitor motif contai ...

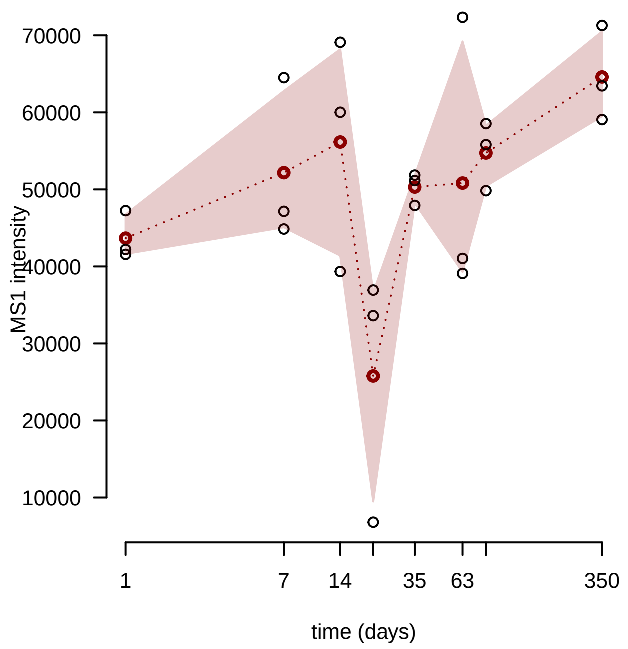

Q8BJZ4
GN=Mrps35
mitochondrial ribosomal protein S35

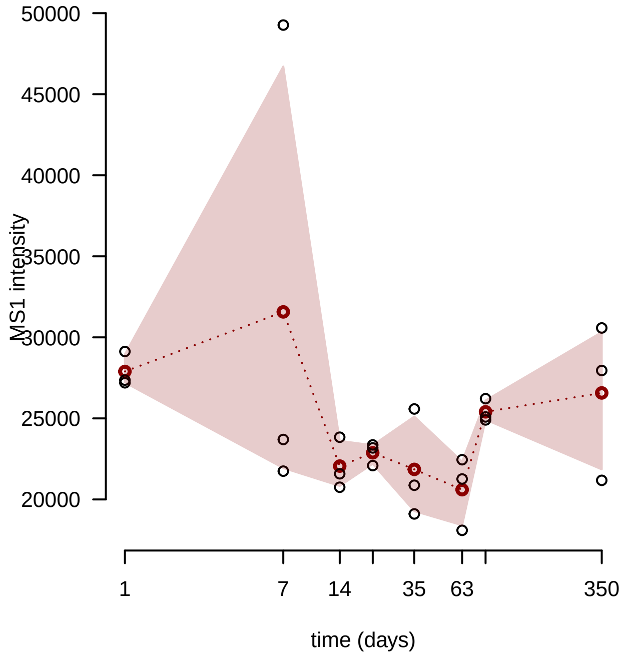

Q8BK03
GN=Miga2
mitoguardin 2

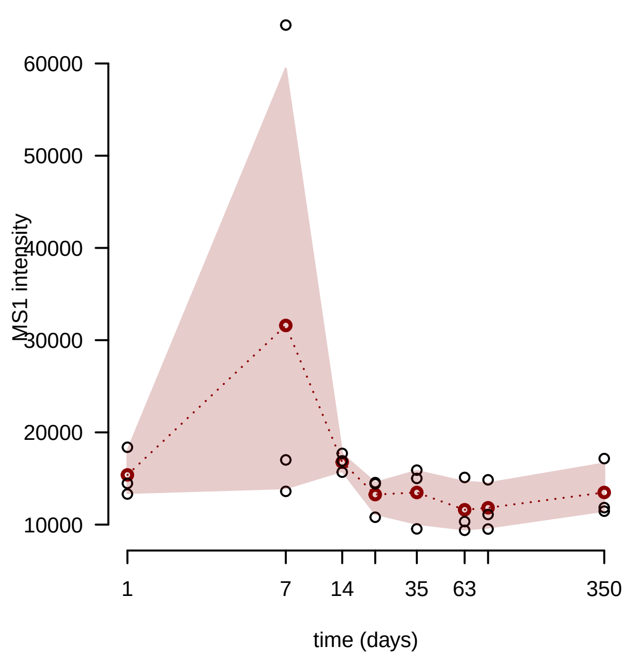

Q8BK08
GN=Tmem11
transmembrane protein 11

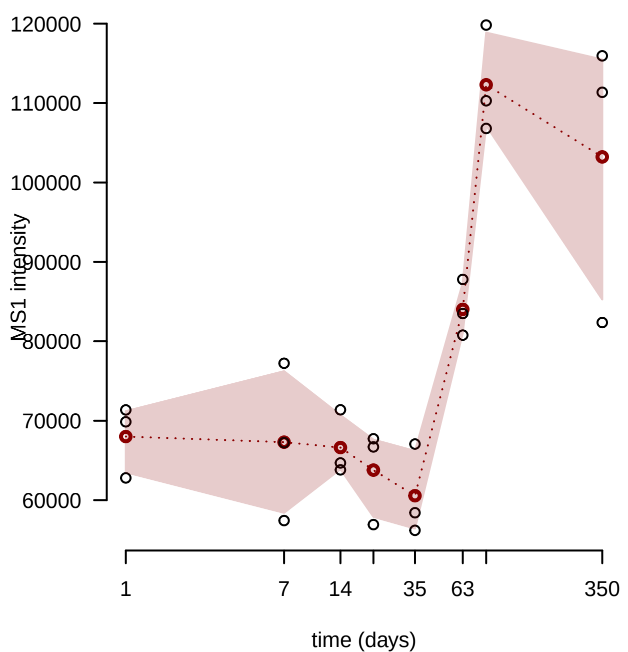

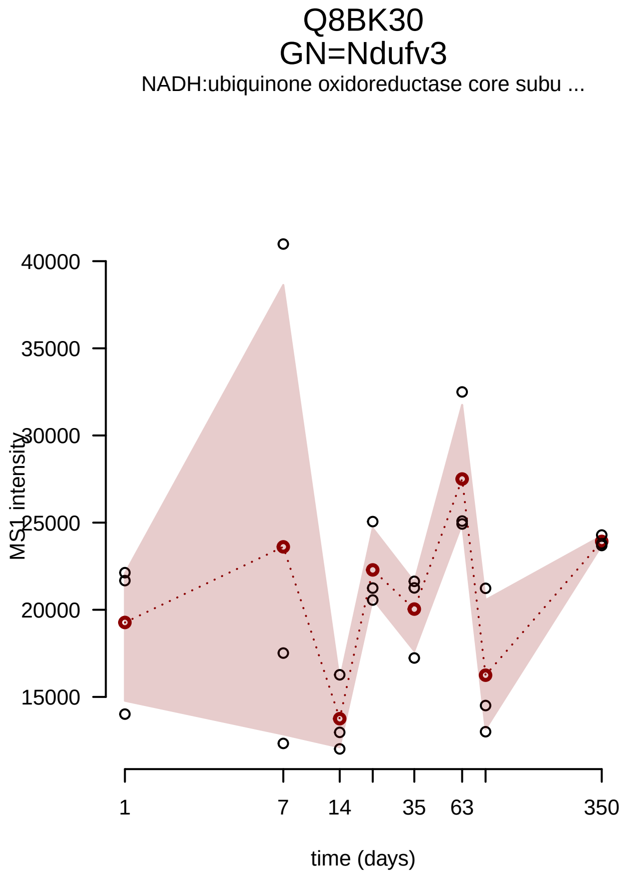
Q8BK30
GN=Ndufv3
NADH:ubiquinone oxidoreductase core subu ...
MS1 intensity
40000
35000
30000
25000
20000
15000
1
7
14
35
63
350
time (days)

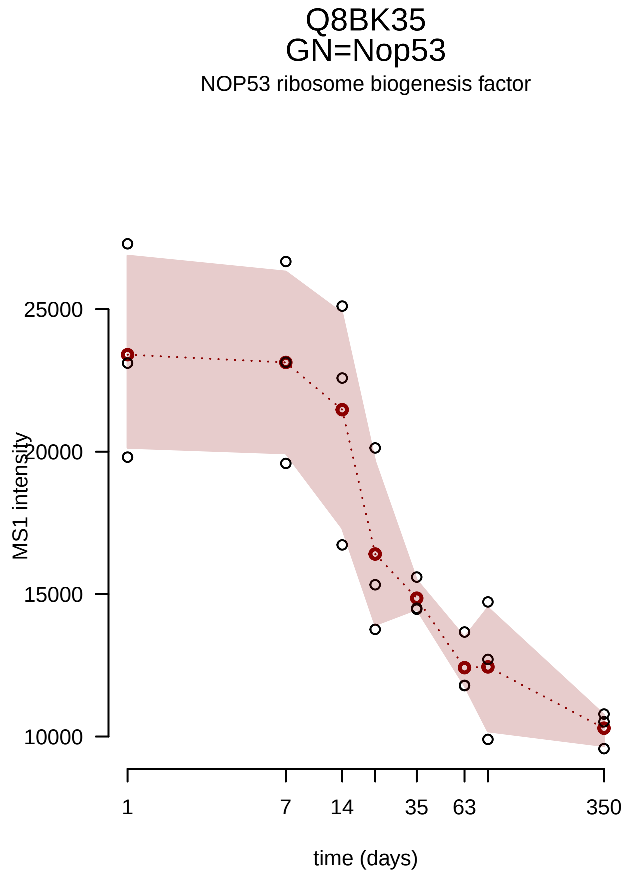
Q8BK35
GN=Nop53
NOP53 ribosome biogenesis factor
MS1 intensity
25000
20000
15000
10000
1
7
14
35
63
350
time (days)

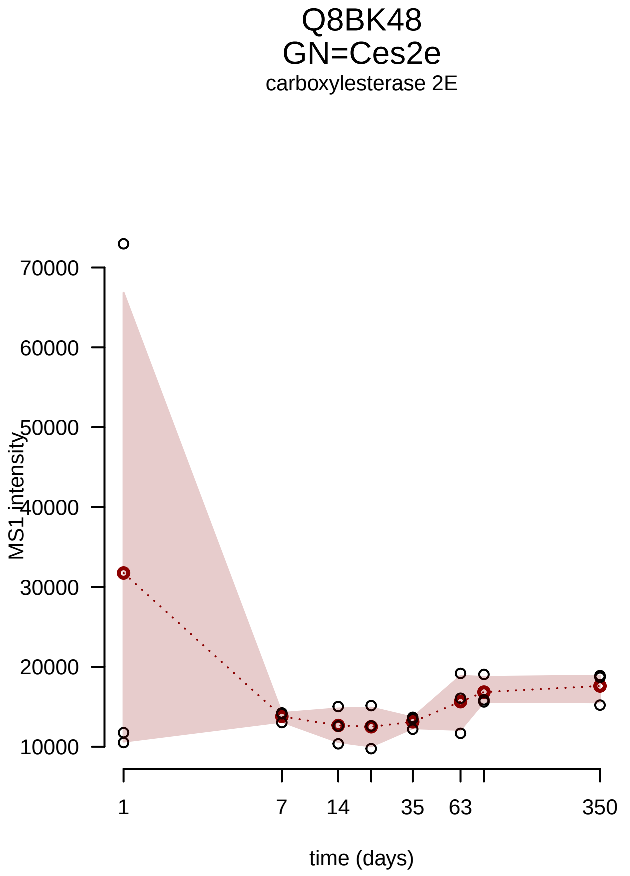
Q8BK48
GN=Ces2e
carboxylesterase 2E
MS1 intensity
70000
60000
50000
40000
30000
20000
10000
1
7
14
35
63
350
time (days)

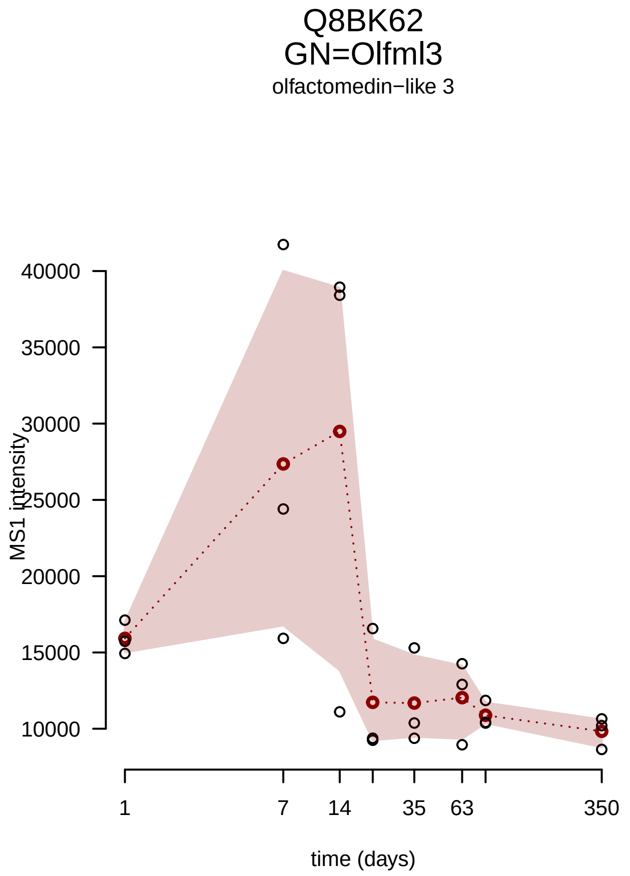
Q8BK62
GN=Olfml3
olfactomedin−like 3
MS1 intensity
40000
35000
30000
25000
20000
15000
10000
1
7
14
35
63
350
time (days)

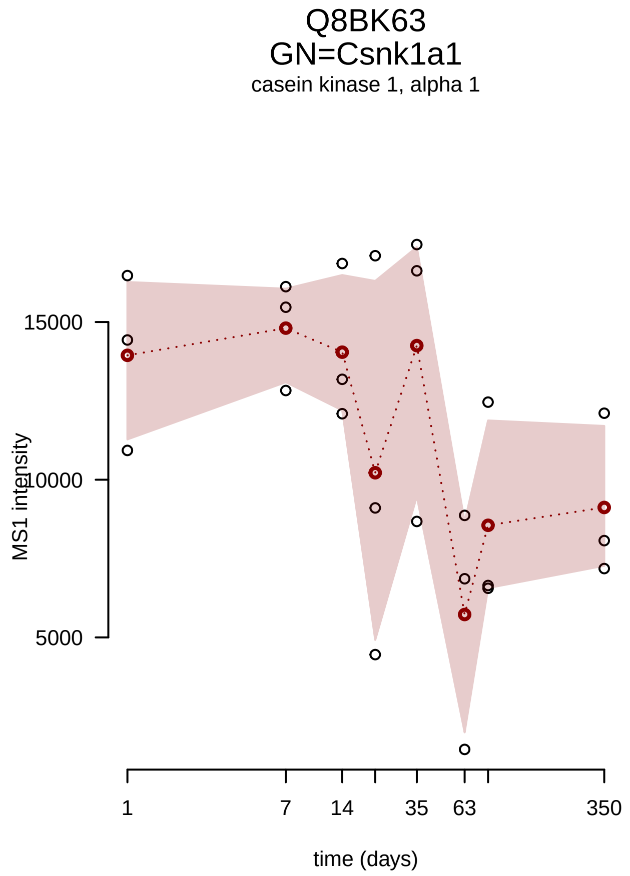
Q8BK63
GN=Csnk1a1
casein kinase 1, alpha 1
MS1 intensity
15000
10000
5000
1
7
14
35
63
350
time (days)

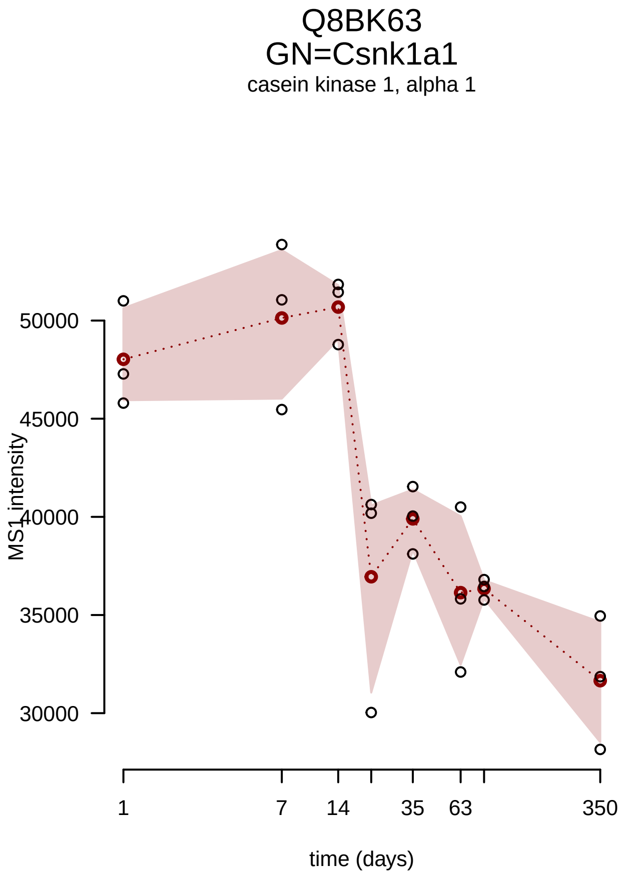
Q8BK63
GN=Csnk1a1
casein kinase 1, alpha 1
MS1 intensity
50000
45000
40000
35000
30000
1
7
14
35
63
350
time (days)

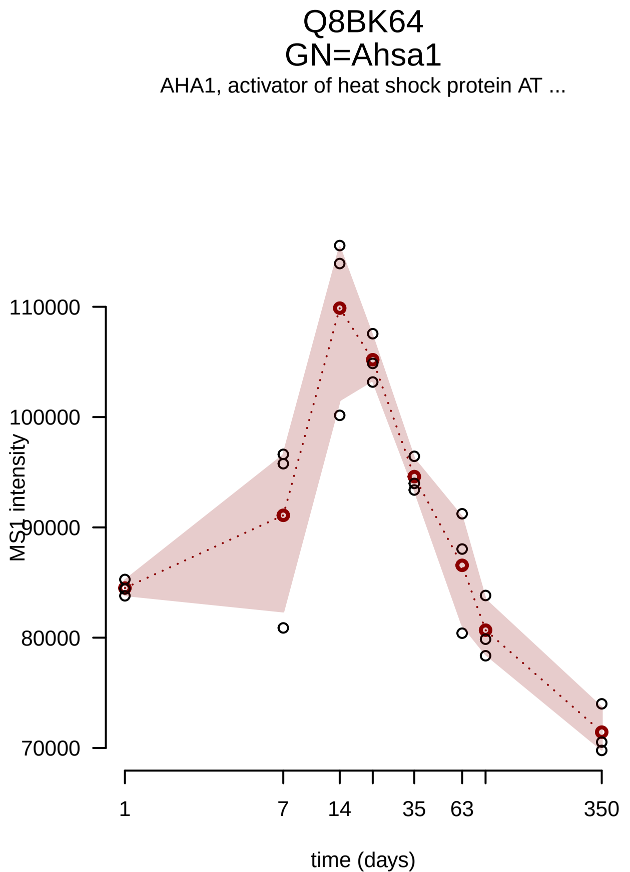
Q8BK64
GN=Ahsa1
AHA1, activator of heat shock protein AT ...
MS1 intensity
110000
100000
90000
80000
70000
1
7
14
35
63
350
time (days)

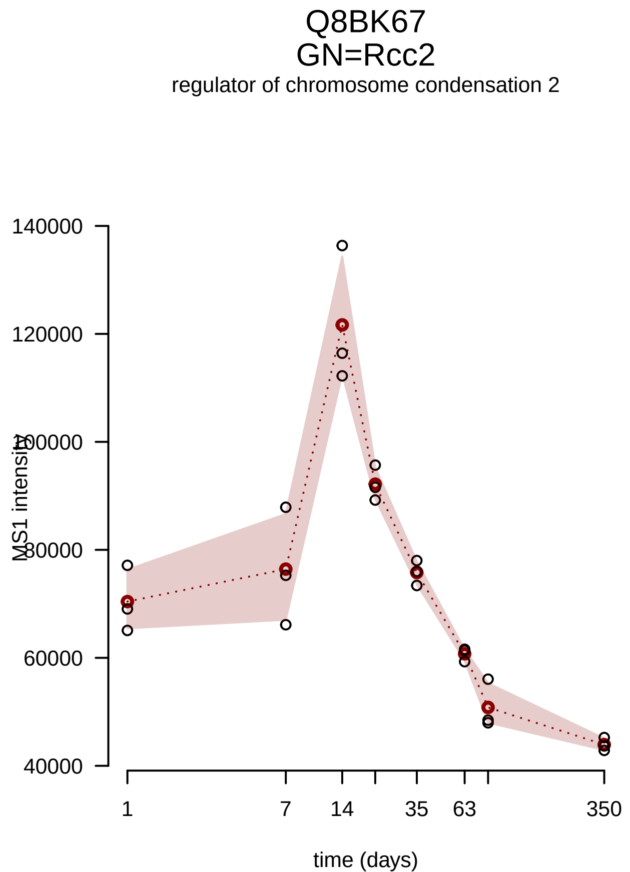
Q8BK67
GN=Rcc2
regulator of chromosome condensation 2
MS1 intensity
140000
120000
100000
80000
60000
40000
1
7
14
35
63
350
time (days)

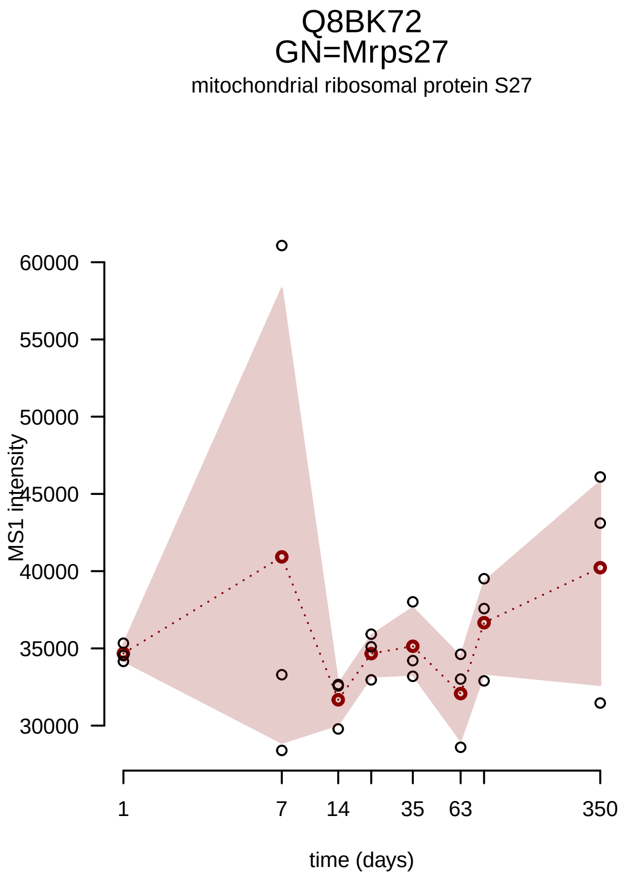
Q8BK72
GN=Mrps27
mitochondrial ribosomal protein S27
MS1 intensity
60000
55000
50000
45000
40000
35000
30000
1
7
14
35
63
350
time (days)

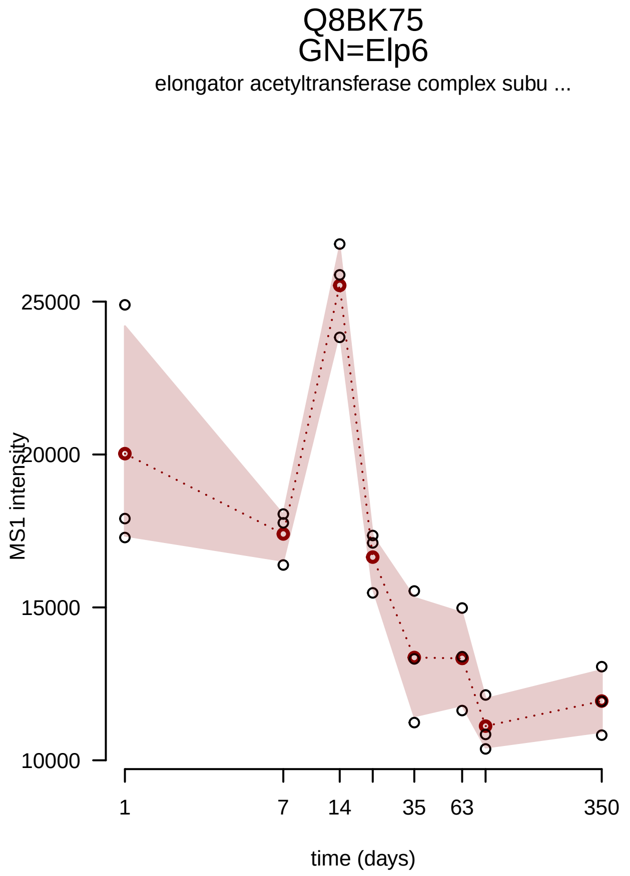
Q8BK75
GN=Elp6
elongator acetyltransferase complex subu ...
MS1 intensity
25000
20000
15000
10000
1
7
14
35
63
350
time (days)

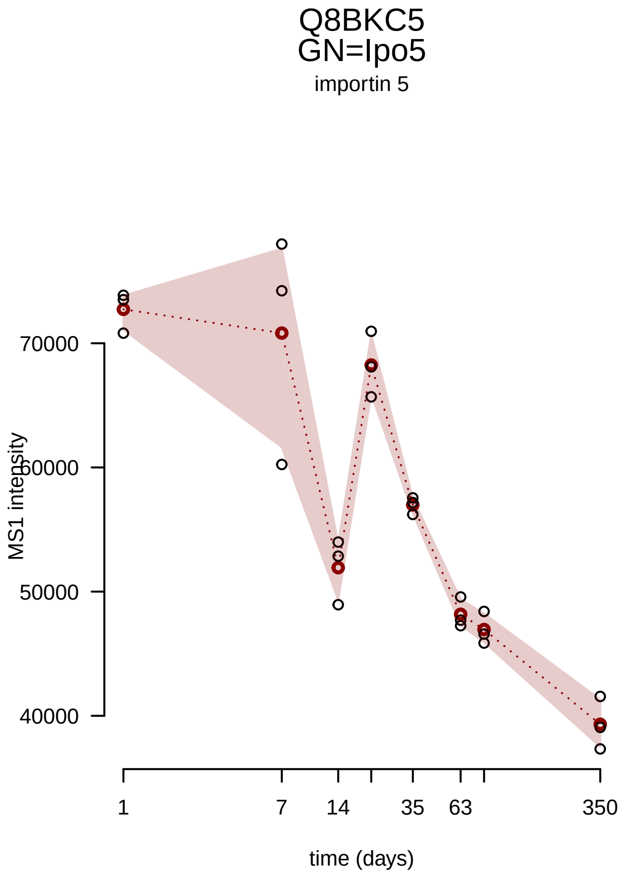
Q8BKC5
GN=Ipo5
importin 5
MS1 intensity
70000
60000
50000
40000
1
7
14
35
63
350
time (days)

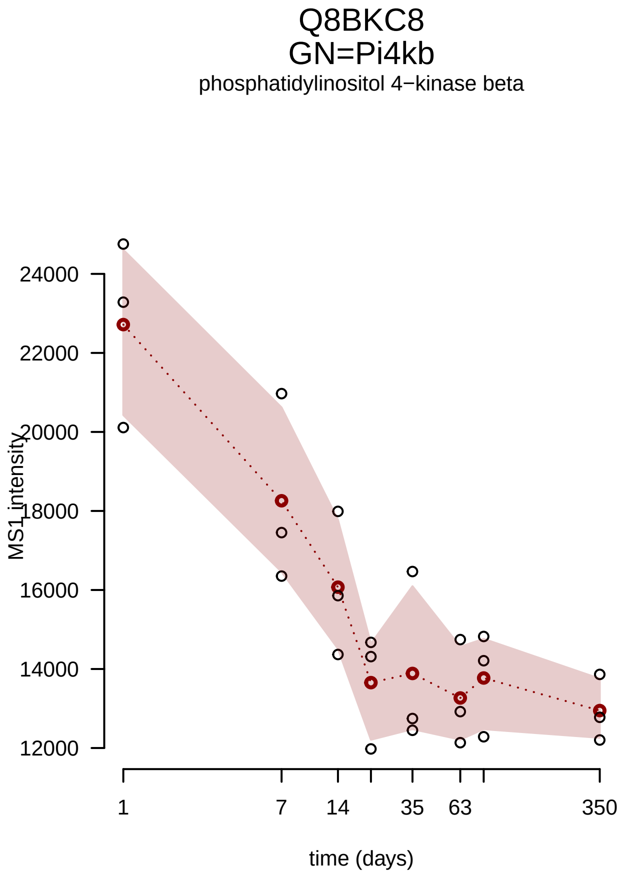
Q8BKC8
GN=Pi4kb
phosphatidylinositol 4−kinase beta
MS1 intensity
24000
22000
20000
18000
16000
14000
12000
1
7
14
35
63
350
time (days)

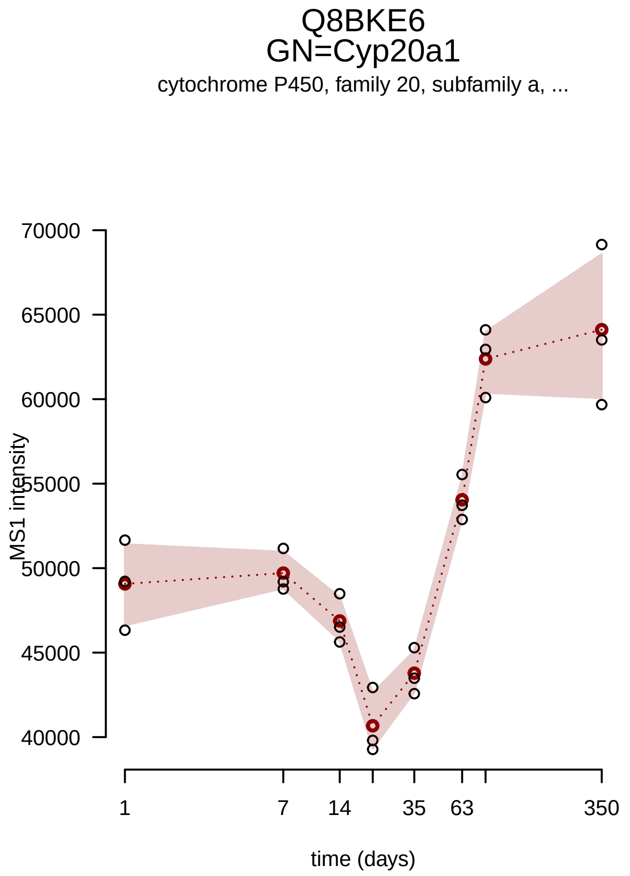
Q8BKE6
GN=Cyp20a1
cytochrome P450, family 20, subfamily a, ...
MS1 intensity
70000
65000
60000
55000
50000
45000
40000
1
7
14
35
63
350
time (days)

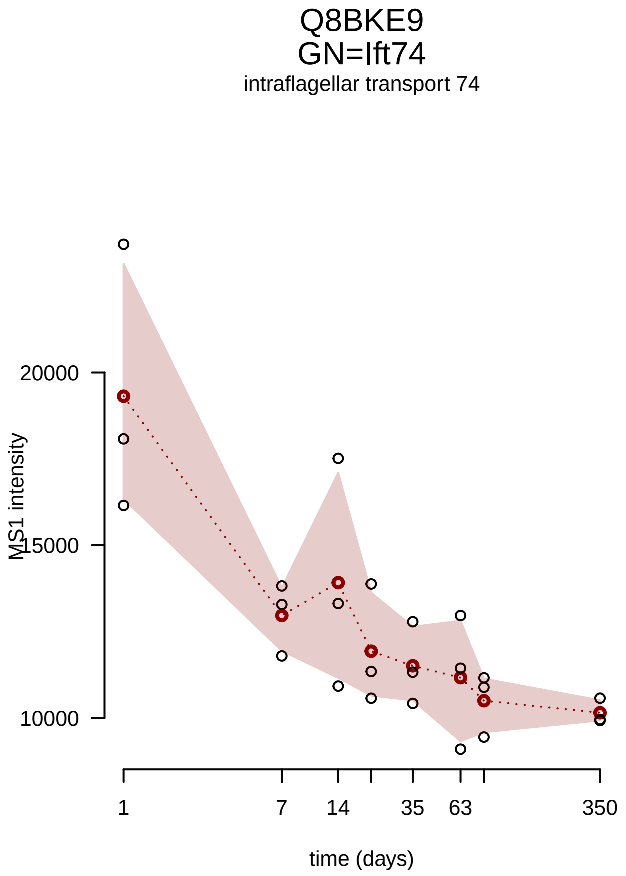
Q8BKE9
GN=Ift74
intraflagellar transport 74
MS1 intensity
20000
15000
10000
1
7
14
35
63
350
time (days)

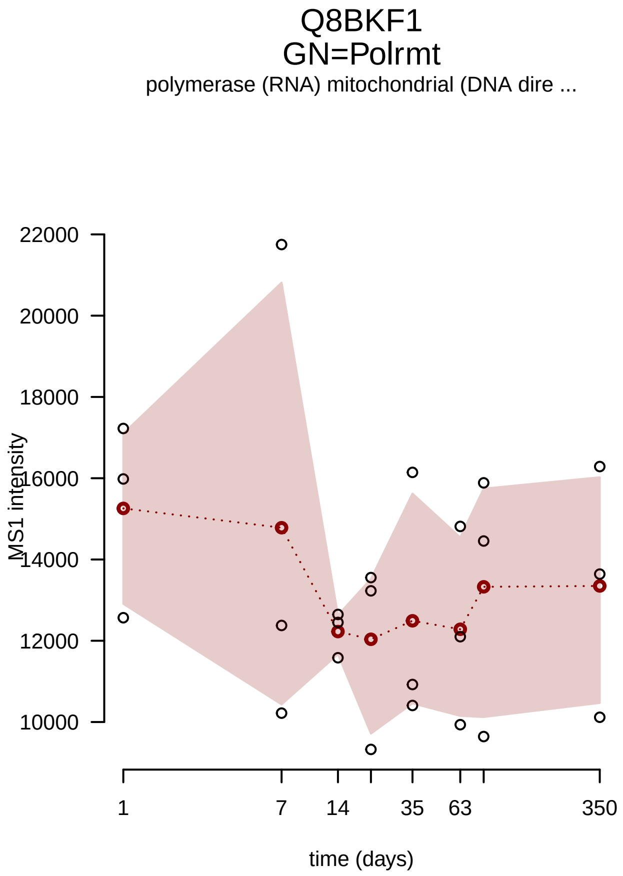
Q8BKF1
GN=Polrmt
polymerase (RNA) mitochondrial (DNA dire ...
MS1 intensity
22000
20000
18000
16000
14000
12000
10000
1
7
14
35
63
350
time (days)

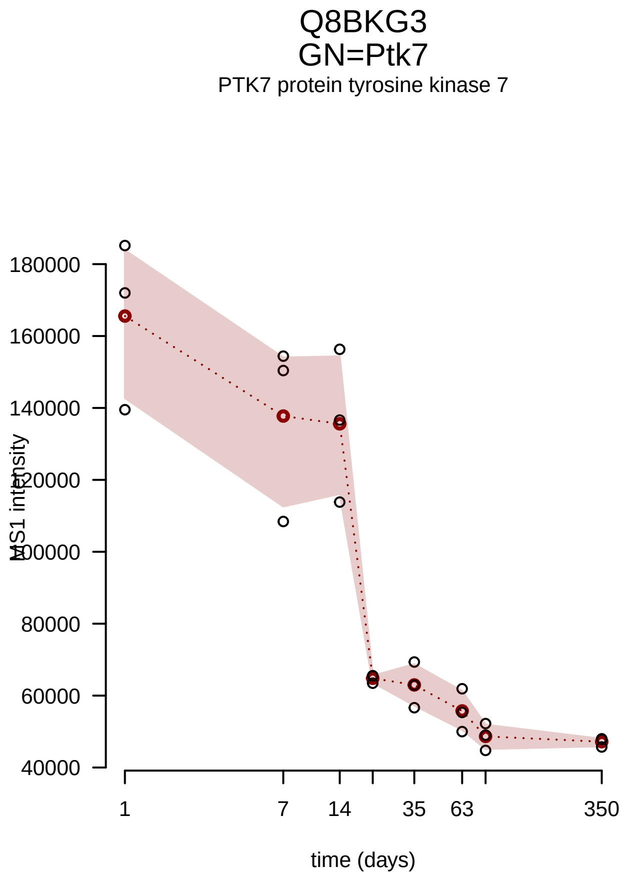
Q8BKG3
GN=Ptk7
PTK7 protein tyrosine kinase 7
MS1 intensity
180000
160000
140000
120000
100000
80000
60000
40000
1
7
14
35
63
350
time (days)

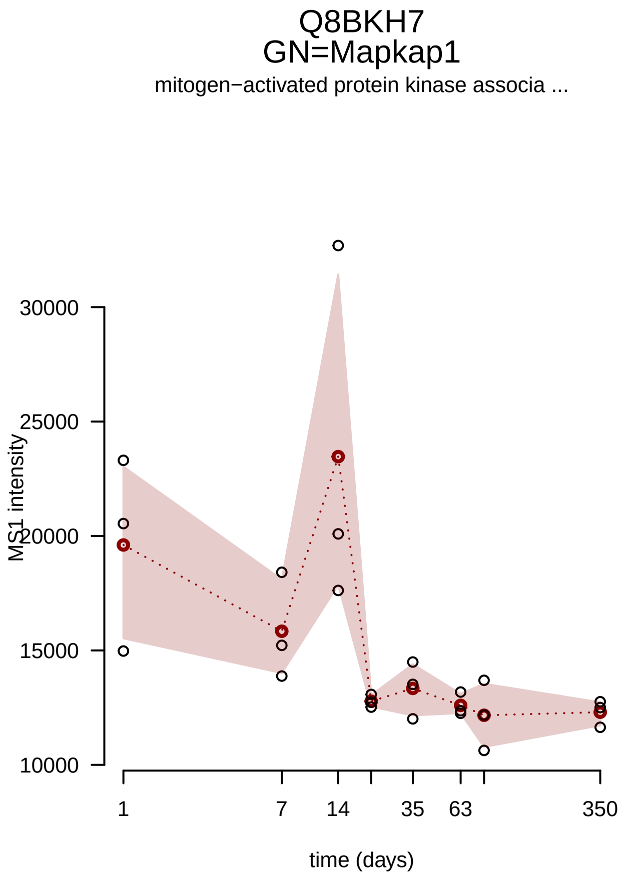
Q8BKH7
GN=Mapkap1
mitogen−activated protein kinase associa ...
MS1 intensity
30000
25000
20000
15000
10000
1
7
14
35
63
350
time (days)

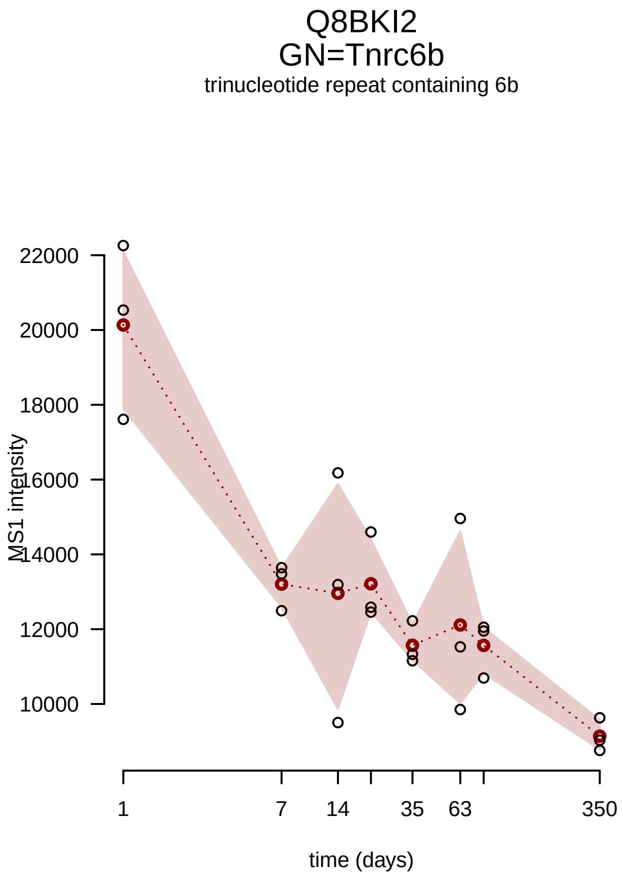
Q8BKI2
GN=Tnrc6b
trinucleotide repeat containing 6b
MS1 intensity
22000
20000
18000
16000
14000
12000
10000
1
7
14
35
63
350
time (days)

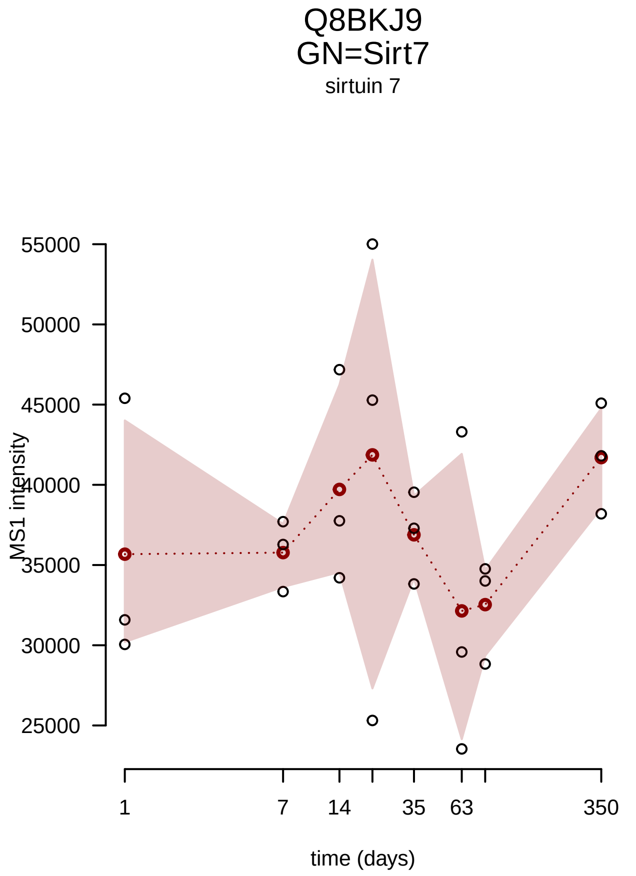
Q8BKJ9
GN=Sirt7
sirtuin 7
MS1 intensity
55000
50000
45000
40000
35000
30000
25000
1
7
14
35
63
350
time (days)

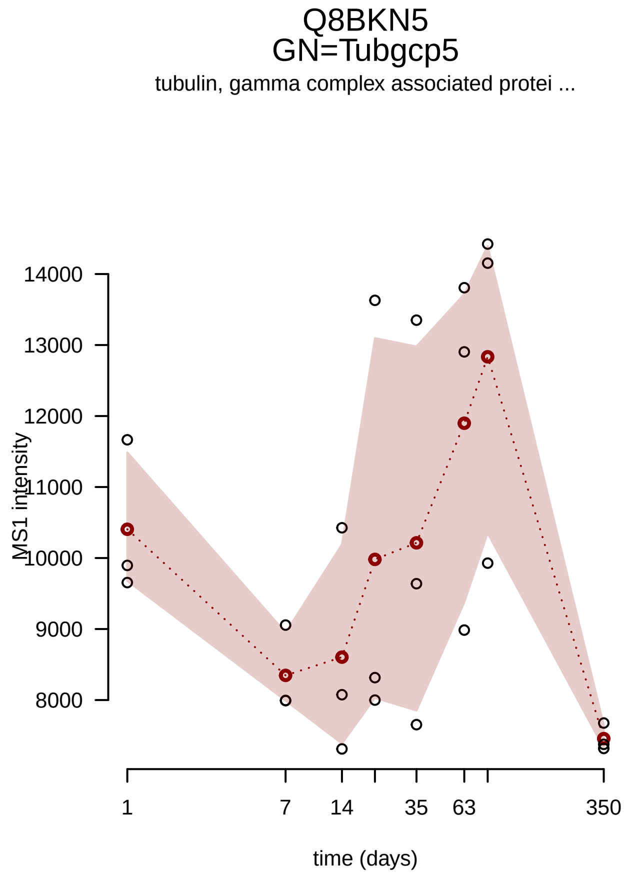
Q8BKN5
GN=Tubgcp5
tubulin, gamma complex associated protei ...
MS1 intensity
14000
13000
12000
11000
10000
9000
8000
1
7
14
35
63
350
time (days)

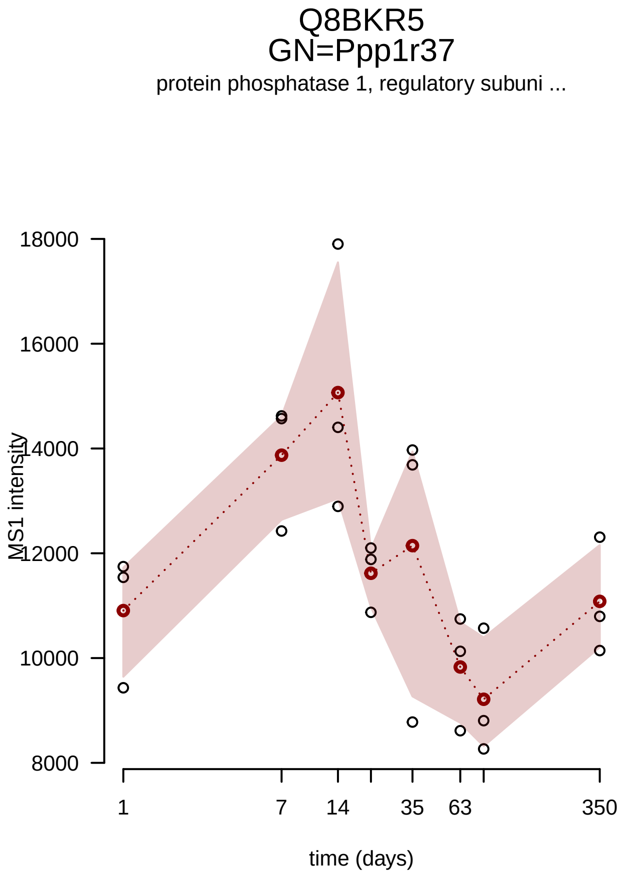
Q8BKR5
GN=Ppp1r37
protein phosphatase 1, regulatory subuni ...
MS1 intensity
18000
16000
14000
12000
10000
8000
1
7
14
35
63
350
time (days)

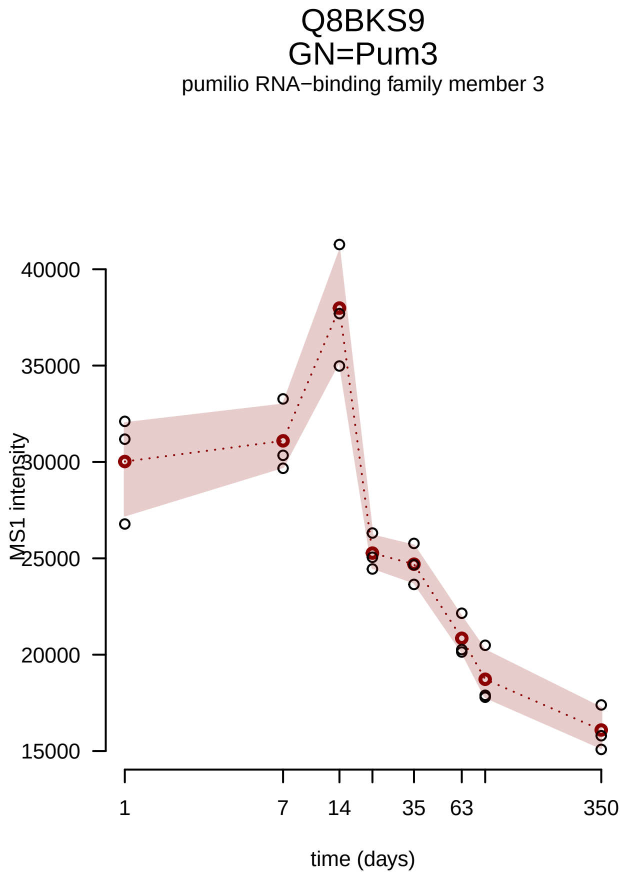
Q8BKS9
GN=Pum3
pumilio RNA−binding family member 3
MS1 intensity
40000
35000
30000
25000
20000
15000
1
7
14
35
63
350
time (days)

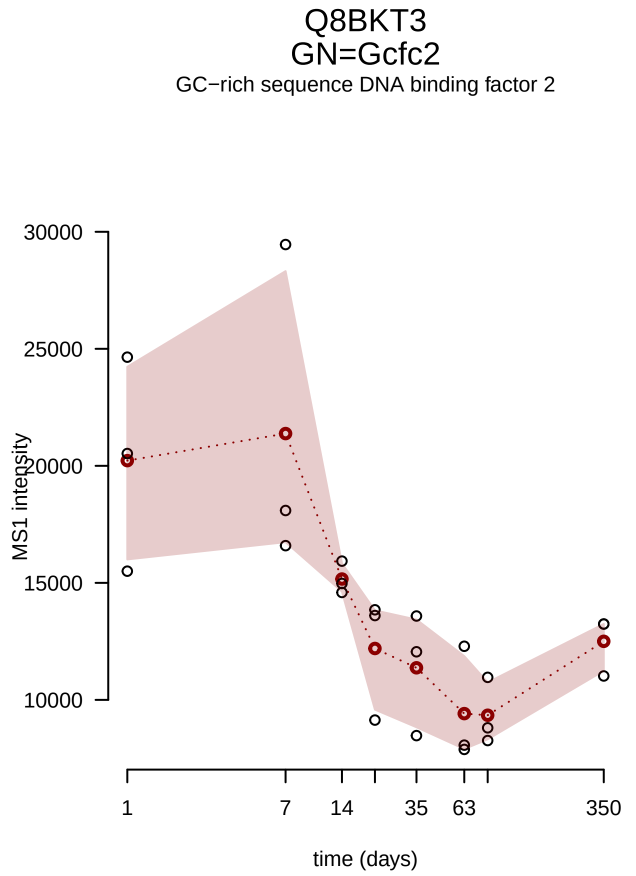
Q8BKT3
GN=Gcfc2
GC−rich sequence DNA binding factor 2
MS1 intensity
30000
25000
20000
15000
10000
1
7
14
35
63
350
time (days)

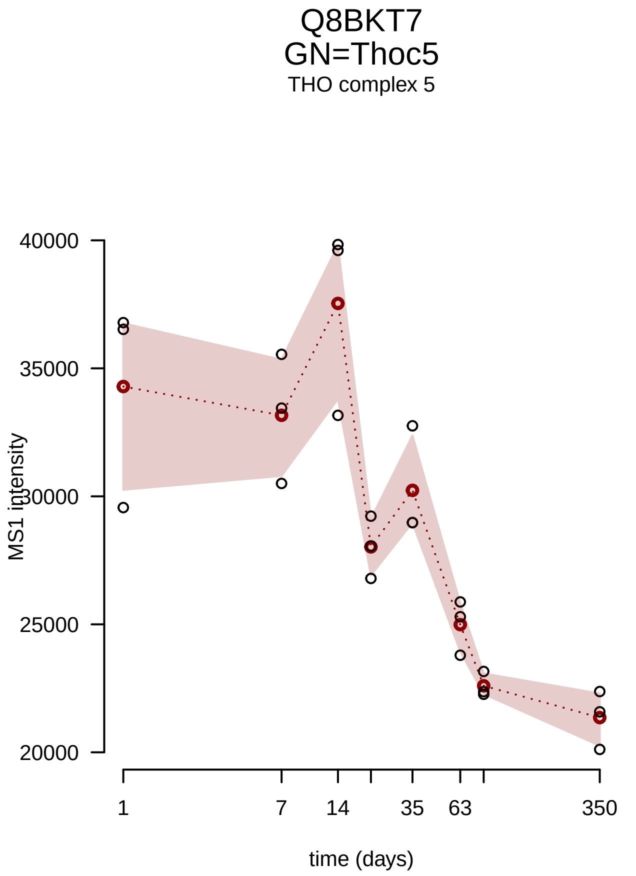
Q8BKT7
GN=Thoc5
THO complex 5
MS1 intensity
40000
35000
30000
25000
20000
1
7
14
35
63
350
time (days)

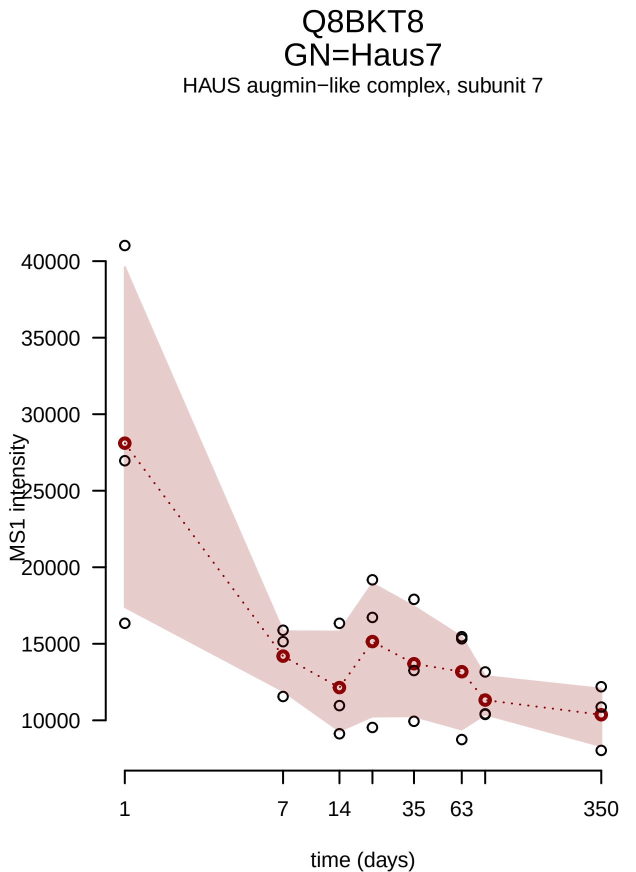
Q8BKT8
GN=Haus7
HAUS augmin−like complex, subunit 7
MS1 intensity
40000
35000
30000
25000
20000
15000
10000
1
7
14
35
63
350
time (days)

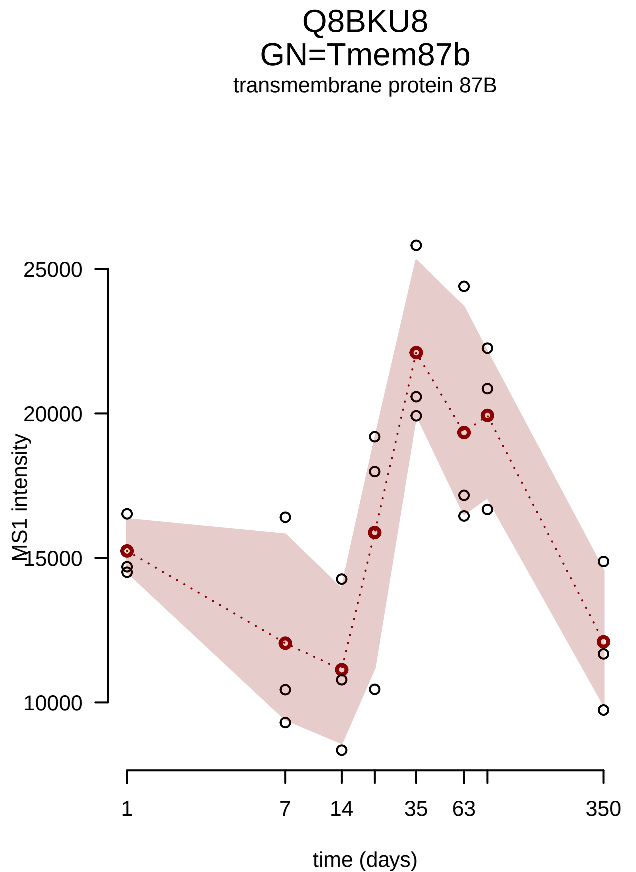
Q8BKU8
GN=Tmem87b
transmembrane protein 87B
MS1 intensity
25000
20000
15000
10000
1
7
14
35
63
350
time (days)

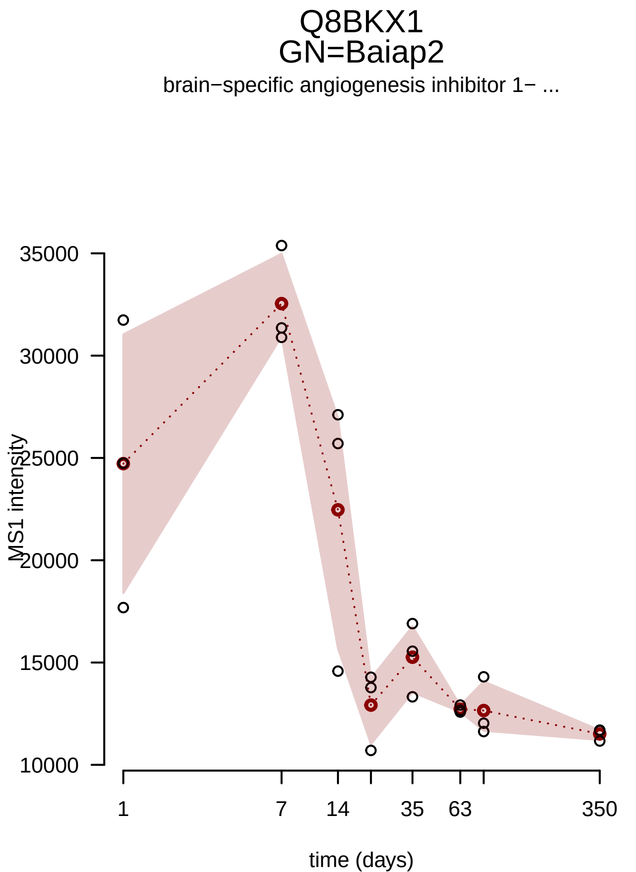
Q8BKX1
GN=Baiap2
brain−specific angiogenesis inhibitor 1− ...
MS1 intensity
35000
30000
25000
20000
15000
10000
1
7
14
35
63
350
time (days)

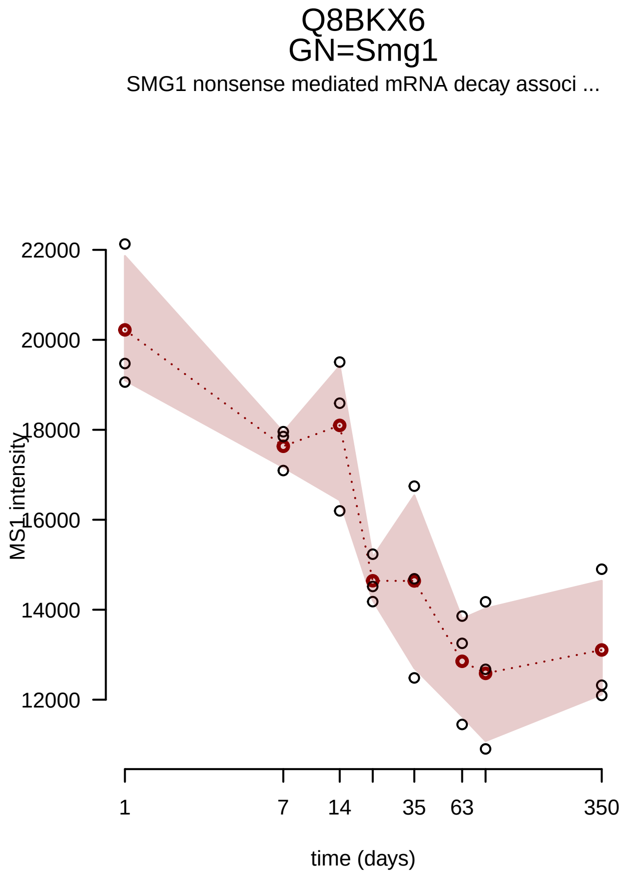
Q8BKX6
GN=Smg1
SMG1 nonsense mediated mRNA decay associ ...
22000
20000
18000
16000
14000
12000
MS1 intensity
1
7
14
35
63
350
time (days)

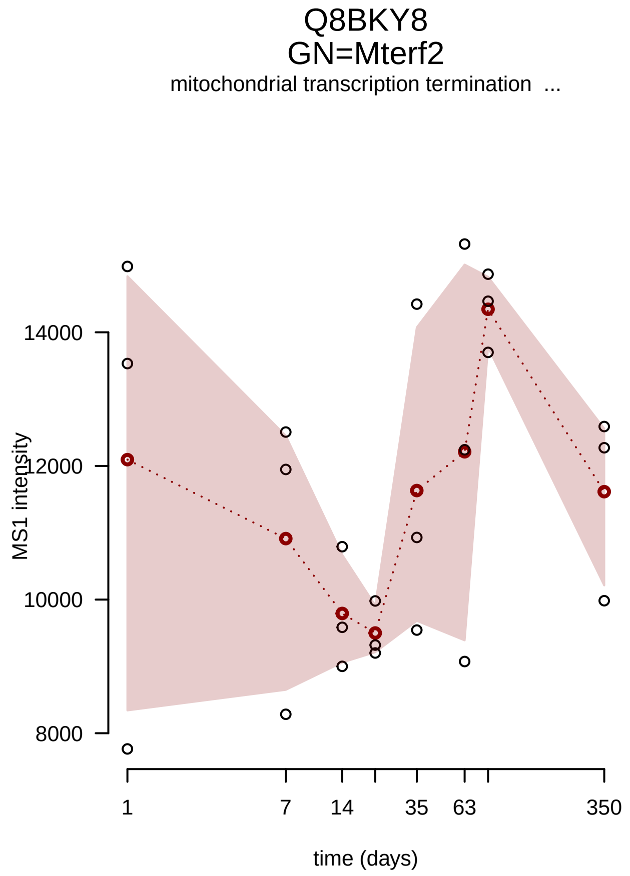
Q8BKY8
GN=Mterf2
mitochondrial transcription termination ...
14000
12000
10000
8000
MS1 intensity
1
7
14
35
63
350
time (days)

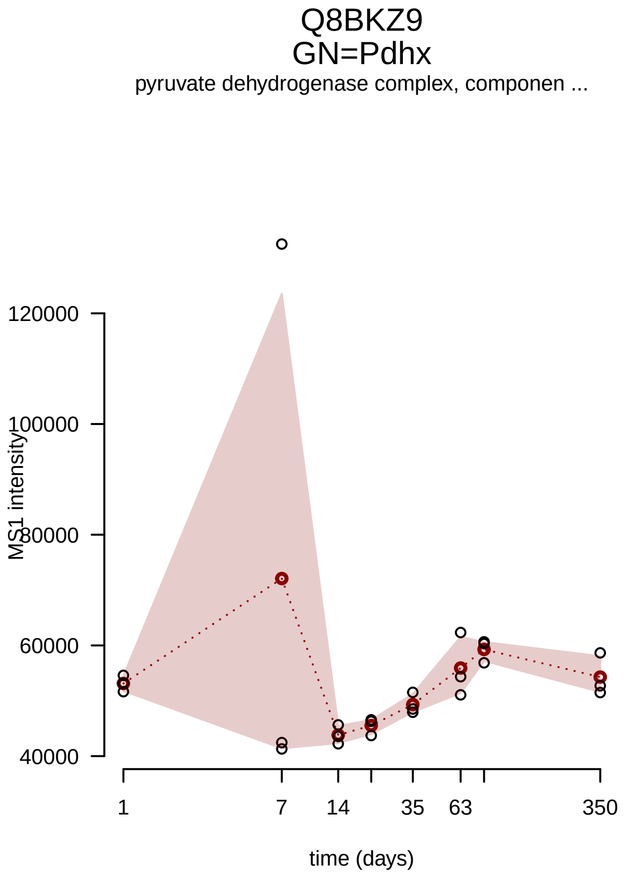
Q8BKZ9
GN=Pdhx
pyruvate dehydrogenase complex, componen ...
120000
100000
80000
60000
40000
MS1 intensity
1
7
14
35
63
350
time (days)

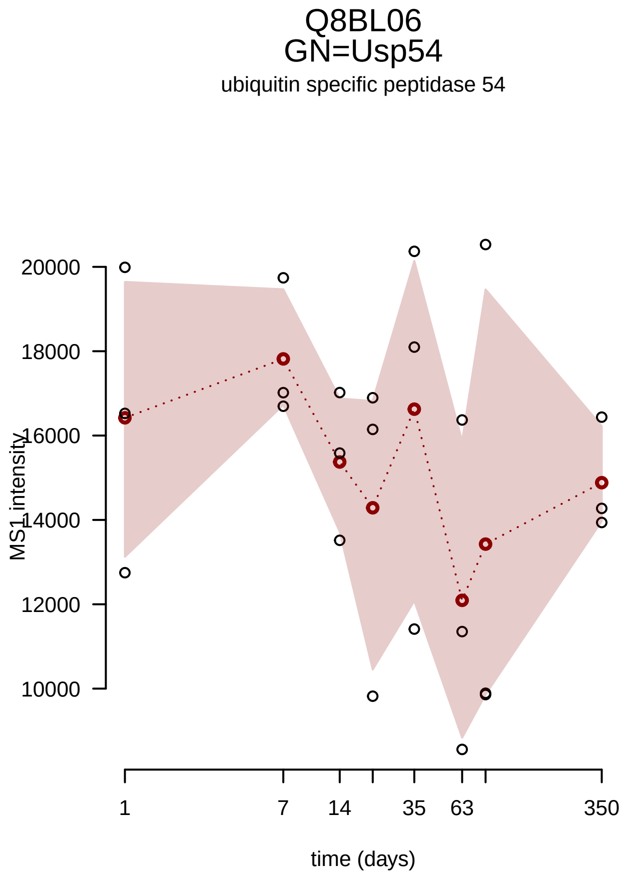
Q8BL06
GN=Usp54
ubiquitin specific peptidase 54
20000
18000
16000
14000
12000
10000
MS1 intensity
1
7
14
35
63
350
time (days)

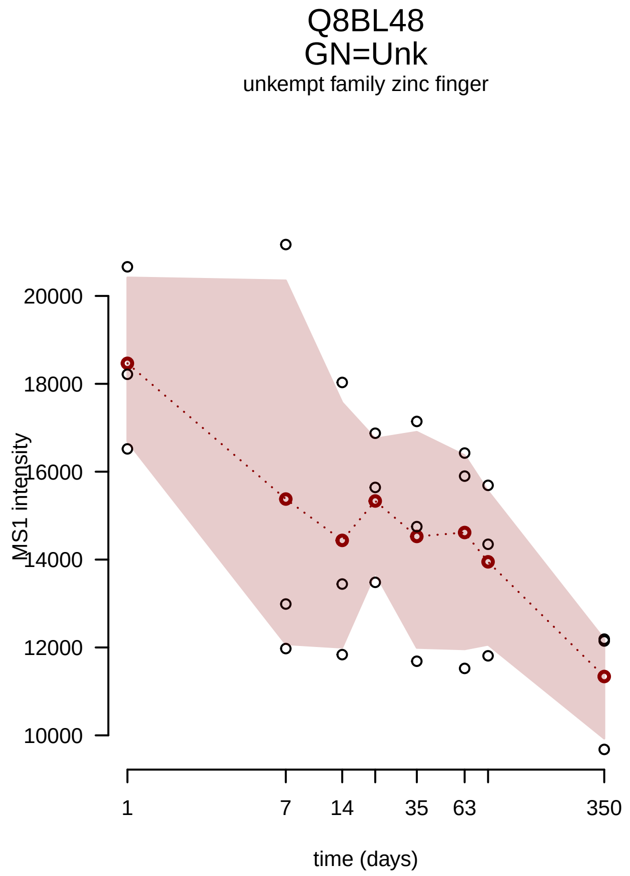
Q8BL48
GN=Unk
unkempt family zinc finger
20000
18000
16000
14000
12000
10000
MS1 intensity
1
7
14
35
63
350
time (days)

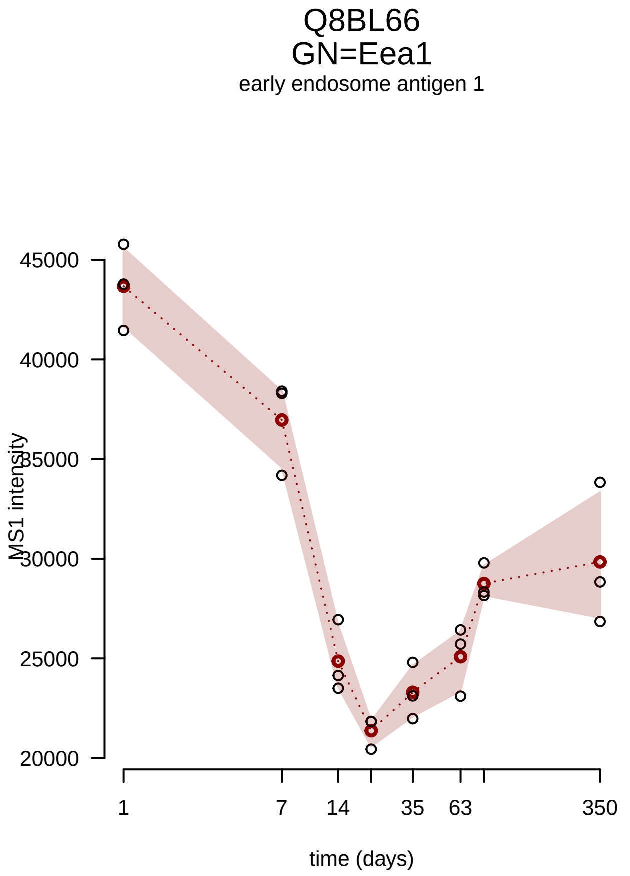
Q8BL66
GN=Eea1
early endosome antigen 1
45000
40000
35000
30000
25000
20000
MS1 intensity
1
7
14
35
63
350
time (days)

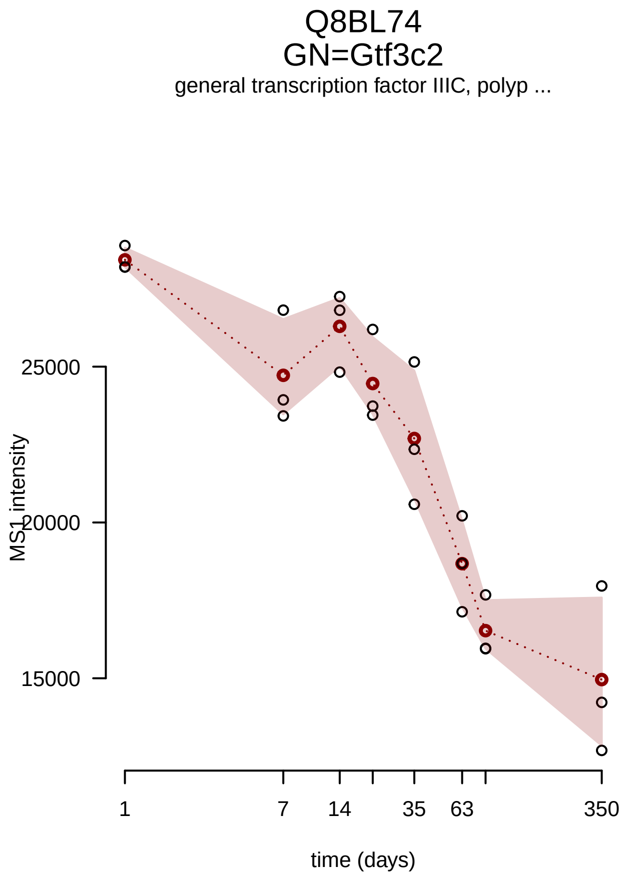
Q8BL74
GN=Gtf3c2
general transcription factor IIIC, polyp ...
25000
20000
15000
MS1 intensity
1
7
14
35
63
350
time (days)

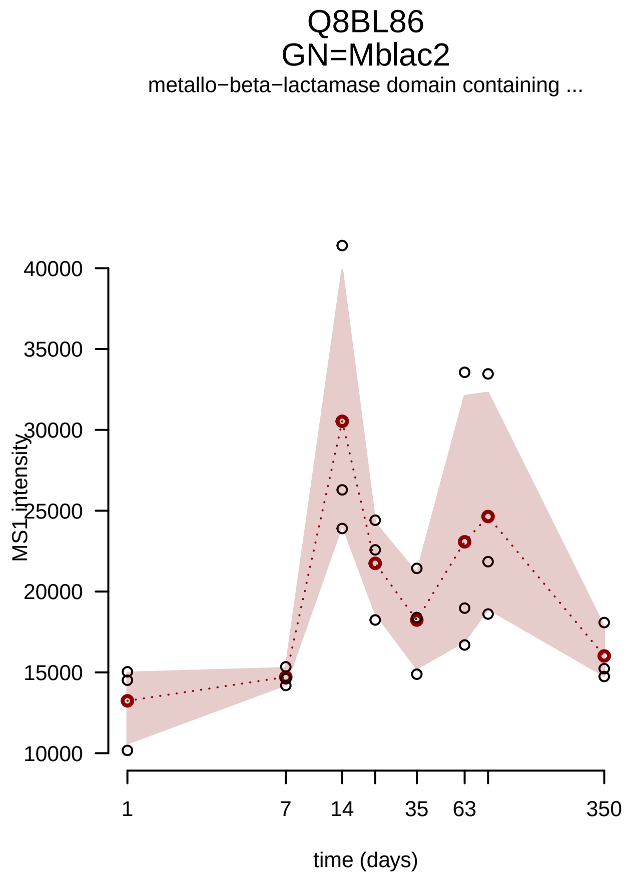
Q8BL86
GN=Mblac2
metallo−beta−lactamase domain containing ...
40000
35000
30000
25000
20000
15000
10000
MS1 intensity
1
7
14
35
63
350
time (days)

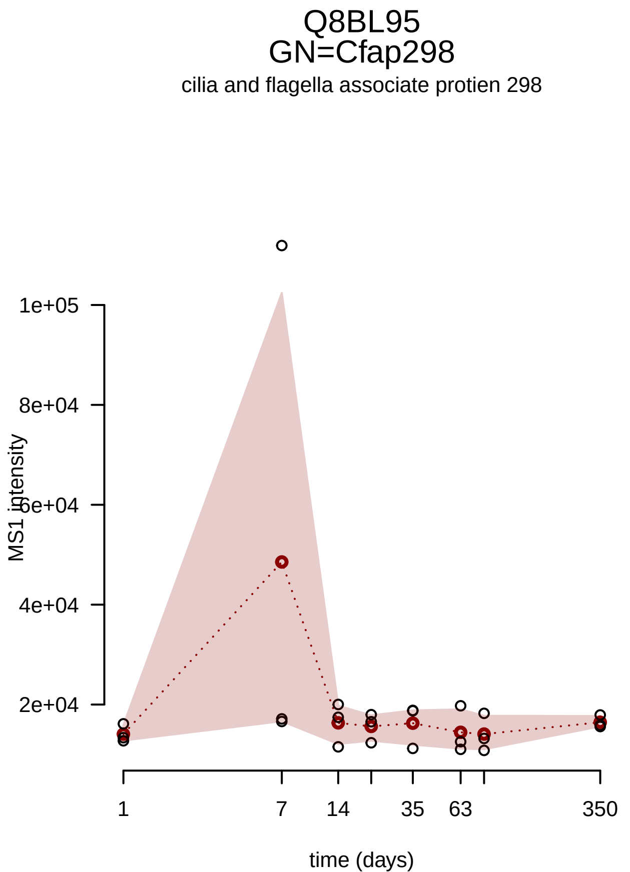
Q8BL95
GN=Cfap298
cilia and flagella associate protien 298
1e+05
8e+04
6e+04
4e+04
2e+04
MS1 intensity
1
7
14
35
63
350
time (days)

Q8BL97
GN=Srsf7
serine and arginine−rich splicing factor ...

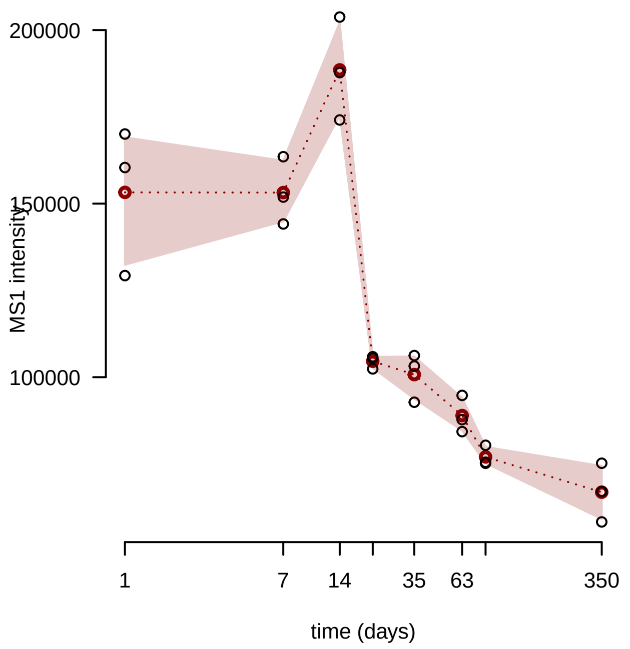

Q8BL99
GN=Dop1a
DOP1 leucine zipper like protein A

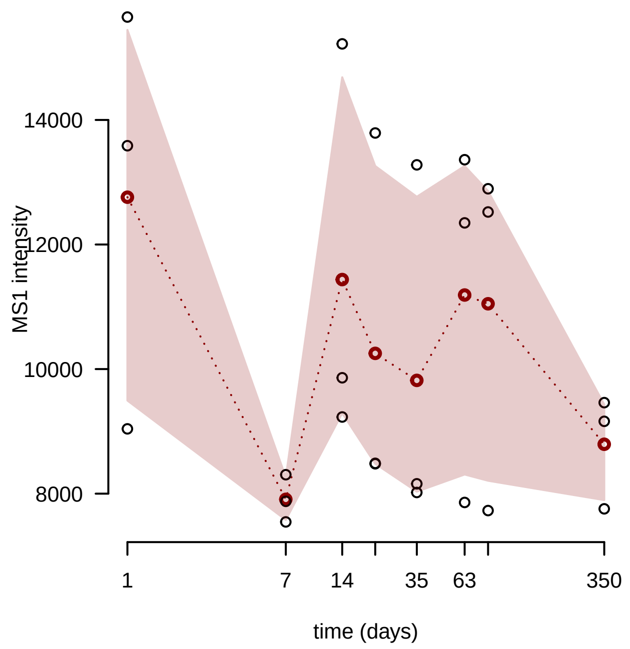

Q8BLB0
GN=Zfp3
zinc finger protein 3

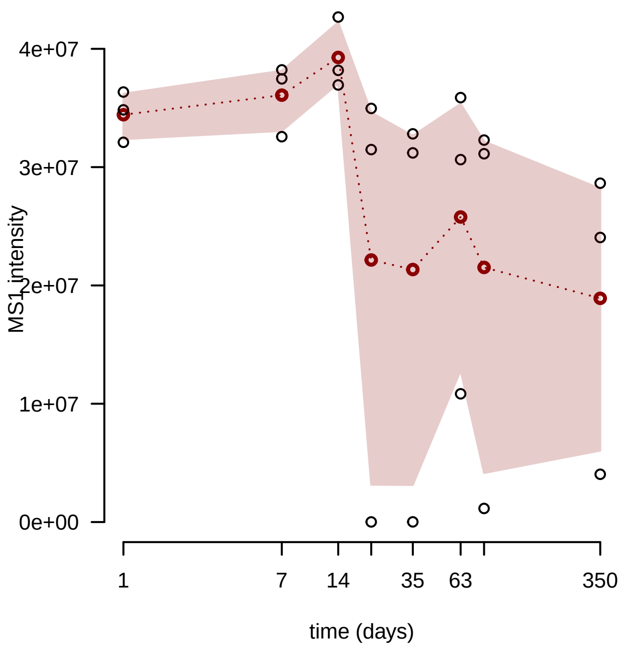

Q8BLB7
GN=L3mbtl3
L3MBTL3 histone methyl−lysine binding pr ...

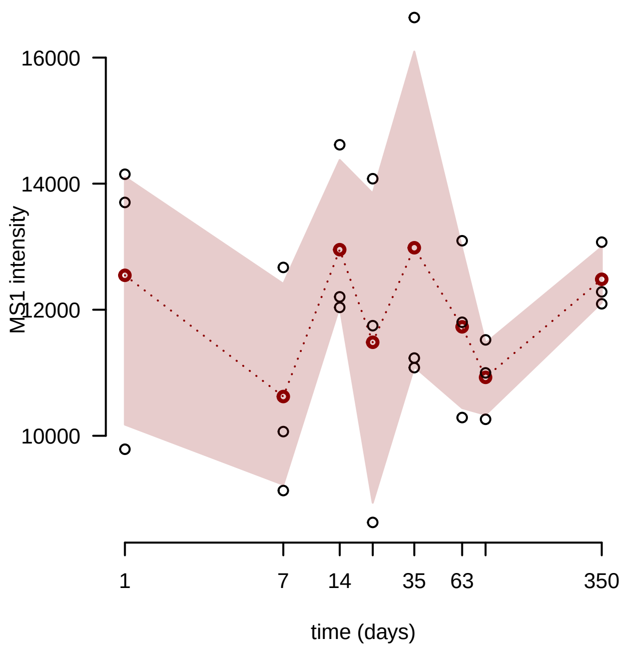

Q8BLF1
GN=Nceh1
neutral cholesterol ester hydrolase 1

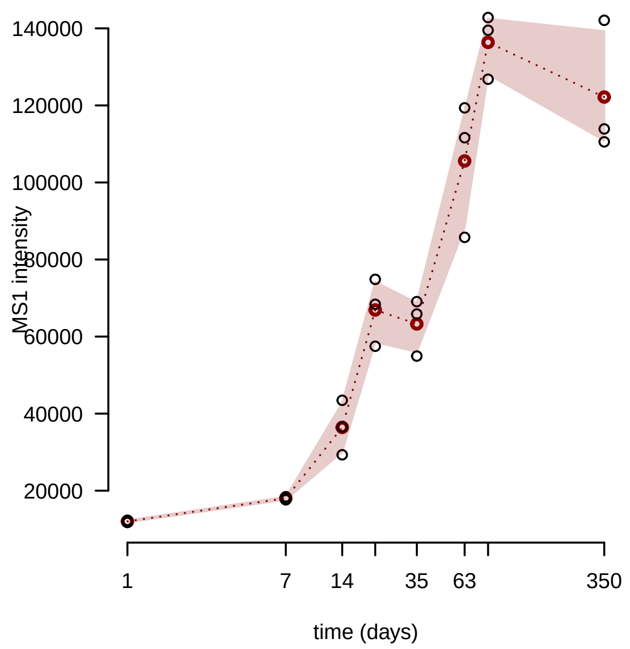

Q8BLG2
GN=Lpar4
lysophosphatidic acid receptor 4

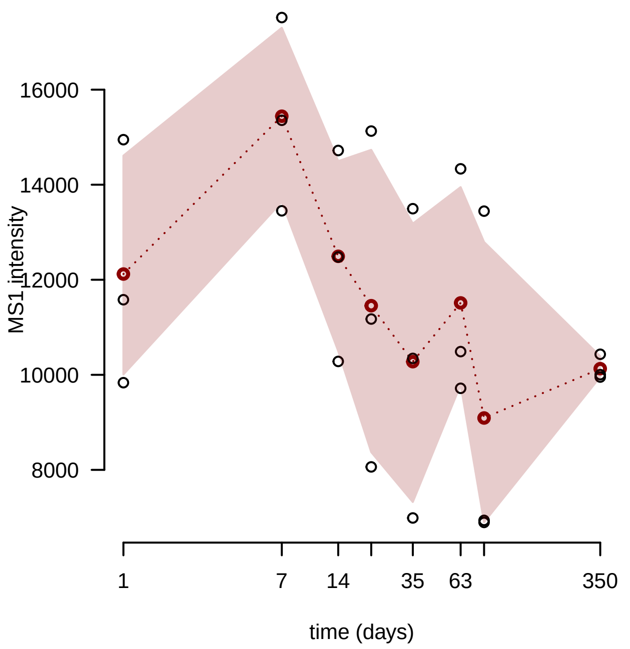

Q8BLH7
GN=Hirip3
HIRA interacting protein 3

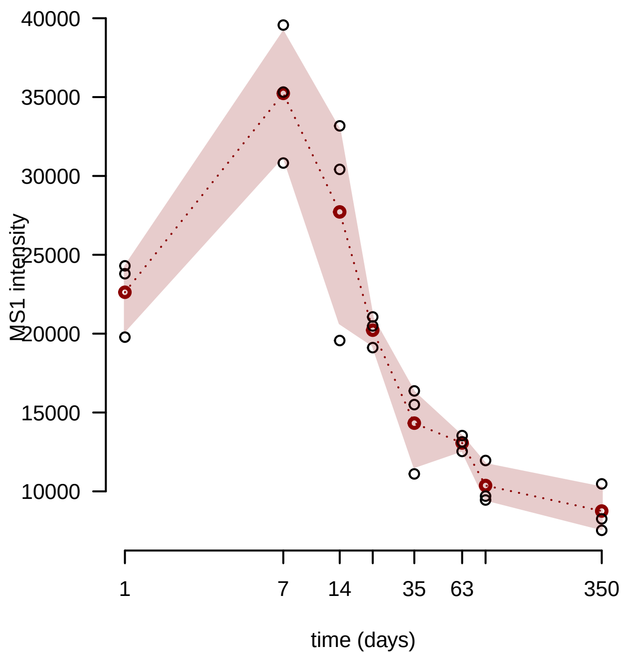

Q8BLK3
GN=Lsamp
limbic system−associated membrane protei ...

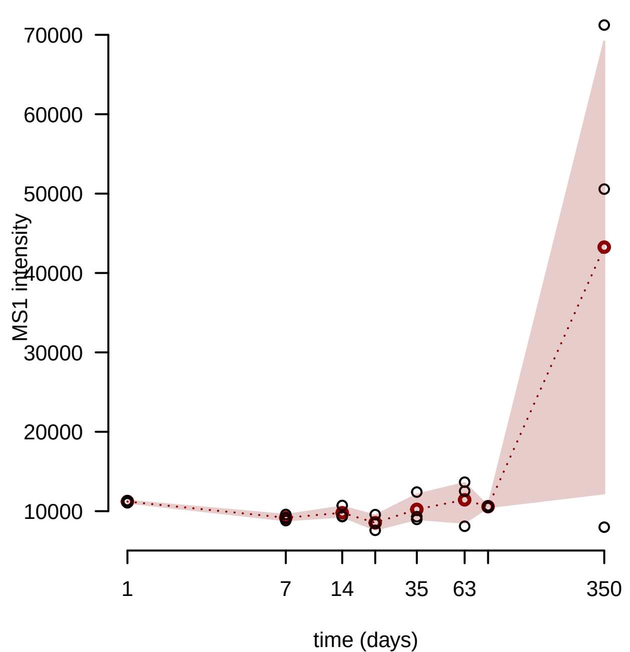

Q8BLN5
GN=Lss
lanosterol synthase

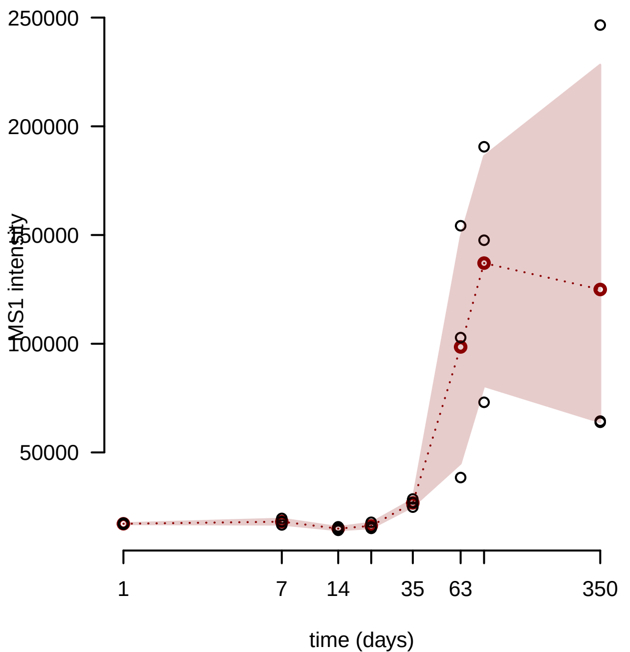

Q8BLR9
GN=Hif1an
hypoxia−inducible factor 1, alpha subuni ...

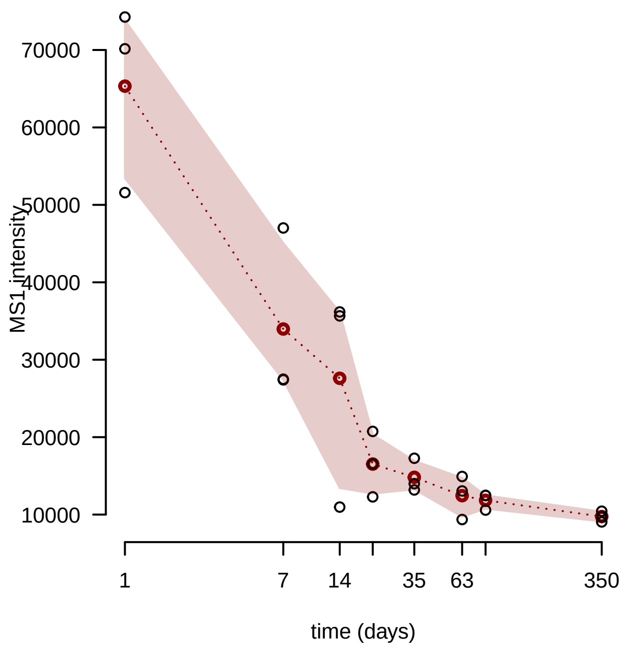

Q8BLU0
GN=Flrt2
fibronectin leucine rich transmembrane p ...

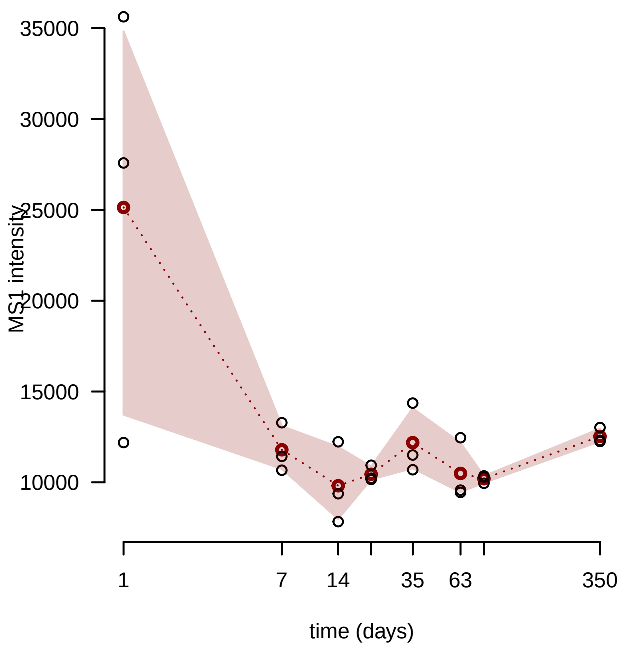

Q8BLV3
GN=Slc9a7
solute carrier family 9 (sodium/hydrogen ...

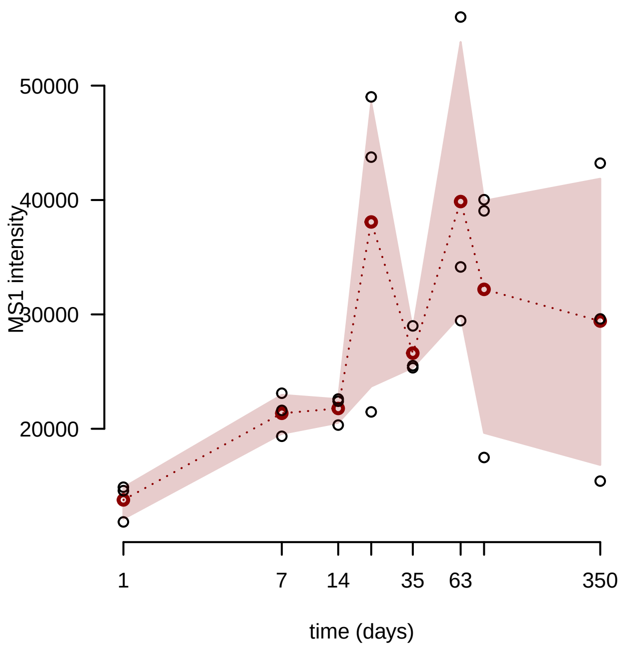

Q8BLX4
GN=Slc35c1
solute carrier family 35, member C1

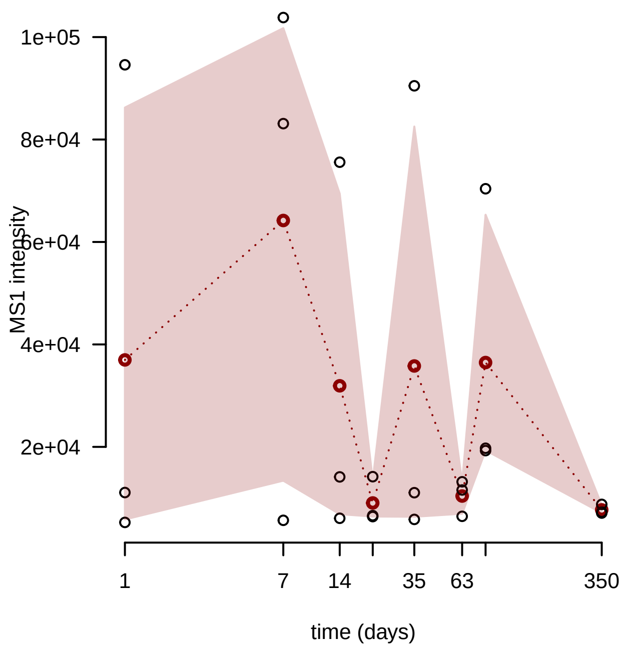

Q8BLX7
GN=Col16a1
collagen, type XVI, alpha 1

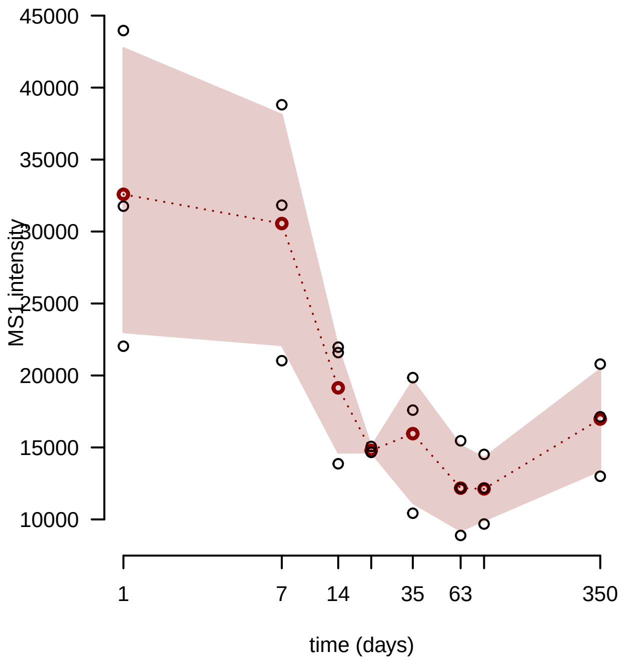

Q8BLY1
GN=Smoc1
SPARC related modular calcium binding 1

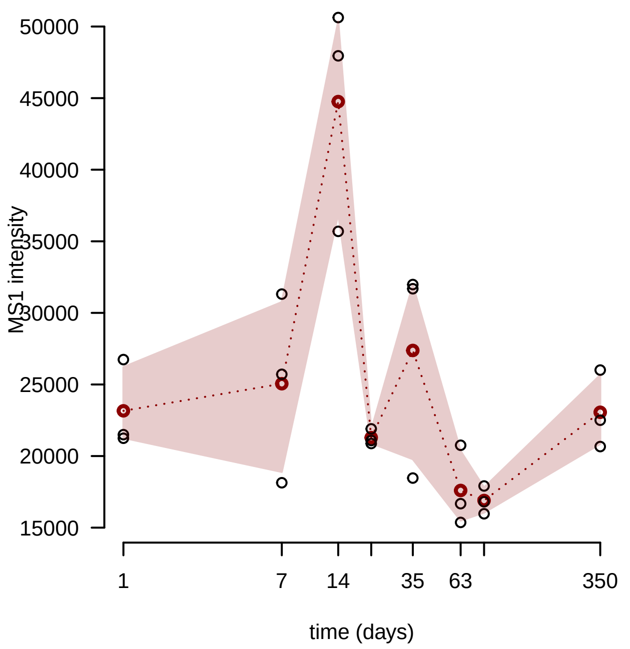

Q8BLY2
GN=Tarsl2
threonyl−tRNA synthetase−like 2

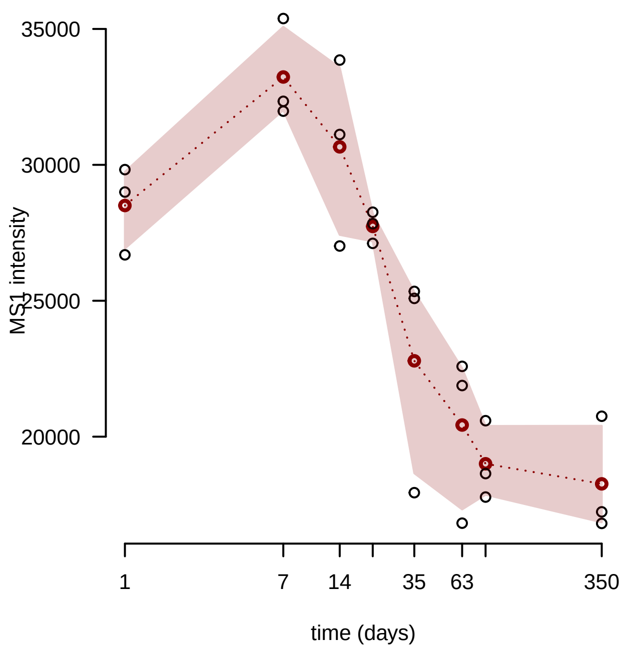

Q8BLY7
GN=Hps6
HPS6, biogenesis of lysosomal organelles ...

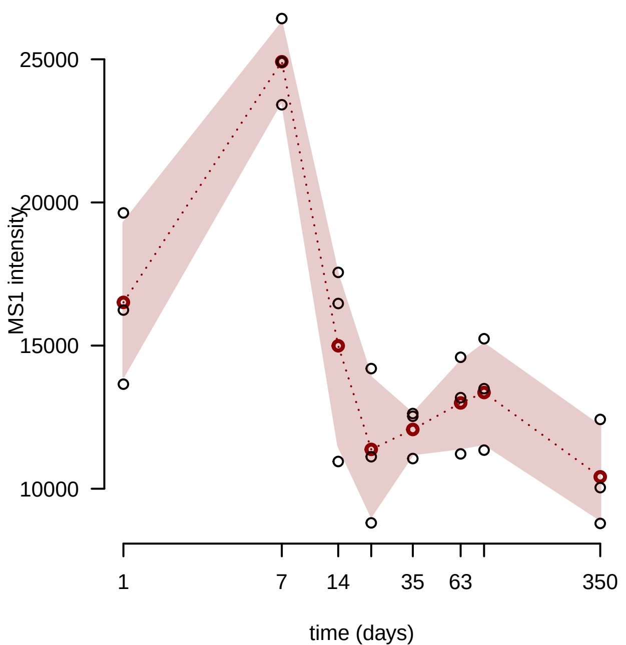

Q8BM39
GN=Prpf18
pre−mRNA processing factor 18

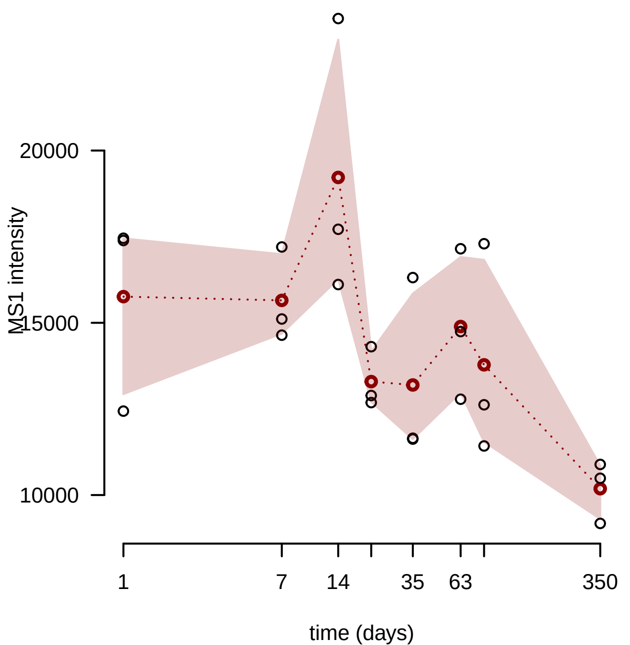

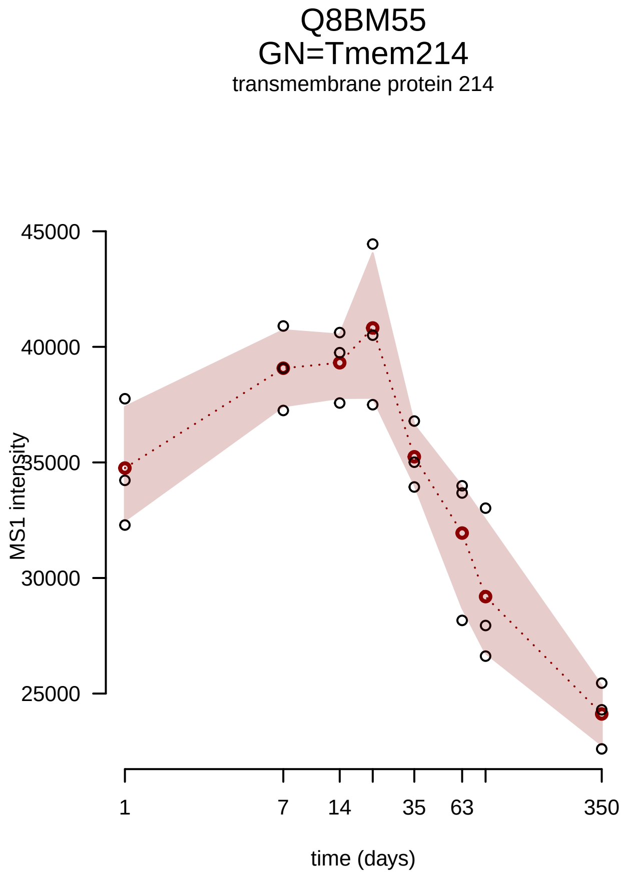
Q8BM55
GN=Tmem214
transmembrane protein 214
45000
40000
35000
30000
25000
MS1 intensity
1
7
14
35
63
350
time (days)

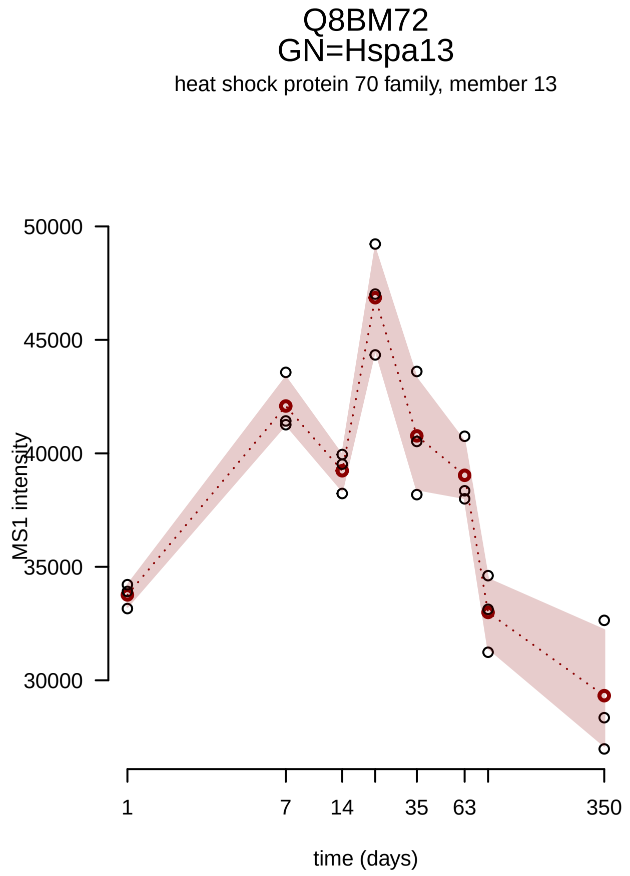
Q8BM72
GN=Hspa13
heat shock protein 70 family, member 13
50000
45000
40000
35000
30000
MS1 intensity
1
7
14
35
63
350
time (days)

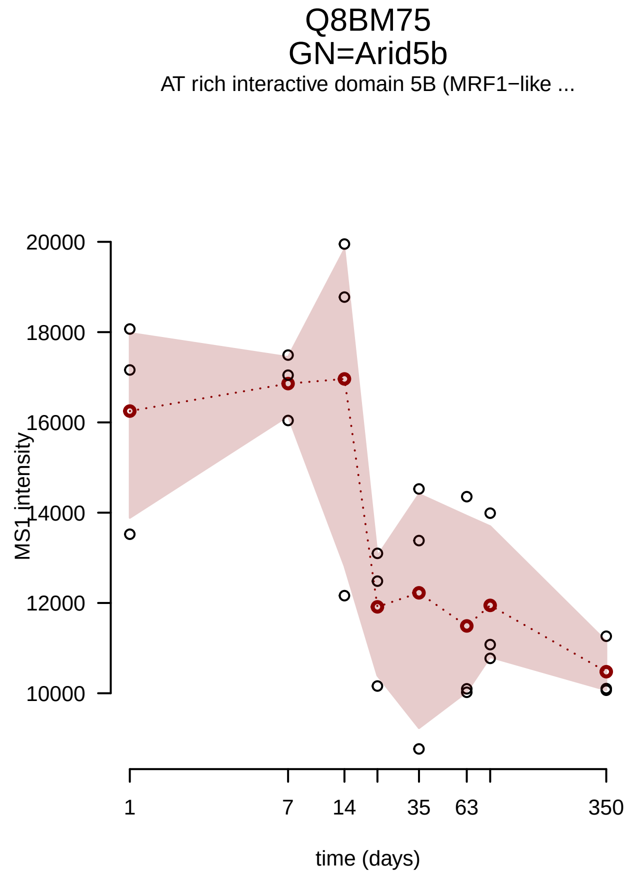
Q8BM75
GN=Arid5b
AT rich interactive domain 5B (MRF1−like ...
20000
18000
16000
14000
12000
10000
MS1 intensity
1
7
14
35
63
350
time (days)

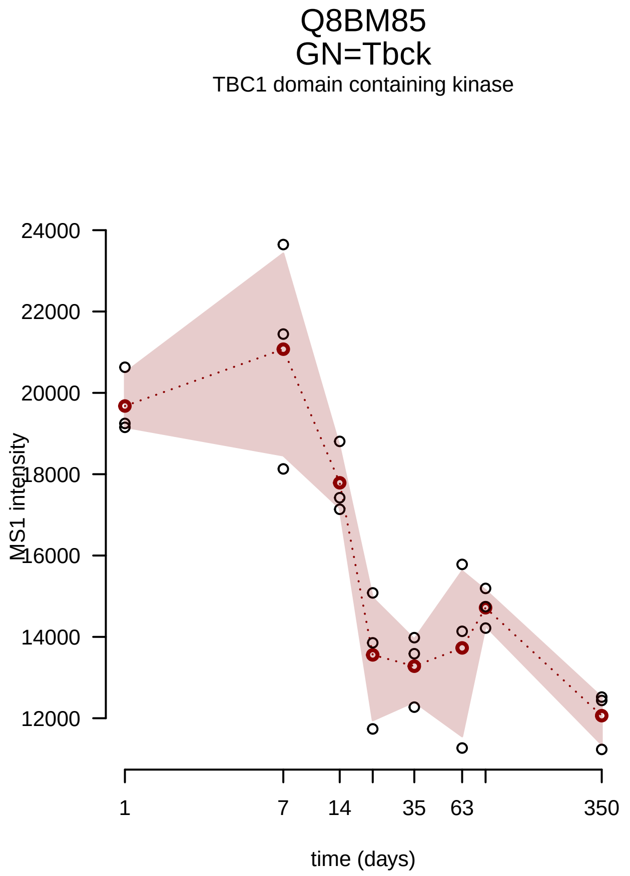
Q8BM85
GN=Tbck
TBC1 domain containing kinase
24000
22000
20000
18000
16000
14000
12000
MS1 intensity
1
7
14
35
63
350
time (days)

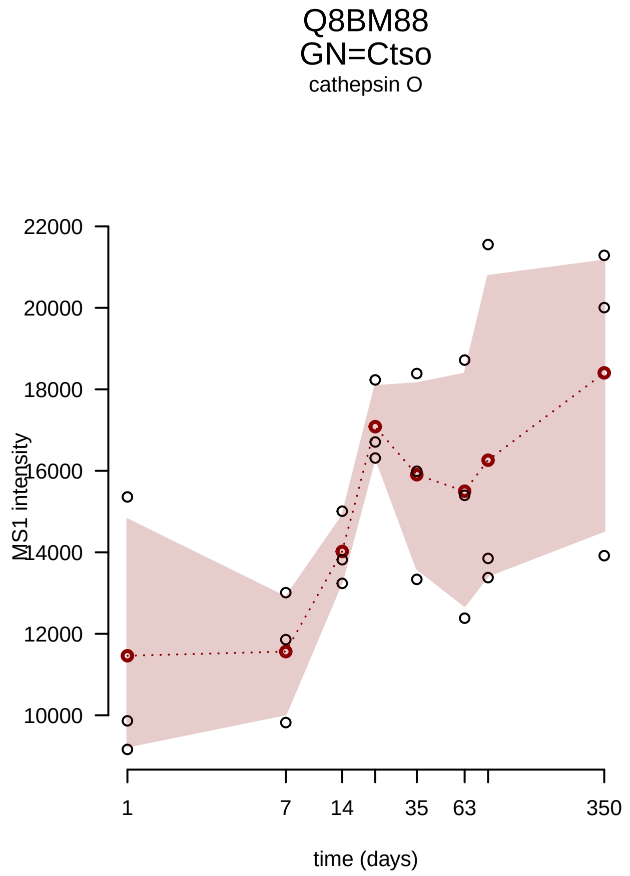
Q8BM88
GN=Ctso
cathepsin O
22000
20000
18000
16000
14000
12000
10000
MS1 intensity
1
7
14
35
63
350
time (days)

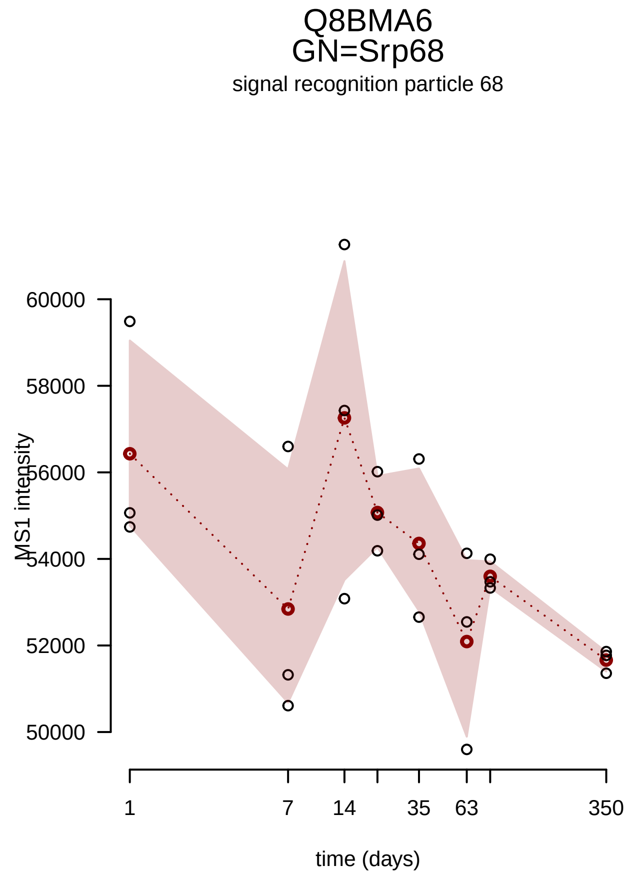
Q8BMA6
GN=Srp68
signal recognition particle 68
60000
58000
56000
54000
52000
50000
MS1 intensity
1
7
14
35
63
350
time (days)

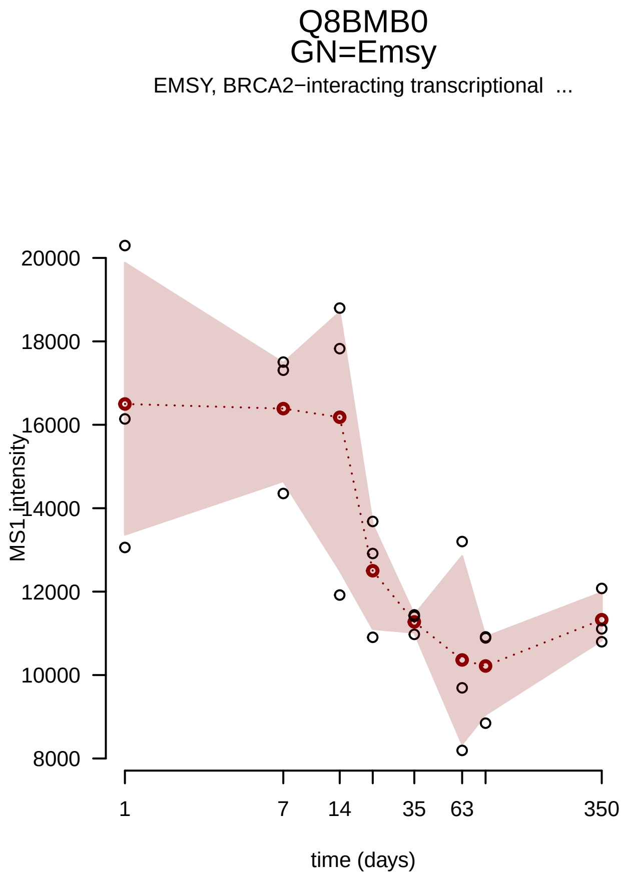
Q8BMB0
GN=Emsy
EMSY, BRCA2−interacting transcriptional ...
20000
18000
16000
14000
12000
10000
8000
MS1 intensity
1
7
14
35
63
350
time (days)

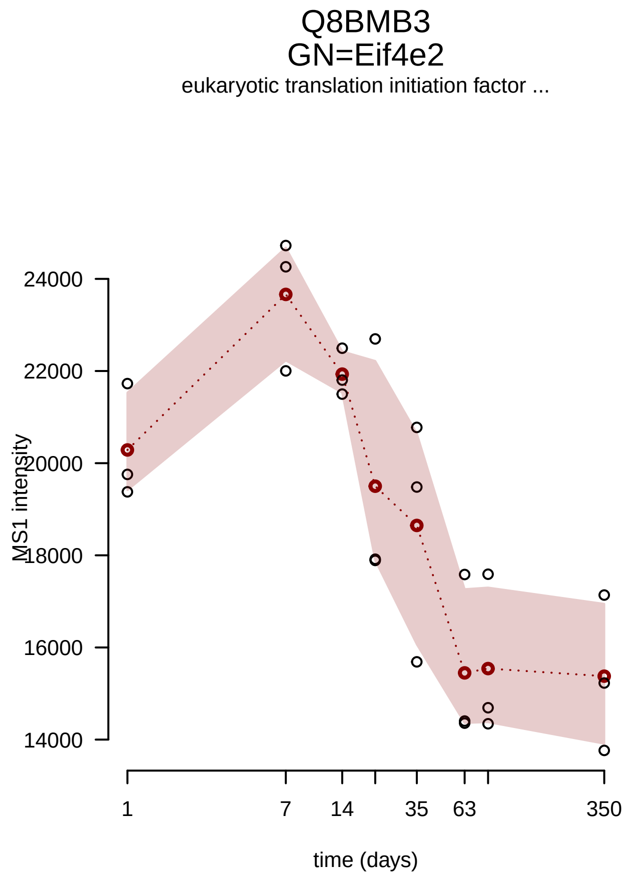
Q8BMB3
GN=Eif4e2
eukaryotic translation initiation factor ...
24000
22000
20000
18000
16000
14000
MS1 intensity
1
7
14
35
63
350
time (days)

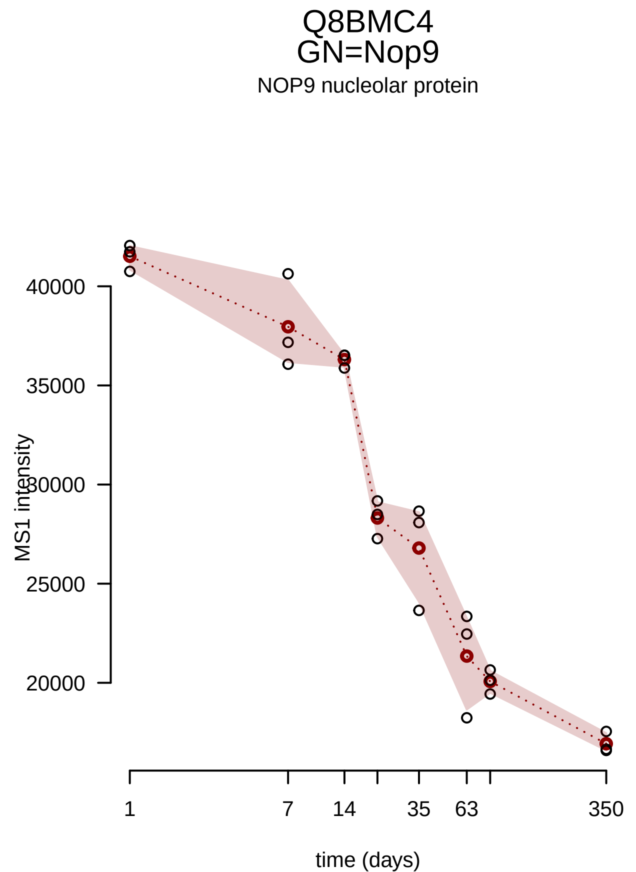
Q8BMC4
GN=Nop9
NOP9 nucleolar protein
40000
35000
30000
25000
20000
MS1 intensity
1
7
14
35
63
350
time (days)

Q8BMD6
GN=Tmem260
transmembrane protein 260

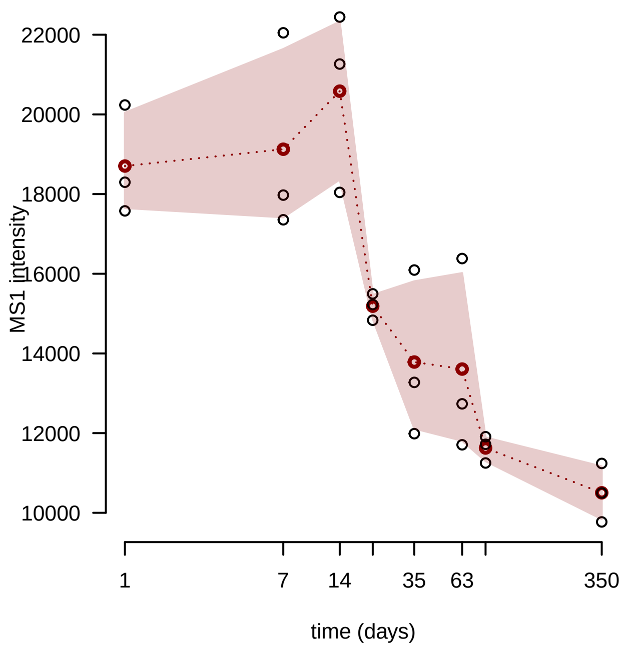

Q8BMD8
GN=Slc25a24
solute carrier family 25 (mitochondrial ...

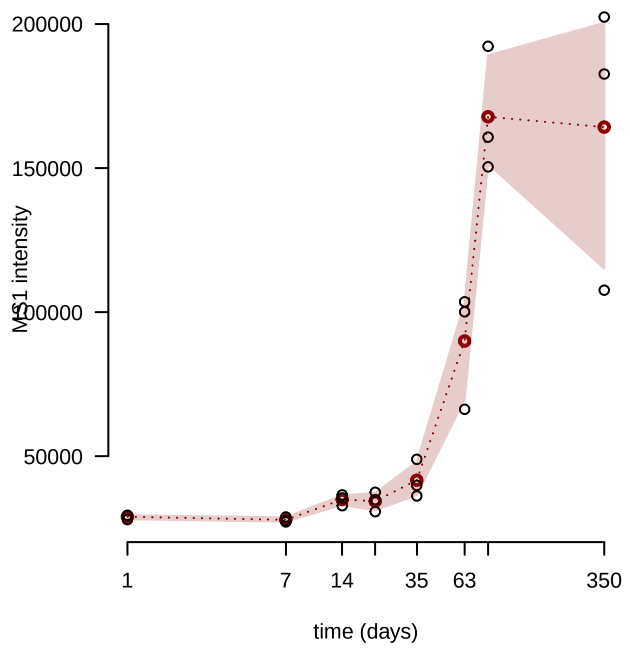

Q8BME9
GN=Cbln4
cerebellin 4 precursor protein

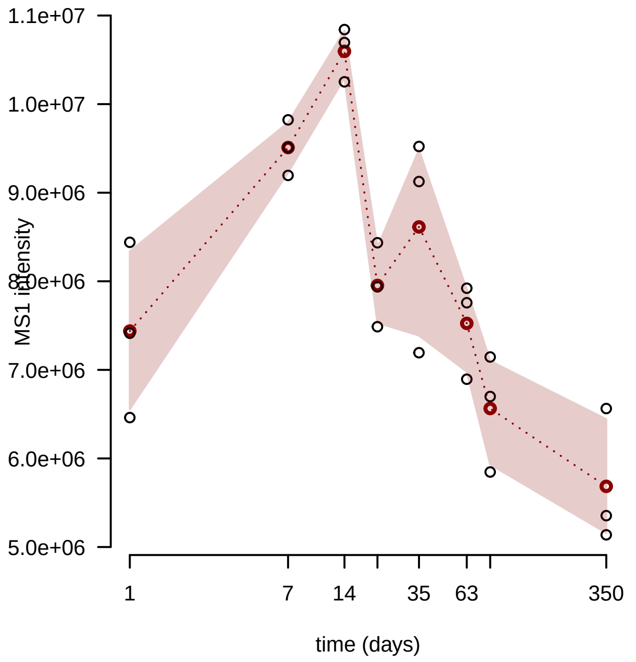

Q8BMF3
GN=Me3
malic enzyme 3, NADP(+)−dependent, mitoc ...

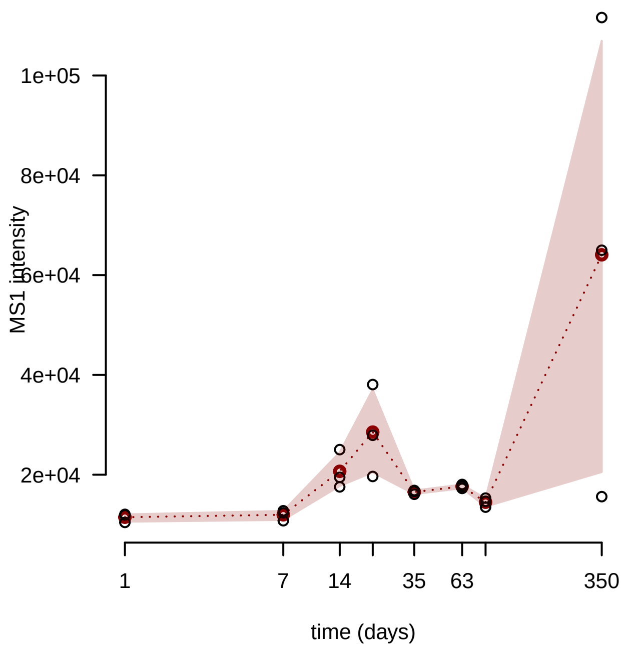

Q8BMF4
GN=Dlat
dihydrolipoamide S−acetyltransferase (E2 ...

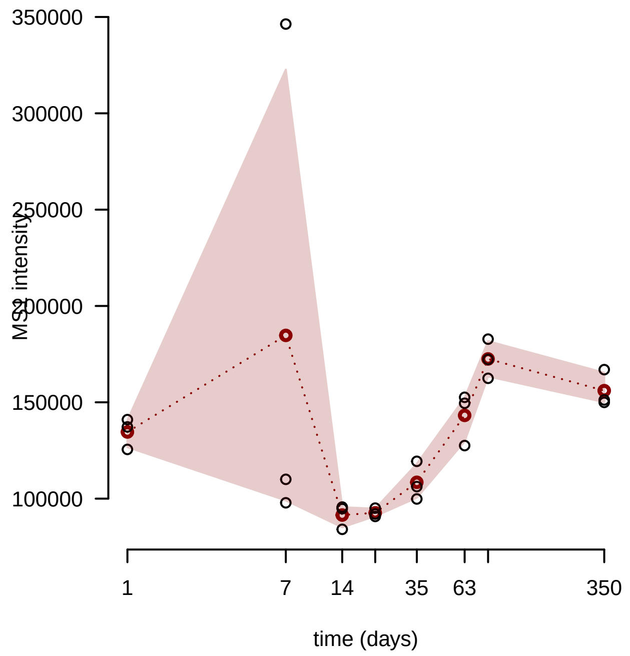

Q8BMG1
GN=Atrip
ATR interacting protein

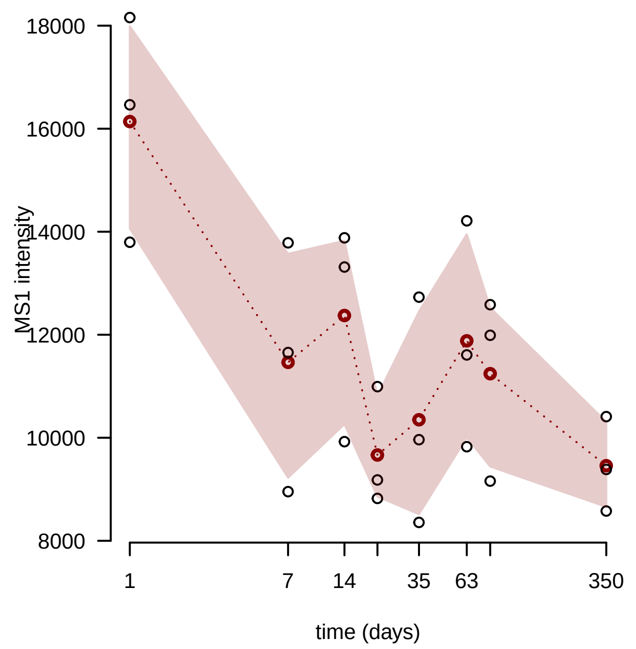

Q8BMG7
GN=Rab3gap2
RAB3 GTPase activating protein subunit 2

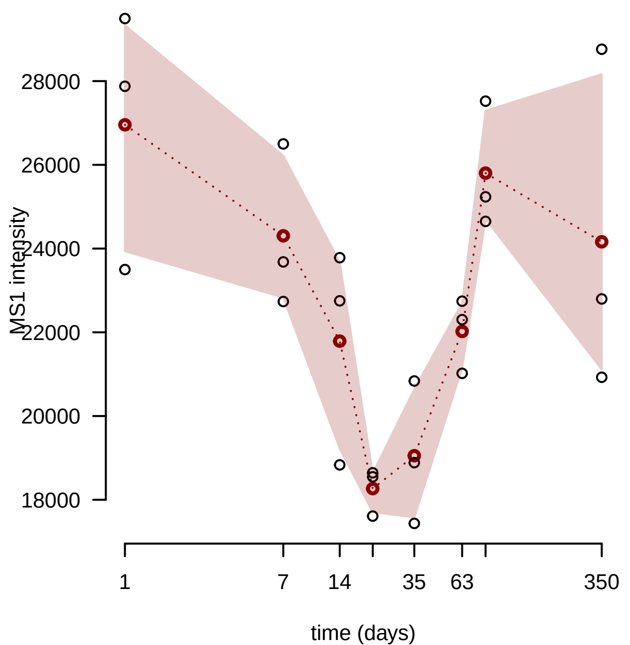

Q8BMG8
GN=Slc25a32
solute carrier family 25, member 32

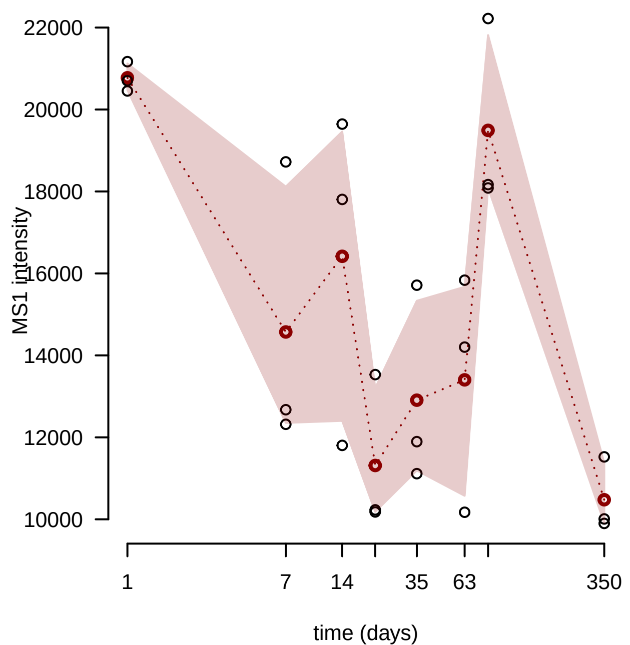

Q8BMI0
GN=Fbxo38
F−box protein 38

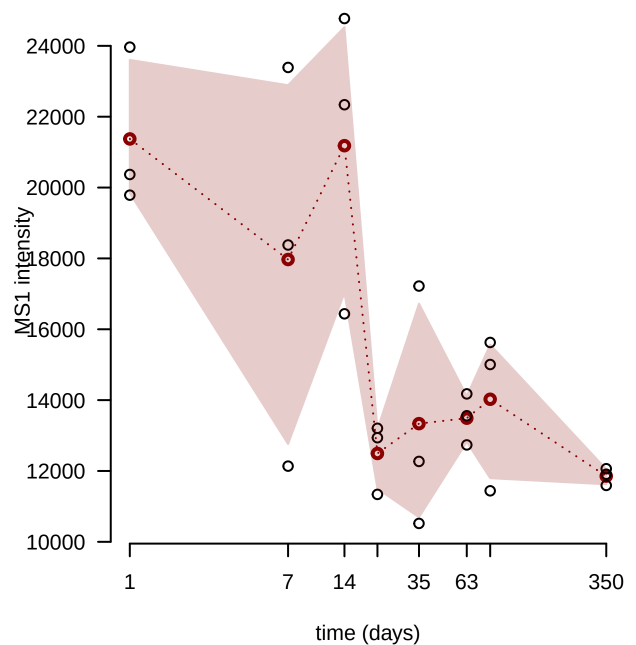

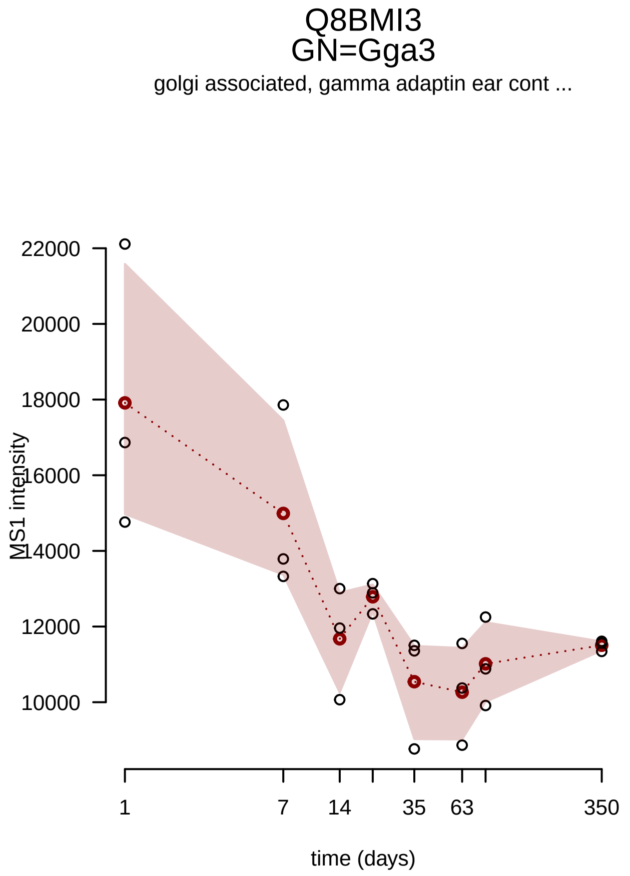
Q8BMI3
GN=Gga3
golgi associated, gamma adaptin ear cont ...
MS1 intensity
22000
20000
18000
16000
14000
12000
10000
1
7
14
35
63
350
time (days)

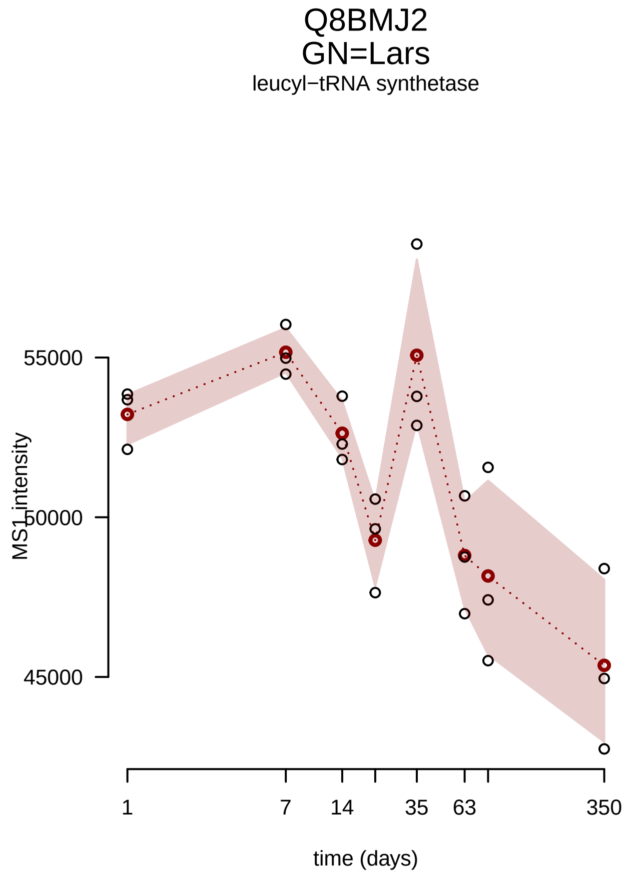
Q8BMJ2
GN=Lars
leucyl−tRNA synthetase
MS1 intensity
55000
50000
45000
1
7
14
35
63
350
time (days)

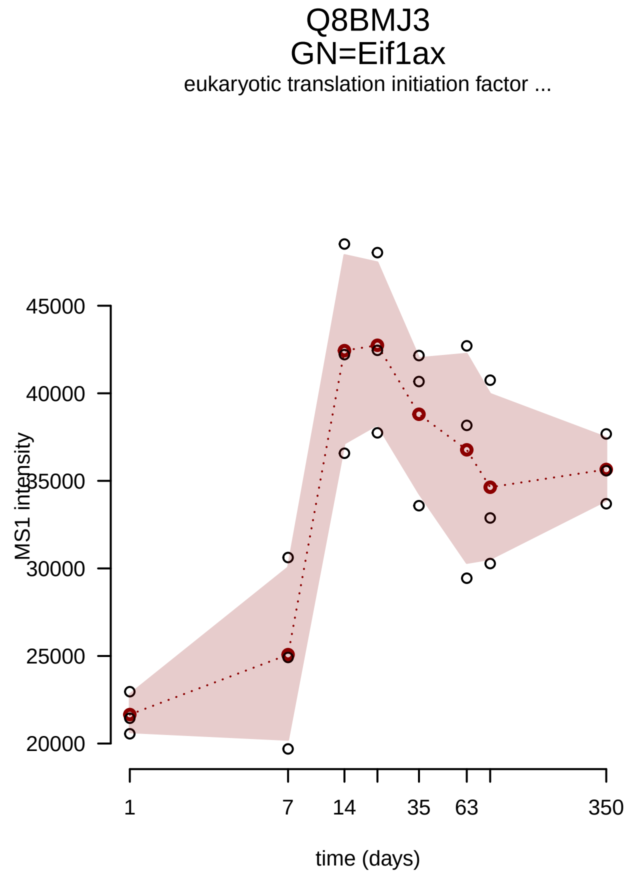
Q8BMJ3
GN=Eif1ax
eukaryotic translation initiation factor ...
MS1 intensity
45000
40000
35000
30000
25000
20000
1
7
14
35
63
350
time (days)

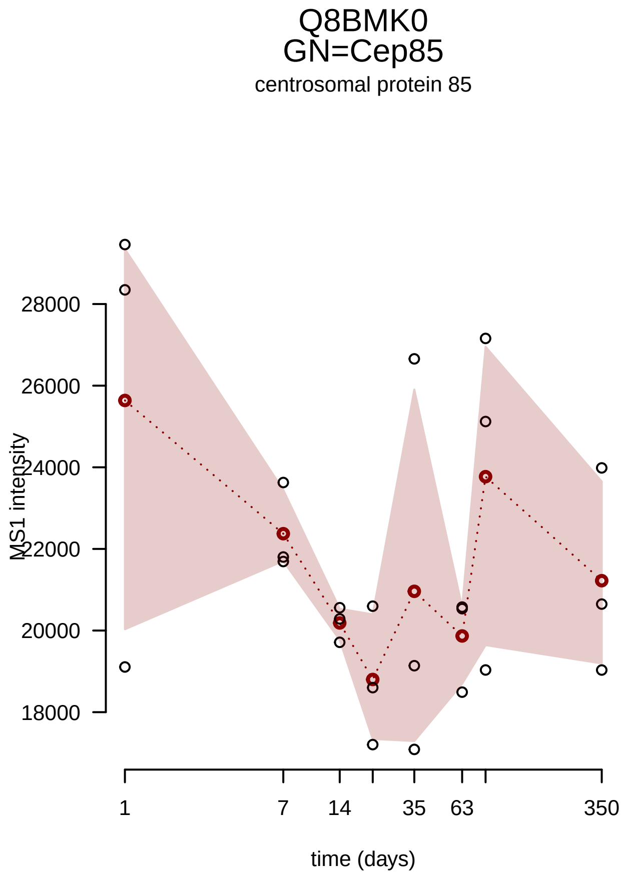
Q8BMK0
GN=Cep85
centrosomal protein 85
MS1 intensity
28000
26000
24000
22000
20000
18000
1
7
14
35
63
350
time (days)

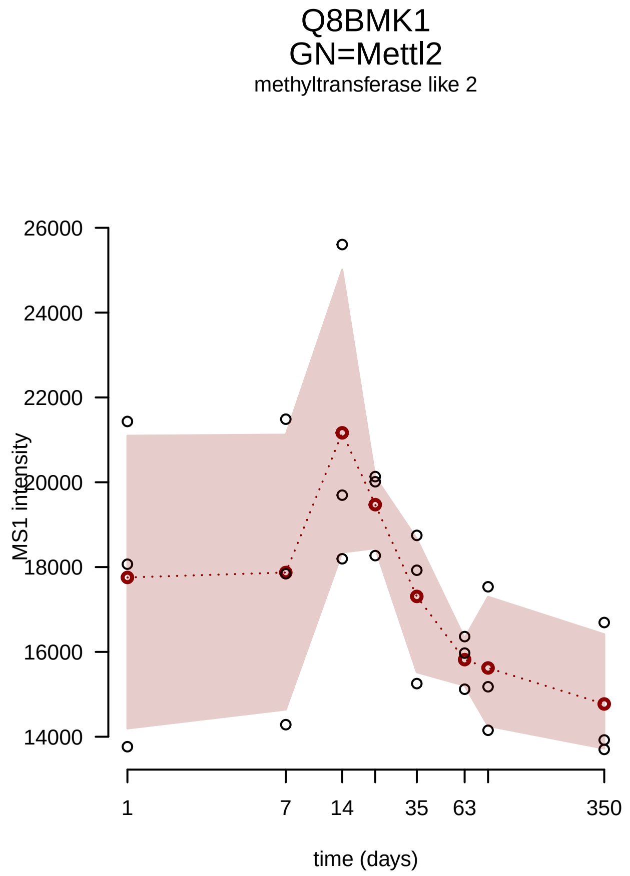
Q8BMK1
GN=Mettl2
methyltransferase like 2
MS1 intensity
26000
24000
22000
20000
18000
16000
14000
1
7
14
35
63
350
time (days)

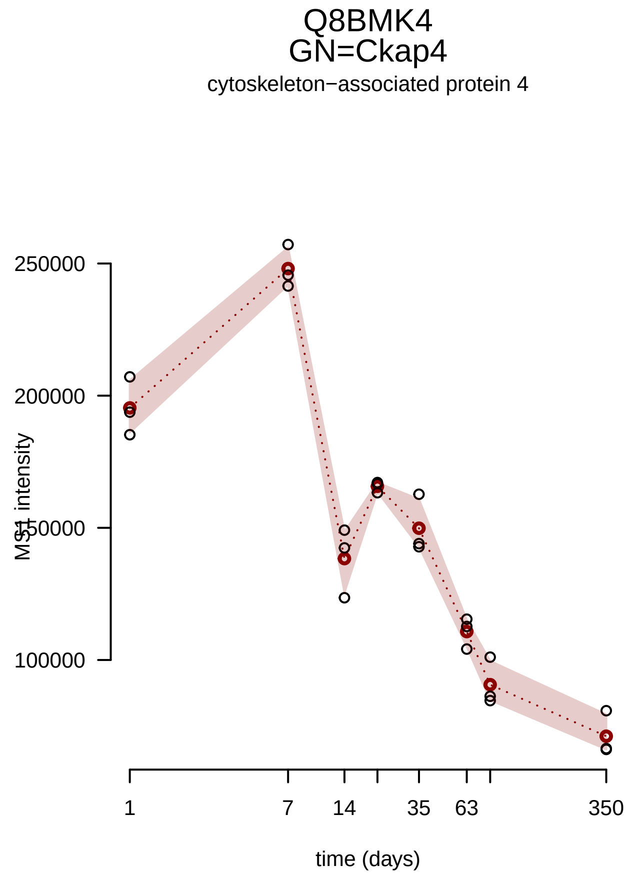
Q8BMK4
GN=Ckap4
cytoskeleton−associated protein 4
MS1 intensity
250000
200000
150000
100000
1
7
14
35
63
350
time (days)

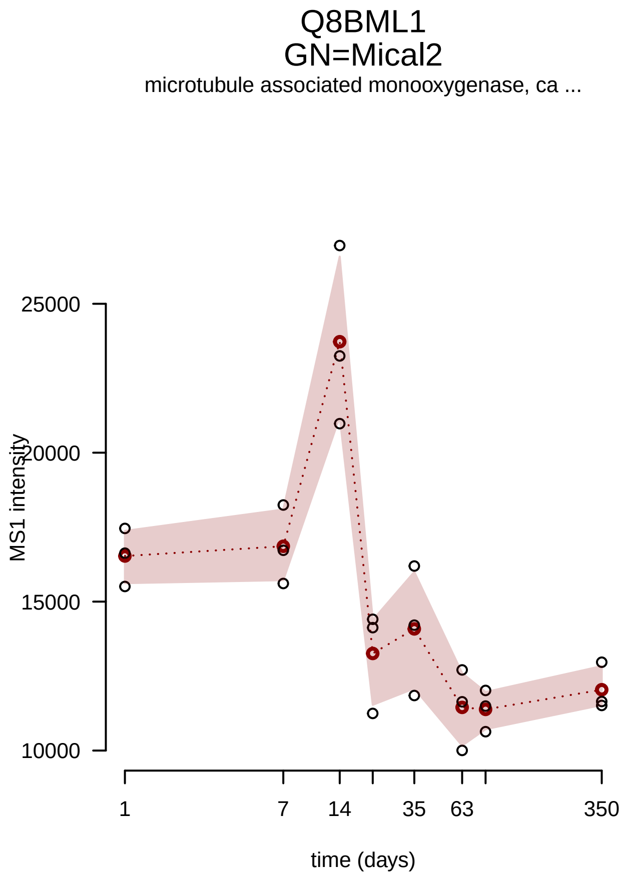
Q8BML1
GN=Mical2
microtubule associated monooxygenase, ca ...
MS1 intensity
25000
20000
15000
10000
1
7
14
35
63
350
time (days)

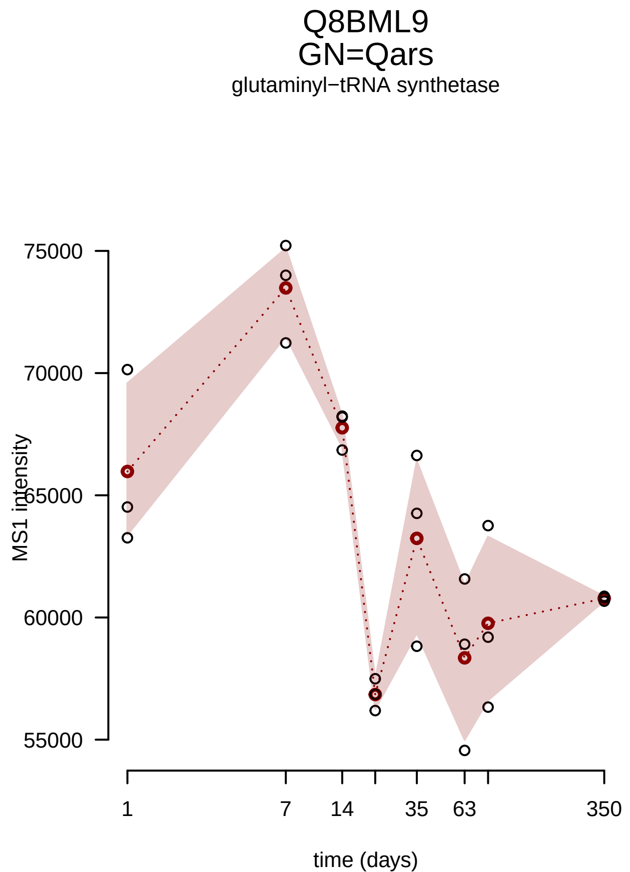
Q8BML9
GN=Qars
glutaminyl−tRNA synthetase
MS1 intensity
75000
70000
65000
60000
55000
1
7
14
35
63
350
time (days)

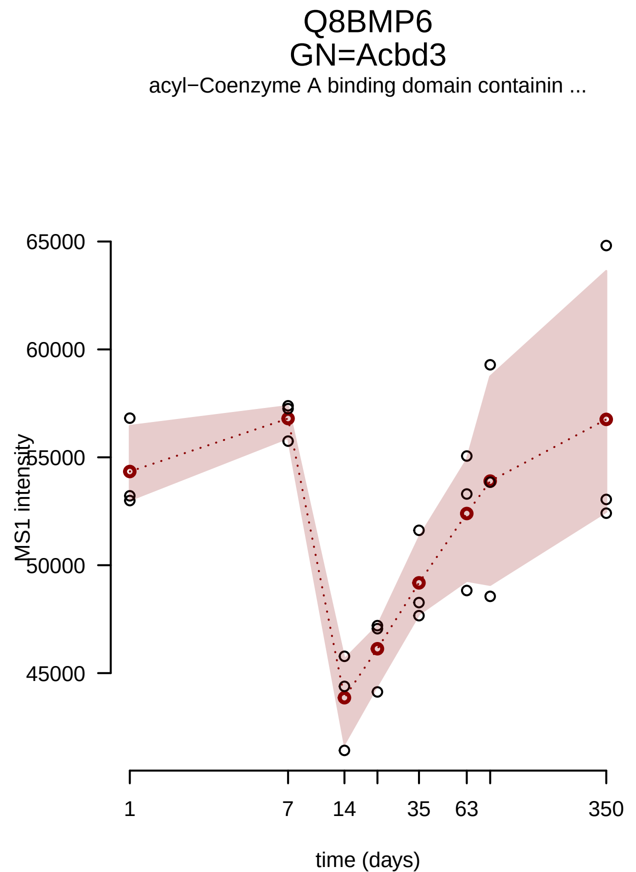
Q8BMP6
GN=Acbd3
acyl−Coenzyme A binding domain containin ...
MS1 intensity
65000
60000
55000
50000
45000
1
7
14
35
63
350
time (days)

Q8BMQ2
GN=Gtf3c4
general transcription factor IIIC, polyp ...

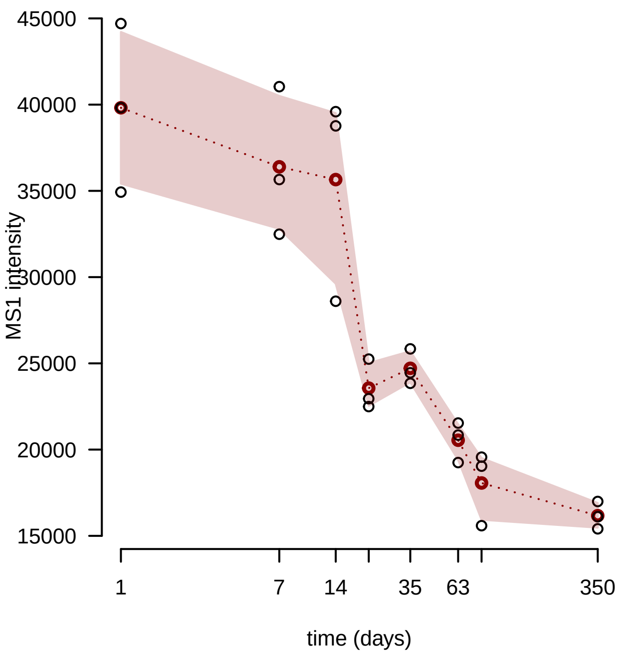

Q8BMQ3
GN=Bnc2
basonuclin 2

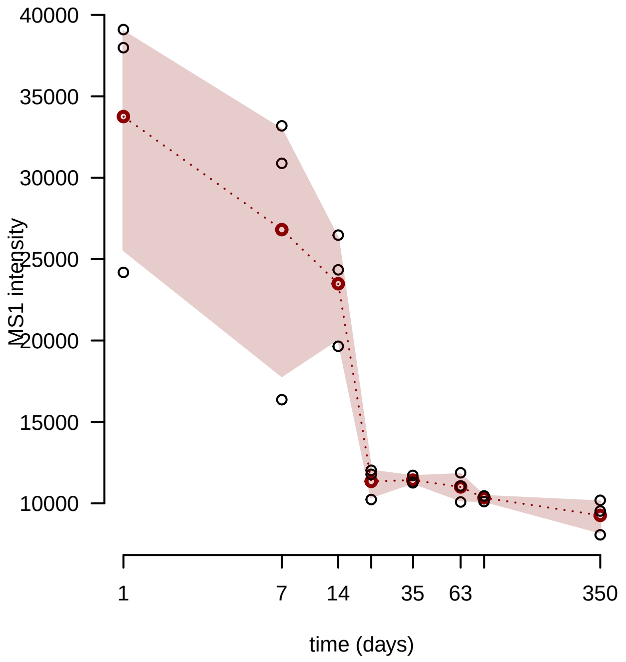

Q8BMQ8
GN=Mon1b
MON1 homolog B, secretory traffciking as ...

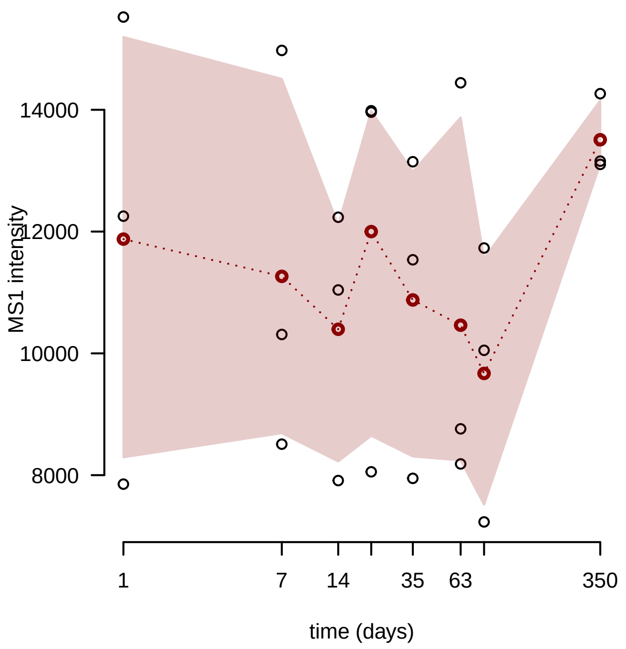

Q8BMS1
GN=Hadha
hydroxyacyl−CoA dehydrogenase trifunctio ...

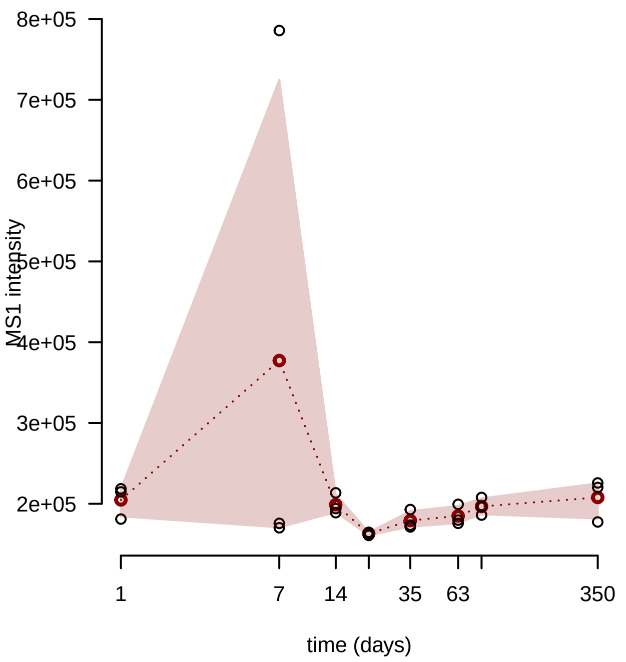

Q8BMS4
GN=Coq3
coenzyme Q3 methyltransferase

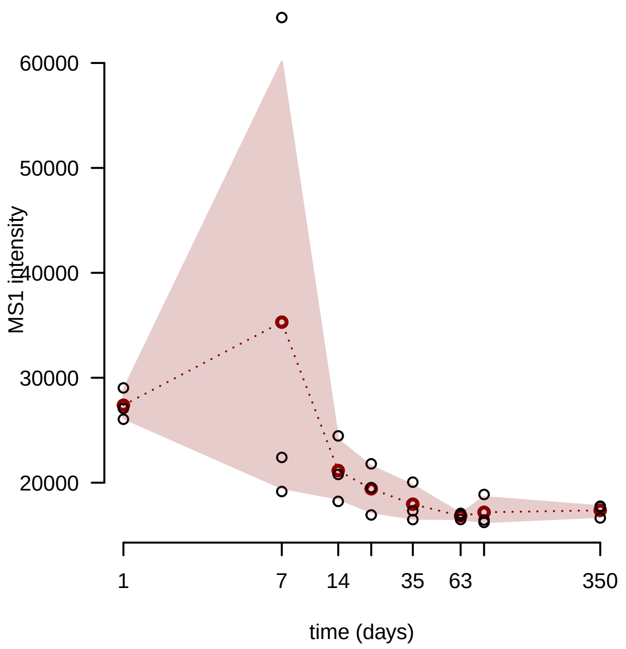

Q8BMS9
GN=Rassf2
Ras association (RalGDS/AF−6) domain fam ...

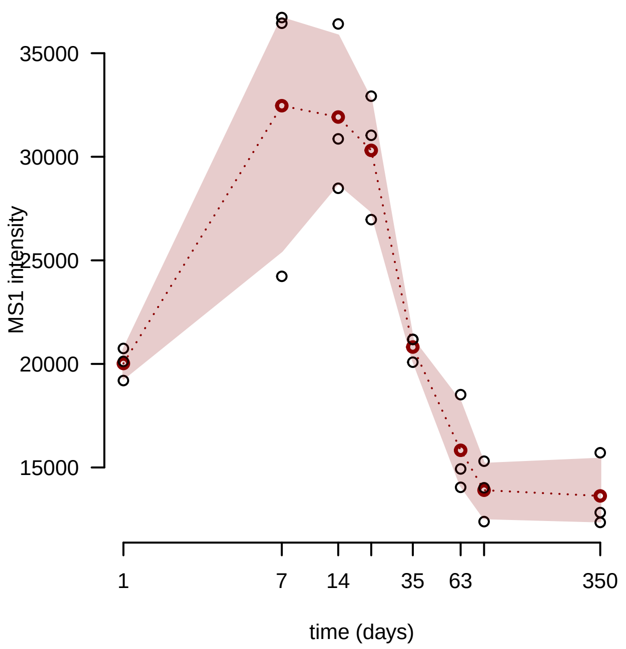

Q8BMT9
GN=Hhat
hedgehog acyltransferase

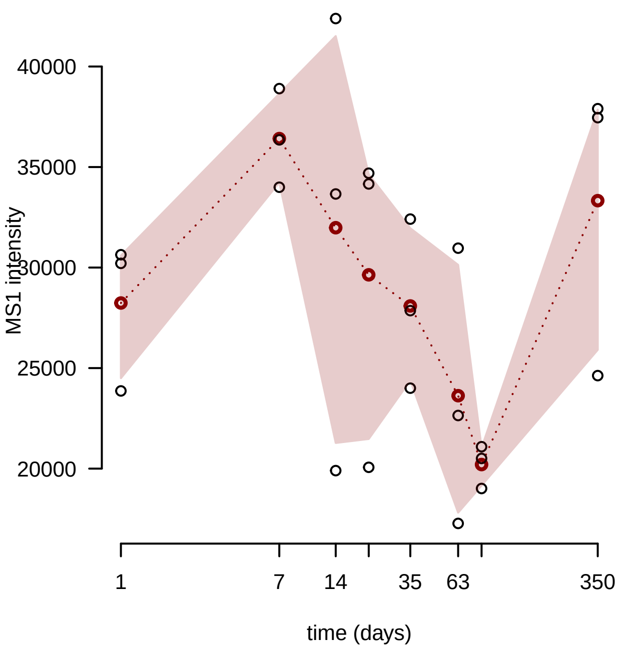

Q8BMZ5
GN=Tsen34
tRNA splicing endonuclease subunit 34

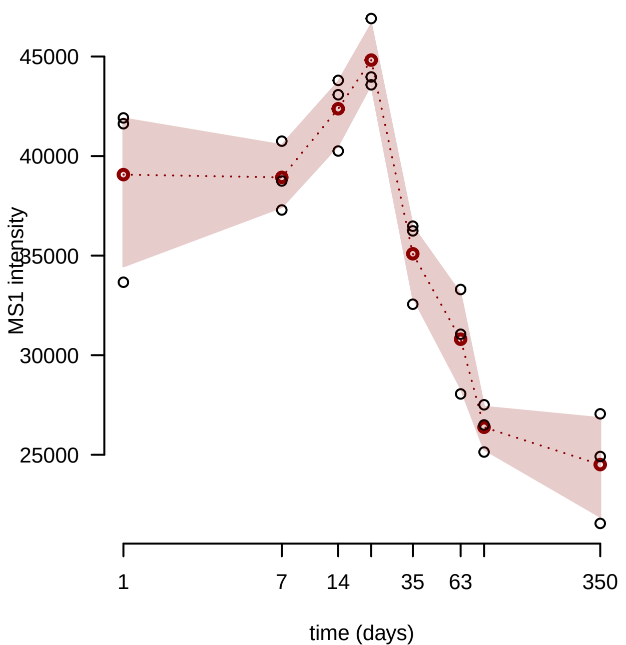

Q8BN21
GN=Vrk2
vaccinia related kinase 2

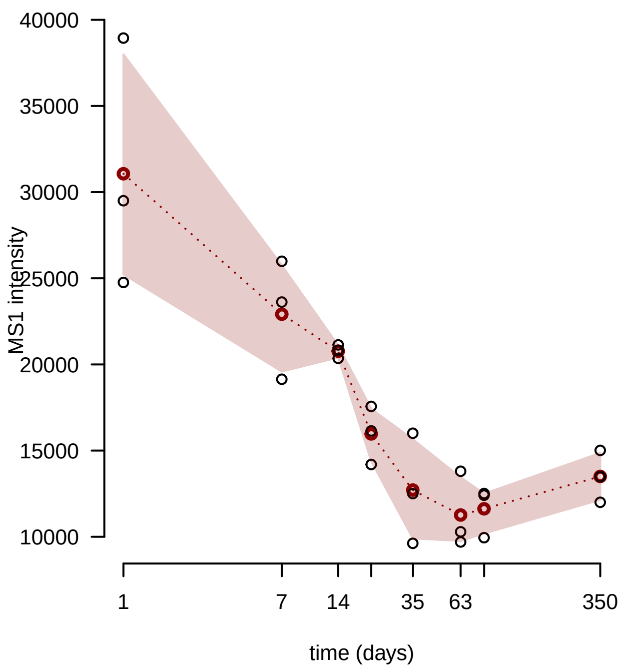

Q8BN57
GN=

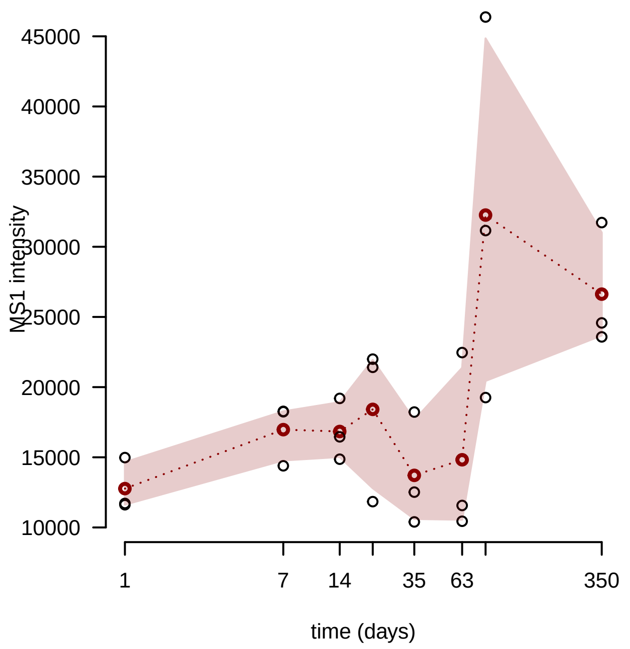

Q8BN58
GN=Arhgap28
Rho GTPase activating protein 28

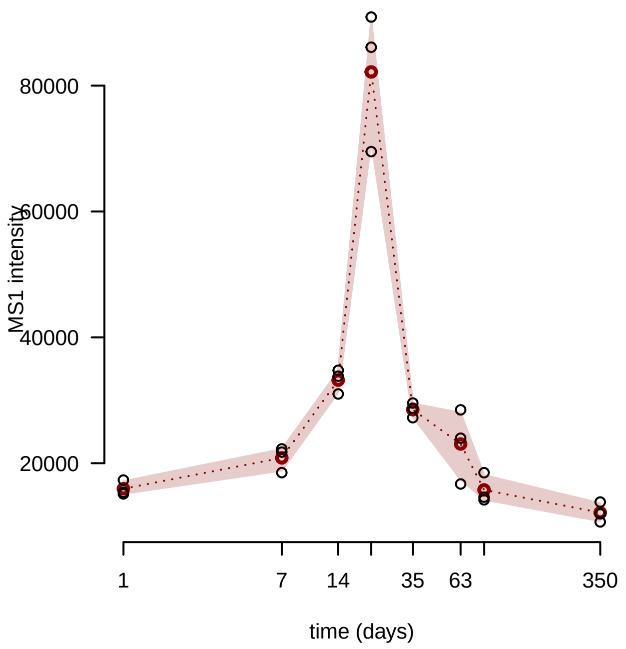

Q8BN78
GN=Zbtb33
zinc finger and BTB domain containing 33

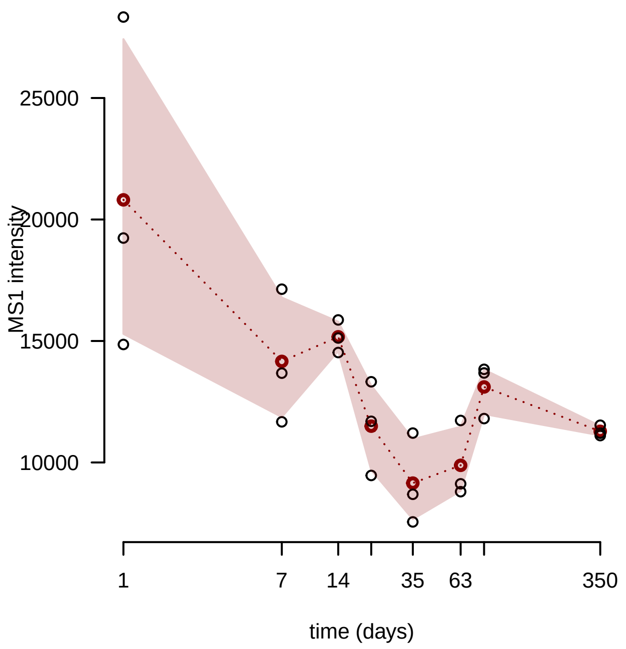

Q8BN82
GN=Slc17a5
solute carrier family 17 (anion/sugar tr ...

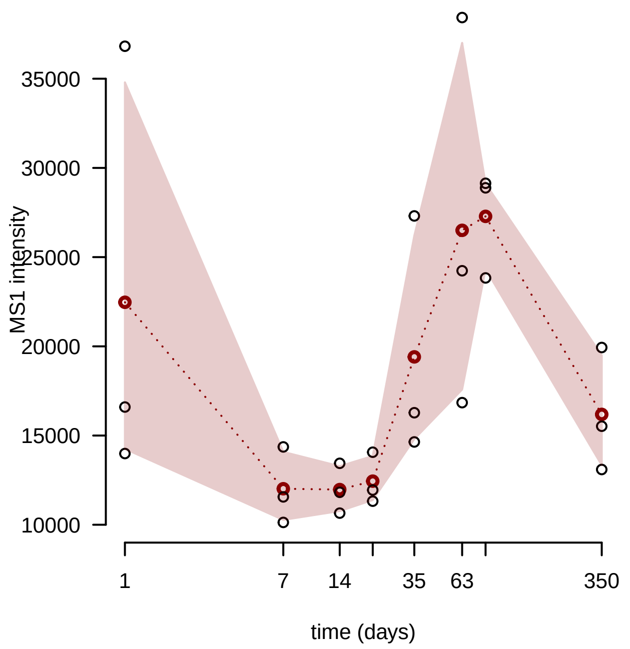

Q8BND3
GN=Wdr35
WD repeat domain 35

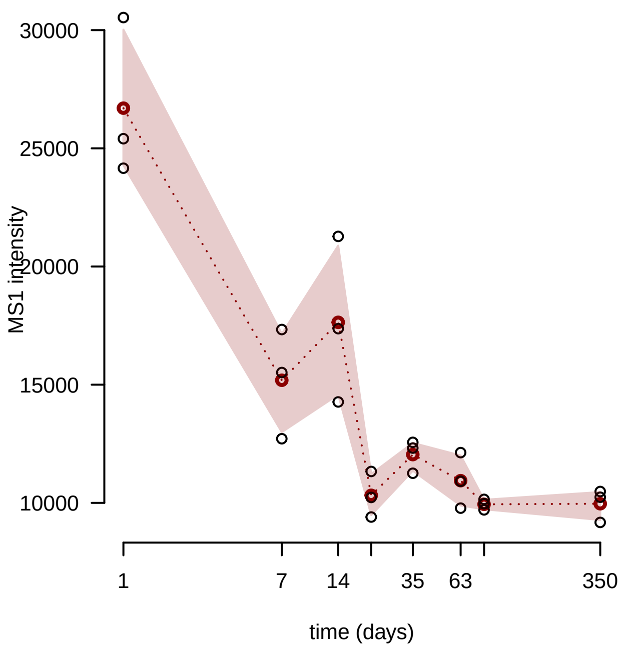

Q8BND4
GN=Ints6l
integrator complex subunit 6 like

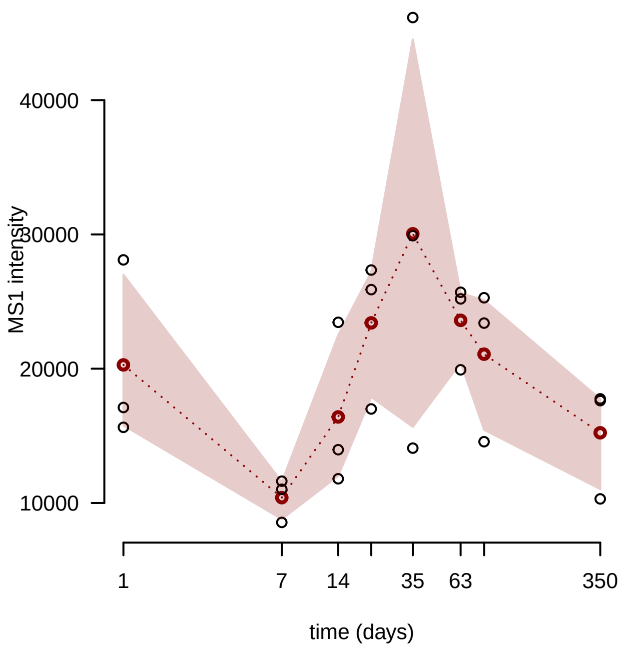

Q8BND5
GN=Qsox1
quiescin Q6 sulfhydryl oxidase 1

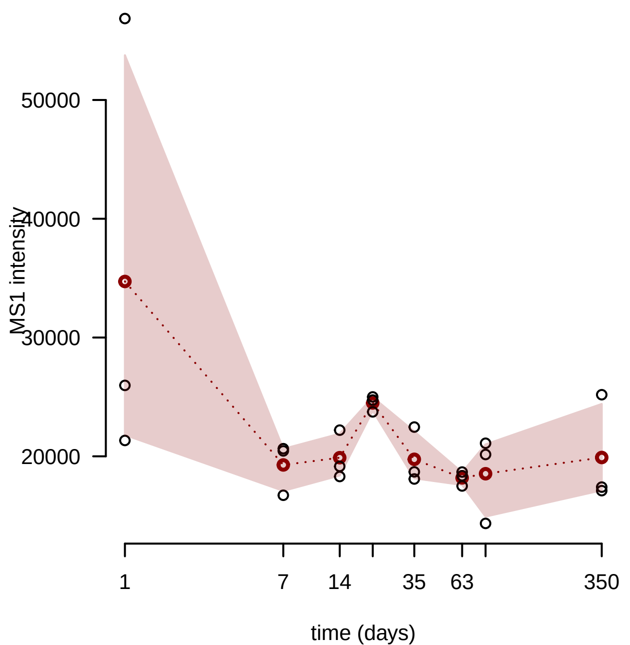

Q8BNE1
GN=Tcaf1
TRPM8 channel−associated factor 1

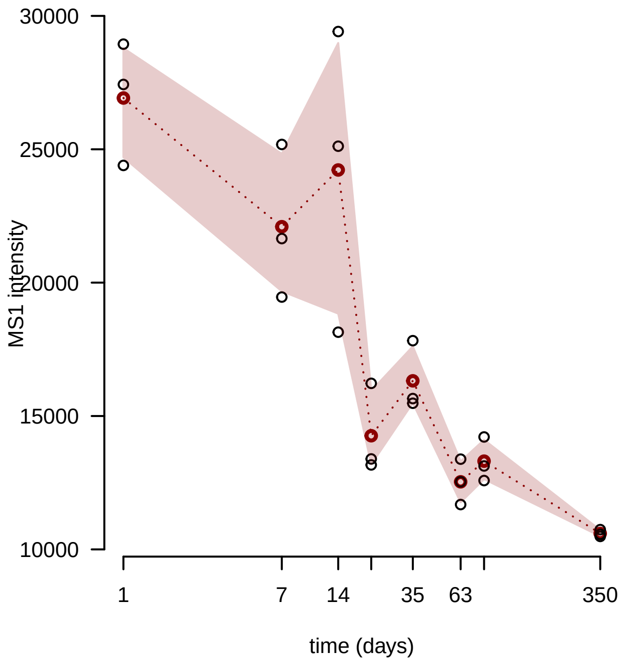

Q8BNI4
GN=Derl2
Der1−like domain family, member 2

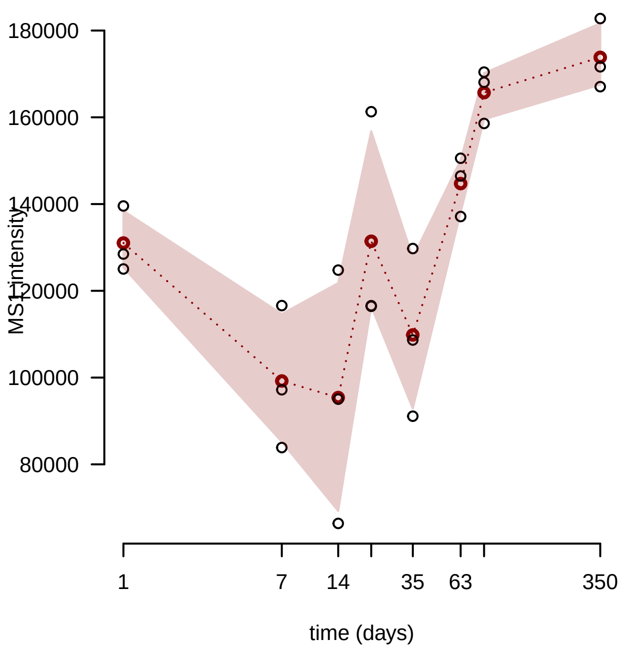

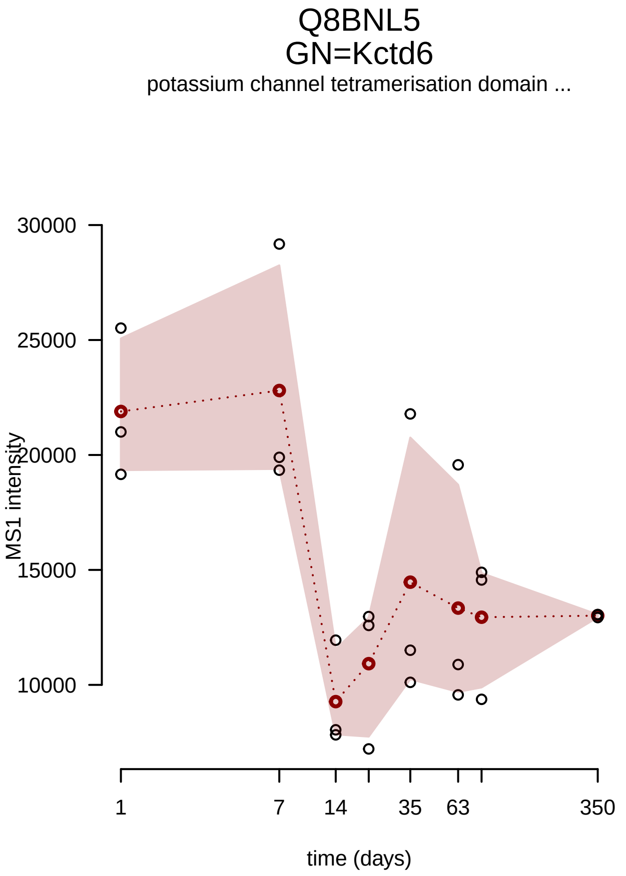
Q8BNL5
GN=Kctd6
potassium channel tetramerisation domain ...
MS1 intensity
30000
25000
20000
15000
10000
1
7
14
35
63
350
time (days)

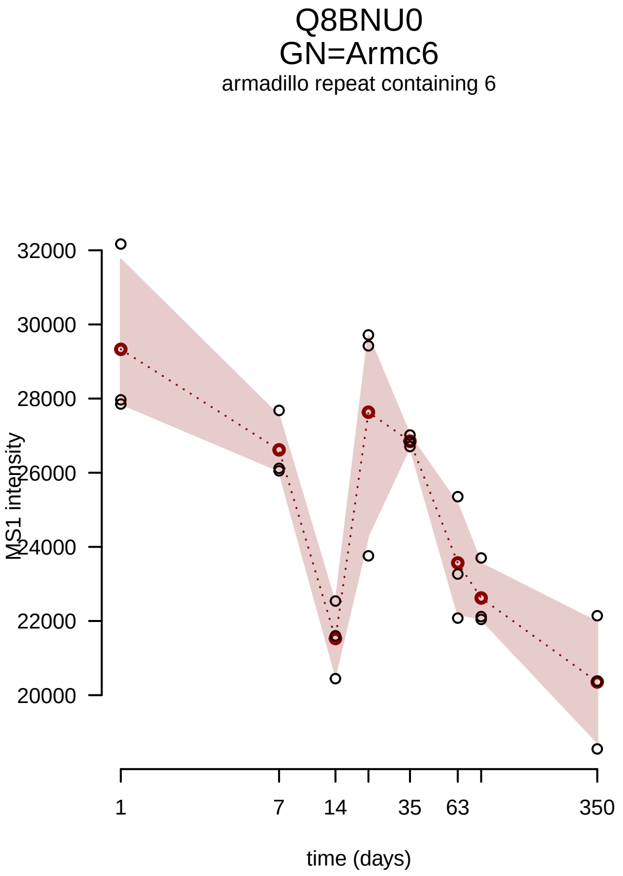
Q8BNU0
GN=Armc6
armadillo repeat containing 6
MS1 intensity
32000
30000
28000
26000
24000
22000
20000
1
7
14
35
63
350
time (days)

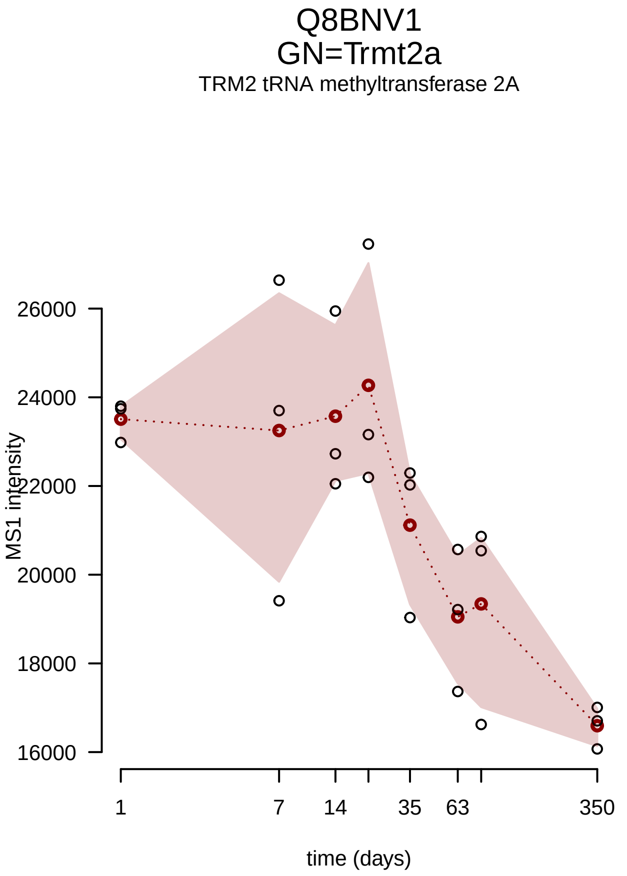
Q8BNV1
GN=Trmt2a
TRM2 tRNA methyltransferase 2A
MS1 intensity
26000
24000
22000
20000
18000
16000
1
7
14
35
63
350
time (days)

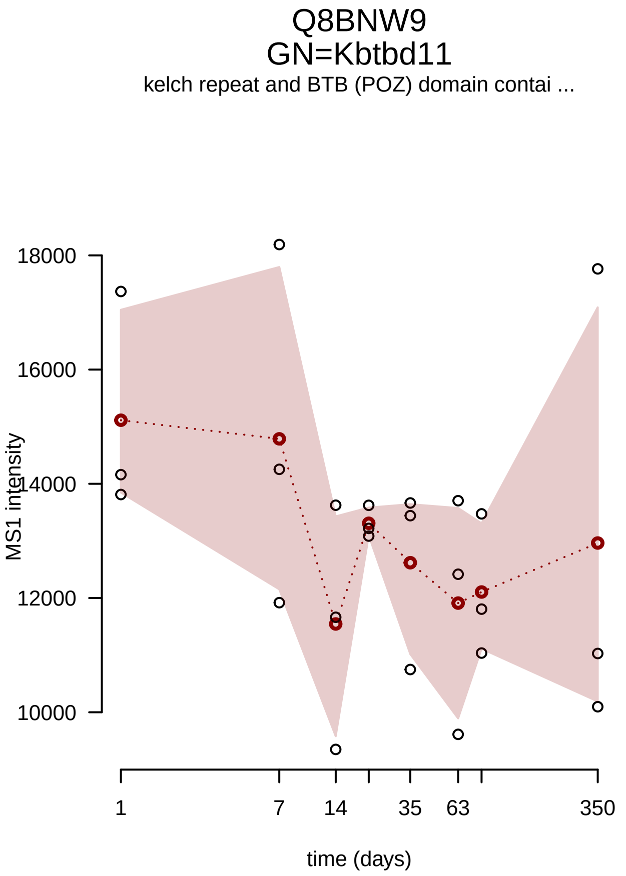
Q8BNW9
GN=Kbtbd11
kelch repeat and BTB (POZ) domain contai ...
MS1 intensity
18000
16000
14000
12000
10000
1
7
14
35
63
350
time (days)

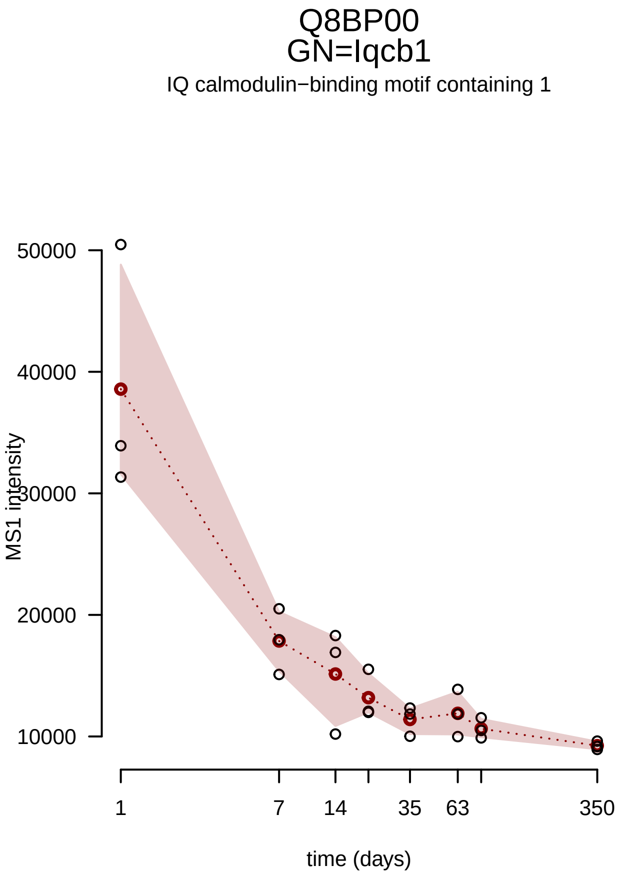
Q8BP00
GN=Iqcb1
IQ calmodulin−binding motif containing 1
MS1 intensity
50000
40000
30000
20000
10000
1
7
14
35
63
350
time (days)

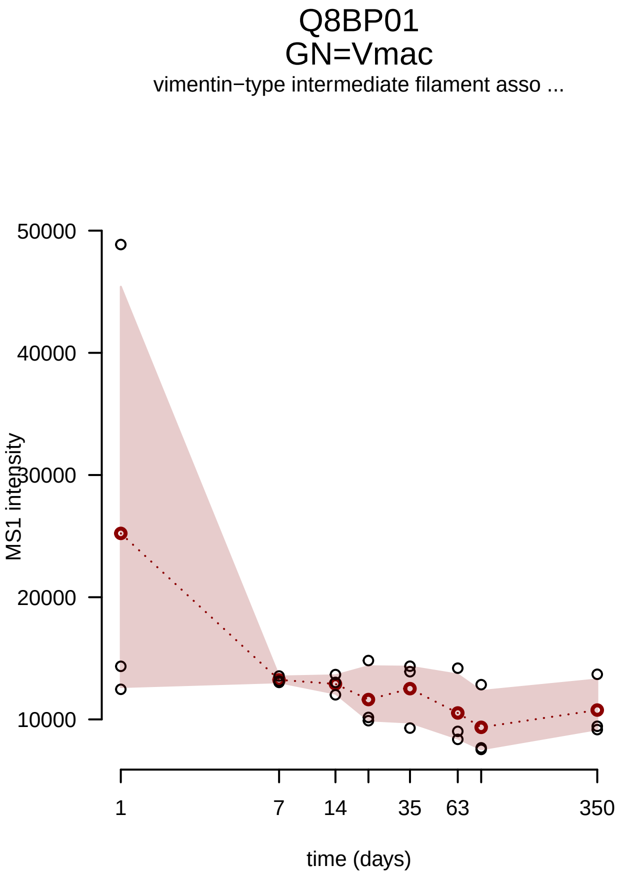
Q8BP01
GN=Vmac
vimentin−type intermediate filament asso ...
MS1 intensity
50000
40000
30000
20000
10000
1
7
14
35
63
350
time (days)

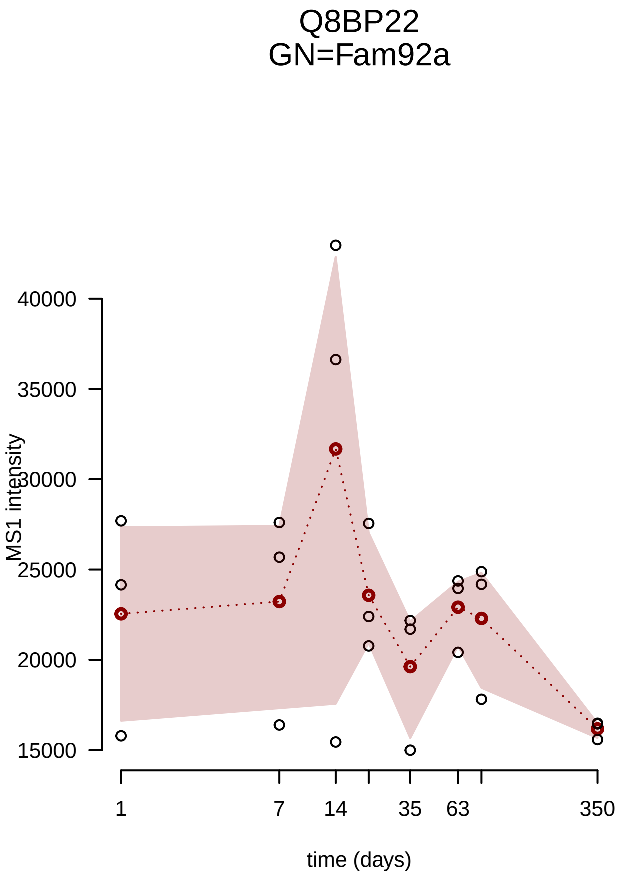
Q8BP22
GN=Fam92a
MS1 intensity
40000
35000
30000
25000
20000
15000
1
7
14
35
63
350
time (days)

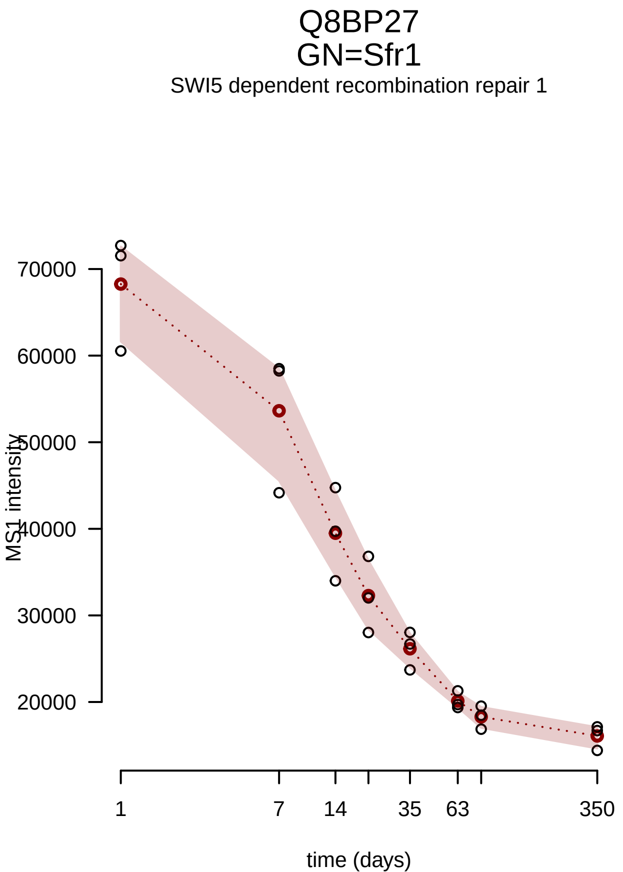
Q8BP27
GN=Sfr1
SWI5 dependent recombination repair 1
MS1 intensity
70000
60000
50000
40000
30000
20000
1
7
14
35
63
350
time (days)

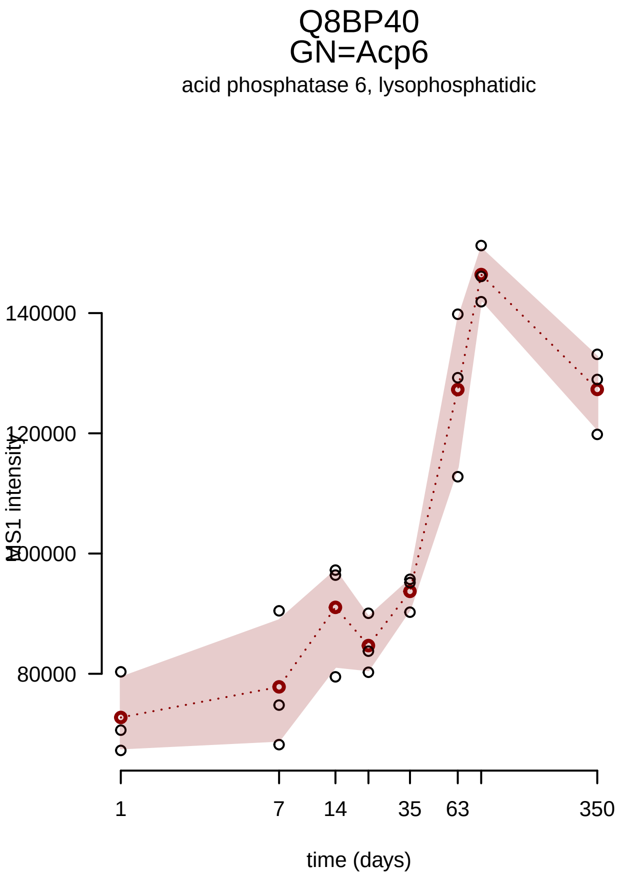
Q8BP40
GN=Acp6
acid phosphatase 6, lysophosphatidic
MS1 intensity
140000
120000
100000
80000
1
7
14
35
63
350
time (days)

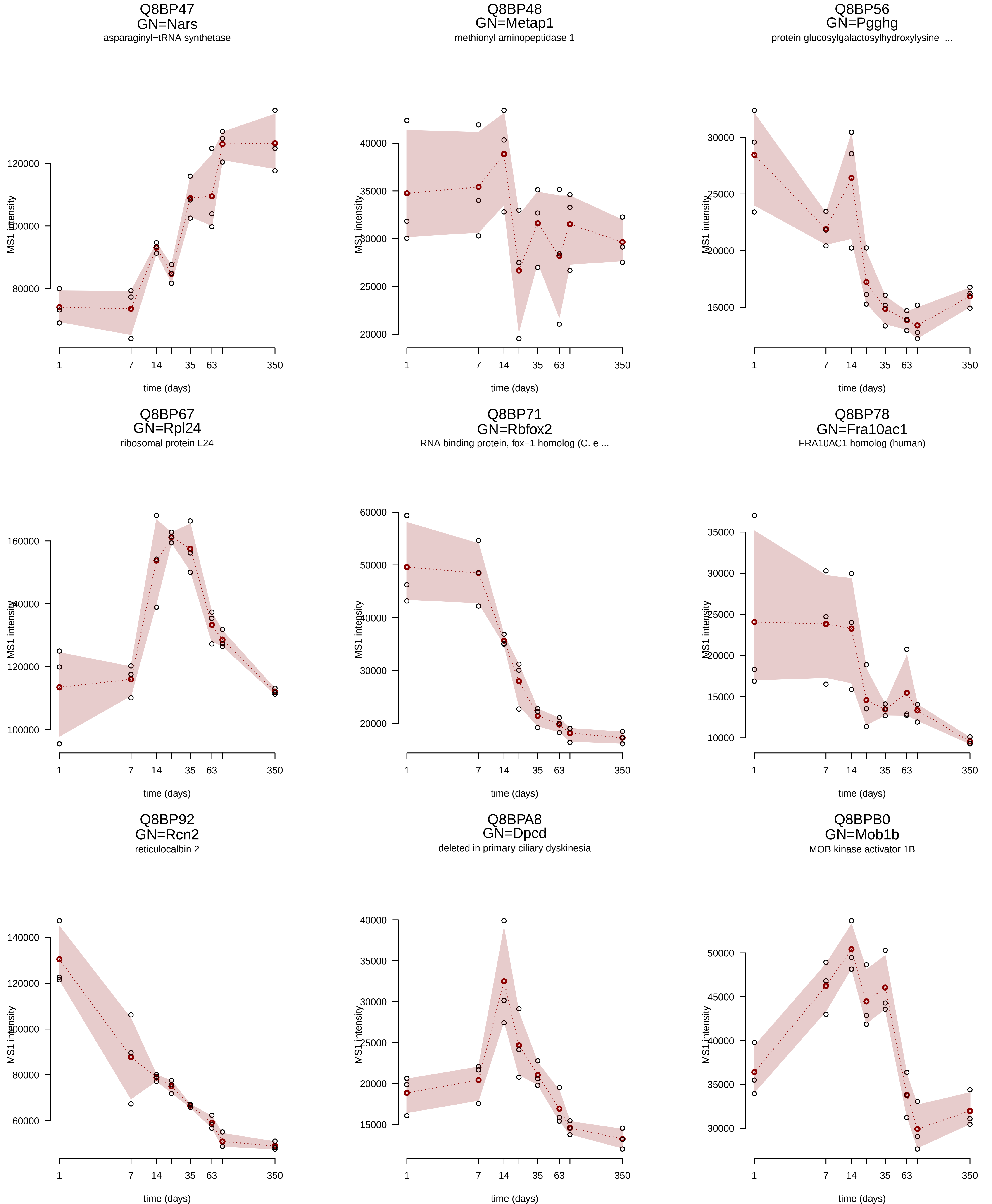
Q8BP47
GN=Nars
asparaginyl−tRNA synthetase
Q8BP48
GN=Metap1
methionyl aminopeptidase 1
Q8BP56
GN=Pgghg
protein glucosylgalactosylhydroxylysine ...
Q8BP67
GN=Rpl24
ribosomal protein L24
Q8BP71
GN=Rbfox2
RNA binding protein, fox−1 homolog (C. e ...
Q8BP78
GN=Fra10ac1
FRA10AC1 homolog (human)
Q8BP92
GN=Rcn2
reticulocalbin 2
Q8BPA8
GN=Dpcd
deleted in primary ciliary dyskinesia
Q8BPB0
GN=Mob1b
MOB kinase activator 1B
MS1 intensity
time (days)
1
7
14
35
63
350

Q8BPB5
GN=Efemp1
epidermal growth factor−containing fibul ...

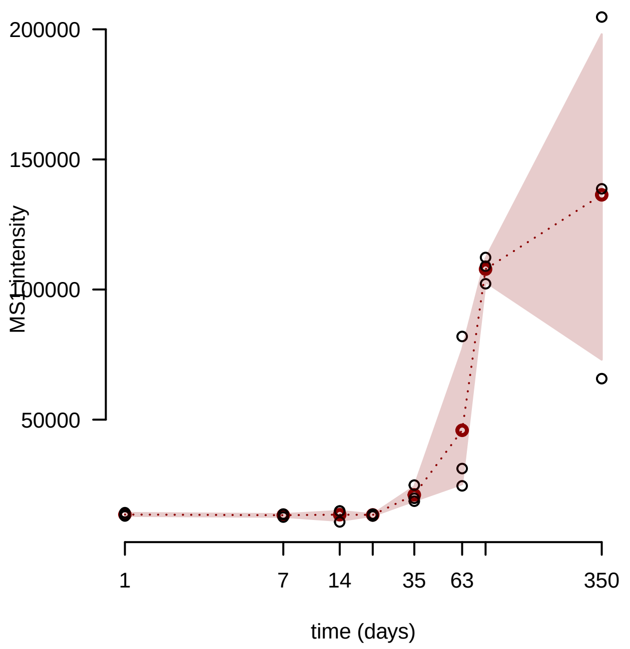

Q8BPE4
GN=Tmem177
transmembrane protein 177

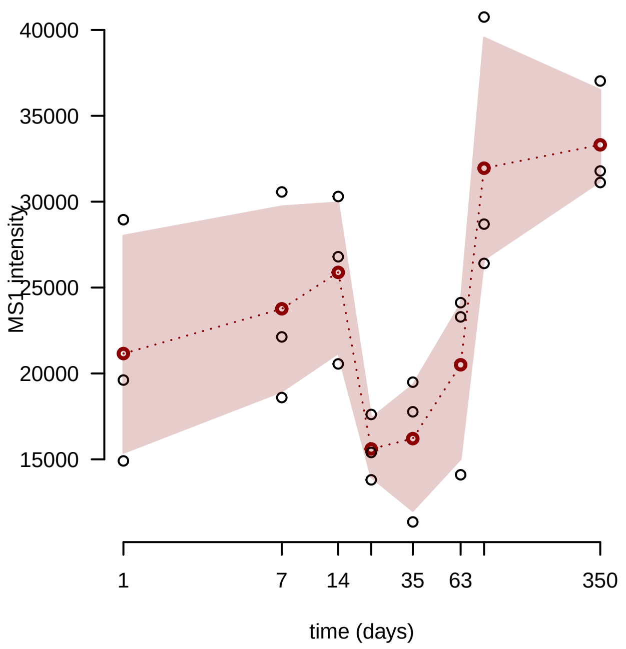

Q8BPG6
GN=Sumf2
sulfatase modifying factor 2

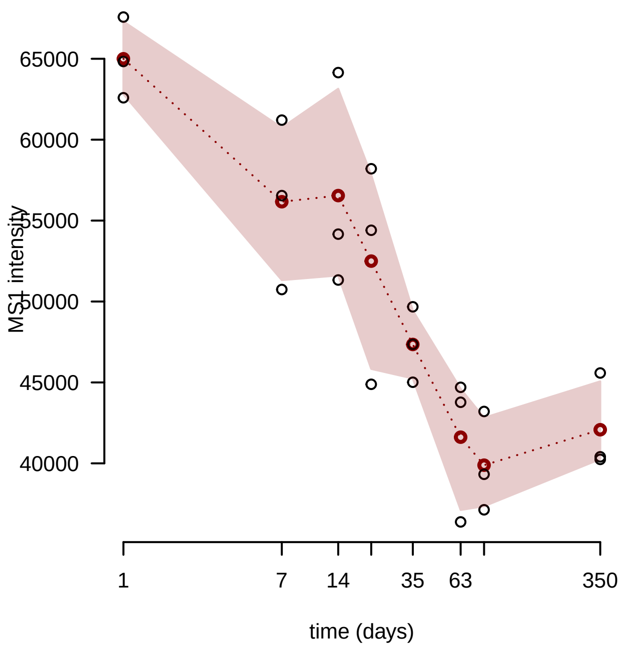

Q8BPK2
GN=Zcchc3
zinc finger, CCHC domain containing 3

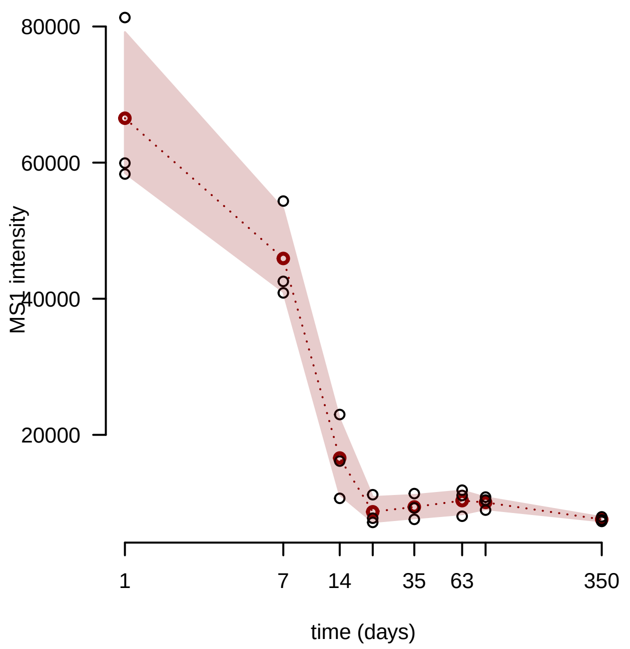

Q8BPM0
GN=Daam1
dishevelled associated activator of morp ...

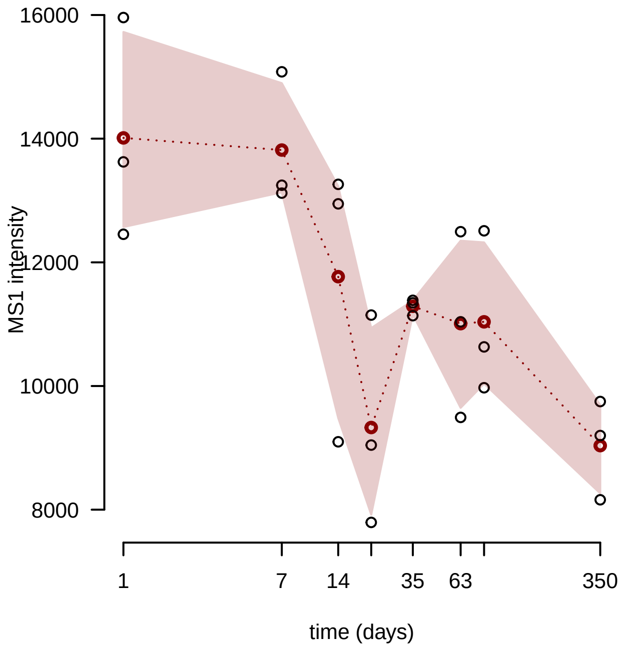

Q8BPM2
GN=Map4k5
mitogen−activated protein kinase kinase ...

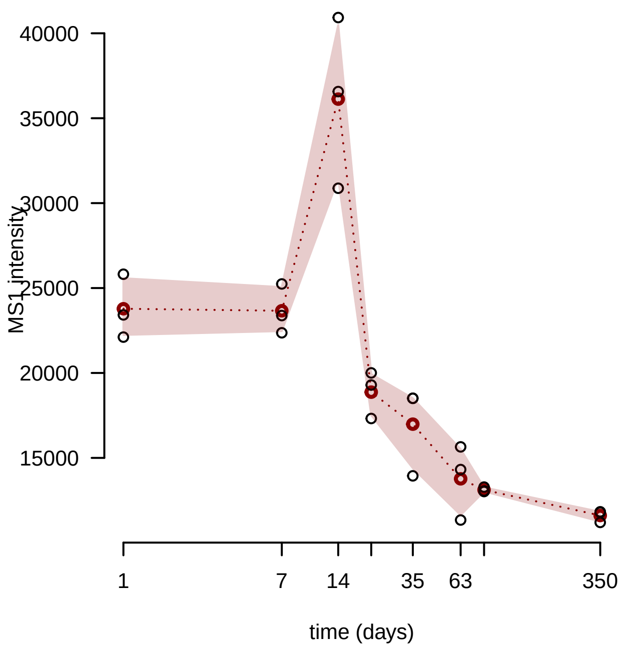

Q8BPN8
GN=Dmxl2
Dmx−like 2

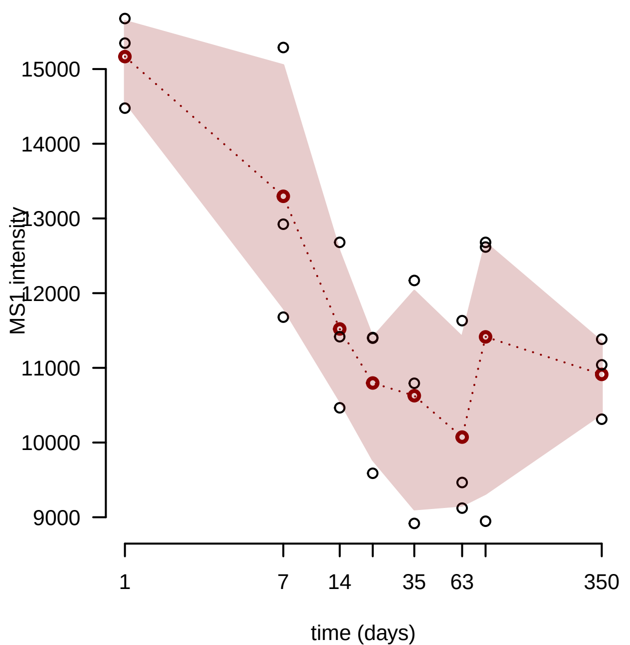

Q8BPP0
GN=Znf436

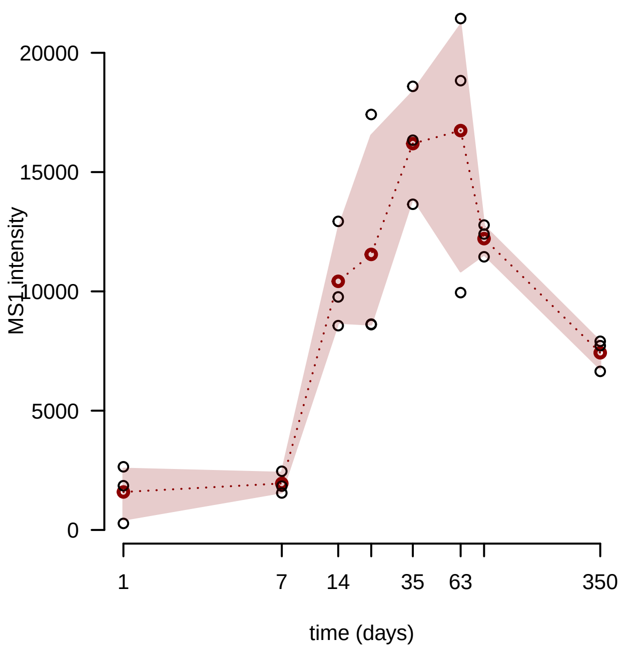

Q8BPS4
GN=Gpr180
G protein−coupled receptor 180

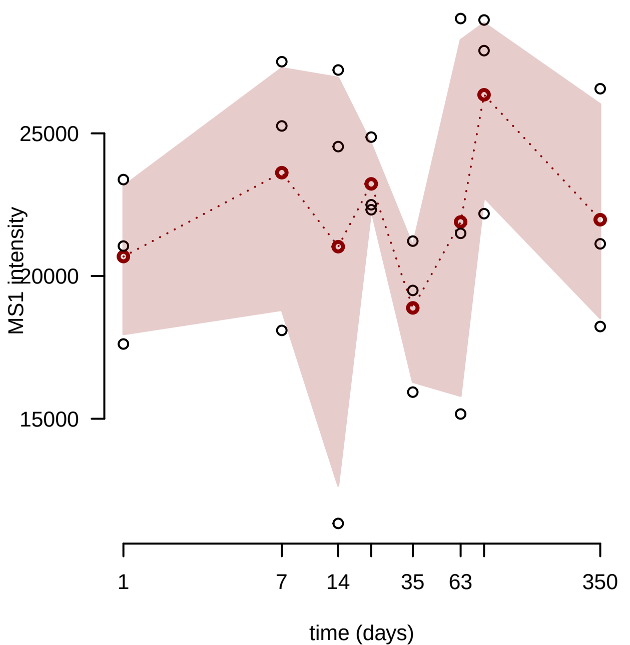

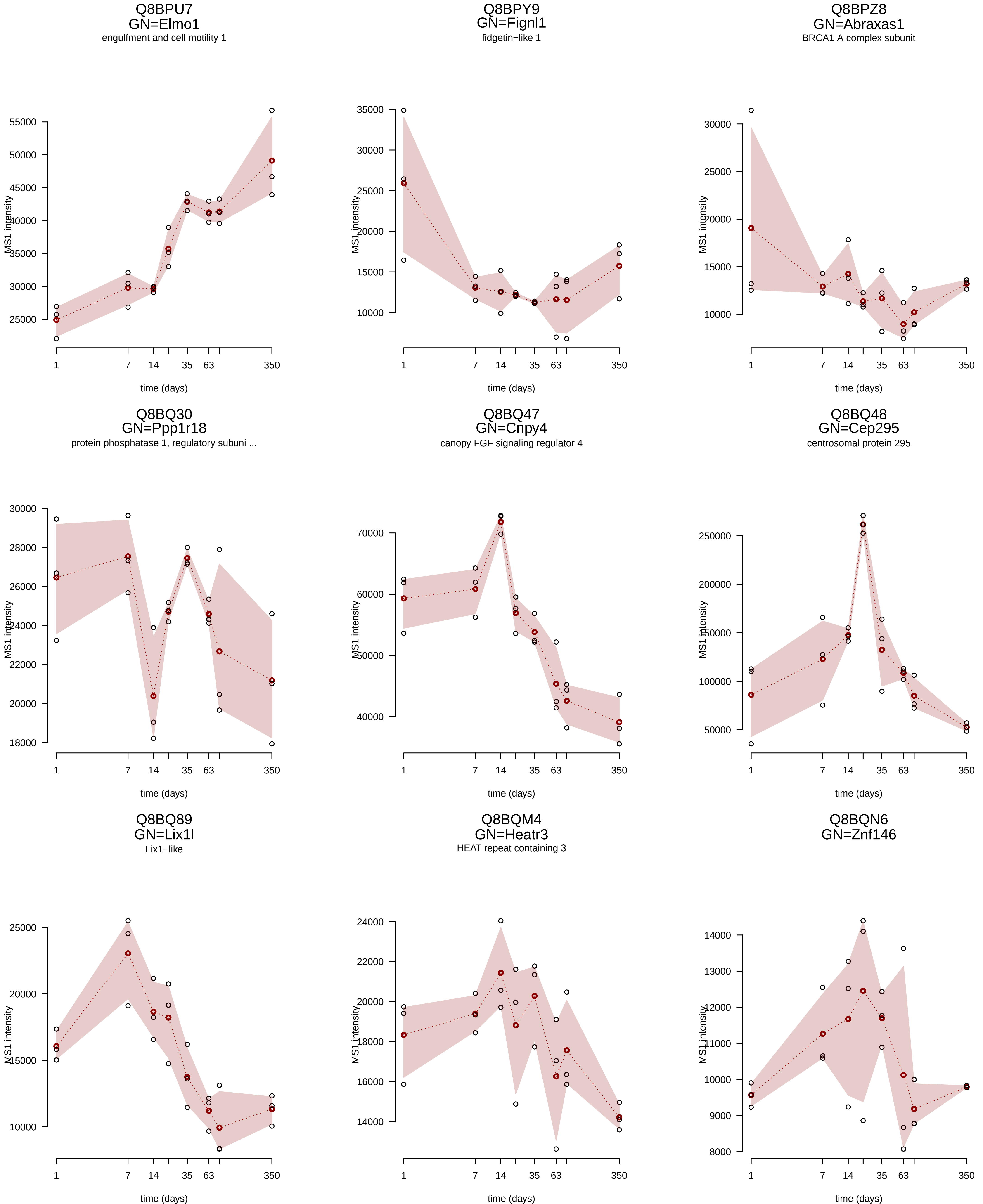

Q8BPU7
GN=Elmo1
engulfment and cell motility 1
Q8BPY9
GN=Fignl1
fidgetin−like 1
Q8BPZ8
GN=Abraxas1
BRCA1 A complex subunit
Q8BQ30
GN=Ppp1r18
protein phosphatase 1, regulatory subuni ...
Q8BQ47
GN=Cnpy4
canopy FGF signaling regulator 4
Q8BQ48
GN=Cep295
centrosomal protein 295
Q8BQ89
GN=Lix1l
Lix1−like
Q8BQM4
GN=Heatr3
HEAT repeat containing 3
Q8BQN6
GN=Znf146
MS1 intensity
time (days)
1
7
14
35
63
350

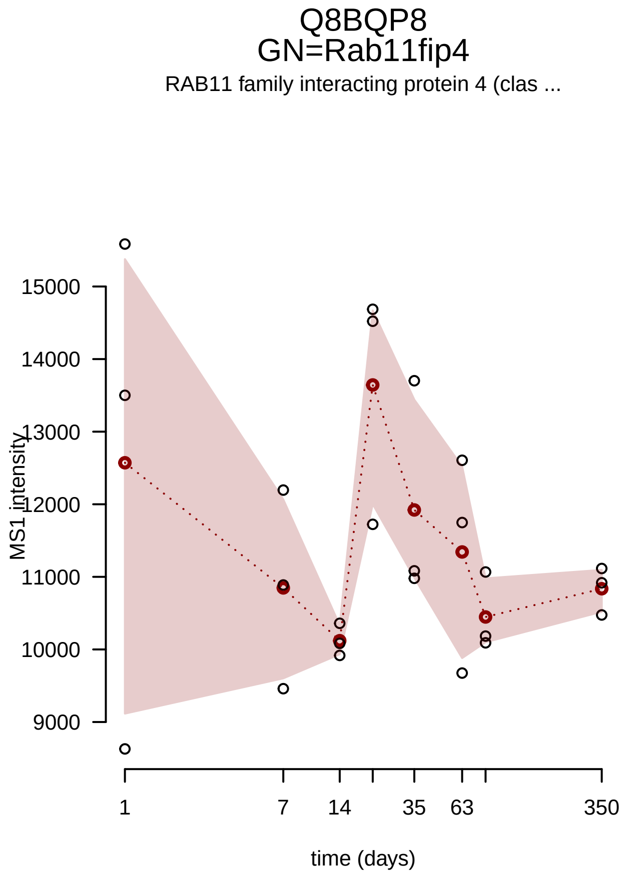
Q8BQP8
GN=Rab11fip4
RAB11 family interacting protein 4 (clas ...
MS1 intensity
15000
14000
13000
12000
11000
10000
9000
1
7
14
35
63
350
time (days)

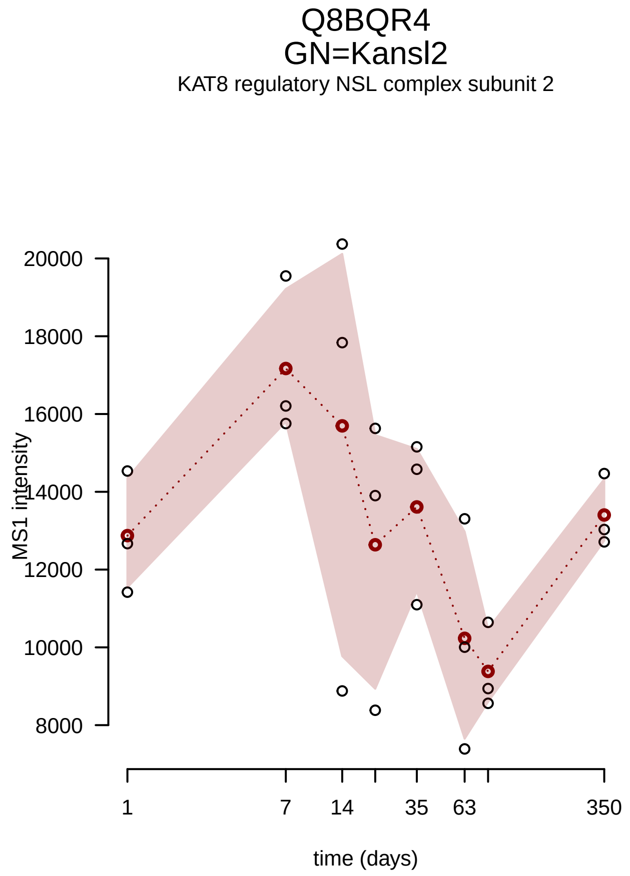
Q8BQR4
GN=Kansl2
KAT8 regulatory NSL complex subunit 2
MS1 intensity
20000
18000
16000
14000
12000
10000
8000
1
7
14
35
63
350
time (days)

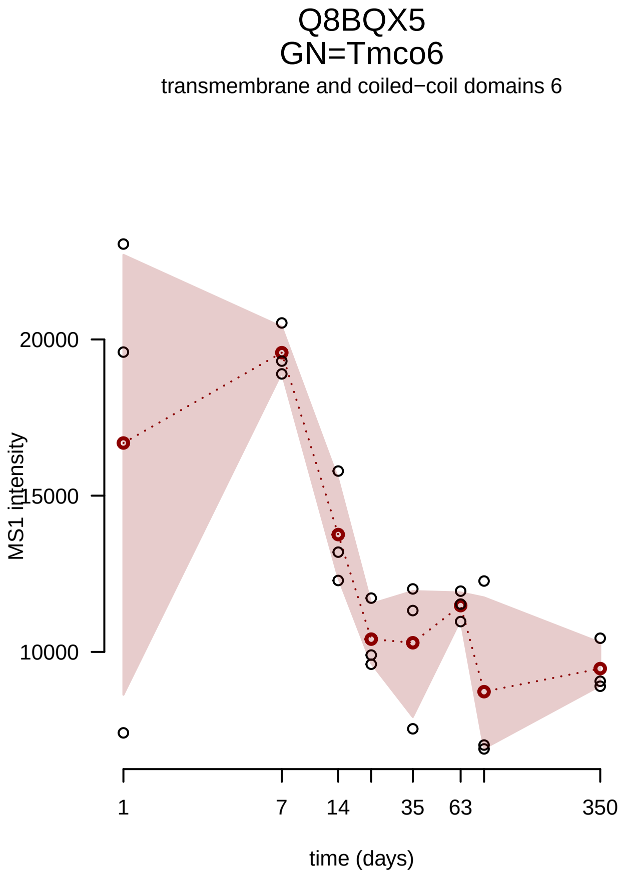
Q8BQX5
GN=Tmco6
transmembrane and coiled−coil domains 6
MS1 intensity
20000
15000
10000
1
7
14
35
63
350
time (days)

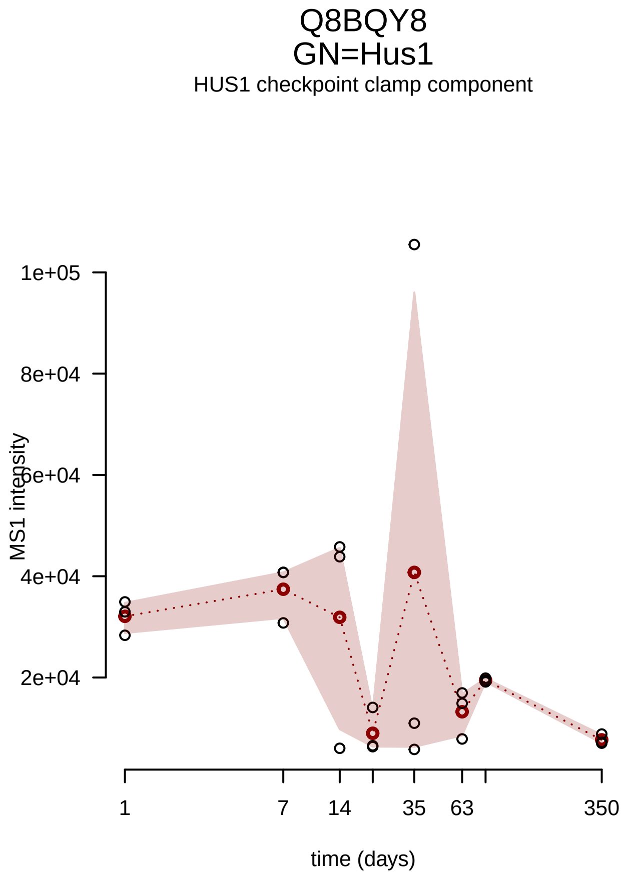
Q8BQY8
GN=Hus1
HUS1 checkpoint clamp component
MS1 intensity
1e+05
8e+04
6e+04
4e+04
2e+04
1
7
14
35
63
350
time (days)

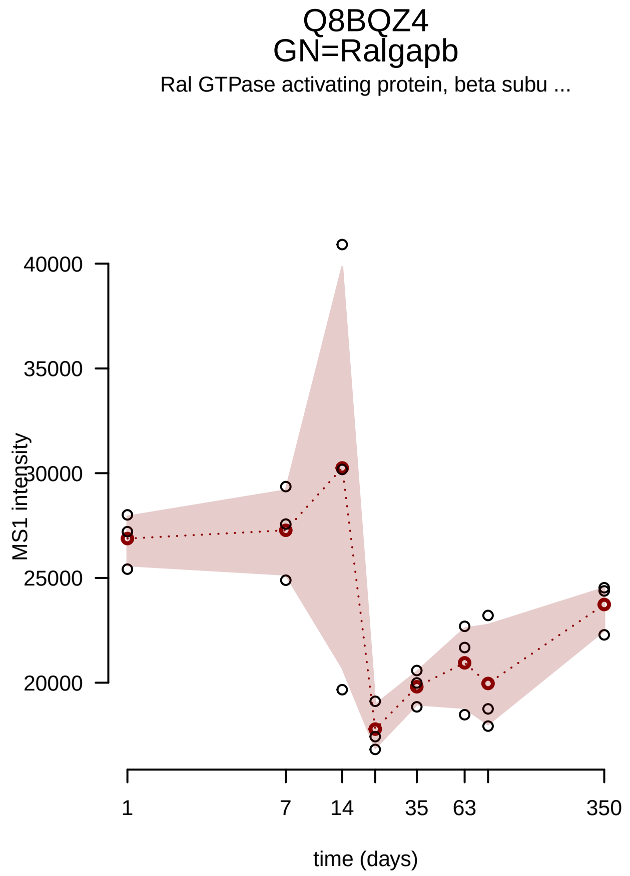
Q8BQZ4
GN=Ralgapb
Ral GTPase activating protein, beta subu ...
MS1 intensity
40000
35000
30000
25000
20000
1
7
14
35
63
350
time (days)

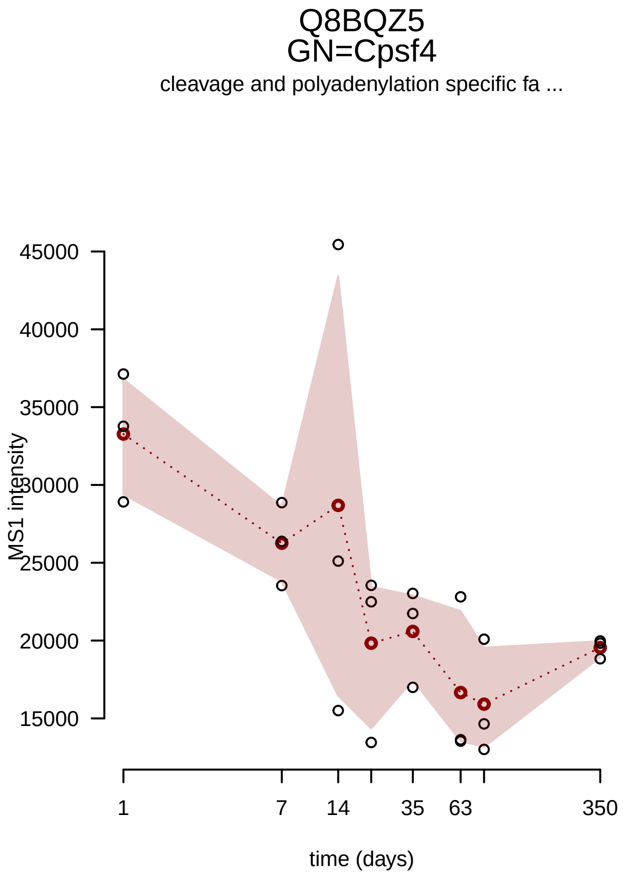
Q8BQZ5
GN=Cpsf4
cleavage and polyadenylation specific fa ...
MS1 intensity
45000
40000
35000
30000
25000
20000
15000
1
7
14
35
63
350
time (days)

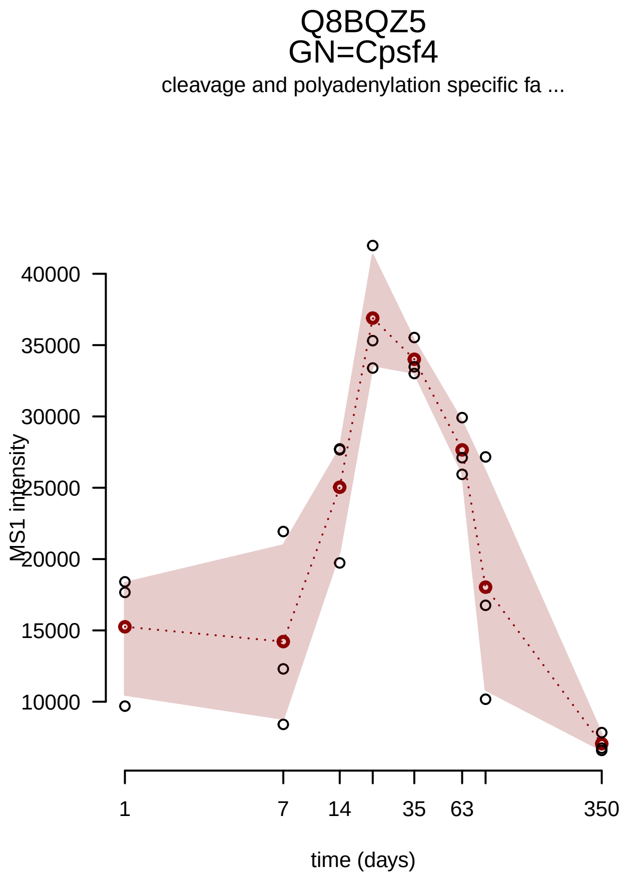
Q8BQZ5
GN=Cpsf4
cleavage and polyadenylation specific fa ...
MS1 intensity
40000
35000
30000
25000
20000
15000
10000
1
7
14
35
63
350
time (days)

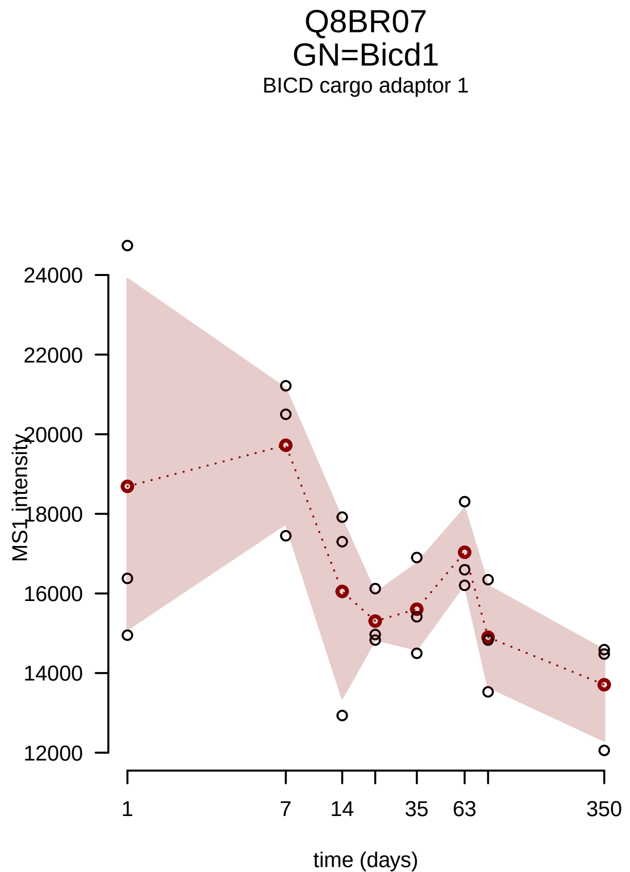
Q8BR07
GN=Bicd1
BICD cargo adaptor 1
MS1 intensity
24000
22000
20000
18000
16000
14000
12000
1
7
14
35
63
350
time (days)

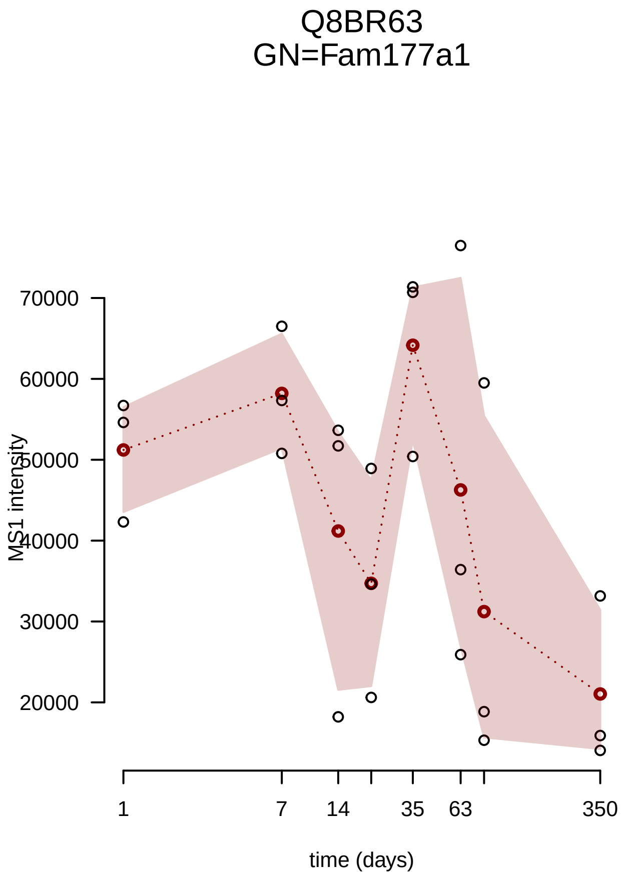
Q8BR63
GN=Fam177a1
MS1 intensity
70000
60000
50000
40000
30000
20000
1
7
14
35
63
350
time (days)

Q8BR65
GN=Suds3
suppressor of defective silencing 3 homo ...

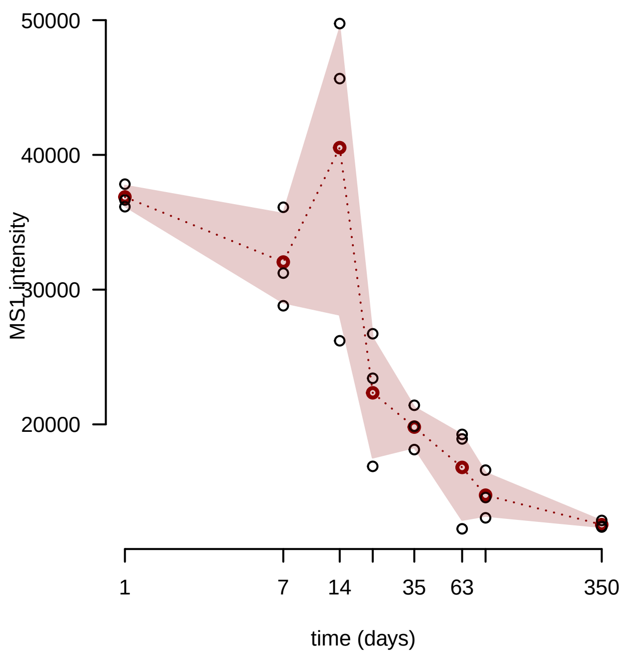

Q8BR70
GN=Yipf6
Yip1 domain family, member 6

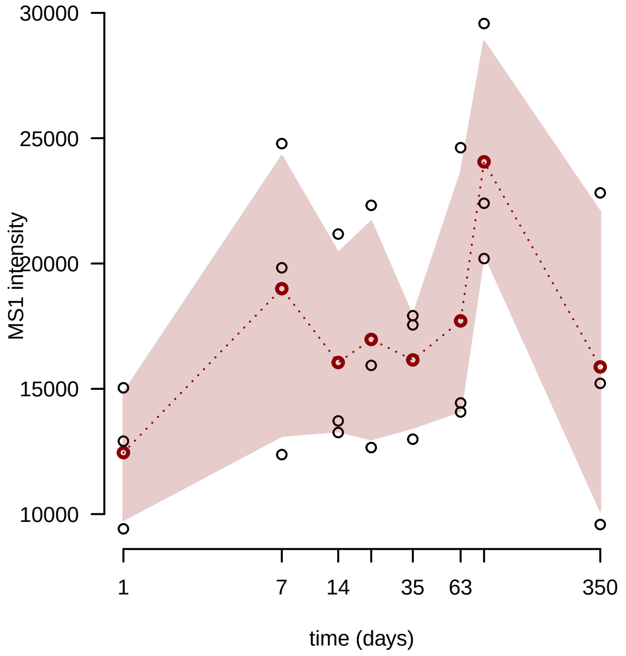

Q8BR76
GN=Tmem67
transmembrane protein 67

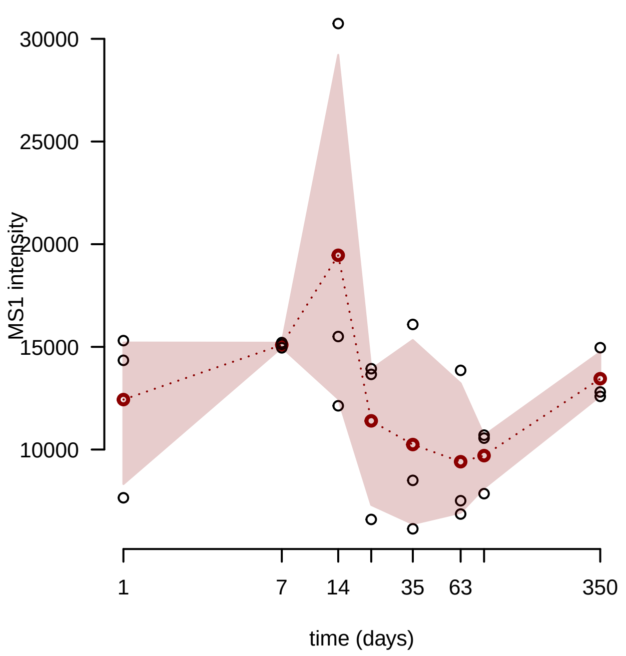

Q8BR90
GN=

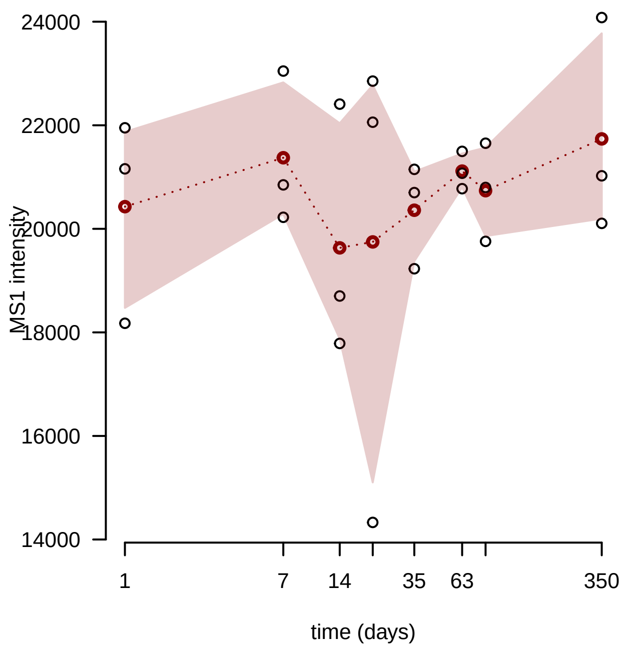

Q8BR92
GN=Palm2

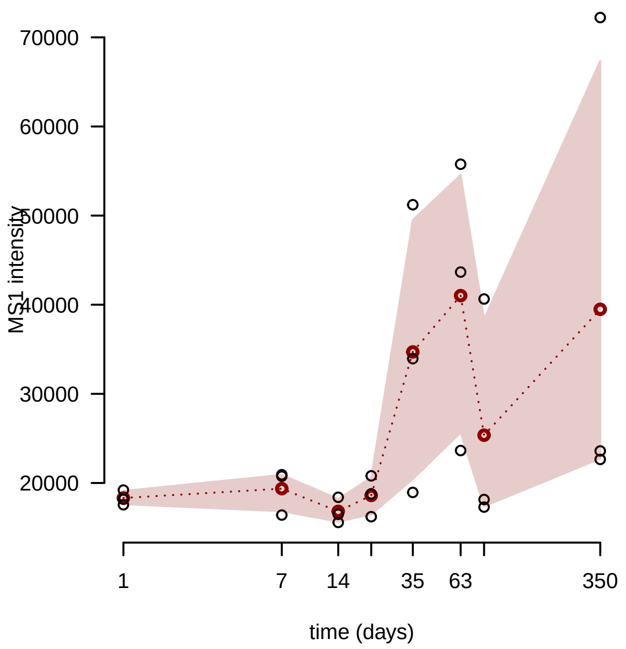

Q8BRF7
GN=Scfd1
Sec1 family domain containing 1

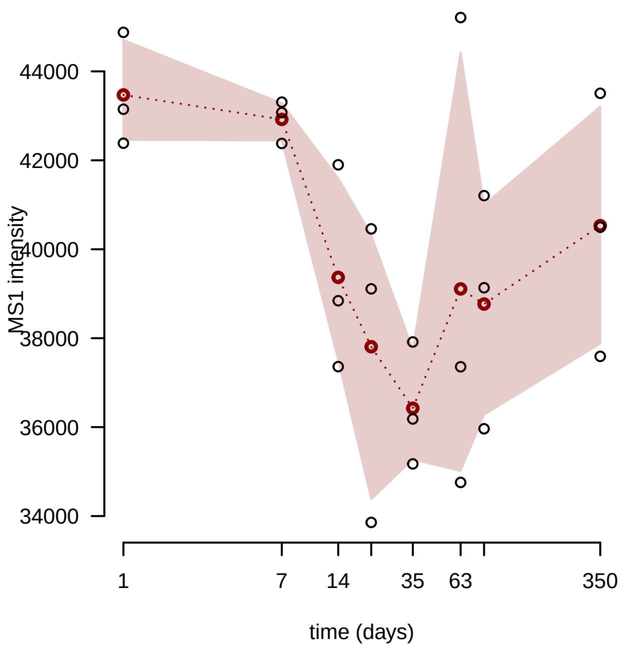

Q8BRG8
GN=Tmem209
transmembrane protein 209

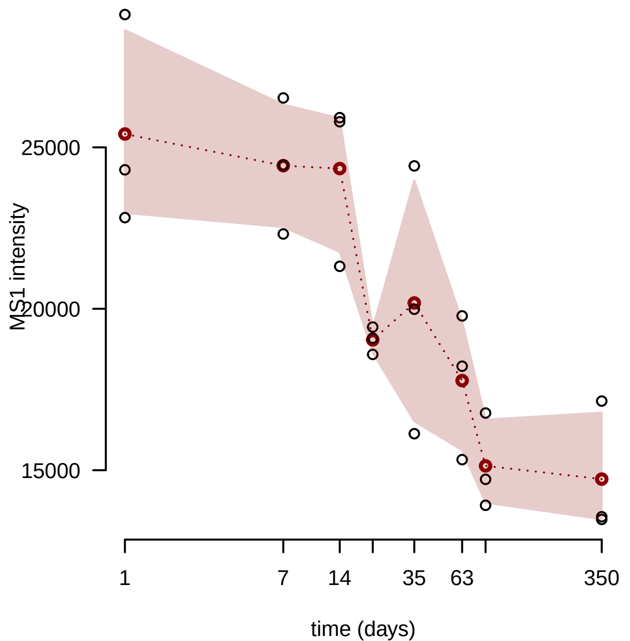

Q8BRH0
GN=Tmtc3
transmembrane and tetratricopeptide repe ...

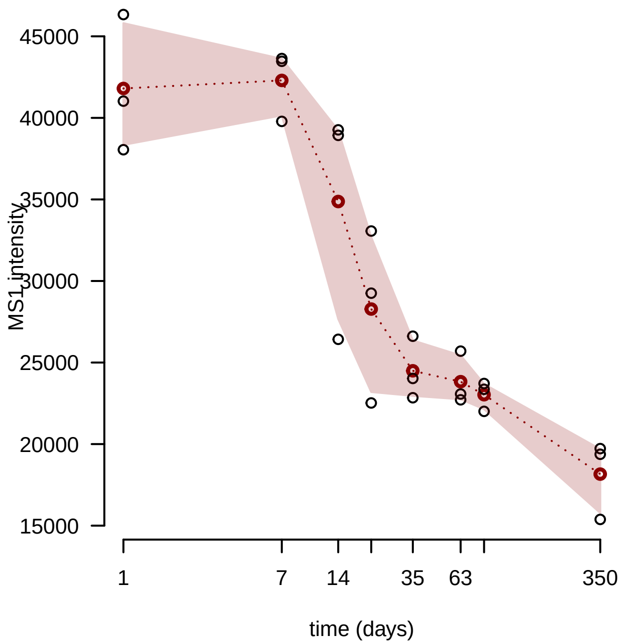

Q8BRH4
GN=Kmt2c
lysine (K)−specific methyltransferase 2C

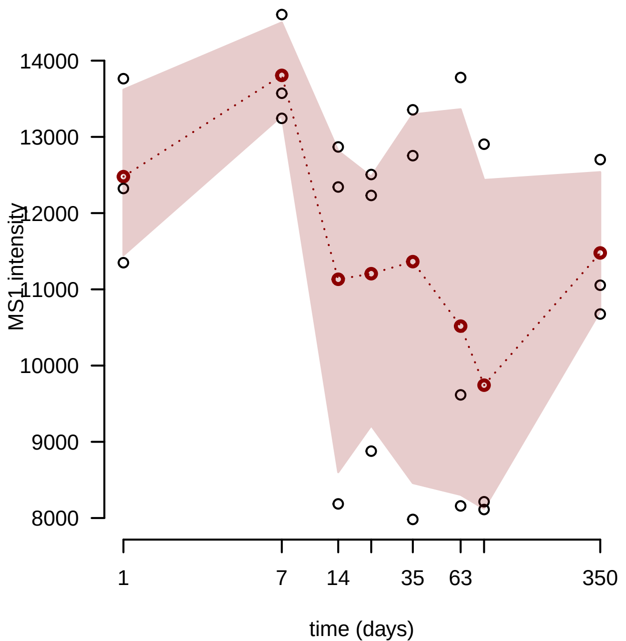

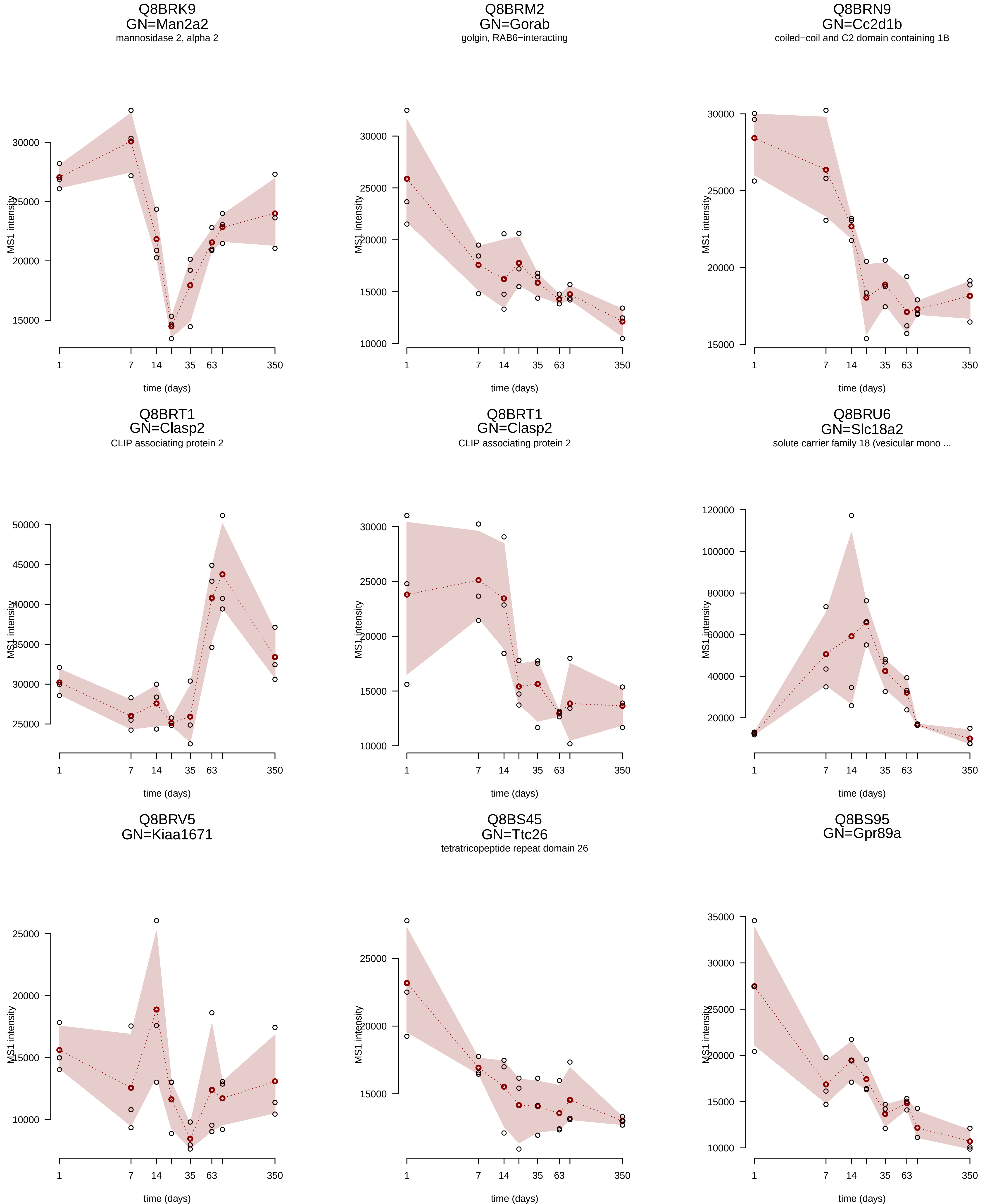
Q8BRK9
GN=Man2a2
mannosidase 2, alpha 2
Q8BRM2
GN=Gorab
golgin, RAB6−interacting
Q8BRN9
GN=Cc2d1b
coiled−coil and C2 domain containing 1B
Q8BRT1
GN=Clasp2
CLIP associating protein 2
Q8BRT1
GN=Clasp2
CLIP associating protein 2
Q8BRU6
GN=Slc18a2
solute carrier family 18 (vesicular mono ...
Q8BRV5
GN=Kiaa1671
Q8BS45
GN=Ttc26
tetratricopeptide repeat domain 26
Q8BS95
GN=Gpr89a
MS1 intensity
time (days)
1
7
14
35
63
350

Q8BSA9
GN=Tyw3
tRNA−yW synthesizing protein 3 homolog (...

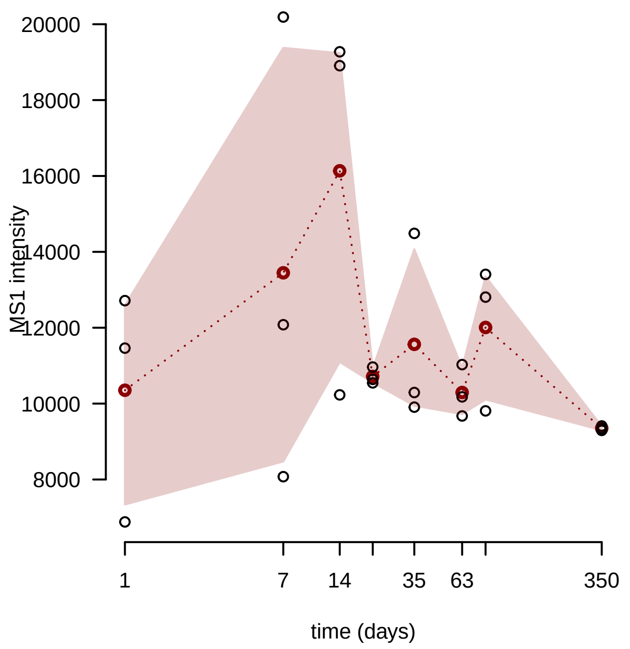

Q8BSD5
GN=She
src homology 2 domain−containing transfo ...

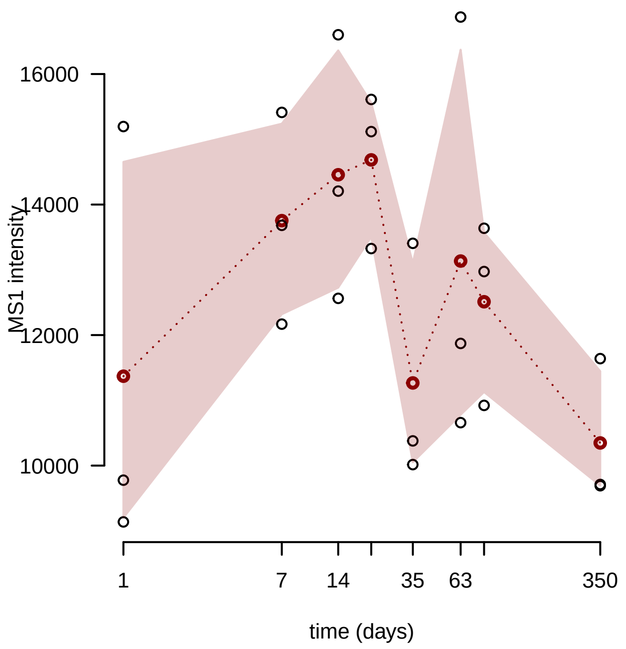

Q8BSE0
GN=Rmdn2
regulator of microtubule dynamics 2

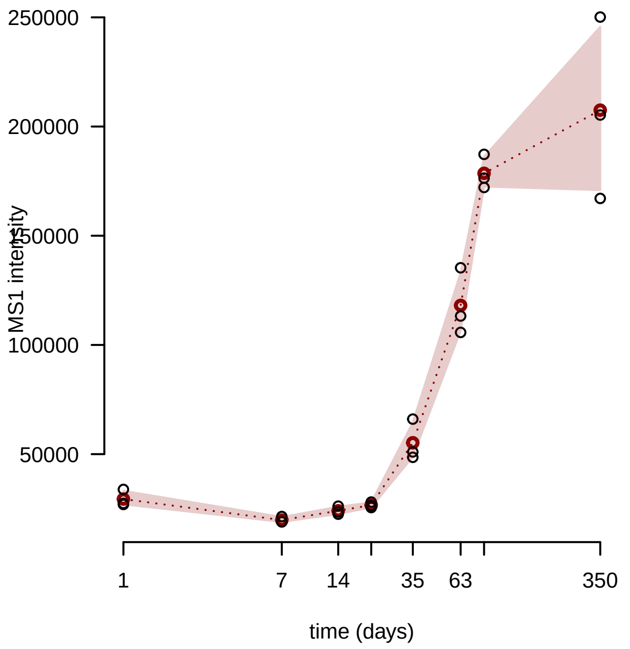

Q8BSF4
GN=Pisd
phosphatidylserine decarboxylase

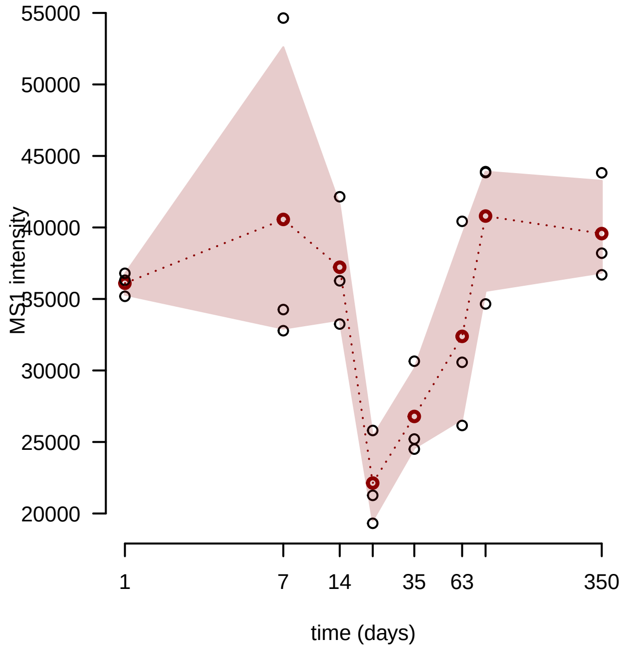

Q8BSI6
GN=R3hcc1
R3H domain and coiled−coil containing 1

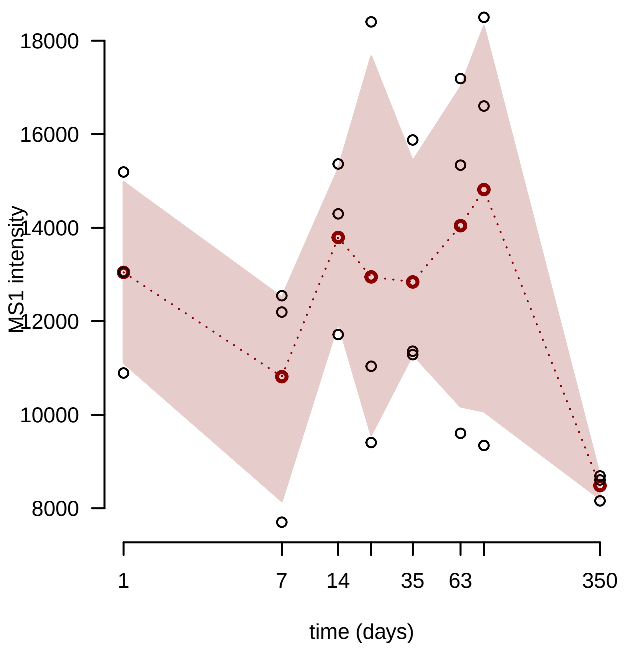

Q8BSK8
GN=Rps6kb1
ribosomal protein S6 kinase, polypeptide ...

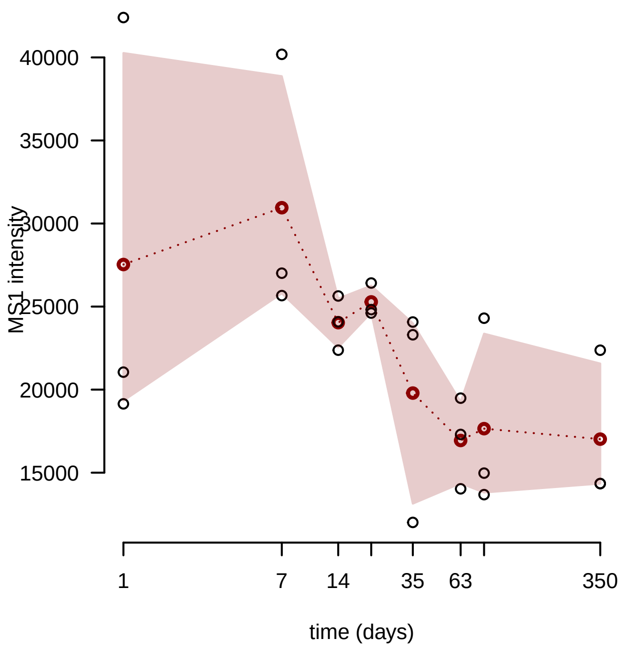

Q8BSL7
GN=Arf2
ADP−ribosylation factor 2

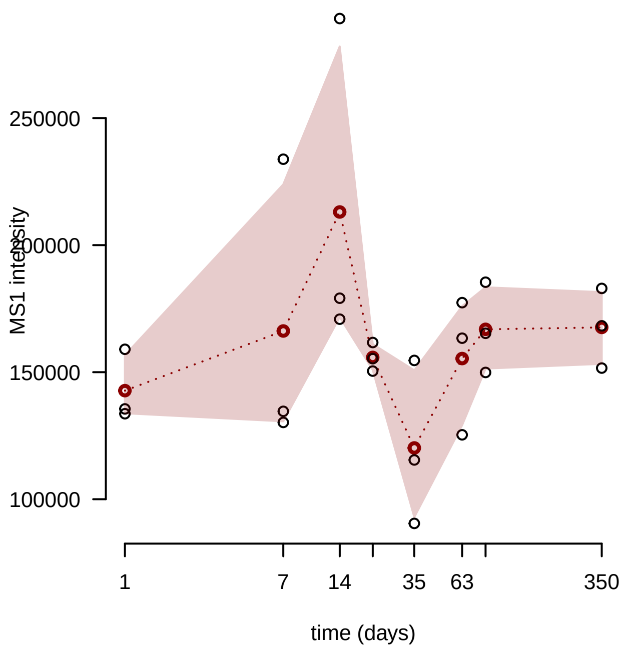

Q8BSM7
GN=Slc43a1
solute carrier family 43, member 1

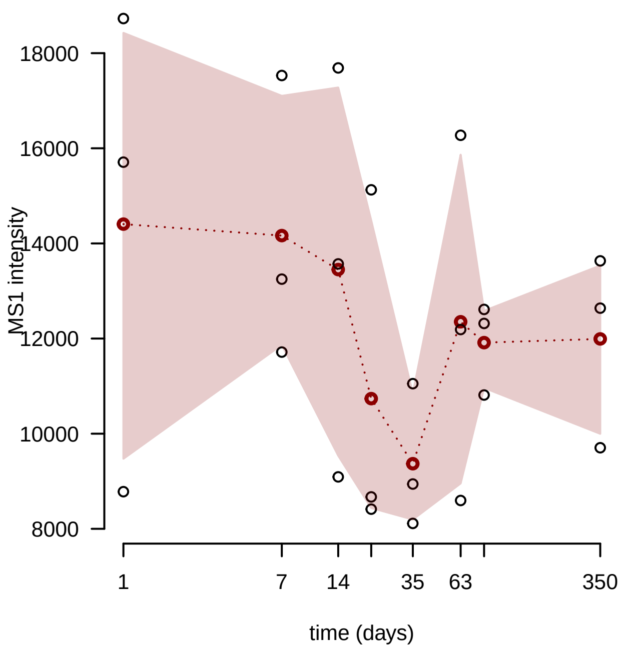

Q8BSN5
GN=Phf23
PHD finger protein 23

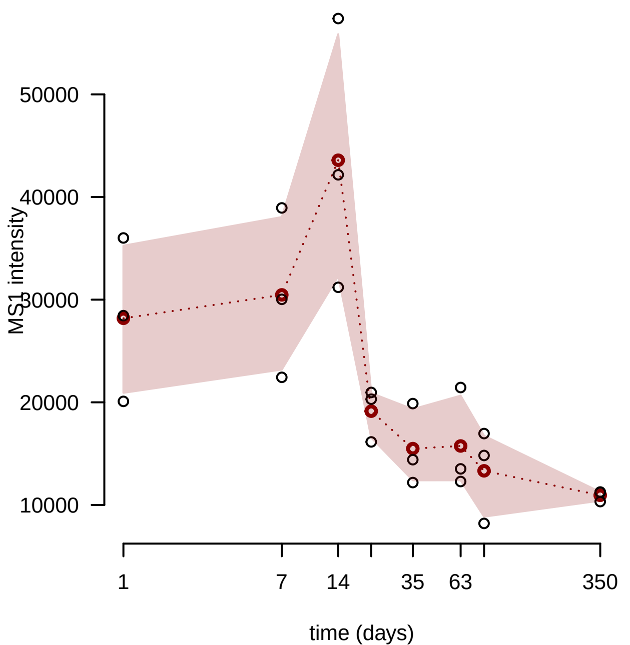

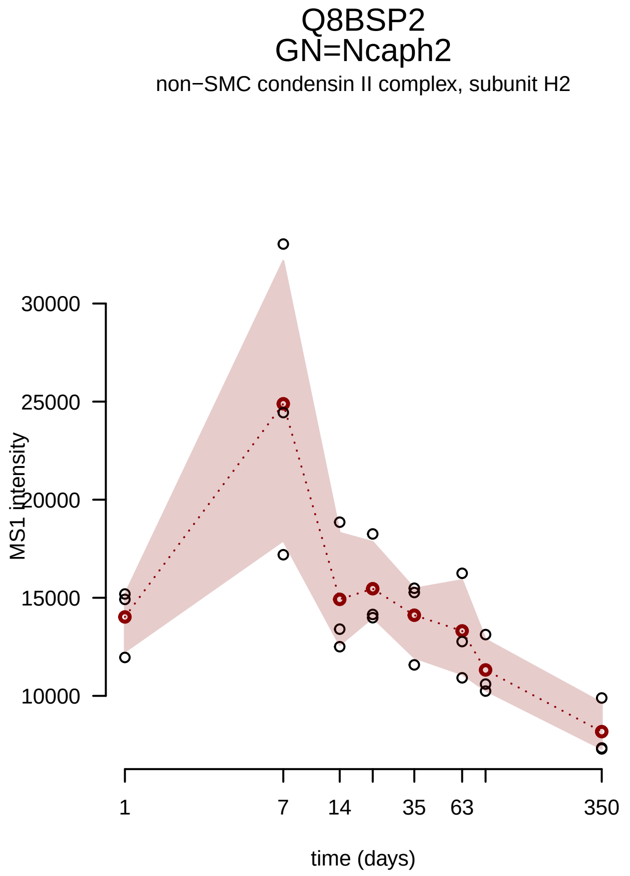
Q8BSP2
GN=Ncaph2
non−SMC condensin II complex, subunit H2
MS1 intensity
30000
25000
20000
15000
10000
1
7
14
35
63
350
time (days)

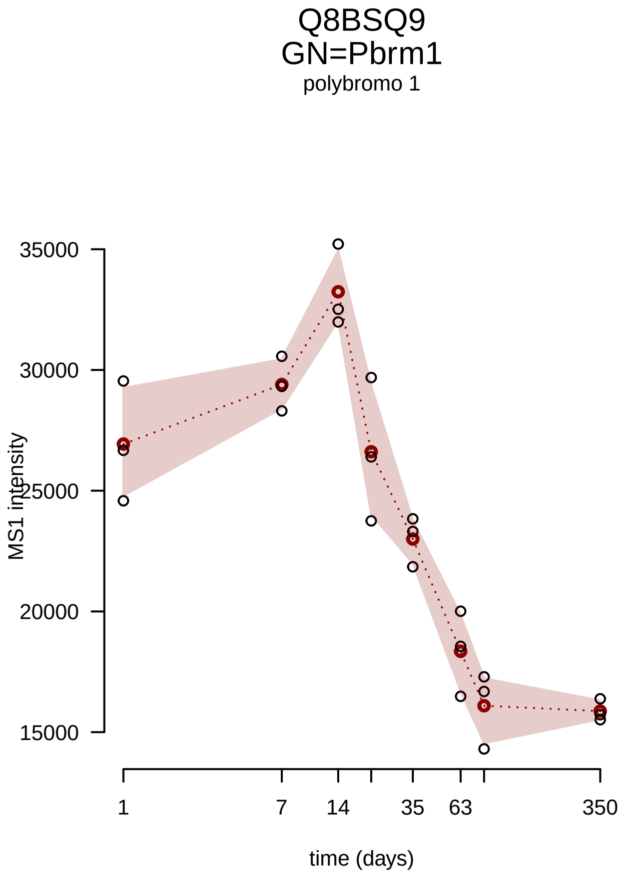
Q8BSQ9
GN=Pbrm1
polybromo 1
MS1 intensity
35000
30000
25000
20000
15000
1
7
14
35
63
350
time (days)

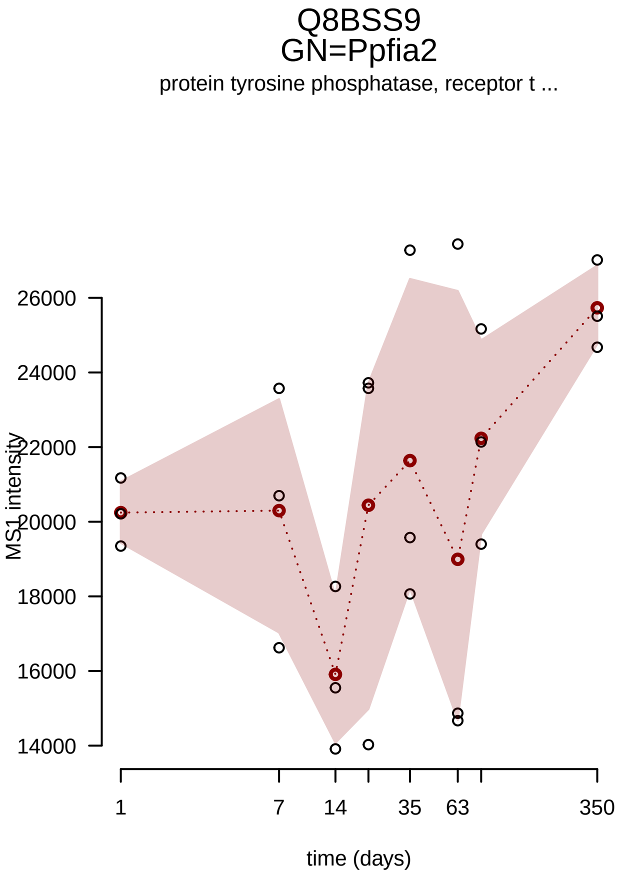
Q8BSS9
GN=Ppfia2
protein tyrosine phosphatase, receptor t ...
MS1 intensity
26000
24000
22000
20000
18000
16000
14000
1
7
14
35
63
350
time (days)

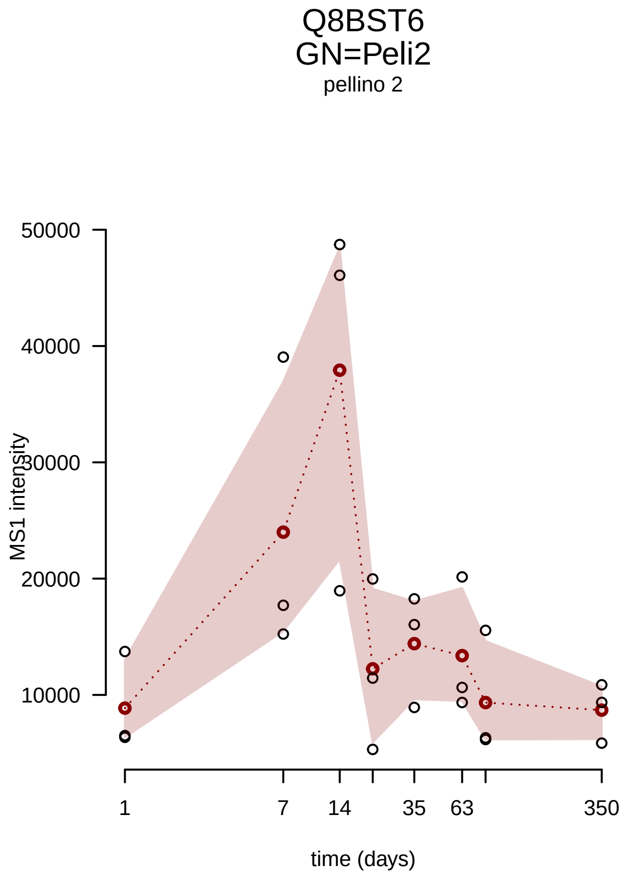
Q8BST6
GN=Peli2
pellino 2
MS1 intensity
50000
40000
30000
20000
10000
1
7
14
35
63
350
time (days)

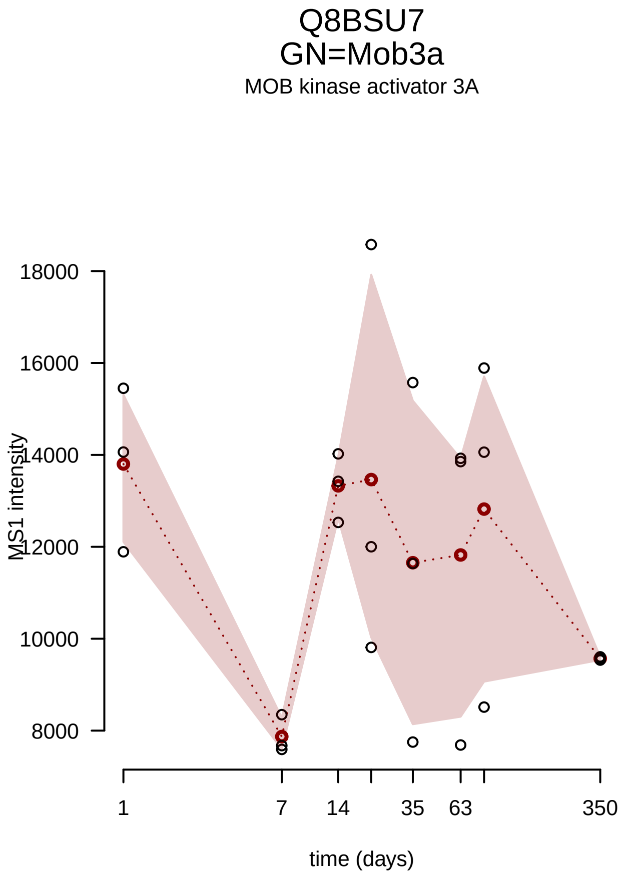
Q8BSU7
GN=Mob3a
MOB kinase activator 3A
MS1 intensity
18000
16000
14000
12000
10000
8000
1
7
14
35
63
350
time (days)

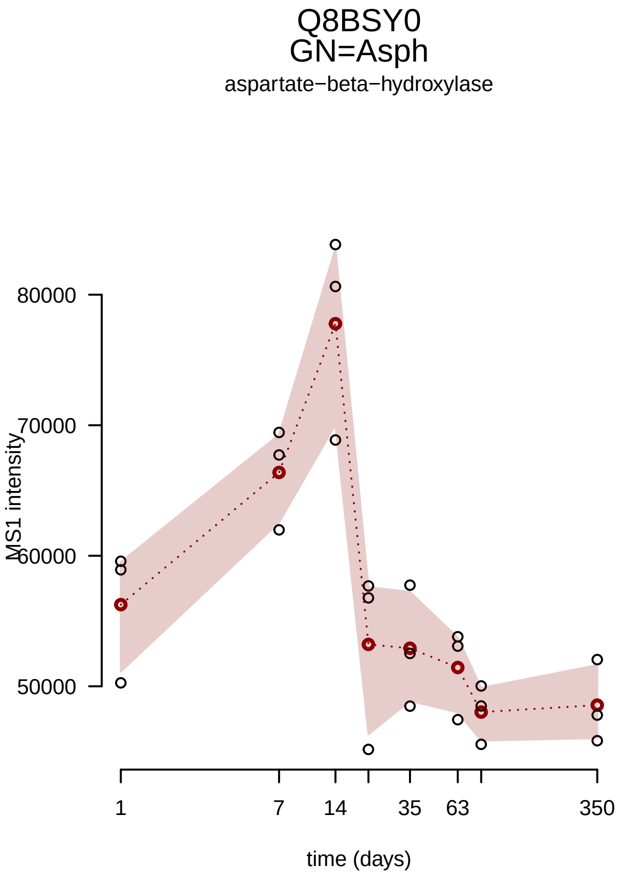
Q8BSY0
GN=Asph
aspartate−beta−hydroxylase
MS1 intensity
80000
70000
60000
50000
1
7
14
35
63
350
time (days)

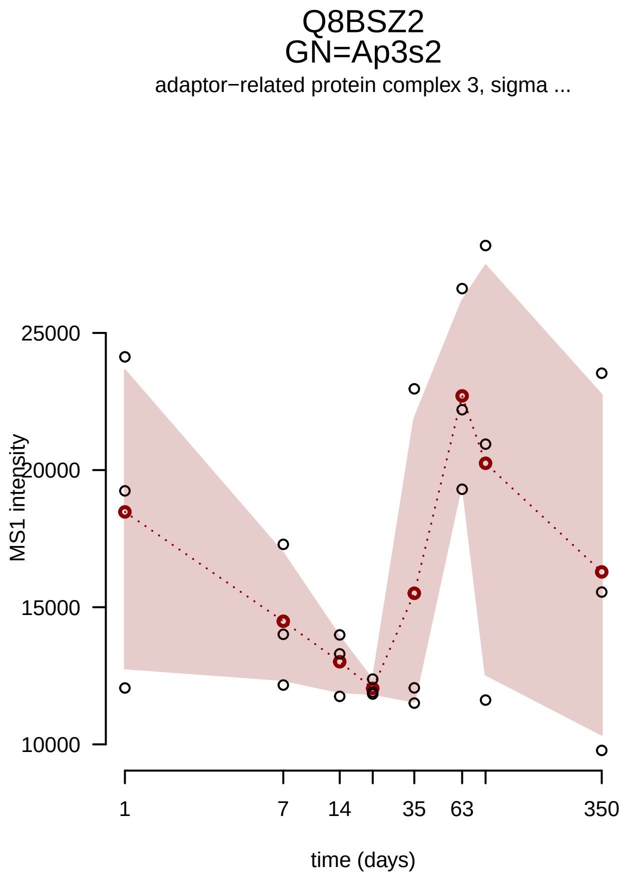
Q8BSZ2
GN=Ap3s2
adaptor−related protein complex 3, sigma ...
MS1 intensity
25000
20000
15000
10000
1
7
14
35
63
350
time (days)

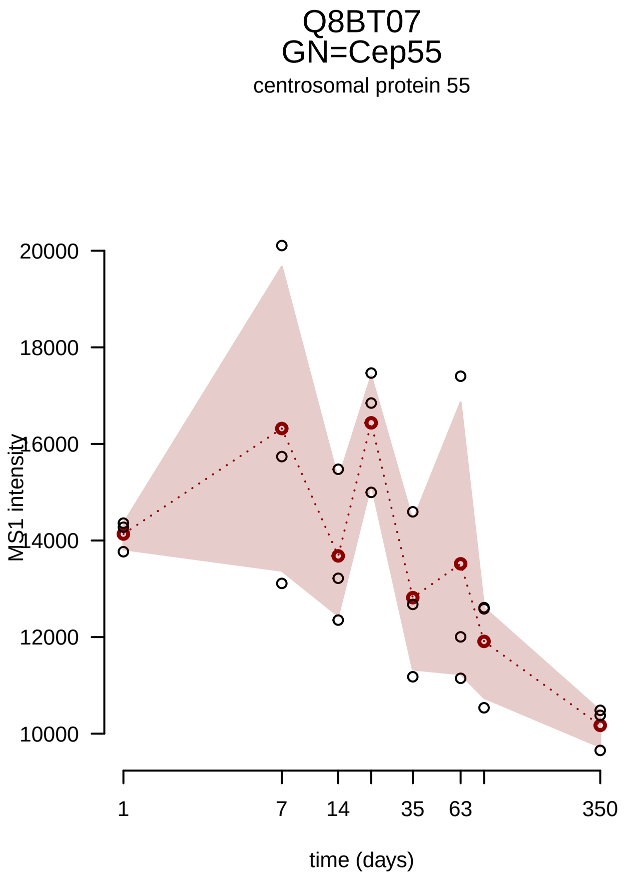
Q8BT07
GN=Cep55
centrosomal protein 55
MS1 intensity
20000
18000
16000
14000
12000
10000
1
7
14
35
63
350
time (days)

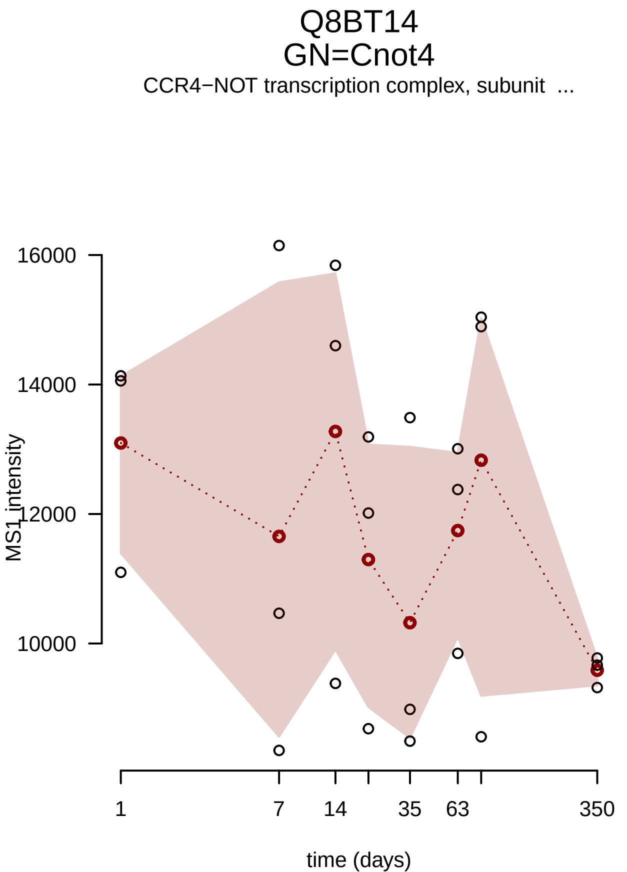
Q8BT14
GN=Cnot4
CCR4−NOT transcription complex, subunit ...
MS1 intensity
16000
14000
12000
10000
1
7
14
35
63
350
time (days)

Q8BT51
GN=COA4

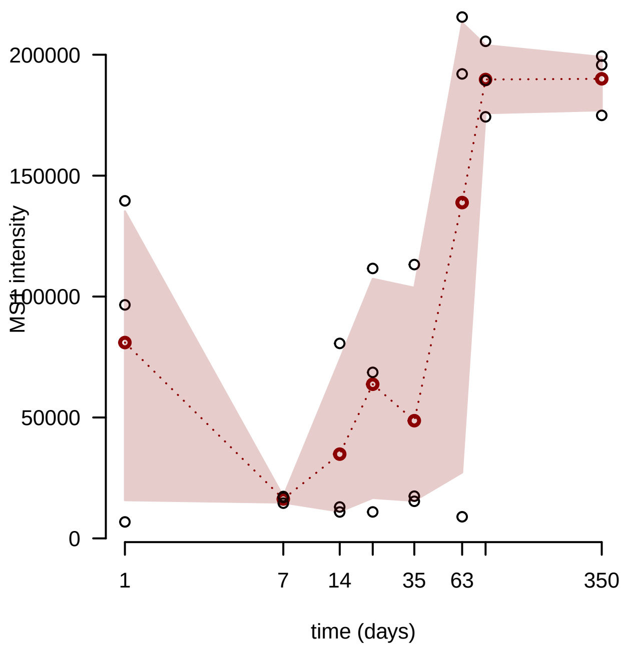

Q8BT60
GN=Cpne3
copine III

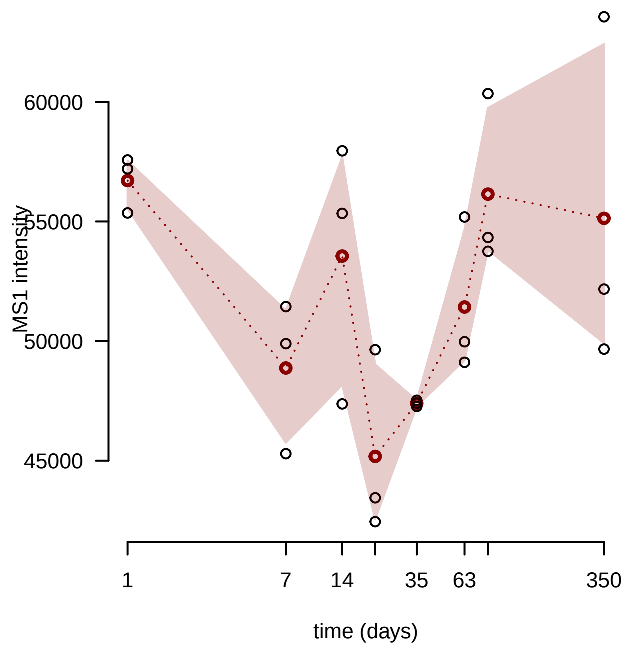

Q8BTE0
GN=Sdhaf4
succinate dehydrogenase complex assembly ...

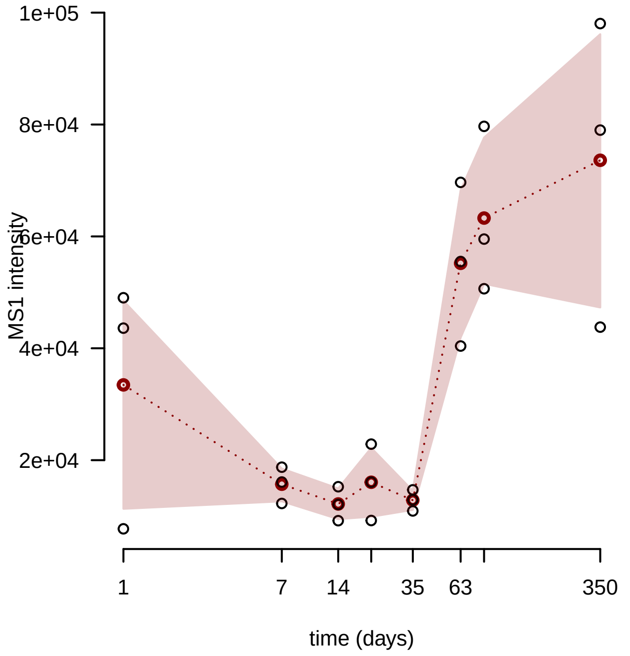

Q8BTE5
GN=Cebpzos
CCAAT/enhancer binding protein (C/EBP), ...

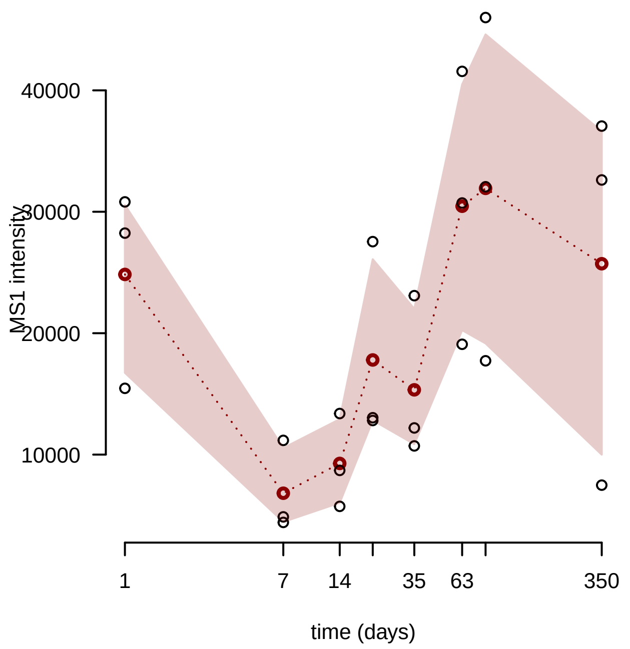

Q8BTG3
GN=Tcp11l1
t−complex 11 like 1

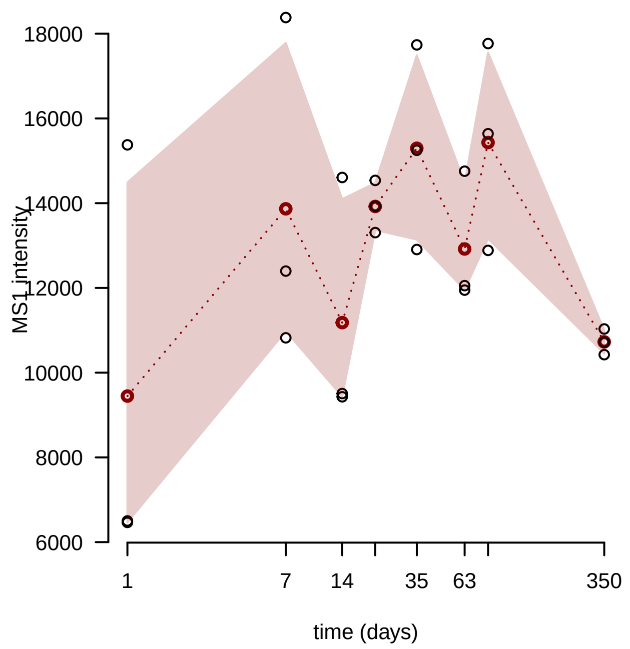

Q8BTG7
GN=Ndrg4
N−myc downstream regulated gene 4

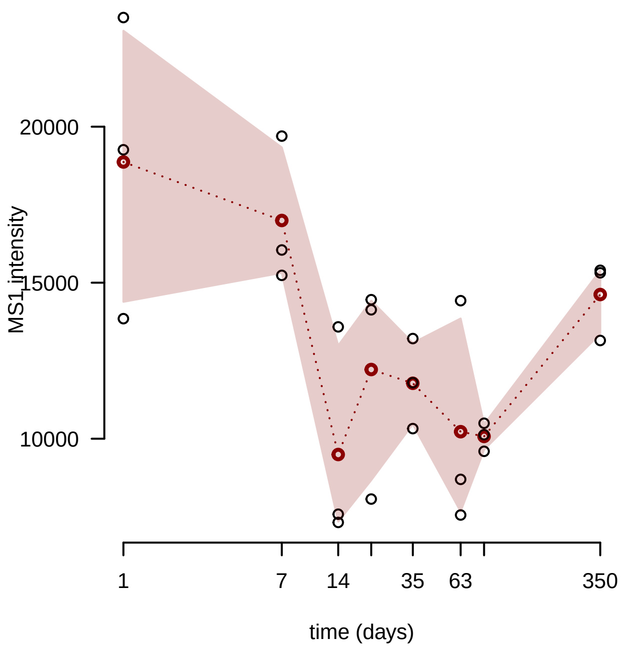

Q8BTH8
GN=Csnk1g1
casein kinase 1, gamma 1

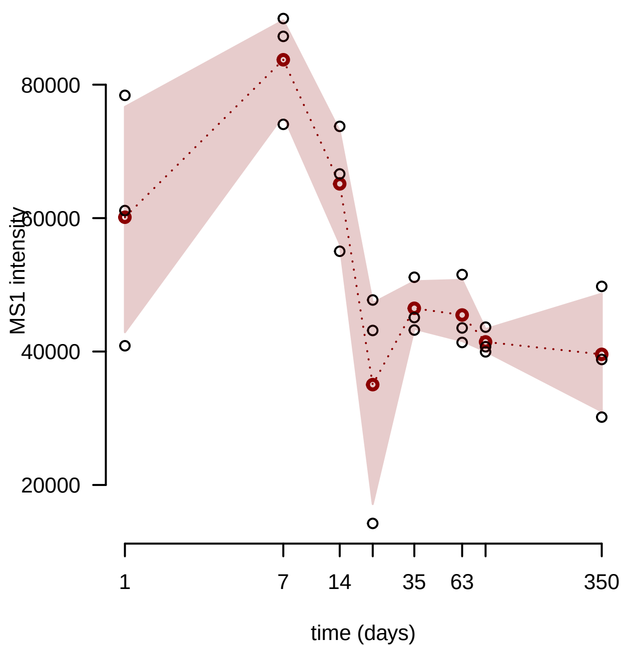

Q8BTI7
GN=Ankrd52
ankyrin repeat domain 52

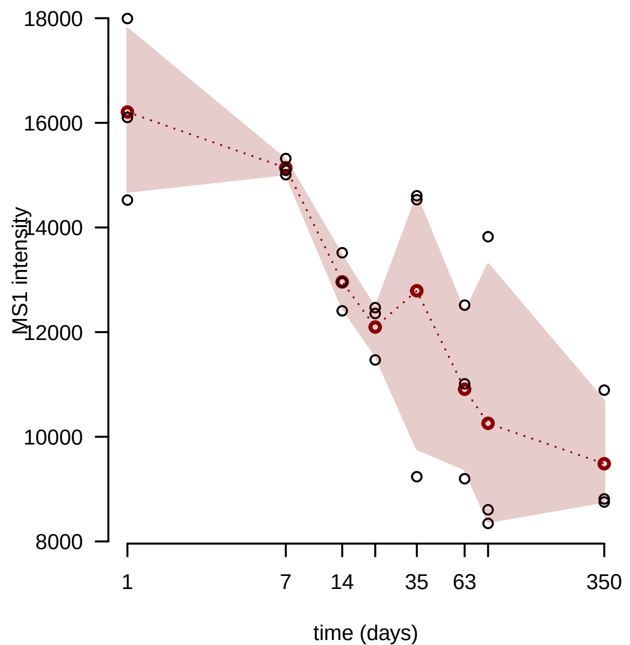

Q8BTI8
GN=Srrm2
serine/arginine repetitive matrix 2

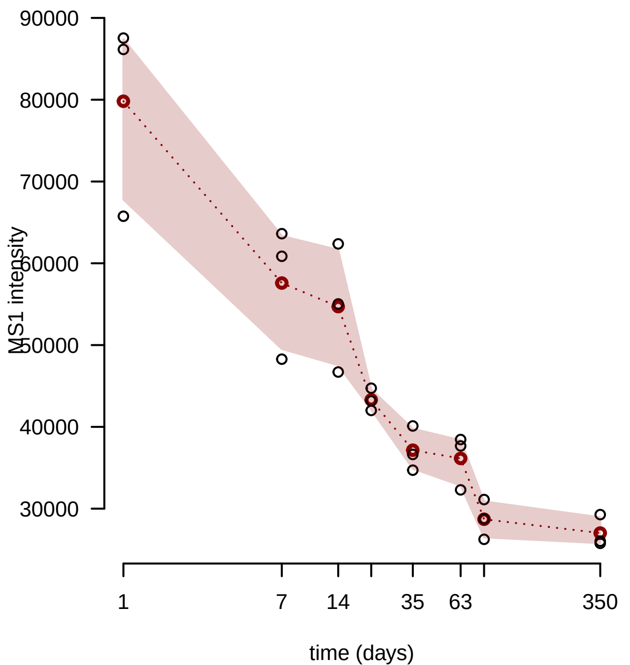

Q8BTJ4
GN=Enpp4
ectonucleotide pyrophosphatase/phosphodi ...

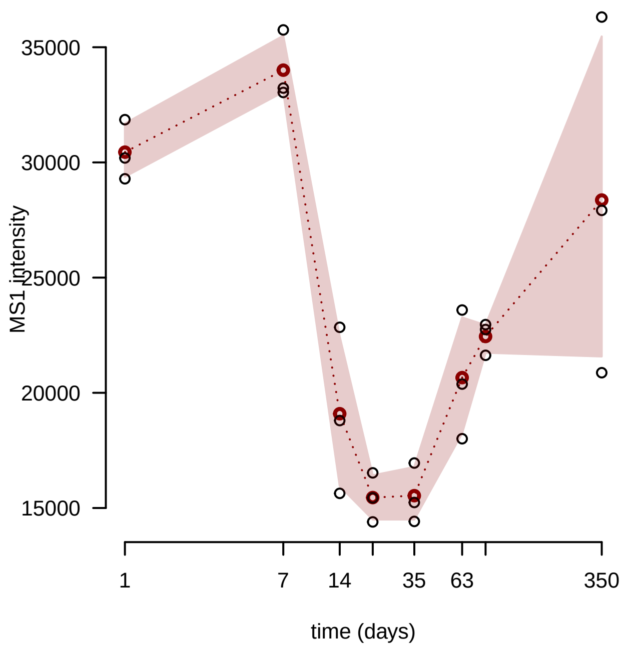

Q8BTK5
GN=Smyd4
SET and MYND domain containing 4

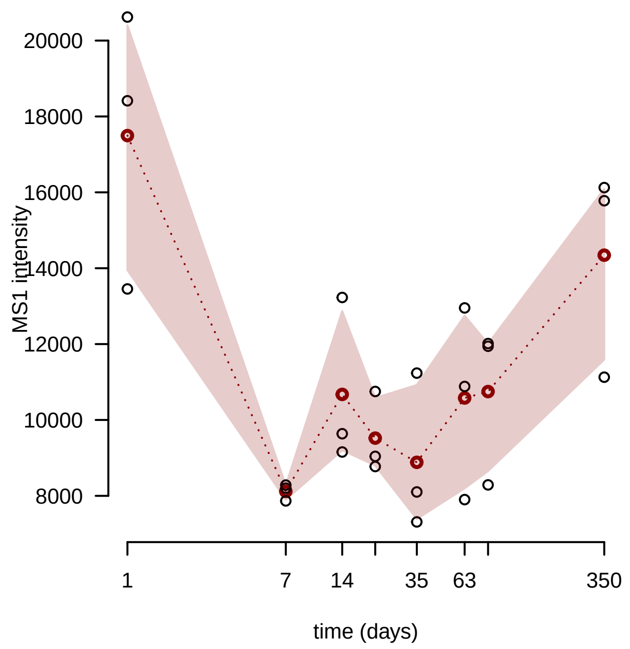

Q8BTM8
GN=Flna
filamin, alpha

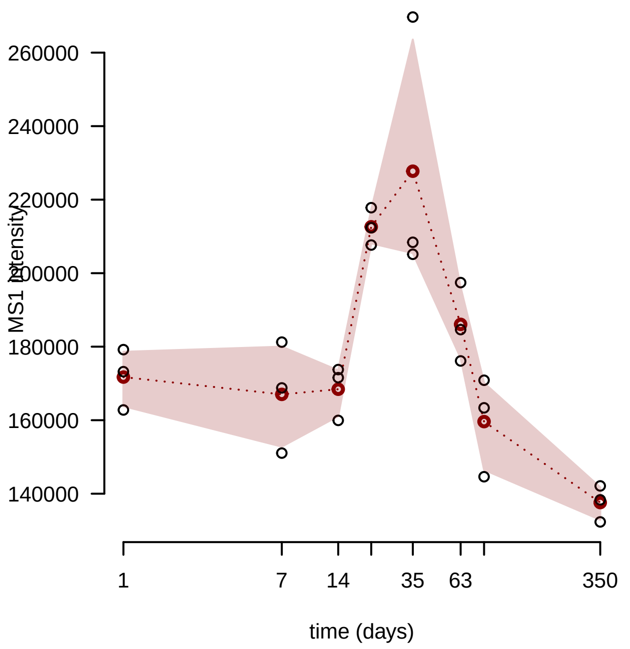

Q8BTR5
GN=Dusp28
dual specificity phosphatase 28

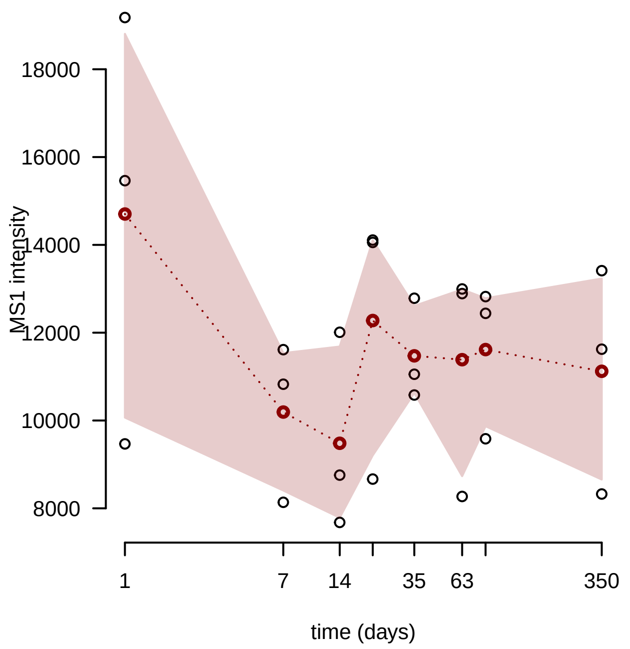

Q8BTS4
GN=Nup54
nucleoporin 54

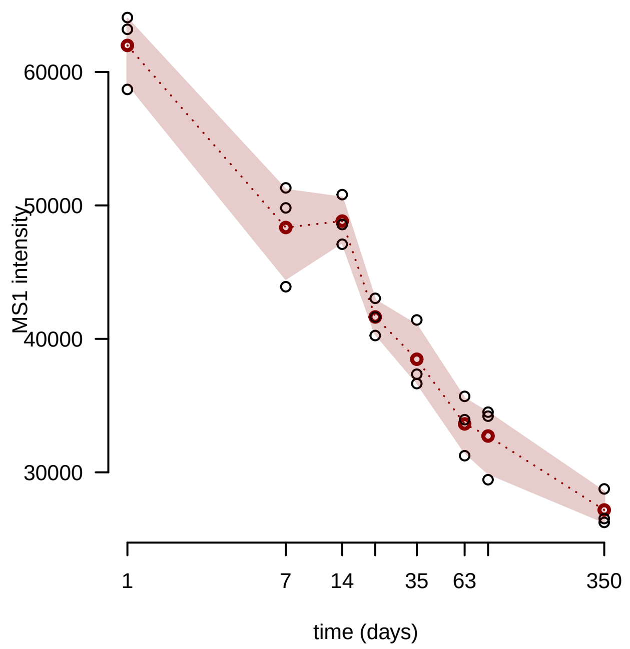

Q8BTT6
GN=Diexf

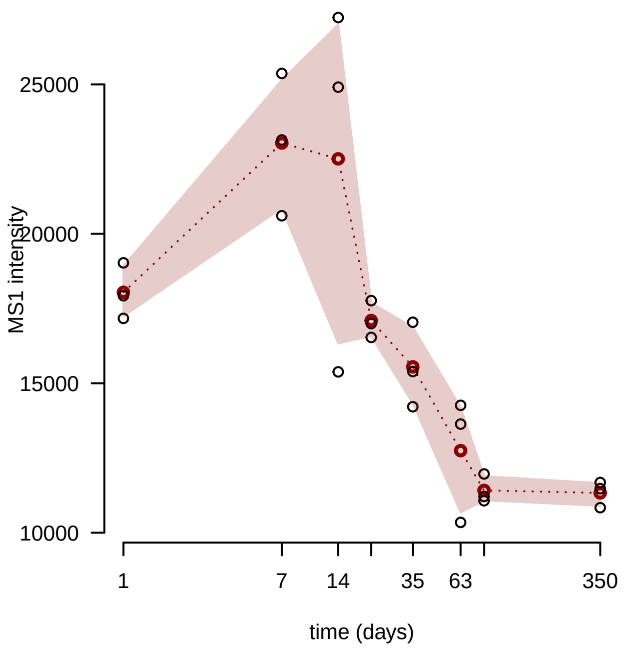

Q8BTU1
GN=Cfap20
cilia and flagella associated protein 20

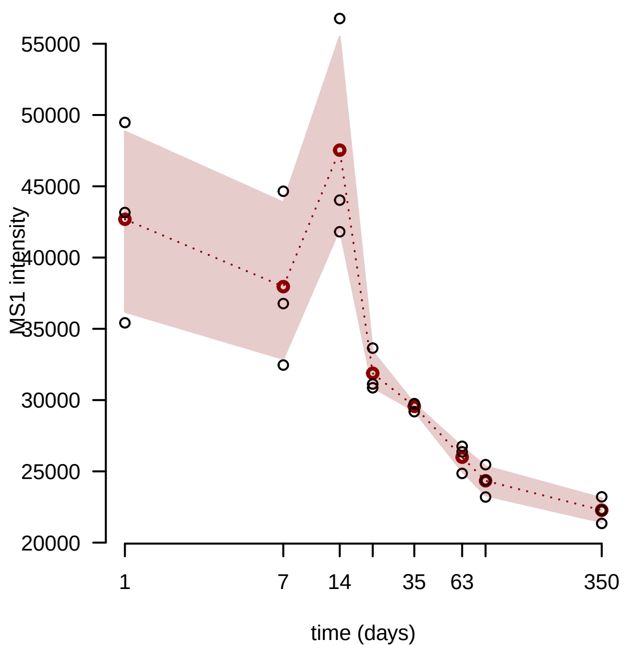

Q8BTV1
GN=Tusc3
tumor suppressor candidate 3

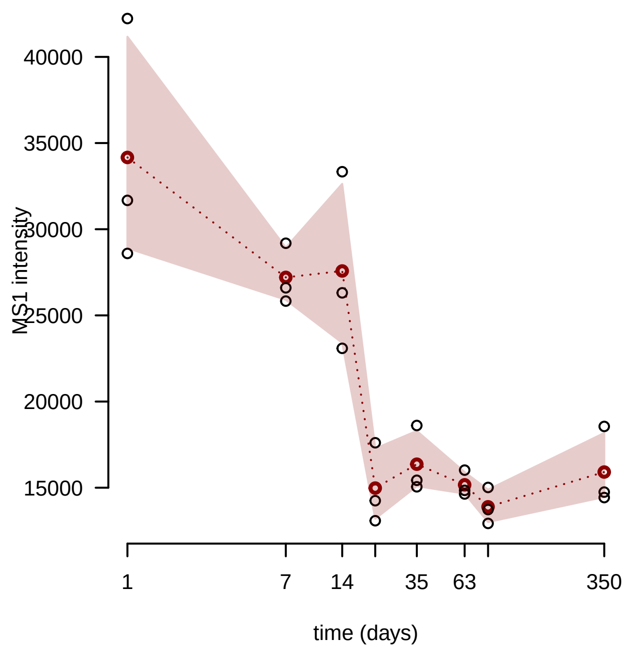

Q8BTV2
GN=Cpsf7
cleavage and polyadenylation specific fa ...

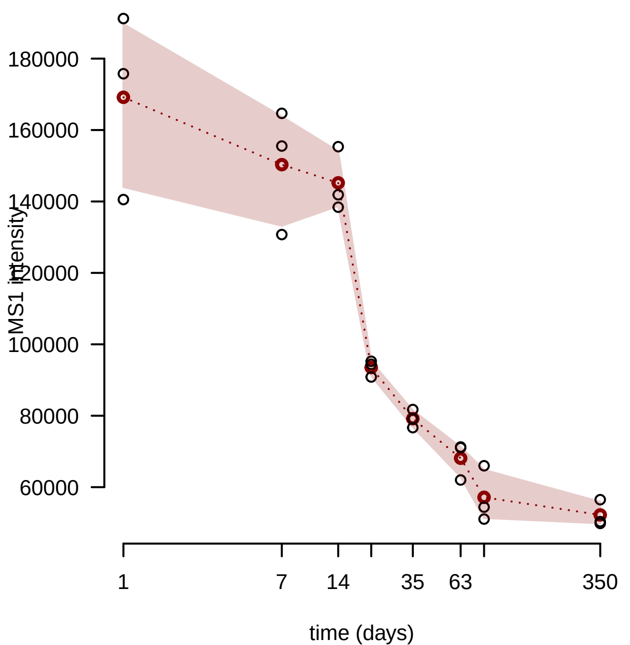

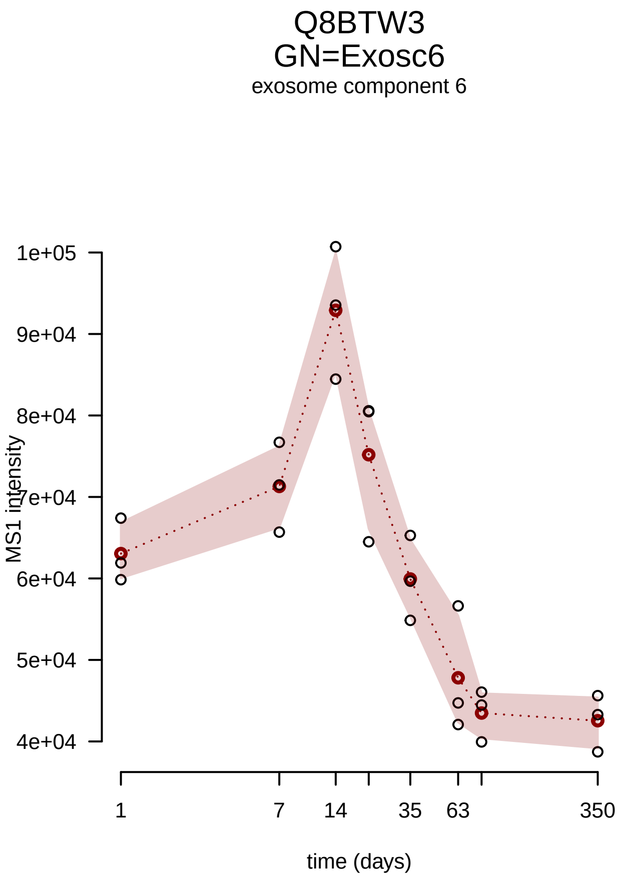
Q8BTW3
GN=Exosc6
exosome component 6
MS1 intensity
1e+05
9e+04
8e+04
7e+04
6e+04
5e+04
4e+04
1
7
14
35
63
350
time (days)

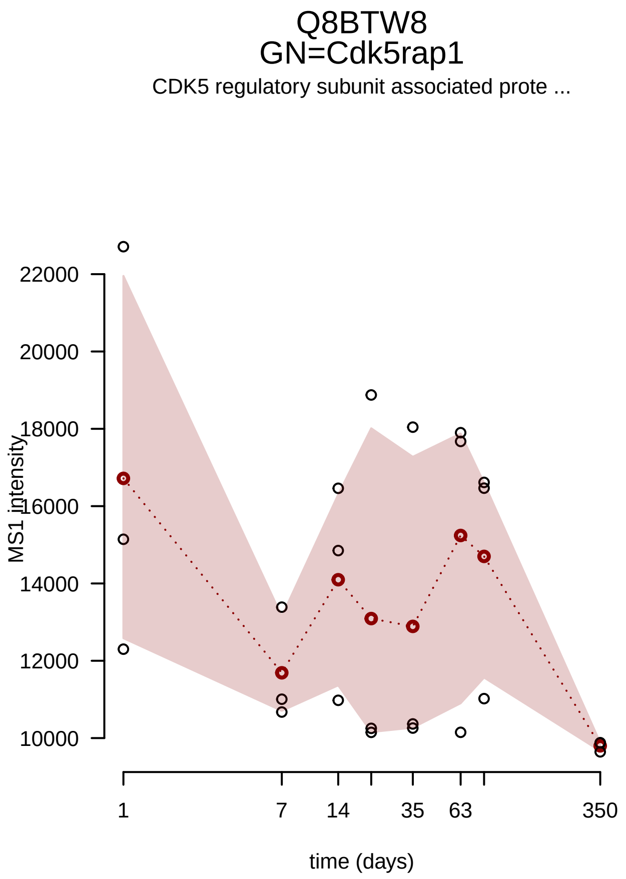
Q8BTW8
GN=Cdk5rap1
CDK5 regulatory subunit associated prote ...
MS1 intensity
22000
20000
18000
16000
14000
12000
10000
1
7
14
35
63
350
time (days)

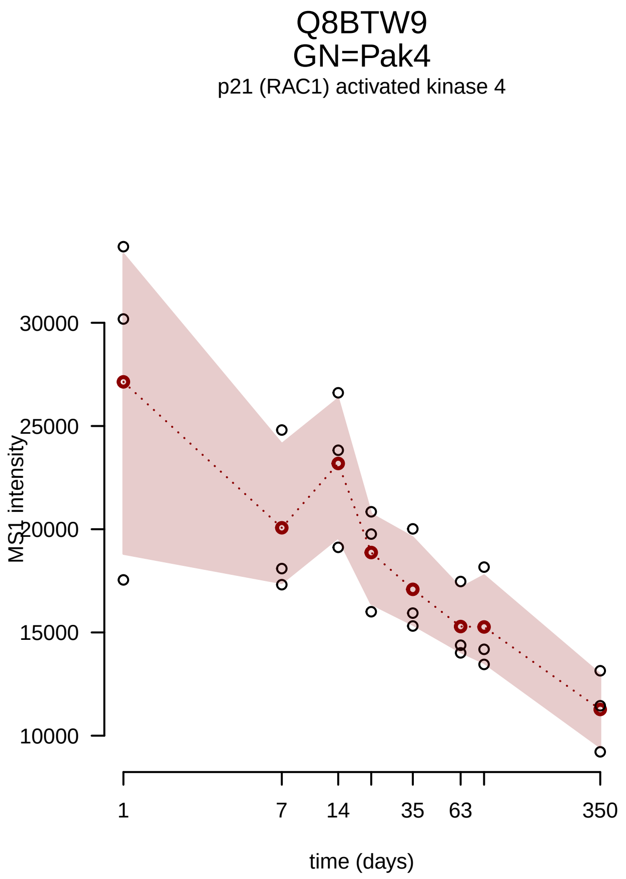
Q8BTW9
GN=Pak4
p21 (RAC1) activated kinase 4
MS1 intensity
30000
25000
20000
15000
10000
1
7
14
35
63
350
time (days)

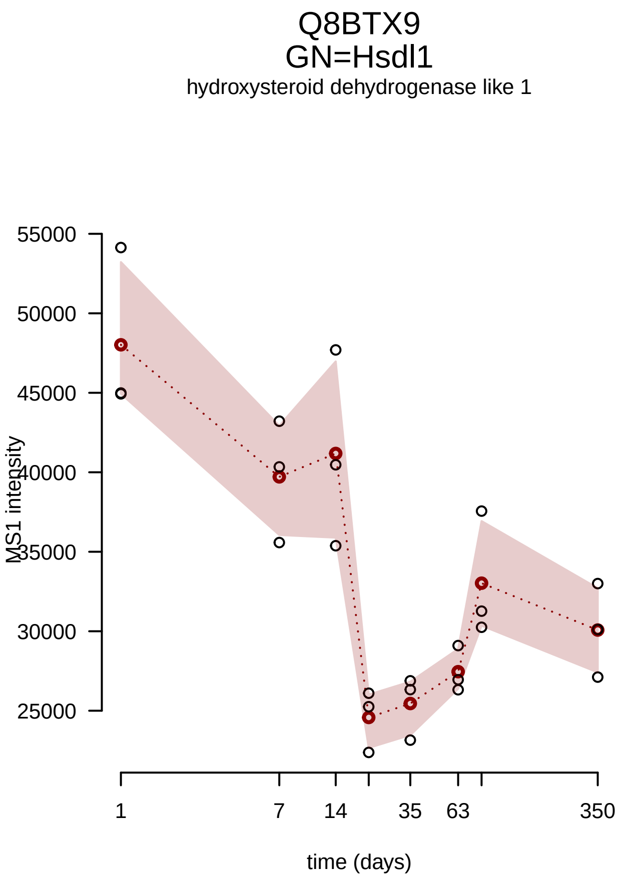
Q8BTX9
GN=Hsdl1
hydroxysteroid dehydrogenase like 1
MS1 intensity
55000
50000
45000
40000
35000
30000
25000
1
7
14
35
63
350
time (days)

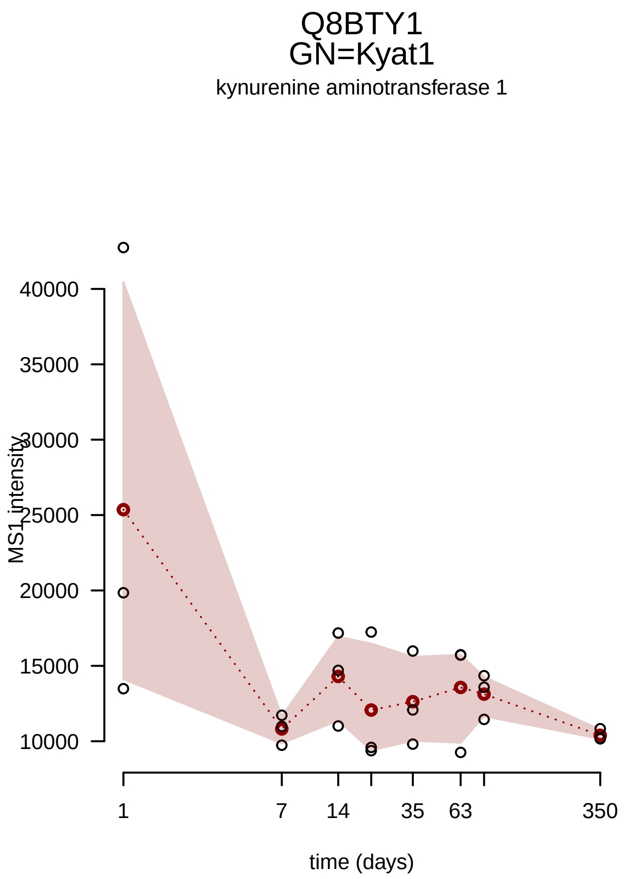
Q8BTY1
GN=Kyat1
kynurenine aminotransferase 1
MS1 intensity
40000
35000
30000
25000
20000
15000
10000
1
7
14
35
63
350
time (days)

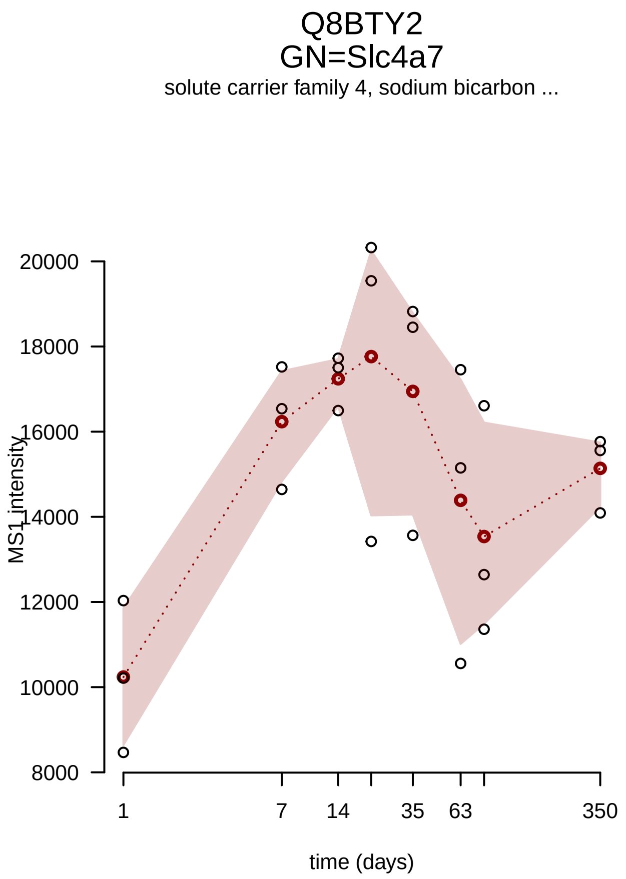
Q8BTY2
GN=Slc4a7
solute carrier family 4, sodium bicarbon ...
MS1 intensity
20000
18000
16000
14000
12000
10000
8000
1
7
14
35
63
350
time (days)

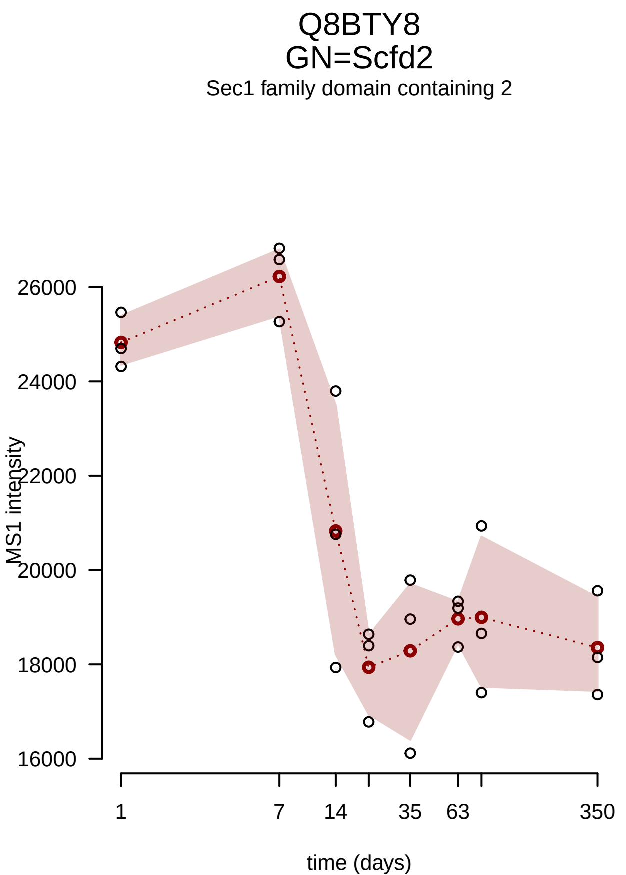
Q8BTY8
GN=Scfd2
Sec1 family domain containing 2
MS1 intensity
26000
24000
22000
20000
18000
16000
1
7
14
35
63
350
time (days)

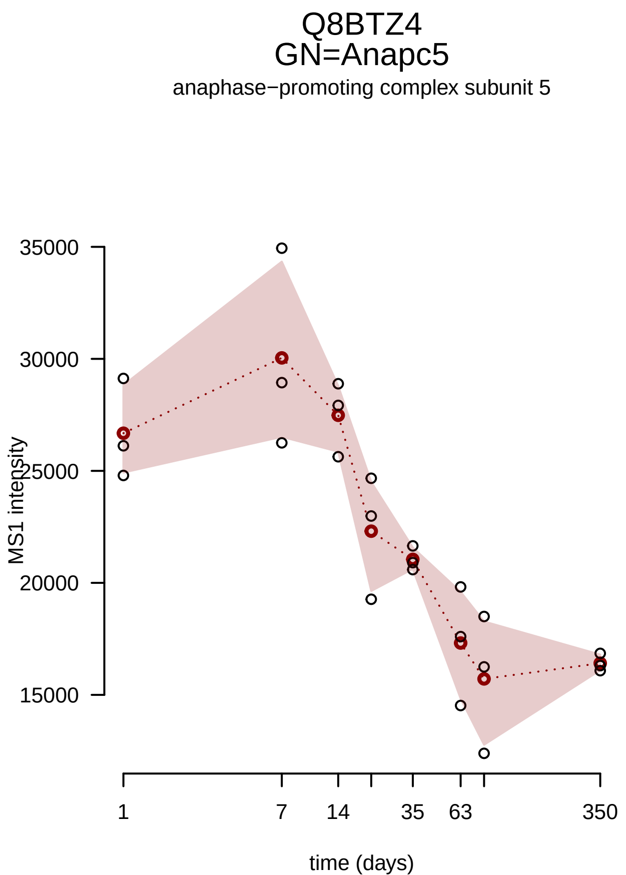
Q8BTZ4
GN=Anapc5
anaphase−promoting complex subunit 5
MS1 intensity
35000
30000
25000
20000
15000
1
7
14
35
63
350
time (days)

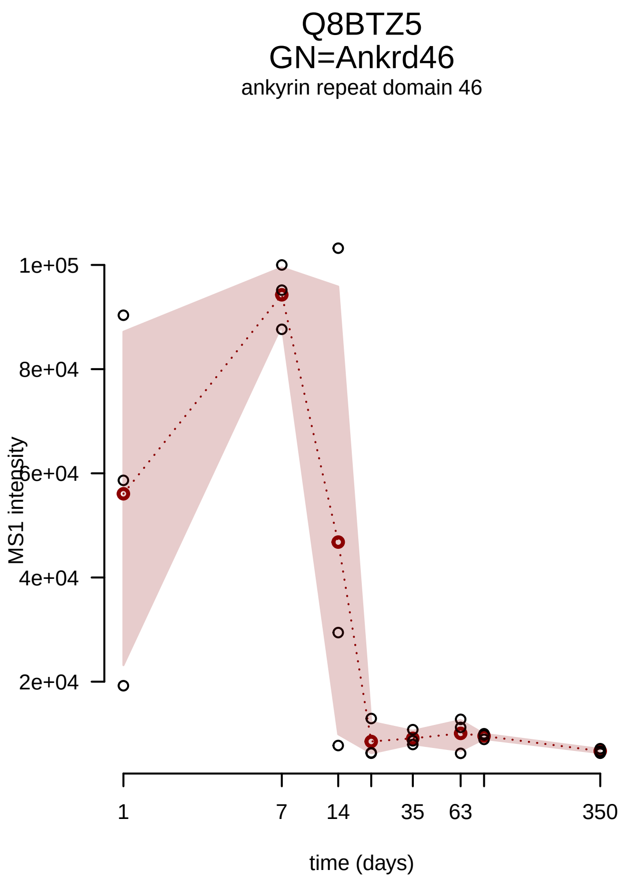
Q8BTZ5
GN=Ankrd46
ankyrin repeat domain 46
MS1 intensity
1e+05
8e+04
6e+04
4e+04
2e+04
1
7
14
35
63
350
time (days)

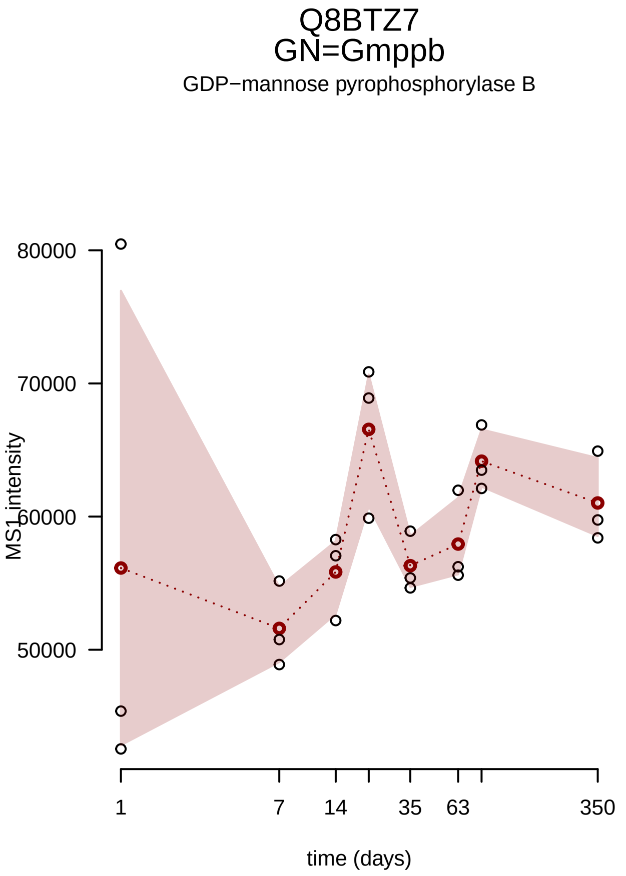
Q8BTZ7
GN=Gmppb
GDP−mannose pyrophosphorylase B
80000
70000
60000
50000
MS1 intensity
1
7
14
35
63
350
time (days)
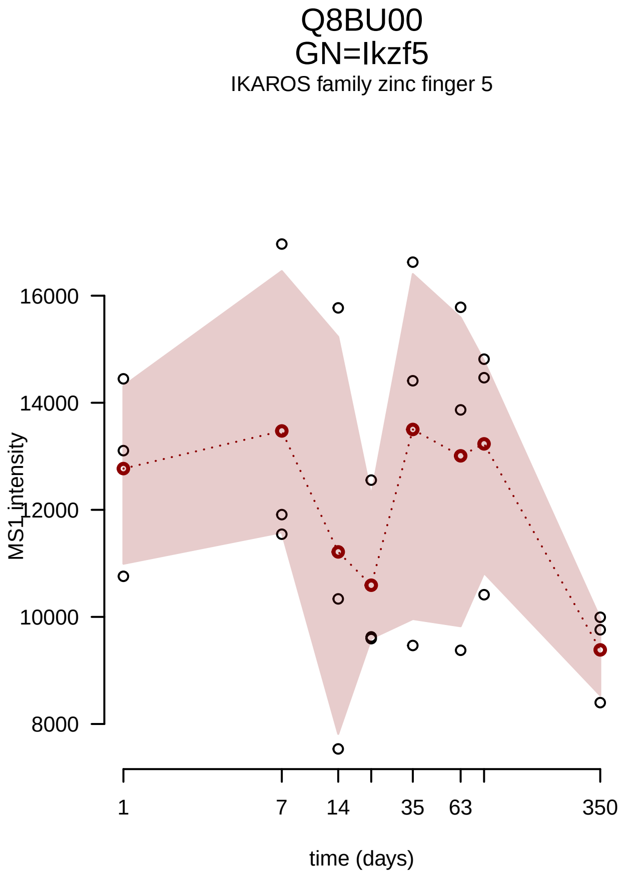
Q8BU00
GN=Ikzf5
IKAROS family zinc finger 5
16000
14000
12000
10000
8000
MS1 intensity
1
7
14
35
63
350
time (days)
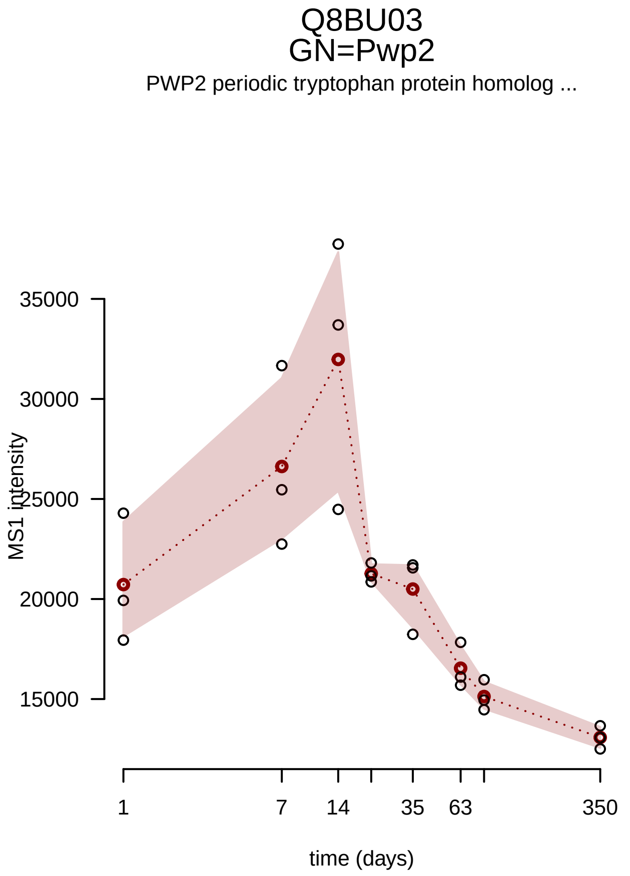
Q8BU03
GN=Pwp2
PWP2 periodic tryptophan protein homolog ...
35000
30000
25000
20000
15000
MS1 intensity
1
7
14
35
63
350
time (days)
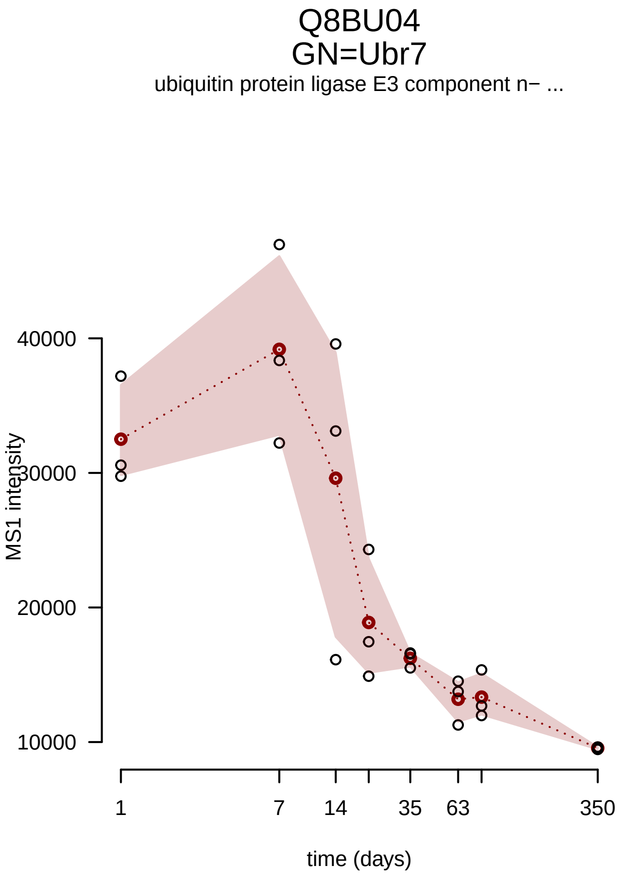
Q8BU04
GN=Ubr7
ubiquitin protein ligase E3 component n− ...
40000
30000
20000
10000
MS1 intensity
1
7
14
35
63
350
time (days)
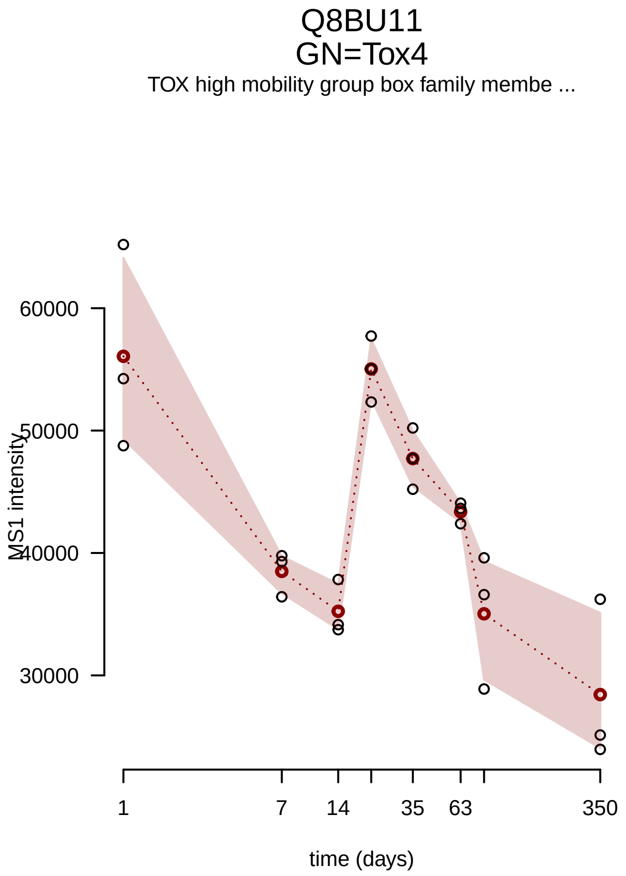
Q8BU11
GN=Tox4
TOX high mobility group box family membe ...
60000
50000
40000
30000
MS1 intensity
1
7
14
35
63
350
time (days)
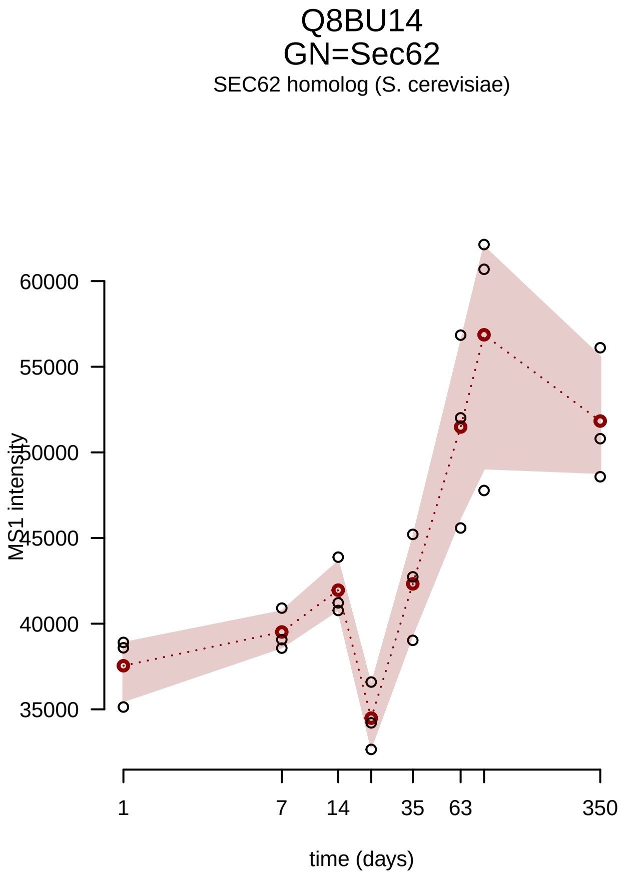
Q8BU14
GN=Sec62
SEC62 homolog (S. cerevisiae)
60000
55000
50000
45000
40000
35000
MS1 intensity
1
7
14
35
63
350
time (days)
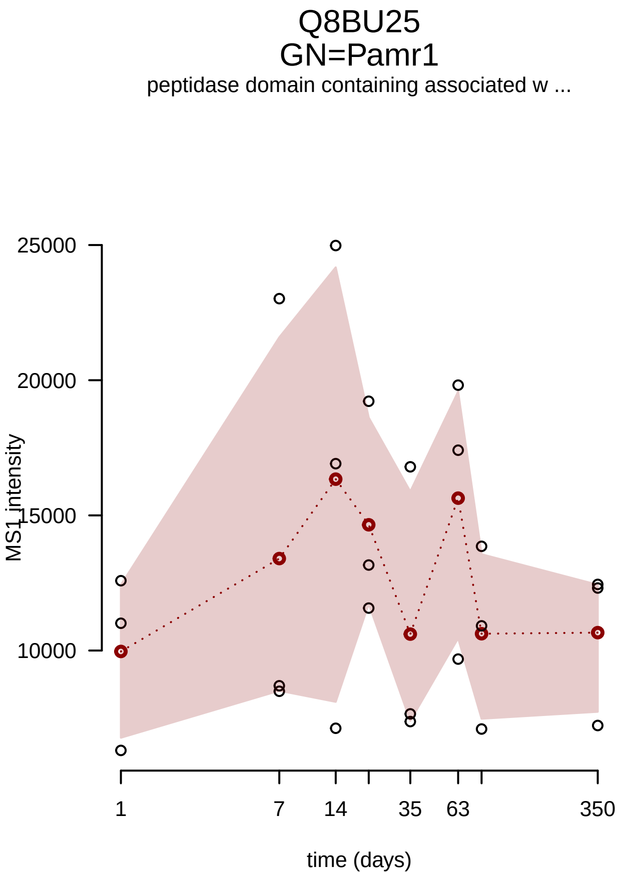
Q8BU25
GN=Pamr1
peptidase domain containing associated w ...
25000
20000
15000
10000
MS1 intensity
1
7
14
35
63
350
time (days)
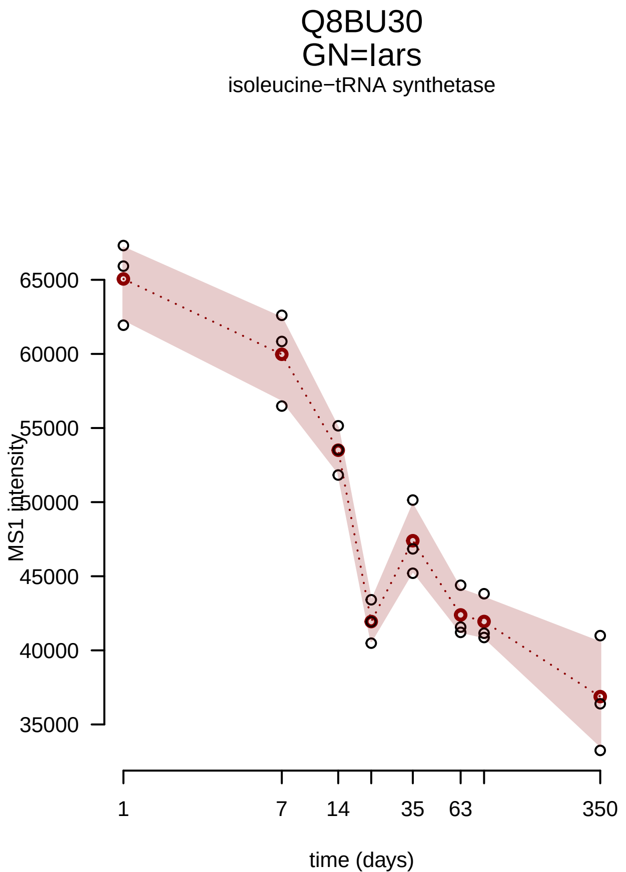
Q8BU30
GN=Iars
isoleucine−tRNA synthetase
65000
60000
55000
50000
45000
40000
35000
MS1 intensity
1
7
14
35
63
350
time (days)
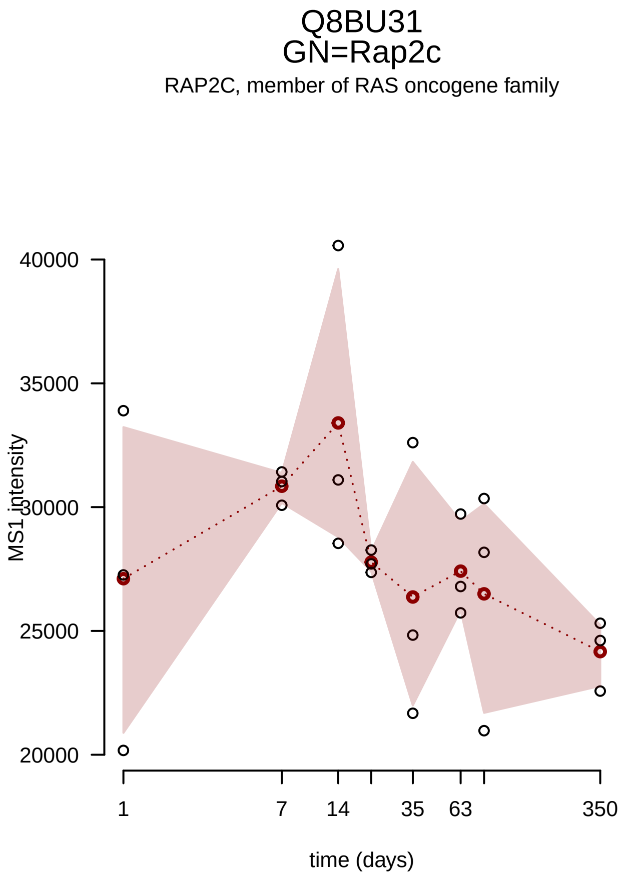
Q8BU31
GN=Rap2c
RAP2C, member of RAS oncogene family
40000
35000
30000
25000
20000
MS1 intensity
1
7
14
35
63
350
time (days)

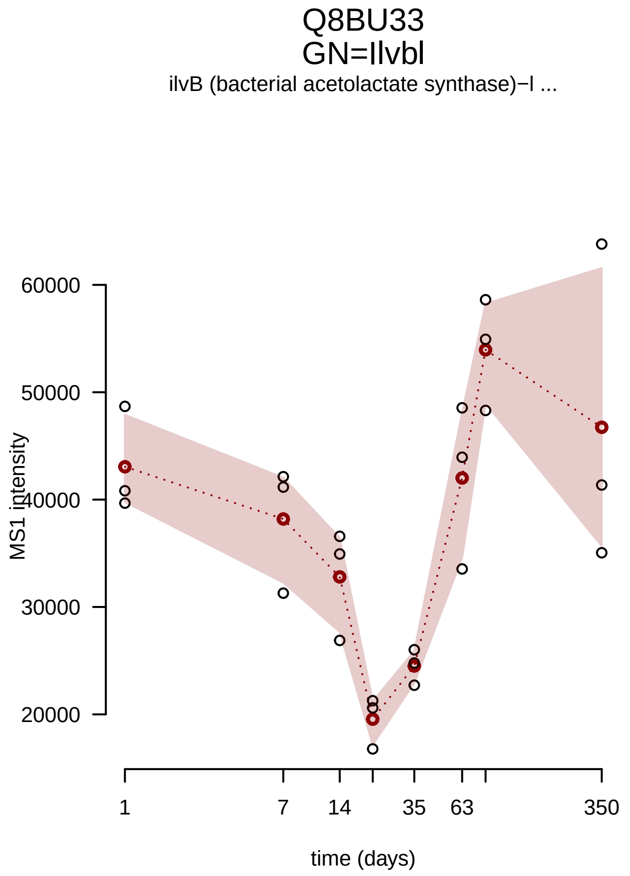
Q8BU33
GN=Ilvbl
ilvB (bacterial acetolactate synthase)−l ...
MS1 intensity
60000
50000
40000
30000
20000
1
7
14
35
63
350
time (days)
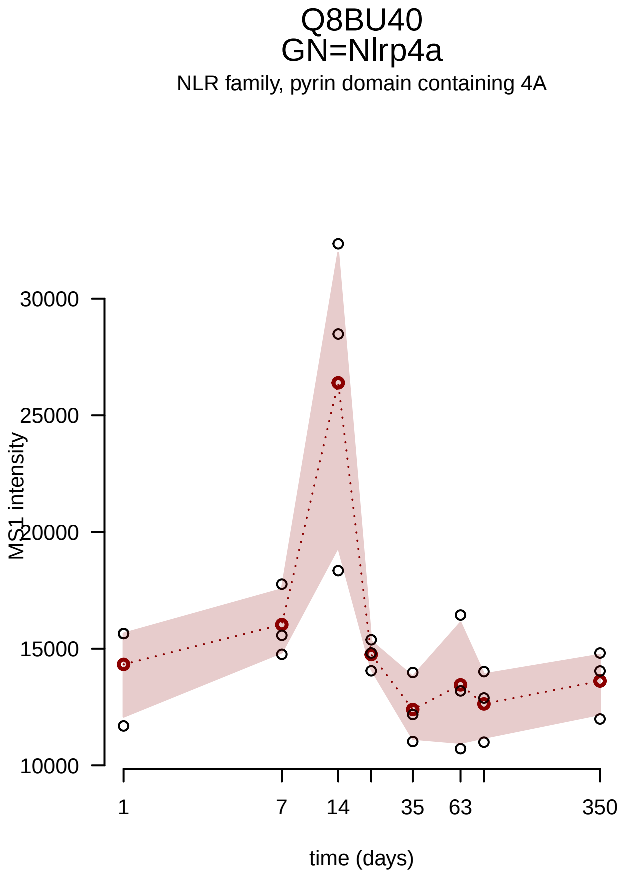
Q8BU40
GN=Nlrp4a
NLR family, pyrin domain containing 4A
MS1 intensity
30000
25000
20000
15000
10000
1
7
14
35
63
350
time (days)
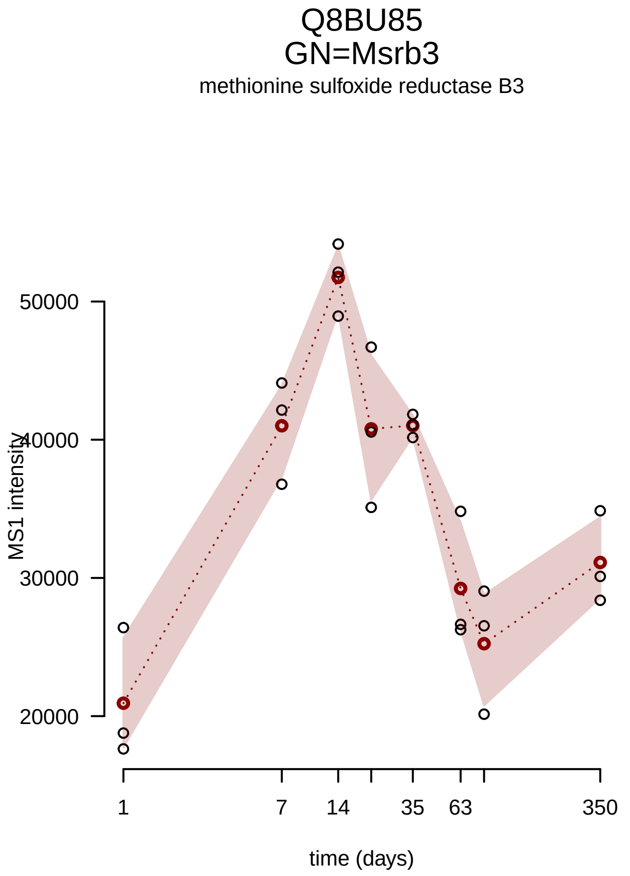
Q8BU85
GN=Msrb3
methionine sulfoxide reductase B3
MS1 intensity
50000
40000
30000
20000
1
7
14
35
63
350
time (days)
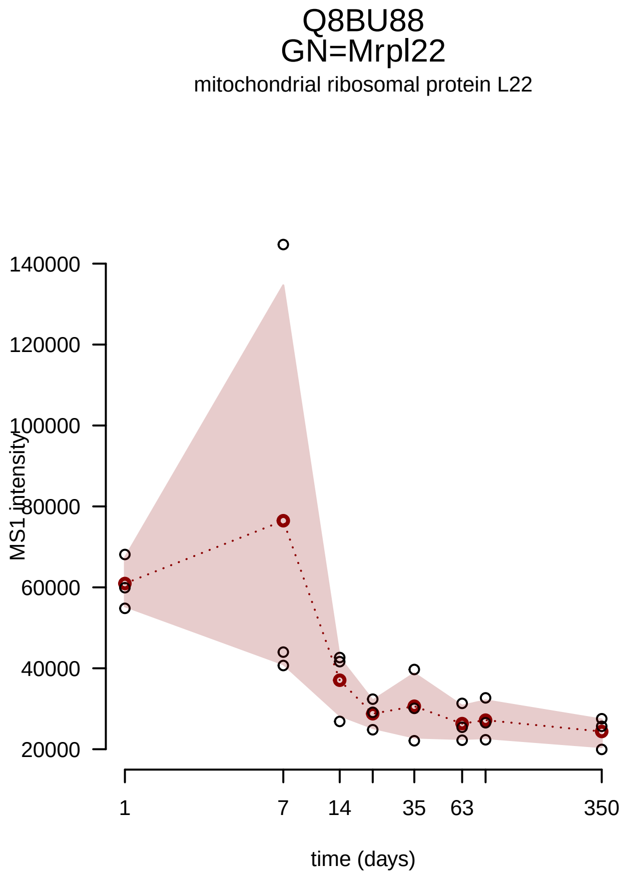
Q8BU88
GN=Mrpl22
mitochondrial ribosomal protein L22
MS1 intensity
140000
120000
100000
80000
60000
40000
20000
1
7
14
35
63
350
time (days)
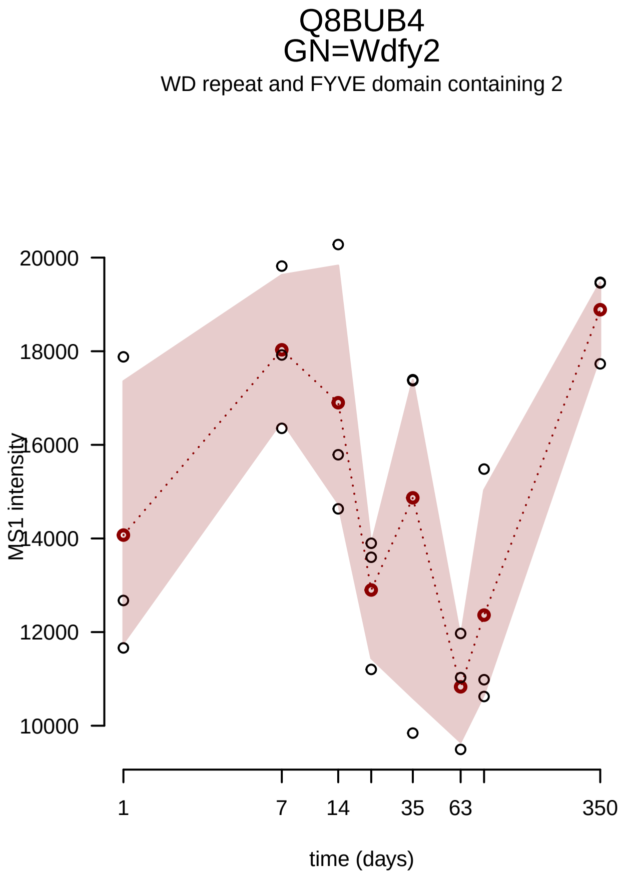
Q8BUB4
GN=Wdfy2
WD repeat and FYVE domain containing 2
MS1 intensity
20000
18000
16000
14000
12000
10000
1
7
14
35
63
350
time (days)
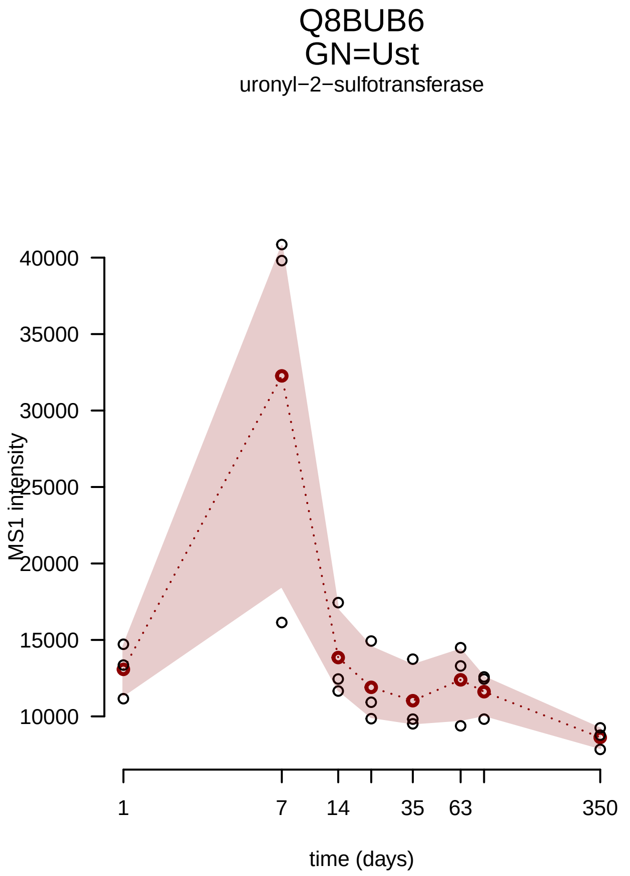
Q8BUB6
GN=Ust
uronyl−2−sulfotransferase
MS1 intensity
40000
35000
30000
25000
20000
15000
10000
1
7
14
35
63
350
time (days)
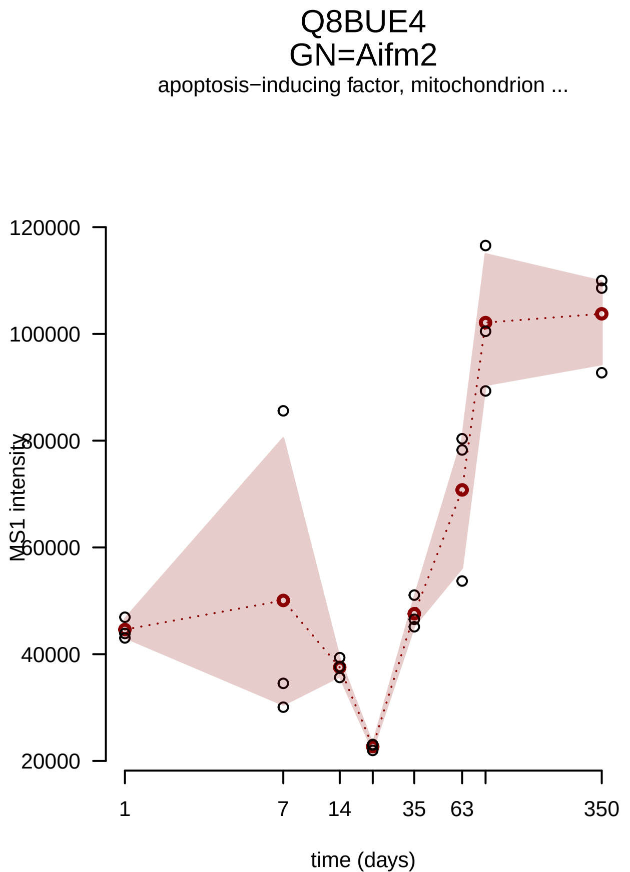
Q8BUE4
GN=Aifm2
apoptosis−inducing factor, mitochondrion ...
MS1 intensity
120000
100000
80000
60000
40000
20000
1
7
14
35
63
350
time (days)
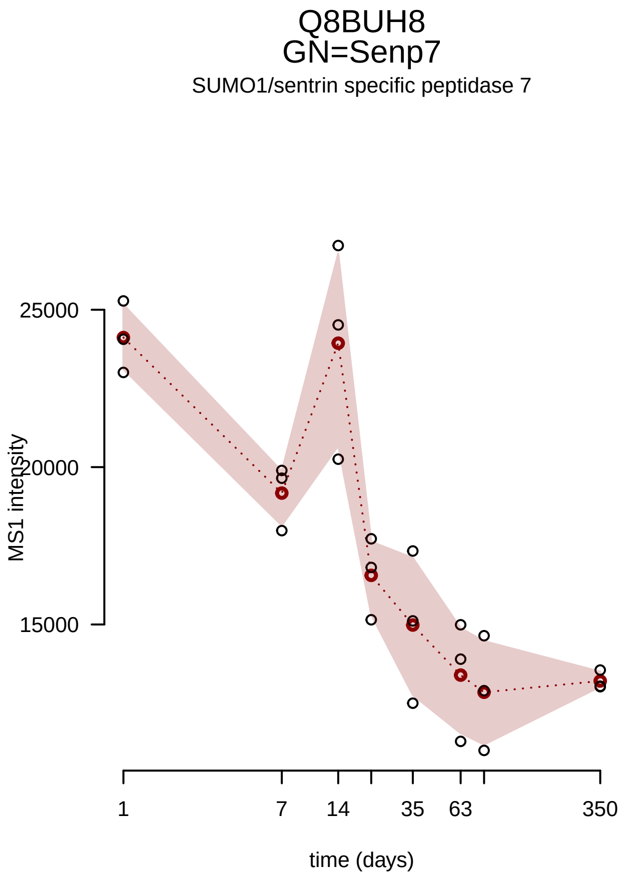
Q8BUH8
GN=Senp7
SUMO1/sentrin specific peptidase 7
MS1 intensity
25000
20000
15000
1
7
14
35
63
350
time (days)
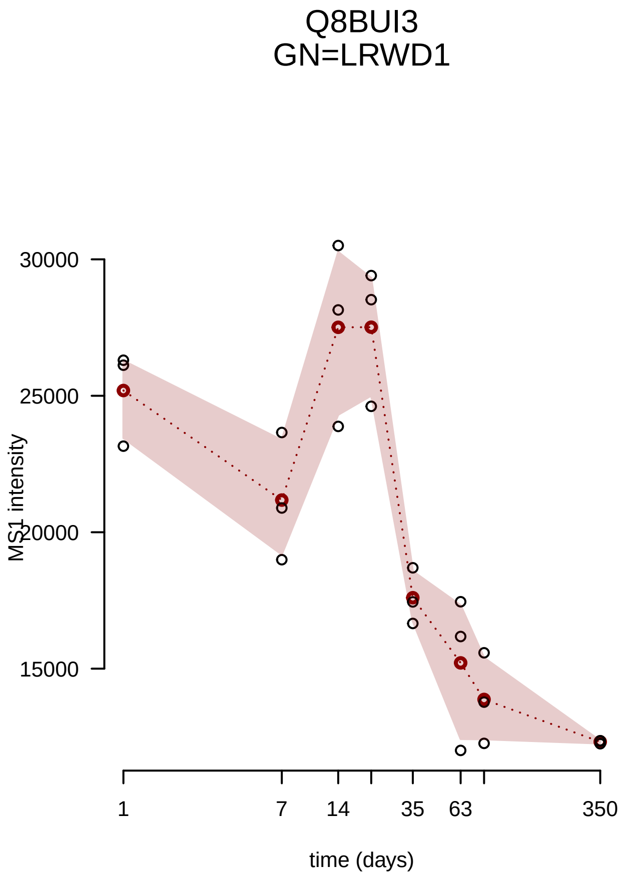
Q8BUI3
GN=LRWD1
MS1 intensity
30000
25000
20000
15000
1
7
14
35
63
350
time (days)

Q8BUK6
GN=Hook3
hook microtubule tethering protein 3

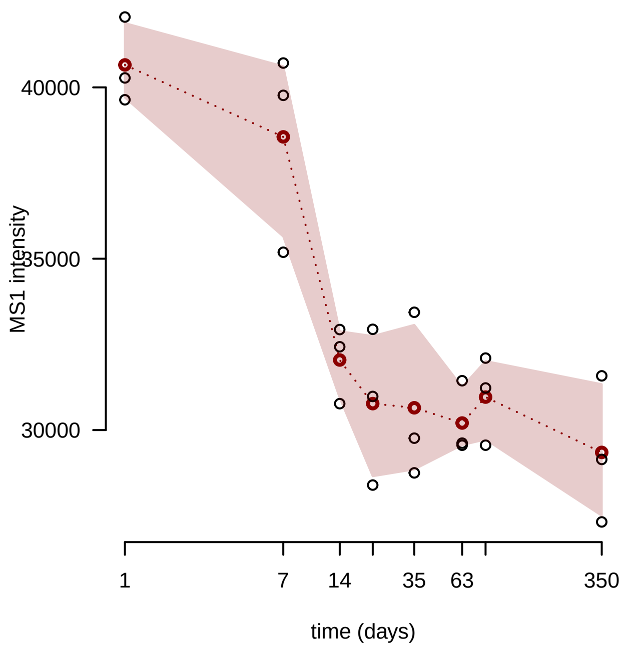

Q8BUL5
GN=Klhl7
kelch−like 7

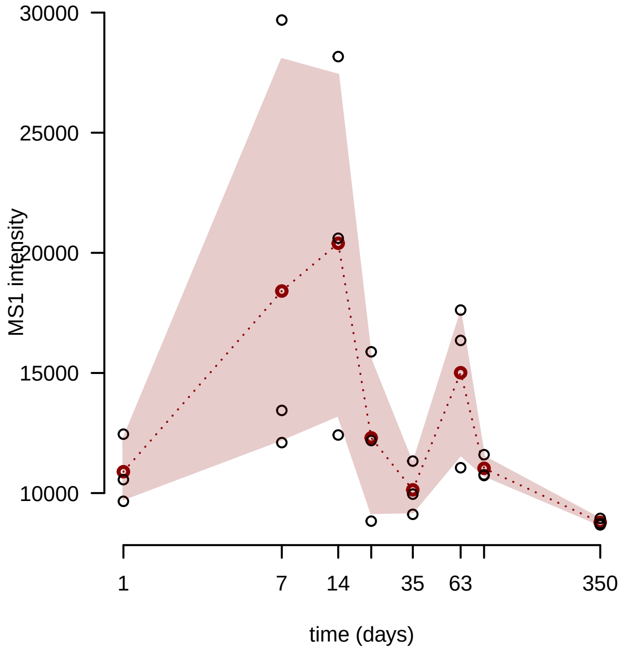

Q8BUL6
GN=Plekha1
pleckstrin homology domain containing, f ...

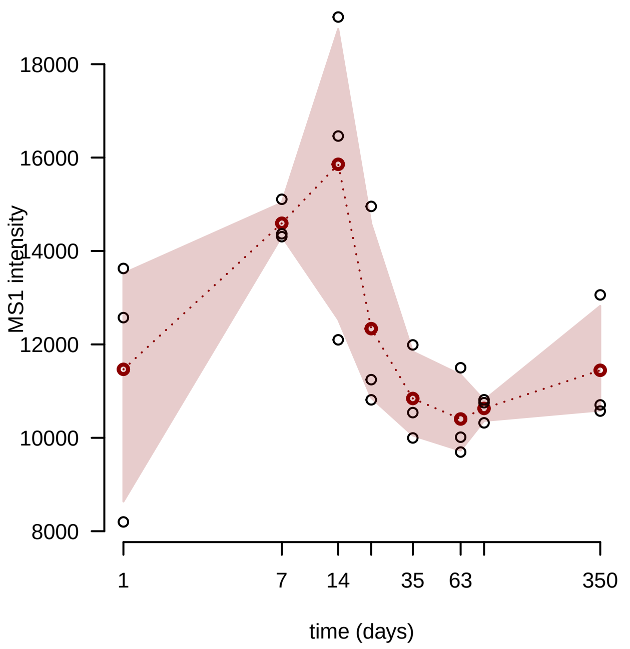

Q8BUN5
GN=Smad3
SMAD family member 3

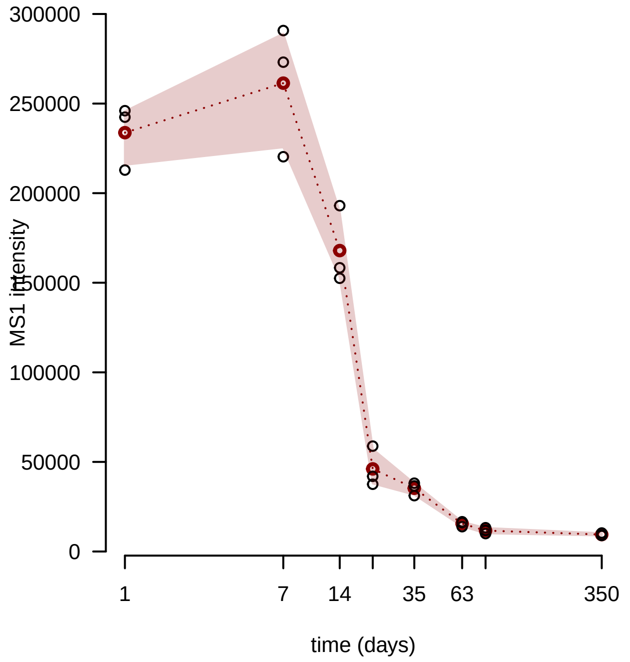

Q8BUR3
GN=Foxj3
forkhead box J3

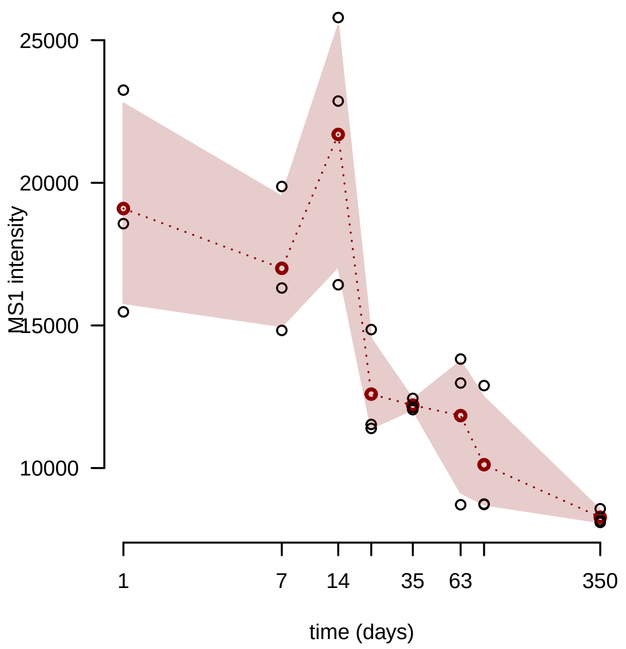

Q8BUR4
GN=Dock1
dedicator of cytokinesis 1

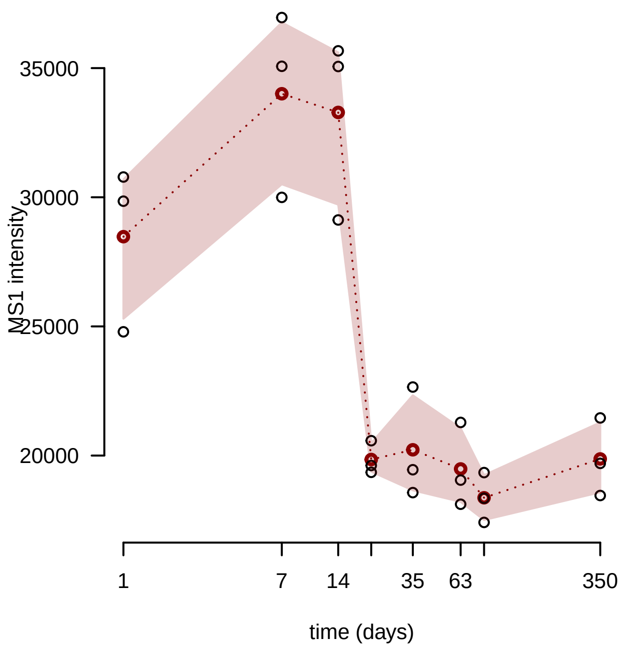

Q8BUV3
GN=Gphn
gephyrin

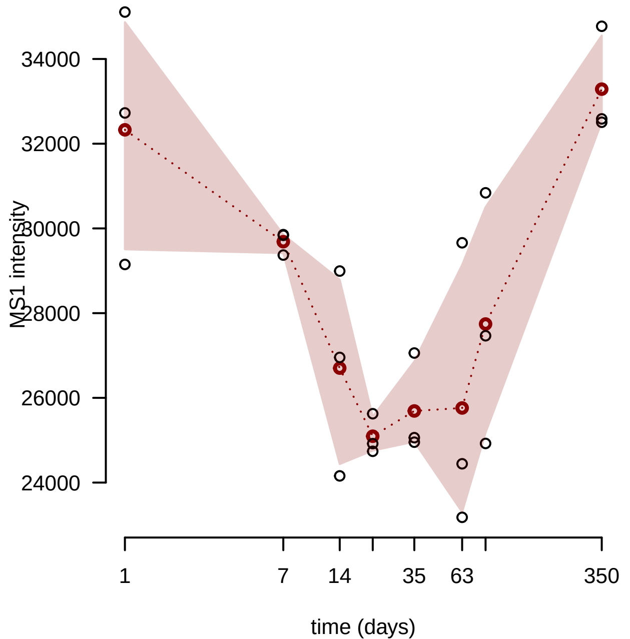

Q8BUV8
GN=Gpr107
G protein−coupled receptor 107

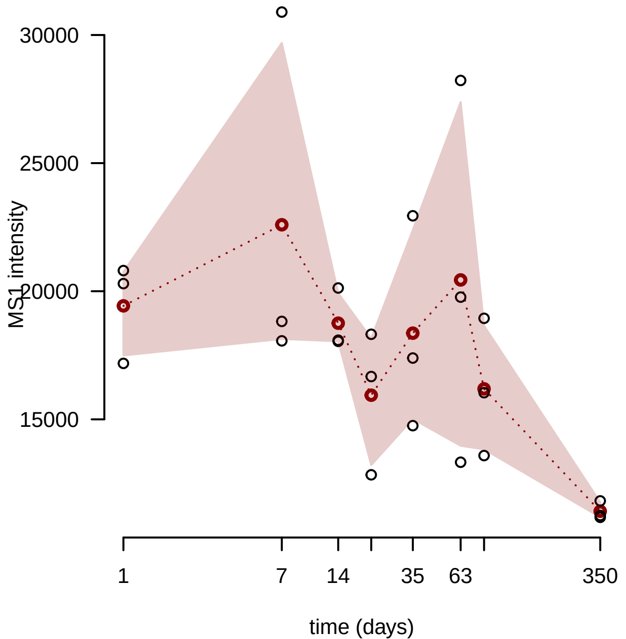

Q8BUY5
GN=Timmdc1
translocase of inner mitochondrial membr ...

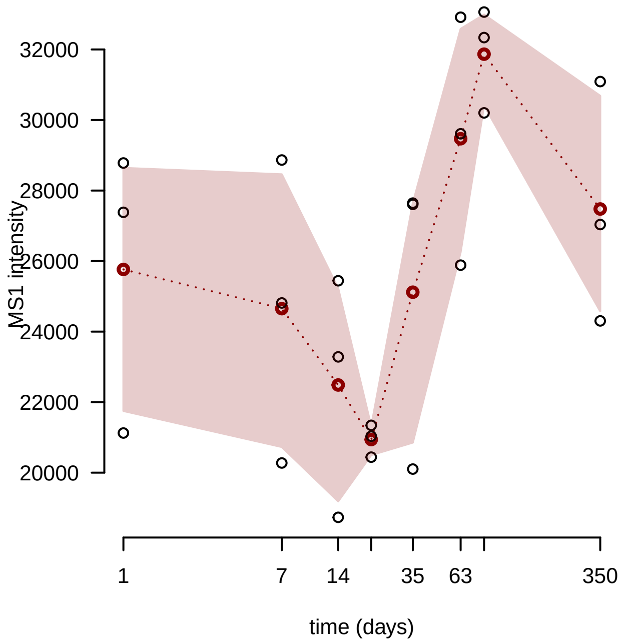

Q8BUY8
GN=Gprasp2
G protein−coupled receptor associated so ...

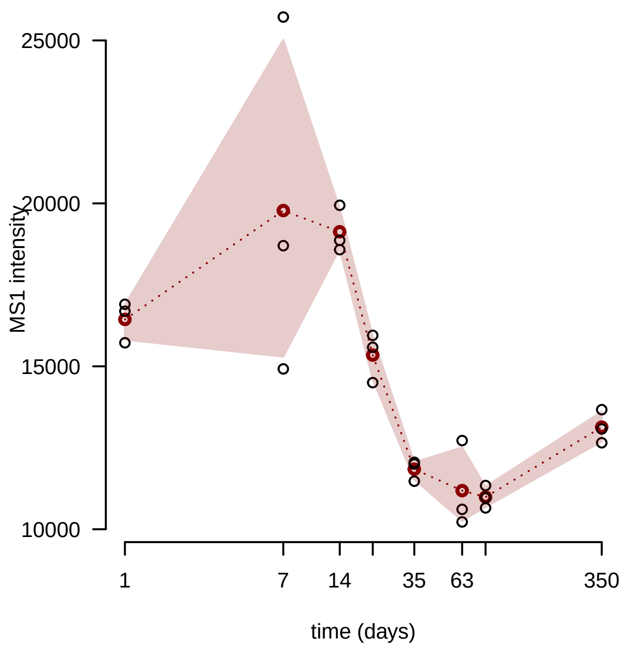

Q8BUY9
GN=Pggt1b
protein geranylgeranyltransferase type I ...

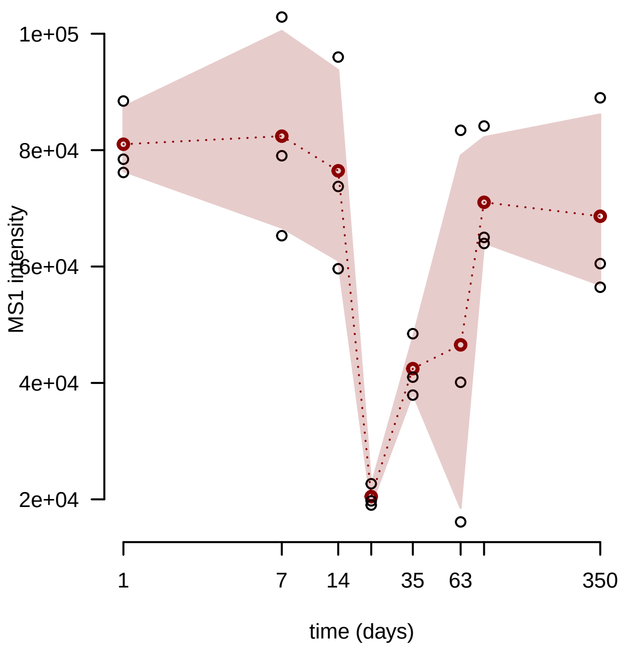

Q8BV13
GN=Cops7b
COP9 signalosome subunit 7B

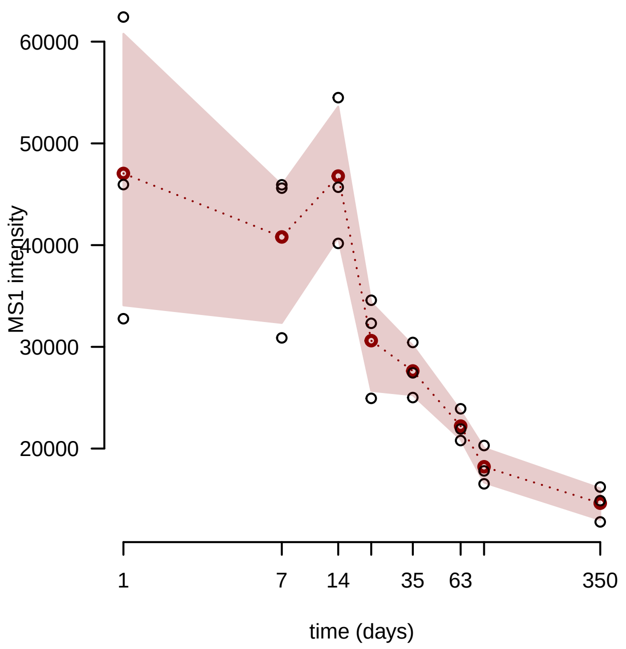

Q8BVA2
GN=Tmem222
transmembrane protein 222

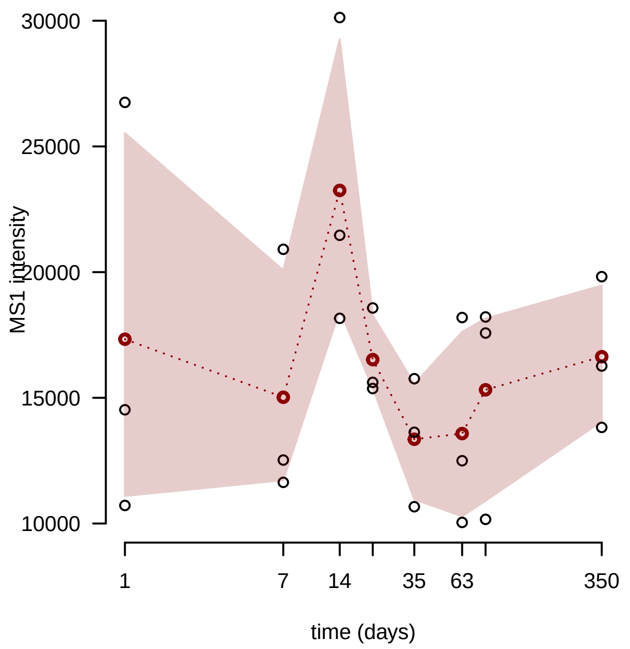

Q8BVA4
GN=Lmod1
leiomodin 1 (smooth muscle)

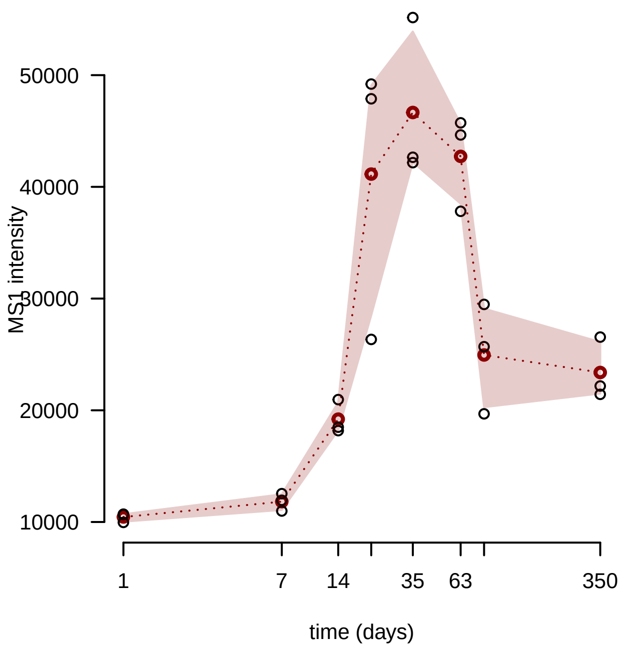

Q8BVA5
GN=Ldah
lipid droplet associated hydrolase

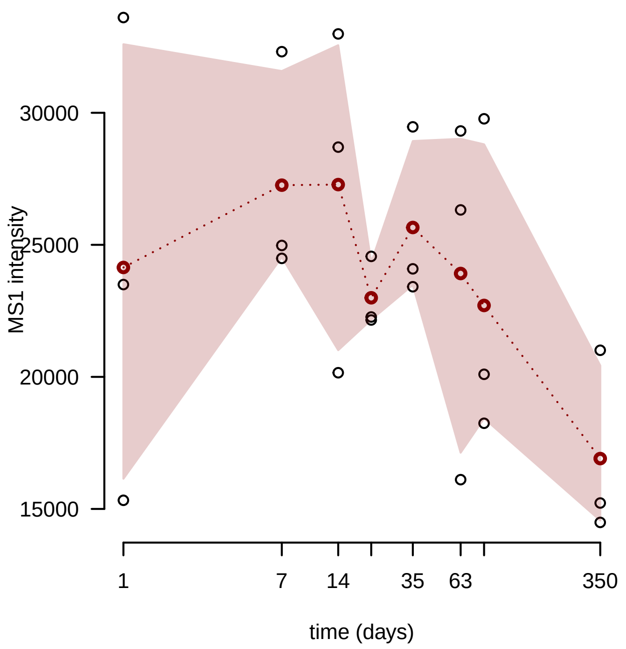

Q8BVD5
GN=Mpp7
membrane protein, palmitoylated 7 (MAGUK ...

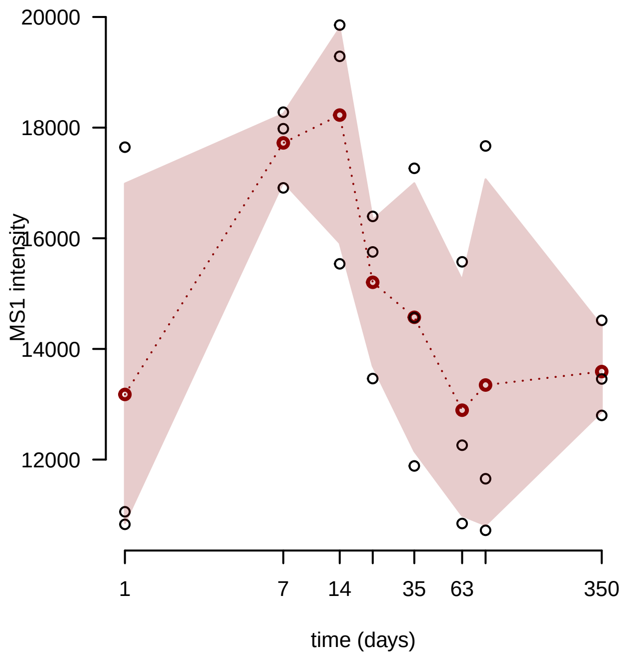

Q8BVD7
GN=C1qtnf7
C1q and tumor necrosis factor related pr ...

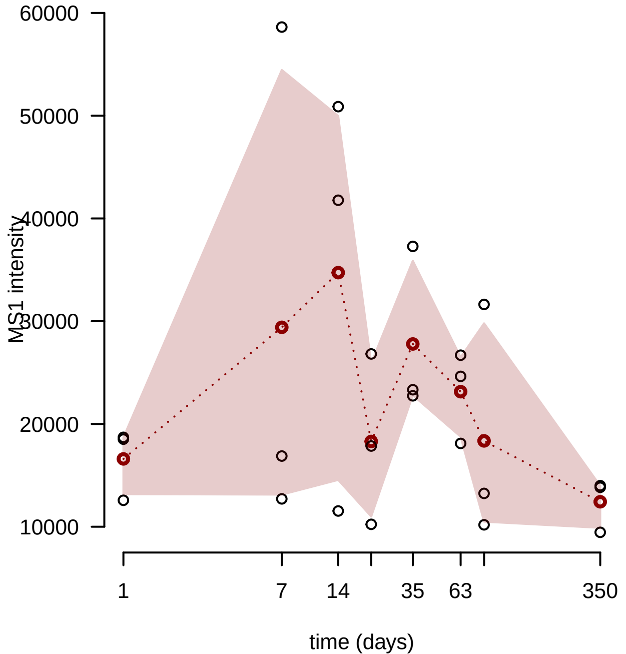

Q8BVE3
GN=Atp6v1h
ATPase, H+ transporting, lysosomal V1 su ...

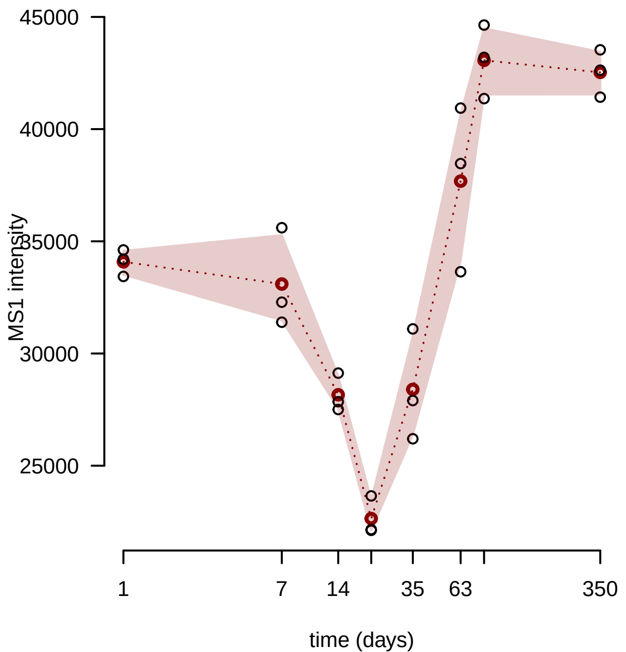

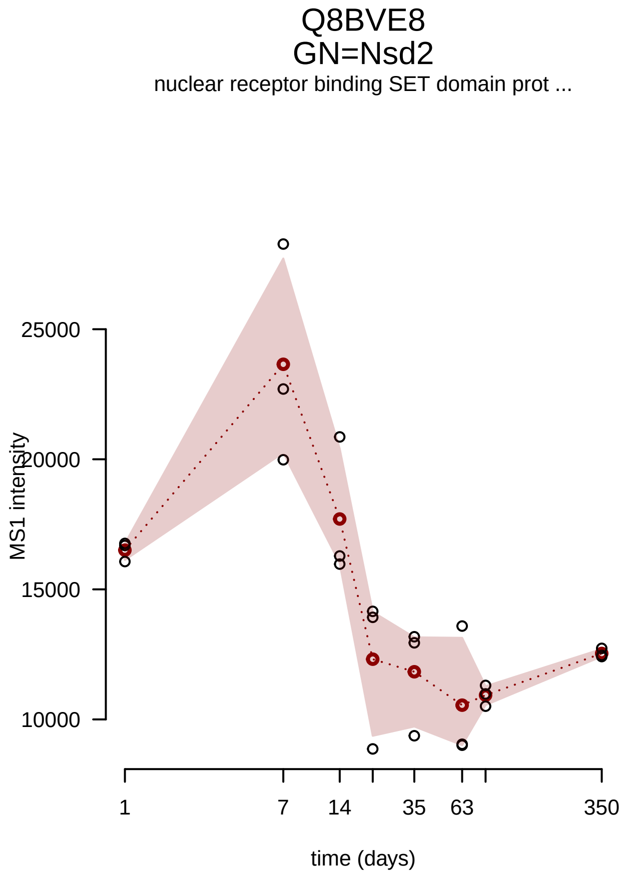
Q8BVE8
GN=Nsd2
nuclear receptor binding SET domain prot ...
25000
20000
15000
10000
MS1 intensity
1
7
14
35
63
350
time (days)

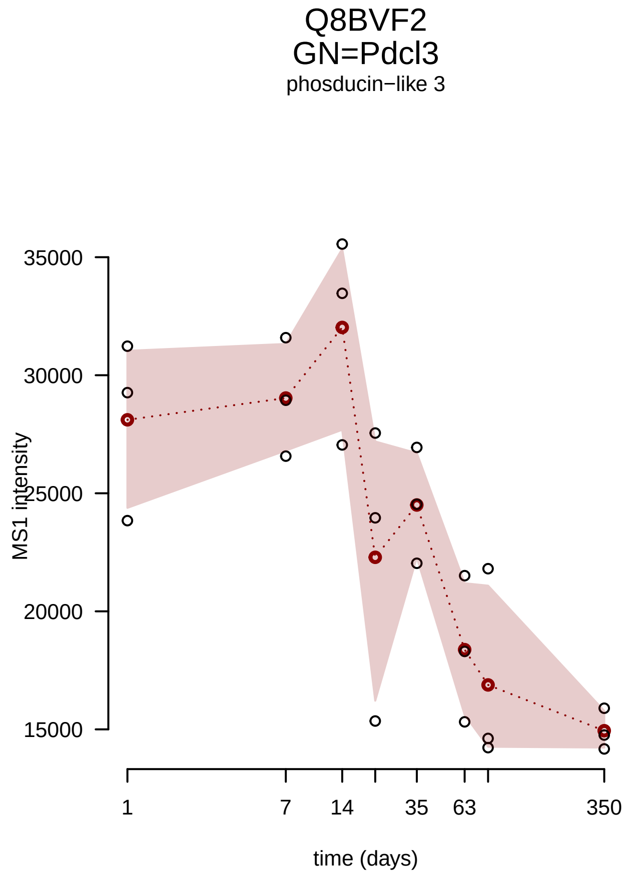
Q8BVF2
GN=Pdcl3
phosducin−like 3
35000
30000
25000
20000
15000
MS1 intensity
1
7
14
35
63
350
time (days)

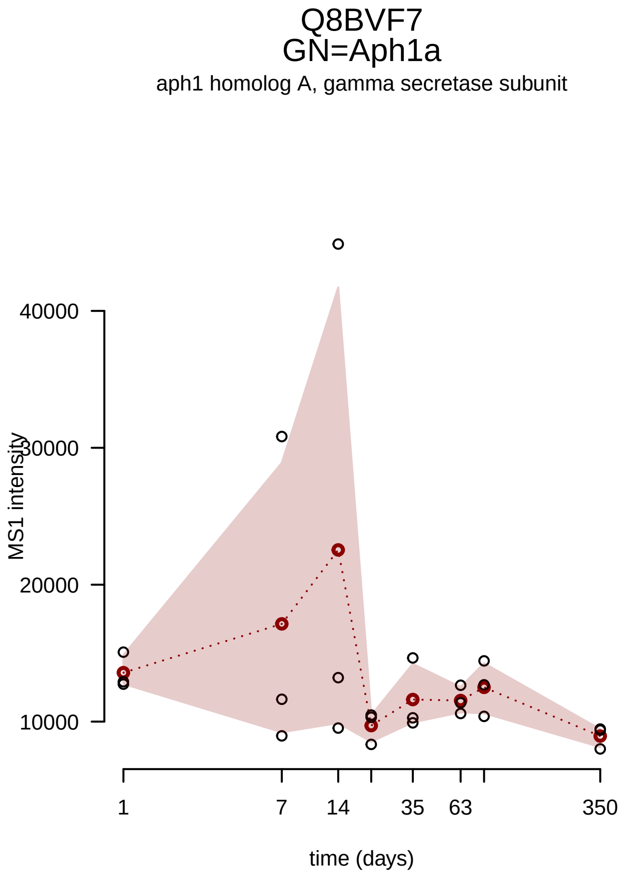
Q8BVF7
GN=Aph1a
aph1 homolog A, gamma secretase subunit
40000
30000
20000
10000
MS1 intensity
1
7
14
35
63
350
time (days)

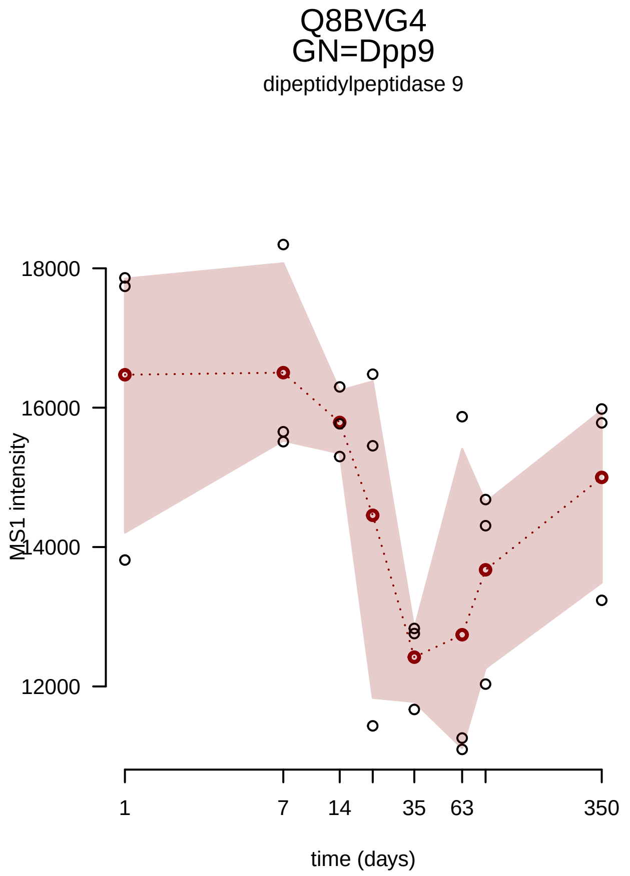
Q8BVG4
GN=Dpp9
dipeptidylpeptidase 9
18000
16000
14000
12000
MS1 intensity
1
7
14
35
63
350
time (days)

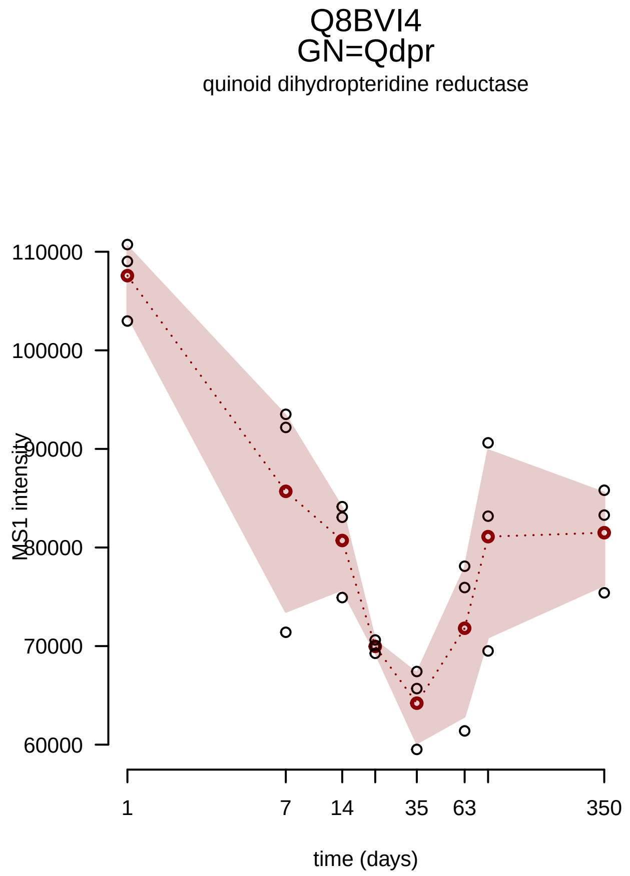
Q8BVI4
GN=Qdpr
quinoid dihydropteridine reductase
110000
100000
90000
80000
70000
60000
MS1 intensity
1
7
14
35
63
350
time (days)

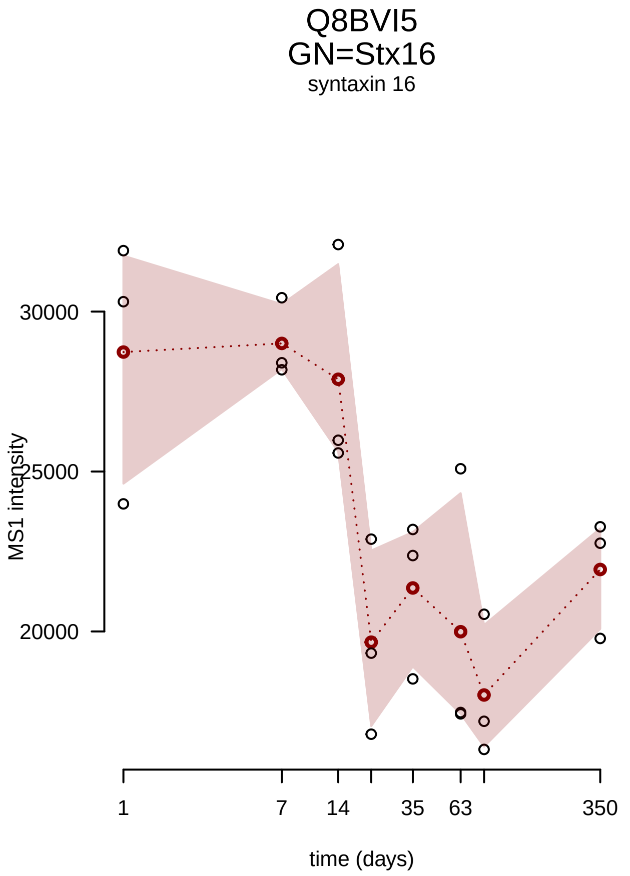
Q8BVI5
GN=Stx16
syntaxin 16
30000
25000
20000
MS1 intensity
1
7
14
35
63
350
time (days)

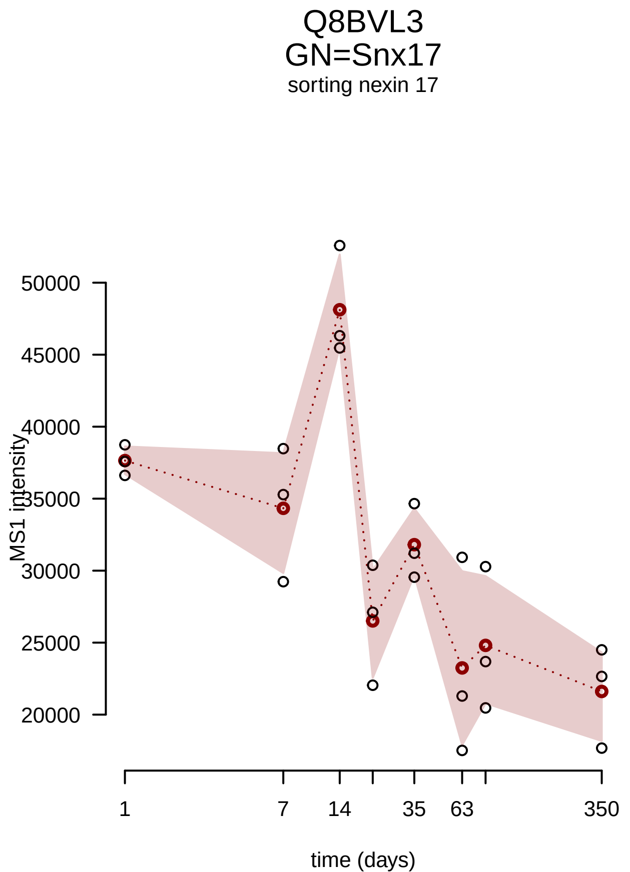
Q8BVL3
GN=Snx17
sorting nexin 17
50000
45000
40000
35000
30000
25000
20000
MS1 intensity
1
7
14
35
63
350
time (days)

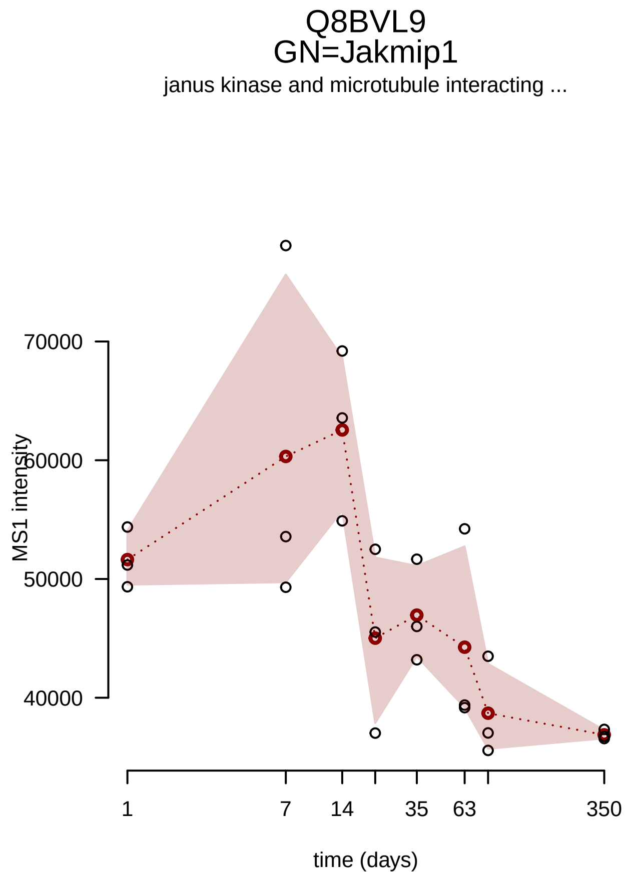
Q8BVL9
GN=Jakmip1
janus kinase and microtubule interacting ...
70000
60000
50000
40000
MS1 intensity
1
7
14
35
63
350
time (days)

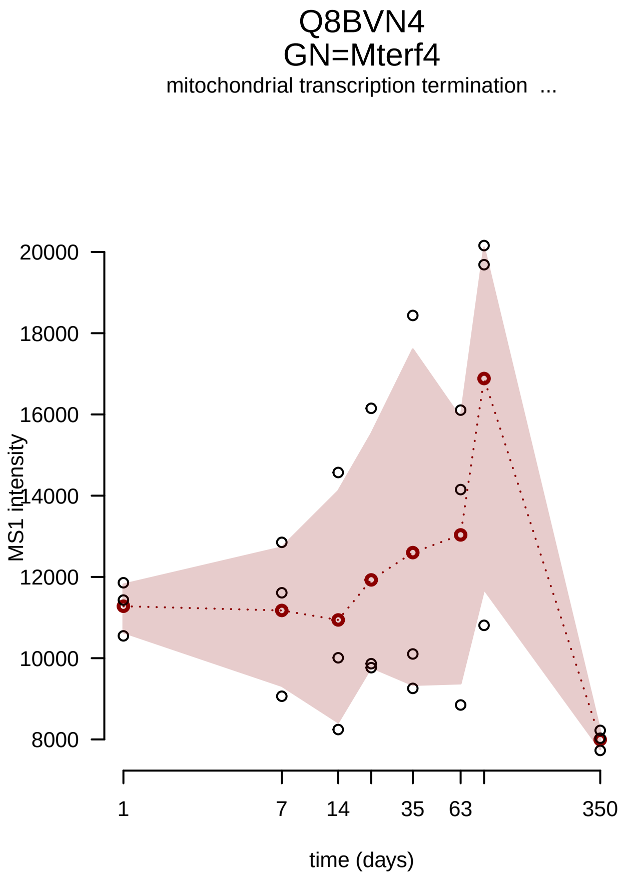
Q8BVN4
GN=Mterf4
mitochondrial transcription termination ...
20000
18000
16000
14000
12000
10000
8000
MS1 intensity
1
7
14
35
63
350
time (days)

Q8BVN9
GN=Mael
maelstrom spermatogenic transposon silen ...

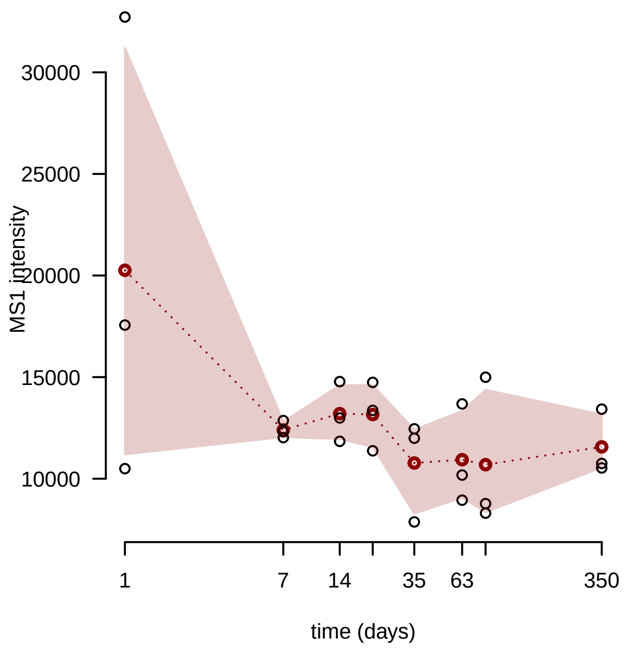

Q8BVQ5
GN=Ppme1
protein phosphatase methylesterase 1

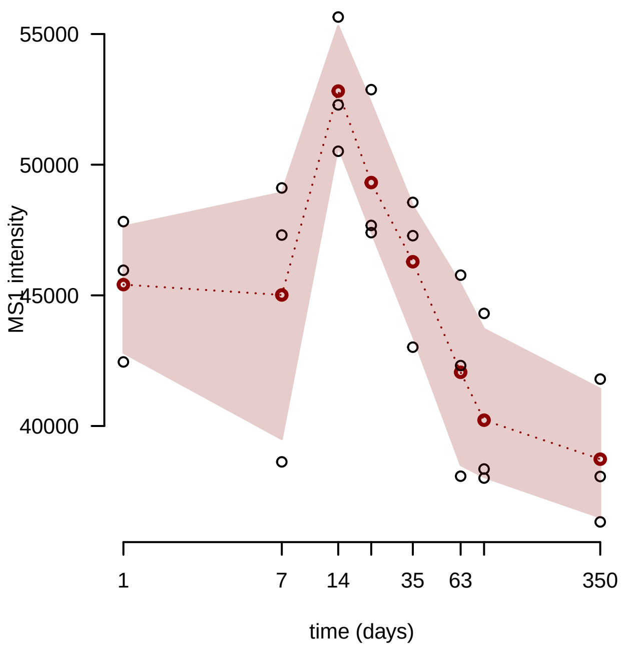

Q8BVR6
GN=Rspry1
ring finger and SPRY domain containing 1

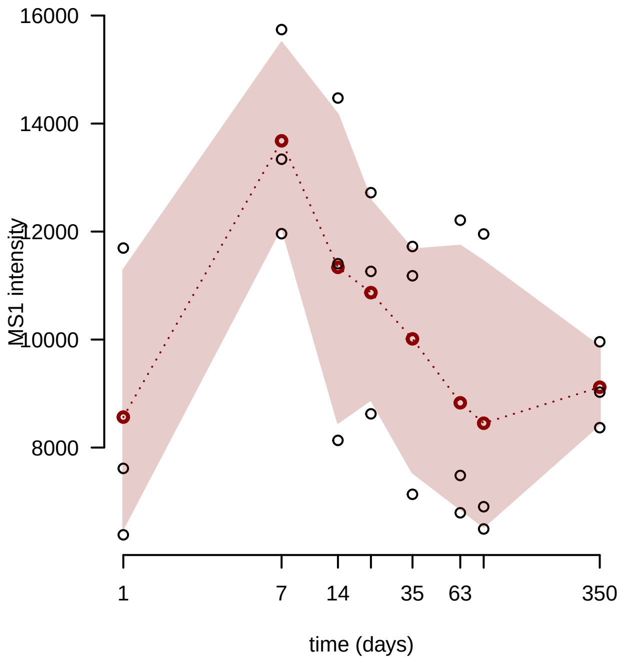

Q8BVU0
GN=Lrch3
leucine−rich repeats and calponin homolo ...

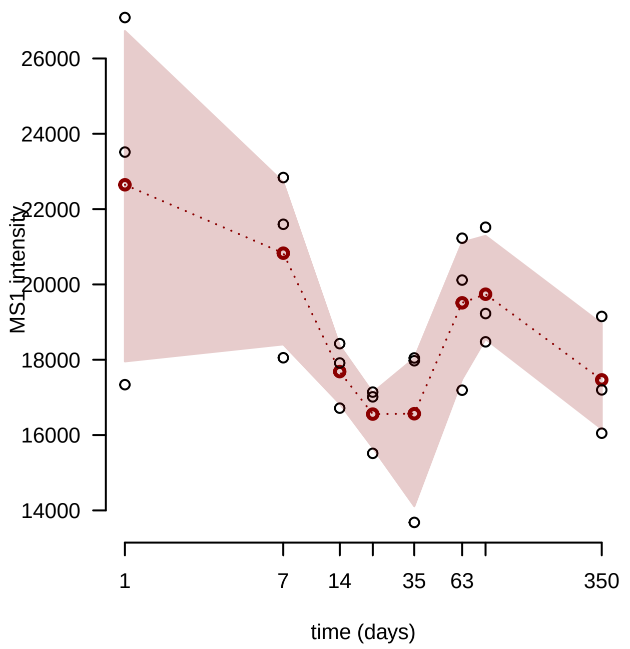

Q8BVU5
GN=Nudt9
nudix (nucleoside diphosphate linked moi ...

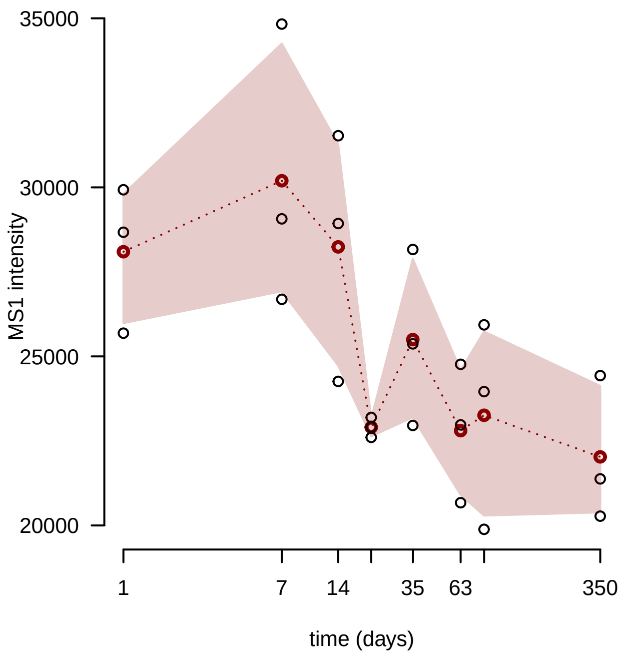

Q8BVW0
GN=Ganc
glucosidase, alpha; neutral C

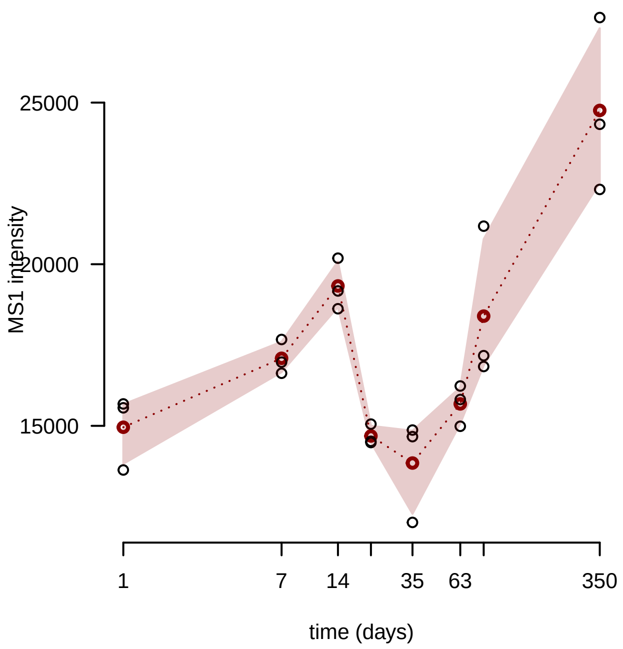

Q8BVW3
GN=Trim14
tripartite motif−containing 14

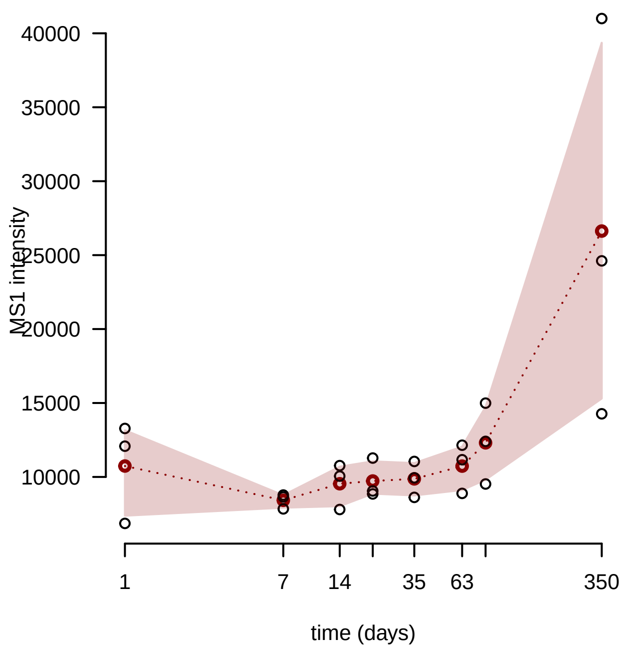

Q8BVY0
GN=Rsl1d1
ribosomal L1 domain containing 1

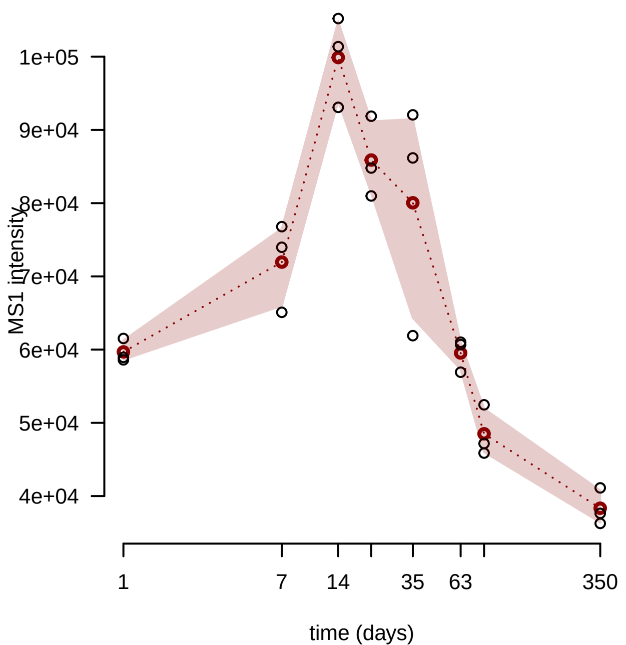

Q8BVZ1
GN=Plin5
perilipin 5

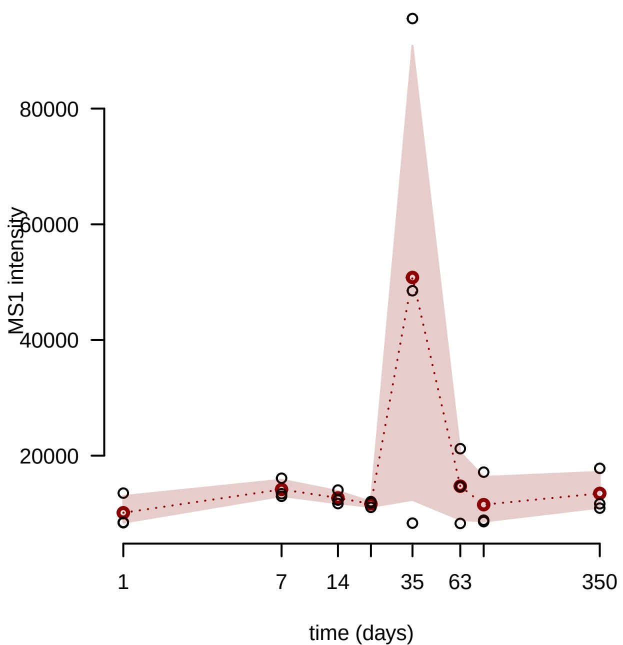

Q8BW10
GN=Nob1
NIN1/RPN12 binding protein 1 homolog

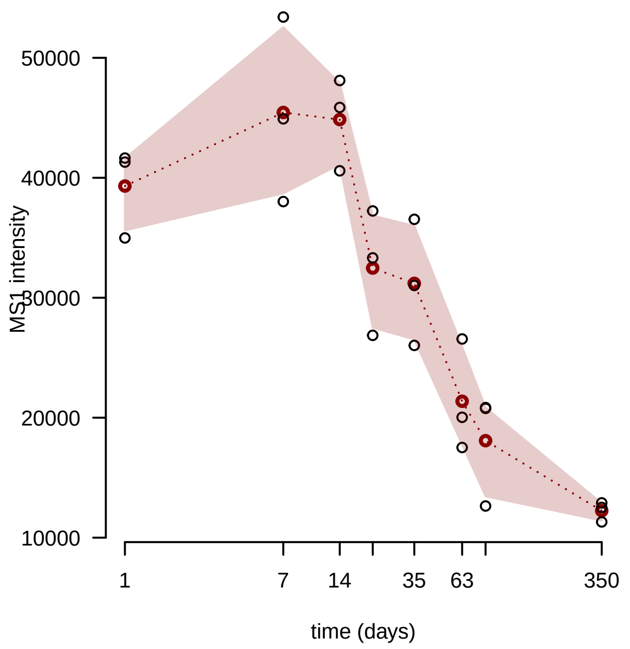

Q8BW41
GN=Pomgnt2
protein O−linked mannose beta 1,4−N−acet ...

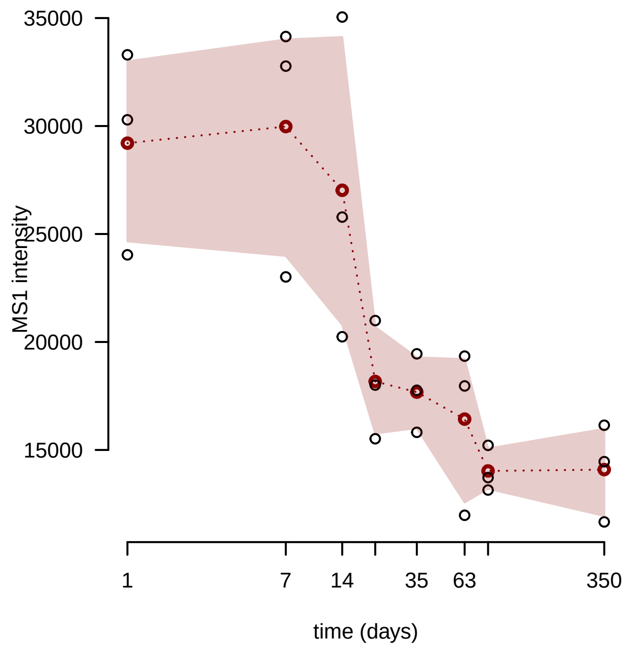

Q8BW56
GN=Gtdc1
glycosyltransferase−like domain containi ...

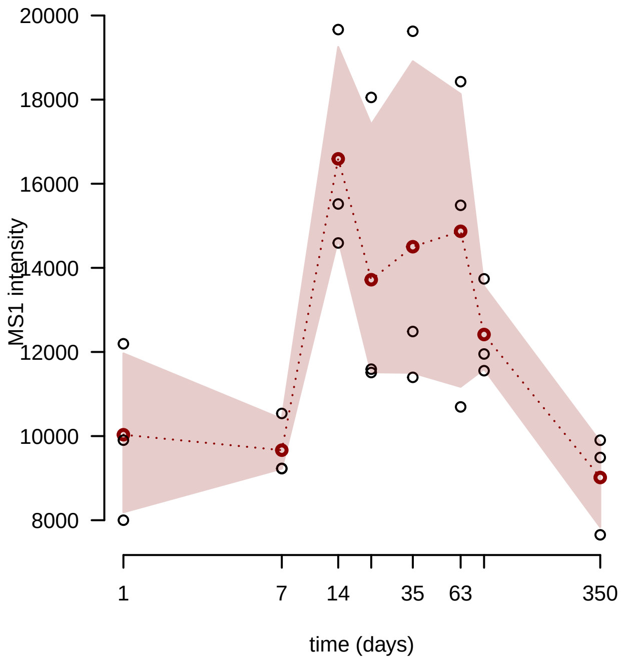

Q8BW70
GN=Usp38
ubiquitin specific peptidase 38

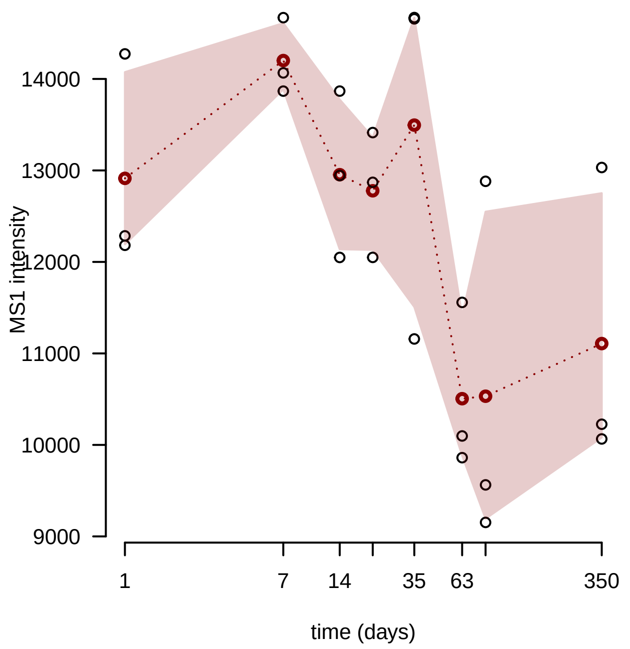

Q8BW72
GN=Kdm4a
lysine (K)−specific demethylase 4A

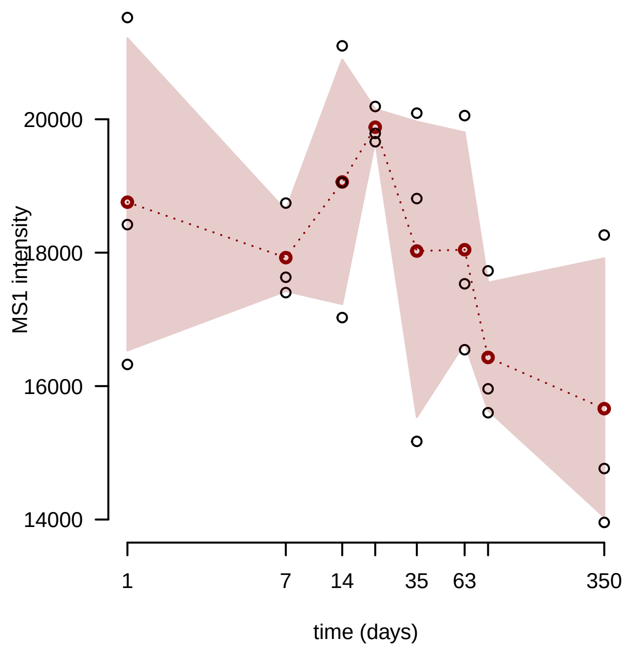

Q8BW75
GN=Maob
monoamine oxidase B

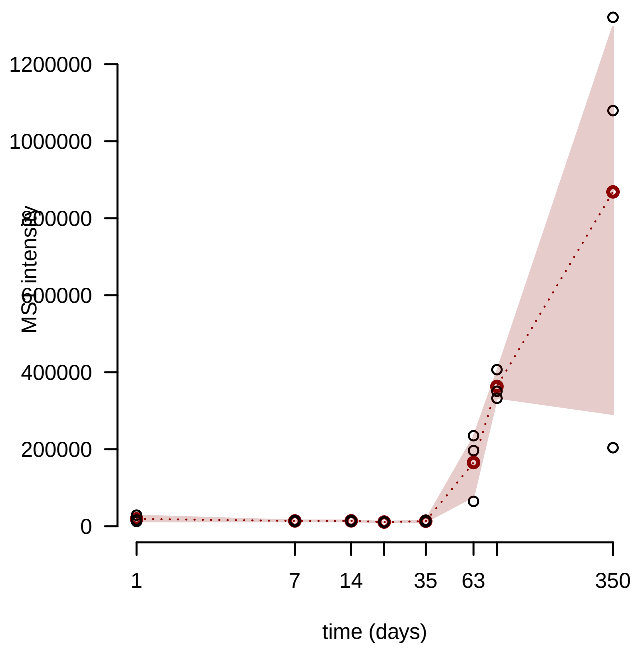

Q8BW96
GN=Camk1d
calcium/calmodulin−dependent protein kin ...

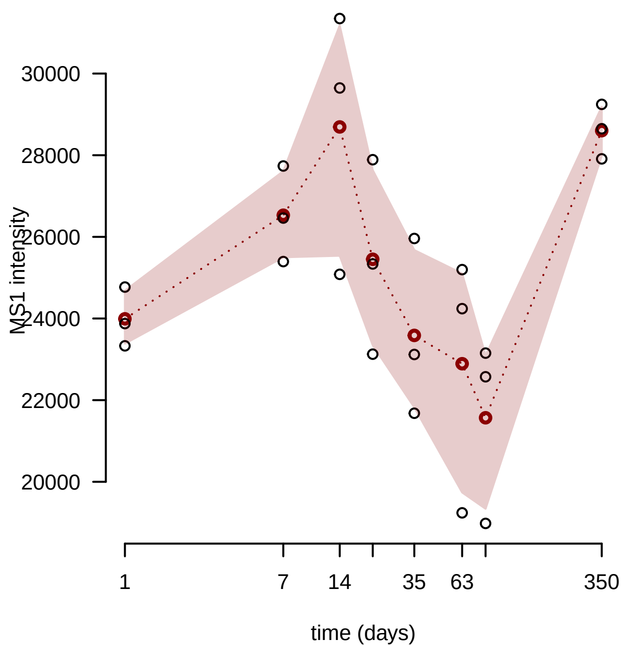

Q8BWD8
GN=Cdk19
cyclin−dependent kinase 19

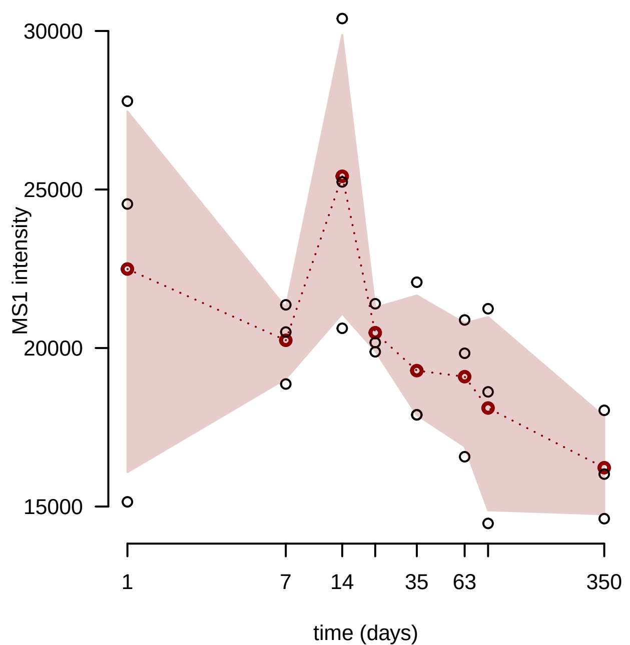

Q8BWF0
GN=Aldh5a1
aldhehyde dehydrogenase family 5, subfam ...

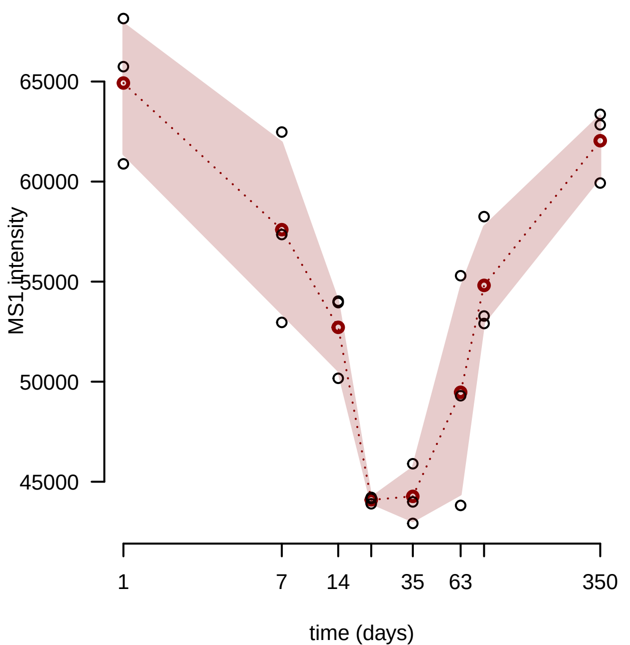

Q8BWG8
GN=Arrb1
arrestin, beta 1

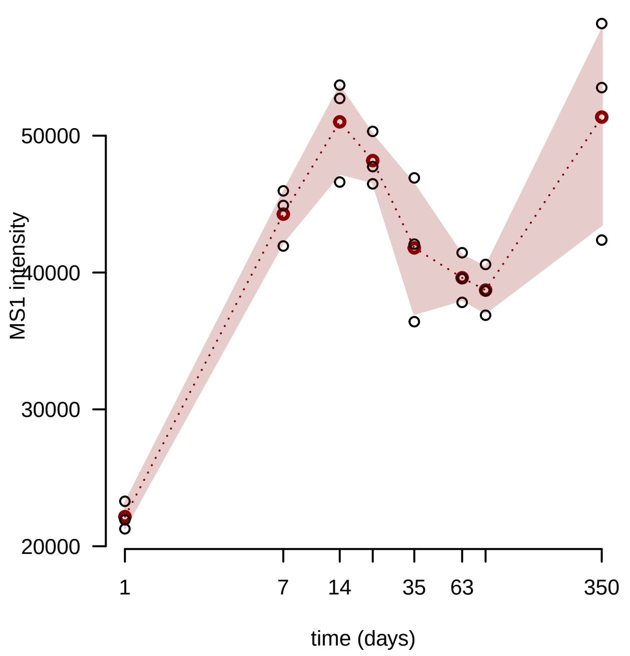

Q8BWG8
GN=Arrb1
arrestin, beta 1

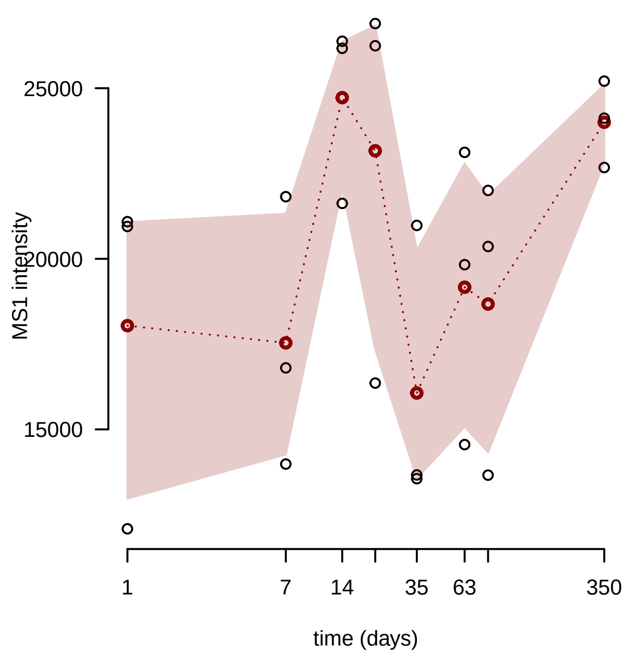

Q8BWH0
GN=Slc38a7
solute carrier family 38, member 7

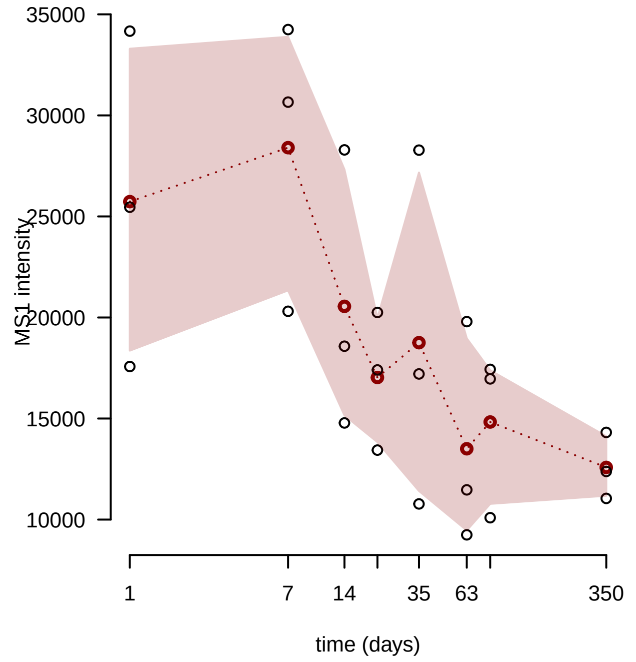

Q8BWJ3
GN=Phka2
phosphorylase kinase alpha 2

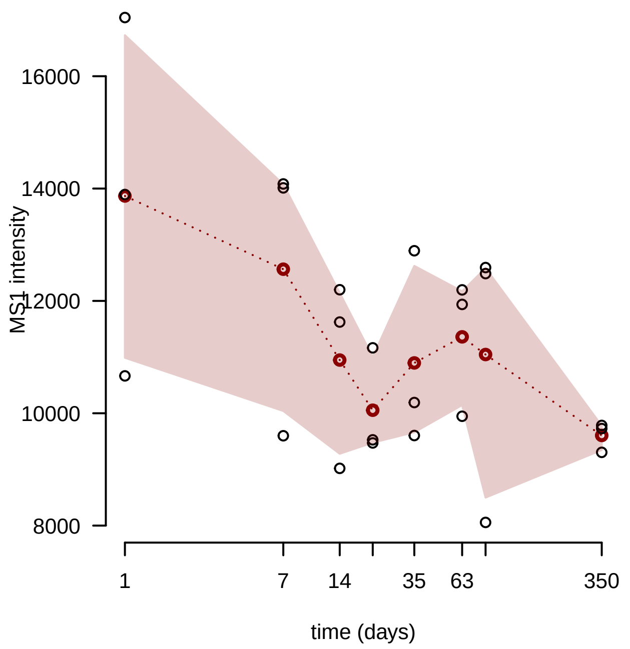

Q8BWL5
GN=Rbms3
RNA binding motif, single stranded inter ...

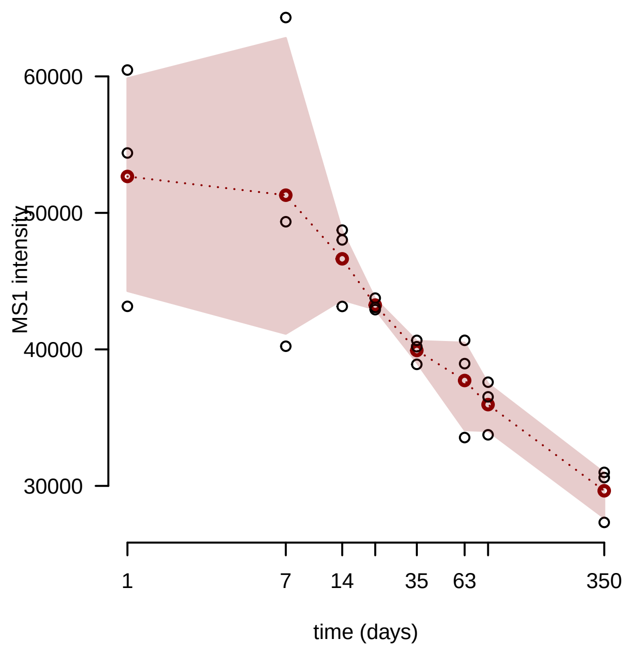

Q8BWM0
GN=Ptges2
prostaglandin E synthase 2

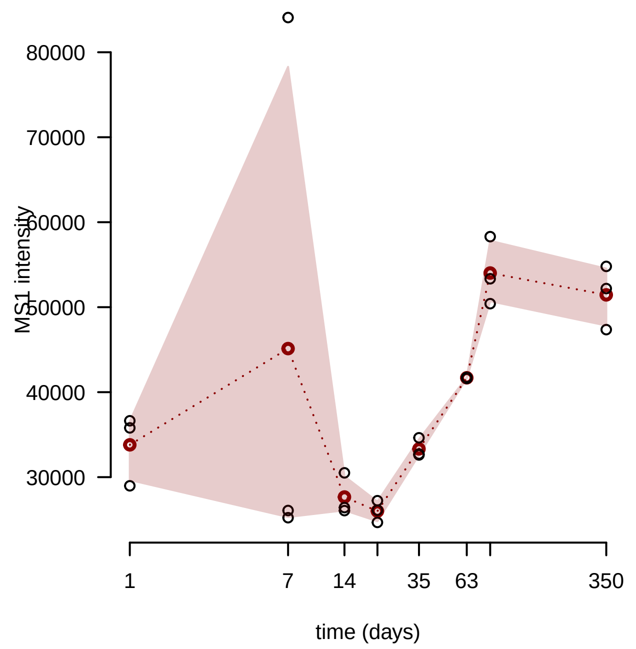

Q8BWN8
GN=Acot4
acyl−CoA thioesterase 4

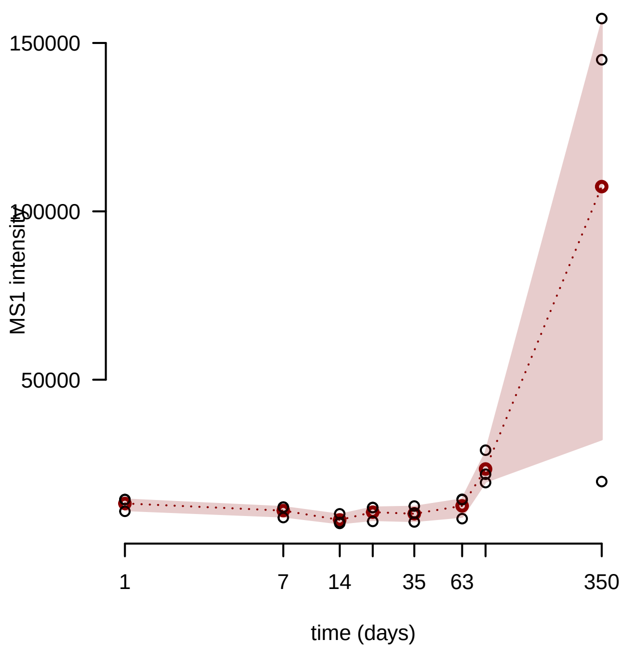

Q8BWP5
GN=Ttpa
tocopherol (alpha) transfer protein

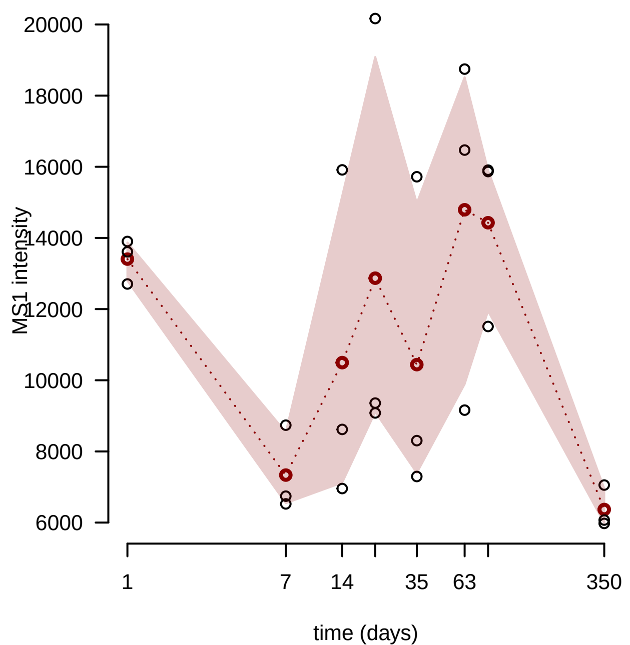

Q8BWP8
GN=B4gat1
beta−1,4−glucuronyltransferase 1

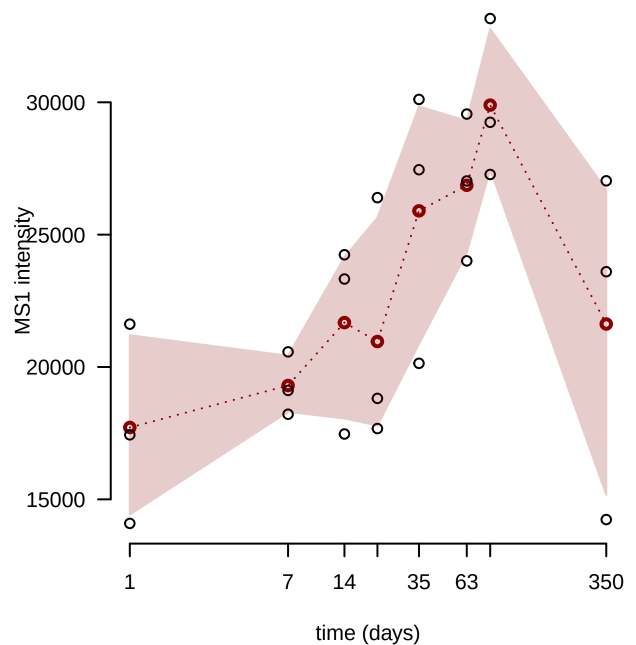

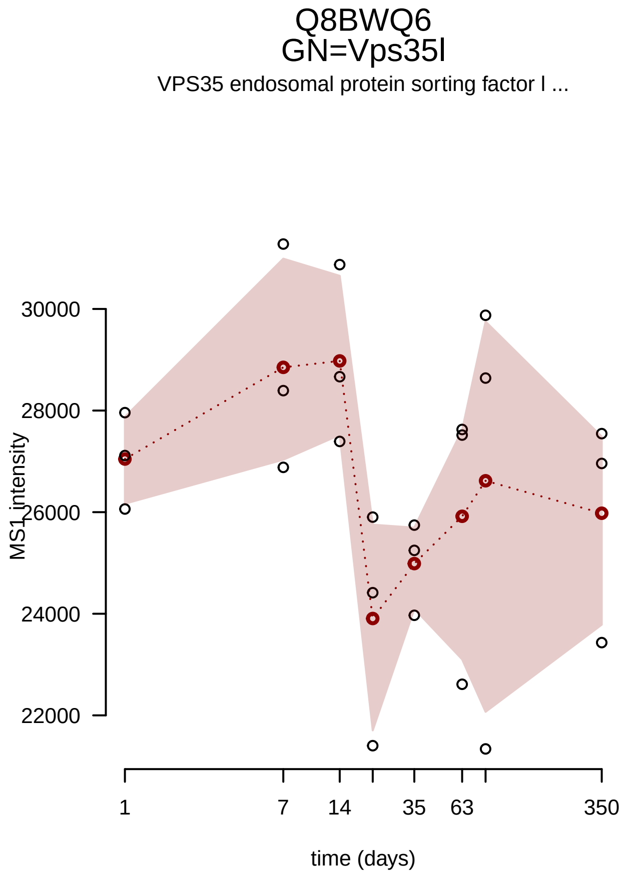
Q8BWQ6
GN=Vps35l
VPS35 endosomal protein sorting factor l ...
MS1 intensity
22000
24000
26000
28000
30000
1
7
14
35
63
350
time (days)

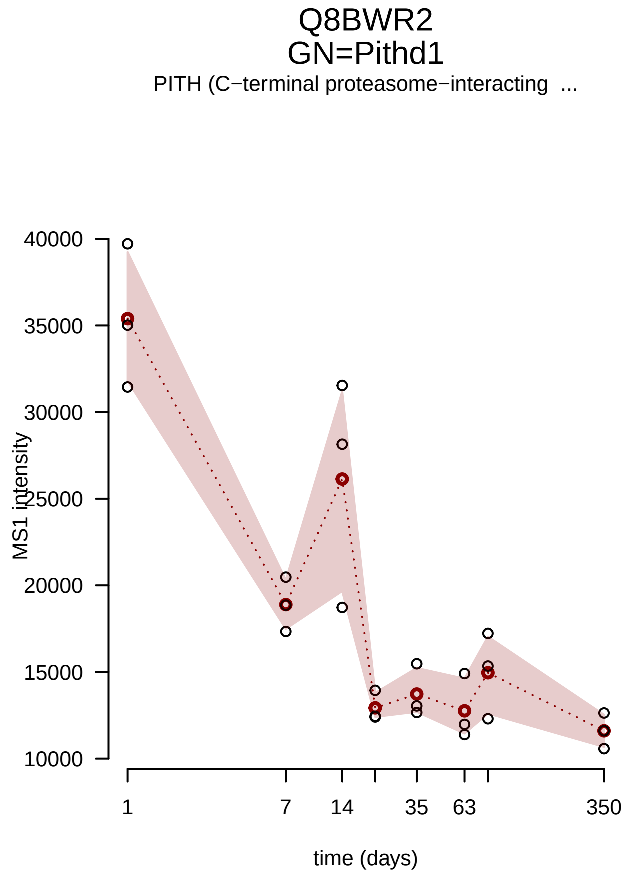
Q8BWR2
GN=Pithd1
PITH (C−terminal proteasome−interacting ...
MS1 intensity
10000
15000
20000
25000
30000
35000
40000
1
7
14
35
63
350
time (days)

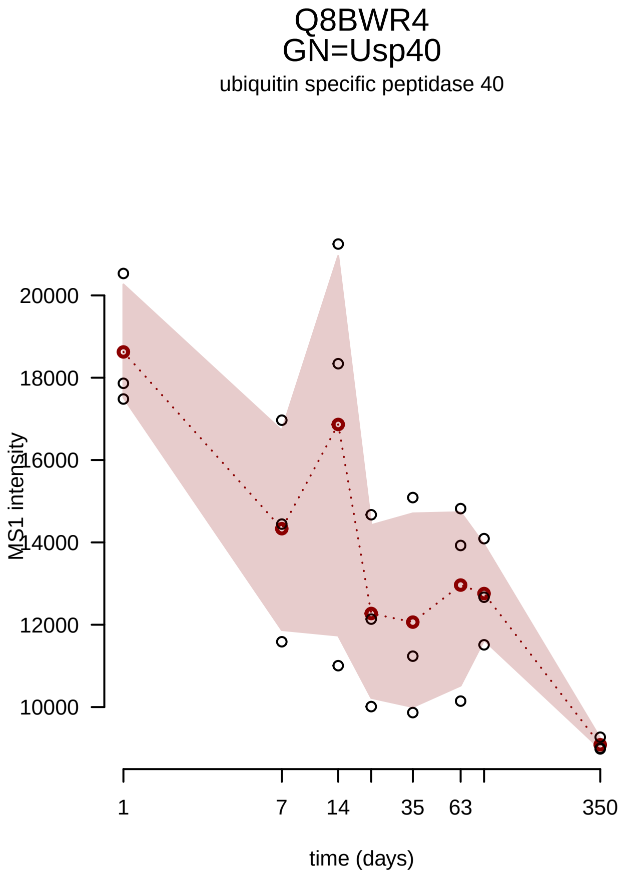
Q8BWR4
GN=Usp40
ubiquitin specific peptidase 40
MS1 intensity
10000
12000
14000
16000
18000
20000
1
7
14
35
63
350
time (days)

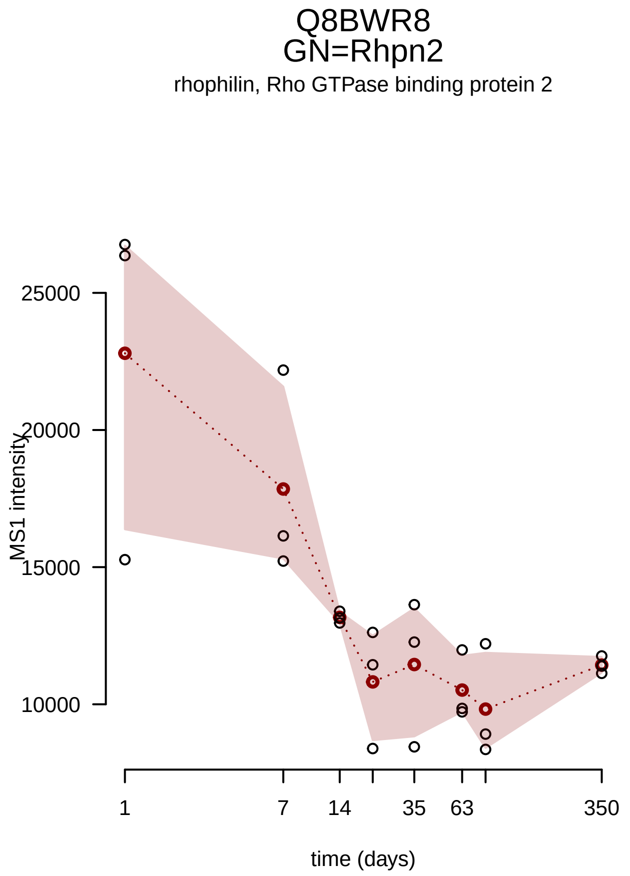
Q8BWR8
GN=Rhpn2
rhophilin, Rho GTPase binding protein 2
MS1 intensity
10000
15000
20000
25000
1
7
14
35
63
350
time (days)

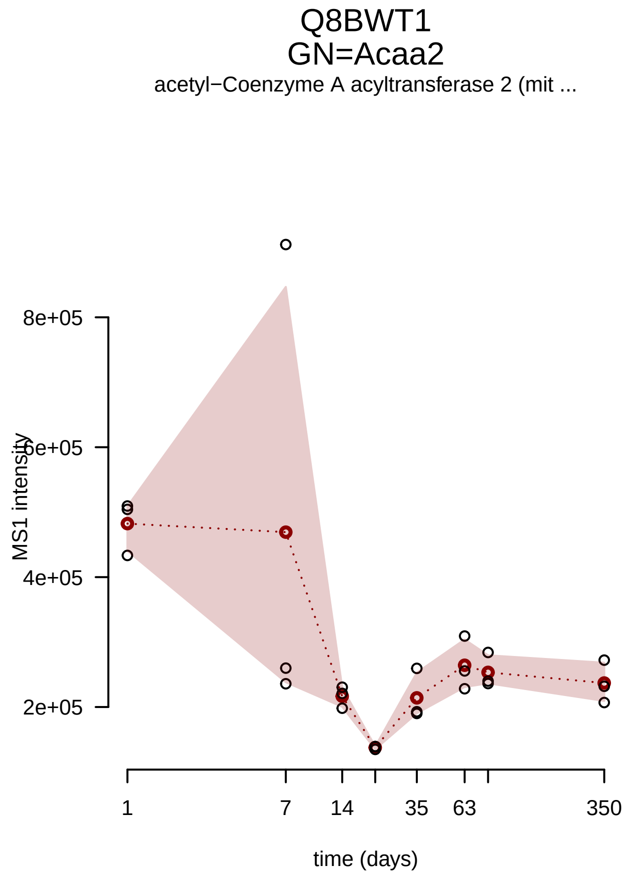
Q8BWT1
GN=Acaa2
acetyl−Coenzyme A acyltransferase 2 (mit ...
MS1 intensity
2e+05
4e+05
6e+05
8e+05
1
7
14
35
63
350
time (days)

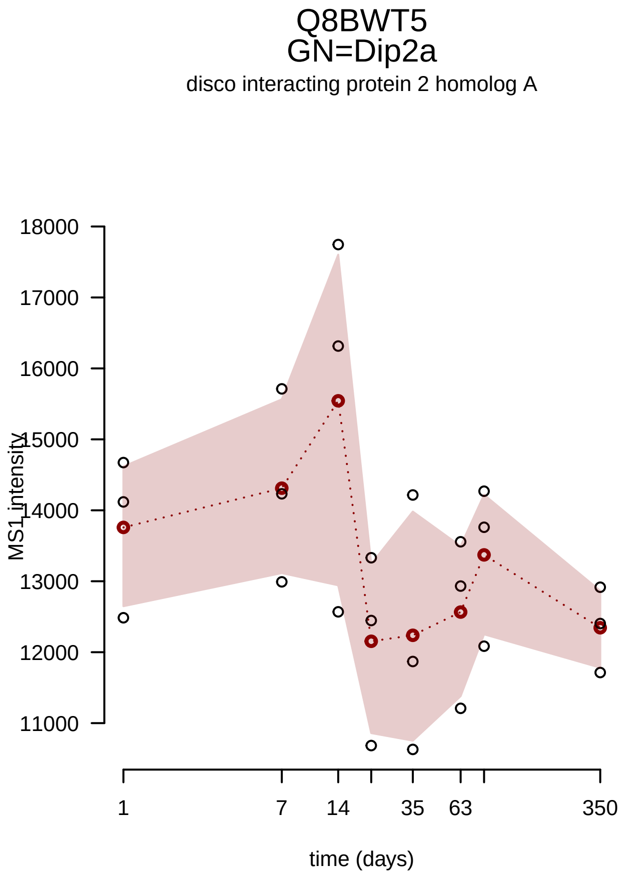
Q8BWT5
GN=Dip2a
disco interacting protein 2 homolog A
MS1 intensity
11000
12000
13000
14000
15000
16000
17000
18000
1
7
14
35
63
350
time (days)

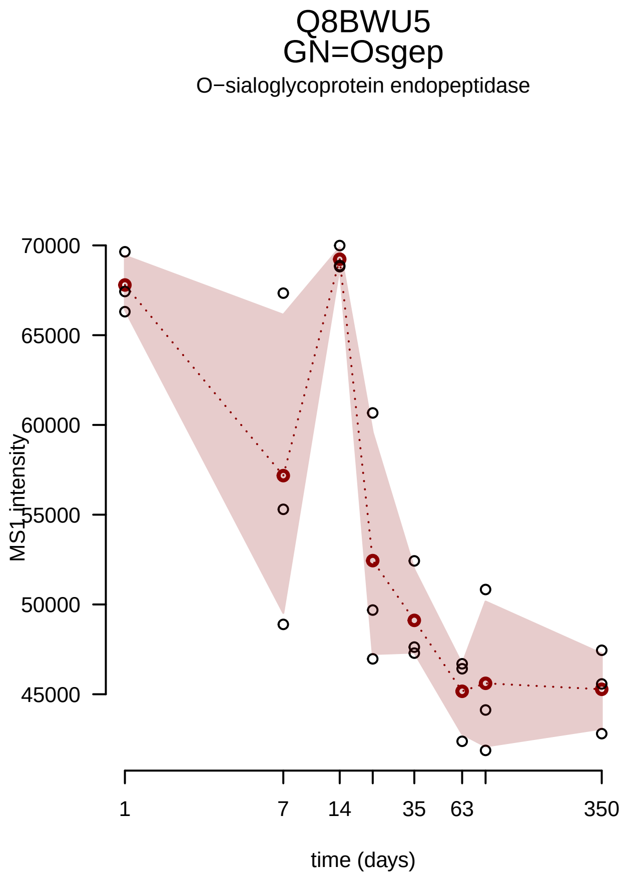
Q8BWU5
GN=Osgep
O−sialoglycoprotein endopeptidase
MS1 intensity
45000
50000
55000
60000
65000
70000
1
7
14
35
63
350
time (days)

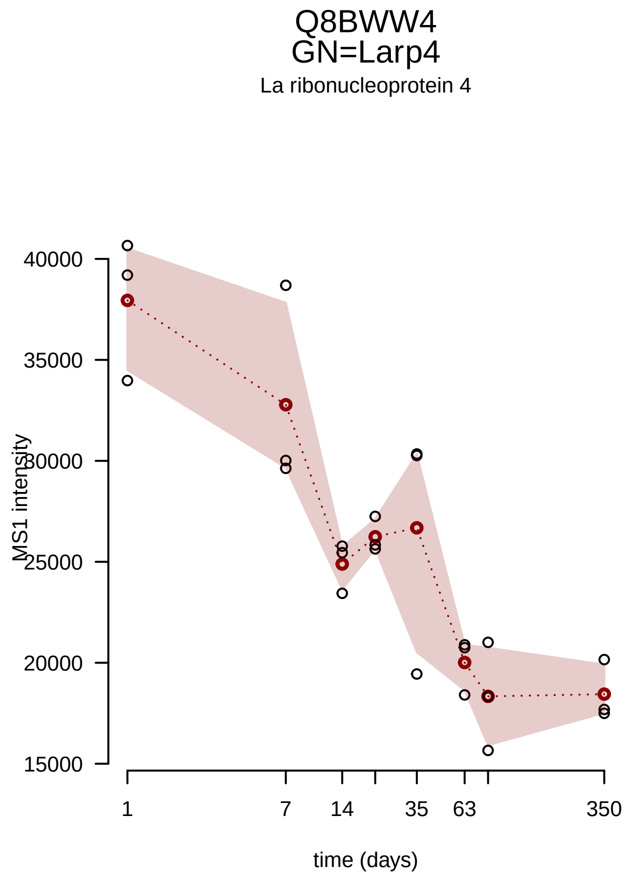
Q8BWW4
GN=Larp4
La ribonucleoprotein 4
MS1 intensity
15000
20000
25000
30000
35000
40000
1
7
14
35
63
350
time (days)

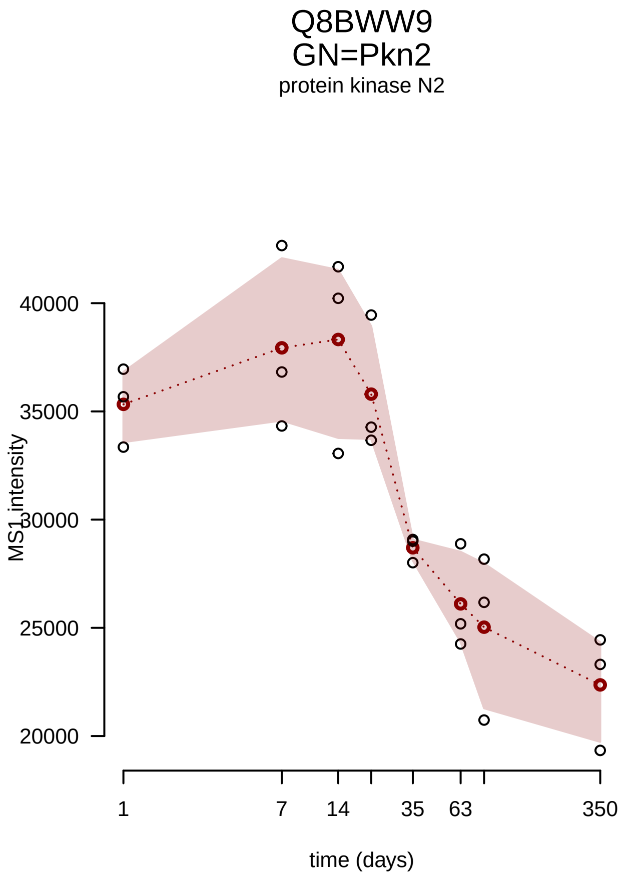
Q8BWW9
GN=Pkn2
protein kinase N2
MS1 intensity
20000
25000
30000
35000
40000
1
7
14
35
63
350
time (days)

Q8BWY2
GN=Clec1a
C−type lectin domain family 1, member a

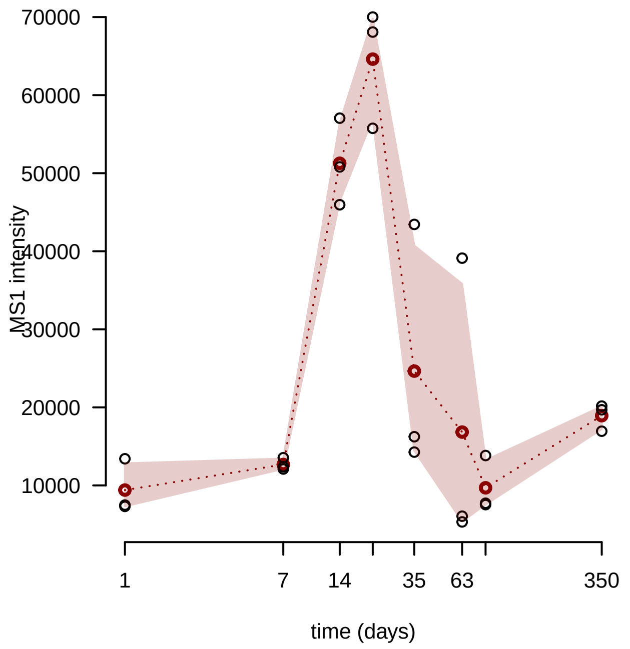

Q8BWY3
GN=Etf1
eukaryotic translation termination facto ...

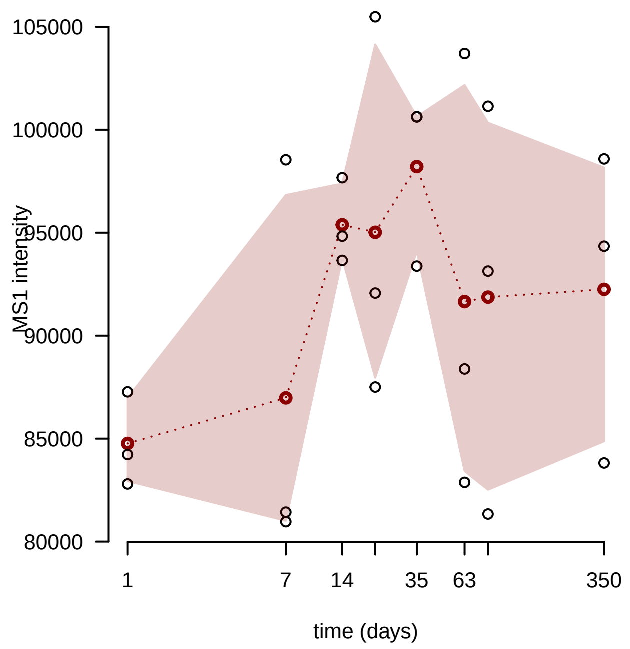

Q8BWY4
GN=Mblac1
metallo−beta−lactamase domain containing ...

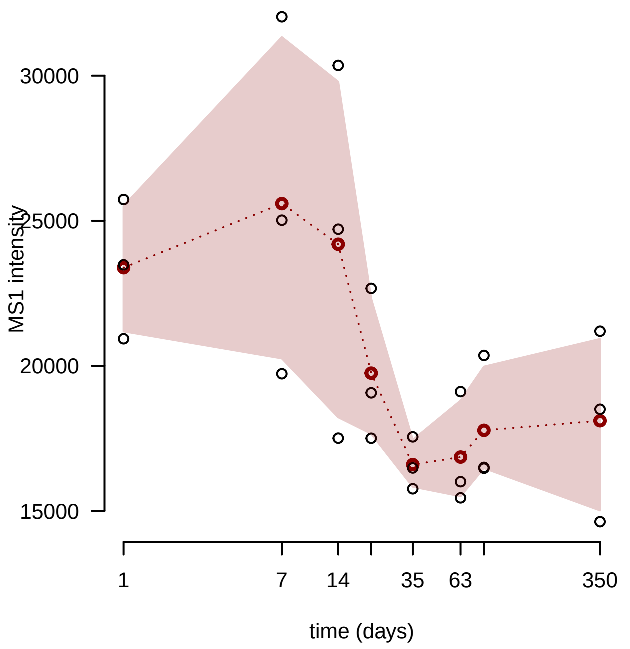

Q8BWY7
GN=Slc39a11
solute carrier family 39 (metal ion tran ...

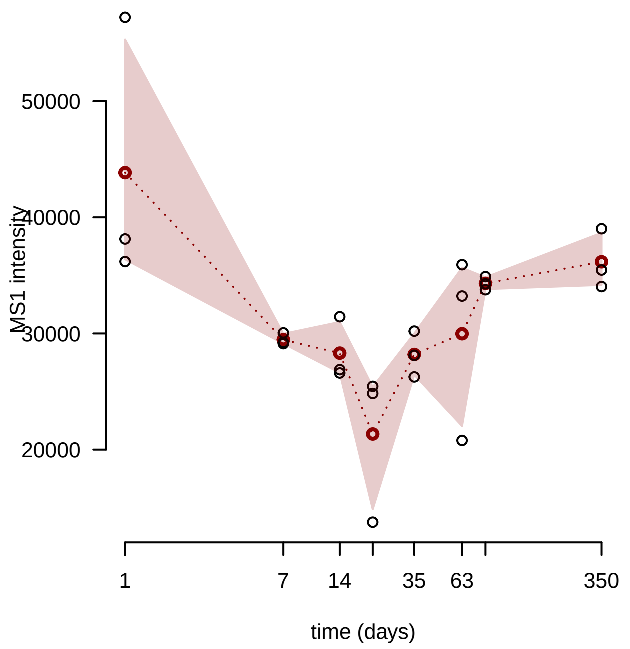

Q8BWY9
GN=Cip2a
cell proliferation regulating inhibitor ...

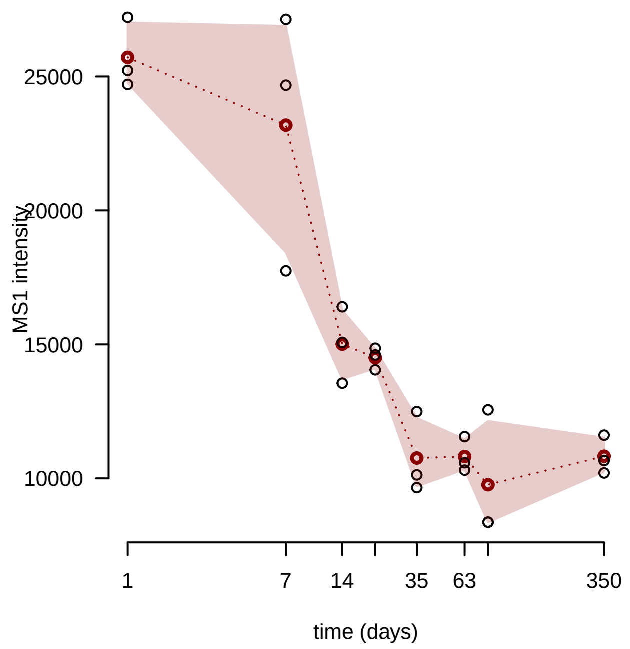

Q8BWZ3
GN=Naa25
N(alpha)−acetyltransferase 25, NatB auxi ...

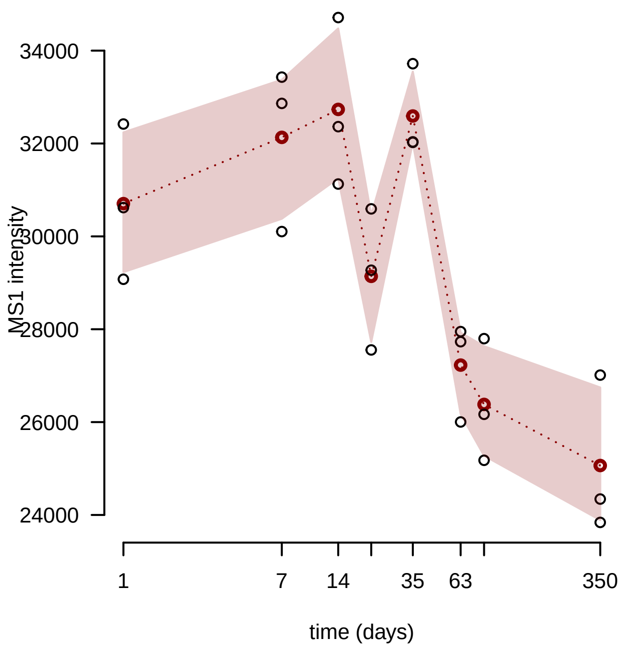

Q8BX02
GN=Kank2
KN motif and ankyrin repeat domains 2

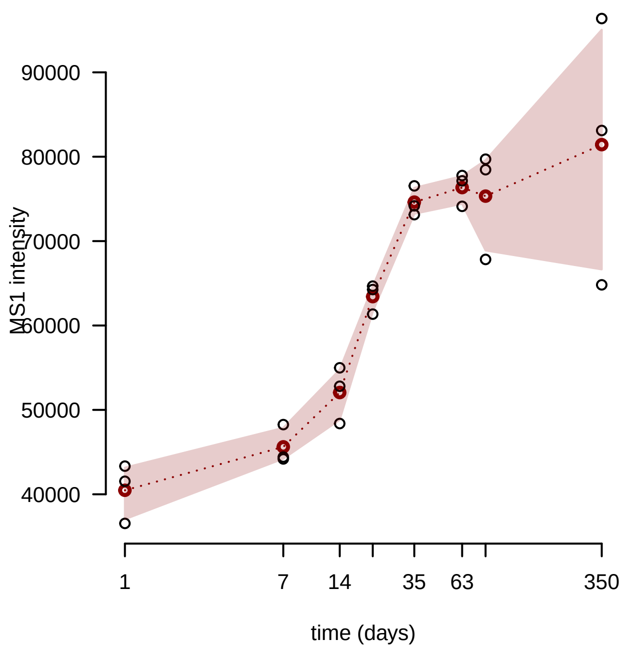

Q8BX05
GN=Gk5
glycerol kinase 5 (putative)

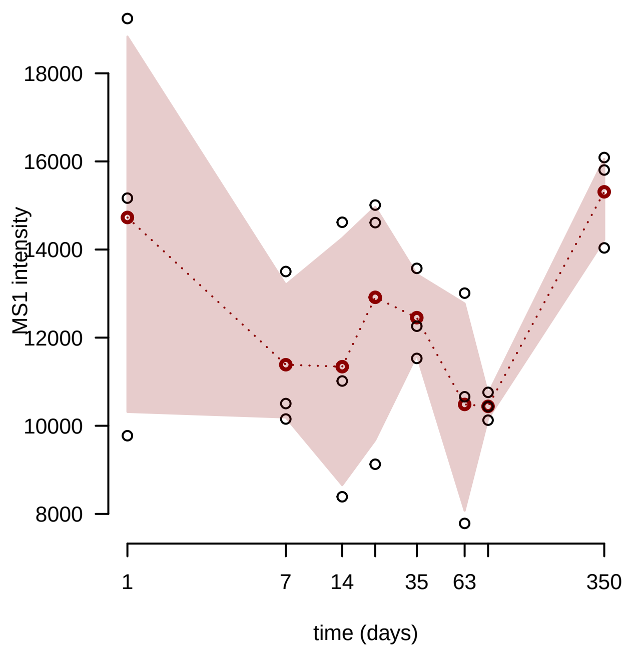

Q8BX07
GN=Ctdsp2
CTD (carboxy−terminal domain, RNA polyme ...

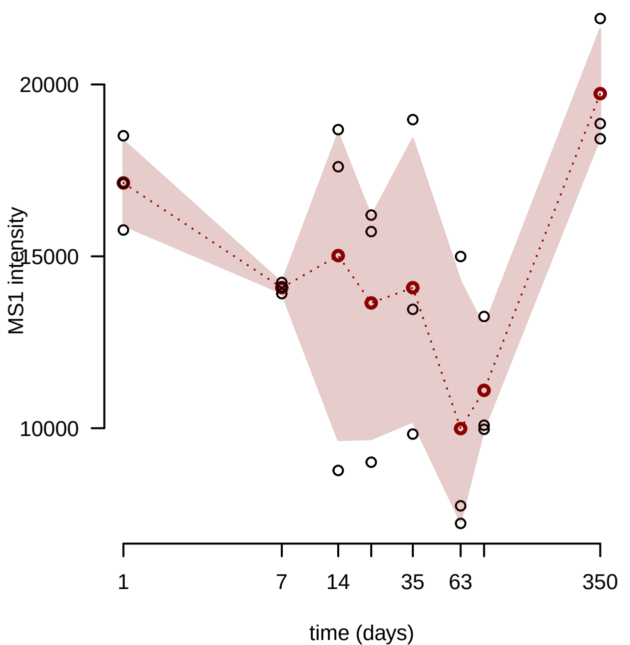

Q8BX09
GN=Rbbp5
retinoblastoma binding protein 5, histon ...

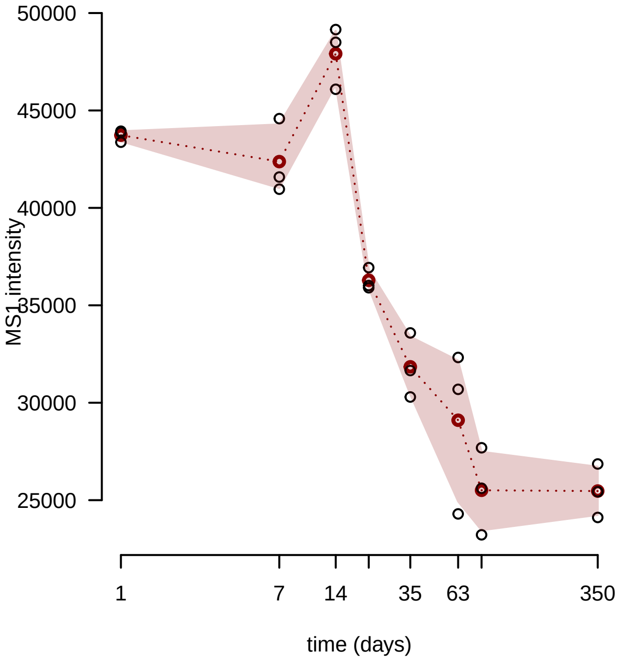

Q8BX10
GN=Pgam5
phosphoglycerate mutase family member 5

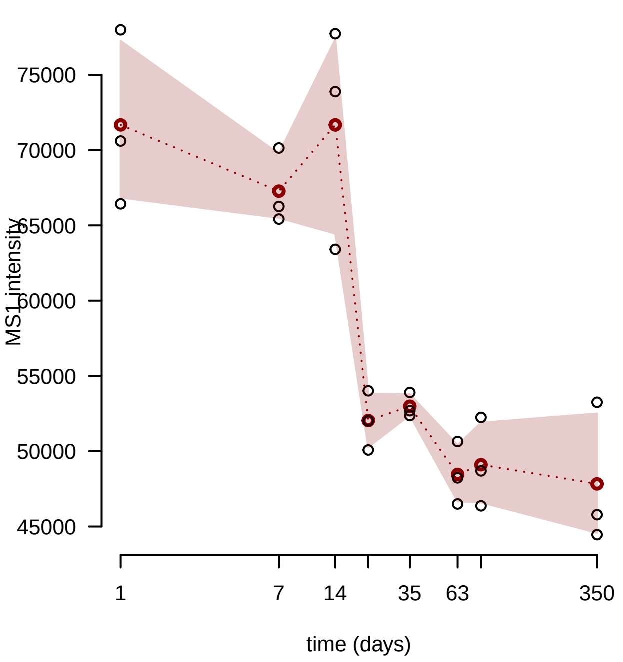

Q8BX13
GN=Ube3d

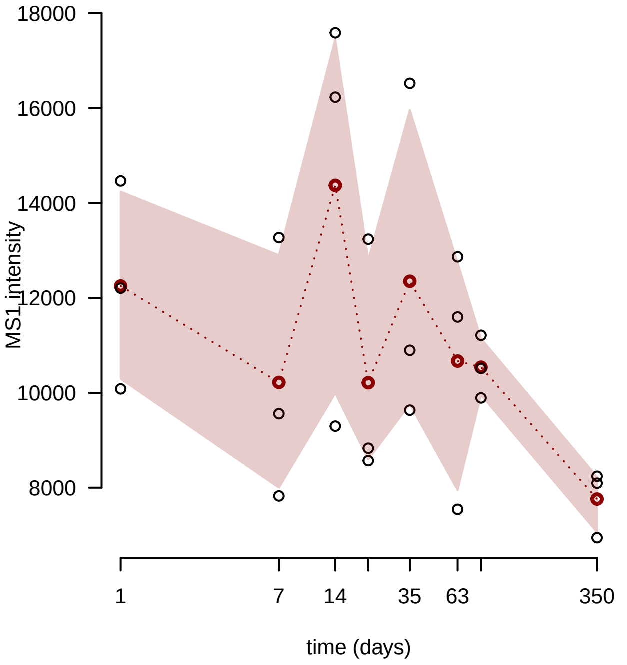

Q8BX17
GN=Gemin5
gem nuclear organelle associated protein ...

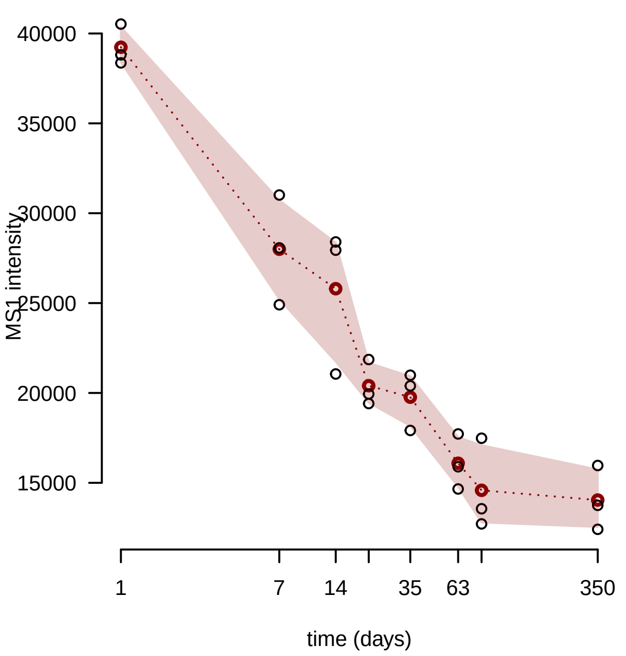

Q8BX57
GN=Pxk
PX domain containing serine/threonine ki ...

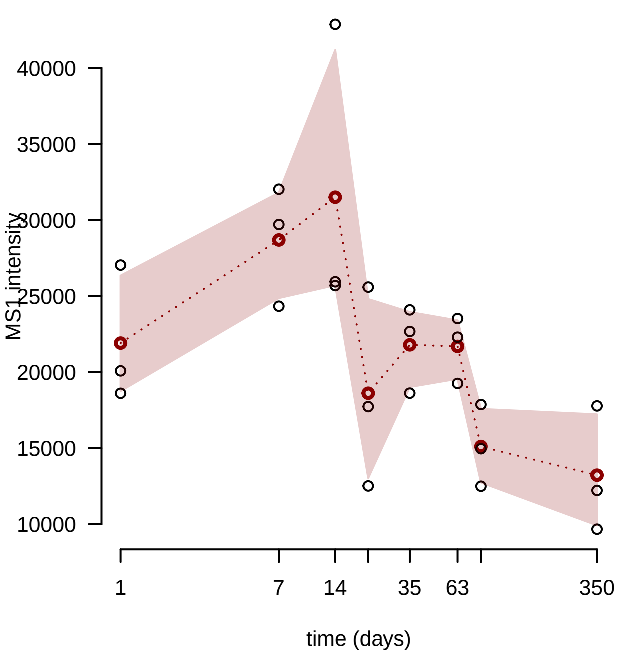

Q8BX70
GN=Vps13c
vacuolar protein sorting 13C

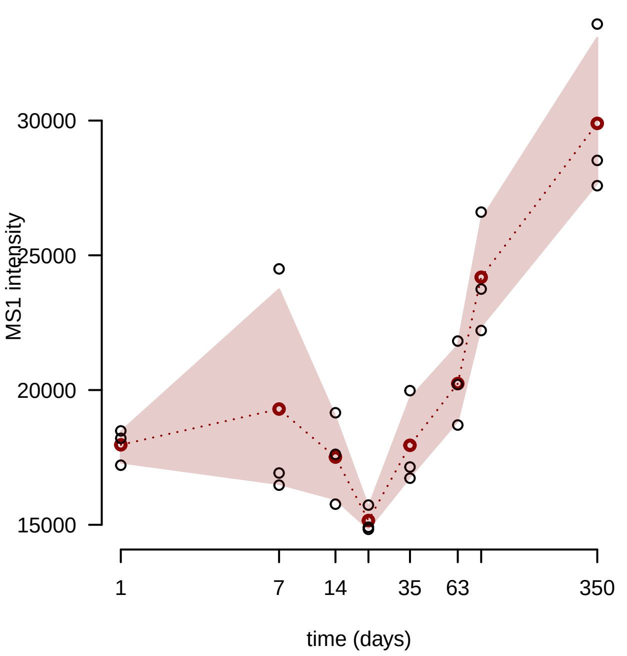

Q8BX80
GN=Engase
endo−beta−N−acetylglucosaminidase

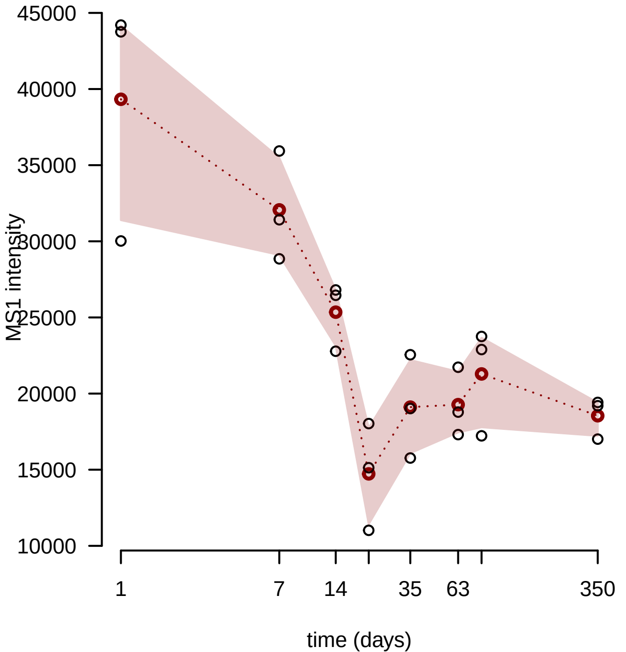

Q8BX90
GN=Fndc3a
fibronectin type III domain containing 3 ...

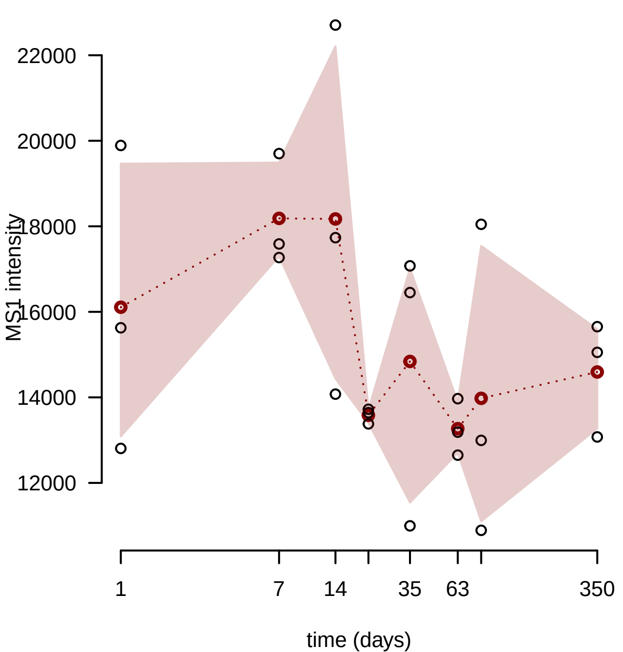

Q8BX94
GN=Osbpl2
oxysterol binding protein−like 2

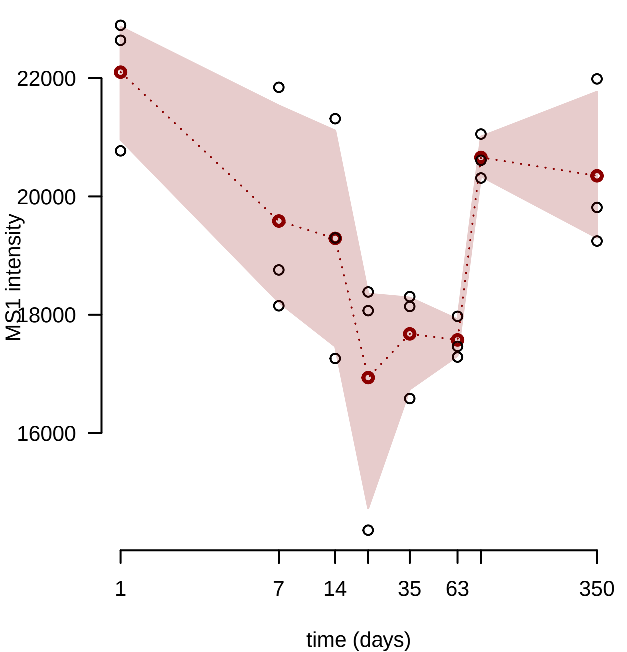

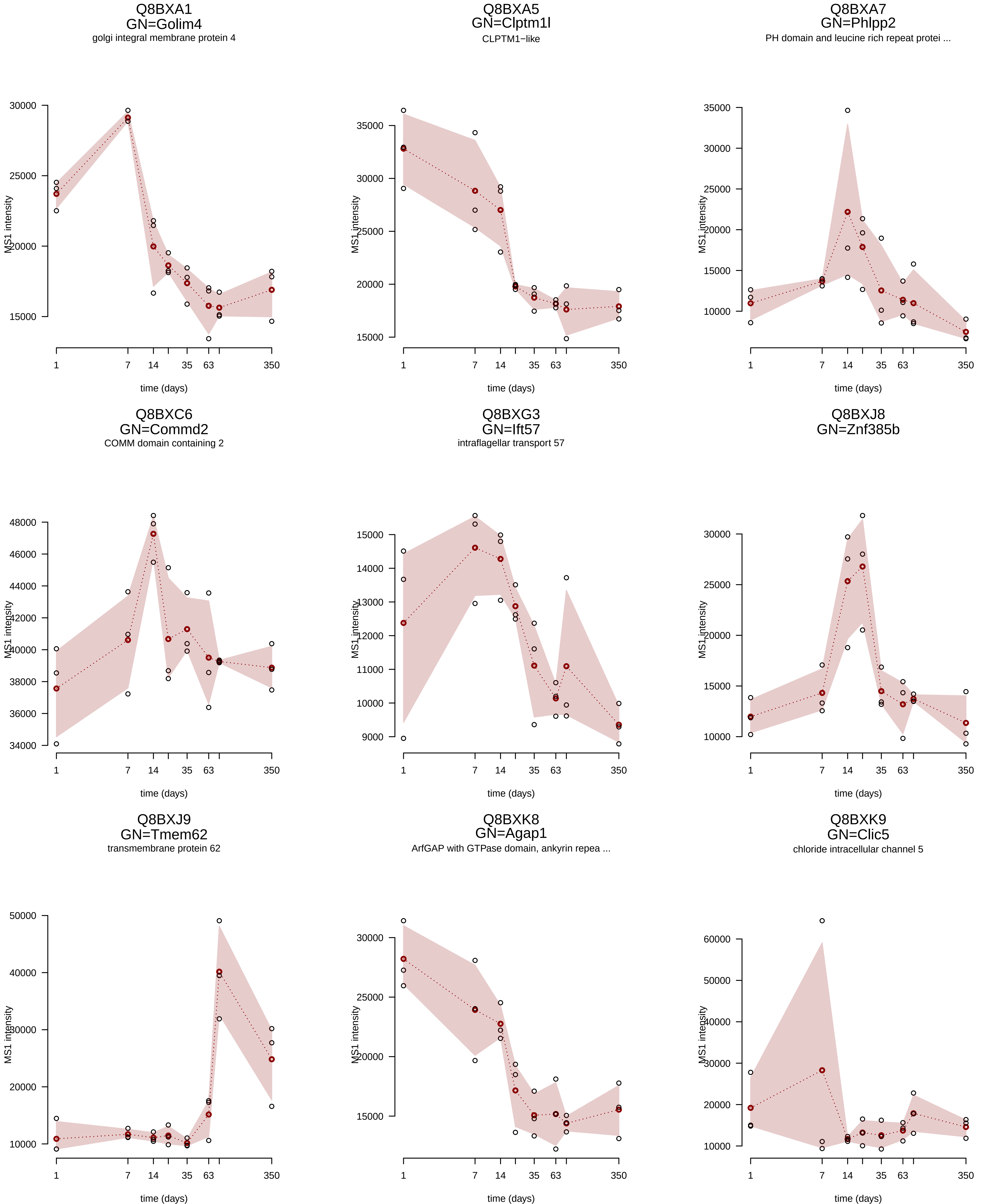
Q8BXA1
GN=Golim4
golgi integral membrane protein 4
Q8BXA5
GN=Clptm1l
CLPTM1−like
Q8BXA7
GN=Phlpp2
PH domain and leucine rich repeat protei ...
Q8BXC6
GN=Commd2
COMM domain containing 2
Q8BXG3
GN=Ift57
intraflagellar transport 57
Q8BXJ8
GN=Znf385b
Q8BXJ9
GN=Tmem62
transmembrane protein 62
Q8BXK8
GN=Agap1
ArfGAP with GTPase domain, ankyrin repea ...
Q8BXK9
GN=Clic5
chloride intracellular channel 5
MS1 intensity
time (days)
1
7
14
35
63
350

Q8BXL7
GN=Arfrp1
ADP−ribosylation factor related protein ...

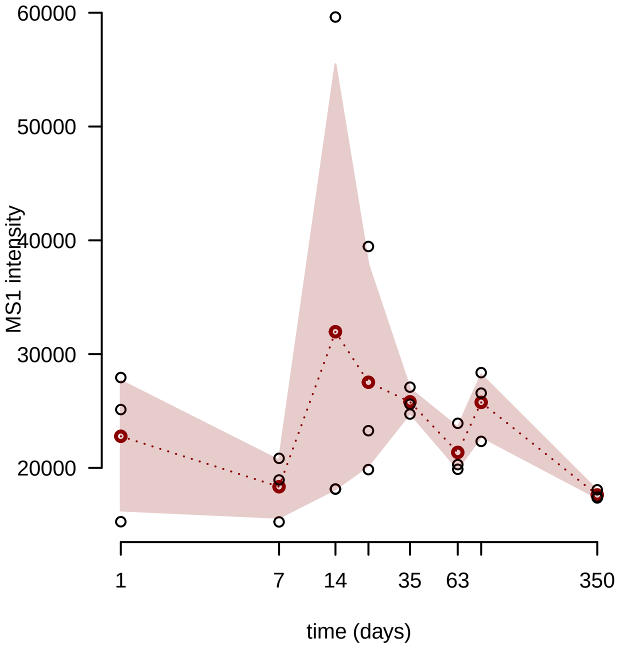

Q8BXN7
GN=Ppm1k
protein phosphatase 1K (PP2C domain cont ...

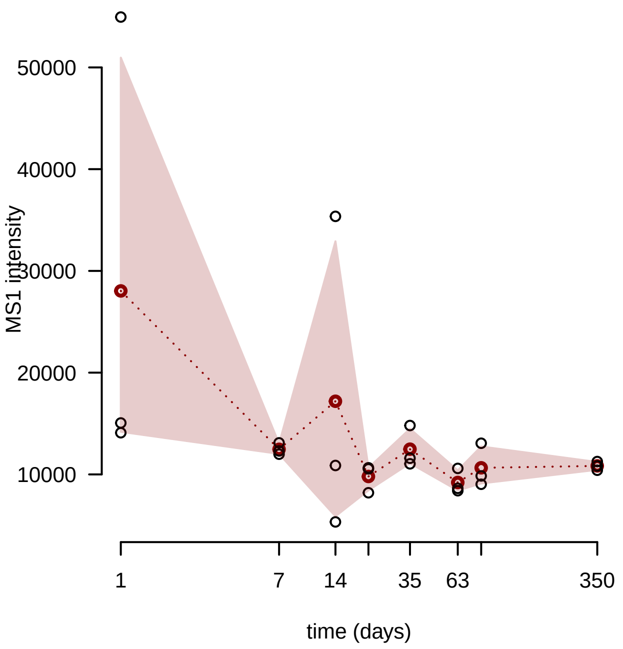

Q8BXN9
GN=Tmem87a
transmembrane protein 87A

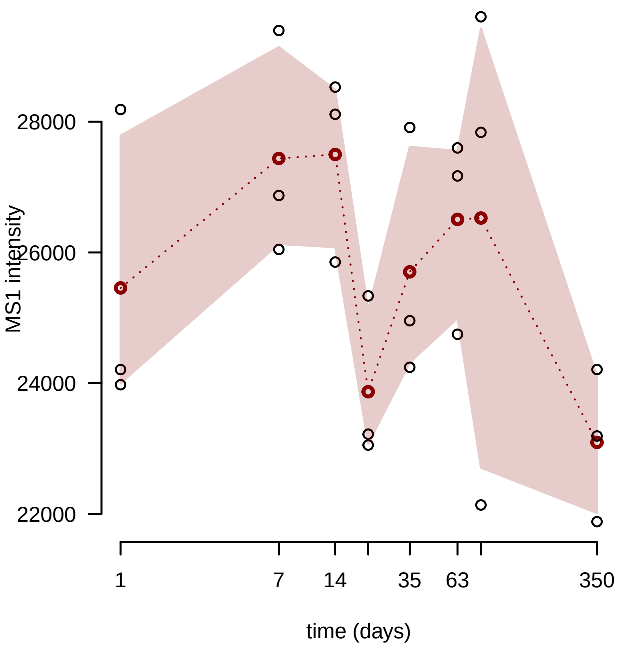

Q8BXQ2
GN=Pigt
phosphatidylinositol glycan anchor biosy ...

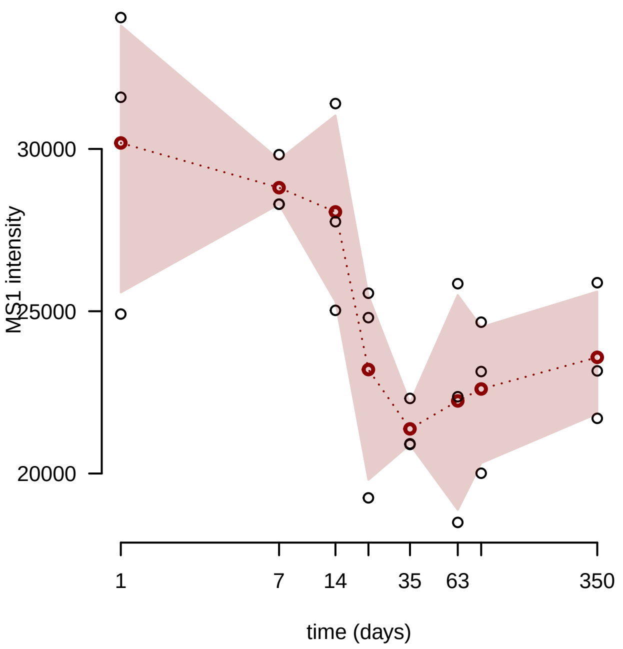

Q8BXR9
GN=Osbpl6
oxysterol binding protein−like 6

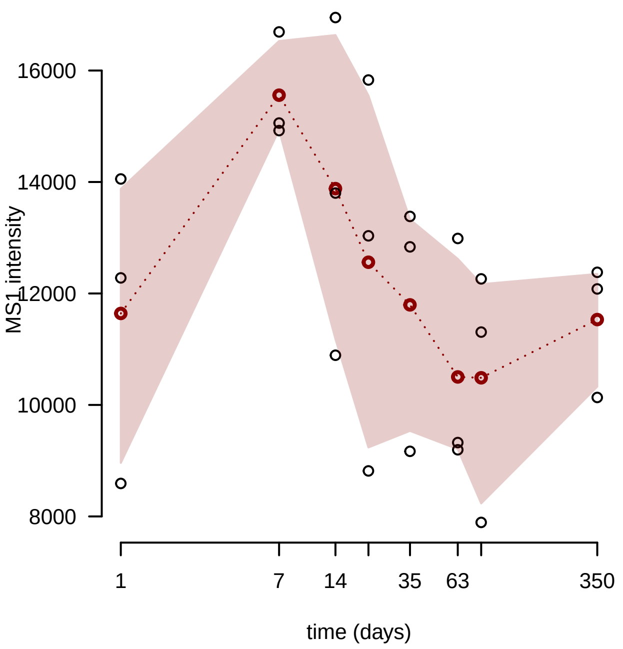

Q8BXV2
GN=Bri3bp
Bri3 binding protein

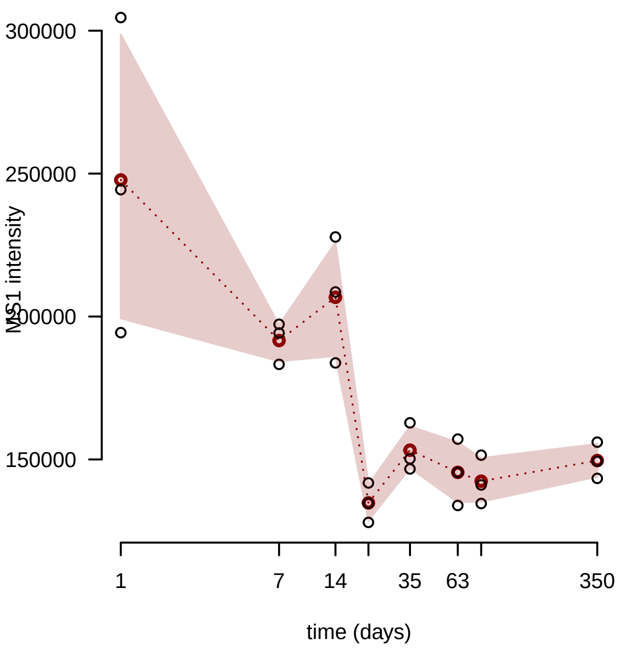

Q8BXZ1
GN=Tmx3
thioredoxin−related transmembrane protei ...

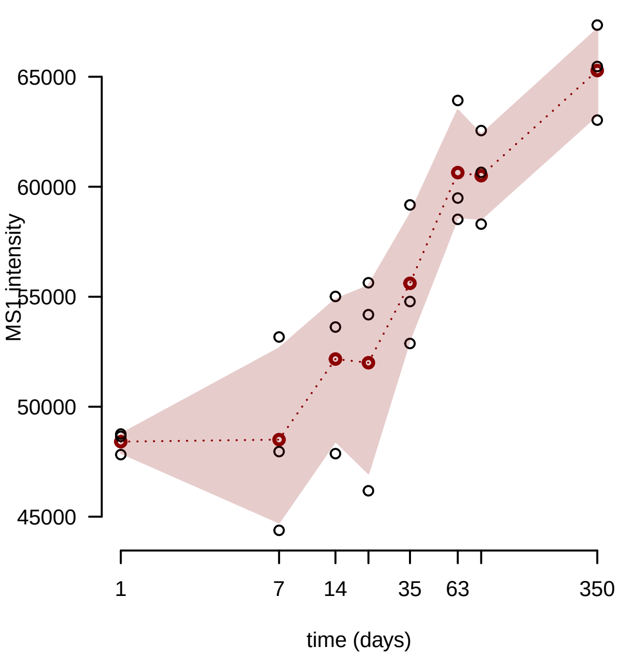

Q8BY02
GN=Nkrf
NF−kappaB repressing factor

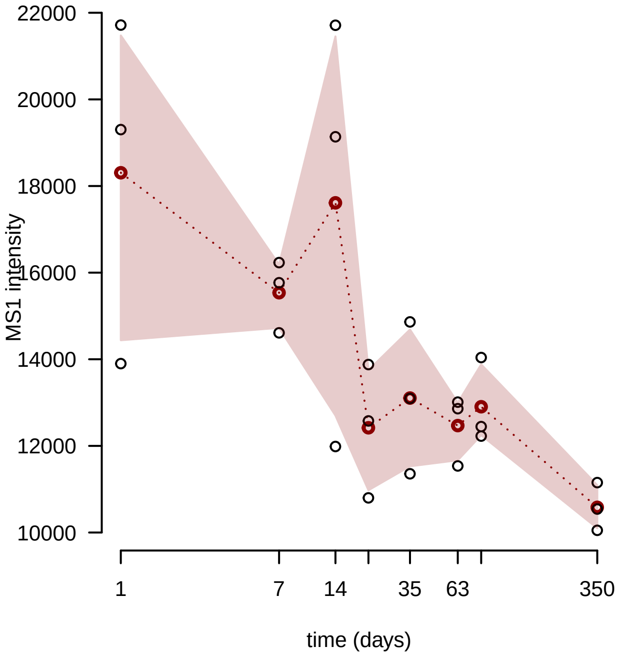

Q8BY46
GN=Znf574

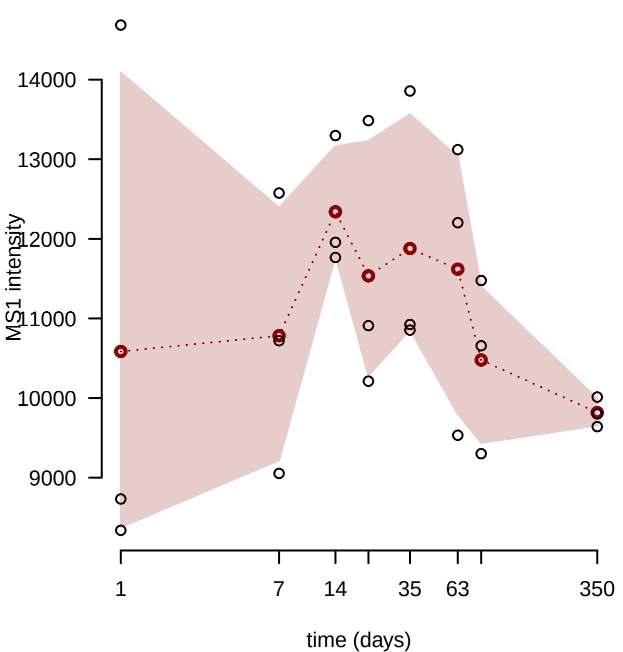

Q8BY71
GN=Hat1
histone aminotransferase 1

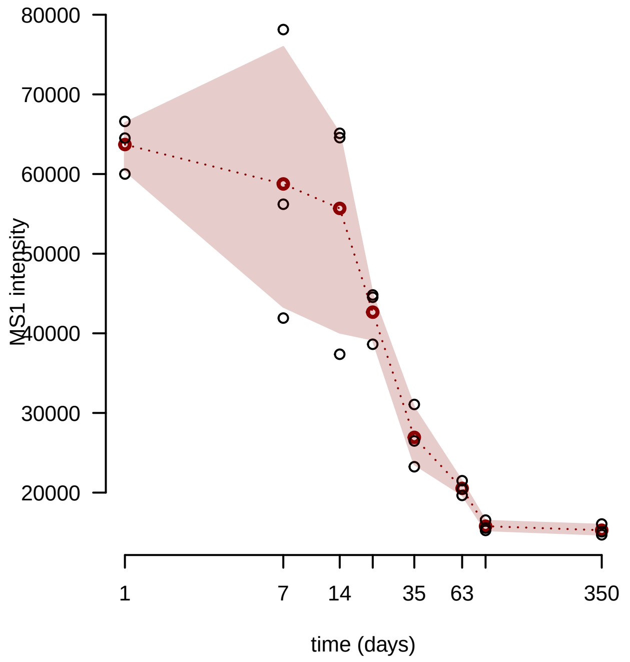

Q8BY87
GN=Usp47
ubiquitin specific peptidase 47

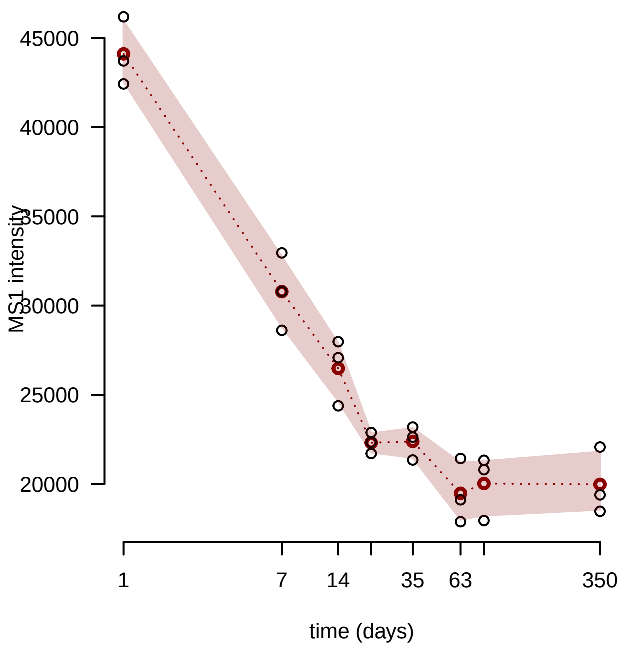

Q8BY89
GN=Slc44a2
solute carrier family 44, member 2

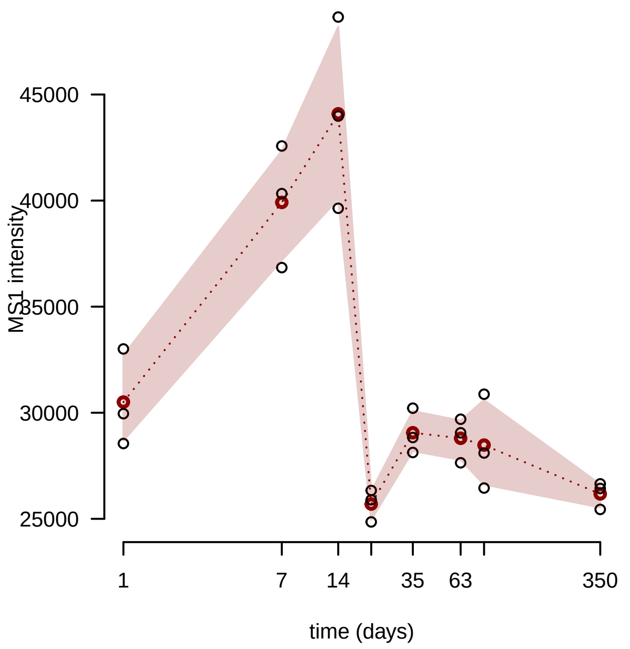

Q8BYA0
GN=Tbcd
tubulin−specific chaperone d

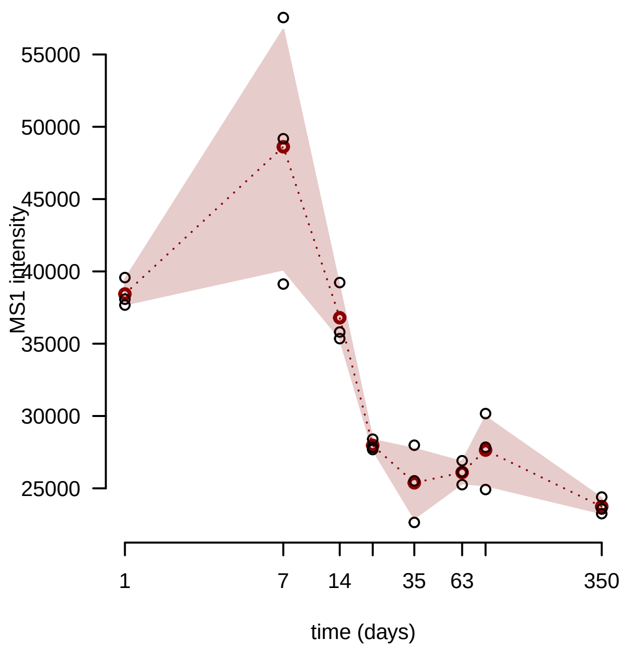

Q8BYB9
GN=Poglut1
protein O−glucosyltransferase 1

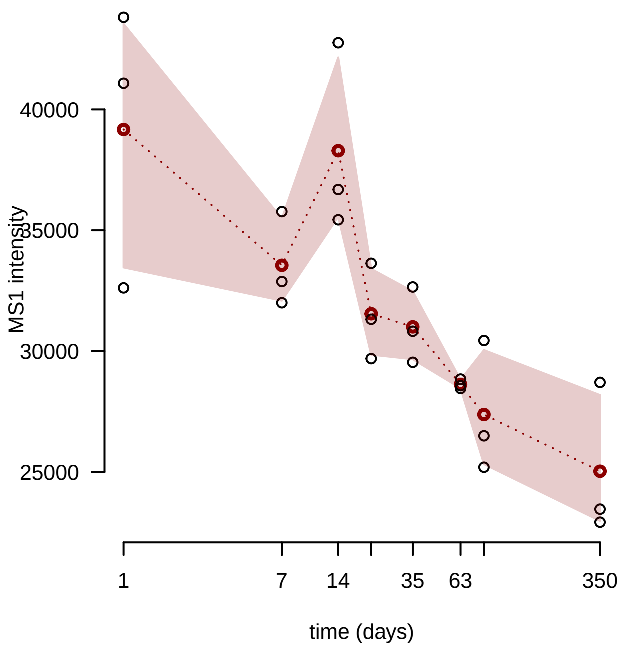

Q8BYC6
GN=Taok3
TAO kinase 3

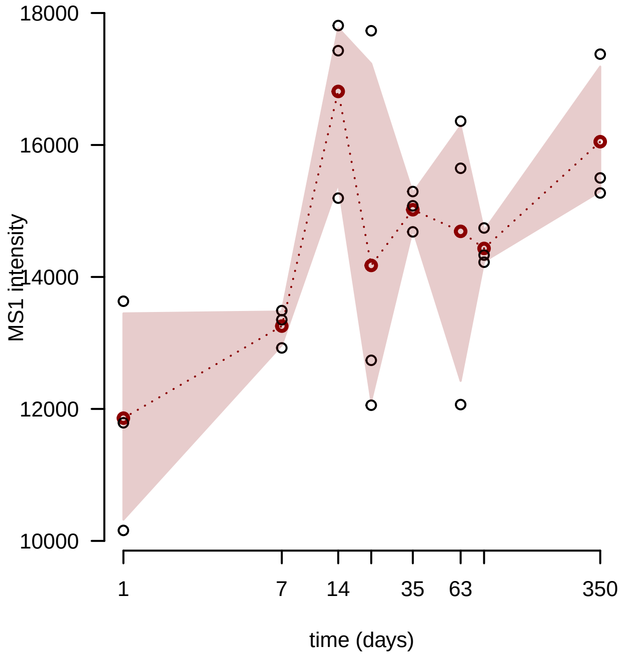

Q8BYH3
GN=Trmt13
tRNA methyltransferase 13

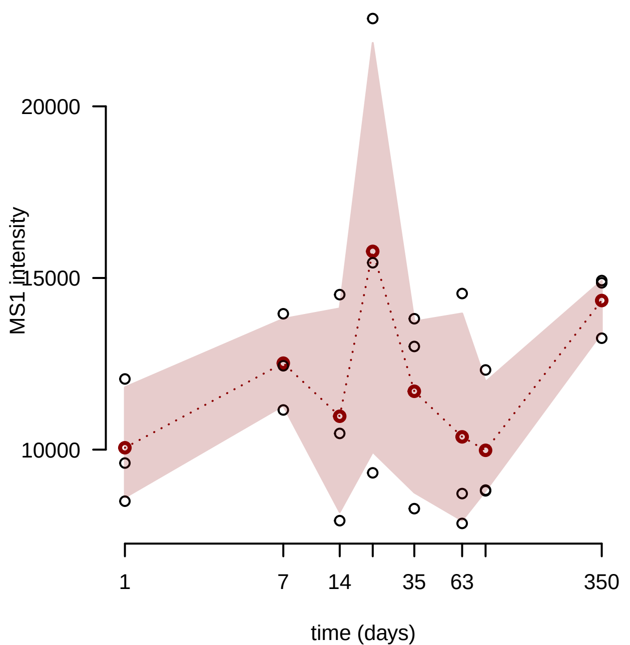

Q8BYH7
GN=Tbc1d17
TBC1 domain family, member 17

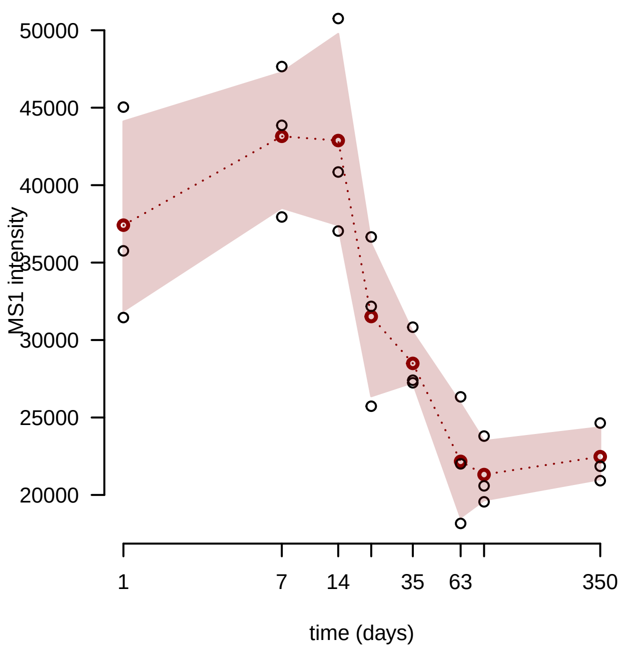

Q8BYH8
GN=Chd9
chromodomain helicase DNA binding protei ...

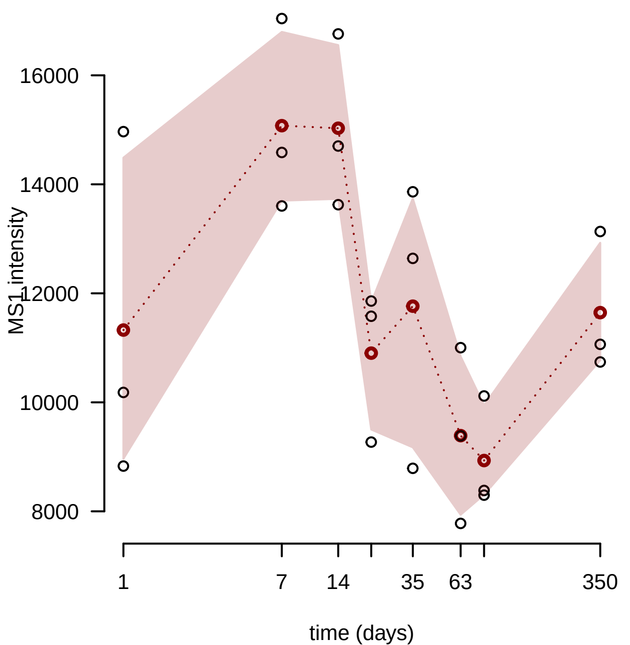

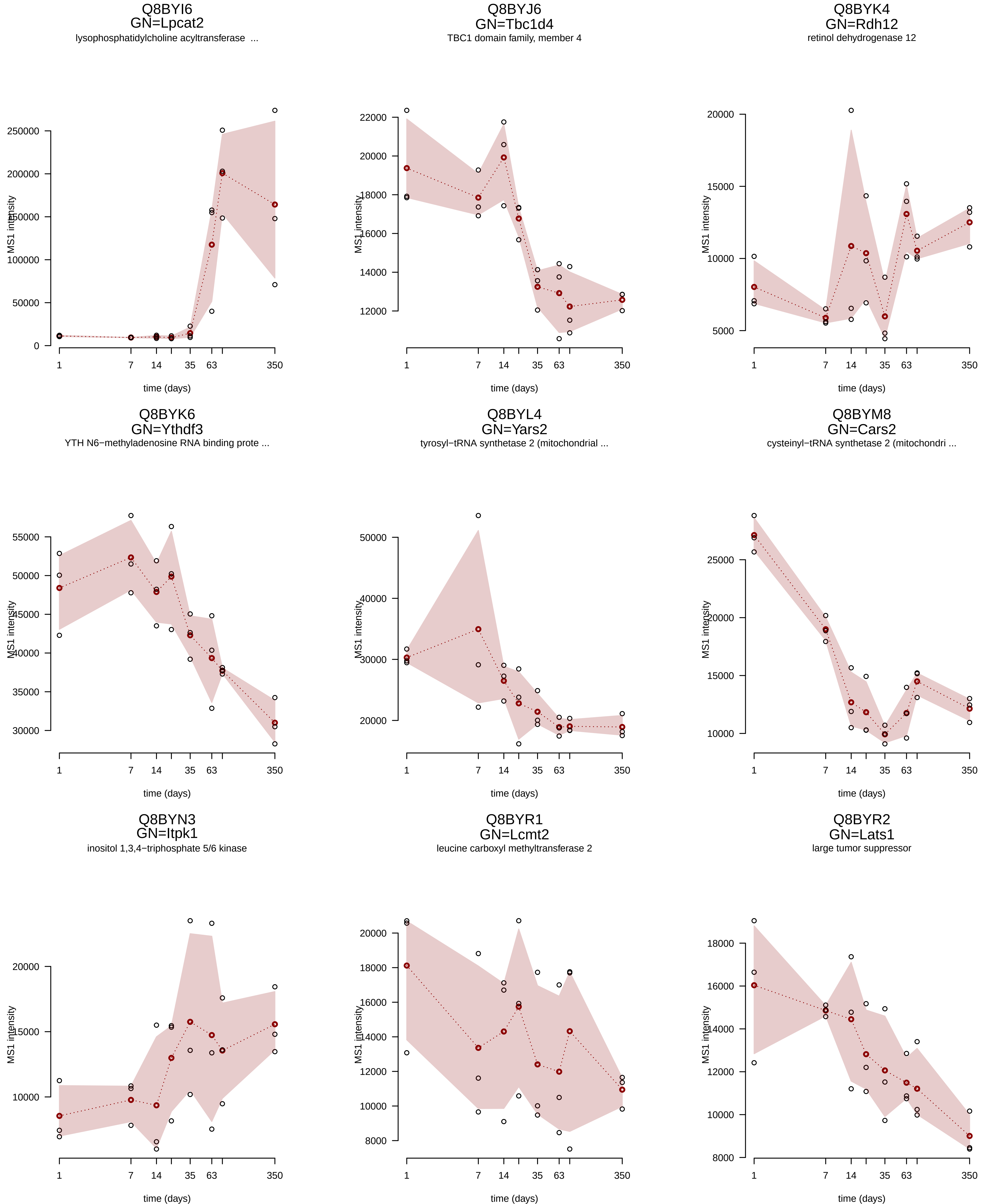
Q8BYI6
GN=Lpcat2
lysophosphatidylcholine acyltransferase ...
Q8BYJ6
GN=Tbc1d4
TBC1 domain family, member 4
Q8BYK4
GN=Rdh12
retinol dehydrogenase 12
Q8BYK6
GN=Ythdf3
YTH N6−methyladenosine RNA binding prote ...
Q8BYL4
GN=Yars2
tyrosyl−tRNA synthetase 2 (mitochondrial ...
Q8BYM8
GN=Cars2
cysteinyl−tRNA synthetase 2 (mitochondri ...
Q8BYN3
GN=Itpk1
inositol 1,3,4−triphosphate 5/6 kinase
Q8BYR1
GN=Lcmt2
leucine carboxyl methyltransferase 2
Q8BYR2
GN=Lats1
large tumor suppressor
MS1 intensity
time (days)
1
7
14
35
63
350

Q8BYU6
GN=Tor1aip2
torsin A interacting protein 2

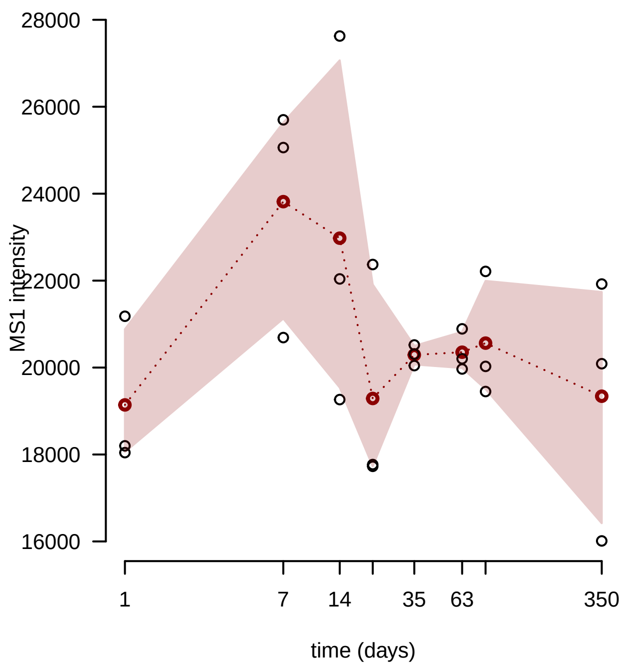

Q8BYW1
GN=Arhgap25
Rho GTPase activating protein 25

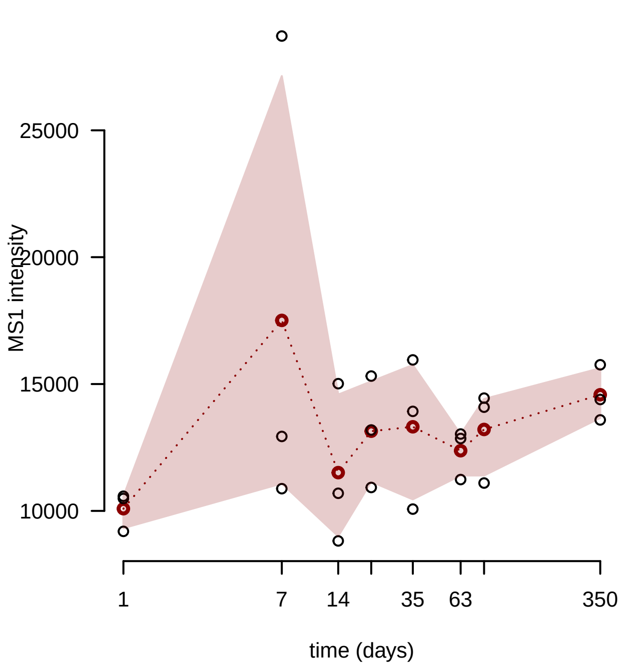

Q8BYW9
GN=Eogt
EGF domain−specific O−linked N−acetylglu ...

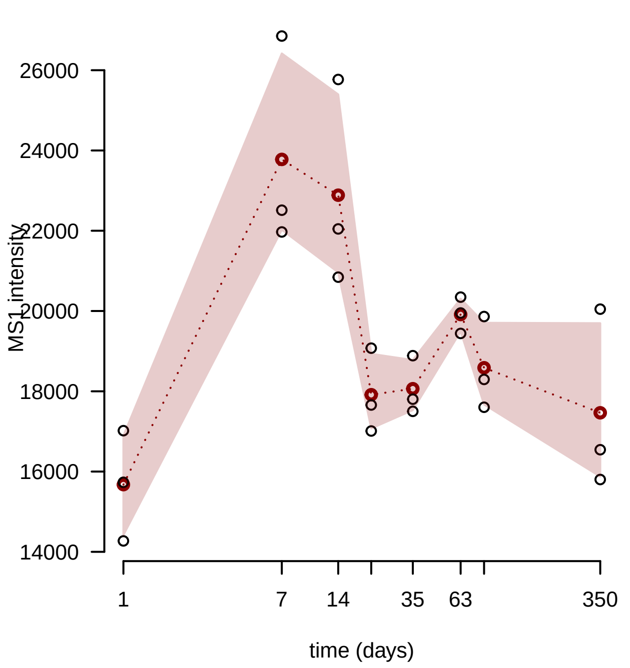

Q8BYY4
GN=Ttc39b
tetratricopeptide repeat domain 39B

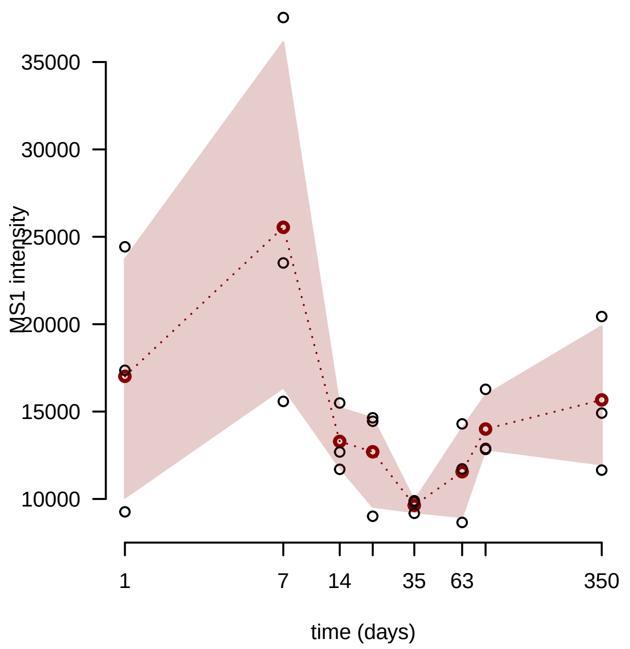

Q8BYZ7
GN=Elmo3
engulfment and cell motility 3

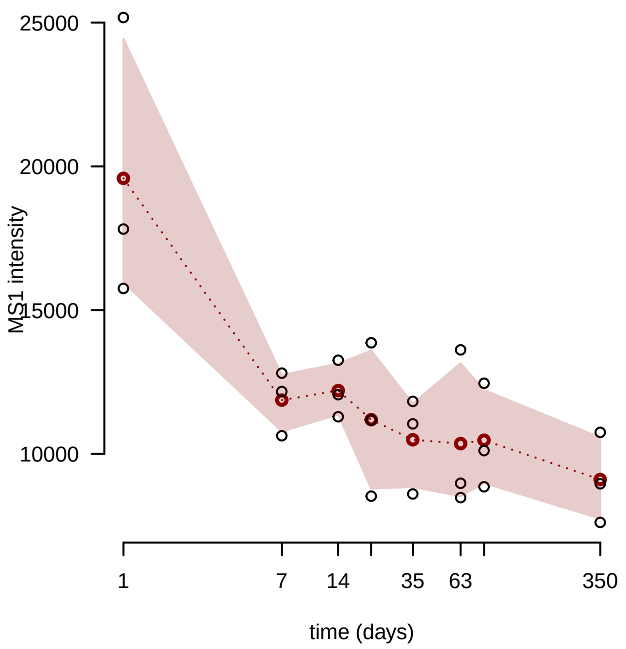

Q8BZ03
GN=Prkd2
protein kinase D2

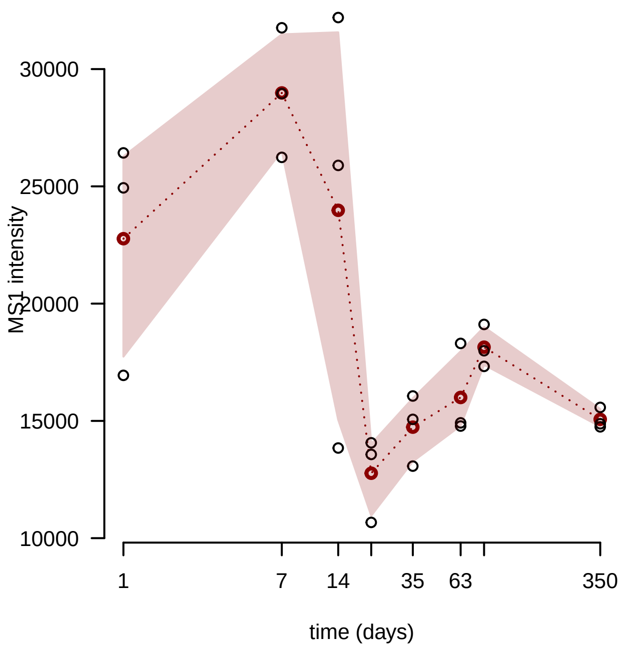

Q8BZ05
GN=Arap2
ArfGAP with RhoGAP domain, ankyrin repea ...

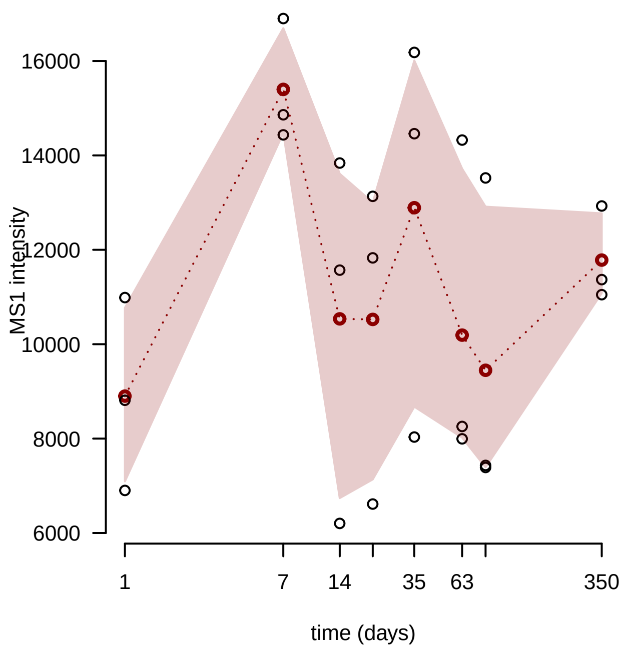

Q8BZ09
GN=Slc25a21
solute carrier family 25 (mitochondrial ...

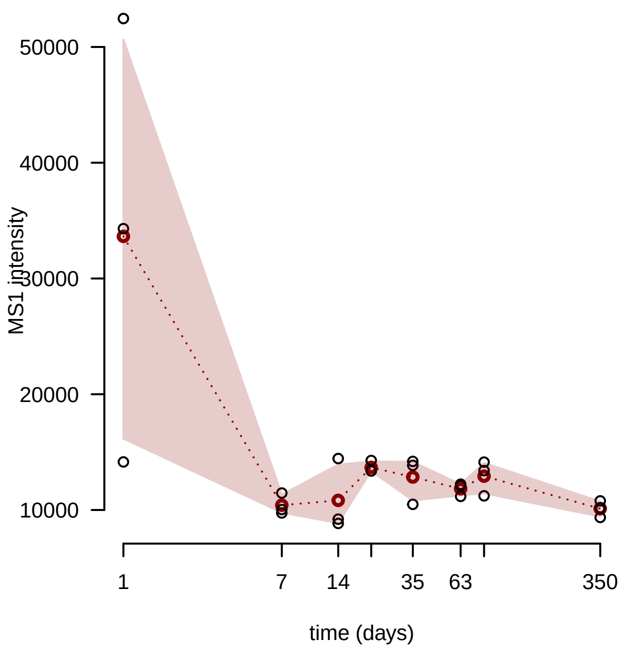

Q8BZ20
GN=Parp12
poly (ADP−ribose) polymerase family, mem ...

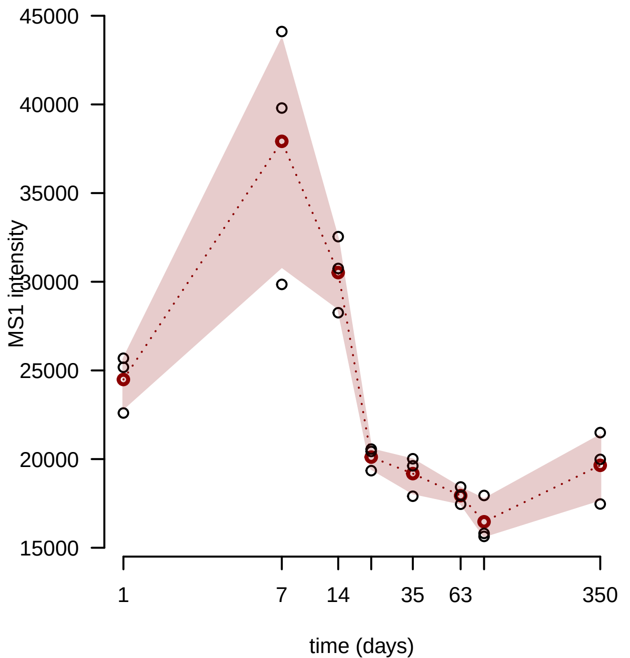

Q8BZ21
GN=Kat6a
K(lysine) acetyltransferase 6A

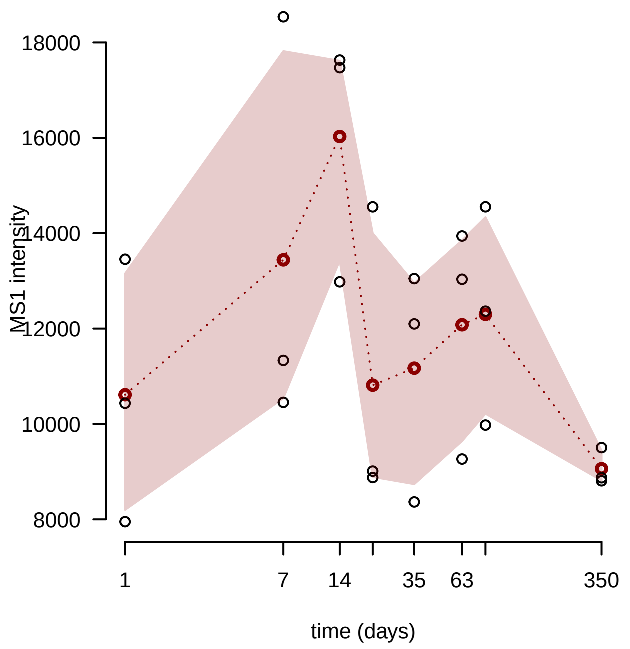

Q8BZ32
GN=Asxl2
ASXL transcriptional regulator 2

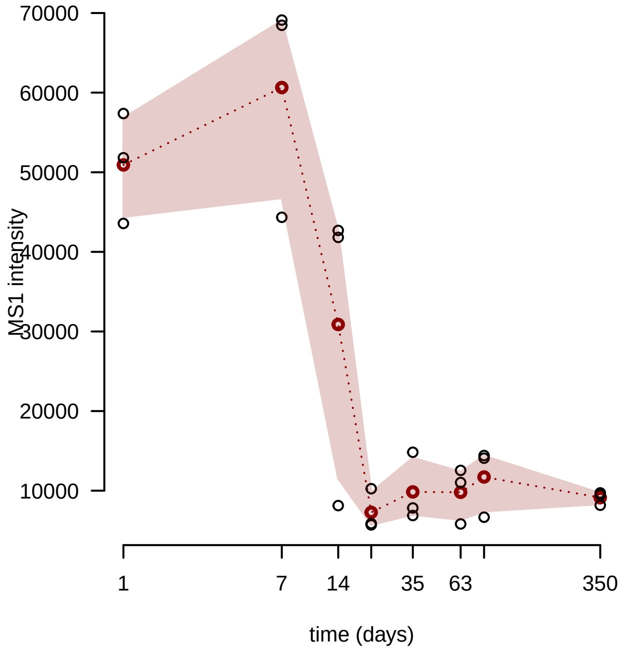

Q8BZ36
GN=Rint1
RAD50 interactor 1

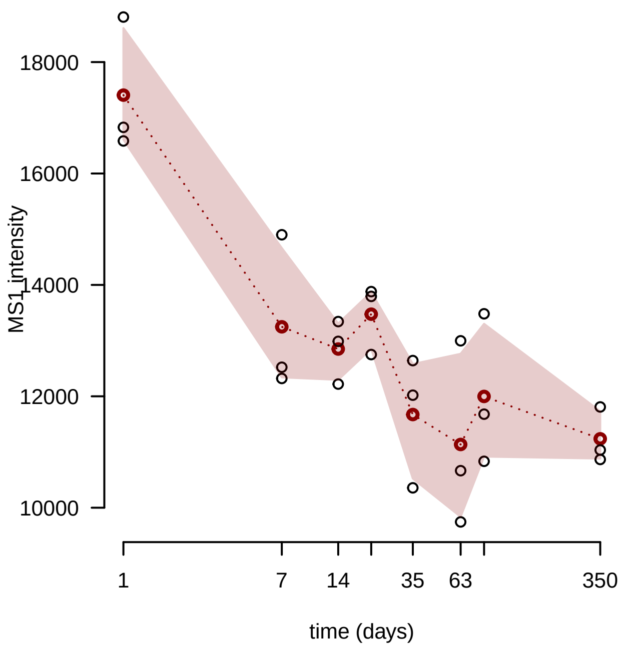

Q8BZ47
GN=Znf609

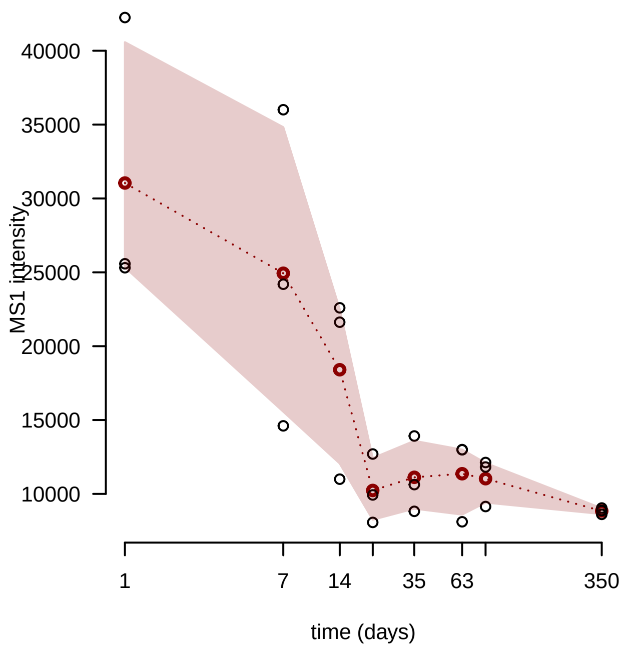

Q8BZ60
GN=Ston2
stonin 2

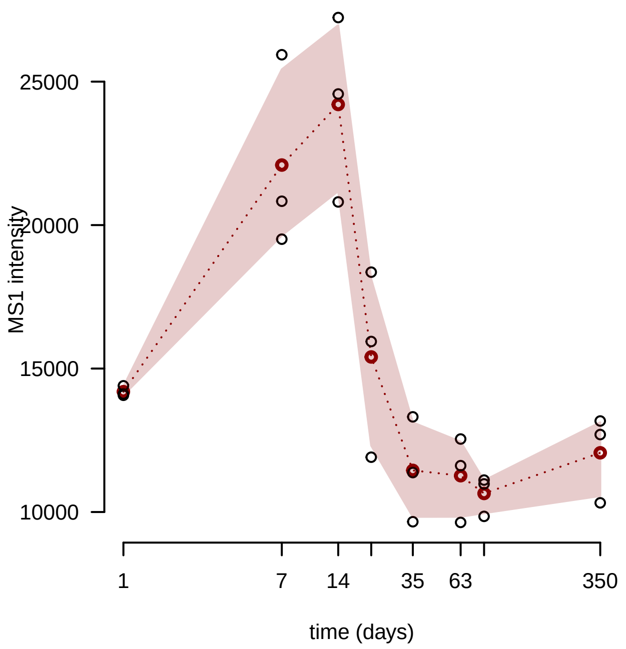

Q8BZ98
GN=Dnm3
dynamin 3

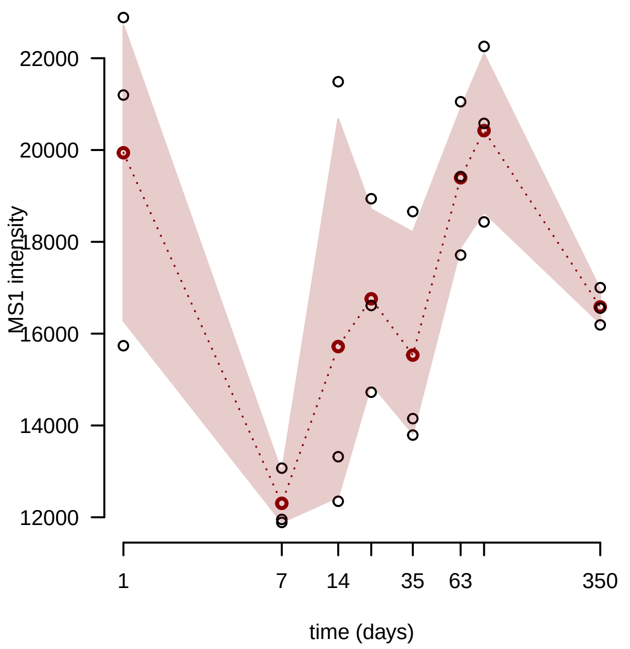

Q8BZA9
GN=Tigar
Trp53 induced glycolysis regulatory phos ...

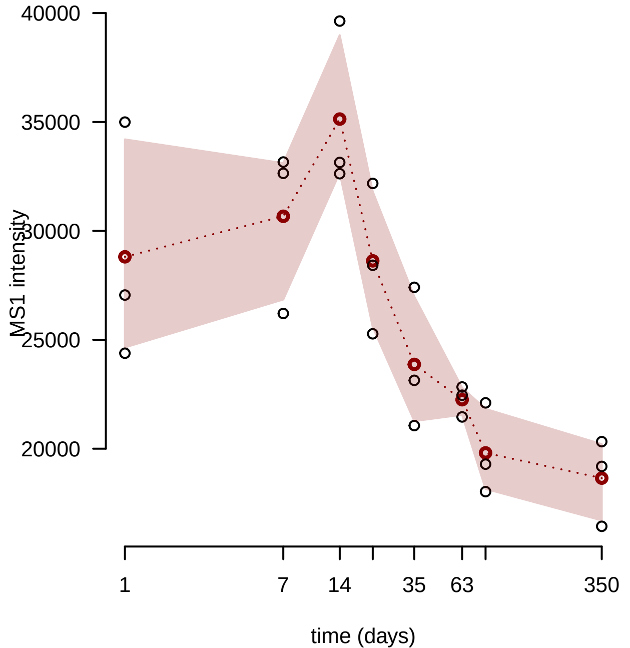

Q8BZB2
GN=Ppcdc
phosphopantothenoylcysteine decarboxylas ...

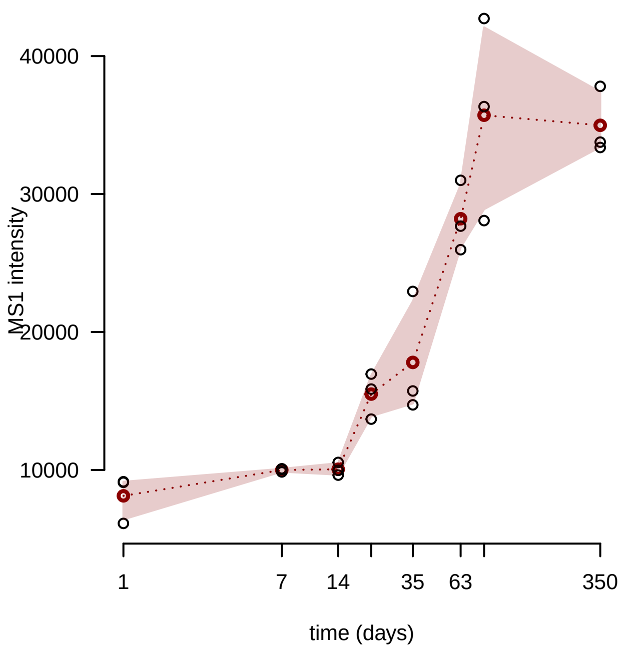

Q8BZF8
GN=Pgm5
phosphoglucomutase 5

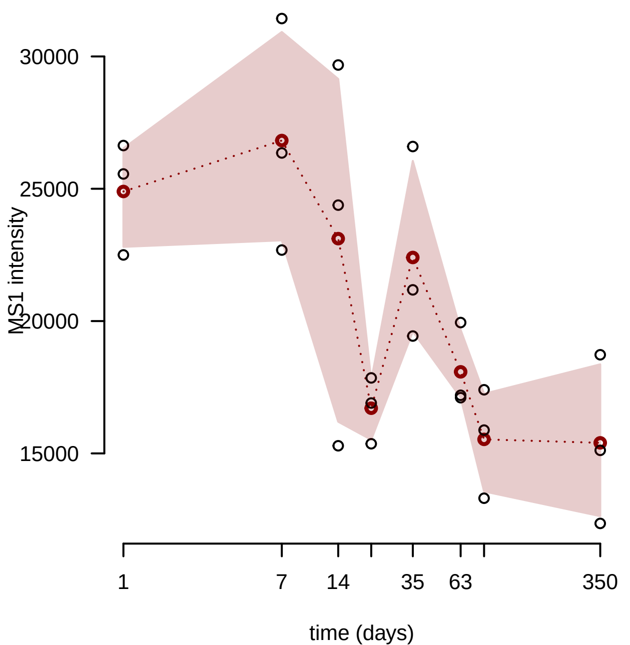

Q8BZH4
GN=Pogz
pogo transposable element with ZNF domai ...

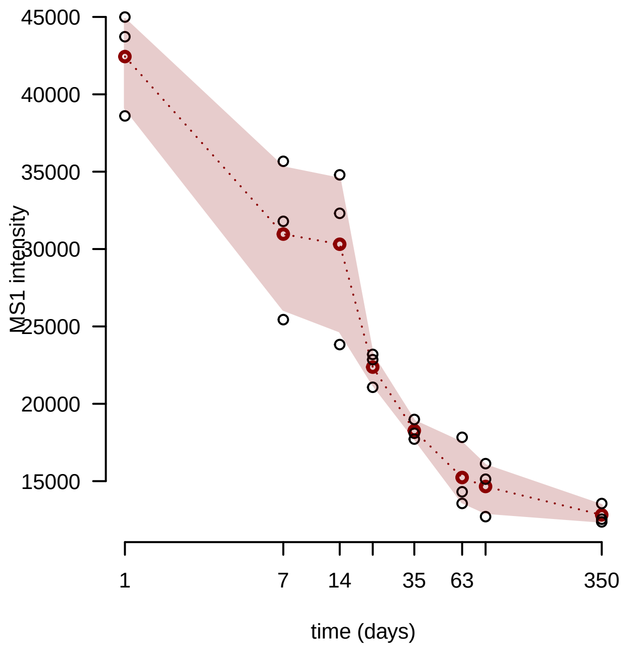

Q8BZJ7
GN=Dcun1d2
DCN1, defective in cullin neddylation 1, ...

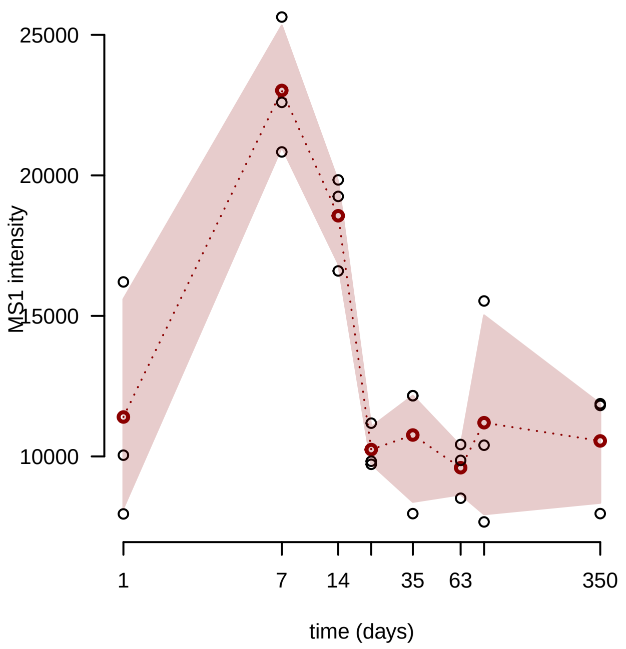

Q8BZM0
GN=Klhl12
kelch−like 12

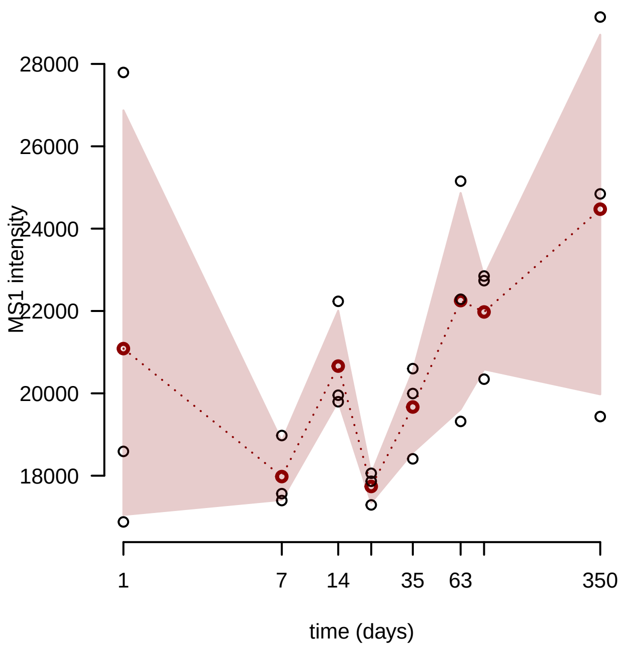

Q8BZM1
GN=Glmn
glomulin, FKBP associated protein

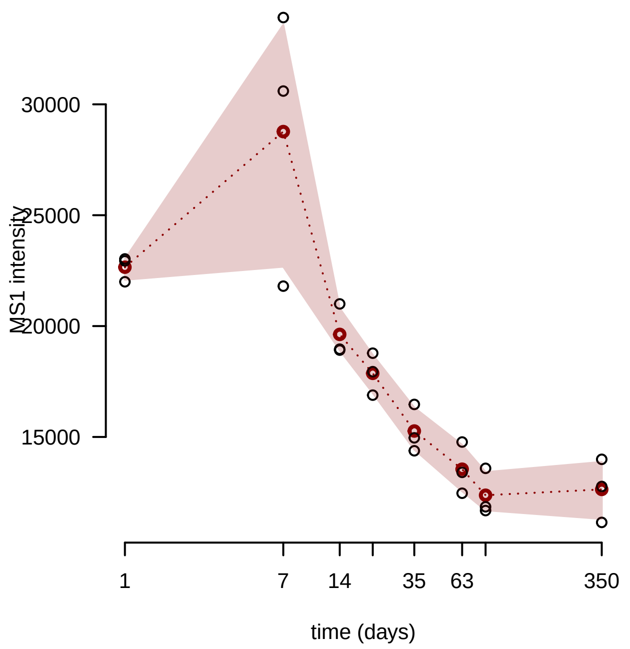

Q8BZN6
GN=Dock10
dedicator of cytokinesis 10

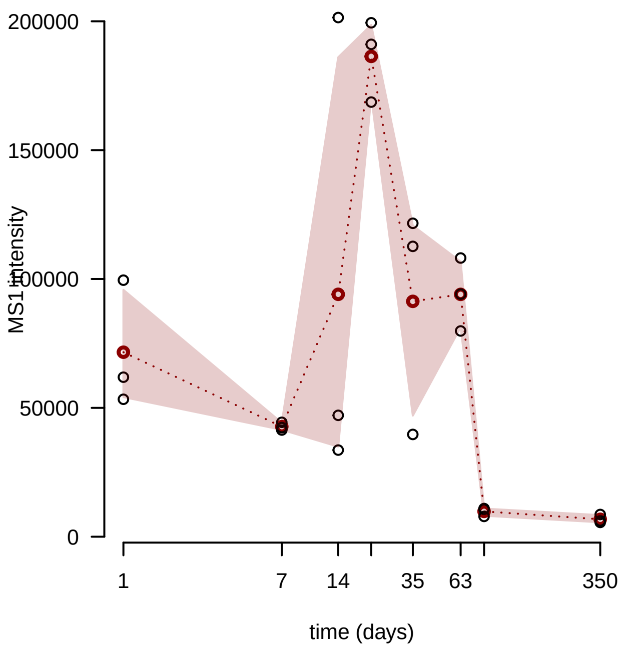

Q8BZQ7
GN=Anapc2
anaphase promoting complex subunit 2

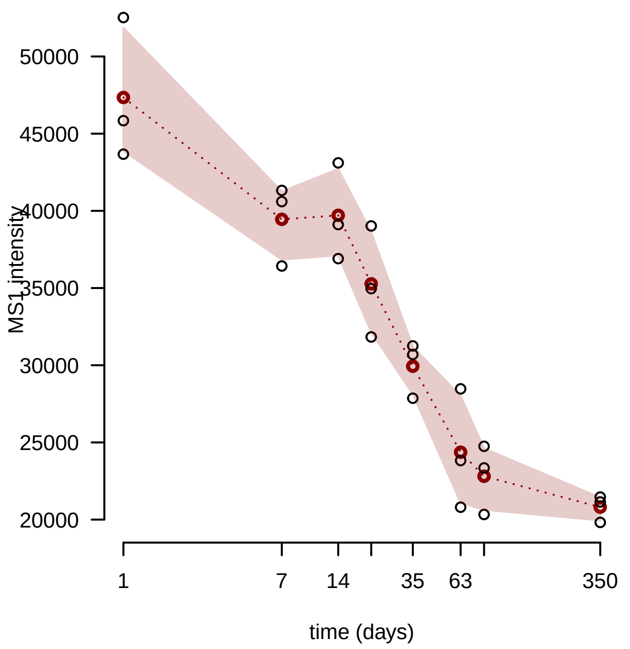

Q8BZR9
GN=Ncbp3
nuclear cap binding subunit 3

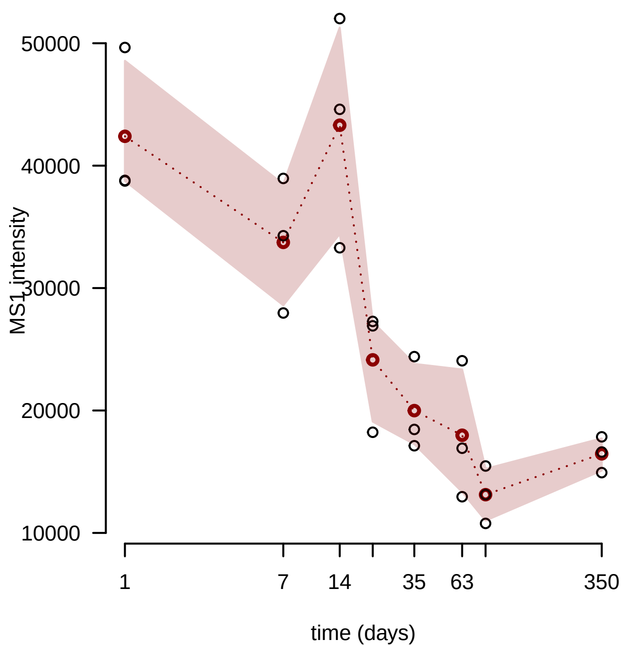

Q8BZS9
GN=Dhx32
DEAH (Asp−Glu−Ala−His) box polypeptide 3 ...

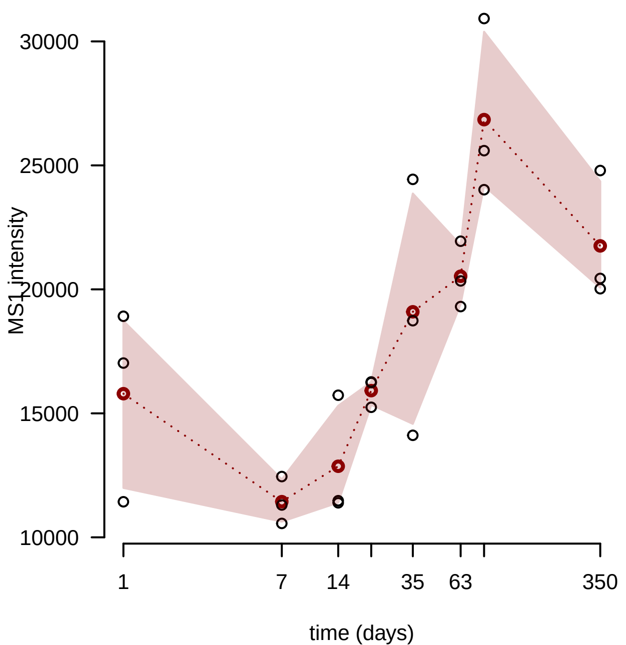

Q8BZW8
GN=Nhlrc2
NHL repeat containing 2

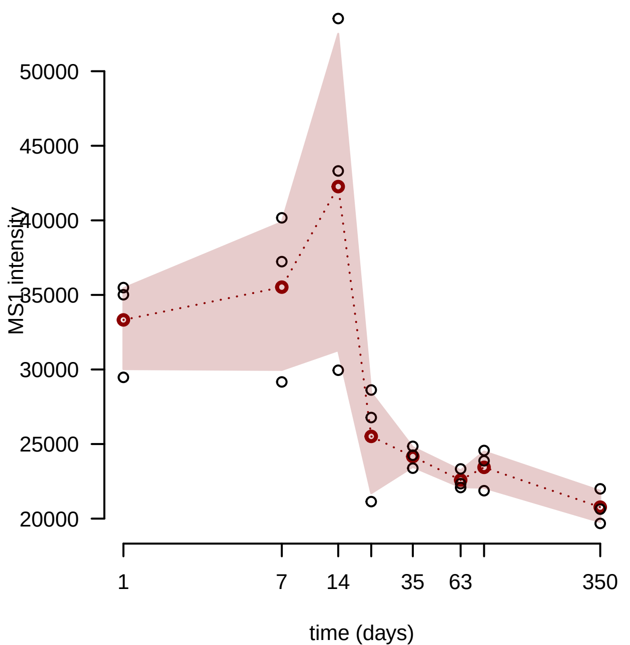

Q8BZX4
GN=Srek1
splicing regulatory glutamine/lysine−ric ...

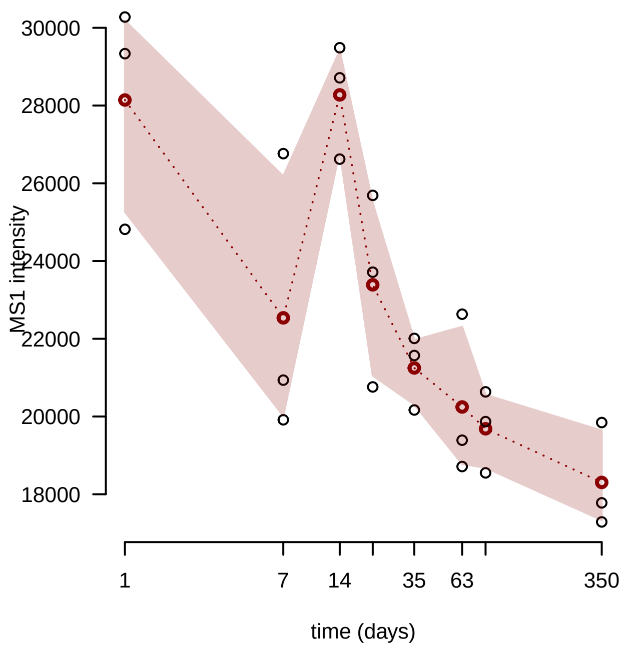

Q8BZZ3
GN=Wwp1
WW domain containing E3 ubiquitin protei ...

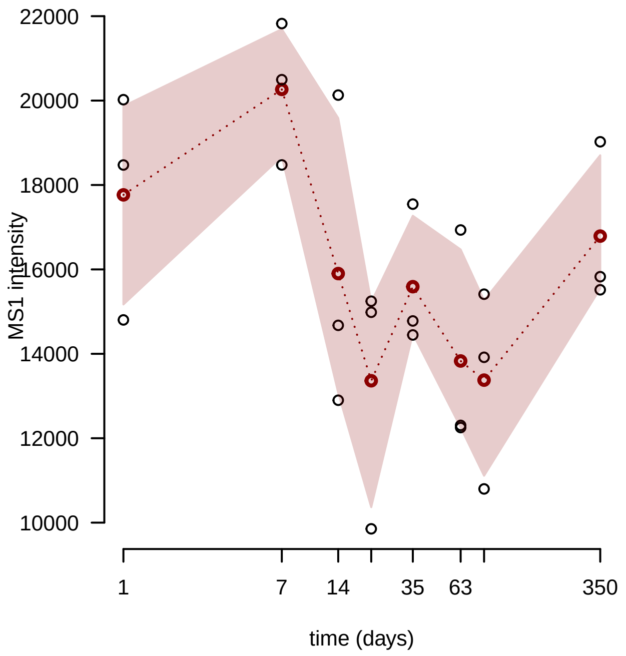

Q8C006
GN=Trim35
tripartite motif−containing 35

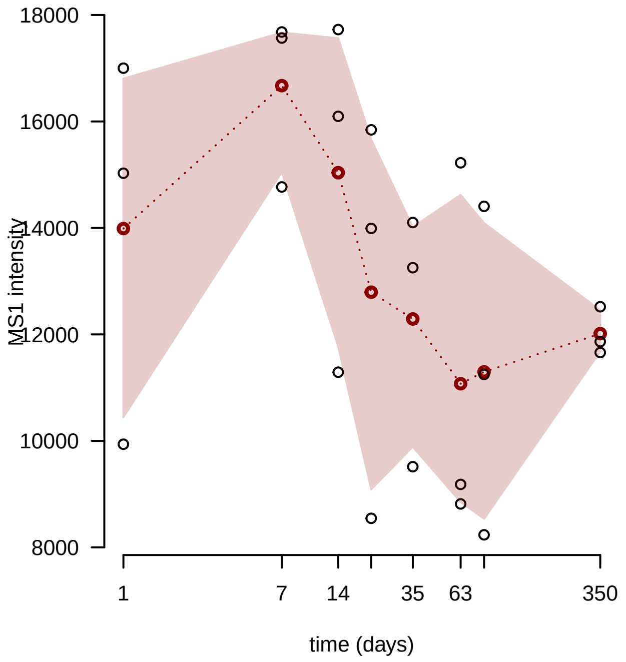

Q8C013
GN=Tpbgl
trophoblast glycoprotein−like

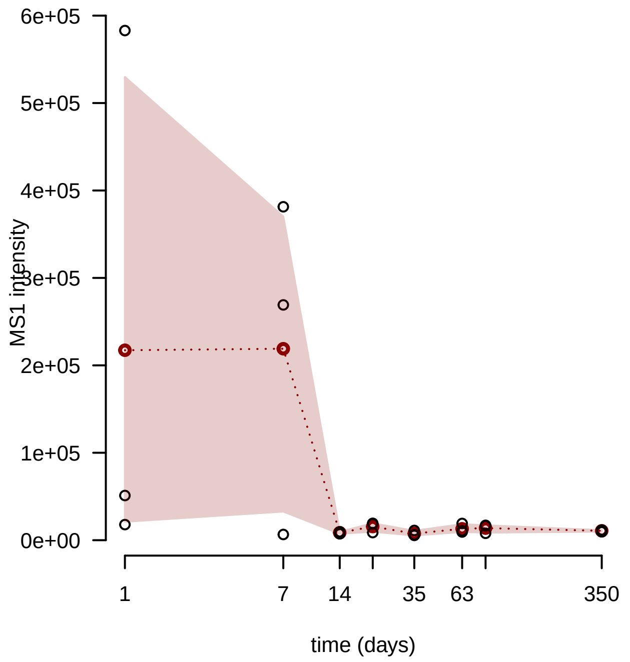

Q8C025
GN=Chpt1
choline phosphotransferase 1

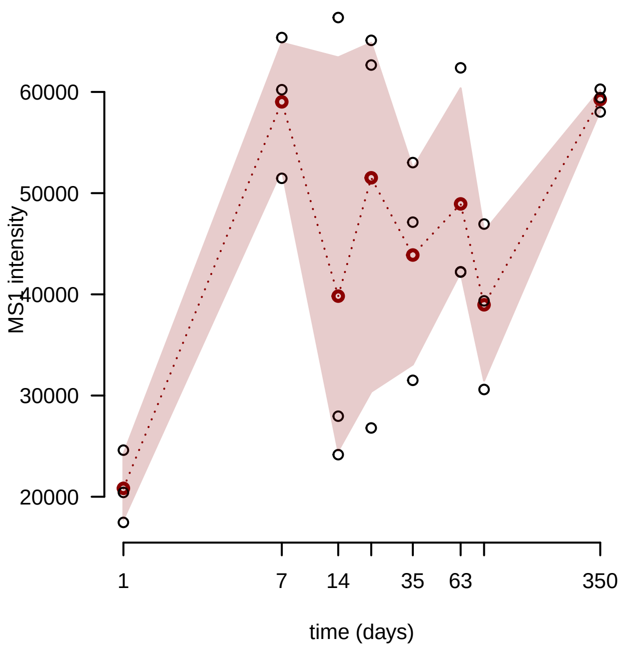

Q8C033
GN=Arhgef10
Rho guanine nucleotide exchange factor (...

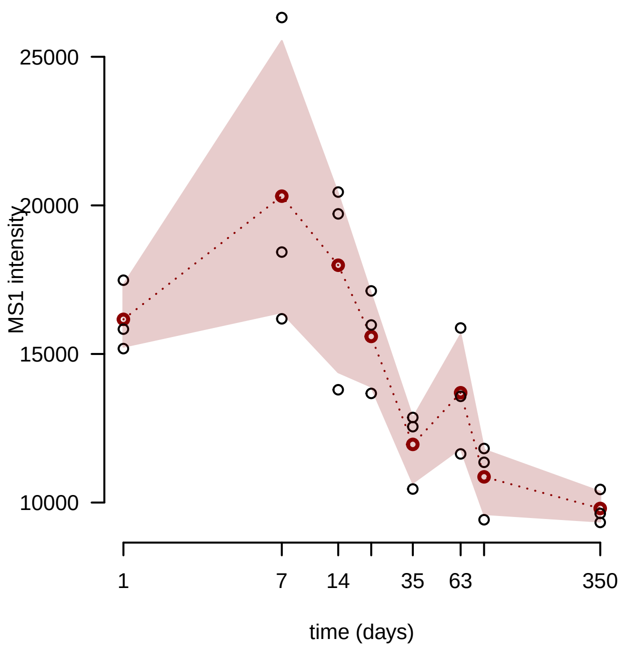

Q8C050
GN=Rps6ka5
ribosomal protein S6 kinase, polypeptide ...

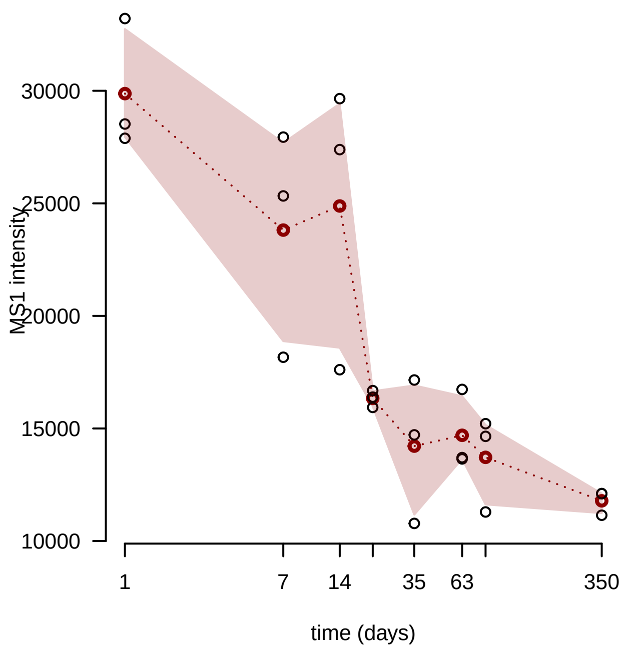

Q8C052
GN=Map1s
microtubule−associated protein 1S

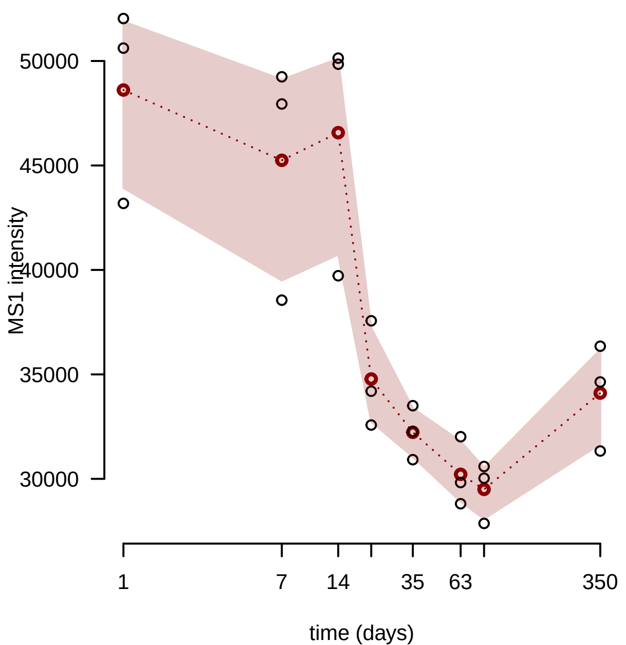

Q8C078
GN=Camkk2
calcium/calmodulin−dependent protein kin ...

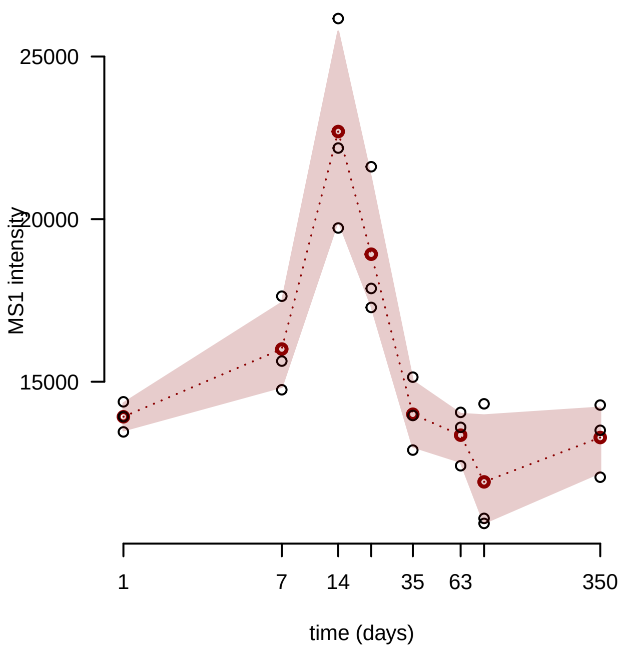

Q8C079
GN=Strip1
striatin interacting protein 1

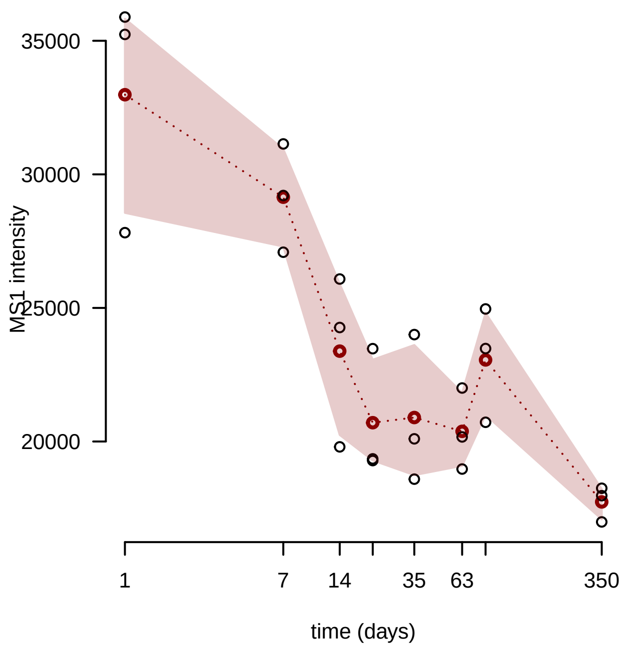

Q8C080
GN=Snx16
sorting nexin 16

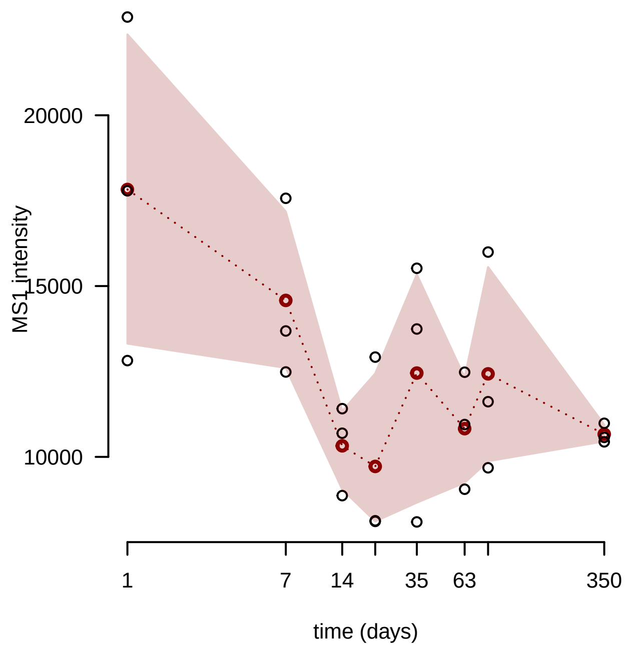

Q8C092
GN=Taf5
TATA−box binding protein associated fact ...

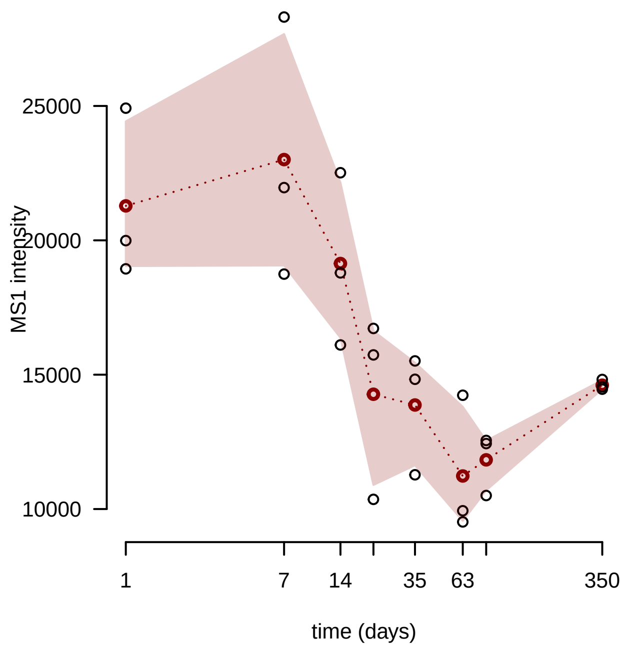

Q8C0C0
GN=Zhx2
zinc fingers and homeoboxes 2

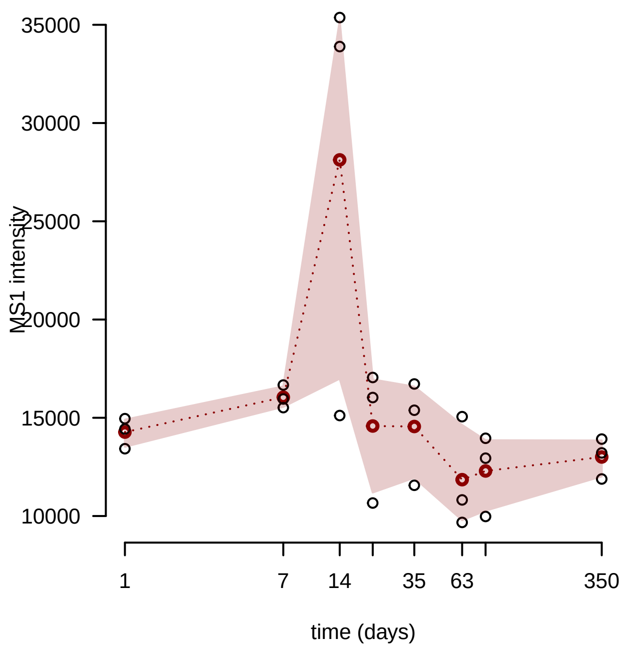

Q8C0C7
GN=Farsa
phenylalanyl−tRNA synthetase, alpha subu ...

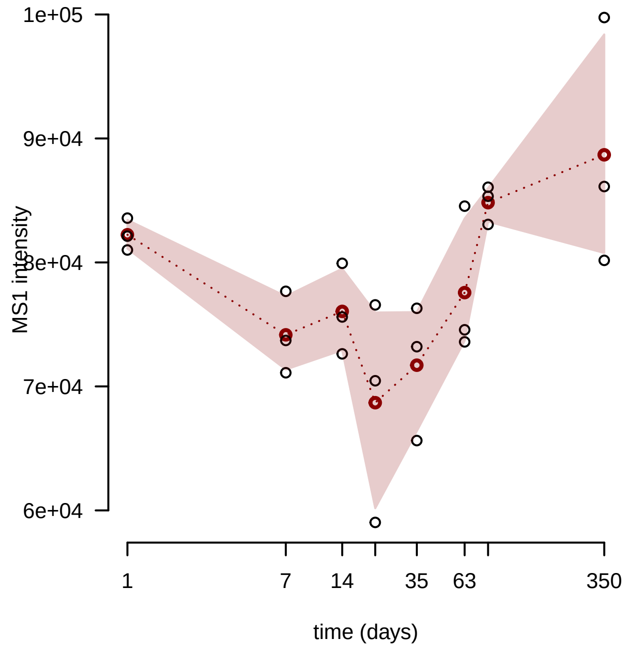

Q8C0D0
GN=Trub1
TruB pseudouridine (psi) synthase family ...

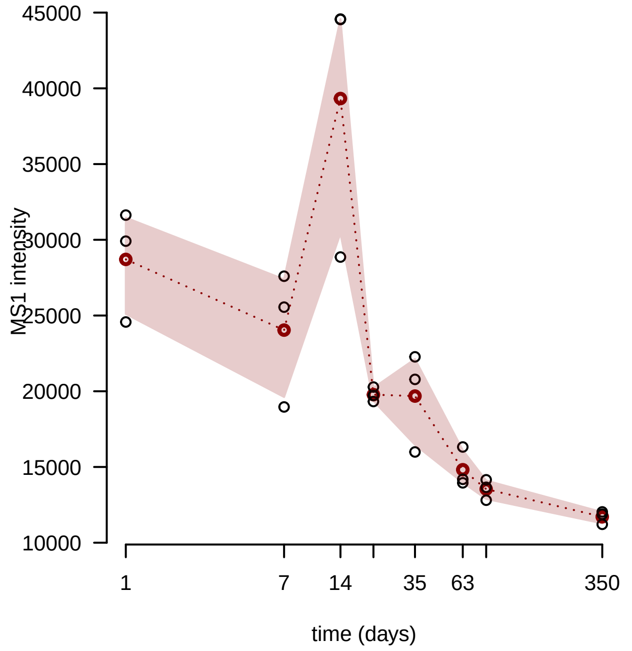

Q8C0D4
GN=Arhgap12
Rho GTPase activating protein 12

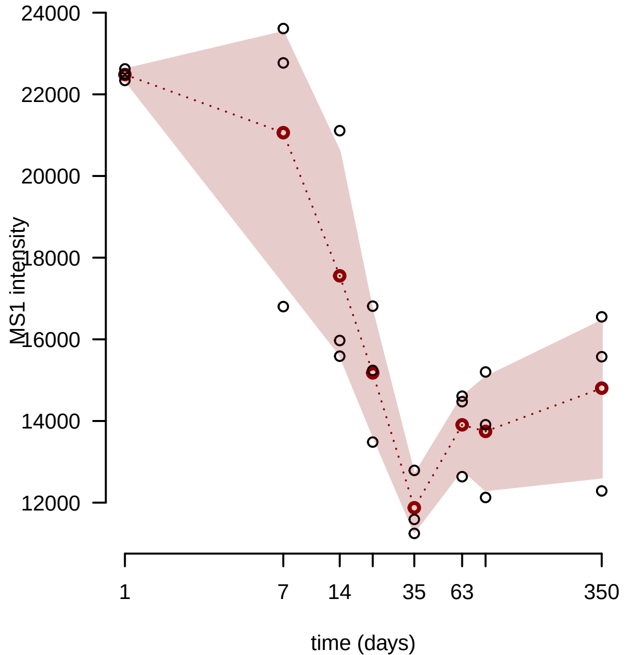

Q8C0D5
GN=Efl1
elongation factor like GTPase 1

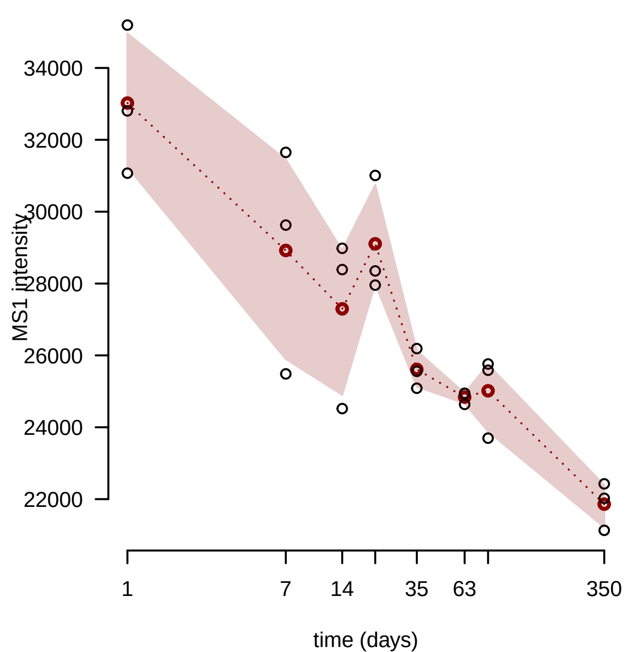

Q8C0D7
GN=Ing4
inhibitor of growth family, member 4

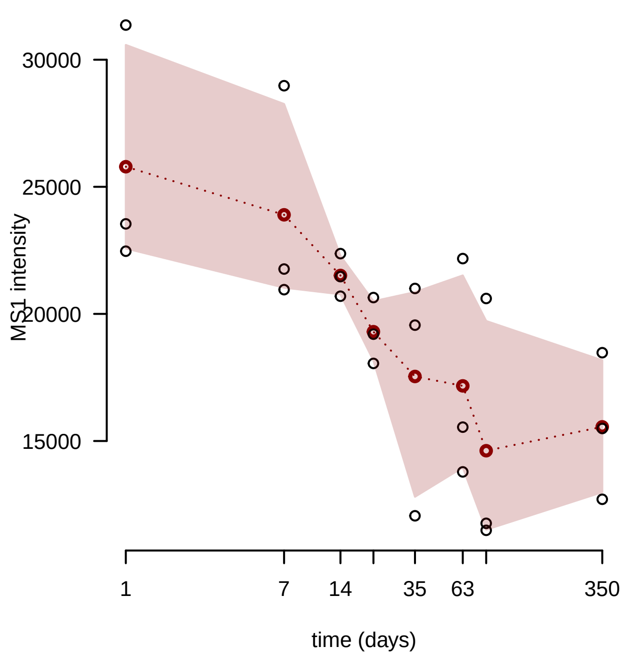

Q8C0D9
GN=Cep68
centrosomal protein 68

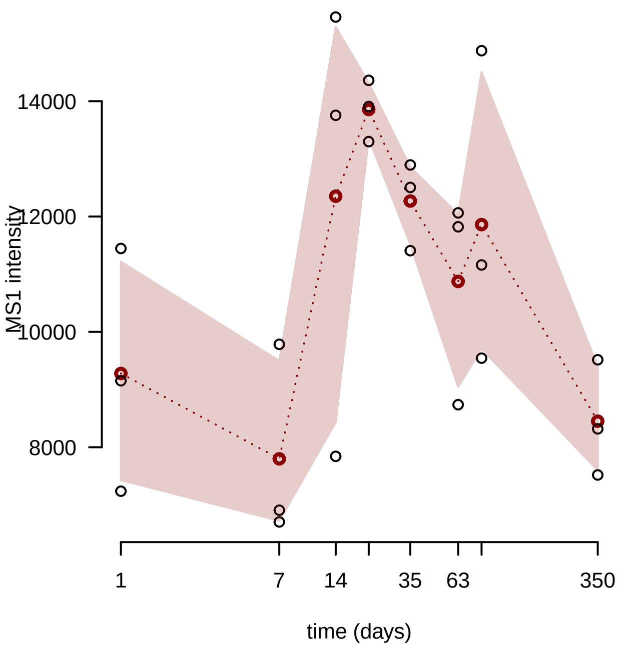

Q8C0E2
GN=Vps26b
VPS26 retromer complex component B

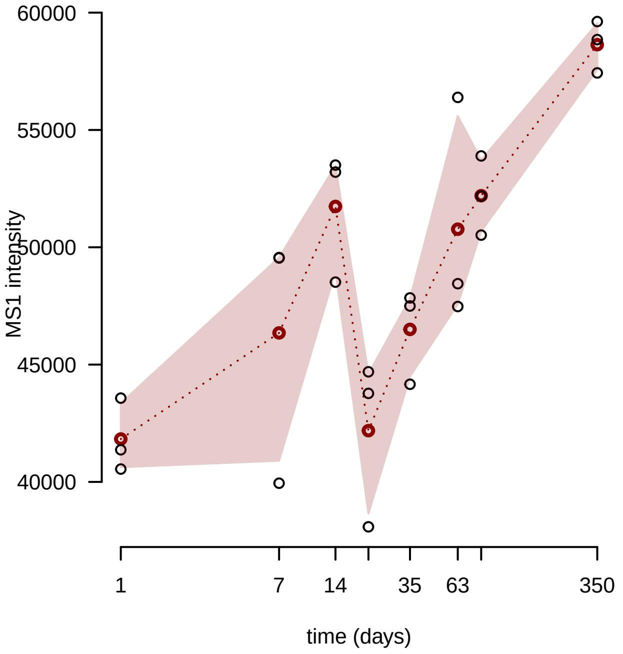

Q8C0E3
GN=Trim47
tripartite motif−containing 47

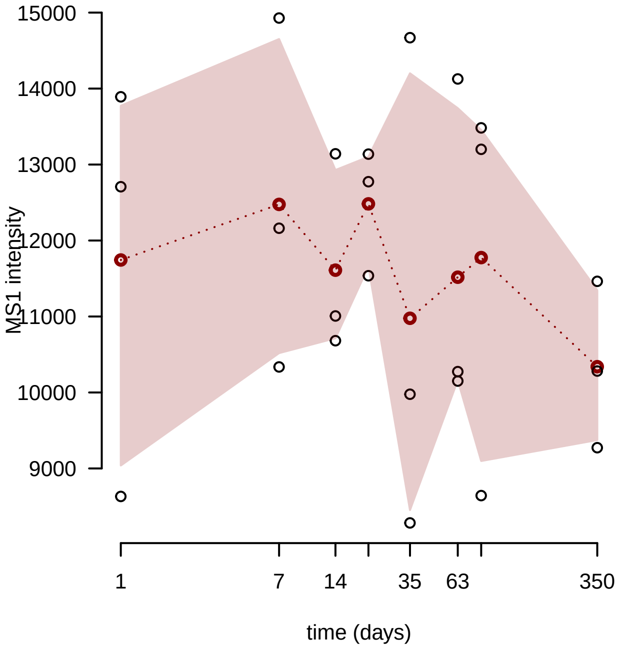

Q8C0F9
GN=Prss35
protease, serine 35

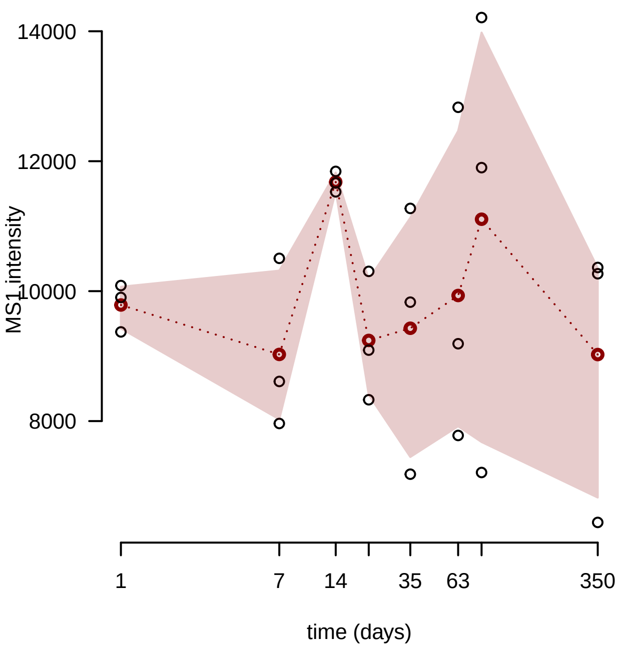

Q8C0I1
GN=Agps
alkylglycerone phosphate synthase

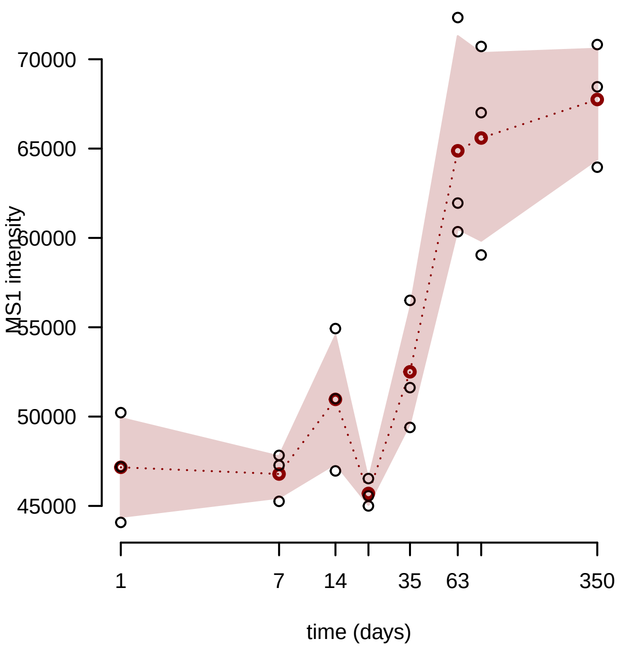

Q8C0J2
GN=Atg16l1
autophagy related 16−like 1 (S. cerevisi ...

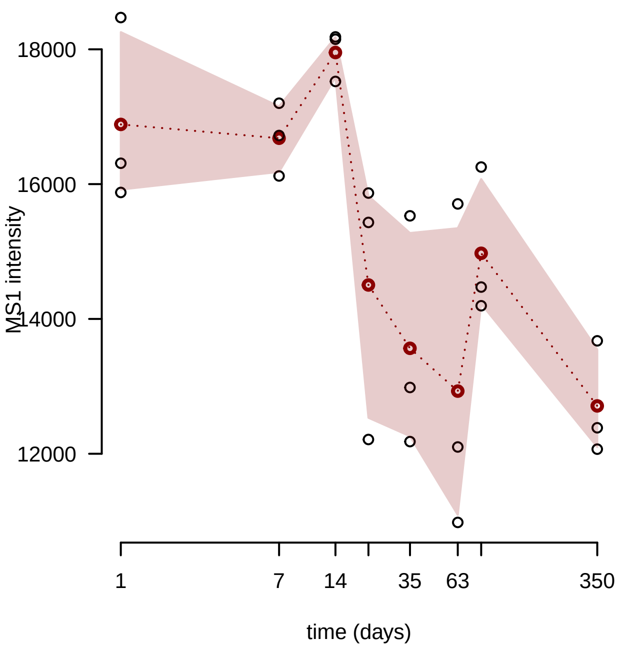

Q8C0J6
GN=Sowahc
sosondowah ankyrin repeat domain family ...

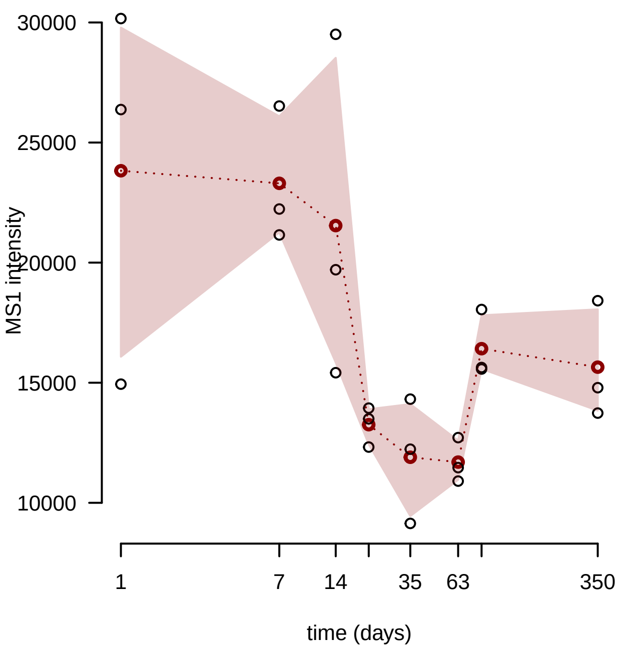

Q8C0K5
GN=Slc25a16
solute carrier family 25 (mitochondrial ...

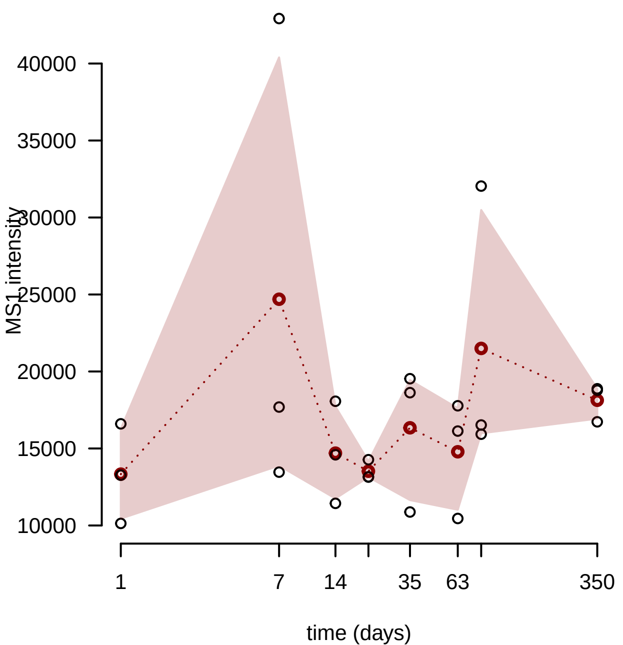

Q8C0L0
GN=Tmx4
thioredoxin−related transmembrane protei ...

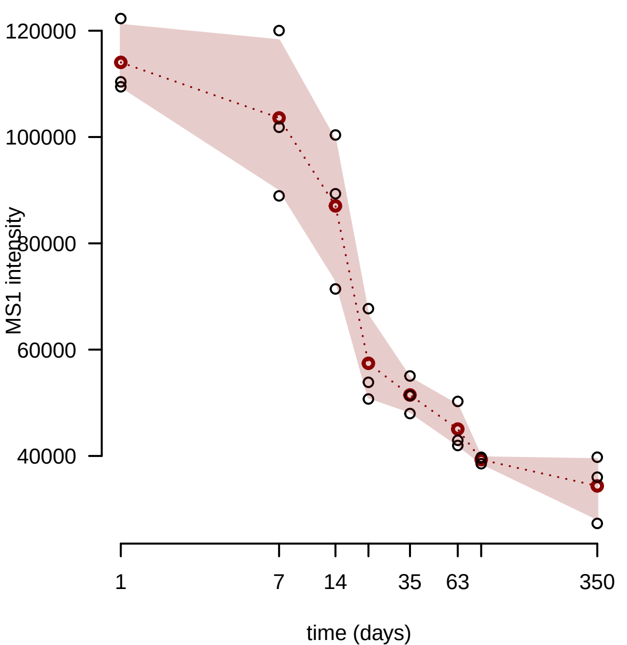

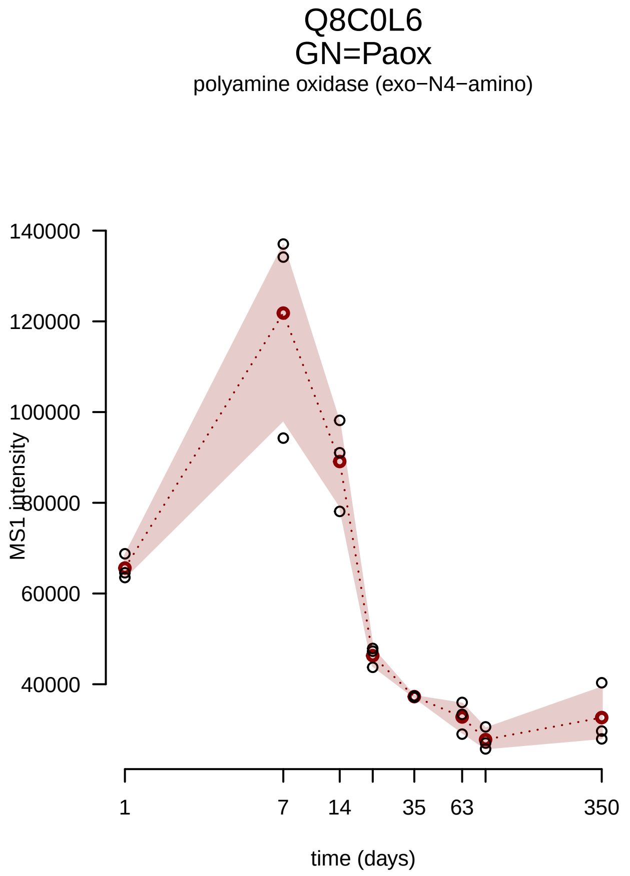
Q8C0L6
GN=Paox
polyamine oxidase (exo−N4−amino)
MS1 intensity
140000
120000
100000
80000
60000
40000
1
7
14
35
63
350
time (days)

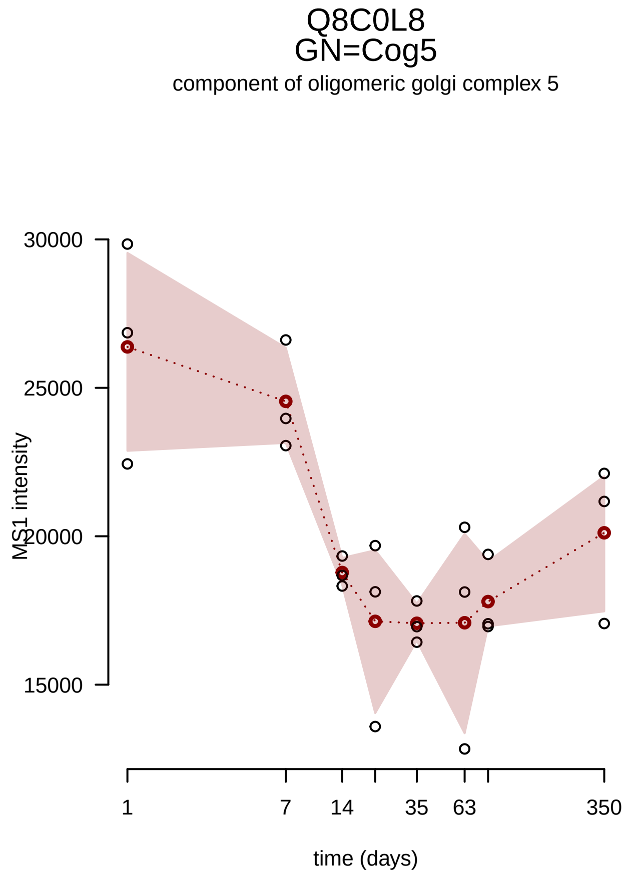
Q8C0L8
GN=Cog5
component of oligomeric golgi complex 5
MS1 intensity
30000
25000
20000
15000
1
7
14
35
63
350
time (days)

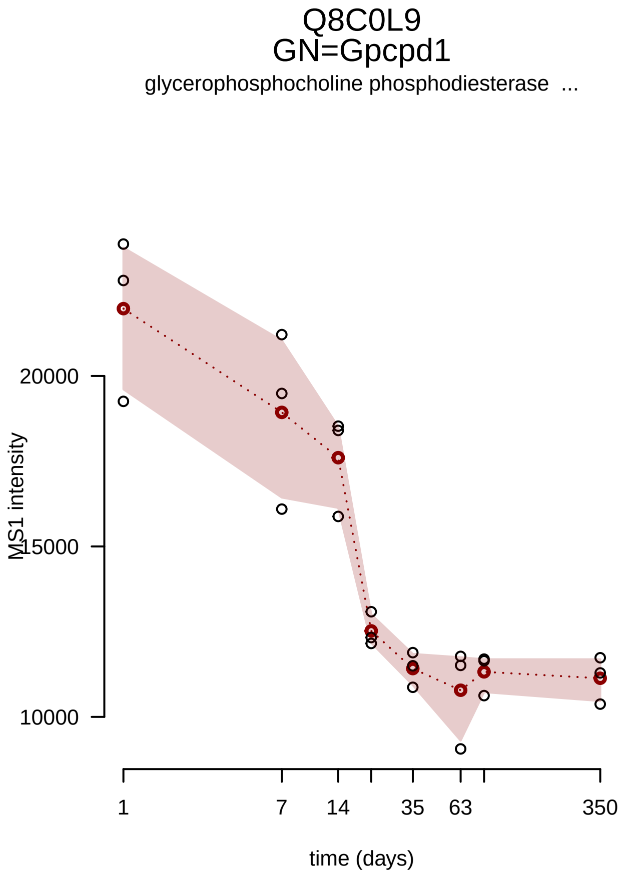
Q8C0L9
GN=Gpcpd1
glycerophosphocholine phosphodiesterase ...
MS1 intensity
20000
15000
10000
1
7
14
35
63
350
time (days)

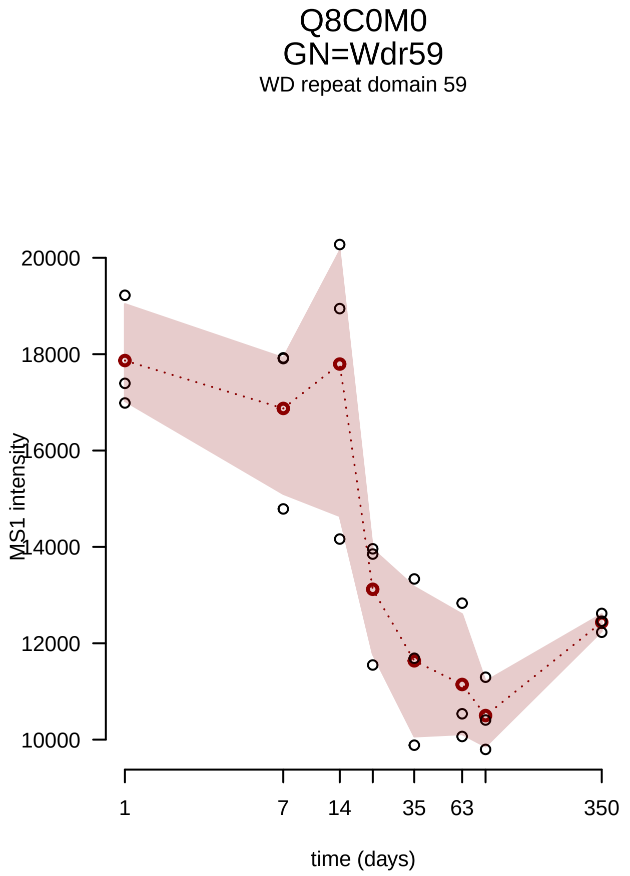
Q8C0M0
GN=Wdr59
WD repeat domain 59
MS1 intensity
20000
18000
16000
14000
12000
10000
1
7
14
35
63
350
time (days)

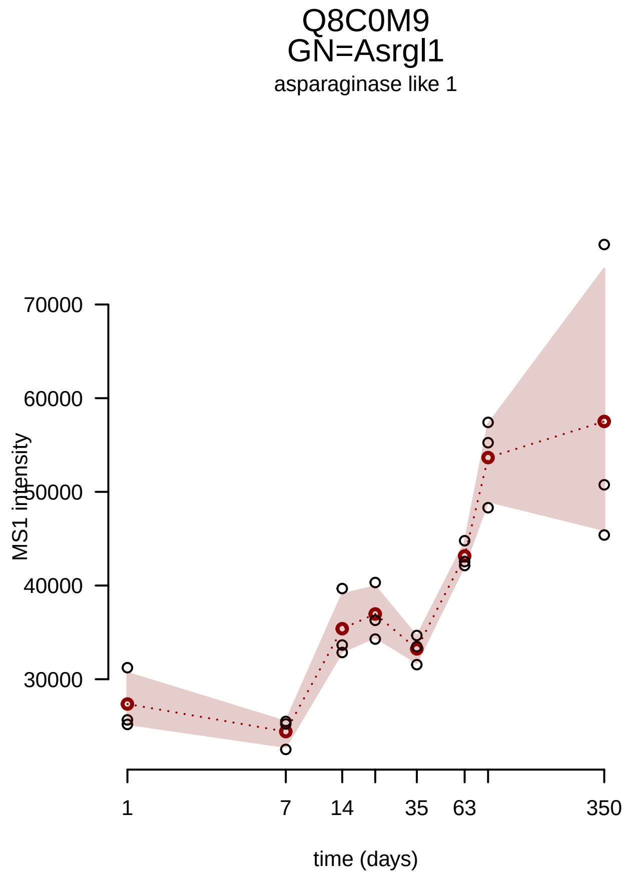
Q8C0M9
GN=Asrgl1
asparaginase like 1
MS1 intensity
70000
60000
50000
40000
30000
1
7
14
35
63
350
time (days)

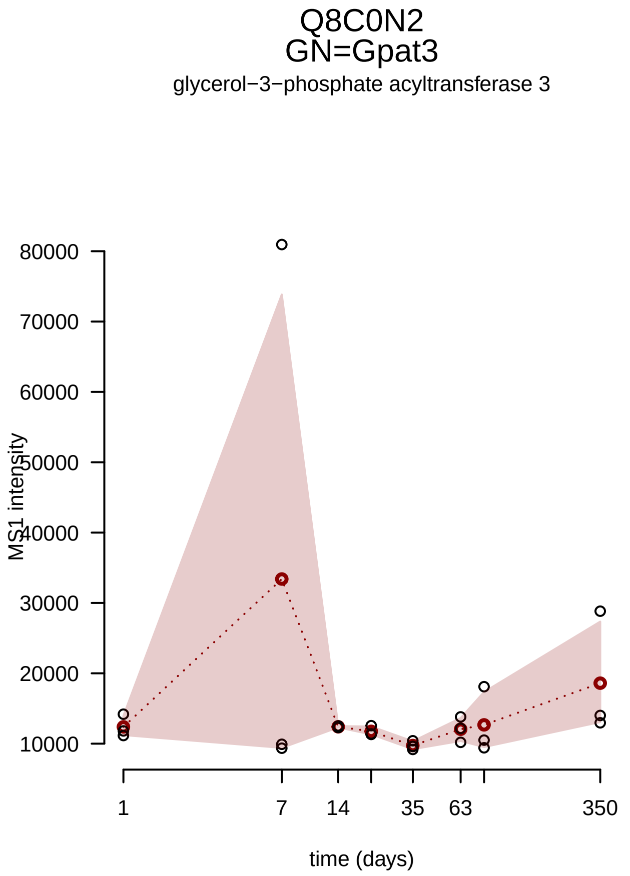
Q8C0N2
GN=Gpat3
glycerol−3−phosphate acyltransferase 3
MS1 intensity
80000
70000
60000
50000
40000
30000
20000
10000
1
7
14
35
63
350
time (days)

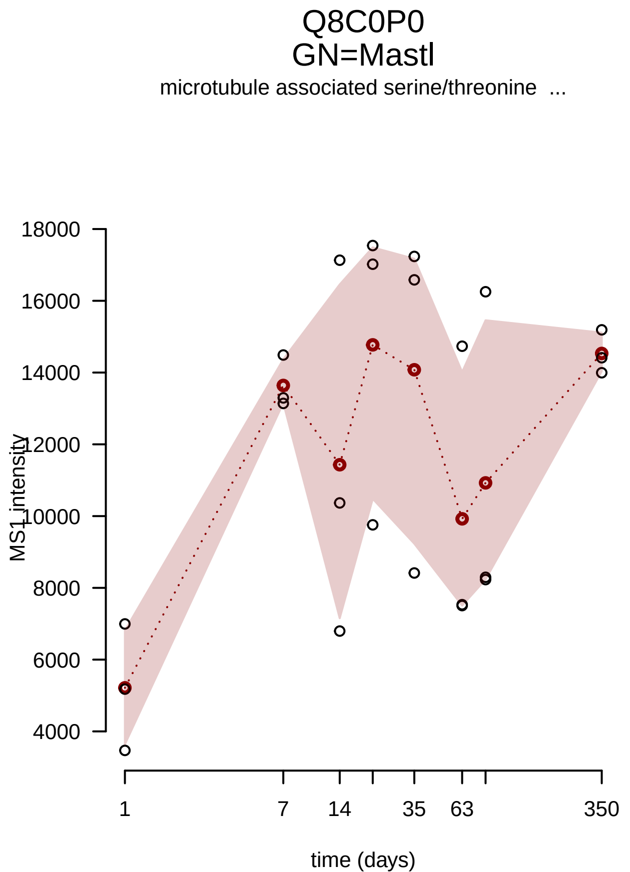
Q8C0P0
GN=Mastl
microtubule associated serine/threonine ...
MS1 intensity
18000
16000
14000
12000
10000
8000
6000
4000
1
7
14
35
63
350
time (days)

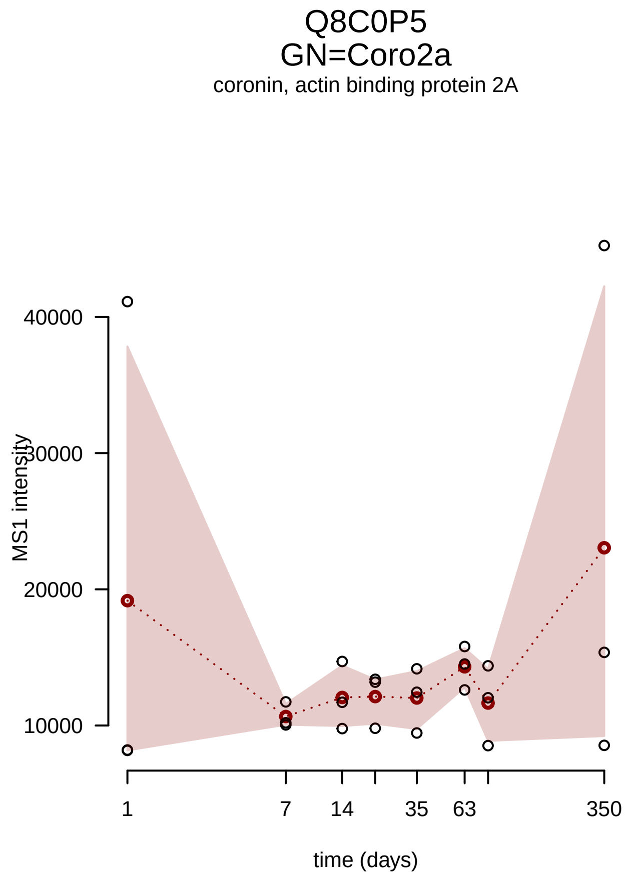
Q8C0P5
GN=Coro2a
coronin, actin binding protein 2A
MS1 intensity
40000
30000
20000
10000
1
7
14
35
63
350
time (days)

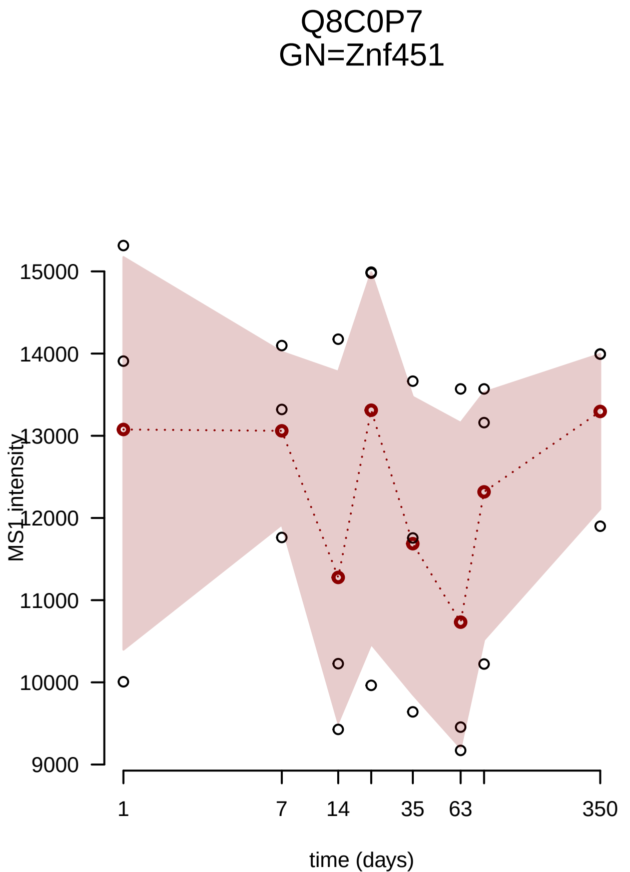
Q8C0P7
GN=Znf451
MS1 intensity
15000
14000
13000
12000
11000
10000
9000
1
7
14
35
63
350
time (days)

Q8C0Q2
GN=Zhx3
zinc fingers and homeoboxes 3

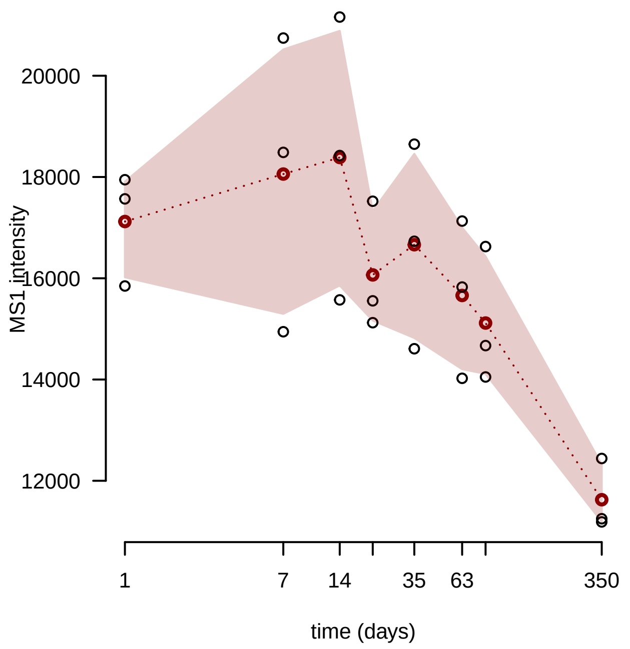

Q8C0Q9
GN=Rapgef5
Rap guanine nucleotide exchange factor (...

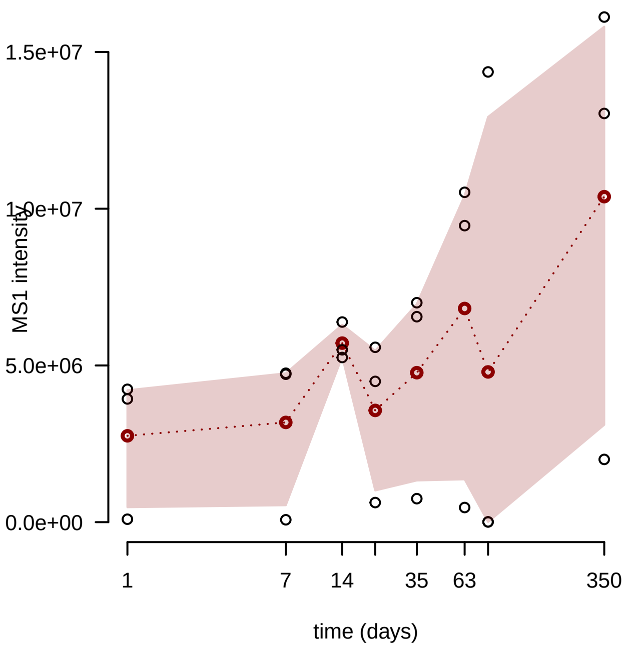

Q8C0R0
GN=Usp37
ubiquitin specific peptidase 37

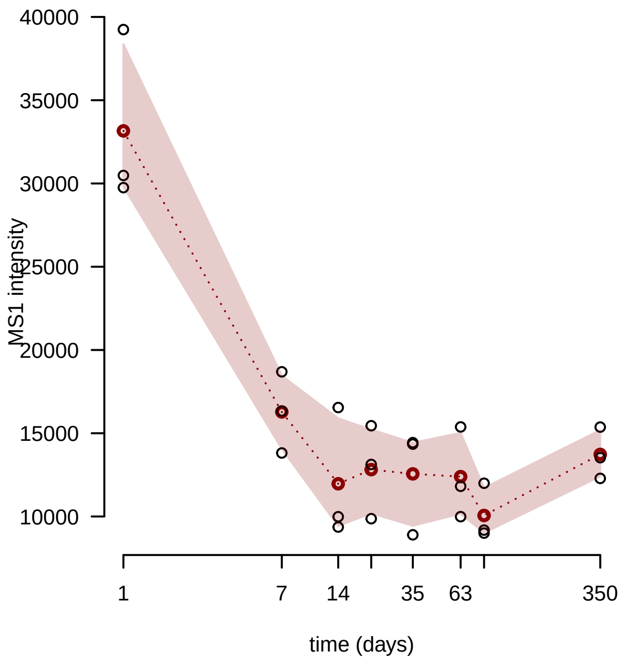

Q8C0S1
GN=Dis3l
DIS3 like exosome 3'−5' exoribonuclease

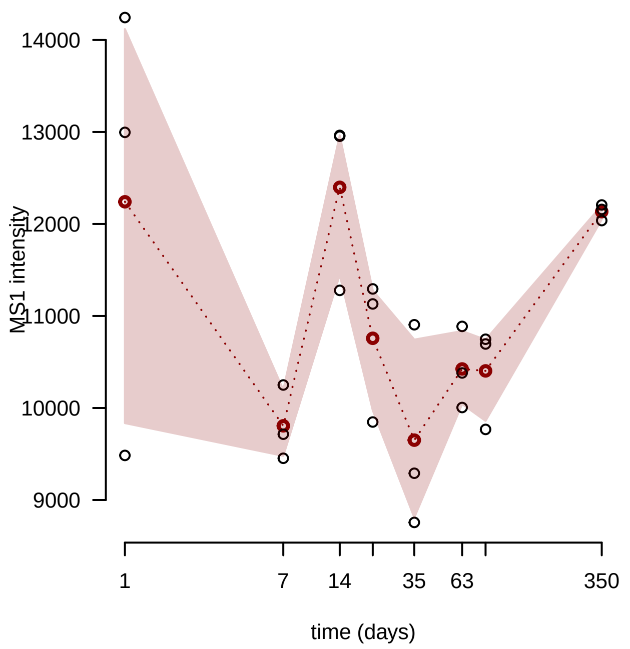

Q8C0V0
GN=Tlk1
tousled−like kinase 1

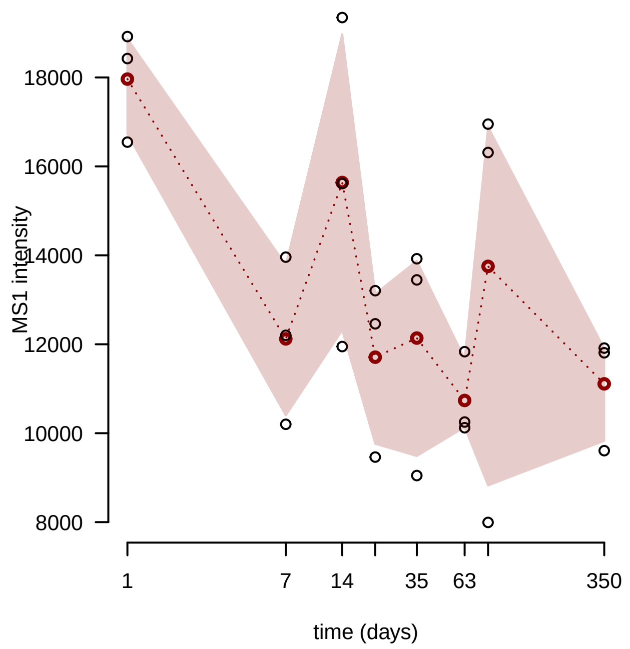

Q8C0V9
GN=Frmd6
FERM domain containing 6

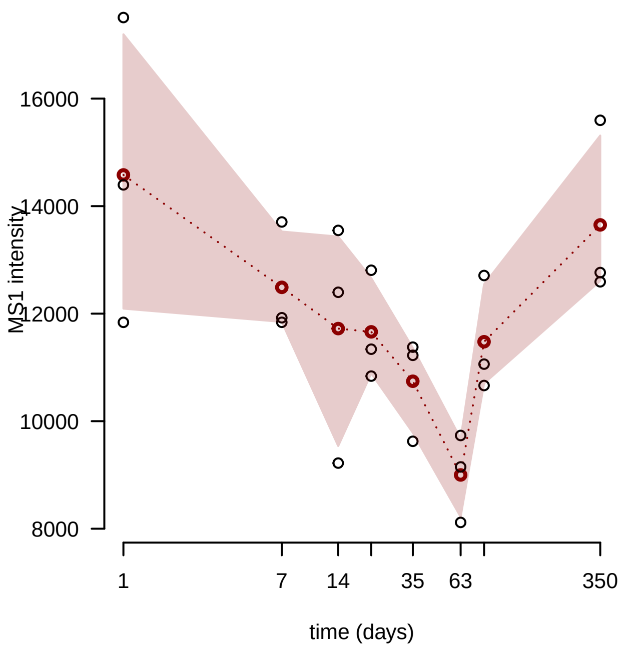

Q8C0Y0
GN=Ppp4r4
protein phosphatase 4, regulatory subuni ...

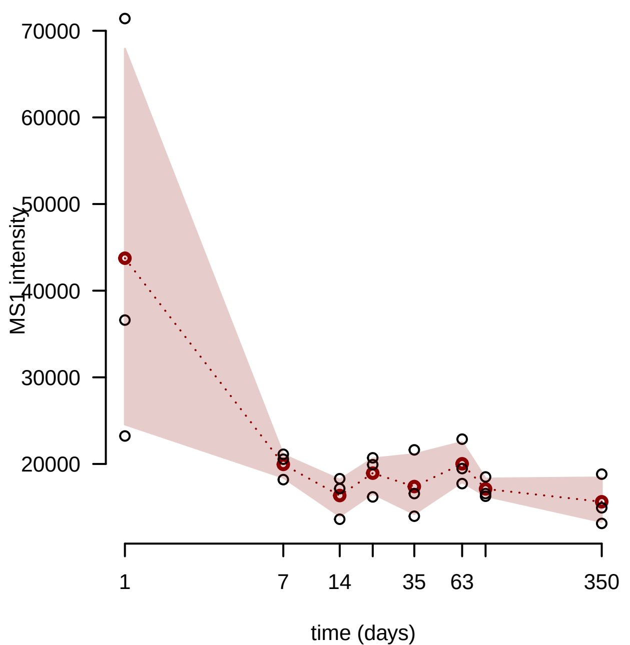

Q8C0Y1
GN=Tgif2
TGFB−induced factor homeobox 2

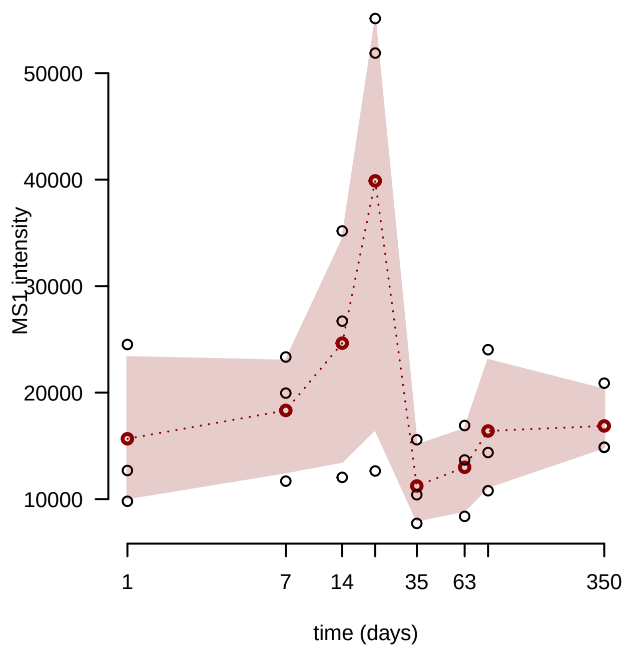

Q8C0Z1
GN=Fam234a
family with sequence similarity 234, mem ...

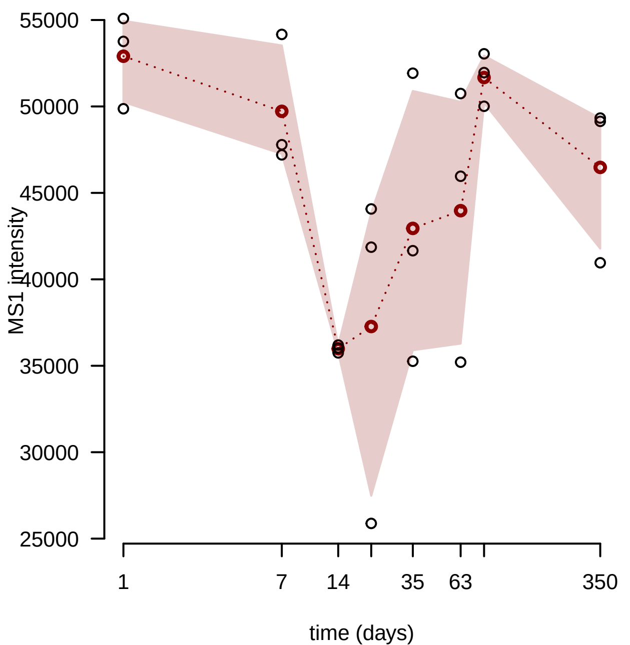

Q8C129
GN=Lnpep
leucyl/cystinyl aminopeptidase

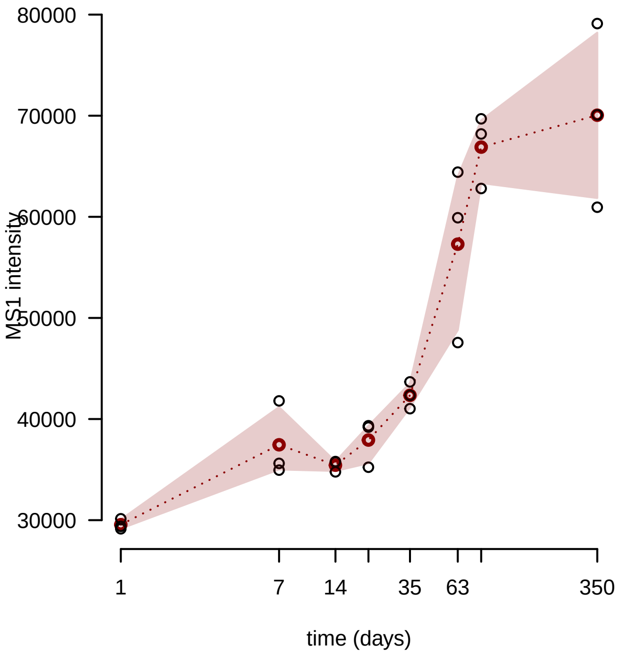

Q8C142
GN=Ldlrap1
low density lipoprotein receptor adaptor ...

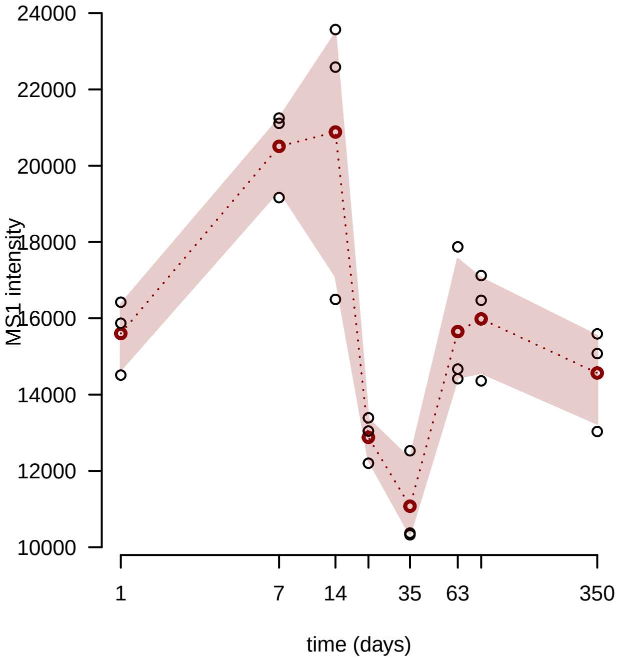

Q8C145
GN=Slc39a6
solute carrier family 39 (metal ion tran ...

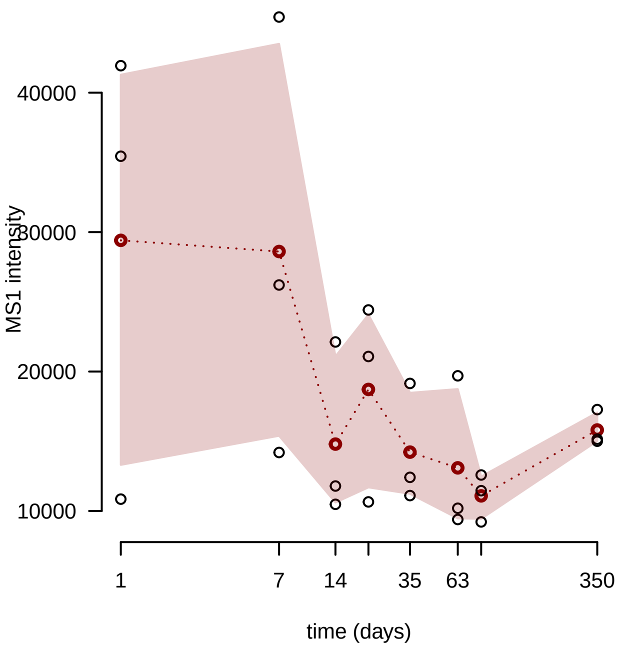

Q8C147
GN=Dock8
dedicator of cytokinesis 8

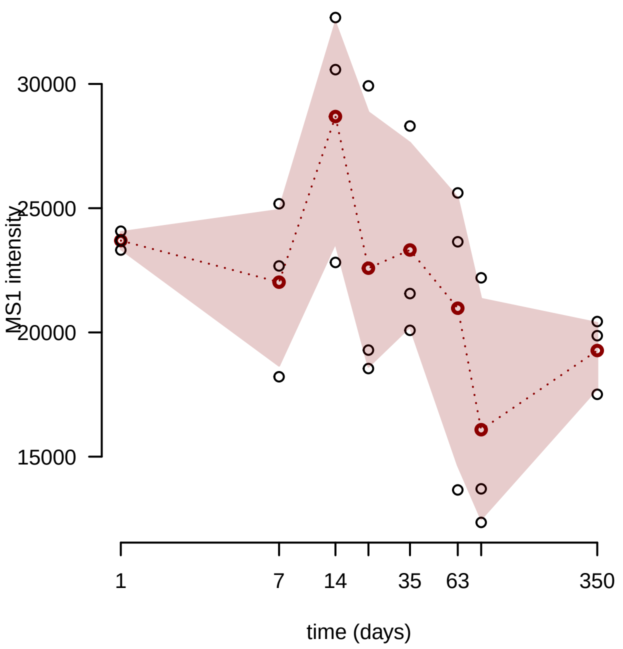

Q8C156
GN=Ncaph
non−SMC condensin I complex, subunit H

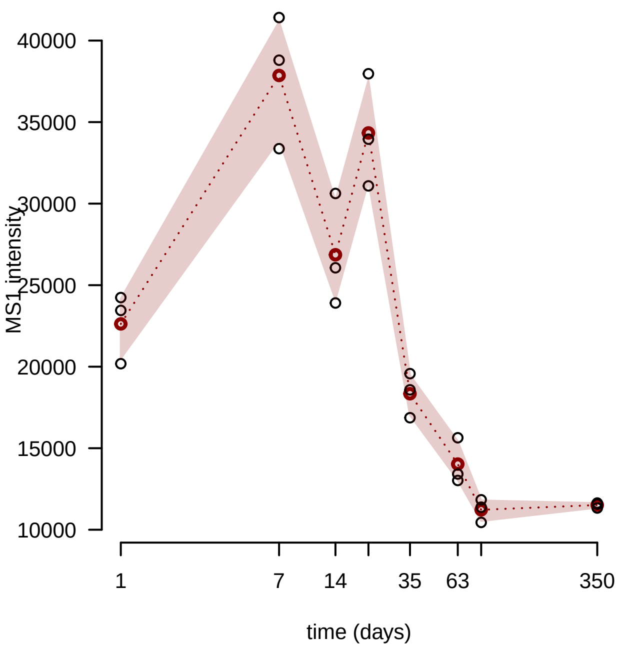

Q8C163
GN=Exog
endo/exonuclease (5'−3'), endonuclease G ...

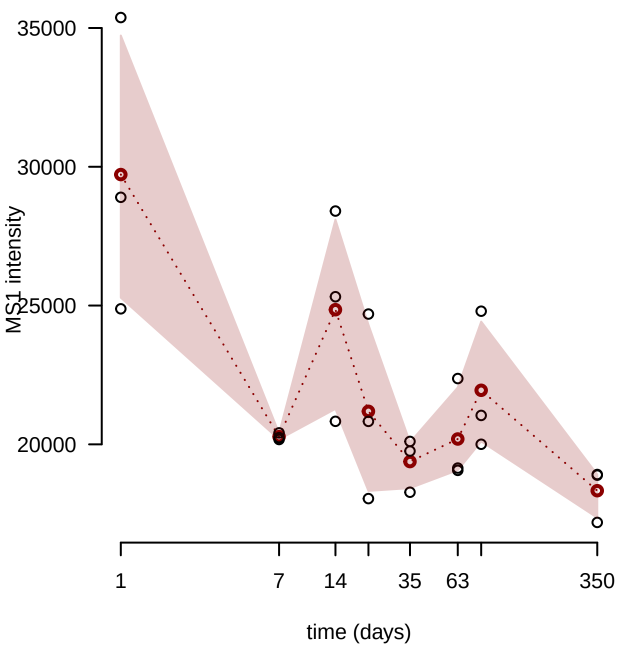

Q8C165
GN=Pm20d1
peptidase M20 domain containing 1

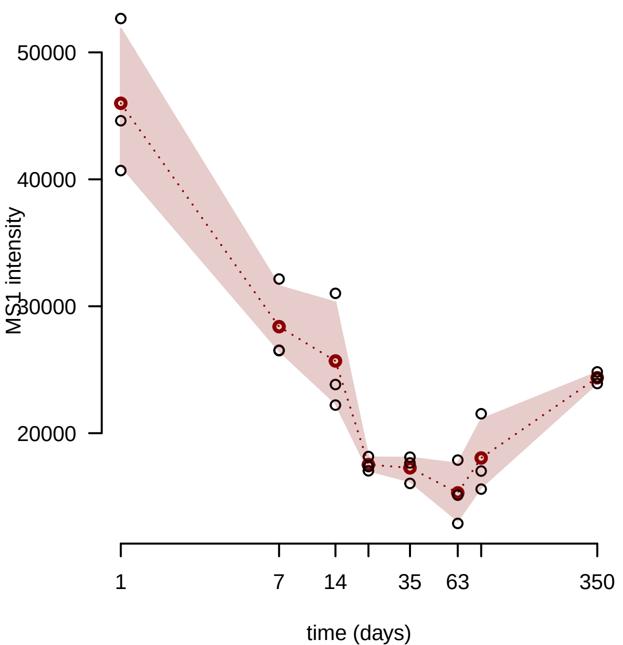

Q8C166
GN=Cpne1
copine I

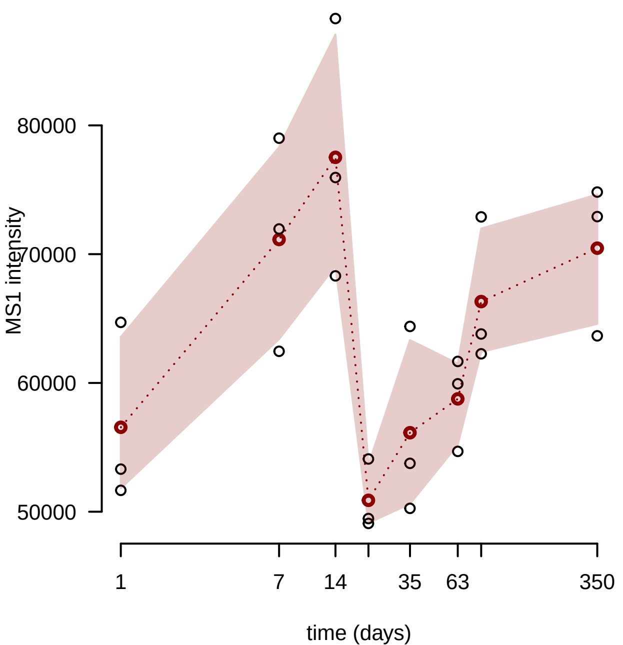

Q8C167
GN=Prepl
prolyl endopeptidase−like

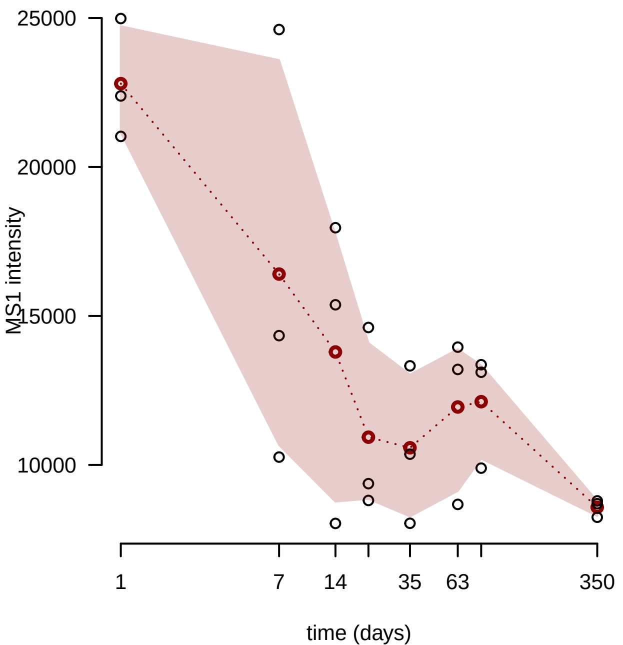

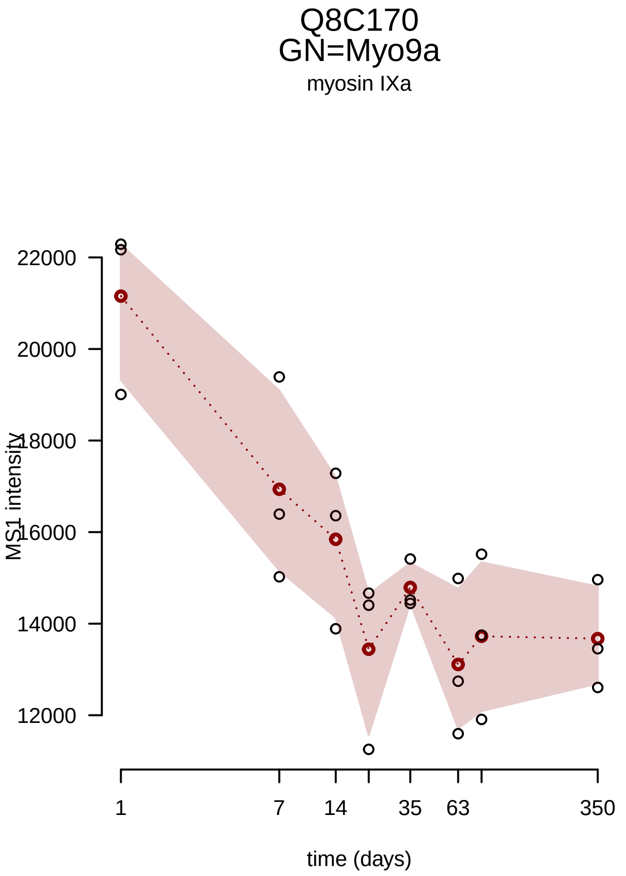
Q8C170
GN=Myo9a
myosin IXa
22000
20000
18000
16000
14000
12000
MS1 intensity
1
7
14
35
63
350
time (days)

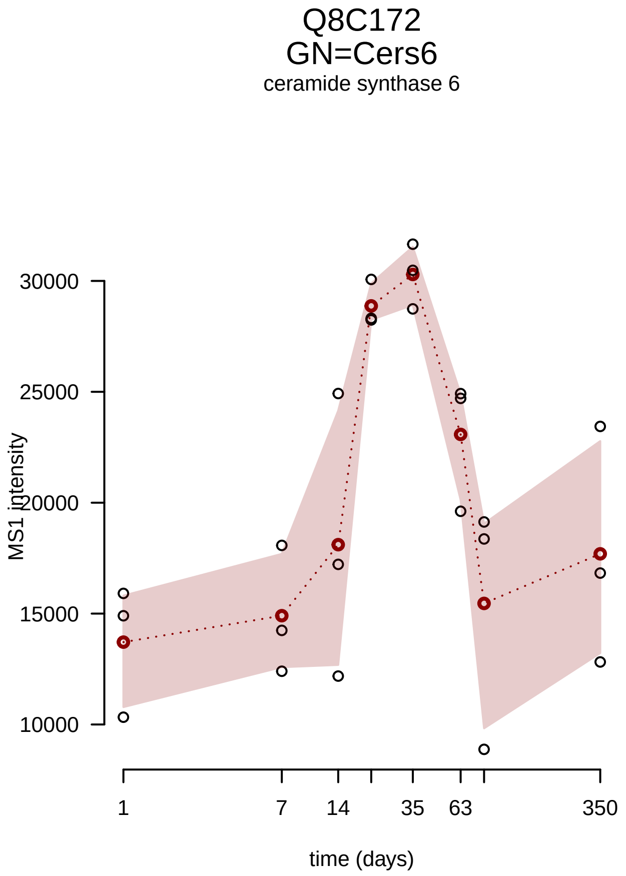
Q8C172
GN=Cers6
ceramide synthase 6
30000
25000
20000
15000
10000
MS1 intensity
1
7
14
35
63
350
time (days)

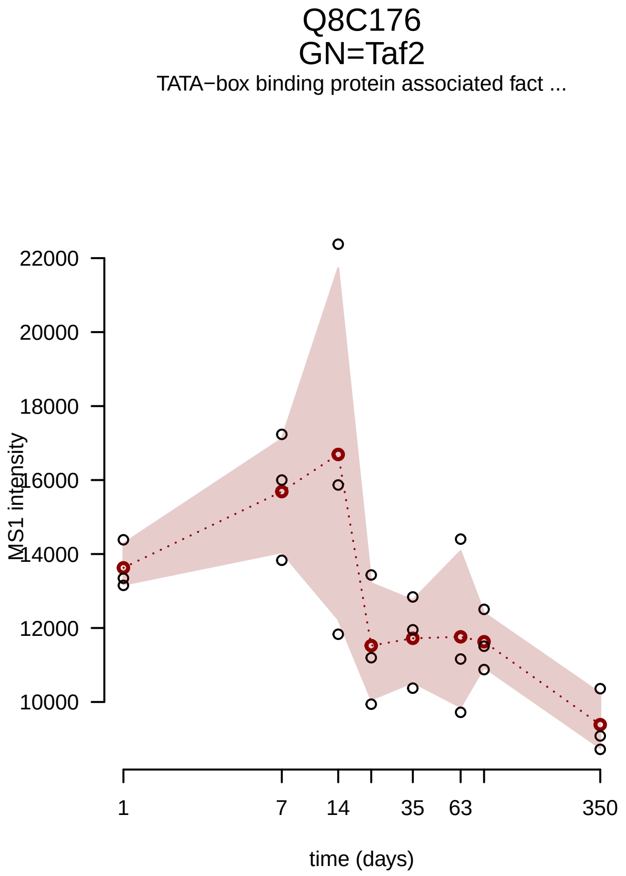
Q8C176
GN=Taf2
TATA−box binding protein associated fact ...
22000
20000
18000
16000
14000
12000
10000
MS1 intensity
1
7
14
35
63
350
time (days)

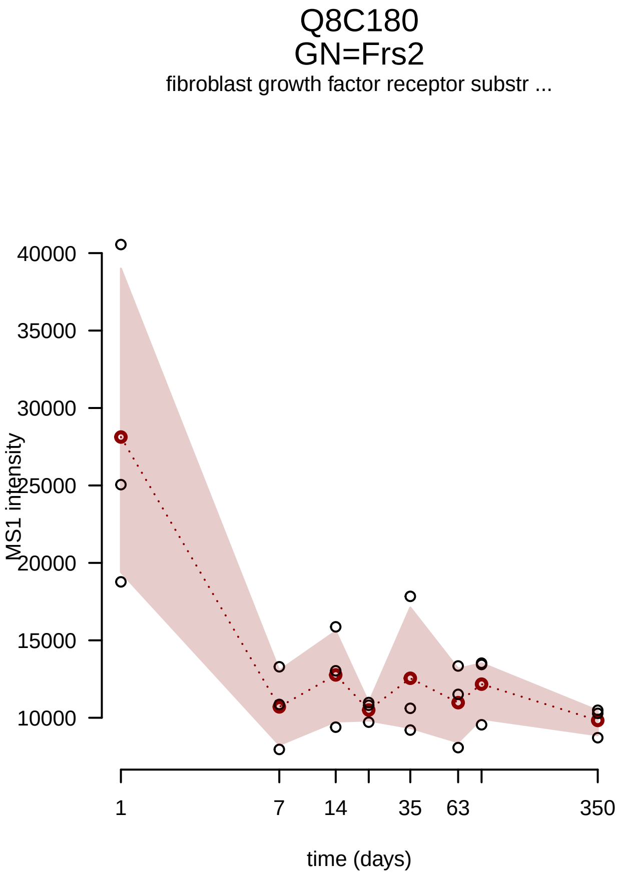
Q8C180
GN=Frs2
fibroblast growth factor receptor substr ...
40000
35000
30000
25000
20000
15000
10000
MS1 intensity
1
7
14
35
63
350
time (days)

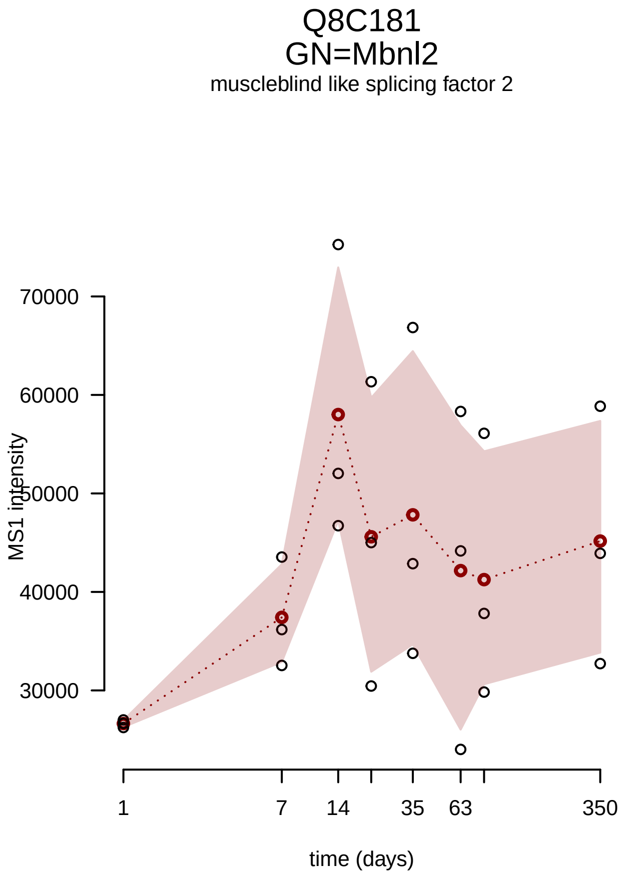
Q8C181
GN=Mbnl2
muscleblind like splicing factor 2
70000
60000
50000
40000
30000
MS1 intensity
1
7
14
35
63
350
time (days)

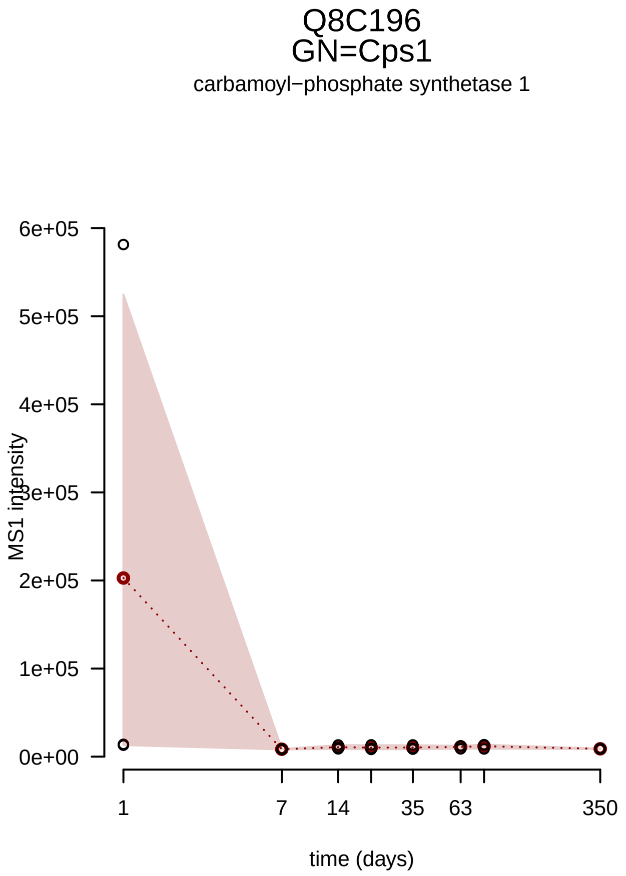
Q8C196
GN=Cps1
carbamoyl−phosphate synthetase 1
6e+05
5e+05
4e+05
3e+05
2e+05
1e+05
0e+00
MS1 intensity
1
7
14
35
63
350
time (days)

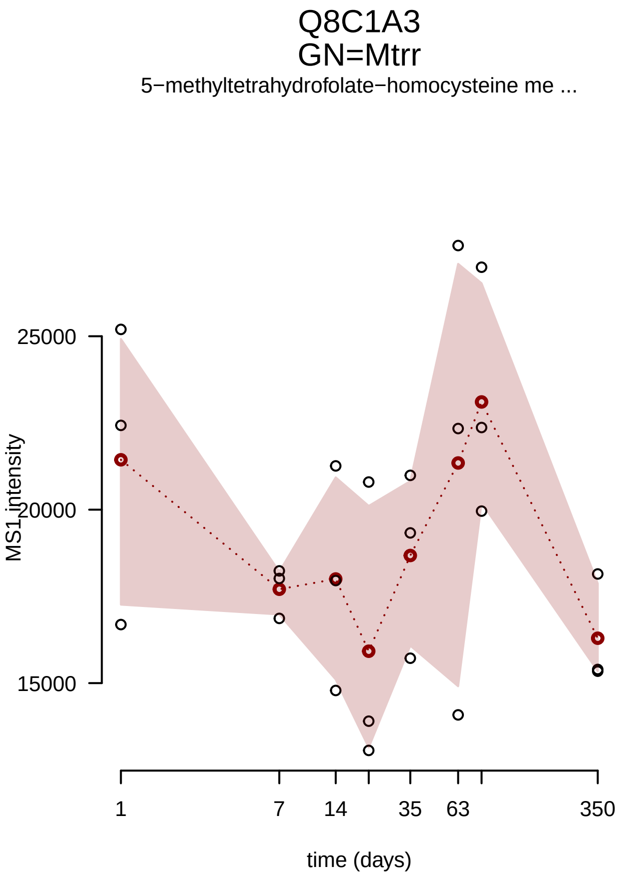
Q8C1A3
GN=Mtrr
5−methyltetrahydrofolate−homocysteine me ...
25000
20000
15000
MS1 intensity
1
7
14
35
63
350
time (days)

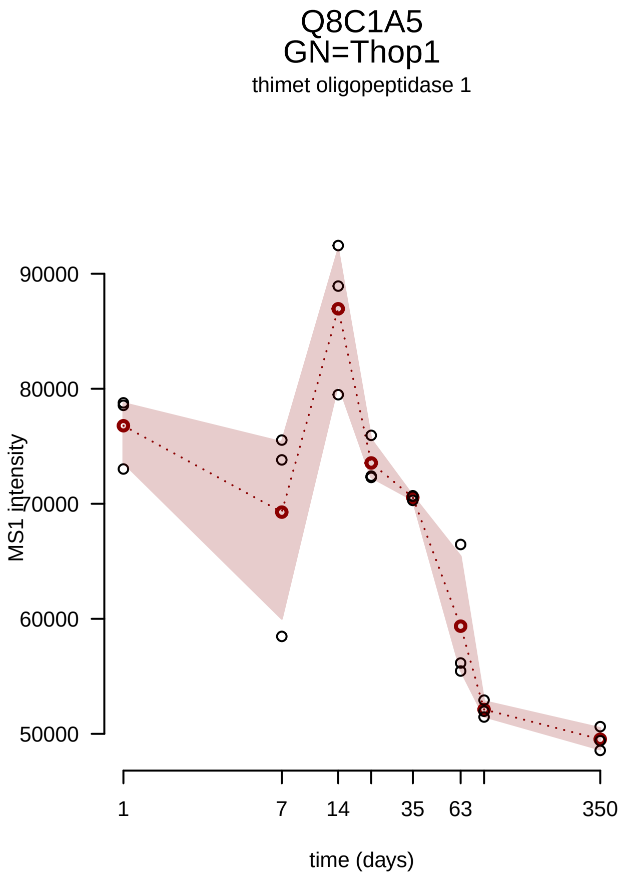
Q8C1A5
GN=Thop1
thimet oligopeptidase 1
90000
80000
70000
60000
50000
MS1 intensity
1
7
14
35
63
350
time (days)

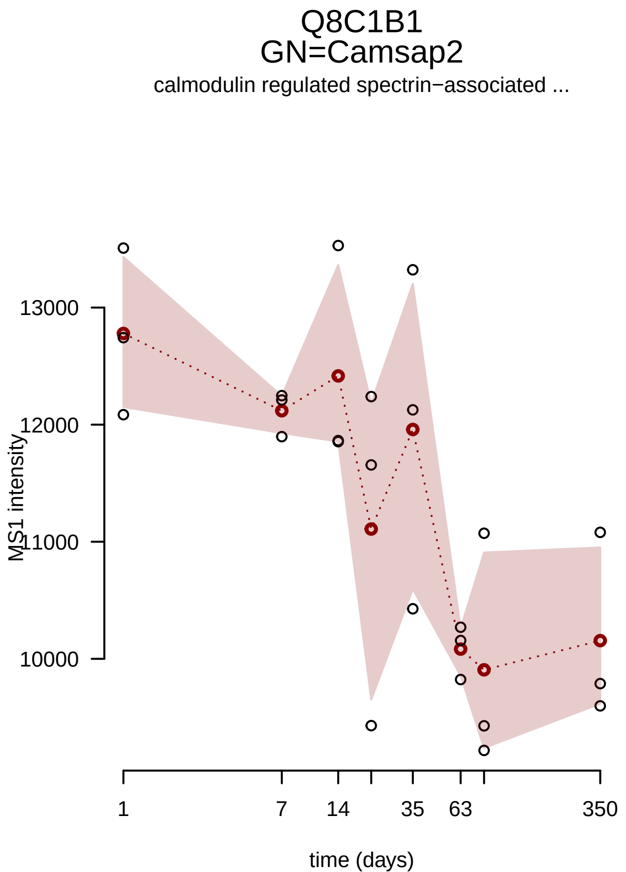
Q8C1B1
GN=Camsap2
calmodulin regulated spectrin−associated ...
13000
12000
11000
10000
MS1 intensity
1
7
14
35
63
350
time (days)

Q8C1B7
GN=Sept11

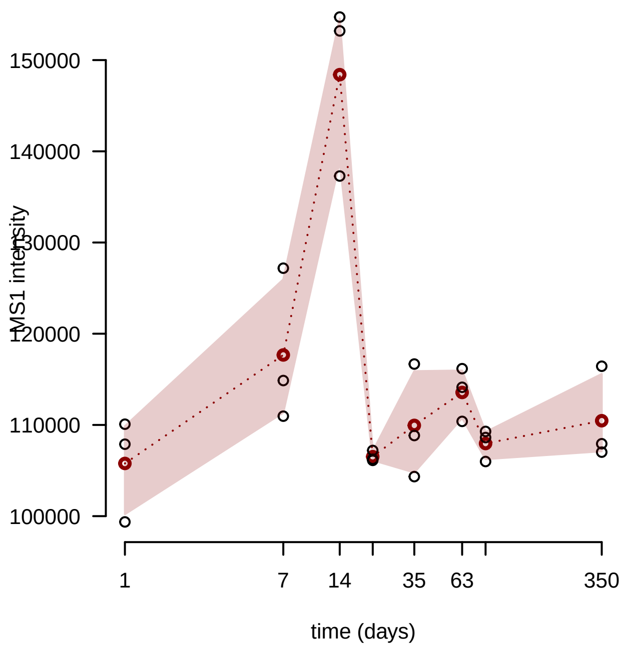

Q8C1D8
GN=Iws1
IWS1, SUPT6 interacting protein

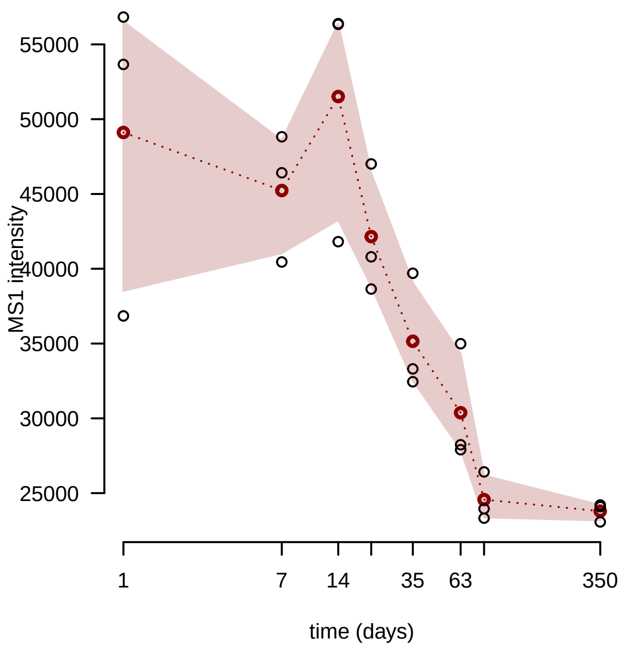

Q8C1E7
GN=Tmem120a
transmembrane protein 120A

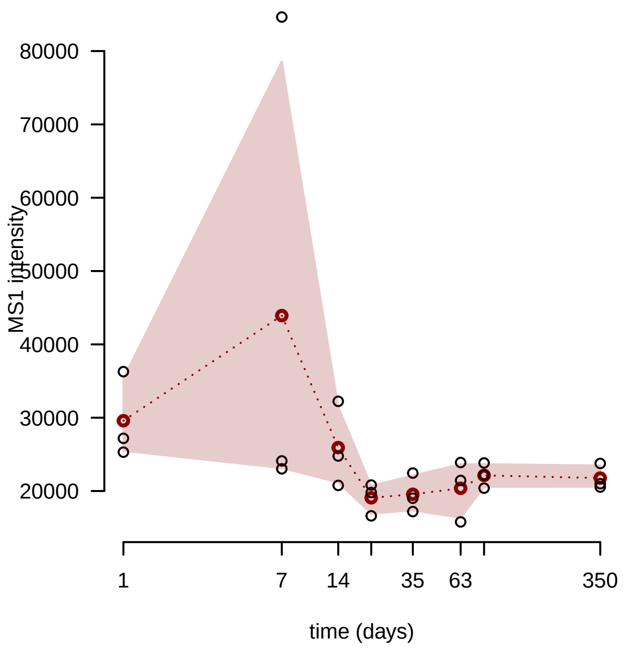

Q8C1F4
GN=Csgalnact2
chondroitin sulfate N−acetylgalactosamin ...

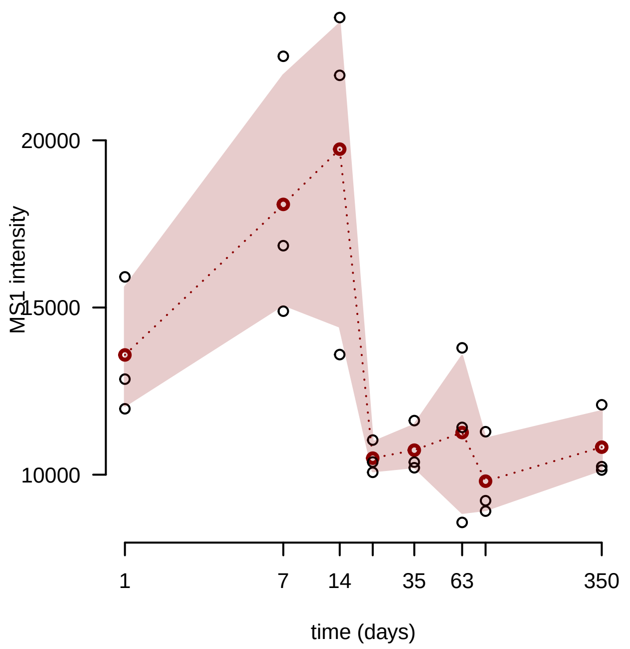

Q8C1M2
GN=Znf428

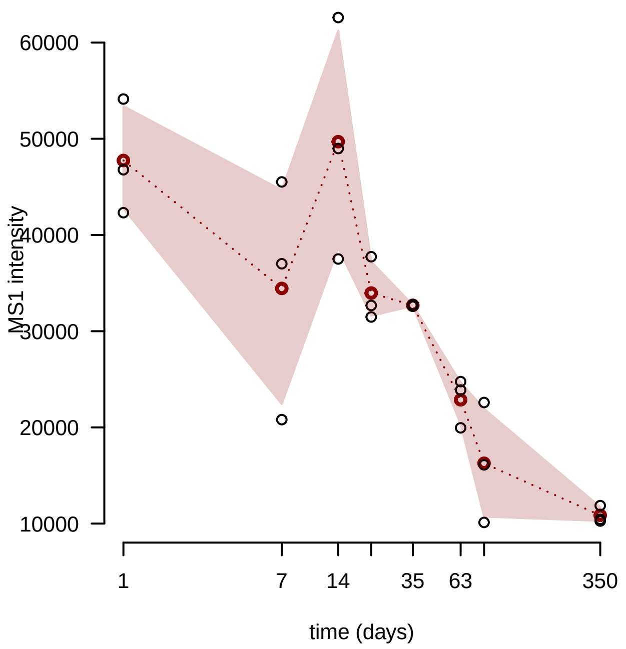

Q8C1Q6
GN=Smim4
small integral membrane protein 4

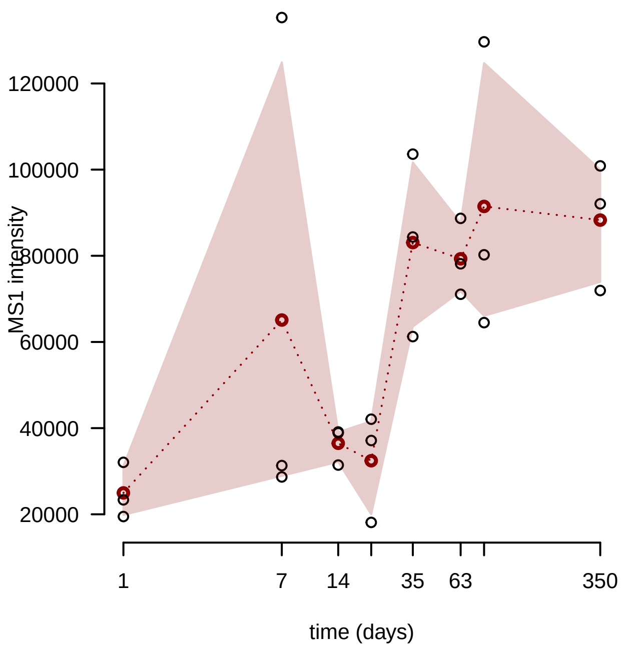

Q8C1S0
GN=Med19
mediator complex subunit 19

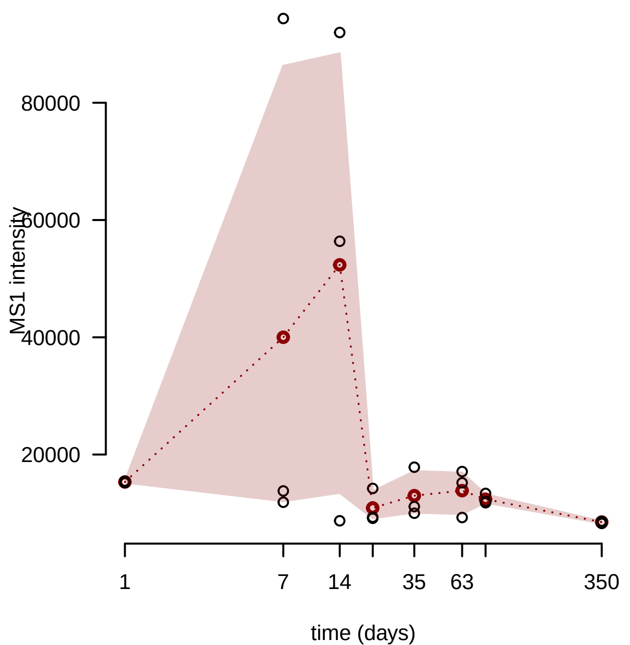

Q8C1Y8
GN=Ccz1
CCZ1 vacuolar protein trafficking and bi ...

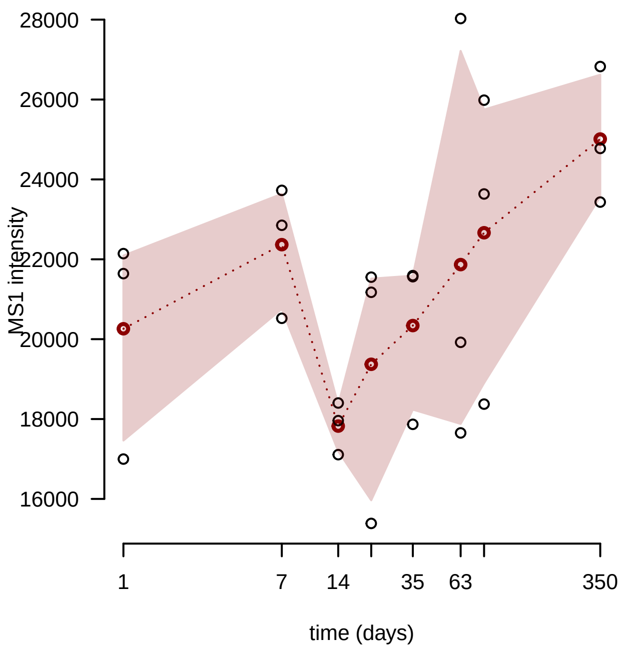

Q8C1Z7
GN=Bbs4
Bardet−Biedl syndrome 4 (human)

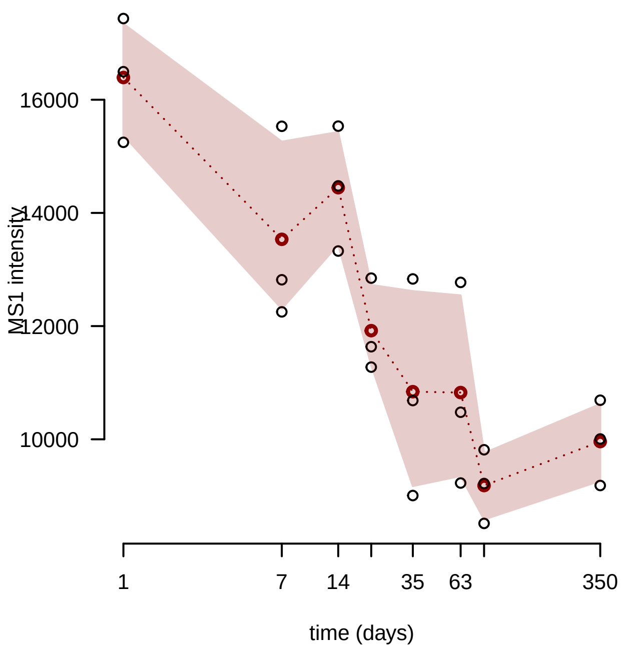

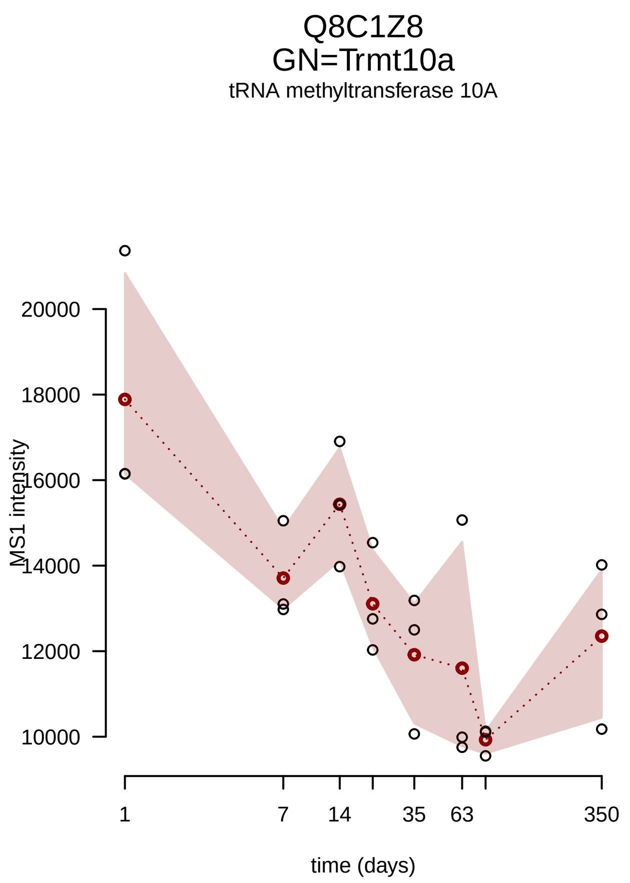
Q8C1Z8
GN=Trmt10a
tRNA methyltransferase 10A
MS1 intensity
time (days)

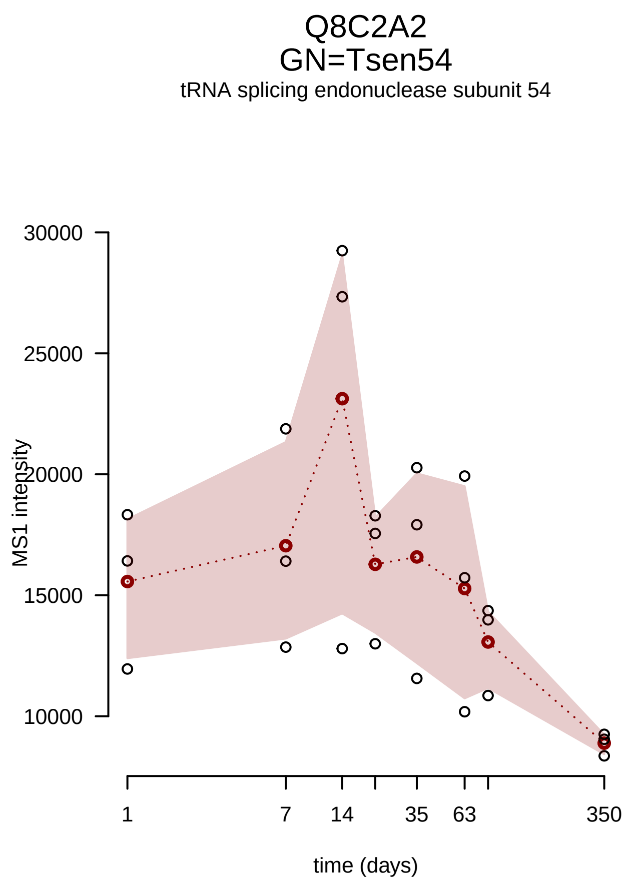
Q8C2A2
GN=Tsen54
tRNA splicing endonuclease subunit 54
MS1 intensity
time (days)

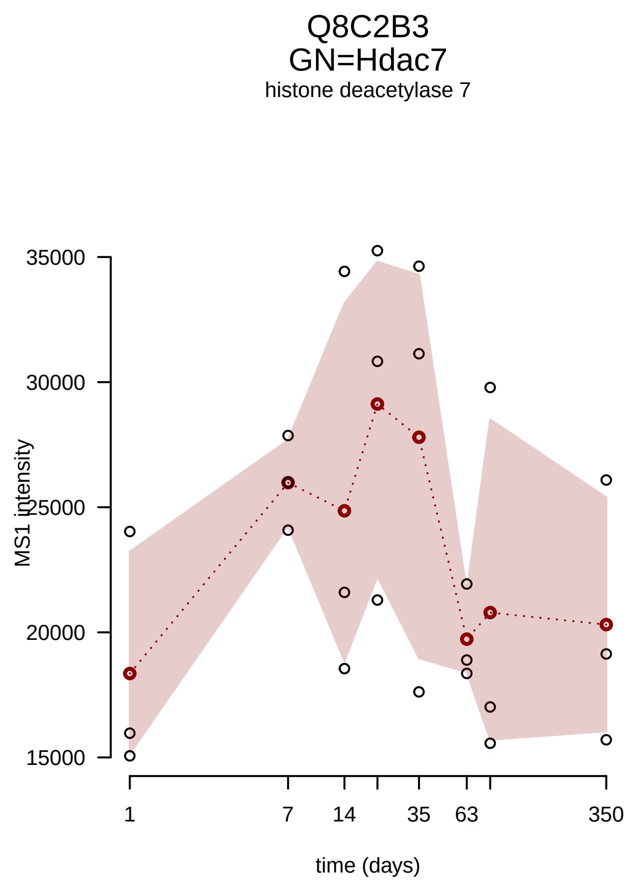
Q8C2B3
GN=Hdac7
histone deacetylase 7
MS1 intensity
time (days)

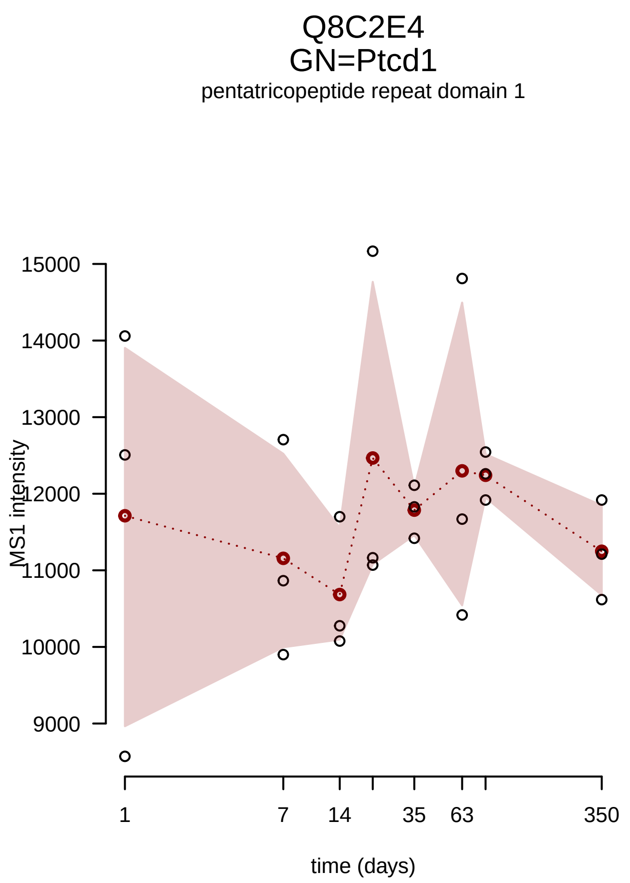
Q8C2E4
GN=Ptcd1
pentatricopeptide repeat domain 1
MS1 intensity
time (days)

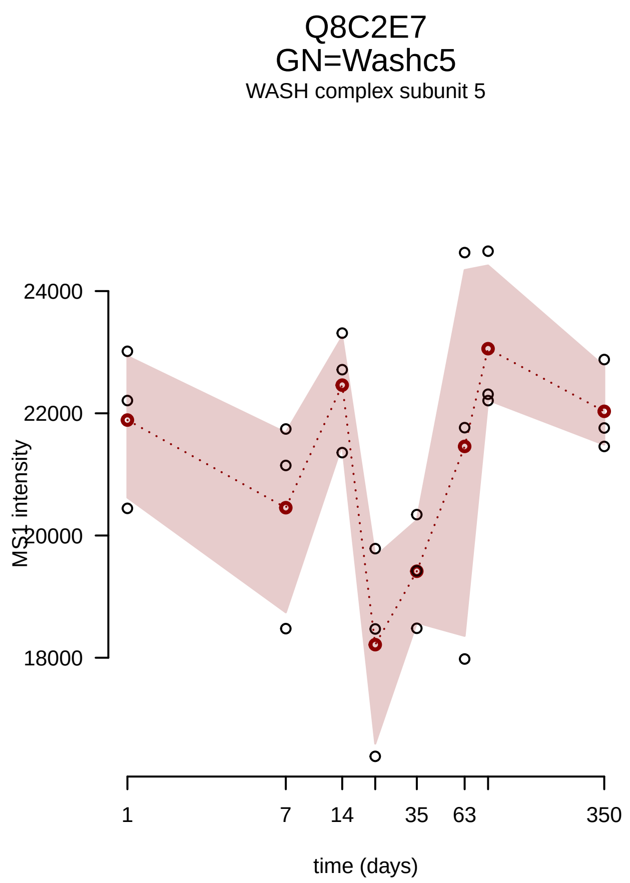
Q8C2E7
GN=Washc5
WASH complex subunit 5
MS1 intensity
time (days)

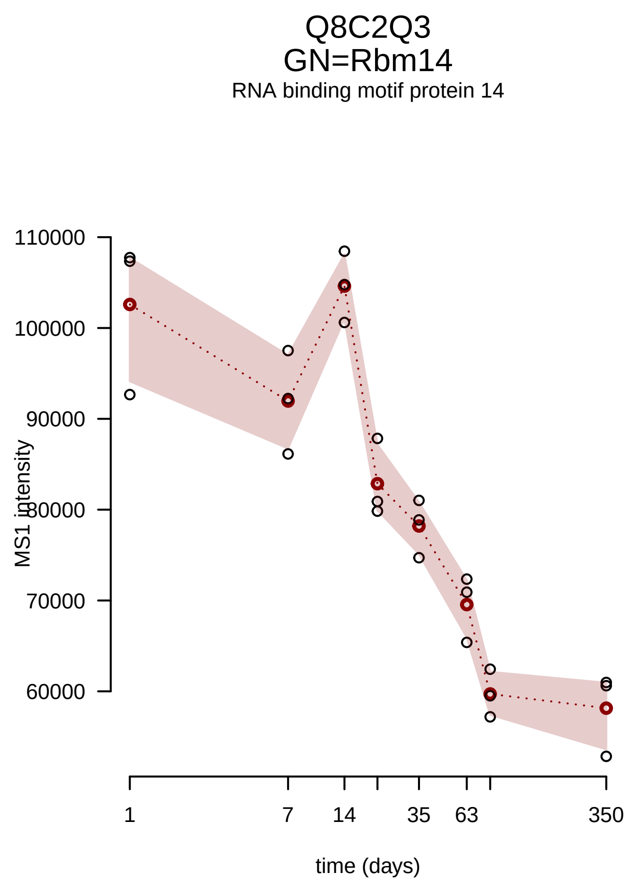
Q8C2Q3
GN=Rbm14
RNA binding motif protein 14
MS1 intensity
time (days)

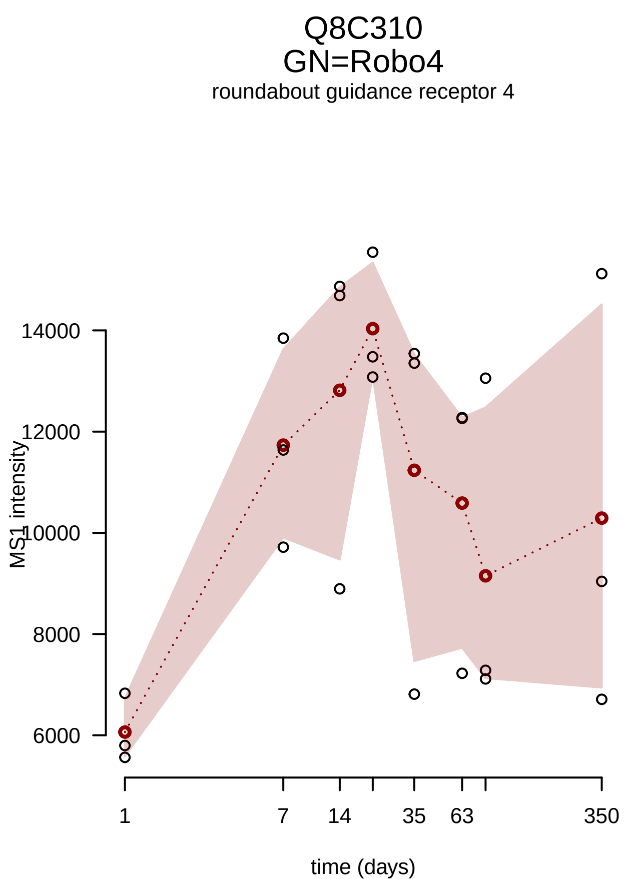
Q8C310
GN=Robo4
roundabout guidance receptor 4
MS1 intensity
time (days)

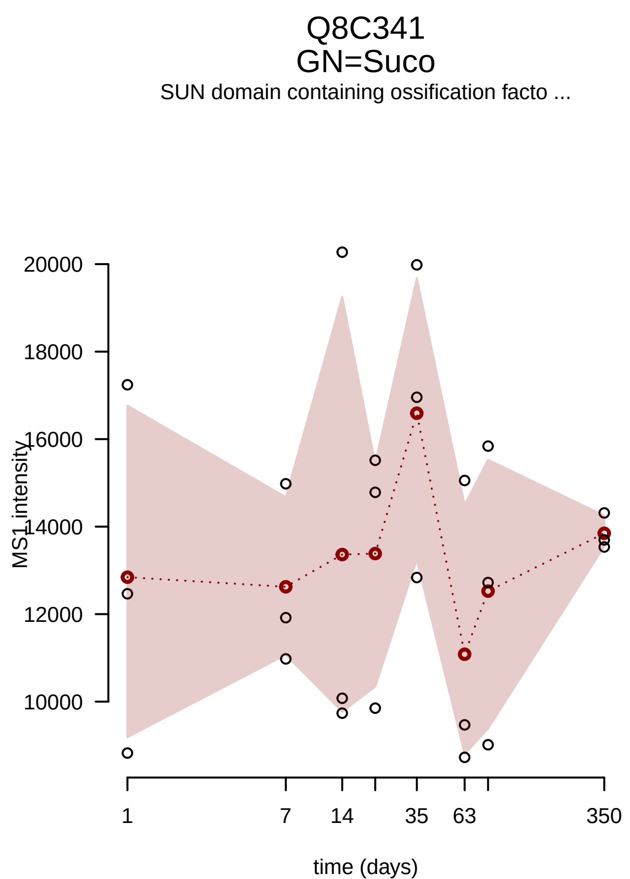
Q8C341
GN=Suco
SUN domain containing ossification facto ...
MS1 intensity
time (days)

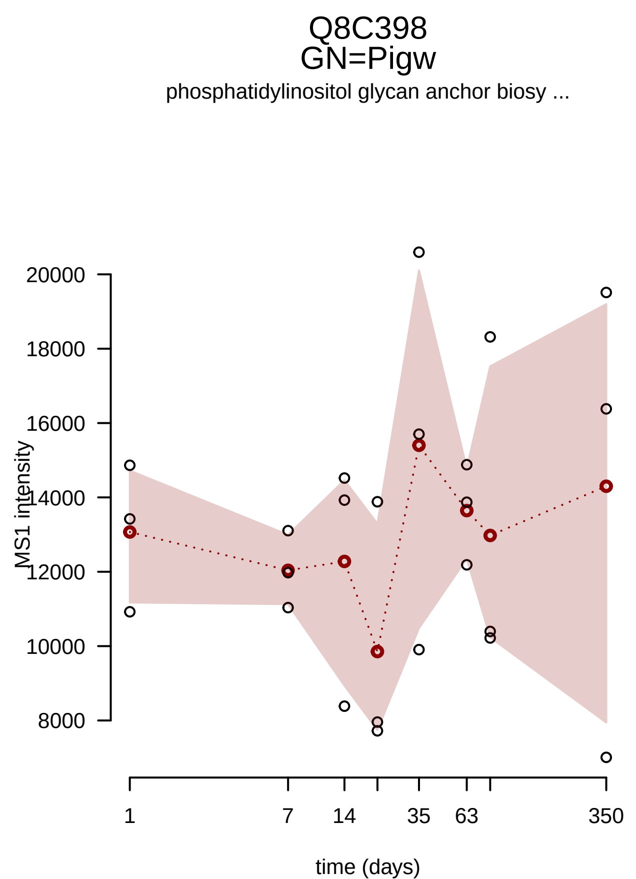
Q8C398
GN=Pigw
phosphatidylinositol glycan anchor biosy ...
MS1 intensity
time (days)

Q8C3B8
GN=Rft1
RFT1 homolog

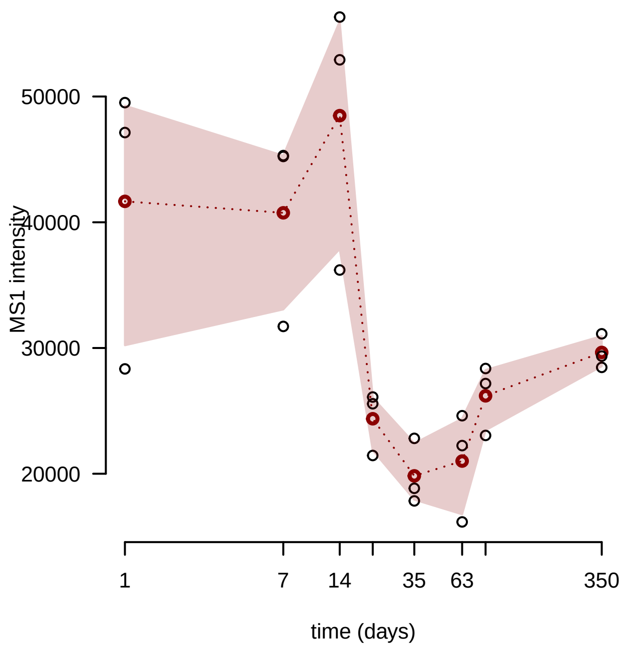

Q8C3F2
GN=Fam120c
family with sequence similarity 120, mem ...

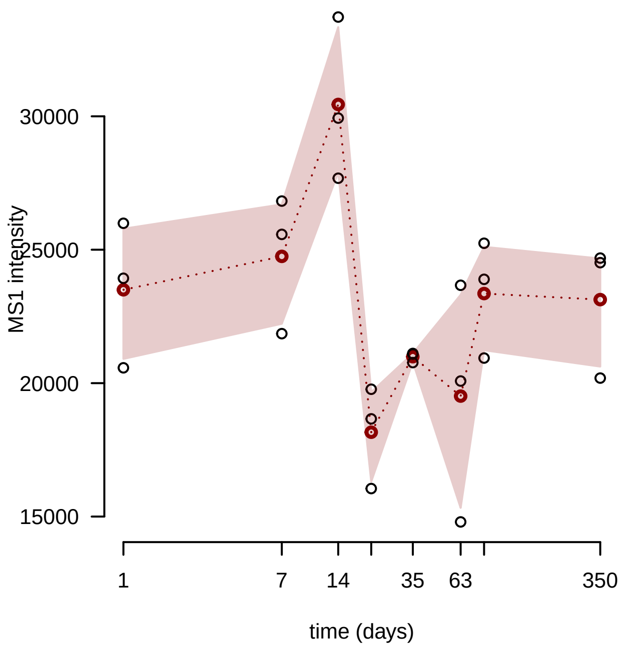

Q8C3I8
GN=Hgh1
HGH1 homolog

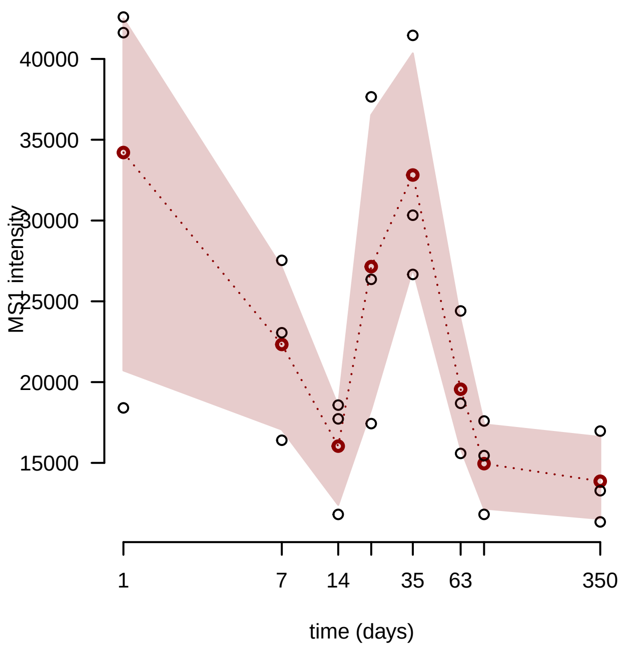

Q8C3J5
GN=Dock2
dedicator of cyto−kinesis 2

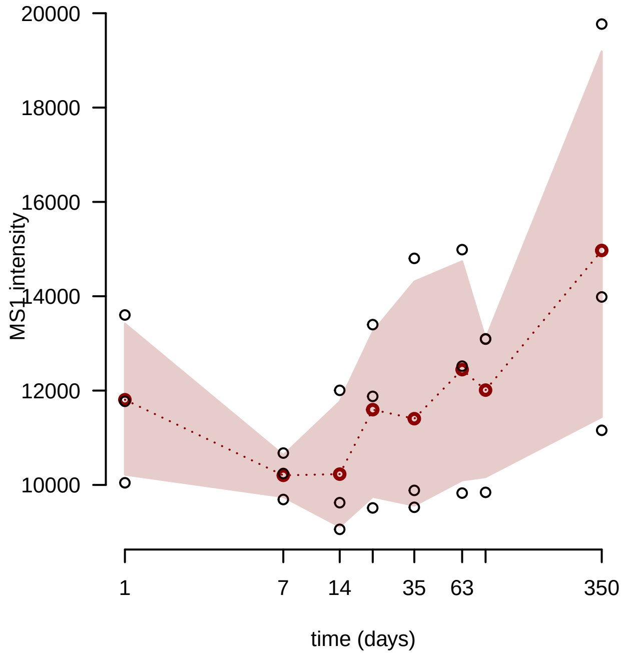

Q8C3P7
GN=Mettl3
methyltransferase like 3

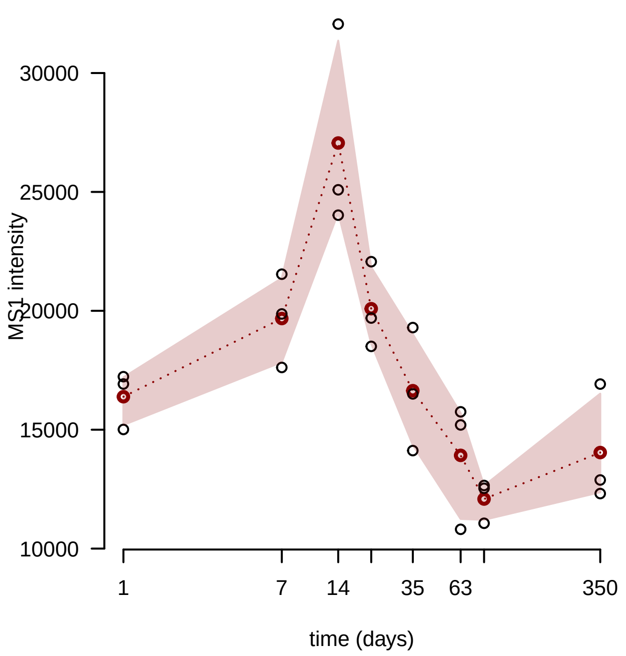

Q8C3Q9
GN=Casp9
caspase 9

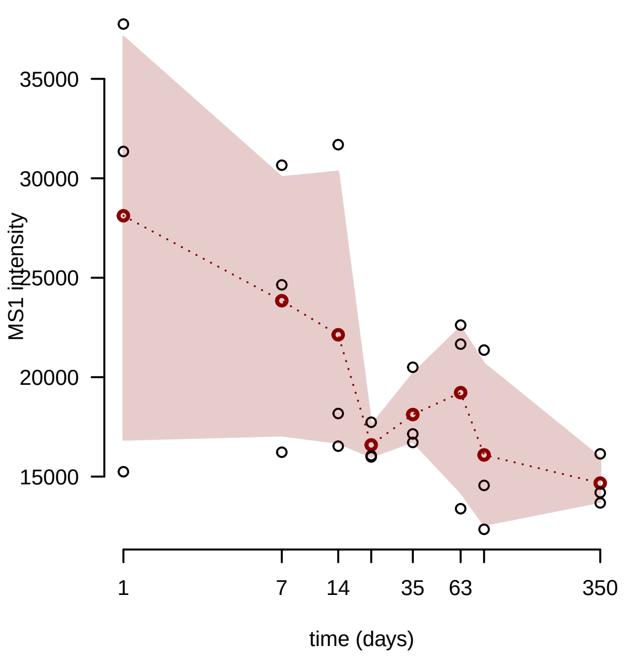

Q8C3R1
GN=Brat1
BRCA1−associated ATM activator 1

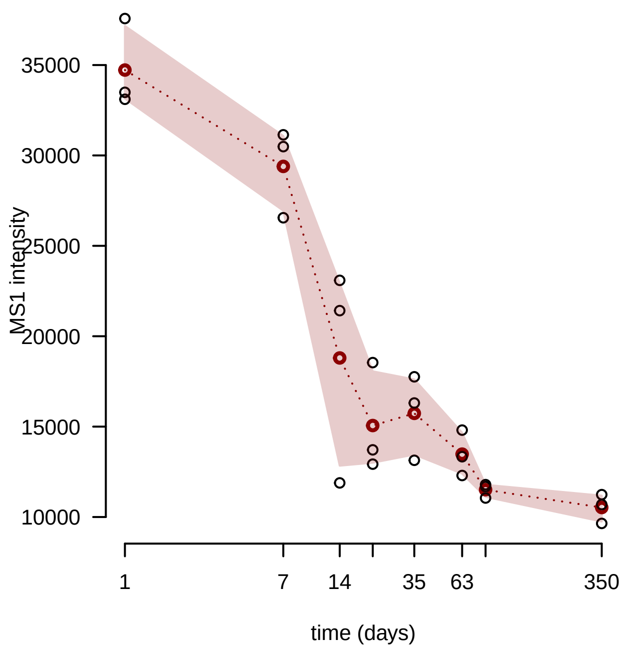

Q8C3S2
GN=Tango6
transport and golgi organization 6

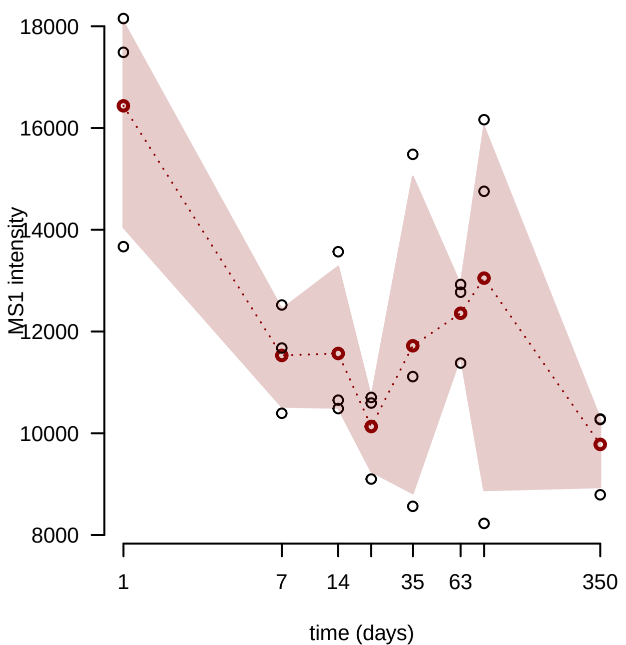

Q8C3W1
GN=

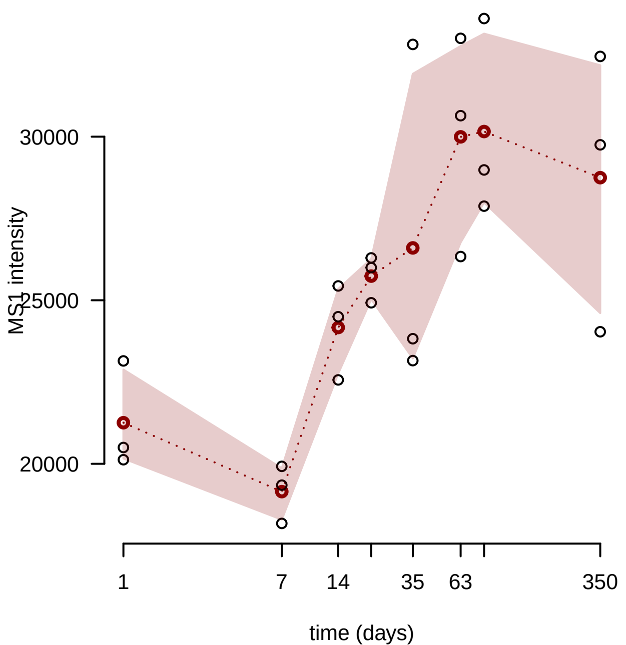

Q8C3X2
GN=Ccdc90b
coiled−coil domain containing 90B

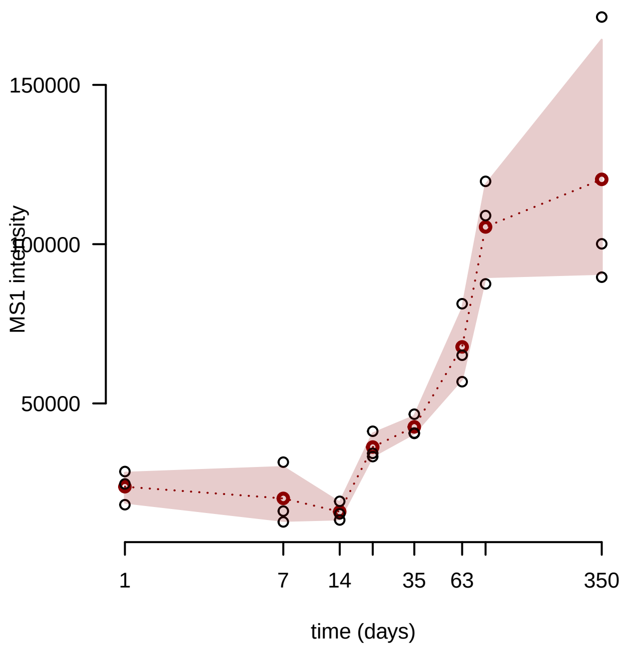

Q8C3X4
GN=Guf1
GUF1 homolog, GTPase

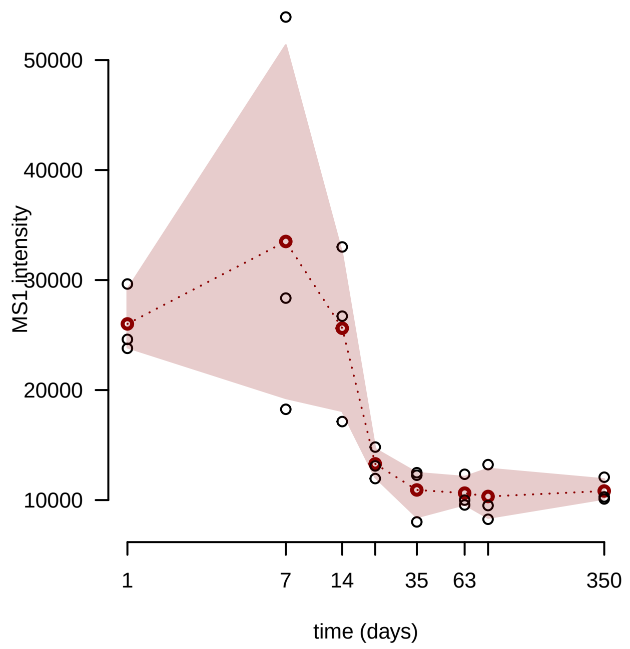

Q8C3X8
GN=Lmf2
lipase maturation factor 2

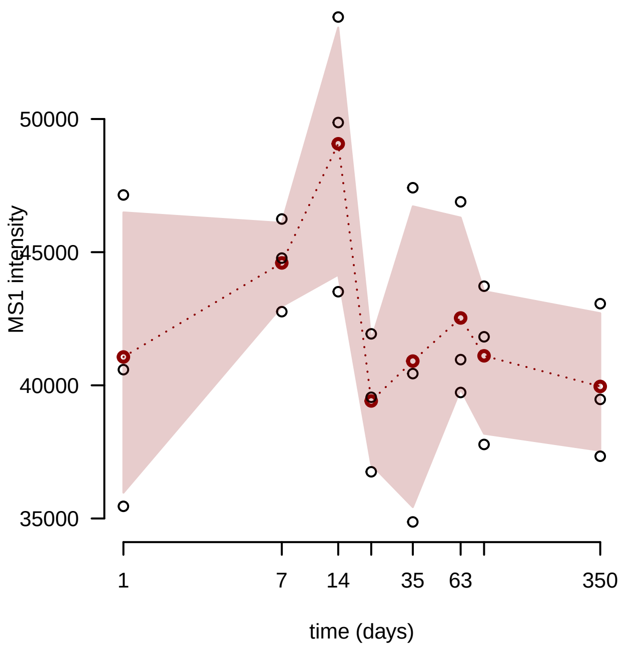

Q8C3Y4
GN=Kntc1
kinetochore associated 1

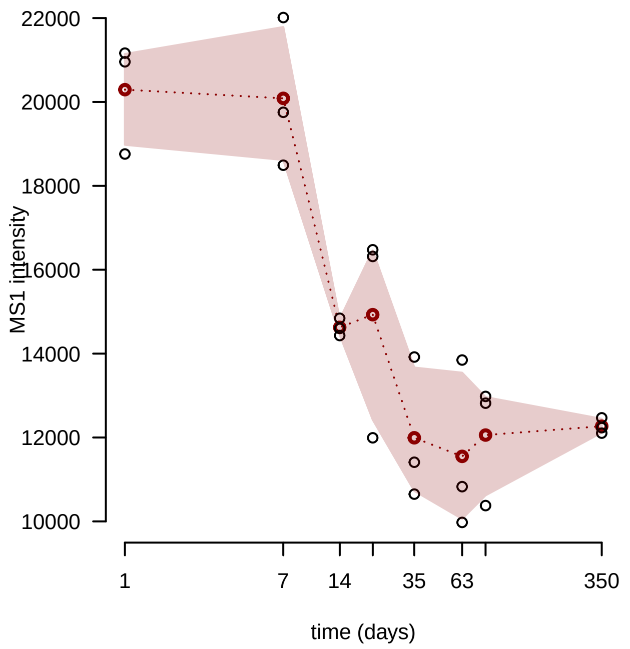

Q8C407
GN=Yipf4
Yip1 domain family, member 4

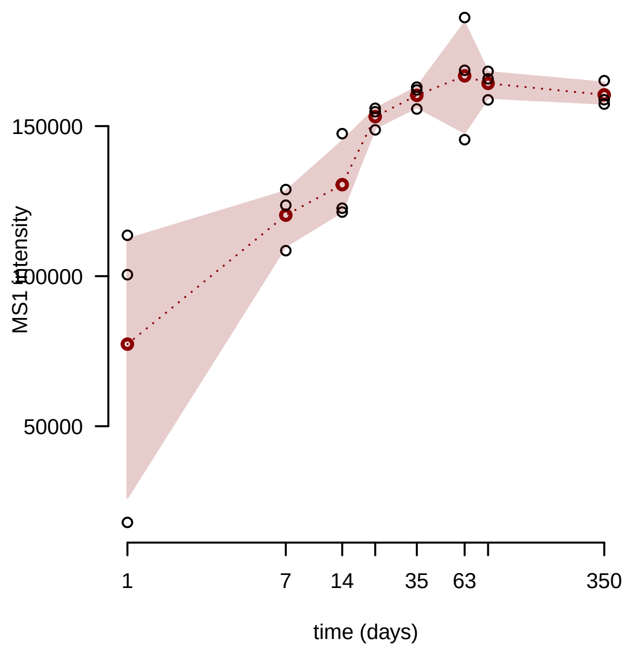

Q8C436
GN=Vcpkmt
valosin containing protein lysine (K) me ...

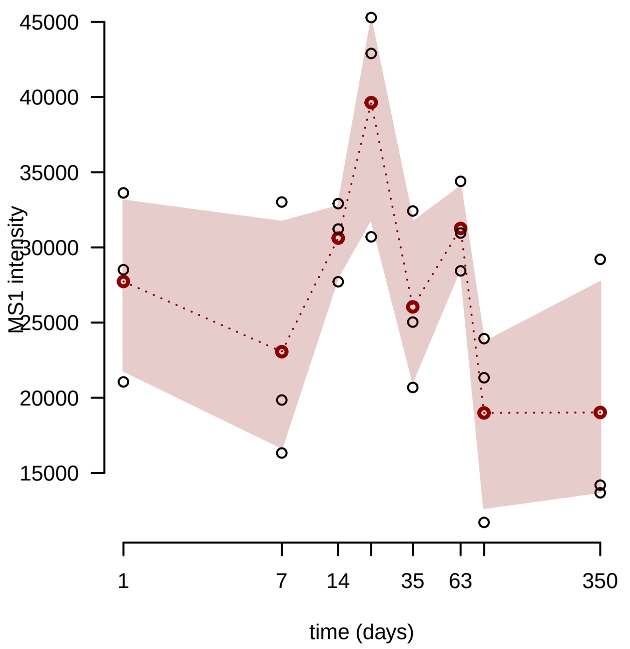

Q8C460
GN=Eri3
exoribonuclease 3

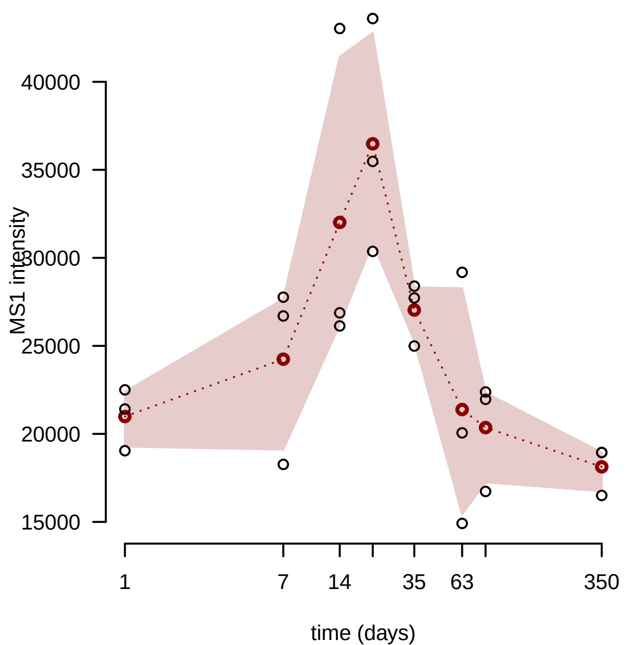

Q8C4A5
GN=Asxl3
ASXL transcriptional regulator 3

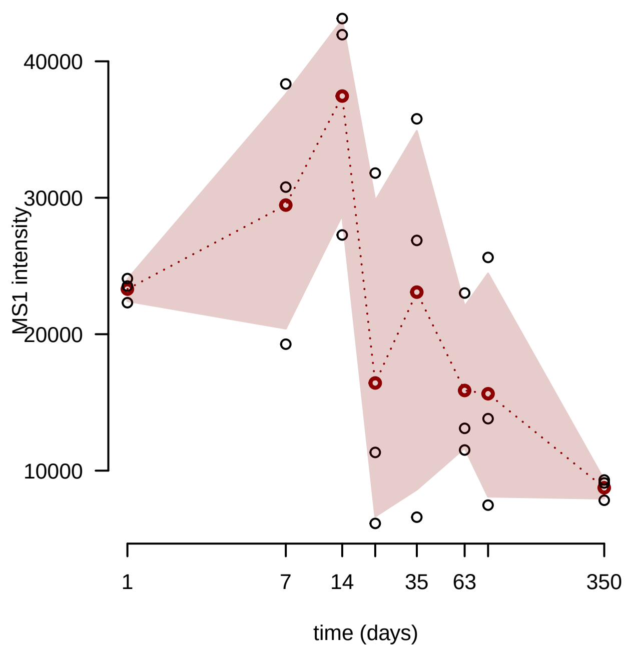

Q8C4B4
GN=Unc119b
unc−119 lipid binding chaperone B

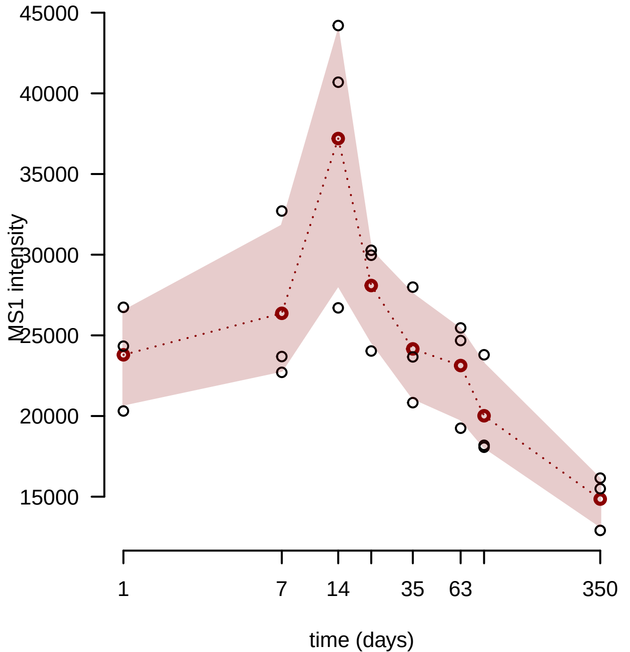

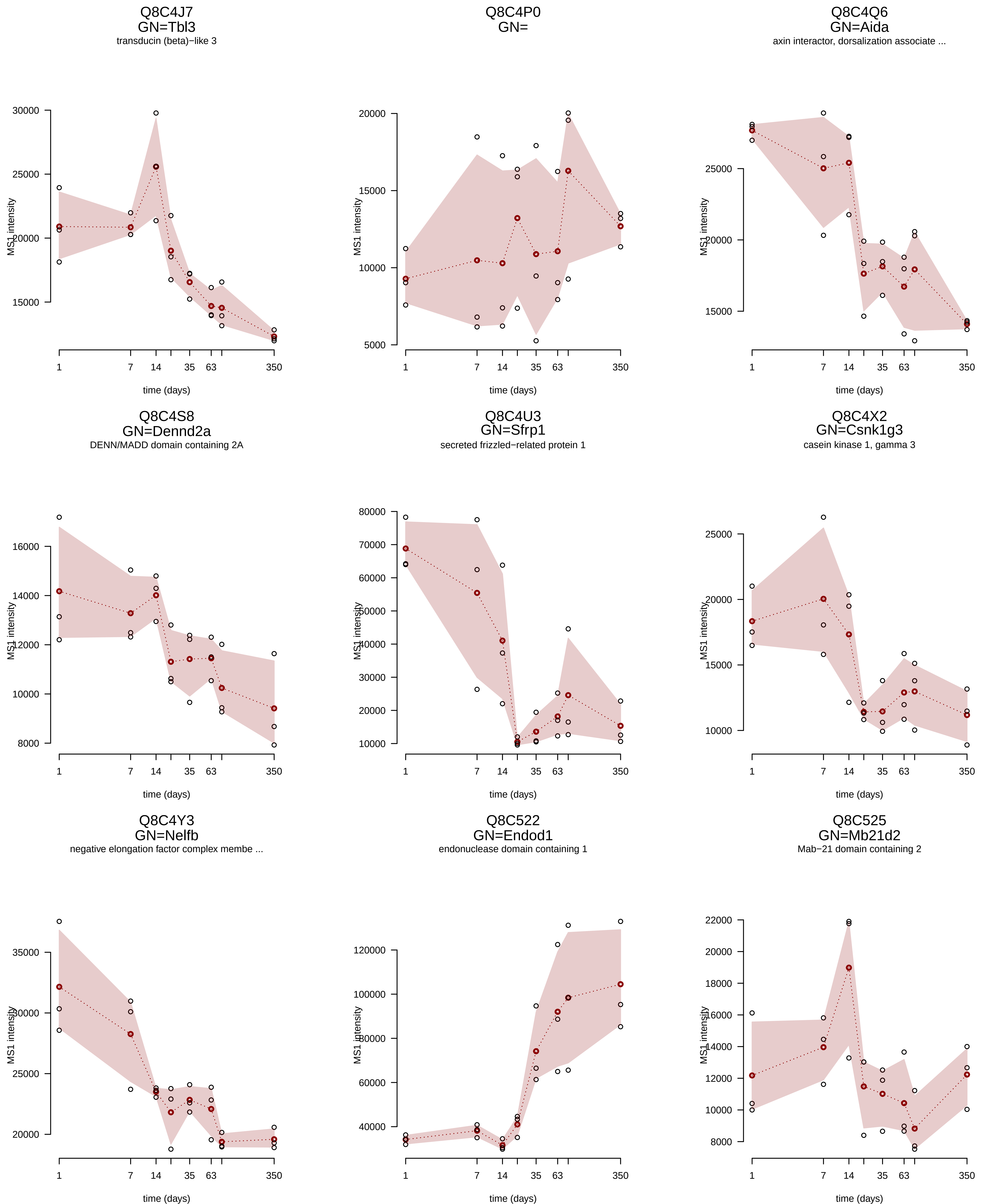
Q8C4J7
GN=Tbl3
transducin (beta)−like 3
MS1 intensity
30000
25000
20000
15000
1
7
14
35
63
350
time (days)
Q8C4P0
GN=
MS1 intensity
20000
15000
10000
5000
1
7
14
35
63
350
time (days)
Q8C4Q6
GN=Aida
axin interactor, dorsalization associate ...
MS1 intensity
25000
20000
15000
1
7
14
35
63
350
time (days)
Q8C4S8
GN=Dennd2a
DENN/MADD domain containing 2A
MS1 intensity
16000
14000
12000
10000
8000
1
7
14
35
63
350
time (days)
Q8C4U3
GN=Sfrp1
secreted frizzled−related protein 1
MS1 intensity
80000
70000
60000
50000
40000
30000
20000
10000
1
7
14
35
63
350
time (days)
Q8C4X2
GN=Csnk1g3
casein kinase 1, gamma 3
MS1 intensity
25000
20000
15000
10000
1
7
14
35
63
350
time (days)
Q8C4Y3
GN=Nelfb
negative elongation factor complex membe ...
MS1 intensity
35000
30000
25000
20000
1
7
14
35
63
350
time (days)
Q8C522
GN=Endod1
endonuclease domain containing 1
MS1 intensity
120000
100000
80000
60000
40000
1
7
14
35
63
350
time (days)
Q8C525
GN=Mb21d2
Mab−21 domain containing 2
MS1 intensity
22000
20000
18000
16000
14000
12000
10000
8000
1
7
14
35
63
350
time (days)

Q8C547
GN=Heatr5b
HEAT repeat containing 5B

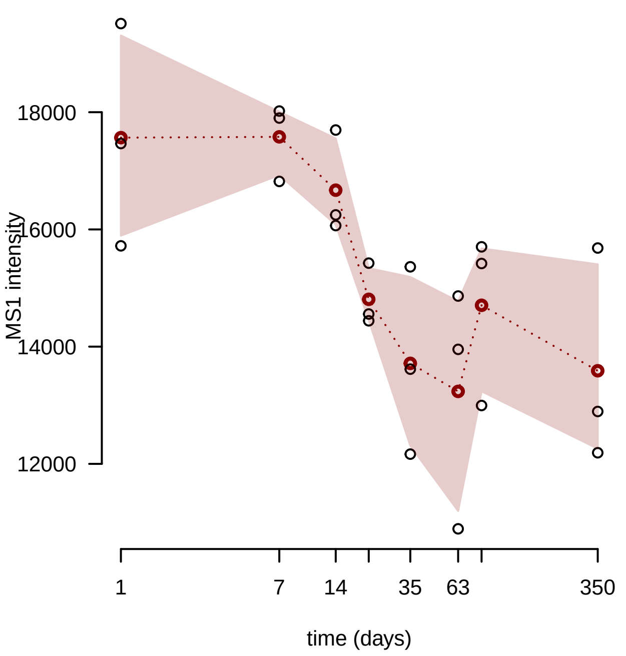

Q8C551
GN=Rad51ap1
RAD51 associated protein 1

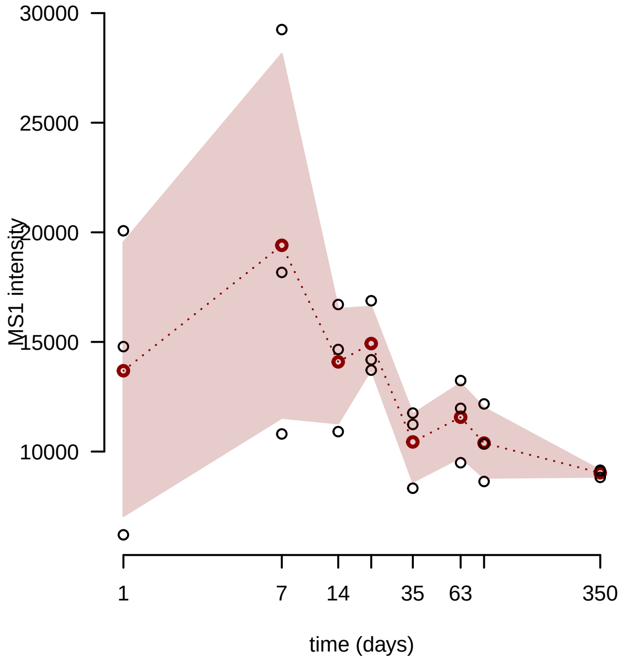

Q8C561
GN=Lmbrd2
LMBR1 domain containing 2

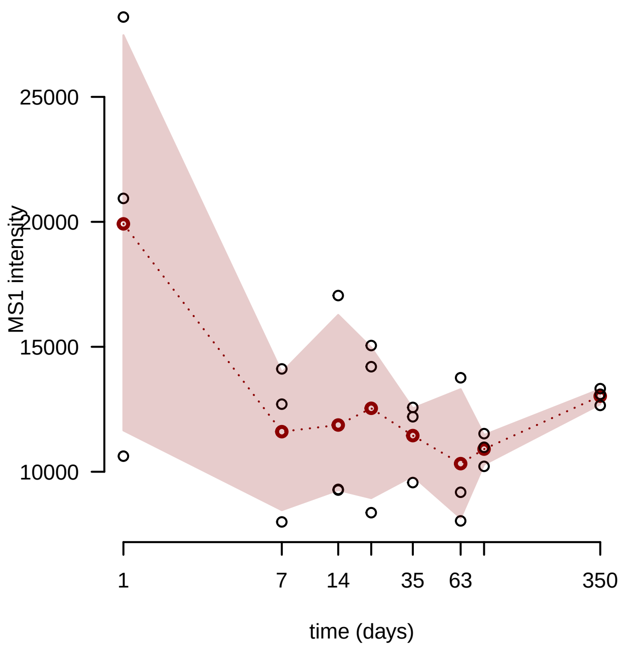

Q8C569
GN=Fam118b
family with sequence similarity 118, mem ...

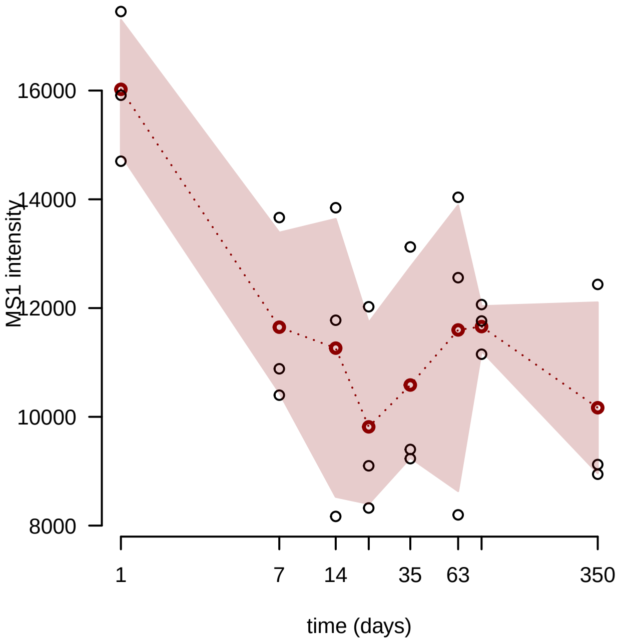

Q8C570
GN=Rae1
ribonucleic acid export 1

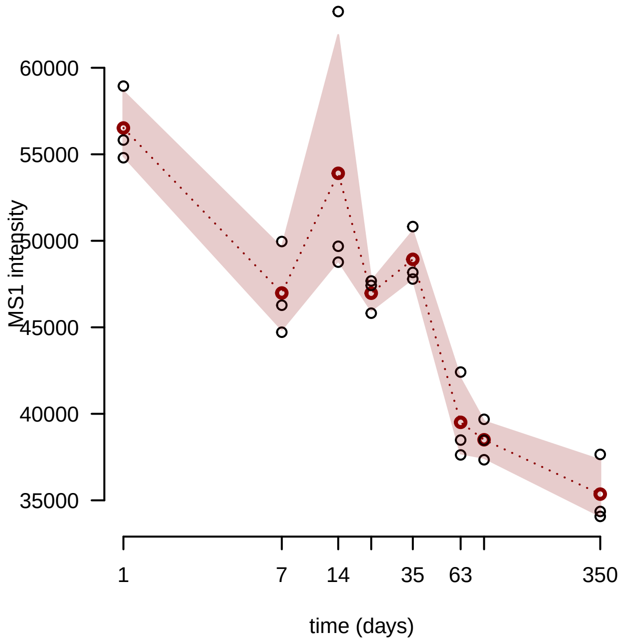

Q8C5D8
GN=Pias2
protein inhibitor of activated STAT 2

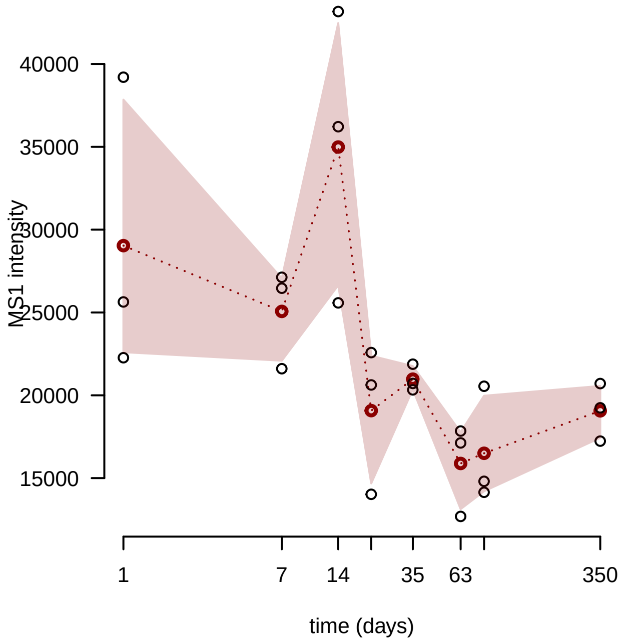

Q8C5H8
GN=Nadk2
NAD kinase 2, mitochondrial

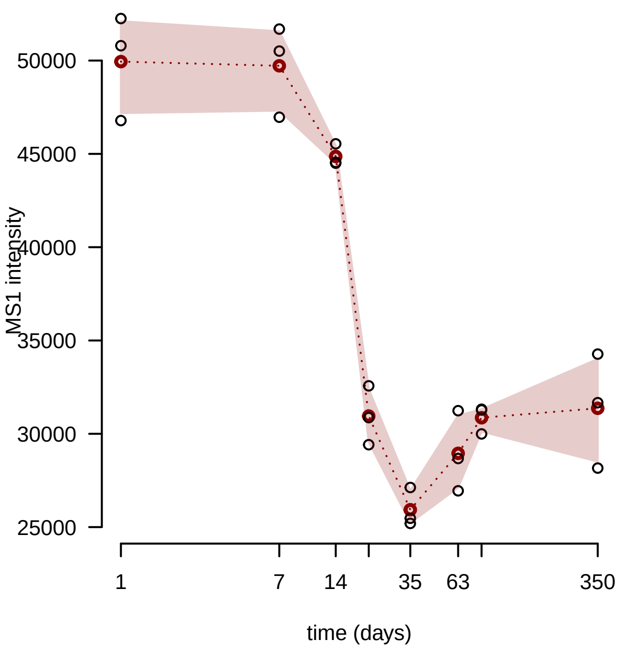

Q8C5K5
GN=

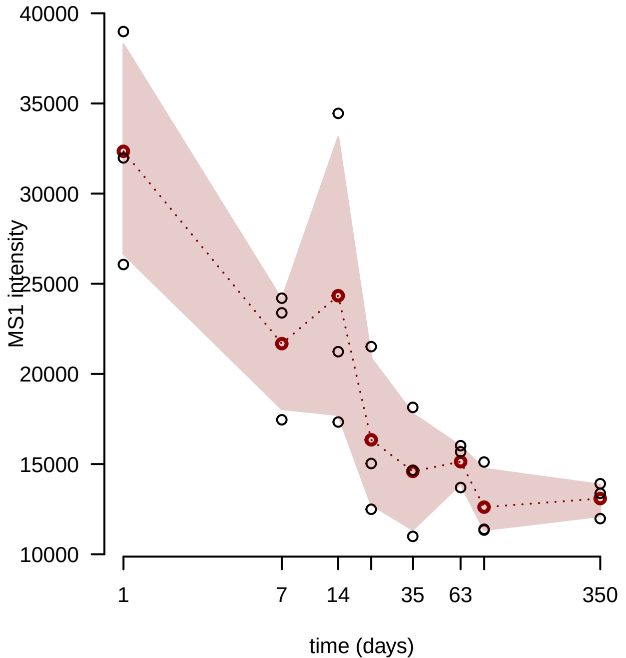

Q8C5L3
GN=Cnot2
CCR4−NOT transcription complex, subunit ...

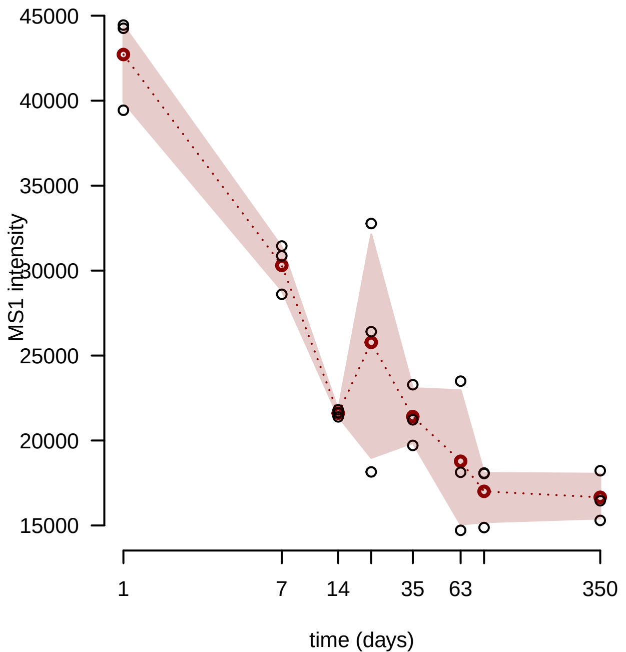

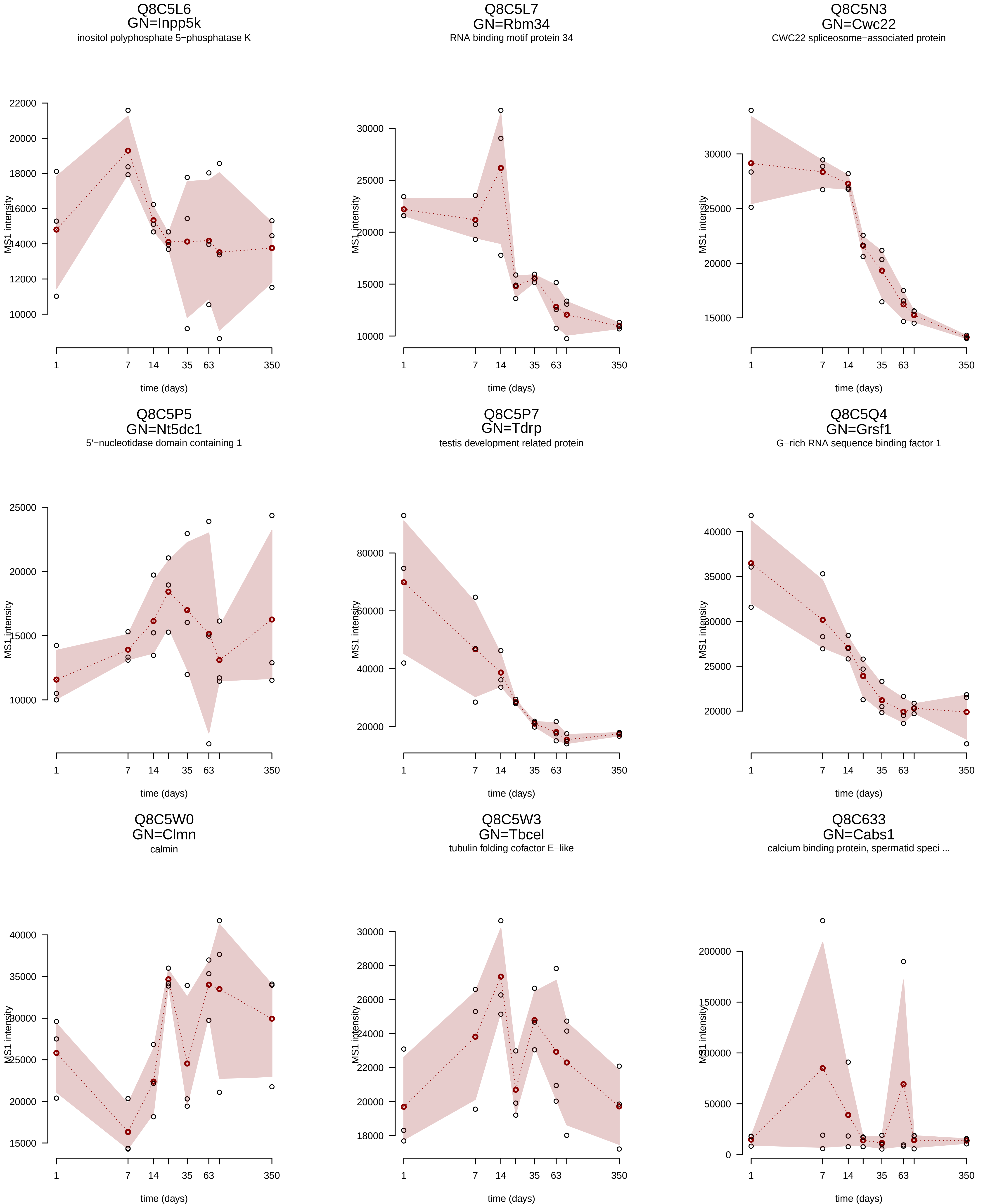
Q8C5L6
GN=Inpp5k
inositol polyphosphate 5−phosphatase K
MS1 intensity
time (days)
Q8C5L7
GN=Rbm34
RNA binding motif protein 34
MS1 intensity
time (days)
Q8C5N3
GN=Cwc22
CWC22 spliceosome−associated protein
MS1 intensity
time (days)
Q8C5P5
GN=Nt5dc1
5'−nucleotidase domain containing 1
MS1 intensity
time (days)
Q8C5P7
GN=Tdrp
testis development related protein
MS1 intensity
time (days)
Q8C5Q4
GN=Grsf1
G−rich RNA sequence binding factor 1
MS1 intensity
time (days)
Q8C5W0
GN=Clmn
calmin
MS1 intensity
time (days)
Q8C5W3
GN=Tbcel
tubulin folding cofactor E−like
MS1 intensity
time (days)
Q8C633
GN=Cabs1
calcium binding protein, spermatid speci ...
MS1 intensity
time (days)
1
7
14
35
63
350

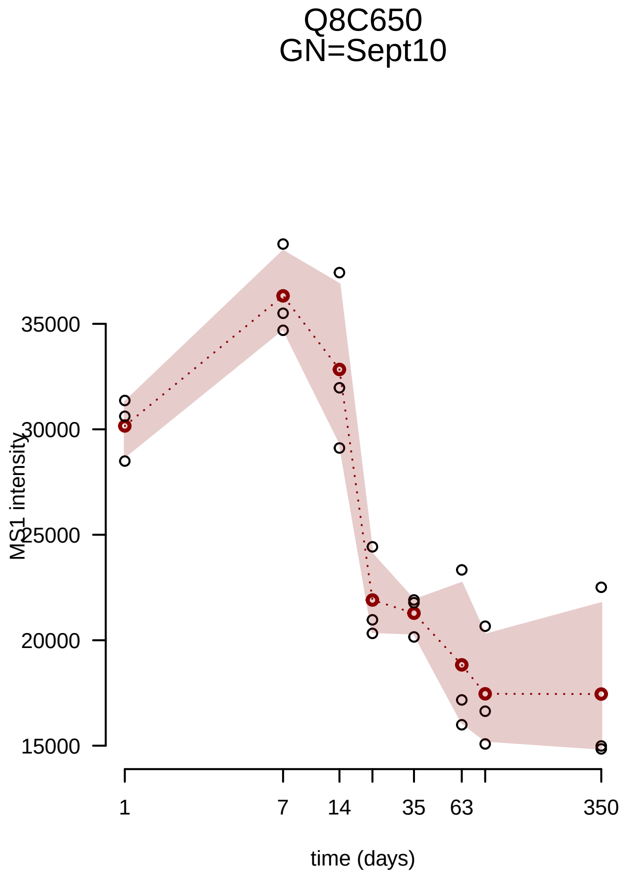
Q8C650
GN=Sept10
MS1 intensity
15000
20000
25000
30000
35000
1
7
14
35
63
350
time (days)

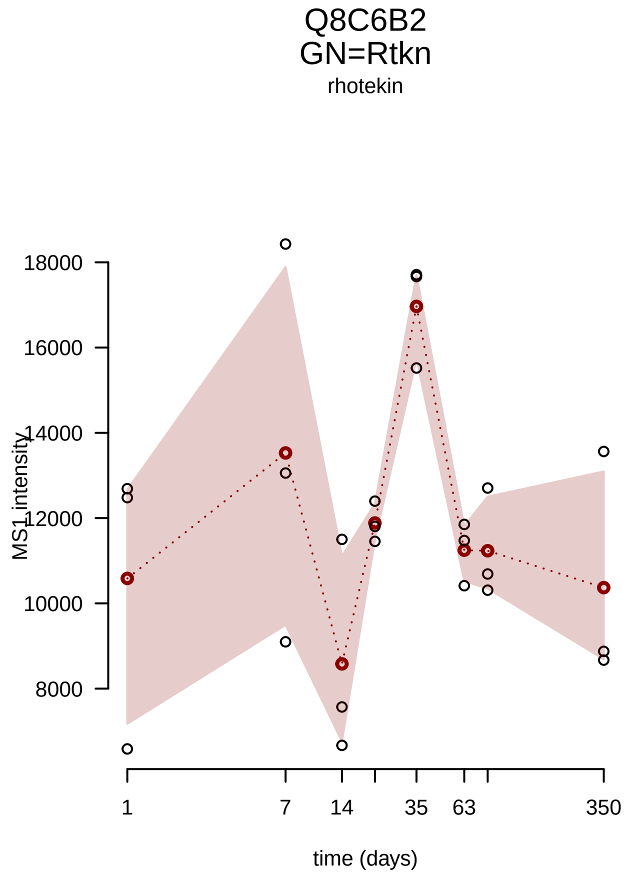
Q8C6B2
GN=Rtkn
rhotekin
MS1 intensity
8000
10000
12000
14000
16000
18000
1
7
14
35
63
350
time (days)

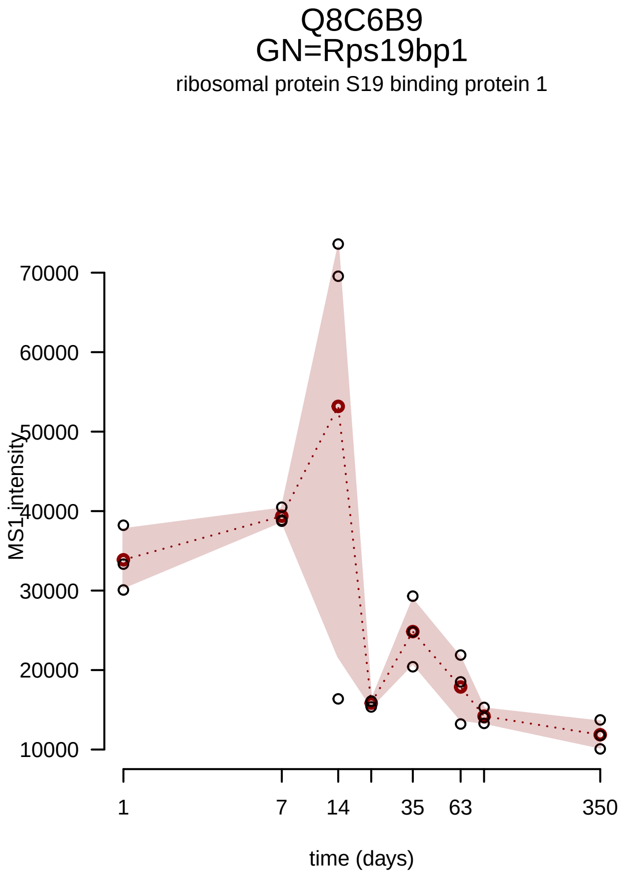
Q8C6B9
GN=Rps19bp1
ribosomal protein S19 binding protein 1
MS1 intensity
10000
20000
30000
40000
50000
60000
70000
1
7
14
35
63
350
time (days)

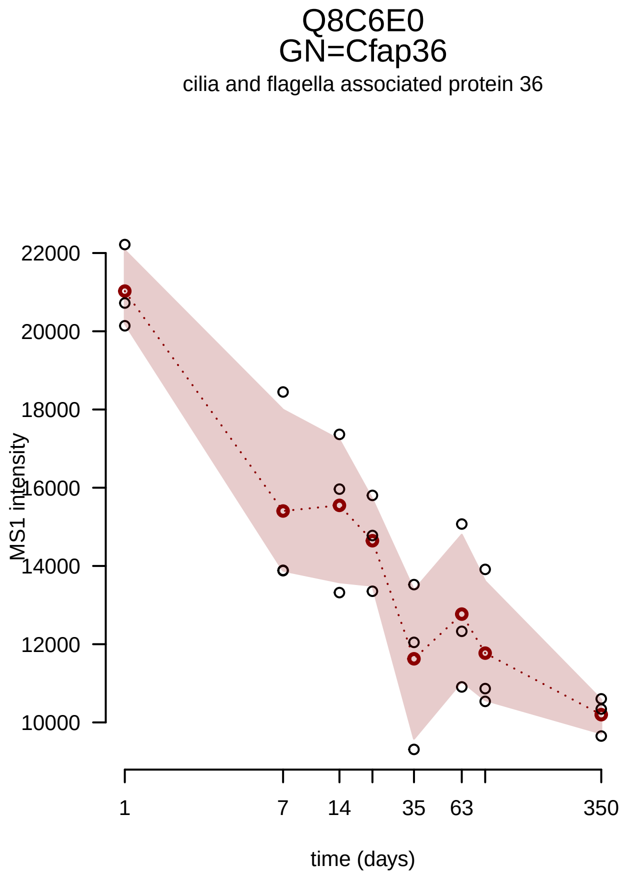
Q8C6E0
GN=Cfap36
cilia and flagella associated protein 36
MS1 intensity
10000
12000
14000
16000
18000
20000
22000
1
7
14
35
63
350
time (days)

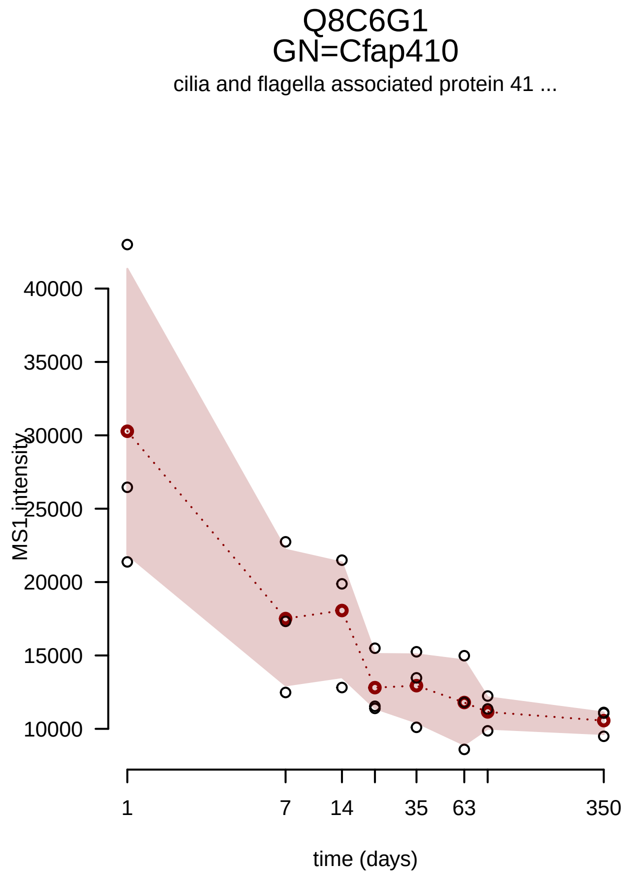
Q8C6G1
GN=Cfap410
cilia and flagella associated protein 41 ...
MS1 intensity
10000
15000
20000
25000
30000
35000
40000
1
7
14
35
63
350
time (days)

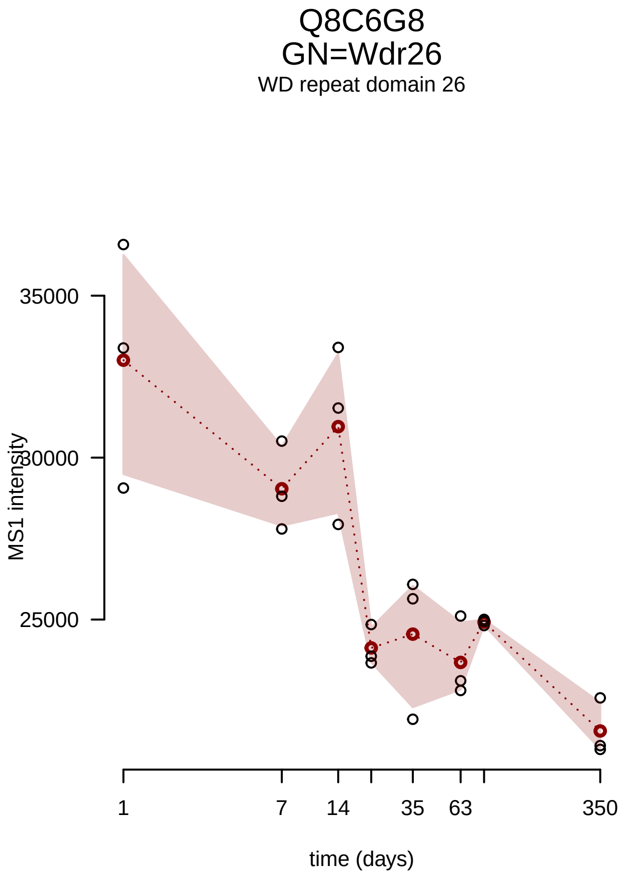
Q8C6G8
GN=Wdr26
WD repeat domain 26
MS1 intensity
25000
30000
35000
1
7
14
35
63
350
time (days)

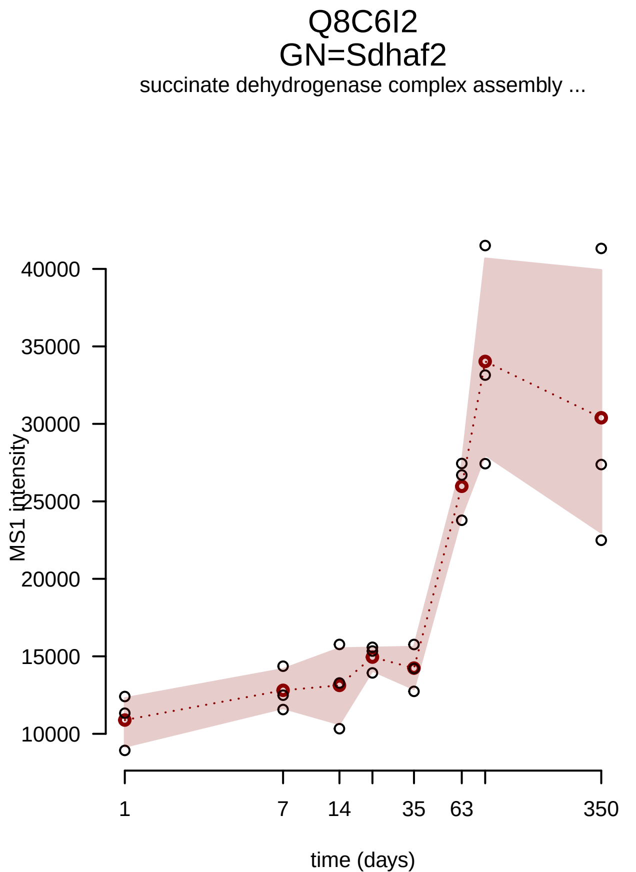
Q8C6I2
GN=Sdhaf2
succinate dehydrogenase complex assembly ...
MS1 intensity
10000
15000
20000
25000
30000
35000
40000
1
7
14
35
63
350
time (days)

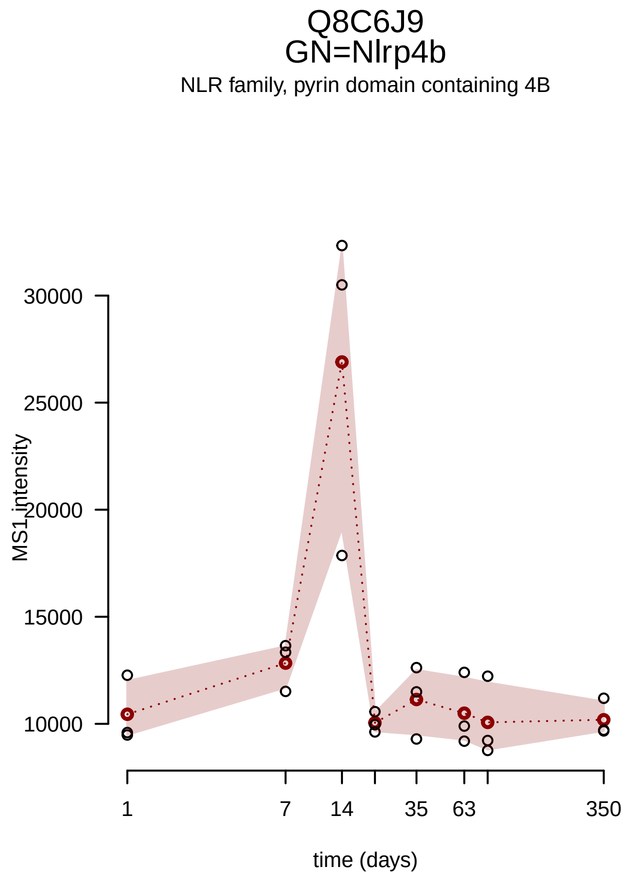
Q8C6J9
GN=Nlrp4b
NLR family, pyrin domain containing 4B
MS1 intensity
10000
15000
20000
25000
30000
1
7
14
35
63
350
time (days)

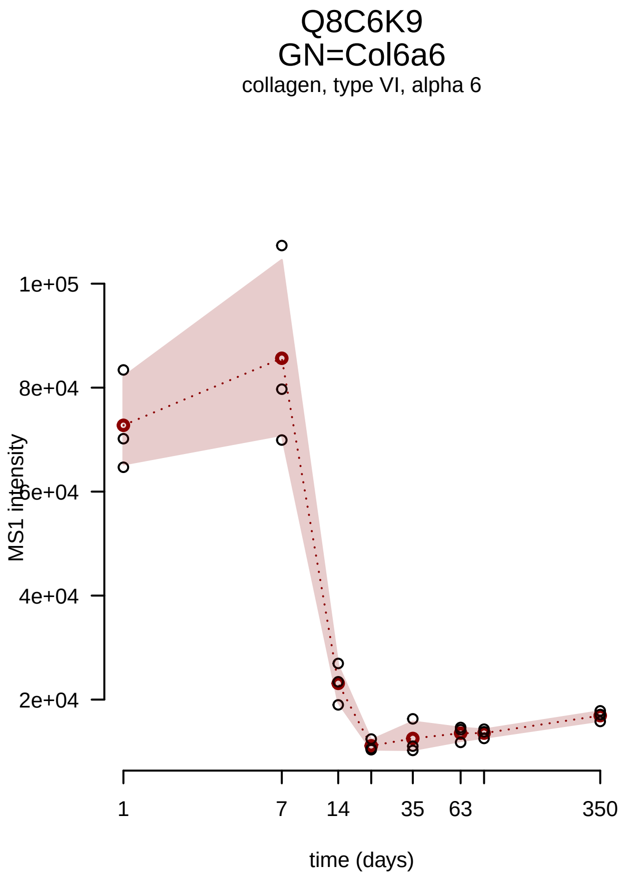
Q8C6K9
GN=Col6a6
collagen, type VI, alpha 6
MS1 intensity
2e+04
4e+04
6e+04
8e+04
1e+05
1
7
14
35
63
350
time (days)

Q8C6L5
GN=Cgas
cyclic GMP−AMP synthase

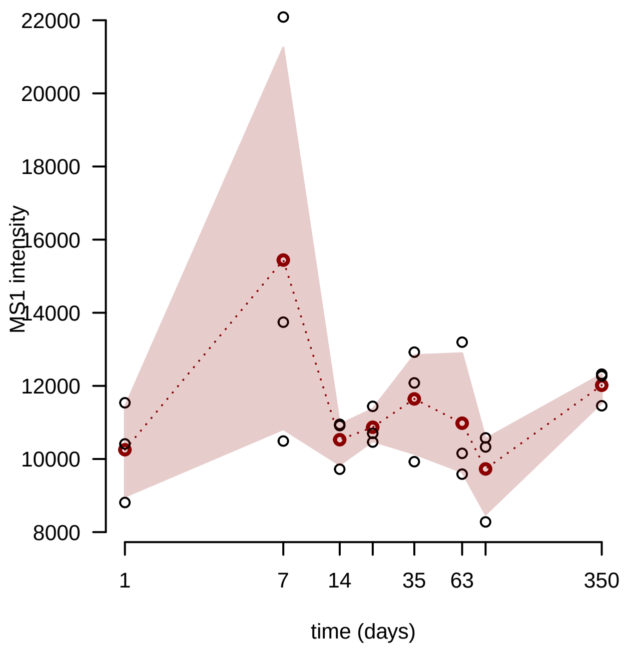

Q8C6P8
GN=Zfp57
zinc finger protein 57

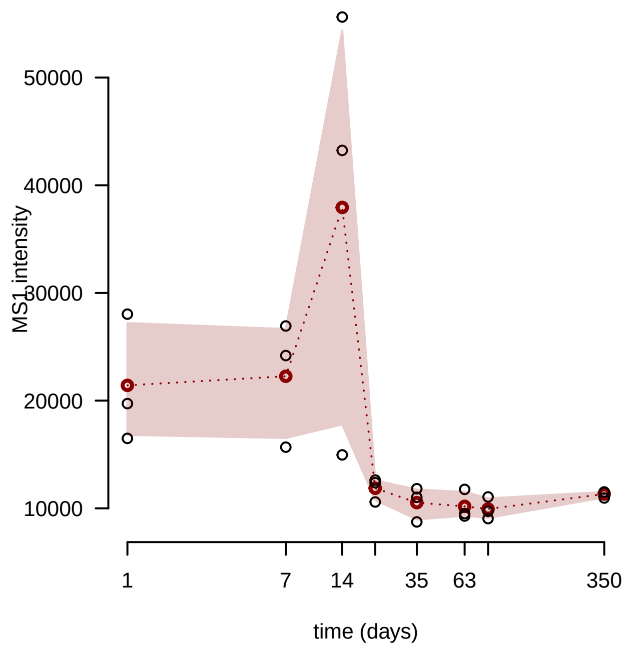

Q8C6U2
GN=Pqlc3
PQ loop repeat containing

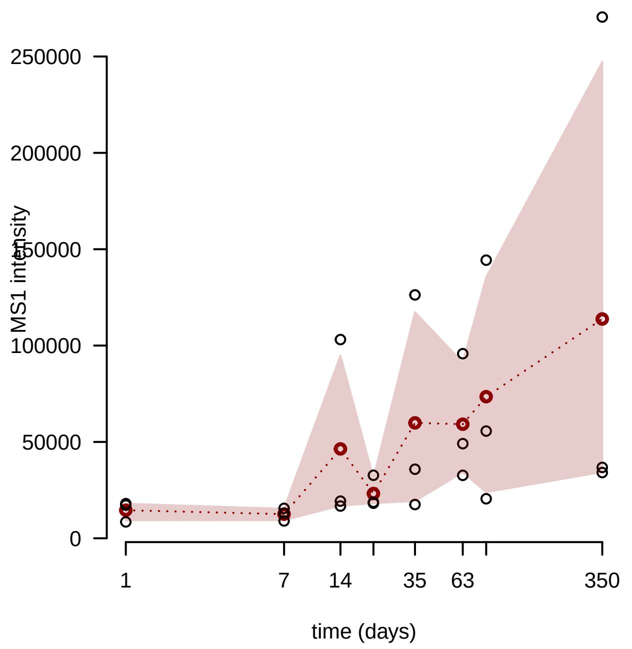

Q8C735
GN=Lin9
lin−9 DREAM MuvB core complex component

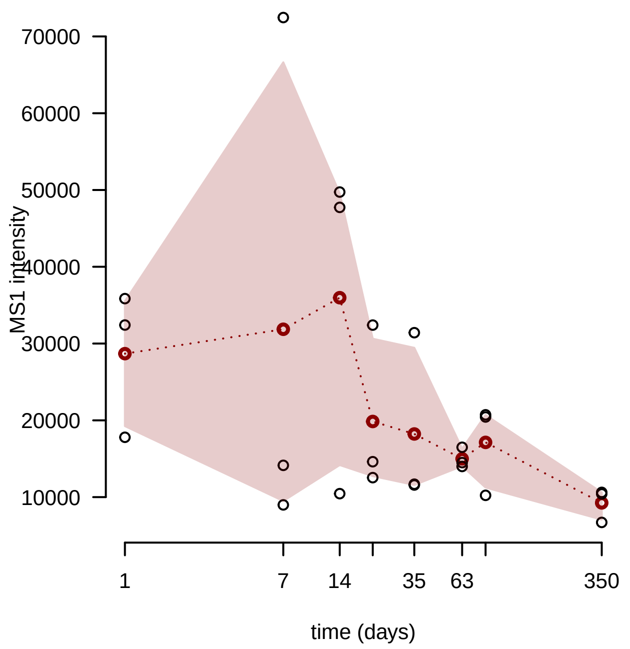

Q8C754
GN=Vps52
VPS52 GARP complex subunit

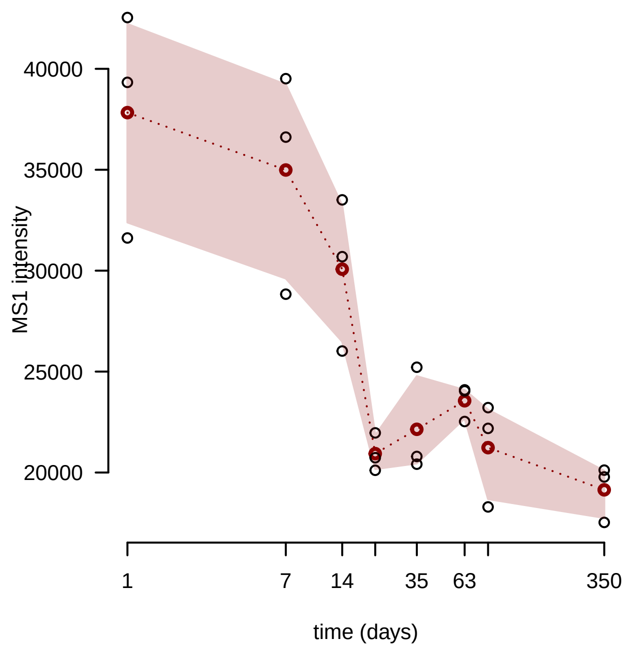

Q8C761
GN=Wdr60

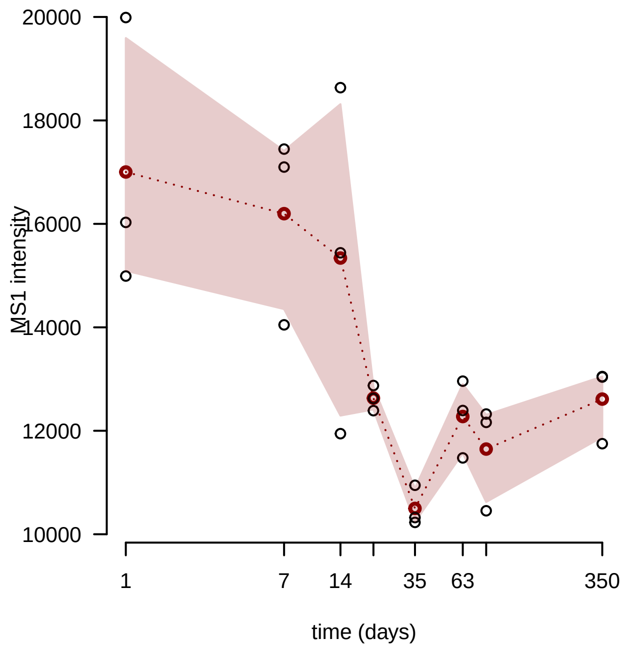

Q8C790
GN=Fam221a
family with sequence similarity 221, mem ...

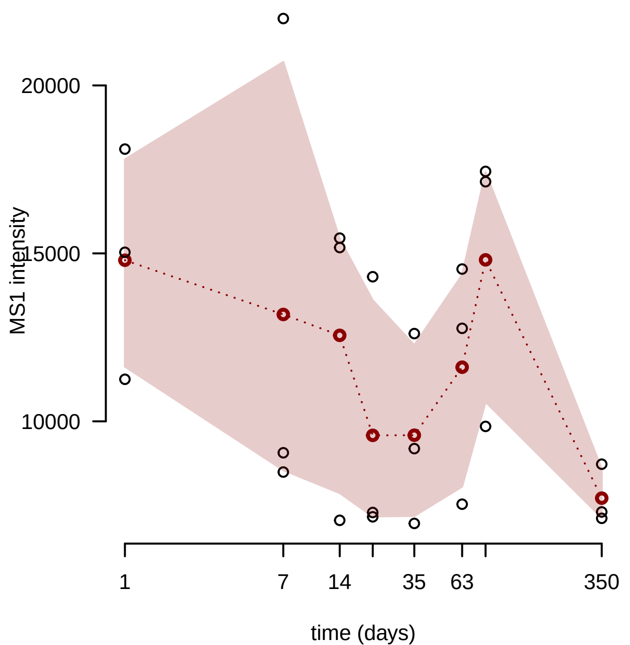

Q8C796
GN=Rcor2
REST corepressor 2

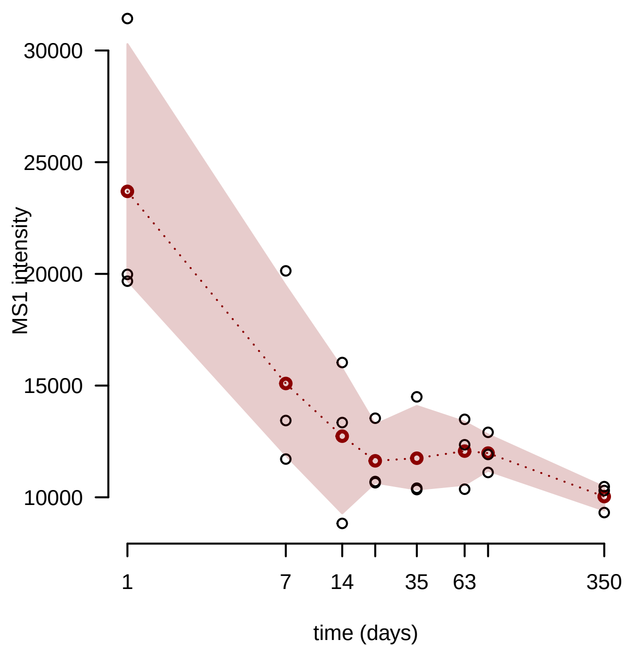

Q8C7D2
GN=Crbn
cereblon

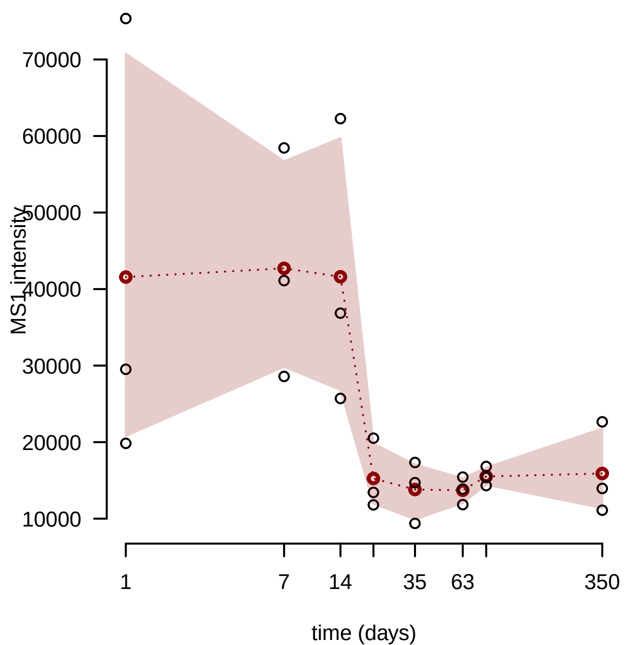

Q8C7E7
GN=Stbd1
starch binding domain 1

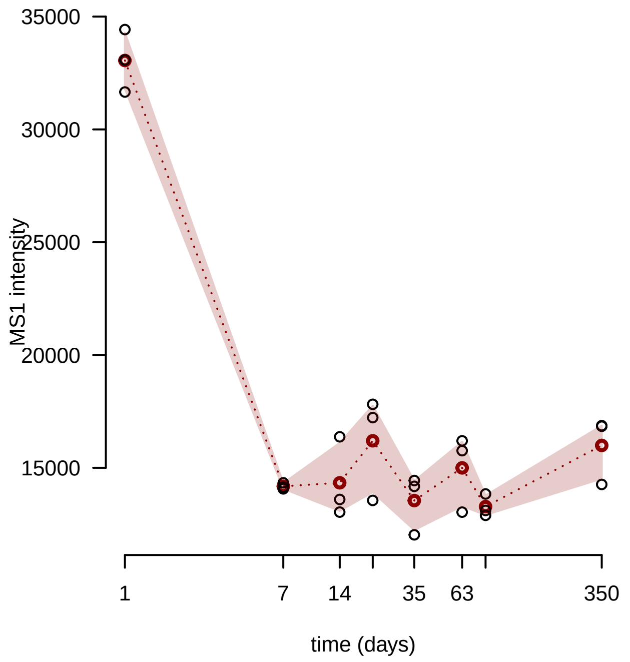

Q8C7E9
GN=Cstf2t
cleavage stimulation factor, 3' pre−RNA ...

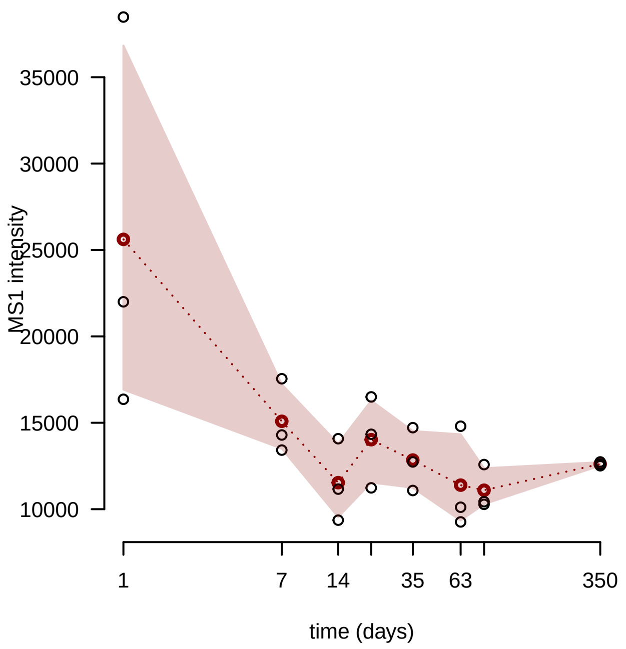

Q8C7H1
GN=Mmaa
methylmalonic aciduria (cobalamin defici ...

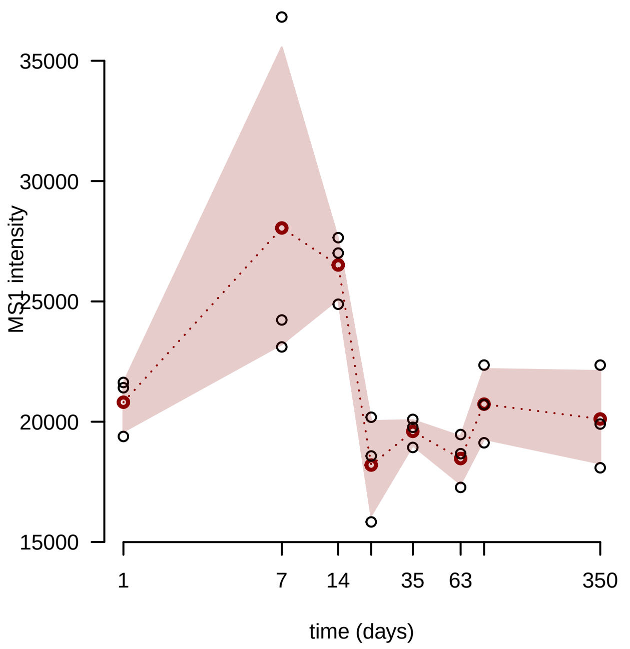

Q8C7K6
GN=Pcyox1l
prenylcysteine oxidase 1 like

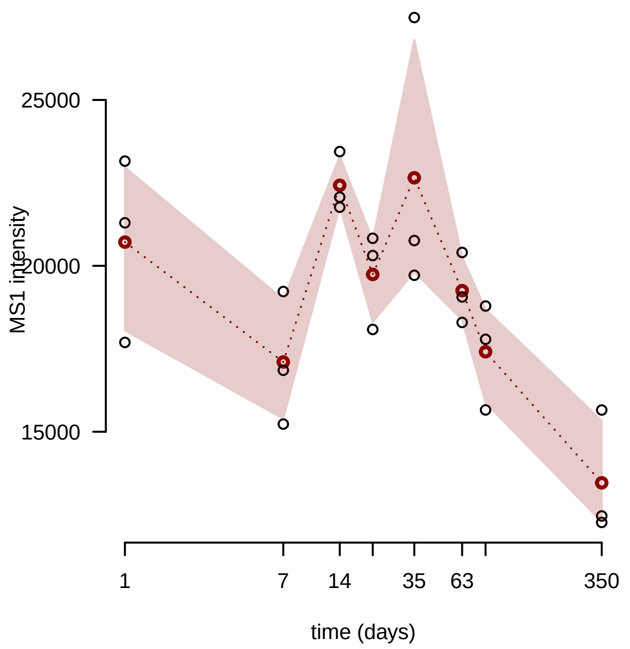

Q8C7Q4
GN=Rbm4
RNA binding motif protein 4

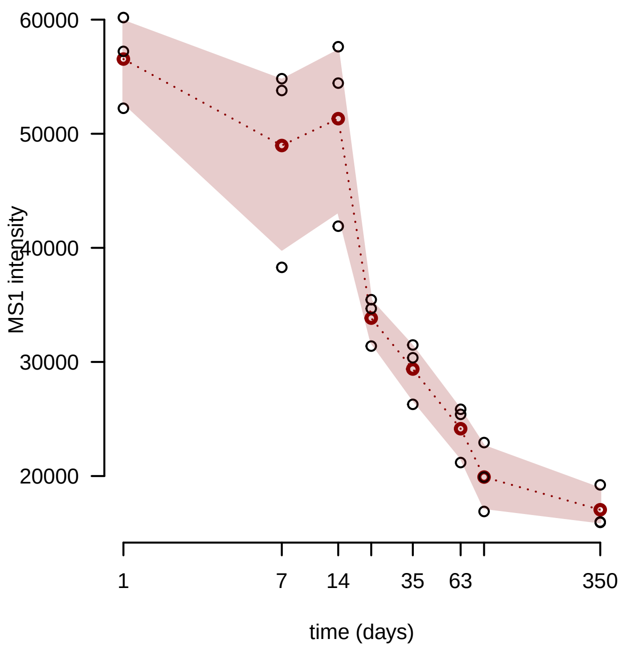

Q8C7R4
GN=Uba6
ubiquitin−like modifier activating enzym ...

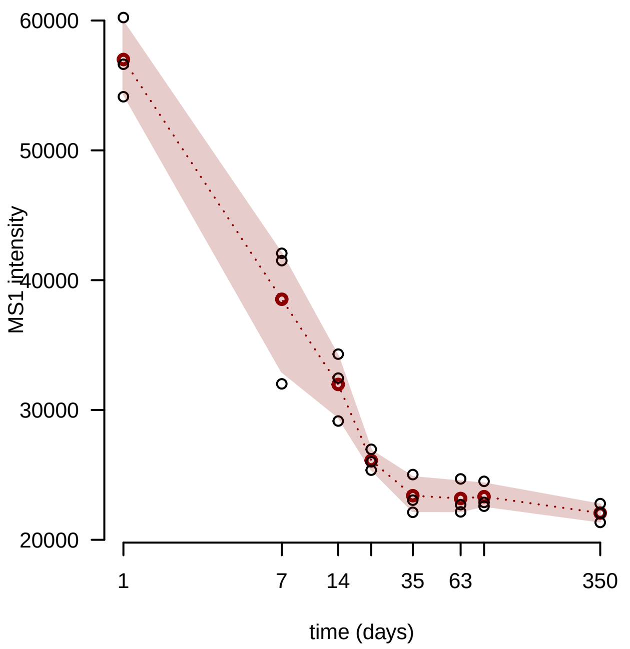

Q8C7U1
GN=N4bp3
NEDD4 binding protein 3

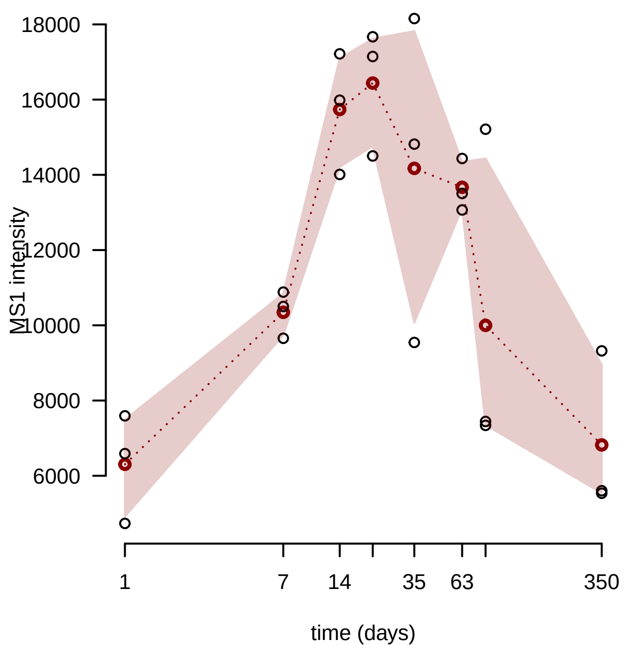

Q8C7V3
GN=Utp15
UTP15 small subunit processome component

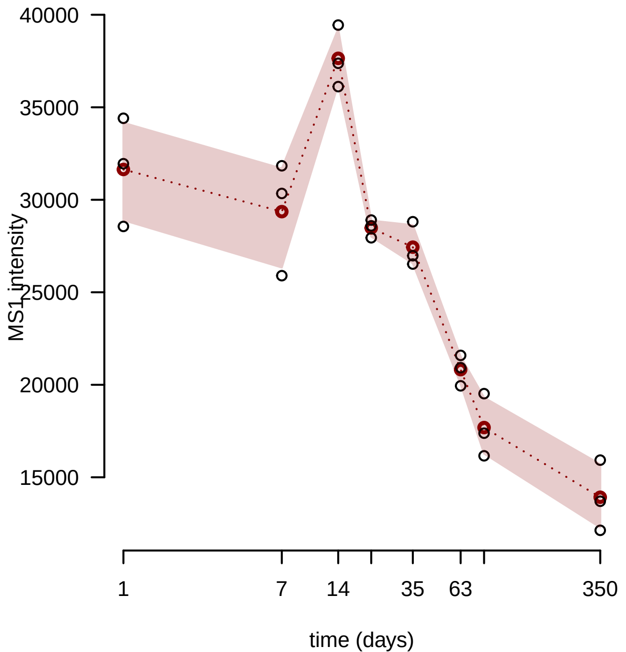

Q8C7V8
GN=Ccdc134
coiled−coil domain containing 134

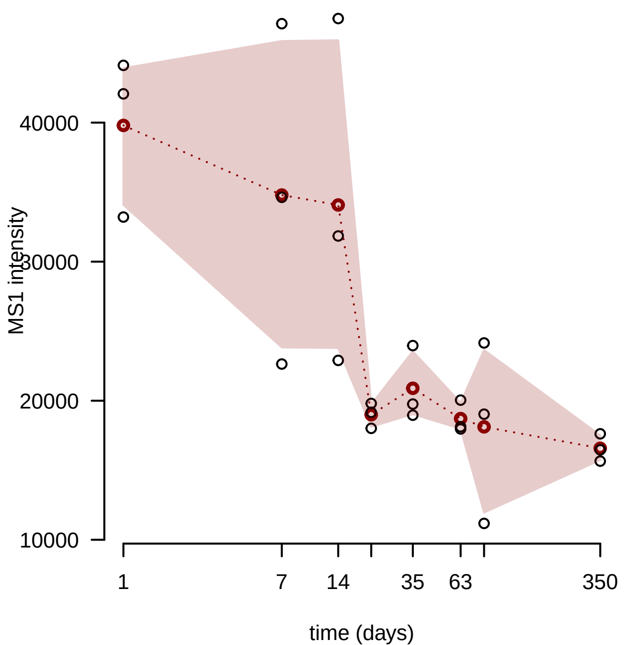

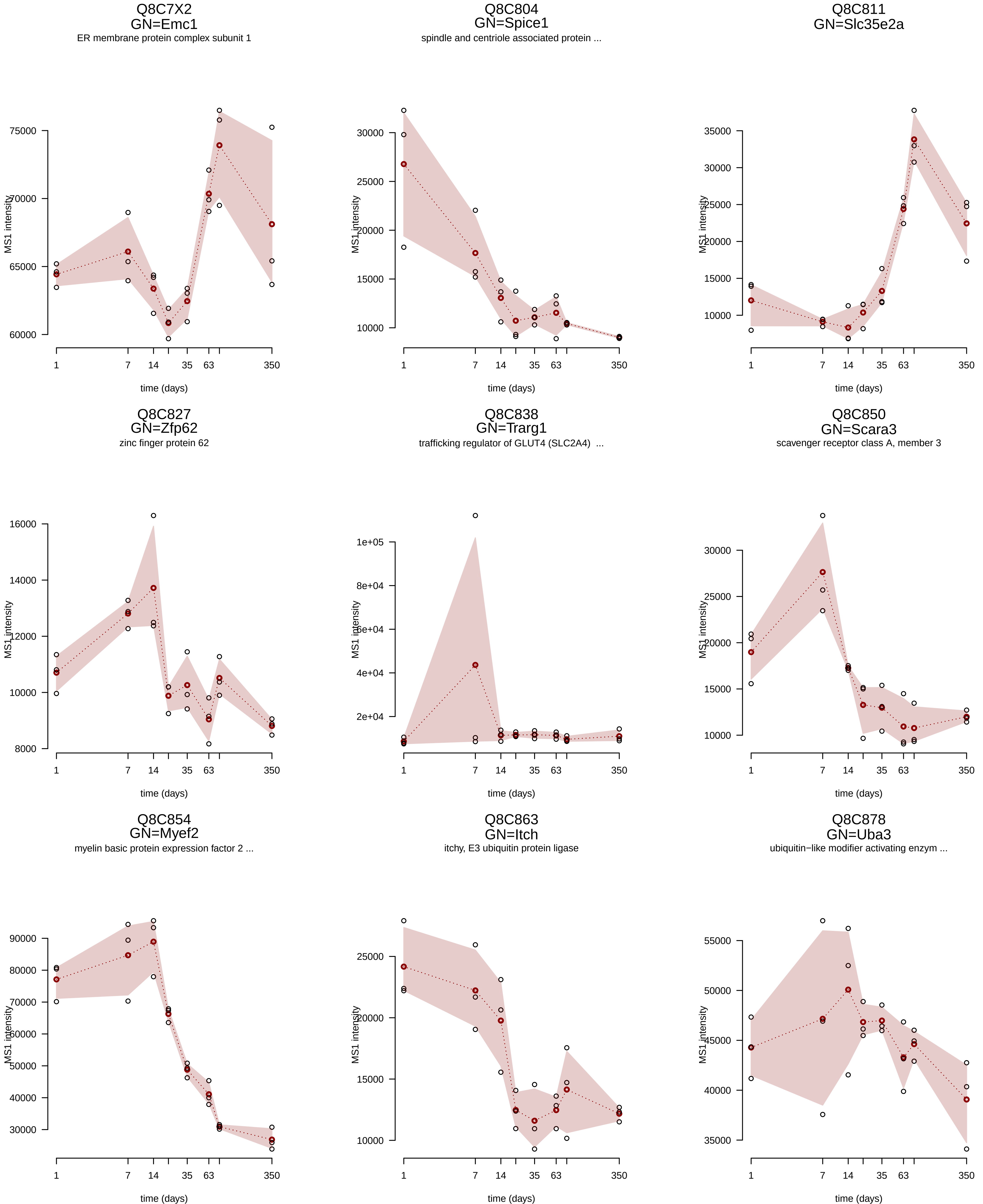
Q8C7X2
GN=Emc1
ER membrane protein complex subunit 1
Q8C804
GN=Spice1
spindle and centriole associated protein ...
Q8C811
GN=Slc35e2a
Q8C827
GN=Zfp62
zinc finger protein 62
Q8C838
GN=Trarg1
trafficking regulator of GLUT4 (SLC2A4) ...
Q8C850
GN=Scara3
scavenger receptor class A, member 3
Q8C854
GN=Myef2
myelin basic protein expression factor 2 ...
Q8C863
GN=Itch
itchy, E3 ubiquitin protein ligase
Q8C878
GN=Uba3
ubiquitin−like modifier activating enzym ...
MS1 intensity
time (days)
1
7
14
35
63
350

Q8C8N2
GN=Scai
suppressor of cancer cell invasion

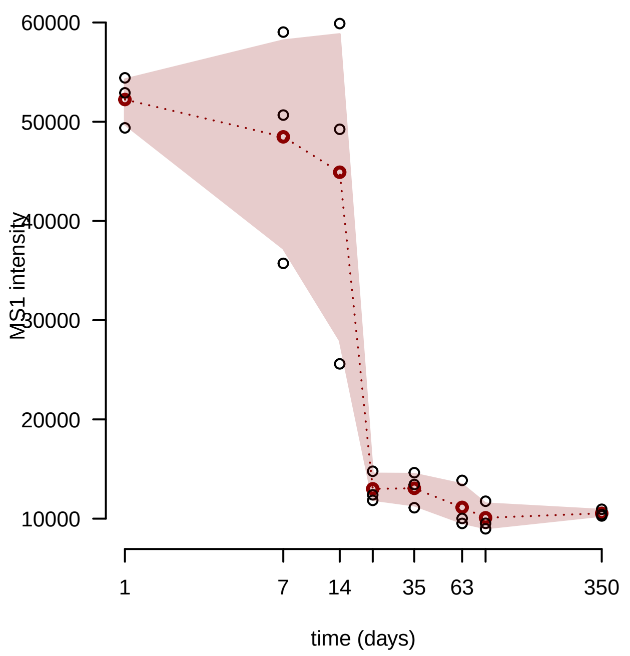

Q8C8R3
GN=Ank2
ankyrin 2, brain

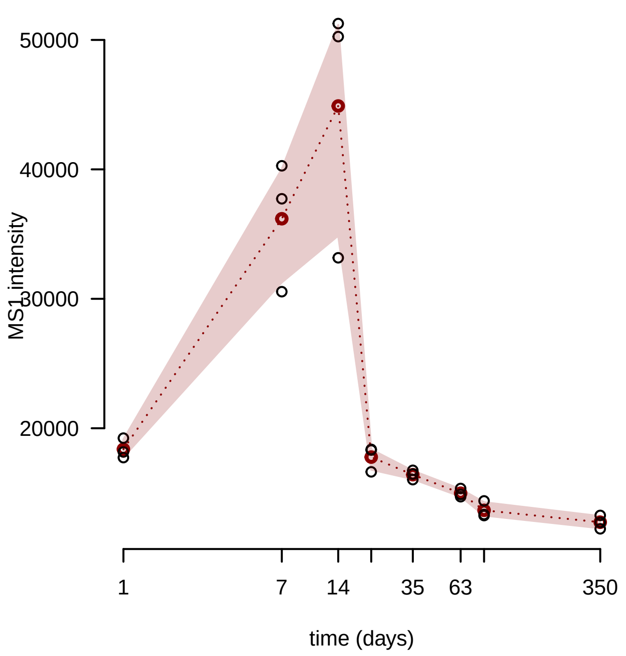

Q8C8R3
GN=Ank2
ankyrin 2, brain

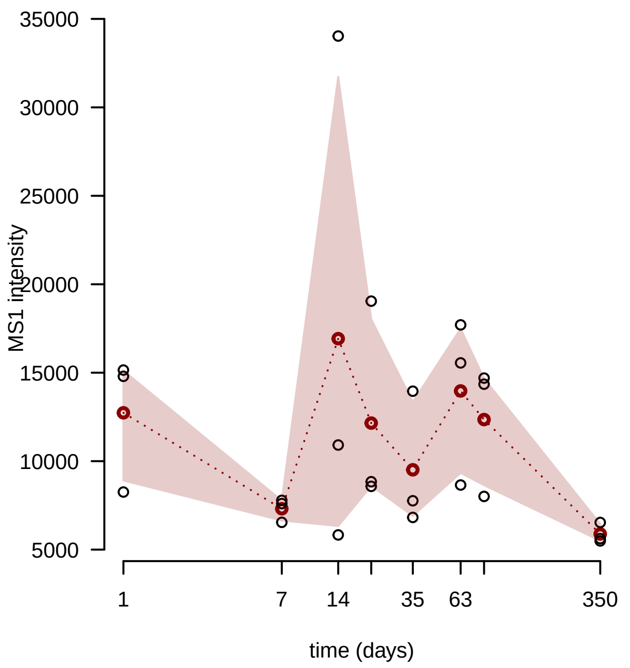

Q8C8T8
GN=Tsr2
TSR2 20S rRNA accumulation

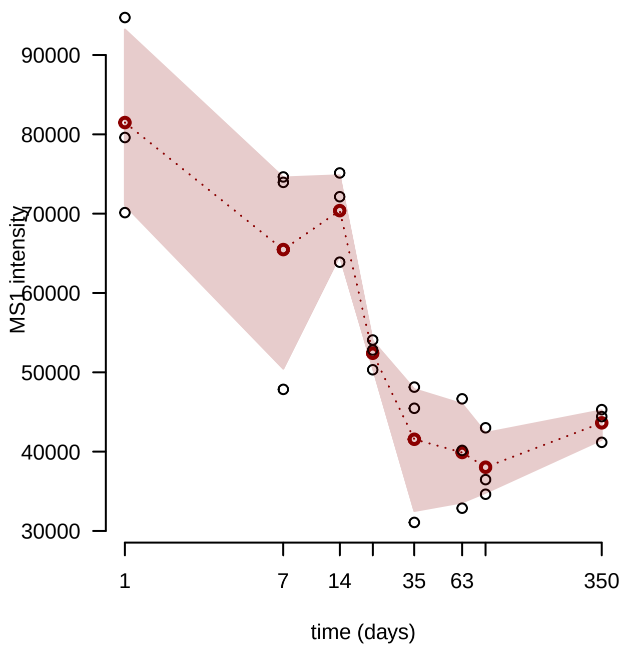

Q8C8U0
GN=Ppfibp1
PTPRF interacting protein, binding prote ...

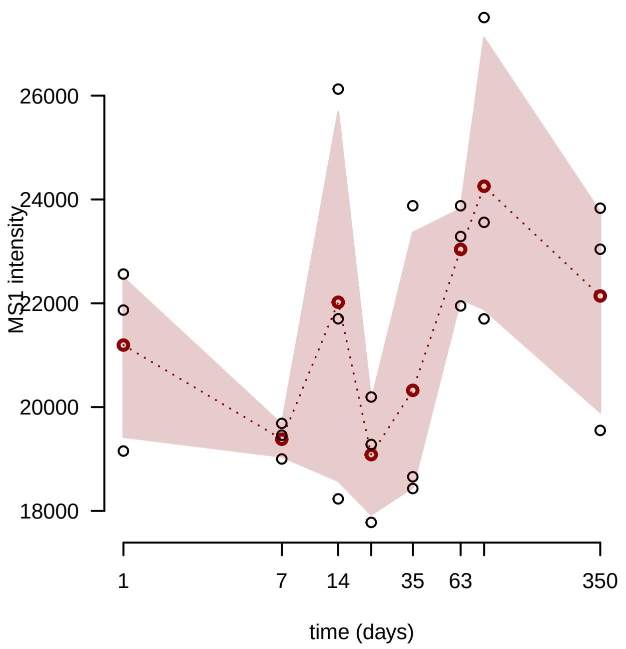

Q8C9A2
GN=Endov
endonuclease V

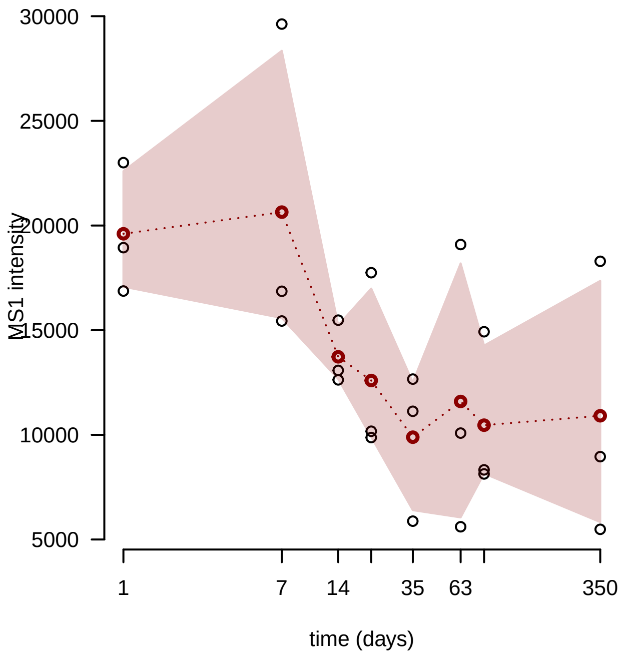

Q8C9B9
GN=Dido1
death inducer−obliterator 1

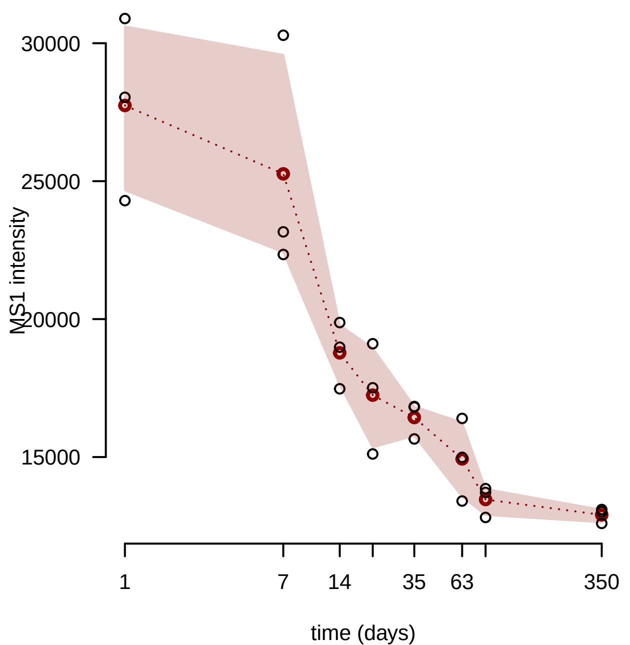

Q8C9M2
GN=Ccdc15
coiled−coil domain containing 15

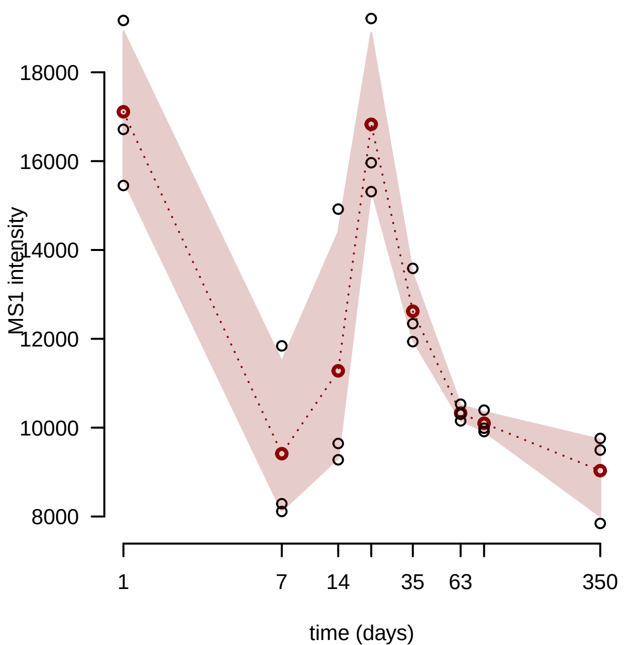

Q8C9S4
GN=Ccdc186
coiled−coil domain containing 186

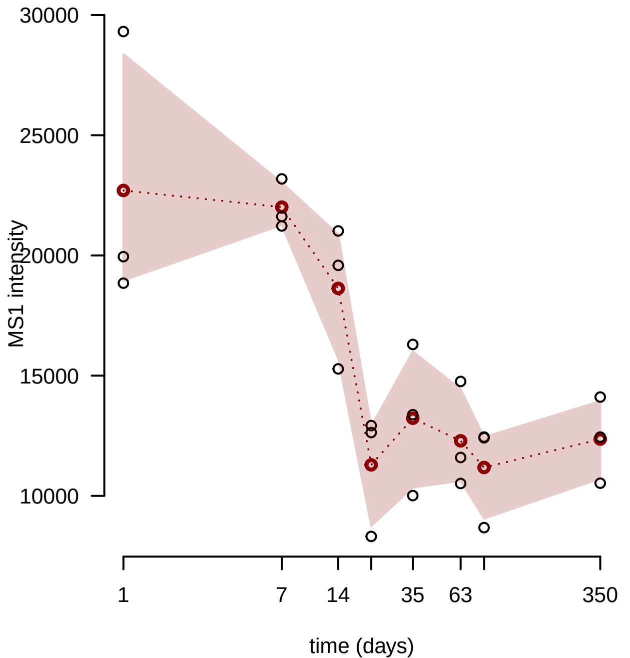

Q8C9S8
GN=Atg4a
autophagy related 4A, cysteine peptidase

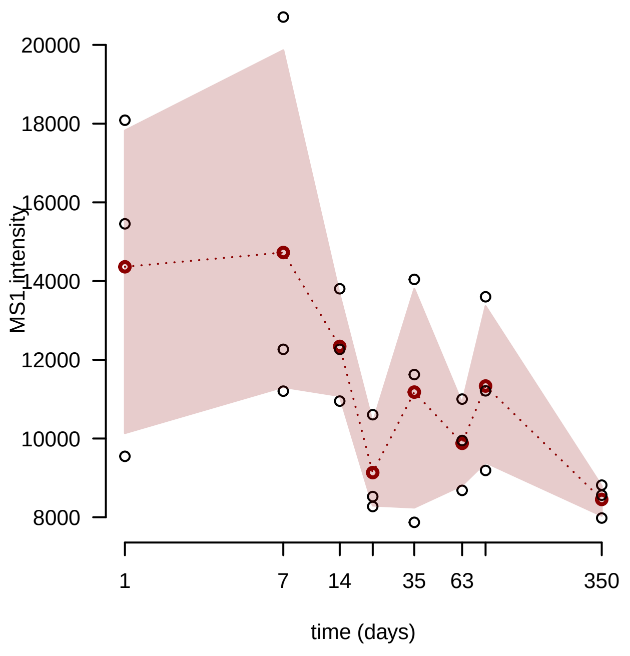

Q8C9X6
GN=Epc1
enhancer of polycomb homolog 1

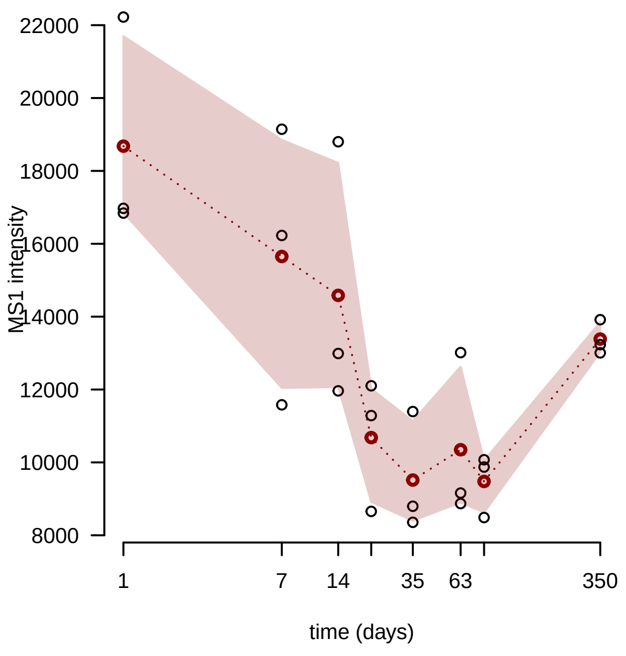

Q8CA71
GN=Shisa4
shisa family member 4

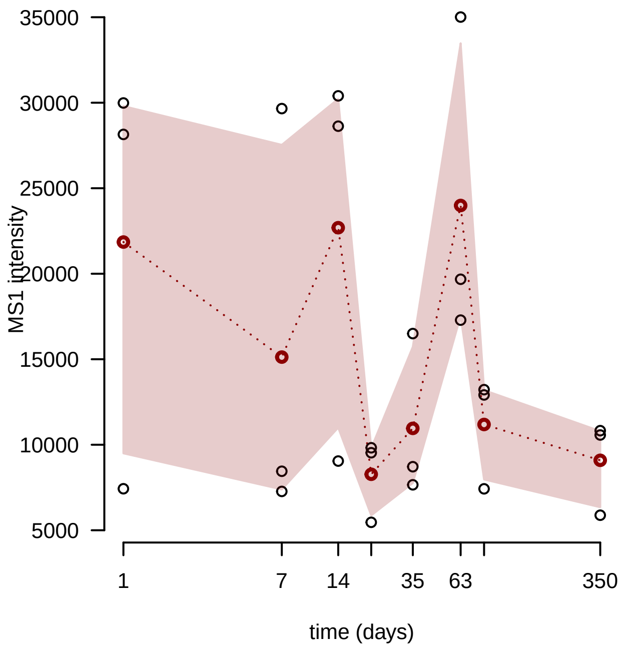

Q8CA72
GN=Gan
giant axonal neuropathy

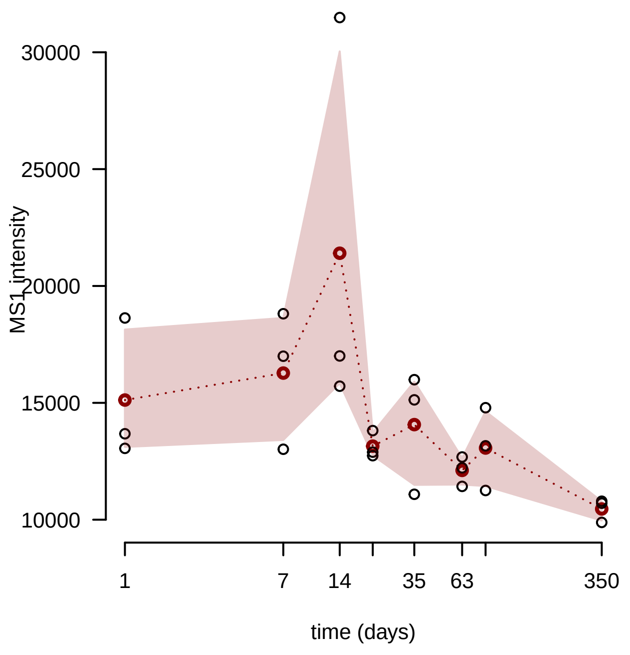

Q8CAA7
GN=Pgm2l1
phosphoglucomutase 2−like 1

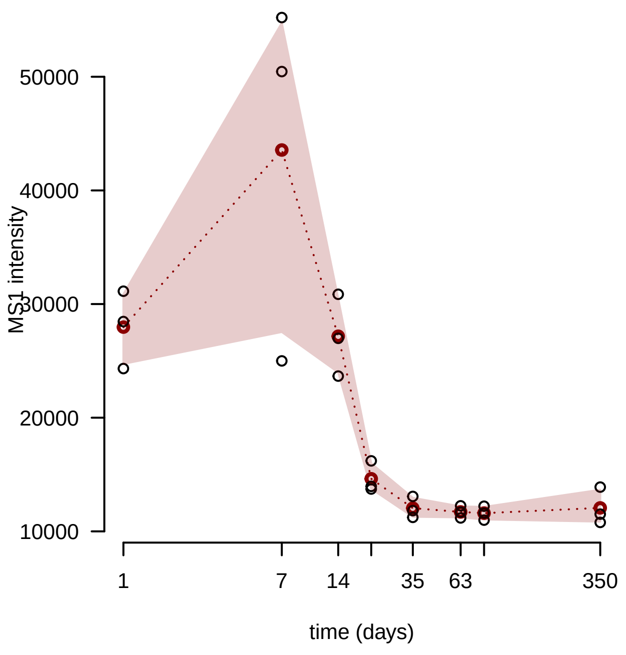

Q8CAB8
GN=Castor2
cytosolic arginine sensor for mTORC1 sub ...

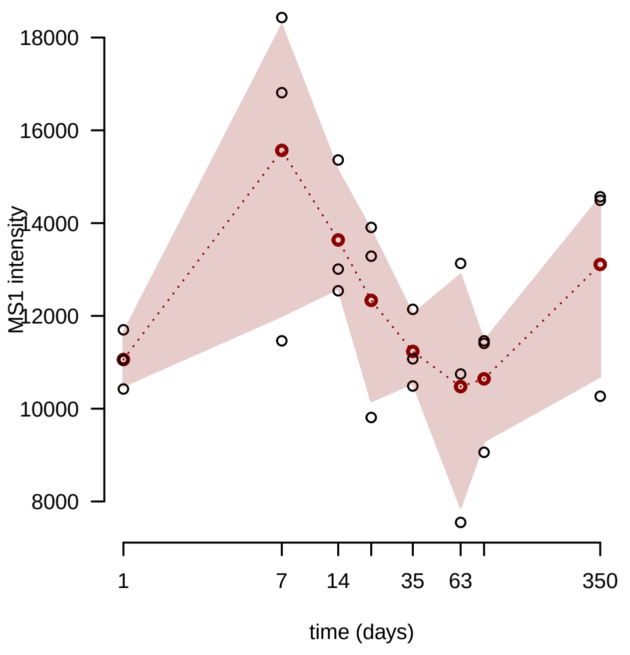

Q8CAK1
GN=Iba57
IBA57 homolog, iron−sulfur cluster assem ...

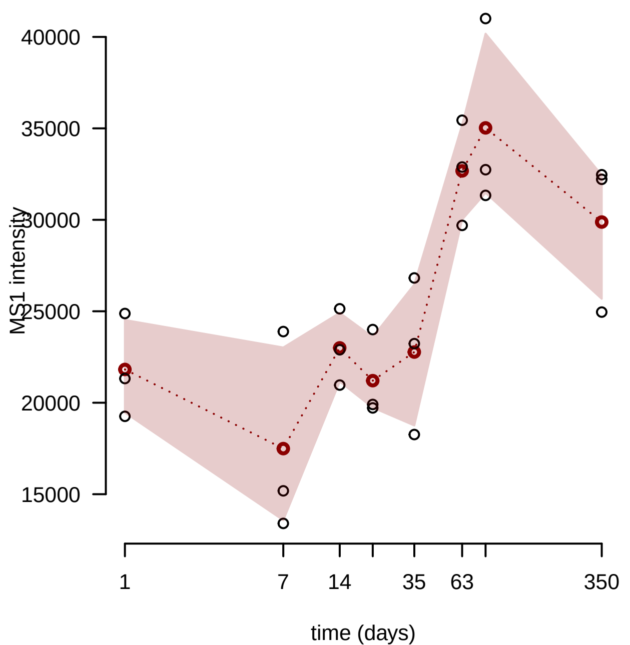

Q8CAK3
GN=Ryden

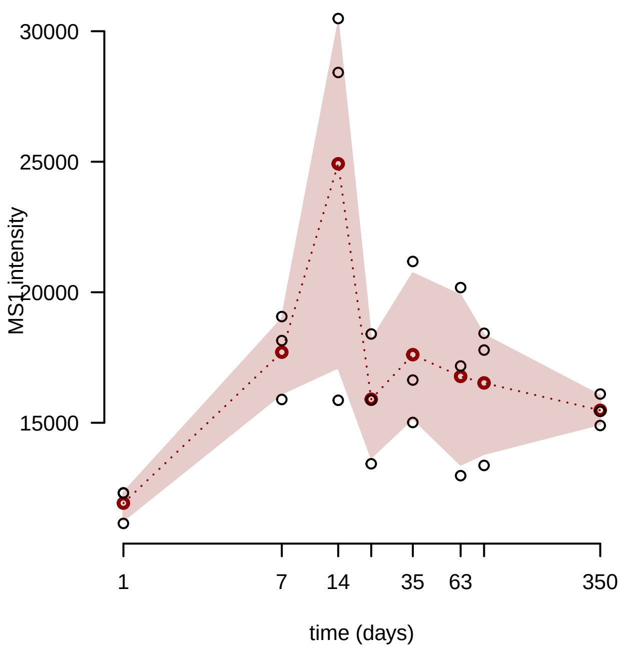

Q8CAM5
GN=Rab36
RAB36, member RAS oncogene family

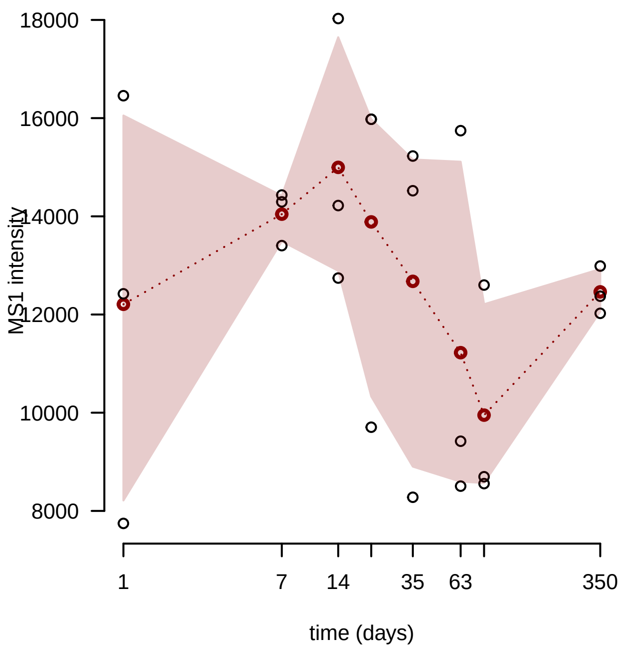

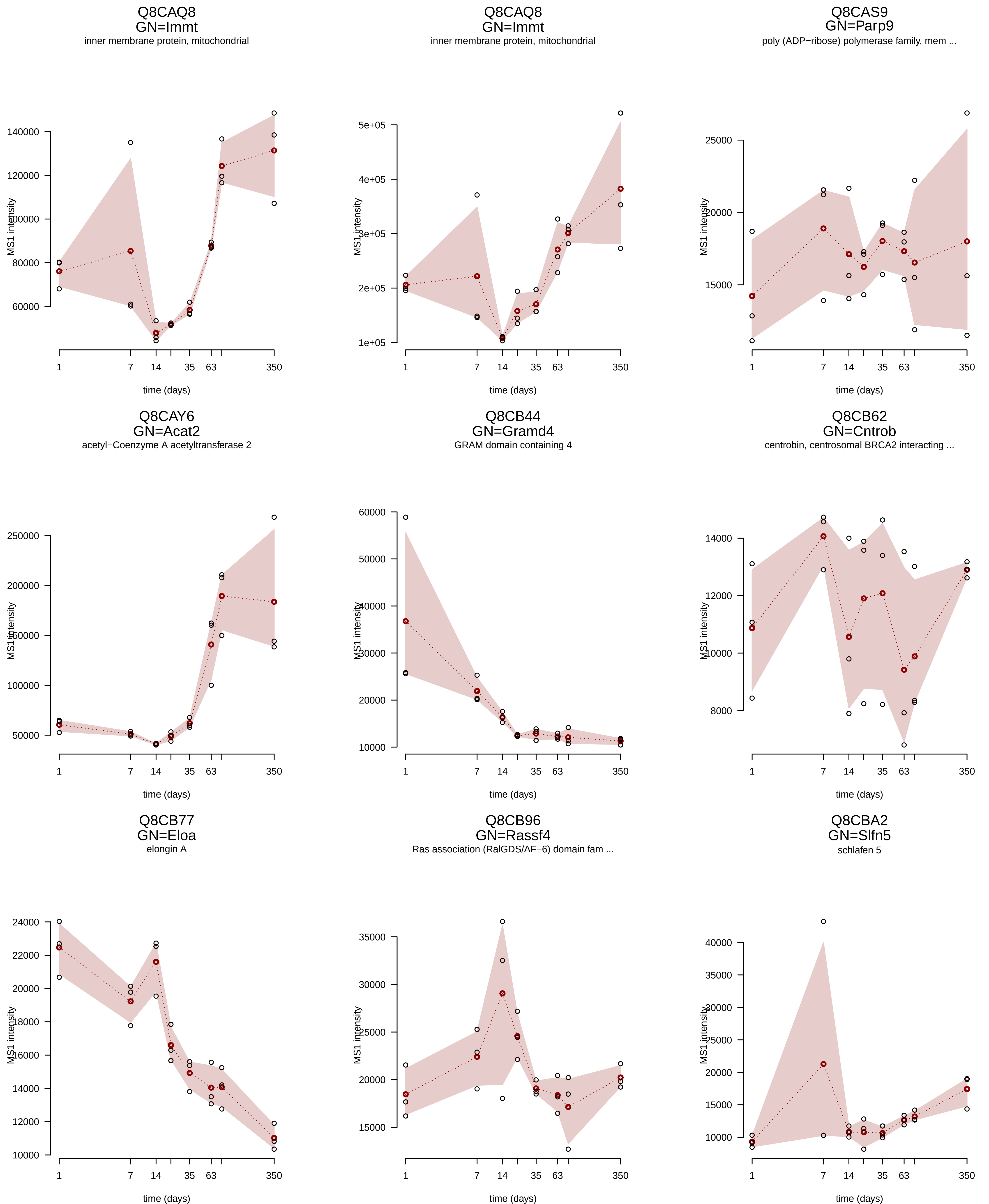

Q8CAQ8
GN=Immt
inner membrane protein, mitochondrial
Q8CAQ8
GN=Immt
inner membrane protein, mitochondrial
Q8CAS9
GN=Parp9
poly (ADP−ribose) polymerase family, mem ...
Q8CAY6
GN=Acat2
acetyl−Coenzyme A acetyltransferase 2
Q8CB44
GN=Gramd4
GRAM domain containing 4
Q8CB62
GN=Cntrob
centrobin, centrosomal BRCA2 interacting ...
Q8CB77
GN=Eloa
elongin A
Q8CB96
GN=Rassf4
Ras association (RalGDS/AF−6) domain fam ...
Q8CBA2
GN=Slfn5
schlafen 5
MS1 intensity
time (days)
1
7
14
35
63
350

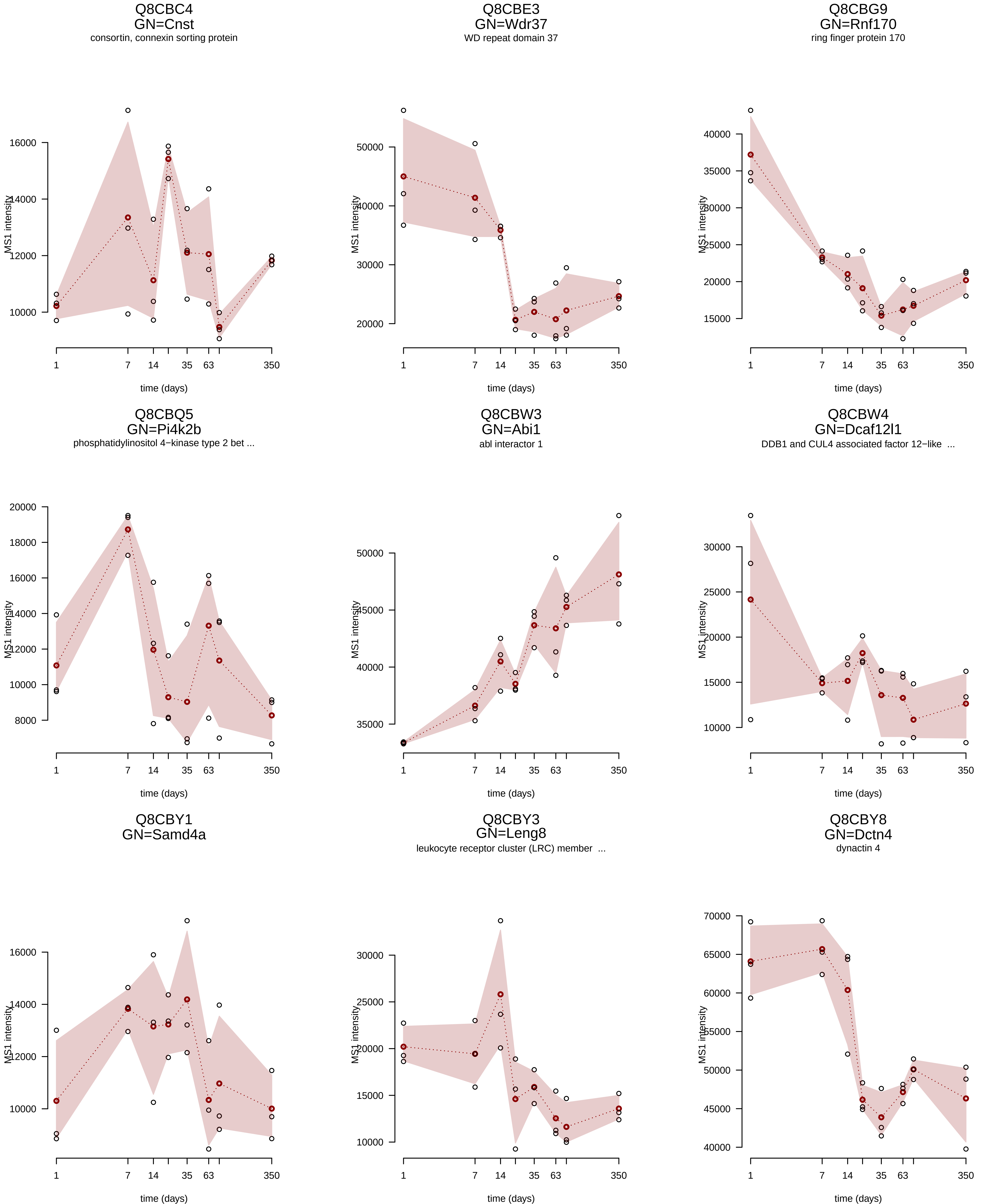
Q8CBC4
GN=Cnst
consortin, connexin sorting protein
MS1 intensity
10000
12000
14000
16000
1
7
14
35
63
350
time (days)
Q8CBE3
GN=Wdr37
WD repeat domain 37
MS1 intensity
20000
30000
40000
50000
time (days)
Q8CBG9
GN=Rnf170
ring finger protein 170
MS1 intensity
15000
20000
25000
30000
35000
40000
time (days)
Q8CBQ5
GN=Pi4k2b
phosphatidylinositol 4−kinase type 2 bet ...
MS1 intensity
8000
10000
12000
14000
16000
18000
20000
time (days)
Q8CBW3
GN=Abi1
abl interactor 1
MS1 intensity
35000
40000
45000
50000
time (days)
Q8CBW4
GN=Dcaf12l1
DDB1 and CUL4 associated factor 12−like ...
MS1 intensity
10000
15000
20000
25000
30000
time (days)
Q8CBY1
GN=Samd4a
MS1 intensity
10000
12000
14000
16000
time (days)
Q8CBY3
GN=Leng8
leukocyte receptor cluster (LRC) member ...
MS1 intensity
10000
15000
20000
25000
30000
time (days)
Q8CBY8
GN=Dctn4
dynactin 4
MS1 intensity
40000
45000
50000
55000
60000
65000
70000
time (days)

Q8CC12
GN=Cdan1
congenital dyserythropoietic anemia, typ ...

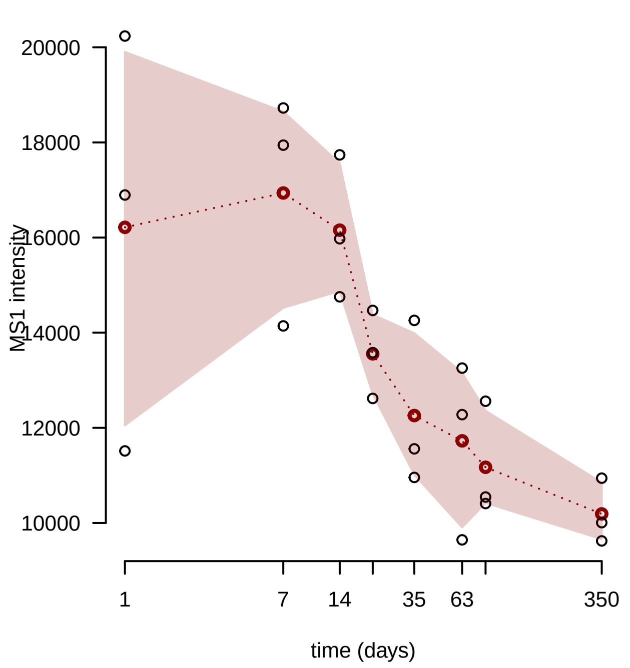

Q8CC21
GN=Ttc19
tetratricopeptide repeat domain 19

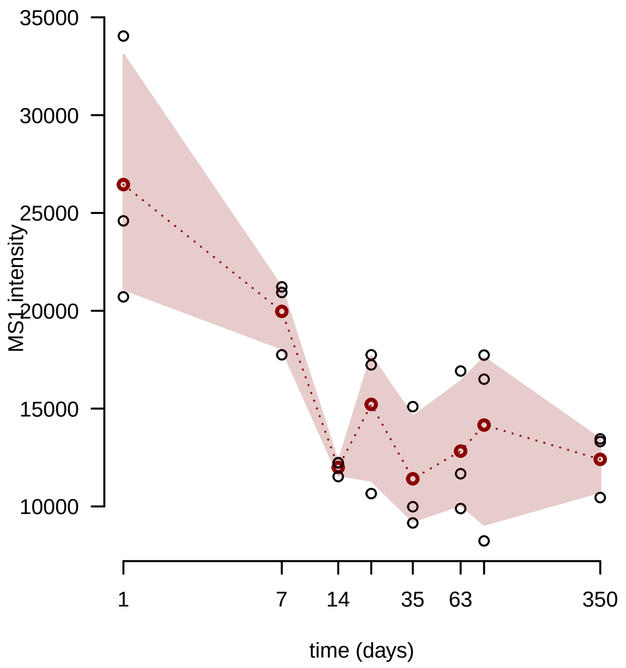

Q8CC27
GN=Cacnb2
calcium channel, voltage−dependent, beta ...

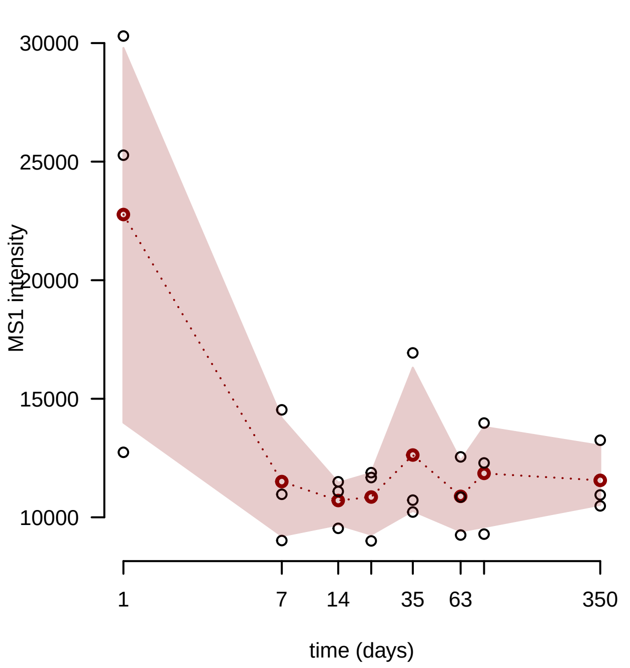

Q8CC35
GN=Synpo
synaptopodin

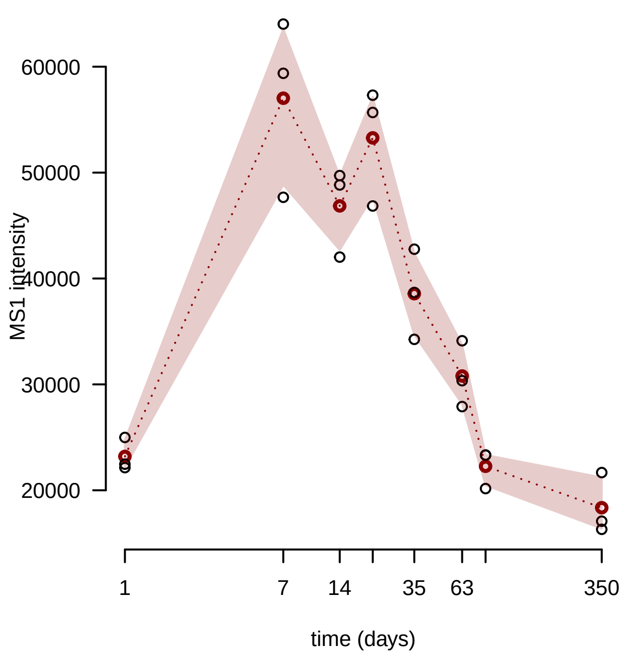

Q8CC35
GN=Synpo
synaptopodin

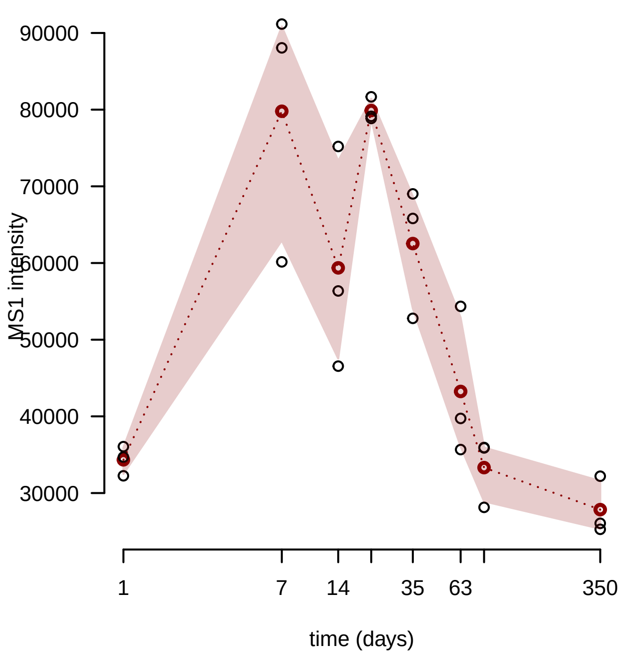

Q8CC70
GN=Cfap300
cilia and flagella associated protein 30 ...

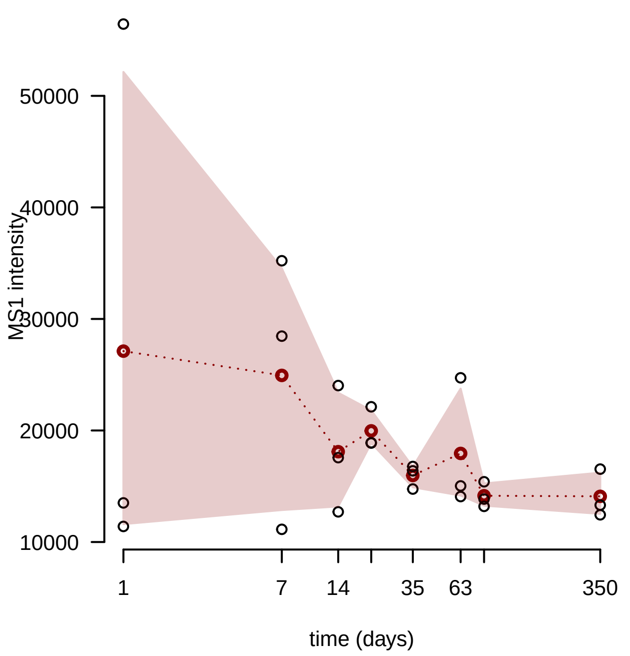

Q8CC86
GN=Naprt
nicotinate phosphoribosyltransferase

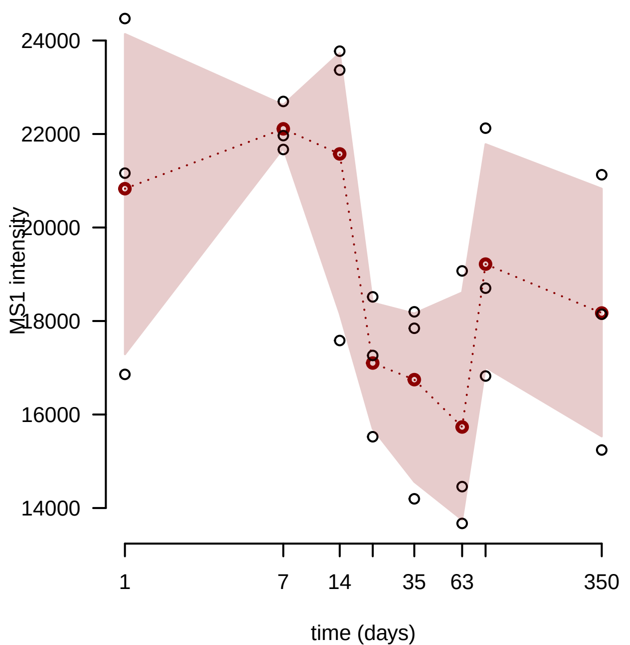

Q8CC88
GN=Vwa8
von Willebrand factor A domain containin ...

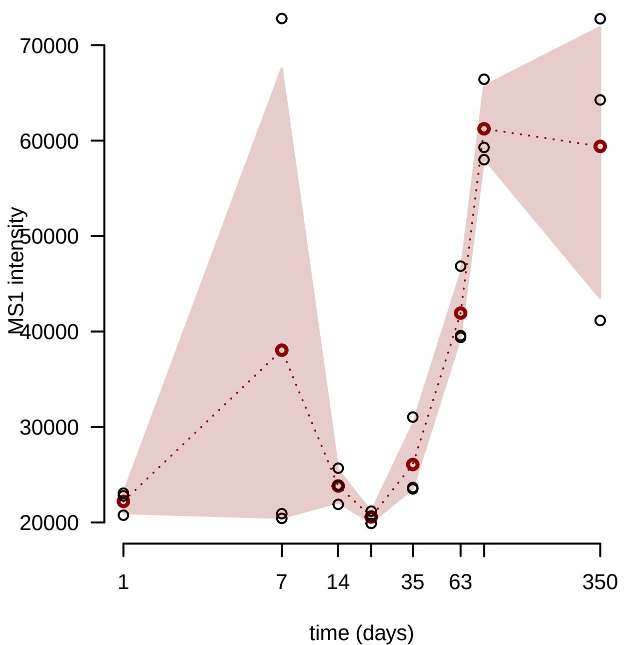

Q8CCA0
GN=Dcun1d4
DCN1, defective in cullin neddylation 1, ...

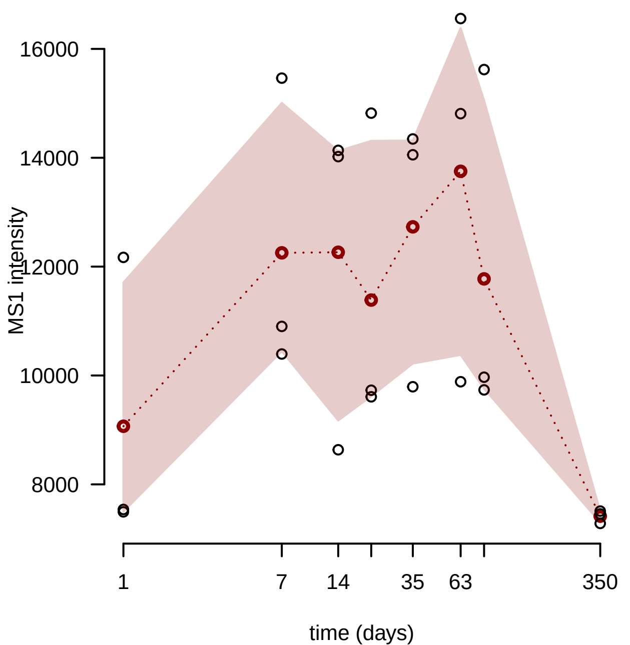

Q8CCB4
GN=Vps53
VPS53 GARP complex subunit

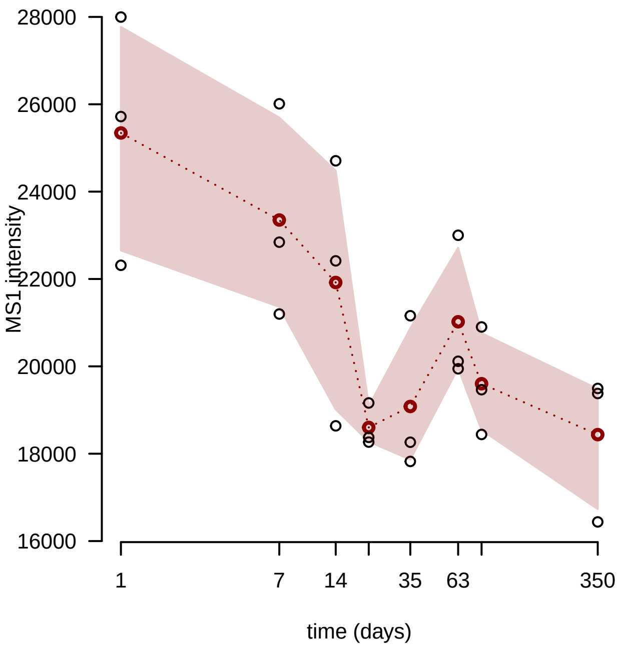

Q8CCB5
GN=Mettl24
methyltransferase like 24

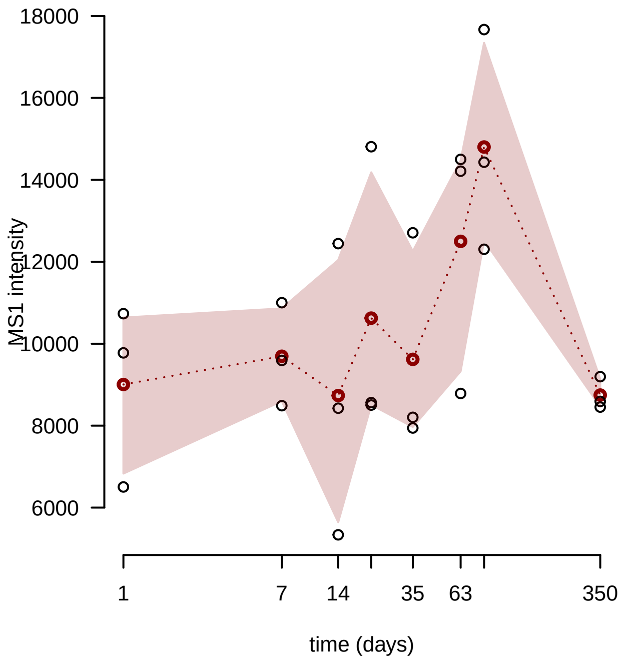

Q8CCF0
GN=Prpf31
pre−mRNA processing factor 31

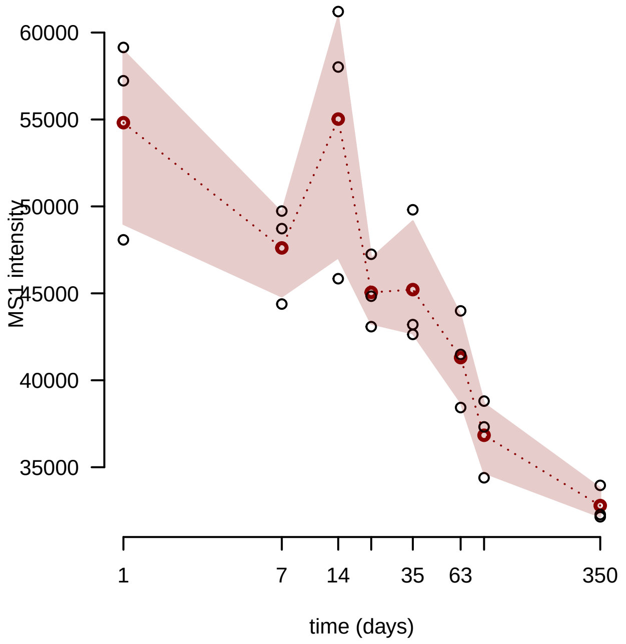

Q8CCH2
GN=Nhlrc3
NHL repeat containing 3

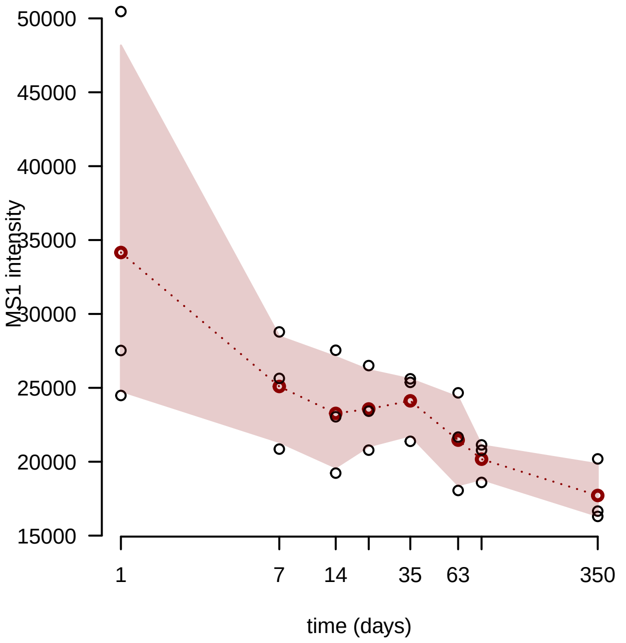

Q8CCH7
GN=Zfpm2
zinc finger protein, multitype 2

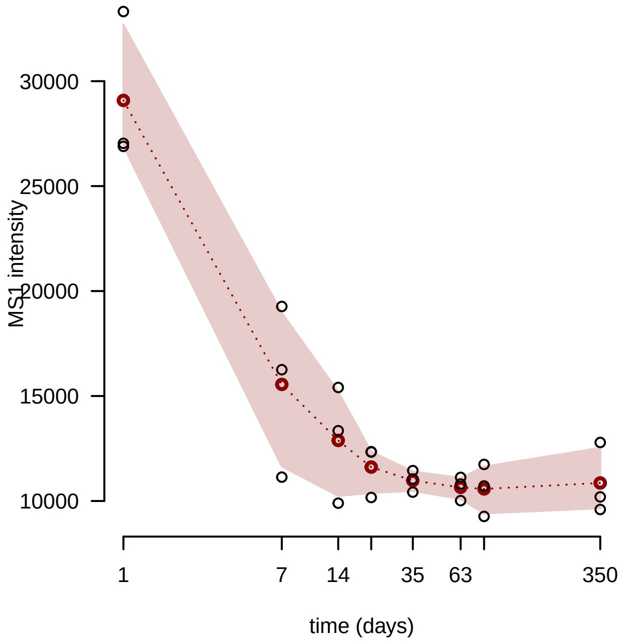

Q8CCI5
GN=Rybp
RING1 and YY1 binding protein

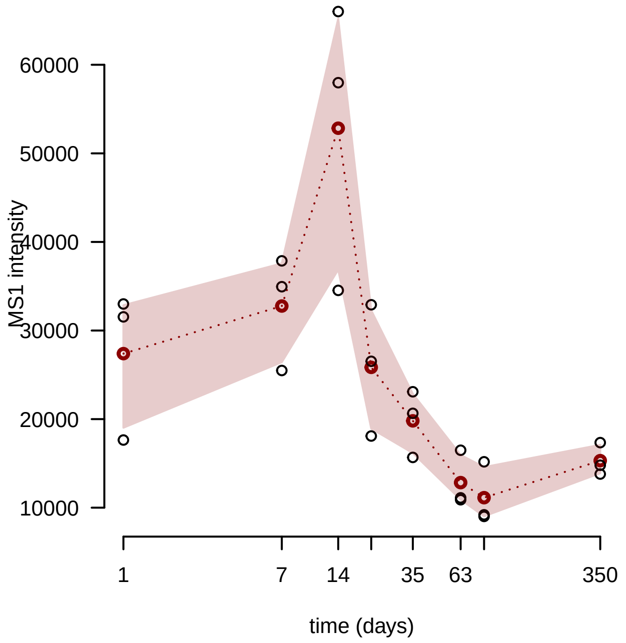

Q8CCJ3
GN=Ufl1
UFM1 specific ligase 1

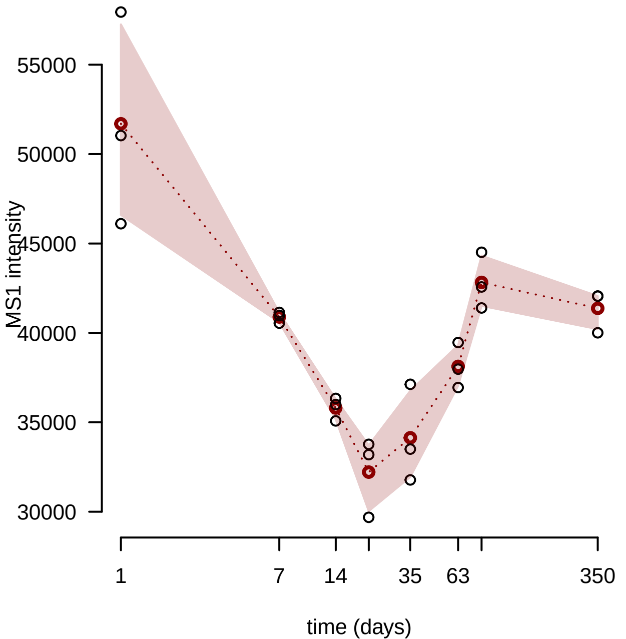

Q8CCJ9
GN=Phf20l1
PHD finger protein 20−like 1

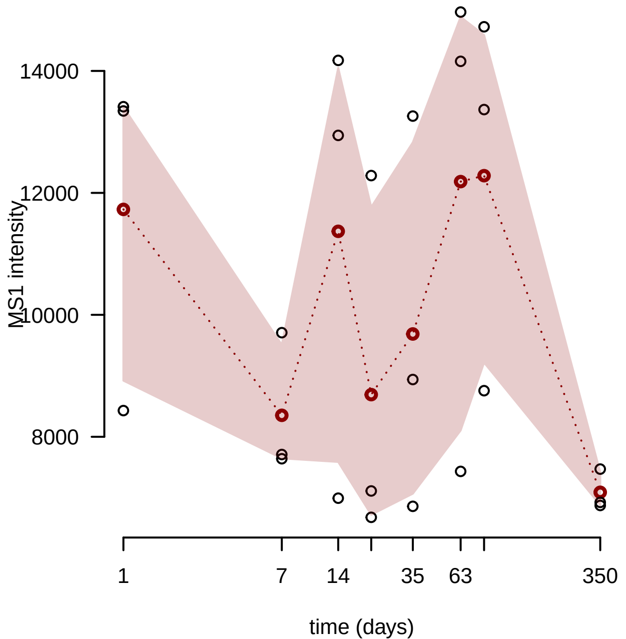

Q8CCK0
GN=H2afy2

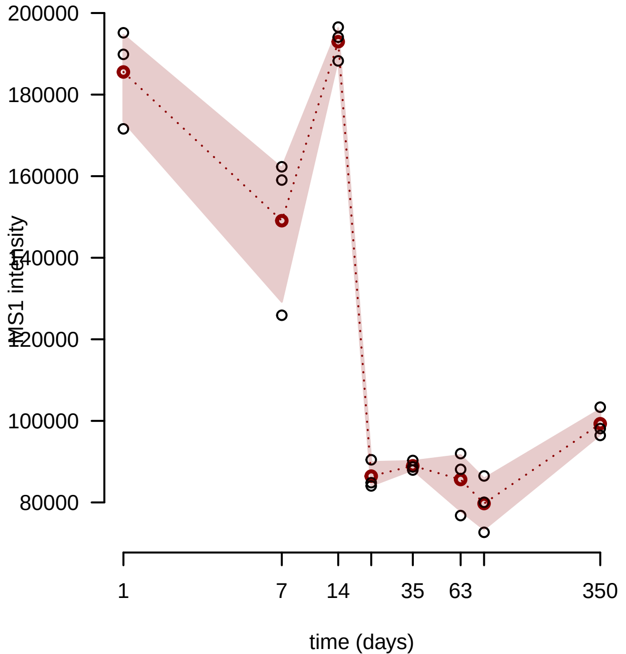

Q8CCM6
GN=Timm21
translocase of inner mitochondrial membr ...

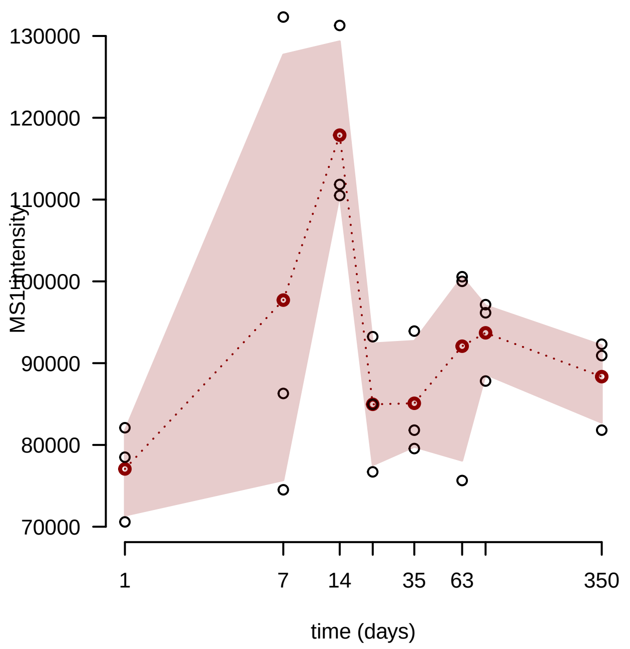

Q8CCN5
GN=Bcas3
BCAS3 microtubule associated cell migrat ...

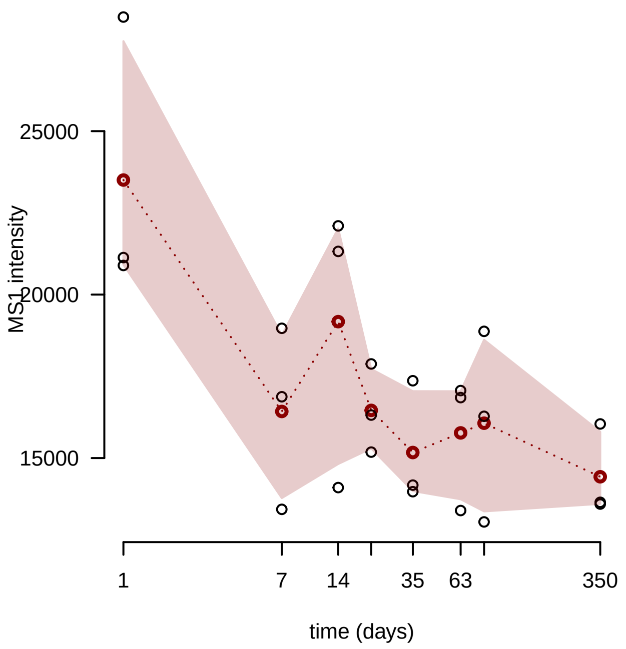

Q8CCP0
GN=Nemf
nuclear export mediator factor

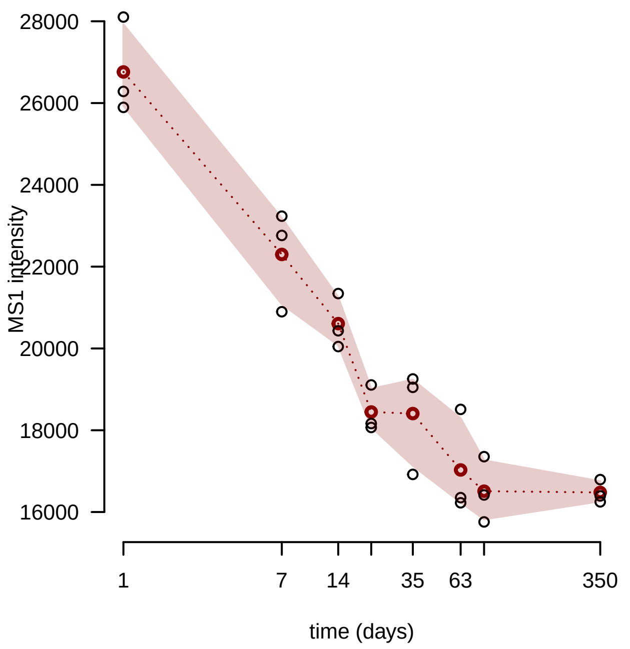

Q8CCS6
GN=Pabpn1
poly(A) binding protein, nuclear 1

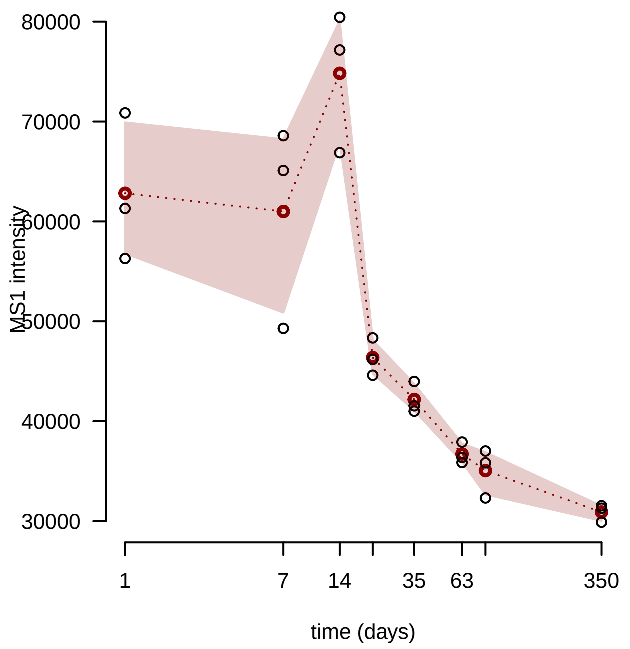

Q8CCT4
GN=Tceal5
transcription elongation factor A (SII)− ...

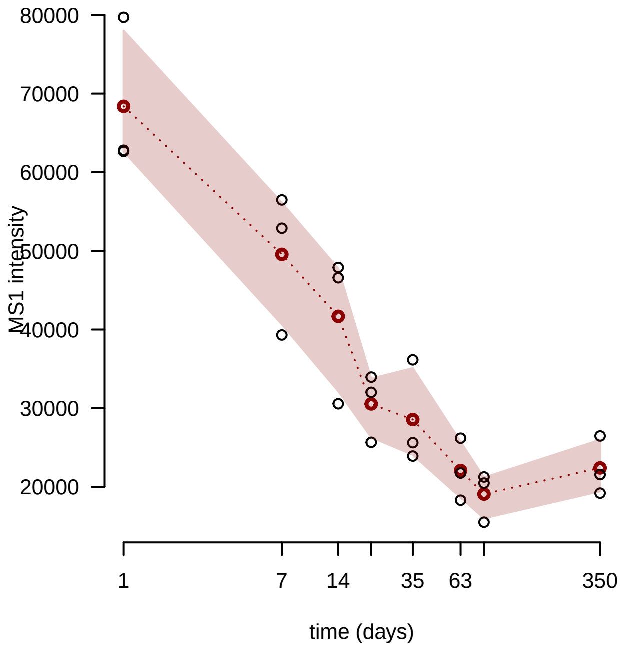

Q8CCX5
GN=Krt222
keratin 222

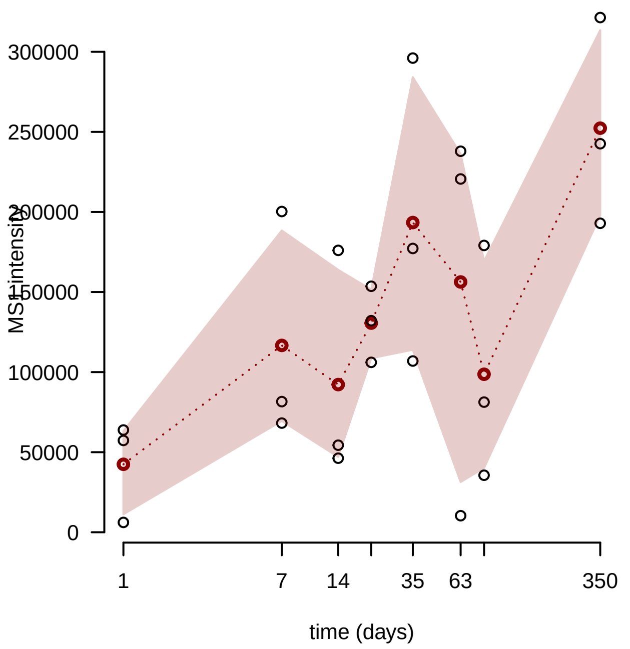

Q8CD10
GN=Micu2
mitochondrial calcium uptake 2

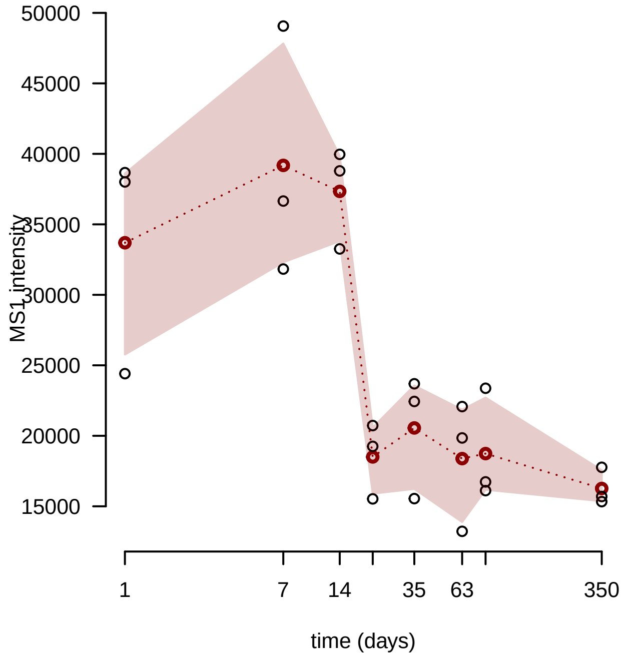

Q8CD15
GN=Riox2
ribosomal oxygenase 2

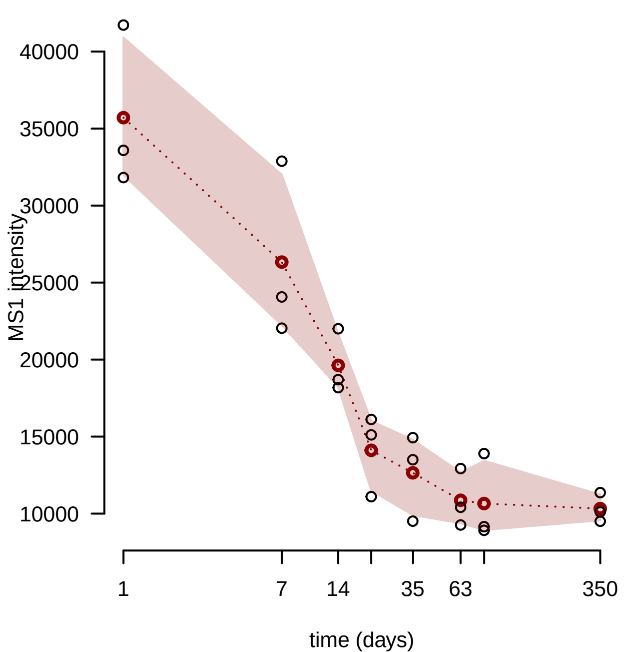

Q8CD19
GN=Lancl3
LanC lantibiotic synthetase component C− ...

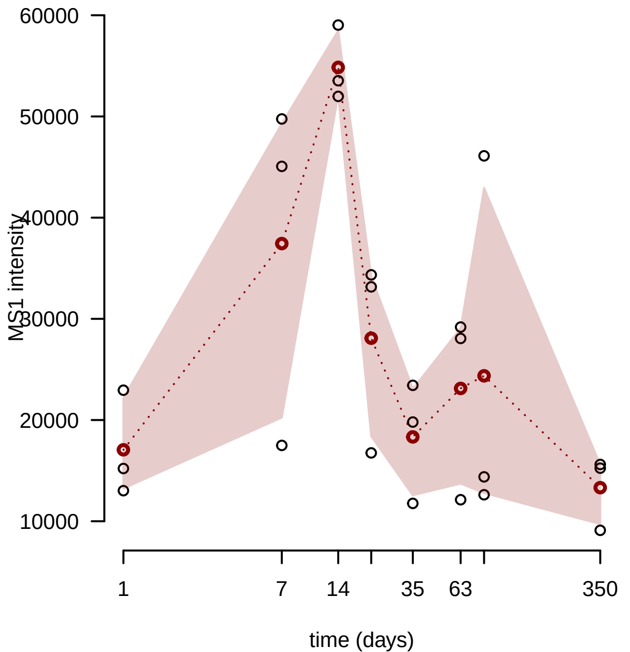

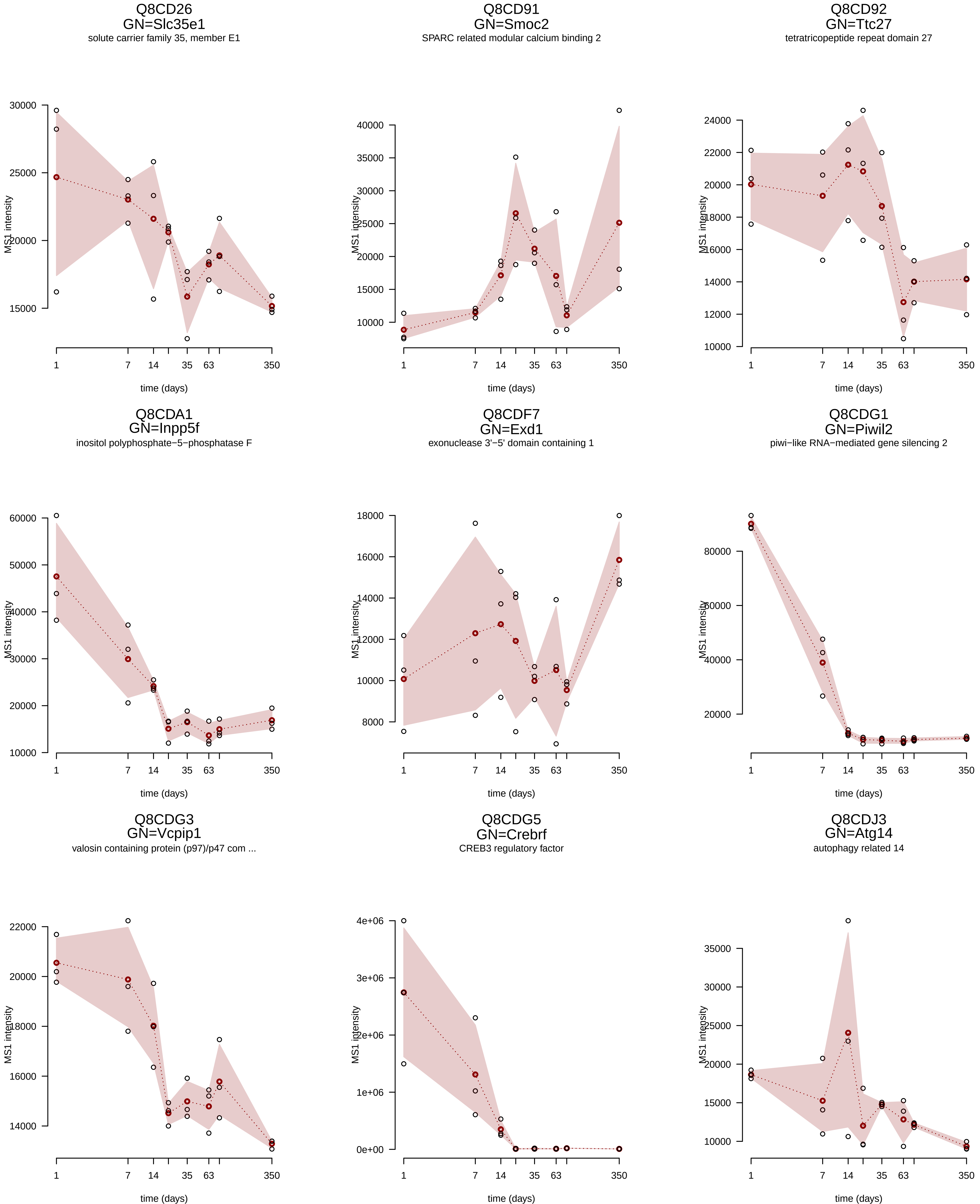
Q8CD26
GN=Slc35e1
solute carrier family 35, member E1
Q8CD91
GN=Smoc2
SPARC related modular calcium binding 2
Q8CD92
GN=Ttc27
tetratricopeptide repeat domain 27
Q8CDA1
GN=Inpp5f
inositol polyphosphate−5−phosphatase F
Q8CDF7
GN=Exd1
exonuclease 3'−5' domain containing 1
Q8CDG1
GN=Piwil2
piwi−like RNA−mediated gene silencing 2
Q8CDG3
GN=Vcpip1
valosin containing protein (p97)/p47 com ...
Q8CDG5
GN=Crebrf
CREB3 regulatory factor
Q8CDJ3
GN=Atg14
autophagy related 14
MS1 intensity
time (days)
1
7
14
35
63
350

Q8CDJ8
GN=Ston1
stonin 1

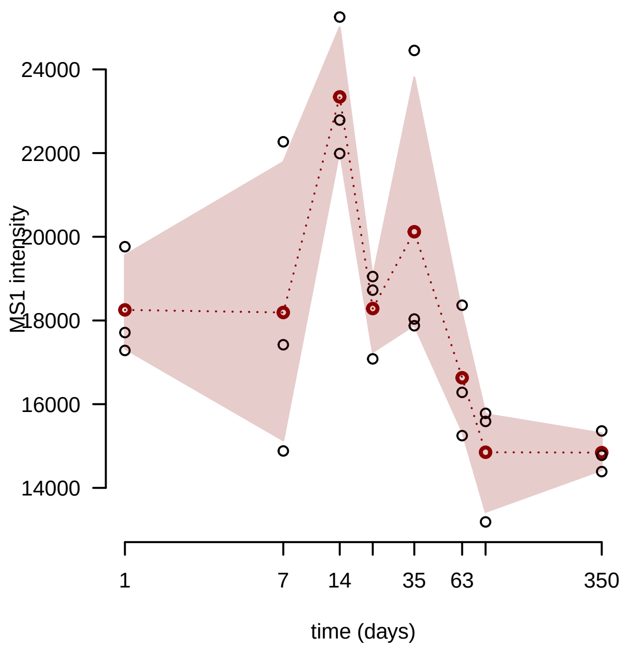

Q8CDM1
GN=Atad2
ATPase family, AAA domain containing 2

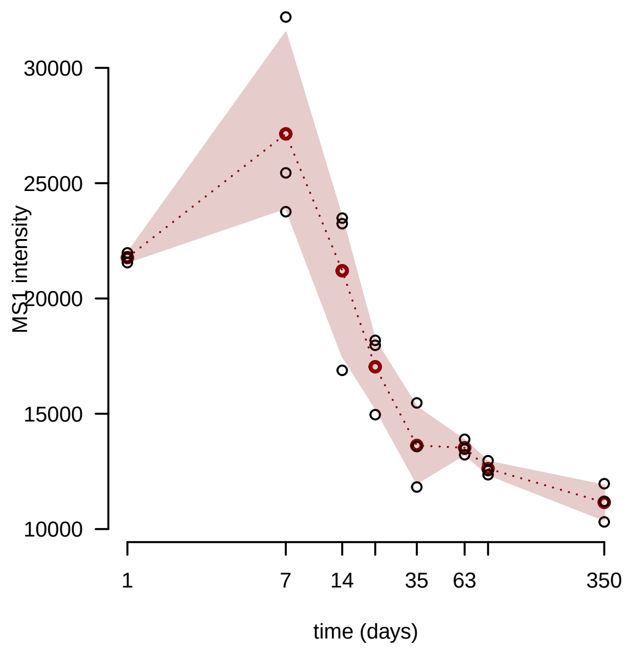

Q8CDM8
GN=Fam160b1

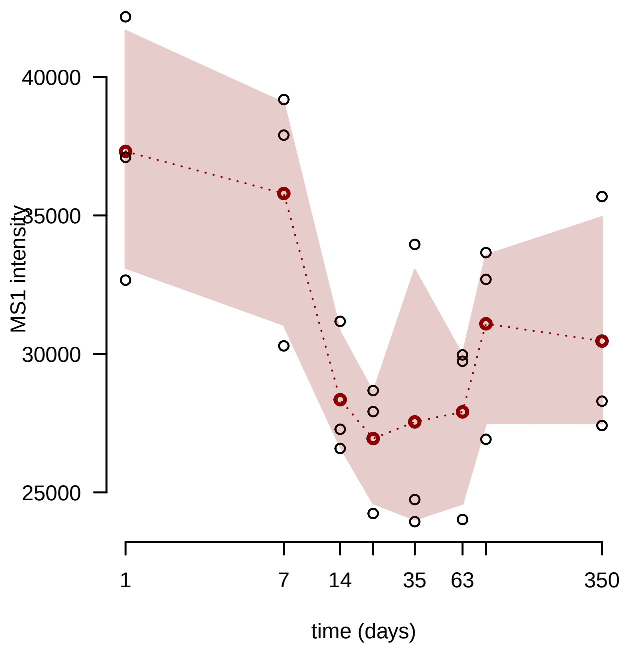

Q8CDN6
GN=Txnl1
thioredoxin−like 1

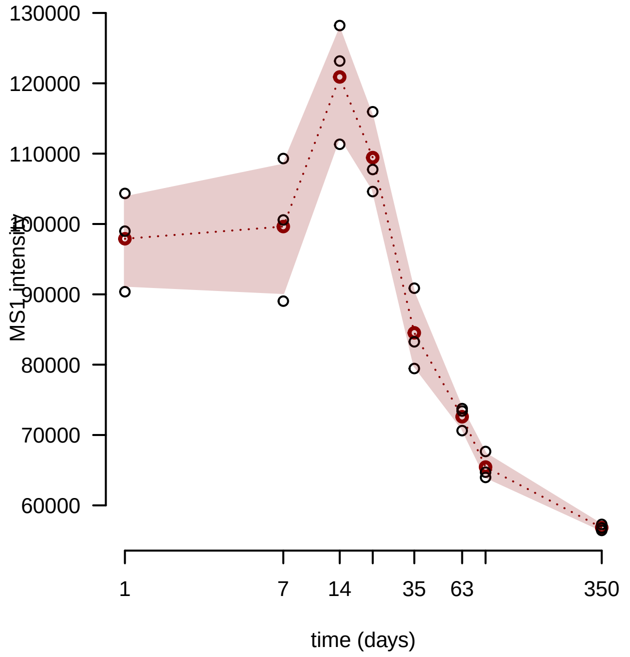

Q8CE33
GN=Klhl11
kelch−like 11

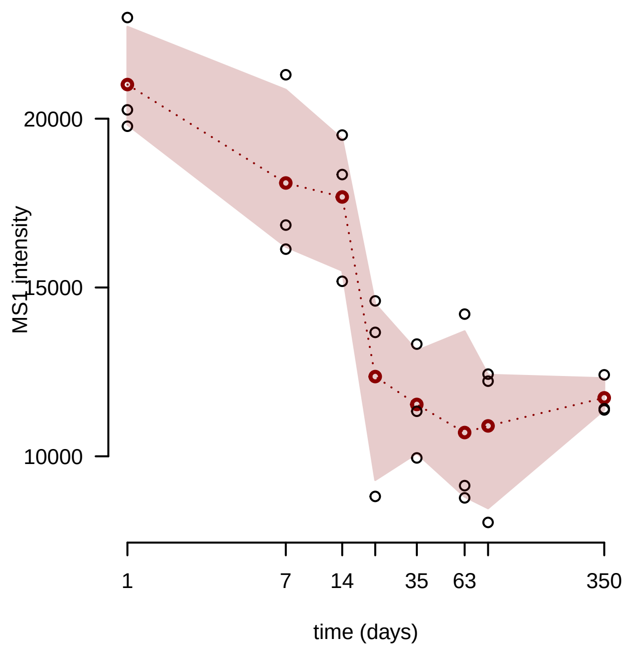

Q8CE46
GN=Pus7l
pseudouridylate synthase 7−like

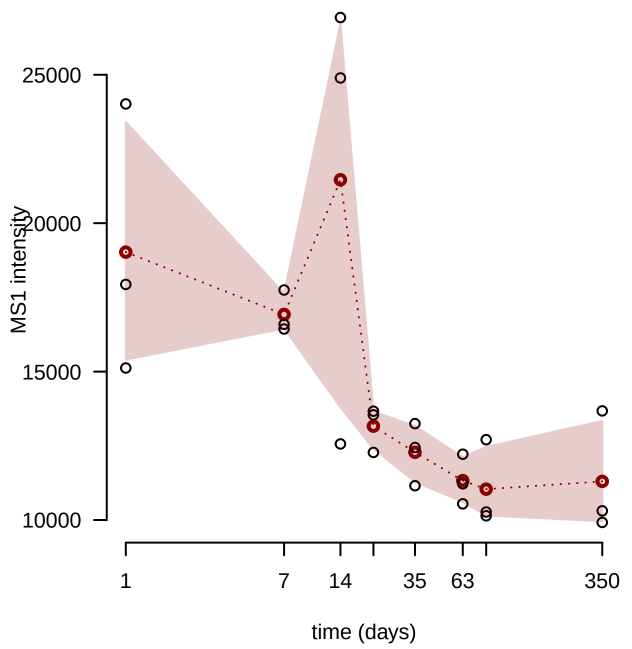

Q8CE50
GN=Snx30
sorting nexin family member 30

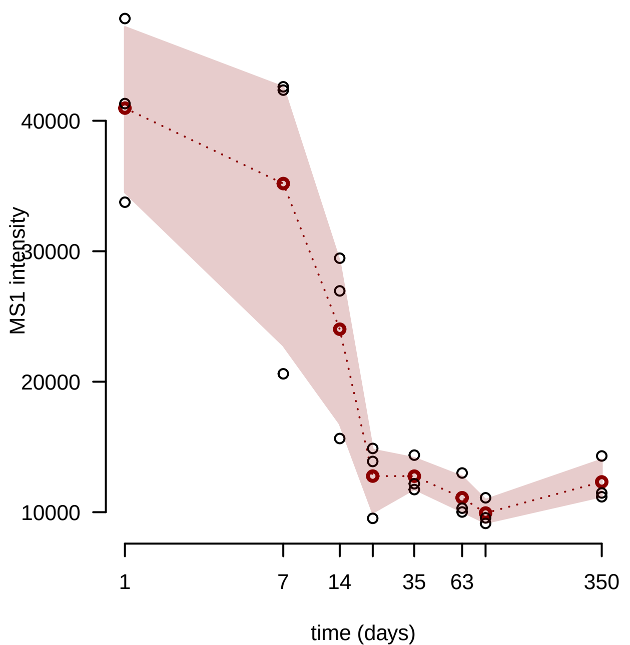

Q8CE64
GN=Znf276

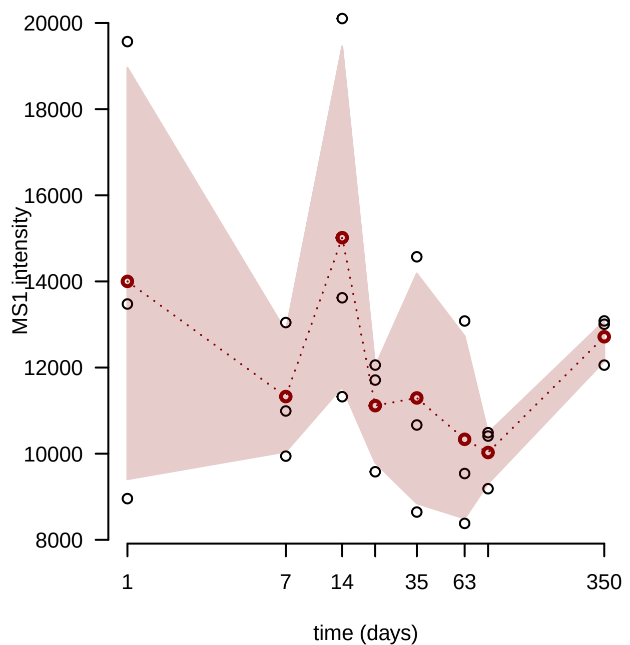

Q8CE90
GN=Map2k7
mitogen−activated protein kinase kinase ...

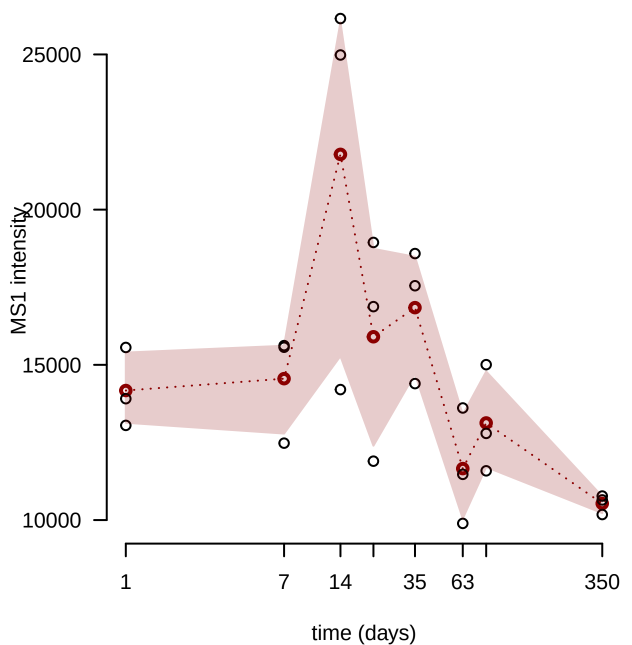

Q8CE96
GN=Trmt6
tRNA methyltransferase 6

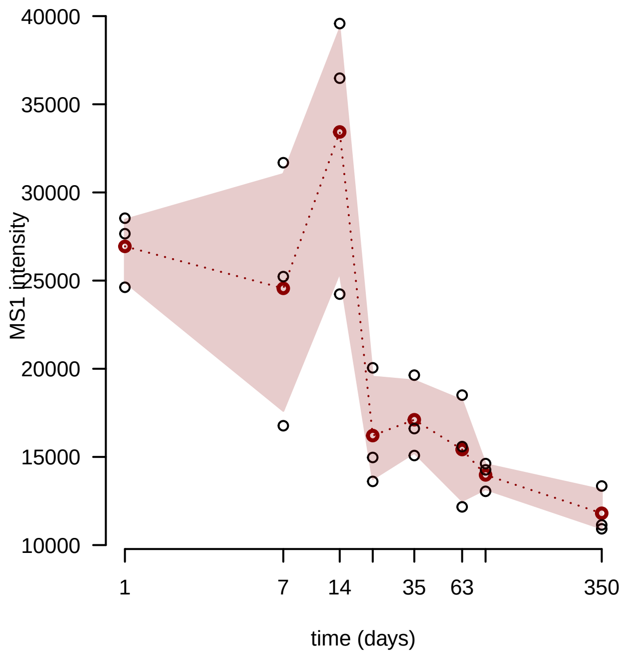

Q8CEC0
GN=Nup88
nucleoporin 88

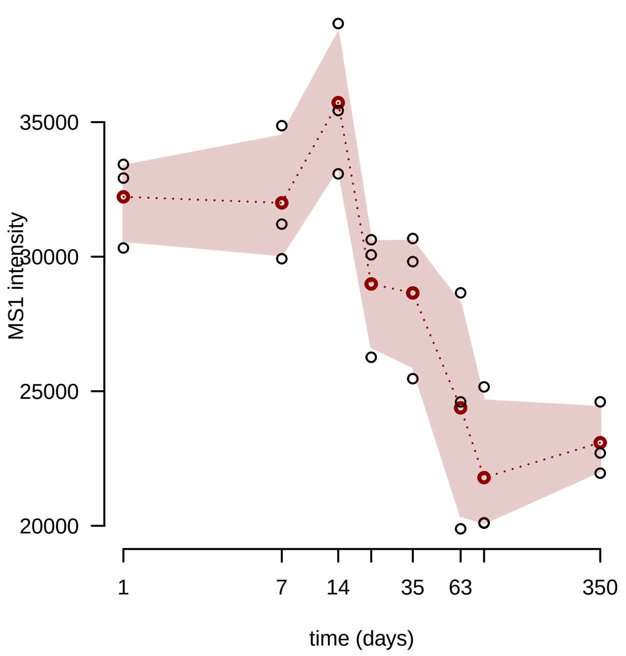

Q8CEC6
GN=Ppwd1
peptidylprolyl isomerase domain and WD r ...

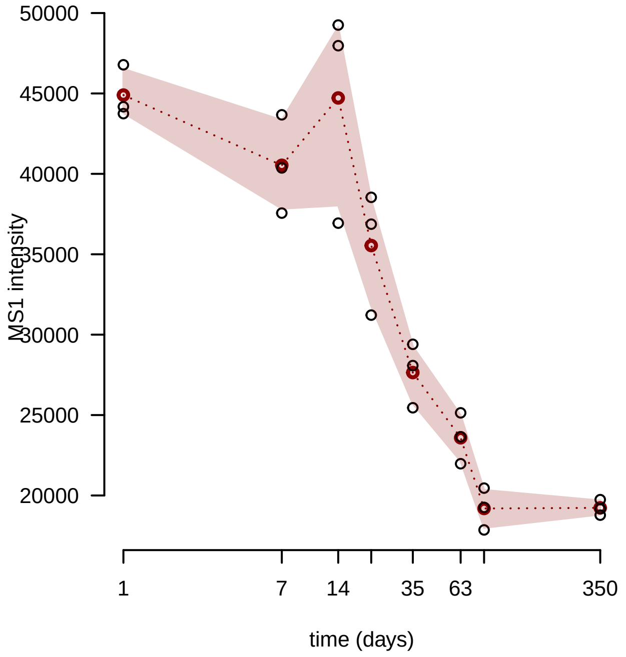

Q8CEE6
GN=Pask
PAS domain containing serine/threonine k ...

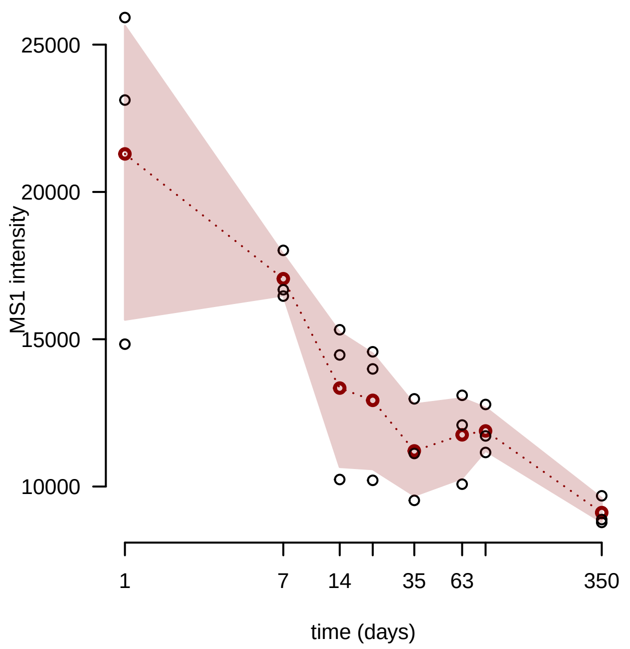

Q8CEE7
GN=Rdh13
retinol dehydrogenase 13 (all−trans and ...

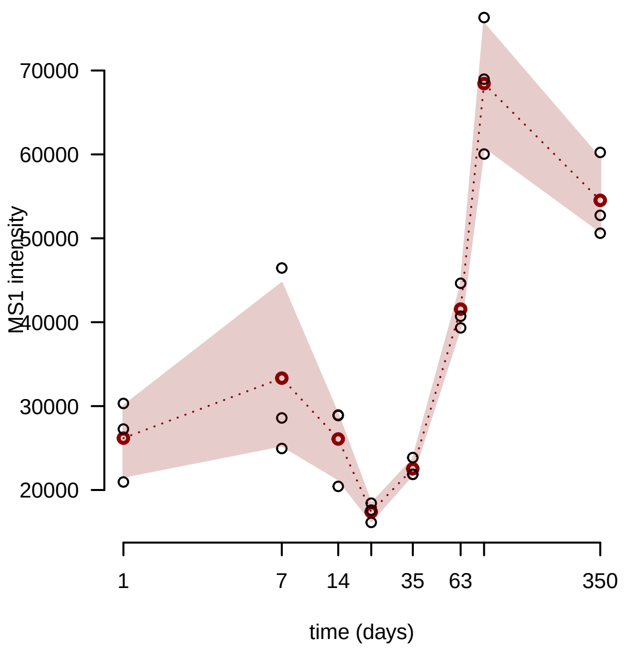

Q8CEG5
GN=Ccdc28b
coiled coil domain containing 28B

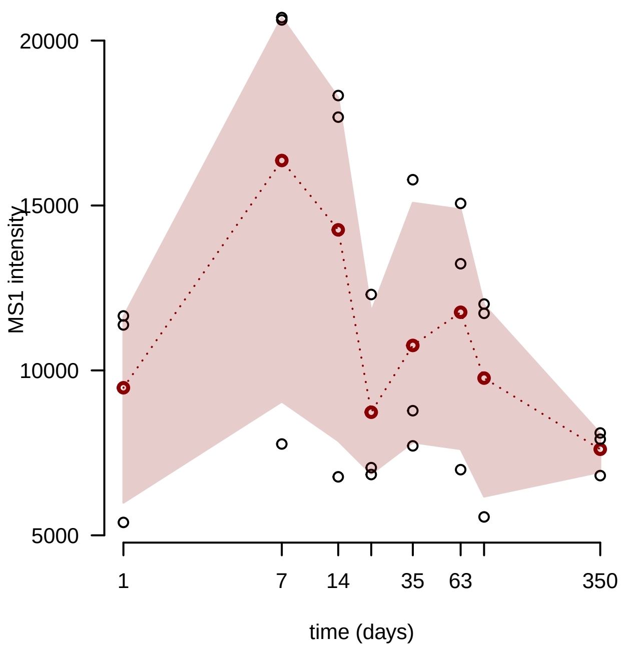

Q8CEI1
GN=Bola3
bolA−like 3 (E. coli)

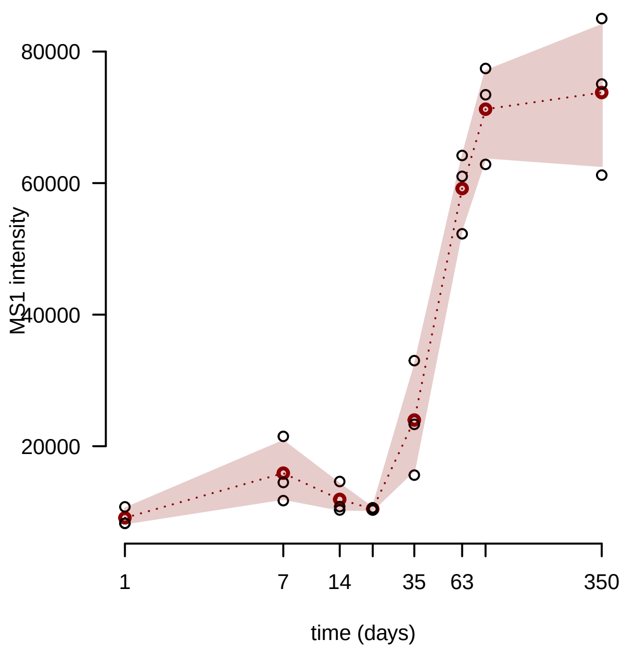

Q8CES0
GN=Naa30
N(alpha)−acetyltransferase 30, NatC cata ...

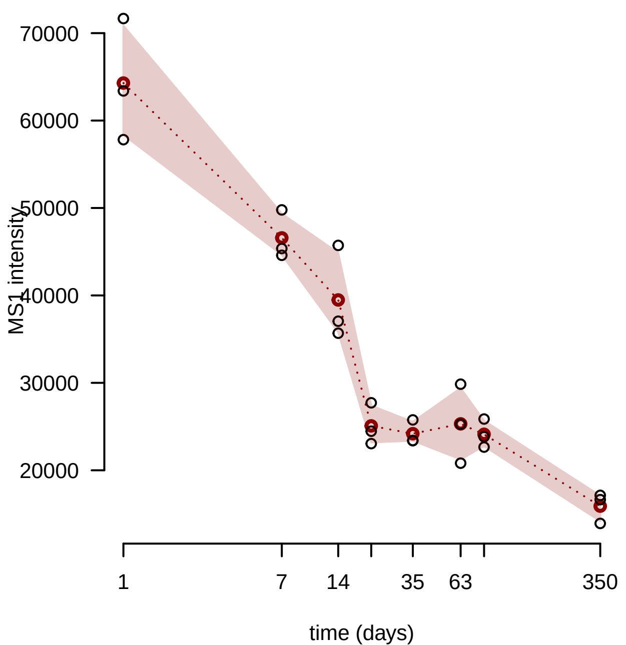

Q8CF60
GN=

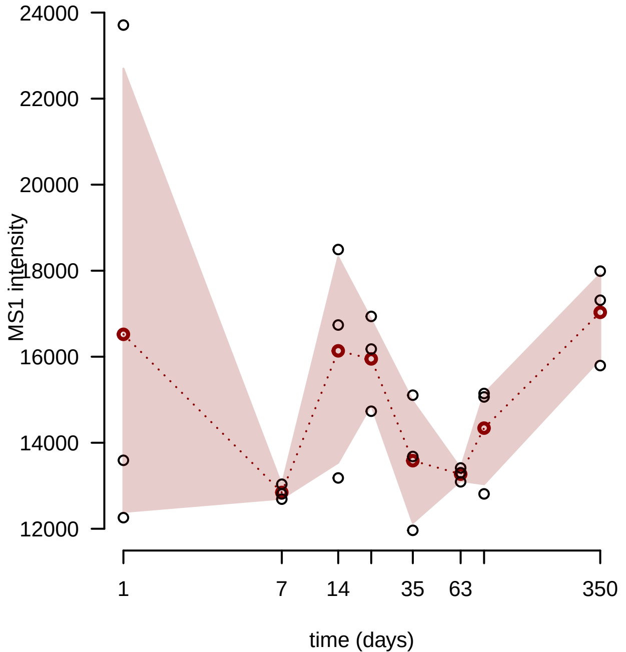

Q8CF66
GN=Lamtor4
late endosomal/lysosomal adaptor, MAPK a ...

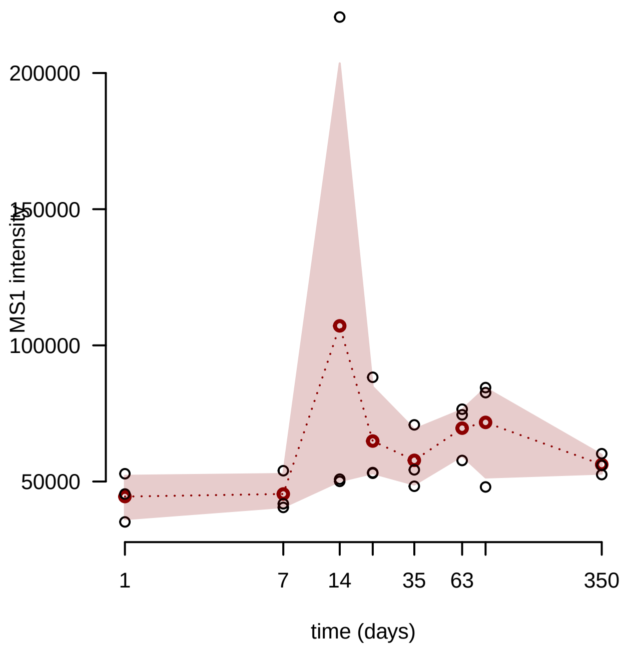

Q8CF89
GN=Tab1
TGF−beta activated kinase 1/MAP3K7 bindi ...

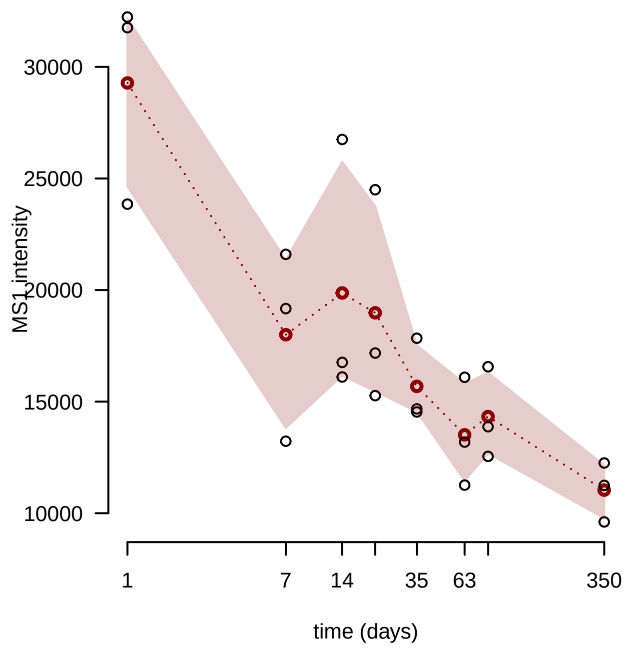

Q8CFA2
GN=Amt
aminomethyltransferase

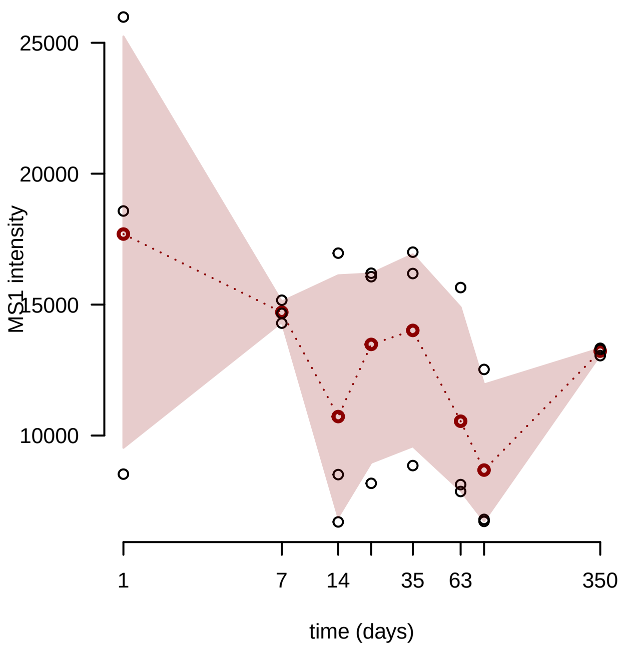

Q8CFC7
GN=Clasrp
CLK4−associating serine/arginine rich pr ...

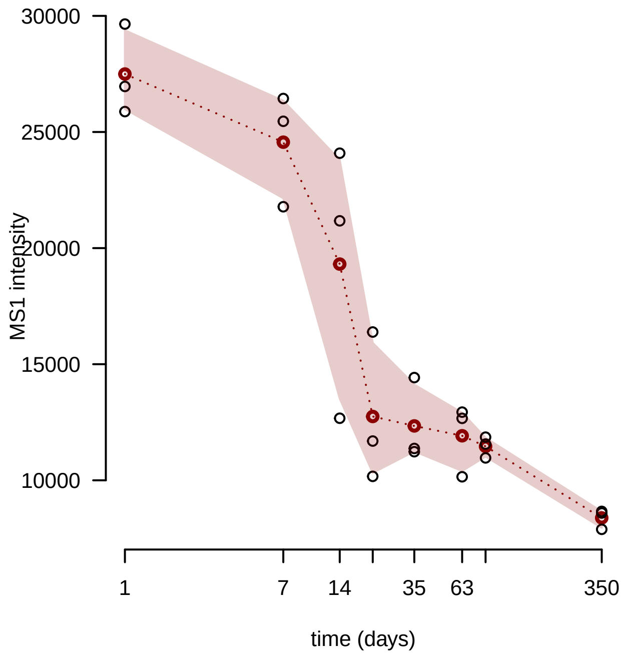

Q8CFD4
GN=Snx8
sorting nexin 8

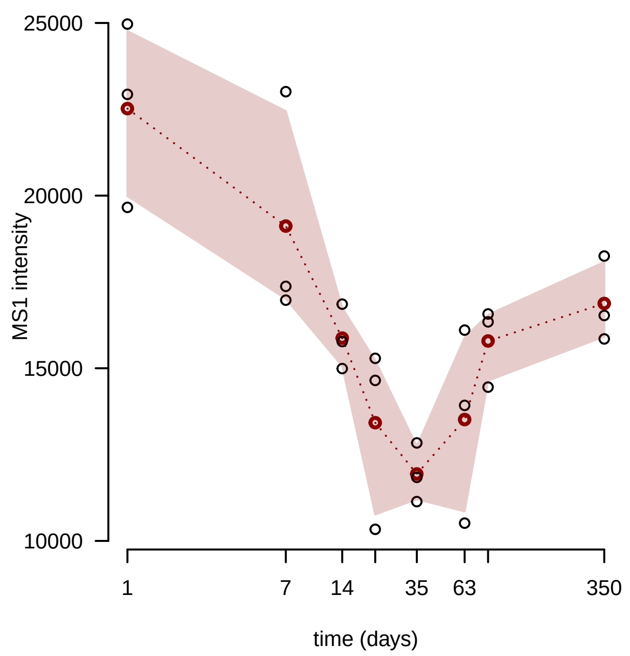

Q8CFD5
GN=Ercc8
excision repaiross−complementing rodent ...

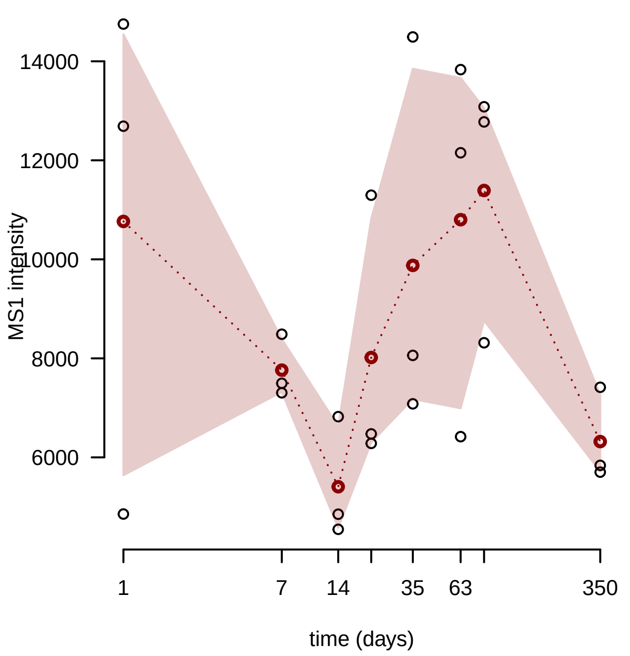

Q8CFE2
GN=Hpf1
histone PARylation factor 1

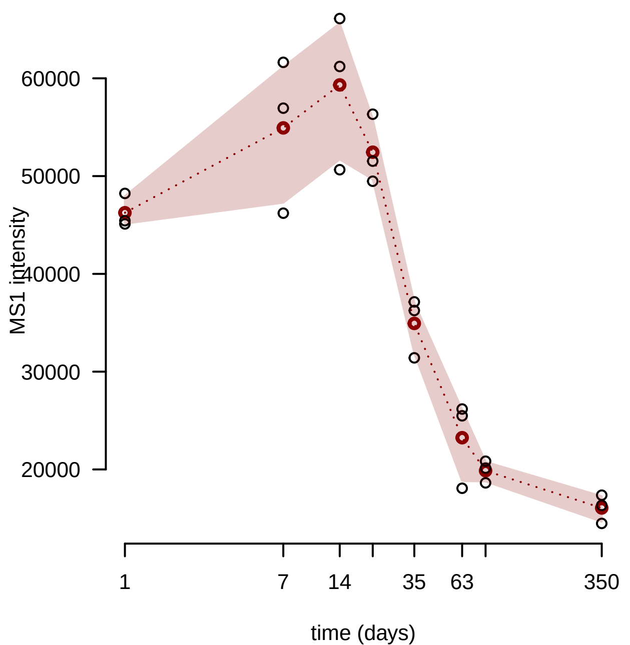

Q8CFE3
GN=Rcor1
REST corepressor 1

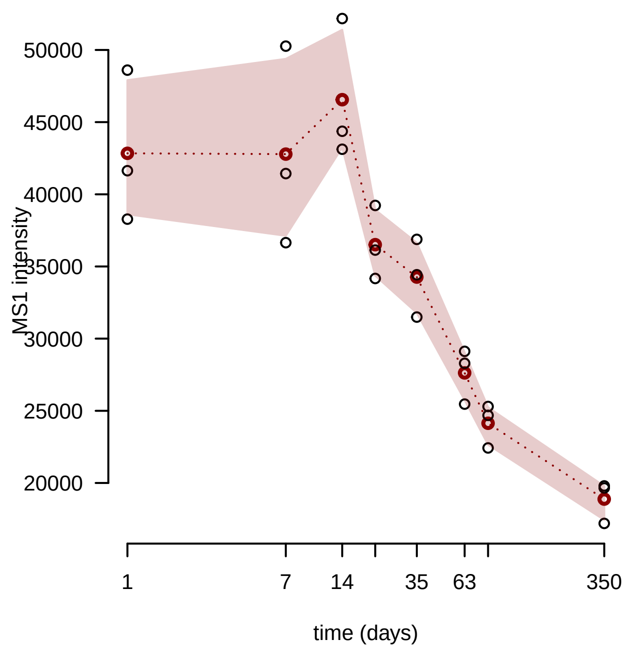

Q8CFE4
GN=Scyl2
SCY1−like 2 (S. cerevisiae)

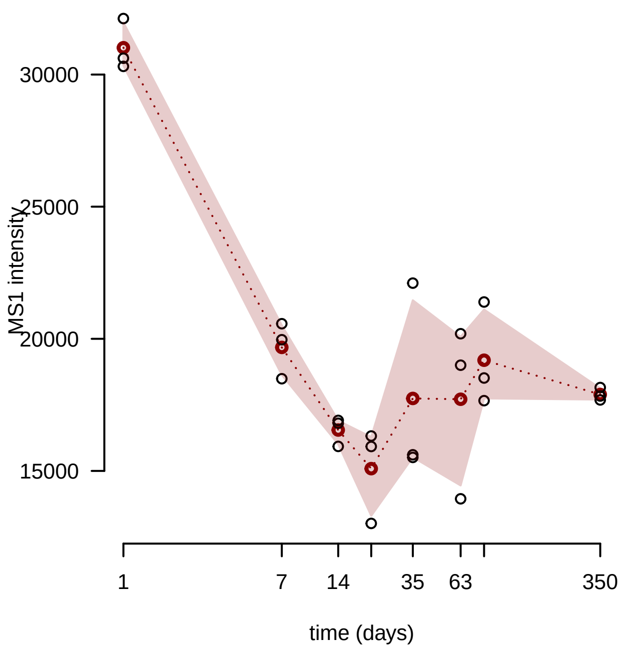

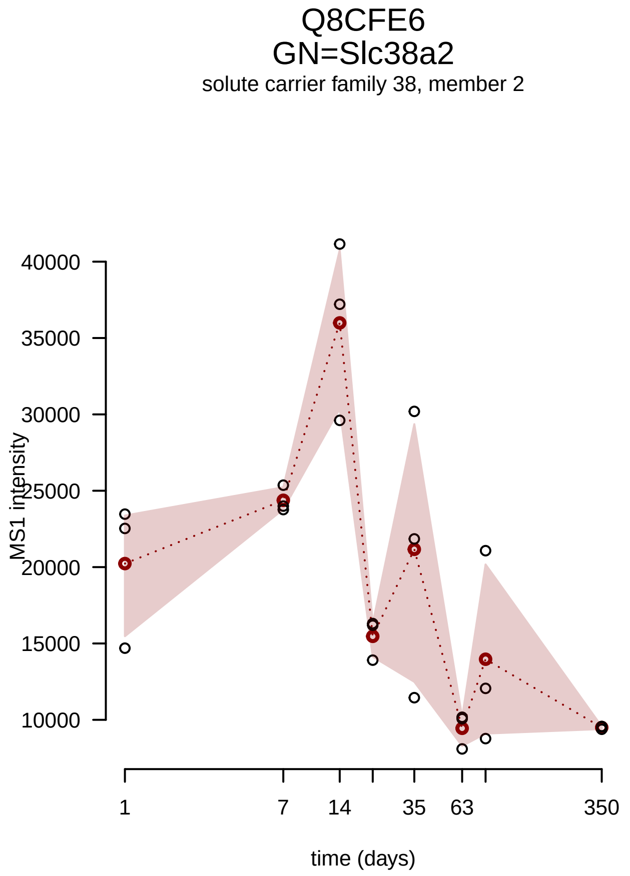
Q8CFE6
GN=Slc38a2
solute carrier family 38, member 2
MS1 intensity
10000 15000 20000 25000 30000 35000 40000
1 7 14 35 63 350
time (days)

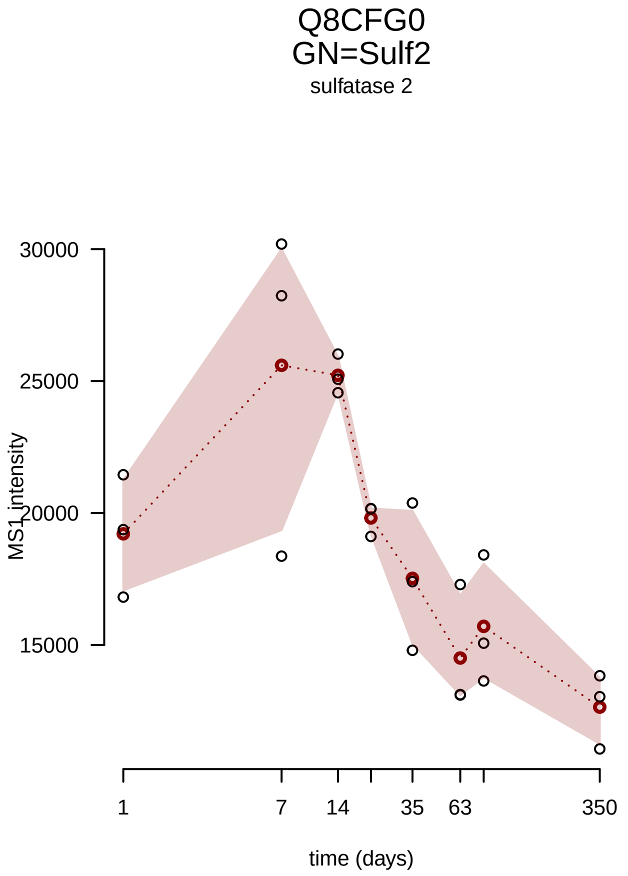
Q8CFG0
GN=Sulf2
sulfatase 2
MS1 intensity
15000 20000 25000 30000
1 7 14 35 63 350
time (days)

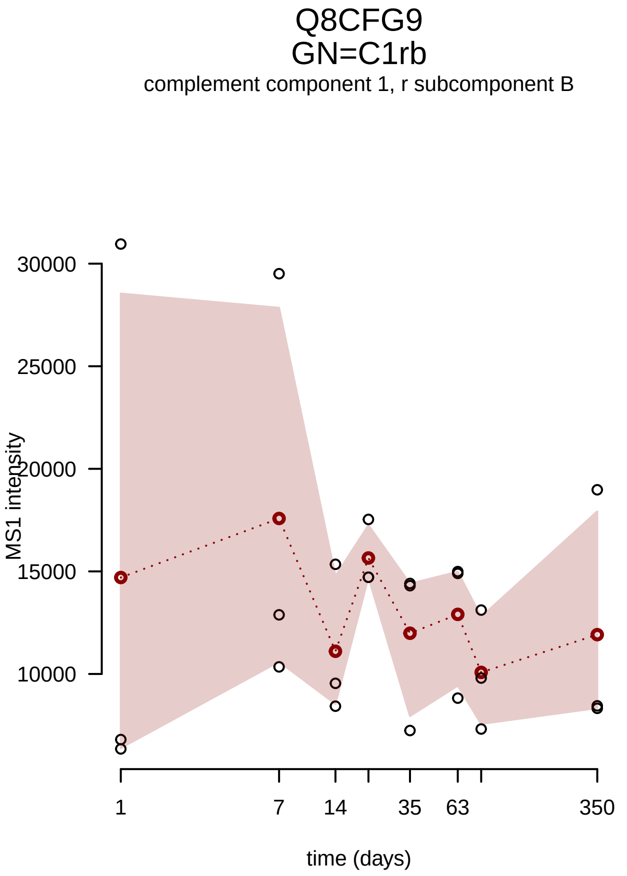
Q8CFG9
GN=C1rb
complement component 1, r subcomponent B
MS1 intensity
10000 15000 20000 25000 30000
1 7 14 35 63 350
time (days)

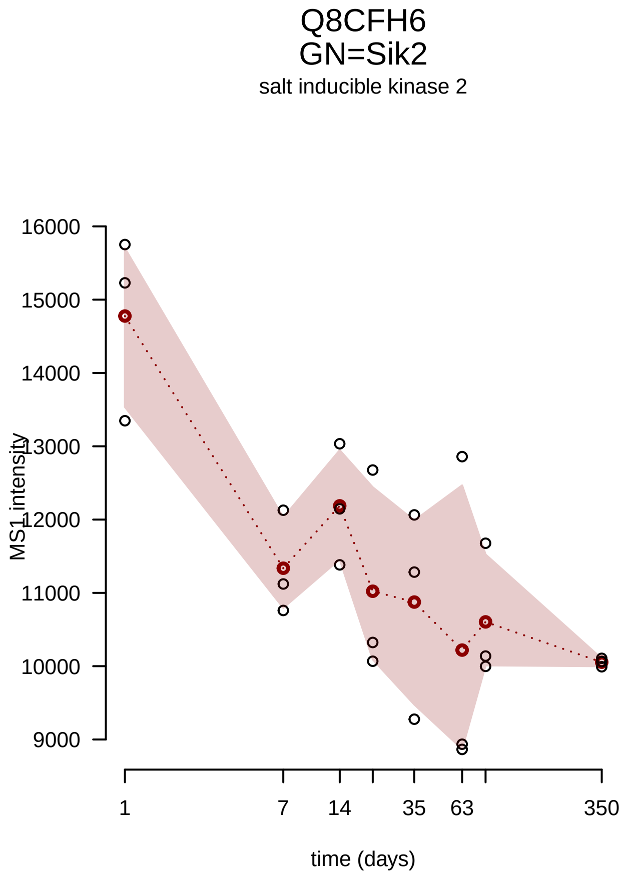
Q8CFH6
GN=Sik2
salt inducible kinase 2
MS1 intensity
9000 10000 11000 12000 13000 14000 15000 16000
1 7 14 35 63 350
time (days)

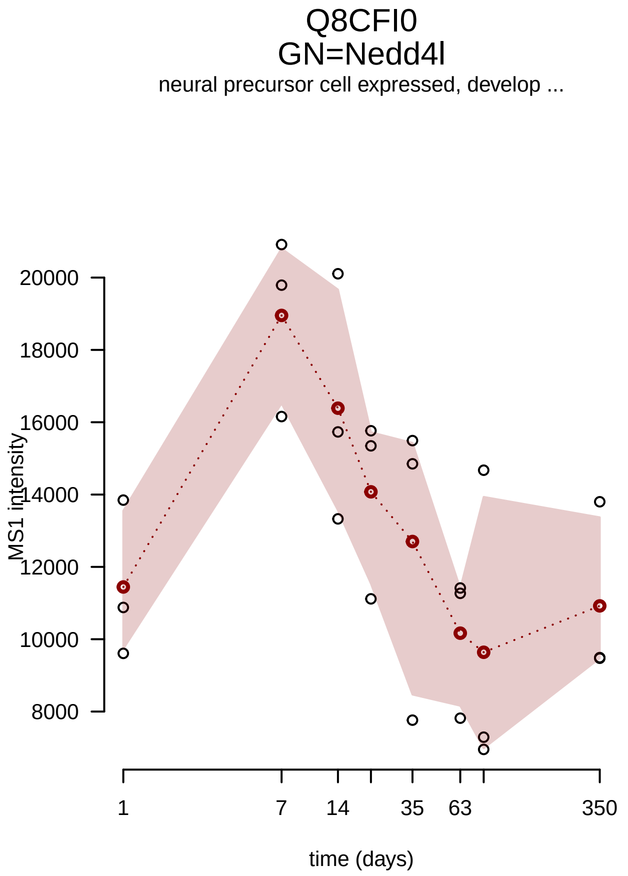
Q8CFI0
GN=Nedd4l
neural precursor cell expressed, develop ...
MS1 intensity
8000 10000 12000 14000 16000 18000 20000
1 7 14 35 63 350
time (days)

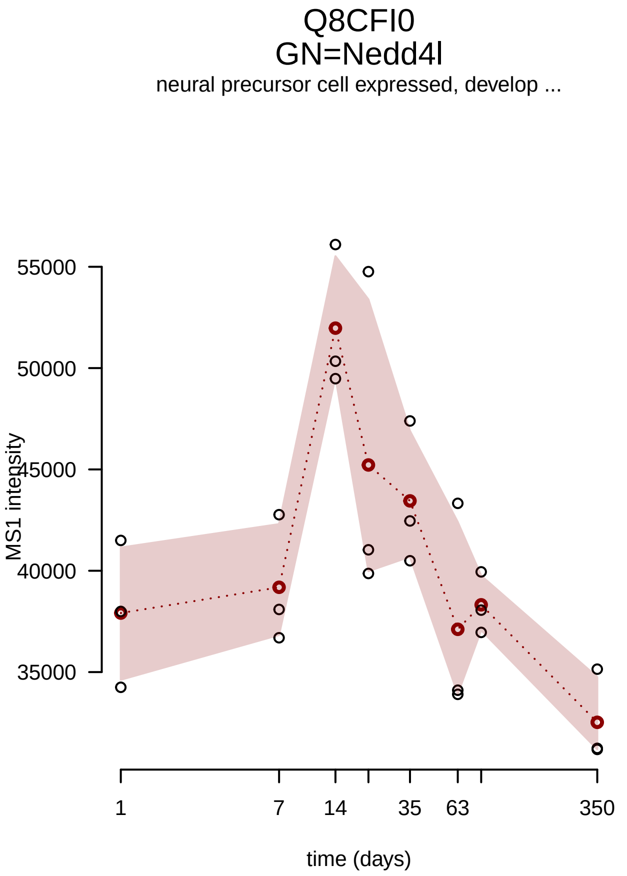
Q8CFI0
GN=Nedd4l
neural precursor cell expressed, develop ...
MS1 intensity
35000 40000 45000 50000 55000
1 7 14 35 63 350
time (days)

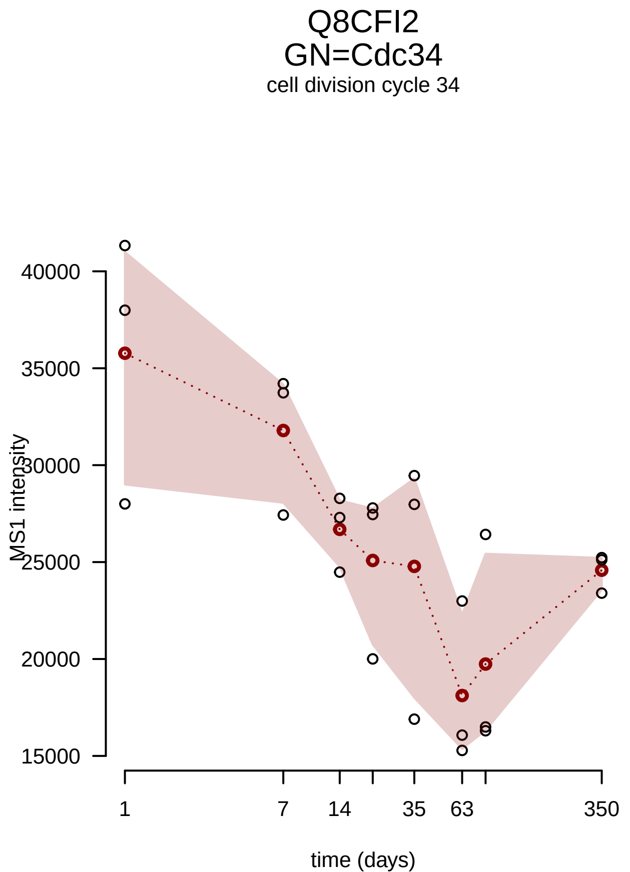
Q8CFI2
GN=Cdc34
cell division cycle 34
MS1 intensity
15000 20000 25000 30000 35000 40000
1 7 14 35 63 350
time (days)

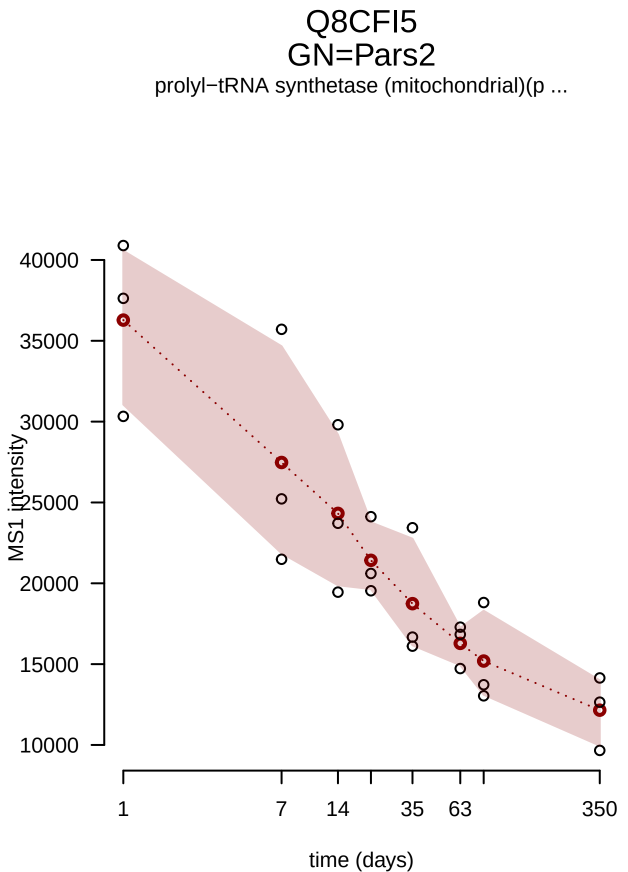
Q8CFI5
GN=Pars2
prolyl−tRNA synthetase (mitochondrial)(p ...
MS1 intensity
10000 15000 20000 25000 30000 35000 40000
1 7 14 35 63 350
time (days)

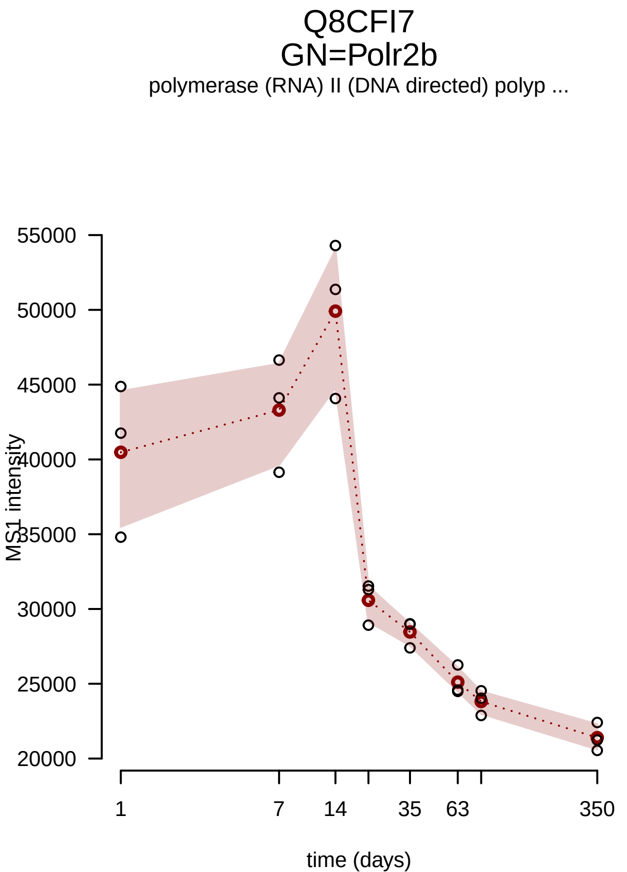
Q8CFI7
GN=Polr2b
polymerase (RNA) II (DNA directed) polyp ...
MS1 intensity
20000 25000 30000 35000 40000 45000 50000 55000
1 7 14 35 63 350
time (days)

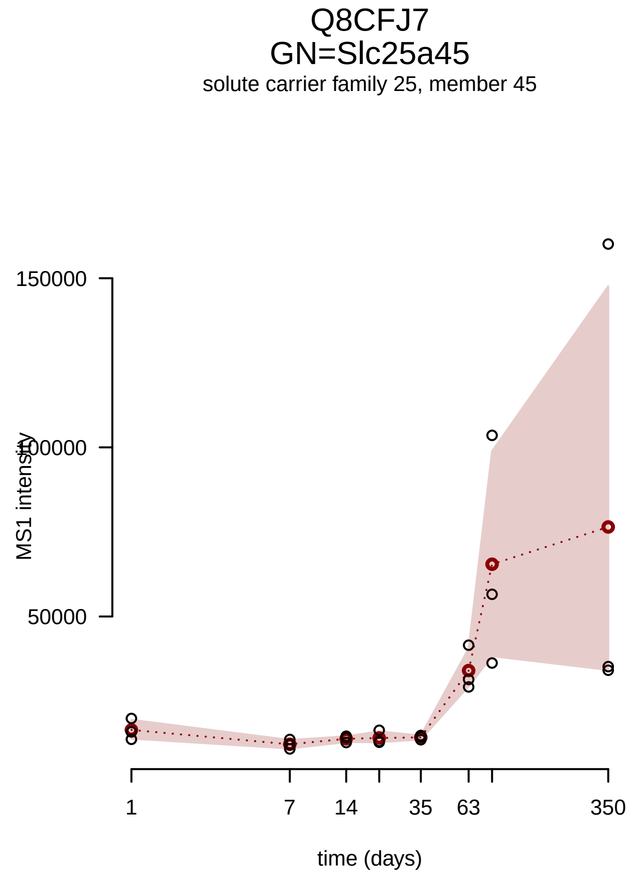
Q8CFJ7
GN=Slc25a45
solute carrier family 25, member 45
MS1 intensity
150000
100000
50000
1
7
14
35
63
350
time (days)

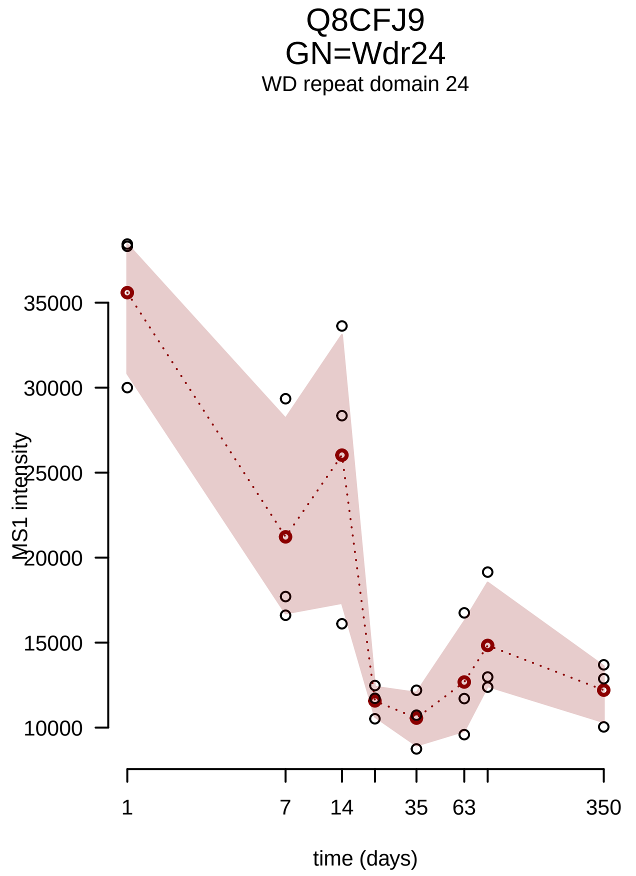
Q8CFJ9
GN=Wdr24
WD repeat domain 24
MS1 intensity
35000
30000
25000
20000
15000
10000
1
7
14
35
63
350
time (days)

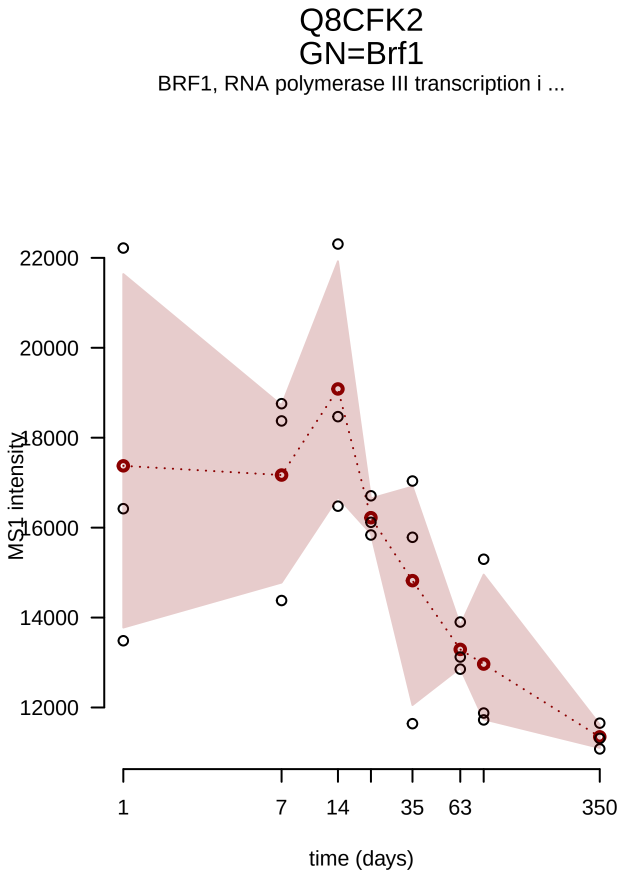
Q8CFK2
GN=Brf1
BRF1, RNA polymerase III transcription i ...
MS1 intensity
22000
20000
18000
16000
14000
12000
1
7
14
35
63
350
time (days)

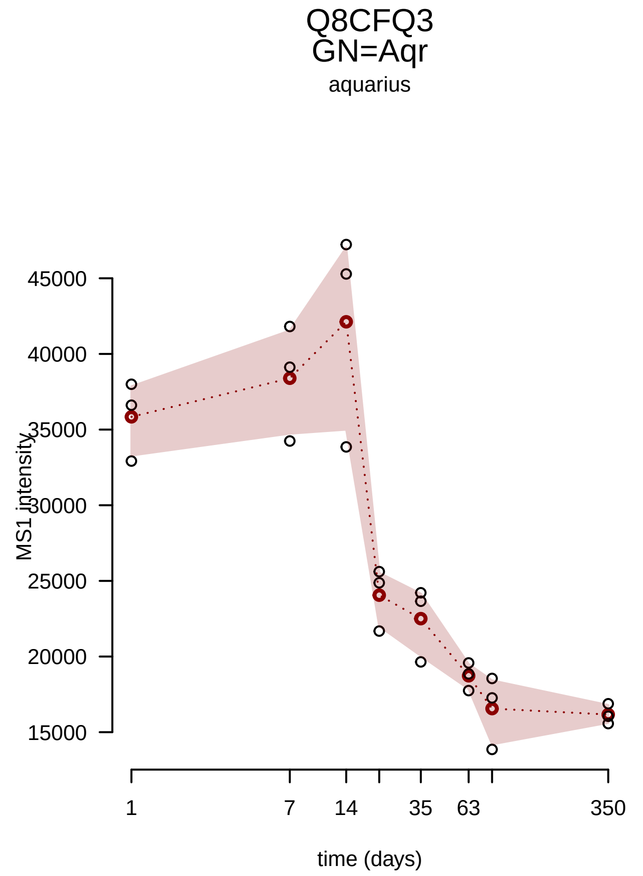
Q8CFQ3
GN=Aqr
aquarius
MS1 intensity
45000
40000
35000
30000
25000
20000
15000
1
7
14
35
63
350
time (days)

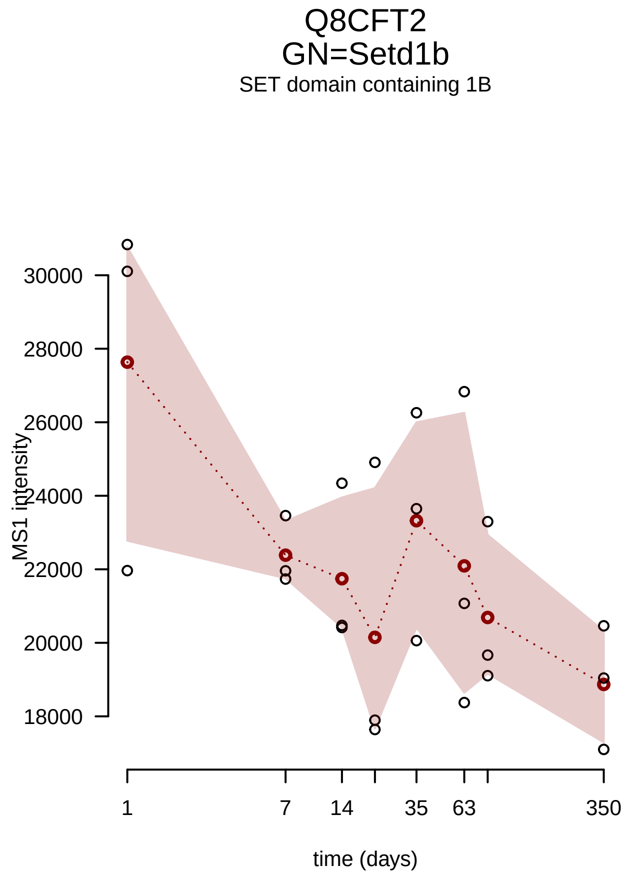
Q8CFT2
GN=Setd1b
SET domain containing 1B
MS1 intensity
30000
28000
26000
24000
22000
20000
18000
1
7
14
35
63
350
time (days)

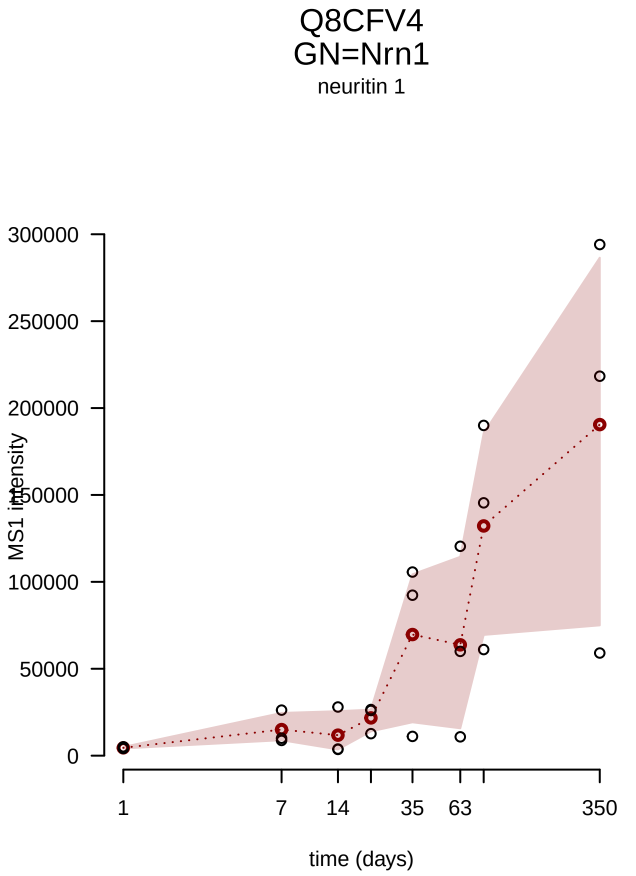
Q8CFV4
GN=Nrn1
neuritin 1
MS1 intensity
300000
250000
200000
150000
100000
50000
0
1
7
14
35
63
350
time (days)

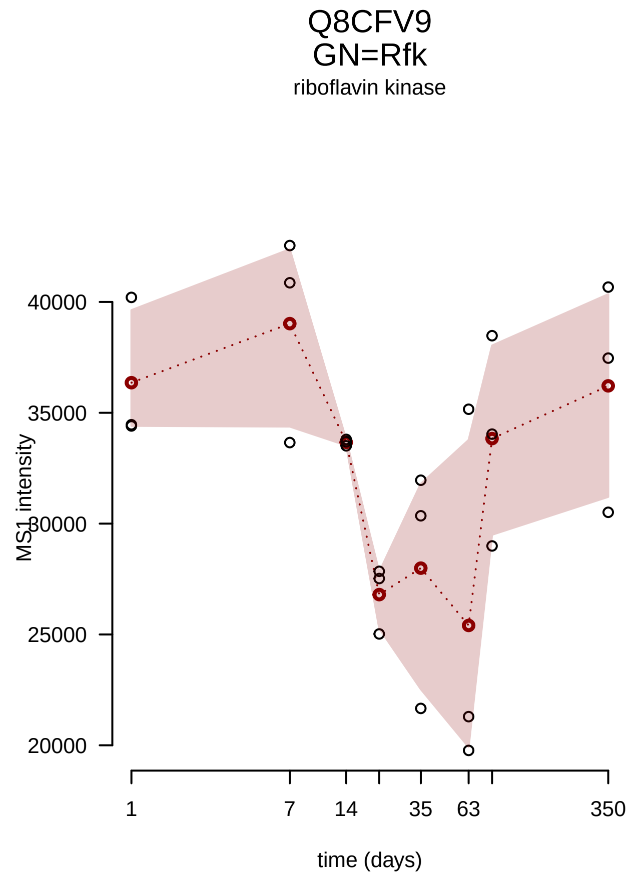
Q8CFV9
GN=Rfk
riboflavin kinase
MS1 intensity
40000
35000
30000
25000
20000
1
7
14
35
63
350
time (days)

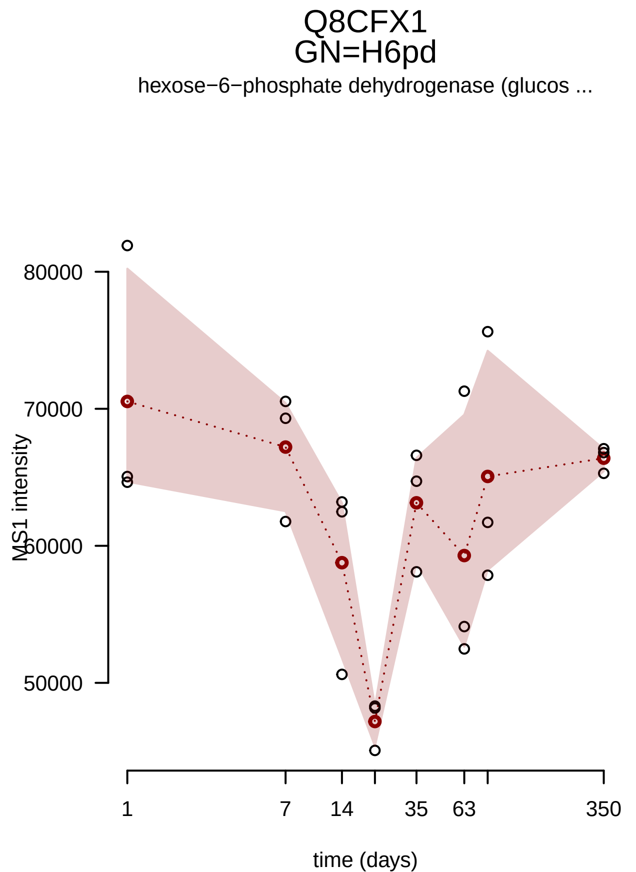
Q8CFX1
GN=H6pd
hexose−6−phosphate dehydrogenase (glucos ...
MS1 intensity
80000
70000
60000
50000
1
7
14
35
63
350
time (days)

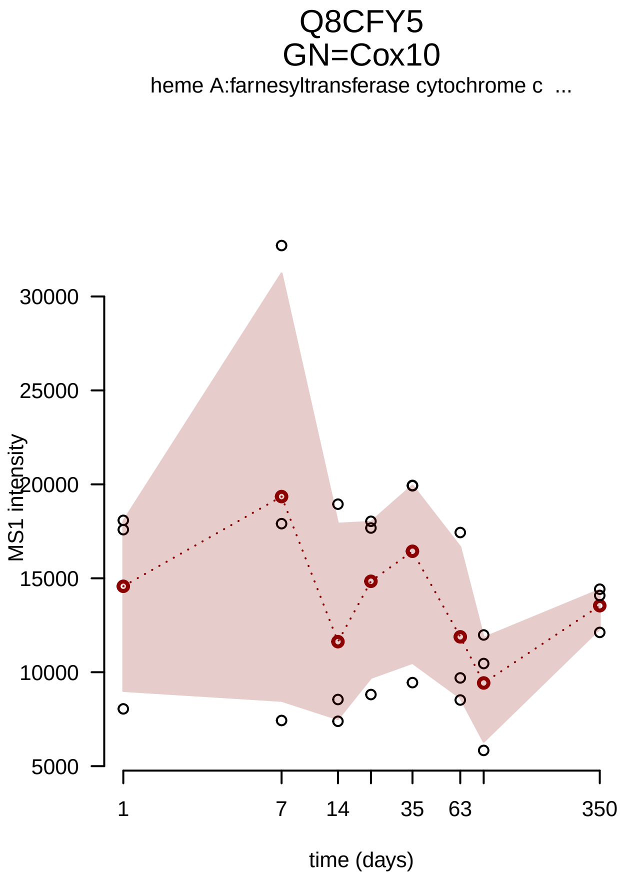
Q8CFY5
GN=Cox10
heme A:farnesyltransferase cytochrome c ...
MS1 intensity
30000
25000
20000
15000
10000
5000
1
7
14
35
63
350
time (days)

Q8CFZ4
GN=Gpc3
glypican 3

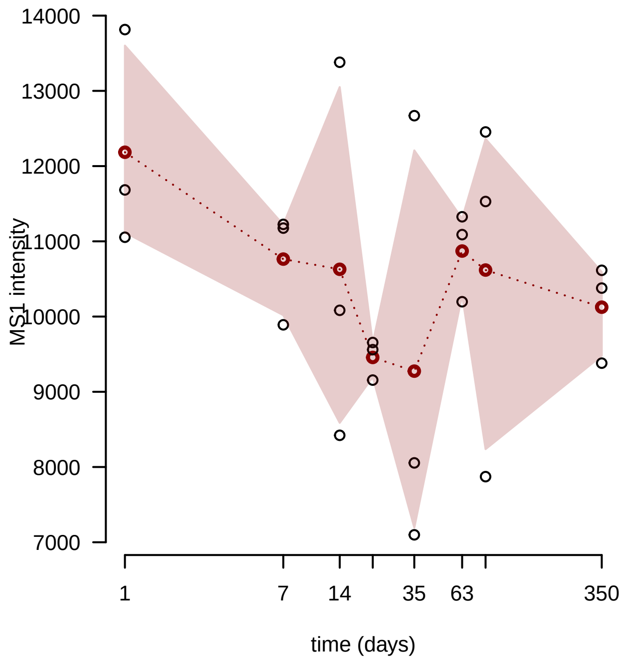

Q8CG03
GN=Pde5a
phosphodiesterase 5A, cGMP−specific

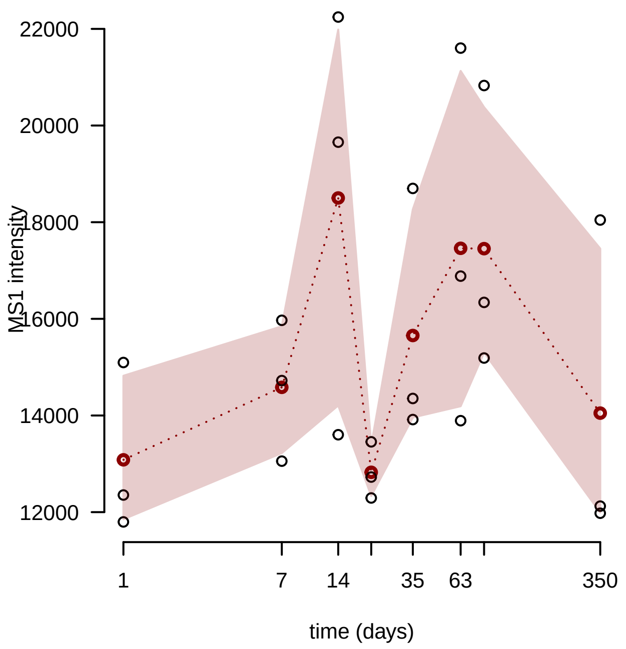

Q8CG14
GN=C1sa

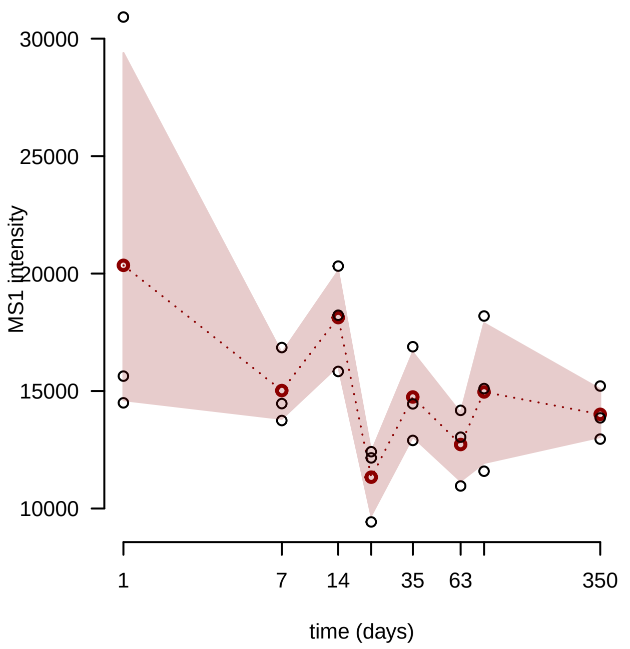

Q8CG19
GN=Ltbp1
latent transforming growth factor beta b ...

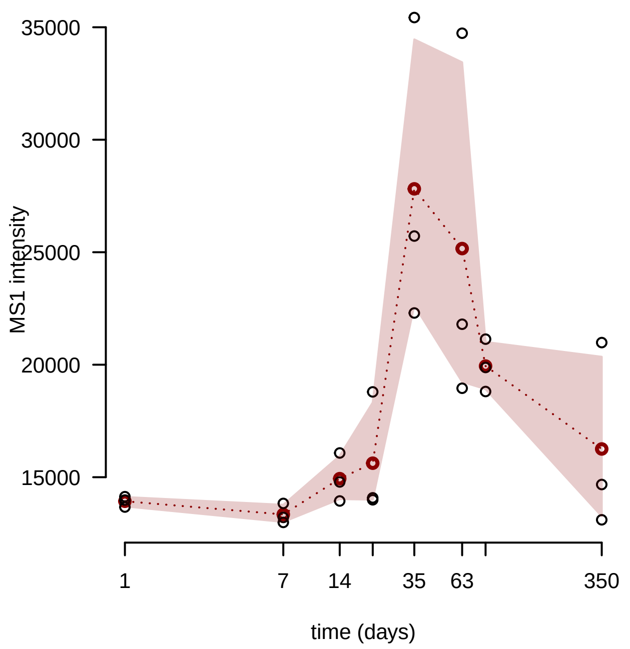

Q8CG46
GN=Smc5
structural maintenance of chromosomes 5

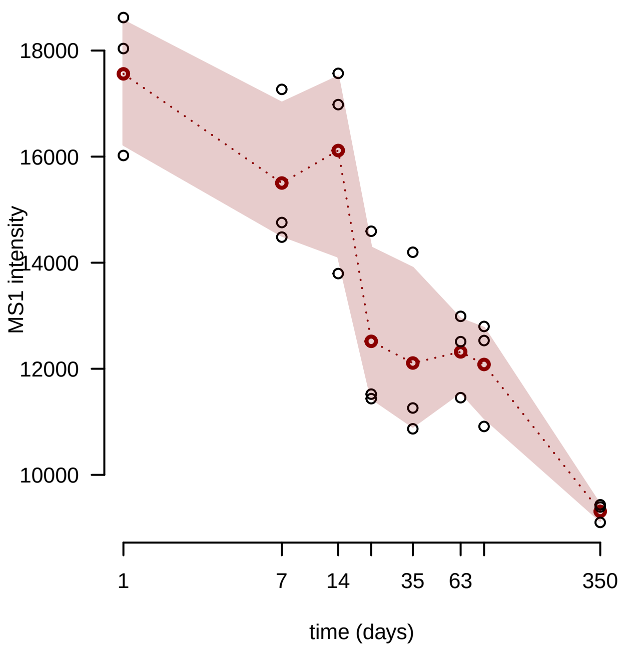

Q8CG47
GN=Smc4
structural maintenance of chromosomes 4

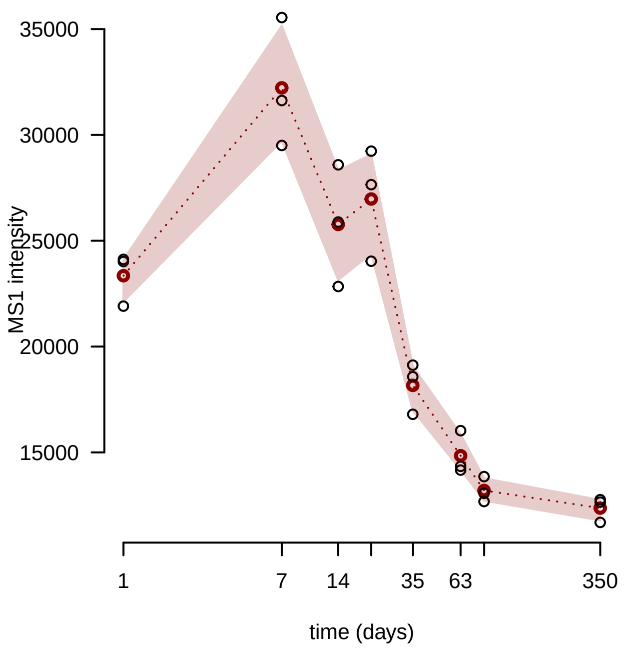

Q8CG48
GN=Smc2
structural maintenance of chromosomes 2

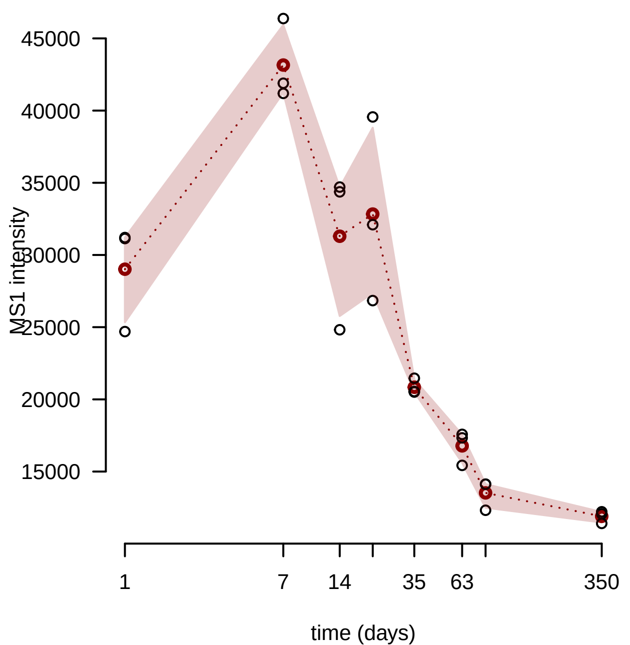

Q8CG50
GN=Rab43
RAB43, member RAS oncogene family

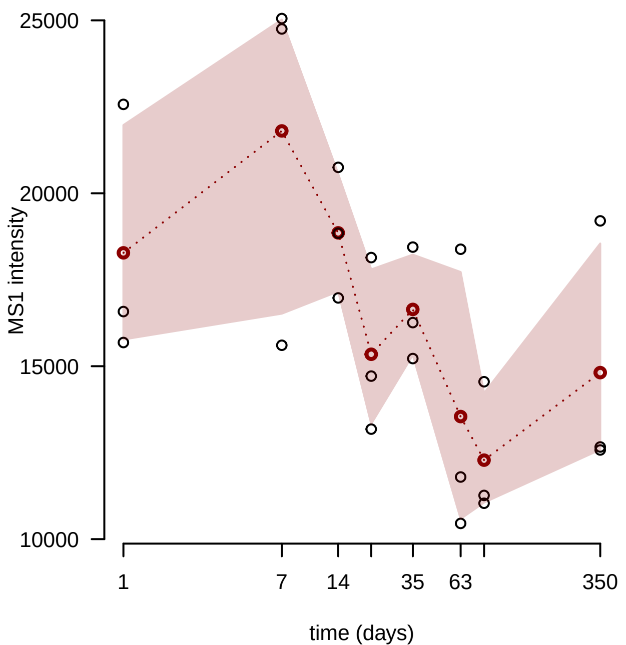

Q8CG70
GN=P3h3
prolyl 3−hydroxylase 3

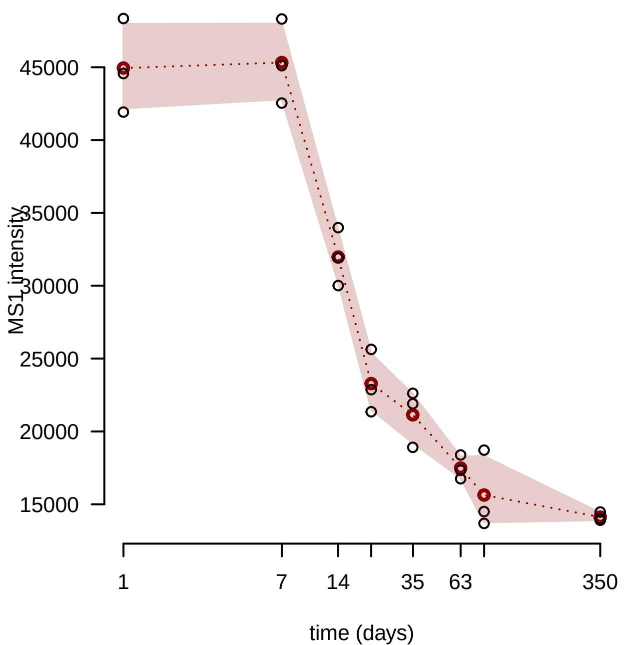

Q8CG71
GN=P3h2
prolyl 3−hydroxylase 2

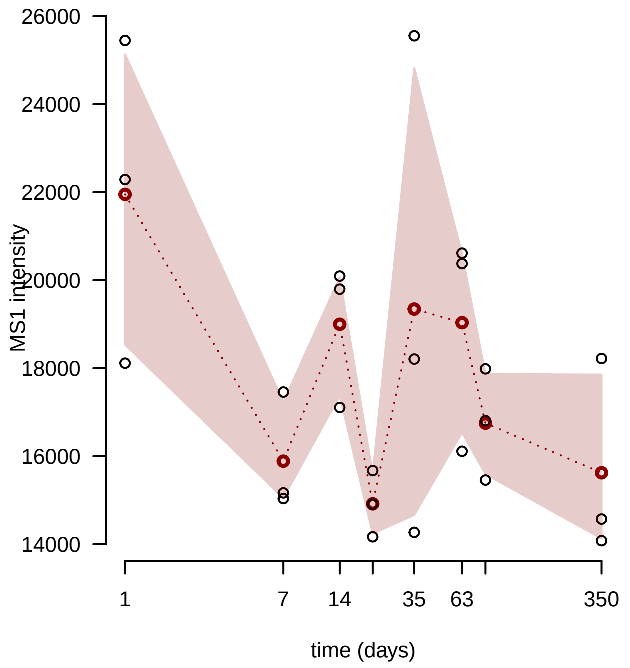

Q8CG72
GN=Adprhl2

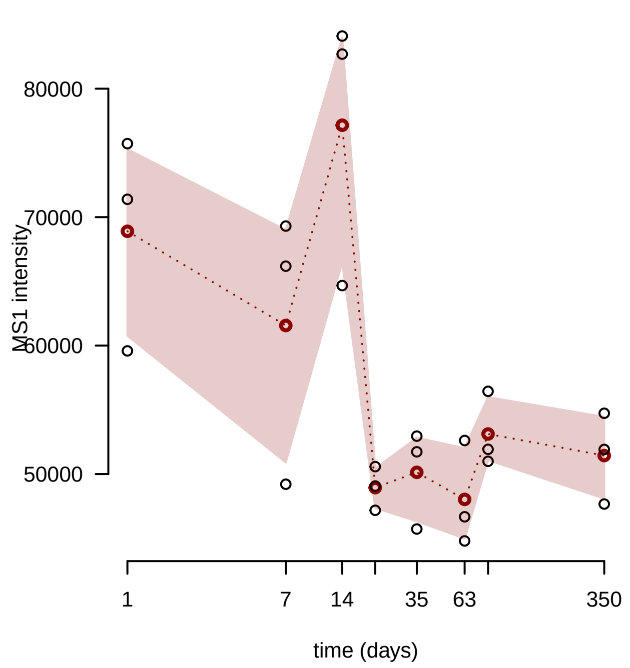

Q8CG76
GN=Akr7a2

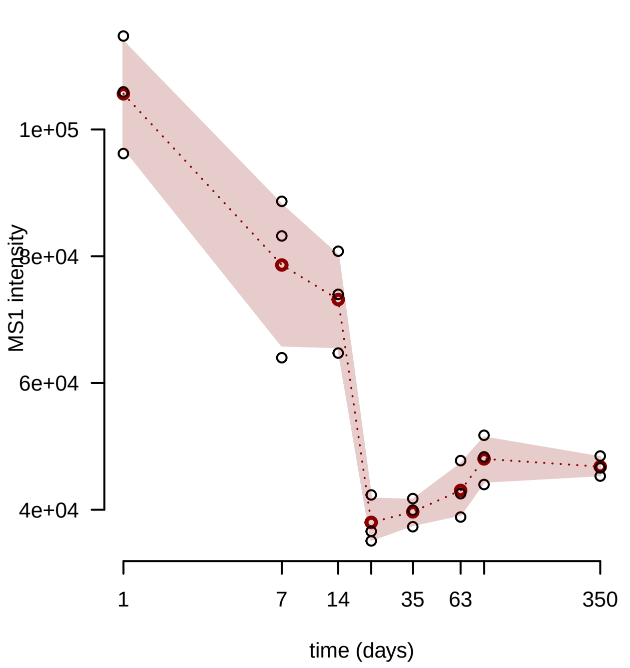

Q8CG79
GN=Tp53bp2

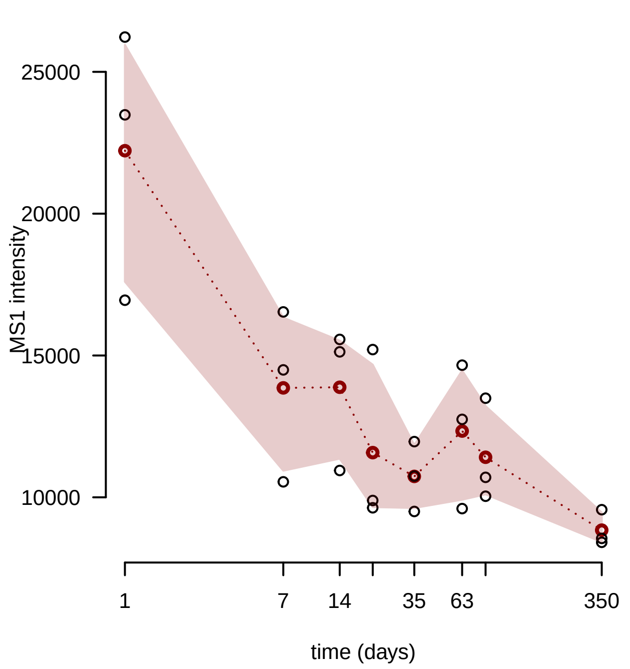

Q8CGA0
GN=Ppm1f
protein phosphatase 1F (PP2C domain cont ...

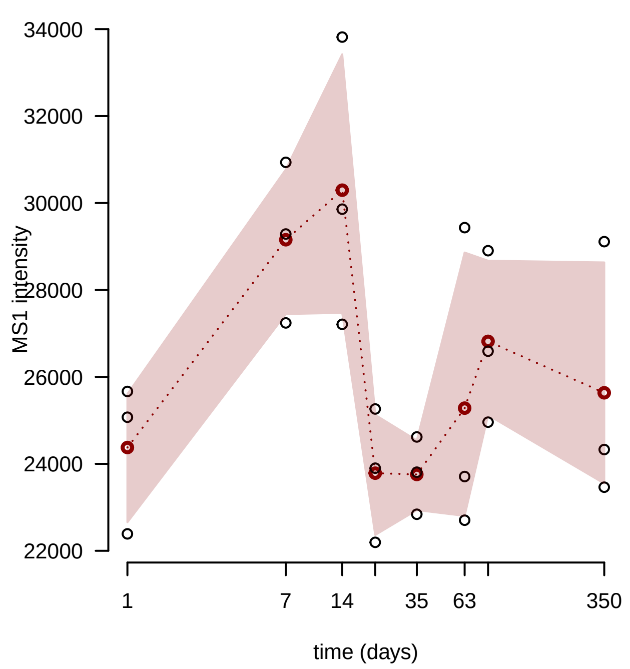

Q8CGA3
GN=Slc43a2
solute carrier family 43, member 2

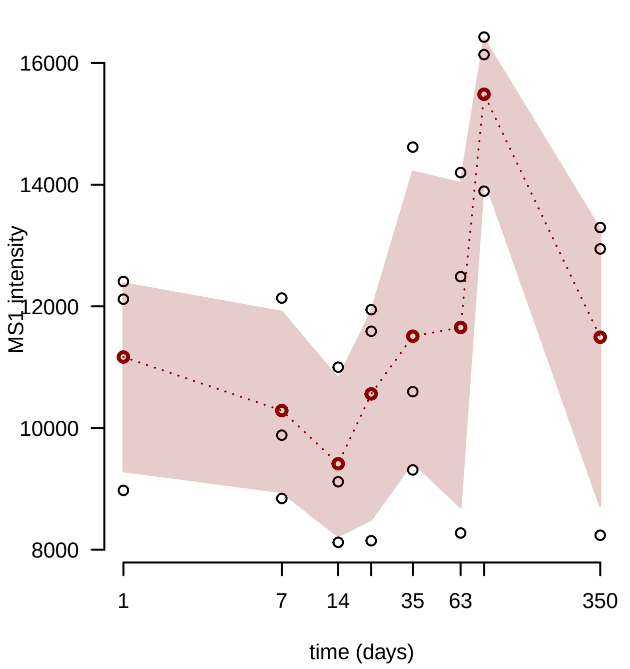

Q8CGA4
GN=Mturn
maturin, neural progenitor differentiati ...

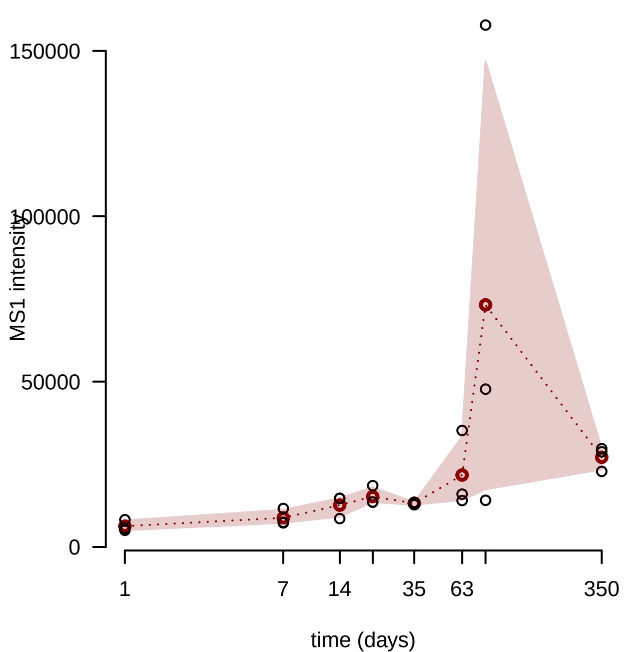

Q8CGB3
GN=Uaca
uveal autoantigen with coiled−coil domai ...

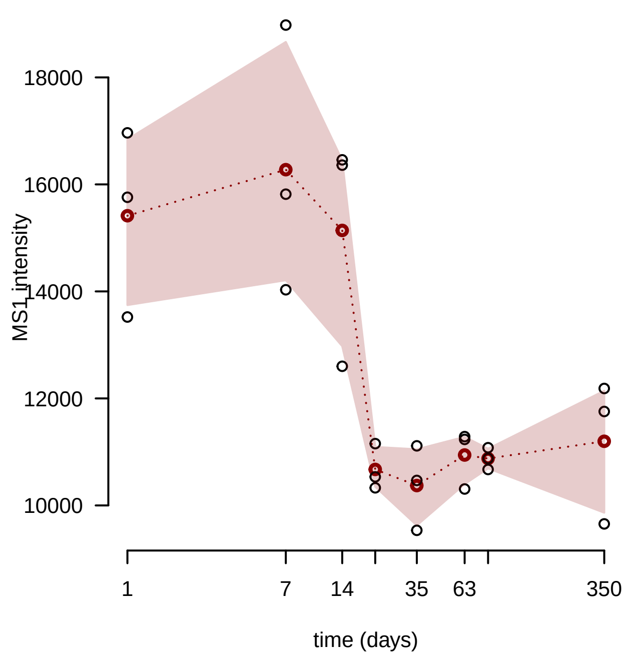

Q8CGB6
GN=Tns2
tensin 2

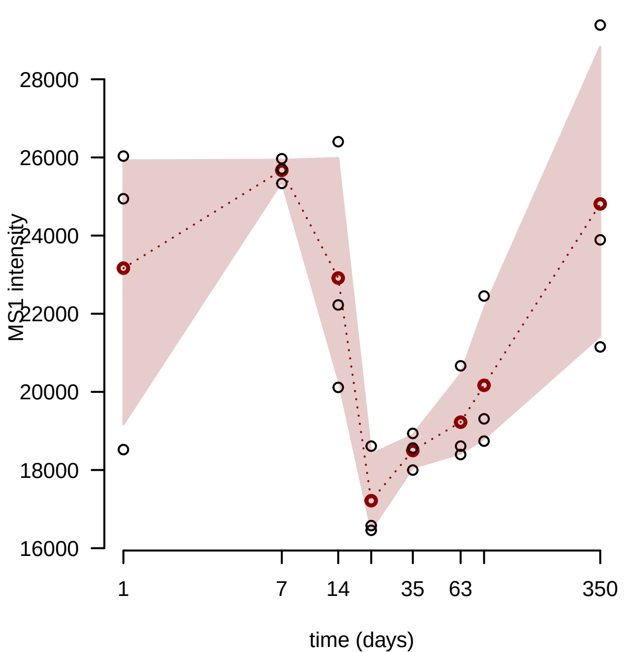

Q8CGC4
GN=Lsm14b
LSM family member 14B

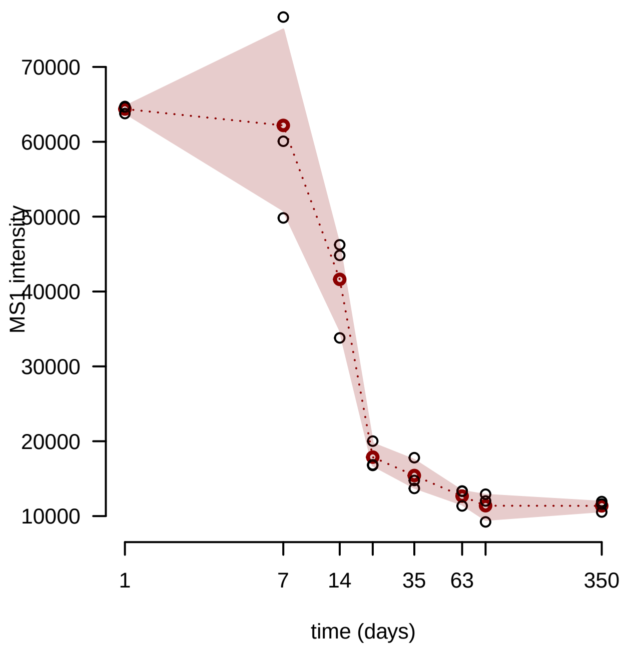

Q8CGC6
GN=Rbm28
RNA binding motif protein 28

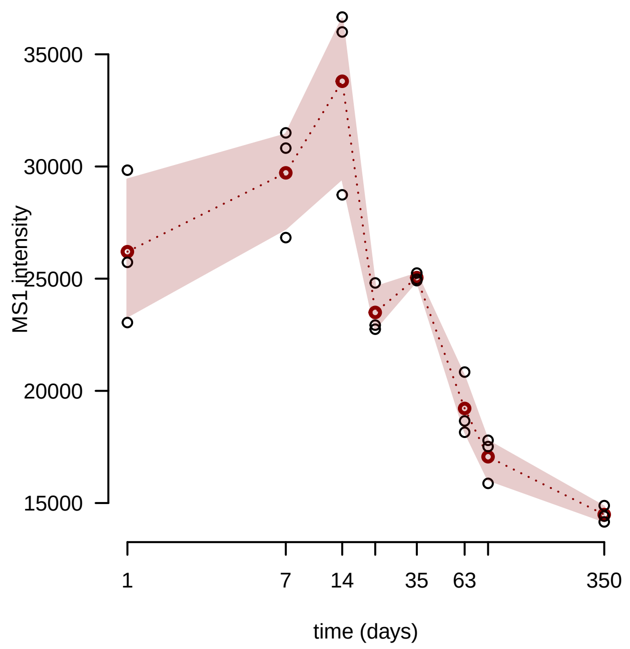

Q8CGC7
GN=Eprs
glutamyl−prolyl−tRNA synthetase

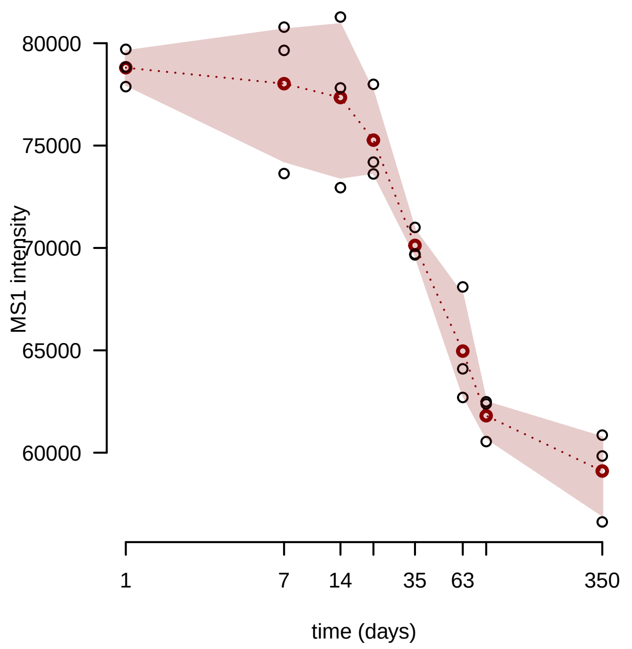

Q8CGE9
GN=Rgs12
regulator of G−protein signaling 12

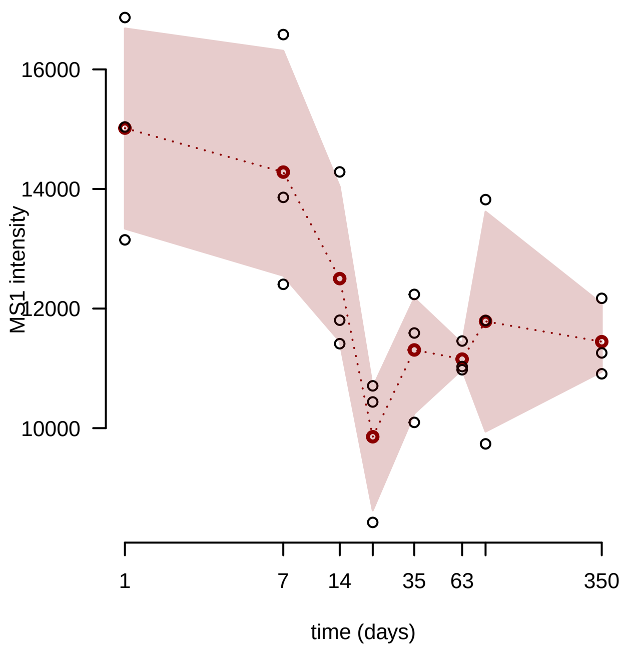

Q8CGF1
GN=Arhgap29
Rho GTPase activating protein 29

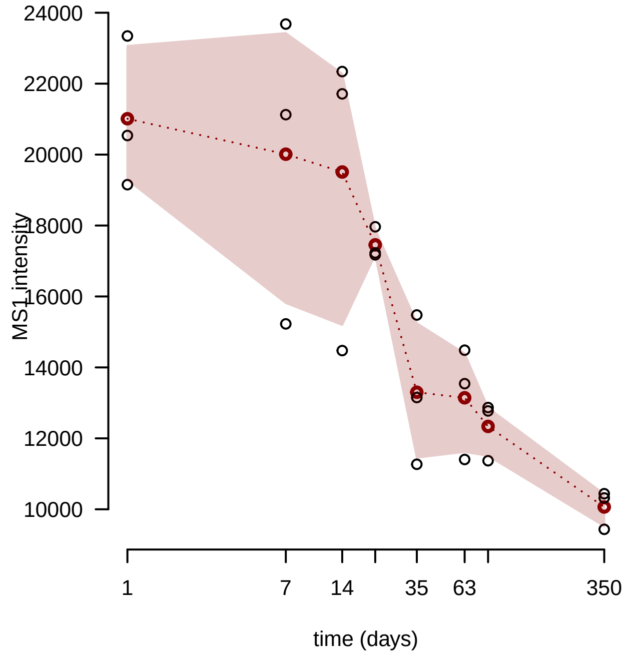

Q8CGF5
GN=Tmem56

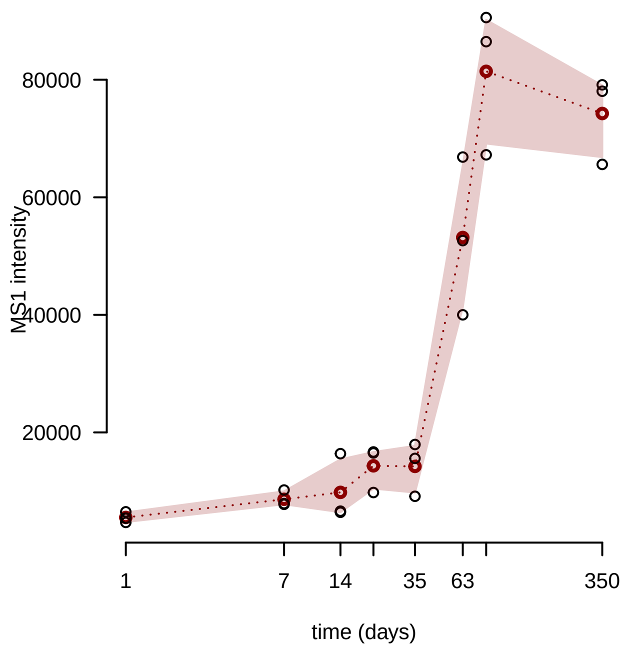

Q8CGF6
GN=Wdr47
WD repeat domain 47

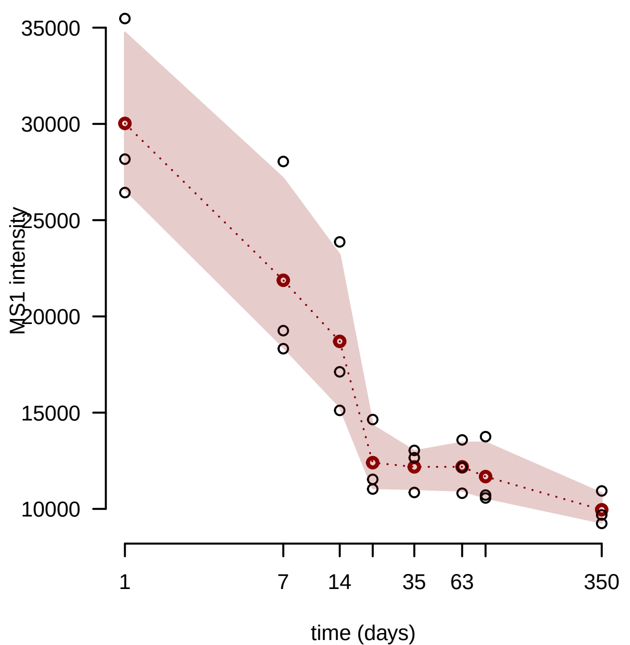

Q8CGF7
GN=Tcerg1
transcription elongation regulator 1 (CA ...

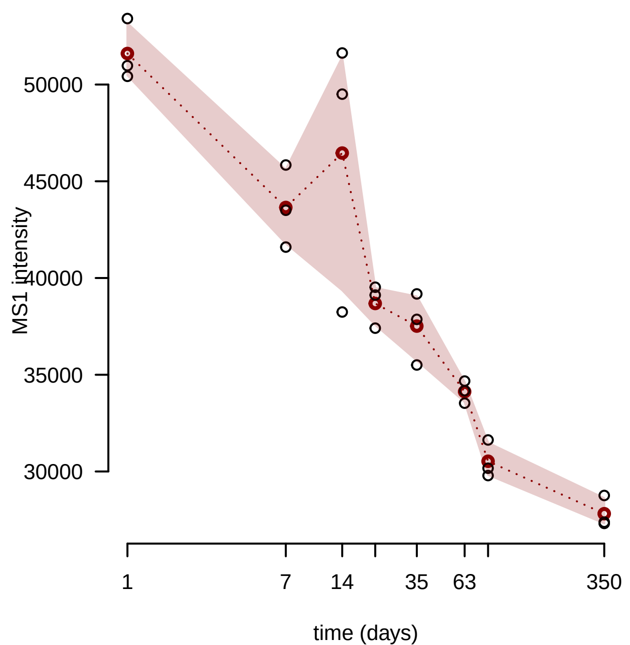

Q8CGI1
GN=Fam193a
family with sequence homology 193, membe ...

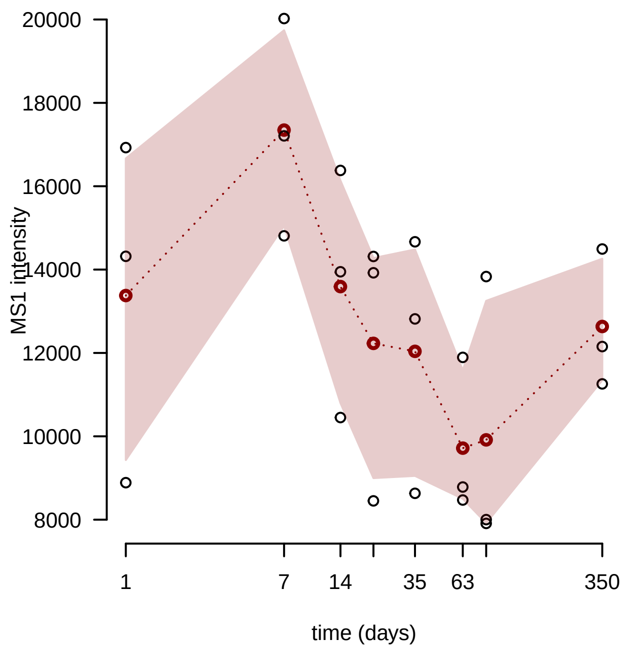

Q8CGK3
GN=Lonp1
lon peptidase 1, mitochondrial

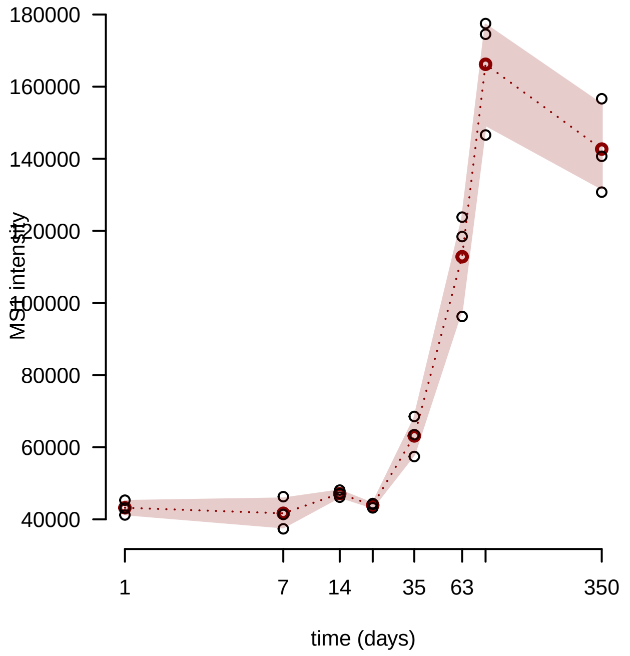

Q8CGN4
GN=Bcor
BCL6 interacting corepressor

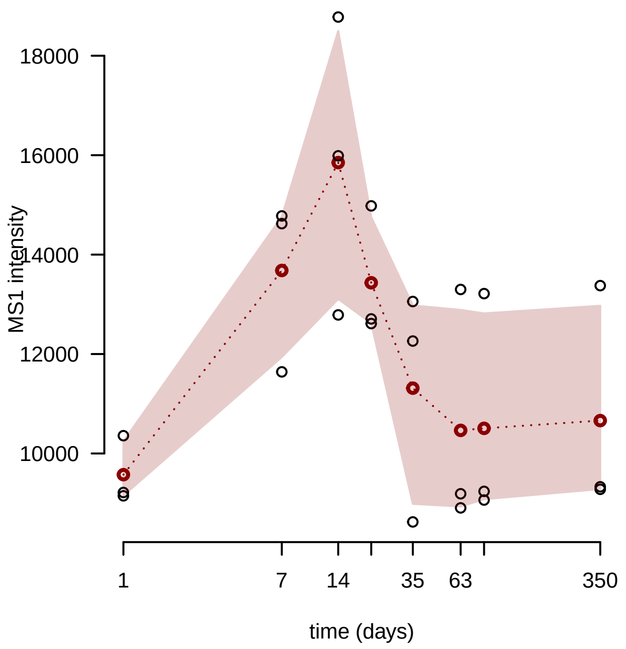

Q8CGN5
GN=Plin1
perilipin 1

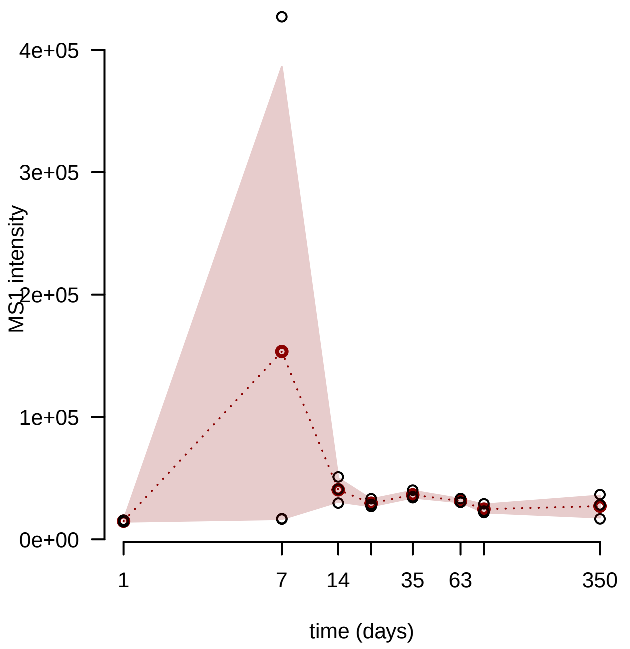

Q8CGP0
GN=Hist3h2bb

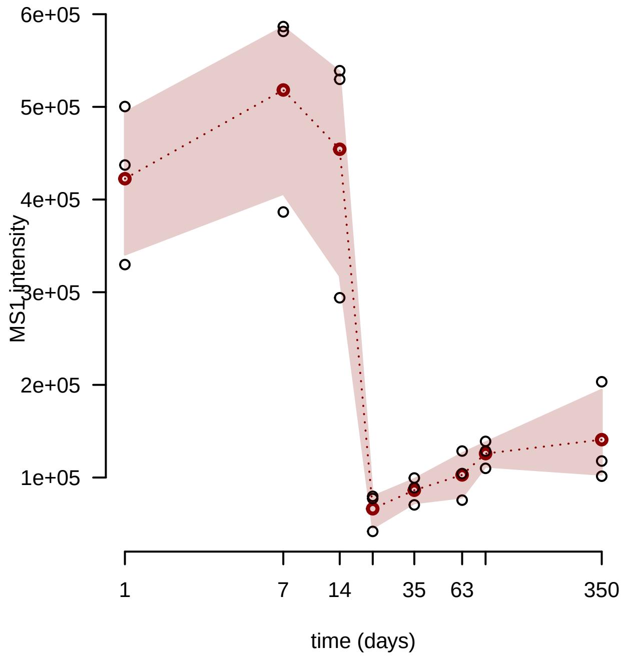

Q8CGU1
GN=Calcoco1
calcium binding and coiled coil domain 1

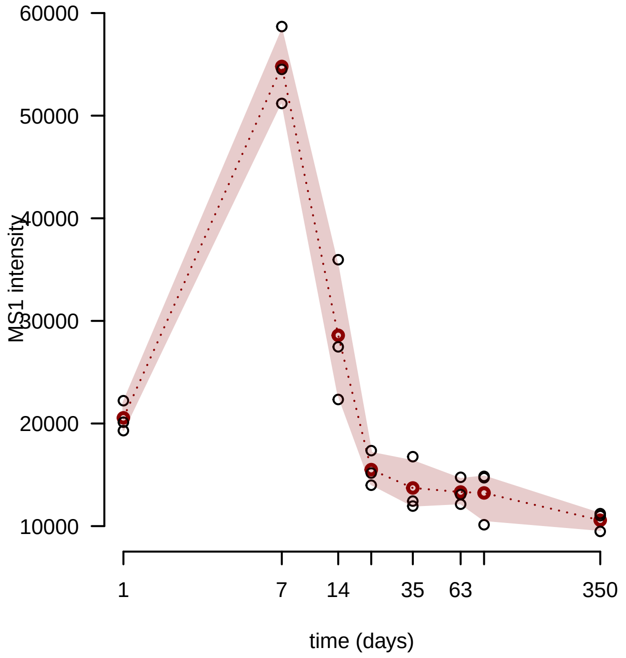

Q8CGY6
GN=Unc45b
unc−45 myosin chaperone B

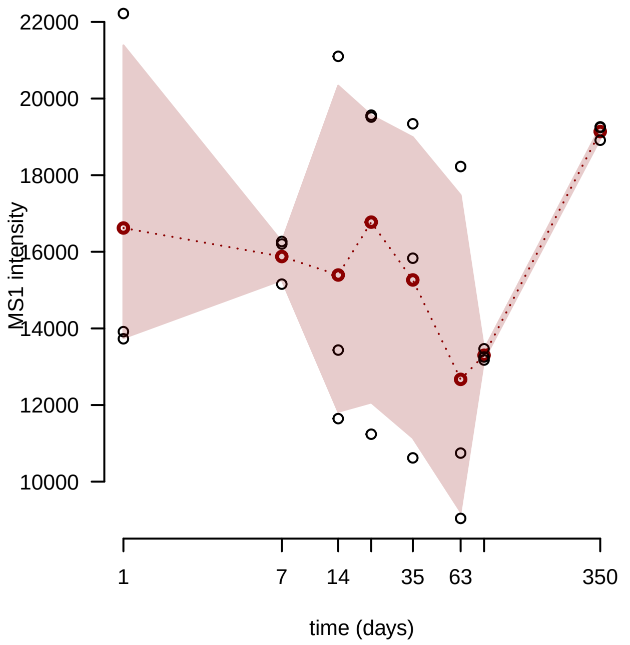

Q8CGY8
GN=Ogt
O−linked N−acetylglucosamine (GlcNAc) tr ...

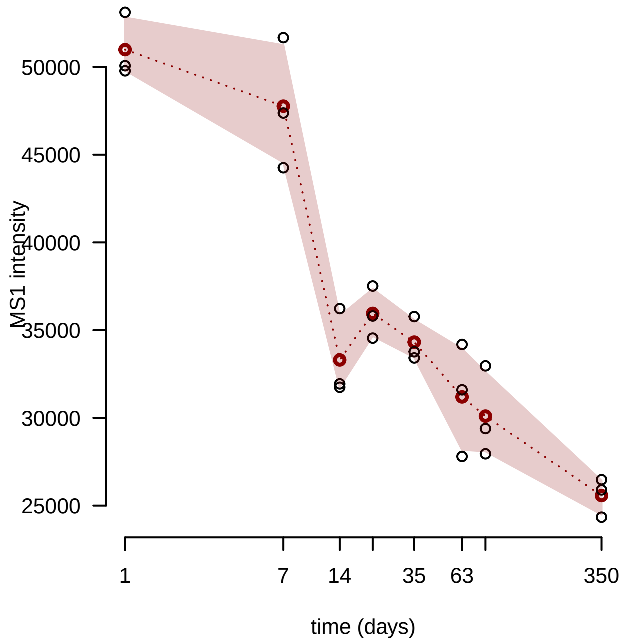

Q8CGY8
GN=Ogt
O−linked N−acetylglucosamine (GlcNAc) tr ...

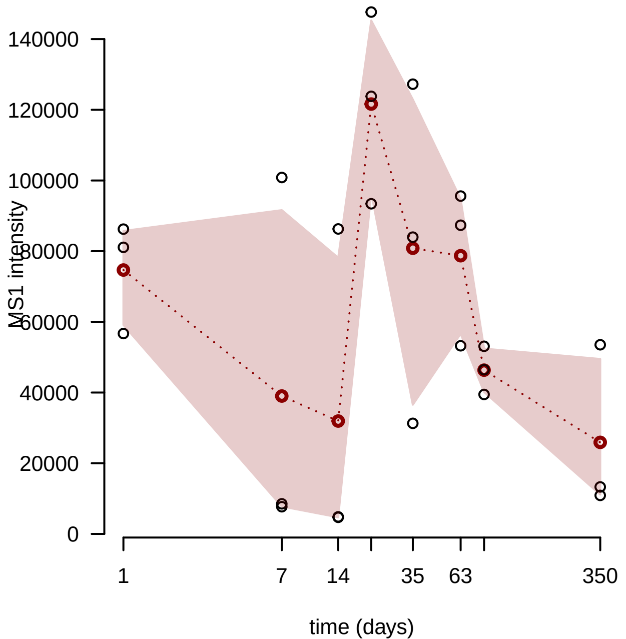

Q8CGZ0
GN=Cherp
calcium homeostasis endoplasmic reticulu ...

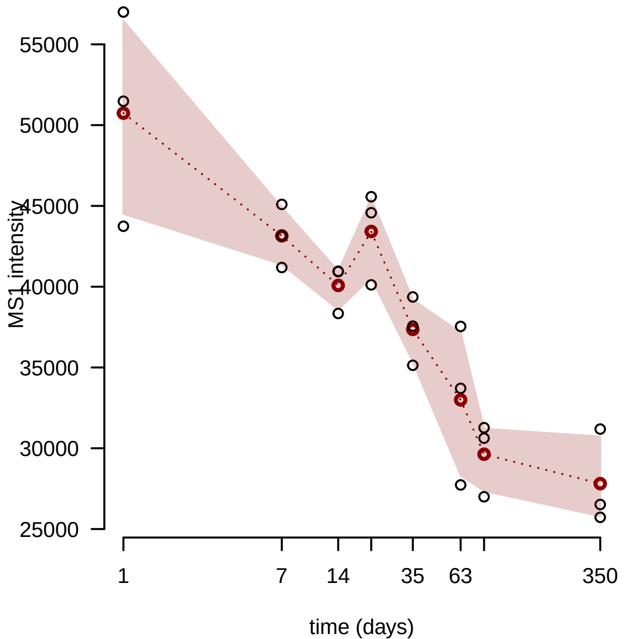

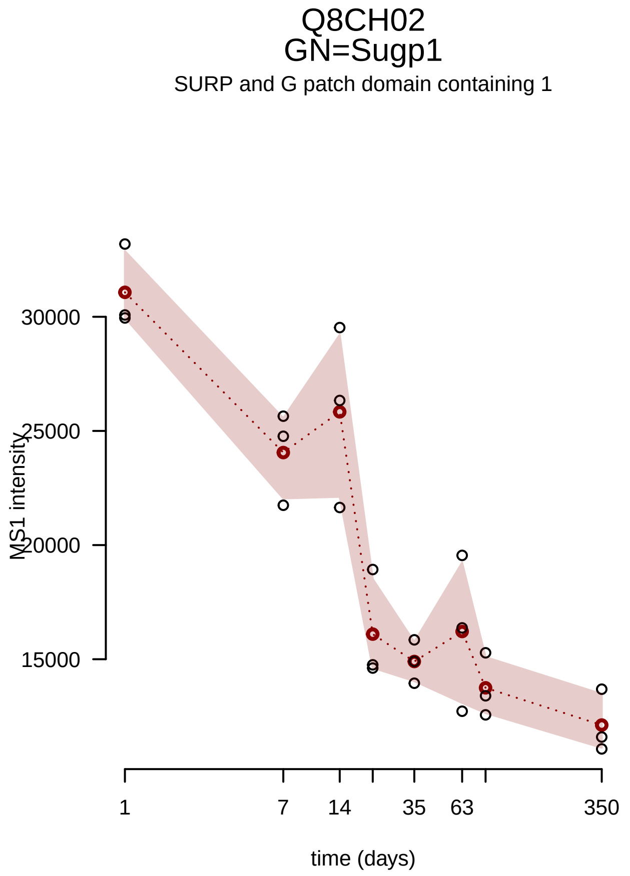
Q8CH02
GN=Sugp1
SURP and G patch domain containing 1
MS1 intensity
30000
25000
20000
15000
1
7
14
35
63
350
time (days)

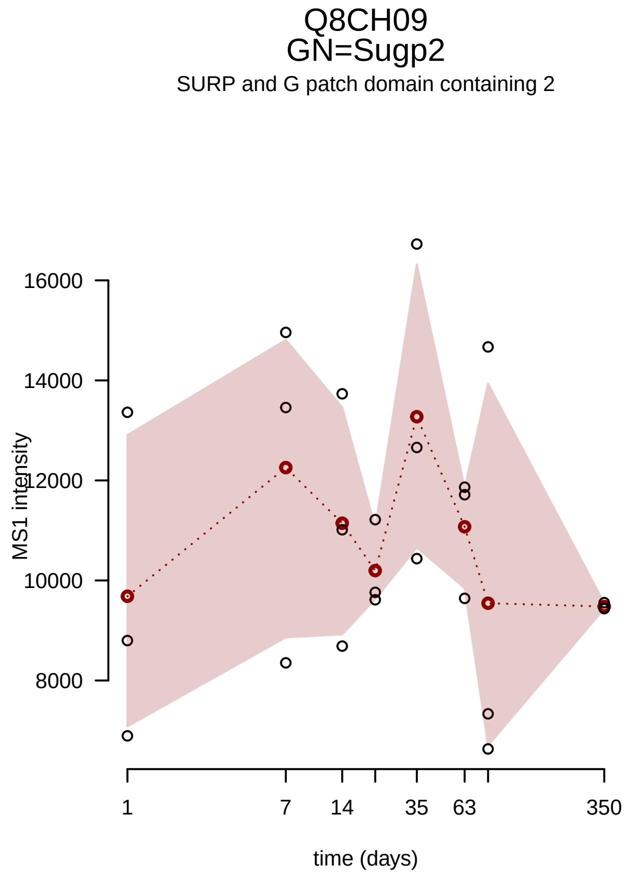
Q8CH09
GN=Sugp2
SURP and G patch domain containing 2
MS1 intensity
16000
14000
12000
10000
8000
1
7
14
35
63
350
time (days)

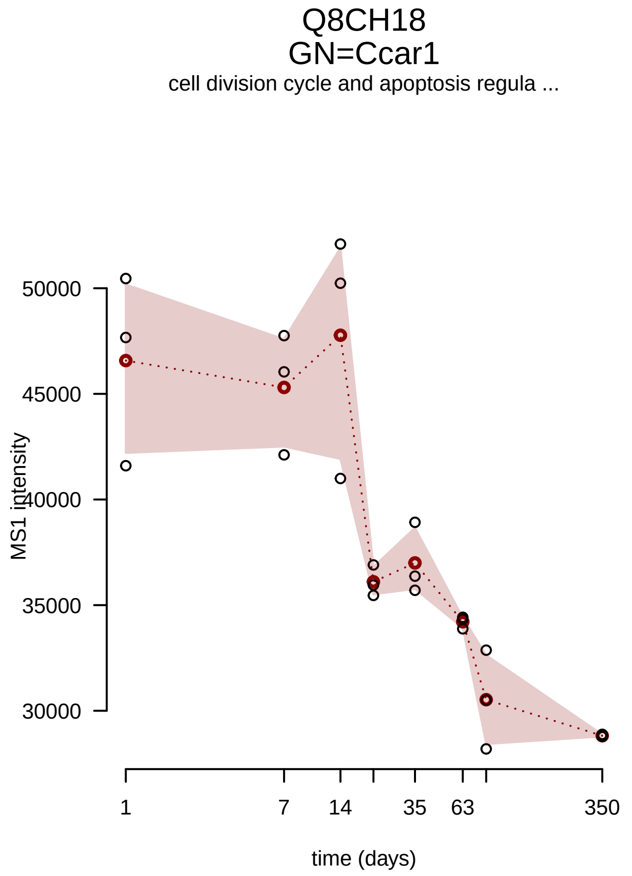
Q8CH18
GN=Ccar1
cell division cycle and apoptosis regula ...
MS1 intensity
50000
45000
40000
35000
30000
1
7
14
35
63
350
time (days)

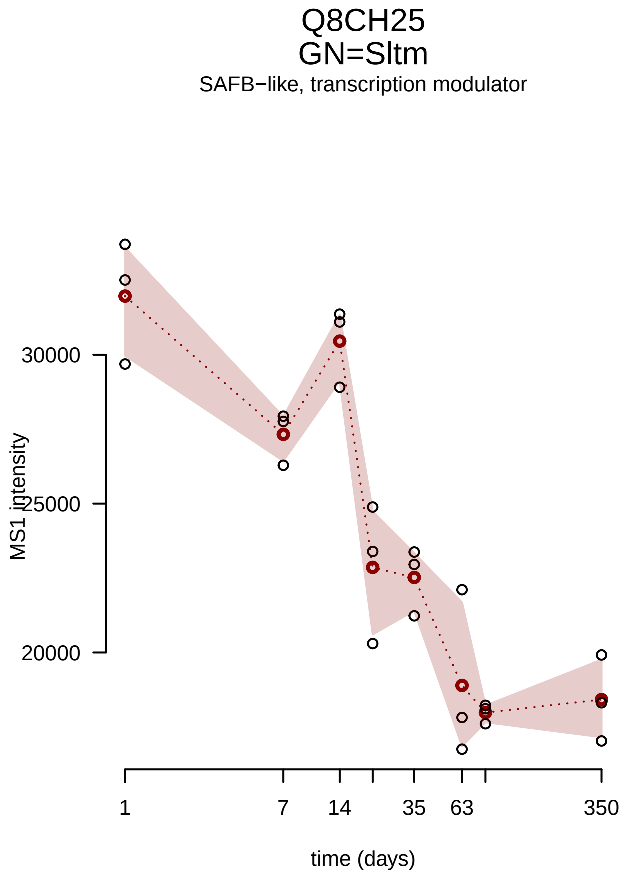
Q8CH25
GN=Sltm
SAFB−like, transcription modulator
MS1 intensity
30000
25000
20000
1
7
14
35
63
350
time (days)

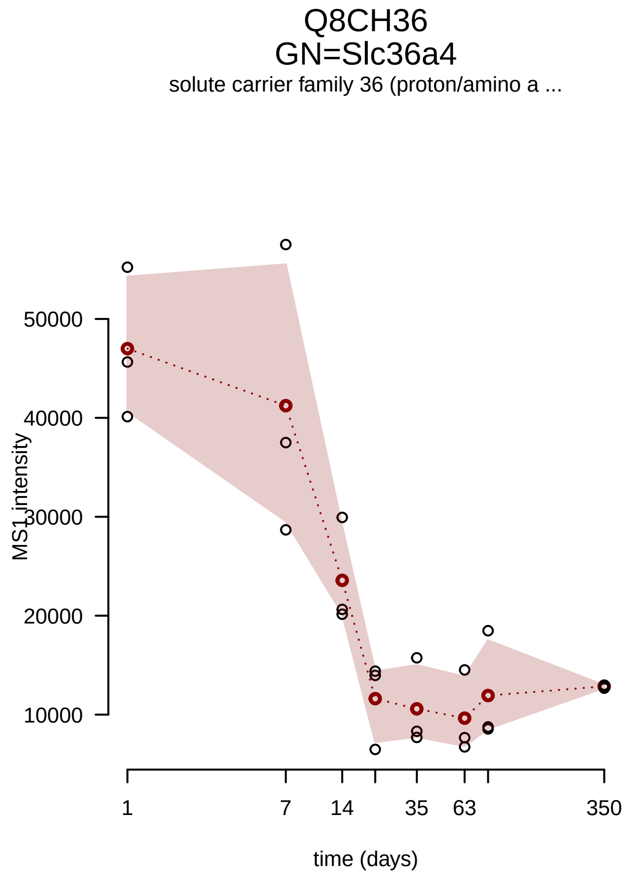
Q8CH36
GN=Slc36a4
solute carrier family 36 (proton/amino a ...
MS1 intensity
50000
40000
30000
20000
10000
1
7
14
35
63
350
time (days)

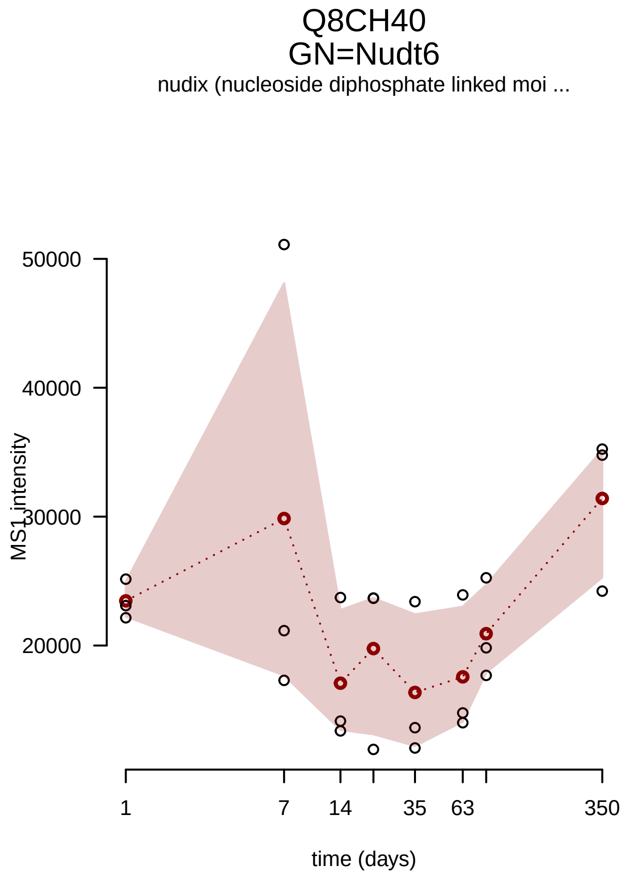
Q8CH40
GN=Nudt6
nudix (nucleoside diphosphate linked moi ...
MS1 intensity
50000
40000
30000
20000
1
7
14
35
63
350
time (days)

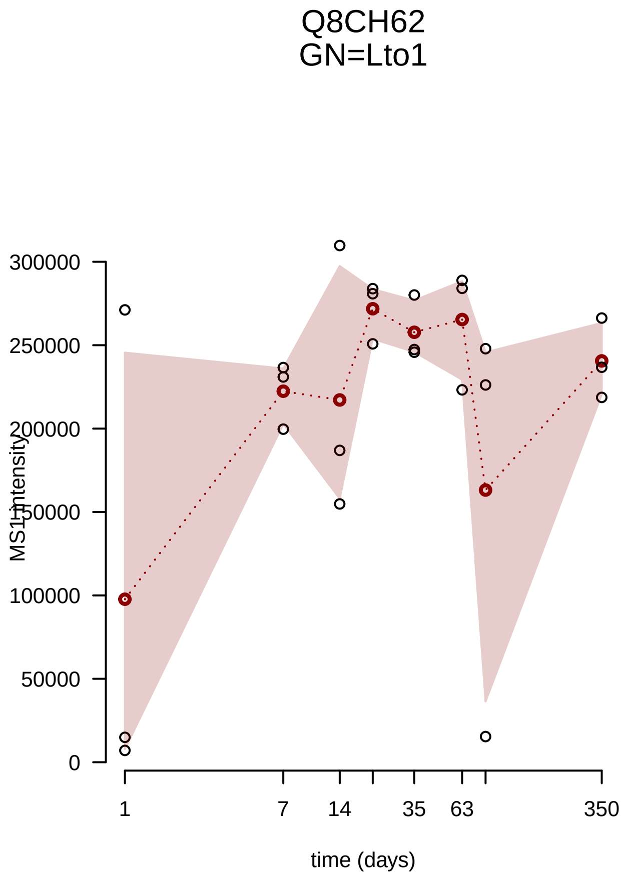
Q8CH62
GN=Lto1
MS1 intensity
300000
250000
200000
150000
100000
50000
0
1
7
14
35
63
350
time (days)

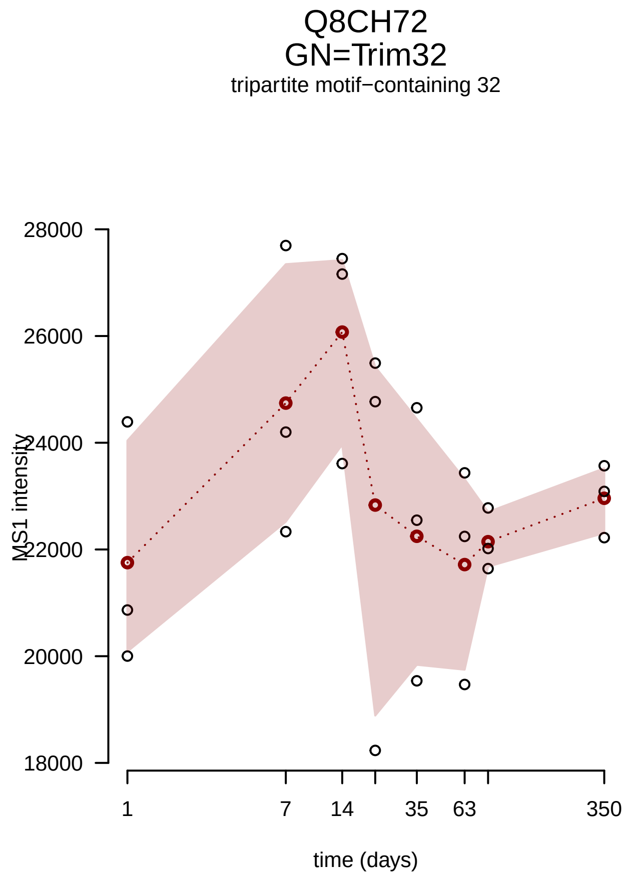
Q8CH72
GN=Trim32
tripartite motif−containing 32
MS1 intensity
28000
26000
24000
22000
20000
18000
1
7
14
35
63
350
time (days)

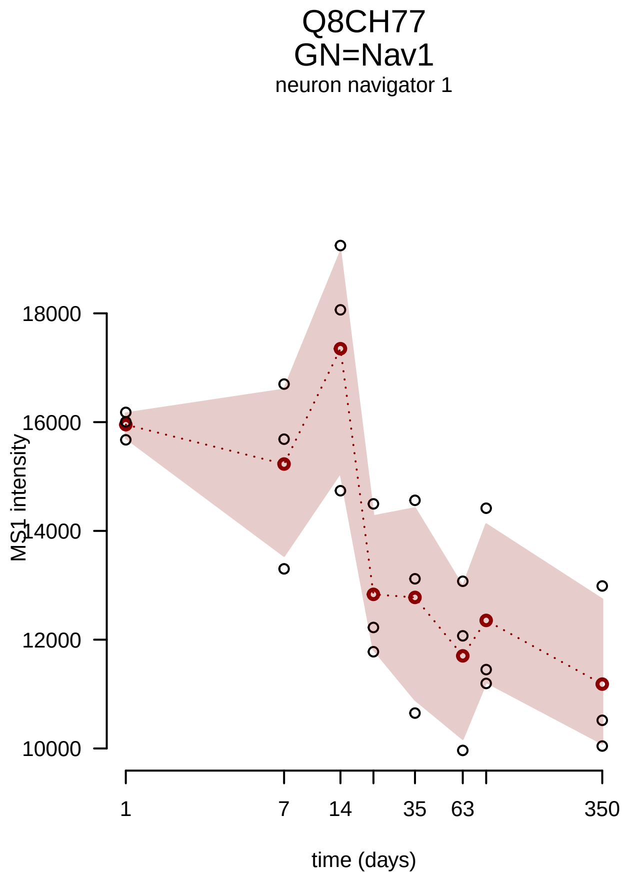
Q8CH77
GN=Nav1
neuron navigator 1
MS1 intensity
18000
16000
14000
12000
10000
1
7
14
35
63
350
time (days)

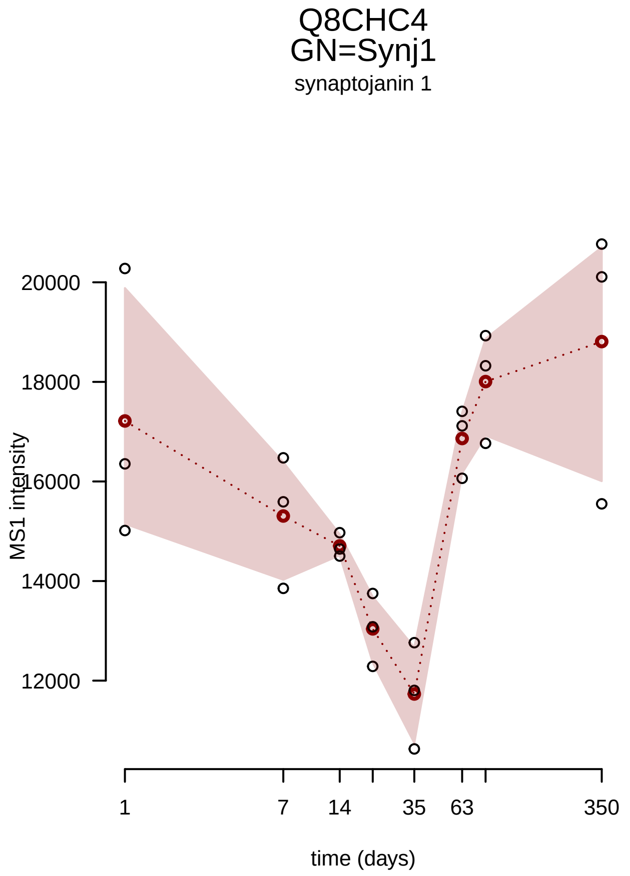
Q8CHC4
GN=Synj1
synaptojanin 1
20000
18000
16000
14000
12000
MS1 intensity
1
7
14
35
63
350
time (days)

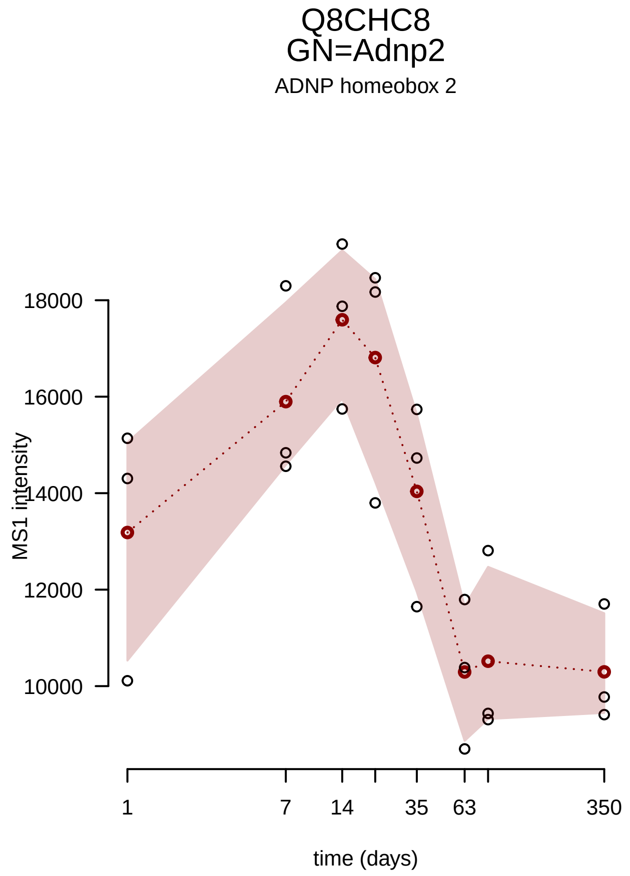
Q8CHC8
GN=Adnp2
ADNP homeobox 2
18000
16000
14000
12000
10000
MS1 intensity
1
7
14
35
63
350
time (days)

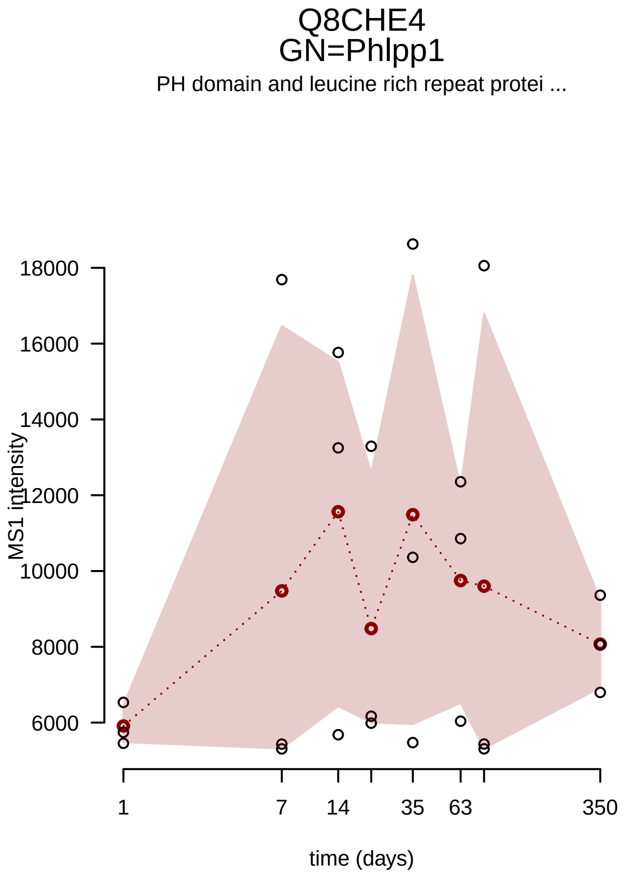
Q8CHE4
GN=Phlpp1
PH domain and leucine rich repeat protei ...
18000
16000
14000
12000
10000
8000
6000
MS1 intensity
1
7
14
35
63
350
time (days)

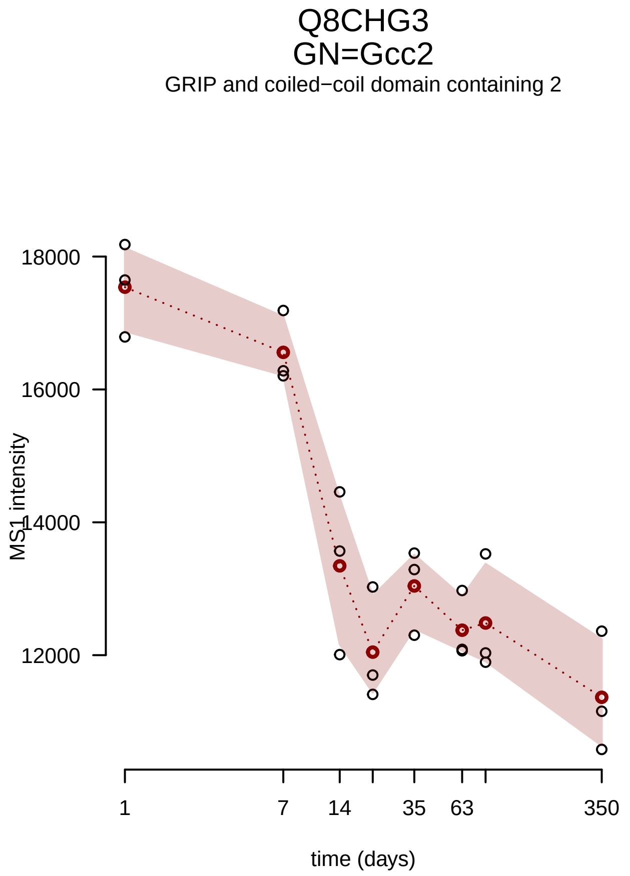
Q8CHG3
GN=Gcc2
GRIP and coiled−coil domain containing 2
18000
16000
14000
12000
MS1 intensity
1
7
14
35
63
350
time (days)

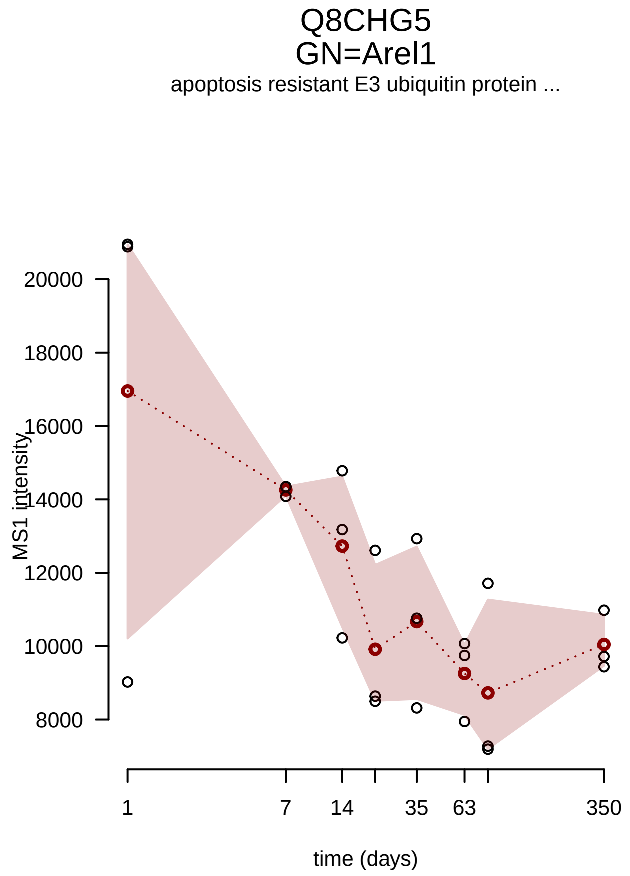
Q8CHG5
GN=Arel1
apoptosis resistant E3 ubiquitin protein ...
20000
18000
16000
14000
12000
10000
8000
MS1 intensity
1
7
14
35
63
350
time (days)

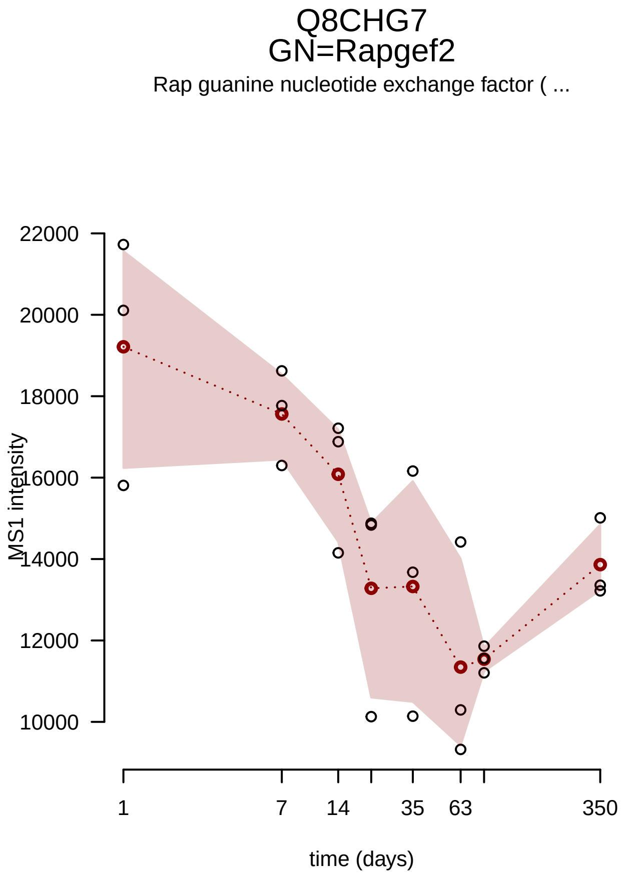
Q8CHG7
GN=Rapgef2
Rap guanine nucleotide exchange factor (...
22000
20000
18000
16000
14000
12000
10000
MS1 intensity
1
7
14
35
63
350
time (days)

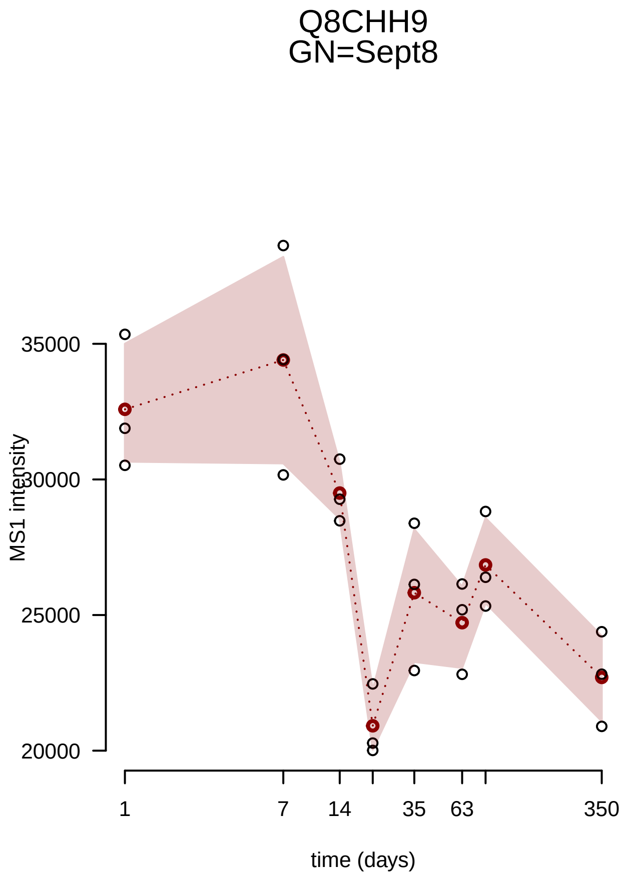
Q8CHH9
GN=Sept8
35000
30000
25000
20000
MS1 intensity
1
7
14
35
63
350
time (days)

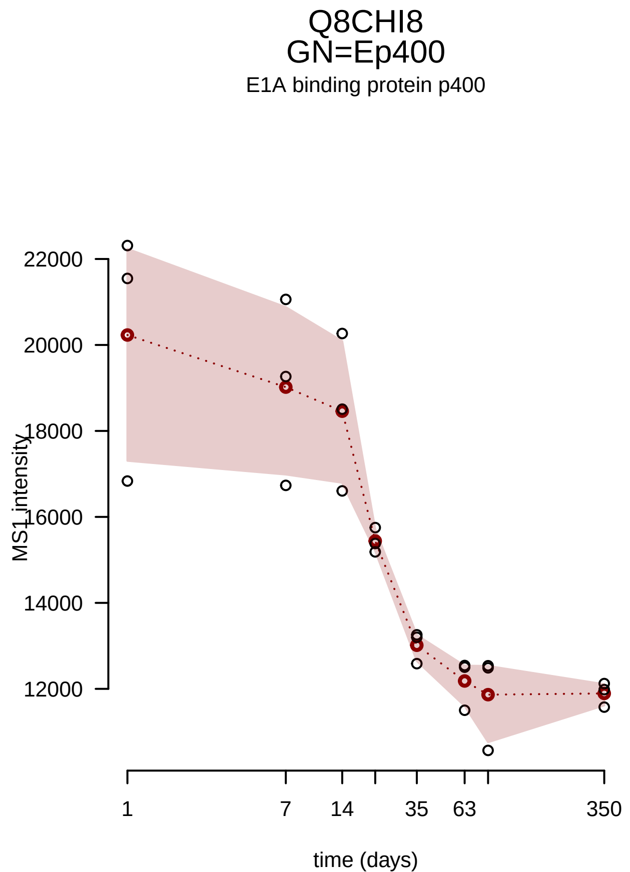
Q8CHI8
GN=Ep400
E1A binding protein p400
22000
20000
18000
16000
14000
12000
MS1 intensity
1
7
14
35
63
350
time (days)

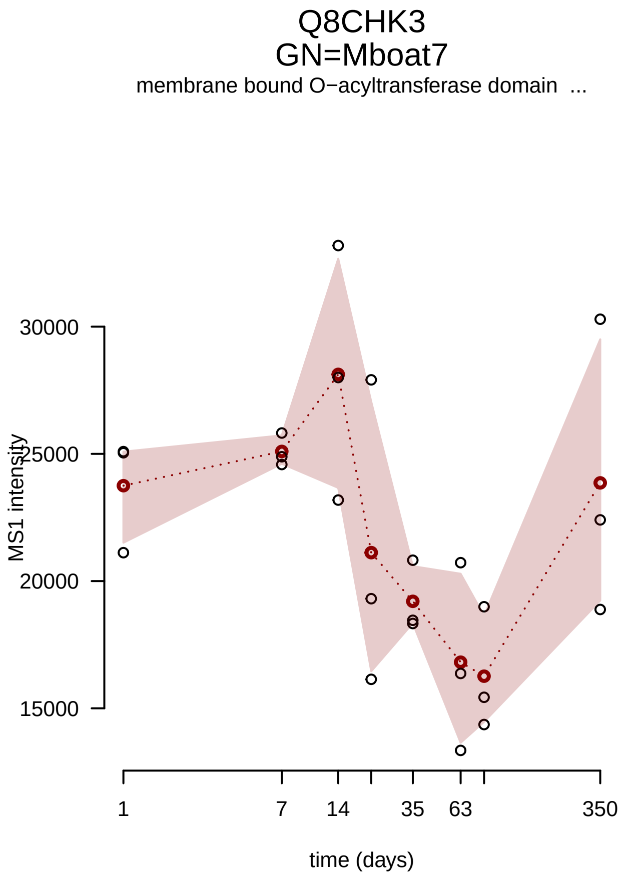
Q8CHK3
GN=Mboat7
membrane bound O−acyltransferase domain ...
30000
25000
20000
15000
MS1 intensity
1
7
14
35
63
350
time (days)

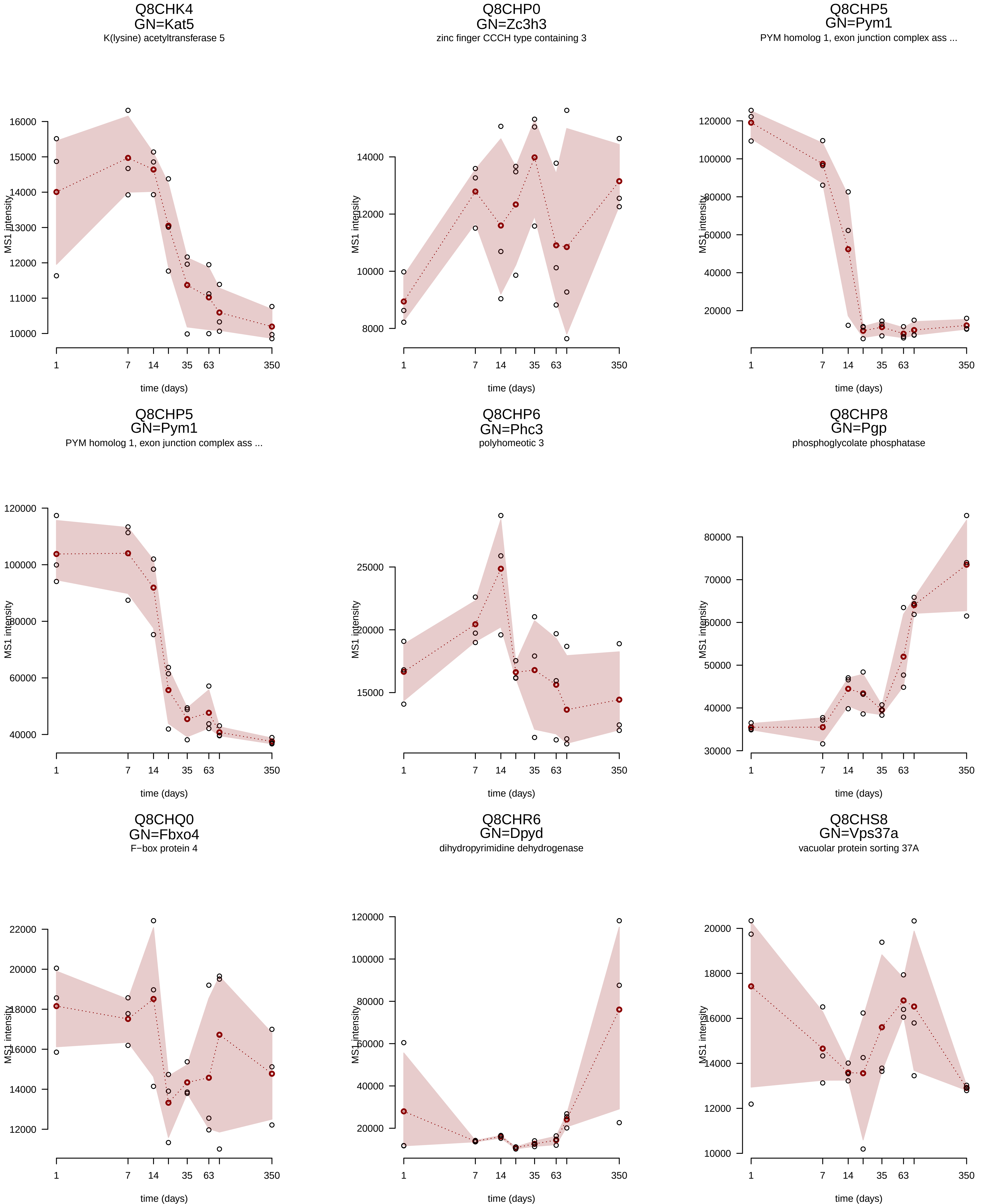
Q8CHK4
GN=Kat5
K(lysine) acetyltransferase 5
MS1 intensity
16000
15000
14000
13000
12000
11000
10000
1
7
14
35
63
350
time (days)
Q8CHP0
GN=Zc3h3
zinc finger CCCH type containing 3
MS1 intensity
14000
12000
10000
8000
1
7
14
35
63
350
time (days)
Q8CHP5
GN=Pym1
PYM homolog 1, exon junction complex ass ...
MS1 intensity
120000
100000
80000
60000
40000
20000
1
7
14
35
63
350
time (days)
Q8CHP5
GN=Pym1
PYM homolog 1, exon junction complex ass ...
MS1 intensity
120000
100000
80000
60000
40000
1
7
14
35
63
350
time (days)
Q8CHP6
GN=Phc3
polyhomeotic 3
MS1 intensity
25000
20000
15000
1
7
14
35
63
350
time (days)
Q8CHP8
GN=Pgp
phosphoglycolate phosphatase
MS1 intensity
80000
70000
60000
50000
40000
30000
1
7
14
35
63
350
time (days)
Q8CHQ0
GN=Fbxo4
F−box protein 4
MS1 intensity
22000
20000
18000
16000
14000
12000
1
7
14
35
63
350
time (days)
Q8CHR6
GN=Dpyd
dihydropyrimidine dehydrogenase
MS1 intensity
120000
100000
80000
60000
40000
20000
1
7
14
35
63
350
time (days)
Q8CHS8
GN=Vps37a
vacuolar protein sorting 37A
MS1 intensity
20000
18000
16000
14000
12000
10000
1
7
14
35
63
350
time (days)

Q8CHT0
GN=Aldh4a1
aldehyde dehydrogenase 4 family, member ...

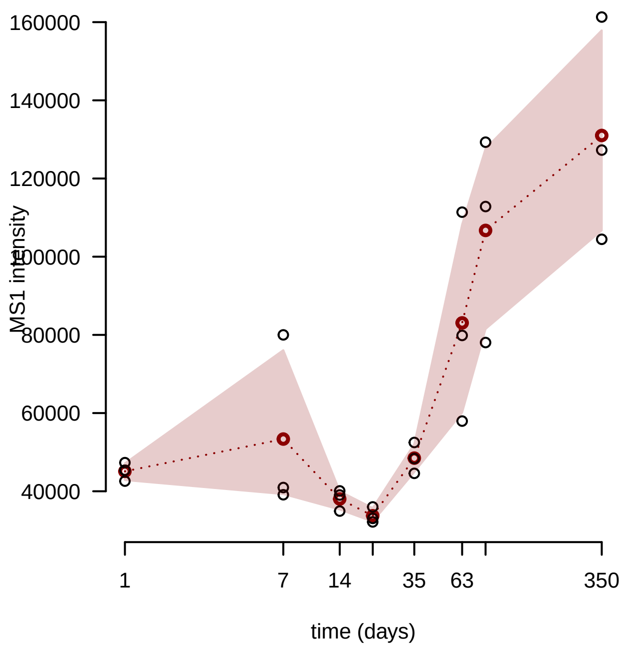

Q8CHT1
GN=Ngef
neuronal guanine nucleotide exchange fac ...

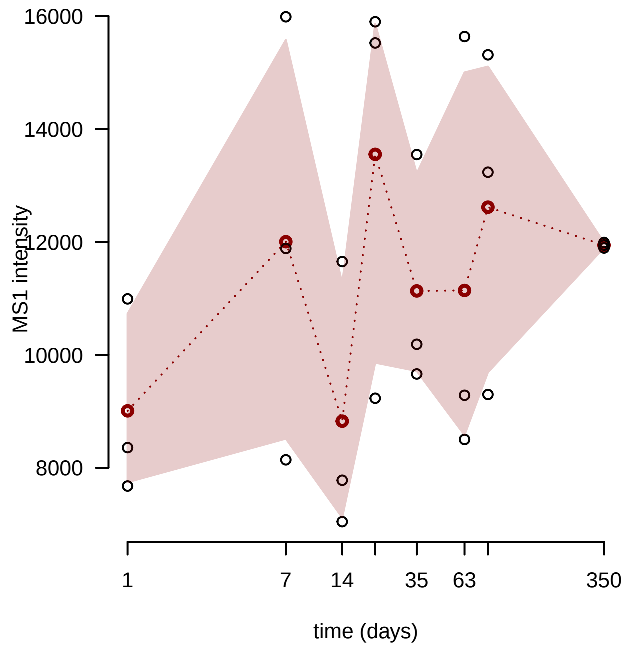

Q8CHT3
GN=Ints5
integrator complex subunit 5

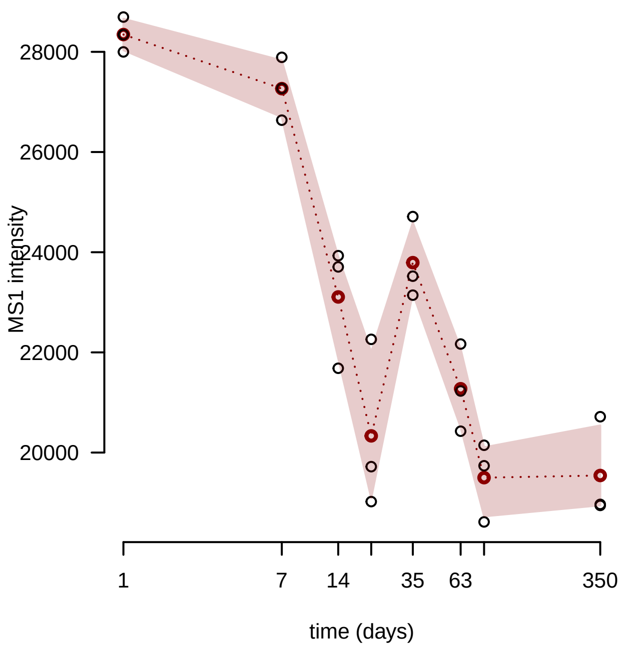

Q8CHU3
GN=Epn2
epsin 2

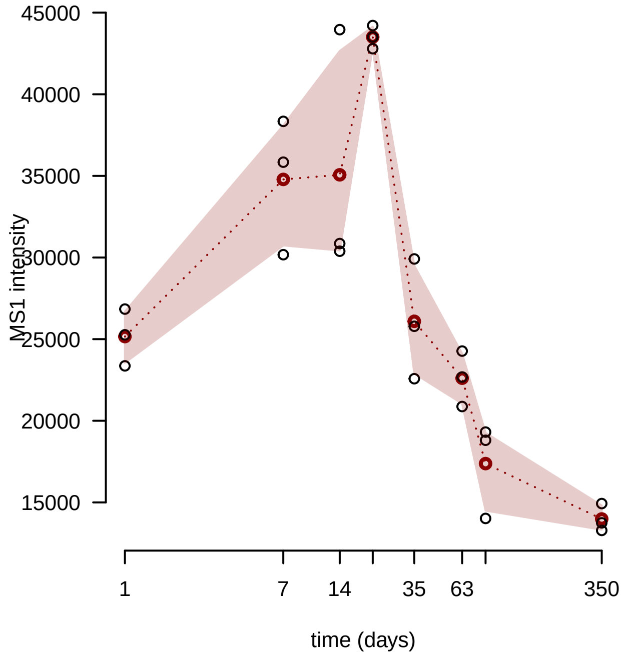

Q8CHV6
GN=Tada2a
transcriptional adaptor 2A

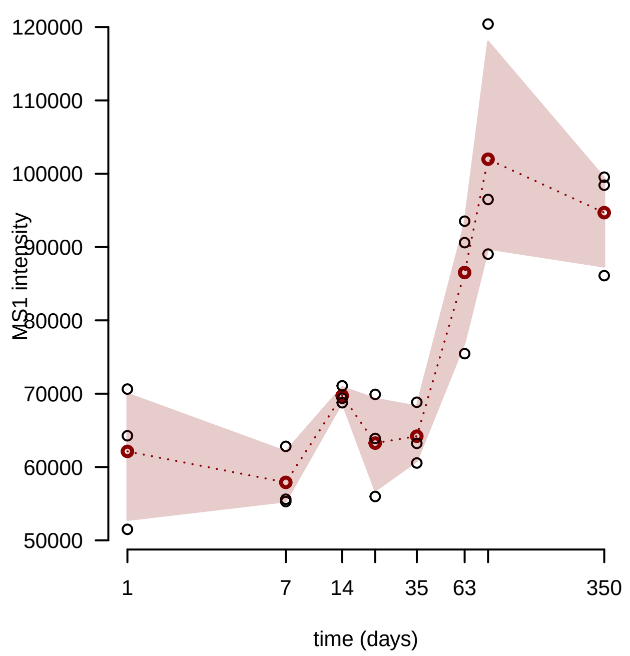

Q8CHW4
GN=Eif2b5
eukaryotic translation initiation factor ...

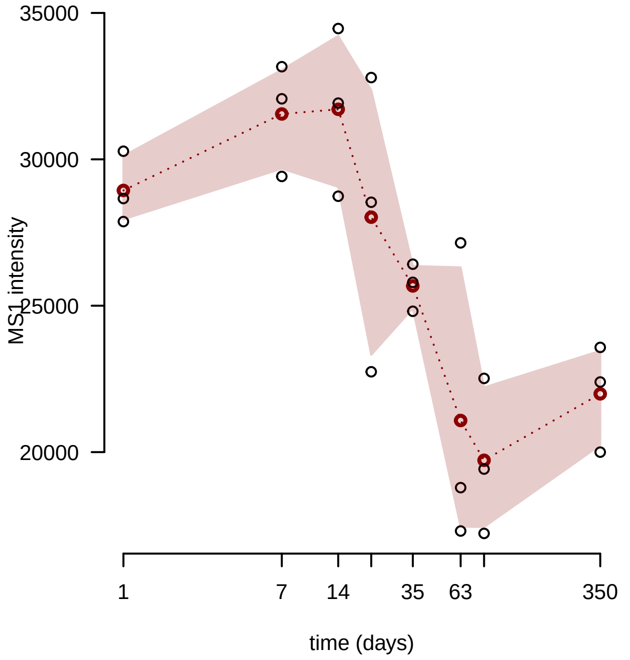

Q8CHX7
GN=Rftn2
raftlin family member 2

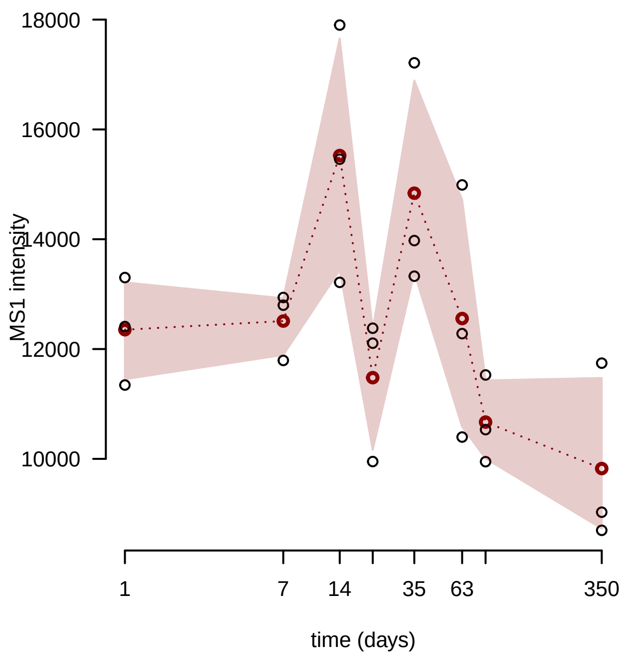

Q8CHY3
GN=Dym
dymeclin

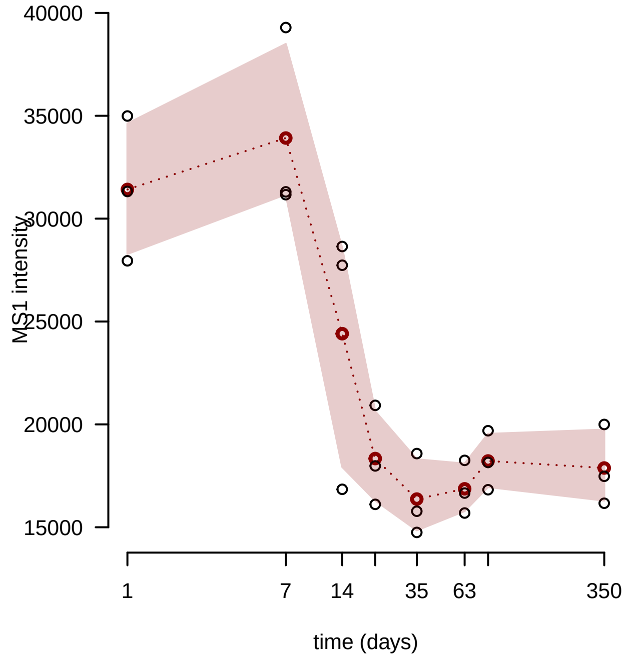

Q8CHY6
GN=Gatad2a
GATA zinc finger domain containing 2A

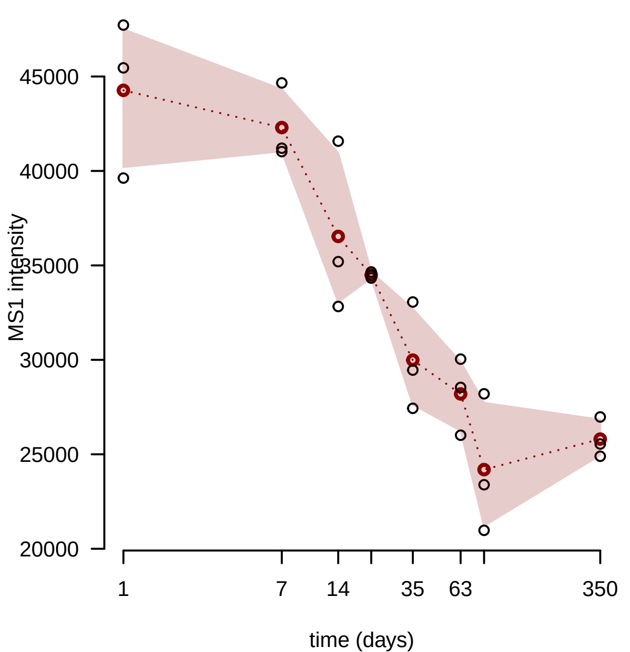

Q8CI04
GN=Cog3
component of oligomeric golgi complex 3

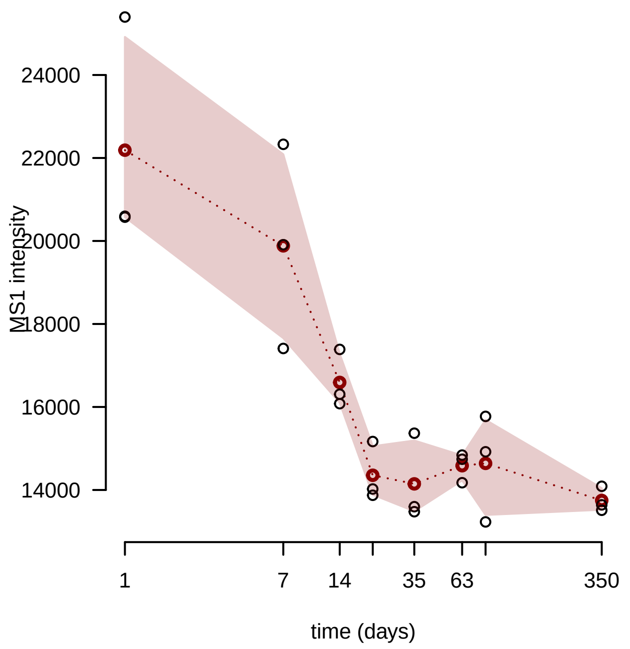

Q8CI08
GN=Slain2
SLAIN motif family, member 2

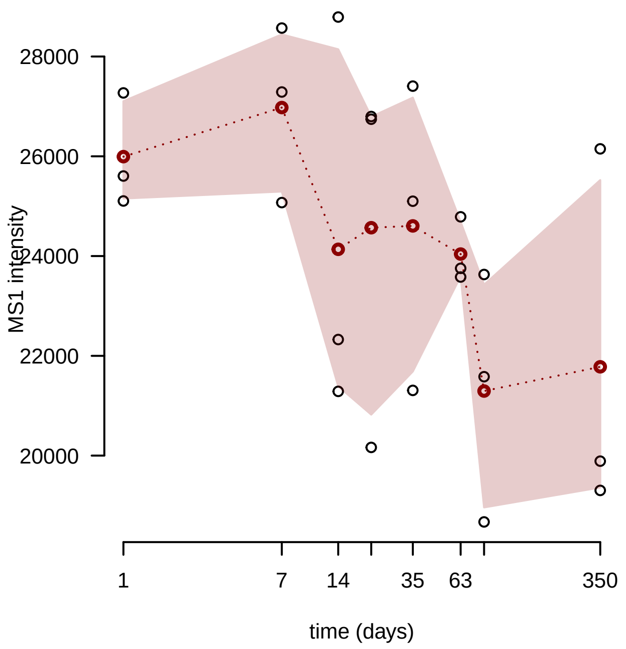

Q8CI11
GN=Gnl3
guanine nucleotide binding protein−like ...

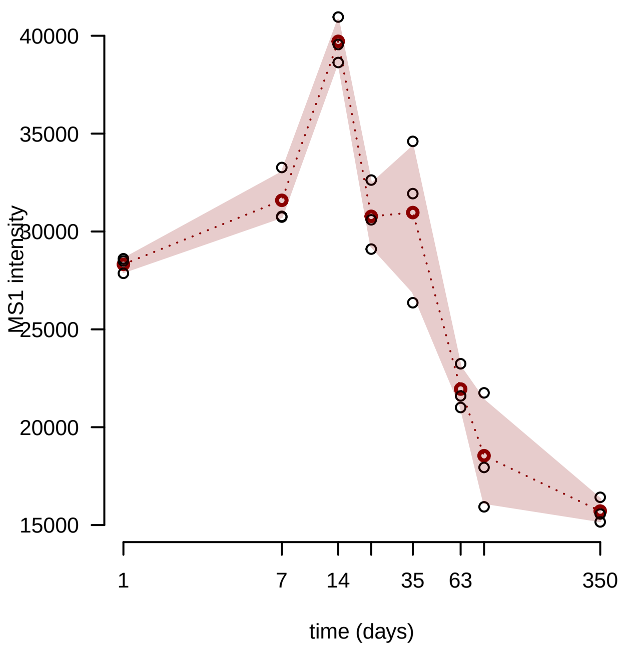

Q8CI12
GN=Smtnl2
smoothelin−like 2

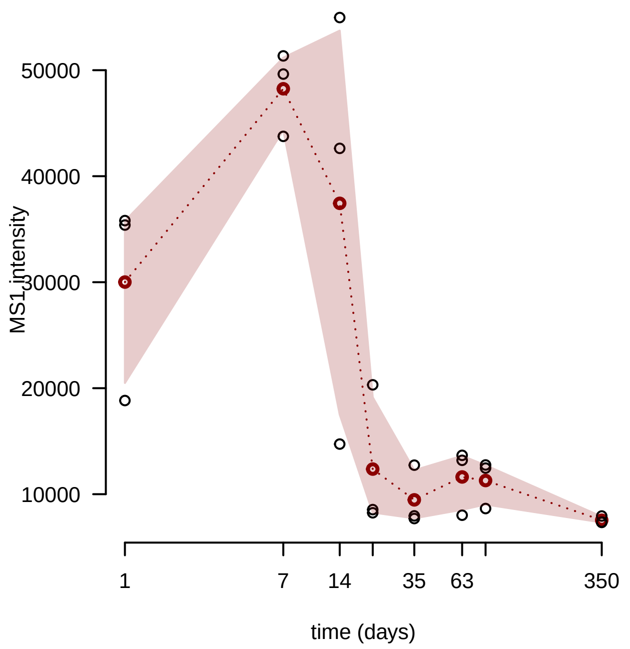

Q8CI32
GN=Bag5
BCL2−associated athanogene 5

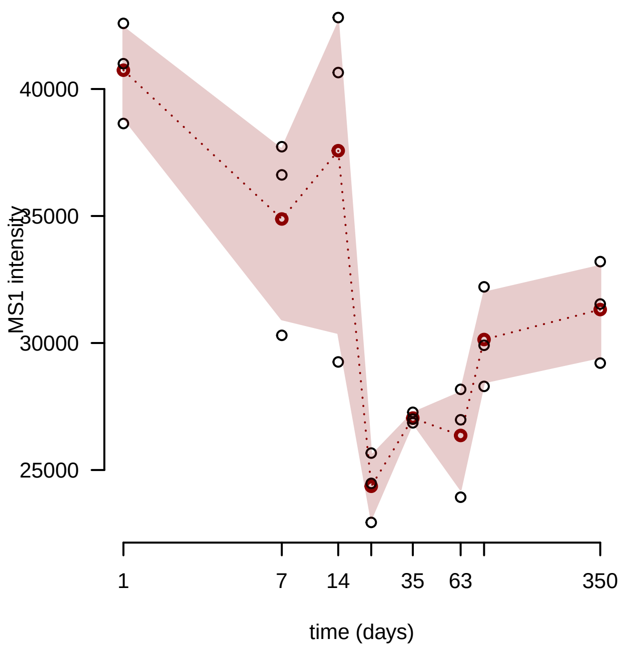

Q8CI33
GN=Cwf19l1
CWF19−like 1, cell cycle control (S. pom ...

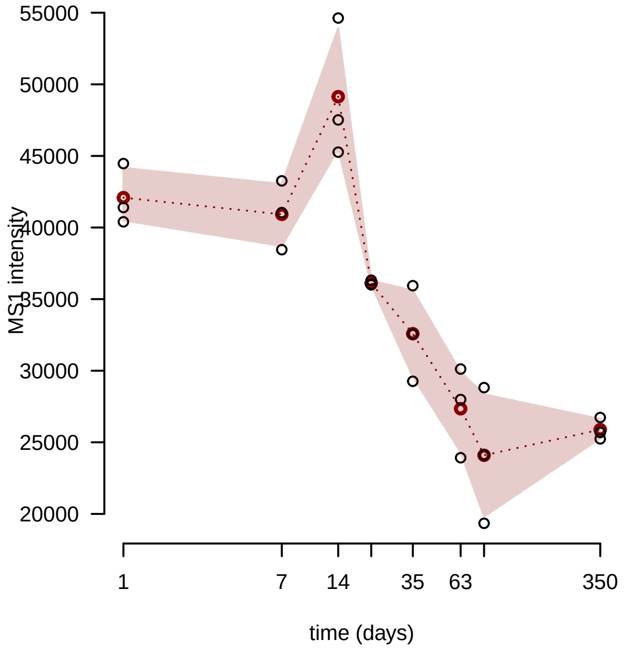

Q8CI43
GN=Myl6b
myosin, light polypeptide 6B

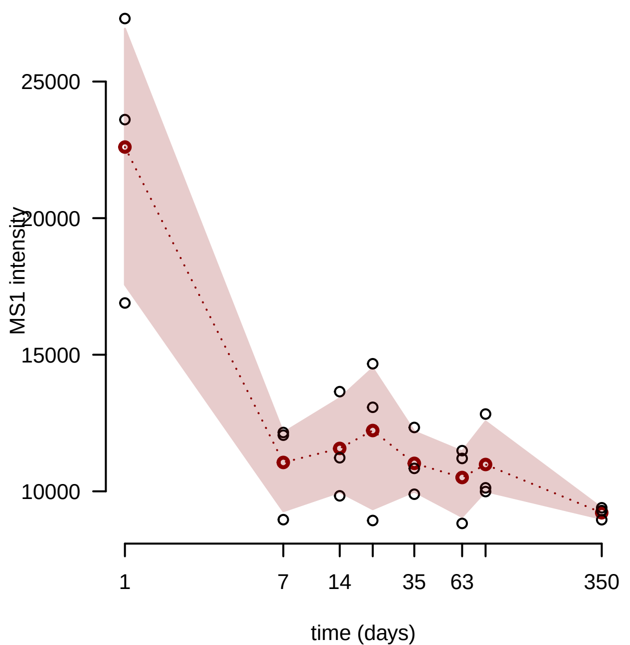

Q8CI51
GN=Pdlim5
PDZ and LIM domain 5

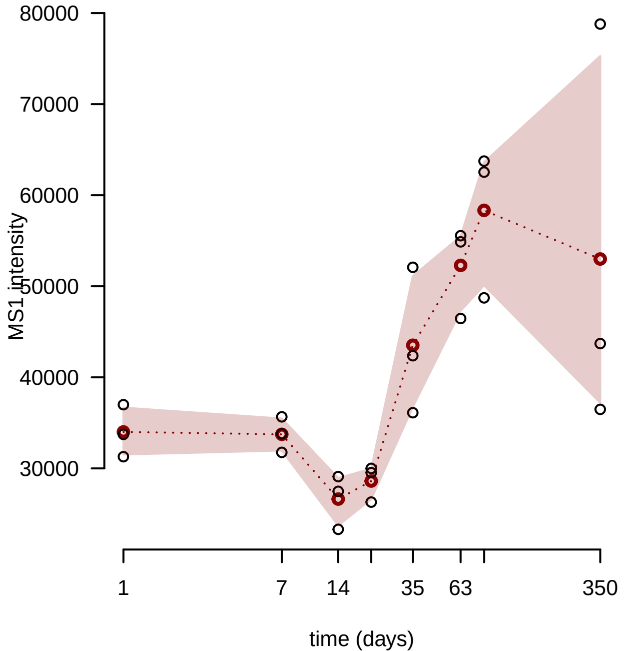

Q8CI51
GN=Pdlim5
PDZ and LIM domain 5

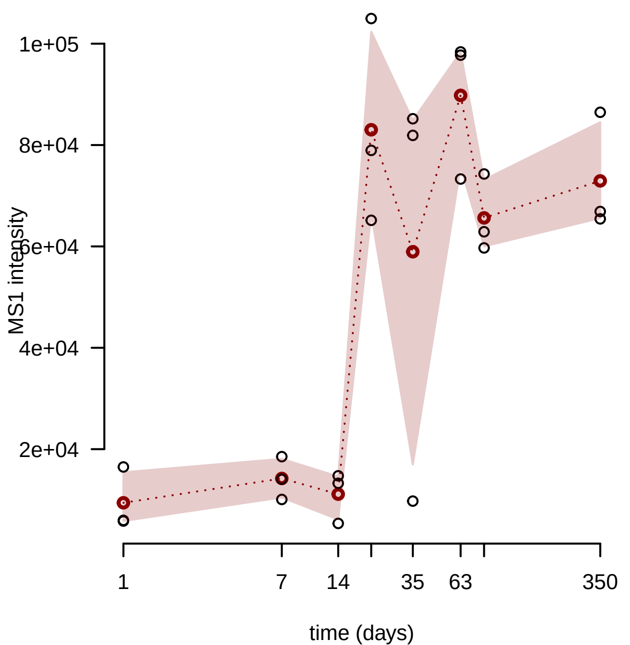

Q8CI59
GN=Steap3
STEAP family member 3

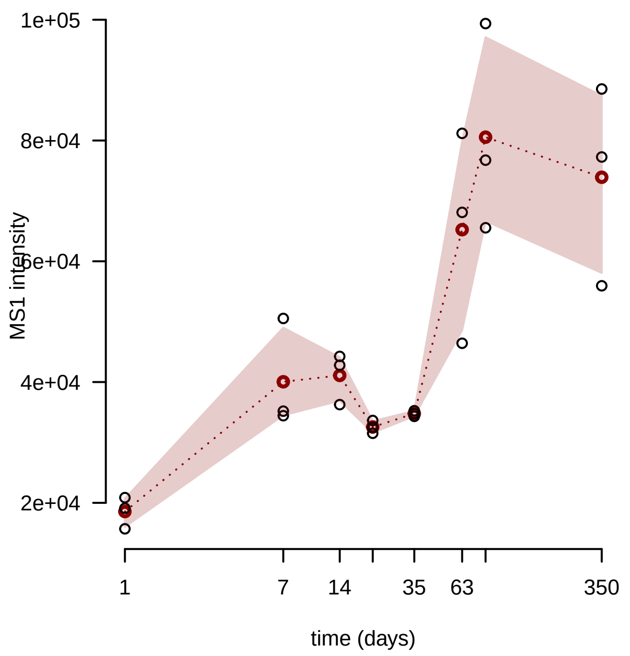

Q8CI59
GN=Steap3
STEAP family member 3

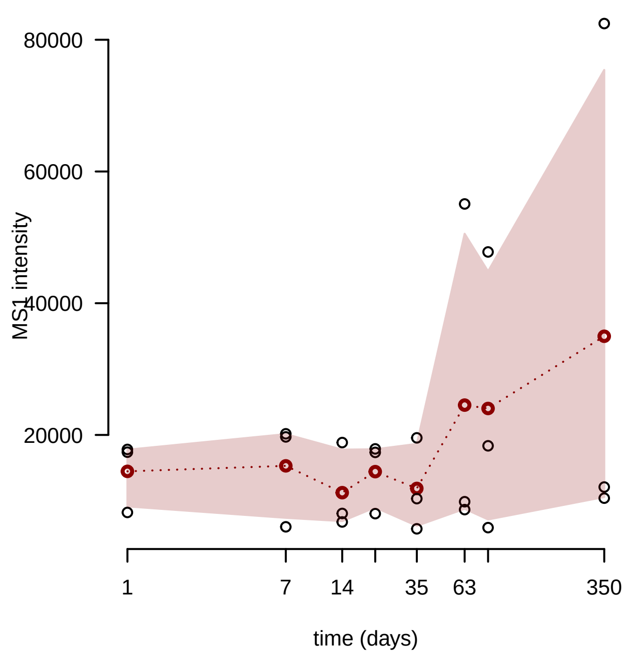

Q8CI61
GN=Bag4
BCL2−associated athanogene 4

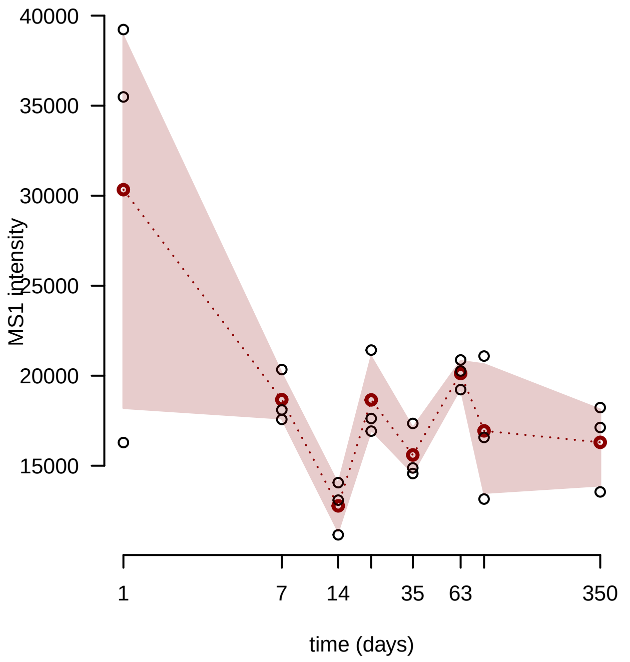

Q8CI70
GN=Lrrc20
leucine rich repeat containing 20

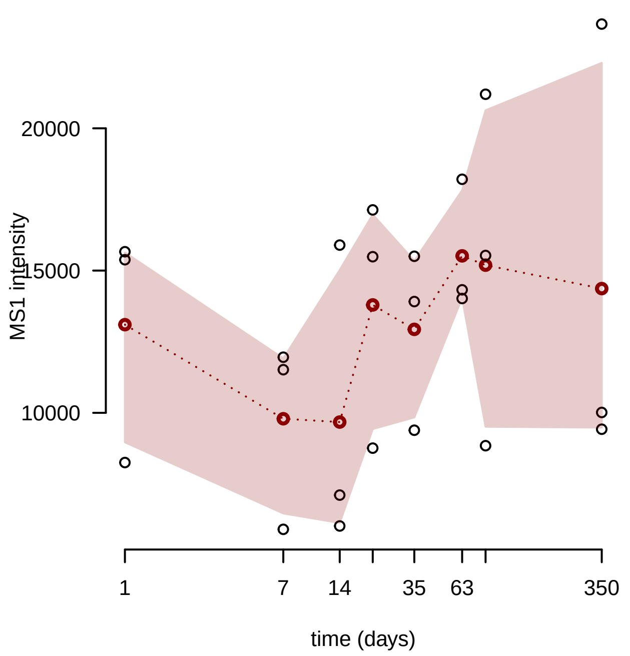

Q8CI71
GN=Vps50
VPS50 EARP/GARPII complex subunit

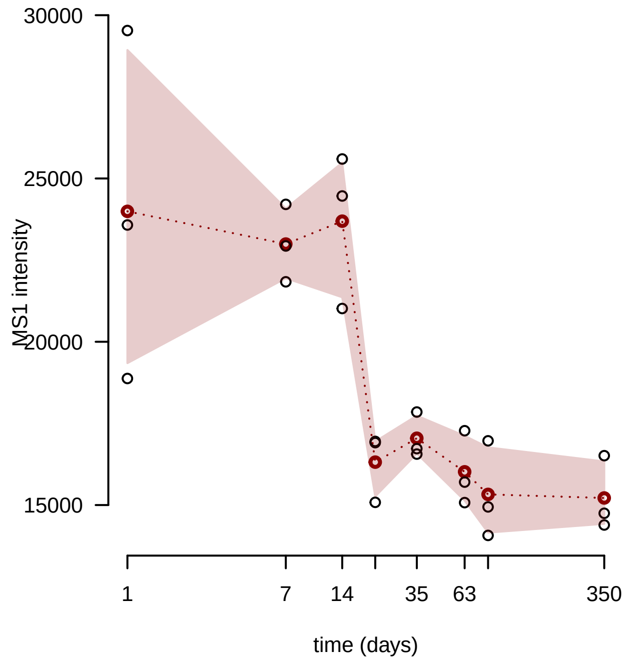

Q8CI75
GN=Dis3l2
DIS3 like 3'−5' exoribonuclease 2

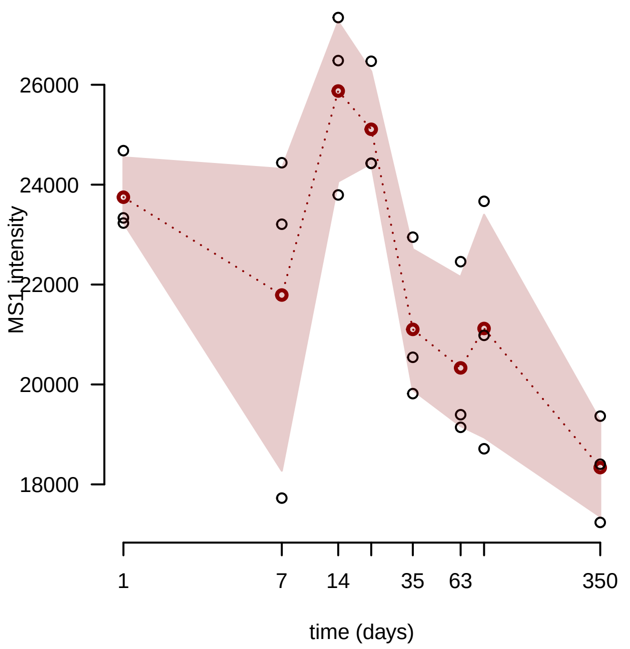

Q8CI78
GN=Rmnd1
required for meiotic nuclear division 1 ...

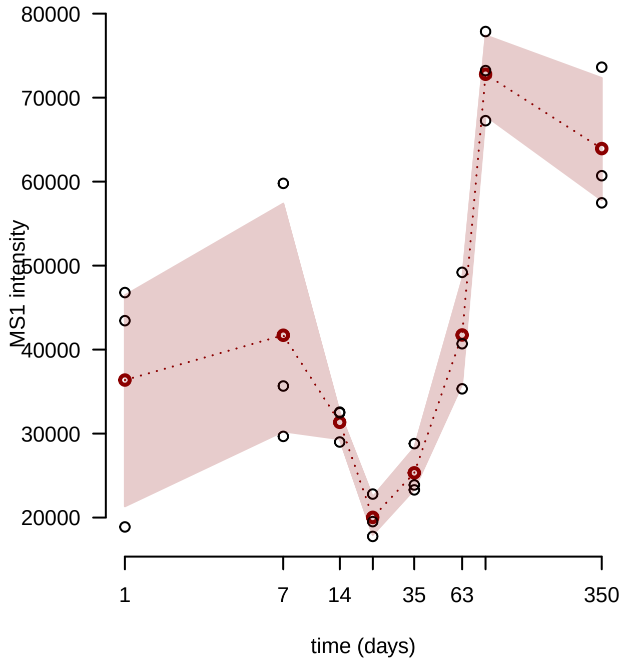

Q8CI94
GN=Pygb
brain glycogen phosphorylase

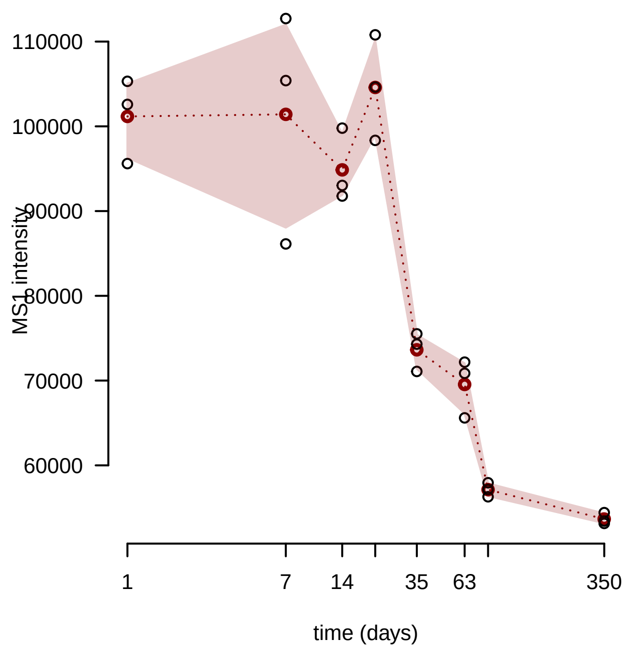

Q8CI95
GN=Osbpl11
oxysterol binding protein−like 11

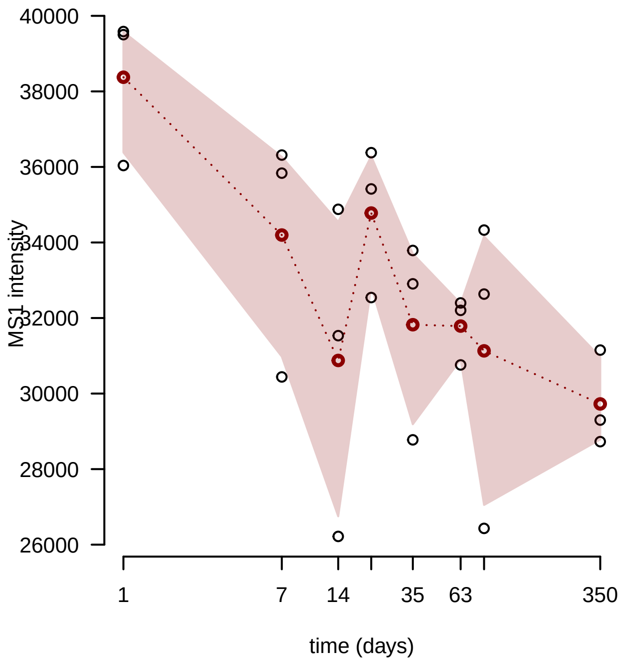

Q8CIA5
GN=Slc35b4
solute carrier family 35, member B4

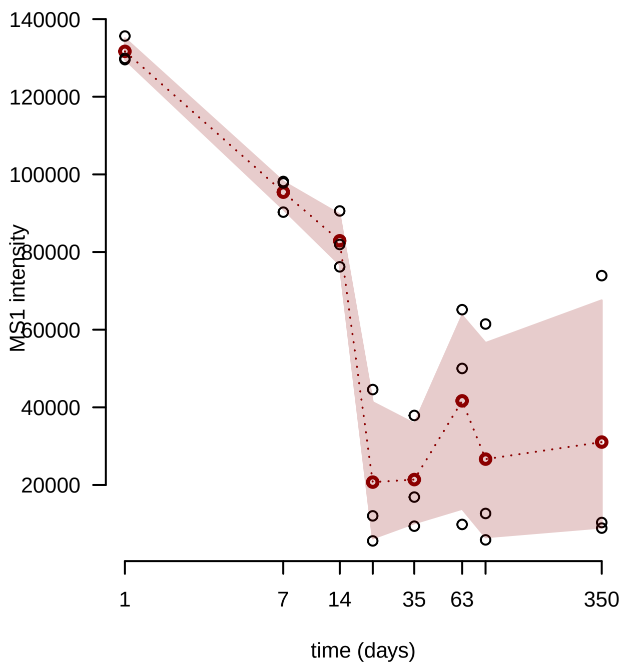

Q8CIB5
GN=Fermt2
fermitin family member 2

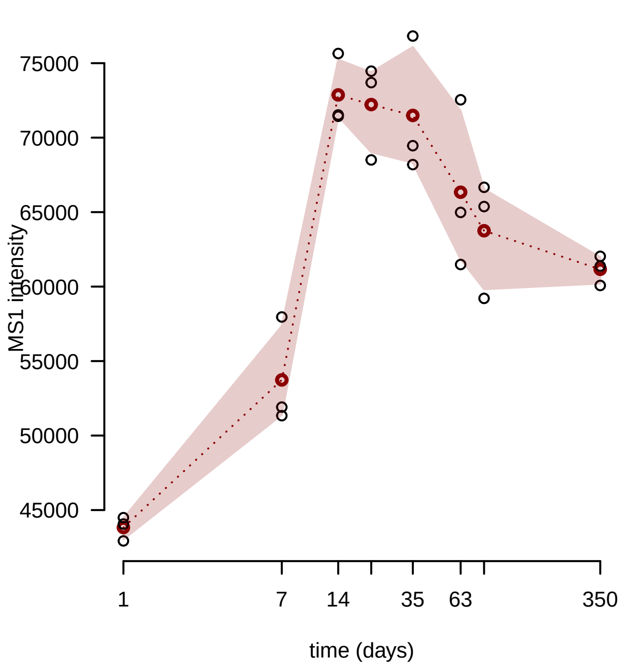

Q8CIB6
GN=Tmem230
transmembrane protein 230

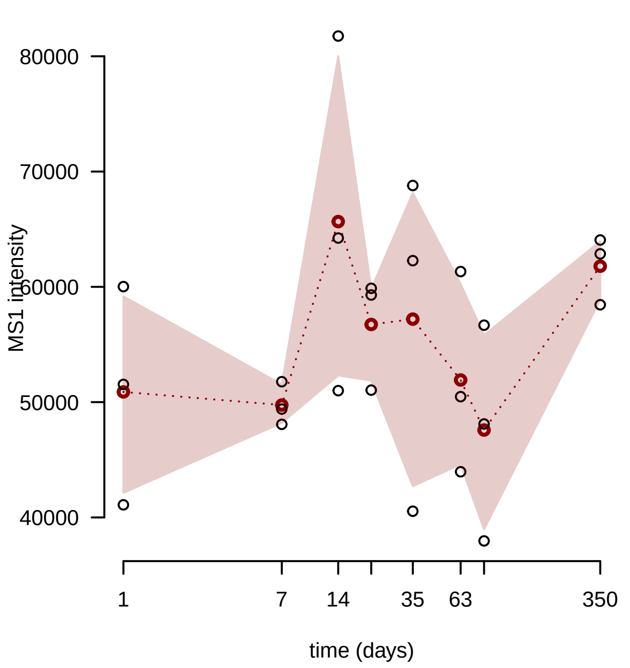

Q8CIB9
GN=Esco2
establishment of sister chromatid cohesi ...

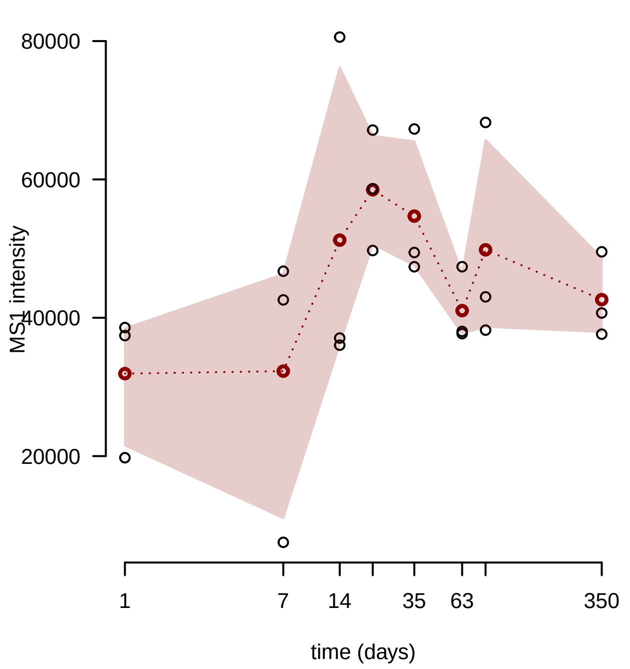

Q8CIC2
GN=Nupl2
nucleoporin like 2

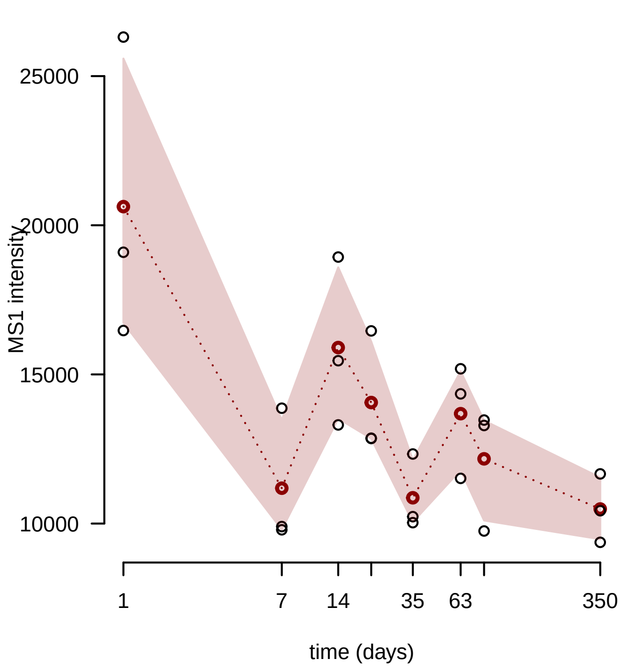

Q8CID0
GN=Kat14
lysine acetyltransferase 14

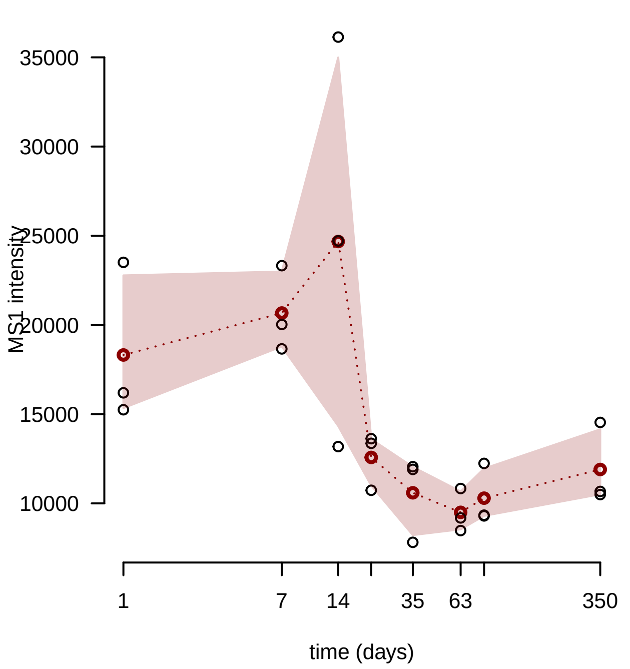

Q8CIE0
GN=Serpina11
serine (or cysteine) peptidase inhibitor ...

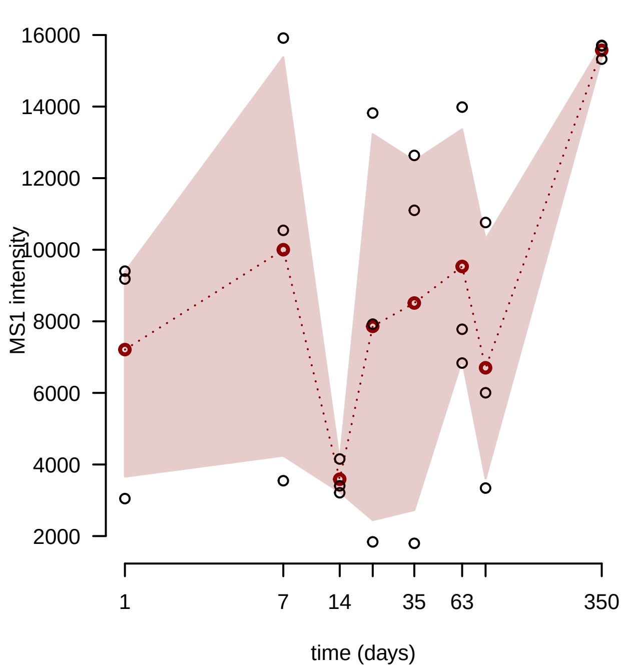

Q8CIE2
GN=Zmiz2
zinc finger, MIZ−type containing 2

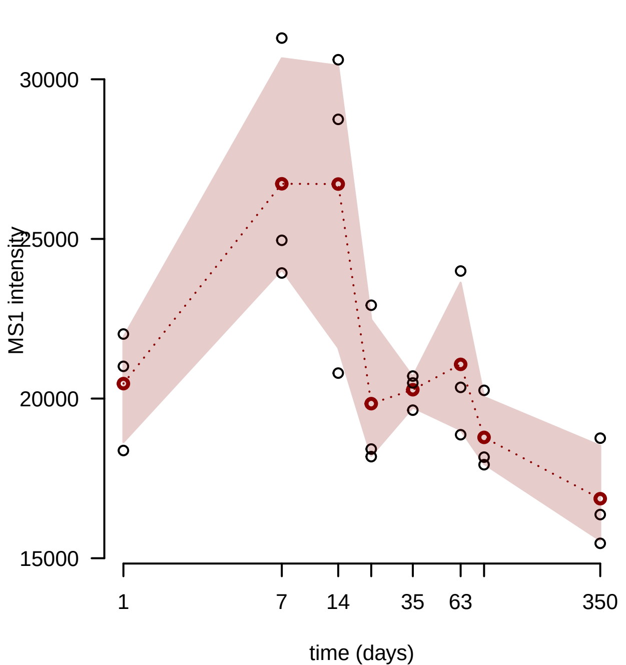

Q8CIE4
GN=Parp10
poly (ADP−ribose) polymerase family, mem ...

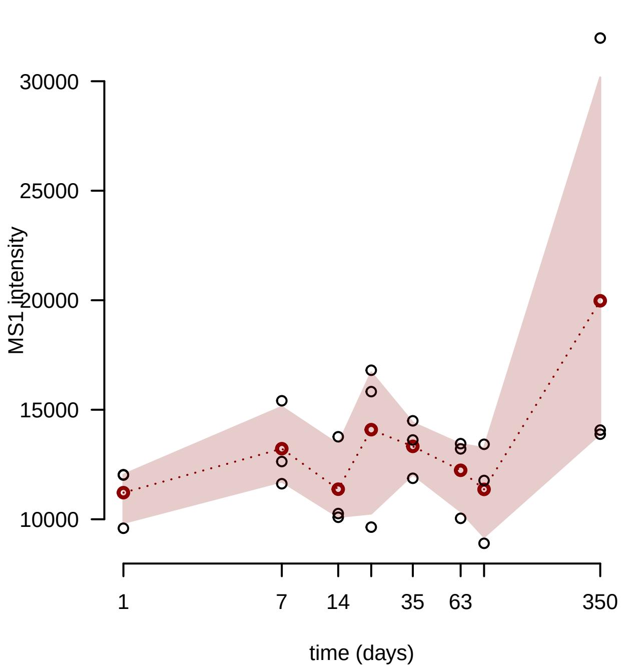

Q8CIE6
GN=Copa
coatomer protein complex subunit alpha

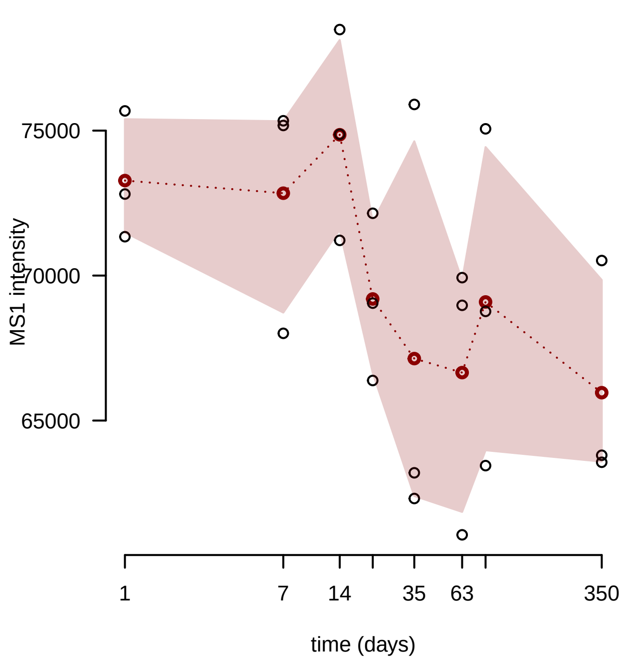

Q8CIF4
GN=Btd
biotinidase

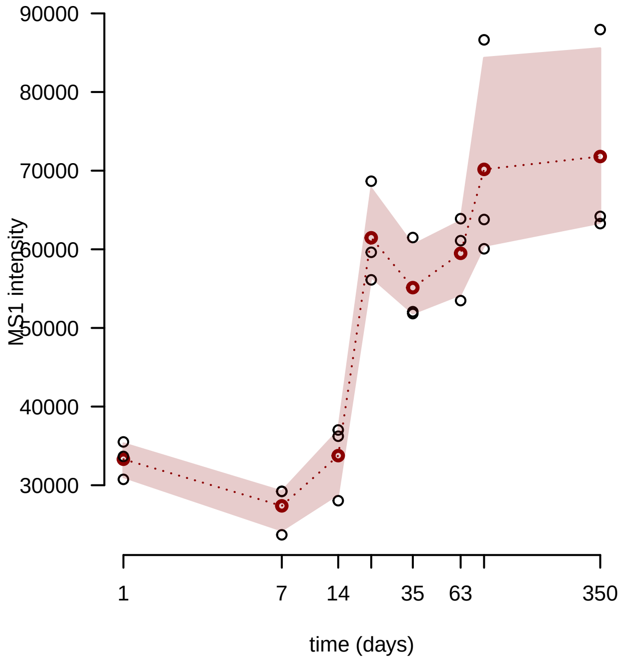

Q8CIF6
GN=Sidt2
SID1 transmembrane family, member 2

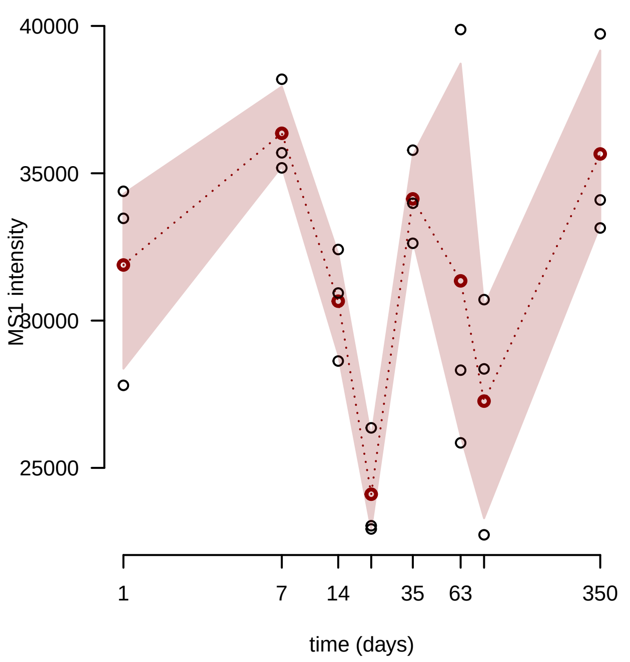

Q8CIG3
GN=Kdm1b
lysine (K)−specific demethylase 1B

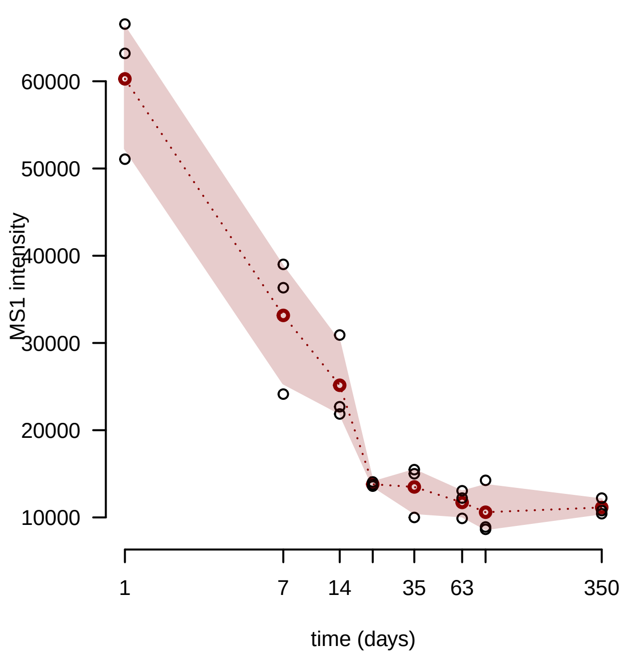

Q8CIG8
GN=Prmt5
protein arginine N−methyltransferase 5

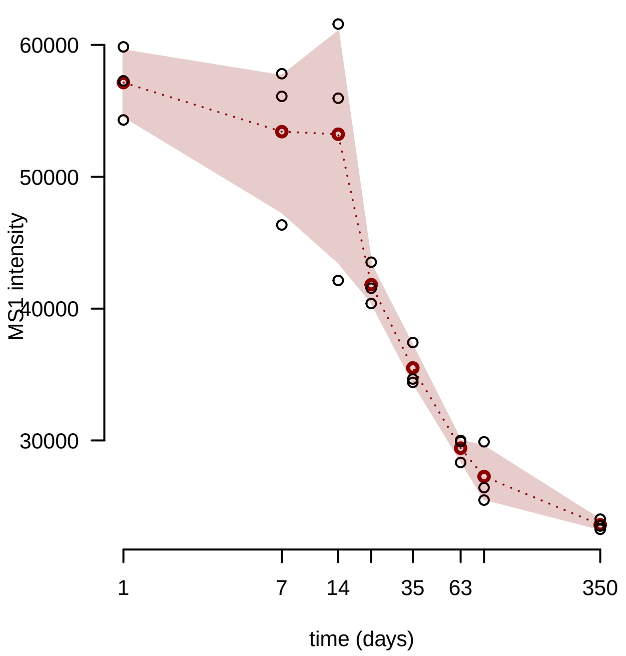

Q8CIG9
GN=Fbxl8
F−box and leucine−rich repeat protein 8

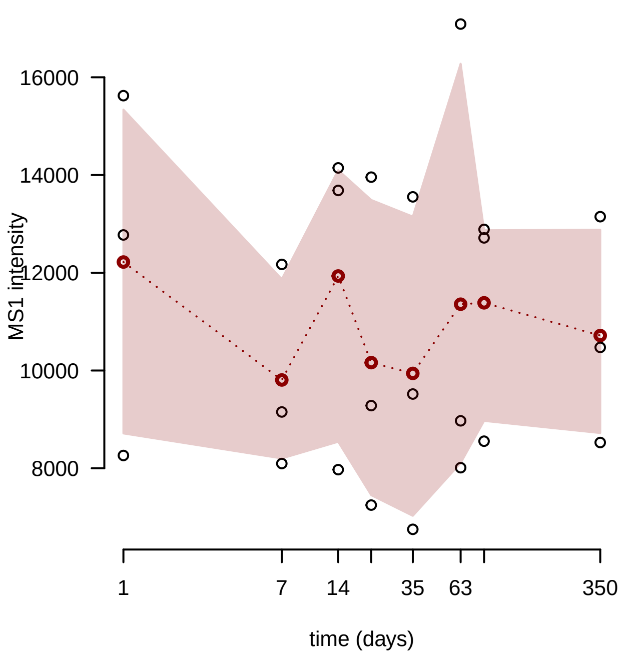

Q8CIH5
GN=Plcg2
phospholipase C, gamma 2

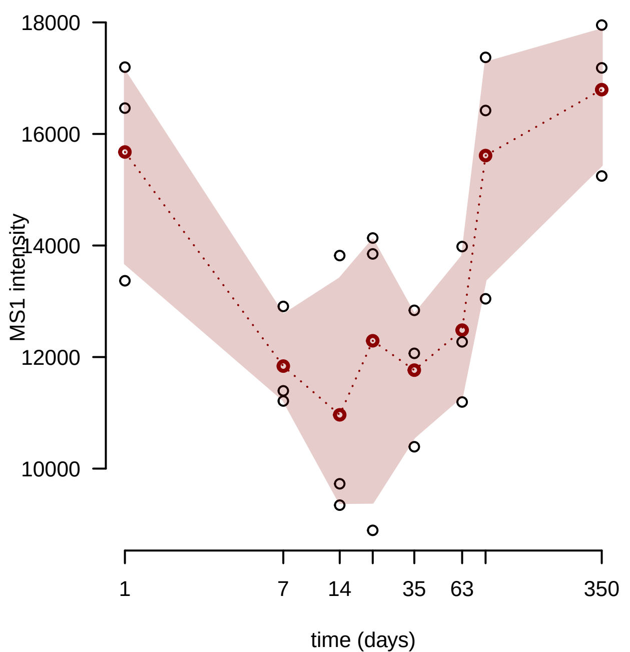

Q8CII2
GN=Cdc123
cell division cycle 123

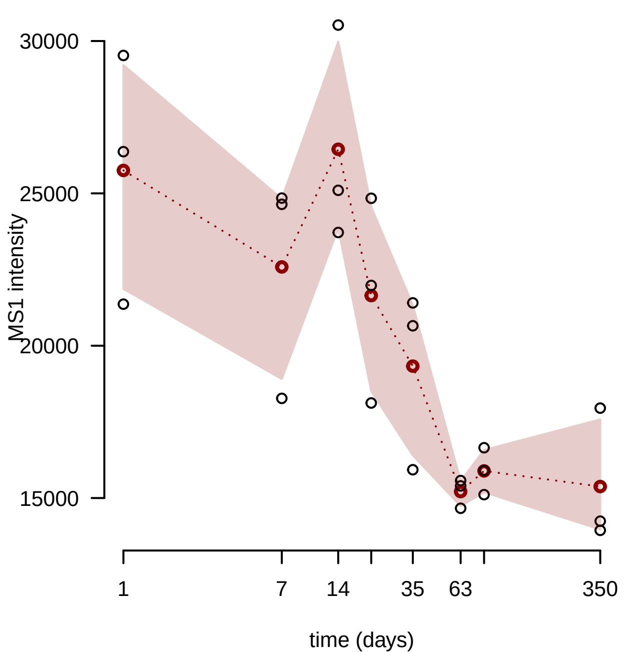

Q8CIL4
GN=

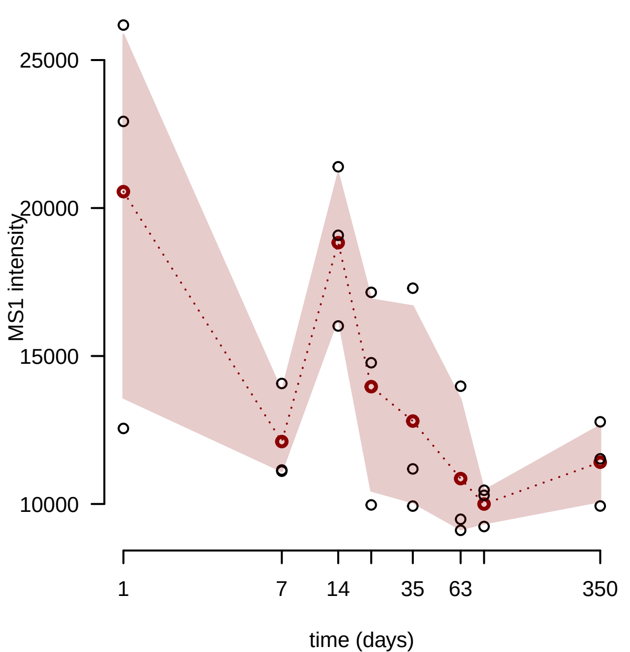

Q8CIM3
GN=D2hgdh
D−2−hydroxyglutarate dehydrogenase

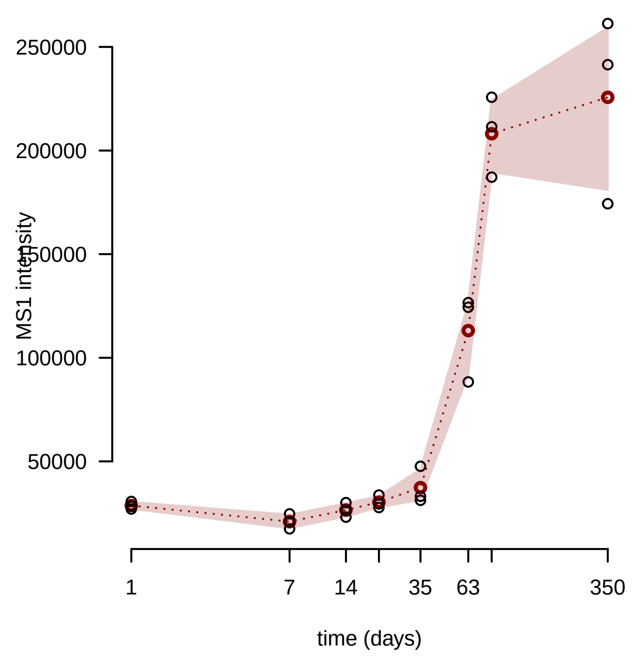

Q8CIM7
GN=Cyp2d26
cytochrome P450, family 2, subfamily d, ...

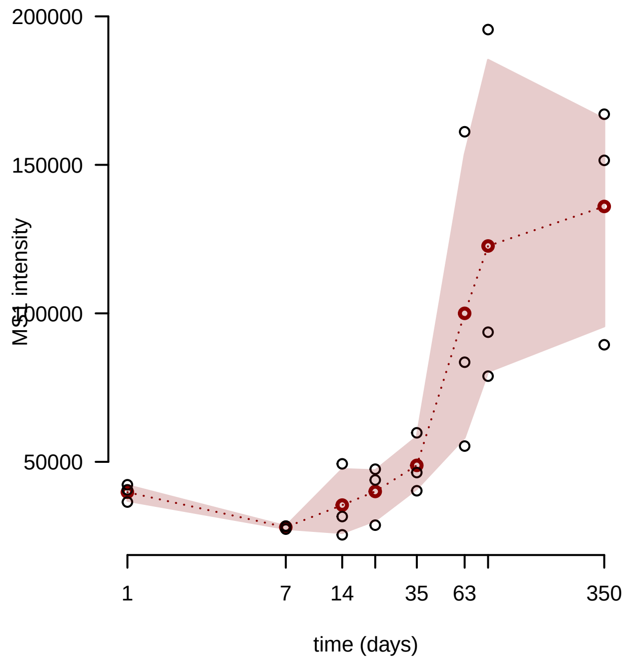

Q8CIM8
GN=Ints4
integrator complex subunit 4

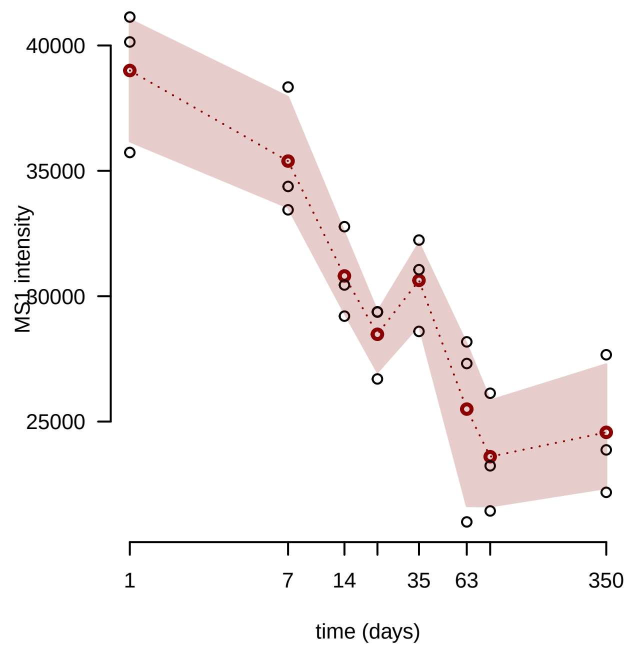

Q8CIN4
GN=Pak2
p21 (RAC1) activated kinase 2

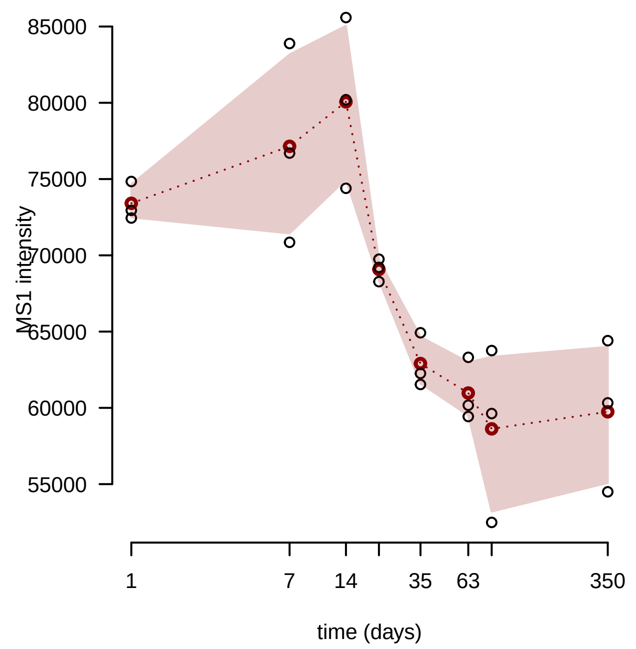

Q8CIP4
GN=Mark4
MAP/microtubule affinity regulating kina ...

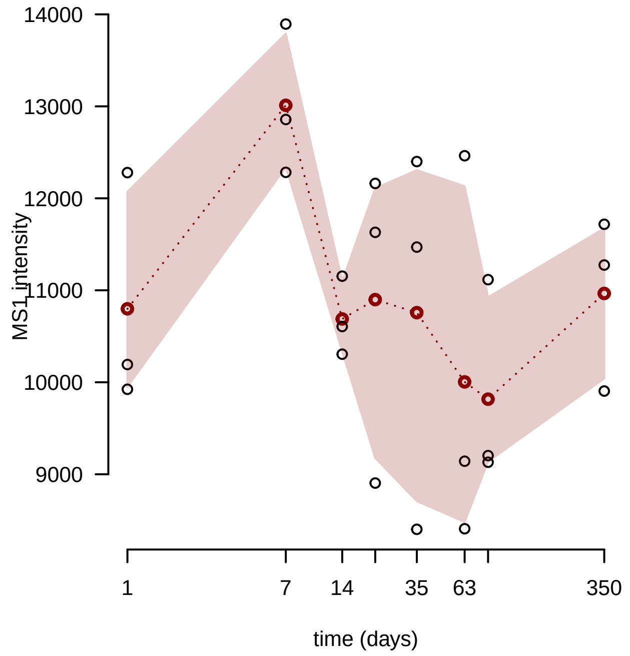

Q8CIQ7
GN=Dock3
dedicator of cyto−kinesis 3

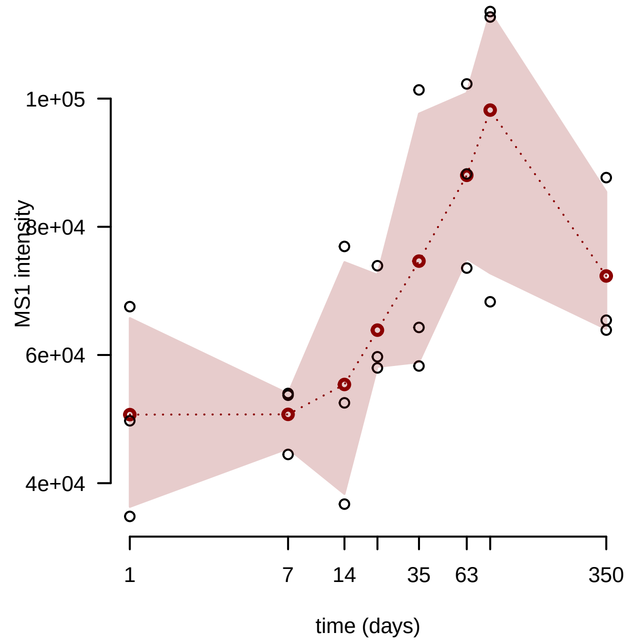

Q8CIR4
GN=Trpm6
transient receptor potential cation chan ...

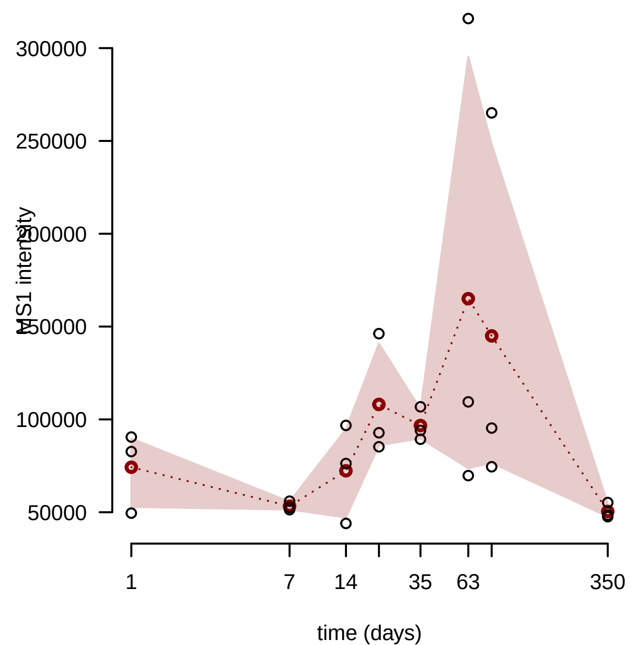

Q8CIT9
GN=Sbsn
suprabasin

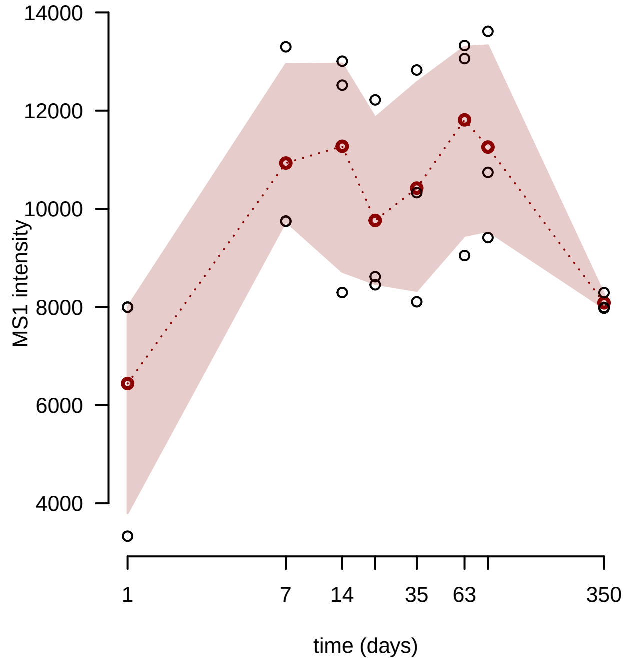

Q8CIV2
GN=Tmem259
transmembrane protein 259

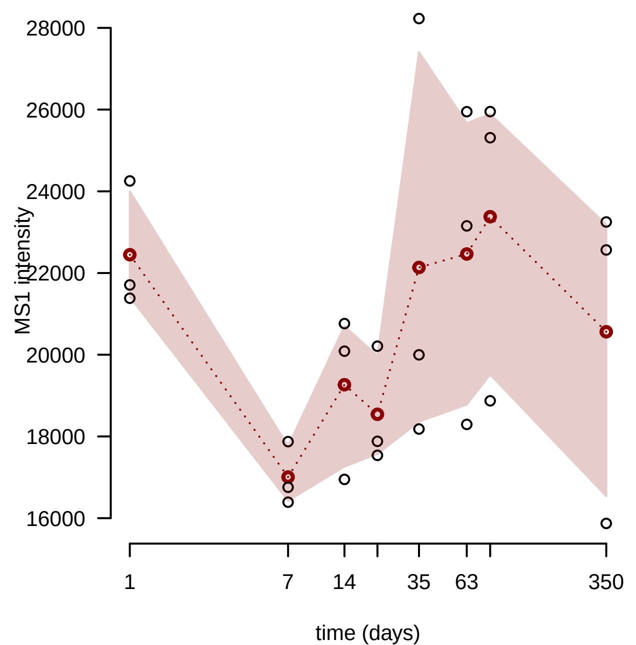

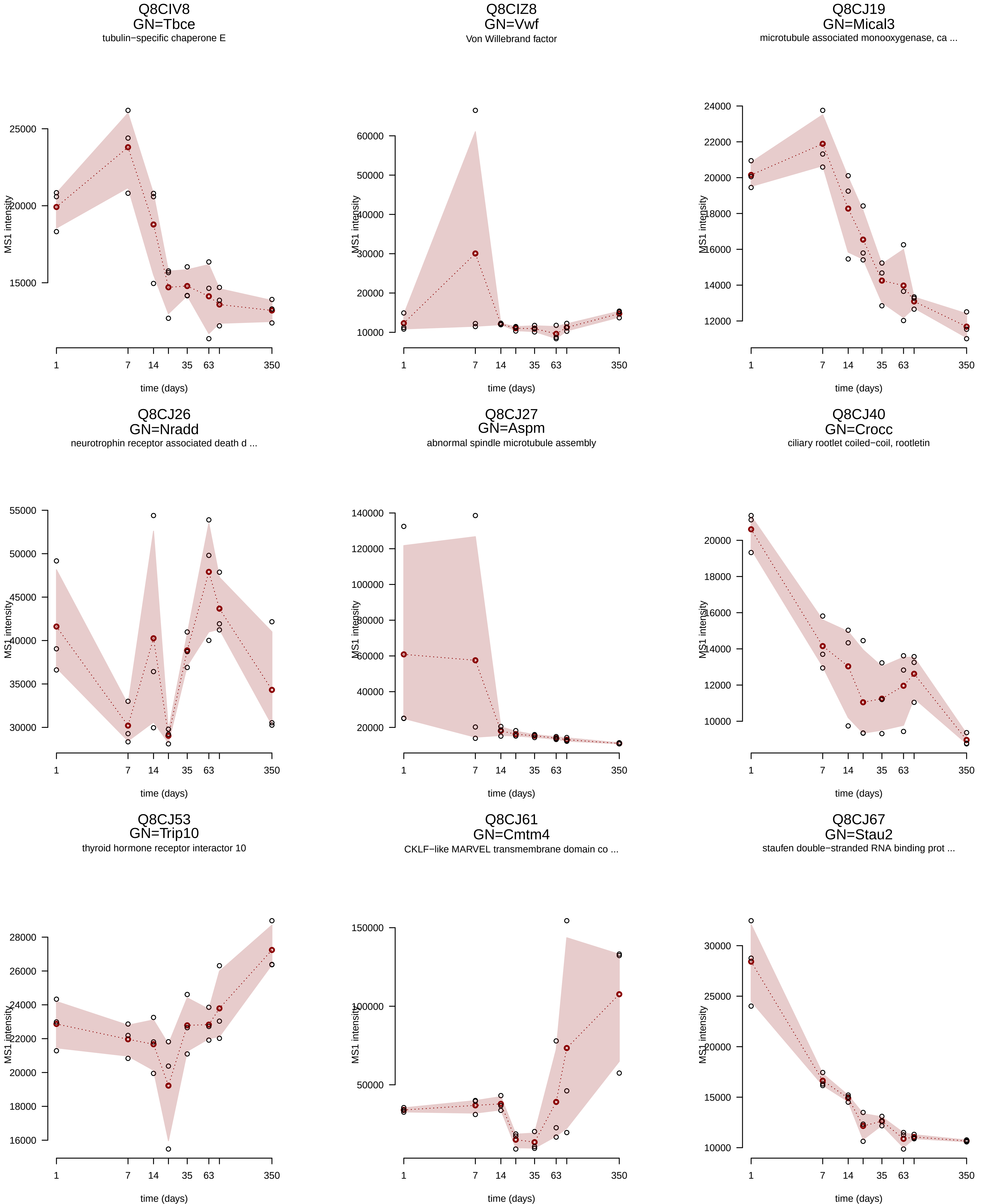
Q8CIV8
GN=Tbce
tubulin−specific chaperone E
MS1 intensity
25000
20000
15000
1
7
14
35
63
350
time (days)
Q8CIZ8
GN=Vwf
Von Willebrand factor
MS1 intensity
60000
50000
40000
30000
20000
10000
1
7
14
35
63
350
time (days)
Q8CJ19
GN=Mical3
microtubule associated monooxygenase, ca ...
MS1 intensity
24000
22000
20000
18000
16000
14000
12000
1
7
14
35
63
350
time (days)
Q8CJ26
GN=Nradd
neurotrophin receptor associated death d ...
MS1 intensity
55000
50000
45000
40000
35000
30000
1
7
14
35
63
350
time (days)
Q8CJ27
GN=Aspm
abnormal spindle microtubule assembly
MS1 intensity
140000
120000
100000
80000
60000
40000
20000
1
7
14
35
63
350
time (days)
Q8CJ40
GN=Crocc
ciliary rootlet coiled−coil, rootletin
MS1 intensity
20000
18000
16000
14000
12000
10000
1
7
14
35
63
350
time (days)
Q8CJ53
GN=Trip10
thyroid hormone receptor interactor 10
MS1 intensity
28000
26000
24000
22000
20000
18000
16000
1
7
14
35
63
350
time (days)
Q8CJ61
GN=Cmtm4
CKLF−like MARVEL transmembrane domain co ...
MS1 intensity
150000
100000
50000
1
7
14
35
63
350
time (days)
Q8CJ67
GN=Stau2
staufen double−stranded RNA binding prot ...
MS1 intensity
30000
25000
20000
15000
10000
1
7
14
35
63
350
time (days)

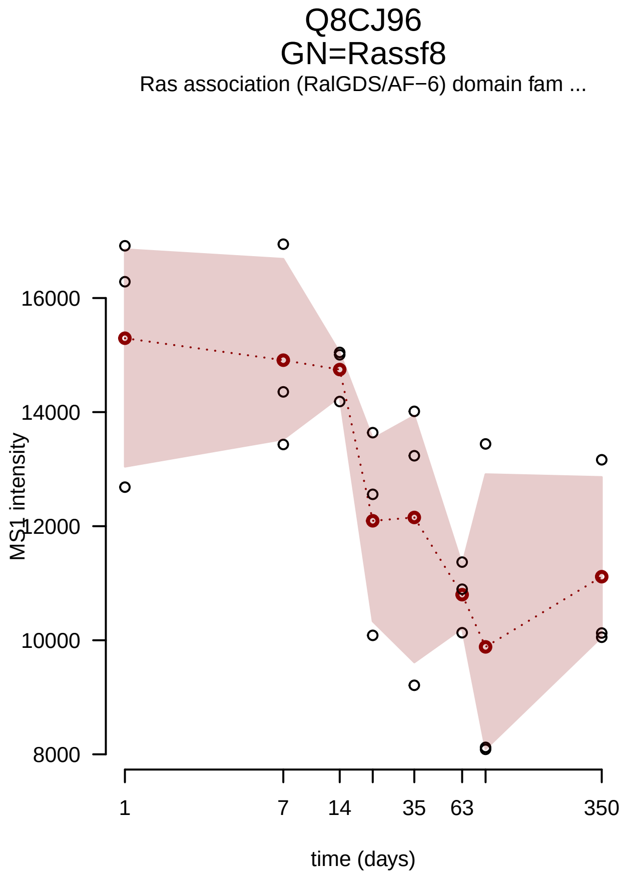
Q8CJ96
GN=Rassf8
Ras association (RalGDS/AF−6) domain fam ...
MS1 intensity
8000
10000
12000
14000
16000
1
7
14
35
63
350
time (days)

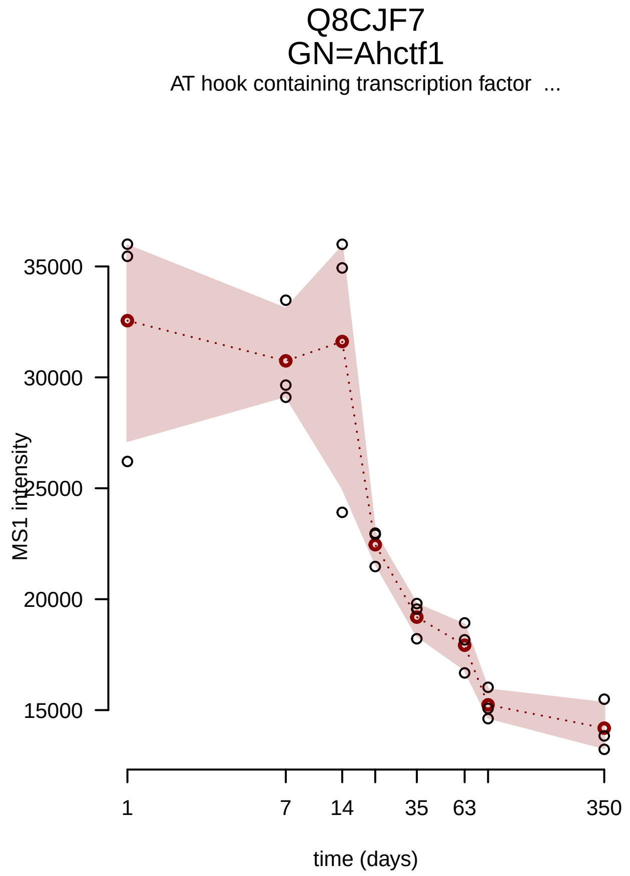
Q8CJF7
GN=Ahctf1
AT hook containing transcription factor ...
MS1 intensity
15000
20000
25000
30000
35000
1
7
14
35
63
350
time (days)

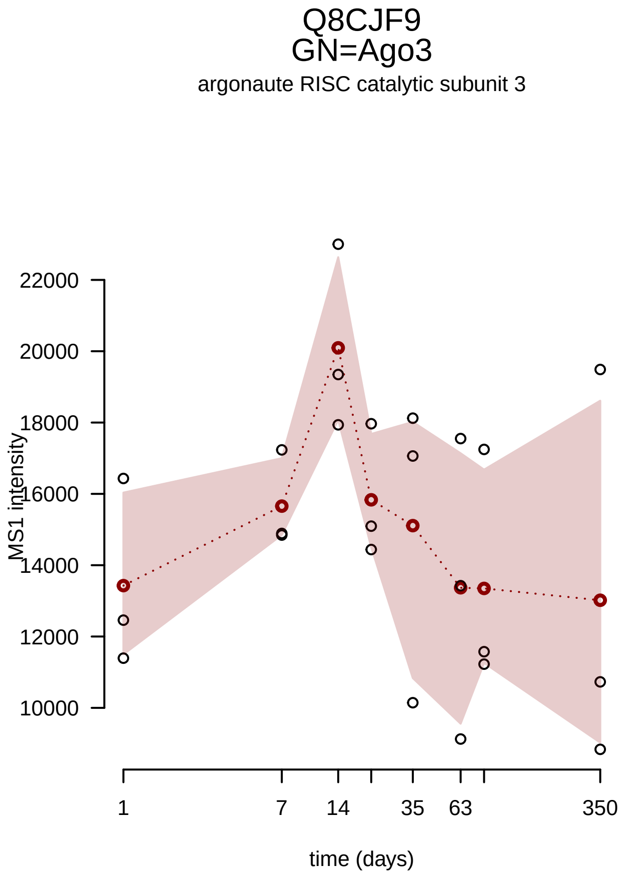
Q8CJF9
GN=Ago3
argonaute RISC catalytic subunit 3
MS1 intensity
10000
12000
14000
16000
18000
20000
22000
1
7
14
35
63
350
time (days)

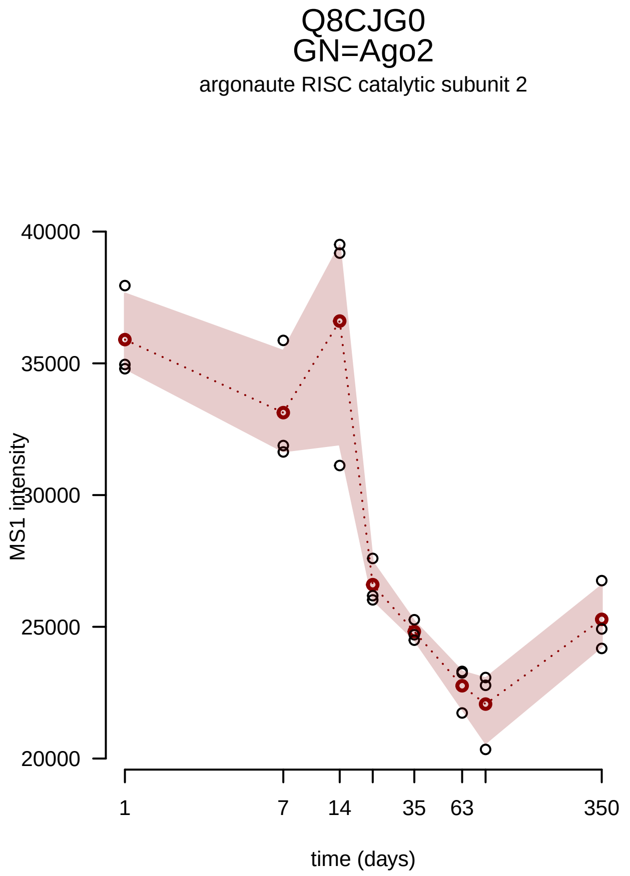
Q8CJG0
GN=Ago2
argonaute RISC catalytic subunit 2
MS1 intensity
20000
25000
30000
35000
40000
1
7
14
35
63
350
time (days)

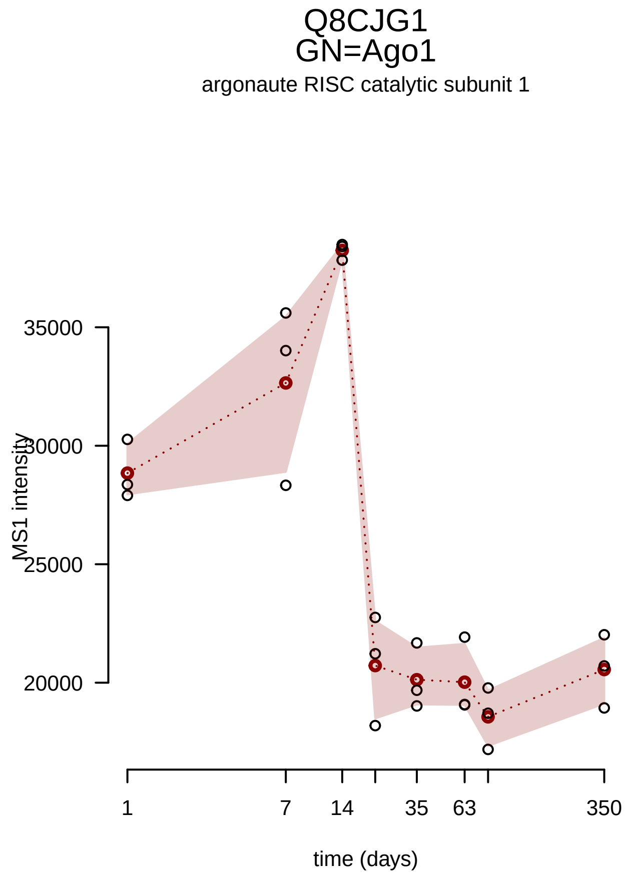
Q8CJG1
GN=Ago1
argonaute RISC catalytic subunit 1
MS1 intensity
20000
25000
30000
35000
1
7
14
35
63
350
time (days)

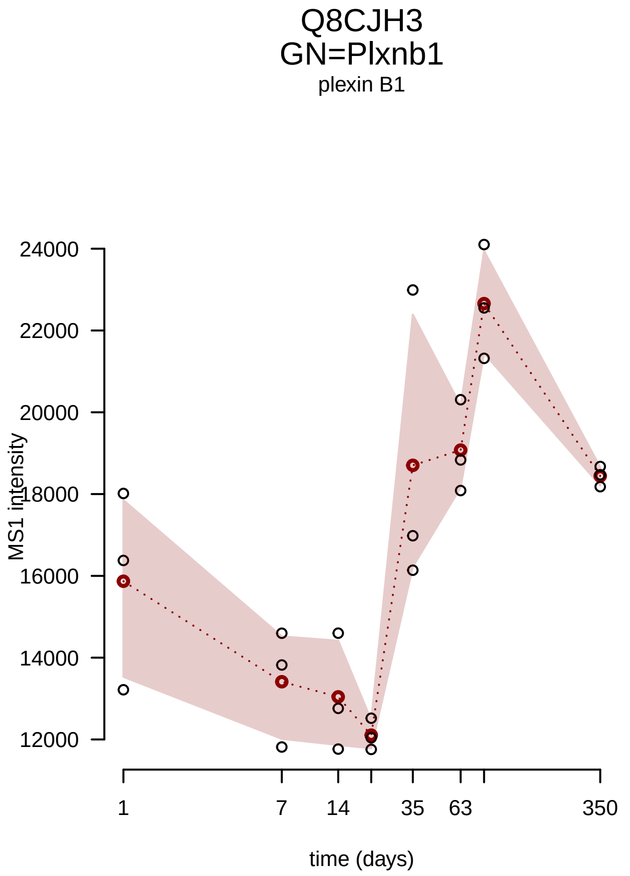
Q8CJH3
GN=Plxnb1
plexin B1
MS1 intensity
12000
14000
16000
18000
20000
22000
24000
1
7
14
35
63
350
time (days)

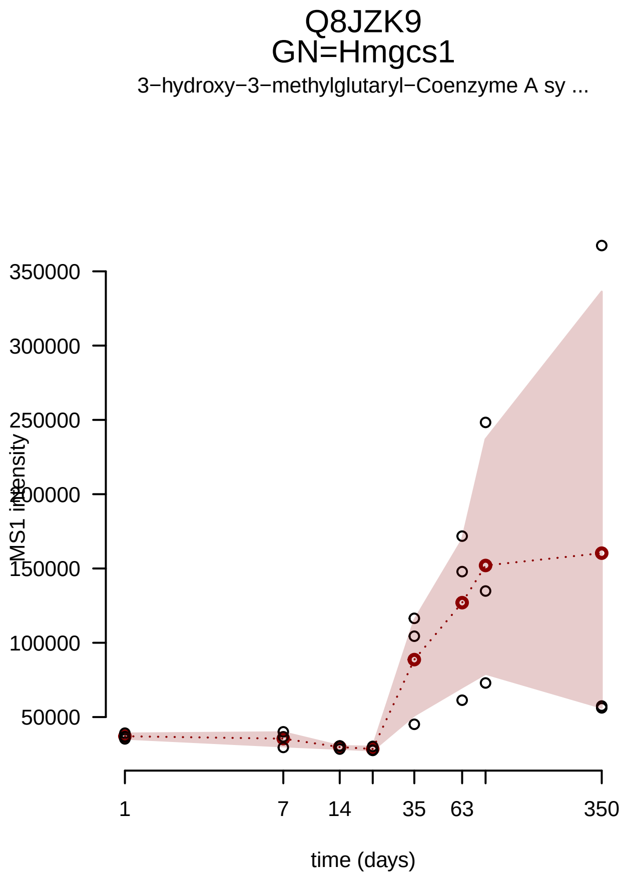
Q8JZK9
GN=Hmgcs1
3−hydroxy−3−methylglutaryl−Coenzyme A sy ...
MS1 intensity
50000
100000
150000
200000
250000
300000
350000
1
7
14
35
63
350
time (days)

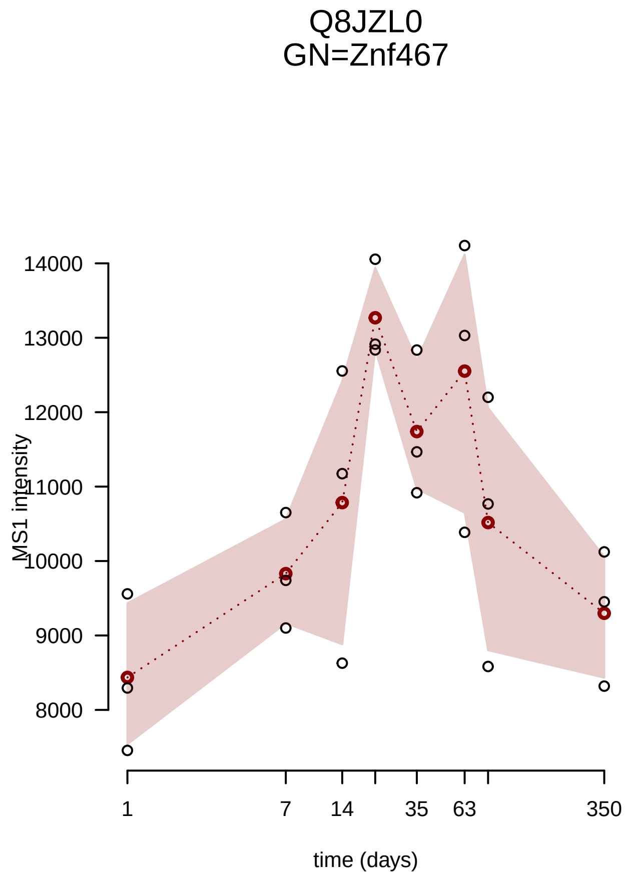
Q8JZL0
GN=Znf467
MS1 intensity
8000
9000
10000
11000
12000
13000
14000
1
7
14
35
63
350
time (days)

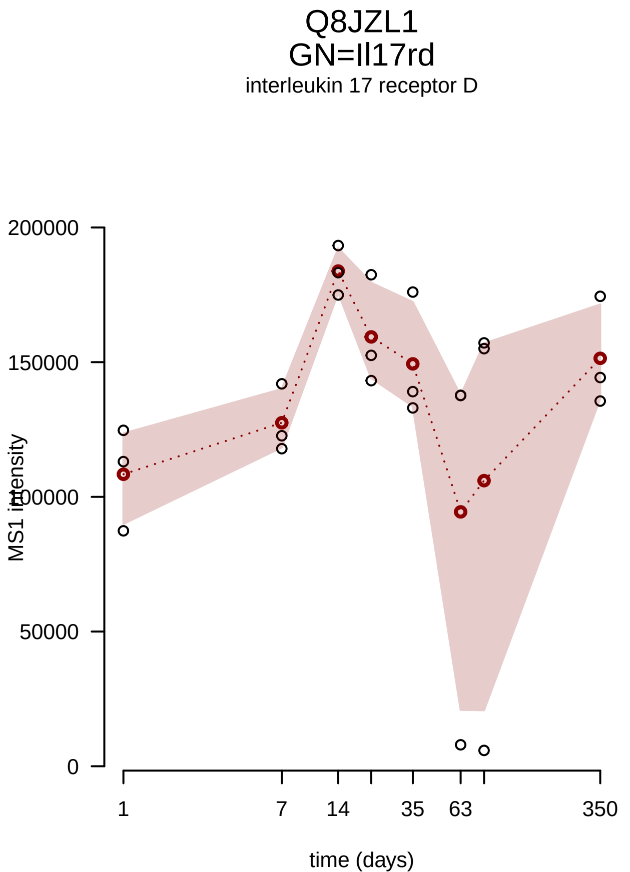
Q8JZL1
GN=Il17rd
interleukin 17 receptor D
MS1 intensity
0
50000
100000
150000
200000
1
7
14
35
63
350
time (days)

Q8JZL3
GN=Thtpa
thiamine triphosphatase

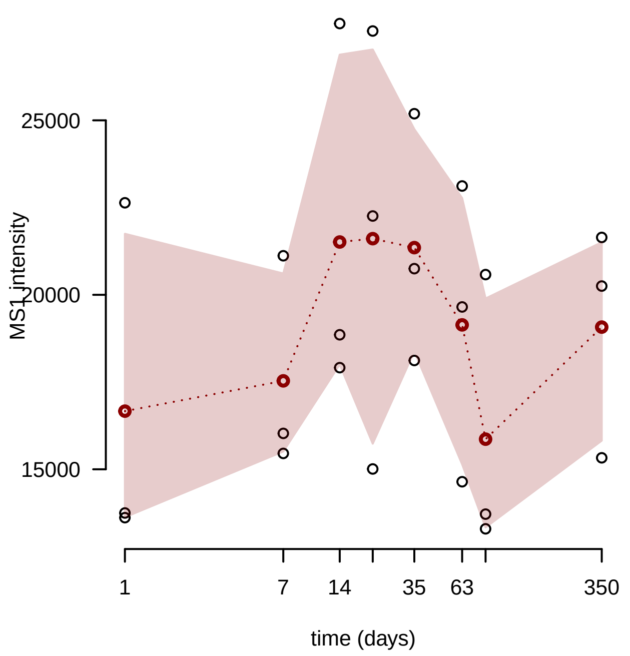

Q8JZM0
GN=Tfb1m
transcription factor B1, mitochondrial

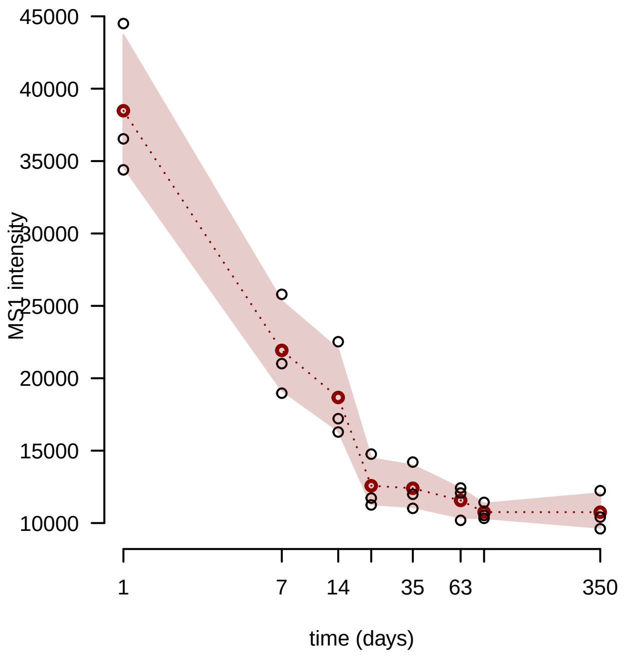

Q8JZM7
GN=Cdc73
cell division cycle 73, Paf1/RNA polymer ...

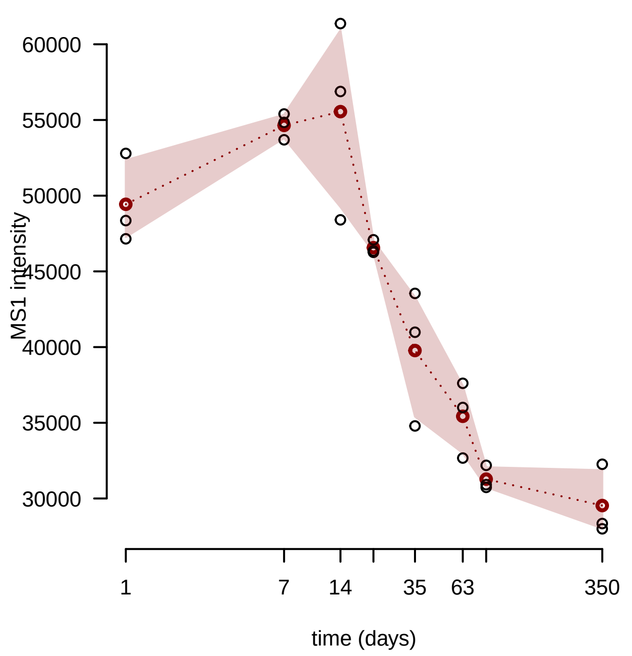

Q8JZN5
GN=Acad9
acyl−Coenzyme A dehydrogenase family, me ...

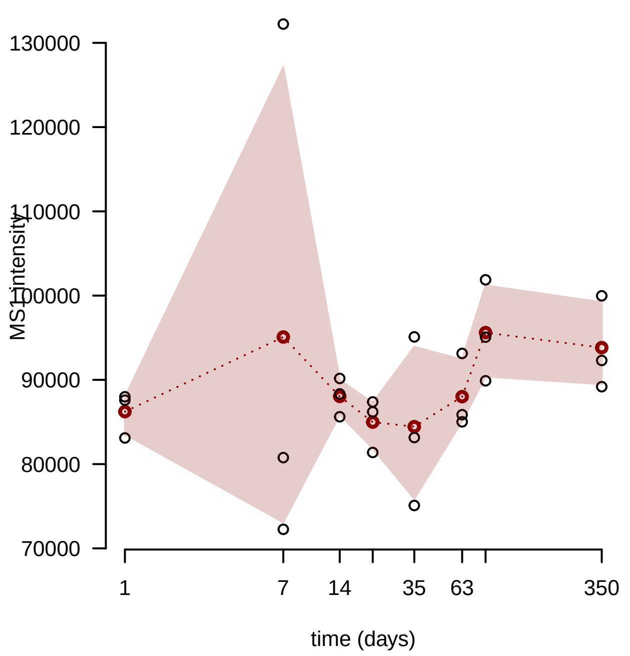

Q8JZN7
GN=Rhot2
ras homolog family member T2

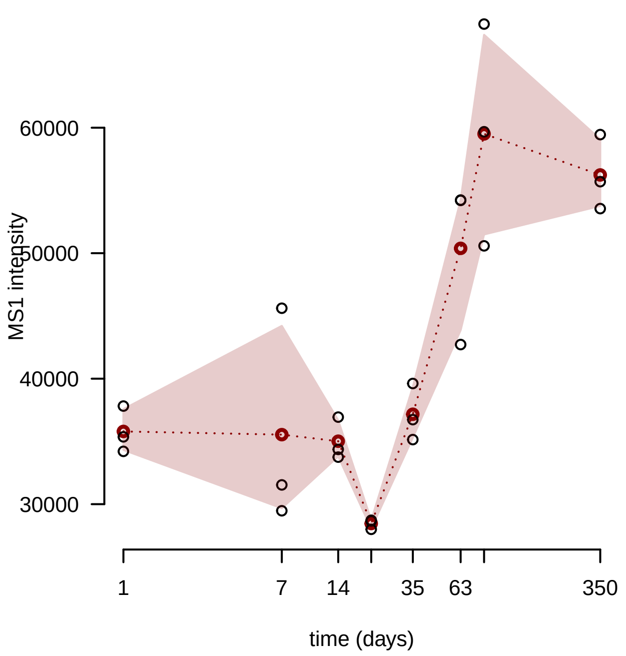

Q8JZP9
GN=Gas2l1
growth arrest−specific 2 like 1

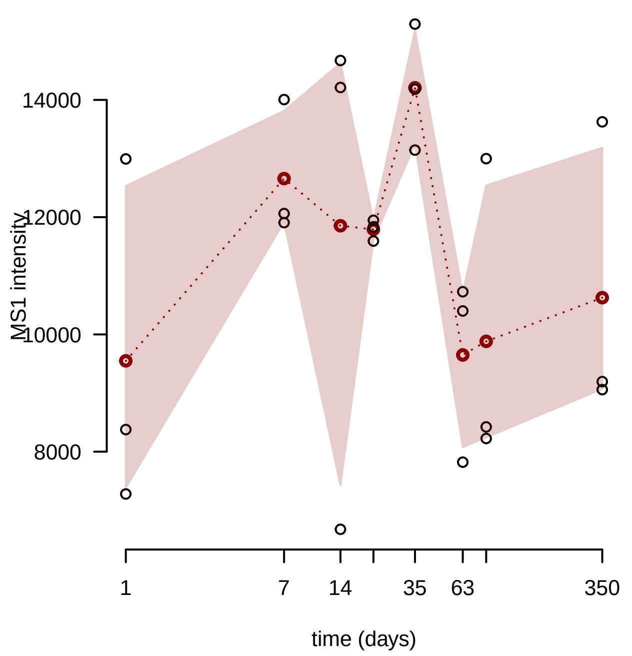

Q8JZQ2
GN=Afg3l2
AFG3−like AAA ATPase 2

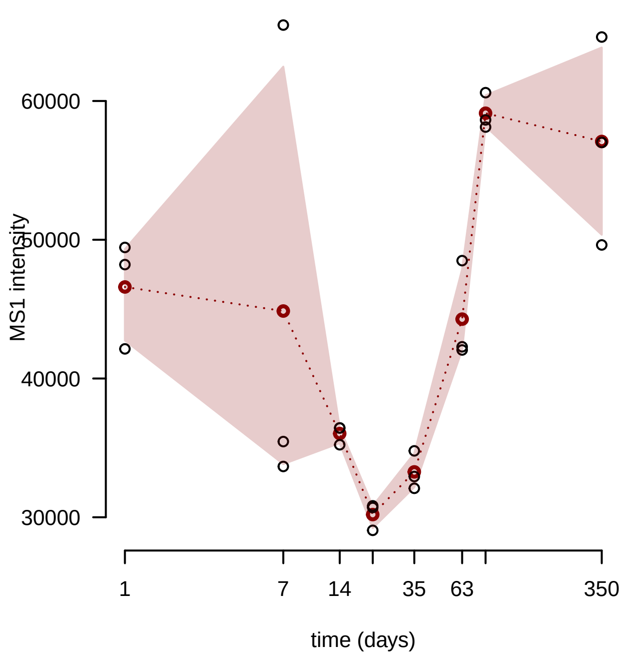

Q8JZQ5
GN=Aoc1
amine oxidase, copper−containing 1

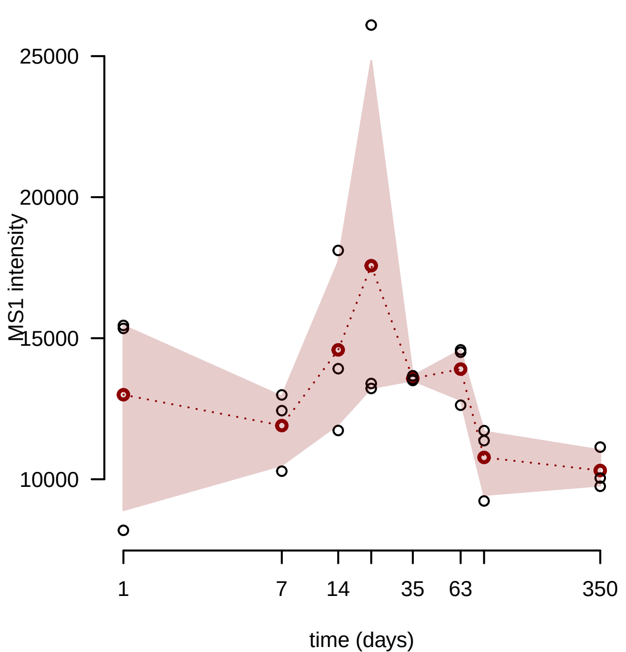

Q8JZQ9
GN=Eif3b
eukaryotic translation initiation factor ...

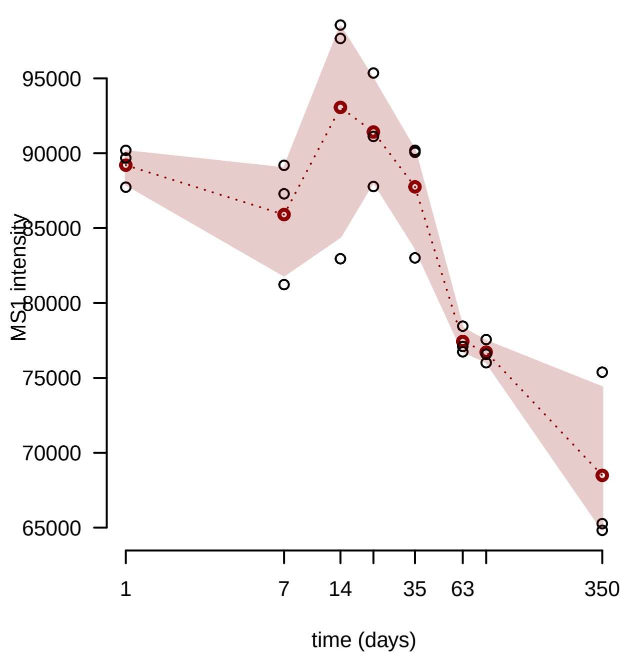

Q8JZR0
GN=Acsl5
acyl−CoA synthetase long−chain family me ...

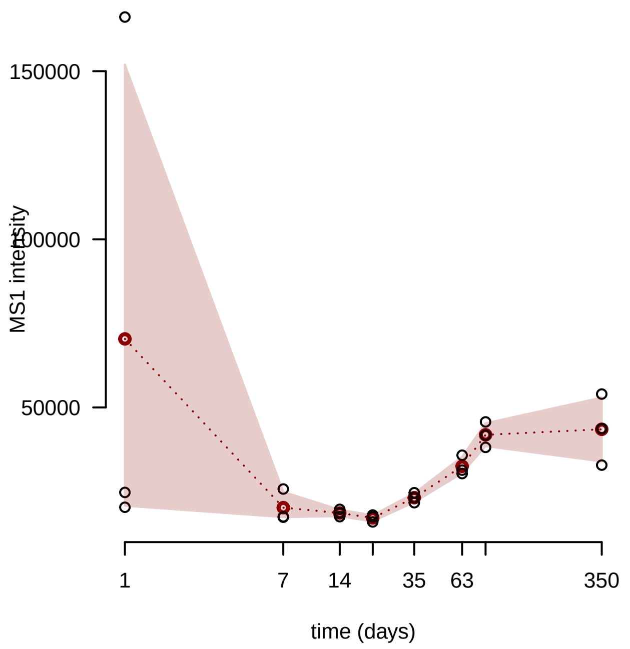

Q8JZR6
GN=Slc4a8
solute carrier family 4 (anion exchanger ...

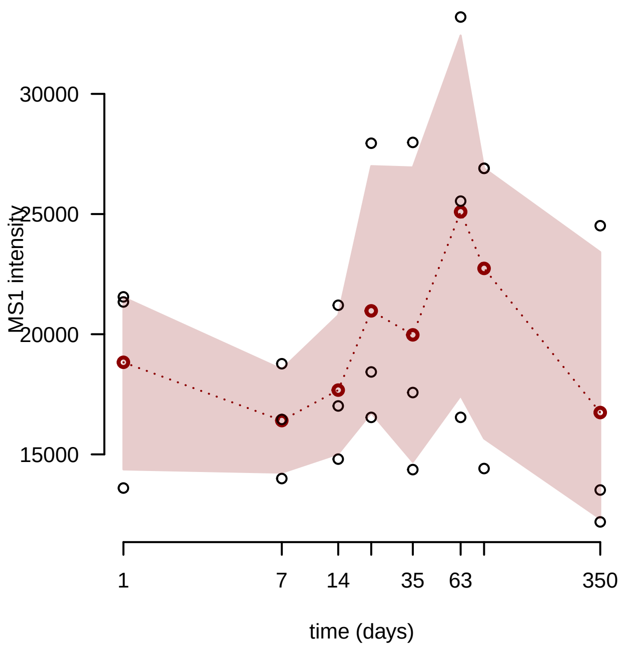

Q8JZS6
GN=N4bp2l2
NEDD4 binding protein 2−like 2

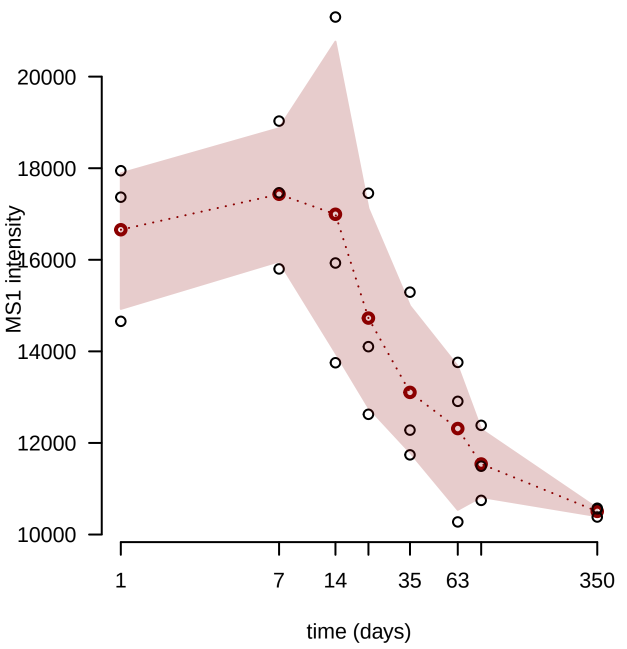

Q8JZS9
GN=Mrpl48
mitochondrial ribosomal protein L48

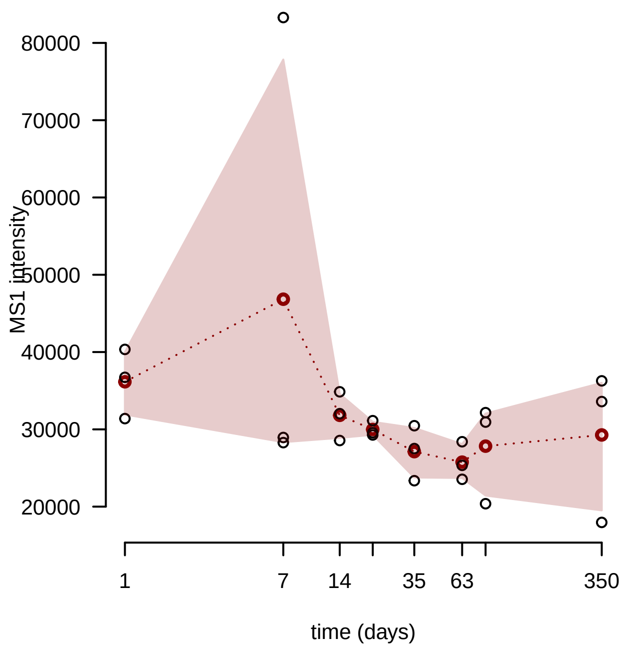

Q8JZU2
GN=Slc25a1
solute carrier family 25 (mitochondrial ...

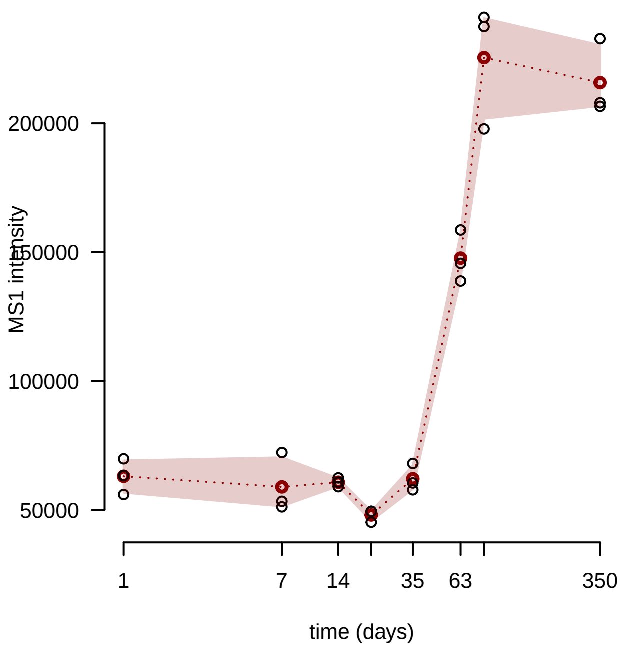

Q8JZV7
GN=Amdhd2
amidohydrolase domain containing 2

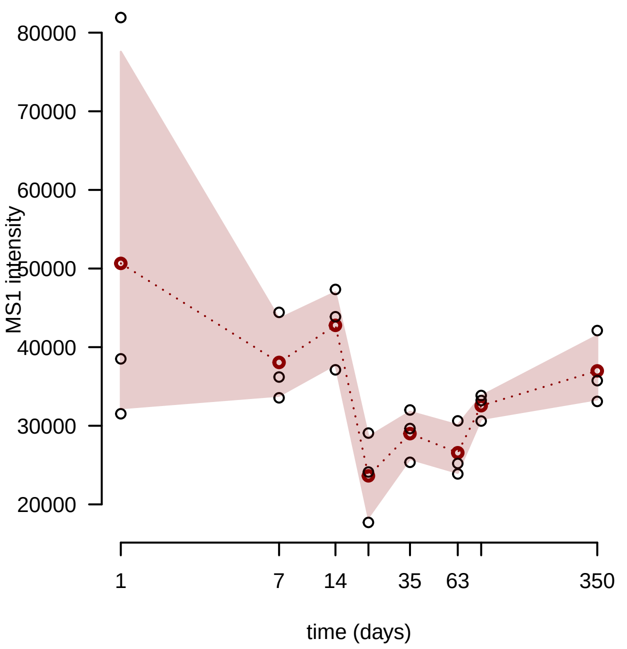

Q8JZV9
GN=Bdh2
3−hydroxybutyrate dehydrogenase, type 2

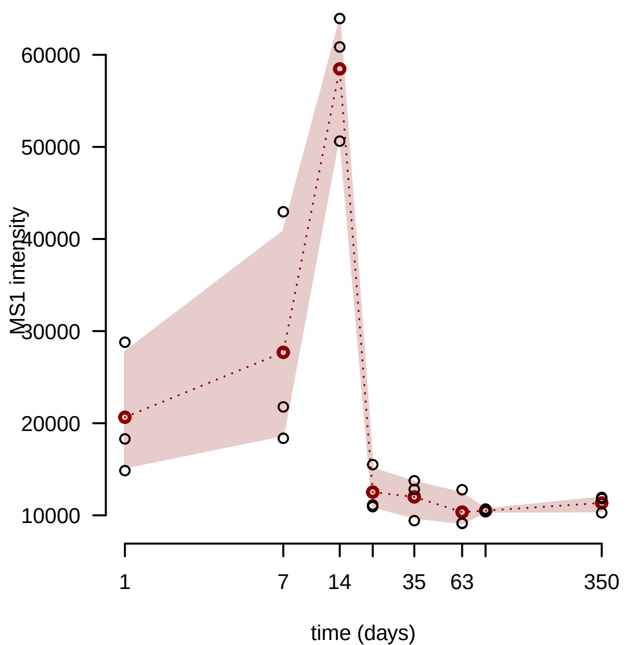

Q8JZX3
GN=Poc1a
POC1 centriolar protein A

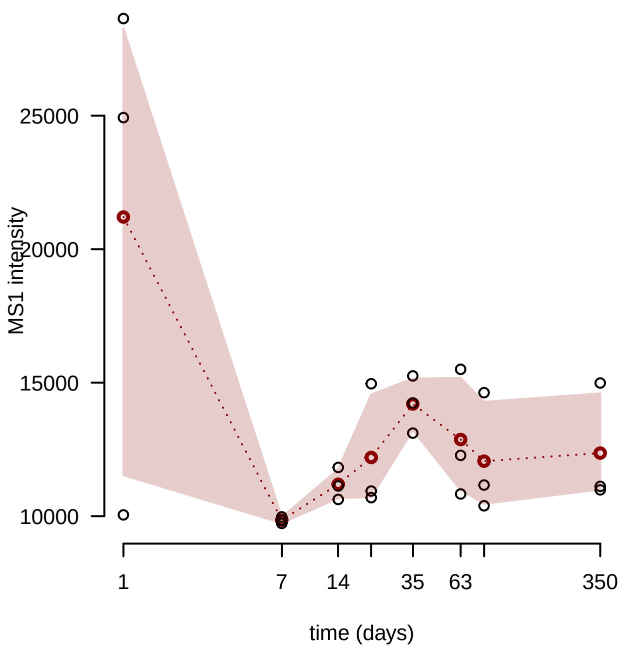

Q8JZX4
GN=Rbm17
RNA binding motif protein 17

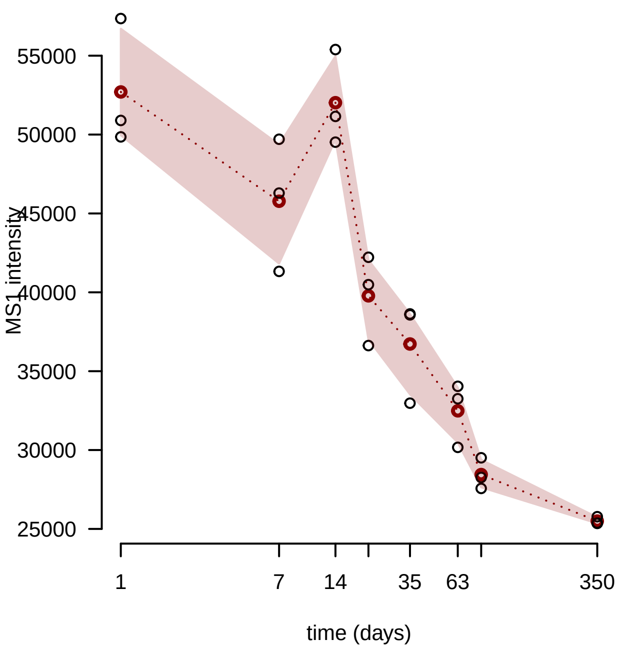

Q8JZX9
GN=Cdc42ep2
CDC42 effector protein (Rho GTPase bindi ...

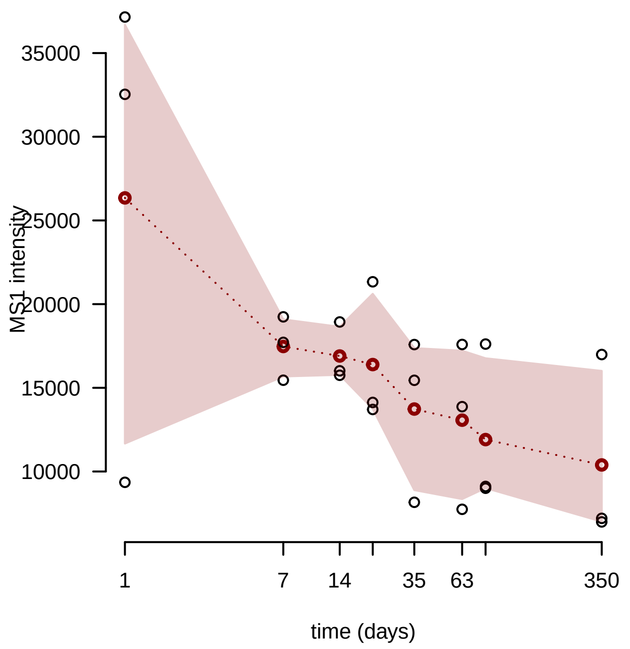

Q8JZY2
GN=Commd10
COMM domain containing 10

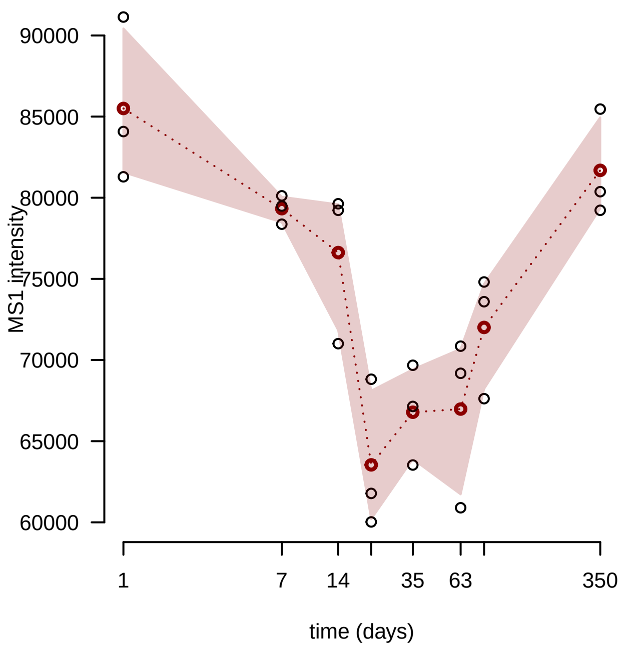

Q8JZY4
GN=Kiaa0391

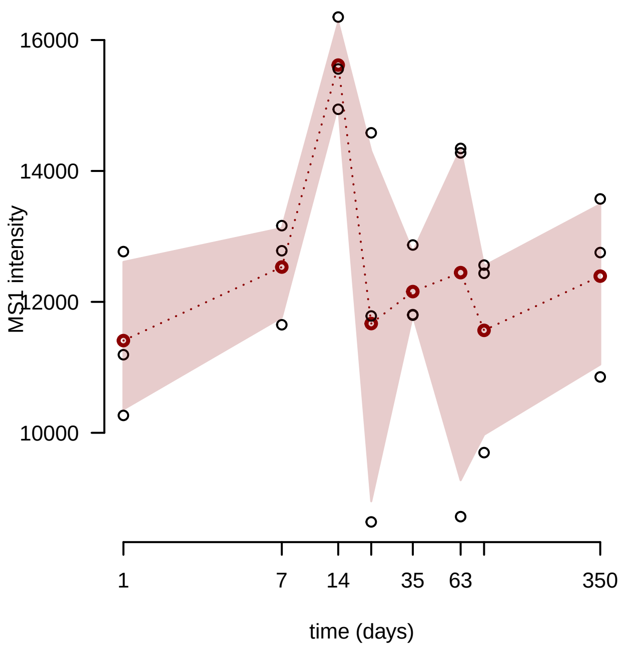

Q8JZZ6
GN=Ammecr1l
AMME chromosomal region gene 1−like

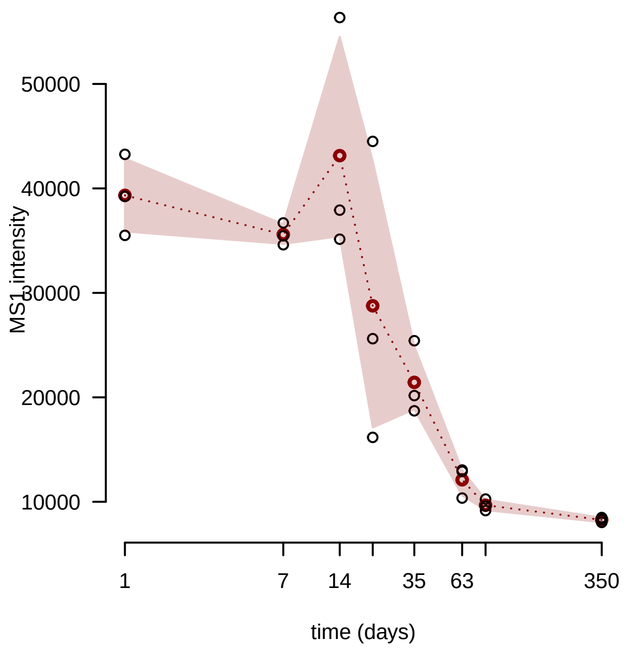

Q8JZZ7
GN=Adgrl2
adhesion G protein−coupled receptor L2

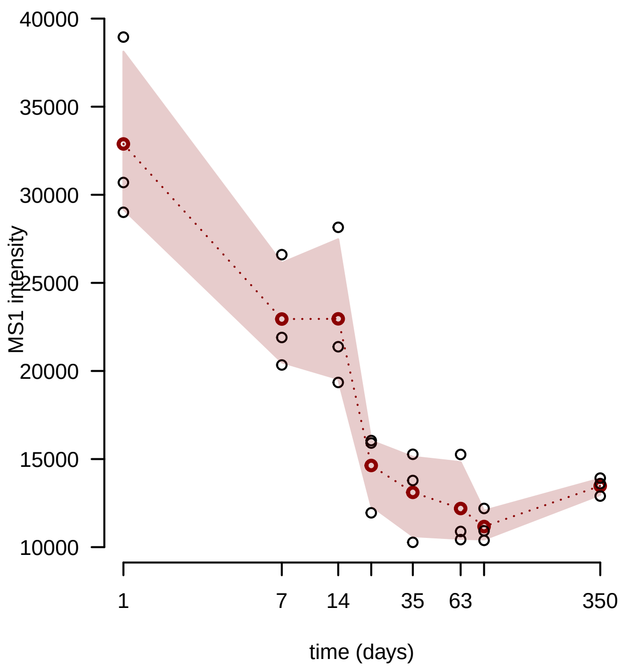

Q8K003
GN=Tma7
translational machinery associated 7

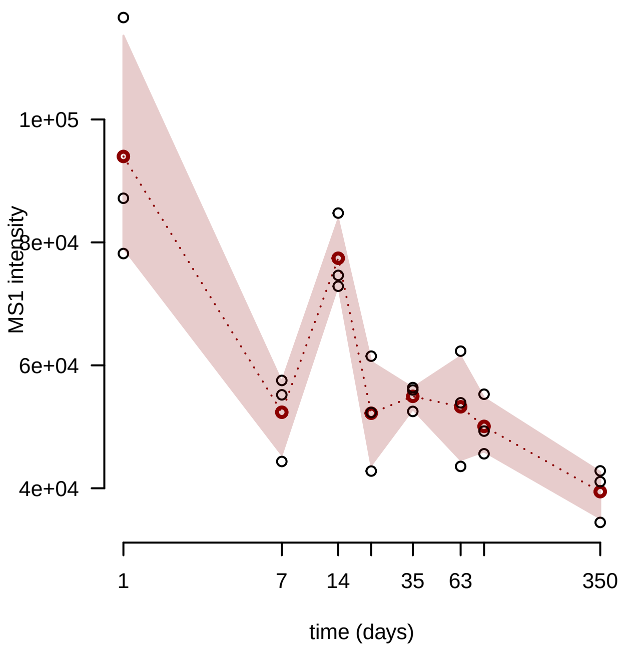

Q8K004
GN=Spata2
spermatogenesis associated 2

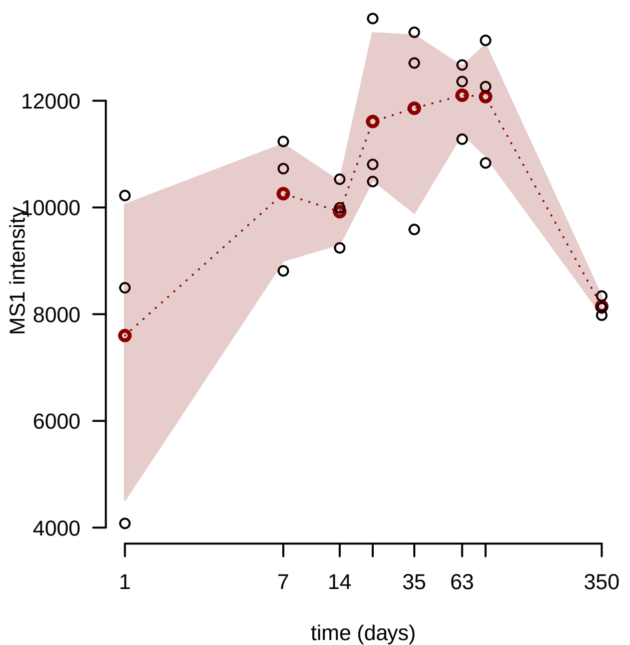

Q8K007
GN=Sulf1
sulfatase 1

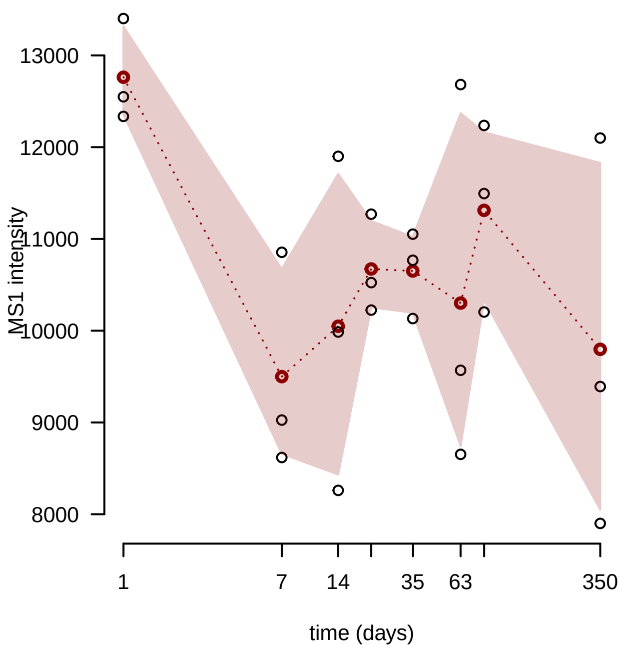

Q8K009
GN=Aldh1l2
aldehyde dehydrogenase 1 family, member ...

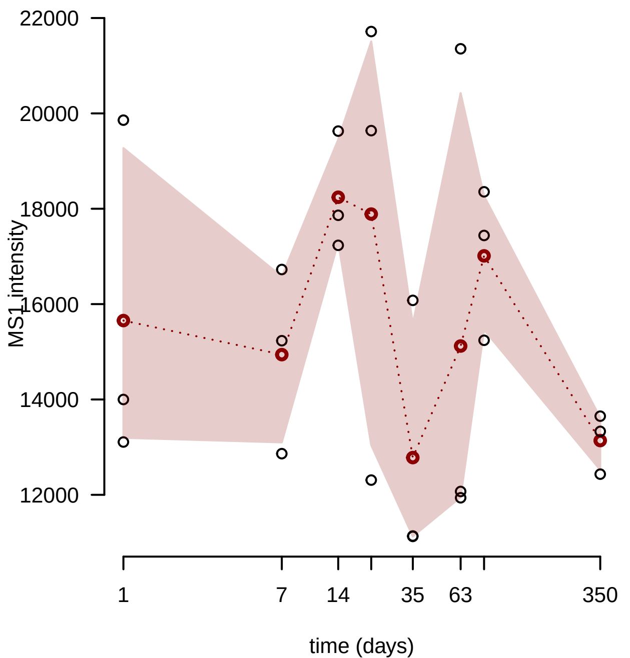

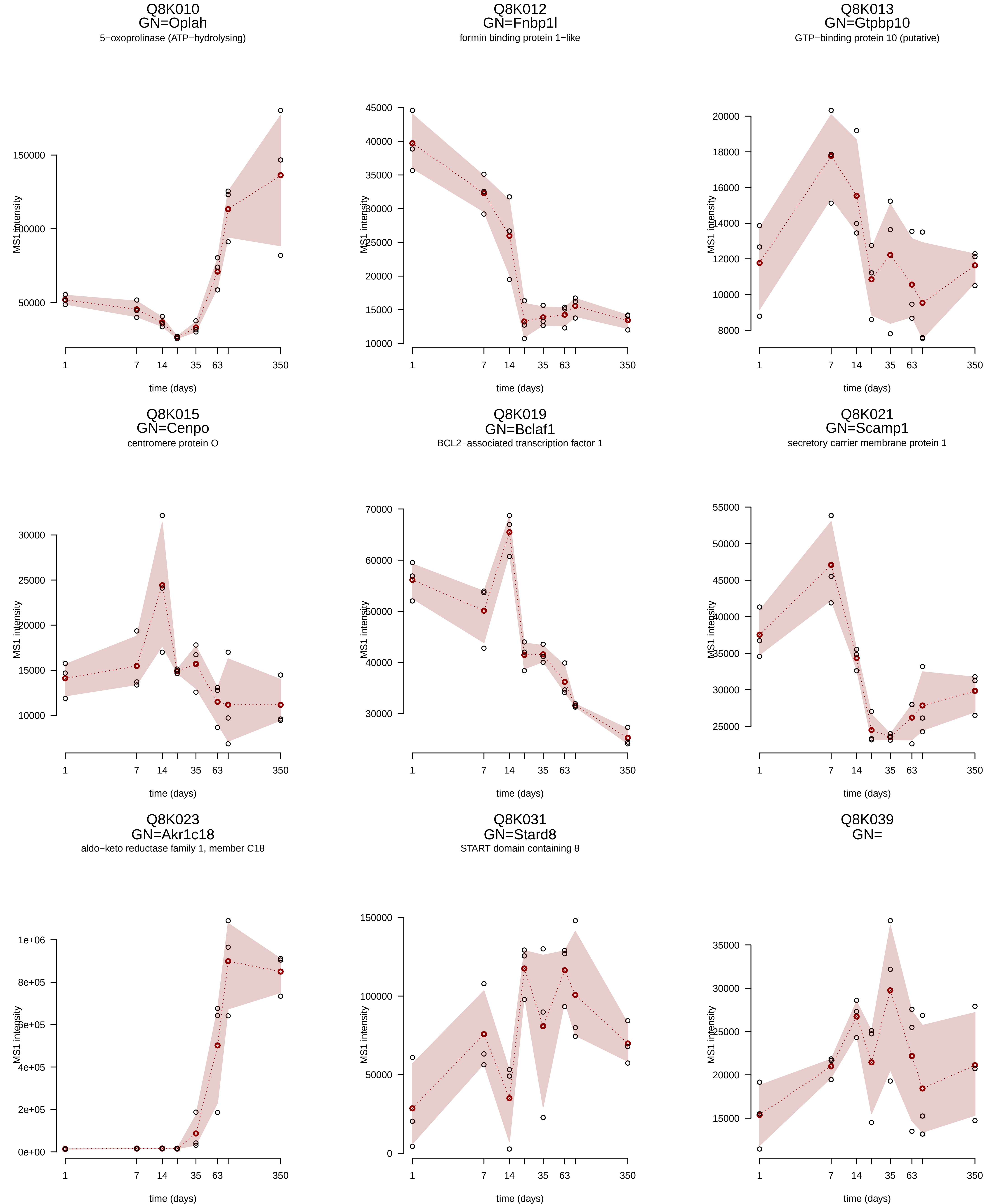
Q8K010
GN=Oplah
5−oxoprolinase (ATP−hydrolysing)
MS1 intensity
150000
100000
50000
1
7
14
35
63
350
time (days)
Q8K012
GN=Fnbp1l
formin binding protein 1−like
MS1 intensity
45000
40000
35000
30000
25000
20000
15000
10000
1
7
14
35
63
350
time (days)
Q8K013
GN=Gtpbp10
GTP−binding protein 10 (putative)
MS1 intensity
20000
18000
16000
14000
12000
10000
8000
1
7
14
35
63
350
time (days)
Q8K015
GN=Cenpo
centromere protein O
MS1 intensity
30000
25000
20000
15000
10000
1
7
14
35
63
350
time (days)
Q8K019
GN=Bclaf1
BCL2−associated transcription factor 1
MS1 intensity
70000
60000
50000
40000
30000
1
7
14
35
63
350
time (days)
Q8K021
GN=Scamp1
secretory carrier membrane protein 1
MS1 intensity
55000
50000
45000
40000
35000
30000
25000
1
7
14
35
63
350
time (days)
Q8K023
GN=Akr1c18
aldo−keto reductase family 1, member C18
MS1 intensity
1e+06
8e+05
6e+05
4e+05
2e+05
0e+00
1
7
14
35
63
350
time (days)
Q8K031
GN=Stard8
START domain containing 8
MS1 intensity
150000
100000
50000
0
1
7
14
35
63
350
time (days)
Q8K039
GN=
MS1 intensity
35000
30000
25000
20000
15000
1
7
14
35
63
350
time (days)

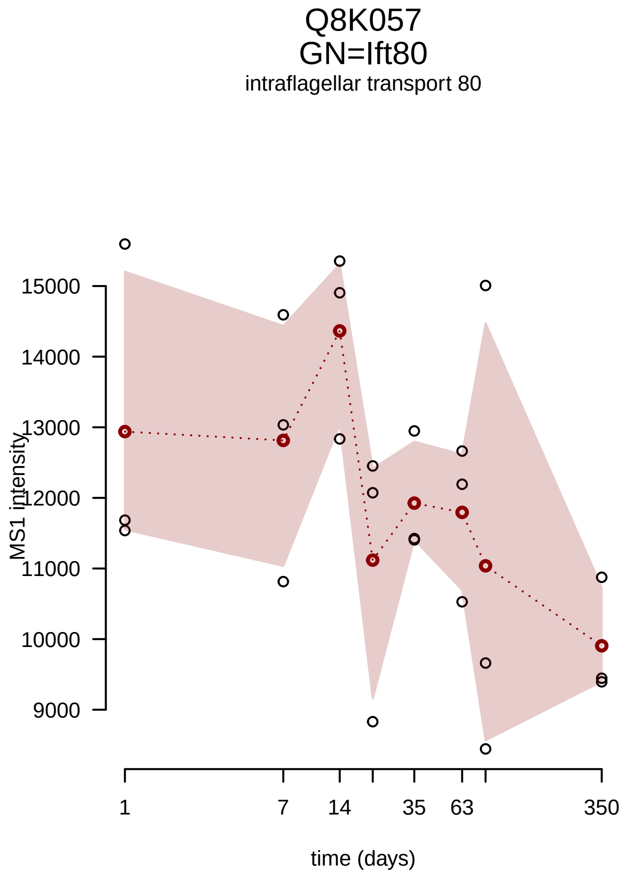
Q8K057
GN=Ift80
intraflagellar transport 80
MS1 intensity
15000
14000
13000
12000
11000
10000
9000
1
7
14
35
63
350
time (days)

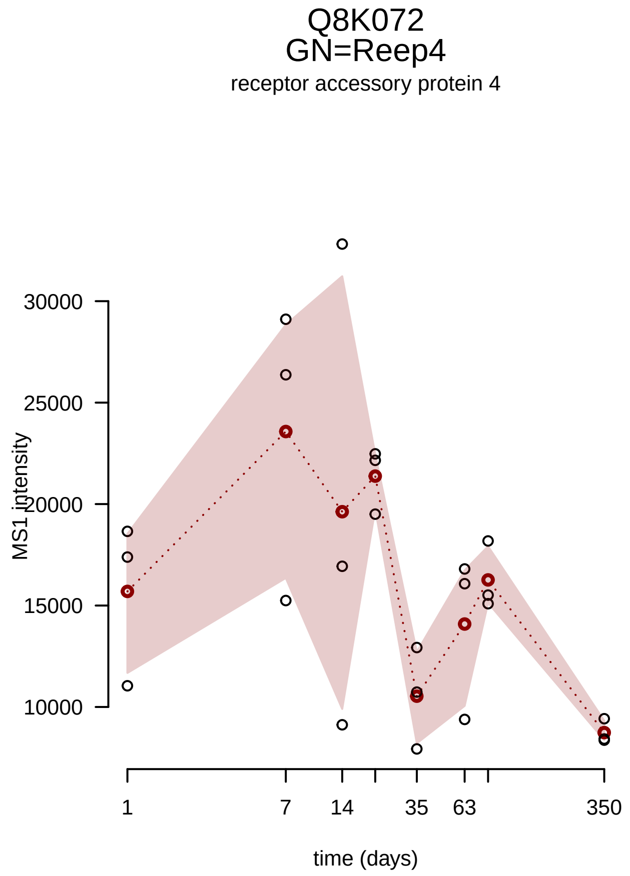
Q8K072
GN=Reep4
receptor accessory protein 4
MS1 intensity
30000
25000
20000
15000
10000
1
7
14
35
63
350
time (days)

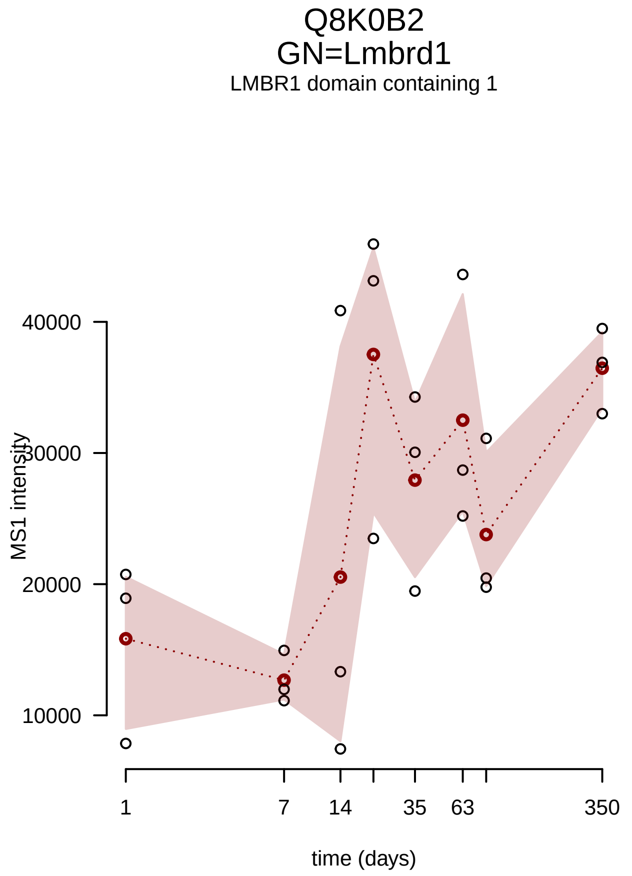
Q8K0B2
GN=Lmbrd1
LMBR1 domain containing 1
MS1 intensity
40000
30000
20000
10000
1
7
14
35
63
350
time (days)

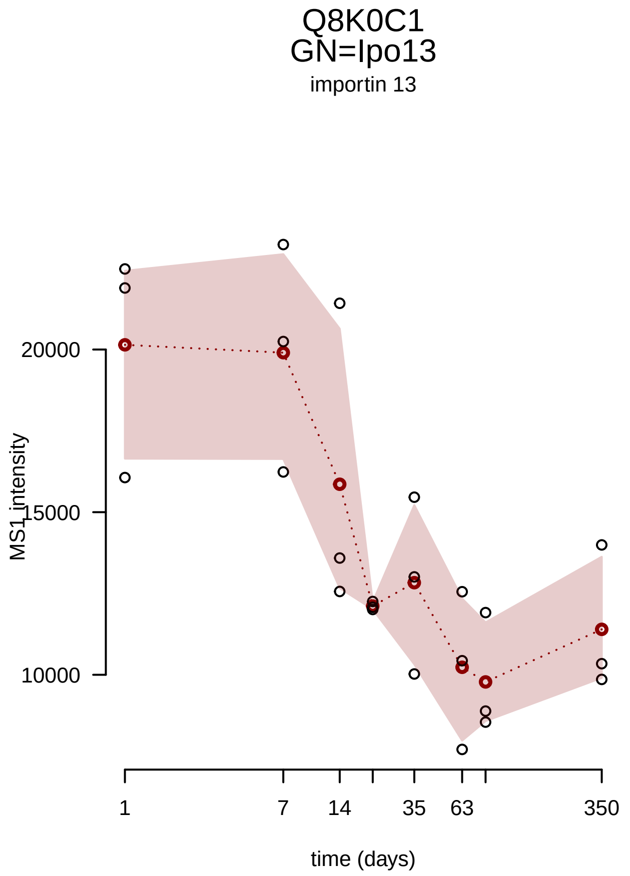
Q8K0C1
GN=Ipo13
importin 13
MS1 intensity
20000
15000
10000
1
7
14
35
63
350
time (days)

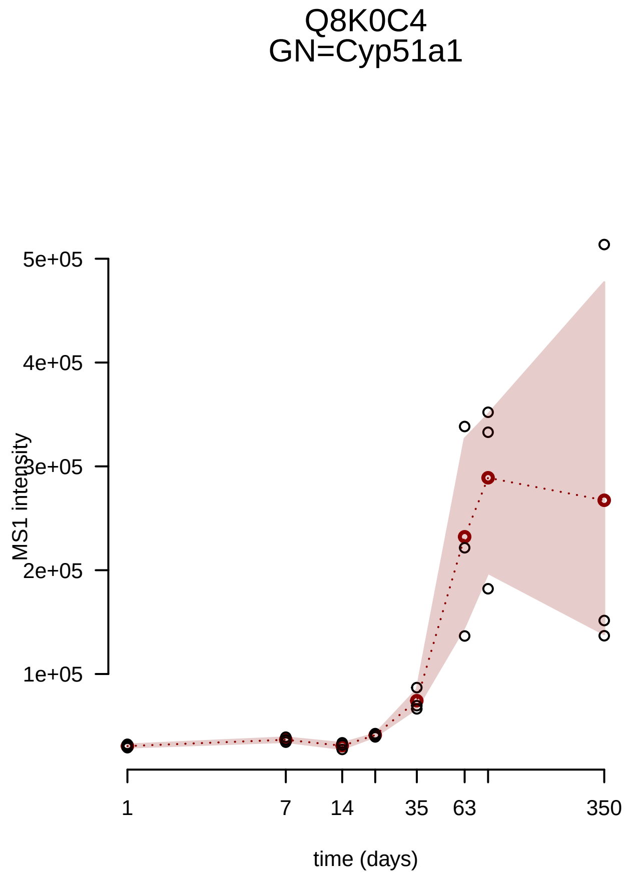
Q8K0C4
GN=Cyp51a1
MS1 intensity
5e+05
4e+05
3e+05
2e+05
1e+05
1
7
14
35
63
350
time (days)

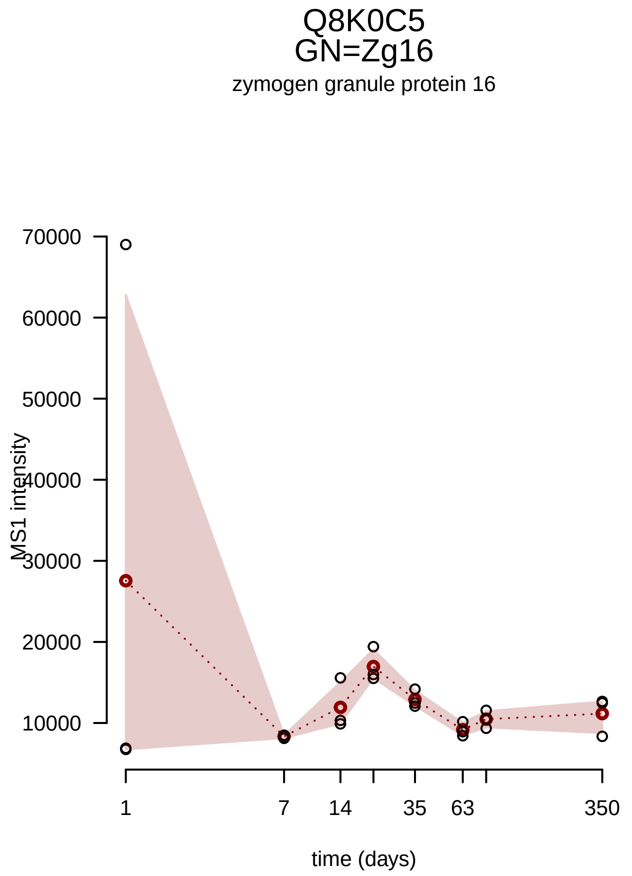
Q8K0C5
GN=Zg16
zymogen granule protein 16
MS1 intensity
70000
60000
50000
40000
30000
20000
10000
1
7
14
35
63
350
time (days)

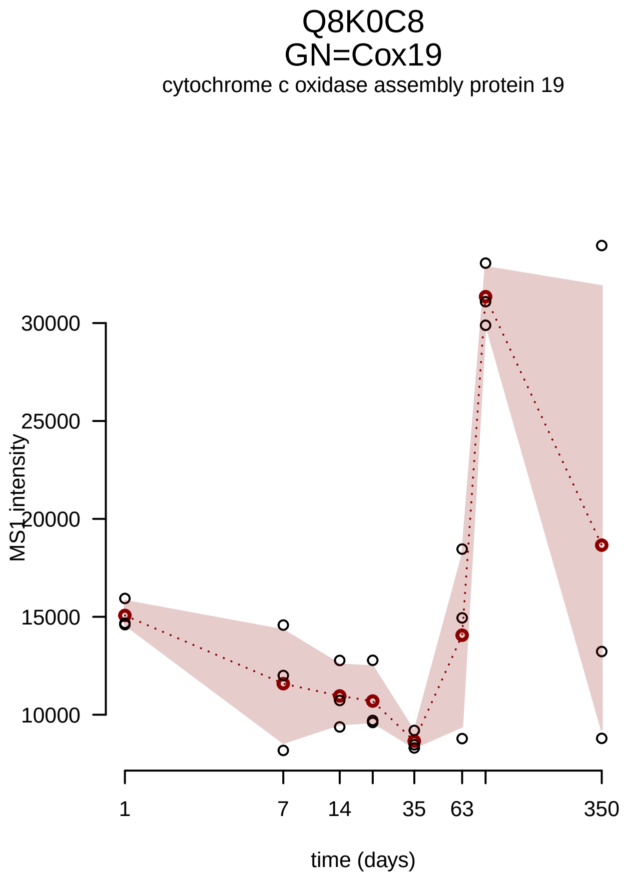
Q8K0C8
GN=Cox19
cytochrome c oxidase assembly protein 19
MS1 intensity
30000
25000
20000
15000
10000
1
7
14
35
63
350
time (days)

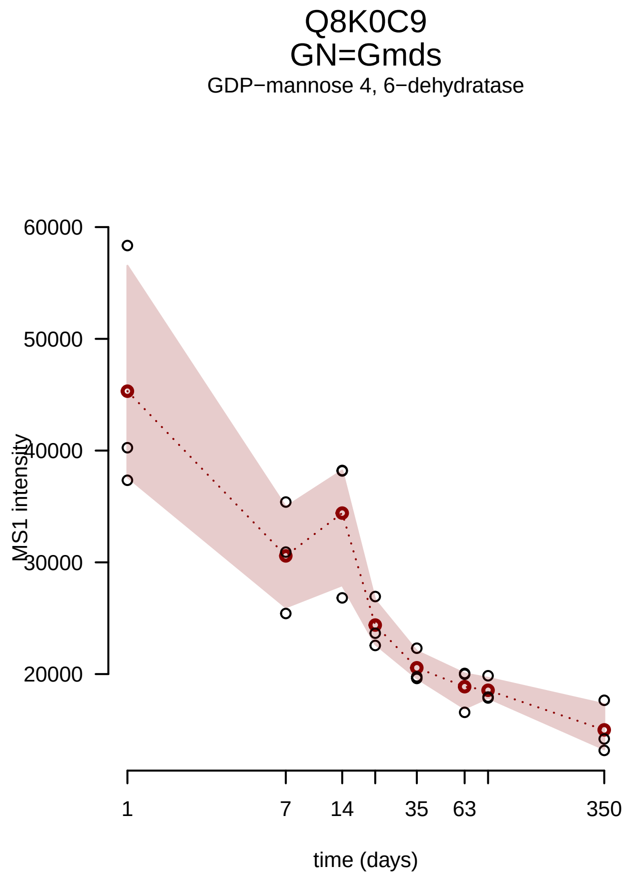
Q8K0C9
GN=Gmds
GDP−mannose 4, 6−dehydratase
MS1 intensity
60000
50000
40000
30000
20000
1
7
14
35
63
350
time (days)

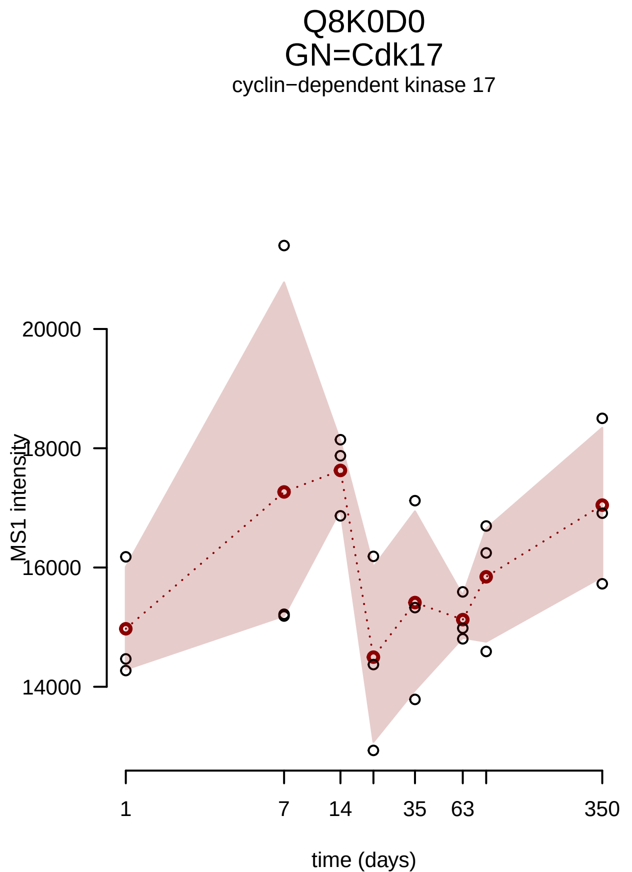
Q8K0D0
GN=Cdk17
cyclin−dependent kinase 17
MS1 intensity
20000
18000
16000
14000
1
7
14
35
63
350
time (days)

Q8K0D5
GN=Gfm1
G elongation factor, mitochondrial 1

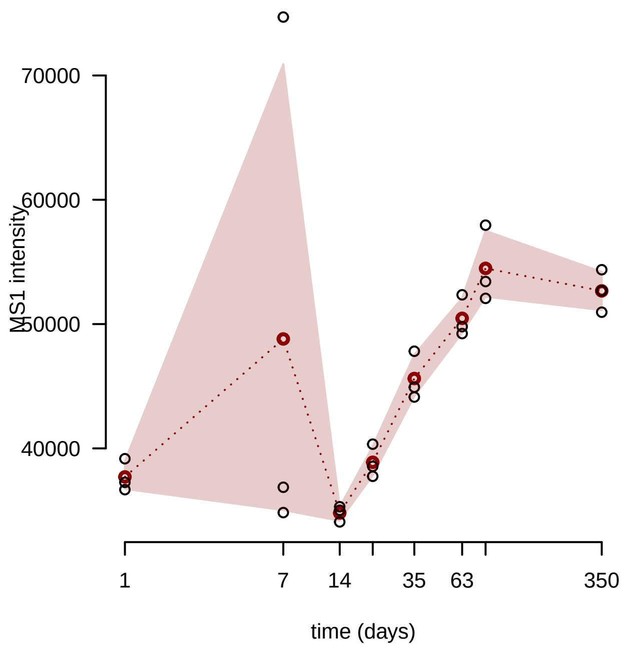

Q8K0D7
GN=Wrb

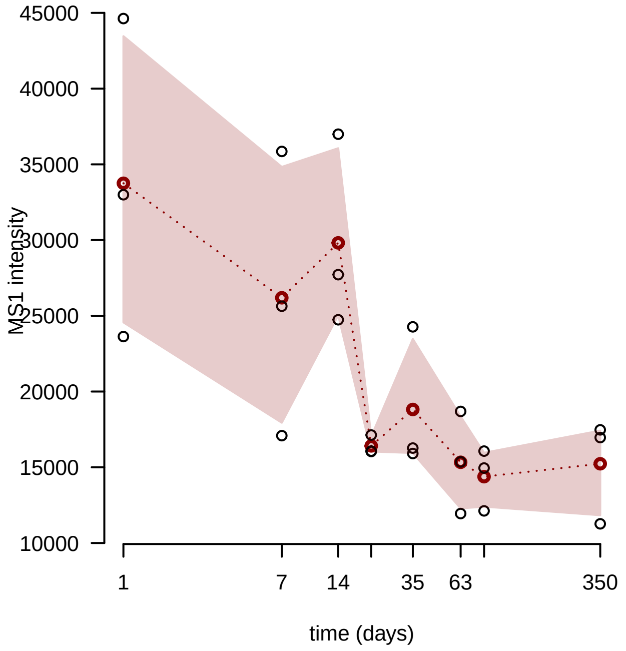

Q8K0E1
GN=Kctd15
potassium channel tetramerisation domain ...

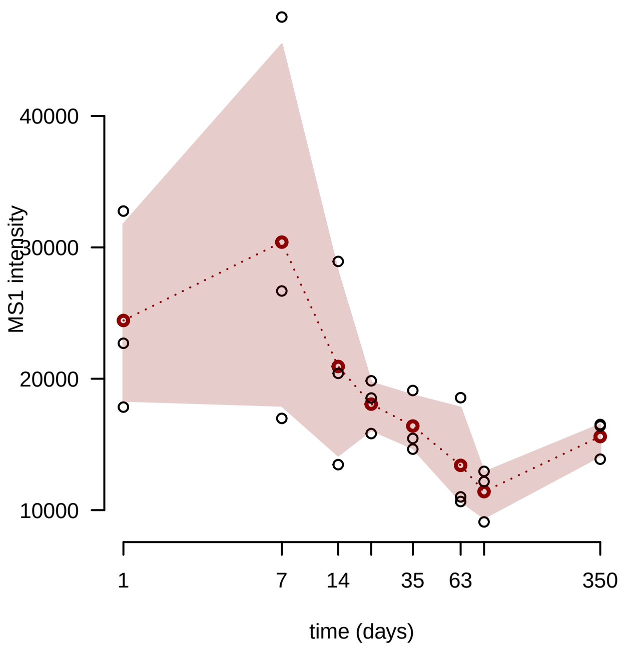

Q8K0E8
GN=Fgb
fibrinogen beta chain

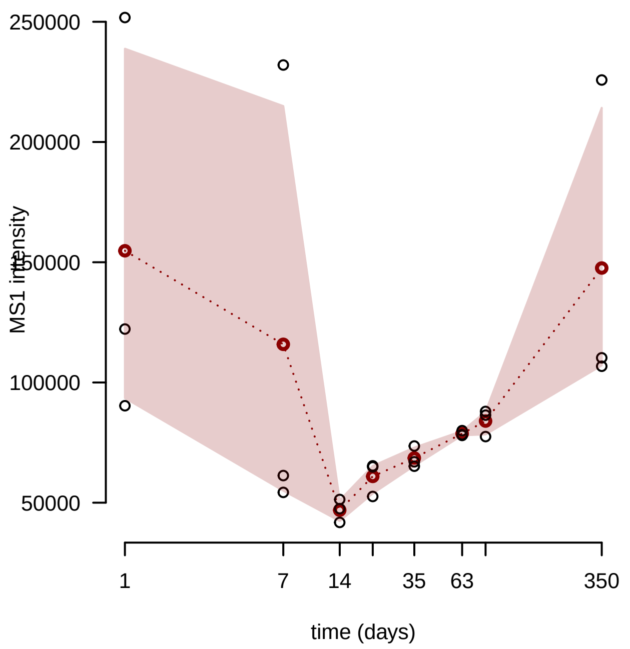

Q8K0F1
GN=Tbc1d23
TBC1 domain family, member 23

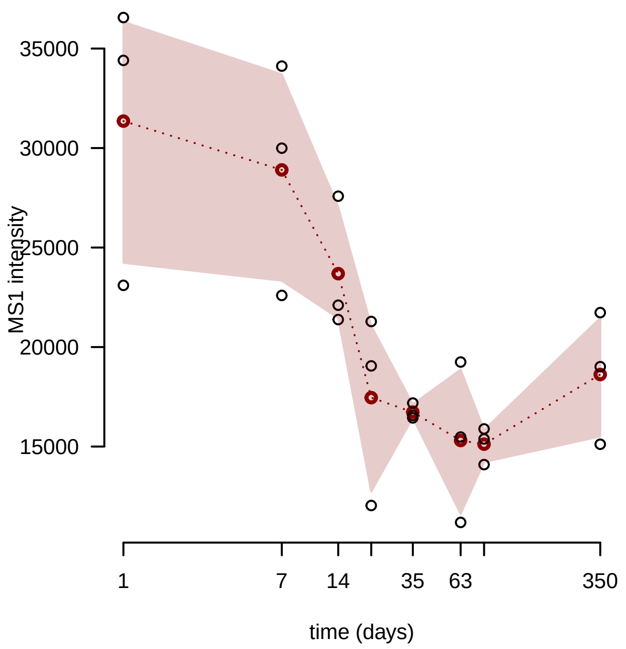

Q8K0G5
GN=Eipr1
EARP complex and GARP complex interactin ...

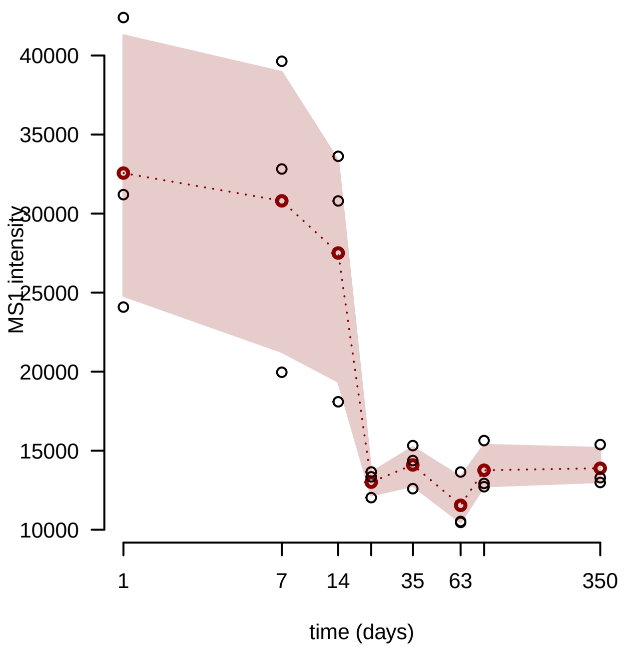

Q8K0G8
GN=Esrp2
epithelial splicing regulatory protein 2

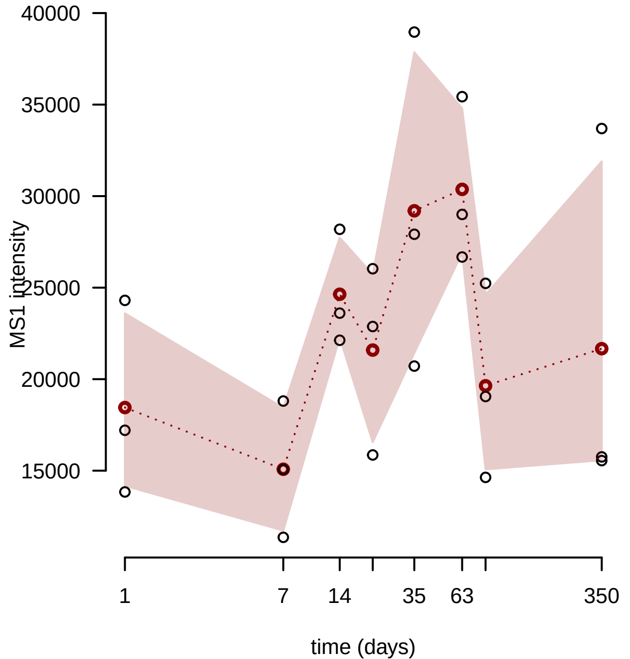

Q8K0H5
GN=Taf10
TATA−box binding protein associated fact ...

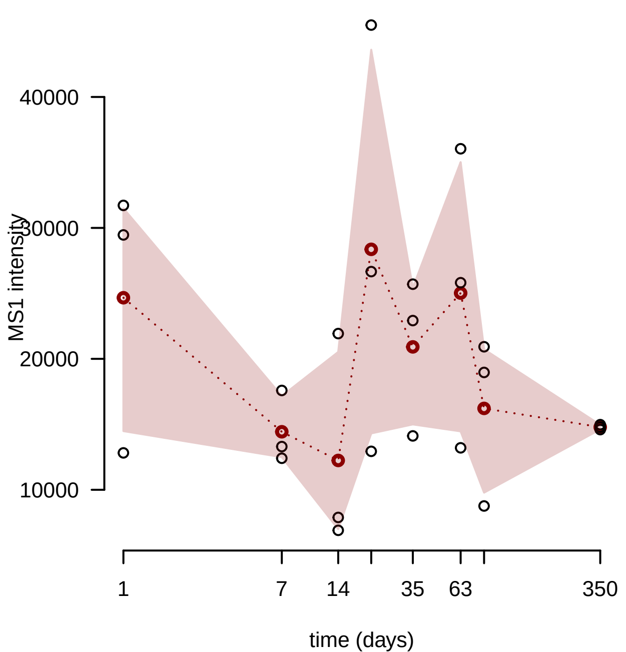

Q8K0H7
GN=Slc45a3
solute carrier family 45, member 3

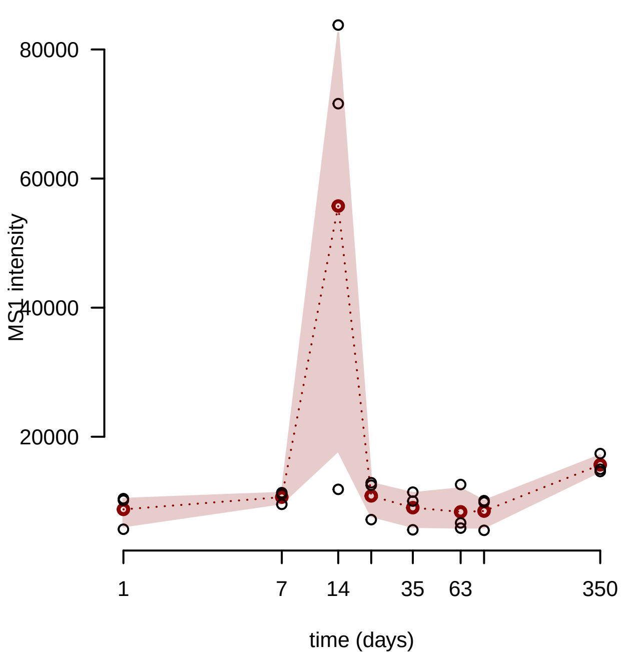

Q8K0L9
GN=Zbtb20
zinc finger and BTB domain containing 20

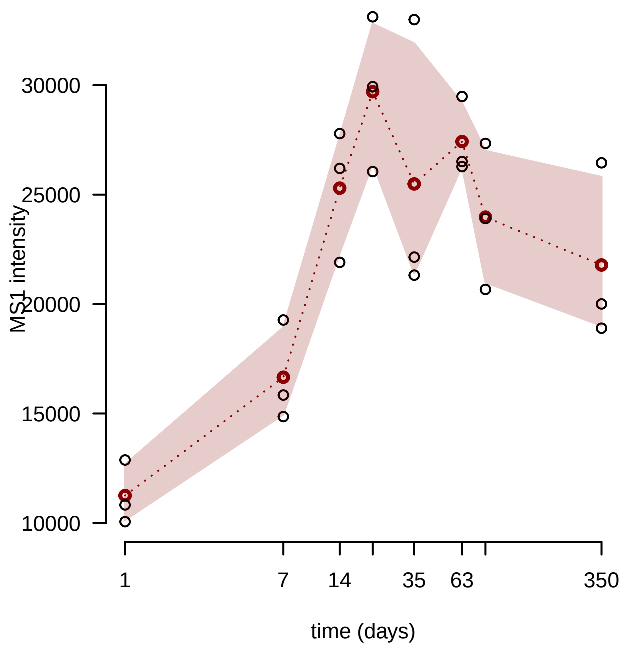

Q8K0P3
GN=Meak7
MTOR associated protein , eak−7 homolog

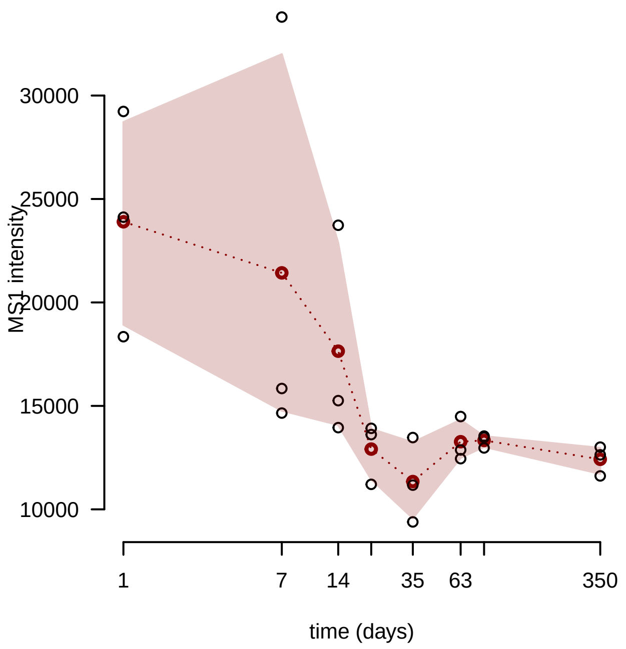

Q8K0Q5
GN=Arhgap18
Rho GTPase activating protein 18

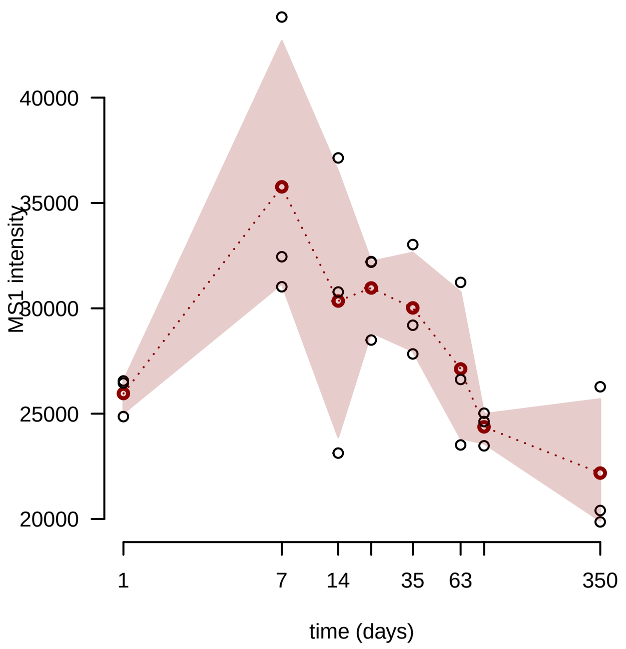

Q8K0T0
GN=Rtn1
reticulon 1

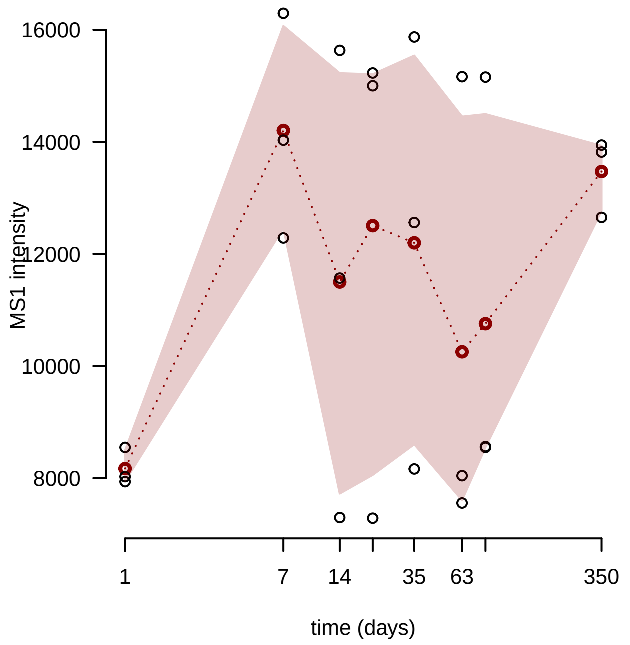

Q8K0T2
GN=Dync2li1
dynein cytoplasmic 2 light intermediate ...

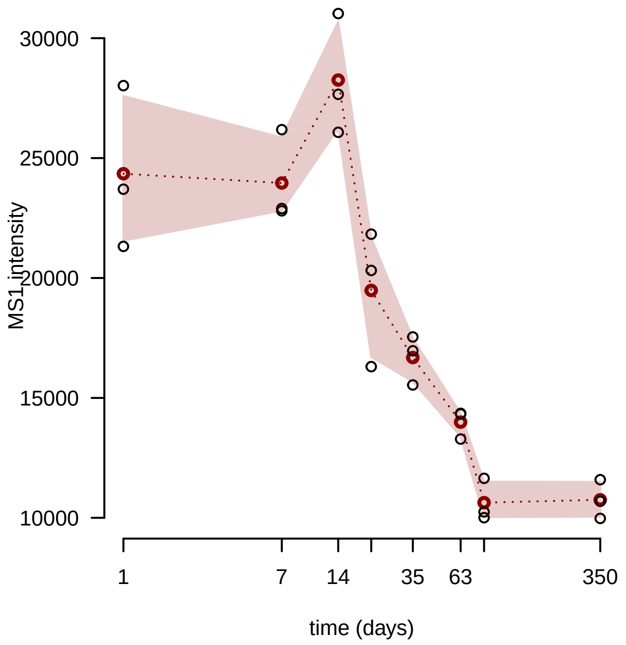

Q8K0T4
GN=Katnal1
katanin p60 subunit A−like 1

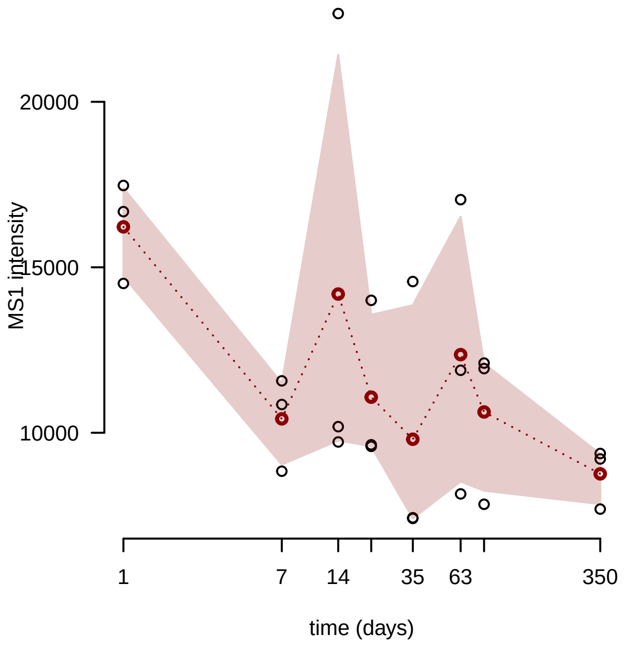

Q8K0U4
GN=Hspa12a
heat shock protein 12A

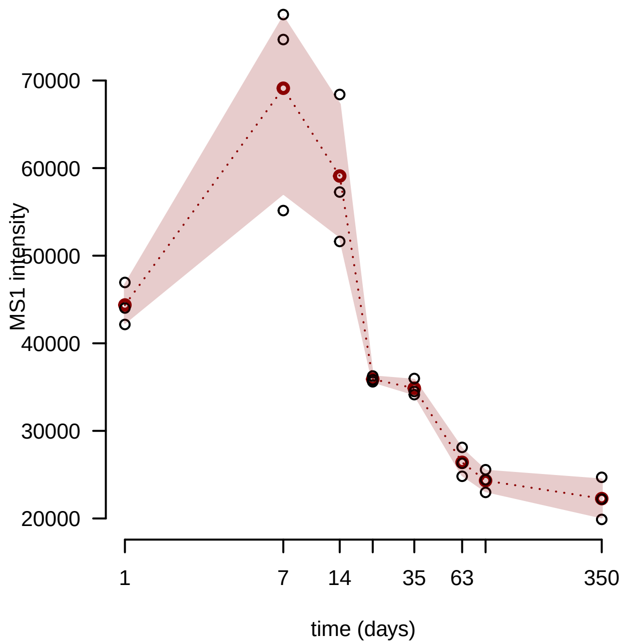

Q8K0V2
GN=Dcun1d3
DCN1, defective in cullin neddylation 1, ...

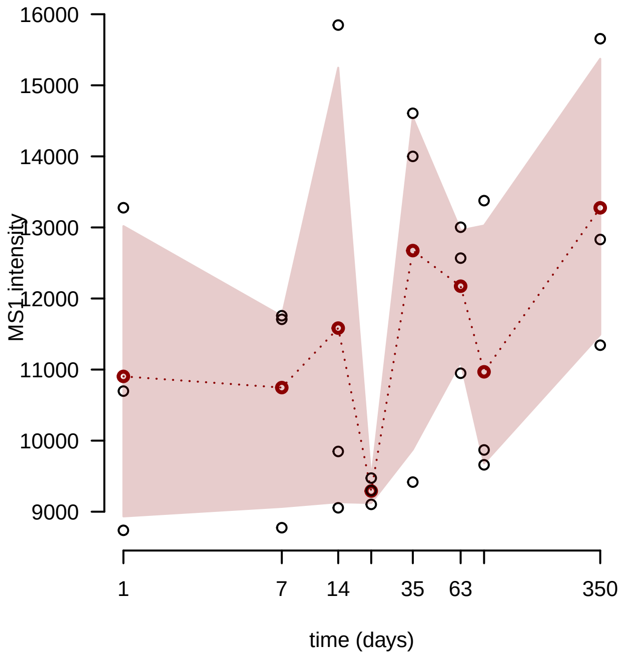

Q8K0V4
GN=Cnot3
CCR4−NOT transcription complex, subunit ...

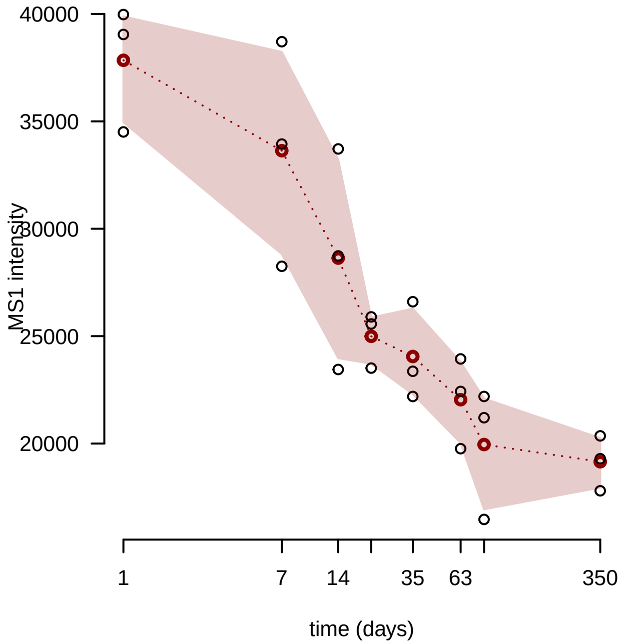

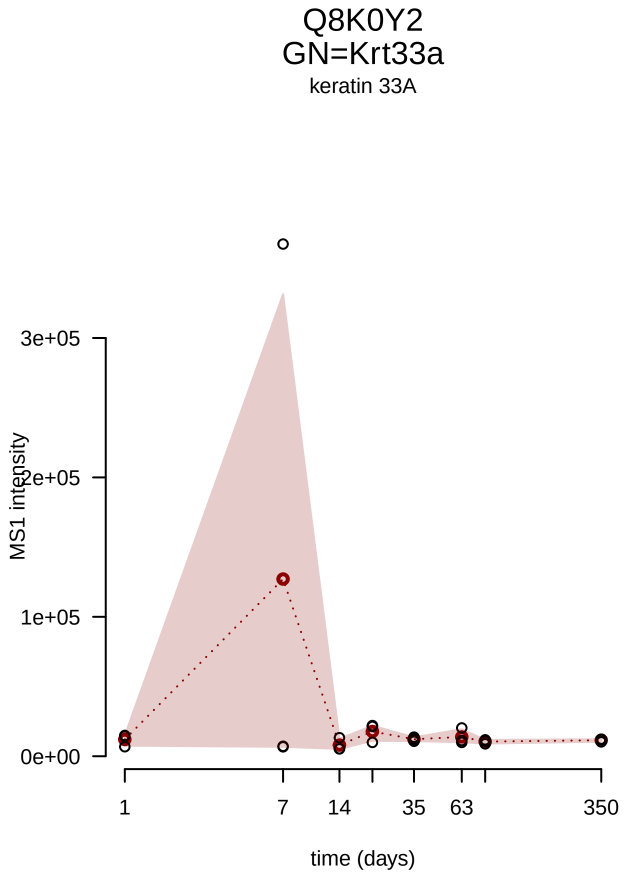
Q8K0Y2
GN=Krt33a
keratin 33A
MS1 intensity
0e+00
1e+05
2e+05
3e+05
1
7
14
35
63
350
time (days)

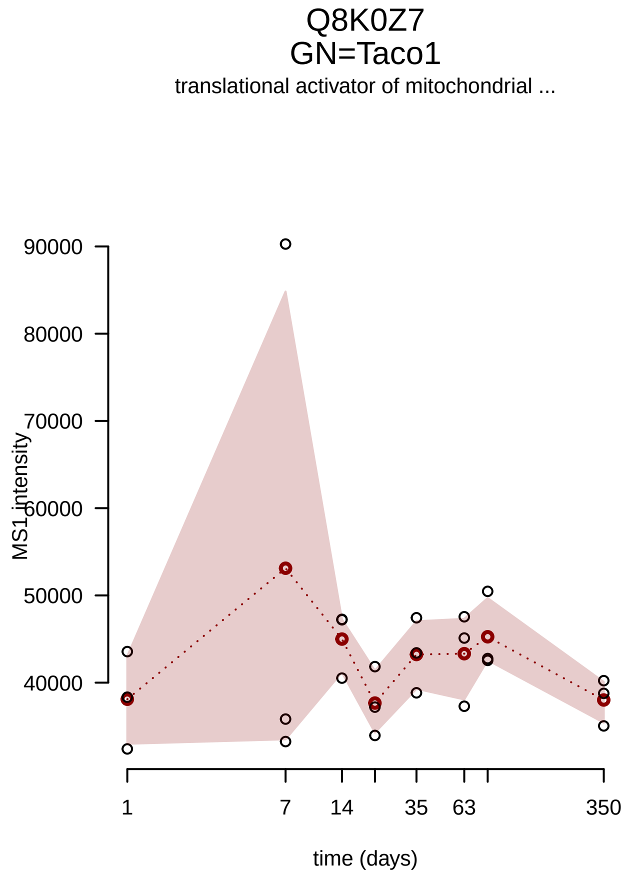
Q8K0Z7
GN=Taco1
translational activator of mitochondrial ...
MS1 intensity
40000
50000
60000
70000
80000
90000
1
7
14
35
63
350
time (days)

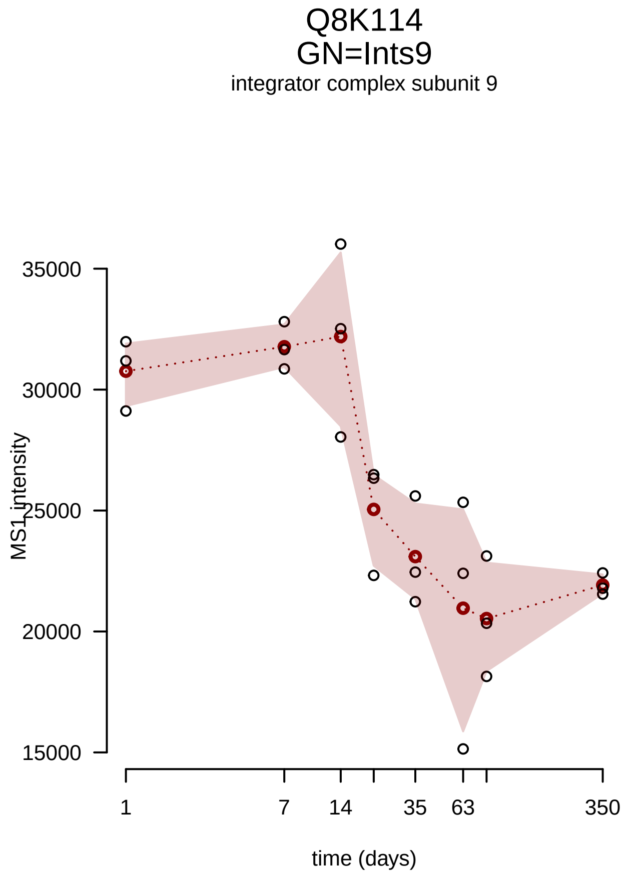
Q8K114
GN=Ints9
integrator complex subunit 9
MS1 intensity
15000
20000
25000
30000
35000
1
7
14
35
63
350
time (days)

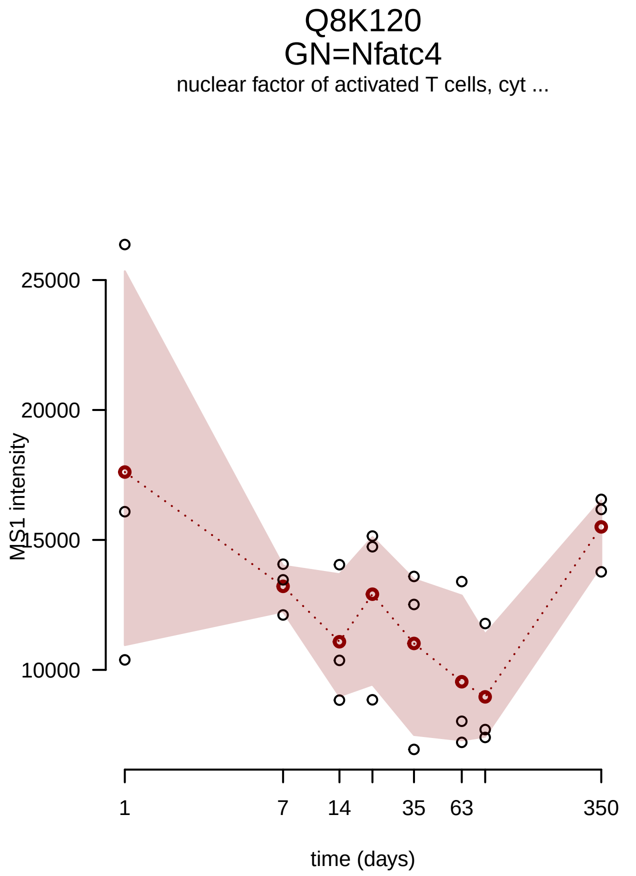
Q8K120
GN=Nfatc4
nuclear factor of activated T cells, cyt ...
MS1 intensity
10000
15000
20000
25000
1
7
14
35
63
350
time (days)

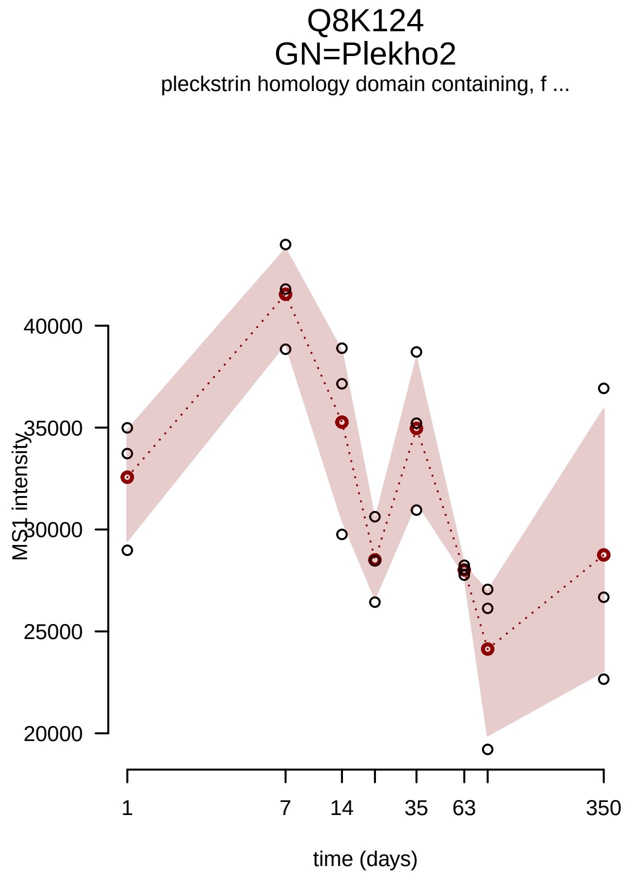
Q8K124
GN=Plekho2
pleckstrin homology domain containing, f ...
MS1 intensity
20000
25000
30000
35000
40000
1
7
14
35
63
350
time (days)

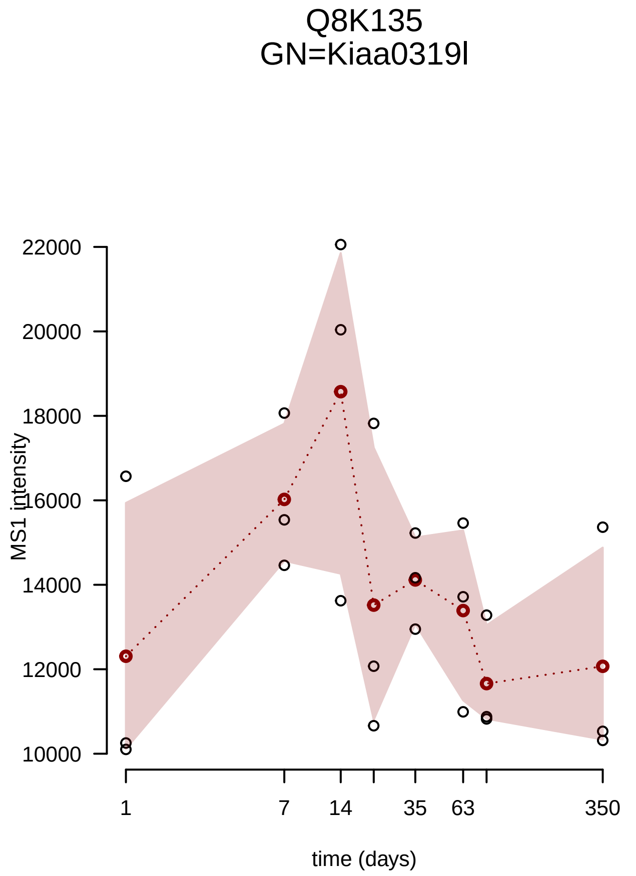
Q8K135
GN=Kiaa0319l
MS1 intensity
10000
12000
14000
16000
18000
20000
22000
1
7
14
35
63
350
time (days)

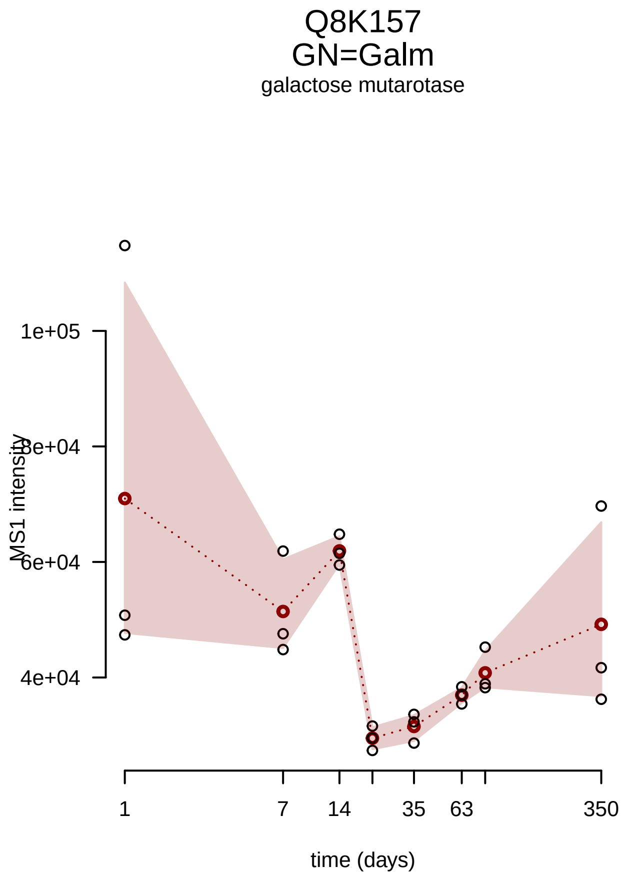
Q8K157
GN=Galm
galactose mutarotase
MS1 intensity
4e+04
6e+04
8e+04
1e+05
1
7
14
35
63
350
time (days)

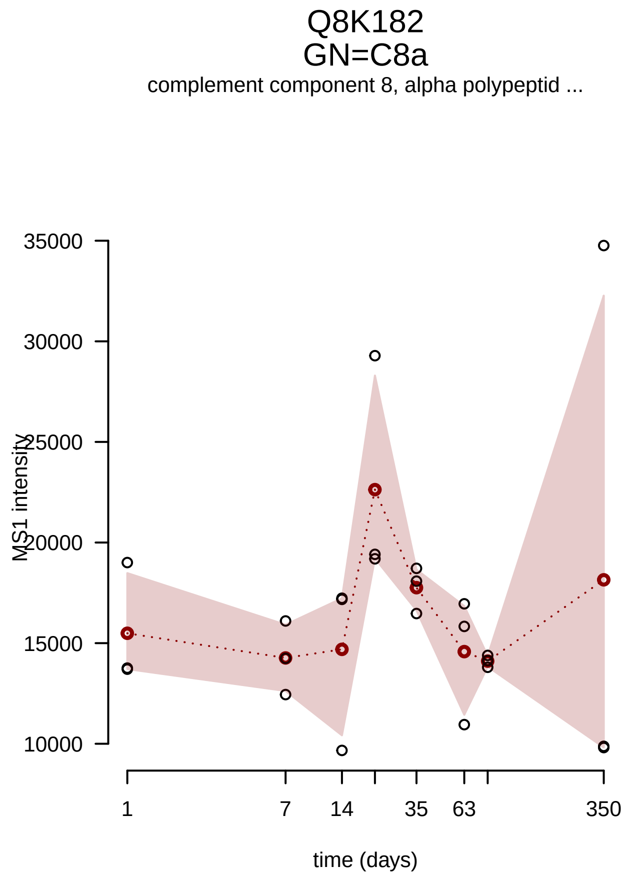
Q8K182
GN=C8a
complement component 8, alpha polypeptid ...
MS1 intensity
10000
15000
20000
25000
30000
35000
1
7
14
35
63
350
time (days)

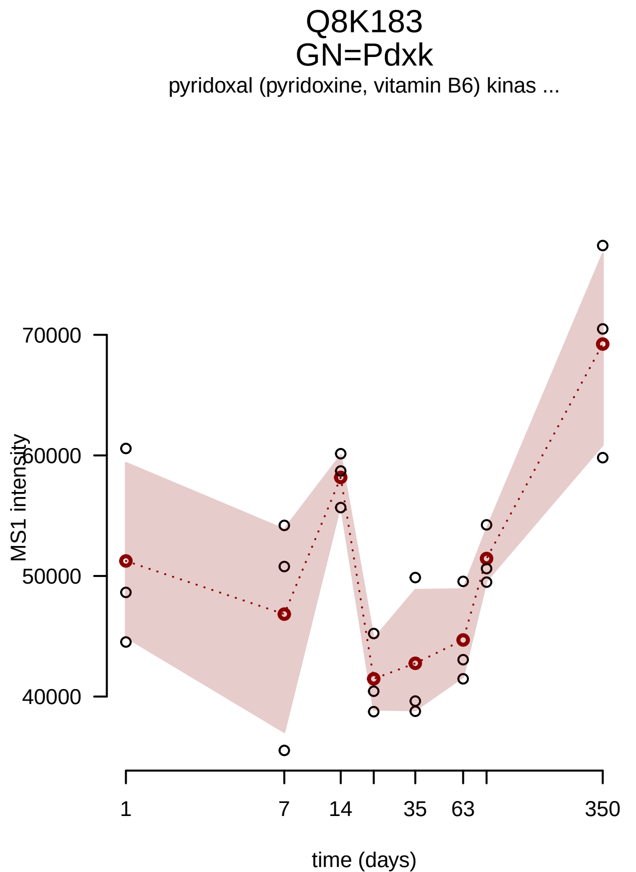
Q8K183
GN=Pdxk
pyridoxal (pyridoxine, vitamin B6) kinas ...
MS1 intensity
40000
50000
60000
70000
1
7
14
35
63
350
time (days)

Q8K190
GN=Saysd1
SAYSVFN motif domain containing 1

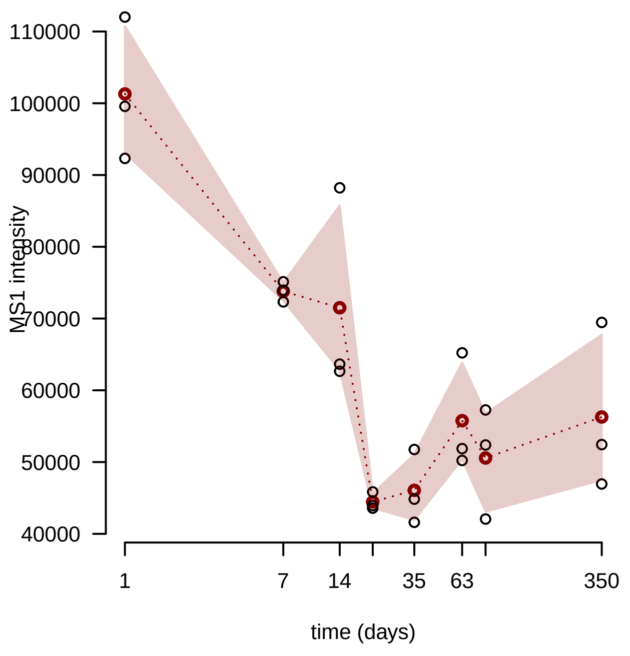

Q8K194
GN=Snrnp27
small nuclear ribonucleoprotein 27 (U4/U ...

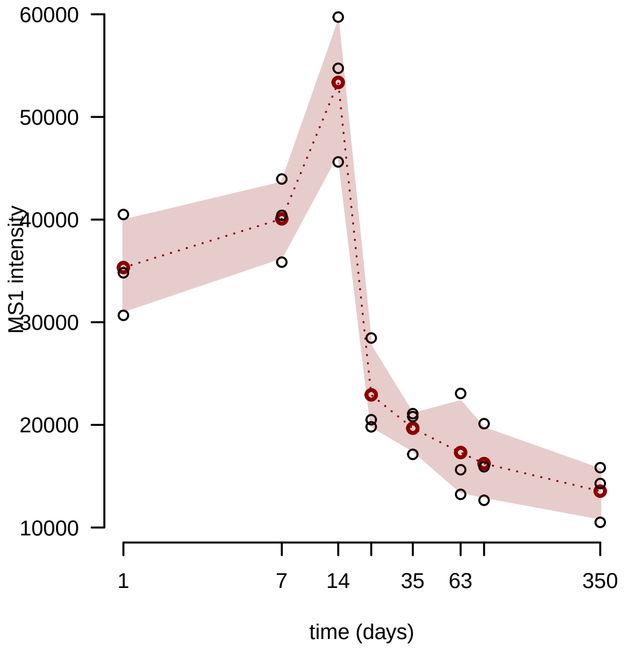

Q8K199
GN=Cmc2
COX assembly mitochondrial protein 2

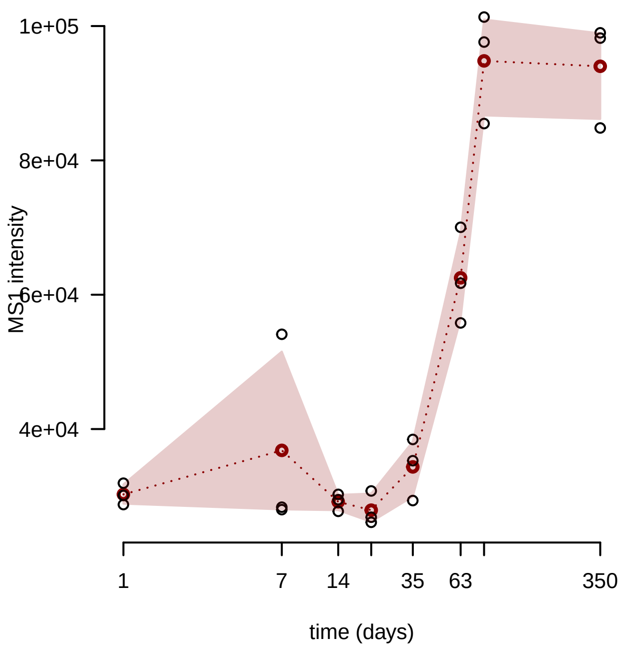

Q8K1A0
GN=Mettl5
methyltransferase like 5

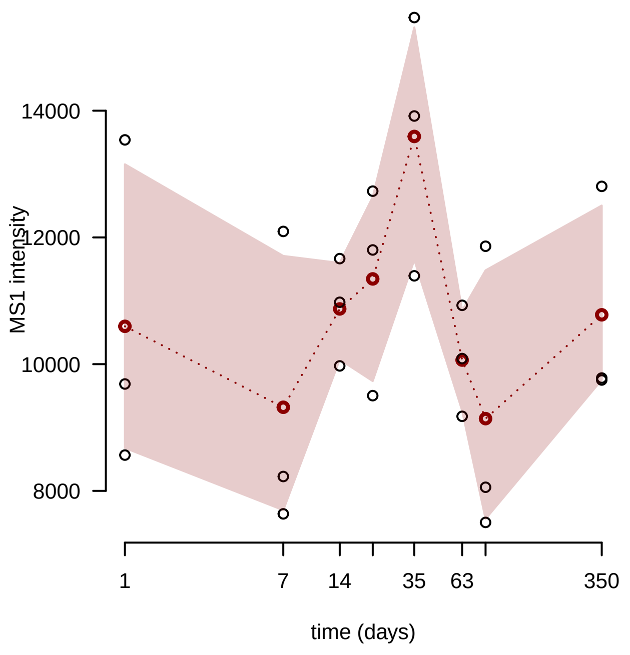

Q8K1A5
GN=Tmem41b
transmembrane protein 41B

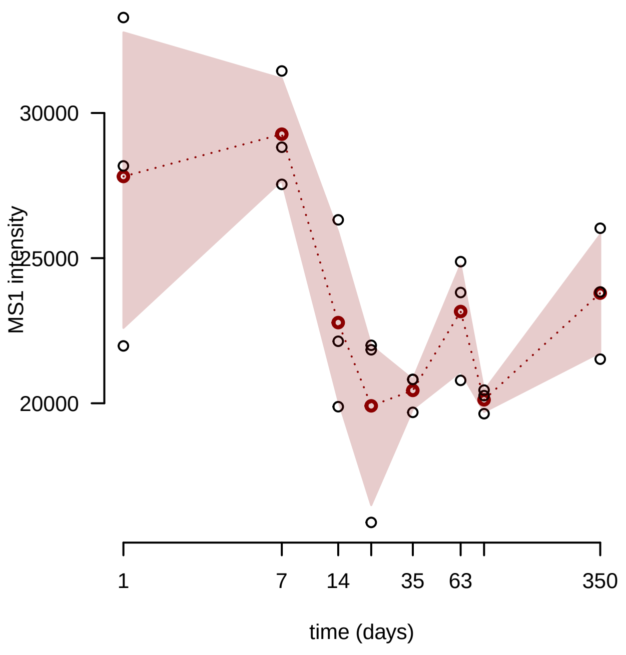

Q8K1A6
GN=Cc2d1a
coiled−coil and C2 domain containing 1A

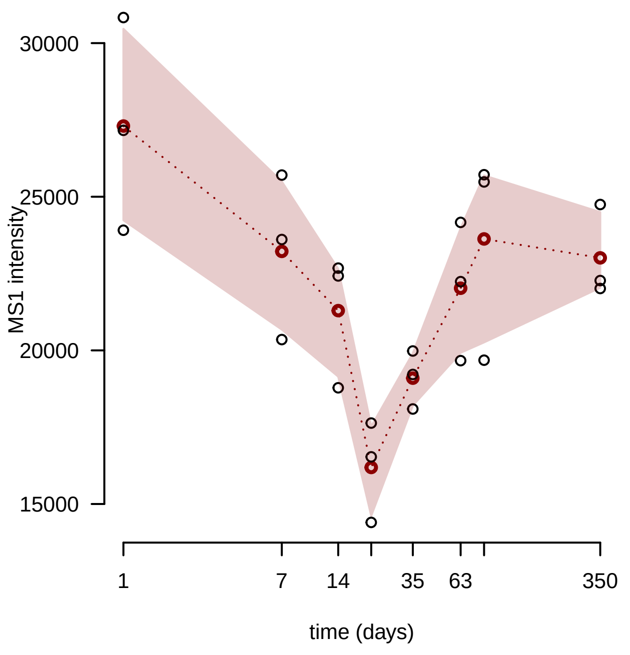

Q8K1B8
GN=Fermt3
fermitin family member 3

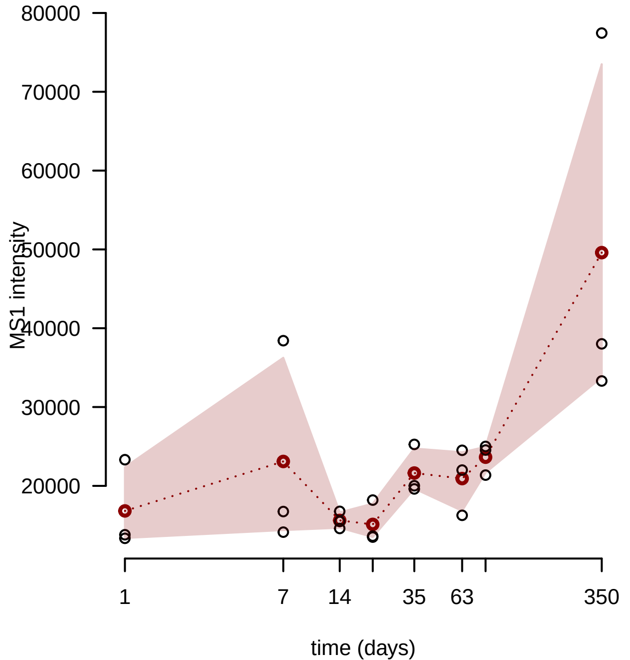

Q8K1B9
GN=Galnt18
polypeptide N−acetylgalactosaminyltransf ...

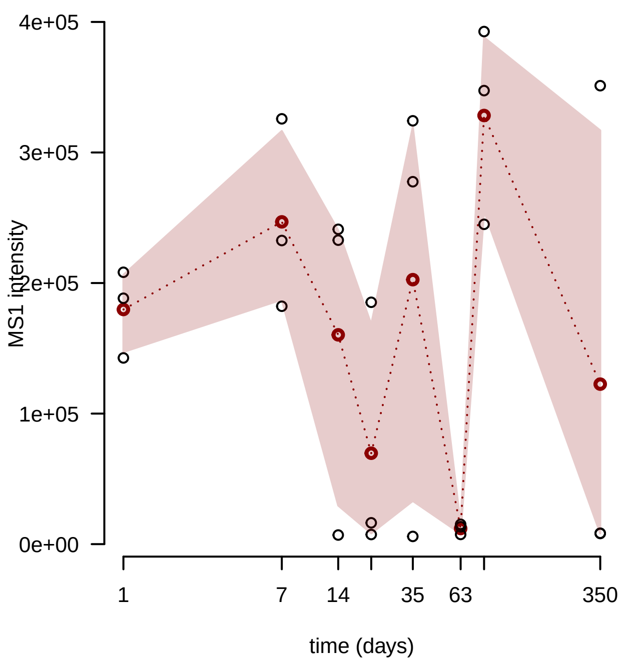

Q8K1C0
GN=Angel2
angel homolog 2

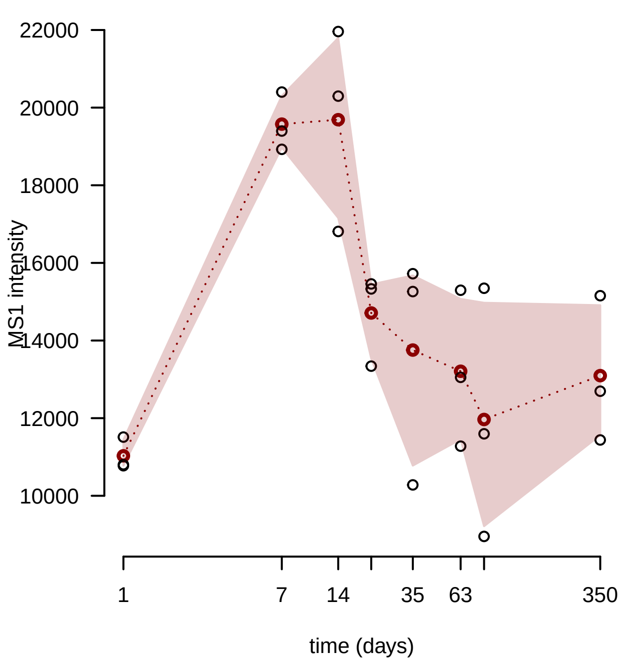

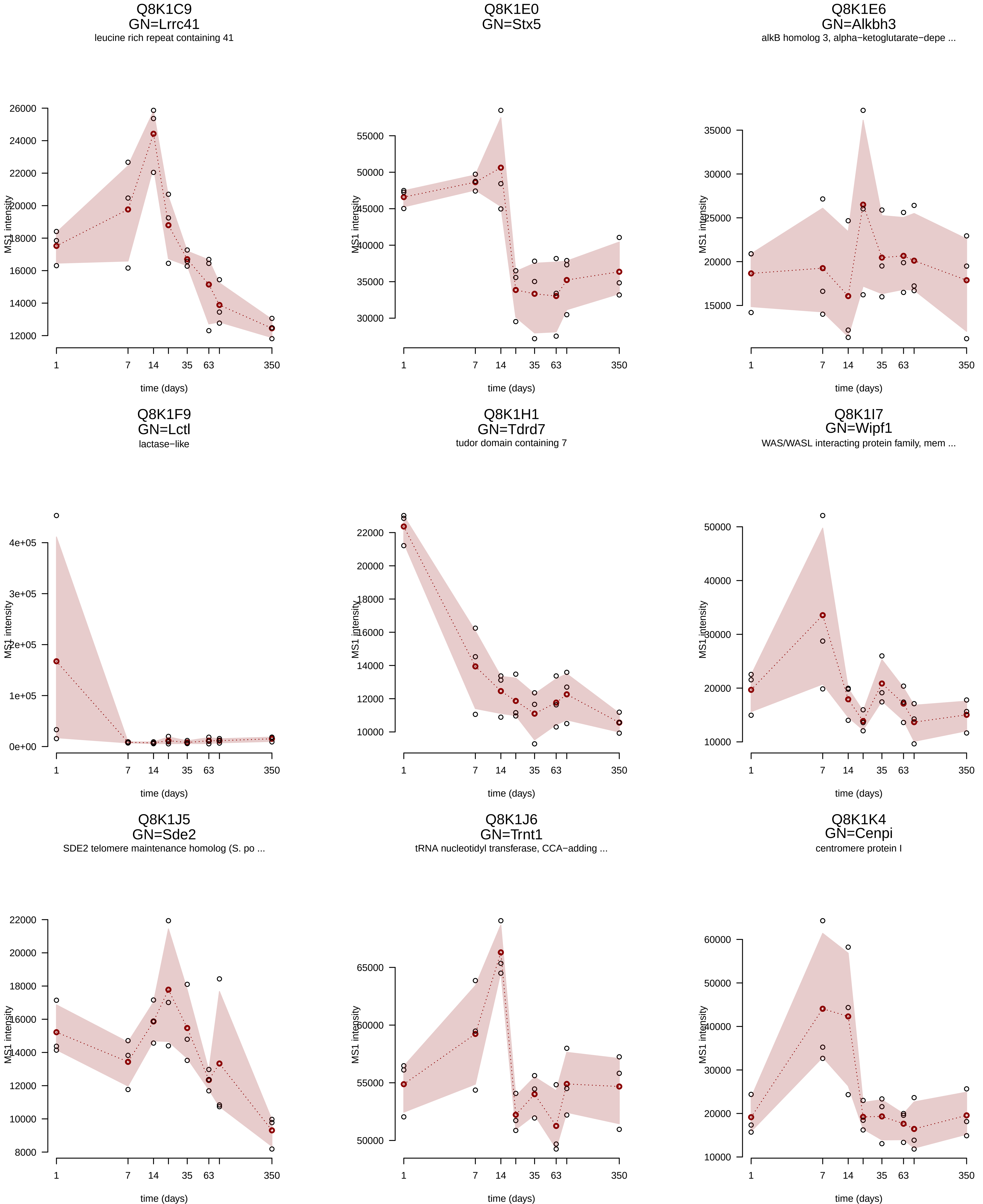
Q8K1C9
GN=Lrrc41
leucine rich repeat containing 41
MS1 intensity
26000
24000
22000
20000
18000
16000
14000
12000
1
7
14
35
63
350
time (days)
Q8K1E0
GN=Stx5
MS1 intensity
55000
50000
45000
40000
35000
30000
1
7
14
35
63
350
time (days)
Q8K1E6
GN=Alkbh3
alkB homolog 3, alpha−ketoglutarate−depe ...
MS1 intensity
35000
30000
25000
20000
15000
1
7
14
35
63
350
time (days)
Q8K1F9
GN=Lctl
lactase−like
MS1 intensity
4e+05
3e+05
2e+05
1e+05
0e+00
1
7
14
35
63
350
time (days)
Q8K1H1
GN=Tdrd7
tudor domain containing 7
MS1 intensity
22000
20000
18000
16000
14000
12000
10000
1
7
14
35
63
350
time (days)
Q8K1I7
GN=Wipf1
WAS/WASL interacting protein family, mem ...
MS1 intensity
50000
40000
30000
20000
10000
1
7
14
35
63
350
time (days)
Q8K1J5
GN=Sde2
SDE2 telomere maintenance homolog (S. po ...
MS1 intensity
22000
20000
18000
16000
14000
12000
10000
8000
1
7
14
35
63
350
time (days)
Q8K1J6
GN=Trnt1
tRNA nucleotidyl transferase, CCA−adding ...
MS1 intensity
65000
60000
55000
50000
1
7
14
35
63
350
time (days)
Q8K1K4
GN=Cenpi
centromere protein I
MS1 intensity
60000
50000
40000
30000
20000
10000
1
7
14
35
63
350
time (days)

Q8K1L2
GN=Spin4
spindlin family, member 4

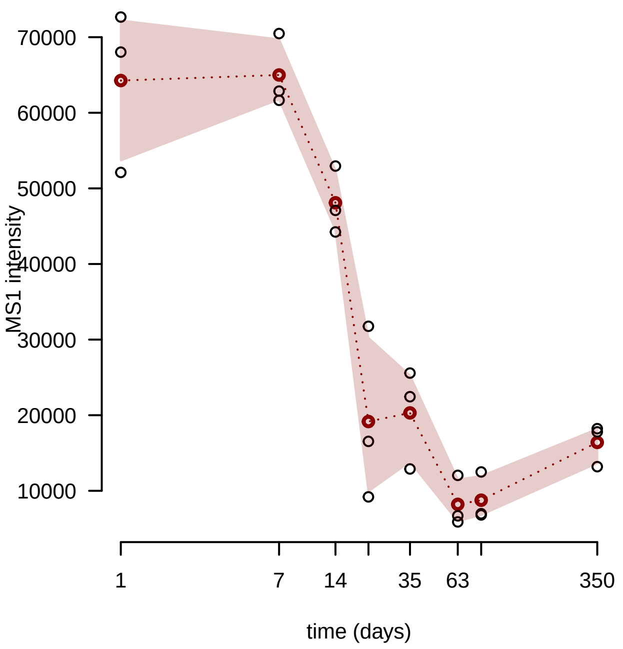

Q8K1L5
GN=Ppp1r11
protein phosphatase 1, regulatory inhibi ...

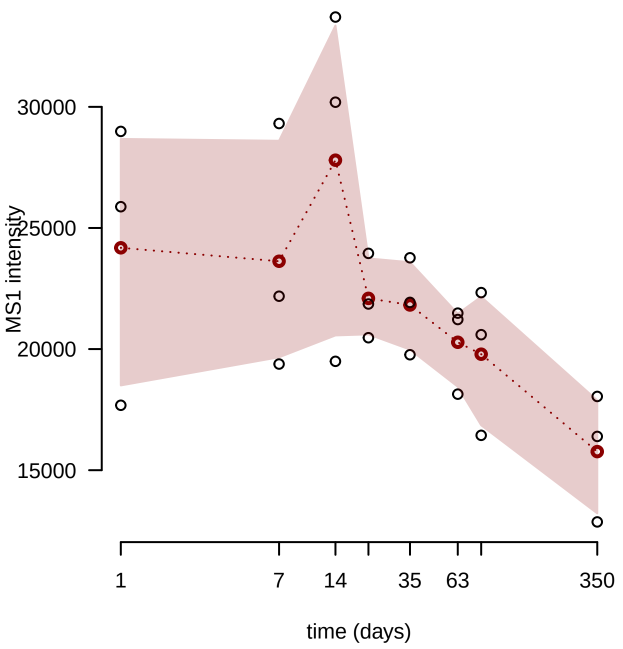

Q8K1M6
GN=Dnm1l
dynamin 1−like

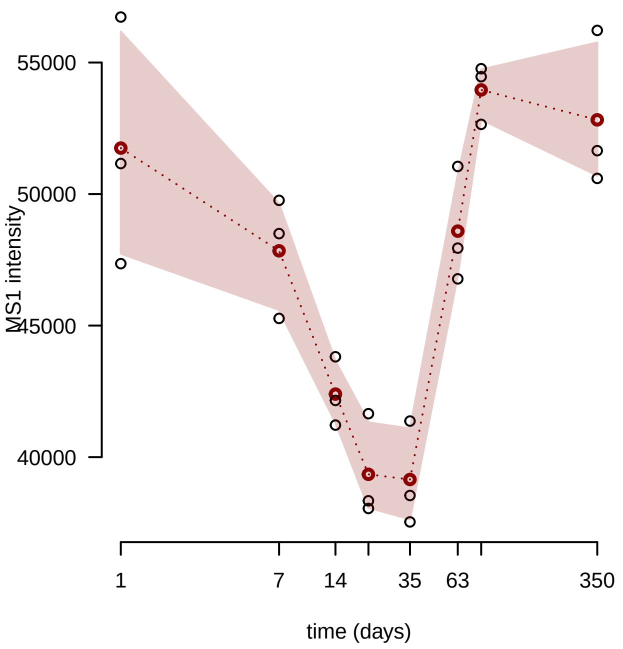

Q8K1M6
GN=Dnm1l
dynamin 1−like

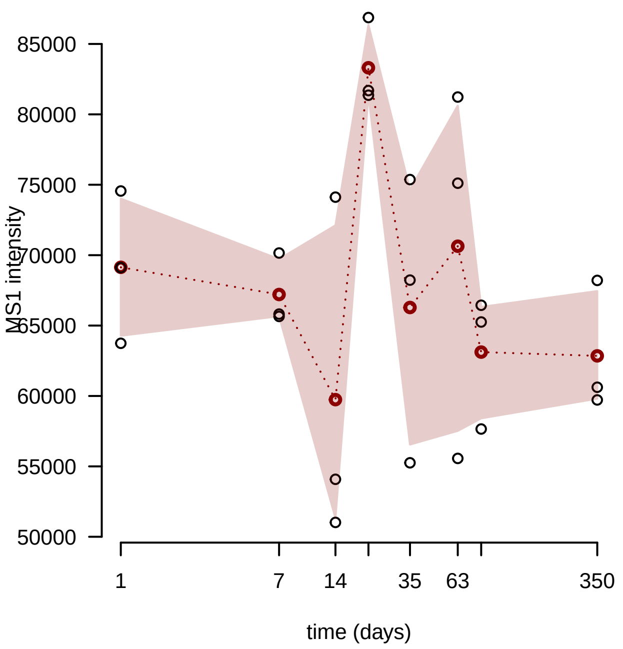

Q8K1N1
GN=Pnpla8
patatin−like phospholipase domain contai ...

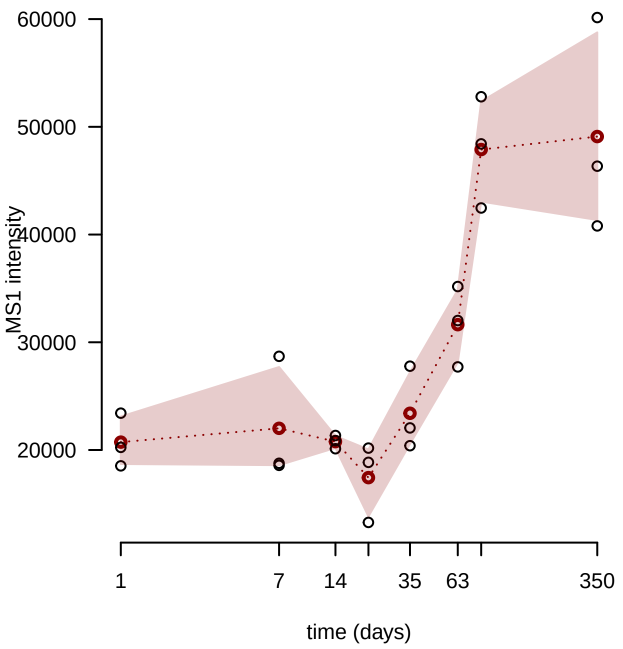

Q8K1N2
GN=Phldb2
pleckstrin homology like domain, family ...

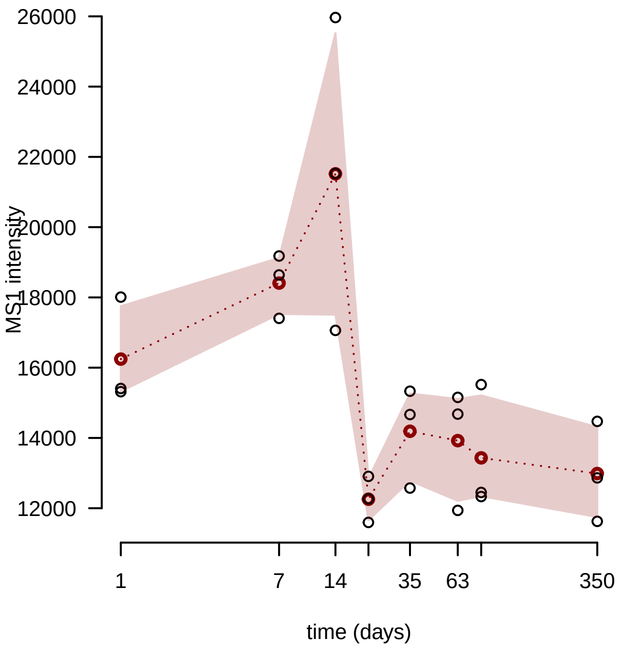

Q8K1N4
GN=Spats2
spermatogenesis associated, serine−rich ...

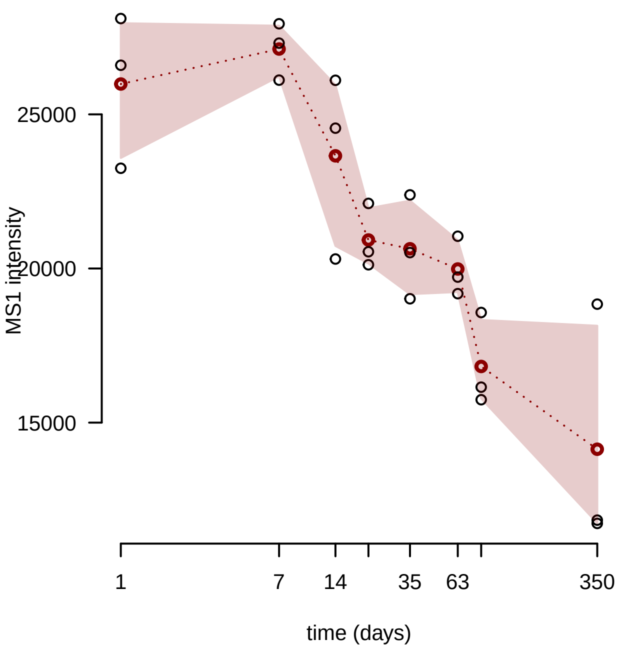

Q8K1R3
GN=Pnpt1
polyribonucleotide nucleotidyltransferas ...

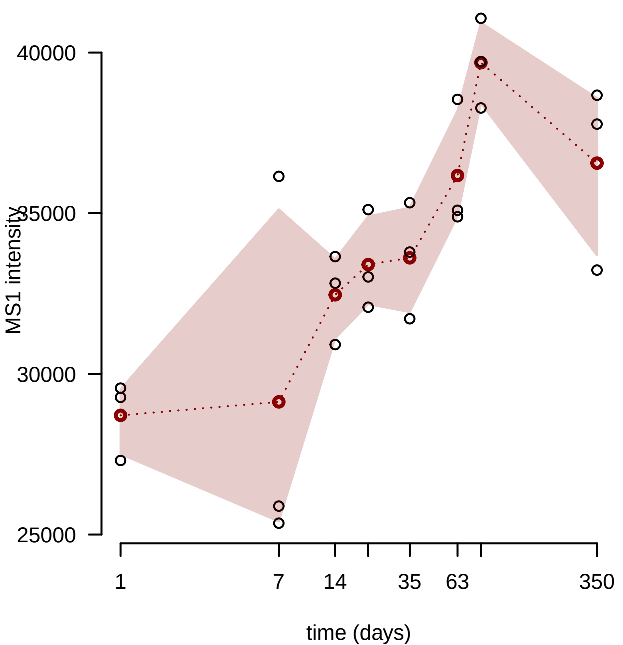

Q8K1R7
GN=Nek9
NIMA (never in mitosis gene a)−related e ...

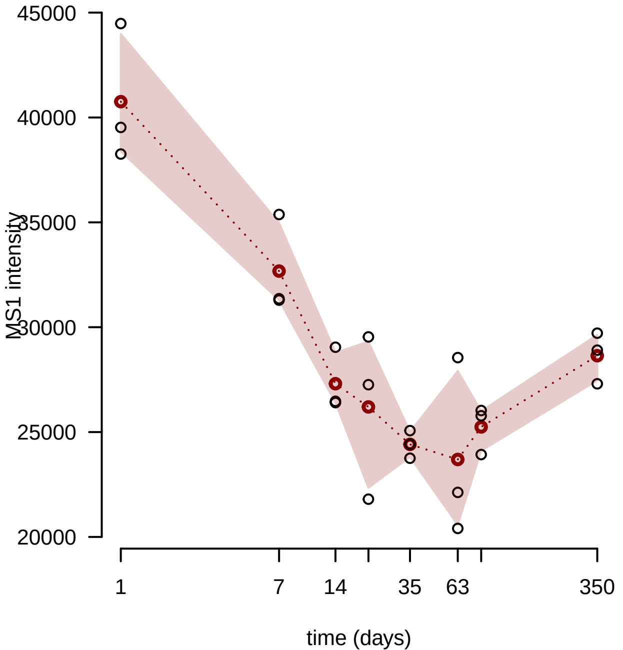

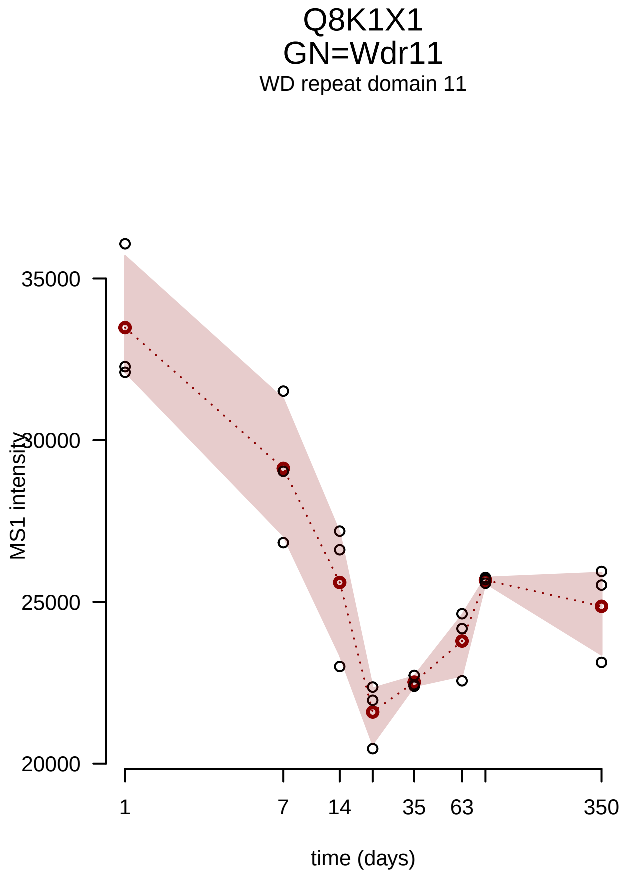
Q8K1X1
GN=Wdr11
WD repeat domain 11
MS1 intensity
35000
30000
25000
20000
1
7
14
35
63
350
time (days)

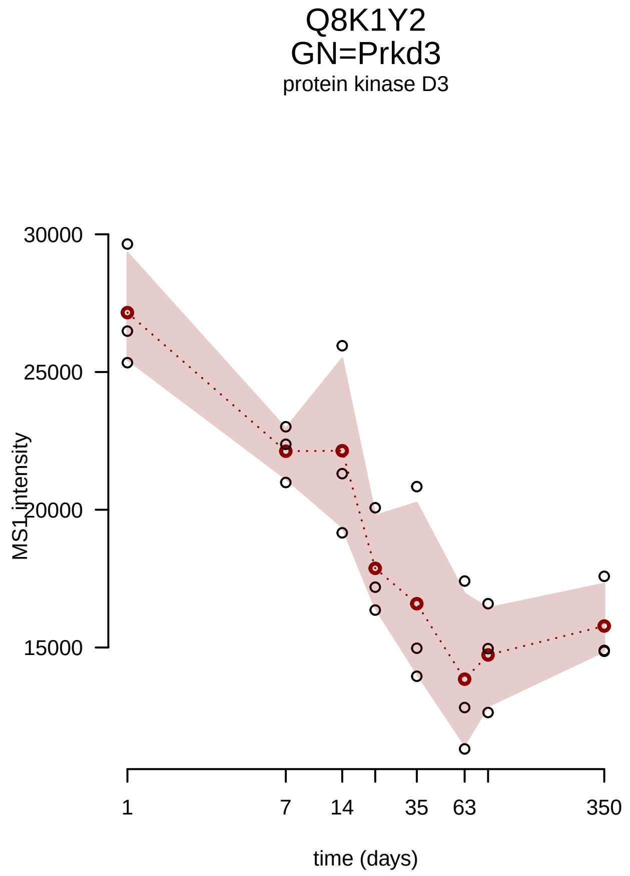
Q8K1Y2
GN=Prkd3
protein kinase D3
MS1 intensity
30000
25000
20000
15000
1
7
14
35
63
350
time (days)

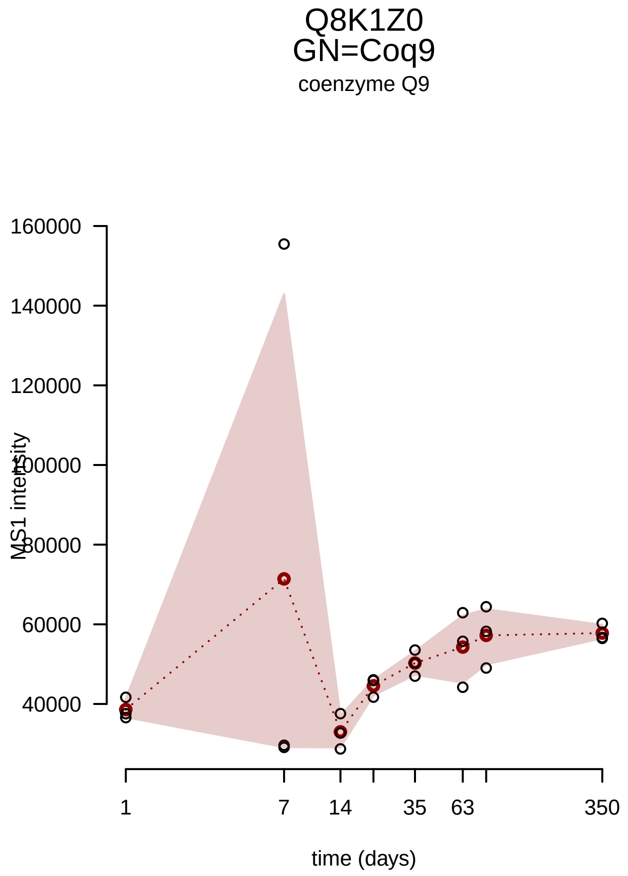
Q8K1Z0
GN=Coq9
coenzyme Q9
MS1 intensity
160000
140000
120000
100000
80000
60000
40000
1
7
14
35
63
350
time (days)

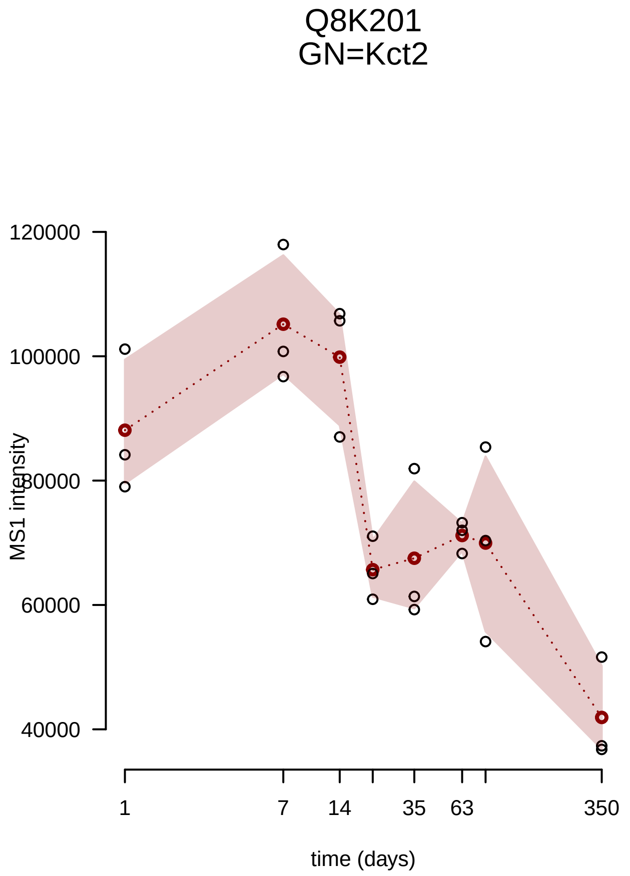
Q8K201
GN=Kct2
MS1 intensity
120000
100000
80000
60000
40000
1
7
14
35
63
350
time (days)

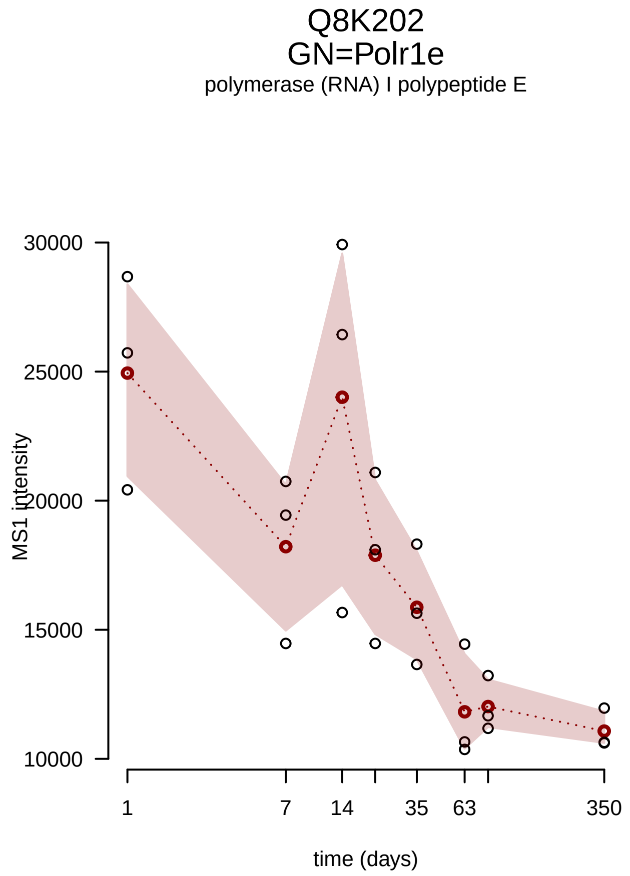
Q8K202
GN=Polr1e
polymerase (RNA) I polypeptide E
MS1 intensity
30000
25000
20000
15000
10000
1
7
14
35
63
350
time (days)

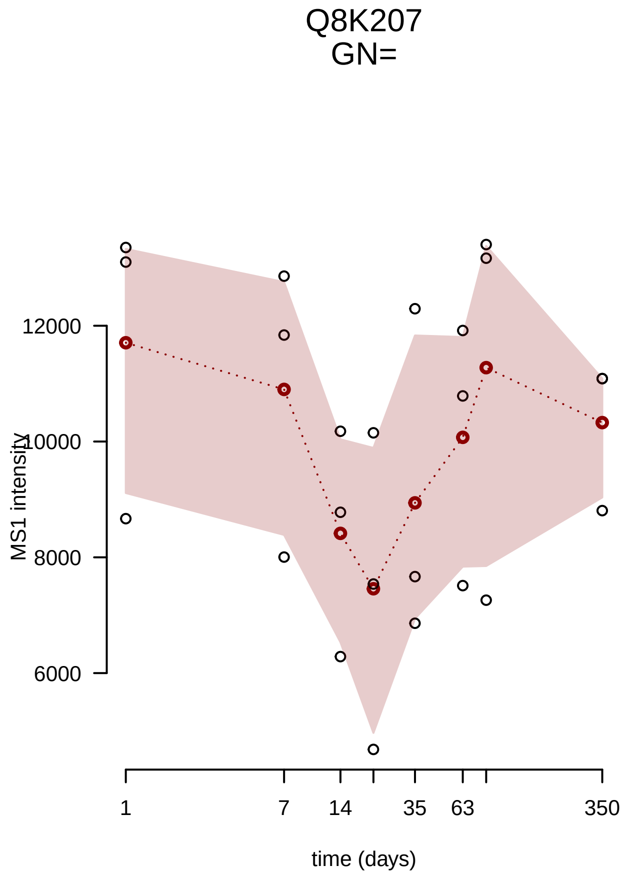
Q8K207
GN=
MS1 intensity
12000
10000
8000
6000
1
7
14
35
63
350
time (days)

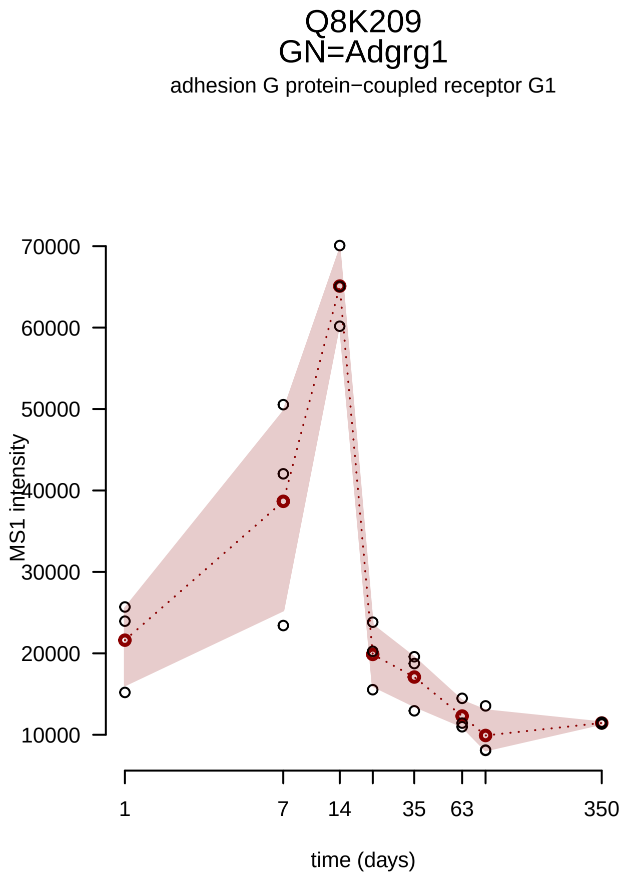
Q8K209
GN=Adgrg1
adhesion G protein−coupled receptor G1
MS1 intensity
70000
60000
50000
40000
30000
20000
10000
1
7
14
35
63
350
time (days)

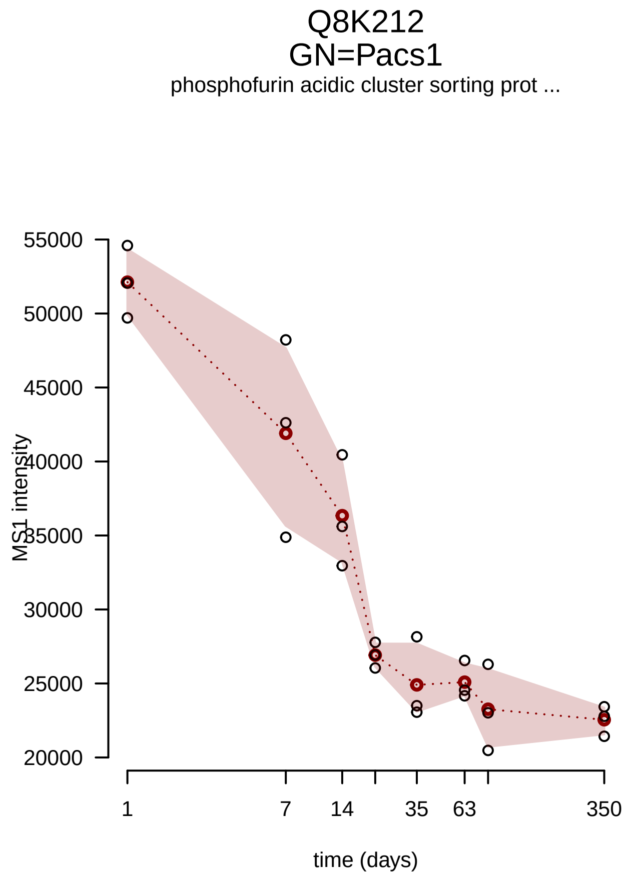
Q8K212
GN=Pacs1
phosphofurin acidic cluster sorting prot ...
MS1 intensity
55000
50000
45000
40000
35000
30000
25000
20000
1
7
14
35
63
350
time (days)

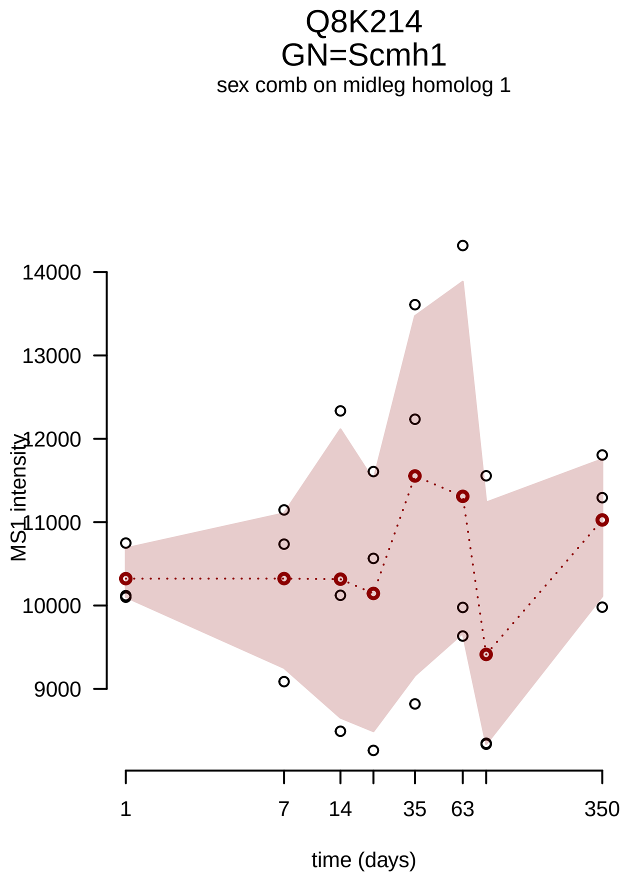
Q8K214
GN=Scmh1
sex comb on midleg homolog 1
MS1 intensity
14000
13000
12000
11000
10000
9000
1
7
14
35
63
350
time (days)

Q8K215
GN=Lyrm4
LYR motif containing 4

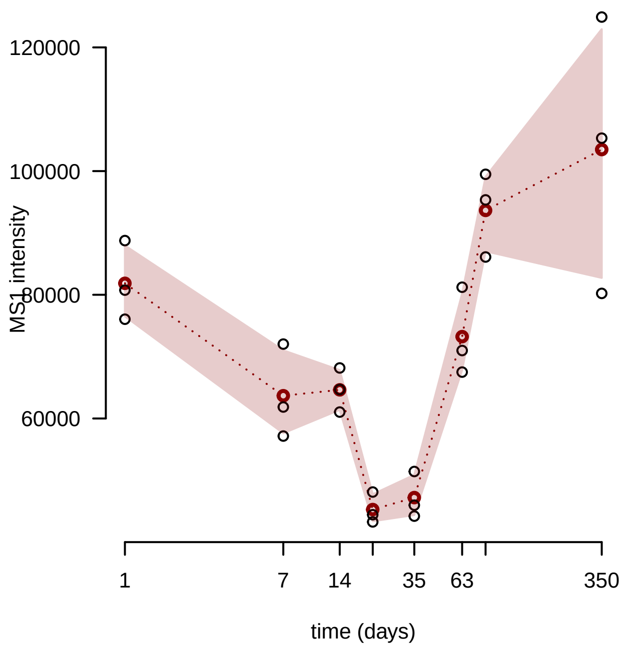

Q8K221
GN=Arfip2
ADP−ribosylation factor interacting prot ...

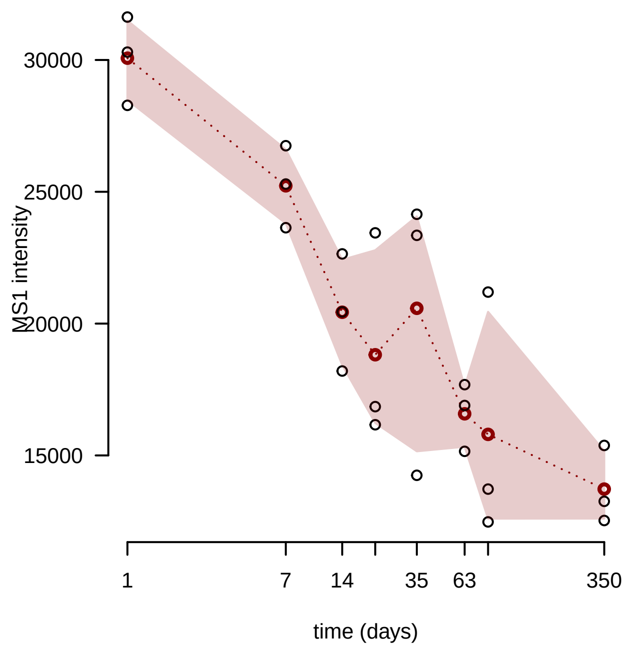

Q8K224
GN=Nat10
N−acetyltransferase 10

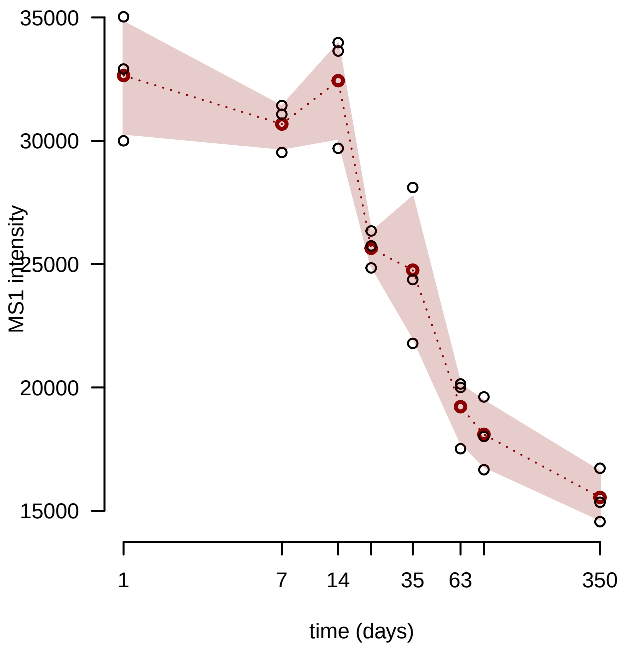

Q8K245
GN=Uvrag
UV radiation resistance associated gene

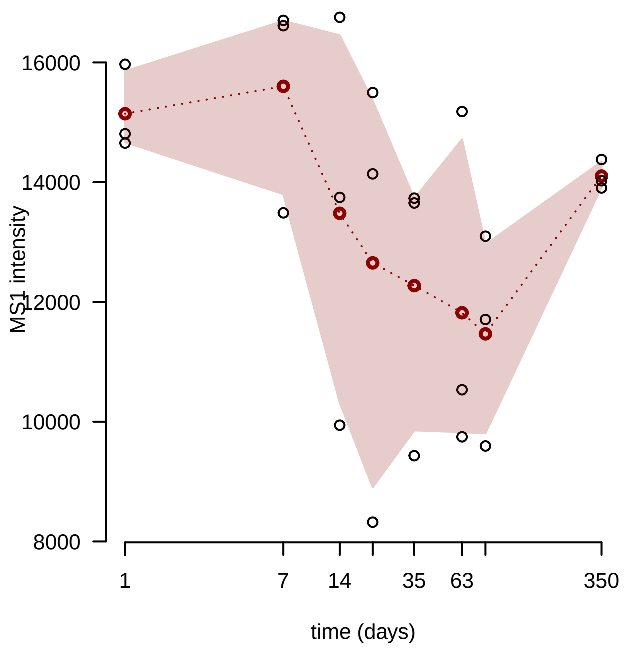

Q8K248
GN=Hpdl
4−hydroxyphenylpyruvate dioxygenase−like

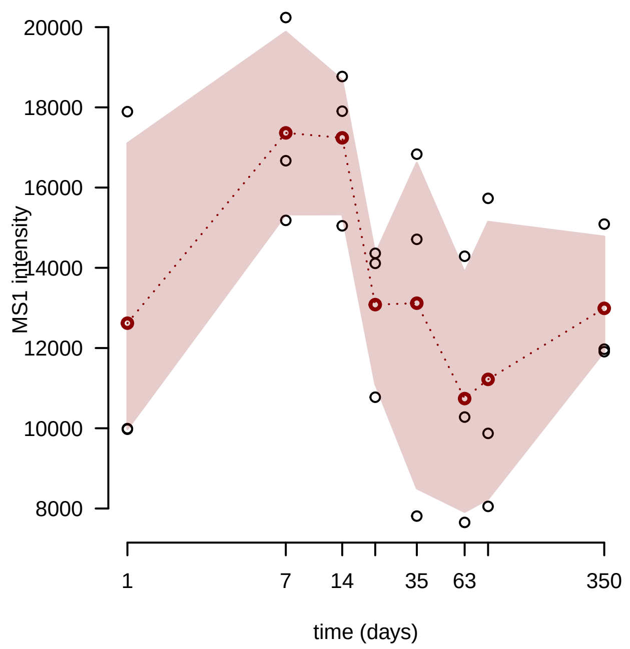

Q8K268
GN=Abcf3
ATP−binding cassette, sub−family F (GCN2 ...

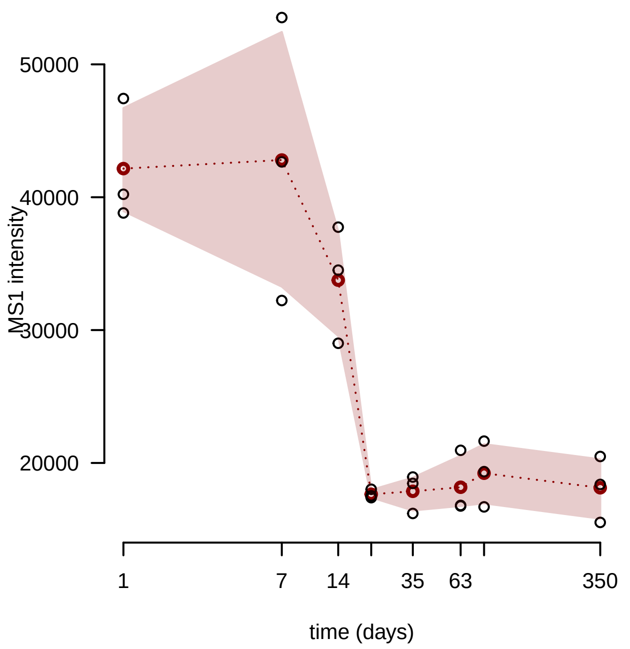

Q8K273
GN=Mmgt1
membrane magnesium transporter 1

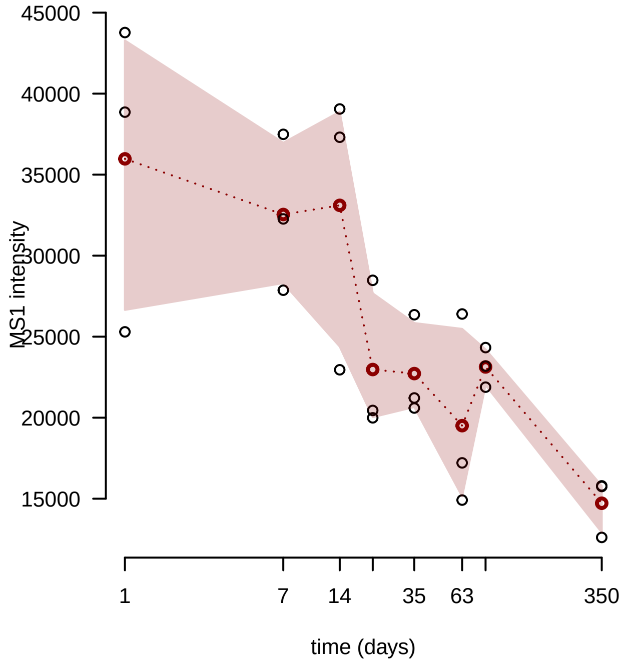

Q8K274
GN=Fn3krp
fructosamine 3 kinase related protein

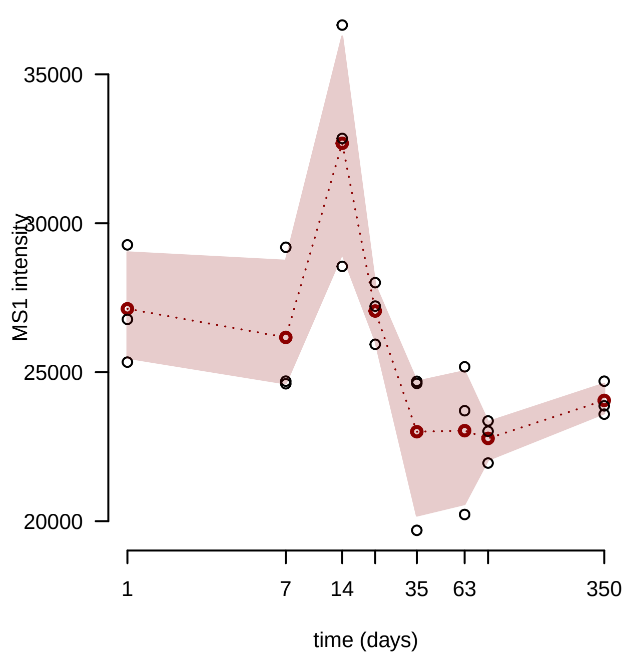

Q8K284
GN=Gtf3c1
general transcription factor III C 1

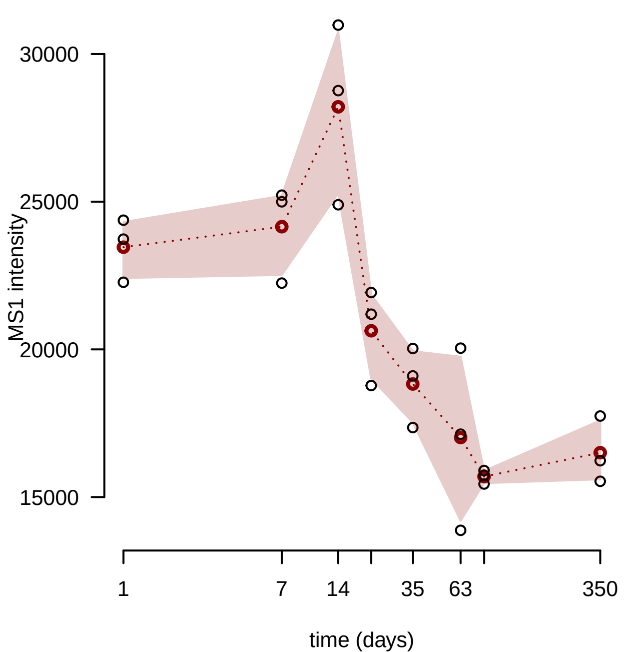

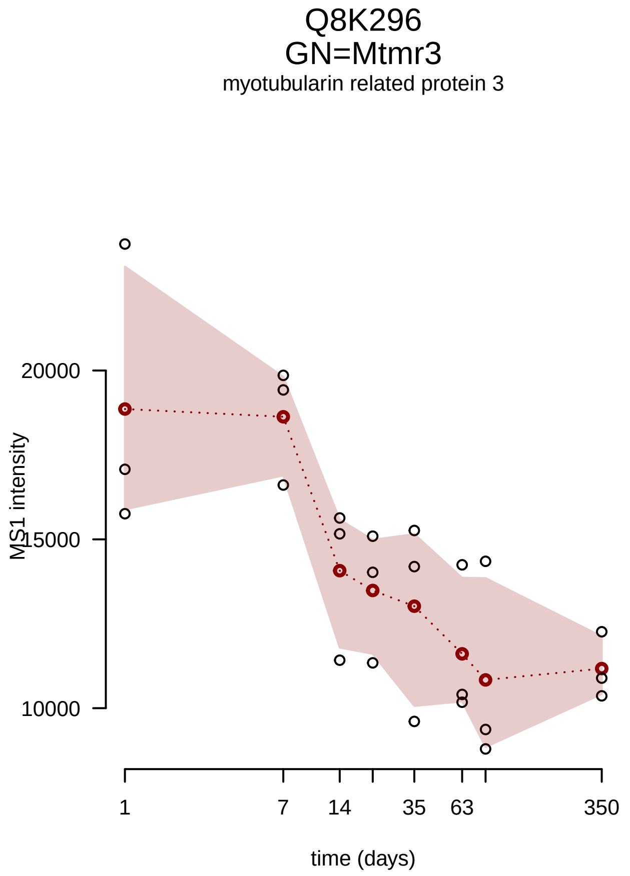
Q8K296
GN=Mtmr3
myotubularin related protein 3
MS1 intensity
20000
15000
10000
1
7
14
35
63
350
time (days)
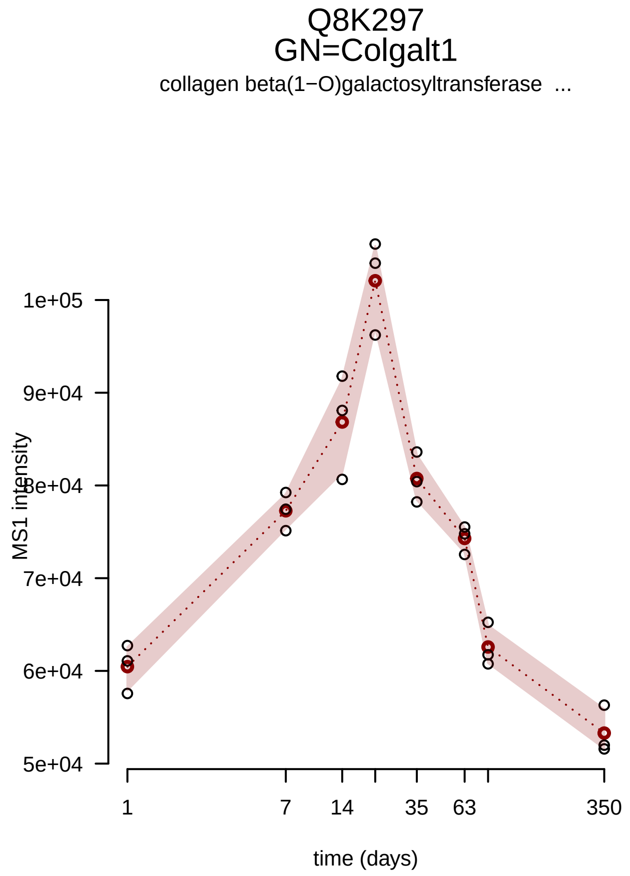
Q8K297
GN=Colgalt1
collagen beta(1−O)galactosyltransferase ...
MS1 intensity
1e+05
9e+04
8e+04
7e+04
6e+04
5e+04
1
7
14
35
63
350
time (days)
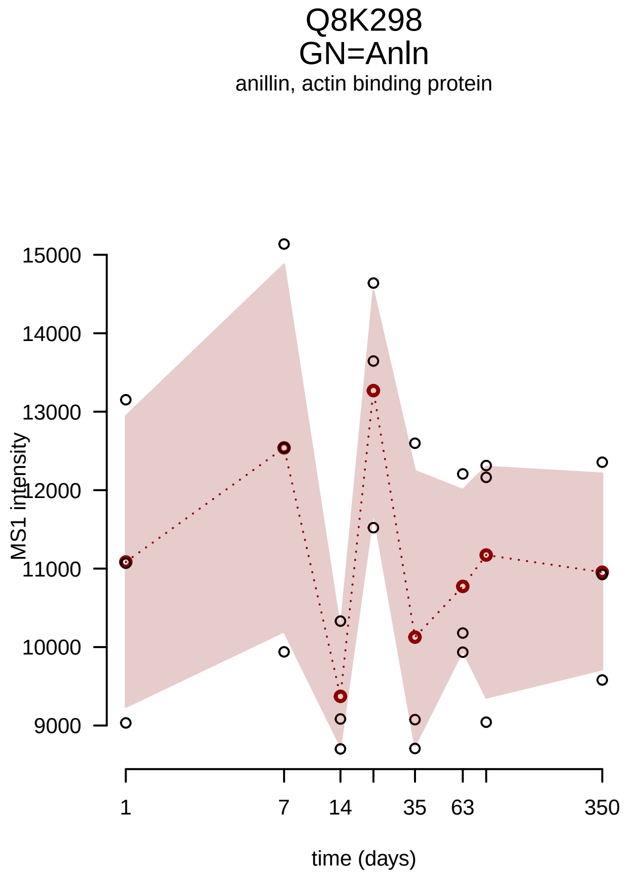
Q8K298
GN=Anln
anillin, actin binding protein
MS1 intensity
15000
14000
13000
12000
11000
10000
9000
1
7
14
35
63
350
time (days)
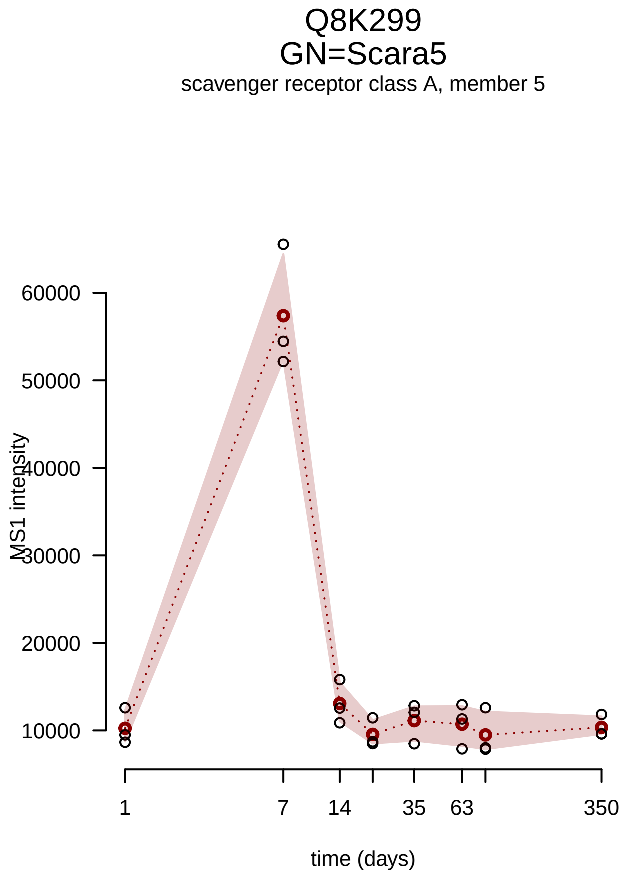
Q8K299
GN=Scara5
scavenger receptor class A, member 5
MS1 intensity
60000
50000
40000
30000
20000
10000
1
7
14
35
63
350
time (days)
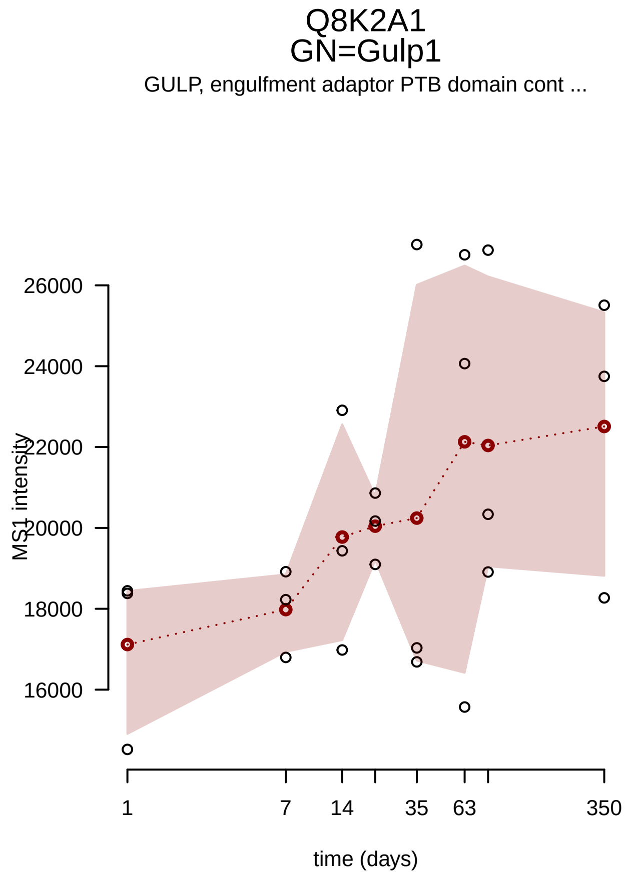
Q8K2A1
GN=Gulp1
GULP, engulfment adaptor PTB domain cont ...
MS1 intensity
26000
24000
22000
20000
18000
16000
1
7
14
35
63
350
time (days)
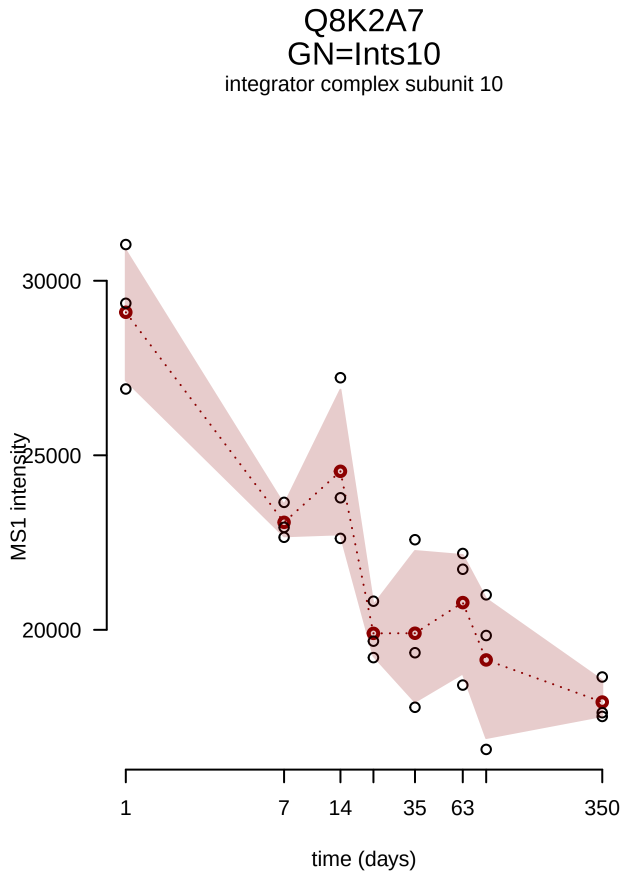
Q8K2A7
GN=Ints10
integrator complex subunit 10
MS1 intensity
30000
25000
20000
1
7
14
35
63
350
time (days)
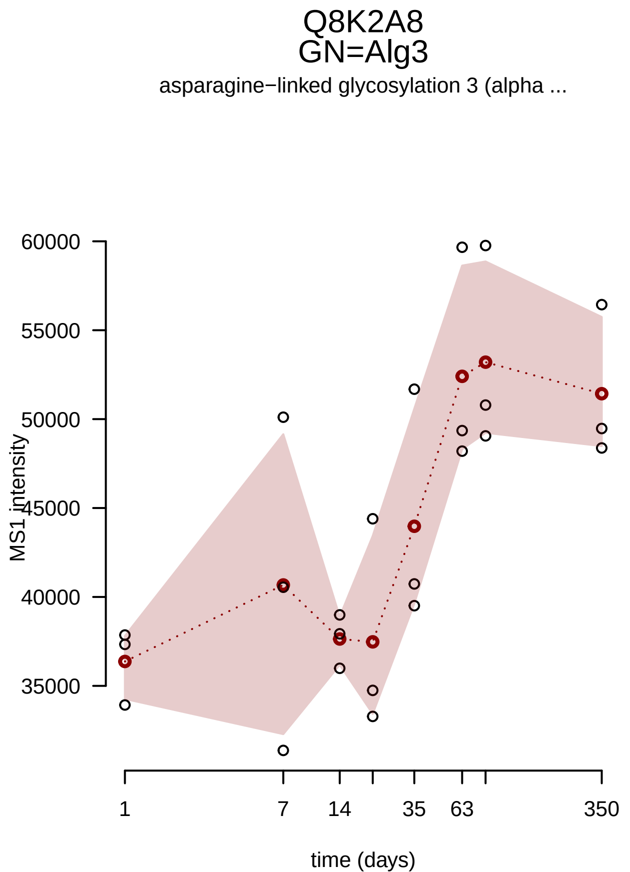
Q8K2A8
GN=Alg3
asparagine−linked glycosylation 3 (alpha ...
MS1 intensity
60000
55000
50000
45000
40000
35000
1
7
14
35
63
350
time (days)
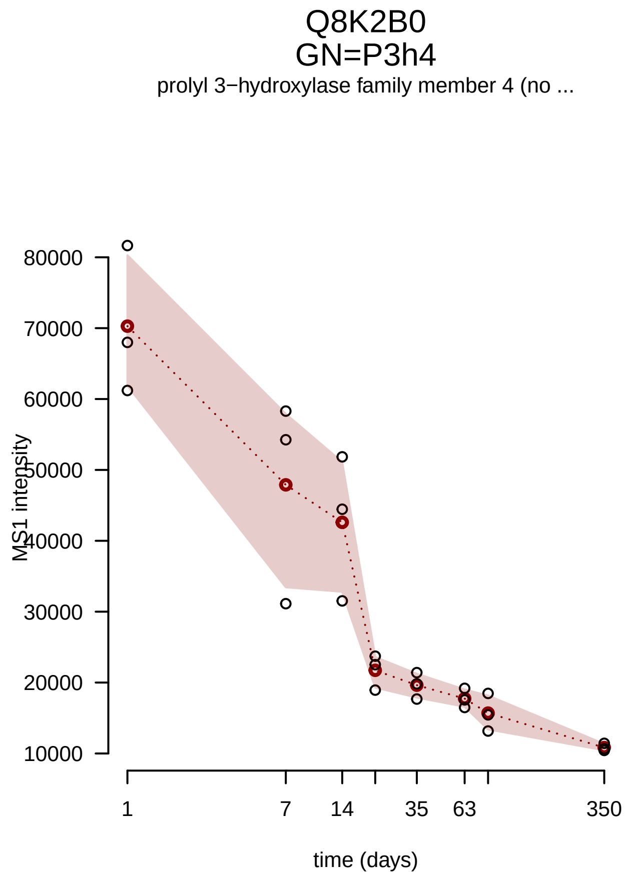
Q8K2B0
GN=P3h4
prolyl 3−hydroxylase family member 4 (no ...
MS1 intensity
80000
70000
60000
50000
40000
30000
20000
10000
1
7
14
35
63
350
time (days)
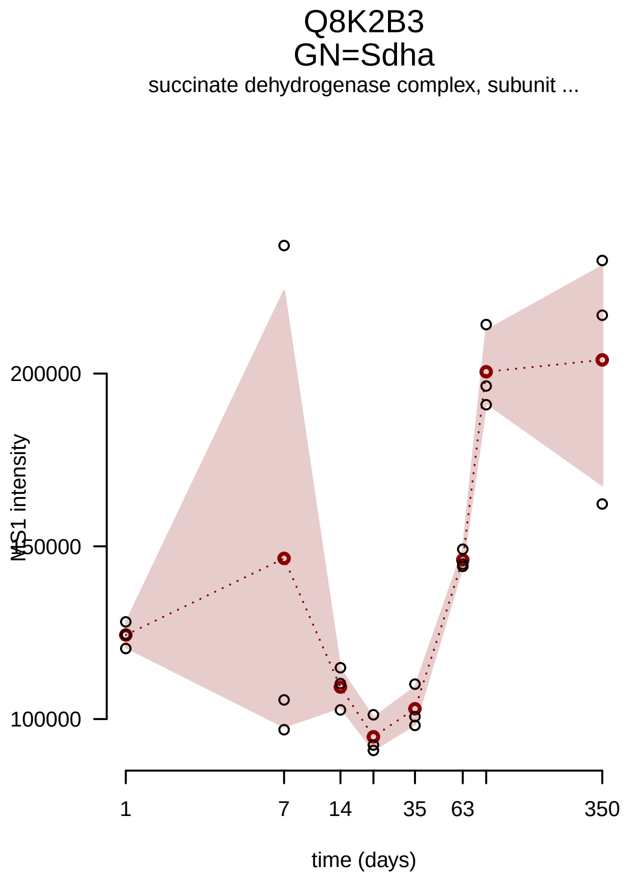
Q8K2B3
GN=Sdha
succinate dehydrogenase complex, subunit ...
MS1 intensity
200000
150000
100000
1
7
14
35
63
350
time (days)

Q8K2C6
GN=Sirt5
sirtuin 5

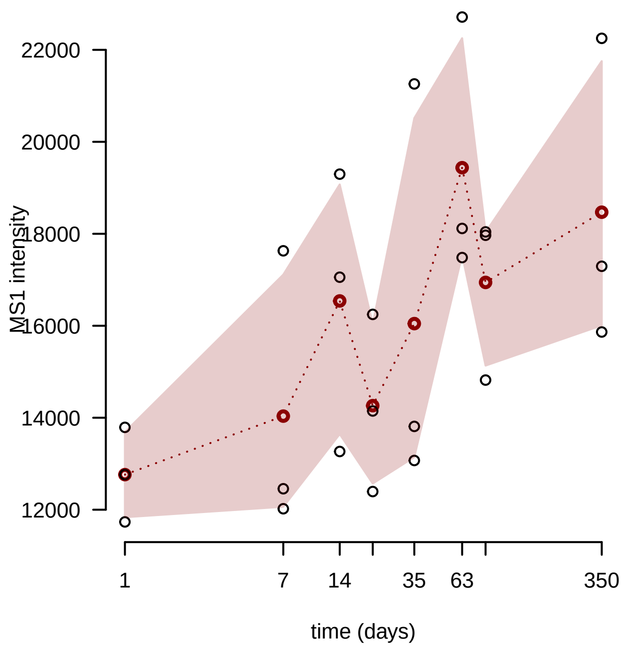

Q8K2C7
GN=Os9
amplified in osteosarcoma

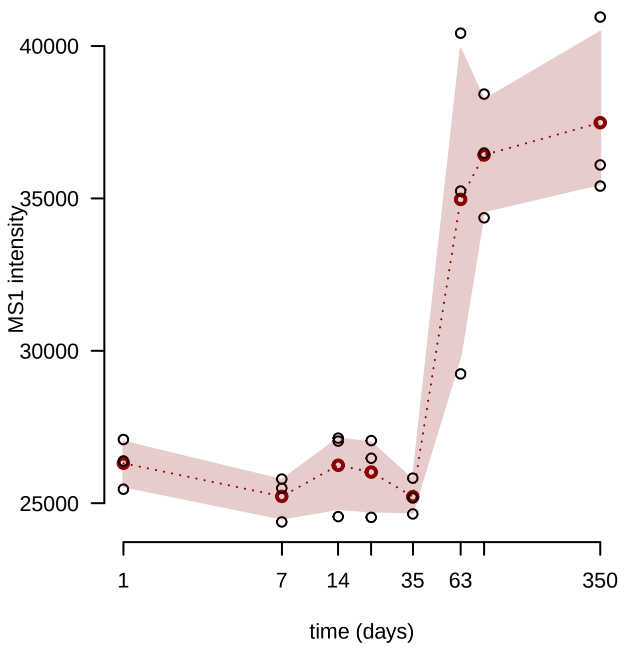

Q8K2C8
GN=Gpat4
glycerol−3−phosphate acyltransferase 4

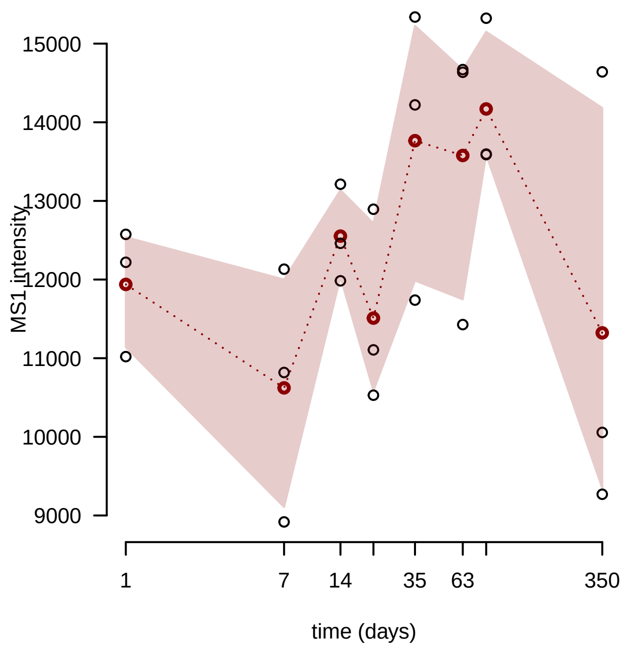

Q8K2C9
GN=Hacd3
3−hydroxyacyl−CoA dehydratase 3

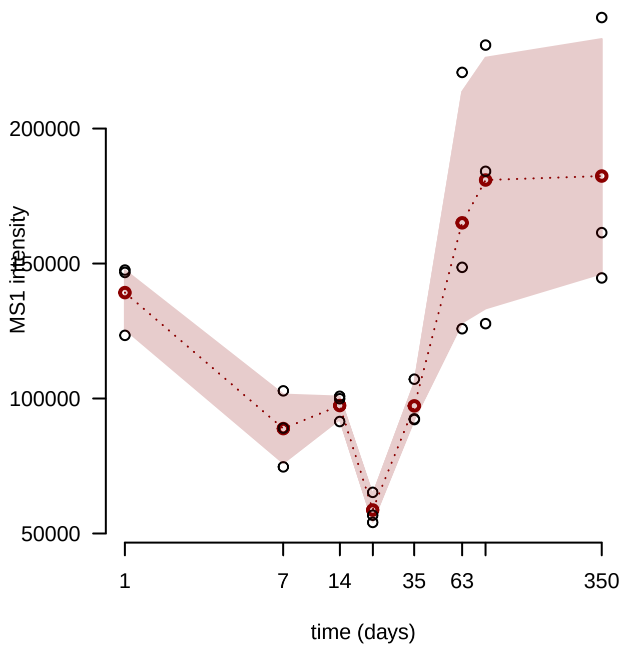

Q8K2D3
GN=Edc3
enhancer of mRNA decapping 3

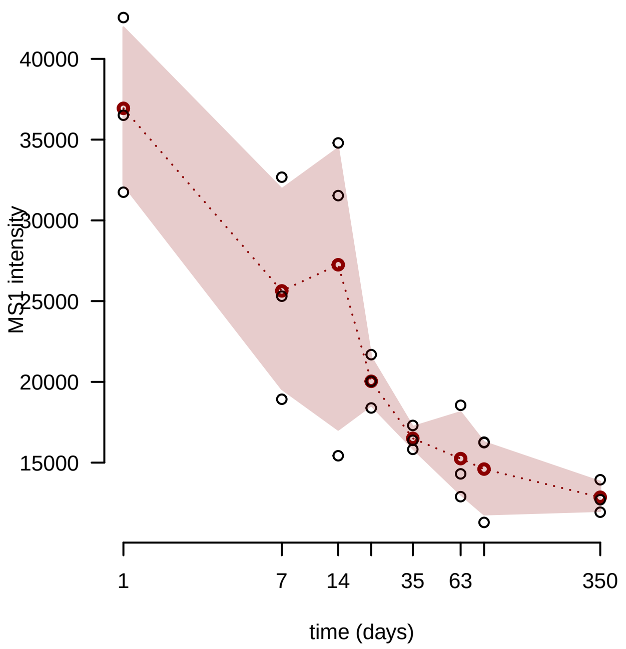

Q8K2D6
GN=Dctd
dCMP deaminase

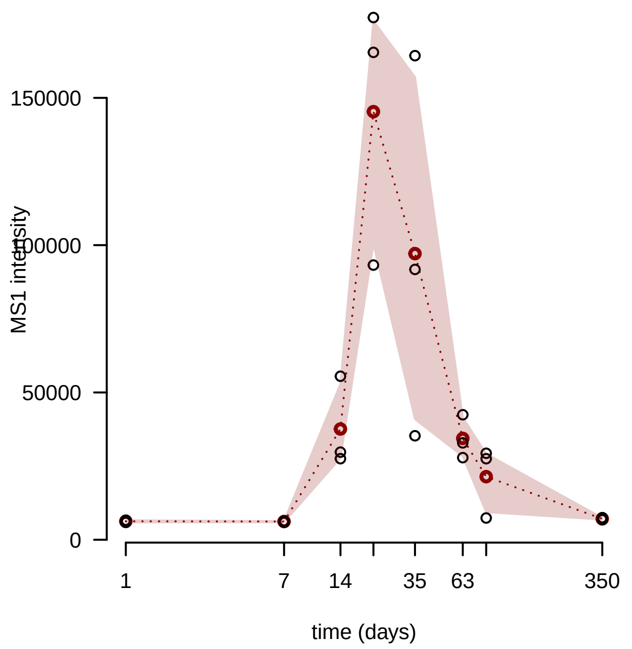

Q8K2F0
GN=Brd3
bromodomain containing 3

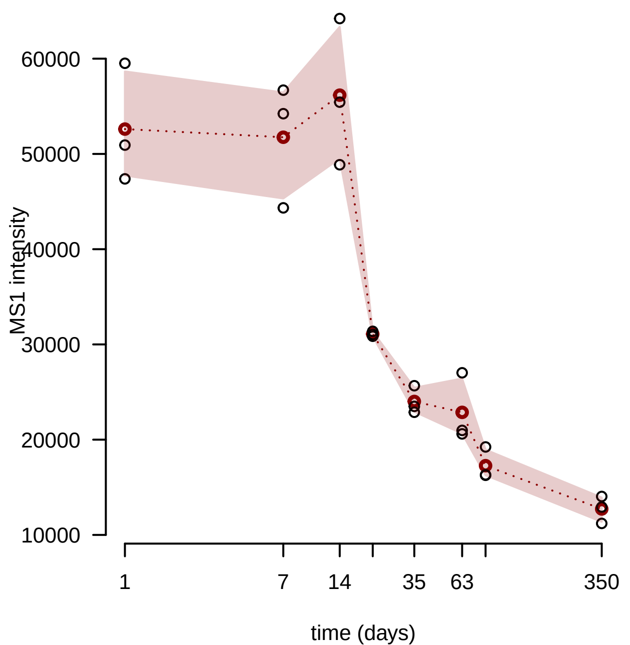

Q8K2F8
GN=Lsm14a
LSM14A mRNA processing body assembly fac ...

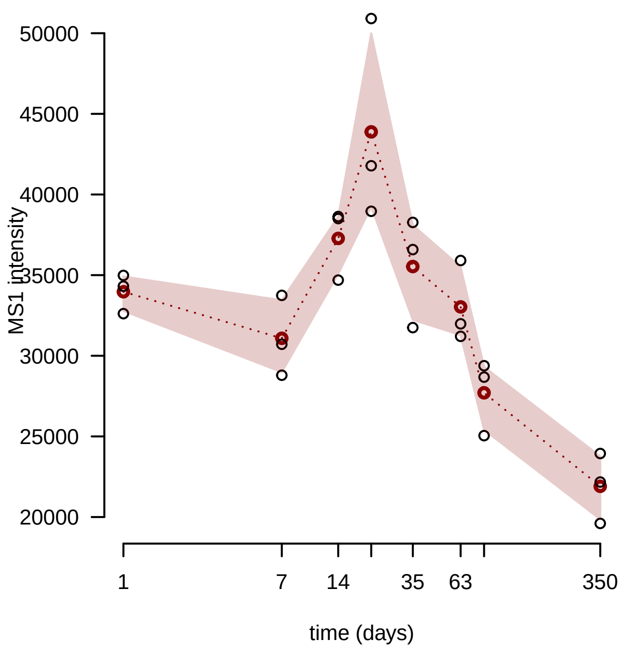

Q8K2G4
GN=Bbs7
Bardet−Biedl syndrome 7 (human)

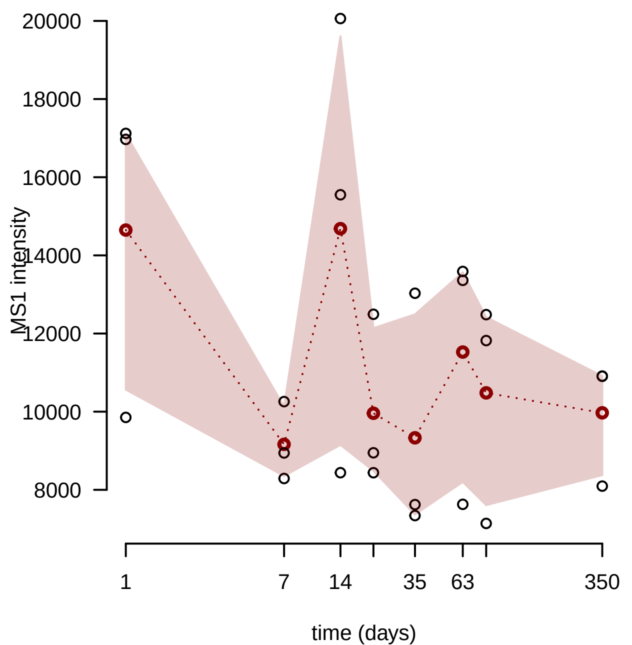

Q8K2H1
GN=Pphln1
periphilin 1

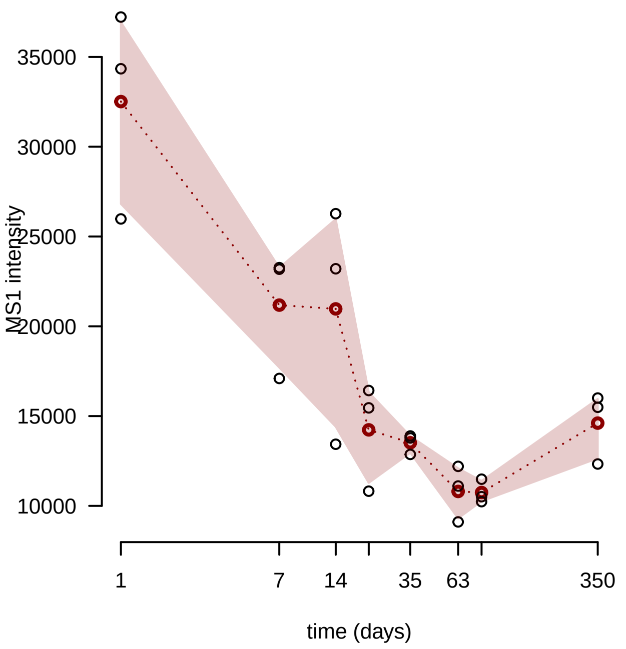

Q8K2H2
GN=Otud6b
OTU domain containing 6B

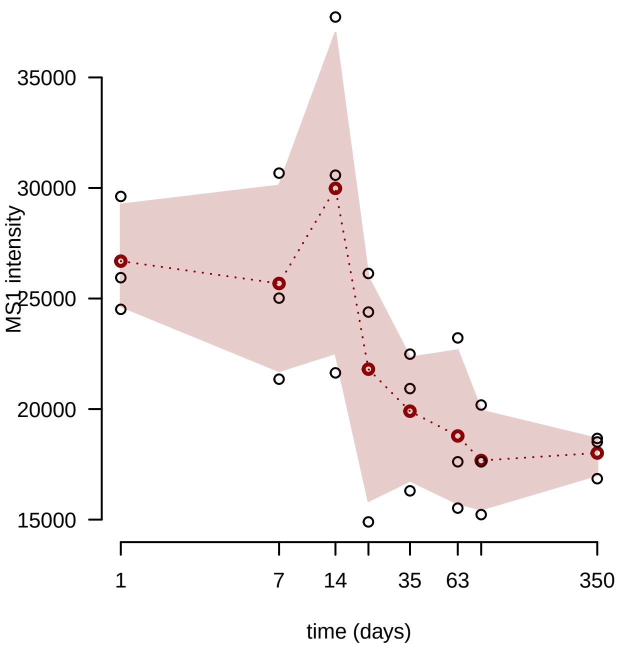

Q8K2H6
GN=Anapc10
anaphase promoting complex subunit 10

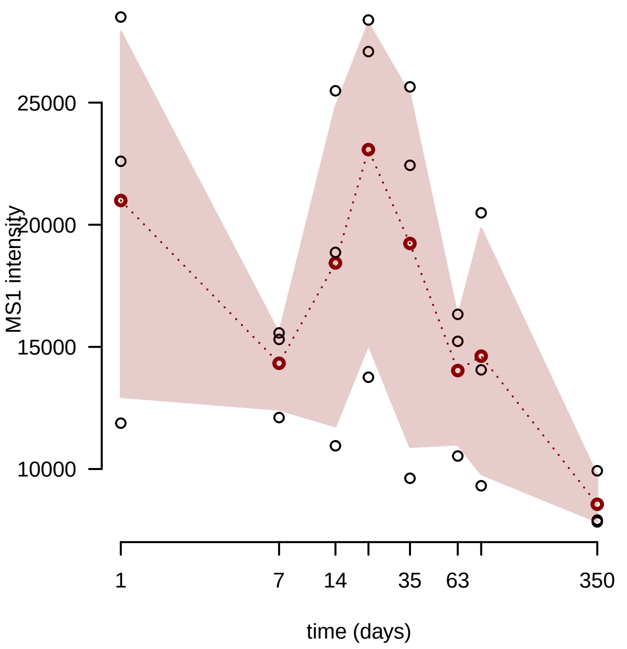

Q8K2I1
GN=Fntb
farnesyltransferase, CAAX box, beta

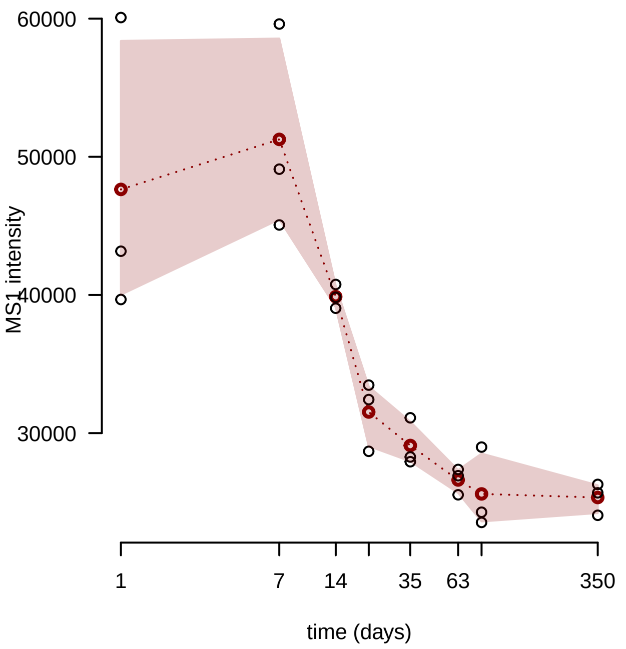

Q8K2I3
GN=Fmo2
flavin containing monooxygenase 2

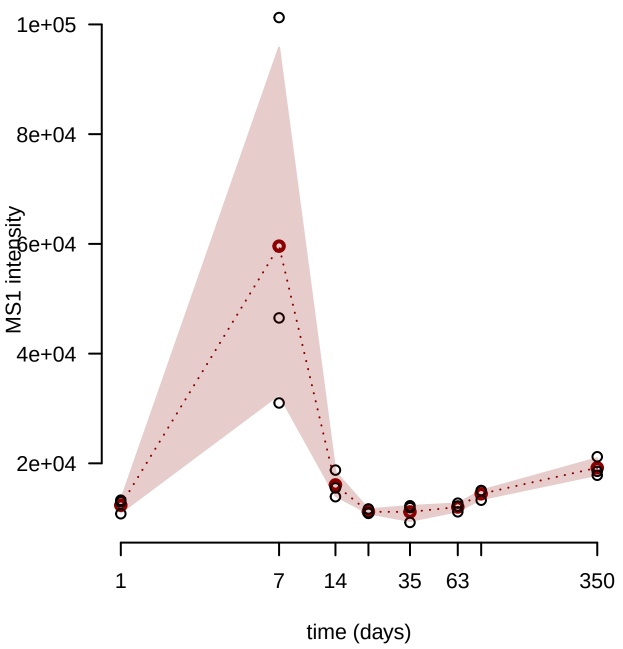

Q8K2I4
GN=Manba
mannosidase, beta A, lysosomal

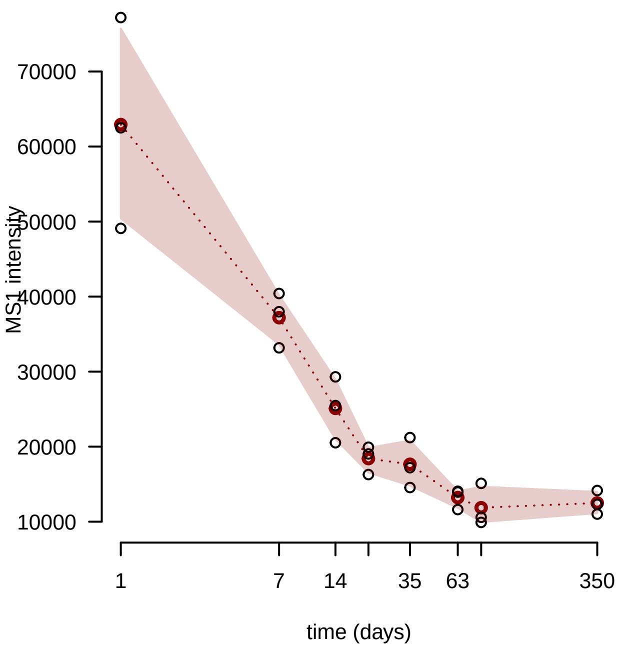

Q8K2I9
GN=Fbh1
F−box DNA helicase 1

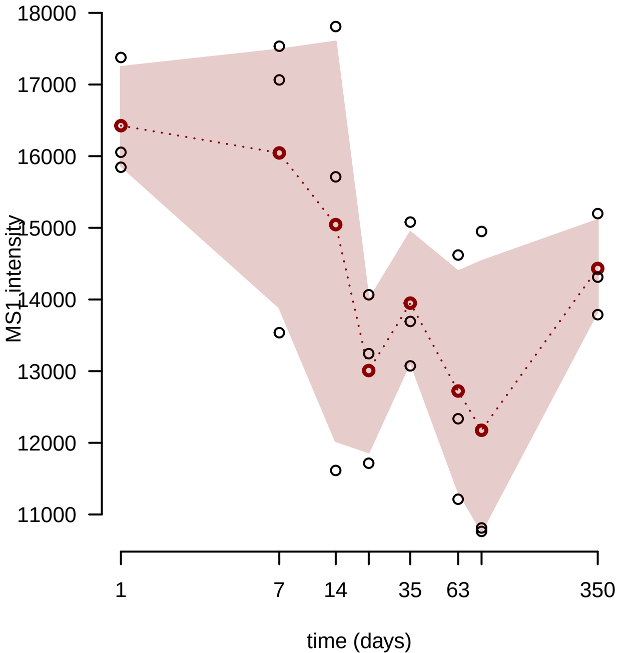

Q8K2J0
GN=Plcd3
phospholipase C, delta 3

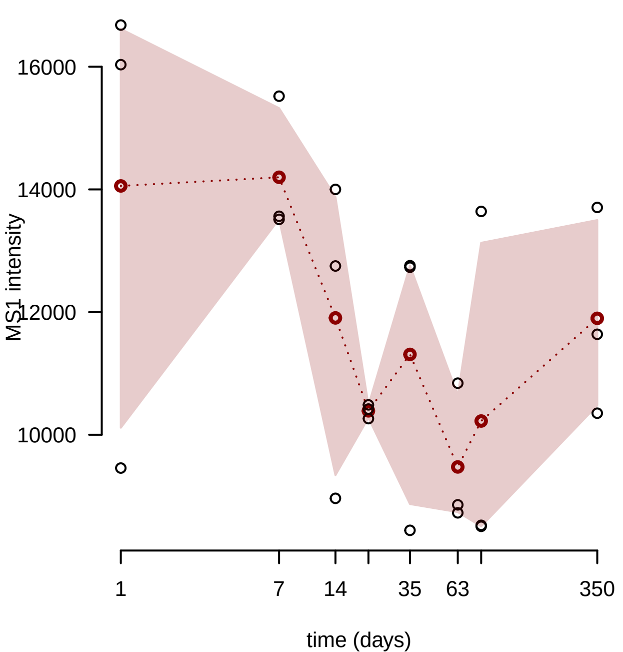

Q8K2K6
GN=Agfg1
ArfGAP with FG repeats 1

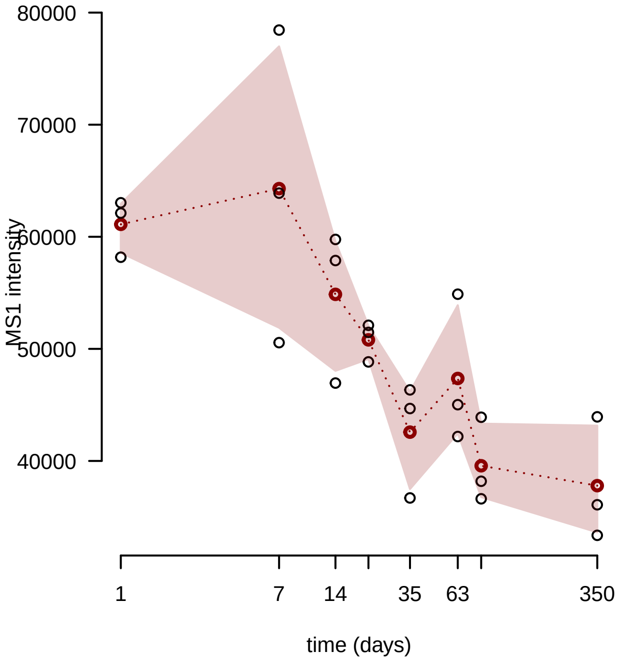

Q8K2L8
GN=Trappc12
trafficking protein particle complex 12

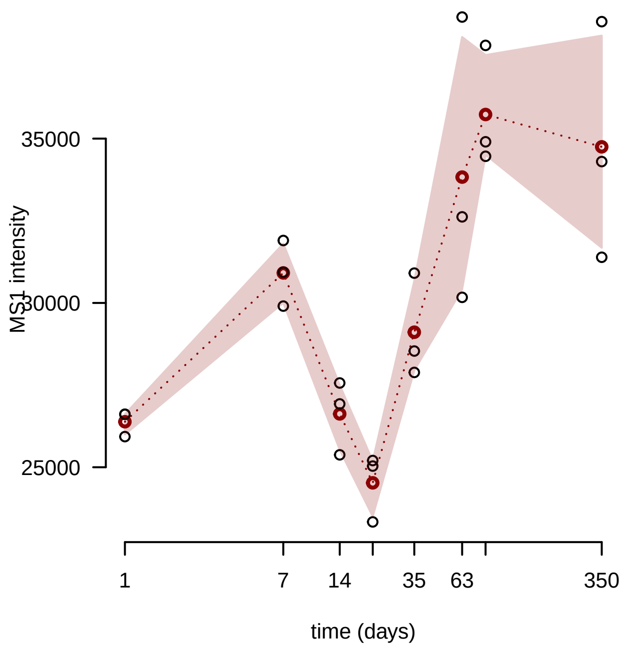

Q8K2M0
GN=Mrpl38
mitochondrial ribosomal protein L38

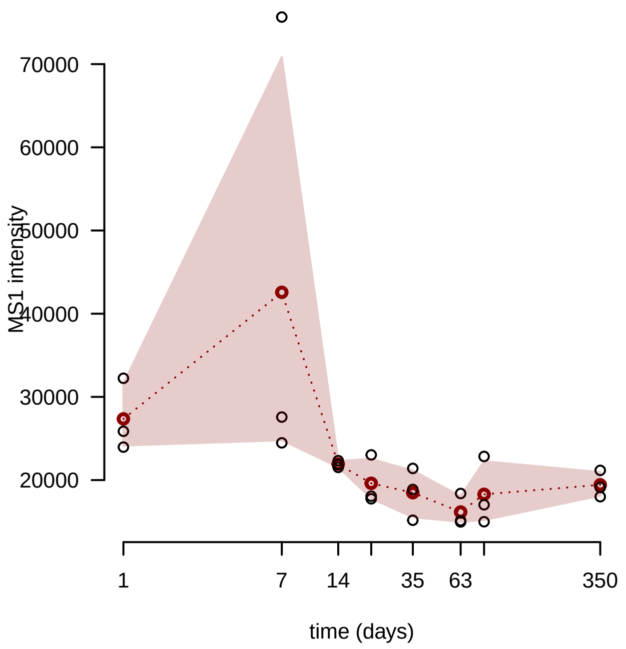

Q8K2Q0
GN=Commd9
COMM domain containing 9

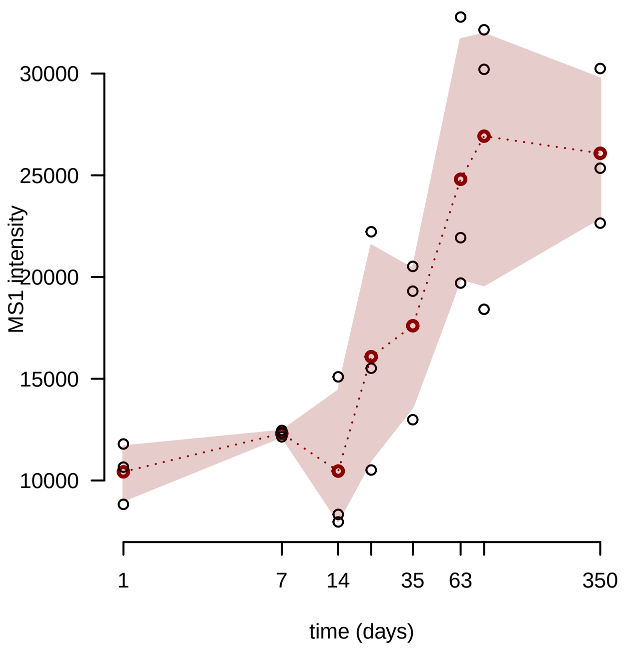

Q8K2Q5
GN=Chchd7
coiled−coil−helix−coiled−coil−helix doma ...

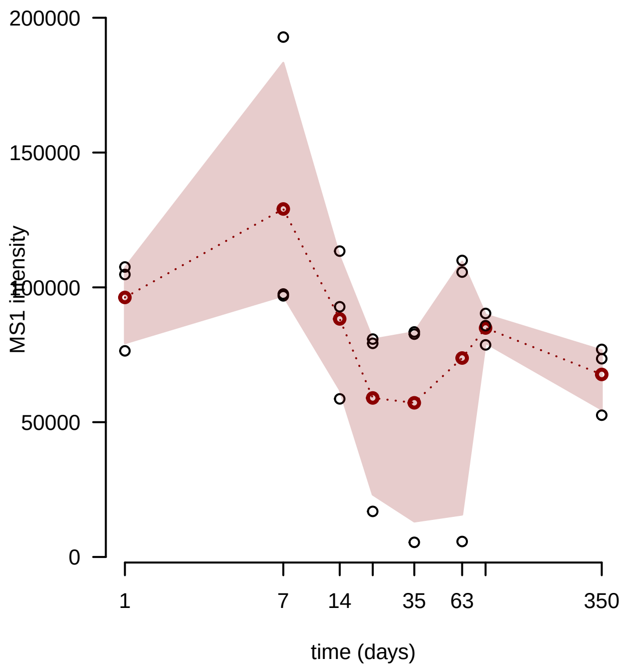

Q8K2Q7
GN=Brox
BRO1 domain and CAAX motif containing

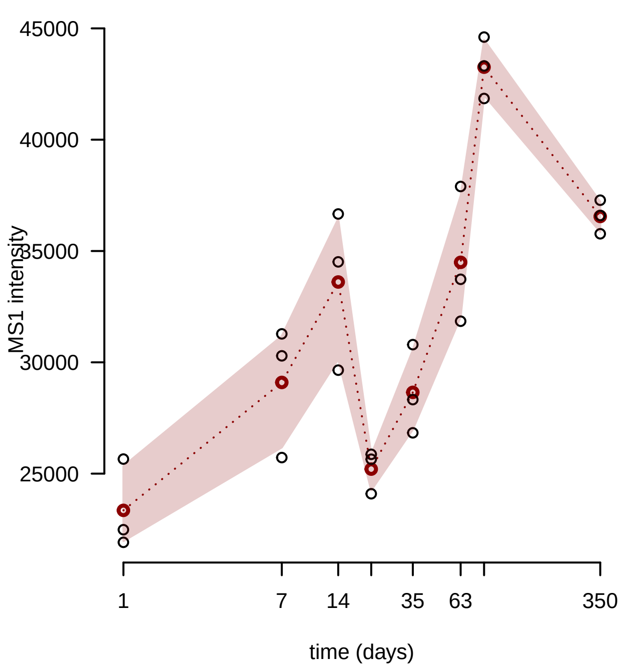

Q8K2Q9
GN=Shtn1
shootin 1

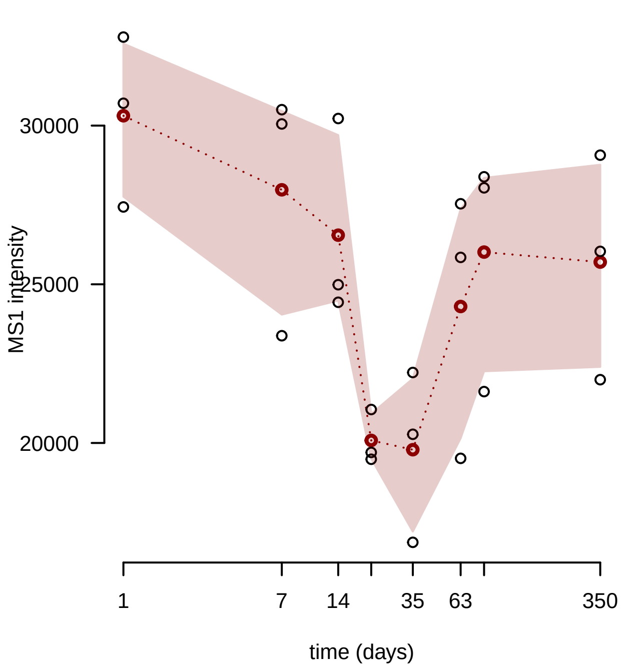

Q8K2R5
GN=Znf668

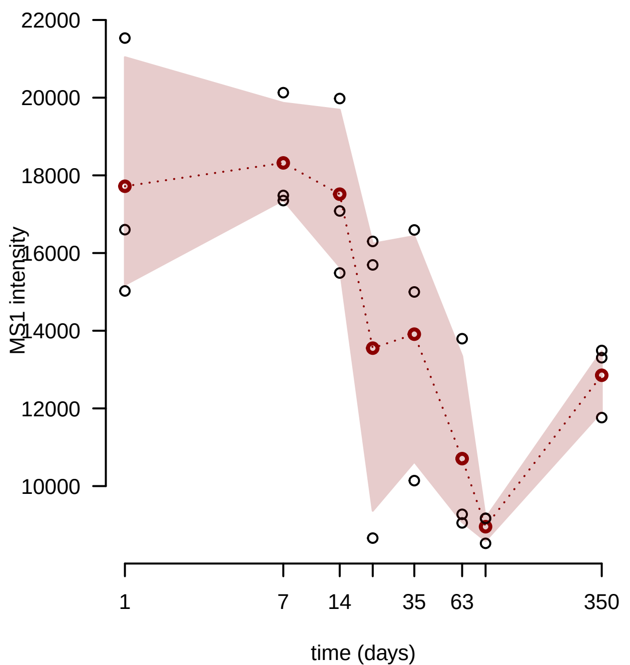

Q8K2T1
GN=Nmral1
NmrA−like family domain containing 1

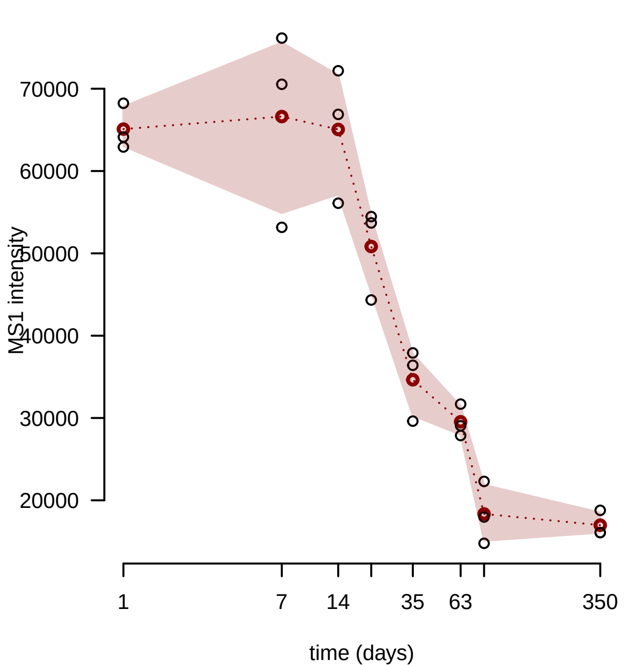

Q8K2T4
GN=Uqcc3
ubiquinol−cytochrome c reductase complex ...

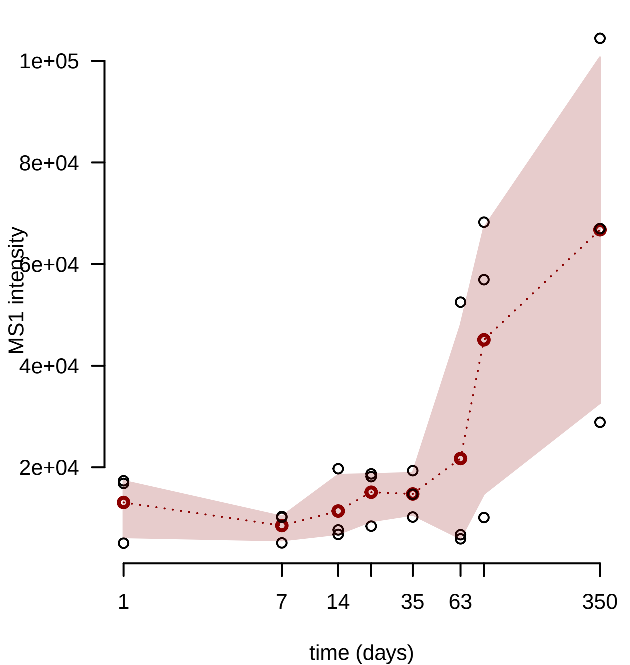

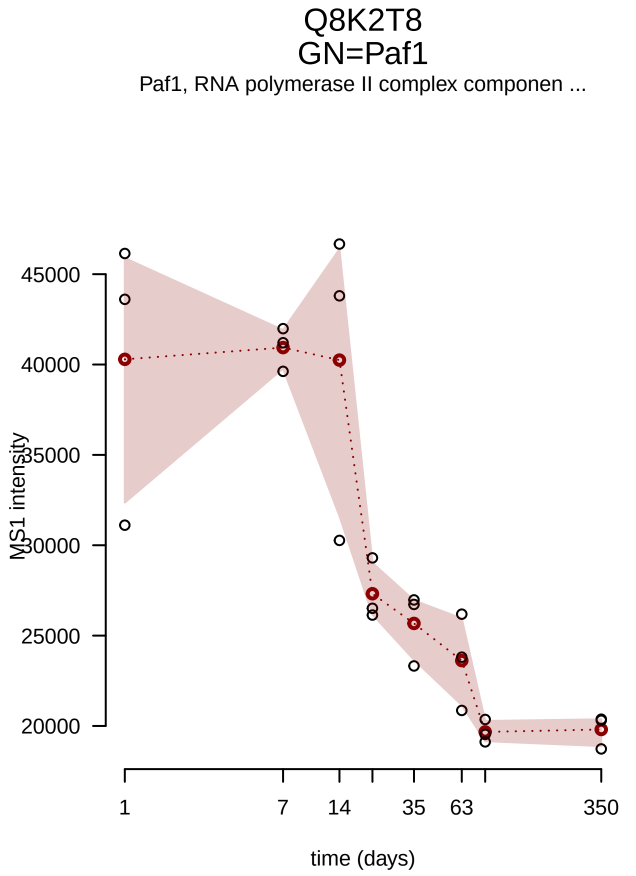
Q8K2T8
GN=Paf1
Paf1, RNA polymerase II complex componen ...
MS1 intensity
45000
40000
35000
30000
25000
20000
1
7
14
35
63
350
time (days)

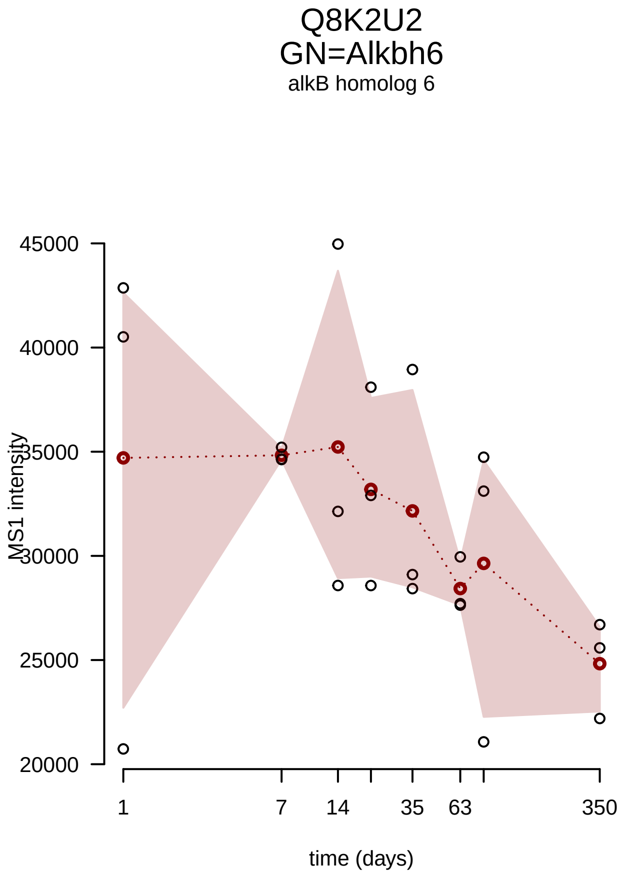
Q8K2U2
GN=Alkbh6
alkB homolog 6
MS1 intensity
45000
40000
35000
30000
25000
20000
1
7
14
35
63
350
time (days)

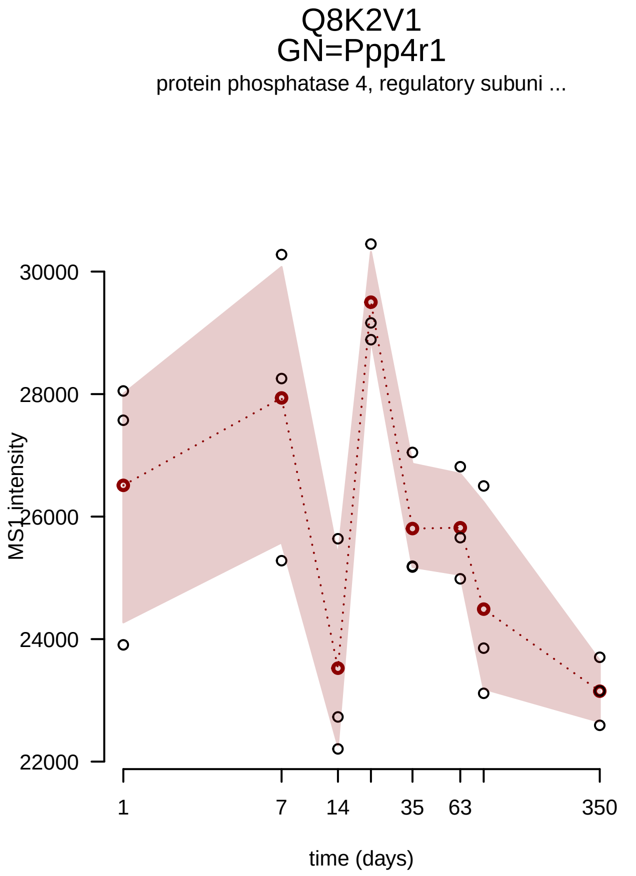
Q8K2V1
GN=Ppp4r1
protein phosphatase 4, regulatory subuni ...
MS1 intensity
30000
28000
26000
24000
22000
1
7
14
35
63
350
time (days)

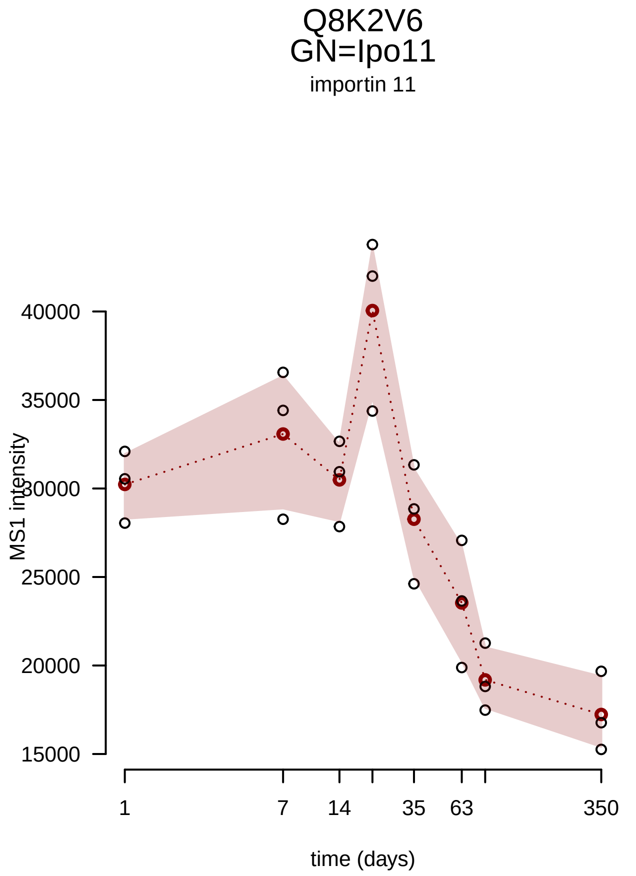
Q8K2V6
GN=Ipo11
importin 11
MS1 intensity
40000
35000
30000
25000
20000
15000
1
7
14
35
63
350
time (days)

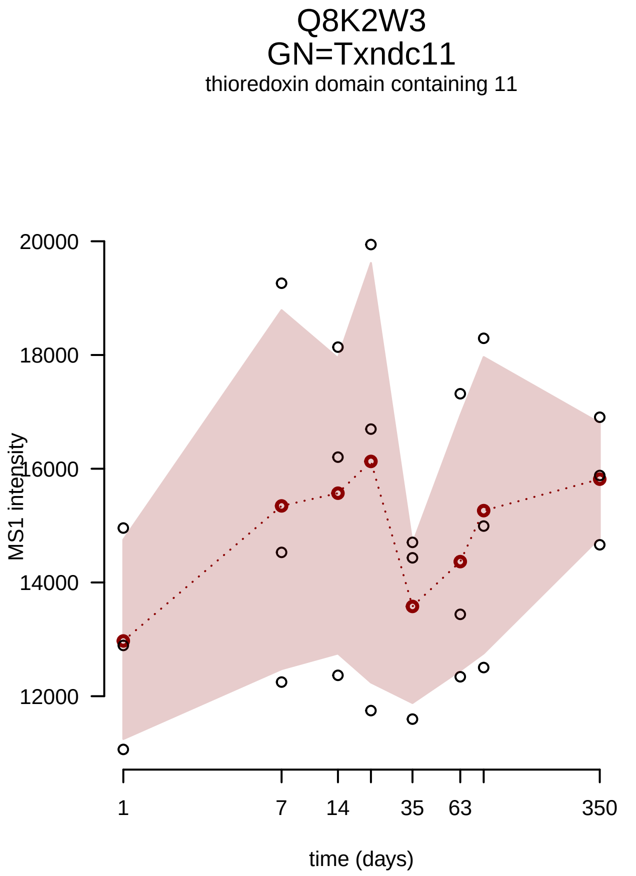
Q8K2W3
GN=Txndc11
thioredoxin domain containing 11
MS1 intensity
20000
18000
16000
14000
12000
1
7
14
35
63
350
time (days)

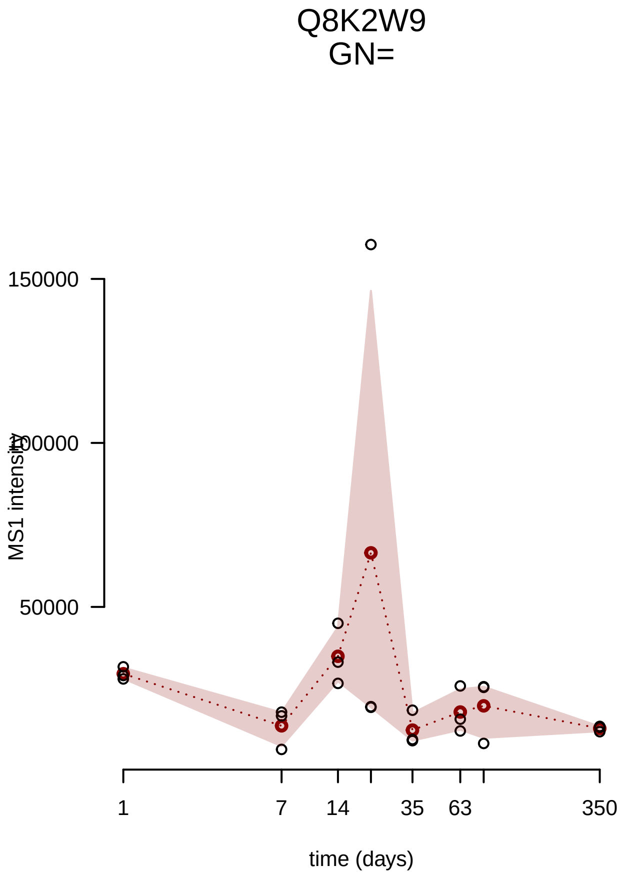
Q8K2W9
GN=
MS1 intensity
150000
100000
50000
1
7
14
35
63
350
time (days)

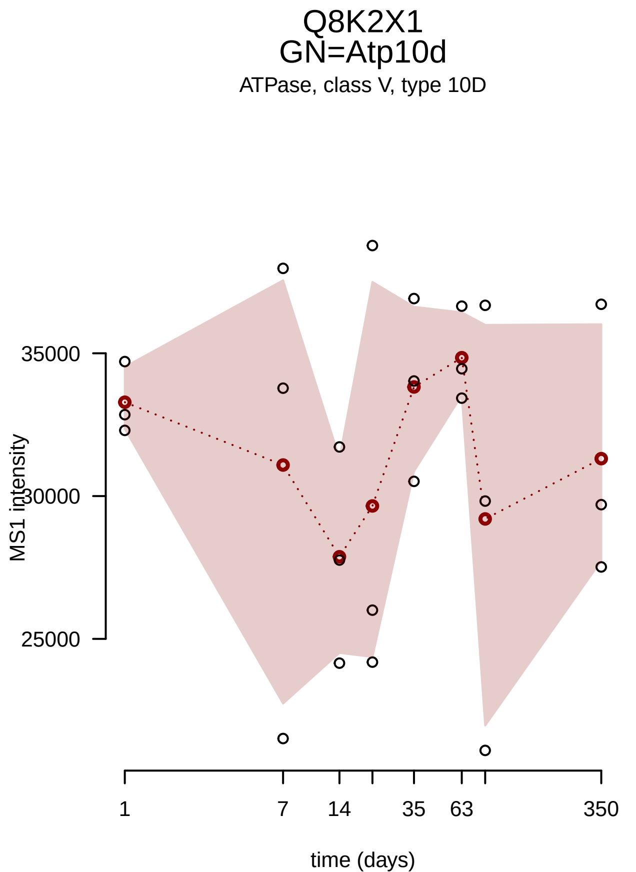
Q8K2X1
GN=Atp10d
ATPase, class V, type 10D
MS1 intensity
35000
30000
25000
1
7
14
35
63
350
time (days)

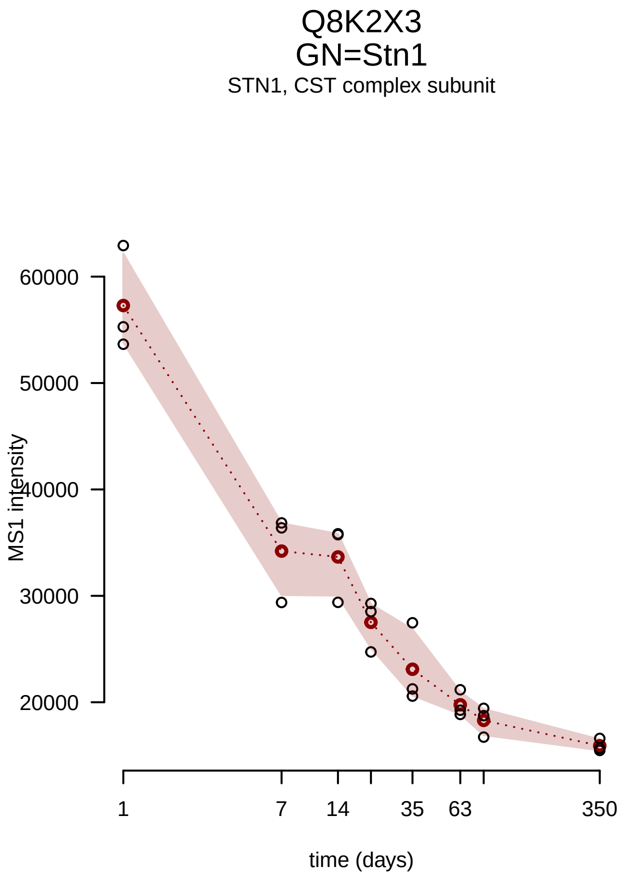
Q8K2X3
GN=Stn1
STN1, CST complex subunit
MS1 intensity
60000
50000
40000
30000
20000
1
7
14
35
63
350
time (days)

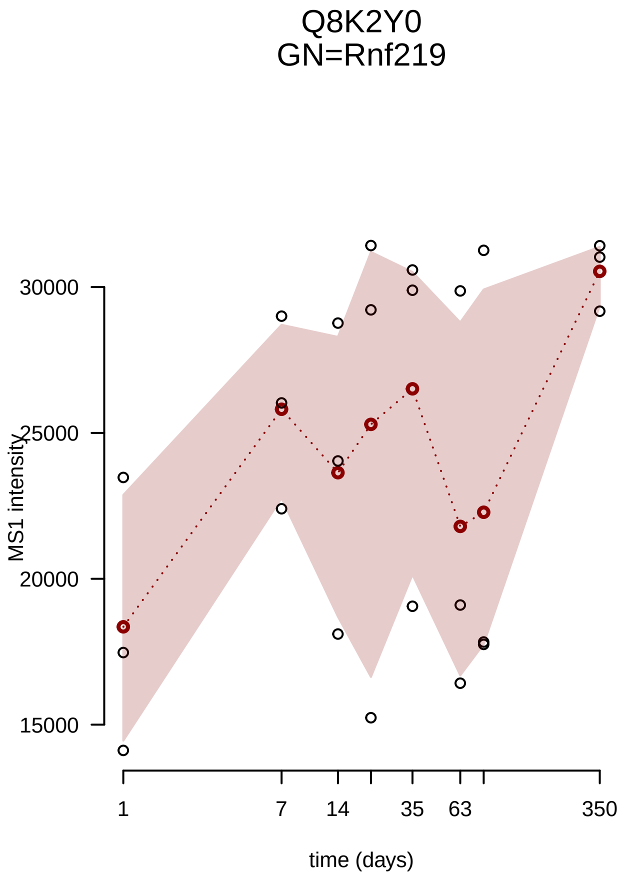
Q8K2Y0
GN=Rnf219
MS1 intensity
30000
25000
20000
15000
1
7
14
35
63
350
time (days)

Q8K2Y3
GN=Eva1b
eva−1 homolog B (C. elegans)

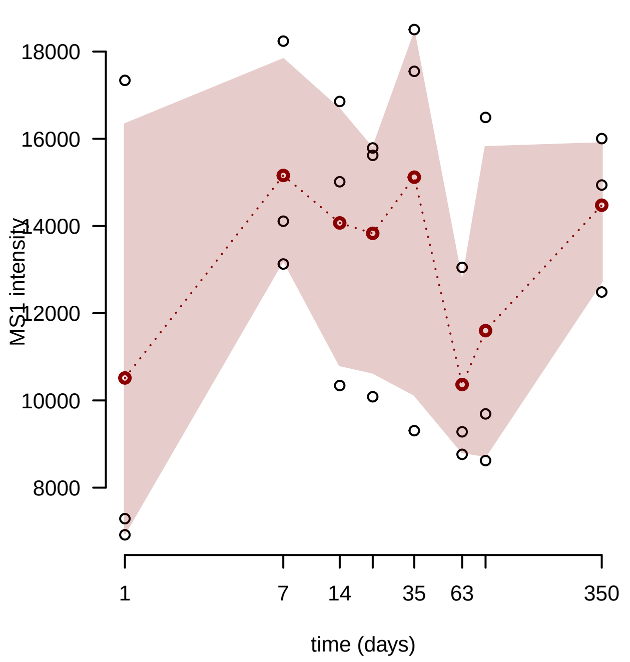

Q8K2Y7
GN=Mrpl47
mitochondrial ribosomal protein L47

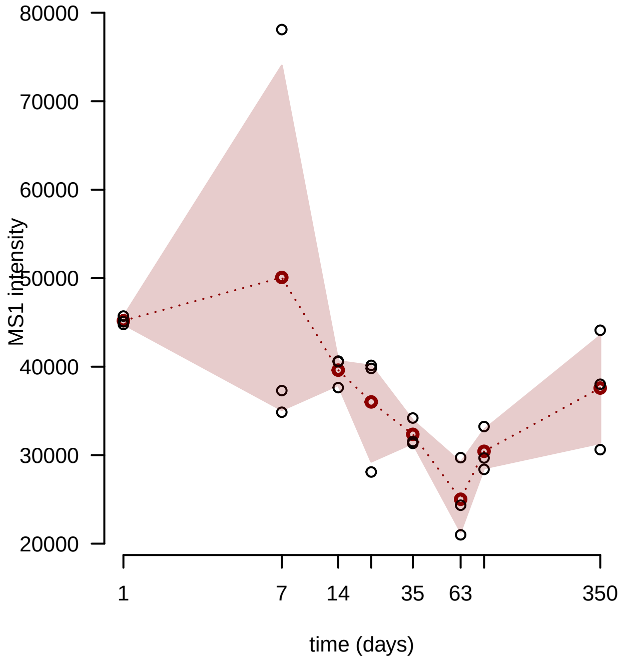

Q8K2Y9
GN=Ccm2
cerebral cavernous malformation 2

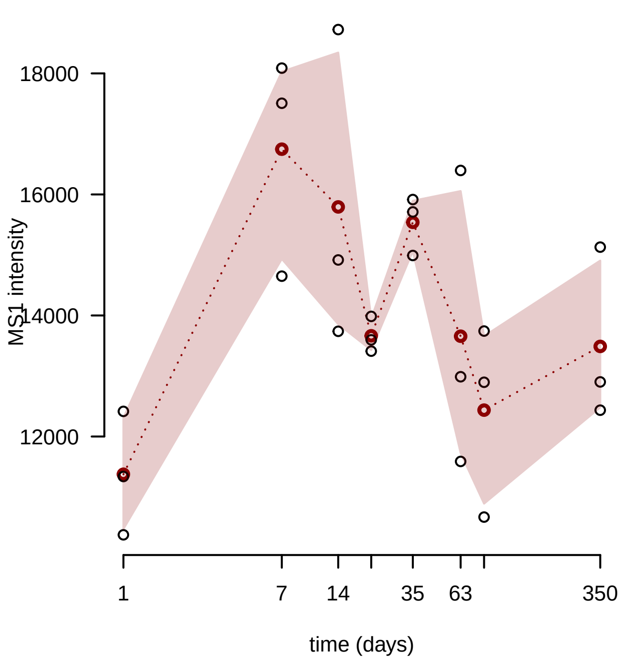

Q8K2Z2
GN=Prpf39
pre−mRNA processing factor 39

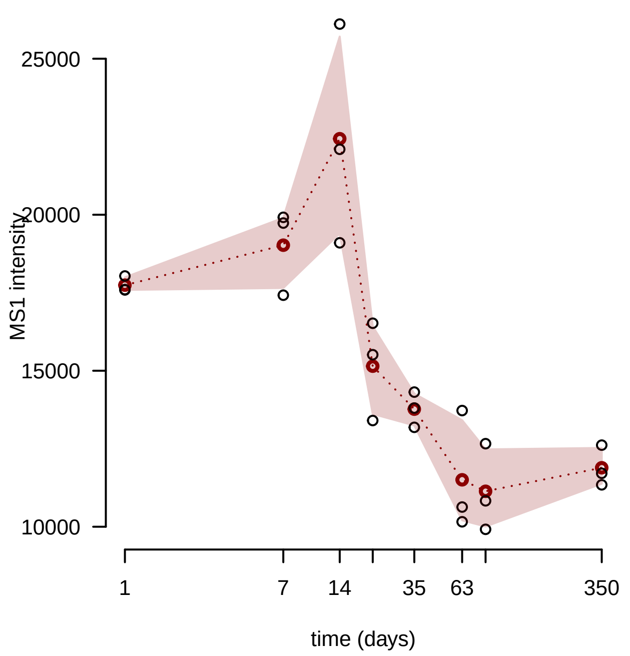

Q8K2Z4
GN=Ncapd2
non−SMC condensin I complex, subunit D2

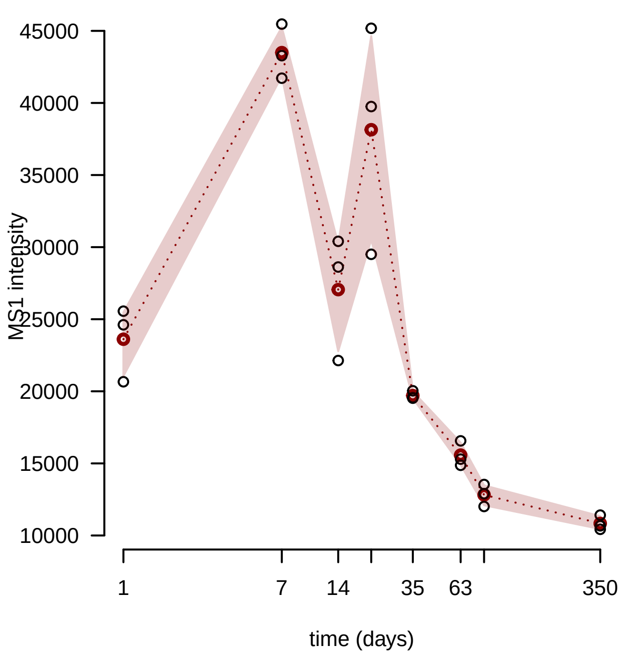

Q8K2Z8
GN=Ube2q2
ubiquitin−conjugating enzyme E2Q family ...

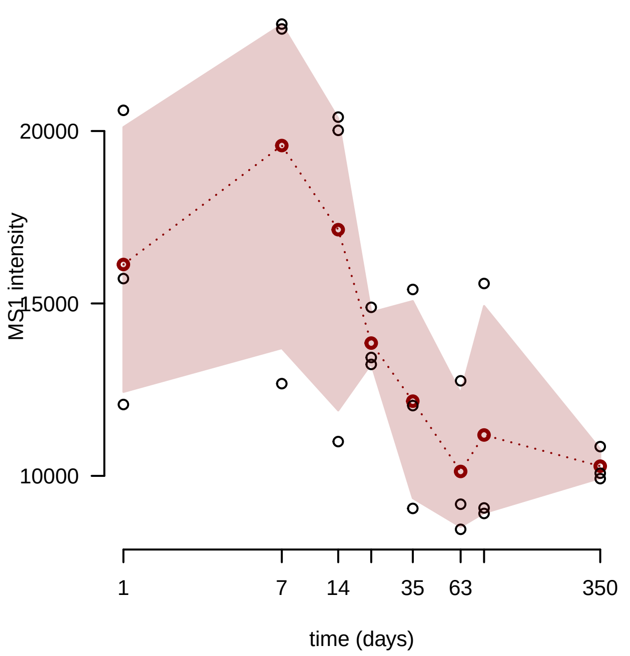

Q8K301
GN=Ddx52
DExD box helicase 52

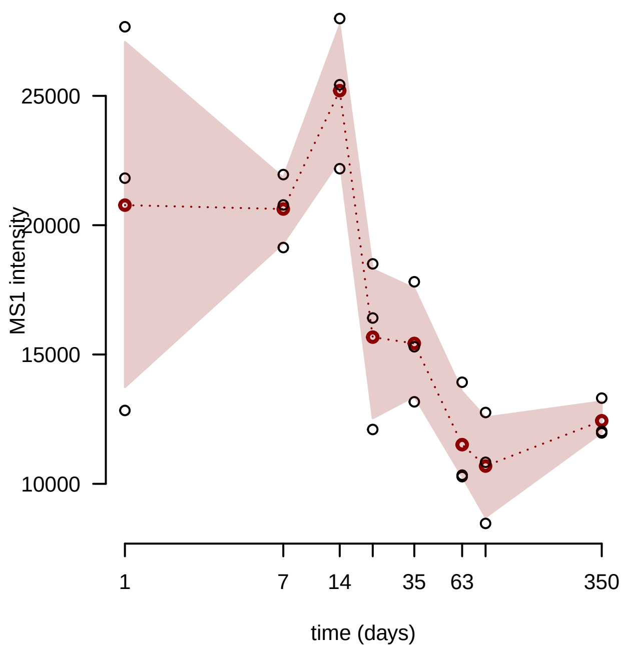

Q8K305
GN=Nsl1
NSL1, MIS12 kinetochore complex componen ...

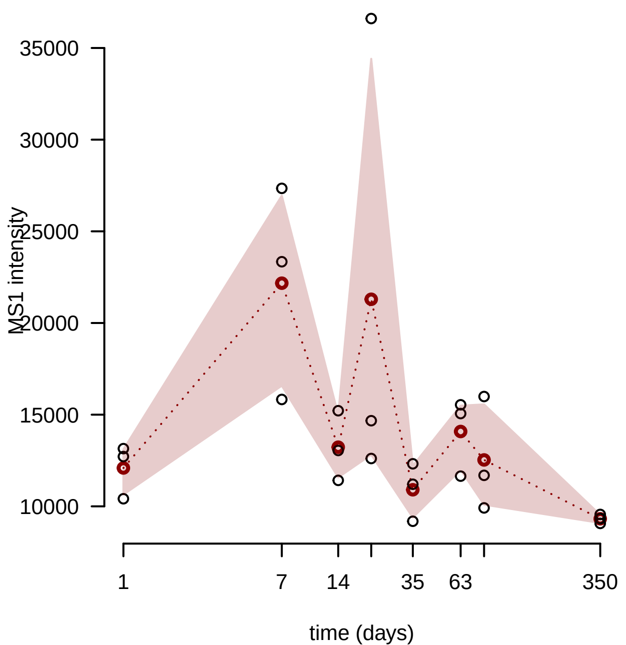

Q8K310
GN=Matr3
matrin 3

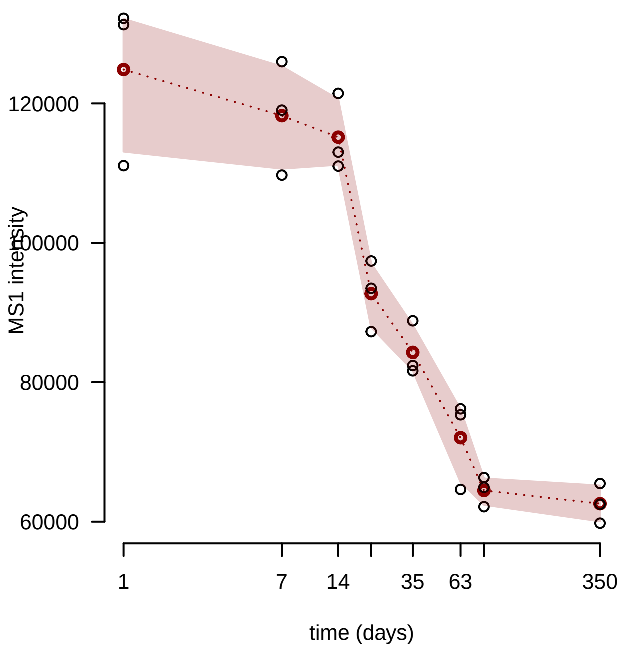

Q8K327
GN=Champ1
chromosome alignment maintaining phospho ...

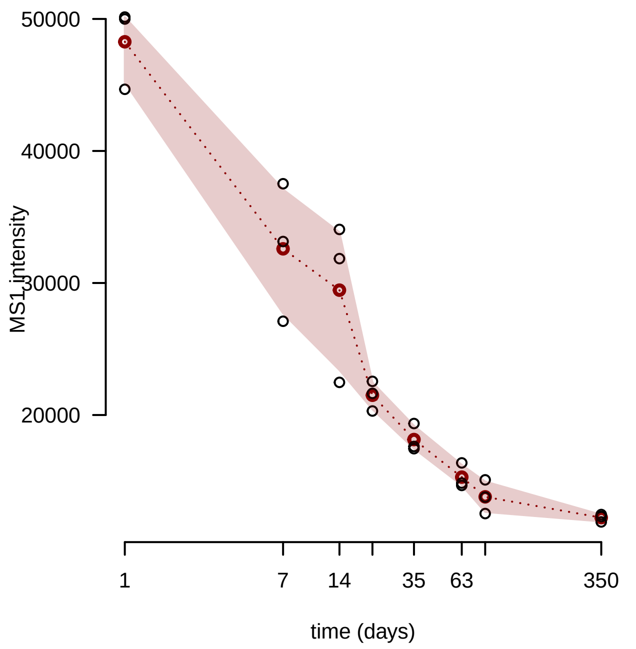

Q8K330
GN=Ssh3
slingshot protein phosphatase 3

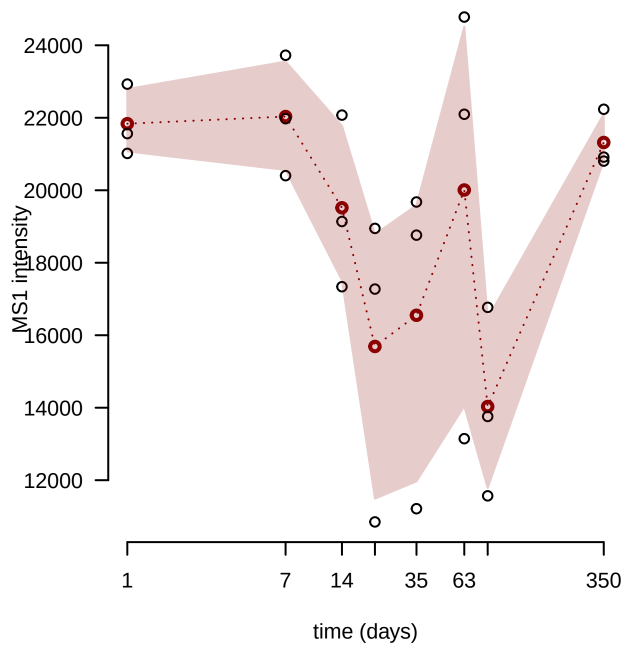

Q8K337
GN=Inpp5b
inositol polyphosphate−5−phosphatase B

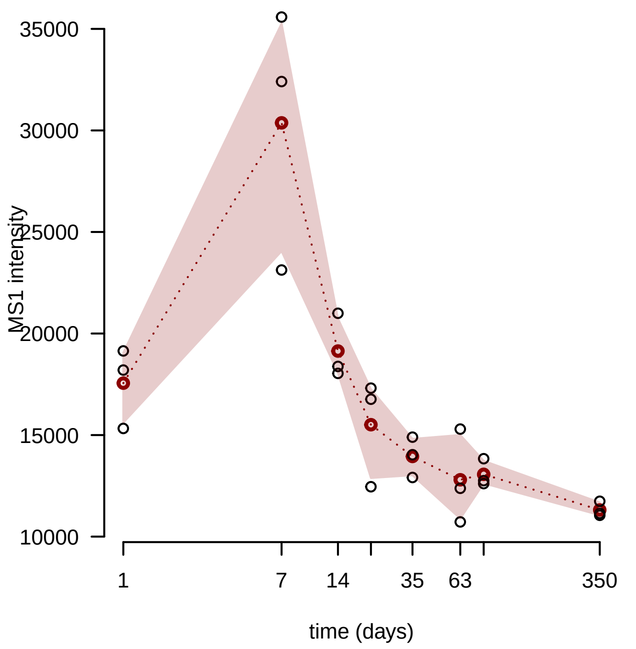

Q8K339
GN=Kin
Kin17 DNA and RNA binding protein

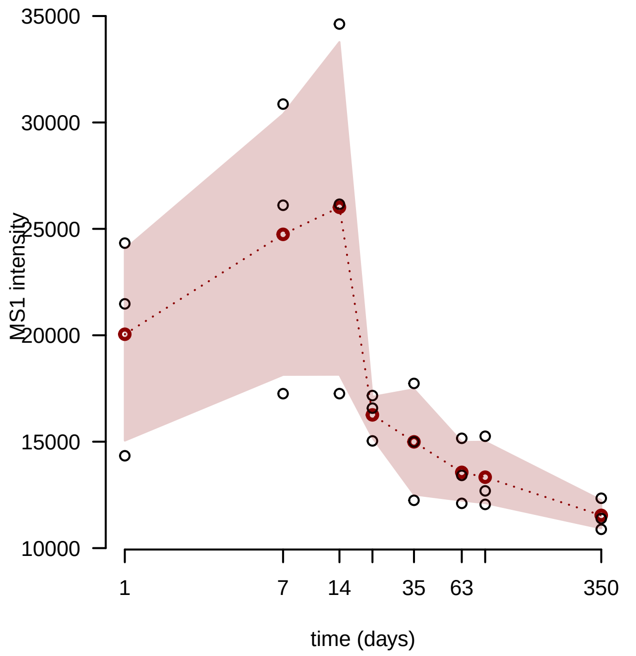

Q8K341
GN=Atat1
alpha tubulin acetyltransferase 1

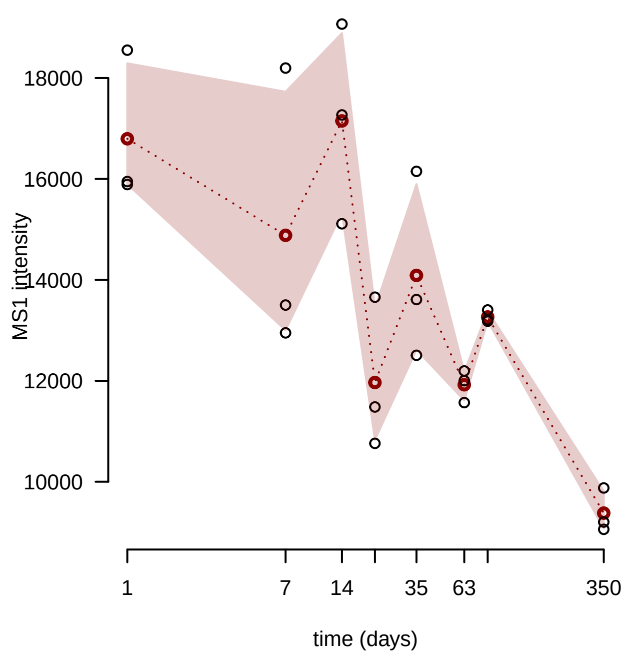

Q8K353
GN=Cystm1
cysteine−rich transmembrane module conta ...

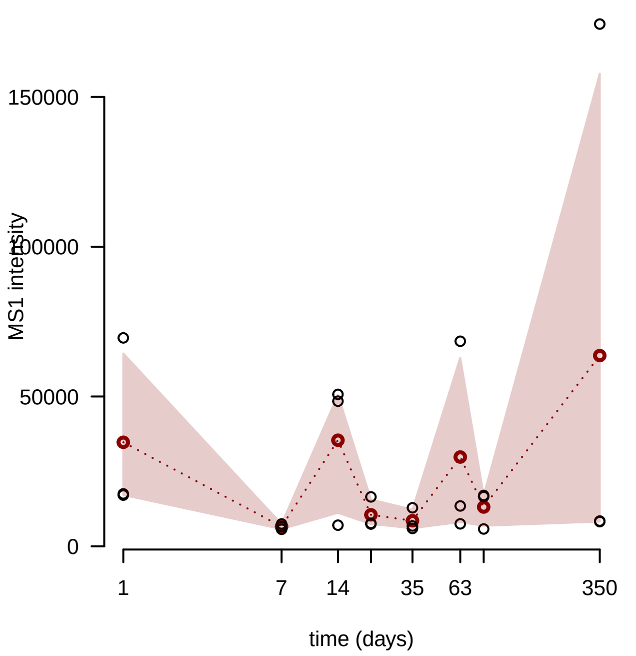

Q8K354
GN=Cbr3
carbonyl reductase 3

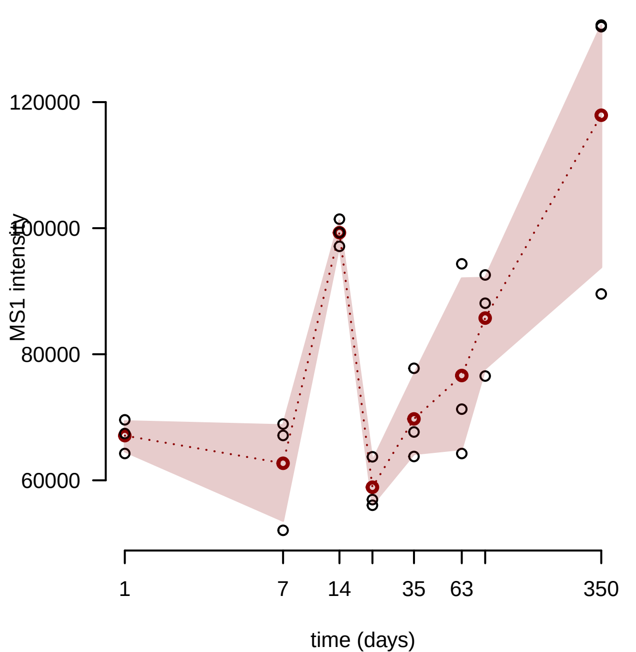

Q8K358
GN=Pigu
phosphatidylinositol glycan anchor biosy ...

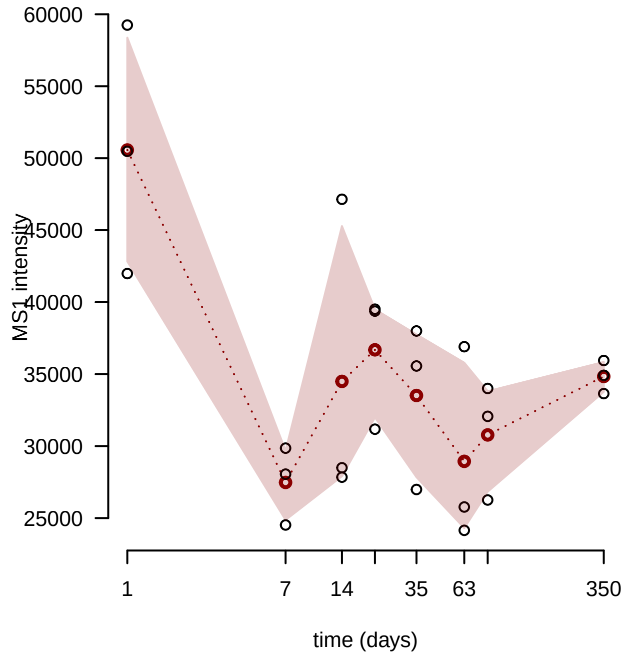

Q8K363
GN=Ddx18
DEAD box helicase 18

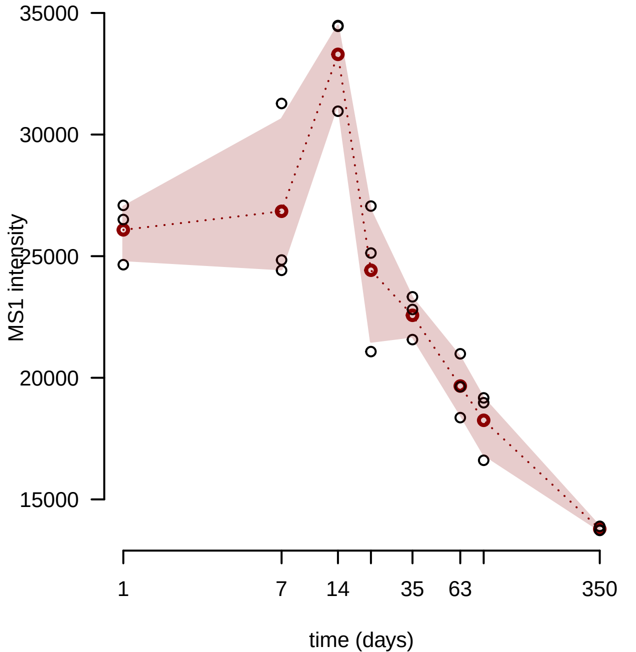

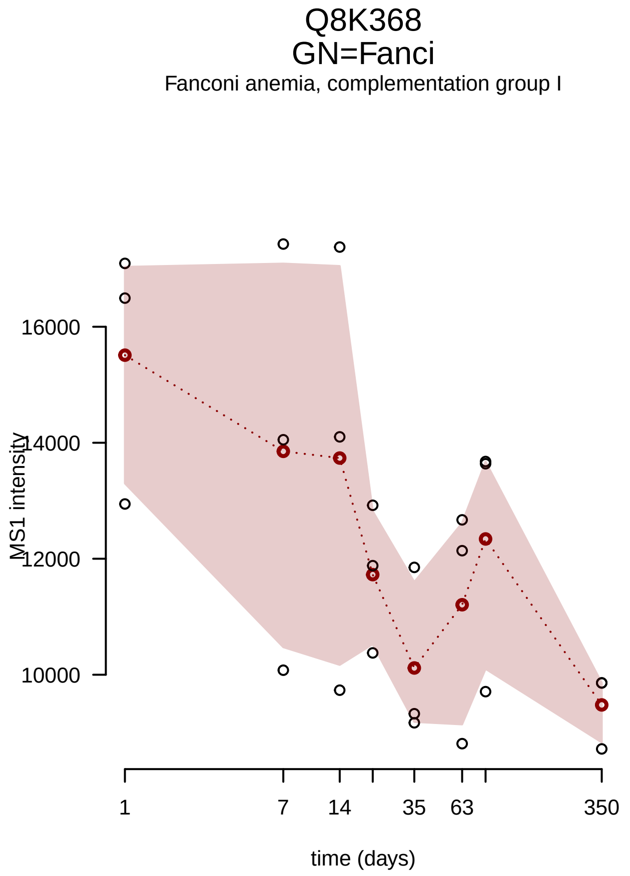
Q8K368
GN=Fanci
Fanconi anemia, complementation group I
MS1 intensity
time (days)

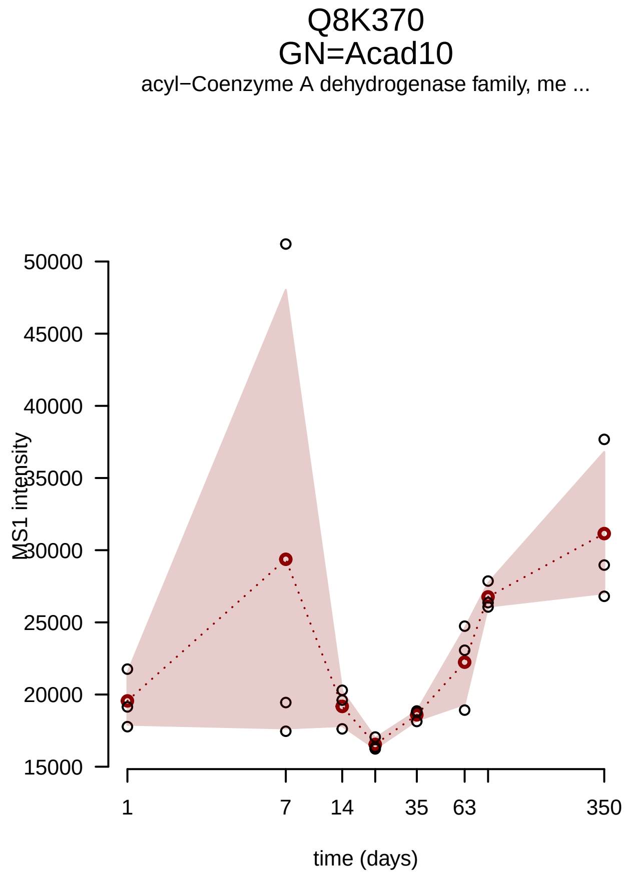
Q8K370
GN=Acad10
acyl−Coenzyme A dehydrogenase family, me ...
MS1 intensity
time (days)

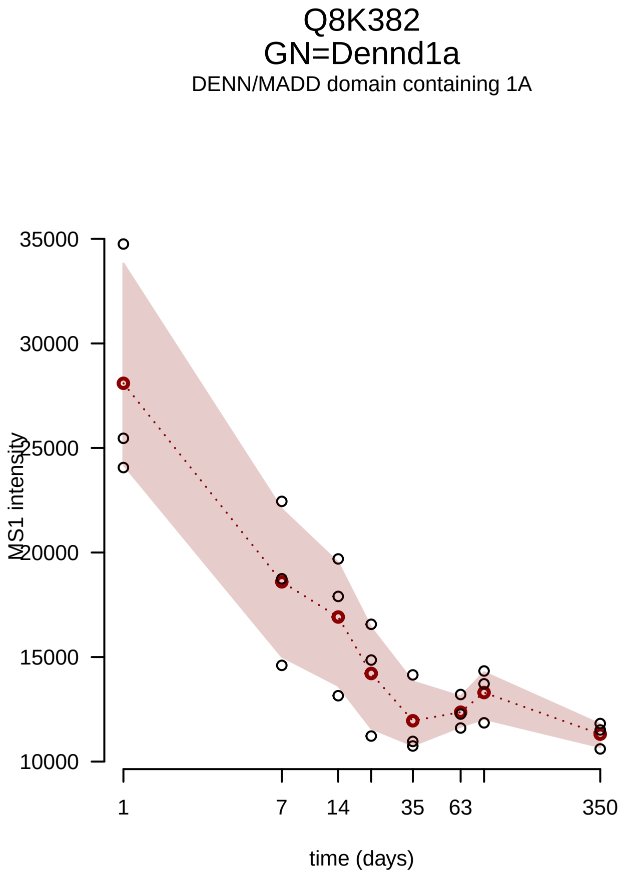
Q8K382
GN=Dennd1a
DENN/MADD domain containing 1A
MS1 intensity
time (days)

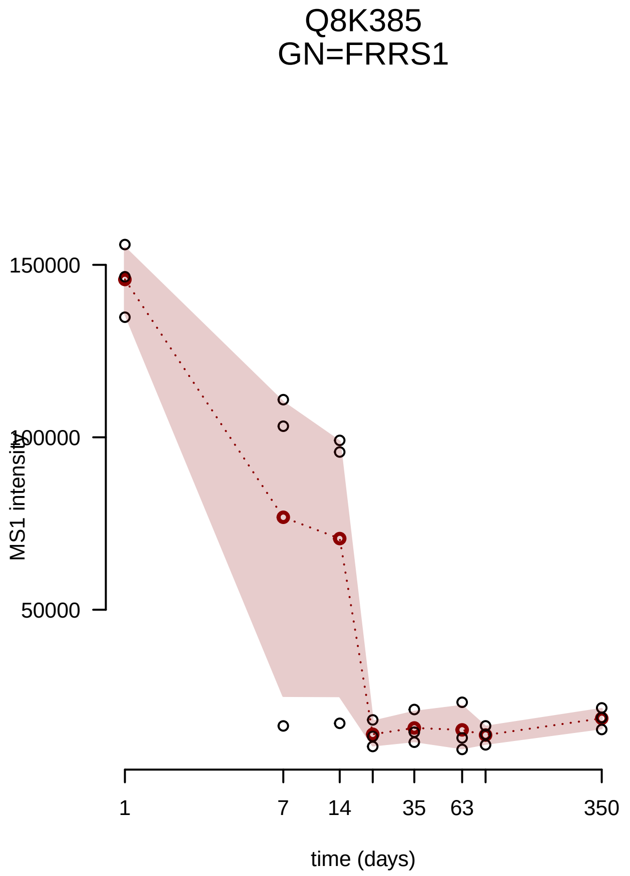
Q8K385
GN=FRRS1
MS1 intensity
time (days)

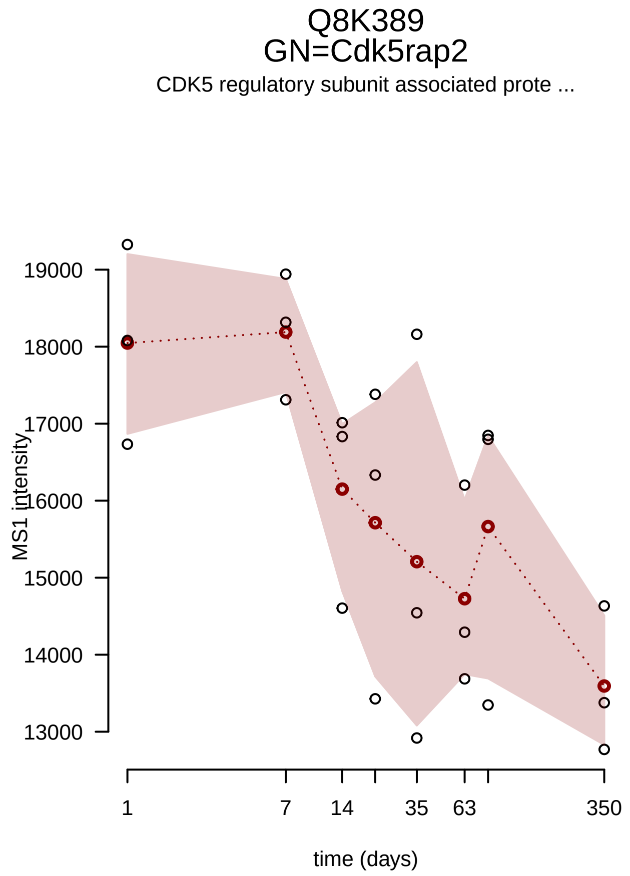
Q8K389
GN=Cdk5rap2
CDK5 regulatory subunit associated prote ...
MS1 intensity
time (days)

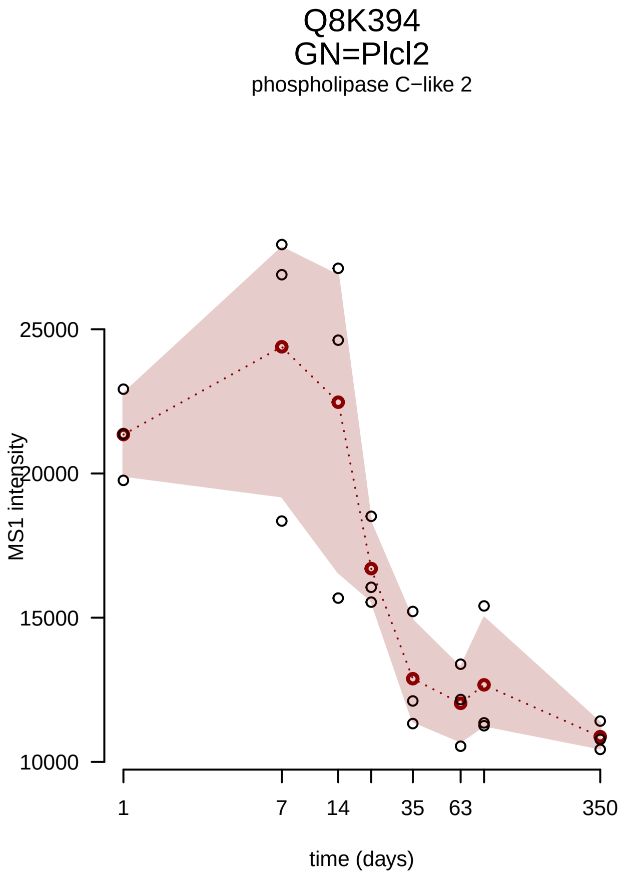
Q8K394
GN=Plcl2
phospholipase C−like 2
MS1 intensity
time (days)

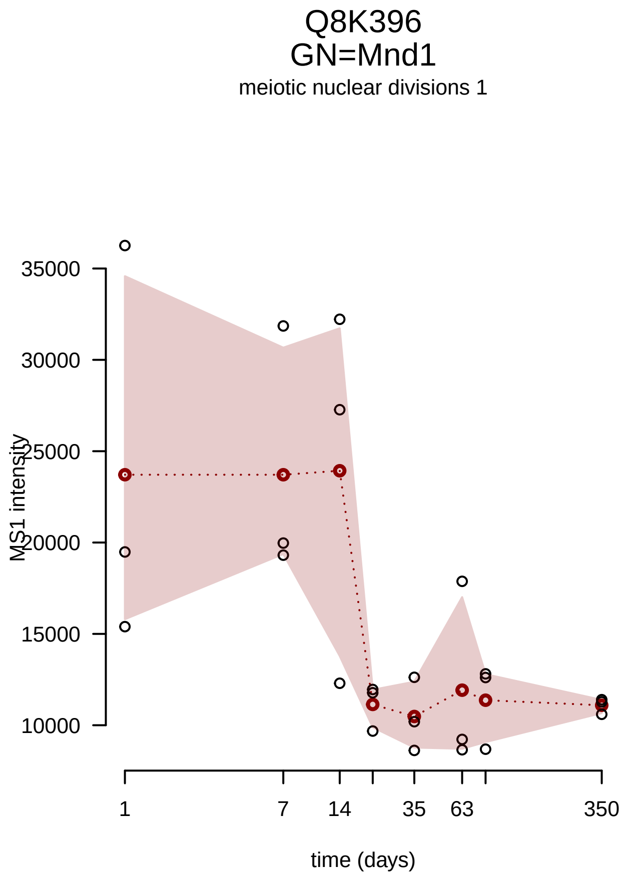
Q8K396
GN=Mnd1
meiotic nuclear divisions 1
MS1 intensity
time (days)

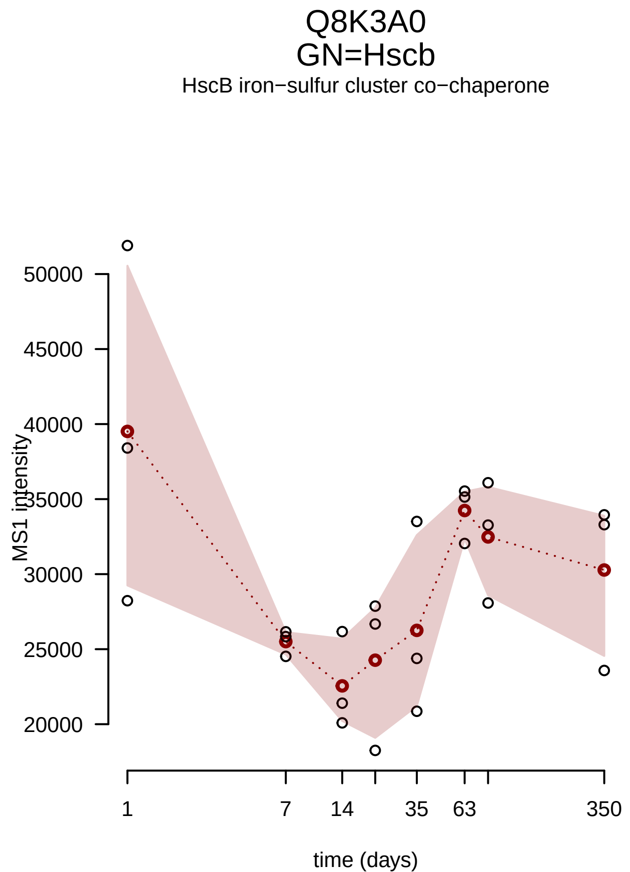
Q8K3A0
GN=Hscb
HscB iron−sulfur cluster co−chaperone
MS1 intensity
time (days)

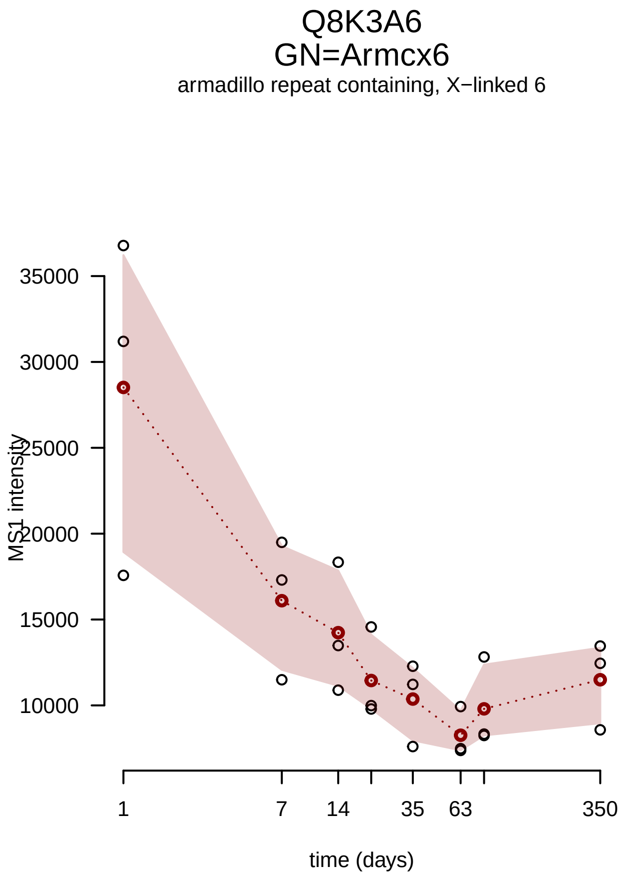
Q8K3A6
GN=Armcx6
armadillo repeat containing, X−linked 6
MS1 intensity
time (days)

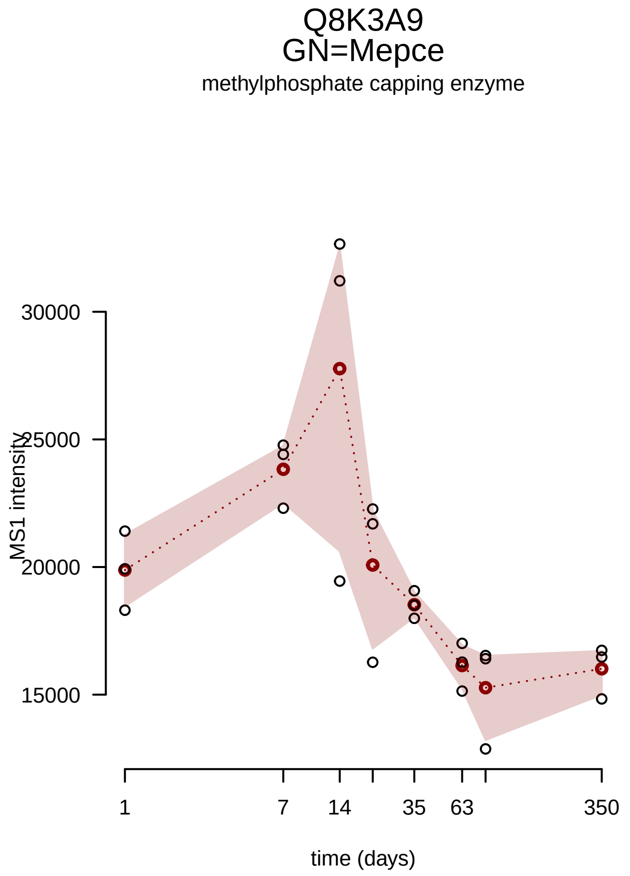
Q8K3A9
GN=Mepce
methylphosphate capping enzyme
MS1 intensity
30000
25000
20000
15000
1
7
14
35
63
350
time (days)

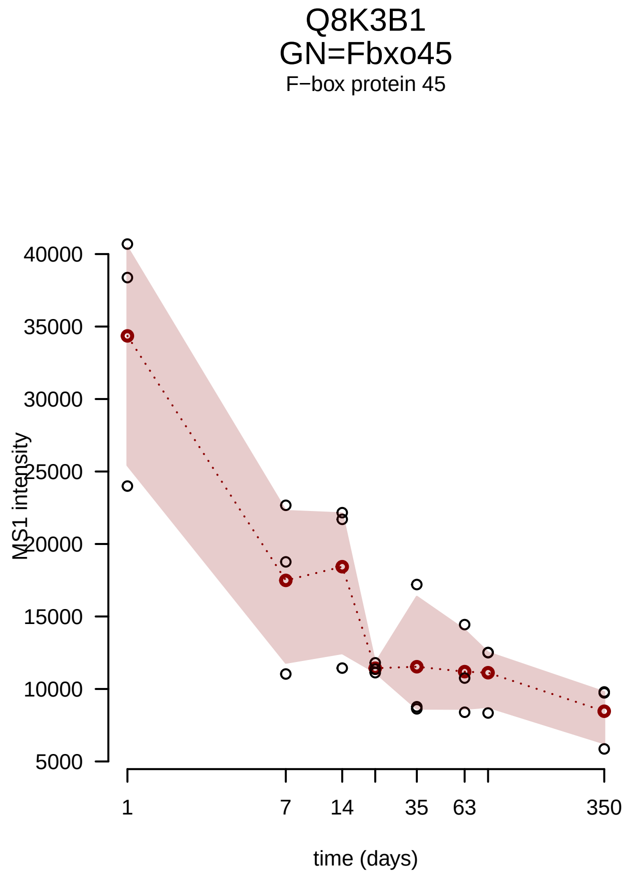
Q8K3B1
GN=Fbxo45
F−box protein 45
MS1 intensity
40000
35000
30000
25000
20000
15000
10000
5000
1
7
14
35
63
350
time (days)

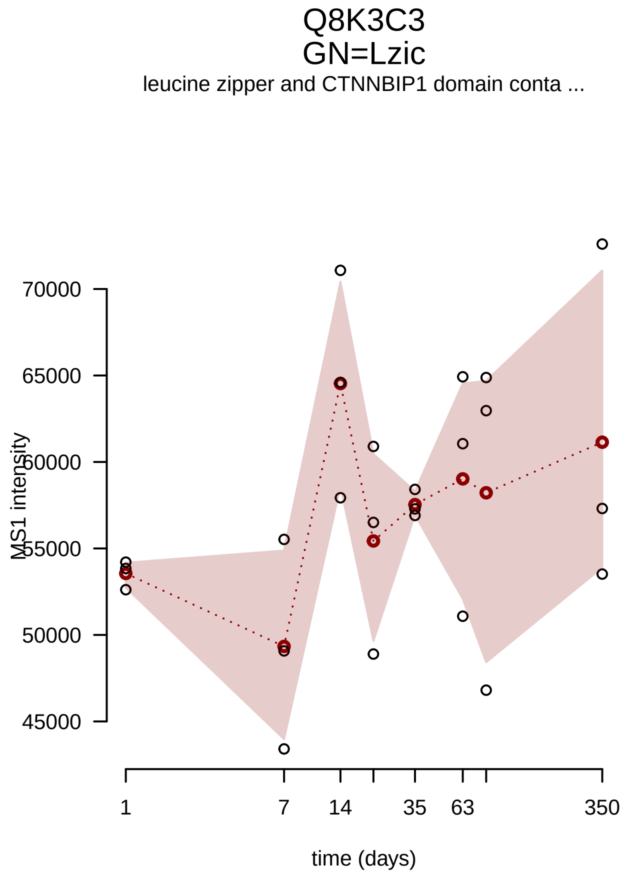
Q8K3C3
GN=Lzic
leucine zipper and CTNNBIP1 domain conta ...
MS1 intensity
70000
65000
60000
55000
50000
45000
1
7
14
35
63
350
time (days)

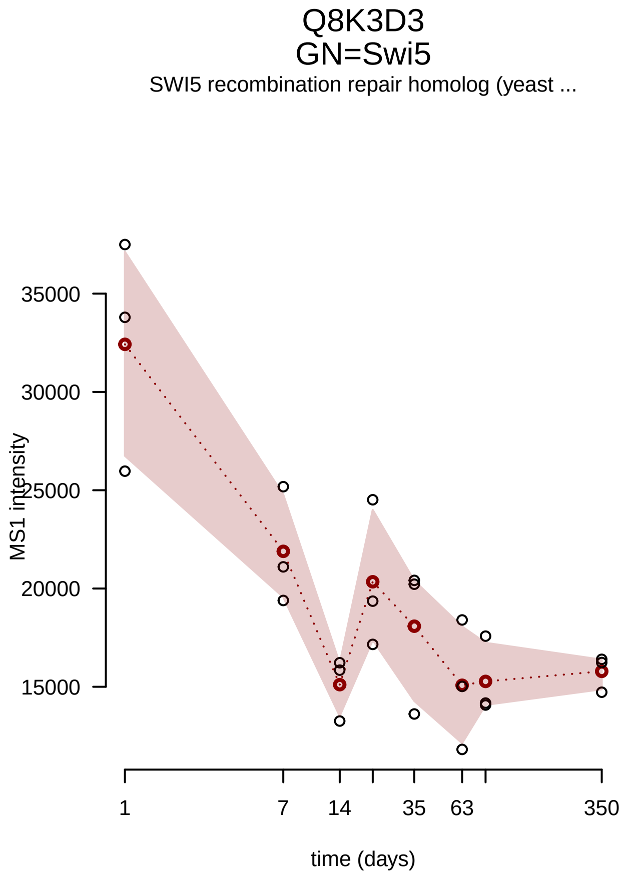
Q8K3D3
GN=Swi5
SWI5 recombination repair homolog (yeast ...
MS1 intensity
35000
30000
25000
20000
15000
1
7
14
35
63
350
time (days)

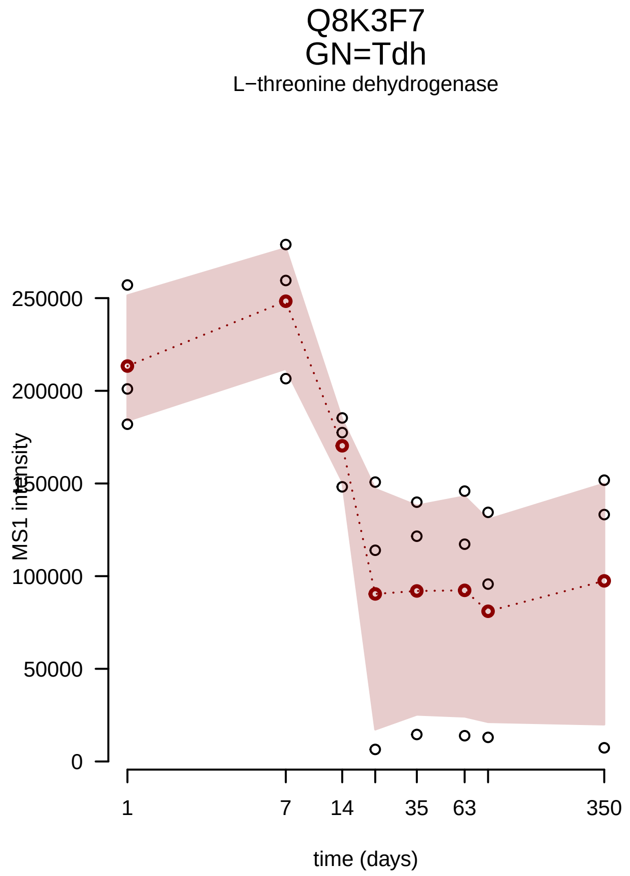
Q8K3F7
GN=Tdh
L−threonine dehydrogenase
MS1 intensity
250000
200000
150000
100000
50000
0
1
7
14
35
63
350
time (days)

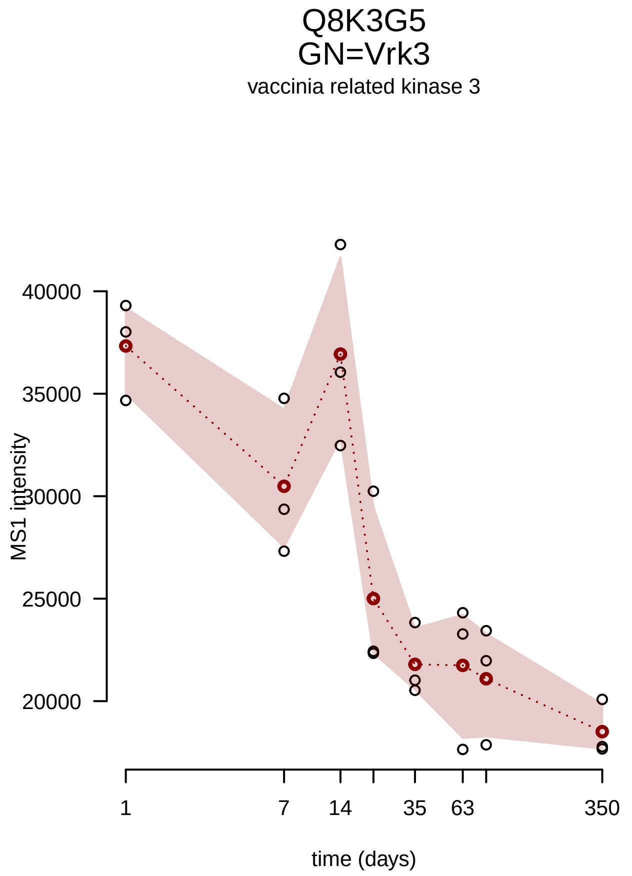
Q8K3G5
GN=Vrk3
vaccinia related kinase 3
MS1 intensity
40000
35000
30000
25000
20000
1
7
14
35
63
350
time (days)

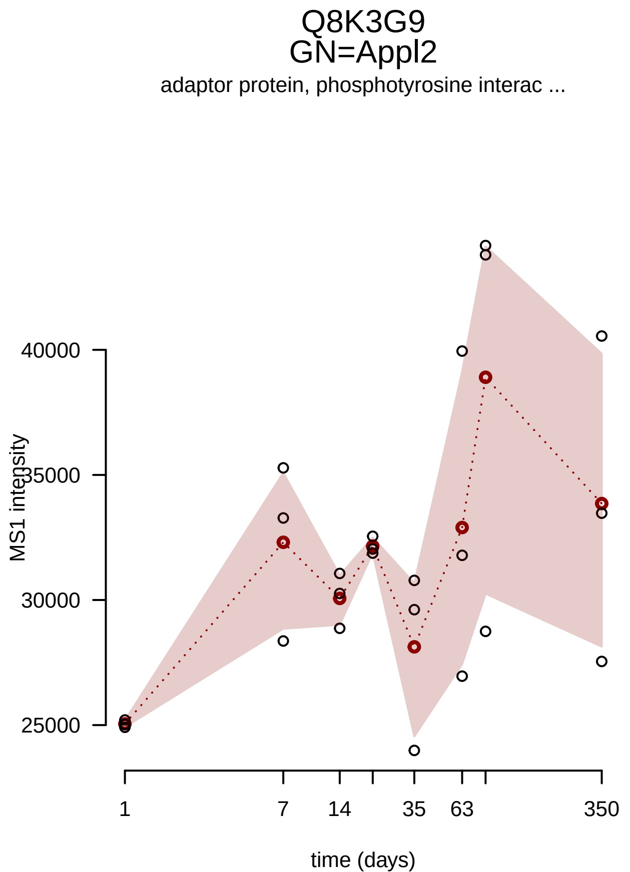
Q8K3G9
GN=Appl2
adaptor protein, phosphotyrosine interac ...
MS1 intensity
40000
35000
30000
25000
1
7
14
35
63
350
time (days)

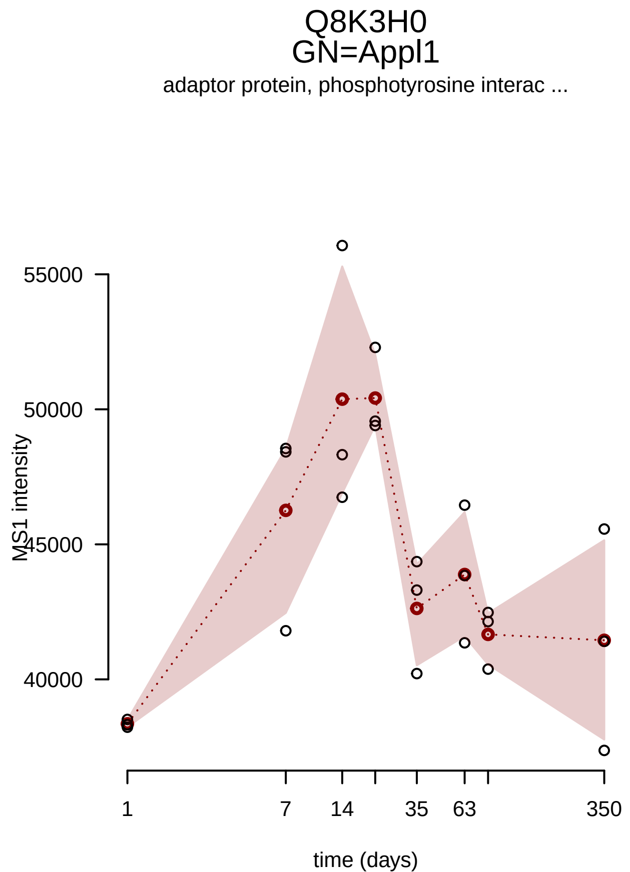
Q8K3H0
GN=Appl1
adaptor protein, phosphotyrosine interac ...
MS1 intensity
55000
50000
45000
40000
1
7
14
35
63
350
time (days)

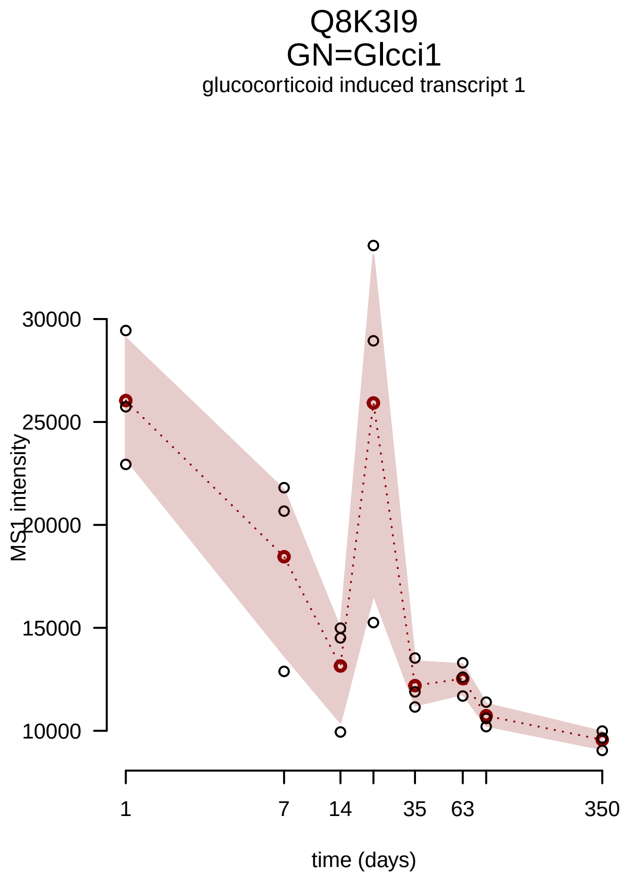
Q8K3I9
GN=Glcci1
glucocorticoid induced transcript 1
MS1 intensity
30000
25000
20000
15000
10000
1
7
14
35
63
350
time (days)

Q8K3J1
GN=Ndufs8
NADH:ubiquinone oxidoreductase core subu ...

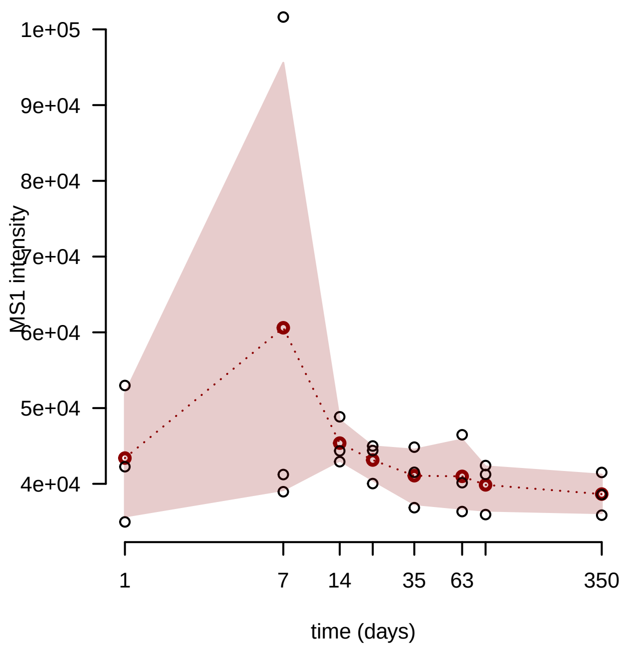

Q8K3J5
GN=Znf131

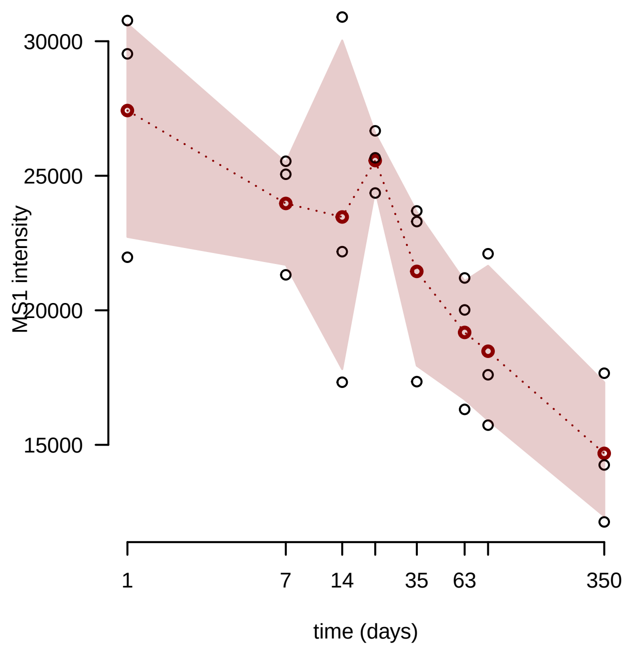

Q8K3J9
GN=Gprc5c
G protein−coupled receptor, family C, gr ...

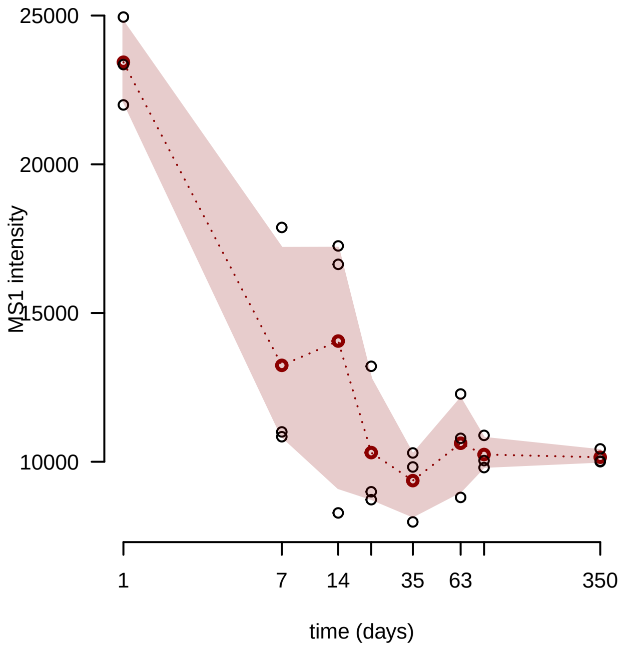

Q8K3K7
GN=Agpat2
1−acylglycerol−3−phosphate O−acyltransfe ...

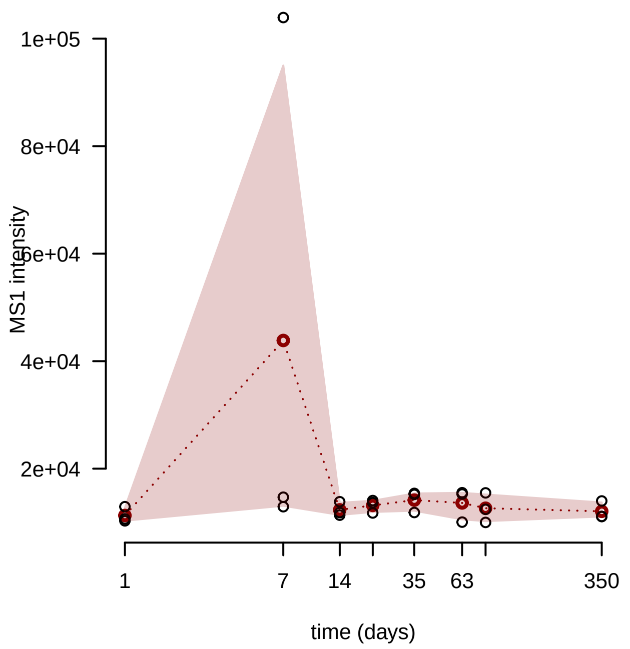

Q8K3K8
GN=Optn
optineurin

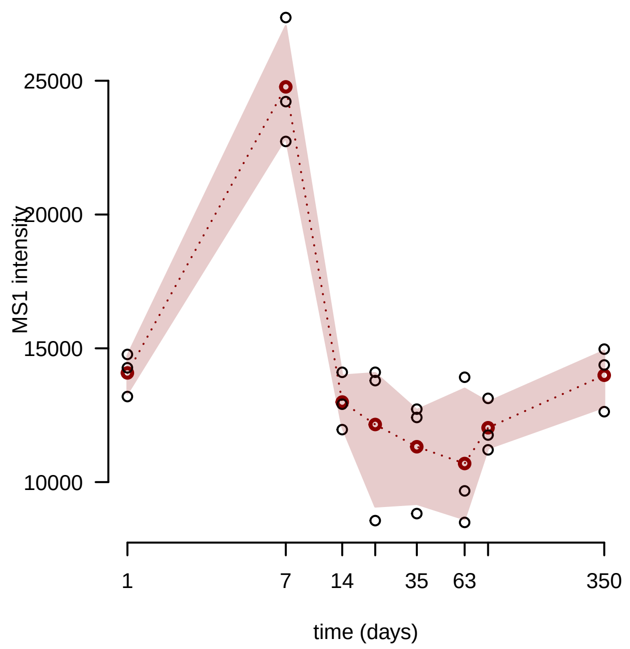

Q8K3P5
GN=Cnot6
CCR4−NOT transcription complex, subunit ...

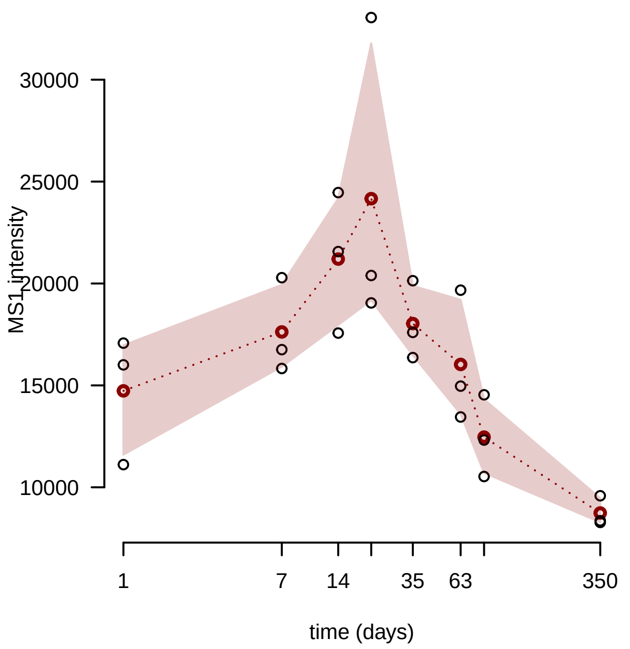

Q8K3V4
GN=Padi6
peptidyl arginine deiminase, type VI

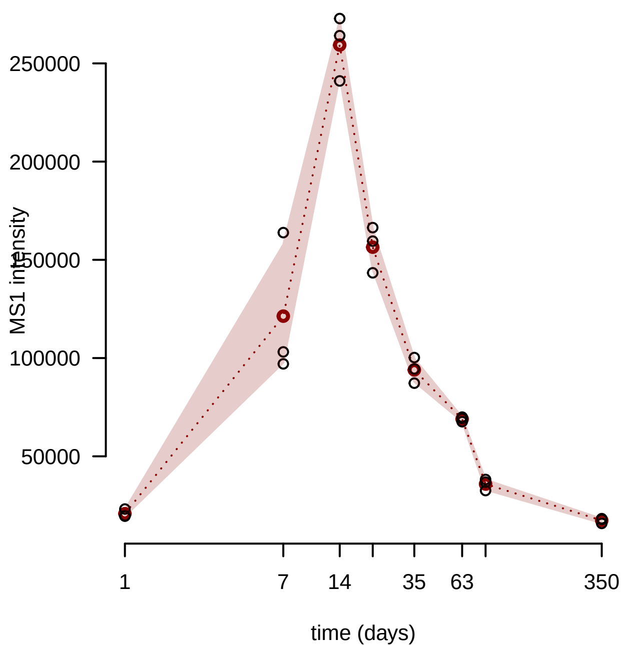

Q8K3W0
GN=Babam2
BRISC and BRCA1 A complex member 2

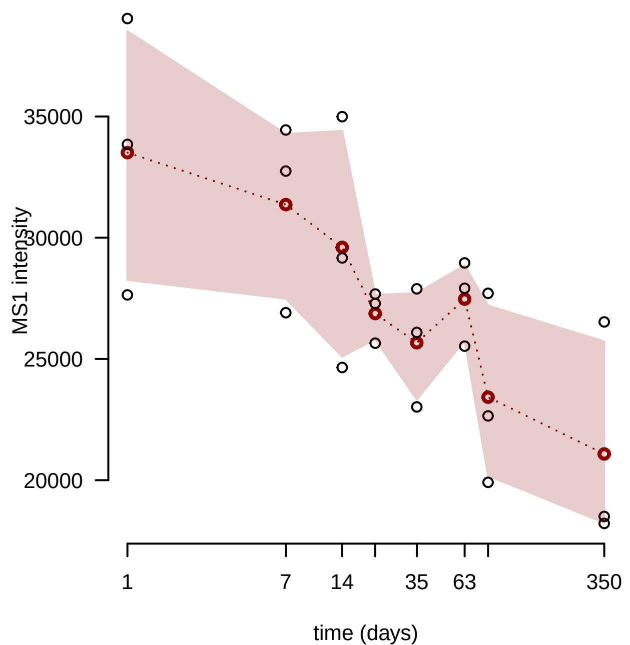

Q8K3W3
GN=Casc3
cancer susceptibility candidate 3

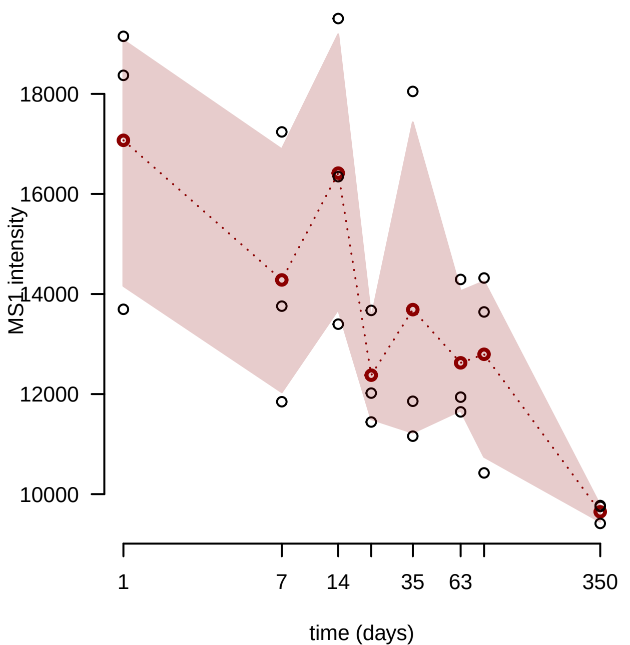

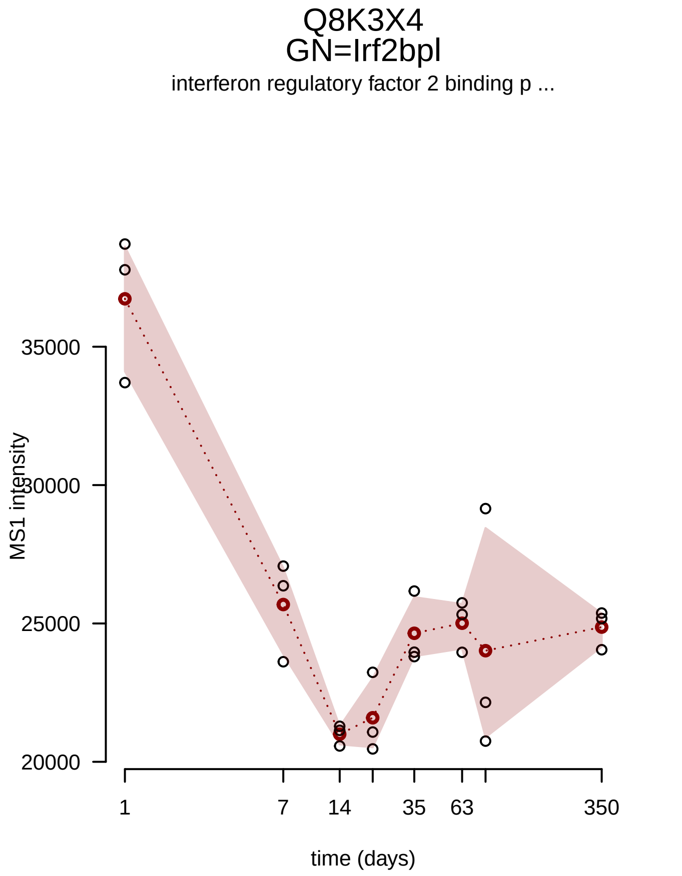
Q8K3X4
GN=Irf2bpl
interferon regulatory factor 2 binding p ...
MS1 intensity
35000
30000
25000
20000
1
7
14
35
63
350
time (days)

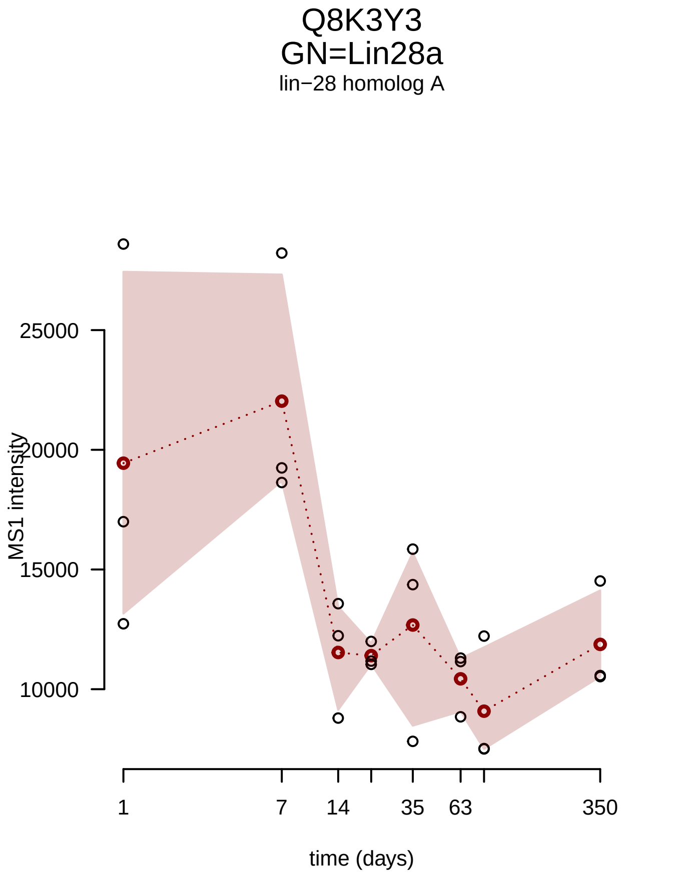
Q8K3Y3
GN=Lin28a
lin−28 homolog A
MS1 intensity
25000
20000
15000
10000
1
7
14
35
63
350
time (days)

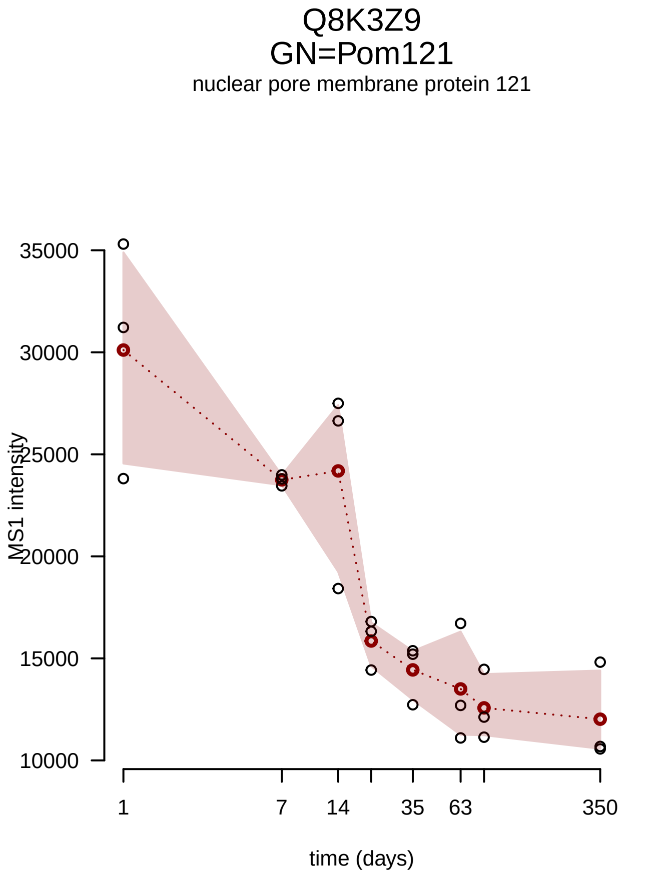
Q8K3Z9
GN=Pom121
nuclear pore membrane protein 121
MS1 intensity
35000
30000
25000
20000
15000
10000
1
7
14
35
63
350
time (days)

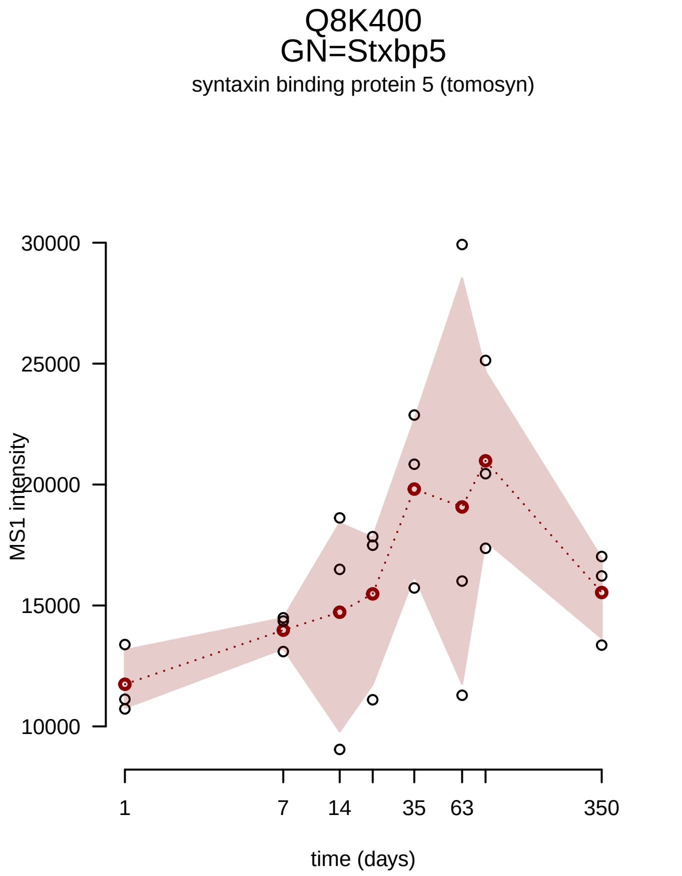
Q8K400
GN=Stxbp5
syntaxin binding protein 5 (tomosyn)
MS1 intensity
30000
25000
20000
15000
10000
1
7
14
35
63
350
time (days)

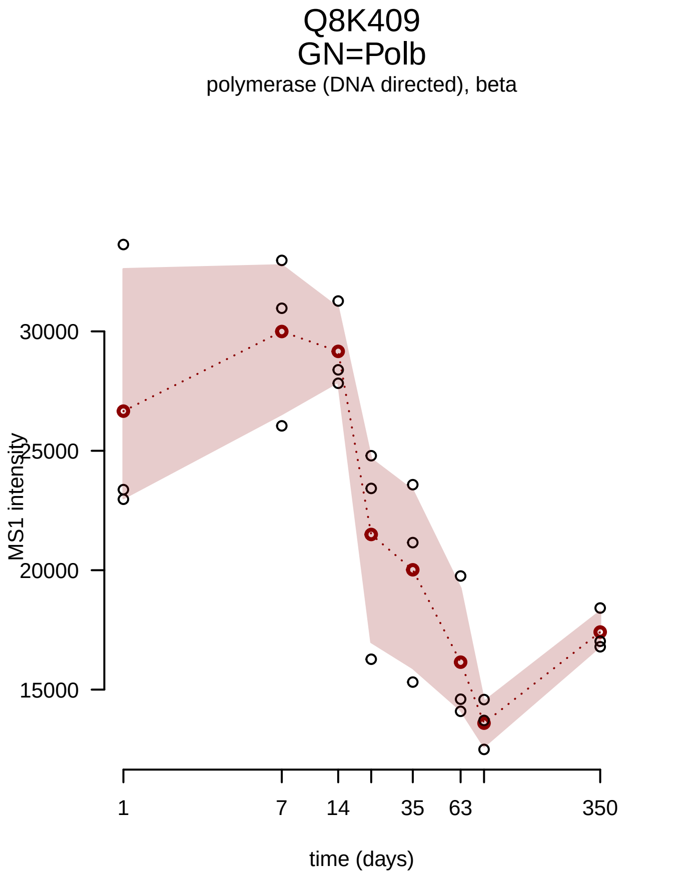
Q8K409
GN=Polb
polymerase (DNA directed), beta
MS1 intensity
30000
25000
20000
15000
1
7
14
35
63
350
time (days)

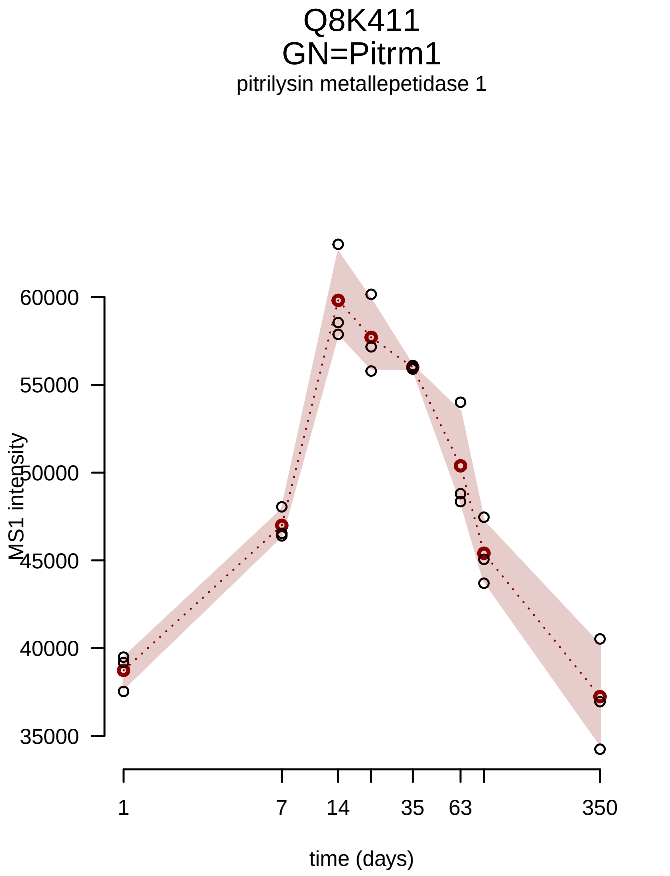
Q8K411
GN=Pitrm1
pitrilysin metallepetidase 1
MS1 intensity
60000
55000
50000
45000
40000
35000
1
7
14
35
63
350
time (days)

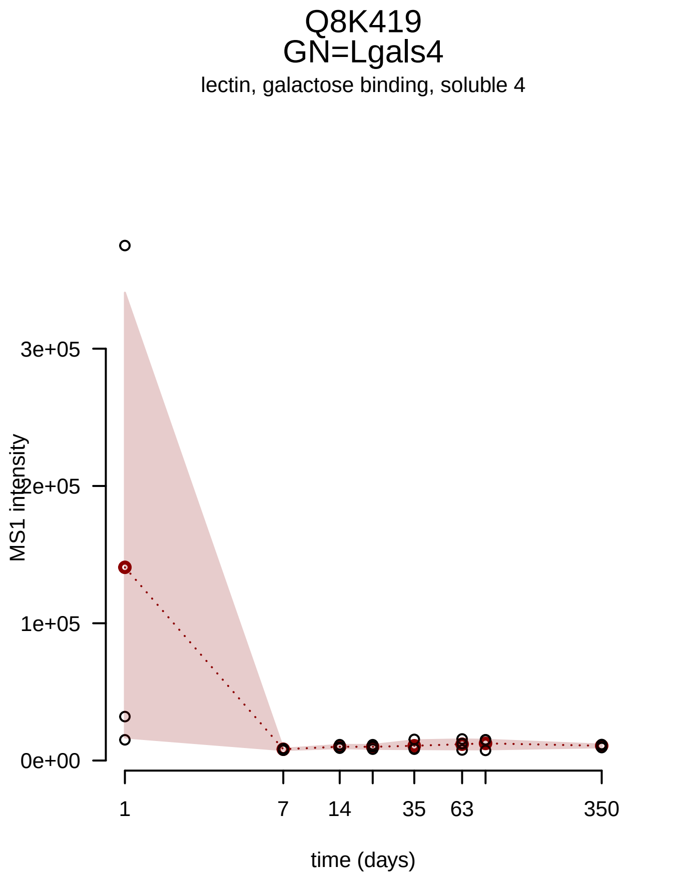
Q8K419
GN=Lgals4
lectin, galactose binding, soluble 4
MS1 intensity
3e+05
2e+05
1e+05
0e+00
1
7
14
35
63
350
time (days)

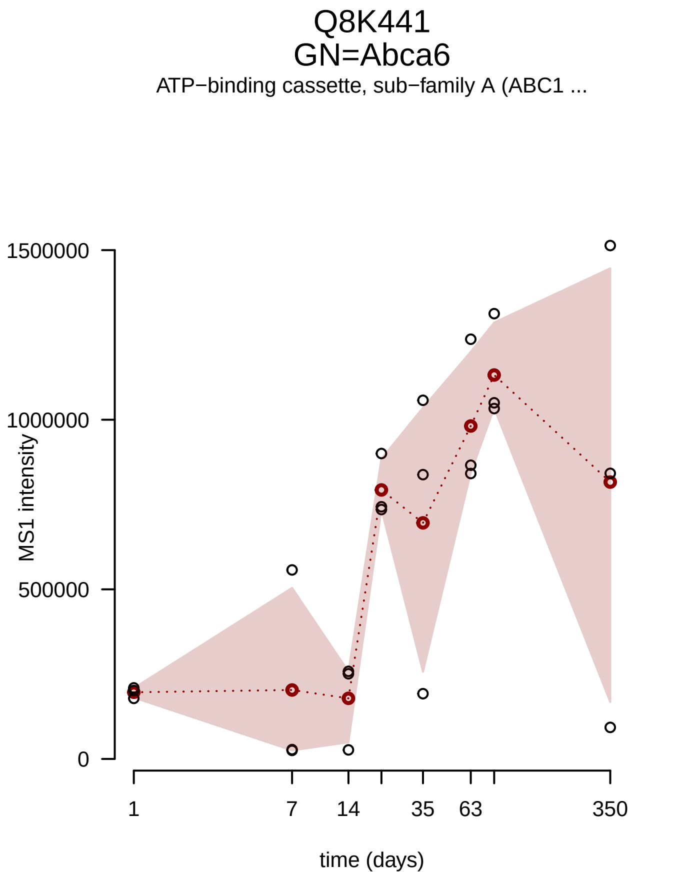
Q8K441
GN=Abca6
ATP−binding cassette, sub−family A (ABC1 ...
MS1 intensity
1500000
1000000
500000
0
1
7
14
35
63
350
time (days)

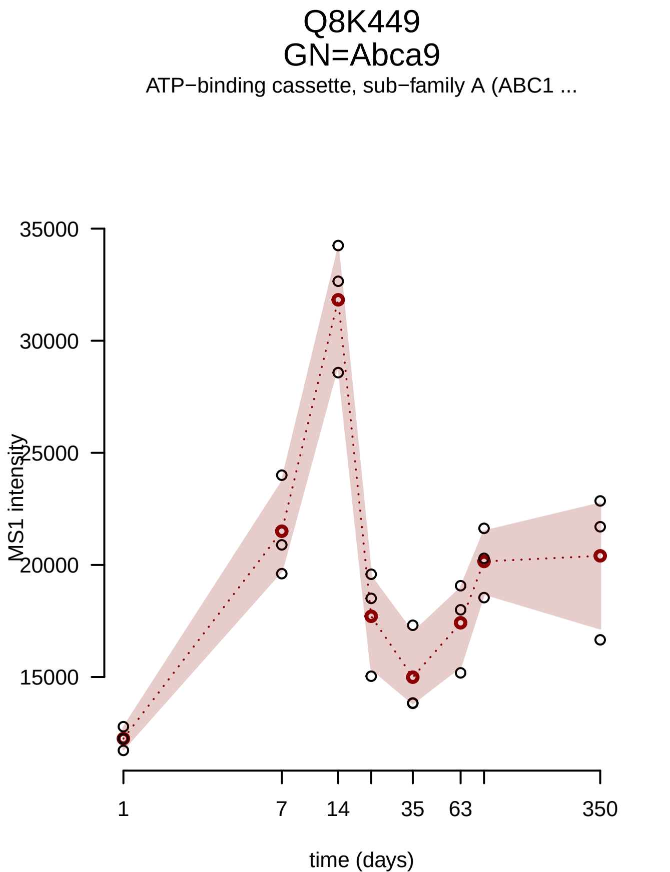
Q8K449
GN=Abca9
ATP−binding cassette, sub−family A (ABC1 ...
MS1 intensity
35000
30000
25000
20000
15000
1
7
14
35
63
350
time (days)

Q8K482
GN=Emilin2
elastin microfibril interfacer 2

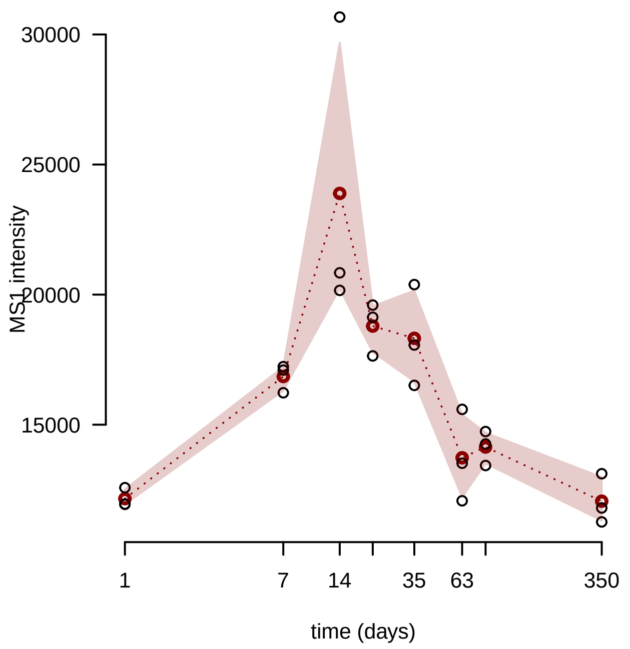

Q8K4B0
GN=Mta1
metastasis associated 1

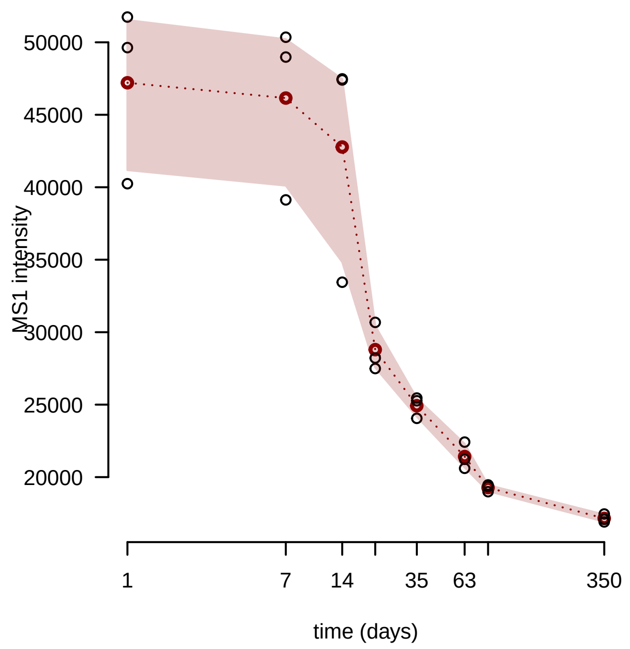

Q8K4D3
GN=Slc36a1
solute carrier family 36 (proton/amino a ...

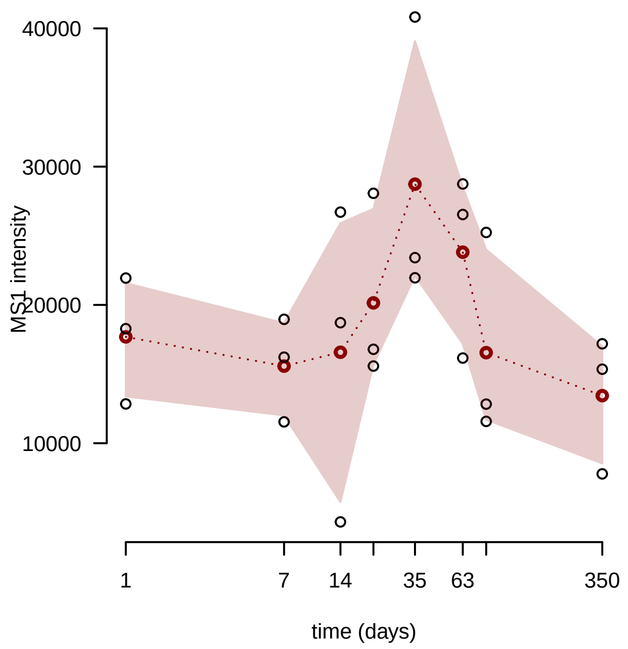

Q8K4E0
GN=Alms1
ALMS1, centrosome and basal body associa ...

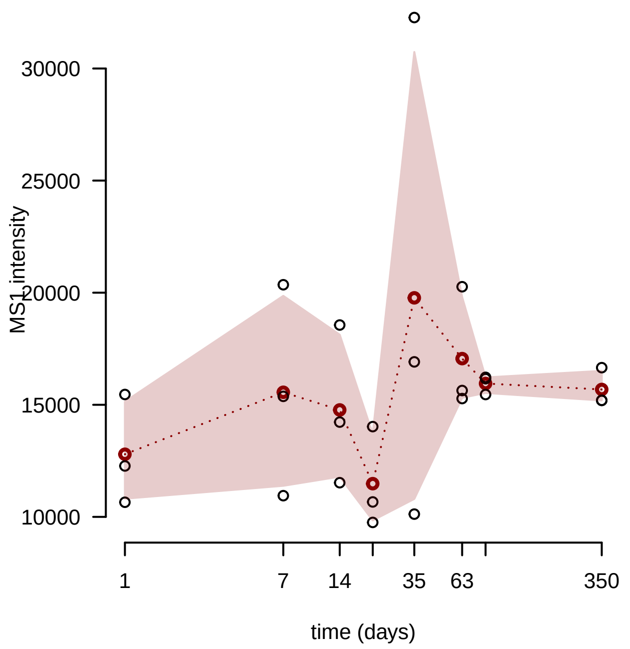

Q8K4F5
GN=Abhd11
abhydrolase domain containing 11

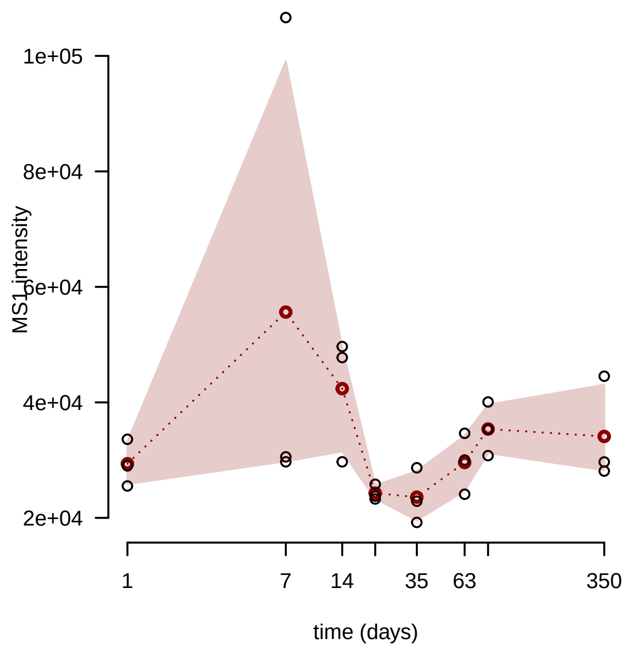

Q8K4F6
GN=Nsun5
NOL1/NOP2/Sun domain family, member 5

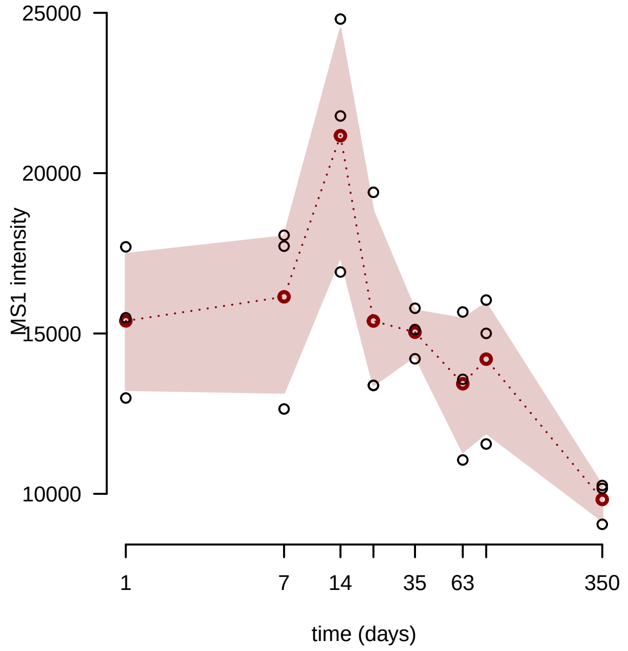

Q8K4G1
GN=Ltbp4
latent transforming growth factor beta b ...

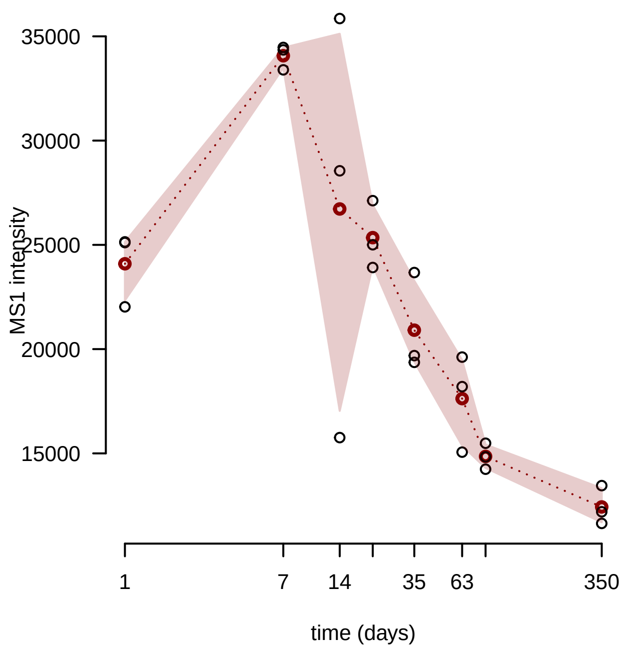

Q8K4G5
GN=Ablim1
actin−binding LIM protein 1

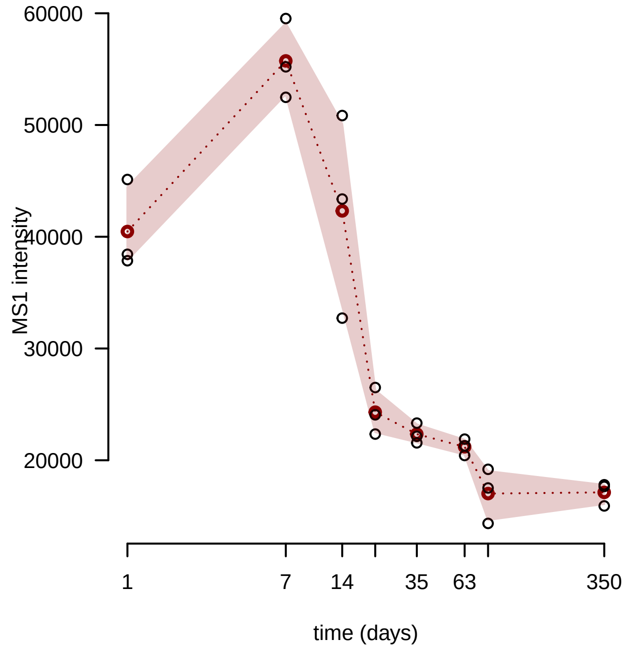

Q8K4H1
GN=Afmid
arylformamidase

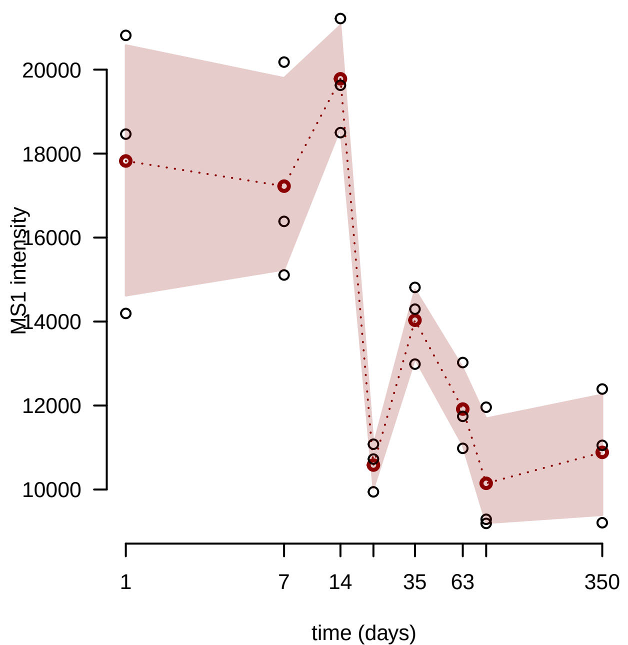

Q8K4I3
GN=Arhgef6
Rac/Cdc42 guanine nucleotide exchange fa ...

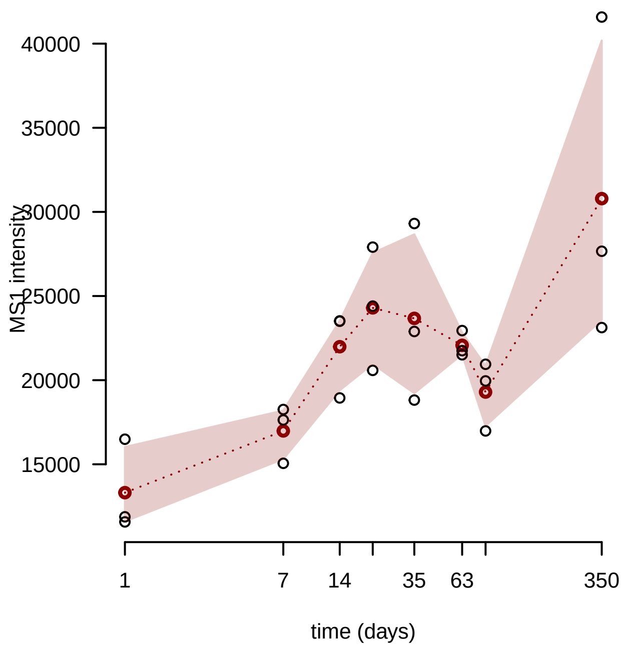

Q8K4J6
GN=Mrtfa
myocardin related transcription factor A

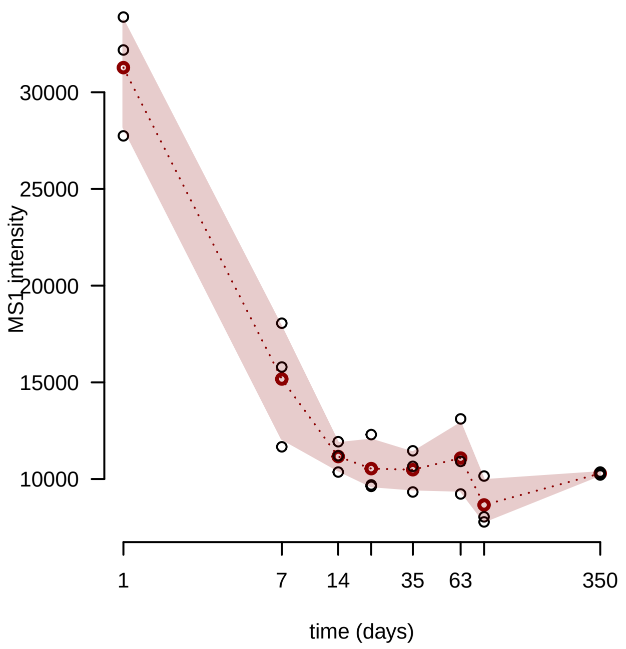

Q8K4K6
GN=Pank1
pantothenate kinase 1

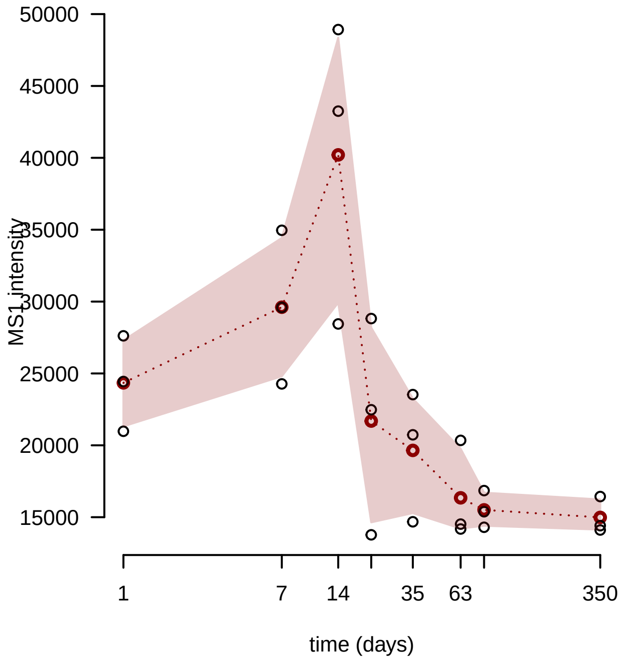

Q8K4L0
GN=Ddx54
DEAD box helicase 54

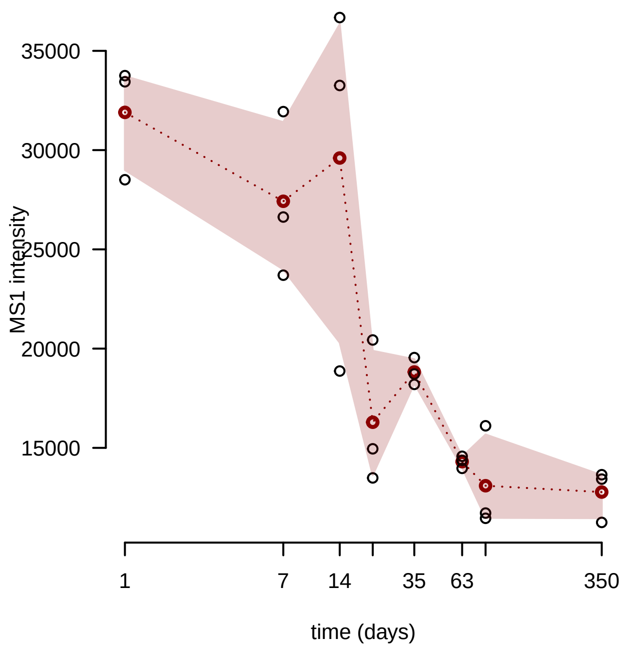

Q8K4L3
GN=Svil
supervillin

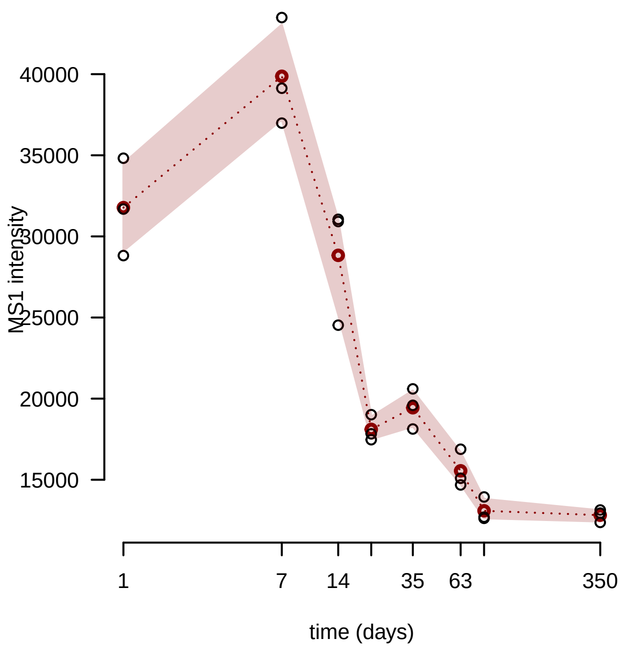

Q8K4L4
GN=Pof1b
premature ovarian failure 1B

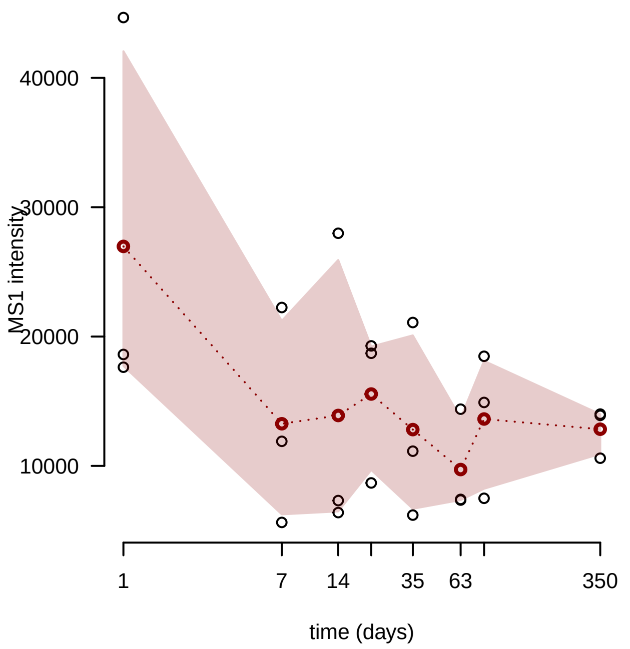

Q8K4M5
GN=Commd1
COMM domain containing 1

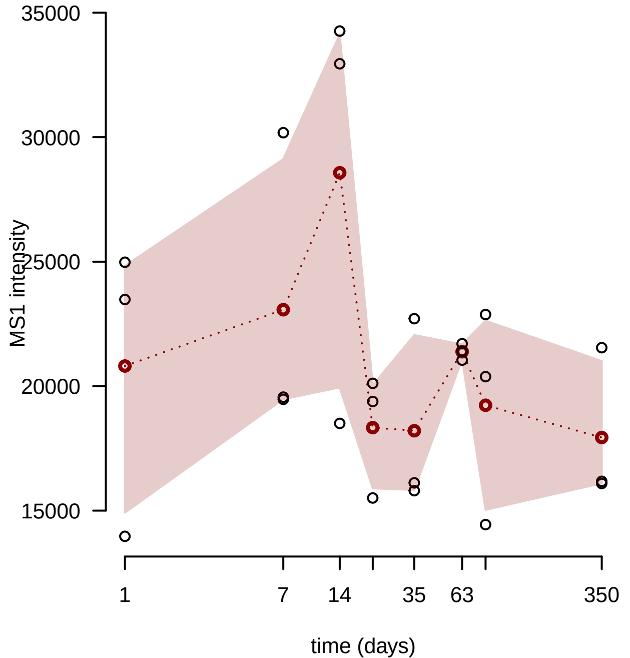

Q8K4P0
GN=Wdr33
WD repeat domain 33

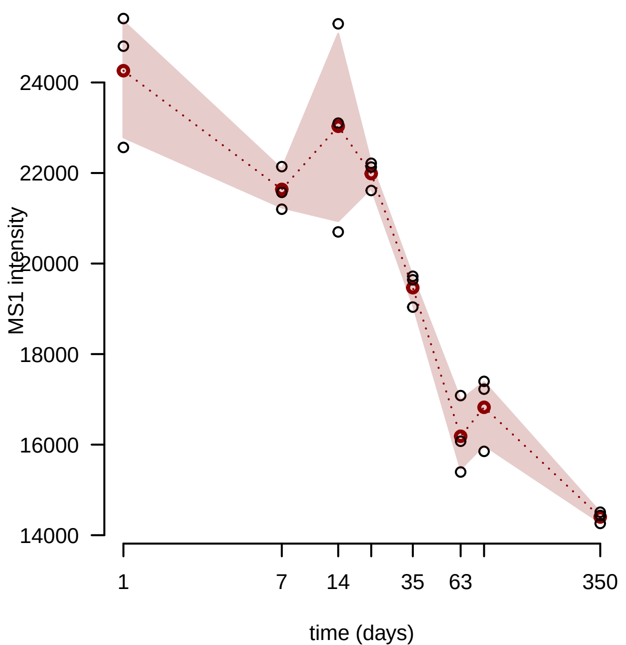

Q8K4P7
GN=St7l
suppression of tumorigenicity 7−like

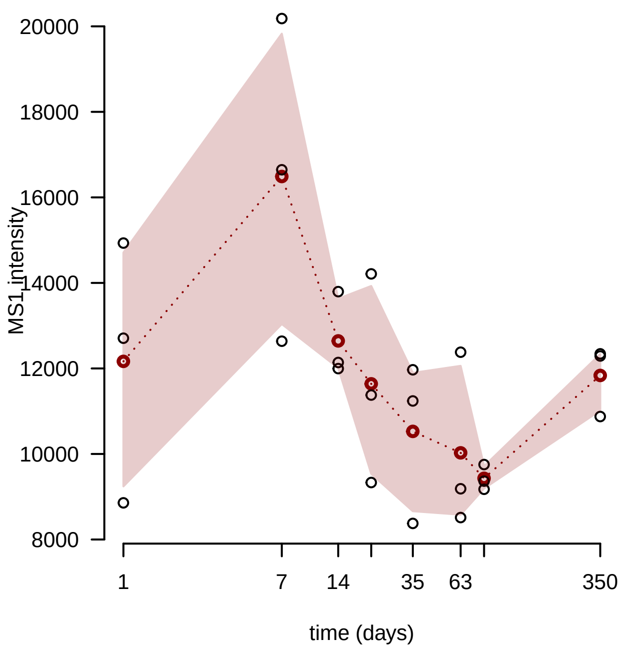

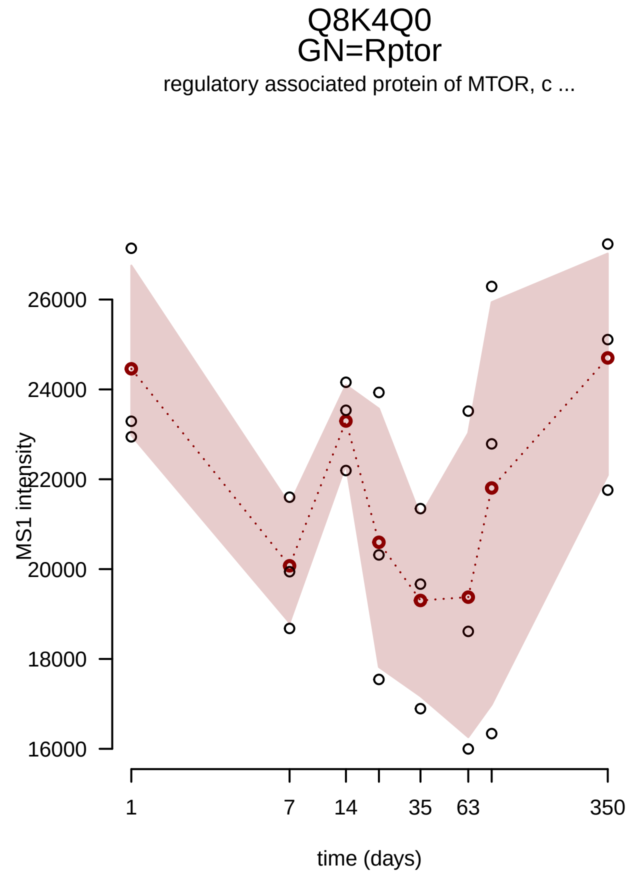
Q8K4Q0
GN=Rptor
regulatory associated protein of MTOR, c ...
26000
24000
22000
20000
18000
16000
MS1 intensity
1
7
14
35
63
350
time (days)

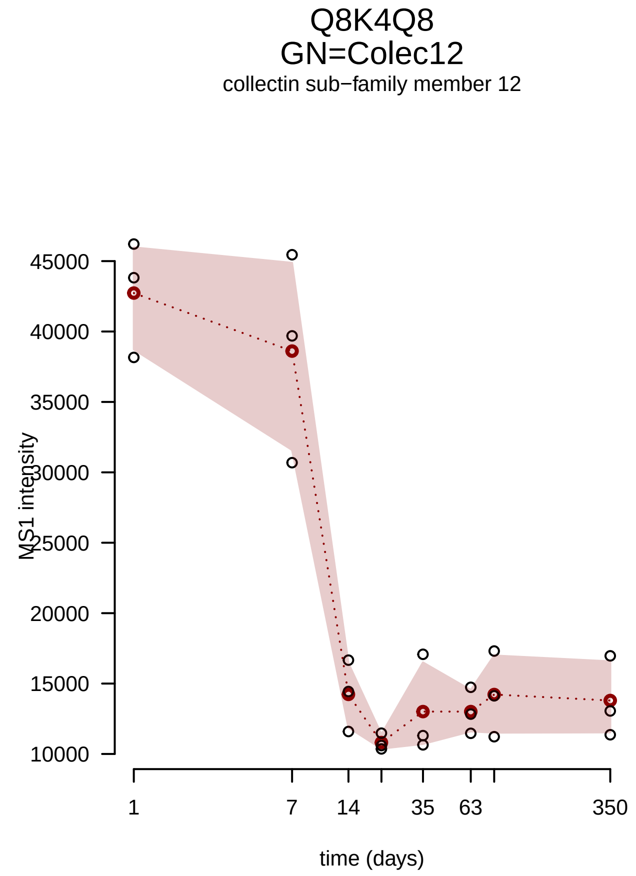
Q8K4Q8
GN=Colec12
collectin sub−family member 12
45000
40000
35000
30000
25000
20000
15000
10000
MS1 intensity
1
7
14
35
63
350
time (days)

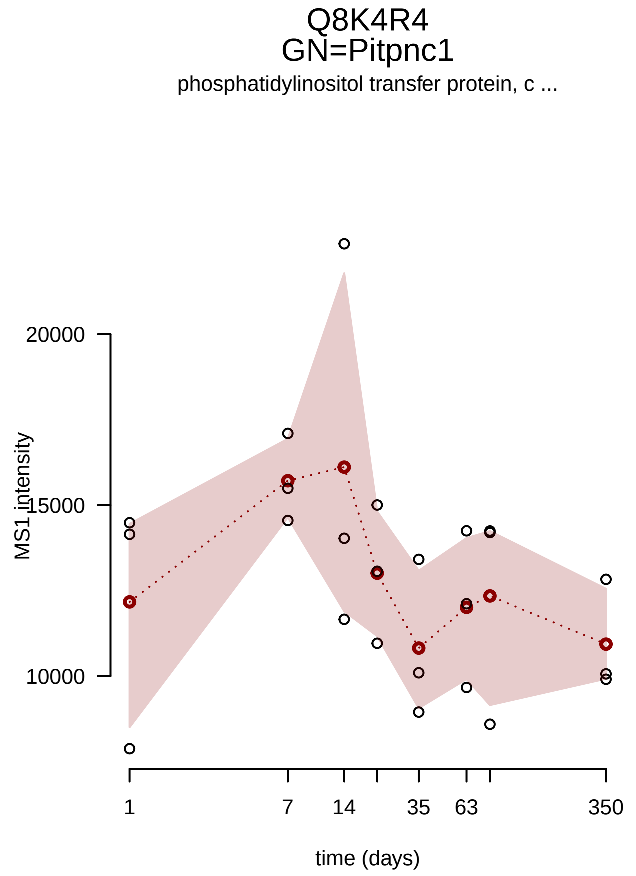
Q8K4R4
GN=Pitpnc1
phosphatidylinositol transfer protein, c ...
20000
15000
10000
MS1 intensity
1
7
14
35
63
350
time (days)

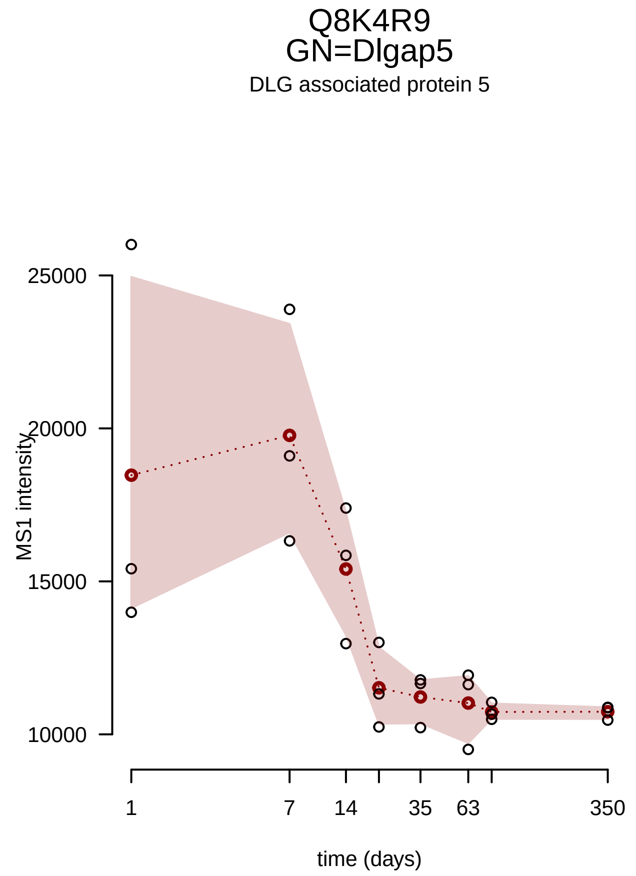
Q8K4R9
GN=Dlgap5
DLG associated protein 5
25000
20000
15000
10000
MS1 intensity
1
7
14
35
63
350
time (days)

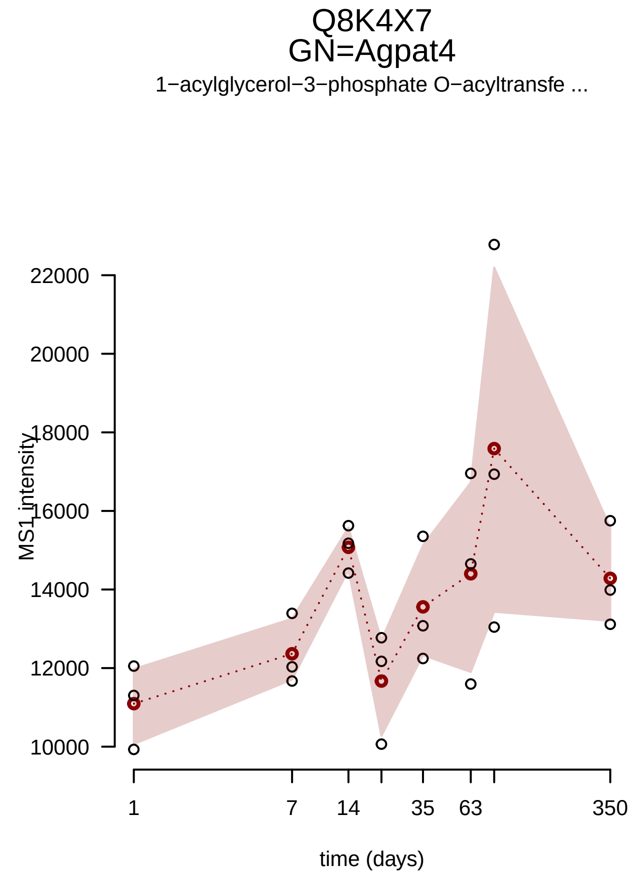
Q8K4X7
GN=Agpat4
1−acylglycerol−3−phosphate O−acyltransfe ...
22000
20000
18000
16000
14000
12000
10000
MS1 intensity
1
7
14
35
63
350
time (days)

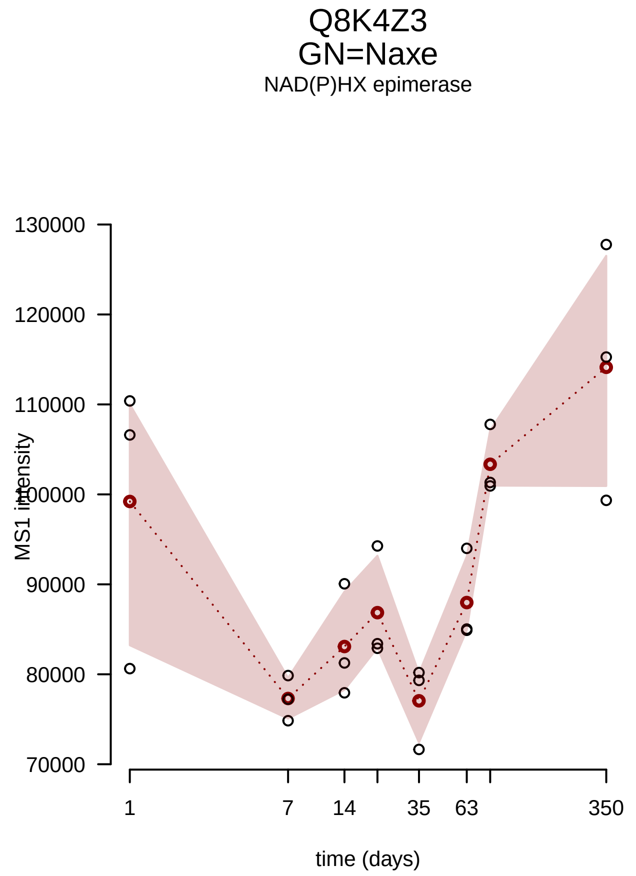
Q8K4Z3
GN=Naxe
NAD(P)HX epimerase
130000
120000
110000
100000
90000
80000
70000
MS1 intensity
1
7
14
35
63
350
time (days)

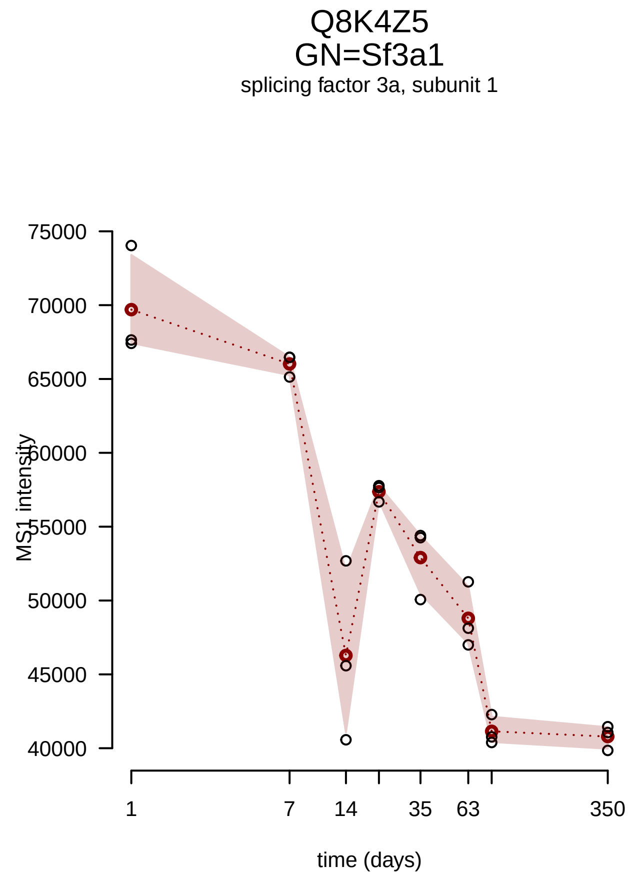
Q8K4Z5
GN=Sf3a1
splicing factor 3a, subunit 1
75000
70000
65000
60000
55000
50000
45000
40000
MS1 intensity
1
7
14
35
63
350
time (days)

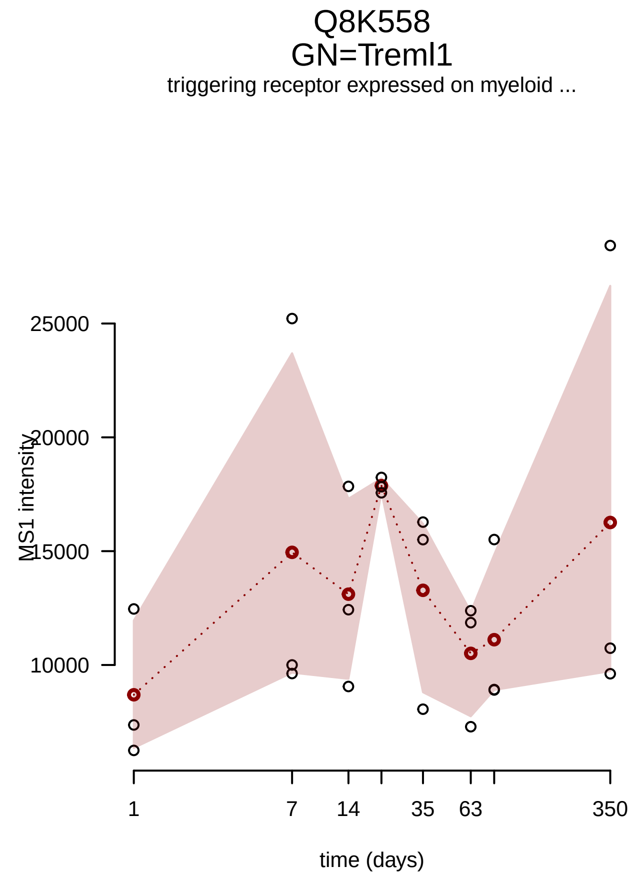
Q8K558
GN=Treml1
triggering receptor expressed on myeloid ...
25000
20000
15000
10000
MS1 intensity
1
7
14
35
63
350
time (days)

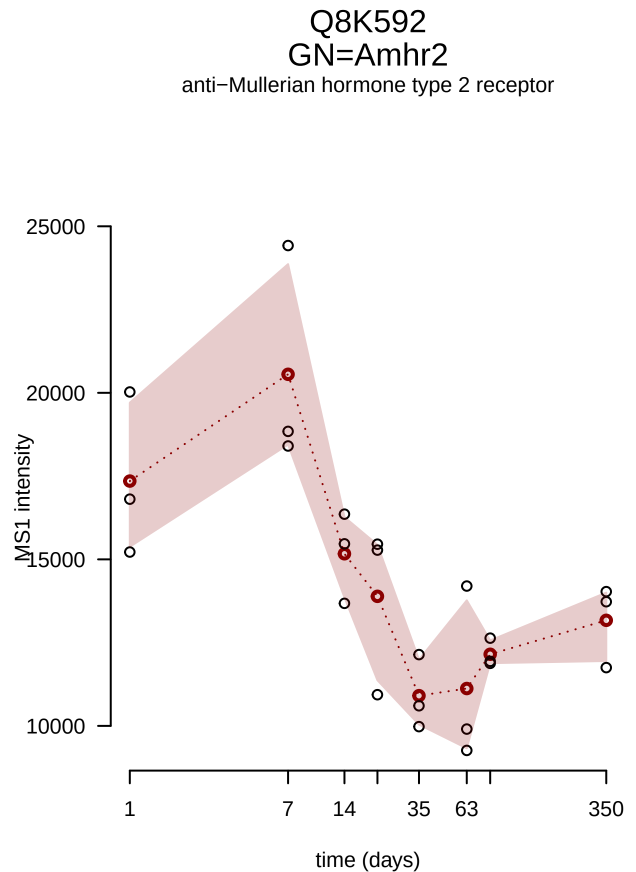
Q8K592
GN=Amhr2
anti−Mullerian hormone type 2 receptor
25000
20000
15000
10000
MS1 intensity
1
7
14
35
63
350
time (days)

Q8K596
GN=Slc8a2
solute carrier family 8 (sodium/calcium ...

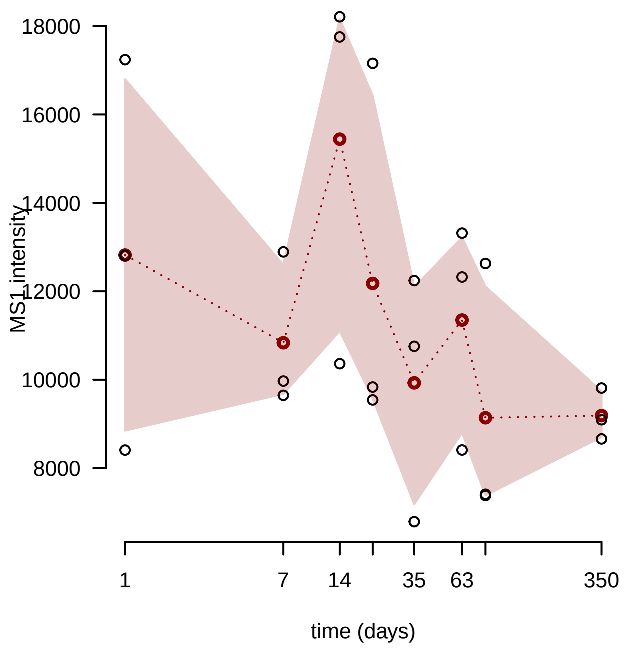

Q8K5B2
GN=Mcfd2
multiple coagulation factor deficiency 2

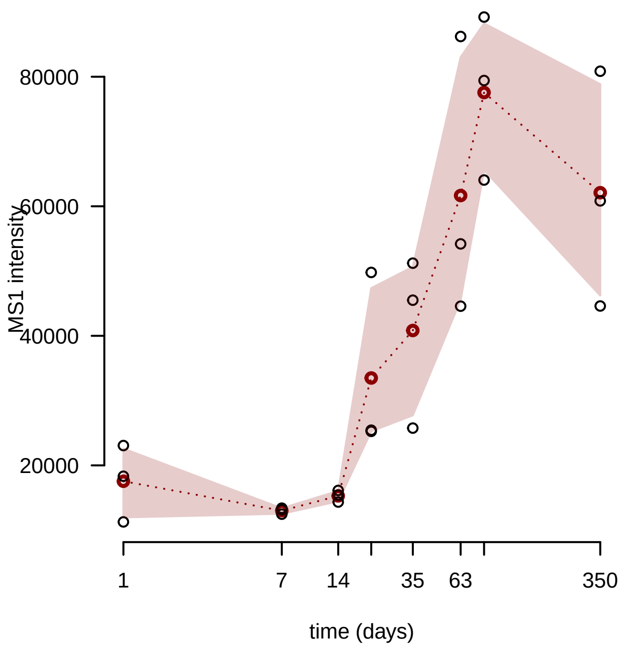

Q8N7N5
GN=Dcaf8
DDB1 and CUL4 associated factor 8

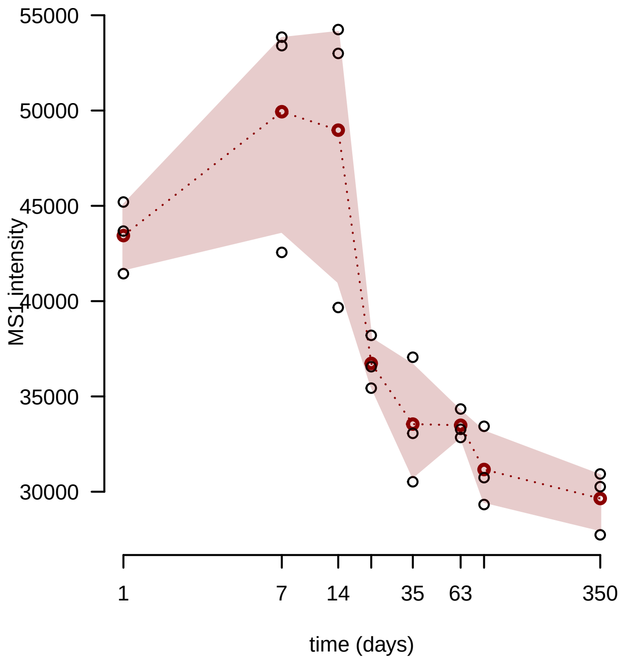

Q8N7N6
GN=

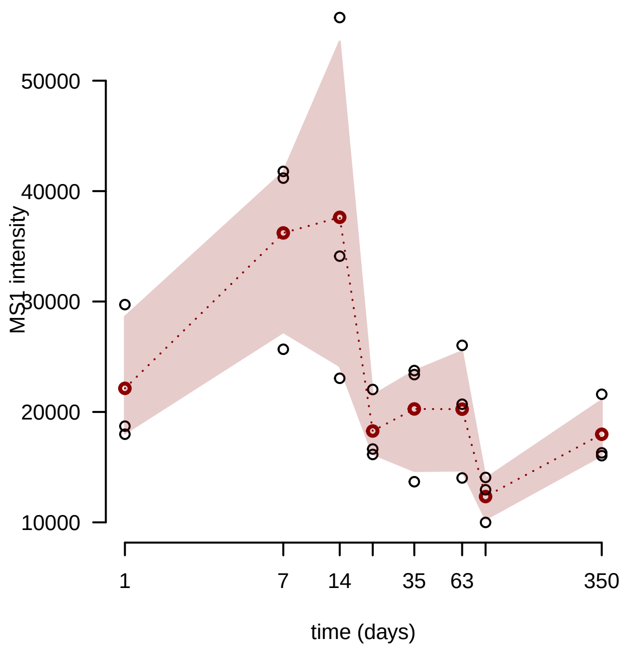

Q8N9S3
GN=Ahsa2
AHA1, activator of heat shock protein AT ...

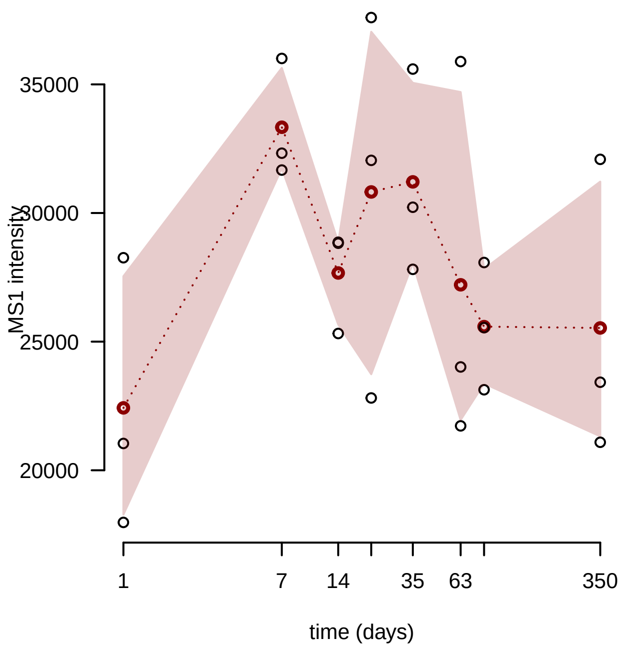

Q8QZR3
GN=Ces2a
carboxylesterase 2A

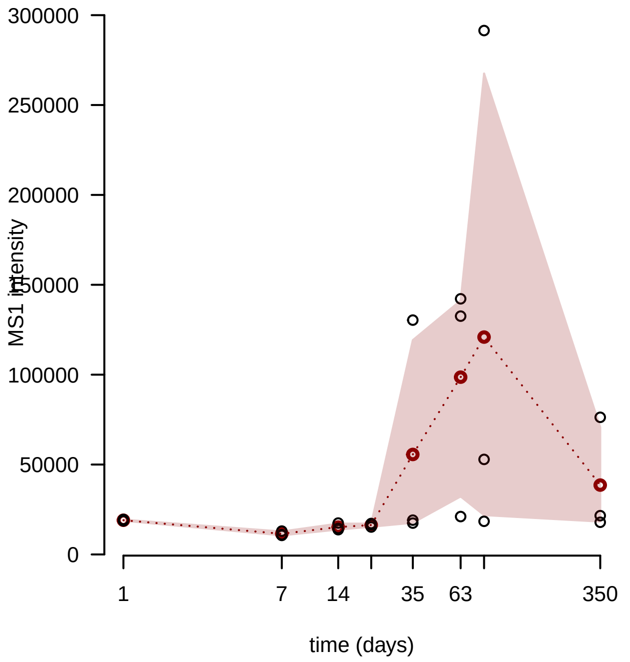

Q8QZR5
GN=Gpt
glutamic pyruvic transaminase, soluble

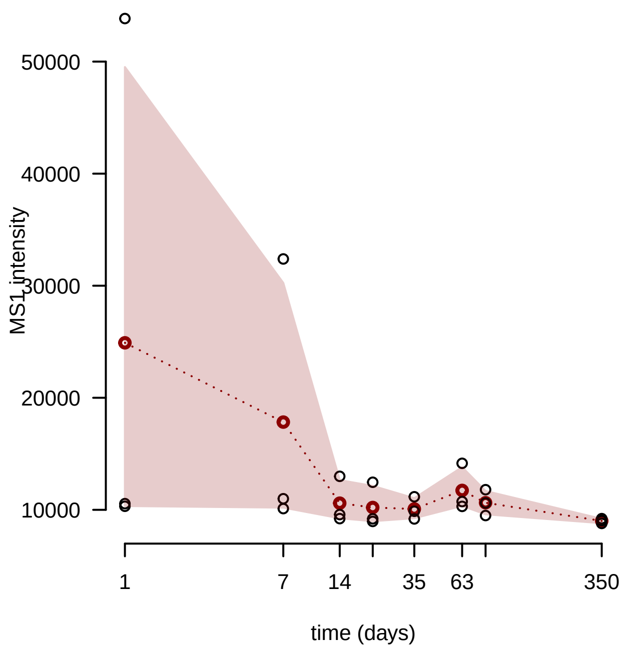

Q8QZS1
GN=Hibch
3−hydroxyisobutyryl−Coenzyme A hydrolase

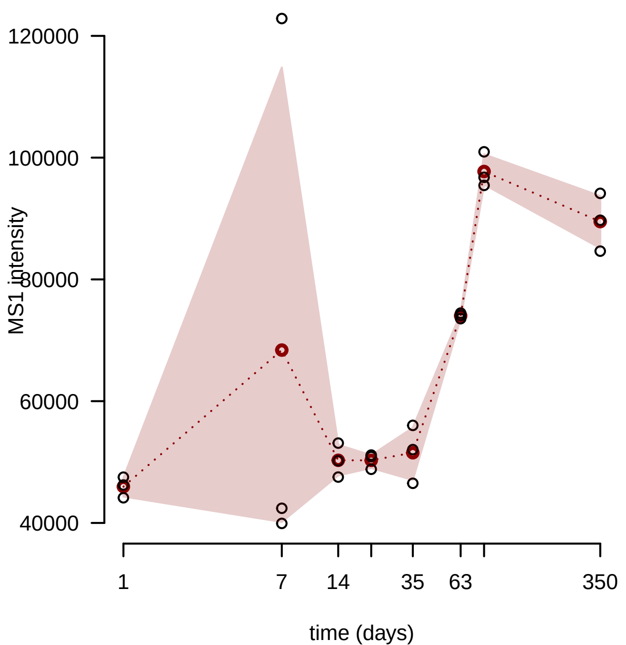

Q8QZS3
GN=Flcn
folliculin

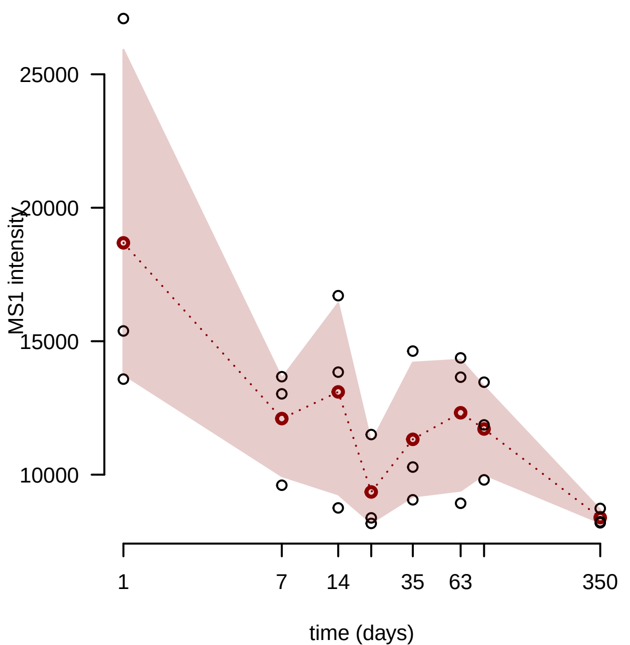

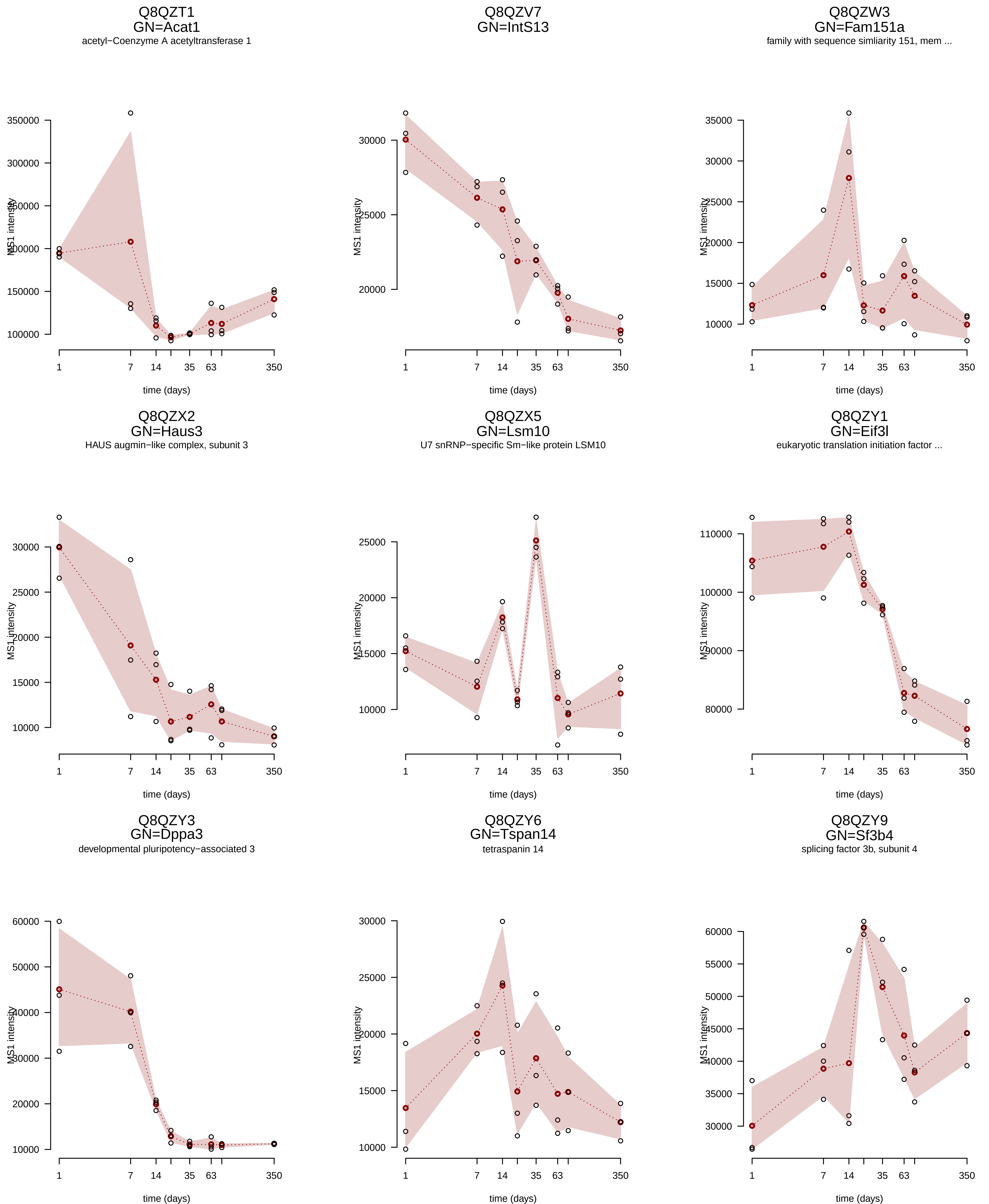
Q8QZT1
GN=Acat1
acetyl−Coenzyme A acetyltransferase 1
MS1 intensity
time (days)
Q8QZV7
GN=IntS13
MS1 intensity
time (days)
Q8QZW3
GN=Fam151a
family with sequence simliarity 151, mem ...
MS1 intensity
time (days)
Q8QZX2
GN=Haus3
HAUS augmin−like complex, subunit 3
MS1 intensity
time (days)
Q8QZX5
GN=Lsm10
U7 snRNP−specific Sm−like protein LSM10
MS1 intensity
time (days)
Q8QZY1
GN=Eif3l
eukaryotic translation initiation factor ...
MS1 intensity
time (days)
Q8QZY3
GN=Dppa3
developmental pluripotency−associated 3
MS1 intensity
time (days)
Q8QZY6
GN=Tspan14
tetraspanin 14
MS1 intensity
time (days)
Q8QZY9
GN=Sf3b4
splicing factor 3b, subunit 4
MS1 intensity
time (days)

Q8QZZ7
GN=Tprkb
Tp53rk binding protein

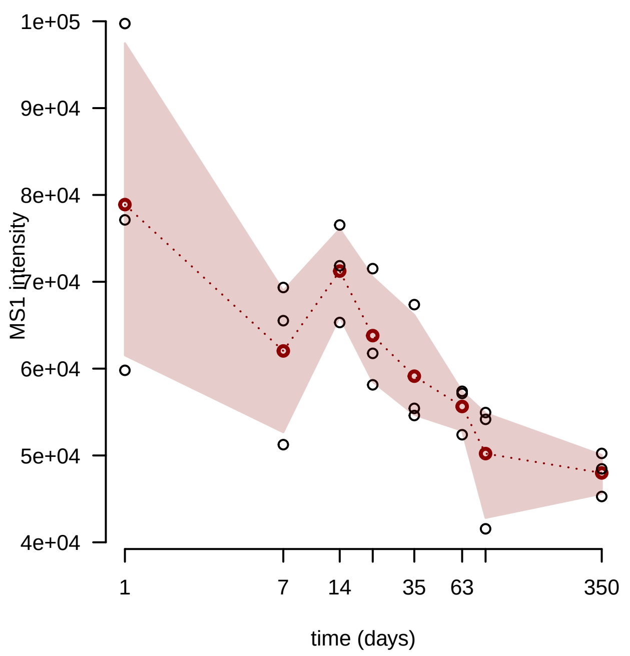

Q8R001
GN=Mapre2
microtubule−associated protein, RP/EB fa ...

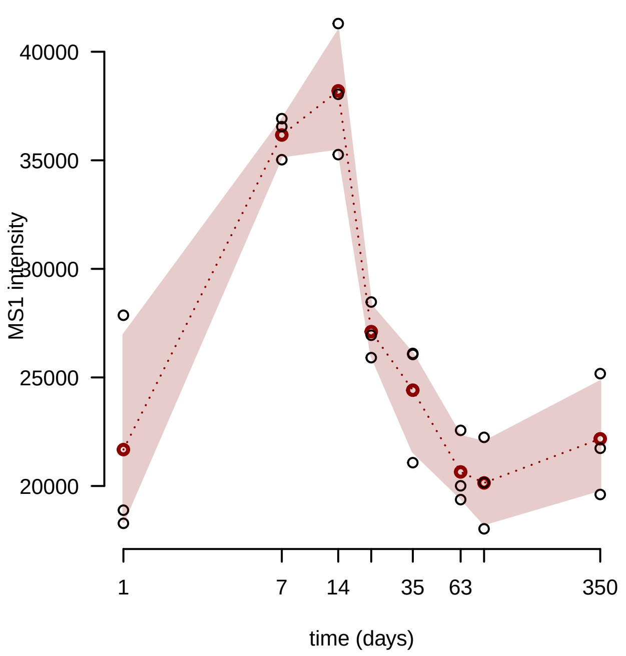

Q8R003
GN=Mbnl3
muscleblind like splicing factor 3

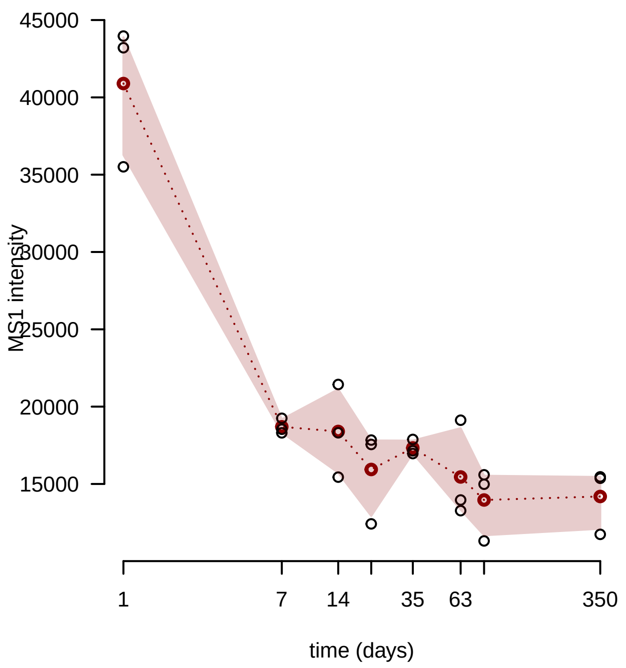

Q8R007
GN=Nectin4
nectin cell adhesion molecule 4

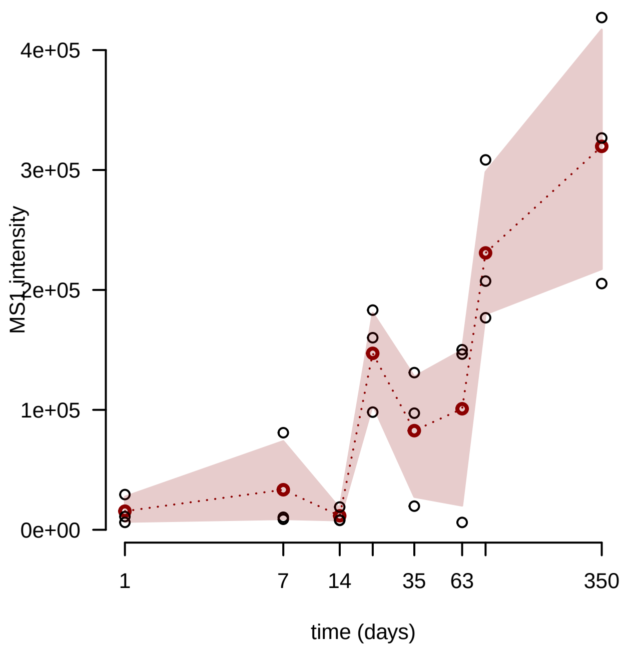

Q8R010
GN=Aimp2
aminoacyl tRNA synthetase complex−intera ...

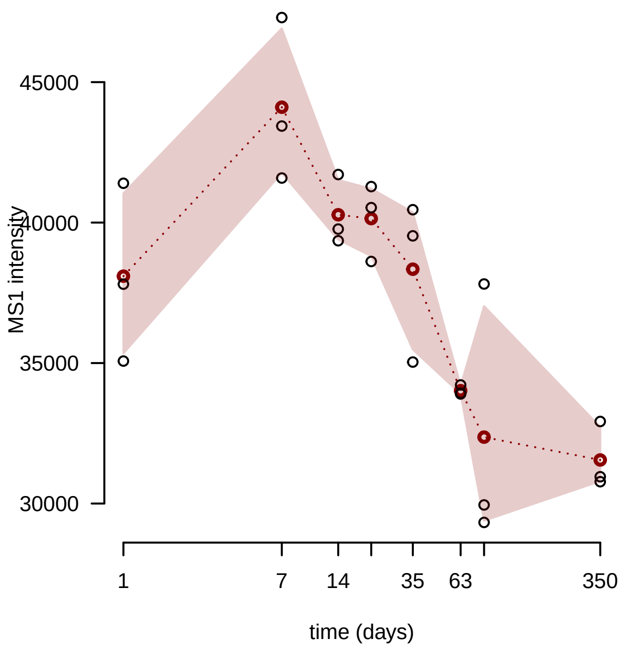

Q8R015
GN=Bloc1s5
biogenesis of lysosomal organelles compl ...

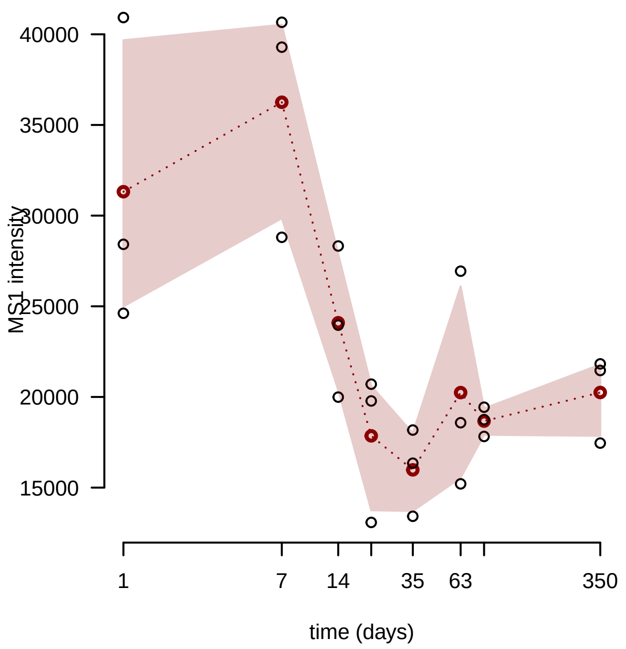

Q8R016
GN=Blmh
bleomycin hydrolase

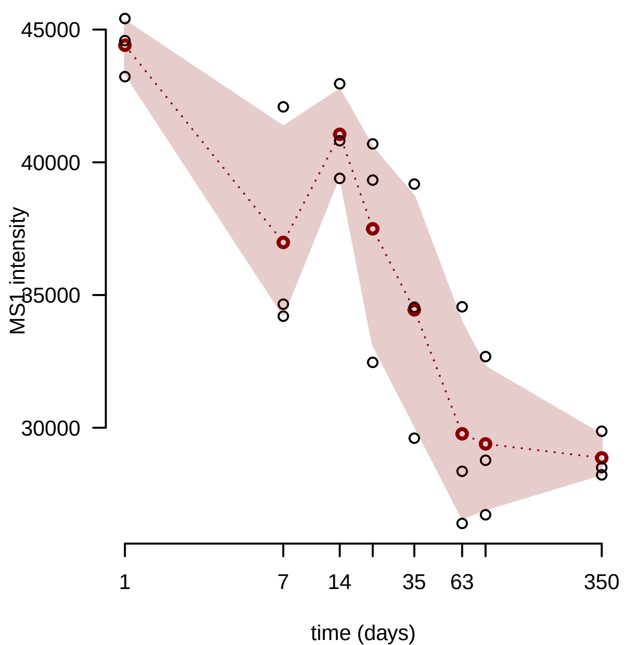

Q8R023
GN=Pcgf1
polycomb group ring finger 1

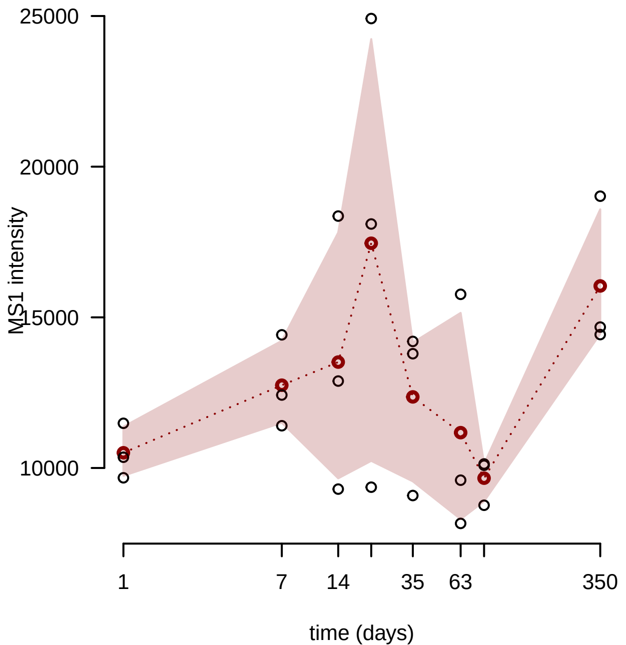

Q8R033
GN=Lyrm2
LYR motif containing 2

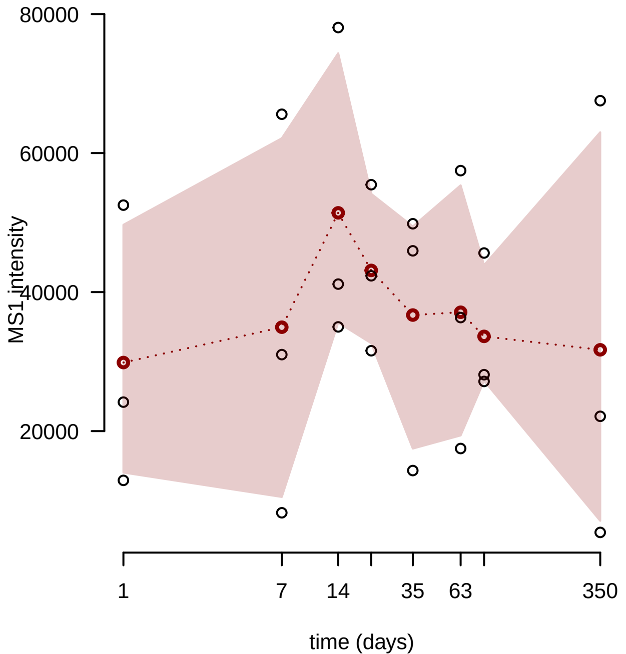

Q8R035
GN=Mrpl58
mitochondrial ribosomal protein L58

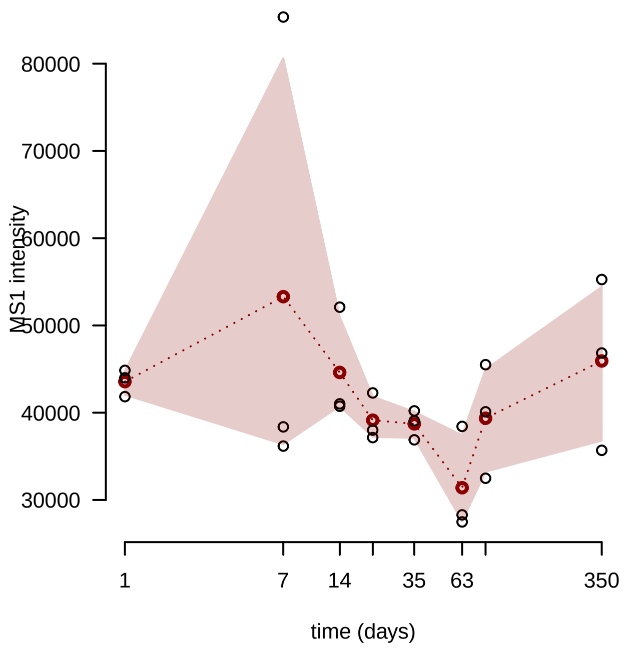

Q8R050
GN=Gspt1
G1 to S phase transition 1

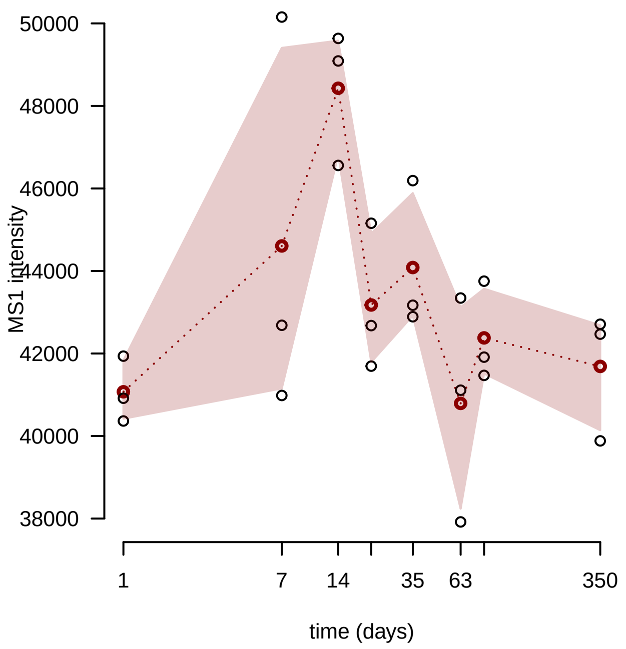

Q8R054
GN=Srpx2
sushi−repeat−containing protein, X−linke ...

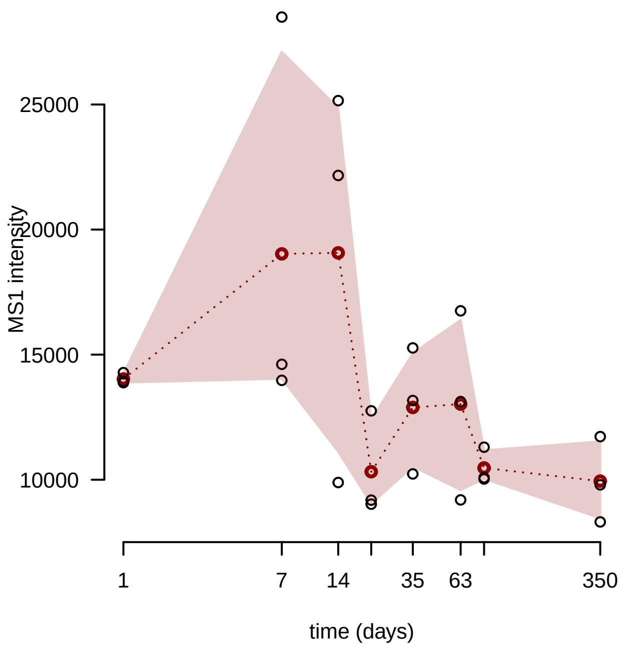

Q8R059
GN=Gale
galactose−4−epimerase, UDP

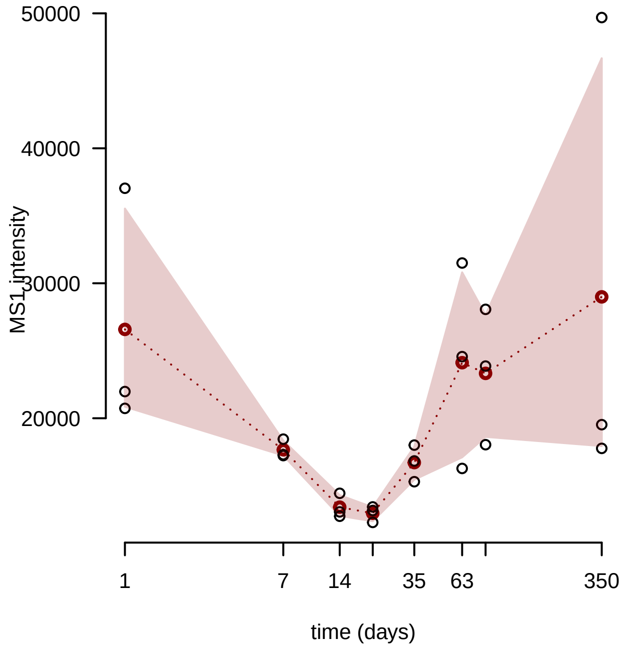

Q8R060
GN=Zwilch
zwilch kinetochore protein

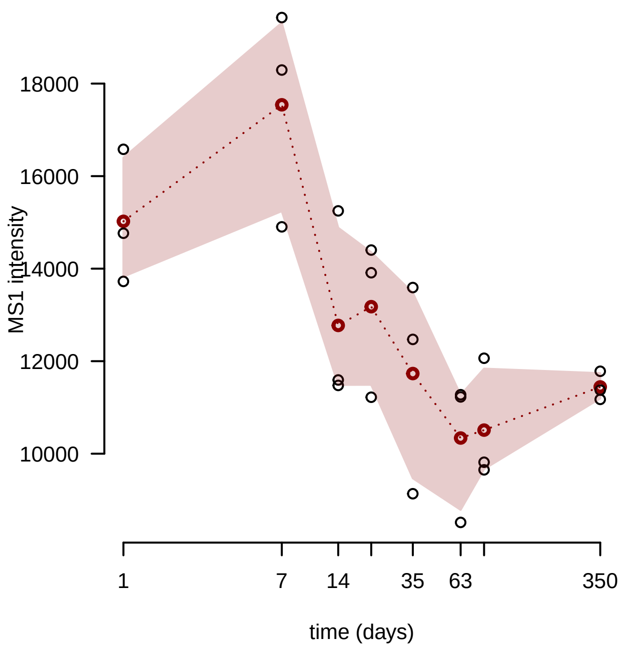

Q8R071
GN=Itpka
inositol 1,4,5−trisphosphate 3−kinase A

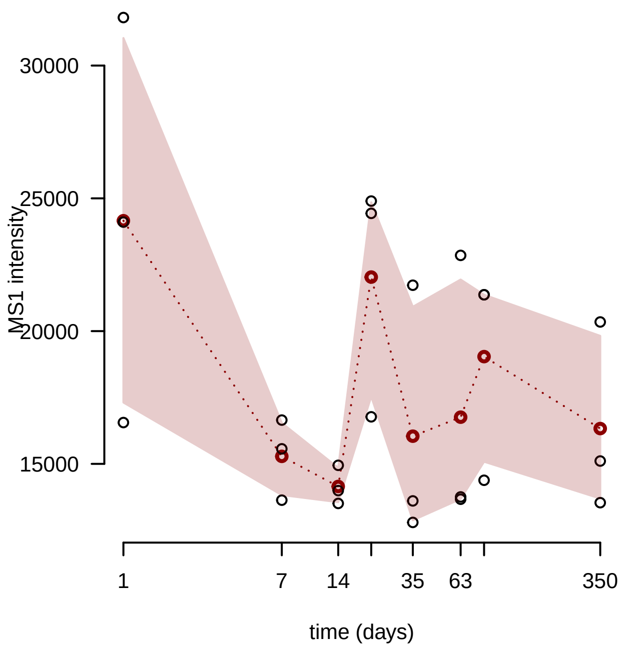

Q8R079
GN=Bfar
bifunctional apoptosis regulator

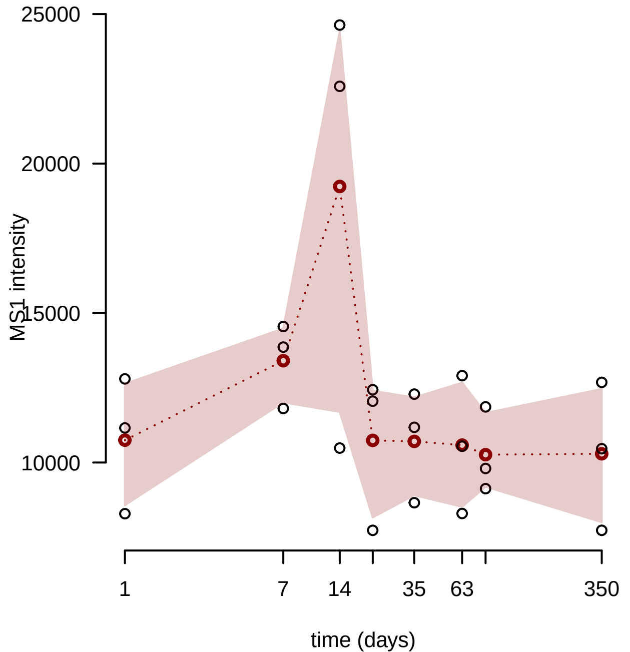

Q8R081
GN=Hnrnpl
heterogeneous nuclear ribonucleoprotein ...

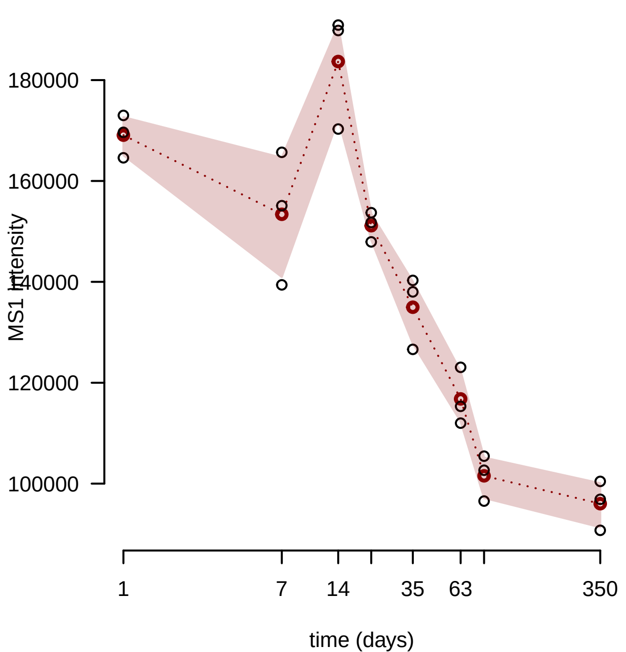

Q8R086
GN=Suox
sulfite oxidase

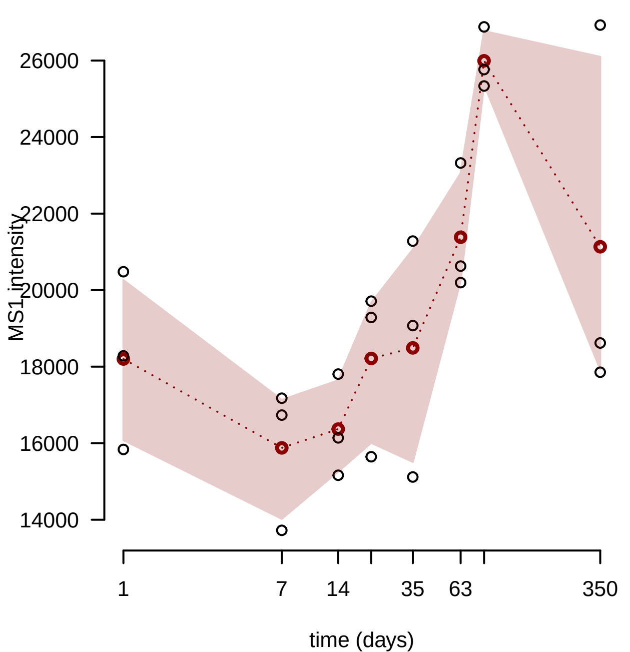

Q8R087
GN=B4galt7
xylosylprotein beta1,4−galactosyltransfe ...

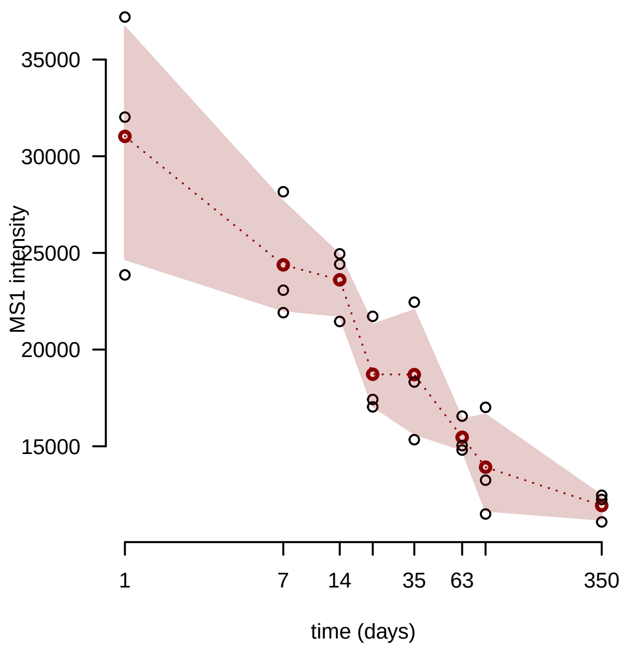

Q8R088
GN=Golph3l
golgi phosphoprotein 3−like

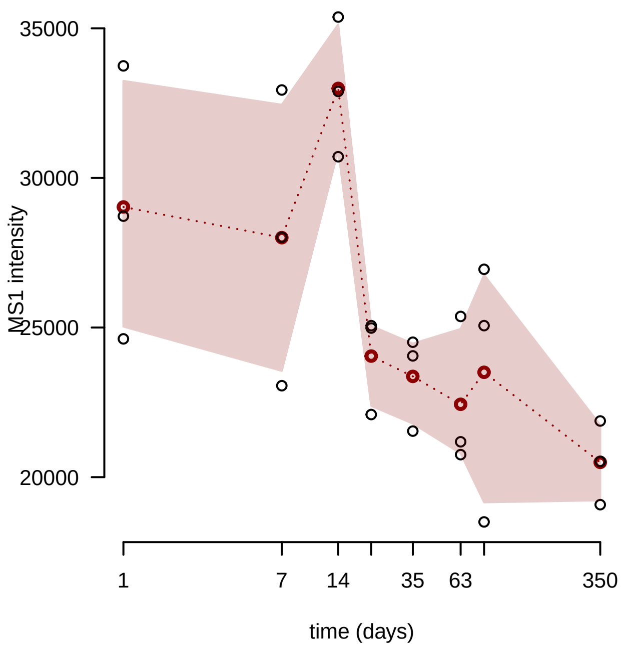

Q8R0A0
GN=Gtf2f2
general transcription factor IIF, polype ...

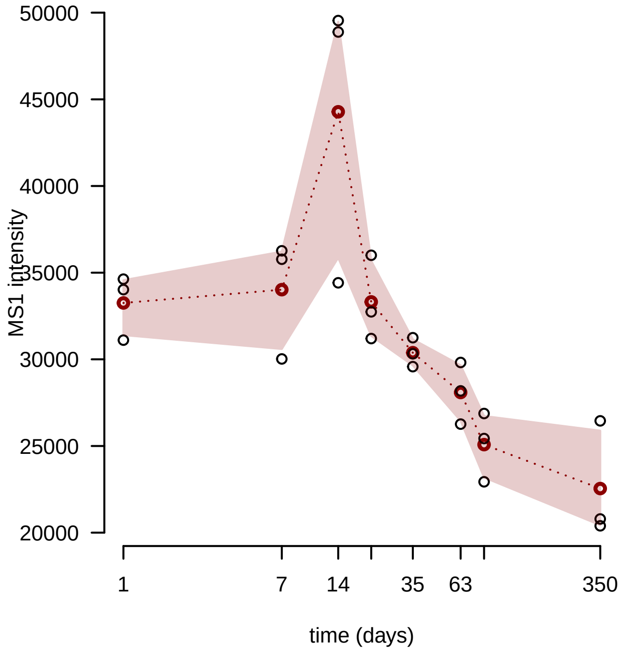

Q8R0A5
GN=Tceal3
transcription elongation factor A (SII)− ...

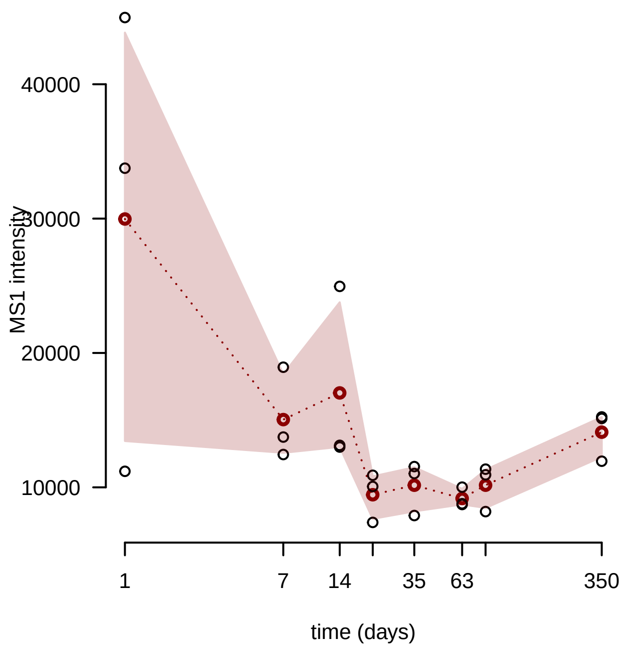

Q8R0A7
GN=Kiaa0513

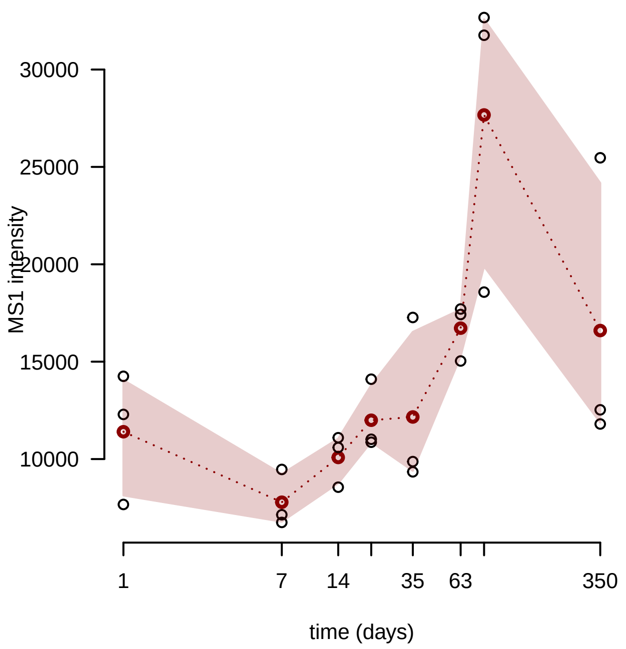

Q8R0F3
GN=Sumf1
sulfatase modifying factor 1

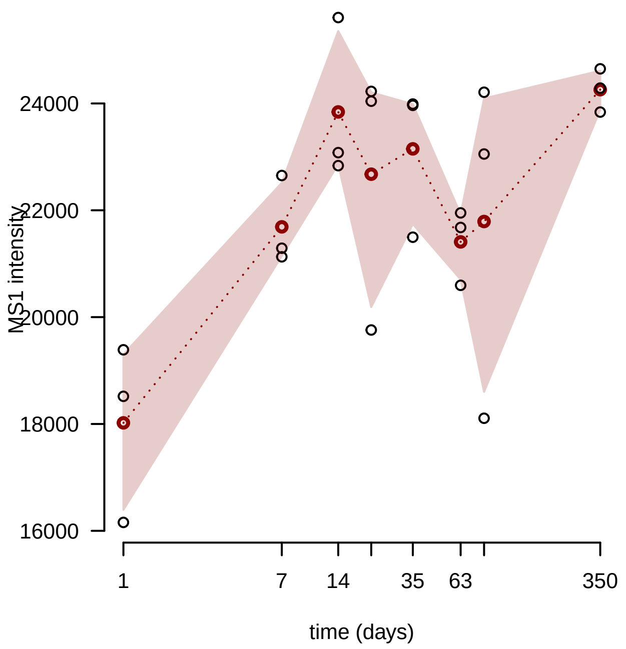

Q8R0F5
GN=Rbmx2
RNA binding motif protein, X−linked 2

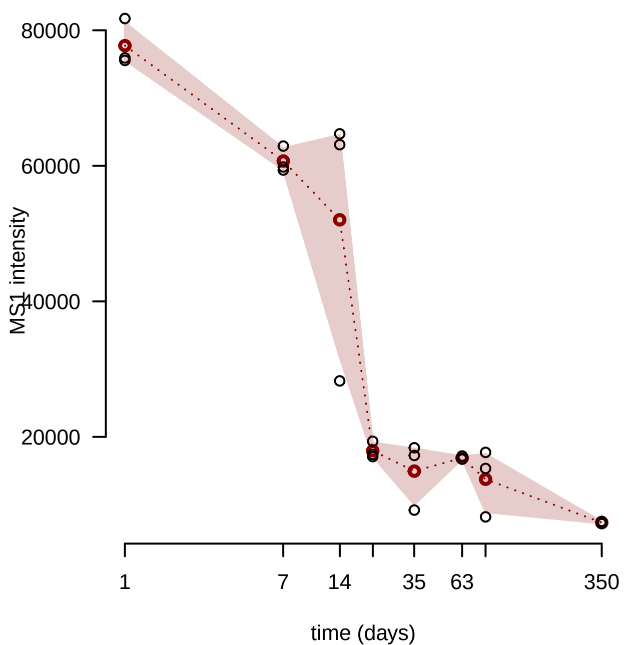

Q8R0F6
GN=Ilkap
integrin−linked kinase−associated serine ...

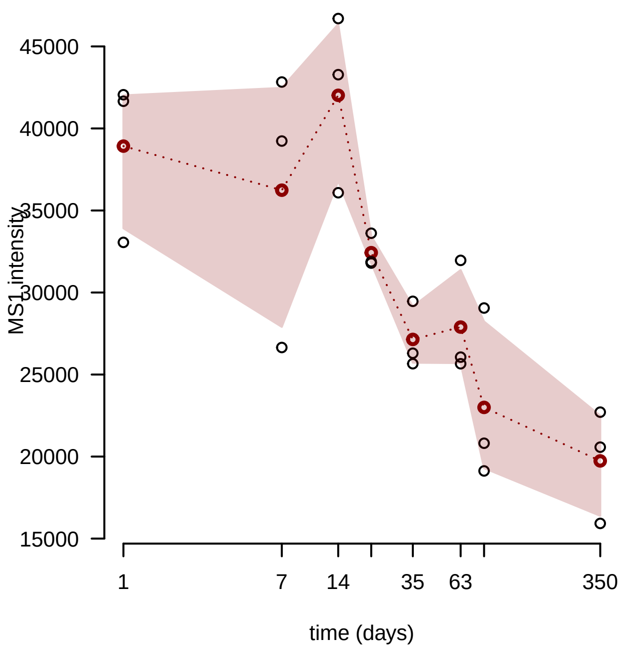

Q8R0F8
GN=Fahd1
fumarylacetoacetate hydrolase domain con ...

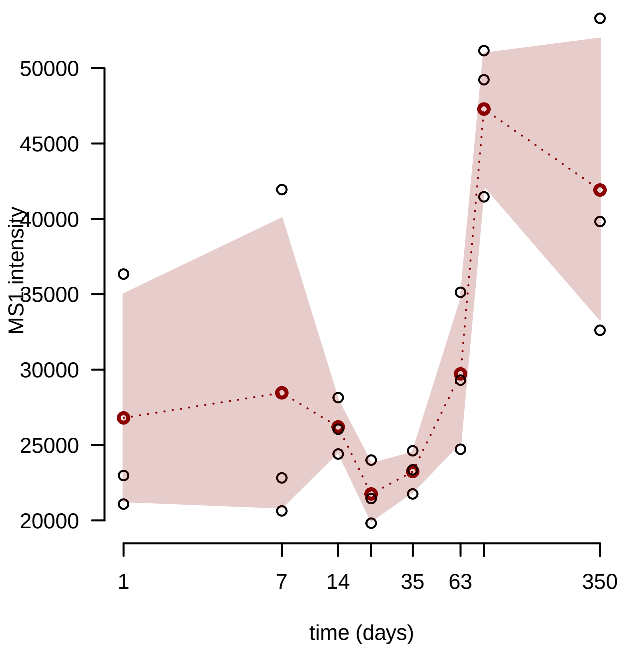

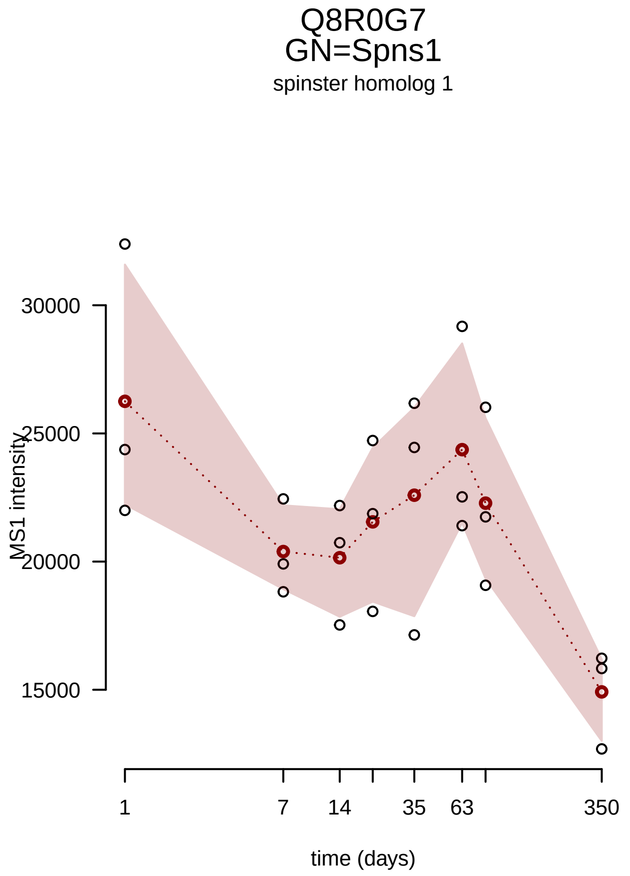
Q8R0G7
GN=Spns1
spinster homolog 1
MS1 intensity
30000
25000
20000
15000
1
7
14
35
63
350
time (days)

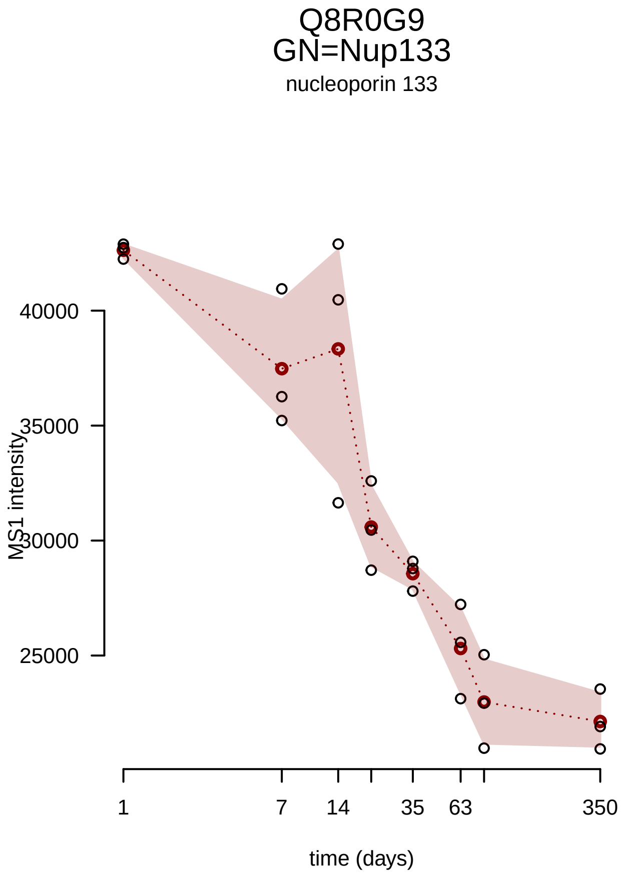
Q8R0G9
GN=Nup133
nucleoporin 133
MS1 intensity
40000
35000
30000
25000
1
7
14
35
63
350
time (days)

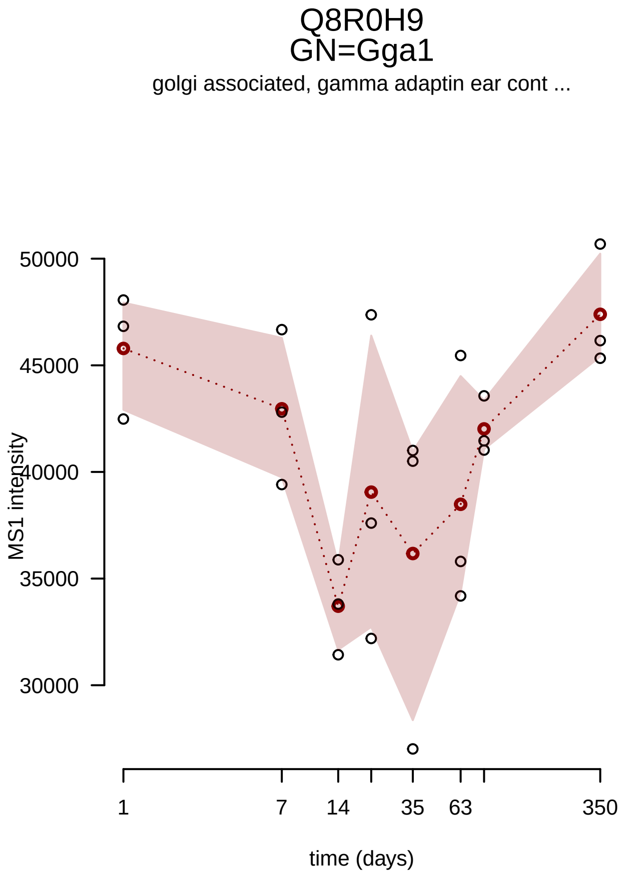
Q8R0H9
GN=Gga1
golgi associated, gamma adaptin ear cont ...
MS1 intensity
50000
45000
40000
35000
30000
1
7
14
35
63
350
time (days)

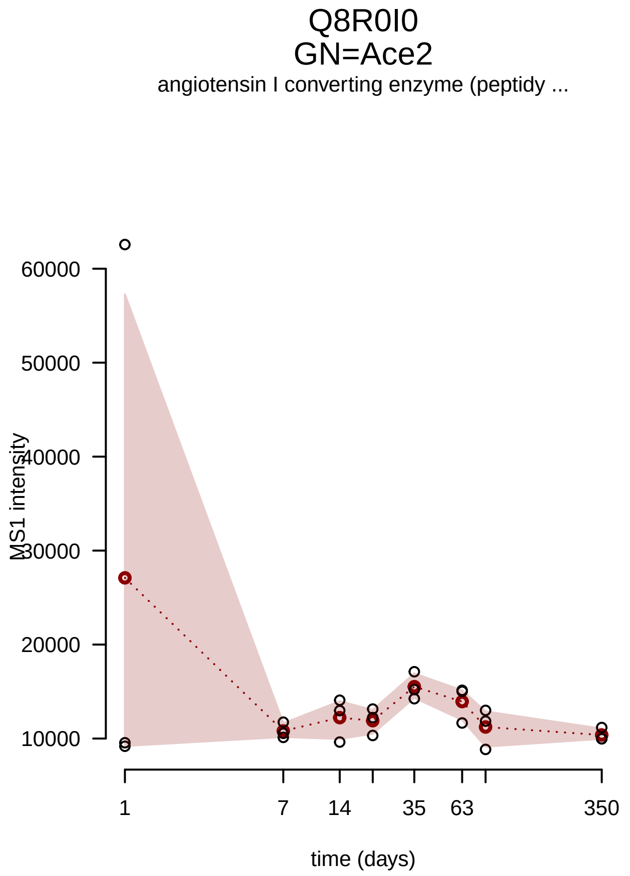
Q8R0I0
GN=Ace2
angiotensin I converting enzyme (peptidy ...
MS1 intensity
60000
50000
40000
30000
20000
10000
1
7
14
35
63
350
time (days)

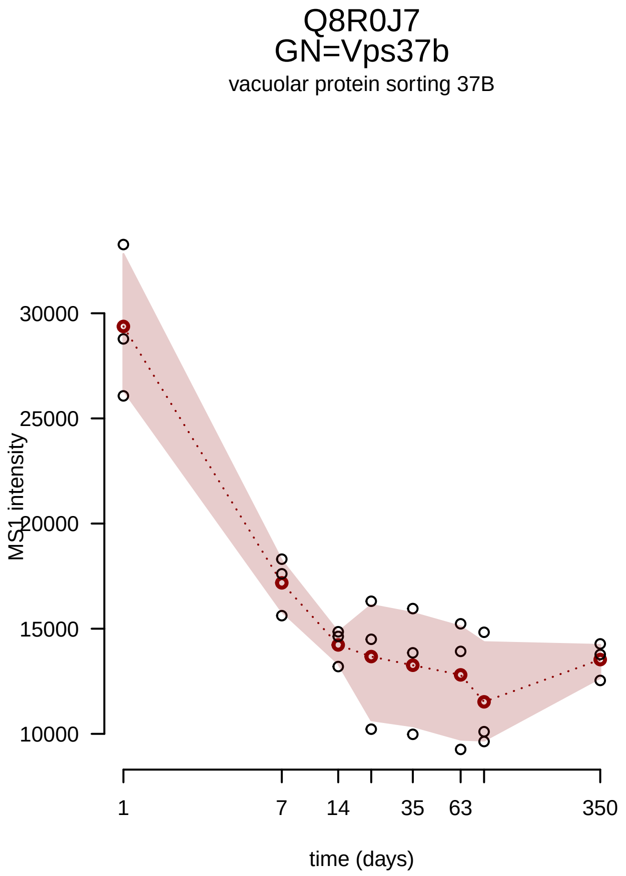
Q8R0J7
GN=Vps37b
vacuolar protein sorting 37B
MS1 intensity
30000
25000
20000
15000
10000
1
7
14
35
63
350
time (days)

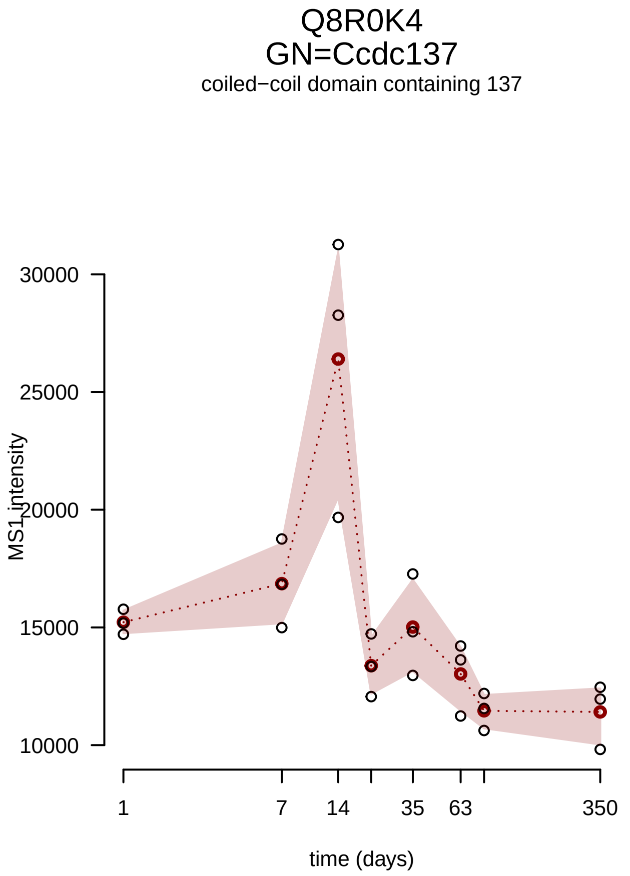
Q8R0K4
GN=Ccdc137
coiled−coil domain containing 137
MS1 intensity
30000
25000
20000
15000
10000
1
7
14
35
63
350
time (days)

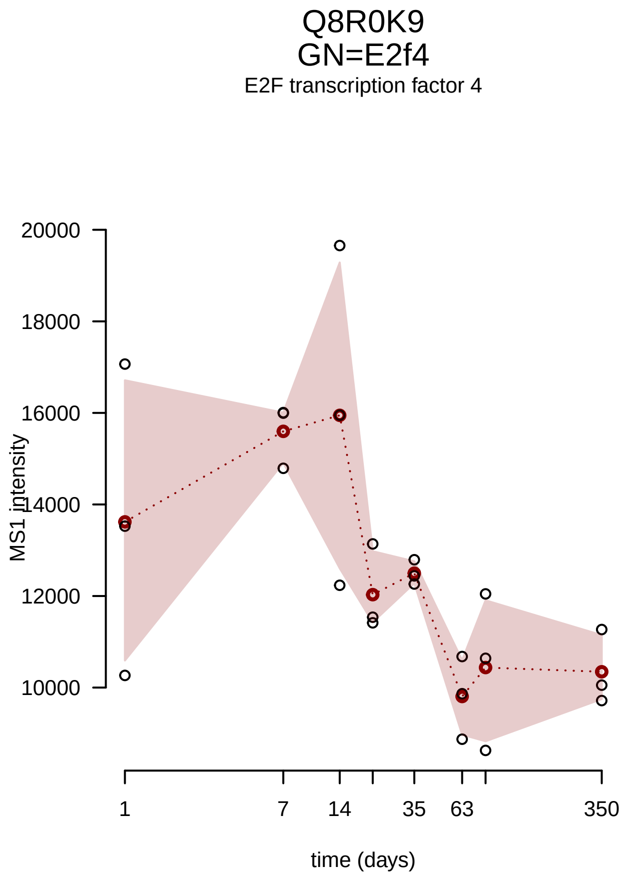
Q8R0K9
GN=E2f4
E2F transcription factor 4
MS1 intensity
20000
18000
16000
14000
12000
10000
1
7
14
35
63
350
time (days)

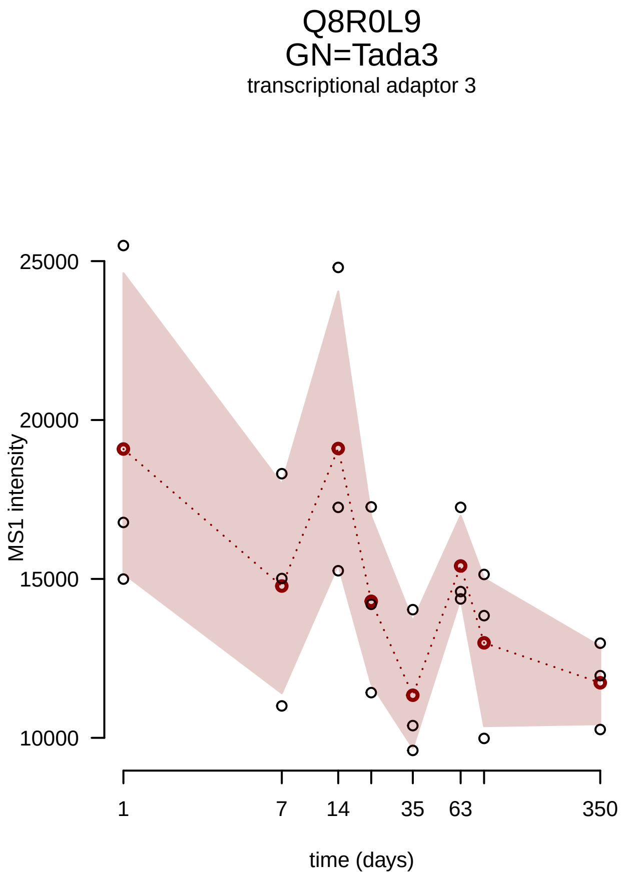
Q8R0L9
GN=Tada3
transcriptional adaptor 3
MS1 intensity
25000
20000
15000
10000
1
7
14
35
63
350
time (days)

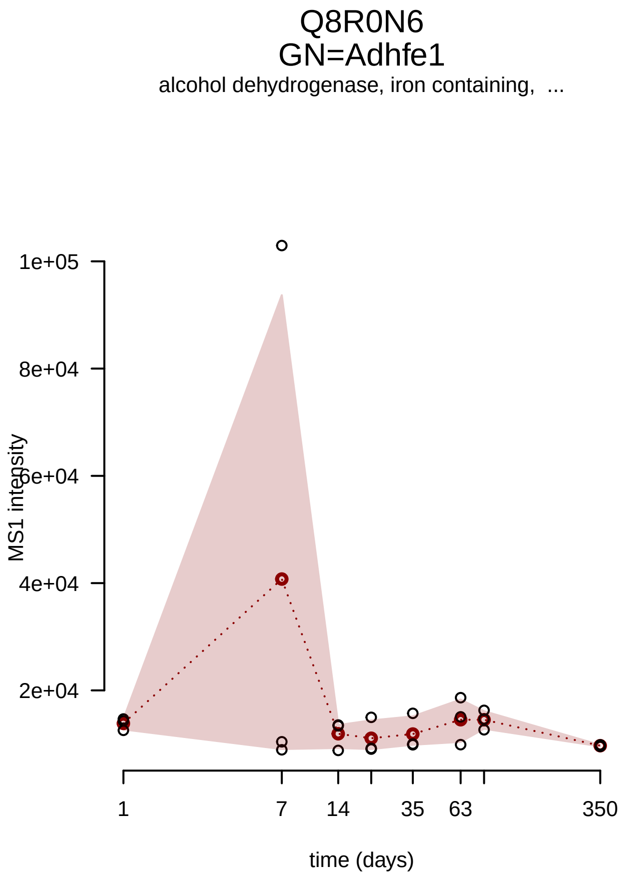
Q8R0N6
GN=Adhfe1
alcohol dehydrogenase, iron containing, ...
MS1 intensity
1e+05
8e+04
6e+04
4e+04
2e+04
1
7
14
35
63
350
time (days)

Q8R0P4
GN=Aamdc
adipogenesis associated Mth938 domain co ...

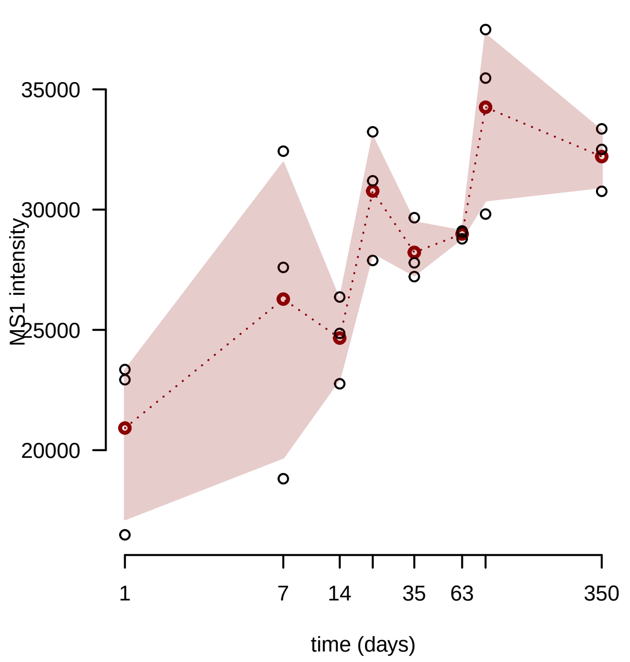

Q8R0S1
GN=Atf7
activating transcription factor 7

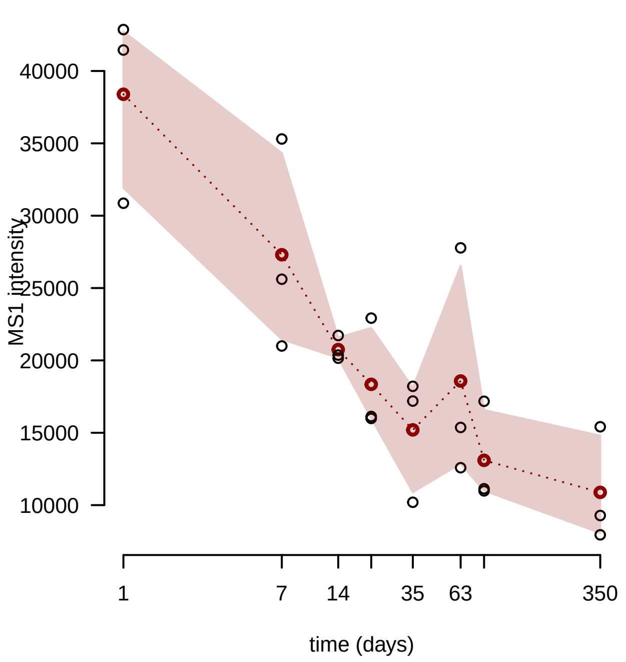

Q8R0S2
GN=Iqsec1
IQ motif and Sec7 domain 1

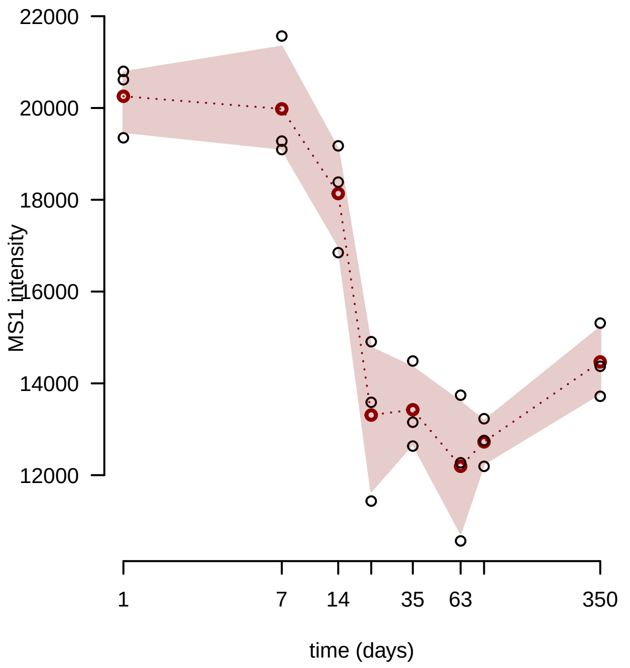

Q8R0T2
GN=Znf768

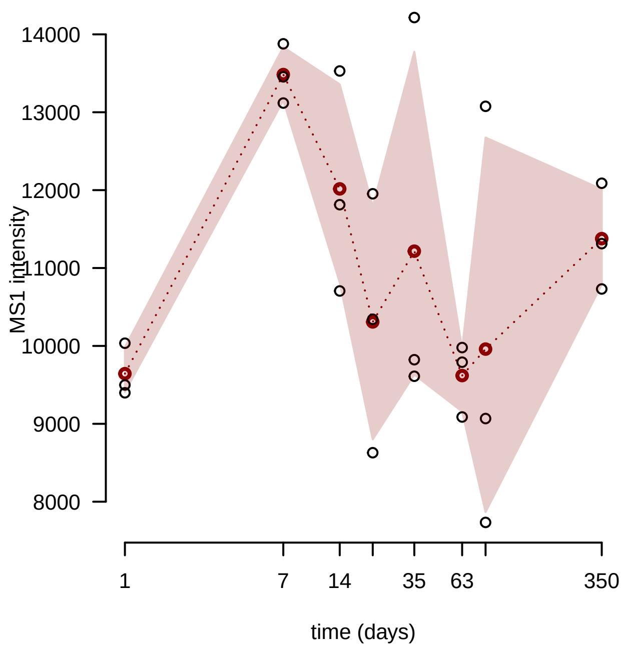

Q8R0W0
GN=Eppk1
epiplakin 1

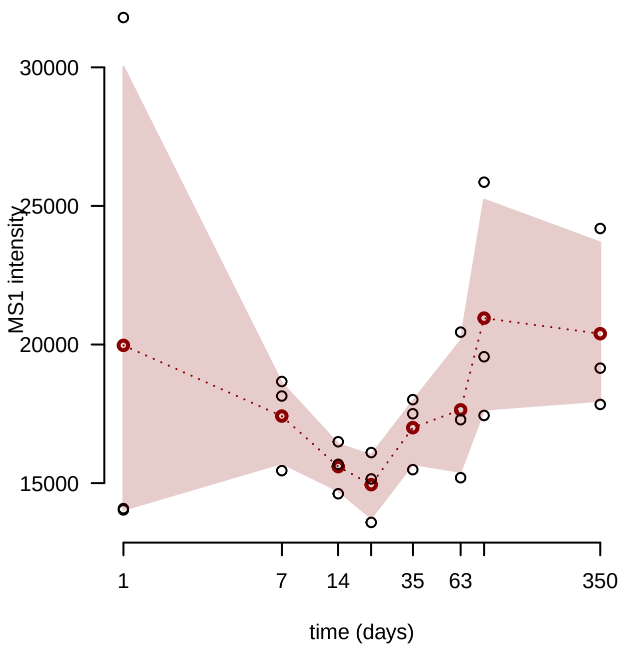

Q8R0X2
GN=Cacul1
CDK2 associated, cullin domain 1

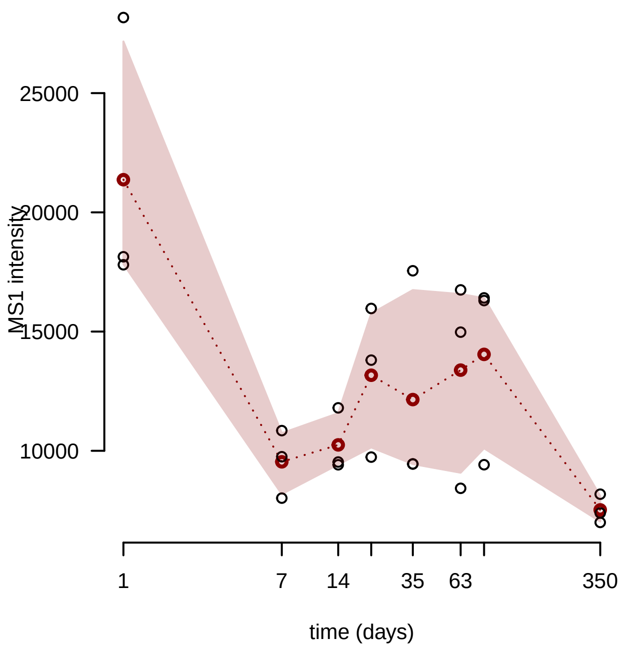

Q8R0X7
GN=Sgpl1
sphingosine phosphate lyase 1

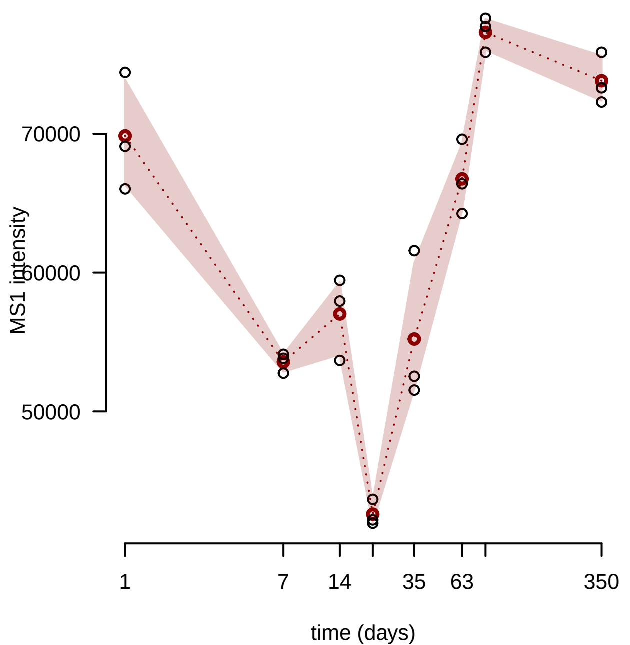

Q8R0Y6
GN=Aldh1l1
aldehyde dehydrogenase 1 family, member ...

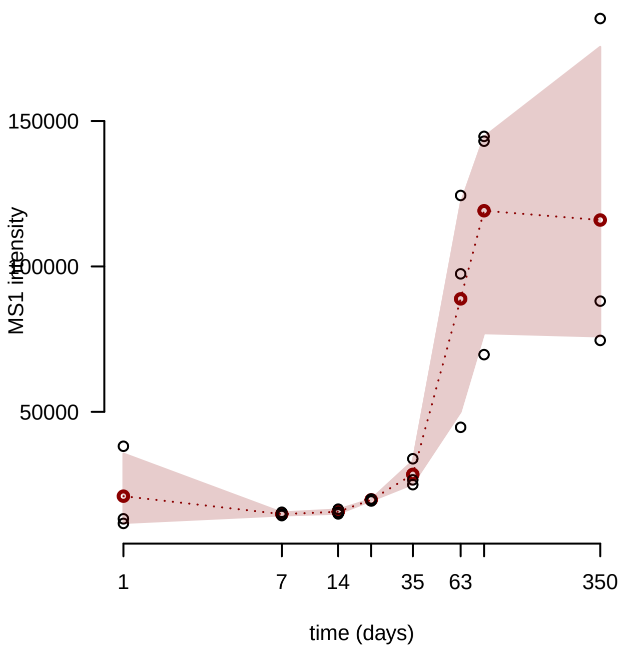

Q8R0Y8
GN=Slc25a42
solute carrier family 25, member 42

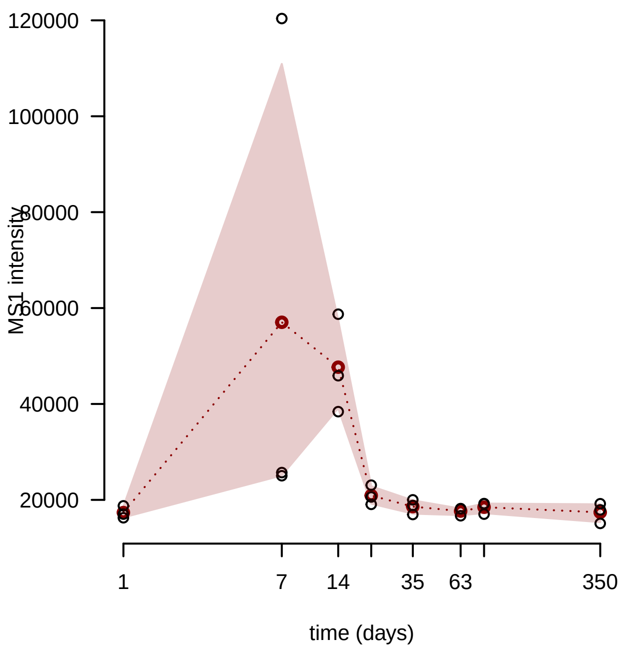

Q8R0Z5
GN=Slc25a28
solute carrier family 25, member 28

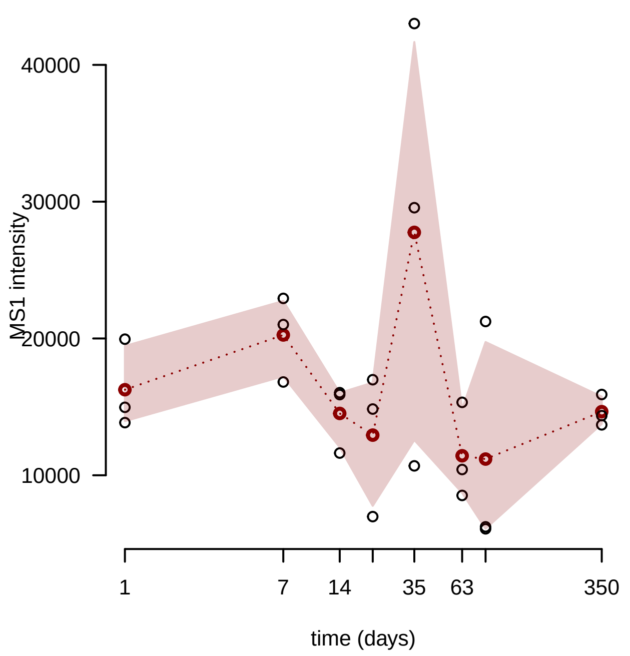

Q8R0Z6
GN=Angptl6
angiopoietin−like 6

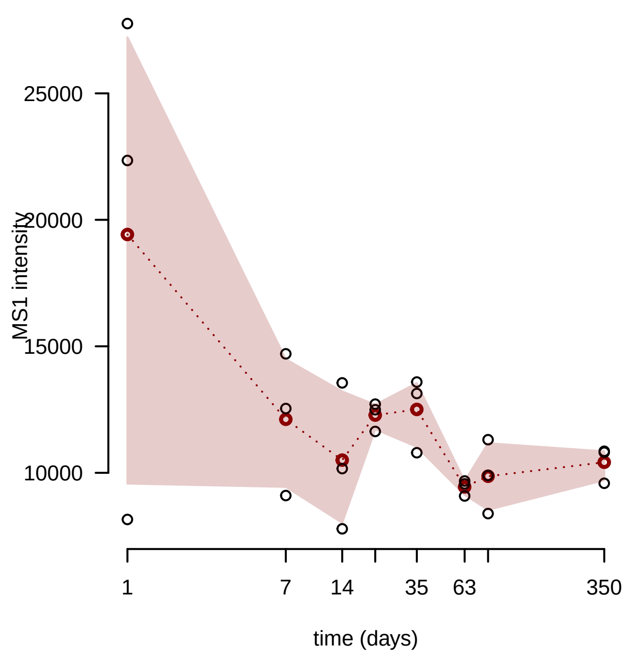

Q8R104
GN=Sirt3
sirtuin 3

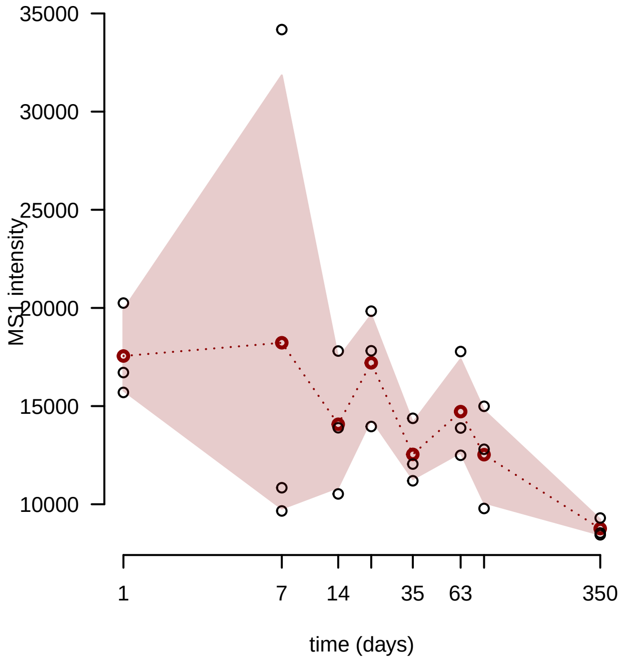

Q8R105
GN=Vps37c
vacuolar protein sorting 37C

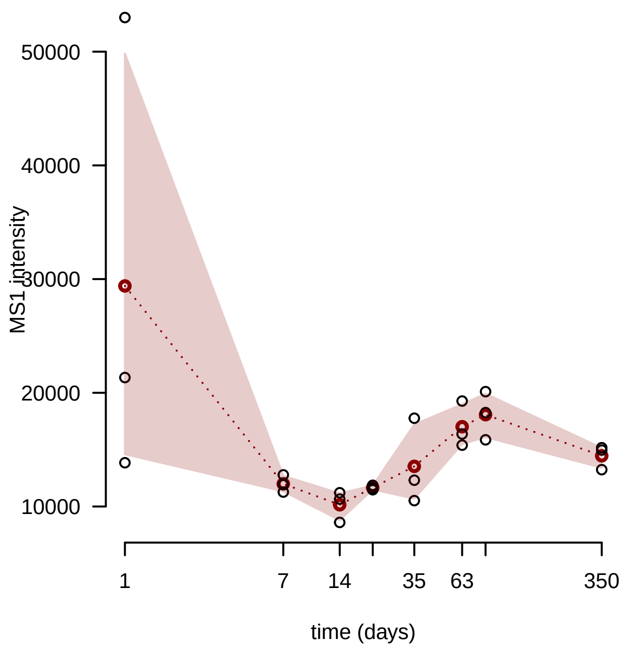

Q8R121
GN=Serpina10
serine (or cysteine) peptidase inhibitor ...

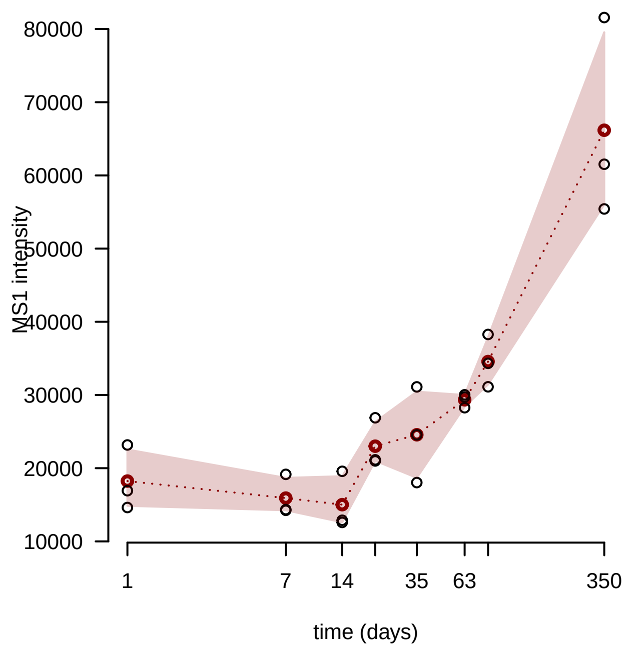

Q8R123
GN=Flad1
flavin adenine dinucleotide synthetase 1

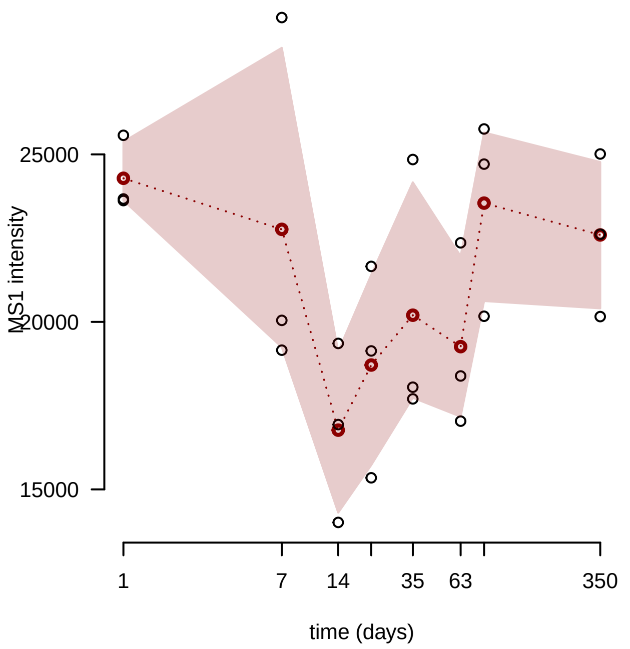

Q8R124
GN=Klhl36
kelch−like 36

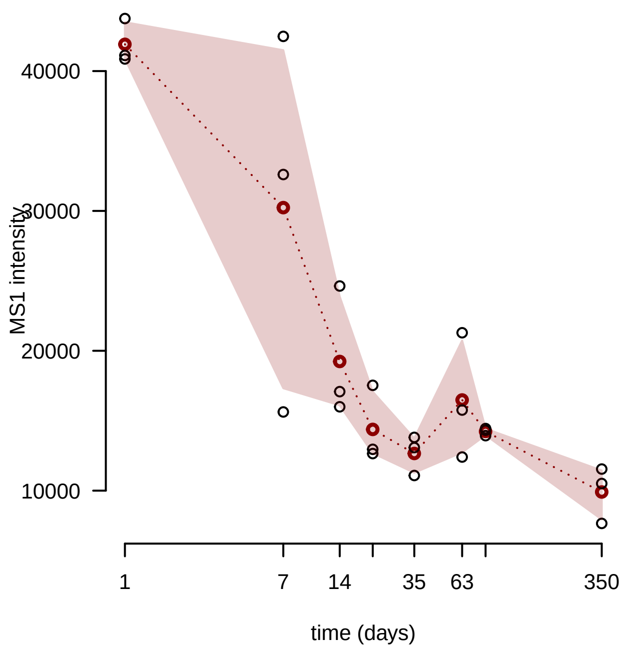

Q8R127
GN=Sccpdh
saccharopine dehydrogenase (putative)

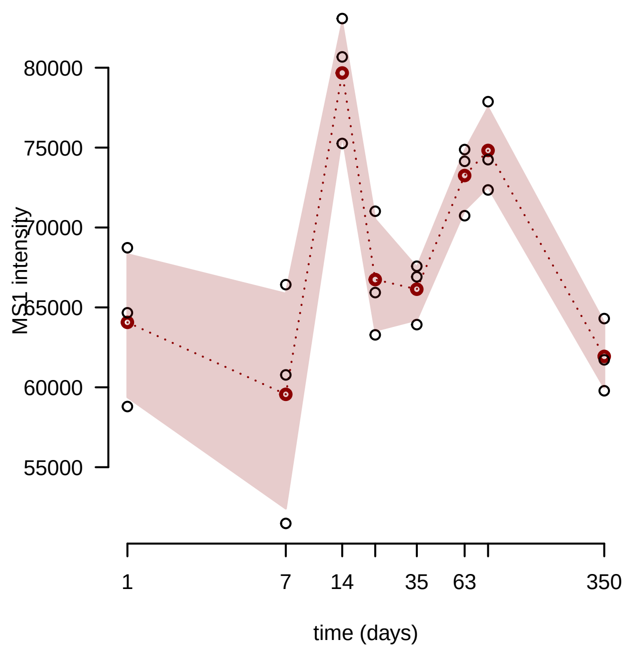

Q8R138
GN=Tmem119
transmembrane protein 119

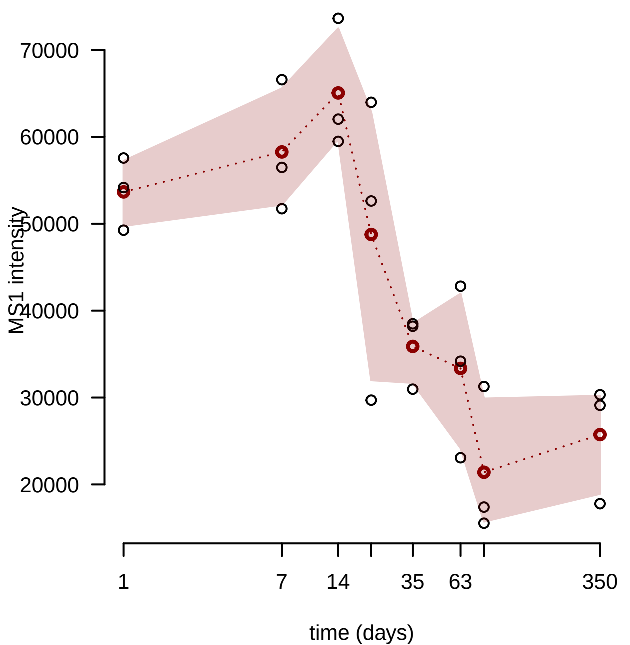

Q8R143
GN=Pttg1ip
pituitary tumor−transforming 1 interacti ...

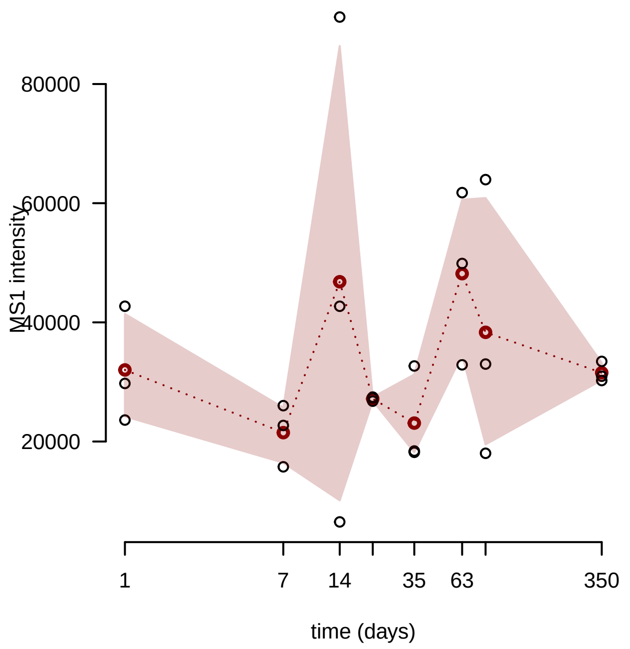

Q8R146
GN=Apeh
acylpeptide hydrolase

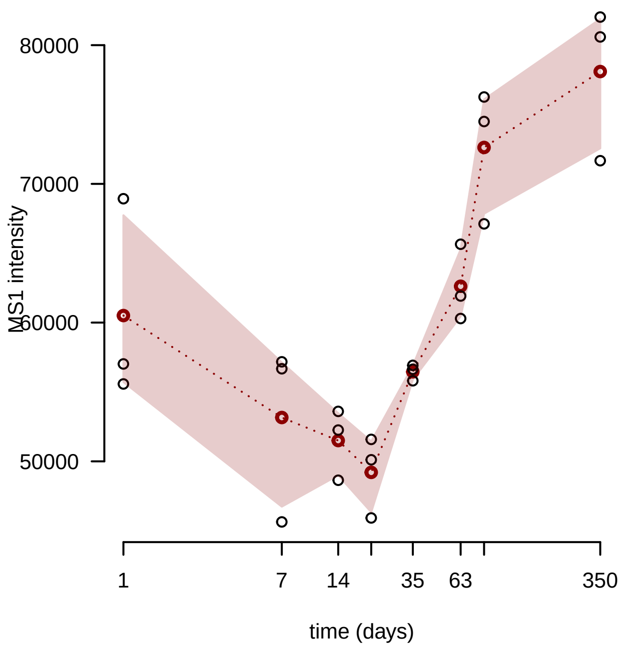

Q8R149
GN=Bud13
BUD13 homolog

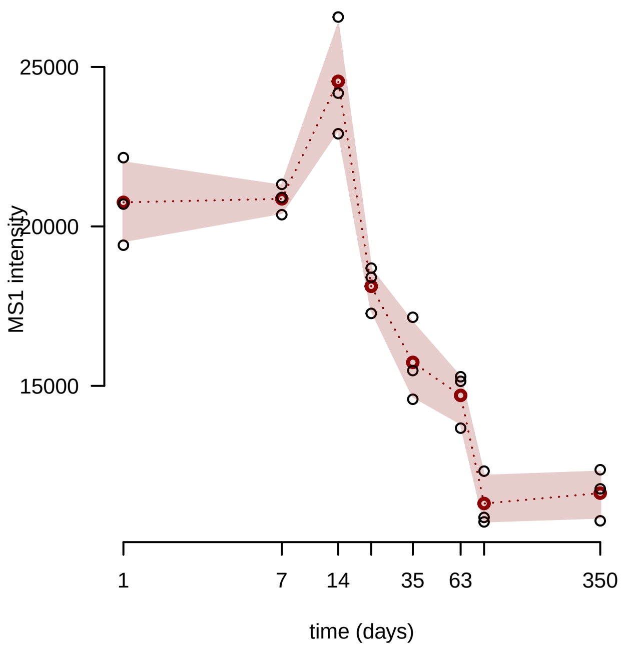

Q8R151
GN=Znfx1
zinc finger, NFX1−type containing 1

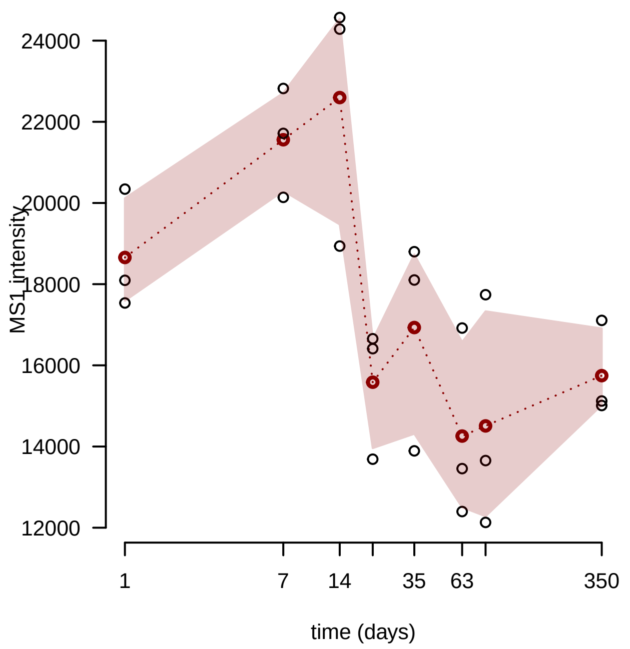

Q8R164
GN=Bphl
biphenyl hydrolase−like (serine hydrolas ...

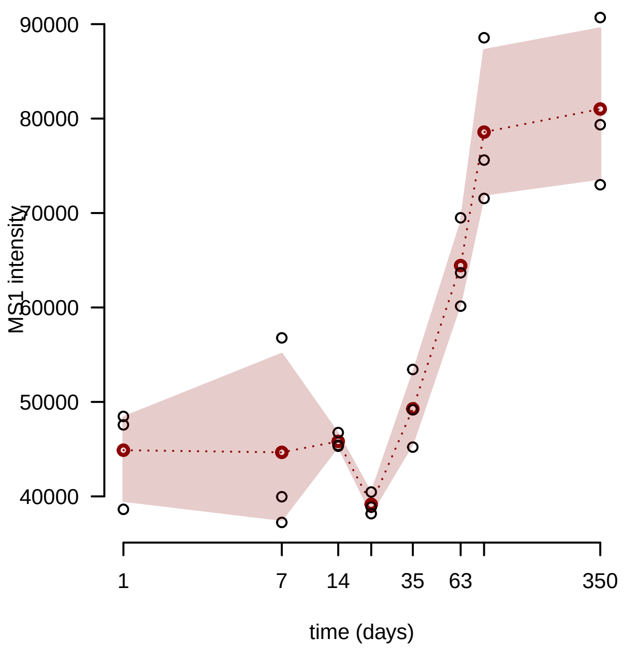

Q8R173
GN=Zdhhc3
zinc finger, DHHC domain containing 3

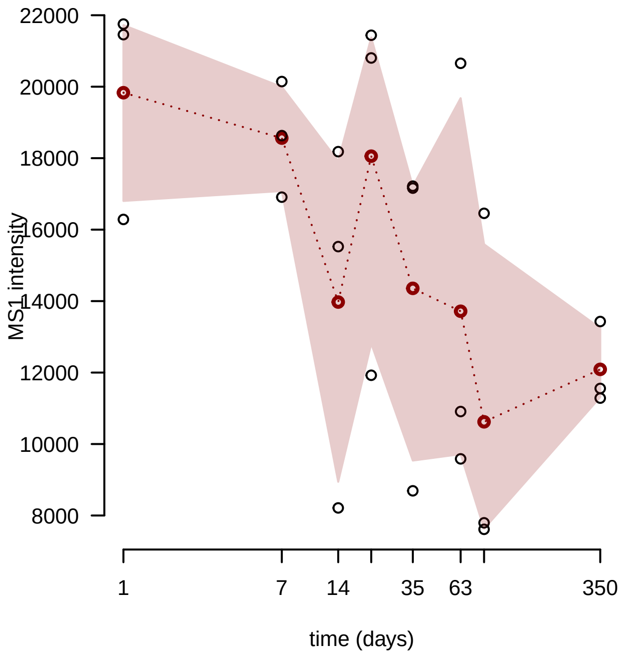

Q8R179
GN=Kbtbd4
kelch repeat and BTB (POZ) domain contai ...

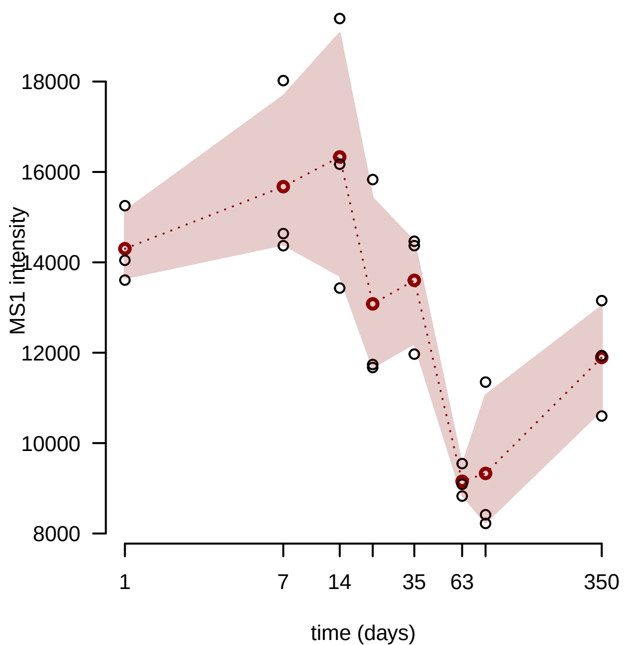

Q8R180
GN=Ero1a
endoplasmic reticulum oxidoreductase 1 a ...

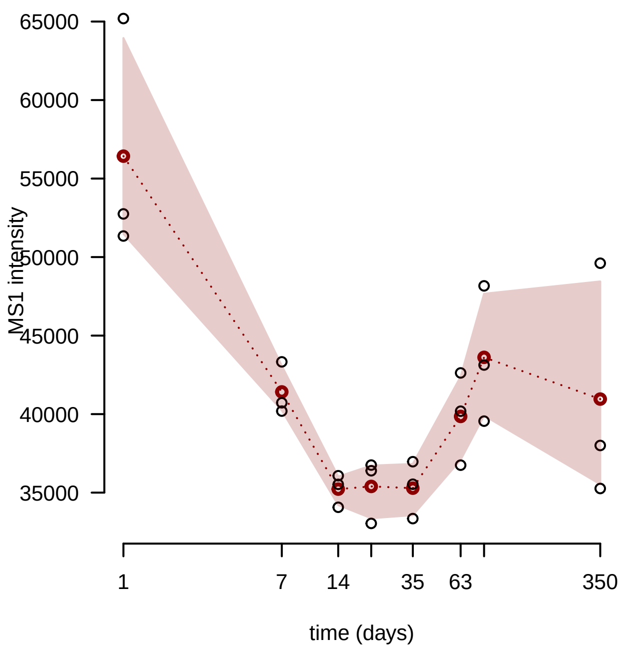

Q8R1A4
GN=Dock7
dedicator of cytokinesis 7

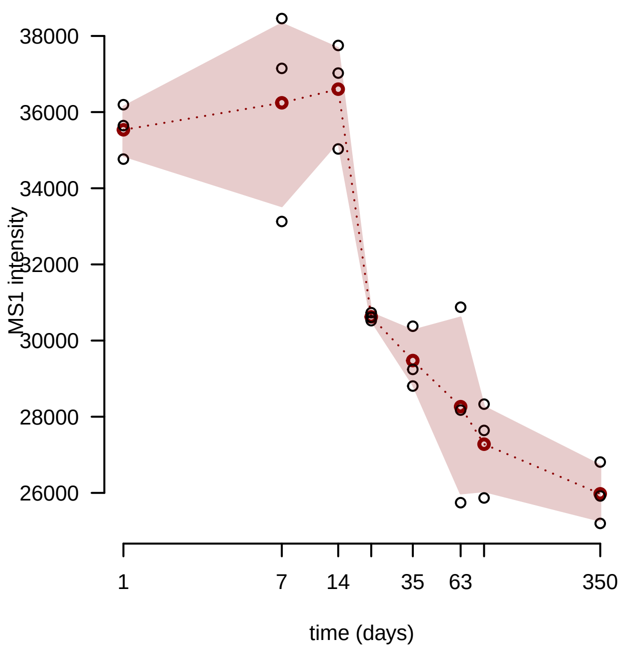

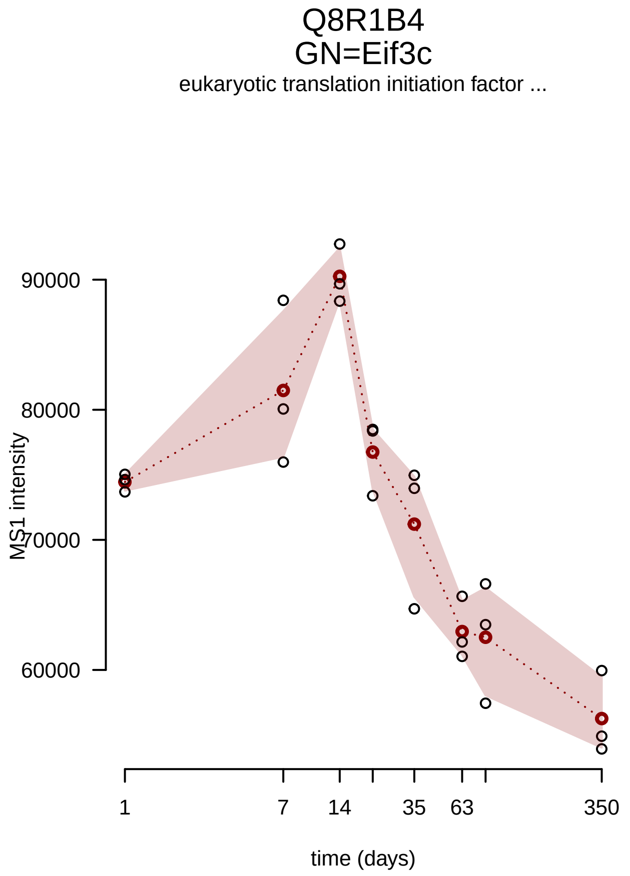
Q8R1B4
GN=Eif3c
eukaryotic translation initiation factor ...
90000
80000
70000
60000
MS1 intensity
1
7
14
35
63
350
time (days)

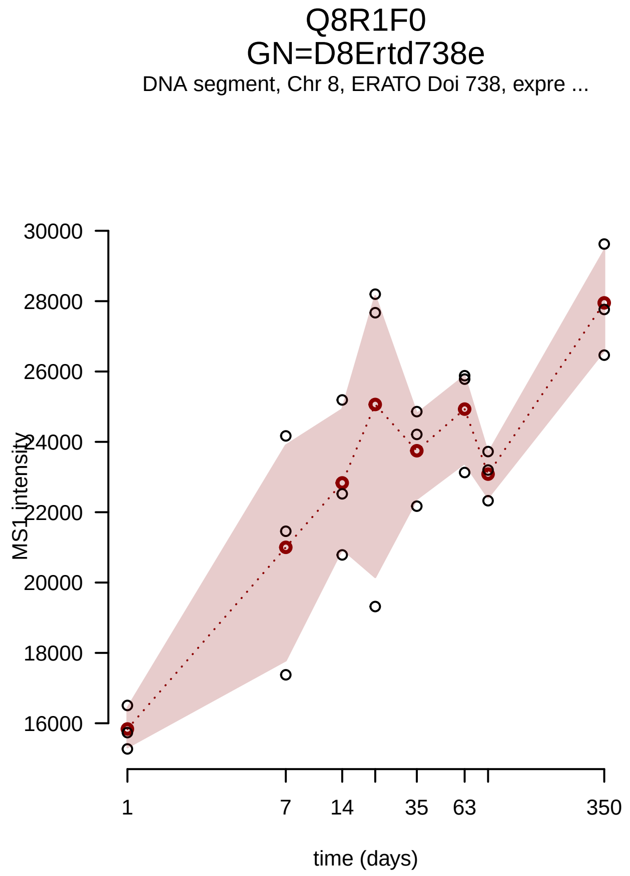
Q8R1F0
GN=D8Ertd738e
DNA segment, Chr 8, ERATO Doi 738, expre ...
30000
28000
26000
24000
22000
20000
18000
16000
MS1 intensity
1
7
14
35
63
350
time (days)

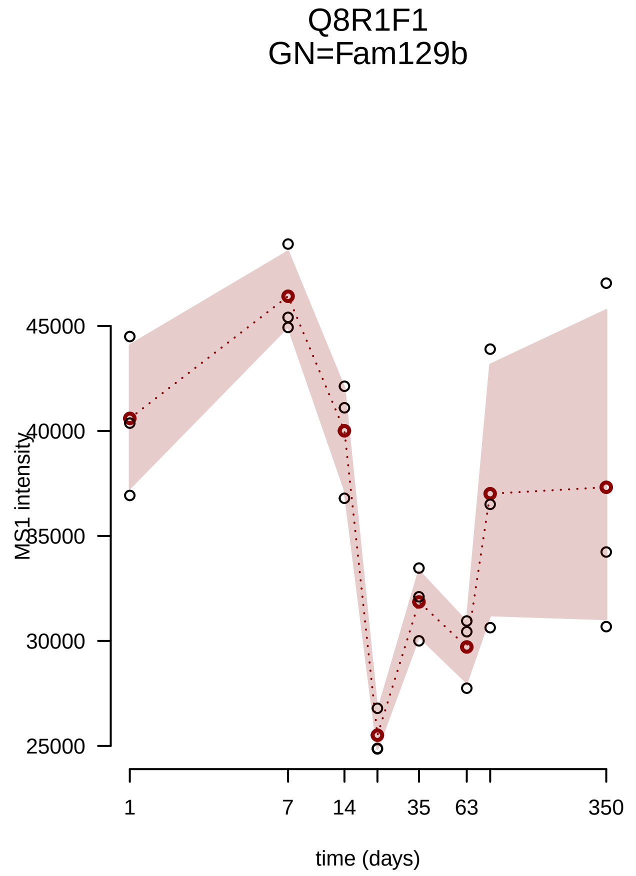
Q8R1F1
GN=Fam129b
45000
40000
35000
30000
25000
MS1 intensity
1
7
14
35
63
350
time (days)

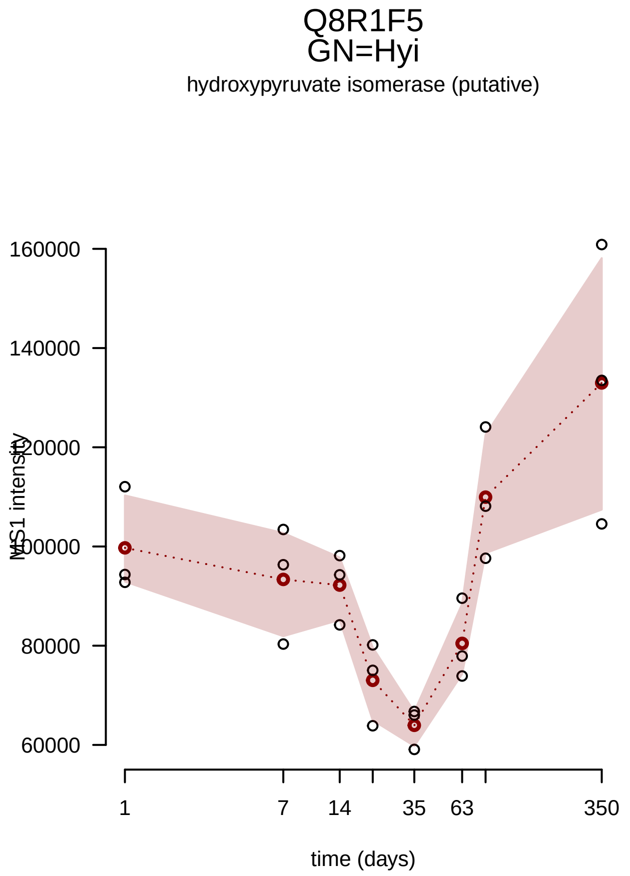
Q8R1F5
GN=Hyi
hydroxypyruvate isomerase (putative)
160000
140000
120000
100000
80000
60000
MS1 intensity
1
7
14
35
63
350
time (days)

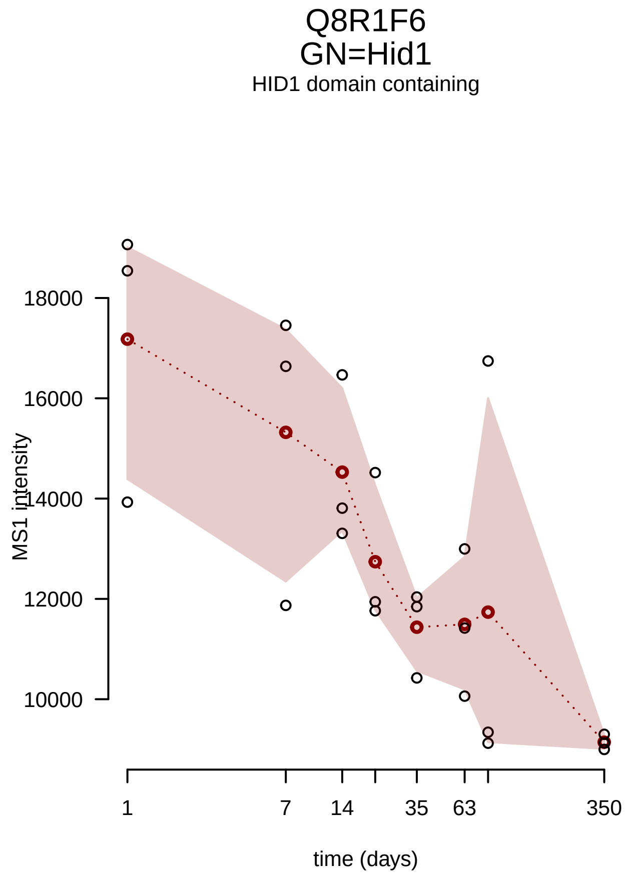
Q8R1F6
GN=Hid1
HID1 domain containing
18000
16000
14000
12000
10000
MS1 intensity
1
7
14
35
63
350
time (days)

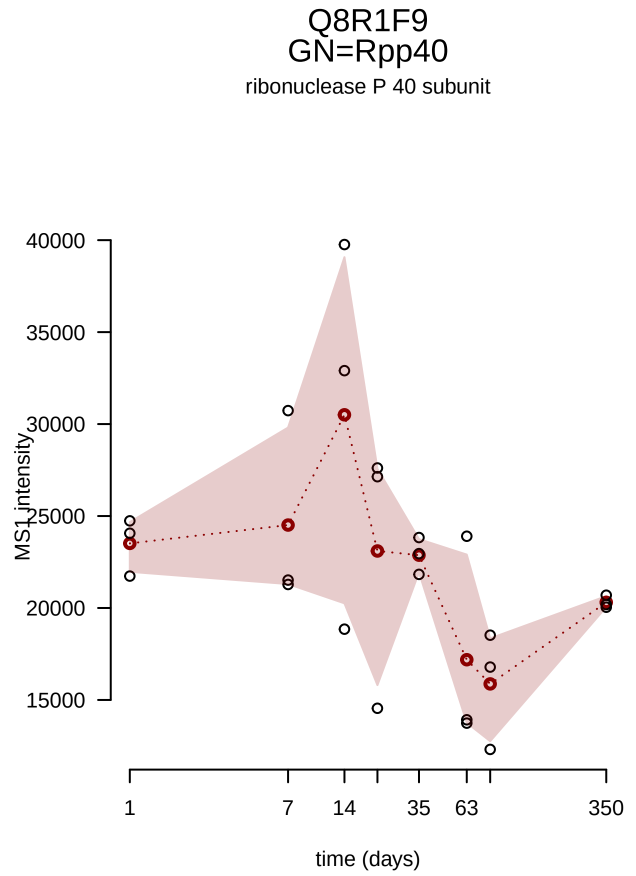
Q8R1F9
GN=Rpp40
ribonuclease P 40 subunit
40000
35000
30000
25000
20000
15000
MS1 intensity
1
7
14
35
63
350
time (days)

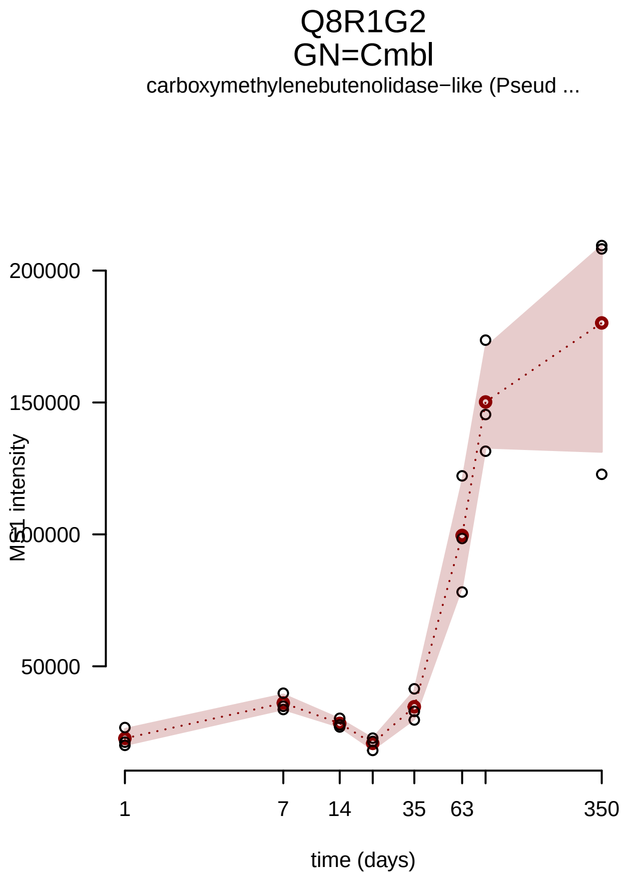
Q8R1G2
GN=Cmbl
carboxymethylenebutenolidase−like (Pseud ...
200000
150000
100000
50000
MS1 intensity
1
7
14
35
63
350
time (days)

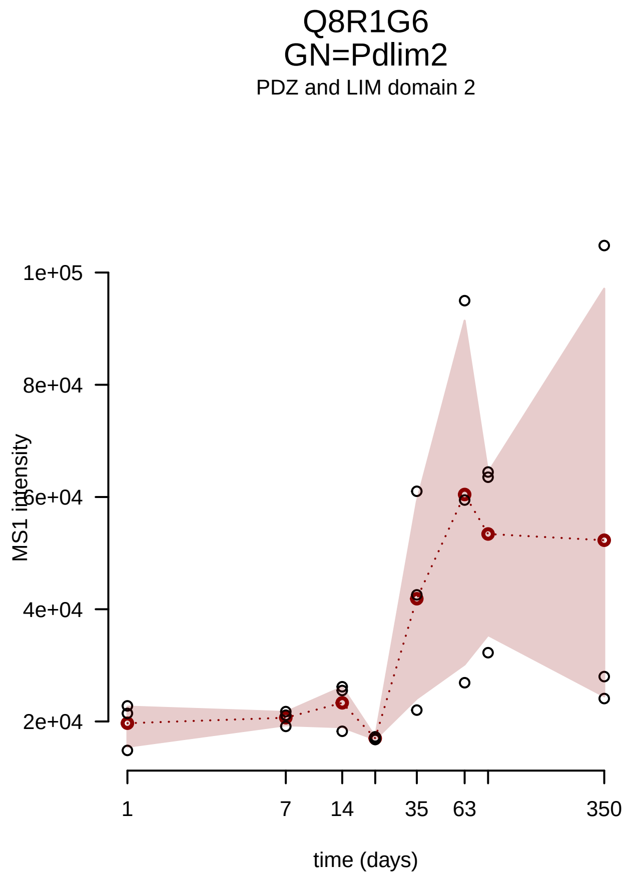
Q8R1G6
GN=Pdlim2
PDZ and LIM domain 2
1e+05
8e+04
6e+04
4e+04
2e+04
MS1 intensity
1
7
14
35
63
350
time (days)

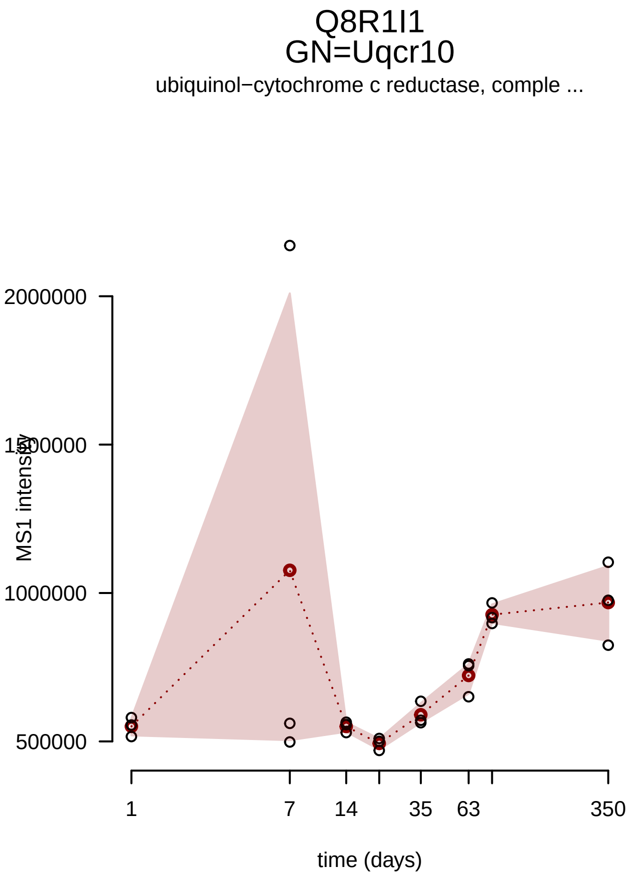
Q8R1I1
GN=Uqcr10
ubiquinol−cytochrome c reductase, comple ...
2000000
1500000
1000000
500000
MS1 intensity
1
7
14
35
63
350
time (days)

Q8R1J3
GN=Zcchc9
zinc finger, CCHC domain containing 9

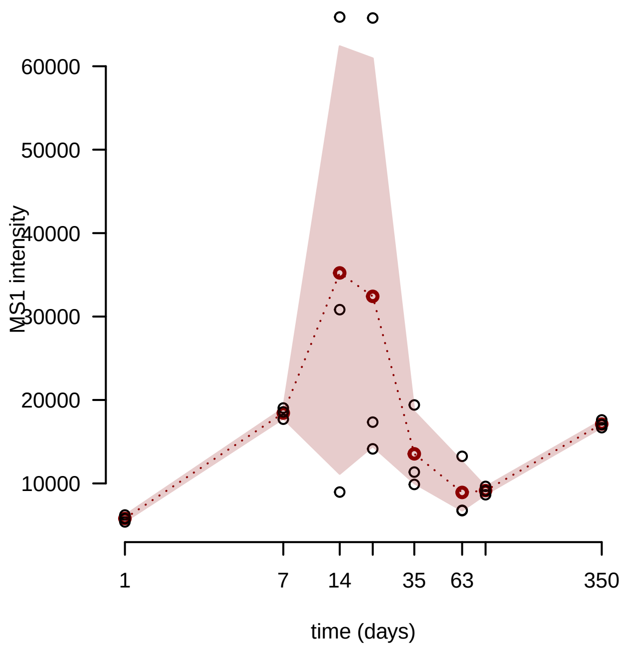

Q8R1J9
GN=Tor2a
torsin family 2, member A

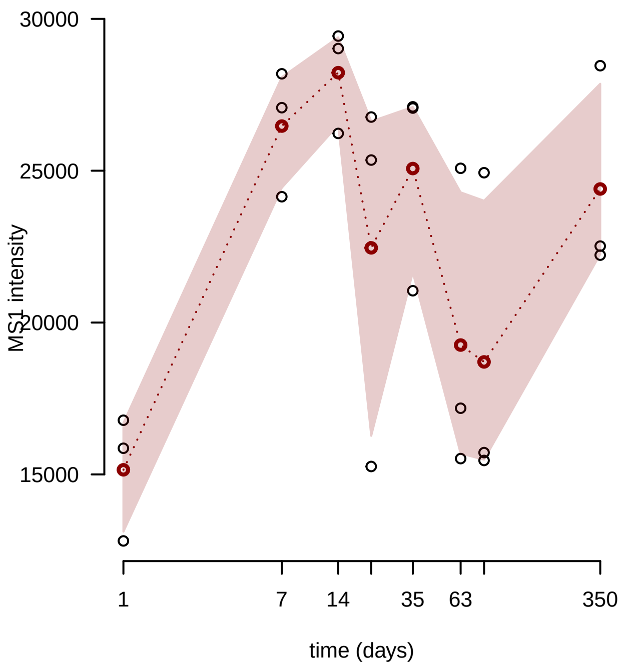

Q8R1K1
GN=Ubac2
ubiquitin associated domain containing 2

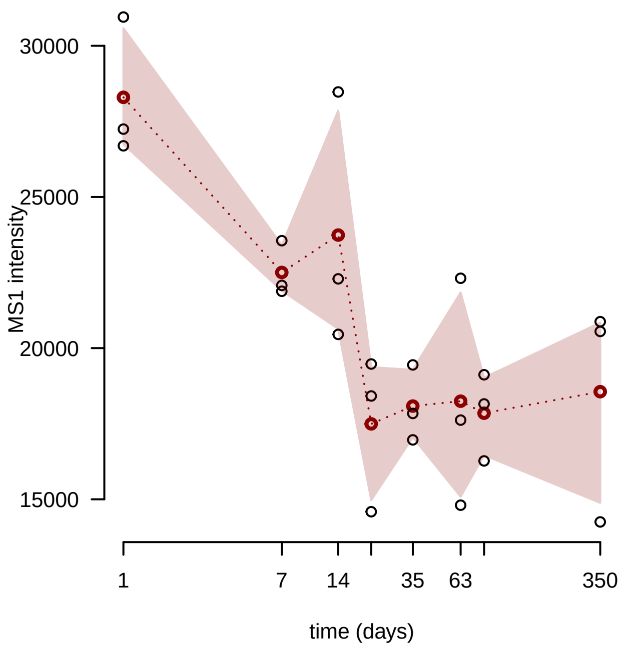

Q8R1K4
GN=Phykpl
5−phosphohydroxy−L−lysine phospholyase

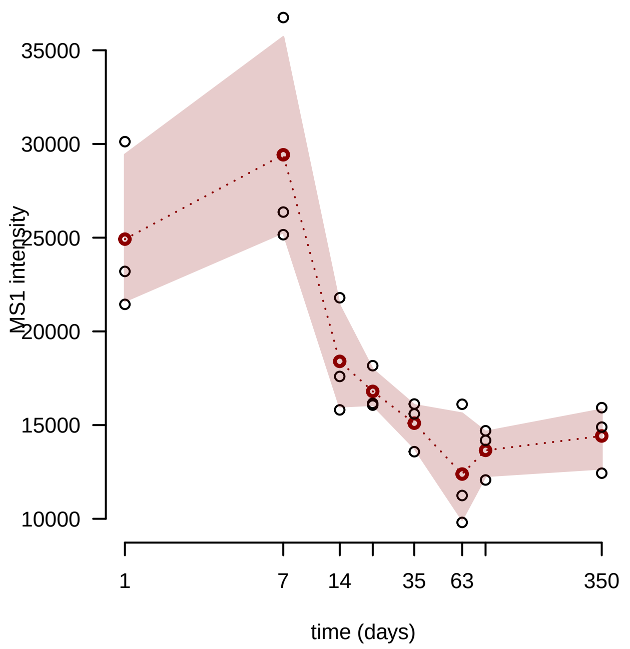

Q8R1L4
GN=Kdelr3
KDEL (Lys−Asp−Glu−Leu) endoplasmic retic ...

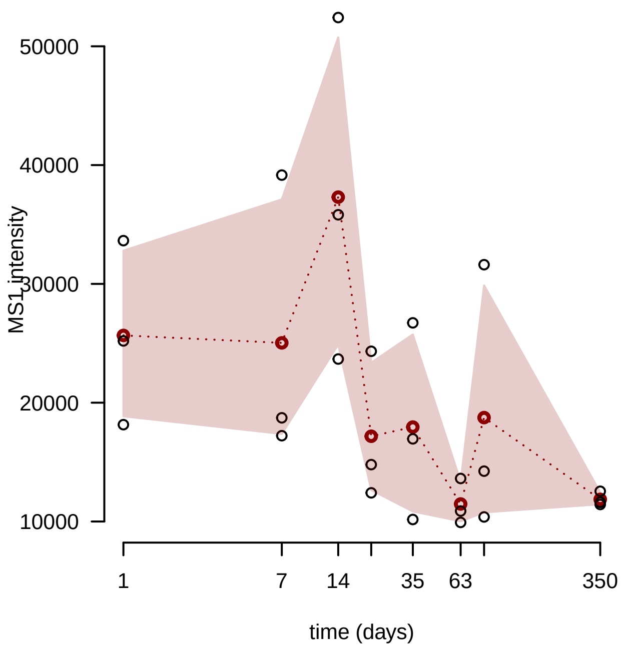

Q8R1M0
GN=Hmces
5−hydroxymethylcytosine (hmC) binding, E ...

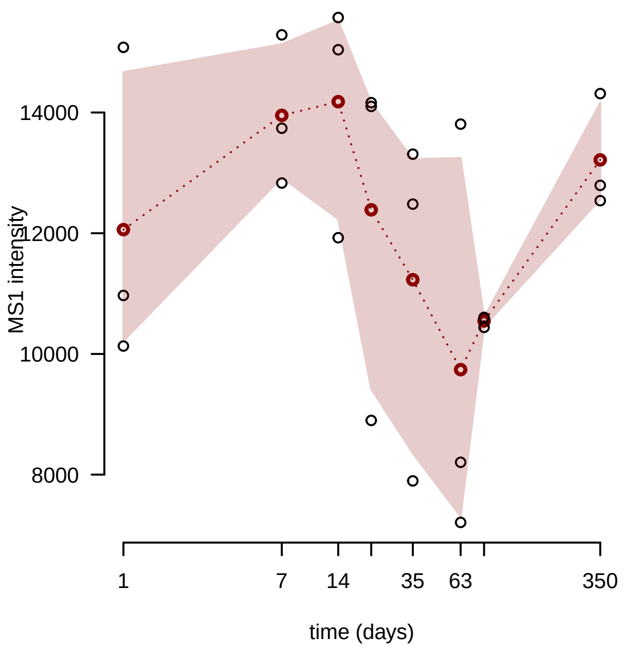

Q8R1M8
GN=Mptx1
mucosal pentraxin 1

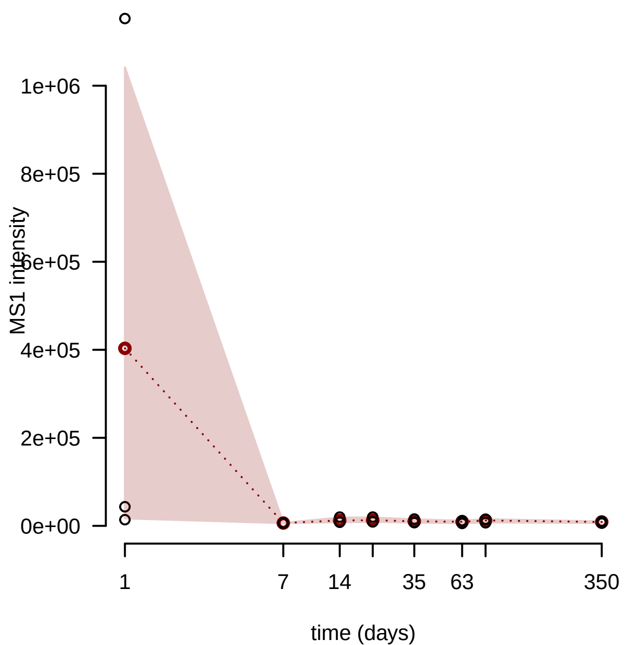

Q8R1N0
GN=Znf830

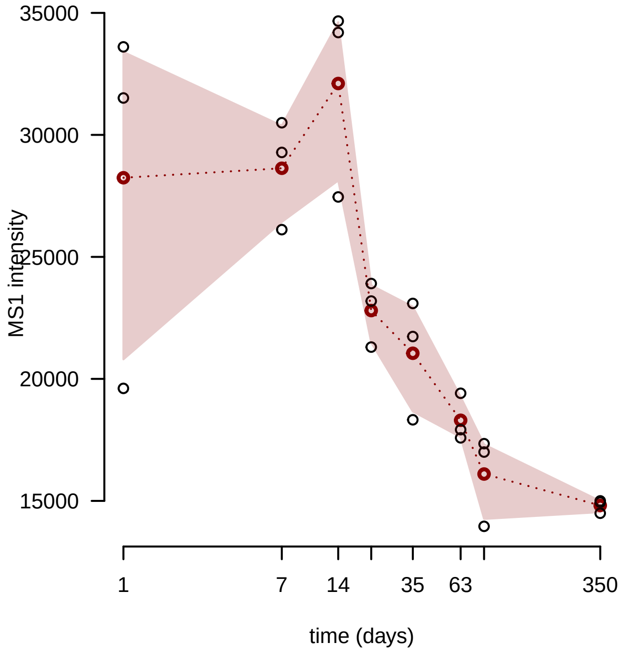

Q8R1N4
GN=Nudcd3
NudC domain containing 3

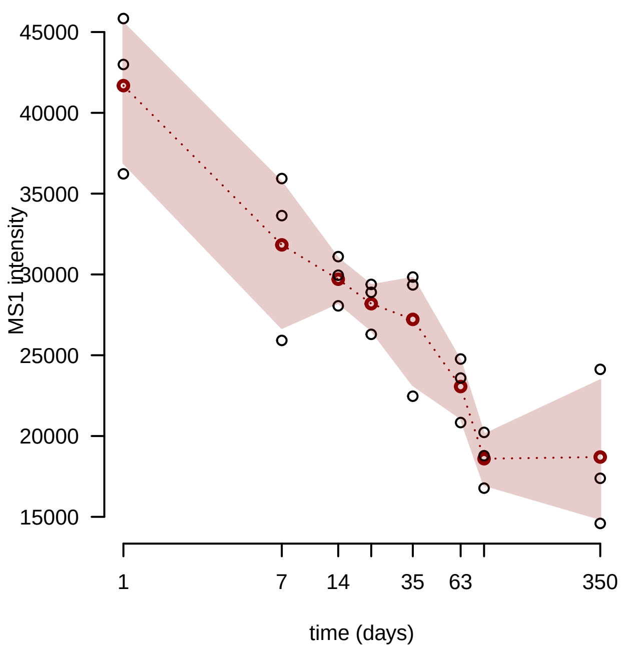

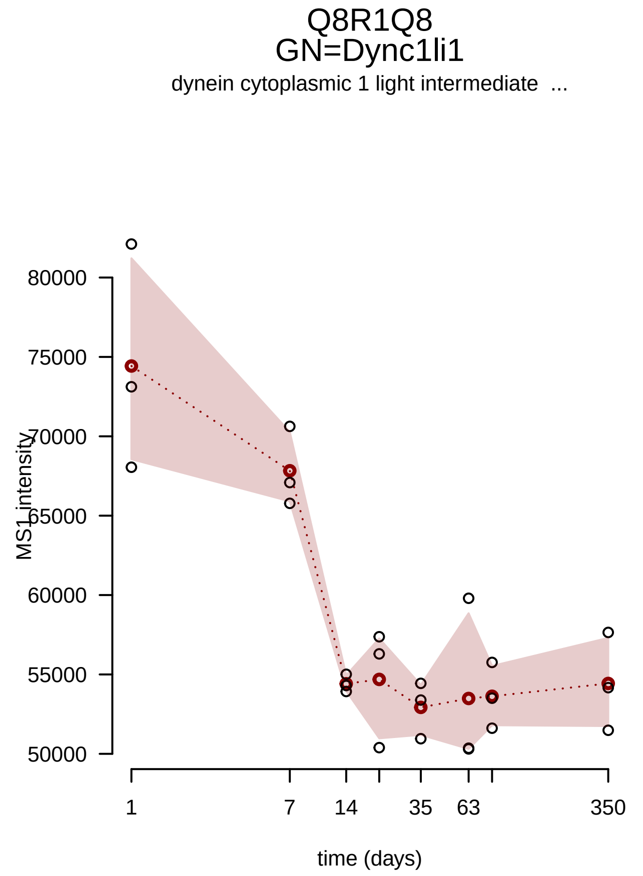
Q8R1Q8
GN=Dync1li1
dynein cytoplasmic 1 light intermediate ...
MS1 intensity
80000
75000
70000
65000
60000
55000
50000
1
7
14
35
63
350
time (days)

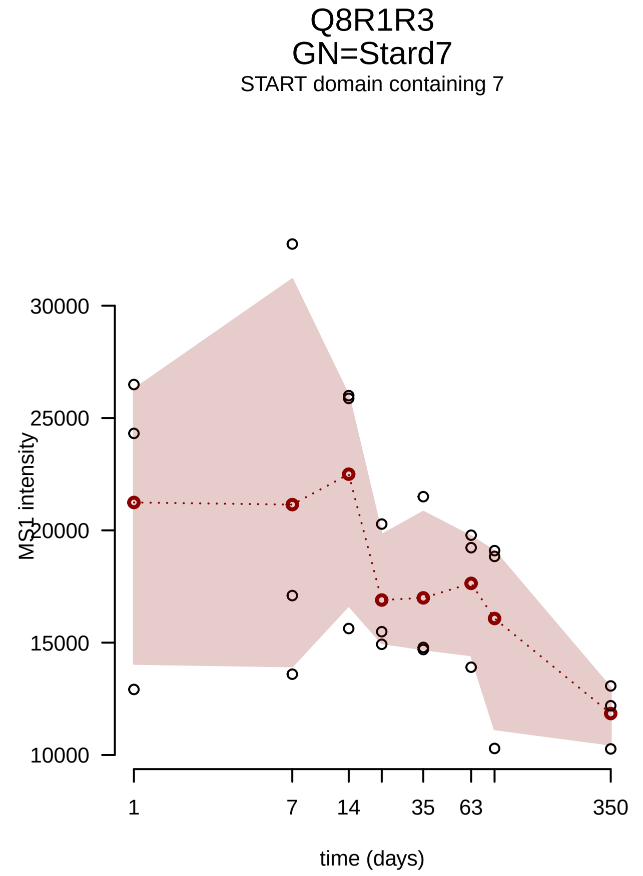
Q8R1R3
GN=Stard7
START domain containing 7
MS1 intensity
30000
25000
20000
15000
10000
1
7
14
35
63
350
time (days)

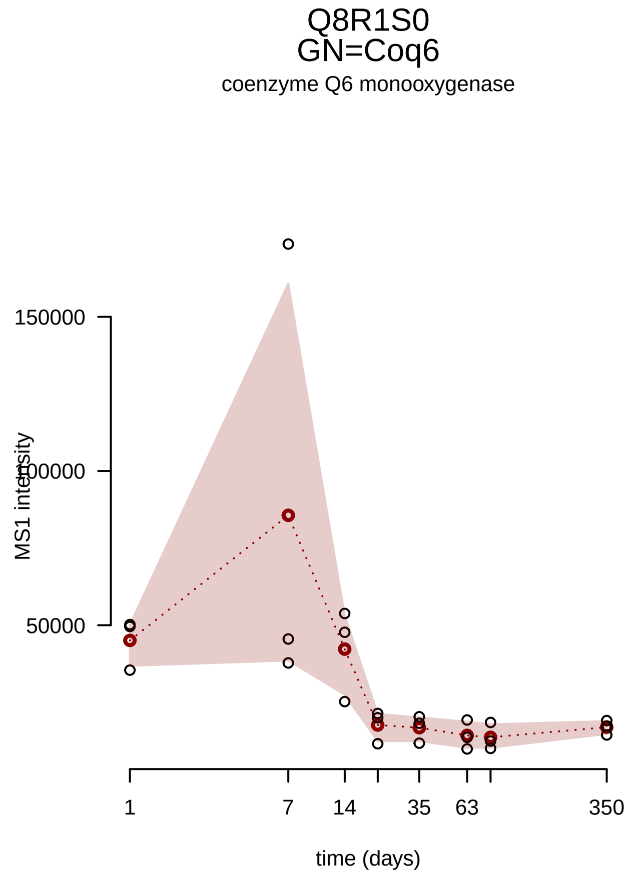
Q8R1S0
GN=Coq6
coenzyme Q6 monooxygenase
MS1 intensity
150000
100000
50000
1
7
14
35
63
350
time (days)

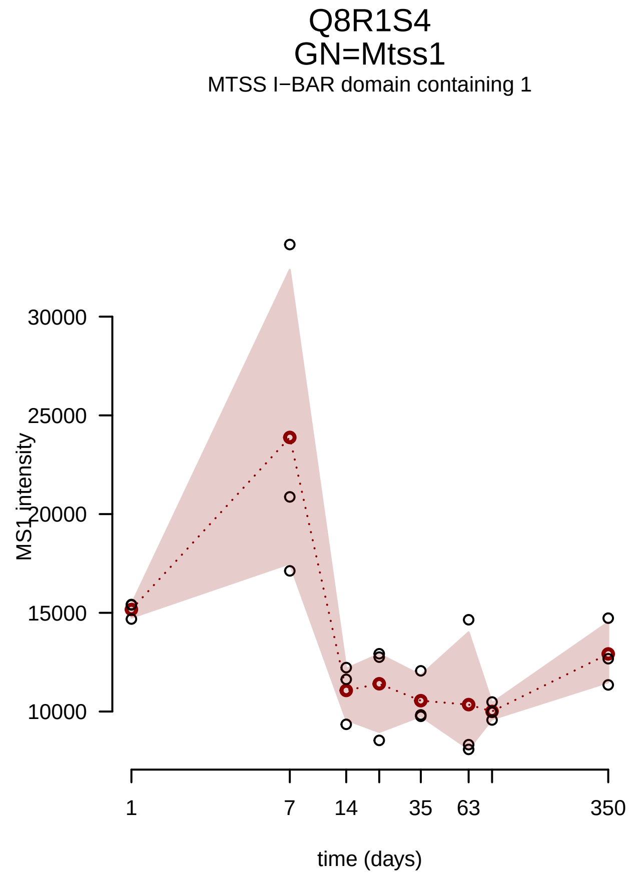
Q8R1S4
GN=Mtss1
MTSS I−BAR domain containing 1
MS1 intensity
30000
25000
20000
15000
10000
1
7
14
35
63
350
time (days)

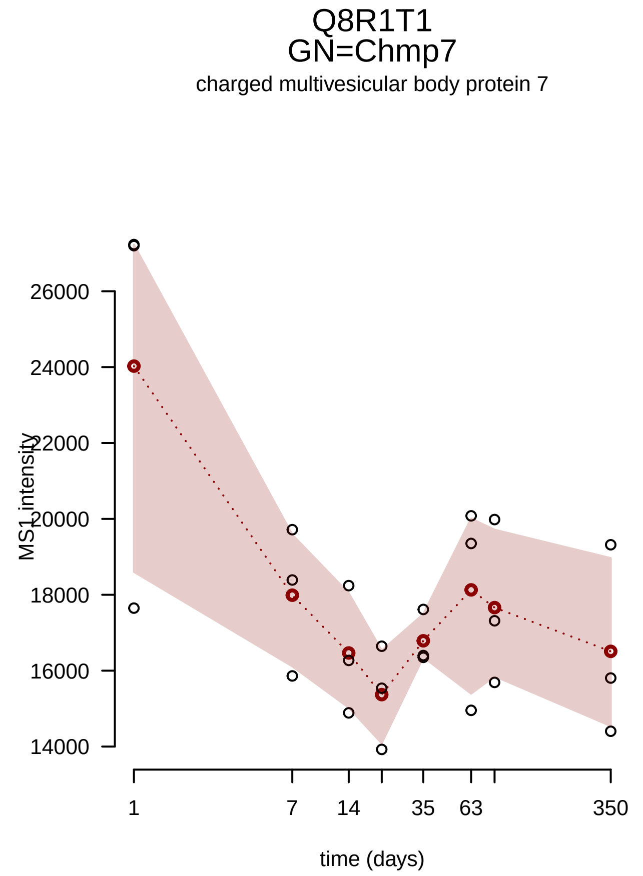
Q8R1T1
GN=Chmp7
charged multivesicular body protein 7
MS1 intensity
26000
24000
22000
20000
18000
16000
14000
1
7
14
35
63
350
time (days)

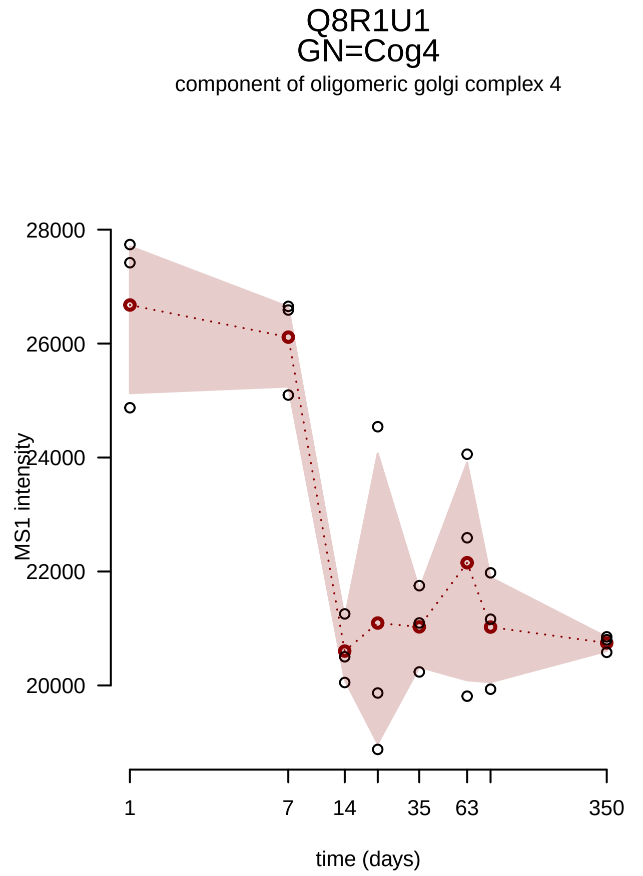
Q8R1U1
GN=Cog4
component of oligomeric golgi complex 4
MS1 intensity
28000
26000
24000
22000
20000
1
7
14
35
63
350
time (days)

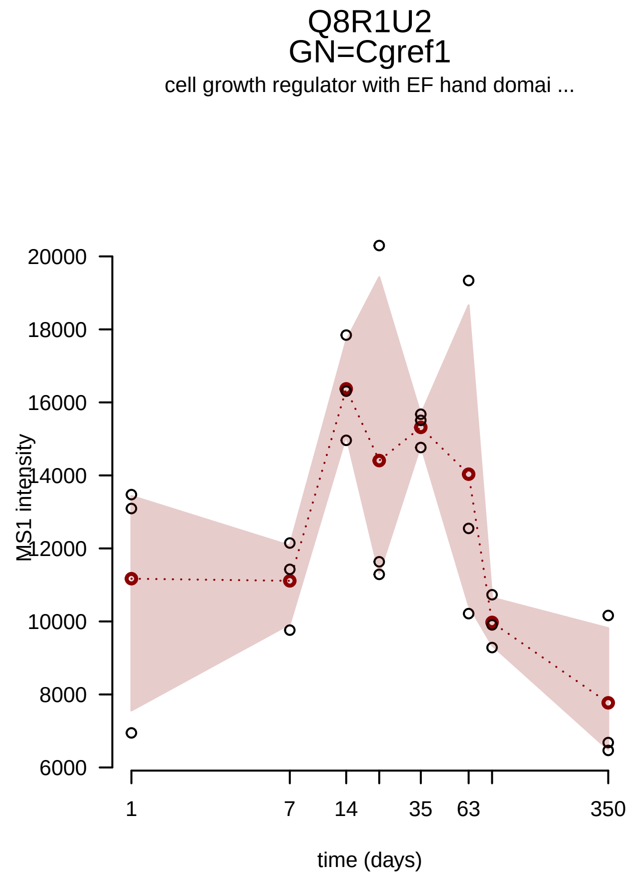
Q8R1U2
GN=Cgref1
cell growth regulator with EF hand domai ...
MS1 intensity
20000
18000
16000
14000
12000
10000
8000
6000
1
7
14
35
63
350
time (days)

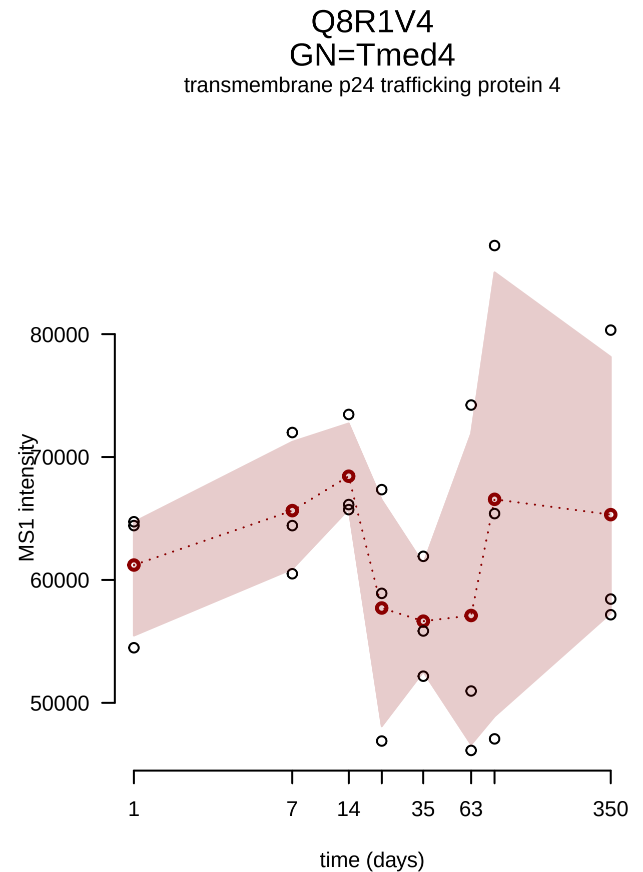
Q8R1V4
GN=Tmed4
transmembrane p24 trafficking protein 4
MS1 intensity
80000
70000
60000
50000
1
7
14
35
63
350
time (days)

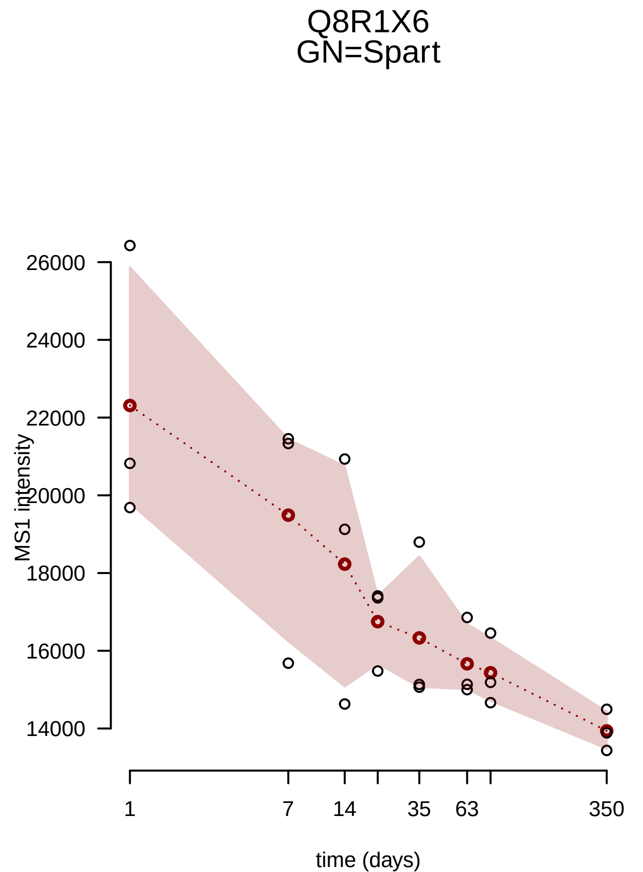
Q8R1X6
GN=Spart
MS1 intensity
26000
24000
22000
20000
18000
16000
14000
1
7
14
35
63
350
time (days)

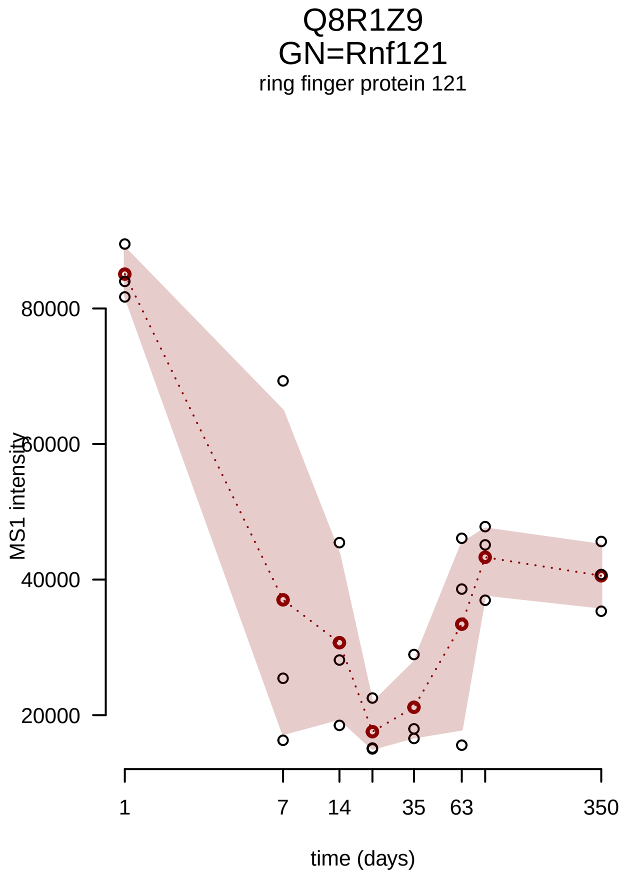
Q8R1Z9
GN=Rnf121
ring finger protein 121
MS1 intensity
20000
40000
60000
80000
1
7
14
35
63
350
time (days)
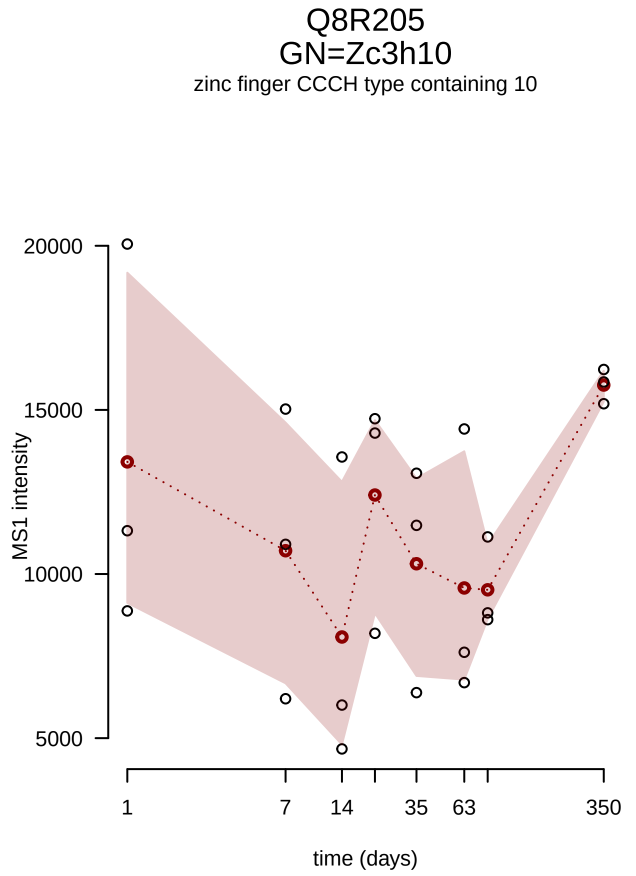
Q8R205
GN=Zc3h10
zinc finger CCCH type containing 10
MS1 intensity
5000
10000
15000
20000
1
7
14
35
63
350
time (days)
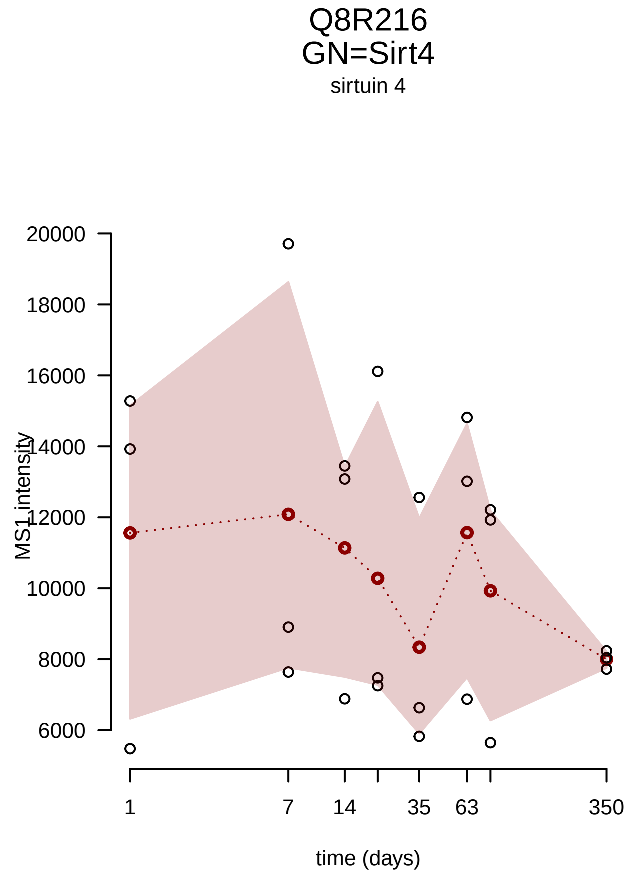
Q8R216
GN=Sirt4
sirtuin 4
MS1 intensity
6000
8000
10000
12000
14000
16000
18000
20000
1
7
14
35
63
350
time (days)
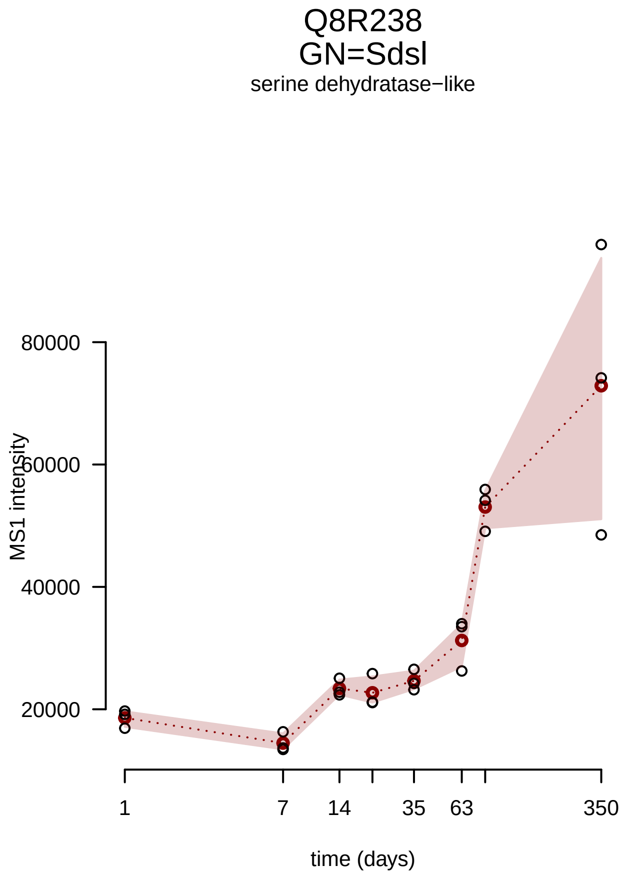
Q8R238
GN=Sdsl
serine dehydratase−like
MS1 intensity
20000
40000
60000
80000
1
7
14
35
63
350
time (days)
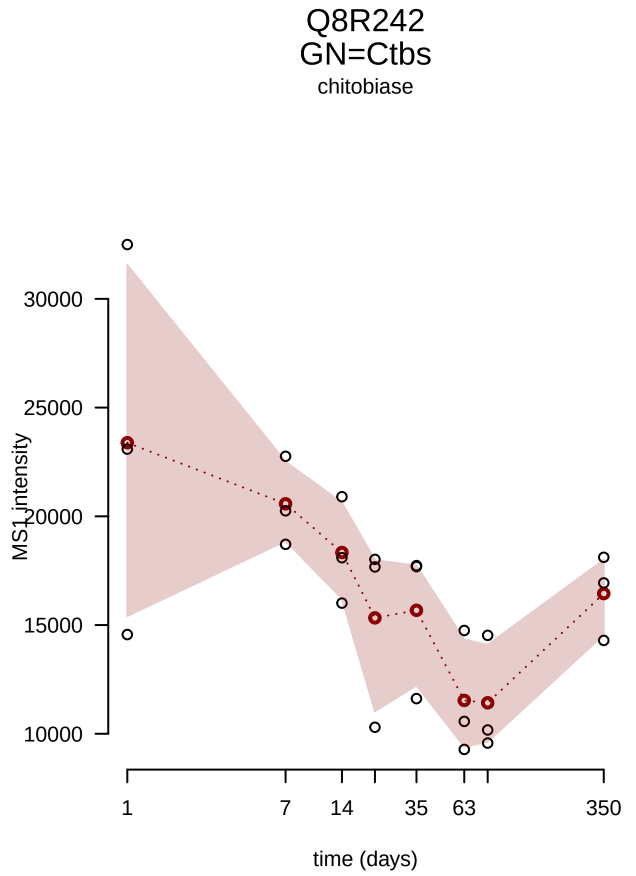
Q8R242
GN=Ctbs
chitobiase
MS1 intensity
10000
15000
20000
25000
30000
1
7
14
35
63
350
time (days)
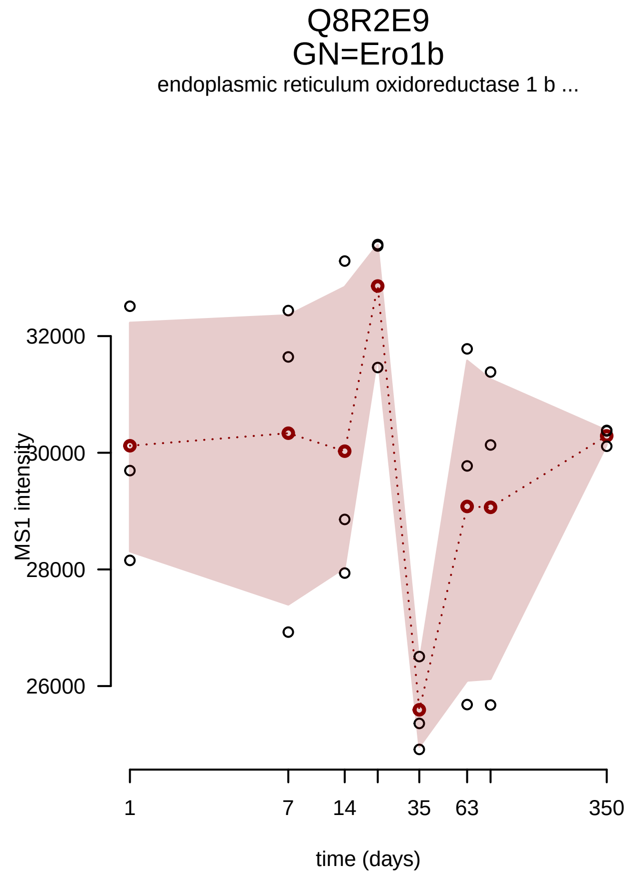
Q8R2E9
GN=Ero1b
endoplasmic reticulum oxidoreductase 1 b ...
MS1 intensity
26000
28000
30000
32000
1
7
14
35
63
350
time (days)
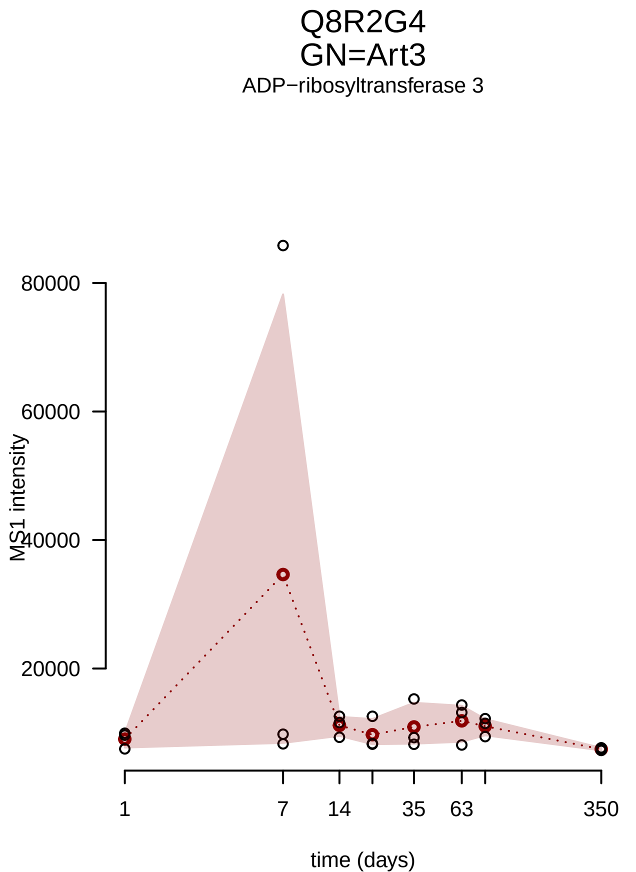
Q8R2G4
GN=Art3
ADP−ribosyltransferase 3
MS1 intensity
20000
40000
60000
80000
1
7
14
35
63
350
time (days)
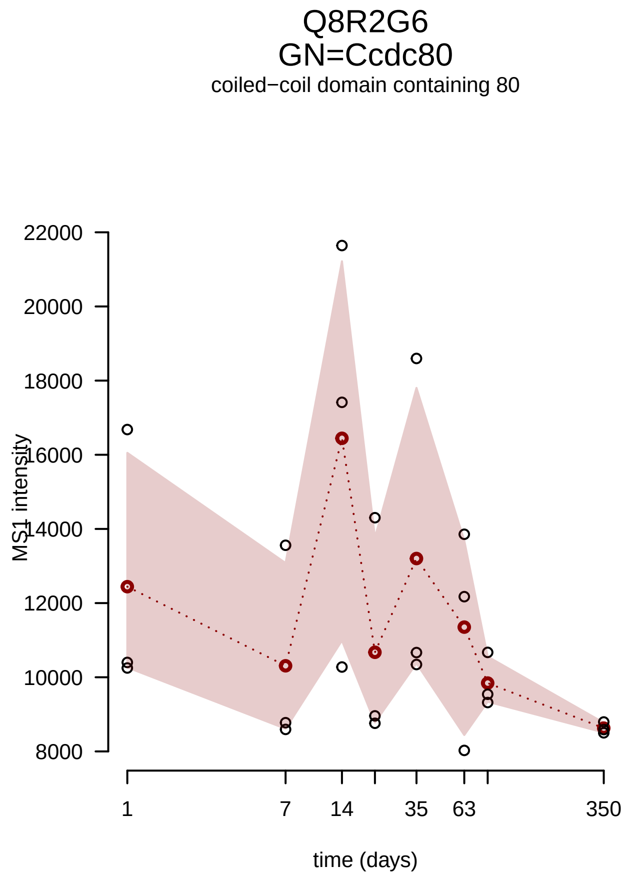
Q8R2G6
GN=Ccdc80
coiled−coil domain containing 80
MS1 intensity
8000
10000
12000
14000
16000
18000
20000
22000
1
7
14
35
63
350
time (days)
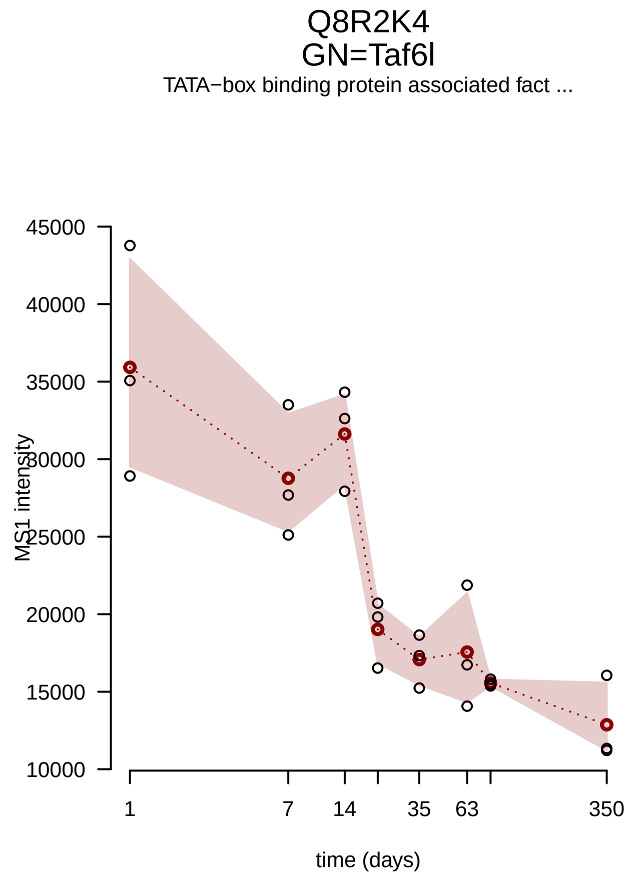
Q8R2K4
GN=Taf6l
TATA−box binding protein associated fact ...
MS1 intensity
10000
15000
20000
25000
30000
35000
40000
45000
1
7
14
35
63
350
time (days)

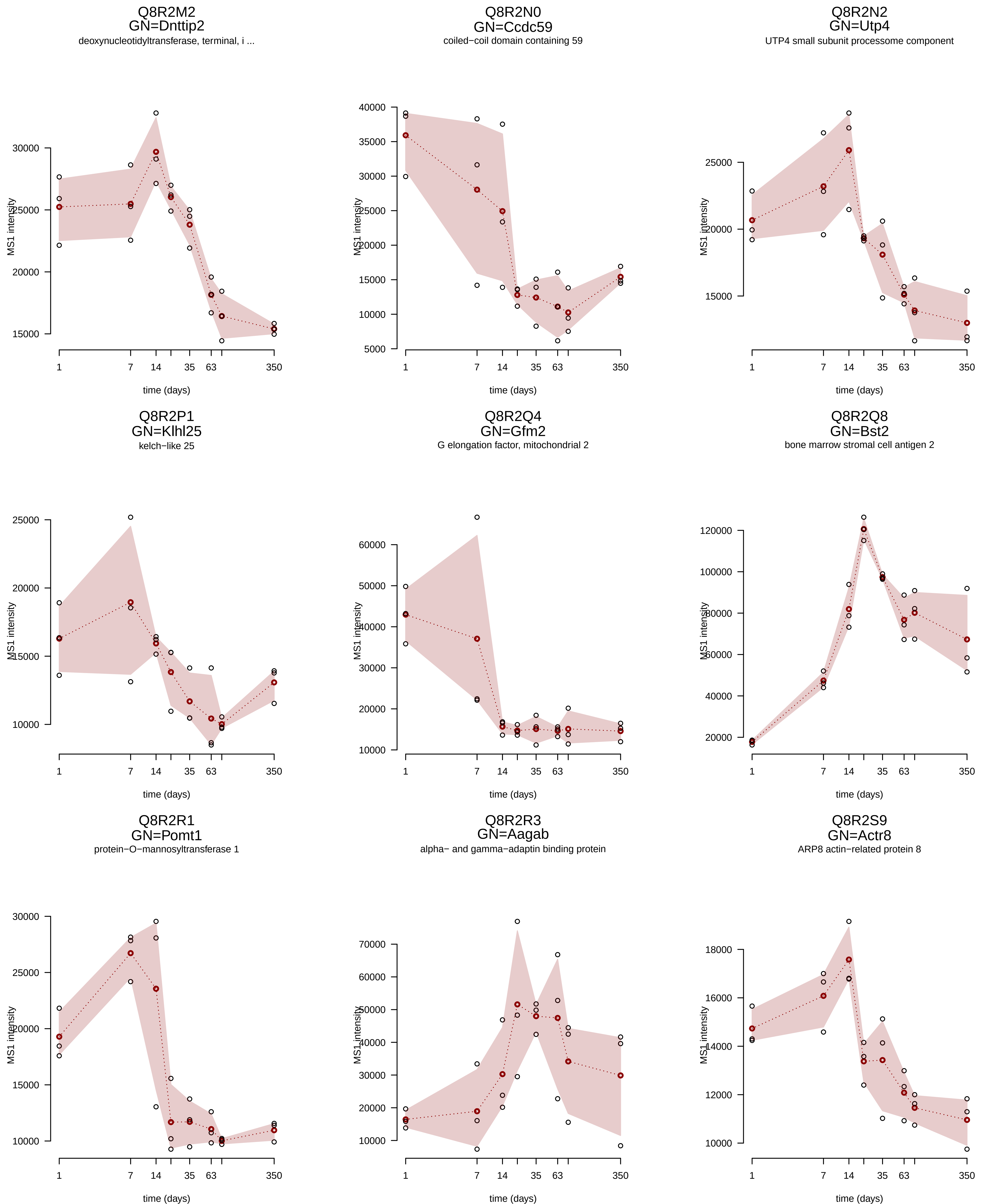
Q8R2M2
GN=Dnttip2
deoxynucleotidyltransferase, terminal, i ...
MS1 intensity
30000
25000
20000
15000
1
7
14
35
63
350
time (days)
Q8R2N0
GN=Ccdc59
coiled−coil domain containing 59
MS1 intensity
40000
35000
30000
25000
20000
15000
10000
5000
1
7
14
35
63
350
time (days)
Q8R2N2
GN=Utp4
UTP4 small subunit processome component
MS1 intensity
25000
20000
15000
1
7
14
35
63
350
time (days)
Q8R2P1
GN=Klhl25
kelch−like 25
MS1 intensity
25000
20000
15000
10000
1
7
14
35
63
350
time (days)
Q8R2Q4
GN=Gfm2
G elongation factor, mitochondrial 2
MS1 intensity
60000
50000
40000
30000
20000
10000
1
7
14
35
63
350
time (days)
Q8R2Q8
GN=Bst2
bone marrow stromal cell antigen 2
MS1 intensity
120000
100000
80000
60000
40000
20000
1
7
14
35
63
350
time (days)
Q8R2R1
GN=Pomt1
protein−O−mannosyltransferase 1
MS1 intensity
30000
25000
20000
15000
10000
1
7
14
35
63
350
time (days)
Q8R2R3
GN=Aagab
alpha− and gamma−adaptin binding protein
MS1 intensity
70000
60000
50000
40000
30000
20000
10000
1
7
14
35
63
350
time (days)
Q8R2S9
GN=Actr8
ARP8 actin−related protein 8
MS1 intensity
18000
16000
14000
12000
10000
1
7
14
35
63
350
time (days)

Q8R2T8
GN=Gtf3c5
general transcription factor IIIC, polyp ...

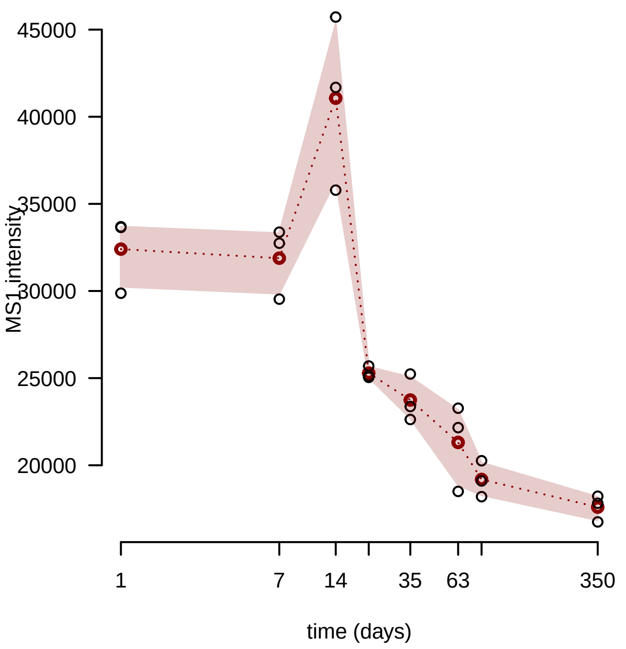

Q8R2U0
GN=Seh1l
SEH1−like (S. cerevisiae

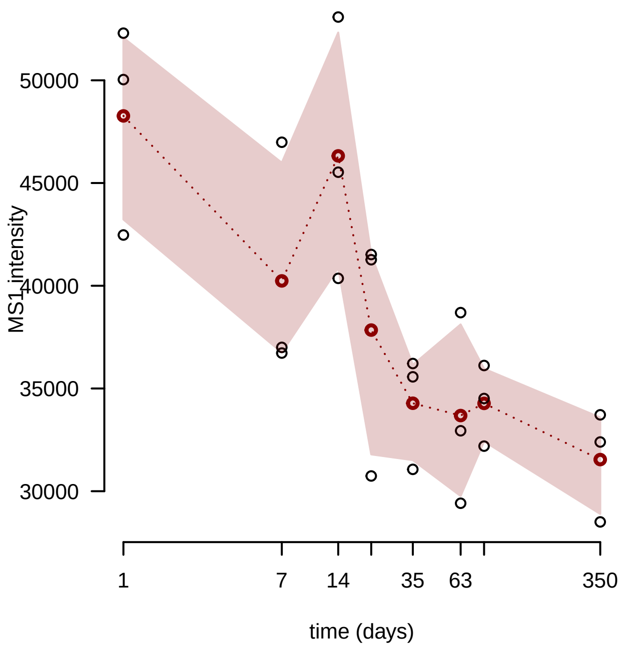

Q8R2U2
GN=Nepro
nucleolus and neural progenitor protein

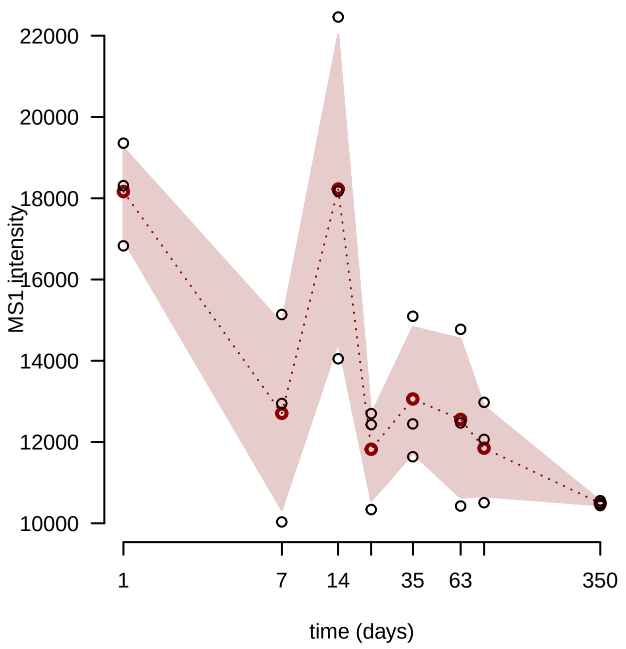

Q8R2U4
GN=Ntmt1
N−terminal Xaa−Pro−Lys N−methyltransfera ...

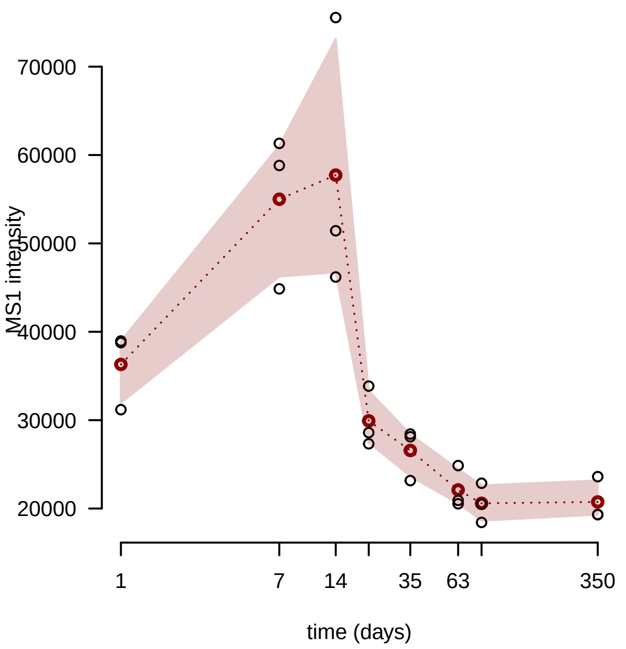

Q8R2U6
GN=Nudt4
nudix (nucleoside diphosphate linked moi ...

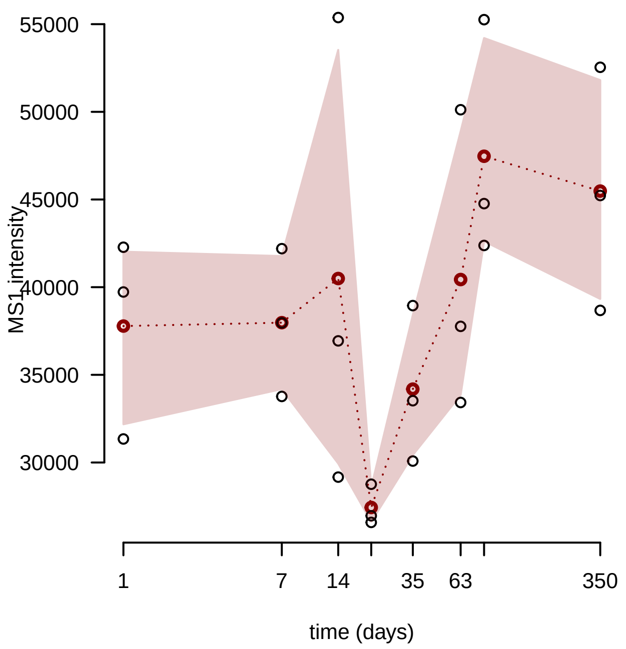

Q8R2U7
GN=Lrrc42
leucine rich repeat containing 42

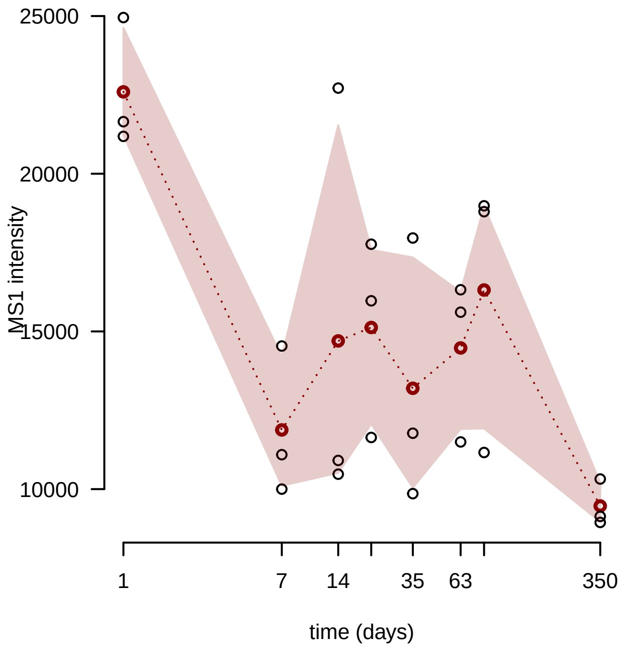

Q8R2V3
GN=Znf445

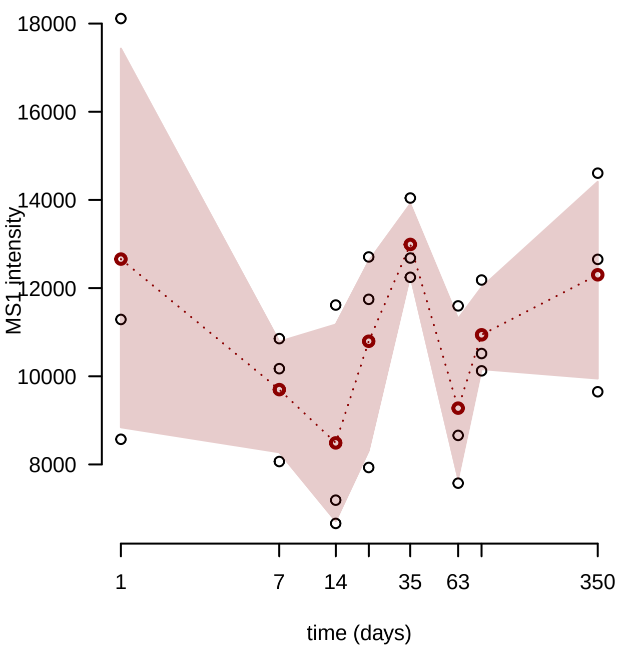

Q8R2V5
GN=Adap2
ArfGAP with dual PH domains 2

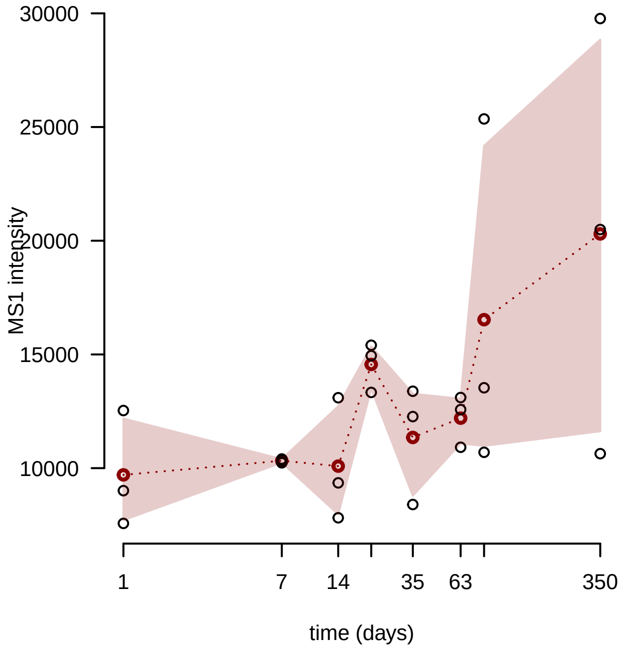

Q8R2W9
GN=Pank3
pantothenate kinase 3

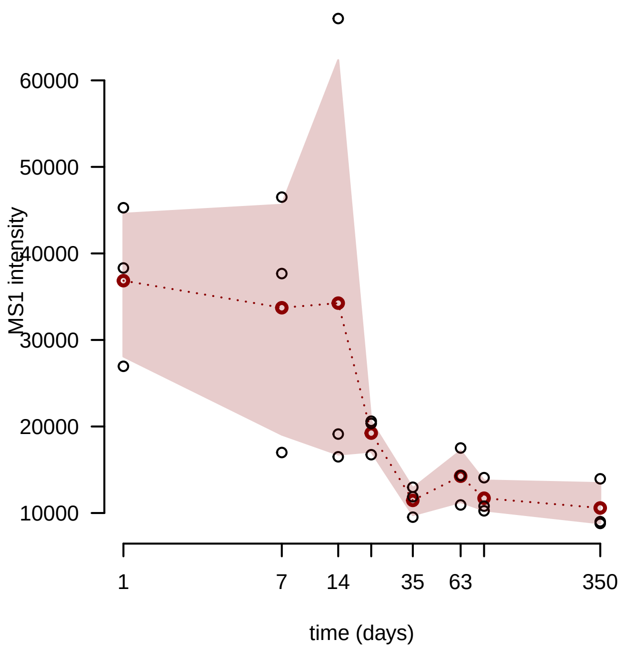

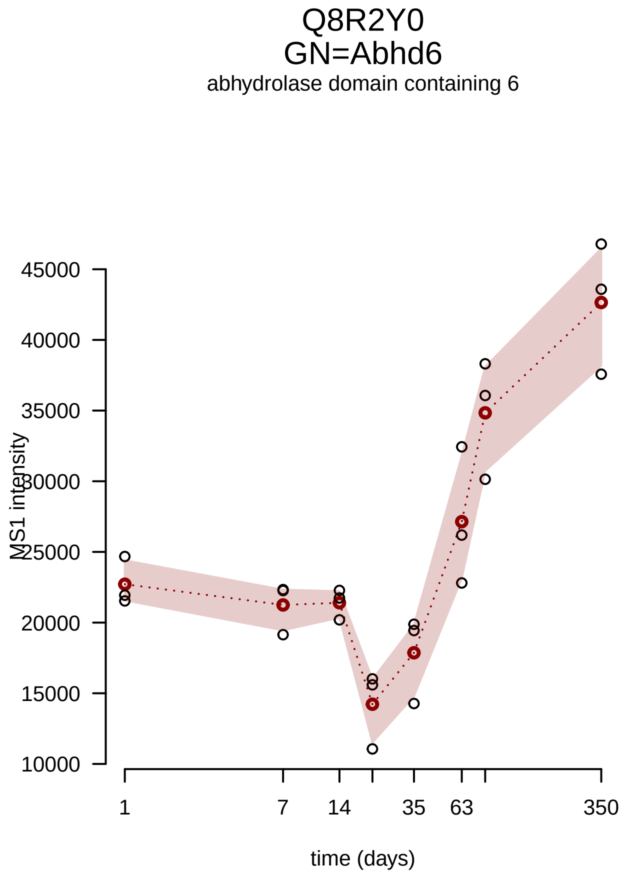
Q8R2Y0
GN=Abhd6
abhydrolase domain containing 6
45000
40000
35000
30000
25000
20000
15000
10000
MS1 intensity
1
7
14
35
63
350
time (days)

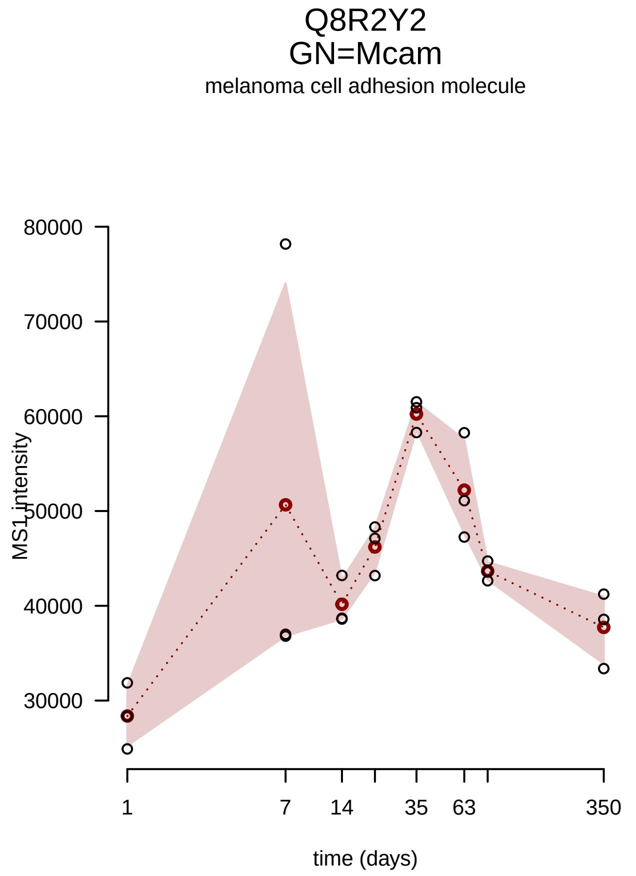
Q8R2Y2
GN=Mcam
melanoma cell adhesion molecule
80000
70000
60000
50000
40000
30000
MS1 intensity
1
7
14
35
63
350
time (days)

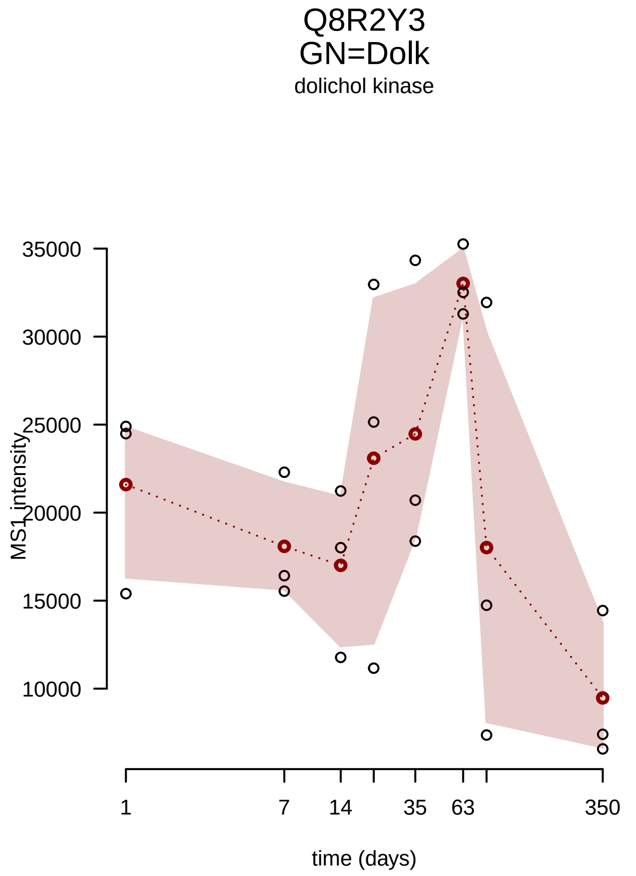
Q8R2Y3
GN=Dolk
dolichol kinase
35000
30000
25000
20000
15000
10000
MS1 intensity
1
7
14
35
63
350
time (days)

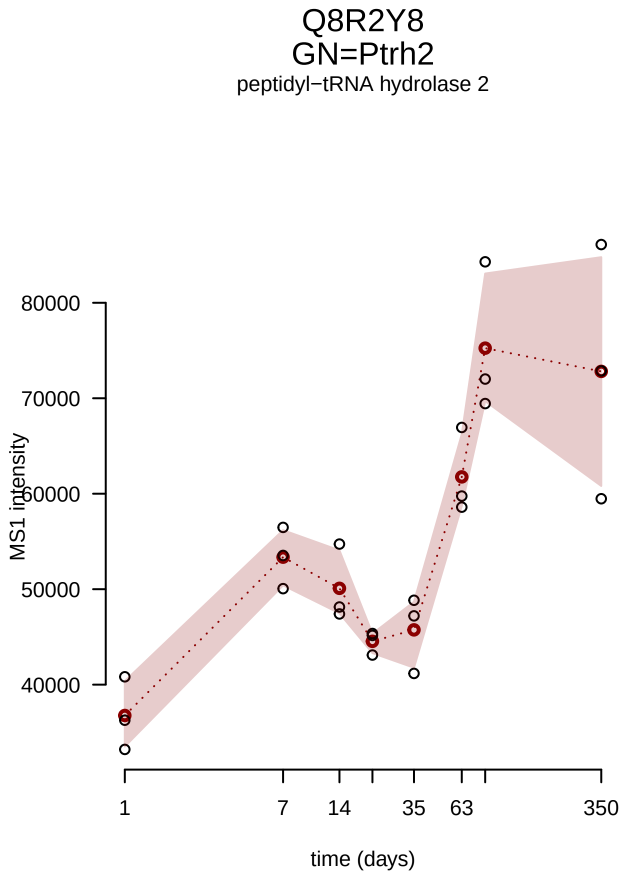
Q8R2Y8
GN=Ptrh2
peptidyl−tRNA hydrolase 2
80000
70000
60000
50000
40000
MS1 intensity
1
7
14
35
63
350
time (days)

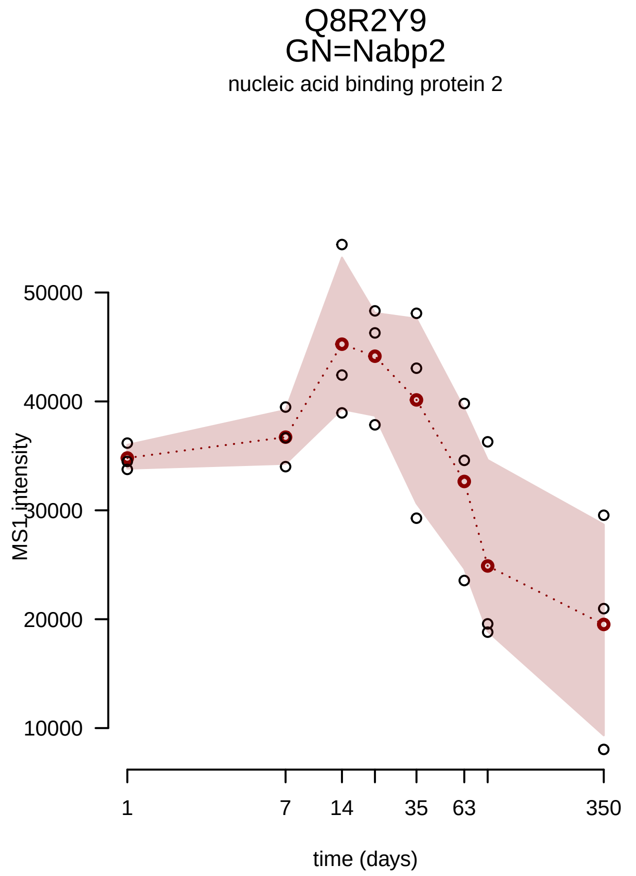
Q8R2Y9
GN=Nabp2
nucleic acid binding protein 2
50000
40000
30000
20000
10000
MS1 intensity
1
7
14
35
63
350
time (days)

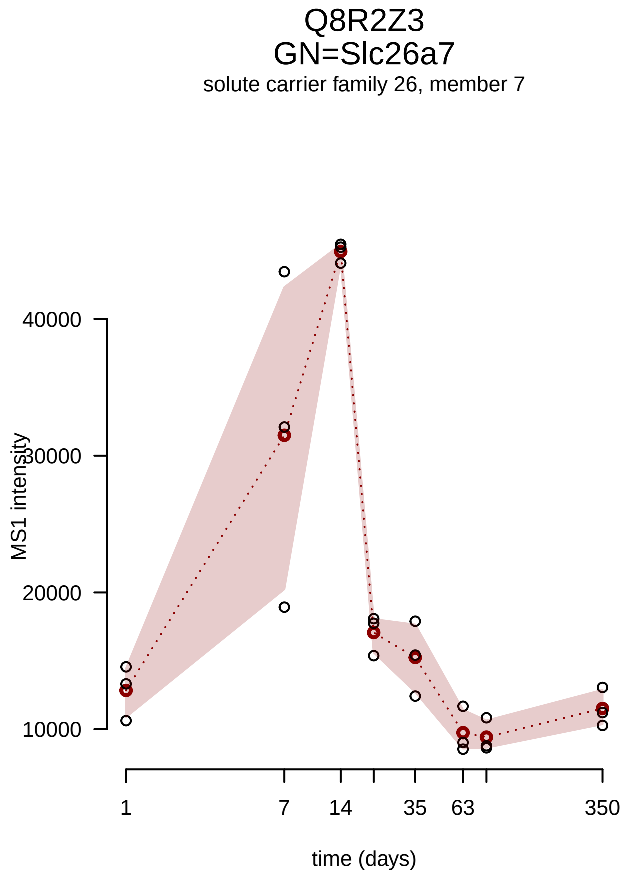
Q8R2Z3
GN=Slc26a7
solute carrier family 26, member 7
40000
30000
20000
10000
MS1 intensity
1
7
14
35
63
350
time (days)

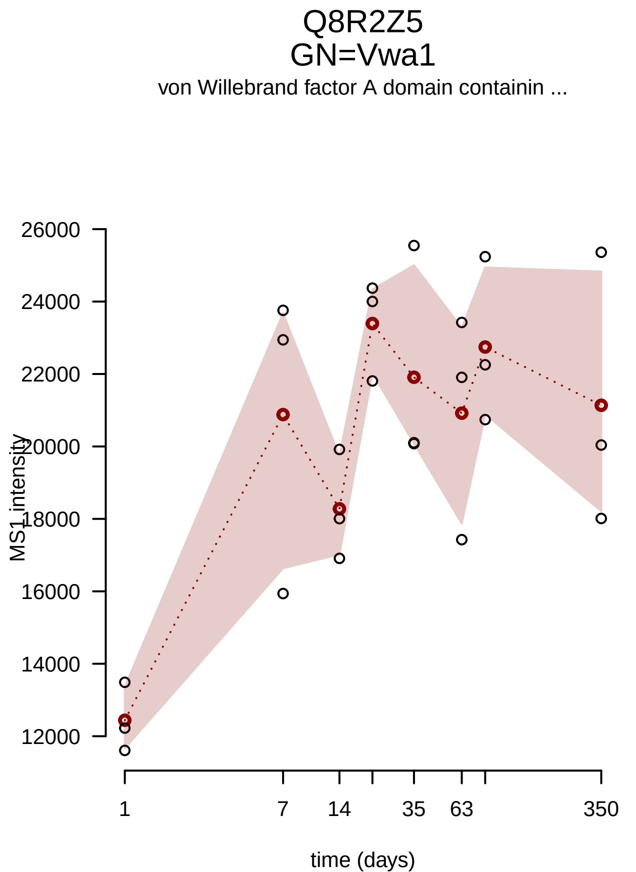
Q8R2Z5
GN=Vwa1
von Willebrand factor A domain containin ...
26000
24000
22000
20000
18000
16000
14000
12000
MS1 intensity
1
7
14
35
63
350
time (days)

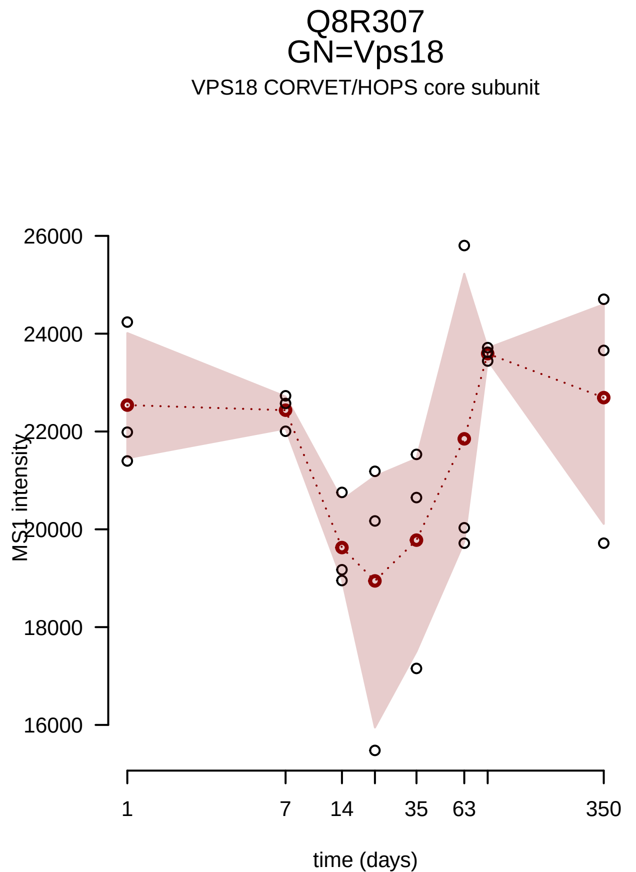
Q8R307
GN=Vps18
VPS18 CORVET/HOPS core subunit
26000
24000
22000
20000
18000
16000
MS1 intensity
1
7
14
35
63
350
time (days)

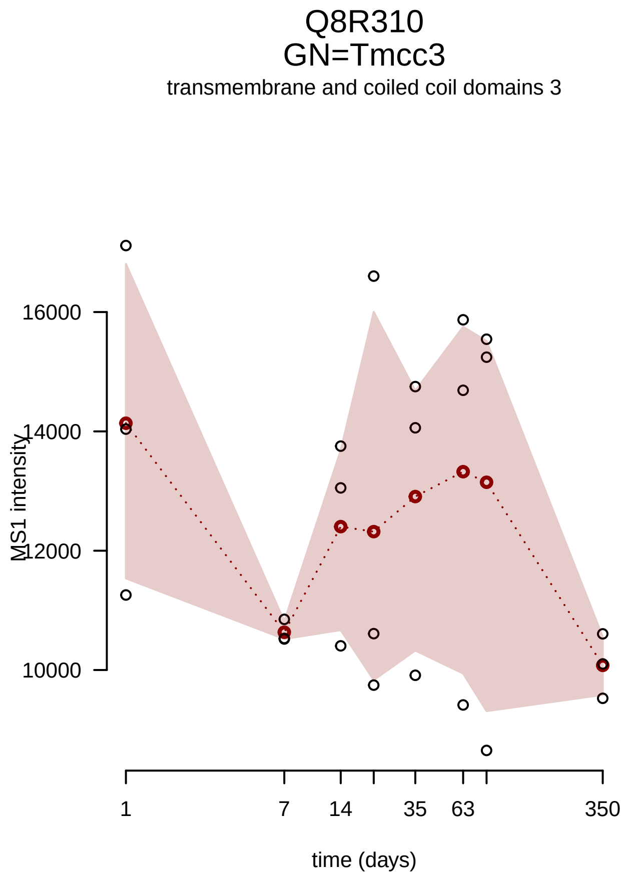
Q8R310
GN=Tmcc3
transmembrane and coiled coil domains 3
16000
14000
12000
10000
MS1 intensity
1
7
14
35
63
350
time (days)

Q8R313
GN=Exoc6
exocyst complex component 6

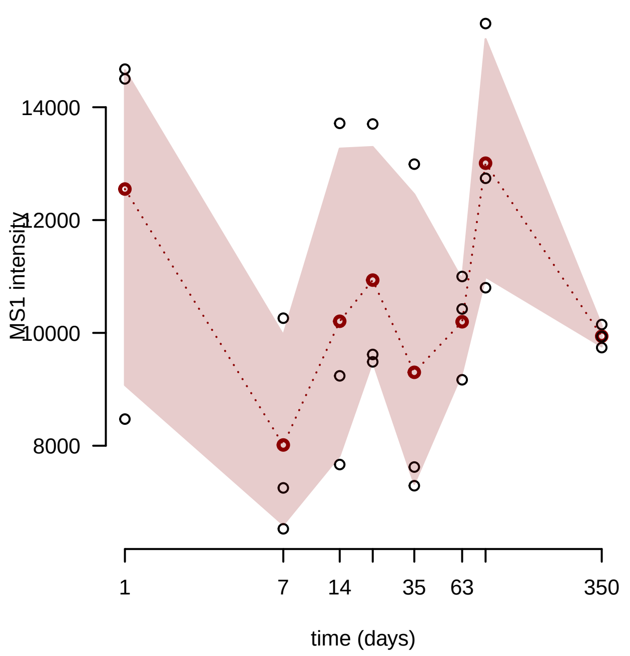

Q8R317
GN=Ubqln1
ubiquilin 1

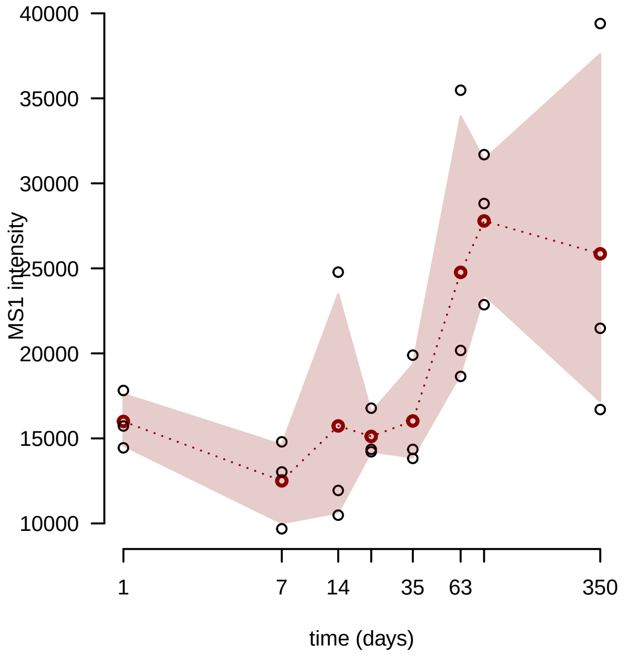

Q8R322
GN=Gle1
GLE1 RNA export mediator (yeast)

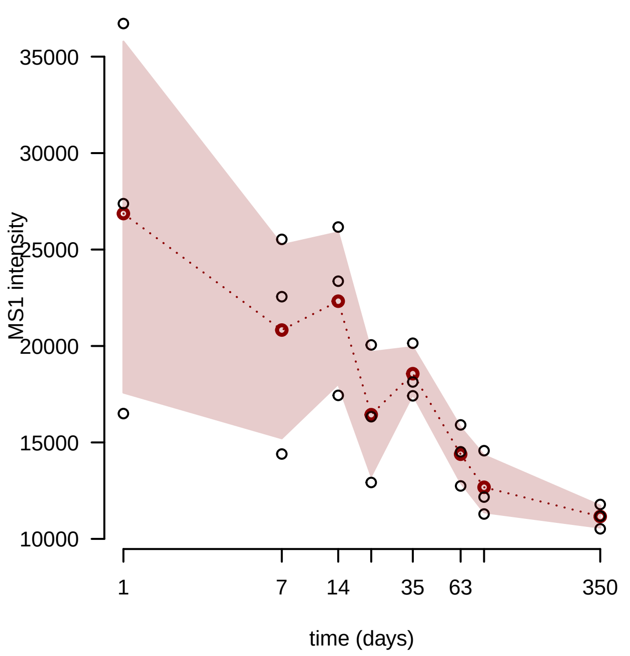

Q8R323
GN=Rfc3
replication factor C (activator 1) 3

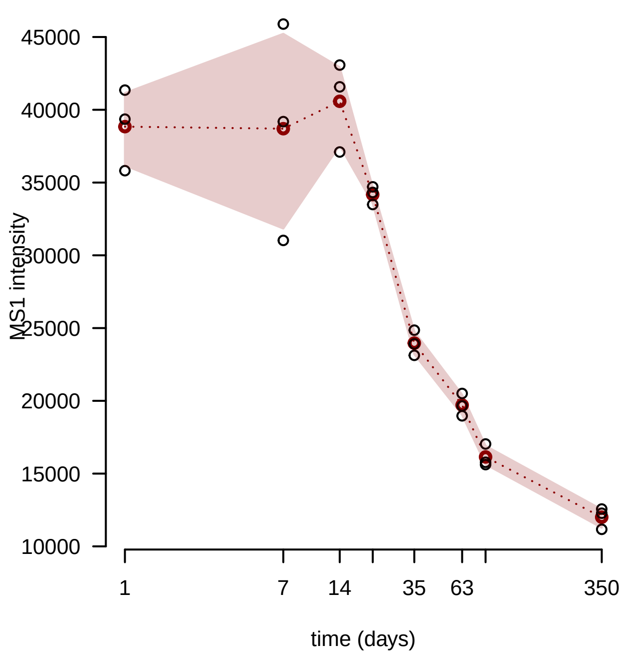

Q8R326
GN=Pspc1
paraspeckle protein 1

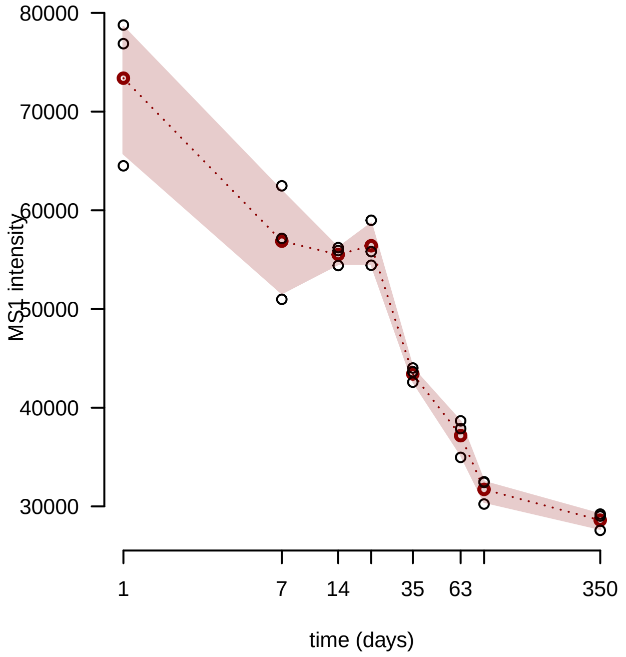

Q8R332
GN=Nup58

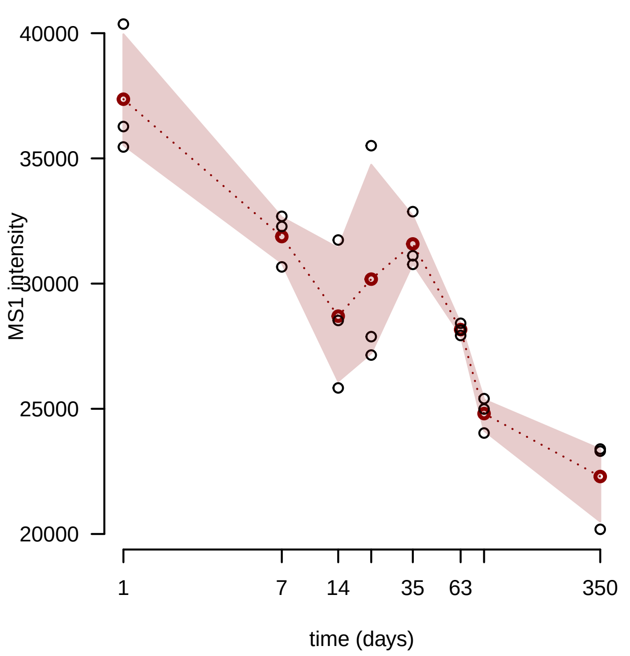

Q8R344
GN=Ccdc12
coiled−coil domain containing 12

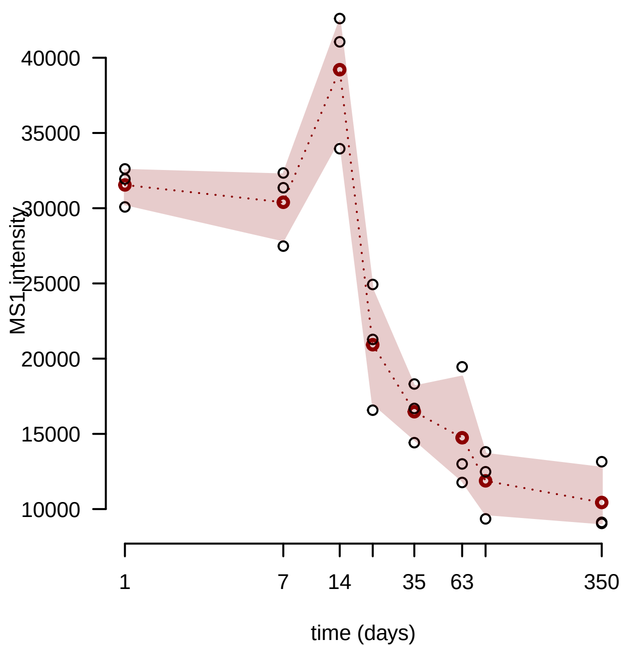

Q8R349
GN=Cdc16
CDC16 cell division cycle 16

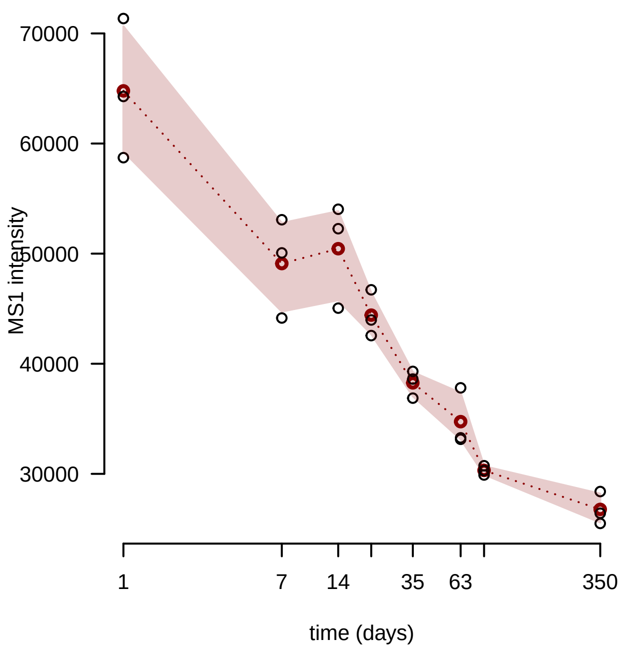

Q8R361
GN=Rab11fip5
RAB11 family interacting protein 5 (clas ...

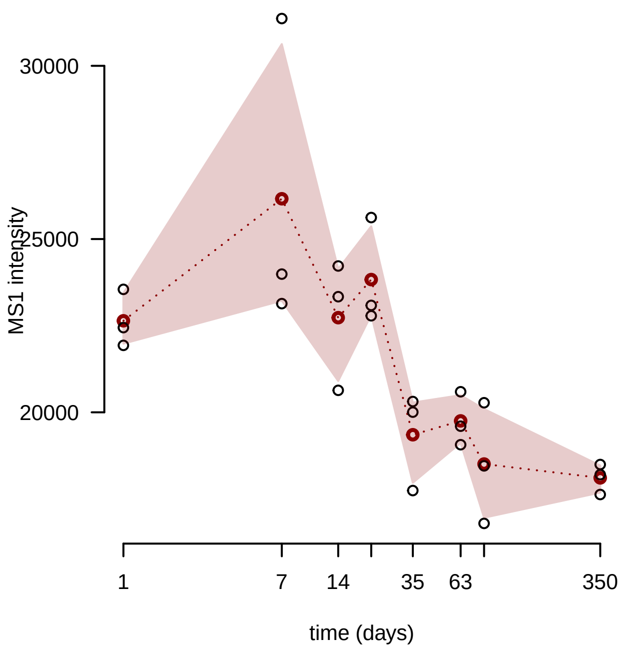

Q8R366
GN=Igsf8
immunoglobulin superfamily, member 8

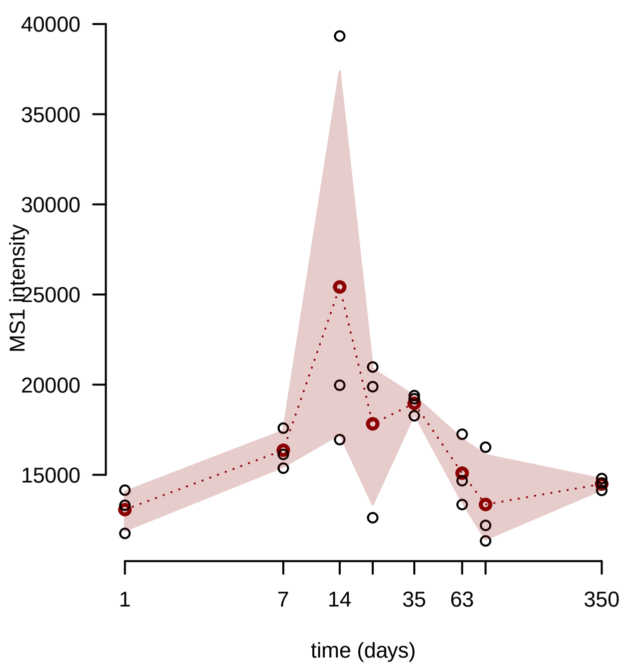

Q8R367
GN=Rerg
RAS−like, estrogen−regulated, growth−inh ...

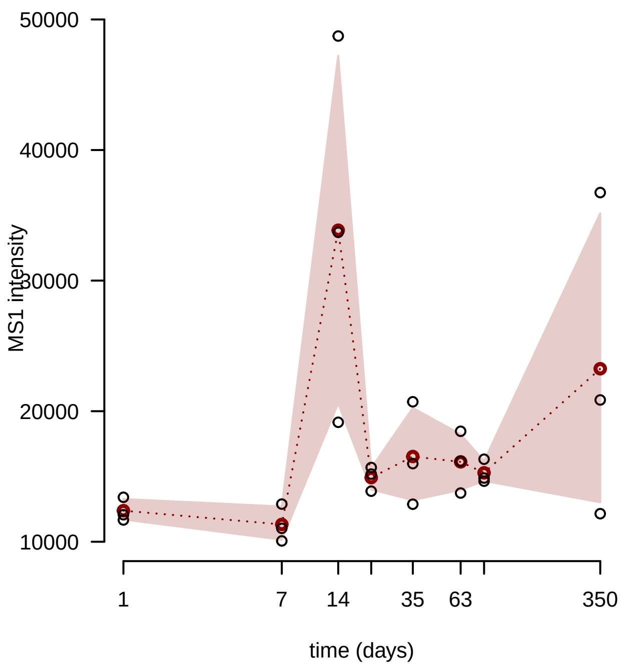

Q8R368
GN=Dnaaf4
dynein axonemal assembly factor 4

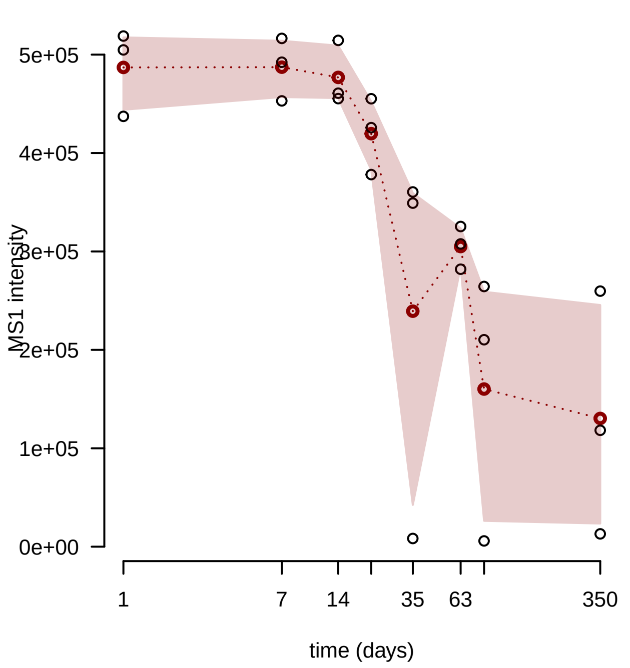

Q8R373
GN=Clmp
CXADR−like membrane protein

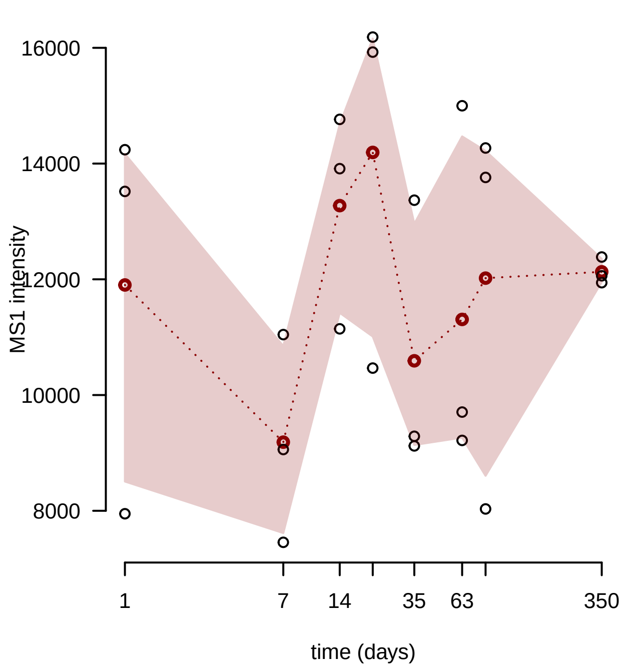

Q8R395
GN=Commd5
COMM domain containing 5

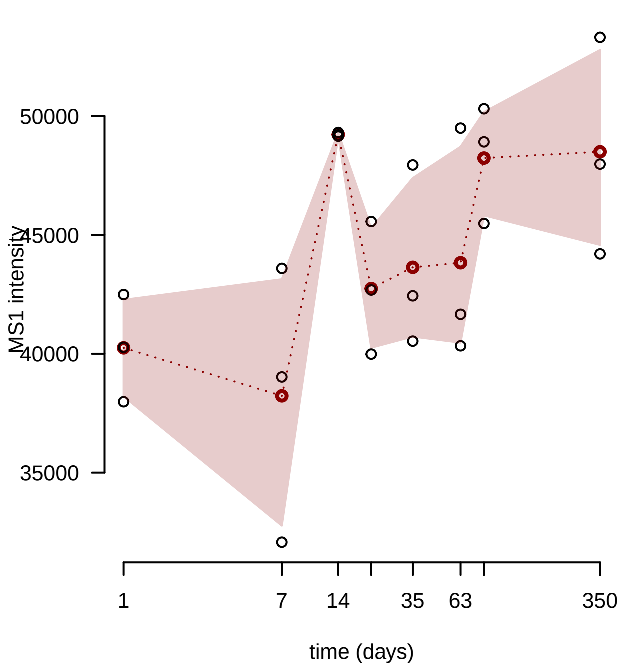

Q8R3B1
GN=Plcd1
phospholipase C, delta 1

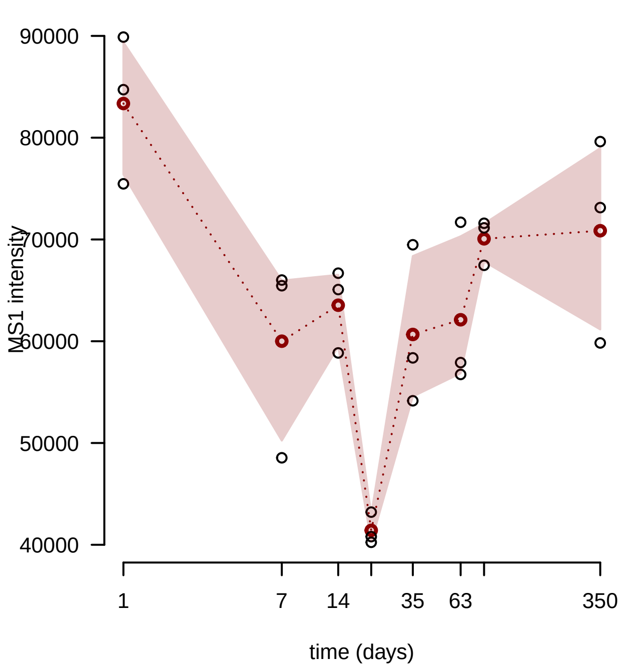

Q8R3B7
GN=Brd8
bromodomain containing 8

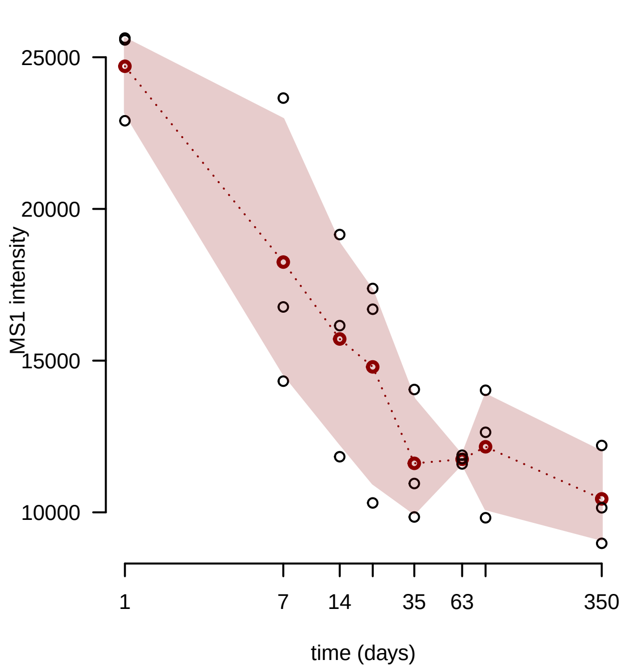

Q8R3C0
GN=Mcmbp
minichromosome maintenance complex bindi ...

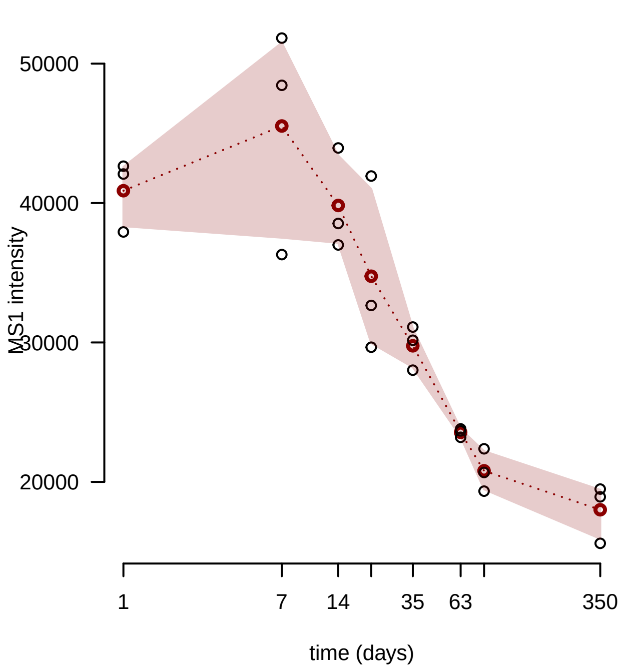

Q8R3C6
GN=Rbm19
RNA binding motif protein 19

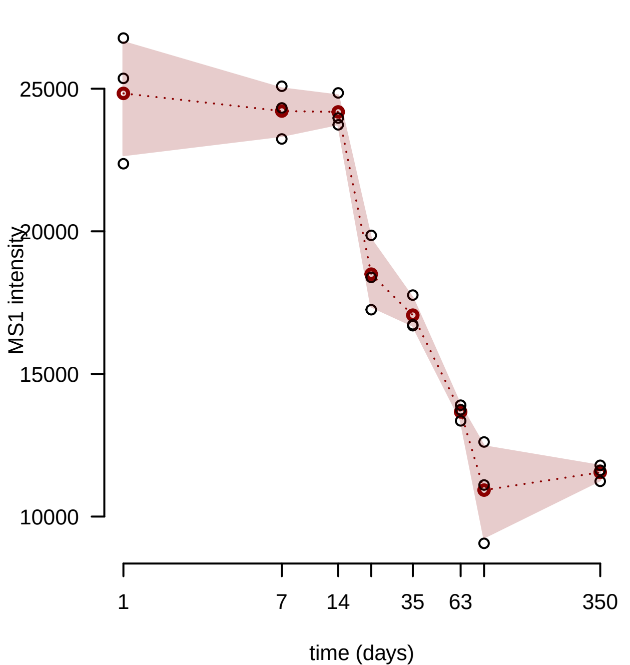

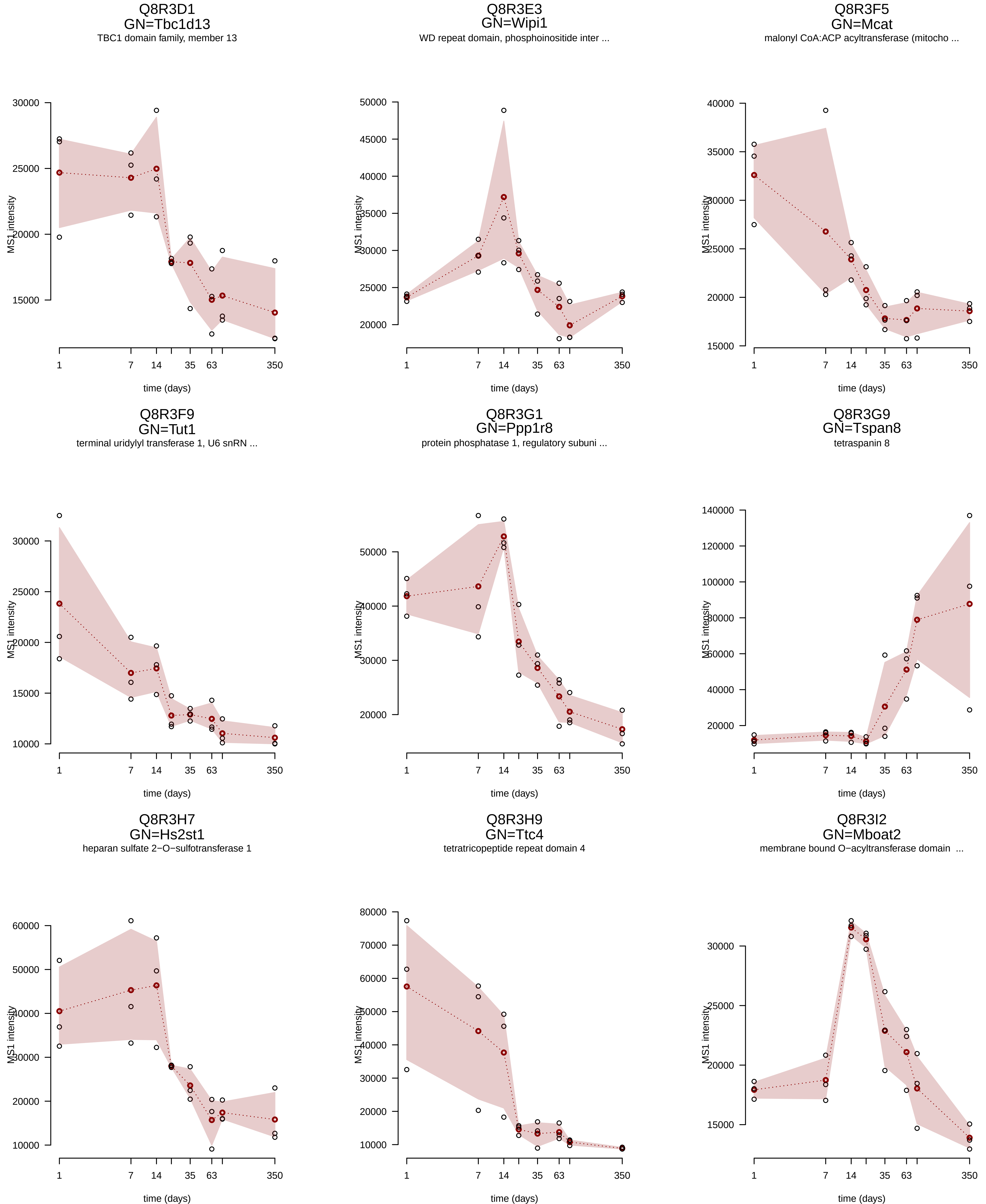
Q8R3D1
GN=Tbc1d13
TBC1 domain family, member 13
Q8R3E3
GN=Wipi1
WD repeat domain, phosphoinositide inter ...
Q8R3F5
GN=Mcat
malonyl CoA:ACP acyltransferase (mitocho ...
Q8R3F9
GN=Tut1
terminal uridylyl transferase 1, U6 snRN ...
Q8R3G1
GN=Ppp1r8
protein phosphatase 1, regulatory subuni ...
Q8R3G9
GN=Tspan8
tetraspanin 8
Q8R3H7
GN=Hs2st1
heparan sulfate 2−O−sulfotransferase 1
Q8R3H9
GN=Ttc4
tetratricopeptide repeat domain 4
Q8R3I2
GN=Mboat2
membrane bound O−acyltransferase domain ...
MS1 intensity
time (days)
1
7
14
35
63
350

Q8R3I3
GN=Cog6
component of oligomeric golgi complex 6

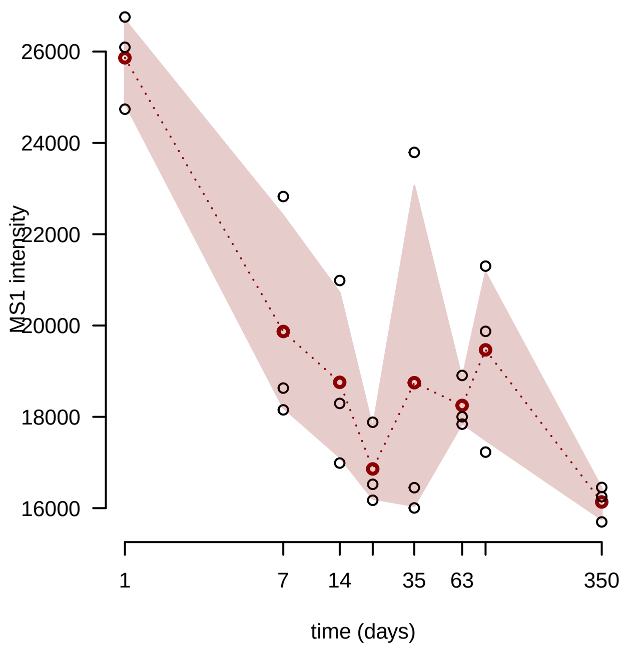

Q8R3K3
GN=Ptcd2
pentatricopeptide repeat domain 2

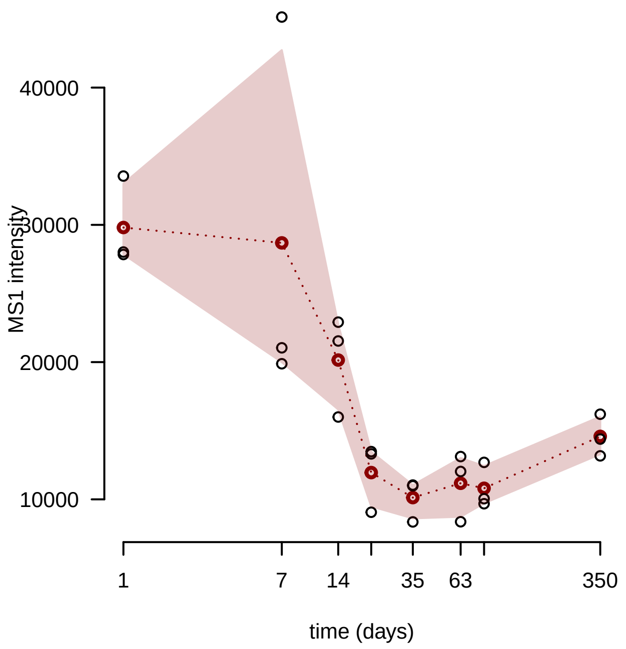

Q8R3L2
GN=Tcf25
transcription factor 25 (basic helix−loo ...

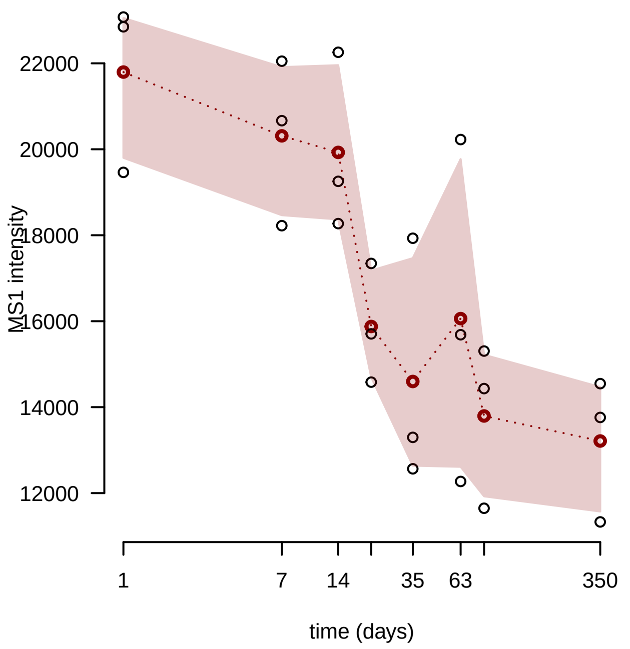

Q8R3L5
GN=Slco3a1
solute carrier organic anion transporter ...

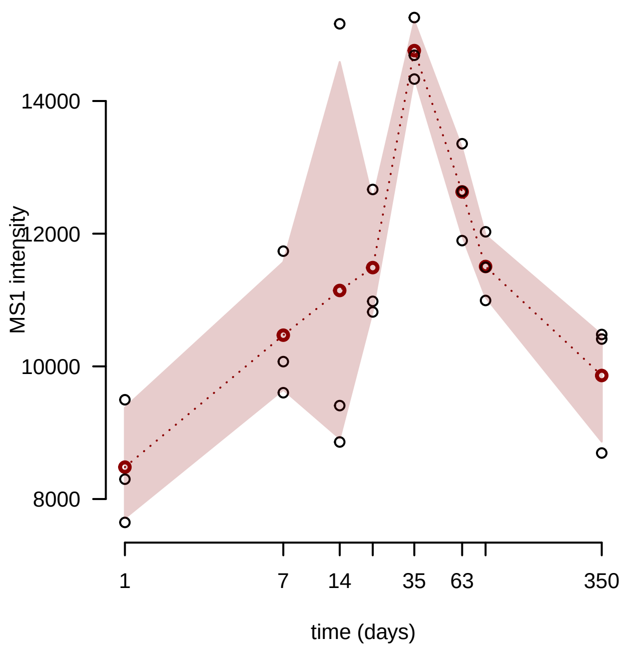

Q8R3N1
GN=Nop14
NOP14 nucleolar protein

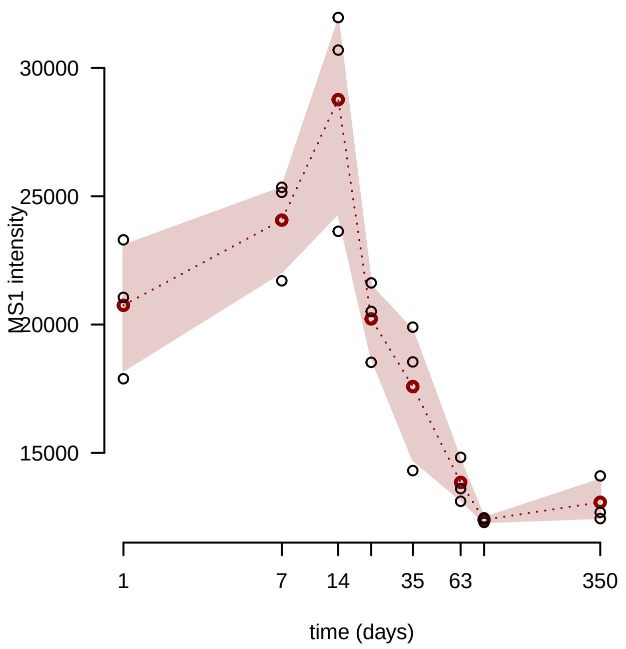

Q8R3N6
GN=Thoc1
THO complex 1

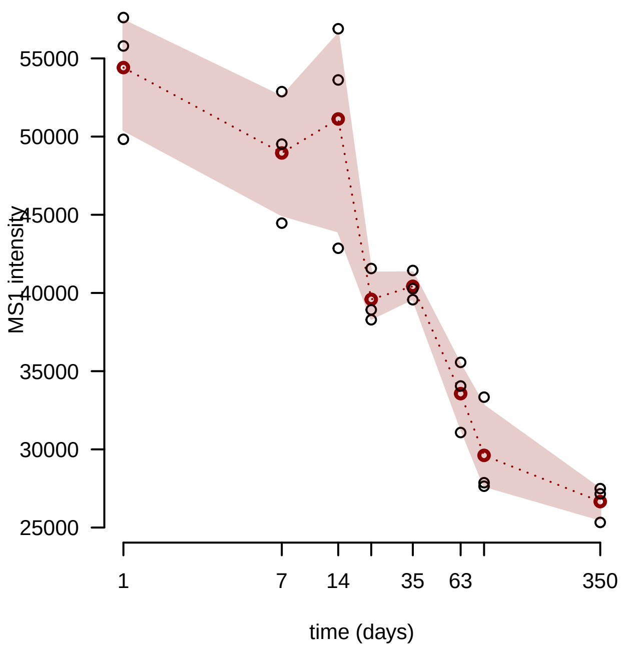

Q8R3P2
GN=Dtx2
deltex 2, E3 ubiquitin ligase

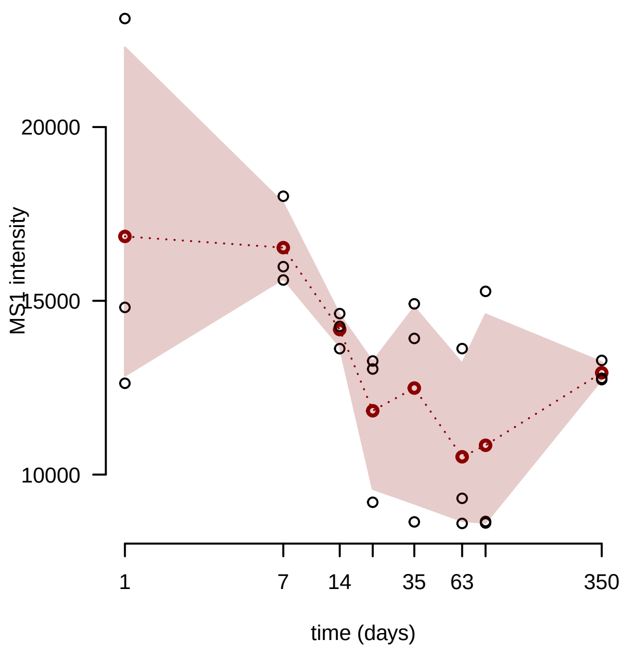

Q8R3P2
GN=Dtx2
deltex 2, E3 ubiquitin ligase

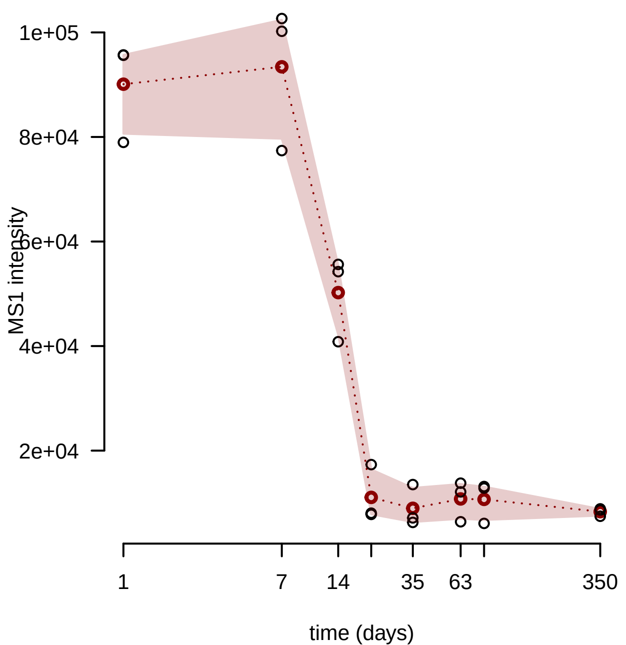

Q8R3P6
GN=Ints14
integrator complex subunit 14

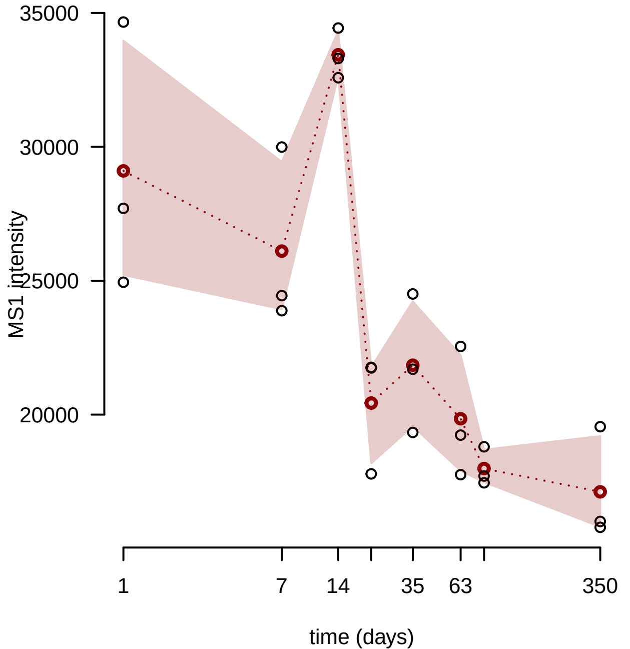

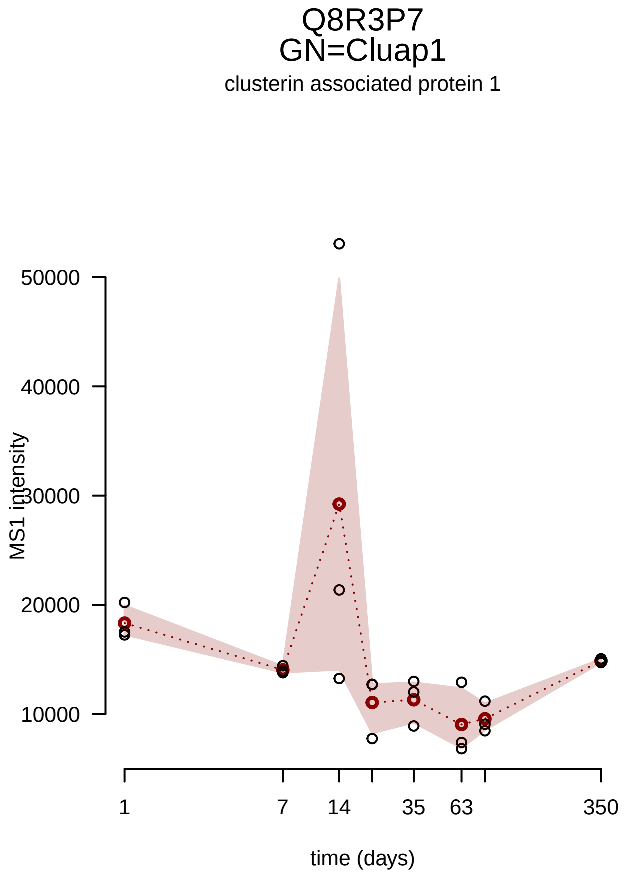
Q8R3P7
GN=Cluap1
clusterin associated protein 1
MS1 intensity
50000
40000
30000
20000
10000
1
7
14
35
63
350
time (days)

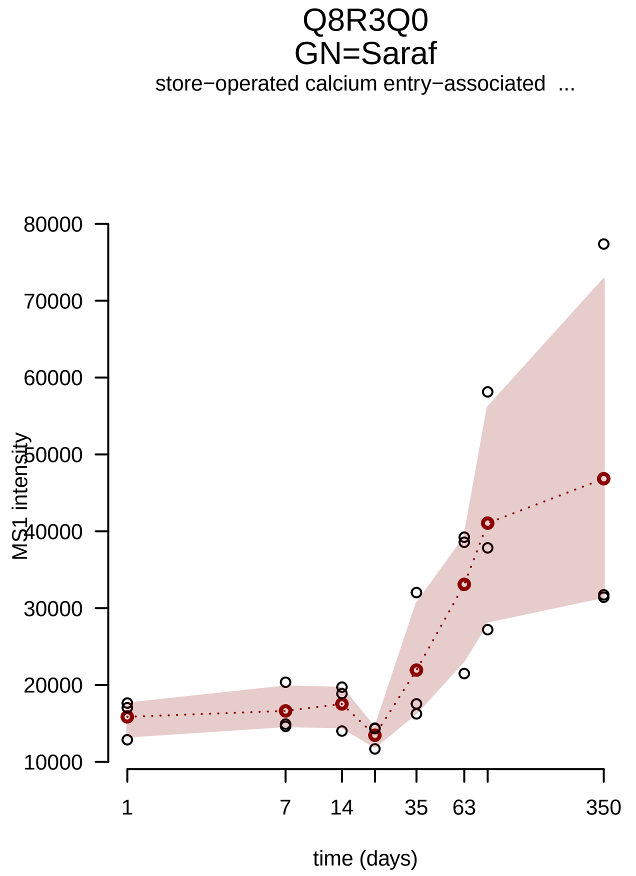
Q8R3Q0
GN=Saraf
store−operated calcium entry−associated ...
MS1 intensity
80000
70000
60000
50000
40000
30000
20000
10000
1
7
14
35
63
350
time (days)

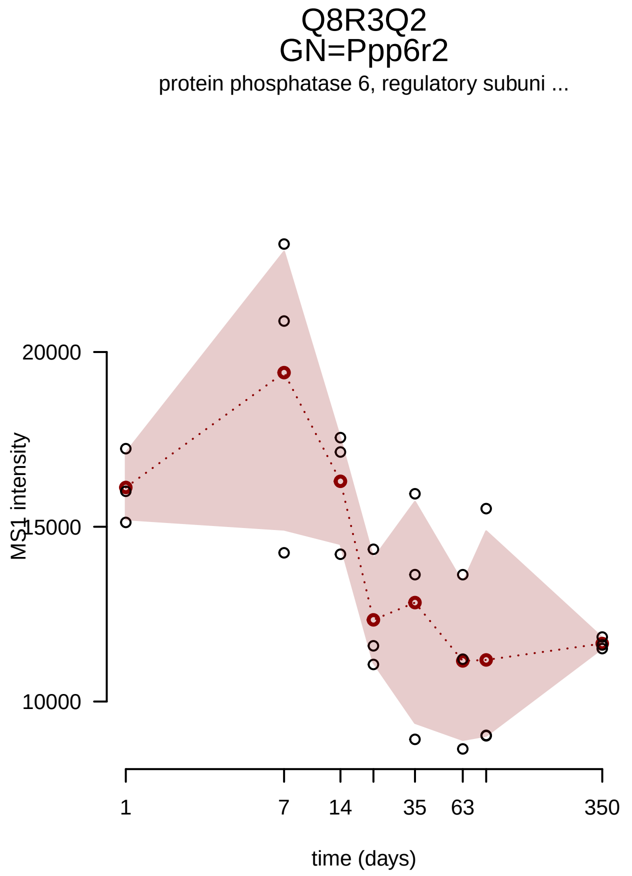
Q8R3Q2
GN=Ppp6r2
protein phosphatase 6, regulatory subuni ...
MS1 intensity
20000
15000
10000
1
7
14
35
63
350
time (days)

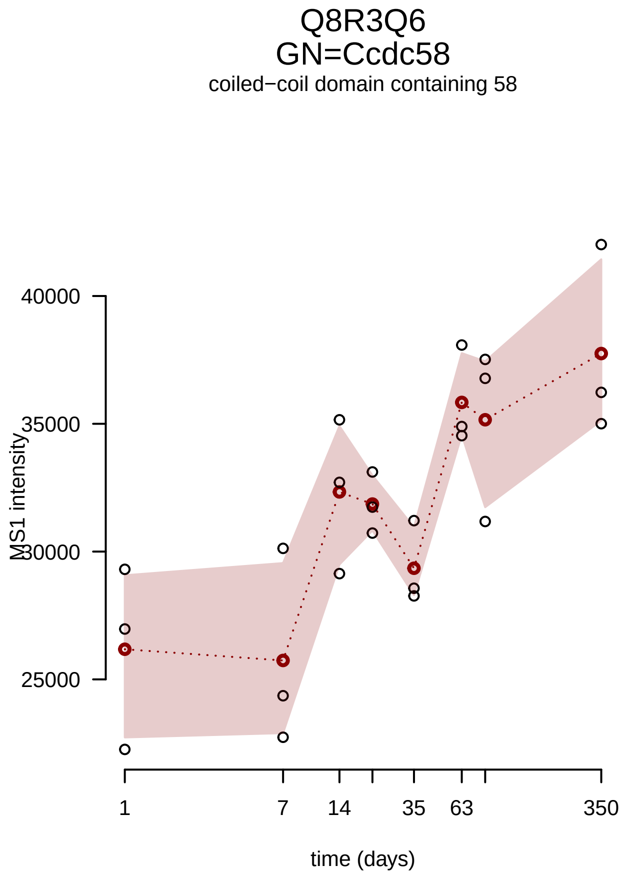
Q8R3Q6
GN=Ccdc58
coiled−coil domain containing 58
MS1 intensity
40000
35000
30000
25000
1
7
14
35
63
350
time (days)

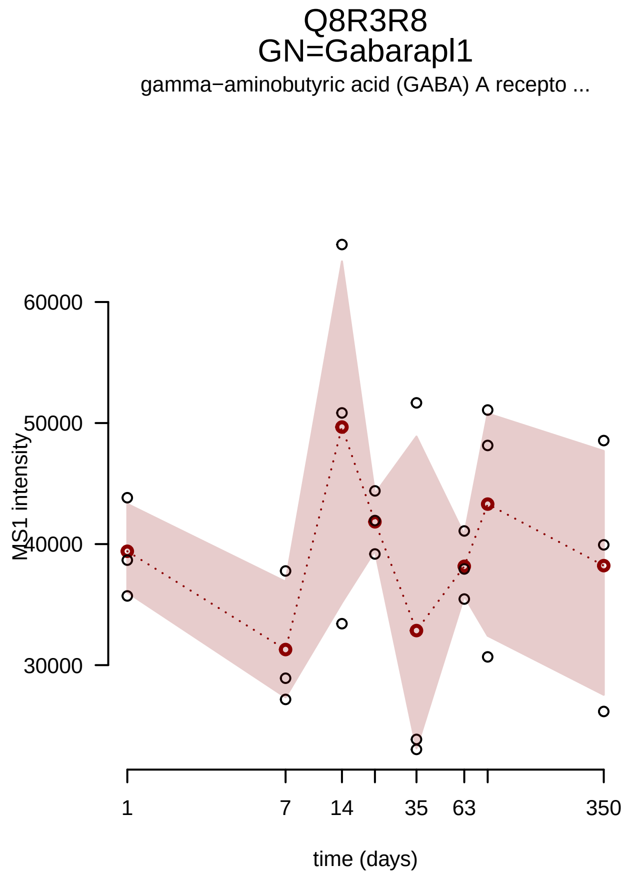
Q8R3R8
GN=Gabarapl1
gamma−aminobutyric acid (GABA) A recepto ...
MS1 intensity
60000
50000
40000
30000
1
7
14
35
63
350
time (days)

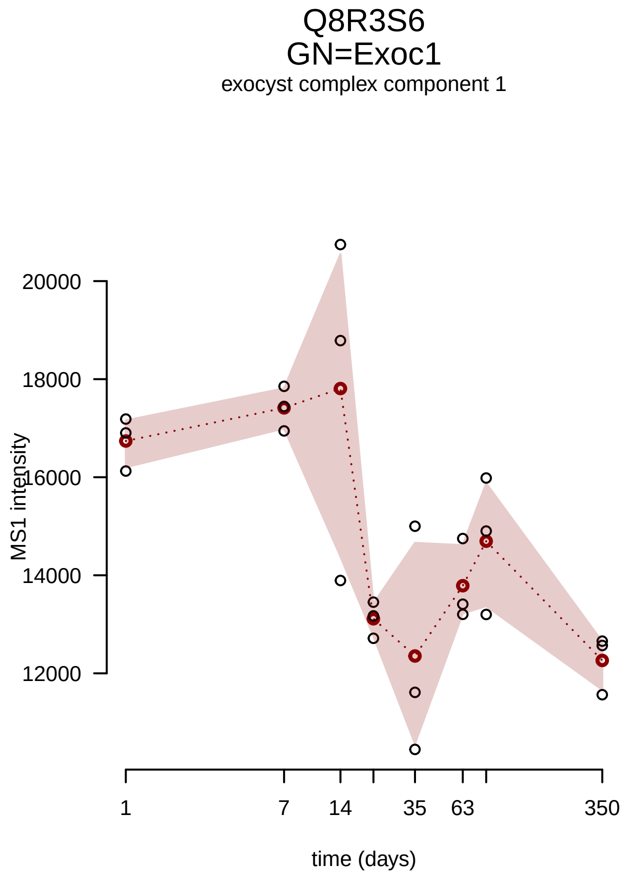
Q8R3S6
GN=Exoc1
exocyst complex component 1
MS1 intensity
20000
18000
16000
14000
12000
1
7
14
35
63
350
time (days)

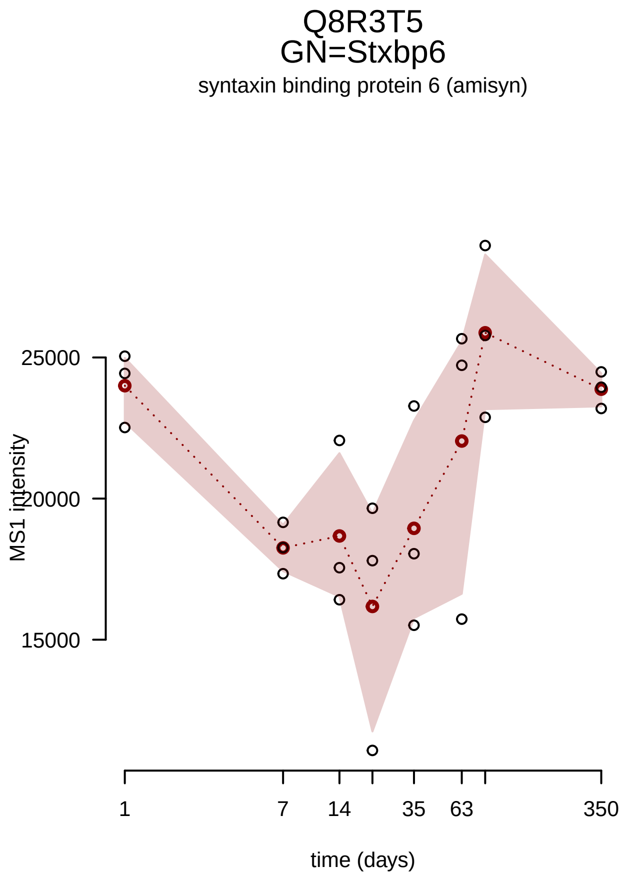
Q8R3T5
GN=Stxbp6
syntaxin binding protein 6 (amisyn)
MS1 intensity
25000
20000
15000
1
7
14
35
63
350
time (days)

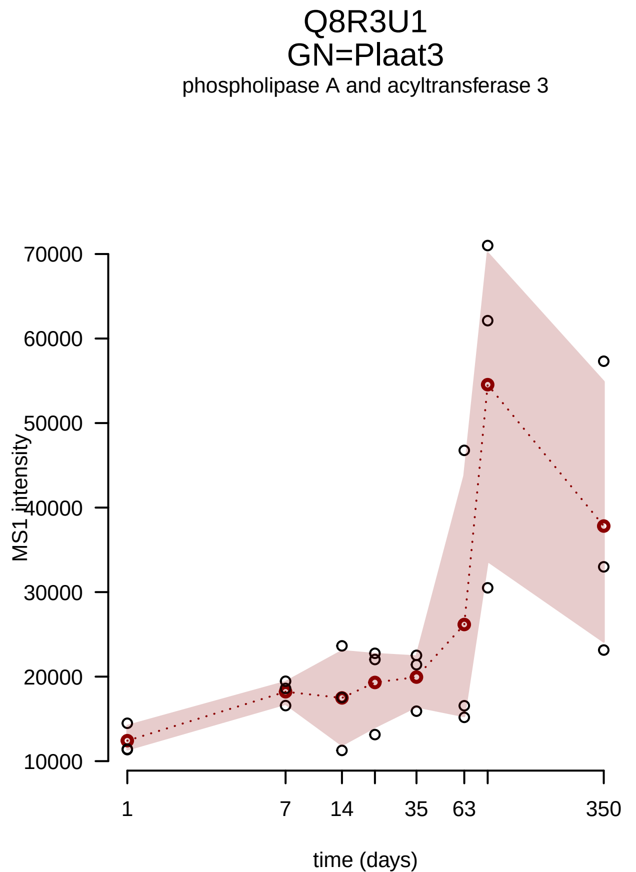
Q8R3U1
GN=Plaat3
phospholipase A and acyltransferase 3
MS1 intensity
70000
60000
50000
40000
30000
20000
10000
1
7
14
35
63
350
time (days)

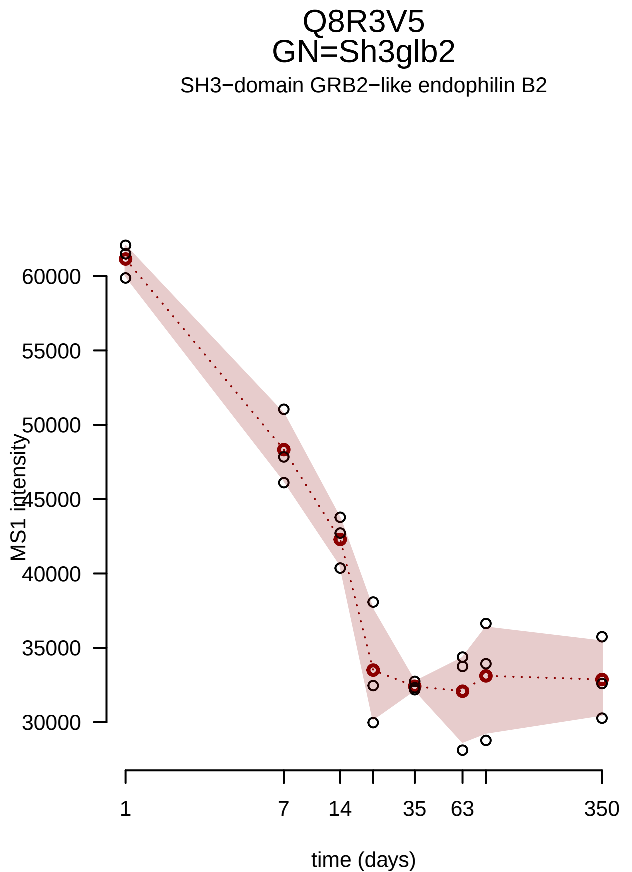
Q8R3V5
GN=Sh3glb2
SH3−domain GRB2−like endophilin B2
MS1 intensity
60000
55000
50000
45000
40000
35000
30000
1
7
14
35
63
350
time (days)

Q8R3V6
GN=Cuedc1
CUE domain containing 1

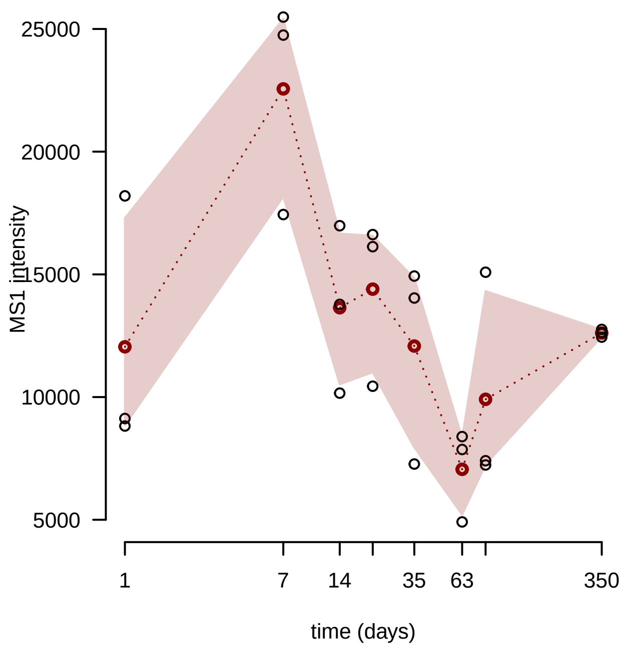

Q8R3W5
GN=Tsen15
tRNA splicing endonuclease subunit 15

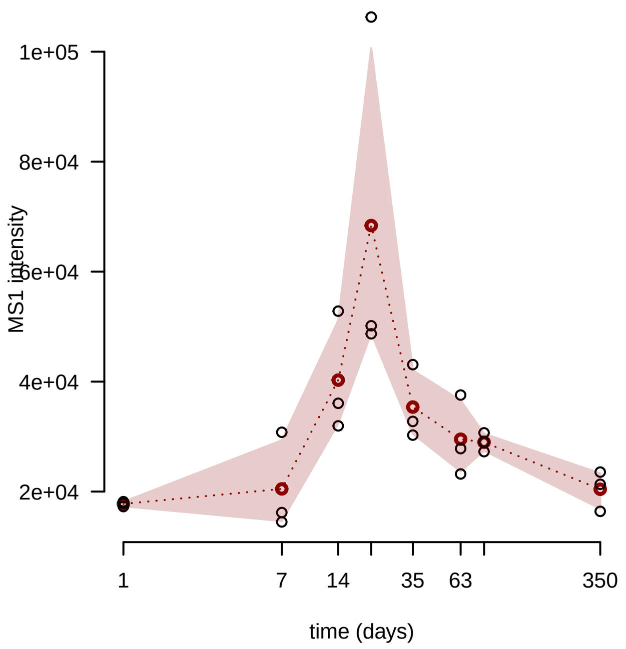

Q8R3W7
GN=Agr3
anterior gradient 3

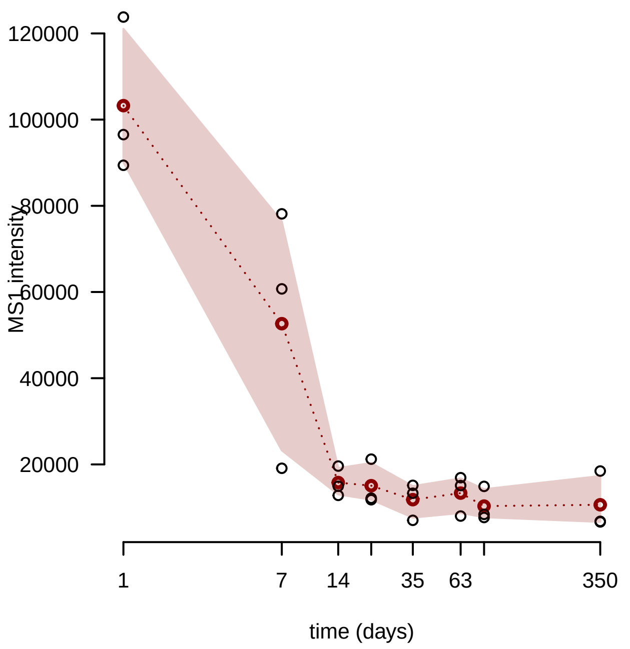

Q8R3Y5
GN=

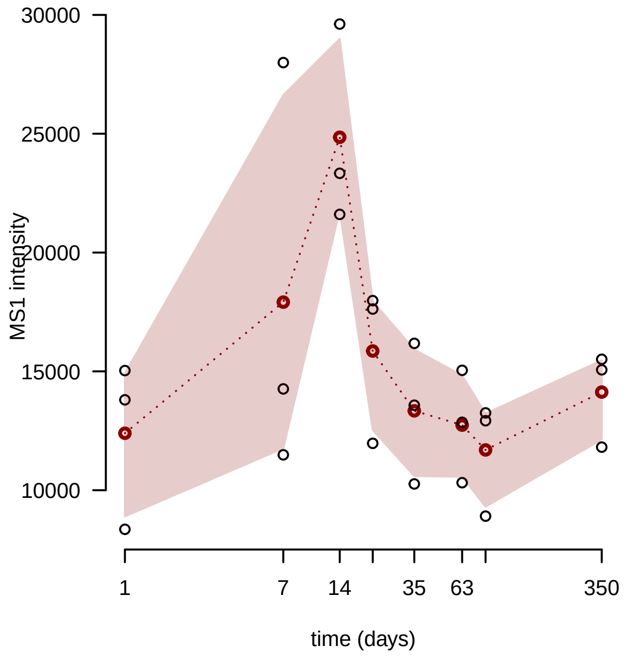

Q8R3Y8
GN=Irf2bp1
interferon regulatory factor 2 binding p ...

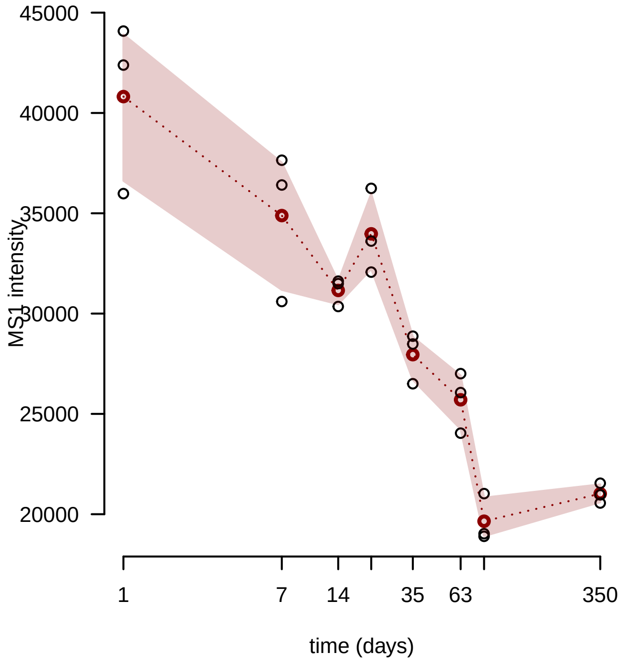

Q8R404
GN=Micos13
mitochondrial contact site and cristae o ...

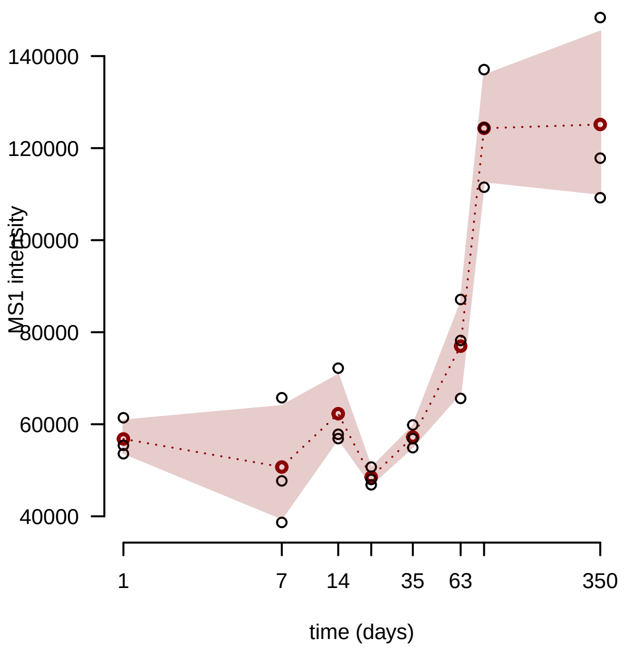

Q8R409
GN=Hexim1
hexamethylene bis−acetamide inducible 1

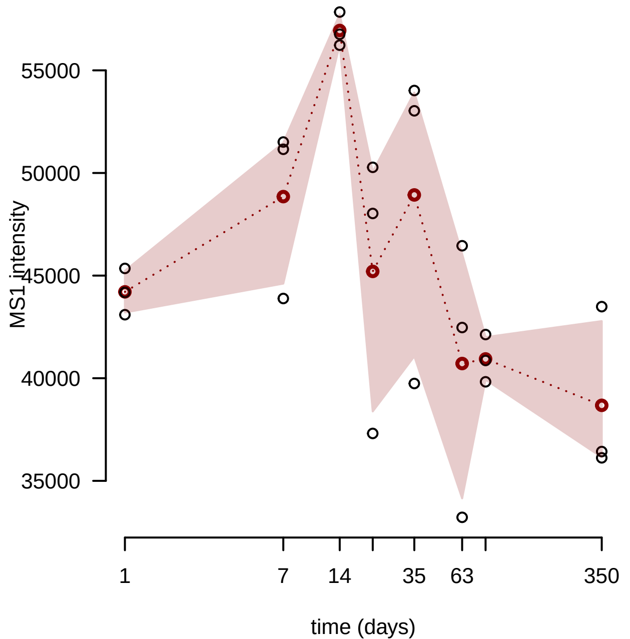

Q8R418
GN=Dicer1
dicer 1, ribonuclease type III

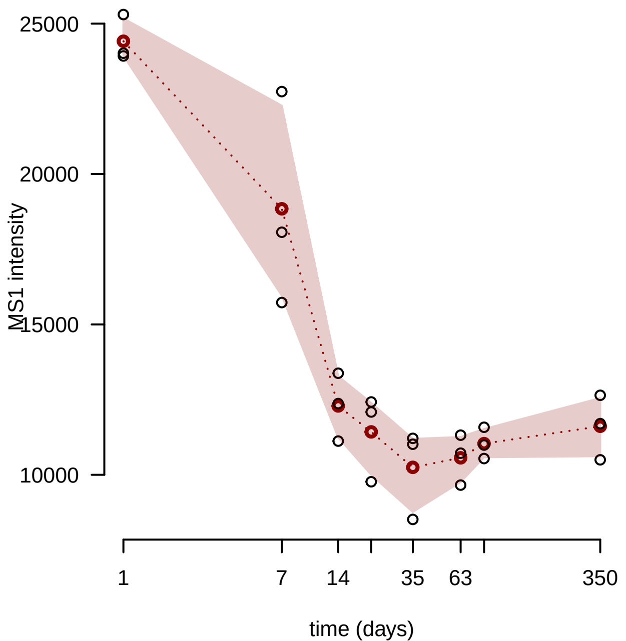

Q8R420
GN=Abca3
ATP−binding cassette, sub−family A (ABC1 ...

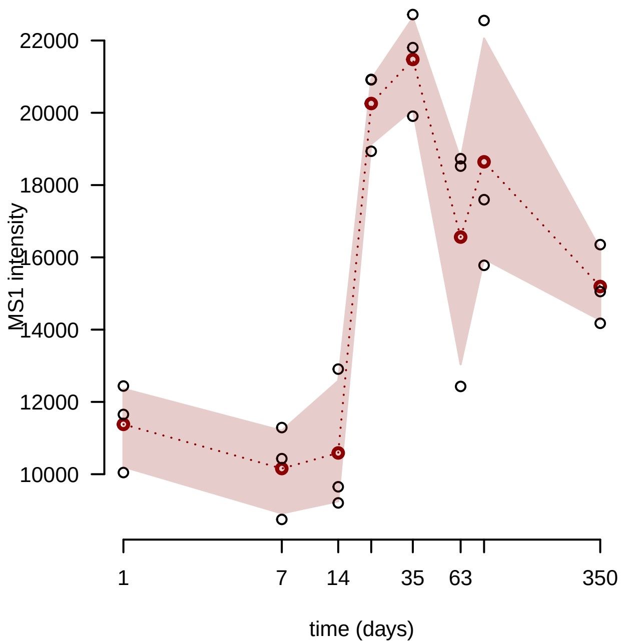

Q8R422
GN=Cd109
CD109 antigen

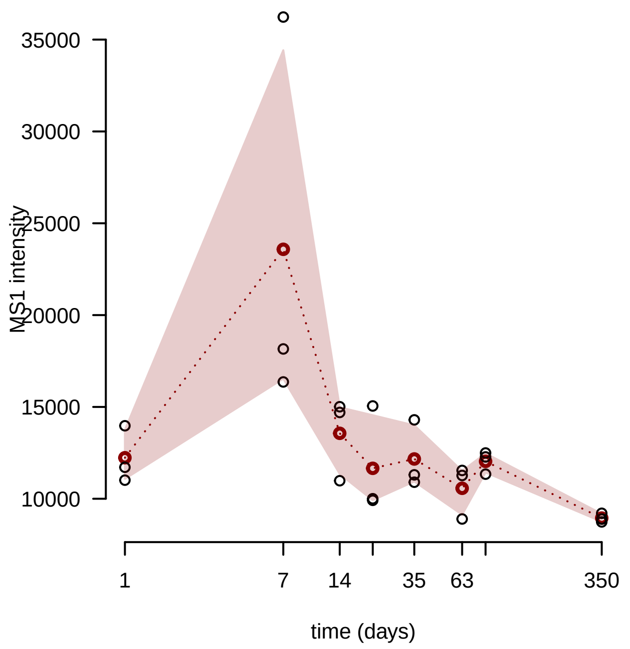

Q8R429
GN=Atp2a1
ATPase, Ca++ transporting, cardiac muscl ...

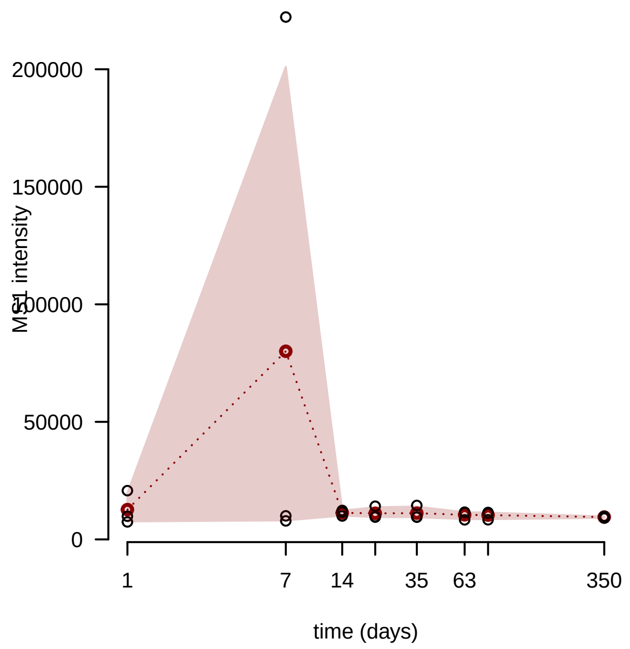

Q8R464
GN=Cadm4
cell adhesion molecule 4

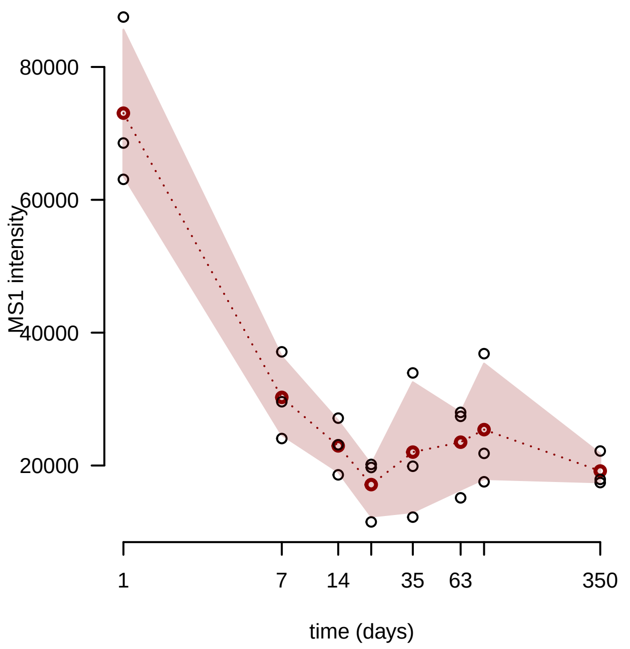

Q8R480
GN=Nup85
nucleoporin 85

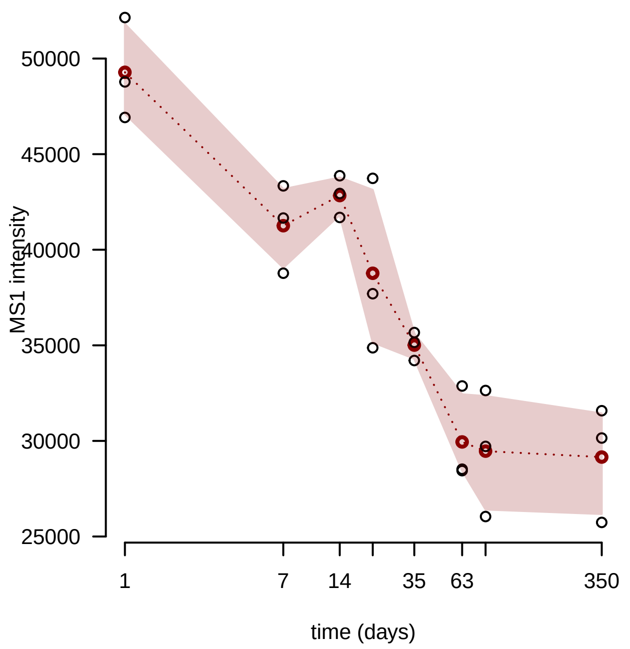

Q8R4C2
GN=Rufy2
RUN and FYVE domain−containing 2

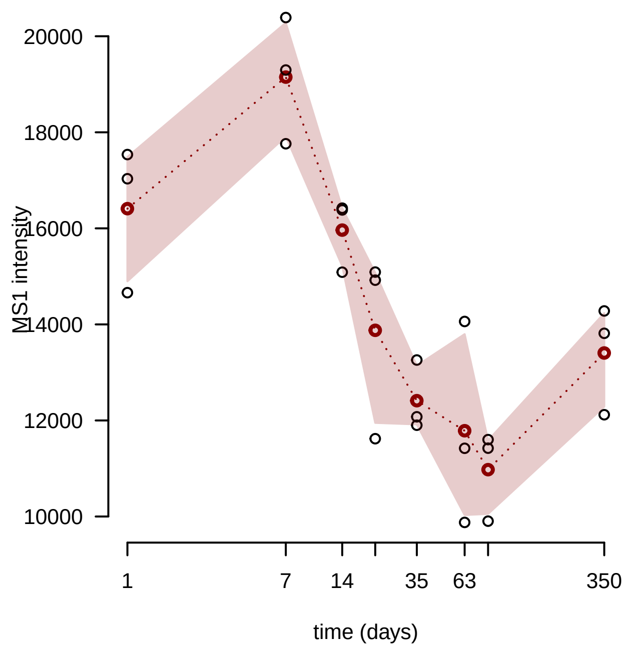

Q8R4D1
GN=Slc9a8
solute carrier family 9 (sodium/hydrogen ...

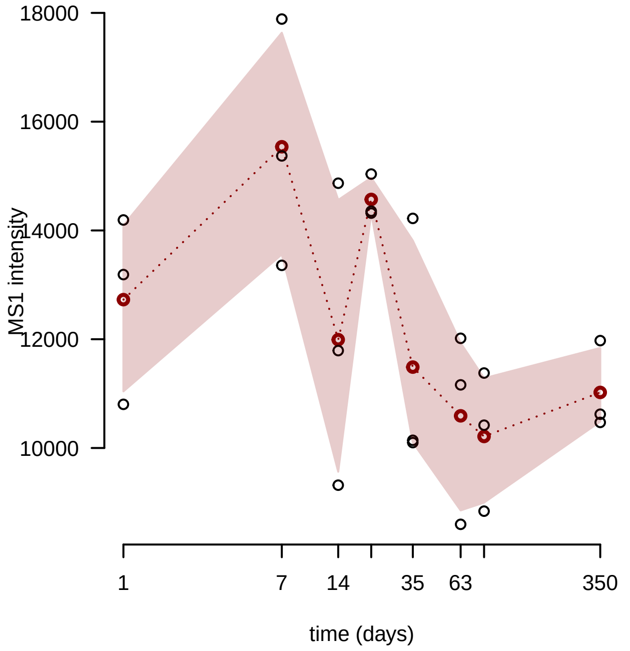

Q8R4E6
GN=Purg
purine−rich element binding protein G

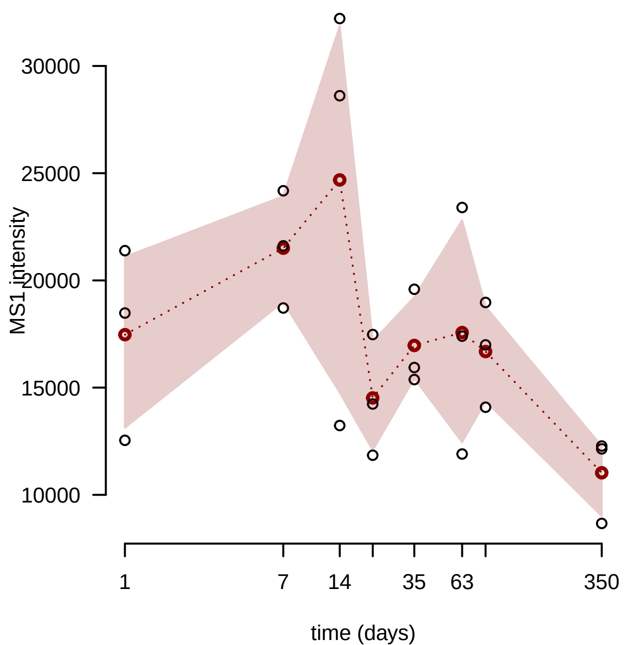

Q8R4G6
GN=Mgat5
mannoside acetylglucosaminyltransferase ...

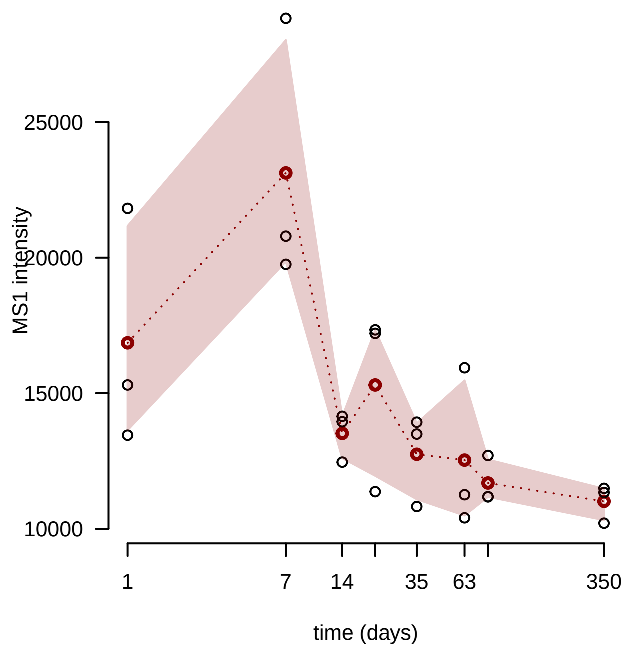

Q8R4H2
GN=Arhgef12
Rho guanine nucleotide exchange factor (...

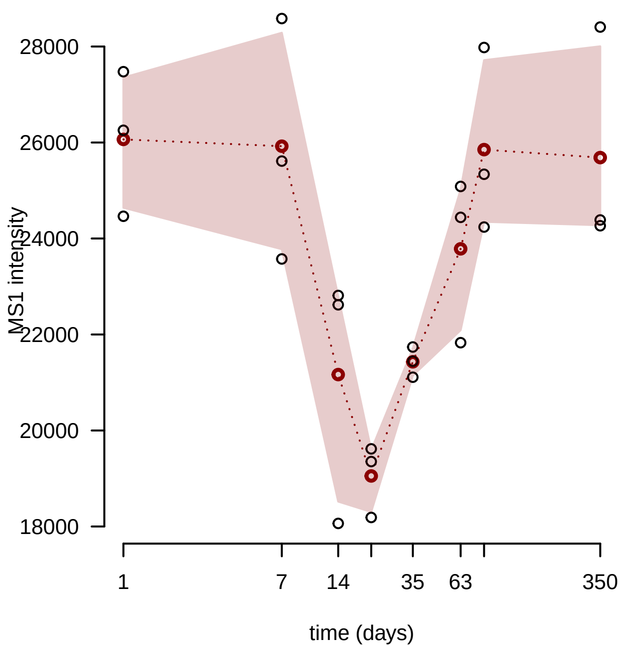

Q8R4H9
GN=Slc30a5
solute carrier family 30 (zinc transport ...

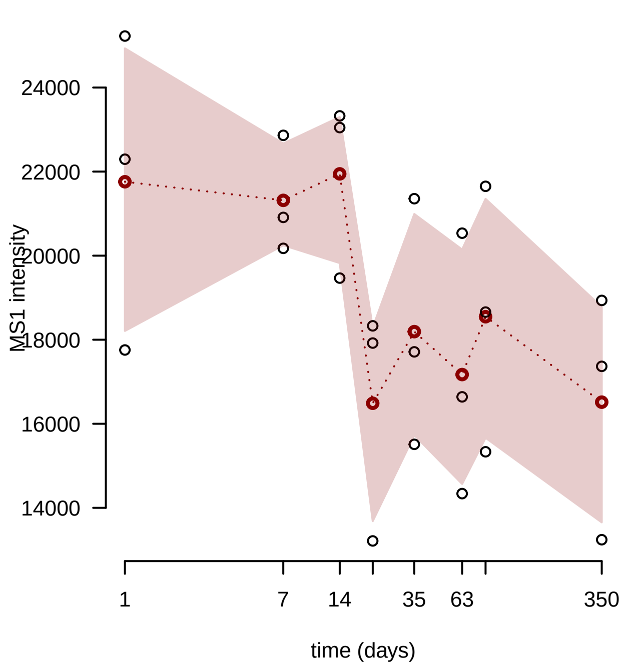

Q8R4N0
GN=Clybl
citrate lyase beta like

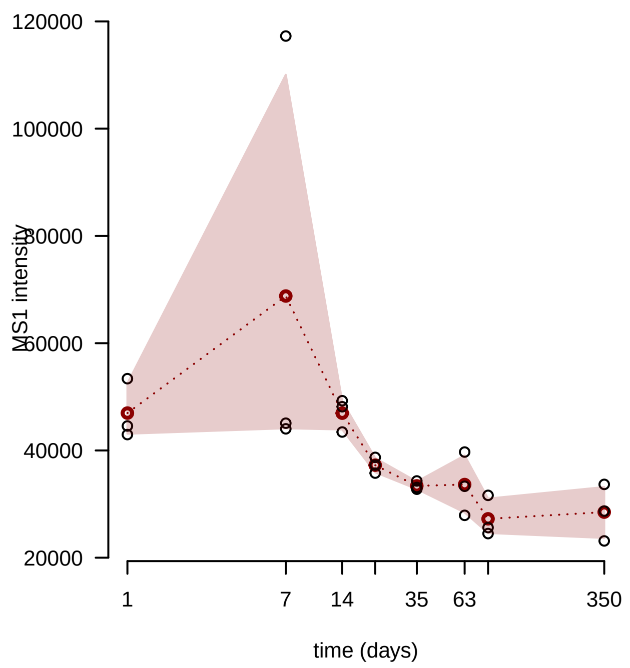

Q8R4P9
GN=Abcc10
ATP−binding cassette, sub−family C (CFTR ...

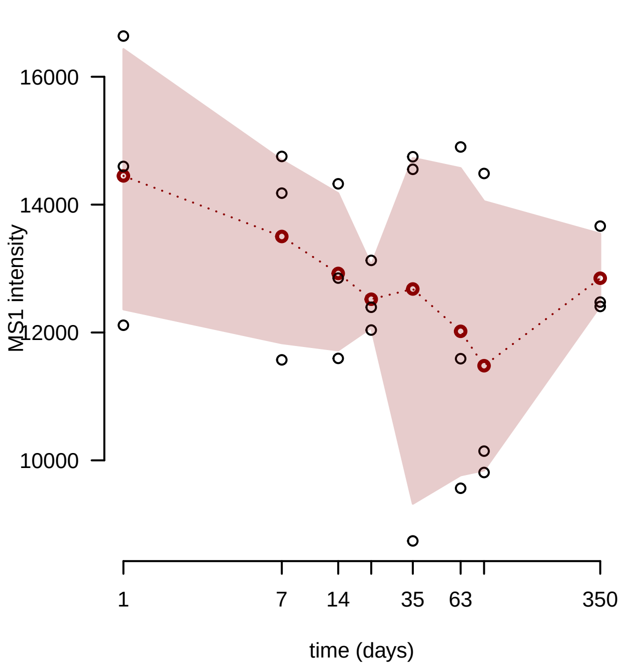

Q8R4R6
GN=Nup35
nucleoporin 35

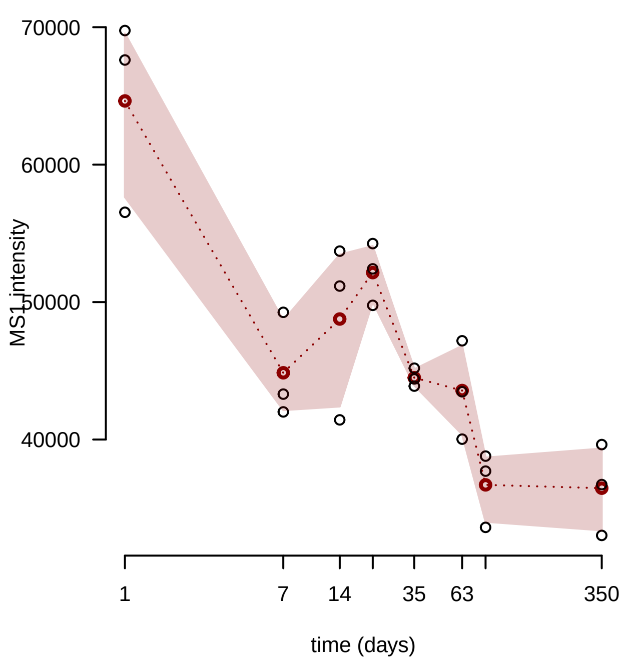

Q8R4U6
GN=Top1mt
DNA topoisomerase 1, mitochondrial

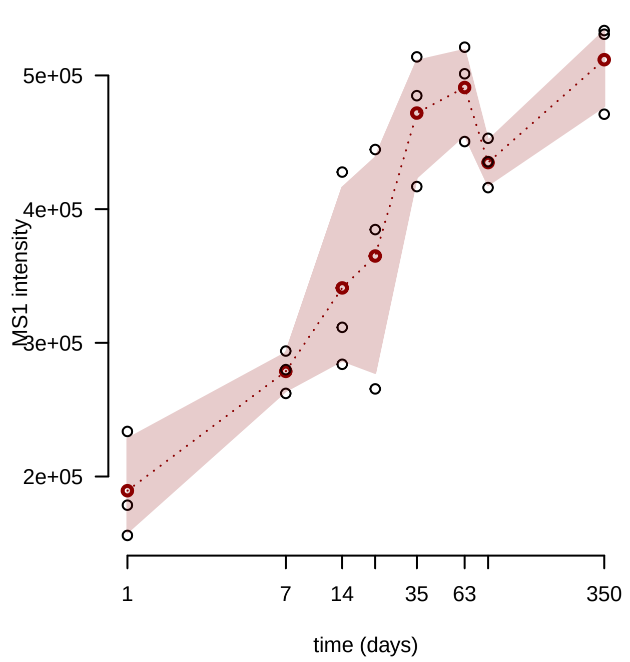

Q8R4U7
GN=Luzp1
leucine zipper protein 1

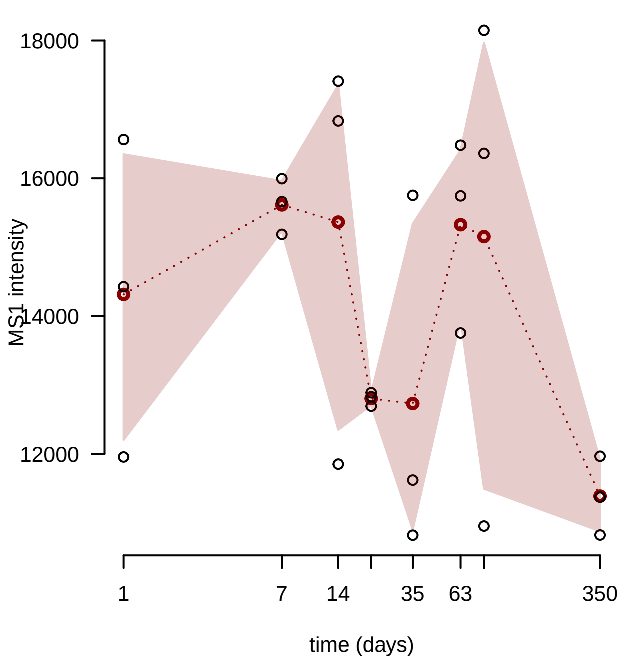

Q8R4V4
GN=Cpz
carboxypeptidase Z

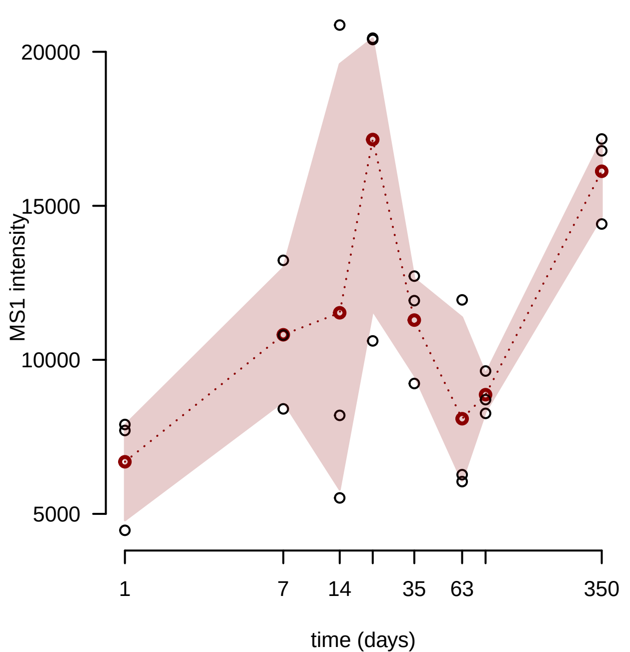

Q8R4X3
GN=Rbm12
RNA binding motif protein 12

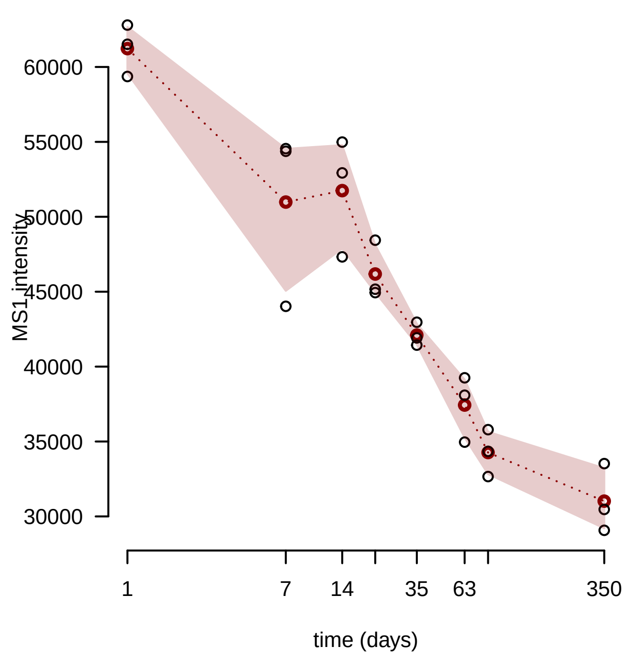

Q8R4Y4
GN=Stab1
stabilin 1

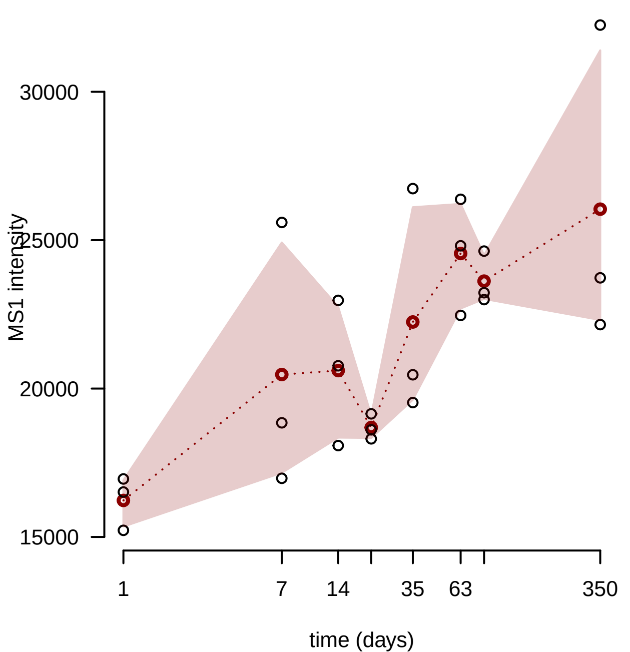

Q8R4Y8
GN=Rttn
rotatin

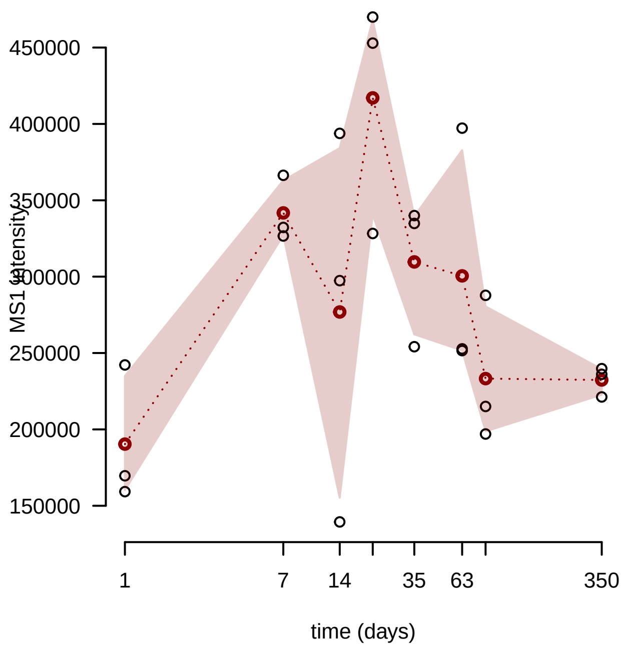

Q8R502
GN=Lrrc8c
leucine rich repeat containing 8 family, ...

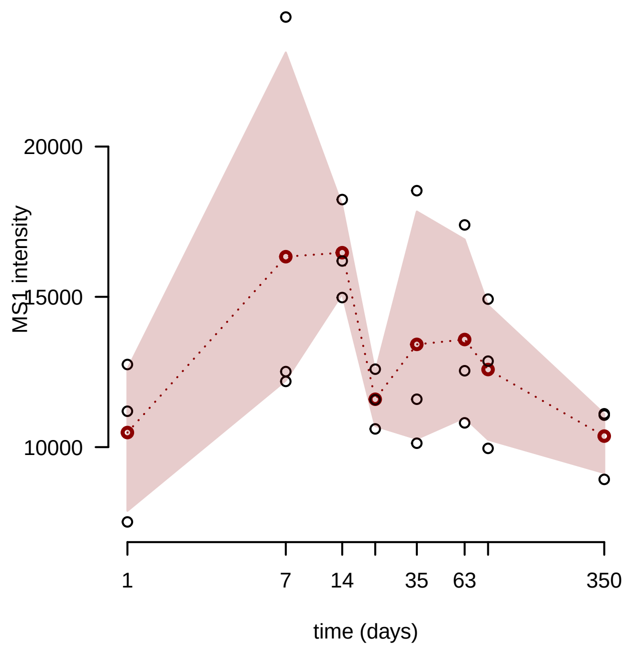

Q8R516
GN=Mib2
mindbomb E3 ubiquitin protein ligase 2

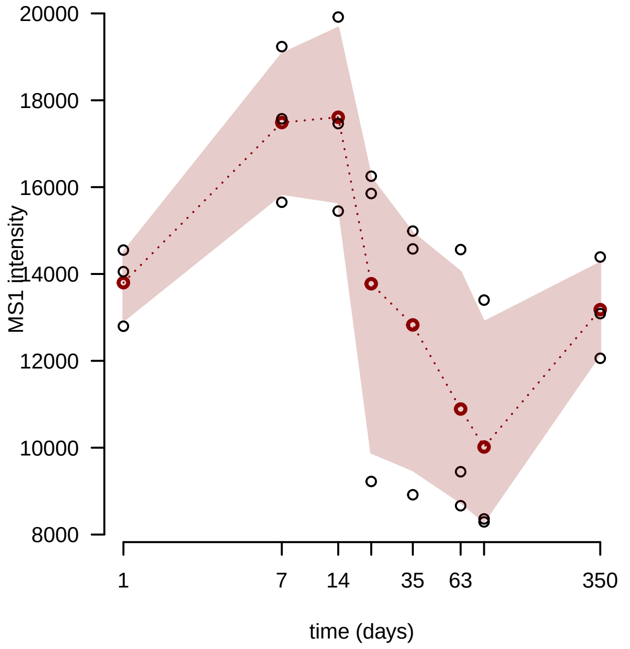

Q8R527
GN=Rhoq
ras homolog family member Q

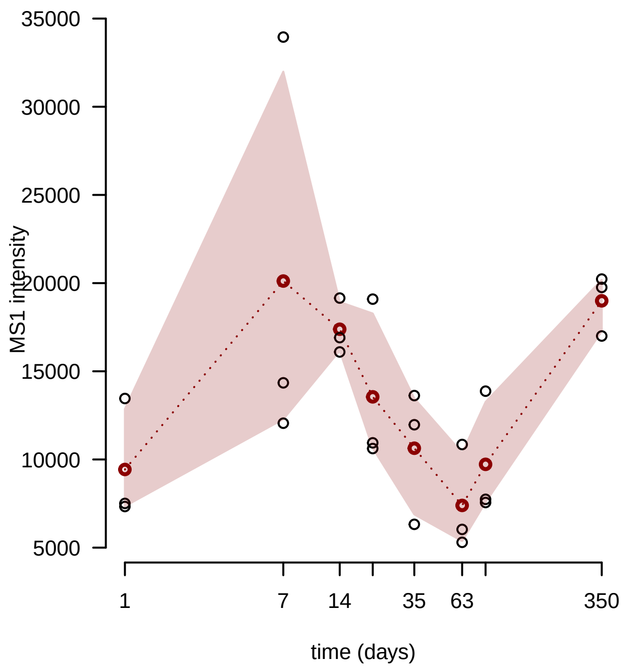

Q8R550
GN=Sh3kbp1
SH3−domain kinase binding protein 1

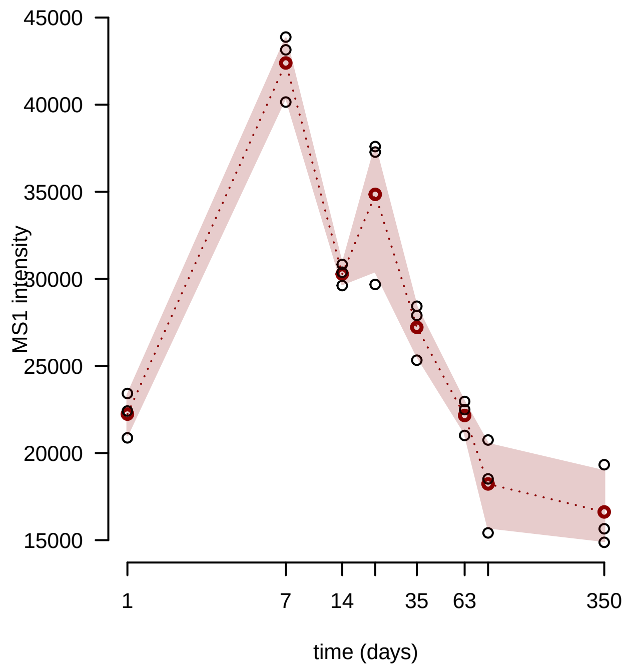

Q8R570
GN=Snap47
synaptosomal−associated protein, 47

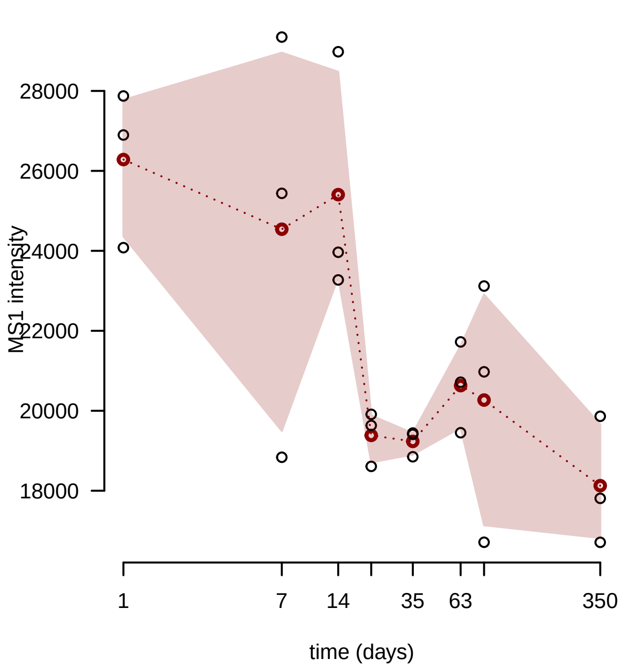

Q8R574
GN=Prpsap2
phosphoribosyl pyrophosphate synthetase− ...

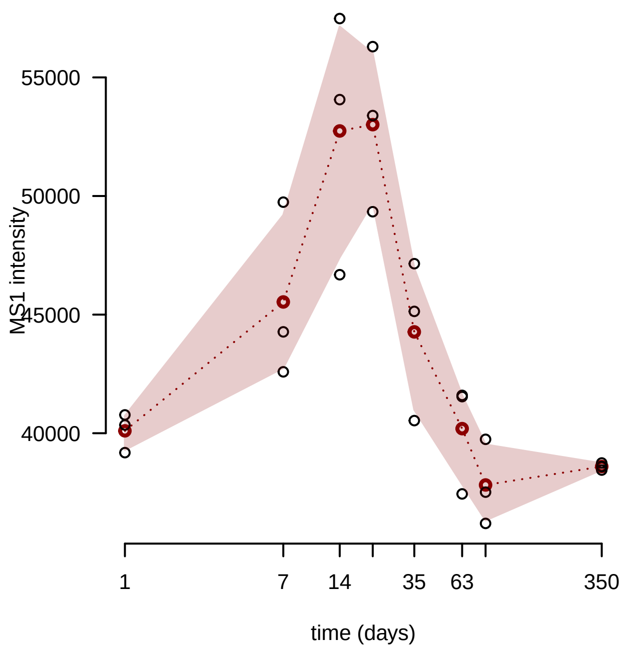

Q8R5A0
GN=Smyd2
SET and MYND domain containing 2

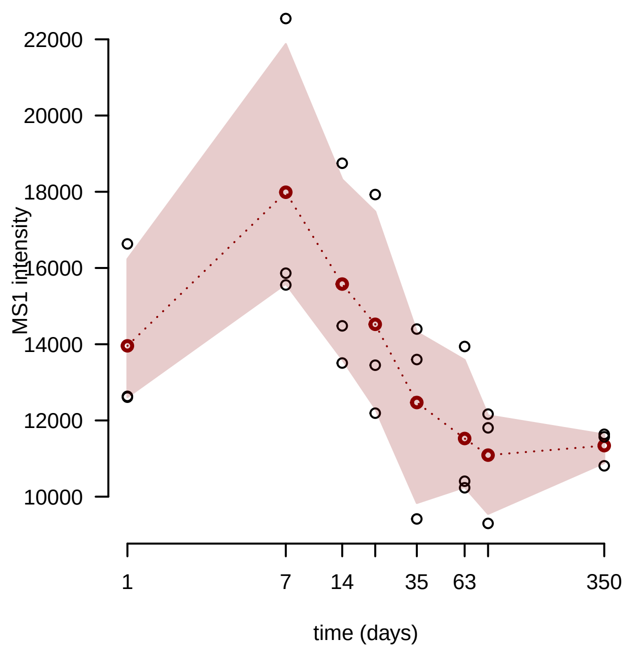

Q8R5A3
GN=Apbb1ip
amyloid beta (A4) precursor protein−bind ...

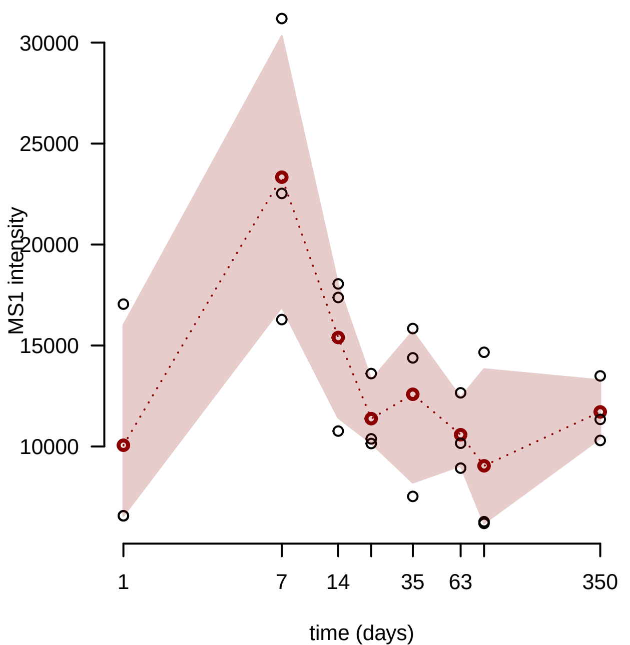

Q8R5A6
GN=Tbc1d22a
TBC1 domain family, member 22a

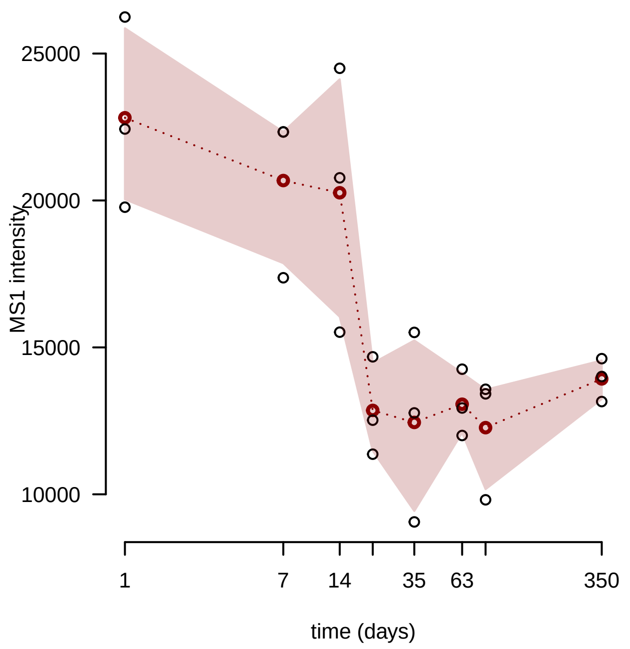

Q8R5C5
GN=Actr1b
ARP1 actin−related protein 1B, centracti ...

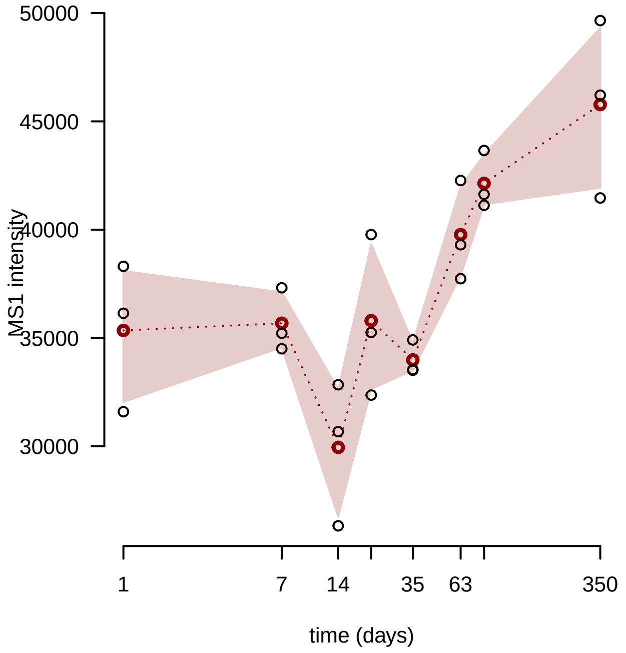

Q8R5C8
GN=Zmynd11
zinc finger, MYND domain containing 11

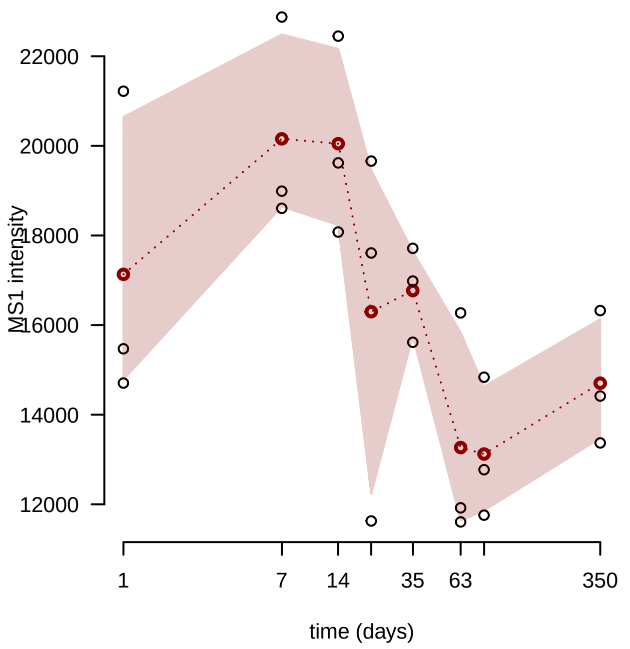

Q8R5F3
GN=Oard1
O−acyl−ADP−ribose deacylase 1

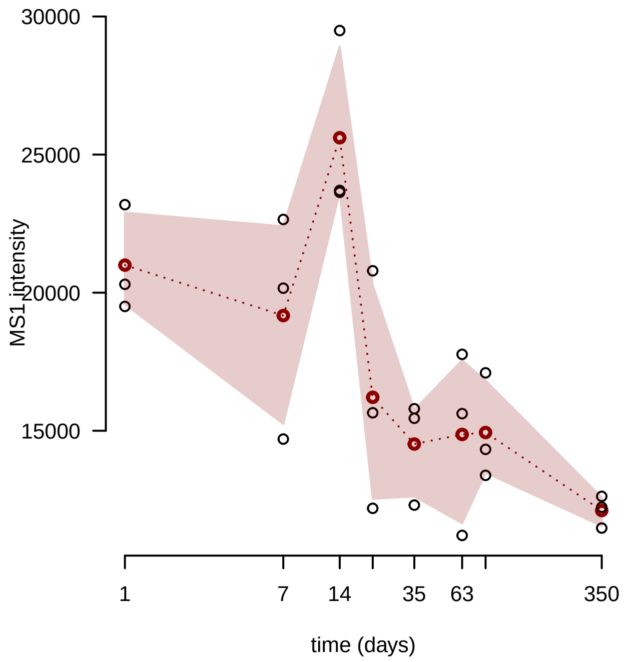

Q8R5F7
GN=Ifih1
interferon induced with helicase C domai ...

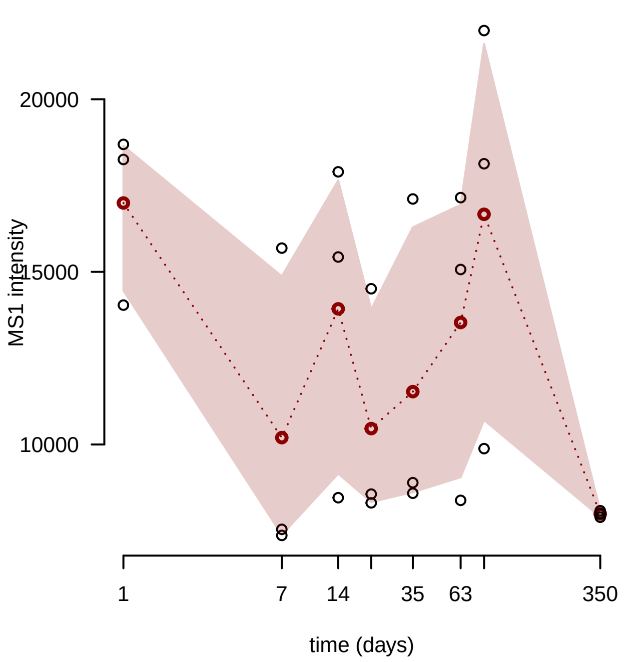

Q8R5G7
GN=Arap3
ArfGAP with RhoGAP domain, ankyrin repea ...

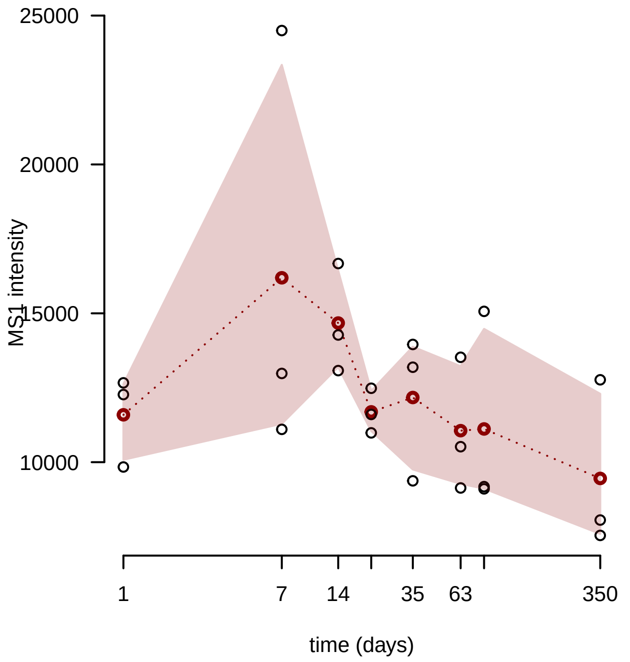

Q8R5H1
GN=Usp15
ubiquitin specific peptidase 15

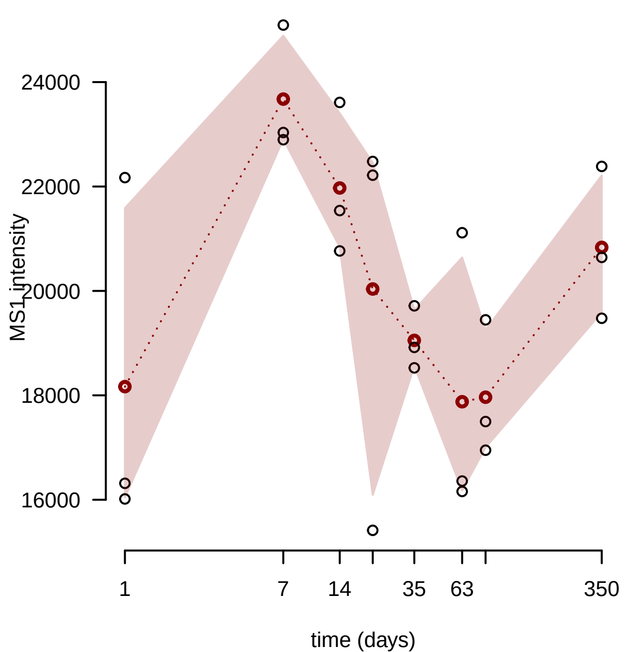

Q8R5H6
GN=Wasf1
WASP family, member 1

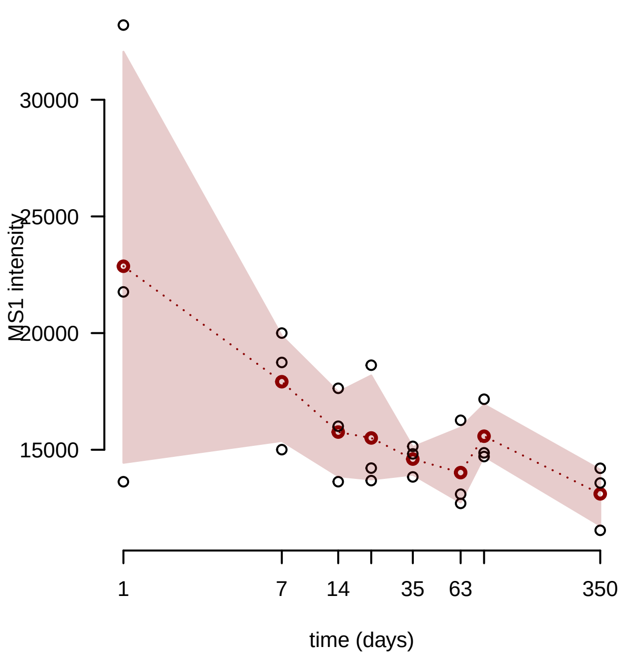

Q8R5I6
GN=

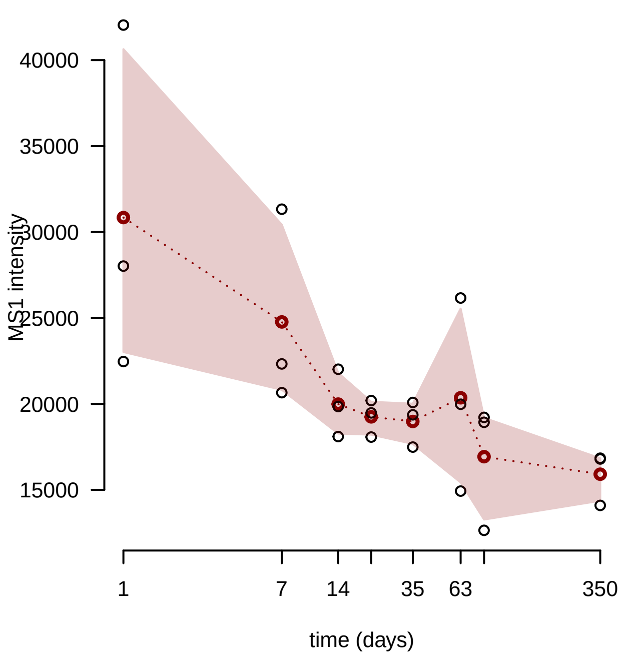

Q8R5J9
GN=Arl6ip5
ADP−ribosylation factor−like 6 interacti ...

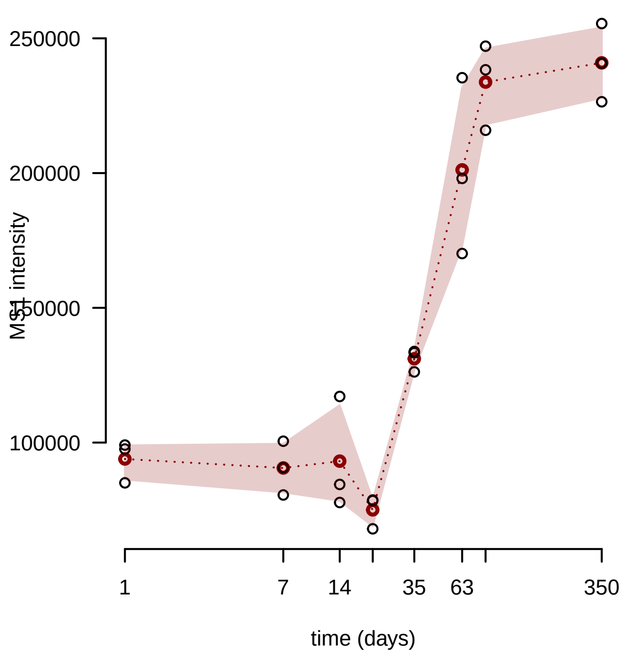

Q8R5K2
GN=Usp33
ubiquitin specific peptidase 33

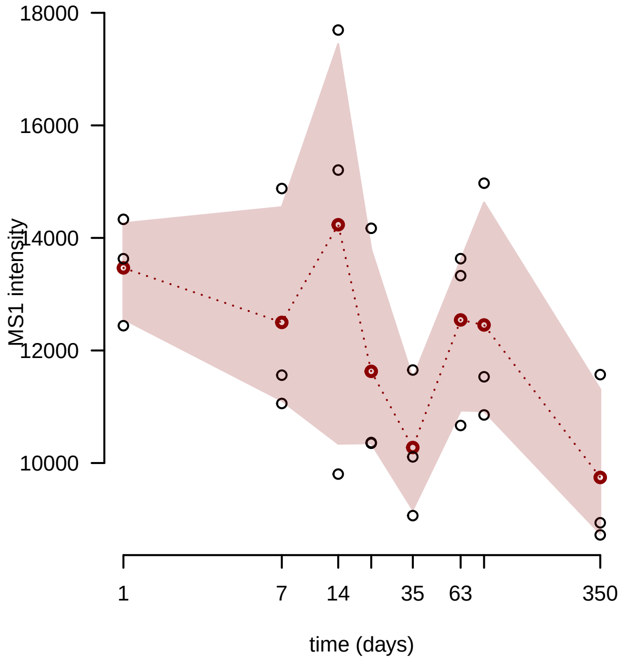

Q8R5K4
GN=Nol6
nucleolar protein family 6 (RNA−associat ...

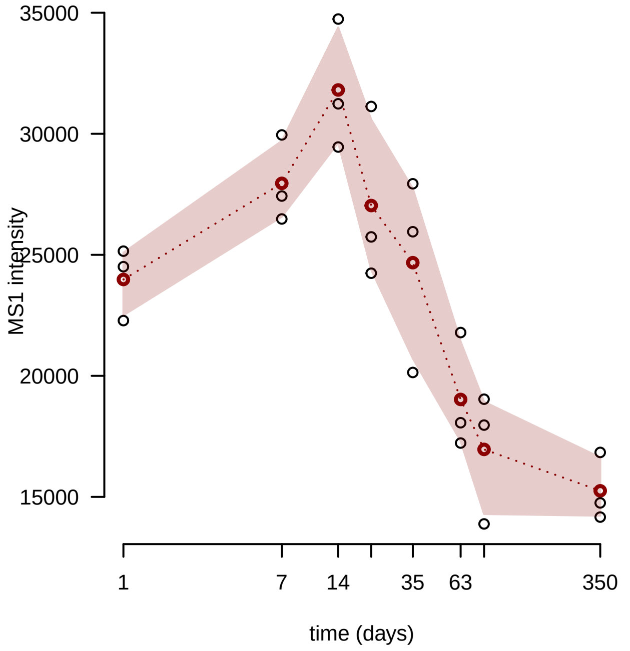

Q8R5L3
GN=Vps39
VPS39 HOPS complex subunit

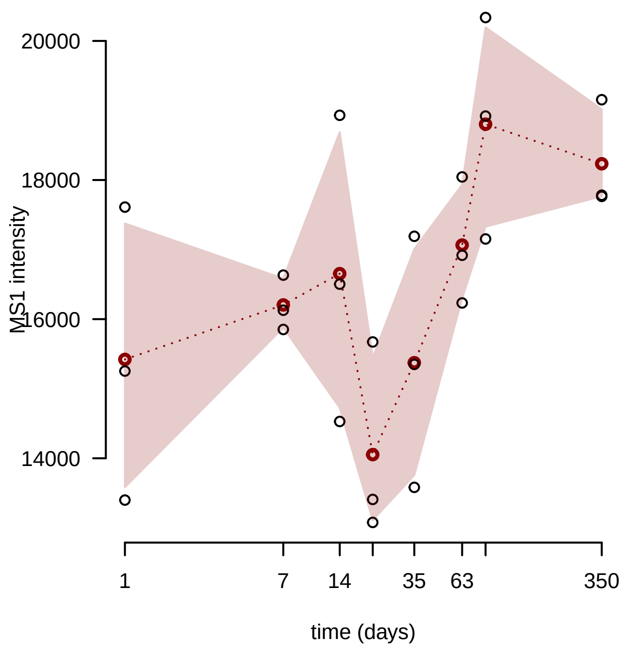

Q8R5M8
GN=Cadm1
cell adhesion molecule 1

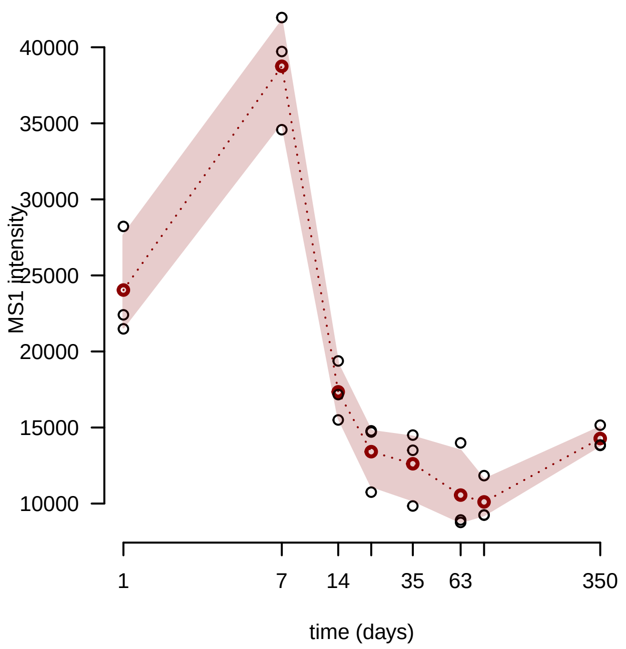

Q8VBT0
GN=Tmx1
thioredoxin−related transmembrane protei ...

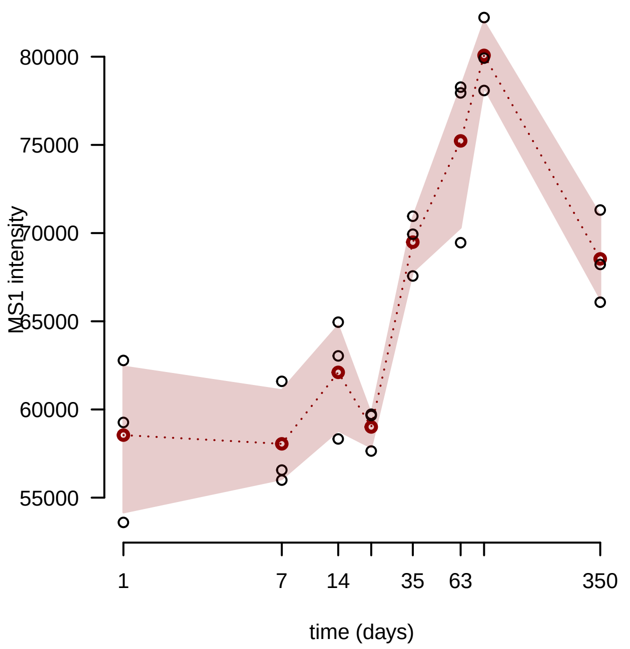

Q8VBT9
GN=Aspscr1
alveolar soft part sarcoma chromosome re ...

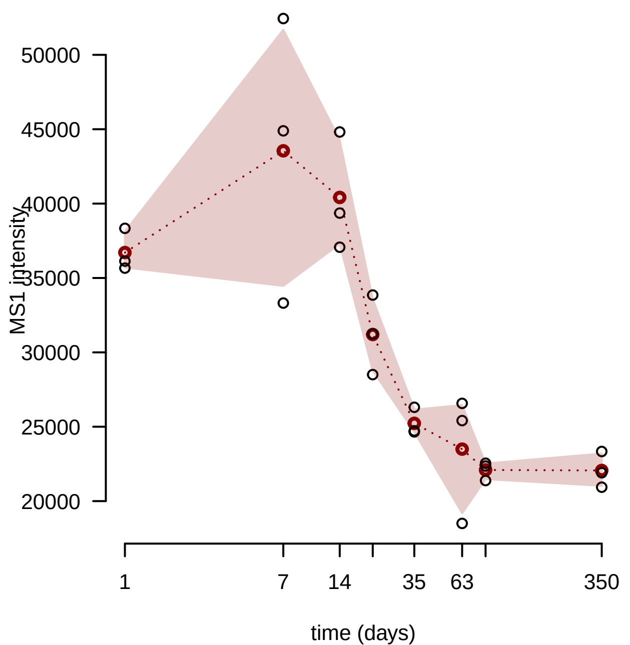

Q8VBU8
GN=Banp
BTG3 associated nuclear protein

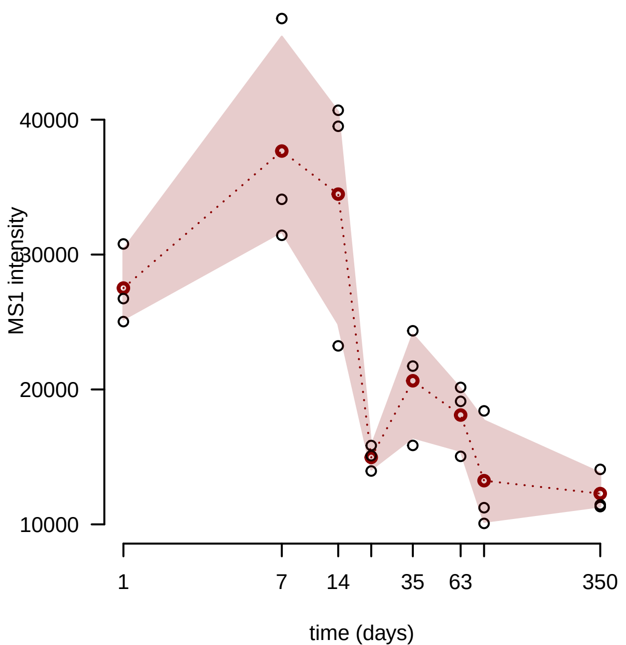

Q8VBV3
GN=Exosc2
exosome component 2

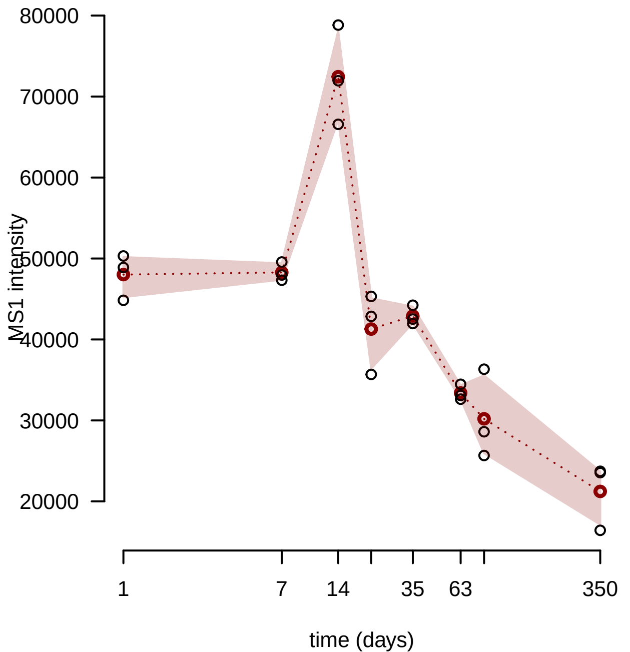

Q8VBV7
GN=Cops8
COP9 signalosome subunit 8

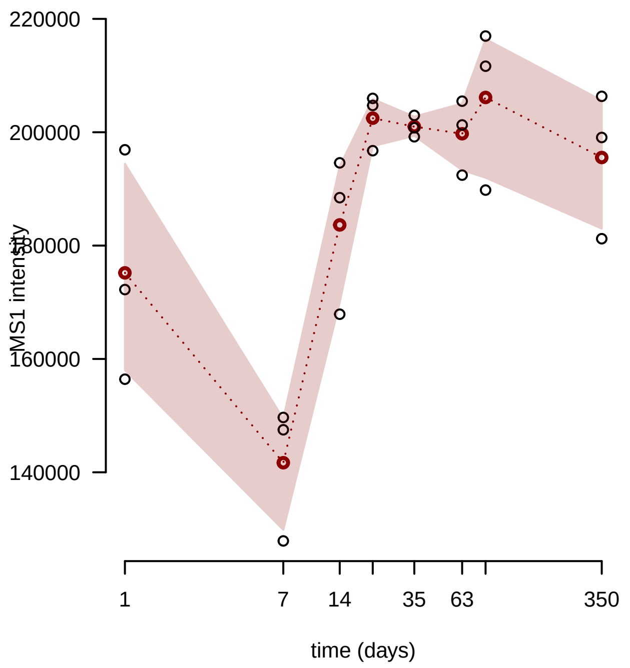

Q8VBW5
GN=Bbx
bobby sox HMG box containing

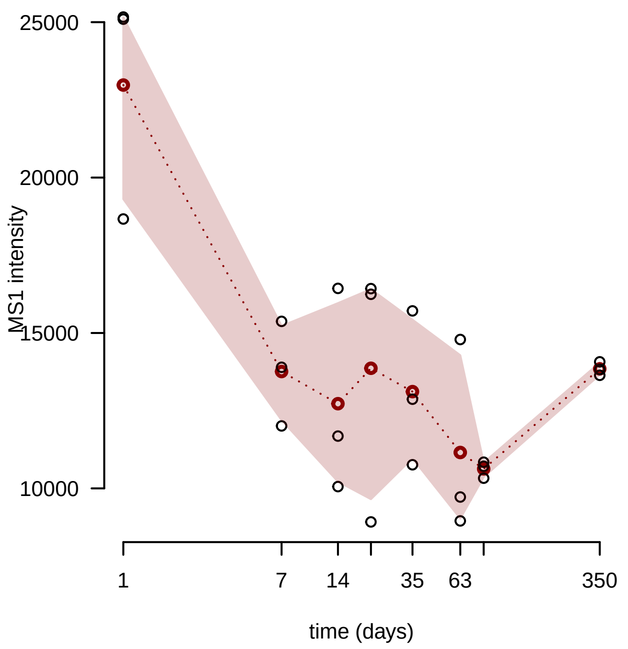

Q8VBW6
GN=Nae1
NEDD8 activating enzyme E1 subunit 1

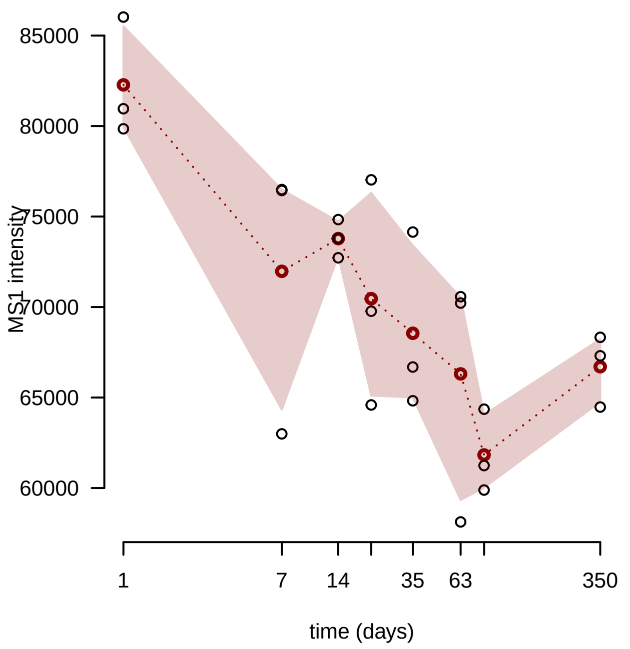

Q8VBX6
GN=Mpdz
multiple PDZ domain crumbs cell polarity ...

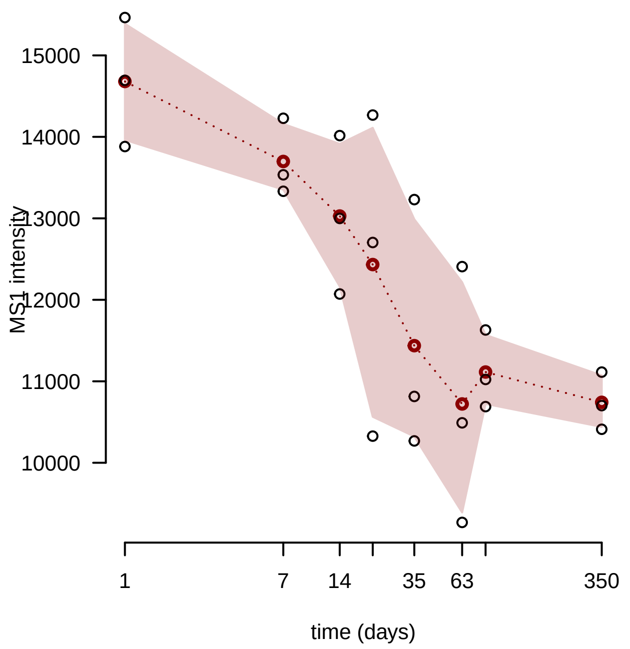

Q8VBZ0
GN=Dhrsx
dehydrogenase/reductase (SDR family) X c ...

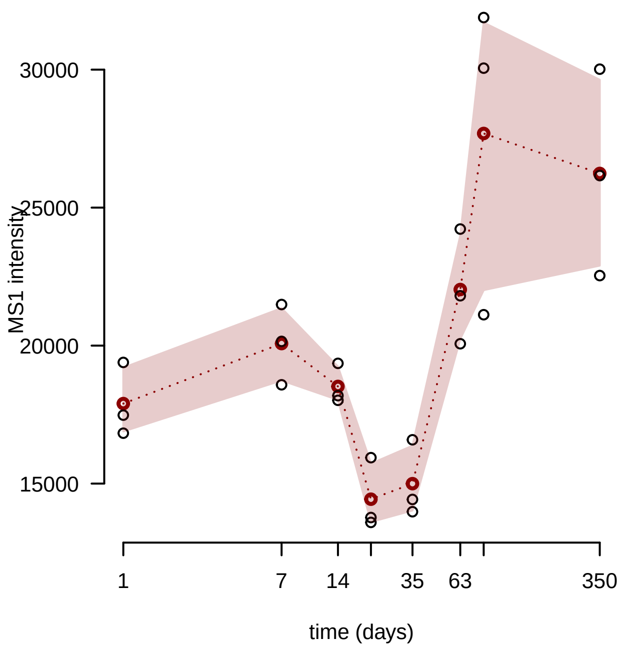

Q8VBZ3
GN=Clptm1
cleft lip and palate associated transmem ...

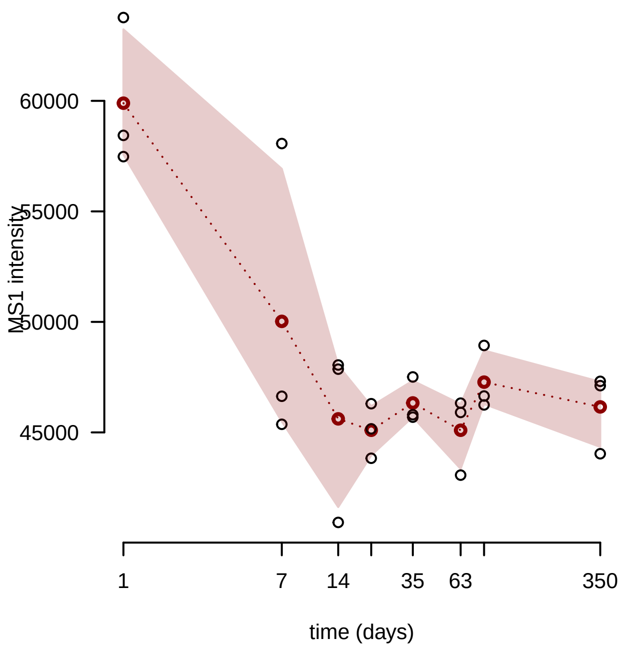

Q8VC03
GN=Eml3
echinoderm microtubule associated protei ...

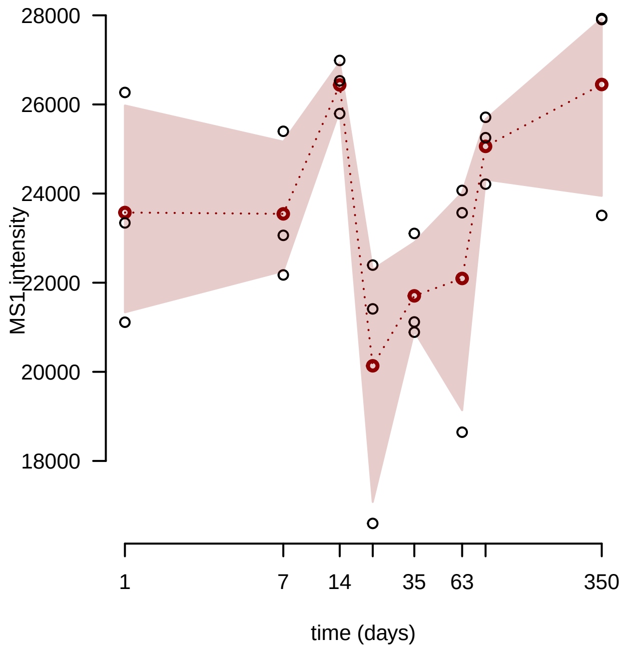

Q8VC12
GN=Uroc1
urocanase domain containing 1

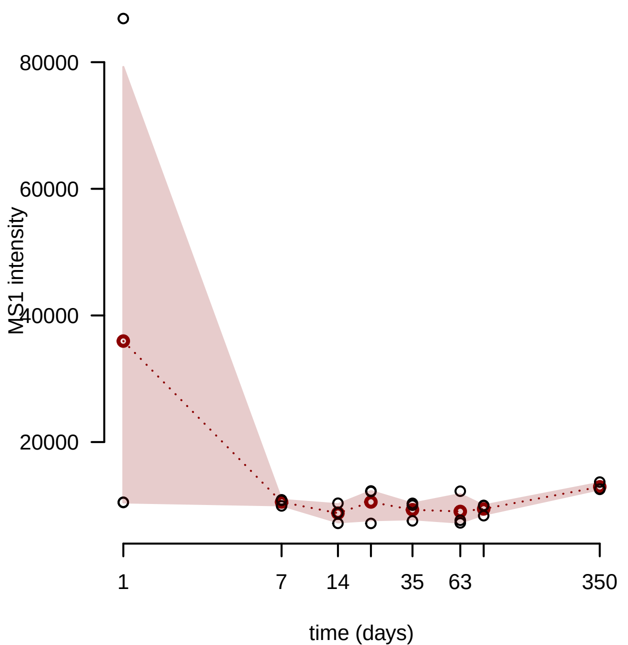

Q8VC16
GN=Lrrc14
leucine rich repeat containing 14

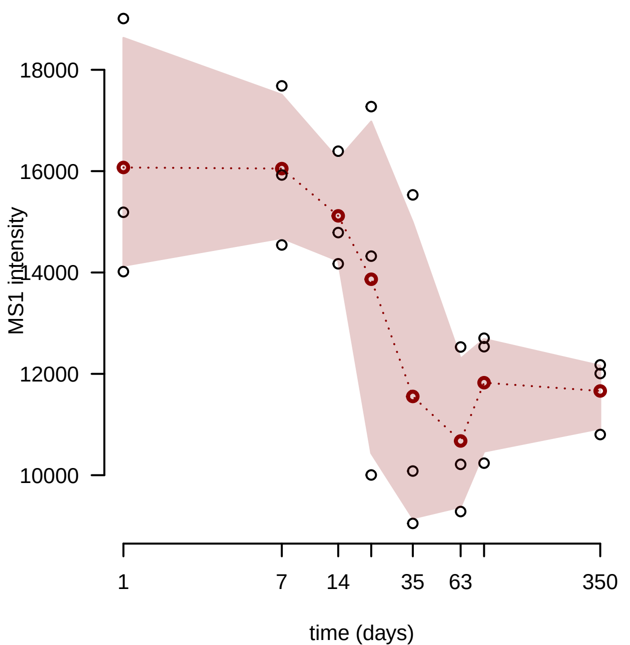

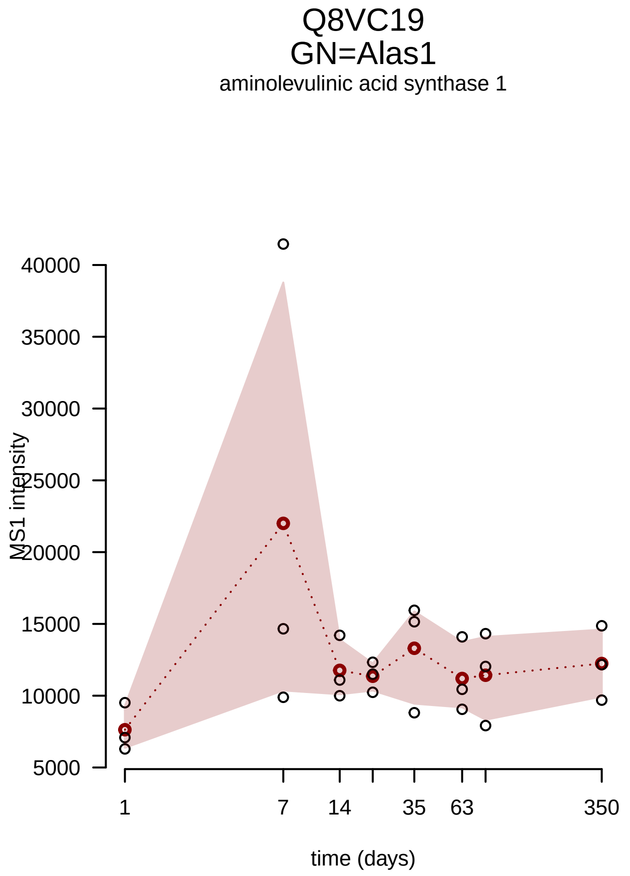
Q8VC19
GN=Alas1
aminolevulinic acid synthase 1
MS1 intensity
40000
35000
30000
25000
20000
15000
10000
5000
1
7
14
35
63
350
time (days)
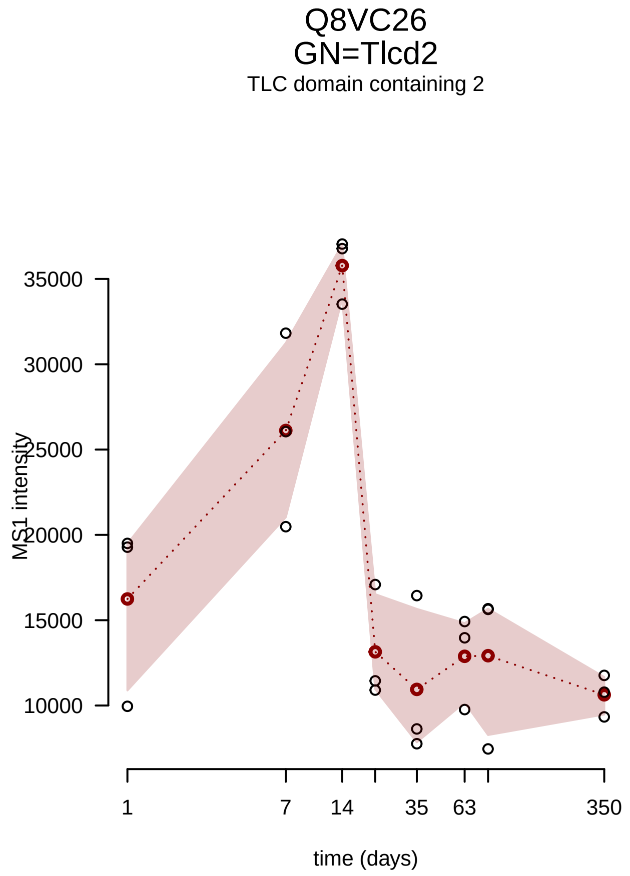
Q8VC26
GN=Tlcd2
TLC domain containing 2
MS1 intensity
35000
30000
25000
20000
15000
10000
1
7
14
35
63
350
time (days)
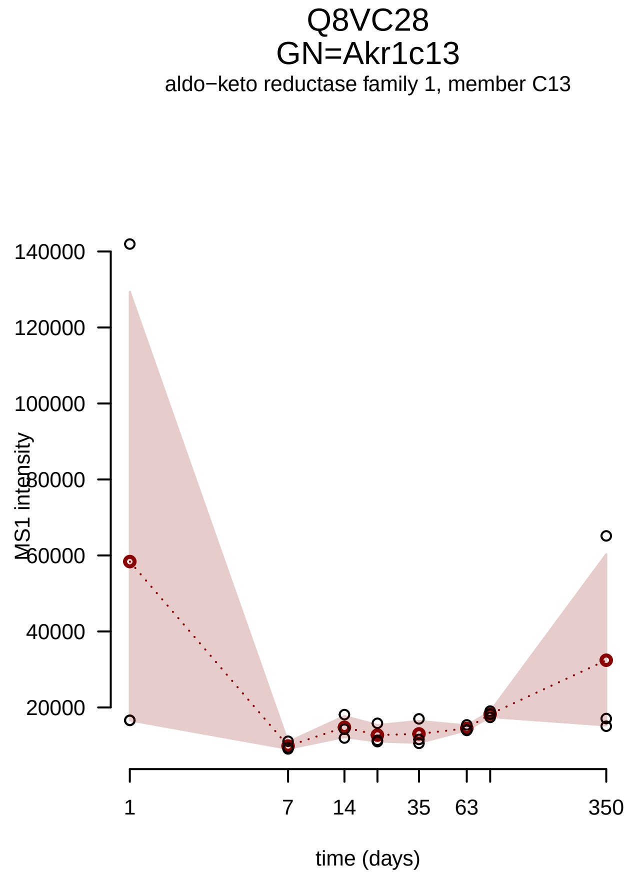
Q8VC28
GN=Akr1c13
aldo−keto reductase family 1, member C13
MS1 intensity
140000
120000
100000
80000
60000
40000
20000
1
7
14
35
63
350
time (days)
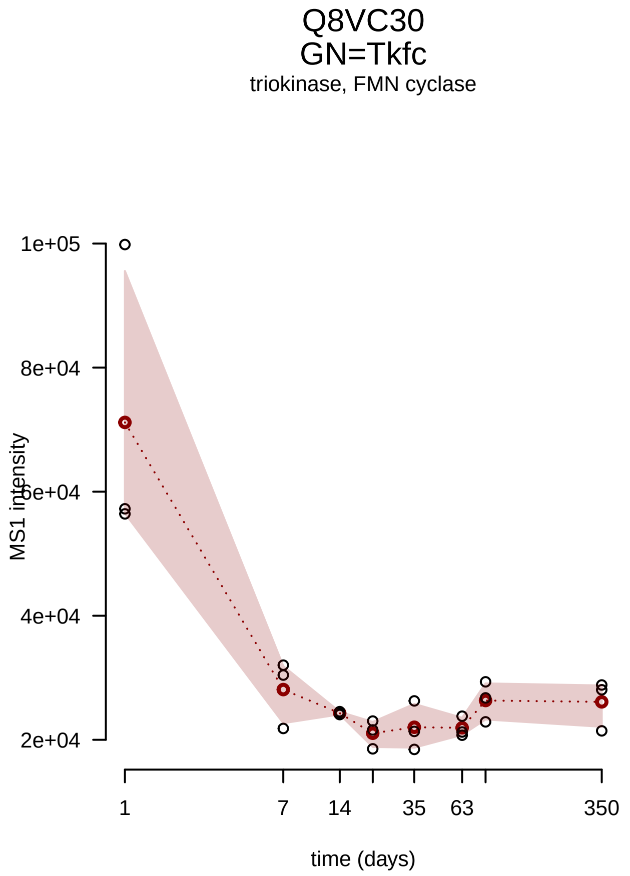
Q8VC30
GN=Tkfc
triokinase, FMN cyclase
MS1 intensity
1e+05
8e+04
6e+04
4e+04
2e+04
1
7
14
35
63
350
time (days)
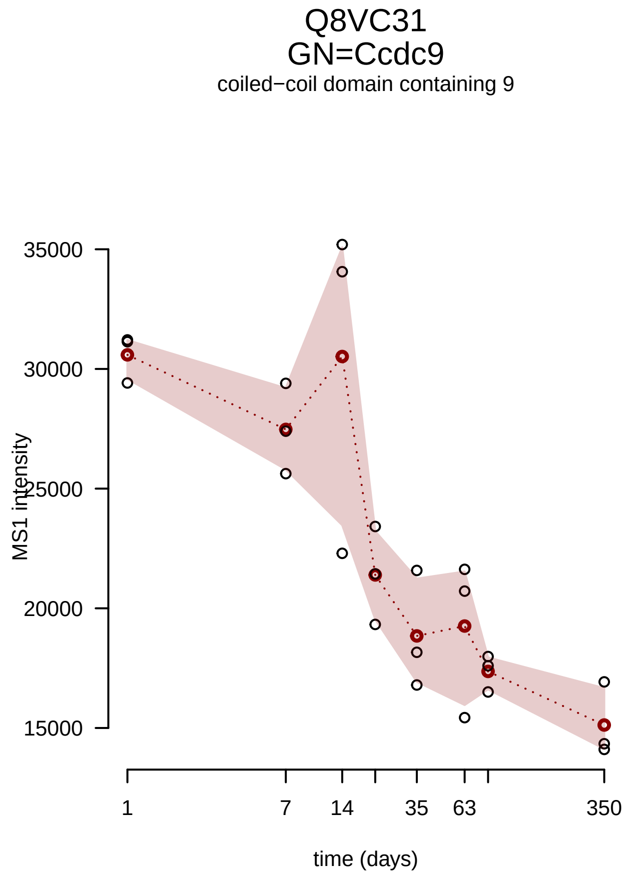
Q8VC31
GN=Ccdc9
coiled−coil domain containing 9
MS1 intensity
35000
30000
25000
20000
15000
1
7
14
35
63
350
time (days)
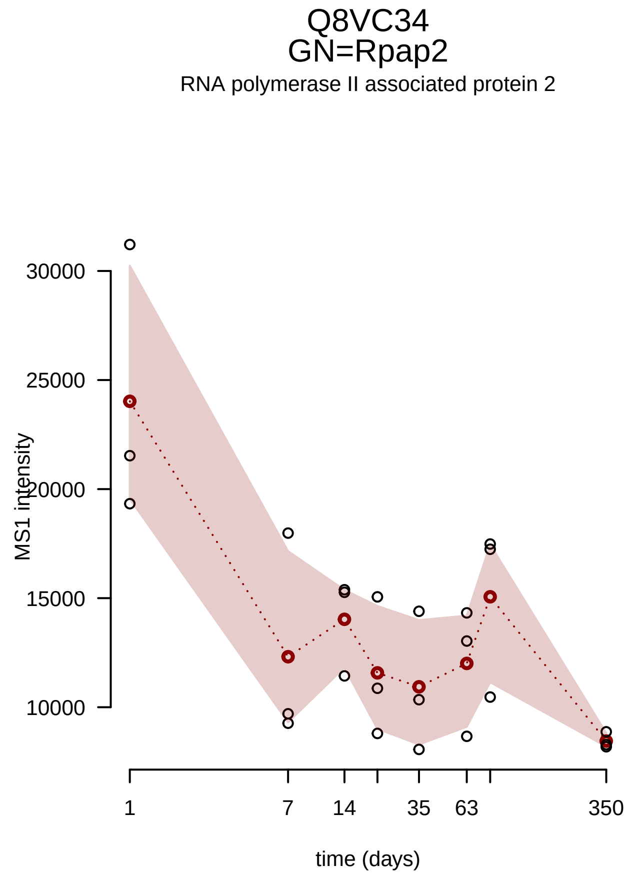
Q8VC34
GN=Rpap2
RNA polymerase II associated protein 2
MS1 intensity
30000
25000
20000
15000
10000
1
7
14
35
63
350
time (days)
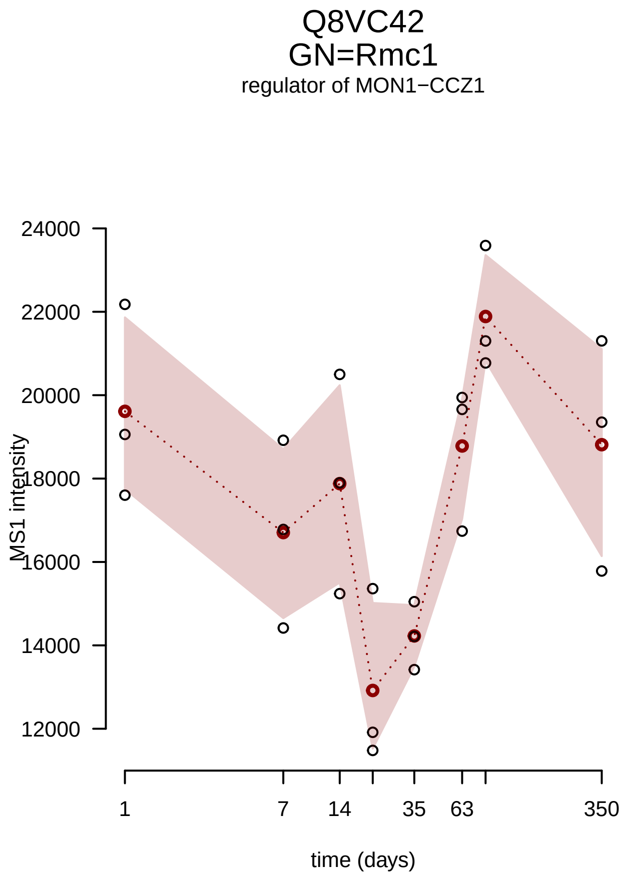
Q8VC42
GN=Rmc1
regulator of MON1−CCZ1
MS1 intensity
24000
22000
20000
18000
16000
14000
12000
1
7
14
35
63
350
time (days)
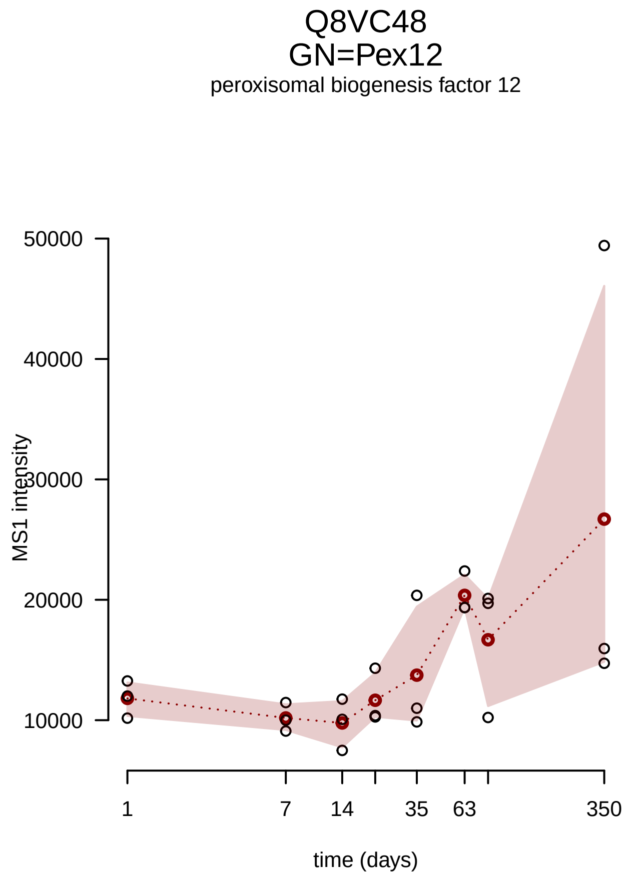
Q8VC48
GN=Pex12
peroxisomal biogenesis factor 12
MS1 intensity
50000
40000
30000
20000
10000
1
7
14
35
63
350
time (days)
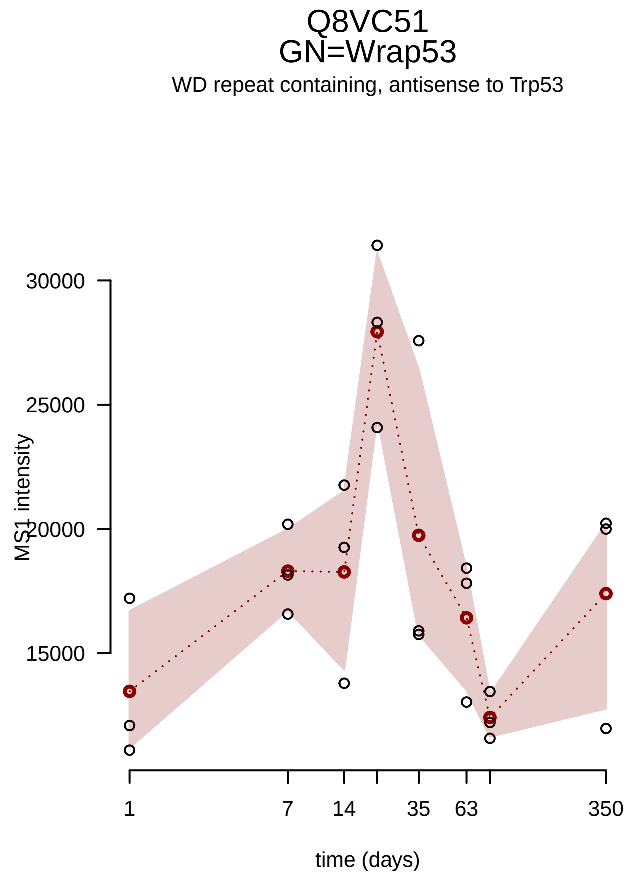
Q8VC51
GN=Wrap53
WD repeat containing, antisense to Trp53
MS1 intensity
30000
25000
20000
15000
1
7
14
35
63
350
time (days)

Q8VC52
GN=Rbpms2
RNA binding protein with multiple splici ...

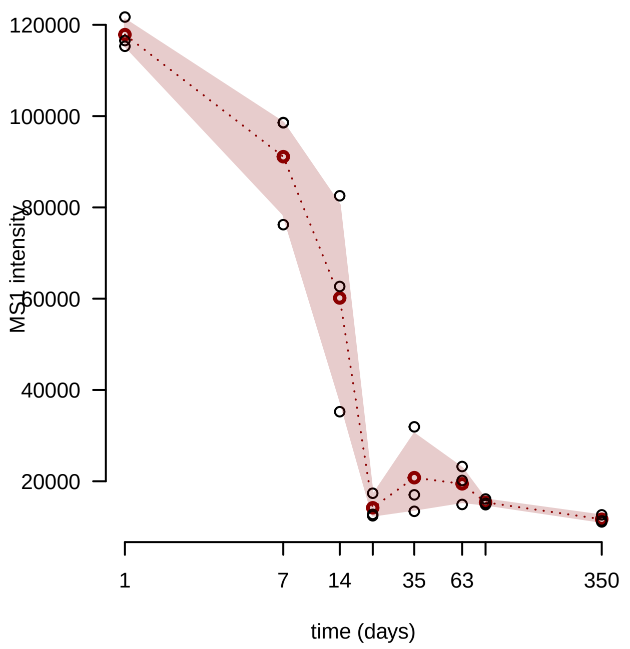

Q8VC57
GN=Kctd5
potassium channel tetramerisation domain ...

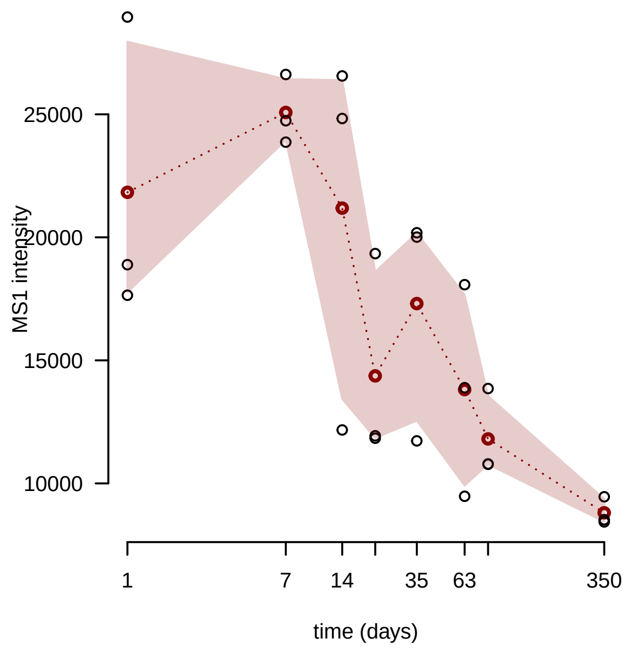

Q8VC60
GN=Glb1l
galactosidase, beta 1−like

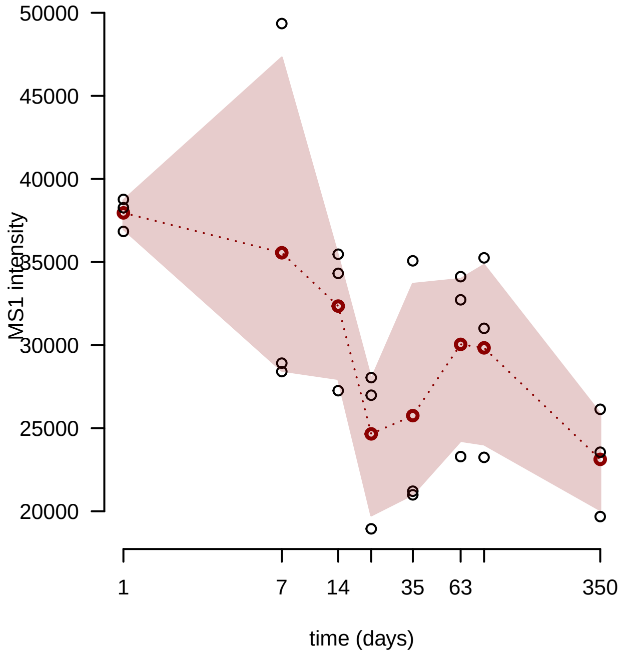

Q8VC65
GN=Nrm
nurim (nuclear envelope membrane protein ...

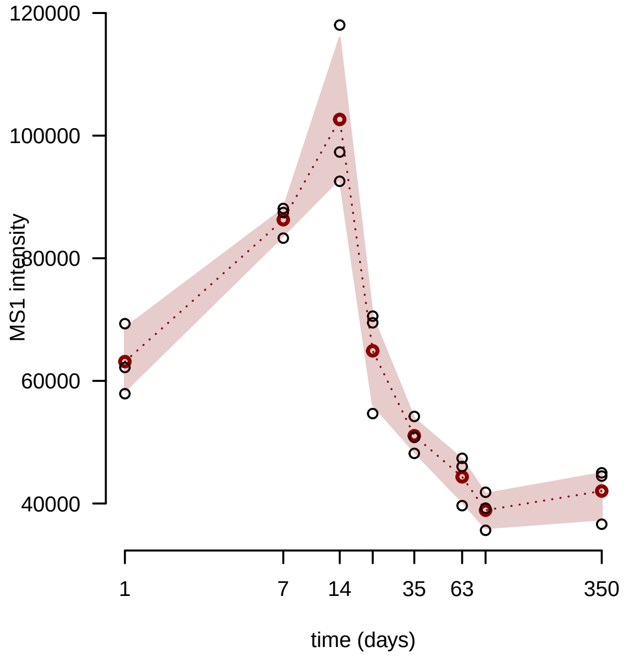

Q8VC70
GN=Rbms2
RNA binding motif, single stranded inter ...

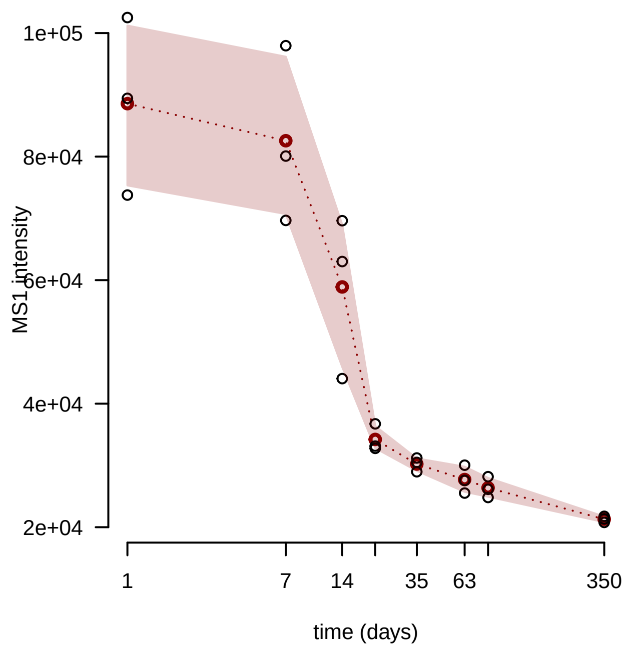

Q8VC74
GN=Cox18
cytochrome c oxidase assembly protein 18

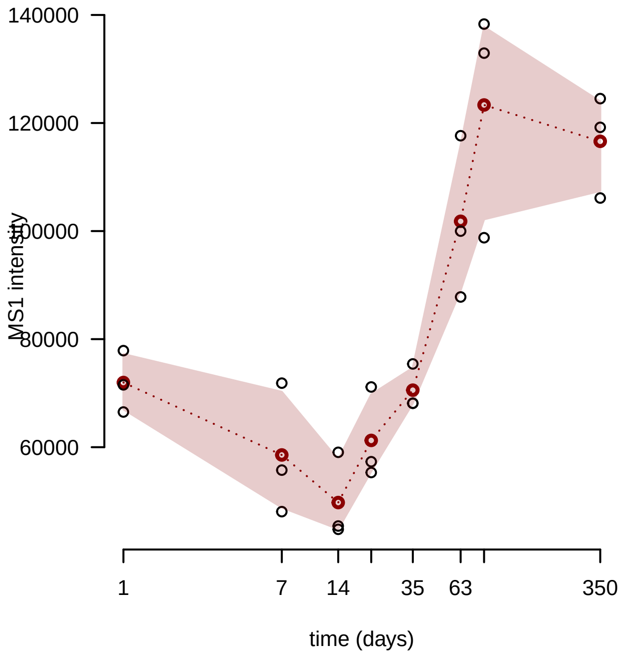

Q8VC85
GN=Lsm1
LSM1 homolog, mRNA degradation associate ...

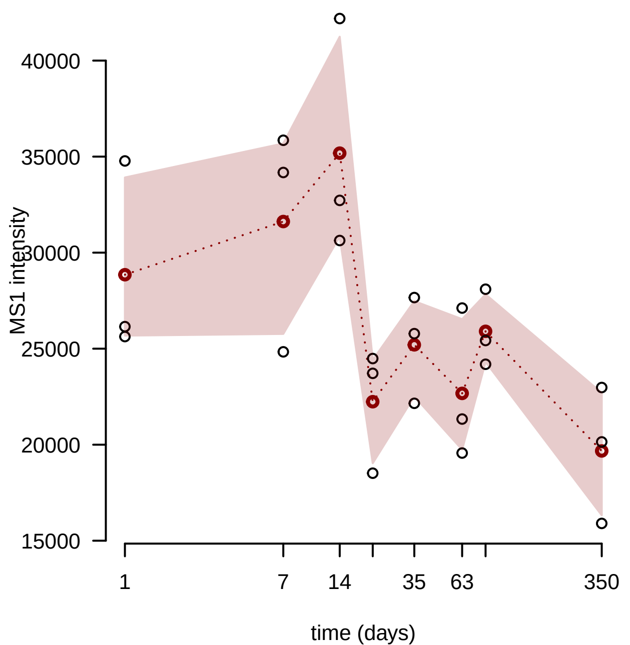

Q8VC88
GN=Gca
grancalcin

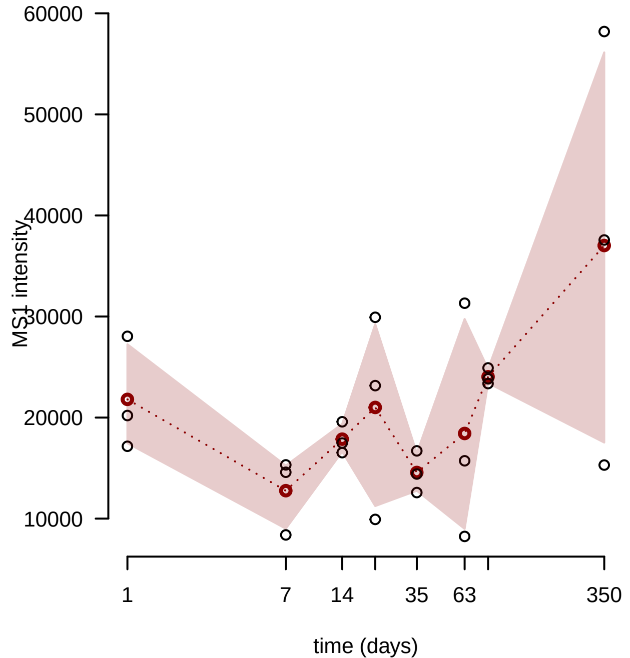

Q8VCA6
GN=Tmem161a
transmembrane protein 161A

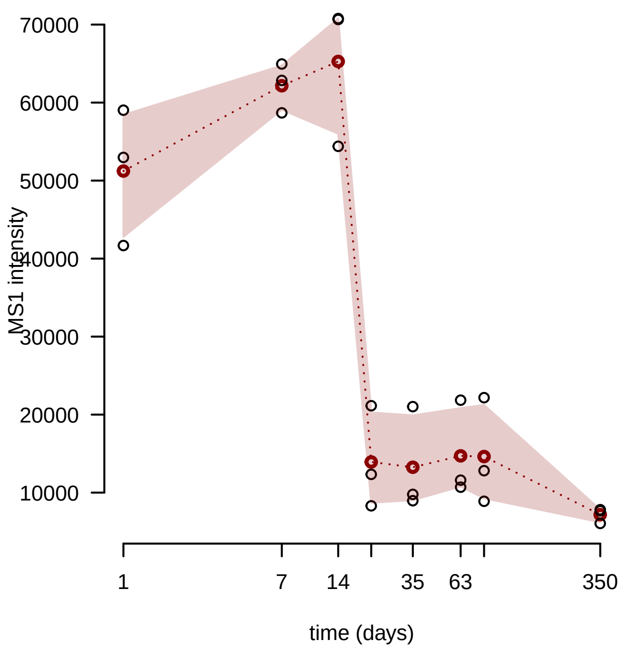

Q8VCA8
GN=Scrn2
secernin 2

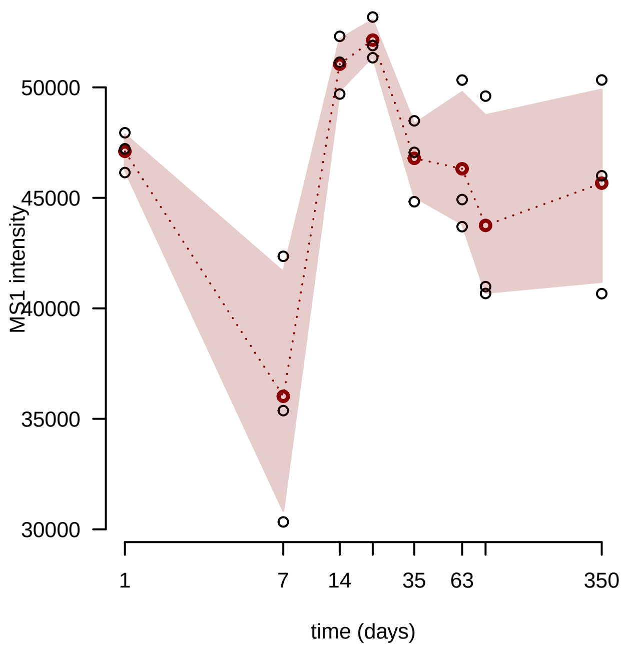

Q8VCB1
GN=Ndc1
NDC1 transmembrane nucleoporin

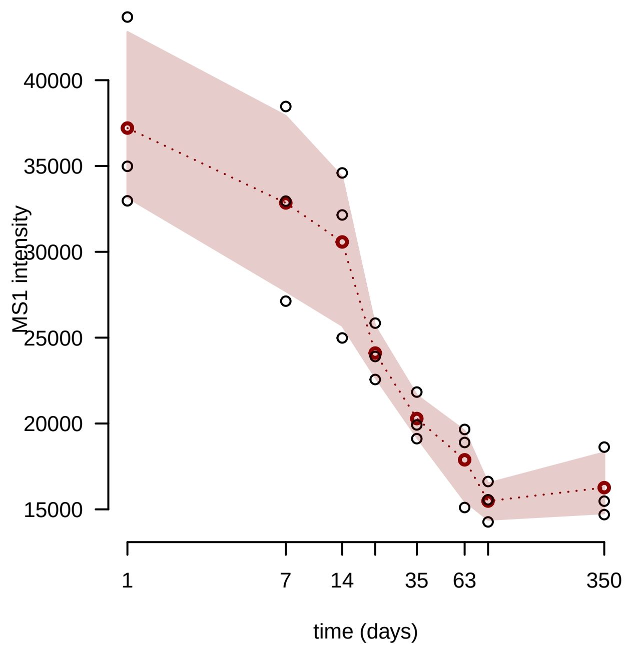

Q8VCB2
GN=Med25
mediator complex subunit 25

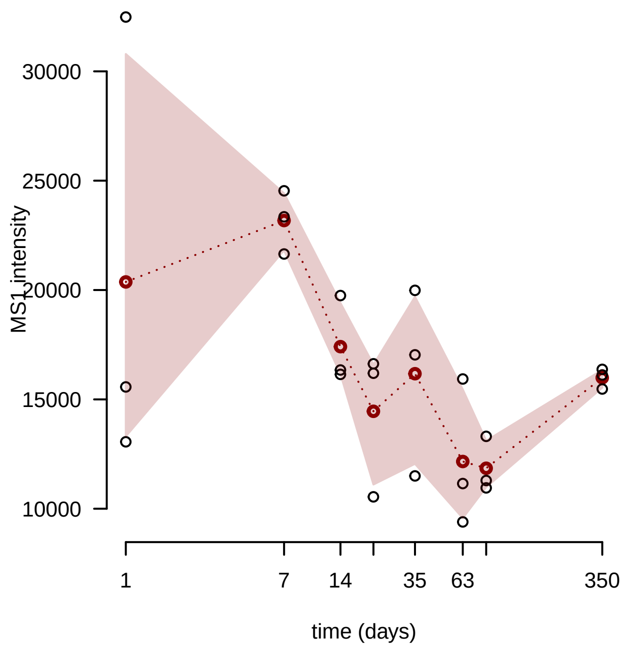

Q8VCC1
GN=Hpgd
hydroxyprostaglandin dehydrogenase 15 (N ...

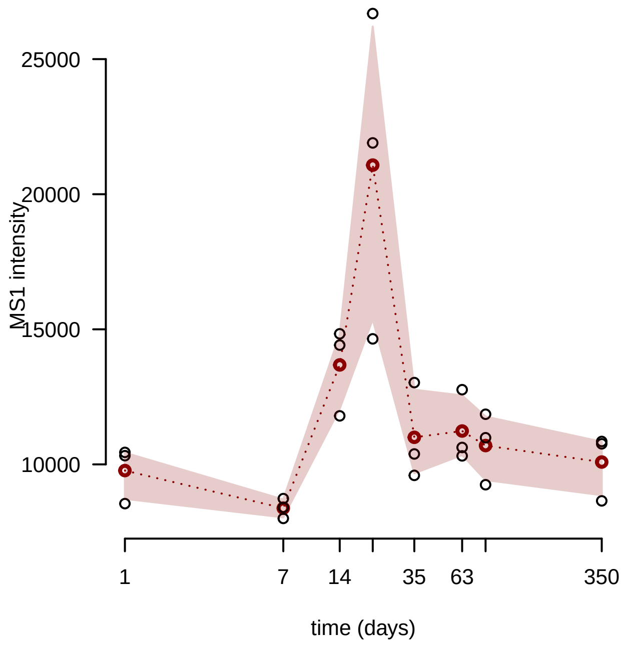

Q8VCC2
GN=Ces1

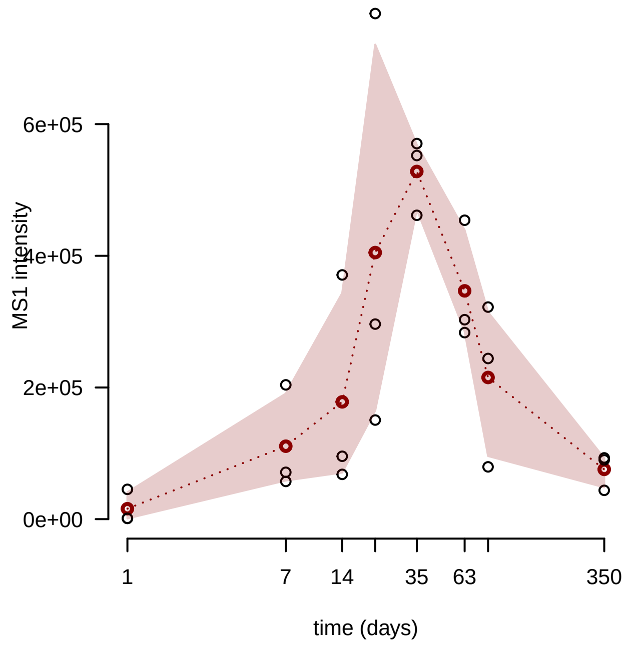

Q8VCC8
GN=Rapgef3
Rap guanine nucleotide exchange factor (...

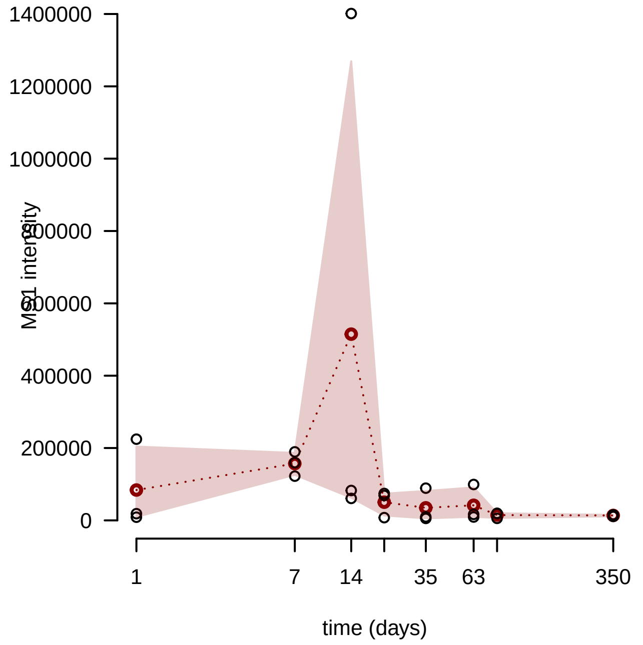

Q8VCC9
GN=Spon1
spondin 1, (f−spondin) extracellular mat ...

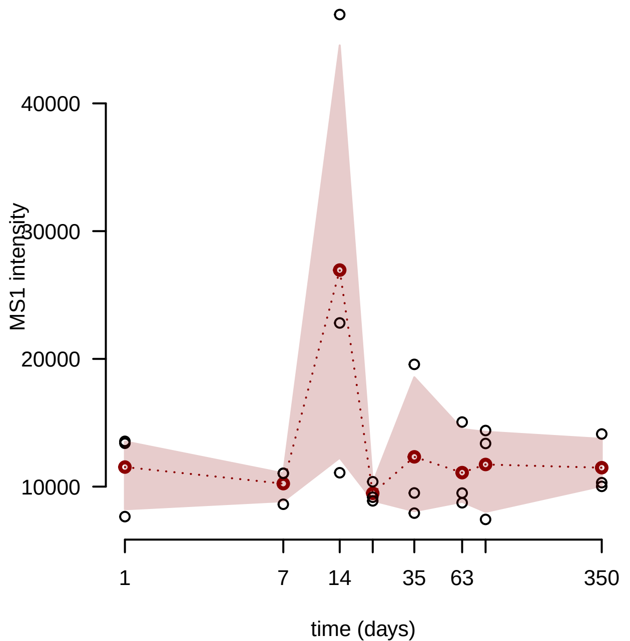

Q8VCD5
GN=Med17
mediator complex subunit 17

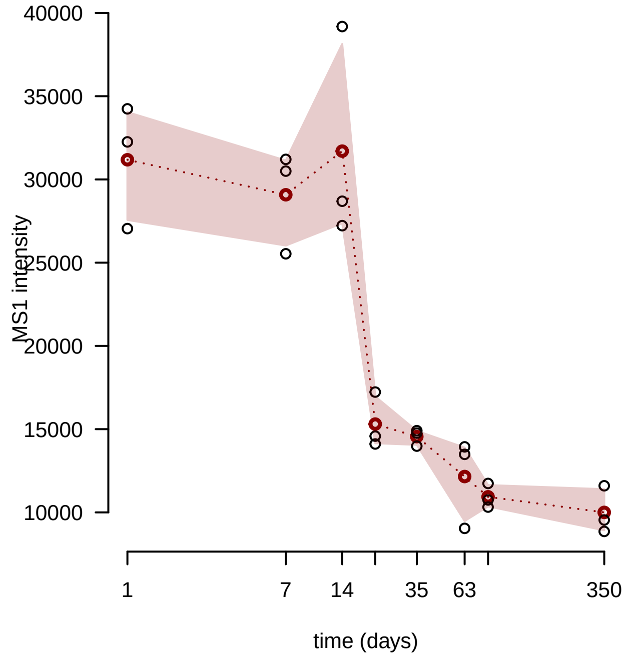

Q8VCD6
GN=Reep2
receptor accessory protein 2

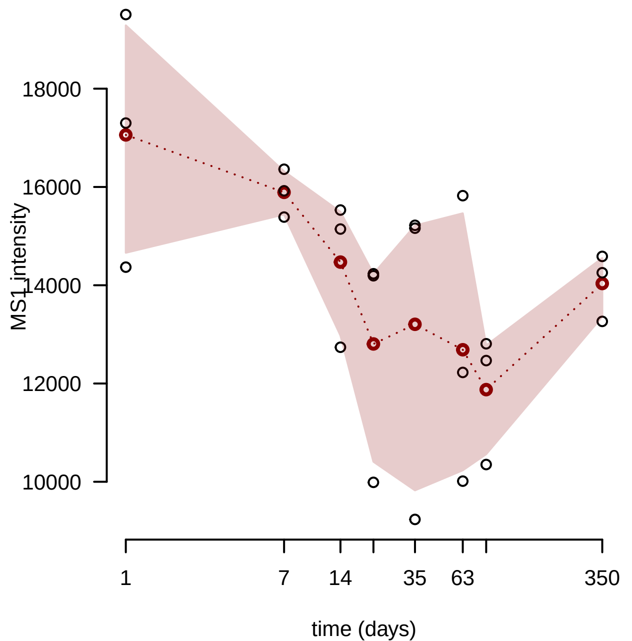

Q8VCE1
GN=Dnajc28
DnaJ heat shock protein family (Hsp40) m ...

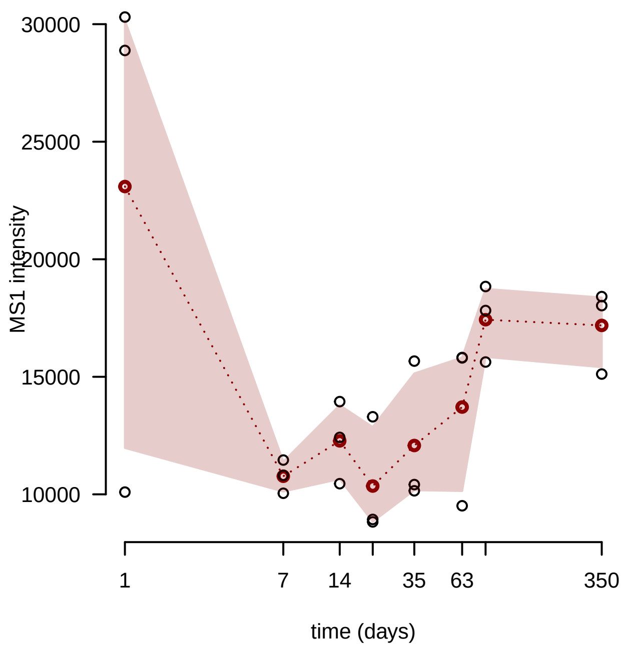

Q8VCE2
GN=Gpn1
GPN−loop GTPase 1

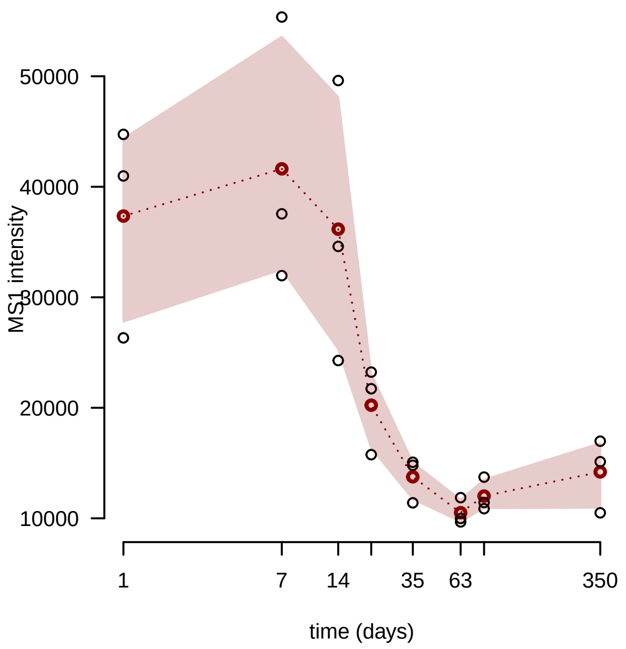

Q8VCE4
GN=

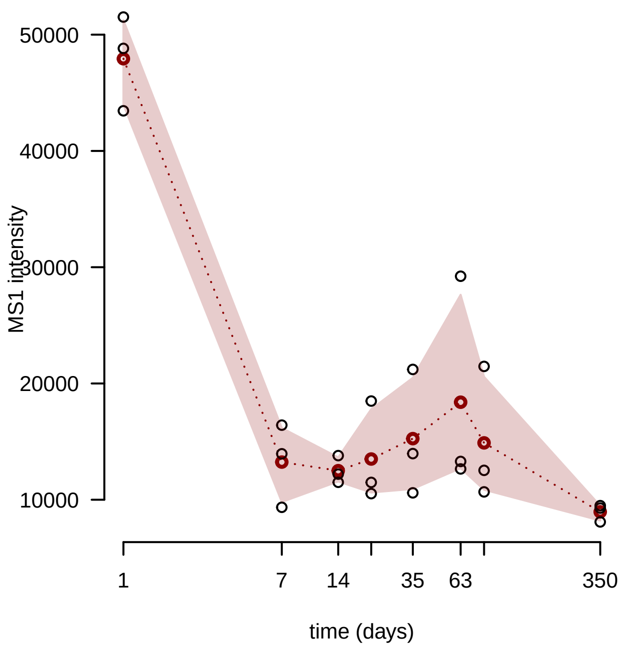

Q8VCE6
GN=Nt5m
5',3'−nucleotidase, mitochondrial

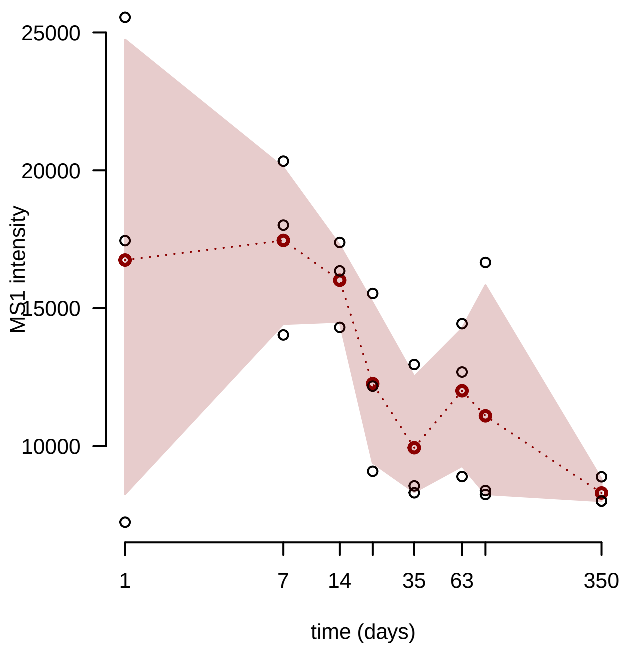

Q8VCE9
GN=Plekhh3
pleckstrin homology domain containing, f ...

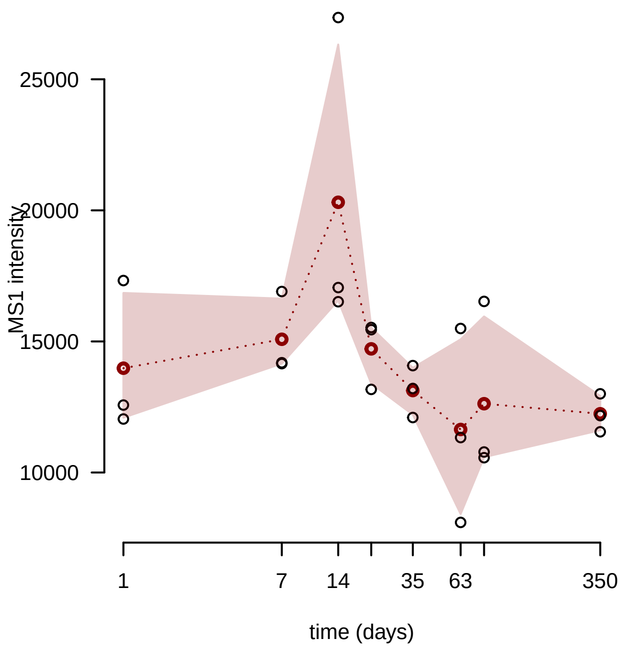

Q8VCF0
GN=Mavs
mitochondrial antiviral signaling protei ...

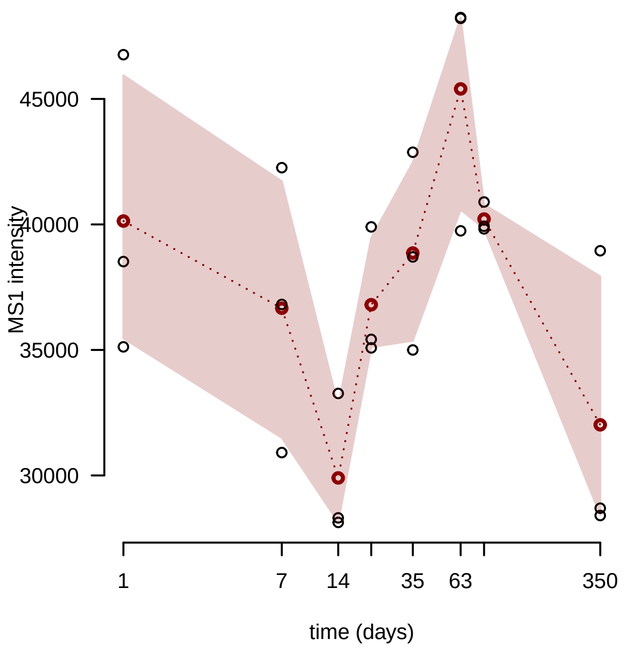

Q8VCF1
GN=Cant1
calcium activated nucleotidase 1

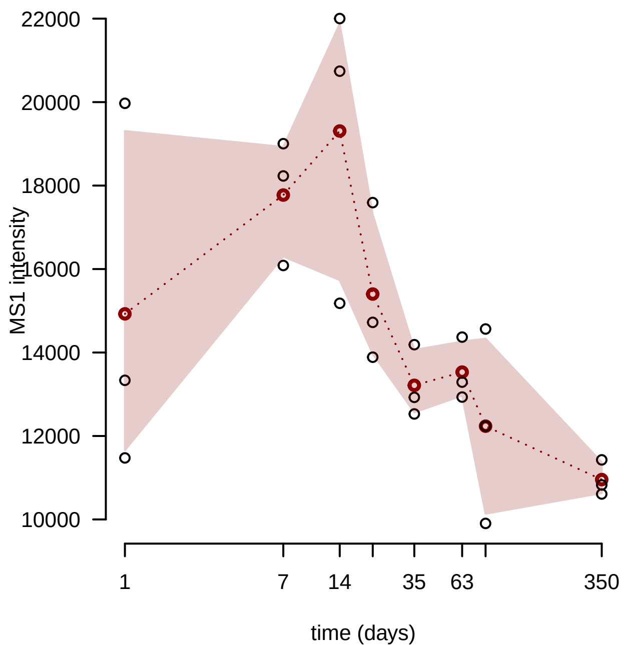

Q8VCG3
GN=Wdr74
WD repeat domain 74

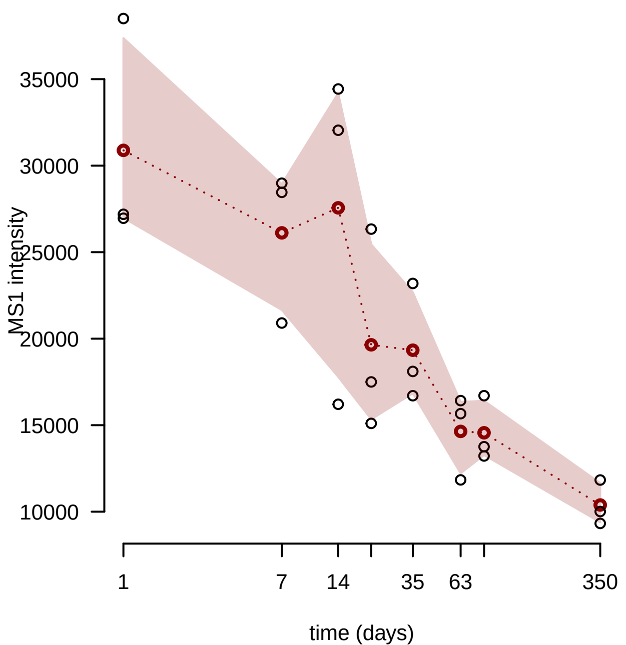

Q8VCG4
GN=C8g
complement component 8, gamma polypeptid ...

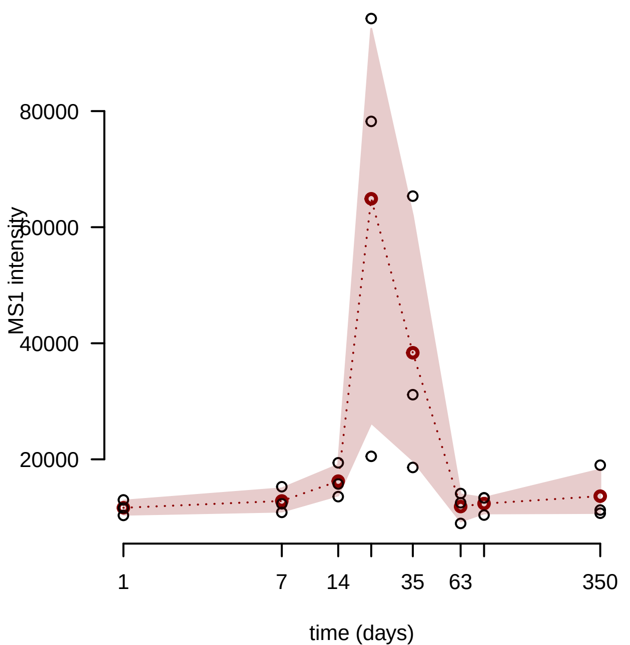

Q8VCG9
GN=Rfxap
regulatory factor X−associated protein

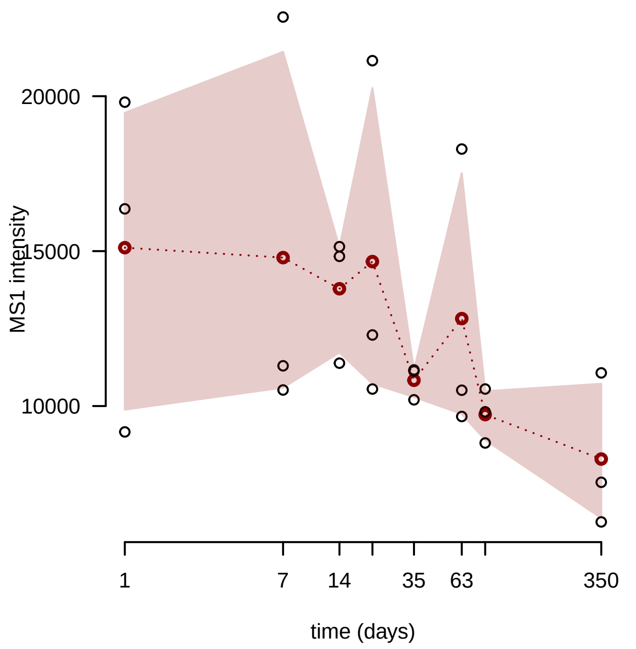

Q8VCH5
GN=Rabepk
Rab9 effector protein with kelch motifs

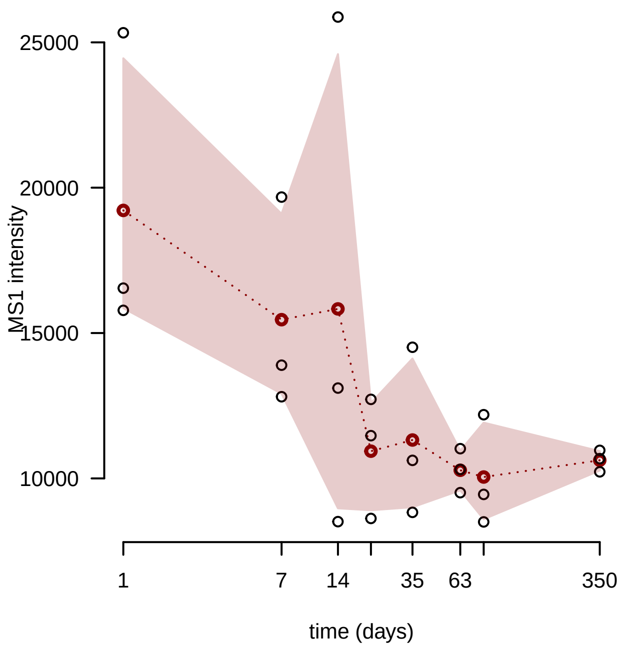

Q8VCH6
GN=Dhcr24
24−dehydrocholesterol reductase

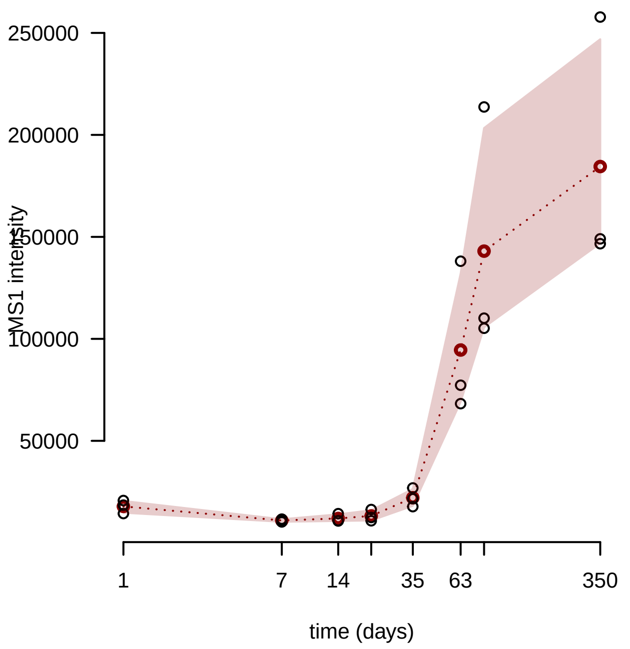

Q8VCH7
GN=Rdh10
retinol dehydrogenase 10 (all−trans)

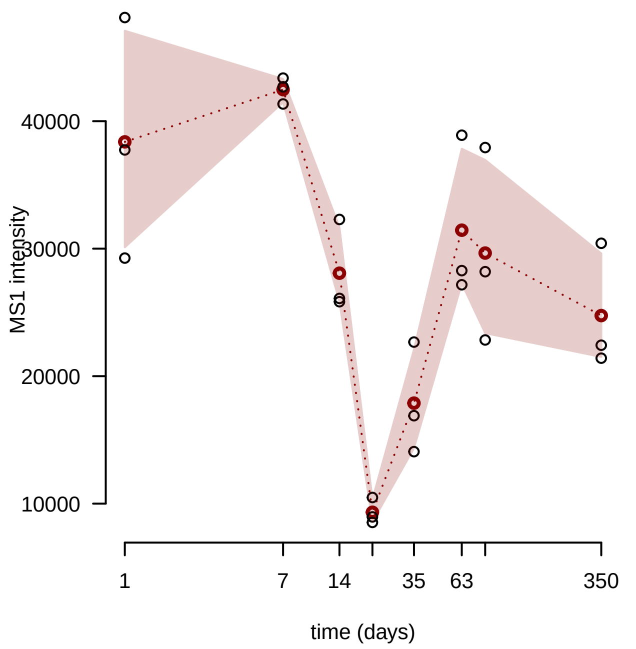

Q8VCH8
GN=Ubxn4
UBX domain protein 4

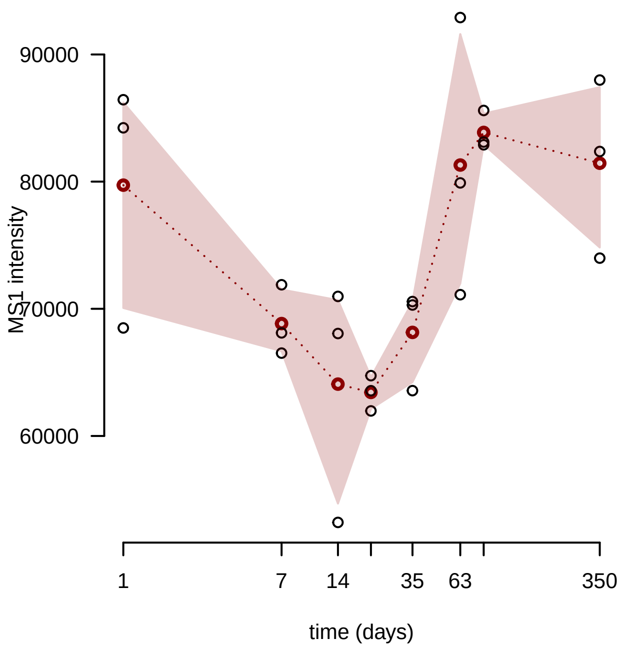

Q8VCI0
GN=Plbd1
phospholipase B domain containing 1

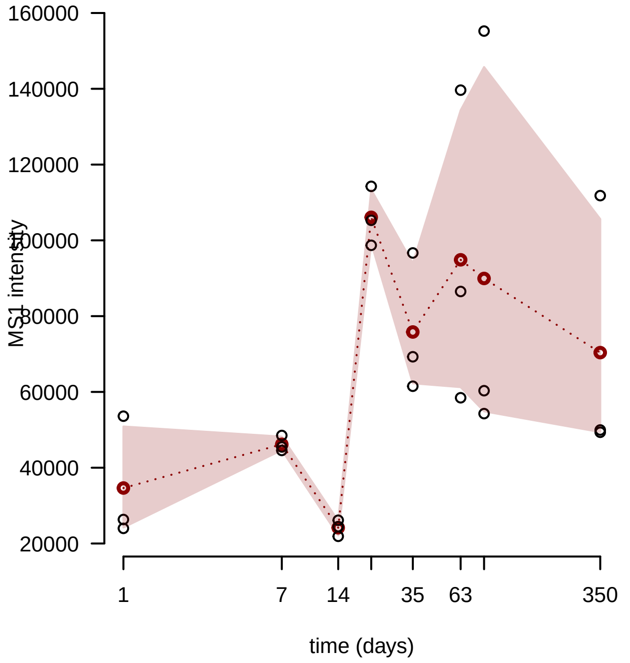

Q8VCI5
GN=Pex19
peroxisomal biogenesis factor 19

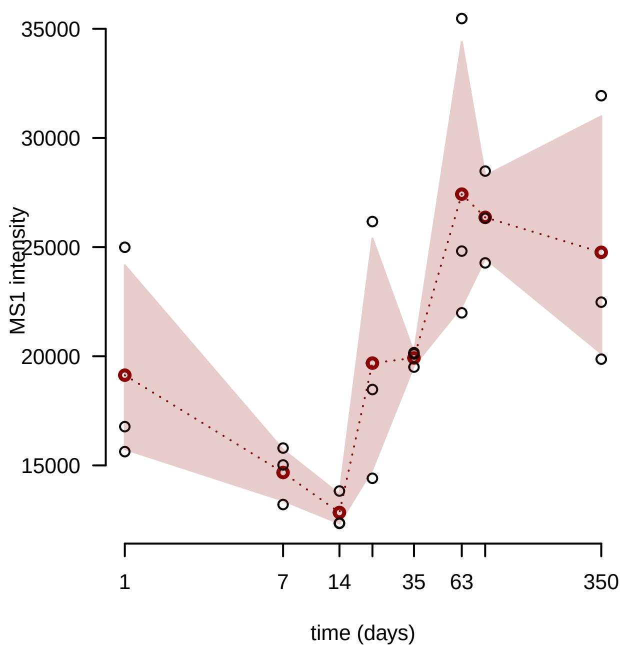

Q8VCK5
GN=Klhl20
kelch−like 20

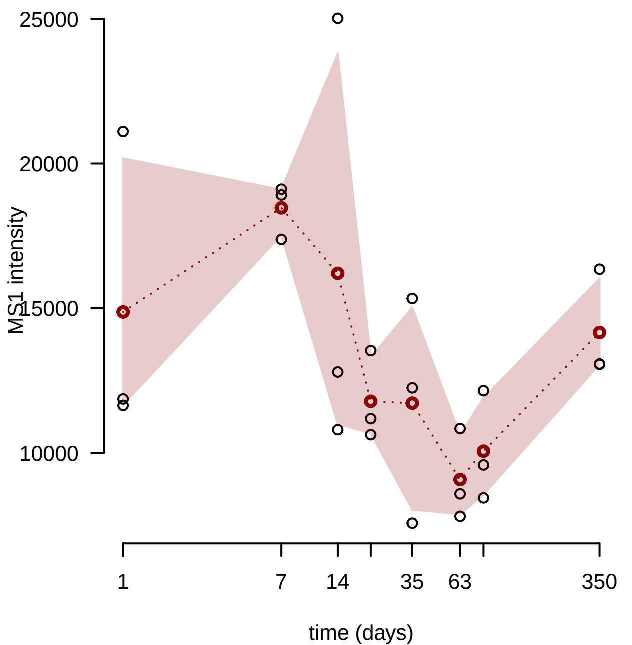

Q8VCL2
GN=Sco2
SCO2 cytochrome c oxidase assembly prote ...

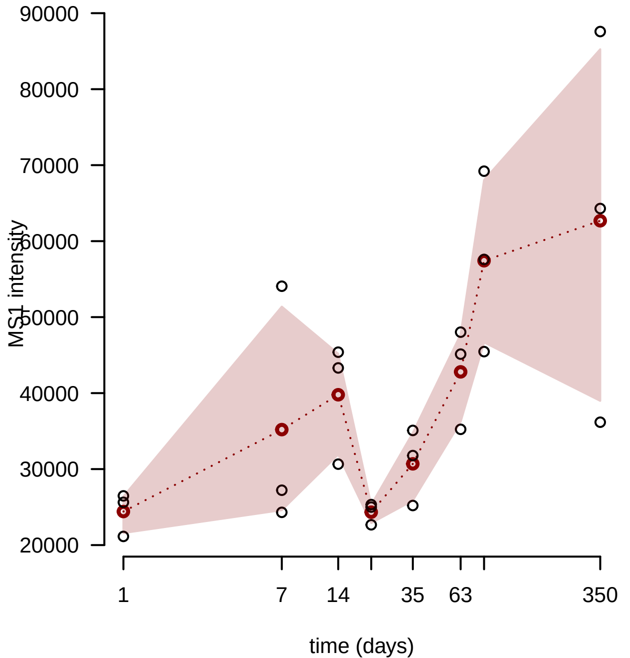

Q8VCM3
GN=Zfyve21
zinc finger, FYVE domain containing 21

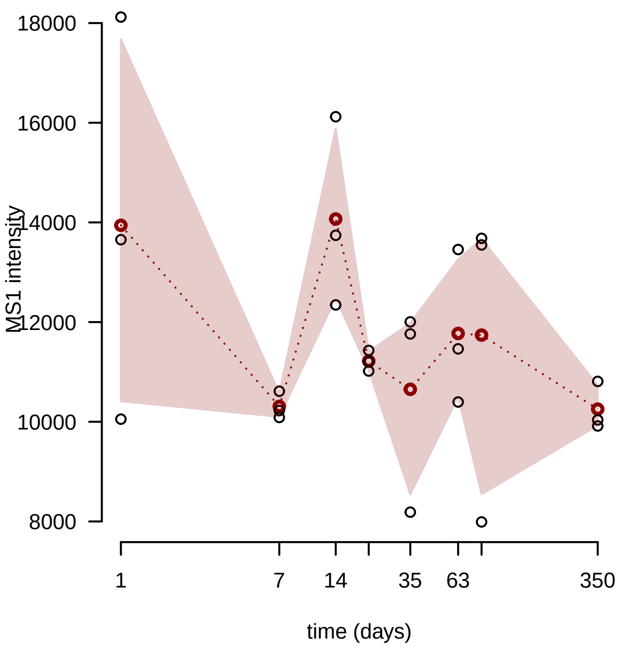

Q8VCM4
GN=Lipt1
lipoyltransferase 1

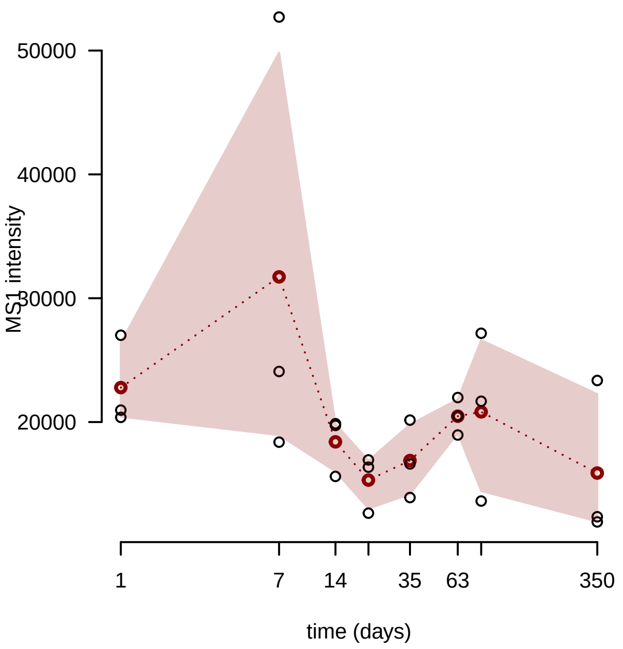

Q8VCM5
GN=Mul1
mitochondrial ubiquitin ligase activator ...

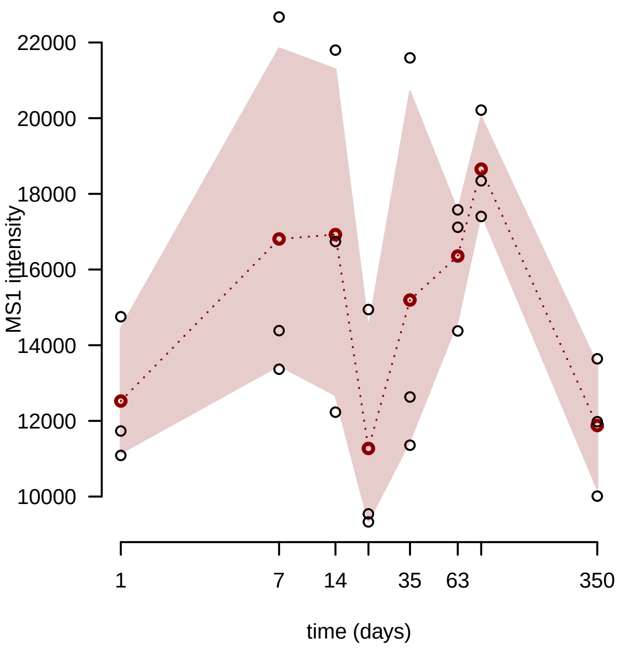

Q8VCM7
GN=Fgg
fibrinogen gamma chain

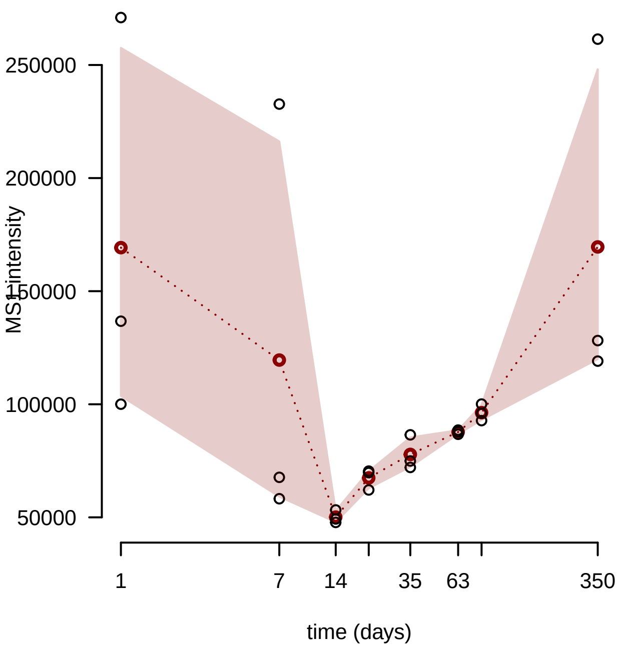

Q8VCM8
GN=Ncln
nicalin

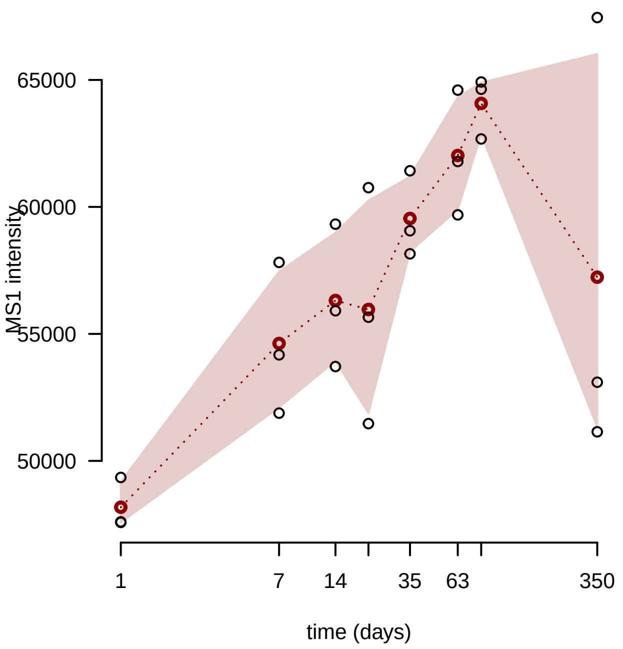

Q8VCN5
GN=Cth
cystathionase (cystathionine gamma−lyase ...

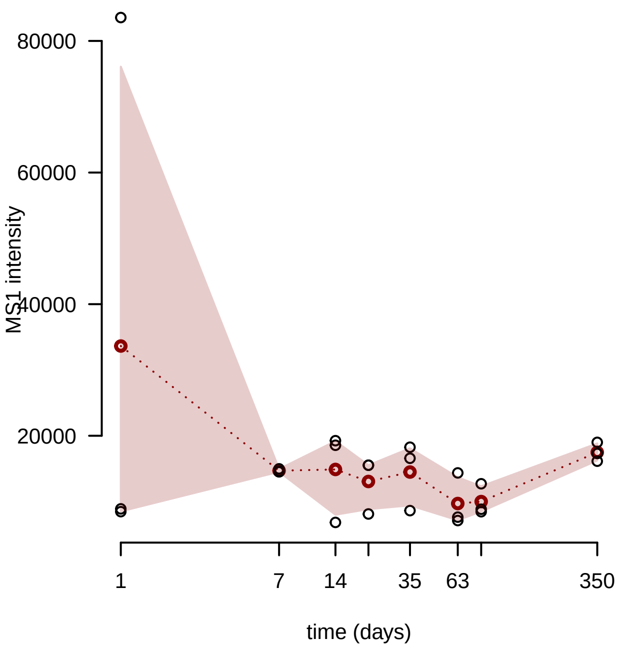

Q8VCN6
GN=Cd99
CD99 antigen

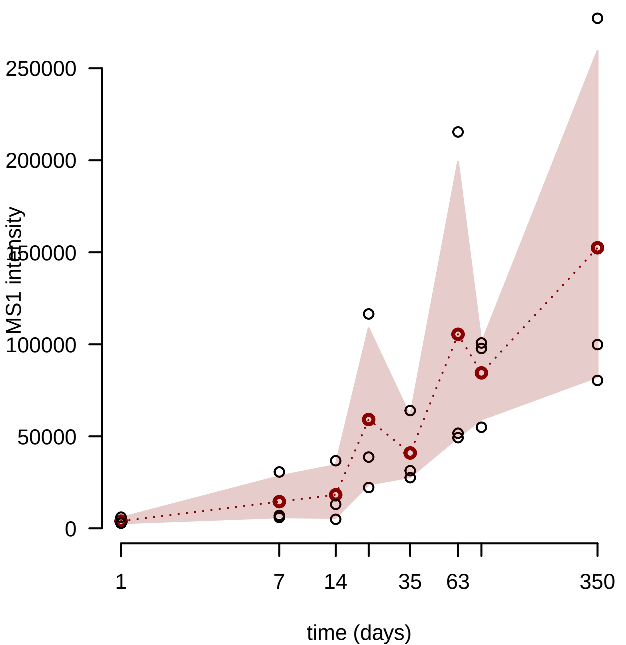

Q8VCN9
GN=Tbcc
tubulin−specific chaperone C

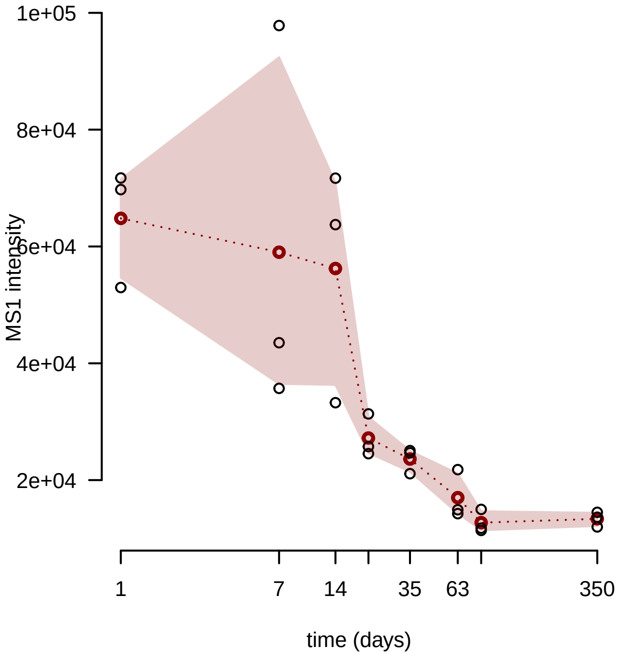

Q8VCP8
GN=Ak6
adenylate kinase 6

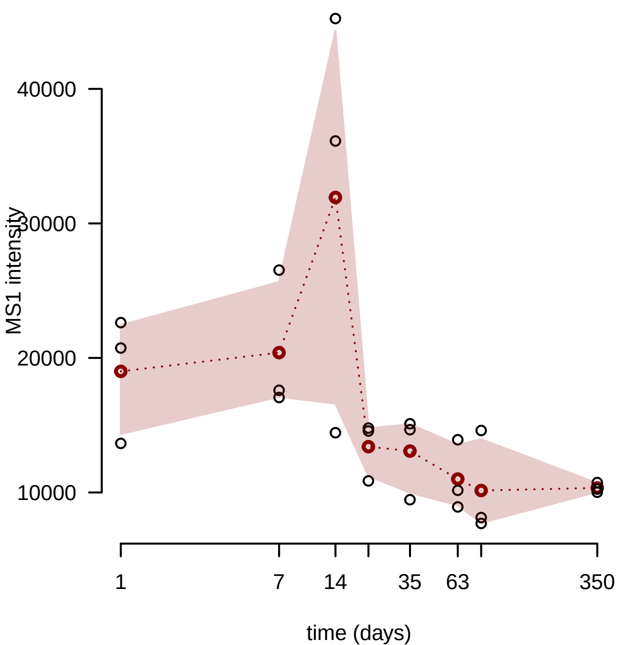

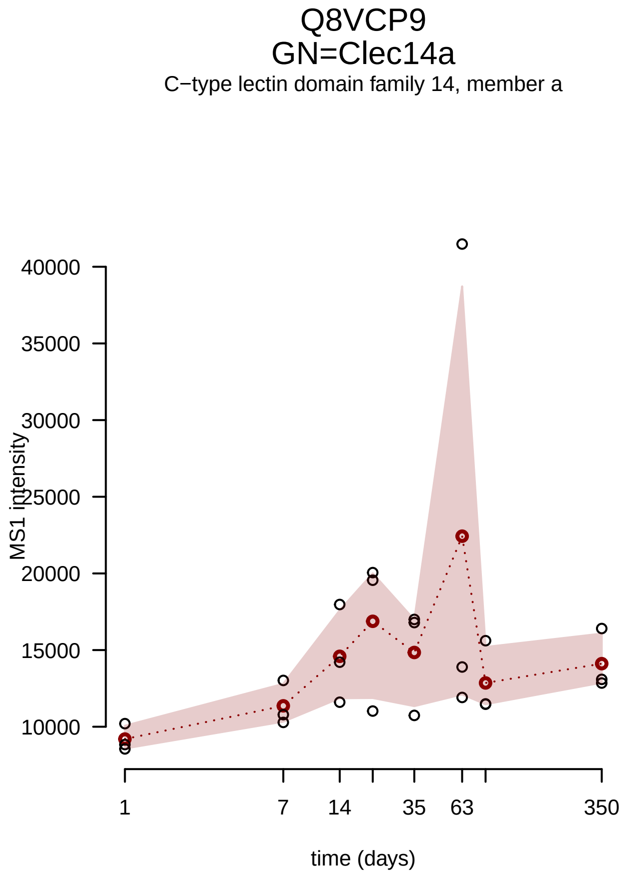
Q8VCP9
GN=Clec14a
C−type lectin domain family 14, member a
MS1 intensity
10000 15000 20000 25000 30000 35000 40000
1 7 14 35 63 350
time (days)

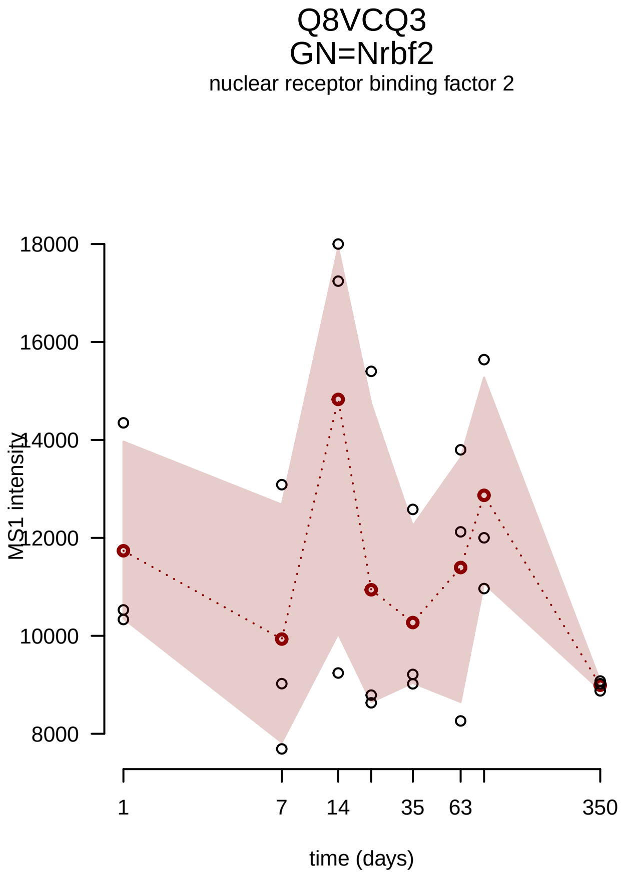
Q8VCQ3
GN=Nrbf2
nuclear receptor binding factor 2
MS1 intensity
8000 10000 12000 14000 16000 18000
1 7 14 35 63 350
time (days)

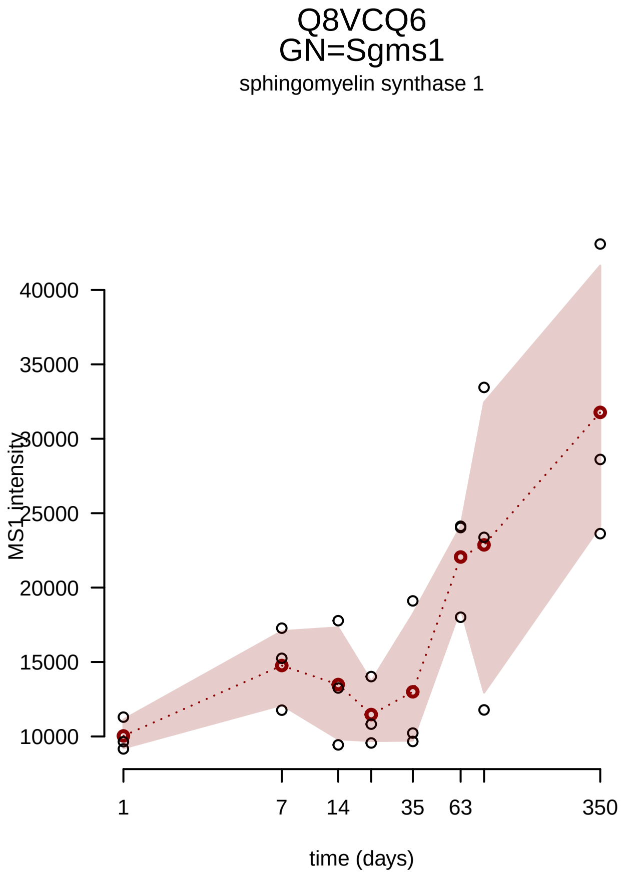
Q8VCQ6
GN=Sgms1
sphingomyelin synthase 1
MS1 intensity
10000 15000 20000 25000 30000 35000 40000
1 7 14 35 63 350
time (days)

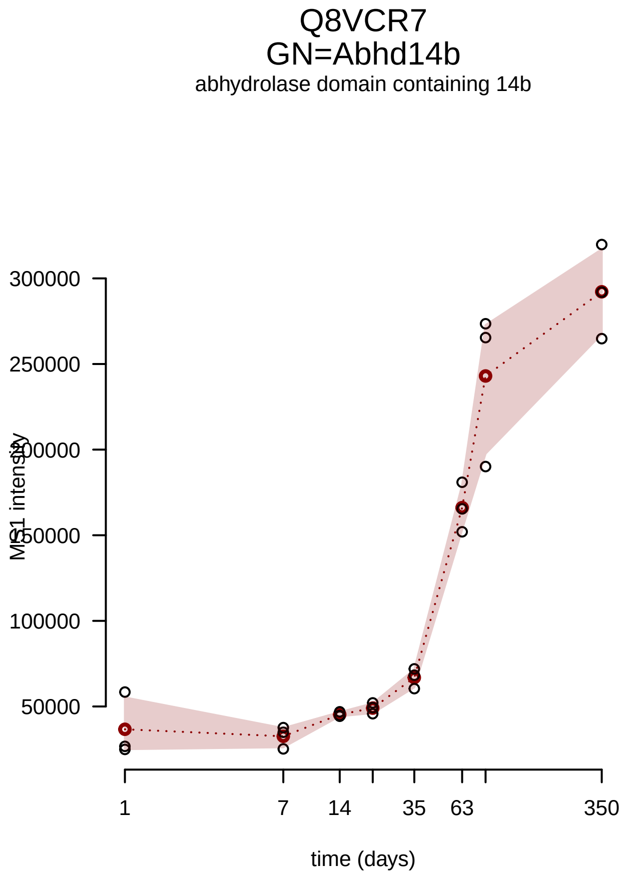
Q8VCR7
GN=Abhd14b
abhydrolase domain containing 14b
MS1 intensity
50000 100000 150000 200000 250000 300000
1 7 14 35 63 350
time (days)

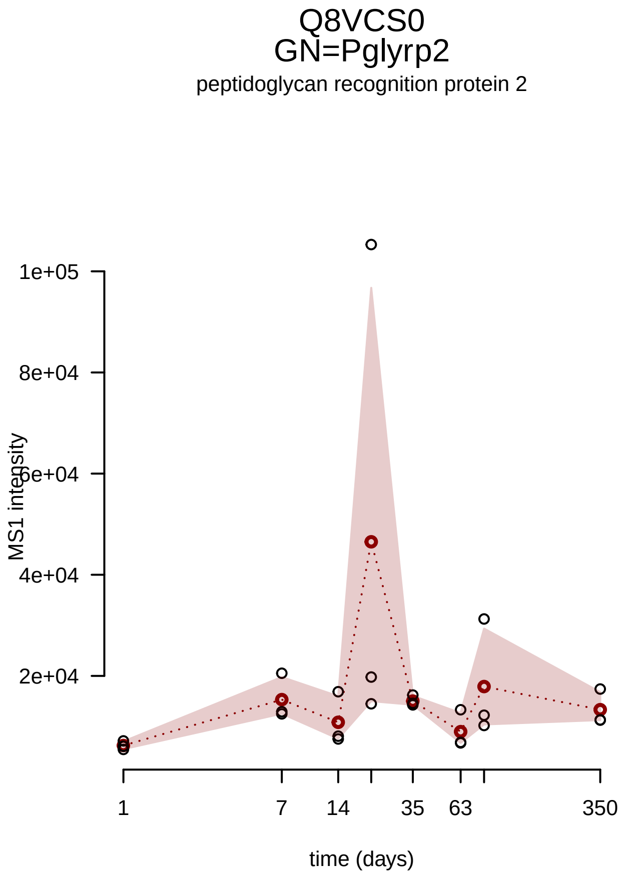
Q8VCS0
GN=Pglyrp2
peptidoglycan recognition protein 2
MS1 intensity
2e+04 4e+04 6e+04 8e+04 1e+05
1 7 14 35 63 350
time (days)

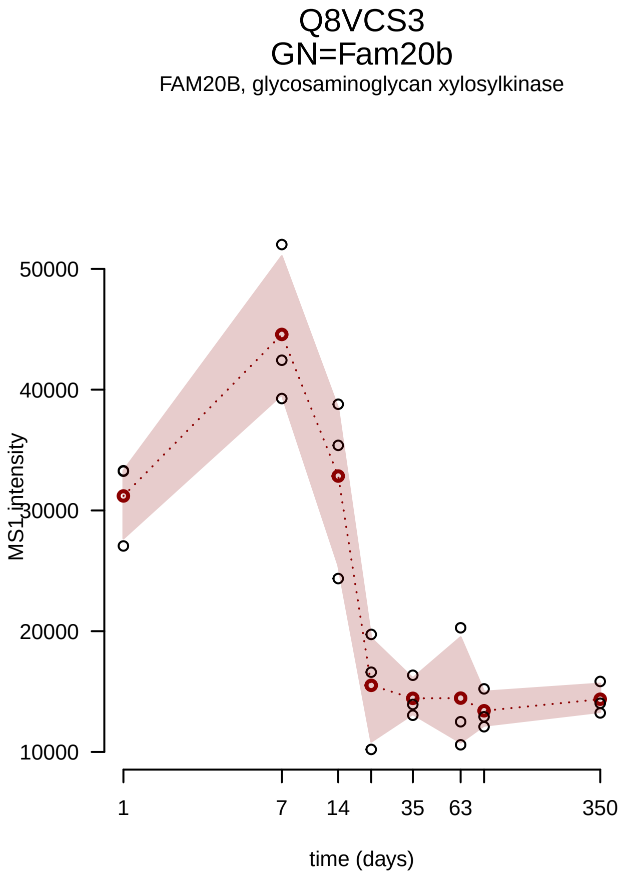
Q8VCS3
GN=Fam20b
FAM20B, glycosaminoglycan xylosylkinase
MS1 intensity
10000 20000 30000 40000 50000
1 7 14 35 63 350
time (days)

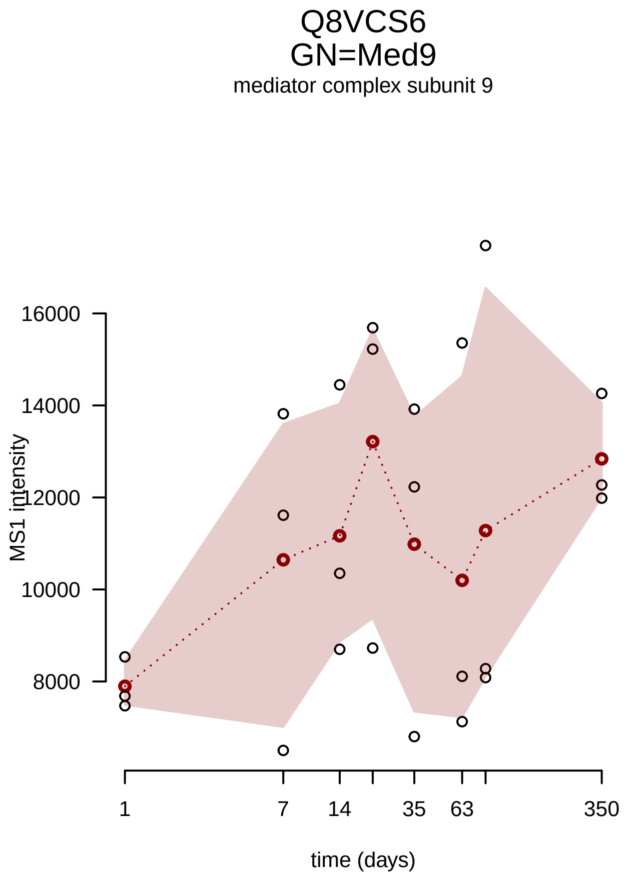
Q8VCS6
GN=Med9
mediator complex subunit 9
MS1 intensity
8000 10000 12000 14000 16000
1 7 14 35 63 350
time (days)

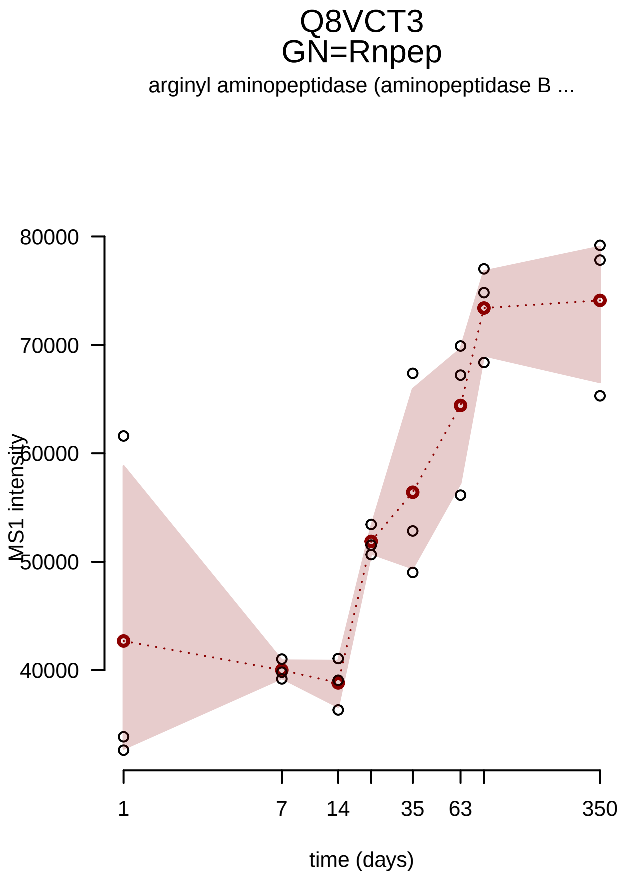
Q8VCT3
GN=Rnpep
arginyl aminopeptidase (aminopeptidase B ...
MS1 intensity
40000 50000 60000 70000 80000
1 7 14 35 63 350
time (days)

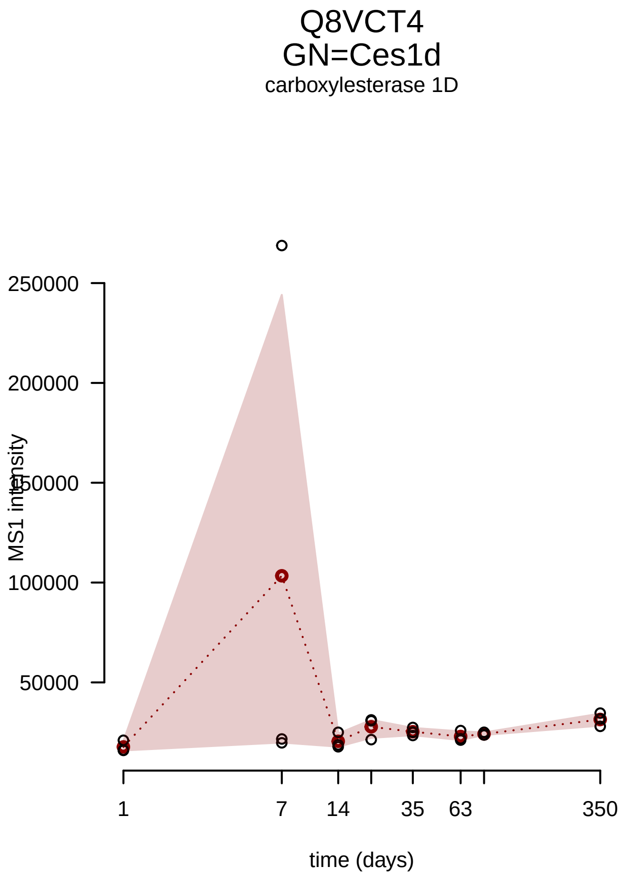
Q8VCT4
GN=Ces1d
carboxylesterase 1D
MS1 intensity
50000 100000 150000 200000 250000
1 7 14 35 63 350
time (days)

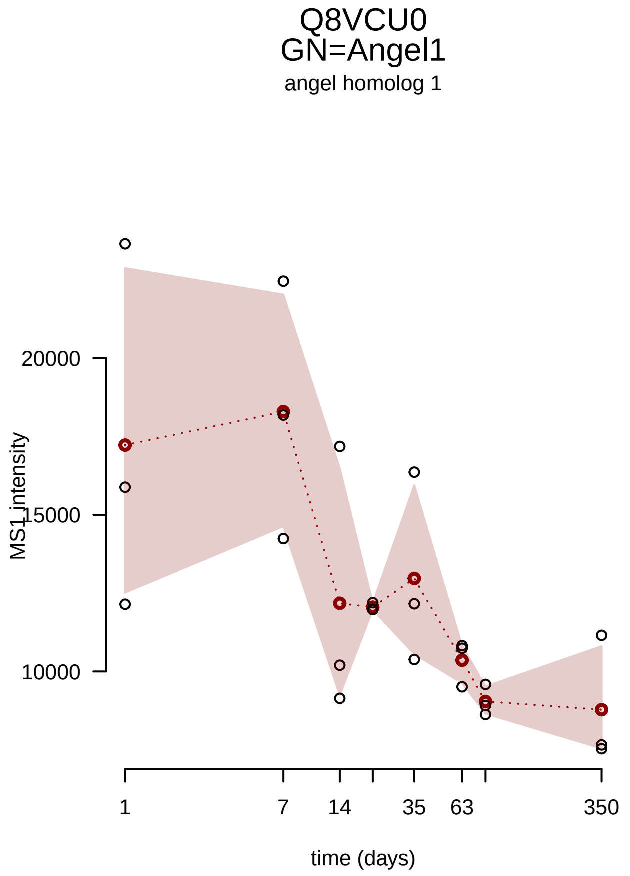
Q8VCU0
GN=Angel1
angel homolog 1
MS1 intensity
time (days)
10000
15000
20000
1
7
14
35
63
350

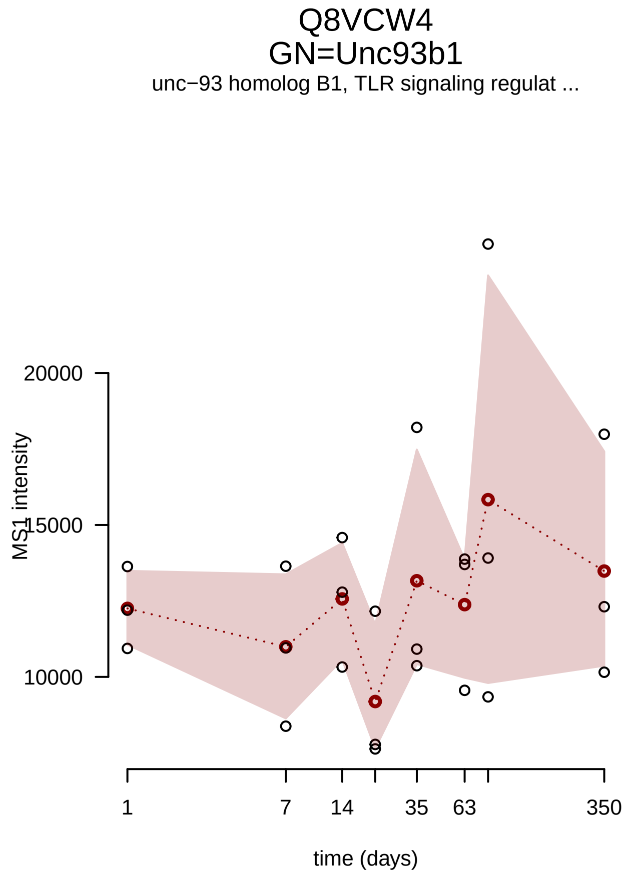
Q8VCW4
GN=Unc93b1
unc−93 homolog B1, TLR signaling regulat ...
MS1 intensity
time (days)
10000
15000
20000
1
7
14
35
63
350

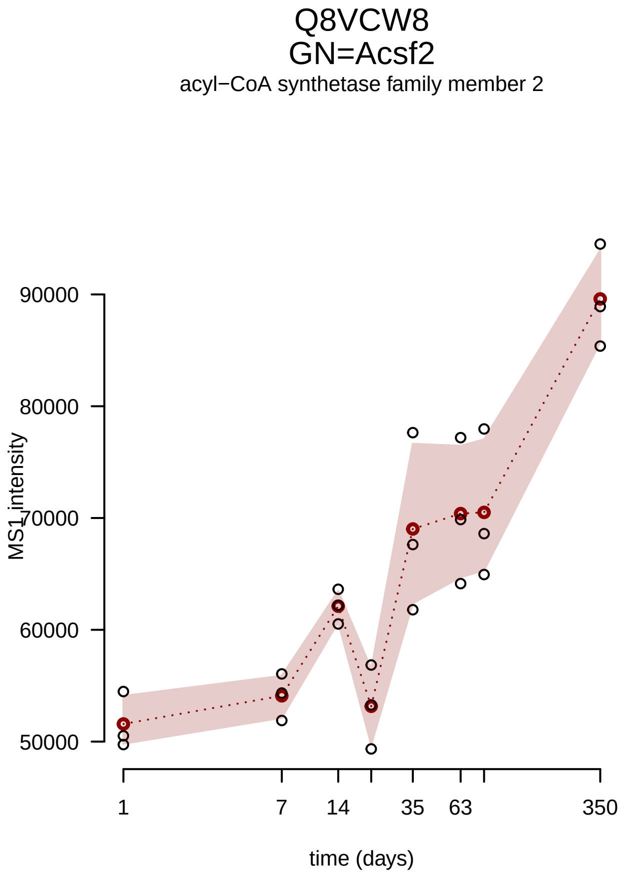
Q8VCW8
GN=Acsf2
acyl−CoA synthetase family member 2
MS1 intensity
time (days)
50000
60000
70000
80000
90000
1
7
14
35
63
350

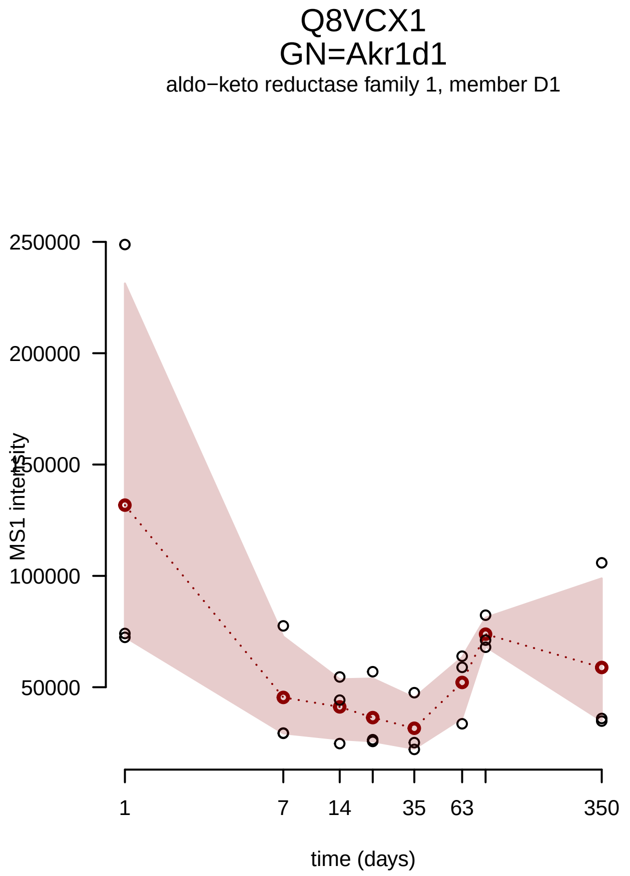
Q8VCX1
GN=Akr1d1
aldo−keto reductase family 1, member D1
MS1 intensity
time (days)
50000
100000
150000
200000
250000
1
7
14
35
63
350

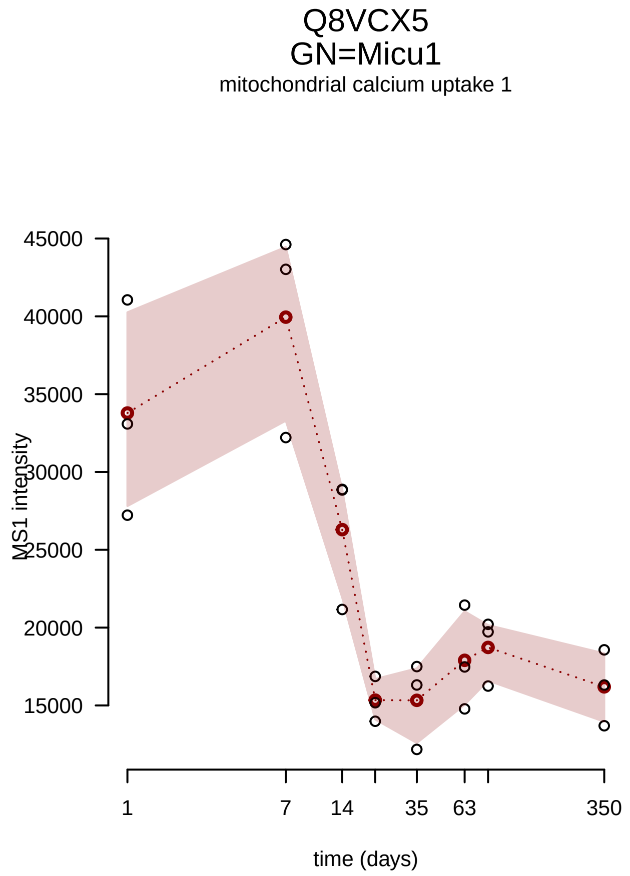
Q8VCX5
GN=Micu1
mitochondrial calcium uptake 1
MS1 intensity
time (days)
15000
20000
25000
30000
35000
40000
45000
1
7
14
35
63
350

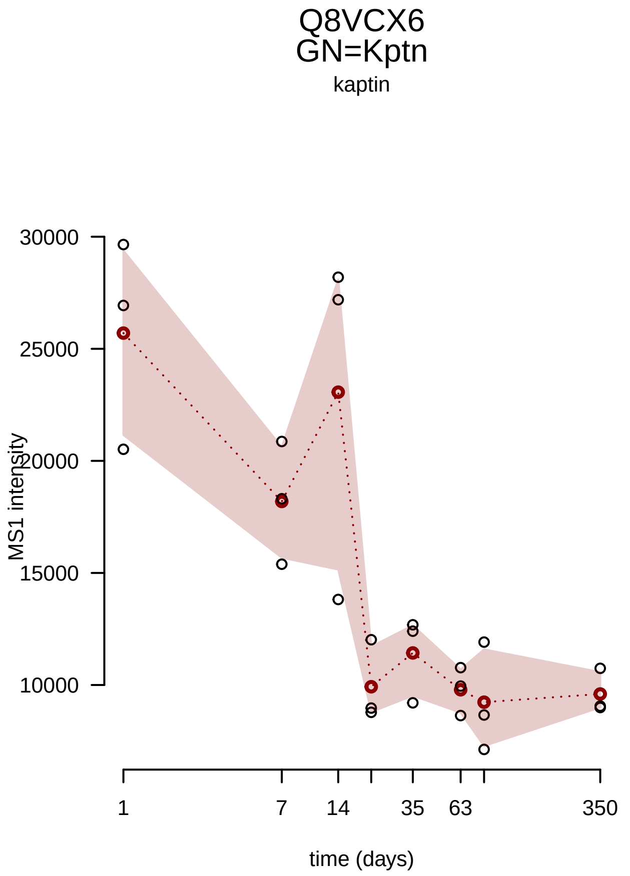
Q8VCX6
GN=Kptn
kaptin
MS1 intensity
time (days)
10000
15000
20000
25000
30000
1
7
14
35
63
350

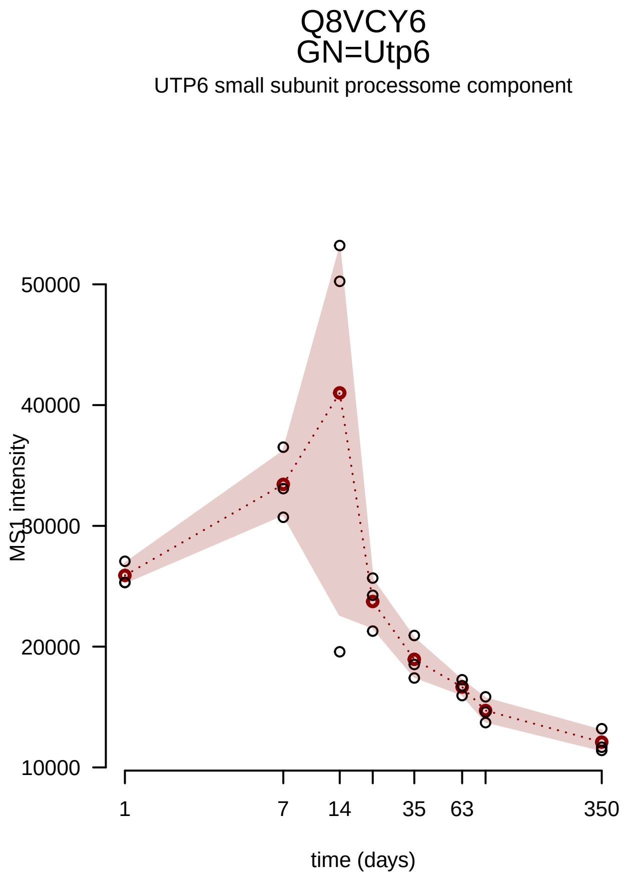
Q8VCY6
GN=Utp6
UTP6 small subunit processome component
MS1 intensity
time (days)
10000
20000
30000
40000
50000
1
7
14
35
63
350

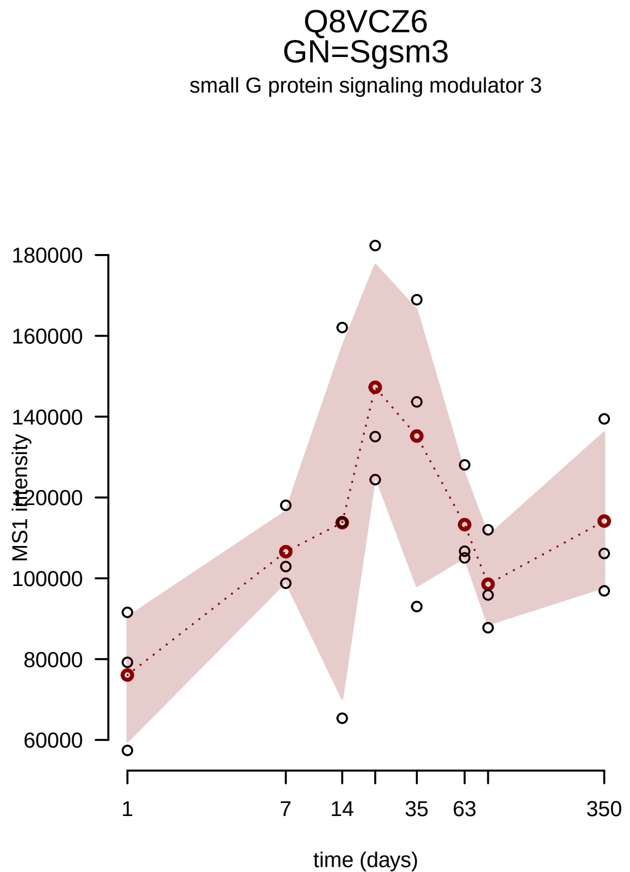
Q8VCZ6
GN=Sgsm3
small G protein signaling modulator 3
MS1 intensity
time (days)
60000
80000
100000
120000
140000
160000
180000
1
7
14
35
63
350

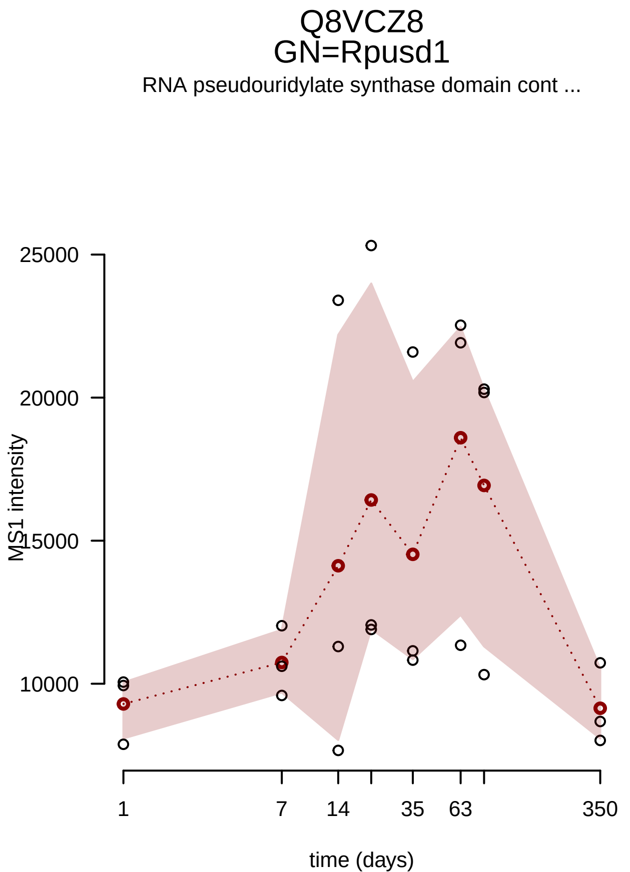
Q8VCZ8
GN=Rpusd1
RNA pseudouridylate synthase domain cont ...
MS1 intensity
time (days)
10000
15000
20000
25000
1
7
14
35
63
350

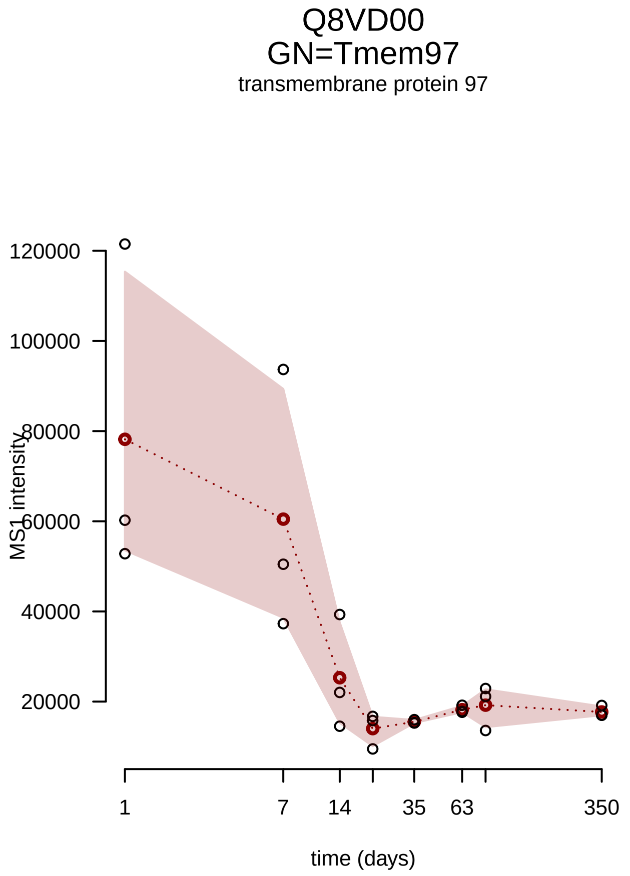
Q8VD00
GN=Tmem97
transmembrane protein 97
MS1 intensity
120000
100000
80000
60000
40000
20000
1
7
14
35
63
350
time (days)

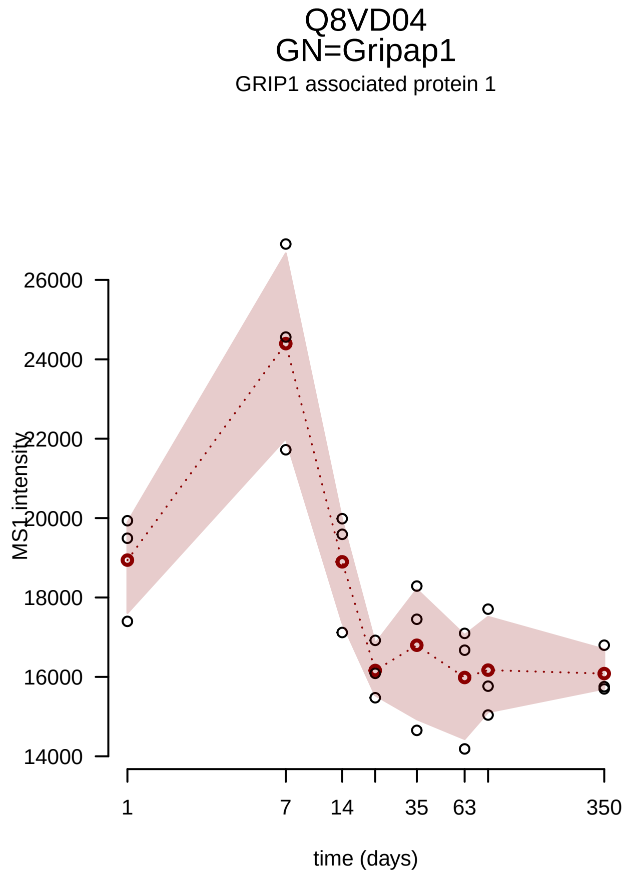
Q8VD04
GN=Gripap1
GRIP1 associated protein 1
MS1 intensity
26000
24000
22000
20000
18000
16000
14000
1
7
14
35
63
350
time (days)

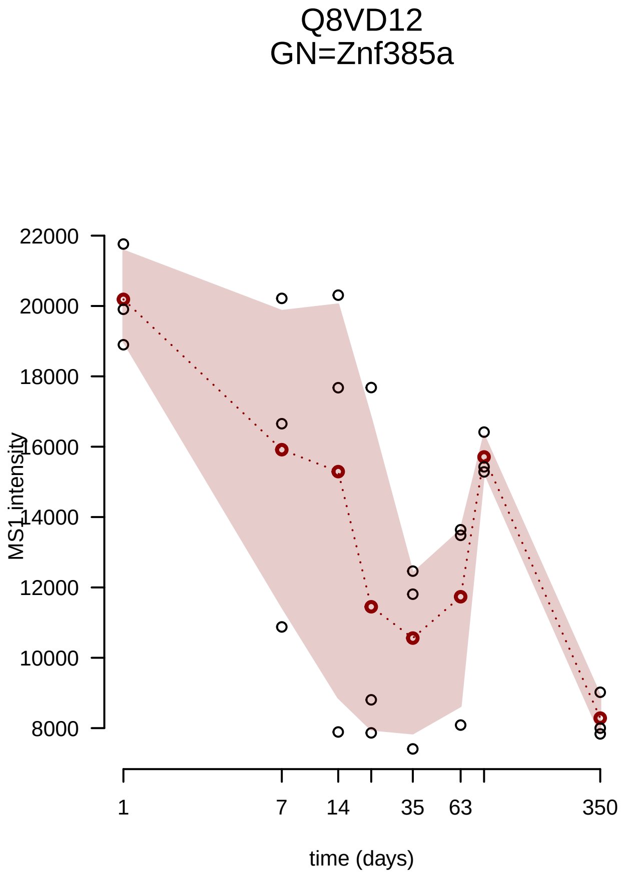
Q8VD12
GN=Znf385a
MS1 intensity
22000
20000
18000
16000
14000
12000
10000
8000
1
7
14
35
63
350
time (days)

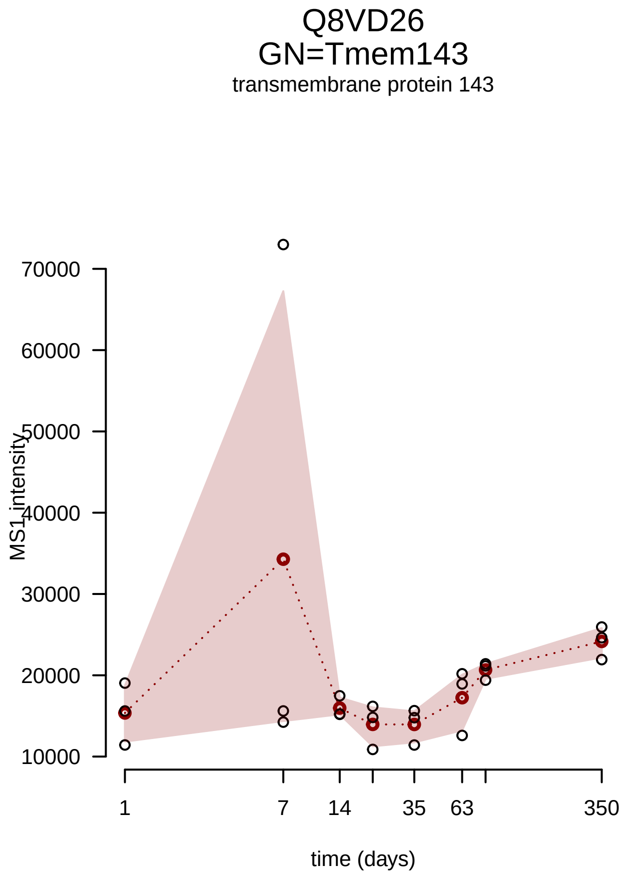
Q8VD26
GN=Tmem143
transmembrane protein 143
MS1 intensity
70000
60000
50000
40000
30000
20000
10000
1
7
14
35
63
350
time (days)

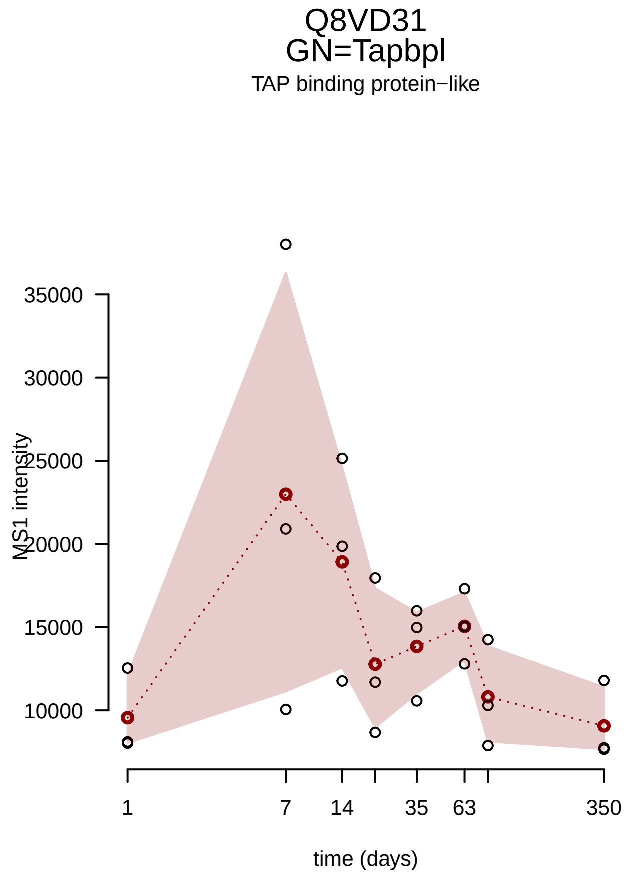
Q8VD31
GN=Tapbpl
TAP binding protein−like
MS1 intensity
35000
30000
25000
20000
15000
10000
1
7
14
35
63
350
time (days)

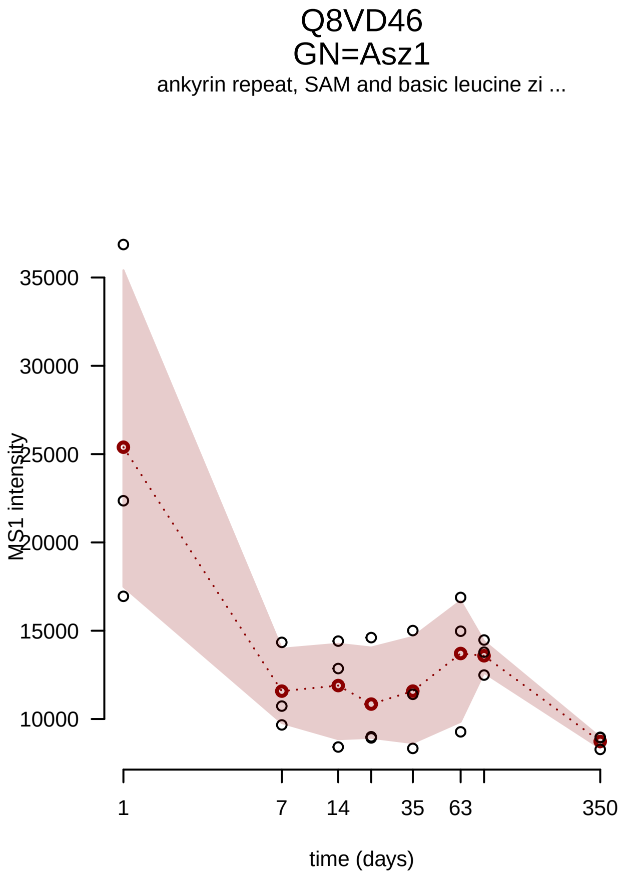
Q8VD46
GN=Asz1
ankyrin repeat, SAM and basic leucine zi ...
MS1 intensity
35000
30000
25000
20000
15000
10000
1
7
14
35
63
350
time (days)

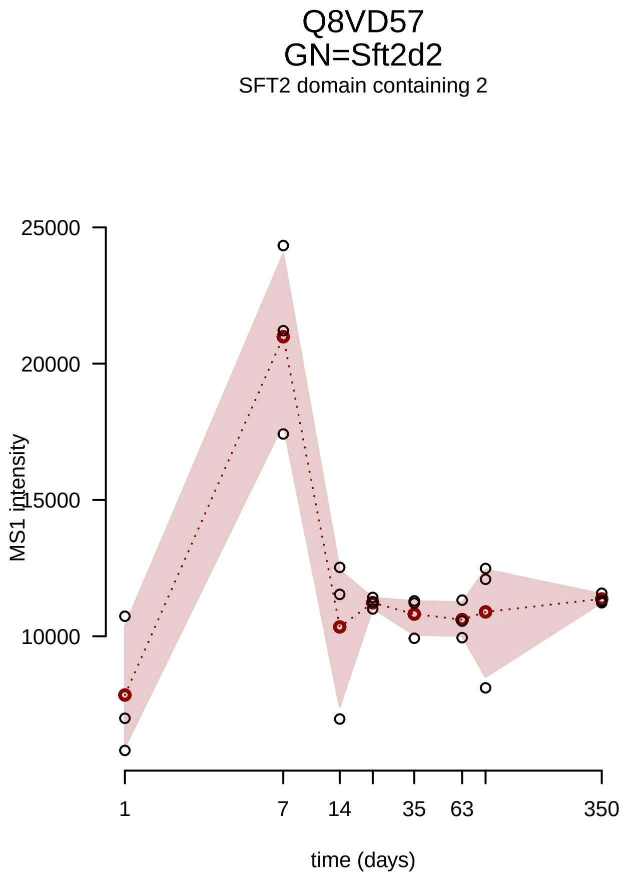
Q8VD57
GN=Sft2d2
SFT2 domain containing 2
MS1 intensity
25000
20000
15000
10000
1
7
14
35
63
350
time (days)

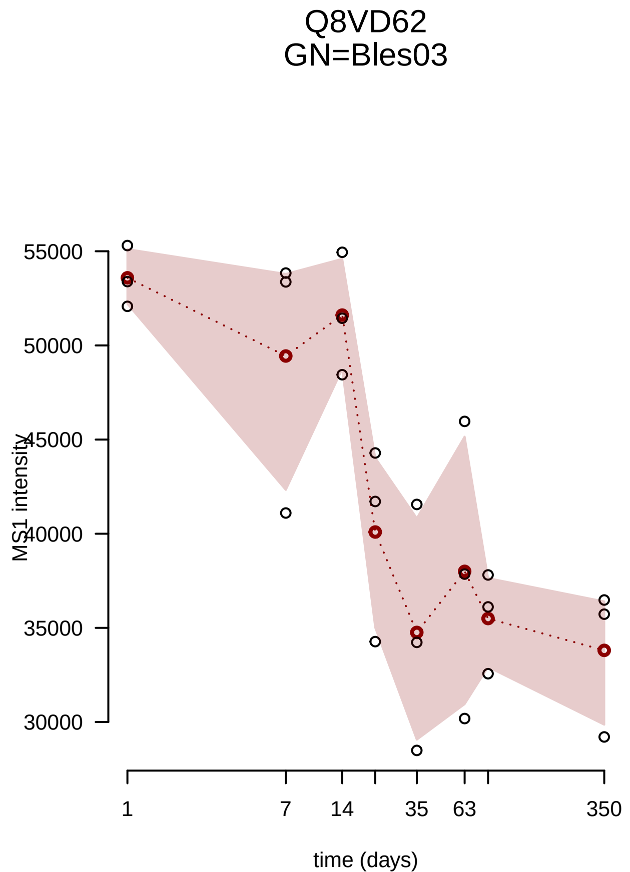
Q8VD62
GN=Bles03
MS1 intensity
55000
50000
45000
40000
35000
30000
1
7
14
35
63
350
time (days)

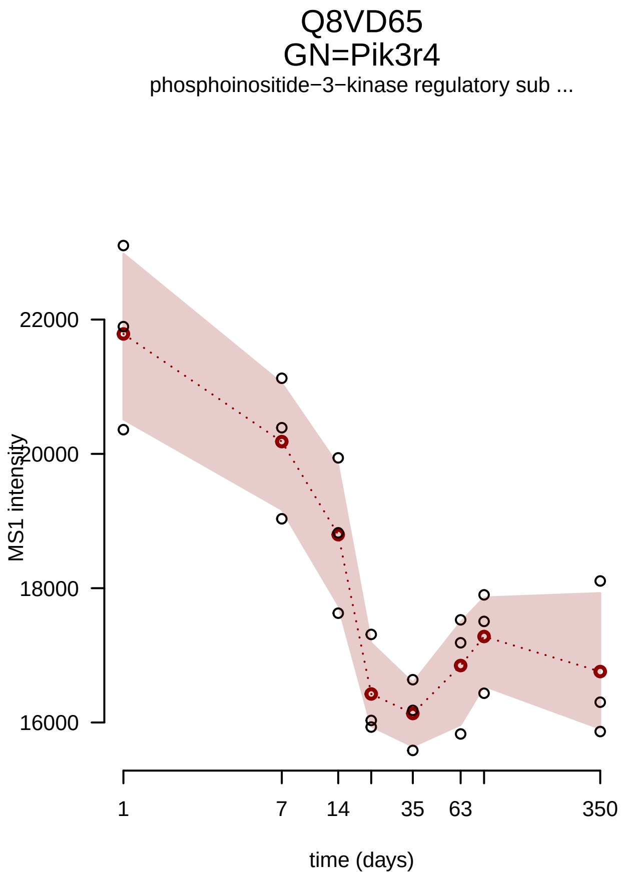
Q8VD65
GN=Pik3r4
phosphoinositide−3−kinase regulatory sub ...
MS1 intensity
22000
20000
18000
16000
1
7
14
35
63
350
time (days)

Q8VD66
GN=Abhd4
abhydrolase domain containing 4

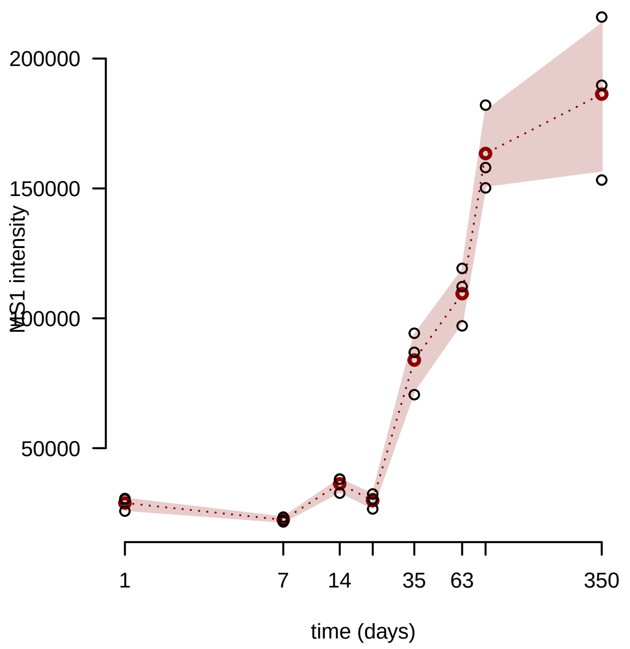

Q8VD72
GN=Ttc8
tetratricopeptide repeat domain 8

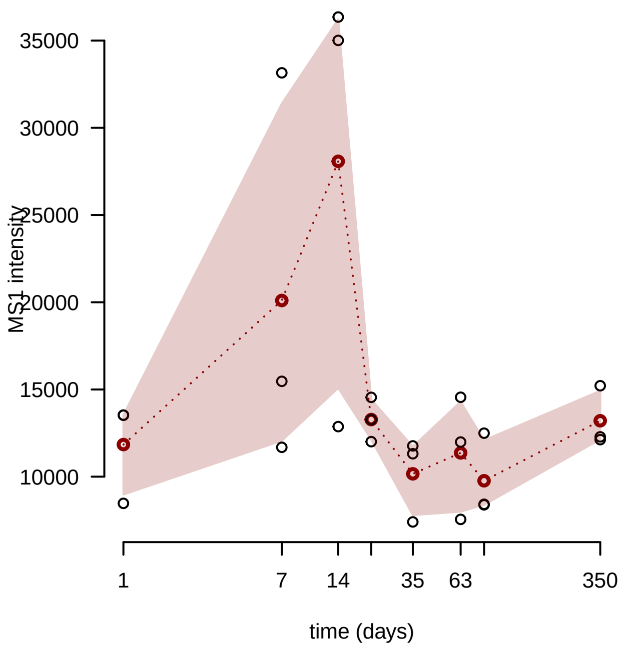

Q8VD75
GN=Hip1
huntingtin interacting protein 1

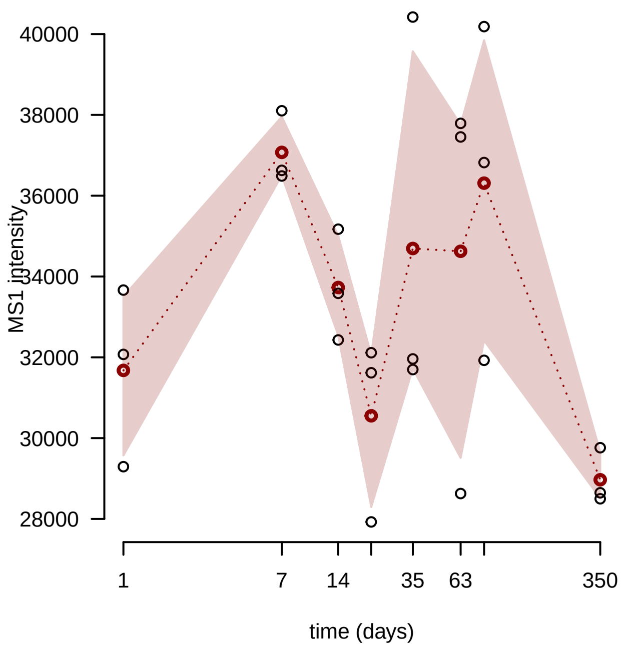

Q8VD76
GN=Gtf2h3
general transcription factor IIH, polype ...

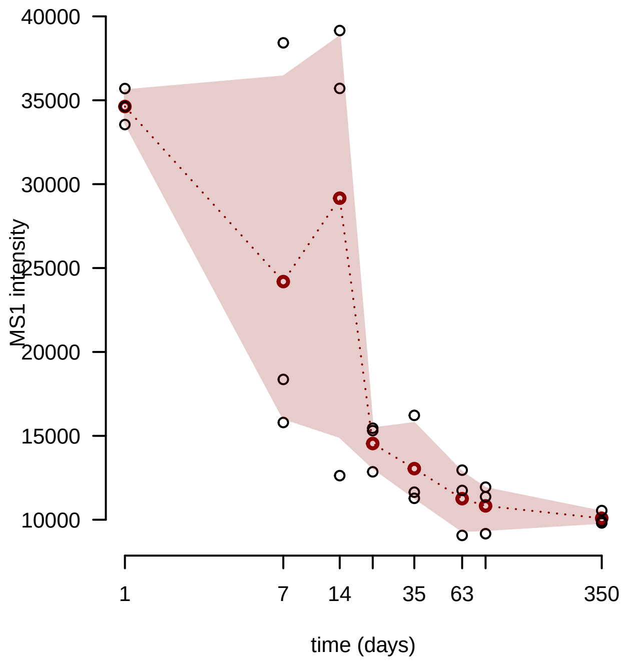

Q8VDB2
GN=Alg12
asparagine−linked glycosylation 12 (alph ...

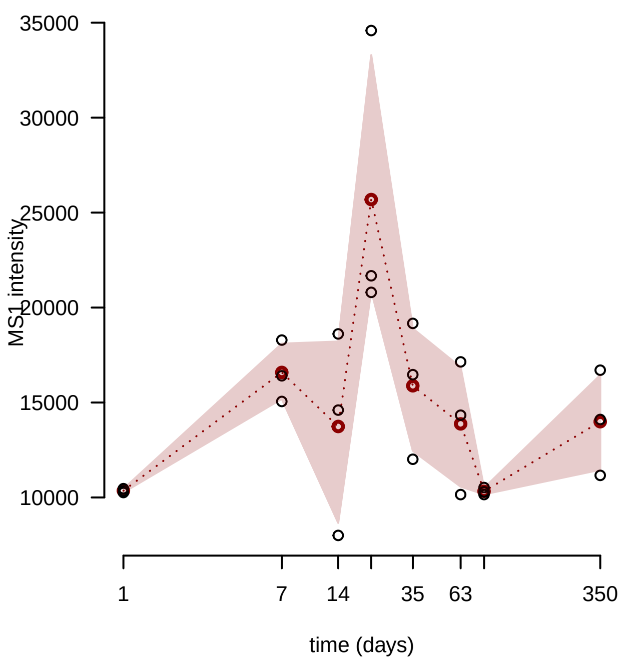

Q8VDC0
GN=Lars2
leucyl−tRNA synthetase, mitochondrial

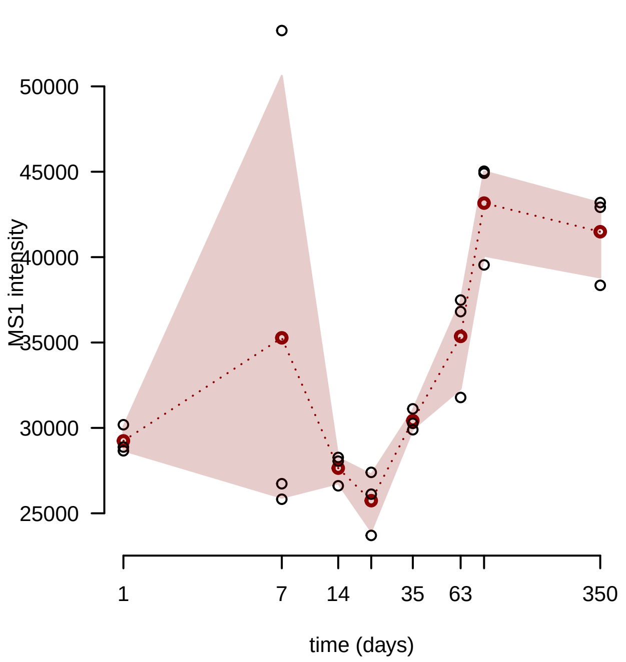

Q8VDC1
GN=Fyco1
FYVE and coiled−coil domain containing 1

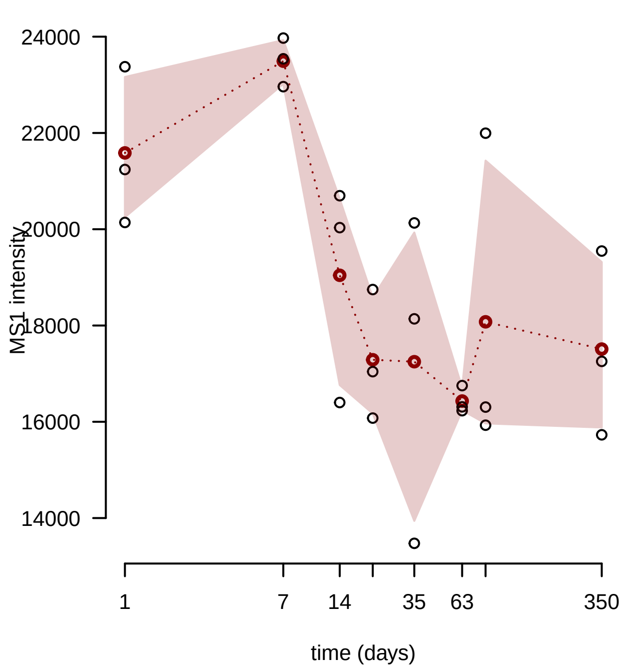

Q8VDD5
GN=Myh9
myosin, heavy polypeptide 9, non−muscle

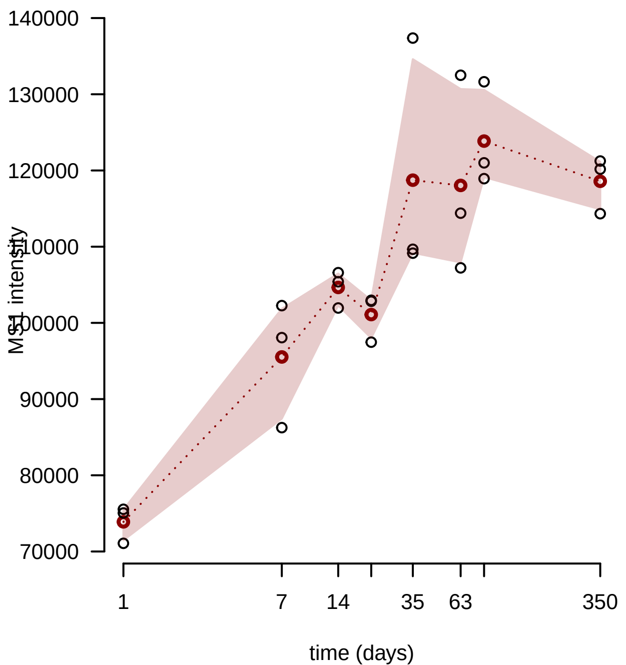

Q8VDD8
GN=Washc1
WASH complex subunit 1

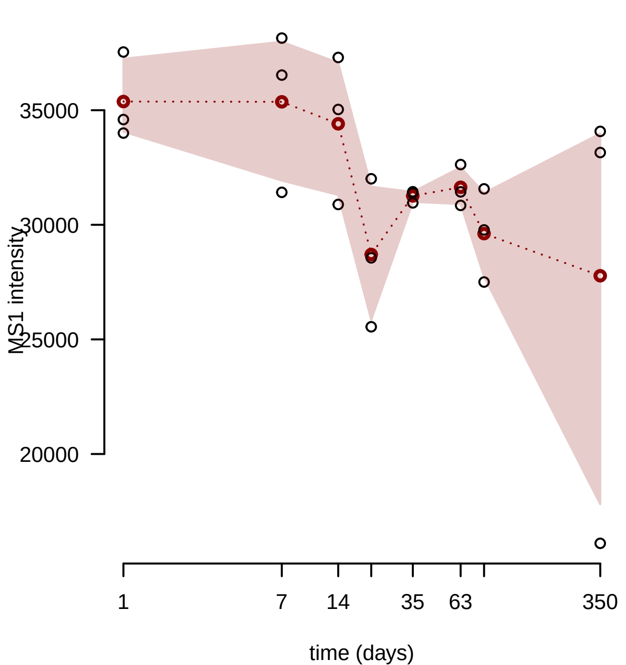

Q8VDD9
GN=Phip
pleckstrin homology domain interacting p ...

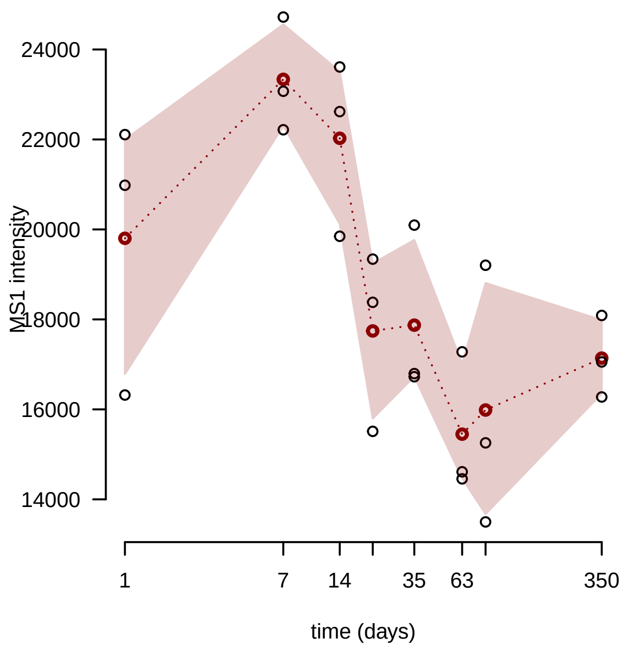

Q8VDF2
GN=Uhrf1
ubiquitin−like, containing PHD and RING ...

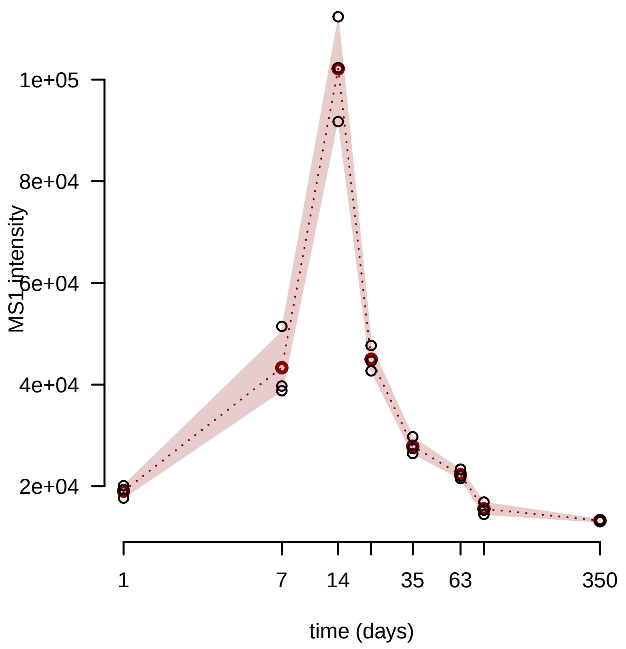

Q8VDG3
GN=Parn
poly(A)−specific ribonuclease (deadenyla ...

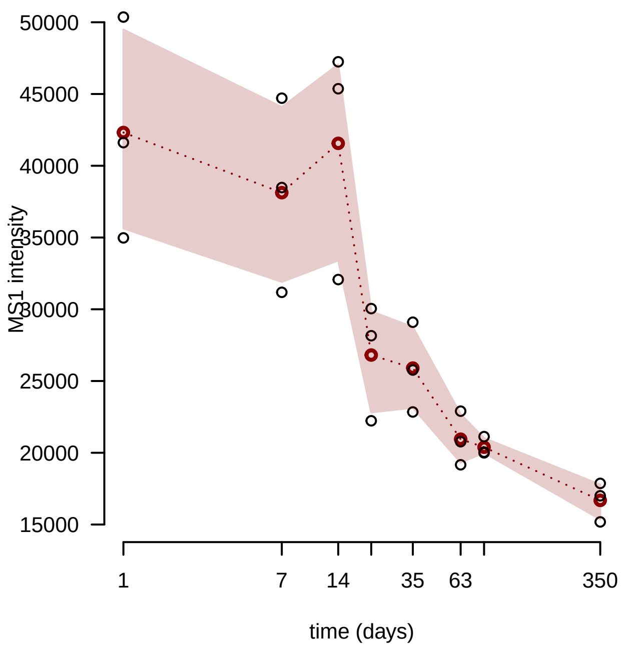

Q8VDG5
GN=Ppcs
phosphopantothenoylcysteine synthetase

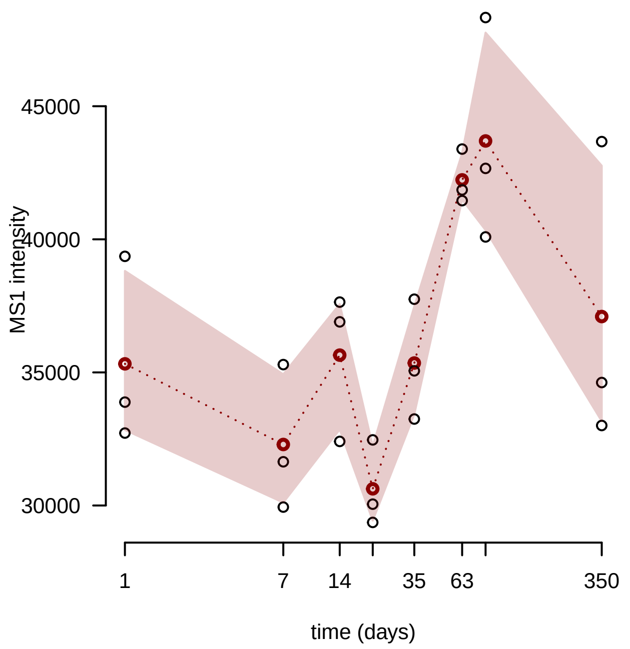

Q8VDG7
GN=Pafah2
platelet−activating factor acetylhydrola ...

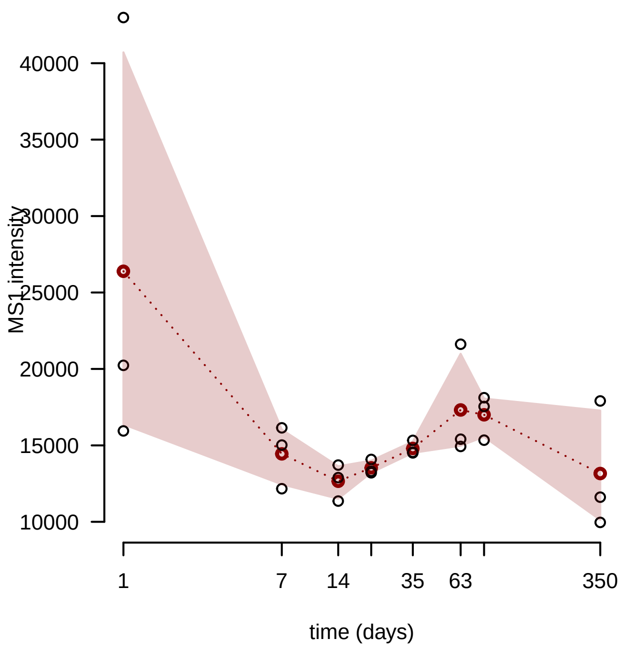

Q8VDH1
GN=Fbxo21
F−box protein 21

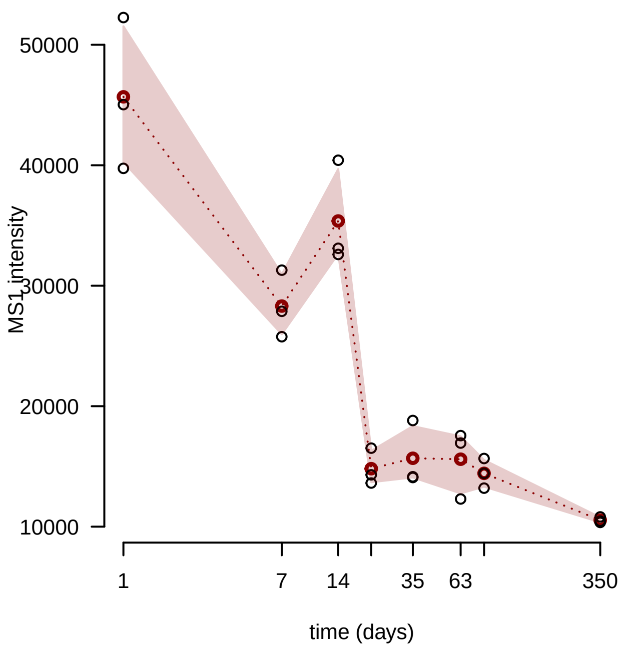

Q8VDI7
GN=Ubac1
ubiquitin associated domain containing 1

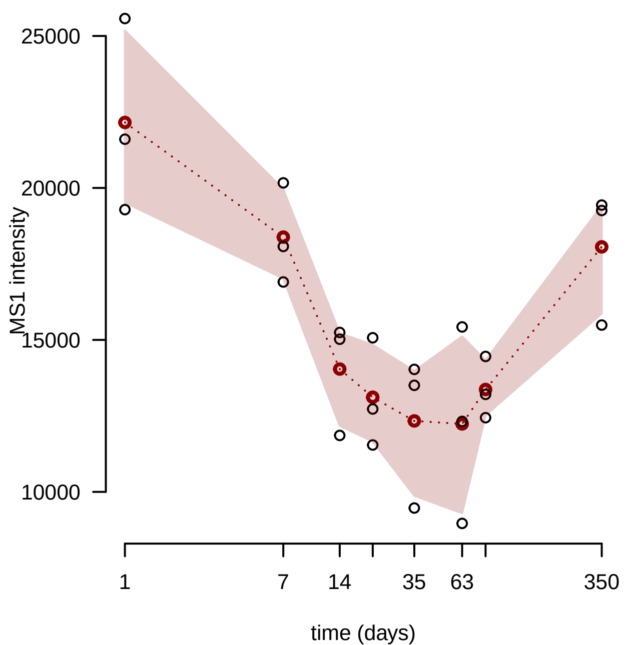

Q8VDI9
GN=Alg9
asparagine−linked glycosylation 9 (alpha ...

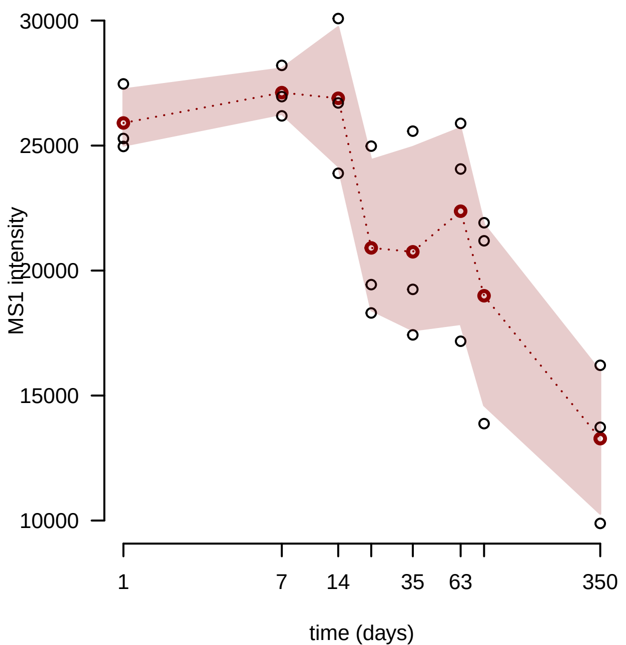

Q8VDJ3
GN=Hdlbp
high density lipoprotein (HDL) binding p ...

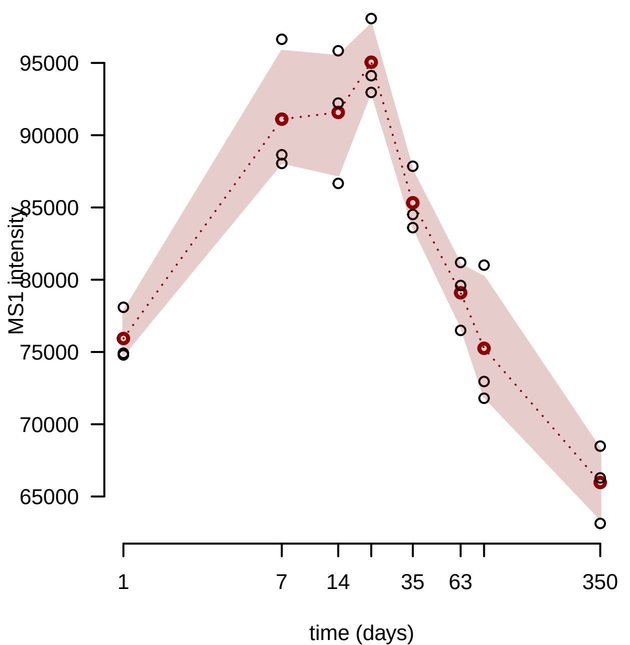

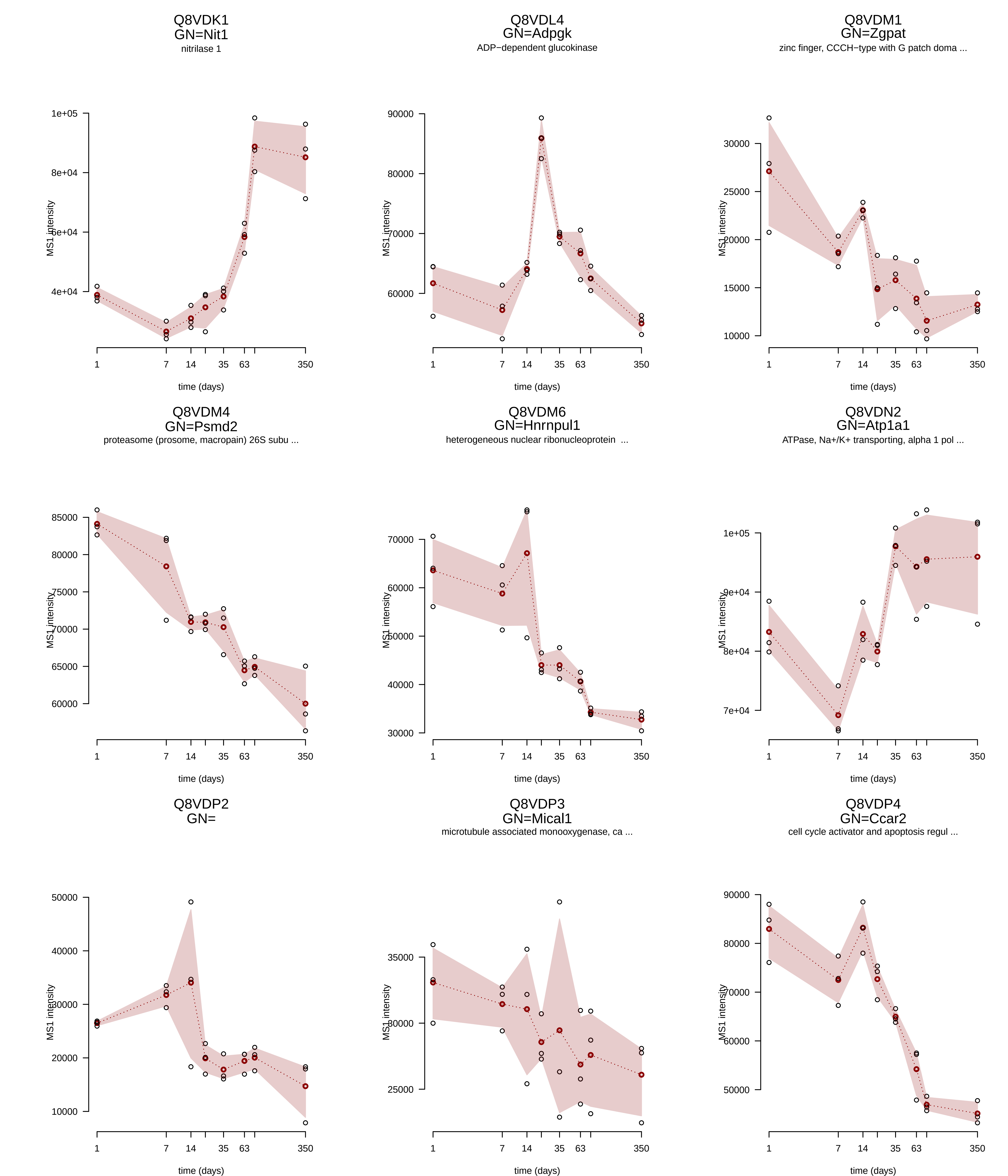
Q8VDK1
GN=Nit1
nitrilase 1
Q8VDL4
GN=Adpgk
ADP−dependent glucokinase
Q8VDM1
GN=Zgpat
zinc finger, CCCH−type with G patch doma ...
Q8VDM4
GN=Psmd2
proteasome (prosome, macropain) 26S subu ...
Q8VDM6
GN=Hnrnpul1
heterogeneous nuclear ribonucleoprotein ...
Q8VDN2
GN=Atp1a1
ATPase, Na+/K+ transporting, alpha 1 pol ...
Q8VDP2
GN=
Q8VDP3
GN=Mical1
microtubule associated monooxygenase, ca ...
Q8VDP4
GN=Ccar2
cell cycle activator and apoptosis regul ...
MS1 intensity
time (days)
1
7
14
35
63
350

Q8VDP6
GN=Cdipt
CDP−diacylglycerol−−inositol 3−phosphati ...

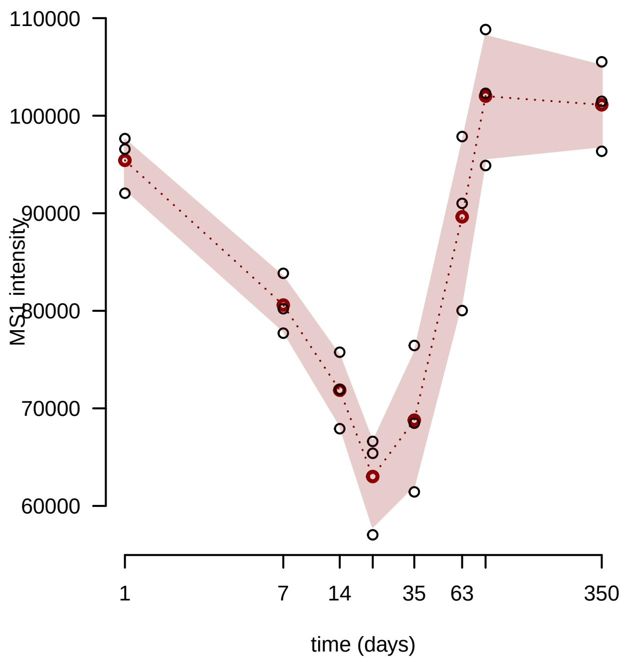

Q8VDQ1
GN=Ptgr2
prostaglandin reductase 2

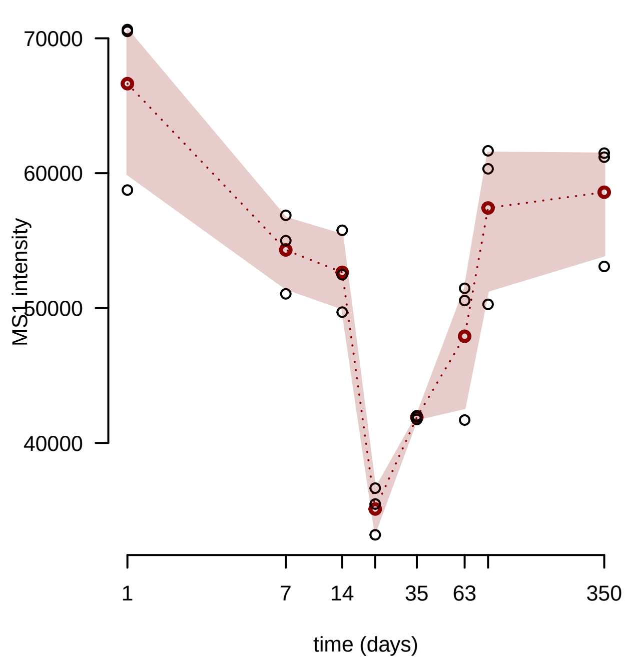

Q8VDQ8
GN=Sirt2
sirtuin 2

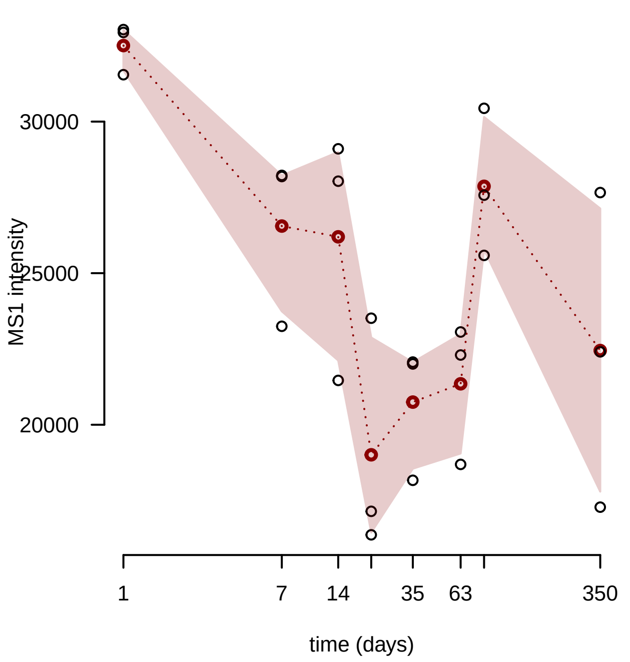

Q8VDQ9
GN=Kri1
KRI1 homolog

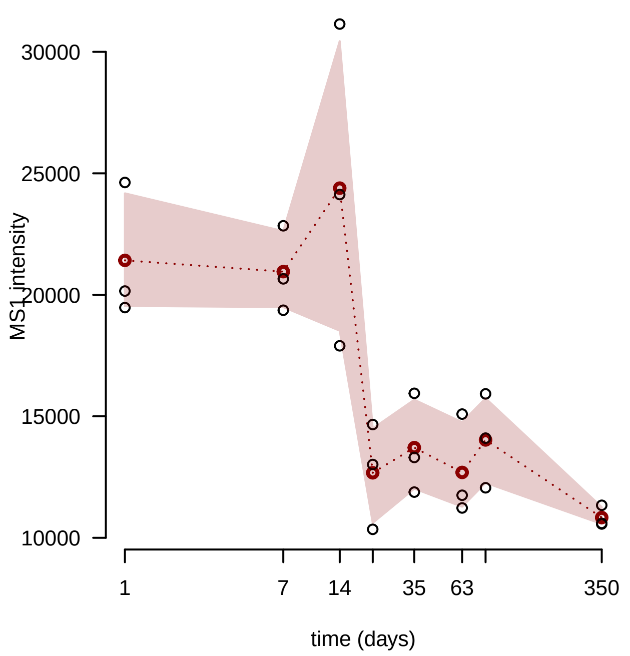

Q8VDR5
GN=Tmem267
transmembrane protein 267

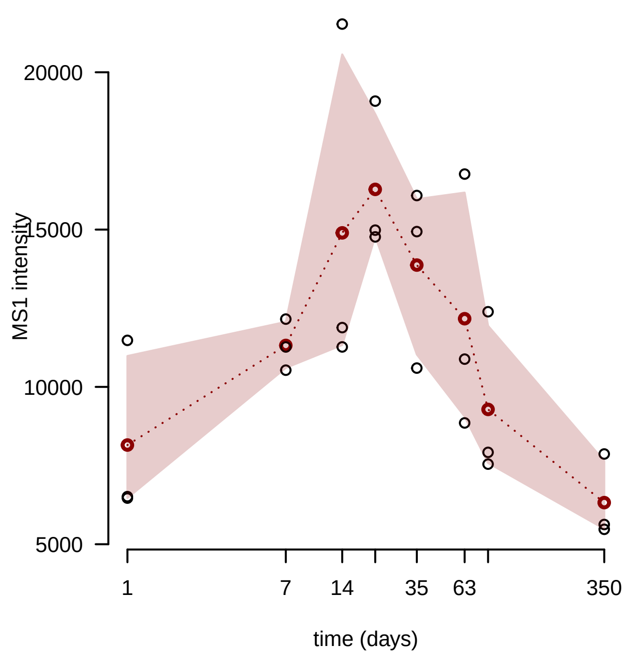

Q8VDR9
GN=Dock6
dedicator of cytokinesis 6

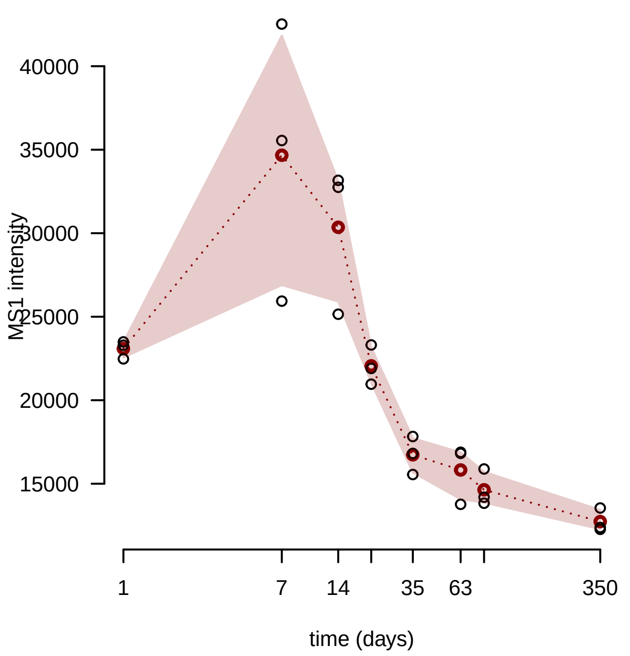

Q8VDS4
GN=Rprd1a
regulation of nuclear pre−mRNA domain co ...

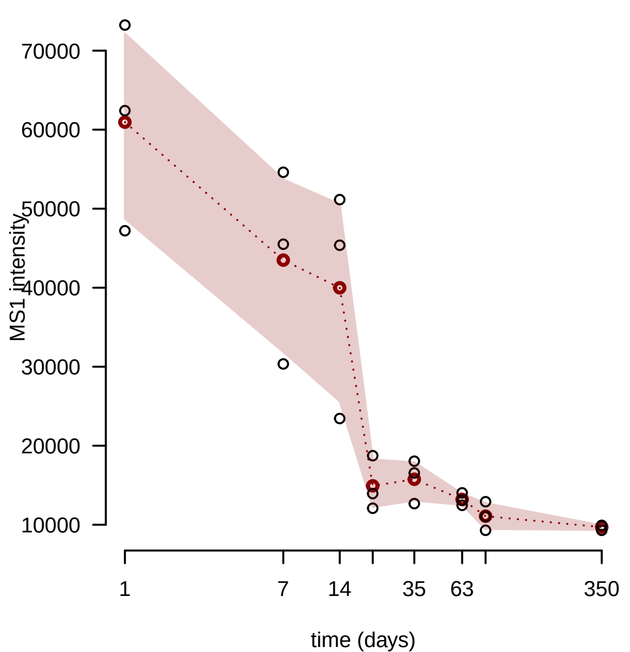

Q8VDS8
GN=Stx18
syntaxin 18

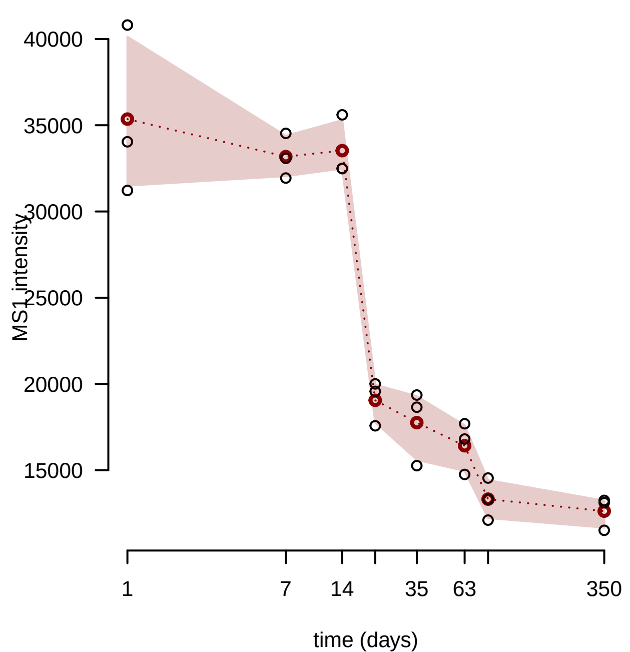

Q8VDT9
GN=Mrpl50
mitochondrial ribosomal protein L50

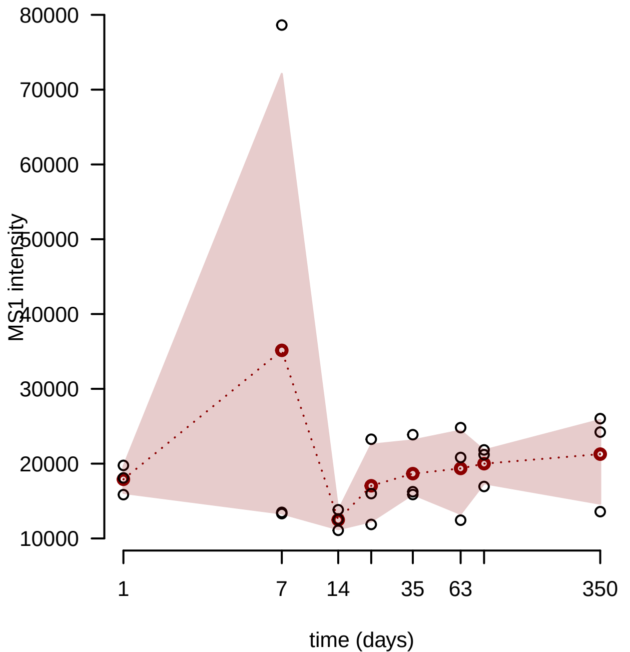

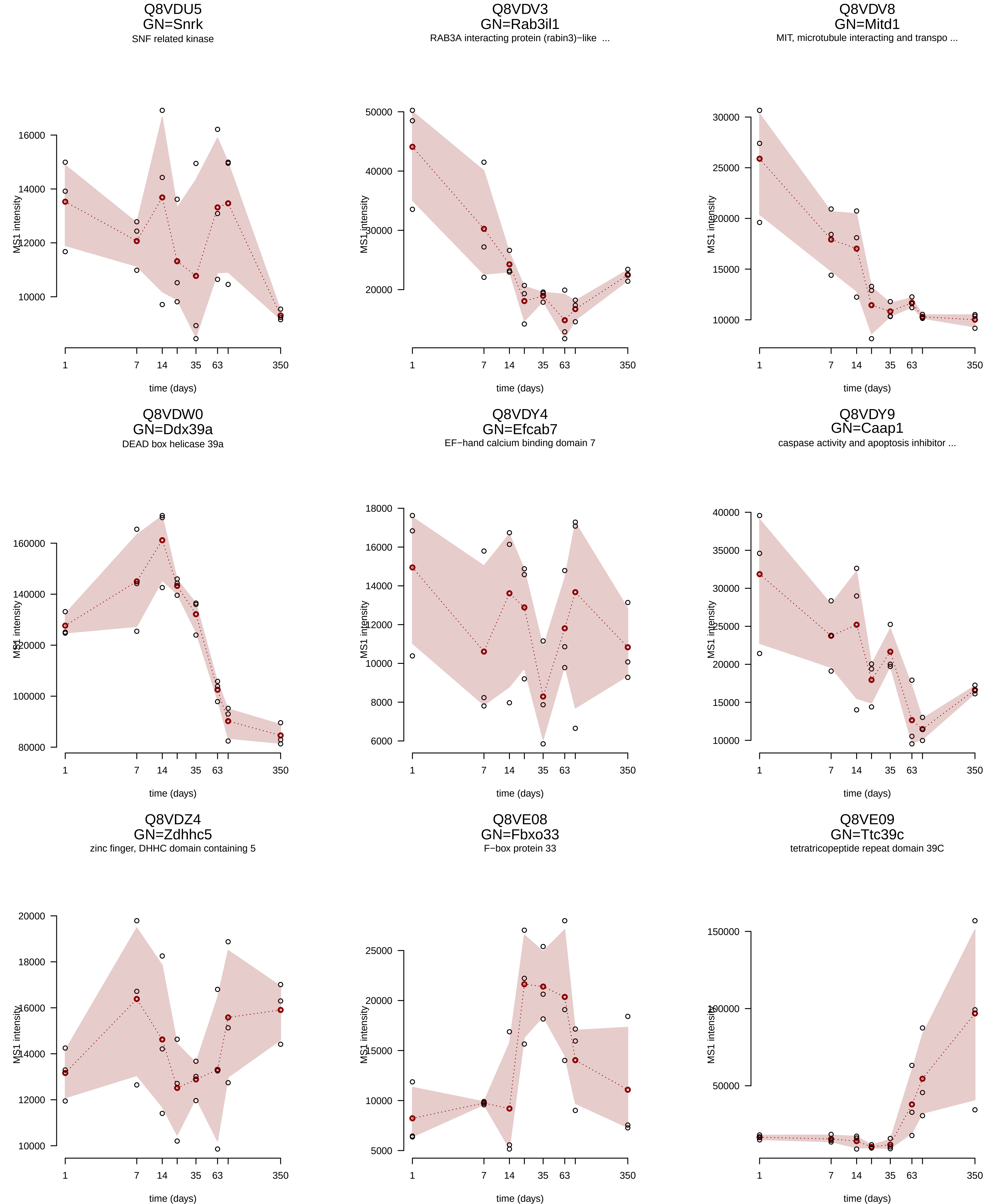
Q8VDU5
GN=Snrk
SNF related kinase
Q8VDV3
GN=Rab3il1
RAB3A interacting protein (rabin3)−like ...
Q8VDV8
GN=Mitd1
MIT, microtubule interacting and transpo ...
Q8VDW0
GN=Ddx39a
DEAD box helicase 39a
Q8VDY4
GN=Efcab7
EF−hand calcium binding domain 7
Q8VDY9
GN=Caap1
caspase activity and apoptosis inhibitor ...
Q8VDZ4
GN=Zdhhc5
zinc finger, DHHC domain containing 5
Q8VE08
GN=Fbxo33
F−box protein 33
Q8VE09
GN=Ttc39c
tetratricopeptide repeat domain 39C
MS1 intensity
time (days)
1
7
14
35
63
350

Q8VE10
GN=Naa40
N(alpha)−acetyltransferase 40, NatD cata ...

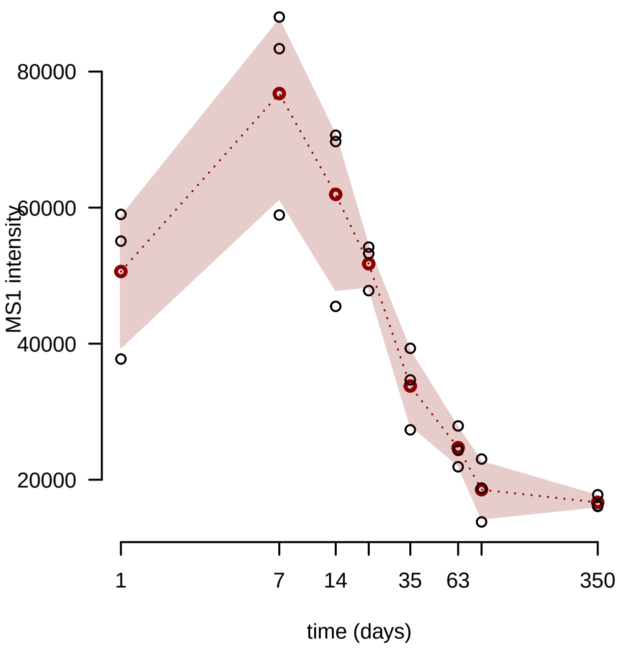

Q8VE11
GN=Mtmr6
myotubularin related protein 6

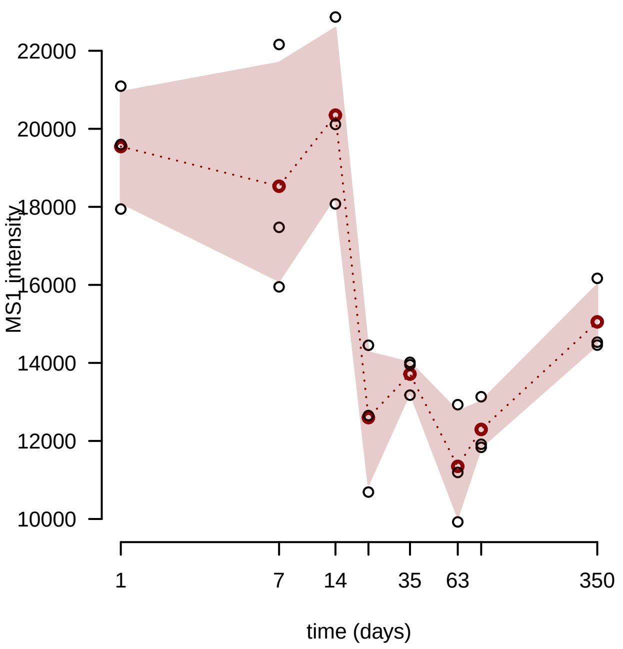

Q8VE18
GN=Smg8
SMG8 nonsense mediated mRNA decay factor

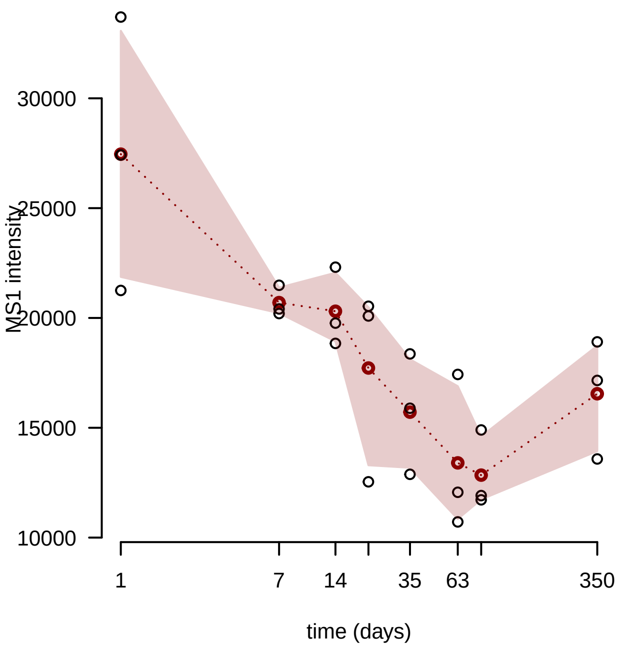

Q8VE19
GN=Mios
meiosis regulator for oocyte development

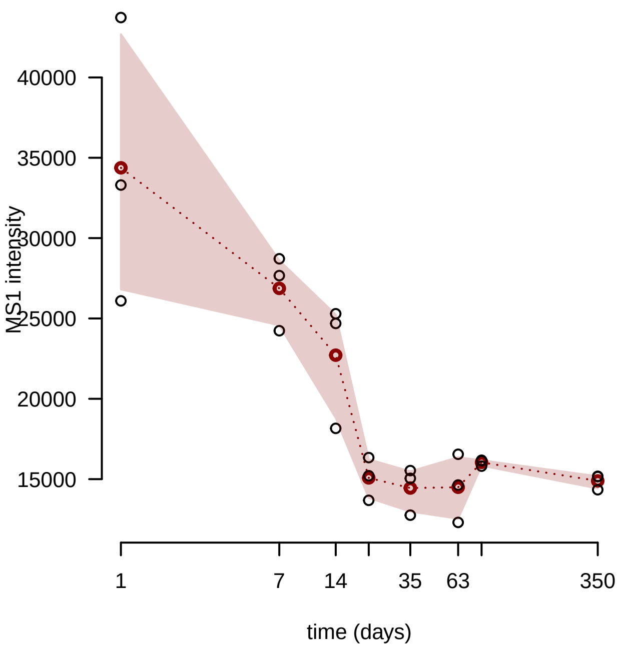

Q8VE22
GN=Mrps23
mitochondrial ribosomal protein S23

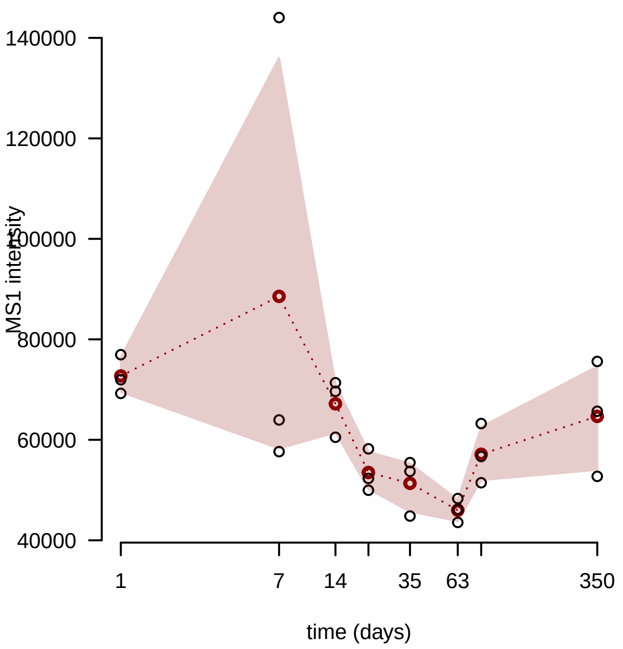

Q8VE37
GN=Rcc1
regulator of chromosome condensation 1

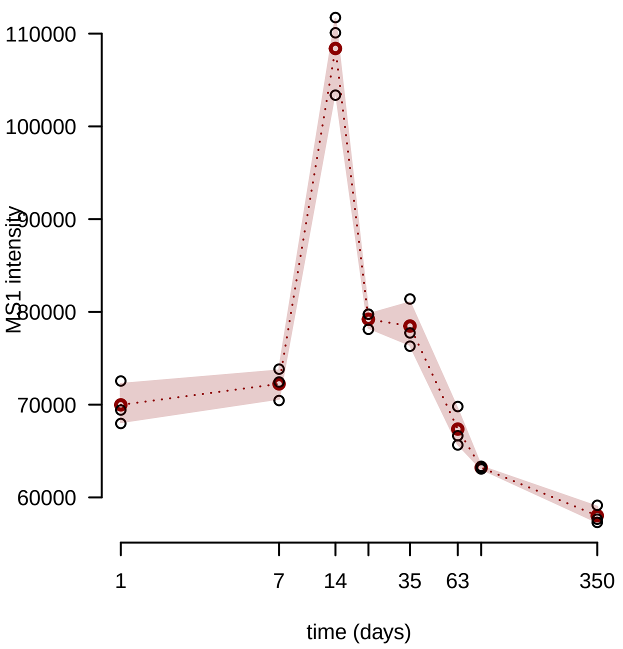

Q8VE38
GN=Oxnad1
oxidoreductase NAD−binding domain contai ...

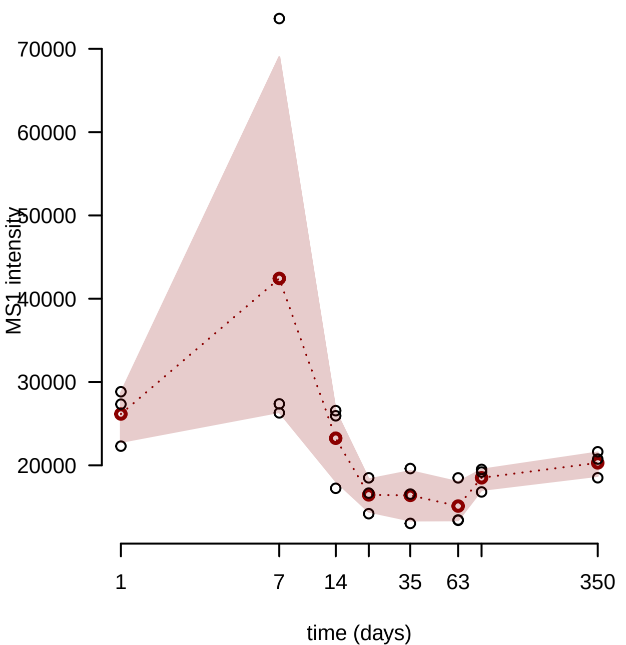

Q8VE42
GN=Ankrd49
ankyrin repeat domain 49

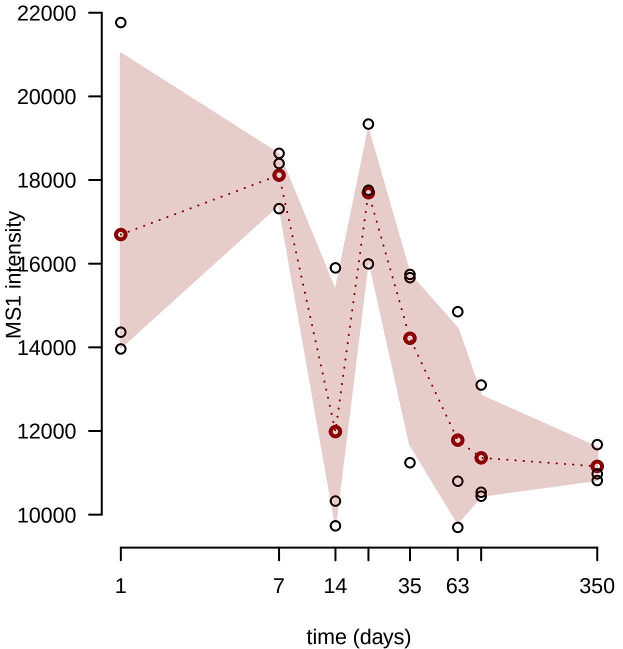

Q8VE47
GN=Uba5
ubiquitin−like modifier activating enzym ...

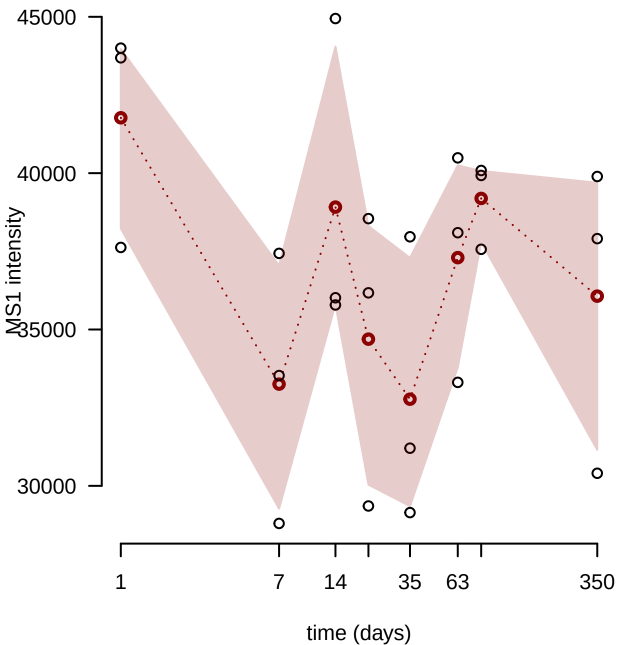

Q8VE62
GN=Paip1
polyadenylate binding protein−interactin ...

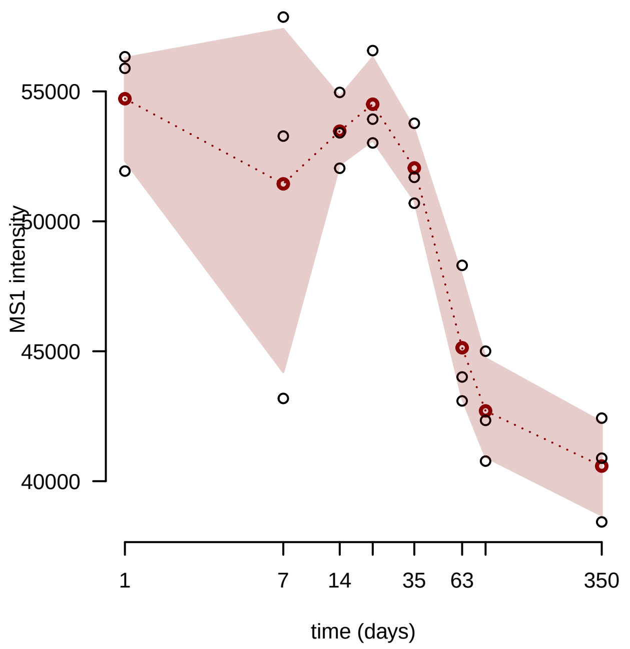

Q8VE65
GN=Taf12
TATA−box binding protein associated fact ...

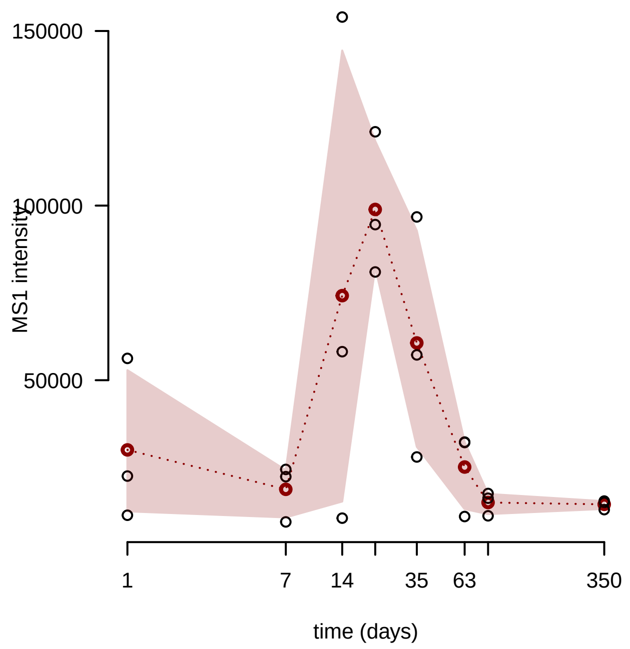

Q8VE70
GN=Pdcd10
programmed cell death 10

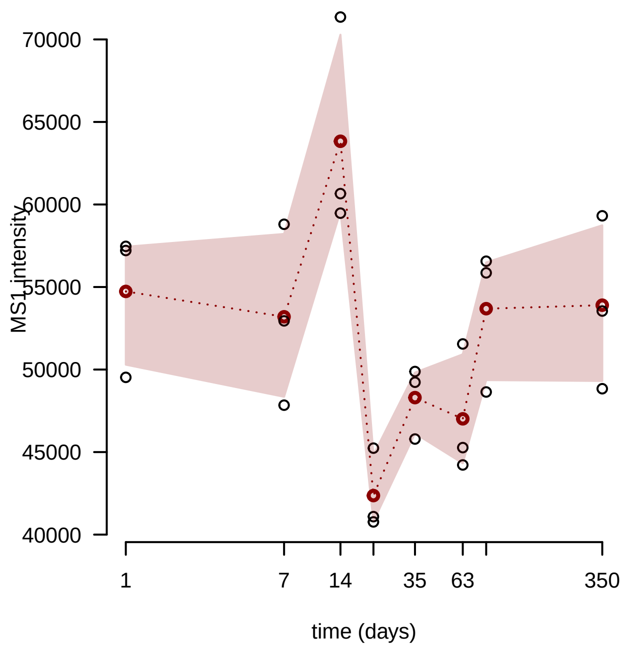

Q8VE73
GN=Cul7
cullin 7

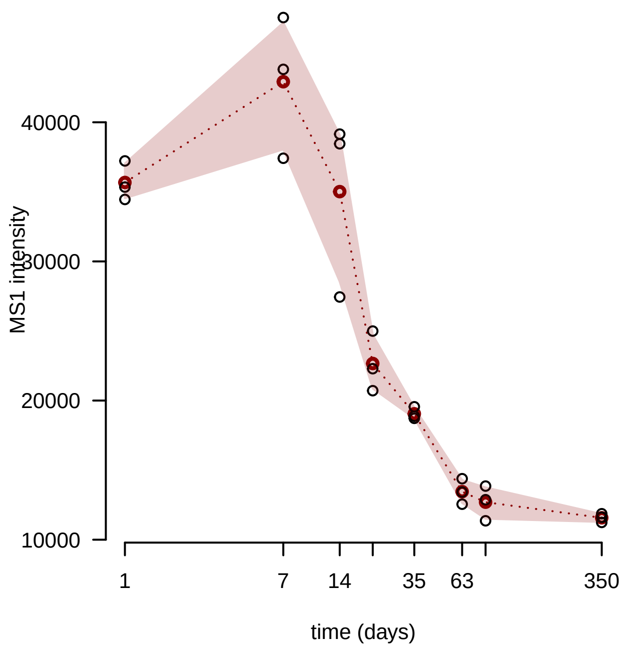

Q8VE80
GN=Thoc3
THO complex 3

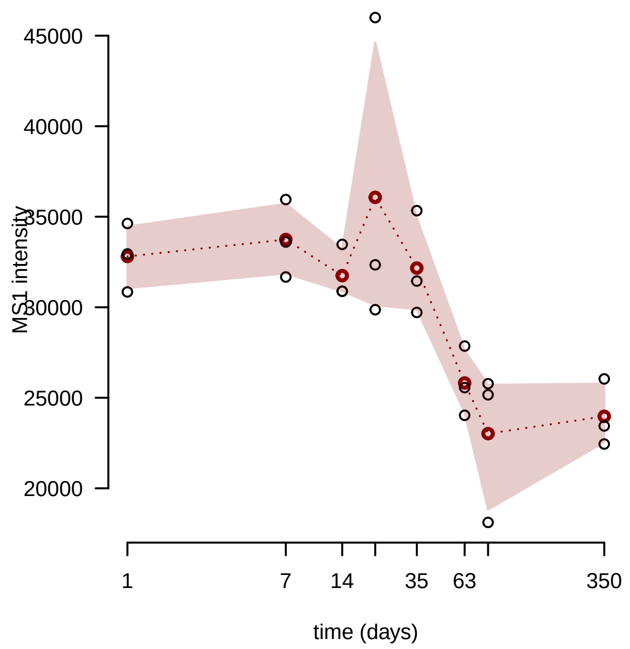

Q8VE88
GN=Fam114a2
family with sequence similarity 114, mem ...

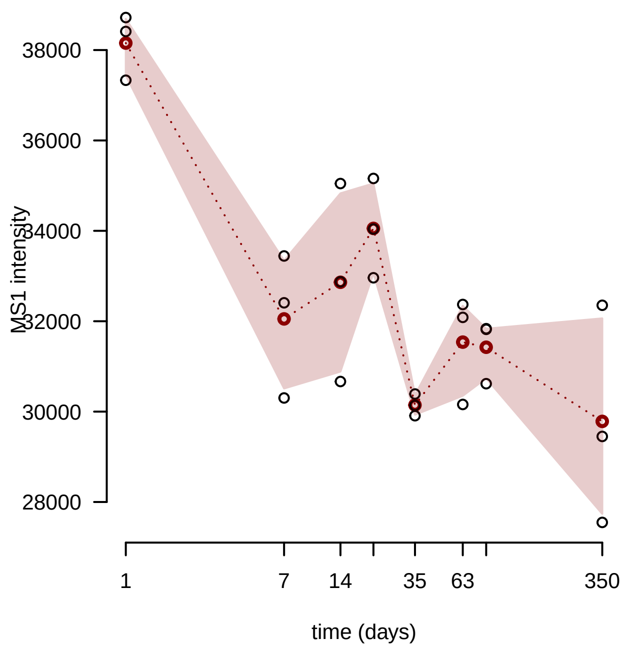

Q8VE91
GN=Retreg1
reticulophagy regulator 1

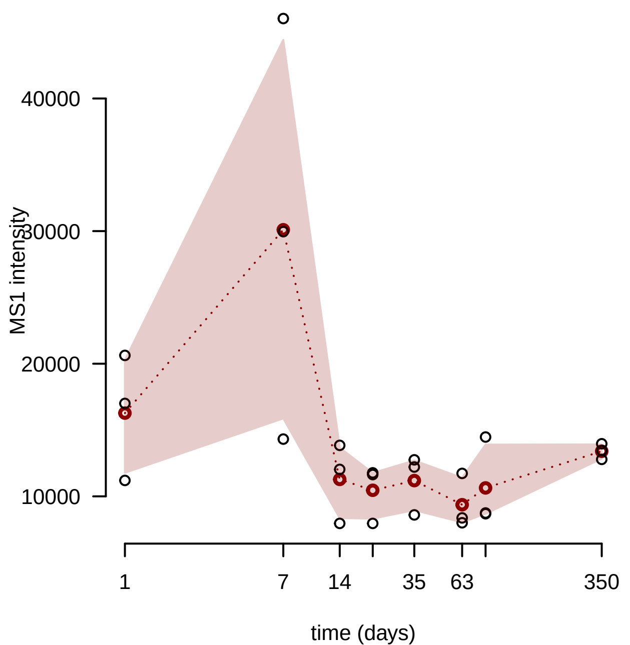

Q8VE92
GN=Rbm4b
RNA binding motif protein 4B

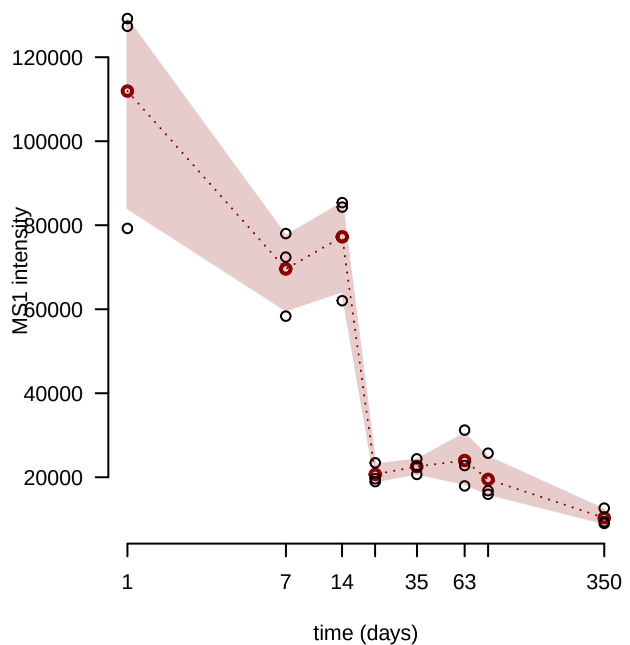

Q8VE94
GN=Fam110c
family with sequence similarity 110, mem ...

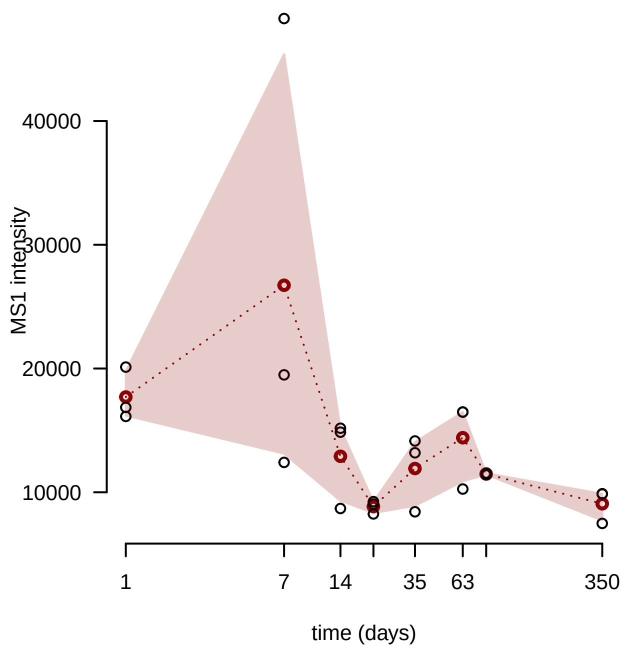

Q8VE95
GN=

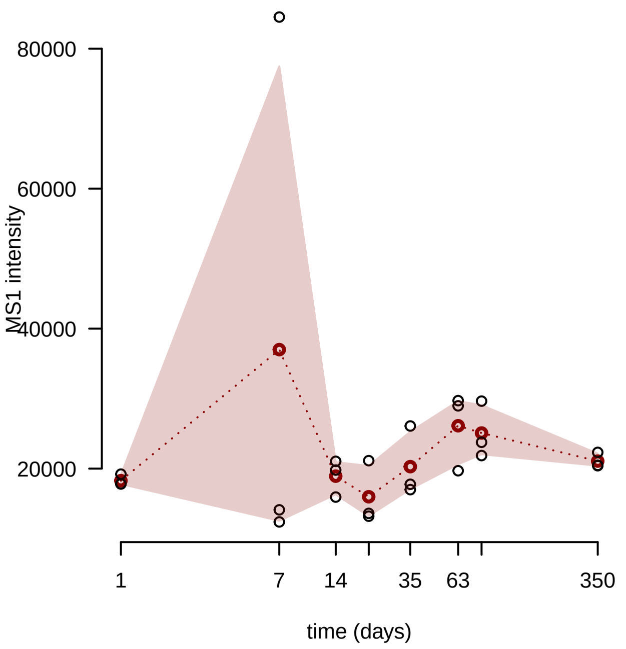

Q8VE96
GN=Slc35f6
solute carrier family 35, member F6

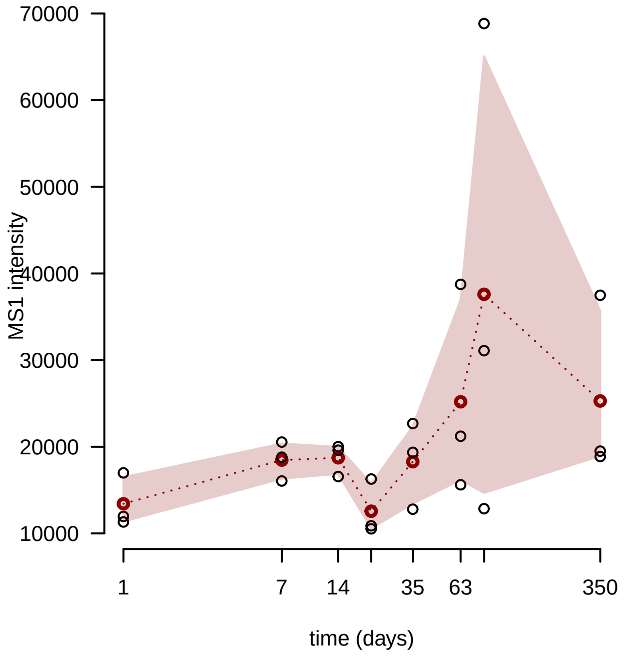

Q8VE97
GN=Srsf4
serine and arginine−rich splicing factor ...

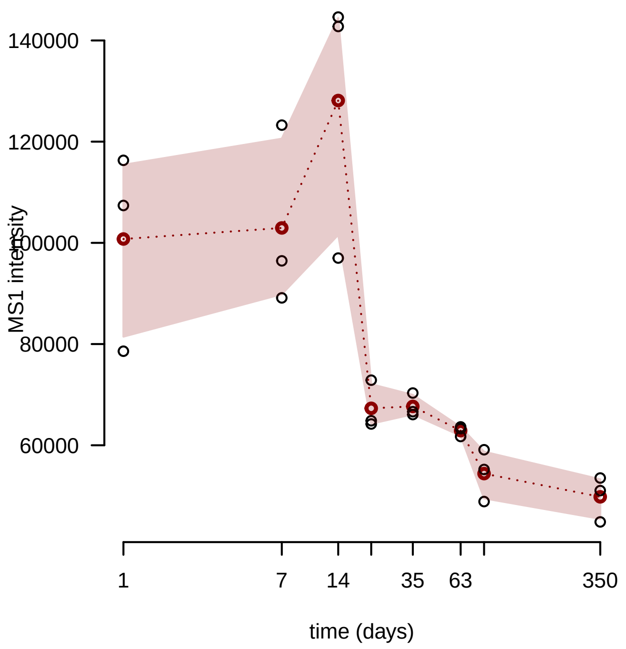

Q8VE98
GN=Cd276
CD276 antigen

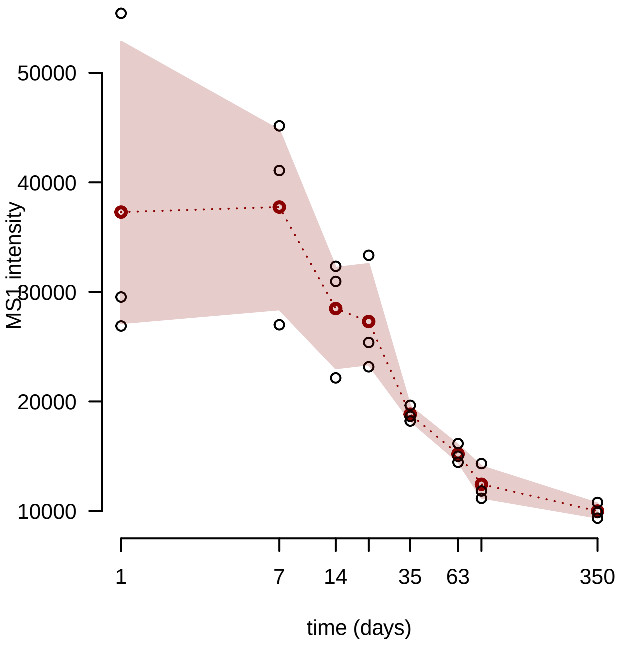

Q8VE99
GN=Ccdc115
coiled−coil domain containing 115

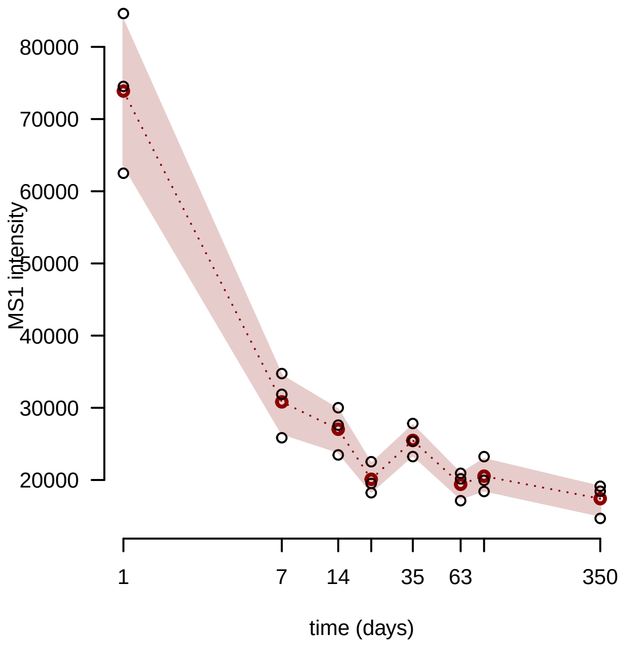

Q8VEA4
GN=Chchd4
coiled−coil−helix−coiled−coil−helix doma ...

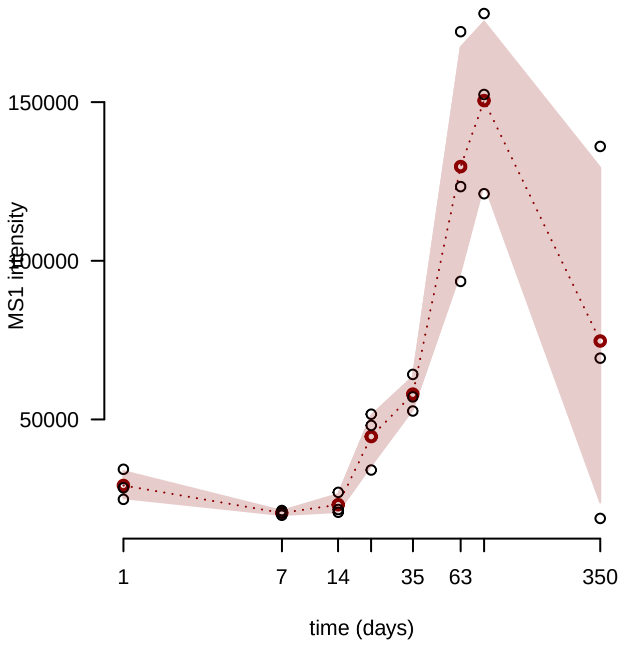

Q8VEB1
GN=Grk5
G protein−coupled receptor kinase 5

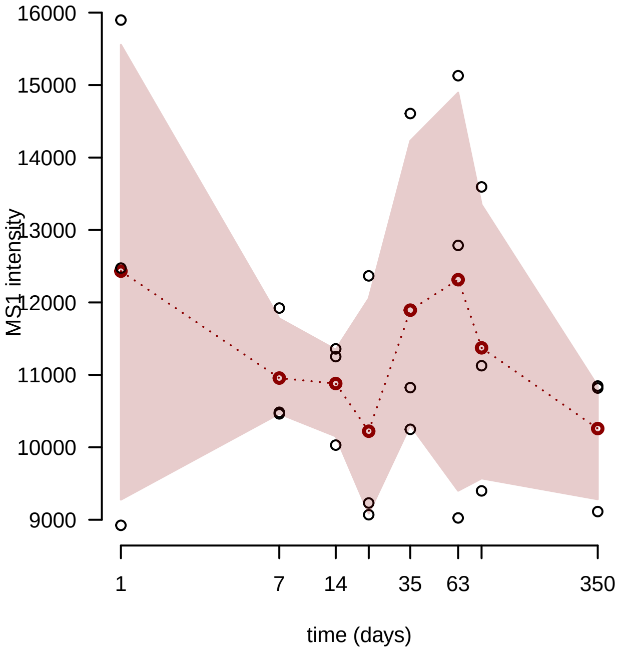

Q8VEB2
GN=Sav1
salvador family WW domain containing 1

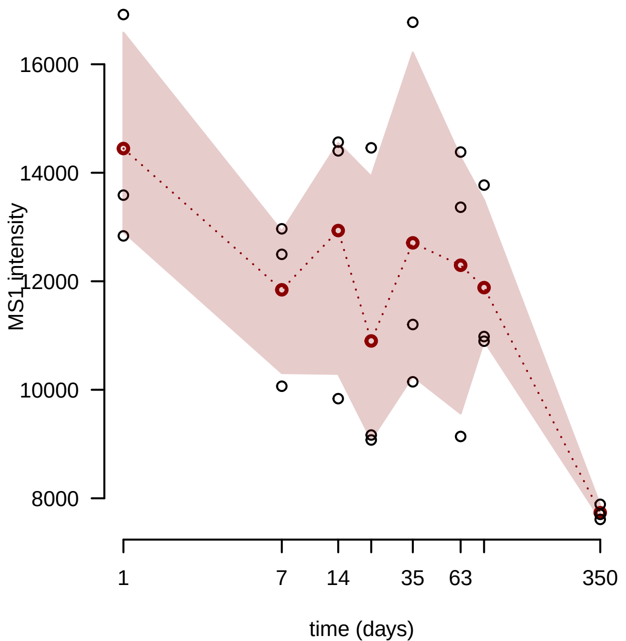

Q8VEB4
GN=Pla2g15
phospholipase A2, group XV

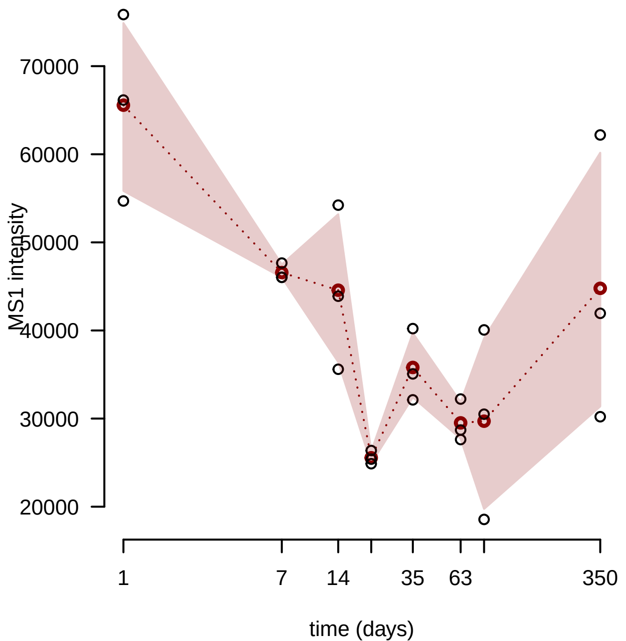

Q8VEB6
GN=Elac1
elaC ribonuclease Z 1

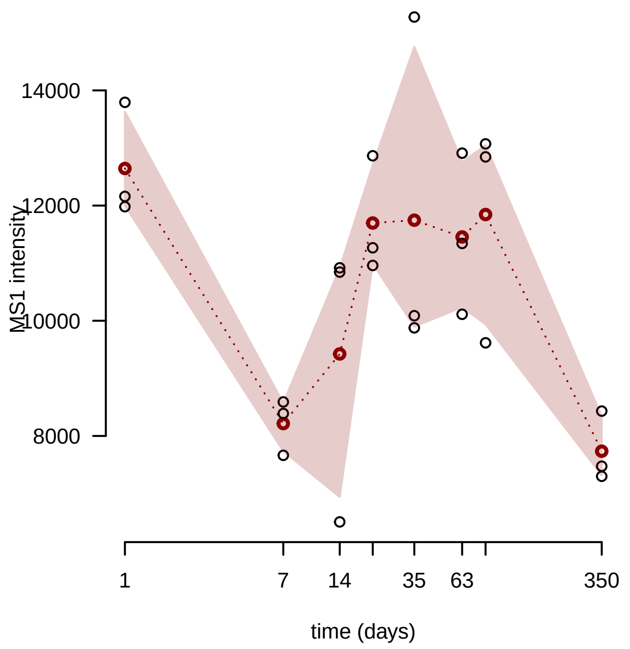

Q8VEC4
GN=Calhm2
calcium homeostasis modulator family mem ...

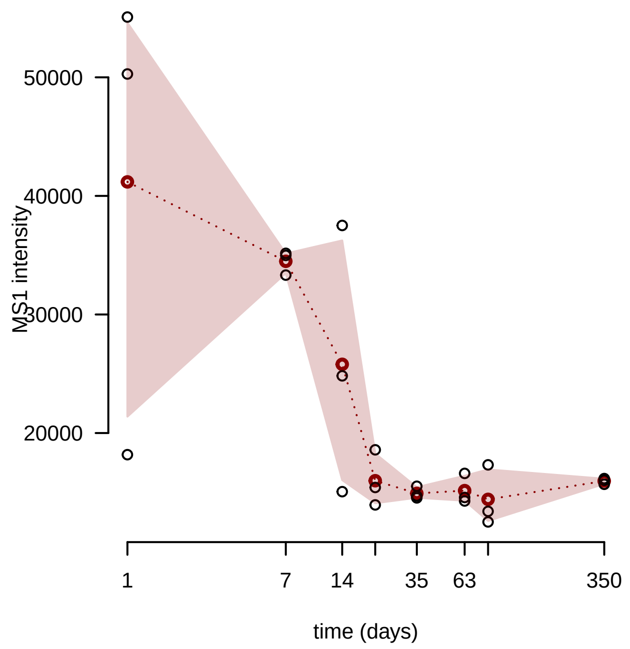

Q8VED2
GN=Bloc1s4
biogenesis of lysosomal organelles compl ...

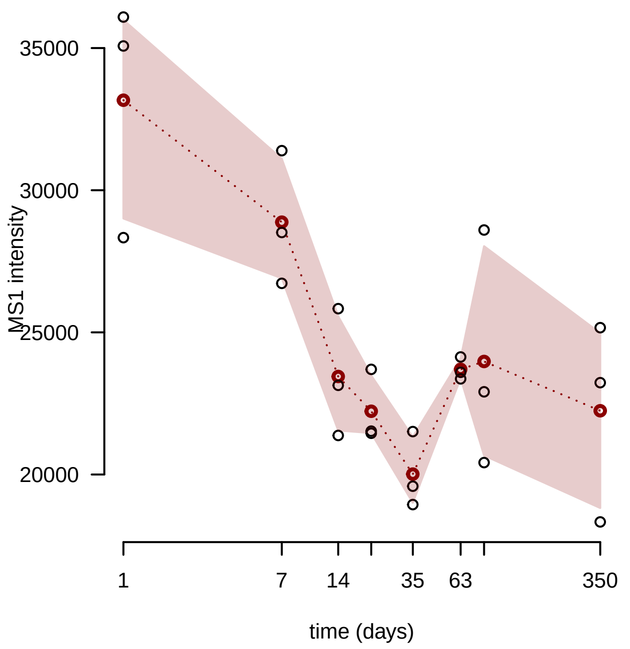

Q8VED5
GN=Krt79
keratin 79

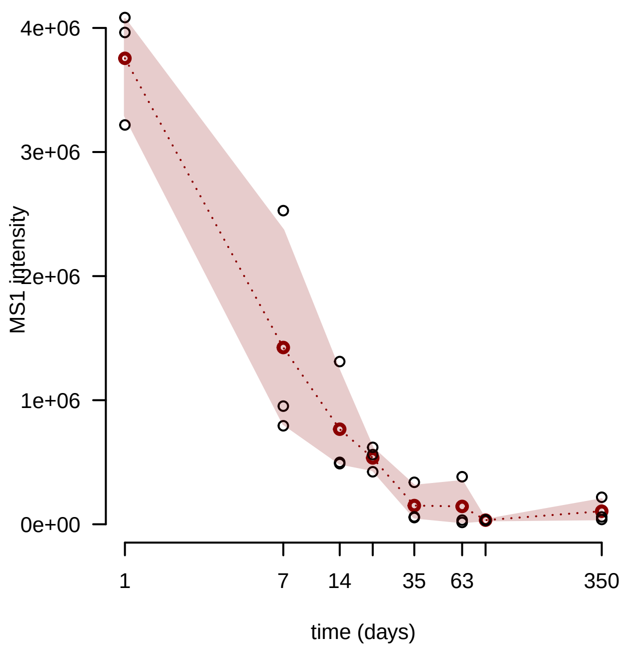

Q8VED9
GN=Lgalsl
galectin like

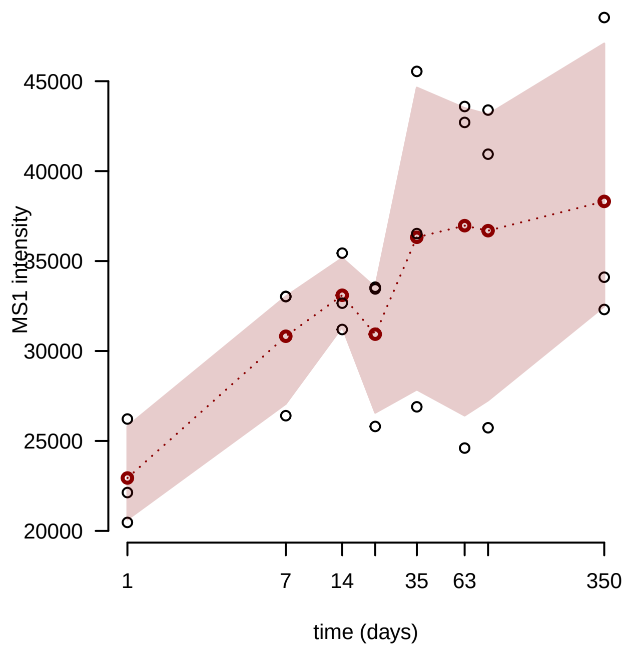

Q8VEE1
GN=Lmcd1
LIM and cysteine−rich domains 1

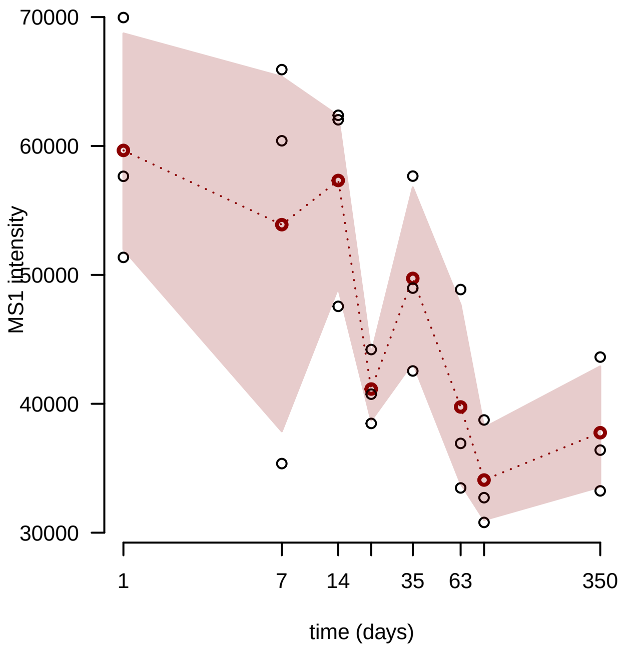

Q8VEE4
GN=Rpa1
replication protein A1

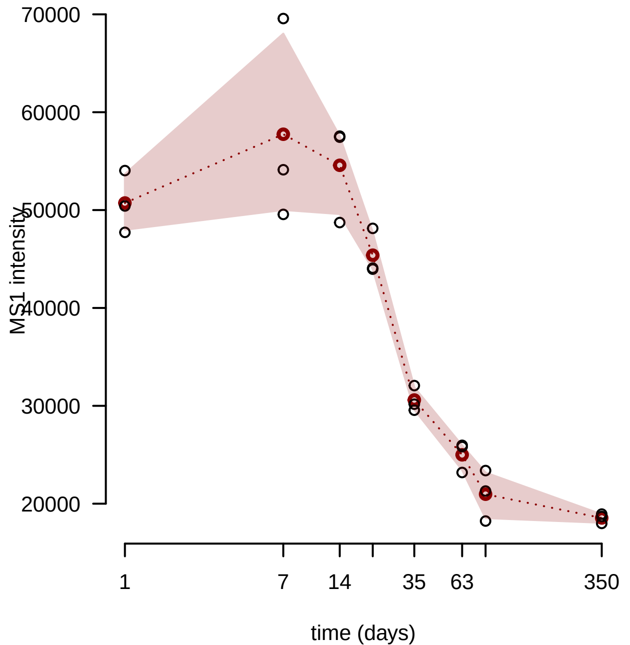

Q8VEF1
GN=Gramd1a
GRAM domain containing 1A

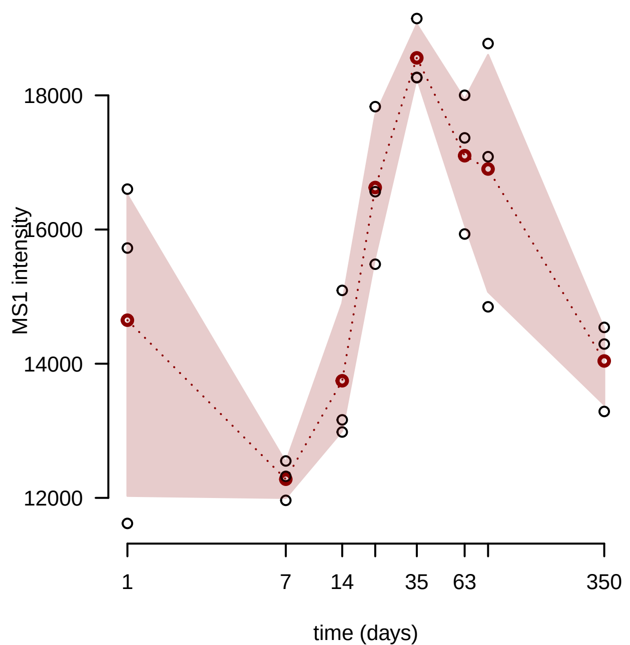

Q8VEG0
GN=Ccdc71
coiled−coil domain containing 71

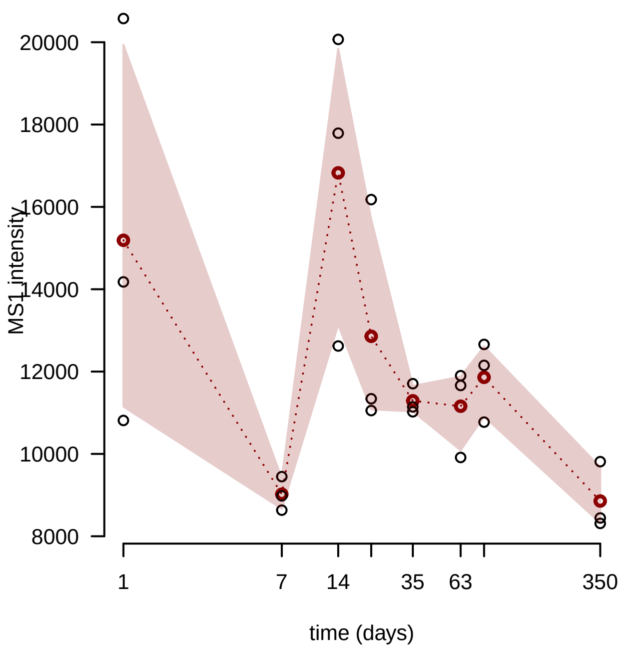

Q8VEG4
GN=Exd2
exonuclease 3'−5' domain containing 2

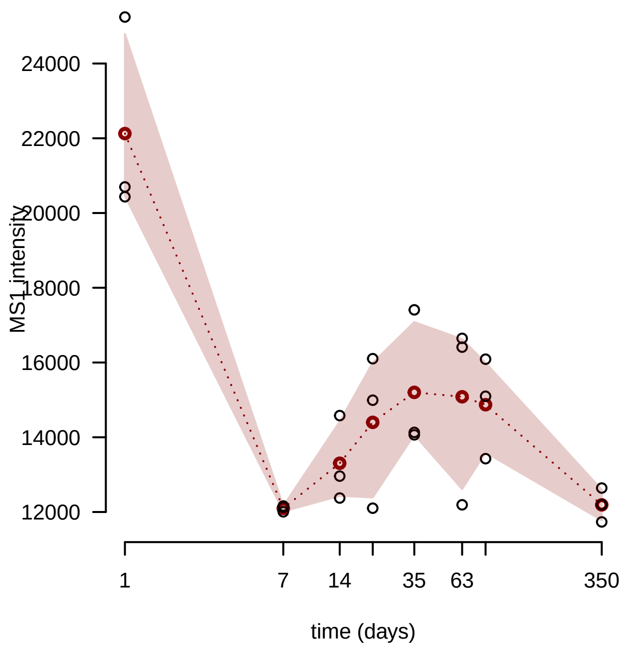

Q8VEG6
GN=Cnot6l
CCR4−NOT transcription complex, subunit ...

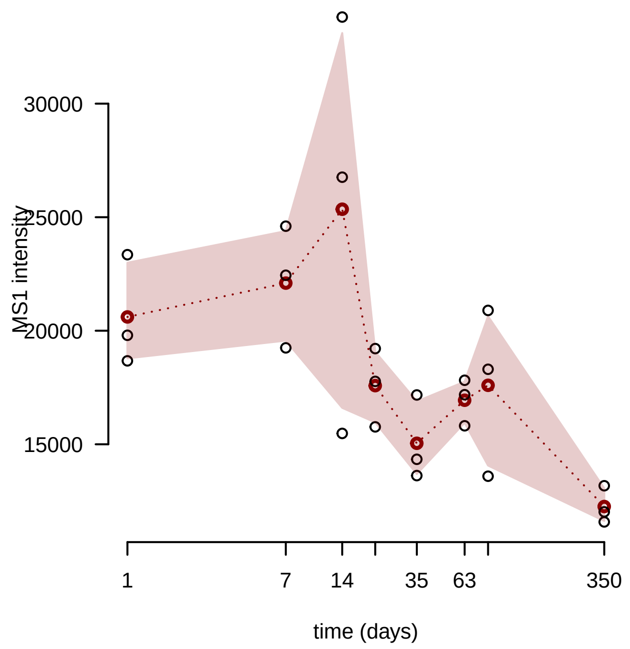

Q8VEH3
GN=Arl8a
ADP−ribosylation factor−like 8A

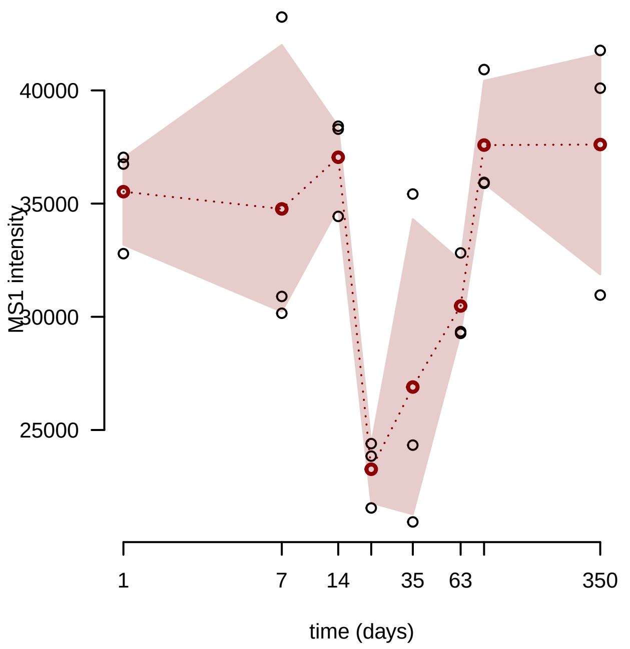

Q8VEH5
GN=Epm2aip1
EPM2A (laforin) interacting protein 1

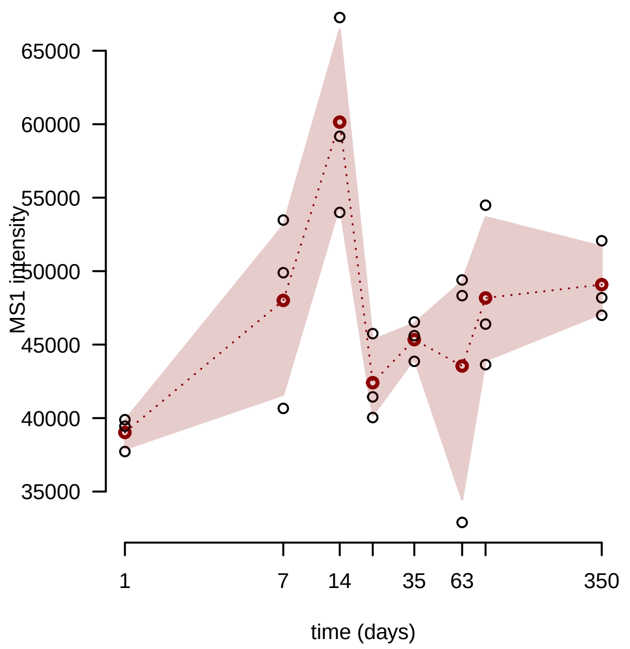

Q8VEH6
GN=Cbwd1
COBW domain containing 1

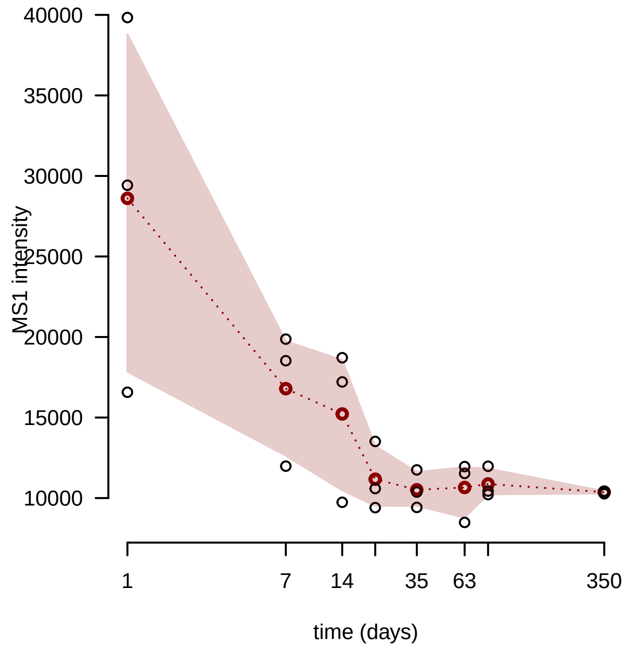

Q8VEH8
GN=Erlec1
endoplasmic reticulum lectin 1

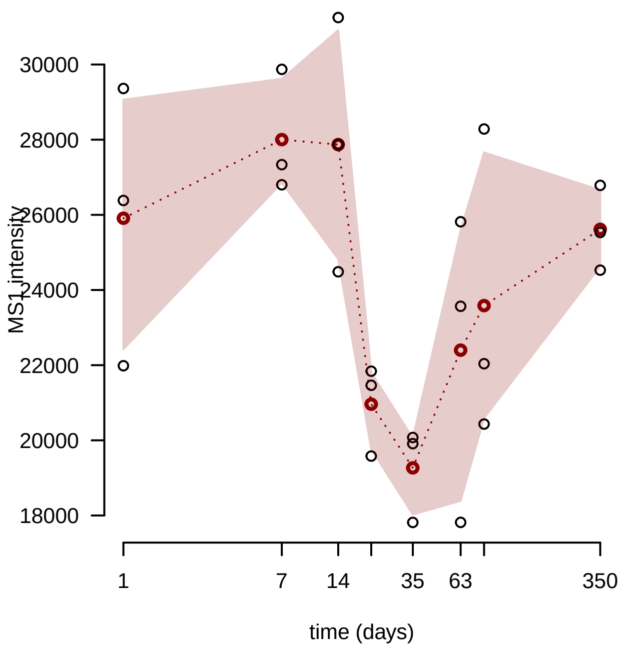

Q8VEJ4
GN=Nle1
notchless homolog 1

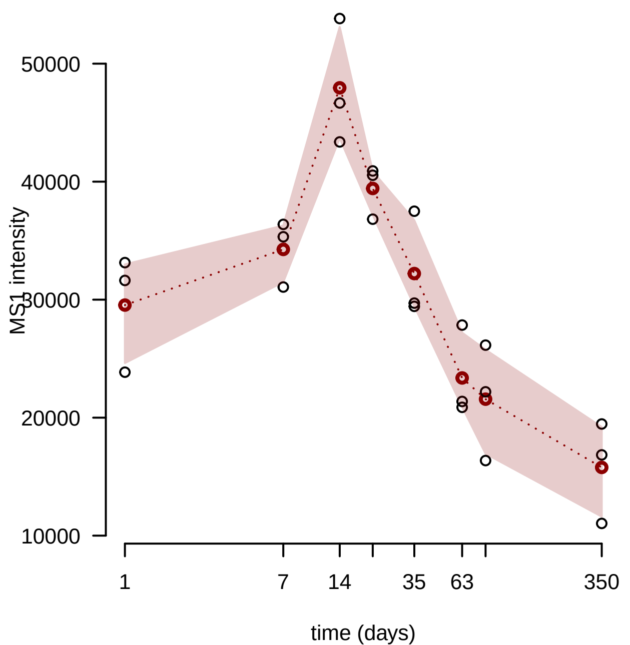

Q8VEJ9
GN=Vps4a
vacuolar protein sorting 4A

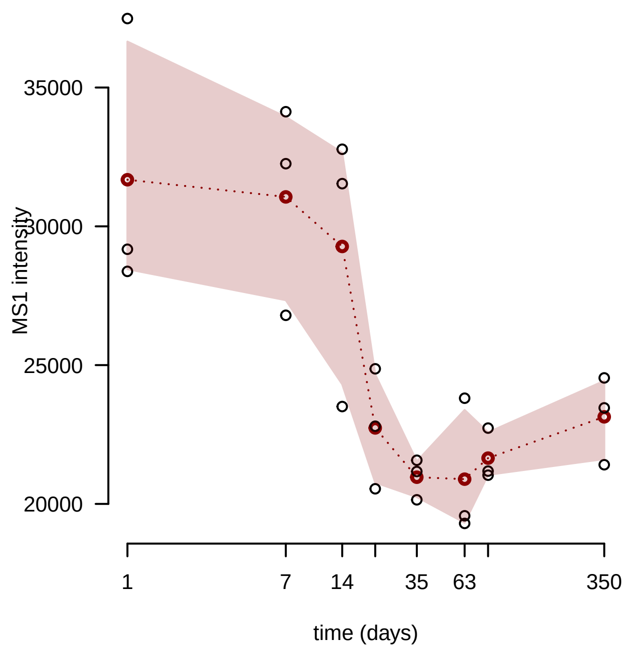

Q8VEK0
GN=Tmem30a
transmembrane protein 30A

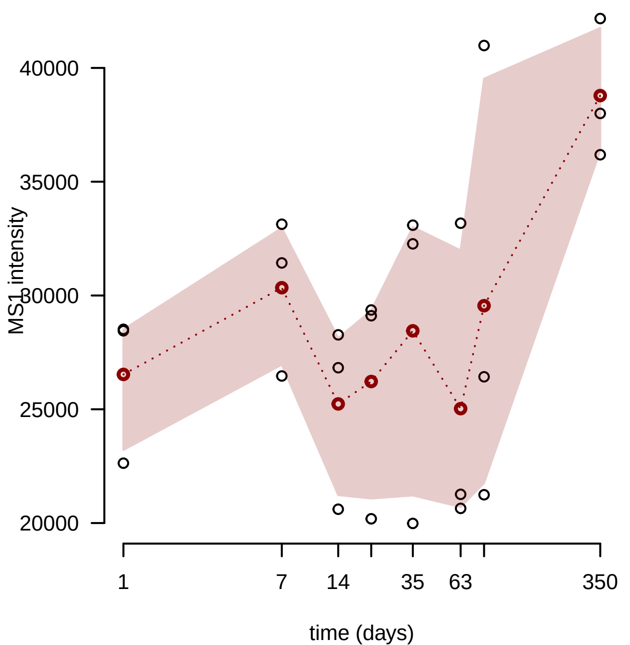

Q8VEK2
GN=Rhbdd2
rhomboid domain containing 2

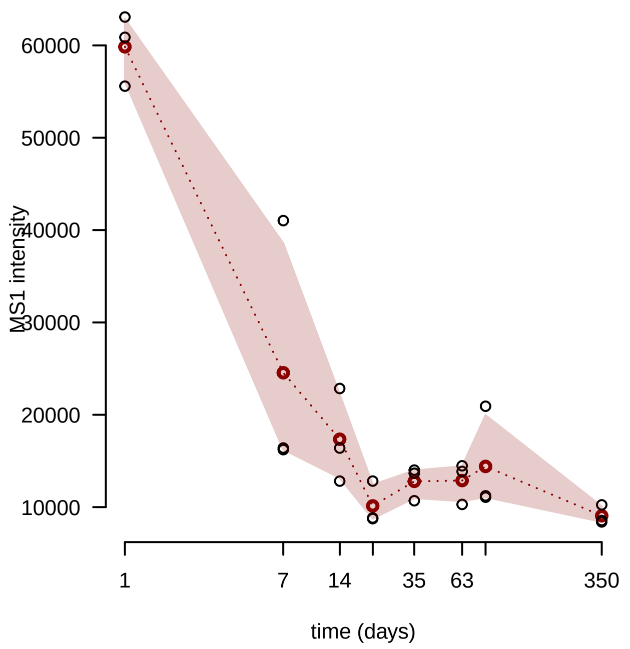

Q8VEK3
GN=Hnrnpu
heterogeneous nuclear ribonucleoprotein ...

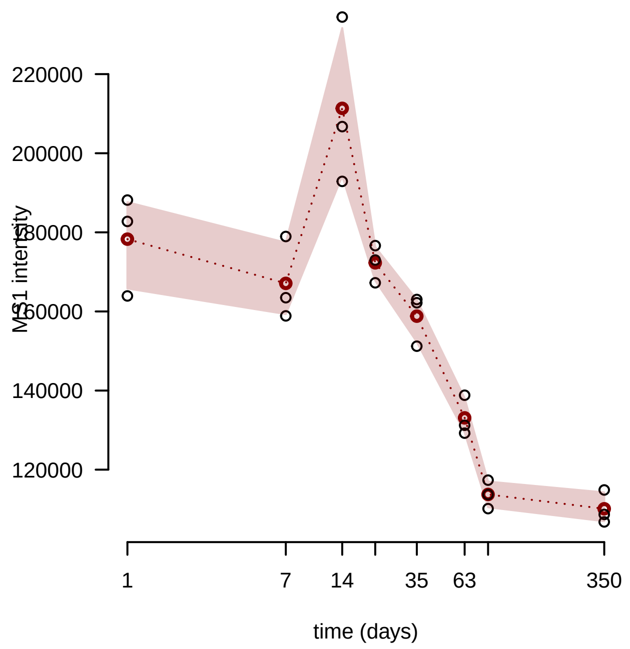

Q8VEK6
GN=Ing3
inhibitor of growth family, member 3

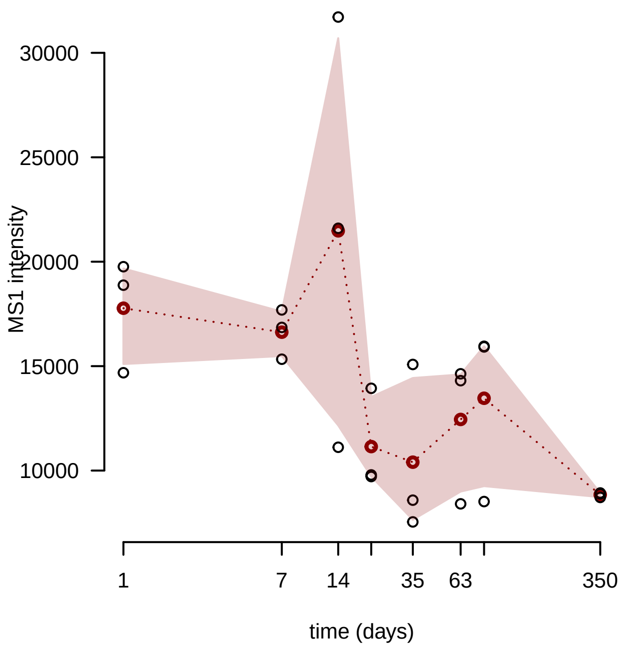

Q8VEL0
GN=Mospd1
motile sperm domain containing 1

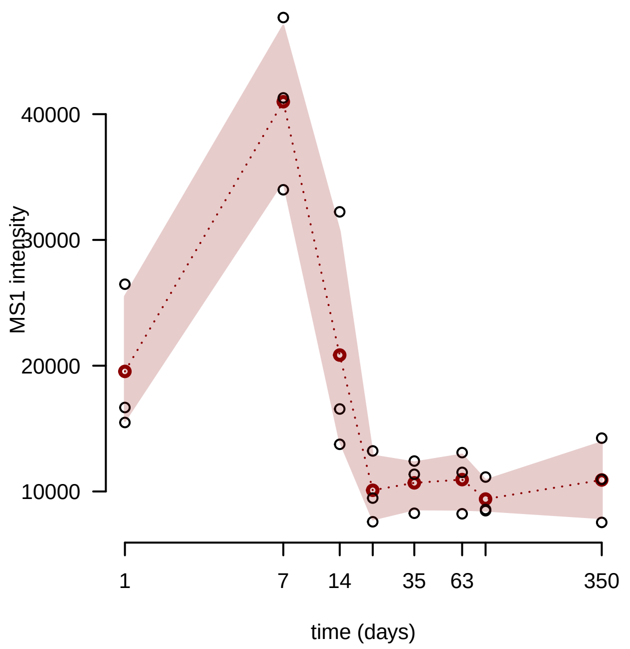

Q8VEL2
GN=Mtmr14
myotubularin related protein 14

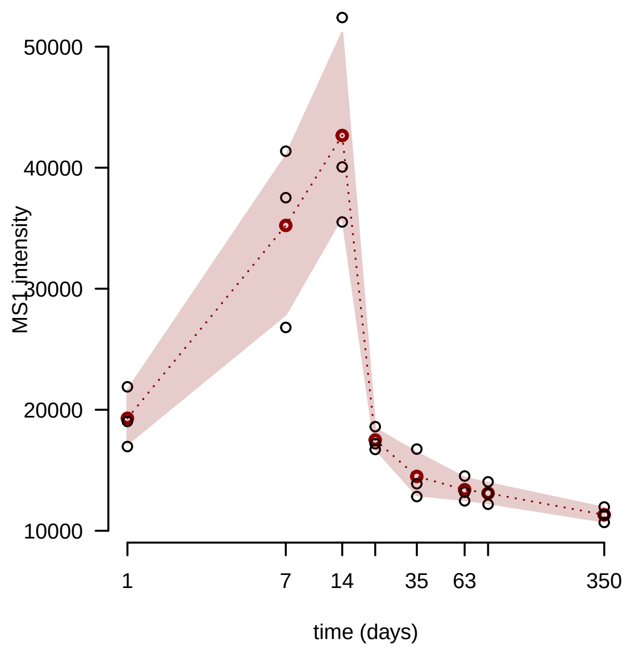

Q8VEM8
GN=Slc25a3
solute carrier family 25 (mitochondrial ...

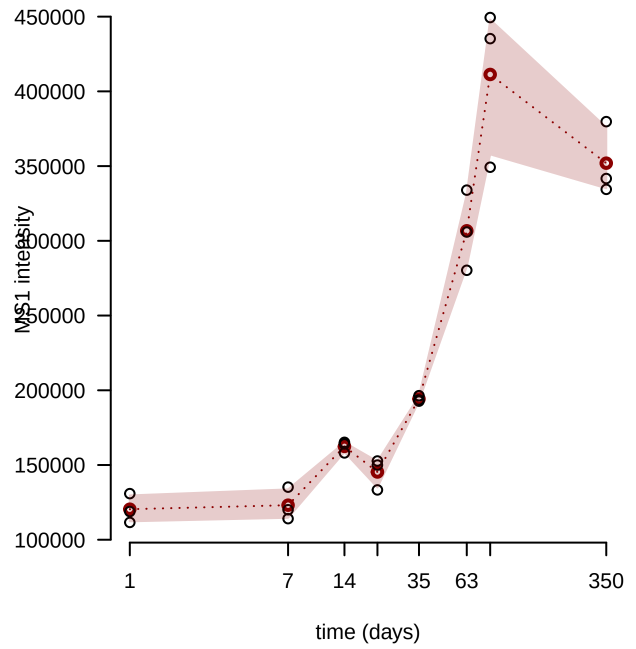

Q8VGD6
GN=Olfr56

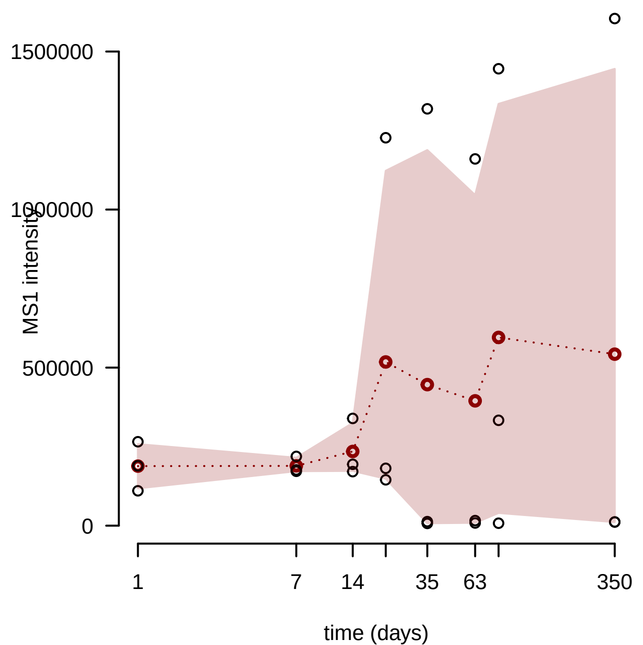

Q8VH37
GN=Hdac8
histone deacetylase 8

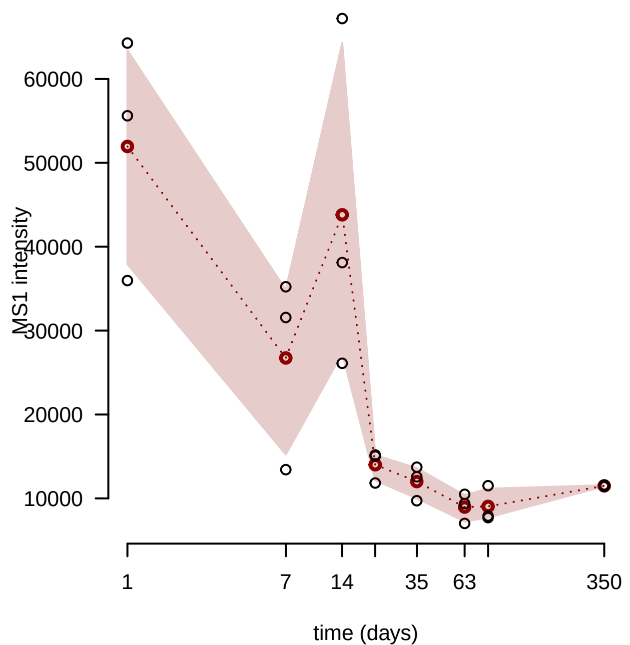

Q8VH51
GN=Rbm39
RNA binding motif protein 39

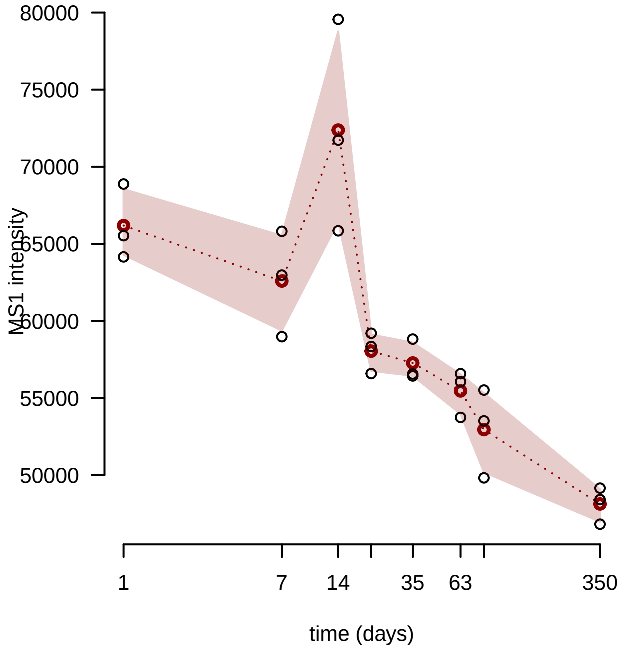

Q8VHC3
GN=Selenom
selenoprotein M

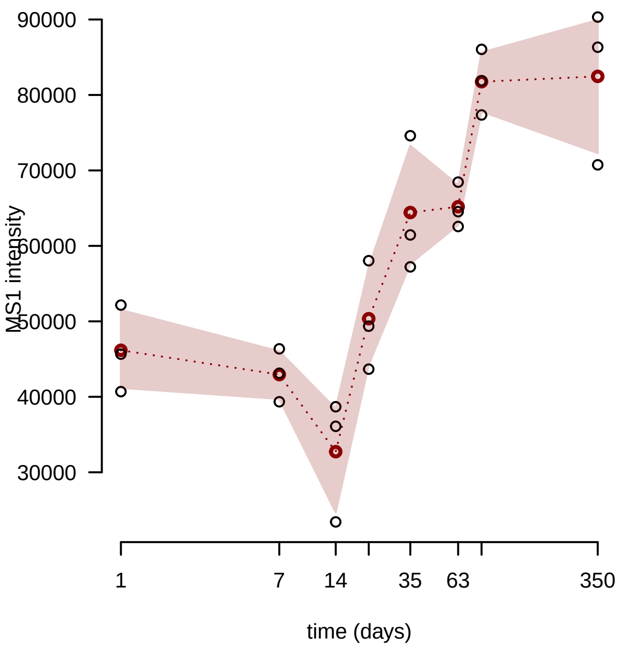

Q8VHD8
GN=Hrnr
hornerin

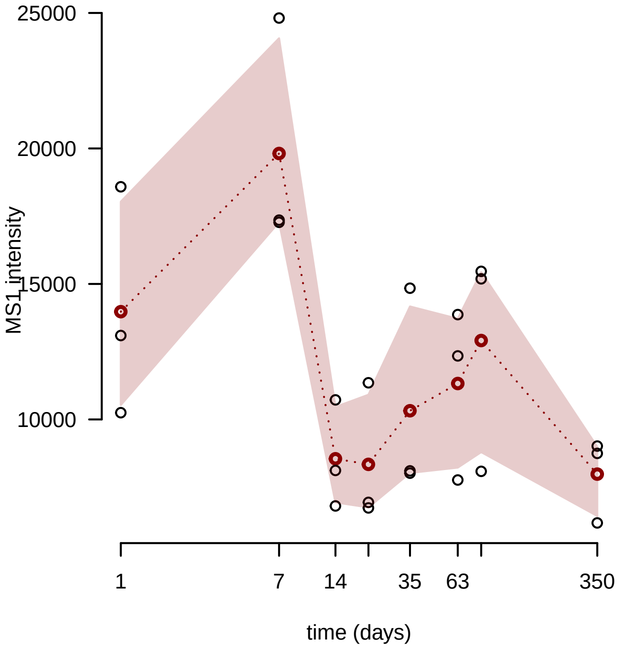

Q8VHE0
GN=Sec63
SEC63−like (S. cerevisiae)

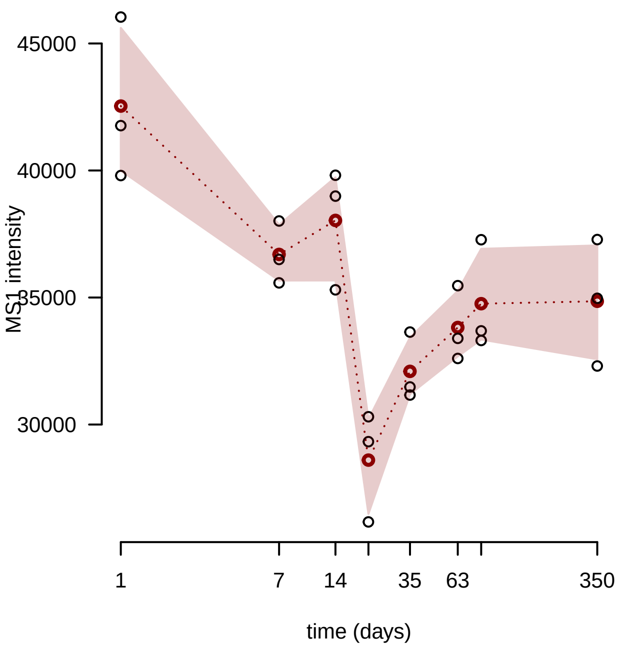

Q8VHG2
GN=Amot
angiomotin

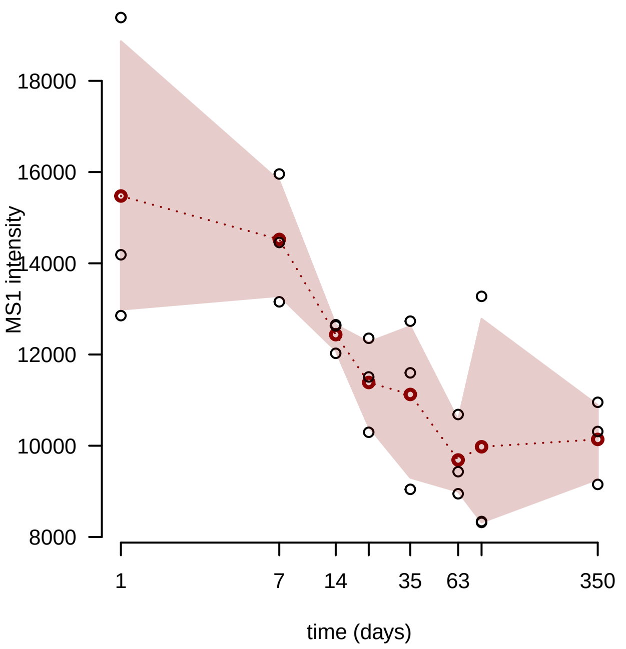

Q8VHH5
GN=Agap3
ArfGAP with GTPase domain, ankyrin repea ...

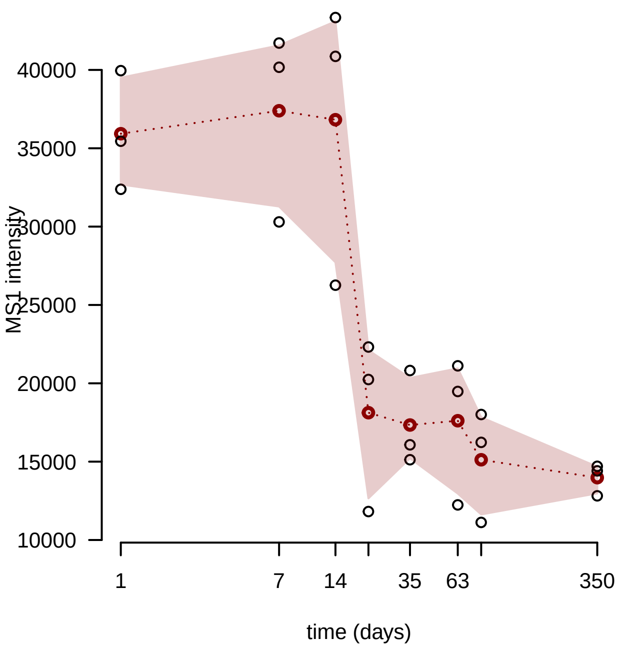

Q8VHH7
GN=Adcy3
adenylate cyclase 3

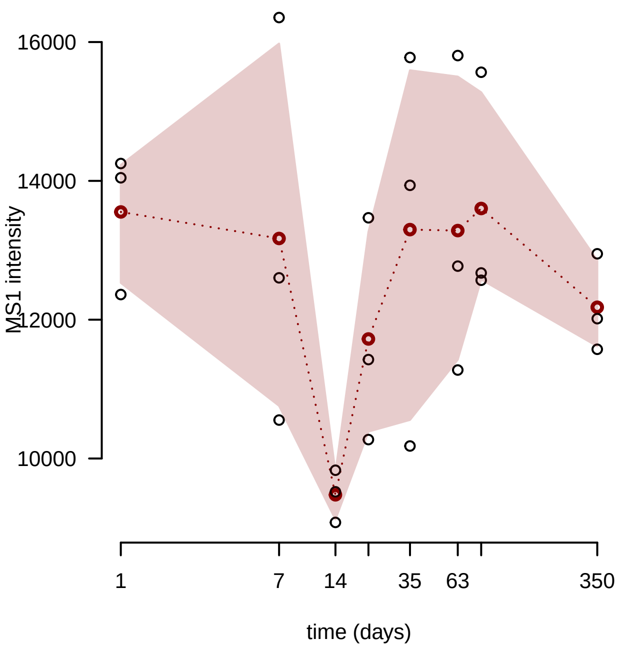

Q8VHI3
GN=Pofut2
protein O−fucosyltransferase 2

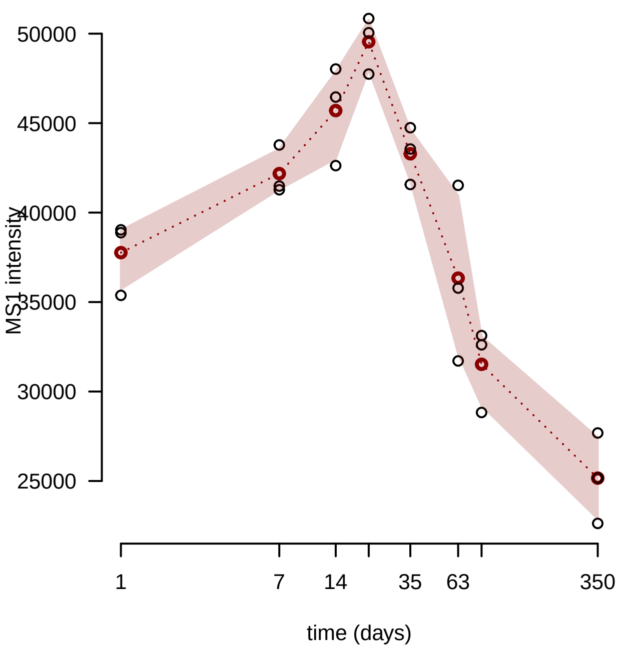

Q8VHJ5
GN=Mark1
MAP/microtubule affinity regulating kina ...

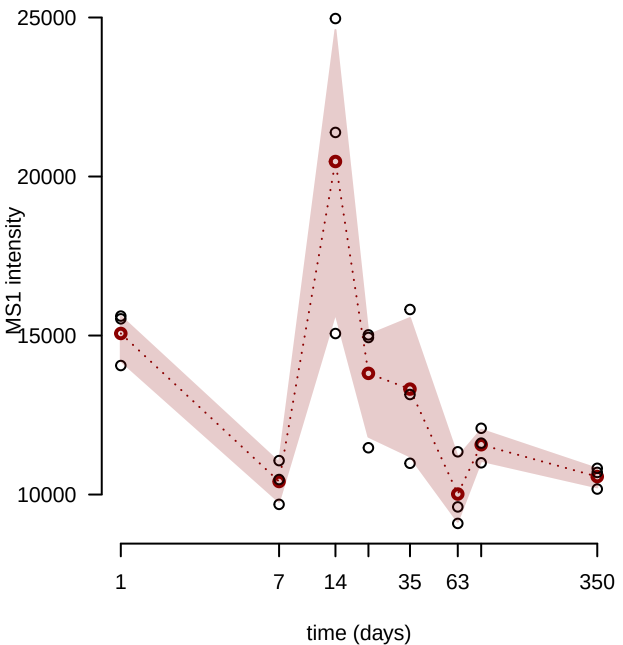

Q8VHK1
GN=Caskin2
CASK−interacting protein 2

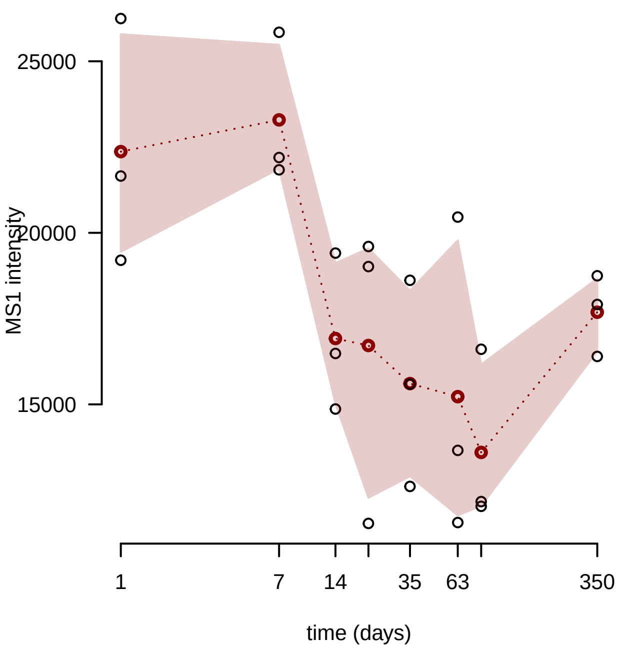

Q8VHK9
GN=Dhx36
DEAH (Asp−Glu−Ala−His) box polypeptide 3 ...

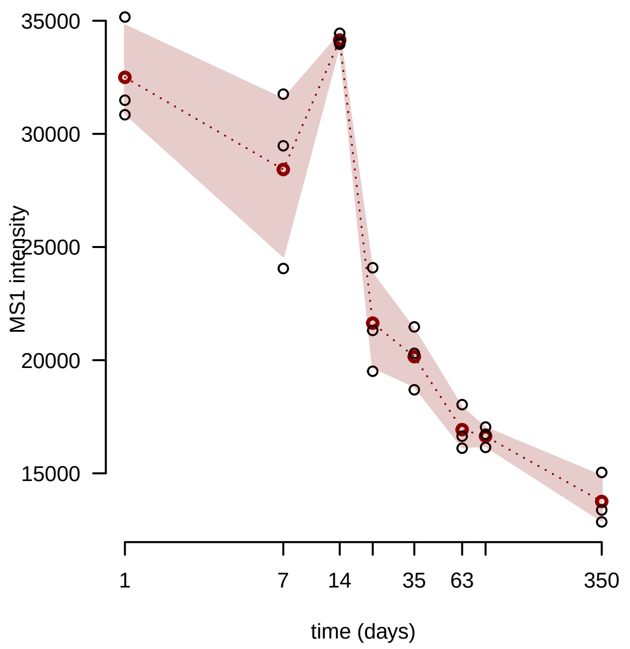

Q8VHL0
GN=Slc14a1
solute carrier family 14 (urea transport ...

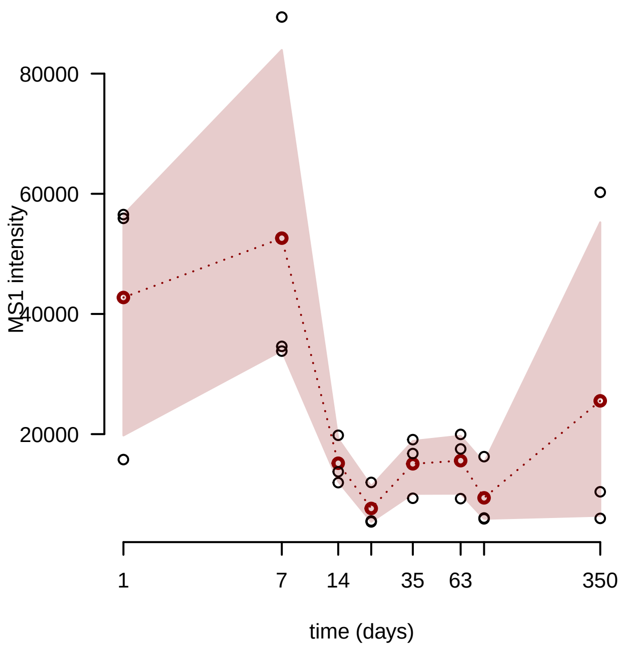

Q8VHL1
GN=Setd7
SET domain containing (lysine methyltran ...

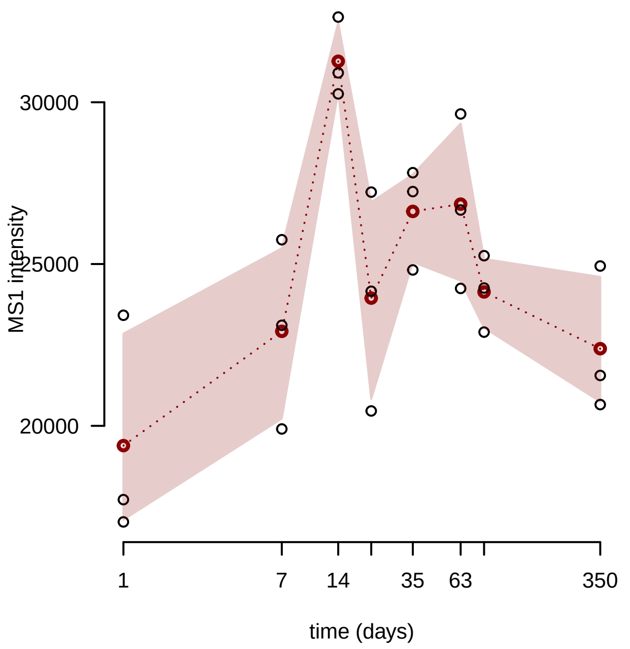

Q8VHN7
GN=Adgrv1
adhesion G protein−coupled receptor V1

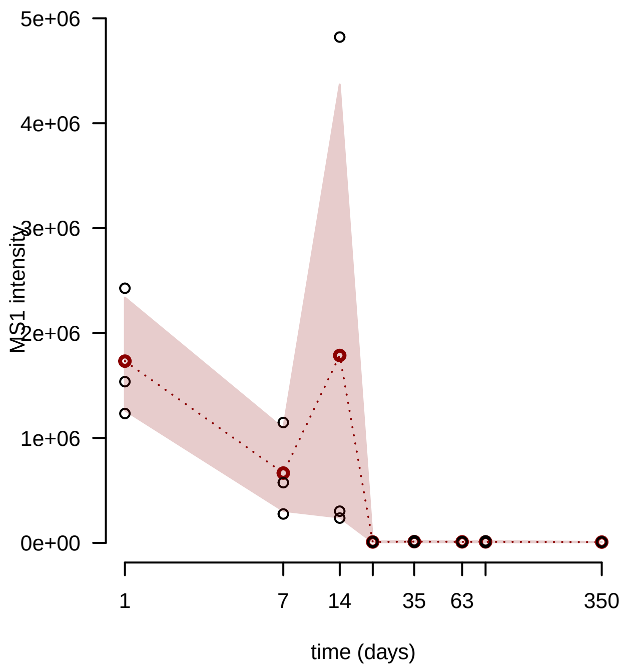

Q8VHN8
GN=Nudt16l1
nudix (nucleoside diphosphate linked moi ...

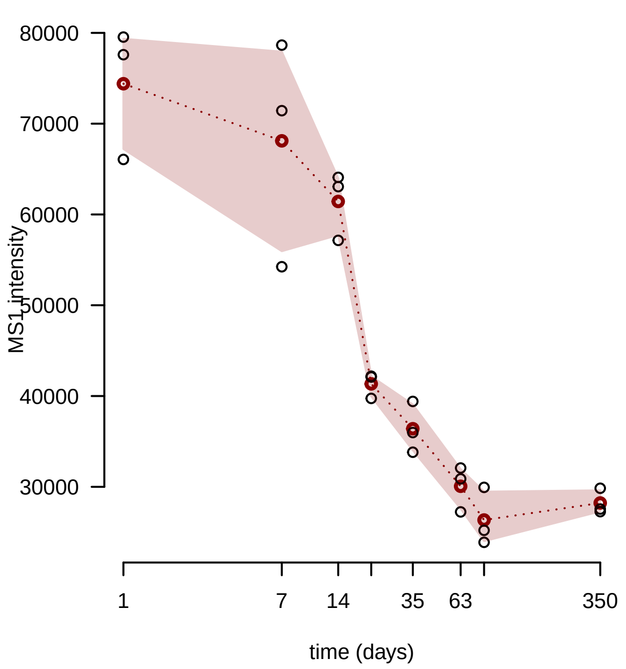

Q8VHQ9
GN=Acot11
acyl−CoA thioesterase 11

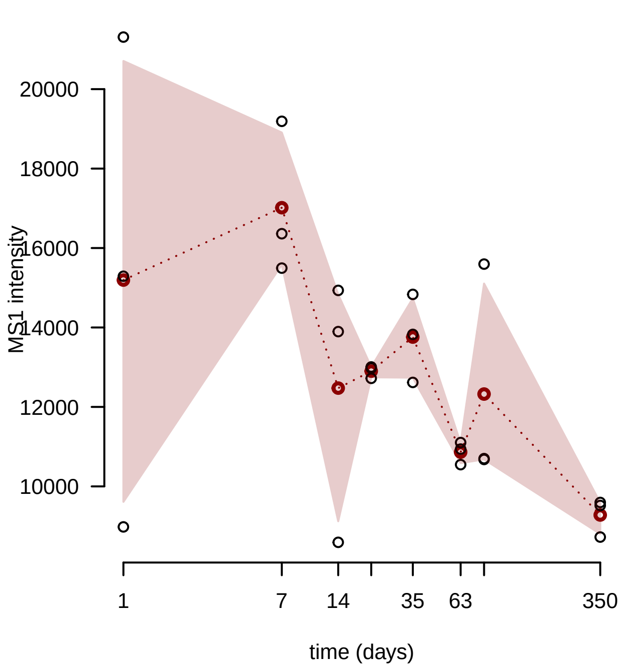

Q8VHR5
GN=Gatad2b
GATA zinc finger domain containing 2B

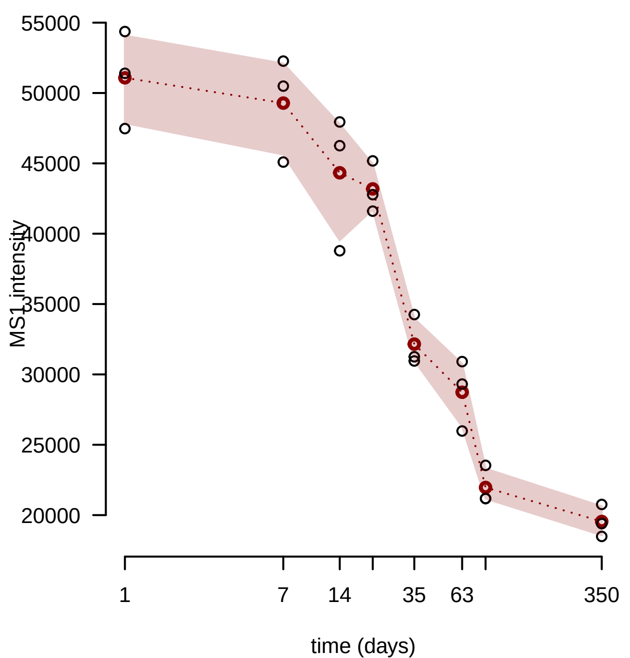

Q8VHX6
GN=Flnc
filamin C, gamma

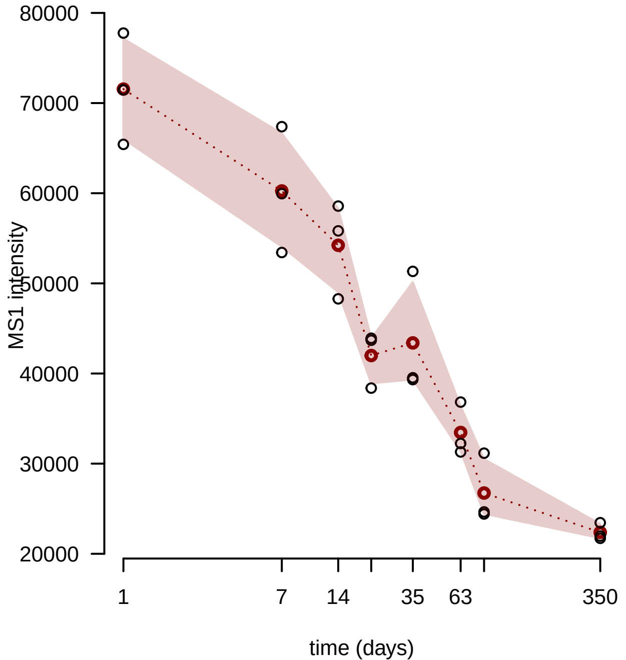

Q8VHY0
GN=Cspg4
chondroitin sulfate proteoglycan 4

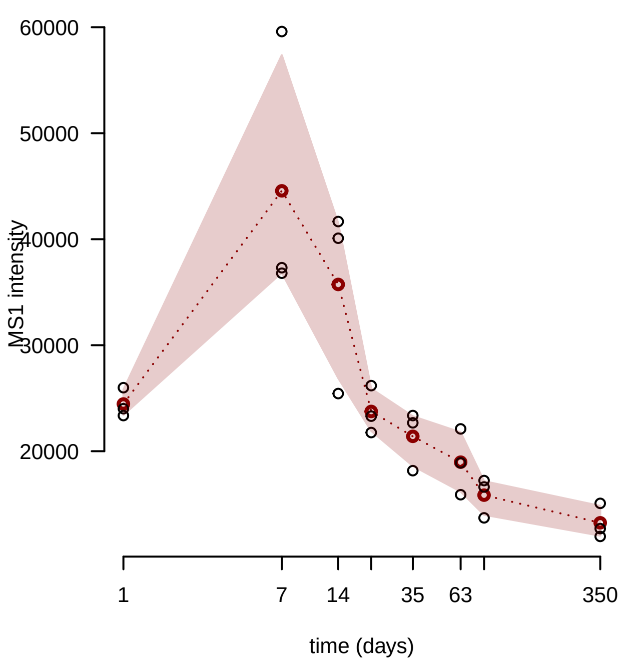

Q8VHZ7
GN=Imp4
IMP4, U3 small nucleolar ribonucleoprote ...

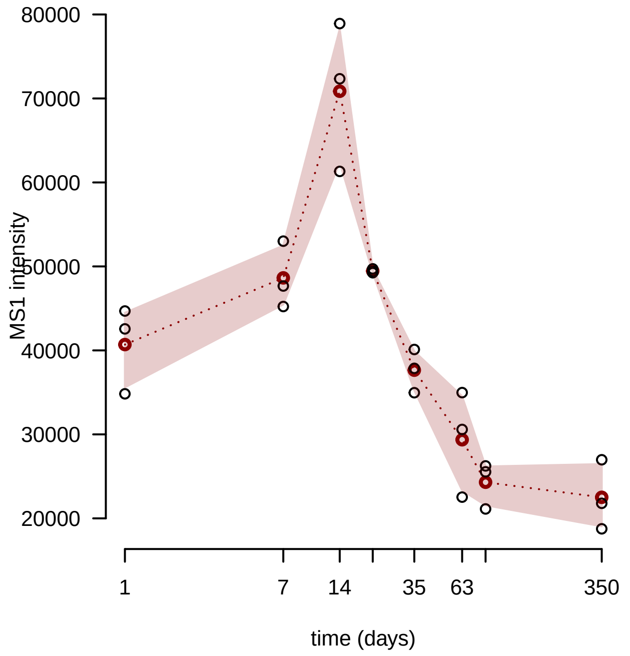

Q8VI33
GN=Taf9
TATA−box binding protein associated fact ...

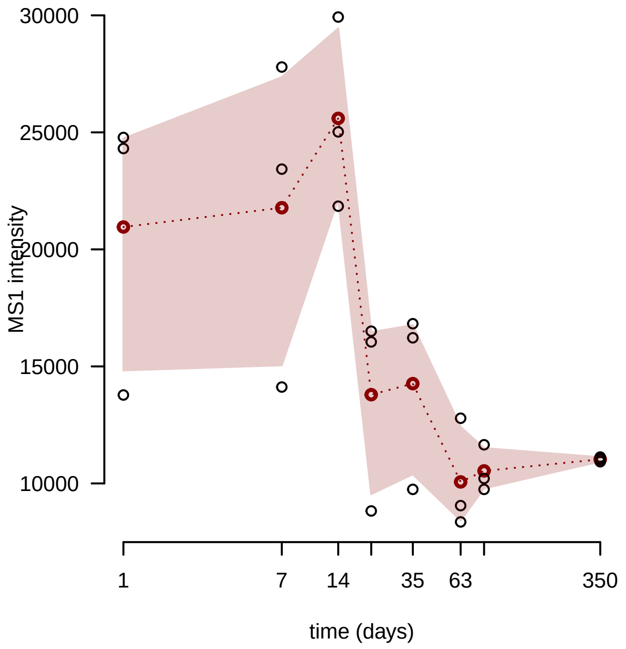

Q8VI36
GN=Pxn
paxillin

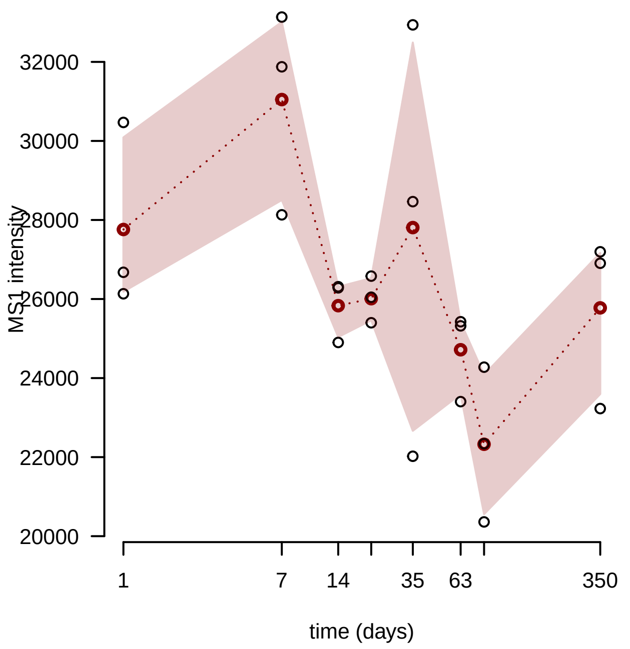

Q8VI59
GN=Pcnx3
pecanex homolog 3

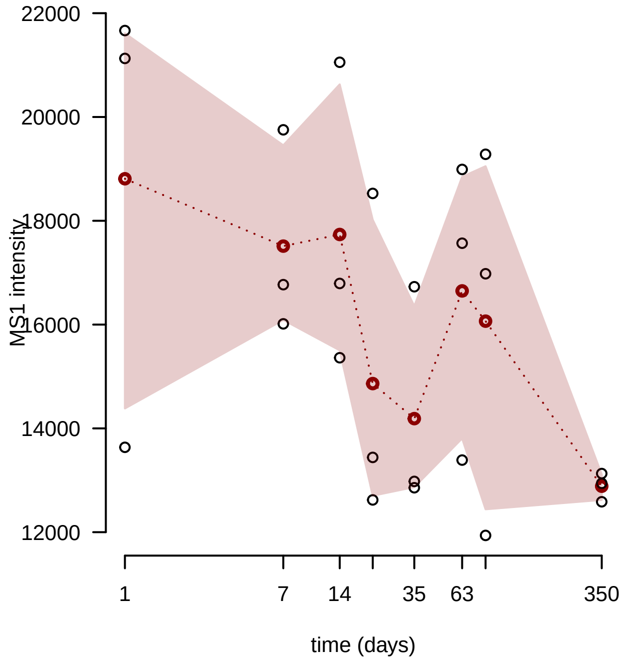

Q8VI63
GN=Mob2
MOB kinase activator 2

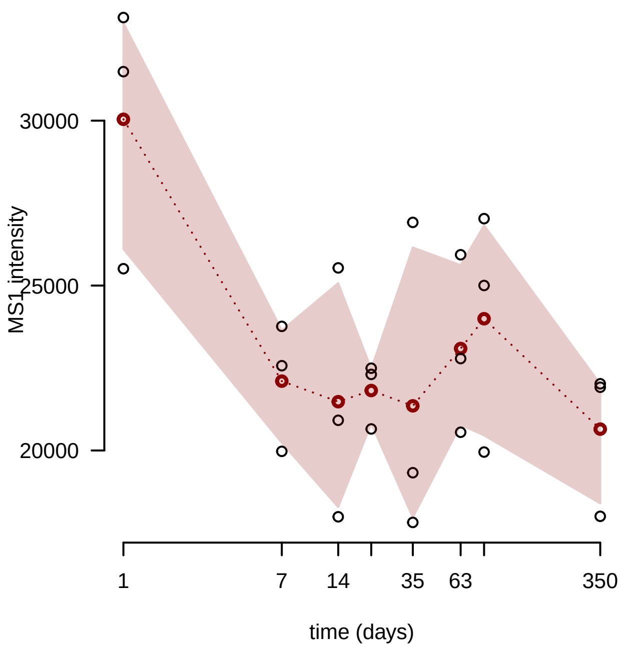

Q8VI64
GN=Mgarp
mitochondria localized glutamic acid ric ...

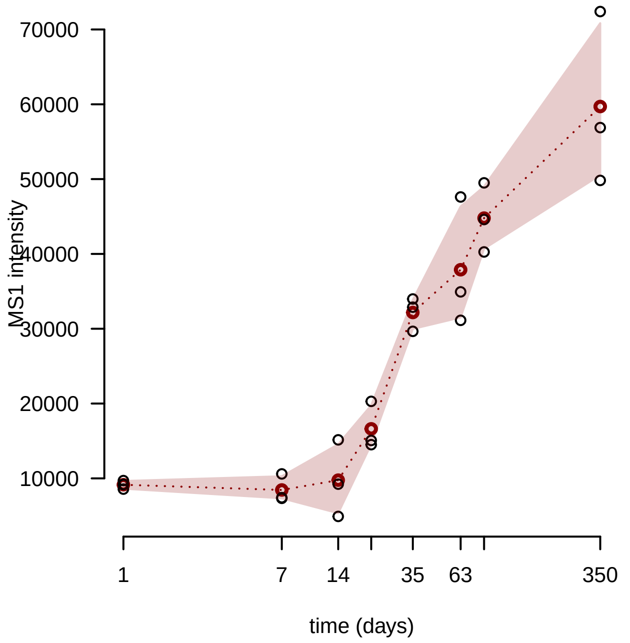

Q8VI75
GN=Ipo4
importin 4

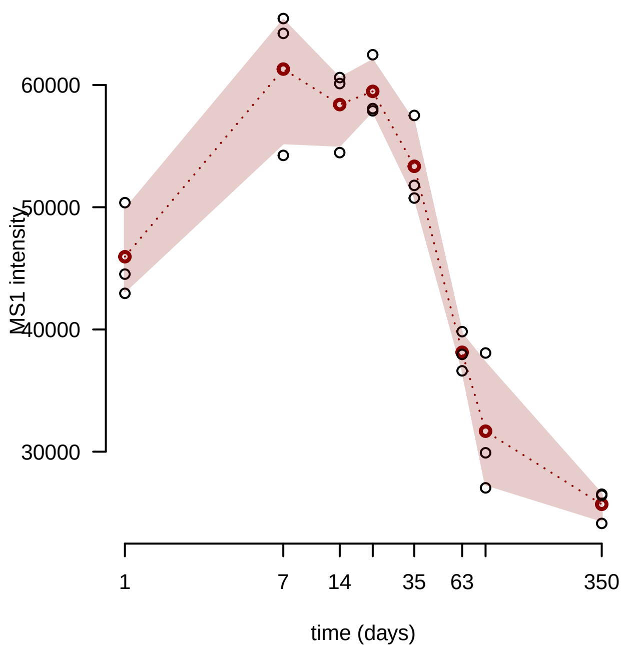

Q8VI84
GN=Noc3l
NOC3 like DNA replication regulator

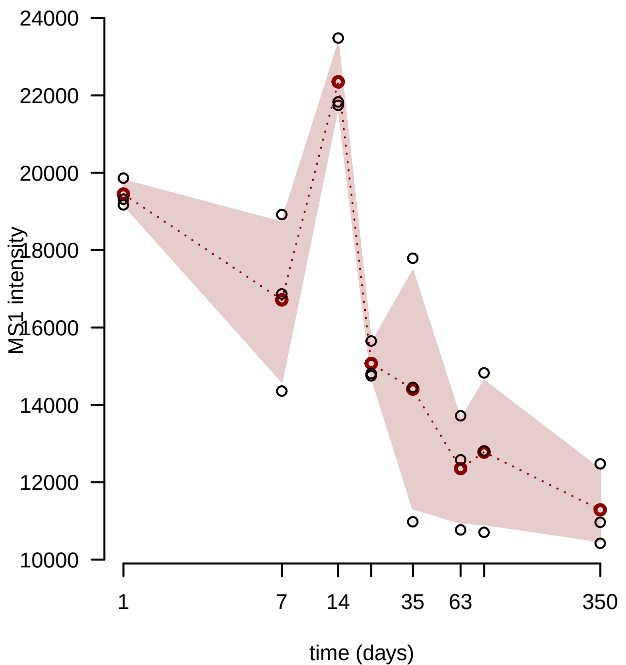

Q8VI95
GN=Oas1d
2'−5' oligoadenylate synthetase 1D

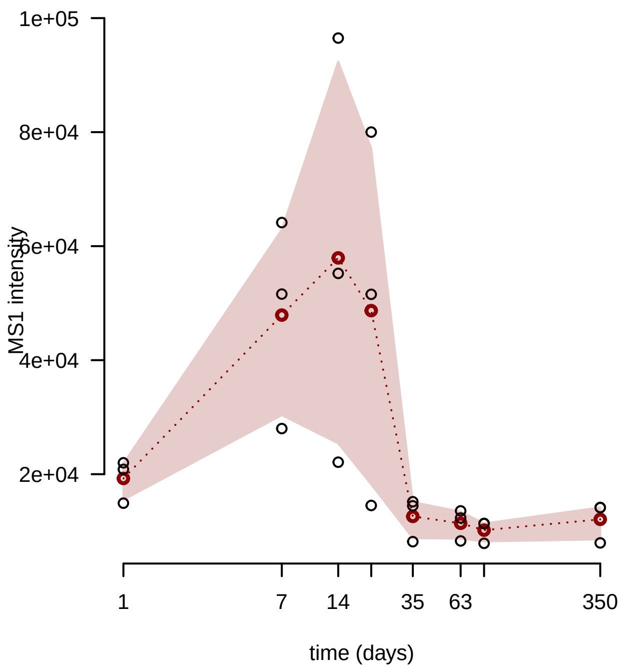

Q8VID5
GN=Recql5
RecQ protein−like 5

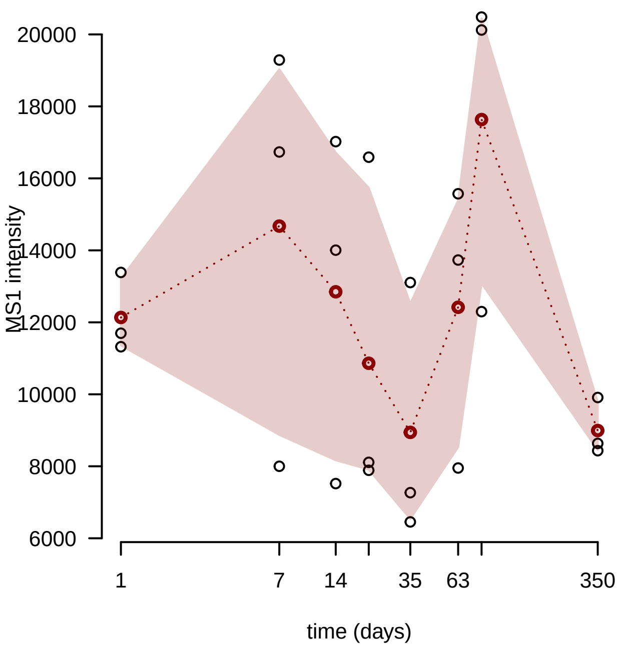

Q8VIG1
GN=Rest
RE1−silencing transcription factor

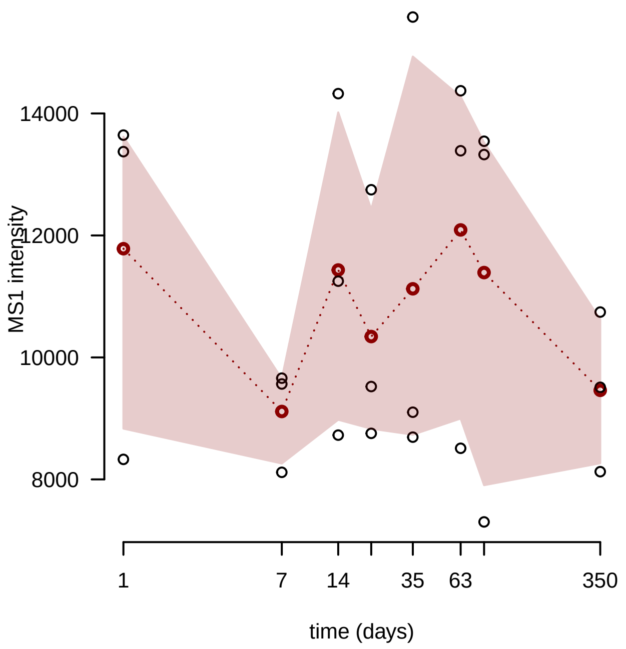

Q8VIH1
GN=Nobox
NOBOX oogenesis homeobox

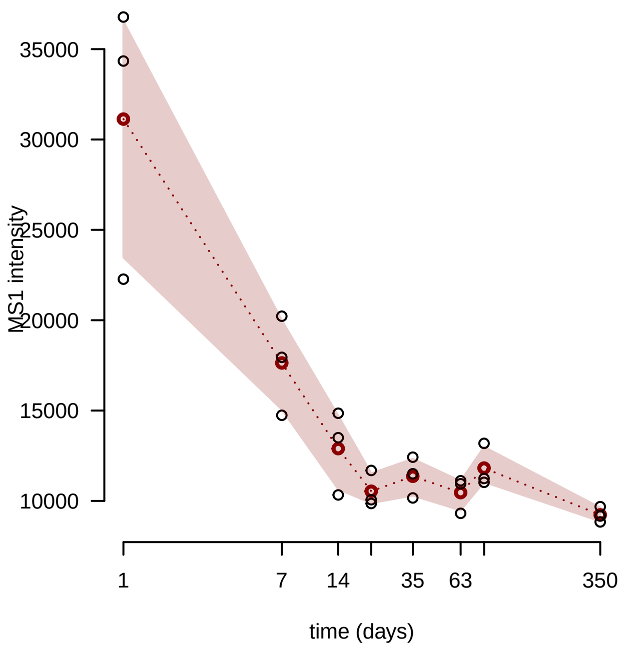

Q8VIJ6
GN=Sfpq
splicing factor proline/glutamine rich (...

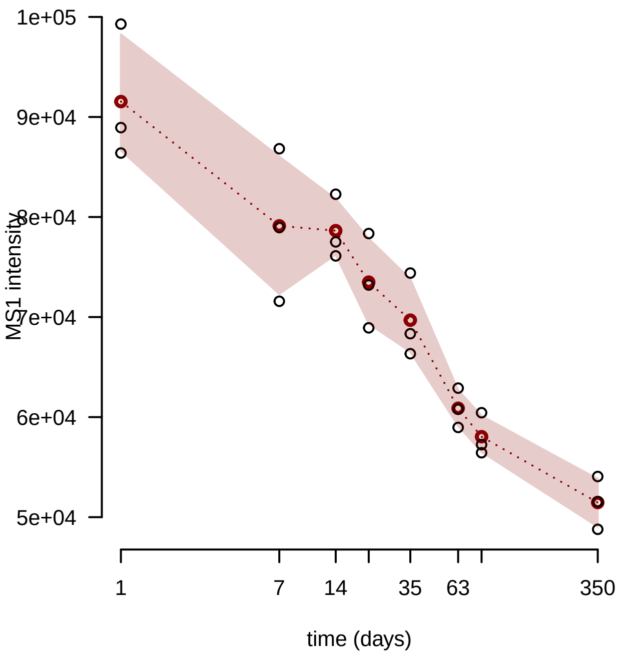

Q8VIJ8
GN=Nprl3
nitrogen permease regulator−like 3

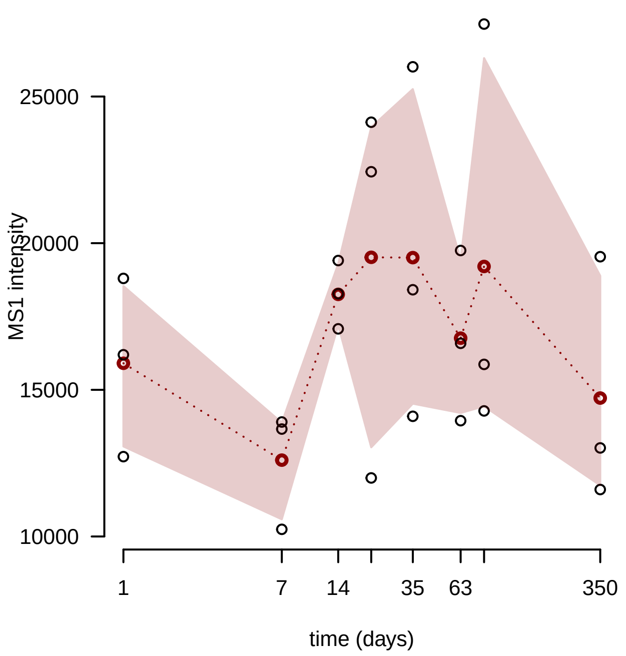

Q8VIK2
GN=Mpv17l2
MPV17 mitochondrial membrane protein−lik ...

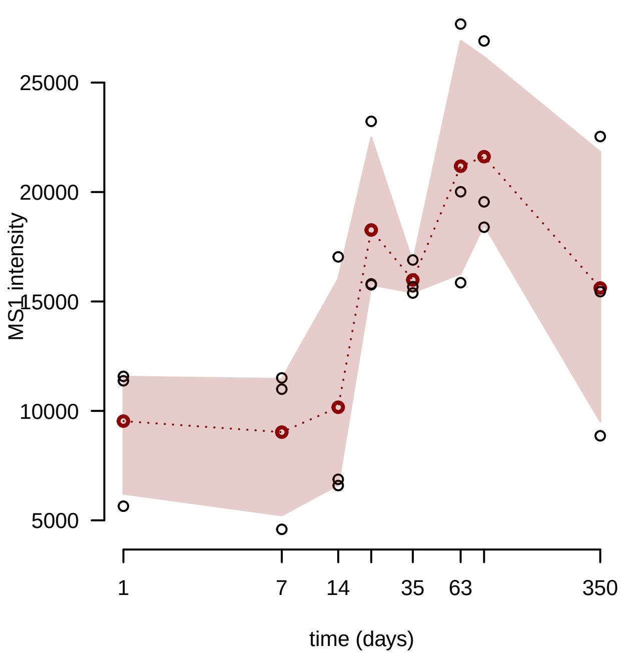

Q8VIK3
GN=H1foo

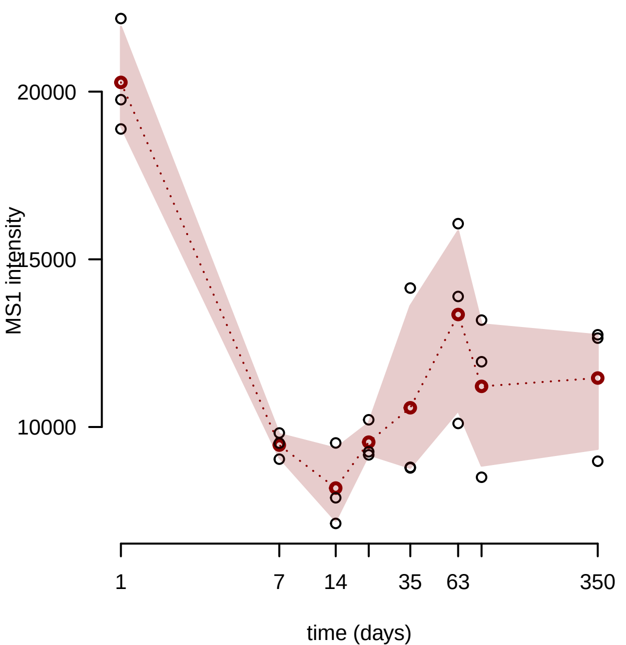

Q8VIL2
GN=Rwdd3
RWD domain containing 3

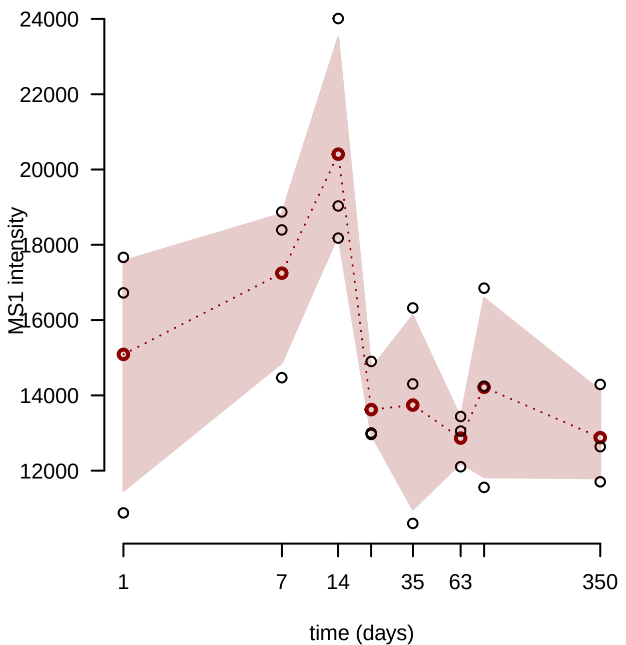

Q8VIM9
GN=Irgq
immunity−related GTPase family, Q

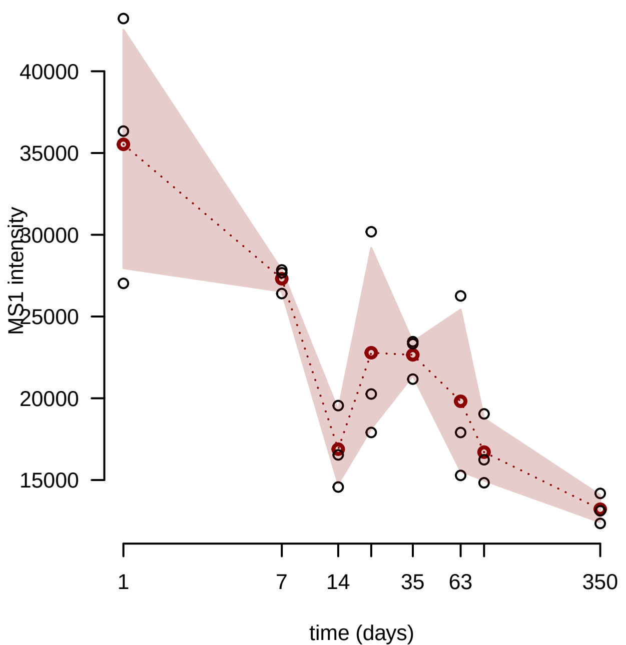

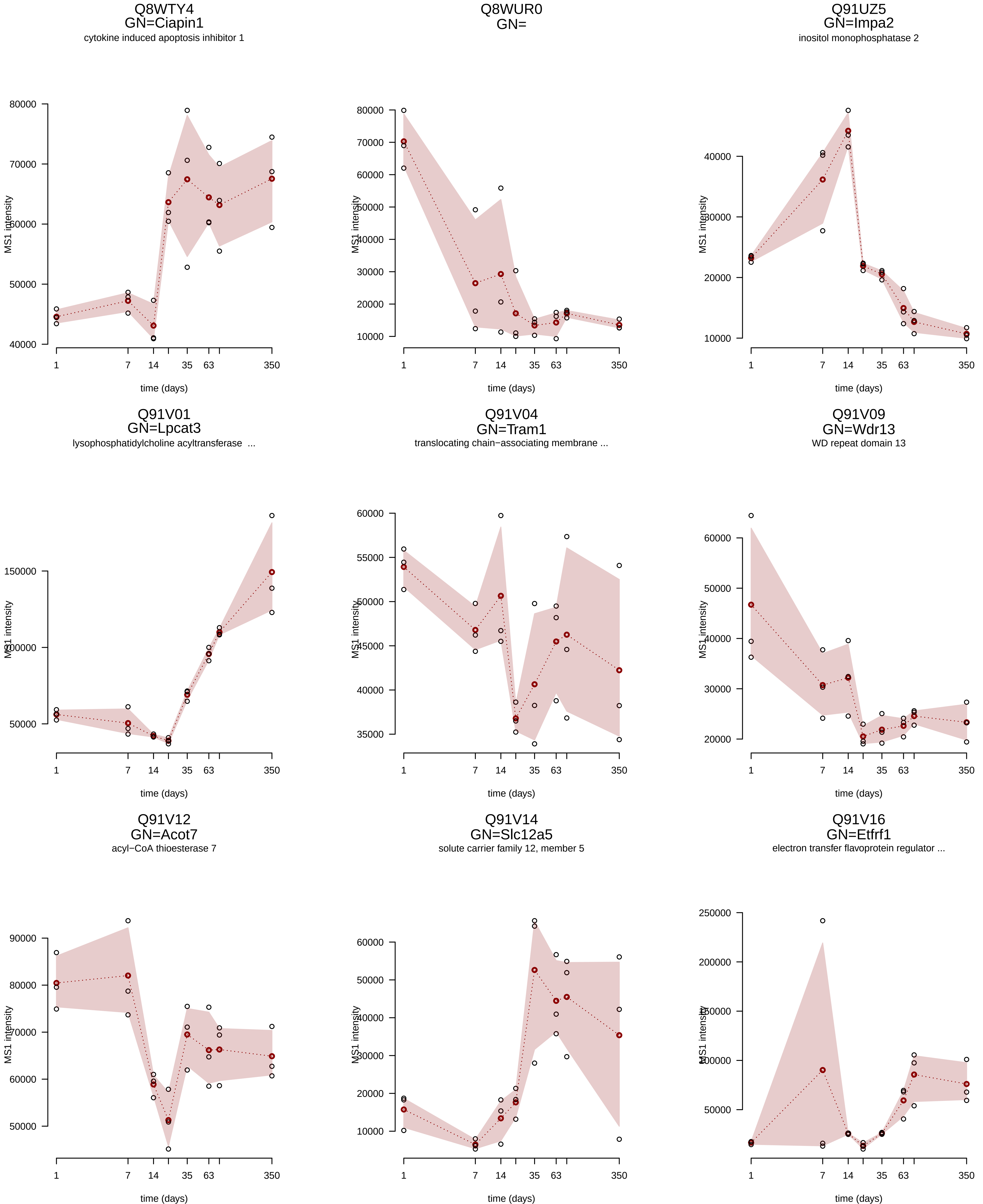
Q8WTY4
GN=Ciapin1
cytokine induced apoptosis inhibitor 1
MS1 intensity
80000
70000
60000
50000
40000
1
7
14
35
63
350
time (days)
Q8WUR0
GN=
MS1 intensity
80000
70000
60000
50000
40000
30000
20000
10000
1
7
14
35
63
350
time (days)
Q91UZ5
GN=Impa2
inositol monophosphatase 2
MS1 intensity
40000
30000
20000
10000
1
7
14
35
63
350
time (days)
Q91V01
GN=Lpcat3
lysophosphatidylcholine acyltransferase ...
MS1 intensity
150000
100000
50000
1
7
14
35
63
350
time (days)
Q91V04
GN=Tram1
translocating chain−associating membrane ...
MS1 intensity
60000
55000
50000
45000
40000
35000
1
7
14
35
63
350
time (days)
Q91V09
GN=Wdr13
WD repeat domain 13
MS1 intensity
60000
50000
40000
30000
20000
1
7
14
35
63
350
time (days)
Q91V12
GN=Acot7
acyl−CoA thioesterase 7
MS1 intensity
90000
80000
70000
60000
50000
1
7
14
35
63
350
time (days)
Q91V14
GN=Slc12a5
solute carrier family 12, member 5
MS1 intensity
60000
50000
40000
30000
20000
10000
1
7
14
35
63
350
time (days)
Q91V16
GN=Etfrf1
electron transfer flavoprotein regulator ...
MS1 intensity
250000
200000
150000
100000
50000
1
7
14
35
63
350
time (days)

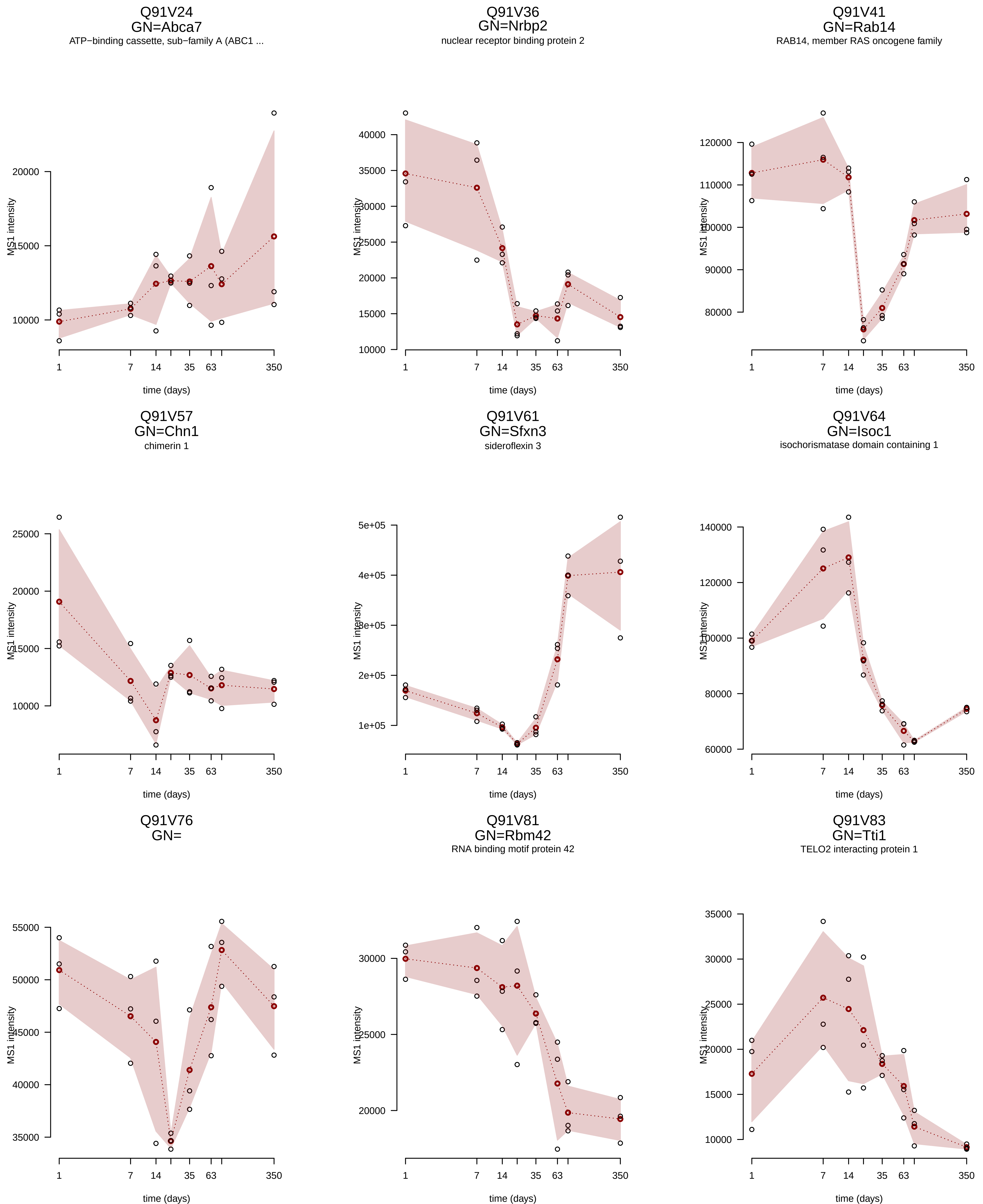
Q91V24
GN=Abca7
ATP−binding cassette, sub−family A (ABC1 ...
Q91V36
GN=Nrbp2
nuclear receptor binding protein 2
Q91V41
GN=Rab14
RAB14, member RAS oncogene family
Q91V57
GN=Chn1
chimerin 1
Q91V61
GN=Sfxn3
sideroflexin 3
Q91V64
GN=Isoc1
isochorismatase domain containing 1
Q91V76
GN=
Q91V81
GN=Rbm42
RNA binding motif protein 42
Q91V83
GN=Tti1
TELO2 interacting protein 1
MS1 intensity
time (days)
1
7
14
35
63
350

Q91V92
GN=Acly
ATP citrate lyase

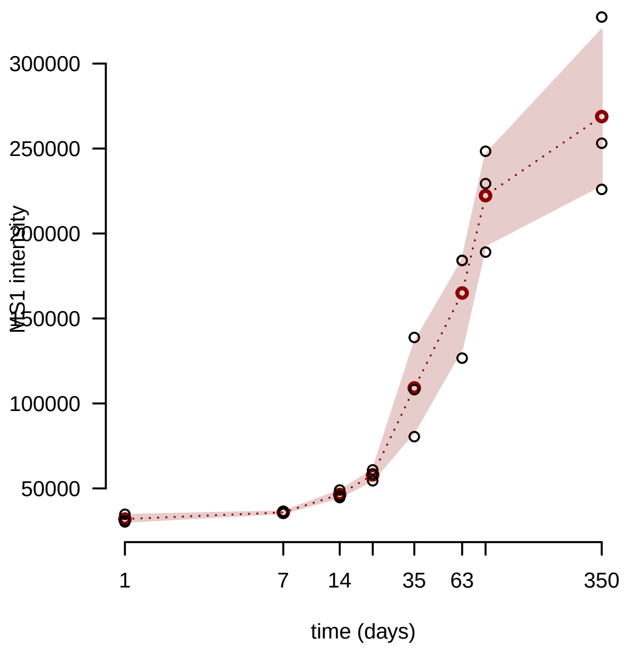

Q91V98
GN=Cd248
CD248 antigen, endosialin

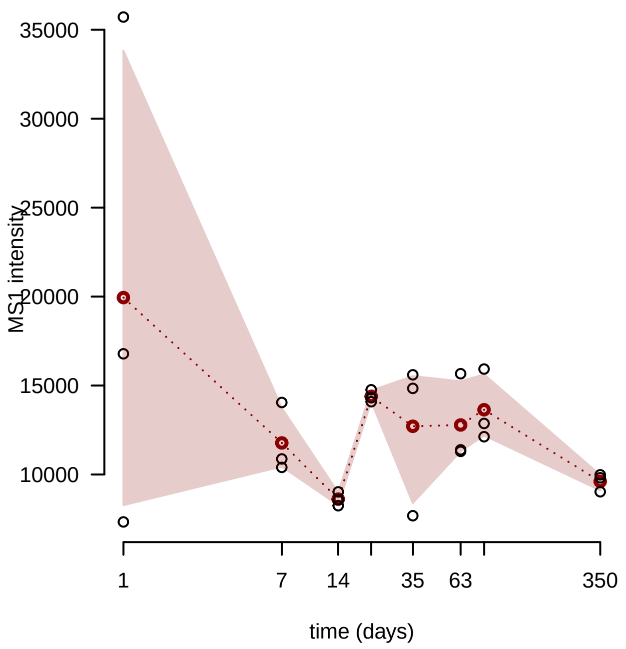

Q91VA6
GN=Poldip2
polymerase (DNA−directed), delta interac ...

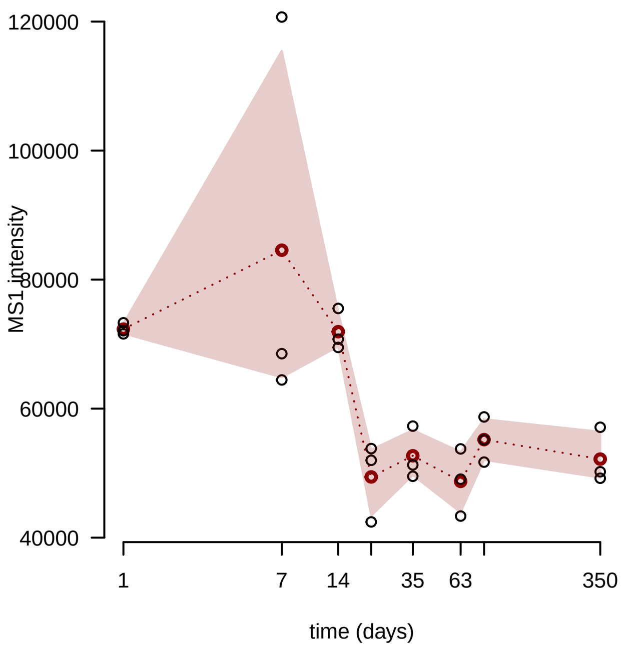

Q91VB4
GN=Hps3
HPS3, biogenesis of lysosomal organelles ...

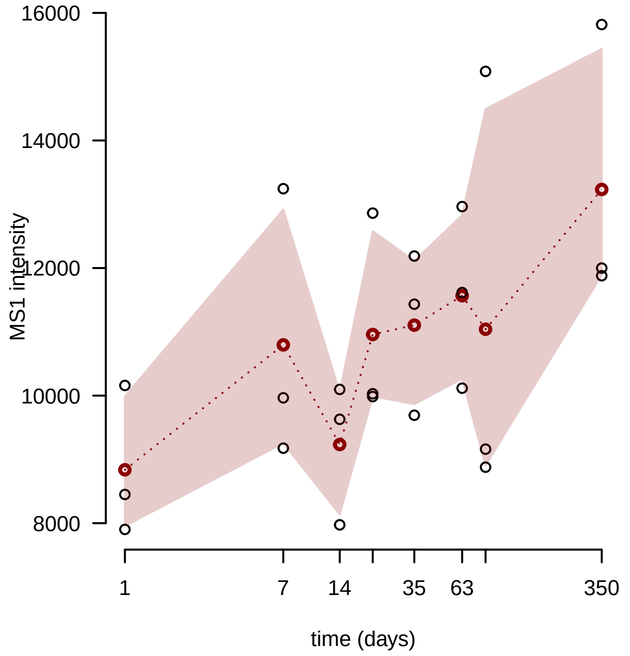

Q91VC3
GN=Eif4a3
eukaryotic translation initiation factor ...

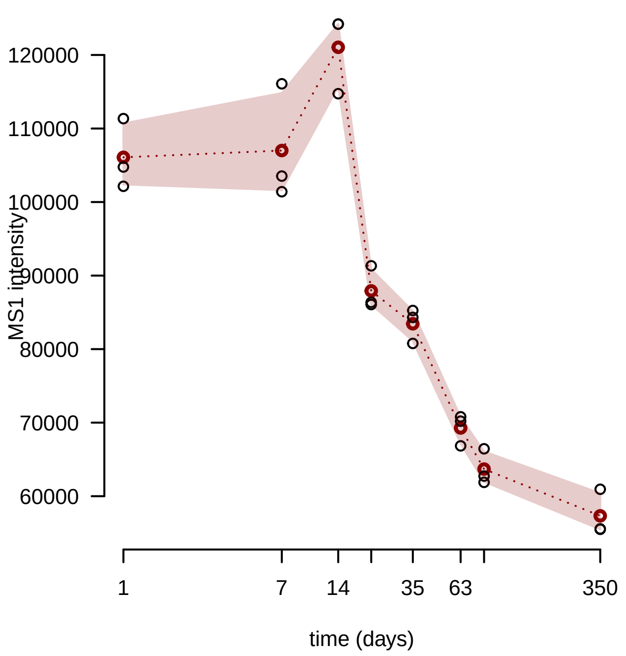

Q91VC4
GN=Plvap
plasmalemma vesicle associated protein

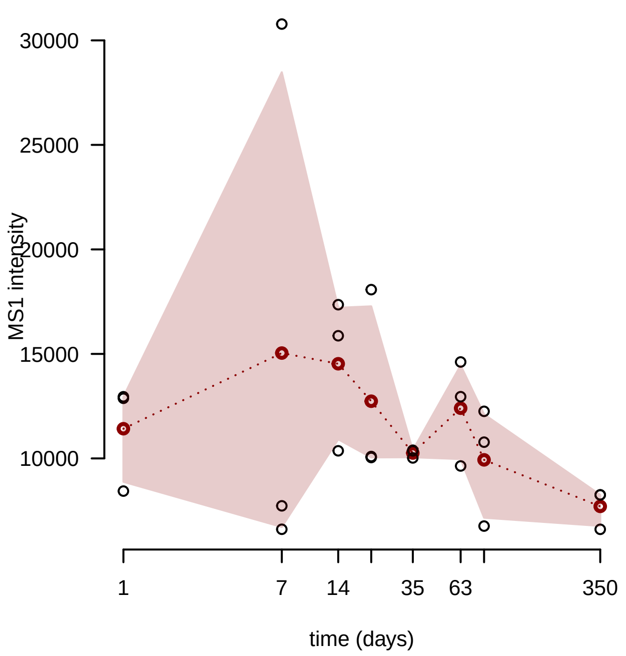

Q91VC7
GN=Ppp1r14a
protein phosphatase 1, regulatory inhibi ...

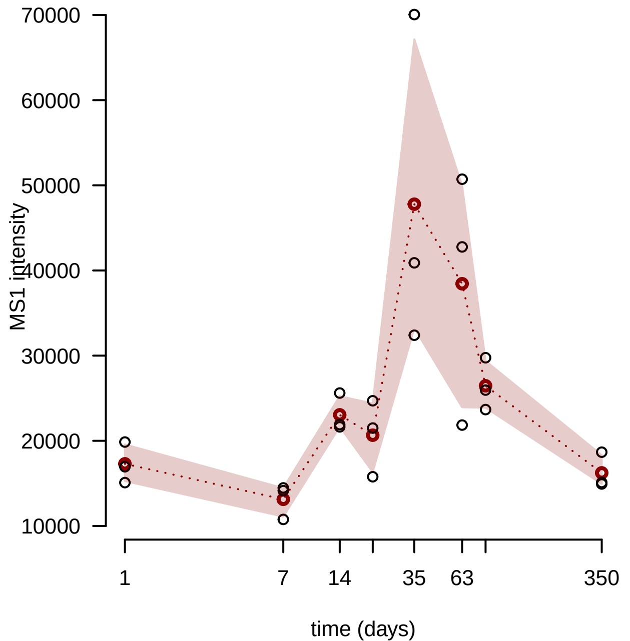

Q91VC9
GN=Ghitm
growth hormone inducible transmembrane p ...

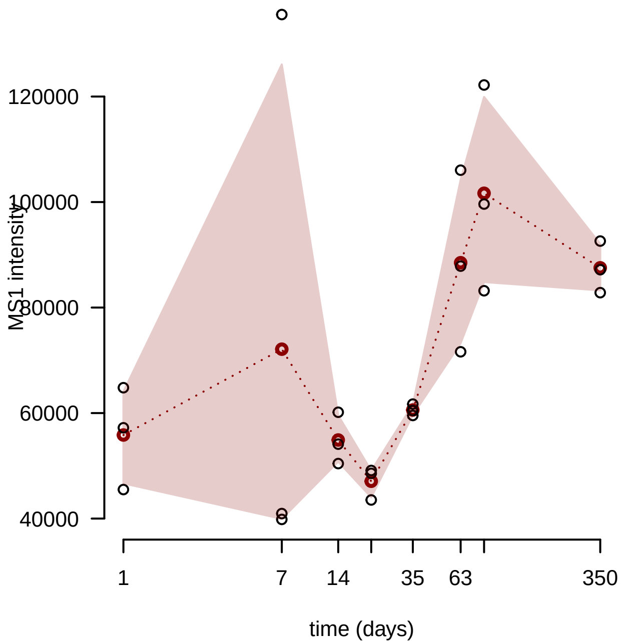

Q91VD9
GN=Ndufs1
NADH:ubiquinone oxidoreductase core subu ...

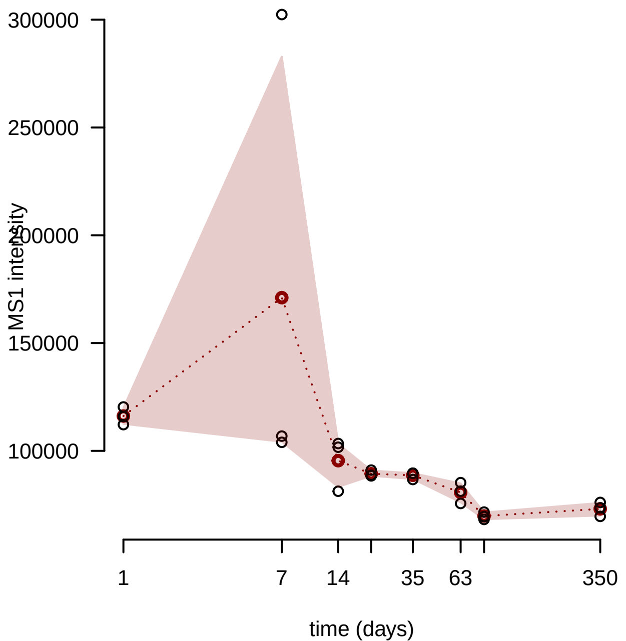

Q91VE0
GN=Slc27a4
solute carrier family 27 (fatty acid tra ...

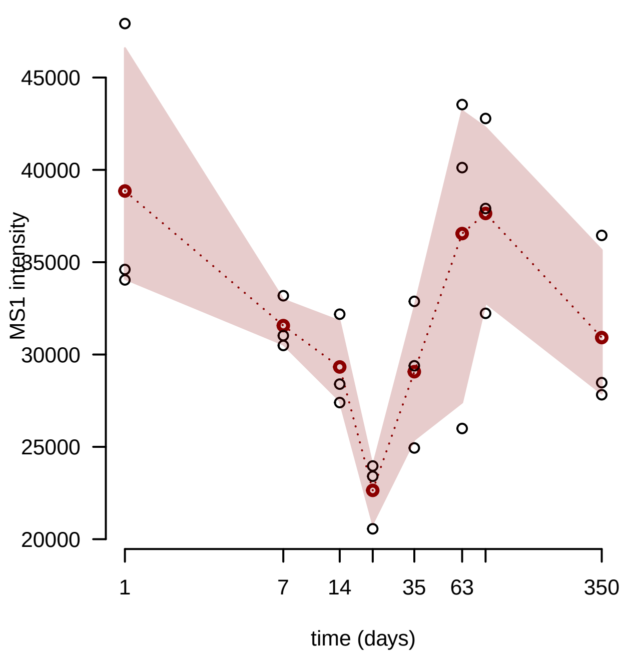

Q91VE6
GN=Nifk
nucleolar protein interacting with the F ...

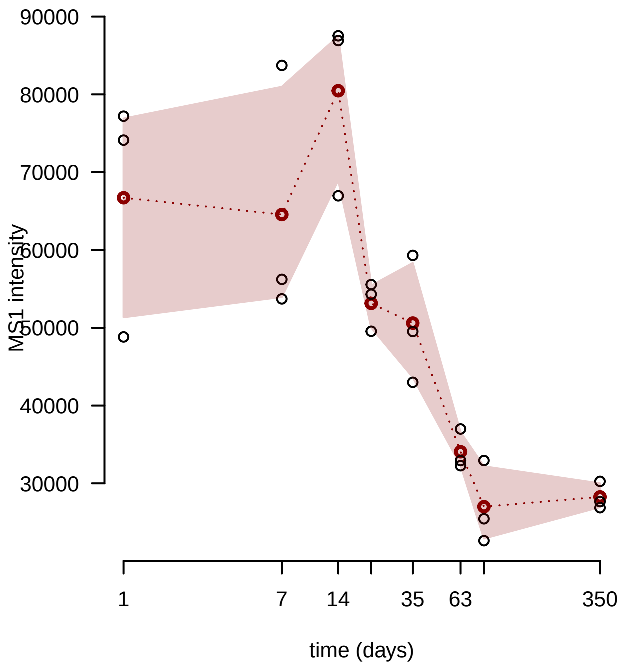

Q91VF2
GN=Hnmt
histamine N−methyltransferase

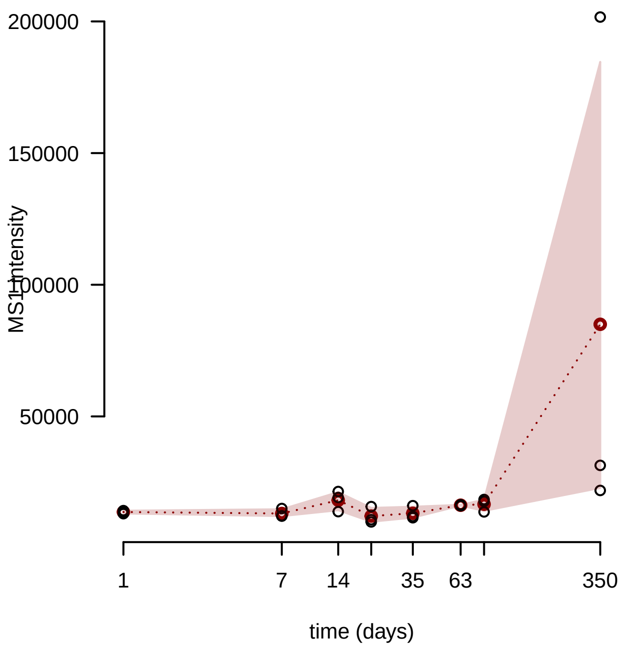

Q91VF6
GN=Col26a1
collagen, type XXVI, alpha 1

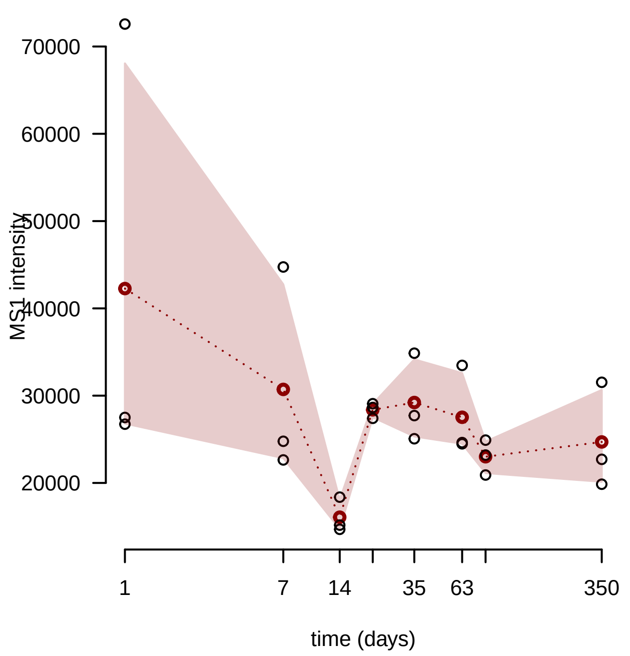

Q91VH2
GN=Snx9
sorting nexin 9

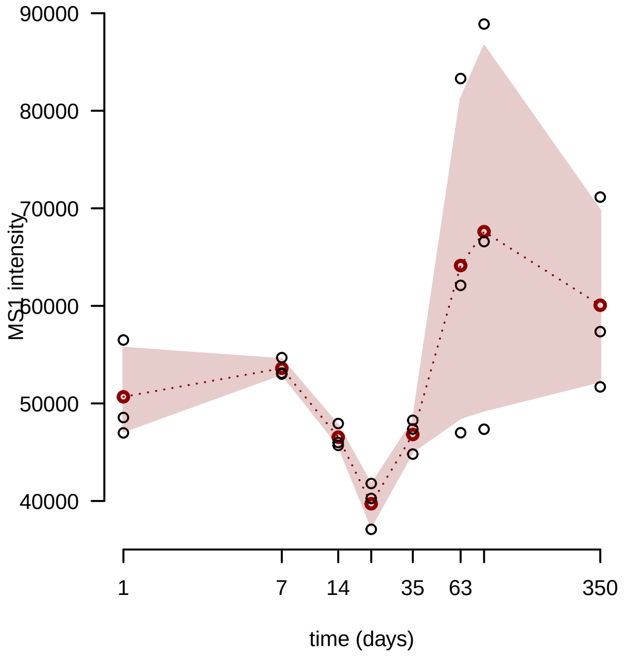

Q91VH6
GN=Memo1
mediator of cell motility 1

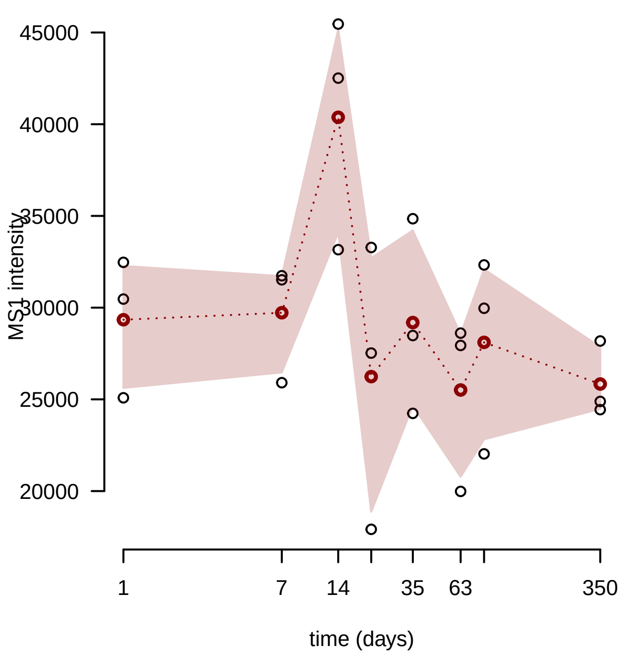

Q91VI7
GN=Rnh1
ribonuclease/angiogenin inhibitor 1

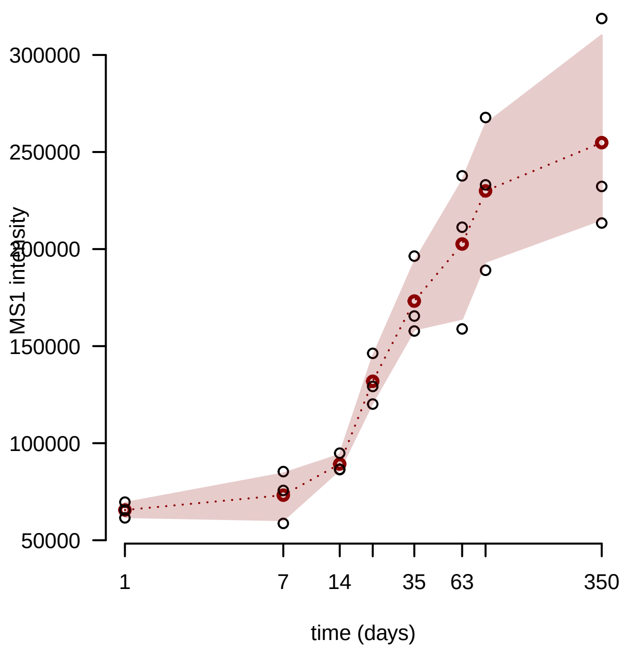

Q91VJ1
GN=Aim2
absent in melanoma 2

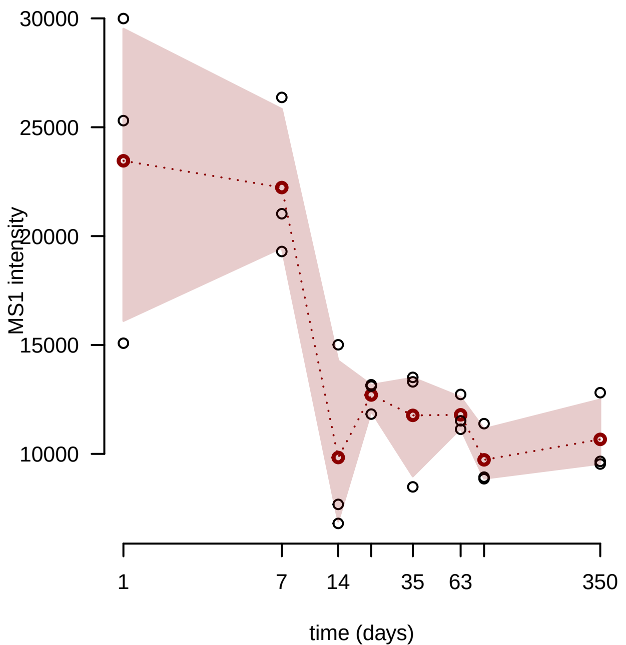

Q91VJ2
GN=Cavin3
caveolae associated 3

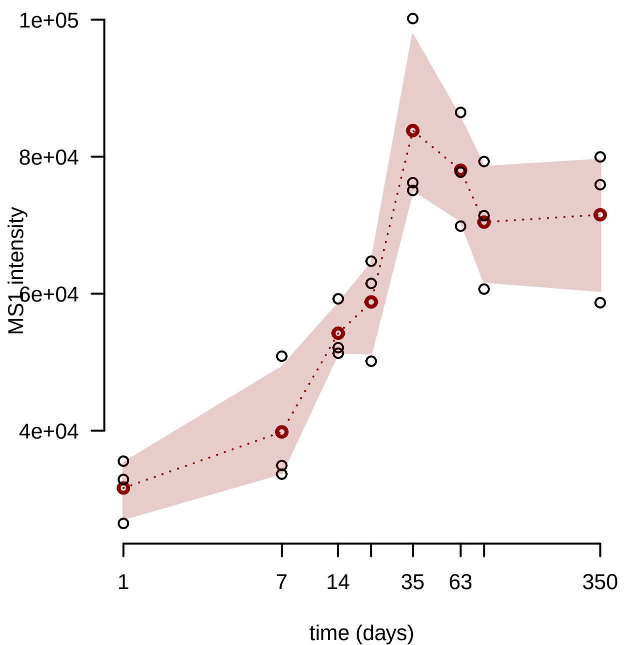

Q91VJ4
GN=Stk38
serine/threonine kinase 38

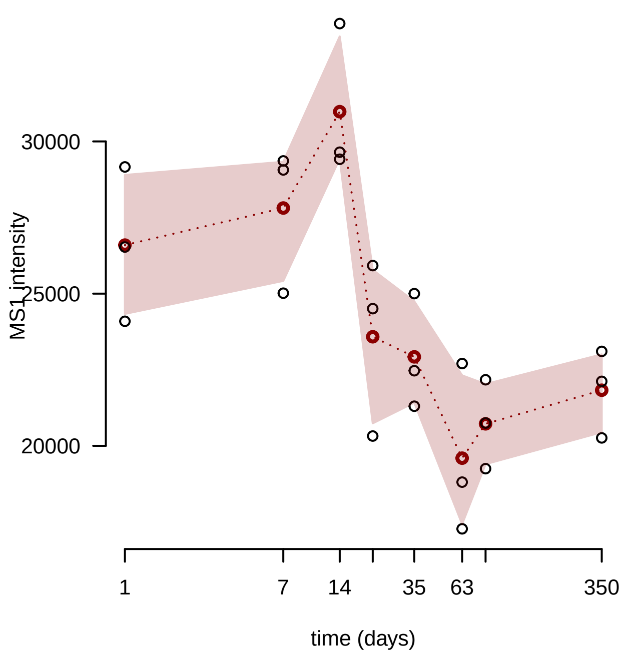

Q91VJ5
GN=Pqbp1
polyglutamine binding protein 1

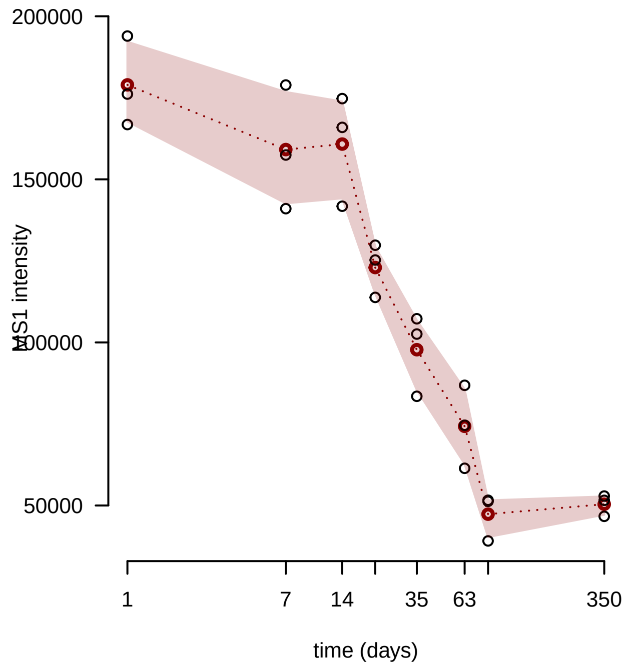

Q91VK1
GN=Bzw2

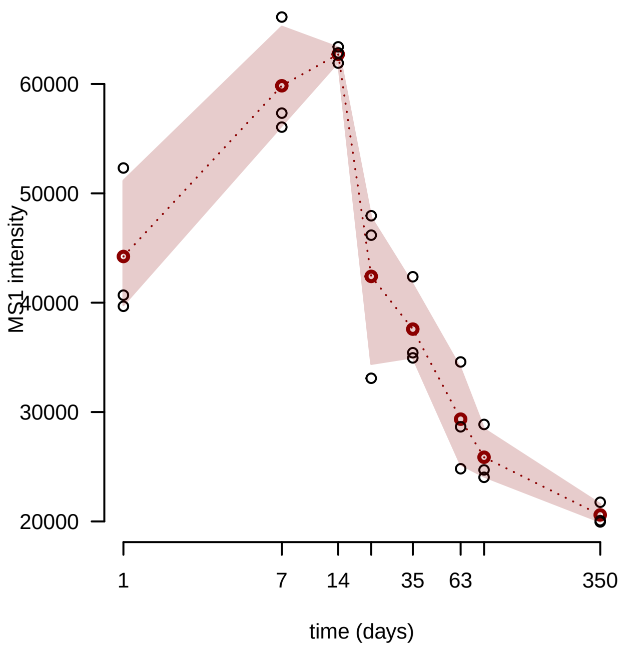

Q91VK4
GN=Itm2c
integral membrane protein 2C

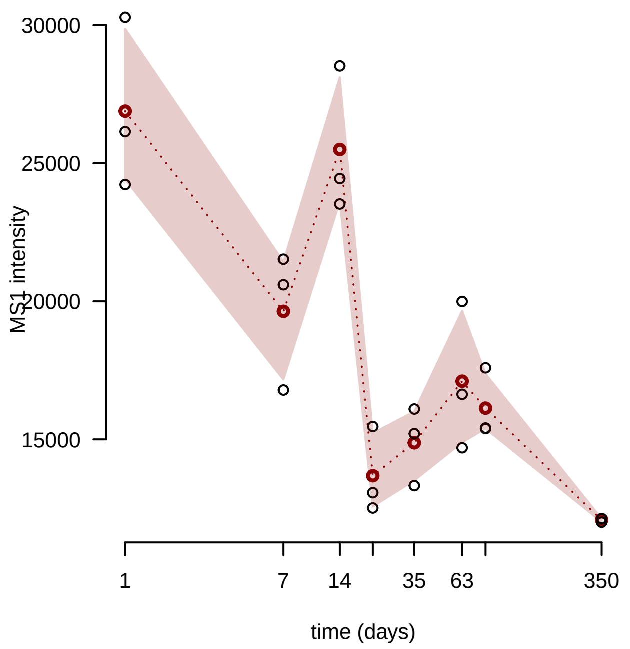

Q91VL8
GN=Terf2ip
telomeric repeat binding factor 2, inter ...

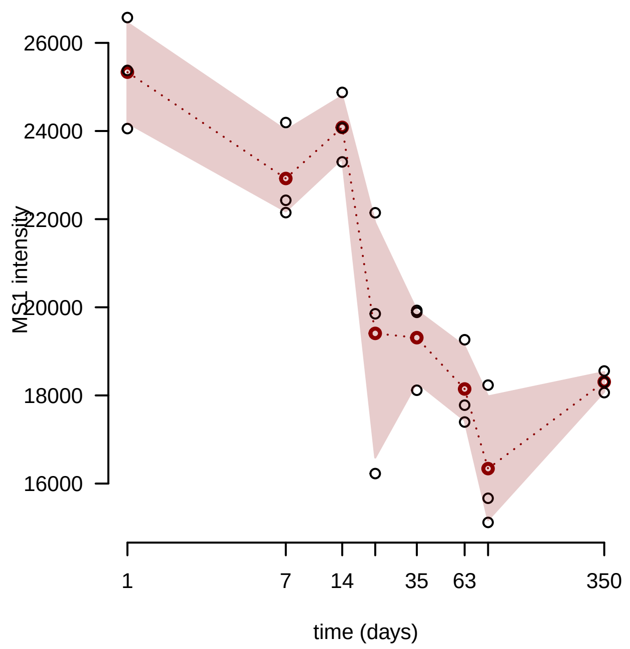

Q91VL9
GN=Zbtb1
zinc finger and BTB domain containing 1

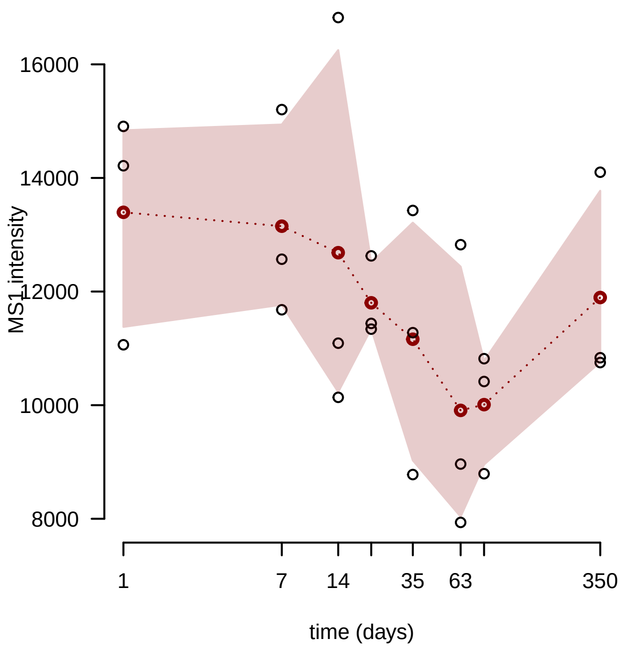

Q91VM3
GN=Wdr45
WD repeat domain 45

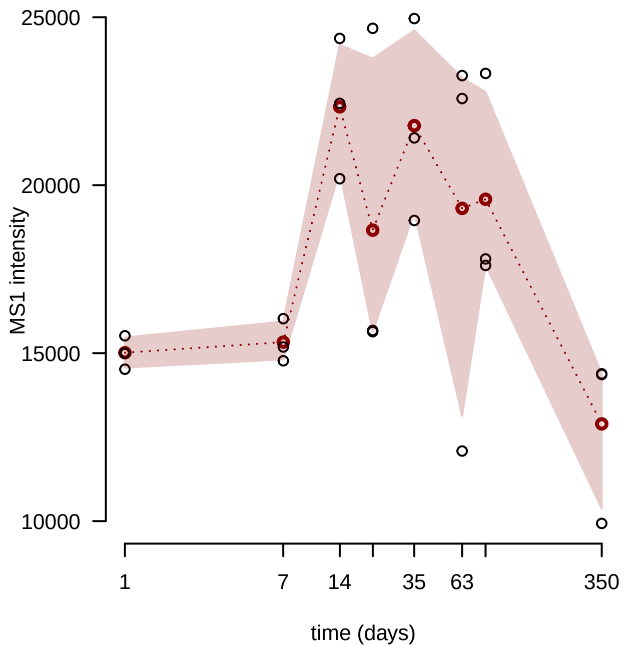

Q91VM5
GN=Rbmxl1
RNA binding motif protein, X−linked like ...

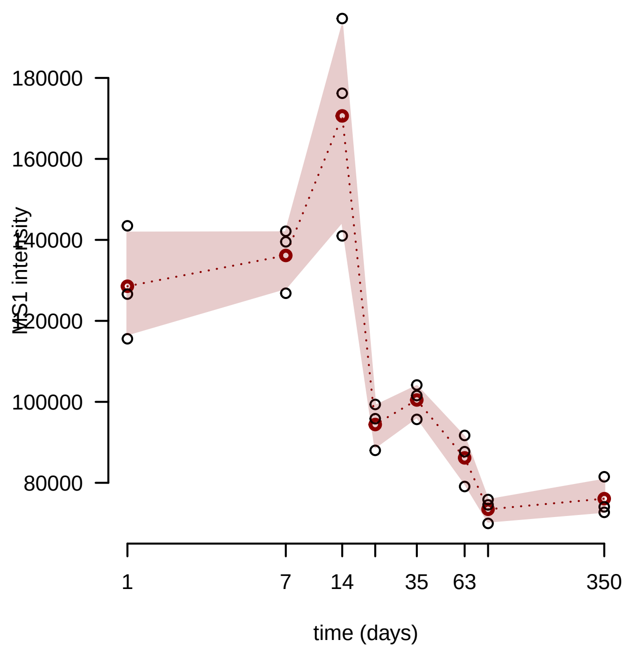

Q91VM9
GN=Ppa2
pyrophosphatase (inorganic) 2

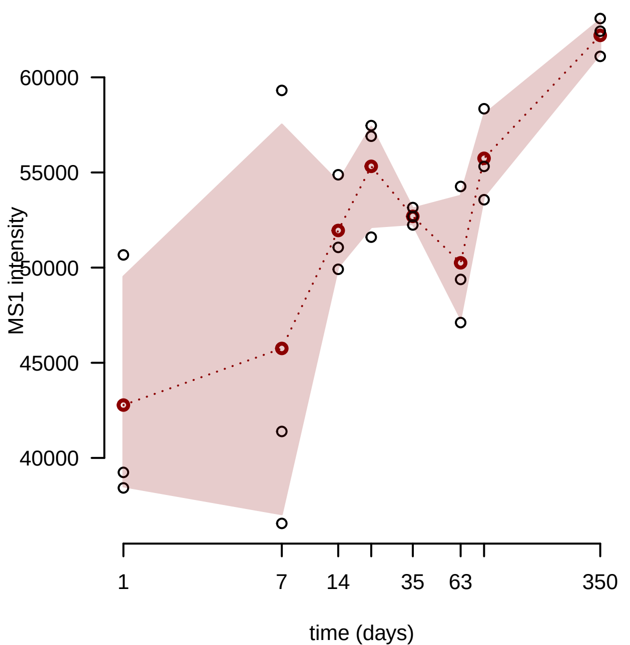

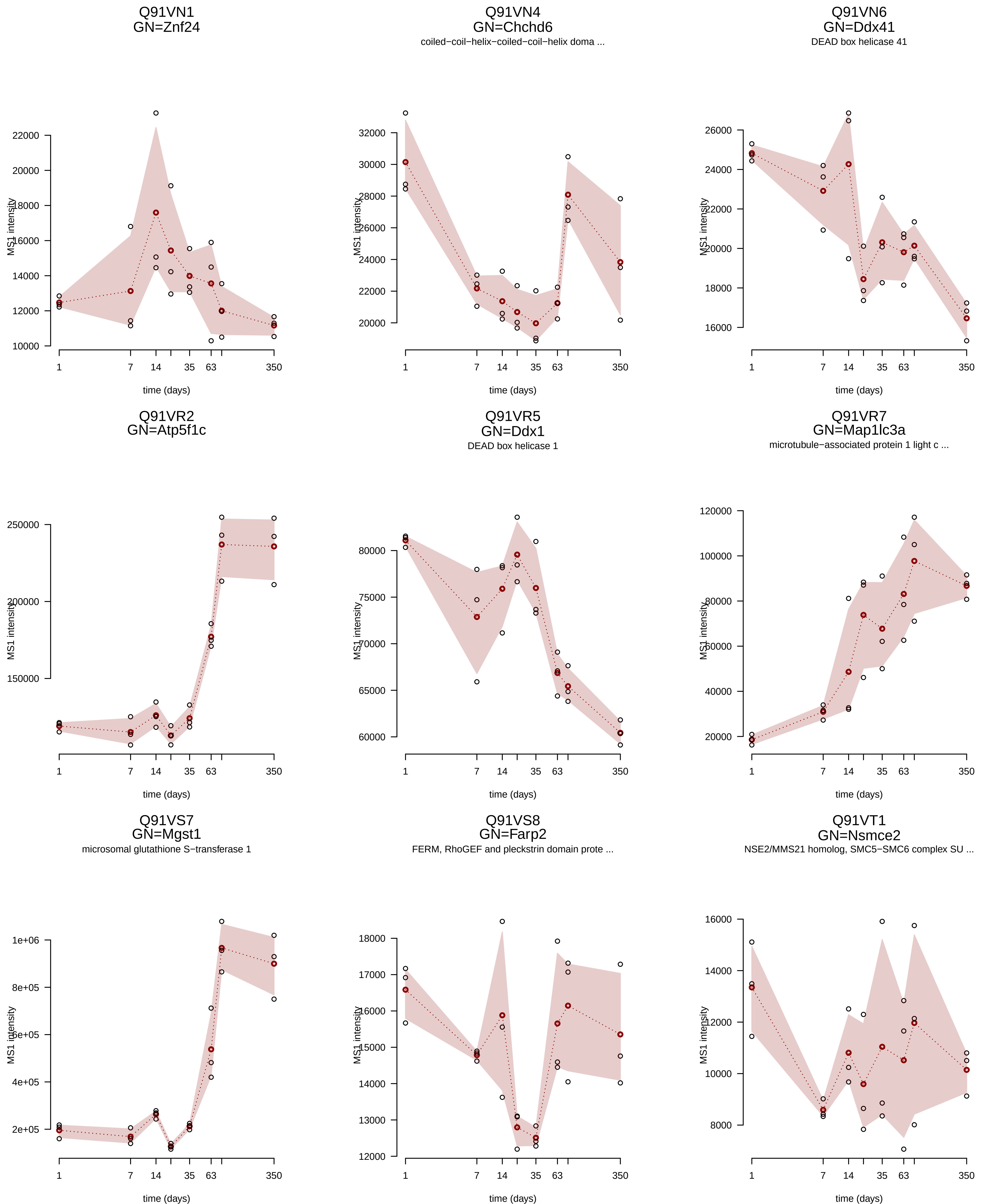
Q91VN1
GN=Znf24
Q91VN4
GN=Chchd6
coiled−coil−helix−coiled−coil−helix doma ...
Q91VN6
GN=Ddx41
DEAD box helicase 41
Q91VR2
GN=Atp5f1c
Q91VR5
GN=Ddx1
DEAD box helicase 1
Q91VR7
GN=Map1lc3a
microtubule−associated protein 1 light c ...
Q91VS7
GN=Mgst1
microsomal glutathione S−transferase 1
Q91VS8
GN=Farp2
FERM, RhoGEF and pleckstrin domain prote ...
Q91VT1
GN=Nsmce2
NSE2/MMS21 homolog, SMC5−SMC6 complex SU ...
MS1 intensity
time (days)
1
7
14
35
63
350

Q91VT4
GN=Cbr4
carbonyl reductase 4

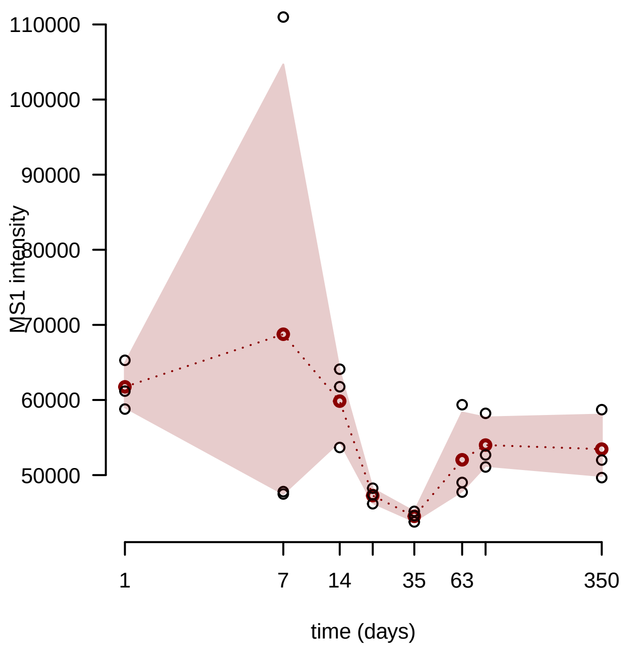

Q91VU0
GN=Fam3c
FAM3 metabolism regulating signaling mol ...

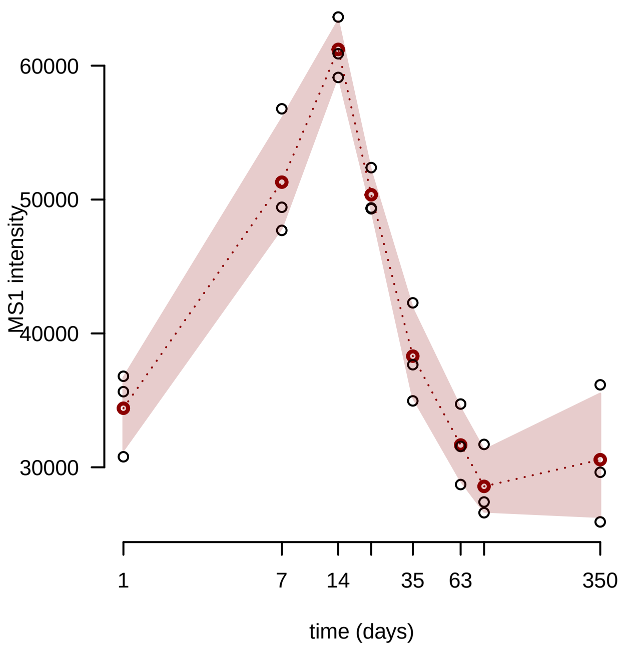

Q91VU1
GN=Yipf1
Yip1 domain family, member 1

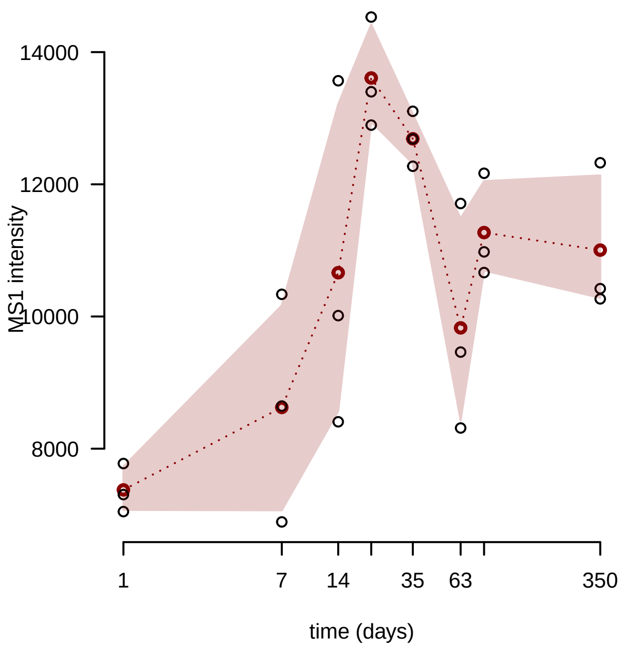

Q91VU6
GN=Dcaf11
DDB1 and CUL4 associated factor 11

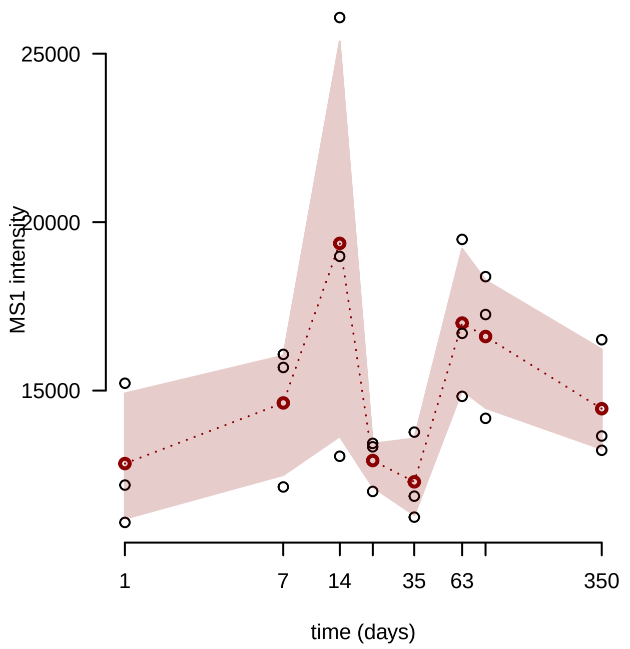

Q91VU7
GN=Pus7
pseudouridylate synthase 7

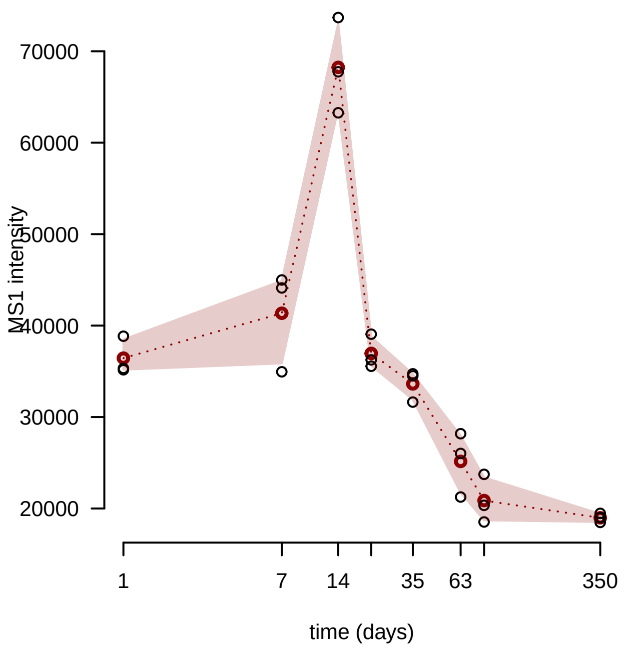

Q91VU8
GN=Ptov1
prostate tumor over expressed gene 1

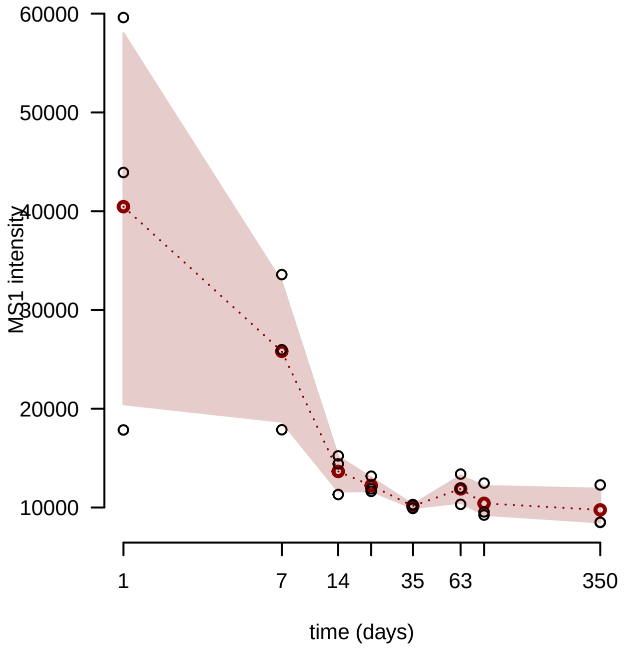

Q91VW3
GN=Sh3bgrl3
SH3 domain binding glutamic acid−rich pr ...

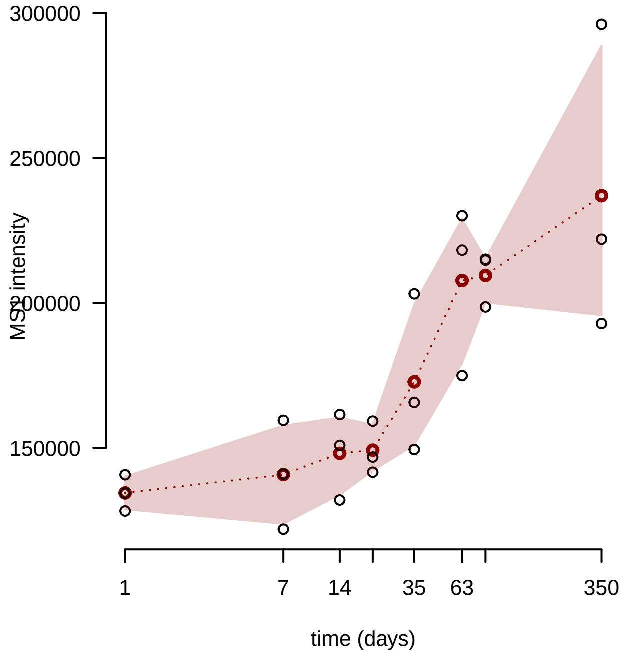

Q91VW5
GN=Golga4
golgi autoantigen, golgin subfamily a, 4

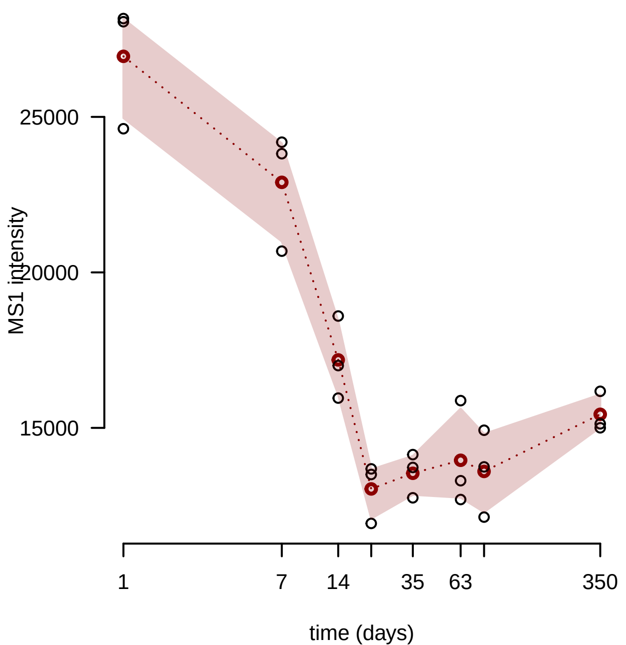

Q91VW9
GN=Zkscan3
zinc finger with KRAB and SCAN domains 3

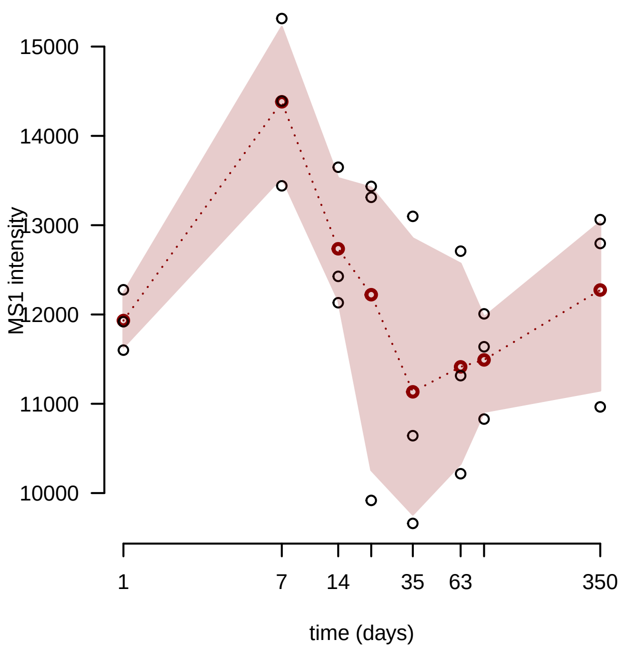

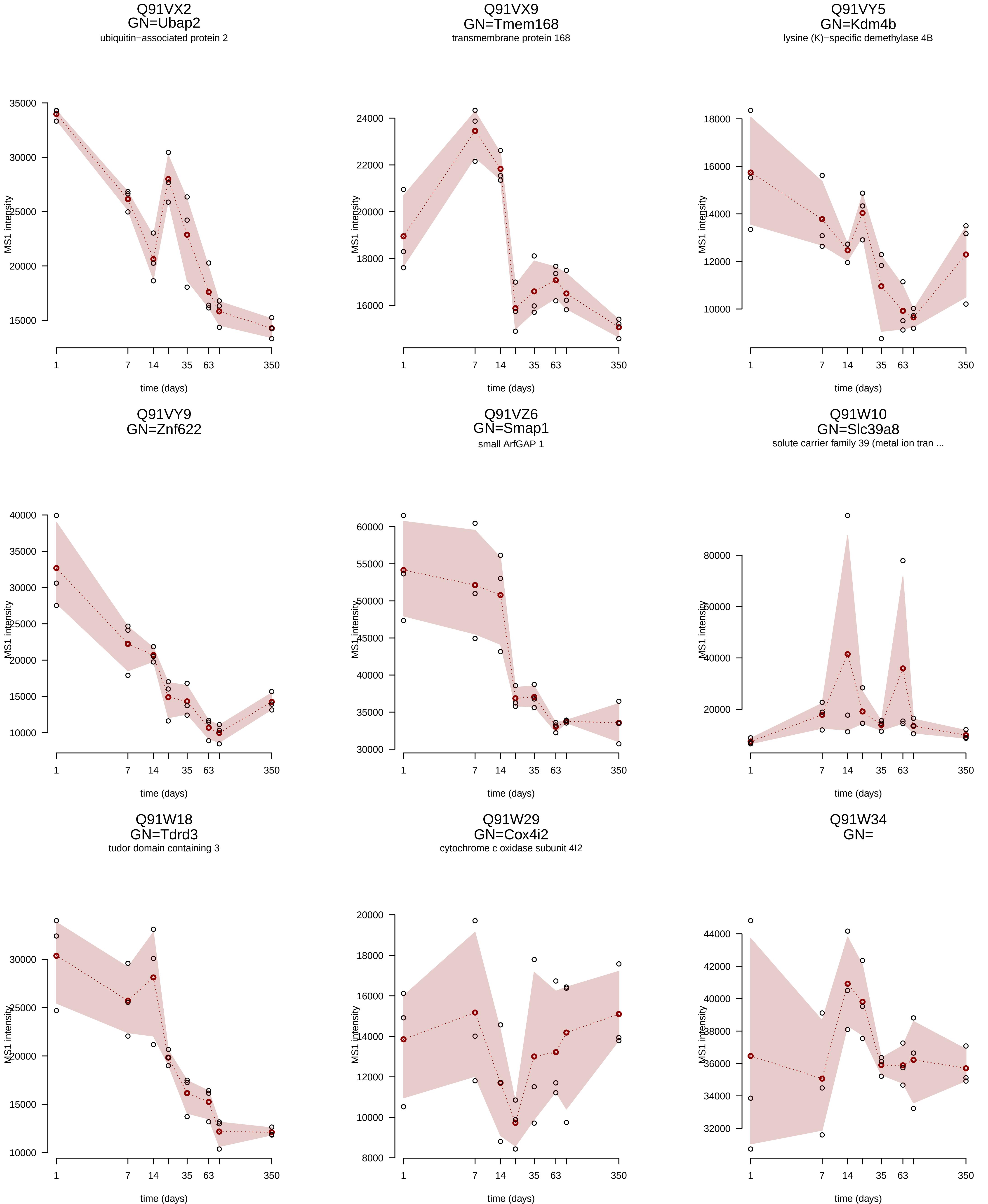
Q91VX2
GN=Ubap2
ubiquitin−associated protein 2
MS1 intensity
35000
30000
25000
20000
15000
1
7
14
35
63
350
time (days)
Q91VX9
GN=Tmem168
transmembrane protein 168
MS1 intensity
24000
22000
20000
18000
16000
1
7
14
35
63
350
time (days)
Q91VY5
GN=Kdm4b
lysine (K)−specific demethylase 4B
MS1 intensity
18000
16000
14000
12000
10000
1
7
14
35
63
350
time (days)
Q91VY9
GN=Znf622
MS1 intensity
40000
35000
30000
25000
20000
15000
10000
1
7
14
35
63
350
time (days)
Q91VZ6
GN=Smap1
small ArfGAP 1
MS1 intensity
60000
55000
50000
45000
40000
35000
30000
1
7
14
35
63
350
time (days)
Q91W10
GN=Slc39a8
solute carrier family 39 (metal ion tran ...
MS1 intensity
80000
60000
40000
20000
1
7
14
35
63
350
time (days)
Q91W18
GN=Tdrd3
tudor domain containing 3
MS1 intensity
30000
25000
20000
15000
10000
1
7
14
35
63
350
time (days)
Q91W29
GN=Cox4i2
cytochrome c oxidase subunit 4I2
MS1 intensity
20000
18000
16000
14000
12000
10000
8000
1
7
14
35
63
350
time (days)
Q91W34
GN=
MS1 intensity
44000
42000
40000
38000
36000
34000
32000
1
7
14
35
63
350
time (days)

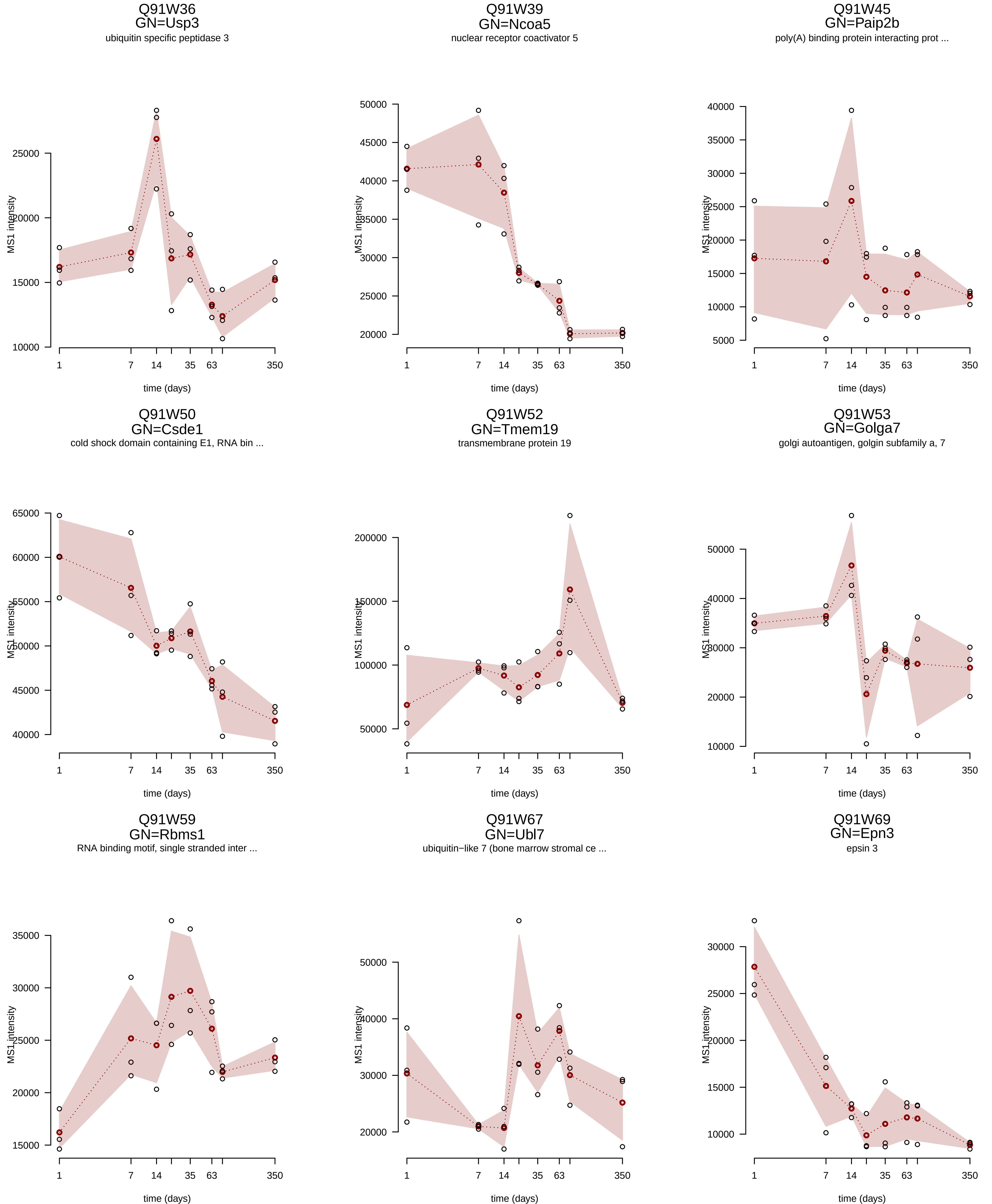
Q91W36
GN=Usp3
ubiquitin specific peptidase 3
Q91W39
GN=Ncoa5
nuclear receptor coactivator 5
Q91W45
GN=Paip2b
poly(A) binding protein interacting prot ...
Q91W50
GN=Csde1
cold shock domain containing E1, RNA bin ...
Q91W52
GN=Tmem19
transmembrane protein 19
Q91W53
GN=Golga7
golgi autoantigen, golgin subfamily a, 7
Q91W59
GN=Rbms1
RNA binding motif, single stranded inter ...
Q91W67
GN=Ubl7
ubiquitin−like 7 (bone marrow stromal ce ...
Q91W69
GN=Epn3
epsin 3
MS1 intensity
time (days)
1
7
14
35
63
350

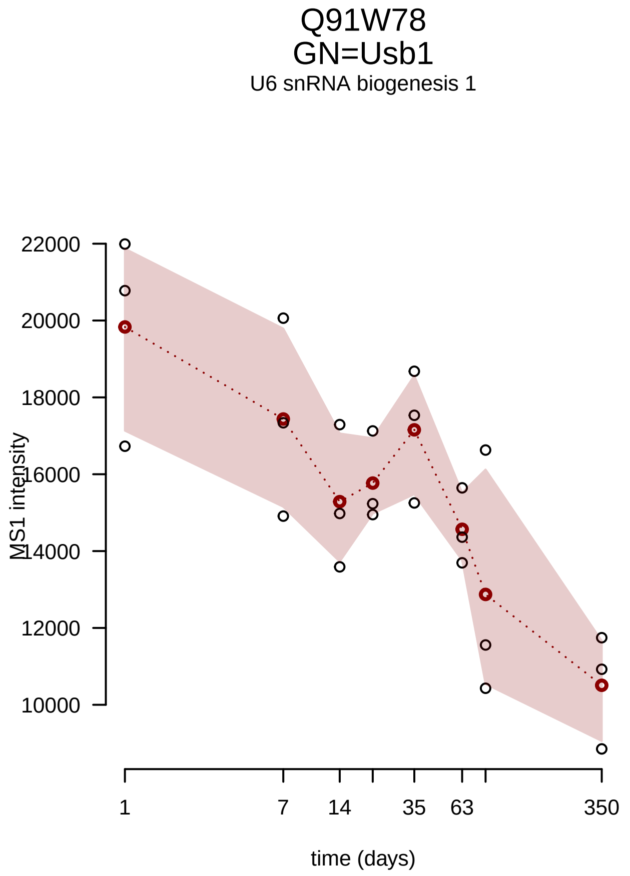
Q91W78
GN=Usb1
U6 snRNA biogenesis 1
MS1 intensity
22000
20000
18000
16000
14000
12000
10000
1
7
14
35
63
350
time (days)

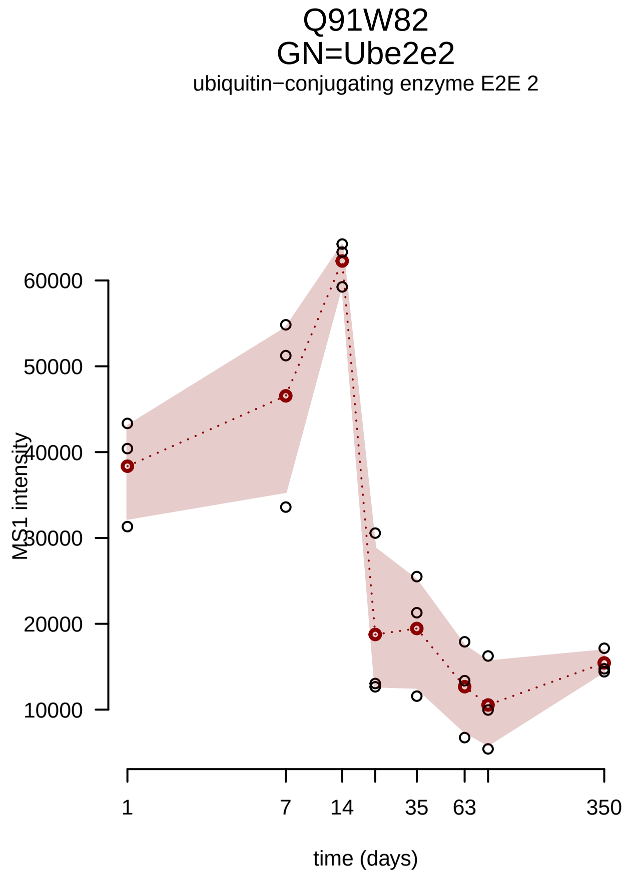
Q91W82
GN=Ube2e2
ubiquitin−conjugating enzyme E2E 2
MS1 intensity
60000
50000
40000
30000
20000
10000
1
7
14
35
63
350
time (days)

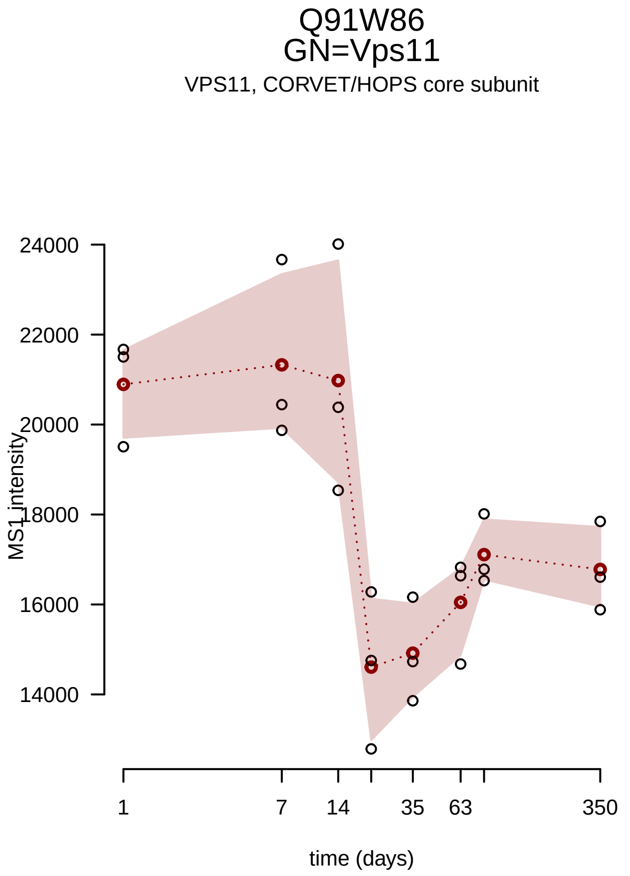
Q91W86
GN=Vps11
VPS11, CORVET/HOPS core subunit
MS1 intensity
24000
22000
20000
18000
16000
14000
1
7
14
35
63
350
time (days)

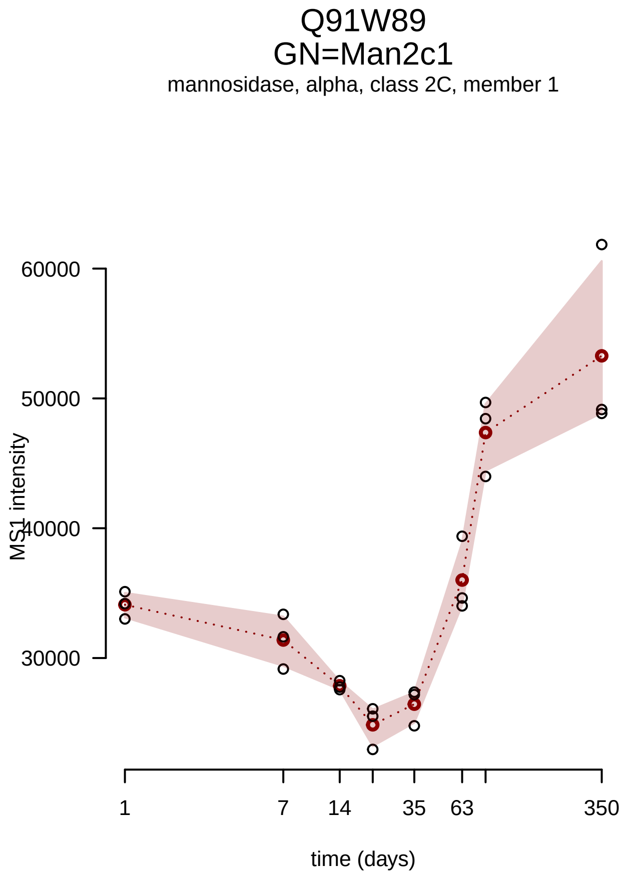
Q91W89
GN=Man2c1
mannosidase, alpha, class 2C, member 1
MS1 intensity
60000
50000
40000
30000
1
7
14
35
63
350
time (days)

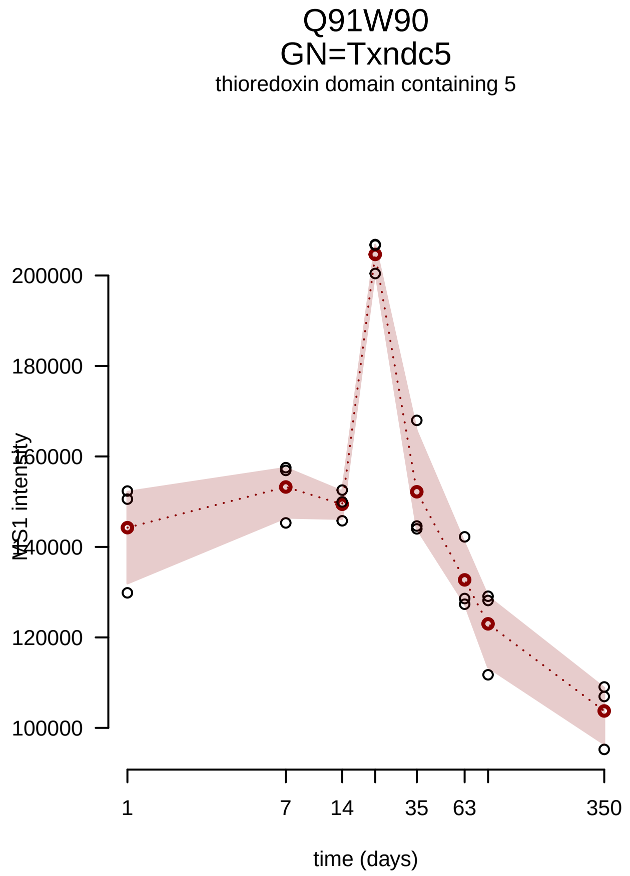
Q91W90
GN=Txndc5
thioredoxin domain containing 5
MS1 intensity
200000
180000
160000
140000
120000
100000
1
7
14
35
63
350
time (days)

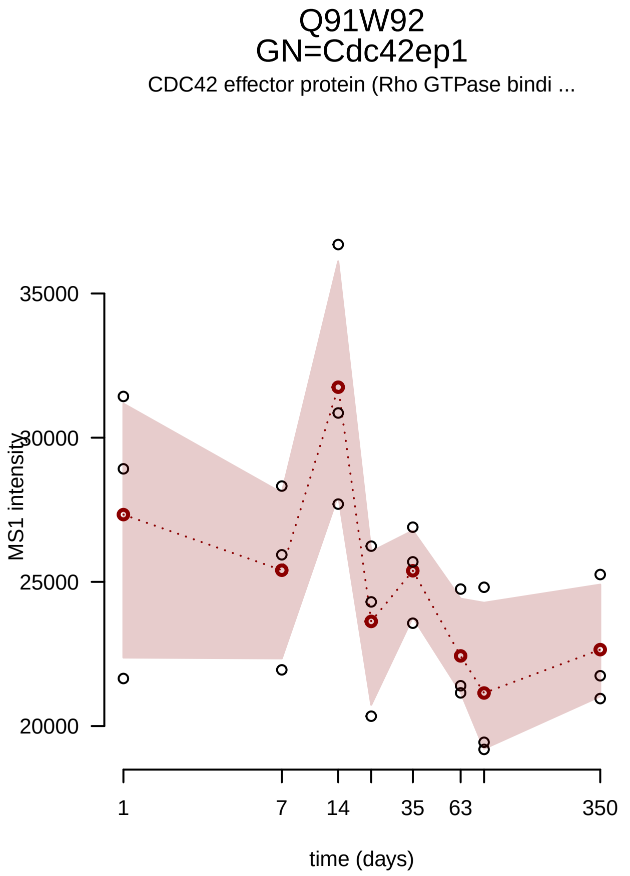
Q91W92
GN=Cdc42ep1
CDC42 effector protein (Rho GTPase bindi ...
MS1 intensity
35000
30000
25000
20000
1
7
14
35
63
350
time (days)

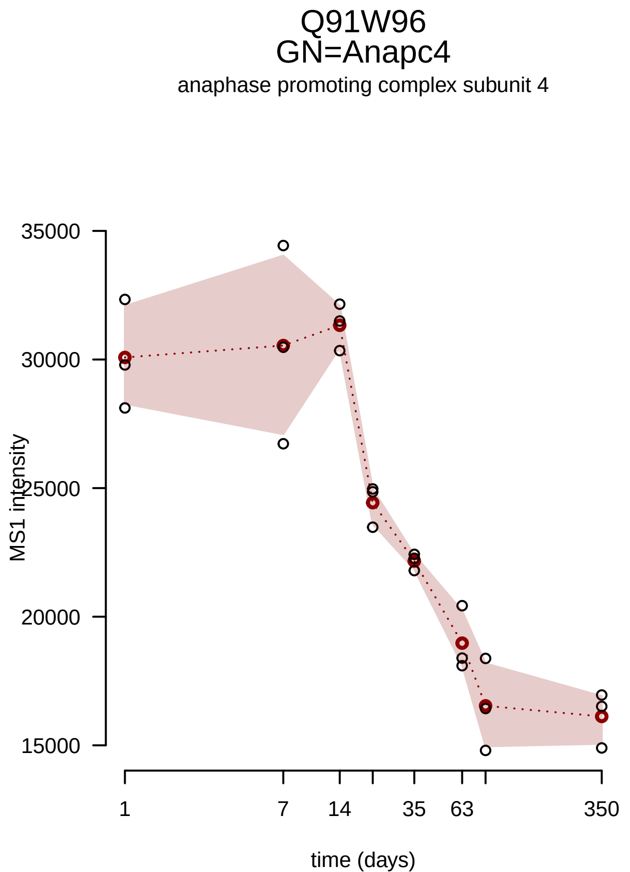
Q91W96
GN=Anapc4
anaphase promoting complex subunit 4
MS1 intensity
35000
30000
25000
20000
15000
1
7
14
35
63
350
time (days)

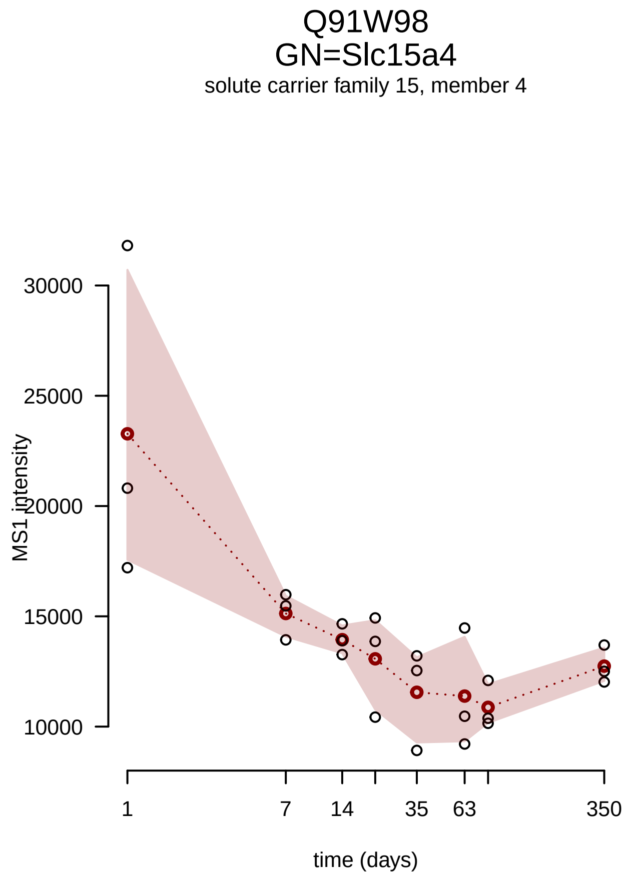
Q91W98
GN=Slc15a4
solute carrier family 15, member 4
MS1 intensity
30000
25000
20000
15000
10000
1
7
14
35
63
350
time (days)

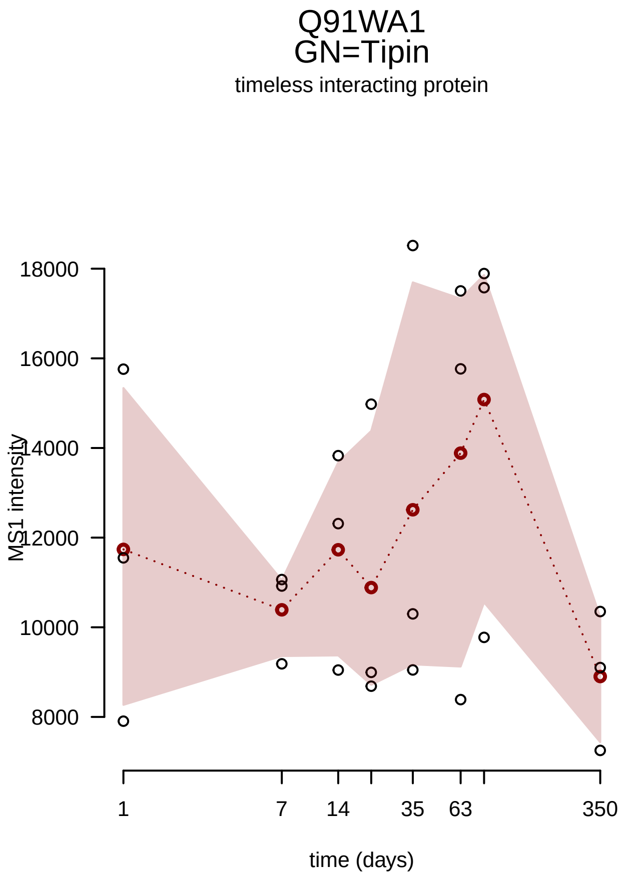
Q91WA1
GN=Tipin
timeless interacting protein
MS1 intensity
18000
16000
14000
12000
10000
8000
1
7
14
35
63
350
time (days)

Q91WA4
GN=Cxxc5
CXXC finger 5

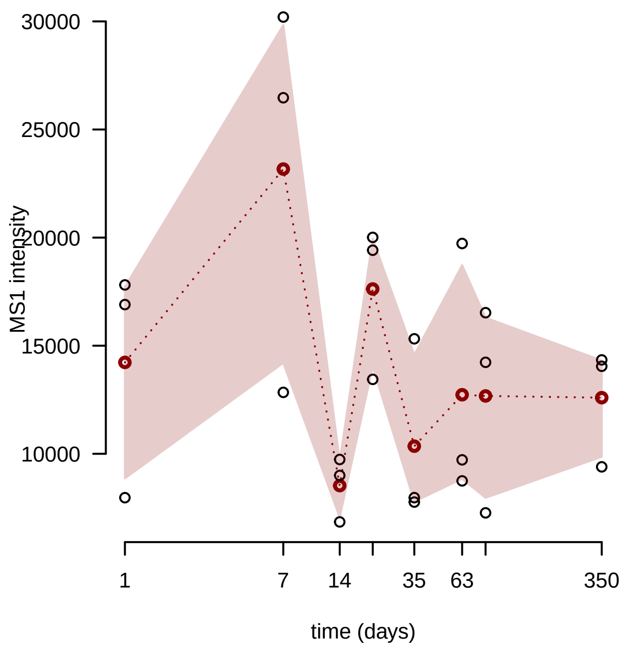

Q91WA6
GN=Sharpin
SHANK−associated RH domain interacting p ...

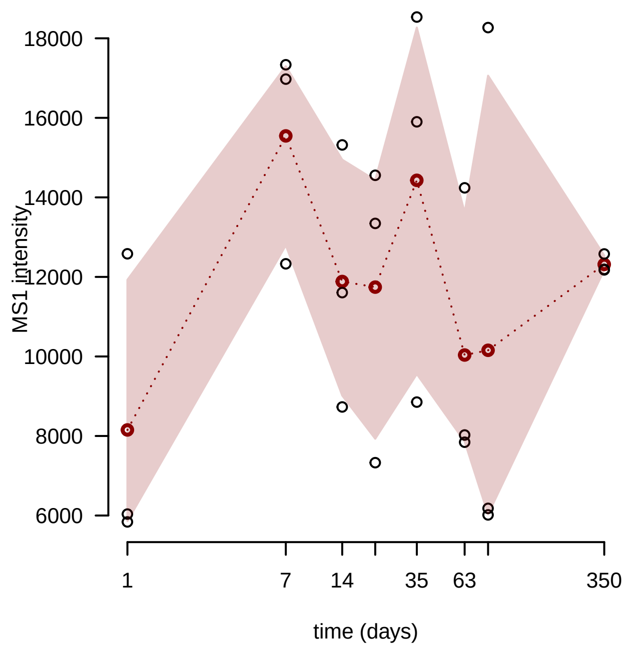

Q91WB4
GN=Plekhf2
pleckstrin homology domain containing, f ...

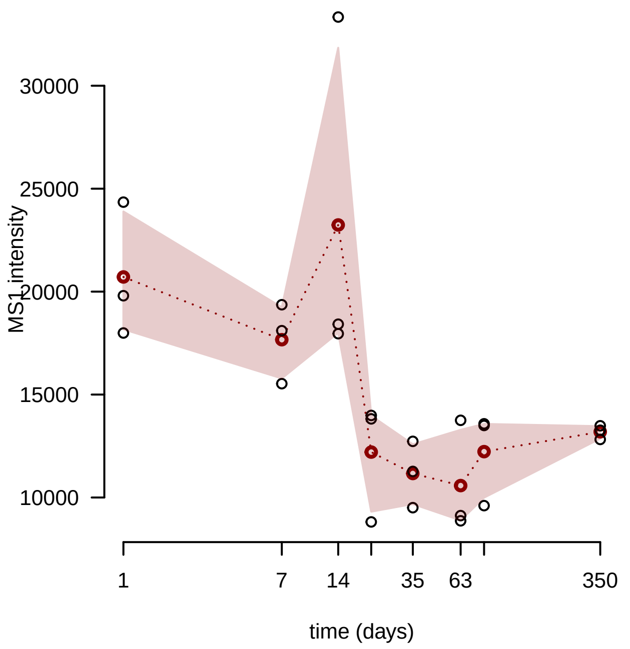

Q91WC0
GN=Setd3
SET domain containing 3

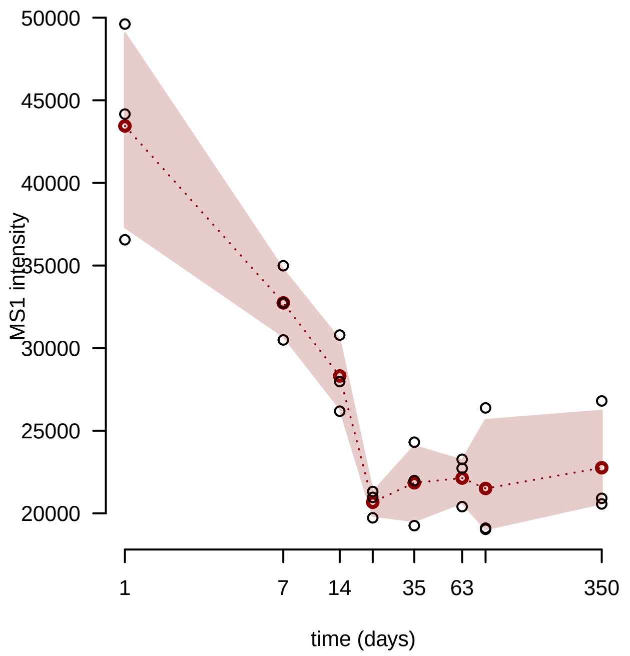

Q91WC3
GN=Acsl6
acyl−CoA synthetase long−chain family me ...

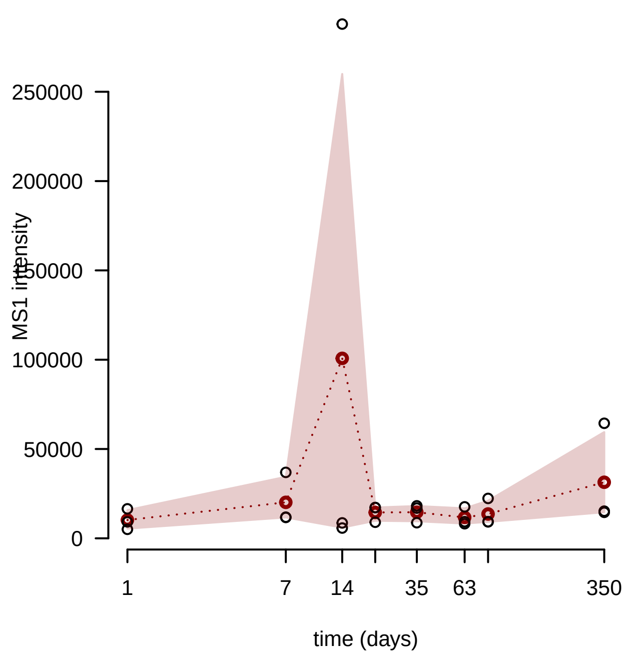

Q91WC9
GN=Daglb
diacylglycerol lipase, beta

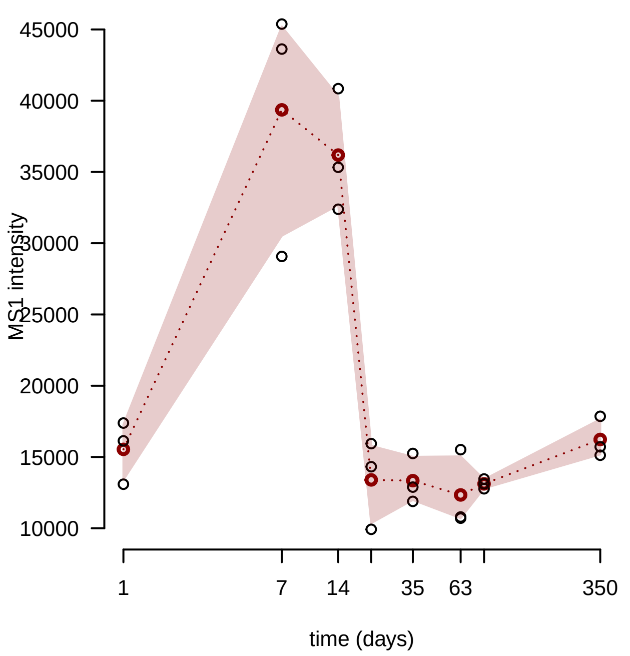

Q91WD0
GN=Gpr108
G protein−coupled receptor 108

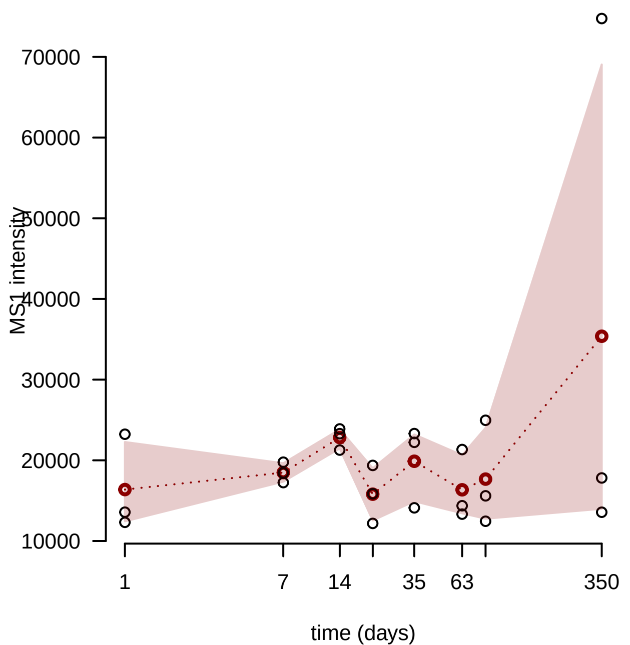

Q91WD1
GN=Polr3d
polymerase (RNA) III (DNA directed) poly ...

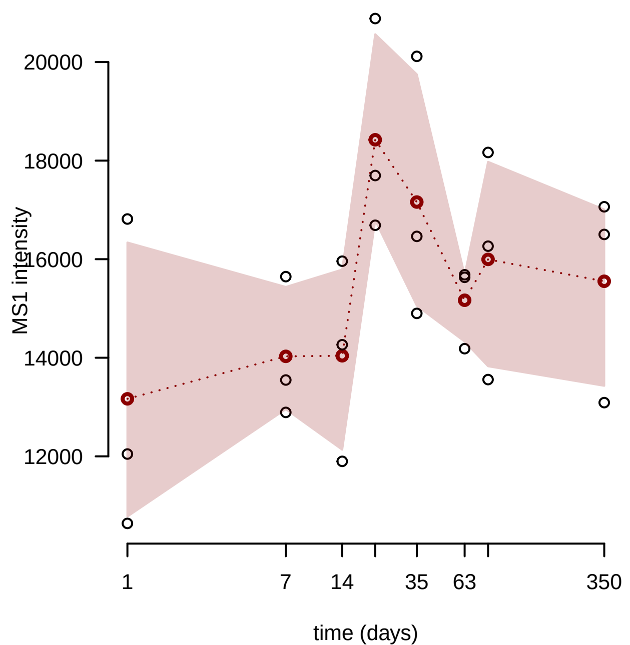

Q91WD5
GN=Ndufs2
NADH:ubiquinone oxidoreductase core subu ...

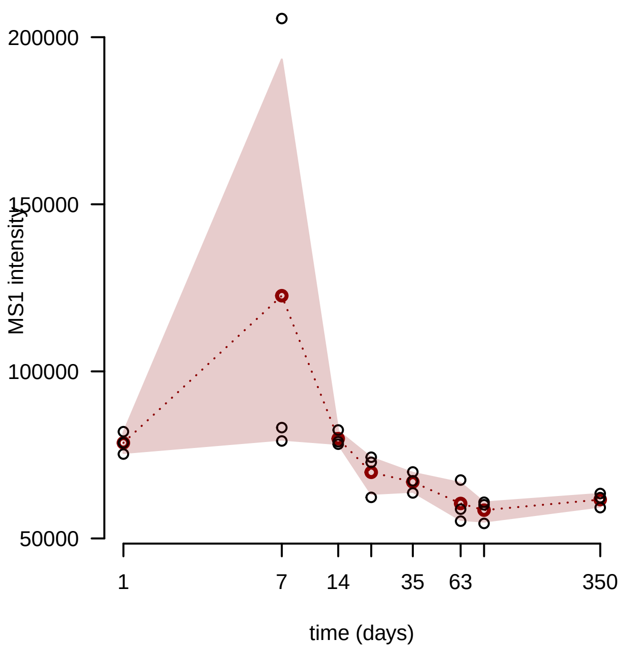

Q91WE1
GN=Snx15
sorting nexin 15

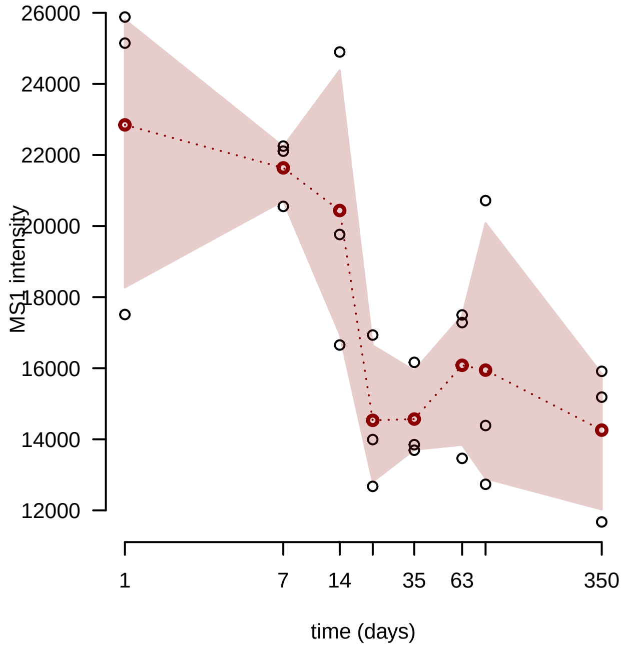

Q91WE2
GN=Fam192a

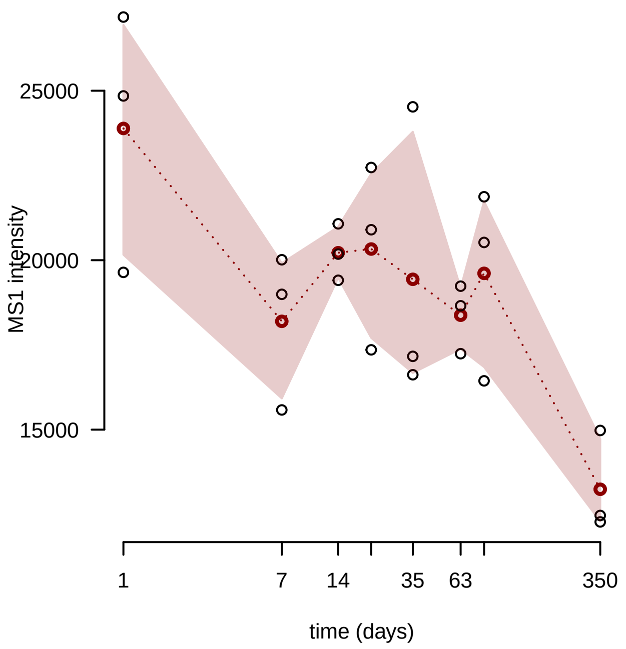

Q91WE3
GN=Rpp25
ribonuclease P/MRP 25 subunit

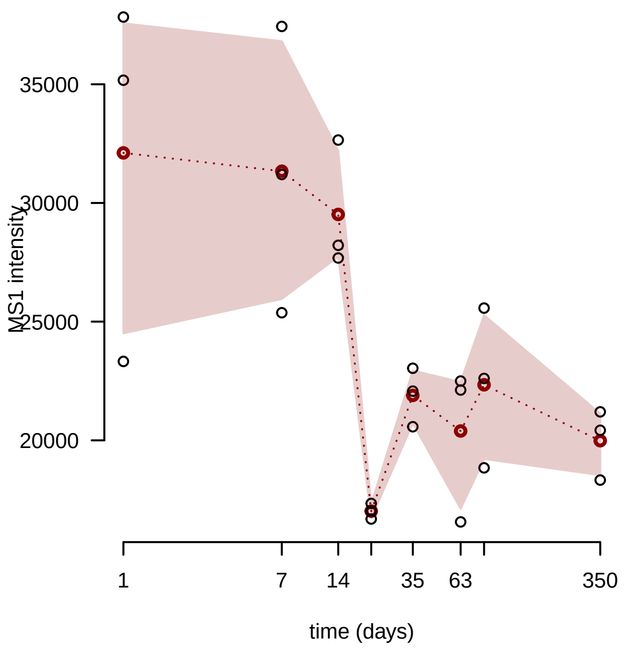

Q91WE4
GN=

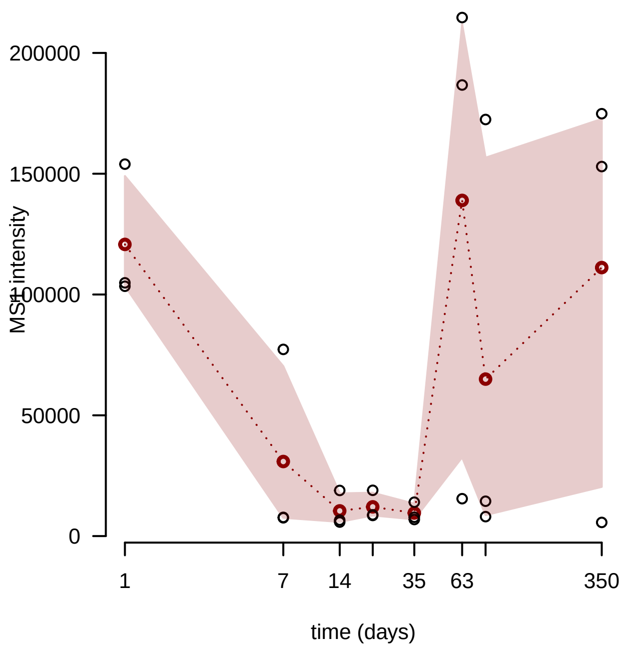

Q91WE6
GN=Cdkal1
CDK5 regulatory subunit associated prote ...

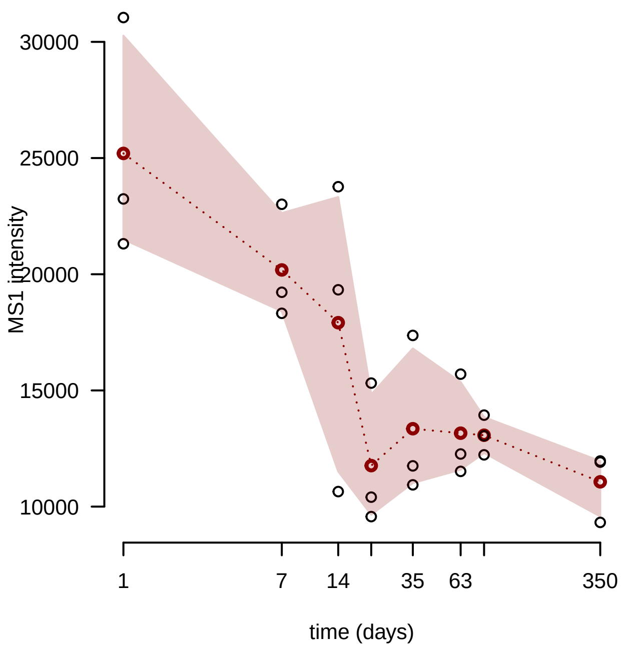

Q91WF0
GN=

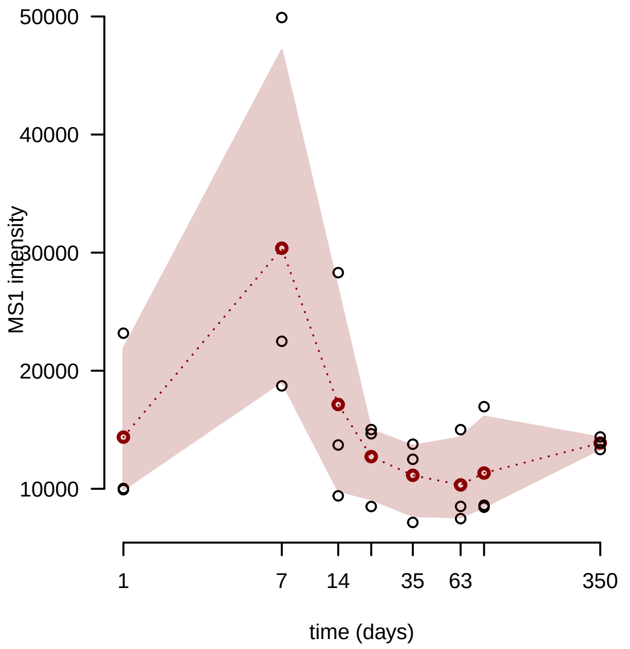

Q91WF7
GN=Fig4
FIG4 phosphoinositide 5−phosphatase

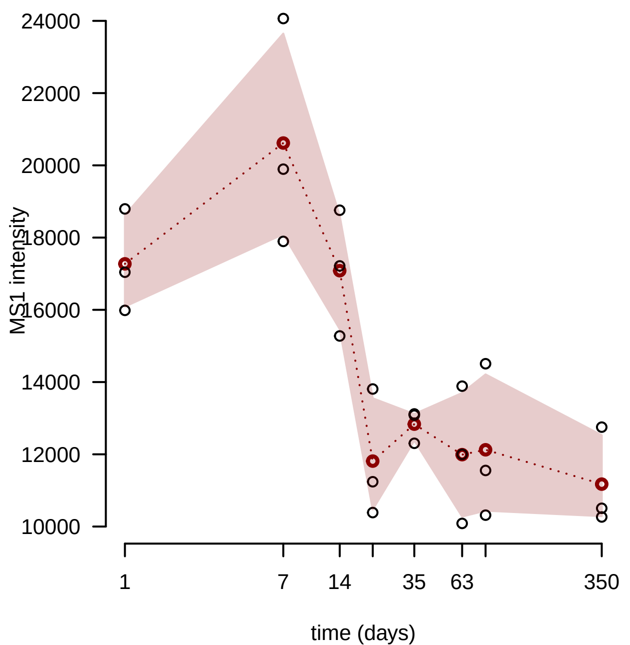

Q91WG0
GN=Ces2c
carboxylesterase 2C

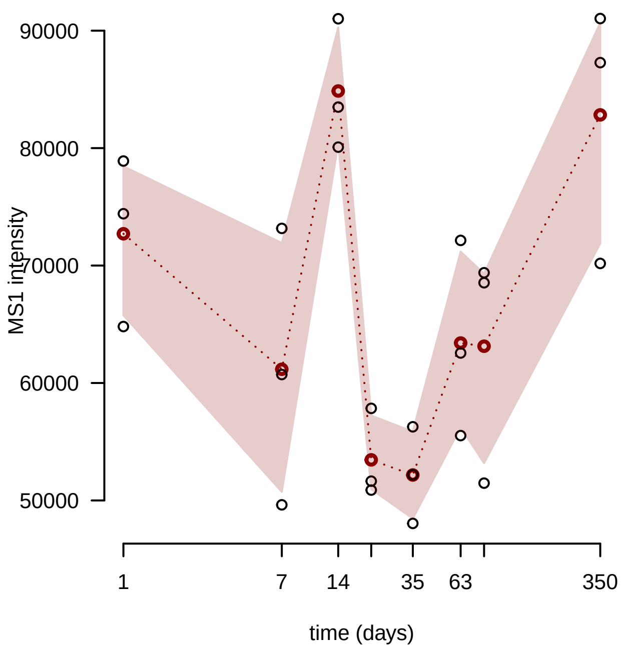

Q91WG2
GN=Rabep2
rabaptin, RAB GTPase binding effector pr ...

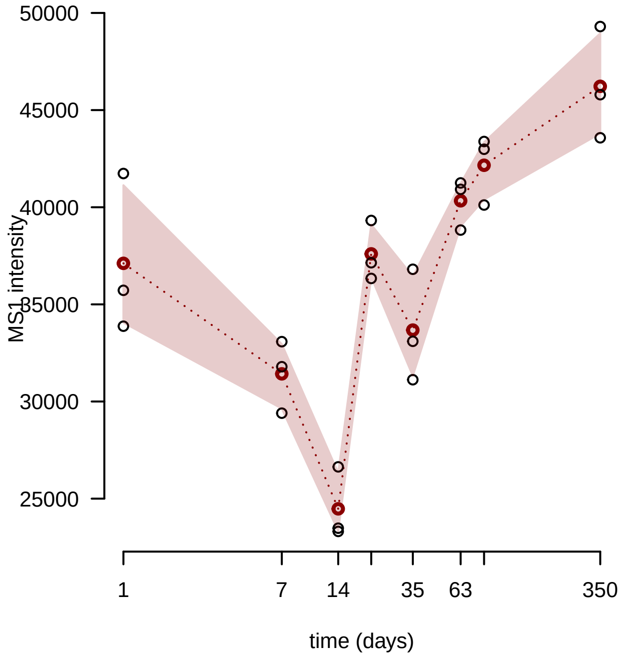

Q91WG4
GN=Elp2
elongator acetyltransferase complex subu ...

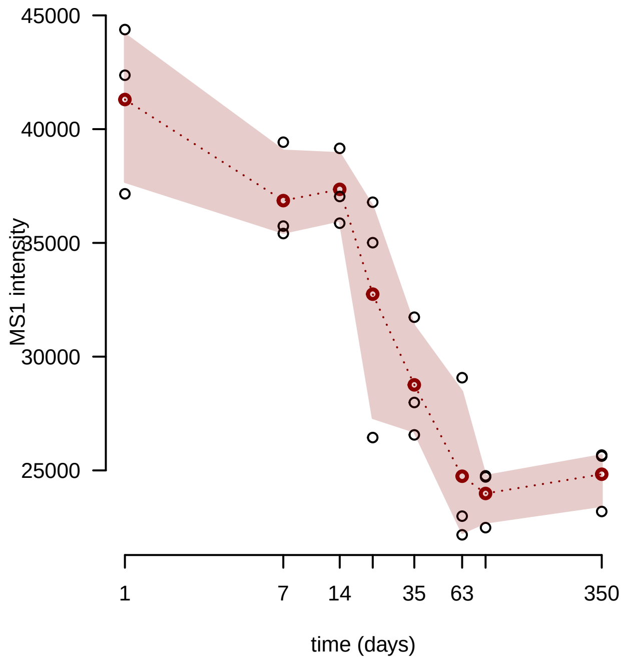

Q91WG5
GN=Prkag2
protein kinase, AMP−activated, gamma 2 n ...

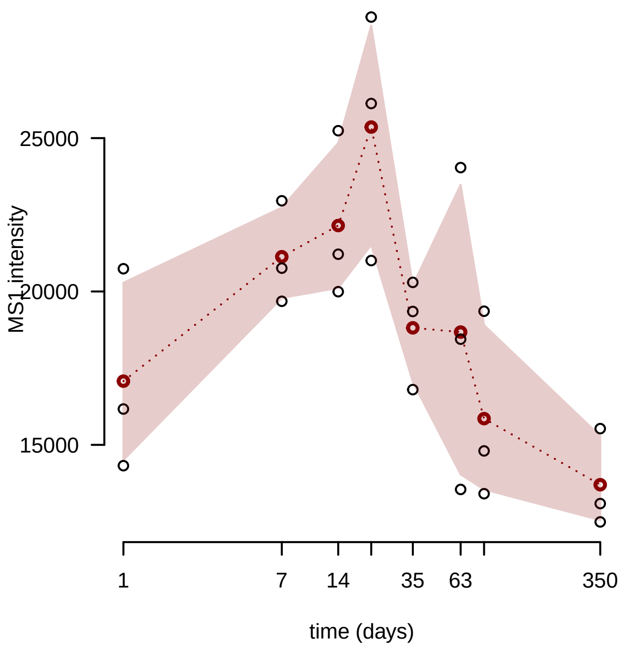

Q91WG8
GN=Gne
glucosamine (UDP−N−acetyl)−2−epimerase/N ...

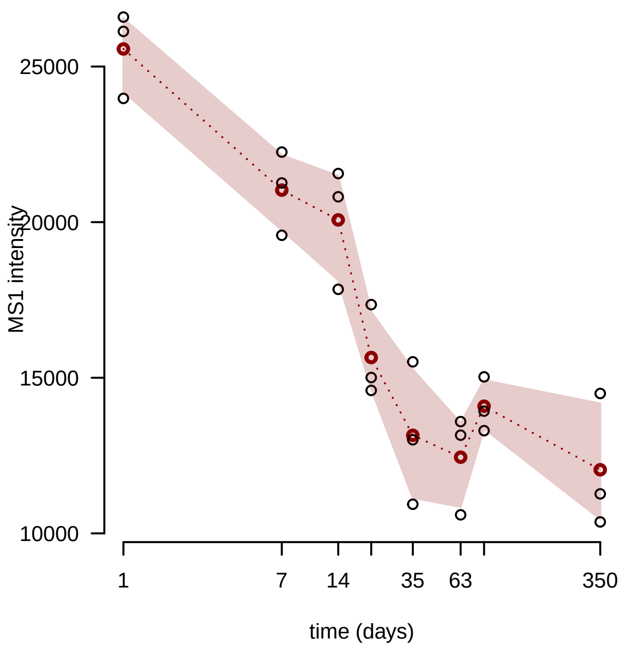

Q91WI7
GN=Itfg2
integrin alpha FG−GAP repeat containing ...

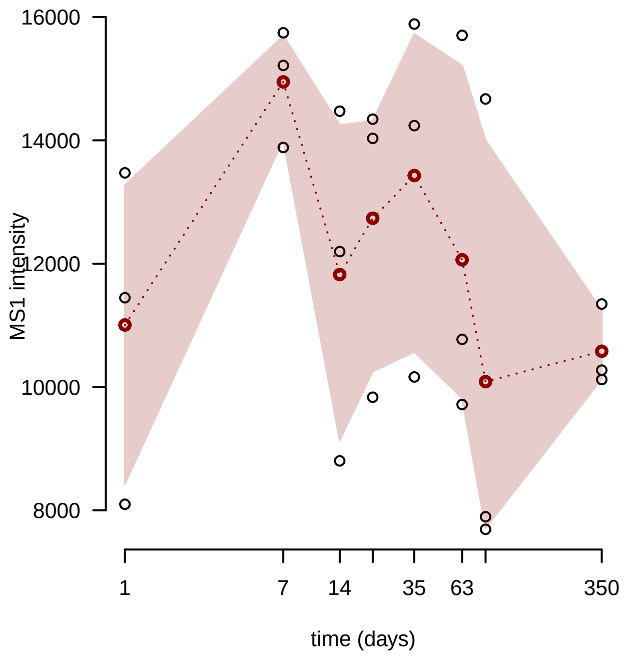

Q91WJ7
GN=Spats2l
spermatogenesis associated, serine−rich ...

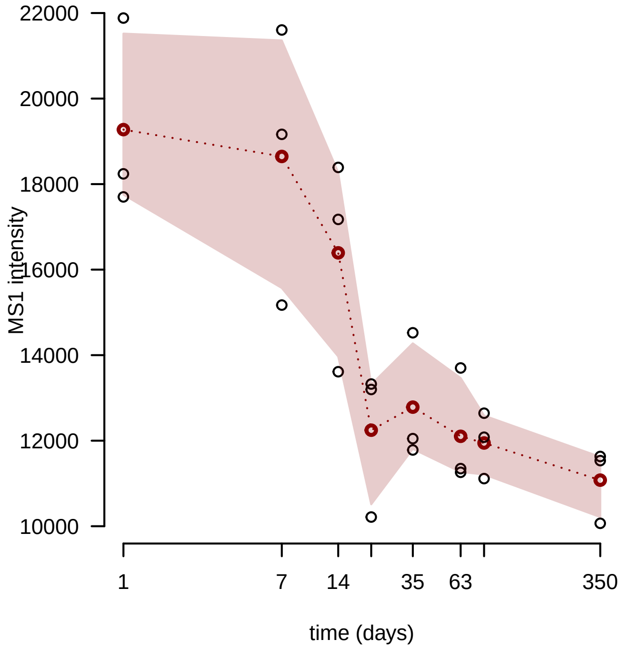

Q91WJ8
GN=Fubp1
far upstream element (FUSE) binding prot ...

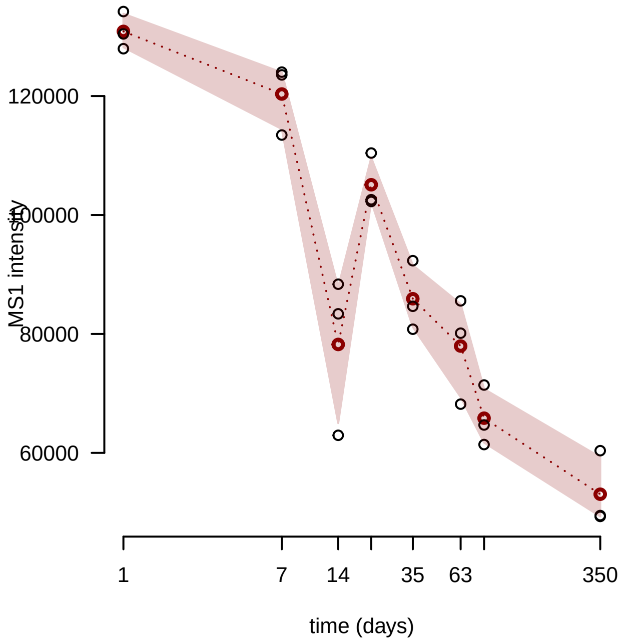

Q91WK0
GN=Lrrfip2
leucine rich repeat (in FLII) interactin ...

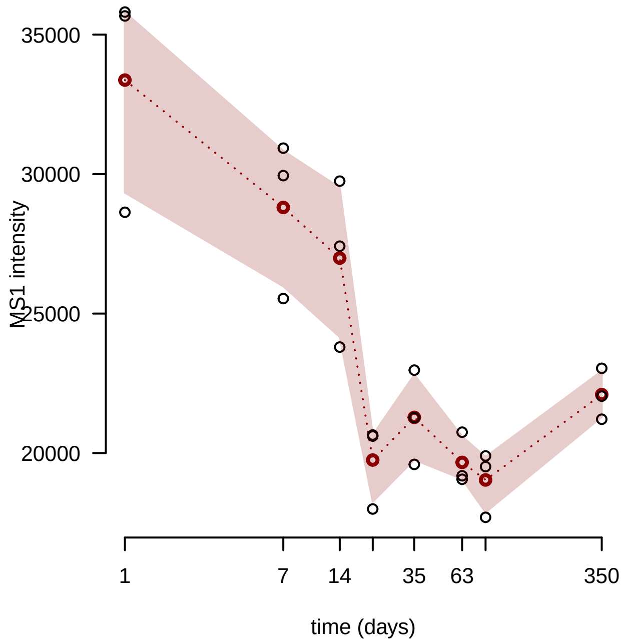

Q91WK1
GN=Spryd4
SPRY domain containing 4

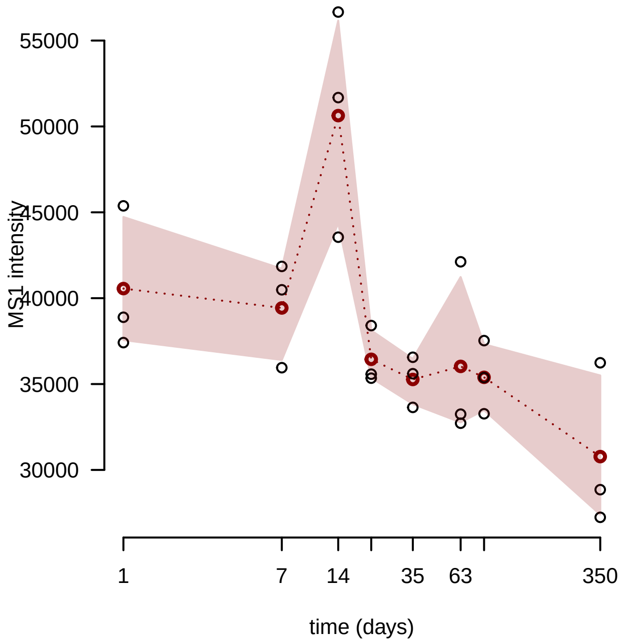

Q91WK2
GN=Eif3h
eukaryotic translation initiation factor ...

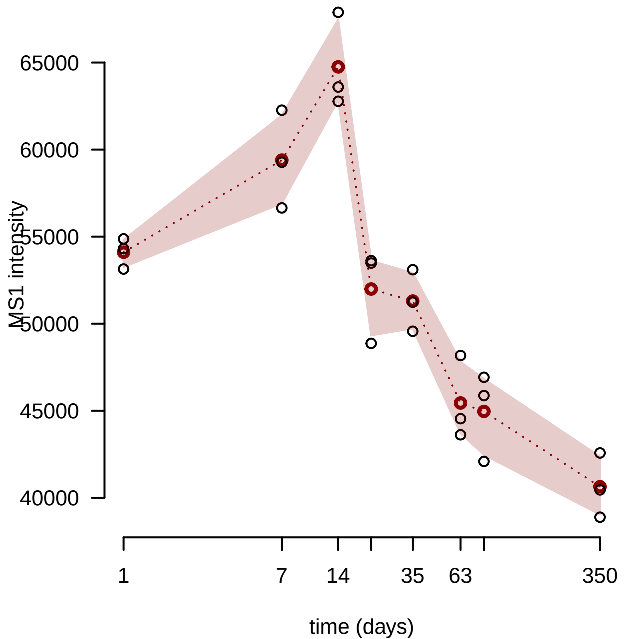

Q91WK5
GN=Gcsh
glycine cleavage system protein H (amino ...

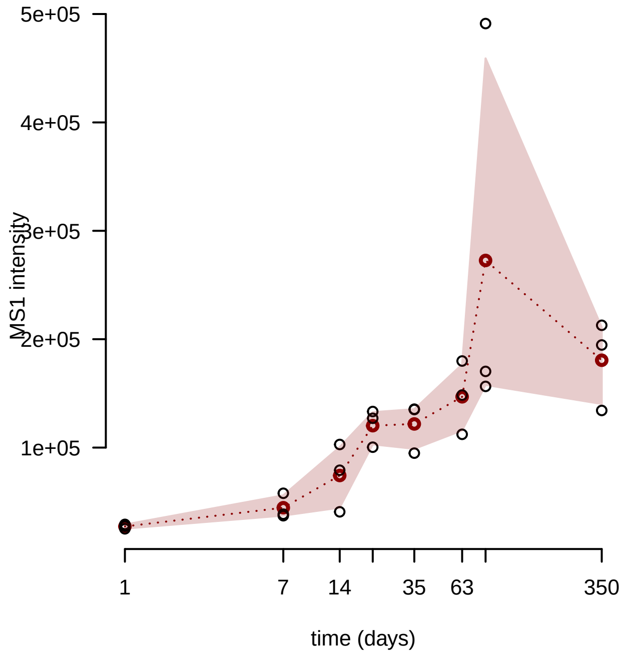

Q91WL0
GN=Eps8l3
EPS8−like 3

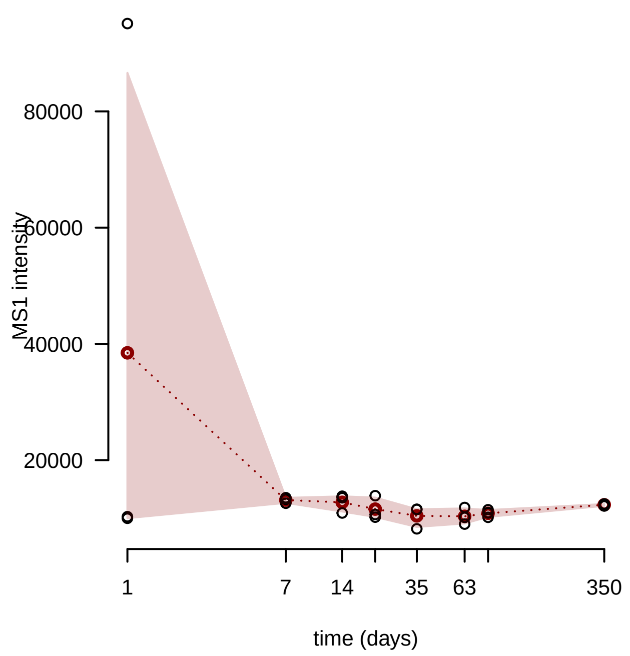

Q91WL6
GN=Snx11
sorting nexin 11

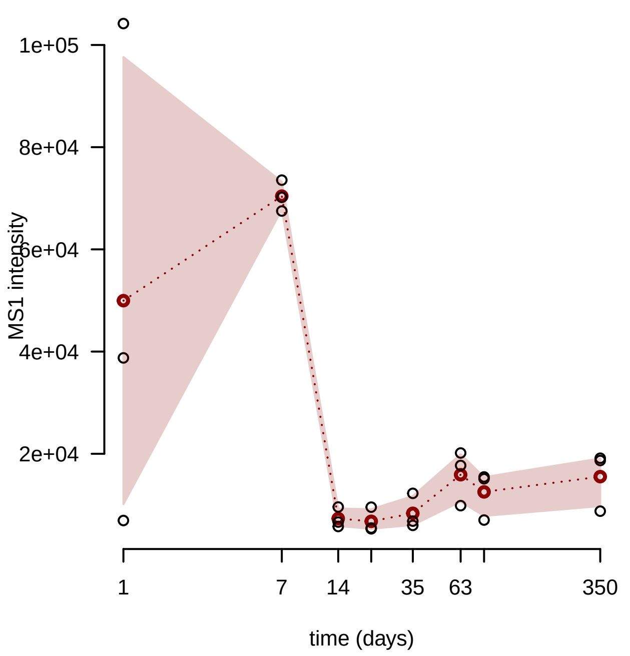

Q91WL8
GN=Wwox
WW domain−containing oxidoreductase

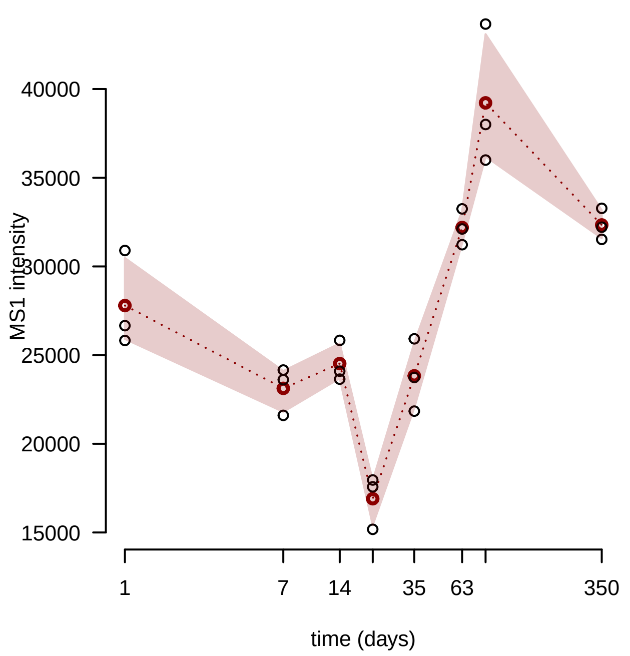

Q91WM1
GN=Strbp
spermatid perinuclear RNA binding protei ...

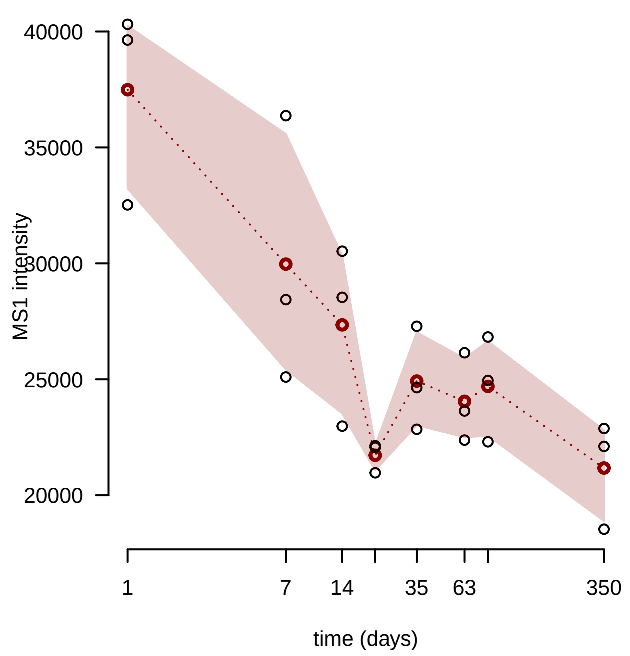

Q91WM2
GN=Hdhd5
haloacid dehalogenase like hydrolase dom ...

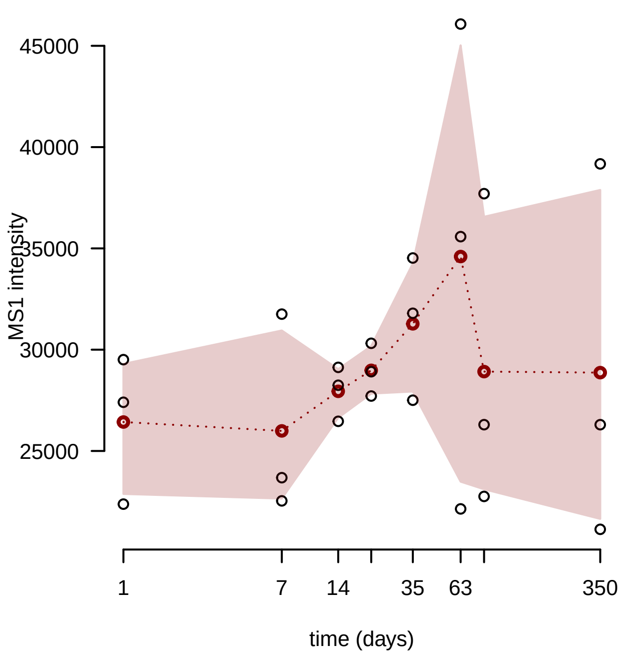

Q91WM3
GN=Rrp9
ribosomal RNA processing 9, U3 small nuc ...

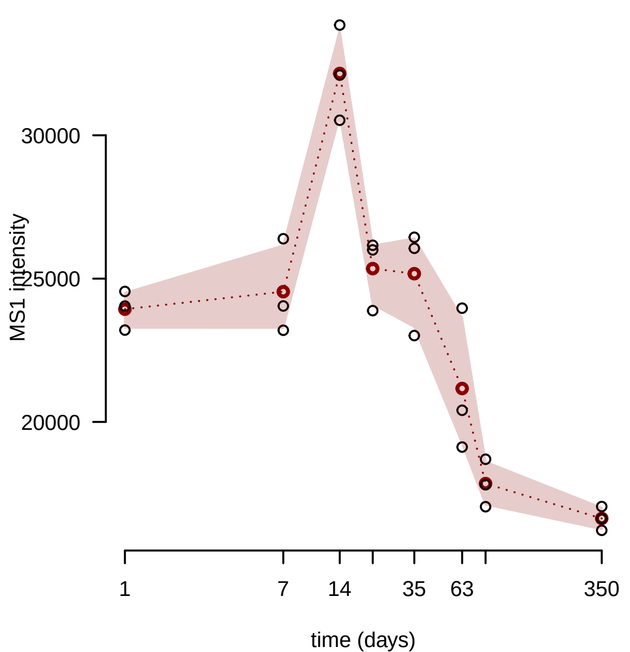

Q91WN1
GN=Dnajc9
DnaJ heat shock protein family (Hsp40) m ...

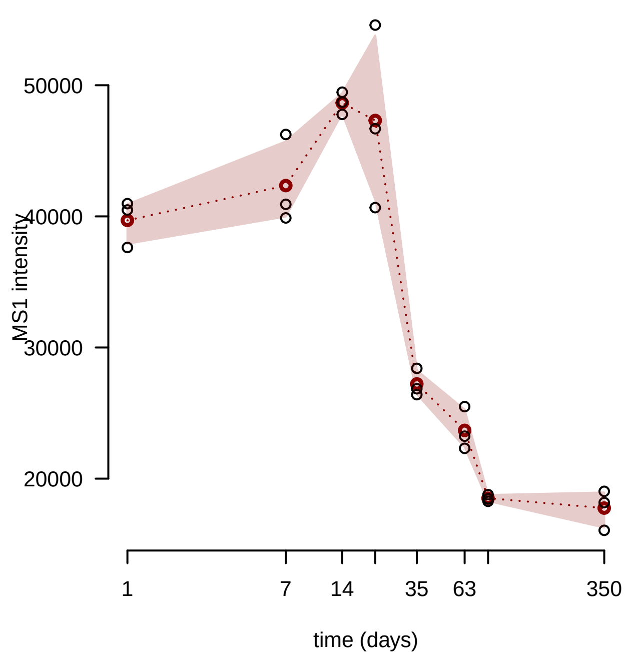

Q91WP0
GN=Masp2
mannan−binding lectin serine peptidase 2

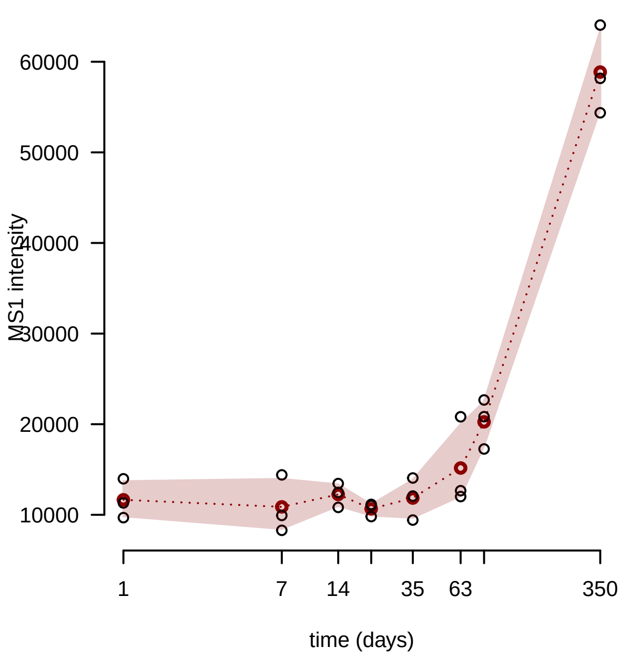

Q91WP6
GN=Serpina3n
serine (or cysteine) peptidase inhibitor ...

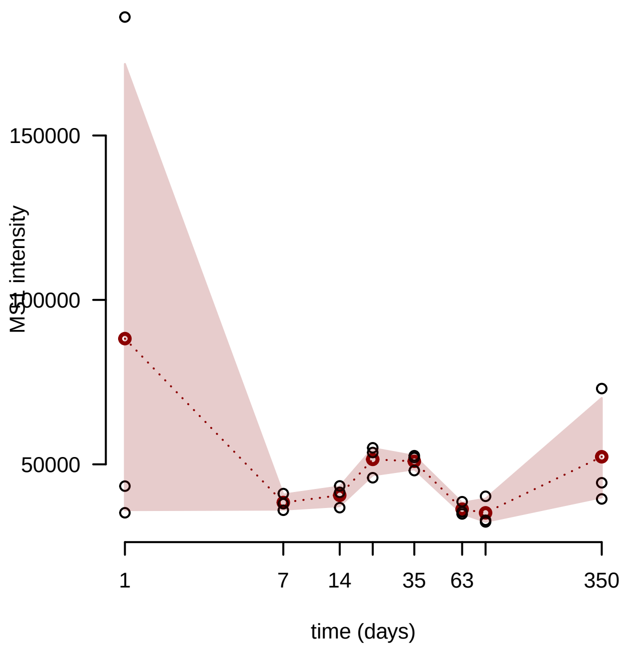

Q91WQ3
GN=Yars
tyrosyl−tRNA synthetase

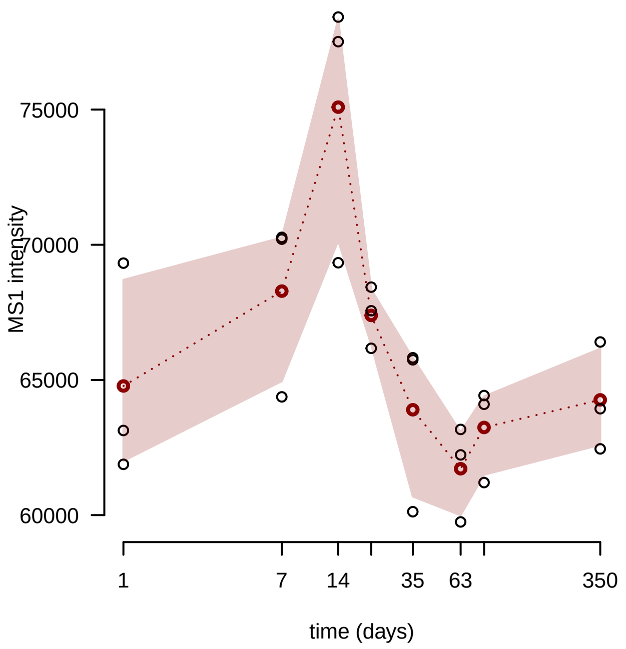

Q91WQ5
GN=Taf5l
TATA−box binding protein associated fact ...

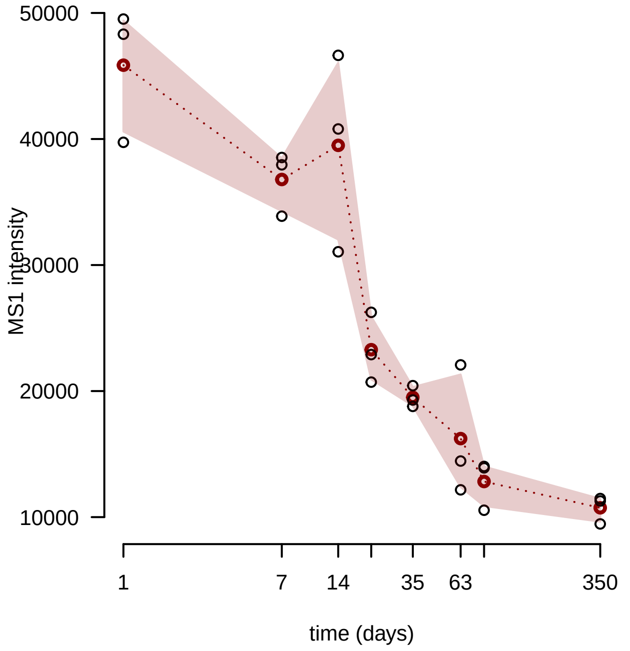

Q91WR3
GN=Ascc2
activating signal cointegrator 1 complex ...

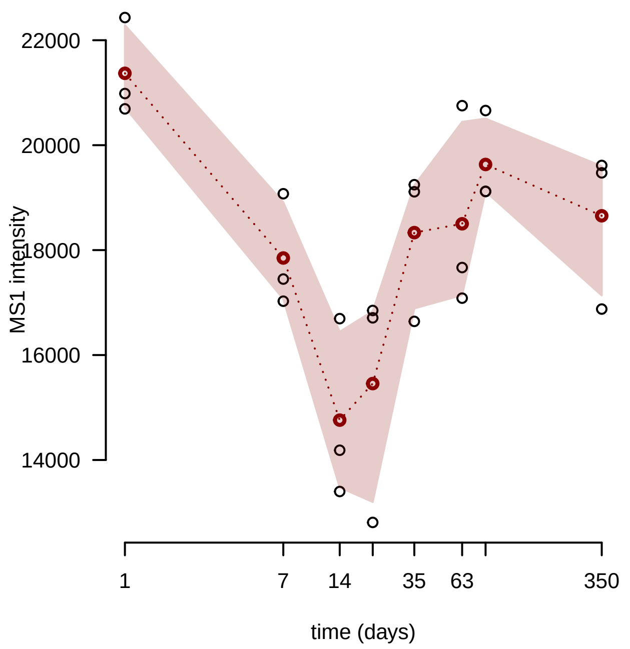

Q91WS0
GN=Cisd1
CDGSH iron sulfur domain 1

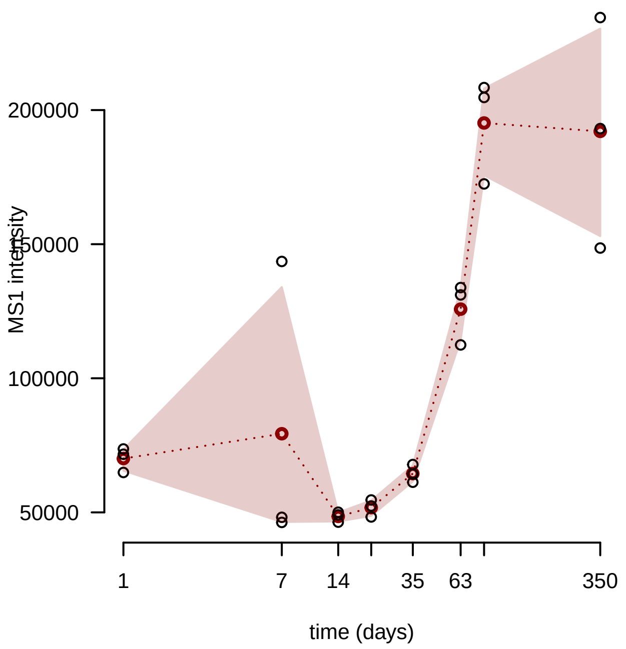

Q91WS4
GN=Bhmt2
betaine−homocysteine methyltransferase 2

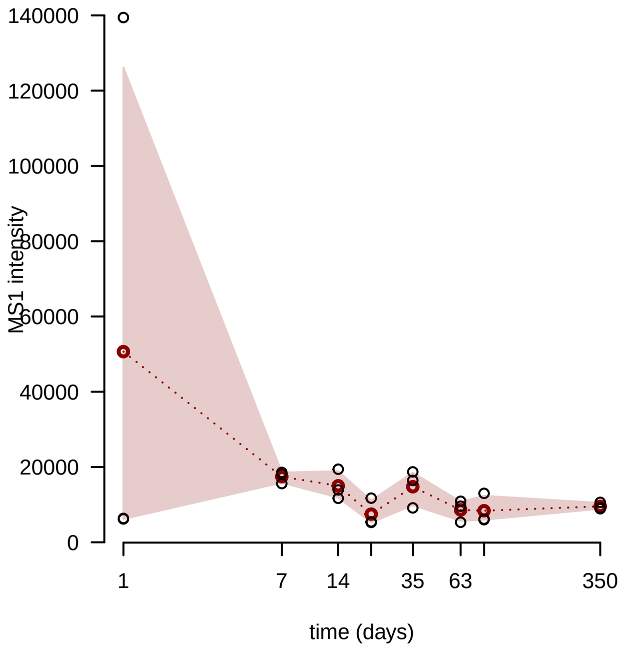

Q91WS7
GN=Depdc7
DEP domain containing 7

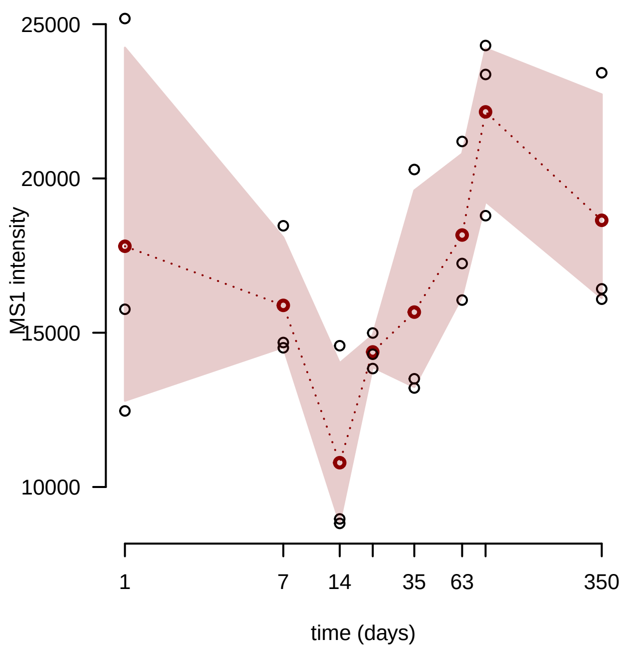

Q91WT4
GN=Dnajc17
DnaJ heat shock protein family (Hsp40) m ...

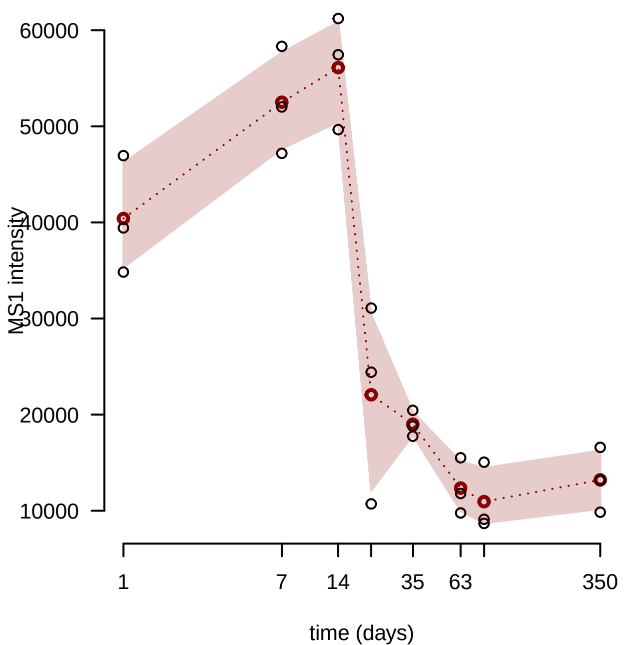

Q91WT8
GN=Rbm47
RNA binding motif protein 47

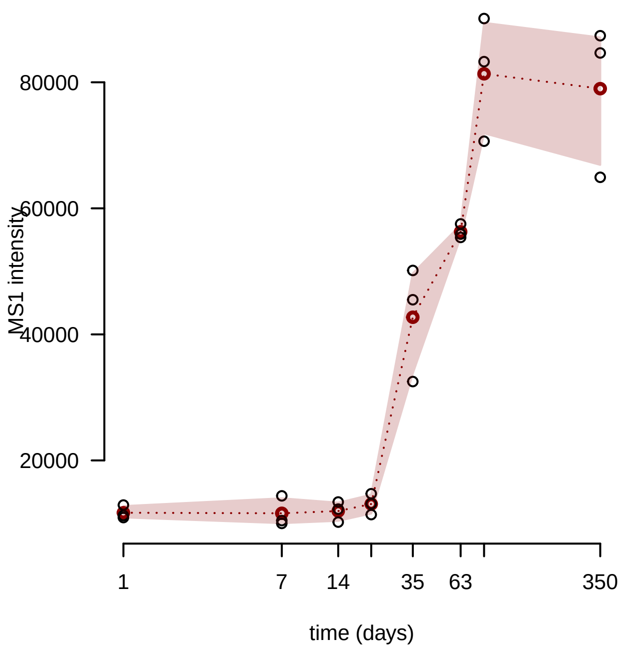

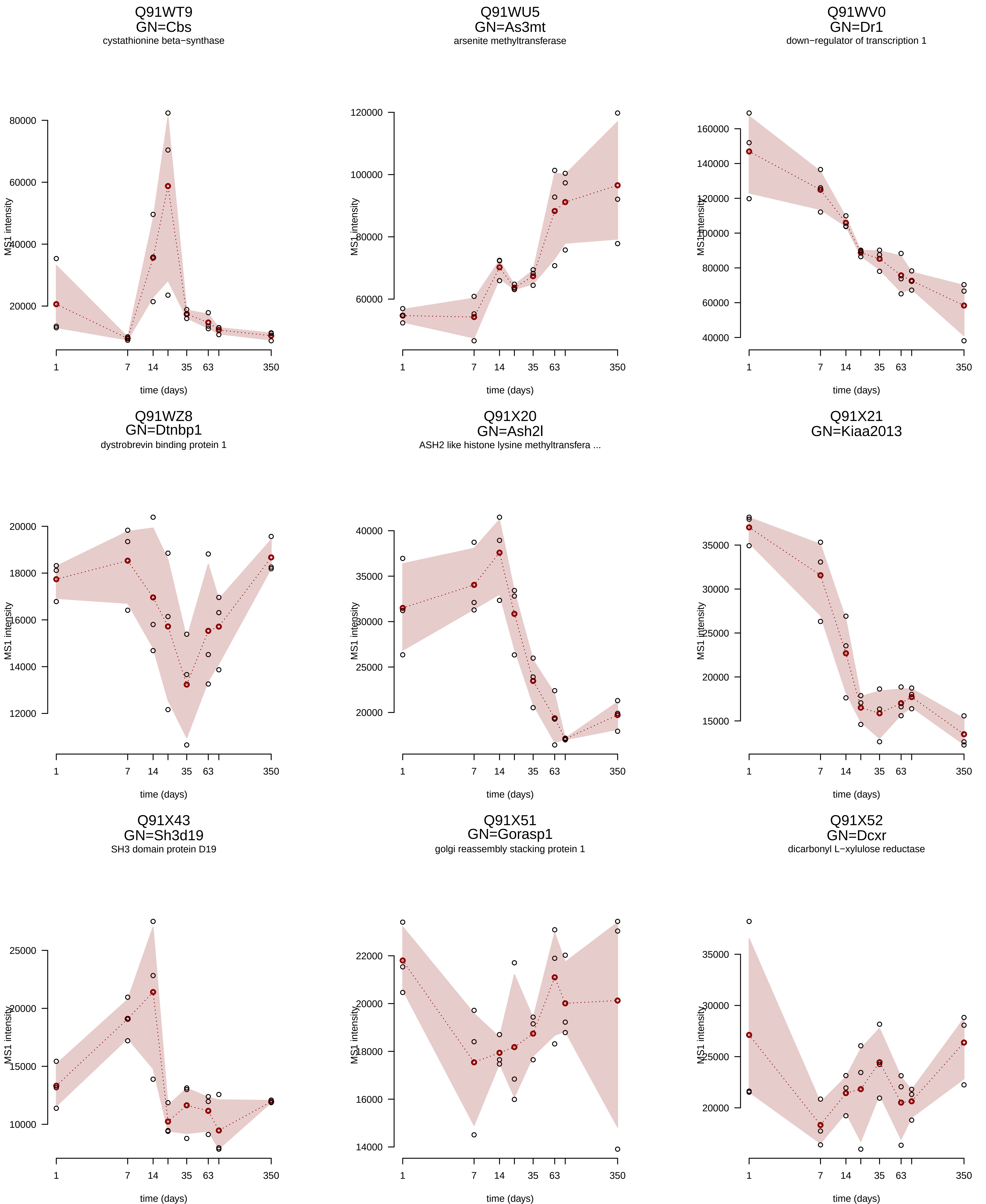
Q91WT9
GN=Cbs
cystathionine beta−synthase
Q91WU5
GN=As3mt
arsenite methyltransferase
Q91WV0
GN=Dr1
down−regulator of transcription 1
Q91WZ8
GN=Dtnbp1
dystrobrevin binding protein 1
Q91X20
GN=Ash2l
ASH2 like histone lysine methyltransfera ...
Q91X21
GN=Kiaa2013
Q91X43
GN=Sh3d19
SH3 domain protein D19
Q91X51
GN=Gorasp1
golgi reassembly stacking protein 1
Q91X52
GN=Dcxr
dicarbonyl L−xylulose reductase
MS1 intensity
time (days)
1
7
14
35
63
350

Q91X58
GN=Zfand2b
zinc finger, AN1 type domain 2B

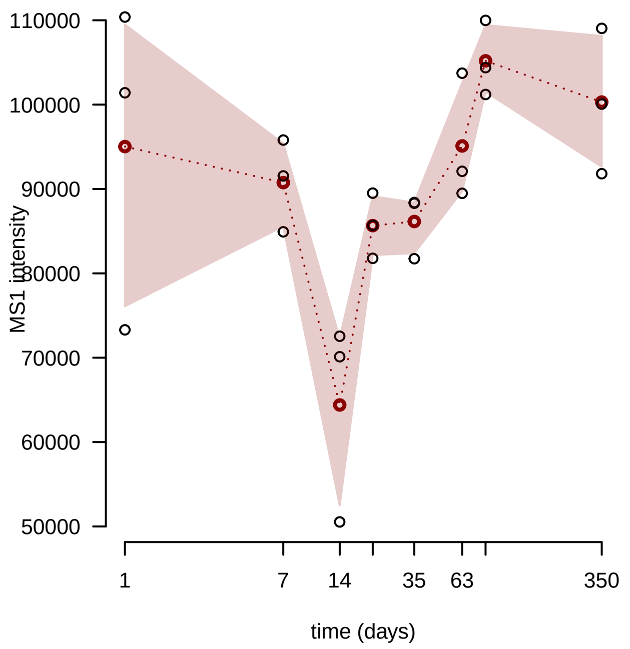

Q91X72
GN=Hpx
hemopexin

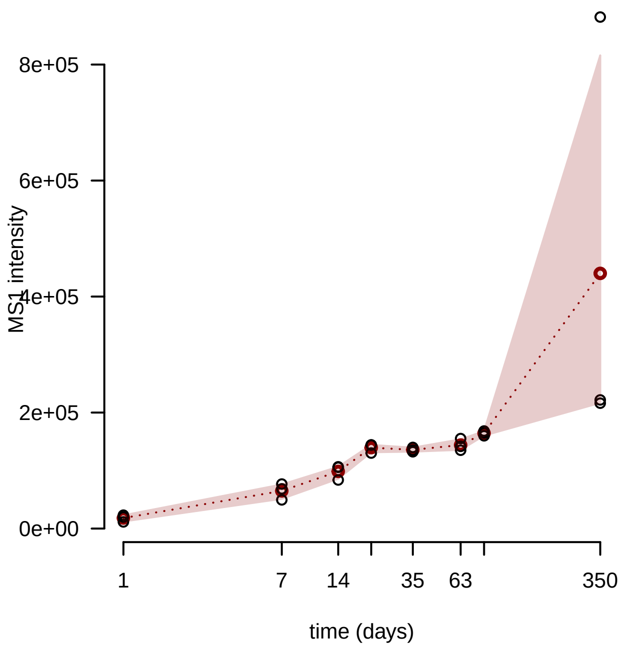

Q91X78
GN=Erlin1
ER lipid raft associated 1

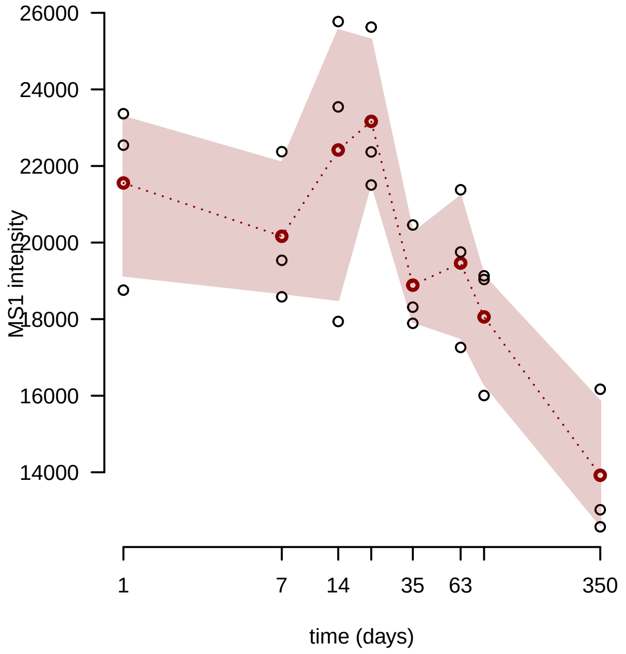

Q91X79
GN=Cela1
chymotrypsin−like elastase family, membe ...

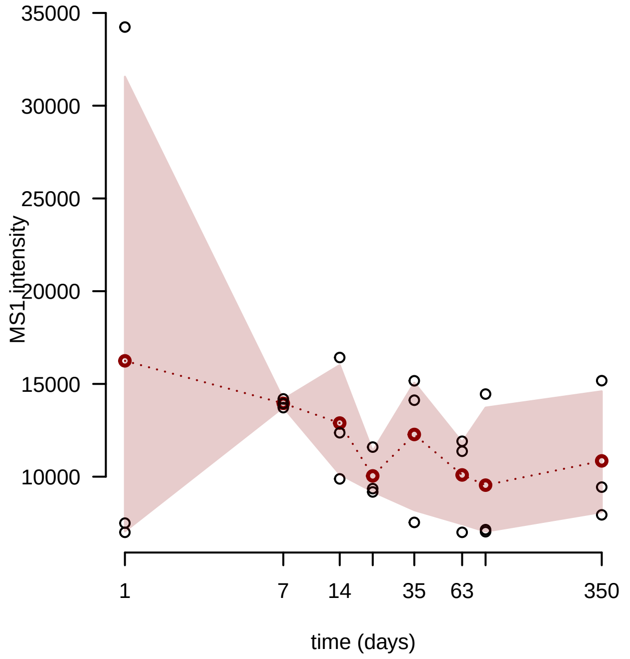

Q91X83
GN=Mat1a
methionine adenosyltransferase I, alpha

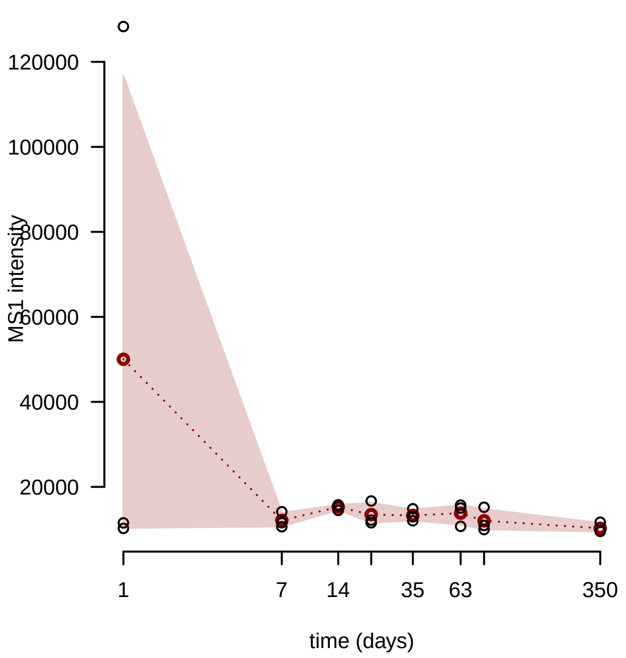

Q91X84
GN=Crtc3
CREB regulated transcription coactivator ...

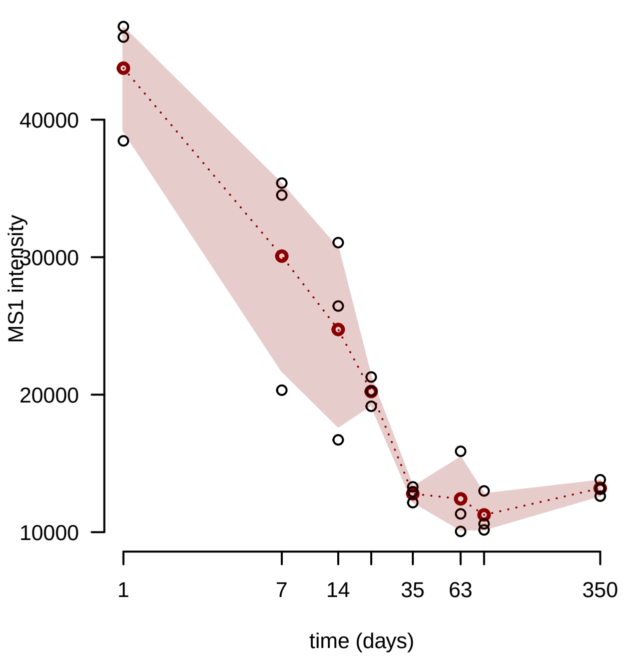

Q91X88
GN=Pomgnt1
protein O−linked mannose beta 1,2−N−acet ...

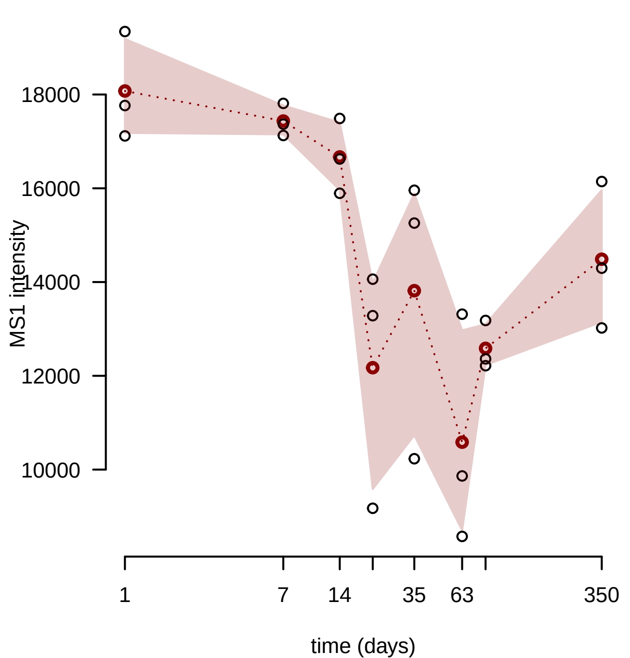

Q91X91
GN=Qprt
quinolinate phosphoribosyltransferase

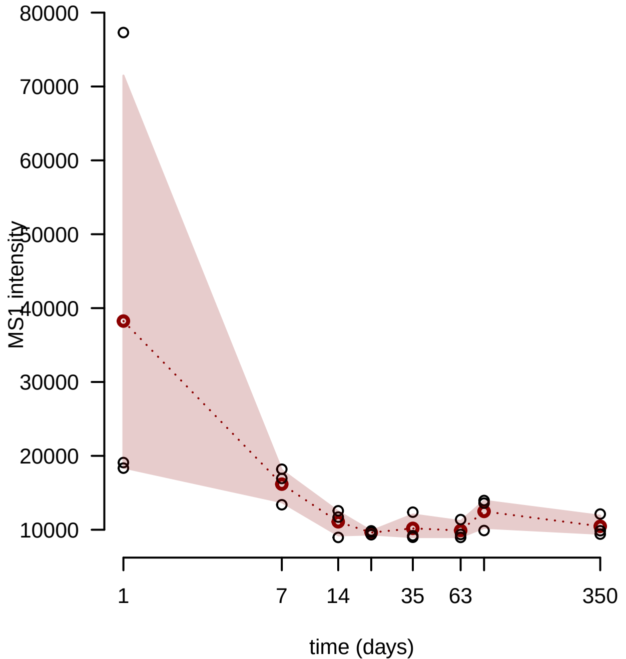

Q91X96
GN=Rabif
RAB interacting factor

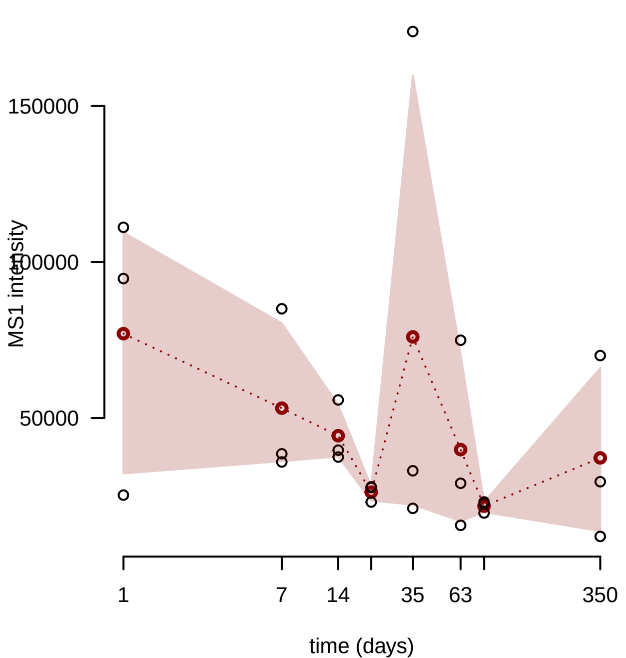

Q91X97
GN=Ncald
neurocalcin delta

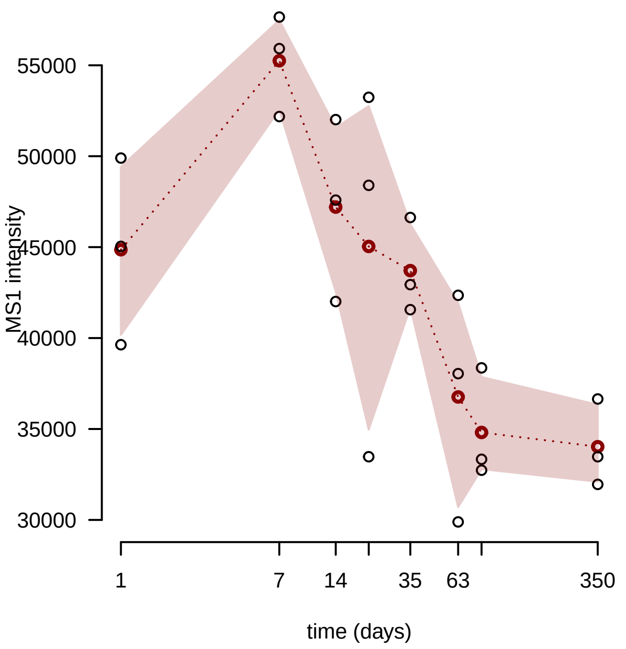

Q91XA2
GN=Golm1
golgi membrane protein 1

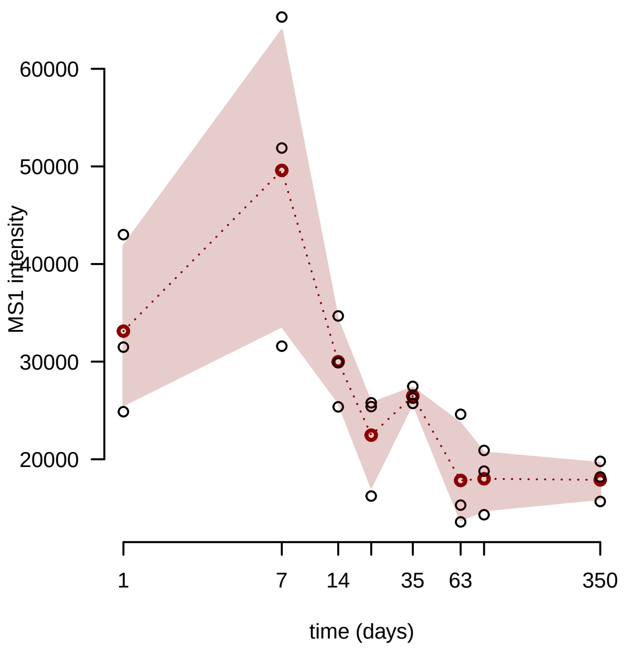

Q91XB0
GN=Trex1
three prime repair exonuclease 1

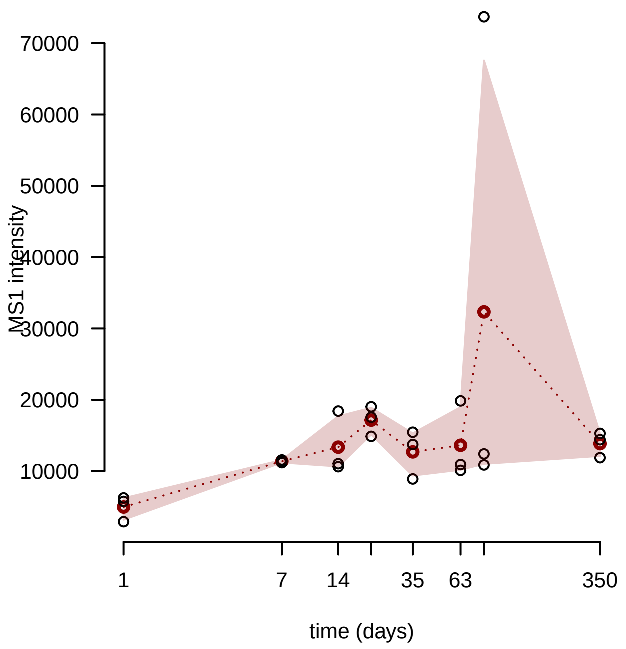

Q91XB7
GN=Yif1a
Yip1 interacting factor homolog A (S. ce ...

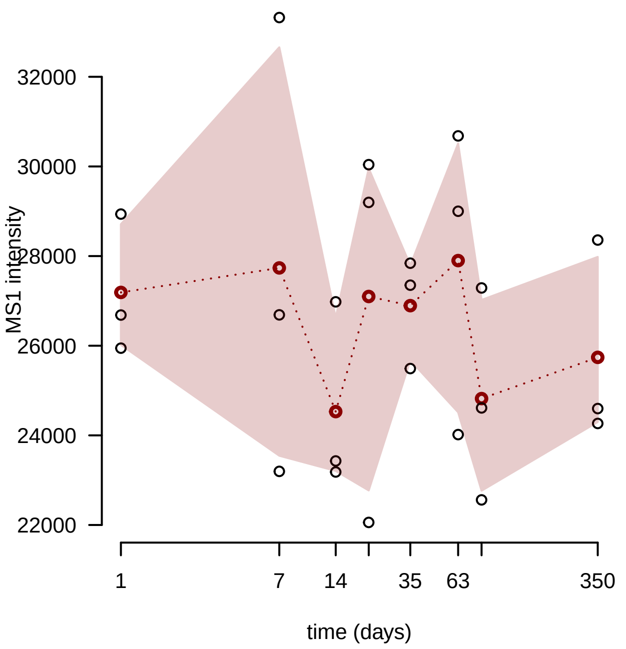

Q91XC8
GN=Dap
death−associated protein

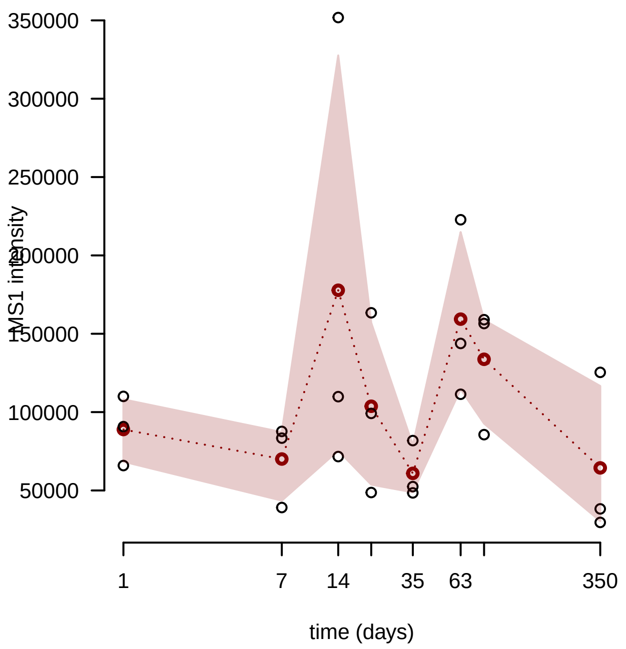

Q91XC9
GN=Pex16
peroxisomal biogenesis factor 16

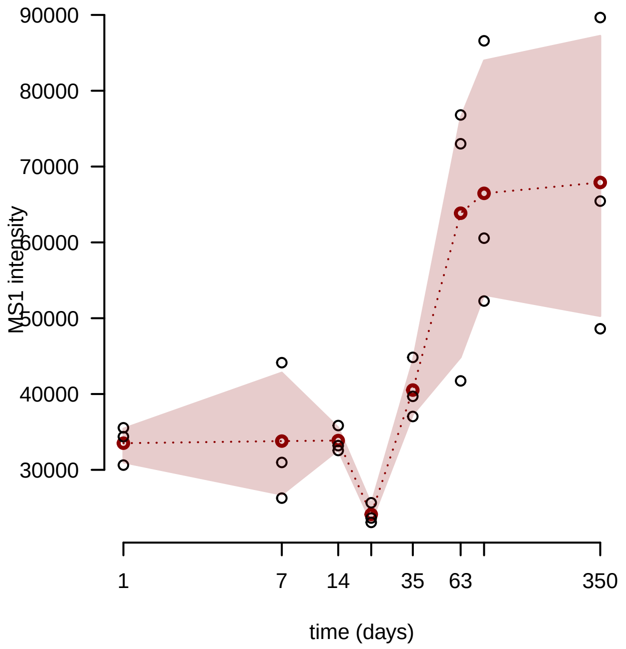

Q91XD4
GN=Ftcd
formiminotransferase cyclodeaminase

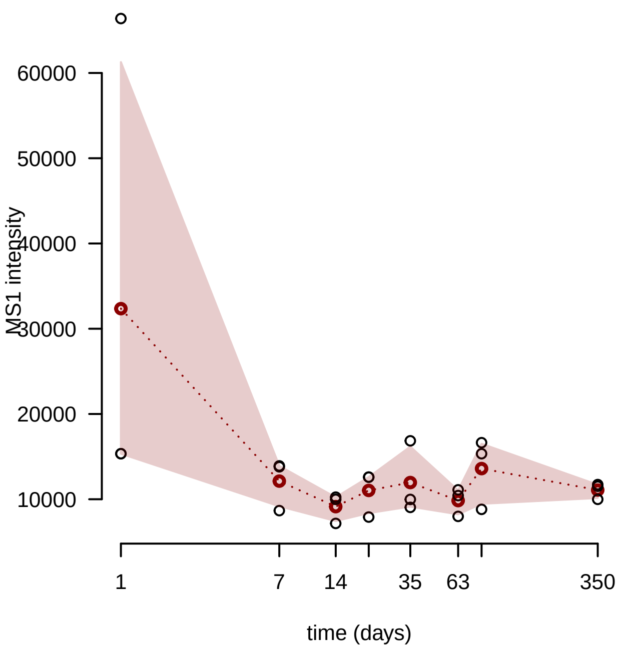

Q91XD6
GN=Vps36
vacuolar protein sorting 36

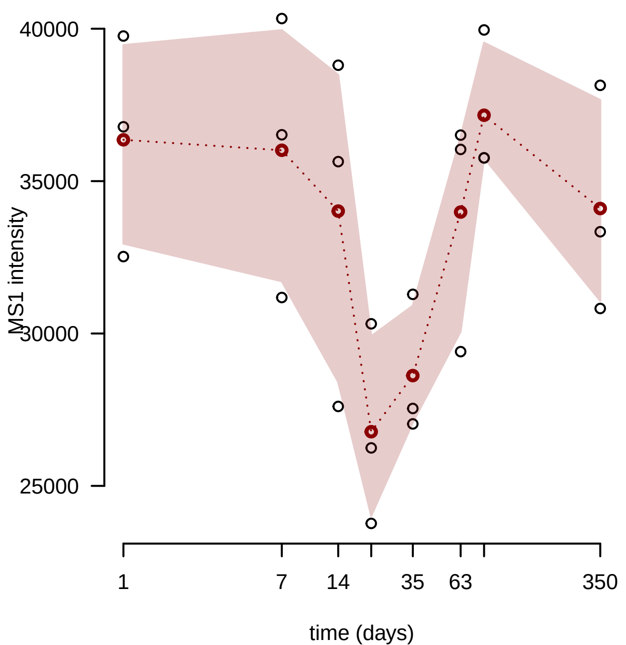

Q91XD7
GN=Creld1
cysteine−rich with EGF−like domains 1

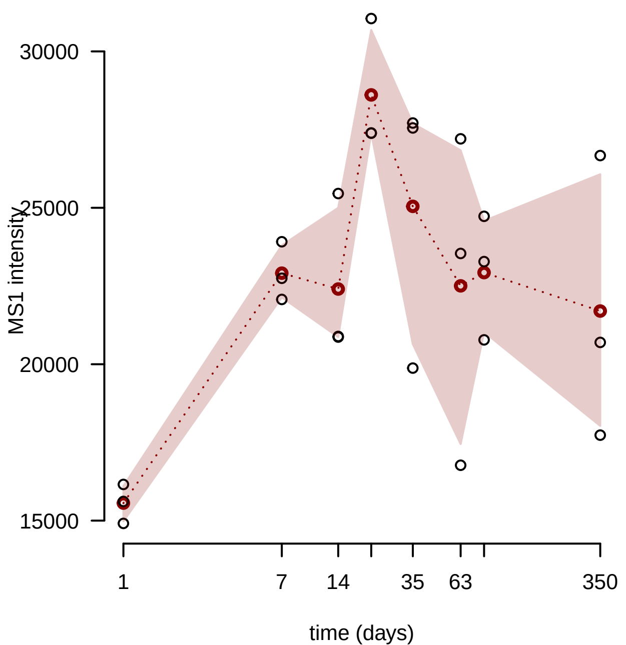

Q91XE4
GN=Acy3
aspartoacylase (aminoacylase) 3

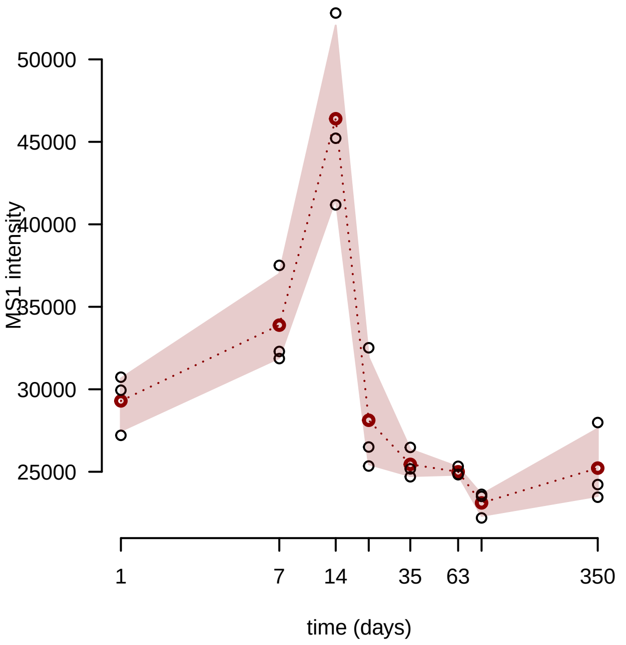

Q91XE8
GN=Tmem205
transmembrane protein 205

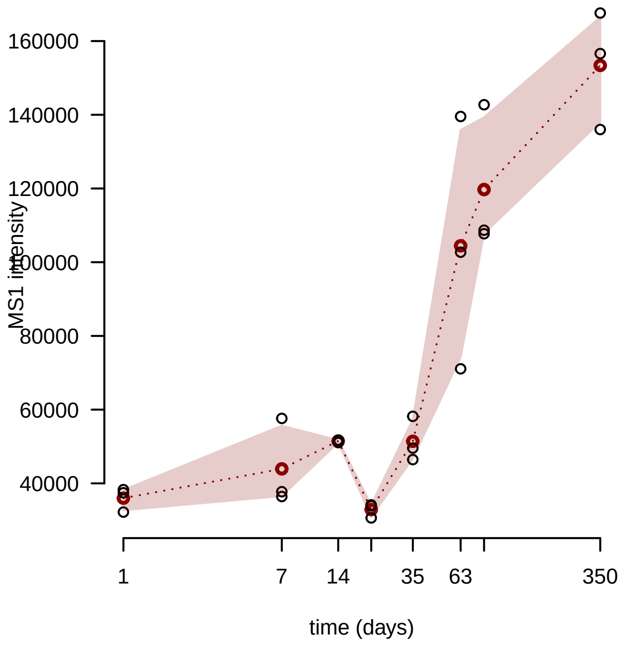

Q91XF0
GN=Pnpo
pyridoxine 5'−phosphate oxidase

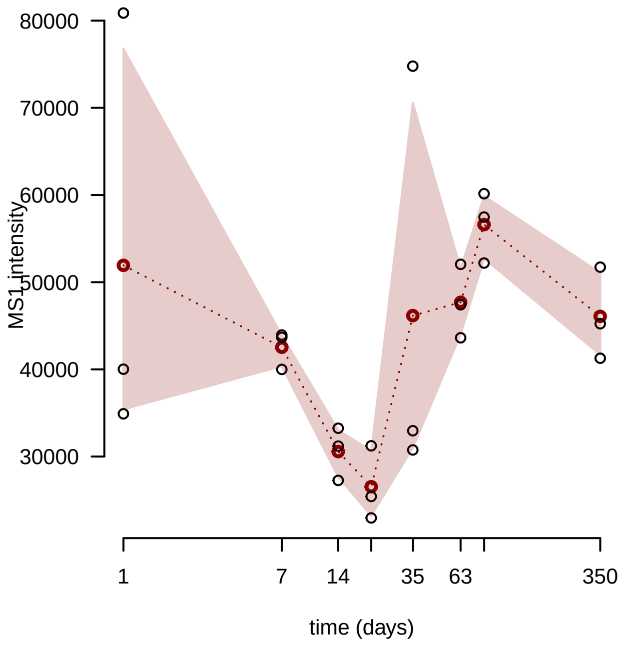

Q91XI1
GN=Dus3l
dihydrouridine synthase 3−like (S. cerev ...

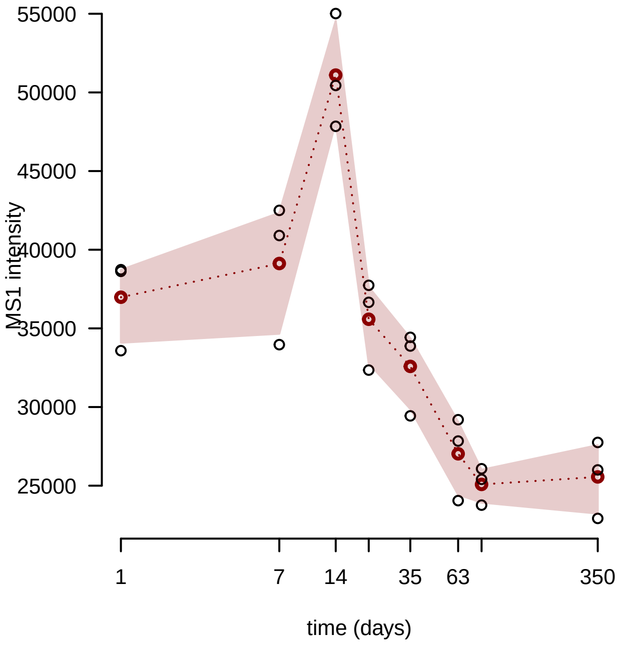

Q91XL3
GN=Uxs1
UDP−glucuronate decarboxylase 1

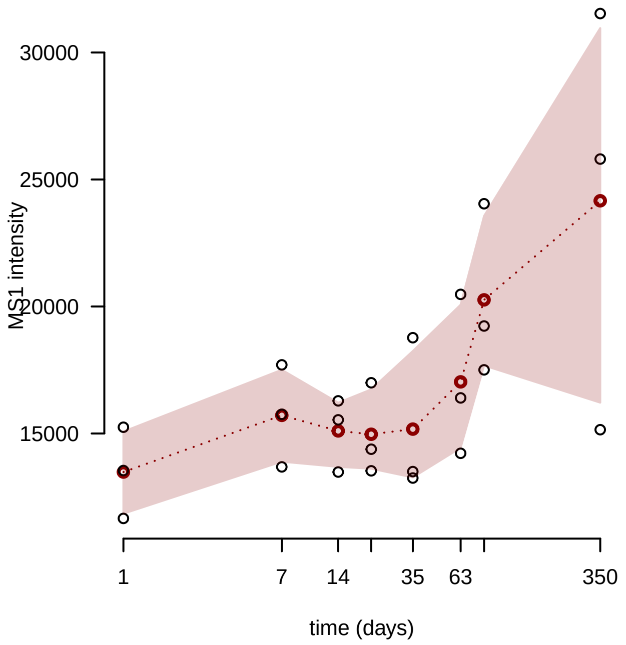

Q91XL9
GN=Osbpl1a
oxysterol binding protein−like 1A

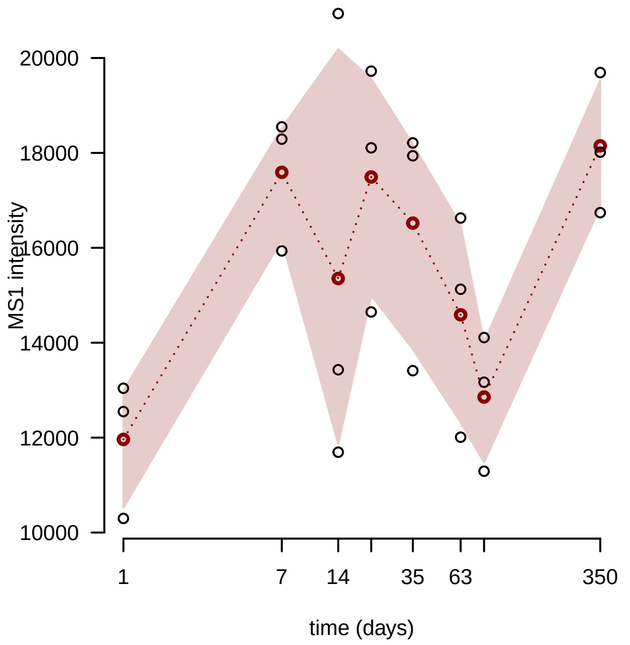

Q91XM9
GN=Dlg2
discs large MAGUK scaffold protein 2

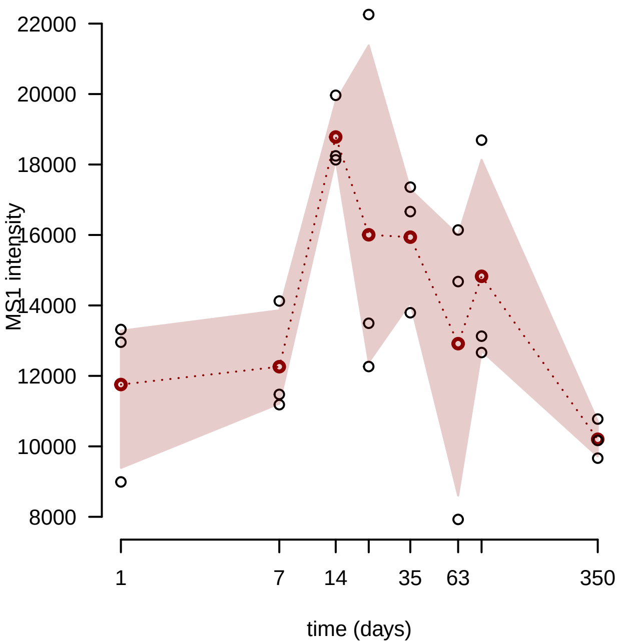

Q91XQ5
GN=Chst15
carbohydrate sulfotransferase 15

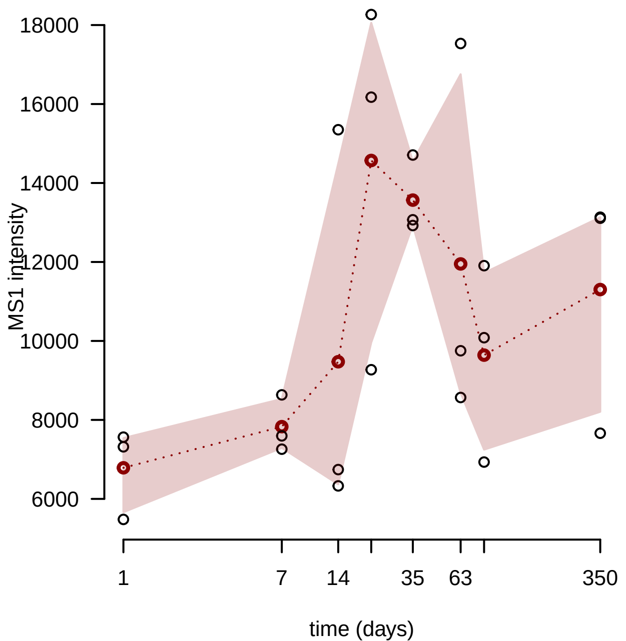

Q91XS1
GN=Mtmr4
myotubularin related protein 4

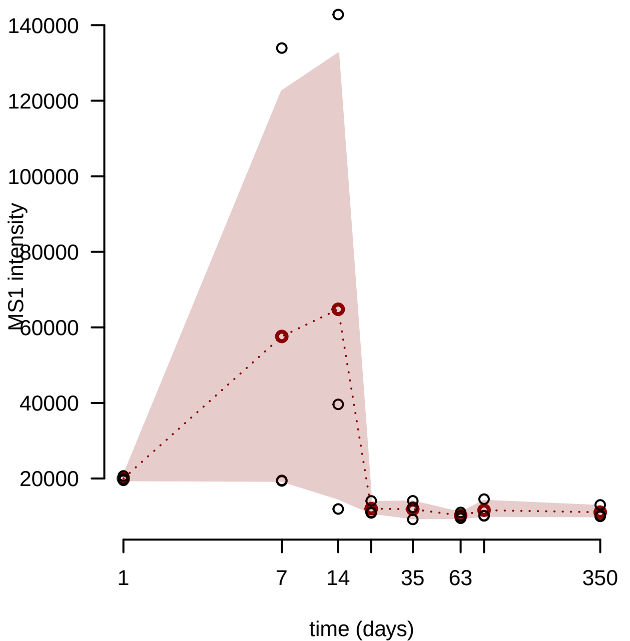

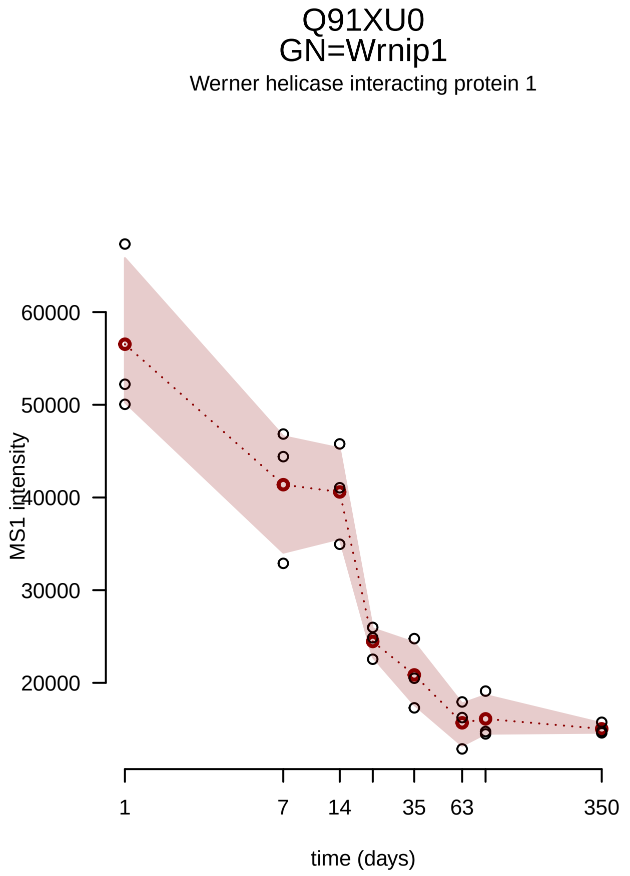
Q91XU0
GN=Wrnip1
Werner helicase interacting protein 1
MS1 intensity
20000
30000
40000
50000
60000
1
7
14
35
63
350
time (days)

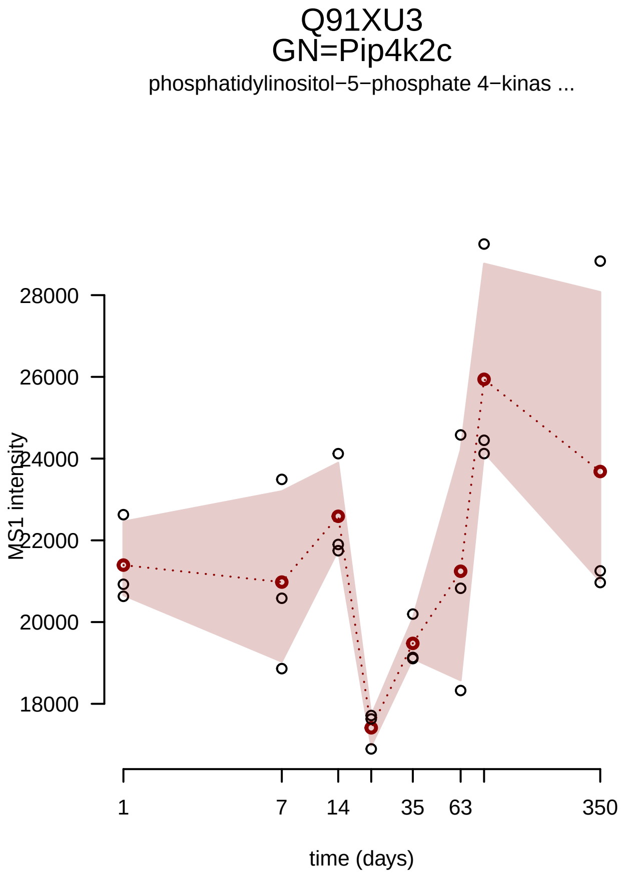
Q91XU3
GN=Pip4k2c
phosphatidylinositol−5−phosphate 4−kinas ...
MS1 intensity
18000
20000
22000
24000
26000
28000
1
7
14
35
63
350
time (days)

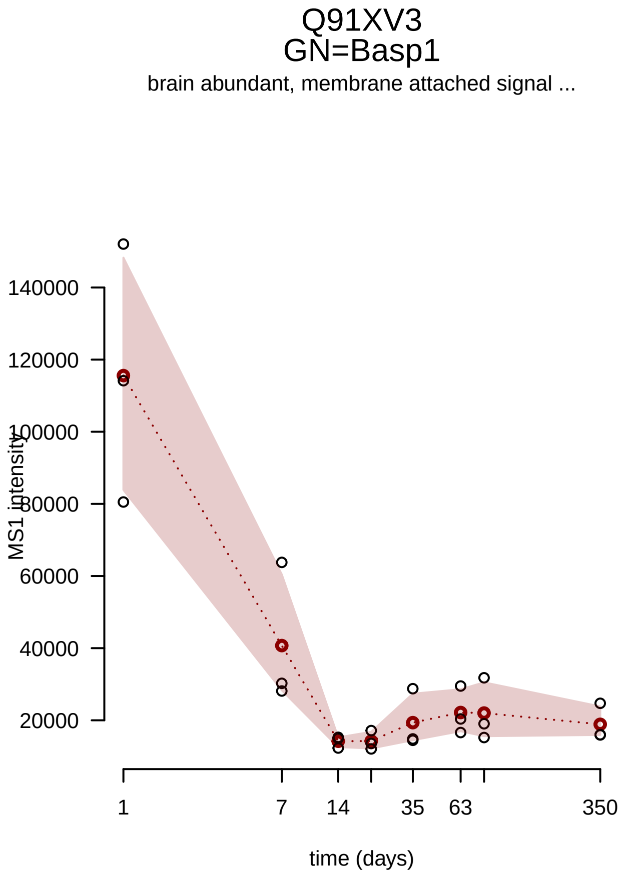
Q91XV3
GN=Basp1
brain abundant, membrane attached signal ...
MS1 intensity
20000
40000
60000
80000
100000
120000
140000
1
7
14
35
63
350
time (days)

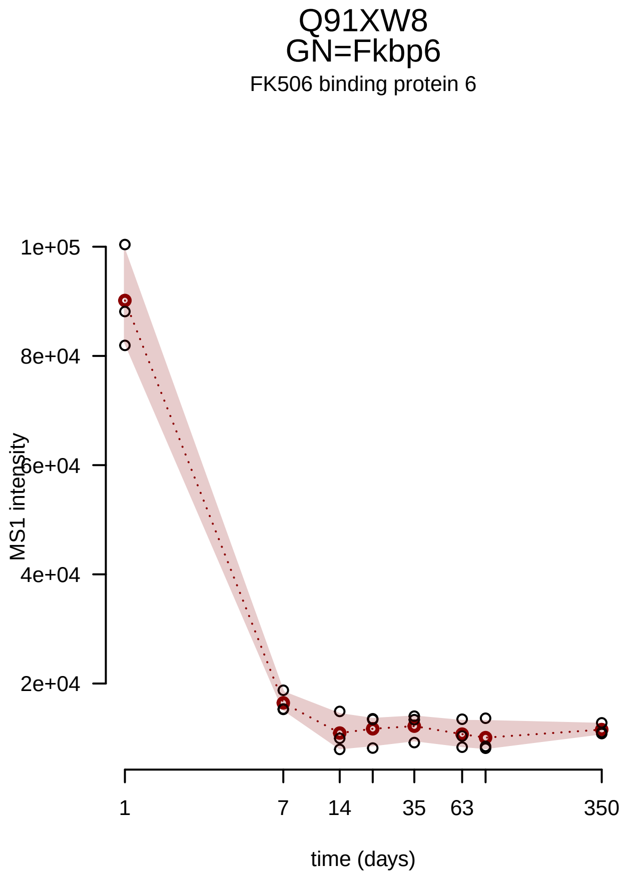
Q91XW8
GN=Fkbp6
FK506 binding protein 6
MS1 intensity
2e+04
4e+04
6e+04
8e+04
1e+05
1
7
14
35
63
350
time (days)

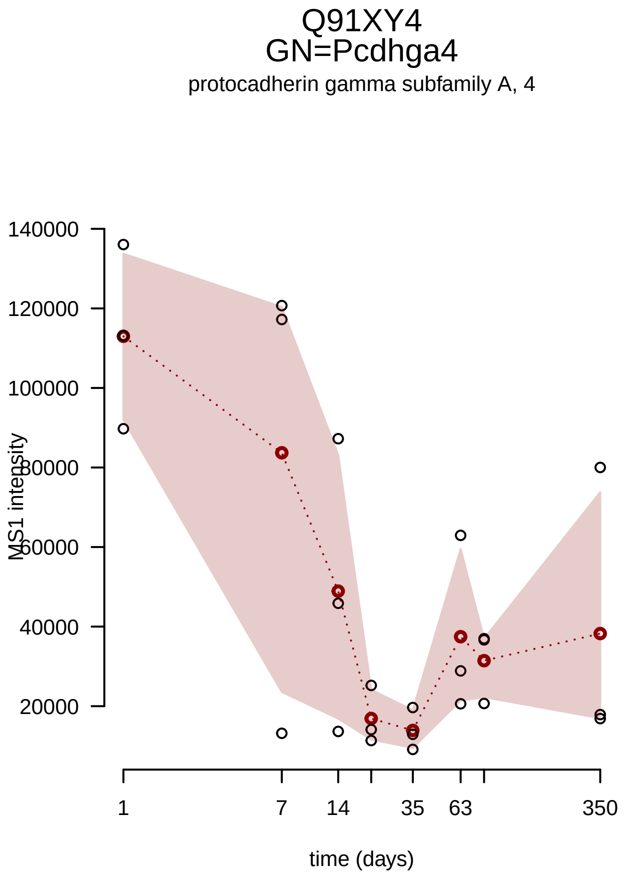
Q91XY4
GN=Pcdhga4
protocadherin gamma subfamily A, 4
MS1 intensity
20000
40000
60000
80000
100000
120000
140000
1
7
14
35
63
350
time (days)

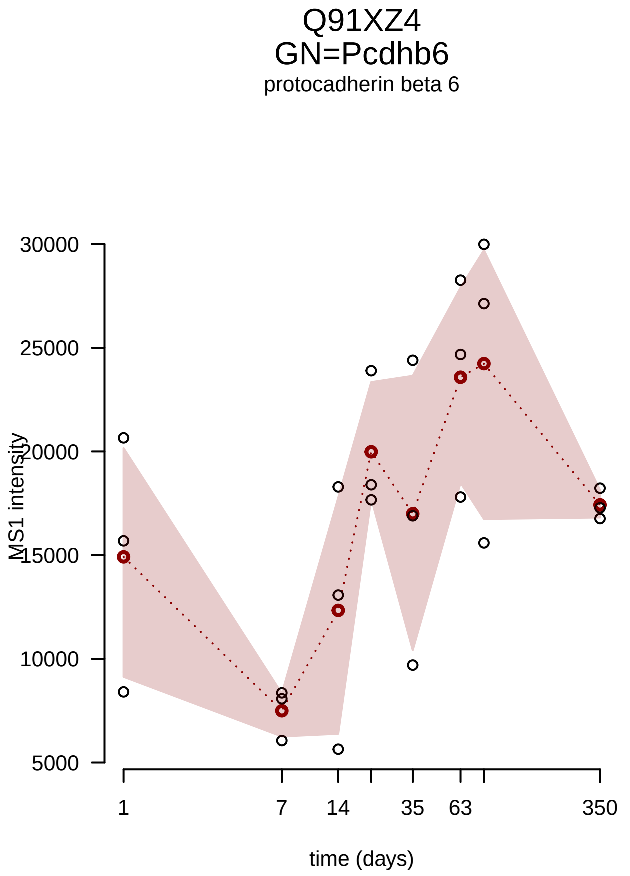
Q91XZ4
GN=Pcdhb6
protocadherin beta 6
MS1 intensity
5000
10000
15000
20000
25000
30000
1
7
14
35
63
350
time (days)

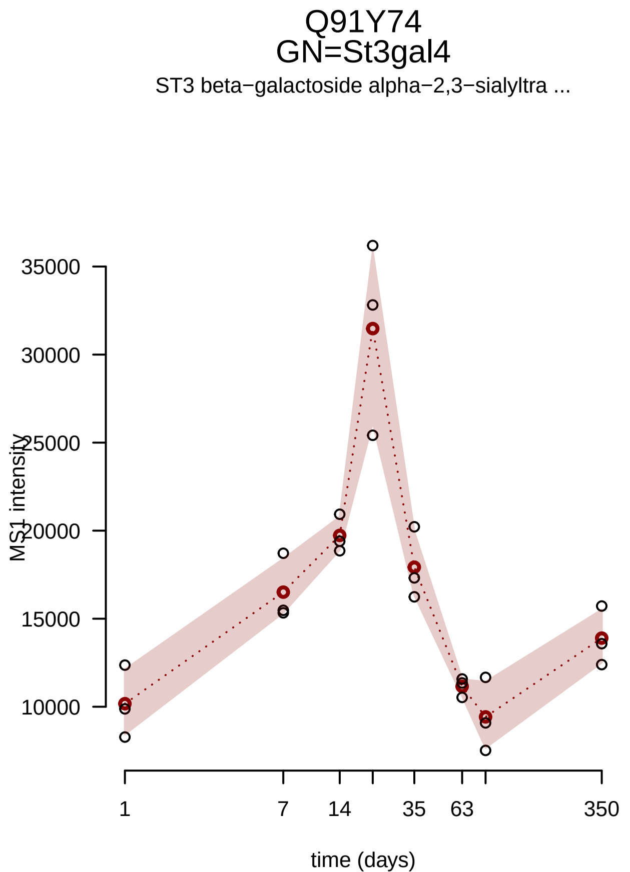
Q91Y74
GN=St3gal4
ST3 beta−galactoside alpha−2,3−sialyltra ...
MS1 intensity
10000
15000
20000
25000
30000
35000
1
7
14
35
63
350
time (days)

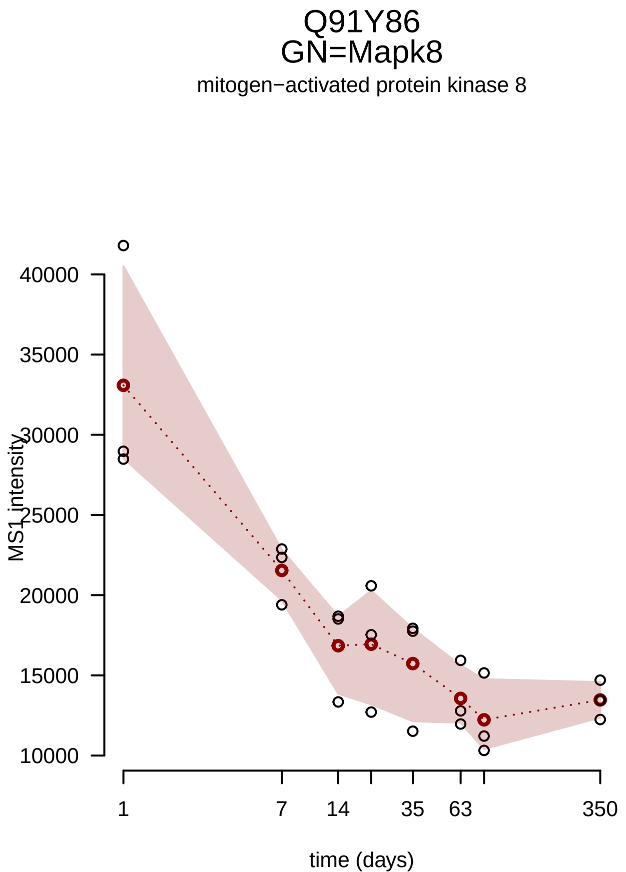
Q91Y86
GN=Mapk8
mitogen−activated protein kinase 8
MS1 intensity
10000
15000
20000
25000
30000
35000
40000
1
7
14
35
63
350
time (days)

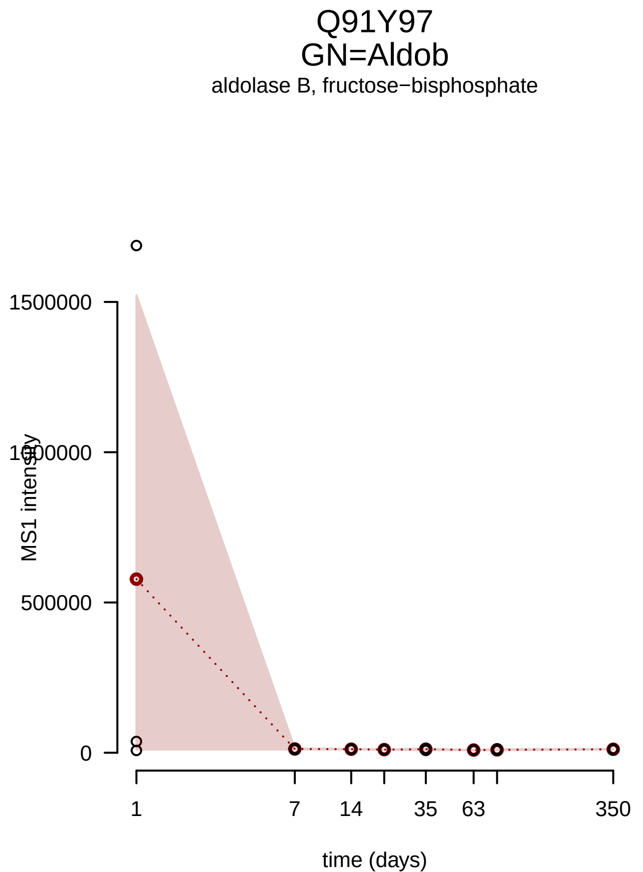
Q91Y97
GN=Aldob
aldolase B, fructose−bisphosphate
MS1 intensity
0
500000
1000000
1500000
1
7
14
35
63
350
time (days)

Q91YD3
GN=Dcp1a
decapping mRNA 1A

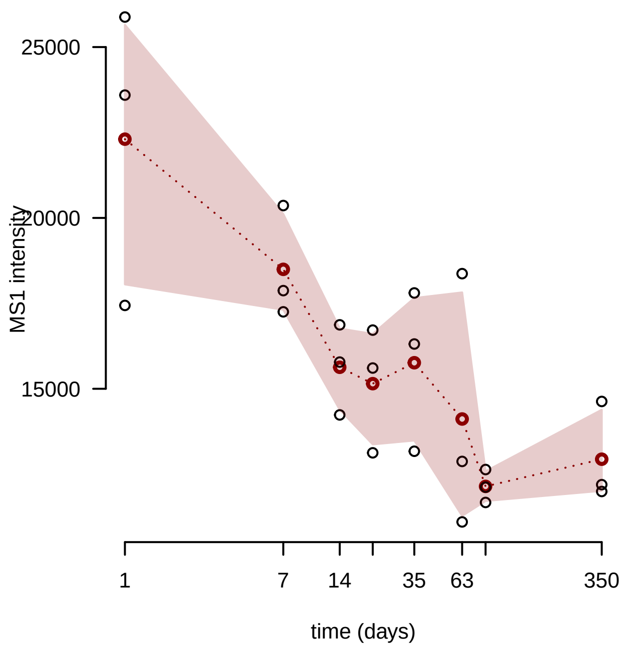

Q91YD9
GN=Wasl
WASP like actin nucleation promoting fac ...

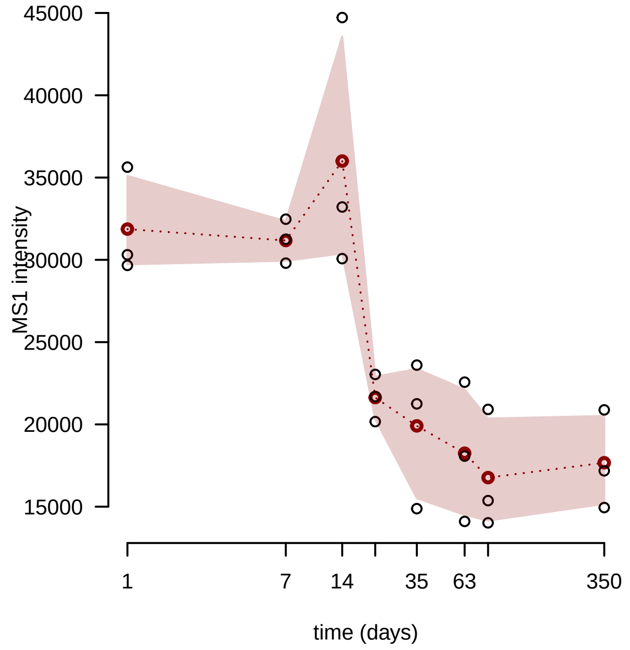

Q91YE3
GN=Egln1
egl−9 family hypoxia−inducible factor 1

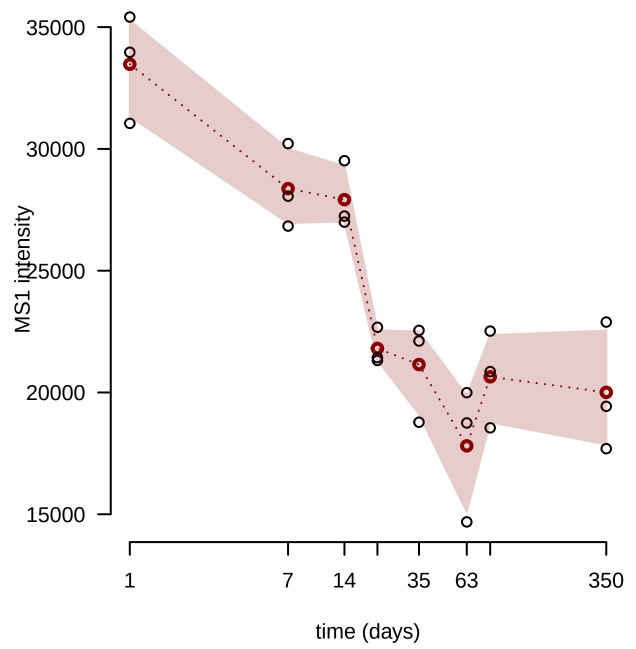

Q91YE5
GN=Baz2a
bromodomain adjacent to zinc finger doma ...

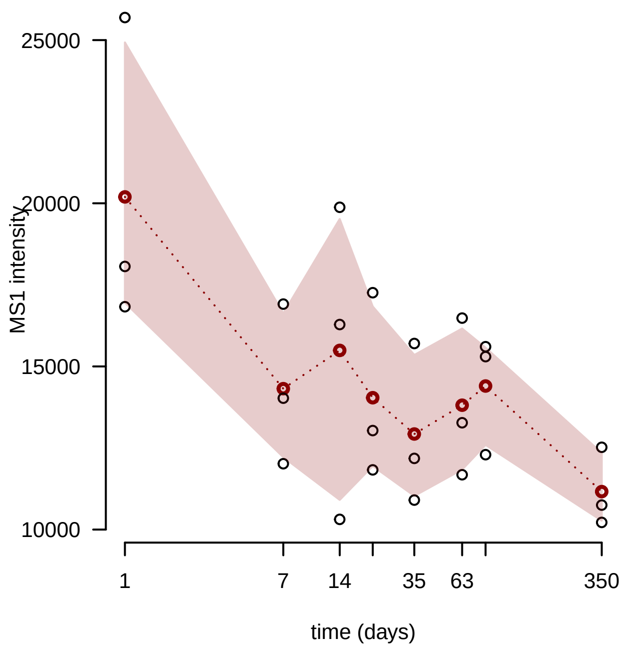

Q91YE6
GN=Ipo9
importin 9

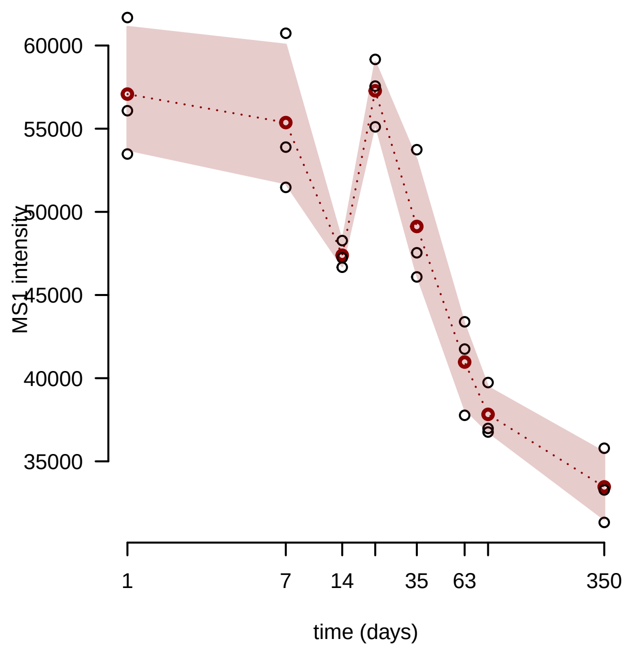

Q91YE7
GN=Rbm5
RNA binding motif protein 5

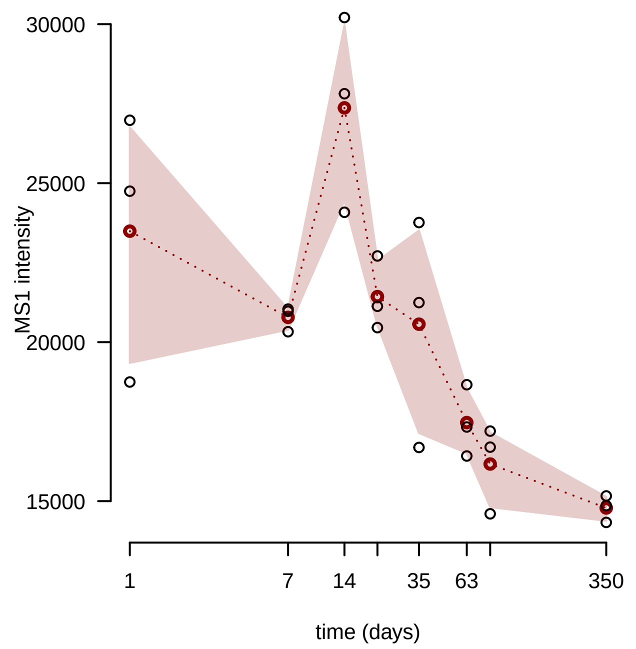

Q91YE8
GN=Synpo2
synaptopodin 2

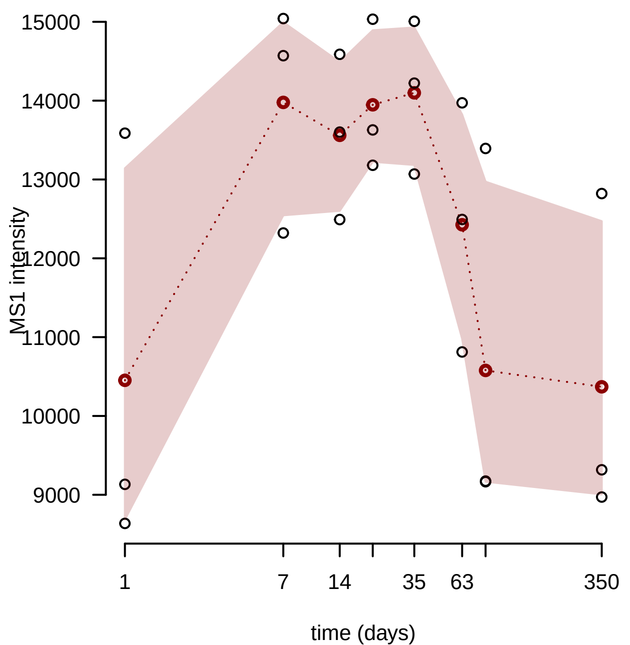

Q91YH5
GN=Atl3
atlastin GTPase 3

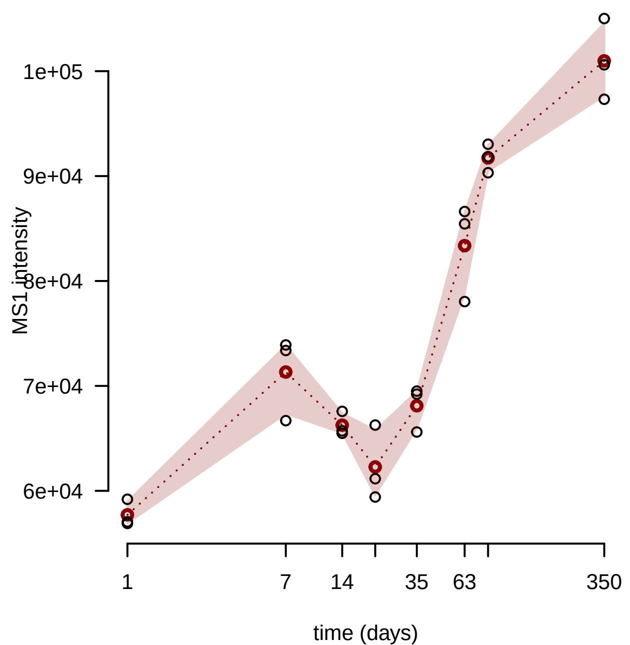

Q91YI0
GN=Asl
argininosuccinate lyase

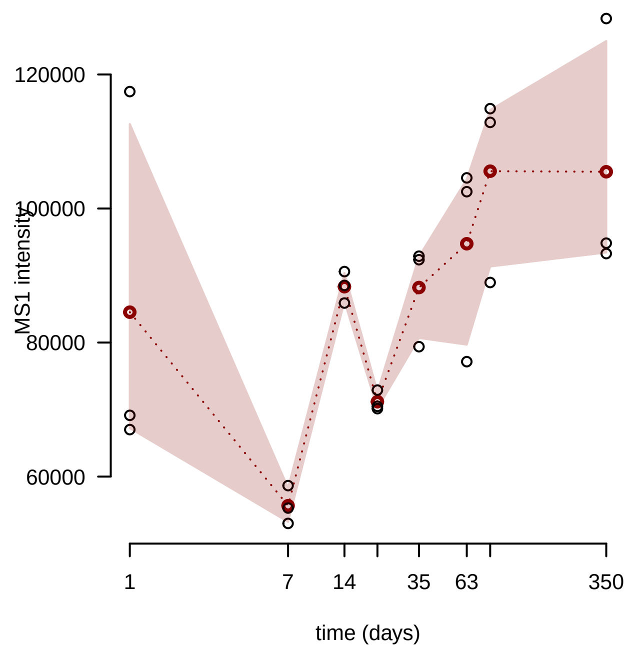

Q91YI1
GN=Atg13
autophagy related 13

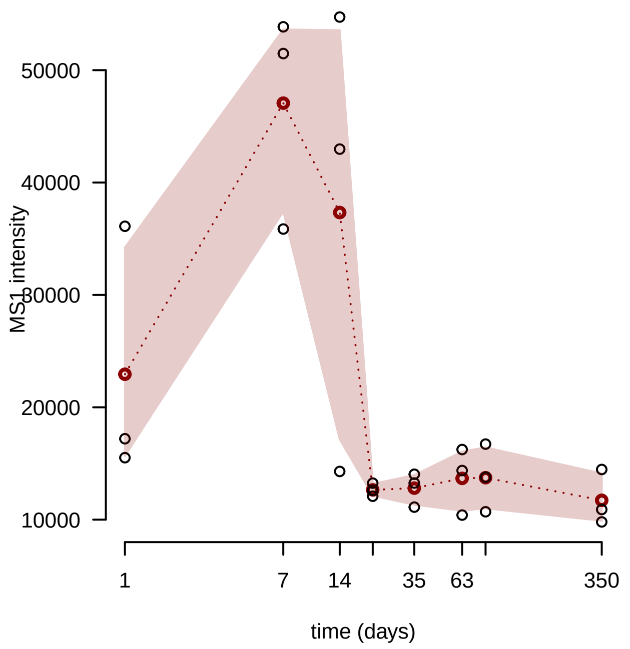

Q91YI4
GN=Arrb2
arrestin, beta 2

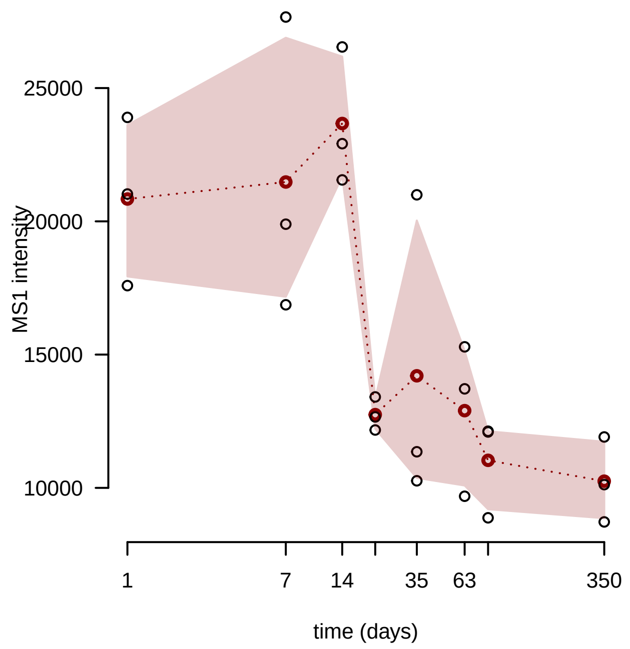

Q91YI6
GN=Tent2
terminal nucleotidyltransferase 2

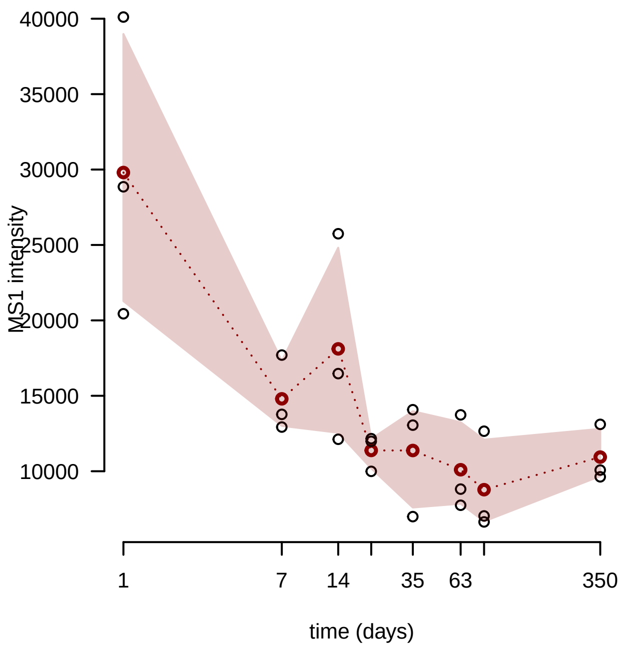

Q91YJ2
GN=Snx4
sorting nexin 4

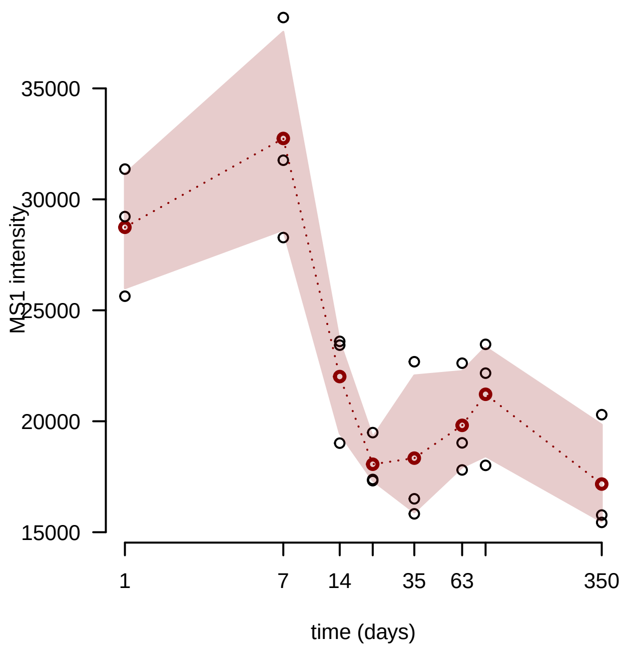

Q91YJ3
GN=Thyn1
thymocyte nuclear protein 1

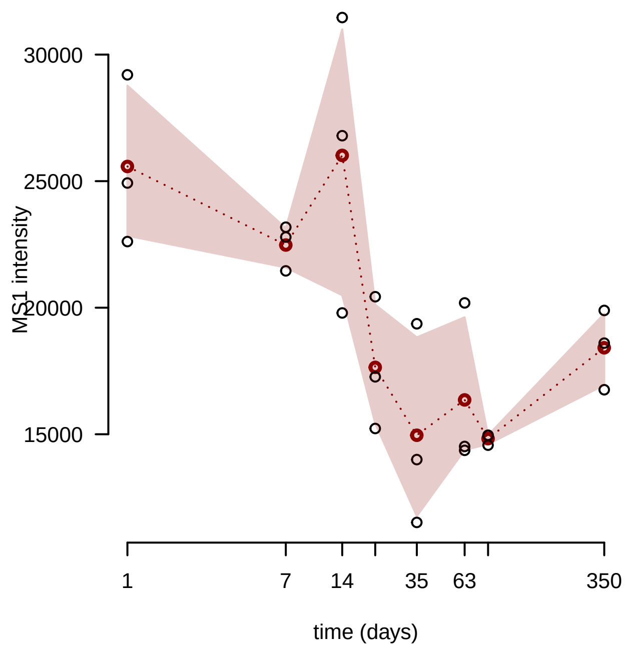

Q91YJ5
GN=Mtif2
mitochondrial translational initiation f ...

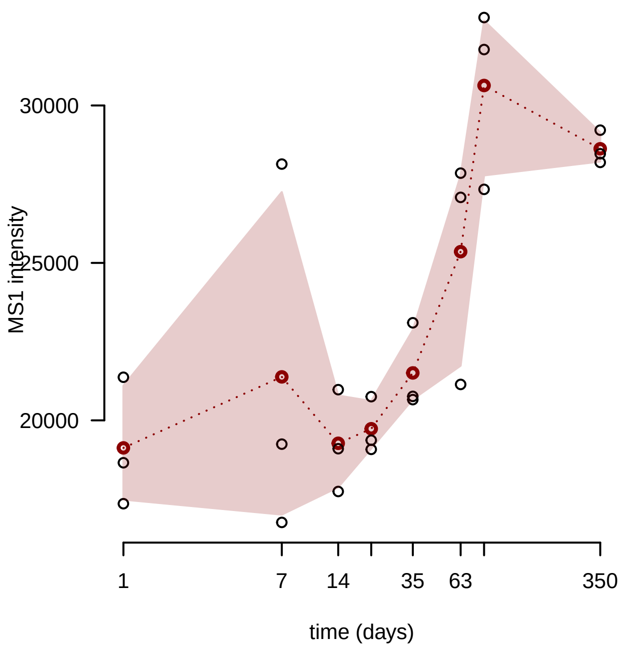

Q91YK0
GN=Lrrc49
leucine rich repeat containing 49

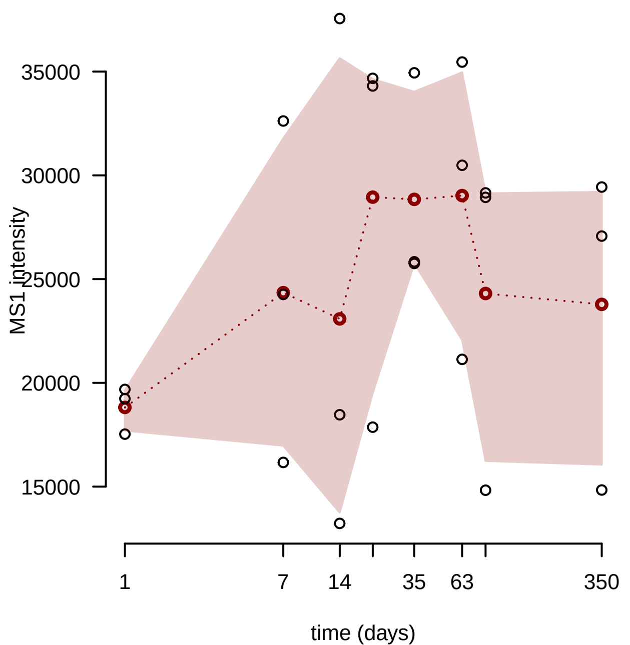

Q91YK2
GN=Rrp1b
ribosomal RNA processing 1B

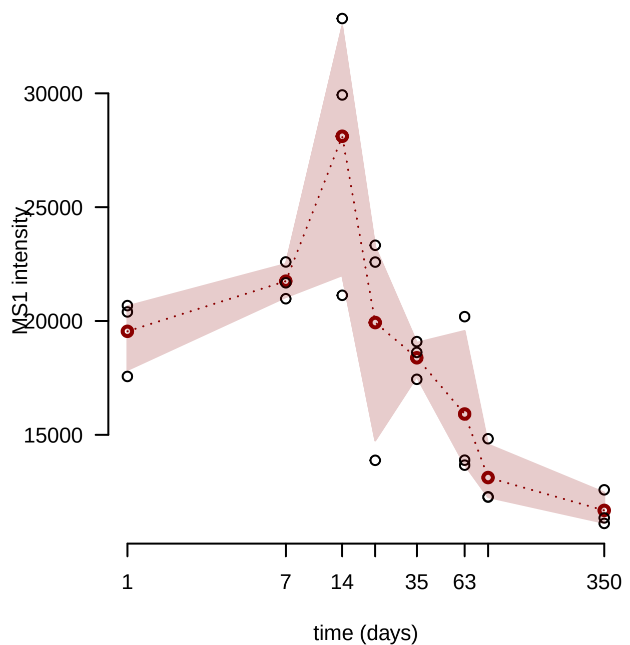

Q91YL2
GN=Rnf126
ring finger protein 126

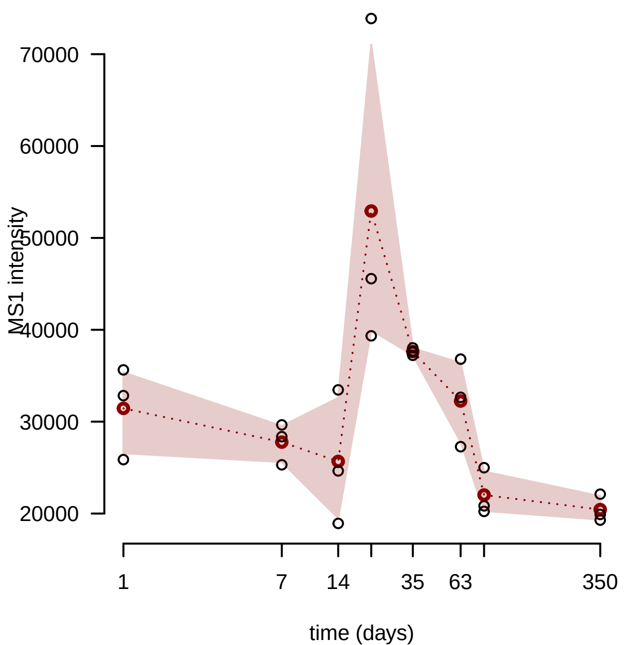

Q91YL3
GN=Uckl1
uridine−cytidine kinase 1−like 1

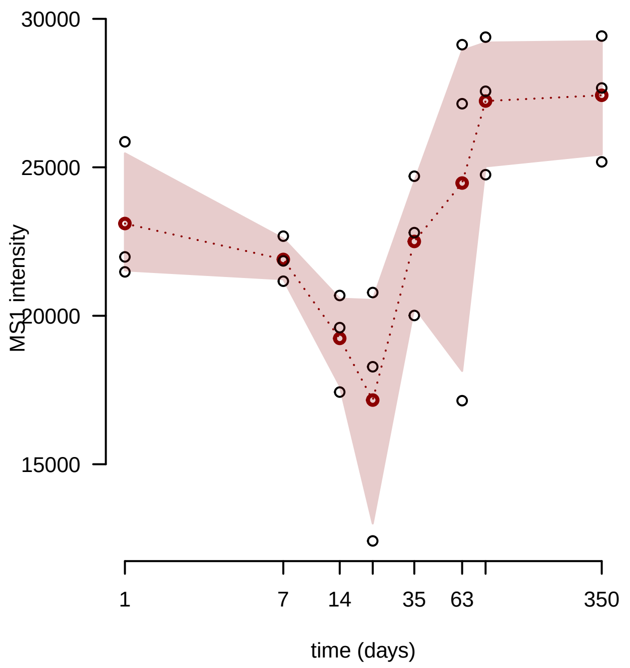

Q91YM2
GN=Arhgap35
Rho GTPase activating protein 35

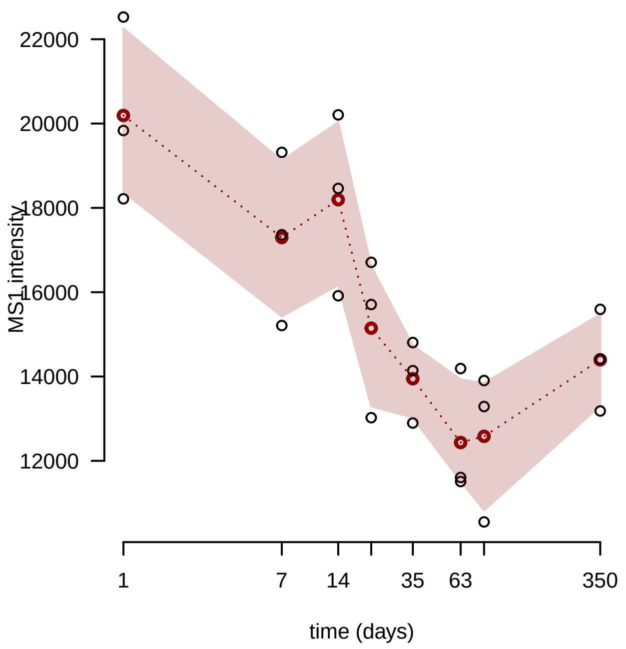

Q91YM4
GN=Tbrg4
transforming growth factor beta regulate ...

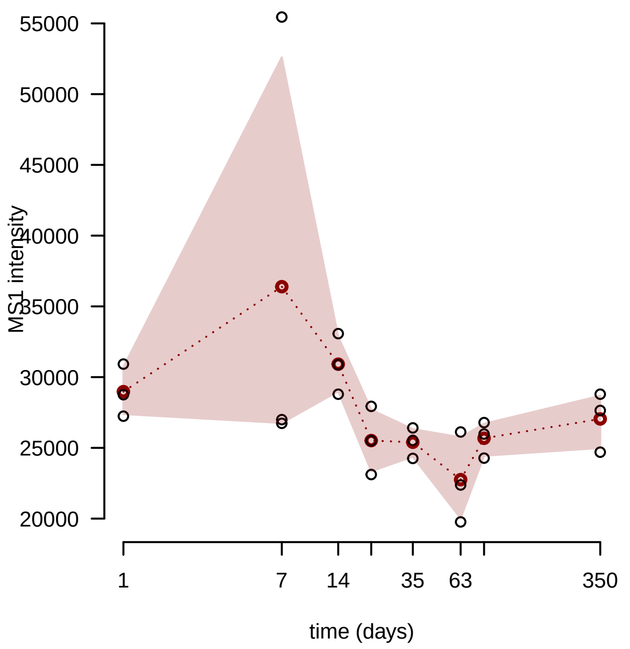

Q91YN0
GN=D6Wsu163e
DNA segment, Chr 6, Wayne State Universi ...

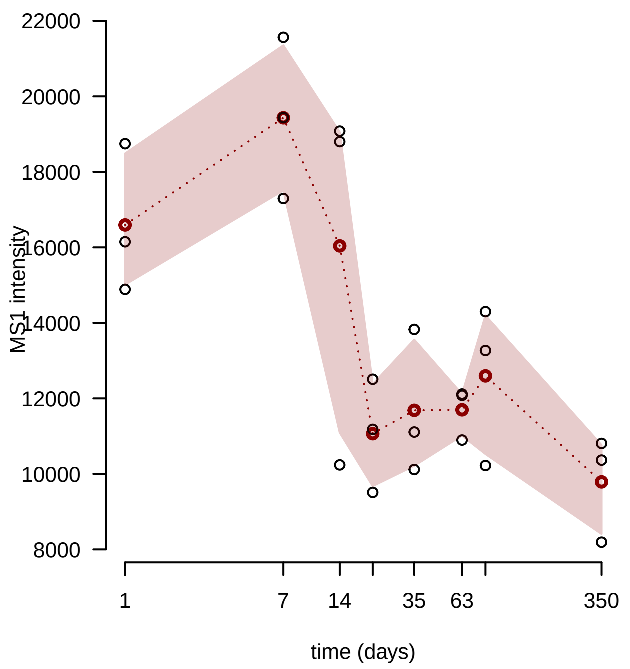

Q91YN1
GN=Fam118a
family with sequence similarity 118, mem ...

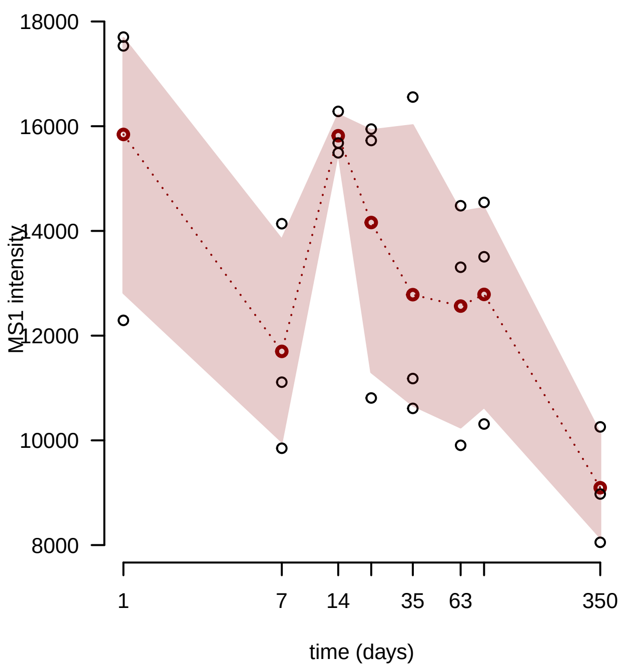

Q91YN5
GN=Uap1
UDP−N−acetylglucosamine pyrophosphorylas ...

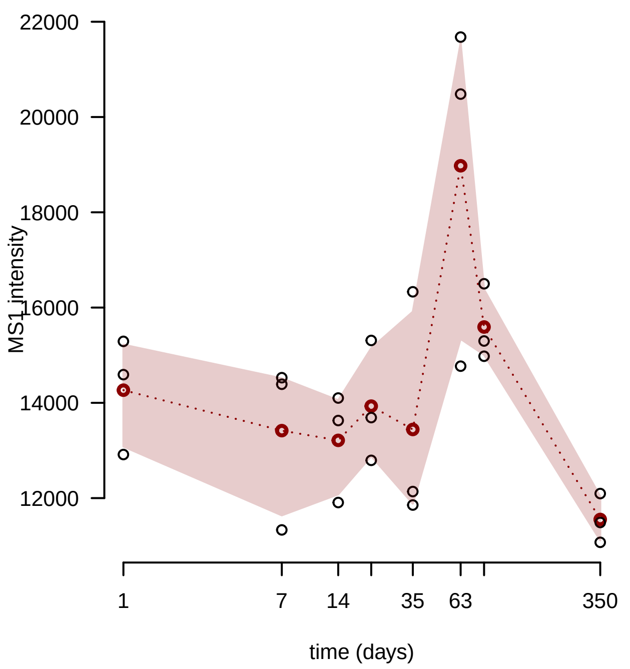

Q91YN9
GN=Bag2
BCL2−associated athanogene 2

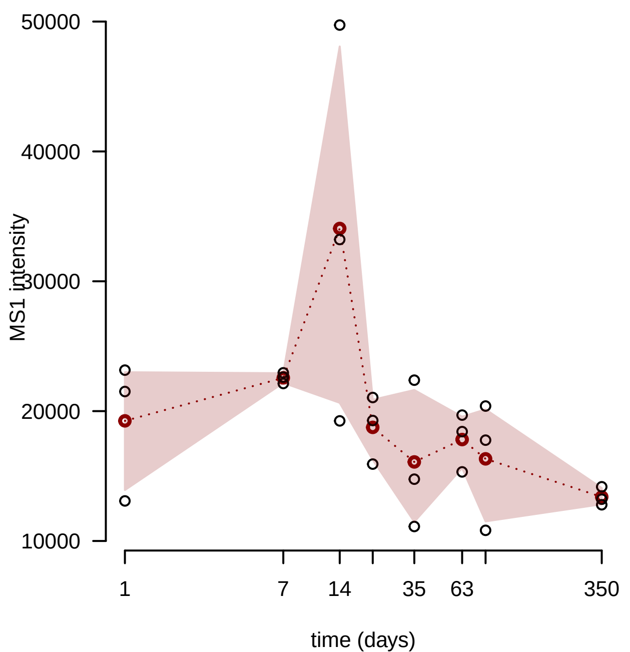

Q91YP0
GN=L2hgdh
L−2−hydroxyglutarate dehydrogenase

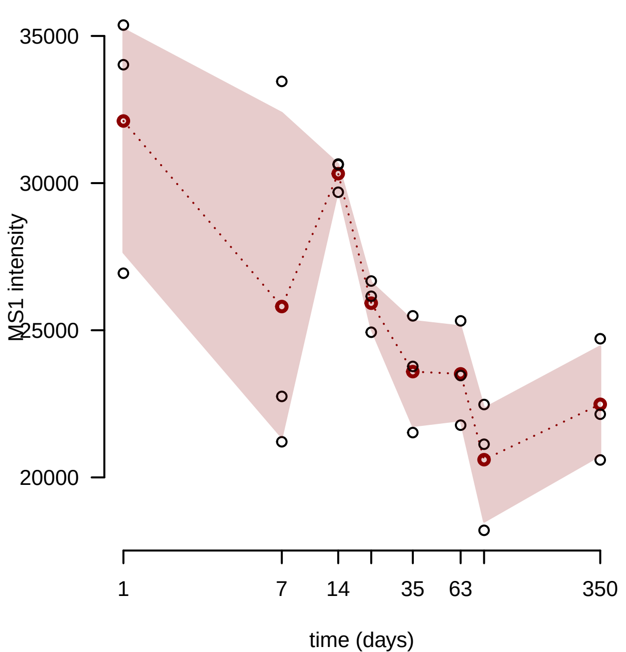

Q91YP1
GN=Bcdin3d
BCDIN3 domain containing

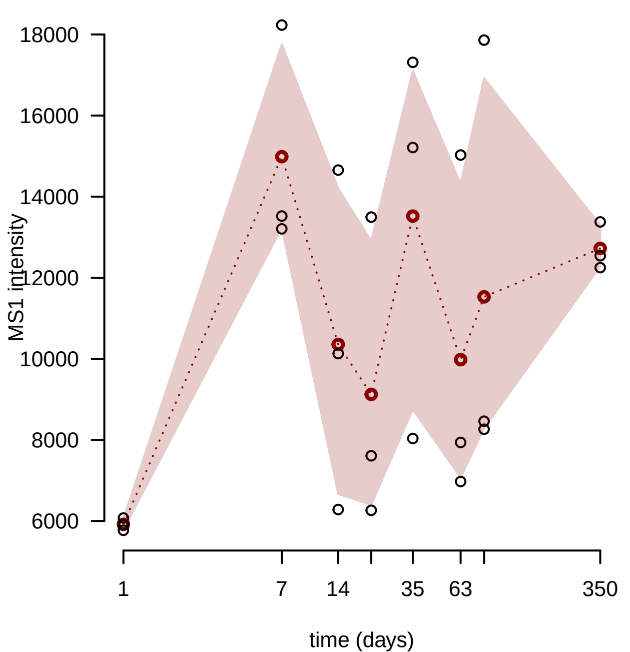

Q91YP2
GN=Nln
neurolysin (metallopeptidase M3 family)

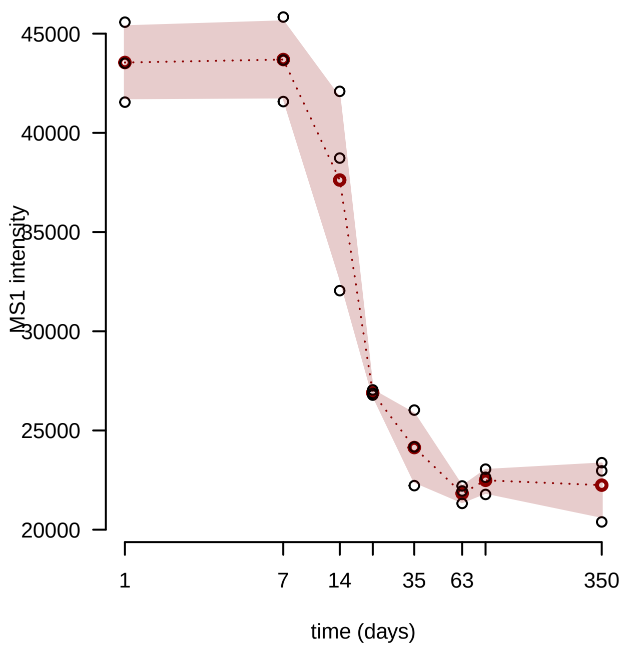

Q91YP3
GN=Dera
deoxyribose−phosphate aldolase (putative ...

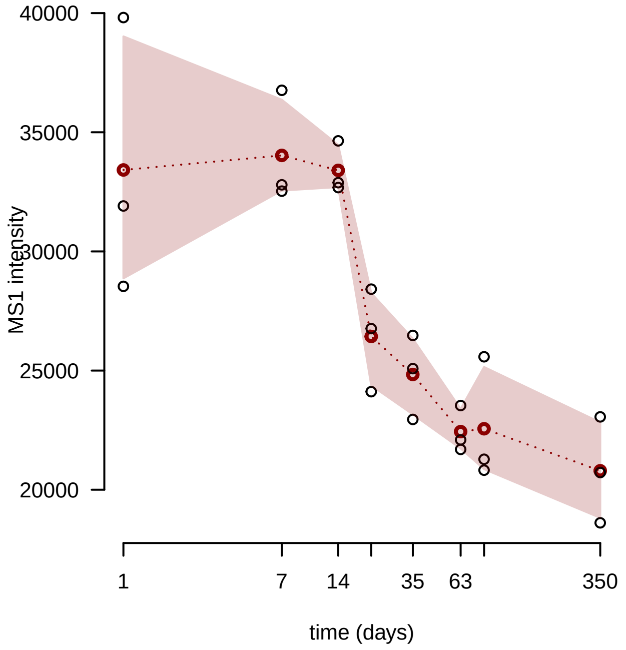

Q91YQ3
GN=Csdc2
cold shock domain containing C2, RNA bin ...

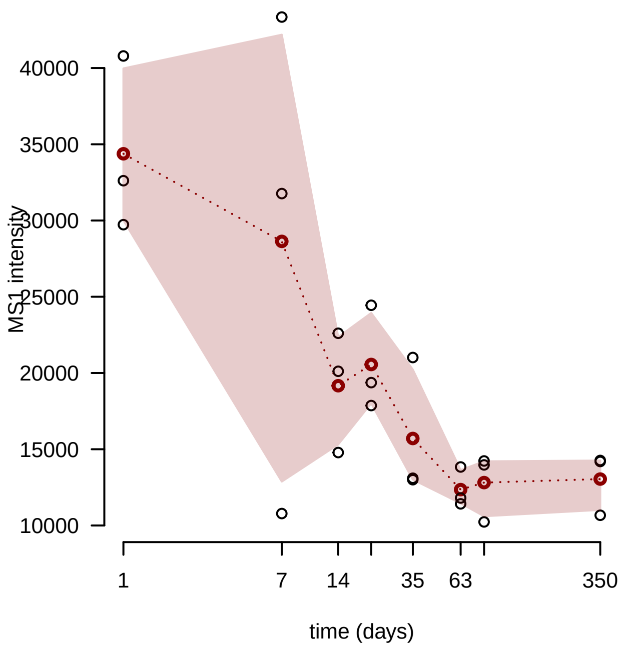

Q91YQ5
GN=Rpn1
ribophorin I

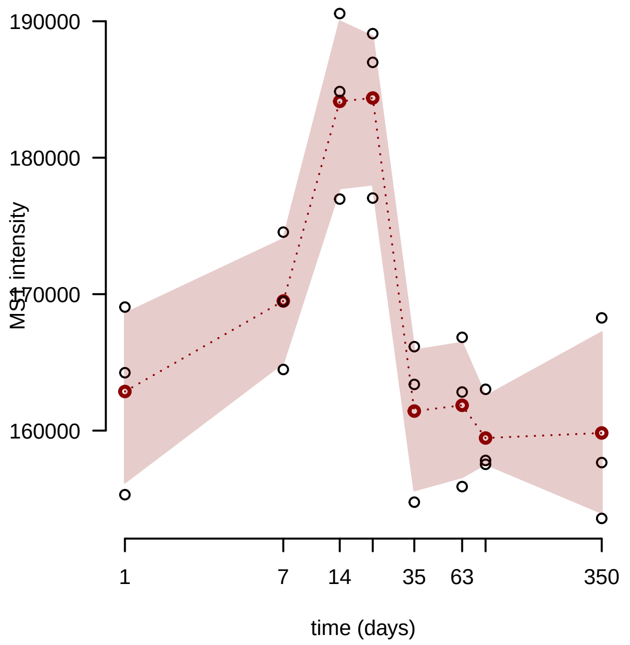

Q91YR1
GN=Twf1
twinfilin actin binding protein 1

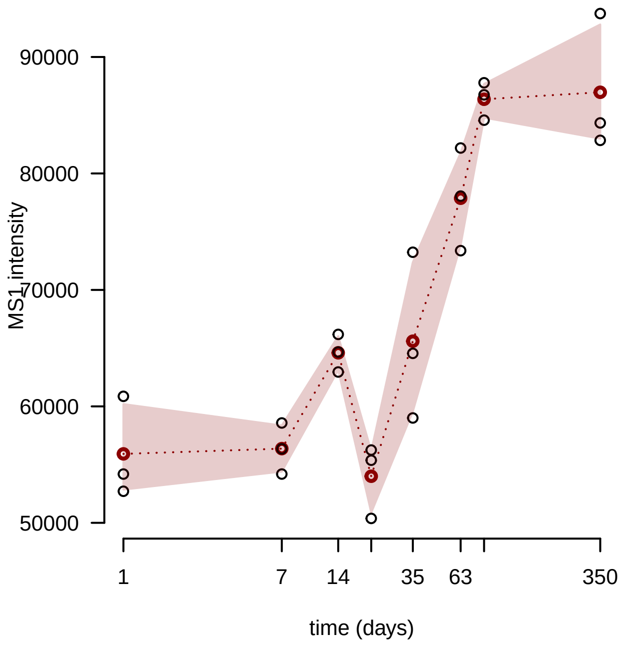

Q91YR5
GN=EEF1AKNMT

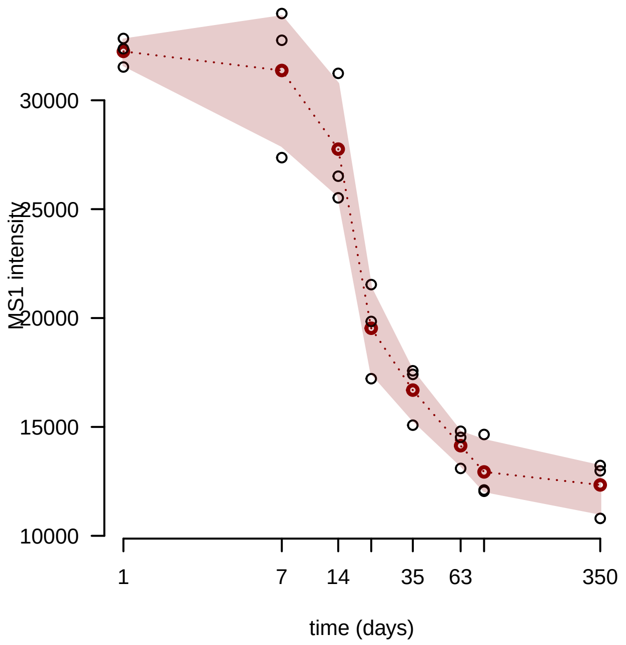

Q91YR7
GN=Prpf6
pre−mRNA splicing factor 6

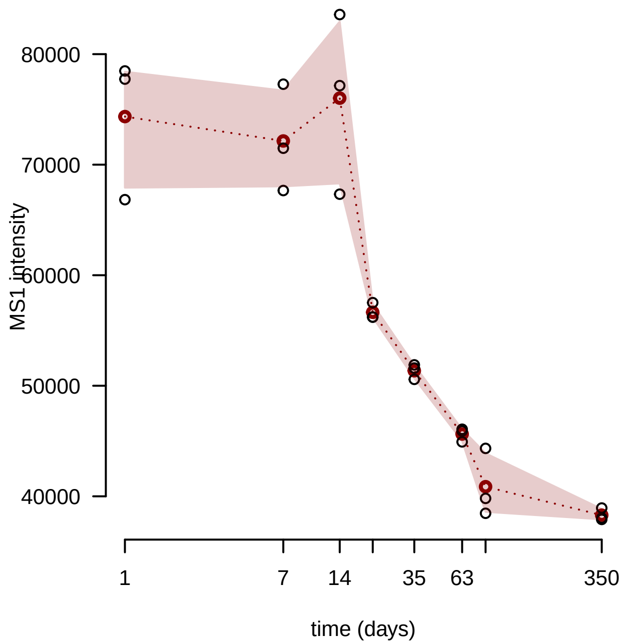

Q91YR9
GN=Ptgr1
prostaglandin reductase 1

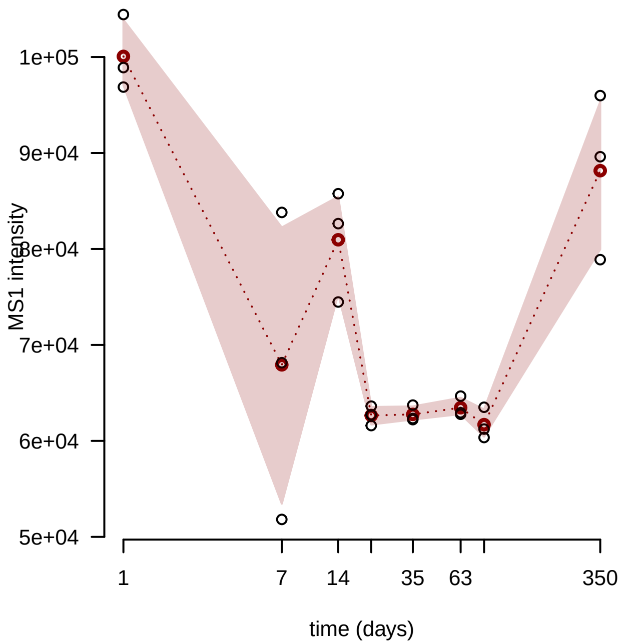

Q91YS8
GN=Camk1
calcium/calmodulin−dependent protein kin ...

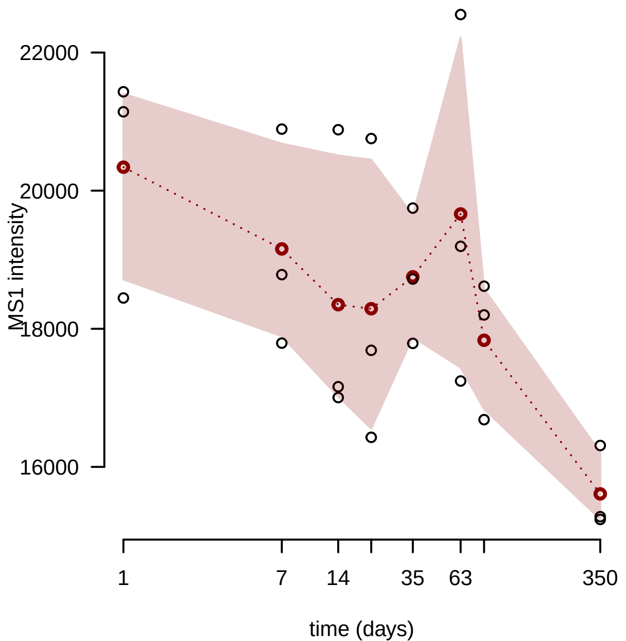

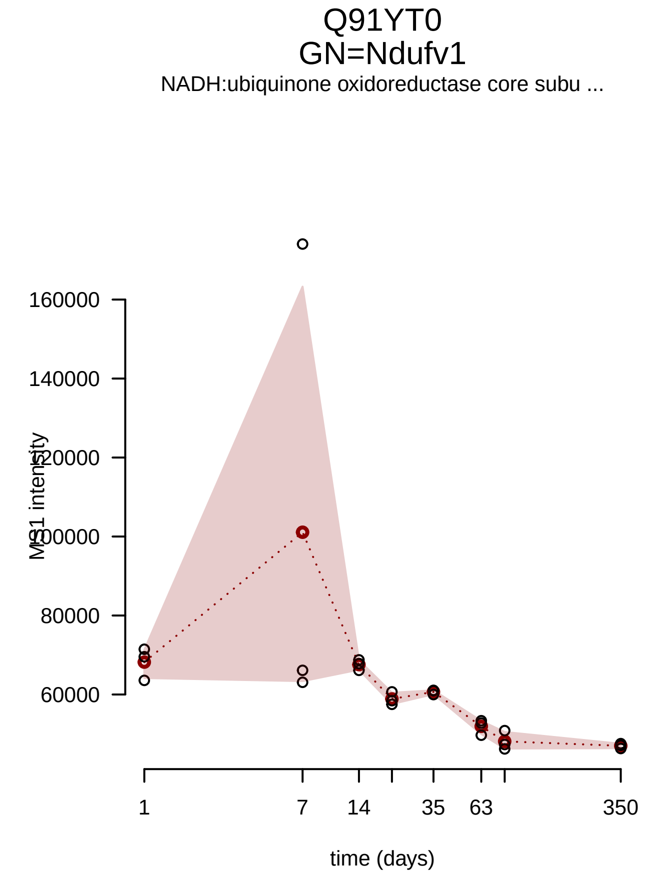
Q91YT0
GN=Ndufv1
NADH:ubiquinone oxidoreductase core subu ...
MS1 intensity
160000
140000
120000
100000
80000
60000
1
7
14
35
63
350
time (days)

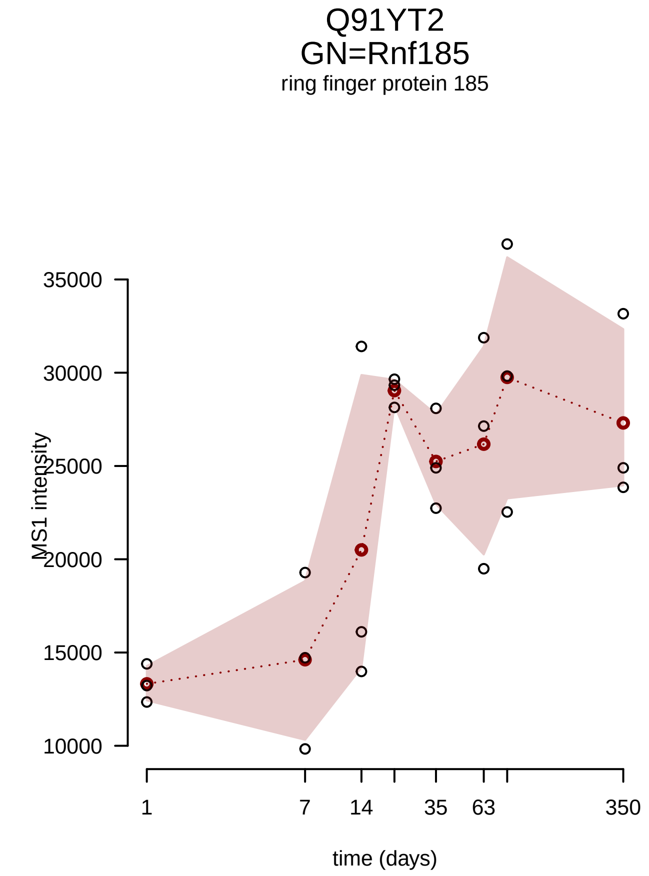
Q91YT2
GN=Rnf185
ring finger protein 185
MS1 intensity
35000
30000
25000
20000
15000
10000
1
7
14
35
63
350
time (days)

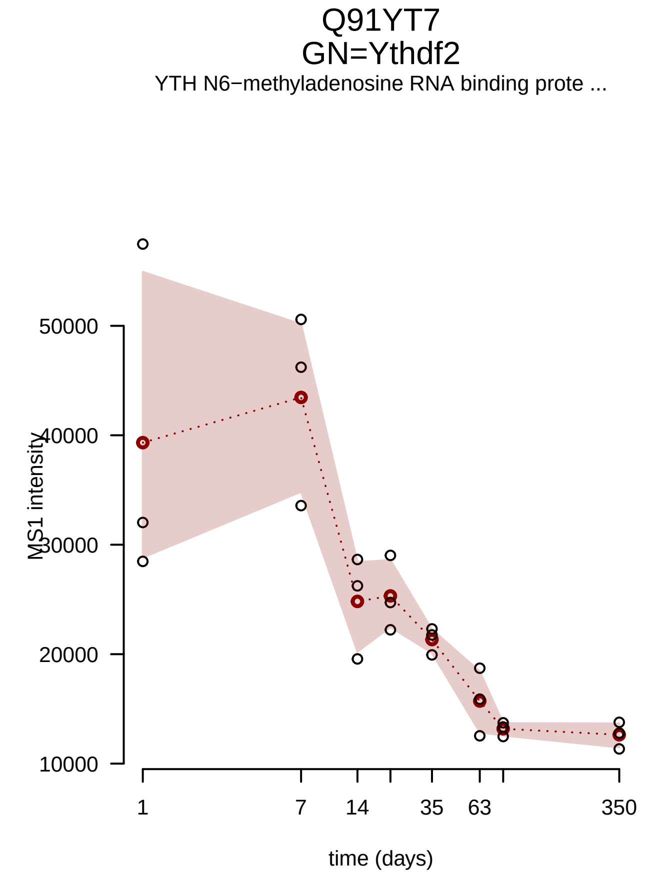
Q91YT7
GN=Ythdf2
YTH N6−methyladenosine RNA binding prote ...
MS1 intensity
50000
40000
30000
20000
10000
1
7
14
35
63
350
time (days)

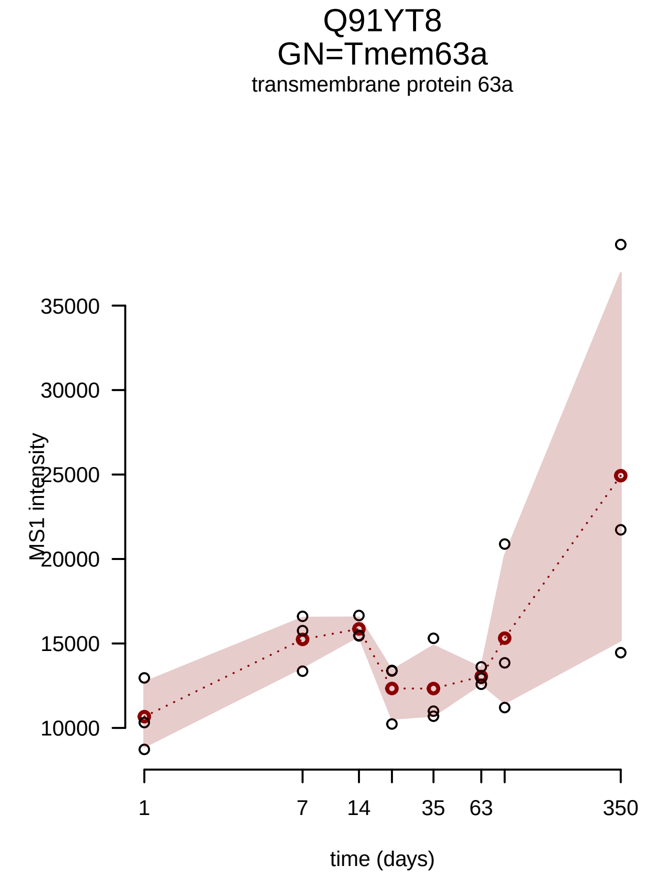
Q91YT8
GN=Tmem63a
transmembrane protein 63a
MS1 intensity
35000
30000
25000
20000
15000
10000
1
7
14
35
63
350
time (days)

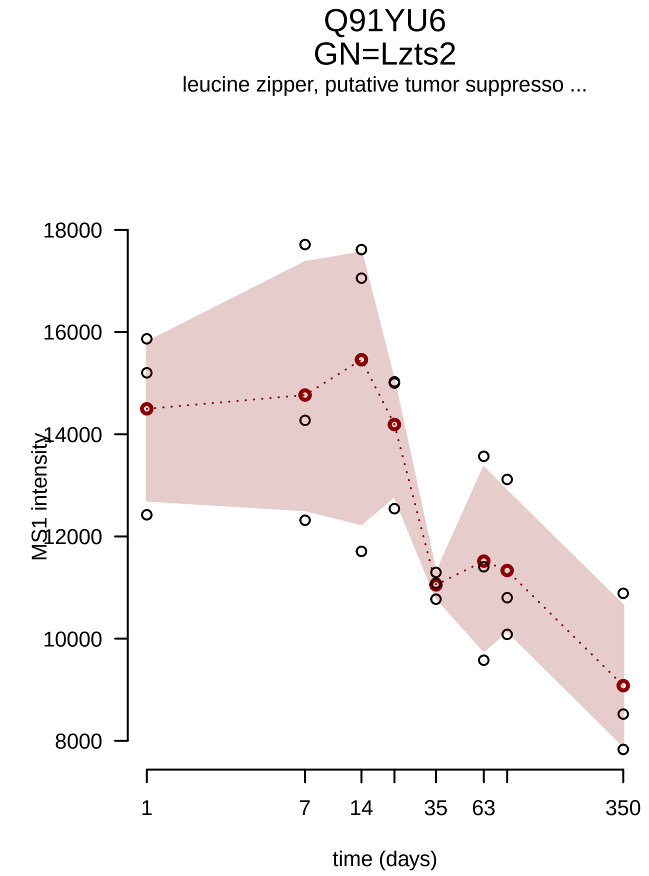
Q91YU6
GN=Lzts2
leucine zipper, putative tumor suppresso ...
MS1 intensity
18000
16000
14000
12000
10000
8000
1
7
14
35
63
350
time (days)

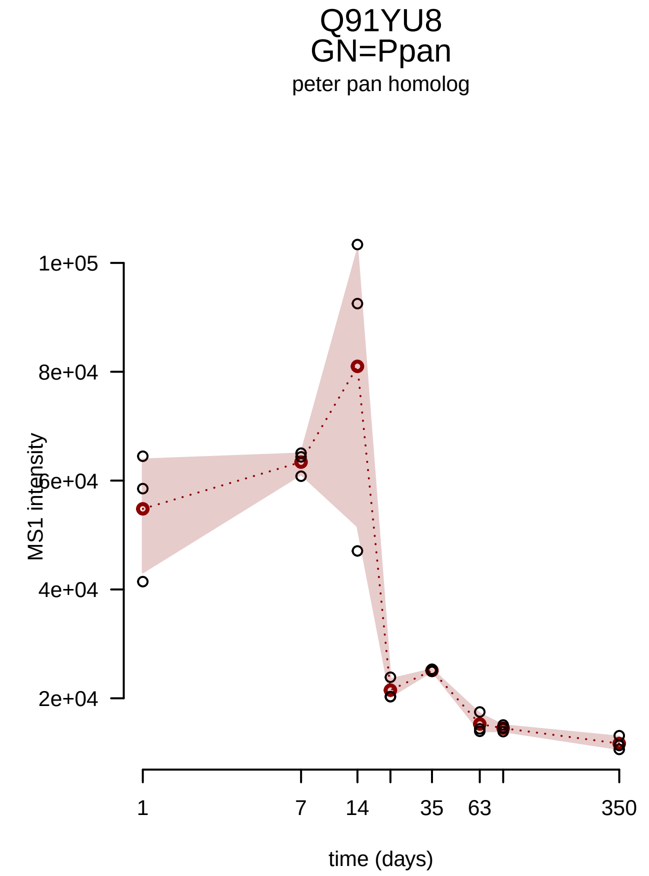
Q91YU8
GN=Ppan
peter pan homolog
MS1 intensity
1e+05
8e+04
6e+04
4e+04
2e+04
1
7
14
35
63
350
time (days)

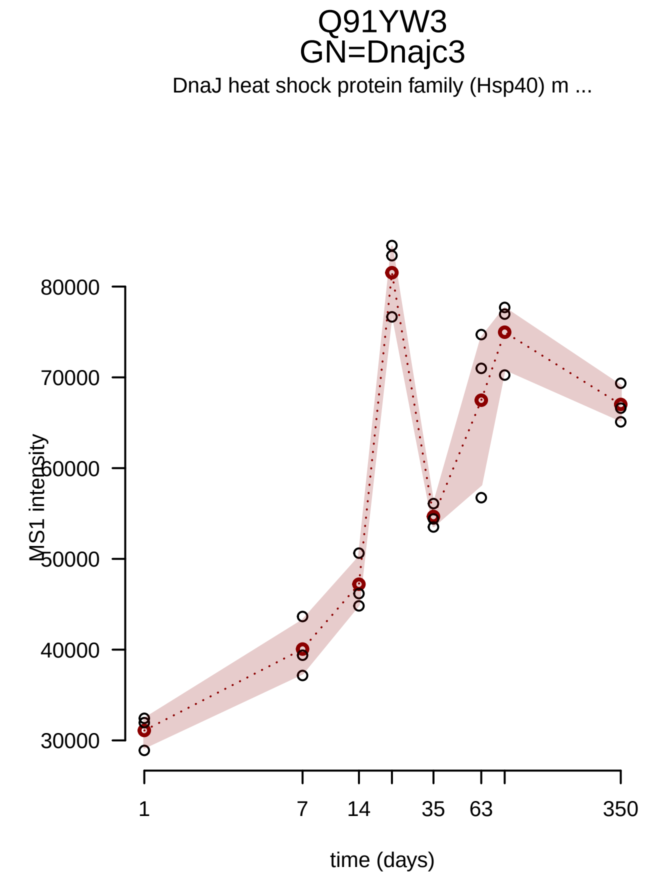
Q91YW3
GN=Dnajc3
DnaJ heat shock protein family (Hsp40) m ...
MS1 intensity
80000
70000
60000
50000
40000
30000
1
7
14
35
63
350
time (days)

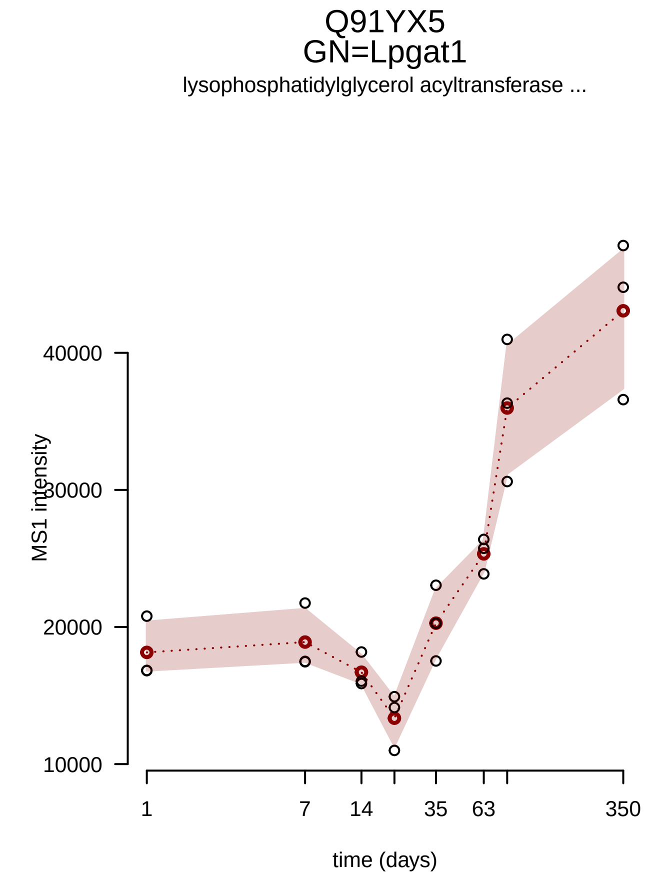
Q91YX5
GN=Lpgat1
lysophosphatidylglycerol acyltransferase ...
MS1 intensity
40000
30000
20000
10000
1
7
14
35
63
350
time (days)

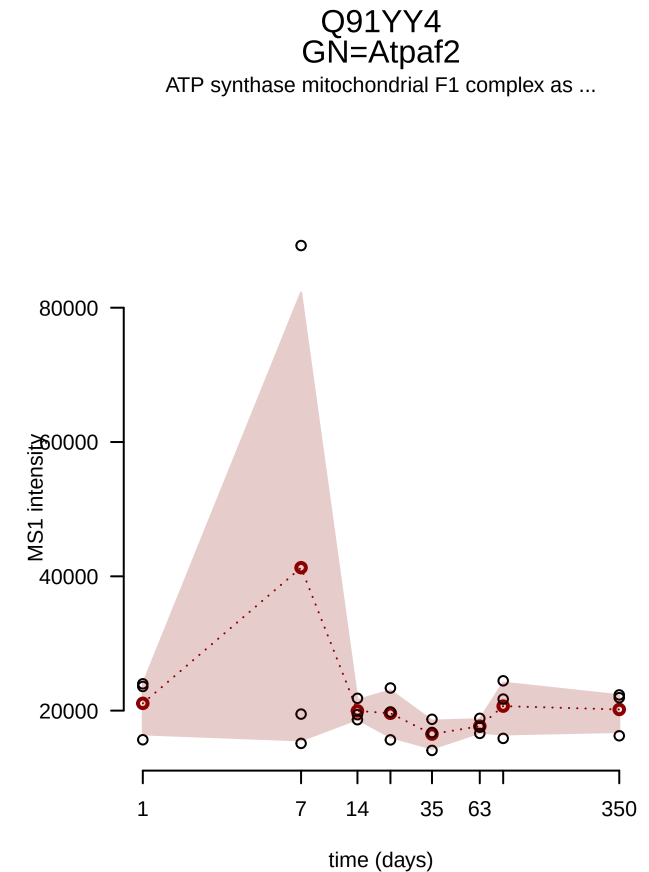
Q91YY4
GN=Atpaf2
ATP synthase mitochondrial F1 complex as ...
MS1 intensity
80000
60000
40000
20000
1
7
14
35
63
350
time (days)

Q91Z31
GN=Ptbp2
polypyrimidine tract binding protein 2

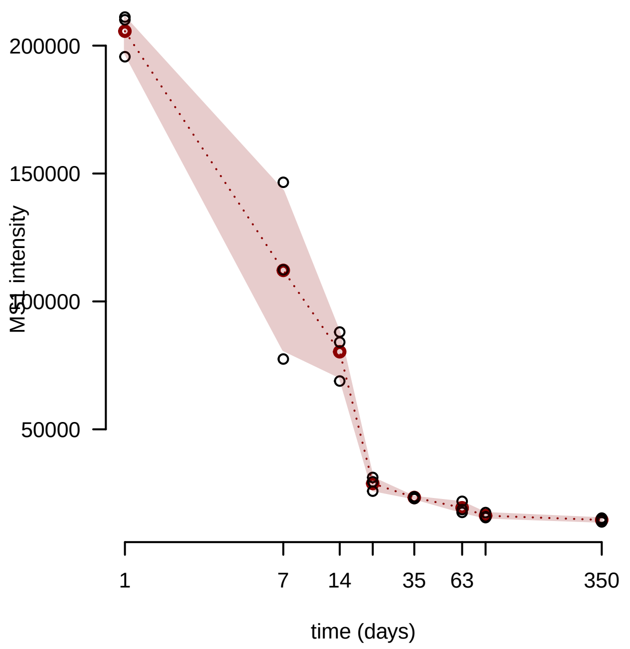

Q91Z38
GN=Ttc1
tetratricopeptide repeat domain 1

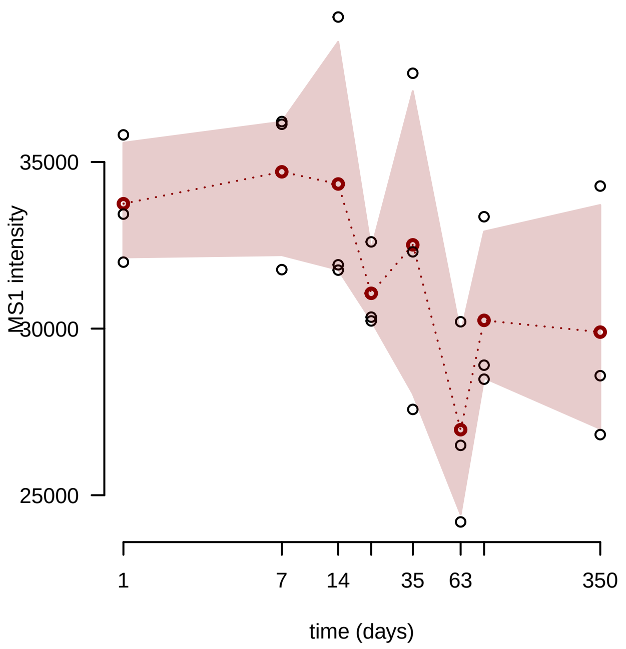

Q91Z49
GN=Fyttd1
forty−two−three domain containing 1

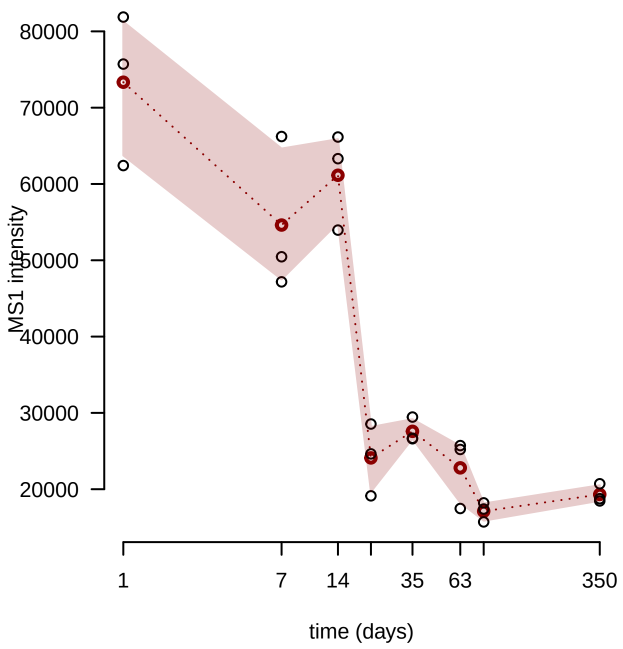

Q91Z53
GN=Grhpr
glyoxylate reductase/hydroxypyruvate red ...

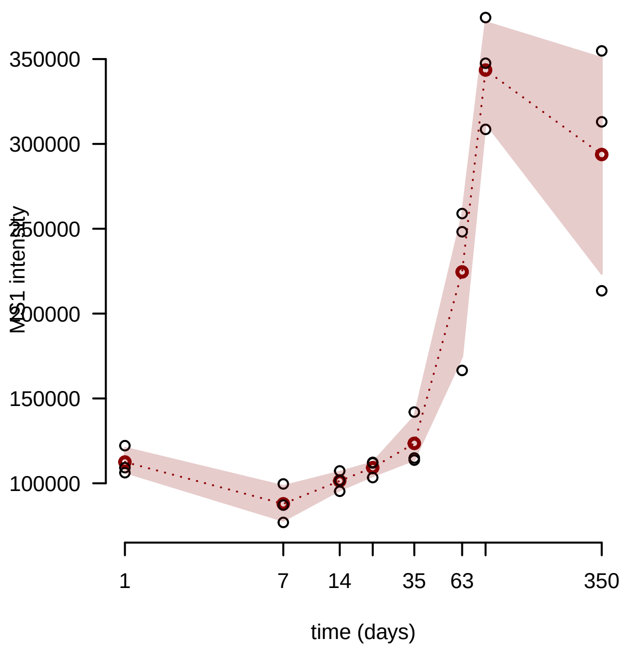

Q91Z67
GN=Srgap2
SLIT−ROBO Rho GTPase activating protein ...

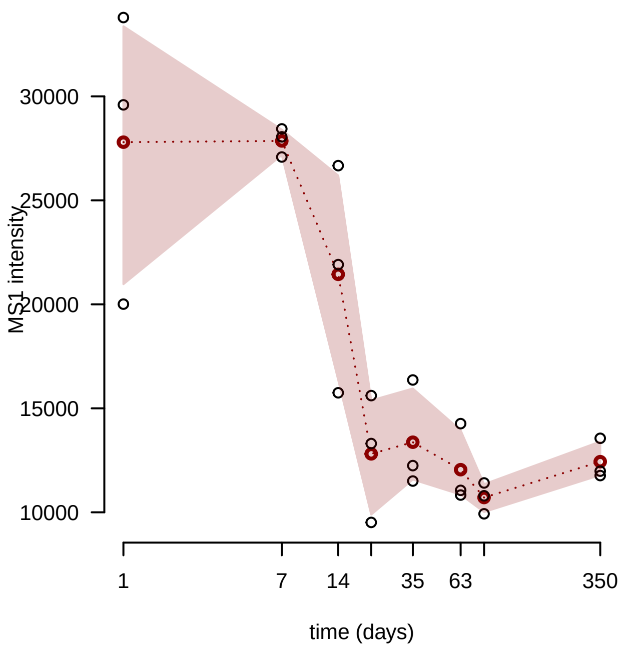

Q91Z69
GN=Srgap1
SLIT−ROBO Rho GTPase activating protein ...

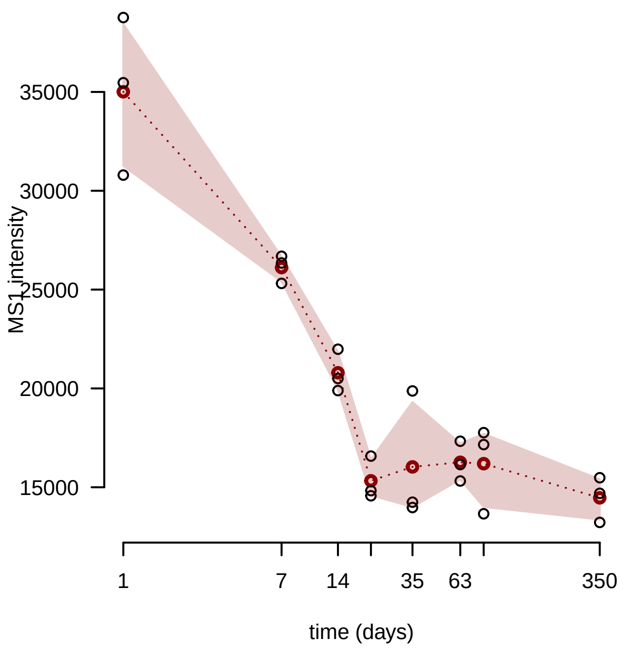

Q91Z92
GN=B3galt6
UDP−Gal:betaGal beta 1,3−galactosyltrans ...

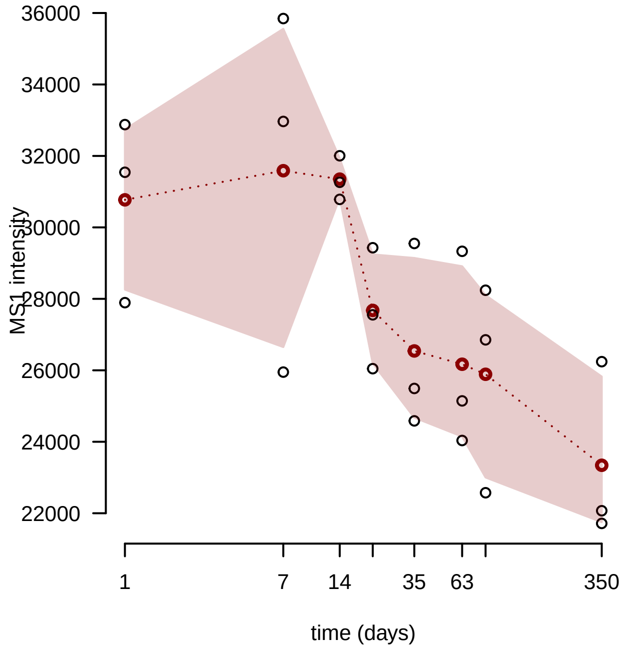

Q91Z96
GN=Bmp2k
BMP2 inducible kinase

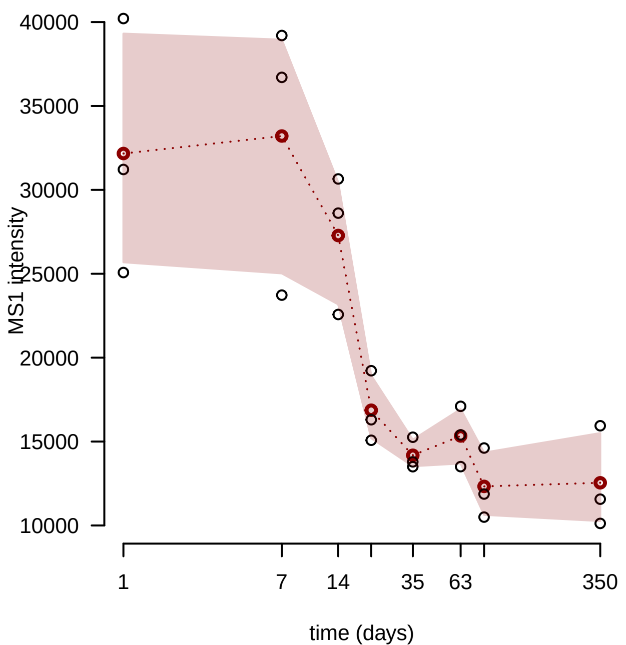

Q91ZA3
GN=Pcca
propionyl−Coenzyme A carboxylase, alpha ...

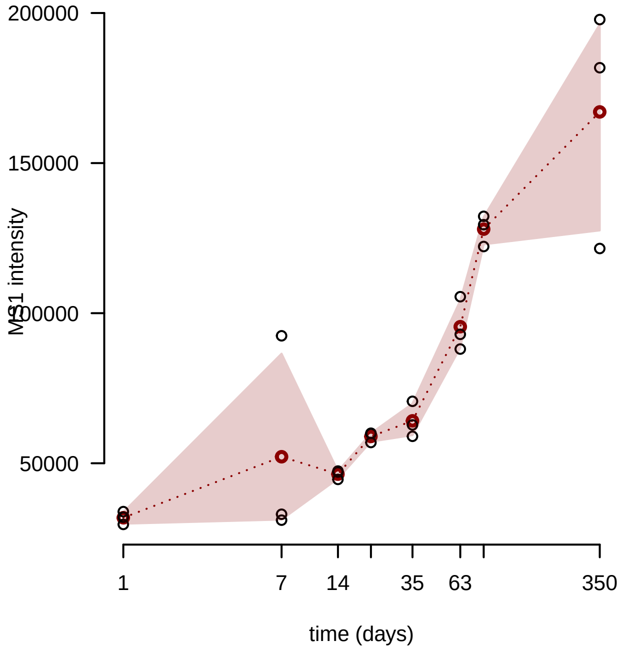

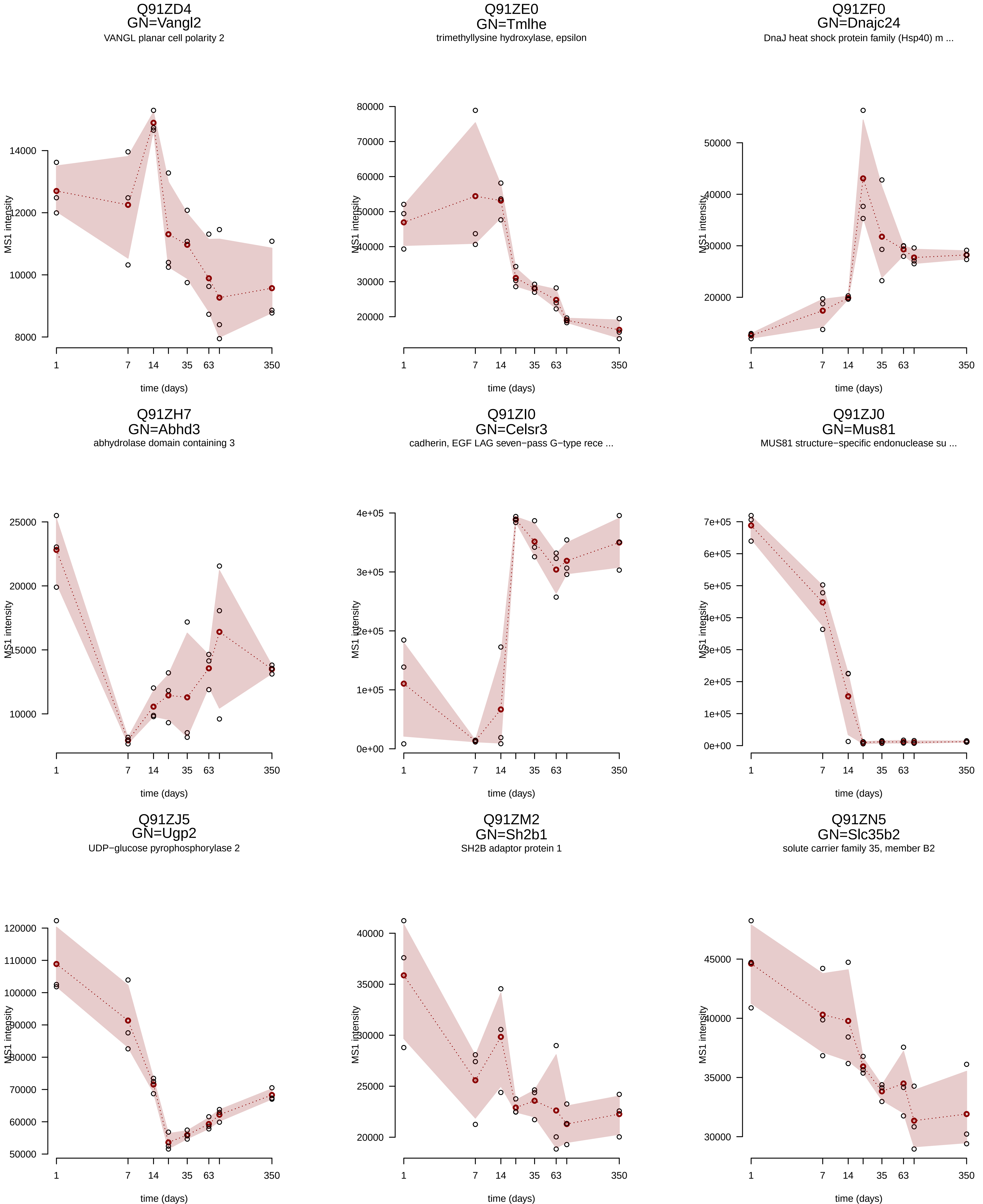

Q91ZD4
GN=Vangl2
VANGL planar cell polarity 2
MS1 intensity
time (days)
Q91ZE0
GN=Tmlhe
trimethyllysine hydroxylase, epsilon
MS1 intensity
time (days)
Q91ZF0
GN=Dnajc24
DnaJ heat shock protein family (Hsp40) m ...
MS1 intensity
time (days)
Q91ZH7
GN=Abhd3
abhydrolase domain containing 3
MS1 intensity
time (days)
Q91ZI0
GN=Celsr3
cadherin, EGF LAG seven−pass G−type rece ...
MS1 intensity
time (days)
Q91ZJ0
GN=Mus81
MUS81 structure−specific endonuclease su ...
MS1 intensity
time (days)
Q91ZJ5
GN=Ugp2
UDP−glucose pyrophosphorylase 2
MS1 intensity
time (days)
Q91ZM2
GN=Sh2b1
SH2B adaptor protein 1
MS1 intensity
time (days)
Q91ZN5
GN=Slc35b2
solute carrier family 35, member B2
MS1 intensity
time (days)
1
7
14
35
63
350

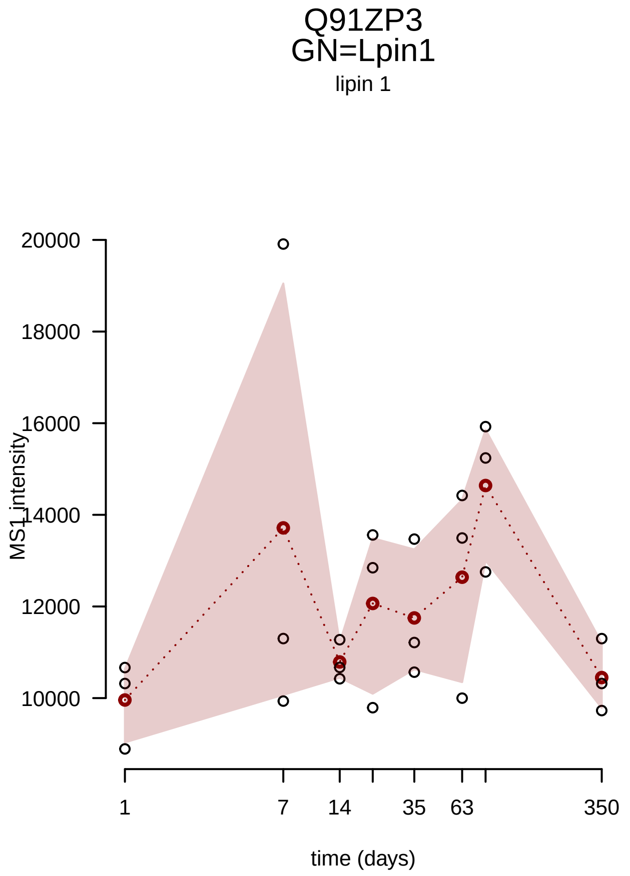
Q91ZP3
GN=Lpin1
lipin 1
MS1 intensity
20000
18000
16000
14000
12000
10000
1
7
14
35
63
350
time (days)

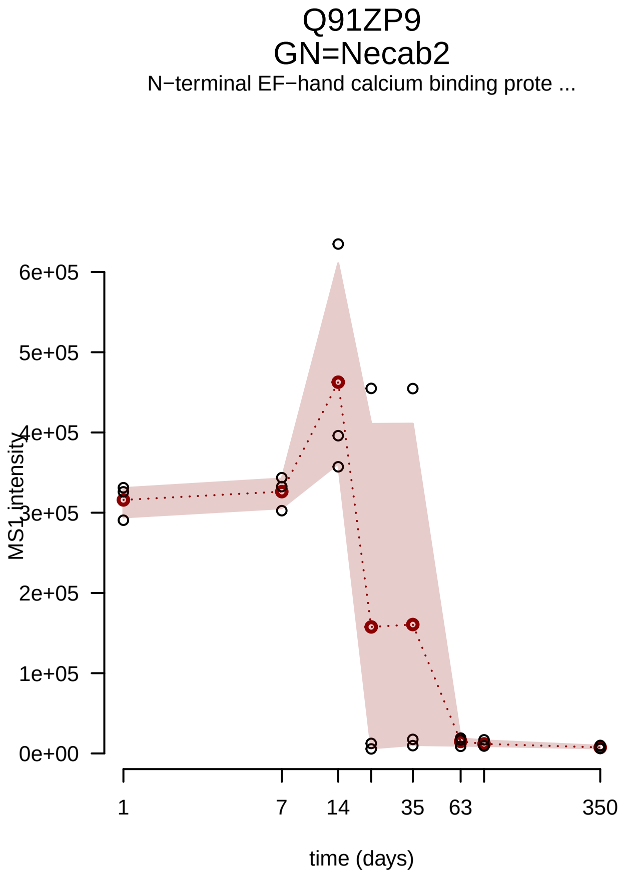
Q91ZP9
GN=Necab2
N−terminal EF−hand calcium binding prote ...
MS1 intensity
6e+05
5e+05
4e+05
3e+05
2e+05
1e+05
0e+00
1
7
14
35
63
350
time (days)

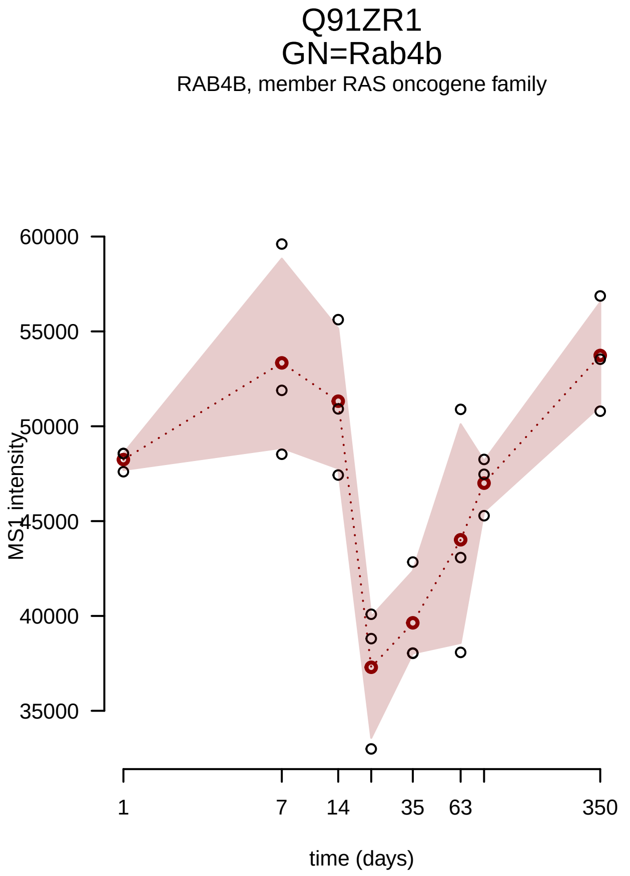
Q91ZR1
GN=Rab4b
RAB4B, member RAS oncogene family
MS1 intensity
60000
55000
50000
45000
40000
35000
1
7
14
35
63
350
time (days)

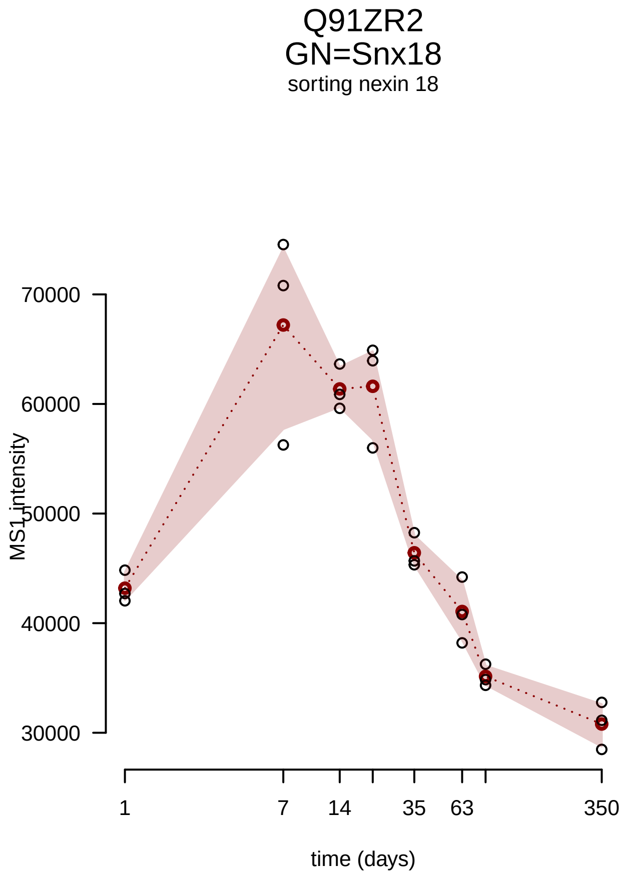
Q91ZR2
GN=Snx18
sorting nexin 18
MS1 intensity
70000
60000
50000
40000
30000
1
7
14
35
63
350
time (days)

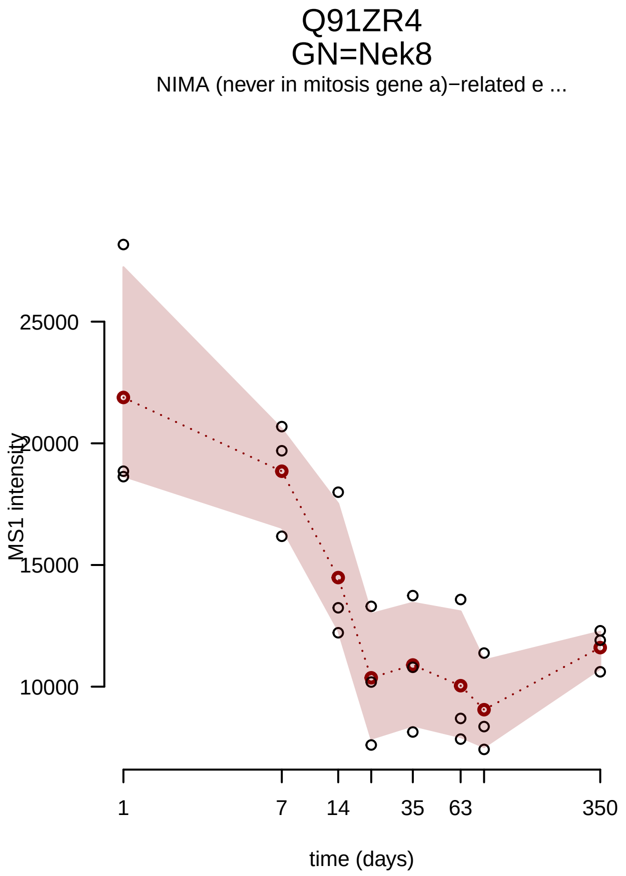
Q91ZR4
GN=Nek8
NIMA (never in mitosis gene a)−related e ...
MS1 intensity
25000
20000
15000
10000
1
7
14
35
63
350
time (days)

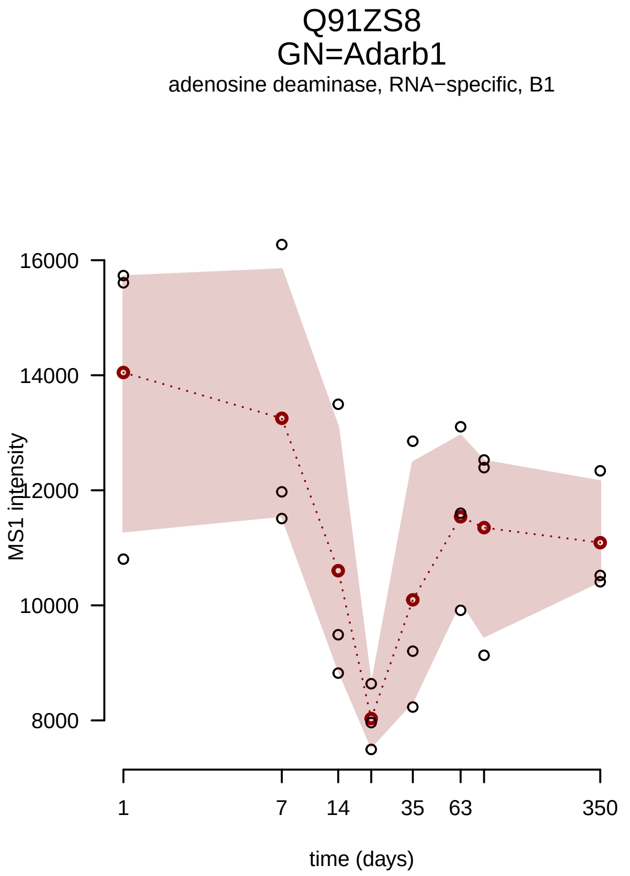
Q91ZS8
GN=Adarb1
adenosine deaminase, RNA−specific, B1
MS1 intensity
16000
14000
12000
10000
8000
1
7
14
35
63
350
time (days)

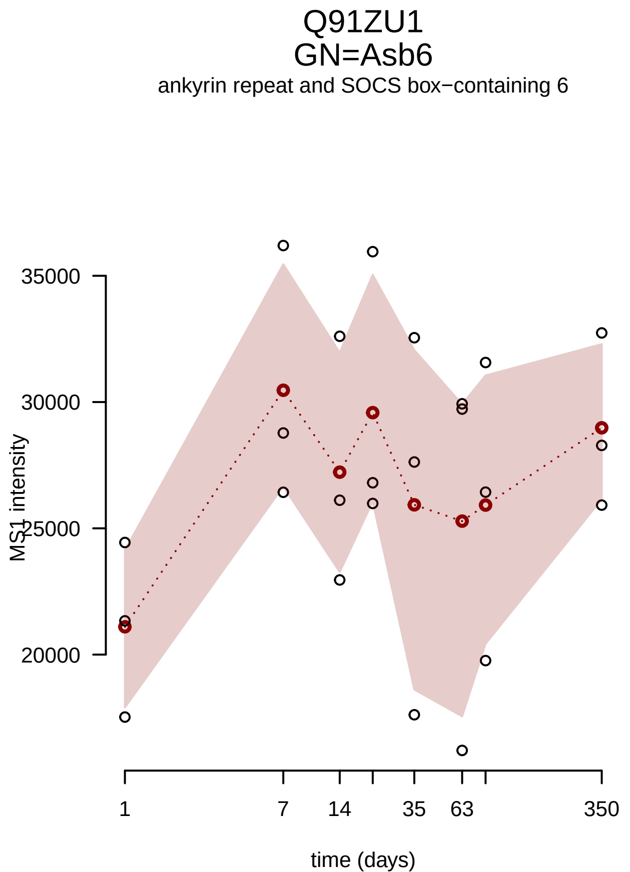
Q91ZU1
GN=Asb6
ankyrin repeat and SOCS box−containing 6
MS1 intensity
35000
30000
25000
20000
1
7
14
35
63
350
time (days)

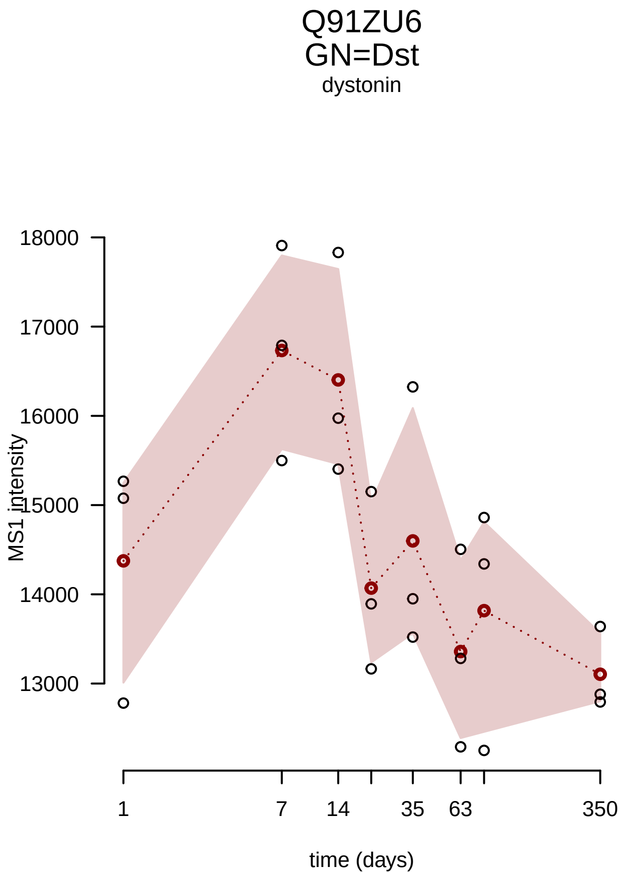
Q91ZU6
GN=Dst
dystonin
MS1 intensity
18000
17000
16000
15000
14000
13000
1
7
14
35
63
350
time (days)

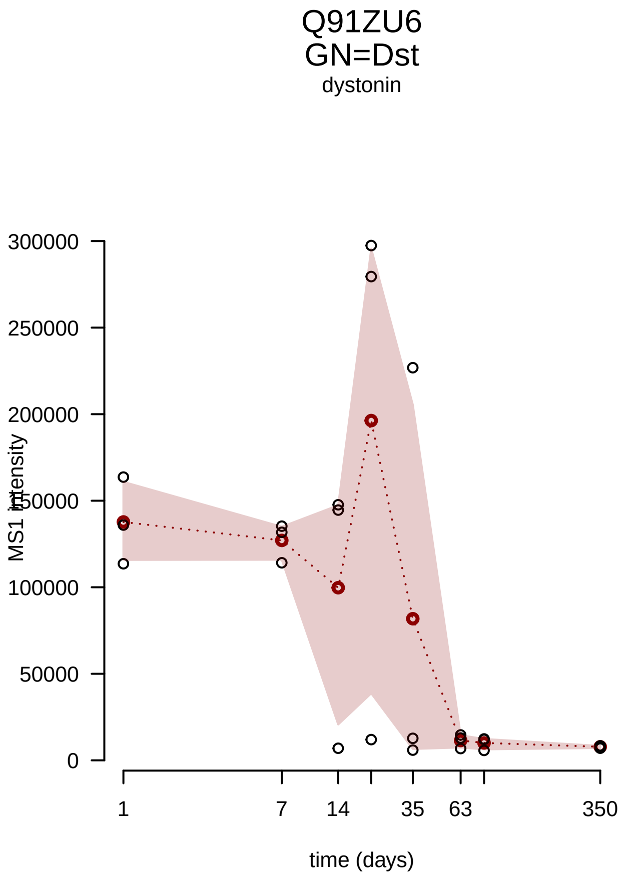
Q91ZU6
GN=Dst
dystonin
MS1 intensity
300000
250000
200000
150000
100000
50000
0
1
7
14
35
63
350
time (days)

Q91ZV0
GN=Mia2
MIA SH3 domain ER export factor 2

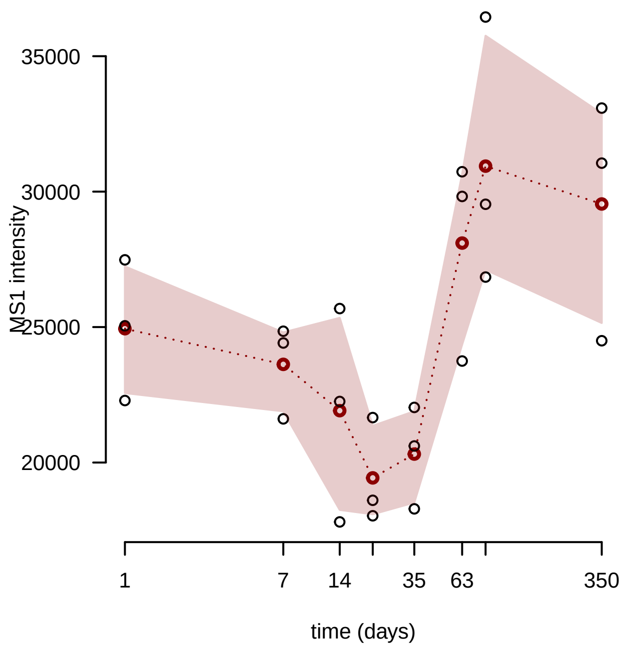

Q91ZV7
GN=Plxdc1
plexin domain containing 1

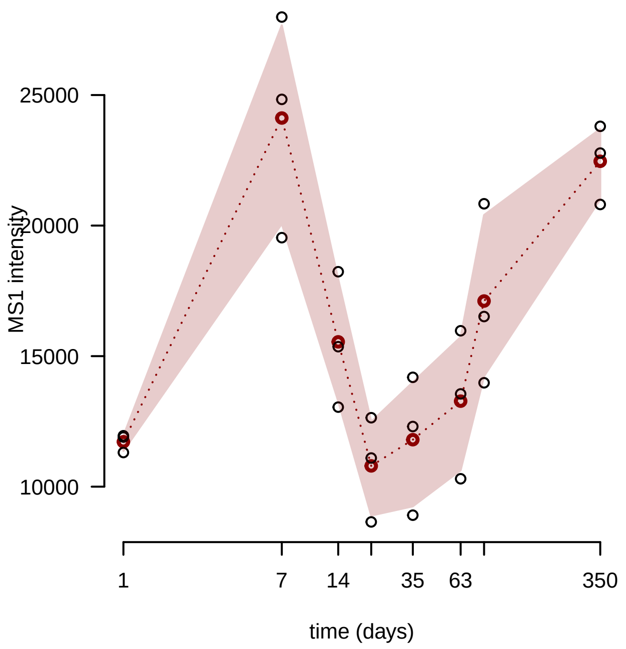

Q91ZV8
GN=Adgra2
adhesion G protein−coupled receptor A2

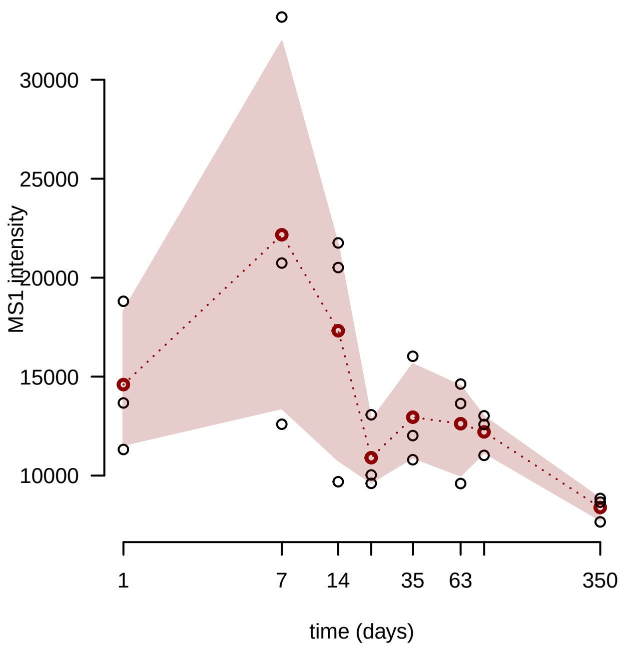

Q91ZW2
GN=Pofut1
protein O−fucosyltransferase 1

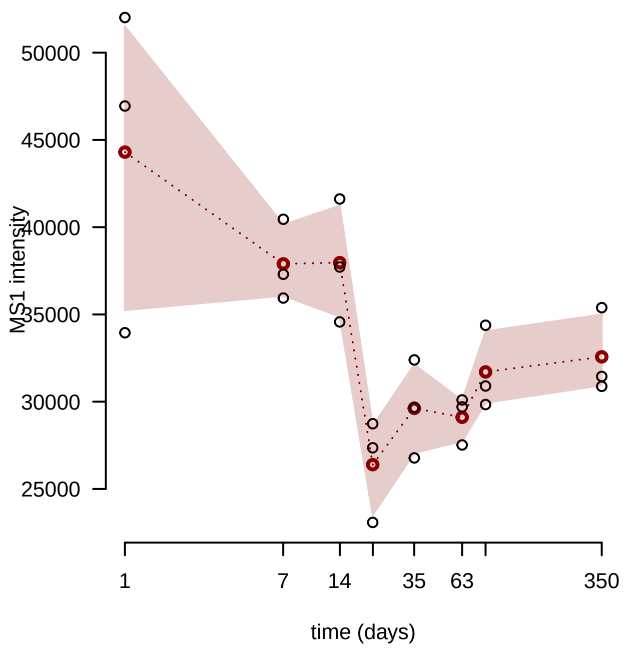

Q91ZW3
GN=Smarca5
SWI/SNF related, matrix associated, acti ...

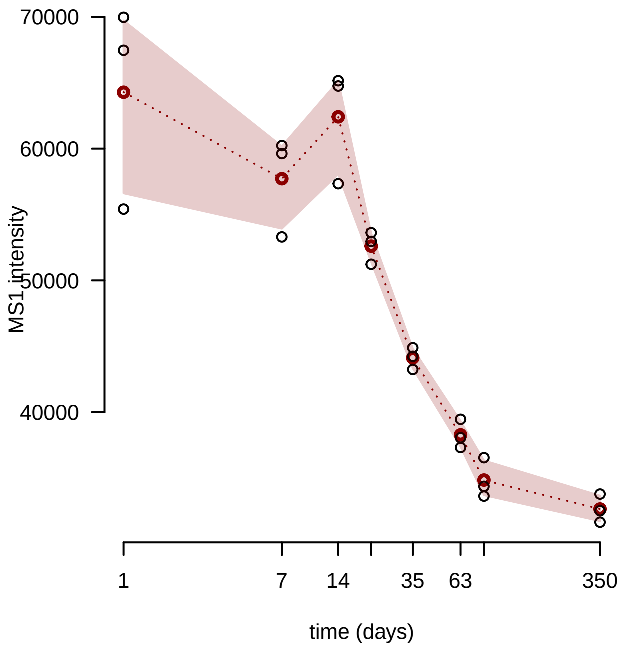

Q91ZX7
GN=Lrp1
low density lipoprotein receptor−related ...

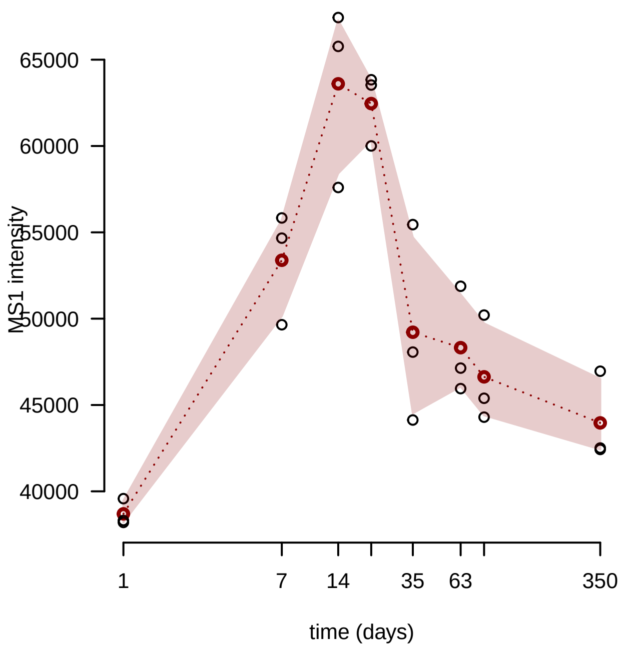

Q920A5
GN=Scpep1
serine carboxypeptidase 1

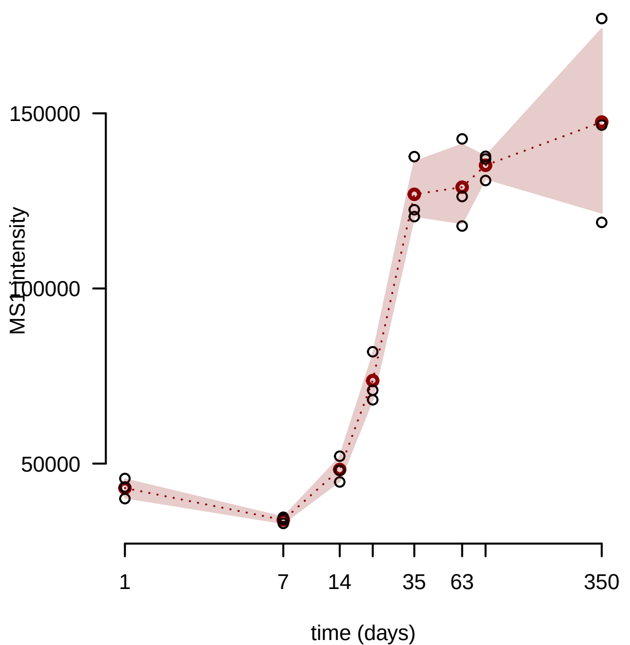

Q920A7
GN=Afg3l1
AFG3−like AAA ATPase 1

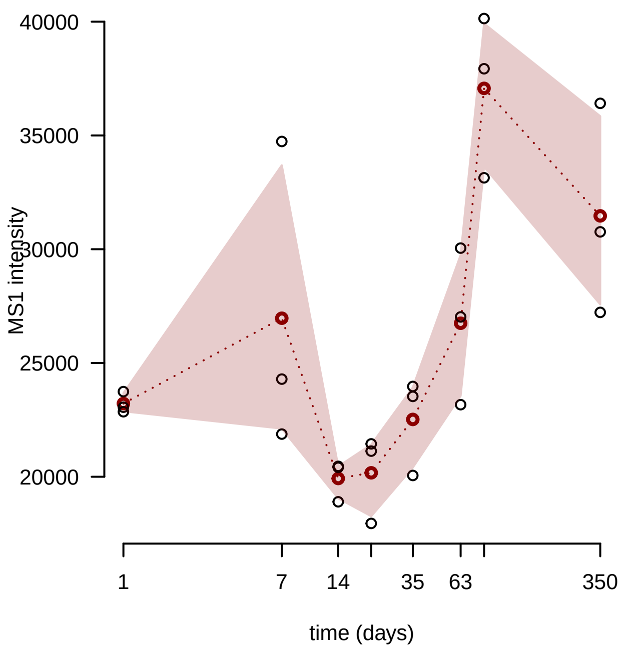

Q920B0
GN=Frmd4b
FERM domain containing 4B

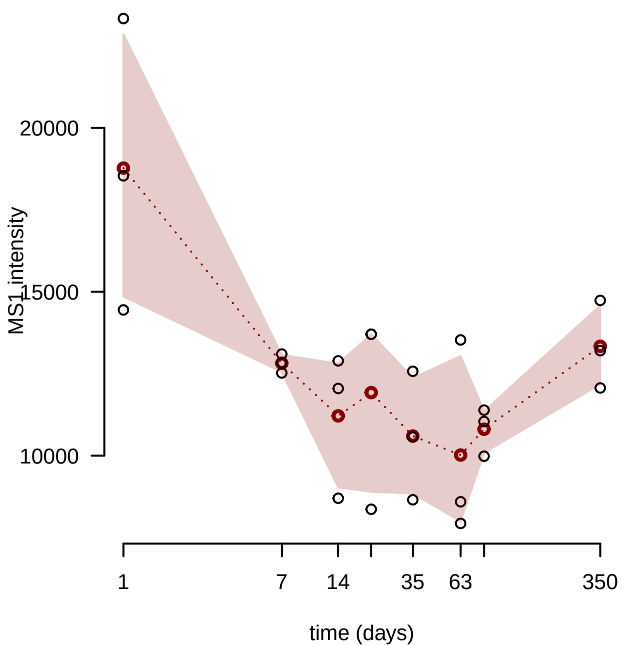

Q920B9
GN=Supt16h

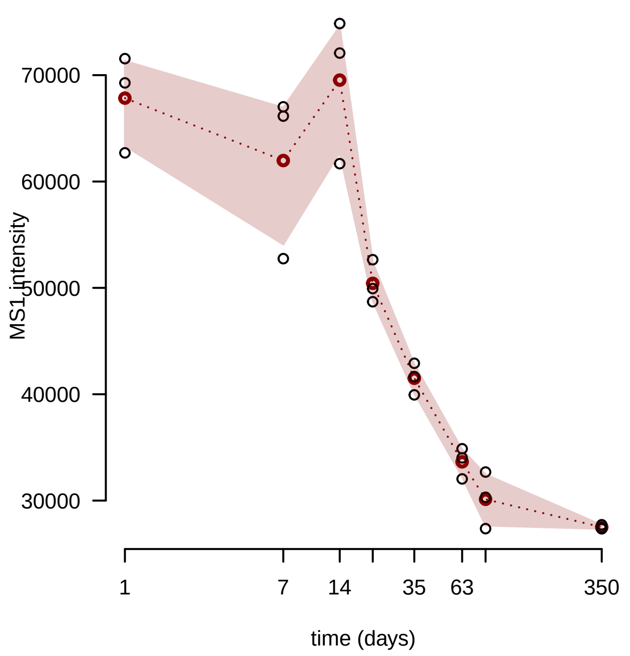

Q920D3
GN=Med28
mediator complex subunit 28

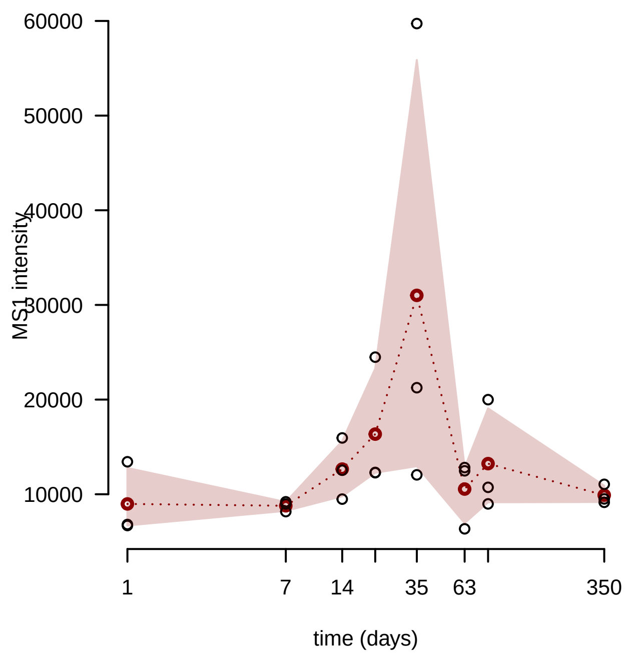

Q920E5
GN=Fdps
farnesyl diphosphate synthetase

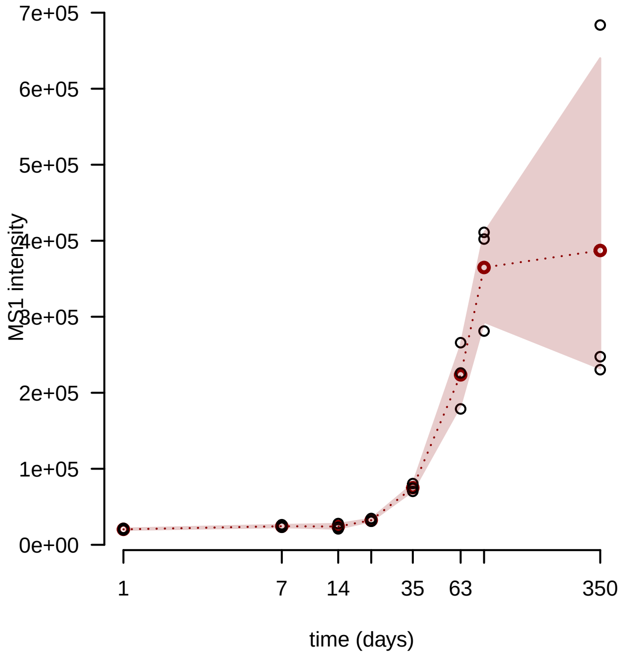

Q920I9
GN=Wdr7
WD repeat domain 7

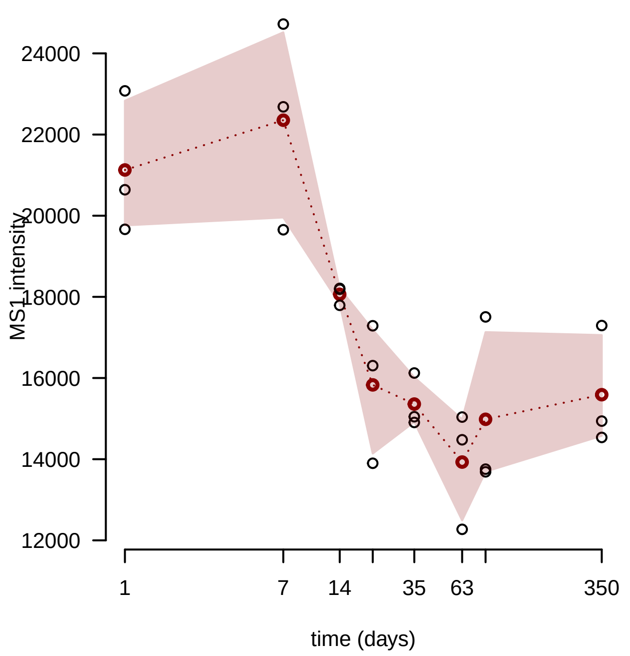

Q920L1
GN=Fads1
fatty acid desaturase 1

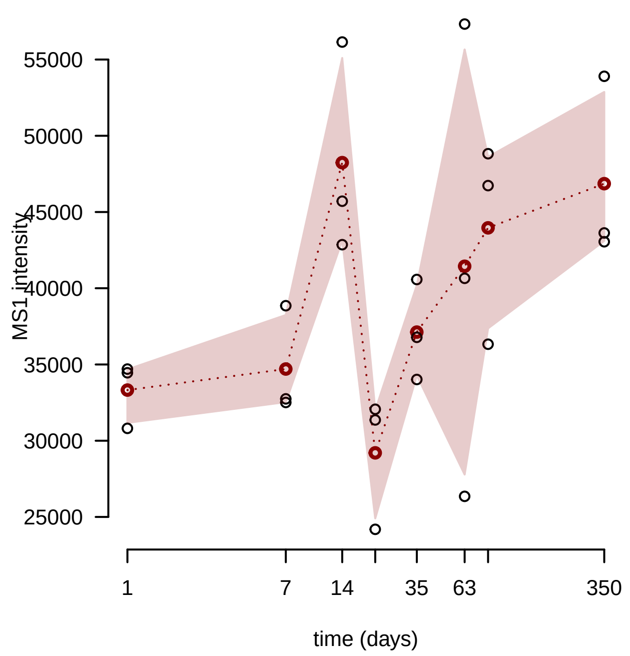

Q920L5
GN=Elovl6
ELOVL family member 6, elongation of lon ...

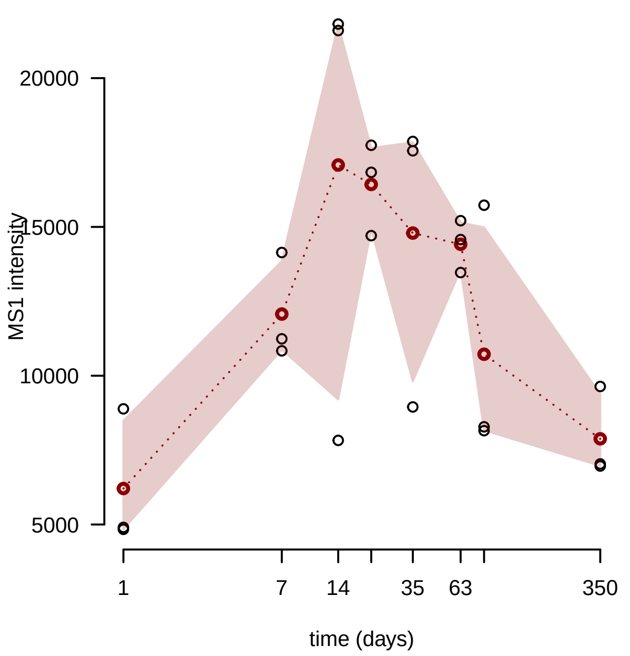

Q920N2
GN=Hlcs
holocarboxylase synthetase (biotin− [pro ...

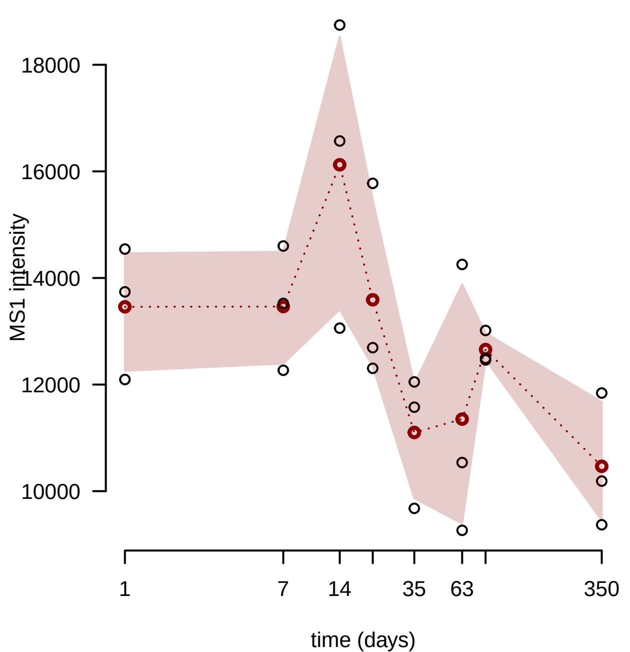

Q920Q4
GN=Vps16
VSP16 CORVET/HOPS core subunit

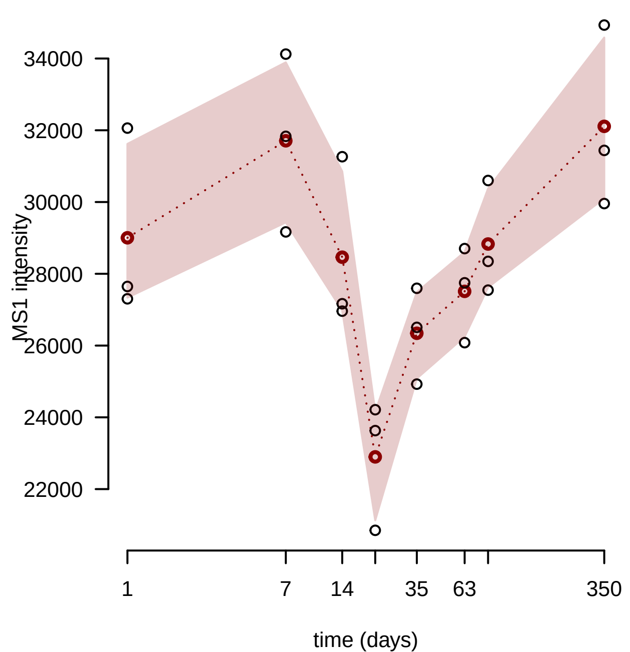

Q920Q6
GN=Msi2
musashi RNA−binding protein 2

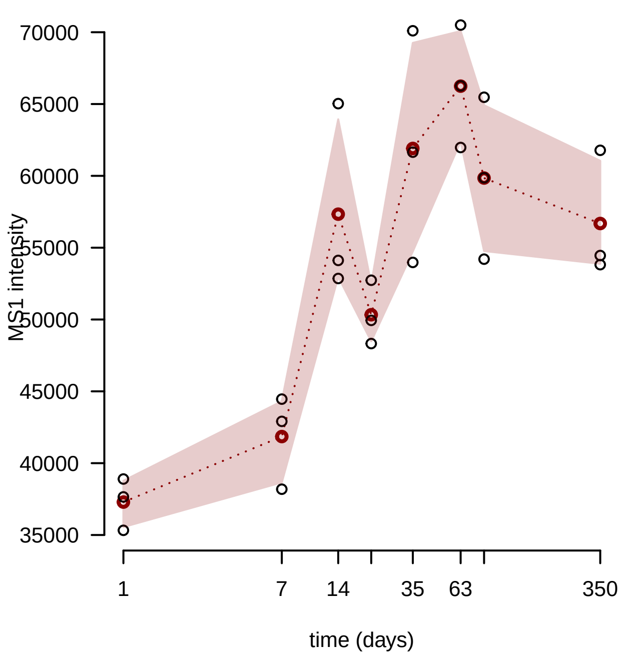

Q920Q8
GN=Ivns1abp
influenza virus NS1A binding protein

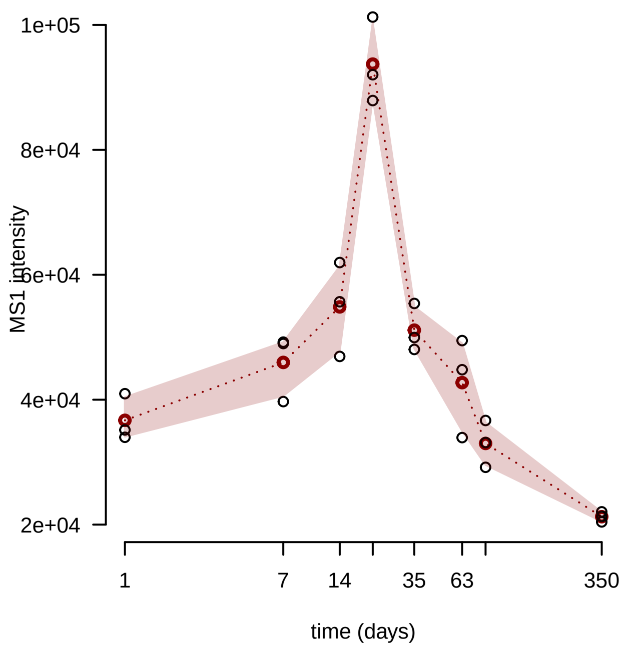

Q920R0
GN=Als2
alsin Rho guanine nucleotide exchange fa ...

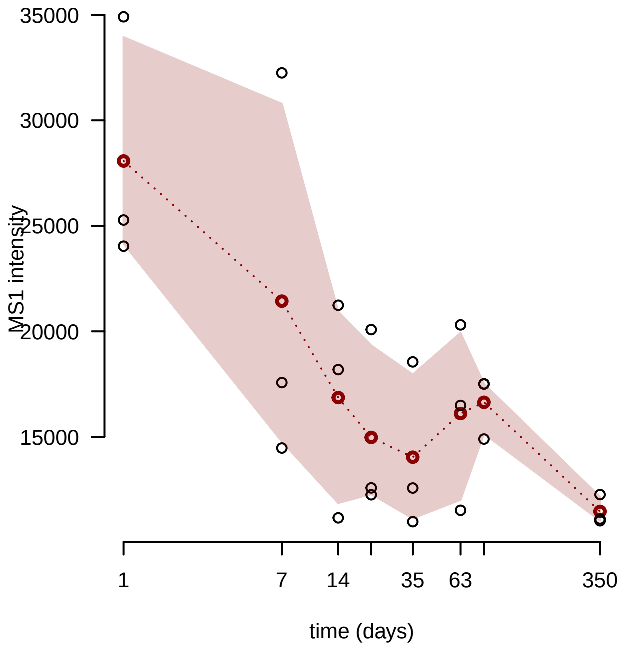

Q920S3
GN=Gatad1
GATA zinc finger domain containing 1

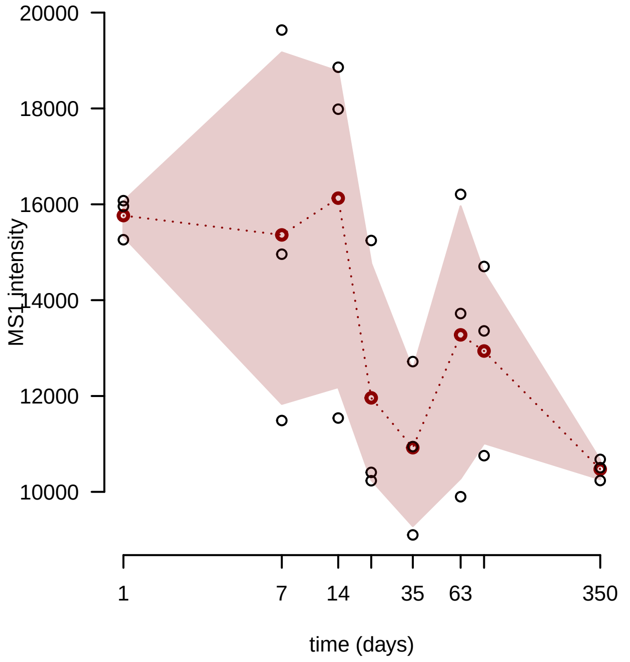

Q920V1
GN=B3galnt1
UDP−GalNAc:betaGlcNAc beta 1,3−galactosa ...

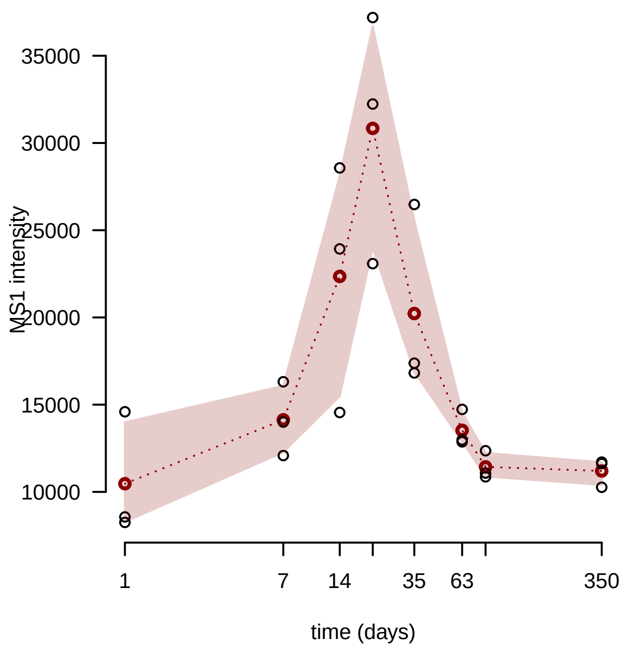

Q921C3
GN=Brwd1
bromodomain and WD repeat domain contain ...

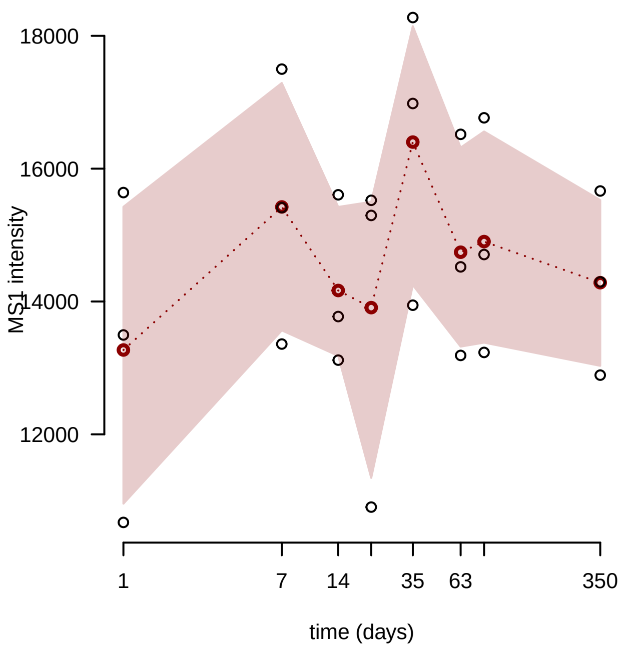

Q921C5
GN=Bicd2
BICD cargo adaptor 2

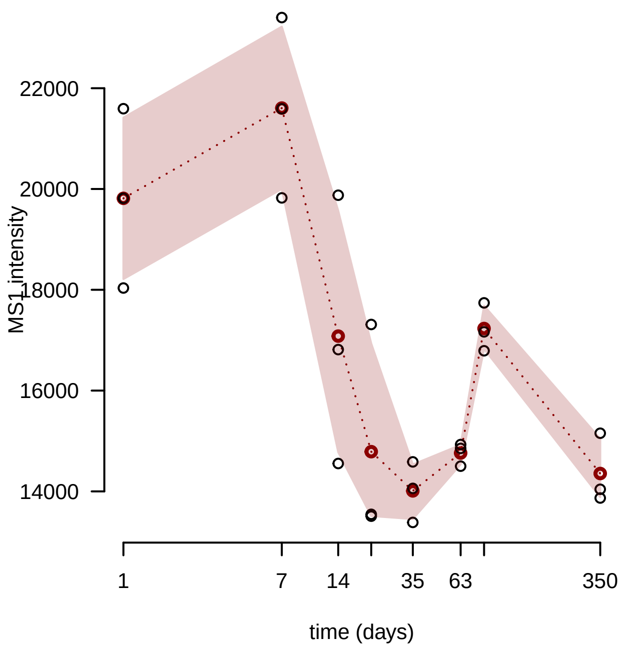

Q921D4
GN=Med6
mediator complex subunit 6

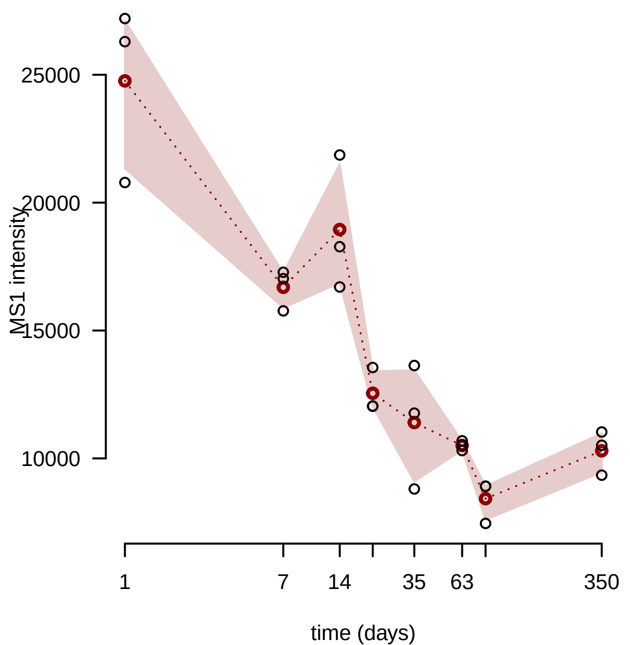

Q921E2
GN=Rab31
RAB31, member RAS oncogene family

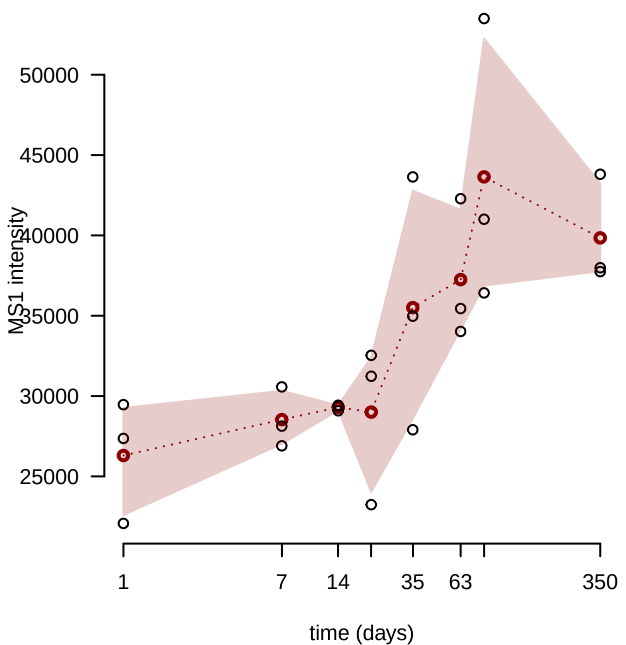

Q921E6
GN=Eed
embryonic ectoderm development

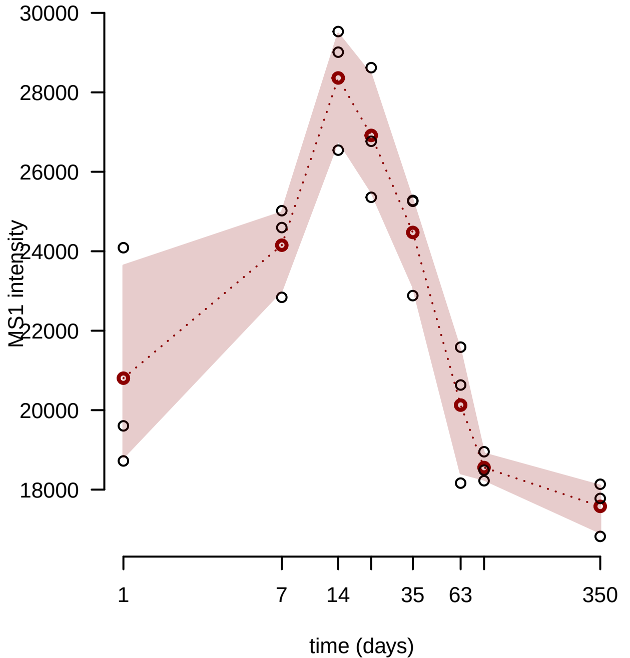

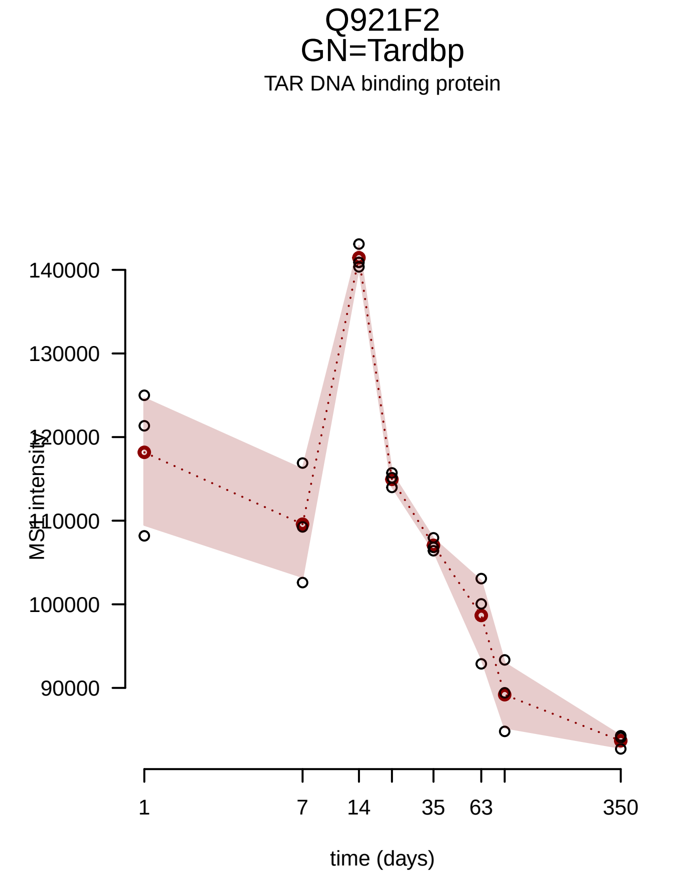
Q921F2
GN=Tardbp
TAR DNA binding protein
MS1 intensity
140000
130000
120000
110000
100000
90000
1
7
14
35
63
350
time (days)

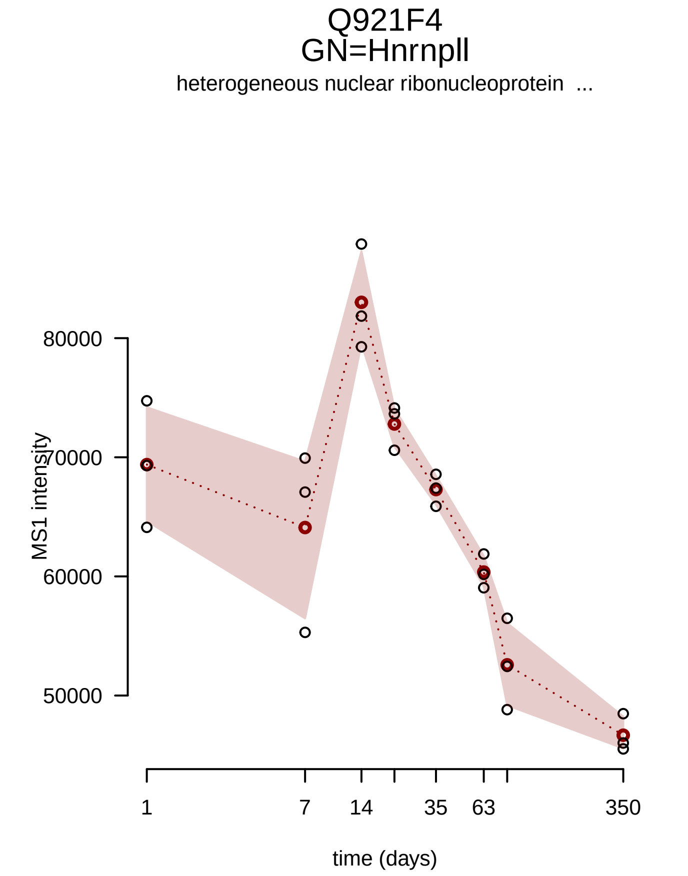
Q921F4
GN=Hnrnpll
heterogeneous nuclear ribonucleoprotein ...
MS1 intensity
80000
70000
60000
50000
1
7
14
35
63
350
time (days)

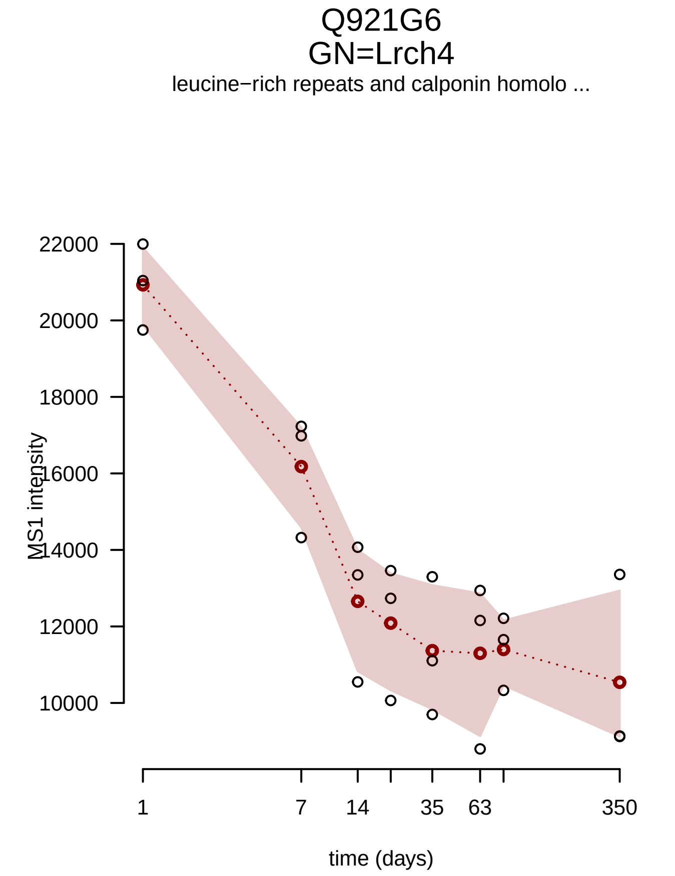
Q921G6
GN=Lrch4
leucine−rich repeats and calponin homolo ...
MS1 intensity
22000
20000
18000
16000
14000
12000
10000
1
7
14
35
63
350
time (days)

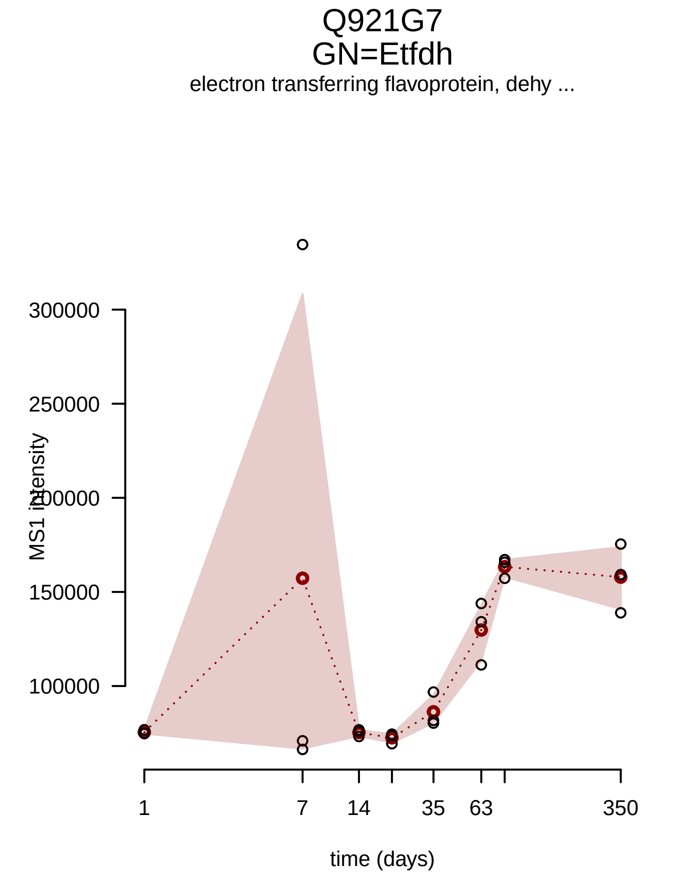
Q921G7
GN=Etfdh
electron transferring flavoprotein, dehy ...
MS1 intensity
300000
250000
200000
150000
100000
1
7
14
35
63
350
time (days)

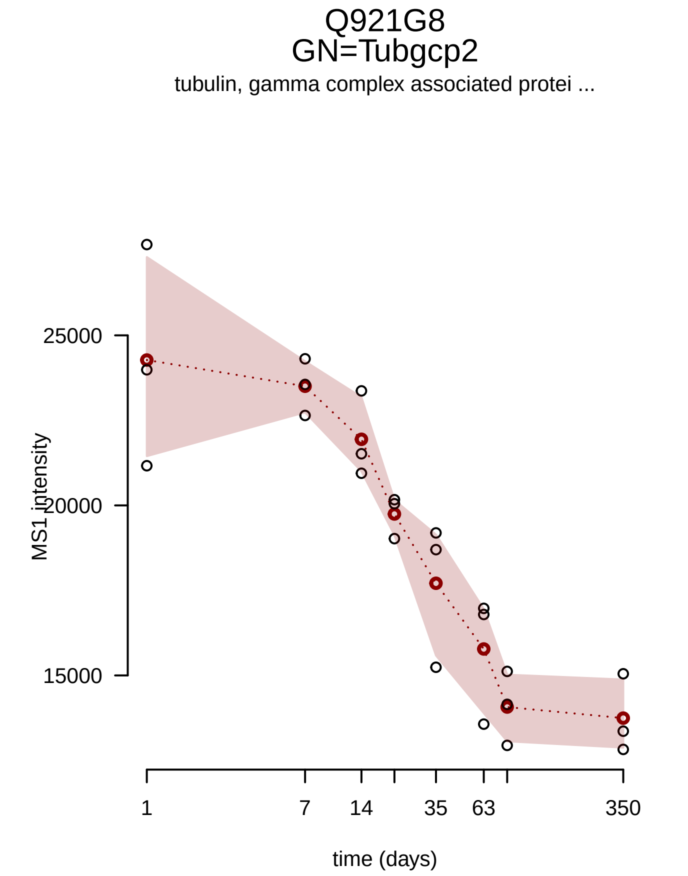
Q921G8
GN=Tubgcp2
tubulin, gamma complex associated protei ...
MS1 intensity
25000
20000
15000
1
7
14
35
63
350
time (days)

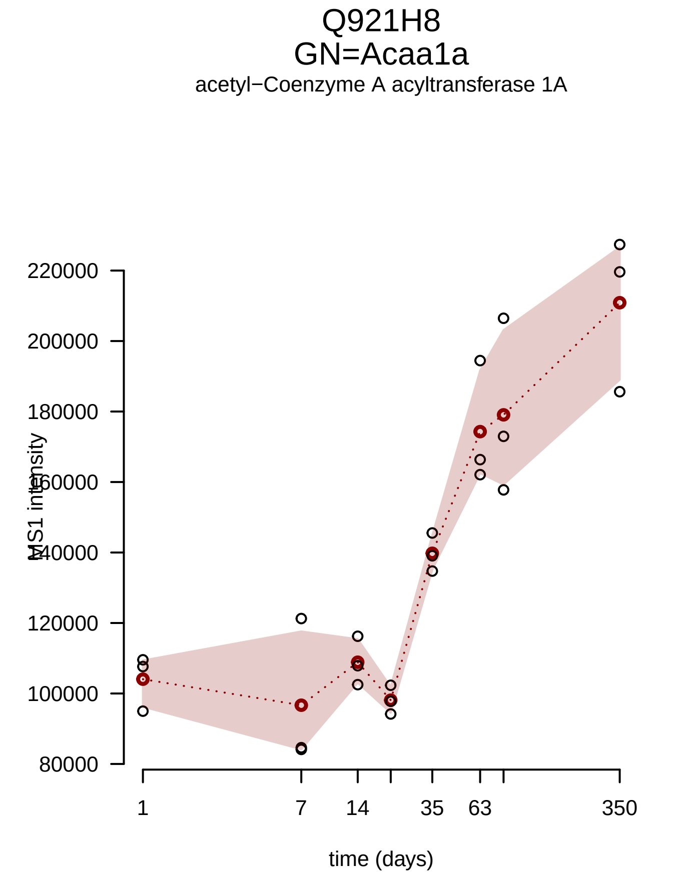
Q921H8
GN=Acaa1a
acetyl−Coenzyme A acyltransferase 1A
MS1 intensity
220000
200000
180000
160000
140000
120000
100000
80000
1
7
14
35
63
350
time (days)

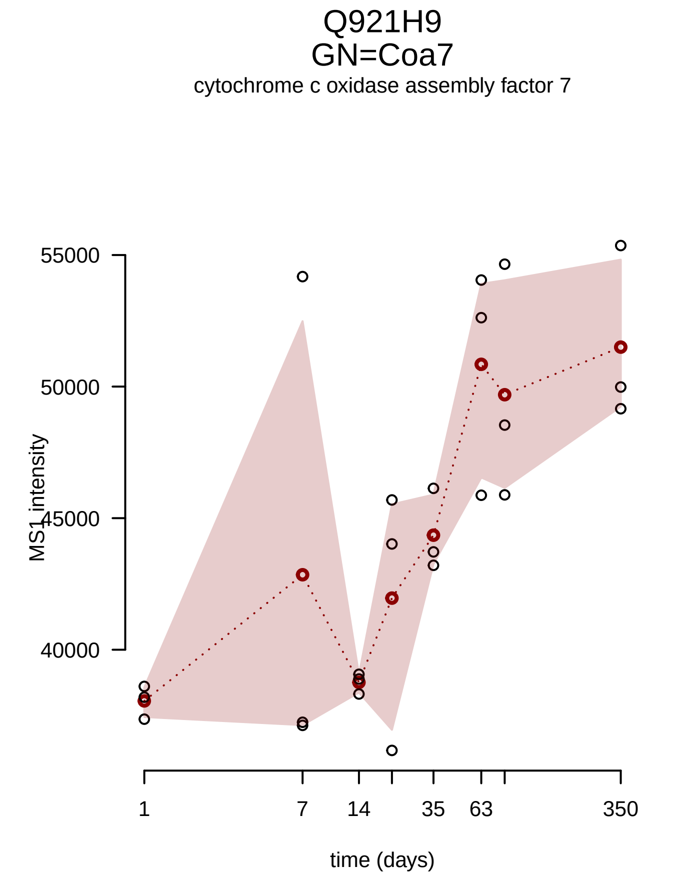
Q921H9
GN=Coa7
cytochrome c oxidase assembly factor 7
MS1 intensity
55000
50000
45000
40000
1
7
14
35
63
350
time (days)

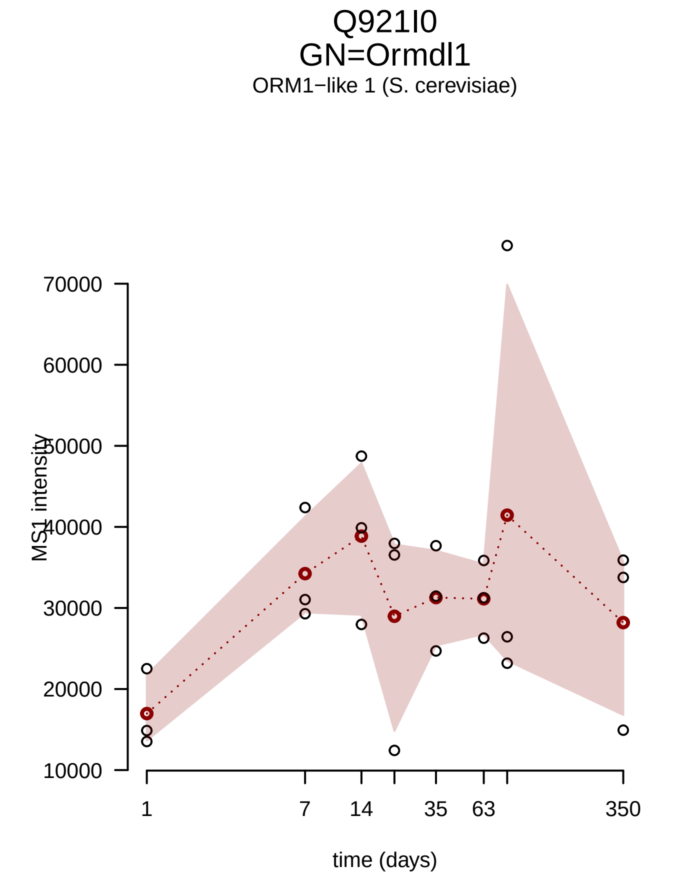
Q921I0
GN=Ormdl1
ORM1−like 1 (S. cerevisiae)
MS1 intensity
70000
60000
50000
40000
30000
20000
10000
1
7
14
35
63
350
time (days)

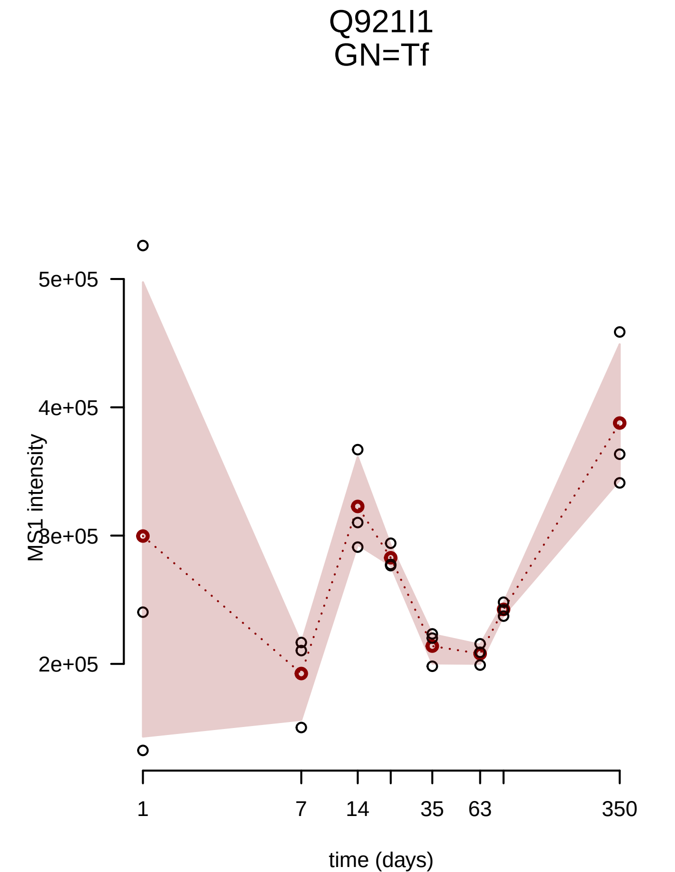
Q921I1
GN=Tf
MS1 intensity
5e+05
4e+05
3e+05
2e+05
1
7
14
35
63
350
time (days)

Q921I2
GN=Klhdc4
kelch domain containing 4

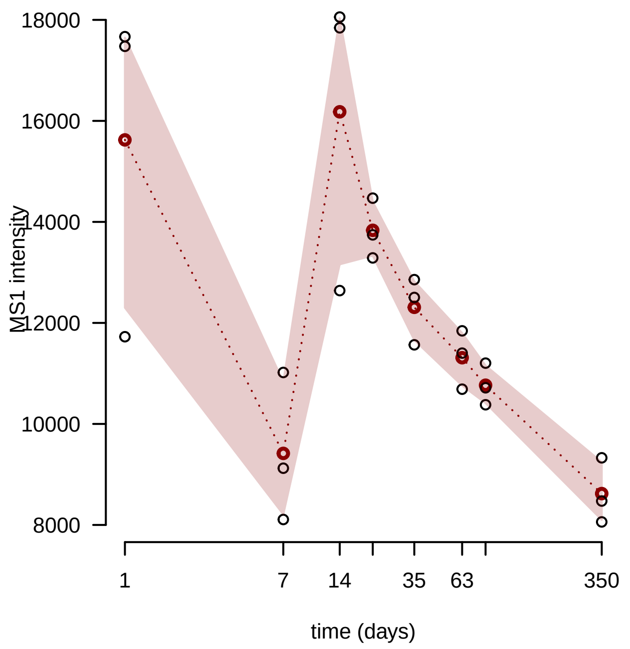

Q921I6
GN=Sh3bp4
SH3−domain binding protein 4

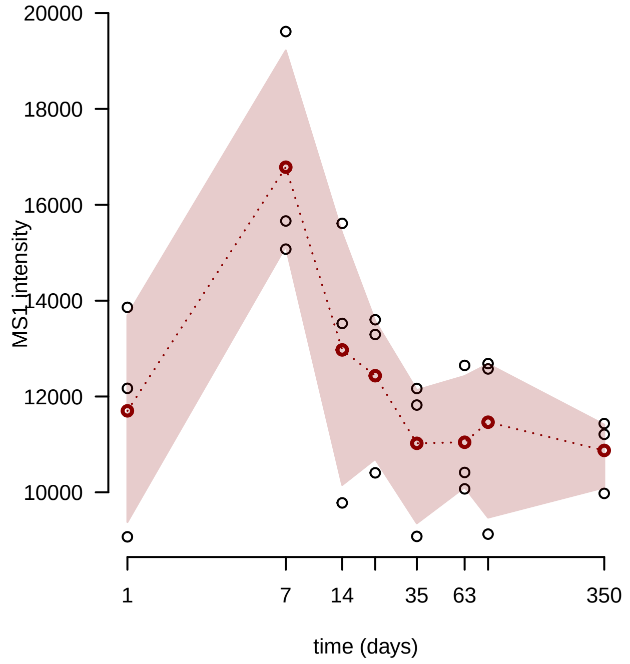

Q921I9
GN=Exosc4
exosome component 4

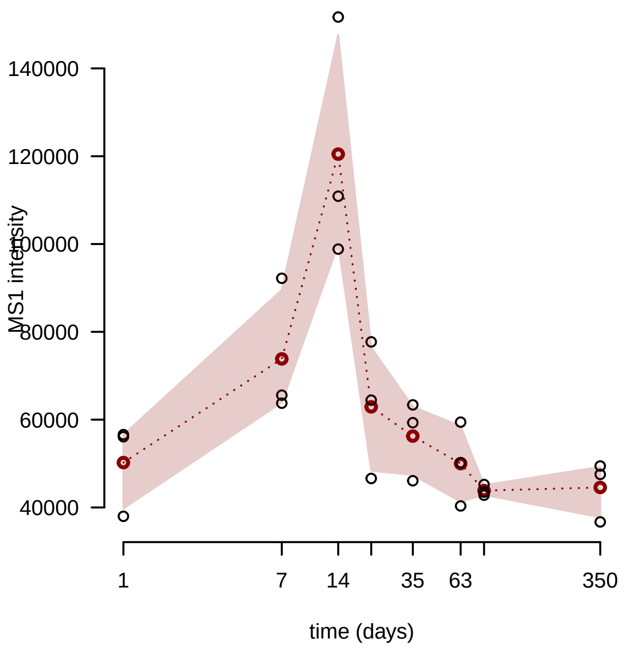

Q921J2
GN=Rheb
Ras homolog enriched in brain

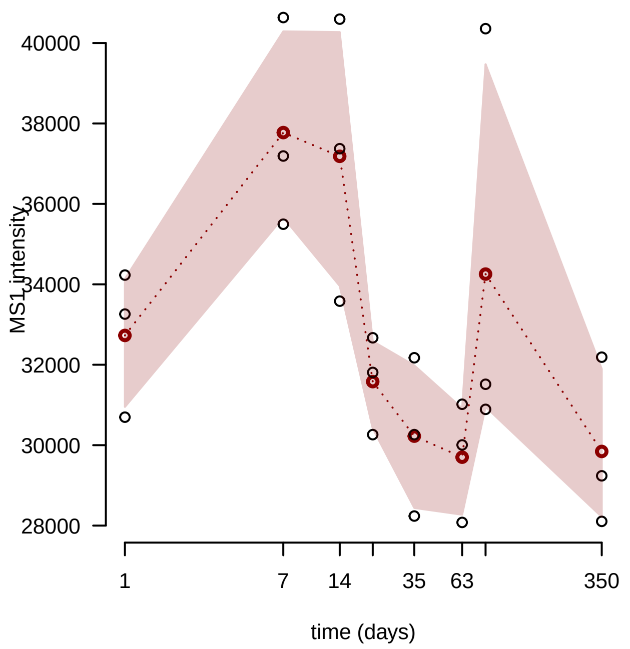

Q921J4
GN=Ube2s
ubiquitin−conjugating enzyme E2S

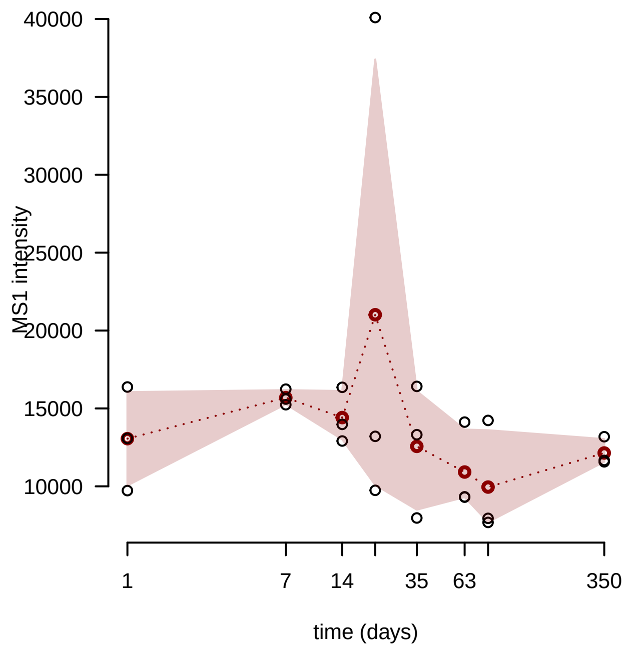

Q921K8
GN=Tcaf2
TRPM8 channel−associated factor 2

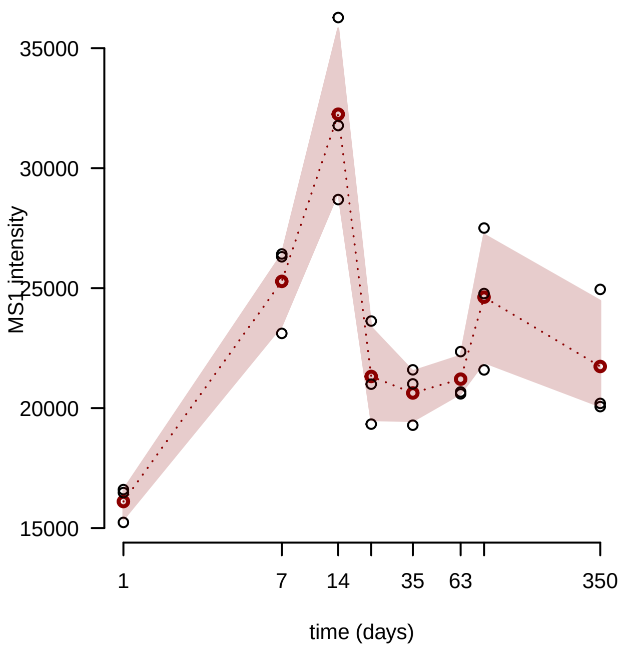

Q921K9
GN=Bcl7b
B cell CLL/lymphoma 7B

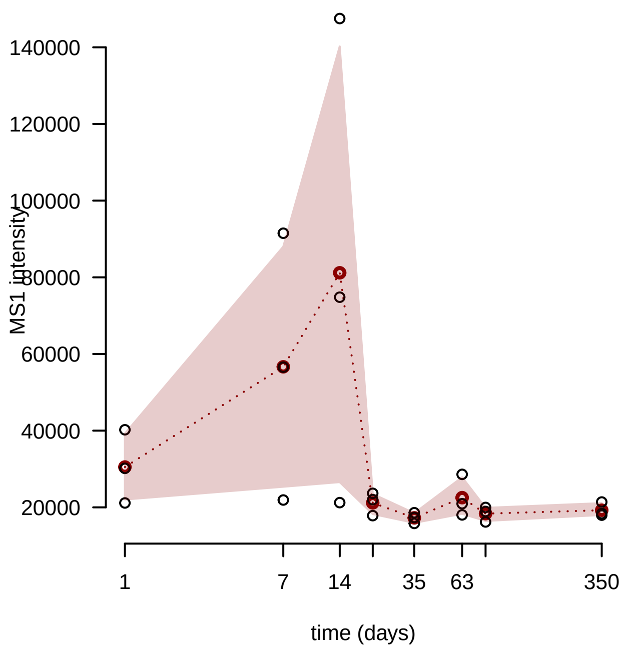

Q921L3
GN=Tmco1
transmembrane and coiled−coil domains 1

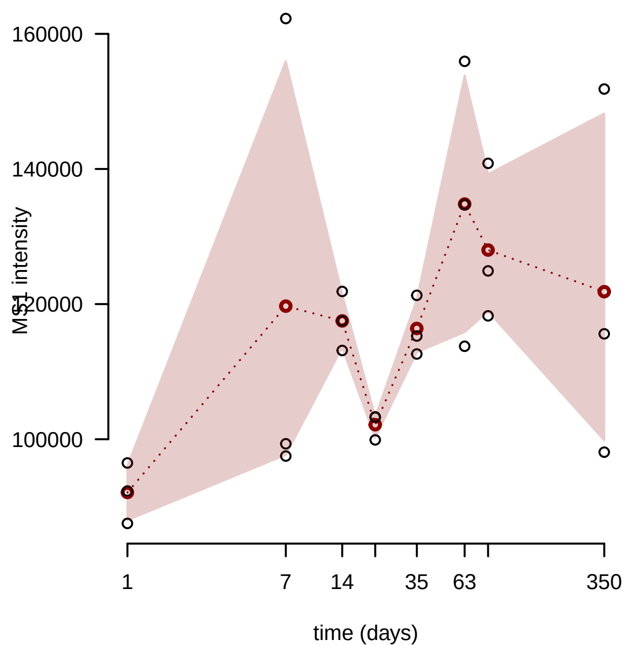

Q921L5
GN=Cog2
component of oligomeric golgi complex 2

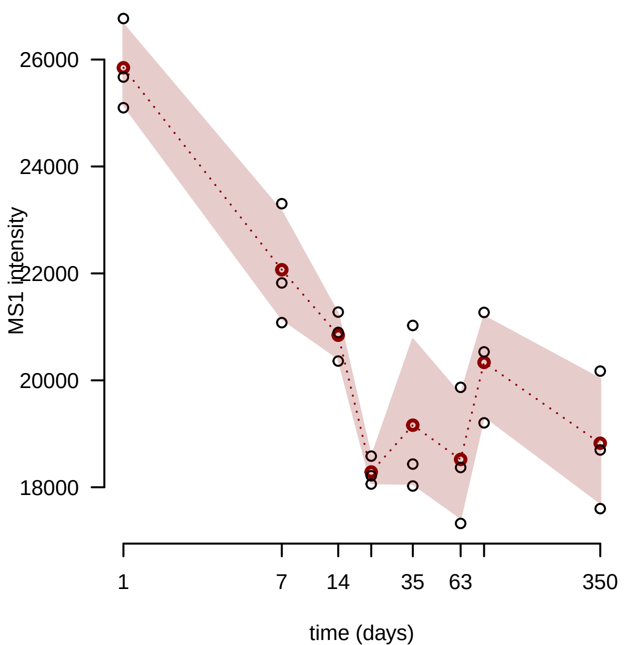

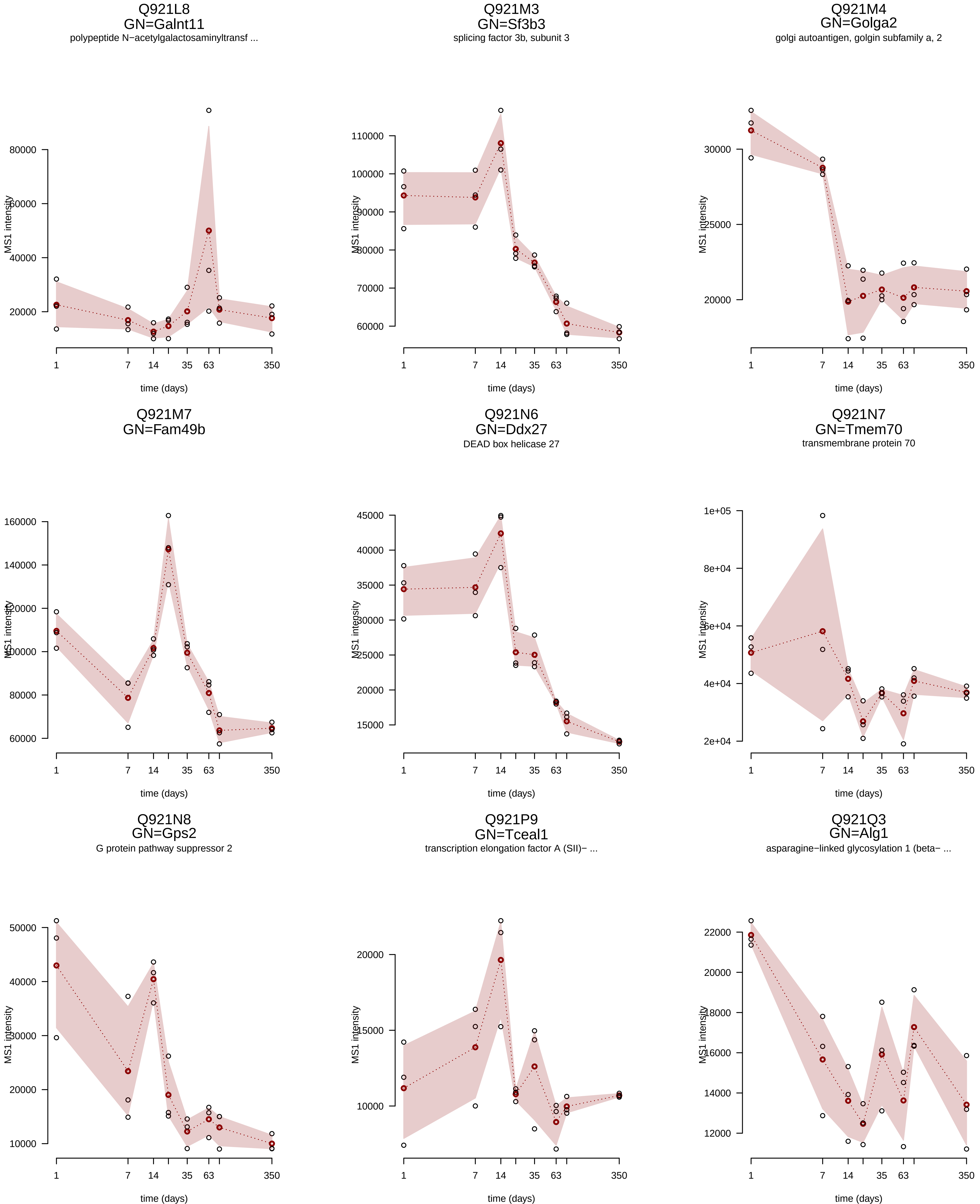
Q921L8
GN=Galnt11
polypeptide N−acetylgalactosaminyltransf ...
MS1 intensity
80000
60000
40000
20000
1
7
14
35
63
350
time (days)
Q921M3
GN=Sf3b3
splicing factor 3b, subunit 3
MS1 intensity
110000
100000
90000
80000
70000
60000
1
7
14
35
63
350
time (days)
Q921M4
GN=Golga2
golgi autoantigen, golgin subfamily a, 2
MS1 intensity
30000
25000
20000
1
7
14
35
63
350
time (days)
Q921M7
GN=Fam49b
MS1 intensity
160000
140000
120000
100000
80000
60000
1
7
14
35
63
350
time (days)
Q921N6
GN=Ddx27
DEAD box helicase 27
MS1 intensity
45000
40000
35000
30000
25000
20000
15000
1
7
14
35
63
350
time (days)
Q921N7
GN=Tmem70
transmembrane protein 70
MS1 intensity
1e+05
8e+04
6e+04
4e+04
2e+04
1
7
14
35
63
350
time (days)
Q921N8
GN=Gps2
G protein pathway suppressor 2
MS1 intensity
50000
40000
30000
20000
10000
1
7
14
35
63
350
time (days)
Q921P9
GN=Tceal1
transcription elongation factor A (SII)− ...
MS1 intensity
20000
15000
10000
1
7
14
35
63
350
time (days)
Q921Q3
GN=Alg1
asparagine−linked glycosylation 1 (beta− ...
MS1 intensity
22000
20000
18000
16000
14000
12000
1
7
14
35
63
350
time (days)

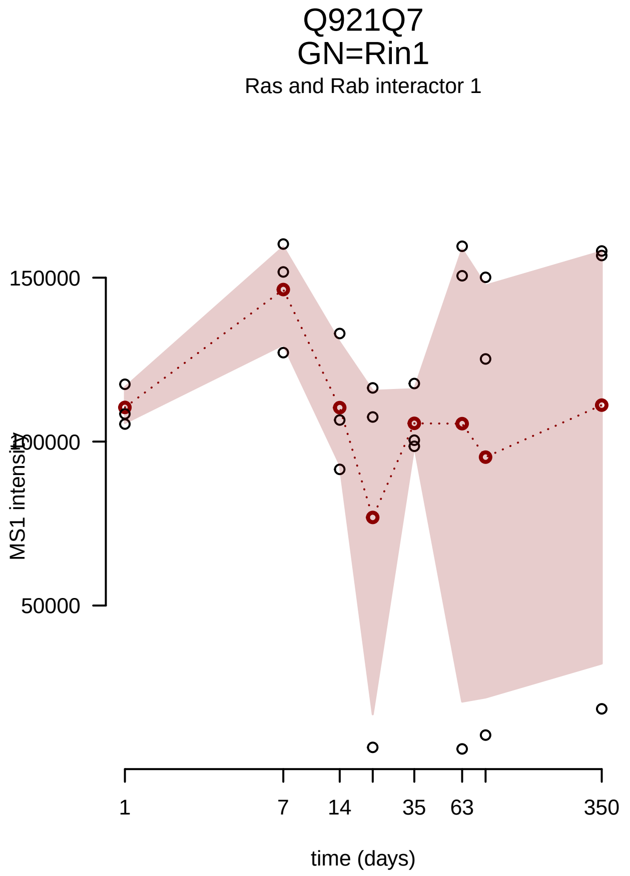
Q921Q7
GN=Rin1
Ras and Rab interactor 1
MS1 intensity
150000
100000
50000
1
7
14
35
63
350
time (days)

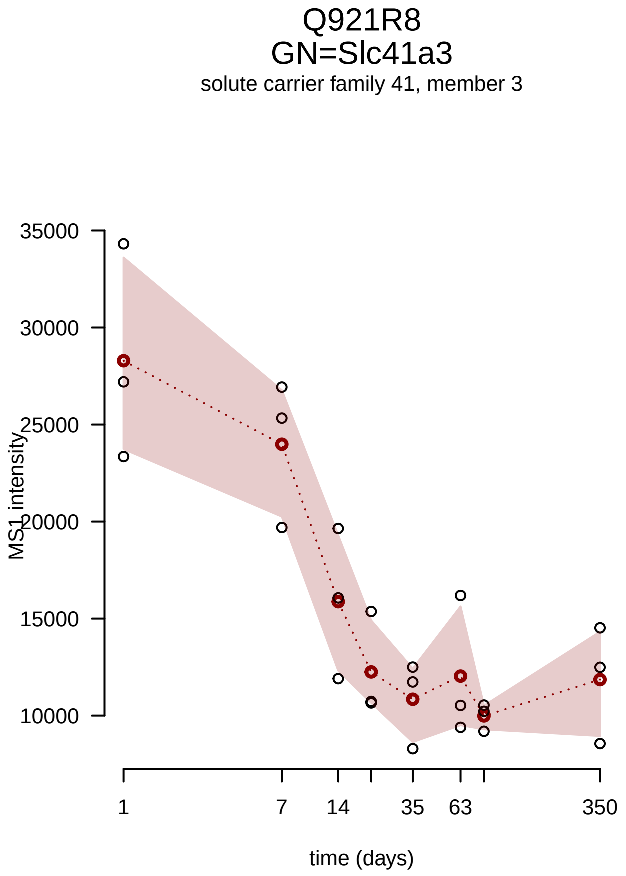
Q921R8
GN=Slc41a3
solute carrier family 41, member 3
MS1 intensity
35000
30000
25000
20000
15000
10000
1
7
14
35
63
350
time (days)

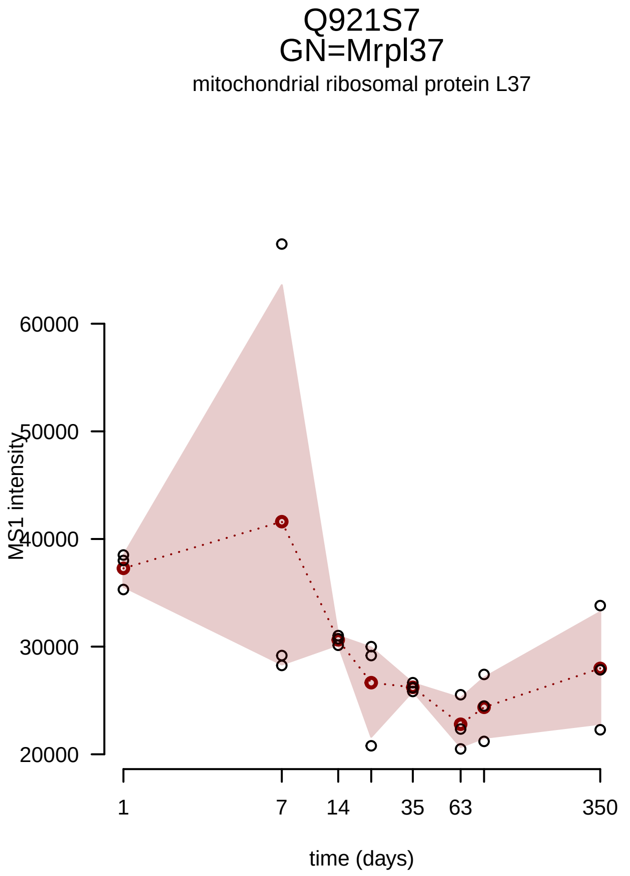
Q921S7
GN=Mrpl37
mitochondrial ribosomal protein L37
MS1 intensity
60000
50000
40000
30000
20000
1
7
14
35
63
350
time (days)

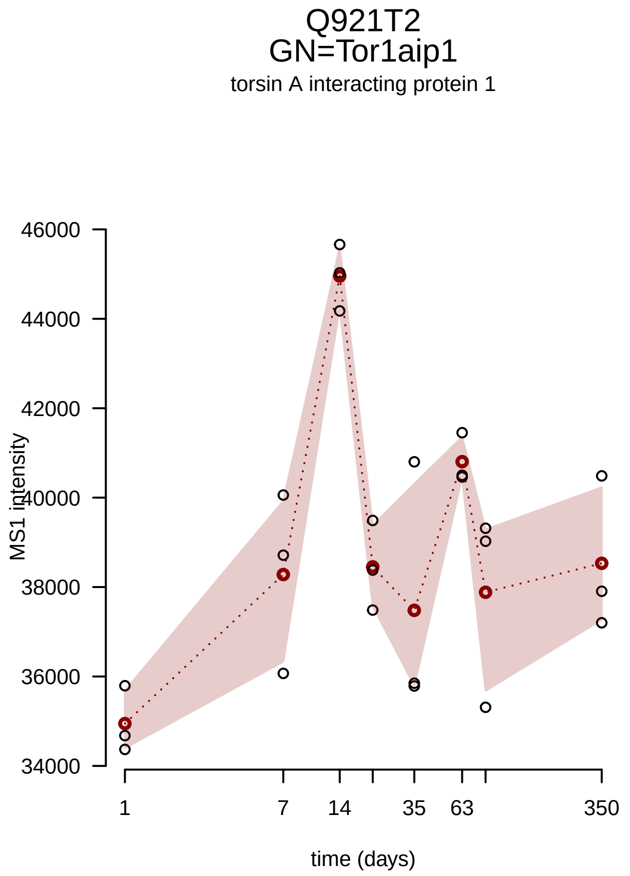
Q921T2
GN=Tor1aip1
torsin A interacting protein 1
MS1 intensity
46000
44000
42000
40000
38000
36000
34000
1
7
14
35
63
350
time (days)

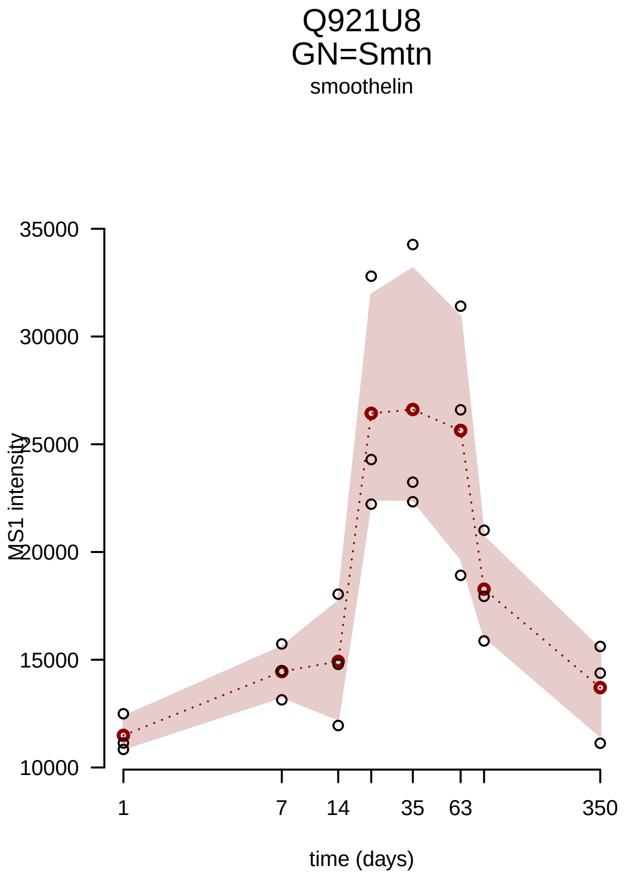
Q921U8
GN=Smtn
smoothelin
MS1 intensity
35000
30000
25000
20000
15000
10000
1
7
14
35
63
350
time (days)

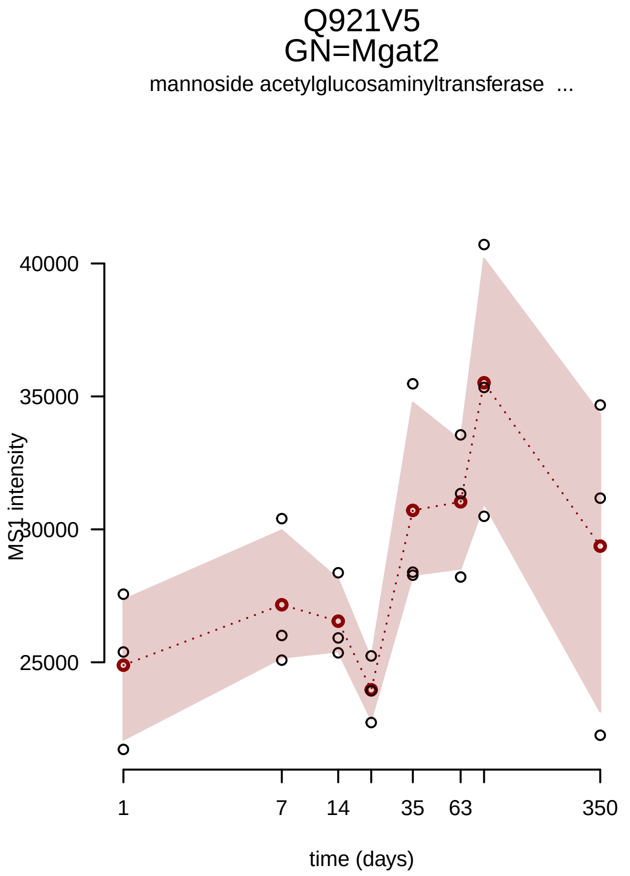
Q921V5
GN=Mgat2
mannoside acetylglucosaminyltransferase ...
MS1 intensity
40000
35000
30000
25000
1
7
14
35
63
350
time (days)

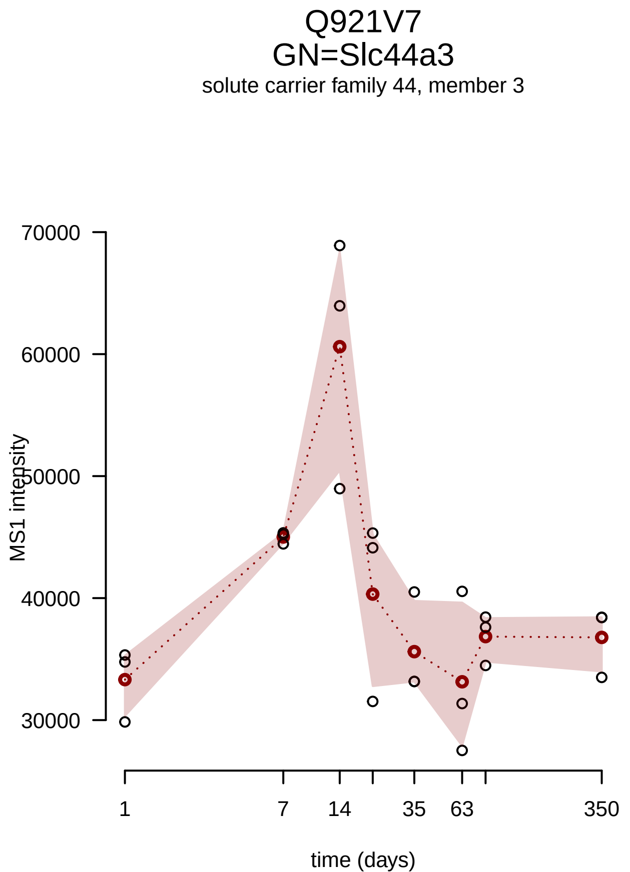
Q921V7
GN=Slc44a3
solute carrier family 44, member 3
MS1 intensity
70000
60000
50000
40000
30000
1
7
14
35
63
350
time (days)

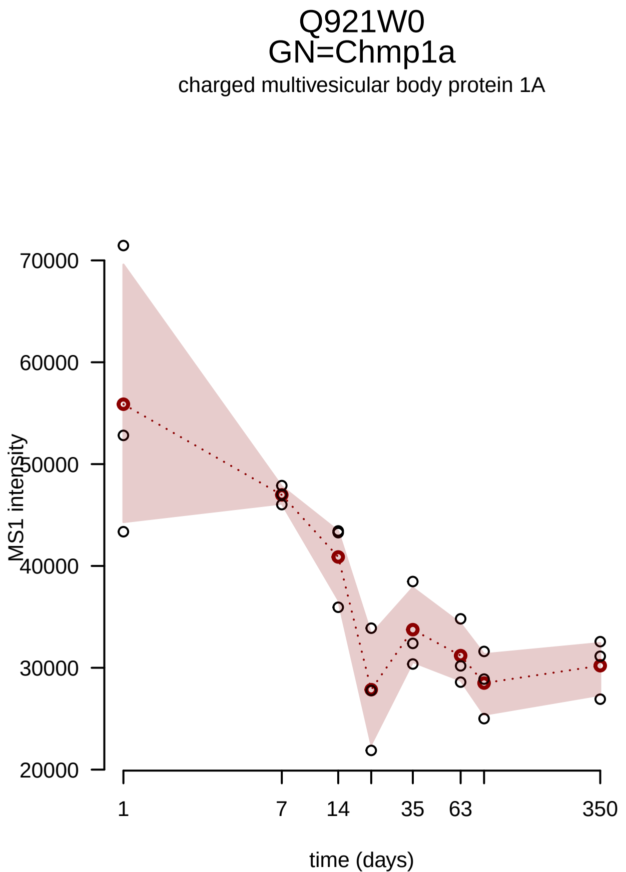
Q921W0
GN=Chmp1a
charged multivesicular body protein 1A
MS1 intensity
70000
60000
50000
40000
30000
20000
1
7
14
35
63
350
time (days)

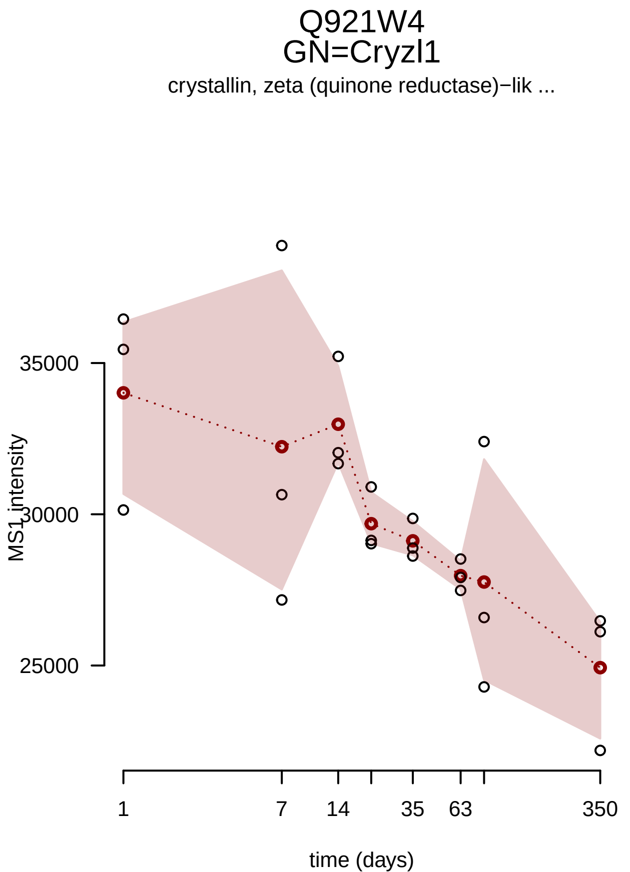
Q921W4
GN=Cryzl1
crystallin, zeta (quinone reductase)−lik ...
MS1 intensity
35000
30000
25000
1
7
14
35
63
350
time (days)

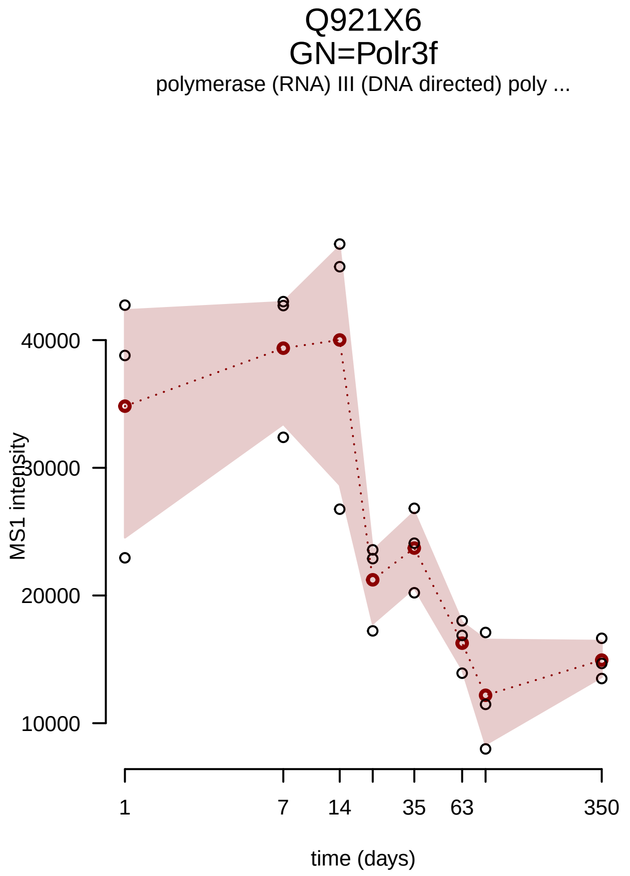
Q921X6
GN=Polr3f
polymerase (RNA) III (DNA directed) poly ...
MS1 intensity
10000
20000
30000
40000
1
7
14
35
63
350
time (days)

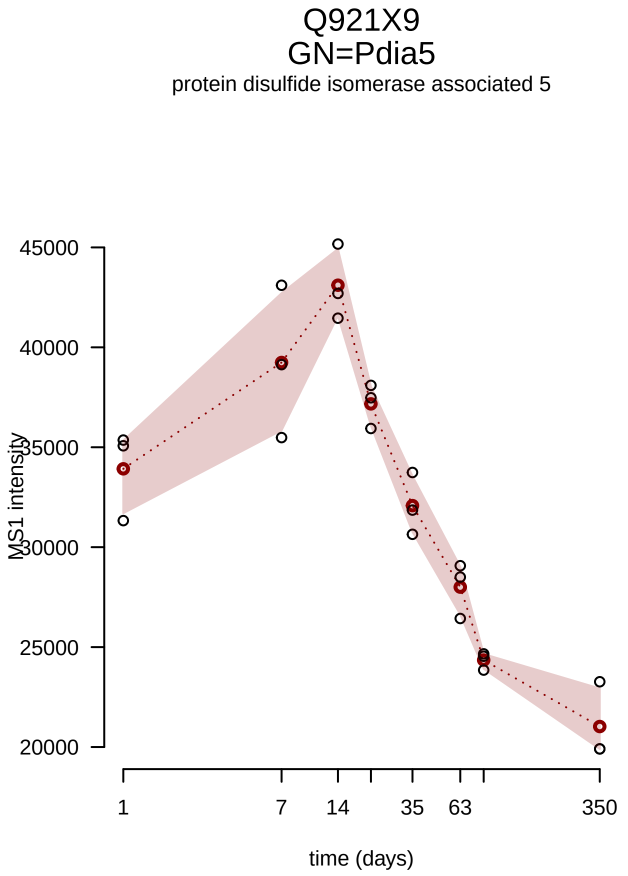
Q921X9
GN=Pdia5
protein disulfide isomerase associated 5
MS1 intensity
20000
25000
30000
35000
40000
45000
1
7
14
35
63
350
time (days)

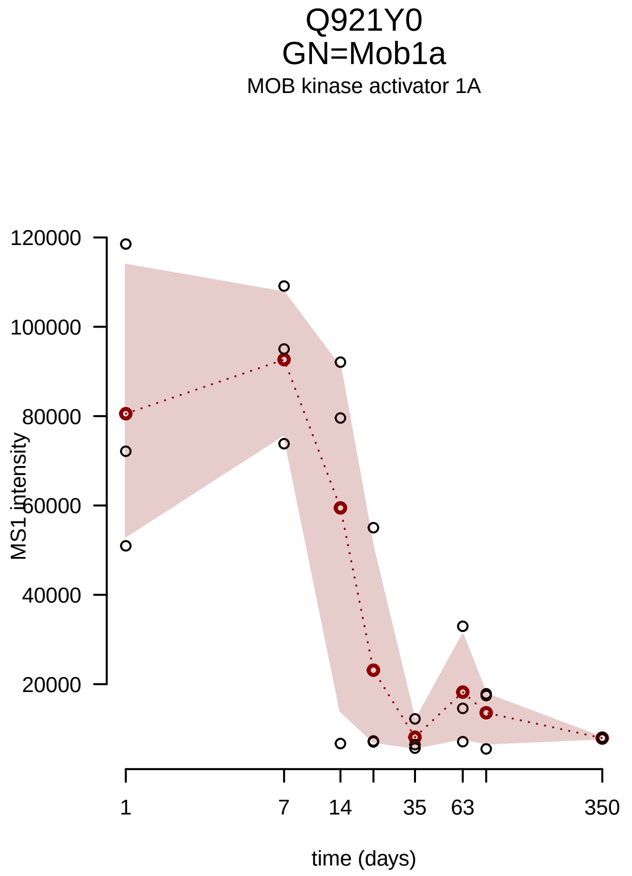
Q921Y0
GN=Mob1a
MOB kinase activator 1A
MS1 intensity
20000
40000
60000
80000
100000
120000
1
7
14
35
63
350
time (days)

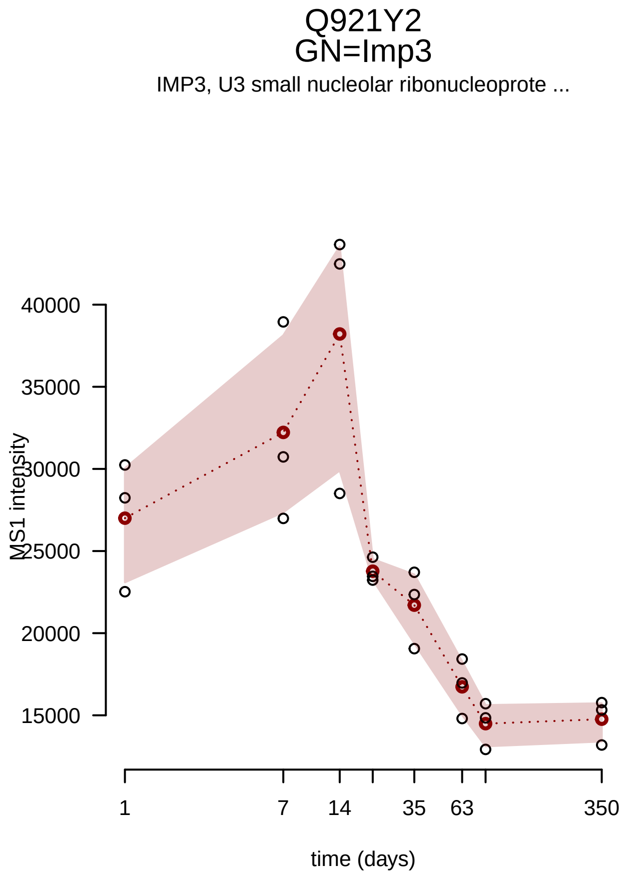
Q921Y2
GN=Imp3
IMP3, U3 small nucleolar ribonucleoprote ...
MS1 intensity
15000
20000
25000
30000
35000
40000
1
7
14
35
63
350
time (days)

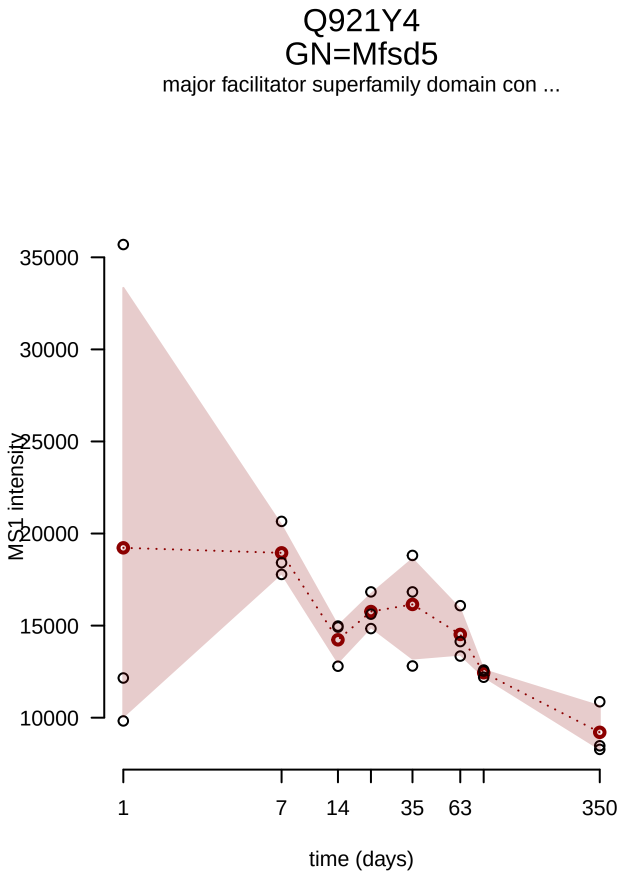
Q921Y4
GN=Mfsd5
major facilitator superfamily domain con ...
MS1 intensity
10000
15000
20000
25000
30000
35000
1
7
14
35
63
350
time (days)

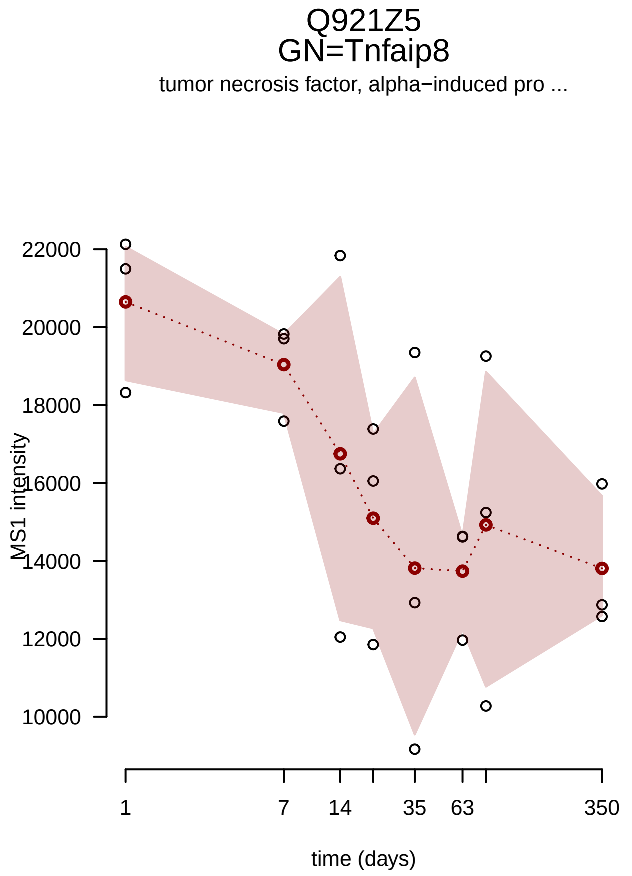
Q921Z5
GN=Tnfaip8
tumor necrosis factor, alpha−induced pro ...
MS1 intensity
10000
12000
14000
16000
18000
20000
22000
1
7
14
35
63
350
time (days)

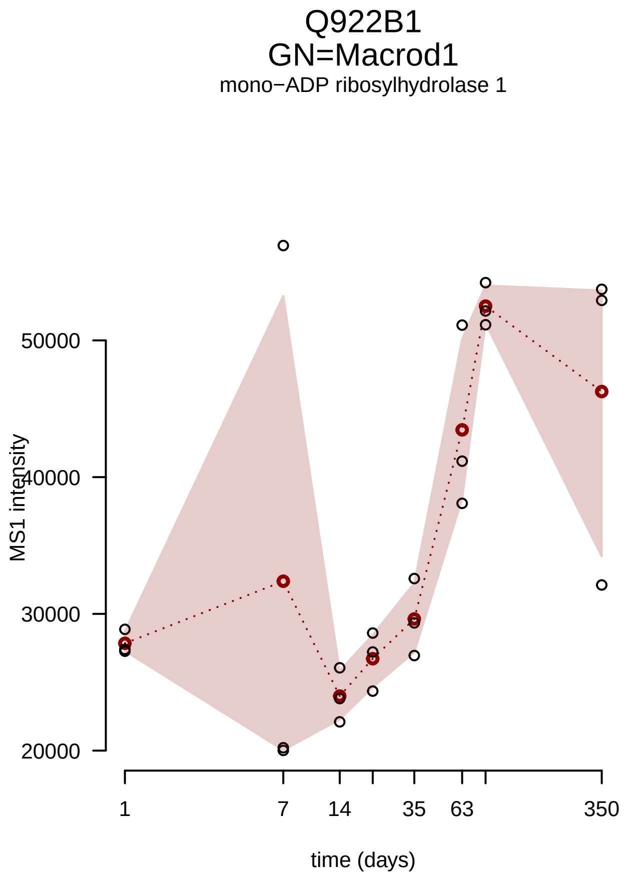
Q922B1
GN=Macrod1
mono−ADP ribosylhydrolase 1
MS1 intensity
20000
30000
40000
50000
1
7
14
35
63
350
time (days)

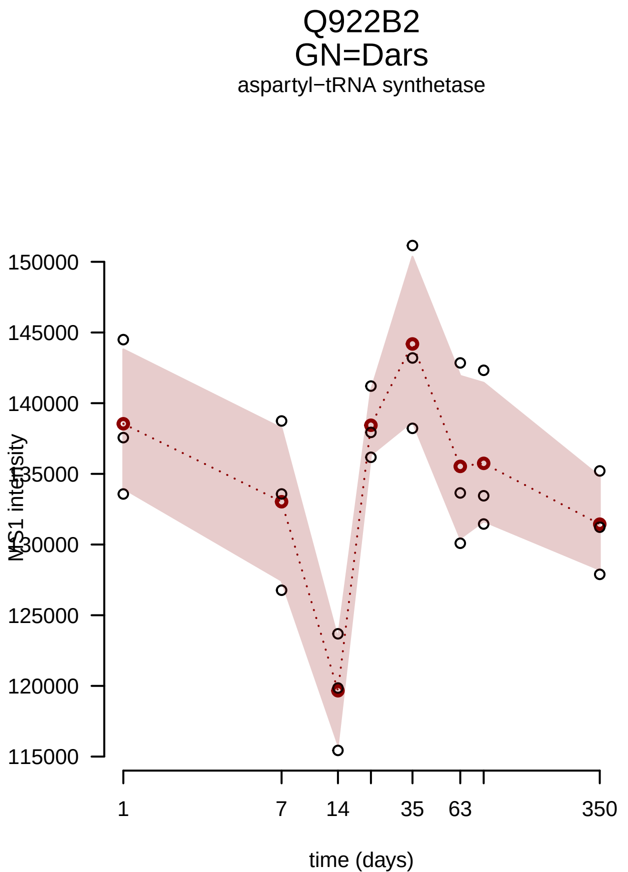
Q922B2
GN=Dars
aspartyl−tRNA synthetase
MS1 intensity
115000
120000
125000
130000
135000
140000
145000
150000
1
7
14
35
63
350
time (days)

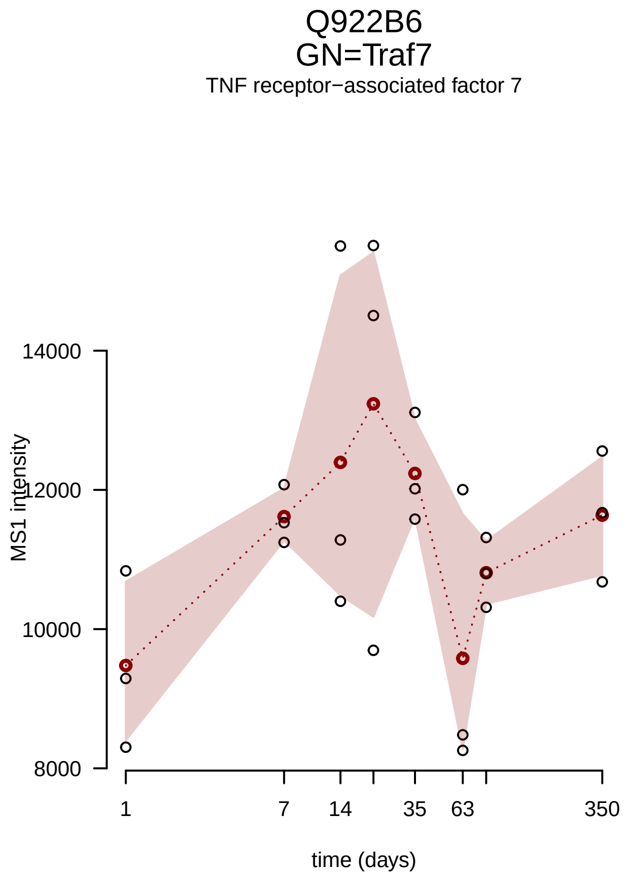
Q922B6
GN=Traf7
TNF receptor−associated factor 7
MS1 intensity
8000
10000
12000
14000
1
7
14
35
63
350
time (days)

Q922B9
GN=Itprid2
ITPR interacting domain containing 2

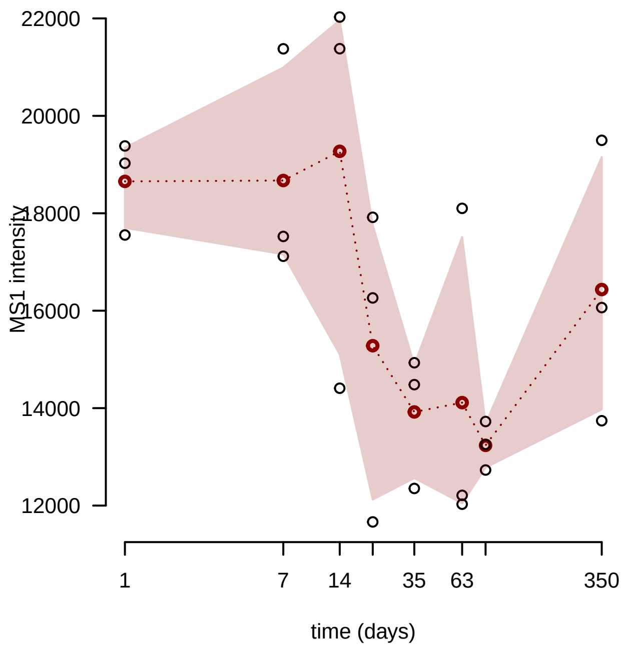

Q922D4
GN=Ppp6r3
protein phosphatase 6, regulatory subuni ...

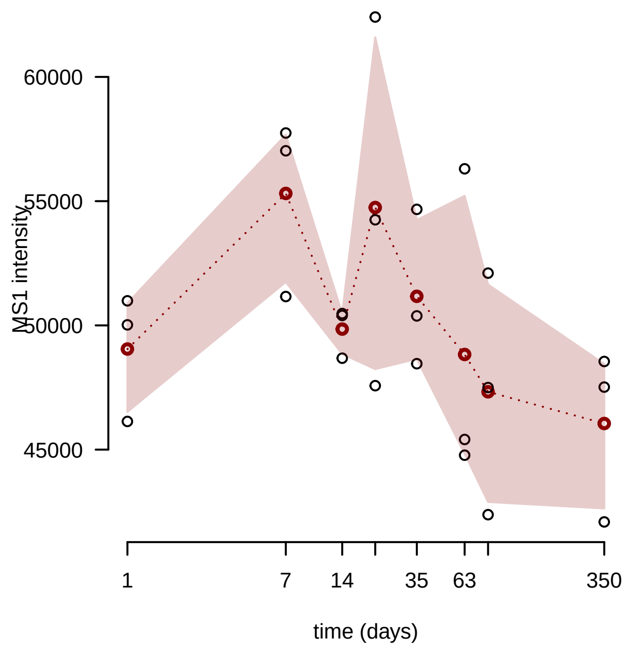

Q922D8
GN=Mthfd1
methylenetetrahydrofolate dehydrogenase ...

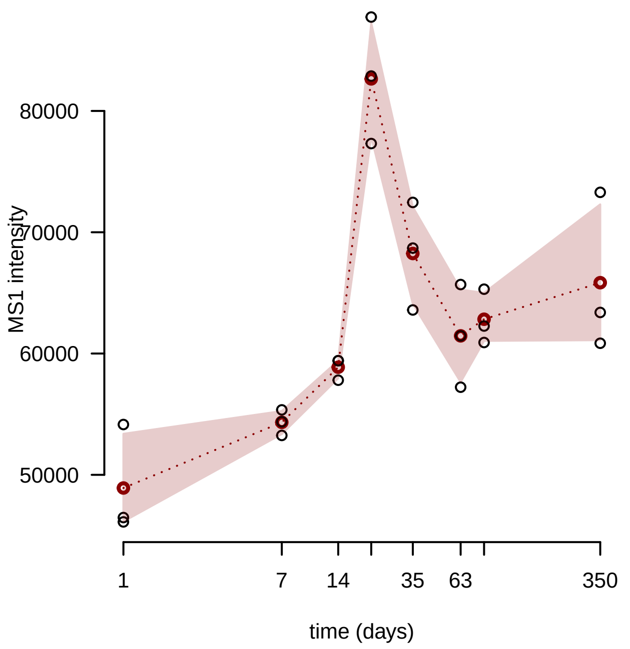

Q922E4
GN=Pcyt2
phosphate cytidylyltransferase 2, ethano ...

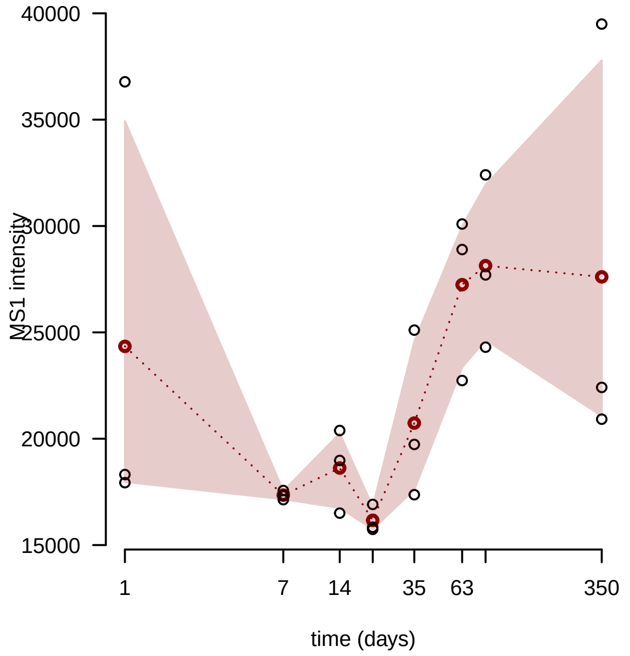

Q922E6
GN=Fastkd2
FAST kinase domains 2

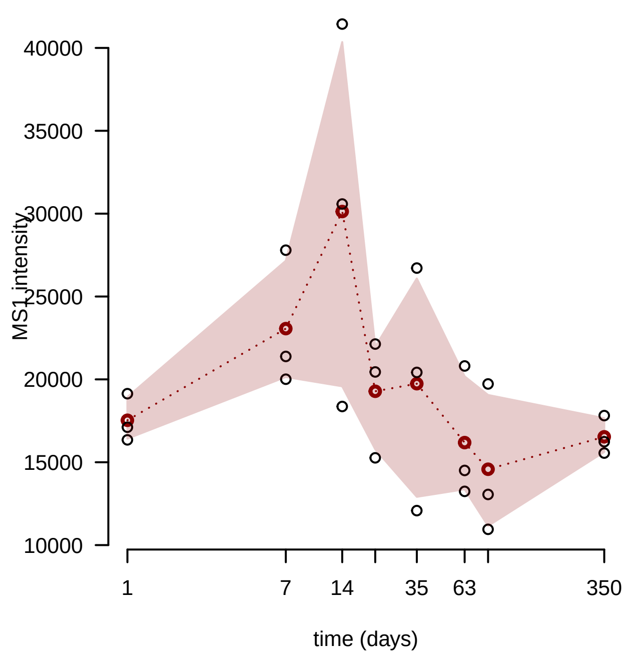

Q922F4
GN=Tubb6
tubulin, beta 6 class V

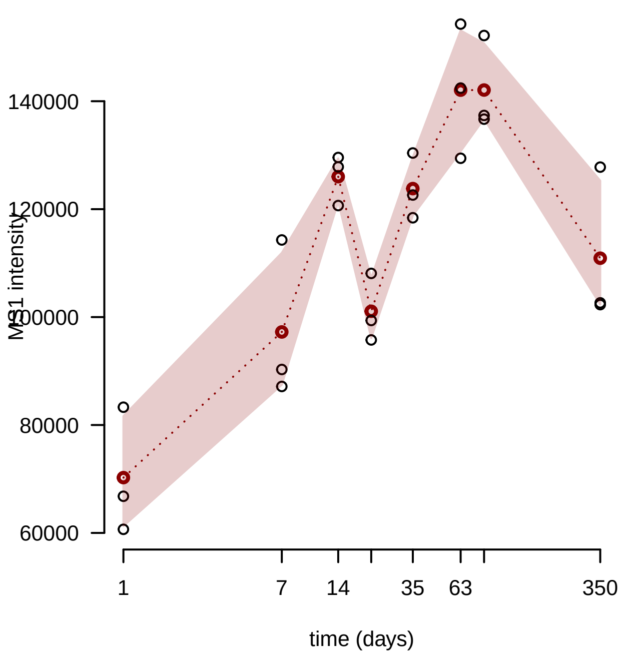

Q922G0
GN=Slc25a36
solute carrier family 25, member 36

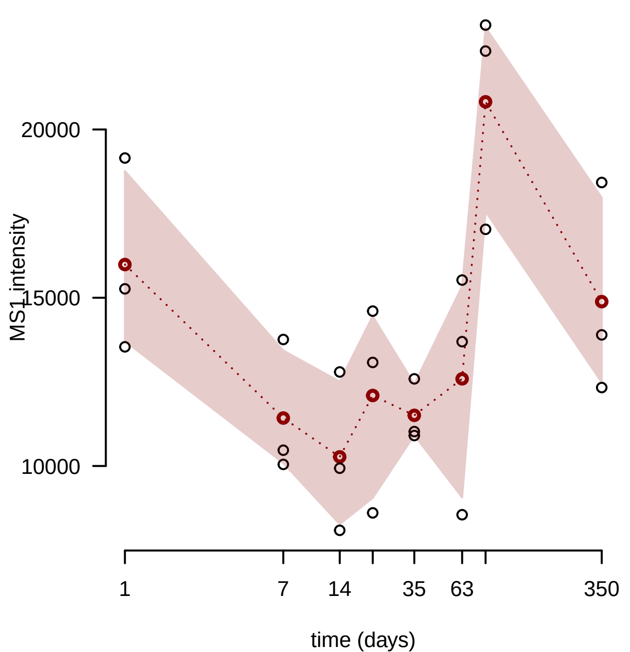

Q922G2
GN=Fam76a
family with sequence similarity 76, memb ...

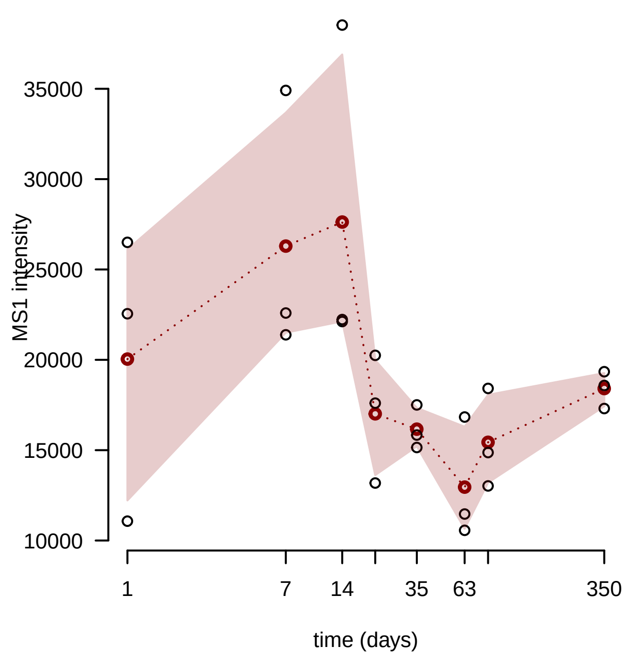

Q922H1
GN=Prmt3
protein arginine N−methyltransferase 3

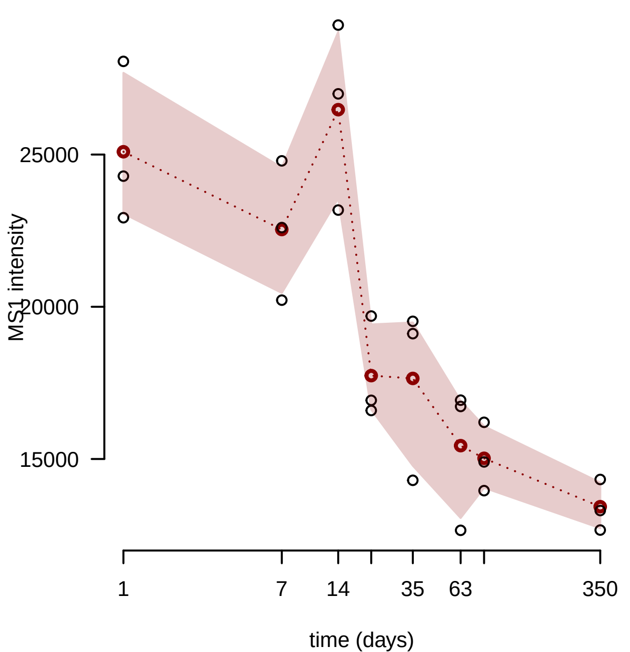

Q922H2
GN=Pdk3
pyruvate dehydrogenase kinase, isoenzyme ...

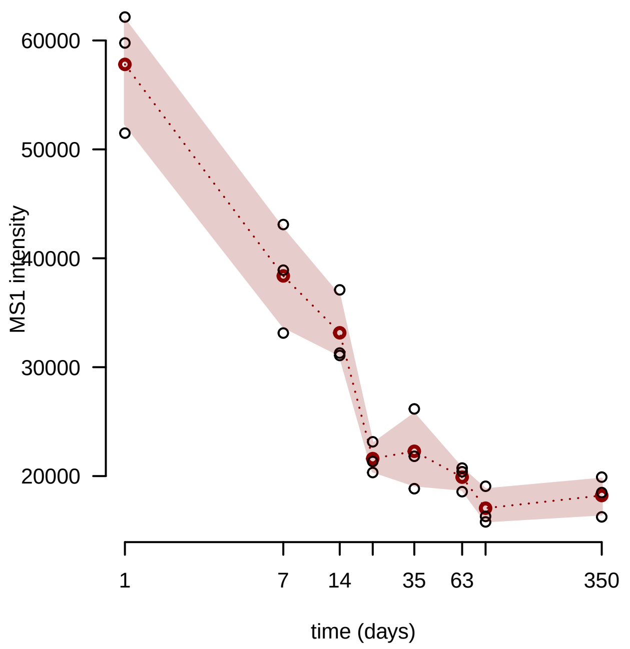

Q922H4
GN=Gmppa
GDP−mannose pyrophosphorylase A

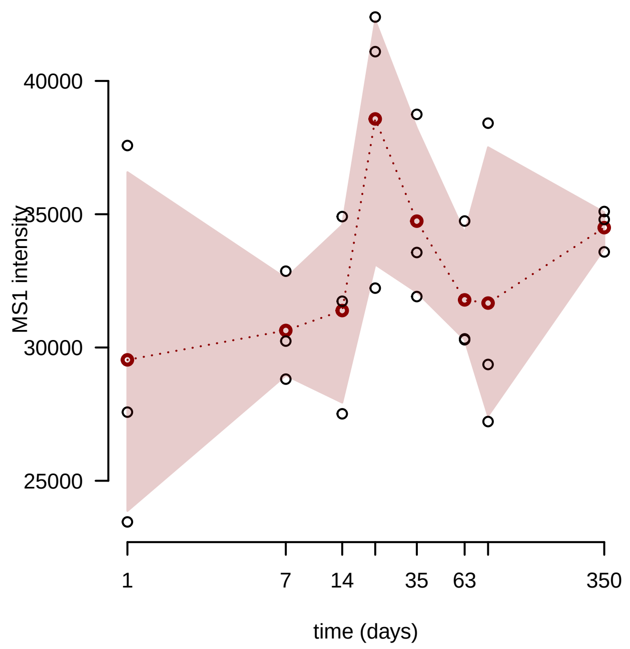

Q922H9
GN=Znf330

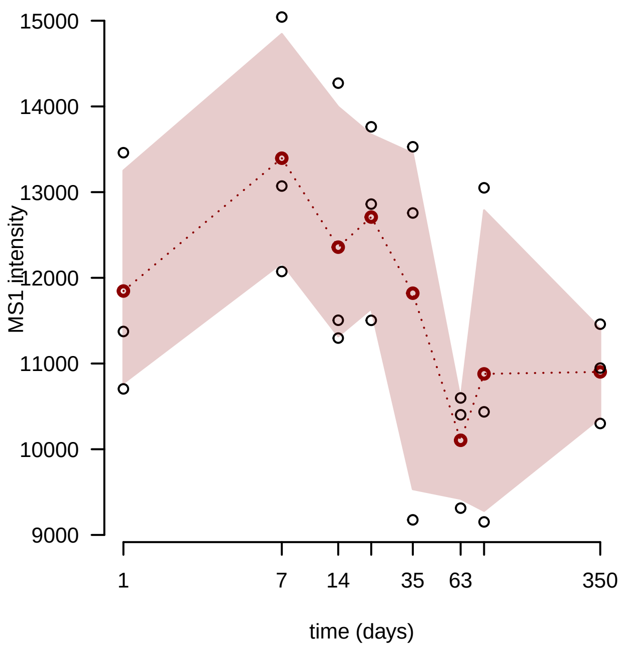

Q922J3
GN=Clip1
CAP−GLY domain containing linker protein ...

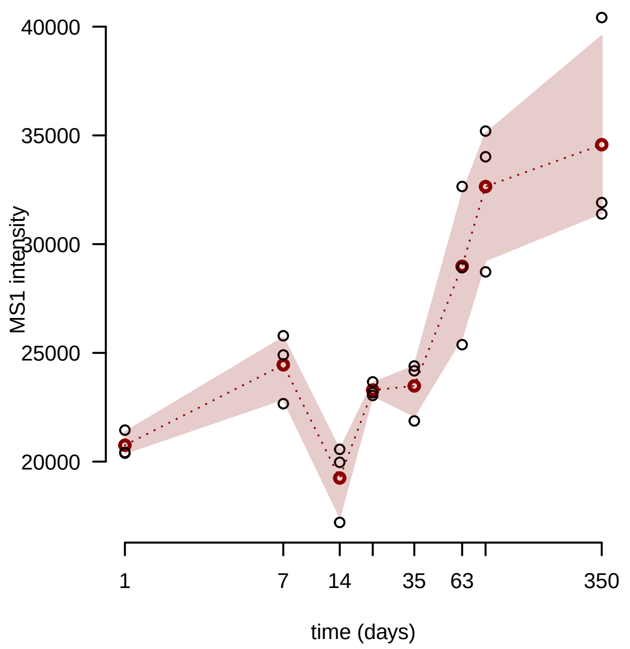

Q922J6
GN=Tspan2
tetraspanin 2

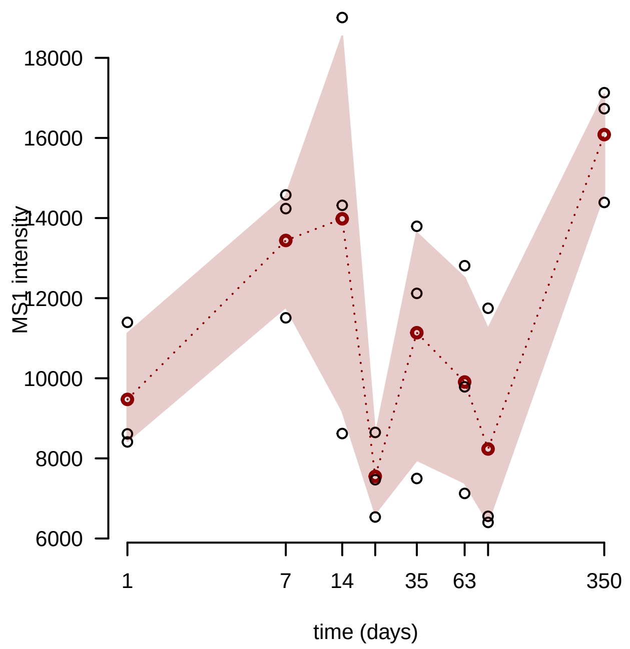

Q922J9
GN=Far1
fatty acyl CoA reductase 1

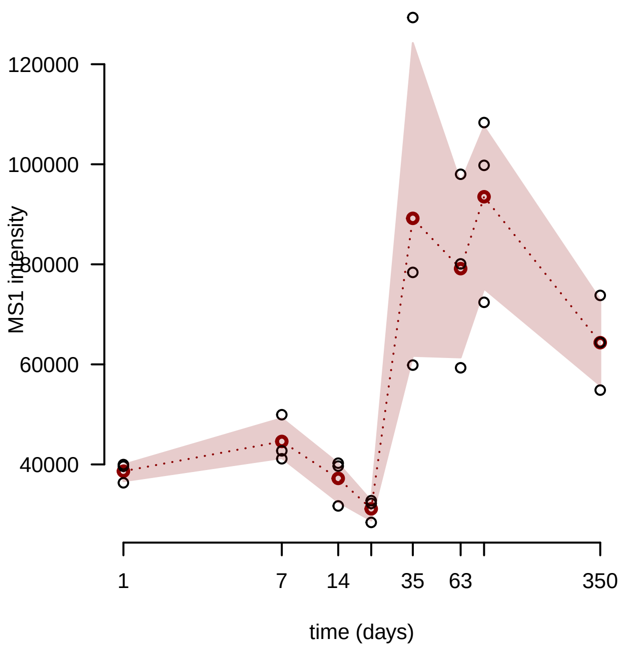

Q922K7
GN=Nop2
NOP2 nucleolar protein

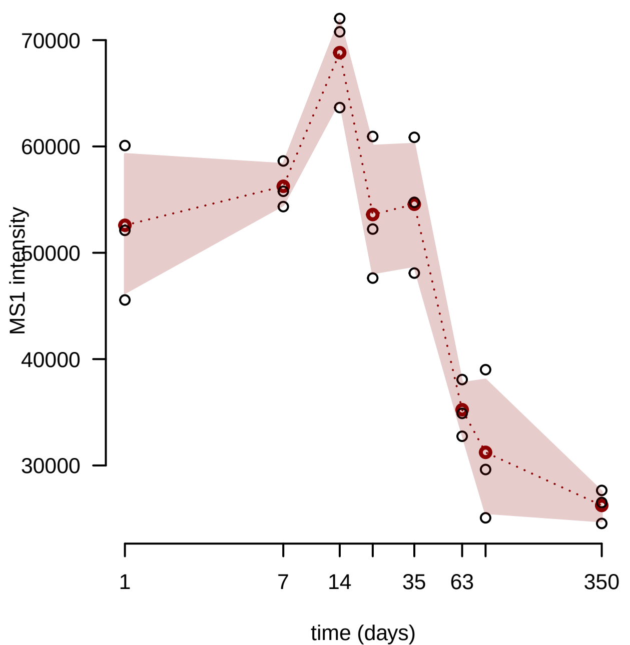

Q922L6
GN=Nelfcd
negative elongation factor complex membe ...

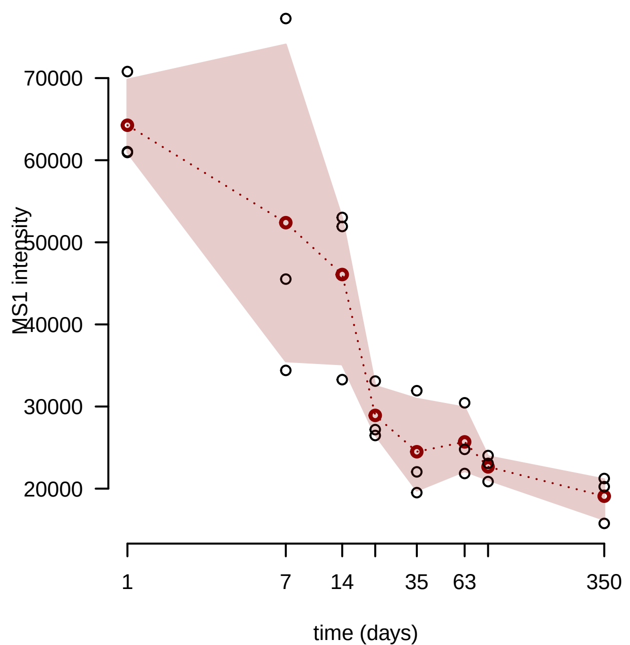

Q922M3
GN=Kctd10
potassium channel tetramerisation domain ...

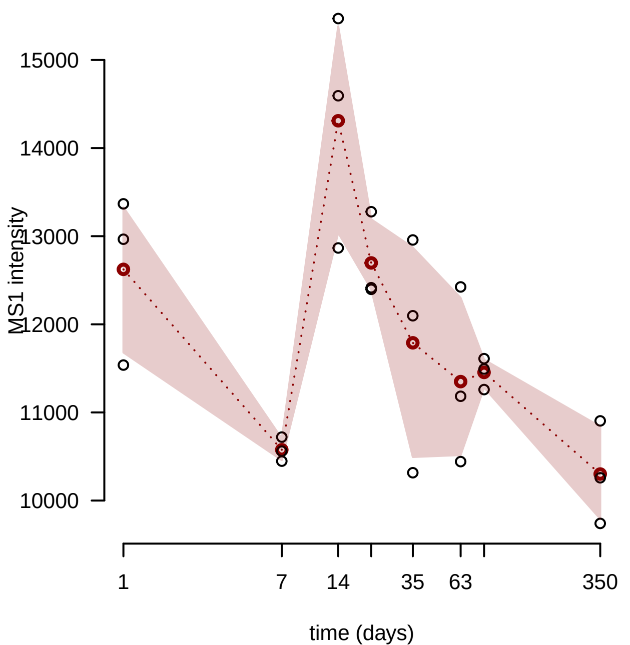

Q922M7
GN=

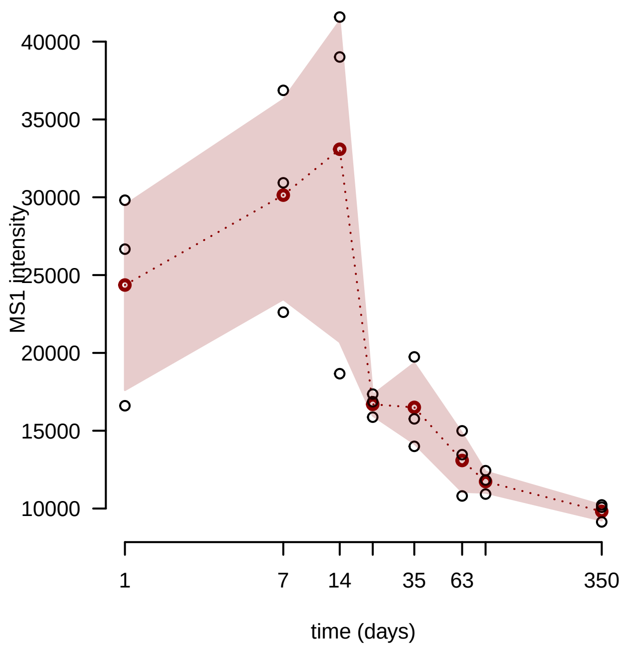

Q922P8
GN=Tmem132a
transmembrane protein 132A

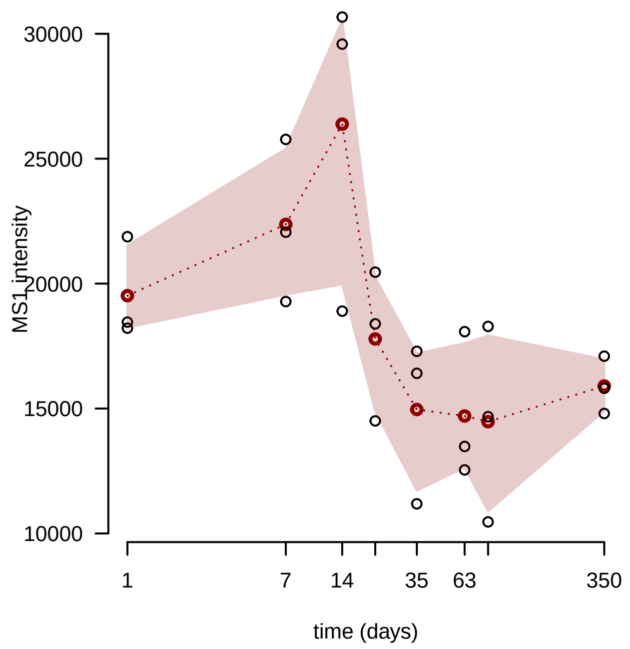

Q922P9
GN=Glyr1
glyoxylate reductase 1 homolog (Arabidop ...

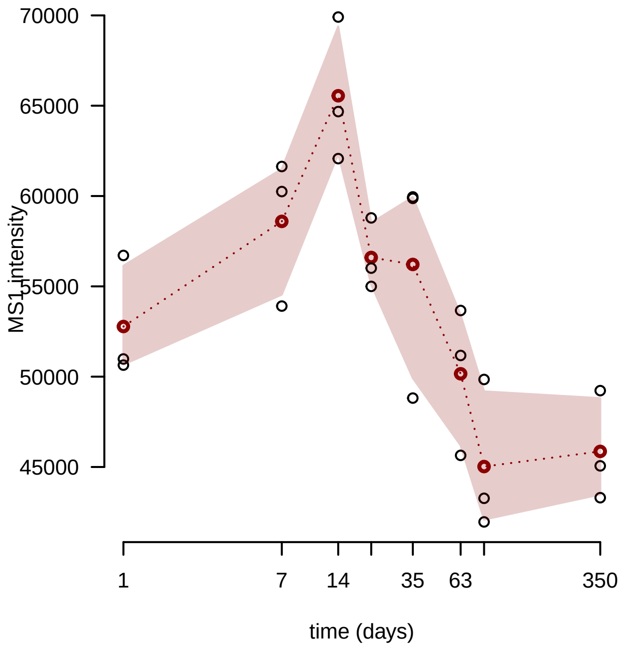

Q922Q1
GN=Marc2

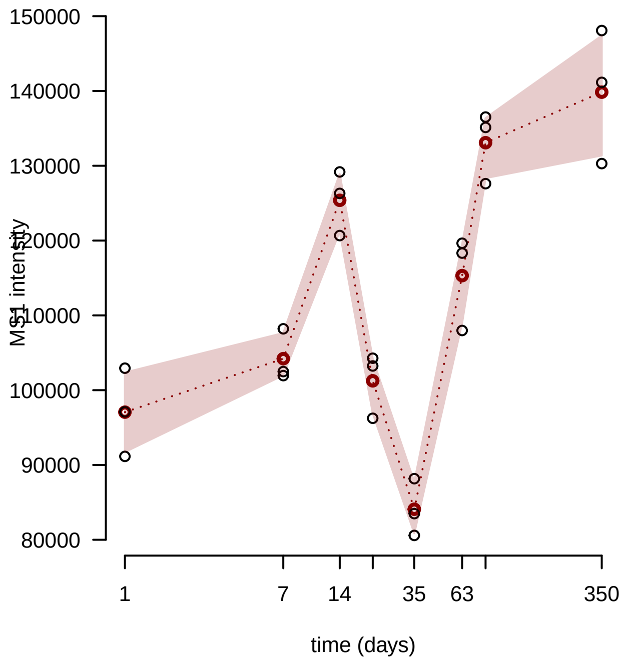

Q922Q2
GN=Riok1
RIO kinase 1

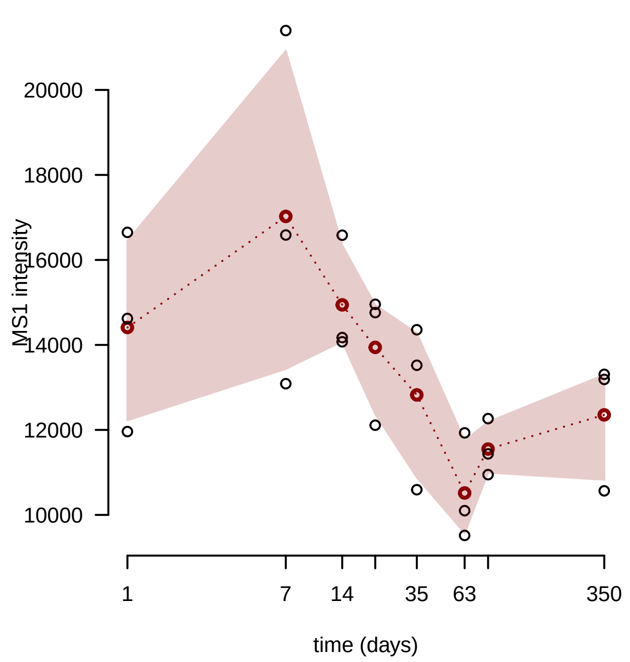

Q922Q4
GN=Pycr2
pyrroline−5−carboxylate reductase family ...

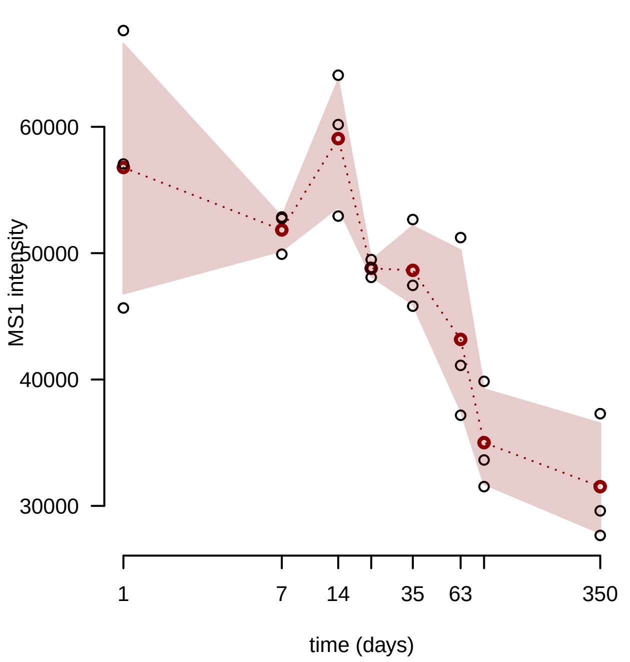

Q922Q5
GN=Slc35b3
solute carrier family 35, member B3

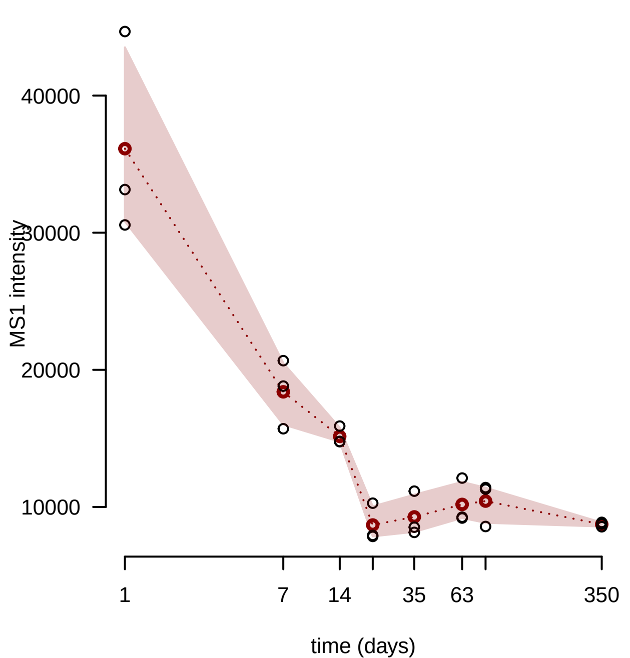

Q922Q8
GN=Lrrc59
leucine rich repeat containing 59

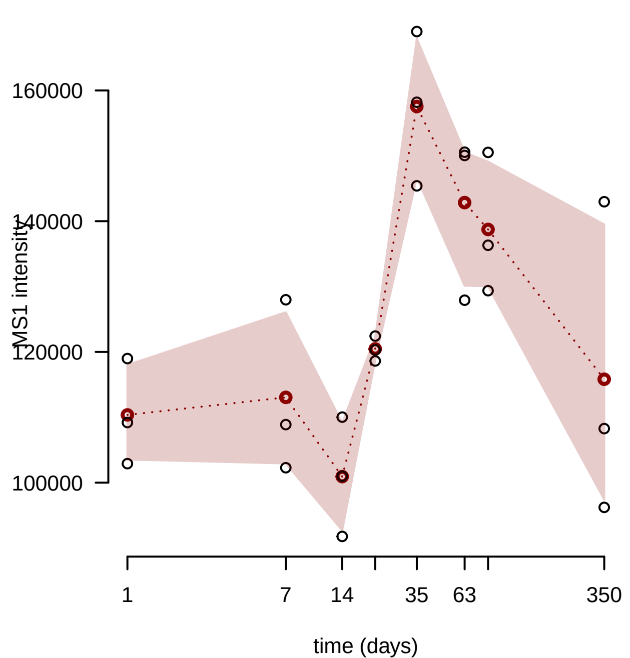

Q922Q9
GN=Chid1
chitinase domain containing 1

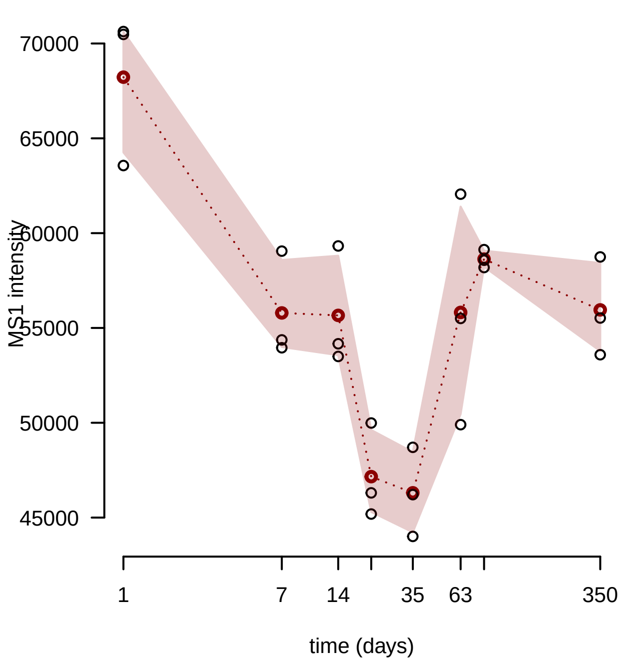

Q922R1
GN=

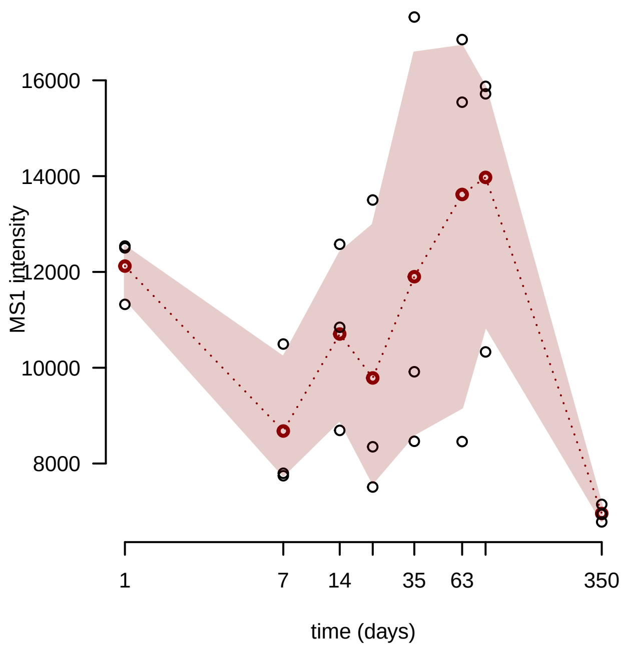

Q922R5
GN=Ppp4r3b
protein phosphatase 4 regulatory subunit ...

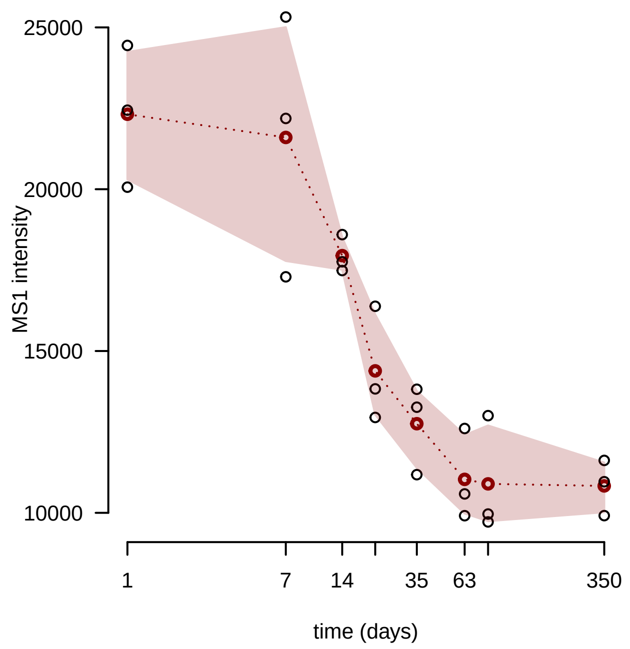

Q922R8
GN=Pdia6
protein disulfide isomerase associated 6

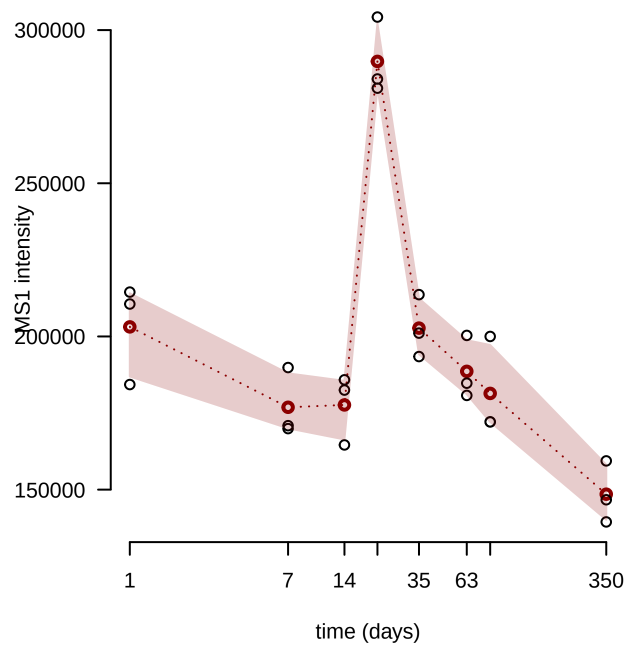

Q922S4
GN=Pde2a
phosphodiesterase 2A, cGMP−stimulated

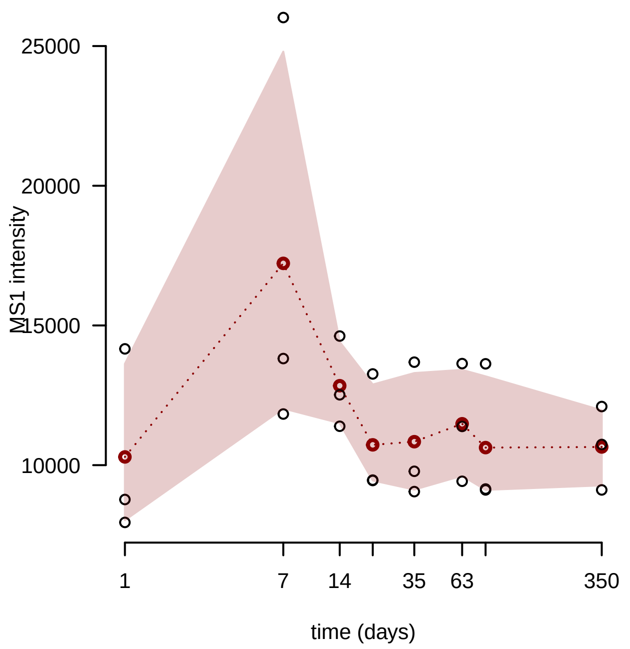

Q922S8
GN=Kif2c
kinesin family member 2C

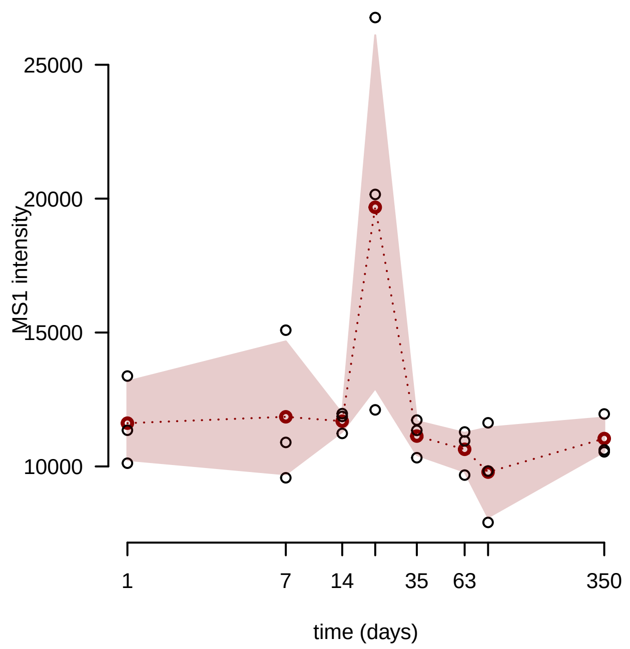

Q922U1
GN=Prpf3
pre−mRNA processing factor 3

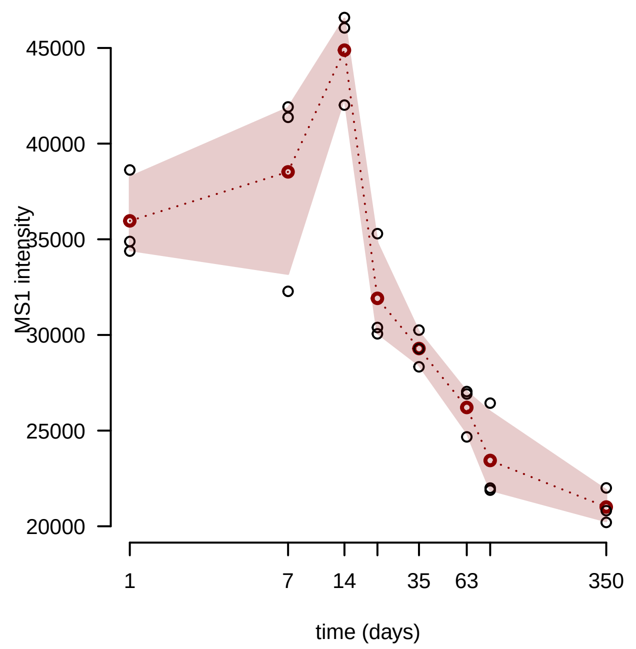

Q922U2
GN=Krt5
keratin 5

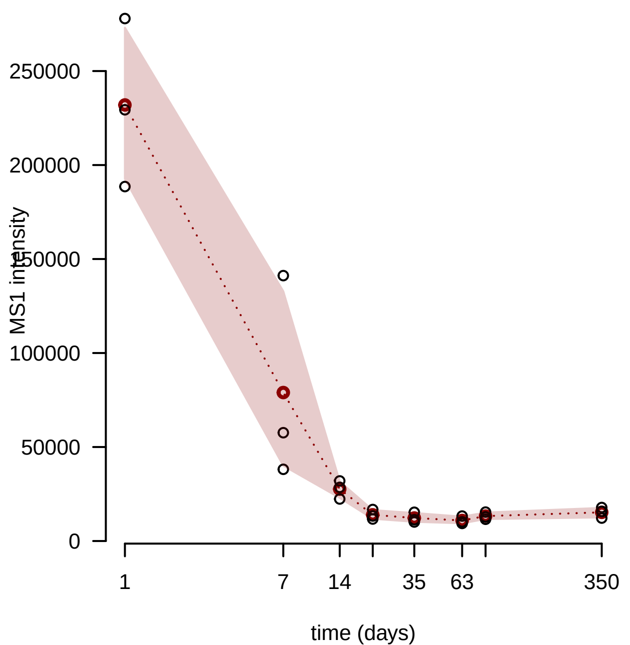

Q922V4
GN=Plrg1
pleiotropic regulator 1

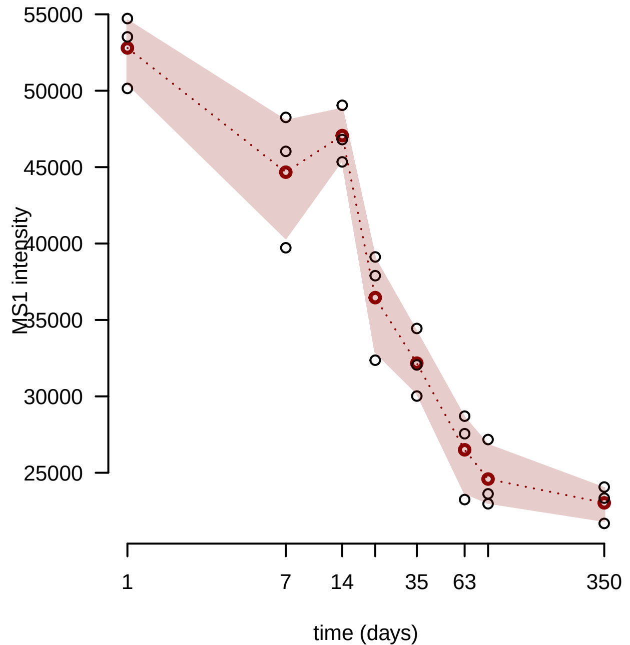

Q922W5
GN=Pycr1
pyrroline−5−carboxylate reductase 1

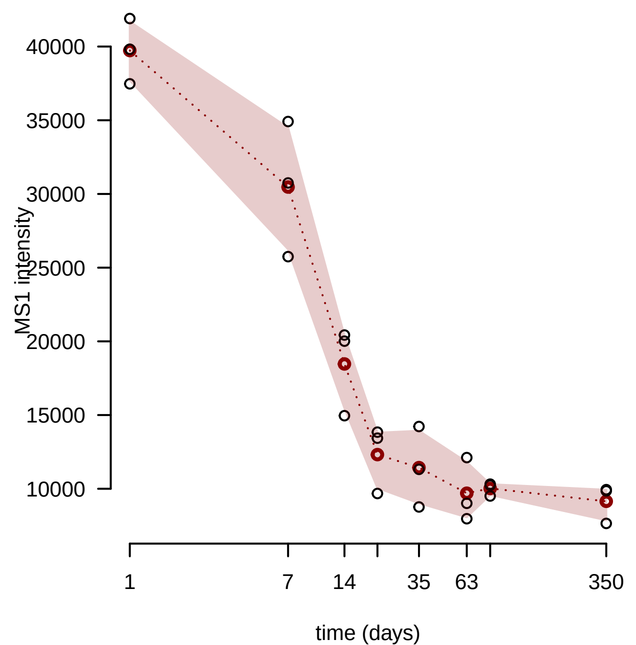

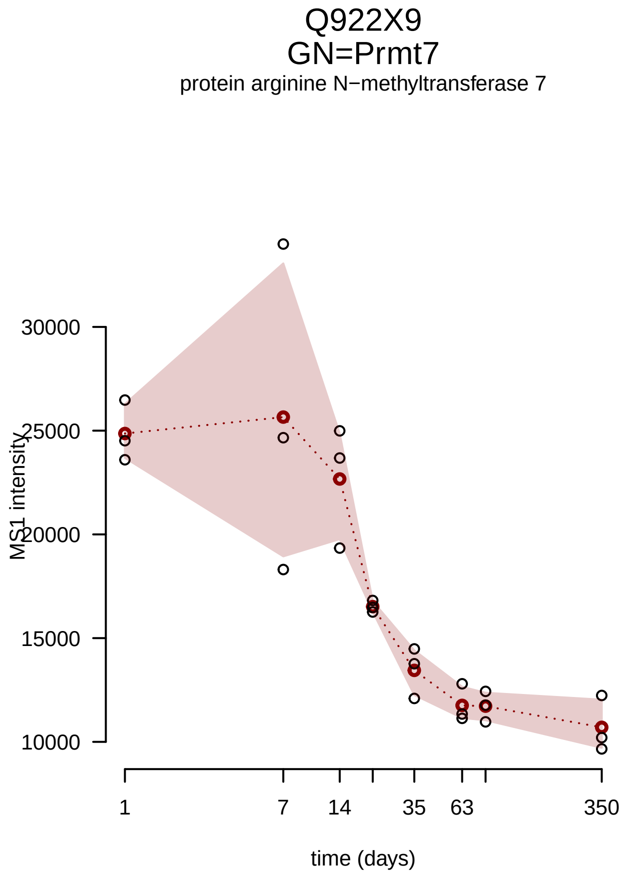
Q922X9
GN=Prmt7
protein arginine N−methyltransferase 7
MS1 intensity
10000
15000
20000
25000
30000
1
7
14
35
63
350
time (days)

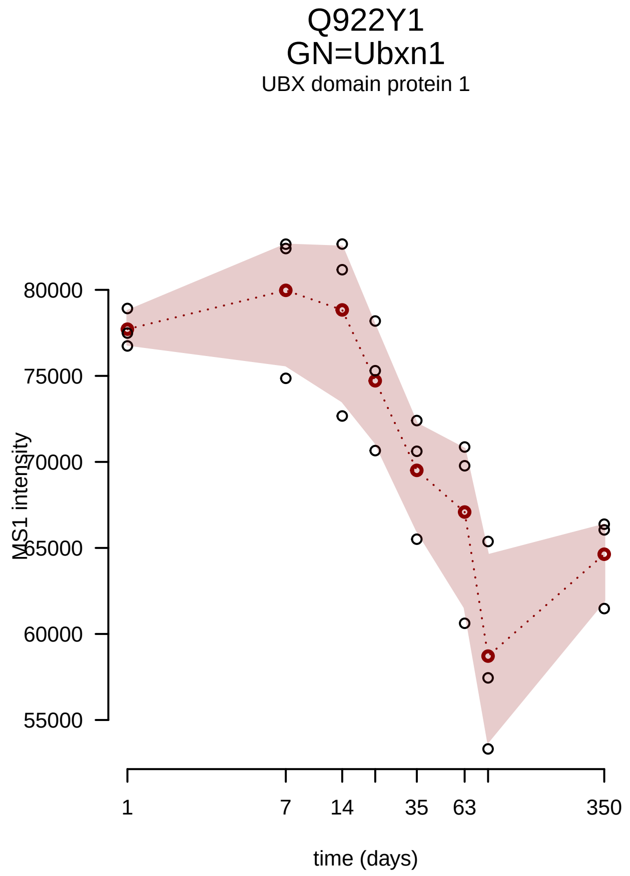
Q922Y1
GN=Ubxn1
UBX domain protein 1
MS1 intensity
55000
60000
65000
70000
75000
80000
1
7
14
35
63
350
time (days)

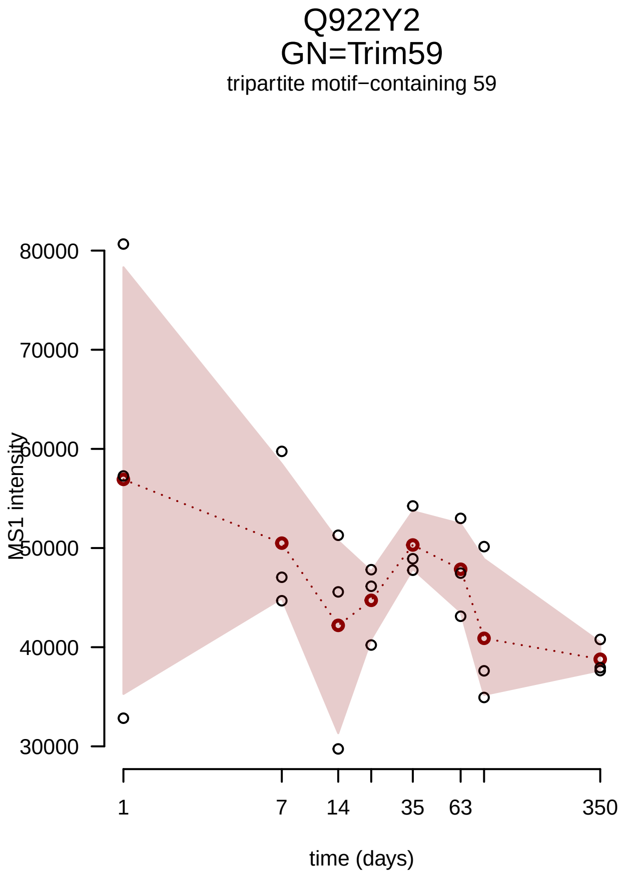
Q922Y2
GN=Trim59
tripartite motif−containing 59
MS1 intensity
30000
40000
50000
60000
70000
80000
1
7
14
35
63
350
time (days)

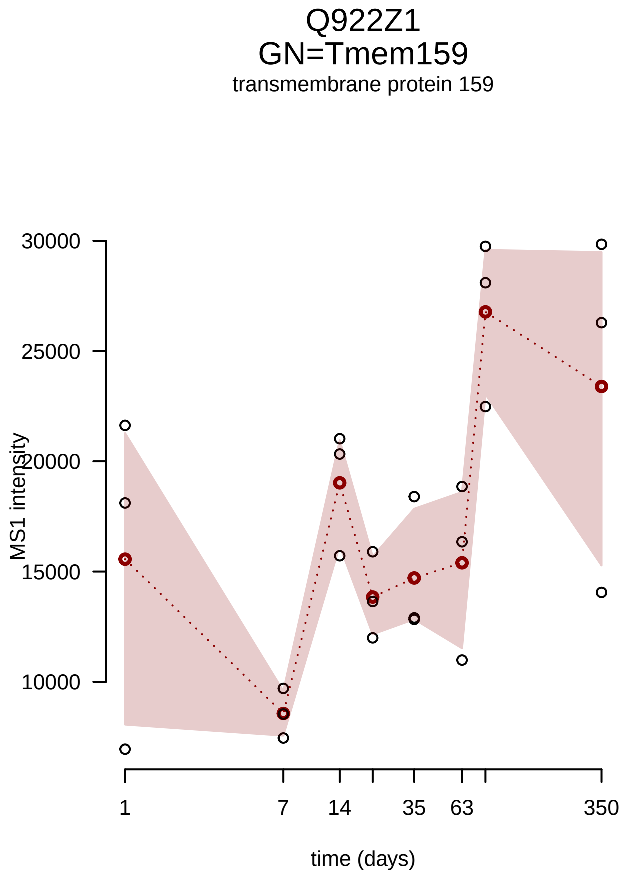
Q922Z1
GN=Tmem159
transmembrane protein 159
MS1 intensity
10000
15000
20000
25000
30000
1
7
14
35
63
350
time (days)

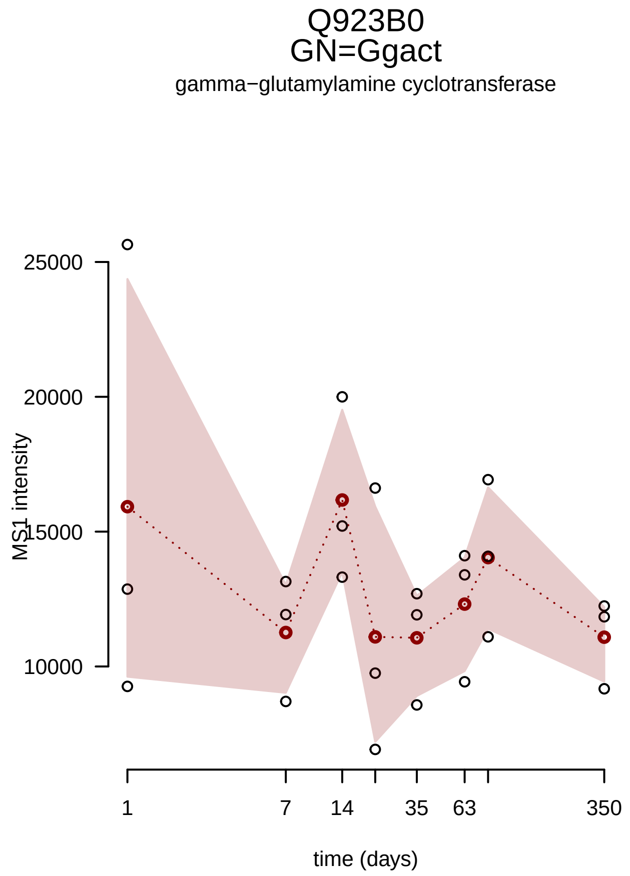
Q923B0
GN=Ggact
gamma−glutamylamine cyclotransferase
MS1 intensity
10000
15000
20000
25000
1
7
14
35
63
350
time (days)

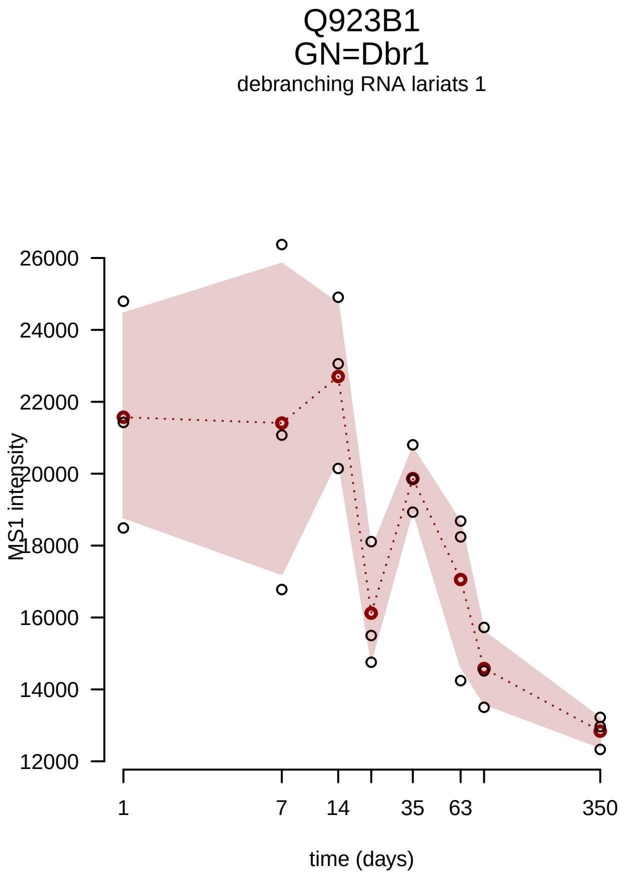
Q923B1
GN=Dbr1
debranching RNA lariats 1
MS1 intensity
12000
14000
16000
18000
20000
22000
24000
26000
1
7
14
35
63
350
time (days)

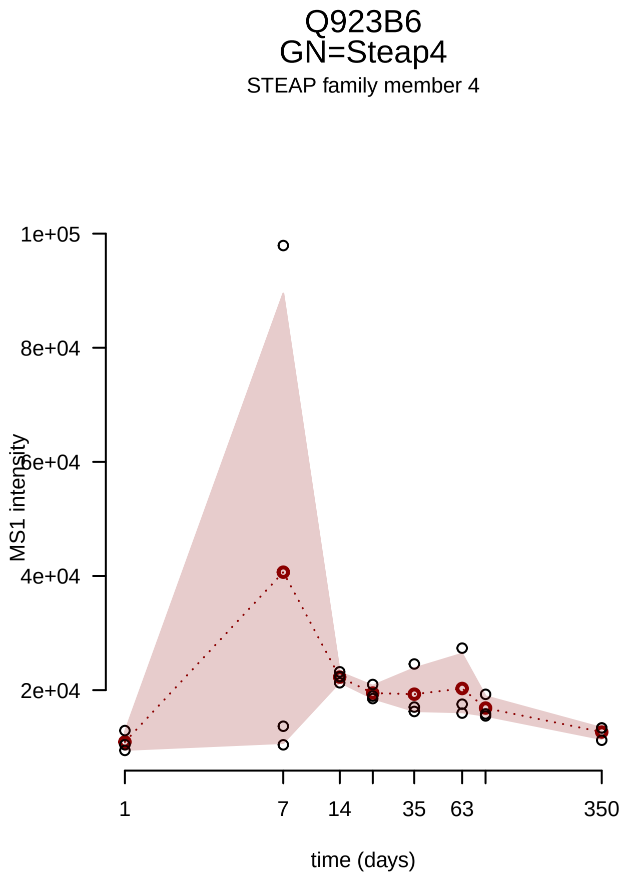
Q923B6
GN=Steap4
STEAP family member 4
MS1 intensity
2e+04
4e+04
6e+04
8e+04
1e+05
1
7
14
35
63
350
time (days)

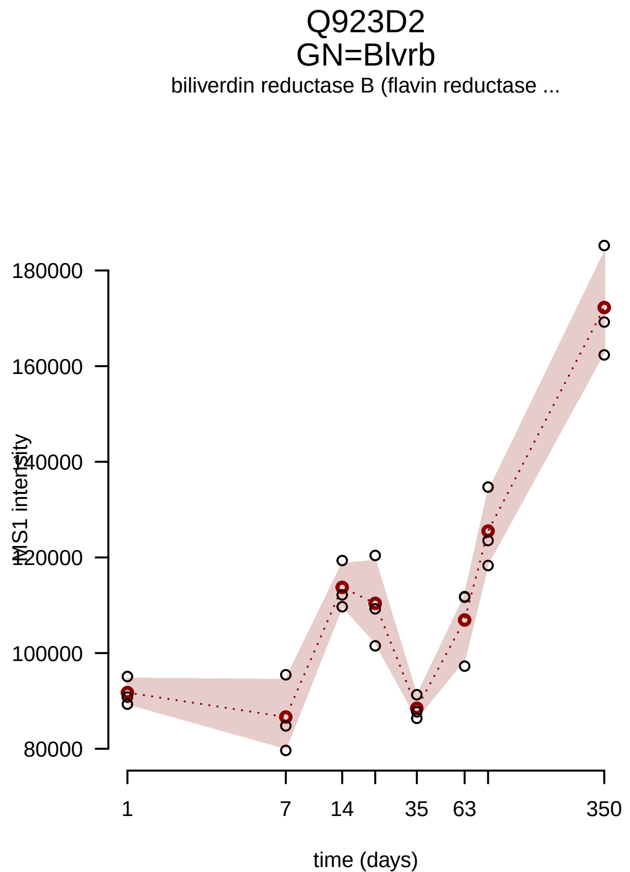
Q923D2
GN=Blvrb
biliverdin reductase B (flavin reductase ...
MS1 intensity
80000
100000
120000
140000
160000
180000
1
7
14
35
63
350
time (days)

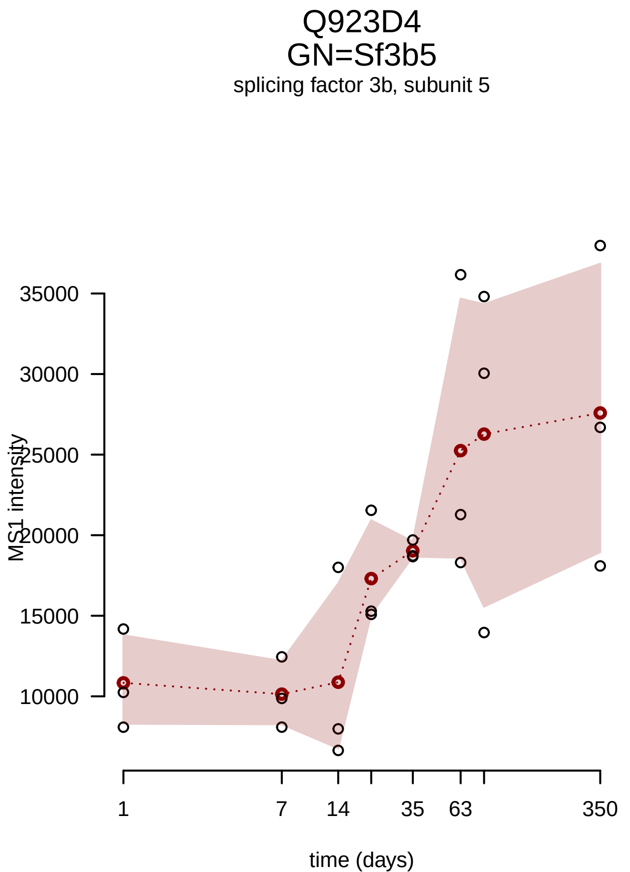
Q923D4
GN=Sf3b5
splicing factor 3b, subunit 5
MS1 intensity
10000
15000
20000
25000
30000
35000
1
7
14
35
63
350
time (days)

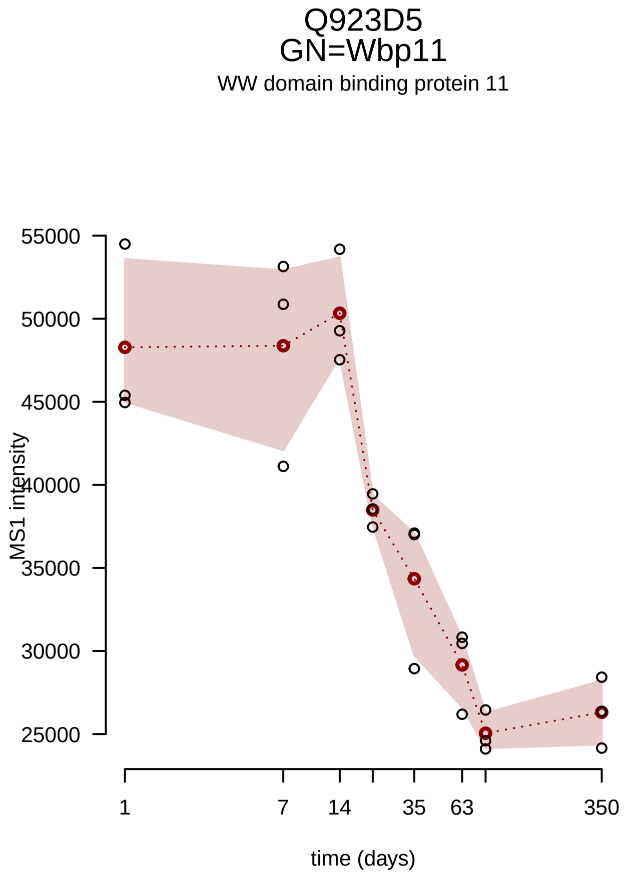
Q923D5
GN=Wbp11
WW domain binding protein 11
MS1 intensity
55000
50000
45000
40000
35000
30000
25000
1
7
14
35
63
350
time (days)
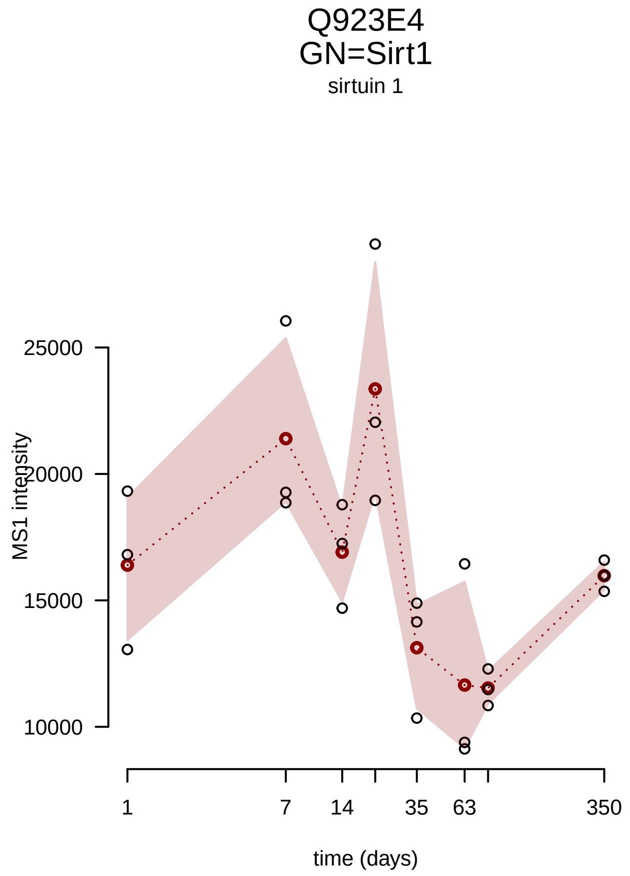
Q923E4
GN=Sirt1
sirtuin 1
MS1 intensity
25000
20000
15000
10000
1
7
14
35
63
350
time (days)
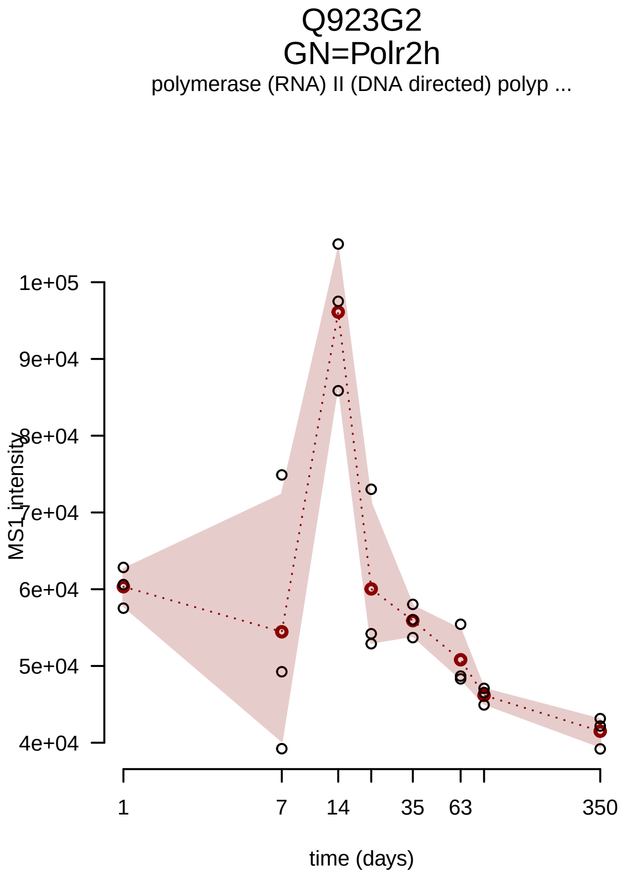
Q923G2
GN=Polr2h
polymerase (RNA) II (DNA directed) polyp ...
MS1 intensity
1e+05
9e+04
8e+04
7e+04
6e+04
5e+04
4e+04
1
7
14
35
63
350
time (days)
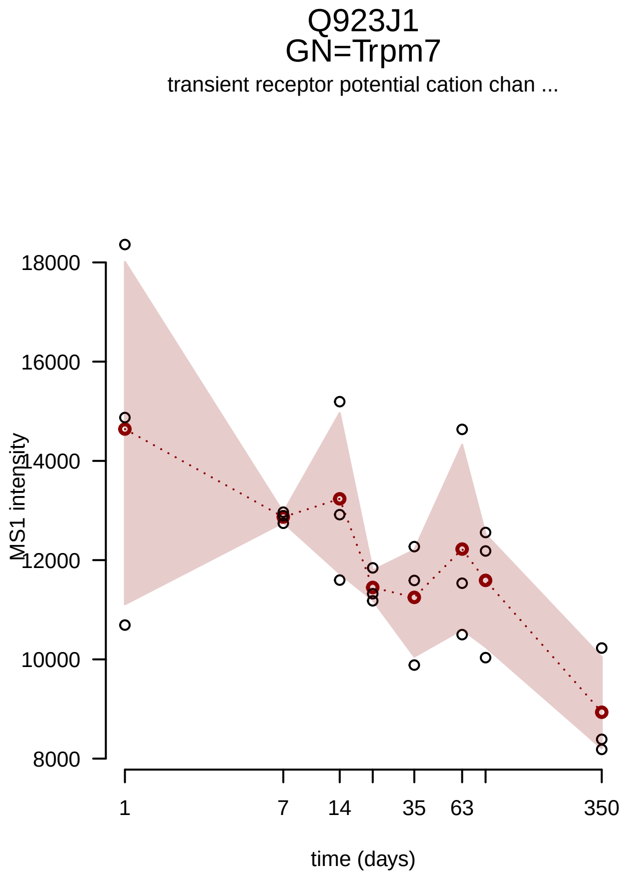
Q923J1
GN=Trpm7
transient receptor potential cation chan ...
MS1 intensity
18000
16000
14000
12000
10000
8000
1
7
14
35
63
350
time (days)
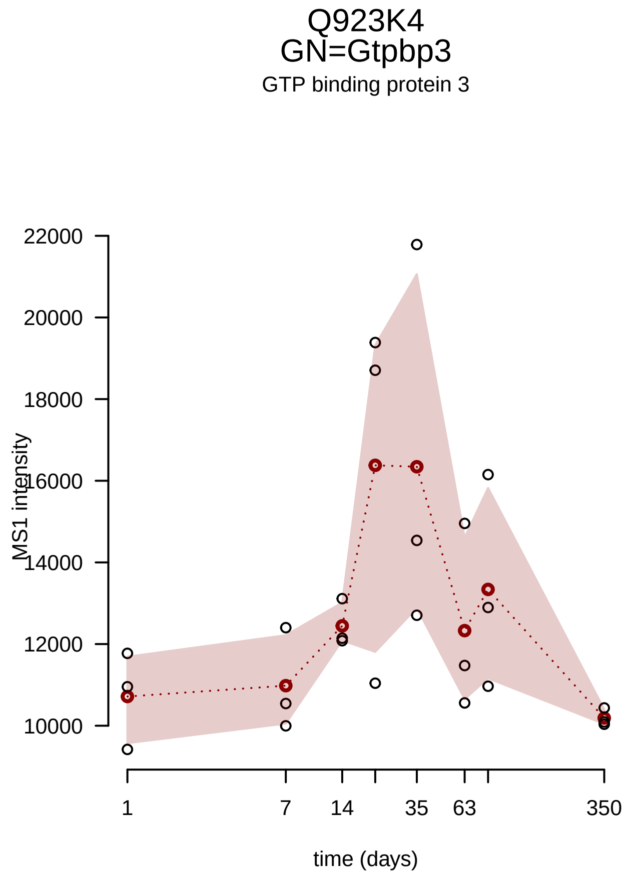
Q923K4
GN=Gtpbp3
GTP binding protein 3
MS1 intensity
22000
20000
18000
16000
14000
12000
10000
1
7
14
35
63
350
time (days)
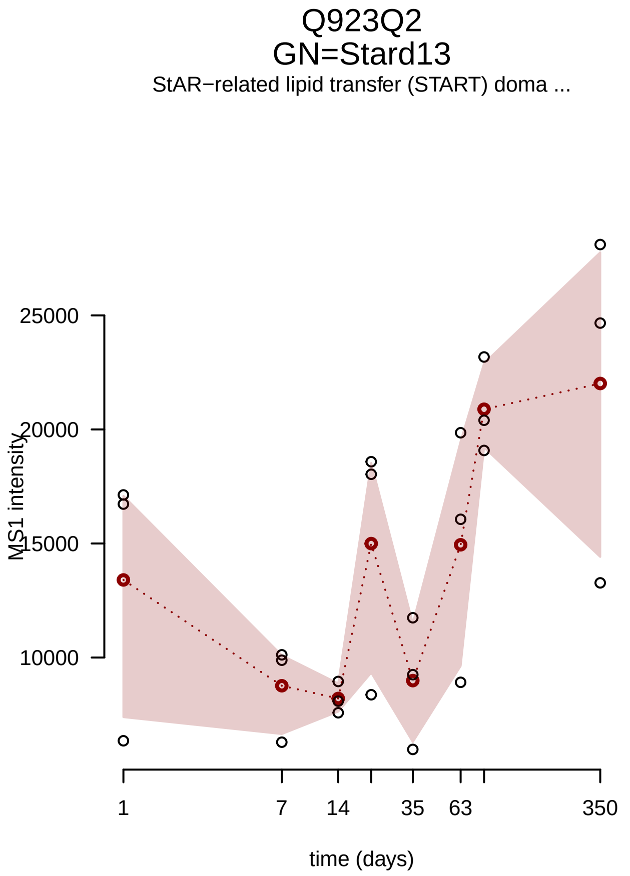
Q923Q2
GN=Stard13
StAR−related lipid transfer (START) doma ...
MS1 intensity
25000
20000
15000
10000
1
7
14
35
63
350
time (days)
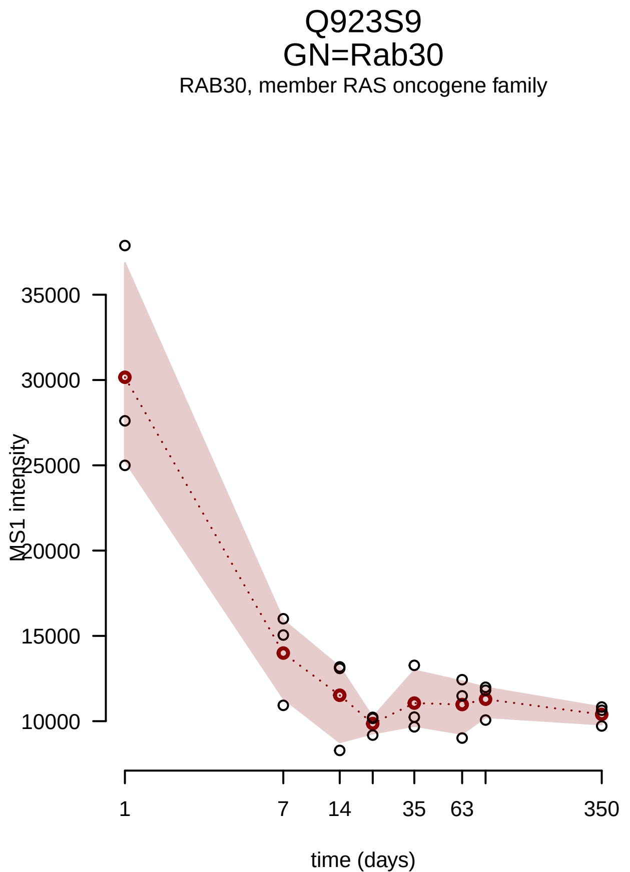
Q923S9
GN=Rab30
RAB30, member RAS oncogene family
MS1 intensity
35000
30000
25000
20000
15000
10000
1
7
14
35
63
350
time (days)
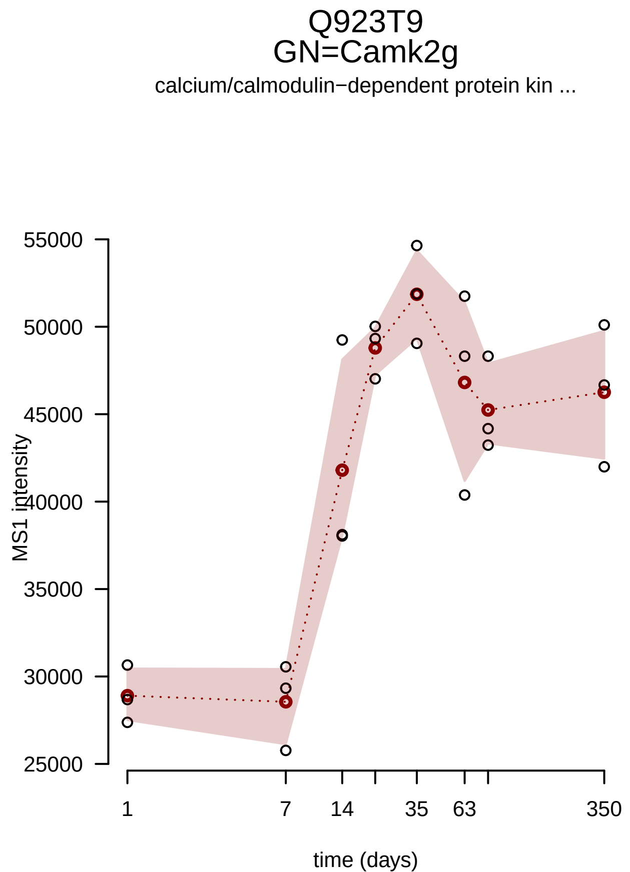
Q923T9
GN=Camk2g
calcium/calmodulin−dependent protein kin ...
MS1 intensity
55000
50000
45000
40000
35000
30000
25000
1
7
14
35
63
350
time (days)
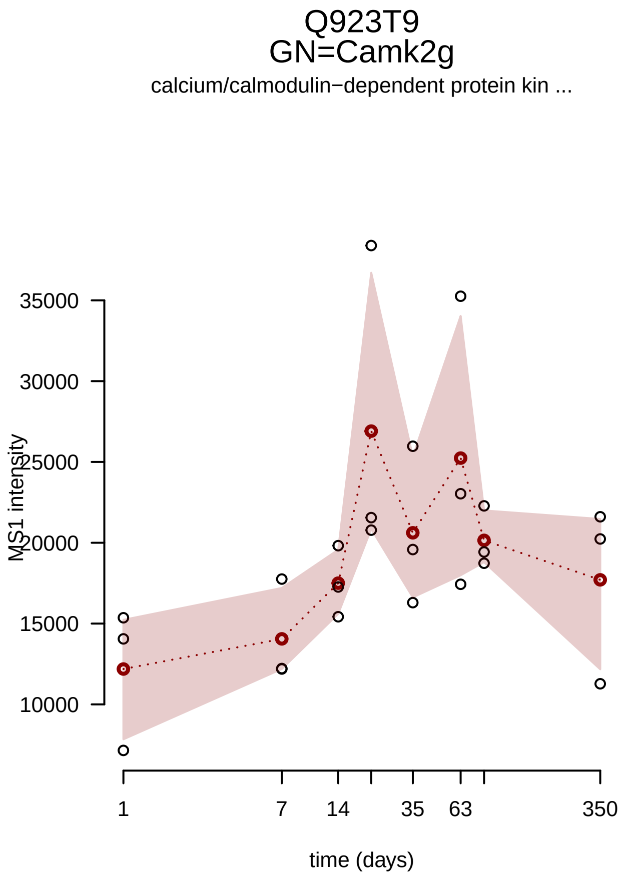
Q923T9
GN=Camk2g
calcium/calmodulin−dependent protein kin ...
MS1 intensity
35000
30000
25000
20000
15000
10000
1
7
14
35
63
350
time (days)

Q923U0
GN=Tom1l1
target of myb1−like 1 (chicken)

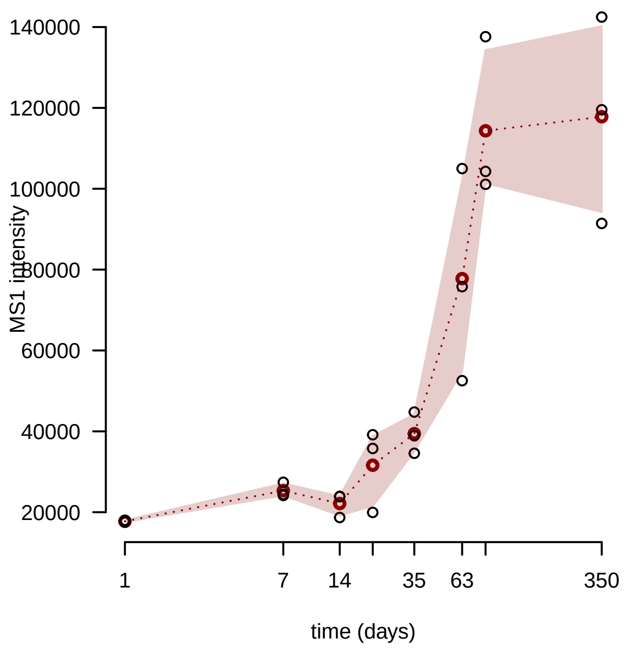

Q923W1
GN=Tgs1
trimethylguanosine synthase 1

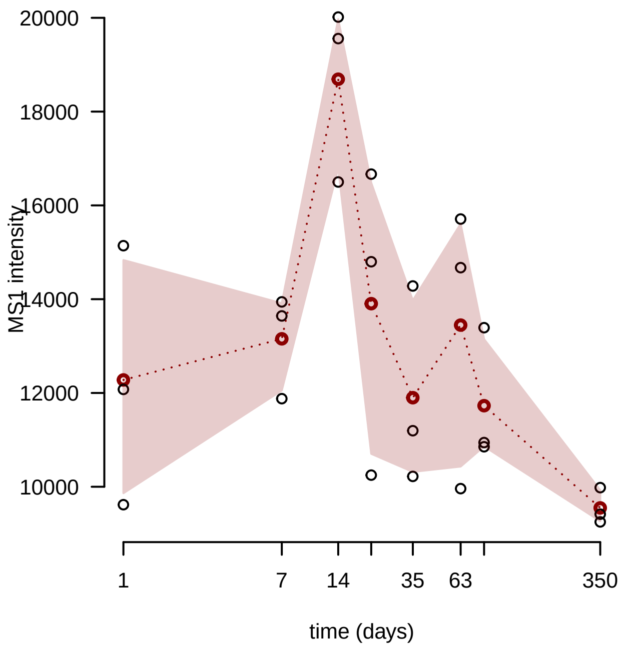

Q923X4
GN=Glrx2
glutaredoxin 2 (thioltransferase)

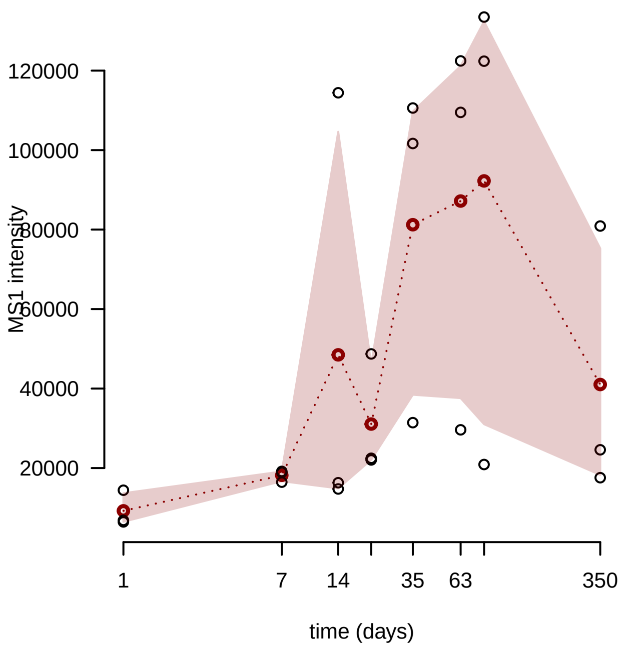

Q923Z0
GN=Gprc5b
G protein−coupled receptor, family C, gr ...

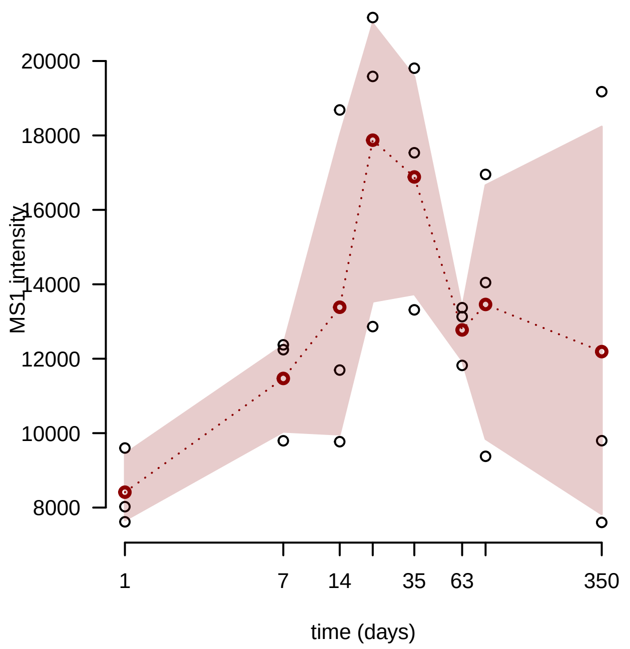

Q923Z3
GN=Mto1
mitochondrial tRNA translation optimizat ...

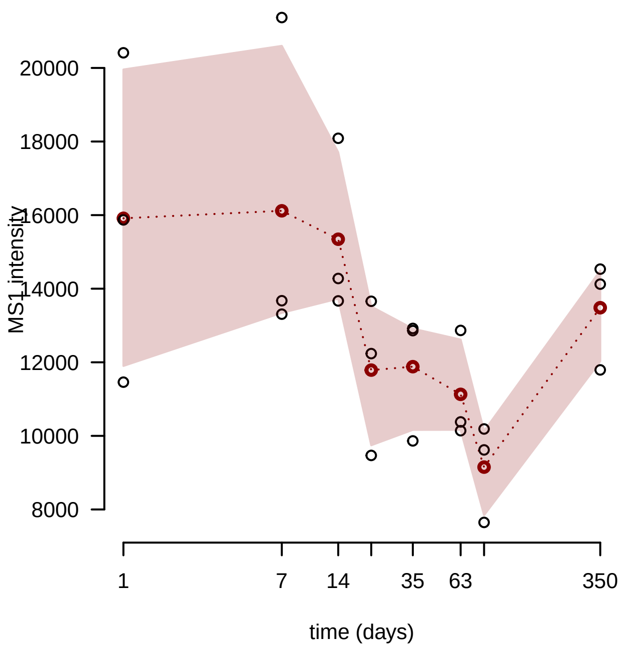

Q924A0
GN=Tcf7l2
transcription factor 7 like 2, T cell sp ...

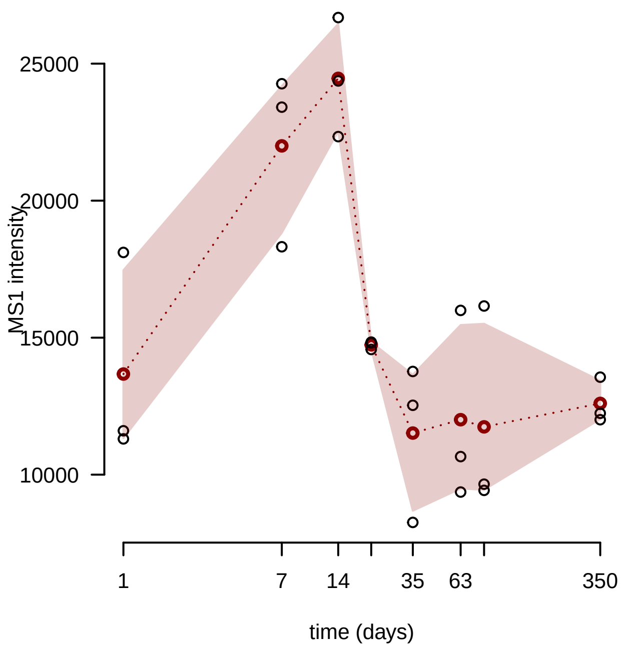

Q924A2
GN=Cic
capicua transcriptional repressor

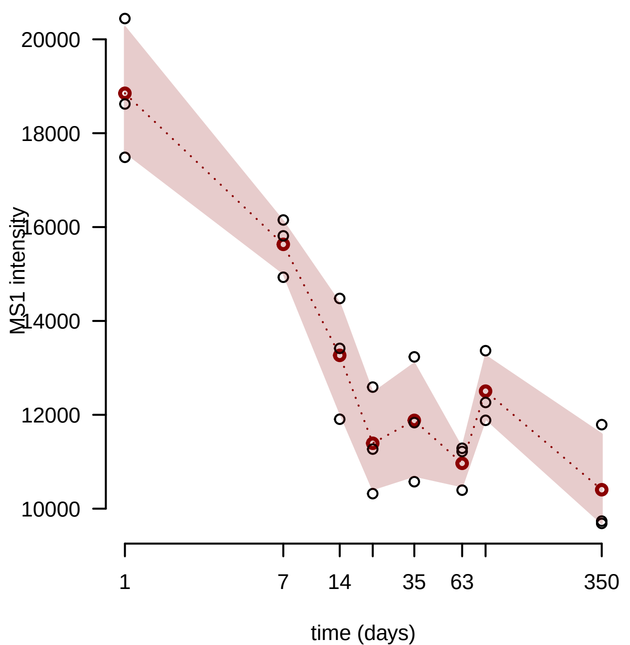

Q924C1
GN=Xpo5
exportin 5

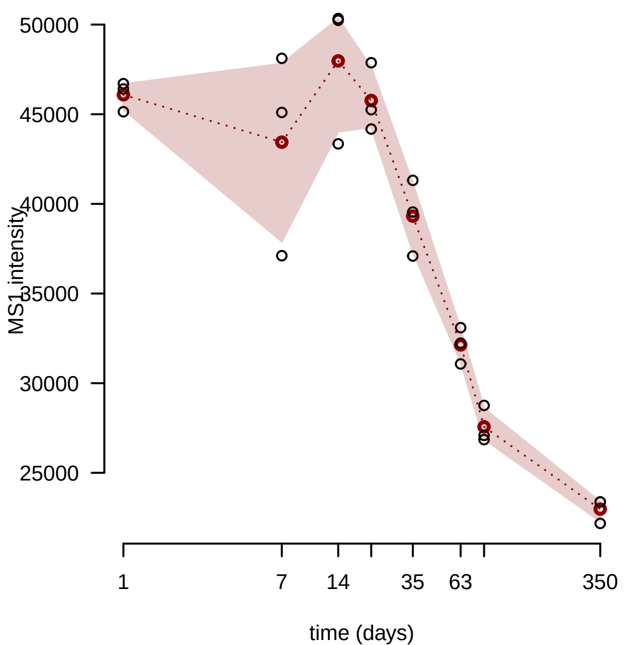

Q924D0
GN=Rtn4ip1
reticulon 4 interacting protein 1

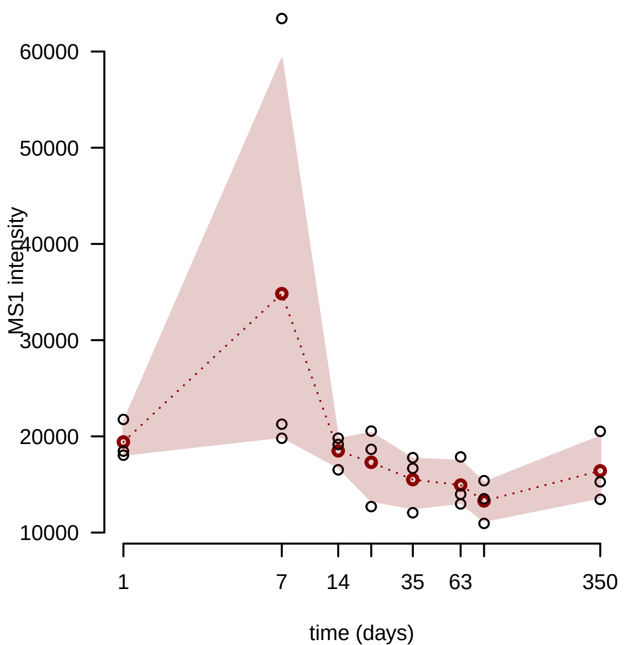

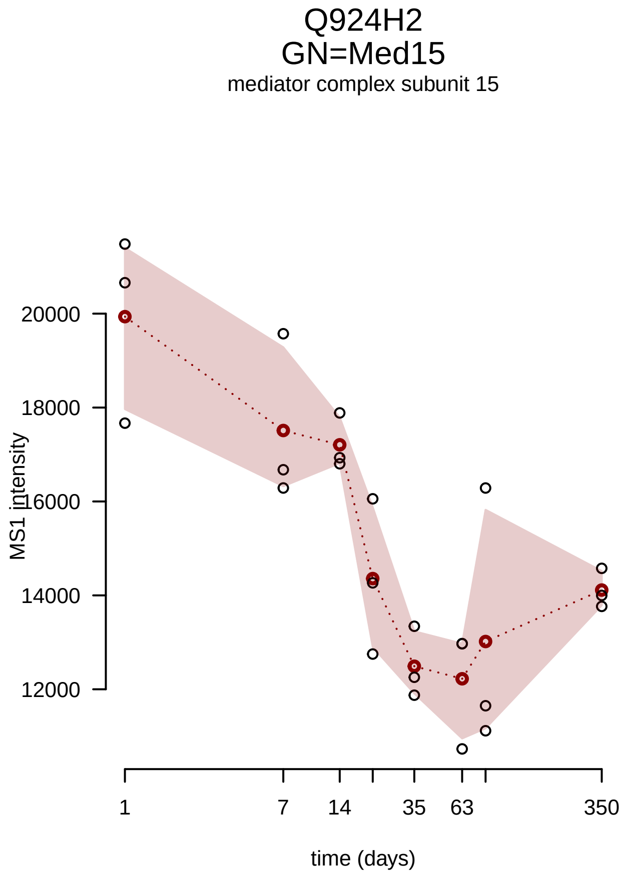
Q924H2
GN=Med15
mediator complex subunit 15
MS1 intensity
20000
18000
16000
14000
12000
1
7
14
35
63
350
time (days)

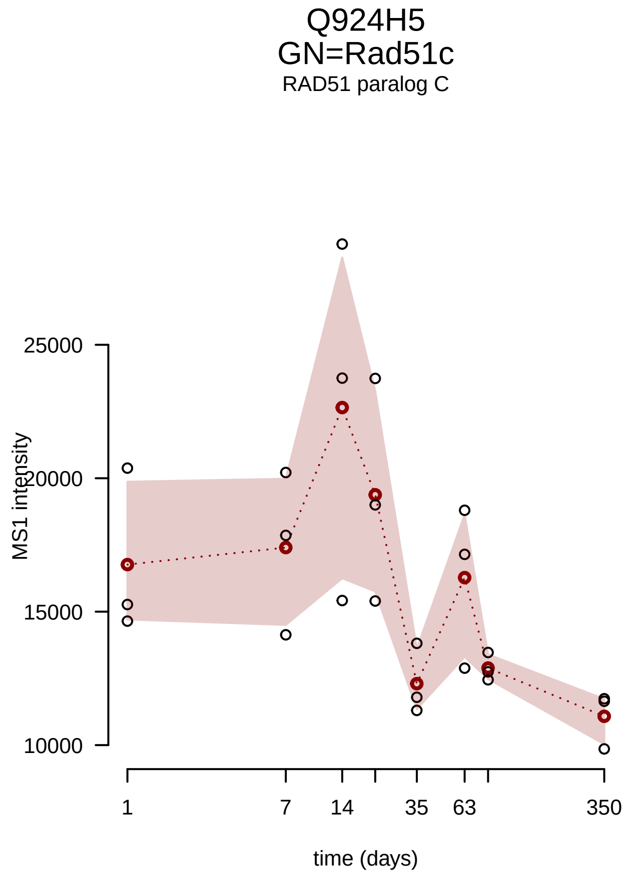
Q924H5
GN=Rad51c
RAD51 paralog C
MS1 intensity
25000
20000
15000
10000
1
7
14
35
63
350
time (days)

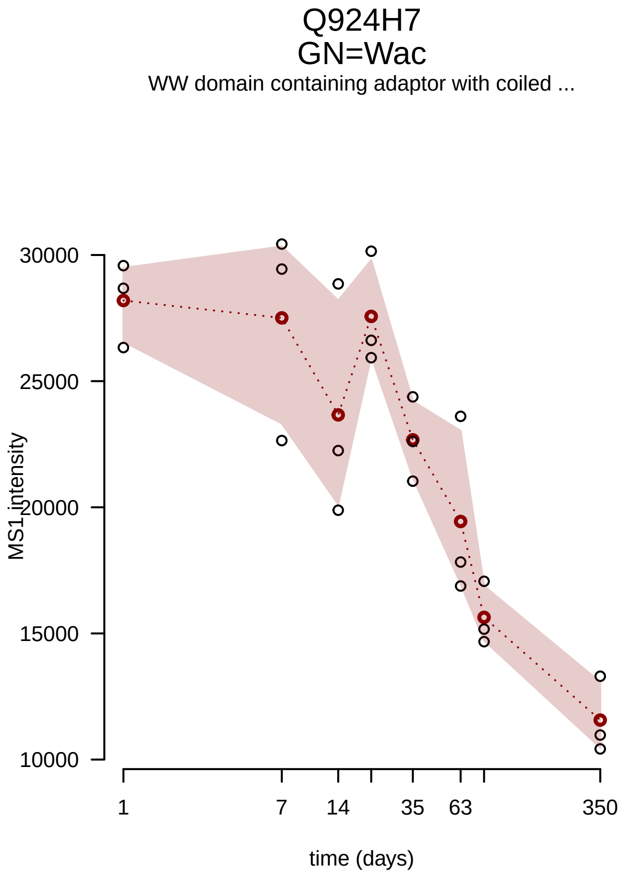
Q924H7
GN=Wac
WW domain containing adaptor with coiled ...
MS1 intensity
30000
25000
20000
15000
10000
1
7
14
35
63
350
time (days)

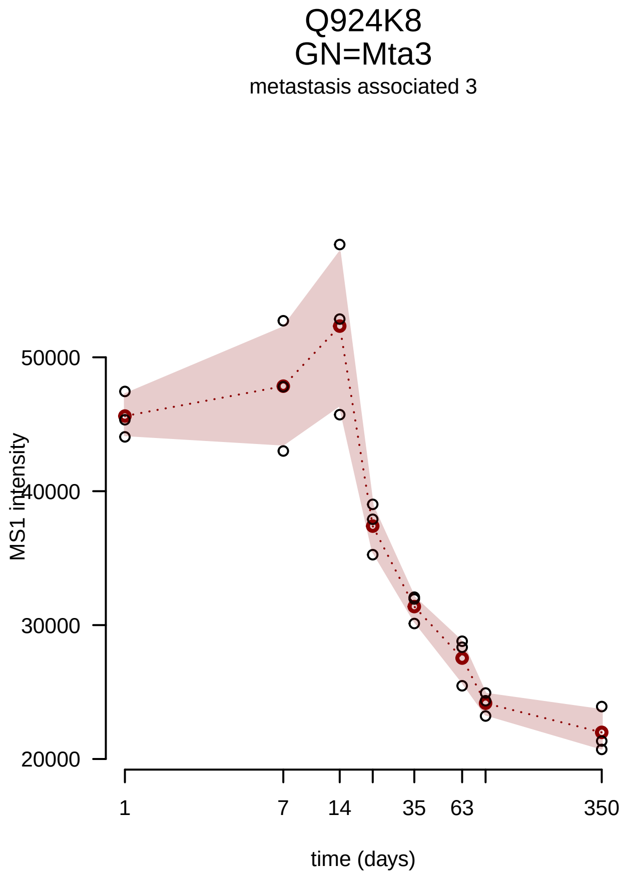
Q924K8
GN=Mta3
metastasis associated 3
MS1 intensity
50000
40000
30000
20000
1
7
14
35
63
350
time (days)

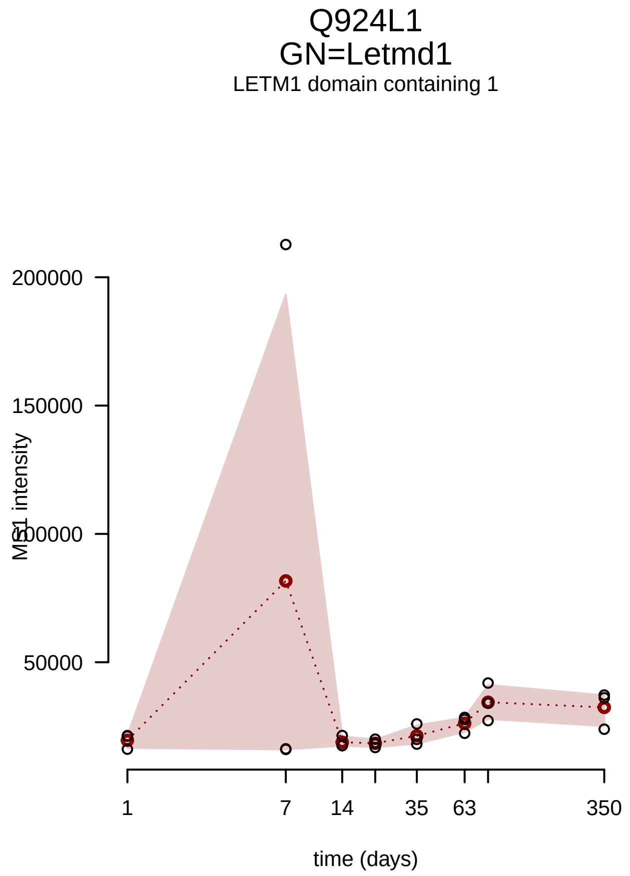
Q924L1
GN=Letmd1
LETM1 domain containing 1
MS1 intensity
200000
150000
100000
50000
1
7
14
35
63
350
time (days)

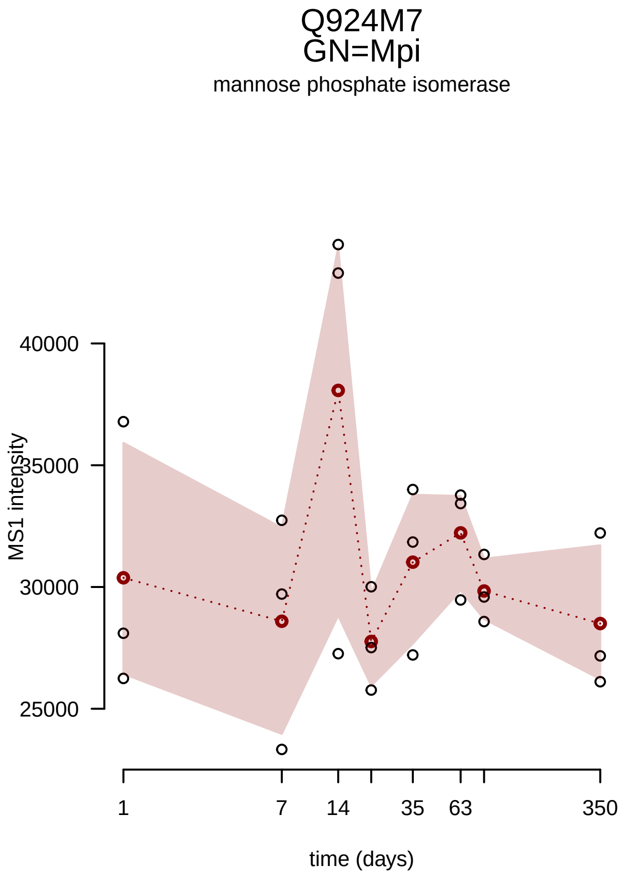
Q924M7
GN=Mpi
mannose phosphate isomerase
MS1 intensity
40000
35000
30000
25000
1
7
14
35
63
350
time (days)

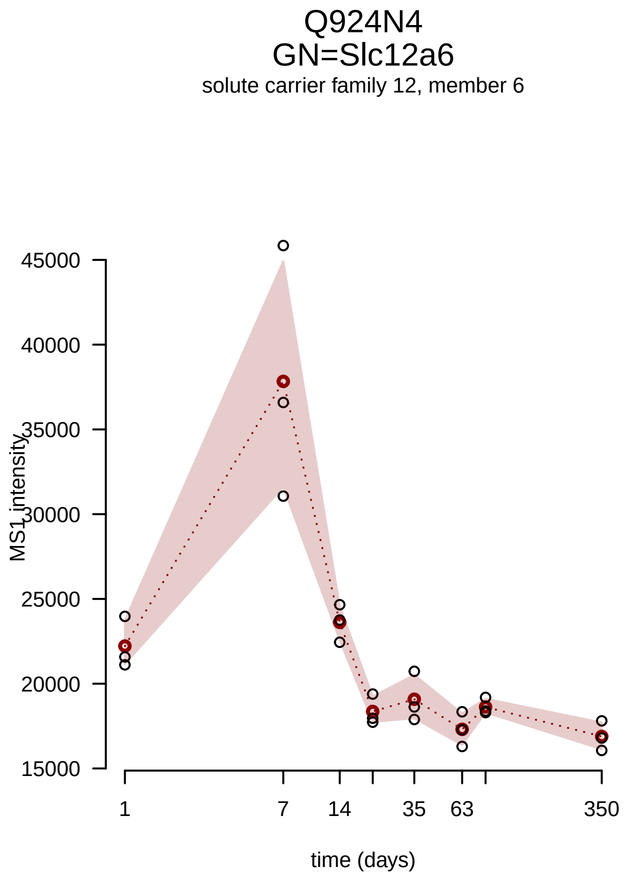
Q924N4
GN=Slc12a6
solute carrier family 12, member 6
MS1 intensity
45000
40000
35000
30000
25000
20000
15000
1
7
14
35
63
350
time (days)

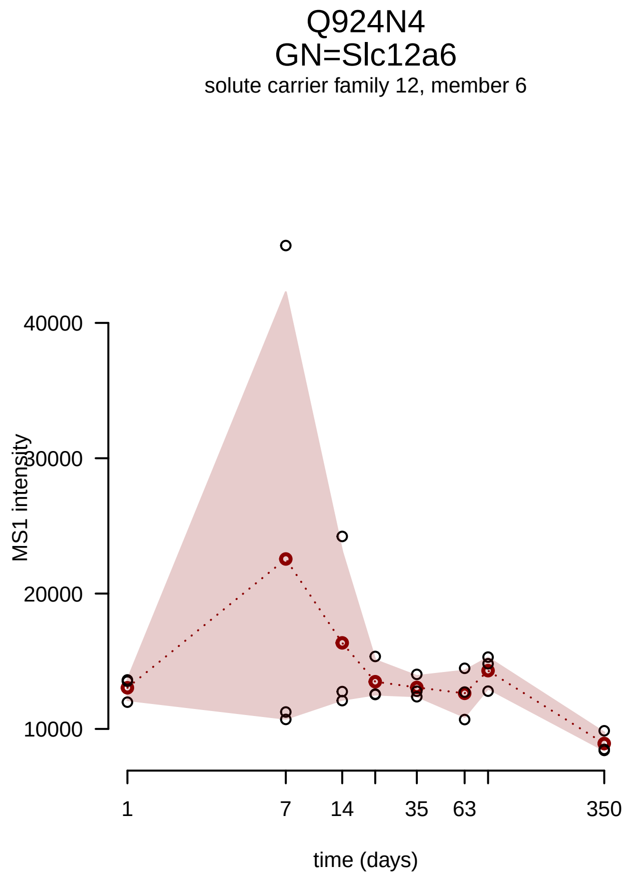
Q924N4
GN=Slc12a6
solute carrier family 12, member 6
MS1 intensity
40000
30000
20000
10000
1
7
14
35
63
350
time (days)

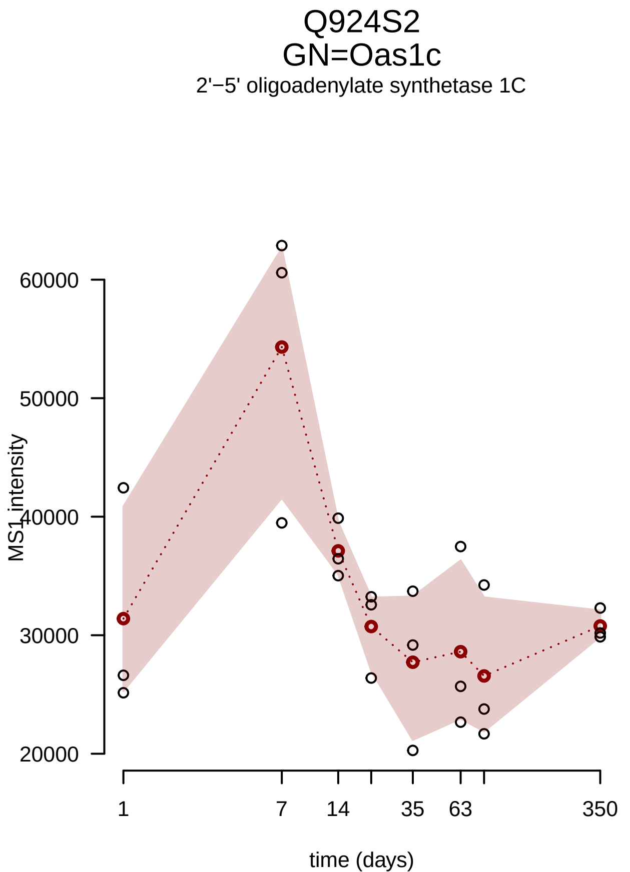
Q924S2
GN=Oas1c
2'−5' oligoadenylate synthetase 1C
MS1 intensity
60000
50000
40000
30000
20000
1
7
14
35
63
350
time (days)

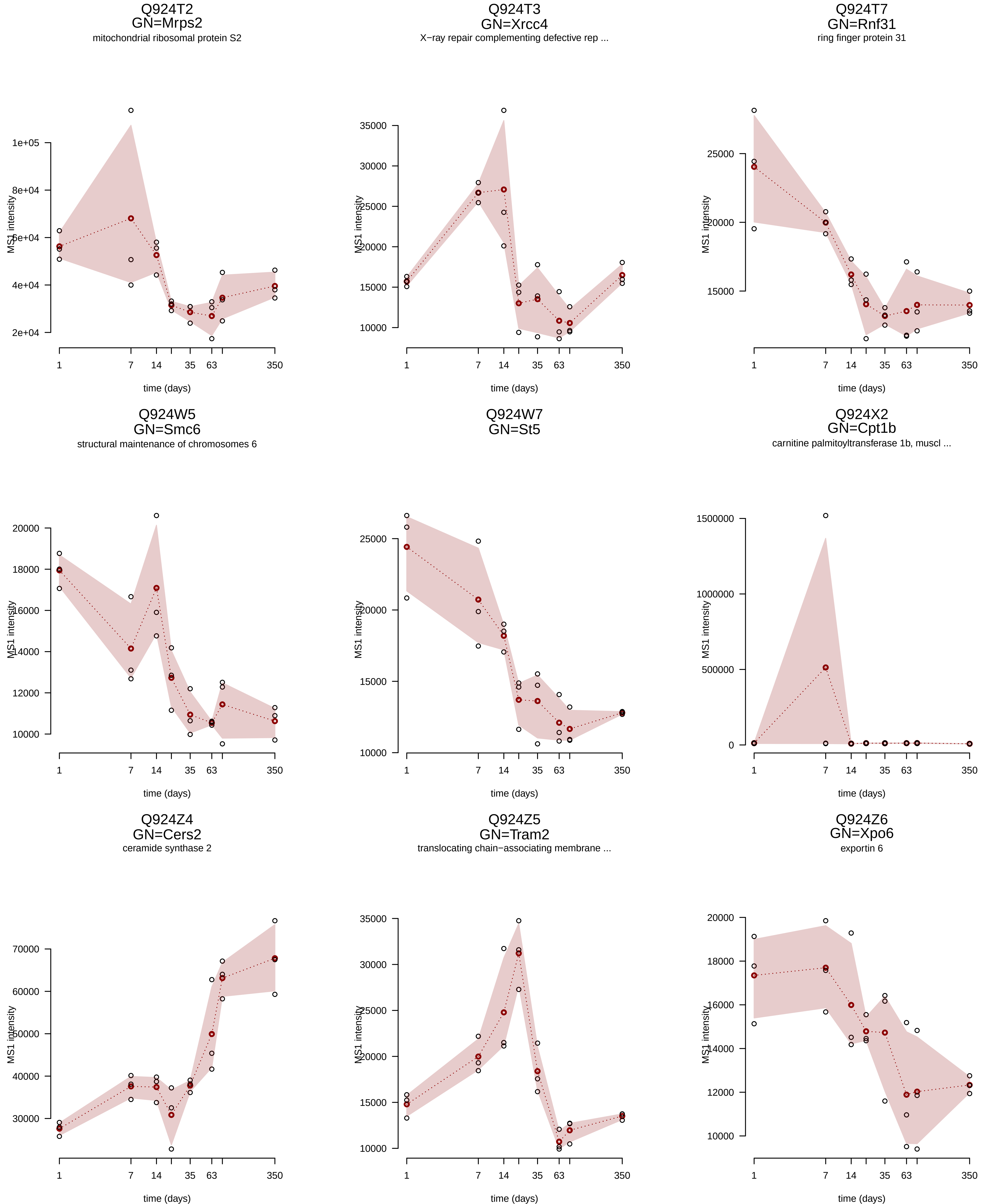
Q924T2
GN=Mrps2
mitochondrial ribosomal protein S2
MS1 intensity
2e+04
4e+04
6e+04
8e+04
1e+05
1
7
14
35
63
350
time (days)
Q924T3
GN=Xrcc4
X−ray repair complementing defective rep ...
MS1 intensity
10000
15000
20000
25000
30000
35000
1
7
14
35
63
350
time (days)
Q924T7
GN=Rnf31
ring finger protein 31
MS1 intensity
15000
20000
25000
1
7
14
35
63
350
time (days)
Q924W5
GN=Smc6
structural maintenance of chromosomes 6
MS1 intensity
10000
12000
14000
16000
18000
20000
1
7
14
35
63
350
time (days)
Q924W7
GN=St5
MS1 intensity
10000
15000
20000
25000
1
7
14
35
63
350
time (days)
Q924X2
GN=Cpt1b
carnitine palmitoyltransferase 1b, muscl ...
MS1 intensity
0
500000
1000000
1500000
1
7
14
35
63
350
time (days)
Q924Z4
GN=Cers2
ceramide synthase 2
MS1 intensity
30000
40000
50000
60000
70000
1
7
14
35
63
350
time (days)
Q924Z5
GN=Tram2
translocating chain−associating membrane ...
MS1 intensity
10000
15000
20000
25000
30000
35000
1
7
14
35
63
350
time (days)
Q924Z6
GN=Xpo6
exportin 6
MS1 intensity
10000
12000
14000
16000
18000
20000
1
7
14
35
63
350
time (days)

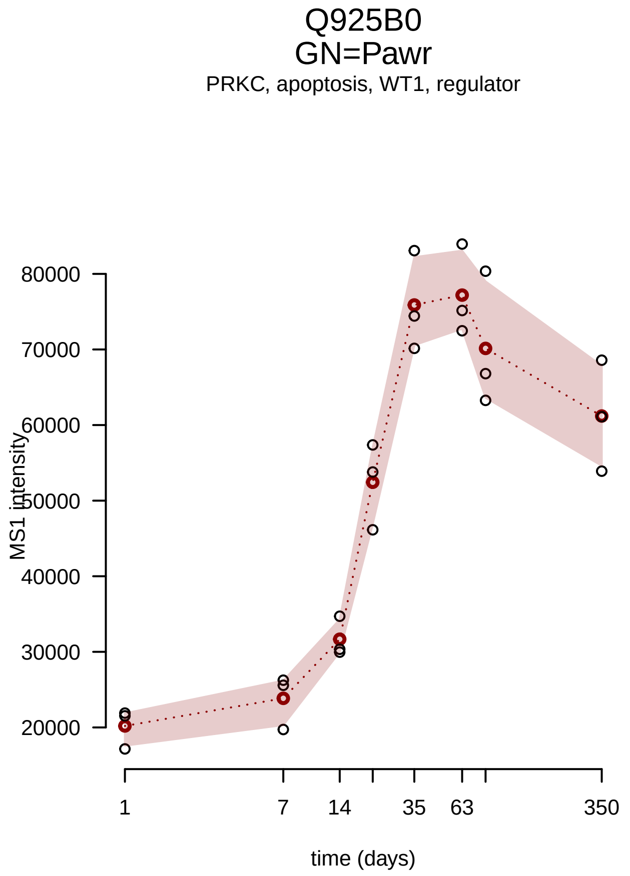
Q925B0
GN=Pawr
PRKC, apoptosis, WT1, regulator
80000
70000
60000
50000
40000
30000
20000
MS1 intensity
1
7
14
35
63
350
time (days)

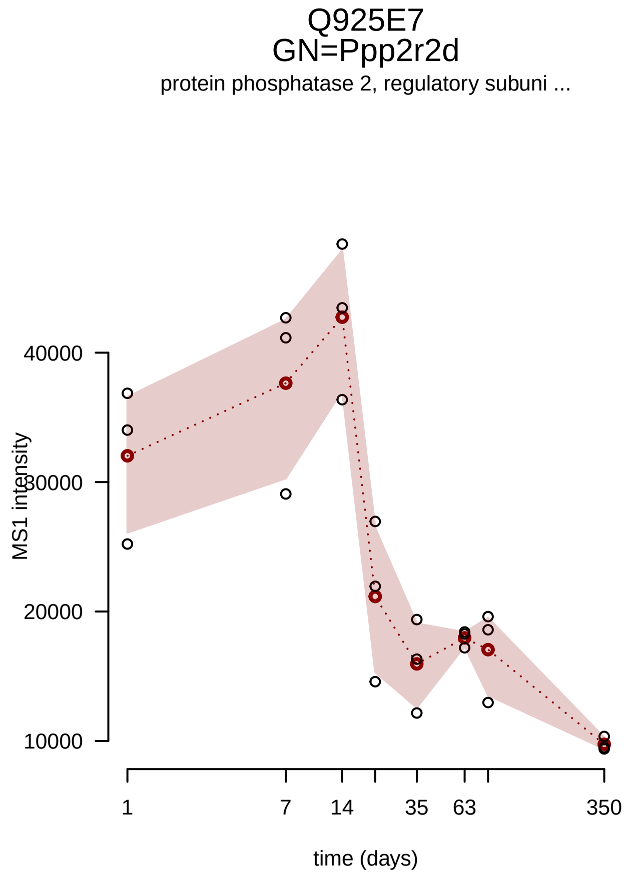
Q925E7
GN=Ppp2r2d
protein phosphatase 2, regulatory subuni ...
40000
30000
20000
10000
MS1 intensity
1
7
14
35
63
350
time (days)

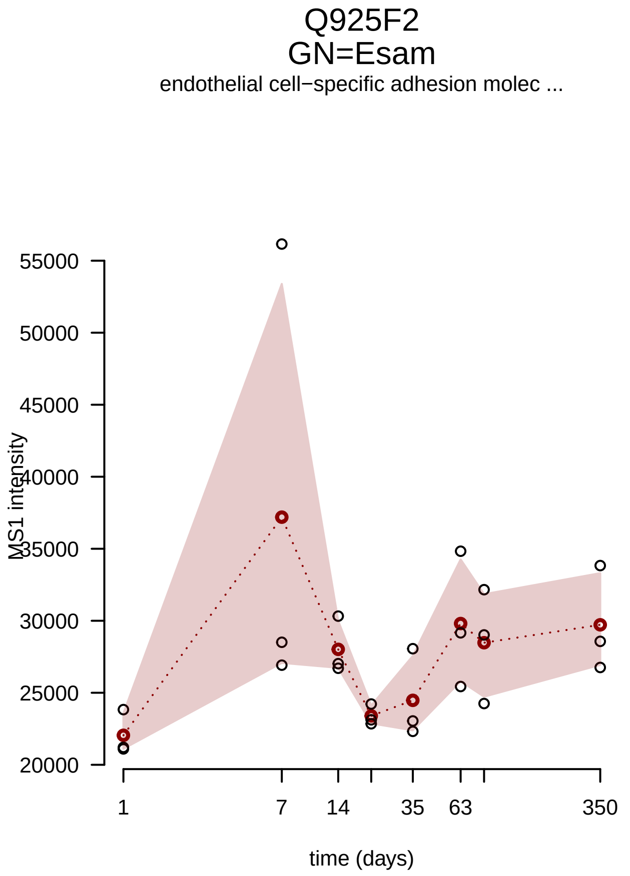
Q925F2
GN=Esam
endothelial cell−specific adhesion molec ...
55000
50000
45000
40000
35000
30000
25000
20000
MS1 intensity
1
7
14
35
63
350
time (days)

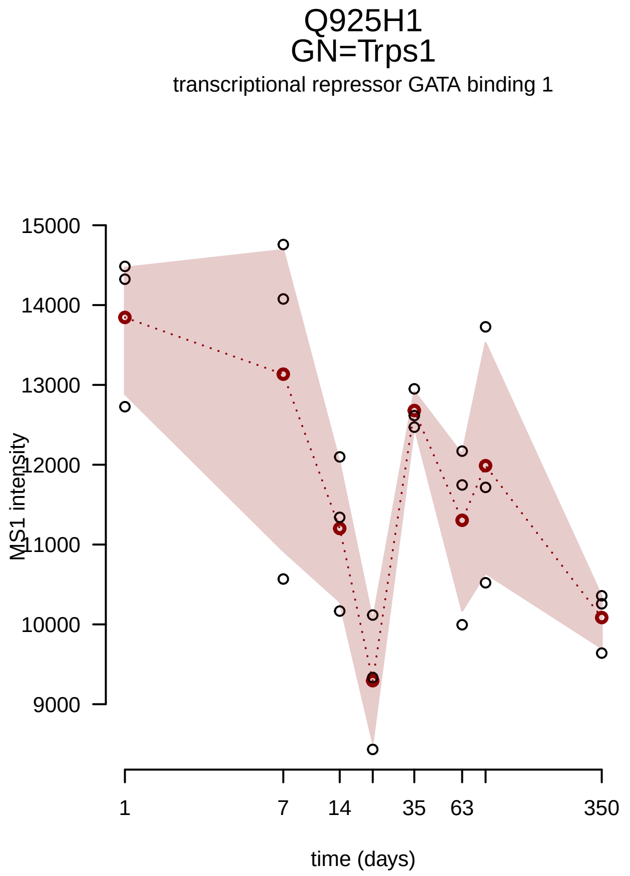
Q925H1
GN=Trps1
transcriptional repressor GATA binding 1
15000
14000
13000
12000
11000
10000
9000
MS1 intensity
1
7
14
35
63
350
time (days)

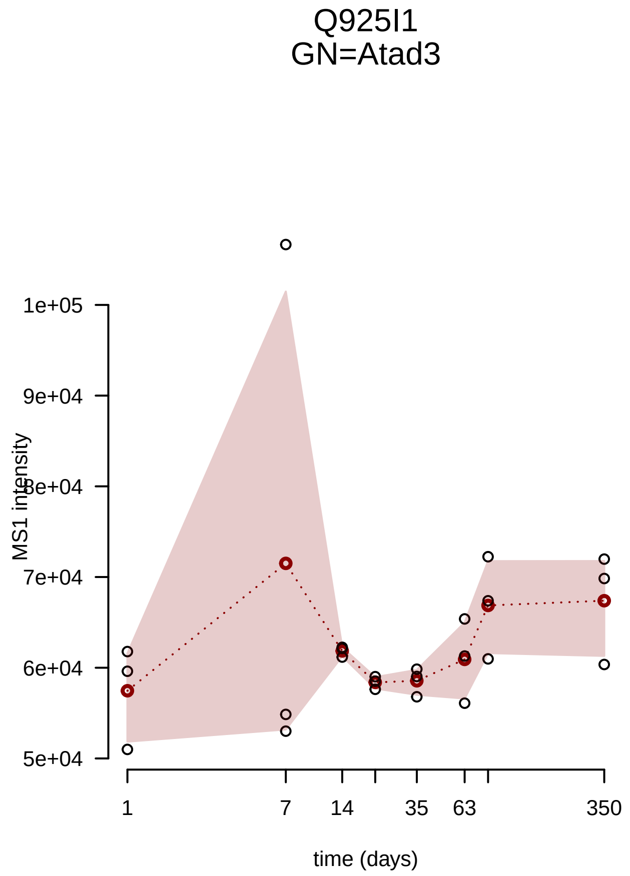
Q925I1
GN=Atad3
1e+05
9e+04
8e+04
7e+04
6e+04
5e+04
MS1 intensity
1
7
14
35
63
350
time (days)

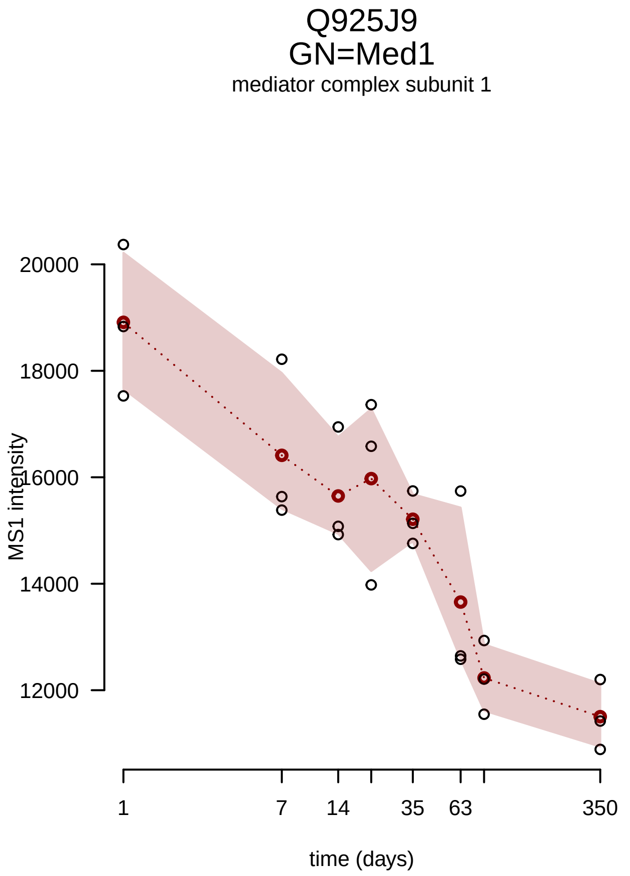
Q925J9
GN=Med1
mediator complex subunit 1
20000
18000
16000
14000
12000
MS1 intensity
1
7
14
35
63
350
time (days)

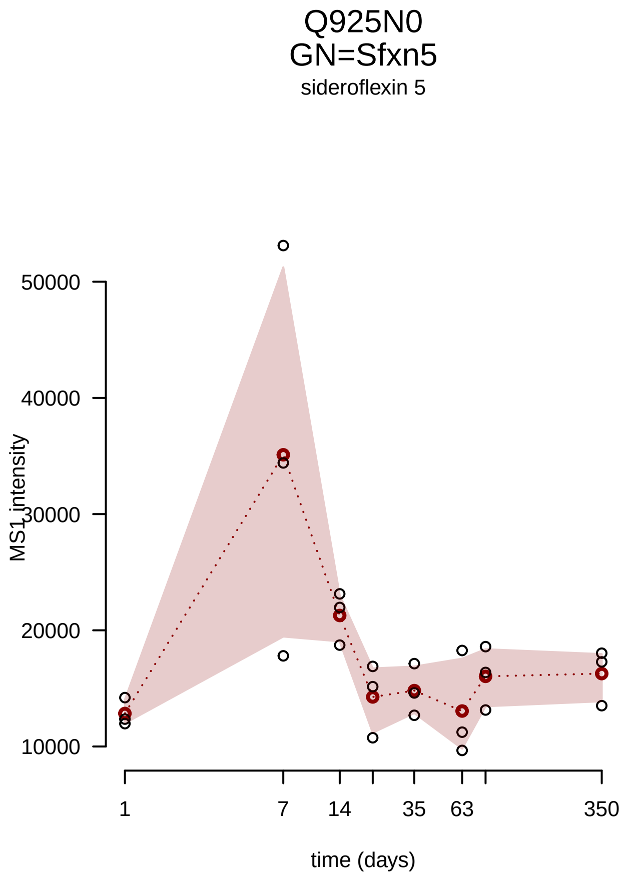
Q925N0
GN=Sfxn5
sideroflexin 5
50000
40000
30000
20000
10000
MS1 intensity
1
7
14
35
63
350
time (days)

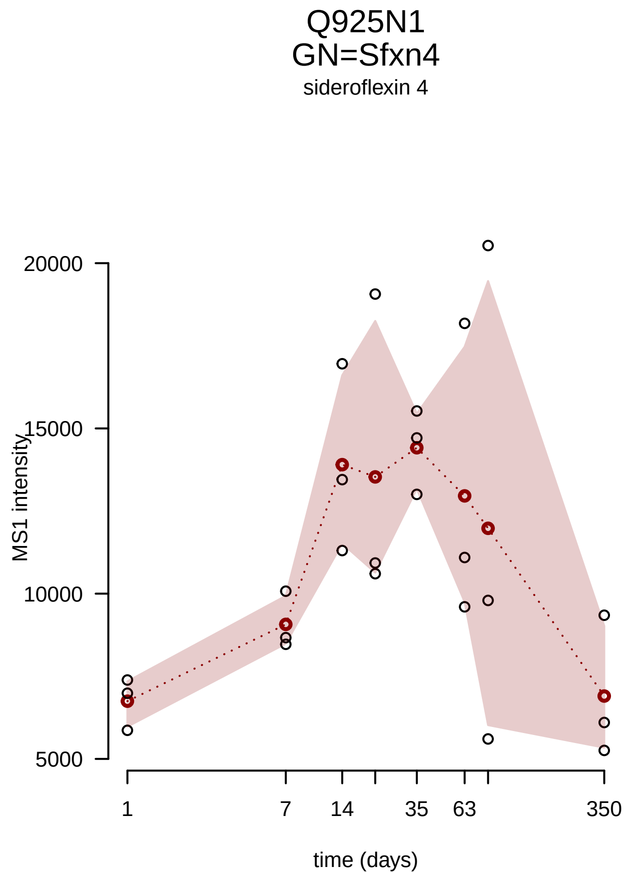
Q925N1
GN=Sfxn4
sideroflexin 4
20000
15000
10000
5000
MS1 intensity
1
7
14
35
63
350
time (days)

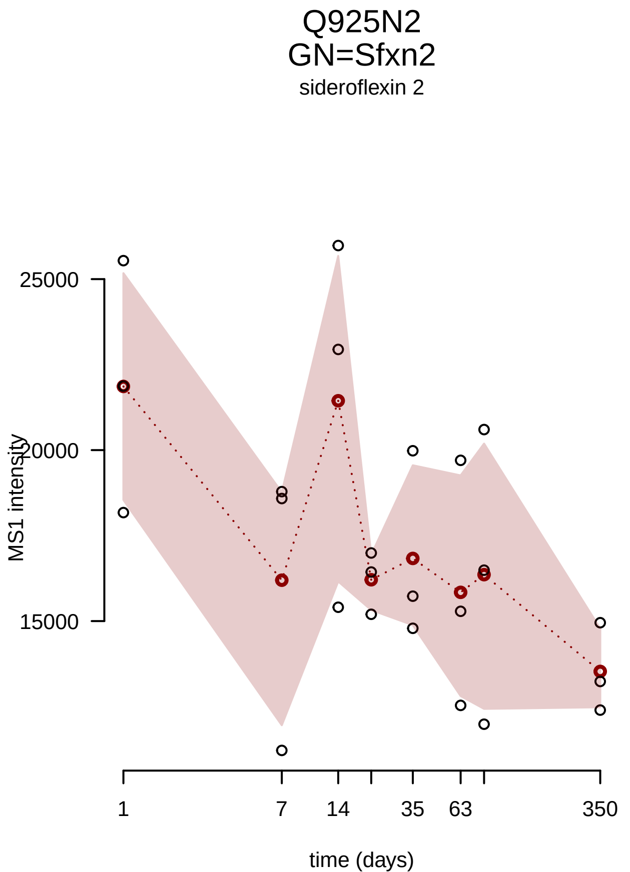
Q925N2
GN=Sfxn2
sideroflexin 2
25000
20000
15000
MS1 intensity
1
7
14
35
63
350
time (days)

Q925T6
GN=Grip1
glutamate receptor interacting protein 1

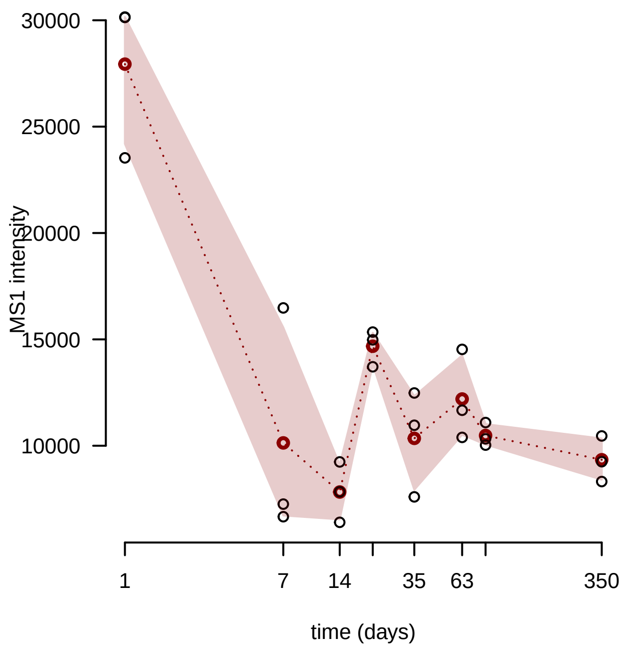

Q925U4
GN=Edem1
ER degradation enhancer, mannosidase alp ...

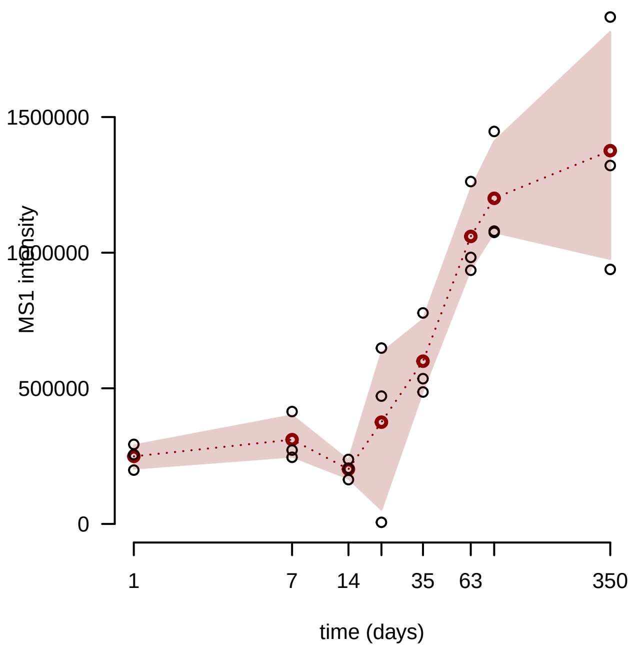

Q93092
GN=Taldo1
transaldolase 1

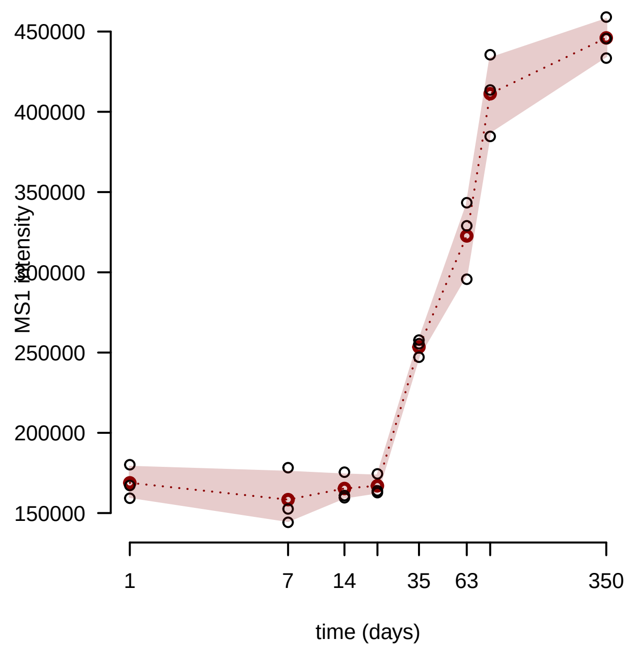

Q99020
GN=Hnrnpab
heterogeneous nuclear ribonucleoprotein ...

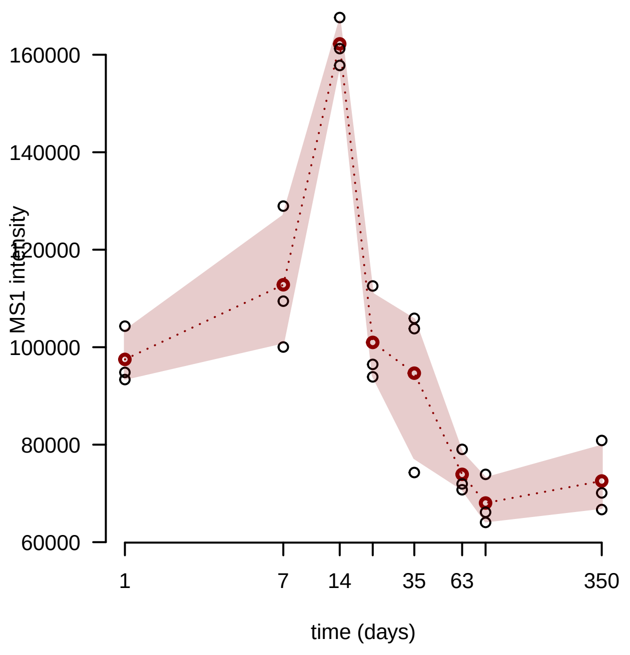

Q99104
GN=Myo5a
myosin VA

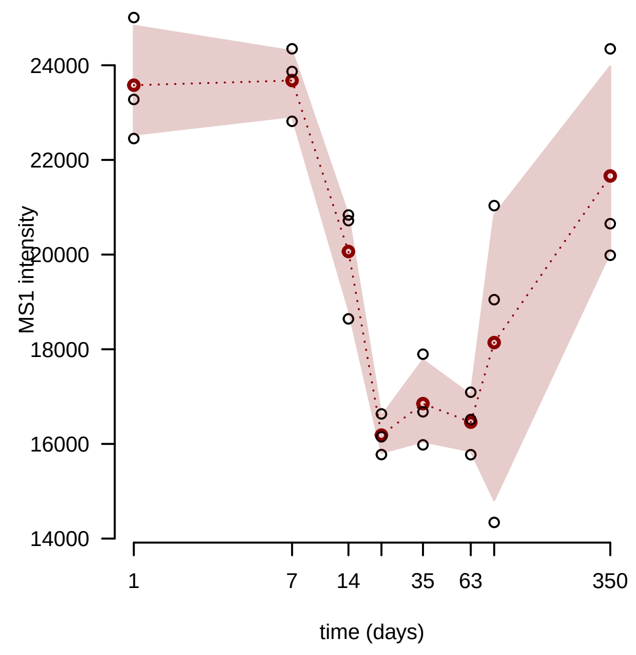

Q99J08
GN=Sec14l2
SEC14−like lipid binding 2

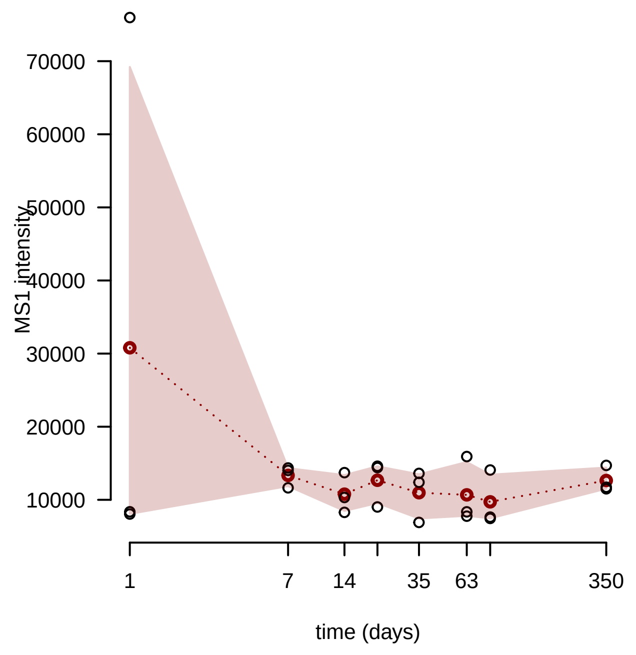

Q99J09
GN=Wdr77
WD repeat domain 77

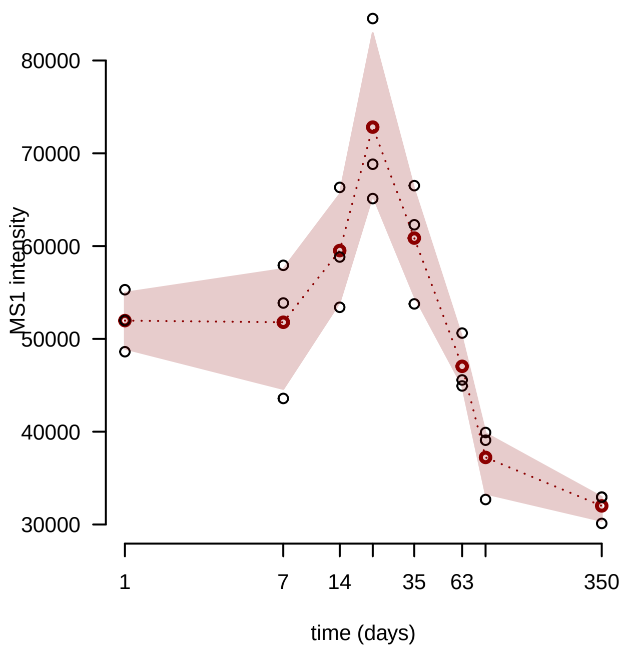

Q99J10
GN=Ctu1
cytosolic thiouridylase subunit 1

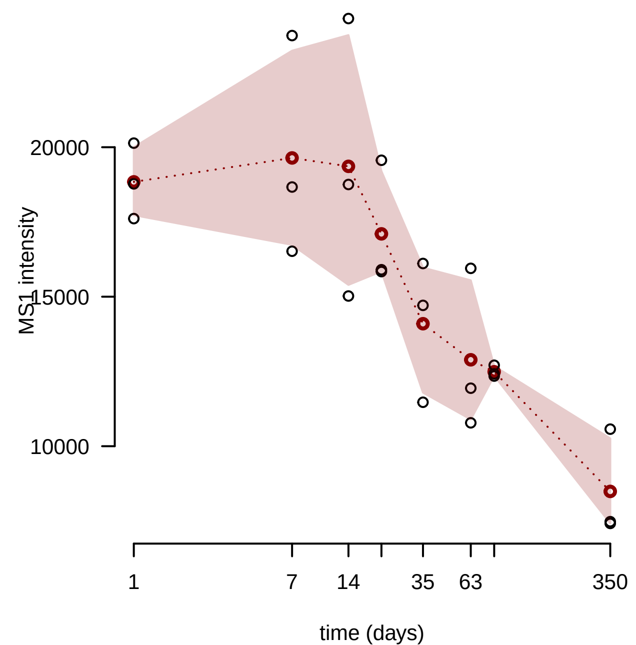

Q99J19
GN=Smim11a

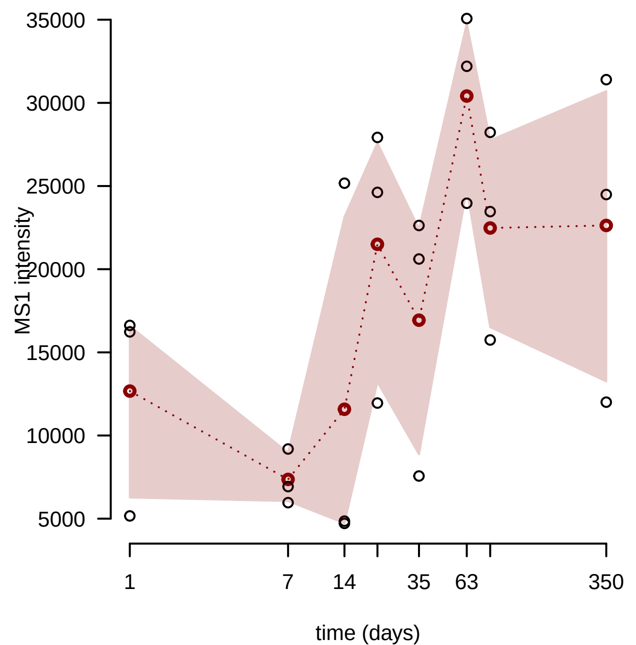

Q99J21
GN=Mcoln1
mucolipin 1

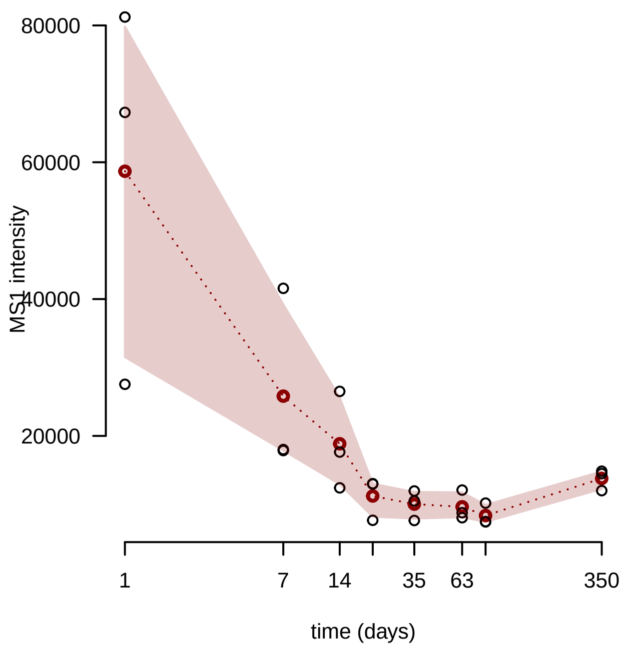

Q99J23
GN=Ghdc
GH3 domain containing

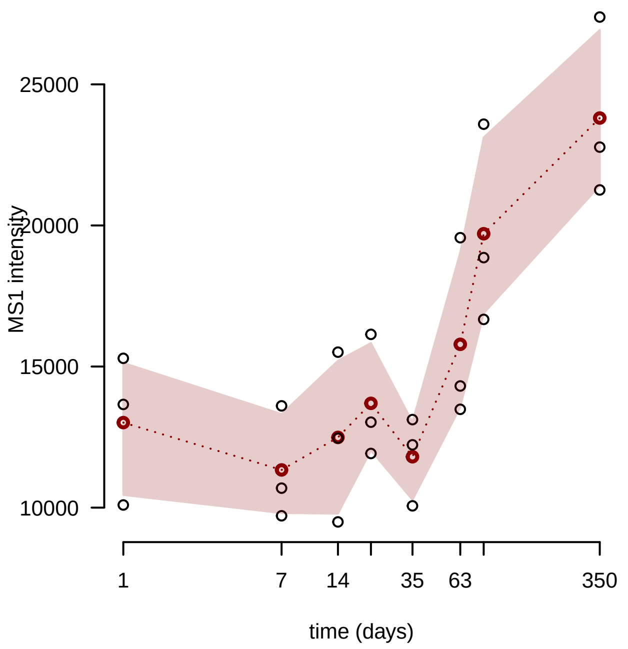

Q99J25
GN=Mrm1
mitochondrial rRNA methyltransferase 1

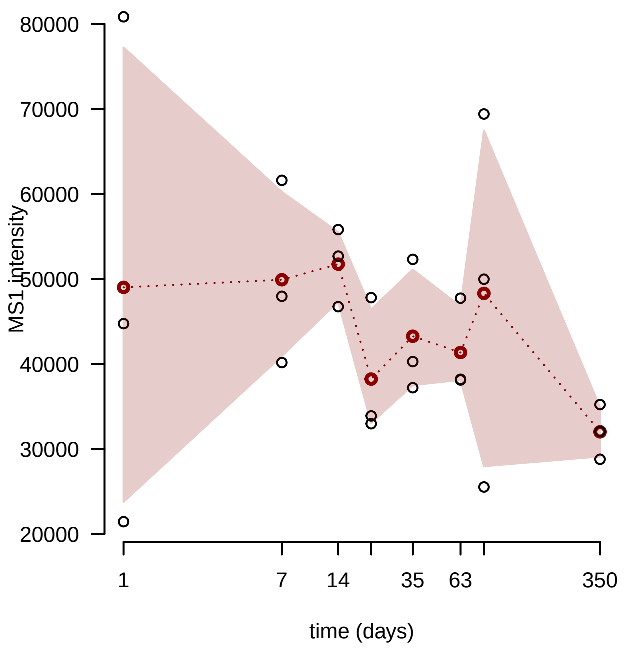

Q99J27
GN=Slc33a1
solute carrier family 33 (acetyl−CoA tra ...

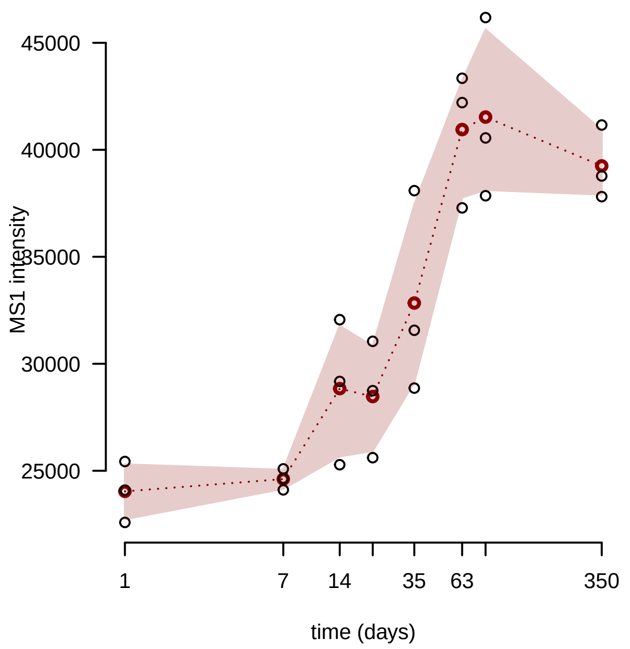

Q99J31
GN=Ophn1
oligophrenin 1

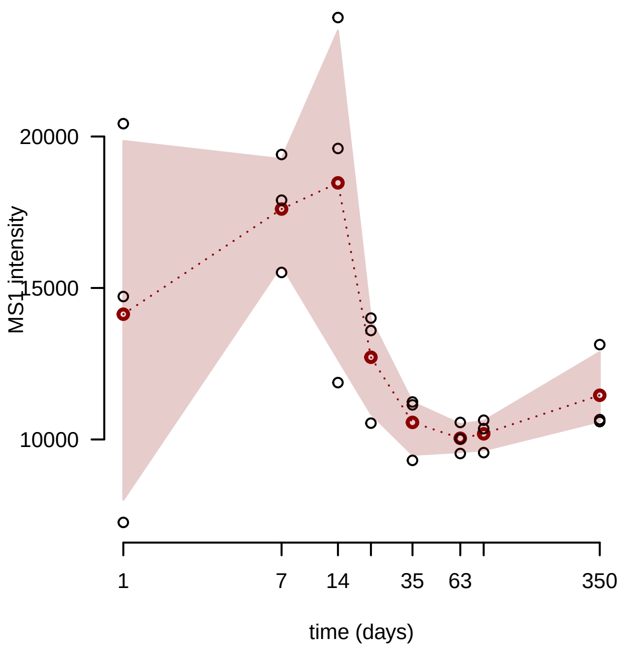

Q99J36
GN=Thumpd1
THUMP domain containing 1

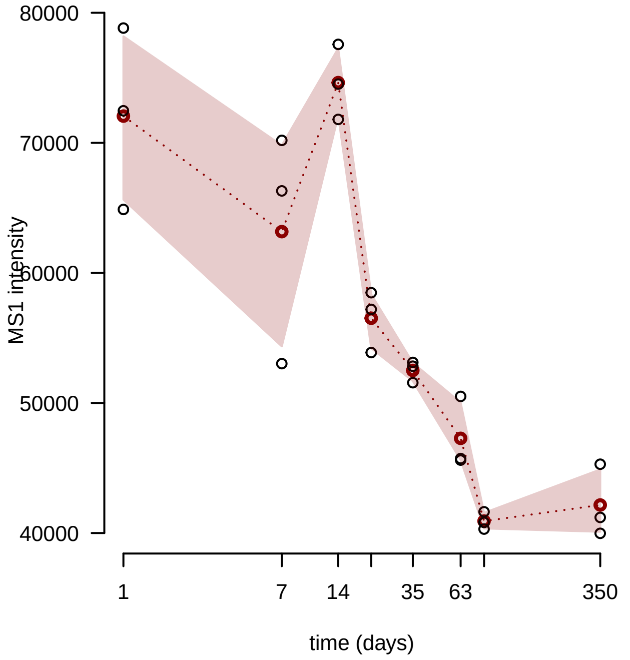

Q99J39
GN=Mlycd
malonyl−CoA decarboxylase

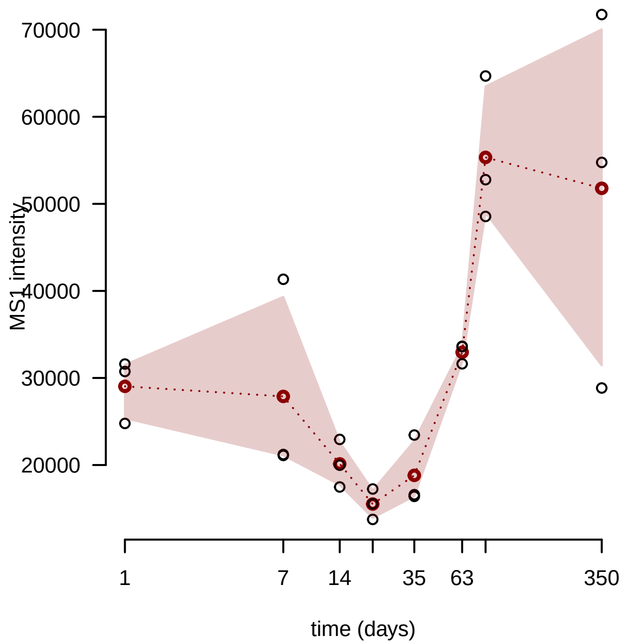

Q99J45
GN=Nrbp1
nuclear receptor binding protein 1

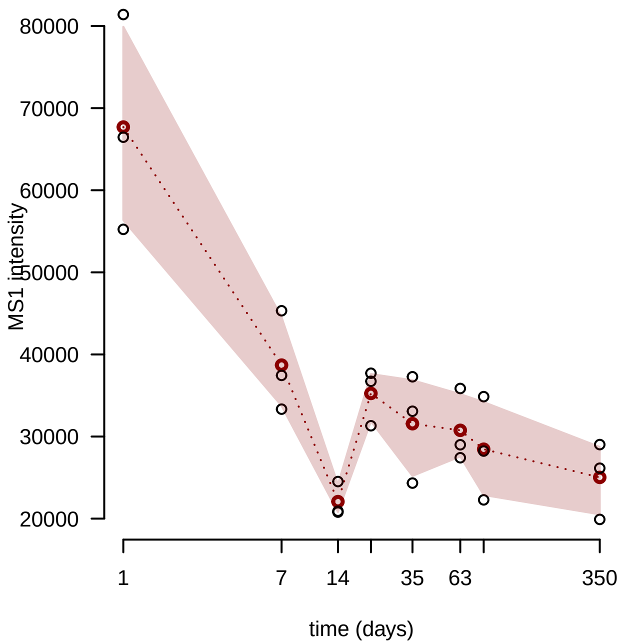

Q99J47
GN=Dhrs7b
dehydrogenase/reductase (SDR family) mem ...

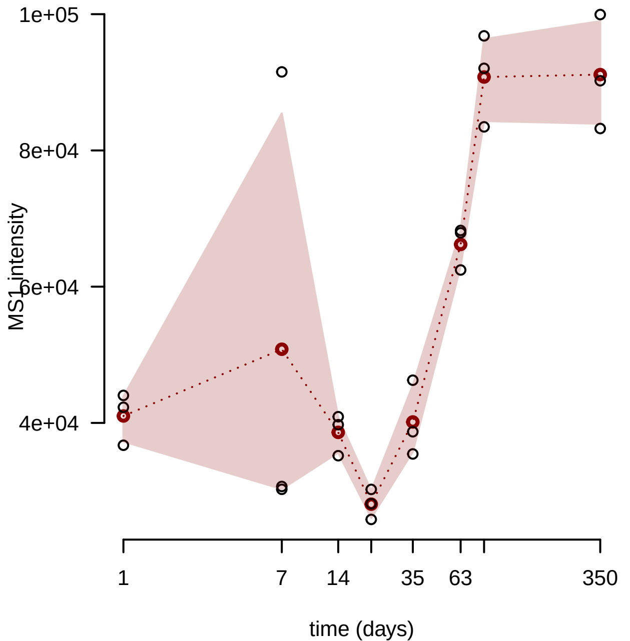

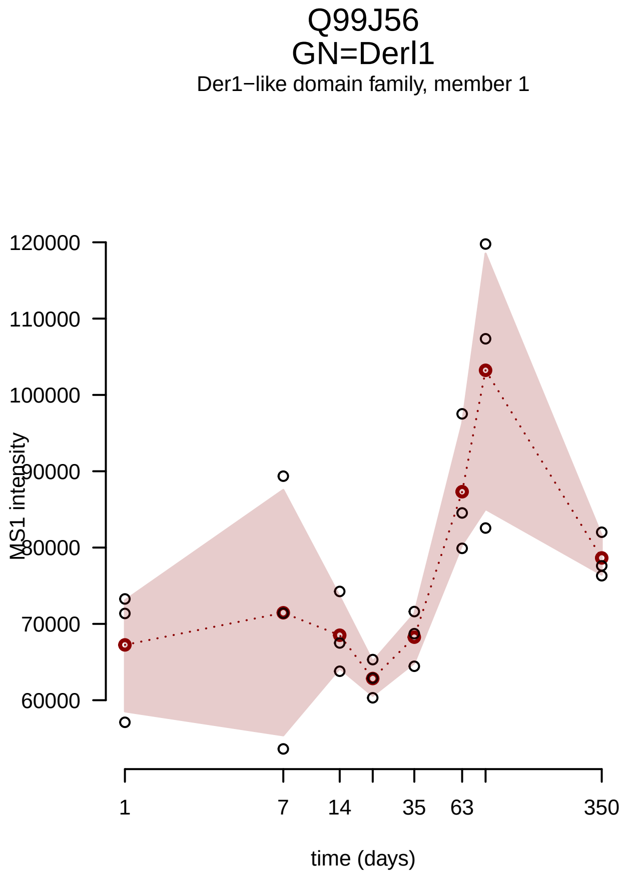
Q99J56
GN=Derl1
Der1−like domain family, member 1
MS1 intensity
60000
70000
80000
90000
100000
110000
120000
time (days)
1
7
14
35
63
350

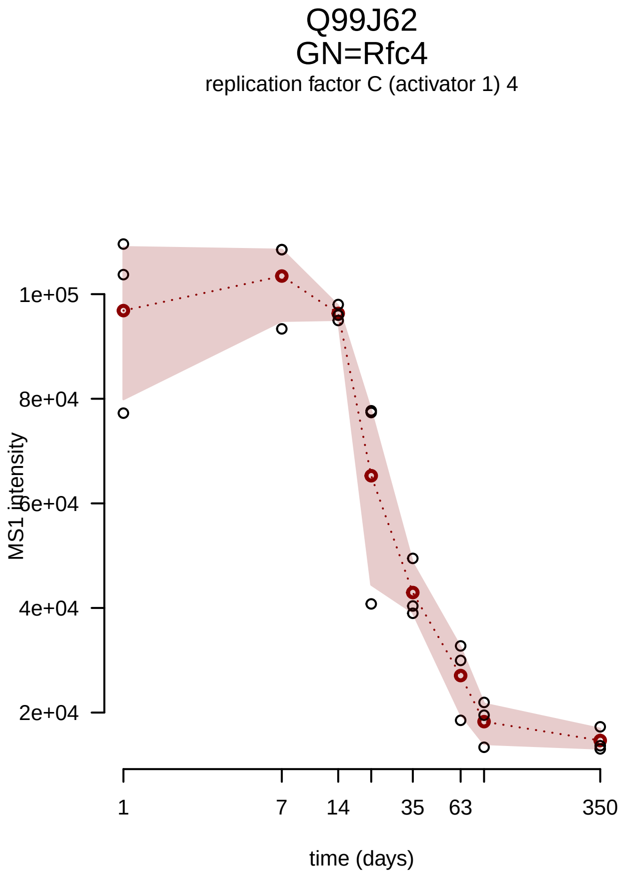
Q99J62
GN=Rfc4
replication factor C (activator 1) 4
MS1 intensity
2e+04
4e+04
6e+04
8e+04
1e+05
time (days)
1
7
14
35
63
350

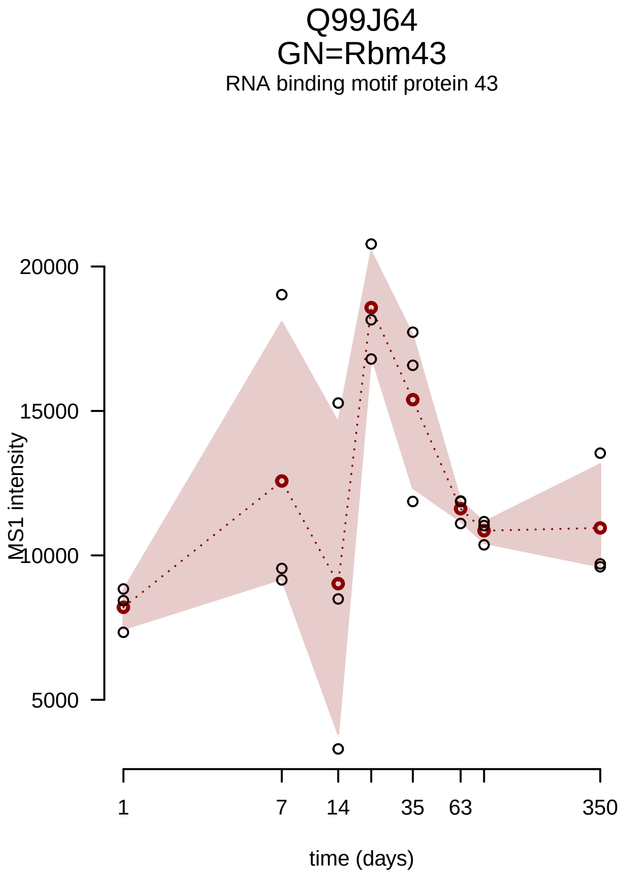
Q99J64
GN=Rbm43
RNA binding motif protein 43
MS1 intensity
5000
10000
15000
20000
time (days)
1
7
14
35
63
350

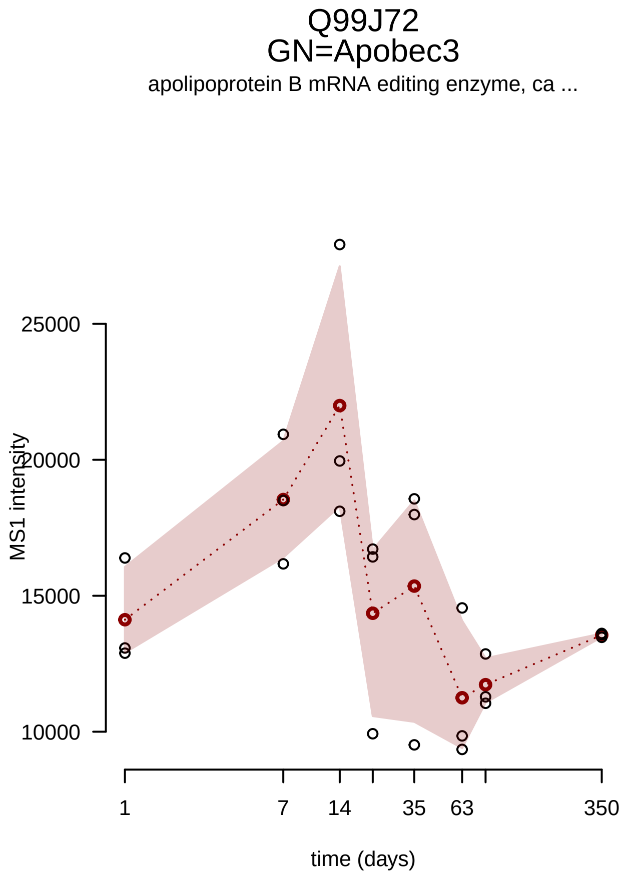
Q99J72
GN=Apobec3
apolipoprotein B mRNA editing enzyme, ca ...
MS1 intensity
10000
15000
20000
25000
time (days)
1
7
14
35
63
350

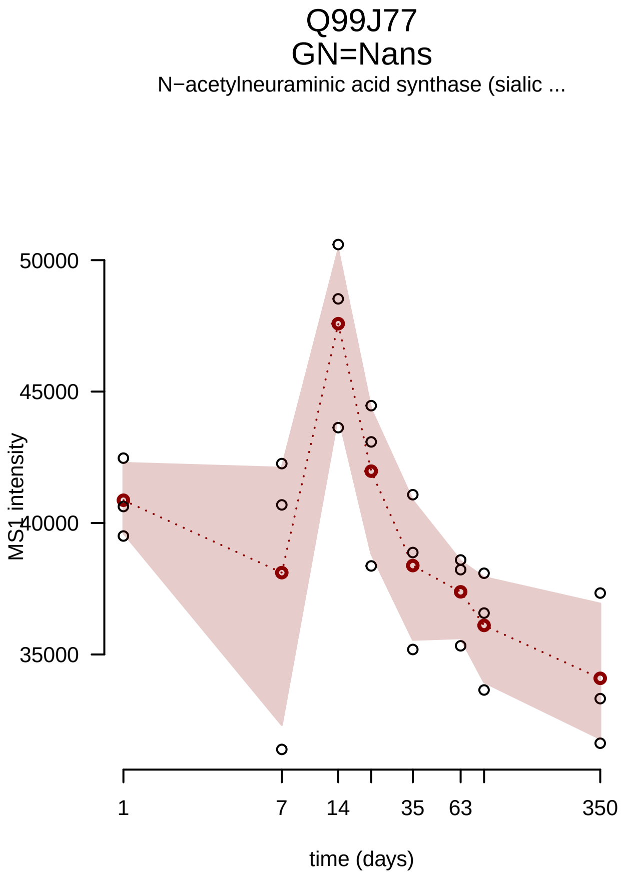
Q99J77
GN=Nans
N−acetylneuraminic acid synthase (sialic ...
MS1 intensity
35000
40000
45000
50000
time (days)
1
7
14
35
63
350

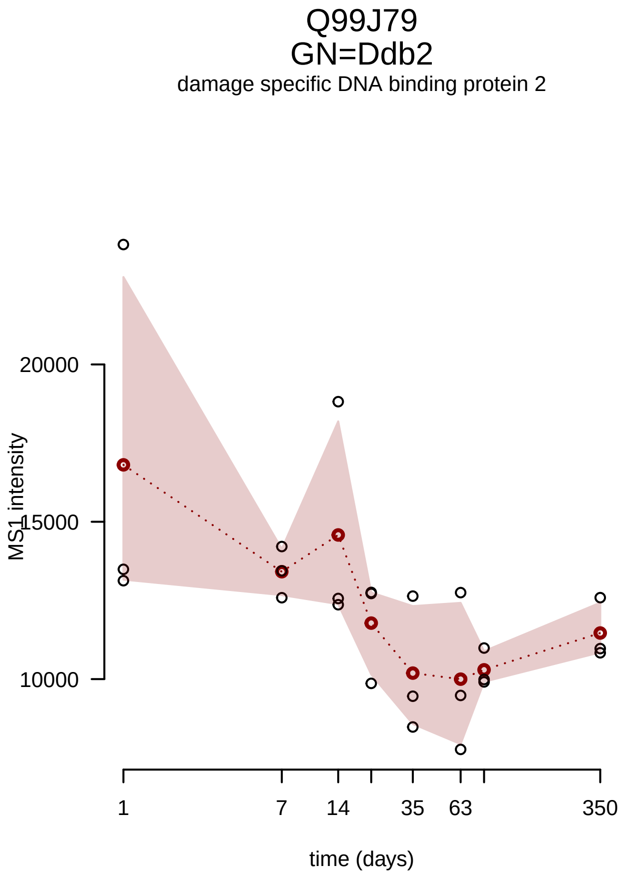
Q99J79
GN=Ddb2
damage specific DNA binding protein 2
MS1 intensity
10000
15000
20000
time (days)
1
7
14
35
63
350

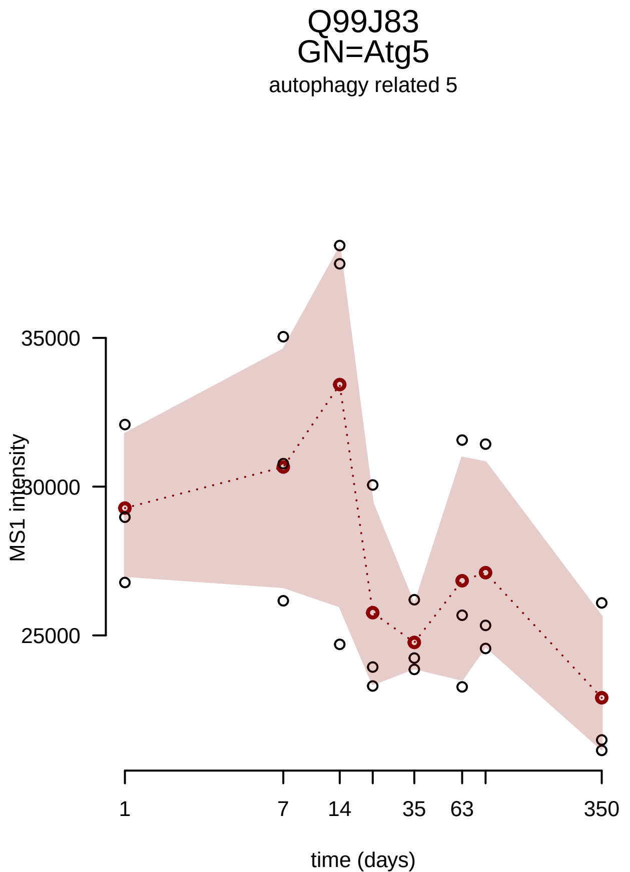
Q99J83
GN=Atg5
autophagy related 5
MS1 intensity
25000
30000
35000
time (days)
1
7
14
35
63
350

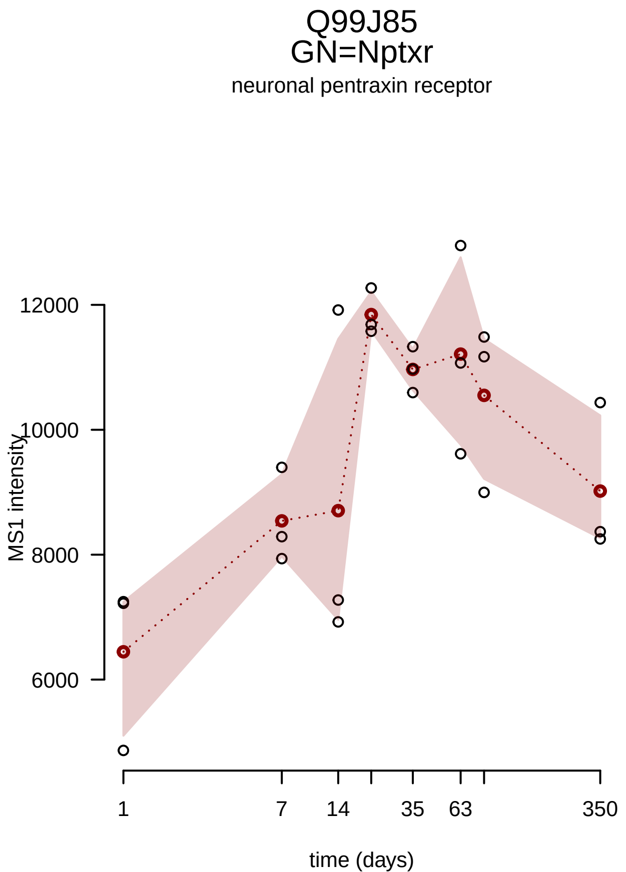
Q99J85
GN=Nptxr
neuronal pentraxin receptor
MS1 intensity
6000
8000
10000
12000
time (days)
1
7
14
35
63
350

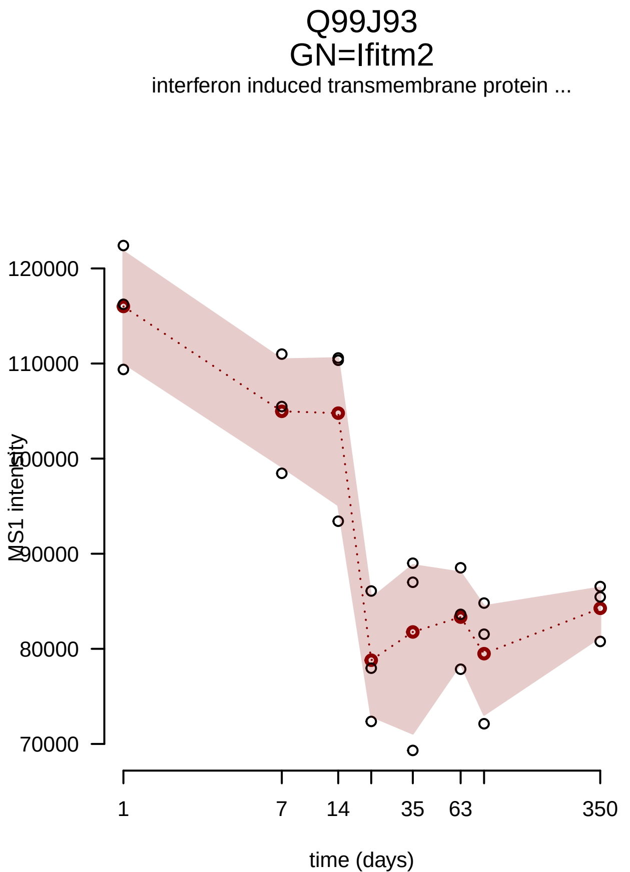
Q99J93
GN=Ifitm2
interferon induced transmembrane protein ...
MS1 intensity
70000
80000
90000
100000
110000
120000
time (days)
1
7
14
35
63
350

Q99J95
GN=Cdk9
cyclin−dependent kinase 9 (CDC2−related ...

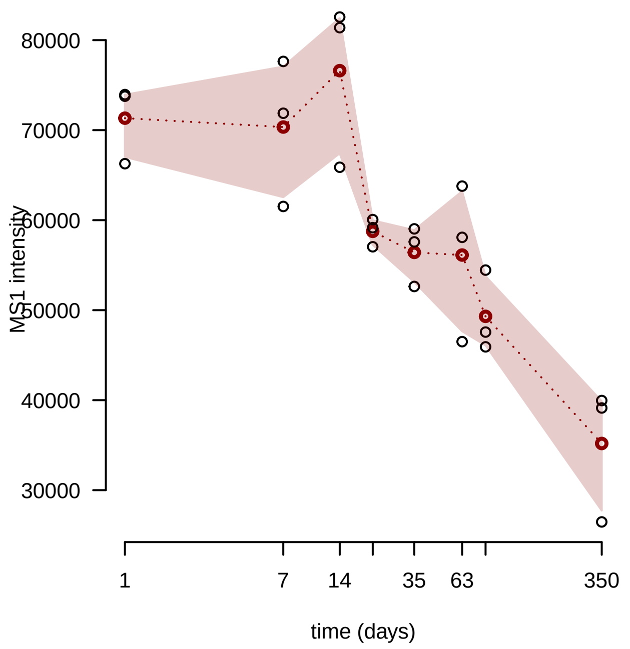

Q99J95
GN=Cdk9
cyclin−dependent kinase 9 (CDC2−related ...

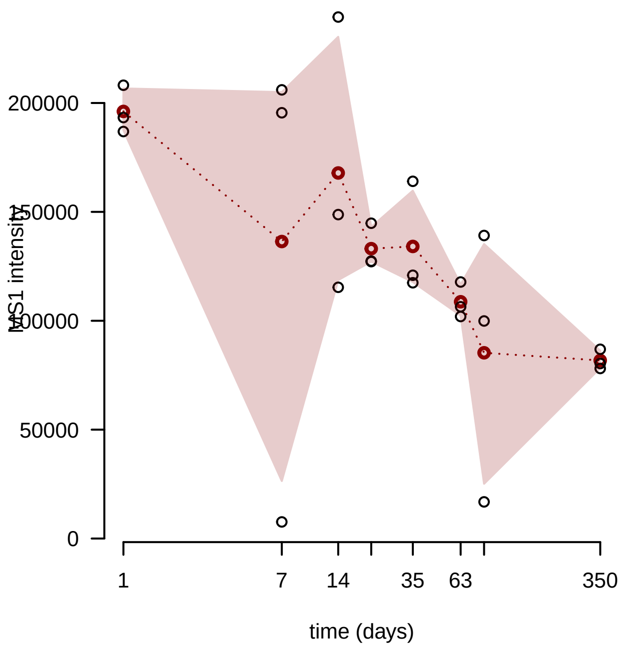

Q99J99
GN=Mpst
mercaptopyruvate sulfurtransferase

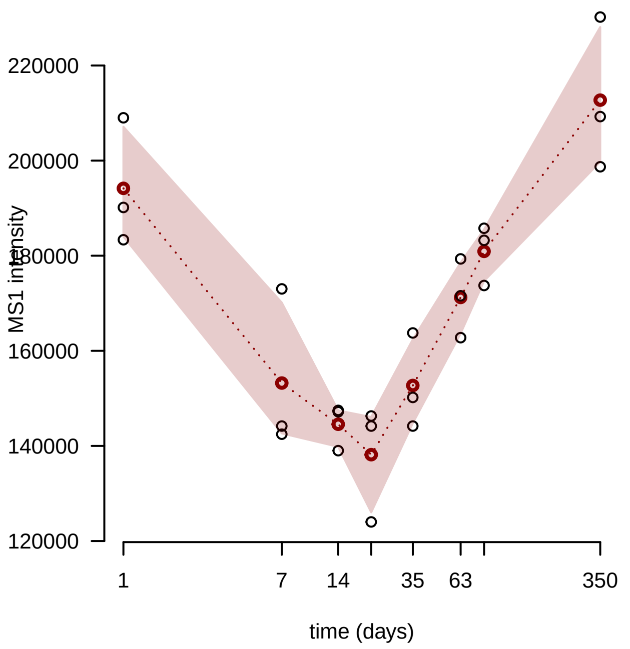

Q99JB2
GN=Stoml2
stomatin (Epb7.2)−like 2

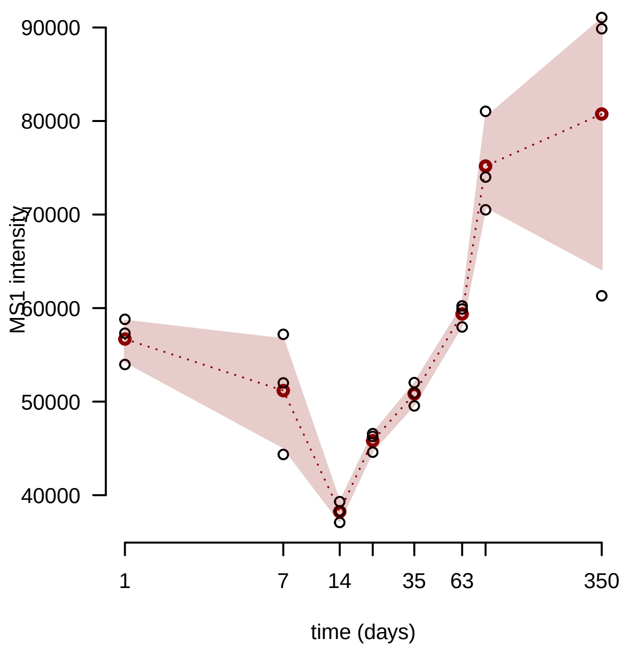

Q99JB8
GN=Pacsin3
protein kinase C and casein kinase subst ...

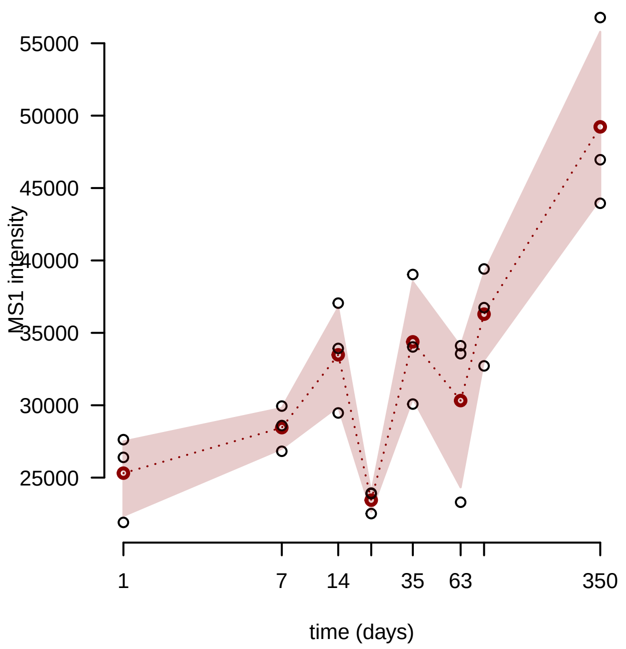

Q99JF5
GN=Mvd
mevalonate (diphospho) decarboxylase

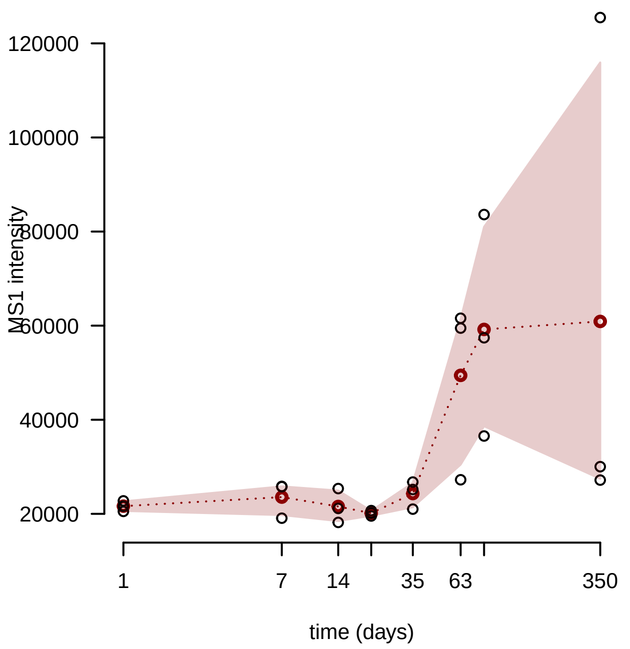

Q99JF8
GN=Psip1
PC4 and SFRS1 interacting protein 1

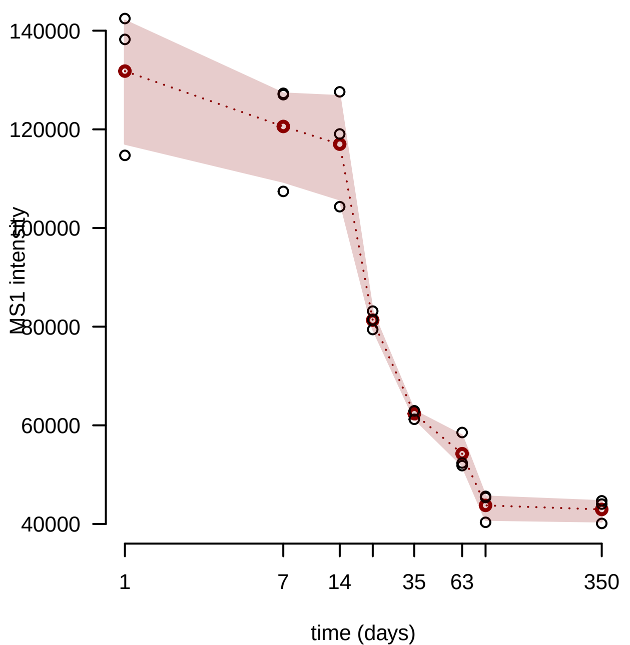

Q99JG3
GN=Anxa13
annexin A13

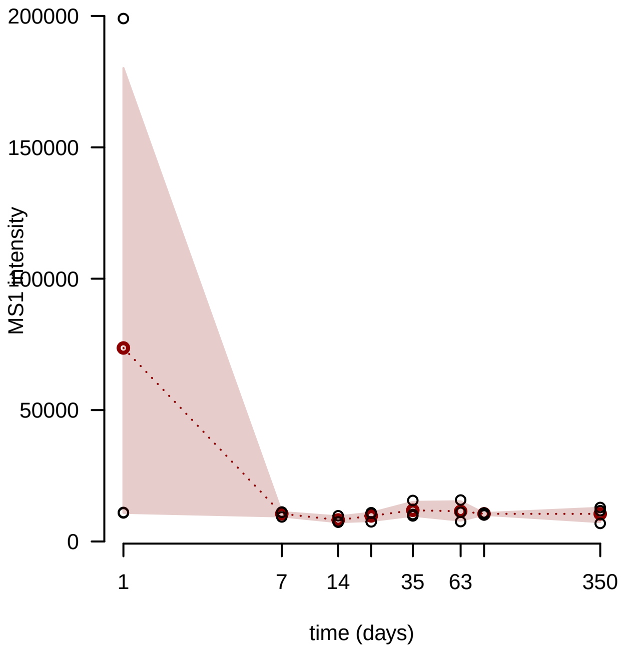

Q99JH1
GN=Rpp25l
ribonuclease P/MRP 25 subunit−like

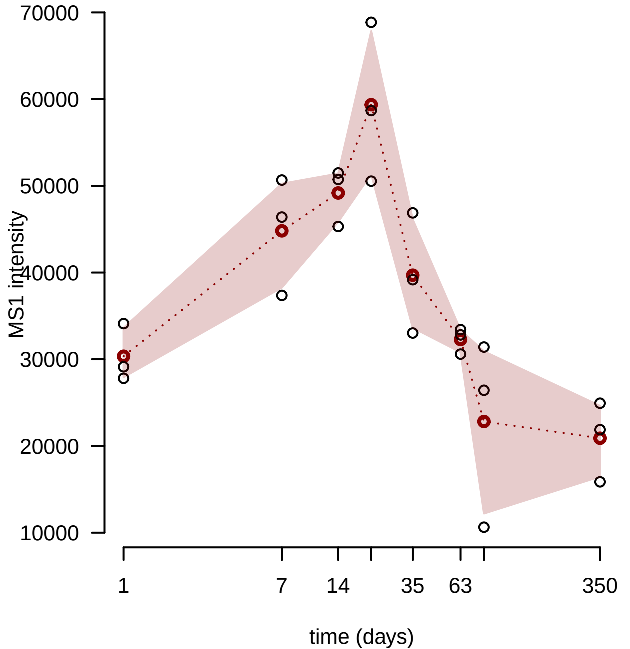

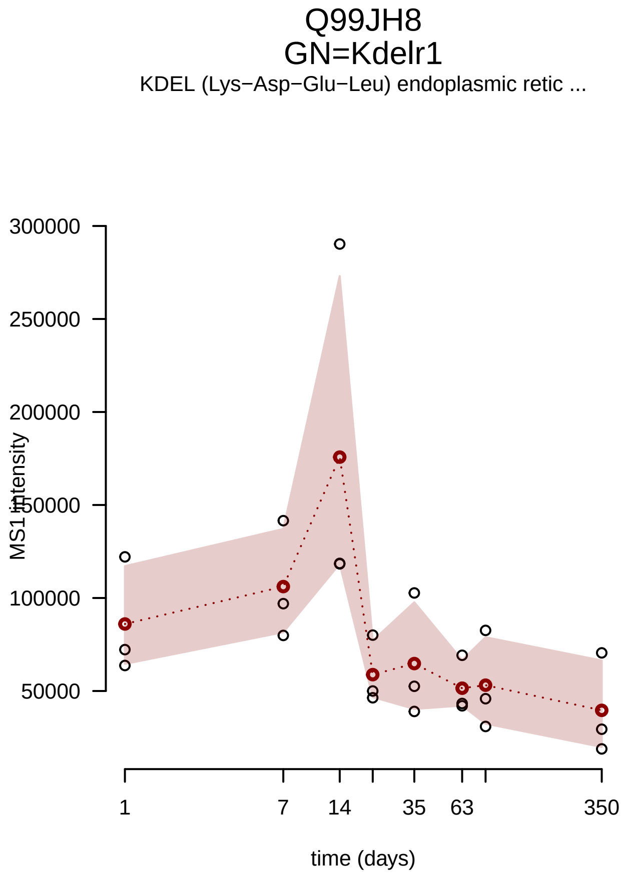
Q99JH8
GN=Kdelr1
KDEL (Lys−Asp−Glu−Leu) endoplasmic retic ...
MS1 intensity
300000
250000
200000
150000
100000
50000
1
7
14
35
63
350
time (days)

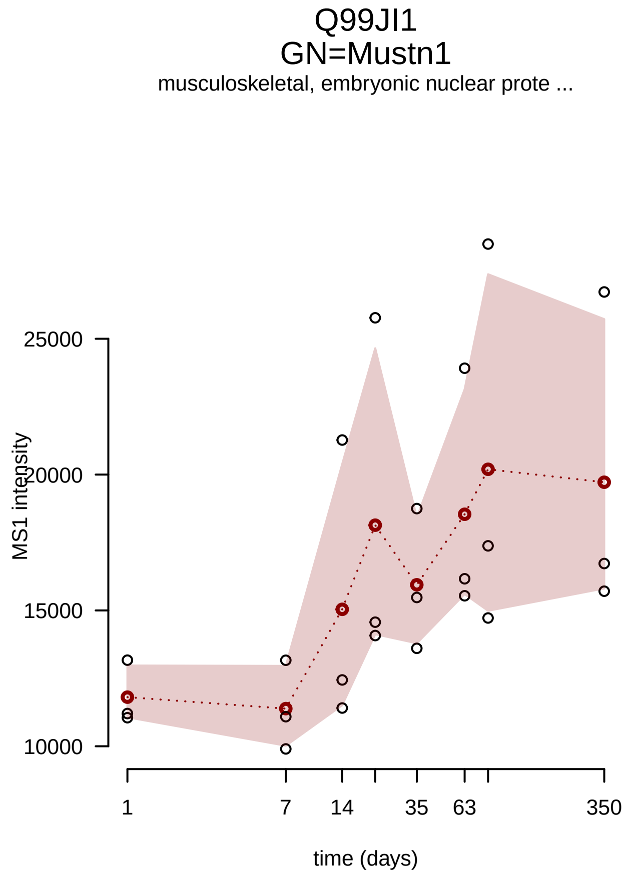
Q99JI1
GN=Mustn1
musculoskeletal, embryonic nuclear prote ...
MS1 intensity
25000
20000
15000
10000
1
7
14
35
63
350
time (days)

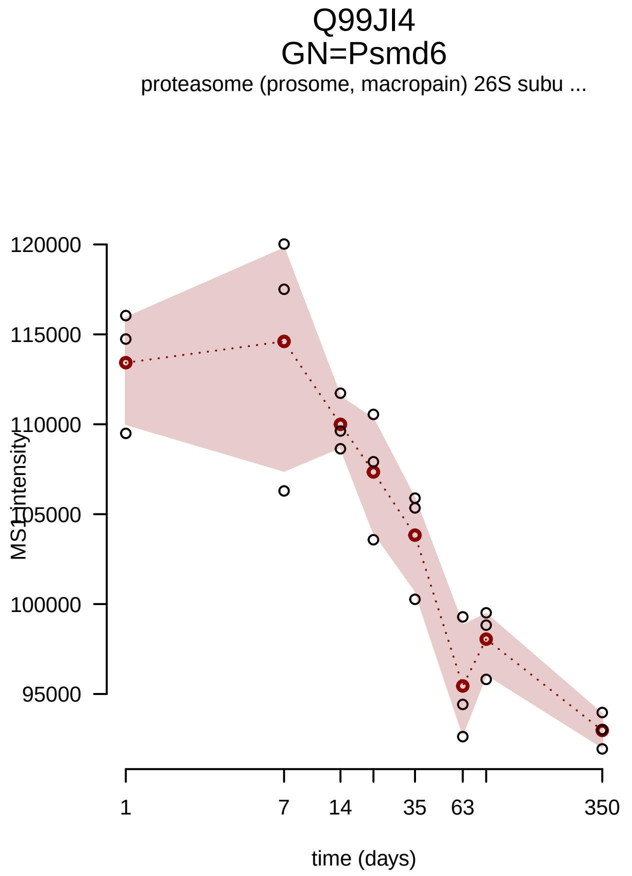
Q99JI4
GN=Psmd6
proteasome (prosome, macropain) 26S subu ...
MS1 intensity
120000
115000
110000
105000
100000
95000
1
7
14
35
63
350
time (days)

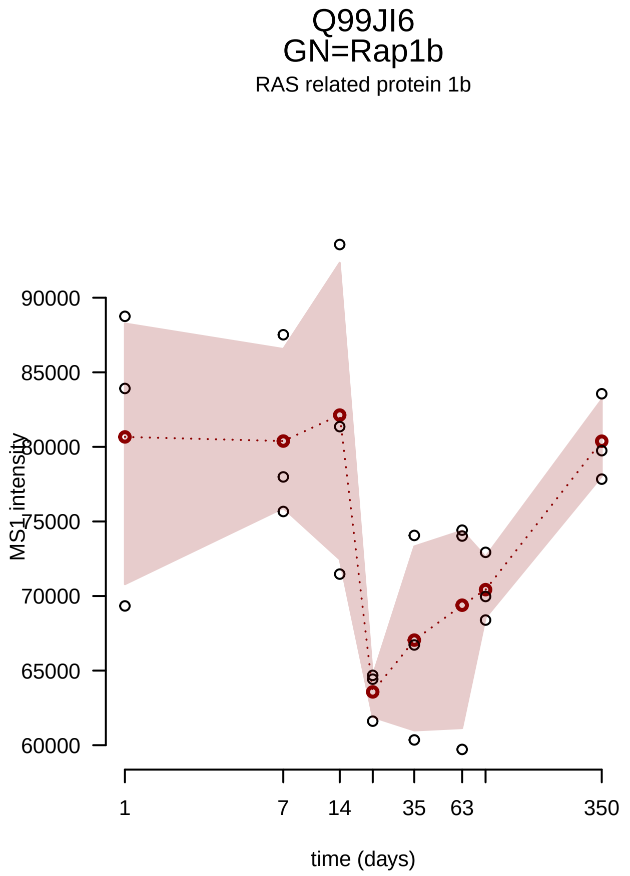
Q99JI6
GN=Rap1b
RAS related protein 1b
MS1 intensity
90000
85000
80000
75000
70000
65000
60000
1
7
14
35
63
350
time (days)

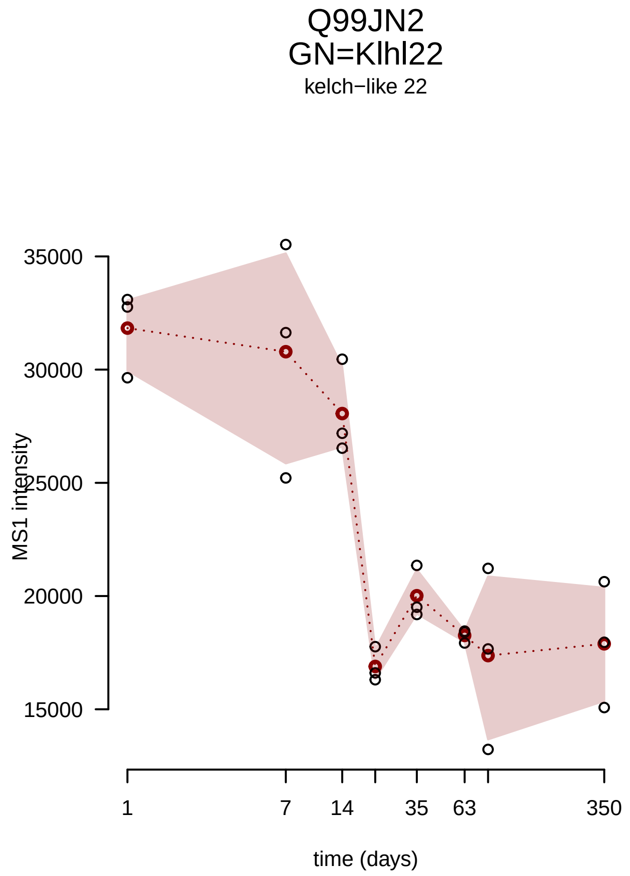
Q99JN2
GN=Klhl22
kelch−like 22
MS1 intensity
35000
30000
25000
20000
15000
1
7
14
35
63
350
time (days)

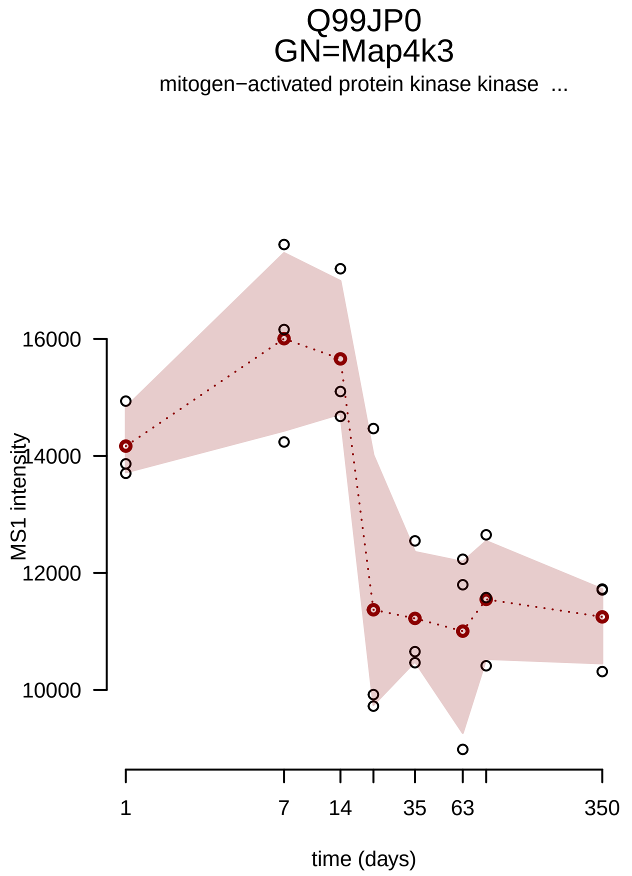
Q99JP0
GN=Map4k3
mitogen−activated protein kinase kinase ...
MS1 intensity
16000
14000
12000
10000
1
7
14
35
63
350
time (days)

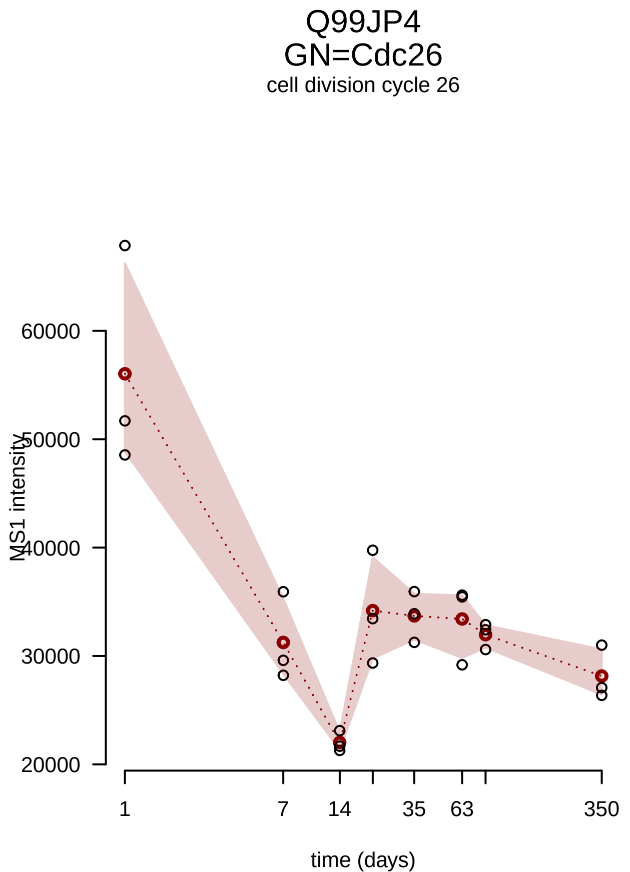
Q99JP4
GN=Cdc26
cell division cycle 26
MS1 intensity
60000
50000
40000
30000
20000
1
7
14
35
63
350
time (days)

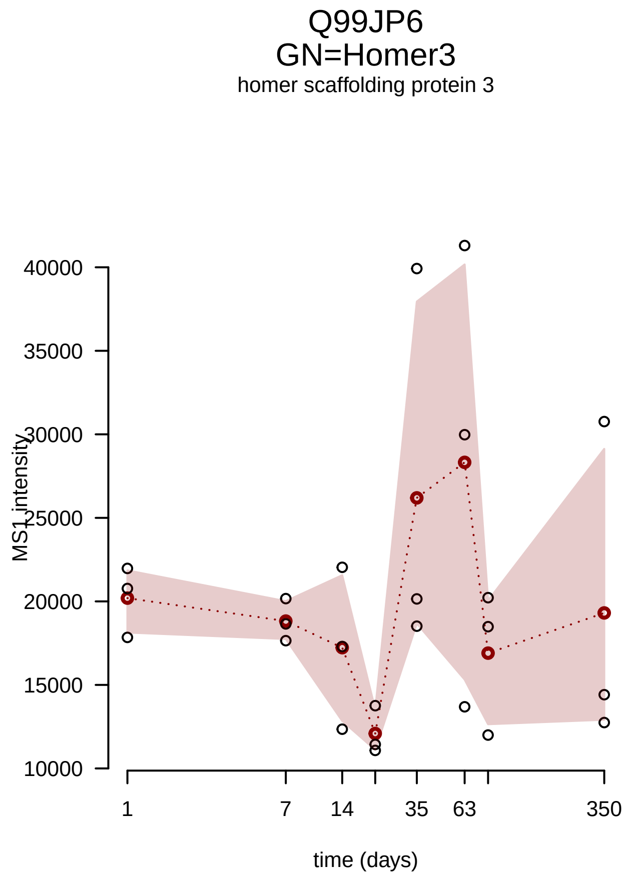
Q99JP6
GN=Homer3
homer scaffolding protein 3
MS1 intensity
40000
35000
30000
25000
20000
15000
10000
1
7
14
35
63
350
time (days)

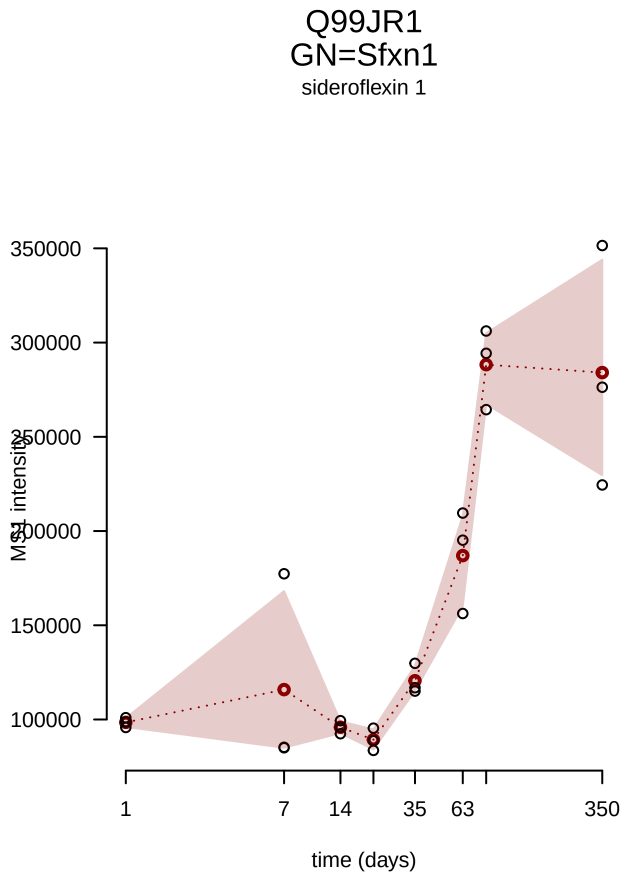
Q99JR1
GN=Sfxn1
sideroflexin 1
MS1 intensity
350000
300000
250000
200000
150000
100000
1
7
14
35
63
350
time (days)

Q99JR5
GN=Tinagl1
tubulointerstitial nephritis antigen−lik ...

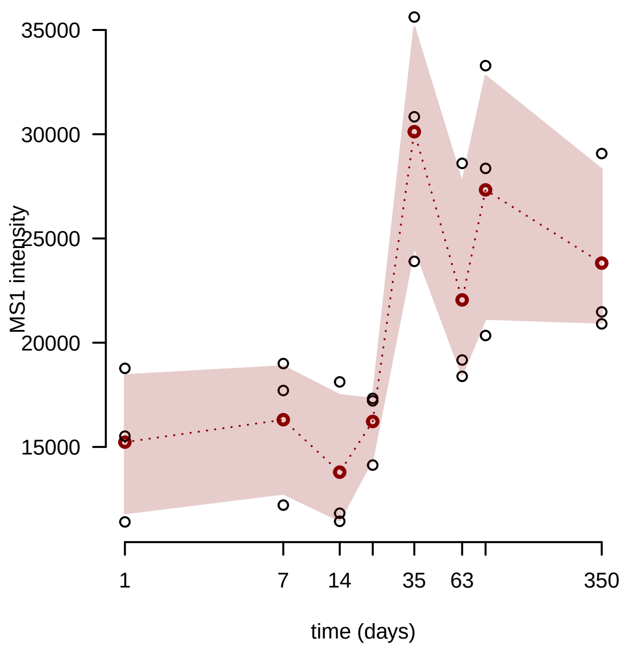

Q99JR6
GN=Nmnat3
nicotinamide nucleotide adenylyltransfer ...

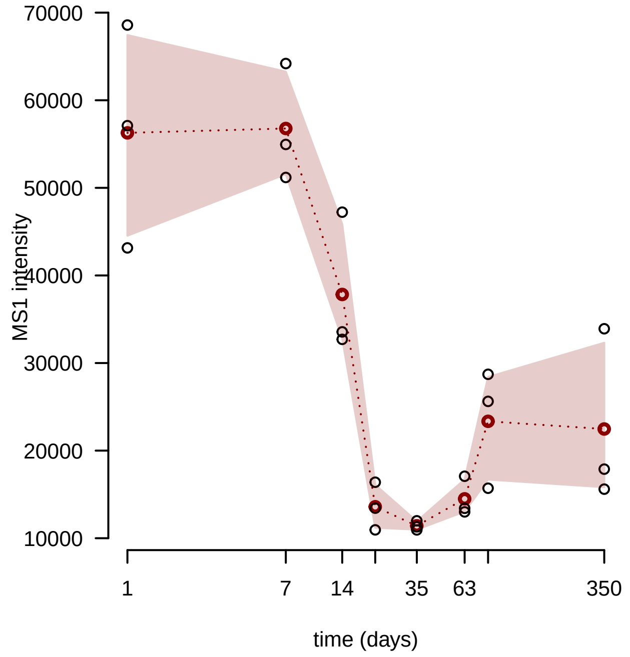

Q99JR8
GN=Smarcd2
SWI/SNF related, matrix associated, acti ...

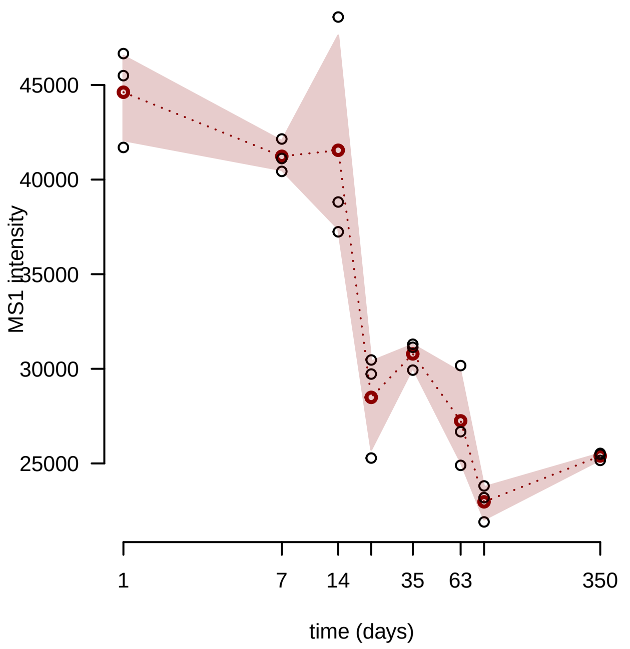

Q99JT1
GN=Gatb
glutamyl−tRNA(Gln) amidotransferase, sub ...

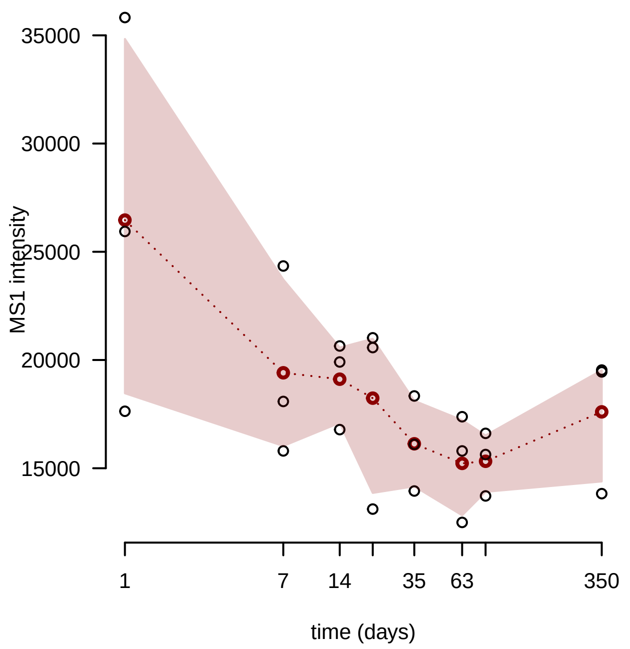

Q99JT2
GN=Stk26
serine/threonine kinase 26

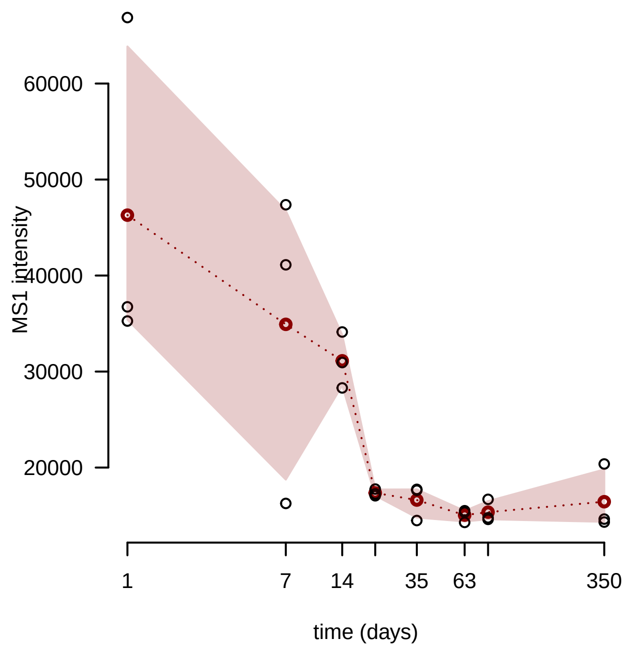

Q99JT6
GN=Tlcd1
TLC domain containing 1

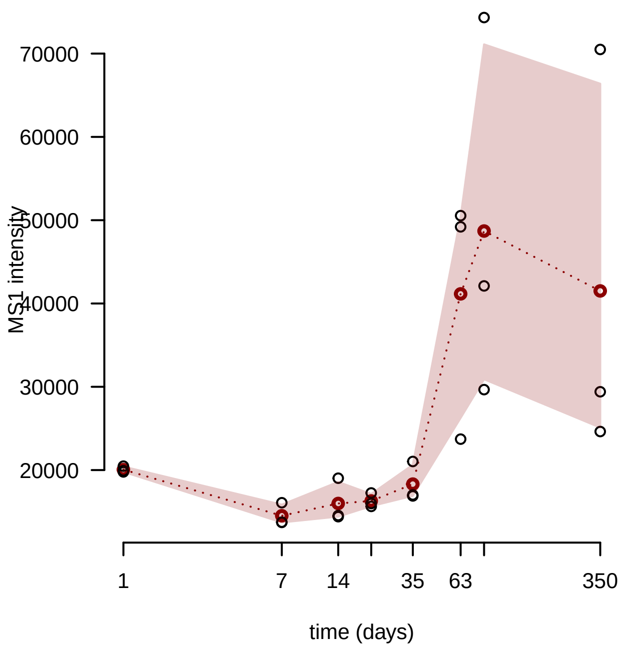

Q99JT9
GN=Adi1
acireductone dioxygenase 1

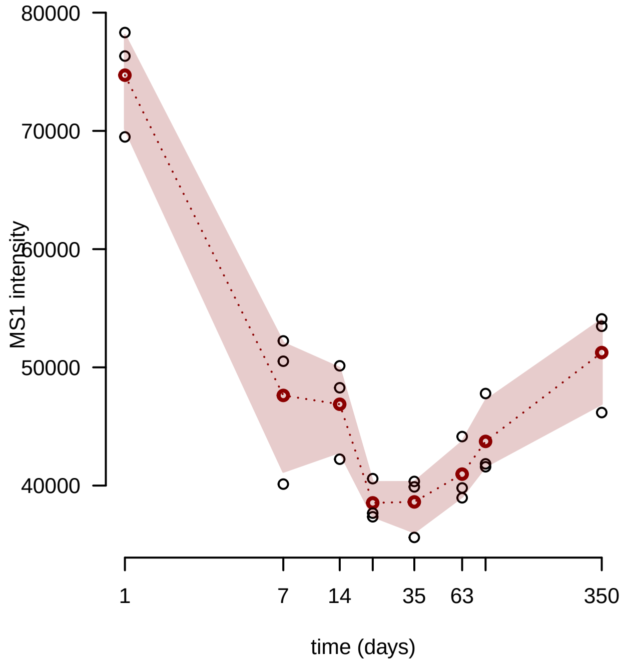

Q99JV5
GN=Stard4
StAR−related lipid transfer (START) doma ...

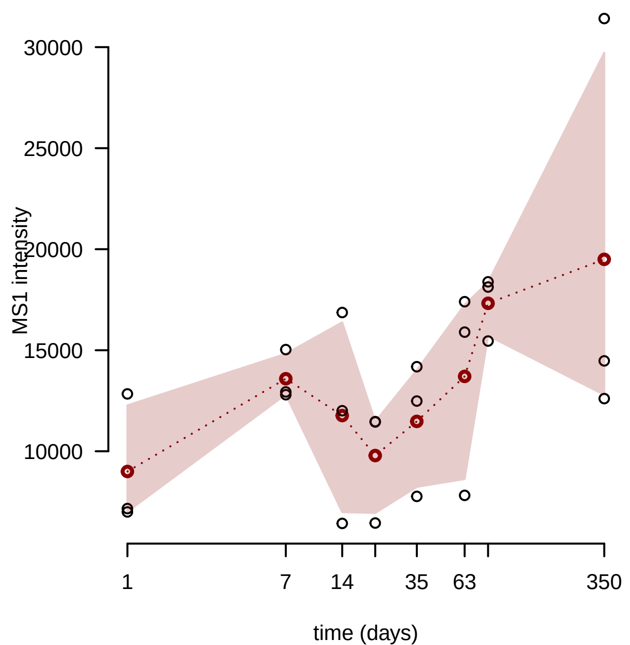

Q99JW2
GN=Acy1
aminoacylase 1

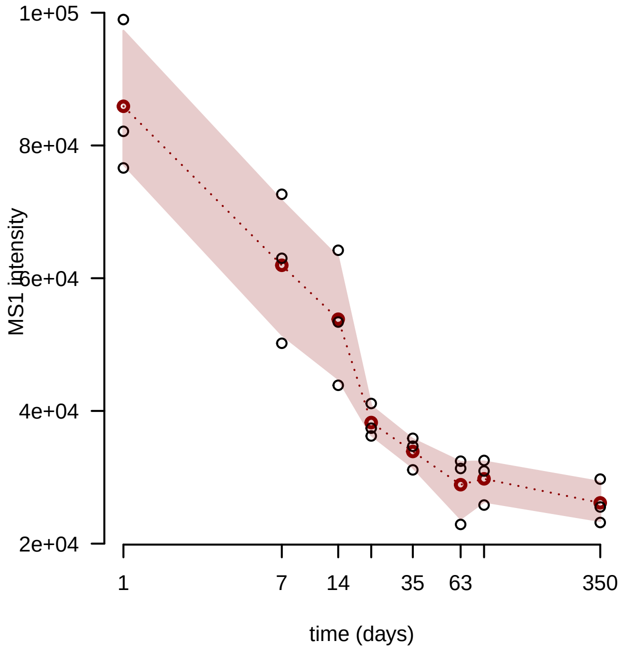

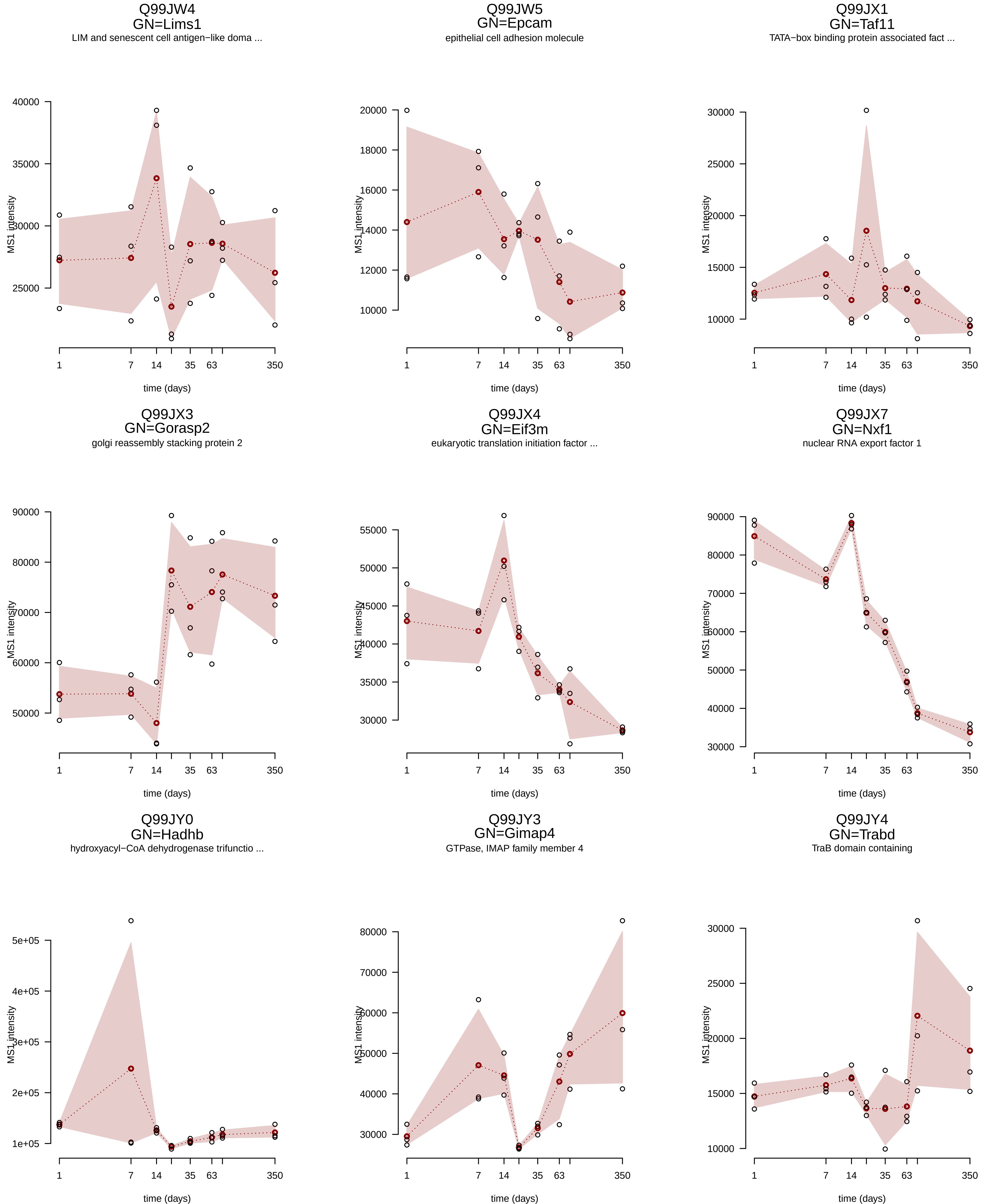
Q99JW4
GN=Lims1
LIM and senescent cell antigen−like doma ...
Q99JW5
GN=Epcam
epithelial cell adhesion molecule
Q99JX1
GN=Taf11
TATA−box binding protein associated fact ...
Q99JX3
GN=Gorasp2
golgi reassembly stacking protein 2
Q99JX4
GN=Eif3m
eukaryotic translation initiation factor ...
Q99JX7
GN=Nxf1
nuclear RNA export factor 1
Q99JY0
GN=Hadhb
hydroxyacyl−CoA dehydrogenase trifunctio ...
Q99JY3
GN=Gimap4
GTPase, IMAP family member 4
Q99JY4
GN=Trabd
TraB domain containing
MS1 intensity
time (days)
1
7
14
35
63
350

Q99JY8
GN=Plpp3
phospholipid phosphatase 3

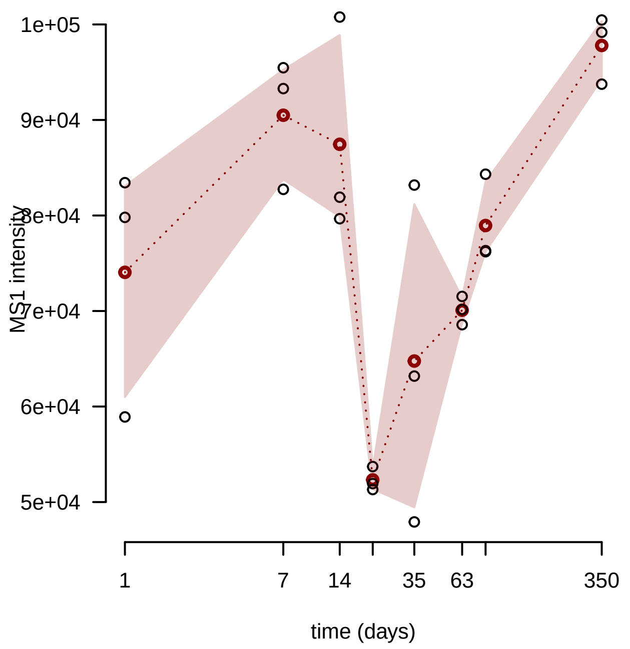

Q99JY9
GN=Actr3
ARP3 actin−related protein 3

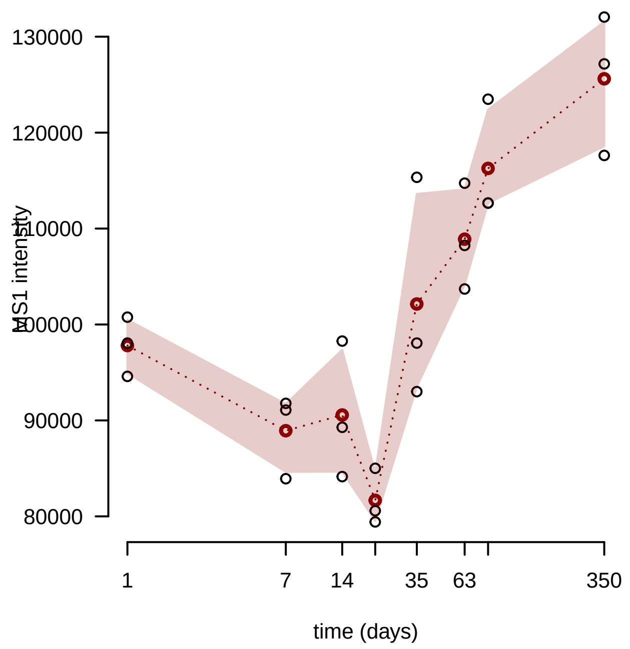

Q99K01
GN=Pdxdc1
pyridoxal−dependent decarboxylase domain ...

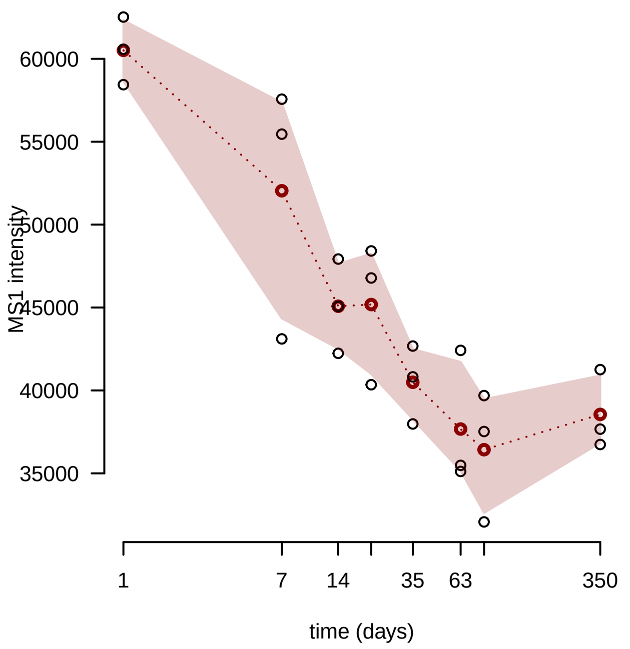

Q99K01
GN=Pdxdc1
pyridoxal−dependent decarboxylase domain ...

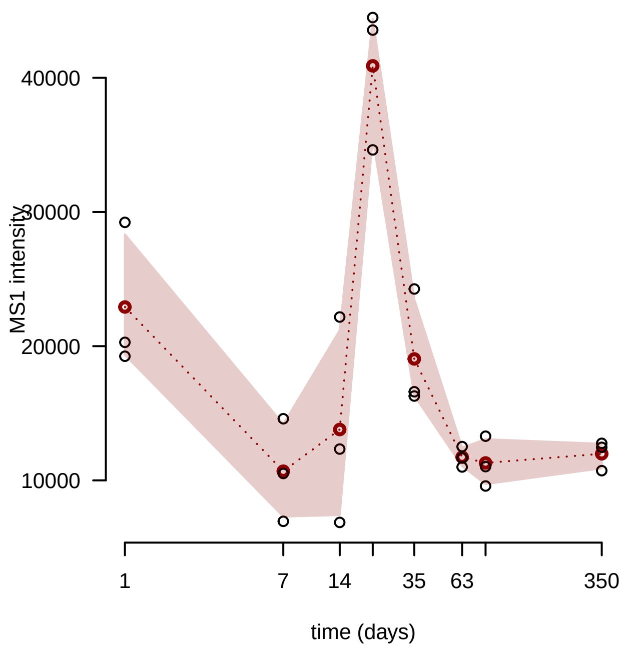

Q99K23
GN=Ufsp2
UFM1−specific peptidase 2

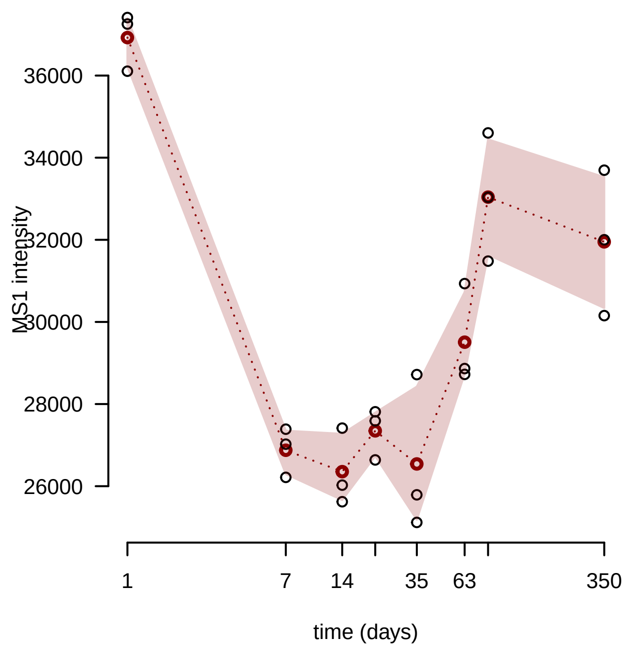

Q99K24
GN=Slc39a3
solute carrier family 39 (zinc transport ...

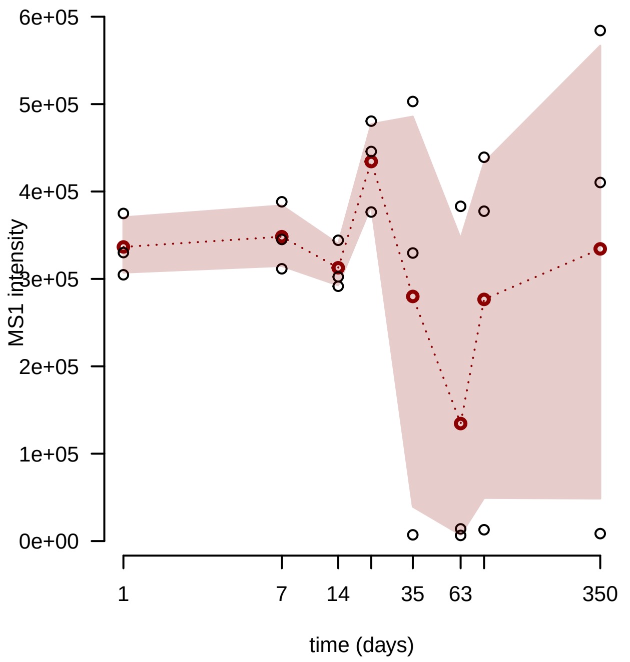

Q99K28
GN=Arfgap2
ADP−ribosylation factor GTPase activatin ...

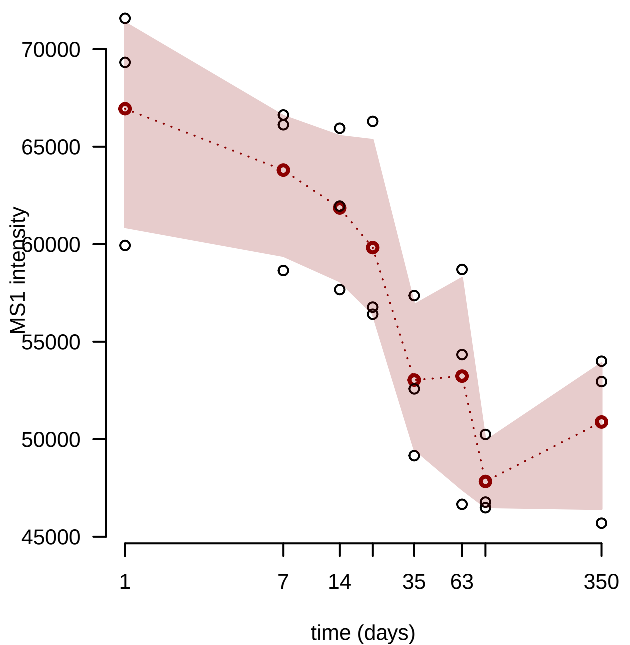

Q99K30
GN=Eps8l2
EPS8−like 2

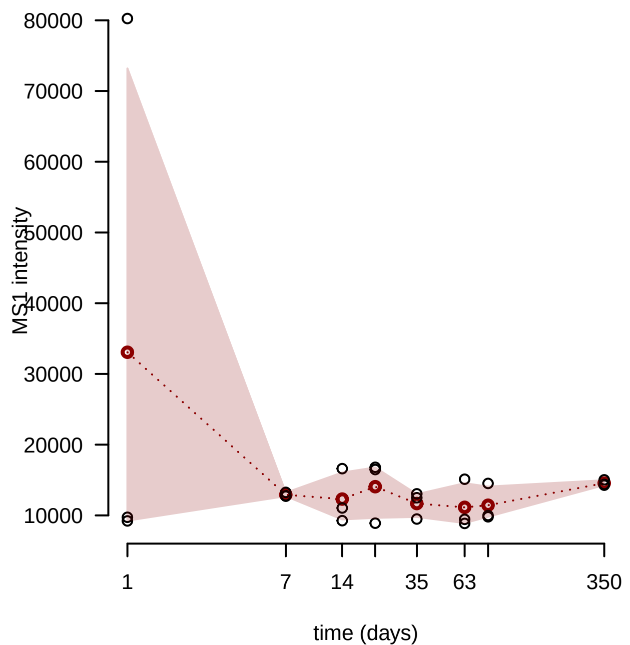

Q99K41
GN=Emilin1
elastin microfibril interfacer 1

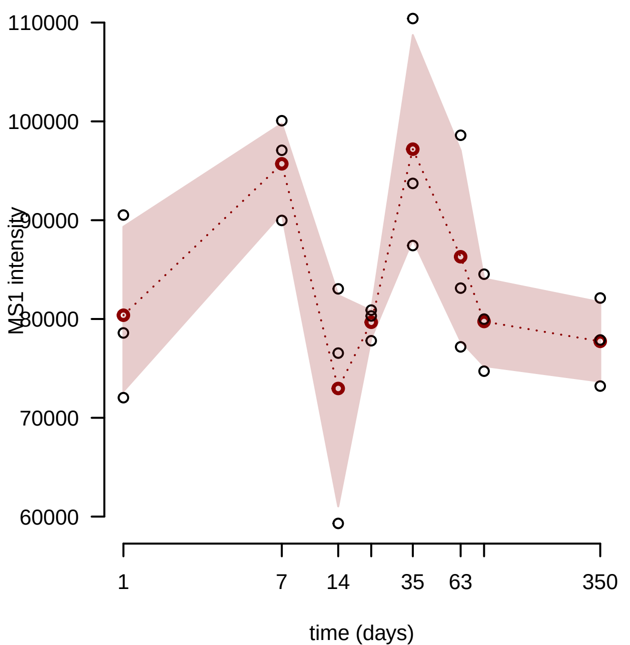

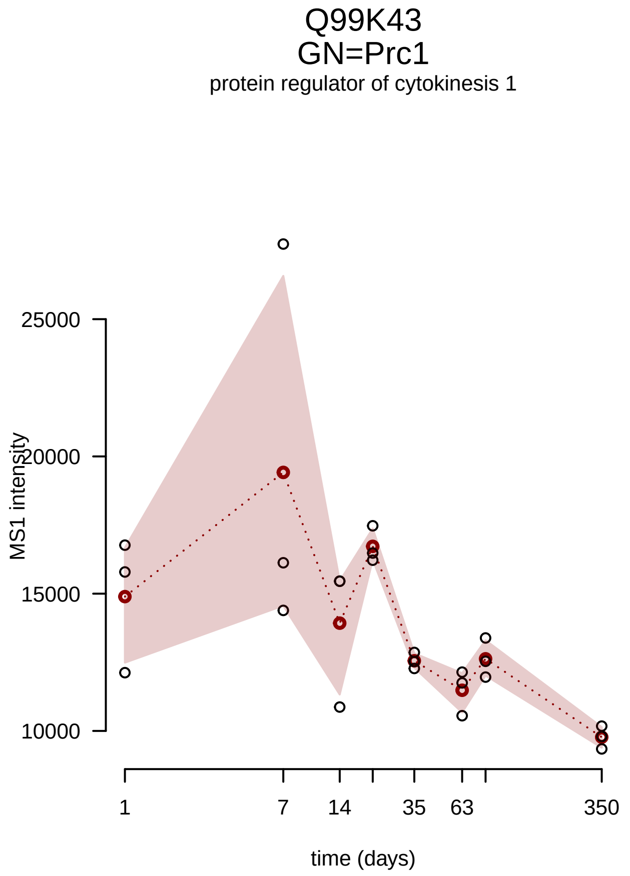
Q99K43
GN=Prc1
protein regulator of cytokinesis 1
MS1 intensity
10000
15000
20000
25000
1
7
14
35
63
350
time (days)
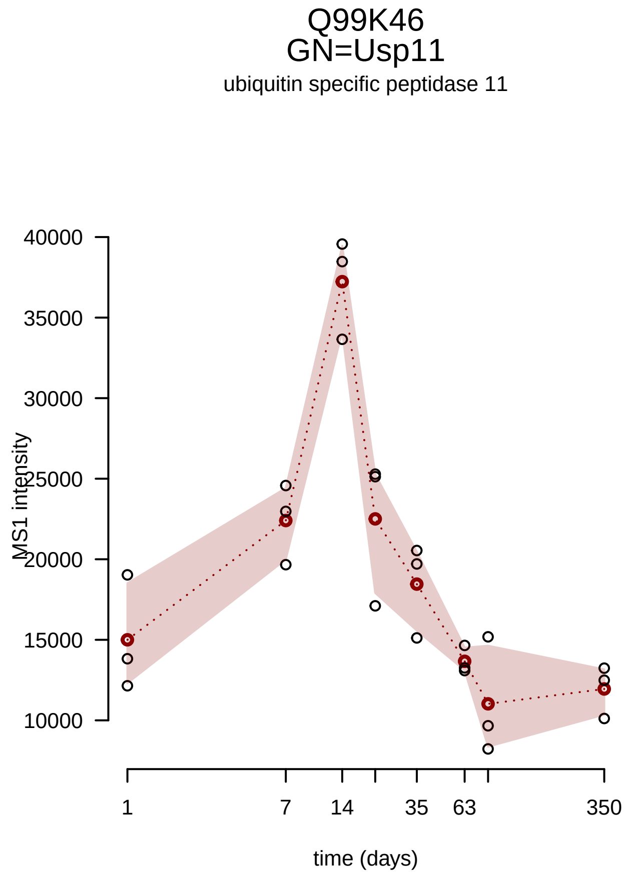
Q99K46
GN=Usp11
ubiquitin specific peptidase 11
MS1 intensity
10000
15000
20000
25000
30000
35000
40000
1
7
14
35
63
350
time (days)
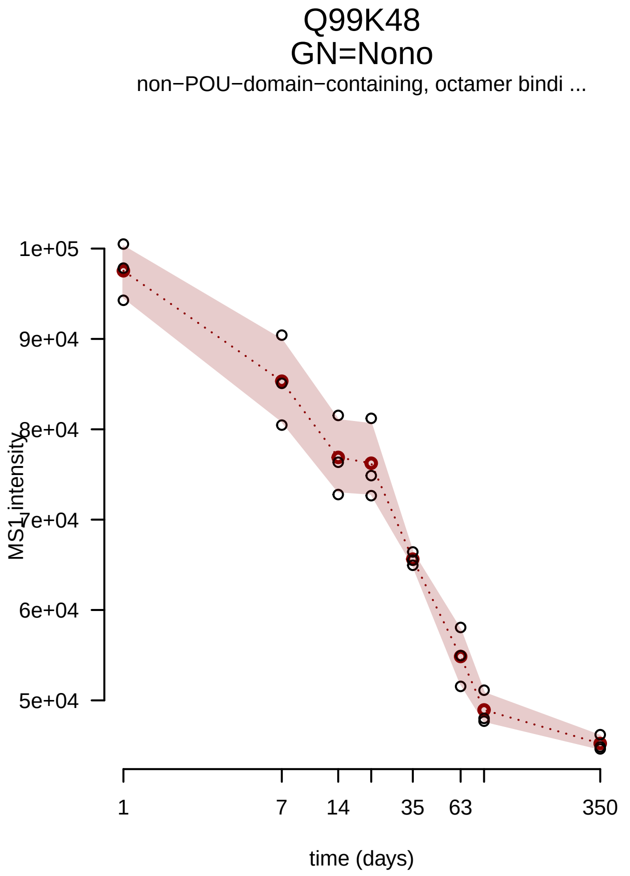
Q99K48
GN=Nono
non−POU−domain−containing, octamer bindi ...
MS1 intensity
5e+04
6e+04
7e+04
8e+04
9e+04
1e+05
1
7
14
35
63
350
time (days)
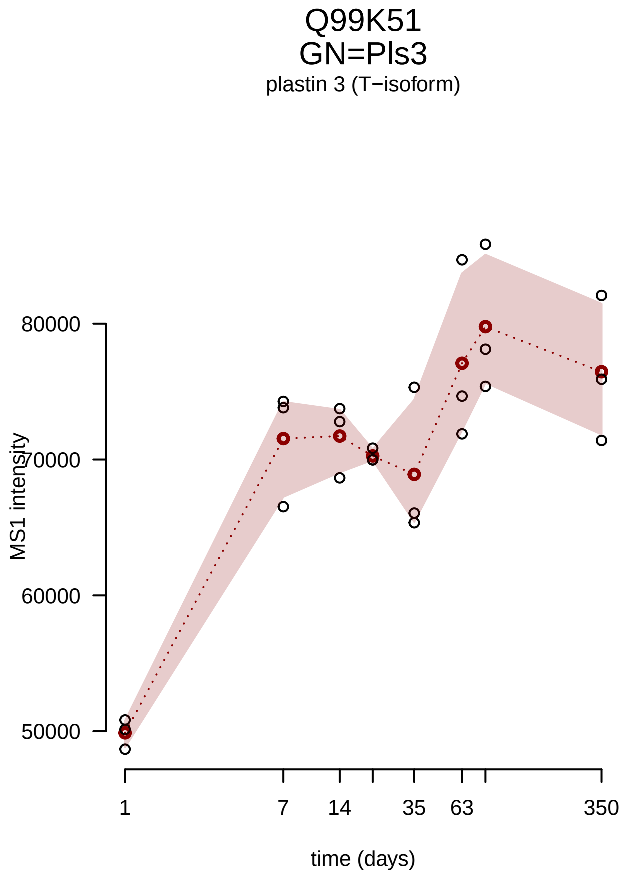
Q99K51
GN=Pls3
plastin 3 (T−isoform)
MS1 intensity
50000
60000
70000
80000
1
7
14
35
63
350
time (days)
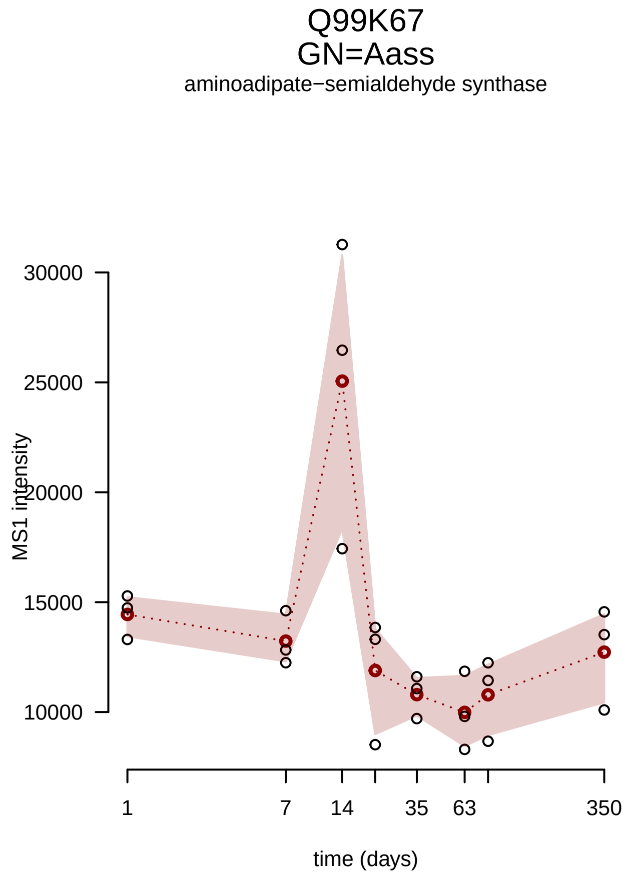
Q99K67
GN=Aass
aminoadipate−semialdehyde synthase
MS1 intensity
10000
15000
20000
25000
30000
1
7
14
35
63
350
time (days)
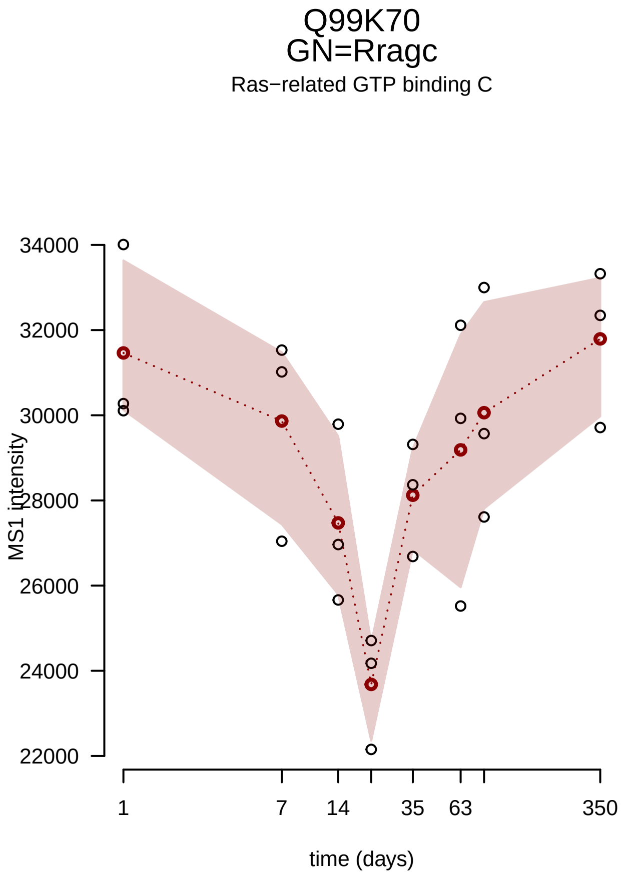
Q99K70
GN=Rragc
Ras−related GTP binding C
MS1 intensity
22000
24000
26000
28000
30000
32000
34000
1
7
14
35
63
350
time (days)
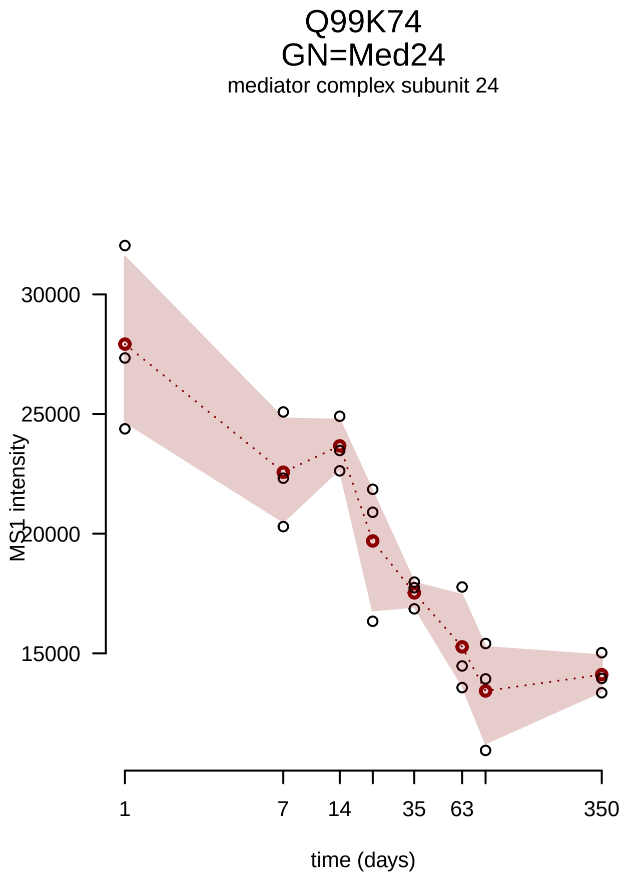
Q99K74
GN=Med24
mediator complex subunit 24
MS1 intensity
15000
20000
25000
30000
1
7
14
35
63
350
time (days)
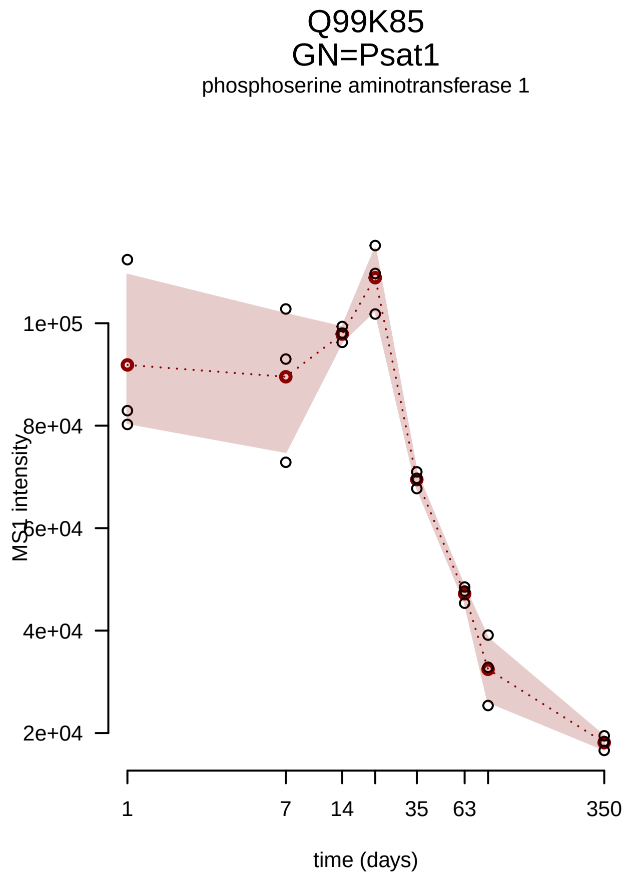
Q99K85
GN=Psat1
phosphoserine aminotransferase 1
MS1 intensity
2e+04
4e+04
6e+04
8e+04
1e+05
1
7
14
35
63
350
time (days)
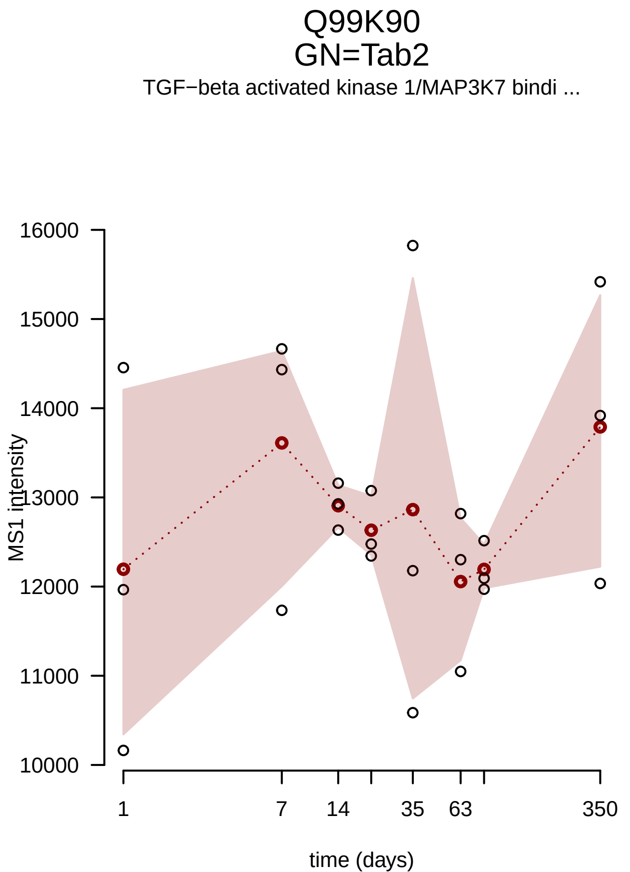
Q99K90
GN=Tab2
TGF−beta activated kinase 1/MAP3K7 bindi ...
MS1 intensity
10000
11000
12000
13000
14000
15000
16000
1
7
14
35
63
350
time (days)

Q99K95
GN=Rtf2
replication termination factor 2

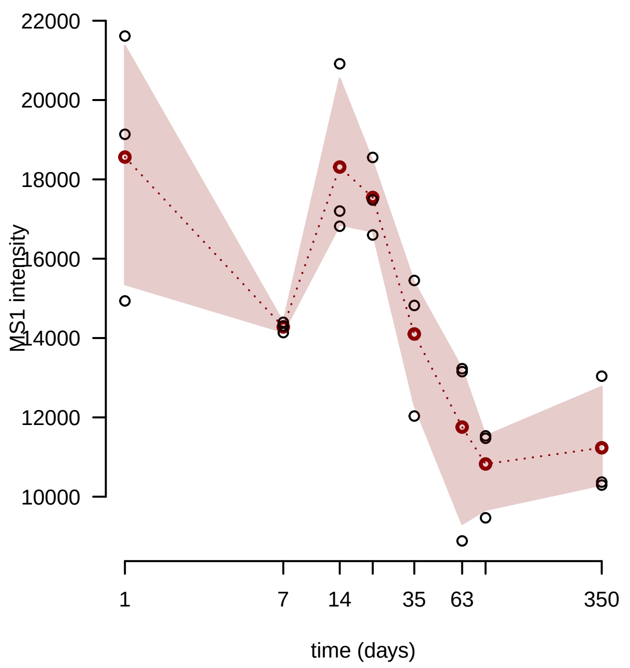

Q99KB8
GN=Hagh
hydroxyacyl glutathione hydrolase

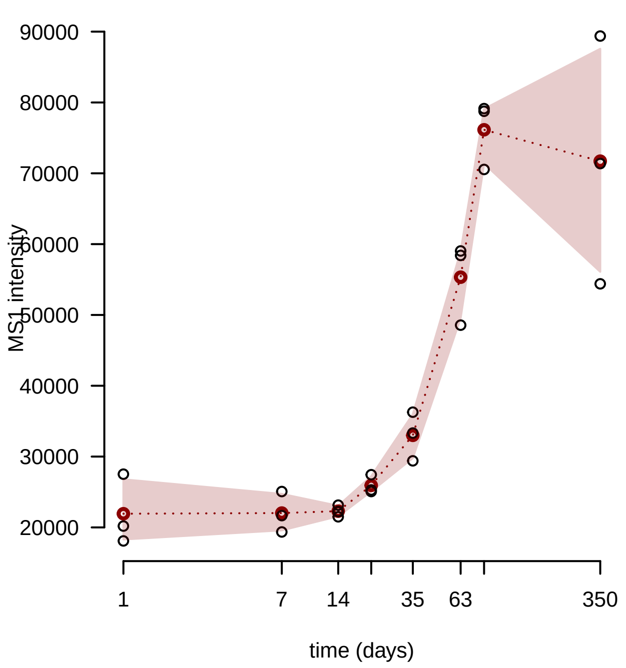

Q99KC8
GN=Vwa5a
von Willebrand factor A domain containin ...

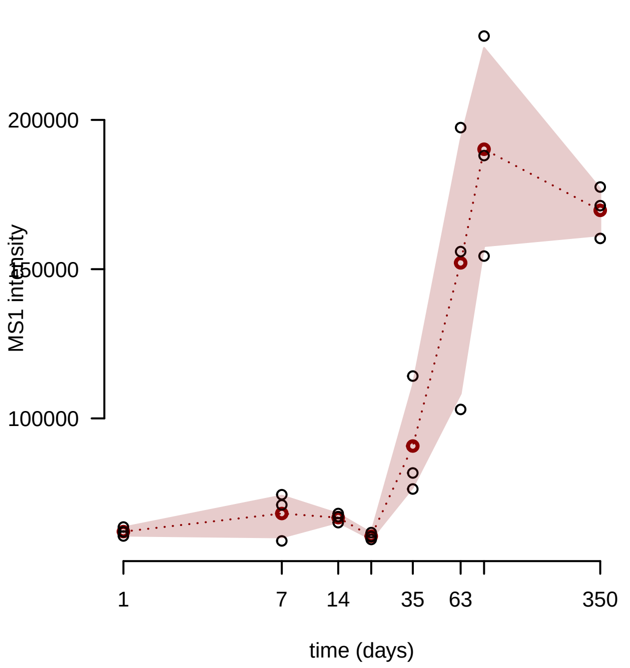

Q99KD5
GN=Unc45a
unc−45 myosin chaperone A

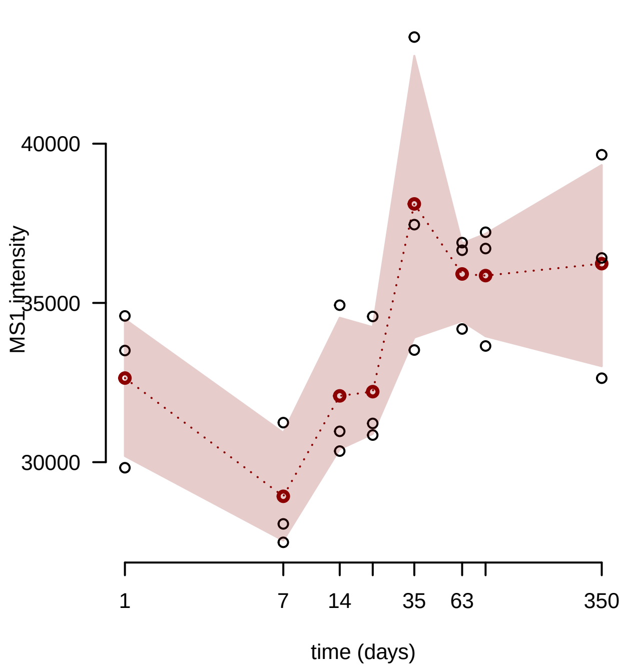

Q99KE1
GN=Me2
malic enzyme 2, NAD(+)−dependent, mitoch ...

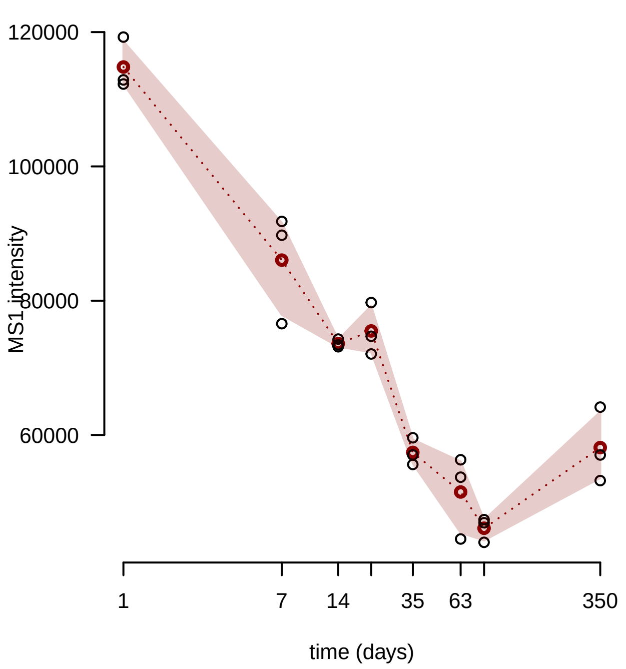

Q99KF1
GN=Tmed9
transmembrane p24 trafficking protein 9

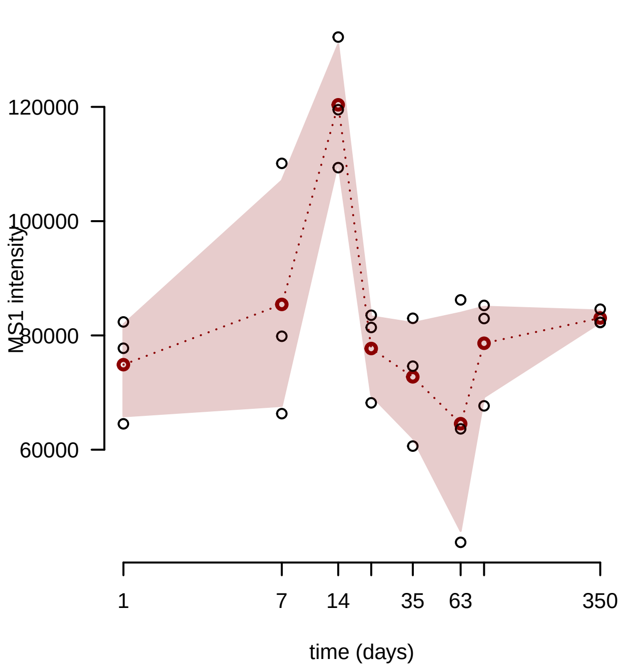

Q99KG3
GN=Rbm10
RNA binding motif protein 10

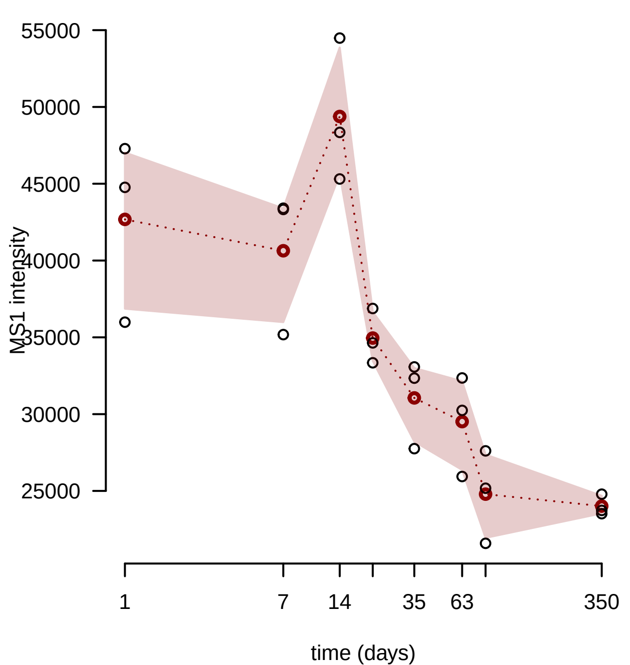

Q99KG3
GN=Rbm10
RNA binding motif protein 10

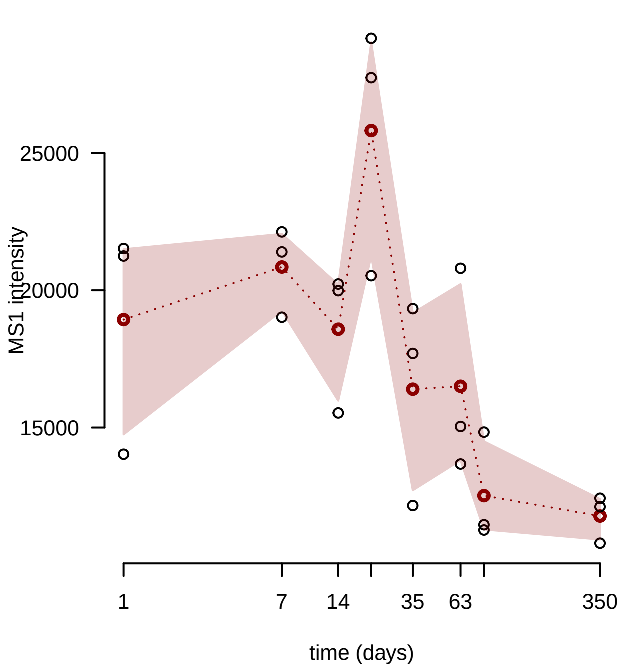

Q99KG5
GN=Lsr
lipolysis stimulated lipoprotein recepto ...

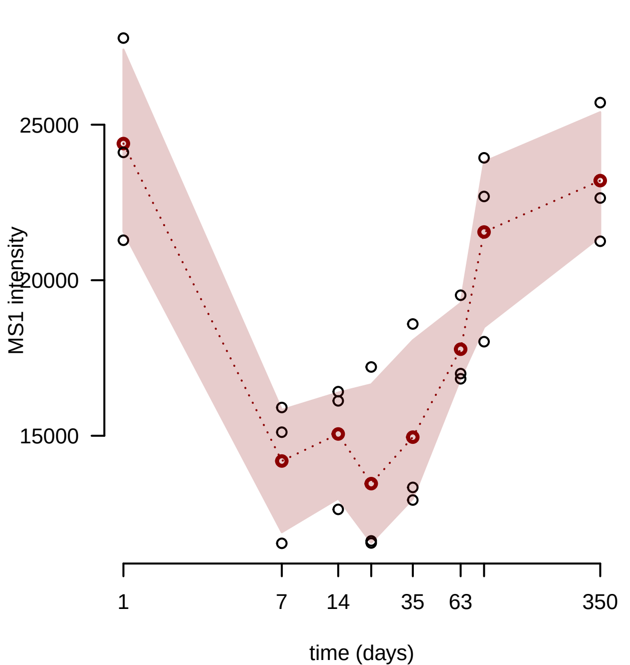

Q99KG7
GN=Hps4
HPS4, biogenesis of lysosomal organelles ...

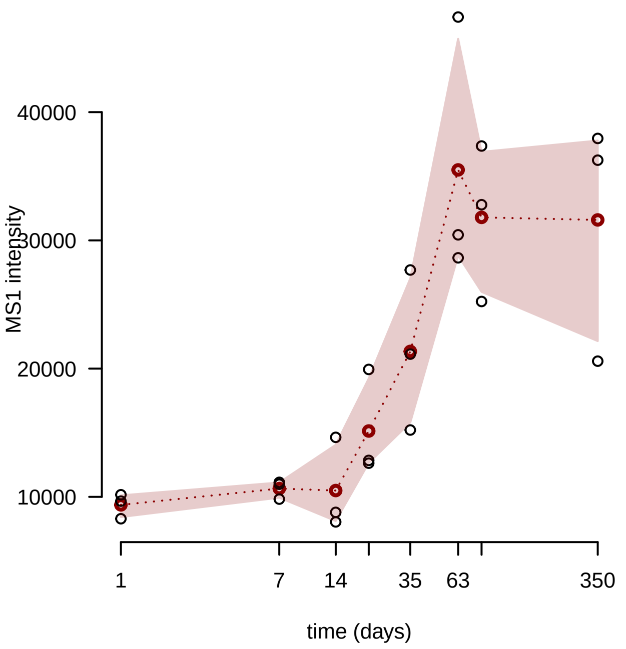

Q99KH8
GN=Stk24
serine/threonine kinase 24

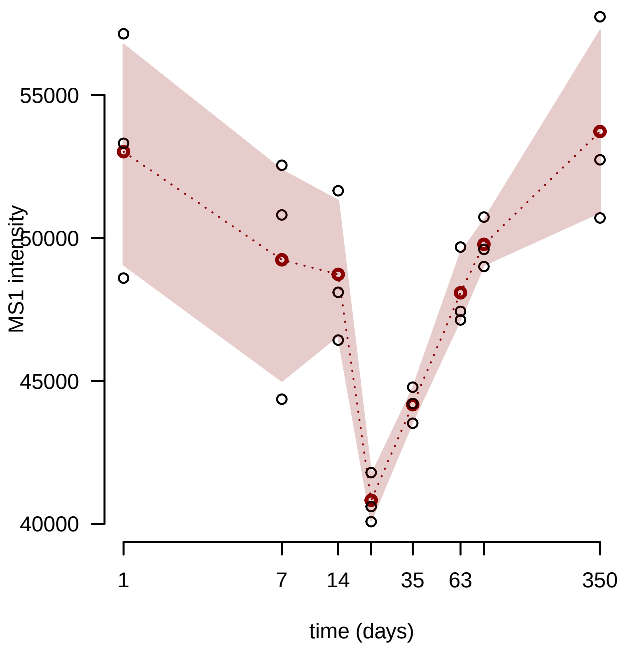

Q99KI0
GN=Aco2
aconitase 2, mitochondrial

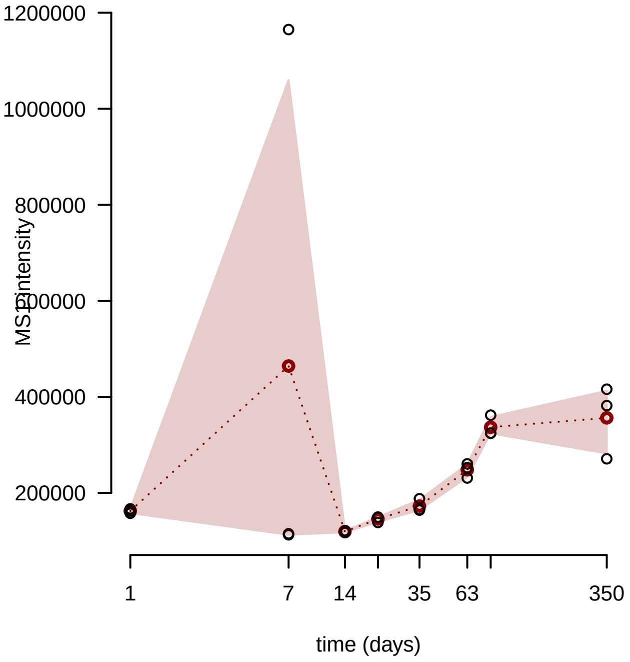

Q99KI3
GN=Emc3
ER membrane protein complex subunit 3

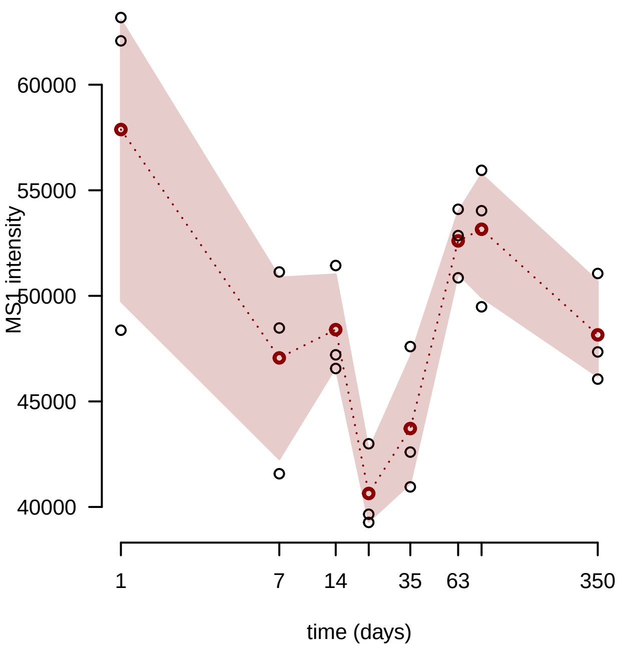

Q99KJ0
GN=Ubxn2a
UBX domain protein 2A

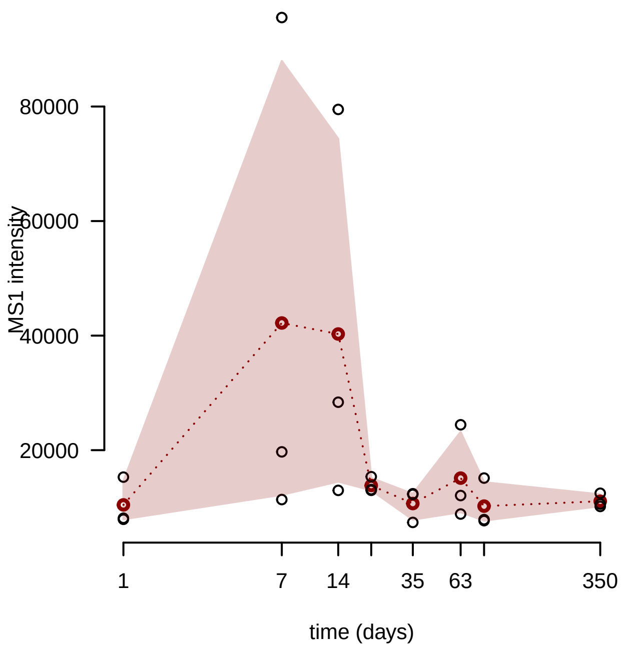

Q99KJ8
GN=Dctn2
dynactin 2

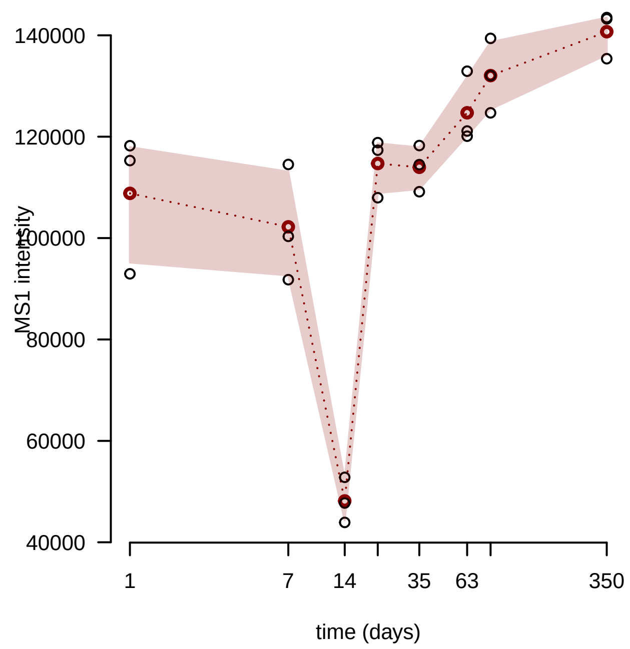

Q99KK1
GN=Reep3
receptor accessory protein 3

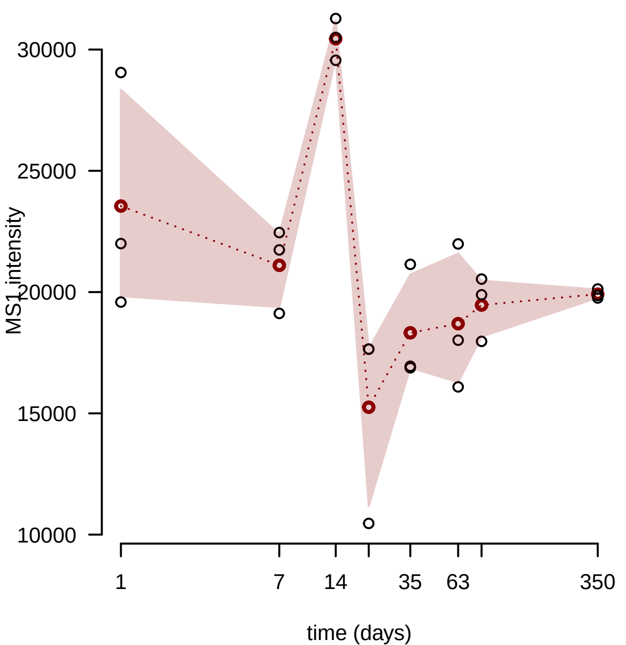

Q99KK2
GN=Cmas
cytidine monophospho−N−acetylneuraminic ...

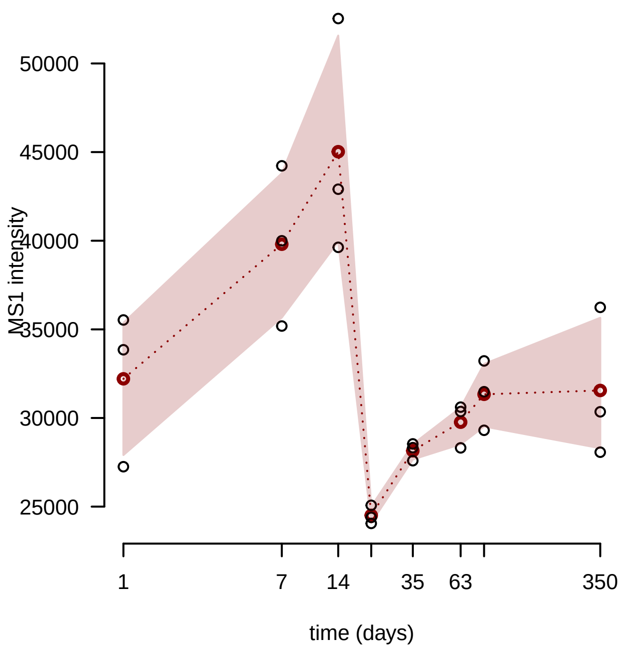

Q99KK7
GN=Dpp3
dipeptidylpeptidase 3

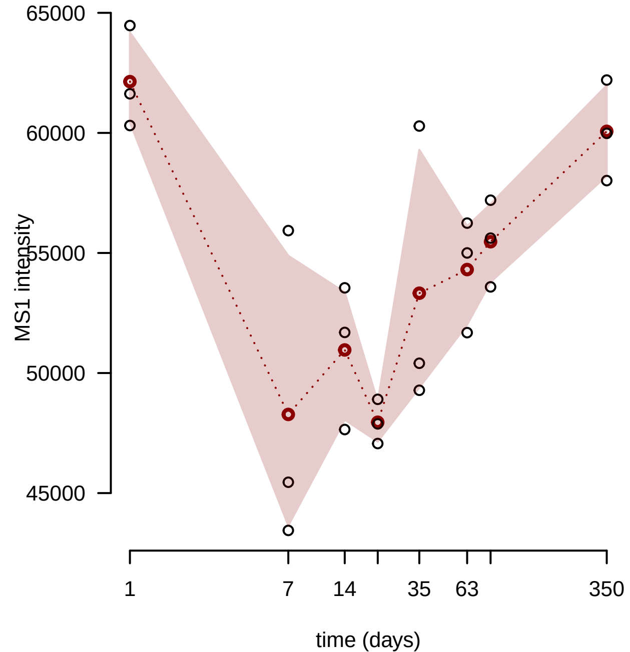

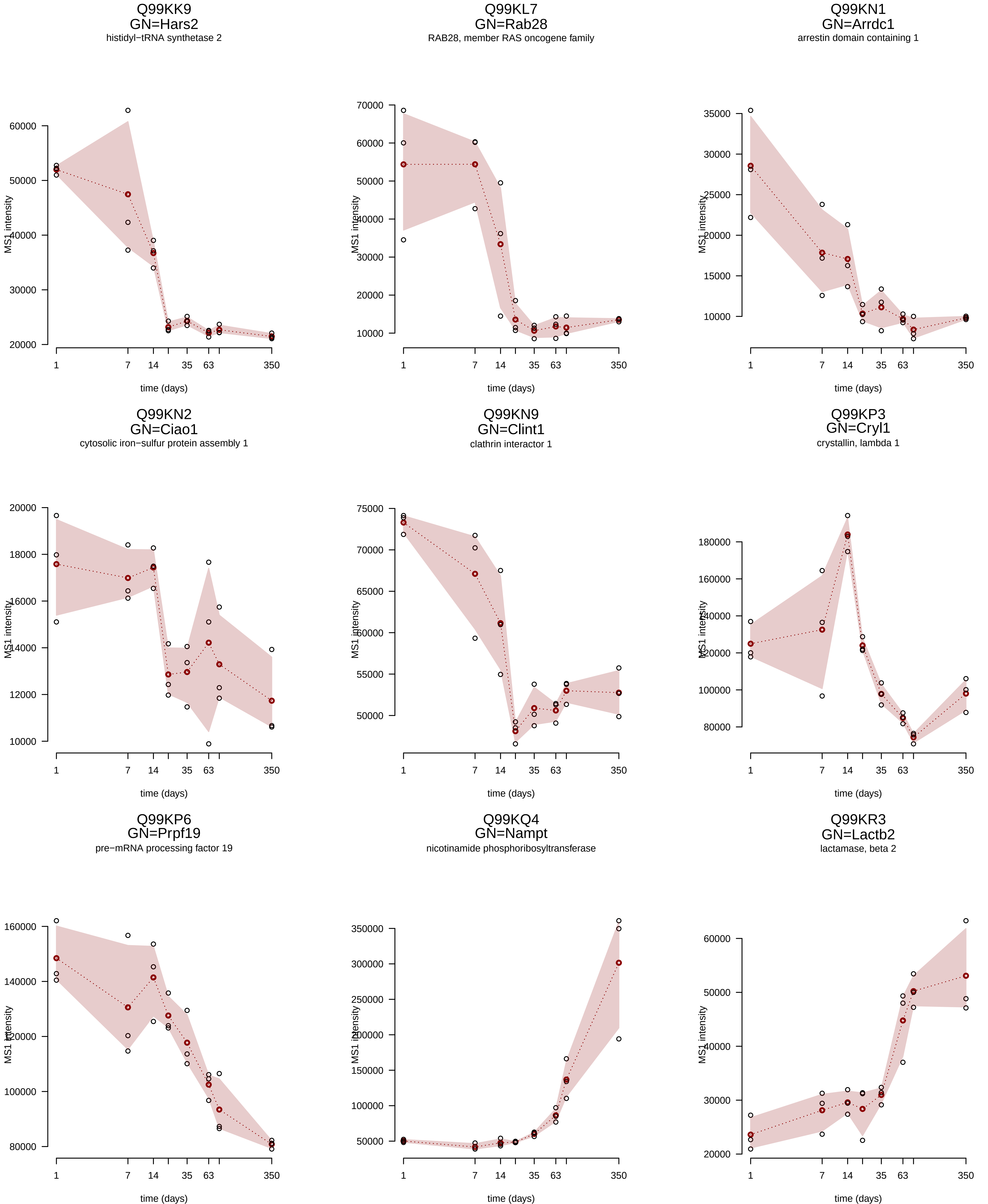
Q99KK9
GN=Hars2
histidyl−tRNA synthetase 2
Q99KL7
GN=Rab28
RAB28, member RAS oncogene family
Q99KN1
GN=Arrdc1
arrestin domain containing 1
Q99KN2
GN=Ciao1
cytosolic iron−sulfur protein assembly 1
Q99KN9
GN=Clint1
clathrin interactor 1
Q99KP3
GN=Cryl1
crystallin, lambda 1
Q99KP6
GN=Prpf19
pre−mRNA processing factor 19
Q99KQ4
GN=Nampt
nicotinamide phosphoribosyltransferase
Q99KR3
GN=Lactb2
lactamase, beta 2
MS1 intensity
time (days)
1
7
14
35
63
350

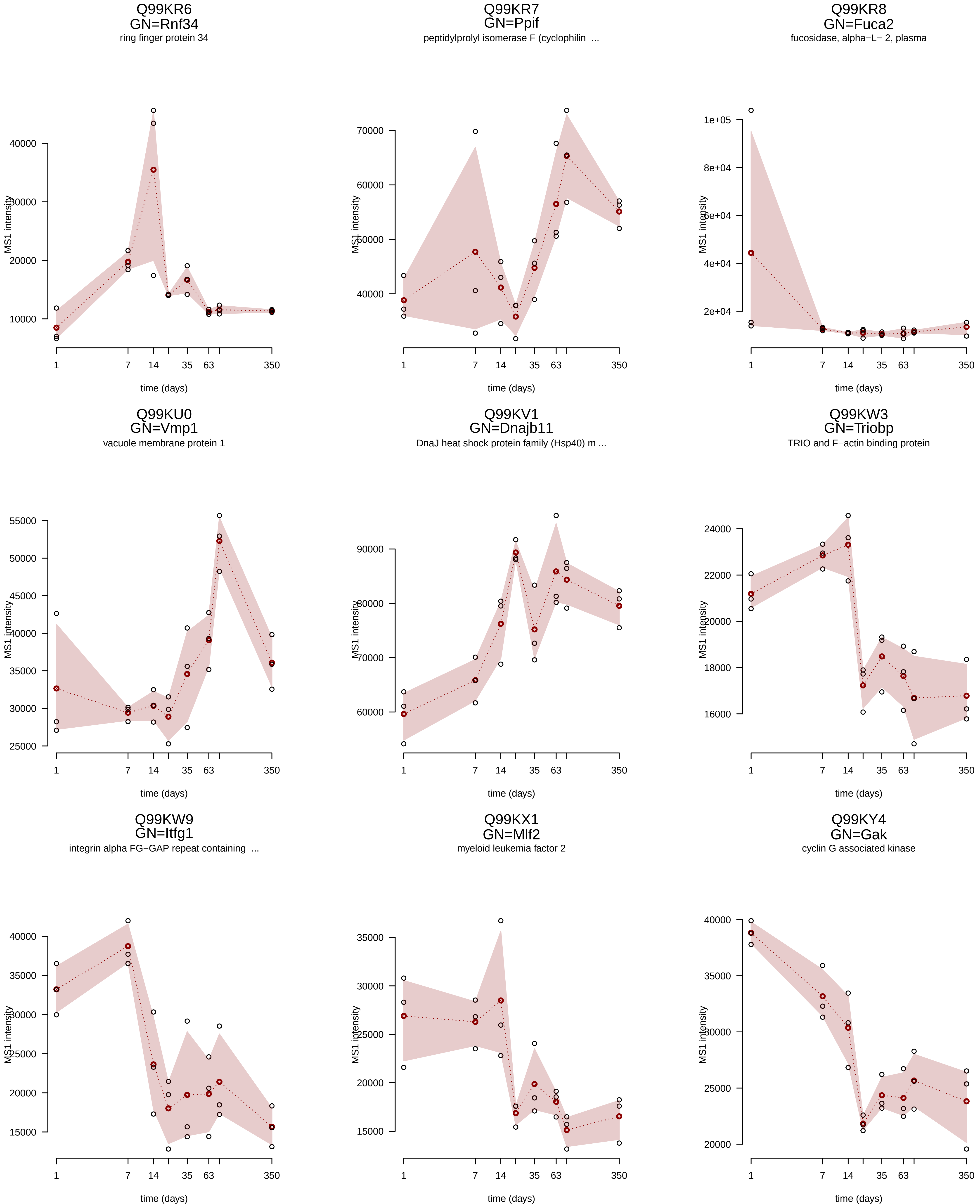
Q99KR6
GN=Rnf34
ring finger protein 34
MS1 intensity
10000
20000
30000
40000
time (days)
1
7
14
35
63
350
Q99KR7
GN=Ppif
peptidylprolyl isomerase F (cyclophilin ...
MS1 intensity
40000
50000
60000
70000
time (days)
1
7
14
35
63
350
Q99KR8
GN=Fuca2
fucosidase, alpha−L− 2, plasma
MS1 intensity
2e+04
4e+04
6e+04
8e+04
1e+05
time (days)
1
7
14
35
63
350
Q99KU0
GN=Vmp1
vacuole membrane protein 1
MS1 intensity
25000
30000
35000
40000
45000
50000
55000
time (days)
1
7
14
35
63
350
Q99KV1
GN=Dnajb11
DnaJ heat shock protein family (Hsp40) m ...
MS1 intensity
60000
70000
80000
90000
time (days)
1
7
14
35
63
350
Q99KW3
GN=Triobp
TRIO and F−actin binding protein
MS1 intensity
16000
18000
20000
22000
24000
time (days)
1
7
14
35
63
350
Q99KW9
GN=Itfg1
integrin alpha FG−GAP repeat containing ...
MS1 intensity
15000
20000
25000
30000
35000
40000
time (days)
1
7
14
35
63
350
Q99KX1
GN=Mlf2
myeloid leukemia factor 2
MS1 intensity
15000
20000
25000
30000
35000
time (days)
1
7
14
35
63
350
Q99KY4
GN=Gak
cyclin G associated kinase
MS1 intensity
20000
25000
30000
35000
40000
time (days)
1
7
14
35
63
350

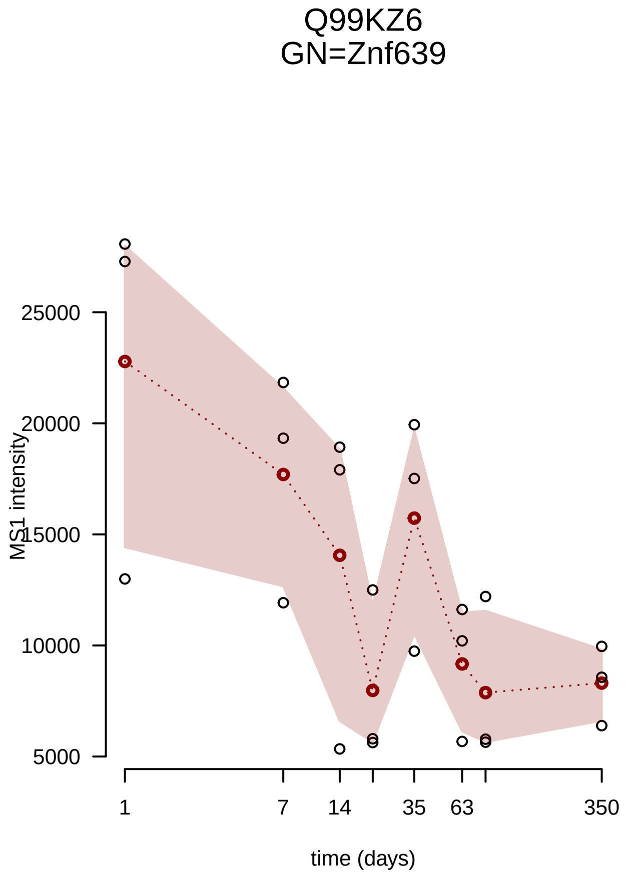
Q99KZ6
GN=Znf639
MS1 intensity
25000
20000
15000
10000
5000
1
7
14
35
63
350
time (days)

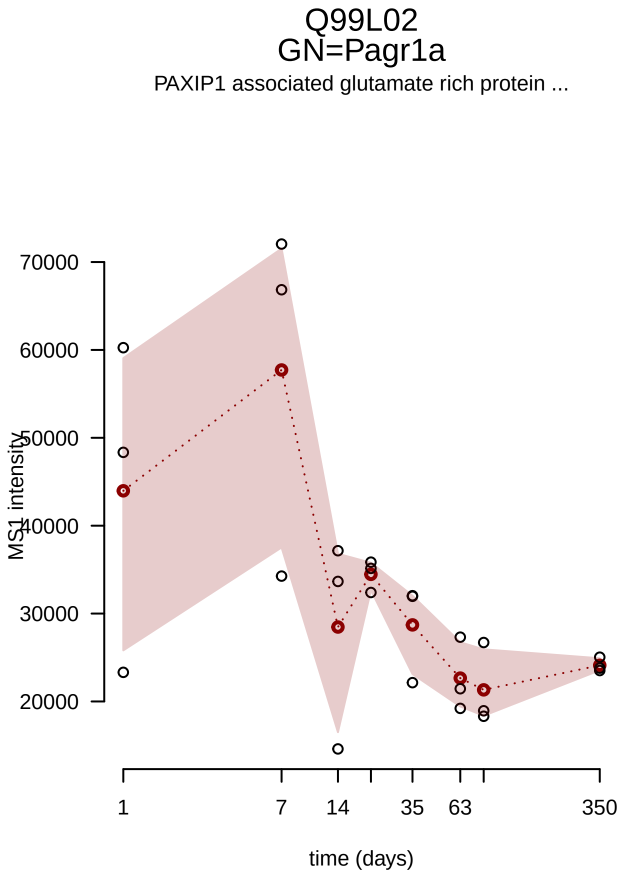
Q99L02
GN=Pagr1a
PAXIP1 associated glutamate rich protein ...
MS1 intensity
70000
60000
50000
40000
30000
20000
1
7
14
35
63
350
time (days)

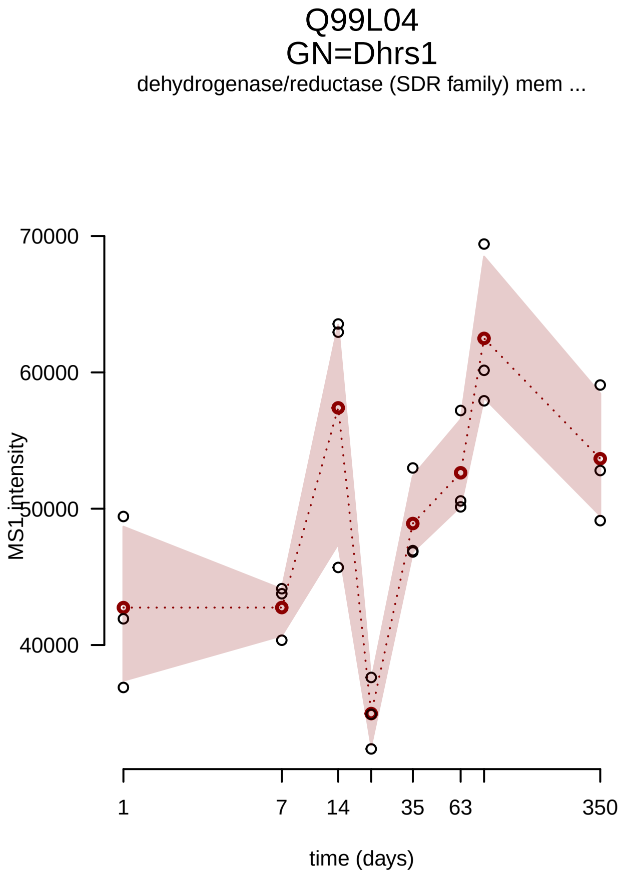
Q99L04
GN=Dhrs1
dehydrogenase/reductase (SDR family) mem ...
MS1 intensity
70000
60000
50000
40000
1
7
14
35
63
350
time (days)

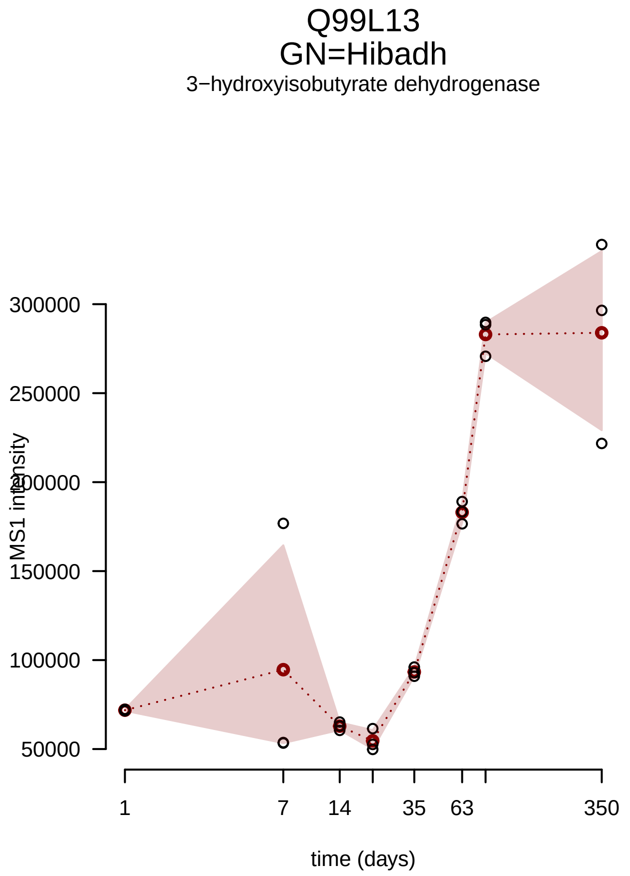
Q99L13
GN=Hibadh
3−hydroxyisobutyrate dehydrogenase
MS1 intensity
300000
250000
200000
150000
100000
50000
1
7
14
35
63
350
time (days)

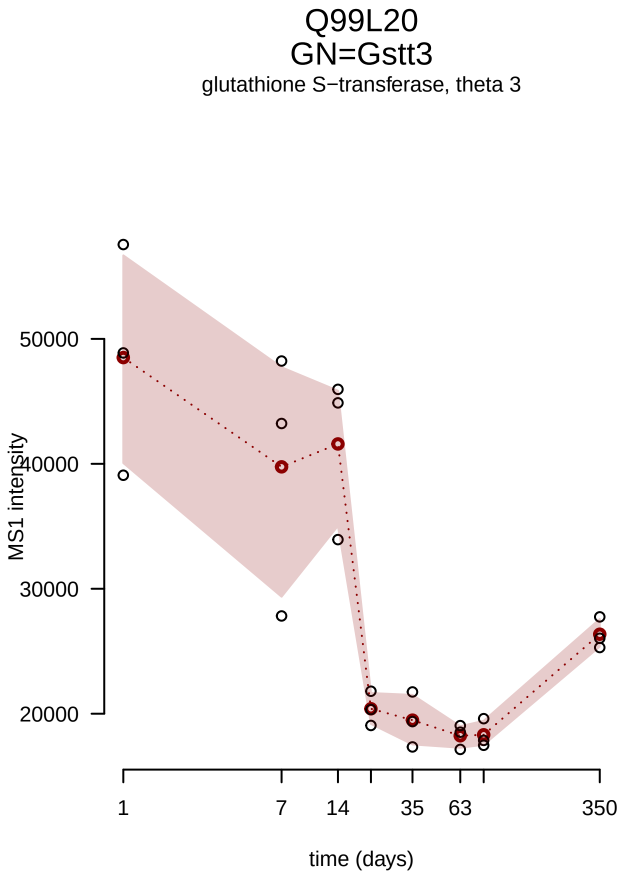
Q99L20
GN=Gstt3
glutathione S−transferase, theta 3
MS1 intensity
50000
40000
30000
20000
1
7
14
35
63
350
time (days)

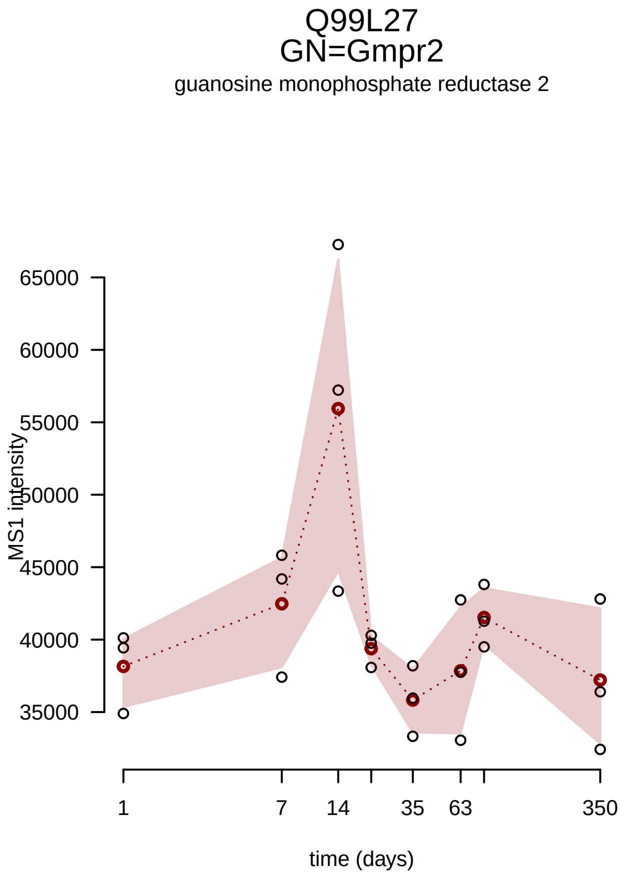
Q99L27
GN=Gmpr2
guanosine monophosphate reductase 2
MS1 intensity
65000
60000
55000
50000
45000
40000
35000
1
7
14
35
63
350
time (days)

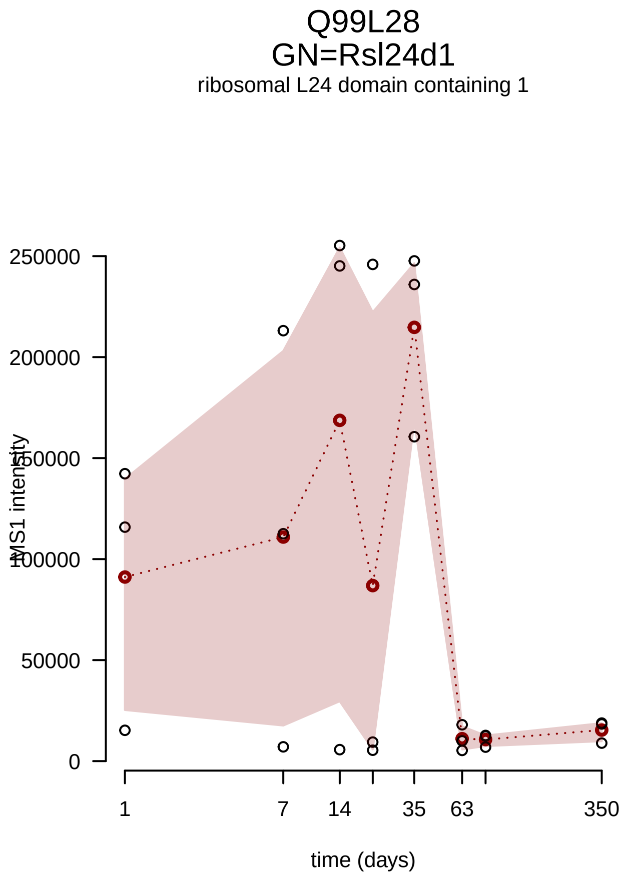
Q99L28
GN=Rsl24d1
ribosomal L24 domain containing 1
MS1 intensity
250000
200000
150000
100000
50000
0
1
7
14
35
63
350
time (days)

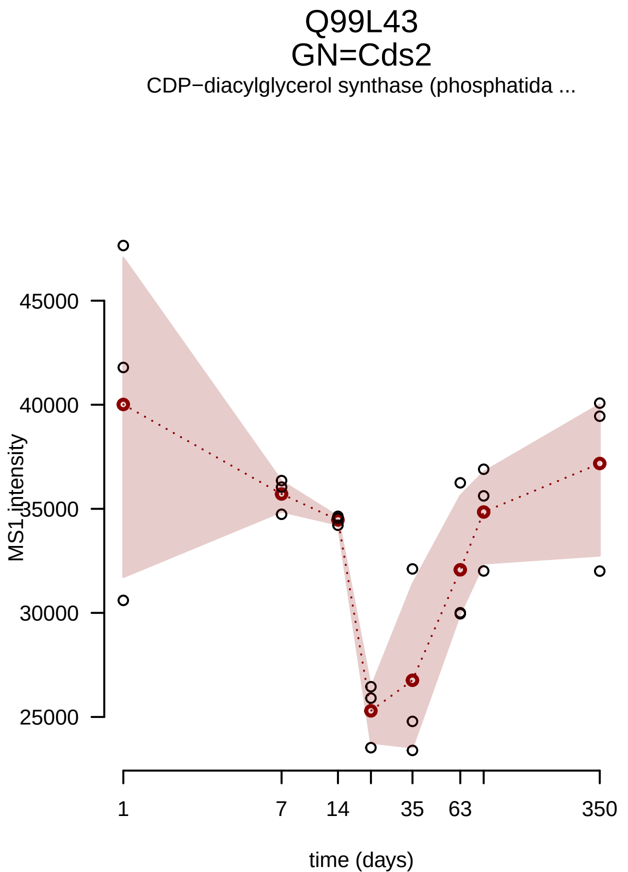
Q99L43
GN=Cds2
CDP−diacylglycerol synthase (phosphatida ...
MS1 intensity
45000
40000
35000
30000
25000
1
7
14
35
63
350
time (days)

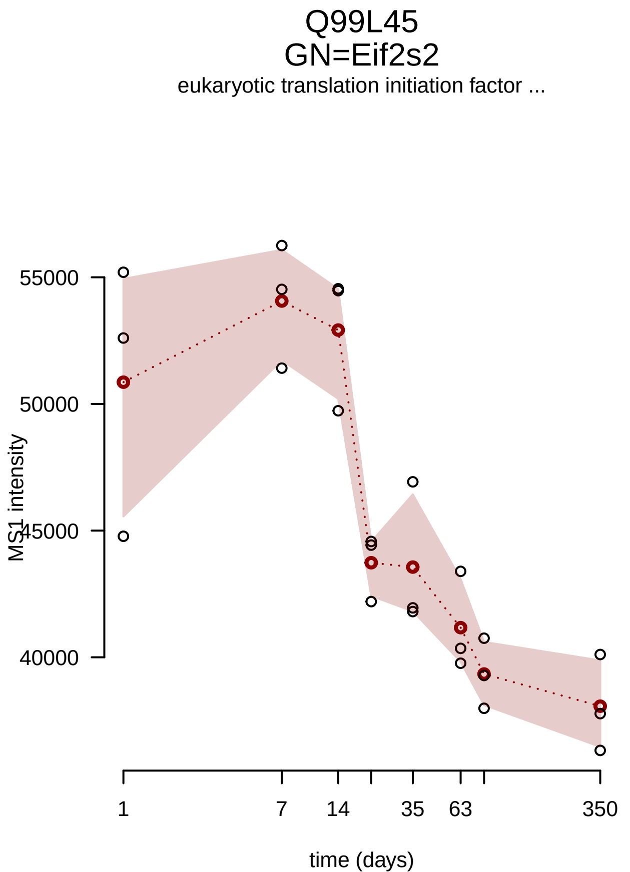
Q99L45
GN=Eif2s2
eukaryotic translation initiation factor ...
MS1 intensity
55000
50000
45000
40000
1
7
14
35
63
350
time (days)

Q99L47
GN=St13
suppression of tumorigenicity 13

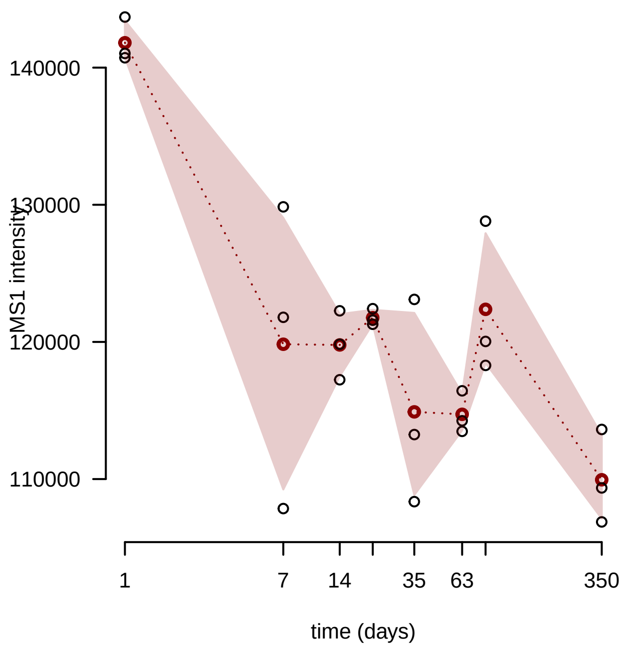

Q99L48
GN=Nmd3
NMD3 ribosome export adaptor

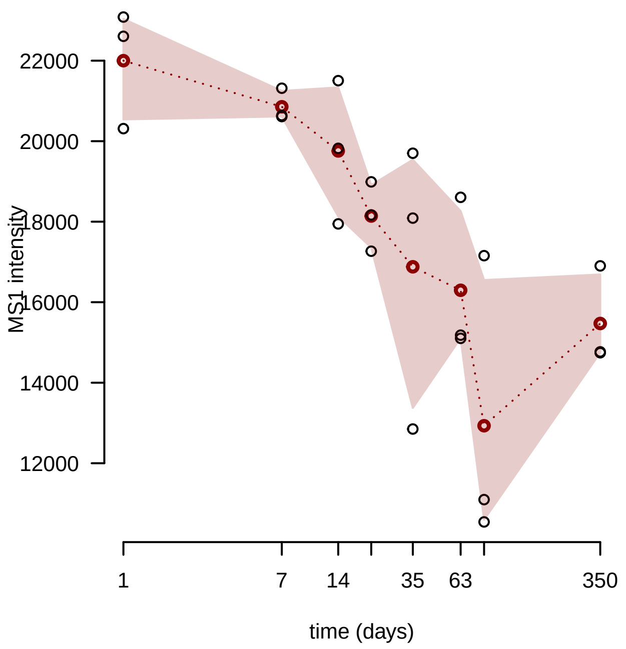

Q99L85
GN=Elp5
elongator acetyltransferase complex subu ...

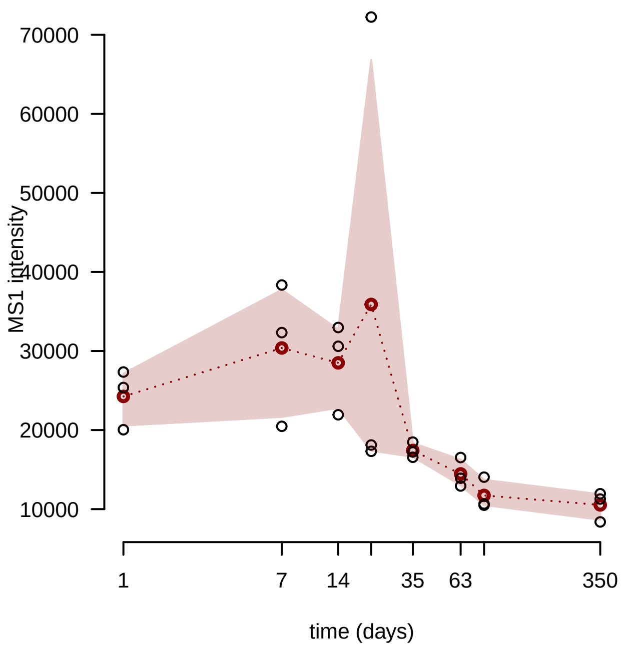

Q99L88
GN=Sntb1
syntrophin, basic 1

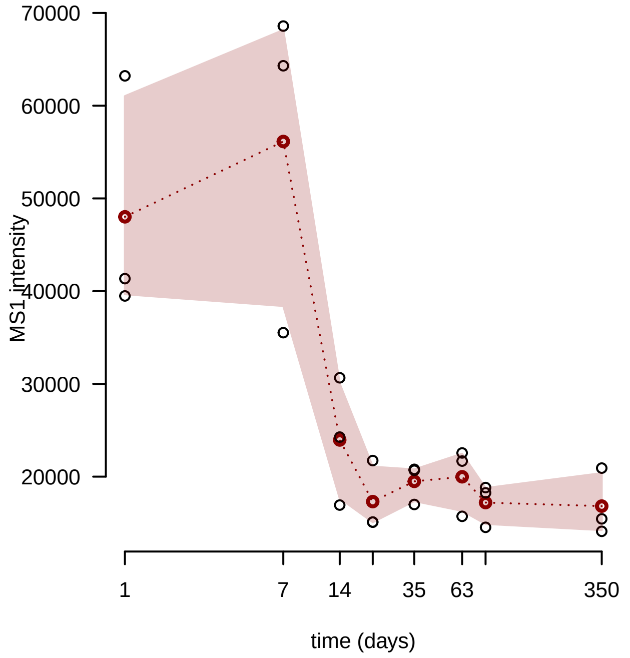

Q99L90
GN=Mcrs1
microspherule protein 1

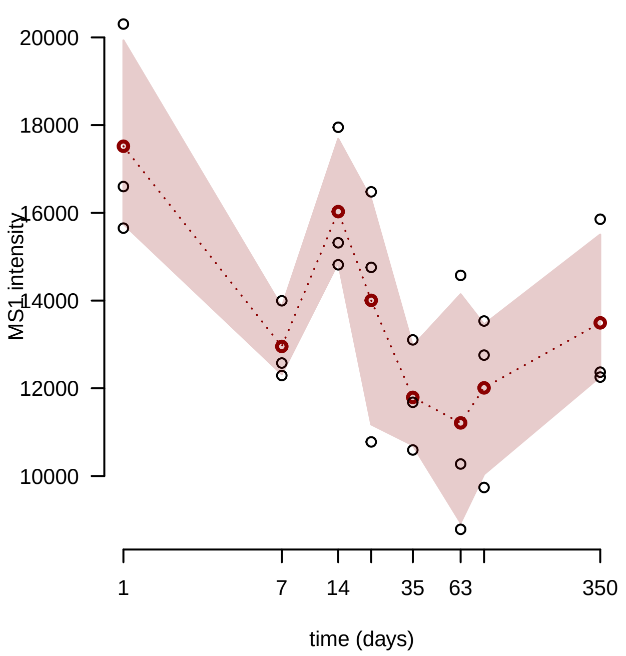

Q99LB0
GN=Dnttip1
deoxynucleotidyltransferase, terminal, i ...

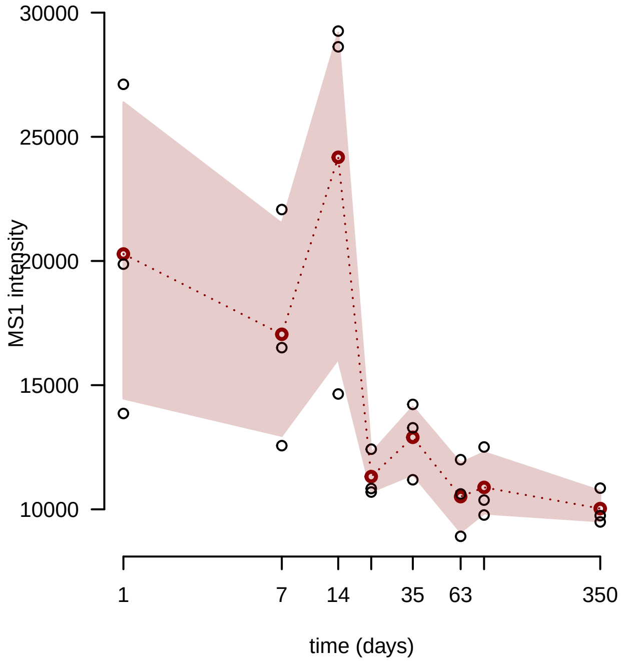

Q99LB2
GN=Dhrs4
dehydrogenase/reductase (SDR family) mem ...

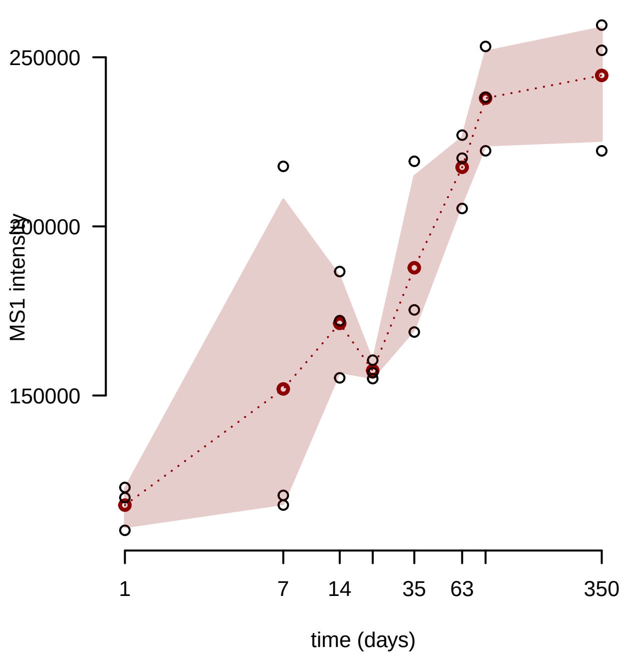

Q99LB6
GN=Mat2b
methionine adenosyltransferase II, beta

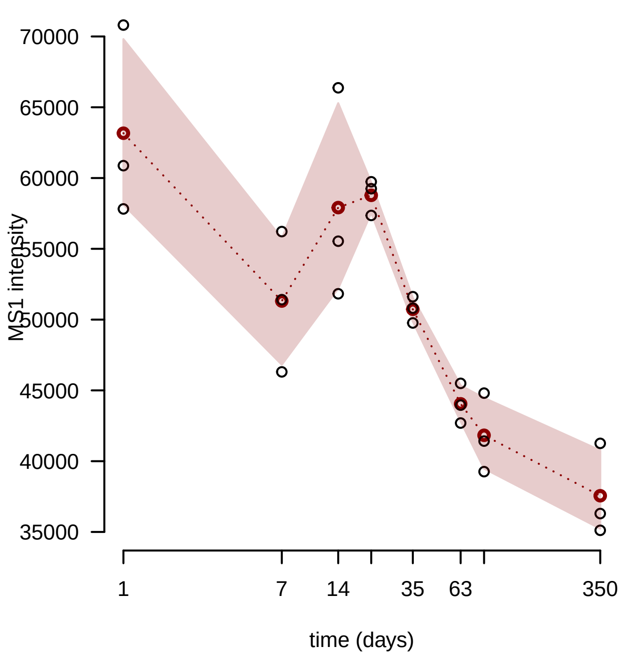

Q99LB7
GN=Sardh
sarcosine dehydrogenase

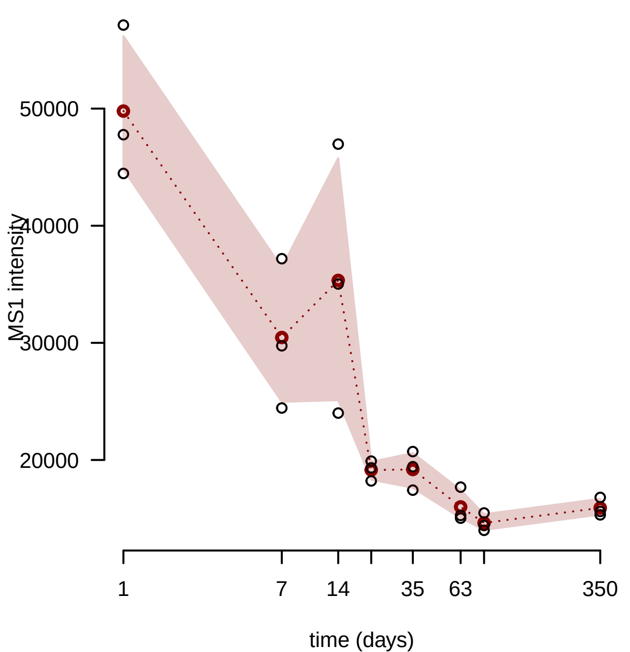

Q99LC2
GN=Cstf1
cleavage stimulation factor, 3' pre−RNA, ...

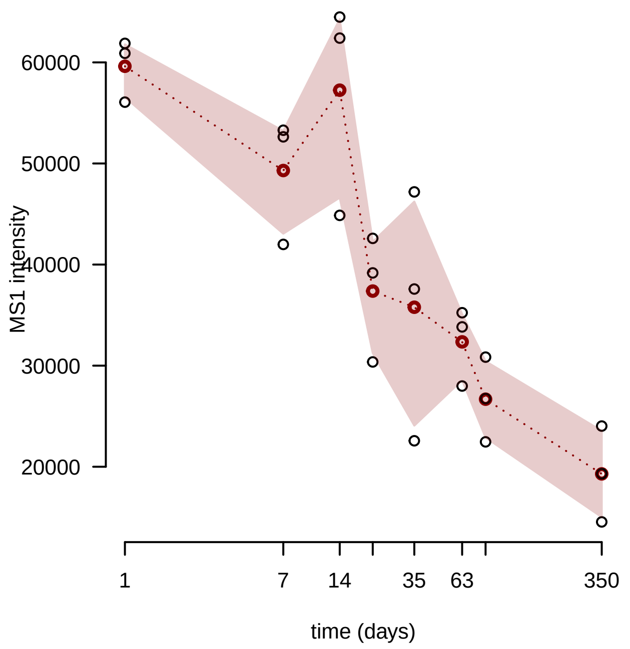

Q99LC3
GN=Ndufa10
NADH:ubiquinone oxidoreductase subunit A ...

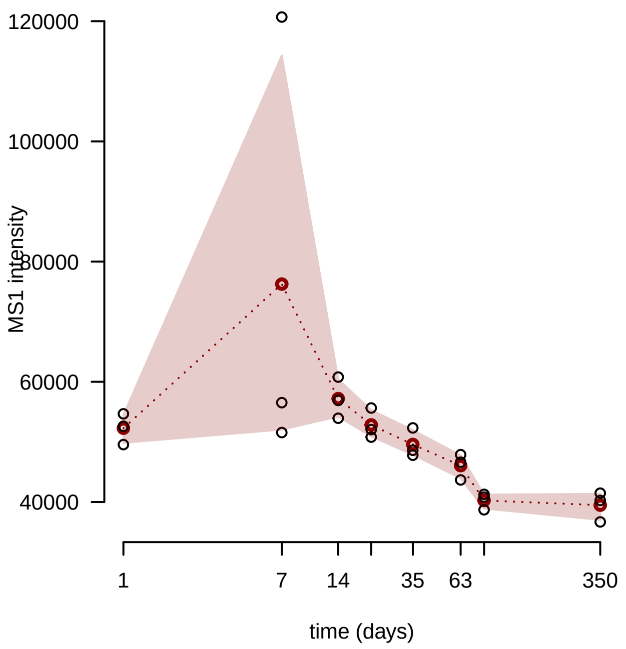

Q99LC5
GN=Etfa
electron transferring flavoprotein, alph ...

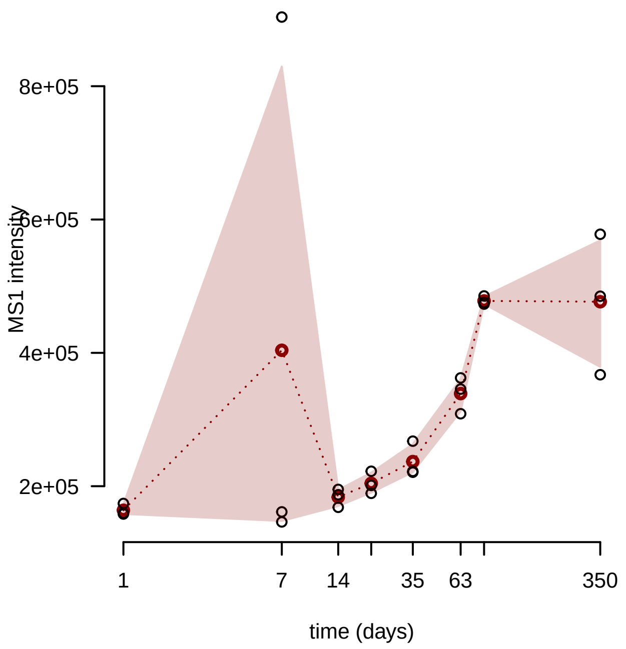

Q99LC8
GN=Eif2b1
eukaryotic translation initiation factor ...

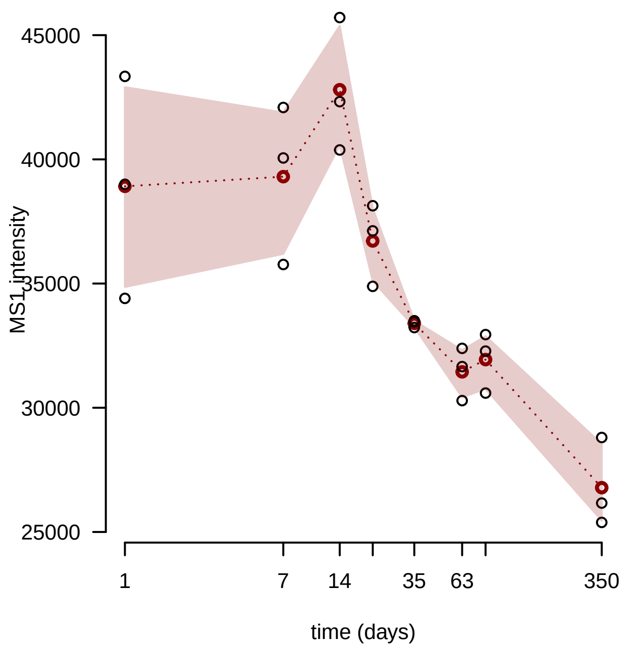

Q99LC9
GN=Pex6
peroxisomal biogenesis factor 6

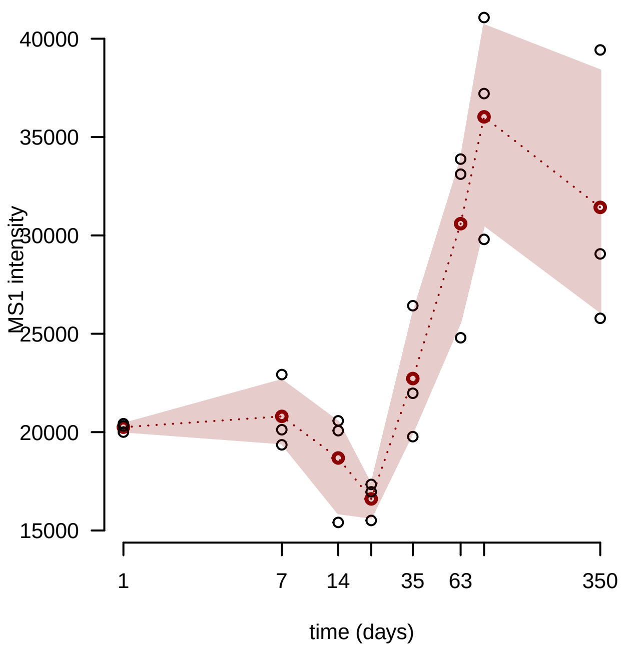

Q99LD4
GN=Gps1
G protein pathway suppressor 1

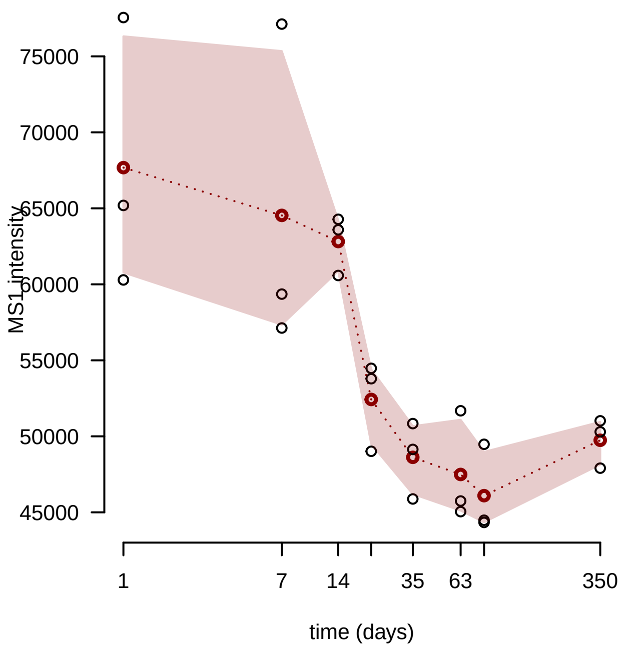

Q99LD8
GN=Ddah2
dimethylarginine dimethylaminohydrolase ...

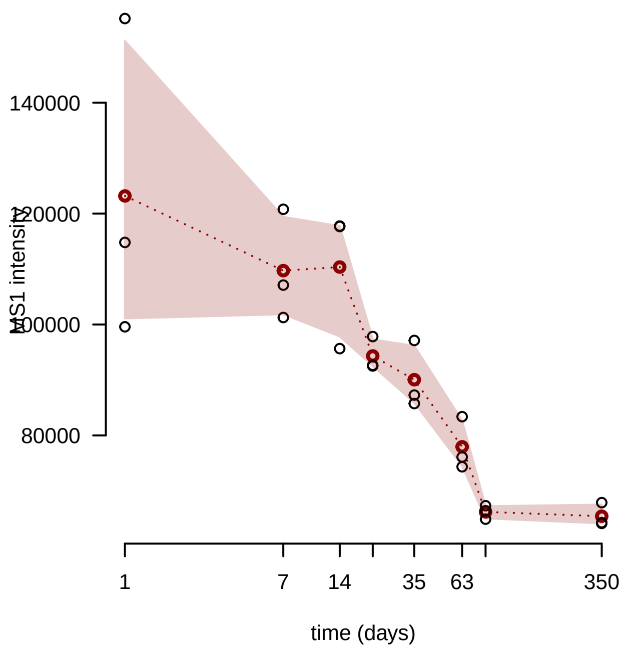

Q99LD9
GN=Eif2b2
eukaryotic translation initiation factor ...

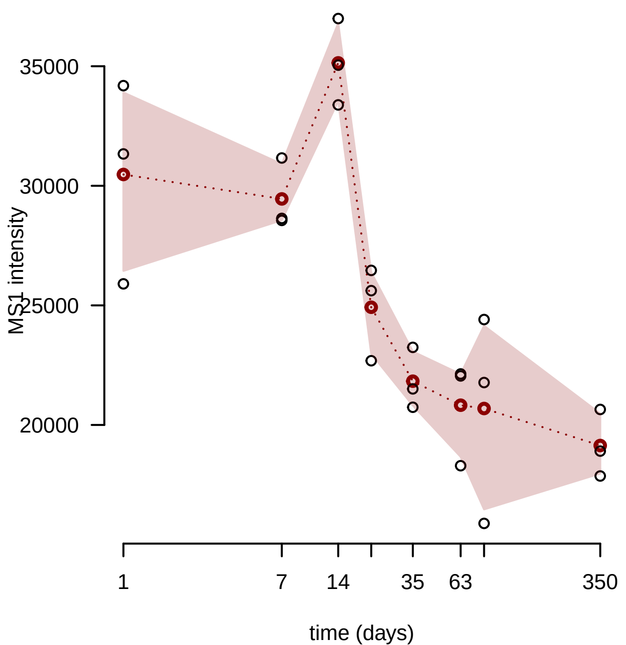

Q99LE1
GN=Rilpl2
Rab interacting lysosomal protein−like 2

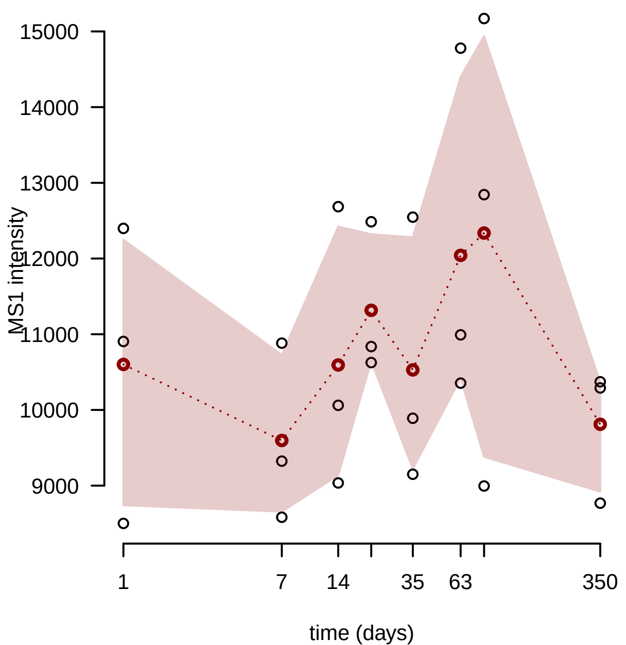

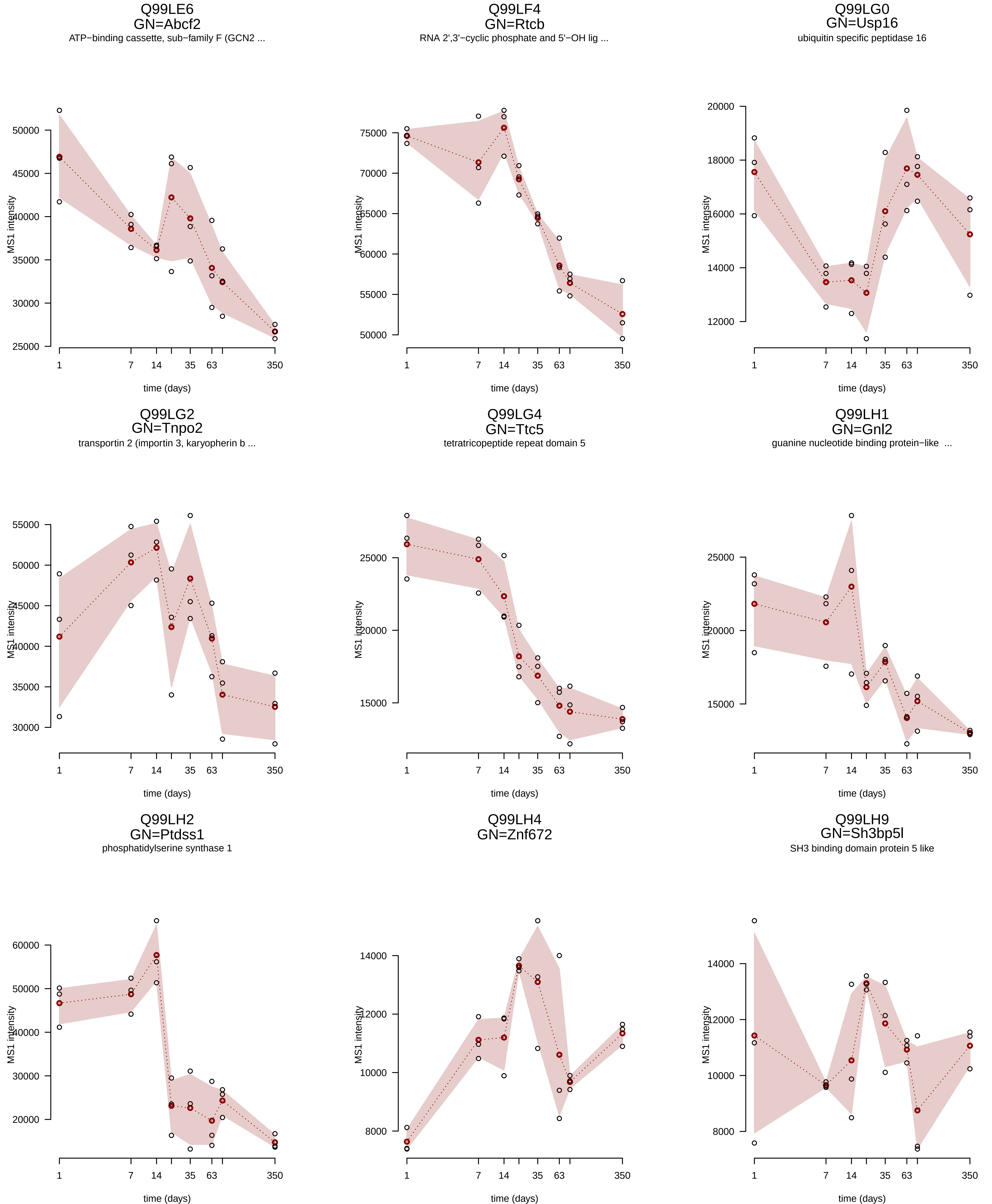
Q99LE6
GN=Abcf2
ATP−binding cassette, sub−family F (GCN2 ...
MS1 intensity
50000
45000
40000
35000
30000
25000
1
7
14
35
63
350
time (days)
Q99LF4
GN=Rtcb
RNA 2',3'−cyclic phosphate and 5'−OH lig ...
MS1 intensity
75000
70000
65000
60000
55000
50000
time (days)
Q99LG0
GN=Usp16
ubiquitin specific peptidase 16
MS1 intensity
20000
18000
16000
14000
12000
time (days)
Q99LG2
GN=Tnpo2
transportin 2 (importin 3, karyopherin b ...
MS1 intensity
55000
50000
45000
40000
35000
30000
time (days)
Q99LG4
GN=Ttc5
tetratricopeptide repeat domain 5
MS1 intensity
25000
20000
15000
time (days)
Q99LH1
GN=Gnl2
guanine nucleotide binding protein−like ...
MS1 intensity
25000
20000
15000
time (days)
Q99LH2
GN=Ptdss1
phosphatidylserine synthase 1
MS1 intensity
60000
50000
40000
30000
20000
time (days)
Q99LH4
GN=Znf672
MS1 intensity
14000
12000
10000
8000
time (days)
Q99LH9
GN=Sh3bp5l
SH3 binding domain protein 5 like
MS1 intensity
14000
12000
10000
8000
time (days)

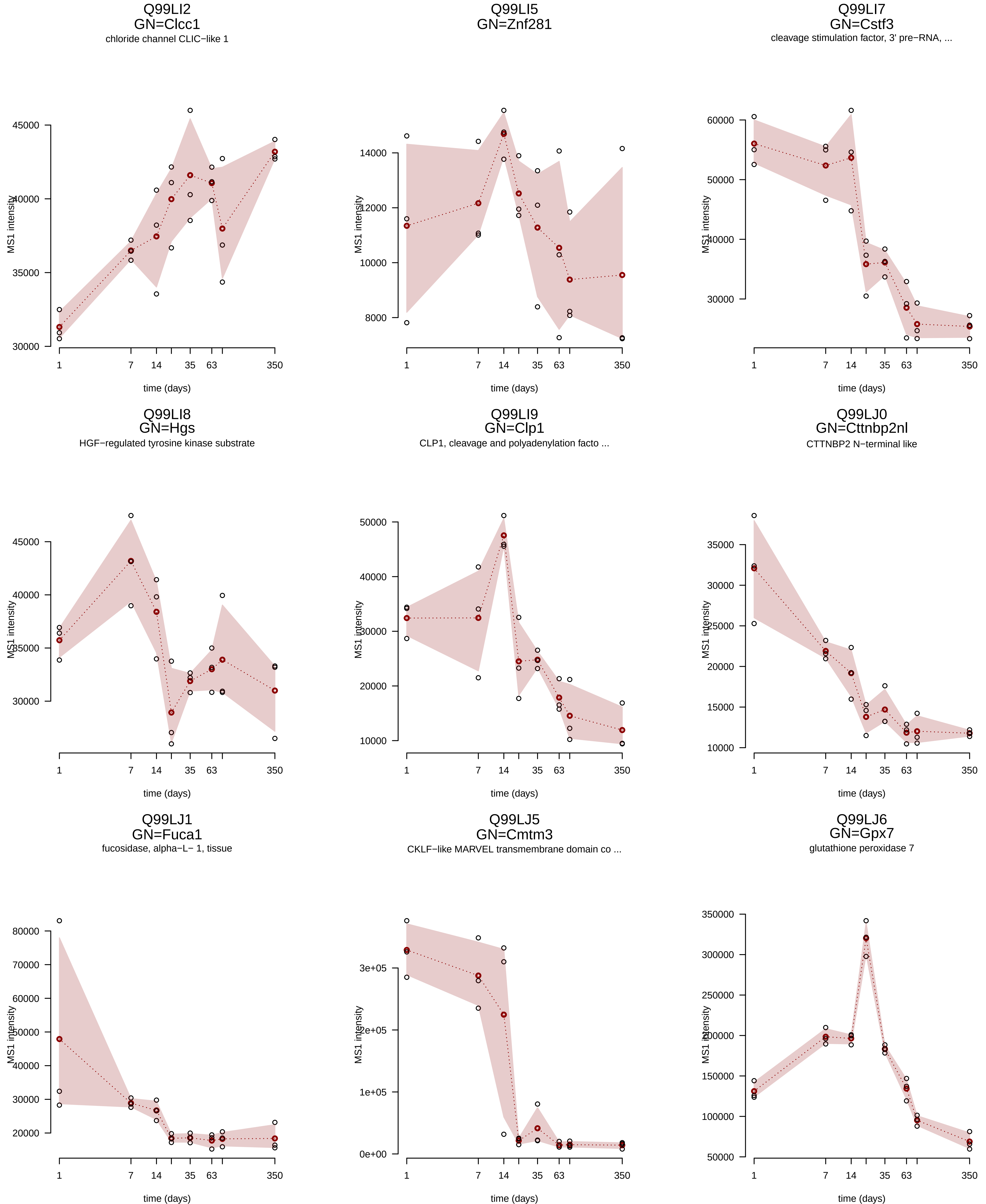
Q99LI2
GN=Clcc1
chloride channel CLIC−like 1
Q99LI5
GN=Znf281
Q99LI7
GN=Cstf3
cleavage stimulation factor, 3' pre−RNA, ...
Q99LI8
GN=Hgs
HGF−regulated tyrosine kinase substrate
Q99LI9
GN=Clp1
CLP1, cleavage and polyadenylation facto ...
Q99LJ0
GN=Cttnbp2nl
CTTNBP2 N−terminal like
Q99LJ1
GN=Fuca1
fucosidase, alpha−L− 1, tissue
Q99LJ5
GN=Cmtm3
CKLF−like MARVEL transmembrane domain co ...
Q99LJ6
GN=Gpx7
glutathione peroxidase 7
MS1 intensity
time (days)
1
7
14
35
63
350

Q99LJ7
GN=Rcbtb2
regulator of chromosome condensation (RC ...

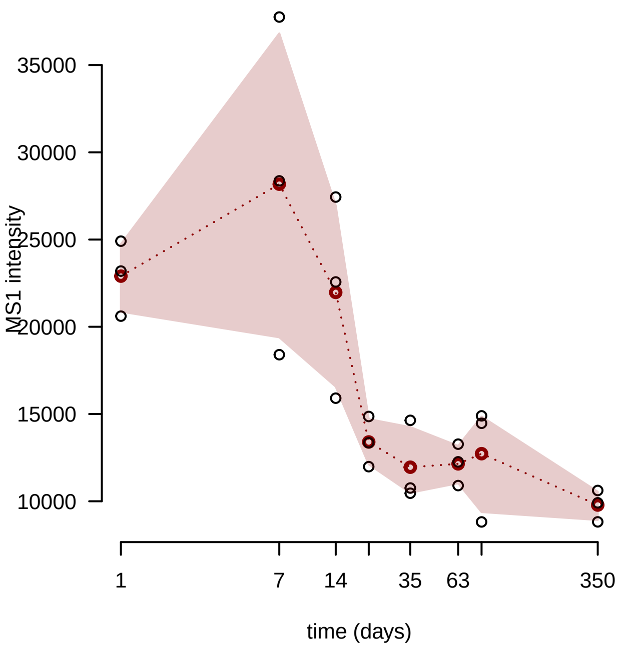

Q99LL5
GN=Pwp1
PWP1 homolog, endonuclein

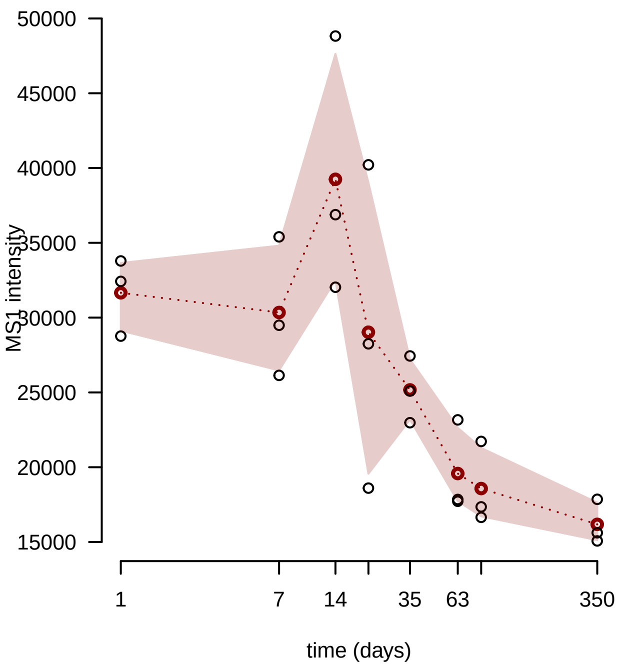

Q99LM2
GN=Cdk5rap3
CDK5 regulatory subunit associated prote ...

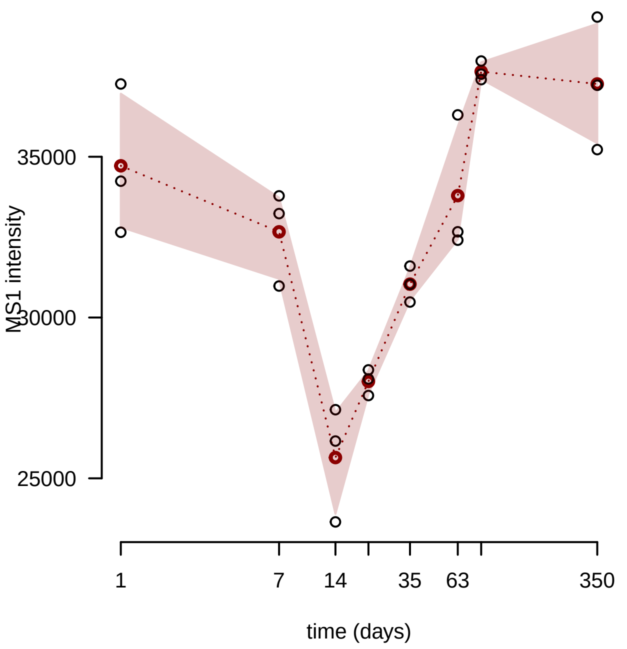

Q99LM9
GN=Tada1
transcriptional adaptor 1

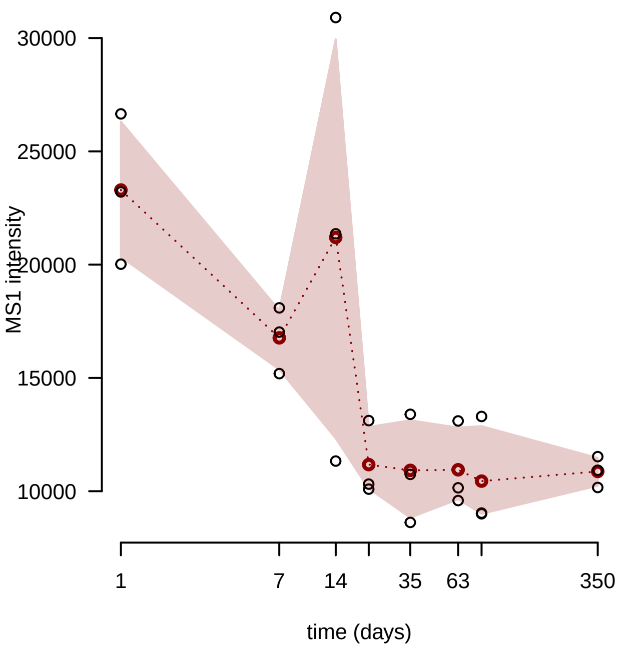

Q99LN9
GN=Dohh
deoxyhypusine hydroxylase/monooxygenase

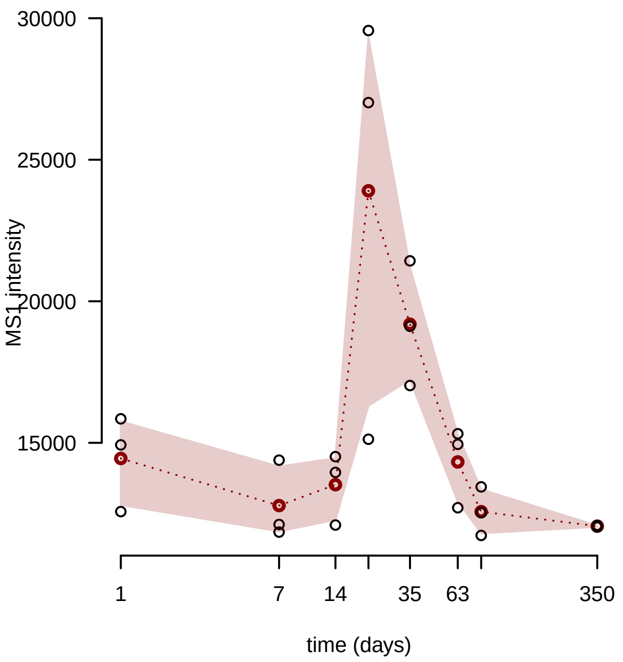

Q99LP6
GN=Grpel1
GrpE−like 1, mitochondrial

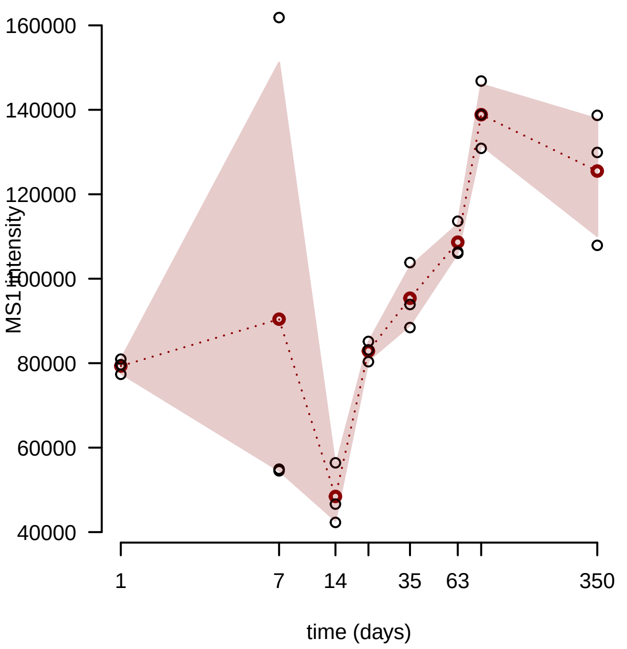

Q99LQ1
GN=Mbip
MAP3K12 binding inhibitory protein 1

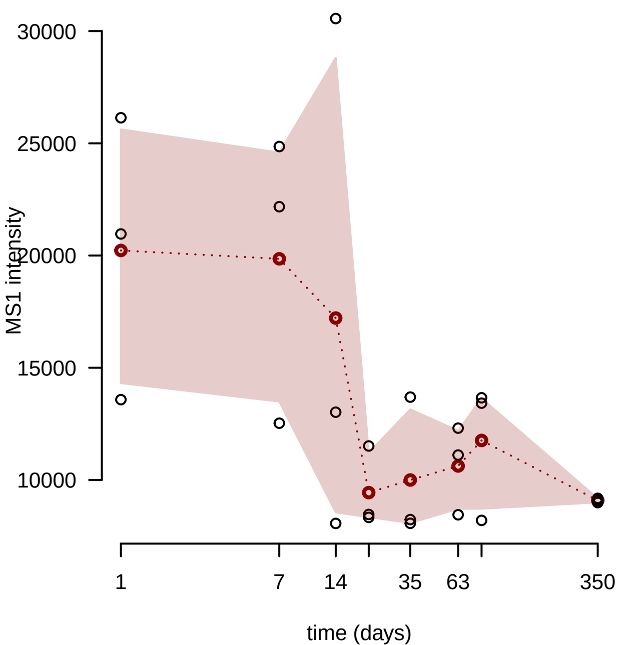

Q99LQ7
GN=Tmem189

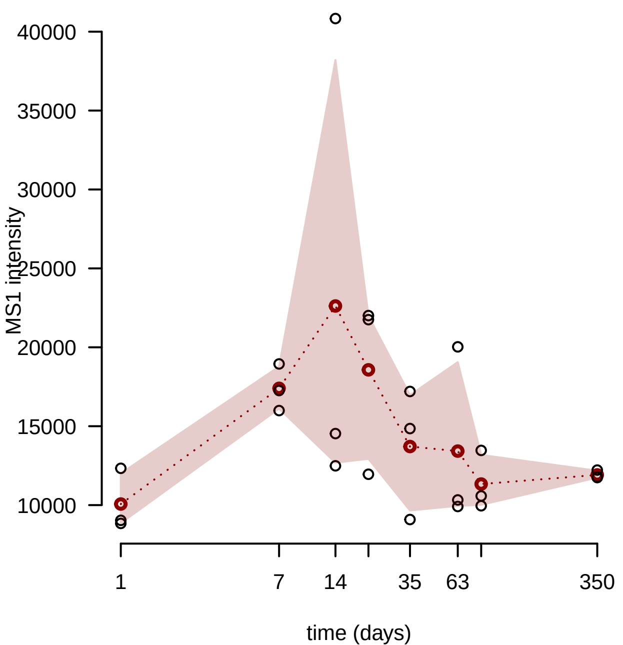

Q99LR1
GN=Abhd12
abhydrolase domain containing 12

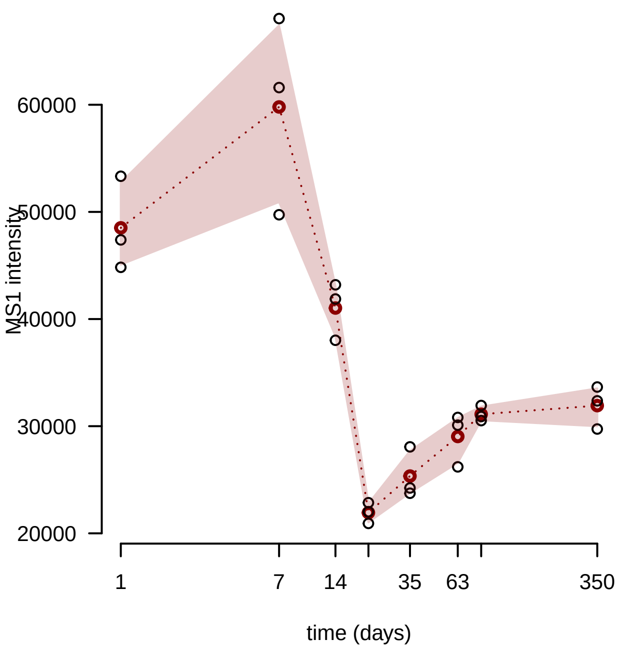

Q99LS3
GN=Psph
phosphoserine phosphatase

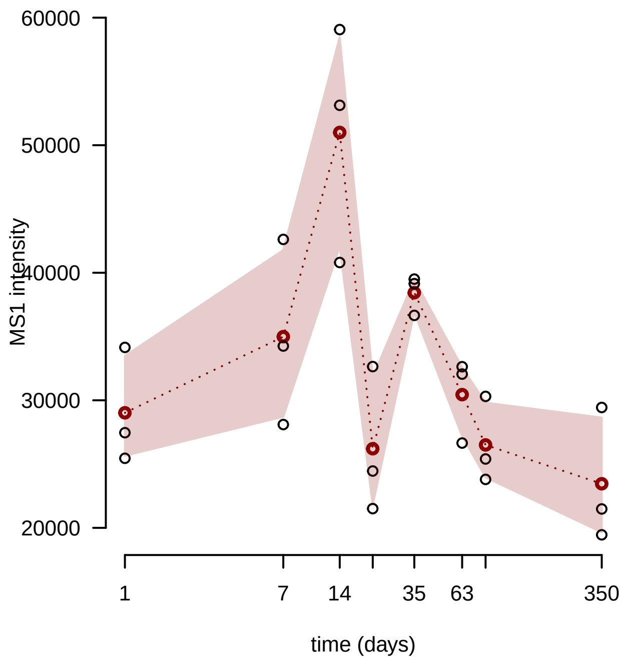

Q99LT0
GN=Dpy30
dpy−30, histone methyltransferase comple ...

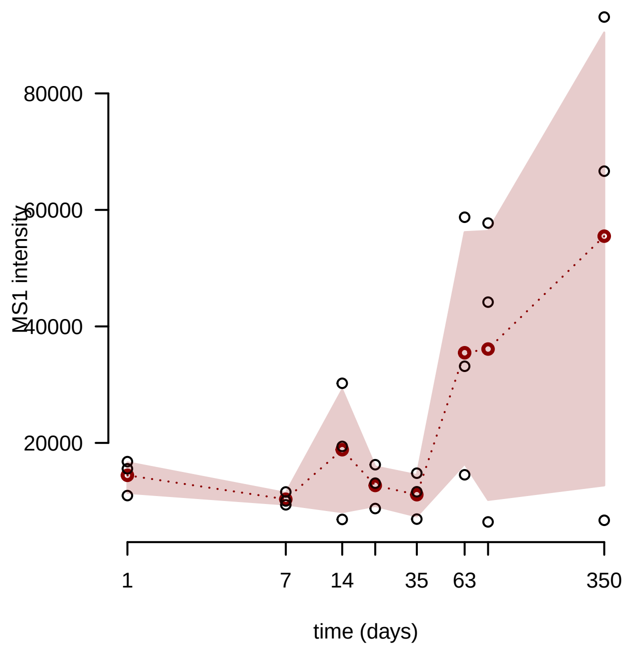

Q99LU0
GN=Chmp1b1

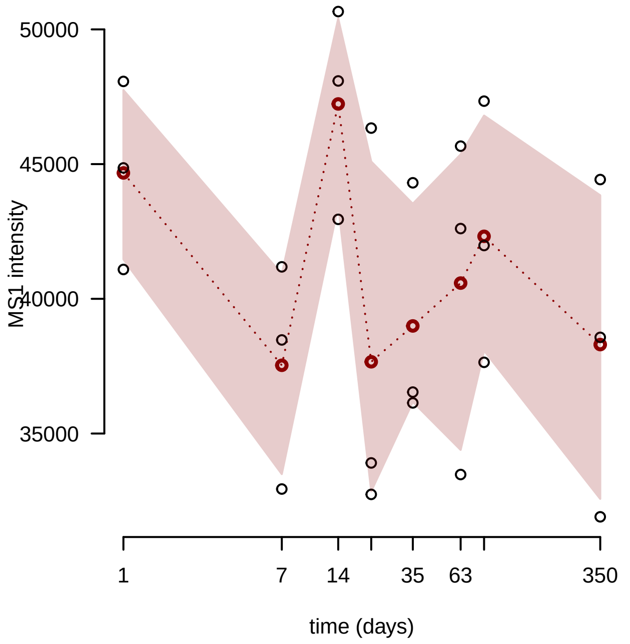

Q99LW0
GN=Ankrd10
ankyrin repeat domain 10

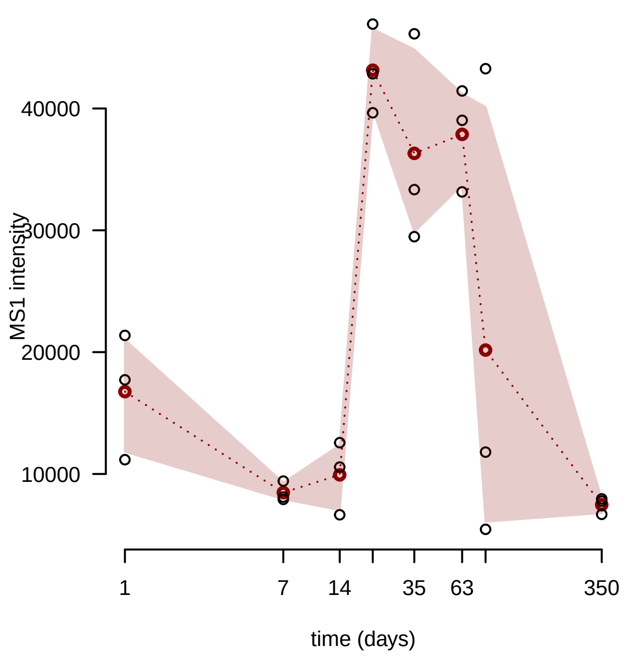

Q99LW6
GN=Yaf2
YY1 associated factor 2

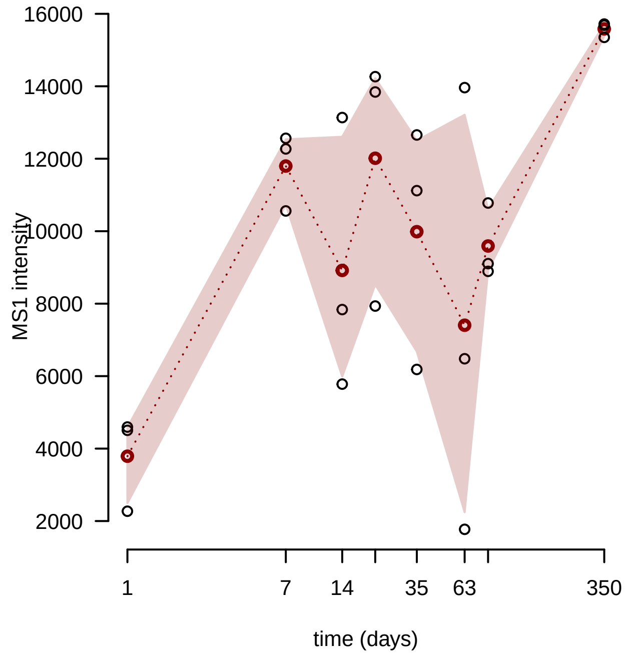

Q99LX0
GN=Park7
Parkinson disease (autosomal recessive, ...

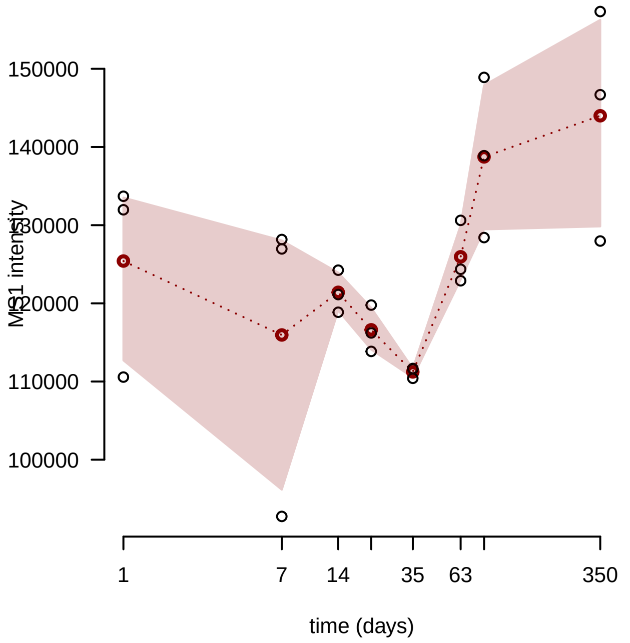

Q99LX5
GN=Mmtag2

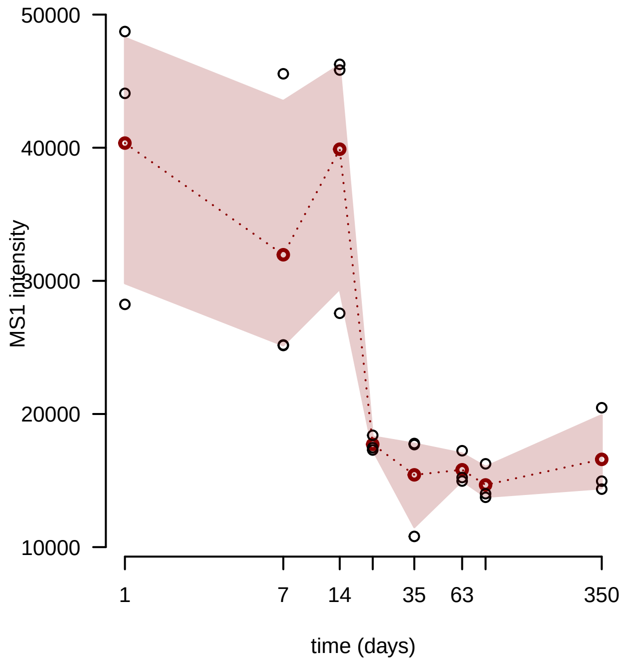

Q99LY9
GN=Ndufs5
NADH:ubiquinone oxidoreductase core subu ...

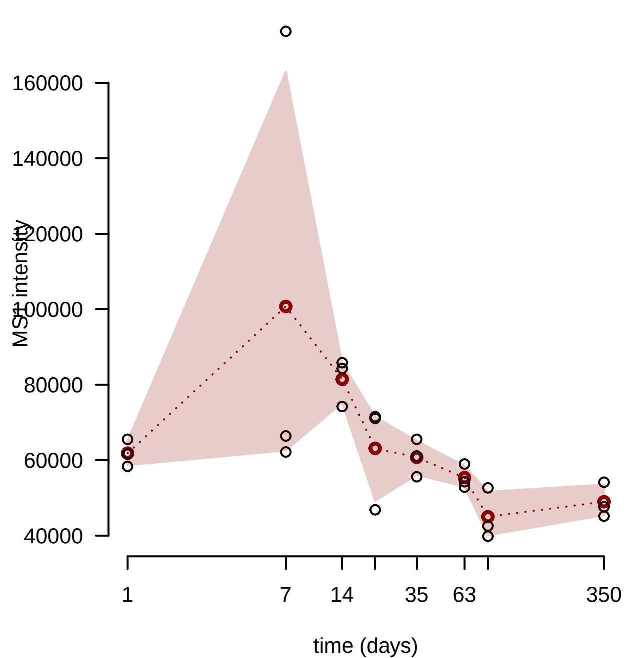

Q99LZ3
GN=Gins4
GINS complex subunit 4 (Sld5 homolog)

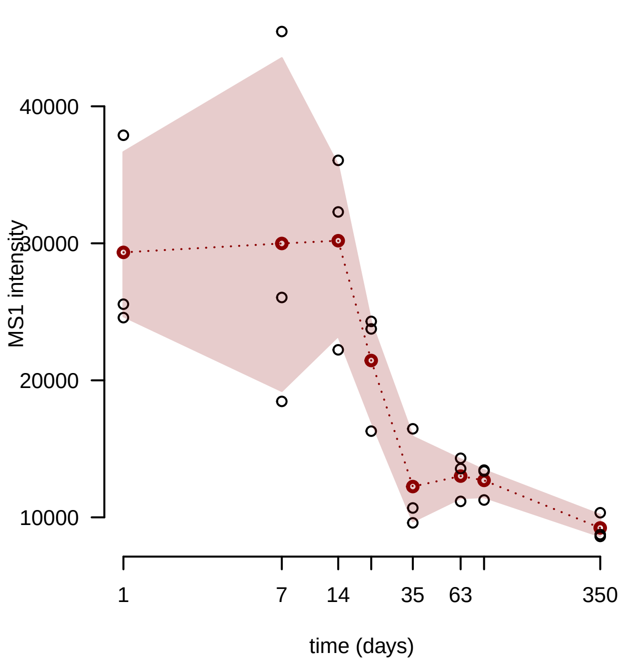

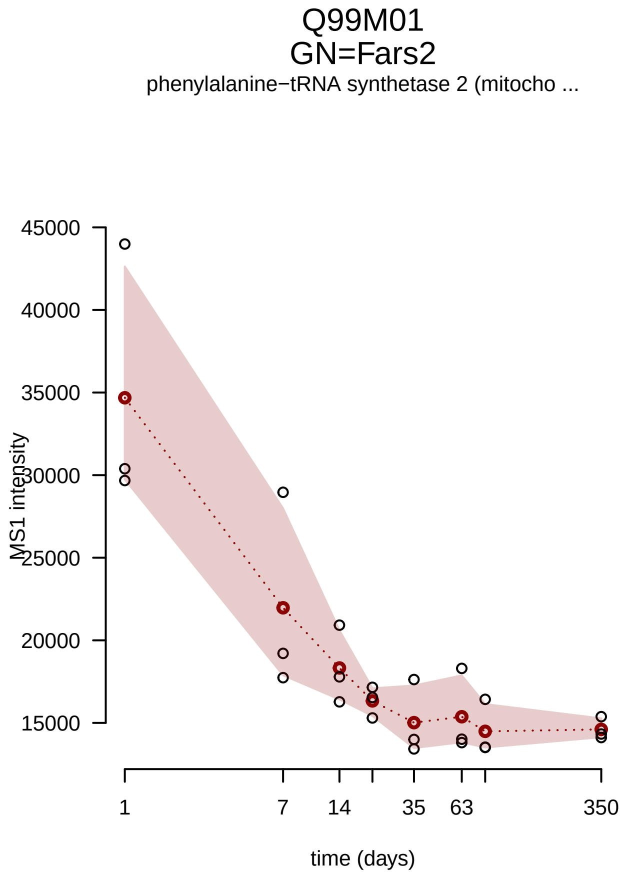
Q99M01
GN=Fars2
phenylalanine−tRNA synthetase 2 (mitocho ...
MS1 intensity
45000
40000
35000
30000
25000
20000
15000
1
7
14
35
63
350
time (days)

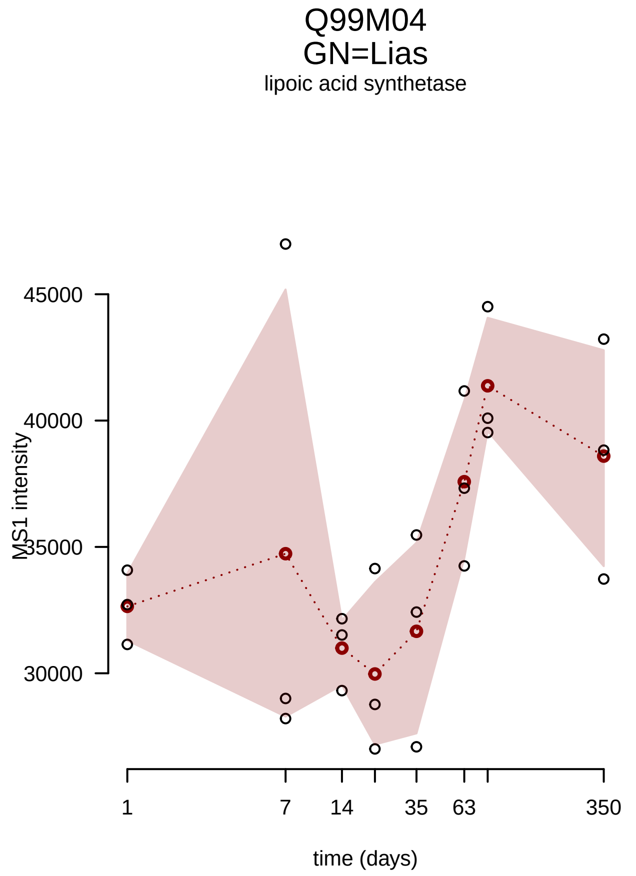
Q99M04
GN=Lias
lipoic acid synthetase
MS1 intensity
45000
40000
35000
30000
1
7
14
35
63
350
time (days)

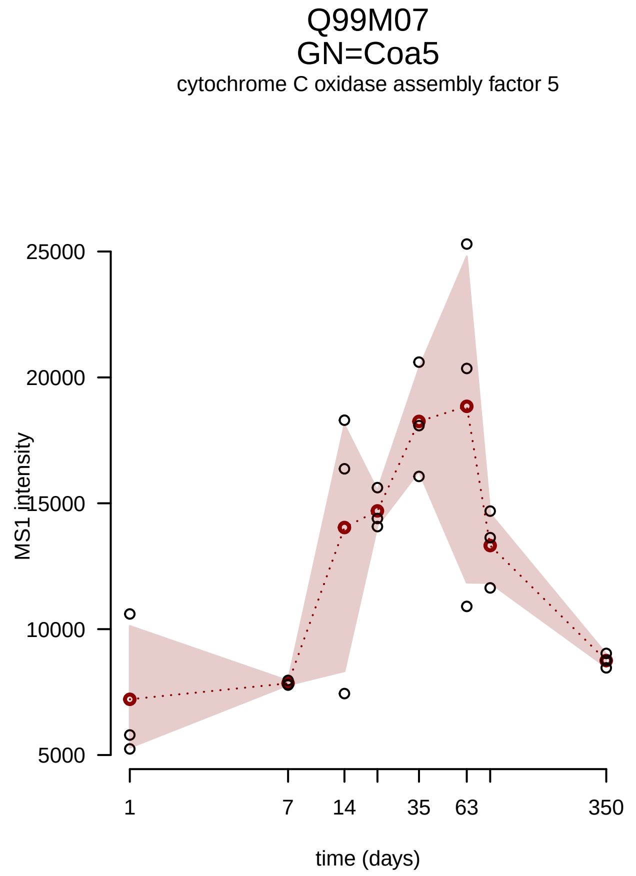
Q99M07
GN=Coa5
cytochrome C oxidase assembly factor 5
MS1 intensity
25000
20000
15000
10000
5000
1
7
14
35
63
350
time (days)

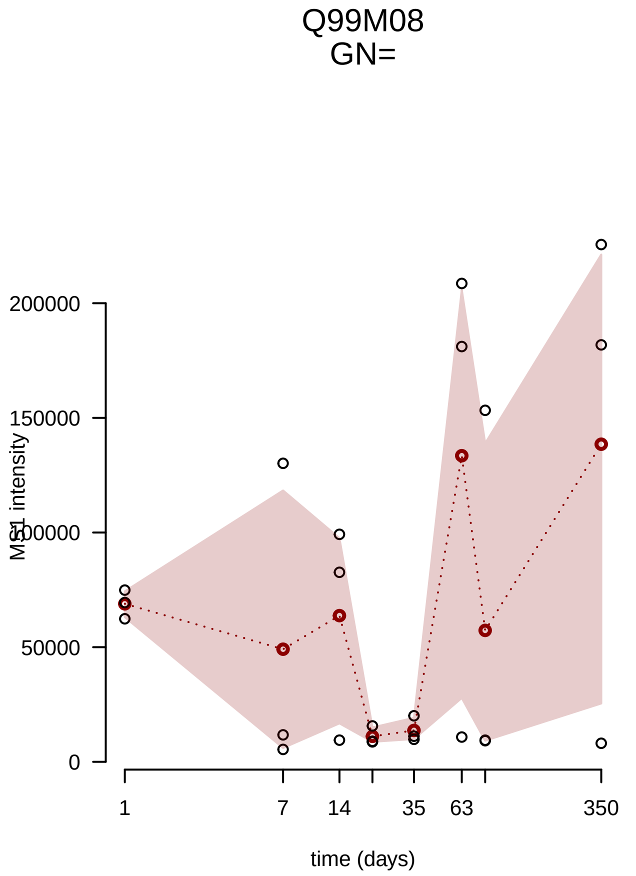
Q99M08
GN=
MS1 intensity
200000
150000
100000
50000
0
1
7
14
35
63
350
time (days)

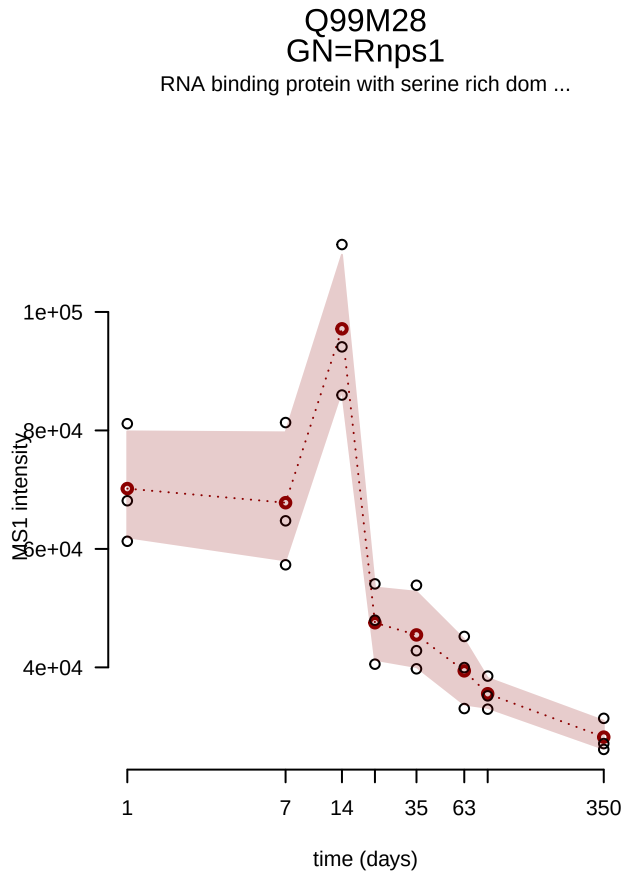
Q99M28
GN=Rnps1
RNA binding protein with serine rich dom ...
MS1 intensity
1e+05
8e+04
6e+04
4e+04
1
7
14
35
63
350
time (days)

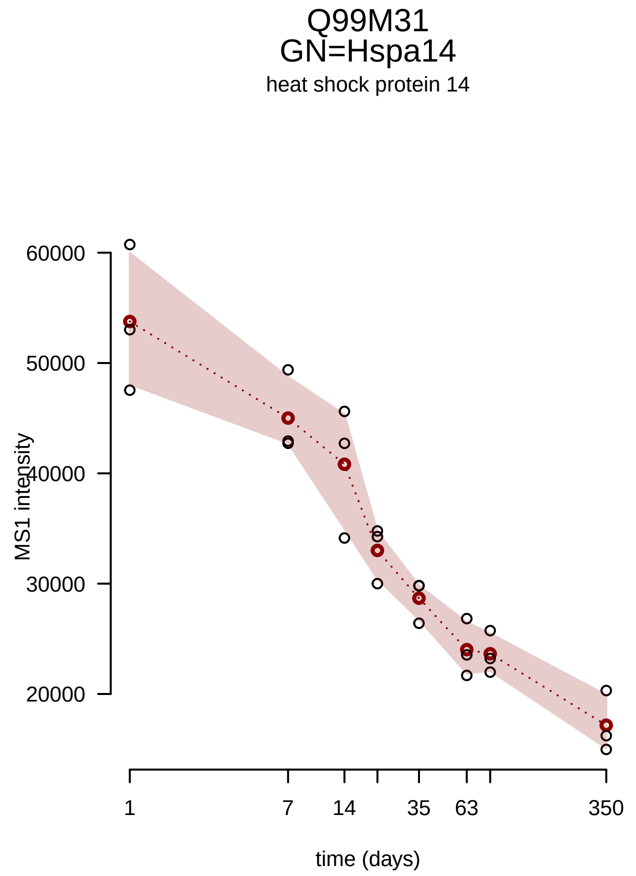
Q99M31
GN=Hspa14
heat shock protein 14
MS1 intensity
60000
50000
40000
30000
20000
1
7
14
35
63
350
time (days)

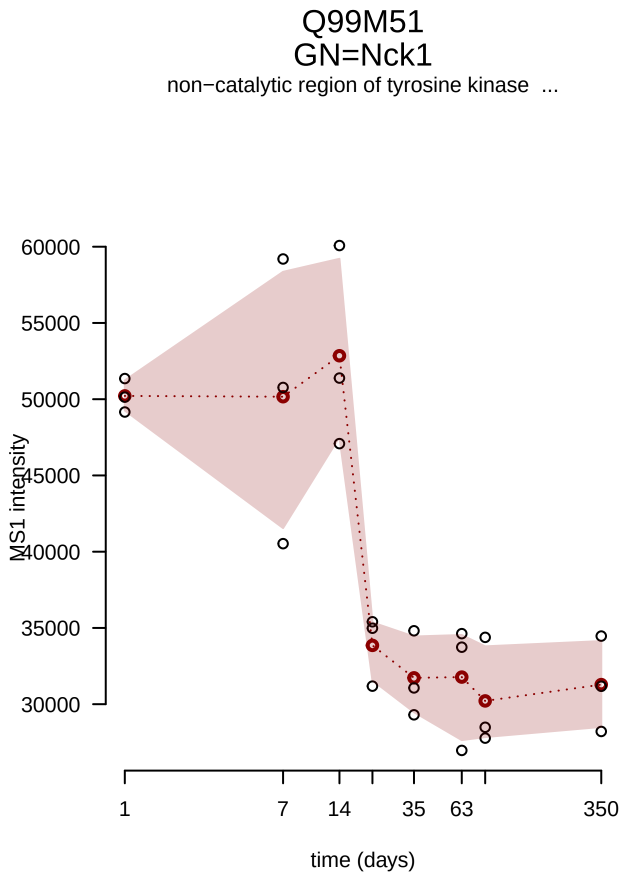
Q99M51
GN=Nck1
non−catalytic region of tyrosine kinase ...
MS1 intensity
60000
55000
50000
45000
40000
35000
30000
1
7
14
35
63
350
time (days)

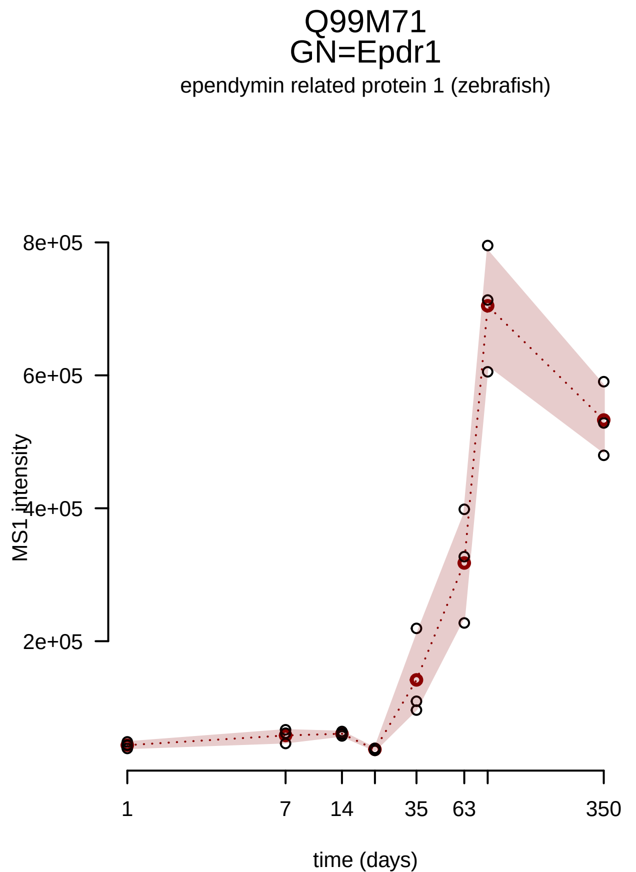
Q99M71
GN=Epdr1
ependymin related protein 1 (zebrafish)
MS1 intensity
8e+05
6e+05
4e+05
2e+05
1
7
14
35
63
350
time (days)

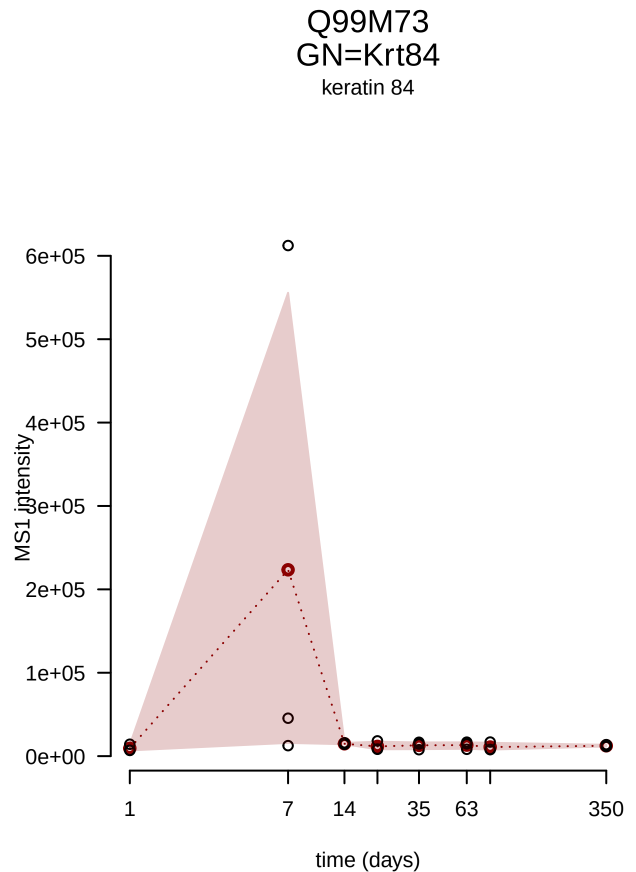
Q99M73
GN=Krt84
keratin 84
MS1 intensity
6e+05
5e+05
4e+05
3e+05
2e+05
1e+05
0e+00
1
7
14
35
63
350
time (days)

Q99M74
GN=Krt82
keratin 82

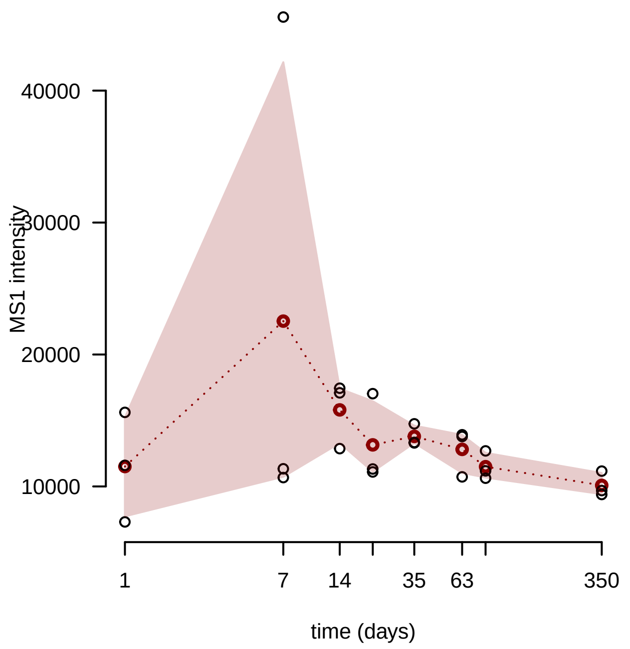

Q99M87
GN=Dnaja3
DnaJ heat shock protein family (Hsp40) m ...

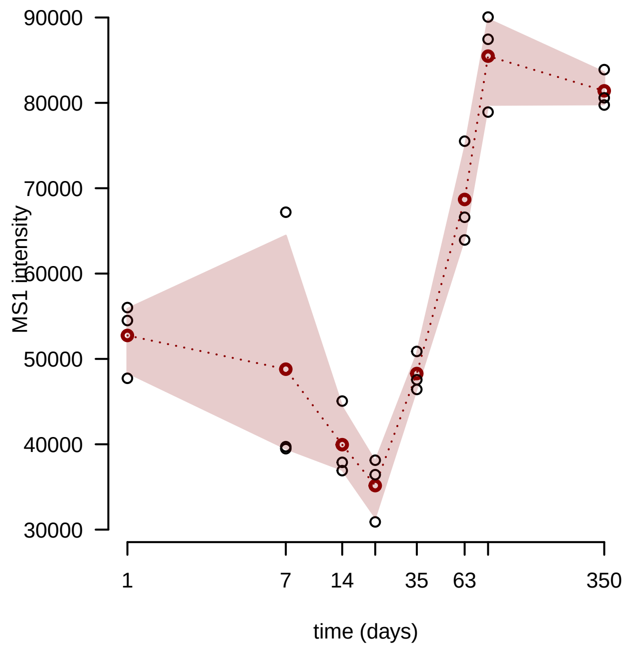

Q99M87
GN=Dnaja3
DnaJ heat shock protein family (Hsp40) m ...

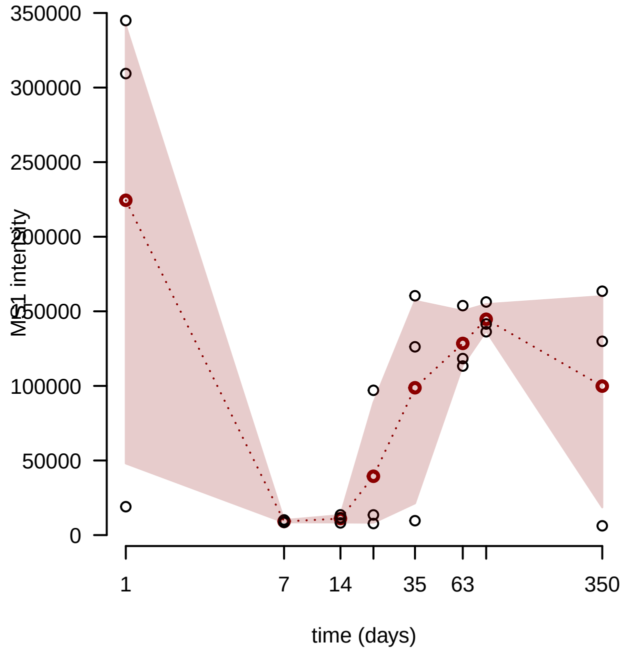

Q99M96
GN=St7
suppression of tumorigenicity 7

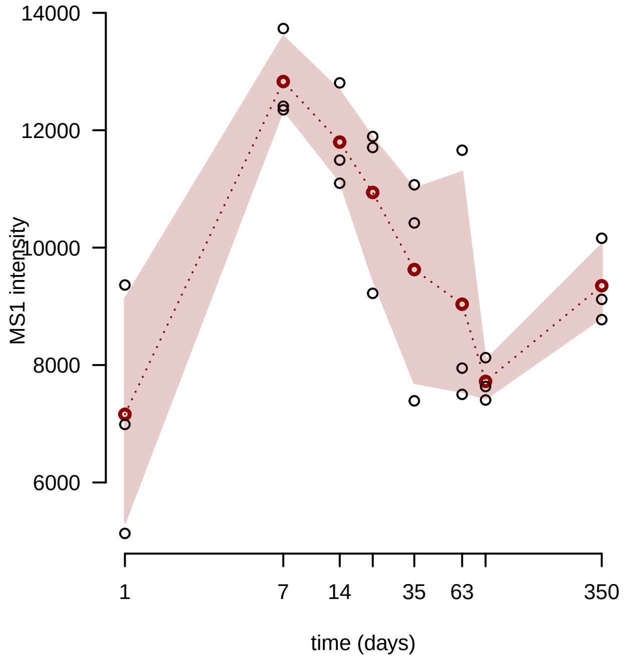

Q99MB7
GN=Rnf141
ring finger protein 141

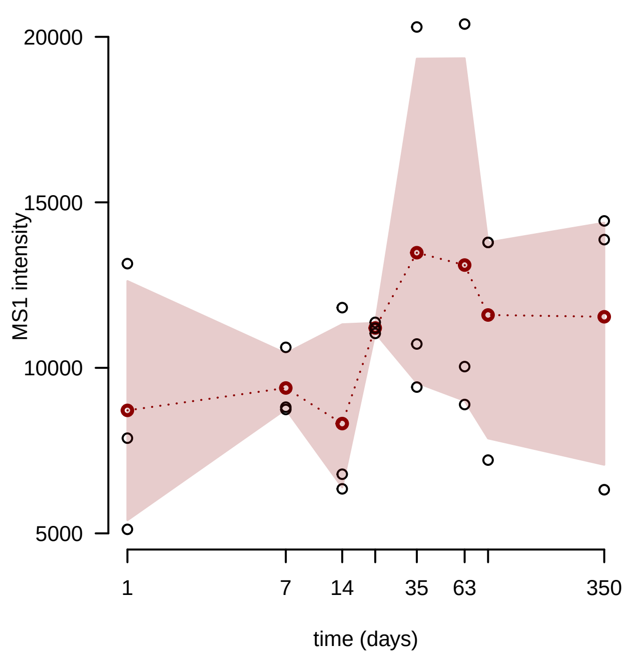

Q99MD6
GN=Txnrd3
thioredoxin reductase 3

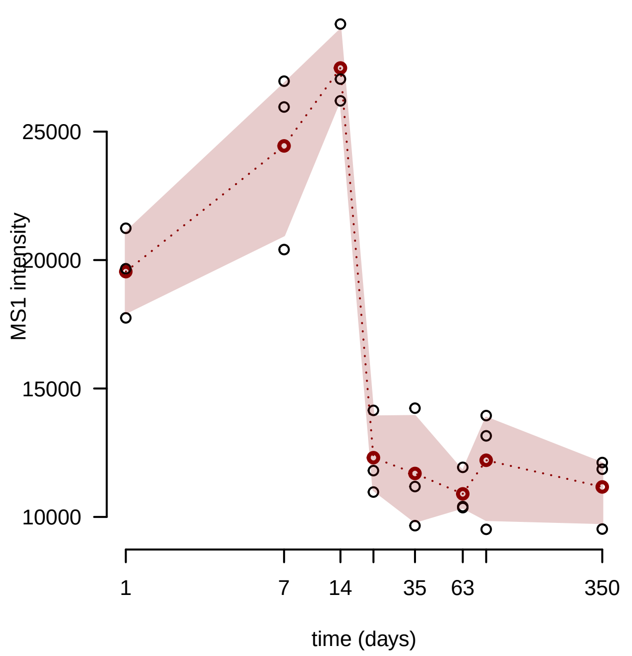

Q99MD8
GN=Mynn
myoneurin

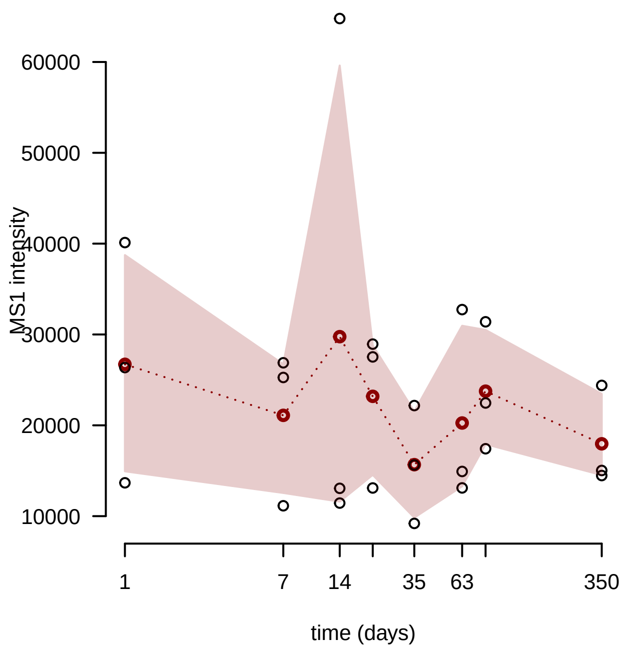

Q99MD9
GN=Nasp
nuclear autoantigenic sperm protein (his ...

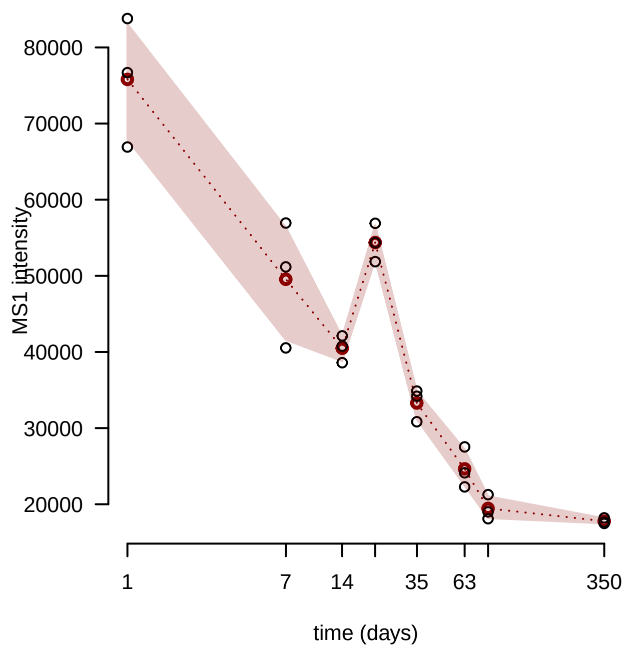

Q99ME2
GN=Wdr6
WD repeat domain 6

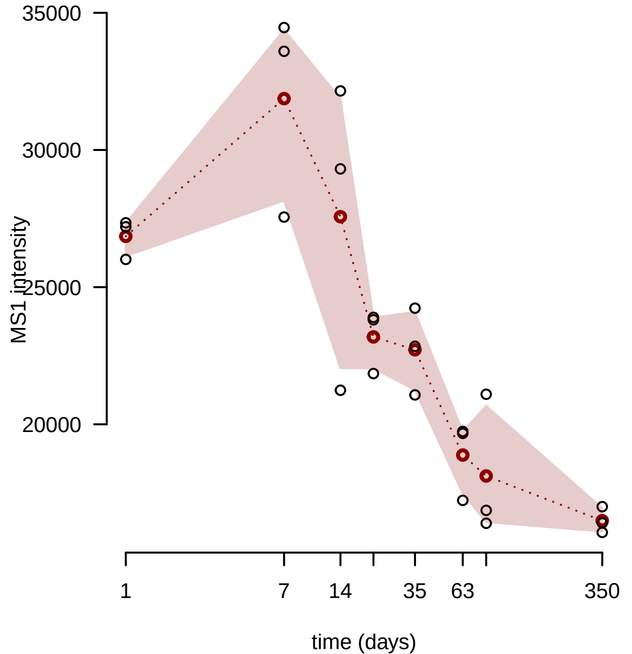

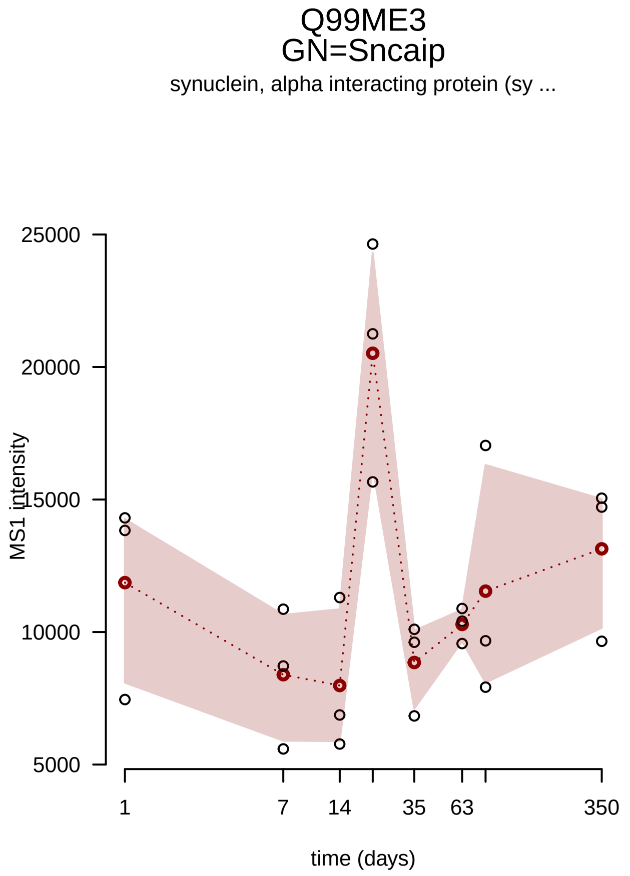
Q99ME3
GN=Sncaip
synuclein, alpha interacting protein (sy ...
MS1 intensity
25000
20000
15000
10000
5000
1
7
14
35
63
350
time (days)

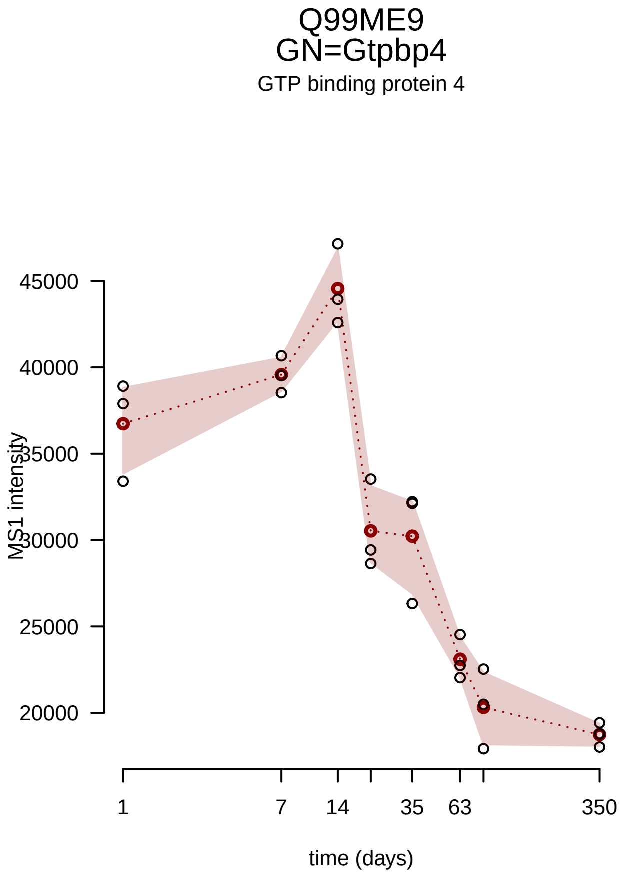
Q99ME9
GN=Gtpbp4
GTP binding protein 4
MS1 intensity
45000
40000
35000
30000
25000
20000
1
7
14
35
63
350
time (days)

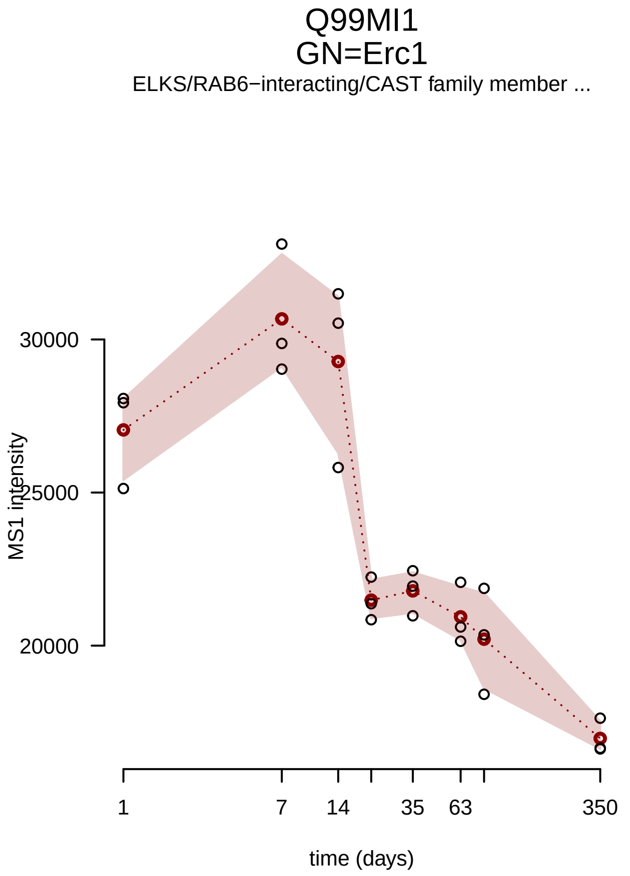
Q99MI1
GN=Erc1
ELKS/RAB6−interacting/CAST family member ...
MS1 intensity
30000
25000
20000
1
7
14
35
63
350
time (days)

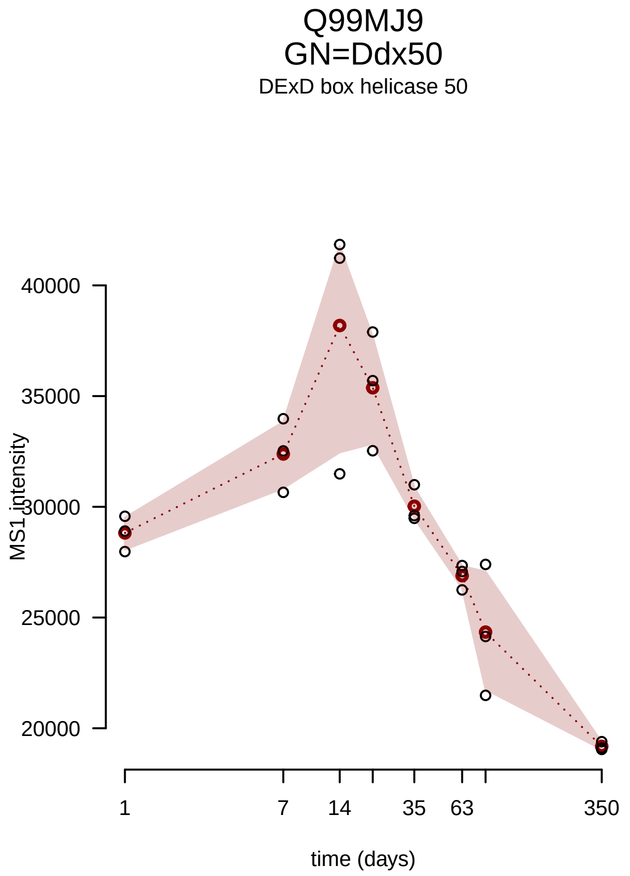
Q99MJ9
GN=Ddx50
DExD box helicase 50
MS1 intensity
40000
35000
30000
25000
20000
1
7
14
35
63
350
time (days)

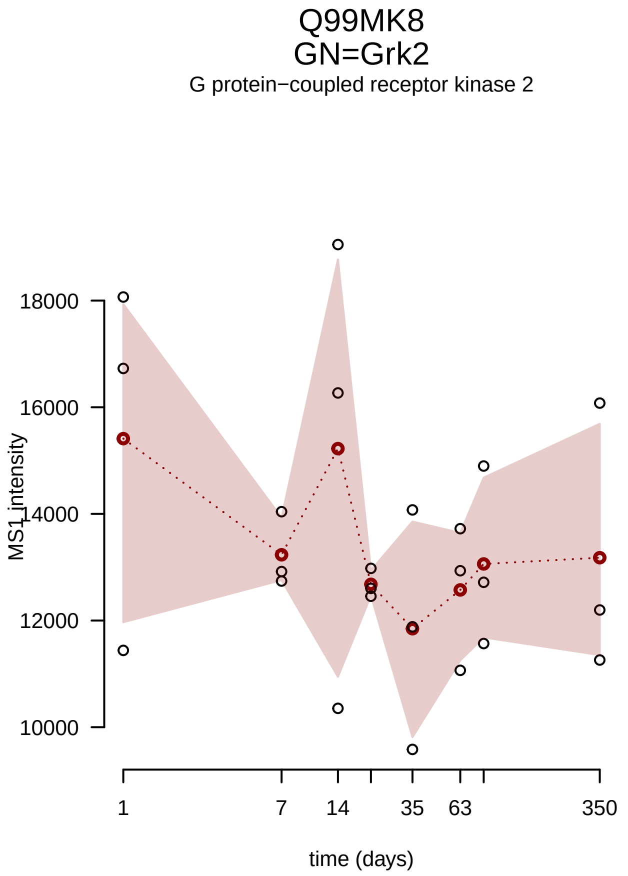
Q99MK8
GN=Grk2
G protein−coupled receptor kinase 2
MS1 intensity
18000
16000
14000
12000
10000
1
7
14
35
63
350
time (days)

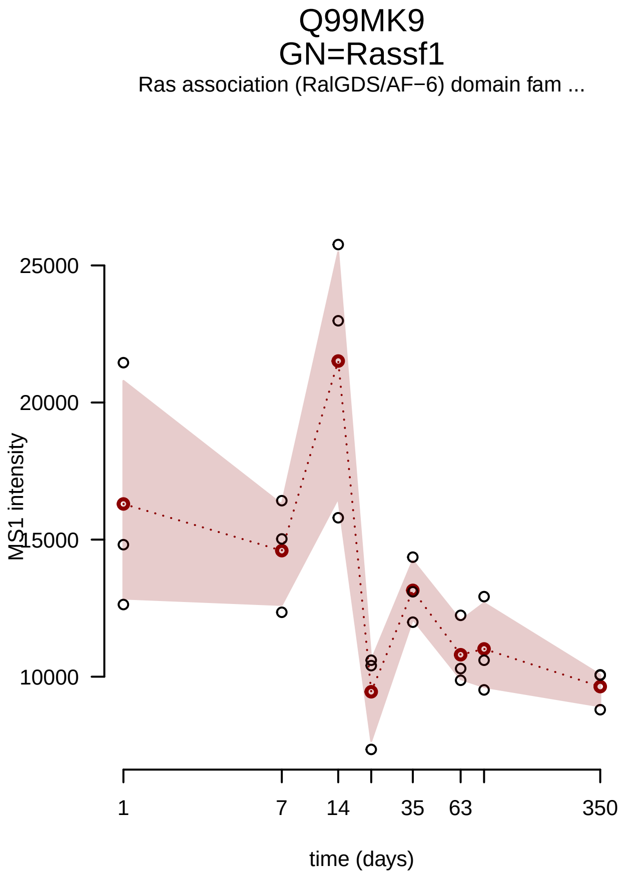
Q99MK9
GN=Rassf1
Ras association (RalGDS/AF−6) domain fam ...
MS1 intensity
25000
20000
15000
10000
1
7
14
35
63
350
time (days)

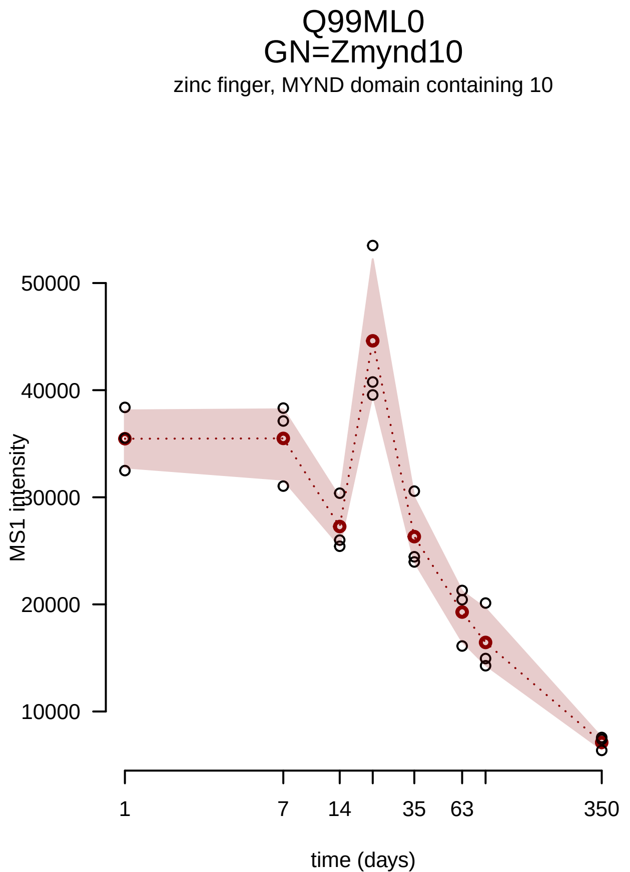
Q99ML0
GN=Zmynd10
zinc finger, MYND domain containing 10
MS1 intensity
50000
40000
30000
20000
10000
1
7
14
35
63
350
time (days)

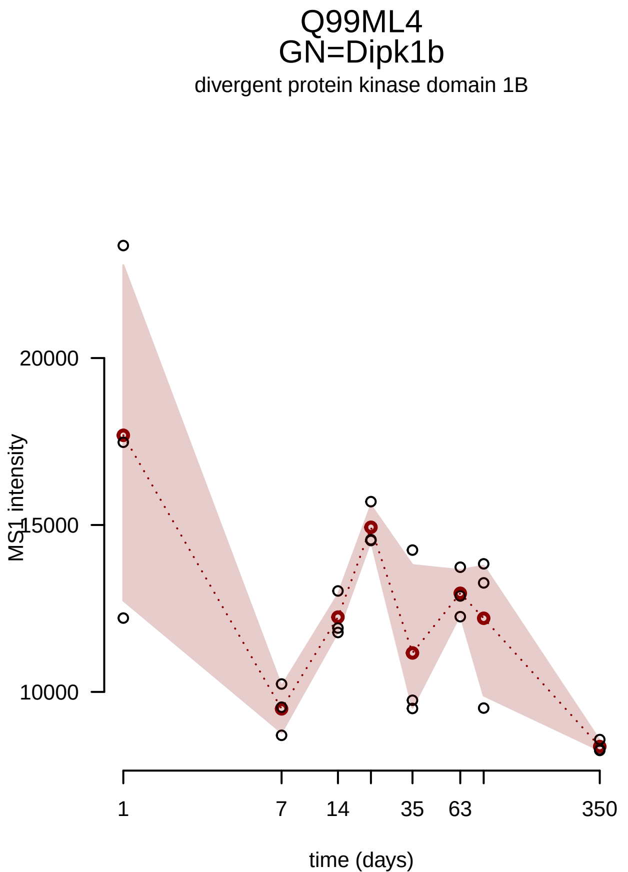
Q99ML4
GN=Dipk1b
divergent protein kinase domain 1B
MS1 intensity
20000
15000
10000
1
7
14
35
63
350
time (days)

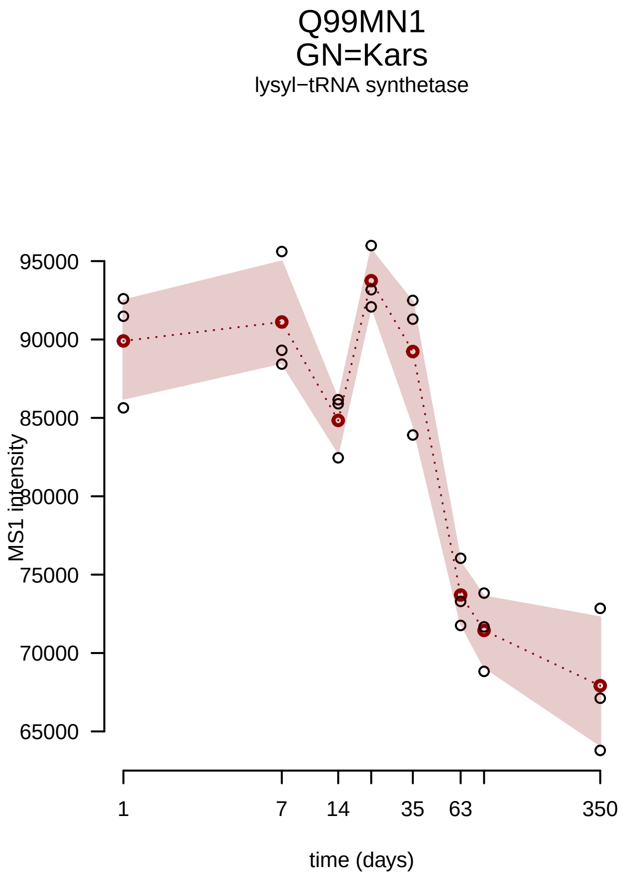
Q99MN1
GN=Kars
lysyl−tRNA synthetase
MS1 intensity
95000
90000
85000
80000
75000
70000
65000
1
7
14
35
63
350
time (days)

Q99MN9
GN=Pccb
propionyl Coenzyme A carboxylase, beta p ...

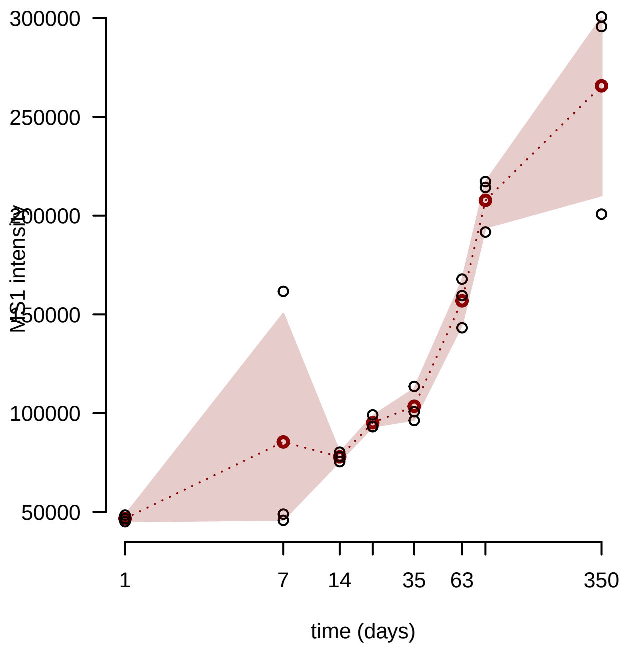

Q99MP8
GN=Brap
BRCA1 associated protein

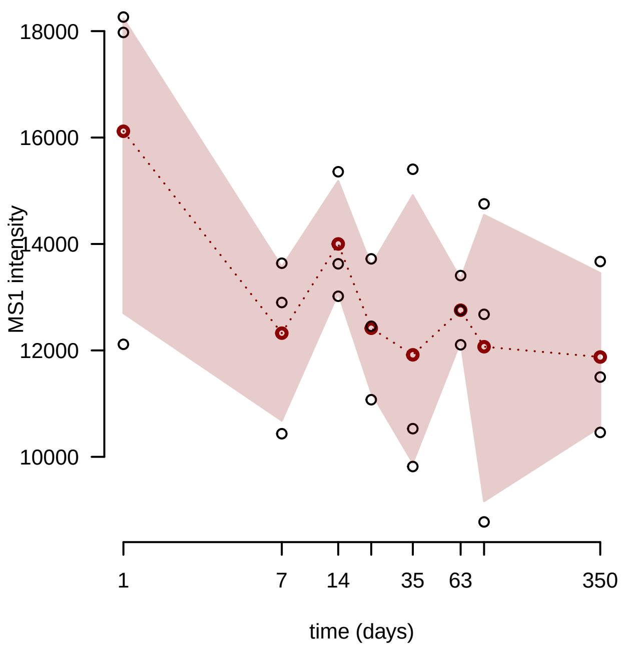

Q99MQ1
GN=Bicc1
BicC family RNA binding protein 1

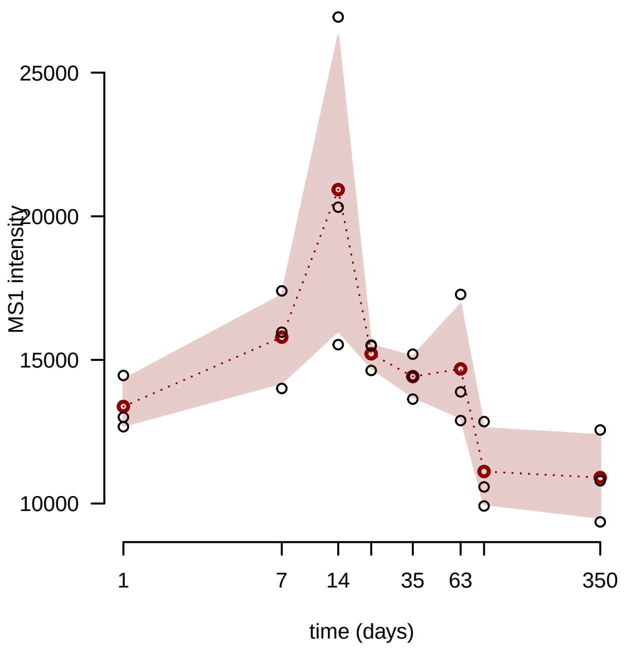

Q99MQ4
GN=Aspn
asporin

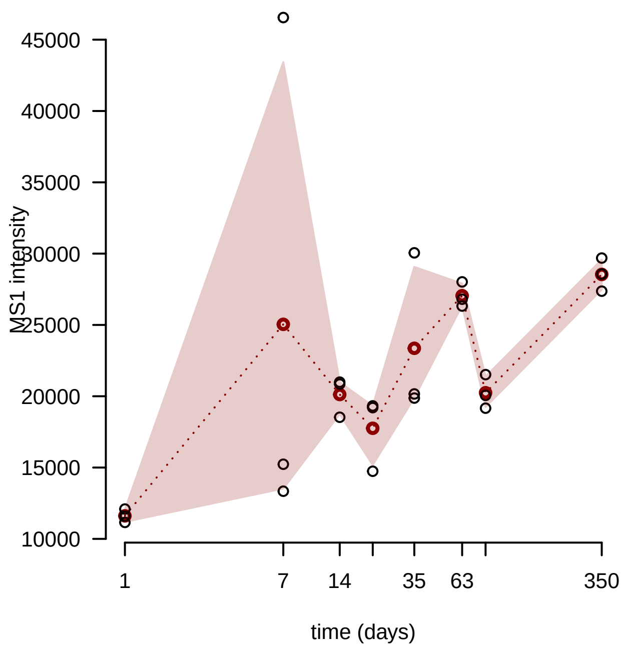

Q99MR1
GN=Gigyf1
GRB10 interacting GYF protein 1

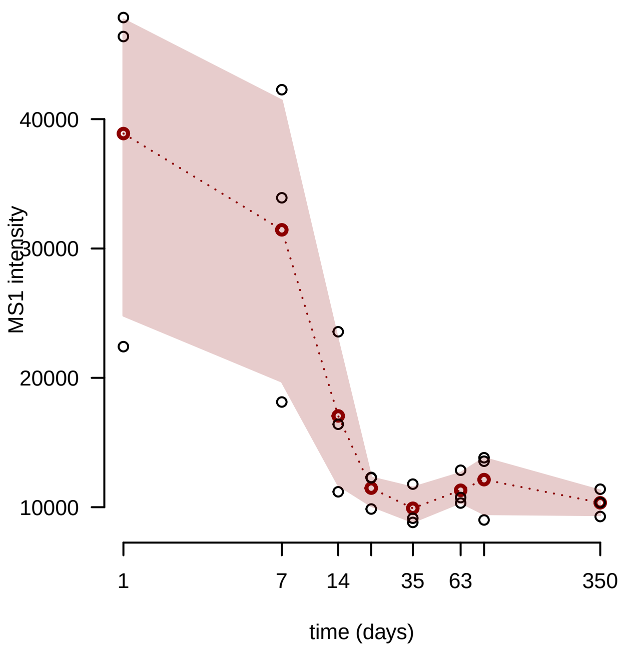

Q99MR3
GN=Slc12a9
solute carrier family 12 (potassium/chlo ...

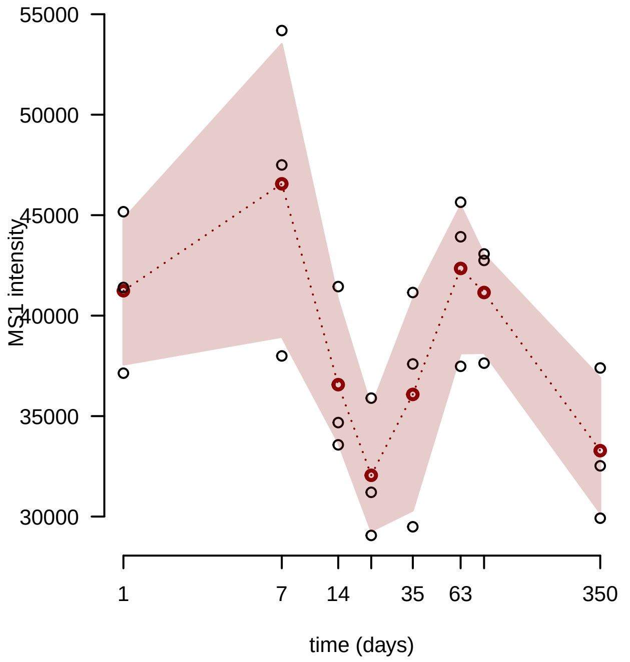

Q99MR6
GN=Srrt
serrate RNA effector molecule homolog (A ...

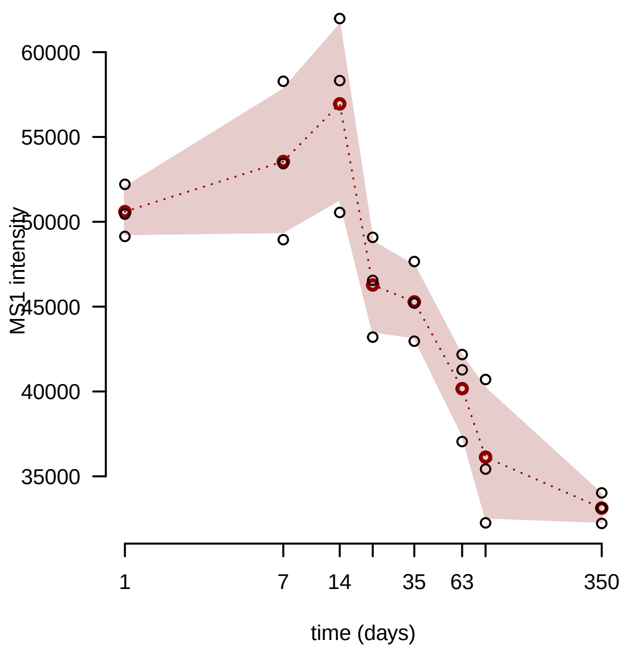

Q99MR8
GN=Mccc1
methylcrotonoyl−Coenzyme A carboxylase 1 ...

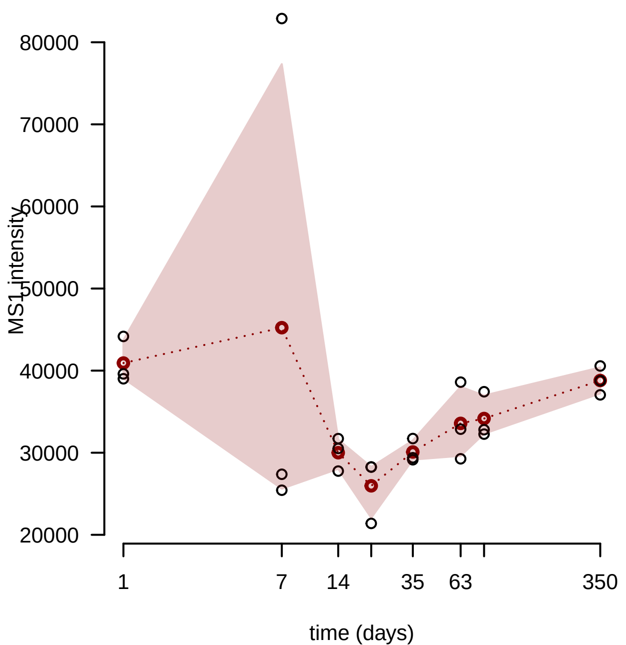

Q99MS7
GN=Ehbp1l1
EH domain binding protein 1−like 1

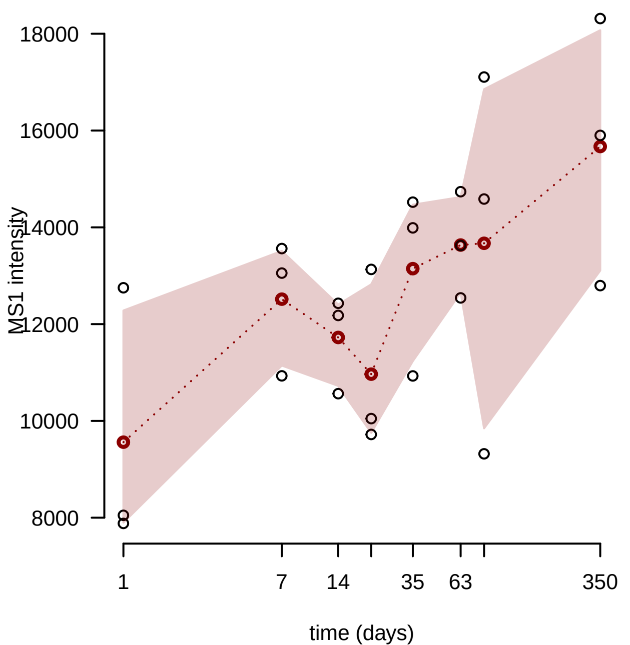

Q99MS8
GN=Tpgs1
tubulin polyglutamylase complex subunit ...

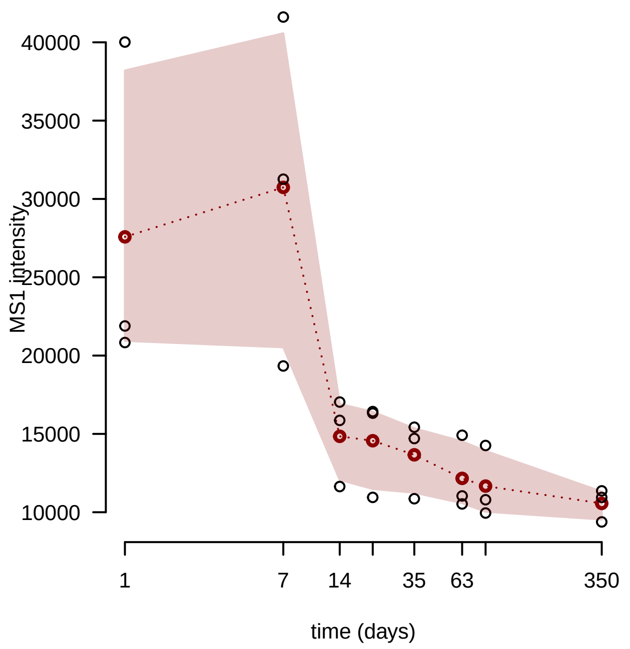

Q99MU3
GN=Adar
adenosine deaminase, RNA−specific

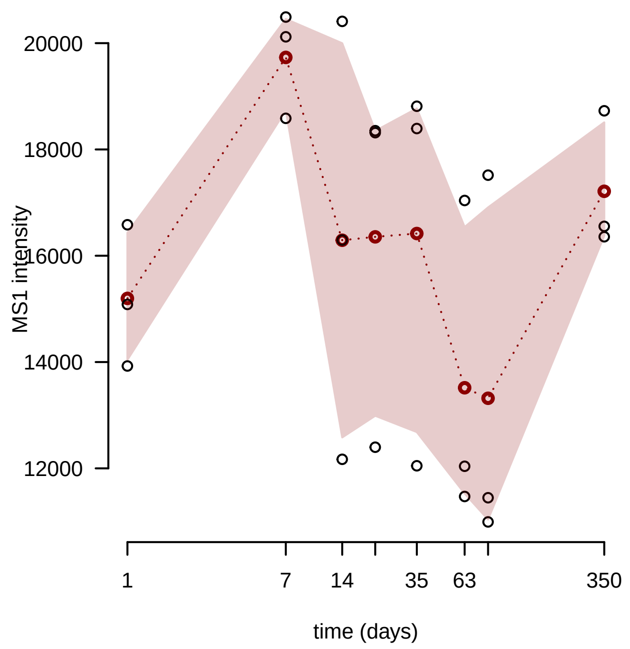

Q99MV1
GN=Tdrd1
tudor domain containing 1

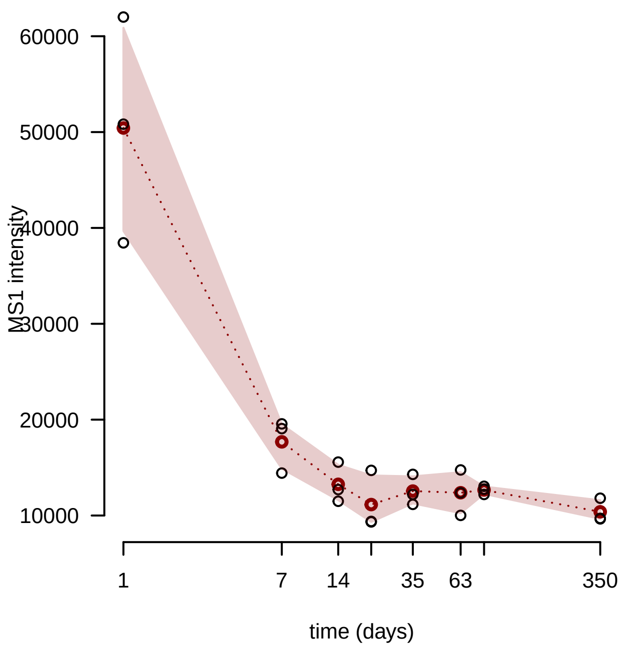

Q99MV5
GN=Mov10l1
Mov10 like RISC complex RNA helicase 1

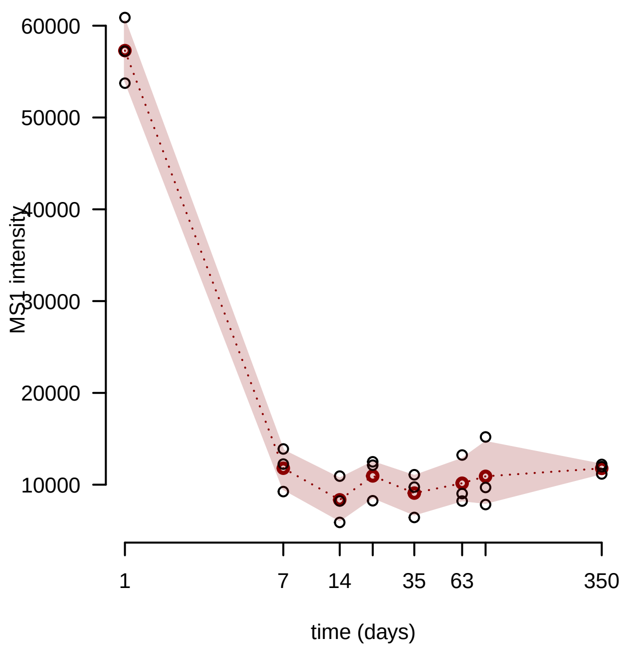

Q99MV7
GN=Rnf17
ring finger protein 17

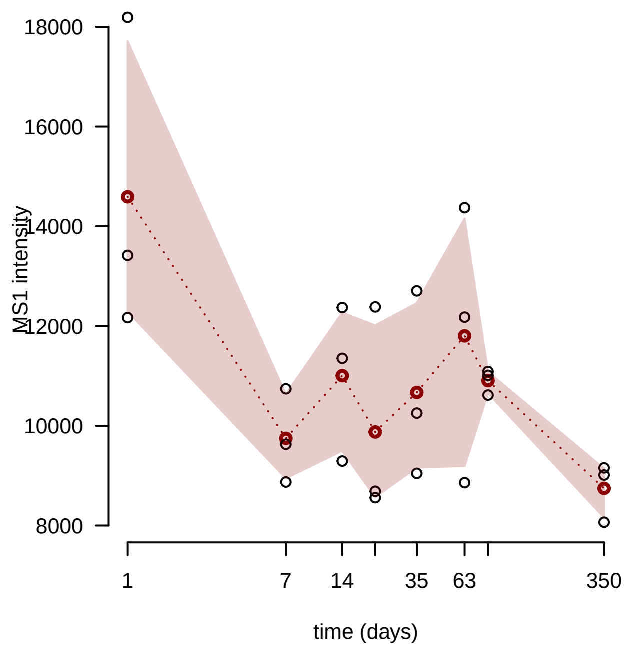

Q99MW1
GN=Stk31
serine threonine kinase 31

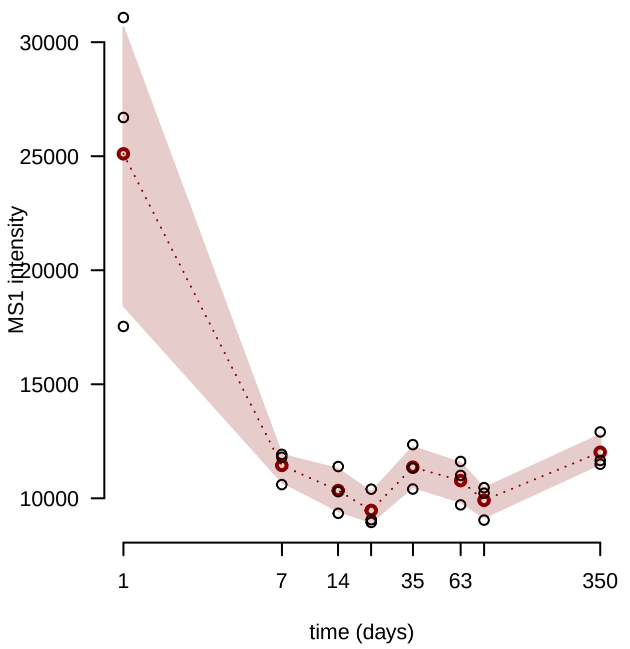

Q99MX0
GN=Tktl1
transketolase−like 1

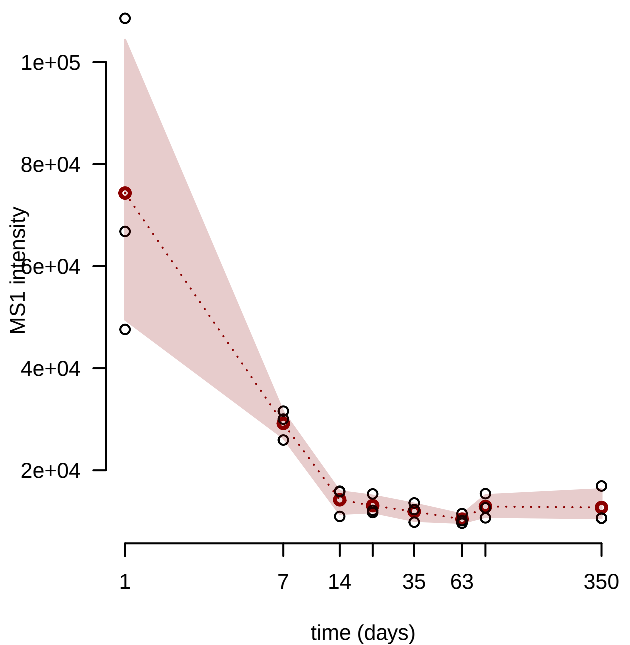

Q99MY8
GN=Ash1l
ASH1 like histone lysine methyltransfera ...

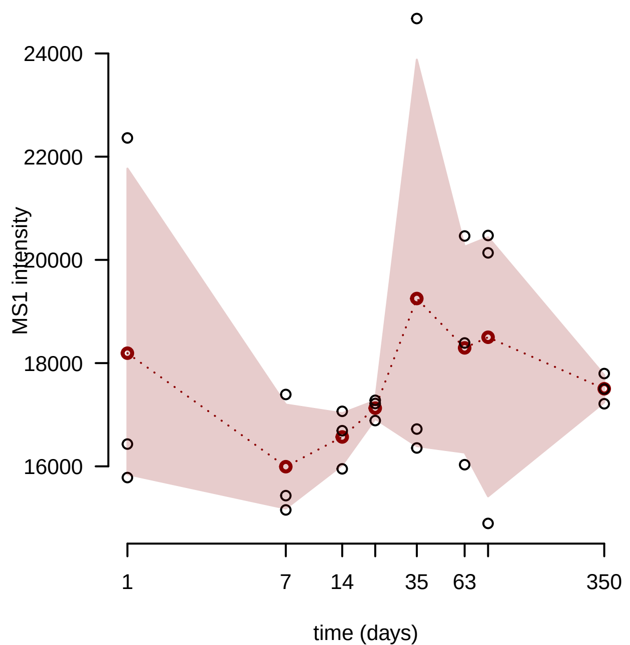

Q99MZ6
GN=Myo7b
myosin VIIB

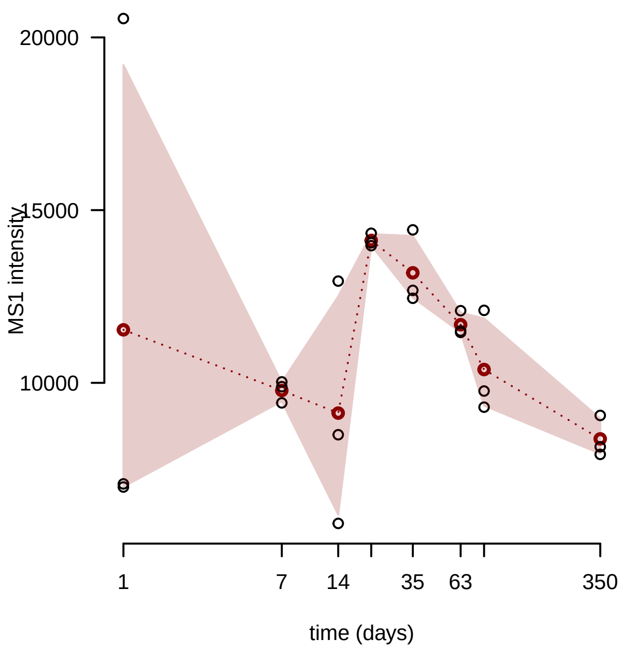

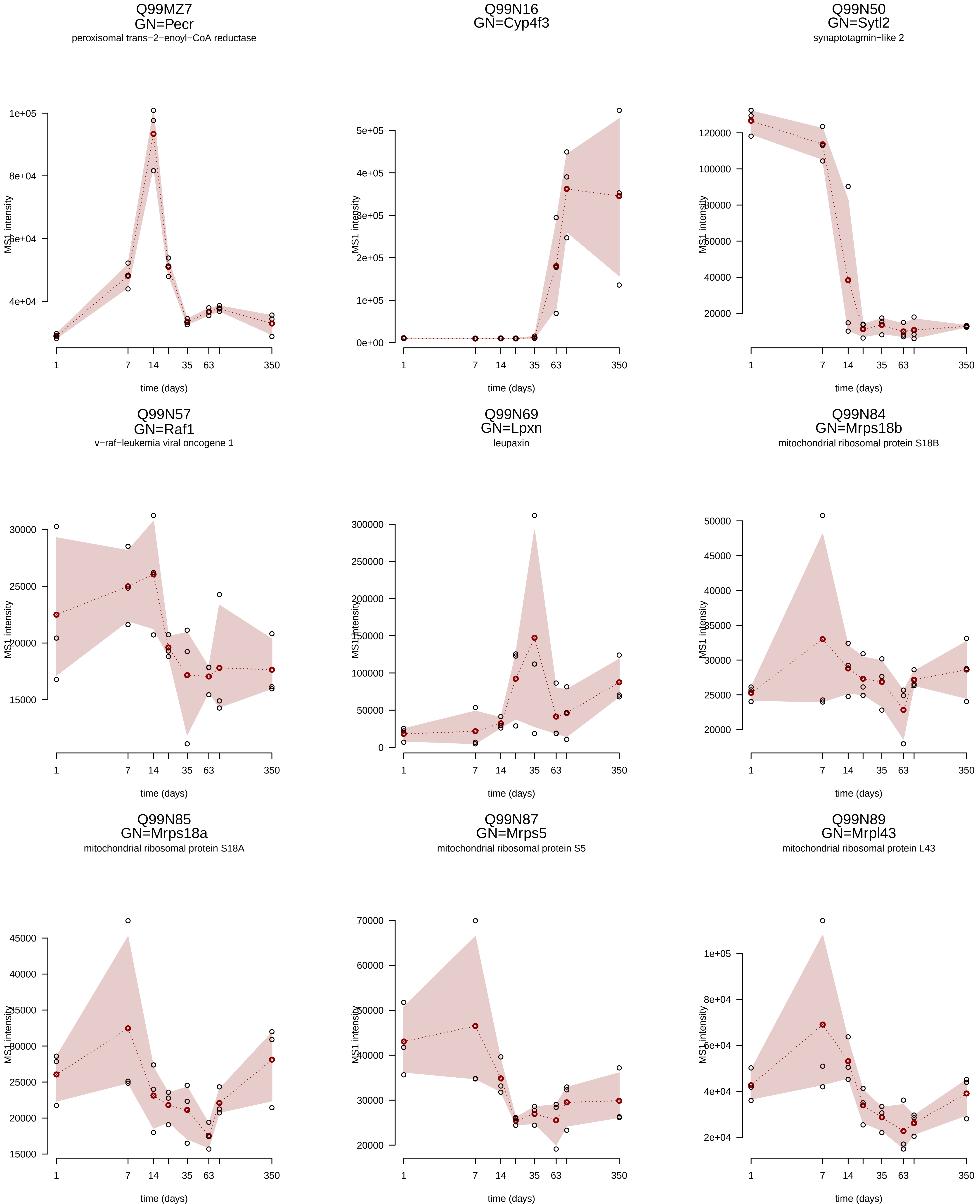
Q99MZ7
GN=Pecr
peroxisomal trans−2−enoyl−CoA reductase
Q99N16
GN=Cyp4f3
Q99N50
GN=Sytl2
synaptotagmin−like 2
Q99N57
GN=Raf1
v−raf−leukemia viral oncogene 1
Q99N69
GN=Lpxn
leupaxin
Q99N84
GN=Mrps18b
mitochondrial ribosomal protein S18B
Q99N85
GN=Mrps18a
mitochondrial ribosomal protein S18A
Q99N87
GN=Mrps5
mitochondrial ribosomal protein S5
Q99N89
GN=Mrpl43
mitochondrial ribosomal protein L43
MS1 intensity
time (days)
1
7
14
35
63
350

Q99N91
GN=Mrpl34
mitochondrial ribosomal protein L34

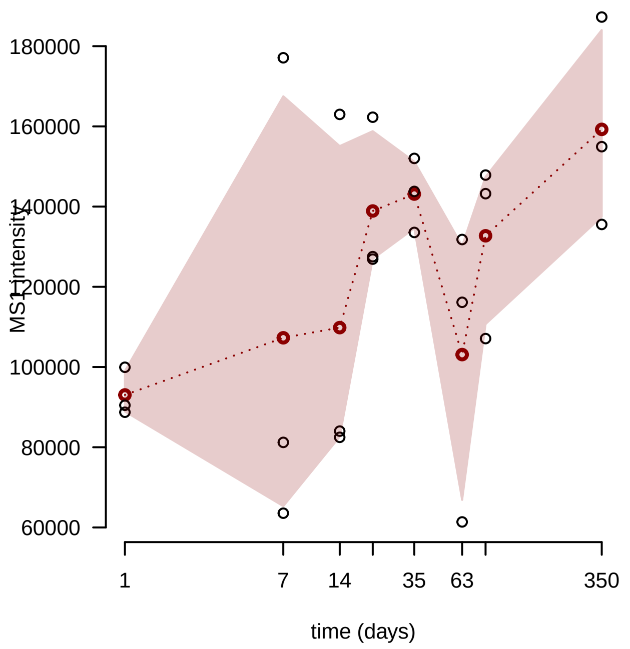

Q99N92
GN=Mrpl27
mitochondrial ribosomal protein L27

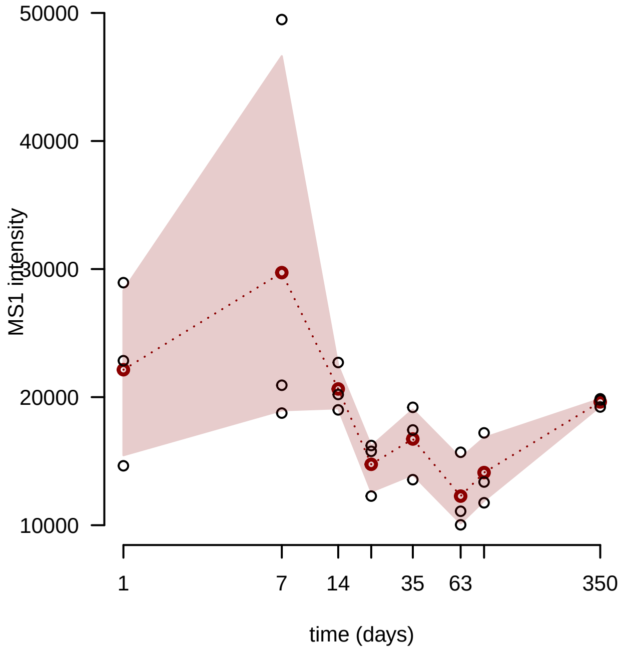

Q99N93
GN=Mrpl16
mitochondrial ribosomal protein L16

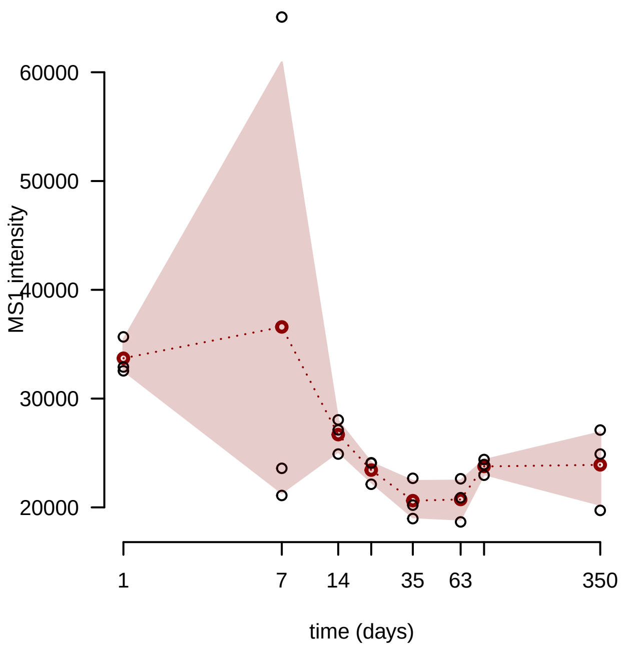

Q99N94
GN=Mrpl9
mitochondrial ribosomal protein L9

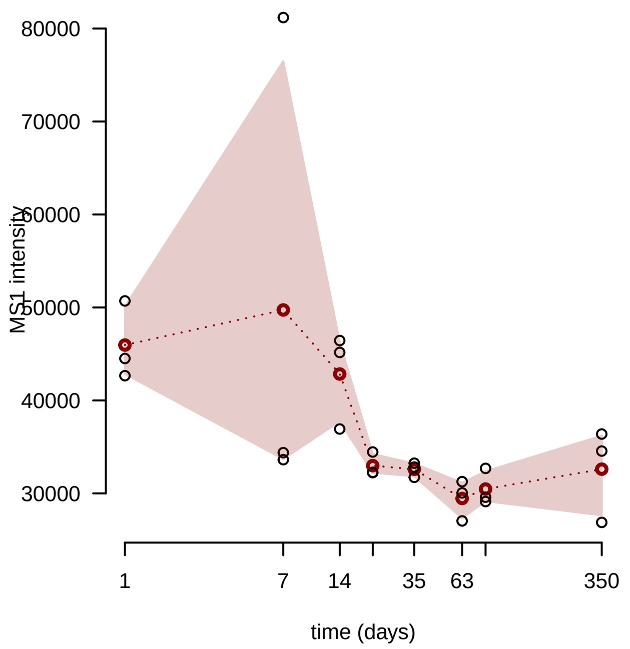

Q99N95
GN=Mrpl3
mitochondrial ribosomal protein L3

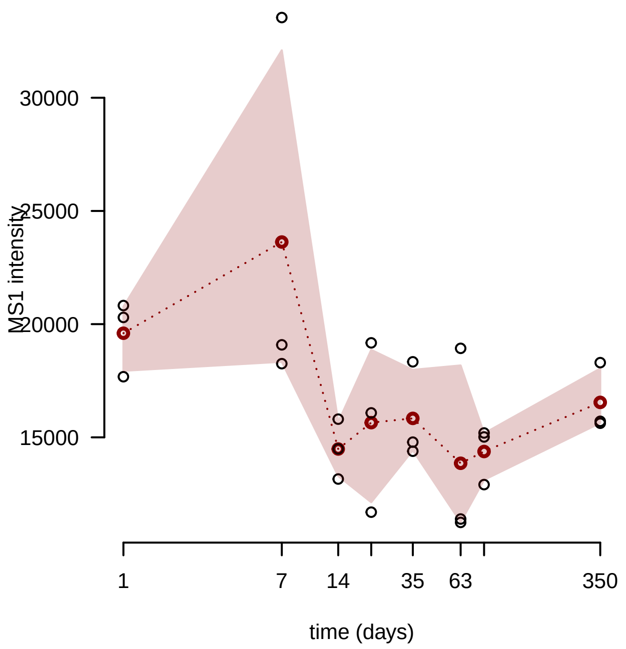

Q99N96
GN=Mrpl1
mitochondrial ribosomal protein L1

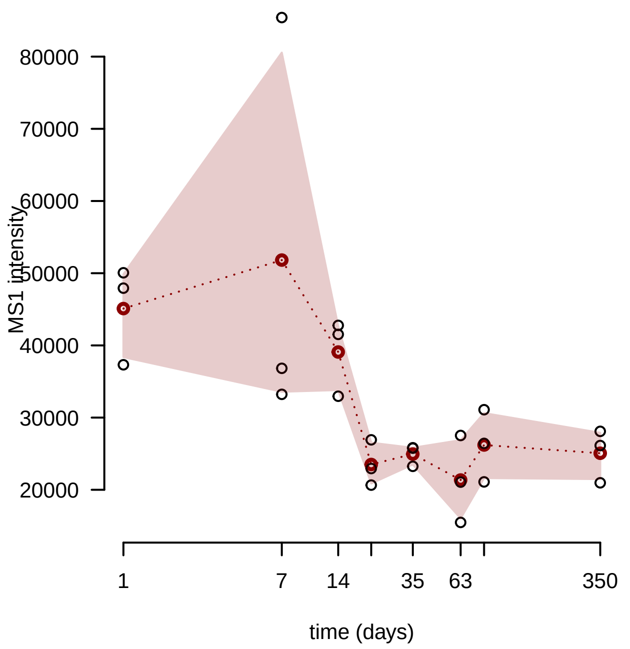

Q99N99
GN=Srd5a2
steroid 5 alpha−reductase 2

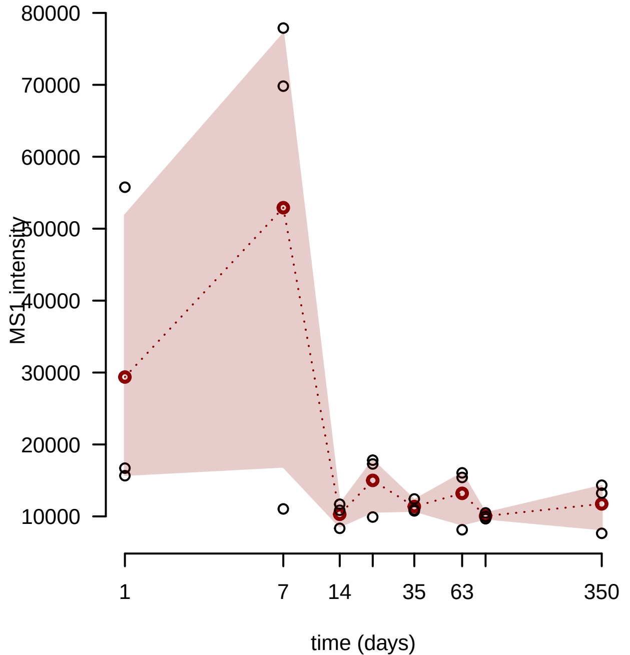

Q99NA9
GN=Pcgf6
polycomb group ring finger 6

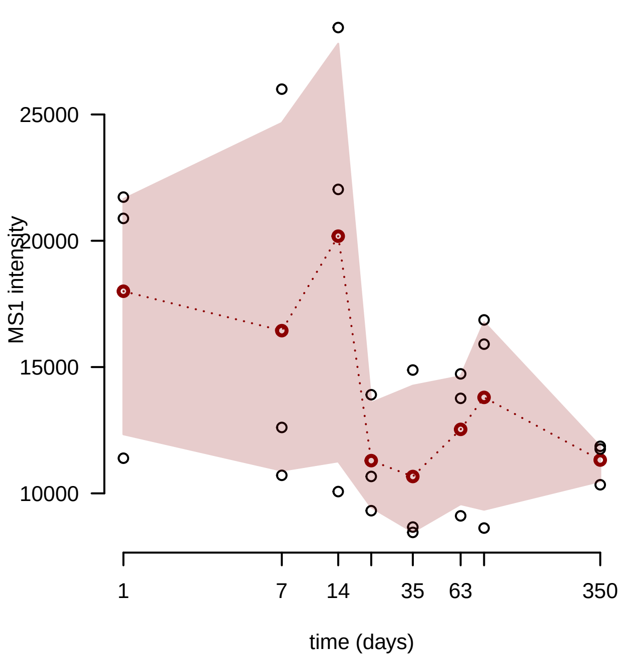

Q99NB1
GN=Acss1
acyl−CoA synthetase short−chain family m ...

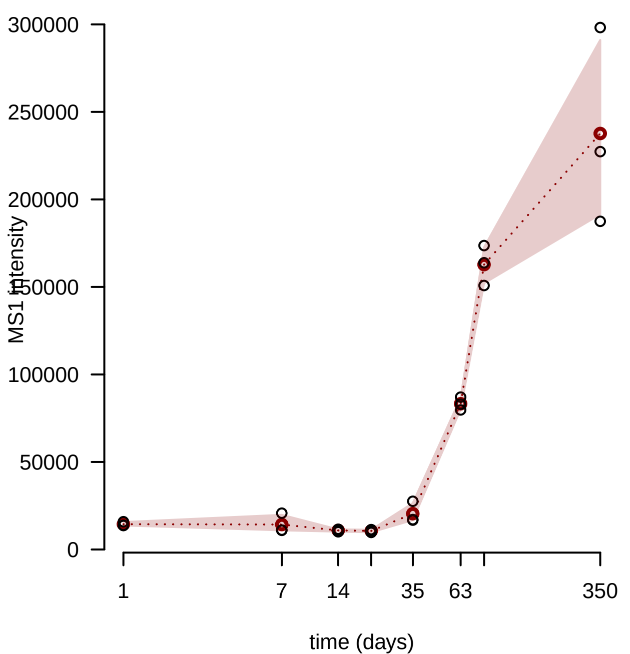

Q99NB8
GN=Ubqln4
ubiquilin 4

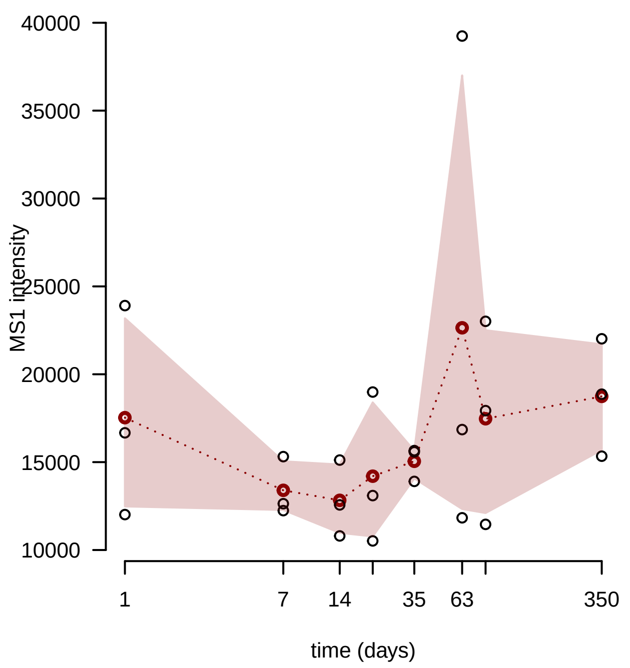

Q99NB9
GN=Sf3b1
splicing factor 3b, subunit 1

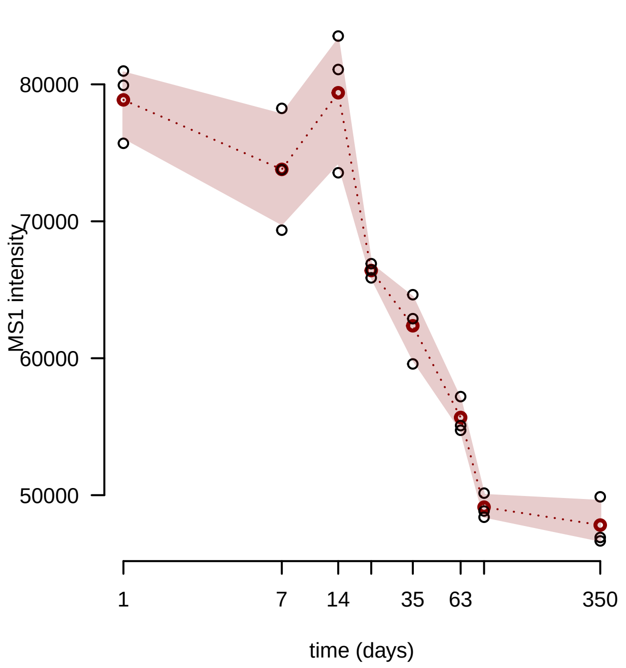

Q99NF3
GN=Cep41
centrosomal protein 41

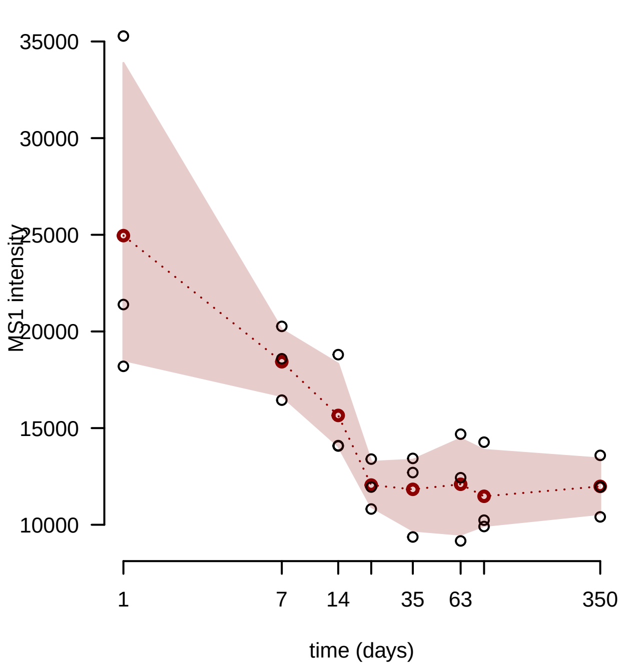

Q99NF8
GN=Ranbp17
RAN binding protein 17

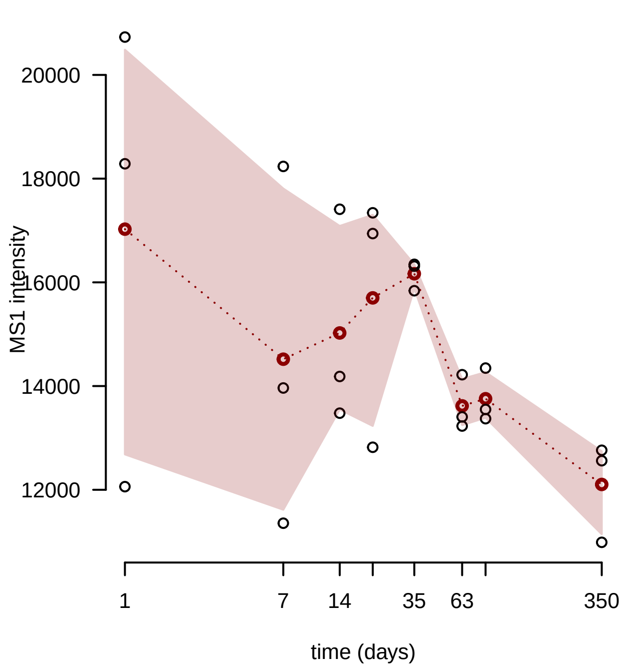

Q99NG0
GN=Rad54l2
RAD54 like 2 (S. cerevisiae)

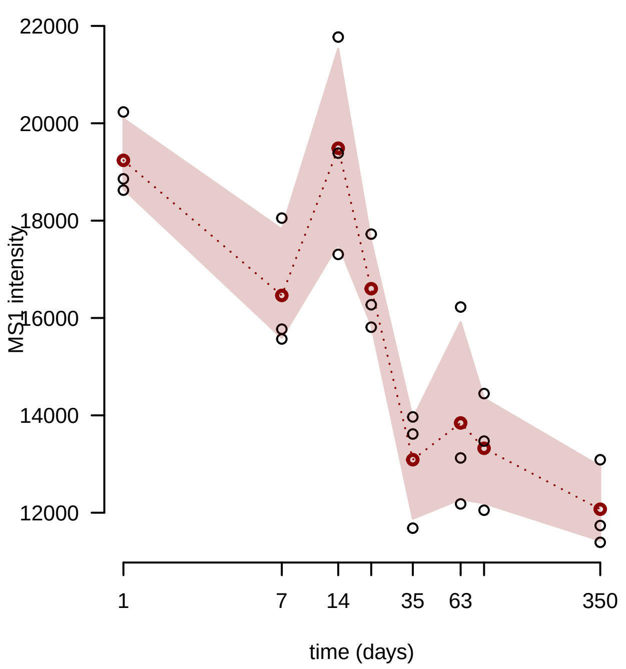

Q99NH0
GN=Ankrd17
ankyrin repeat domain 17

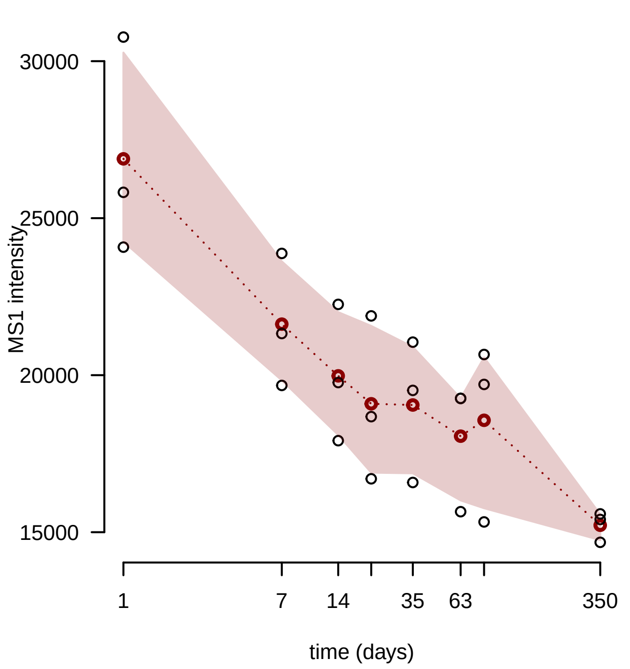

Q99NH2
GN=Pard3
par−3 family cell polarity regulator

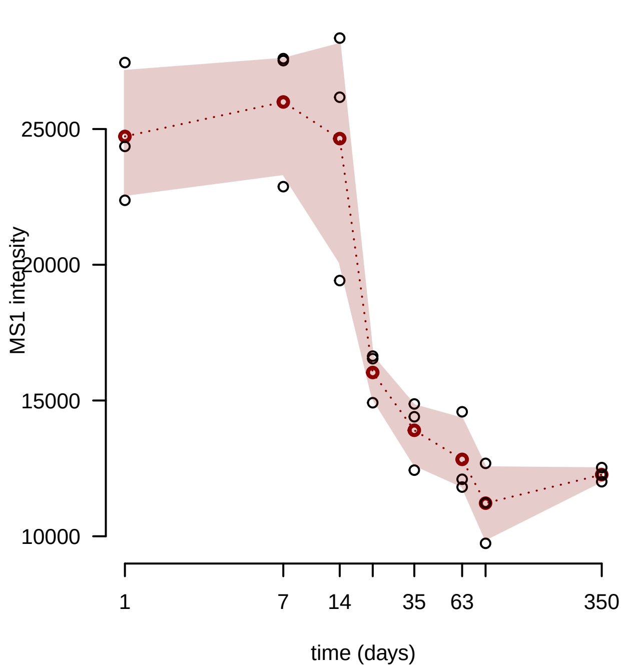

Q99P30
GN=Nudt7
nudix (nucleoside diphosphate linked moi ...

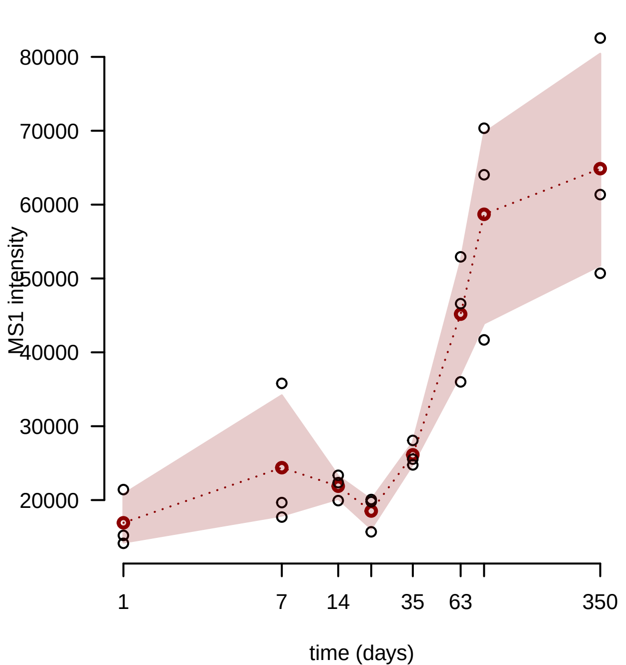

Q99P31
GN=Hspbp1
HSPA (heat shock 70kDa) binding protein, ...

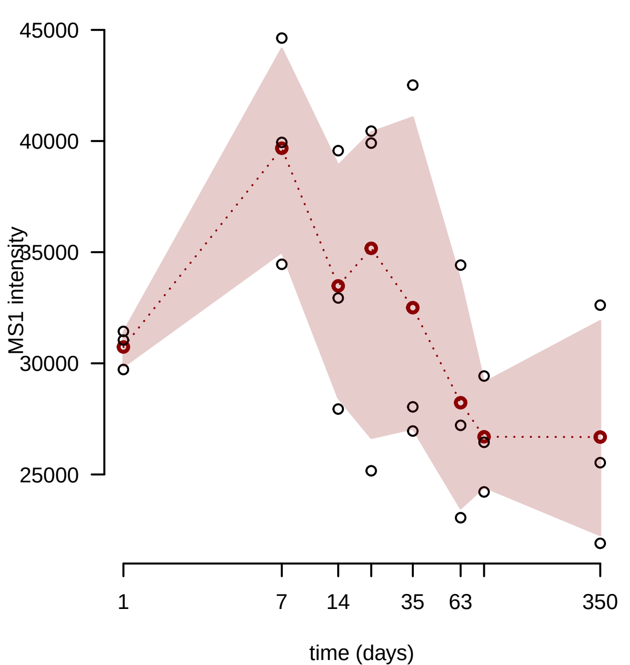

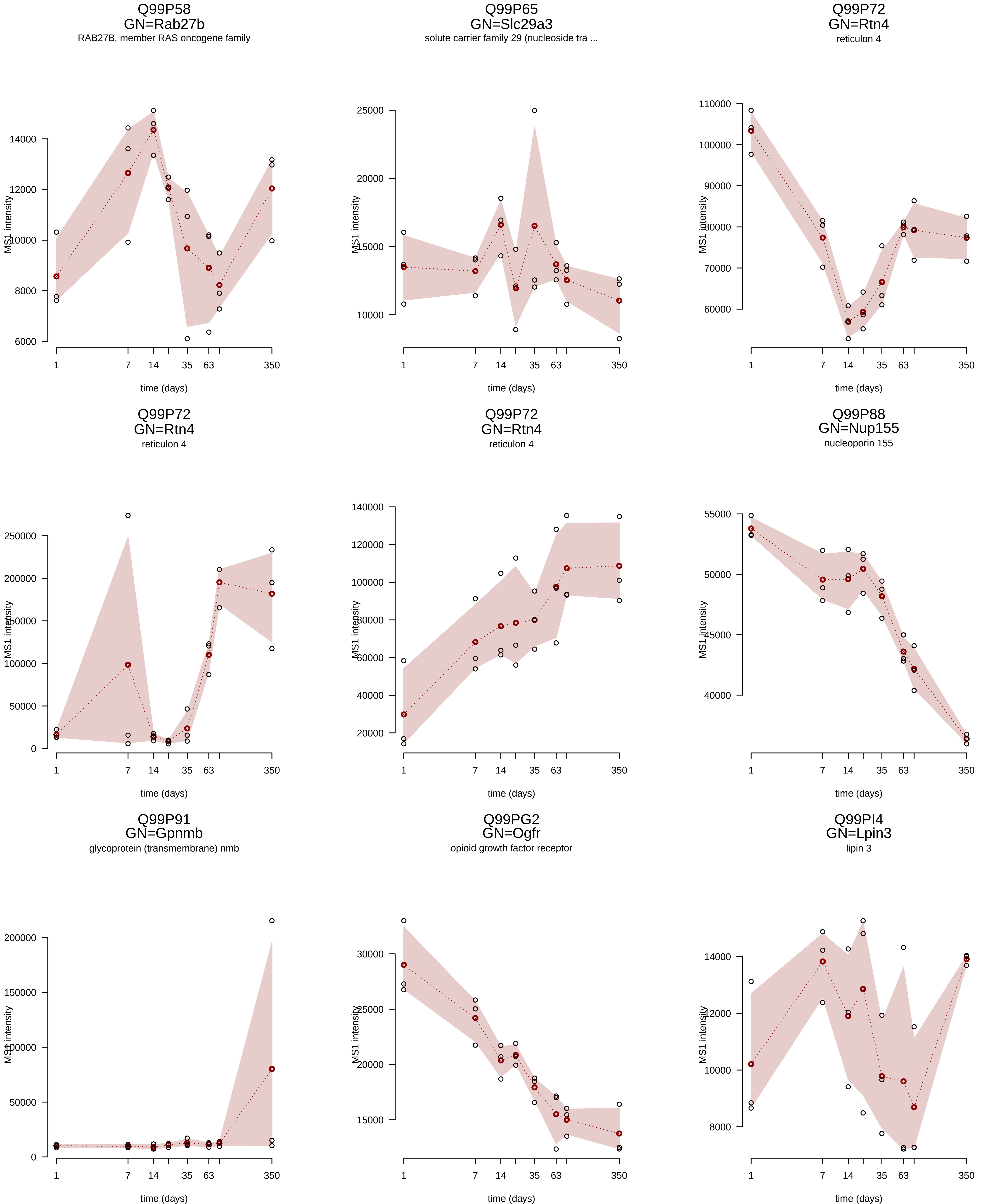
Q99P58
GN=Rab27b
RAB27B, member RAS oncogene family
MS1 intensity
6000
8000
10000
12000
14000
1
7
14
35
63
350
time (days)
Q99P65
GN=Slc29a3
solute carrier family 29 (nucleoside tra ...
MS1 intensity
10000
15000
20000
25000
1
7
14
35
63
350
time (days)
Q99P72
GN=Rtn4
reticulon 4
MS1 intensity
60000
70000
80000
90000
100000
110000
1
7
14
35
63
350
time (days)
Q99P72
GN=Rtn4
reticulon 4
MS1 intensity
0
50000
100000
150000
200000
250000
1
7
14
35
63
350
time (days)
Q99P72
GN=Rtn4
reticulon 4
MS1 intensity
20000
40000
60000
80000
100000
120000
140000
1
7
14
35
63
350
time (days)
Q99P88
GN=Nup155
nucleoporin 155
MS1 intensity
40000
45000
50000
55000
1
7
14
35
63
350
time (days)
Q99P91
GN=Gpnmb
glycoprotein (transmembrane) nmb
MS1 intensity
0
50000
100000
150000
200000
1
7
14
35
63
350
time (days)
Q99PG2
GN=Ogfr
opioid growth factor receptor
MS1 intensity
15000
20000
25000
30000
1
7
14
35
63
350
time (days)
Q99PI4
GN=Lpin3
lipin 3
MS1 intensity
8000
10000
12000
14000
1
7
14
35
63
350
time (days)

Q99PI5
GN=Lpin2
lipin 2

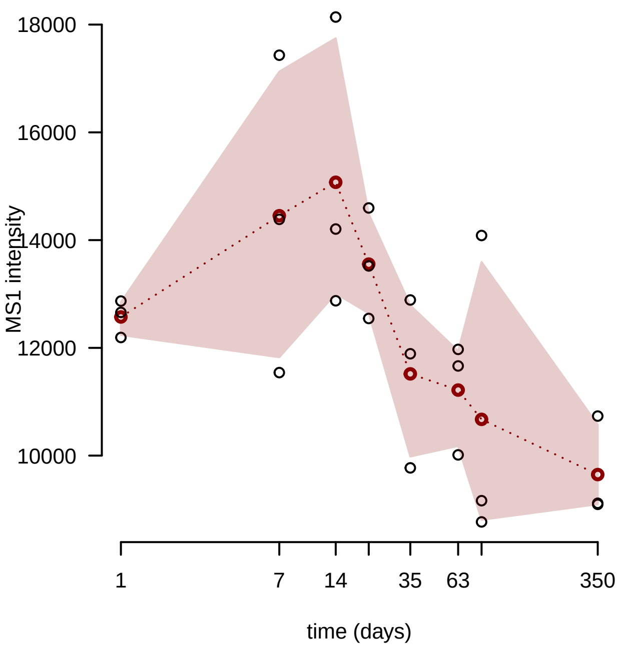

Q99PL5
GN=Rrbp1
ribosome binding protein 1

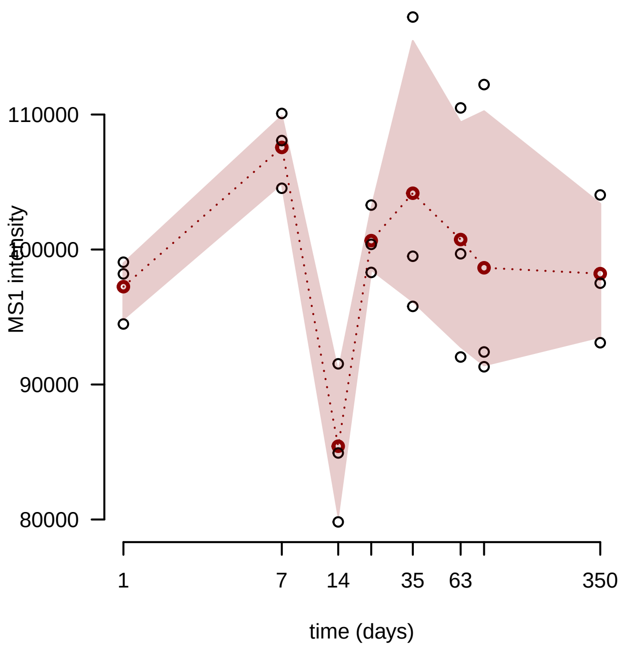

Q99PL6
GN=Ubxn6
UBX domain protein 6

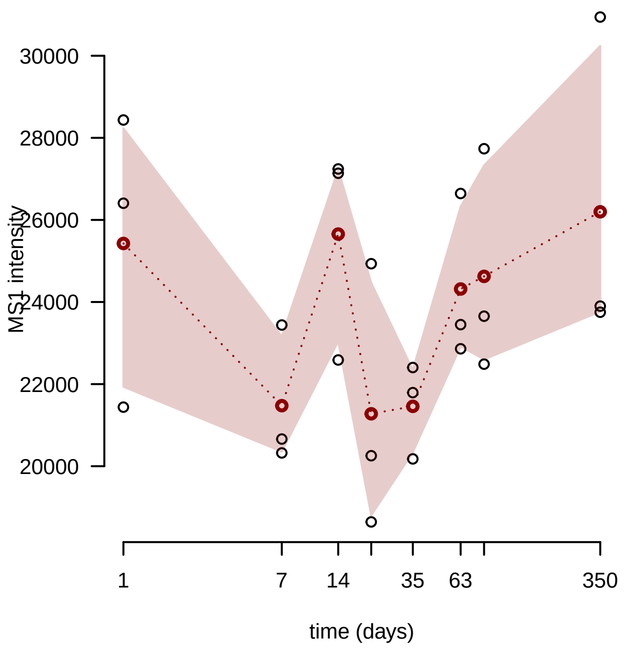

Q99PM3
GN=Gtf2a1
general transcription factor II A, 1

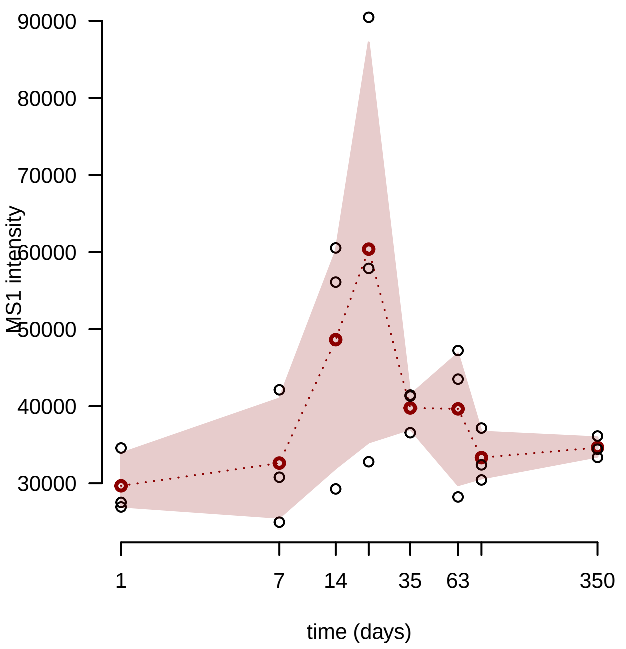

Q99PM9
GN=Uck2
uridine−cytidine kinase 2

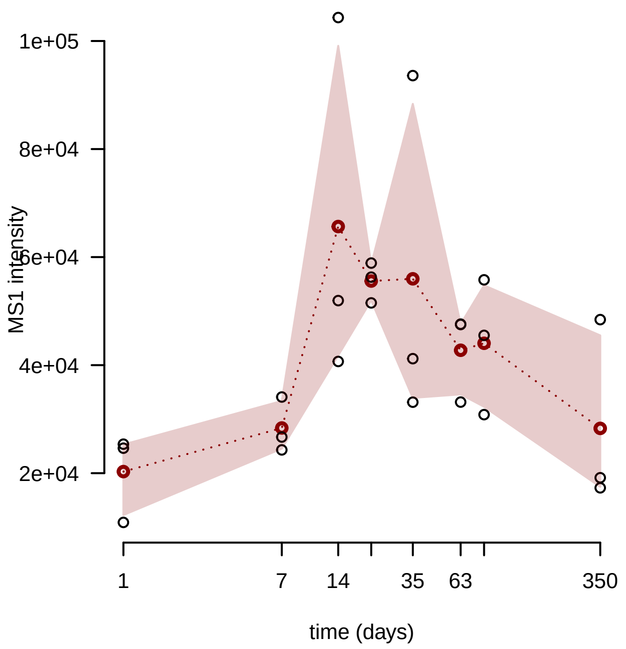

Q99PN3
GN=Trim26
tripartite motif−containing 26

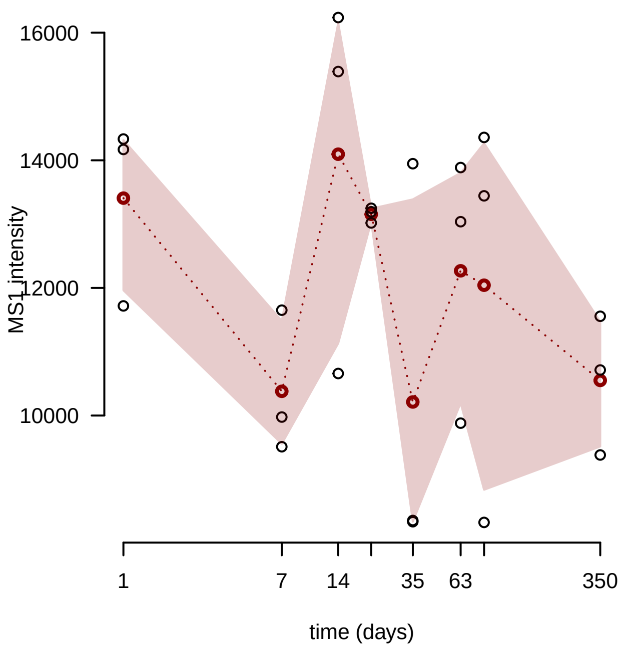

Q99PP2
GN=Znf318

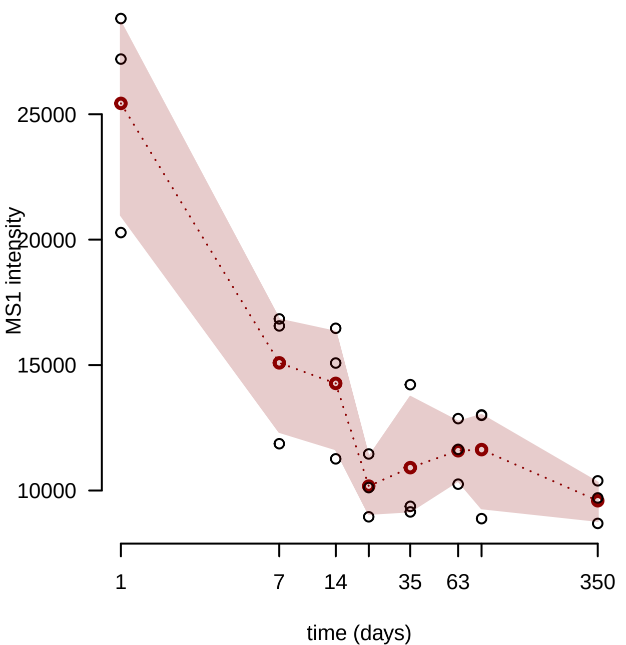

Q99PP7
GN=Trim33
tripartite motif−containing 33

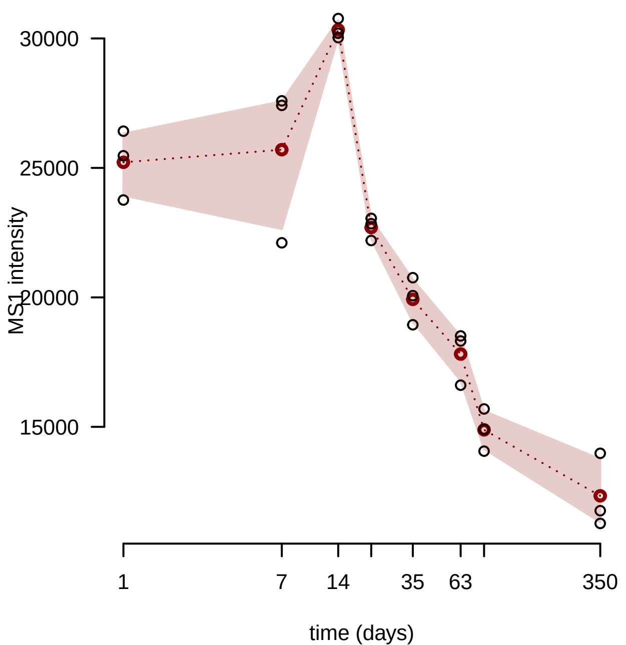

Q99PP7
GN=Trim33
tripartite motif−containing 33

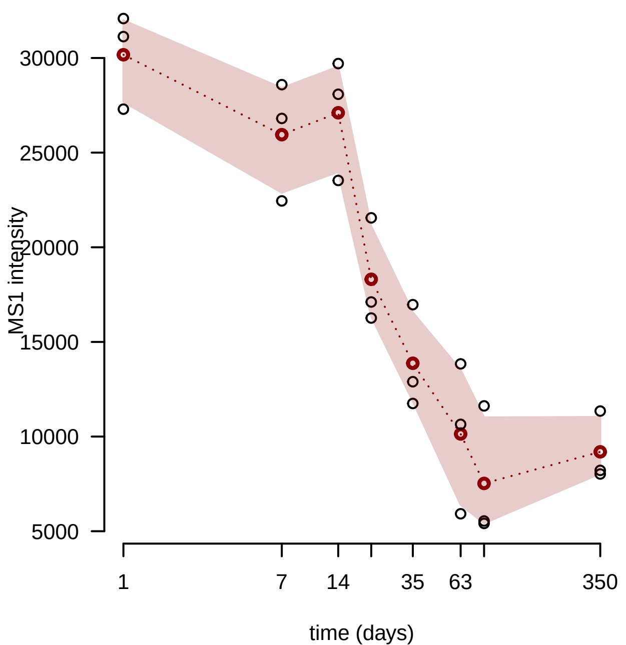

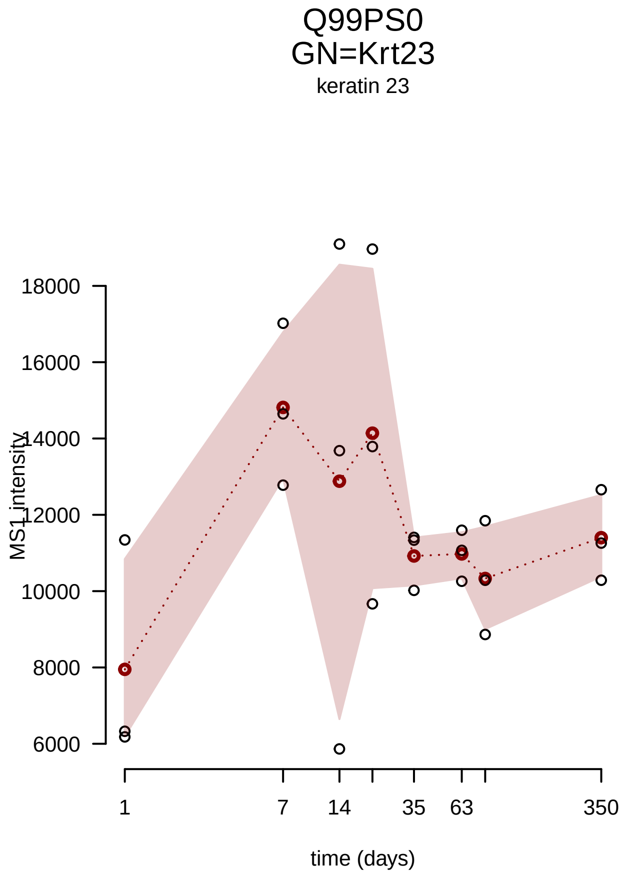
Q99PS0
GN=Krt23
keratin 23
MS1 intensity
6000
8000
10000
12000
14000
16000
18000
1
7
14
35
63
350
time (days)

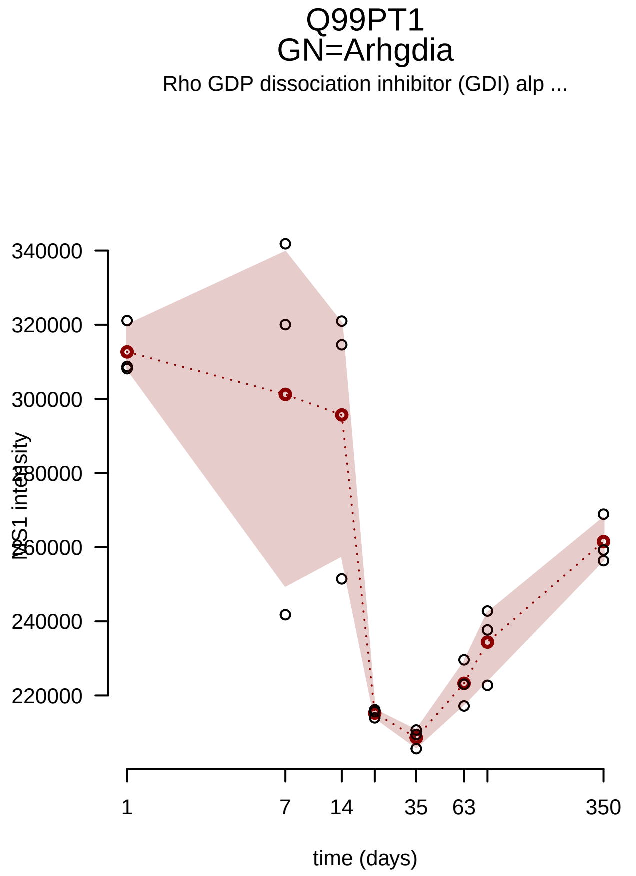
Q99PT1
GN=Arhgdia
Rho GDP dissociation inhibitor (GDI) alp ...
MS1 intensity
220000
240000
260000
280000
300000
320000
340000
1
7
14
35
63
350
time (days)

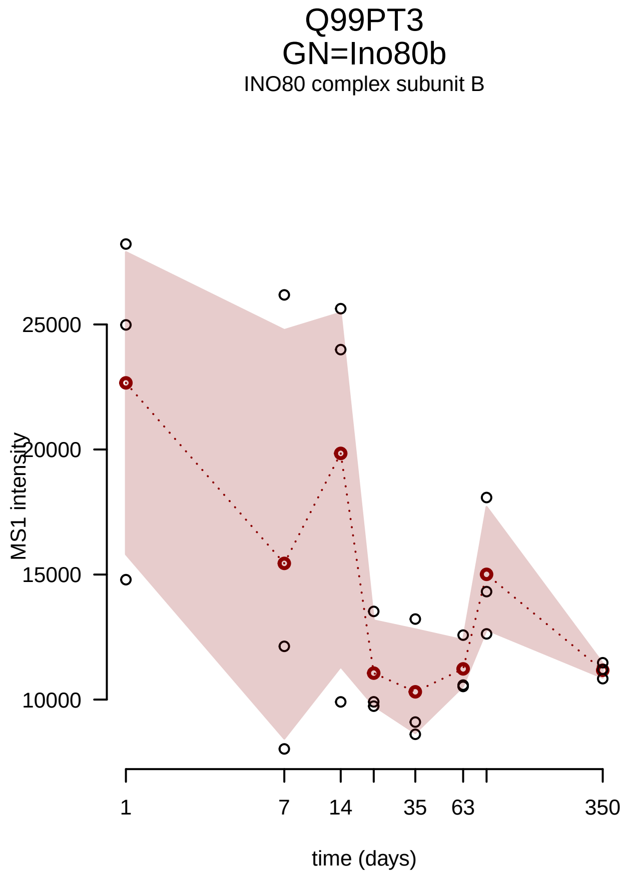
Q99PT3
GN=Ino80b
INO80 complex subunit B
MS1 intensity
10000
15000
20000
25000
1
7
14
35
63
350
time (days)

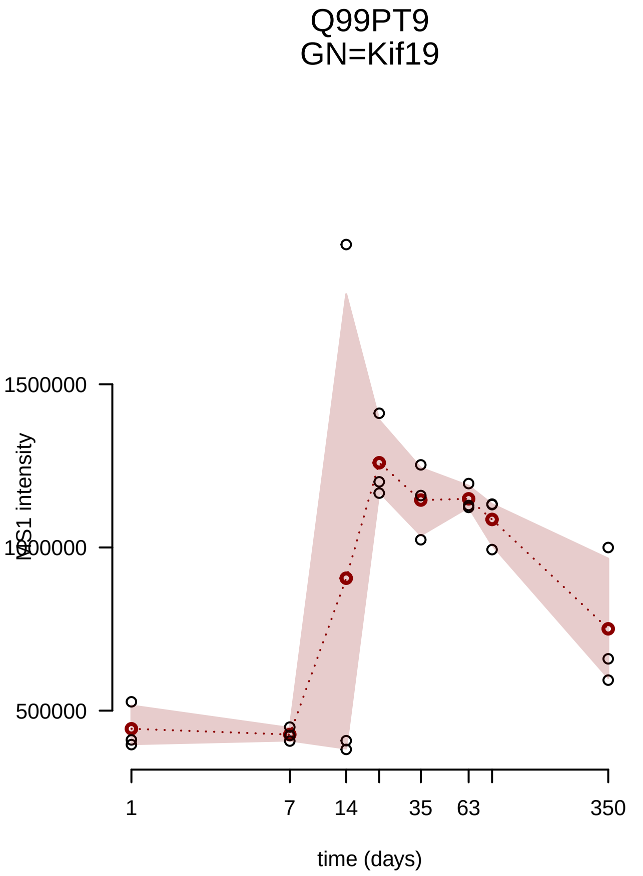
Q99PT9
GN=Kif19
MS1 intensity
500000
1000000
1500000
1
7
14
35
63
350
time (days)

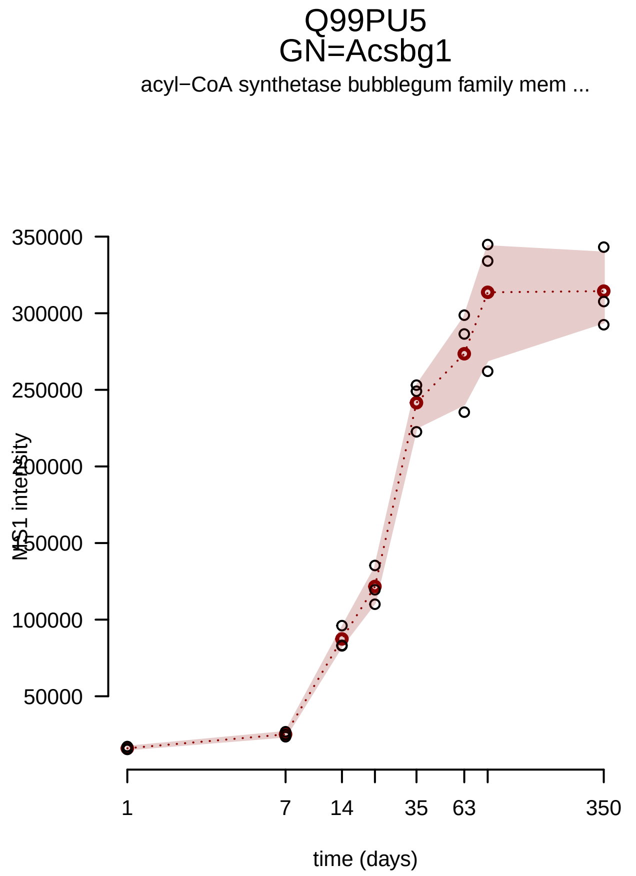
Q99PU5
GN=Acsbg1
acyl−CoA synthetase bubblegum family mem ...
MS1 intensity
50000
100000
150000
200000
250000
300000
350000
1
7
14
35
63
350
time (days)

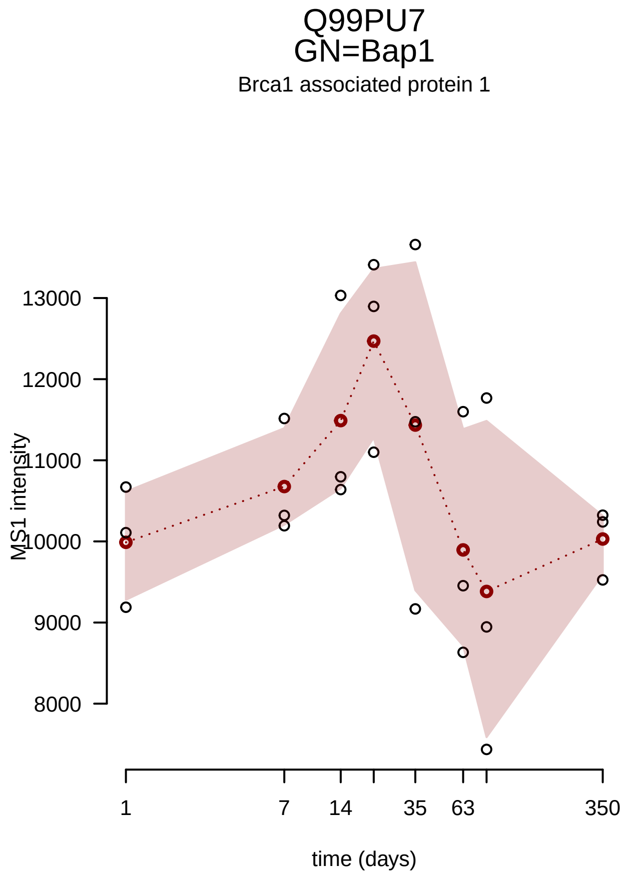
Q99PU7
GN=Bap1
Brca1 associated protein 1
MS1 intensity
8000
9000
10000
11000
12000
13000
1
7
14
35
63
350
time (days)

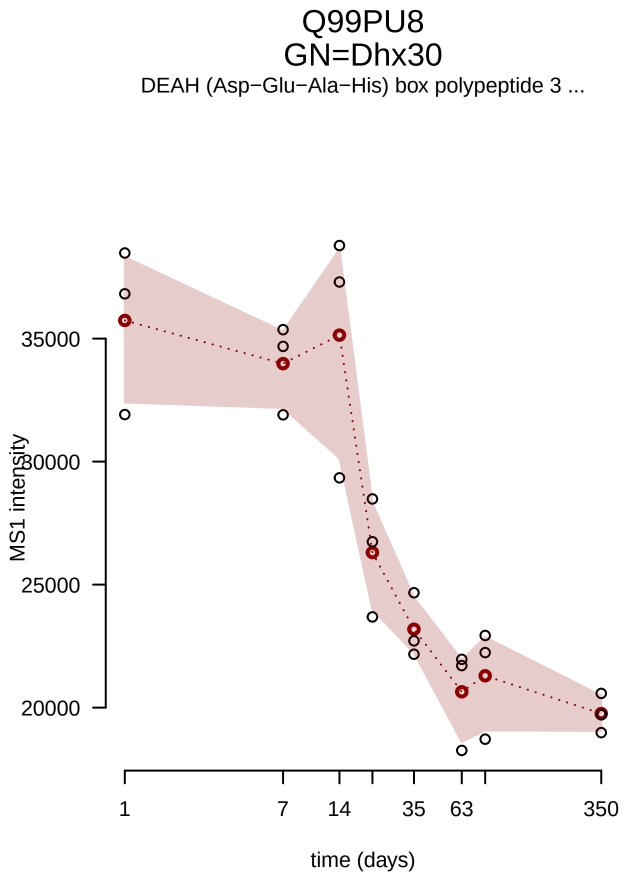
Q99PU8
GN=Dhx30
DEAH (Asp−Glu−Ala−His) box polypeptide 3 ...
MS1 intensity
20000
25000
30000
35000
1
7
14
35
63
350
time (days)

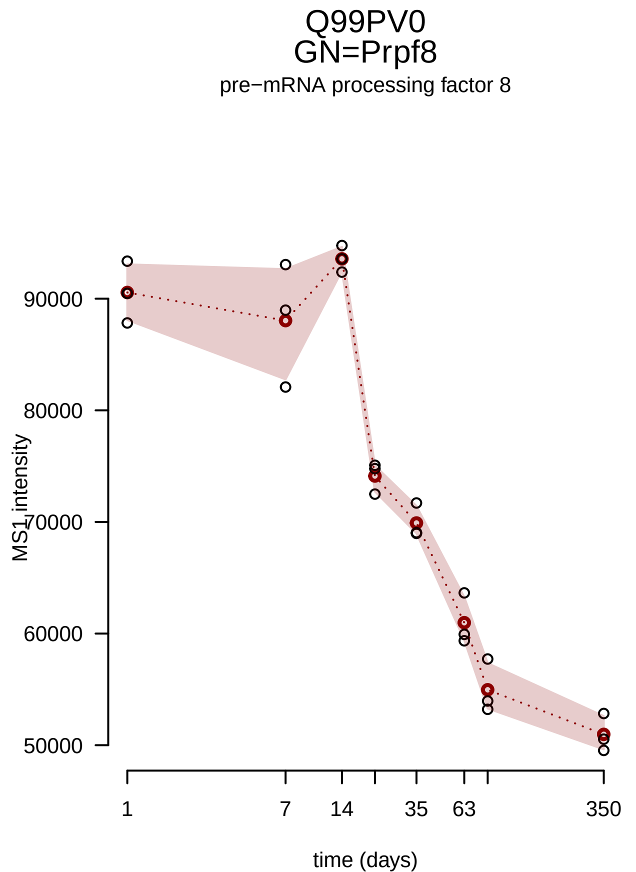
Q99PV0
GN=Prpf8
pre−mRNA processing factor 8
MS1 intensity
50000
60000
70000
80000
90000
1
7
14
35
63
350
time (days)

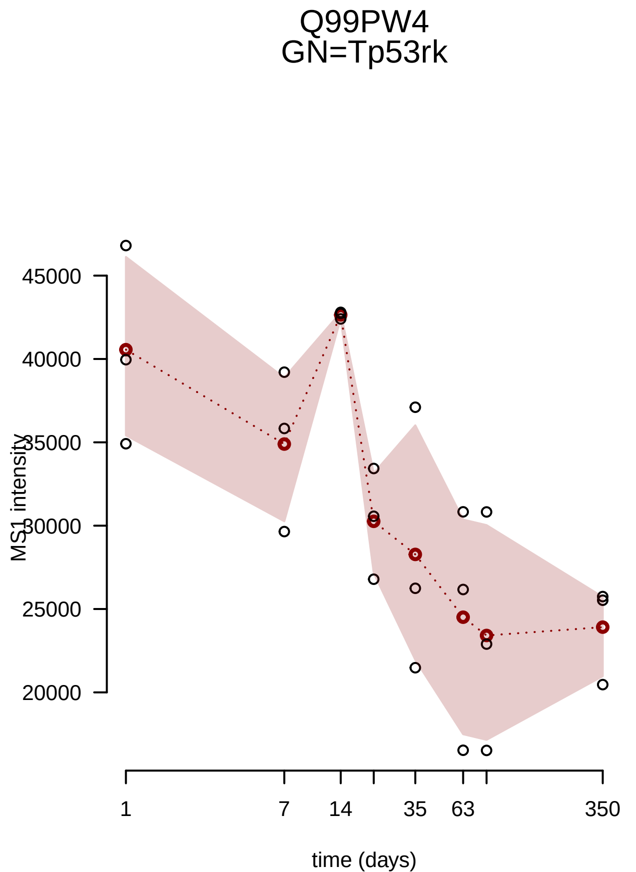
Q99PW4
GN=Tp53rk
MS1 intensity
20000
25000
30000
35000
40000
45000
1
7
14
35
63
350
time (days)

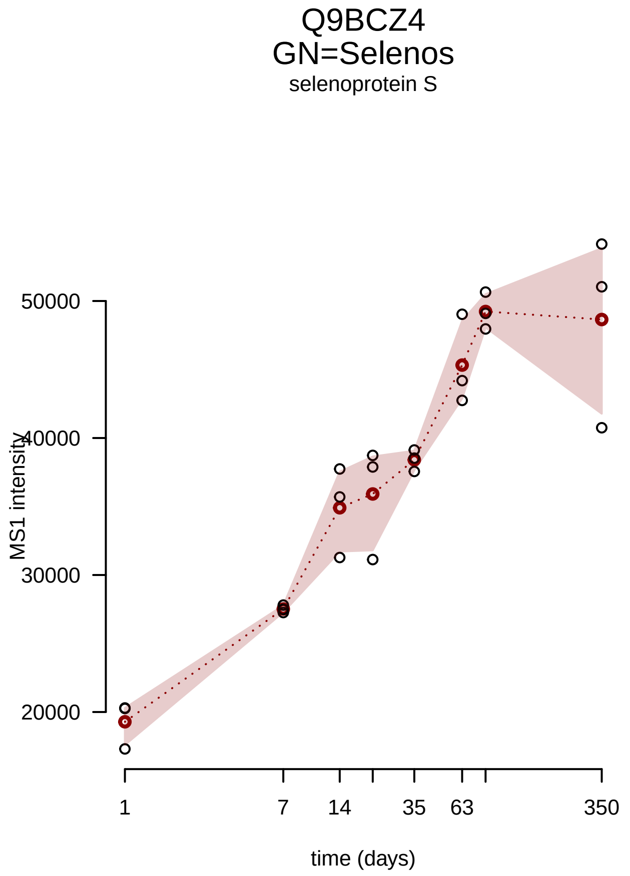
Q9BCZ4
GN=Selenos
selenoprotein S
MS1 intensity
50000
40000
30000
20000
1
7
14
35
63
350
time (days)

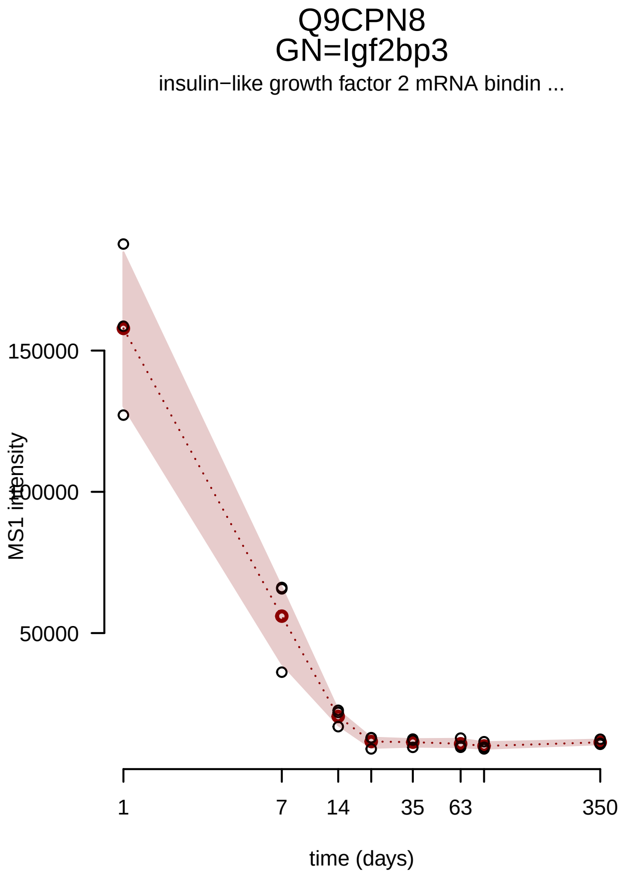
Q9CPN8
GN=Igf2bp3
insulin−like growth factor 2 mRNA bindin ...
MS1 intensity
150000
100000
50000
1
7
14
35
63
350
time (days)

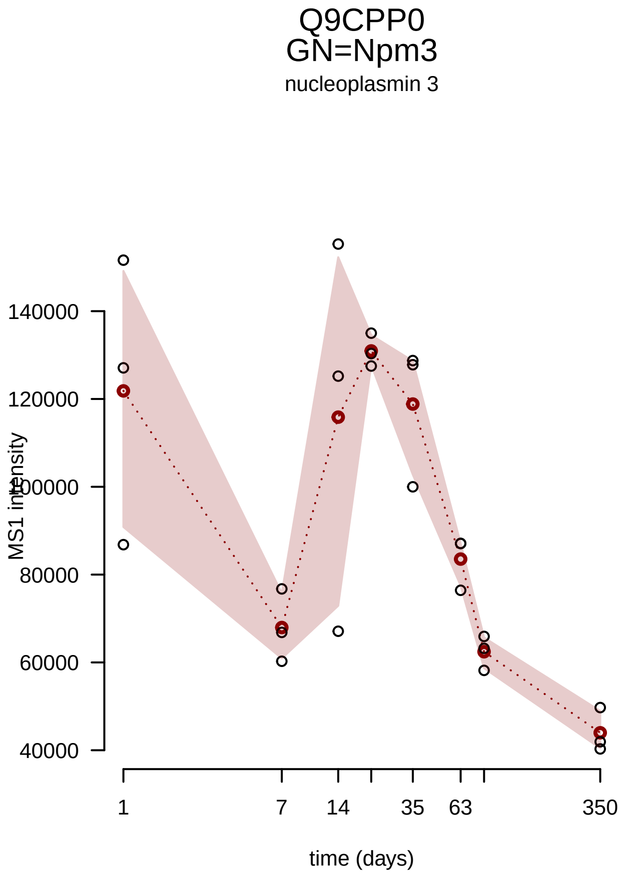
Q9CPP0
GN=Npm3
nucleoplasmin 3
MS1 intensity
140000
120000
100000
80000
60000
40000
1
7
14
35
63
350
time (days)

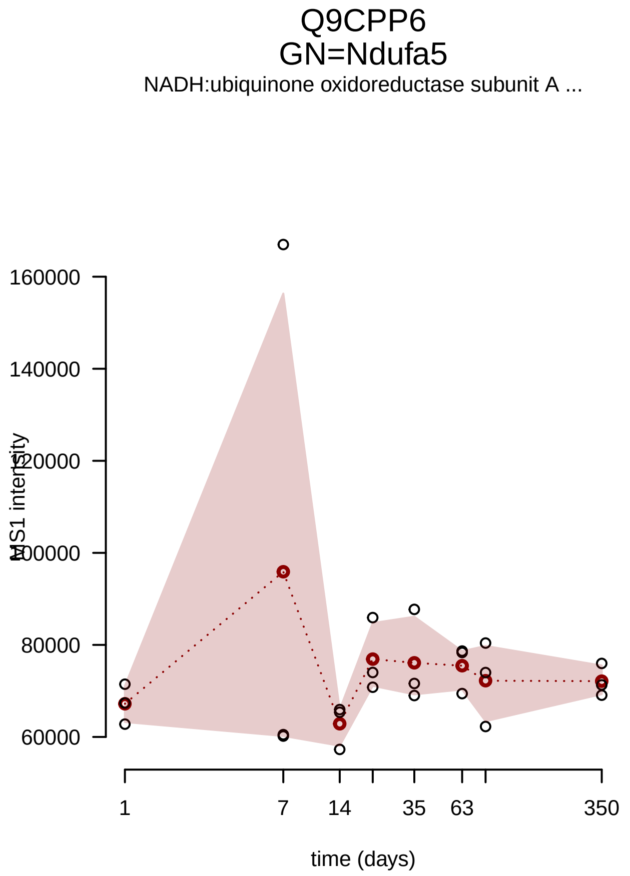
Q9CPP6
GN=Ndufa5
NADH:ubiquinone oxidoreductase subunit A ...
MS1 intensity
160000
140000
120000
100000
80000
60000
1
7
14
35
63
350
time (days)

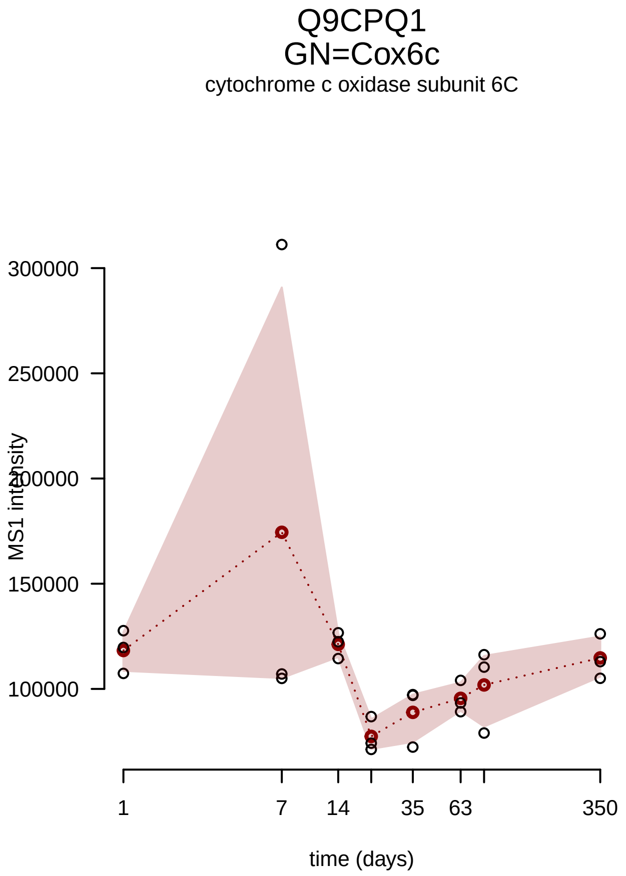
Q9CPQ1
GN=Cox6c
cytochrome c oxidase subunit 6C
MS1 intensity
300000
250000
200000
150000
100000
1
7
14
35
63
350
time (days)

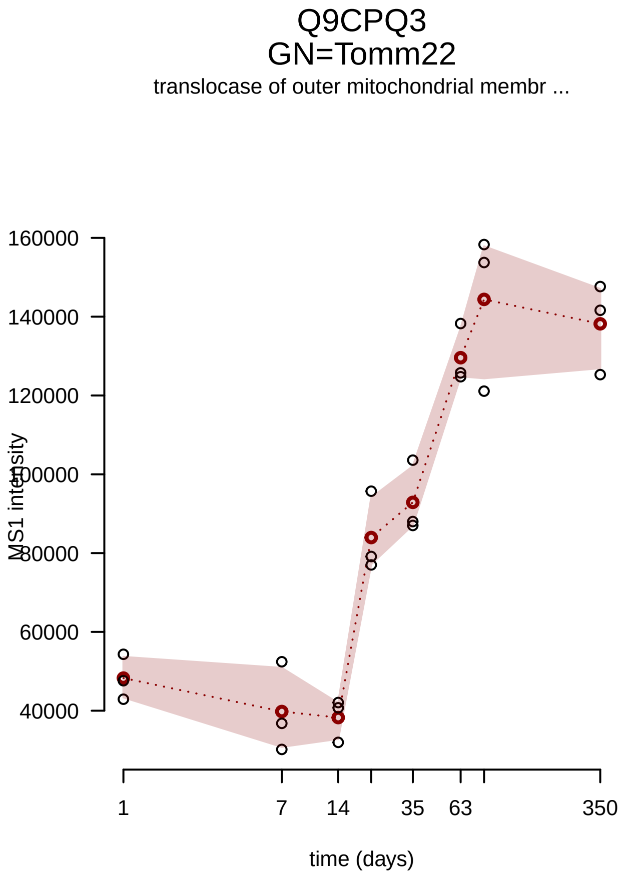
Q9CPQ3
GN=Tomm22
translocase of outer mitochondrial membr ...
MS1 intensity
160000
140000
120000
100000
80000
60000
40000
1
7
14
35
63
350
time (days)

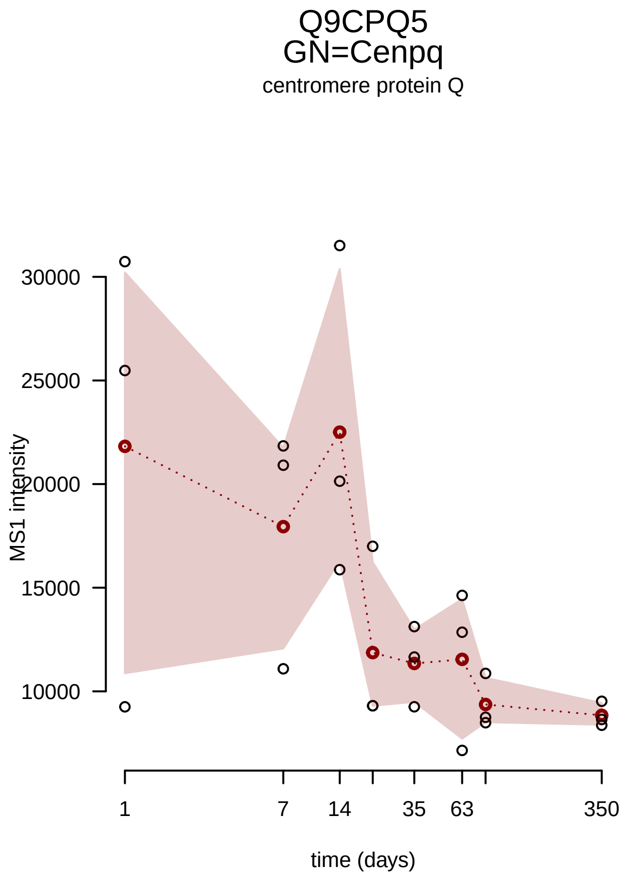
Q9CPQ5
GN=Cenpq
centromere protein Q
MS1 intensity
30000
25000
20000
15000
10000
1
7
14
35
63
350
time (days)

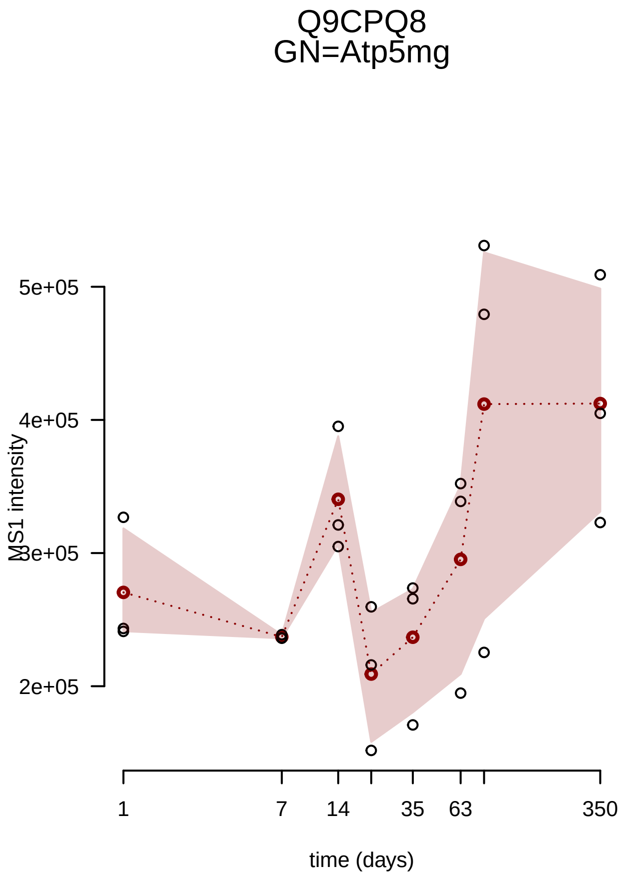
Q9CPQ8
GN=Atp5mg
MS1 intensity
5e+05
4e+05
3e+05
2e+05
1
7
14
35
63
350
time (days)

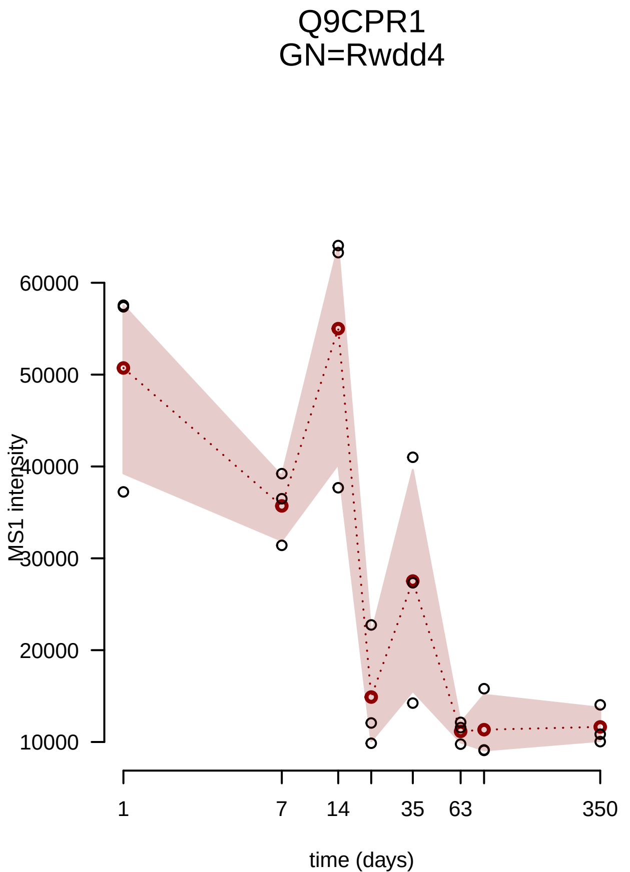
Q9CPR1
GN=Rwdd4
MS1 intensity
60000
50000
40000
30000
20000
10000
1
7
14
35
63
350
time (days)

Q9CPR4
GN=Rpl17
ribosomal protein L17

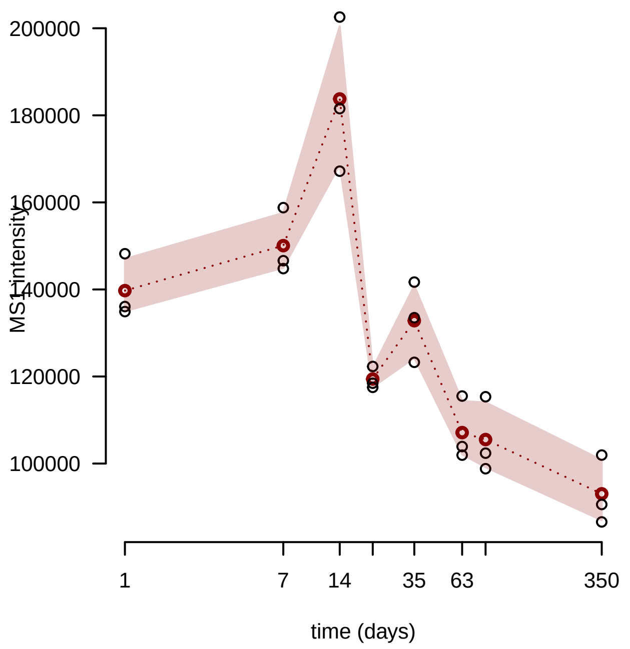

Q9CPR5
GN=Mrpl15
mitochondrial ribosomal protein L15

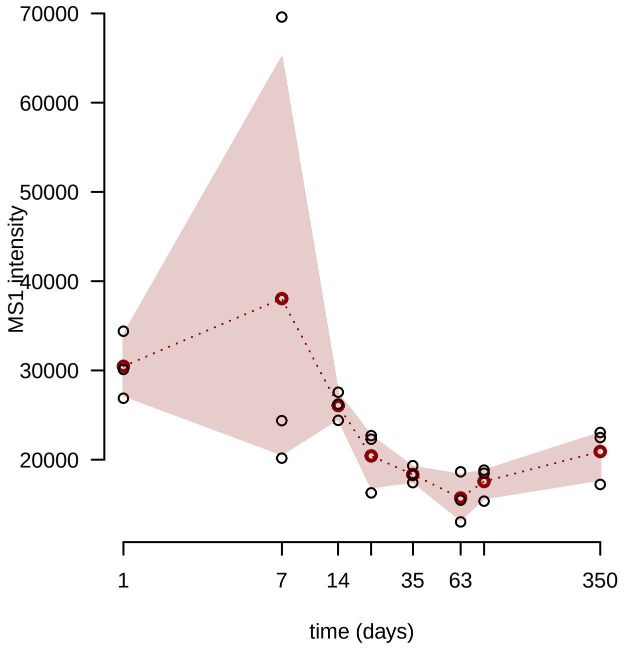

Q9CPR7
GN=Sike1
suppressor of IKBKE 1

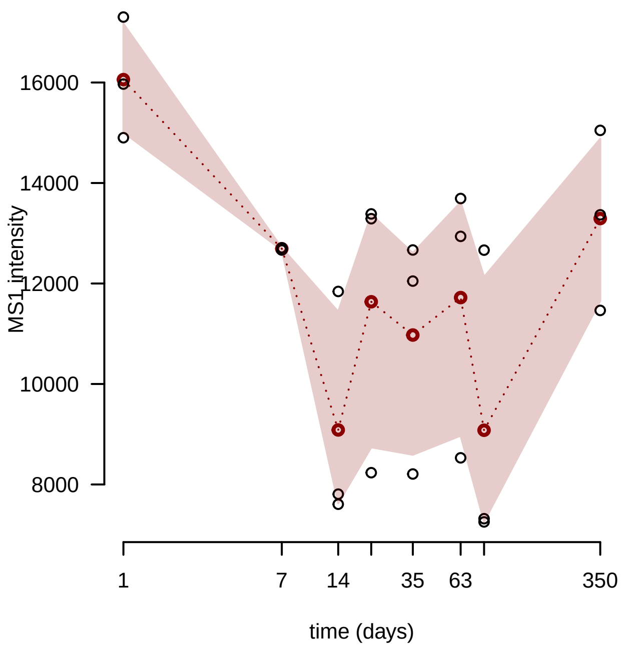

Q9CPR8
GN=Nsmce3
NSE3 homolog, SMC5−SMC6 complex componen ...

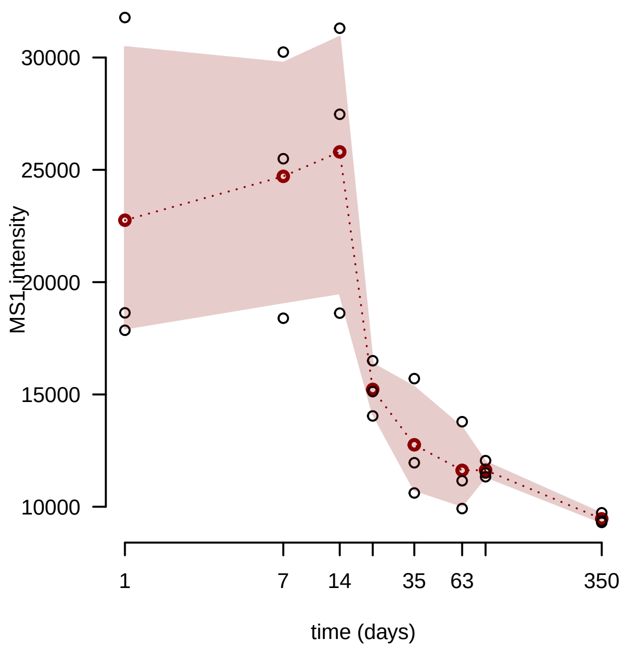

Q9CPS6
GN=Hint3
histidine triad nucleotide binding prote ...

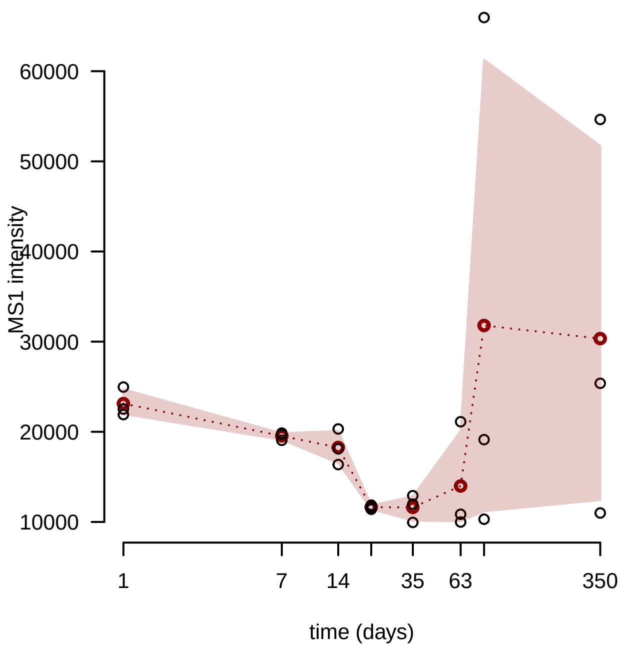

Q9CPS7
GN=Pno1
partner of NOB1 homolog

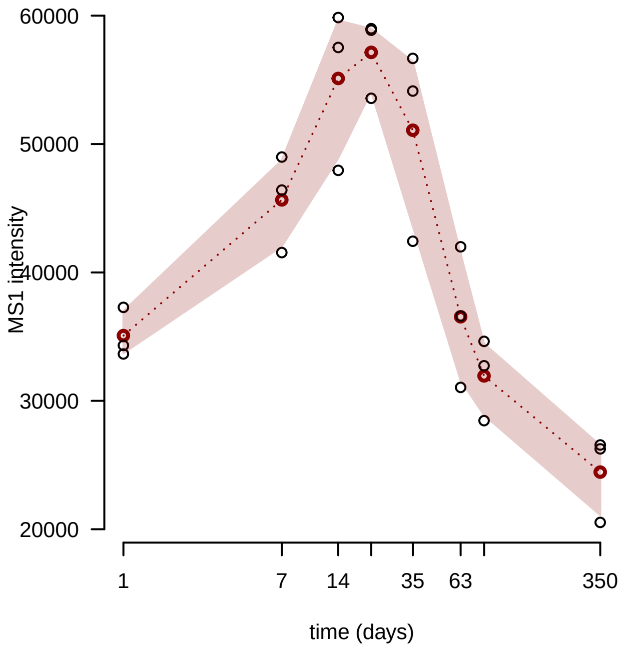

Q9CPT3
GN=Nanp
N−acetylneuraminic acid phosphatase

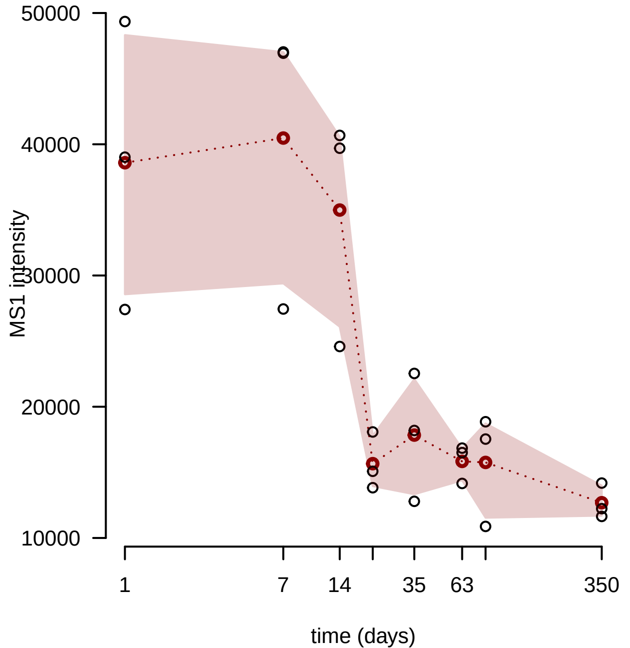

Q9CPT4
GN=Mydgf
myeloid derived growth factor

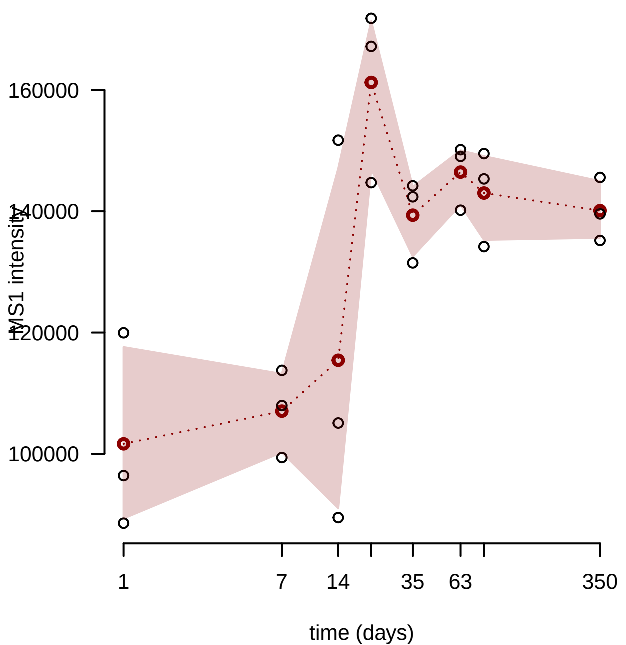

Q9CPT5
GN=Nop16
NOP16 nucleolar protein

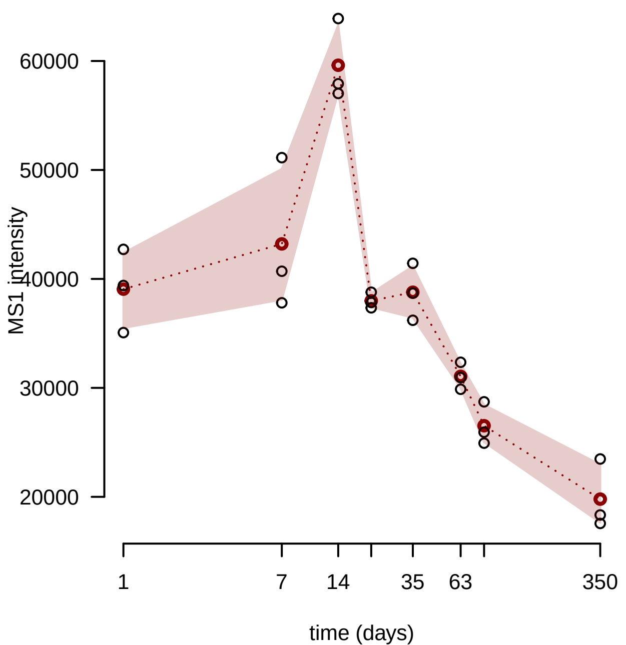

Q9CPU0
GN=Glo1
glyoxalase 1

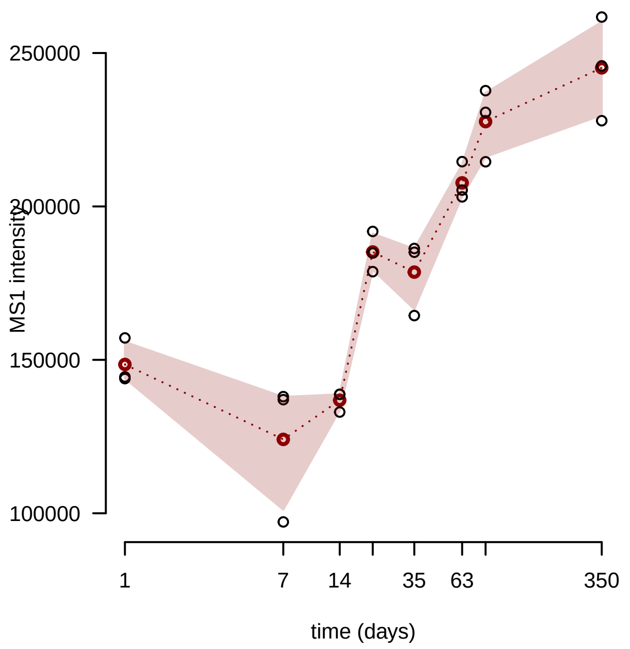

Q9CPU2
GN=Ndufb2
NADH:ubiquinone oxidoreductase subunit B ...

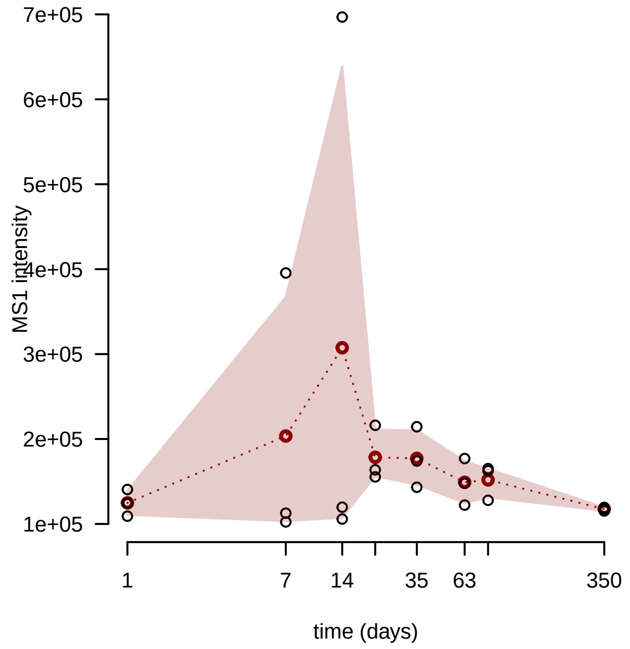

Q9CPU4
GN=Mgst3
microsomal glutathione S−transferase 3

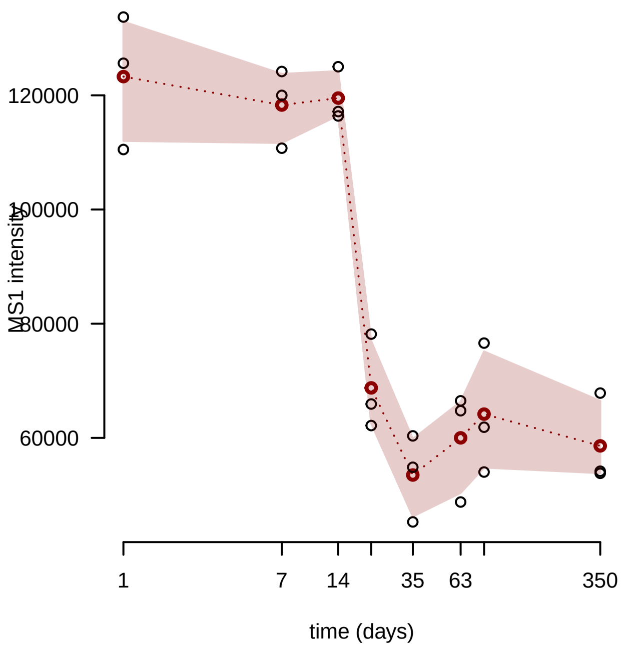

Q9CPV3
GN=Mrpl42
mitochondrial ribosomal protein L42

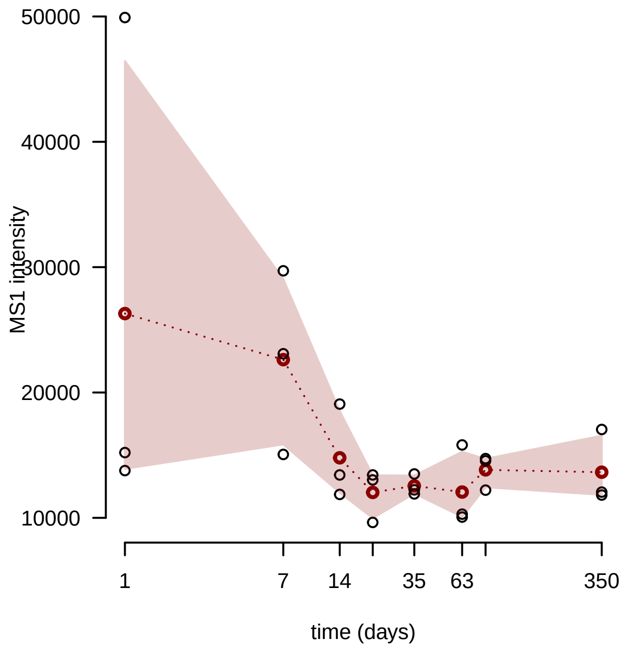

Q9CPV4
GN=Glod4
glyoxalase domain containing 4

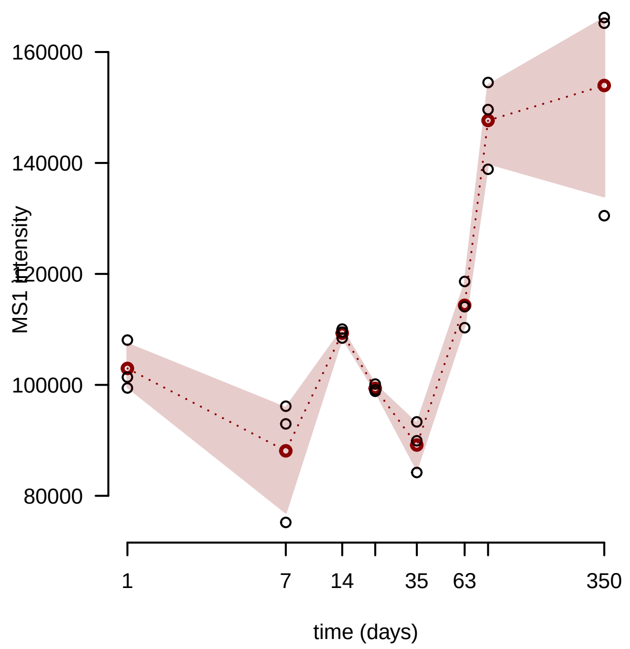

Q9CPV5
GN=Pmf1
polyamine−modulated factor 1

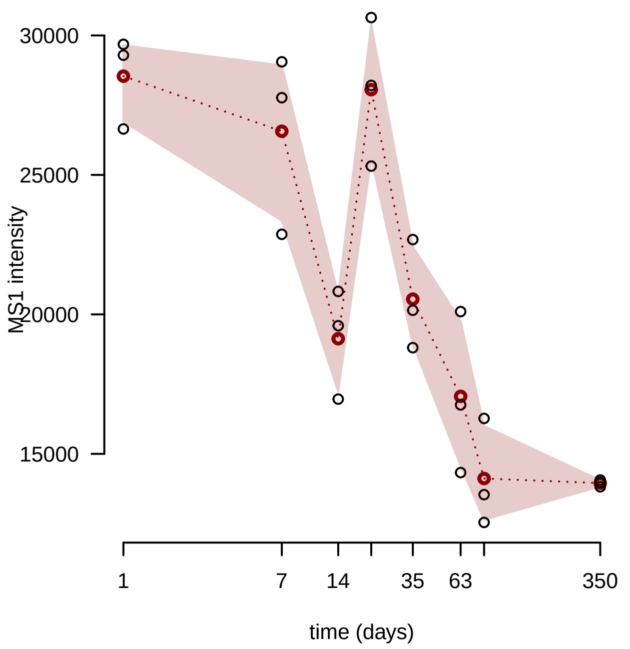

Q9CPV7
GN=Zdhhc6
zinc finger, DHHC domain containing 6

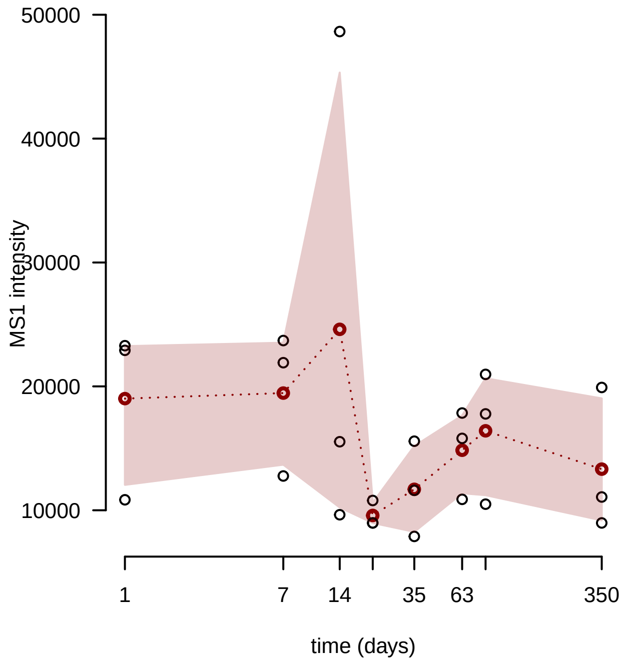

Q9CPW2
GN=Fdx2
ferredoxin 2

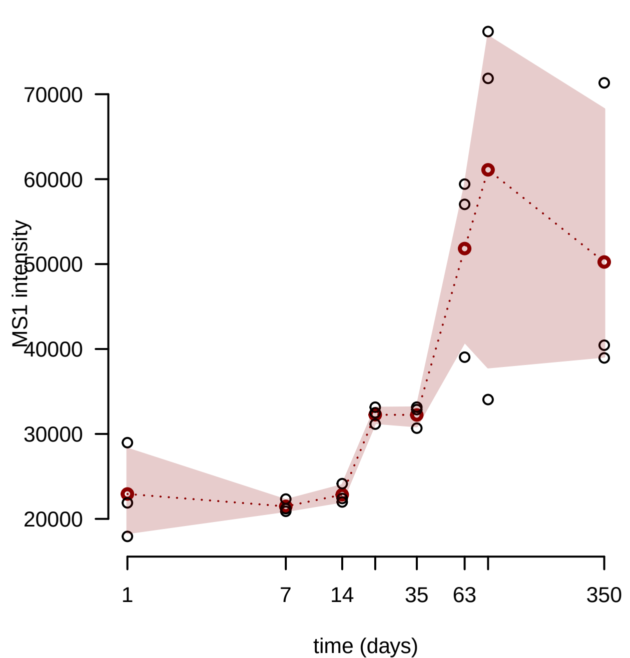

Q9CPW3
GN=Mrpl54
mitochondrial ribosomal protein L54

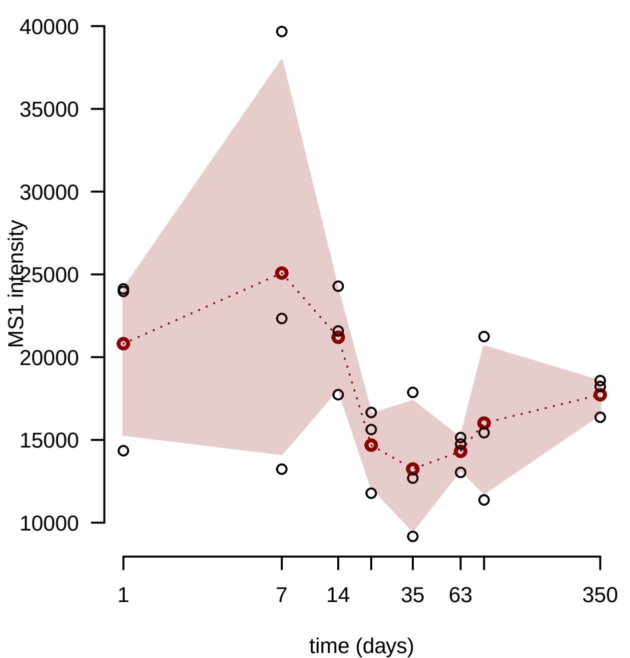

Q9CPW4
GN=Arpc5
actin related protein 2/3 complex, subun ...

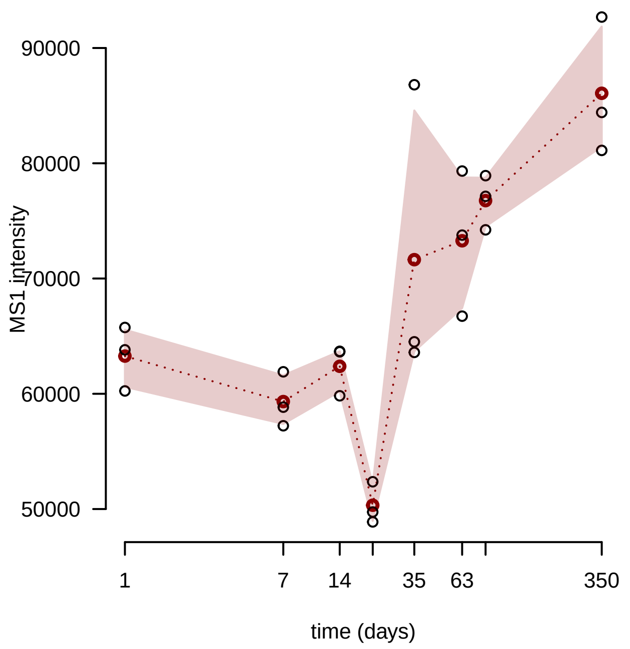

Q9CPW7
GN=Zmat2
zinc finger, matrin type 2

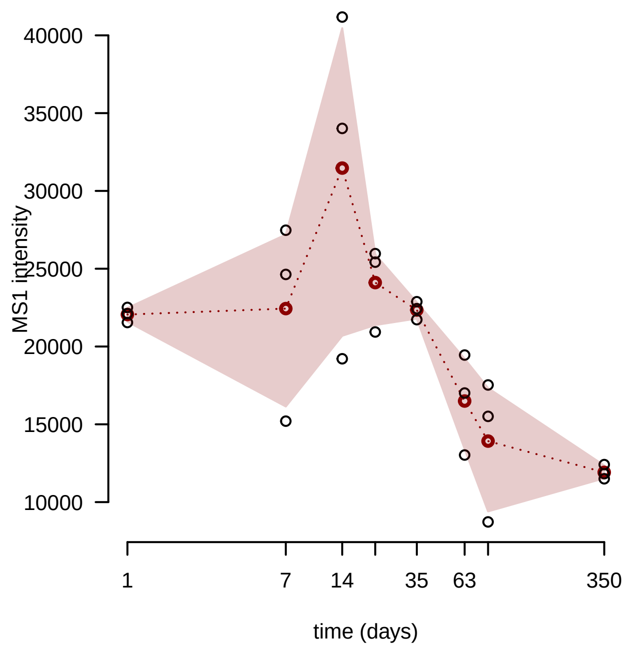

Q9CPW9
GN=Metap1d
methionyl aminopeptidase type 1D (mitoch ...

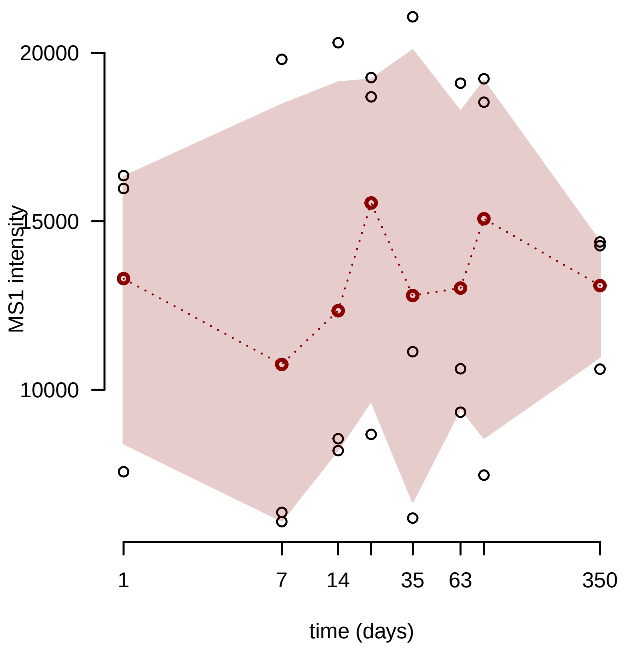

Q9CPX6
GN=Atg3
autophagy related 3

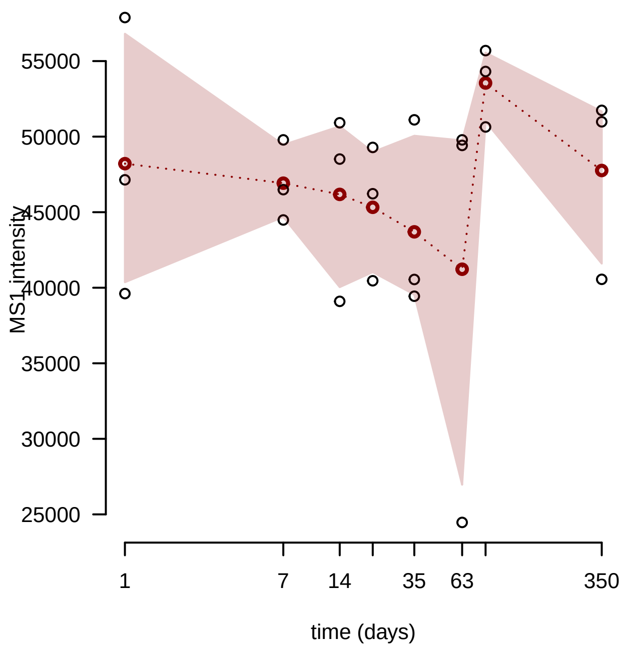

Q9CPX7
GN=Mrps16
mitochondrial ribosomal protein S16

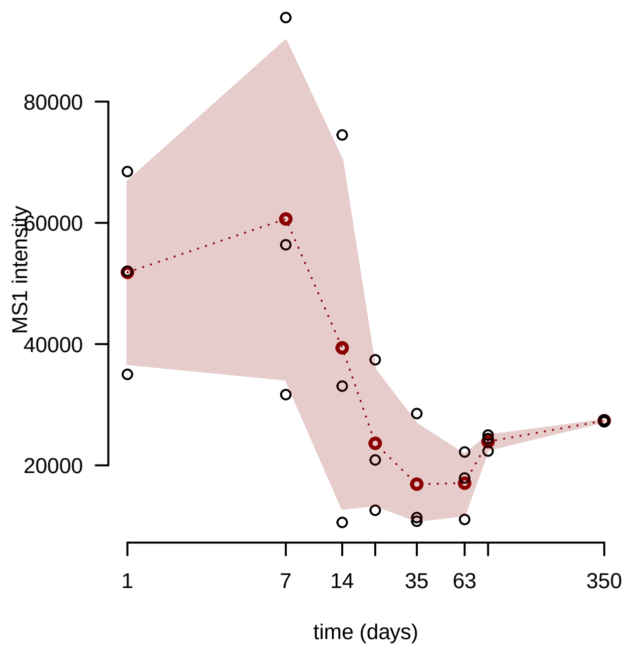

Q9CPX8
GN=Uqcr11
ubiquinol−cytochrome c reductase, comple ...

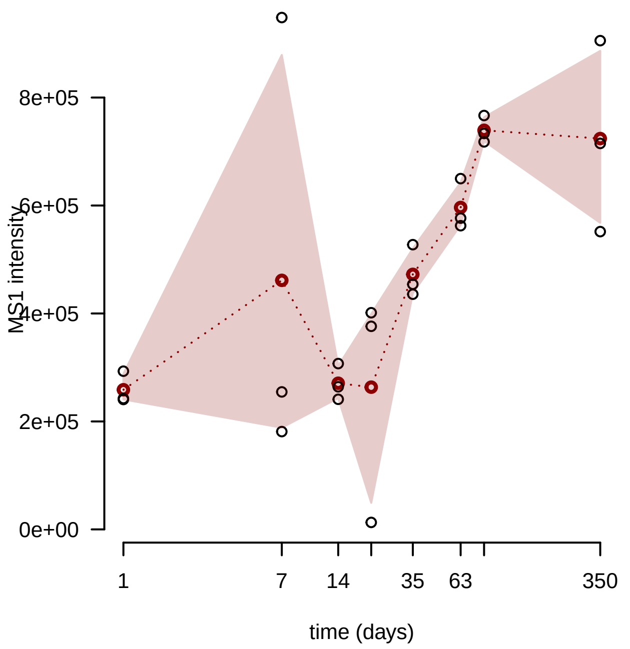

Q9CPY0
GN=Mrm2
mitochondrial rRNA methyltransferase 2

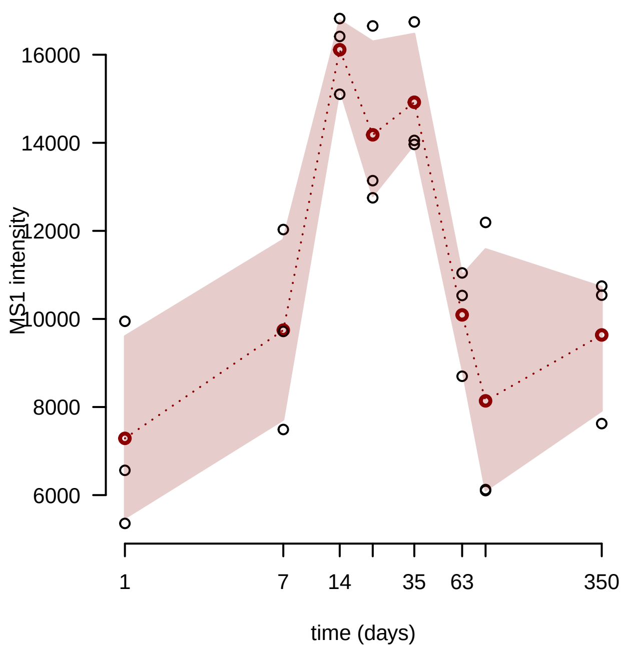

Q9CPY1
GN=Mrpl51
mitochondrial ribosomal protein L51

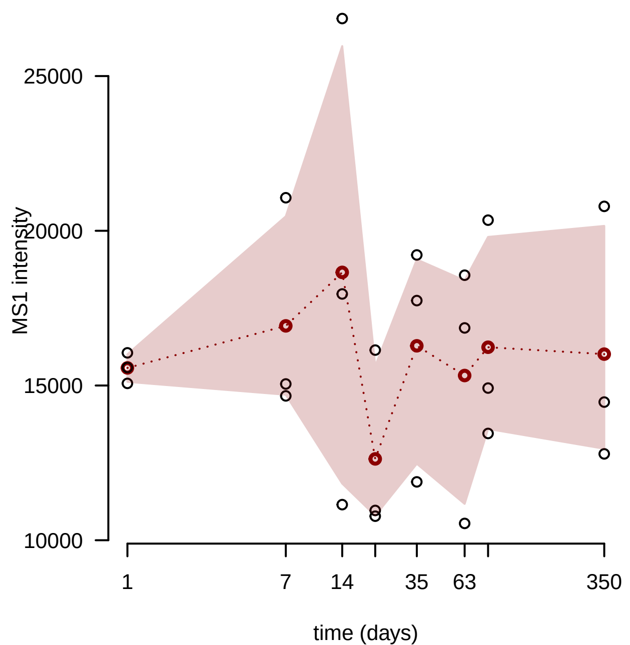

Q9CPY3
GN=Cdca5
cell division cycle associated 5

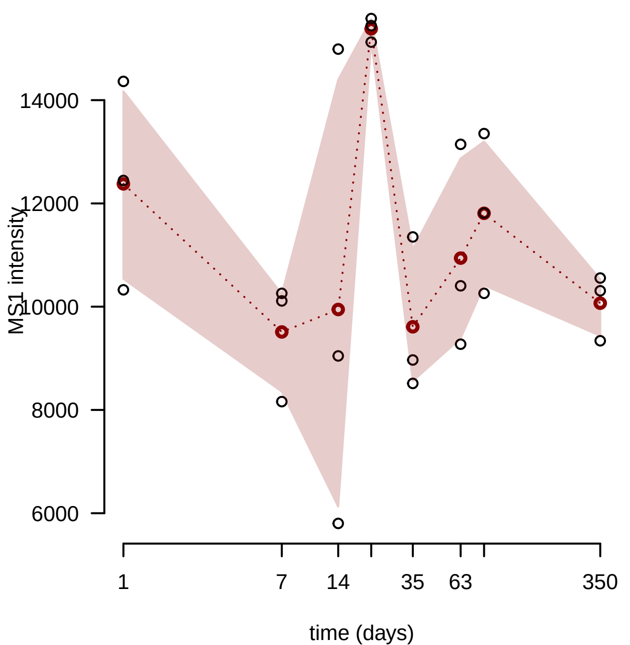

Q9CPY7
GN=Lap3
leucine aminopeptidase 3

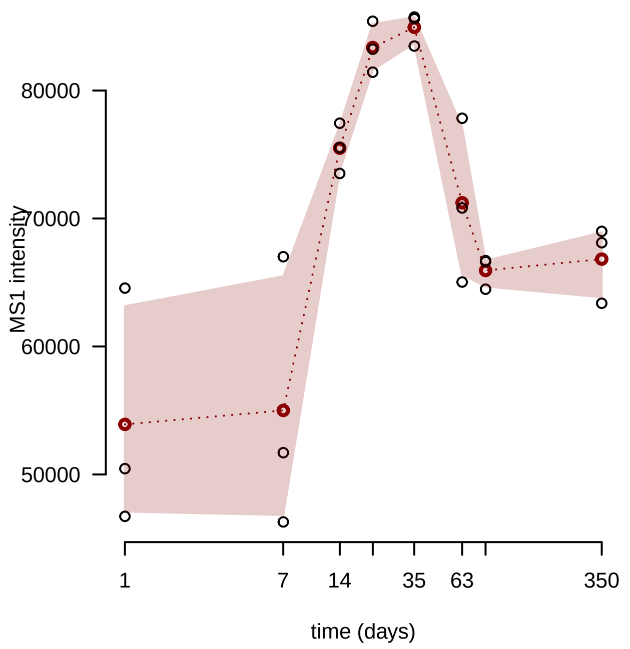

Q9CPZ6
GN=Ormdl3
ORM1−like 3 (S. cerevisiae)

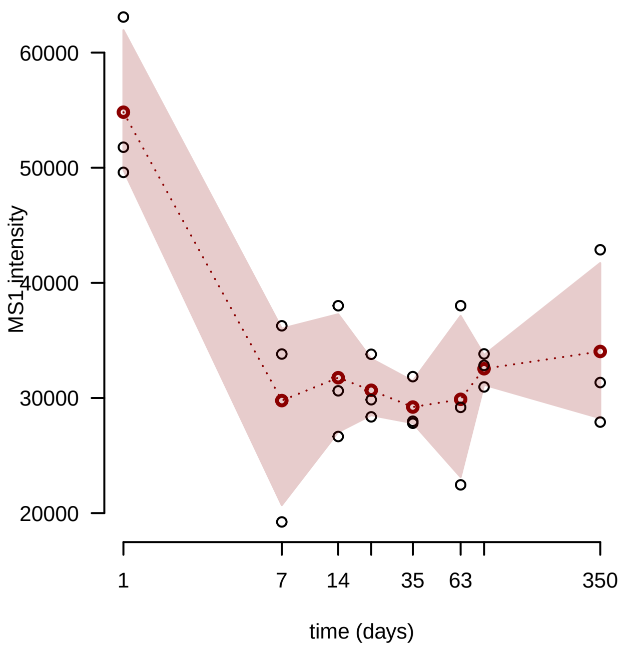

Q9CPZ8
GN=Cmc1
COX assembly mitochondrial protein 1

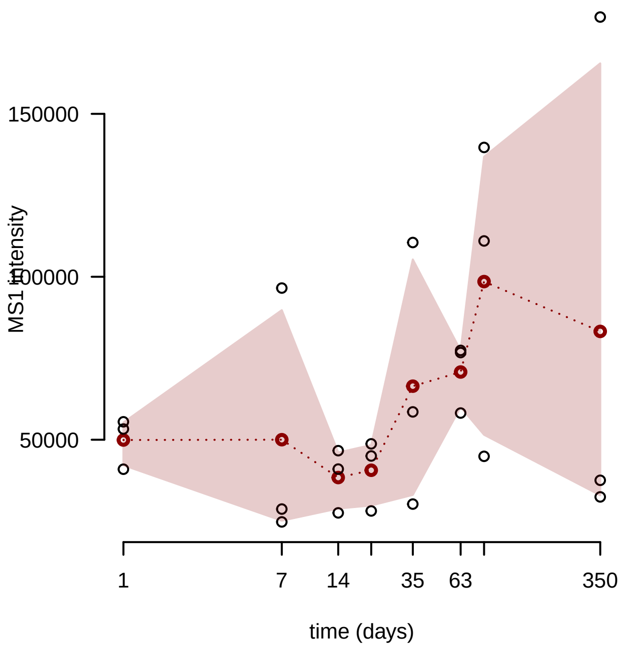

Q9CQ00
GN=Dmac1
distal membrane arm assembly complex 1

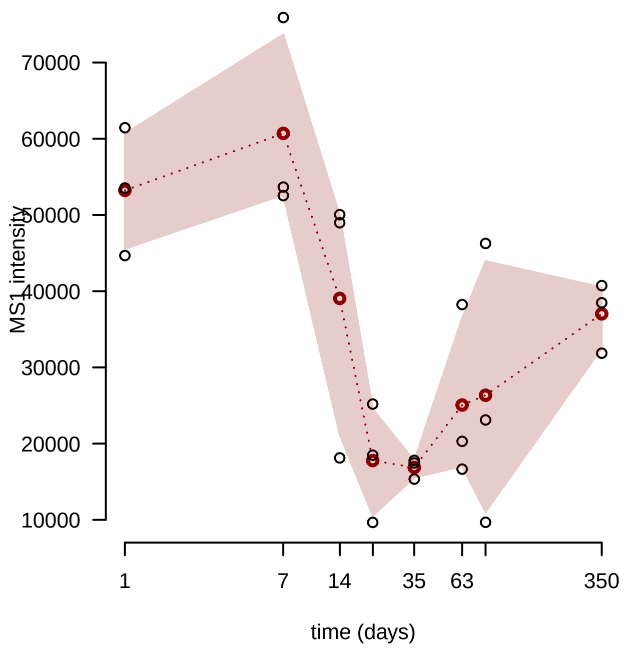

Q9CQ02
GN=Commd4
COMM domain containing 4

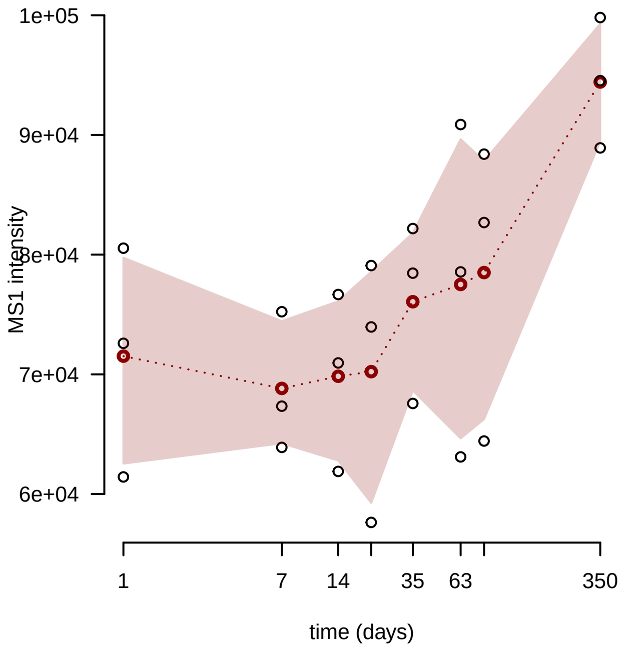

Q9CQ04
GN=Ogfod2
2−oxoglutarate and iron−dependent oxygen ...

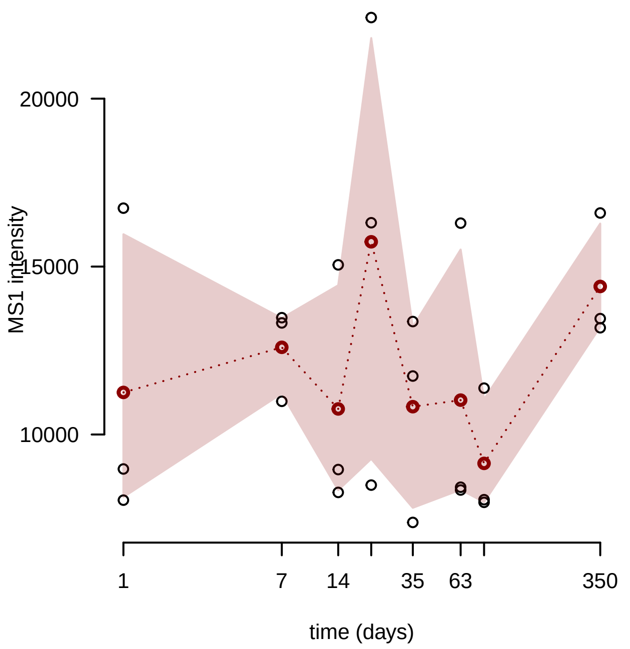

Q9CQ06
GN=Mrpl24
mitochondrial ribosomal protein L24

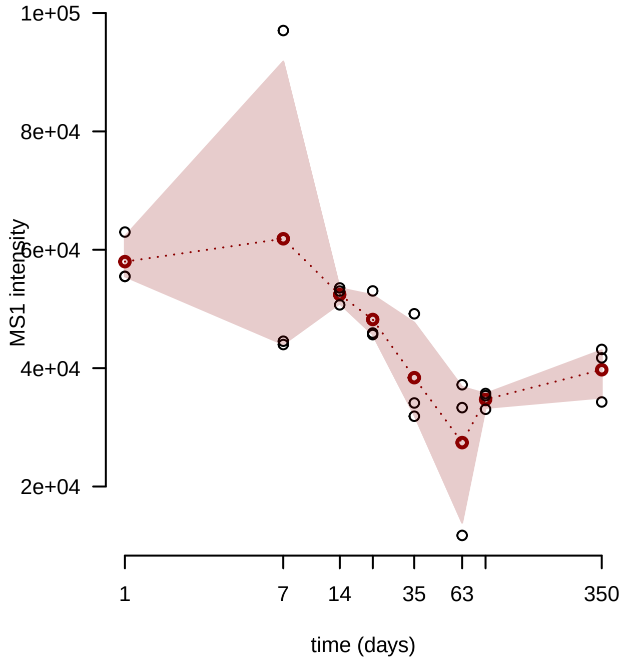

Q9CQ10
GN=Chmp3
charged multivesicular body protein 3

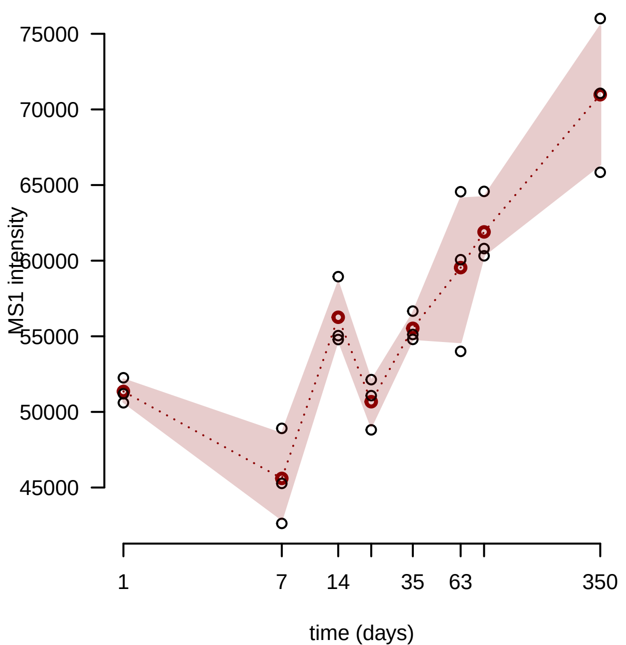

Q9CQ13
GN=Coprs
coordinator of PRMT5, differentiation st ...

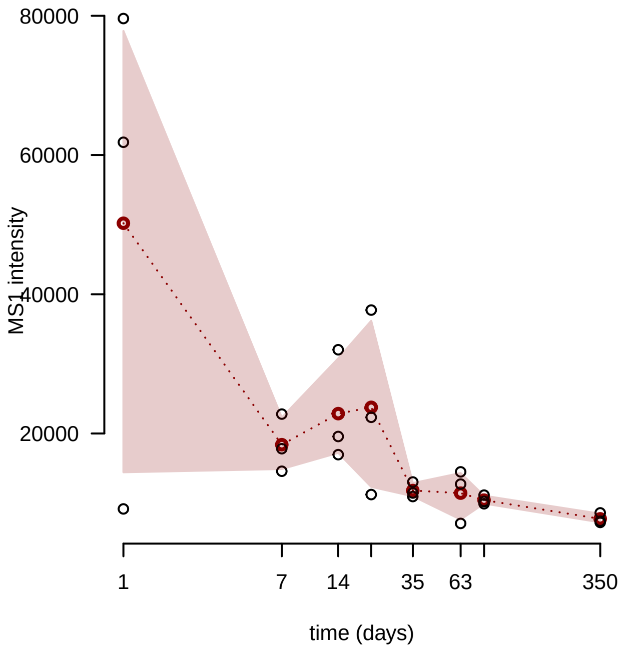

Q9CQ18
GN=Rnaseh2c
ribonuclease H2, subunit C

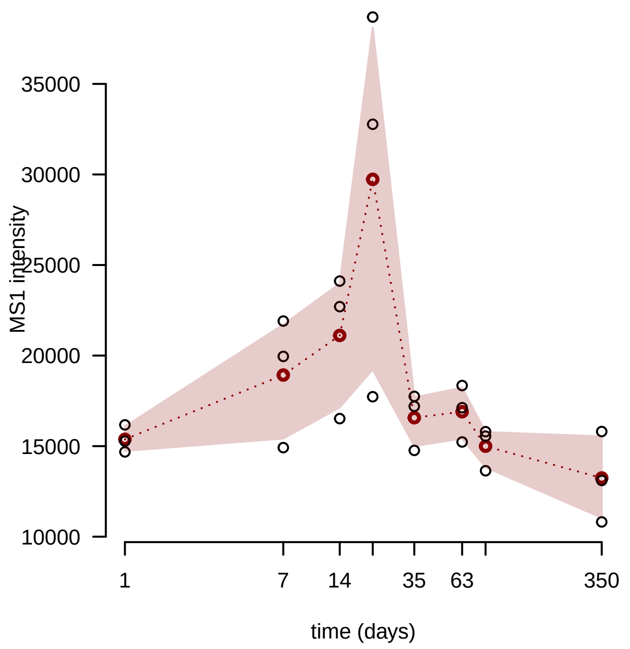

Q9CQ19
GN=Myl9
myosin, light polypeptide 9, regulatory

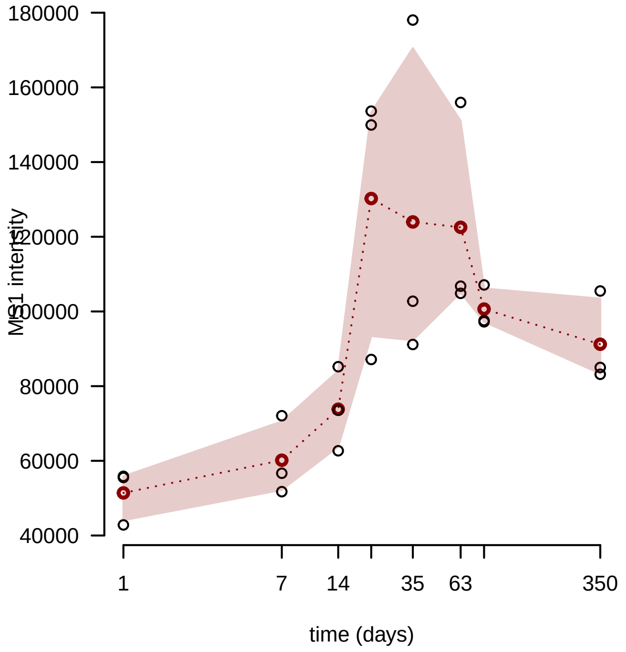

Q9CQ20
GN=Mid1ip1
Mid1 interacting protein 1 (gastrulation ...

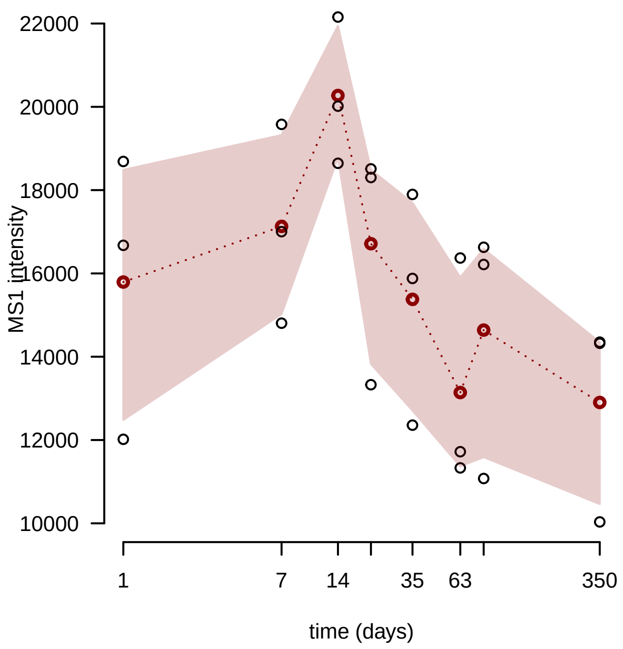

Q9CQ21
GN=Mcts2
malignant T cell amplified sequence 2

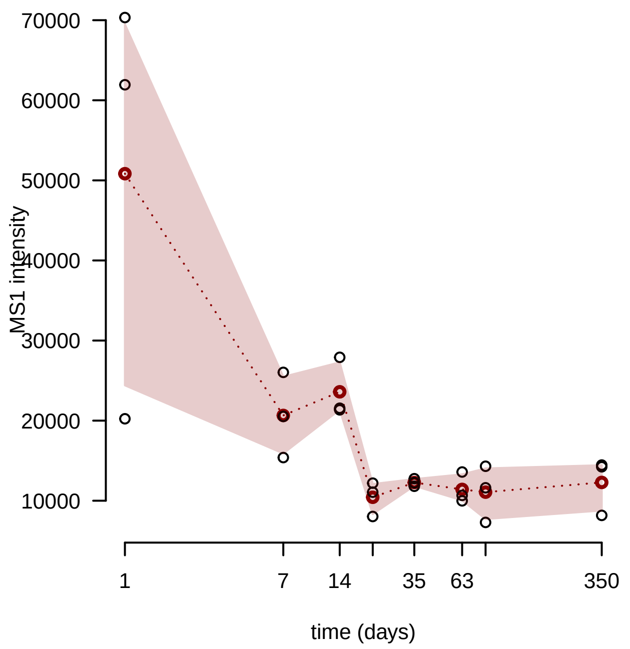

Q9CQ22
GN=Lamtor1
late endosomal/lysosomal adaptor, MAPK a ...

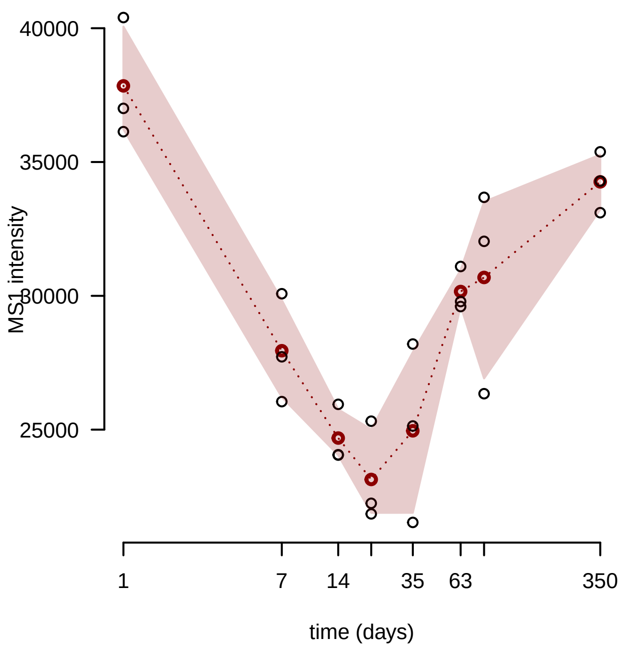

Q9CQ26
GN=Stambp
STAM binding protein

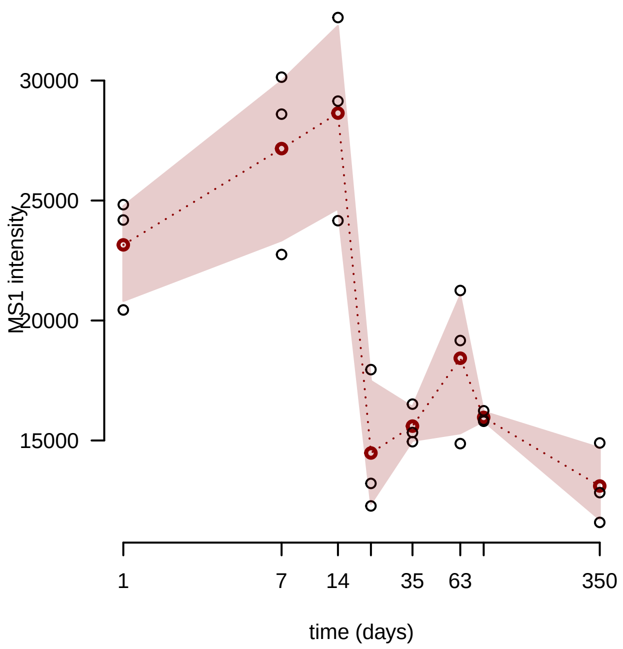

Q9CQ28
GN=Dph6
diphthamine biosynthesis 6

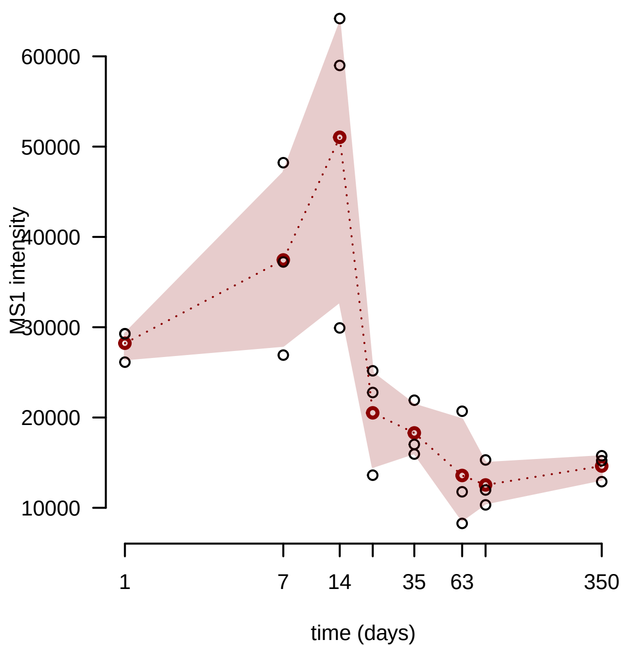

Q9CQ36
GN=Pole4
polymerase (DNA−directed), epsilon 4 (p1 ...

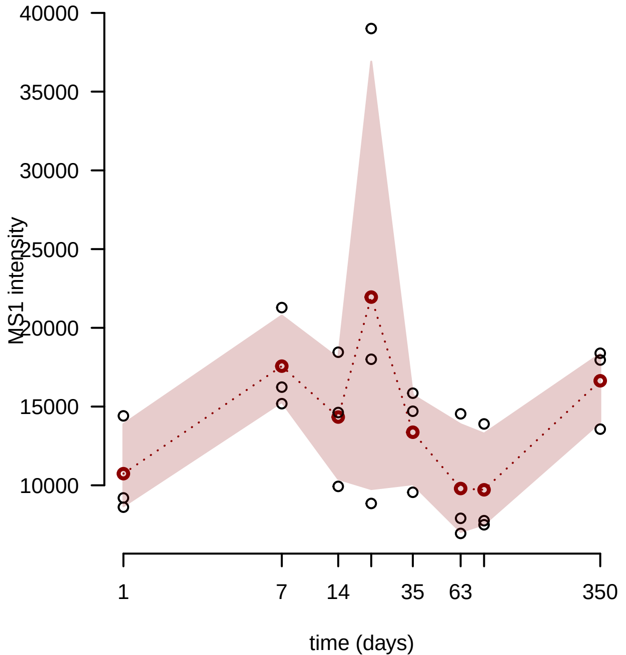

Q9CQ37
GN=Ube2t
ubiquitin−conjugating enzyme E2T

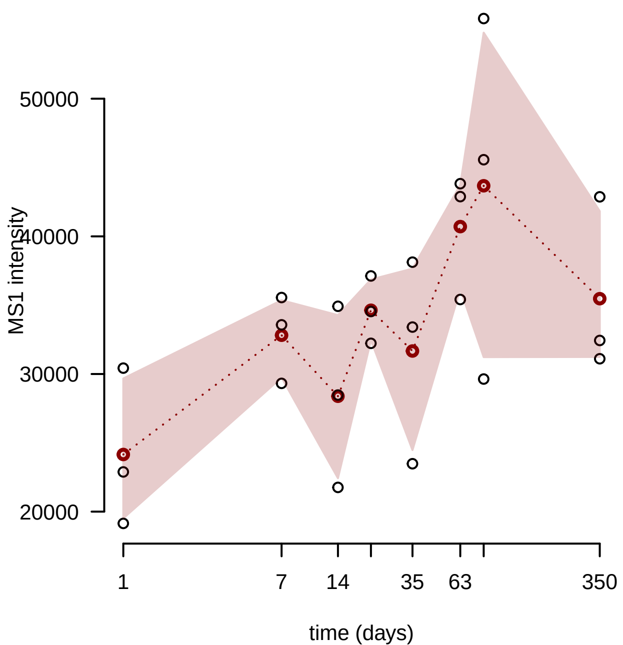

Q9CQ40
GN=Mrpl49
mitochondrial ribosomal protein L49

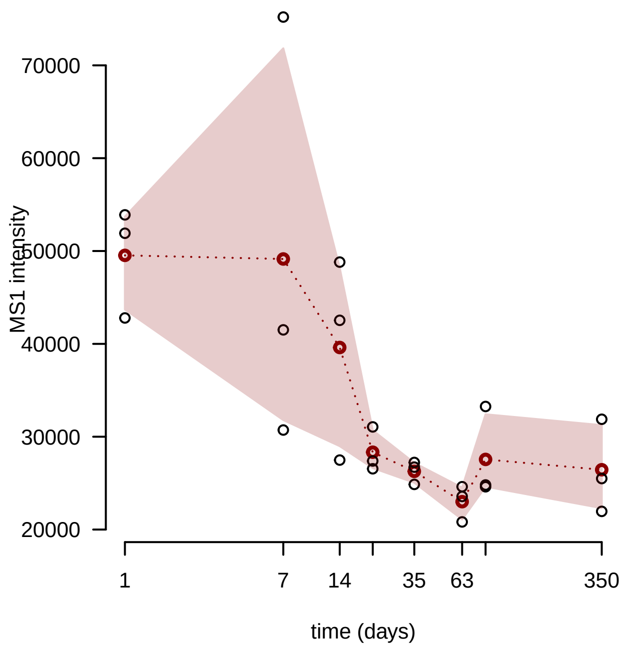

Q9CQ45
GN=Nenf
neuron derived neurotrophic factor

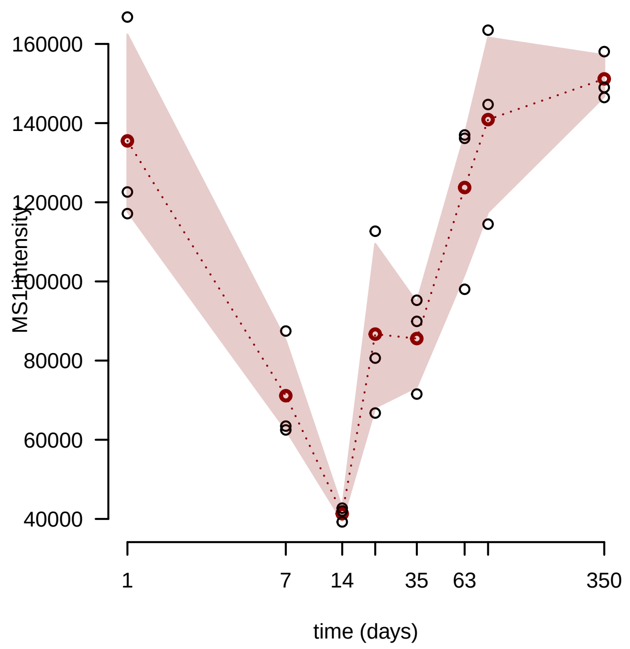

Q9CQ48
GN=Nudcd2
NudC domain containing 2

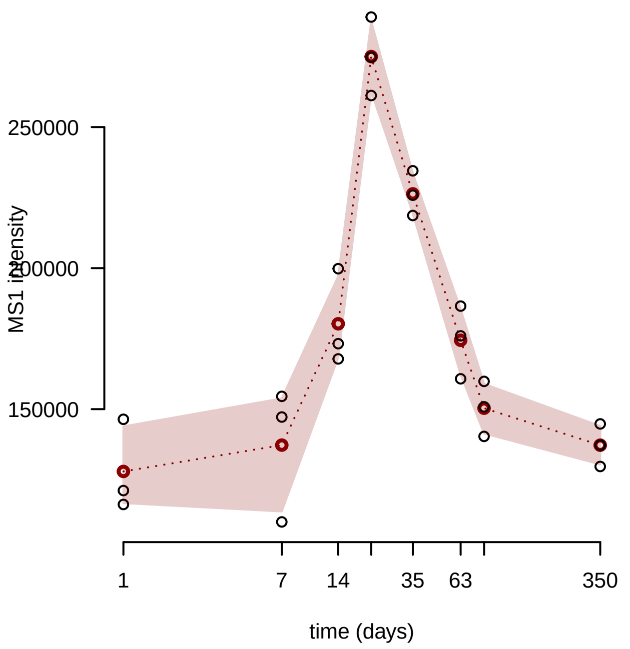

Q9CQ49
GN=Ncbp2
nuclear cap binding protein subunit 2

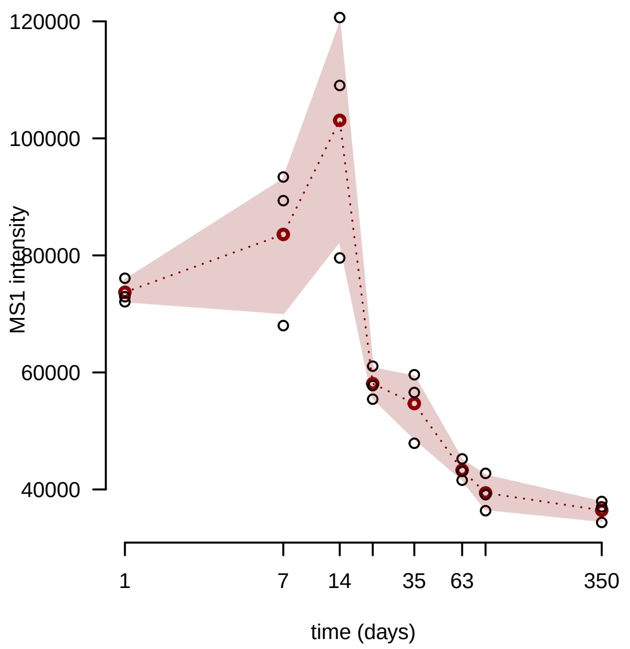

Q9CQ52
GN=Cela3b
chymotrypsin−like elastase family, membe ...

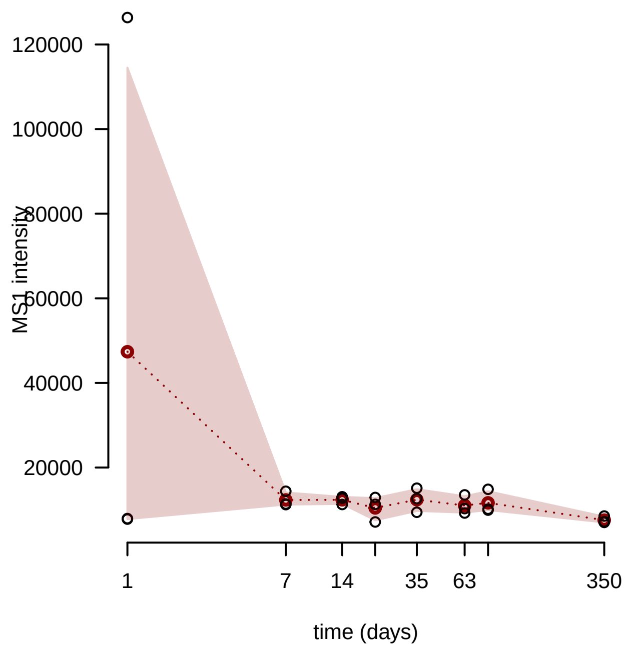

Q9CQ54
GN=Ndufc2
NADH:ubiquinone oxidoreductase subunit C ...

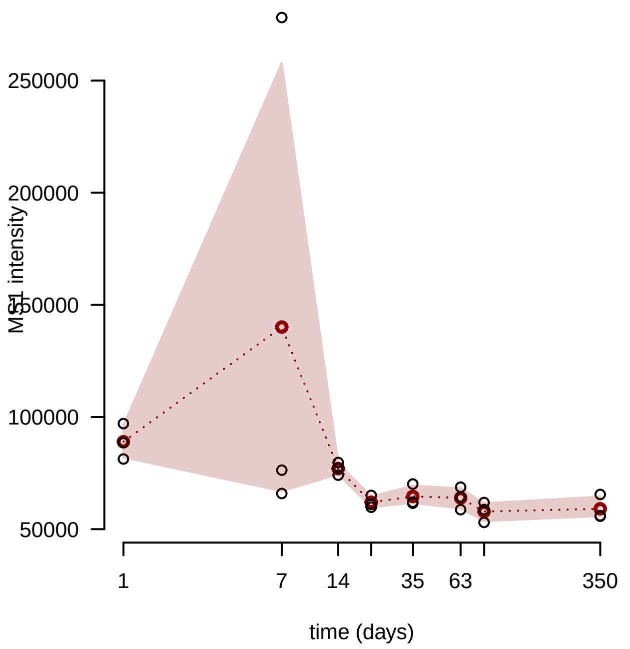

Q9CQ56
GN=Use1
unconventional SNARE in the ER 1 homolog ...

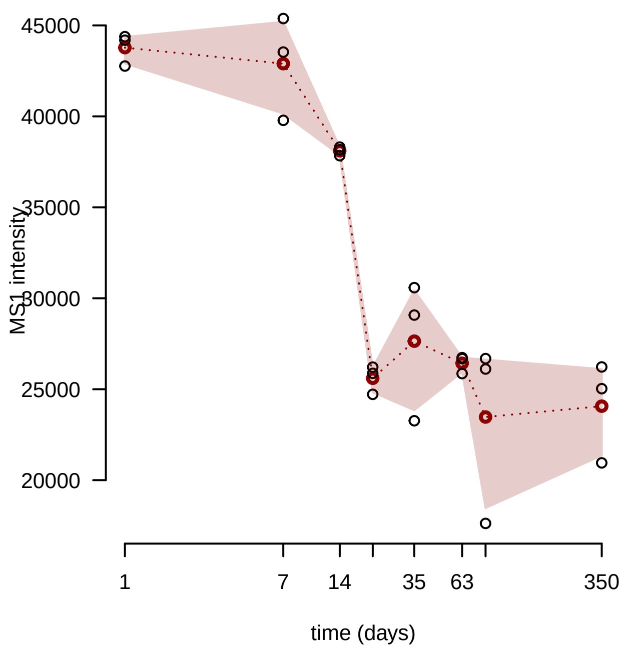

Q9CQ60
GN=Pgls
6−phosphogluconolactonase

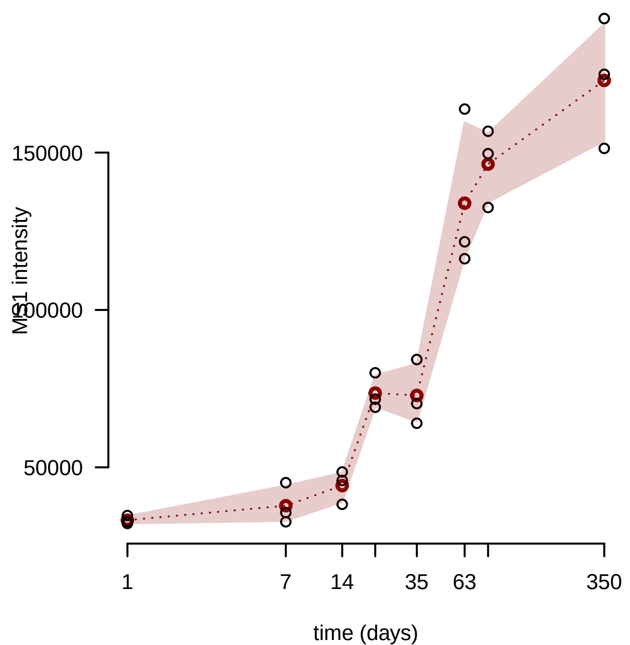

Q9CQ62
GN=Decr1
2,4−dienoyl CoA reductase 1, mitochondri ...

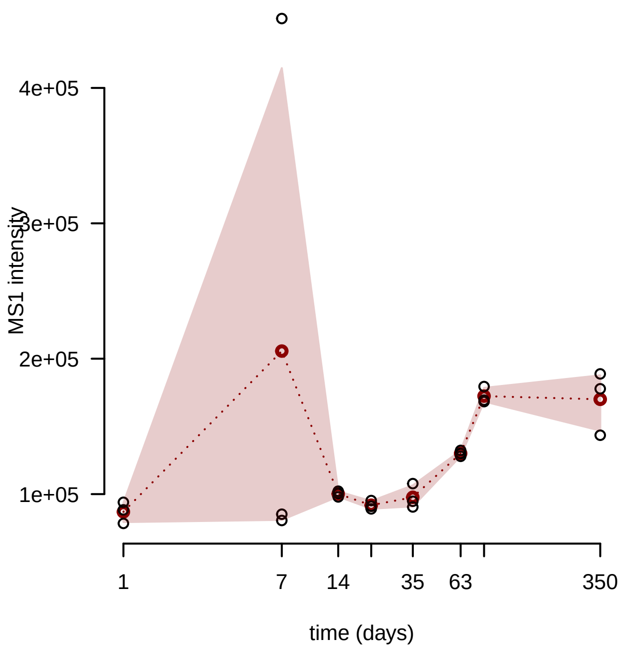

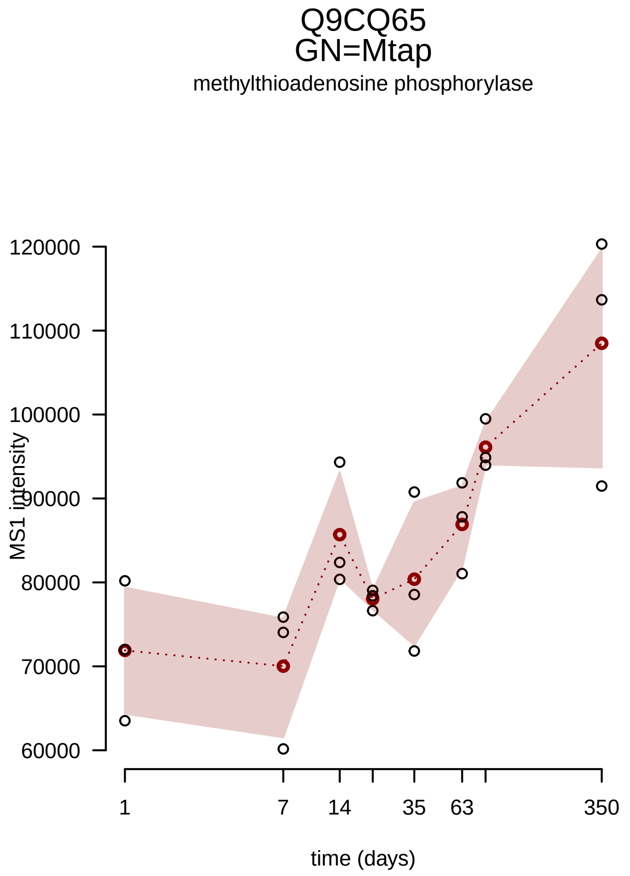
Q9CQ65
GN=Mtap
methylthioadenosine phosphorylase
MS1 intensity
120000
110000
100000
90000
80000
70000
60000
time (days)
1
7
14
35
63
350

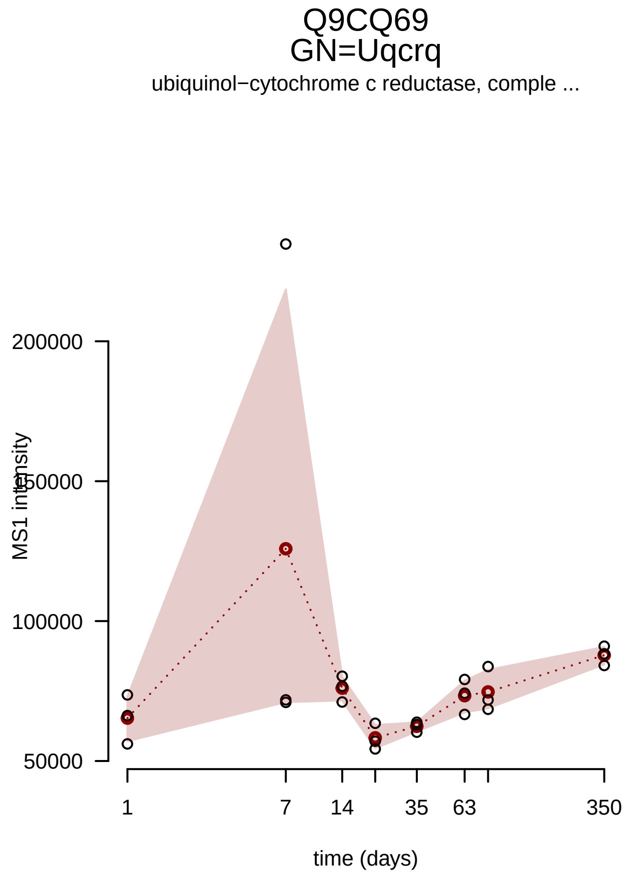
Q9CQ69
GN=Uqcrq
ubiquinol−cytochrome c reductase, comple ...
MS1 intensity
200000
150000
100000
50000
time (days)
1
7
14
35
63
350

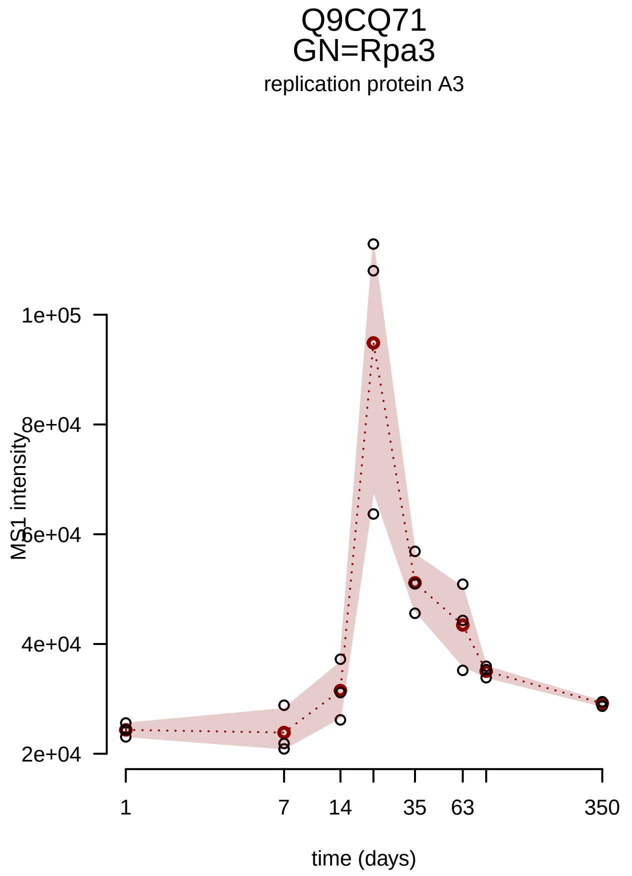
Q9CQ71
GN=Rpa3
replication protein A3
MS1 intensity
1e+05
8e+04
6e+04
4e+04
2e+04
time (days)
1
7
14
35
63
350

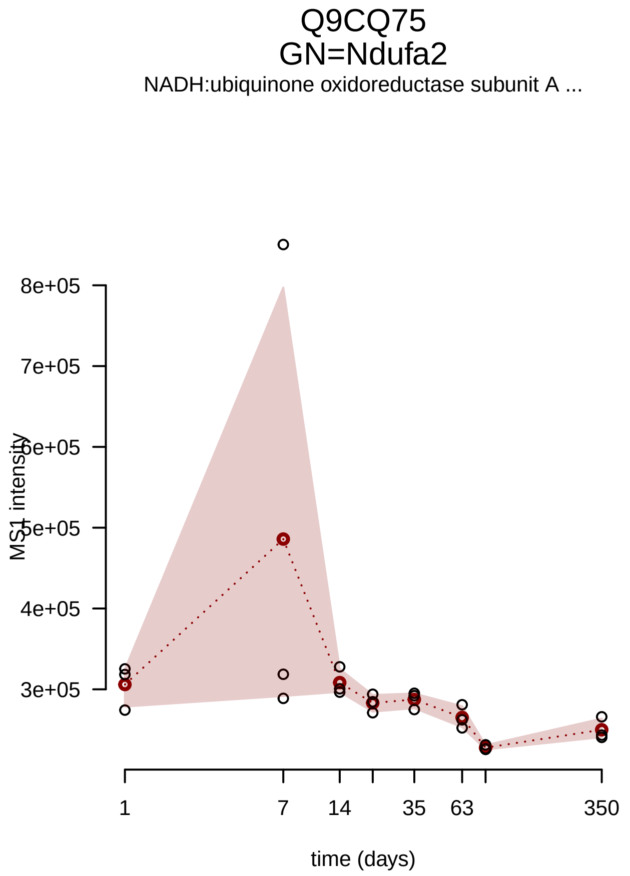
Q9CQ75
GN=Ndufa2
NADH:ubiquinone oxidoreductase subunit A ...
MS1 intensity
8e+05
7e+05
6e+05
5e+05
4e+05
3e+05
time (days)
1
7
14
35
63
350

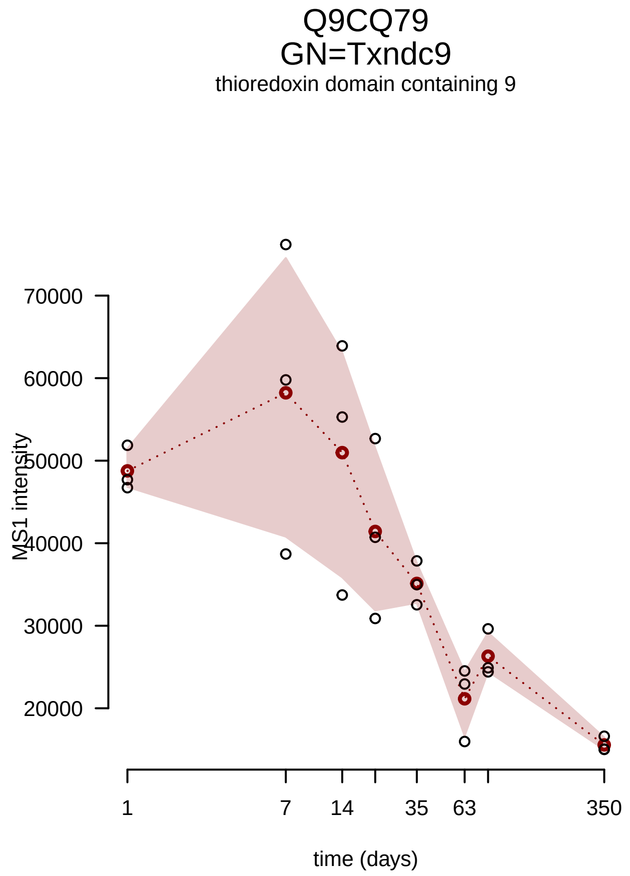
Q9CQ79
GN=Txndc9
thioredoxin domain containing 9
MS1 intensity
70000
60000
50000
40000
30000
20000
time (days)
1
7
14
35
63
350

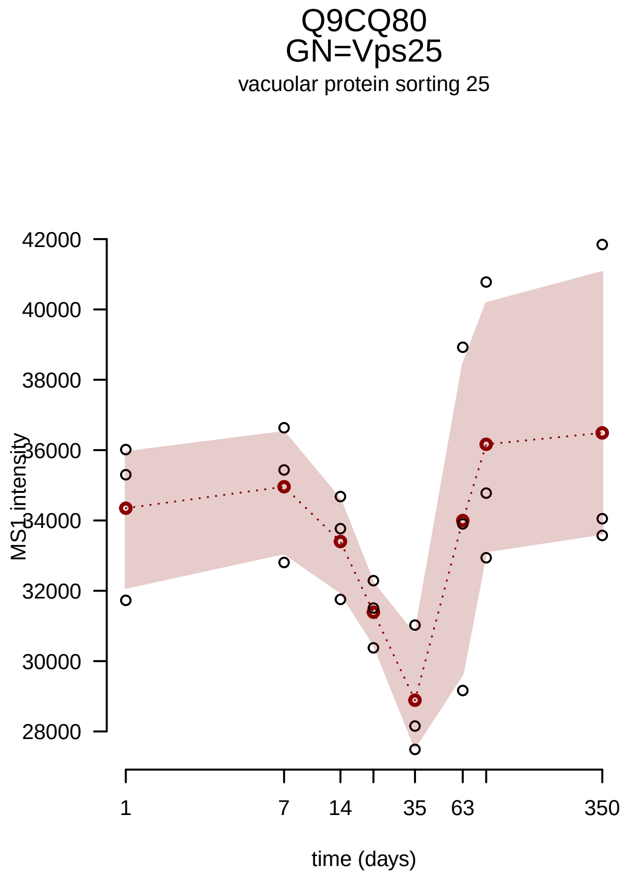
Q9CQ80
GN=Vps25
vacuolar protein sorting 25
MS1 intensity
42000
40000
38000
36000
34000
32000
30000
28000
time (days)
1
7
14
35
63
350

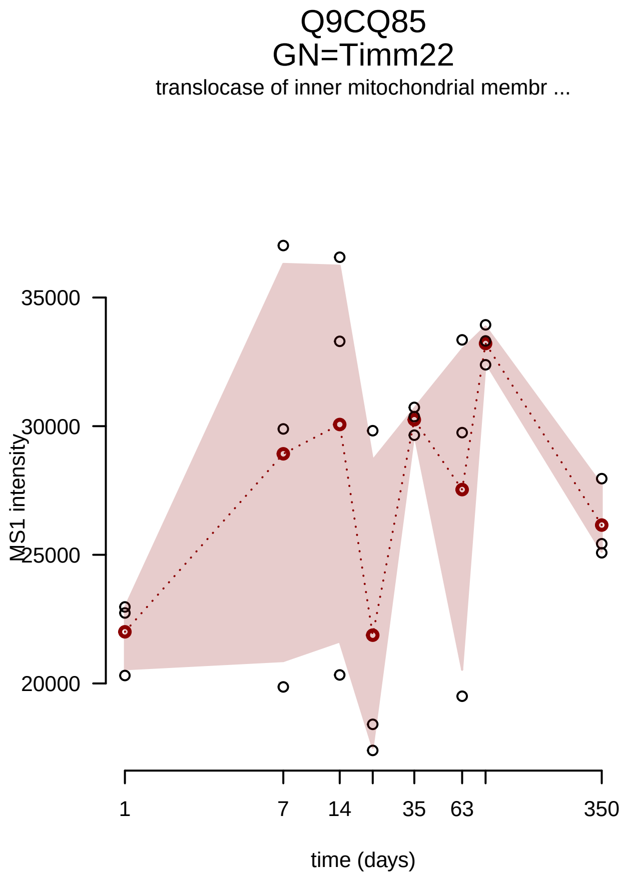
Q9CQ85
GN=Timm22
translocase of inner mitochondrial membr ...
MS1 intensity
35000
30000
25000
20000
time (days)
1
7
14
35
63
350

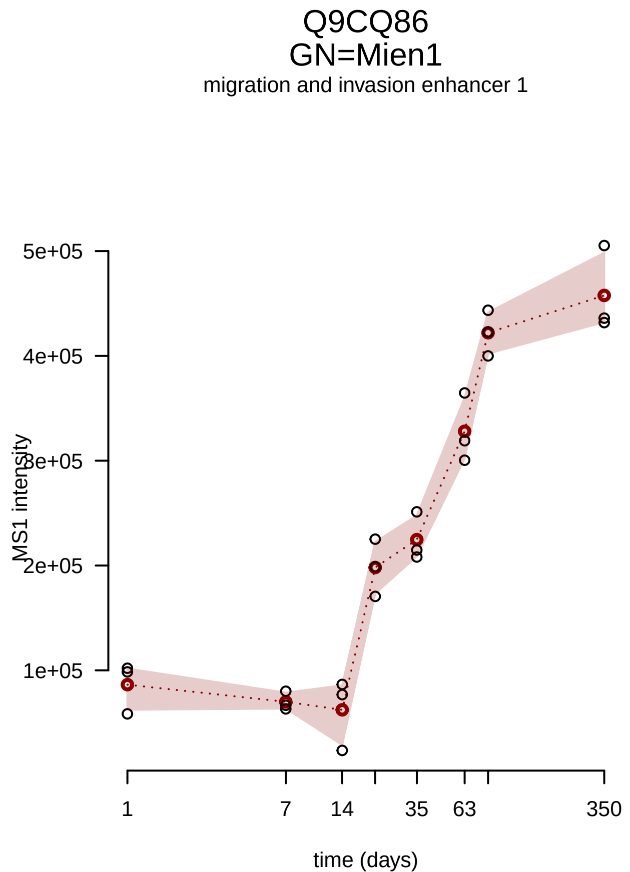
Q9CQ86
GN=Mien1
migration and invasion enhancer 1
MS1 intensity
5e+05
4e+05
3e+05
2e+05
1e+05
time (days)
1
7
14
35
63
350

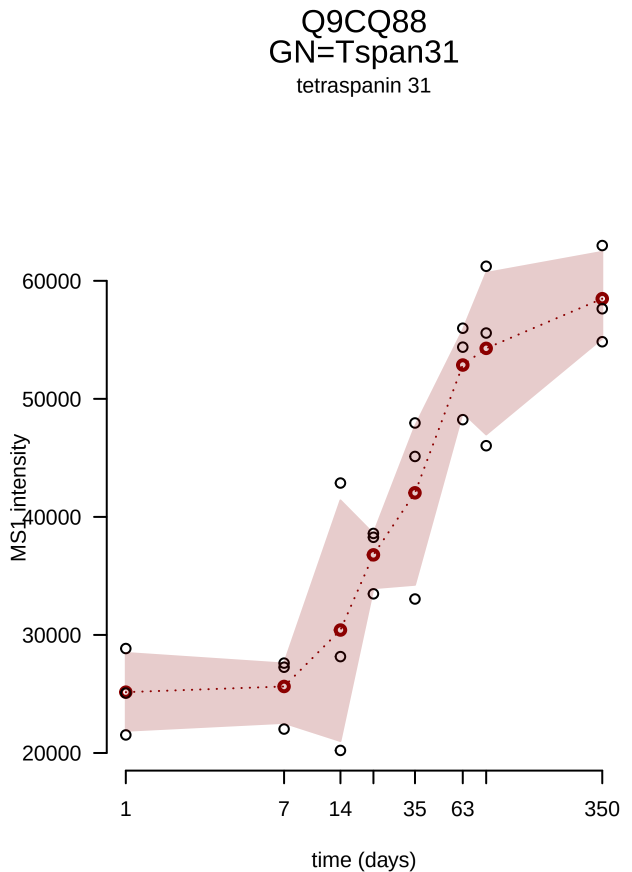
Q9CQ88
GN=Tspan31
tetraspanin 31
MS1 intensity
60000
50000
40000
30000
20000
time (days)
1
7
14
35
63
350

Q9CQ89
GN=Cuta
cutA divalent cation tolerance homolog

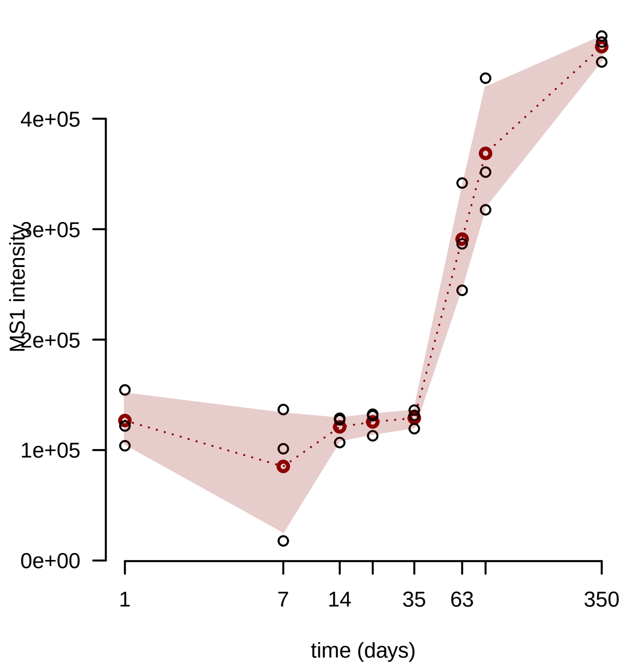

Q9CQ90
GN=

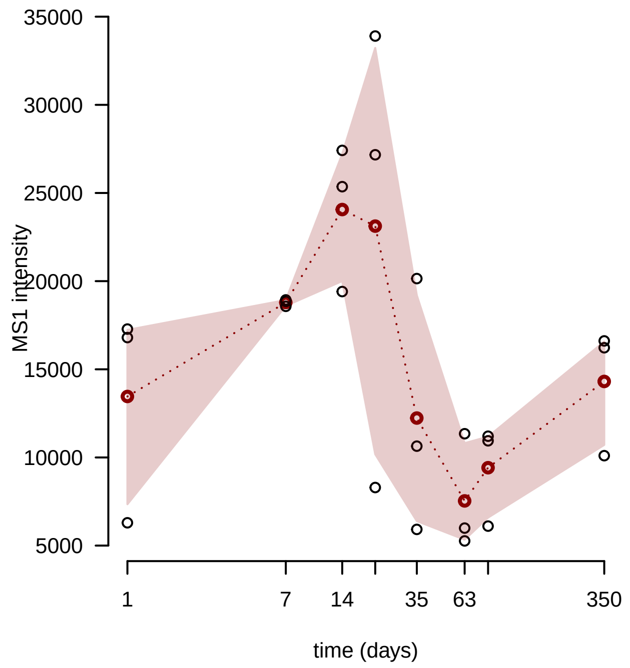

Q9CQ91
GN=Ndufa3
NADH:ubiquinone oxidoreductase subunit A ...

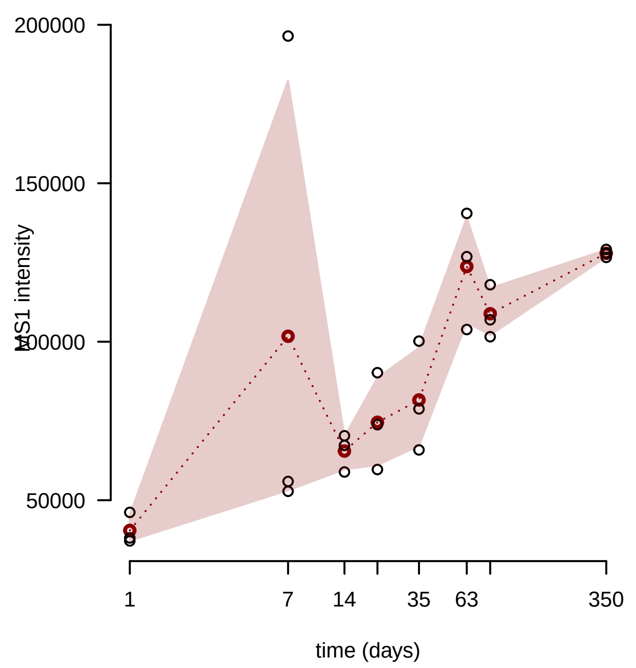

Q9CQ92
GN=Fis1
fission, mitochondrial 1

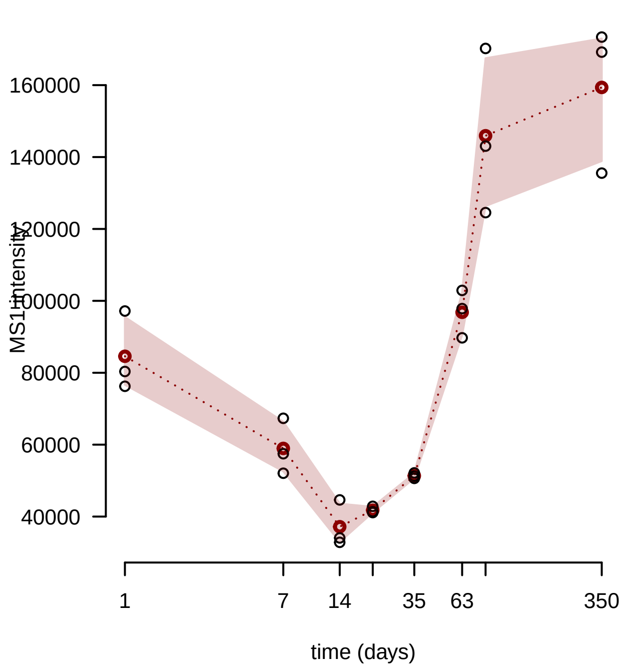

Q9CQA1
GN=Trappc5
trafficking protein particle complex 5

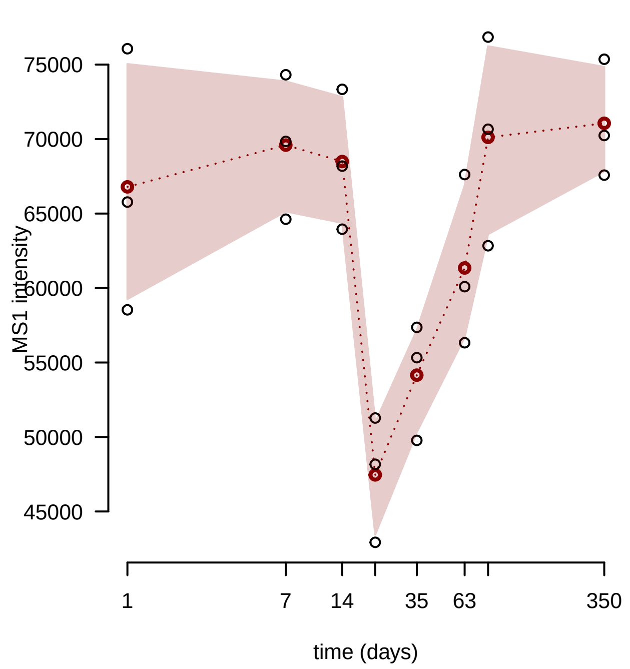

Q9CQA3
GN=Sdhb
succinate dehydrogenase complex, subunit ...

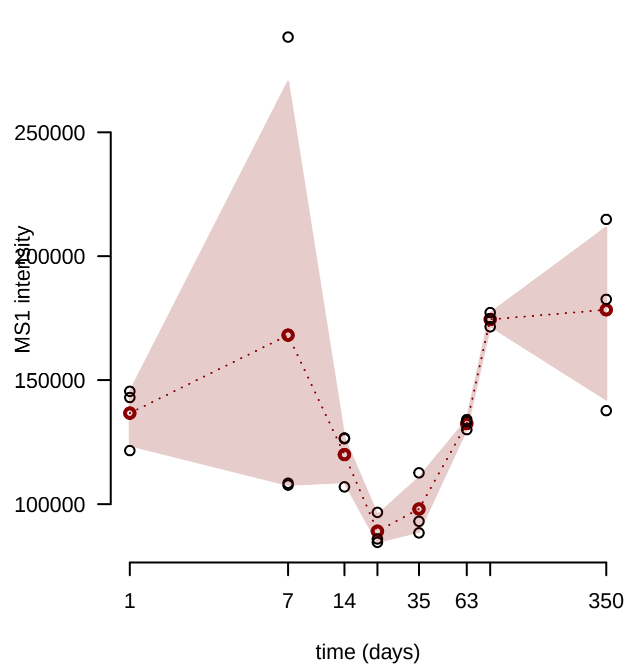

Q9CQA5
GN=Med4
mediator complex subunit 4

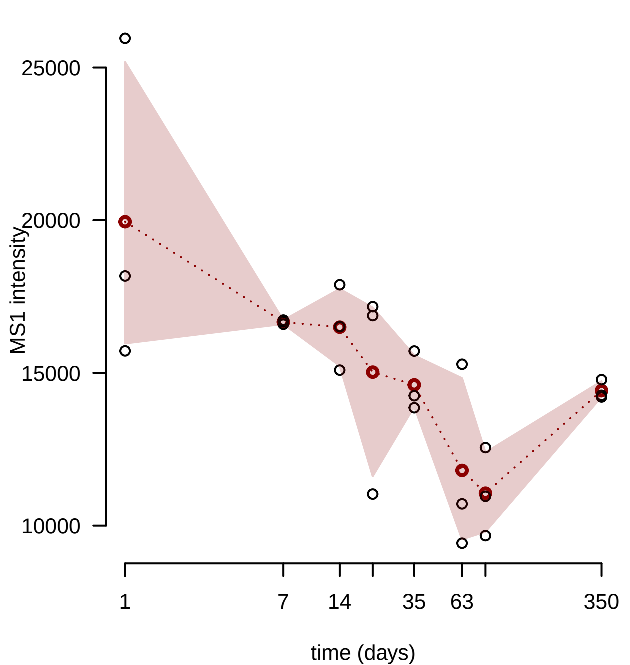

Q9CQA6
GN=Chchd1
coiled−coil−helix−coiled−coil−helix doma ...

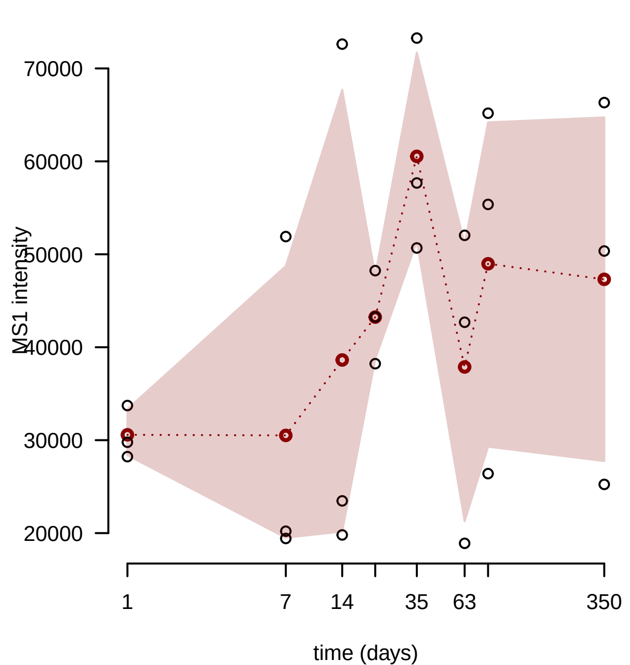

Q9CQA9
GN=Ntpcr
nucleoside−triphosphatase, cancer−relate ...

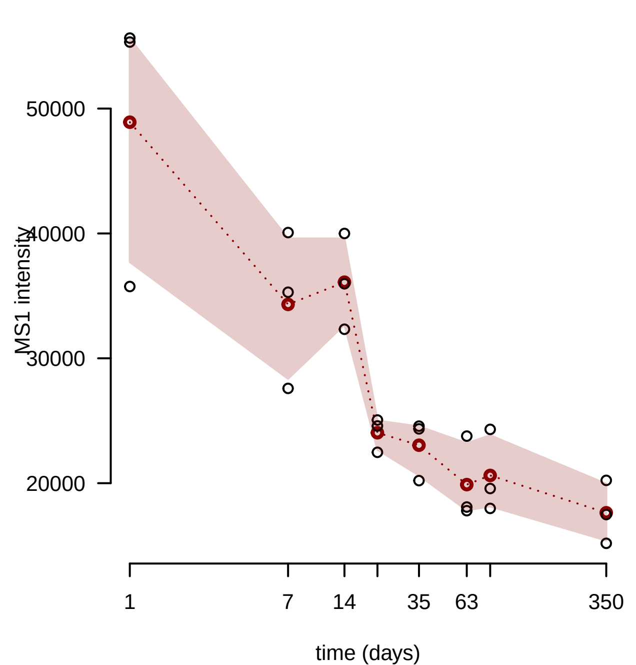

Q9CQB2
GN=Mcrip2
MAPK regulated corepressor interacting p ...

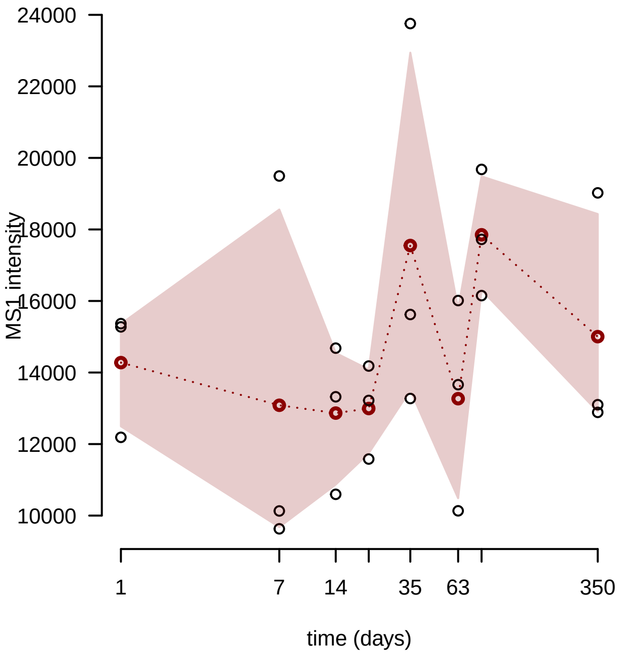

Q9CQB5
GN=Cisd2
CDGSH iron sulfur domain 2

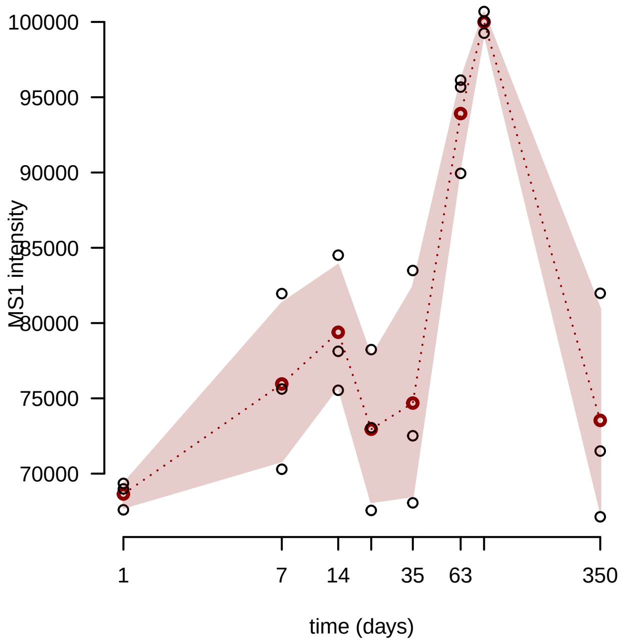

Q9CQC6
GN=Bzw1
basic leucine zipper and W2 domains 1

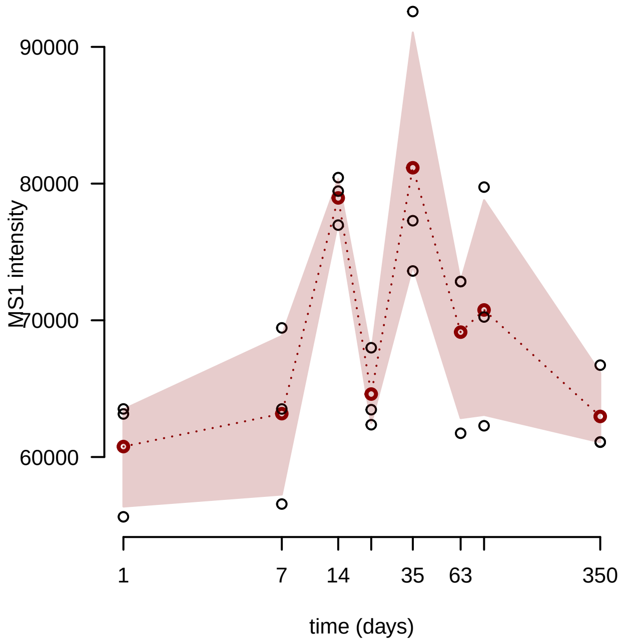

Q9CQC7
GN=Ndufb4
NADH:ubiquinone oxidoreductase subunit B ...

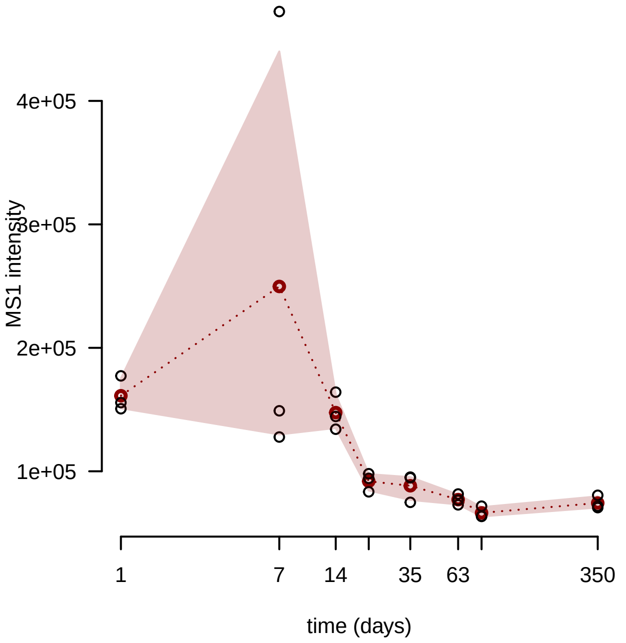

Q9CQC8
GN=Spg21
SPG21, maspardin

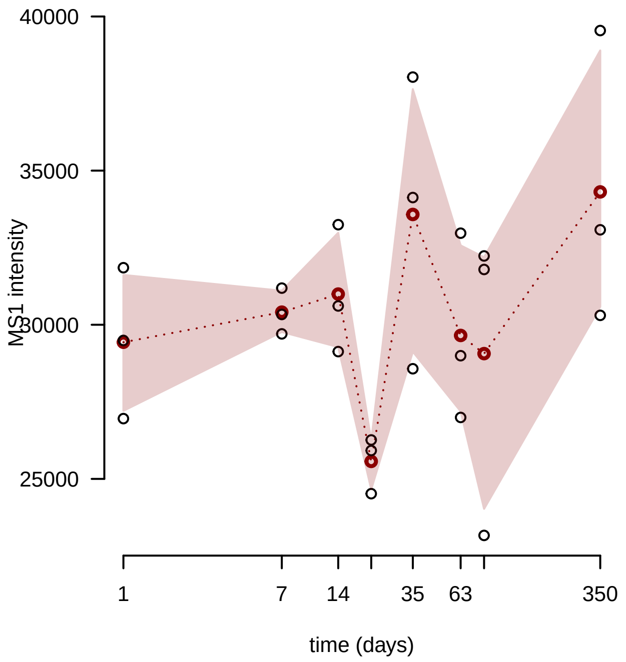

Q9CQC9
GN=Sar1b
secretion associated Ras related GTPase ...

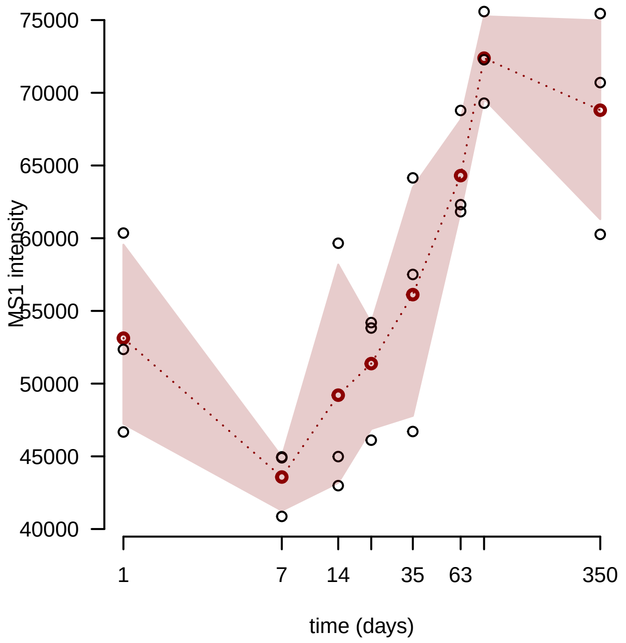

Q9CQD1
GN=Rab5a
RAB5A, member RAS oncogene family

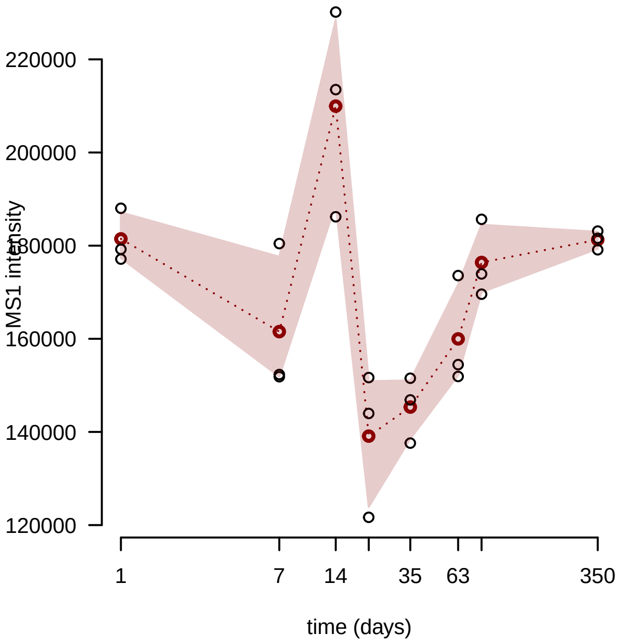

Q9CQD4
GN=Chmp1b2
charged multivesicular body protein 1B2

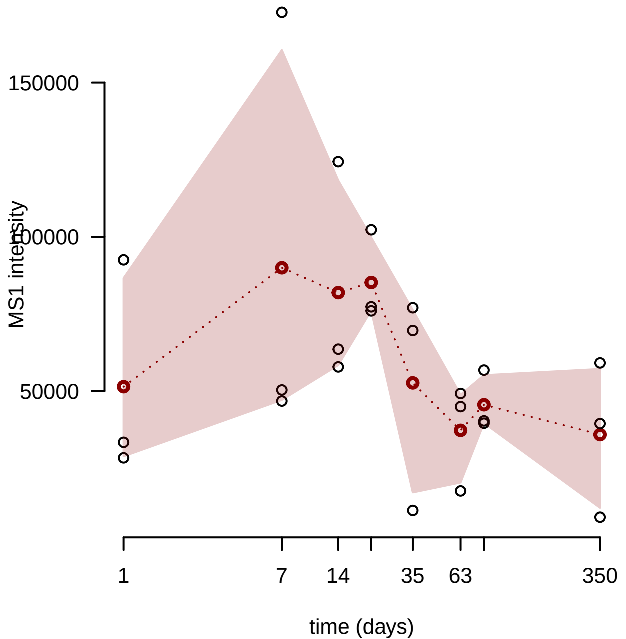

Q9CQE0
GN=Rnf138
ring finger protein 138

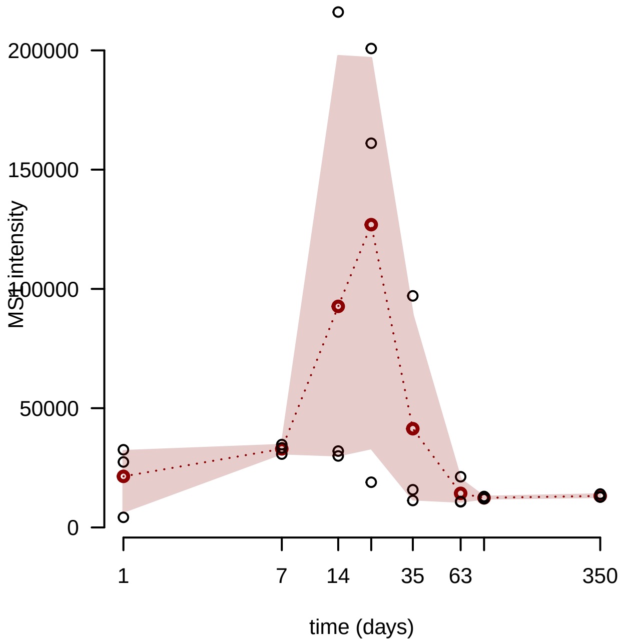

Q9CQE1
GN=Nipsnap3b
nipsnap homolog 3B

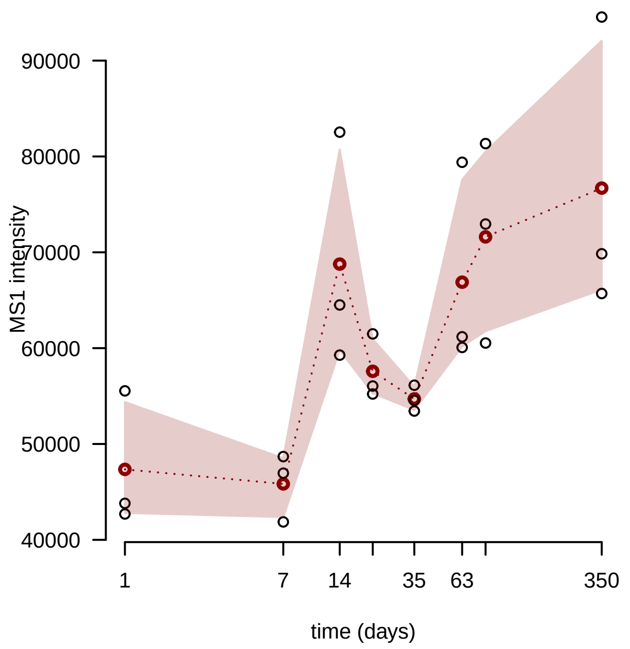

Q9CQE2
GN=Tmem223
transmembrane protein 223

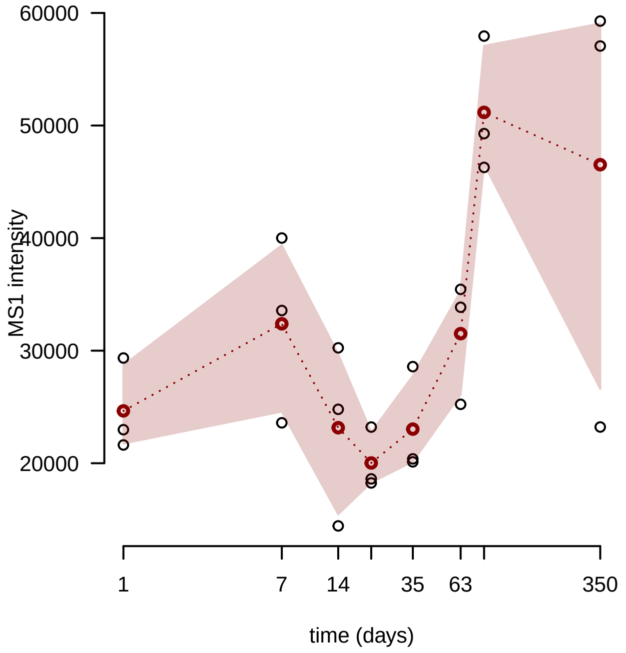

Q9CQE3
GN=Mrps17
mitochondrial ribosomal protein S17

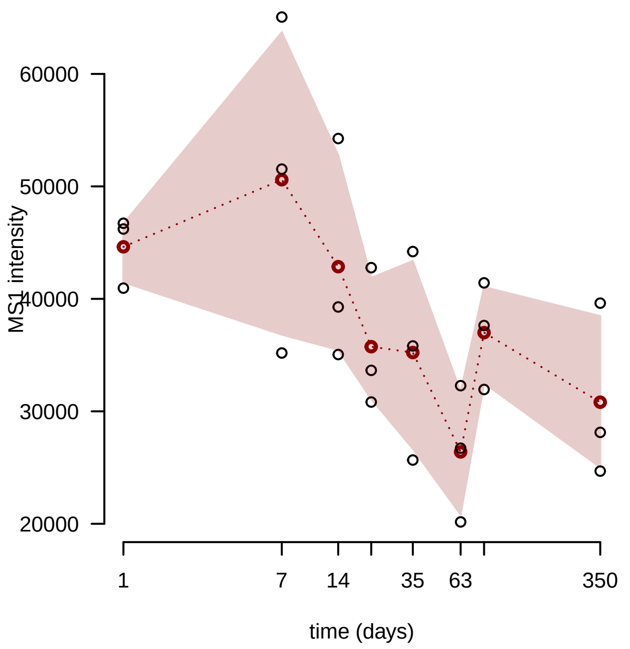

Q9CQE5
GN=Rgs10
regulator of G−protein signalling 10

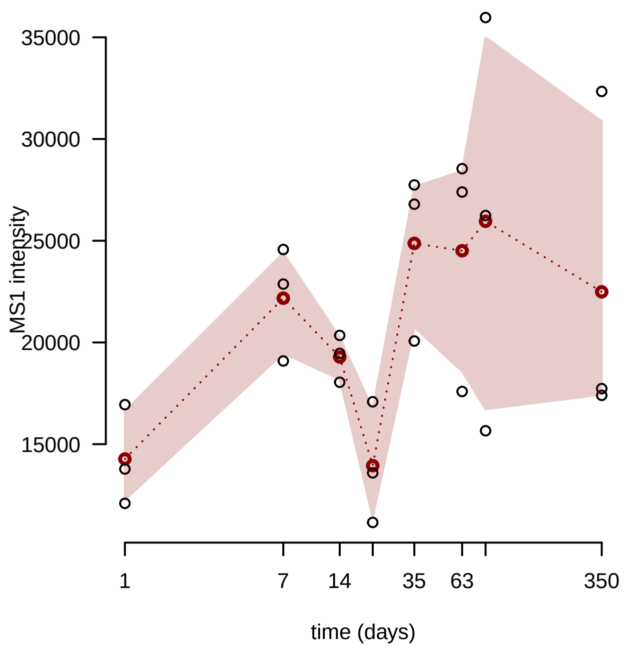

Q9CQE6
GN=Asf1a
anti−silencing function 1A histone chape ...

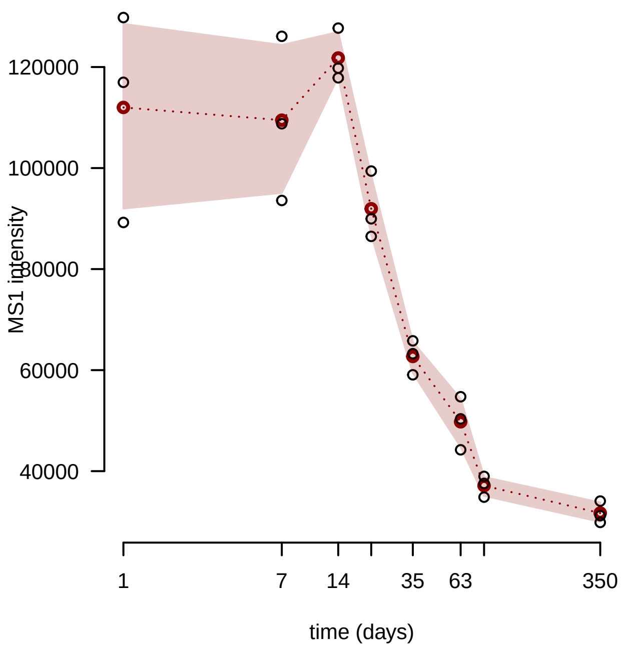

Q9CQE7
GN=Ergic3
ERGIC and golgi 3

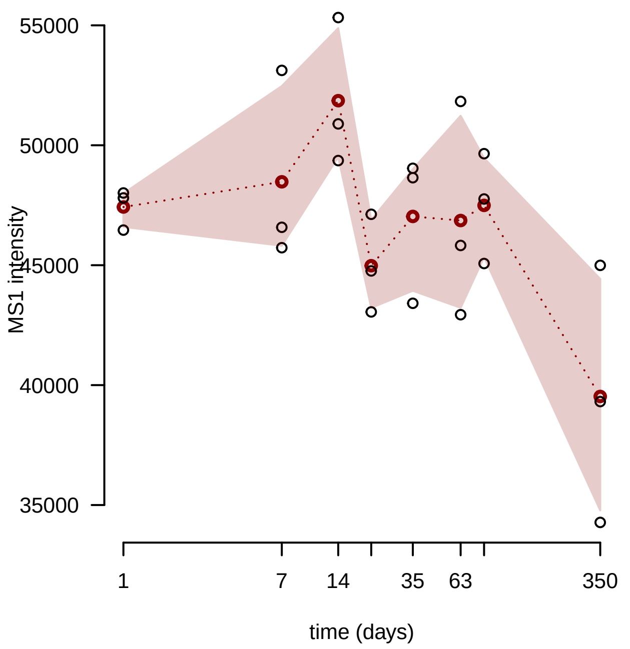

Q9CQE8
GN=RTRAF

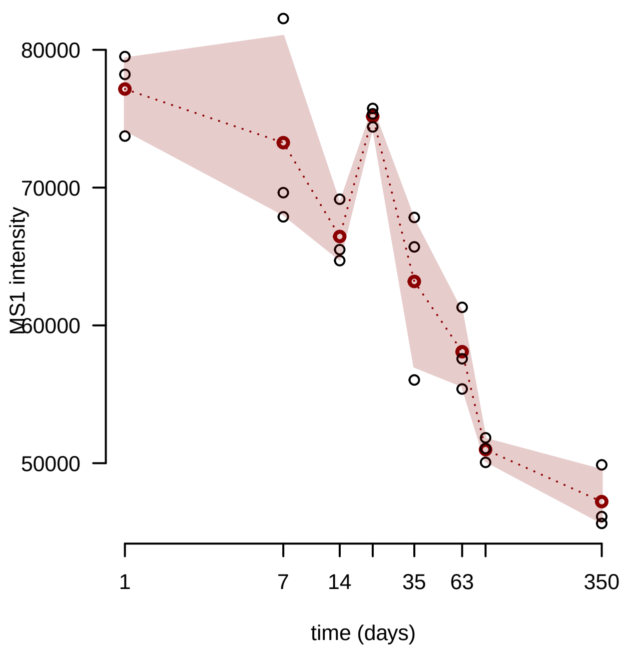

Q9CQE9
GN=Flywch2
FLYWCH family member 2

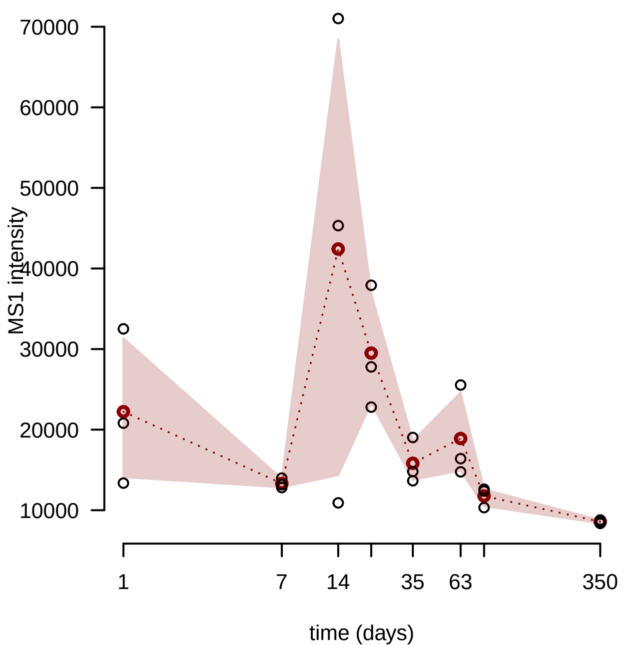

Q9CQF0
GN=Mrpl11
mitochondrial ribosomal protein L11

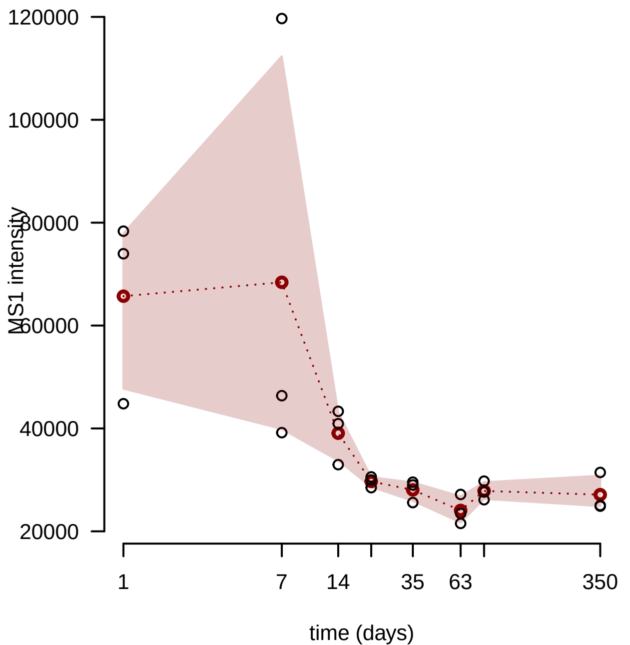

Q9CQF3
GN=Nudt21
nudix (nucleoside diphosphate linked moi ...

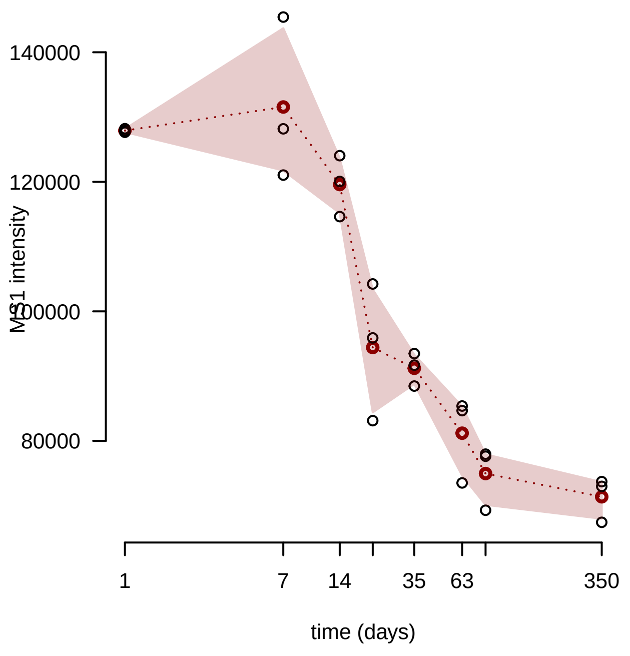

Q9CQF4
GN=

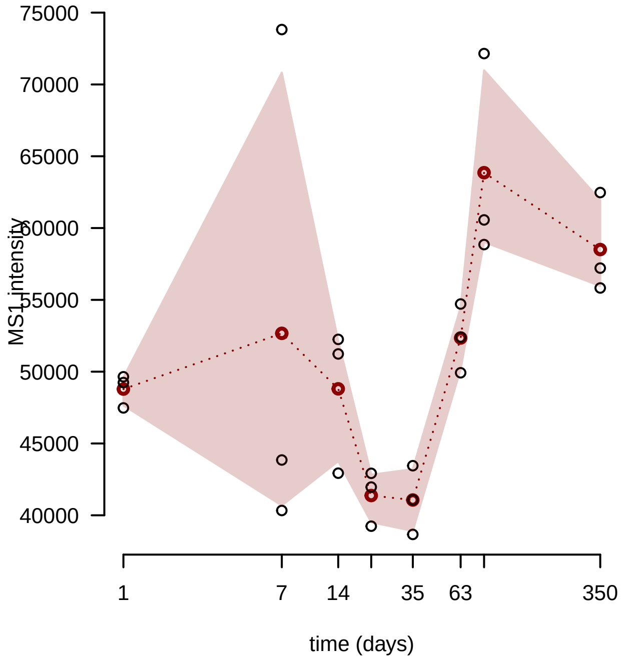

Q9CQF6
GN=Aasdhppt
aminoadipate−semialdehyde dehydrogenase− ...

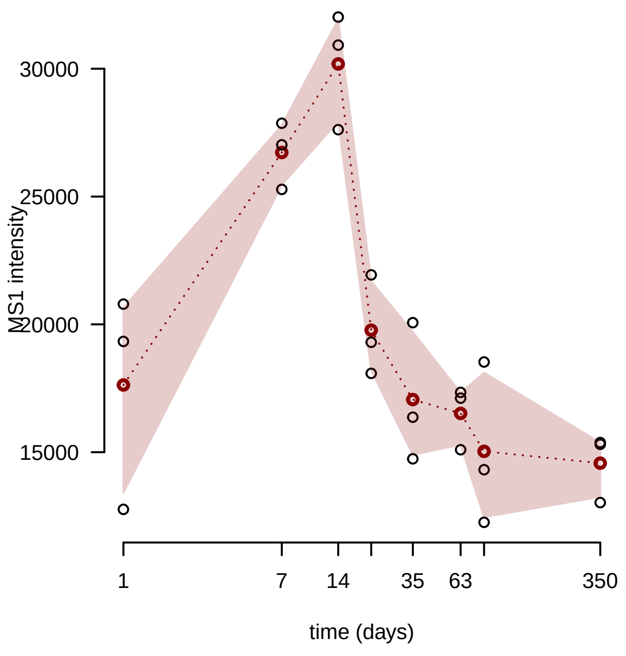

Q9CQF8
GN=Mrpl57
mitochondrial ribosomal protein L57

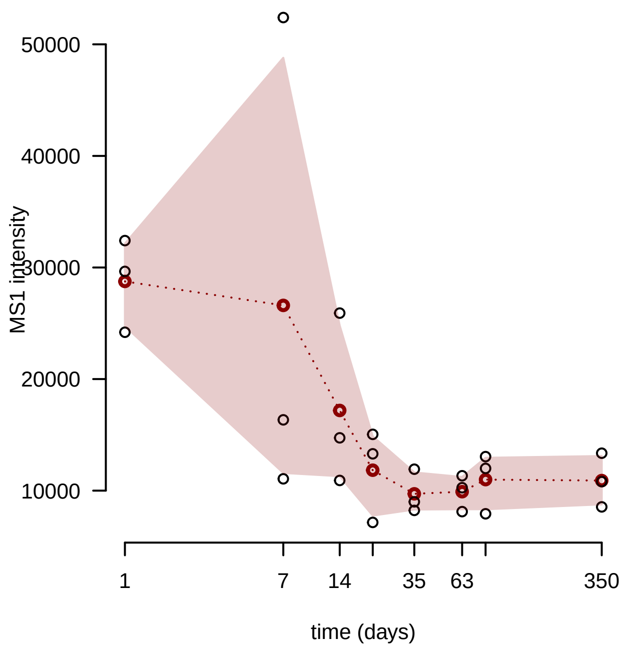

Q9CQF9
GN=Pcyox1
prenylcysteine oxidase 1

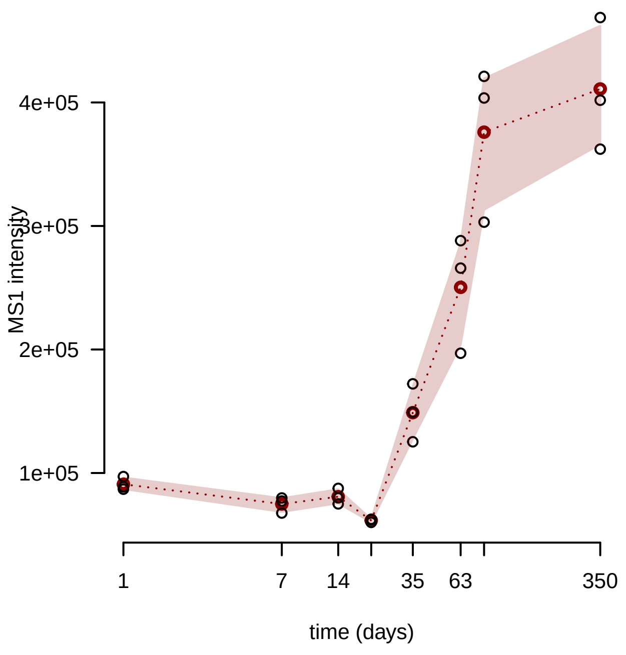

Q9CQG2
GN=Mettl16
methyltransferase like 16

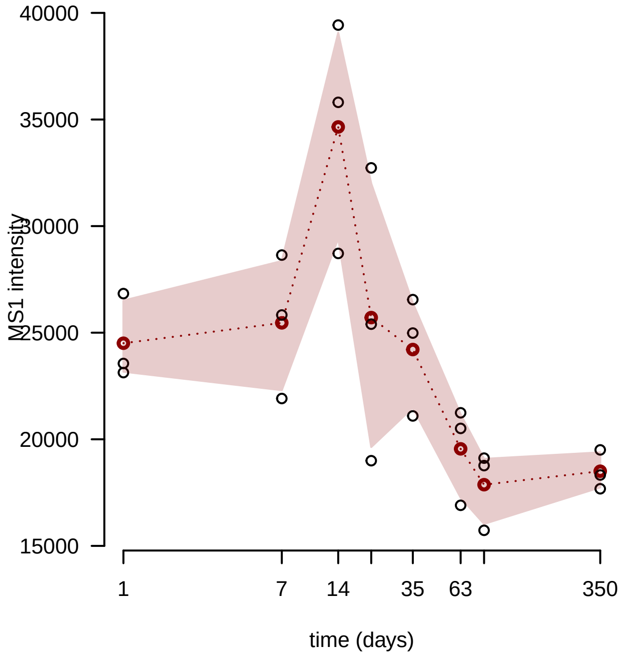

Q9CQG6
GN=Tmem147
transmembrane protein 147

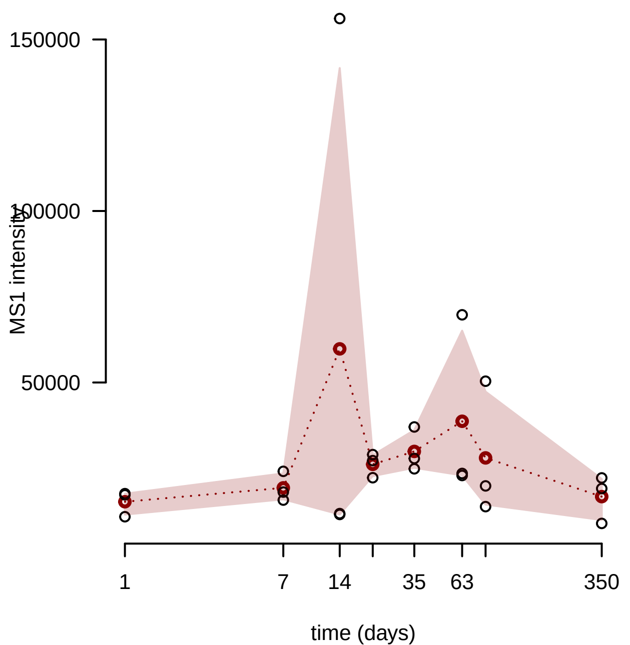

Q9CQG9
GN=Tmem100
transmembrane protein 100

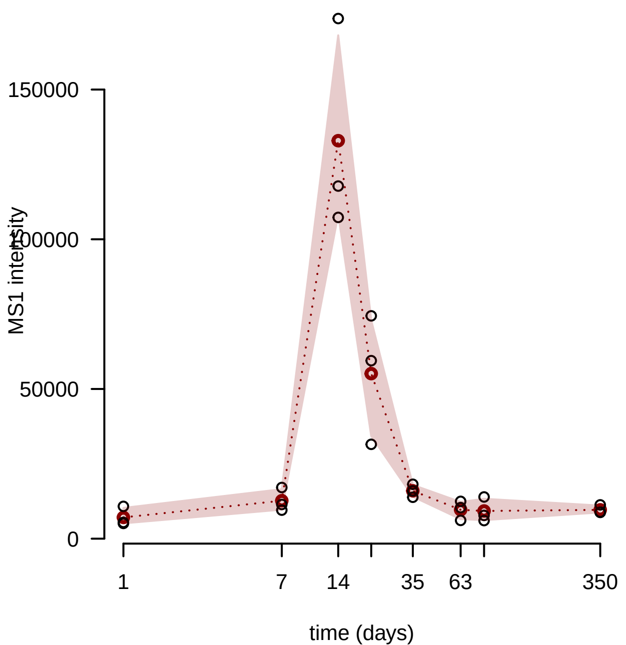

Q9CQH3
GN=Ndufb5
NADH:ubiquinone oxidoreductase subunit B ...

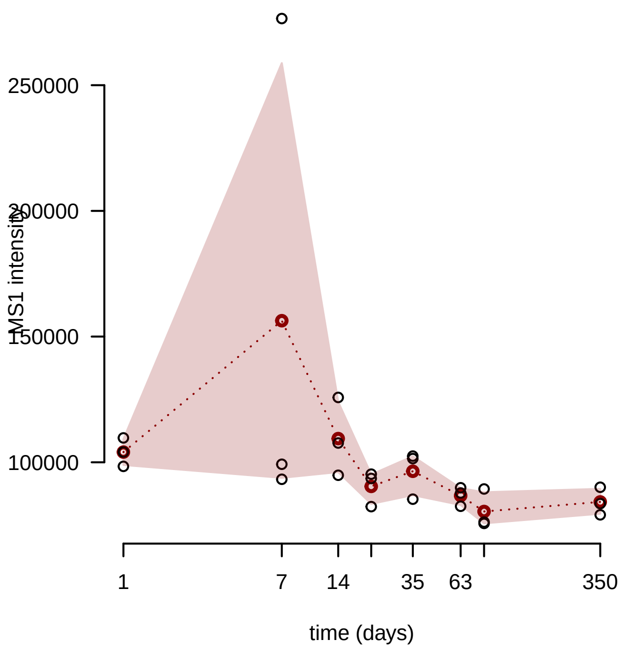

Q9CQH7
GN=Btf3l4
basic transcription factor 3−like 4

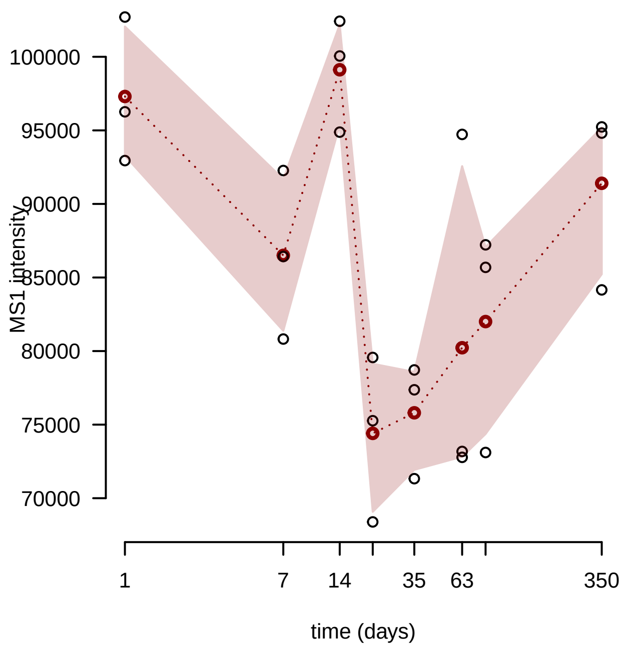

Q9CQH8
GN=Rpp14
ribonuclease P 14 subunit

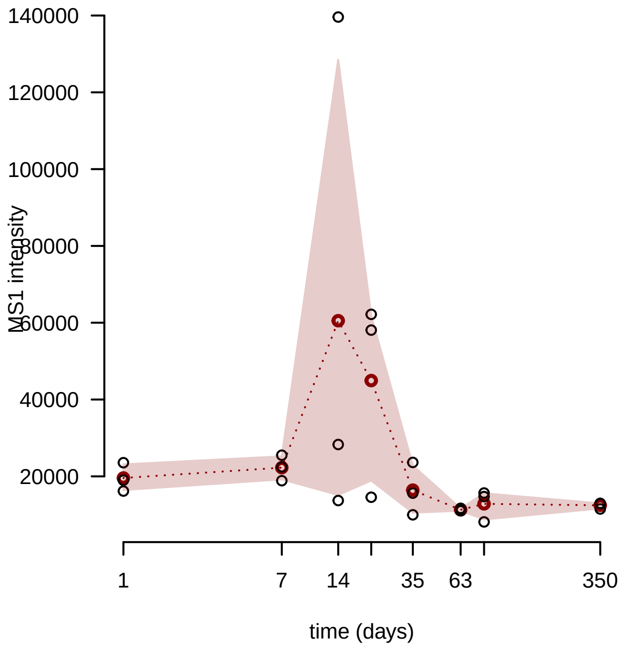

Q9CQI3
GN=Gmfb
glia maturation factor, beta

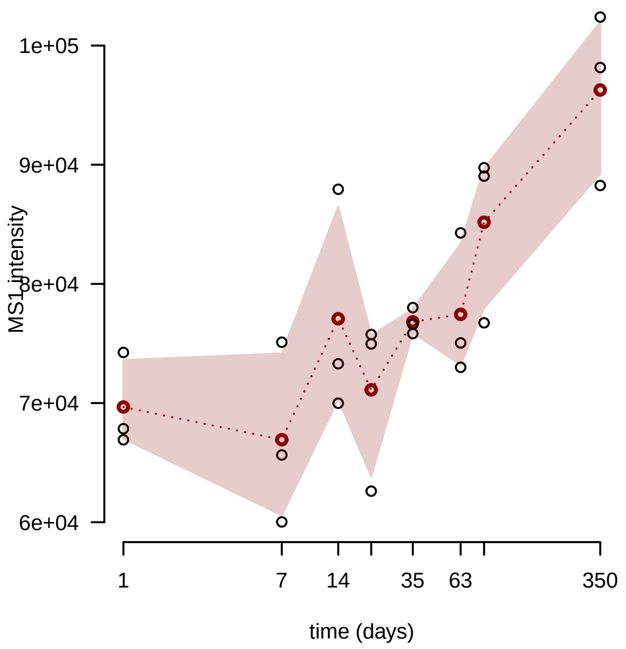

Q9CQI6
GN=Cotl1
coactosin−like 1 (Dictyostelium)

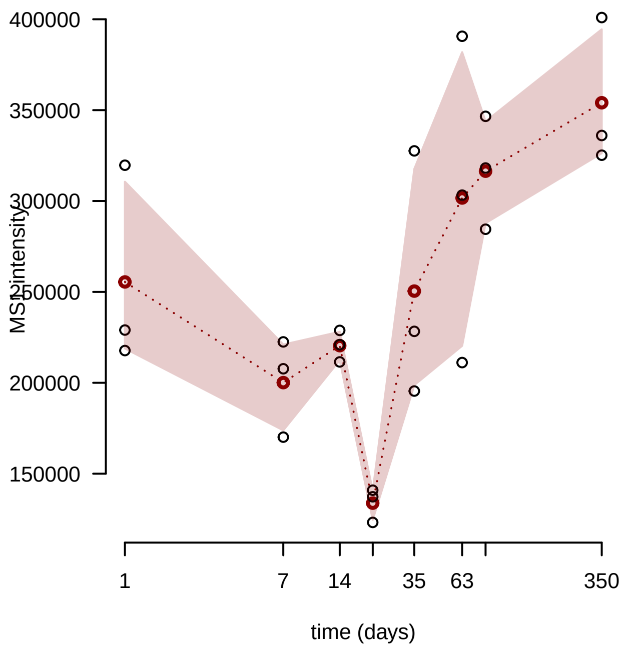

Q9CQI7
GN=Snrpb2
U2 small nuclear ribonucleoprotein B

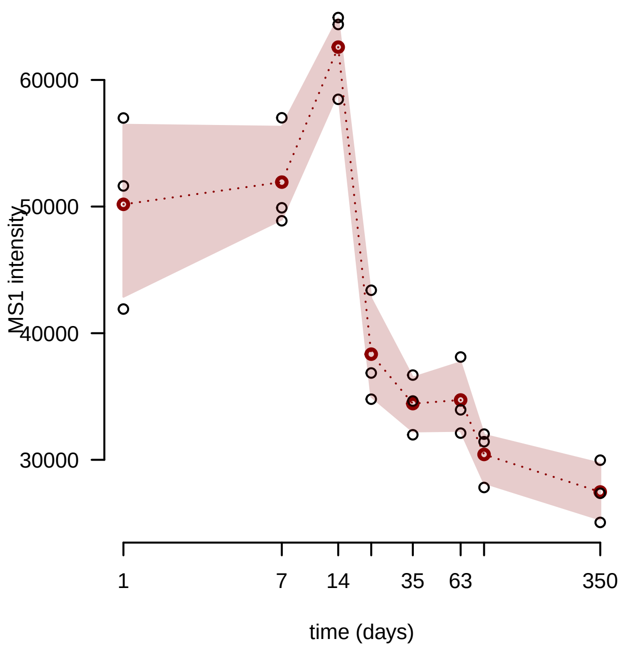

Q9CQI9
GN=Med30
mediator complex subunit 30

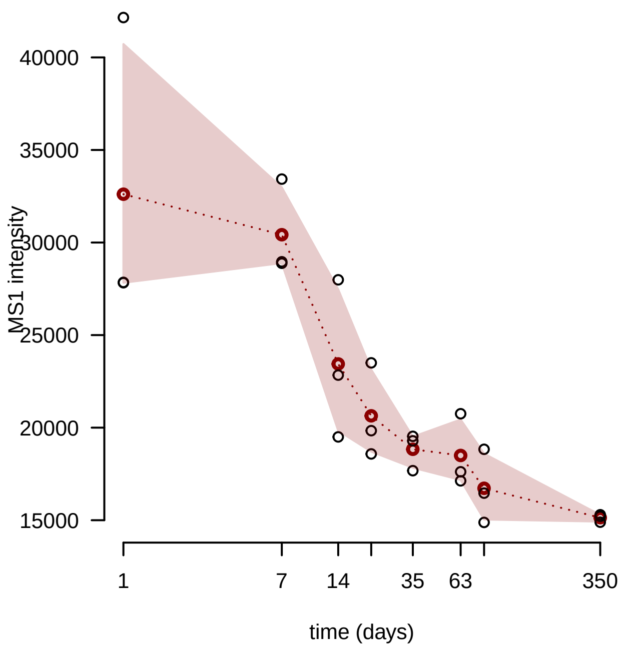

Q9CQJ2
GN=Pih1d1
PIH1 domain containing 1

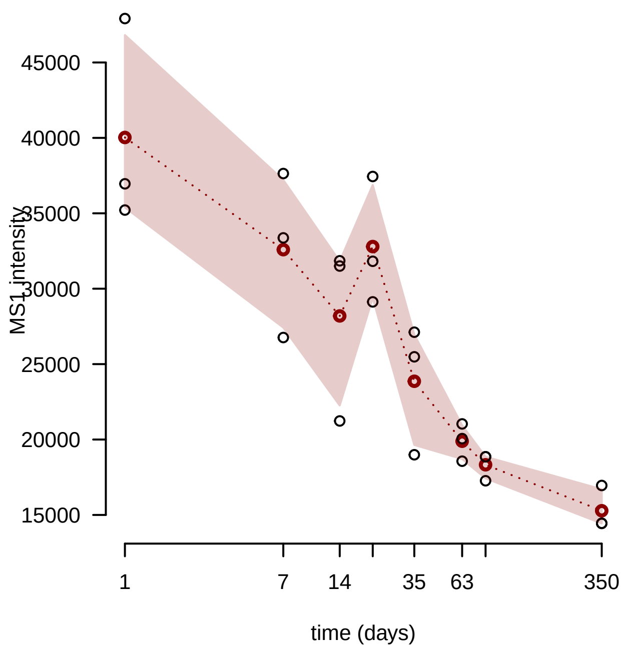

Q9CQJ4
GN=Rnf2
ring finger protein 2

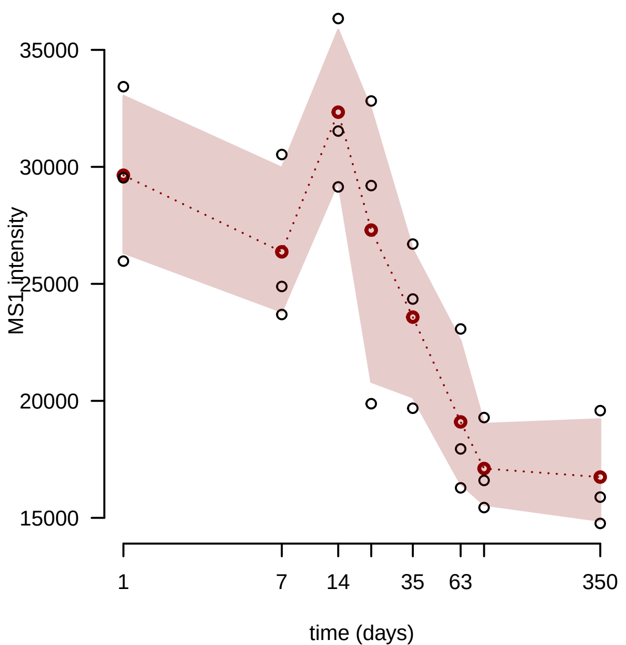

Q9CQJ6
GN=Denr
density−regulated protein

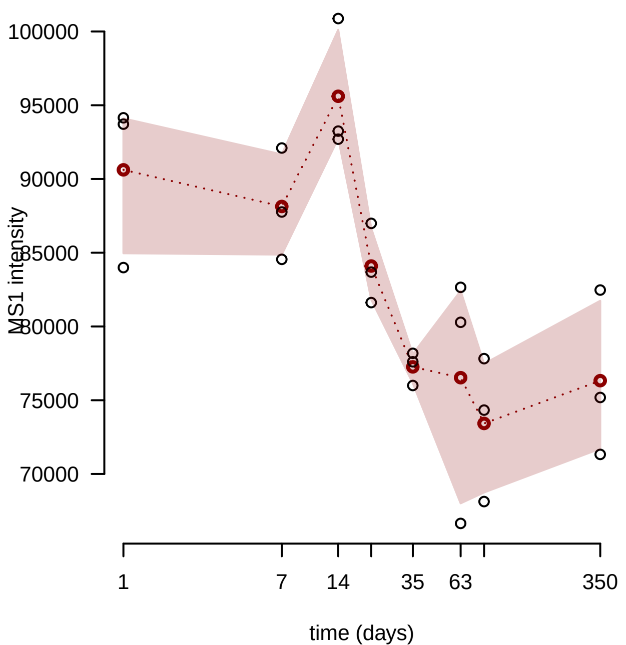

Q9CQJ7
GN=Pttg1
pituitary tumor−transforming gene 1

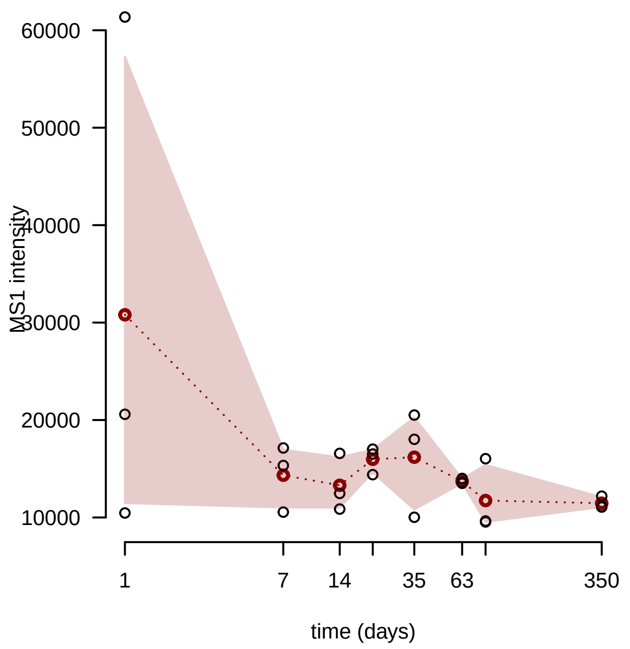

Q9CQJ8
GN=Ndufb9
NADH:ubiquinone oxidoreductase subunit B ...

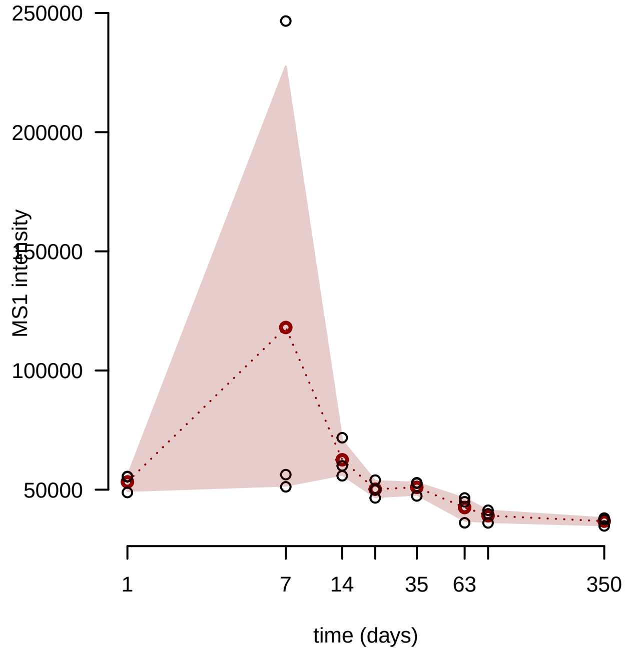

Q9CQK7
GN=Rwdd1
RWD domain containing 1

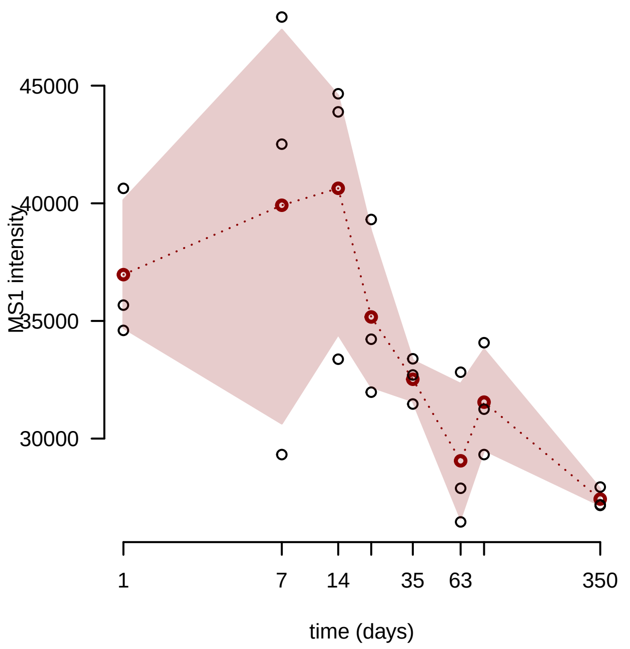

Q9CQL0
GN=Mettl21A

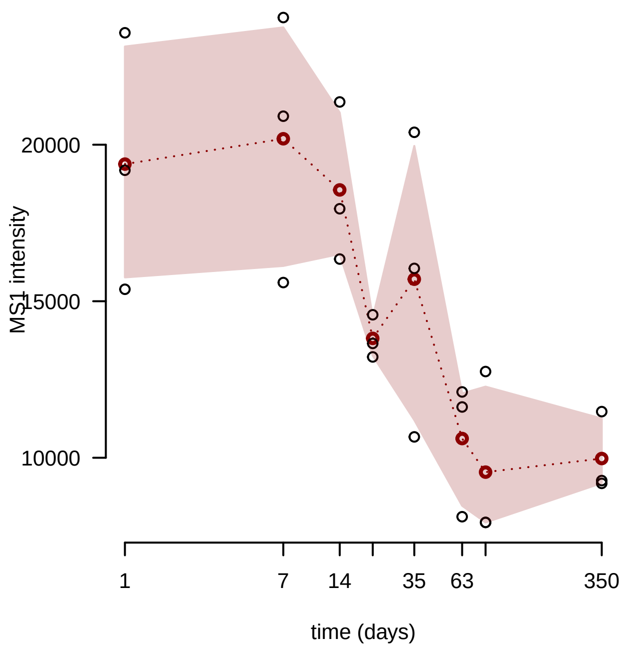

Q9CQL4
GN=Mrpl20
mitochondrial ribosomal protein L20

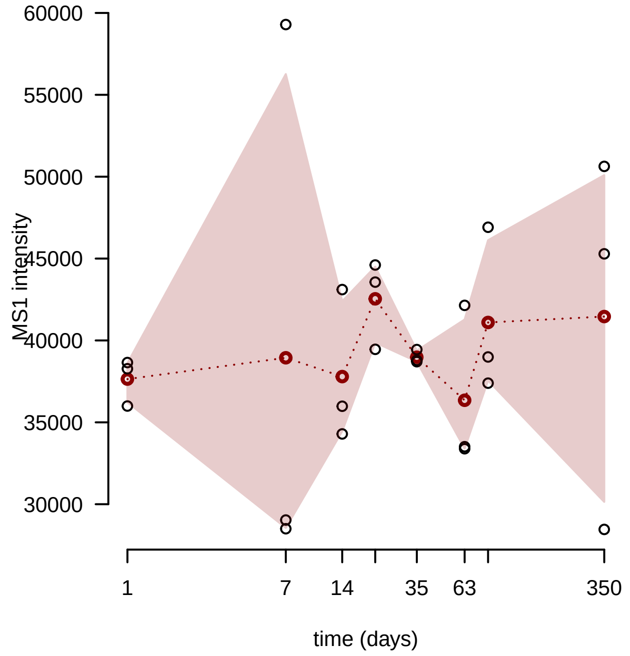

Q9CQL5
GN=Mrpl18
mitochondrial ribosomal protein L18

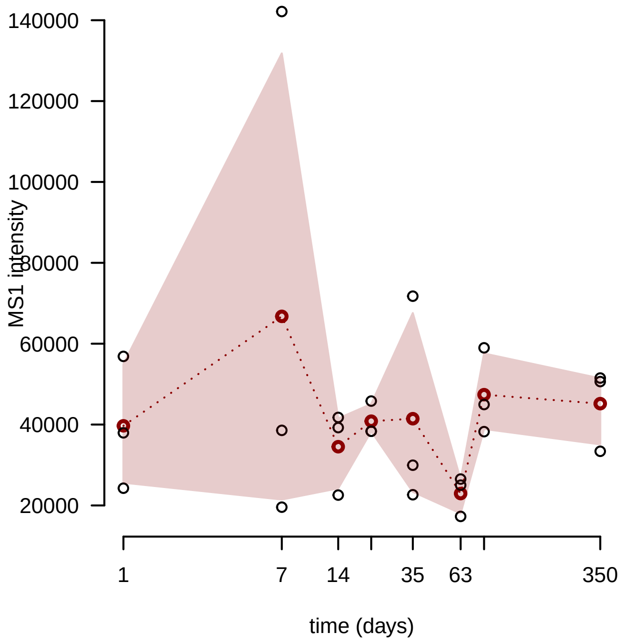

Q9CQL7
GN=Mrfap1
Morf4 family associated protein 1

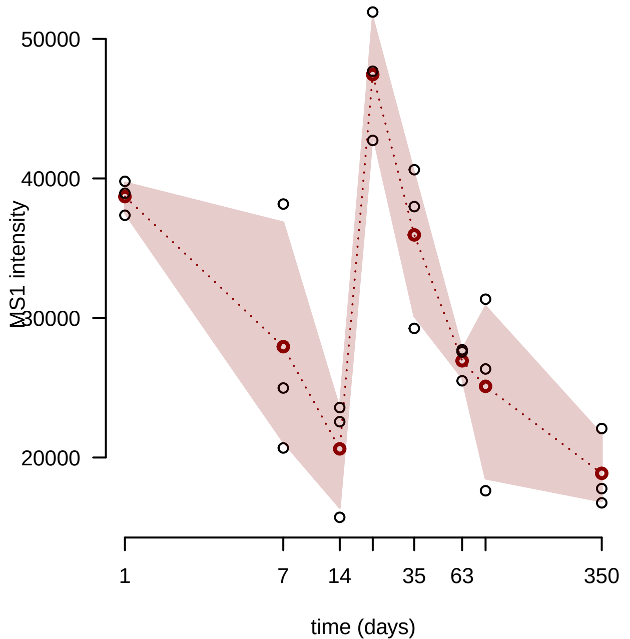

Q9CQM5
GN=Txndc17
thioredoxin domain containing 17

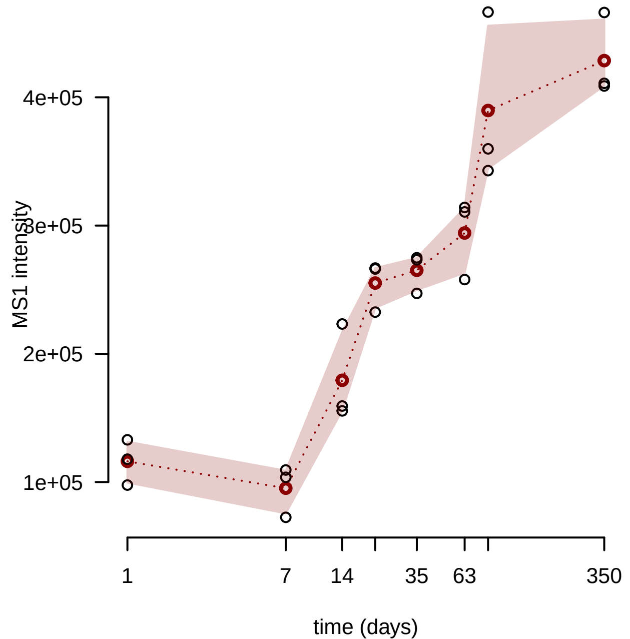

Q9CQM9
GN=Glrx3
glutaredoxin 3

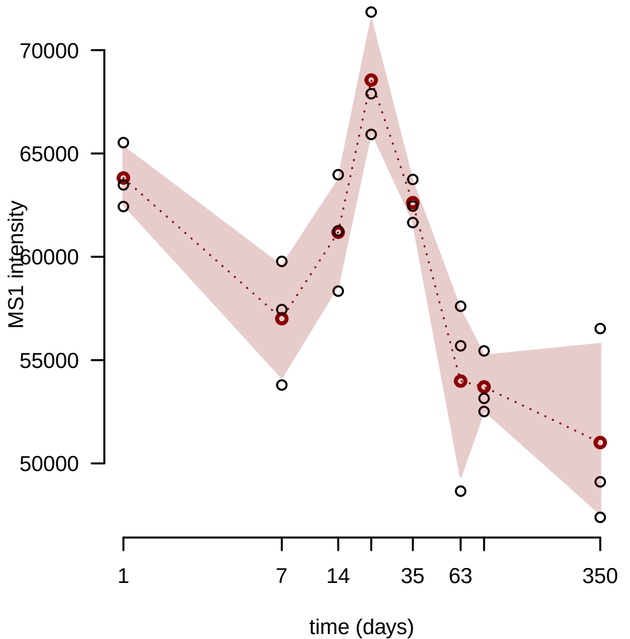

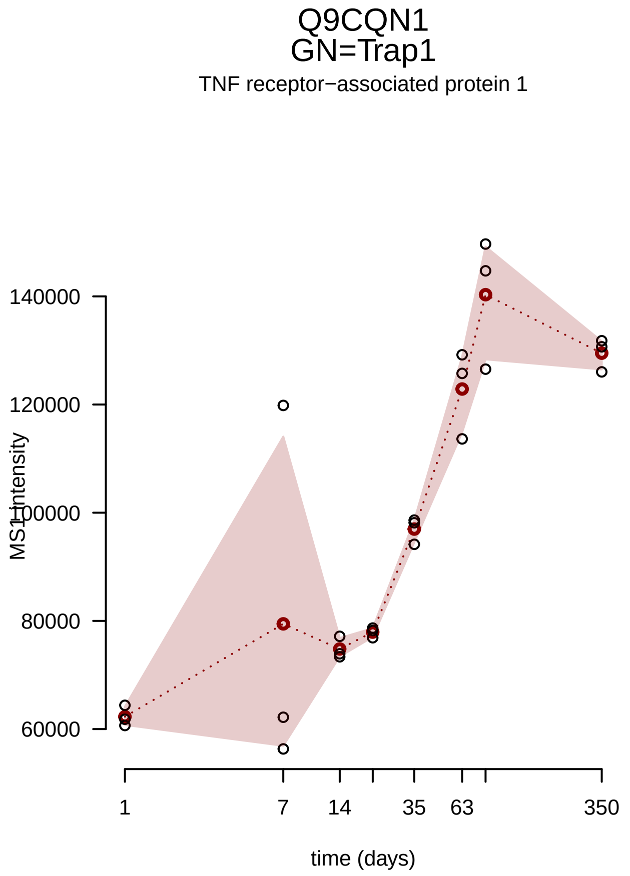
Q9CQN1
GN=Trap1
TNF receptor−associated protein 1
MS1 intensity
60000
80000
100000
120000
140000
1
7
14
35
63
350
time (days)

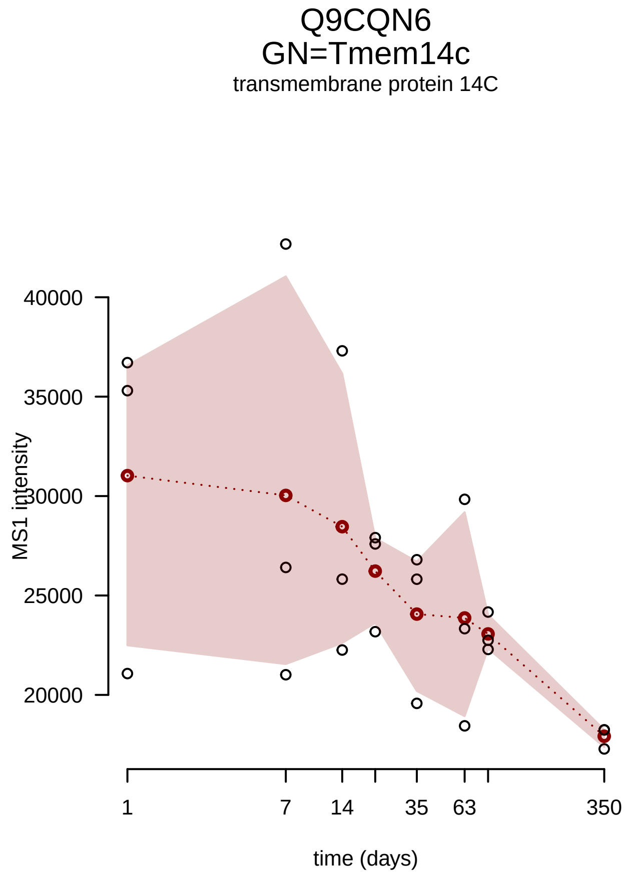
Q9CQN6
GN=Tmem14c
transmembrane protein 14C
MS1 intensity
20000
25000
30000
35000
40000
1
7
14
35
63
350
time (days)

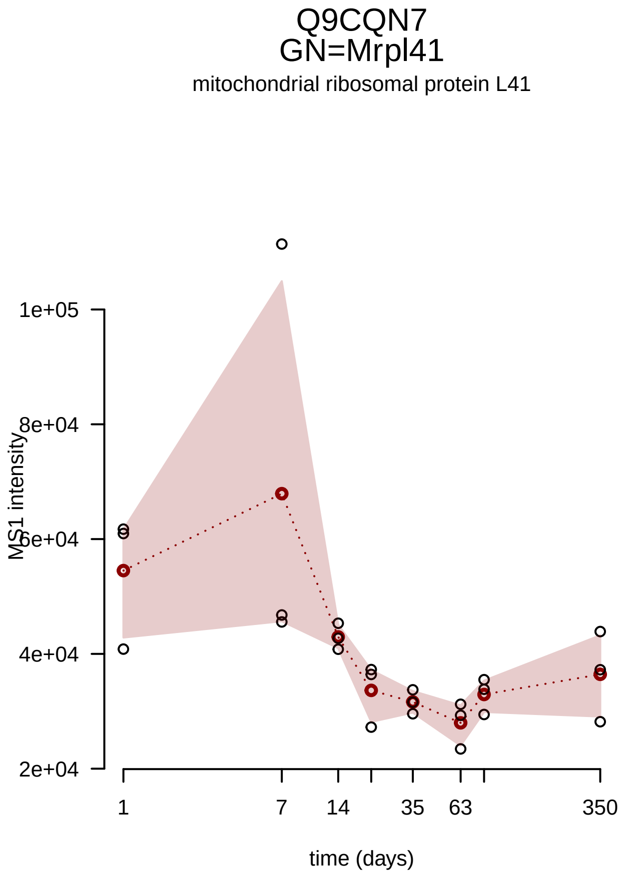
Q9CQN7
GN=Mrpl41
mitochondrial ribosomal protein L41
MS1 intensity
2e+04
4e+04
6e+04
8e+04
1e+05
1
7
14
35
63
350
time (days)

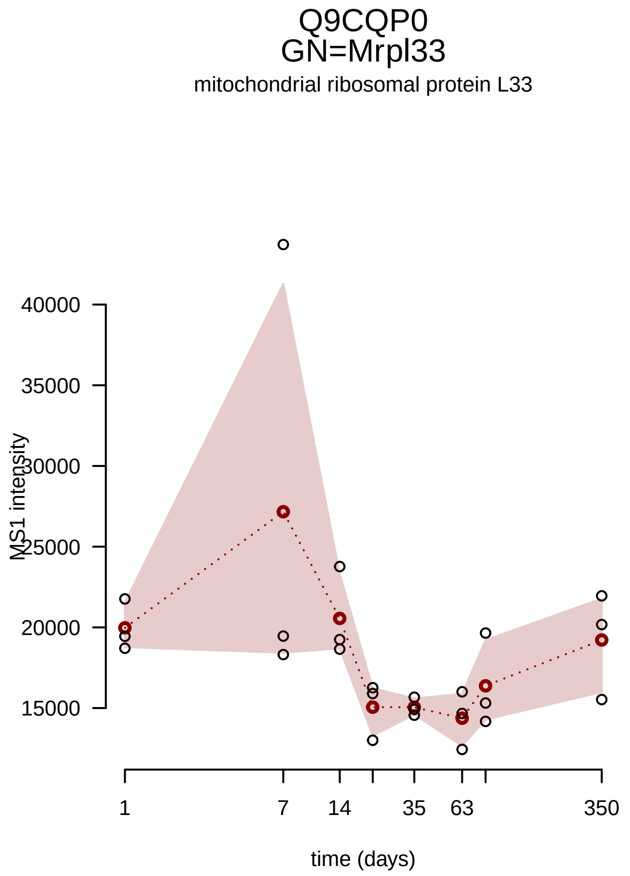
Q9CQP0
GN=Mrpl33
mitochondrial ribosomal protein L33
MS1 intensity
15000
20000
25000
30000
35000
40000
1
7
14
35
63
350
time (days)

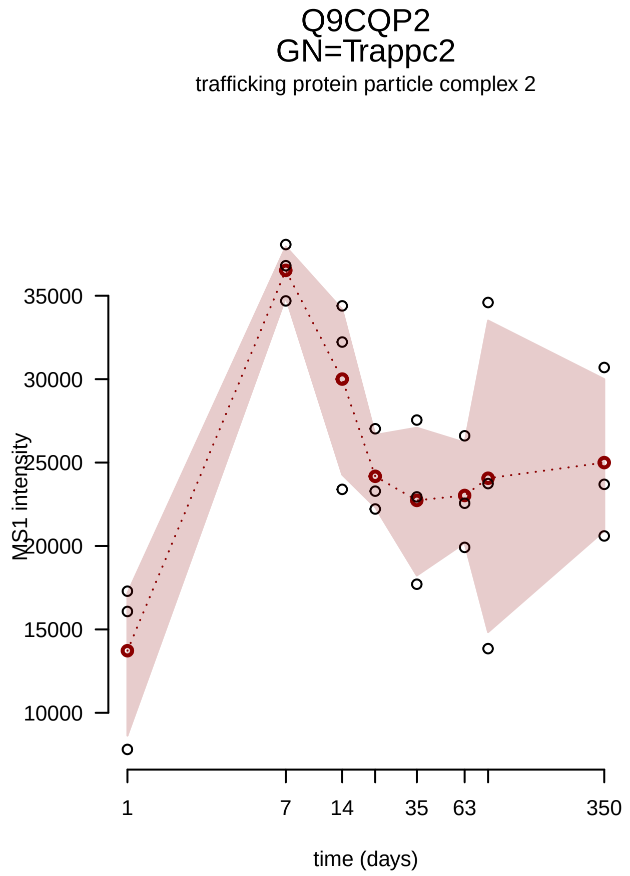
Q9CQP2
GN=Trappc2
trafficking protein particle complex 2
MS1 intensity
10000
15000
20000
25000
30000
35000
1
7
14
35
63
350
time (days)

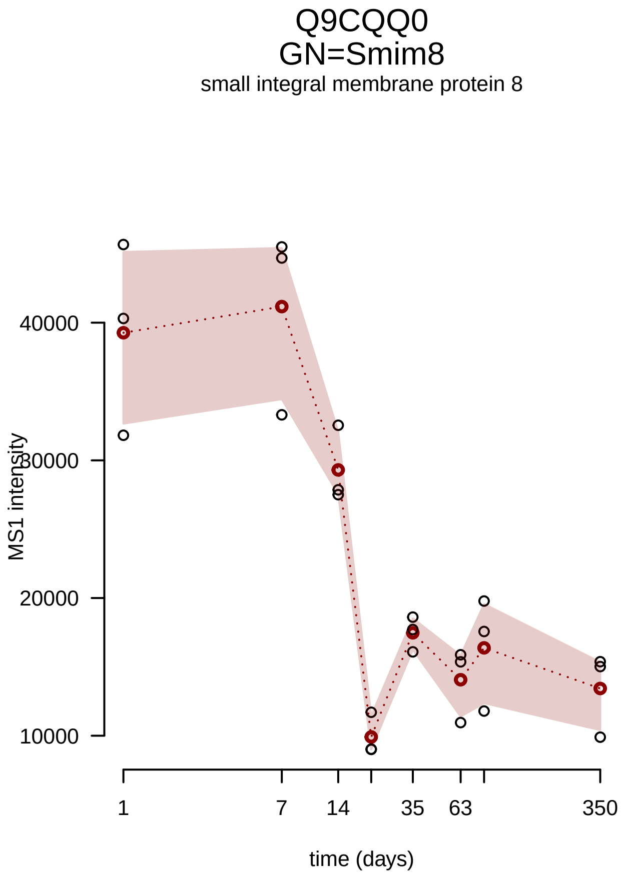
Q9CQQ0
GN=Smim8
small integral membrane protein 8
MS1 intensity
10000
20000
30000
40000
1
7
14
35
63
350
time (days)

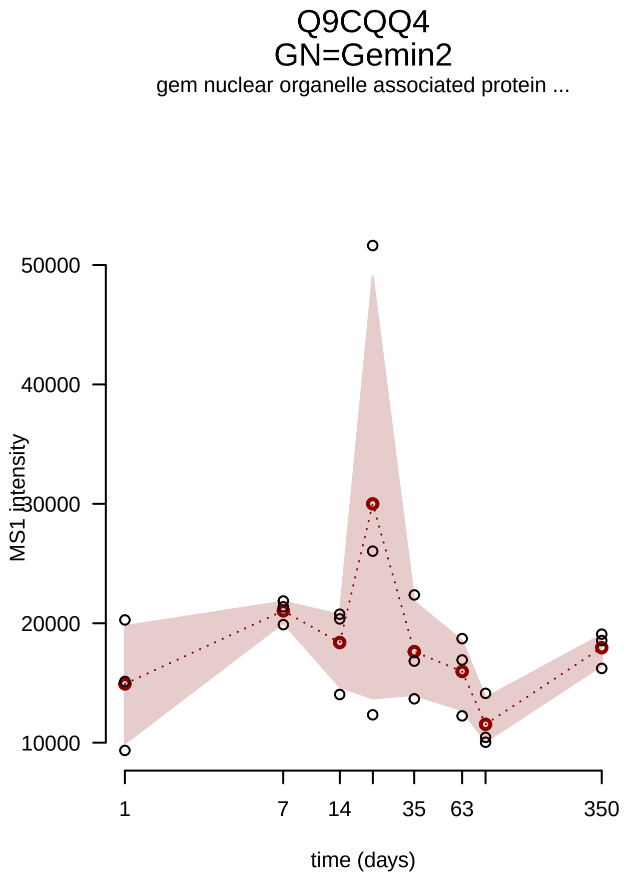
Q9CQQ4
GN=Gemin2
gem nuclear organelle associated protein ...
MS1 intensity
10000
20000
30000
40000
50000
1
7
14
35
63
350
time (days)

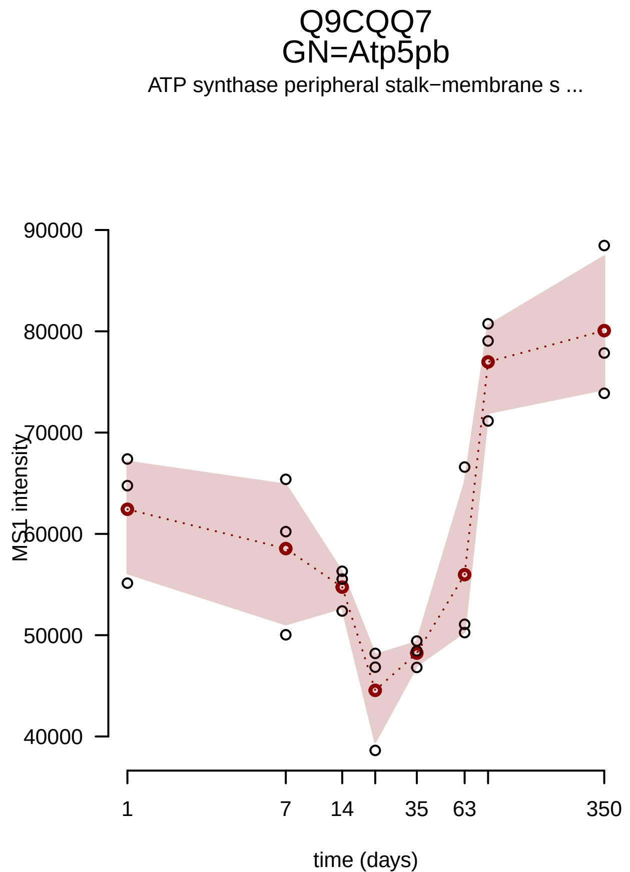
Q9CQQ7
GN=Atp5pb
ATP synthase peripheral stalk−membrane s ...
MS1 intensity
40000
50000
60000
70000
80000
90000
1
7
14
35
63
350
time (days)

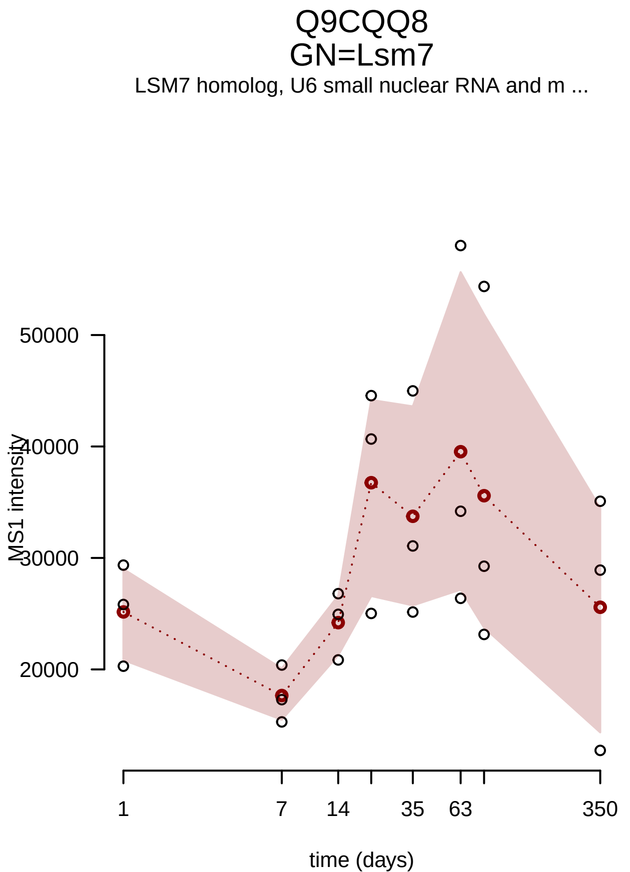
Q9CQQ8
GN=Lsm7
LSM7 homolog, U6 small nuclear RNA and m ...
MS1 intensity
20000
30000
40000
50000
1
7
14
35
63
350
time (days)

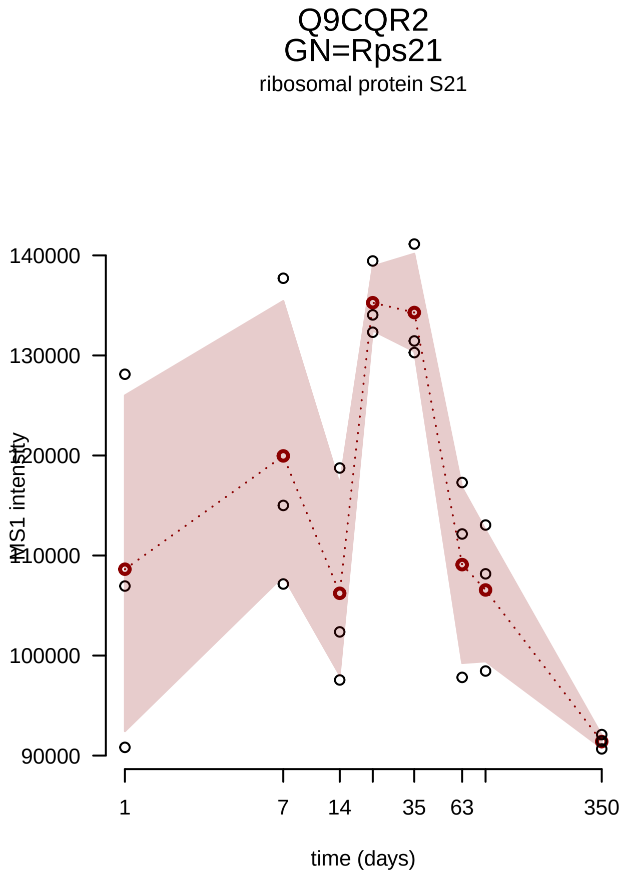
Q9CQR2
GN=Rps21
ribosomal protein S21
MS1 intensity
90000
100000
110000
120000
130000
140000
1
7
14
35
63
350
time (days)

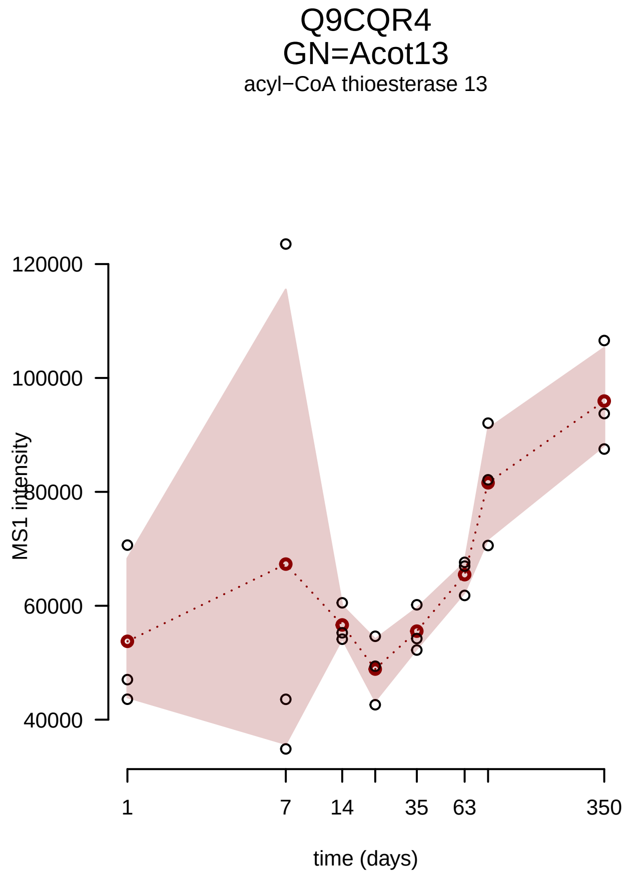
Q9CQR4
GN=Acot13
acyl−CoA thioesterase 13
MS1 intensity
40000
60000
80000
100000
120000
1
7
14
35
63
350
time (days)

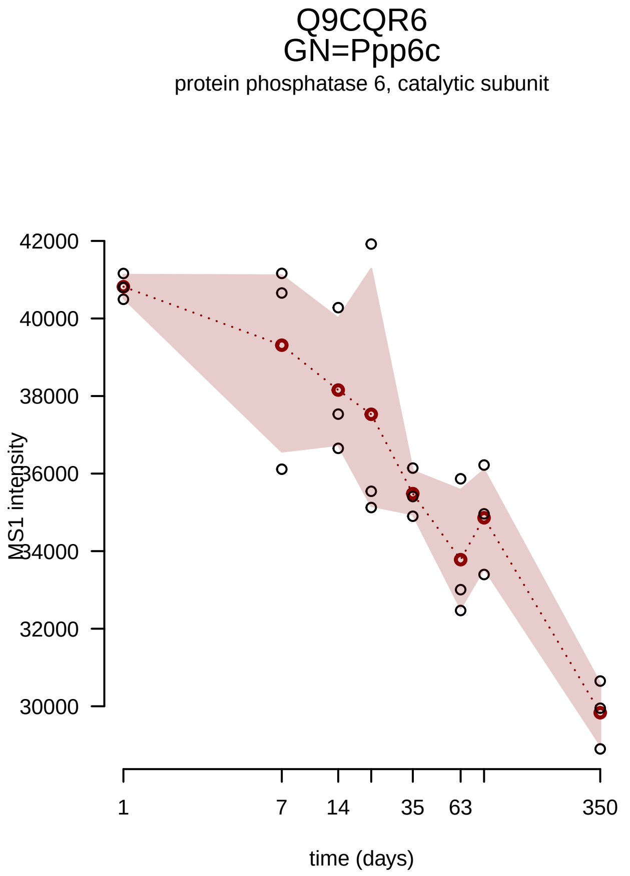
Q9CQR6
GN=Ppp6c
protein phosphatase 6, catalytic subunit
MS1 intensity
30000
32000
34000
36000
38000
40000
42000
1
7
14
35
63
350
time (days)

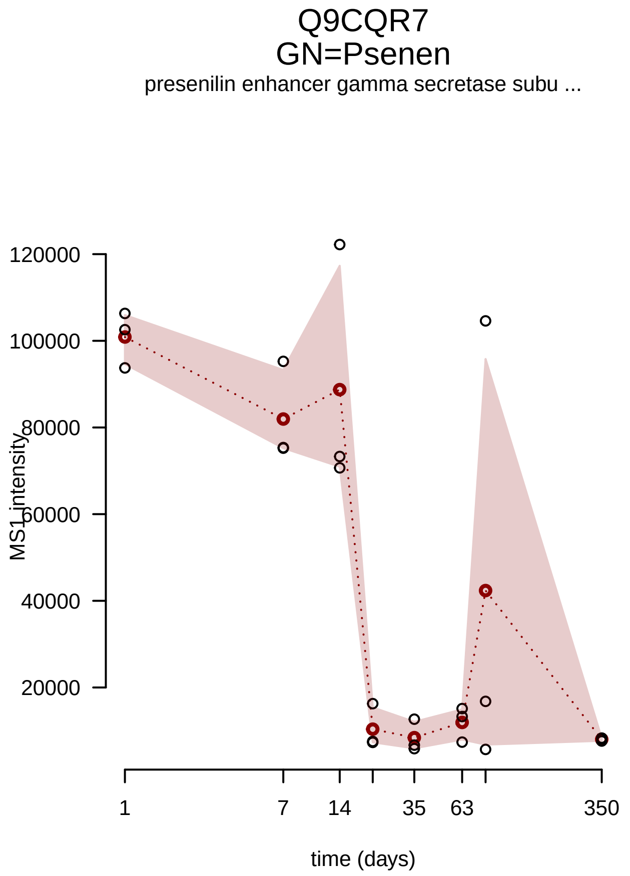
Q9CQR7
GN=Psenen
presenilin enhancer gamma secretase subu ...
MS1 intensity
20000
40000
60000
80000
100000
120000
1
7
14
35
63
350
time (days)

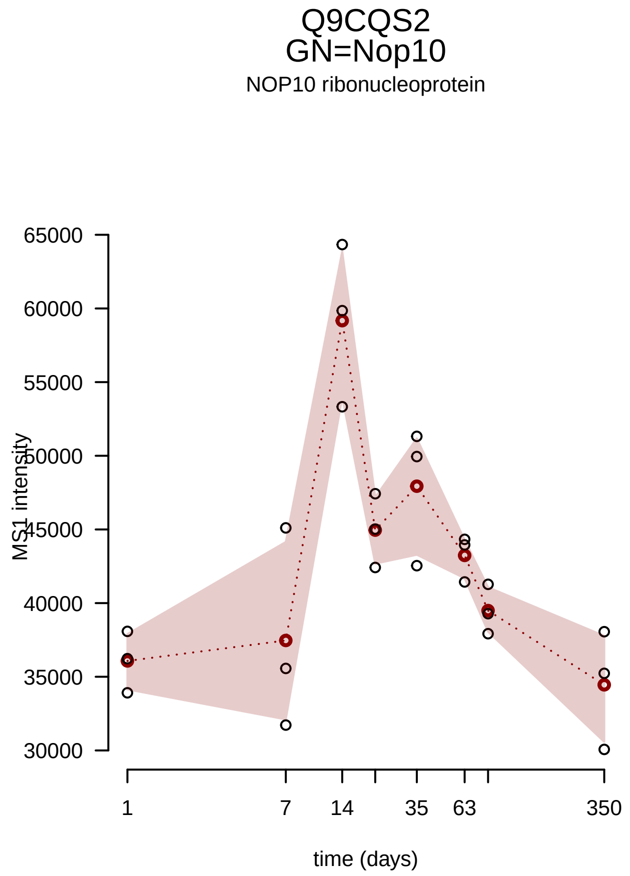
Q9CQS2
GN=Nop10
NOP10 ribonucleoprotein
MS1 intensity
30000
35000
40000
45000
50000
55000
60000
65000
1
7
14
35
63
350
time (days)

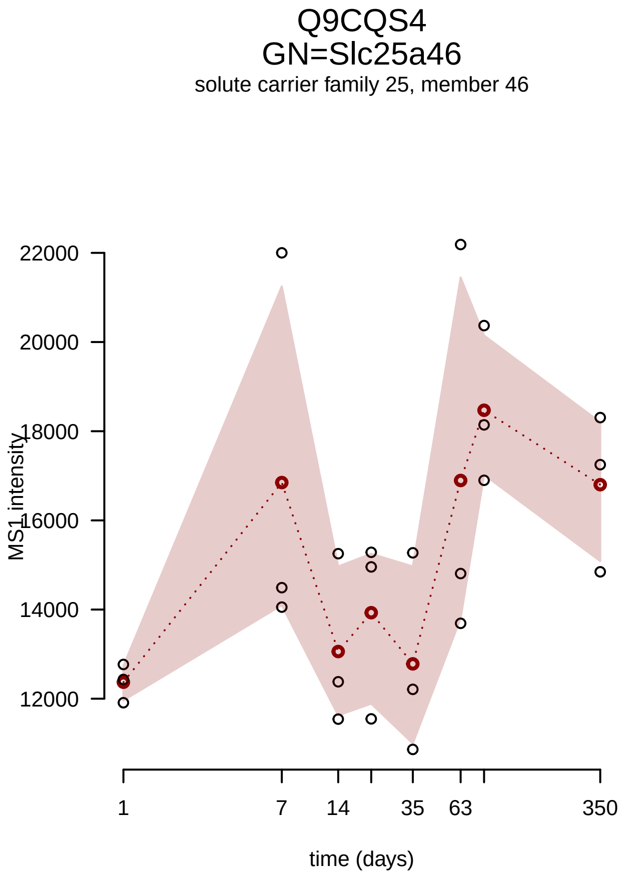
Q9CQS4
GN=Slc25a46
solute carrier family 25, member 46
MS1 intensity
12000
14000
16000
18000
20000
22000
1
7
14
35
63
350
time (days)

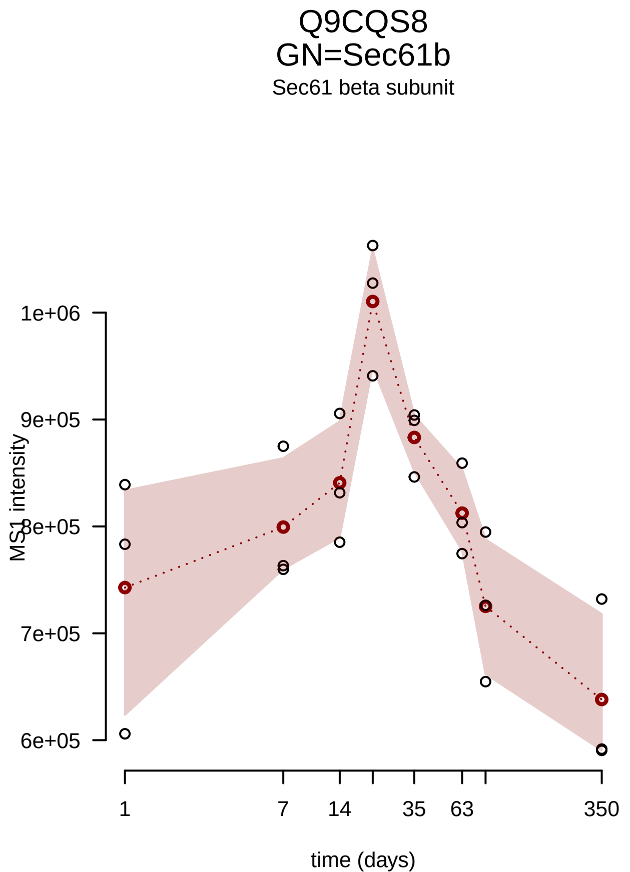
Q9CQS8
GN=Sec61b
Sec61 beta subunit
MS1 intensity
6e+05
7e+05
8e+05
9e+05
1e+06
1
7
14
35
63
350
time (days)

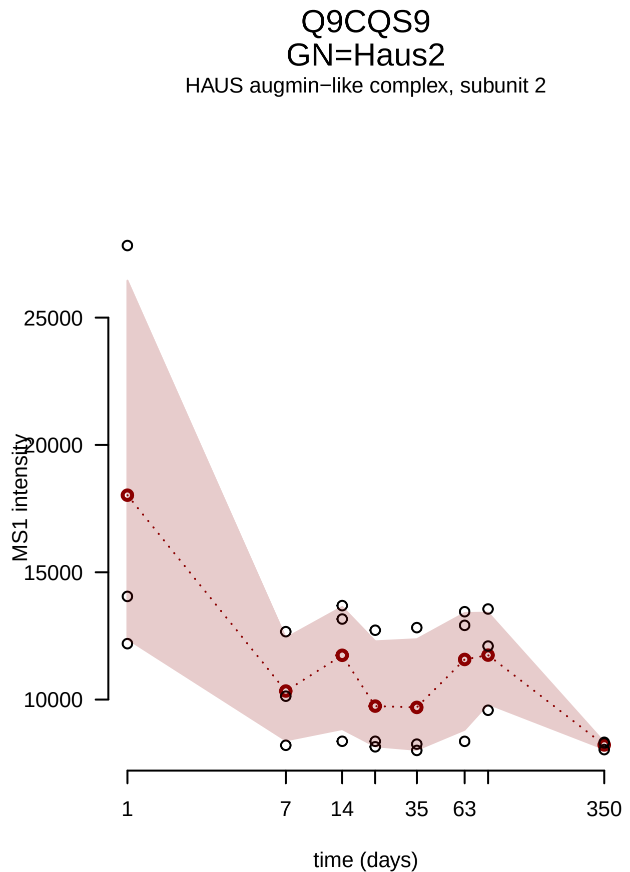
Q9CQS9
GN=Haus2
HAUS augmin−like complex, subunit 2
MS1 intensity
10000
15000
20000
25000
1
7
14
35
63
350
time (days)

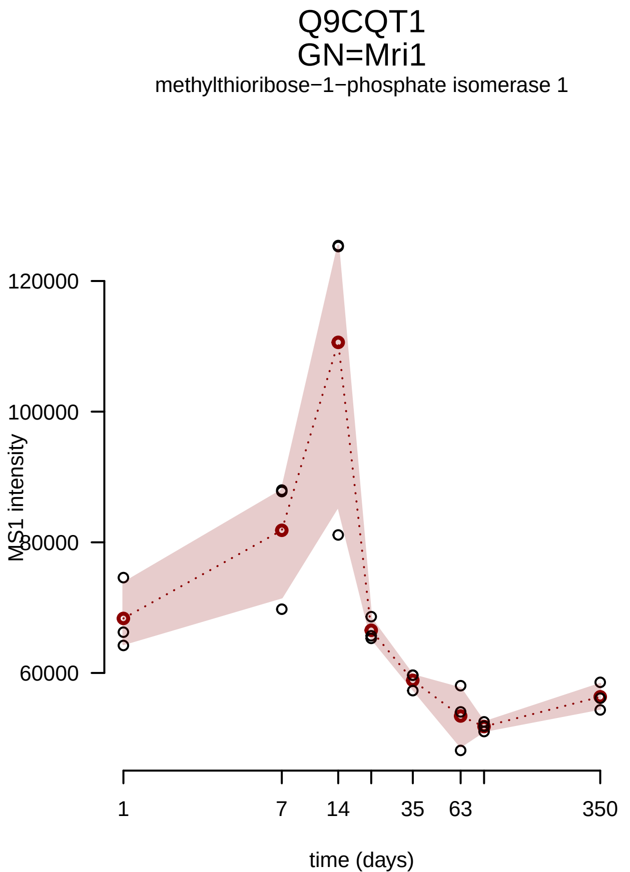
Q9CQT1
GN=Mri1
methylthioribose−1−phosphate isomerase 1
MS1 intensity
60000
80000
100000
120000
1
7
14
35
63
350
time (days)

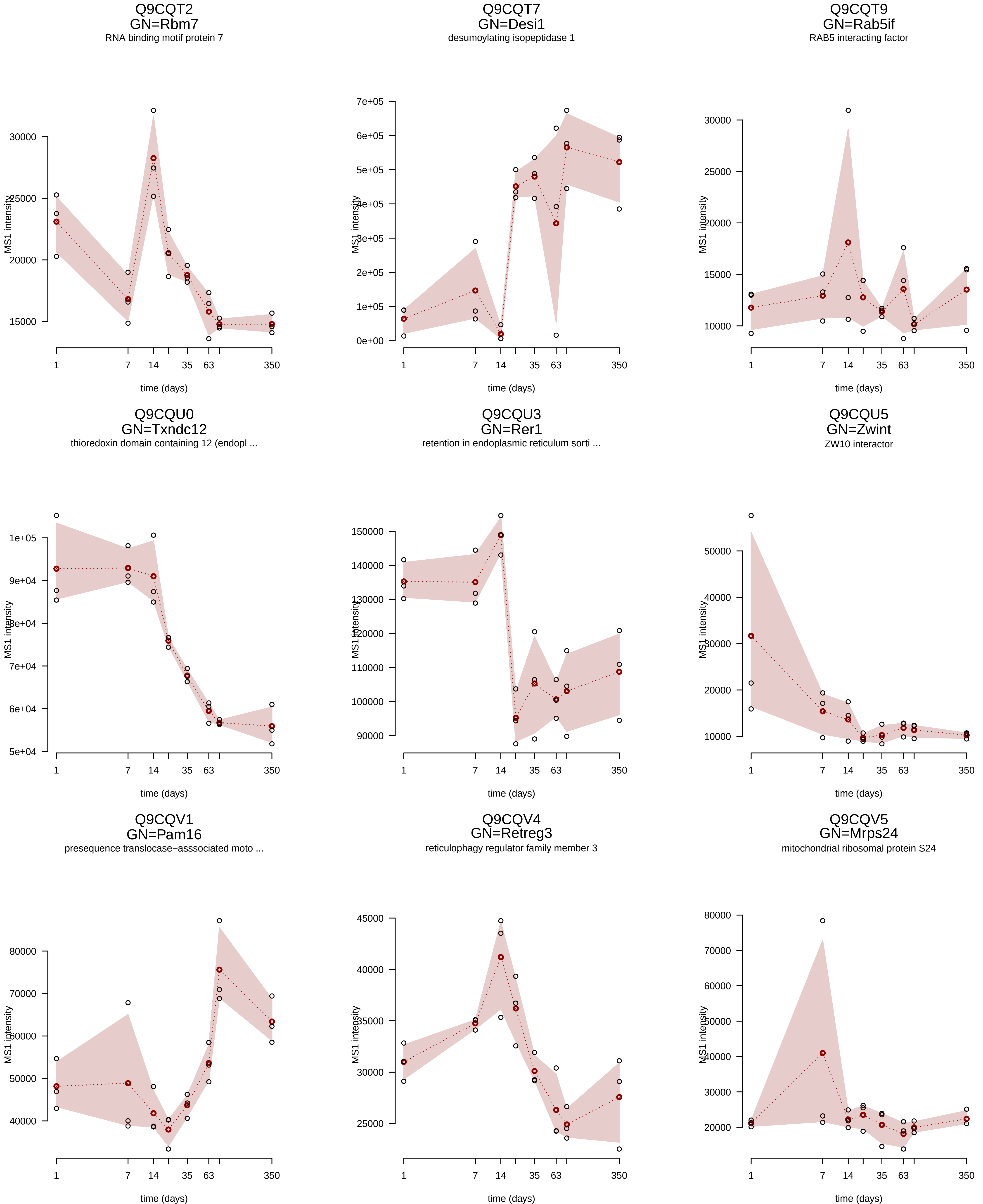
Q9CQT2
GN=Rbm7
RNA binding motif protein 7
Q9CQT7
GN=Desi1
desumoylating isopeptidase 1
Q9CQT9
GN=Rab5if
RAB5 interacting factor
Q9CQU0
GN=Txndc12
thioredoxin domain containing 12 (endopl ...
Q9CQU3
GN=Rer1
retention in endoplasmic reticulum sorti ...
Q9CQU5
GN=Zwint
ZW10 interactor
Q9CQV1
GN=Pam16
presequence translocase−asssociated moto ...
Q9CQV4
GN=Retreg3
reticulophagy regulator family member 3
Q9CQV5
GN=Mrps24
mitochondrial ribosomal protein S24
MS1 intensity
time (days)
1
7
14
35
63
350

Q9CQV6
GN=Map1lc3b
microtubule−associated protein 1 light c ...

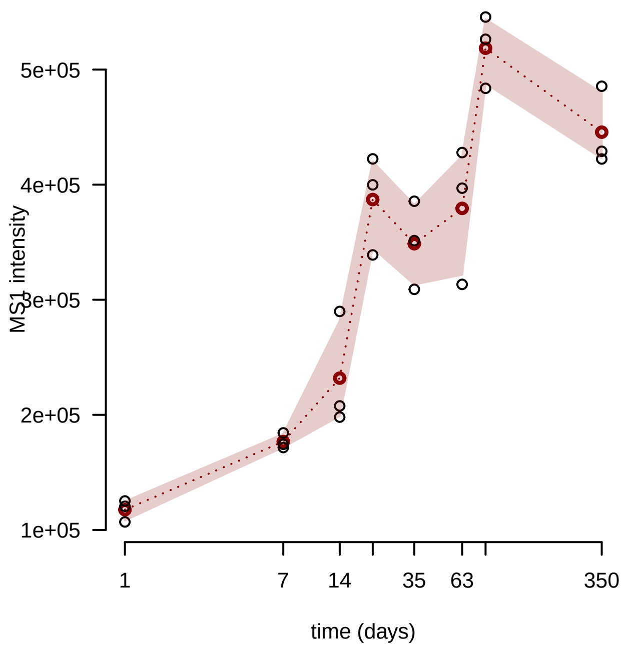

Q9CQV7
GN=Dnajc19
DnaJ heat shock protein family (Hsp40) m ...

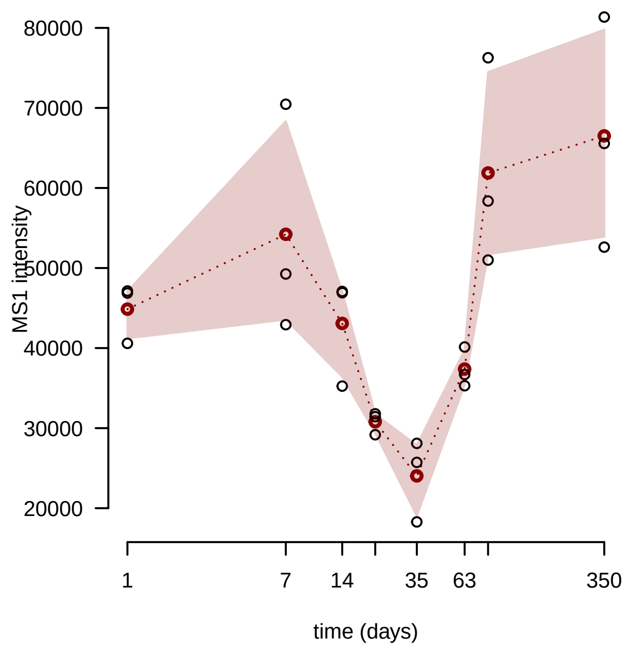

Q9CQV8
GN=Ywhab
tyrosine 3−monooxygenase/tryptophan 5−mo ...

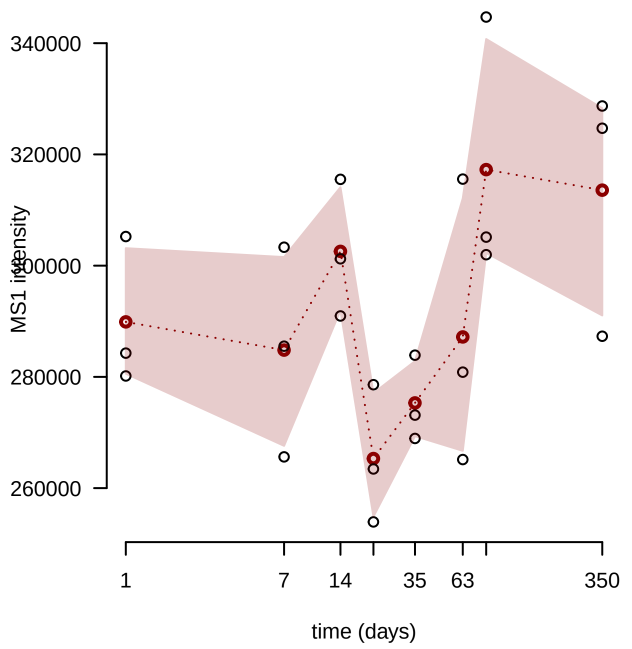

Q9CQW0
GN=Emc6
ER membrane protein complex subunit 6

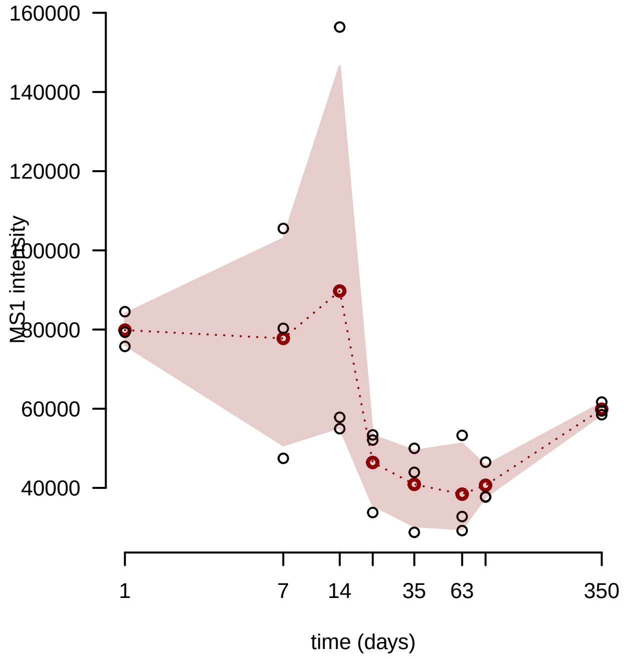

Q9CQW1
GN=Ykt6
YKT6 v−SNARE homolog (S. cerevisiae)

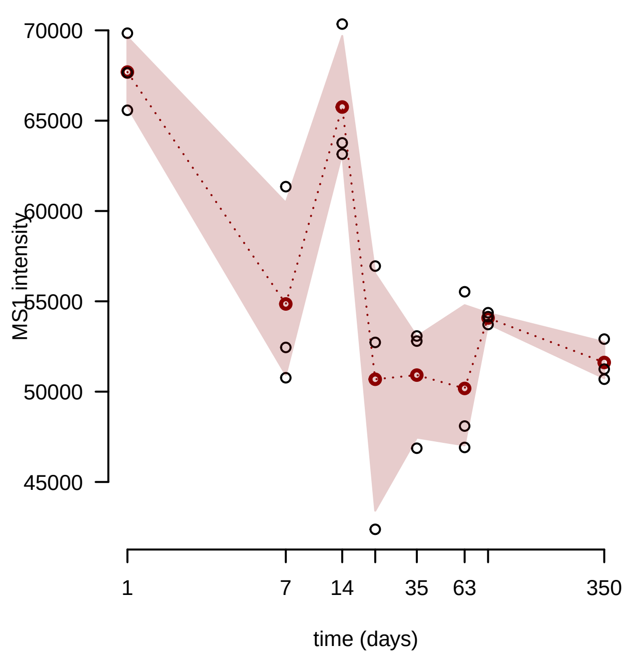

Q9CQW2
GN=Arl8b
ADP−ribosylation factor−like 8B

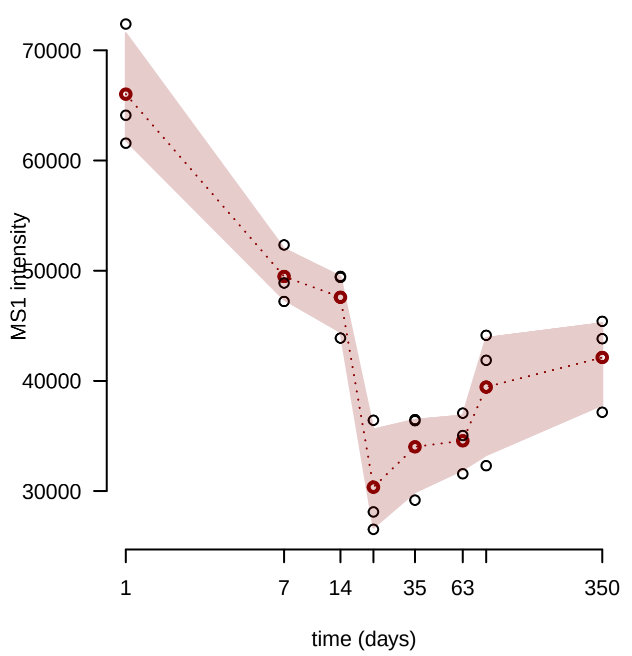

Q9CQW5
GN=Lgals2
lectin, galactose−binding, soluble 2

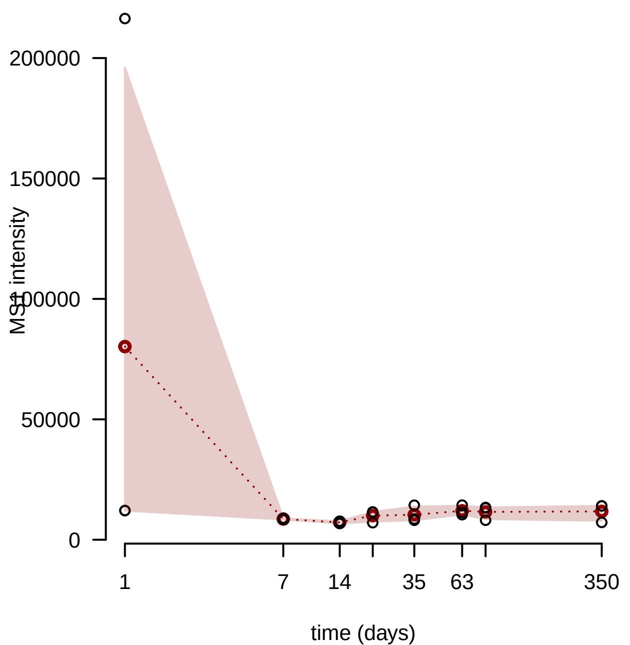

Q9CQW9
GN=Ifitm3
interferon induced transmembrane protein ...

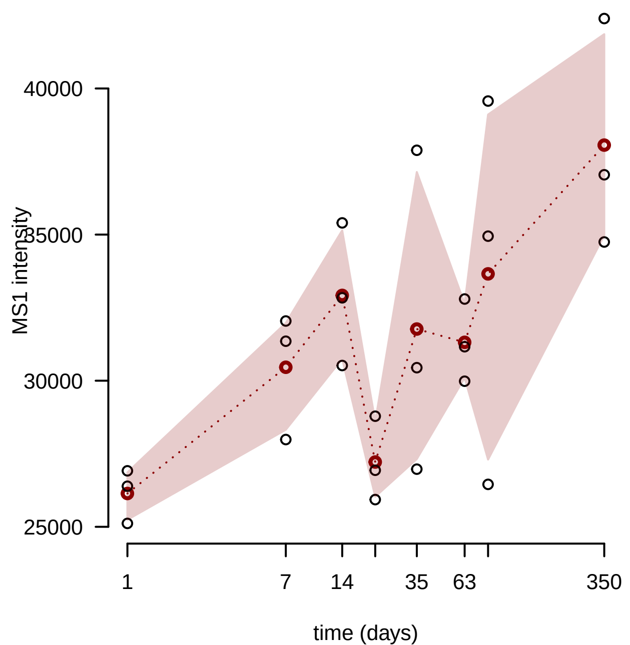

Q9CQX0
GN=Otub2
OTU domain, ubiquitin aldehyde binding 2

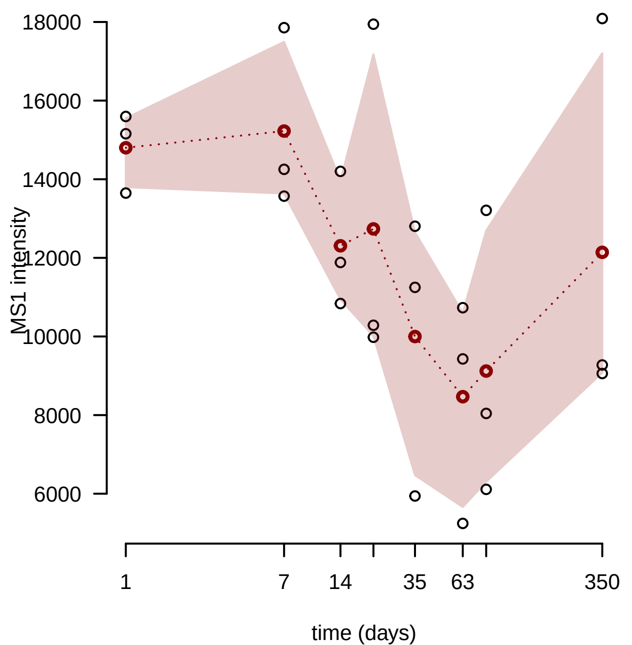

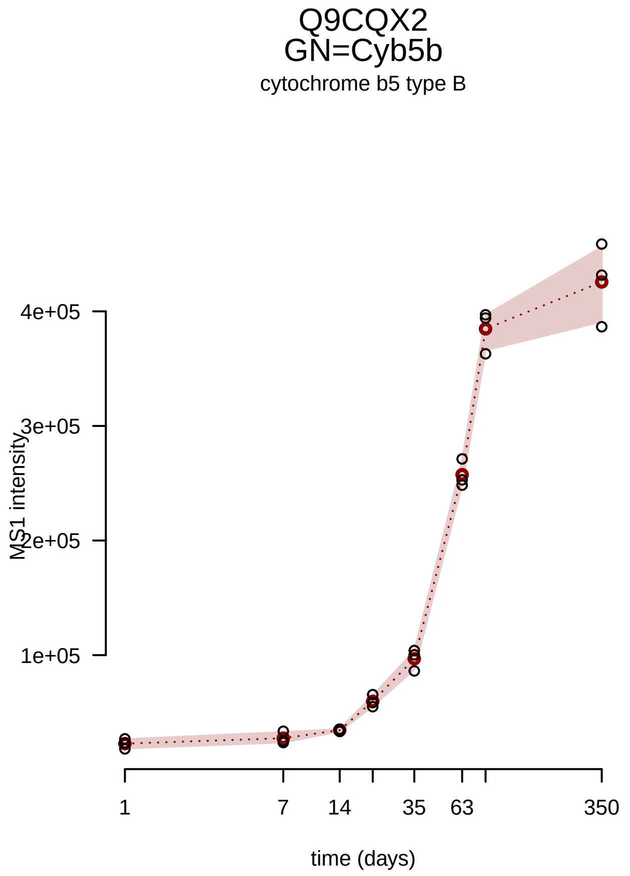
Q9CQX2
GN=Cyb5b
cytochrome b5 type B
MS1 intensity
1e+05
2e+05
3e+05
4e+05
1
7
14
35
63
350
time (days)

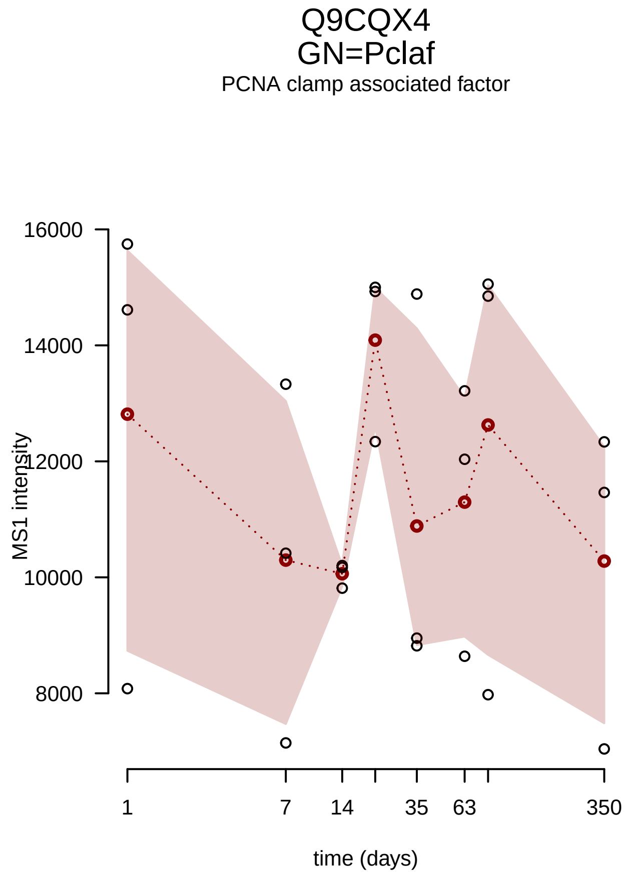
Q9CQX4
GN=Pclaf
PCNA clamp associated factor
MS1 intensity
8000
10000
12000
14000
16000
1
7
14
35
63
350
time (days)

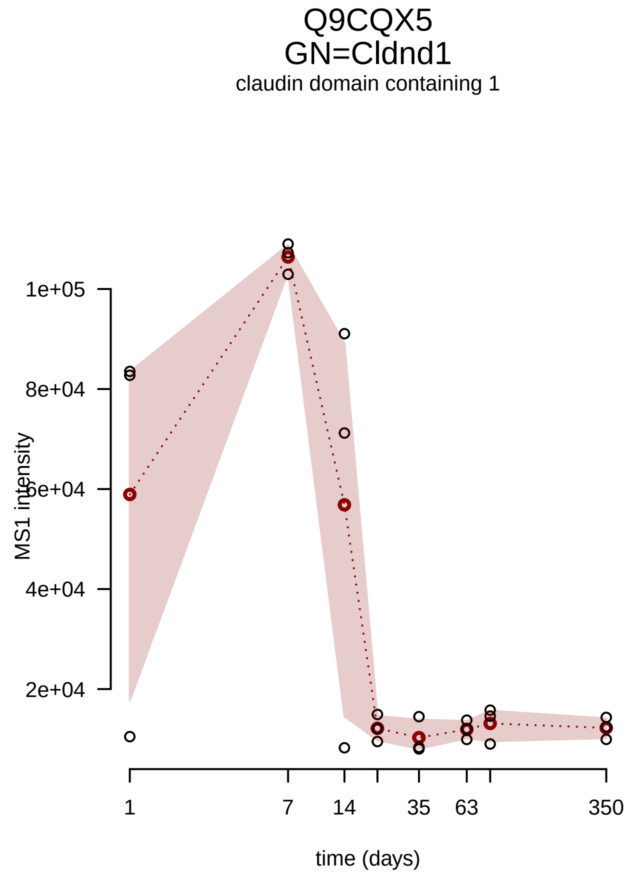
Q9CQX5
GN=Cldnd1
claudin domain containing 1
MS1 intensity
2e+04
4e+04
6e+04
8e+04
1e+05
1
7
14
35
63
350
time (days)

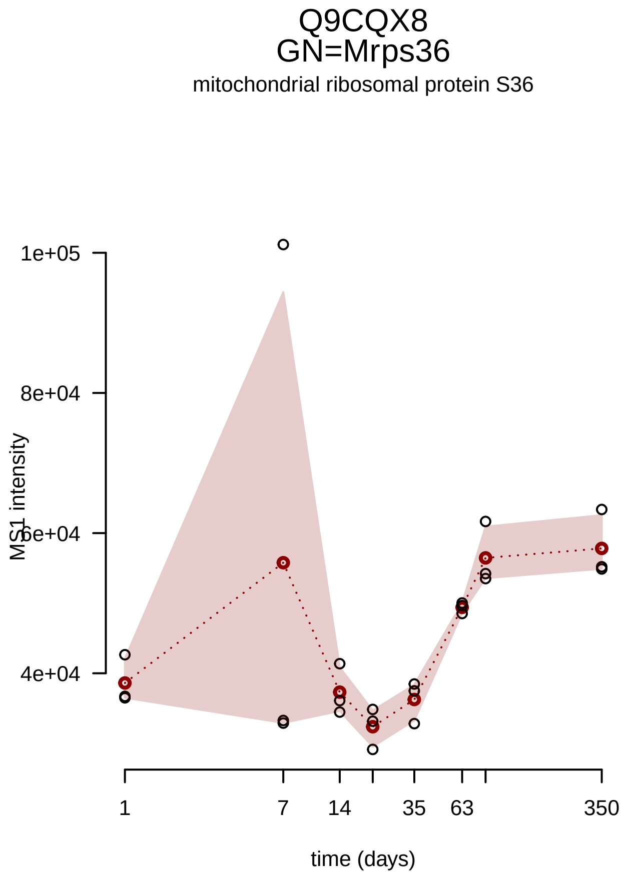
Q9CQX8
GN=Mrps36
mitochondrial ribosomal protein S36
MS1 intensity
4e+04
6e+04
8e+04
1e+05
1
7
14
35
63
350
time (days)

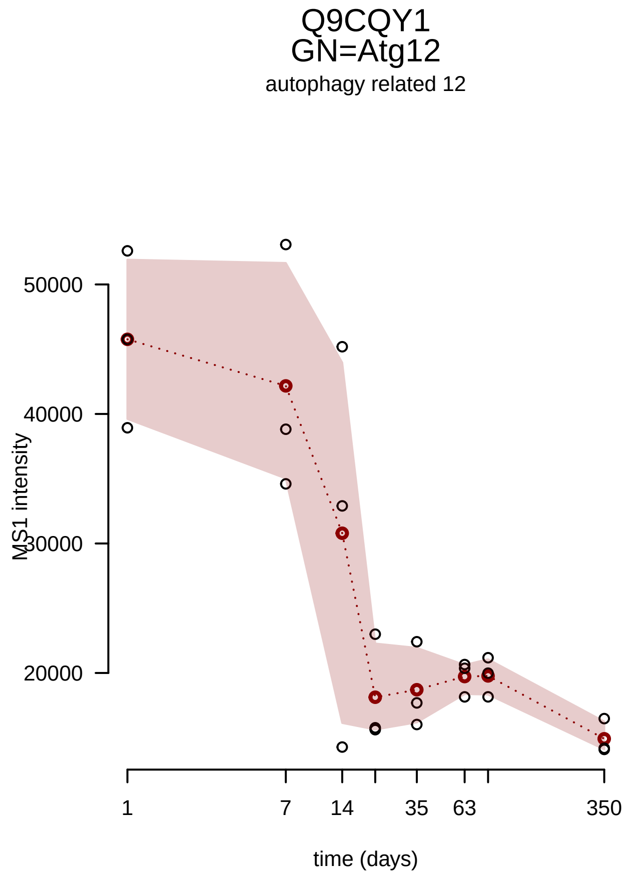
Q9CQY1
GN=Atg12
autophagy related 12
MS1 intensity
20000
30000
40000
50000
1
7
14
35
63
350
time (days)

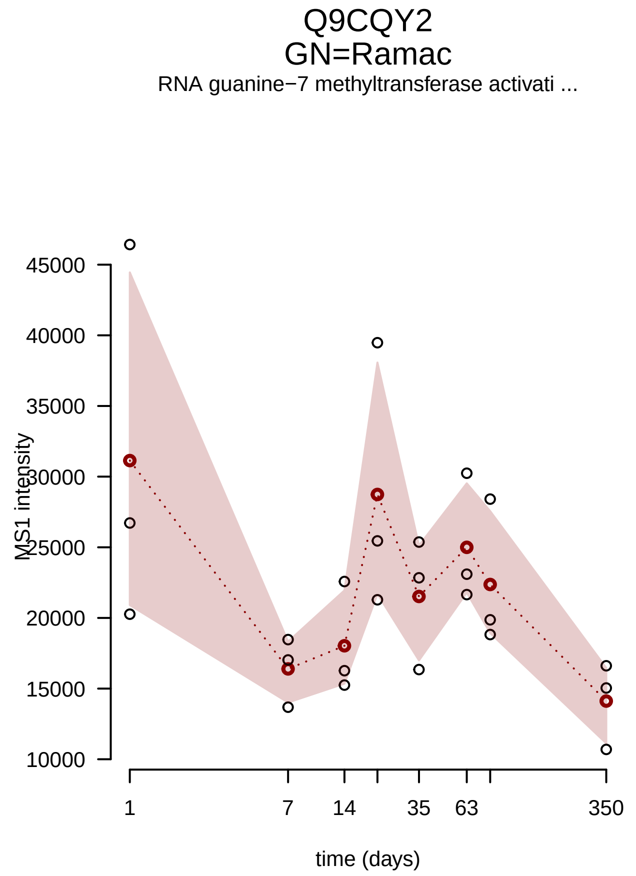
Q9CQY2
GN=Ramac
RNA guanine−7 methyltransferase activati ...
MS1 intensity
10000
15000
20000
25000
30000
35000
40000
45000
1
7
14
35
63
350
time (days)

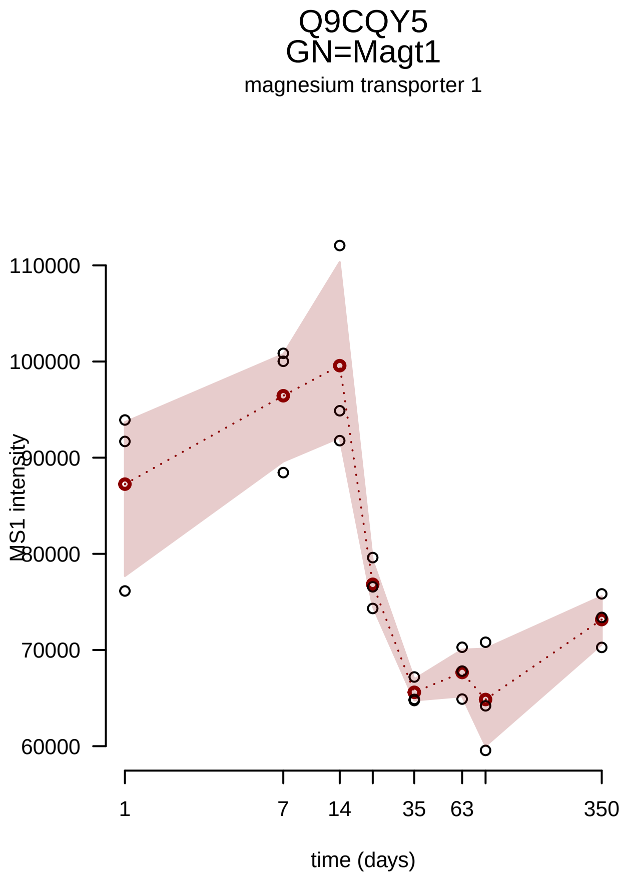
Q9CQY5
GN=Magt1
magnesium transporter 1
MS1 intensity
60000
70000
80000
90000
100000
110000
1
7
14
35
63
350
time (days)

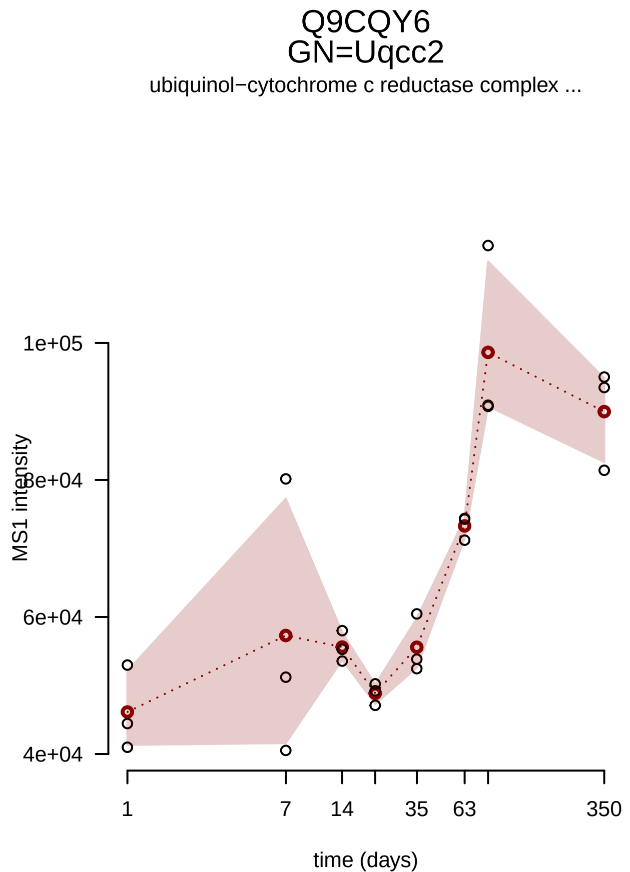
Q9CQY6
GN=Uqcc2
ubiquinol−cytochrome c reductase complex ...
MS1 intensity
4e+04
6e+04
8e+04
1e+05
1
7
14
35
63
350
time (days)

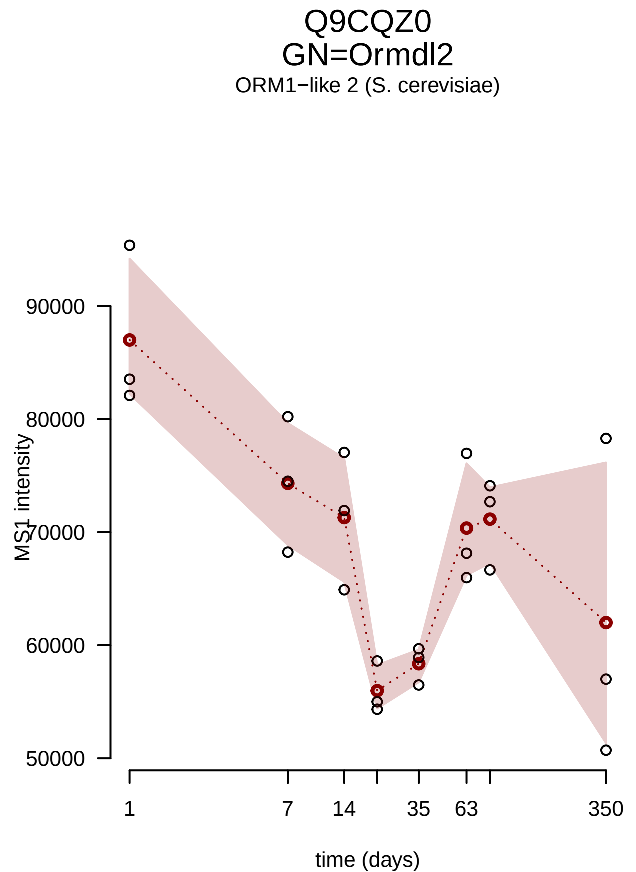
Q9CQZ0
GN=Ormdl2
ORM1−like 2 (S. cerevisiae)
MS1 intensity
50000
60000
70000
80000
90000
1
7
14
35
63
350
time (days)

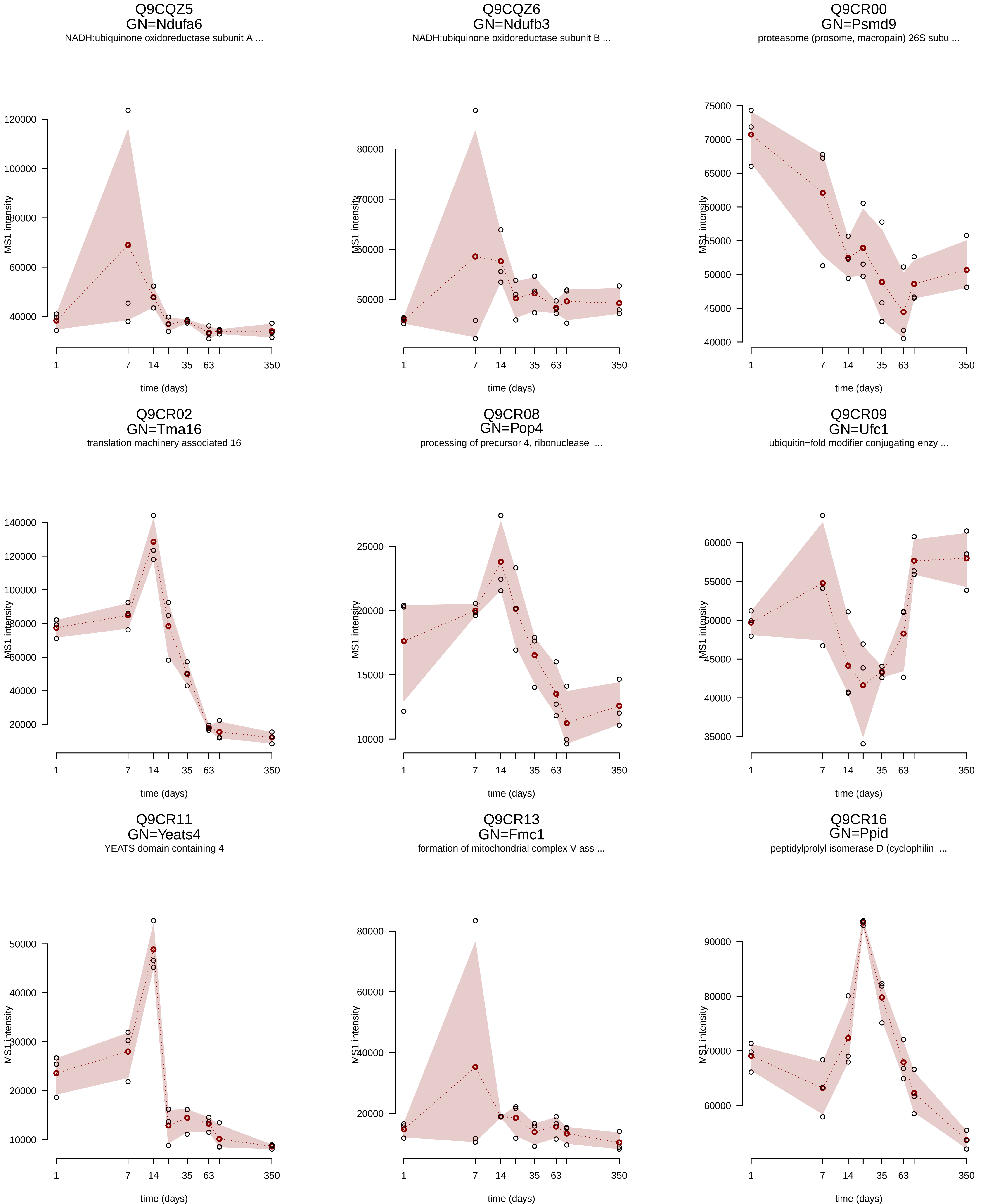
Q9CQZ5
GN=Ndufa6
NADH:ubiquinone oxidoreductase subunit A ...
MS1 intensity
120000
100000
80000
60000
40000
1
7
14
35
63
350
time (days)
Q9CQZ6
GN=Ndufb3
NADH:ubiquinone oxidoreductase subunit B ...
MS1 intensity
80000
70000
60000
50000
1
7
14
35
63
350
time (days)
Q9CR00
GN=Psmd9
proteasome (prosome, macropain) 26S subu ...
MS1 intensity
75000
70000
65000
60000
55000
50000
45000
40000
1
7
14
35
63
350
time (days)
Q9CR02
GN=Tma16
translation machinery associated 16
MS1 intensity
140000
120000
100000
80000
60000
40000
20000
1
7
14
35
63
350
time (days)
Q9CR08
GN=Pop4
processing of precursor 4, ribonuclease ...
MS1 intensity
25000
20000
15000
10000
1
7
14
35
63
350
time (days)
Q9CR09
GN=Ufc1
ubiquitin−fold modifier conjugating enzy ...
MS1 intensity
60000
55000
50000
45000
40000
35000
1
7
14
35
63
350
time (days)
Q9CR11
GN=Yeats4
YEATS domain containing 4
MS1 intensity
50000
40000
30000
20000
10000
1
7
14
35
63
350
time (days)
Q9CR13
GN=Fmc1
formation of mitochondrial complex V ass ...
MS1 intensity
80000
60000
40000
20000
1
7
14
35
63
350
time (days)
Q9CR16
GN=Ppid
peptidylprolyl isomerase D (cyclophilin ...
MS1 intensity
90000
80000
70000
60000
1
7
14
35
63
350
time (days)

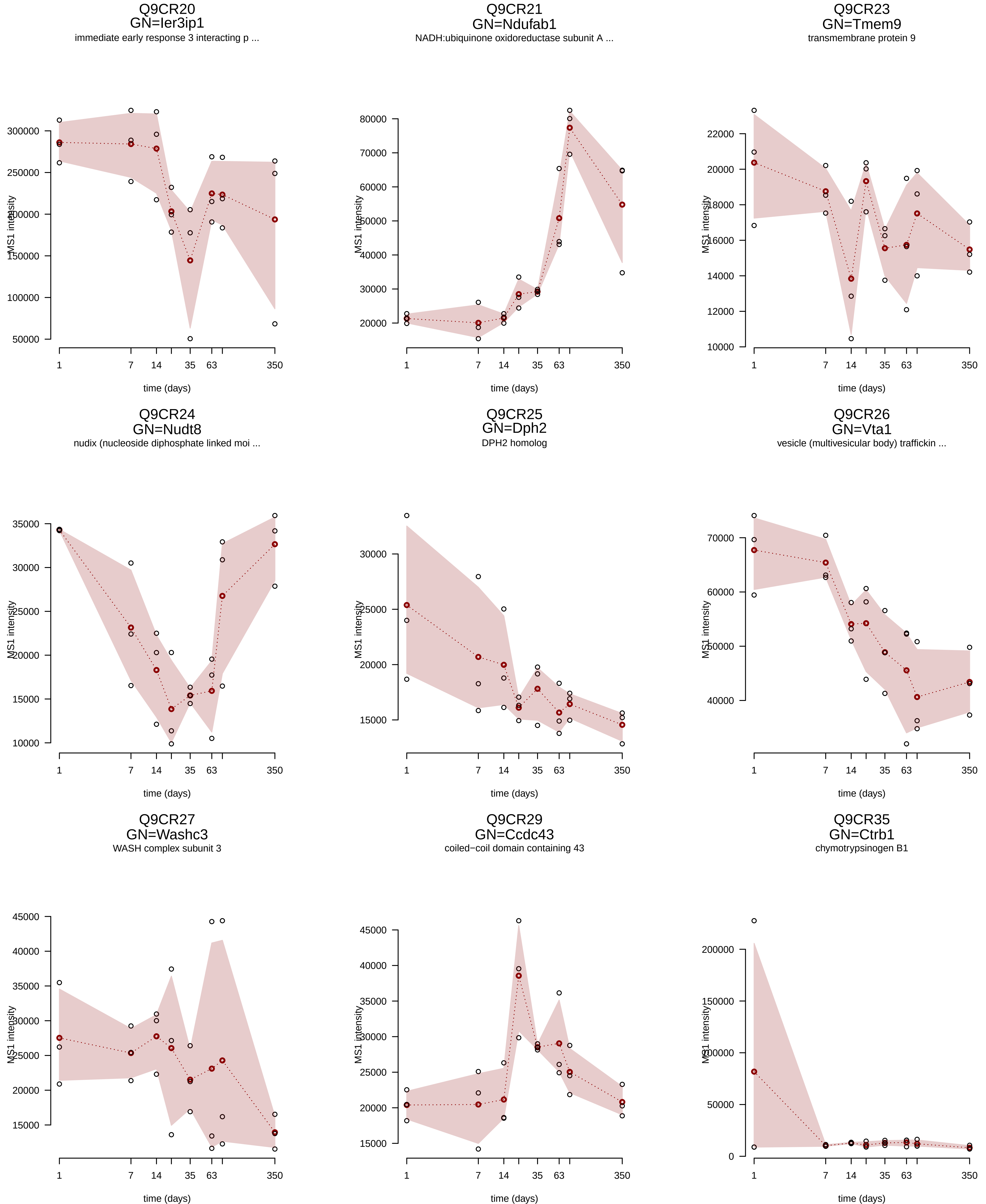

Q9CR20
GN=Ier3ip1
immediate early response 3 interacting p ...
Q9CR21
GN=Ndufab1
NADH:ubiquinone oxidoreductase subunit A ...
Q9CR23
GN=Tmem9
transmembrane protein 9
Q9CR24
GN=Nudt8
nudix (nucleoside diphosphate linked moi ...
Q9CR25
GN=Dph2
DPH2 homolog
Q9CR26
GN=Vta1
vesicle (multivesicular body) traffickin ...
Q9CR27
GN=Washc3
WASH complex subunit 3
Q9CR29
GN=Ccdc43
coiled−coil domain containing 43
Q9CR35
GN=Ctrb1
chymotrypsinogen B1
MS1 intensity
time (days)
1
7
14
35
63
350

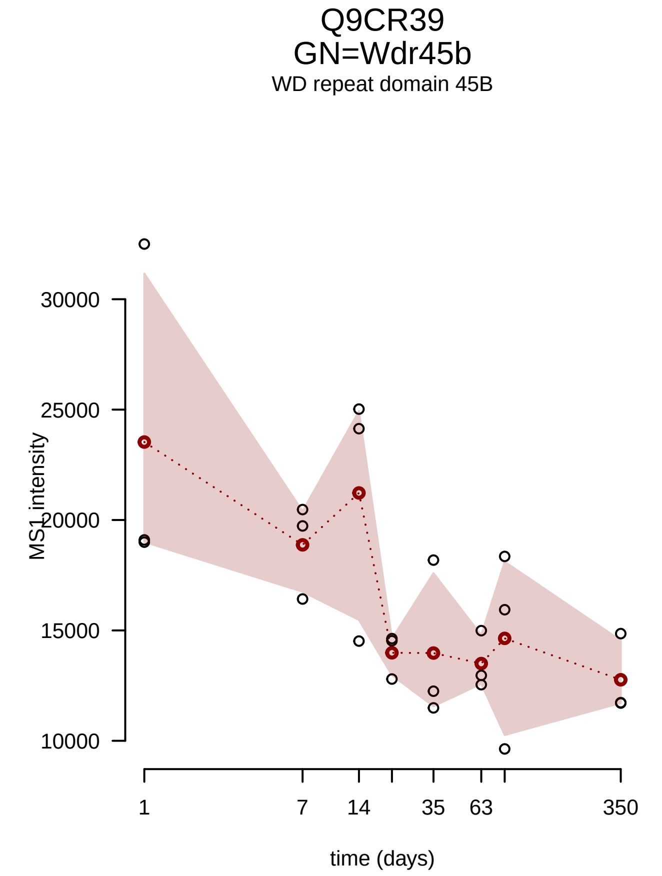
Q9CR39
GN=Wdr45b
WD repeat domain 45B
MS1 intensity
10000
15000
20000
25000
30000
1
7
14
35
63
350
time (days)
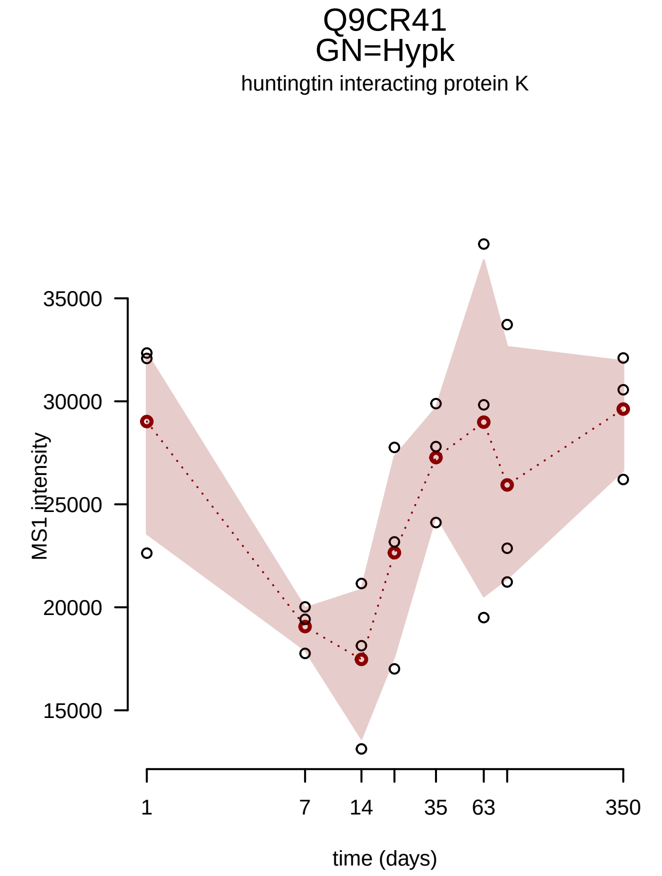
Q9CR41
GN=Hypk
huntingtin interacting protein K
MS1 intensity
15000
20000
25000
30000
35000
1
7
14
35
63
350
time (days)
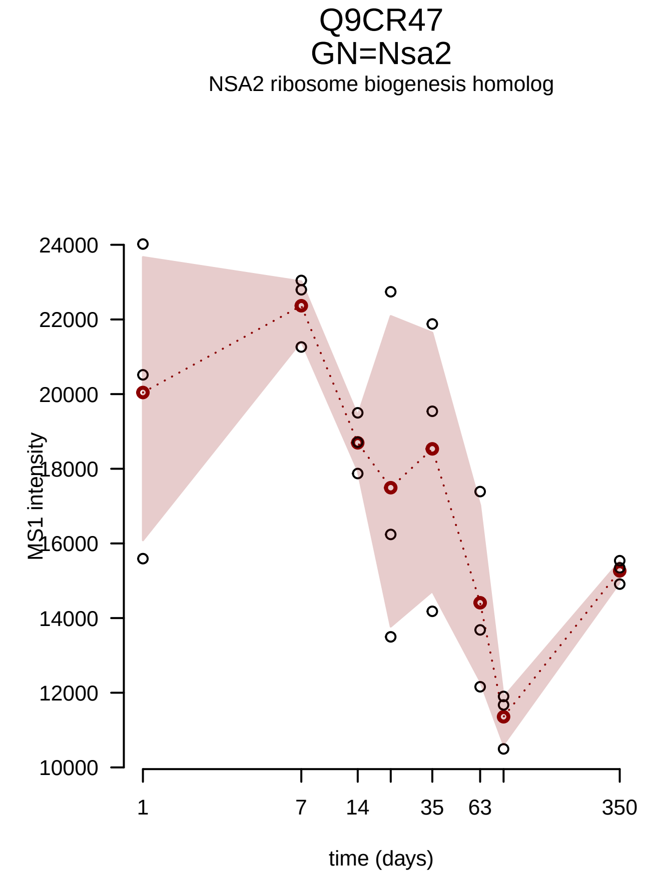
Q9CR47
GN=Nsa2
NSA2 ribosome biogenesis homolog
MS1 intensity
10000
12000
14000
16000
18000
20000
22000
24000
1
7
14
35
63
350
time (days)
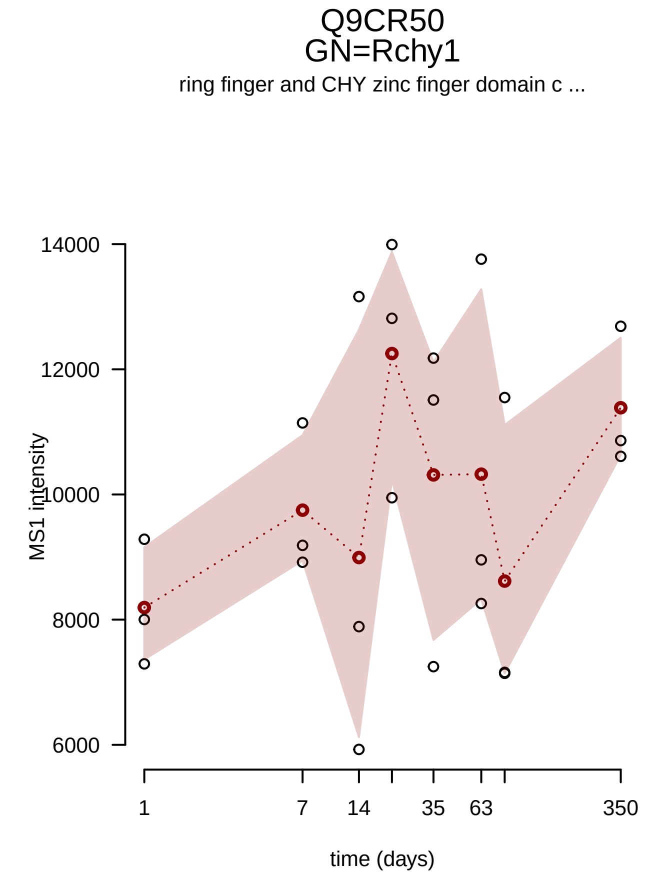
Q9CR50
GN=Rchy1
ring finger and CHY zinc finger domain c ...
MS1 intensity
6000
8000
10000
12000
14000
1
7
14
35
63
350
time (days)
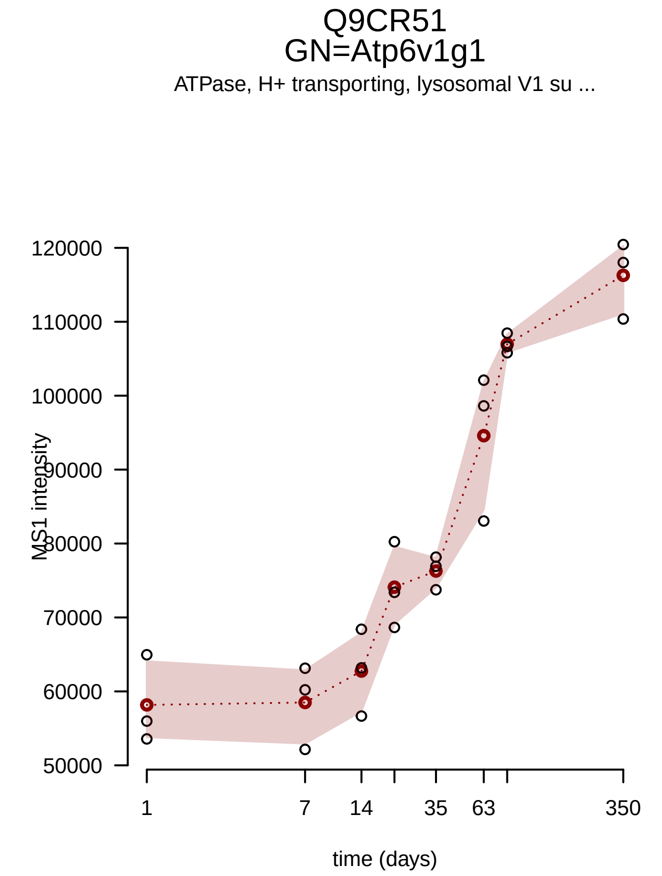
Q9CR51
GN=Atp6v1g1
ATPase, H+ transporting, lysosomal V1 su ...
MS1 intensity
50000
60000
70000
80000
90000
100000
110000
120000
1
7
14
35
63
350
time (days)
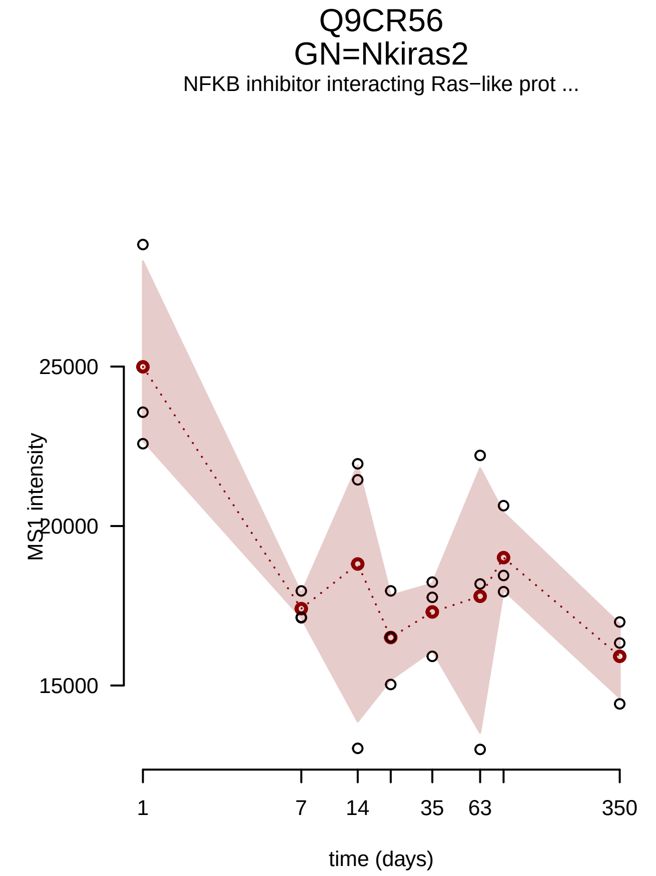
Q9CR56
GN=Nkiras2
NFKB inhibitor interacting Ras−like prot ...
MS1 intensity
15000
20000
25000
1
7
14
35
63
350
time (days)
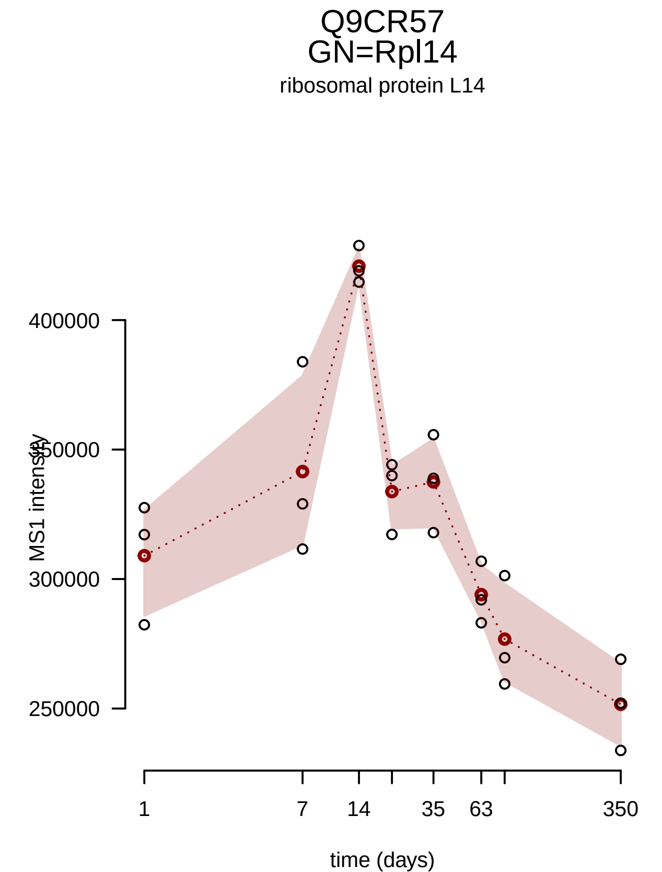
Q9CR57
GN=Rpl14
ribosomal protein L14
MS1 intensity
250000
300000
350000
400000
1
7
14
35
63
350
time (days)
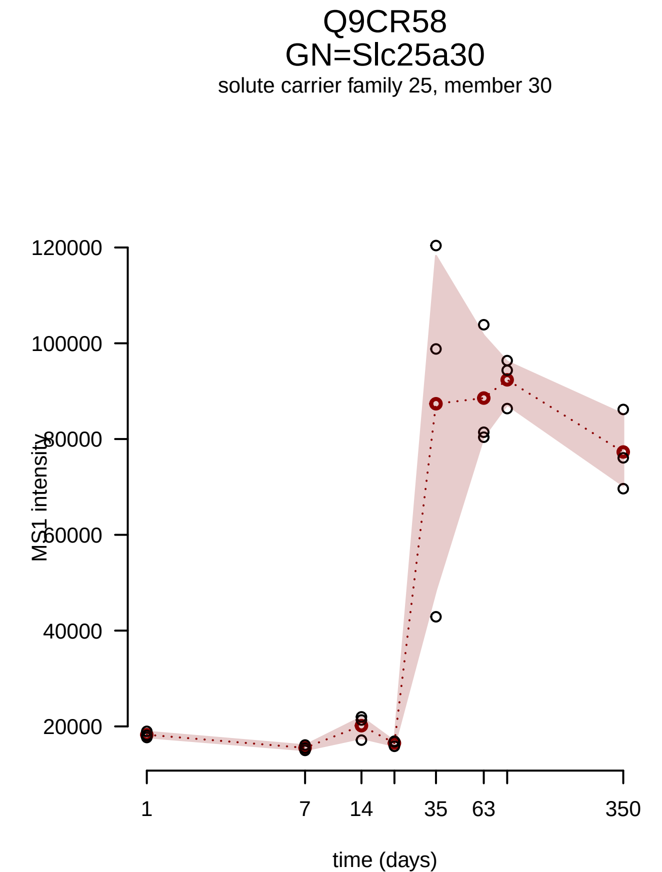
Q9CR58
GN=Slc25a30
solute carrier family 25, member 30
MS1 intensity
20000
40000
60000
80000
100000
120000
1
7
14
35
63
350
time (days)
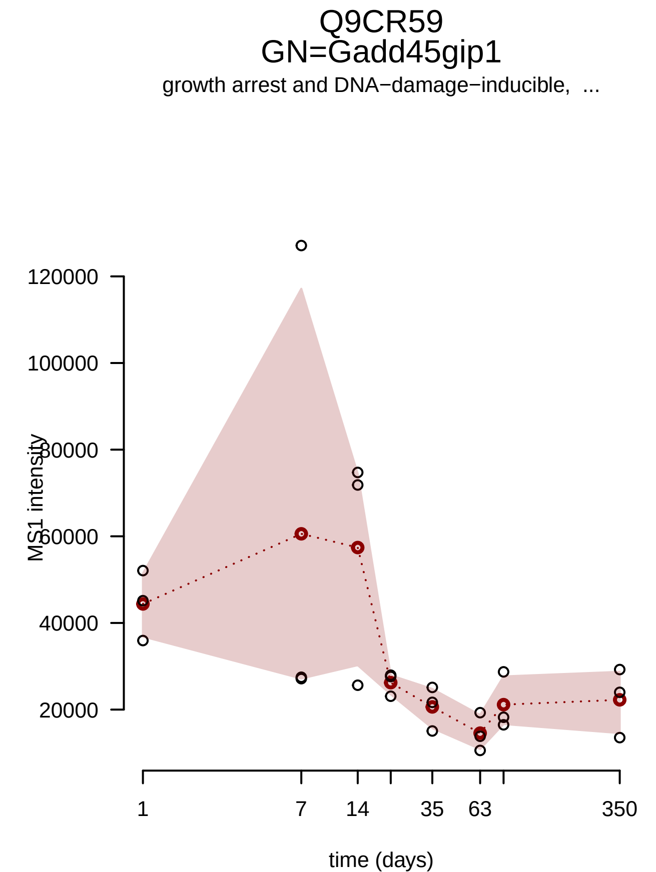
Q9CR59
GN=Gadd45gip1
growth arrest and DNA−damage−inducible, ...
MS1 intensity
20000
40000
60000
80000
100000
120000
1
7
14
35
63
350
time (days)

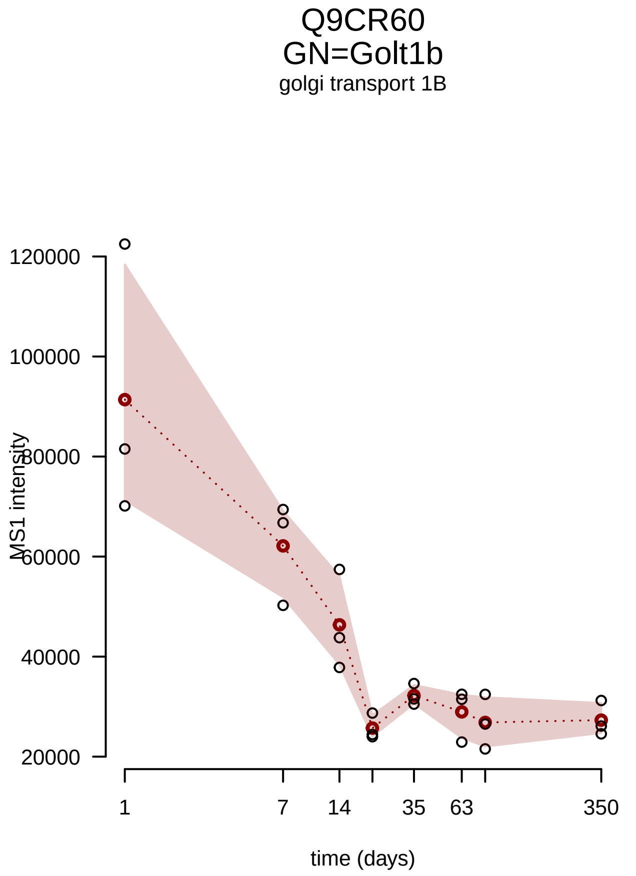
Q9CR60
GN=Golt1b
golgi transport 1B
MS1 intensity
120000
100000
80000
60000
40000
20000
1
7
14
35
63
350
time (days)
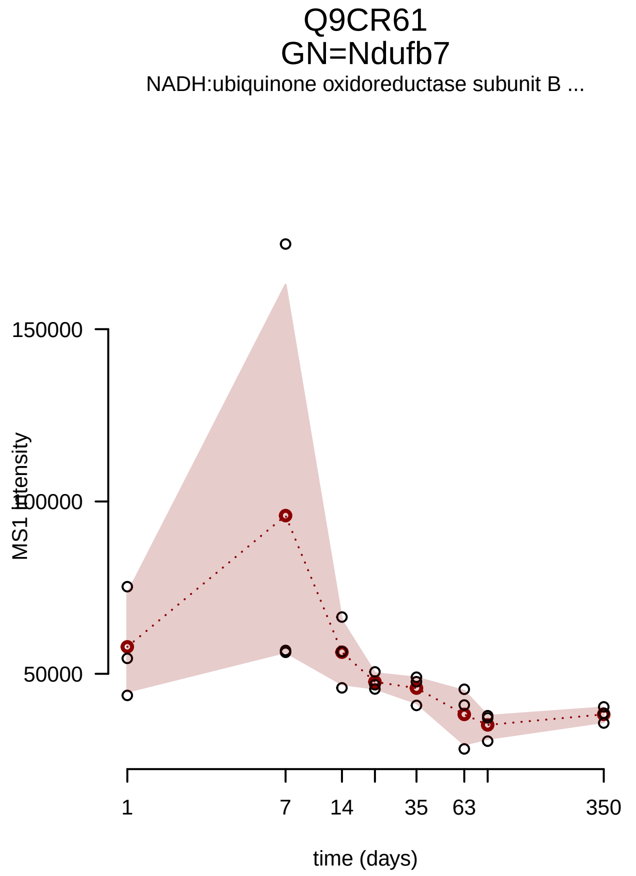
Q9CR61
GN=Ndufb7
NADH:ubiquinone oxidoreductase subunit B ...
MS1 intensity
150000
100000
50000
1
7
14
35
63
350
time (days)
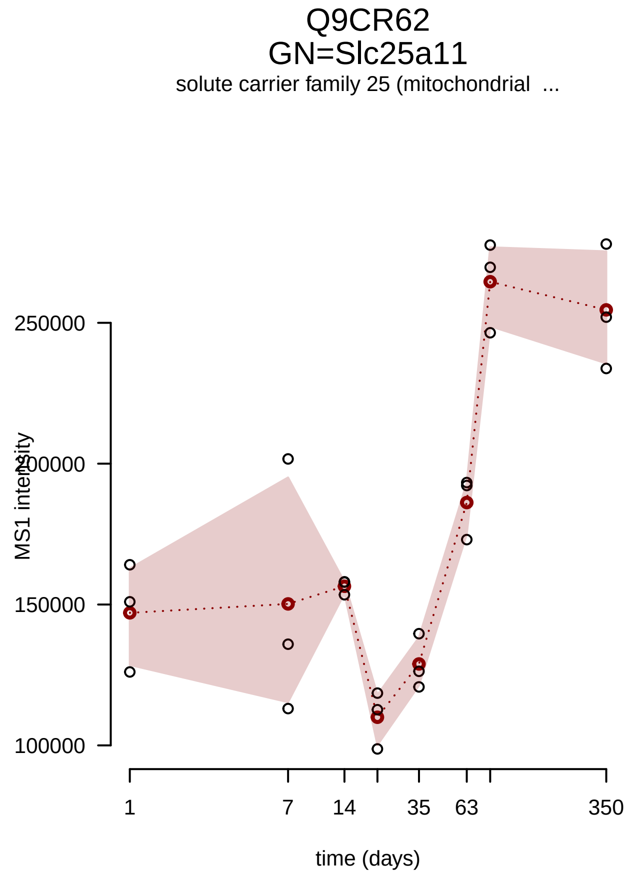
Q9CR62
GN=Slc25a11
solute carrier family 25 (mitochondrial ...
MS1 intensity
250000
200000
150000
100000
1
7
14
35
63
350
time (days)
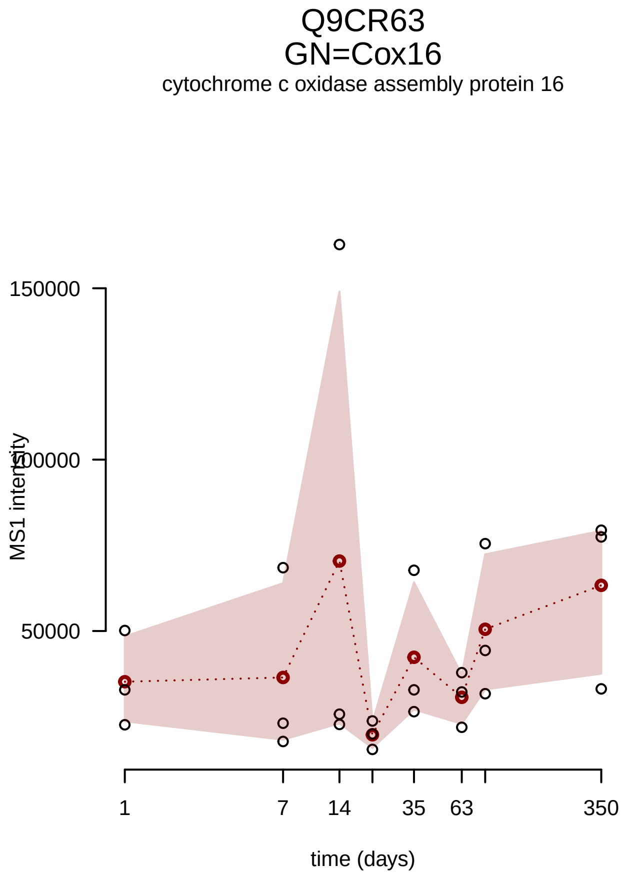
Q9CR63
GN=Cox16
cytochrome c oxidase assembly protein 16
MS1 intensity
150000
100000
50000
1
7
14
35
63
350
time (days)
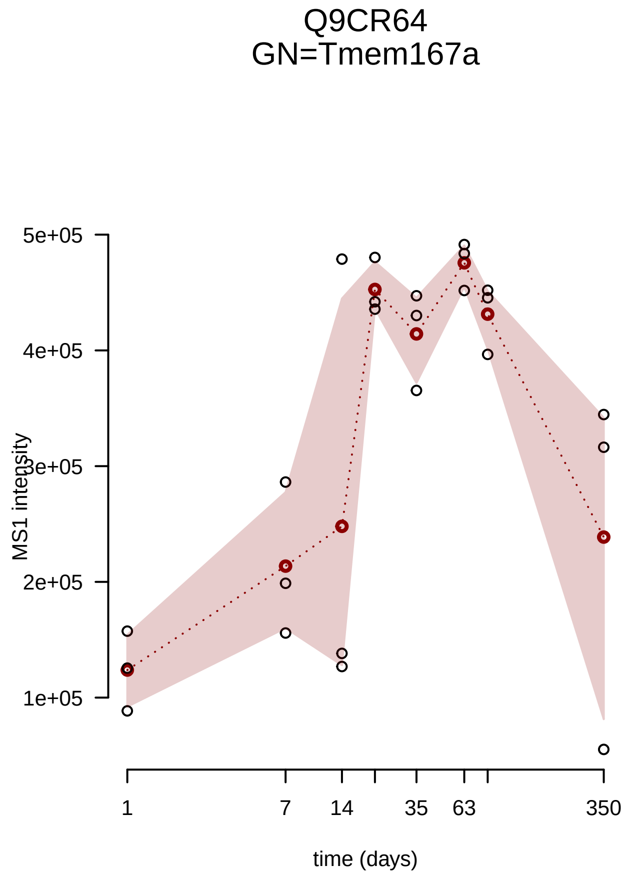
Q9CR64
GN=Tmem167a
MS1 intensity
5e+05
4e+05
3e+05
2e+05
1e+05
1
7
14
35
63
350
time (days)
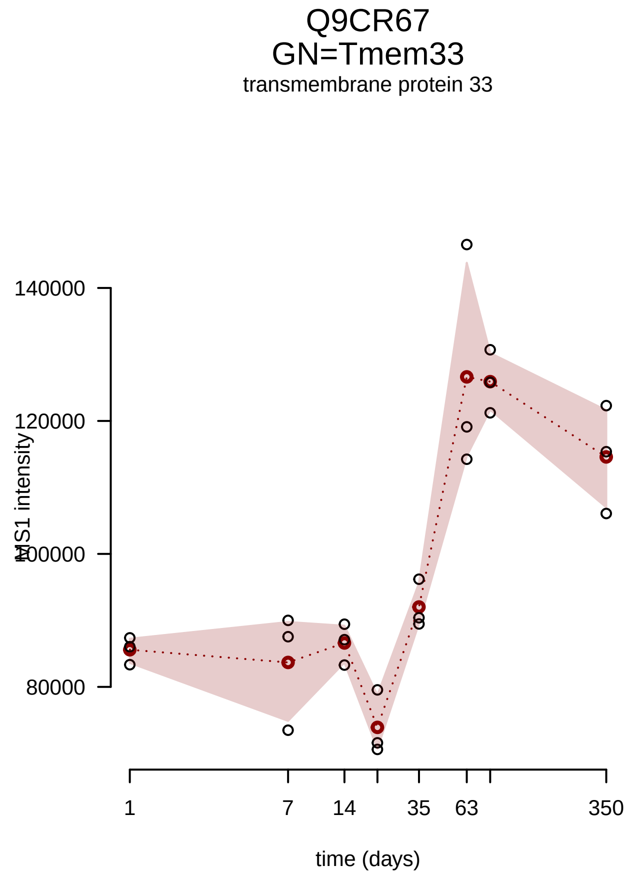
Q9CR67
GN=Tmem33
transmembrane protein 33
MS1 intensity
140000
120000
100000
80000
1
7
14
35
63
350
time (days)
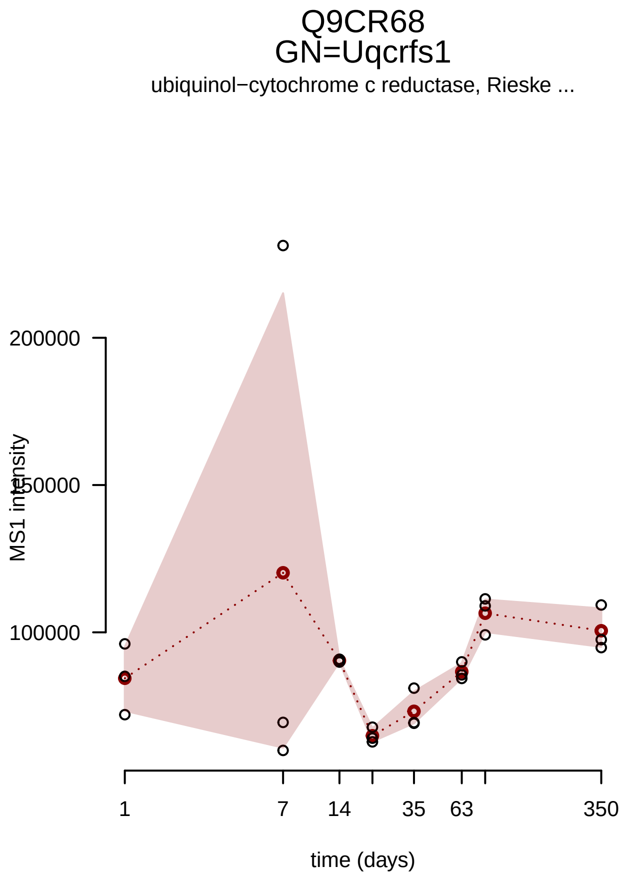
Q9CR68
GN=Uqcrfs1
ubiquinol−cytochrome c reductase, Rieske ...
MS1 intensity
200000
150000
100000
1
7
14
35
63
350
time (days)
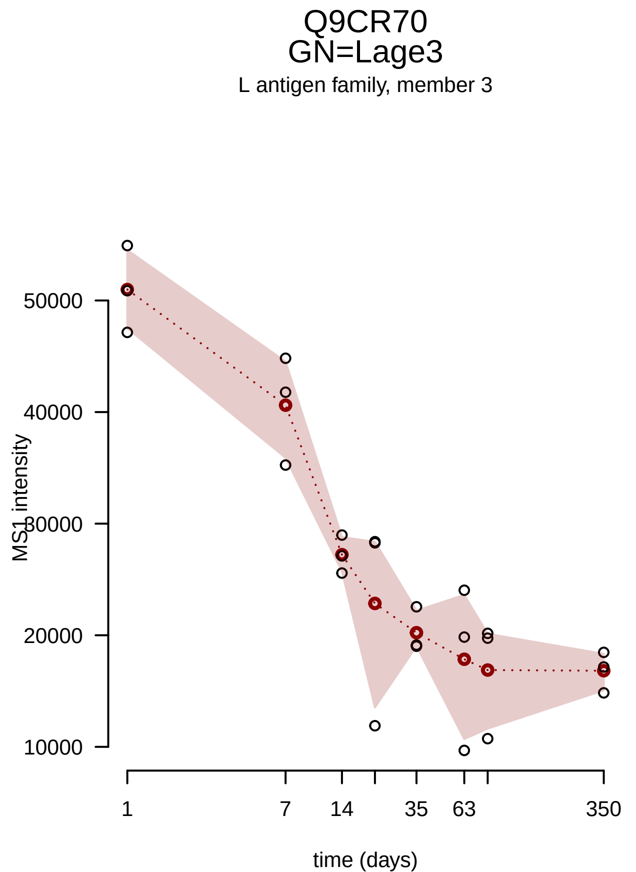
Q9CR70
GN=Lage3
L antigen family, member 3
MS1 intensity
50000
40000
30000
20000
10000
1
7
14
35
63
350
time (days)
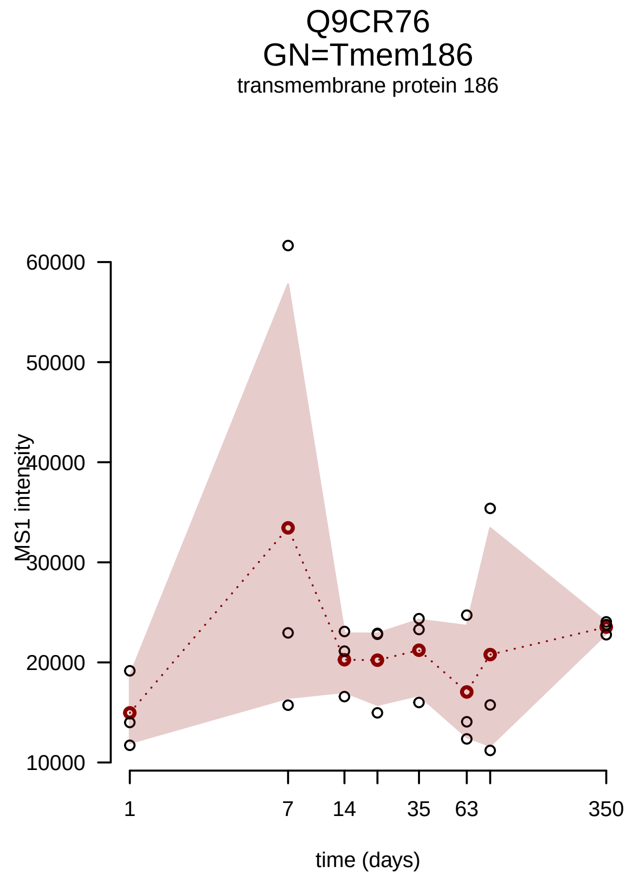
Q9CR76
GN=Tmem186
transmembrane protein 186
MS1 intensity
60000
50000
40000
30000
20000
10000
1
7
14
35
63
350
time (days)

Q9CR78
GN=Msantd3
Myb/SANT−like DNA−binding domain contain ...

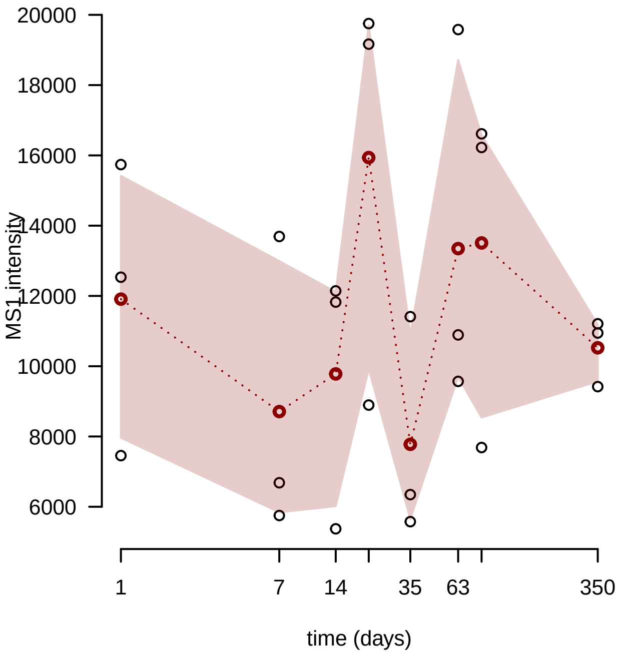

Q9CR80
GN=Fam32a
family with sequence similarity 32, memb ...

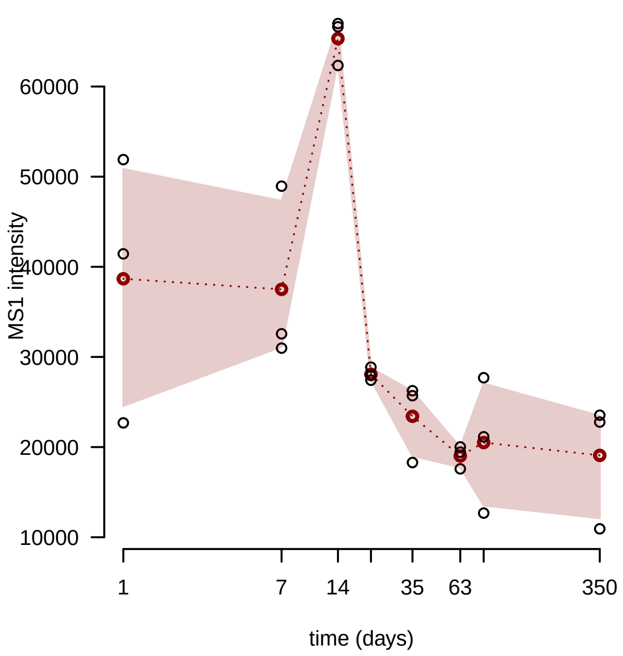

Q9CR86
GN=Carhsp1
calcium regulated heat stable protein 1

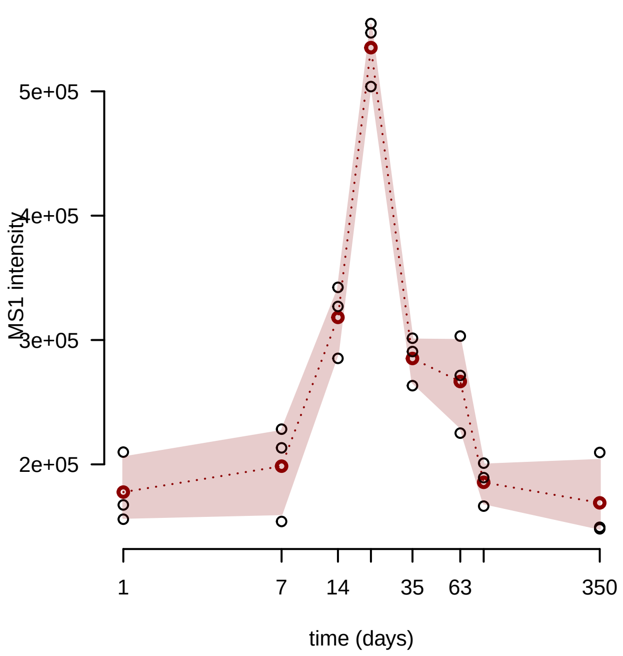

Q9CR88
GN=Mrps14
mitochondrial ribosomal protein S14

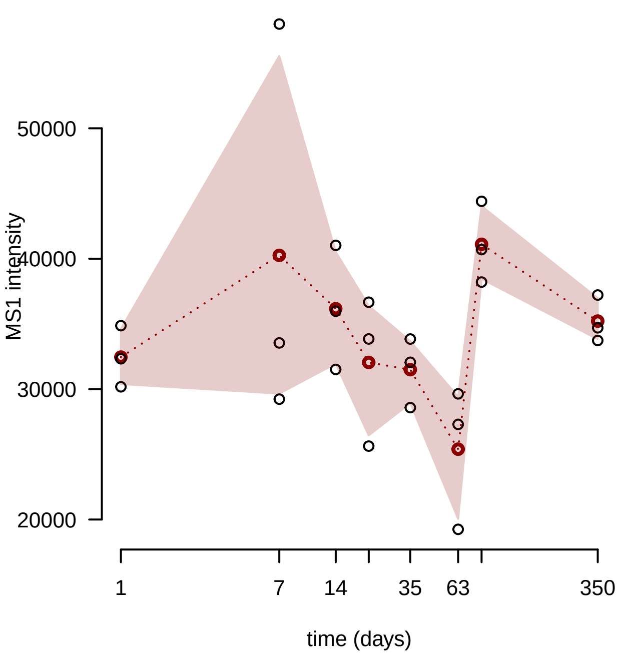

Q9CR89
GN=Ergic2
ERGIC and golgi 2

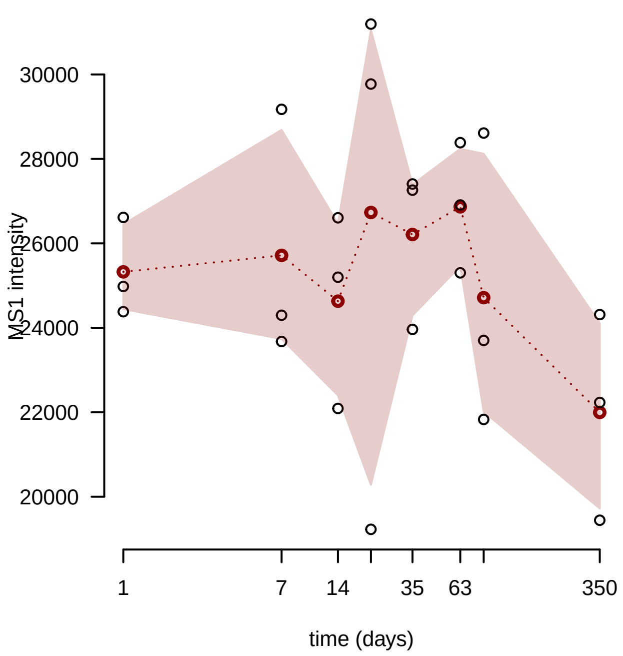

Q9CR95
GN=Necap1
NECAP endocytosis associated 1

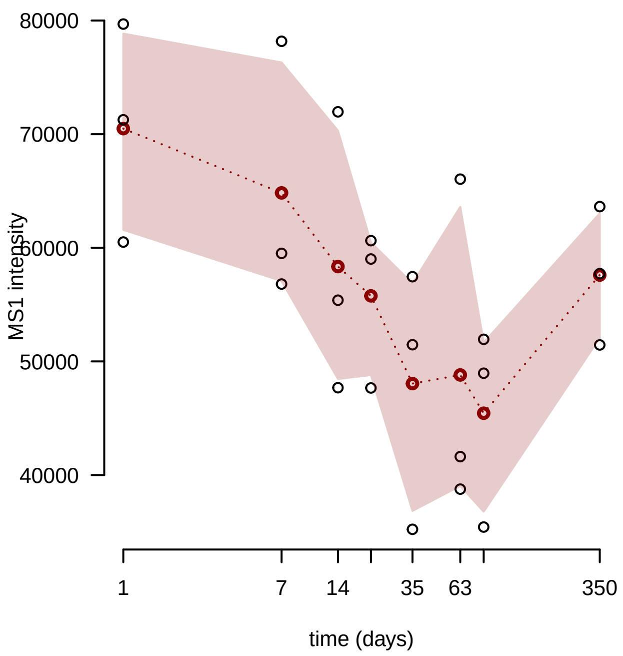

Q9CR96
GN=Tmem208
transmembrane protein 208

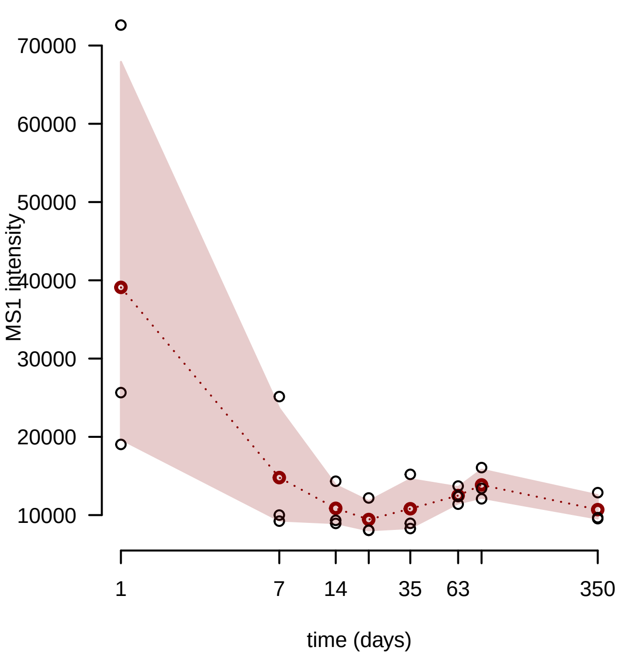

Q9CR98
GN=Fam136a
family with sequence similarity 136, mem ...

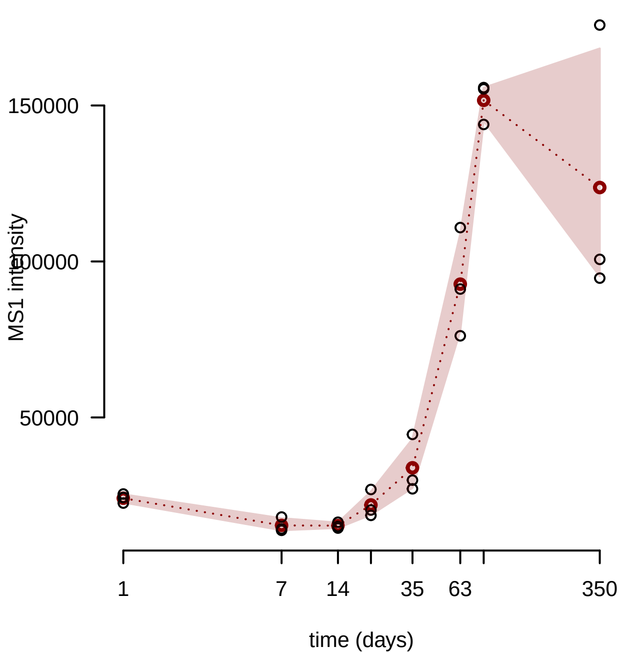

Q9CRA0
GN=Art4
ADP−ribosyltransferase 4

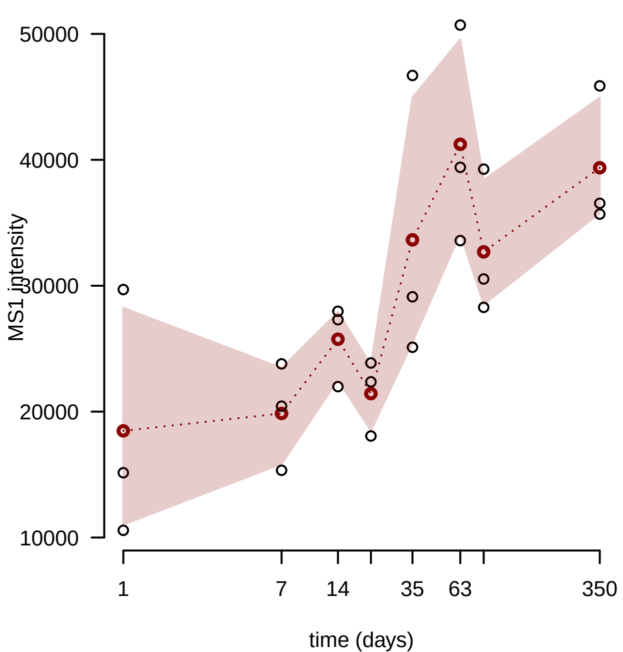

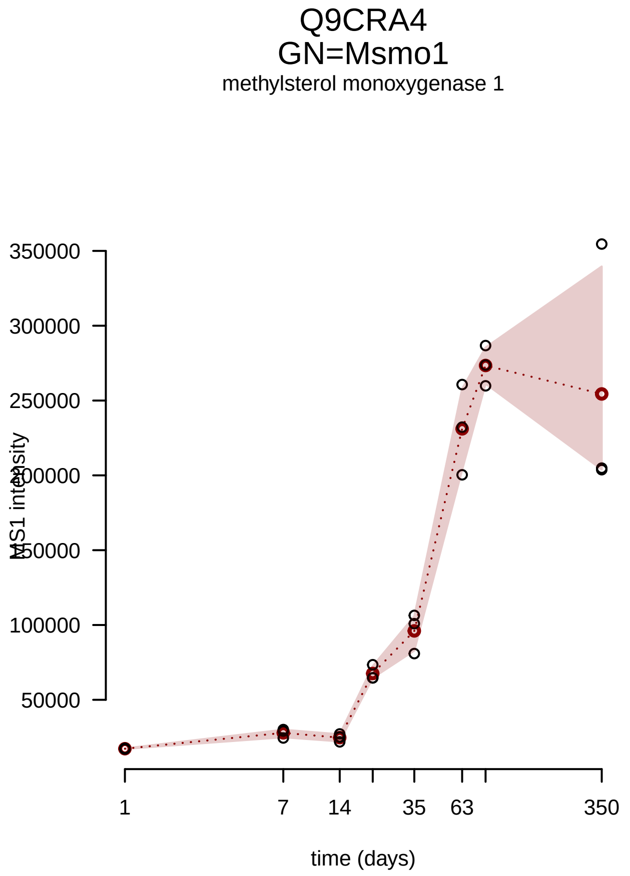
Q9CRA4
GN=Msmo1
methylsterol monoxygenase 1
MS1 intensity
350000
300000
250000
200000
150000
100000
50000
1
7
14
35
63
350
time (days)

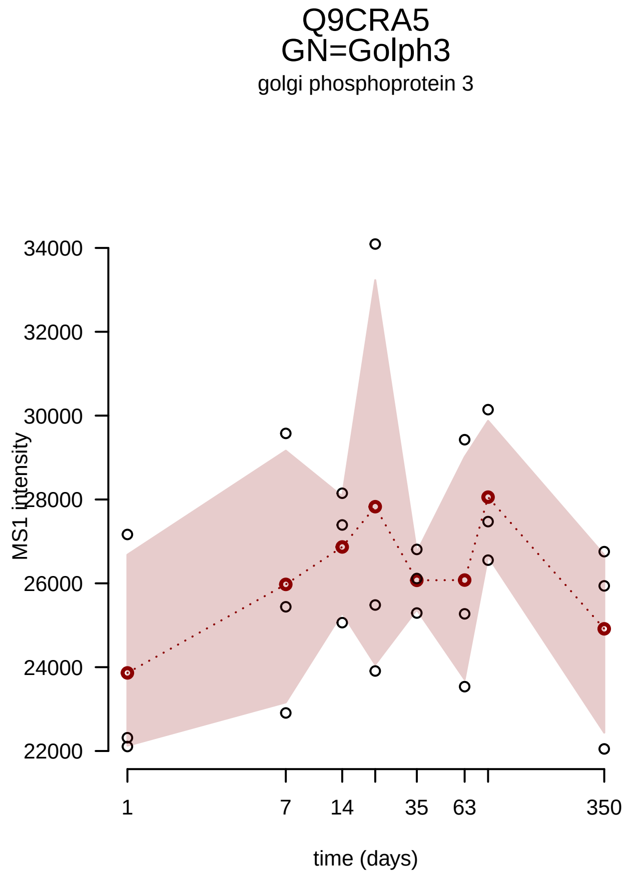
Q9CRA5
GN=Golph3
golgi phosphoprotein 3
MS1 intensity
34000
32000
30000
28000
26000
24000
22000
1
7
14
35
63
350
time (days)

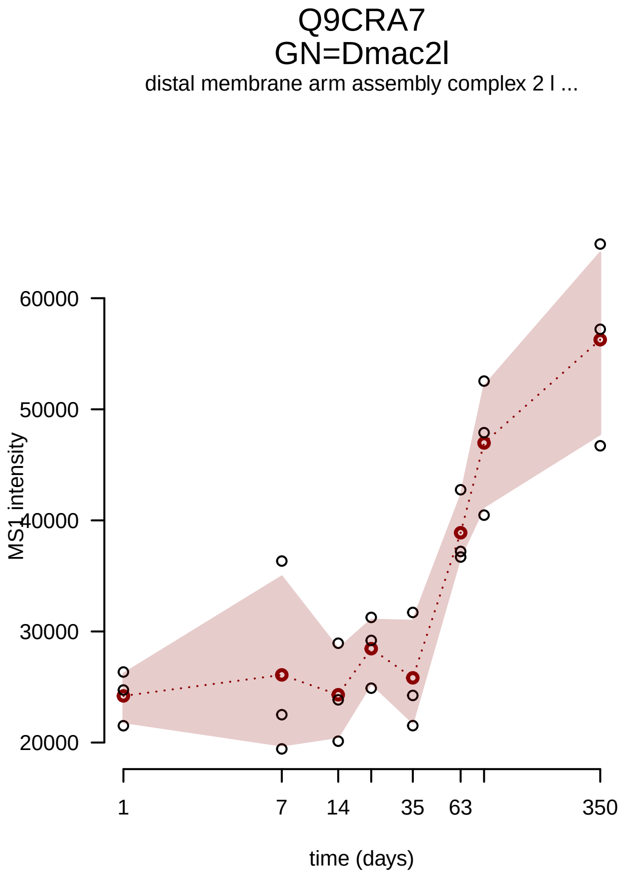
Q9CRA7
GN=Dmac2l
distal membrane arm assembly complex 2 l ...
MS1 intensity
60000
50000
40000
30000
20000
1
7
14
35
63
350
time (days)

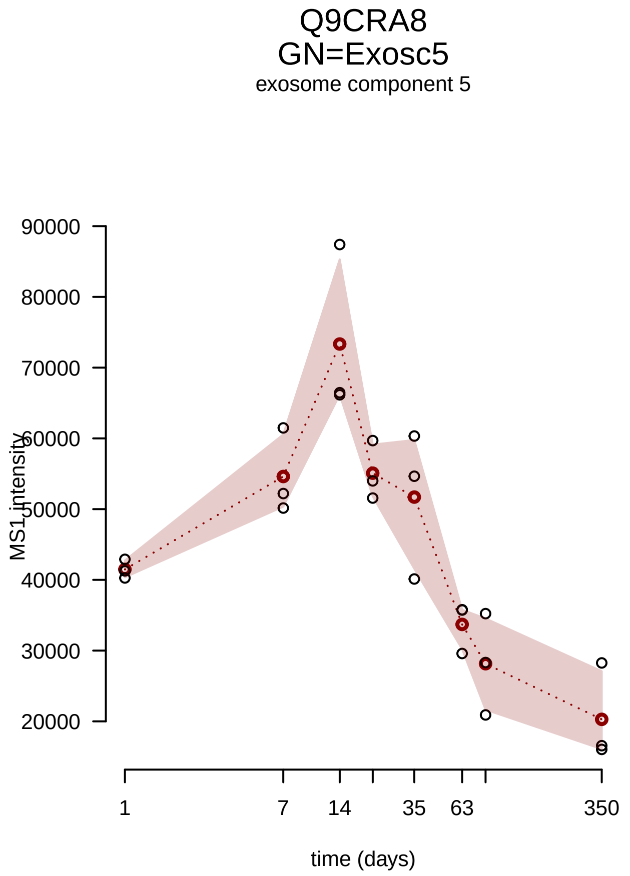
Q9CRA8
GN=Exosc5
exosome component 5
MS1 intensity
90000
80000
70000
60000
50000
40000
30000
20000
1
7
14
35
63
350
time (days)

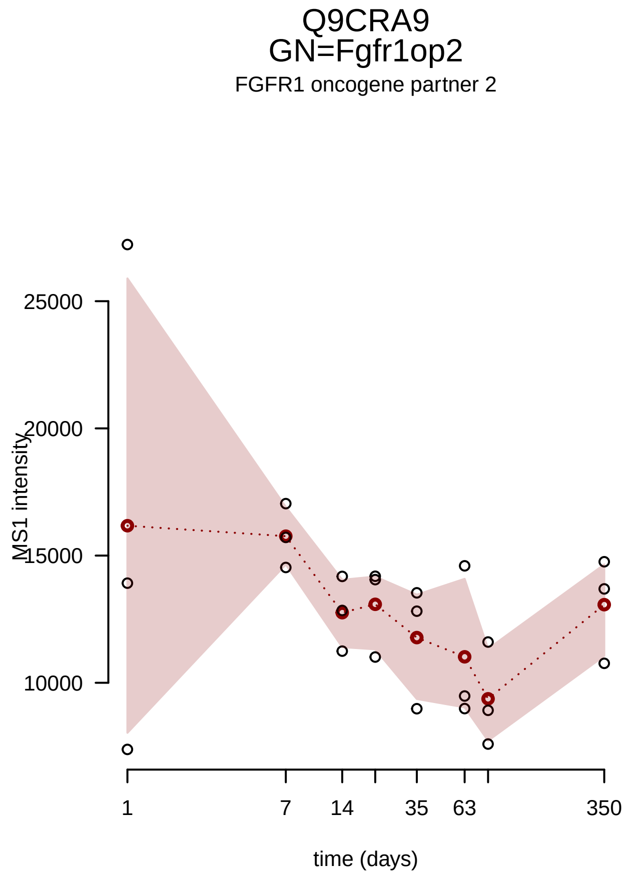
Q9CRA9
GN=Fgfr1op2
FGFR1 oncogene partner 2
MS1 intensity
25000
20000
15000
10000
1
7
14
35
63
350
time (days)

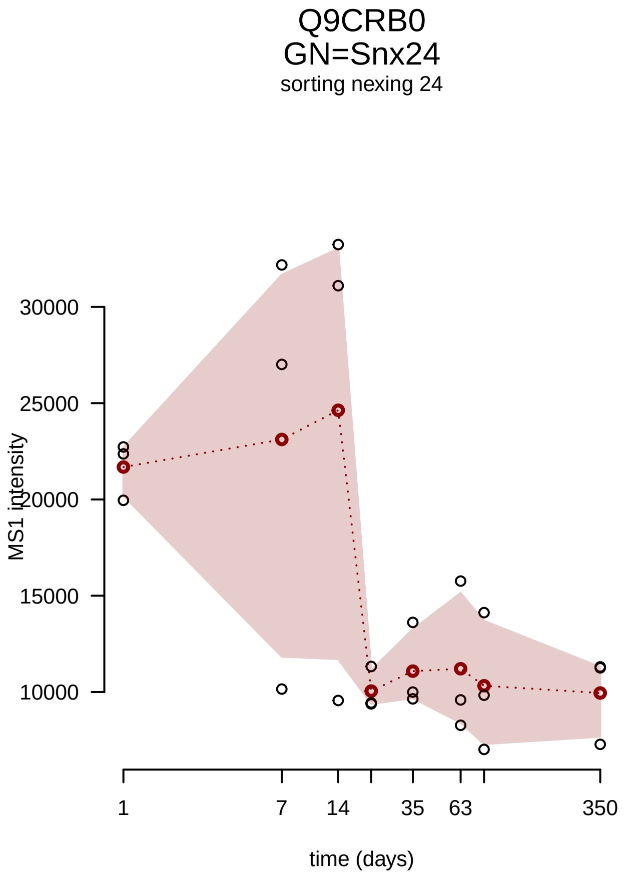
Q9CRB0
GN=Snx24
sorting nexing 24
MS1 intensity
30000
25000
20000
15000
10000
1
7
14
35
63
350
time (days)

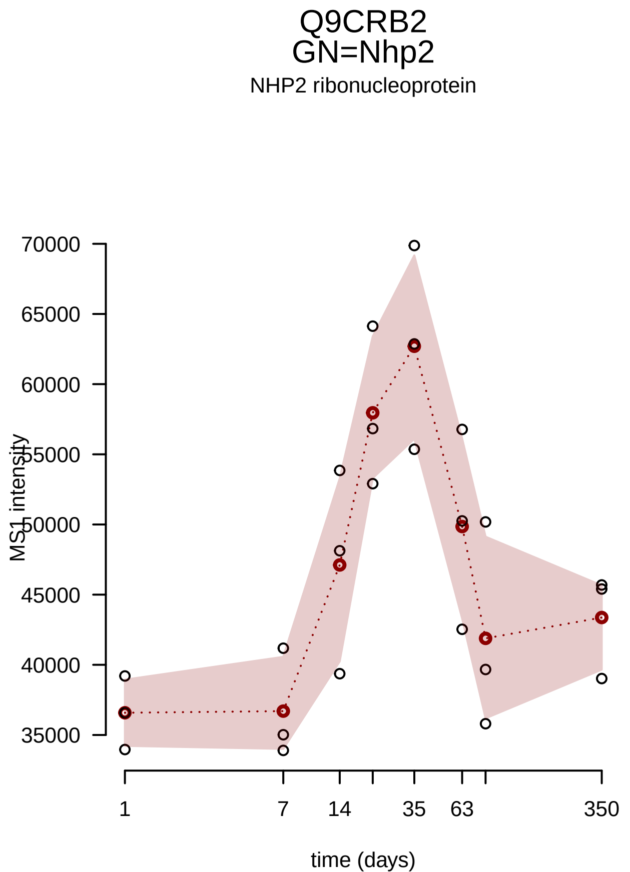
Q9CRB2
GN=Nhp2
NHP2 ribonucleoprotein
MS1 intensity
70000
65000
60000
55000
50000
45000
40000
35000
1
7
14
35
63
350
time (days)

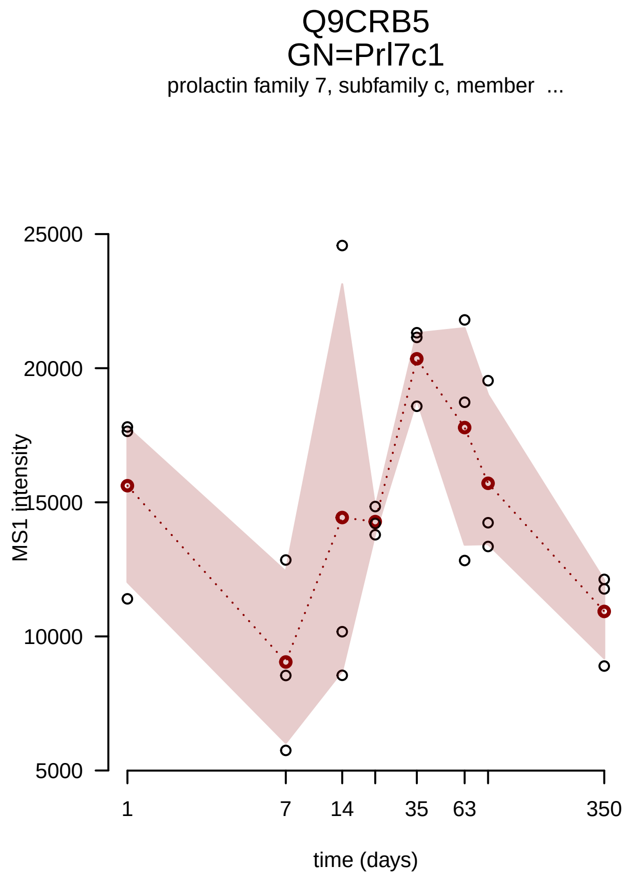
Q9CRB5
GN=Prl7c1
prolactin family 7, subfamily c, member ...
MS1 intensity
25000
20000
15000
10000
5000
1
7
14
35
63
350
time (days)

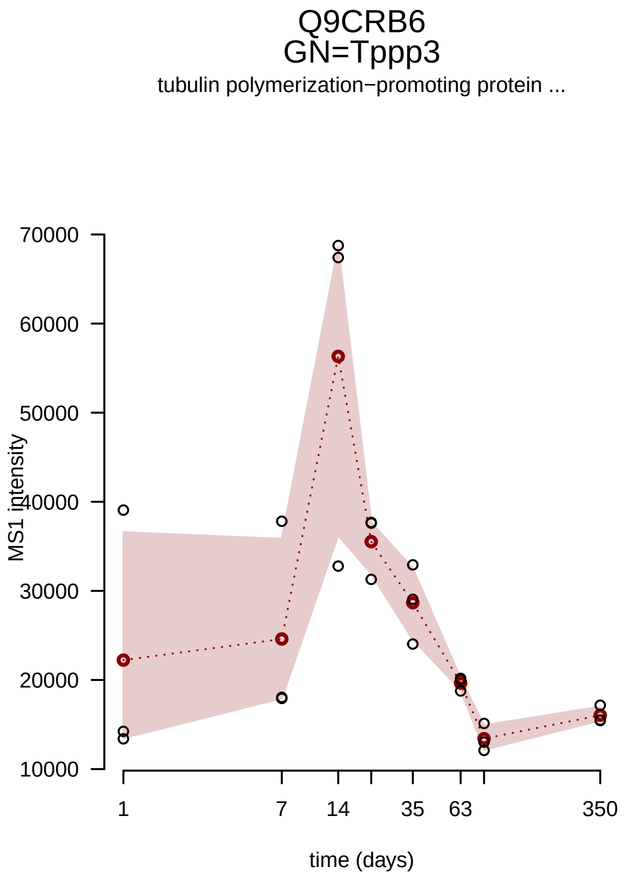
Q9CRB6
GN=Tppp3
tubulin polymerization−promoting protein ...
MS1 intensity
70000
60000
50000
40000
30000
20000
10000
1
7
14
35
63
350
time (days)

Q9CRB8
GN=Mtfp1
mitochondrial fission process 1

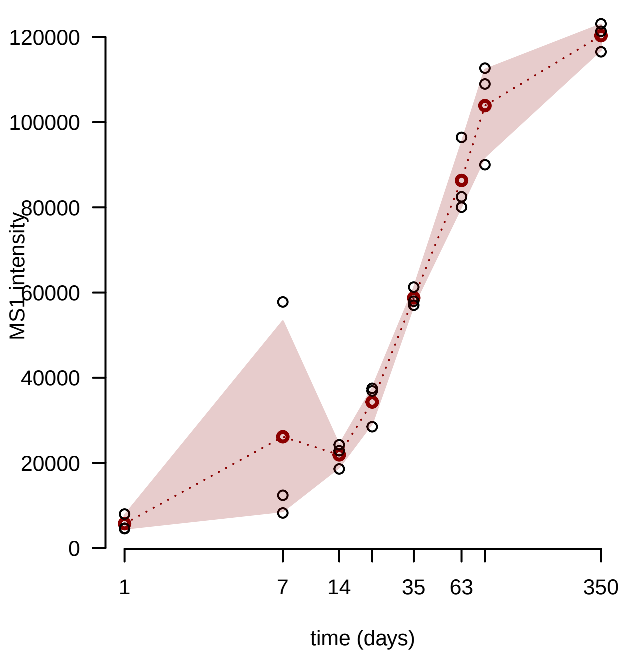

Q9CRB9
GN=Chchd3
coiled−coil−helix−coiled−coil−helix doma ...

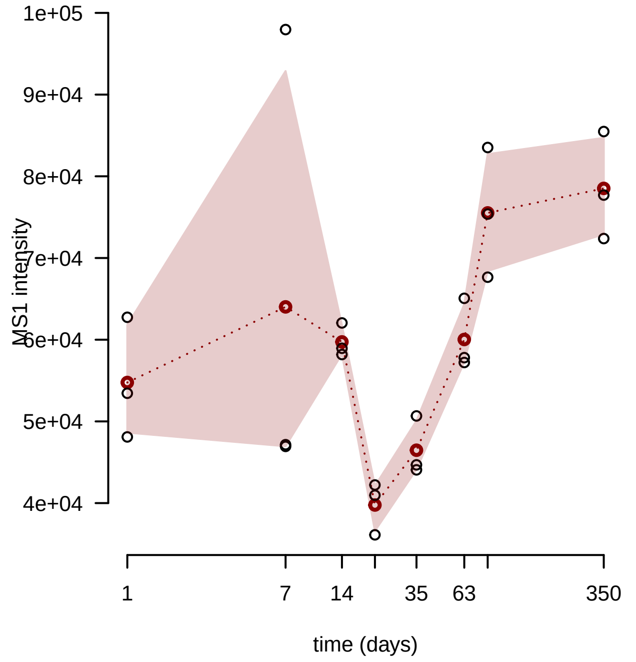

Q9CRC0
GN=Vkorc1
vitamin K epoxide reductase complex, sub ...

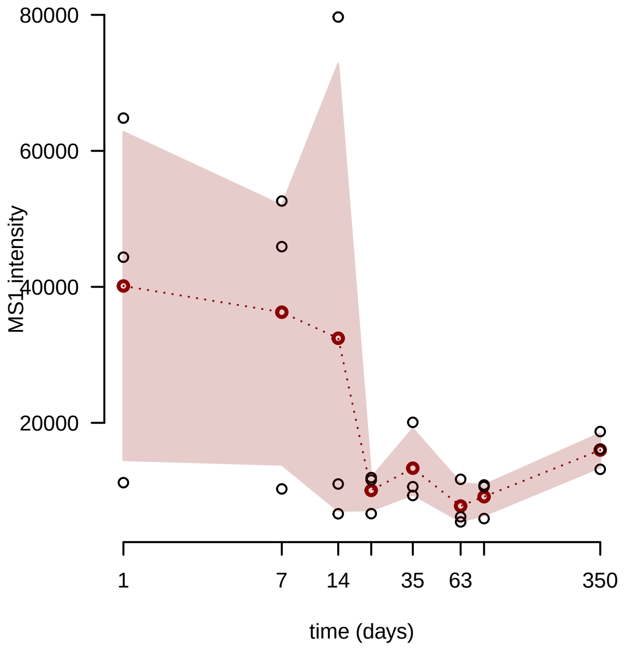

Q9CRC3
GN=

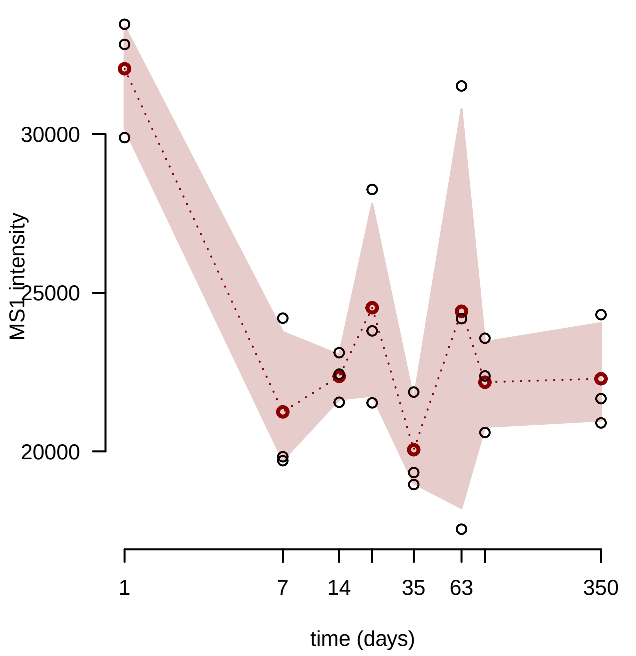

Q9CRC8
GN=Lrrc40
leucine rich repeat containing 40

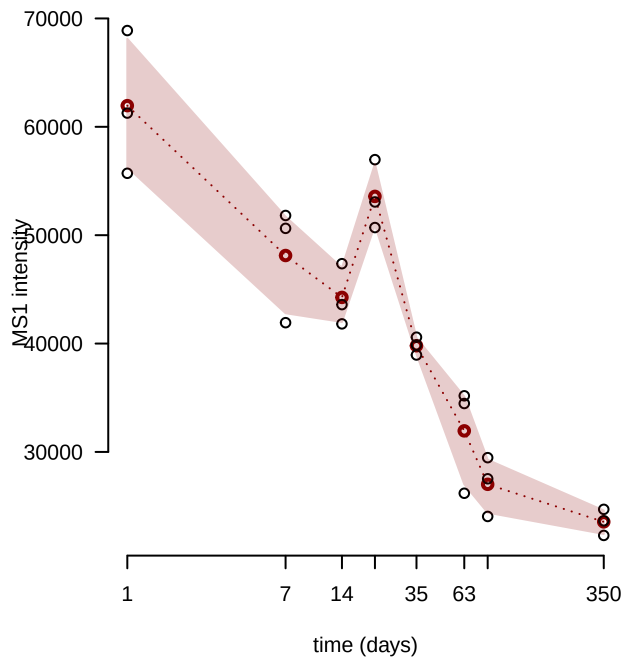

Q9CRC9
GN=Gnpda2
glucosamine−6−phosphate deaminase 2

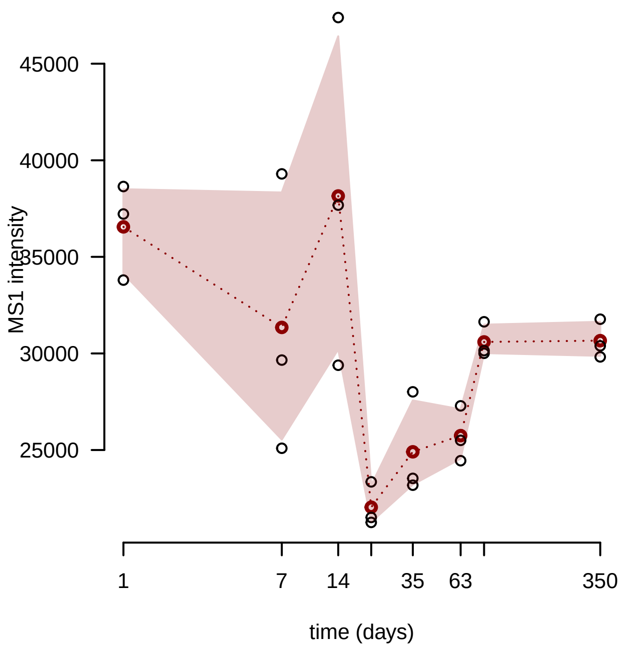

Q9CRD0
GN=Ociad1
OCIA domain containing 1

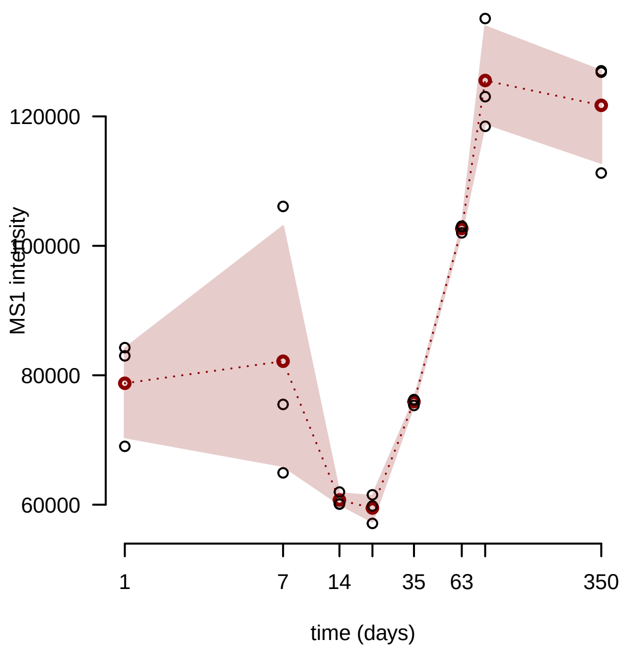

Q9CRD2
GN=Emc2
ER membrane protein complex subunit 2

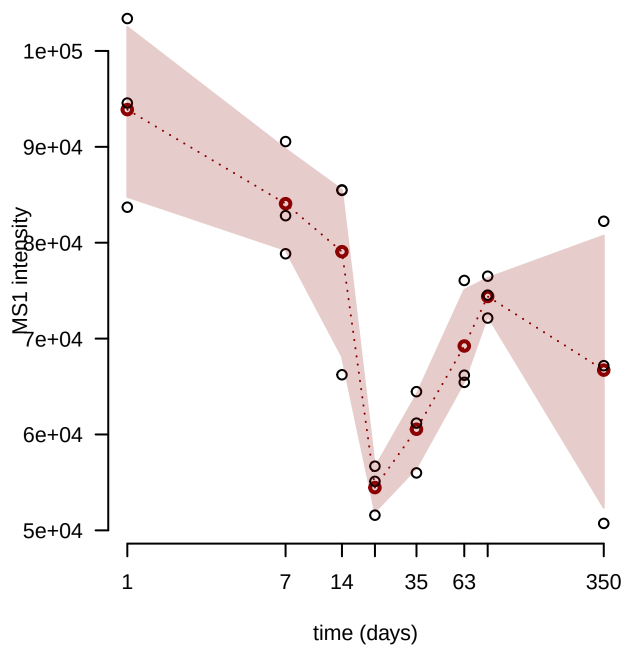

Q9CRG1
GN=Tm7sf3
transmembrane 7 superfamily member 3

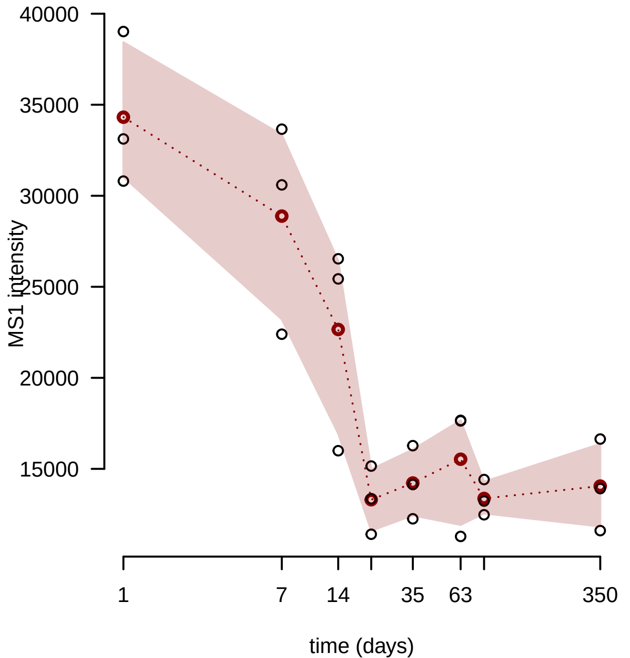

Q9CRT8
GN=Xpot
exportin, tRNA (nuclear export receptor ...

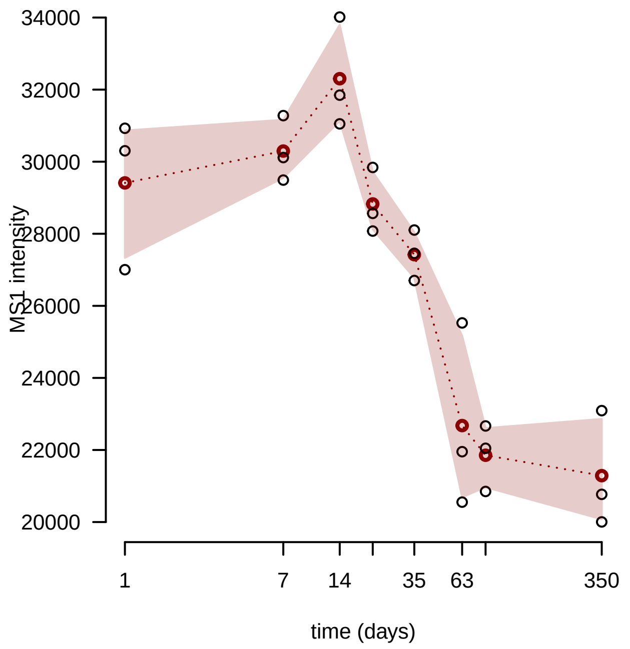

Q9CRW3
GN=

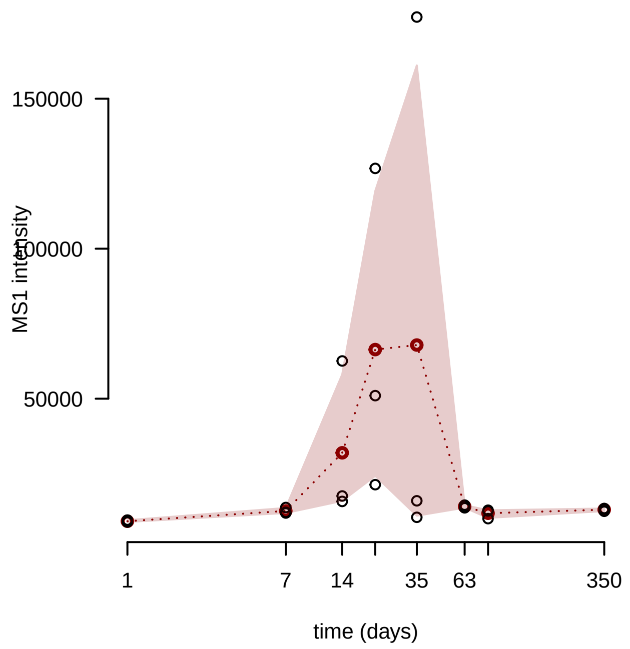

Q9CRY7
GN=Gdpd1
glycerophosphodiester phosphodiesterase ...

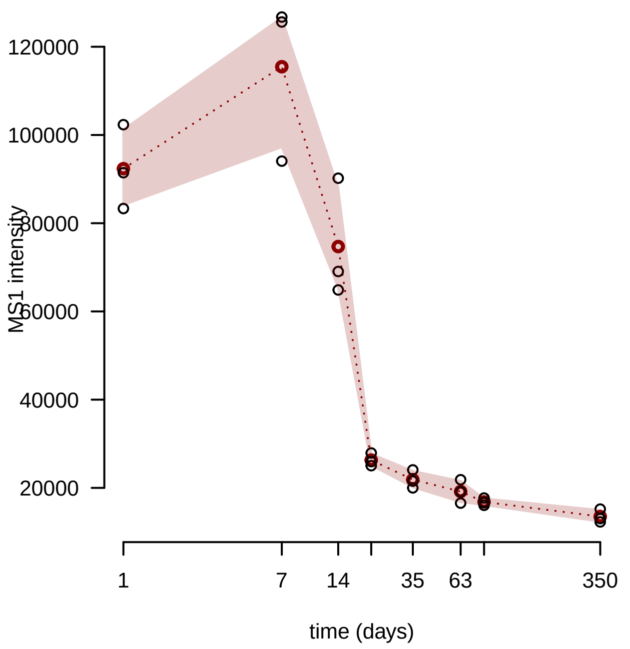

Q9CS00
GN=Cactin
cactin, spliceosome C complex subunit

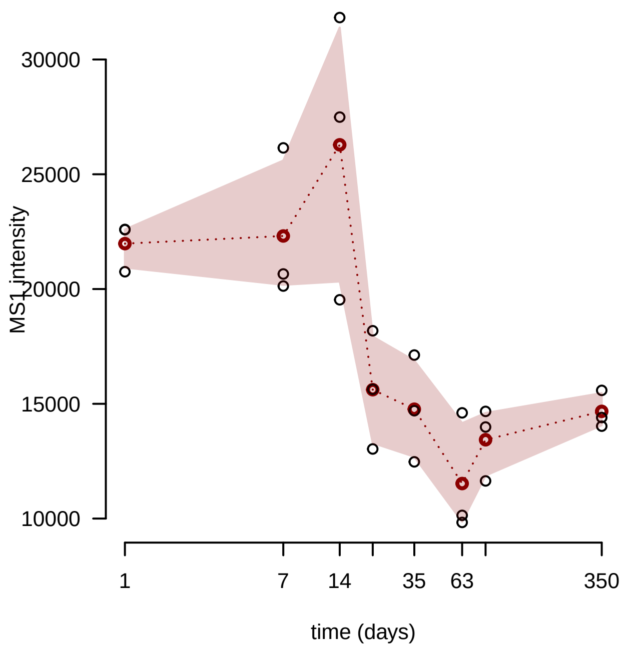

Q9CS42
GN=Prps2
phosphoribosyl pyrophosphate synthetase ...

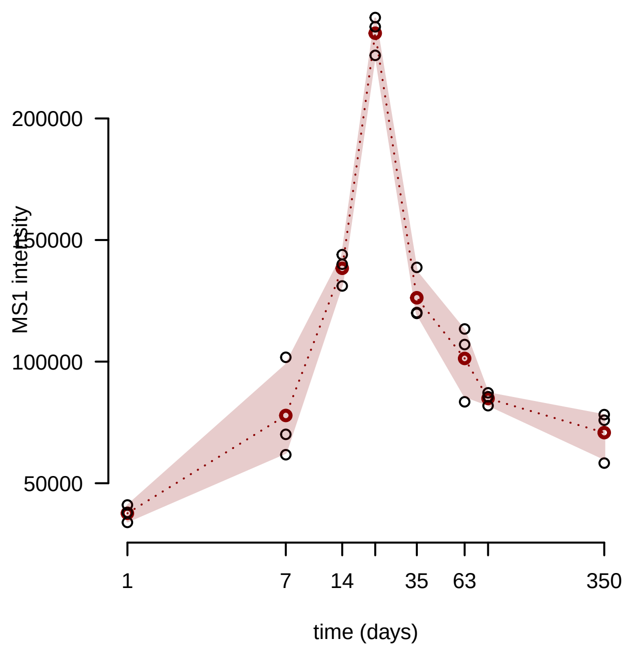

Q9CS74
GN=Ecd
ecdysoneless cell cycle regulator

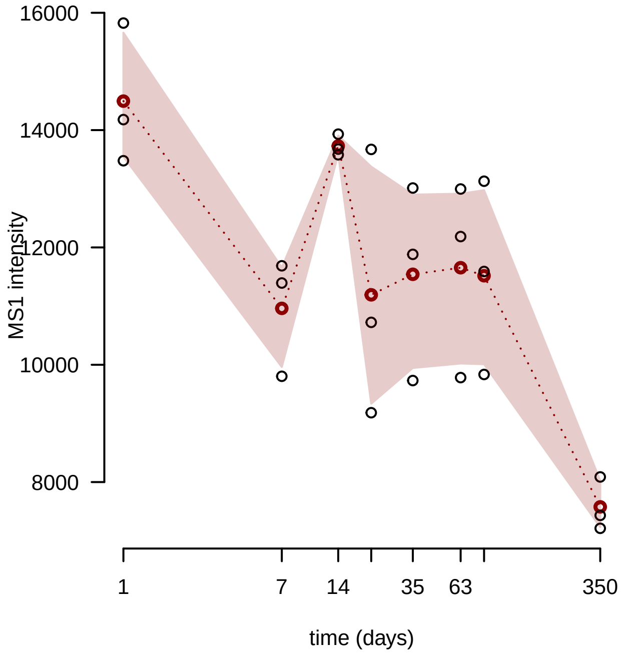

Q9CSB4
GN=Pard3b
par−3 family cell polarity regulator bet ...

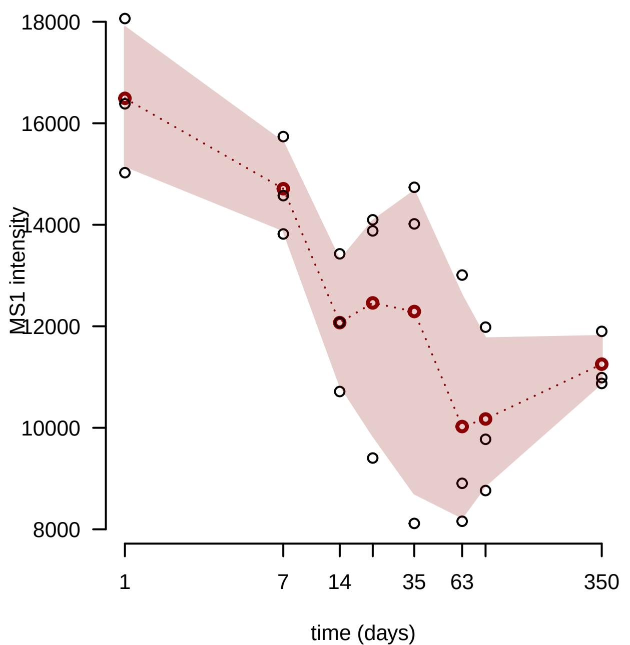

Q9CSH3
GN=Dis3
DIS3 homolog, exosome endoribonuclease a ...

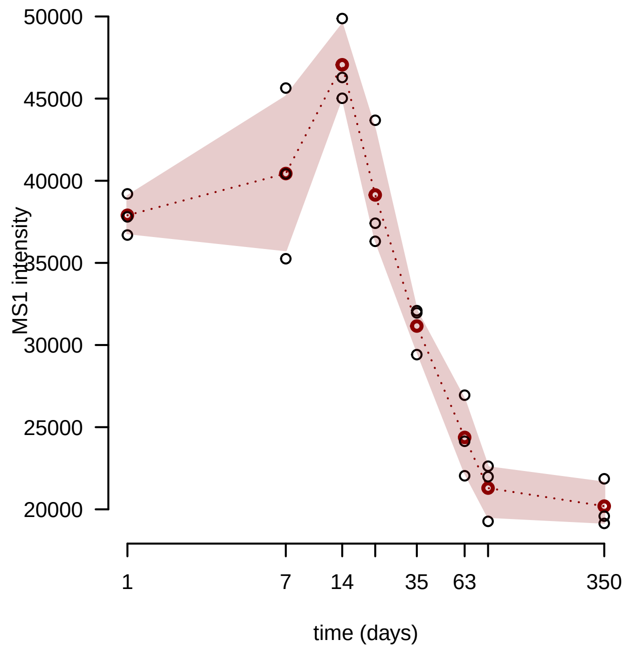

Q9CSN1
GN=Snw1
SNW domain containing 1

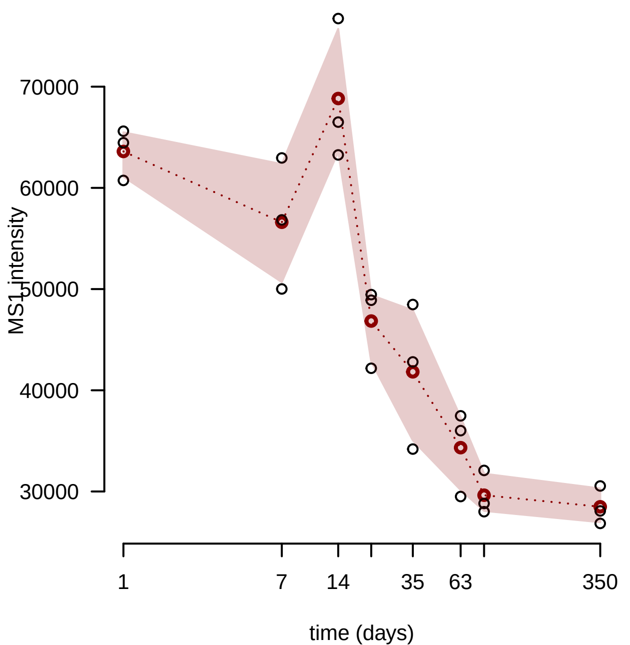

Q9CSU0
GN=Rprd1b
regulation of nuclear pre−mRNA domain co ...

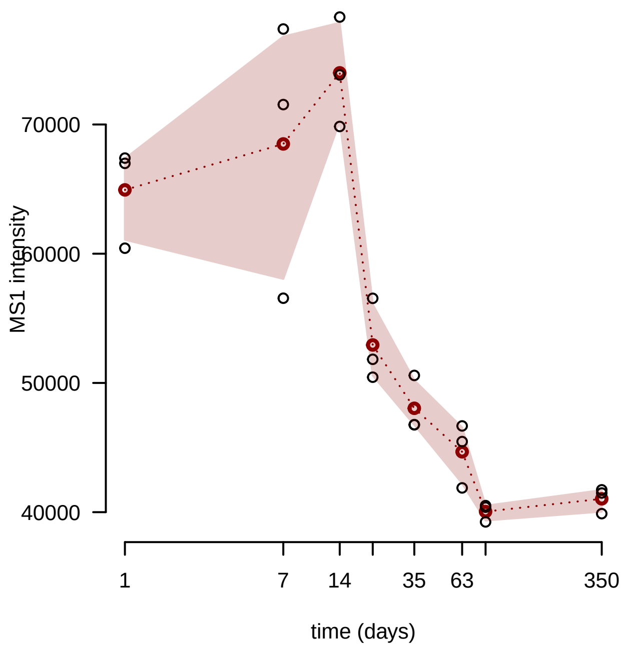

Q9CSV6
GN=Sft2d3
SFT2 domain containing 3

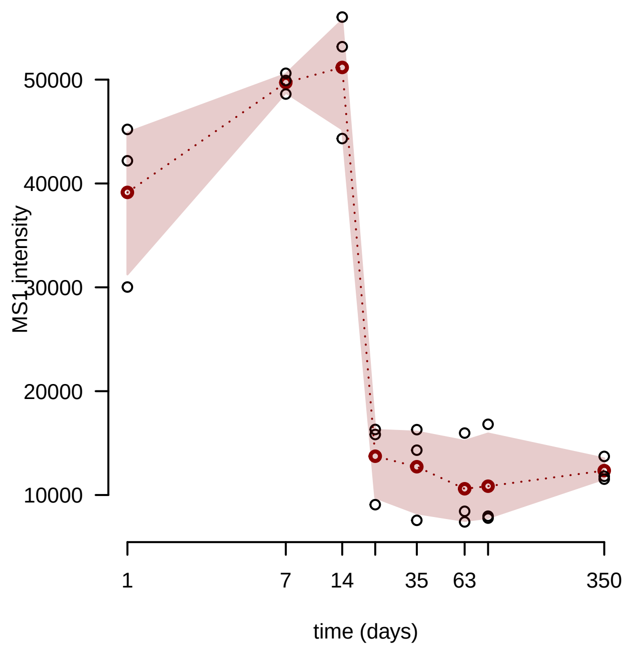

Q9CT10
GN=Ranbp3
RAN binding protein 3

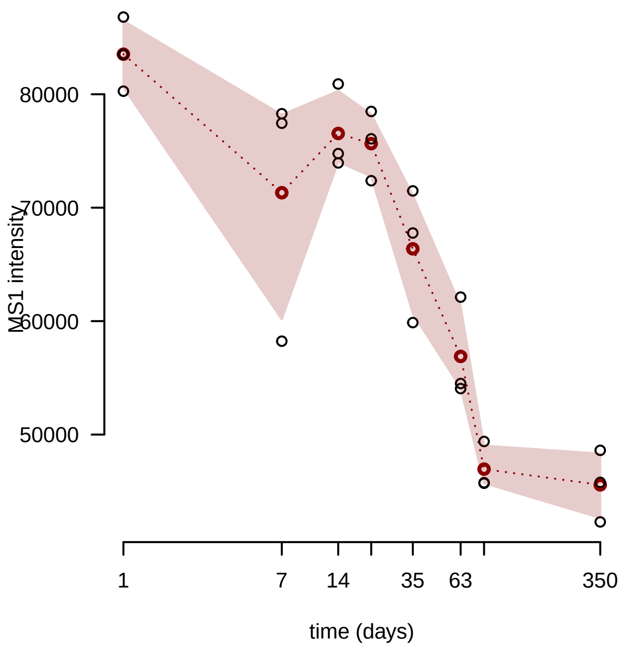

Q9CTH6
GN=Fcf1
FCF1 rRNA processing protein

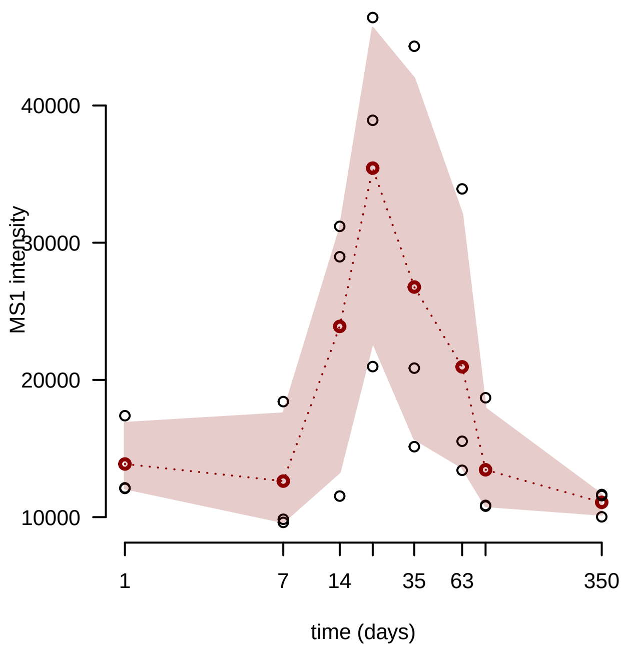

Q9CTN4
GN=Rhobtb3
Rho−related BTB domain containing 3

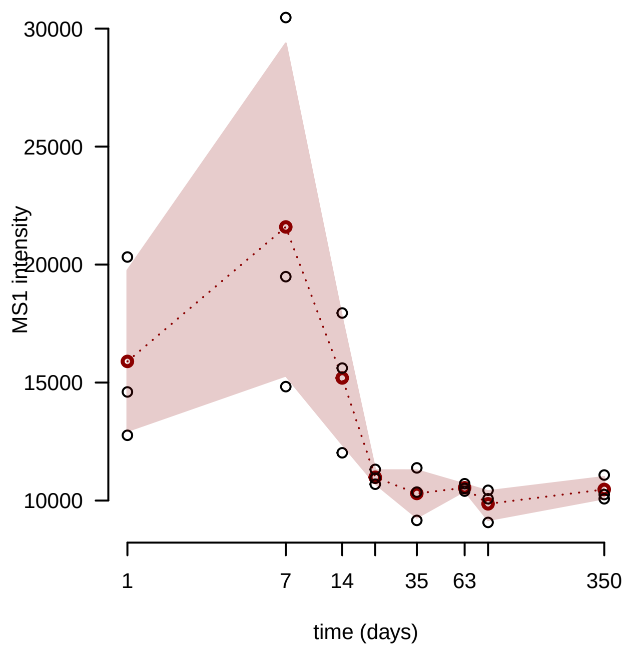

Q9CTY5
GN=Micu3
mitochondrial calcium uptake family, mem ...

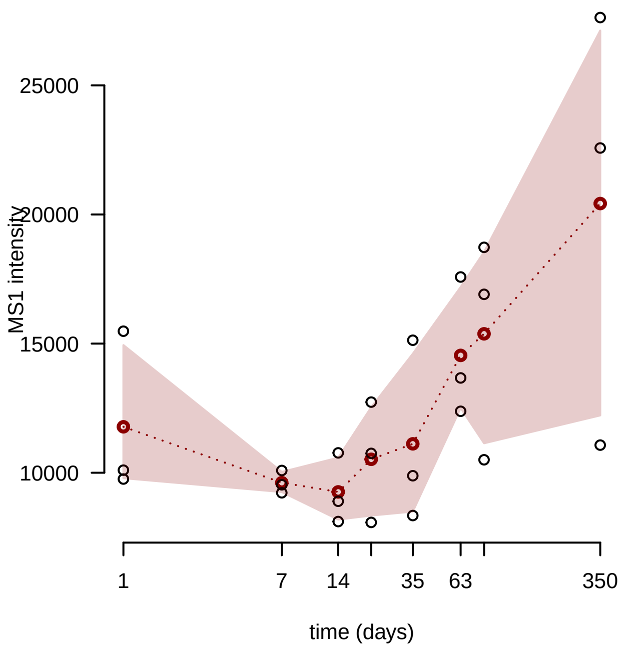

Q9CU62
GN=Smc1a
structural maintenance of chromosomes 1A

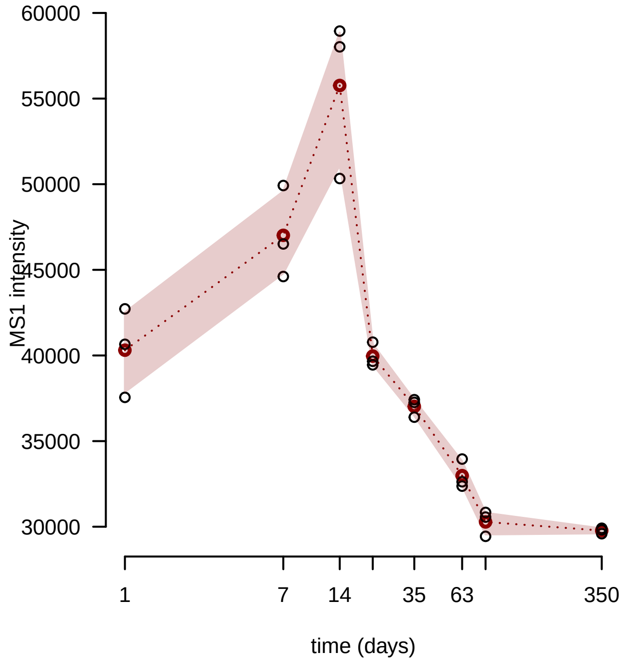

Q9CU65
GN=Zmym2
zinc finger, MYM−type 2

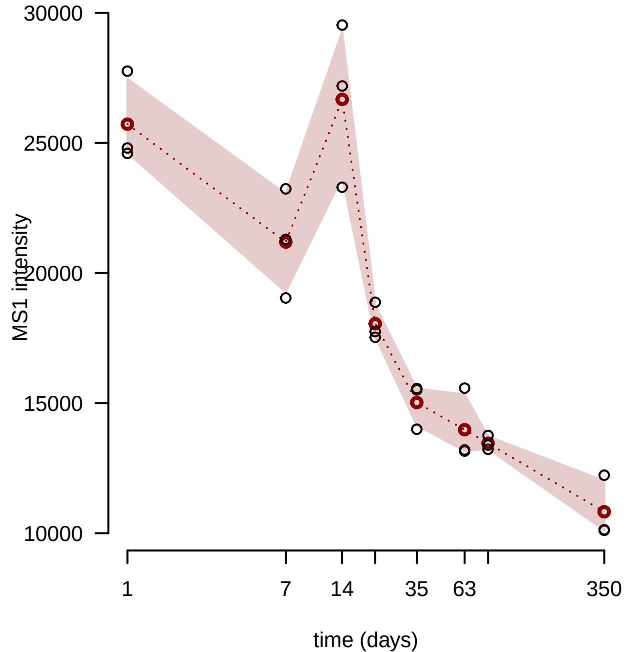

Q9CUN6
GN=Smurf1
SMAD specific E3 ubiquitin protein ligas ...

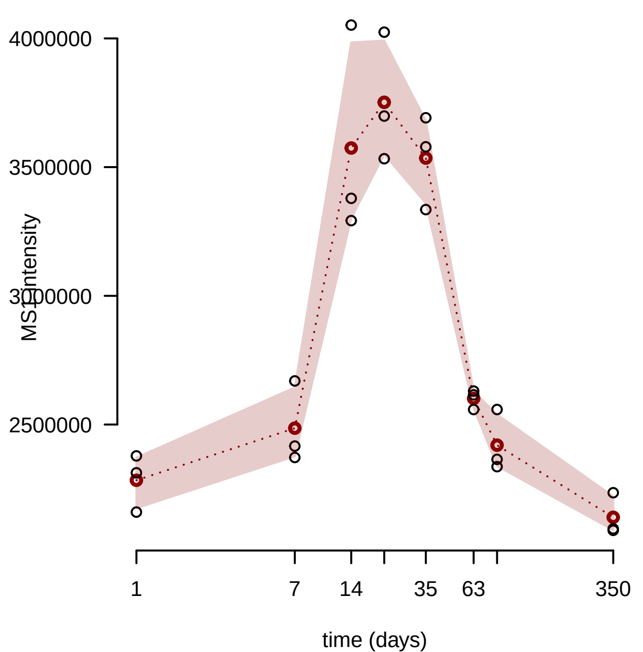

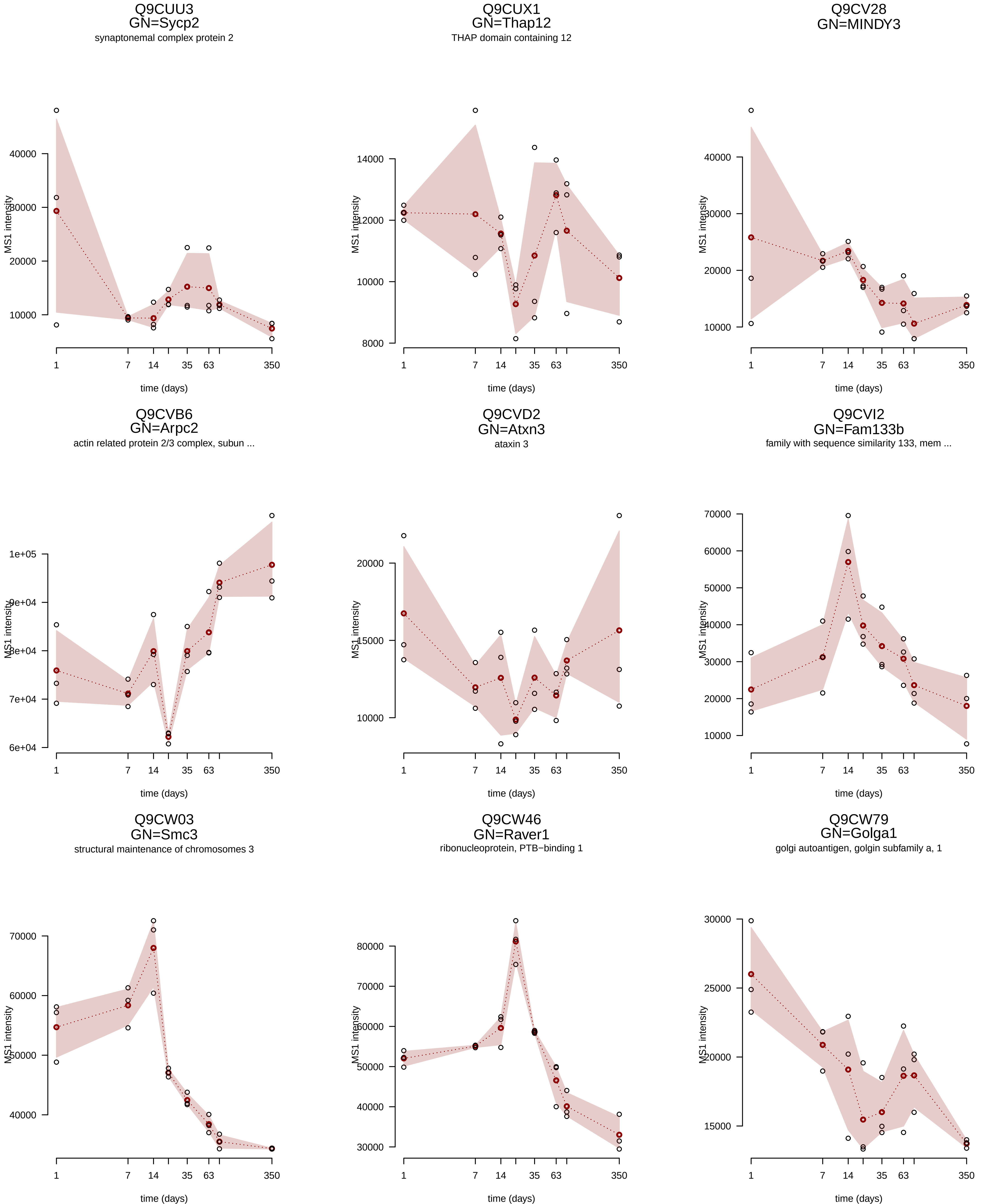
Q9CUU3
GN=Sycp2
synaptonemal complex protein 2
Q9CUX1
GN=Thap12
THAP domain containing 12
Q9CV28
GN=MINDY3
Q9CVB6
GN=Arpc2
actin related protein 2/3 complex, subun ...
Q9CVD2
GN=Atxn3
ataxin 3
Q9CVI2
GN=Fam133b
family with sequence similarity 133, mem ...
Q9CW03
GN=Smc3
structural maintenance of chromosomes 3
Q9CW46
GN=Raver1
ribonucleoprotein, PTB−binding 1
Q9CW79
GN=Golga1
golgi autoantigen, golgin subfamily a, 1
MS1 intensity
time (days)
1
7
14
35
63
350

Q9CWB7
GN=

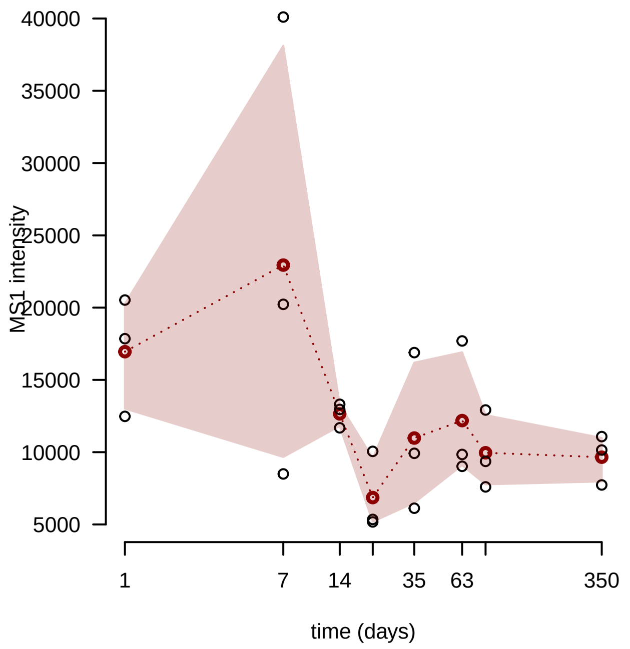

Q9CWD3
GN=Nudt17
nudix (nucleoside diphosphate linked moi ...

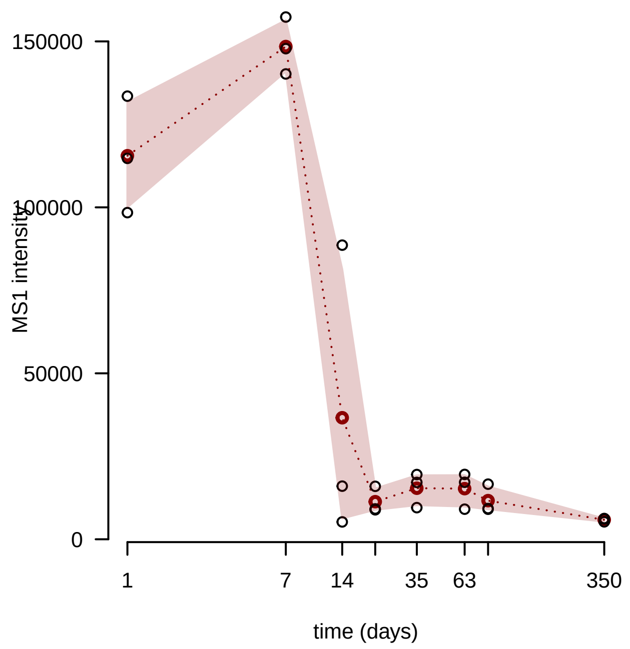

Q9CWD8
GN=Nubpl
nucleotide binding protein−like

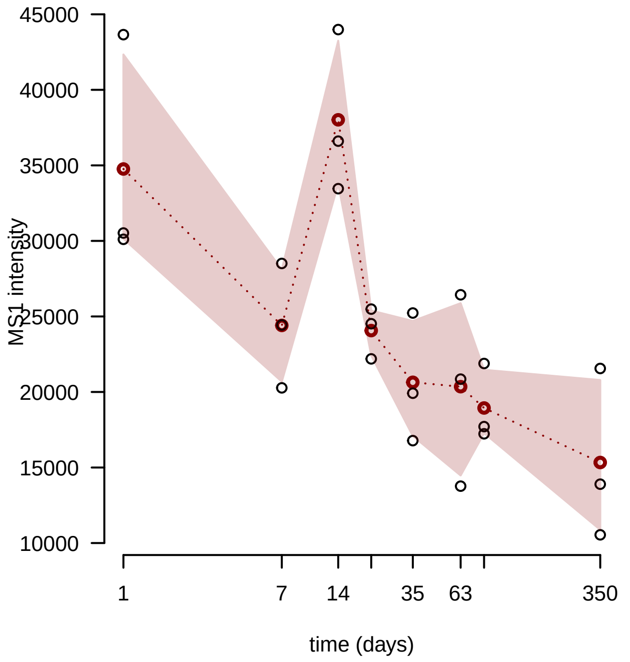

Q9CWE0
GN=Mtfr1l
mitochondrial fission regulator 1−like

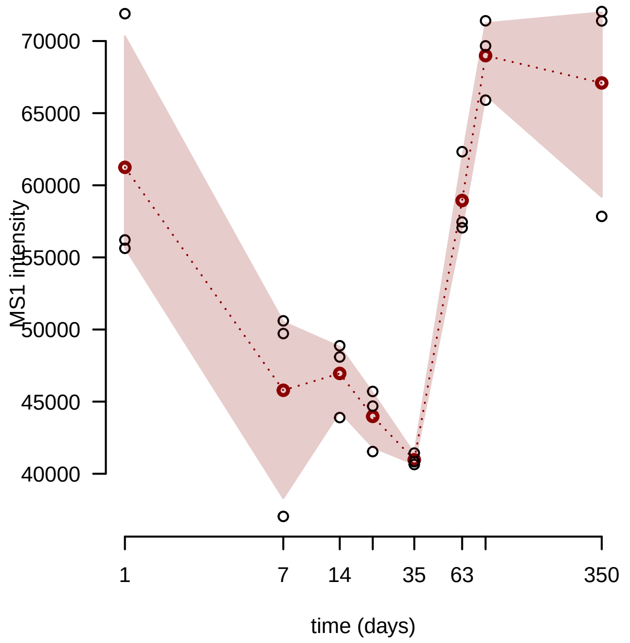

Q9CWE6
GN=Ooep
oocyte expressed protein

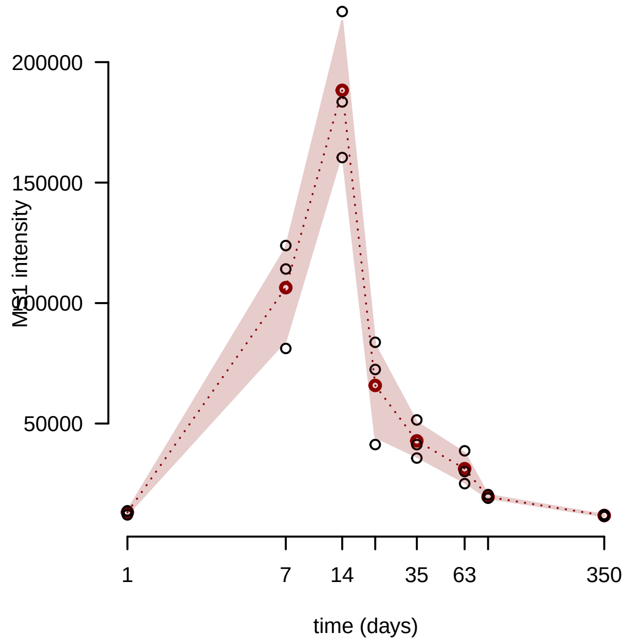

Q9CWF2
GN=Tubb2b
tubulin, beta 2B class IIB

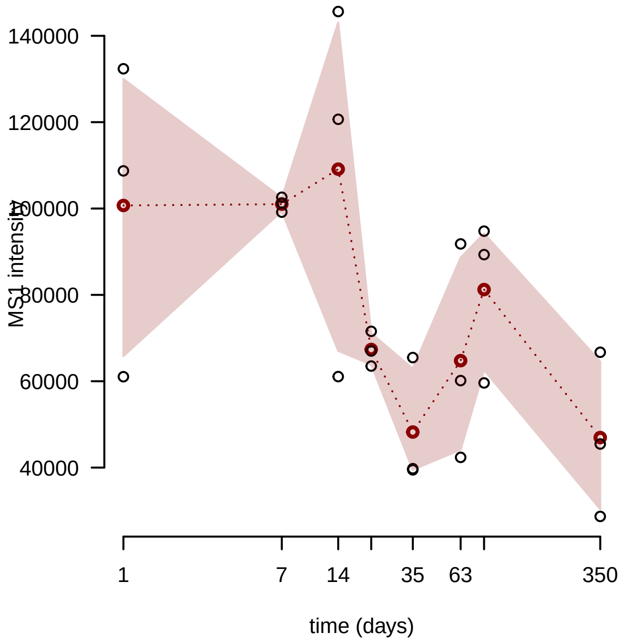

Q9CWF6
GN=Bbs2
Bardet−Biedl syndrome 2 (human)

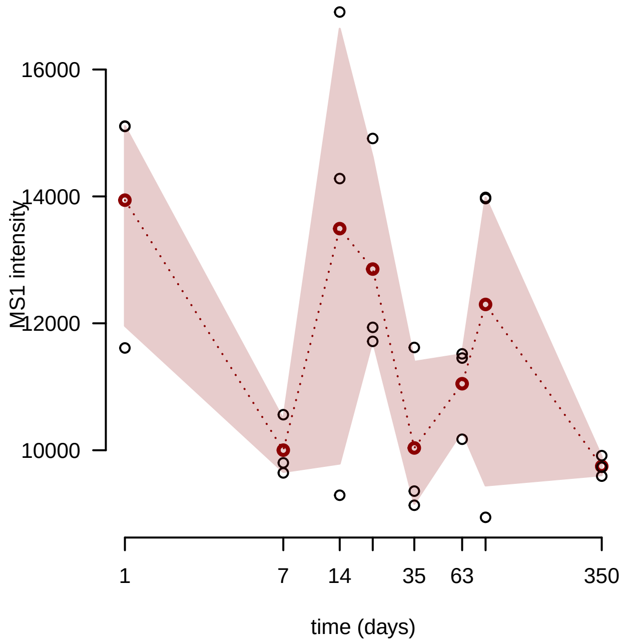

Q9CWG8
GN=Ndufaf7
NADH:ubiquinone oxidoreductase complex a ...

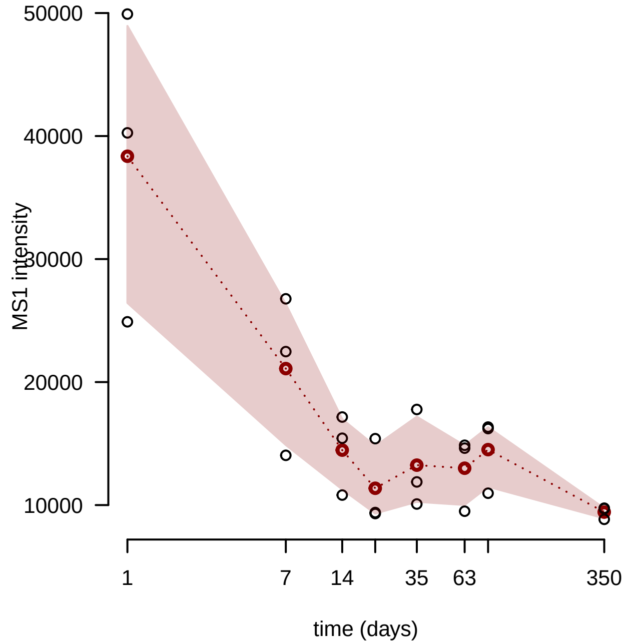

Q9CWG9
GN=Bloc1s2
biogenesis of lysosomal organelles compl ...

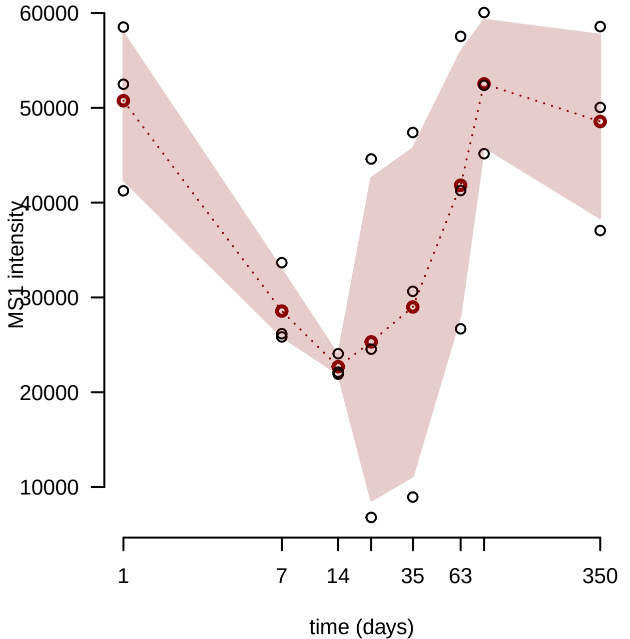

Q9CWH5
GN=Trmt11
tRNA methyltransferase 11

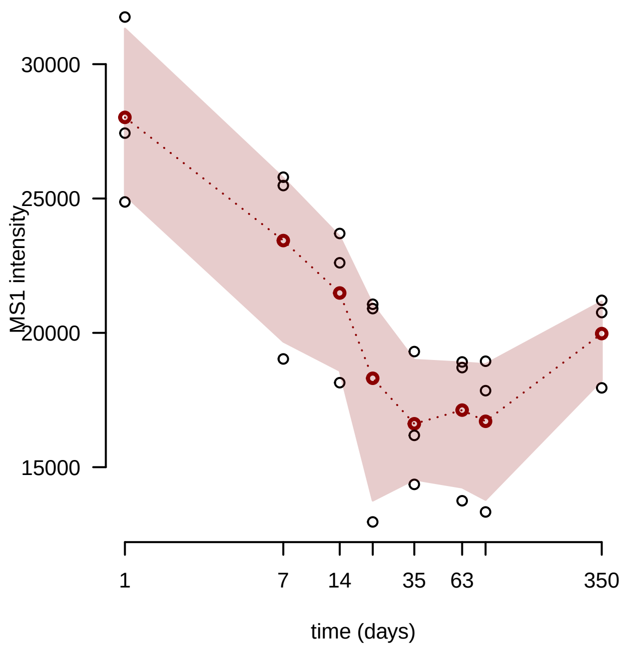

Q9CWI3
GN=Bccip
BRCA2 and CDKN1A interacting protein

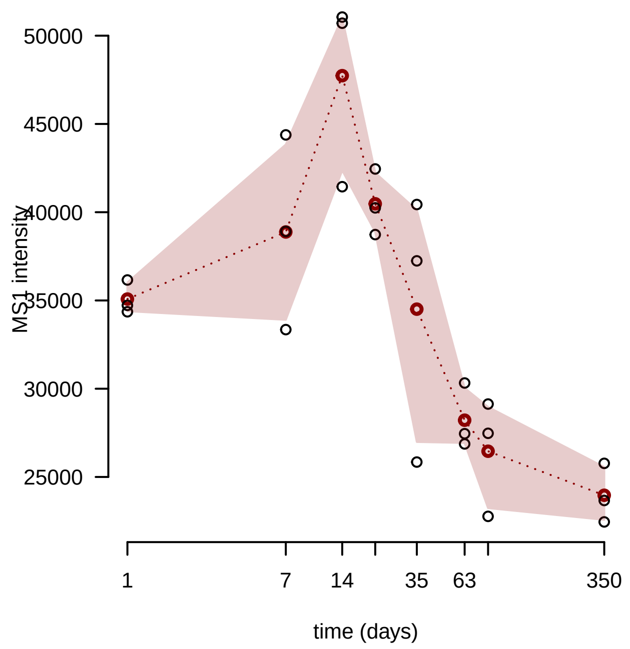

Q9CWJ9
GN=Atic
5−aminoimidazole−4−carboxamide ribonucle ...

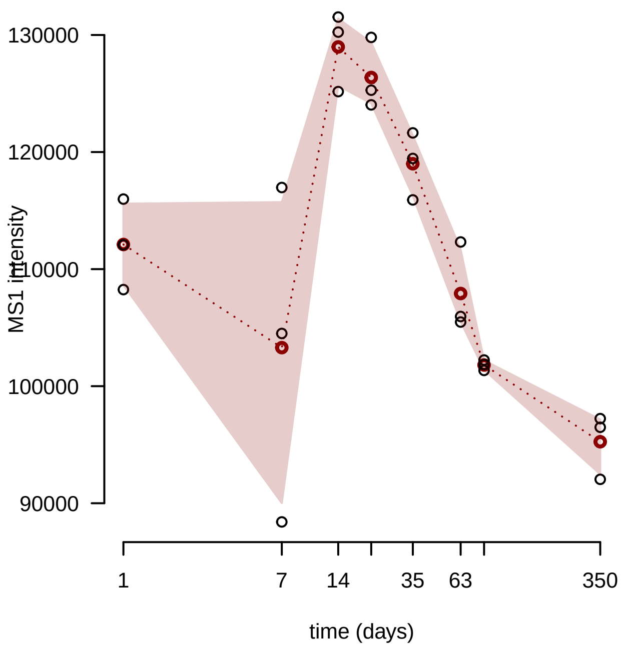

Q9CWK3
GN=Cd2bp2
CD2 cytoplasmic tail binding protein 2

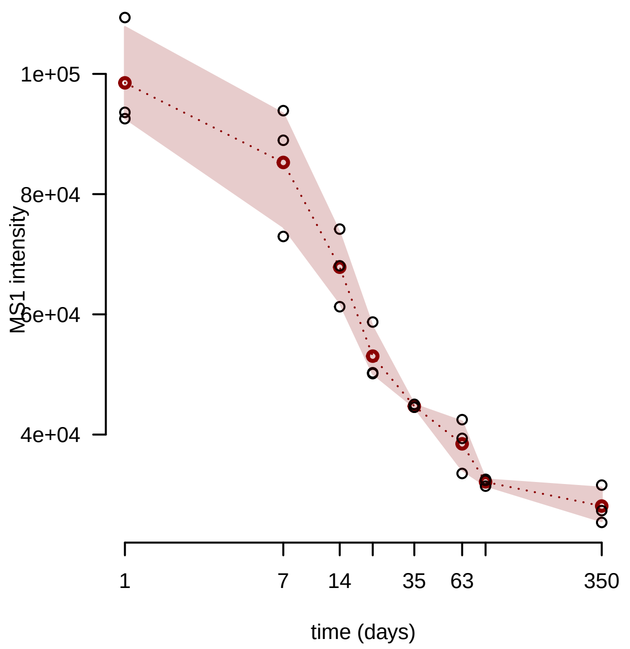

Q9CWK8
GN=Snx2
sorting nexin 2

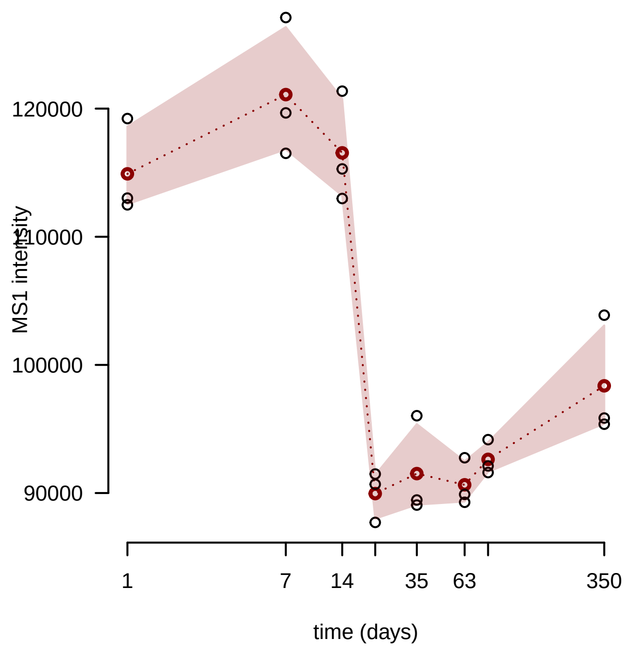

Q9CWL8
GN=Ctnnbl1
catenin, beta like 1

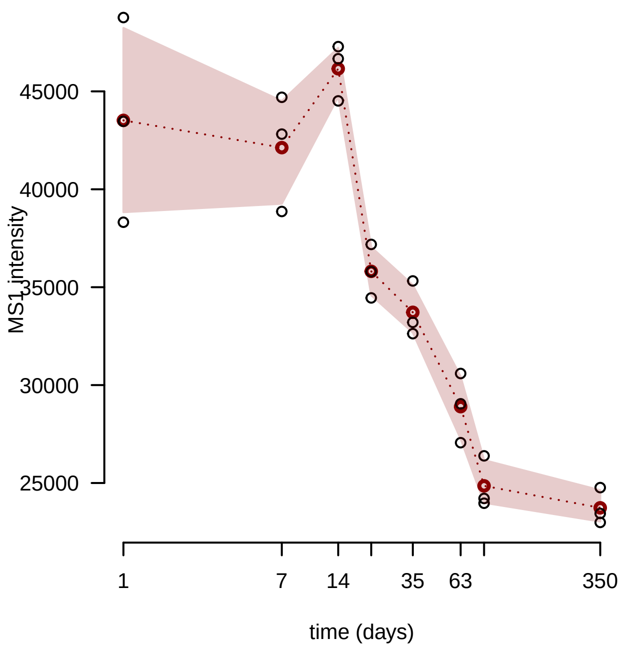

Q9CWM4
GN=Pfdn1
prefoldin 1

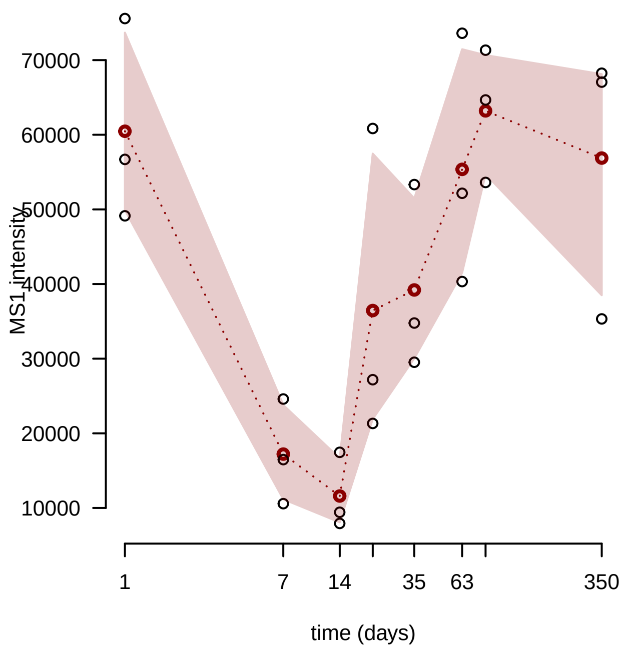

Q9CWN7
GN=Cnot11
CCR4−NOT transcription complex, subunit ...

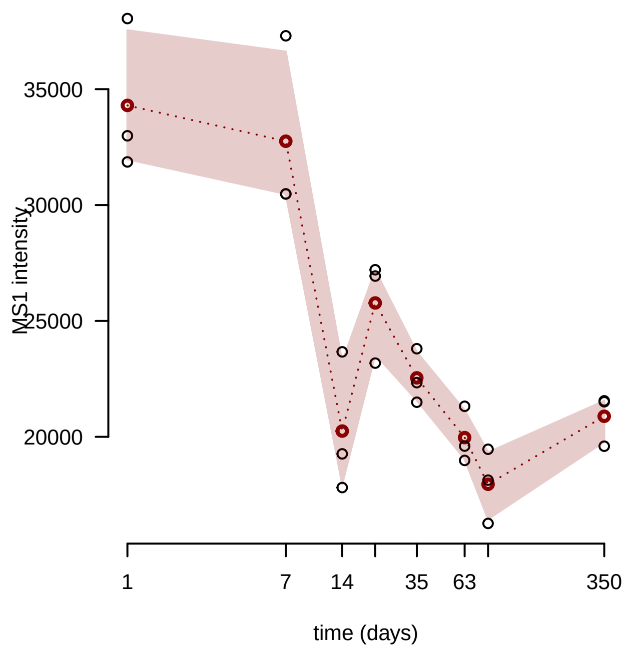

Q9CWP6
GN=Mospd2
motile sperm domain containing 2

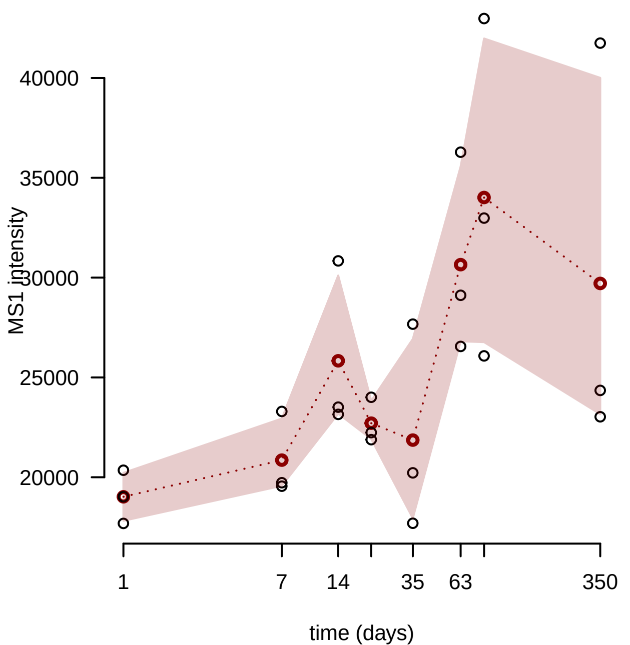

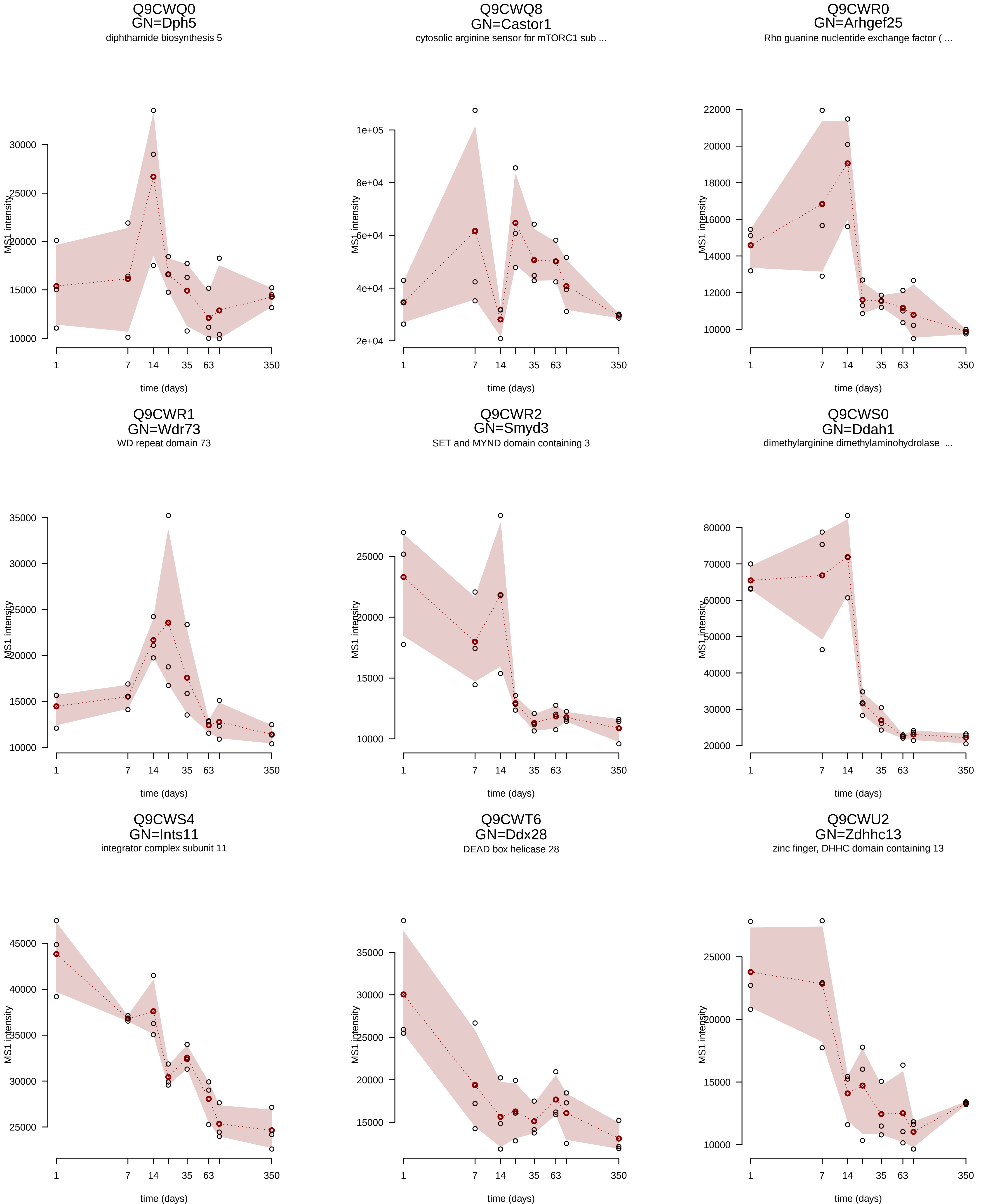

Q9CWQ0
GN=Dph5
diphthamide biosynthesis 5
Q9CWQ8
GN=Castor1
cytosolic arginine sensor for mTORC1 sub ...
Q9CWR0
GN=Arhgef25
Rho guanine nucleotide exchange factor (...
Q9CWR1
GN=Wdr73
WD repeat domain 73
Q9CWR2
GN=Smyd3
SET and MYND domain containing 3
Q9CWS0
GN=Ddah1
dimethylarginine dimethylaminohydrolase ...
Q9CWS4
GN=Ints11
integrator complex subunit 11
Q9CWT6
GN=Ddx28
DEAD box helicase 28
Q9CWU2
GN=Zdhhc13
zinc finger, DHHC domain containing 13
MS1 intensity
time (days)
1
7
14
35
63
350

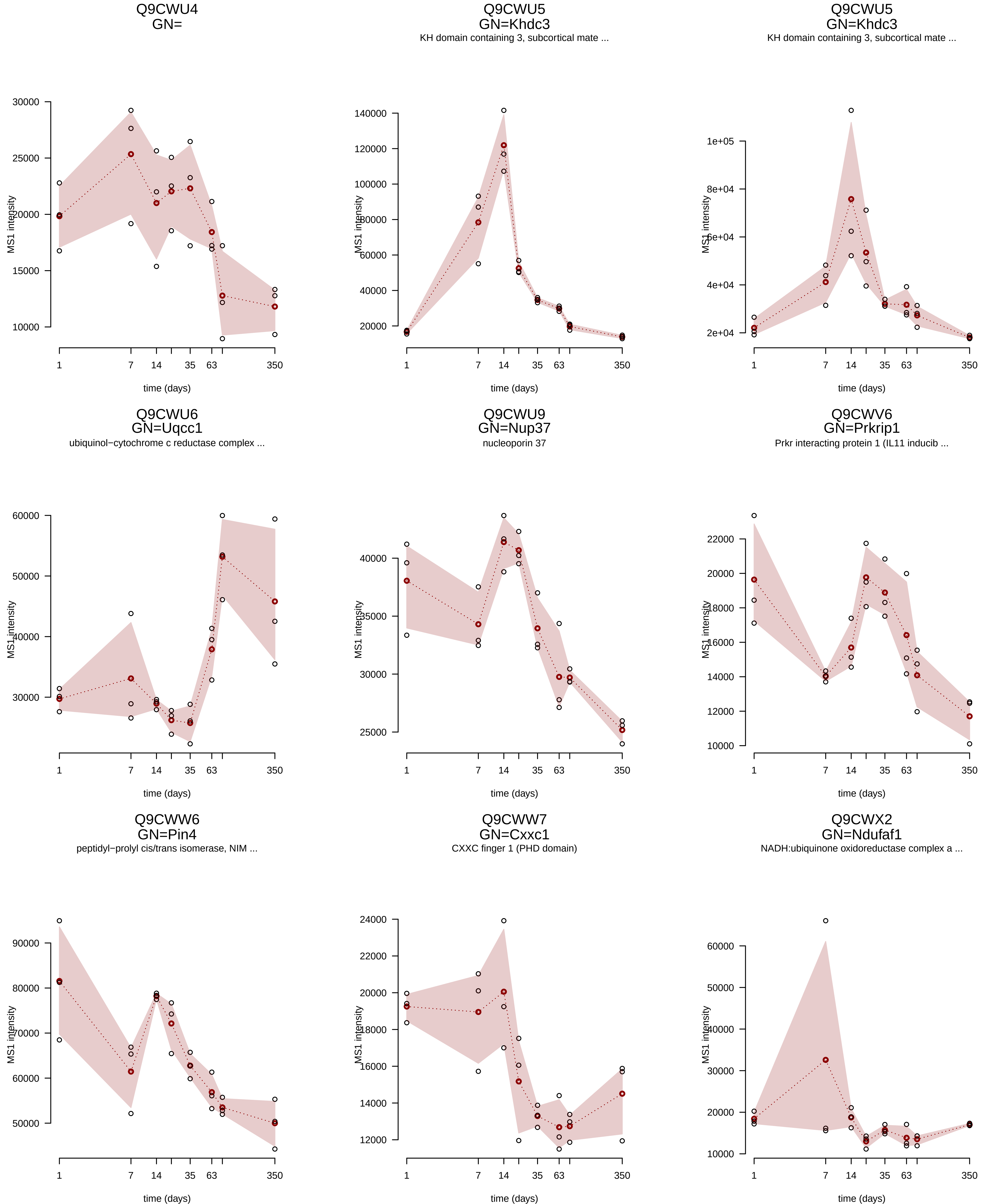
Q9CWU4
GN=
Q9CWU5
GN=Khdc3
KH domain containing 3, subcortical mate ...
Q9CWU5
GN=Khdc3
KH domain containing 3, subcortical mate ...
Q9CWU6
GN=Uqcc1
ubiquinol−cytochrome c reductase complex ...
Q9CWU9
GN=Nup37
nucleoporin 37
Q9CWV6
GN=Prkrip1
Prkr interacting protein 1 (IL11 inducib ...
Q9CWW6
GN=Pin4
peptidyl−prolyl cis/trans isomerase, NIM ...
Q9CWW7
GN=Cxxc1
CXXC finger 1 (PHD domain)
Q9CWX2
GN=Ndufaf1
NADH:ubiquinone oxidoreductase complex a ...
MS1 intensity
time (days)
1
7
14
35
63
350

Q9CWX4
GN=Rpusd4
RNA pseudouridylate synthase domain cont ...

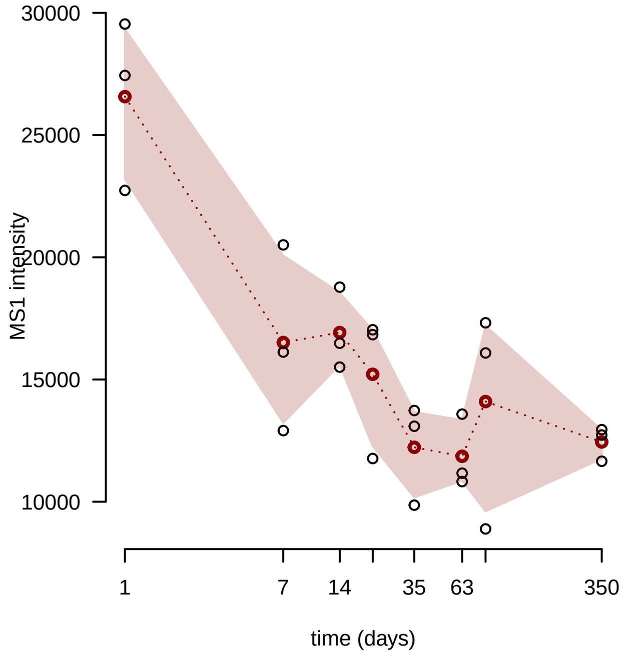

Q9CWX9
GN=Ddx47
DEAD box helicase 47

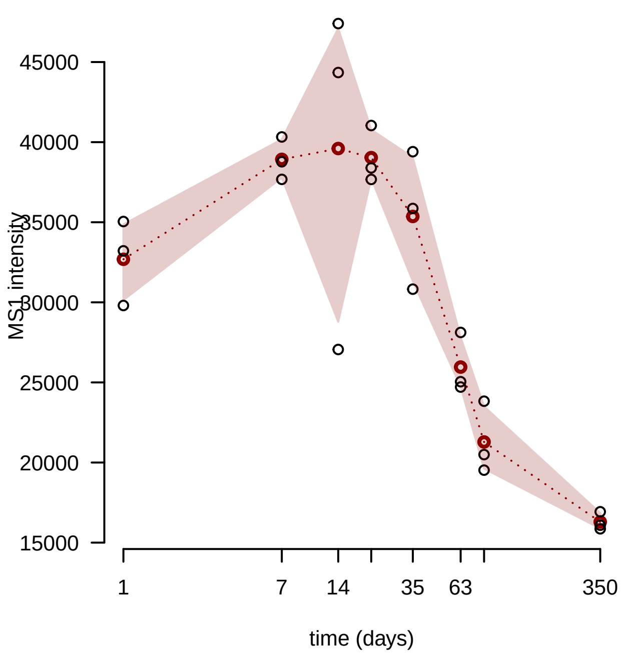

Q9CWY3
GN=Setd6
SET domain containing 6

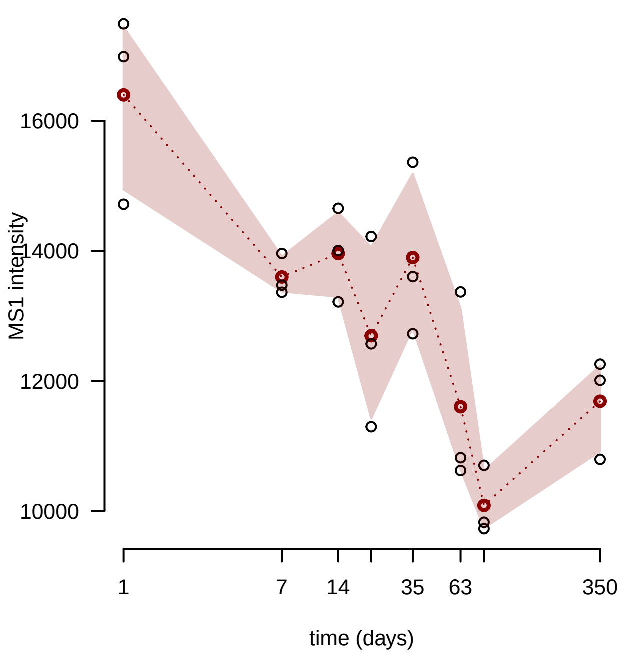

Q9CWY8
GN=Rnaseh2a
ribonuclease H2, large subunit

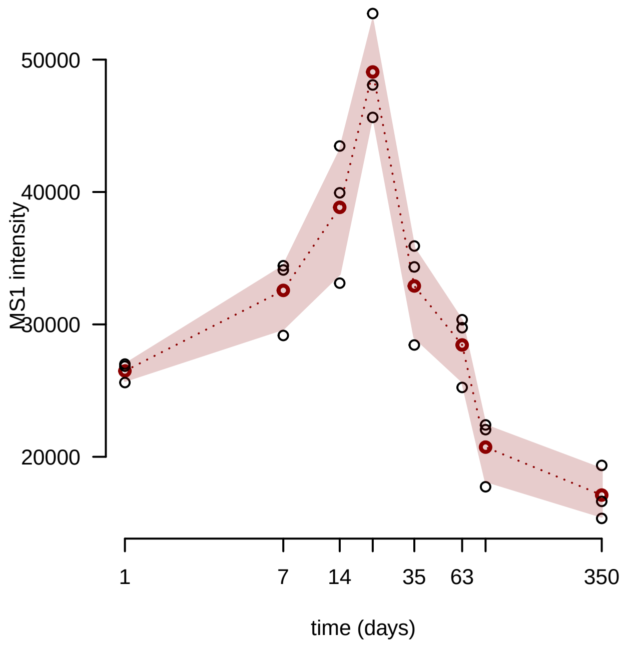

Q9CWZ3
GN=Rbm8a
RNA binding motif protein 8a

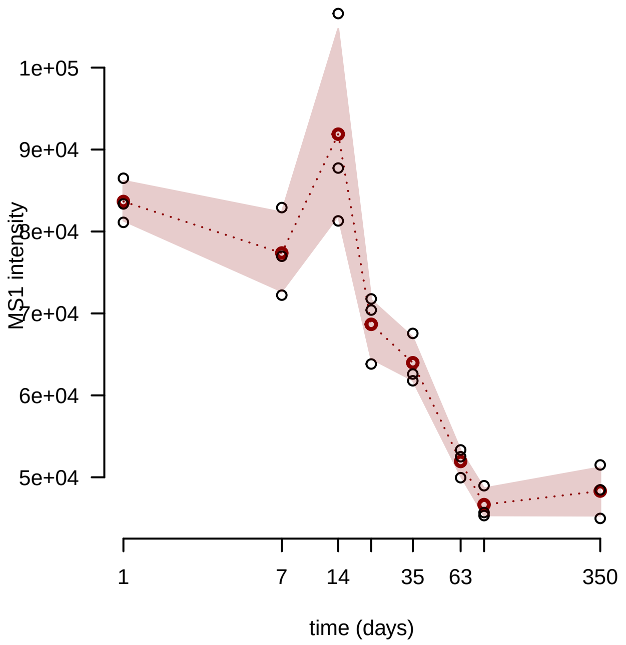

Q9CWZ7
GN=Napg
N−ethylmaleimide sensitive fusion protei ...

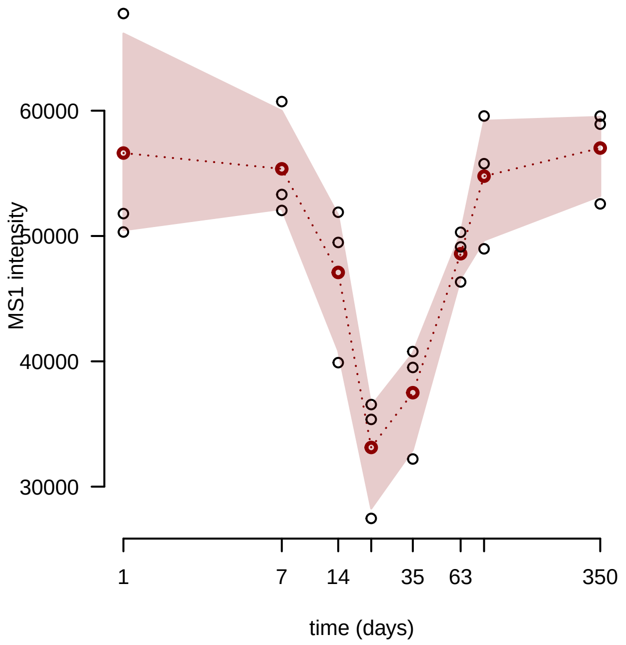

Q9CX00
GN=Ist1
increased sodium tolerance 1 homolog (ye ...

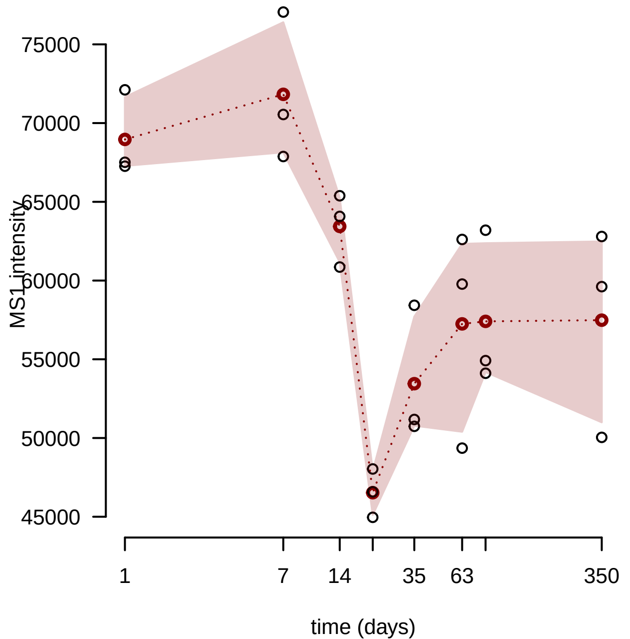

Q9CX11
GN=Utp23
UTP23 small subunit processome component

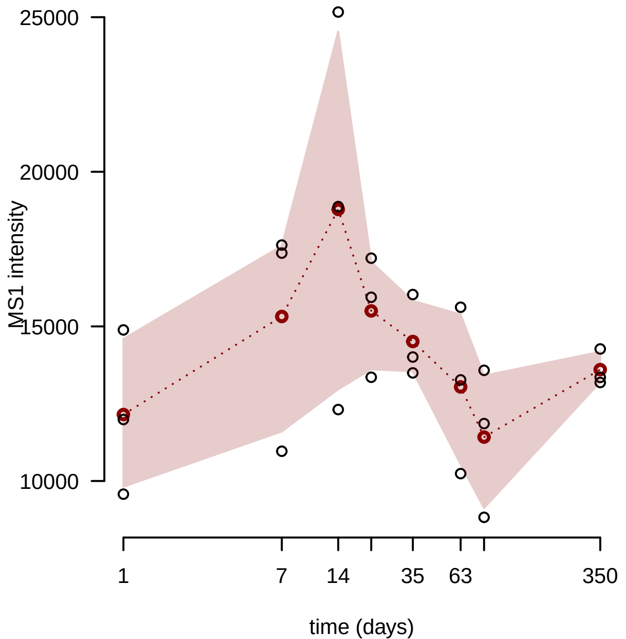

Q9CX13
GN=Cnih4
cornichon family AMPA receptor auxiliary ...

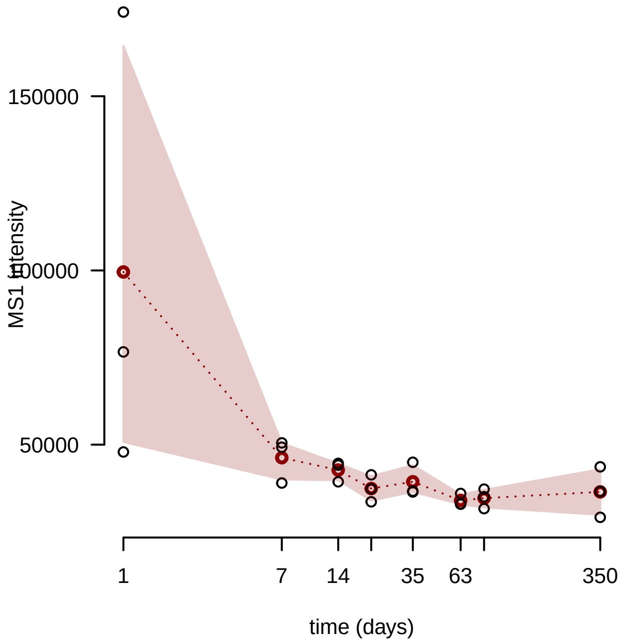

Q9CX30
GN=Yif1b
Yip1 interacting factor homolog B (S. ce ...

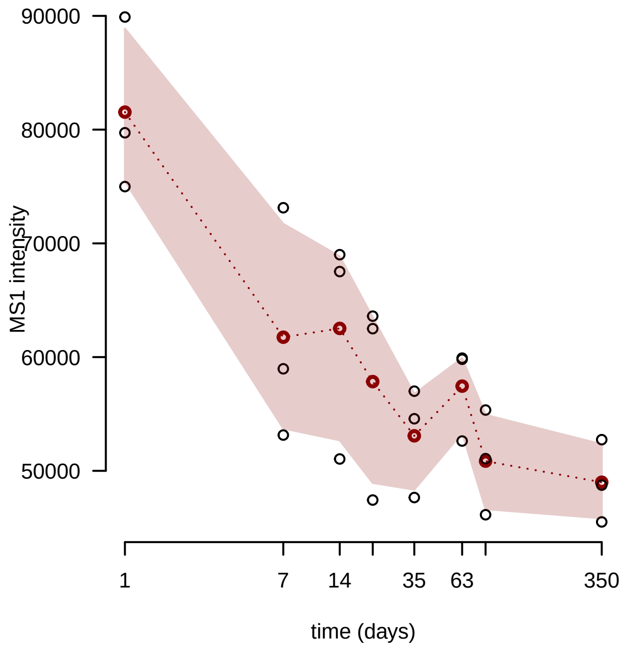

Q9CX34
GN=Sugt1
SGT1, suppressor of G2 allele of SKP1 (S ...

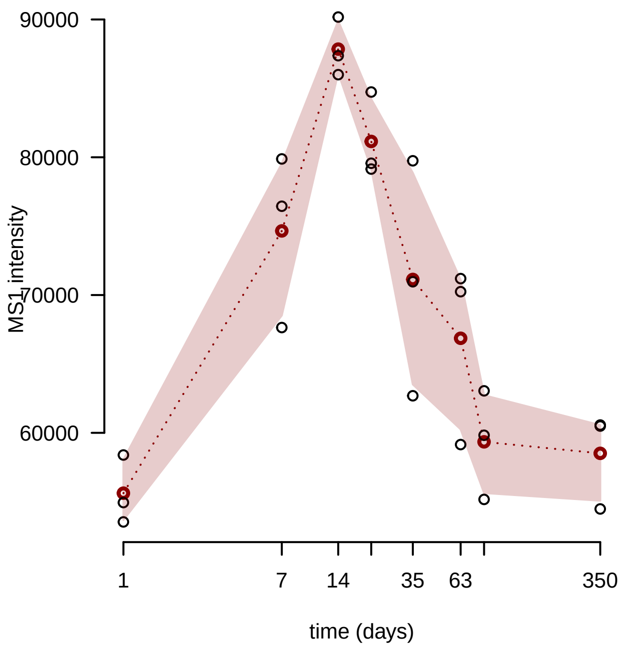

Q9CX48
GN=Zcchc10
zinc finger, CCHC domain containing 10

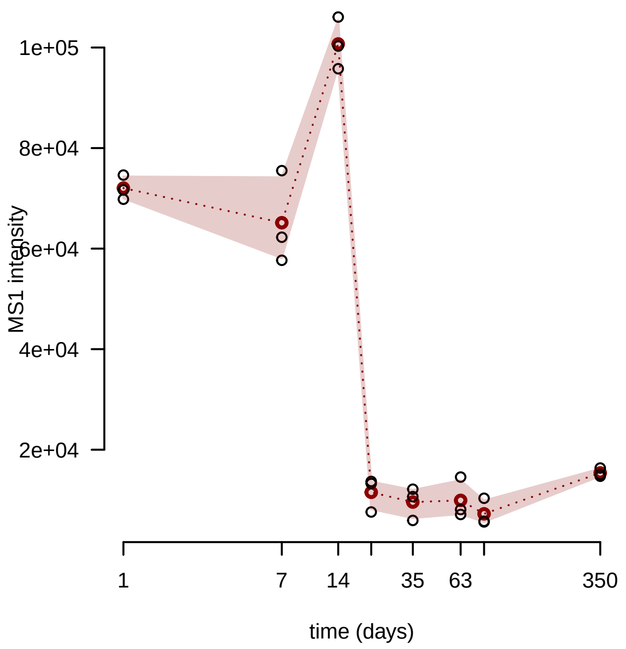

Q9CX53
GN=Gemin6
gem nuclear organelle associated protein ...

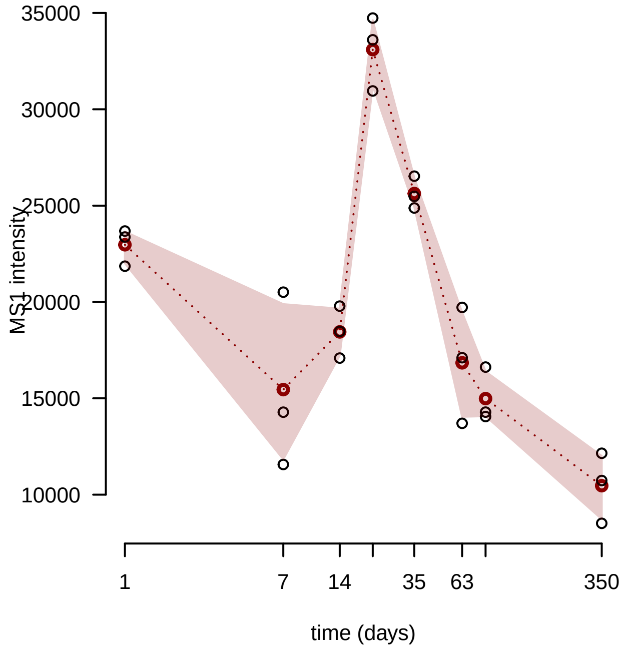

Q9CX56
GN=Psmd8
proteasome (prosome, macropain) 26S subu ...

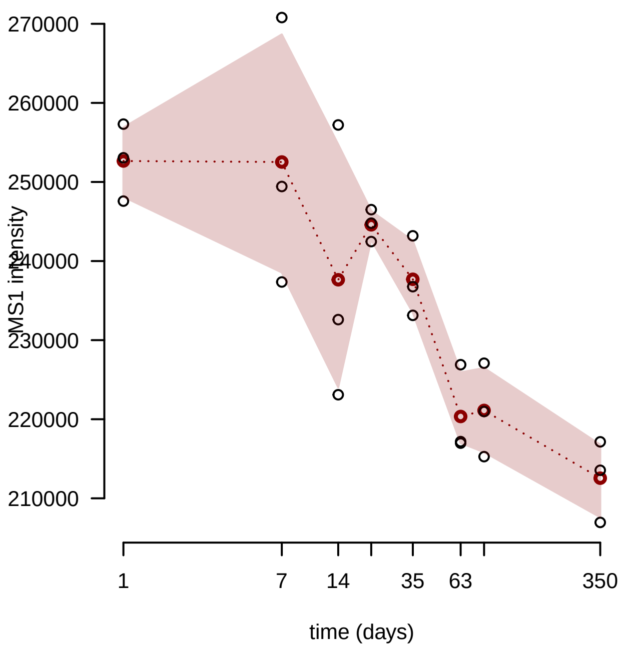

Q9CX60
GN=Lbh
limb−bud and heart

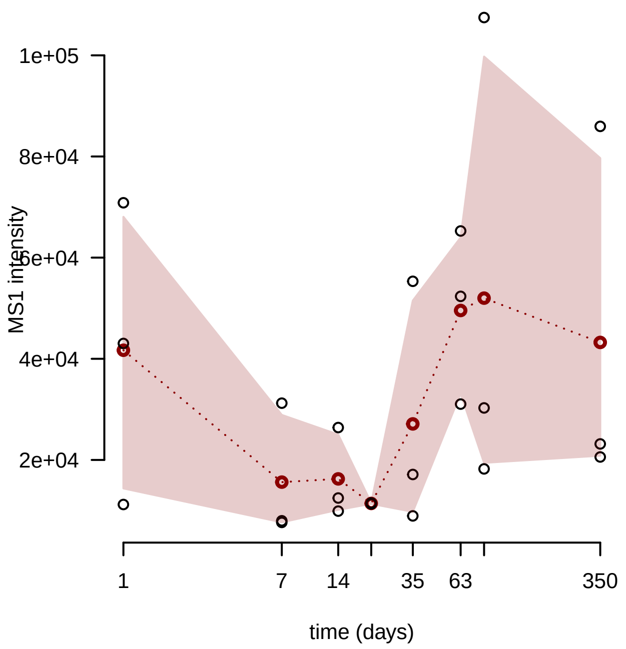

Q9CX66
GN=D10Wsu102e

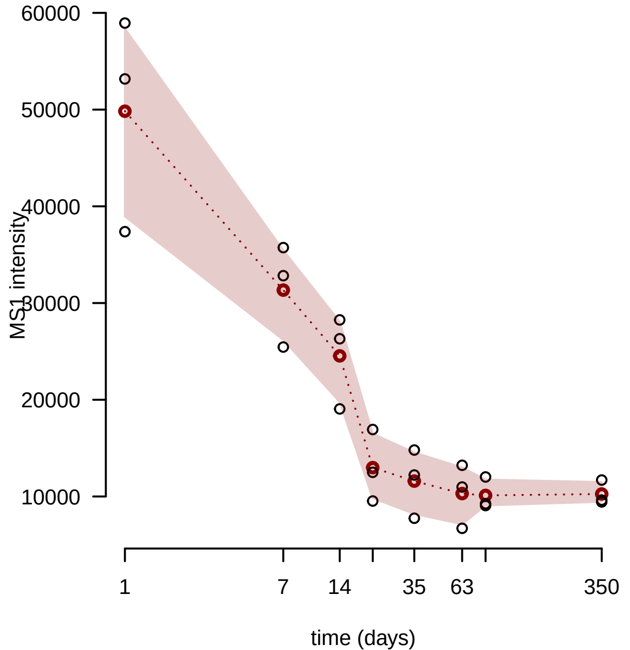

Q9CX80
GN=Cygb
cytoglobin

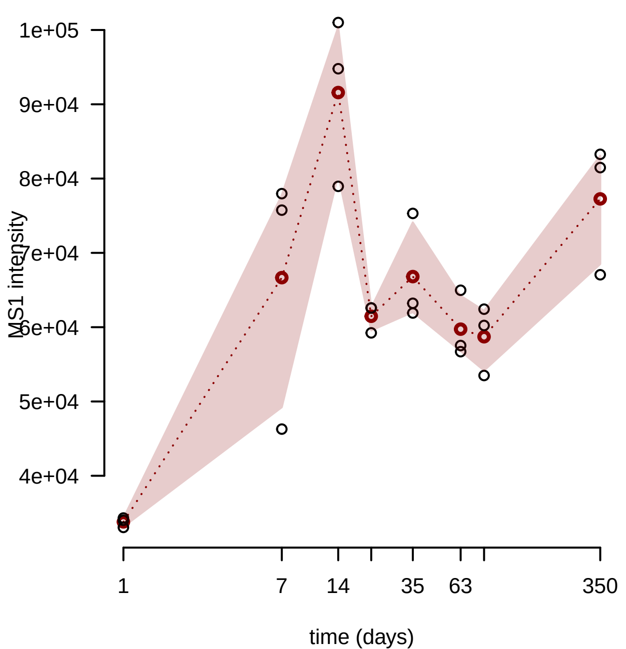

Q9CX83
GN=Armcx1
armadillo repeat containing, X−linked 1

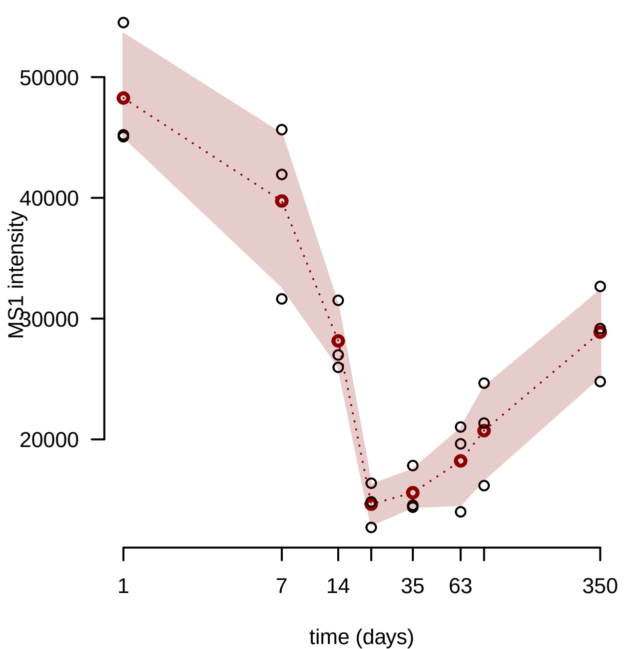

Q9CX86
GN=Hnrnpa0
heterogeneous nuclear ribonucleoprotein ...

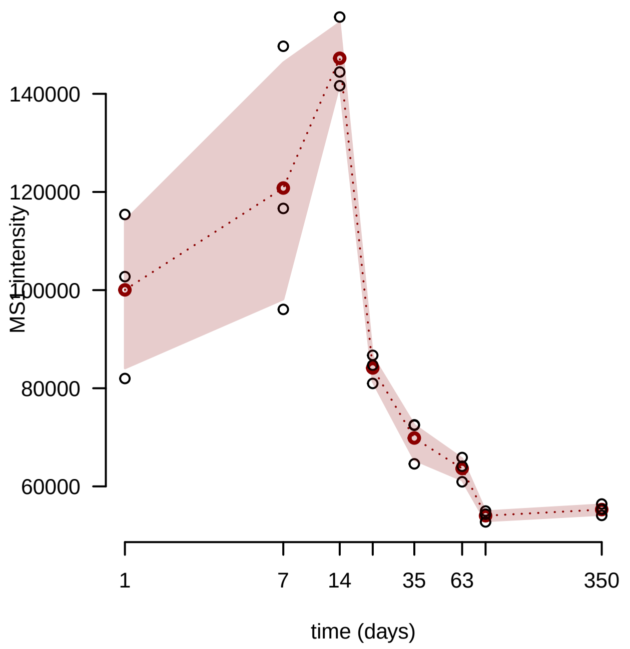

Q9CX97
GN=Wdr55
WD repeat domain 55

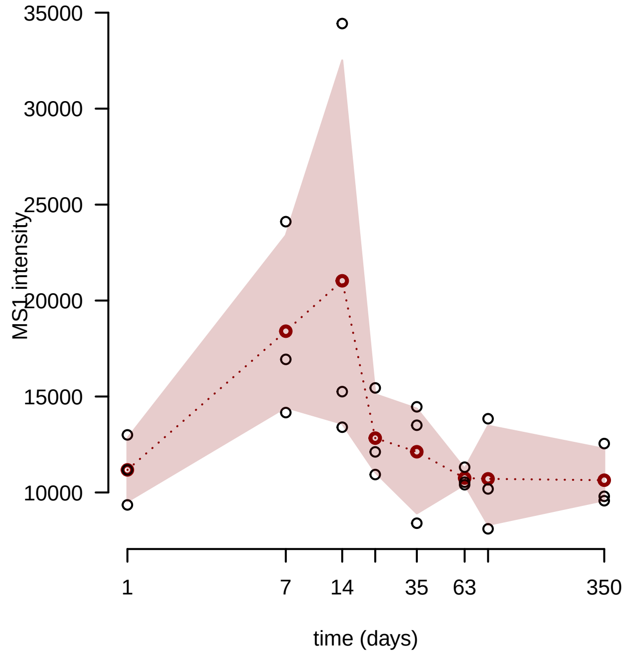

Q9CXA2
GN=L3hypdh
L−3−hydroxyproline dehydratase (trans−)

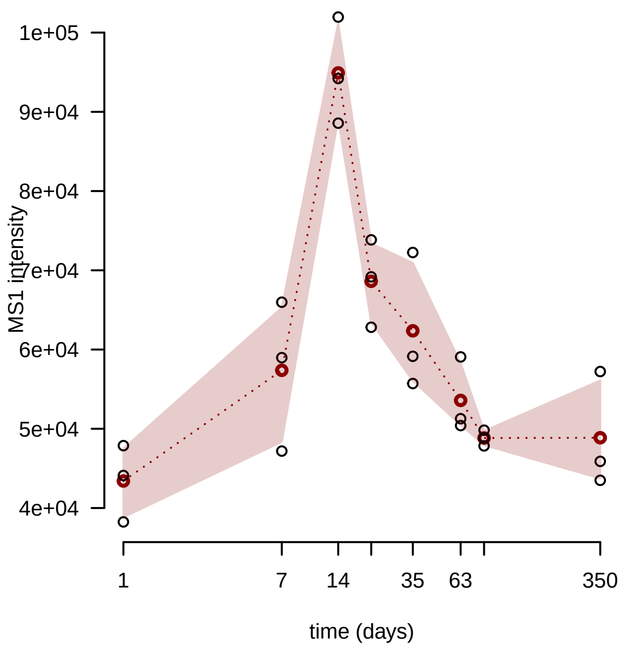

Q9CXC3
GN=Mgme1
mitochondrial genome maintenance exonucl ...

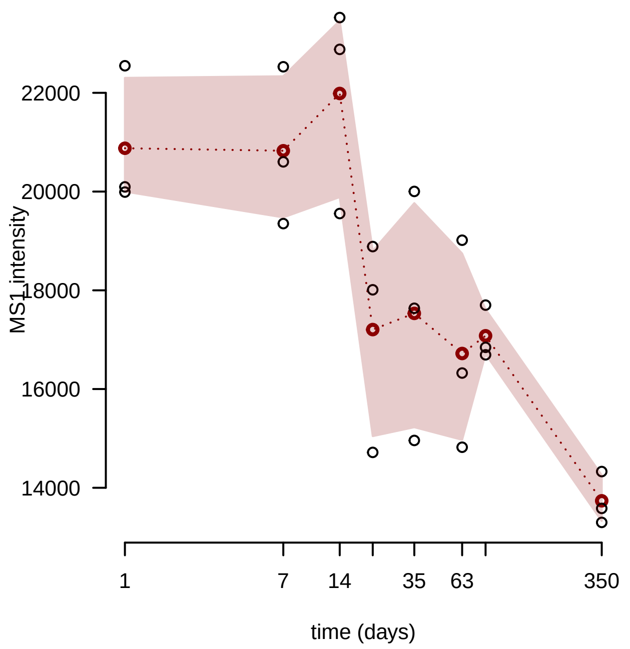

Q9CXD6
GN=Mcur1
mitochondrial calcium uniporter regulato ...

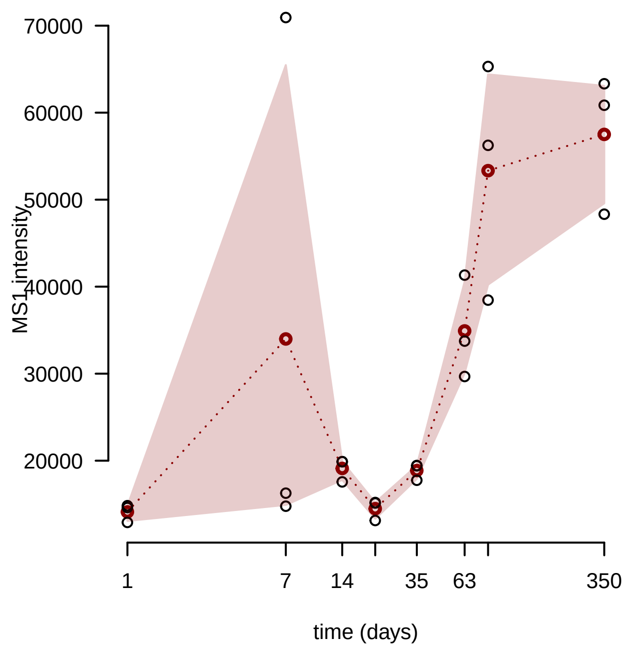

Q9CXD9
GN=Lrrc17
leucine rich repeat containing 17

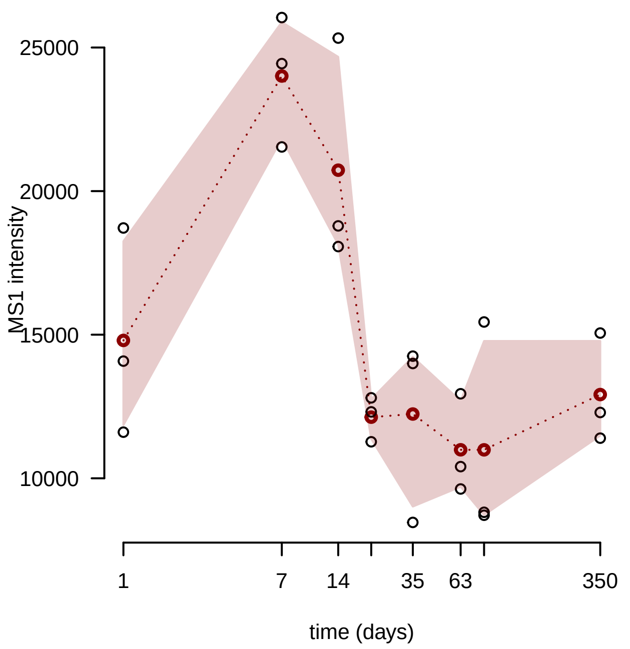

Q9CXE0
GN=Prdm5
PR domain containing 5

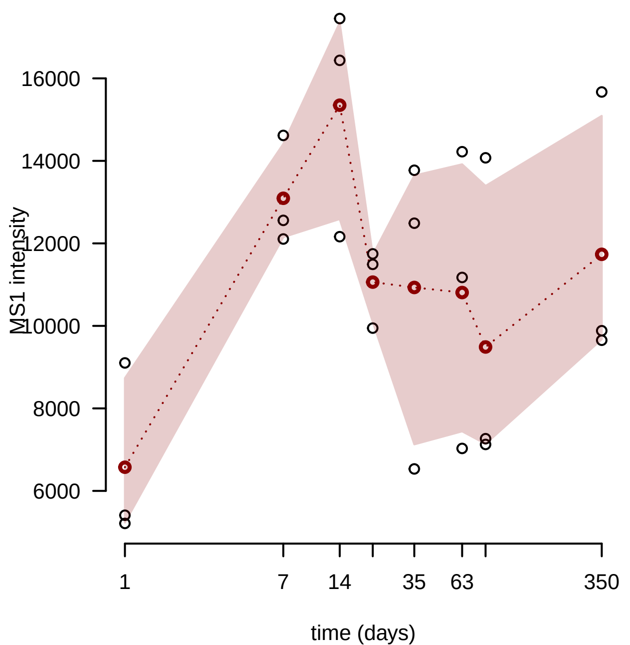

Q9CXE2
GN=Bcl7a
B cell CLL/lymphoma 7A

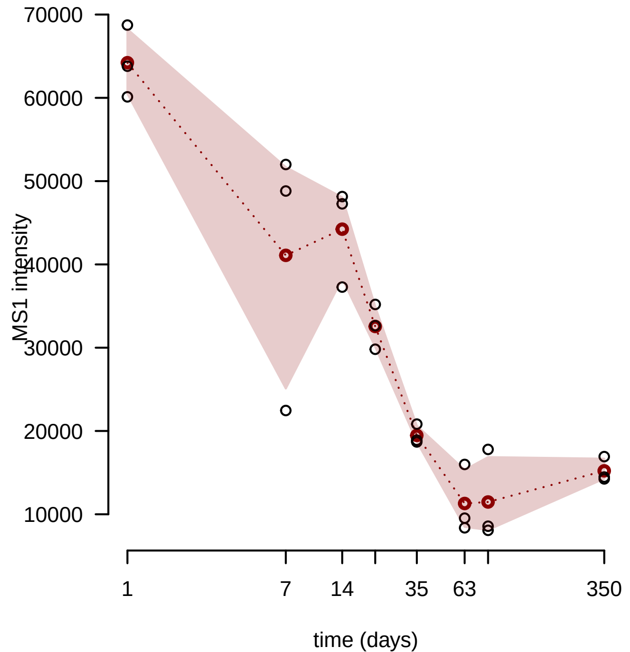

Q9CXE7
GN=Tmed5
transmembrane p24 trafficking protein 5

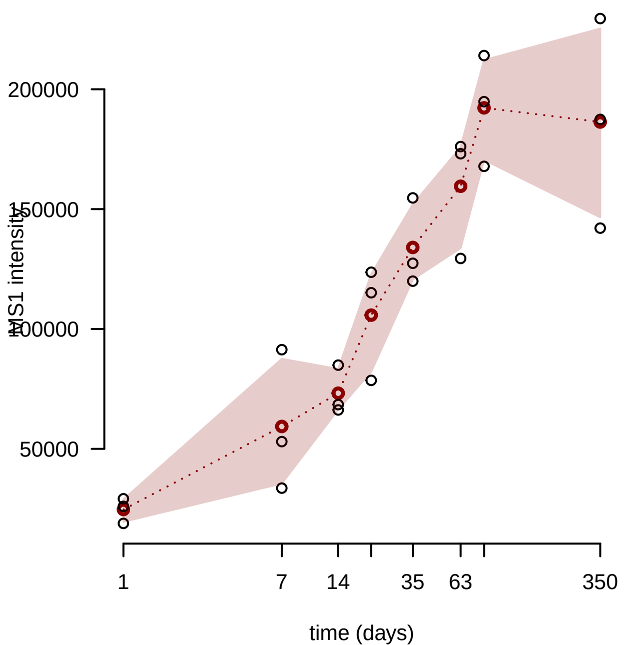

Q9CXF4
GN=Tbc1d15
TBC1 domain family, member 15

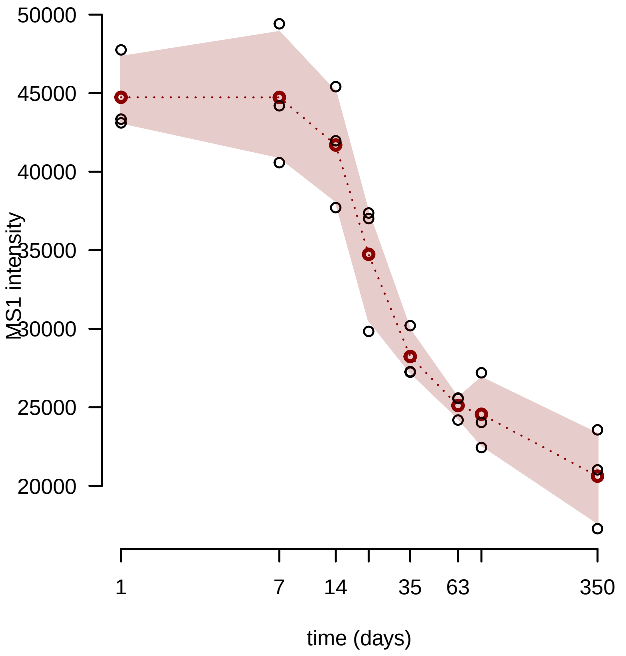

Q9CXF7
GN=Chd1l
chromodomain helicase DNA binding protei ...

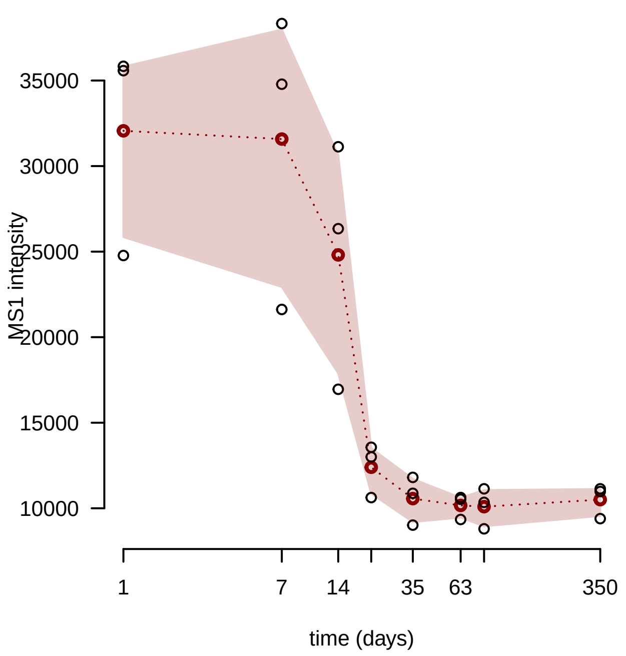

Q9CXG3
GN=Ppil4
peptidylprolyl isomerase (cyclophilin)−l ...

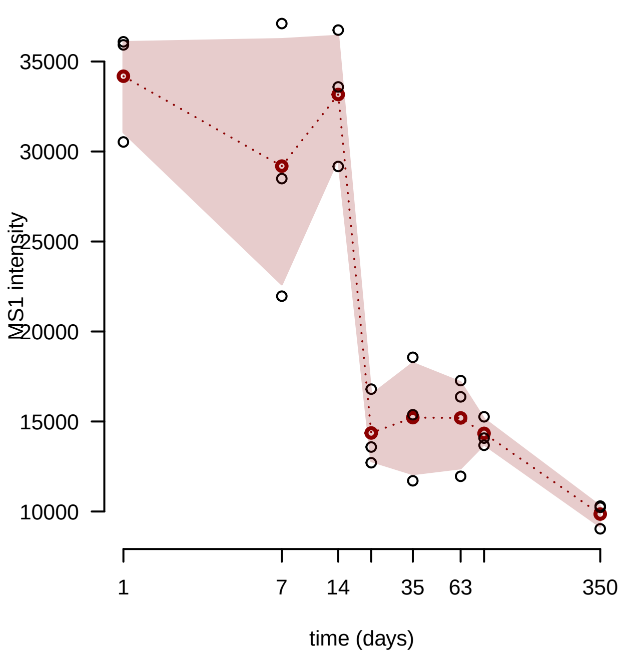

Q9CXI0
GN=Coq5
coenzyme Q5 methyltransferase

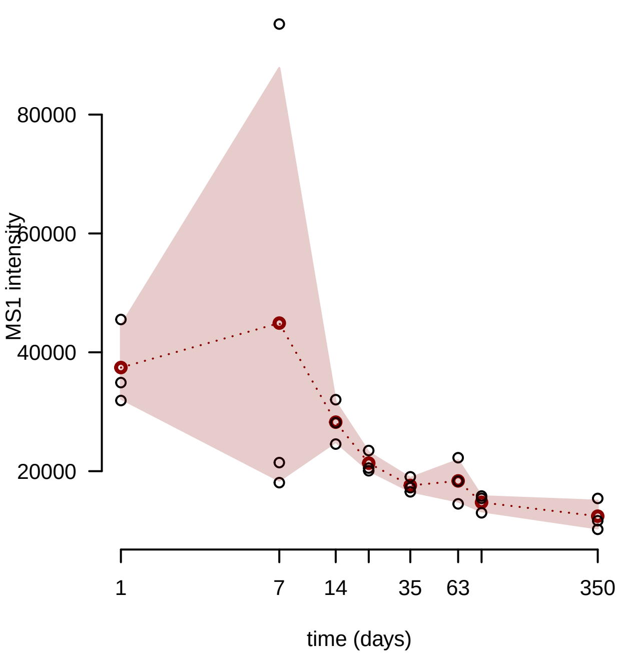

Q9CXI3
GN=Moxd1
monooxygenase, DBH−like 1

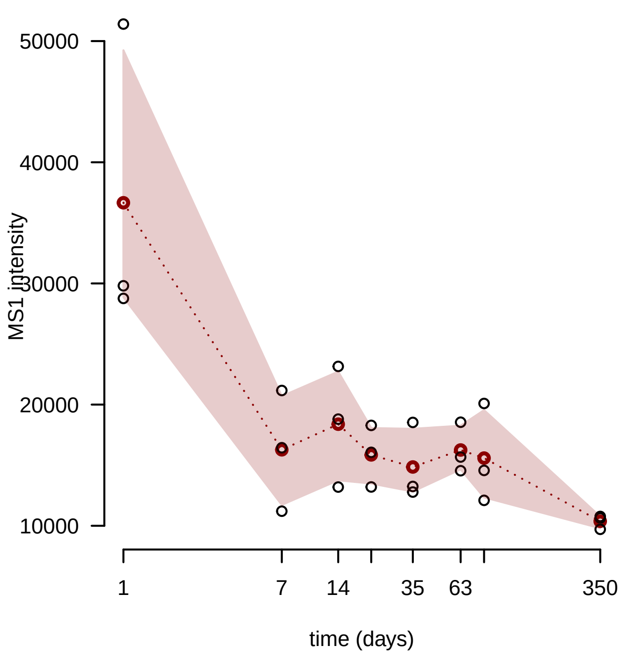

Q9CXI5
GN=Manf
mesencephalic astrocyte−derived neurotro ...

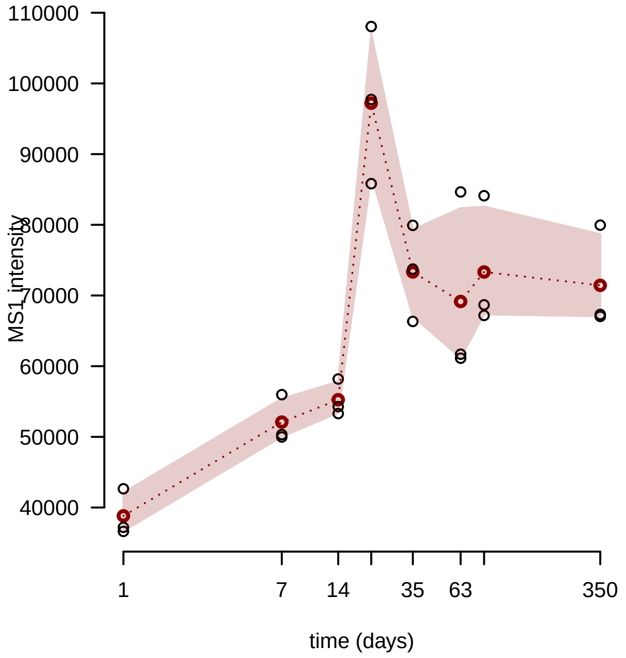

Q9CXJ1
GN=Ears2
glutamyl−tRNA synthetase 2, mitochondria ...

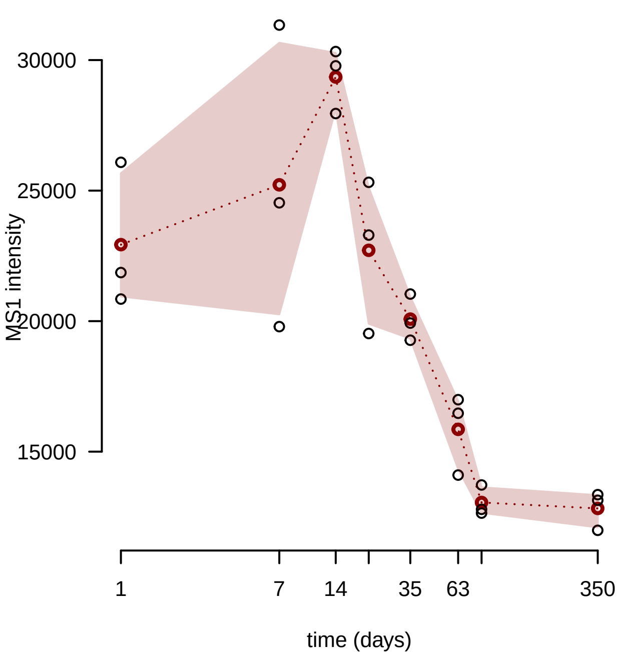

Q9CXJ4
GN=Abcb8
ATP−binding cassette, sub−family B (MDR/ ...

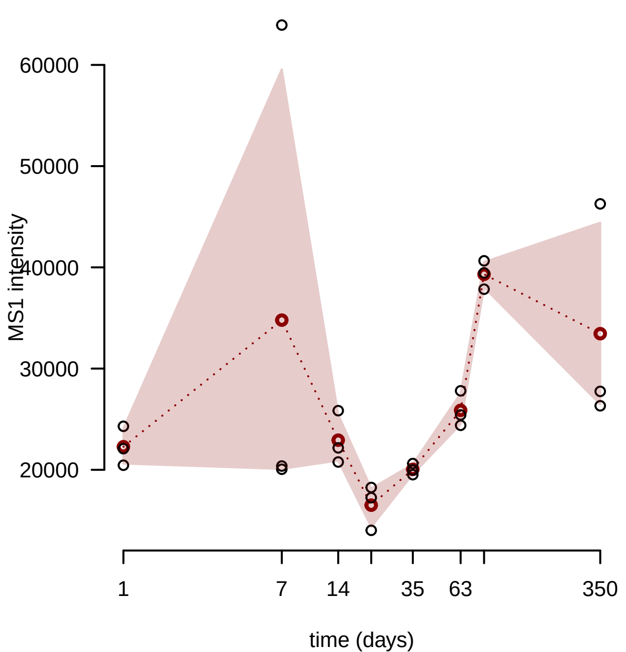

Q9CXK8
GN=Nip7
NIP7, nucleolar pre−rRNA processing prot ...

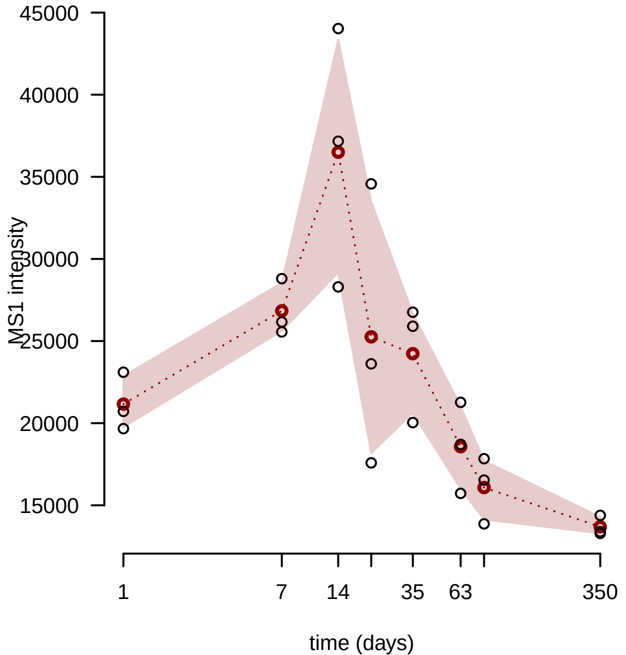

Q9CXK9
GN=Rbm33
RNA binding motif protein 33

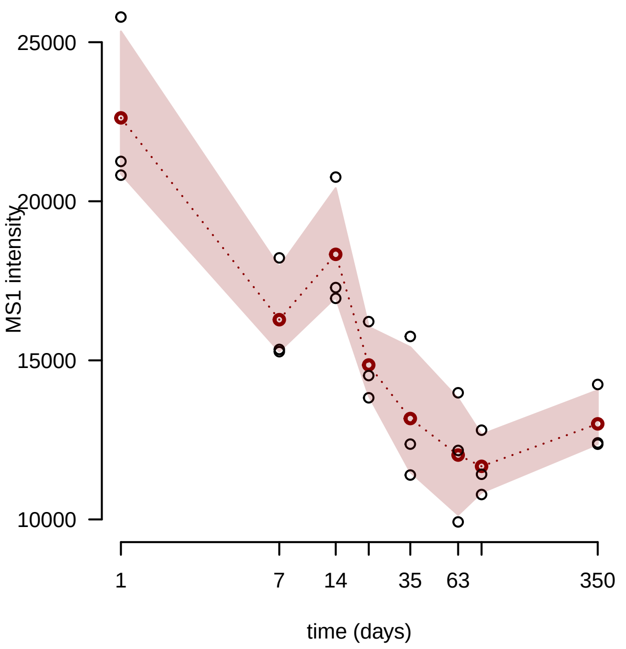

Q9CXL3
GN=

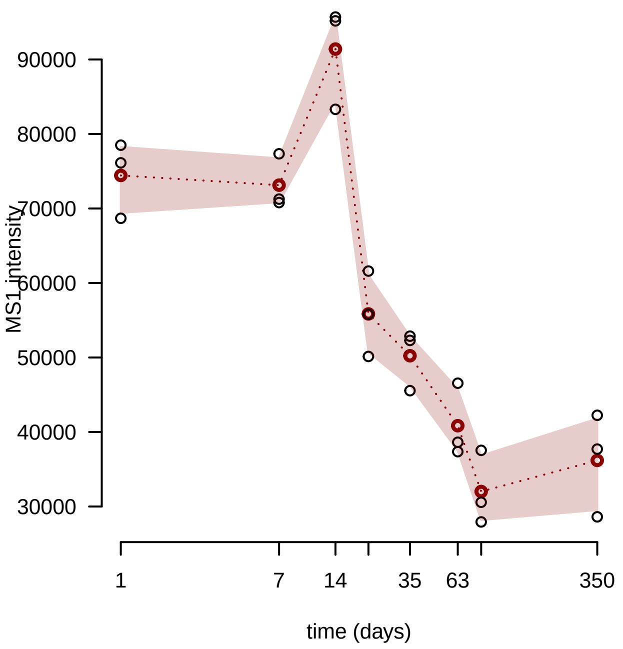

Q9CXN7
GN=Pbld2
phenazine biosynthesis−like protein doma ...

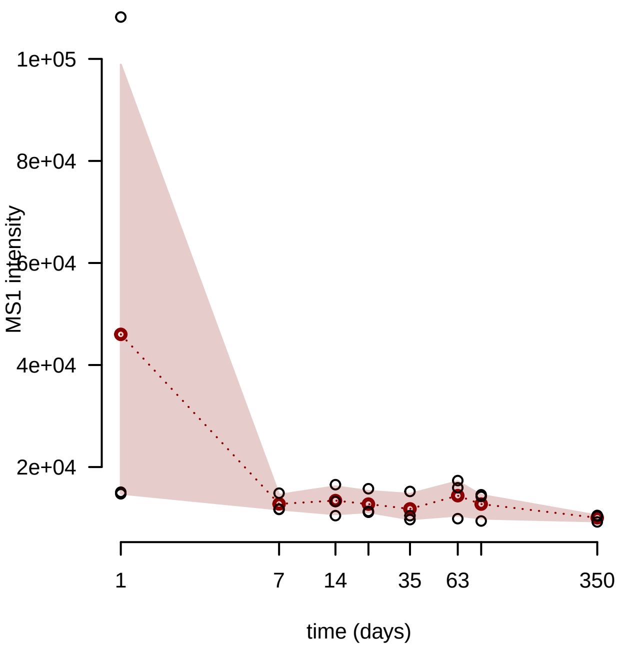

Q9CXR1
GN=Dhrs7
dehydrogenase/reductase (SDR family) mem ...

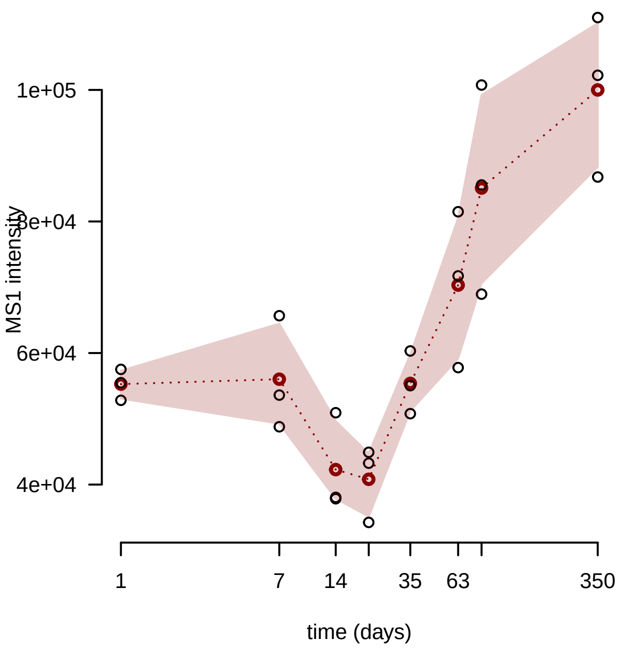

Q9CXS4
GN=Cenpv
centromere protein V

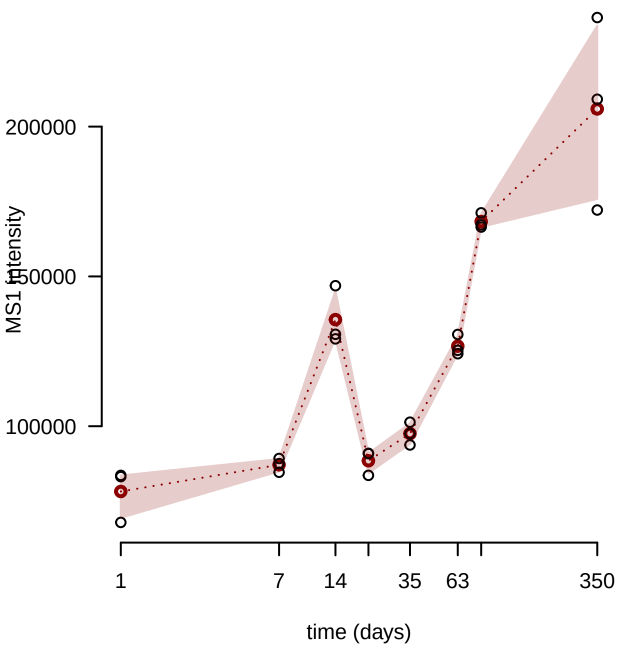

Q9CXT7
GN=Tmem192
transmembrane protein 192

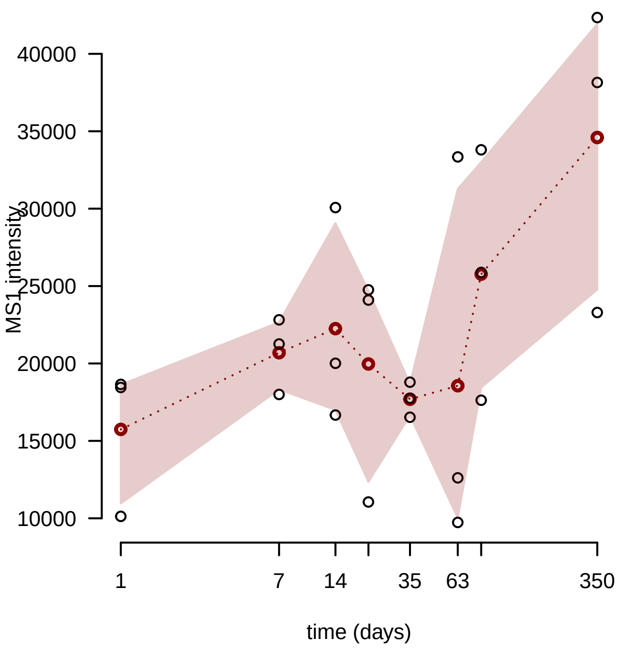

Q9CXT8
GN=Pmpcb
peptidase (mitochondrial processing) bet ...

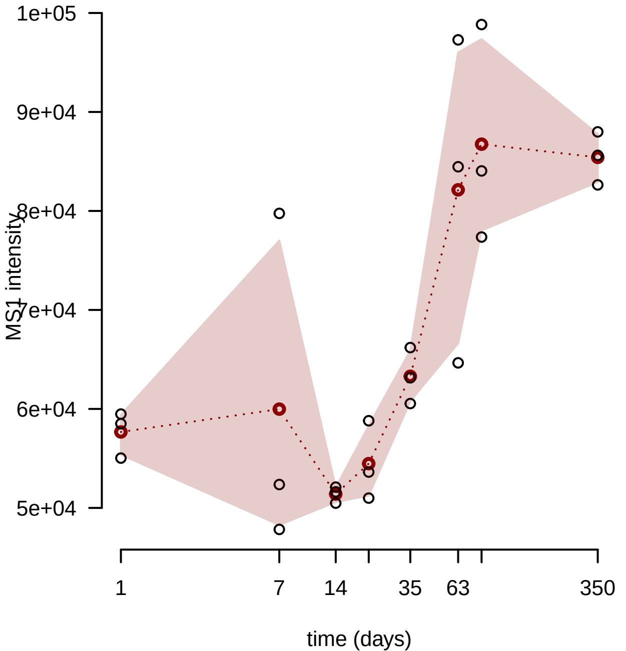

Q9CXU0
GN=Med10
mediator complex subunit 10

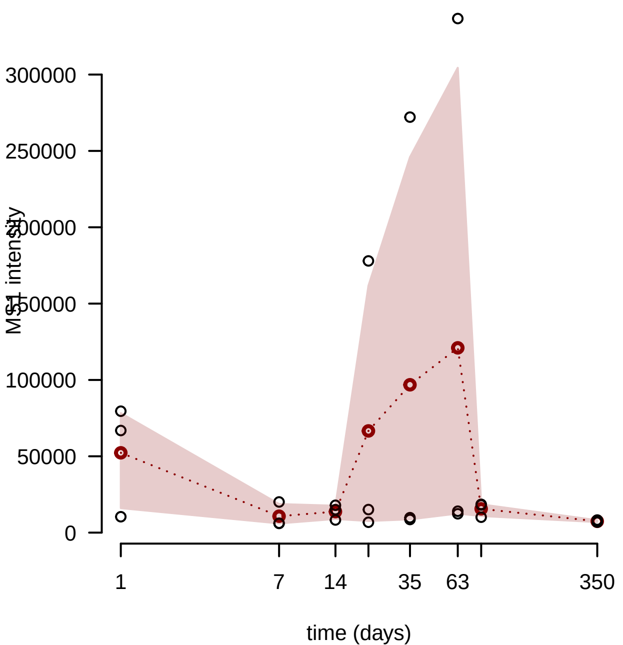

Q9CXU1
GN=Med31
mediator complex subunit 31

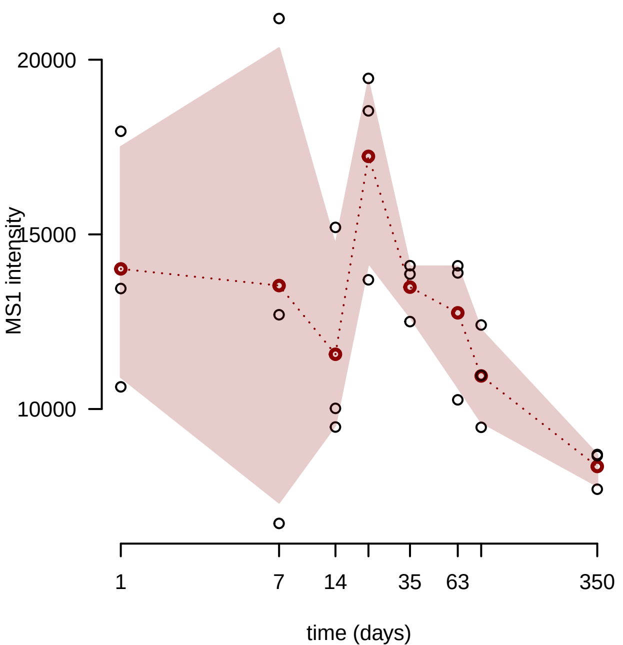

Q9CXU9
GN=Eif1b
eukaryotic translation initiation factor ...

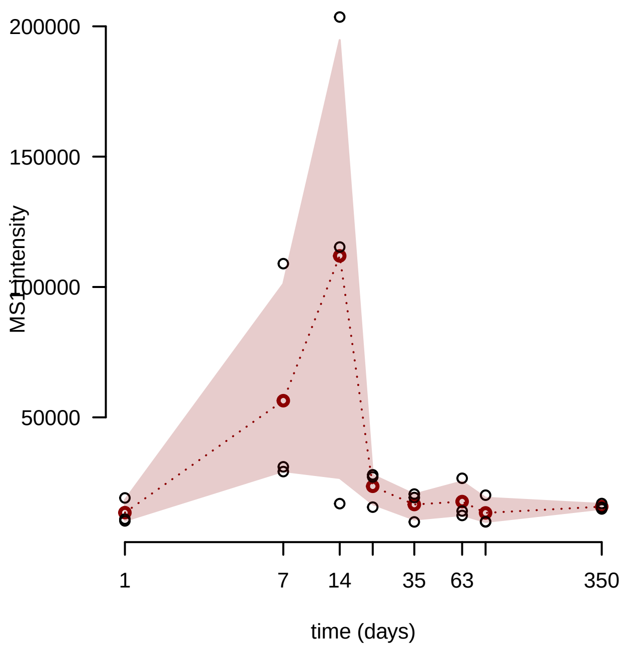

Q9CXV1
GN=Sdhd
succinate dehydrogenase complex, subunit ...

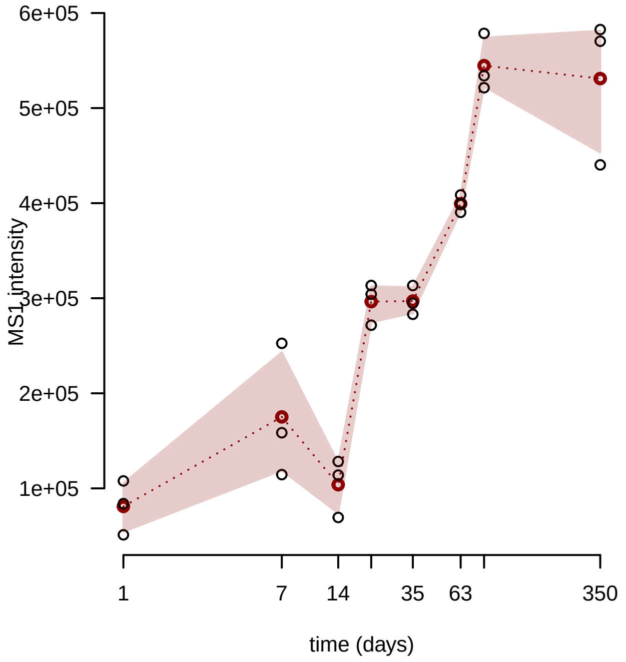

Q9CXV9
GN=Dcun1d5
DCN1, defective in cullin neddylation 1, ...

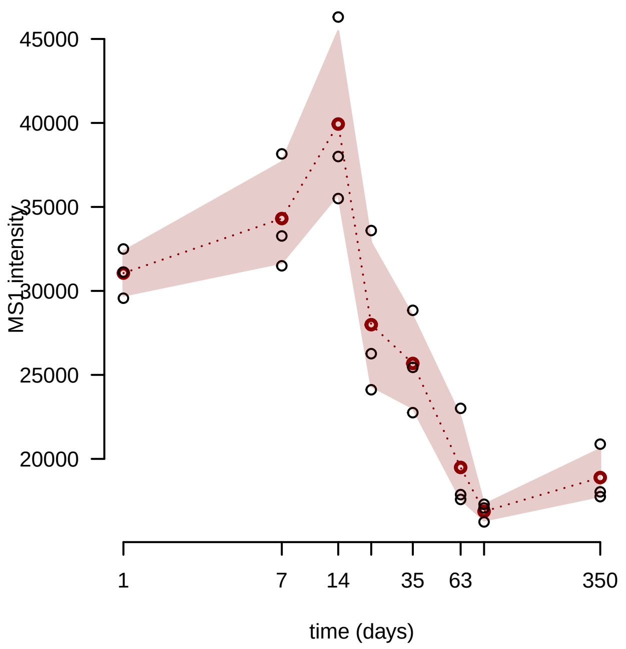

Q9CXW2
GN=Mrps22
mitochondrial ribosomal protein S22

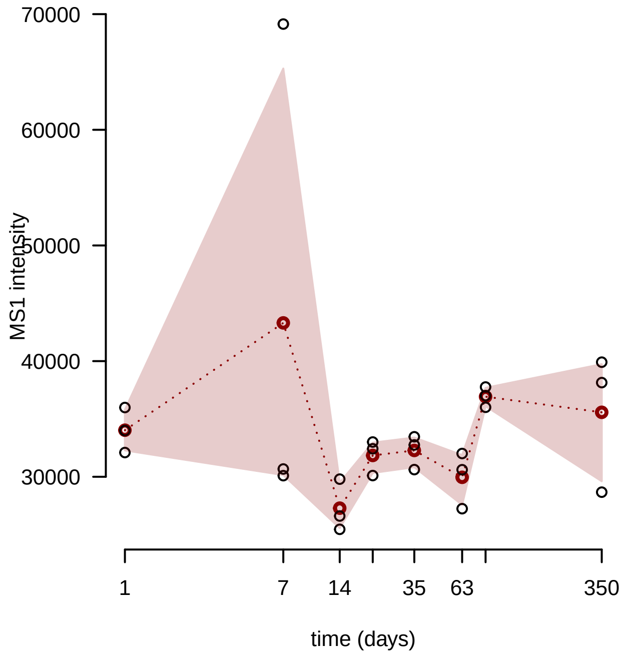

Q9CXW3
GN=Cacybp
calcyclin binding protein

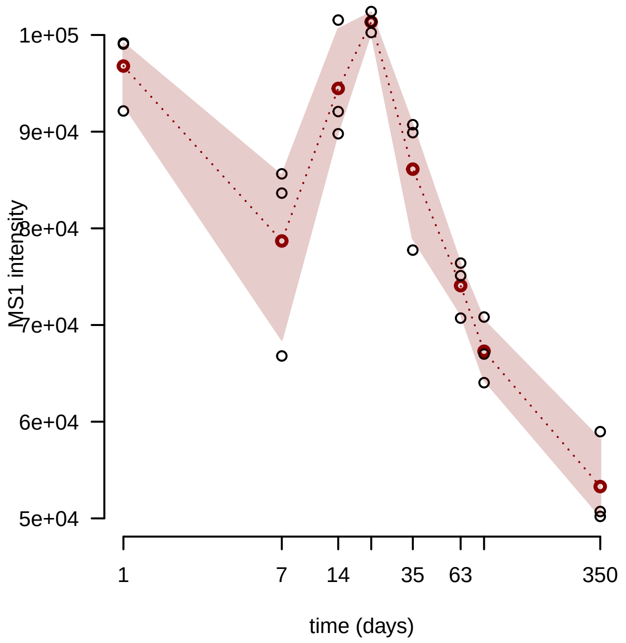

Q9CXW4
GN=Rpl11
ribosomal protein L11

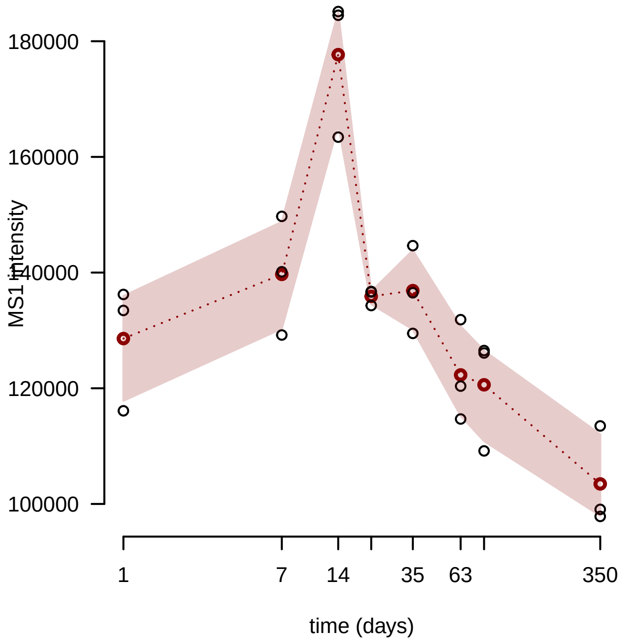

Q9CXX9
GN=Cuedc2
CUE domain containing 2

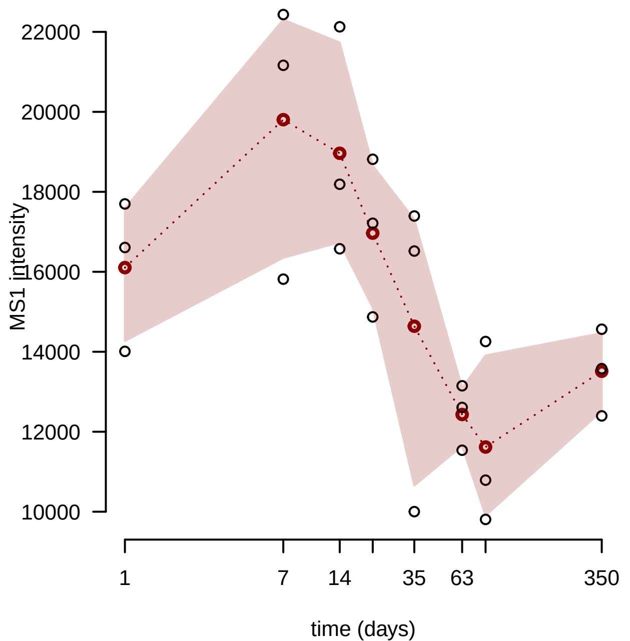

Q9CXY1
GN=Tmem175
transmembrane protein 175

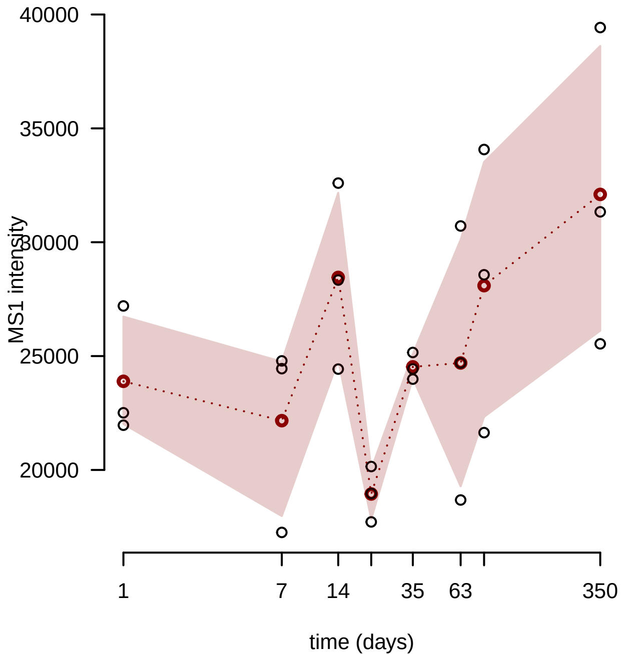

Q9CXY6
GN=Ilf2
interleukin enhancer binding factor 2

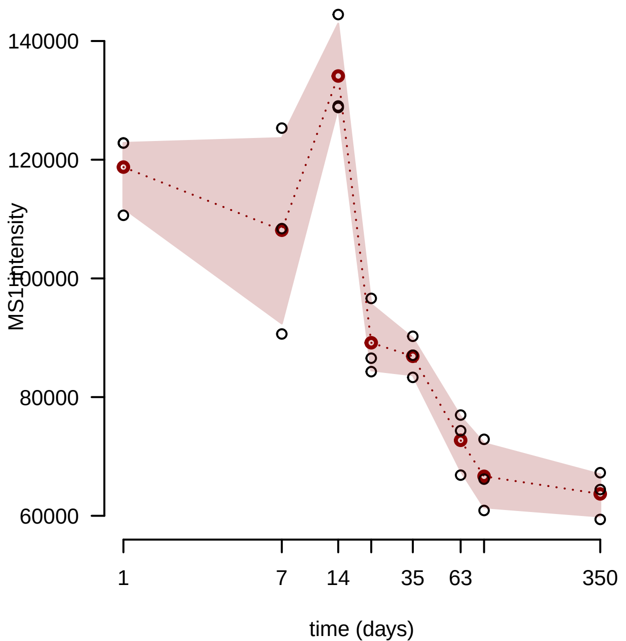

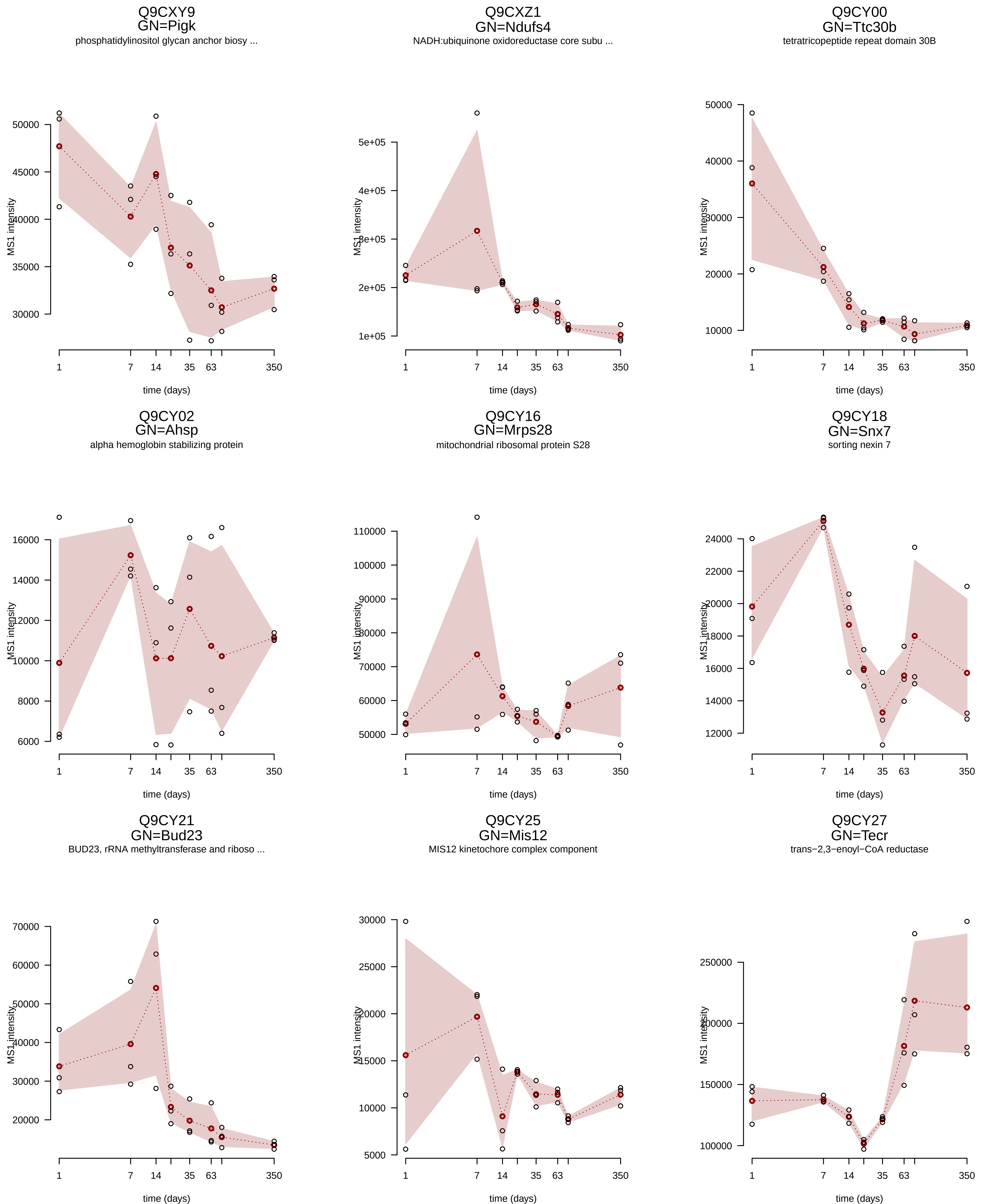
Q9CXY9
GN=Pigk
phosphatidylinositol glycan anchor biosy ...
MS1 intensity
50000
45000
40000
35000
30000
1
7
14
35
63
350
time (days)
Q9CXZ1
GN=Ndufs4
NADH:ubiquinone oxidoreductase core subu ...
MS1 intensity
5e+05
4e+05
3e+05
2e+05
1e+05
1
7
14
35
63
350
time (days)
Q9CY00
GN=Ttc30b
tetratricopeptide repeat domain 30B
MS1 intensity
50000
40000
30000
20000
10000
1
7
14
35
63
350
time (days)
Q9CY02
GN=Ahsp
alpha hemoglobin stabilizing protein
MS1 intensity
16000
14000
12000
10000
8000
6000
1
7
14
35
63
350
time (days)
Q9CY16
GN=Mrps28
mitochondrial ribosomal protein S28
MS1 intensity
110000
100000
90000
80000
70000
60000
50000
1
7
14
35
63
350
time (days)
Q9CY18
GN=Snx7
sorting nexin 7
MS1 intensity
24000
22000
20000
18000
16000
14000
12000
1
7
14
35
63
350
time (days)
Q9CY21
GN=Bud23
BUD23, rRNA methyltransferase and riboso ...
MS1 intensity
70000
60000
50000
40000
30000
20000
1
7
14
35
63
350
time (days)
Q9CY25
GN=Mis12
MIS12 kinetochore complex component
MS1 intensity
30000
25000
20000
15000
10000
5000
1
7
14
35
63
350
time (days)
Q9CY27
GN=Tecr
trans−2,3−enoyl−CoA reductase
MS1 intensity
250000
200000
150000
100000
1
7
14
35
63
350
time (days)

Q9CY28
GN=Gtpbp8
GTP−binding protein 8 (putative)

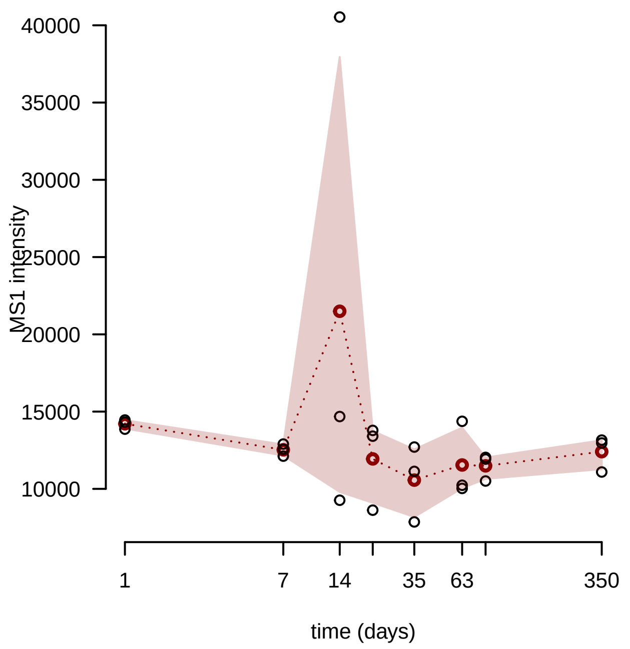

Q9CY34
GN=Ube2f
ubiquitin−conjugating enzyme E2F (putati ...

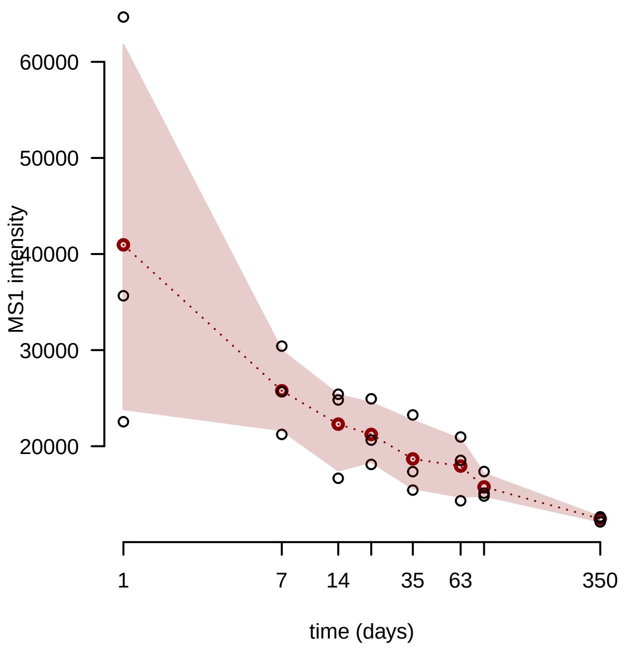

Q9CY45
GN=Eef1akmt1
EEF1A alpha lysine methyltransferase 1

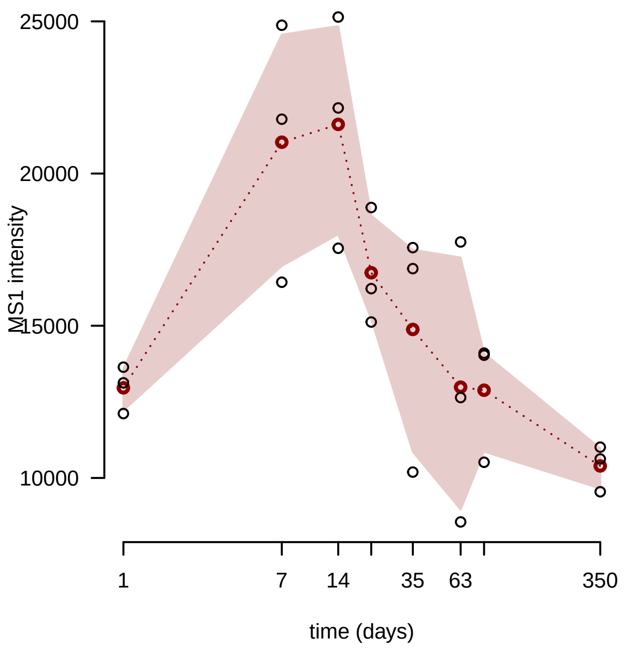

Q9CY50
GN=Ssr1
signal sequence receptor, alpha

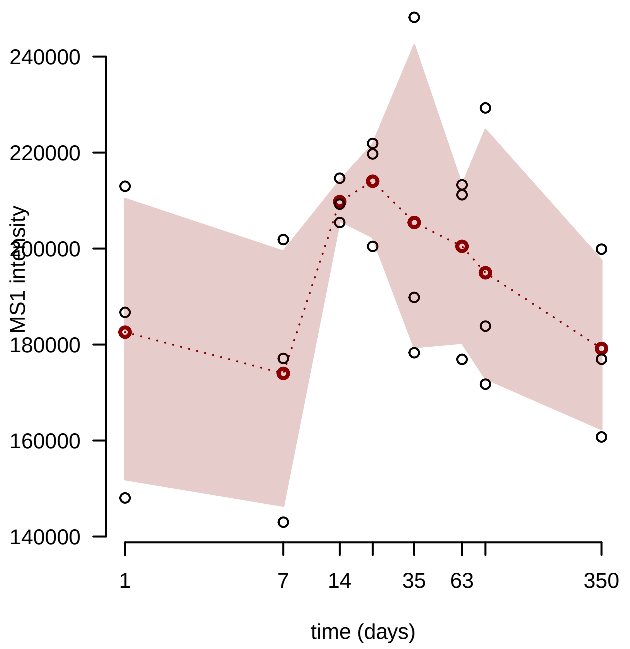

Q9CY52
GN=Thg1l
tRNA−histidine guanylyltransferase 1−lik ...

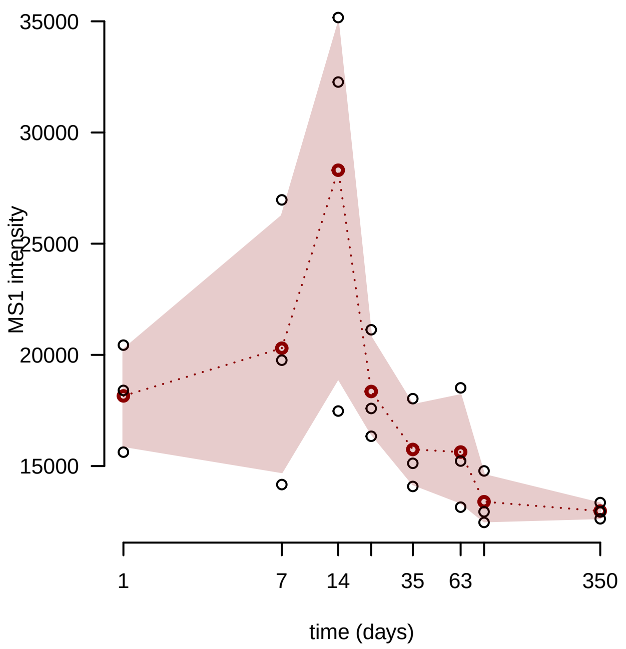

Q9CY57
GN=Chtop
chromatin target of PRMT1

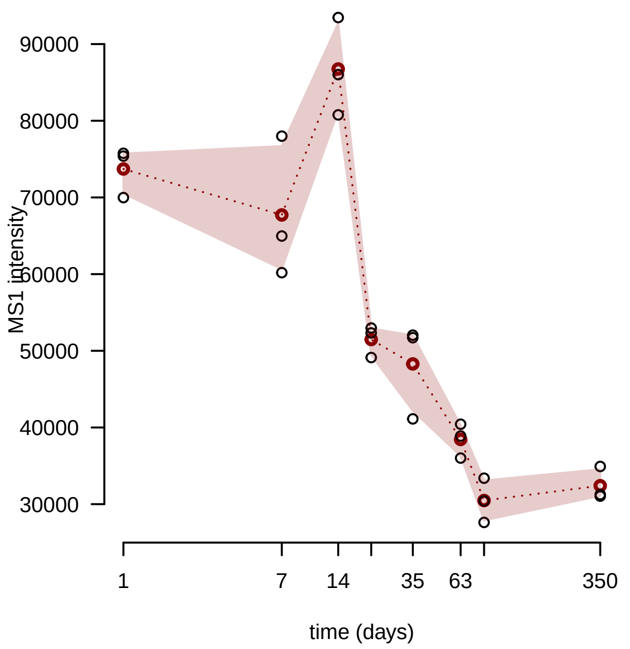

Q9CY58
GN=Serbp1
serpine1 mRNA binding protein 1

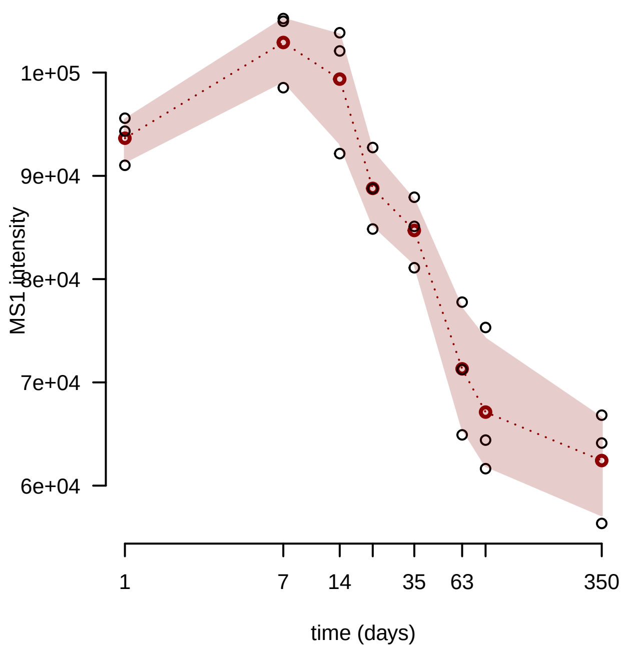

Q9CY58
GN=Serbp1
serpine1 mRNA binding protein 1

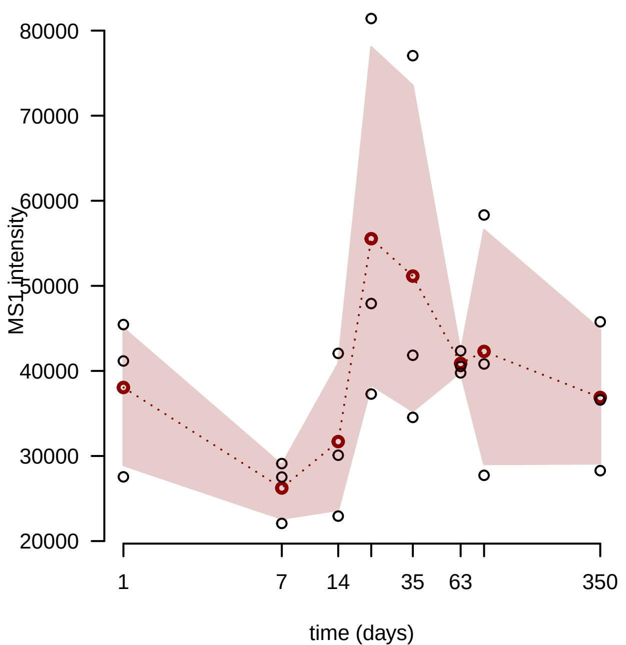

Q9CY62
GN=Rnf181
ring finger protein 181

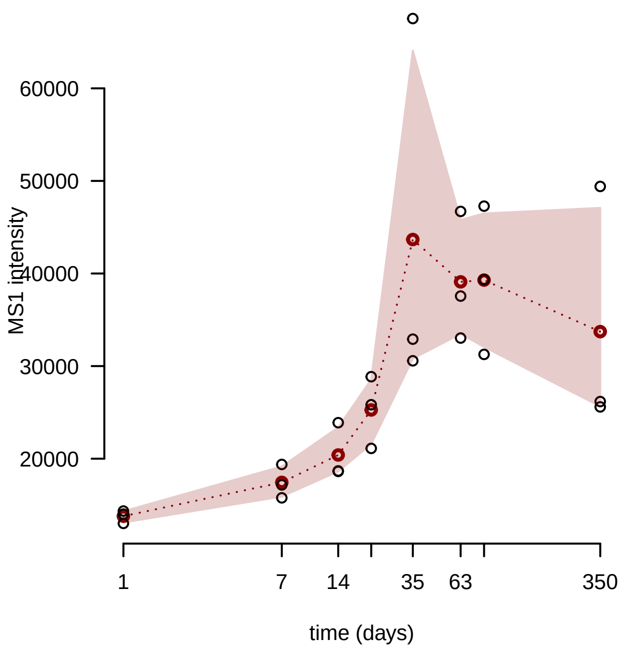

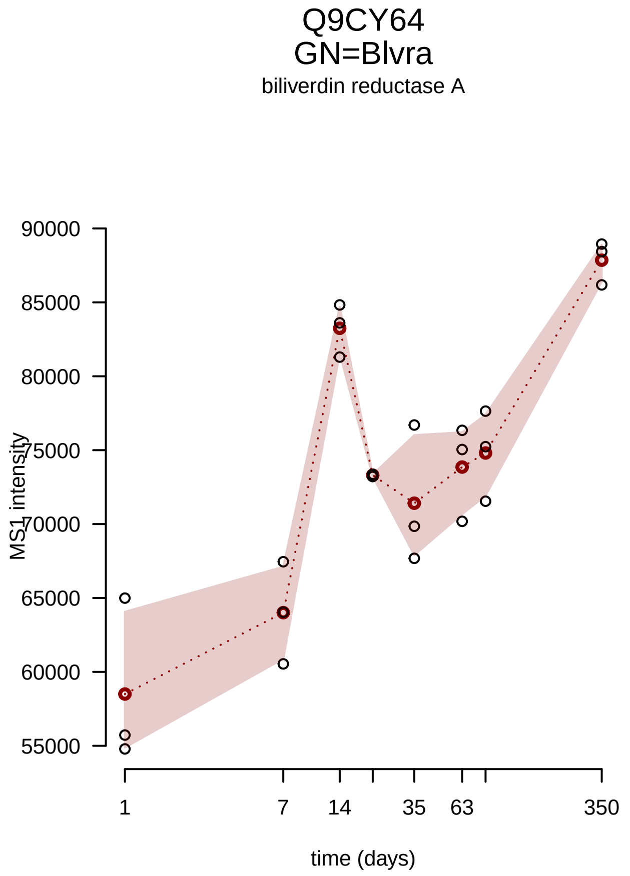
Q9CY64
GN=Blvra
biliverdin reductase A
MS1 intensity
time (days)

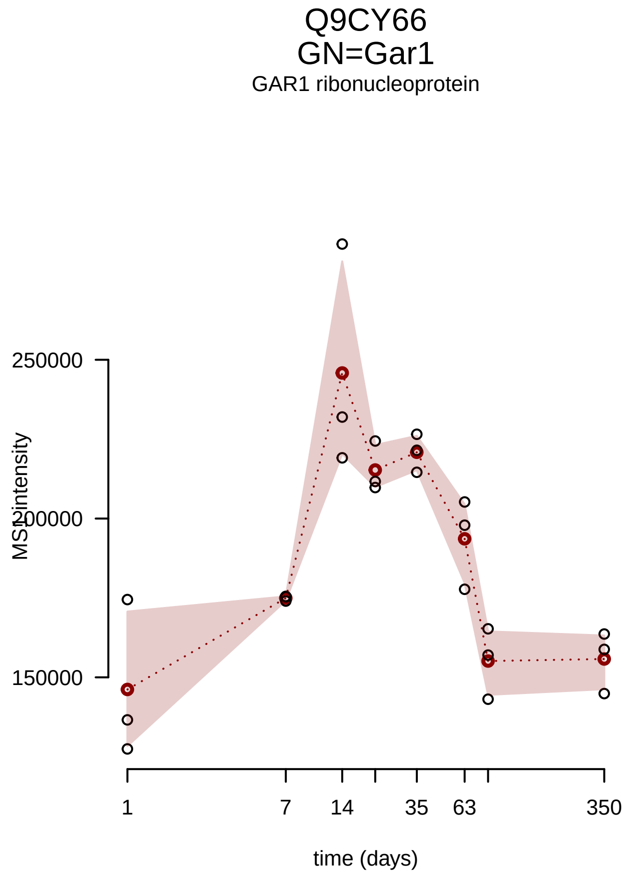
Q9CY66
GN=Gar1
GAR1 ribonucleoprotein
MS1 intensity
time (days)

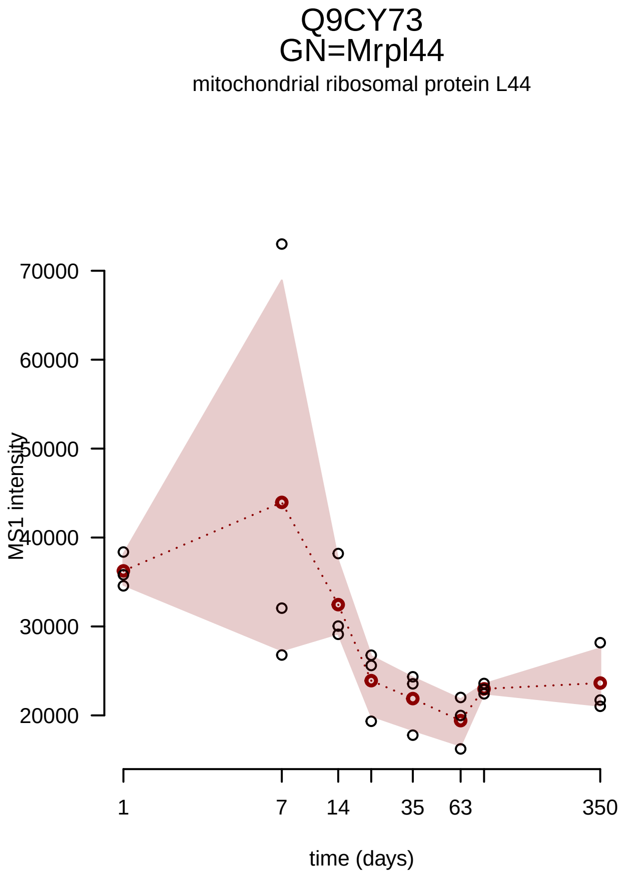
Q9CY73
GN=Mrpl44
mitochondrial ribosomal protein L44
MS1 intensity
time (days)

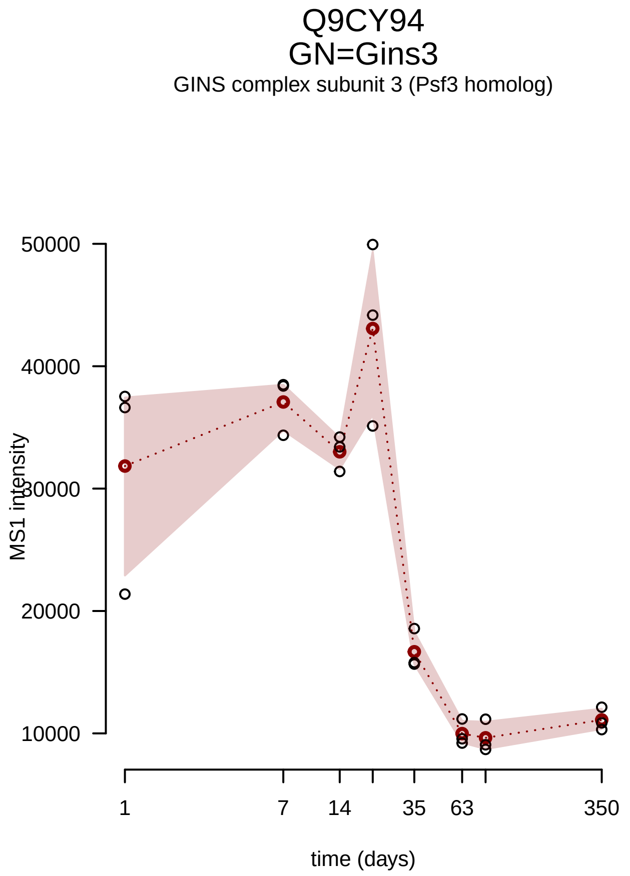
Q9CY94
GN=Gins3
GINS complex subunit 3 (Psf3 homolog)
MS1 intensity
time (days)

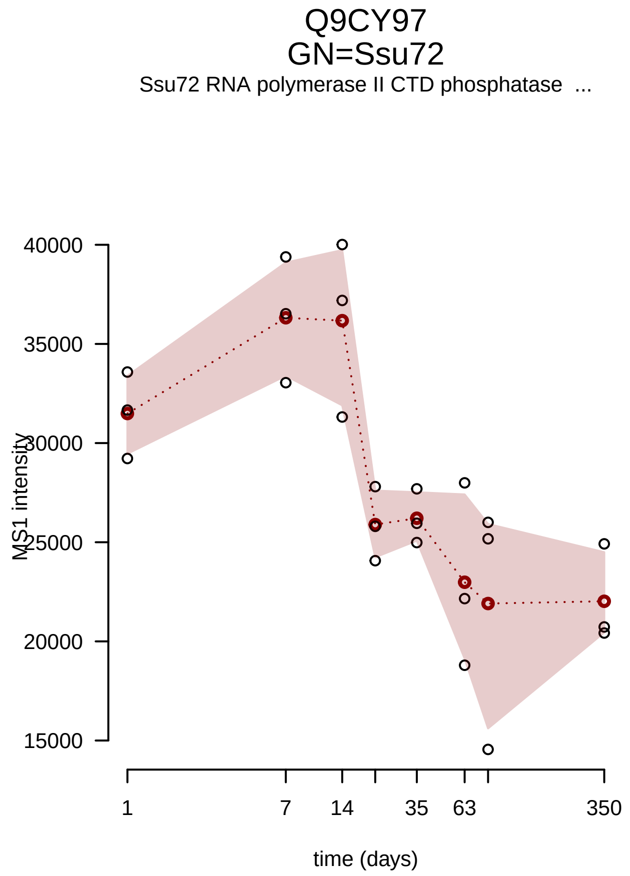
Q9CY97
GN=Ssu72
Ssu72 RNA polymerase II CTD phosphatase ...
MS1 intensity
time (days)

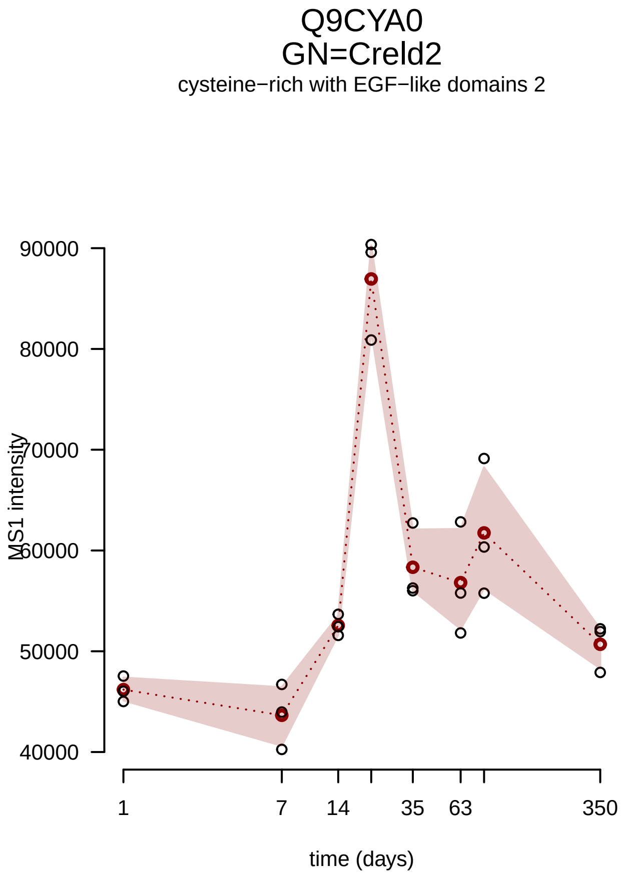
Q9CYA0
GN=Creld2
cysteine−rich with EGF−like domains 2
MS1 intensity
time (days)

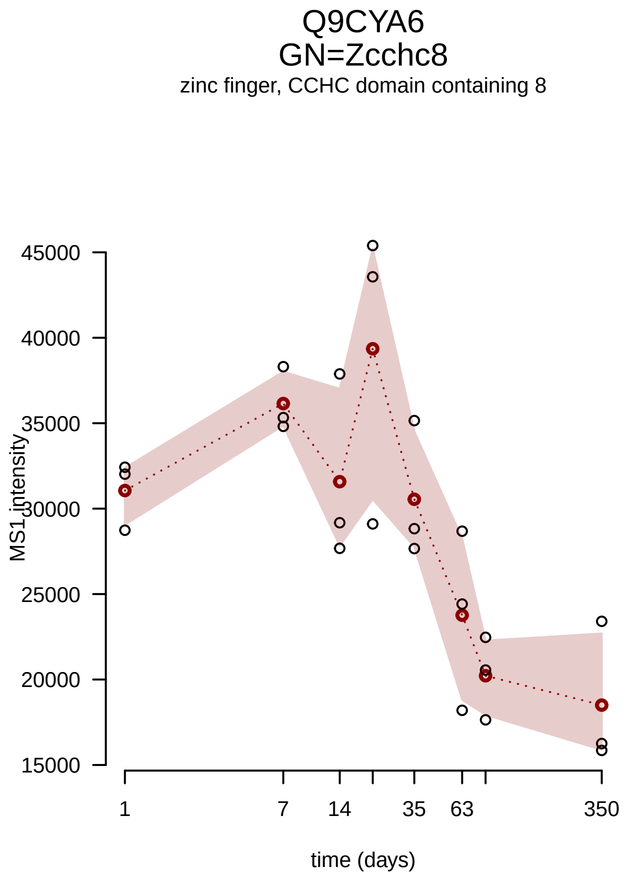
Q9CYA6
GN=Zcchc8
zinc finger, CCHC domain containing 8
MS1 intensity
time (days)

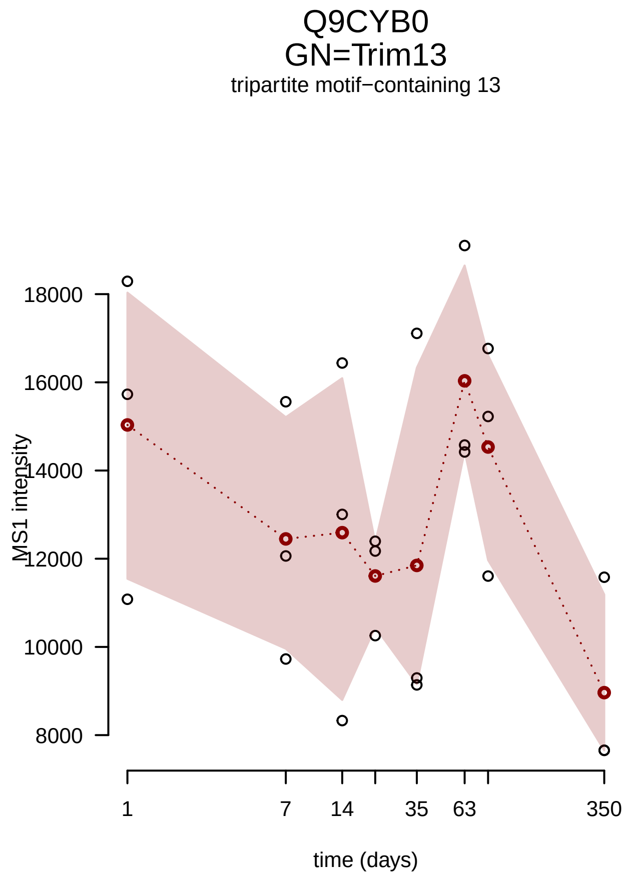
Q9CYB0
GN=Trim13
tripartite motif−containing 13
MS1 intensity
time (days)

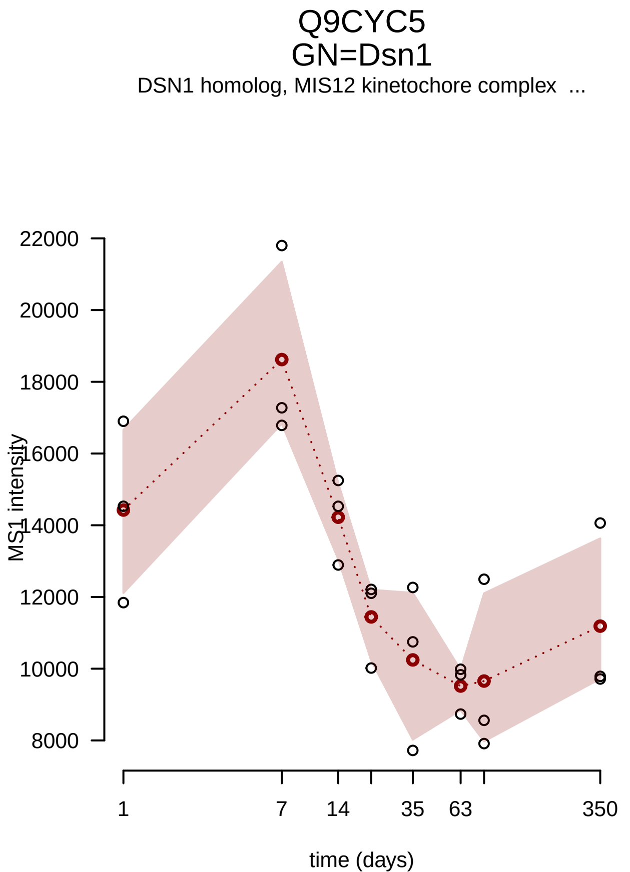
Q9CYC5
GN=Dsn1
DSN1 homolog, MIS12 kinetochore complex ...
MS1 intensity
time (days)

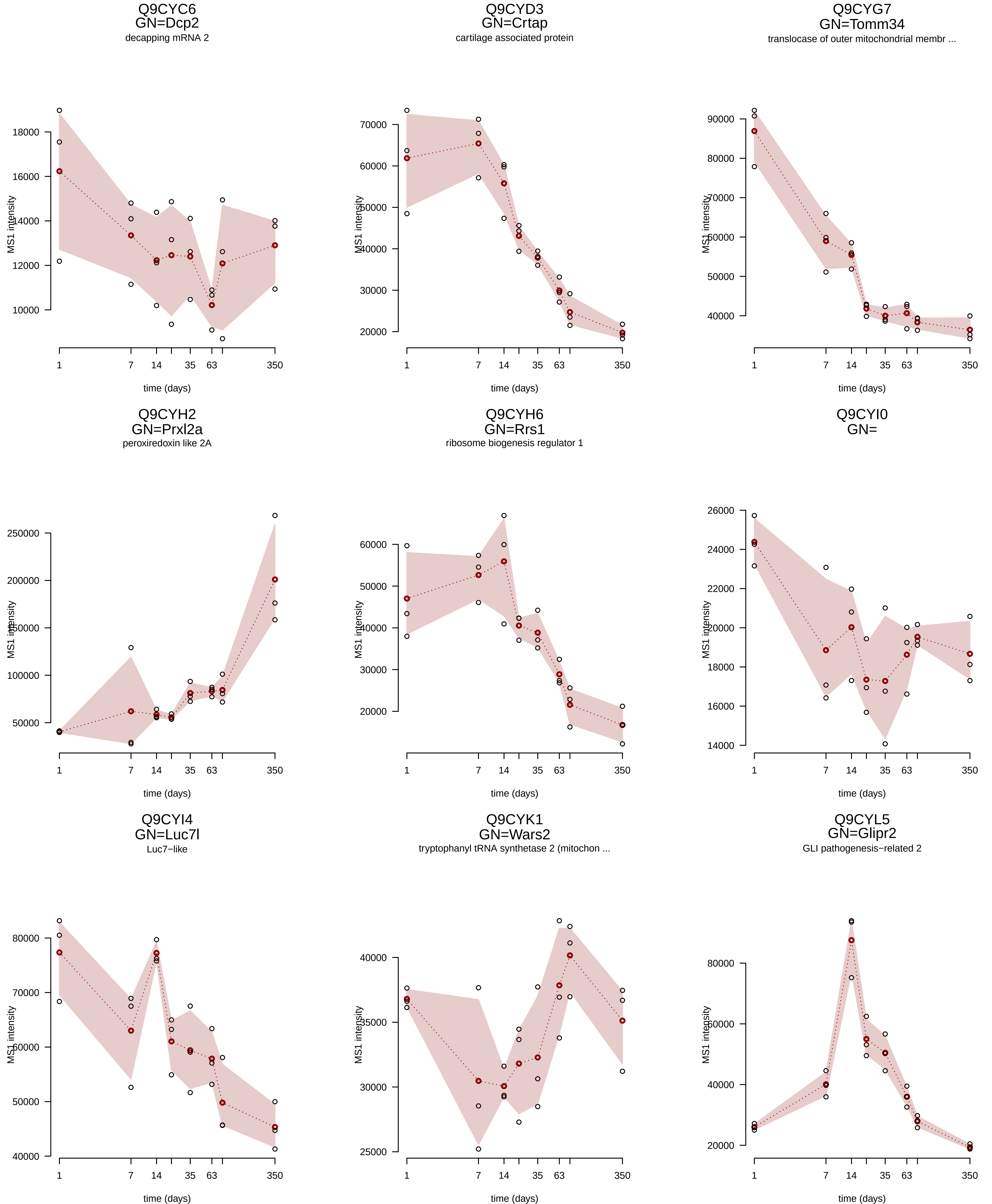

Q9CYC6
GN=Dcp2
decapping mRNA 2
MS1 intensity
time (days)
Q9CYD3
GN=Crtap
cartilage associated protein
MS1 intensity
time (days)
Q9CYG7
GN=Tomm34
translocase of outer mitochondrial membr ...
MS1 intensity
time (days)
Q9CYH2
GN=Prxl2a
peroxiredoxin like 2A
MS1 intensity
time (days)
Q9CYH6
GN=Rrs1
ribosome biogenesis regulator 1
MS1 intensity
time (days)
Q9CYI0
GN=
MS1 intensity
time (days)
Q9CYI4
GN=Luc7l
Luc7−like
MS1 intensity
time (days)
Q9CYK1
GN=Wars2
tryptophanyl tRNA synthetase 2 (mitochon ...
MS1 intensity
time (days)
Q9CYL5
GN=Glipr2
GLI pathogenesis−related 2
MS1 intensity
time (days)

Q9CYN2
GN=Spcs2
signal peptidase complex subunit 2 homol ...

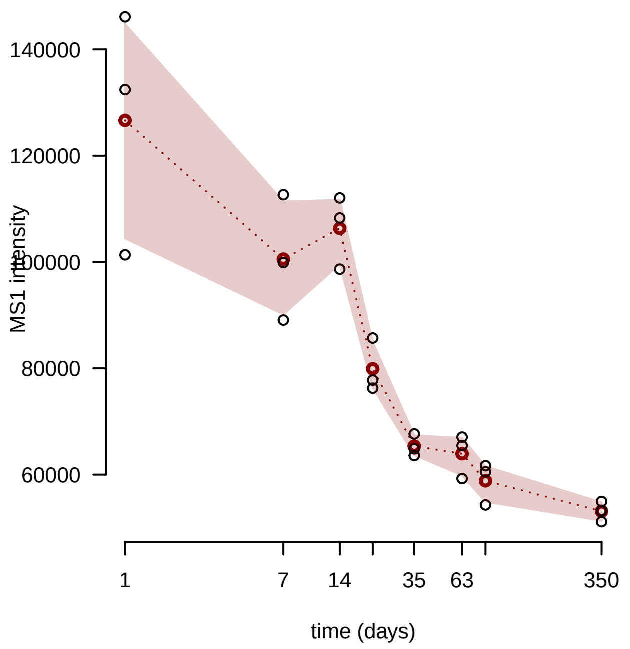

Q9CYN9
GN=Atp6ap2
ATPase, H+ transporting, lysosomal acces ...

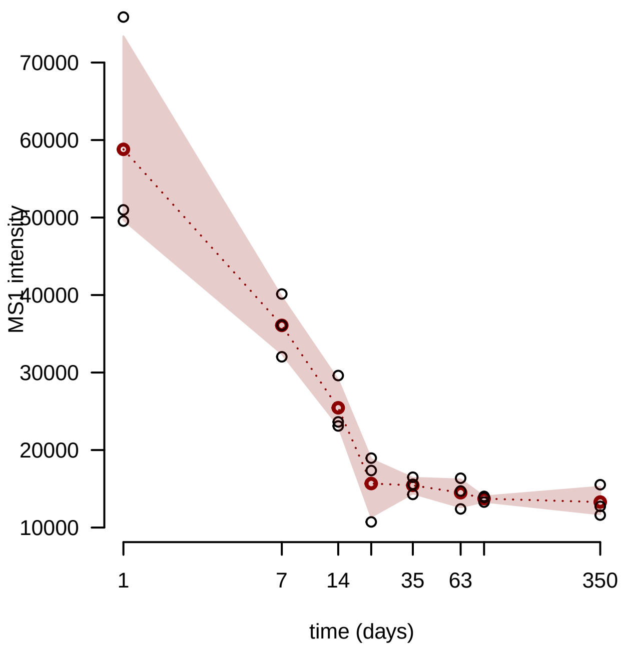

Q9CYP7
GN=Sesn3
sestrin 3

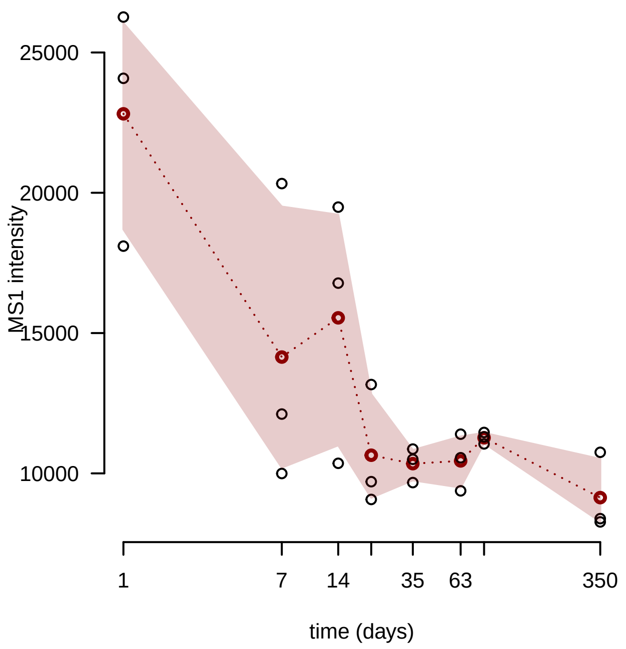

Q9CYR0
GN=Ssbp1
single−stranded DNA binding protein 1

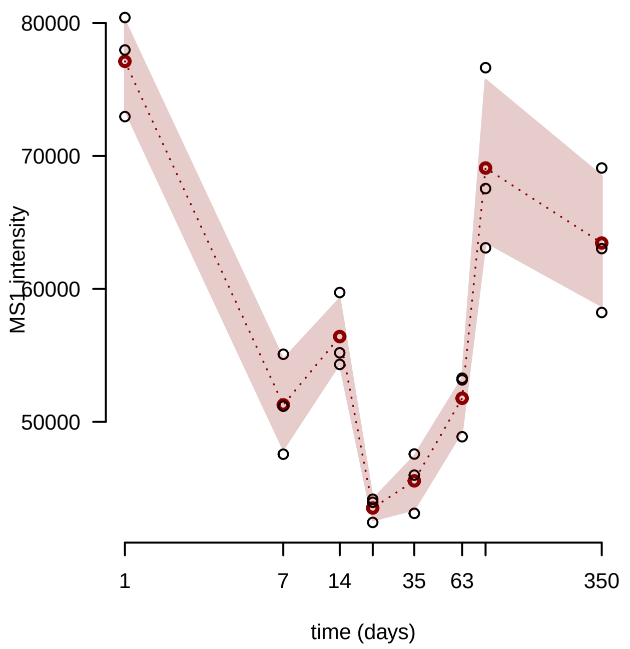

Q9CYR6
GN=Pgm3
phosphoglucomutase 3

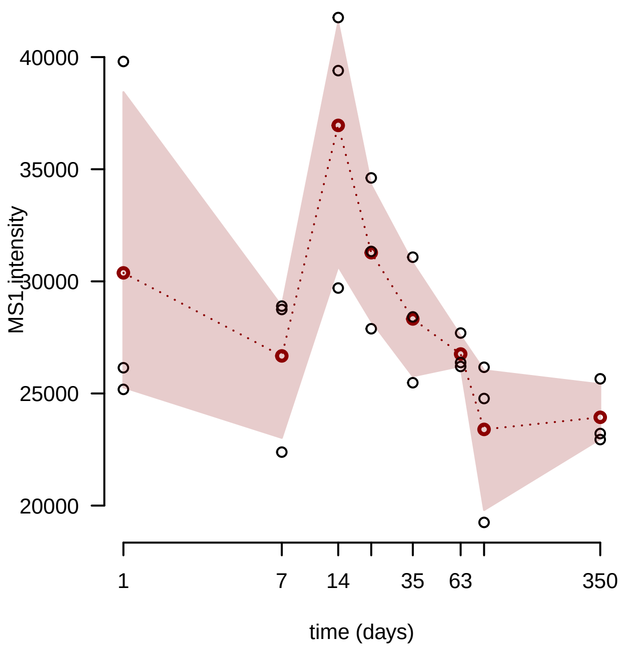

Q9CYT6
GN=Cap2
CAP, adenylate cyclase−associated protei ...

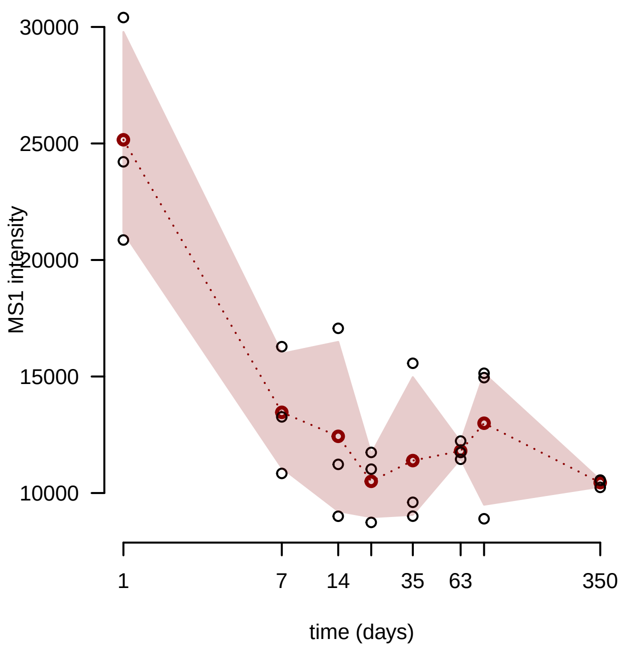

Q9CYU6
GN=Dph7
diphthamine biosynthesis 7

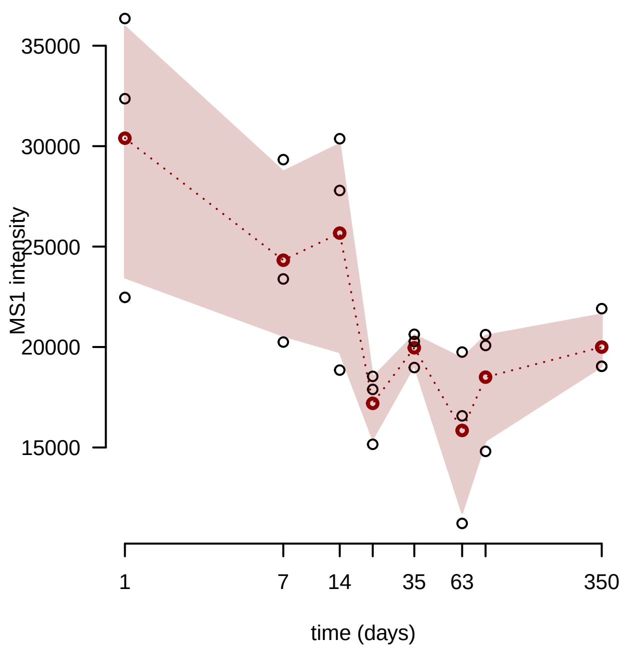

Q9CYV5
GN=Tmem135
transmembrane protein 135

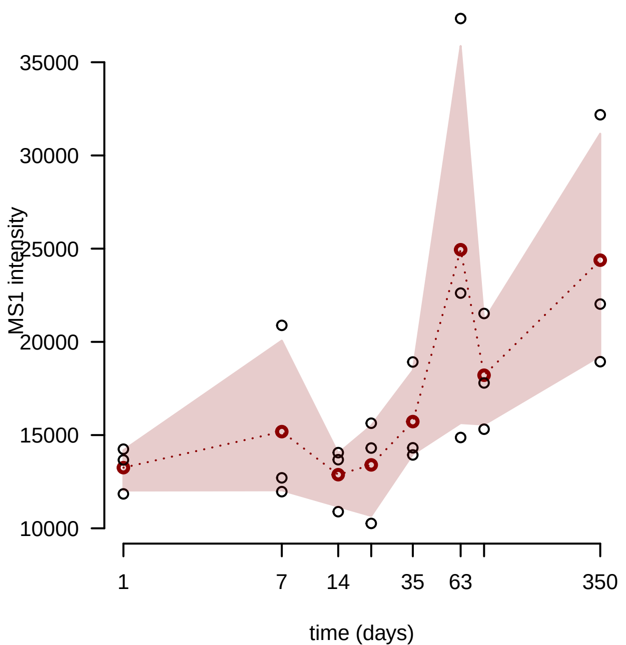

Q9CYW4
GN=Hdhd3
haloacid dehalogenase−like hydrolase dom ...

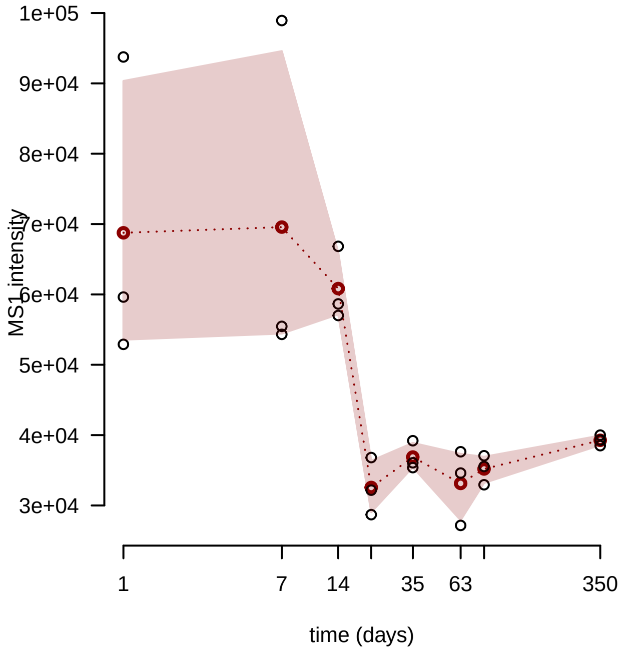

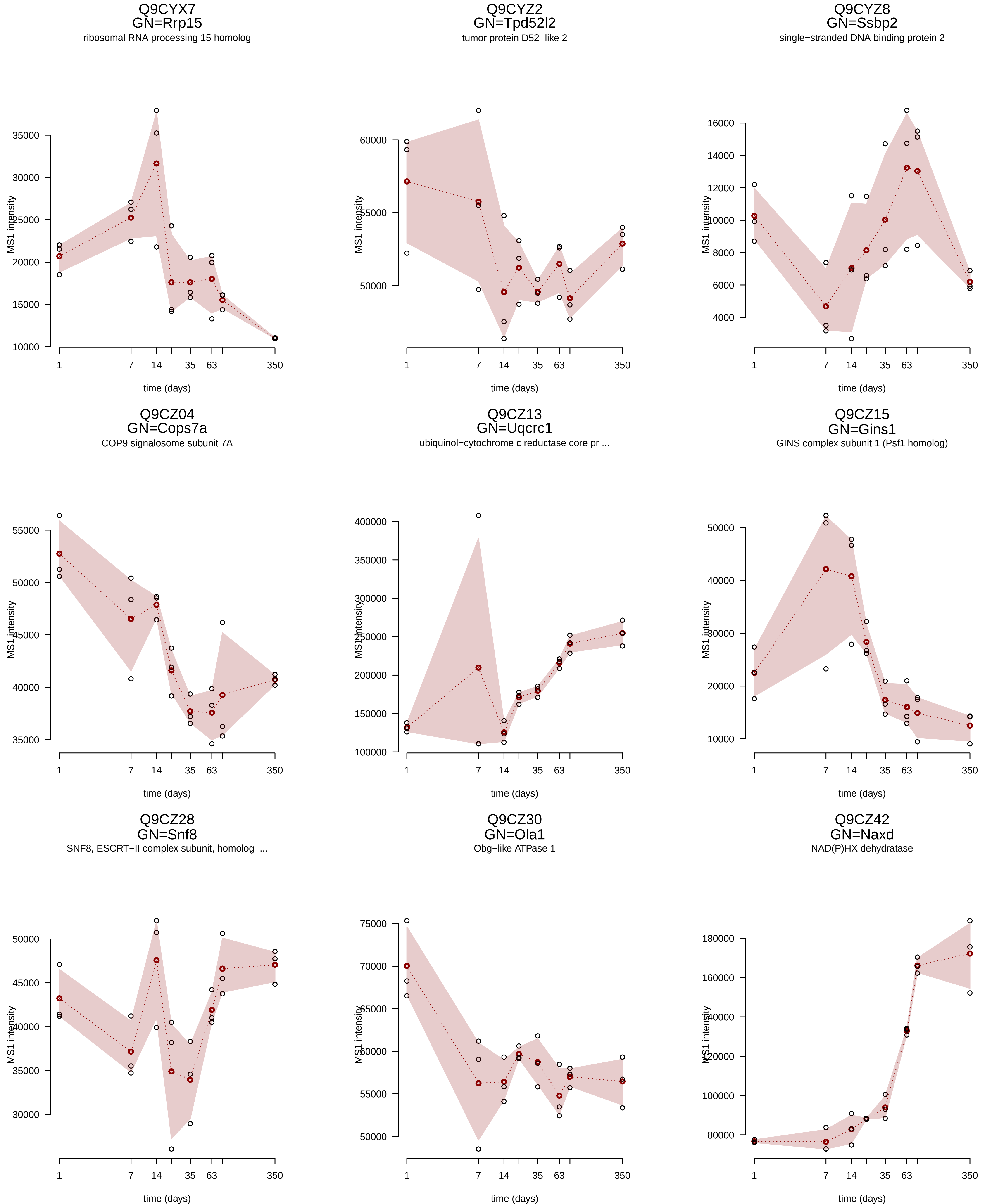
Q9CYX7
GN=Rrp15
ribosomal RNA processing 15 homolog
MS1 intensity
10000
15000
20000
25000
30000
35000
1
7
14
35
63
350
time (days)
Q9CYZ2
GN=Tpd52l2
tumor protein D52−like 2
MS1 intensity
50000
55000
60000
1
7
14
35
63
350
time (days)
Q9CYZ8
GN=Ssbp2
single−stranded DNA binding protein 2
MS1 intensity
4000
6000
8000
10000
12000
14000
16000
1
7
14
35
63
350
time (days)
Q9CZ04
GN=Cops7a
COP9 signalosome subunit 7A
MS1 intensity
35000
40000
45000
50000
55000
1
7
14
35
63
350
time (days)
Q9CZ13
GN=Uqcrc1
ubiquinol−cytochrome c reductase core pr ...
MS1 intensity
100000
150000
200000
250000
300000
350000
400000
1
7
14
35
63
350
time (days)
Q9CZ15
GN=Gins1
GINS complex subunit 1 (Psf1 homolog)
MS1 intensity
10000
20000
30000
40000
50000
1
7
14
35
63
350
time (days)
Q9CZ28
GN=Snf8
SNF8, ESCRT−II complex subunit, homolog ...
MS1 intensity
30000
35000
40000
45000
50000
1
7
14
35
63
350
time (days)
Q9CZ30
GN=Ola1
Obg−like ATPase 1
MS1 intensity
50000
55000
60000
65000
70000
75000
1
7
14
35
63
350
time (days)
Q9CZ42
GN=Naxd
NAD(P)HX dehydratase
MS1 intensity
80000
100000
120000
140000
160000
180000
1
7
14
35
63
350
time (days)

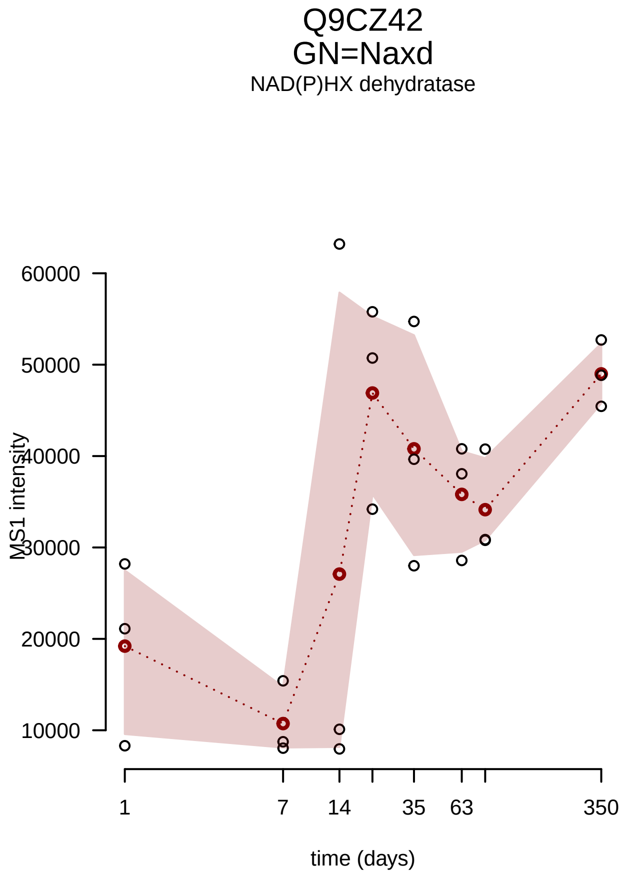
Q9CZ42
GN=Naxd
NAD(P)HX dehydratase
10000
20000
30000
40000
50000
60000
MS1 intensity
1
7
14
35
63
350
time (days)

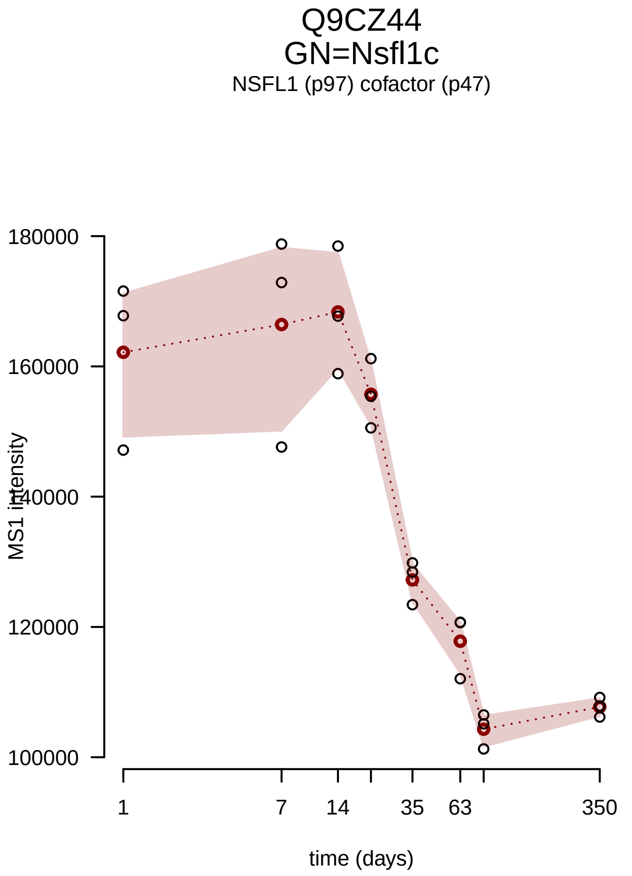
Q9CZ44
GN=Nsfl1c
NSFL1 (p97) cofactor (p47)
100000
120000
140000
160000
180000
MS1 intensity
1
7
14
35
63
350
time (days)

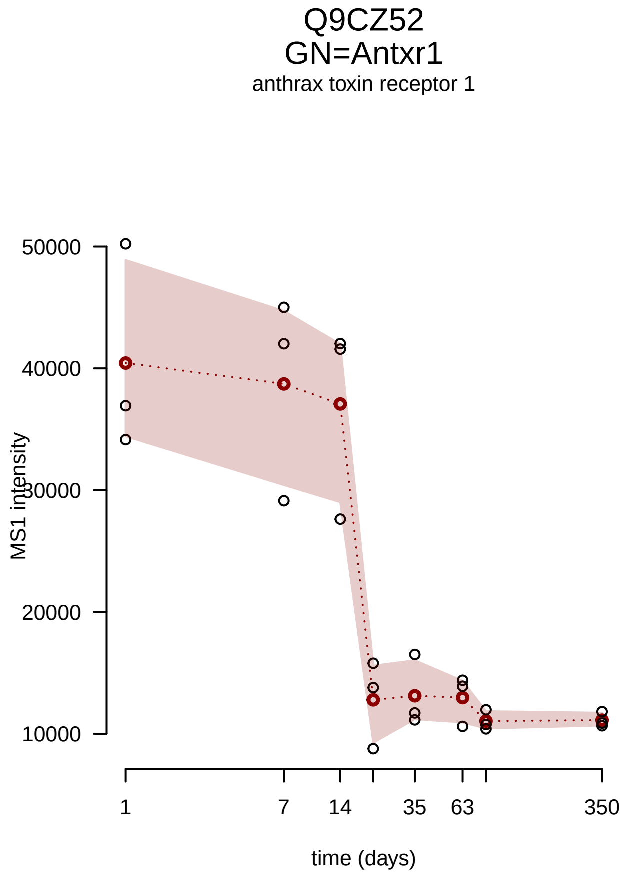
Q9CZ52
GN=Antxr1
anthrax toxin receptor 1
10000
20000
30000
40000
50000
MS1 intensity
1
7
14
35
63
350
time (days)

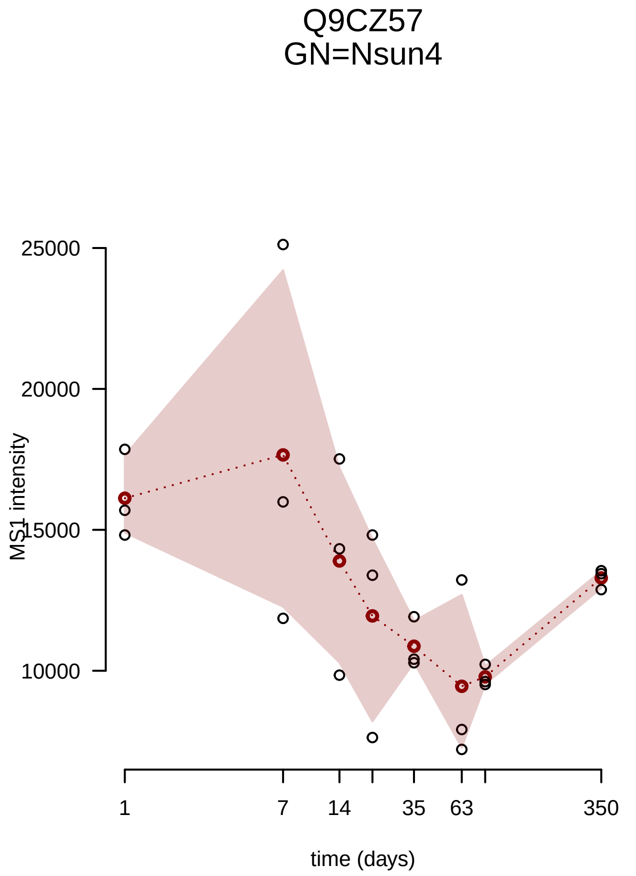
Q9CZ57
GN=Nsun4
10000
15000
20000
25000
MS1 intensity
1
7
14
35
63
350
time (days)

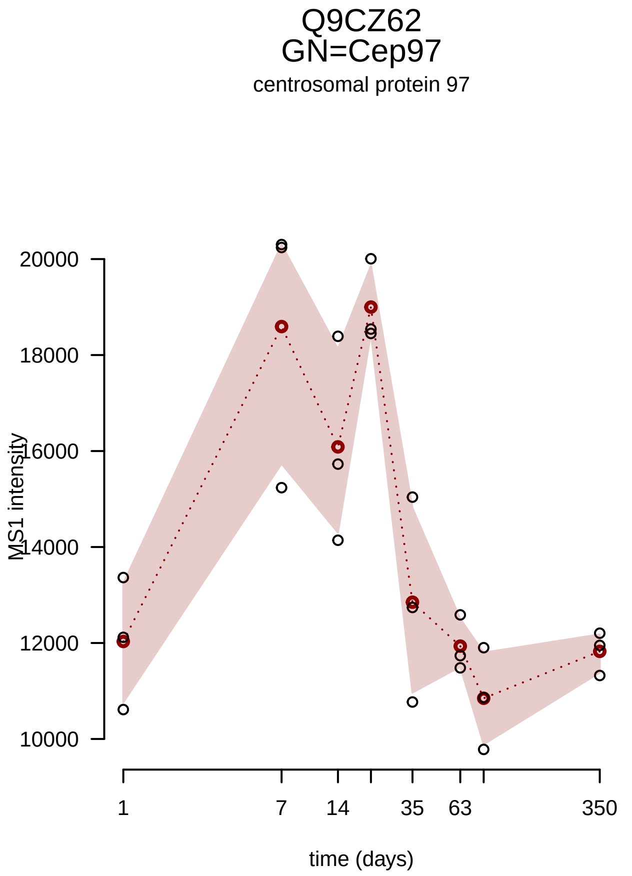
Q9CZ62
GN=Cep97
centrosomal protein 97
10000
12000
14000
16000
18000
20000
MS1 intensity
1
7
14
35
63
350
time (days)

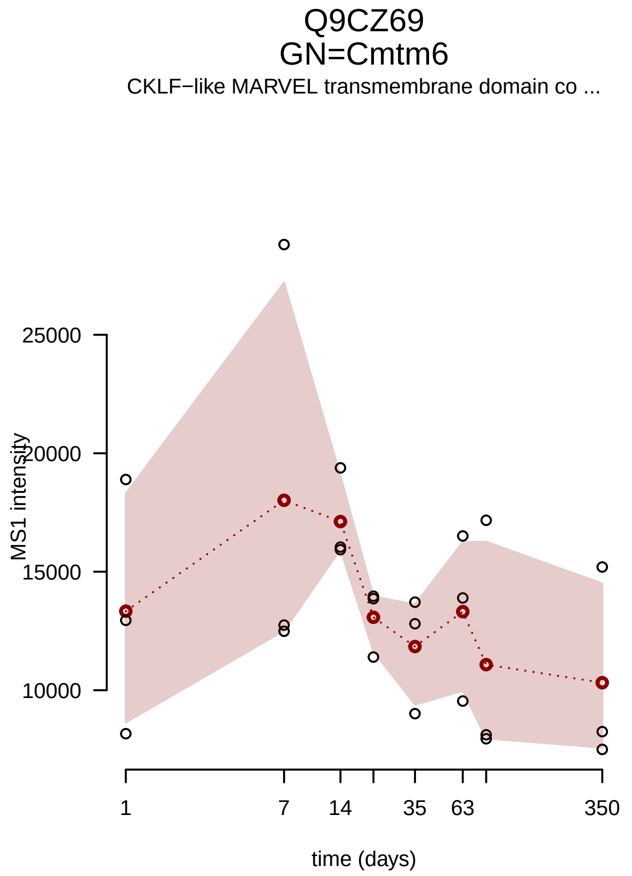
Q9CZ69
GN=Cmtm6
CKLF−like MARVEL transmembrane domain co ...
10000
15000
20000
25000
MS1 intensity
1
7
14
35
63
350
time (days)

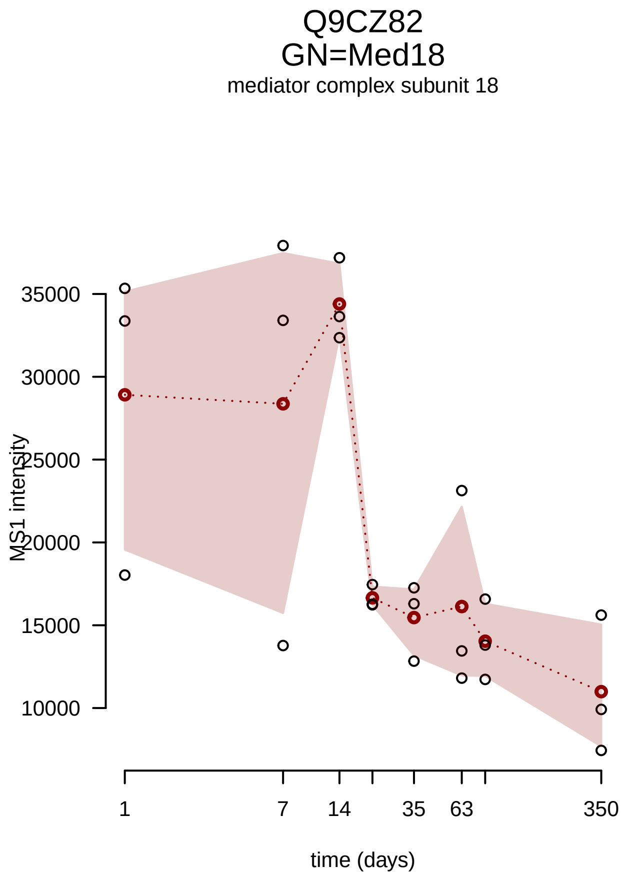
Q9CZ82
GN=Med18
mediator complex subunit 18
10000
15000
20000
25000
30000
35000
MS1 intensity
1
7
14
35
63
350
time (days)

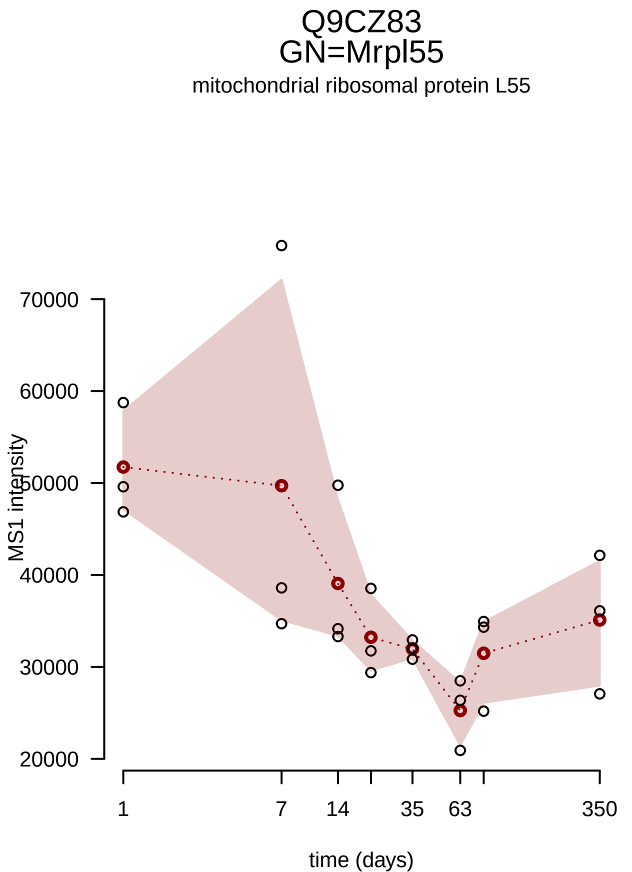
Q9CZ83
GN=Mrpl55
mitochondrial ribosomal protein L55
20000
30000
40000
50000
60000
70000
MS1 intensity
1
7
14
35
63
350
time (days)

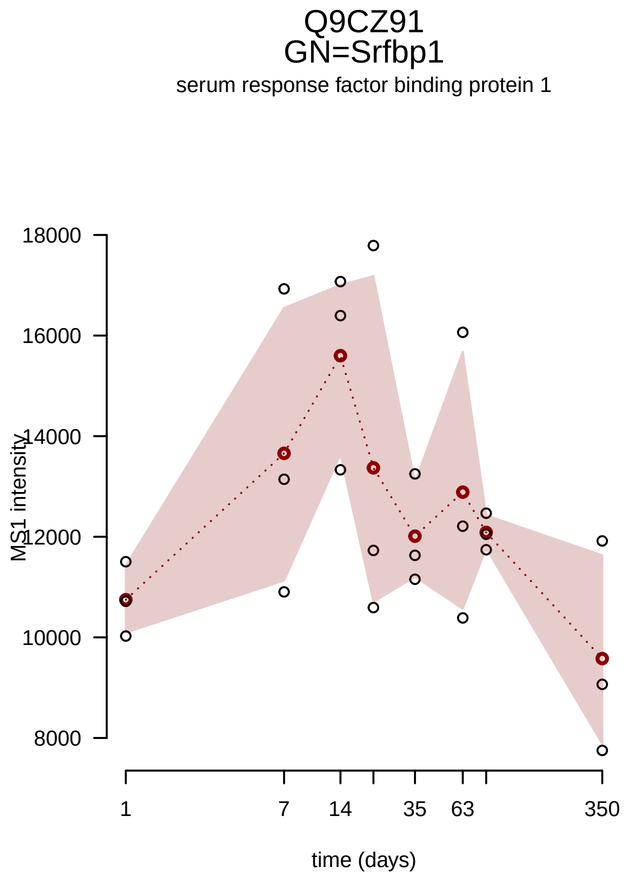
Q9CZ91
GN=Srfbp1
serum response factor binding protein 1
8000
10000
12000
14000
16000
18000
MS1 intensity
1
7
14
35
63
350
time (days)

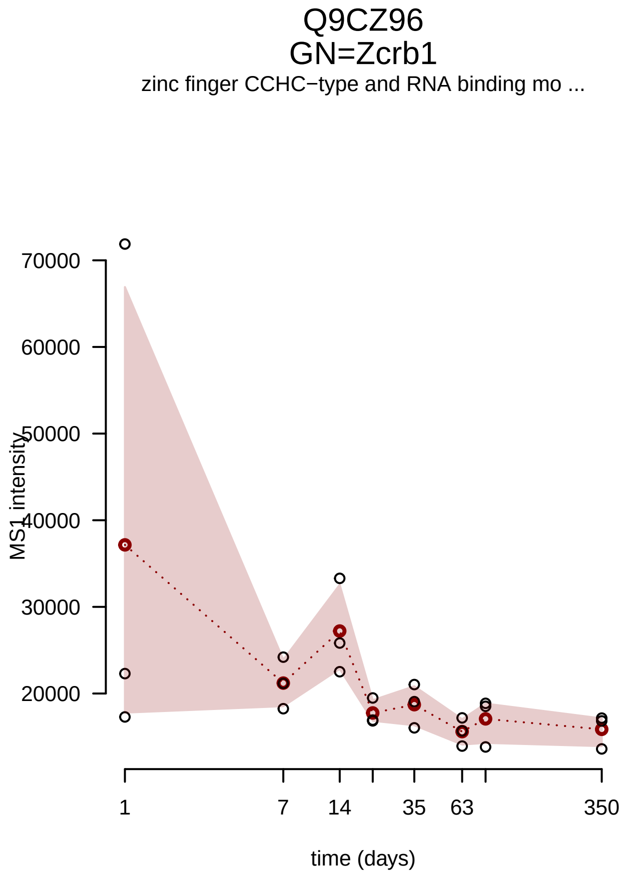
Q9CZ96
GN=Zcrb1
zinc finger CCHC−type and RNA binding mo ...
MS1 intensity
time (days)

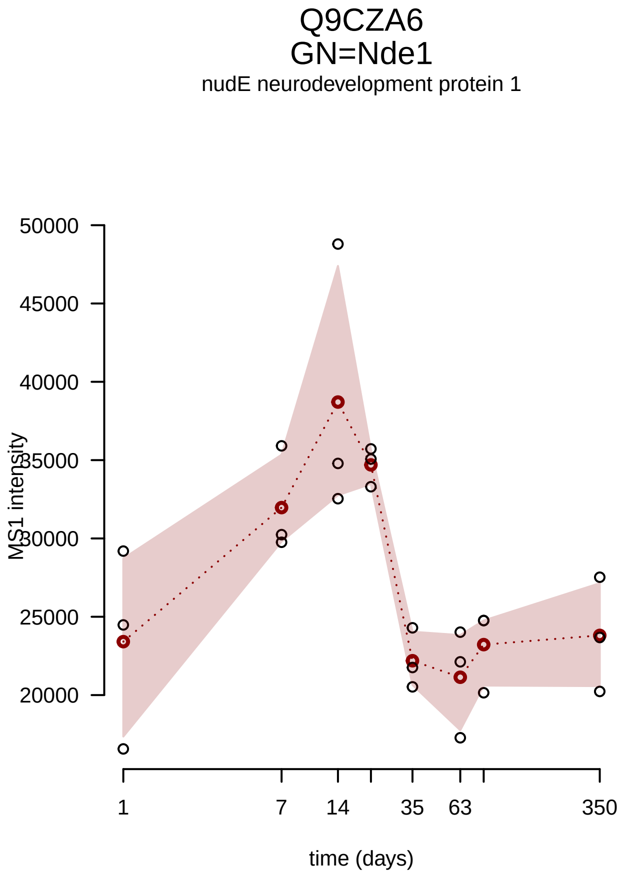
Q9CZA6
GN=Nde1
nudE neurodevelopment protein 1
MS1 intensity
time (days)

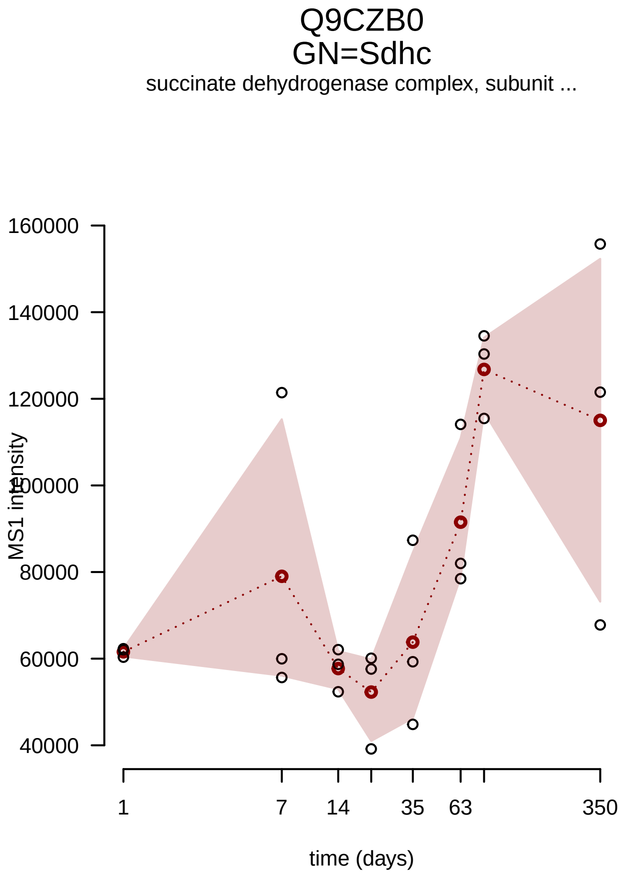
Q9CZB0
GN=Sdhc
succinate dehydrogenase complex, subunit ...
MS1 intensity
time (days)

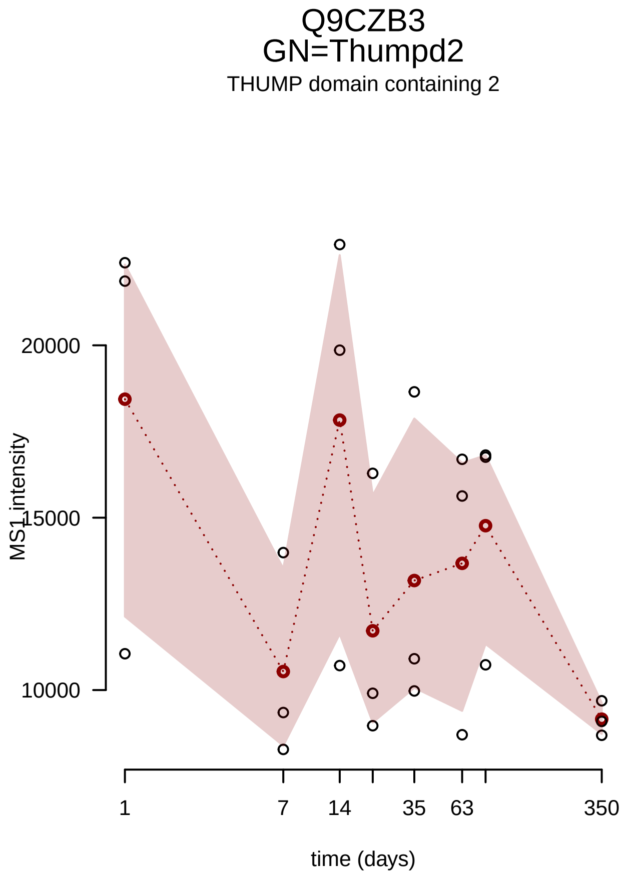
Q9CZB3
GN=Thumpd2
THUMP domain containing 2
MS1 intensity
time (days)

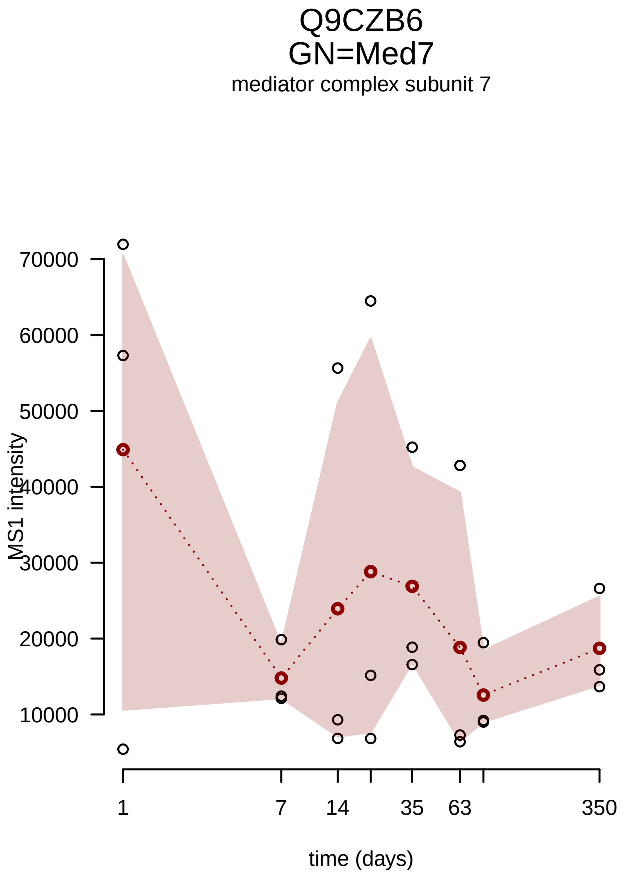
Q9CZB6
GN=Med7
mediator complex subunit 7
MS1 intensity
time (days)

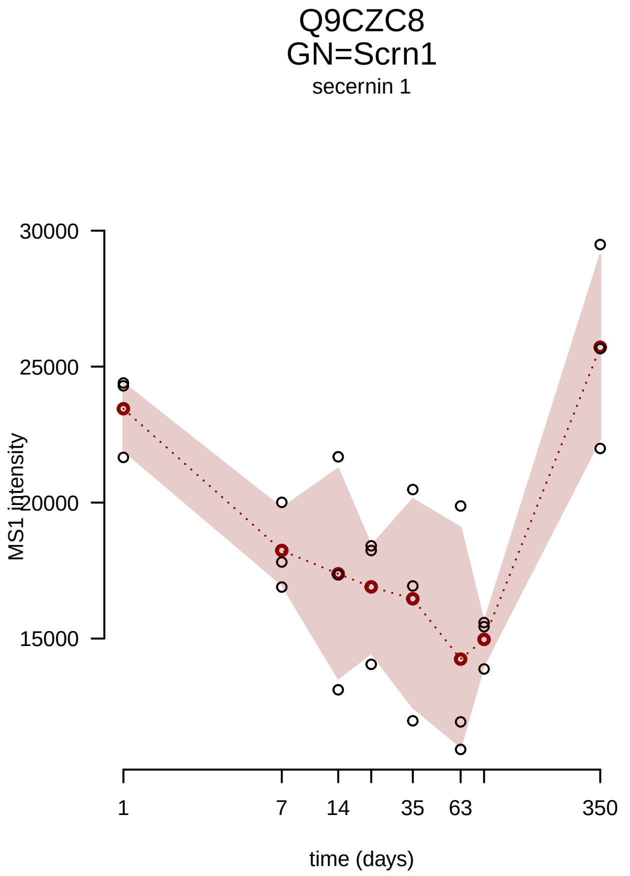
Q9CZC8
GN=Scrn1
secernin 1
MS1 intensity
time (days)

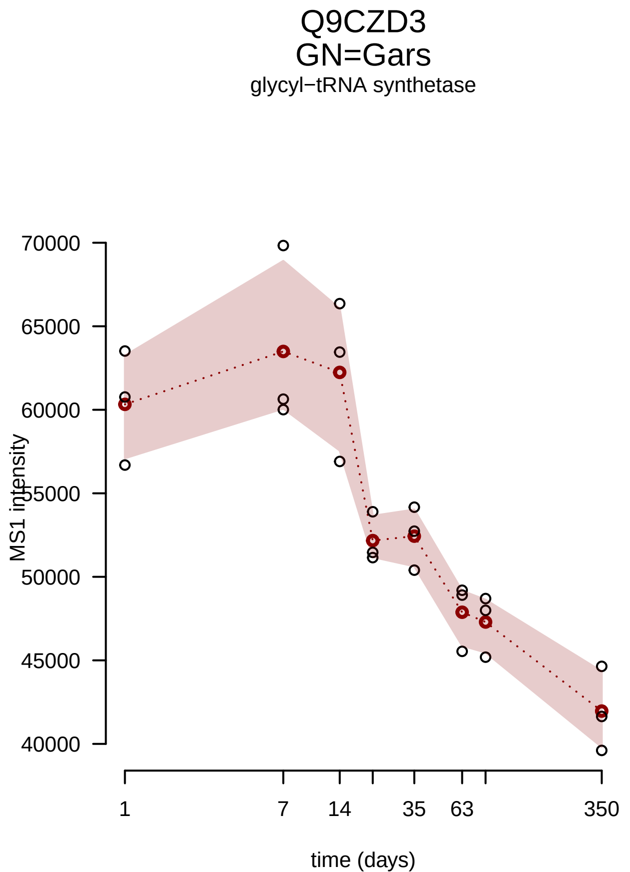
Q9CZD3
GN=Gars
glycyl−tRNA synthetase
MS1 intensity
time (days)

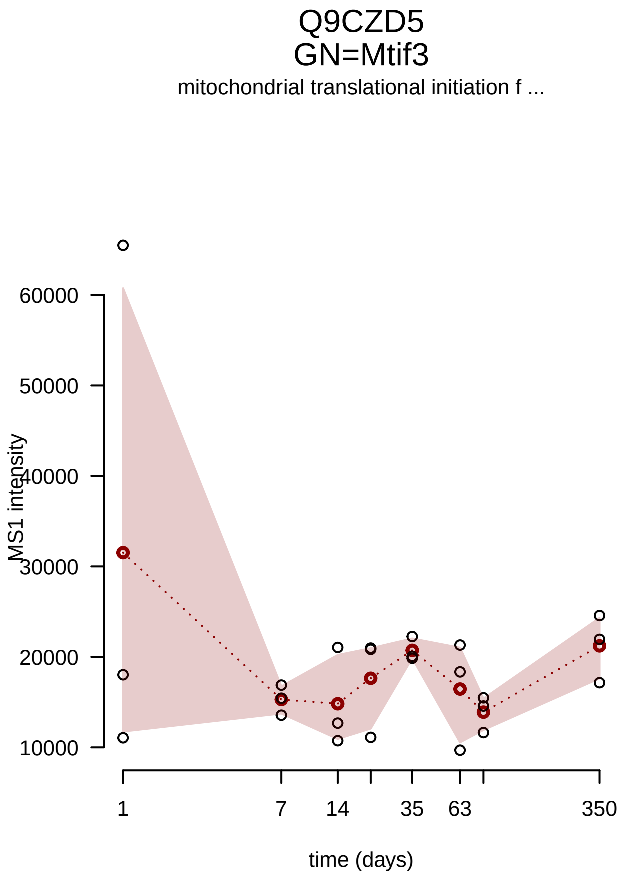
Q9CZD5
GN=Mtif3
mitochondrial translational initiation f ...
MS1 intensity
time (days)

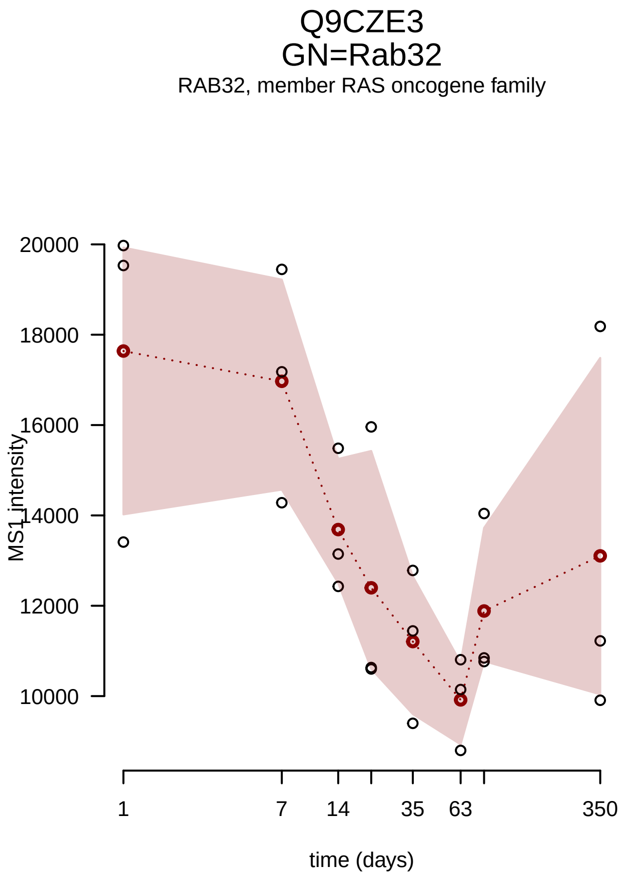
Q9CZE3
GN=Rab32
RAB32, member RAS oncogene family
MS1 intensity
time (days)

Q9CZG3
GN=Commd8
COMM domain containing 8

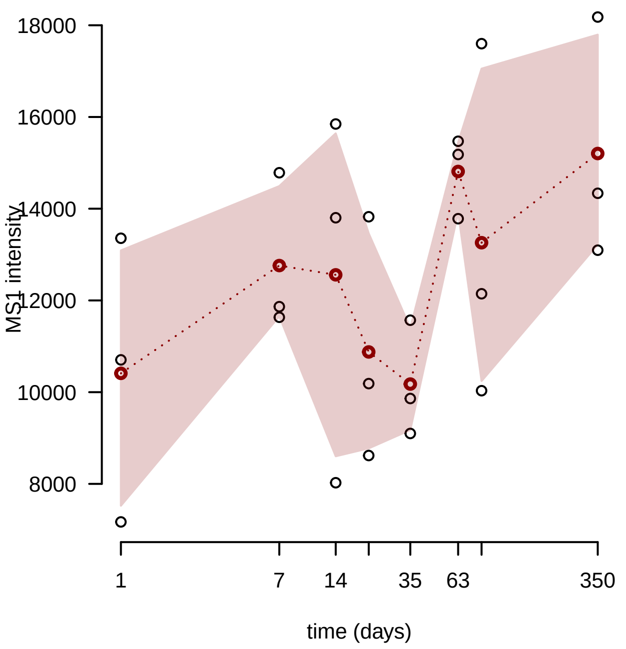

Q9CZG9
GN=Pdzd11
PDZ domain containing 11

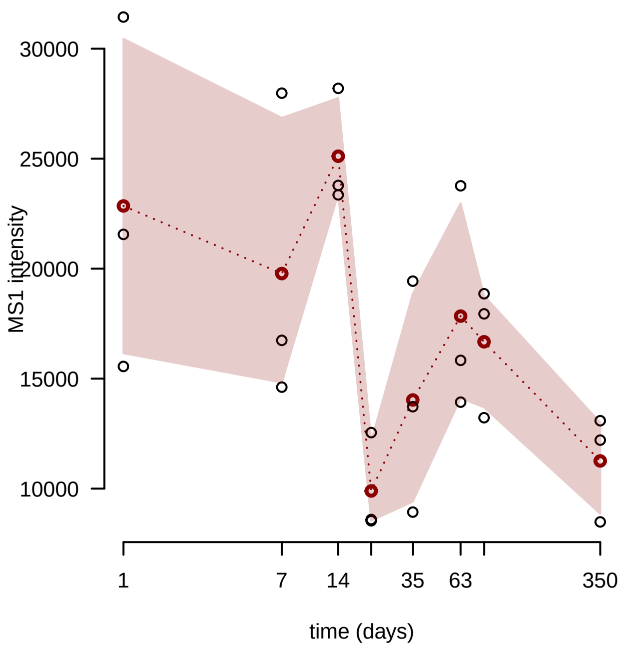

Q9CZH3
GN=Psmg3
proteasome (prosome, macropain) assembly ...

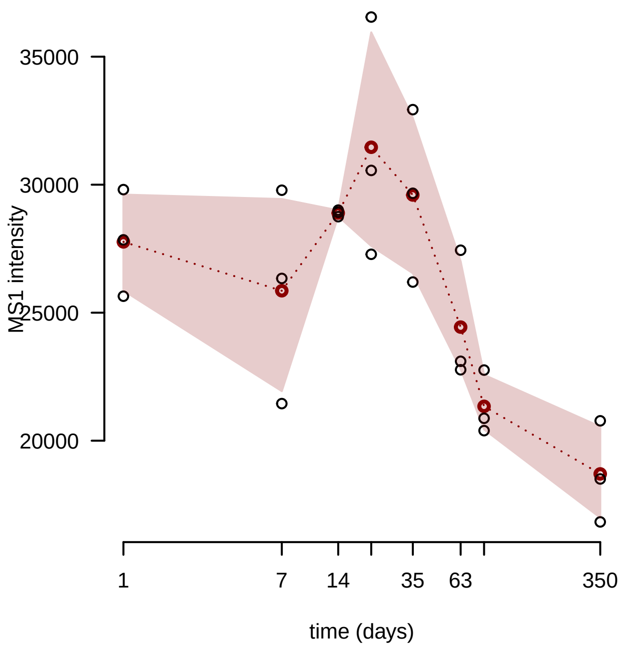

Q9CZH7
GN=Mxra7
matrix−remodelling associated 7

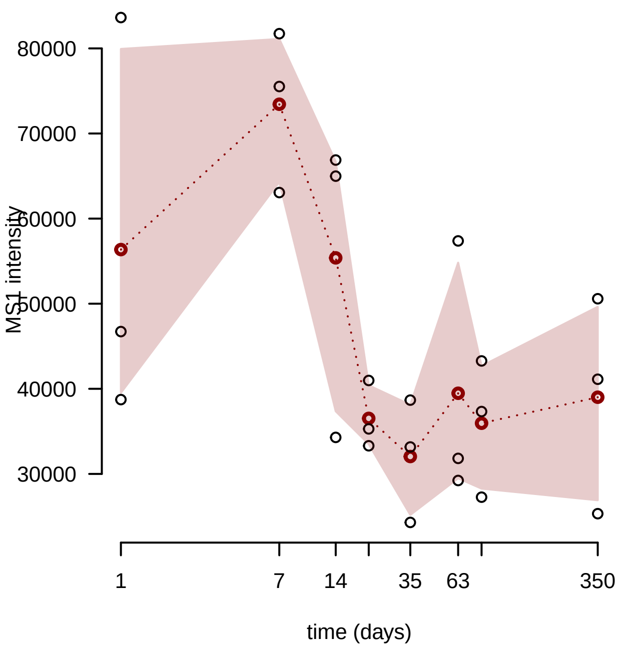

Q9CZI9
GN=Aen
apoptosis enhancing nuclease

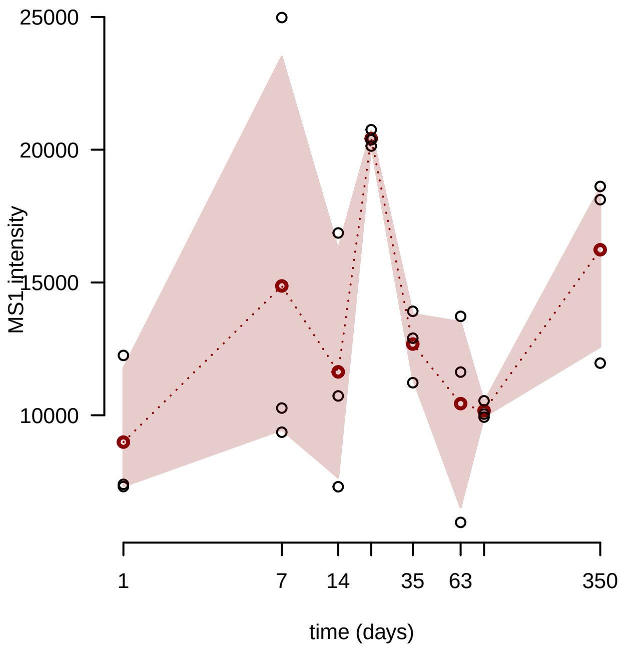

Q9CZJ1
GN=Utp11
UTP11 small subunit processome component

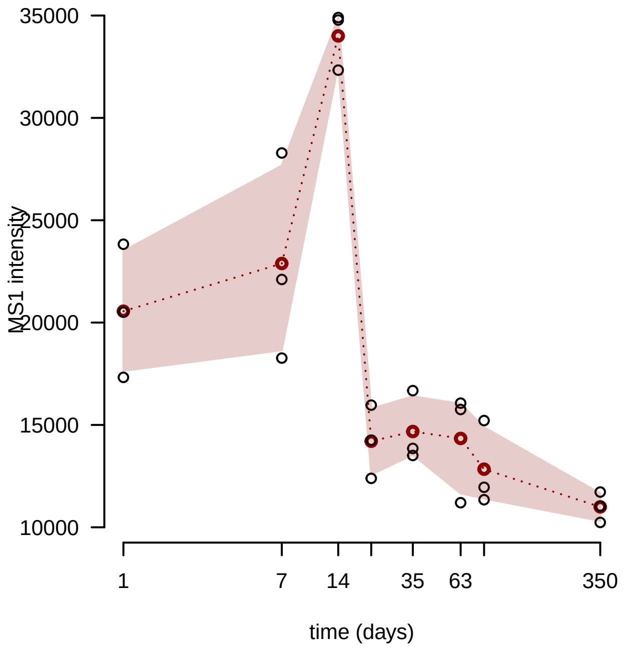

Q9CZJ2
GN=Hspa12b
heat shock protein 12B

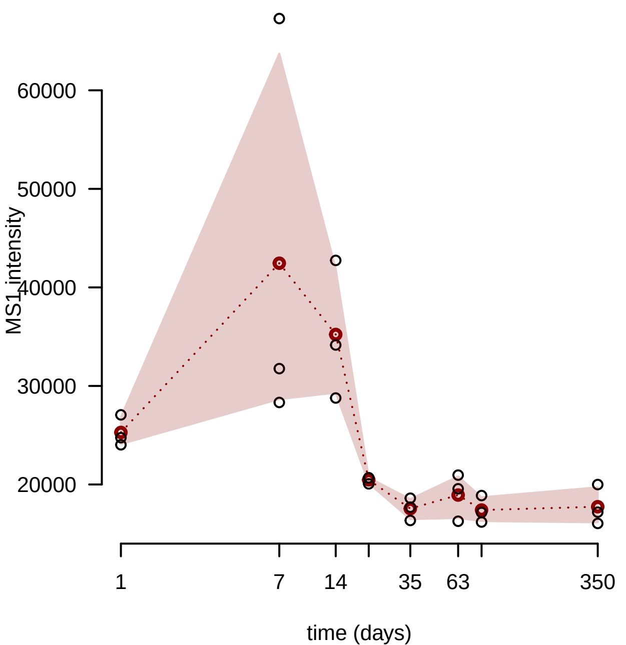

Q9CZK6
GN=Anks3
ankyrin repeat and sterile alpha motif d ...

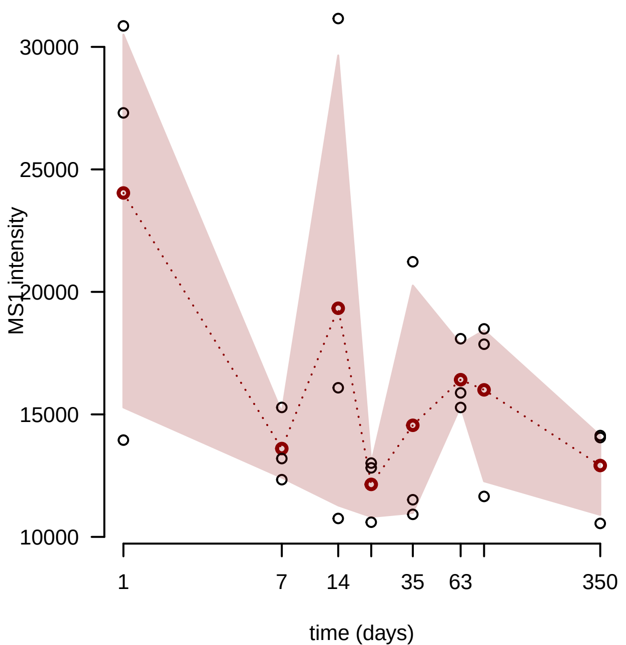

Q9CZL2
GN=Fam241a
family with sequence similarity 241, mem ...

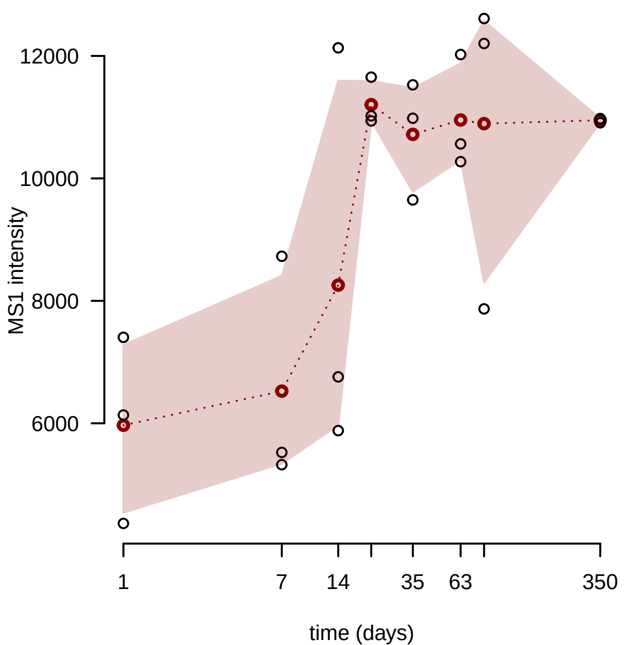

Q9CZL5
GN=Pcbd2
pterin 4 alpha carbinolamine dehydratase ...

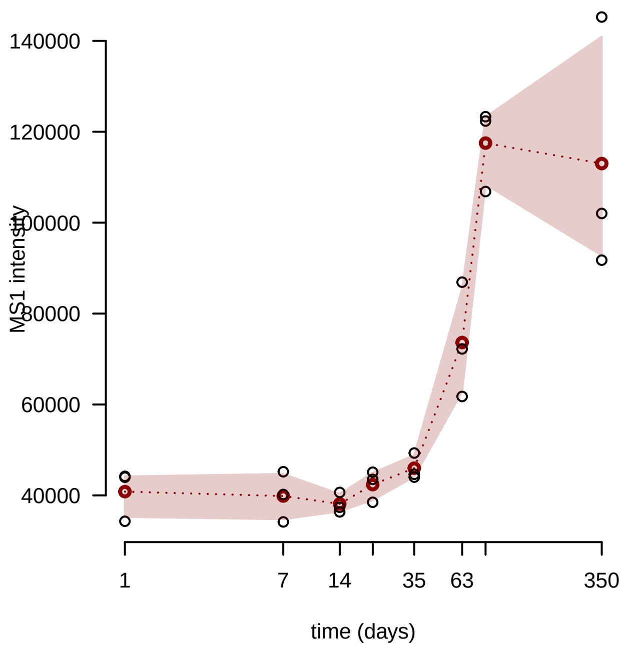

Q9CZM2
GN=Rpl15
ribosomal protein L15

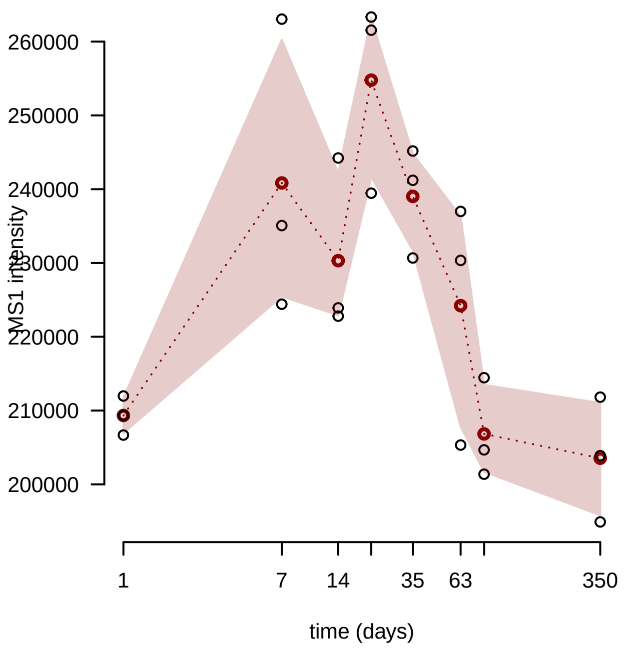

Q9CZN7
GN=Shmt2
serine hydroxymethyltransferase 2 (mitoc ...

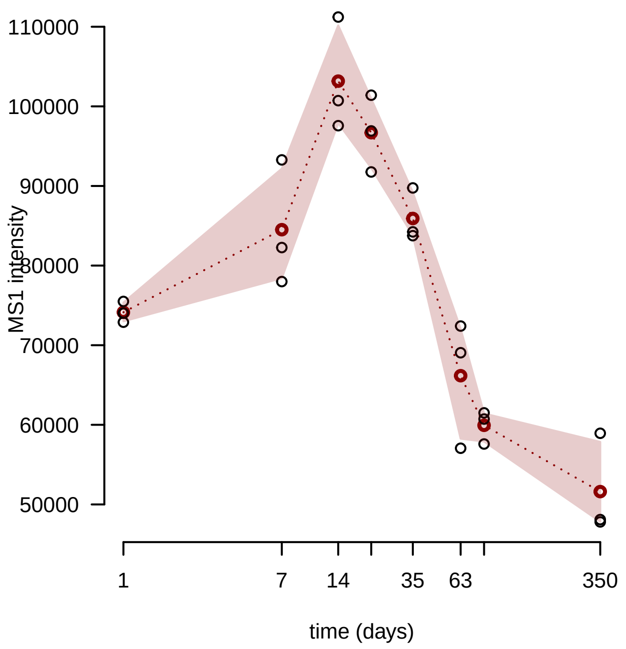

Q9CZN8
GN=Qrsl1
glutaminyl−tRNA synthase (glutamine−hydr ...

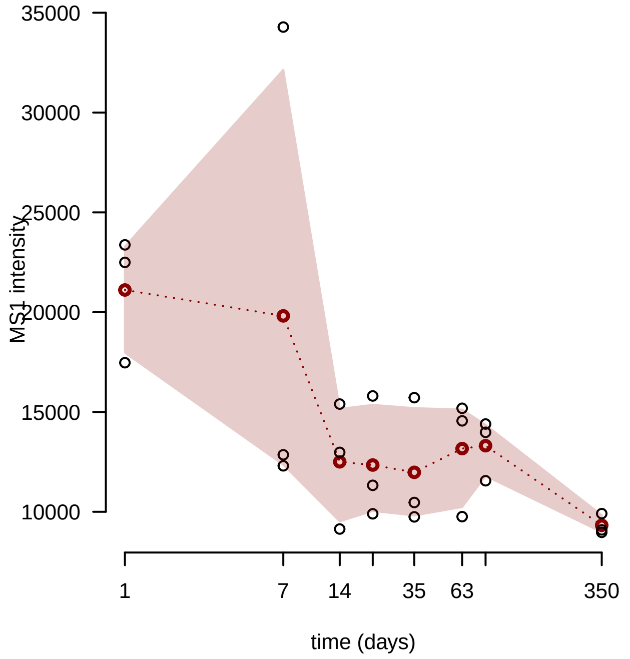

Q9CZP5
GN=Bcs1l
BCS1−like (yeast)

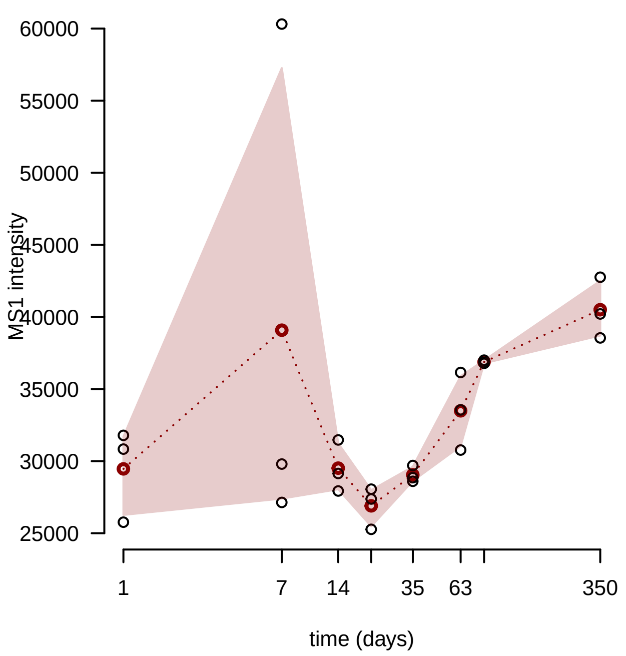

Q9CZP7
GN=Cdc37l1
cell division cycle 37−like 1

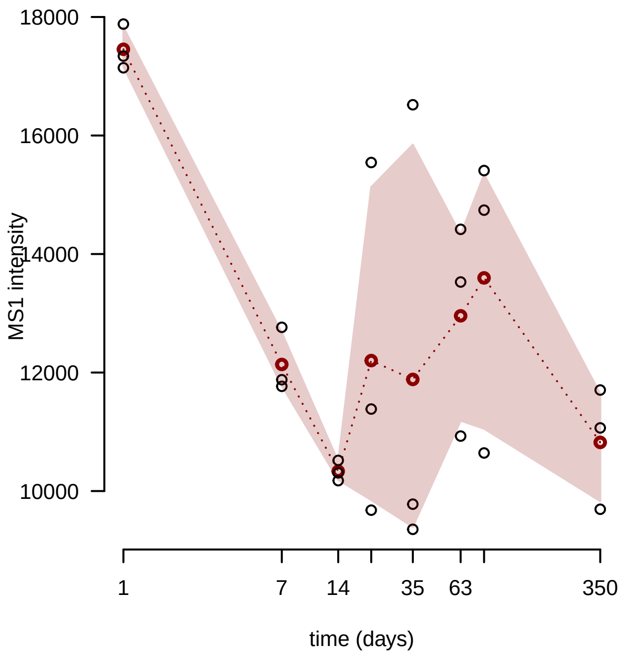

Q9CZR2
GN=Naalad2
N−acetylated alpha−linked acidic dipepti ...

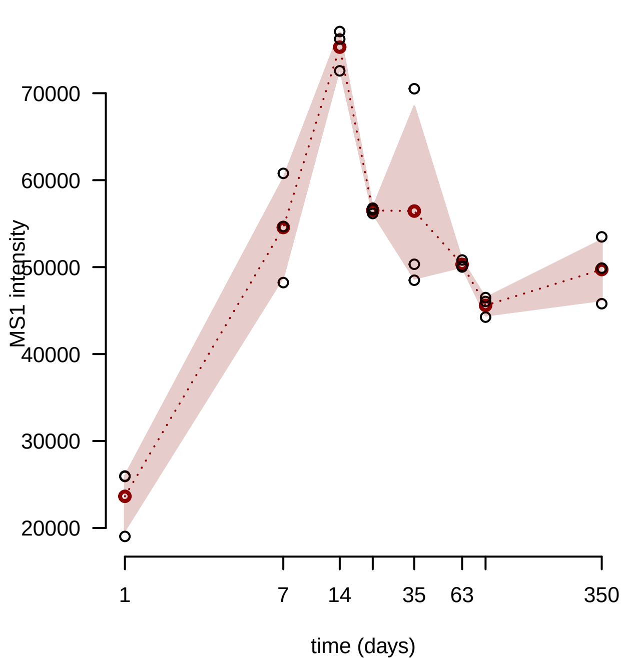

Q9CZR3
GN=Tomm40l
translocase of outer mitochondrial membr ...

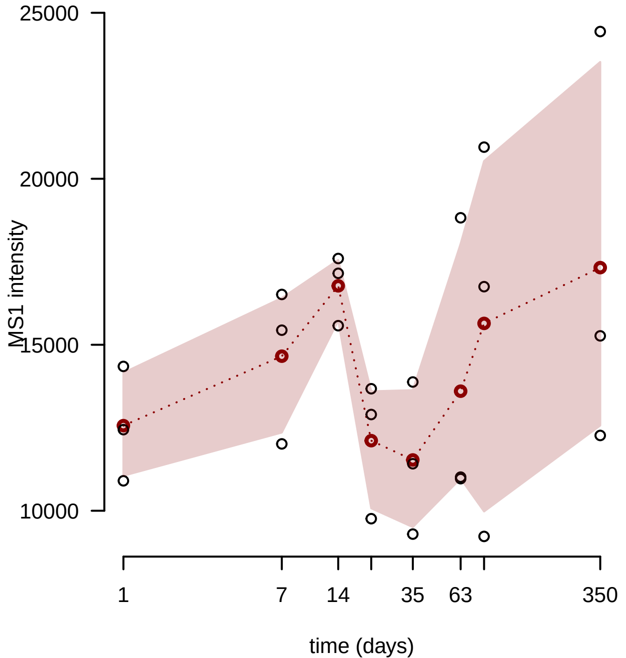

Q9CZR8
GN=Tsfm
Ts translation elongation factor, mitoch ...

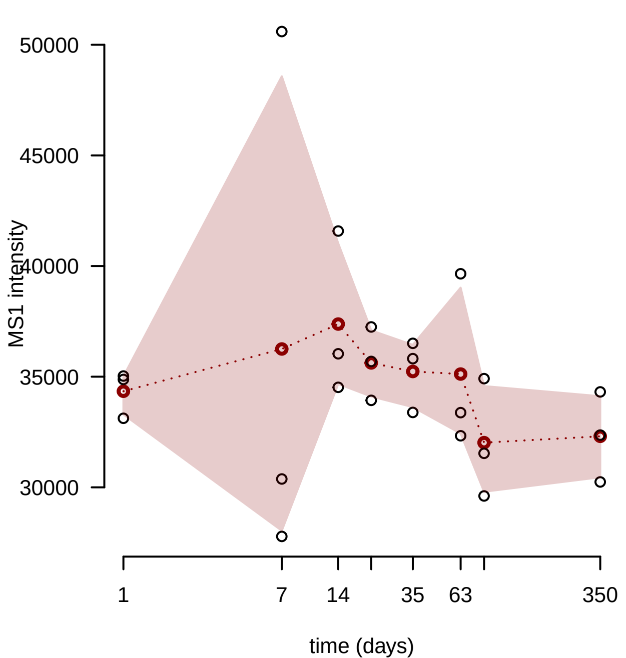

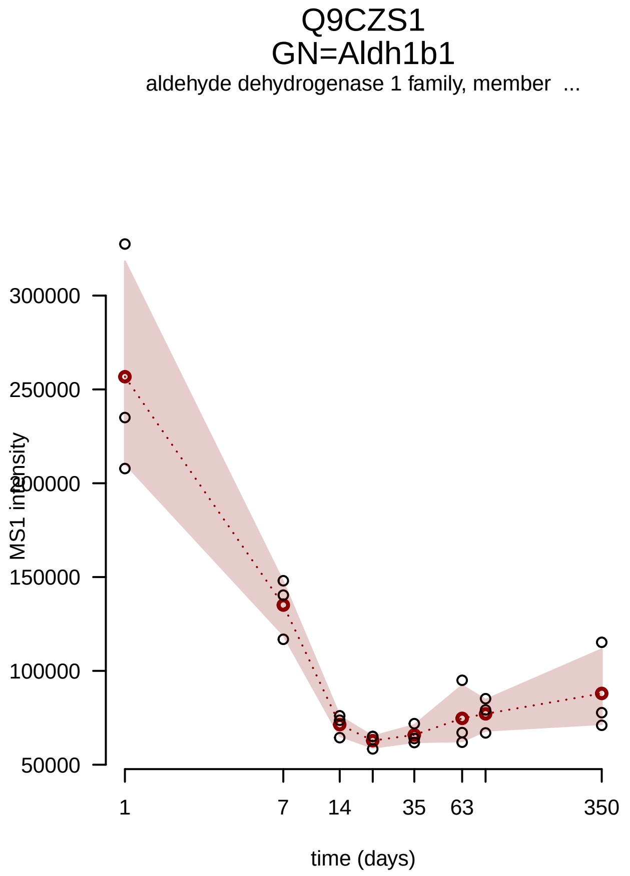
Q9CZS1
GN=Aldh1b1
aldehyde dehydrogenase 1 family, member ...
300000
250000
200000
150000
100000
50000
MS1 intensity
1
7
14
35
63
350
time (days)

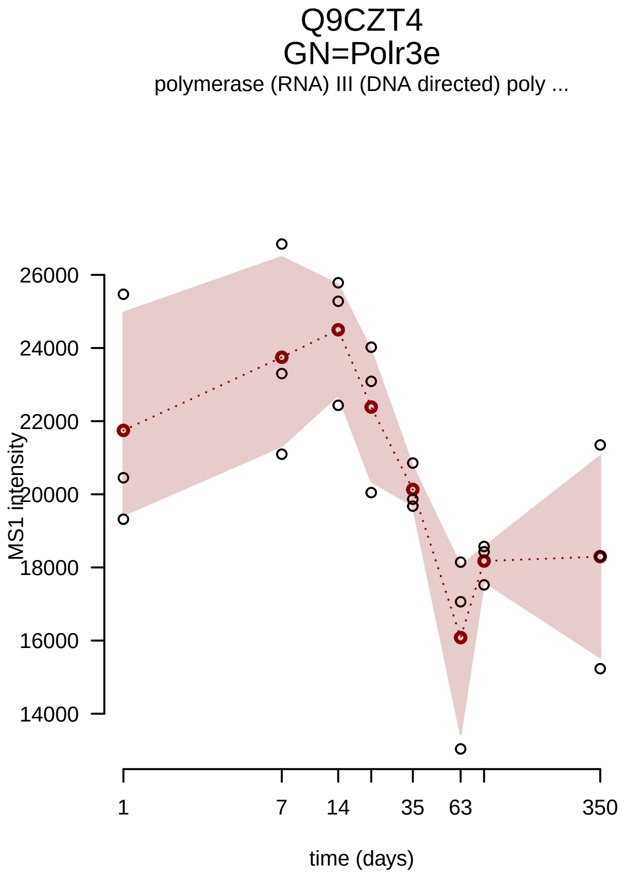
Q9CZT4
GN=Polr3e
polymerase (RNA) III (DNA directed) poly ...
26000
24000
22000
20000
18000
16000
14000
MS1 intensity
1
7
14
35
63
350
time (days)

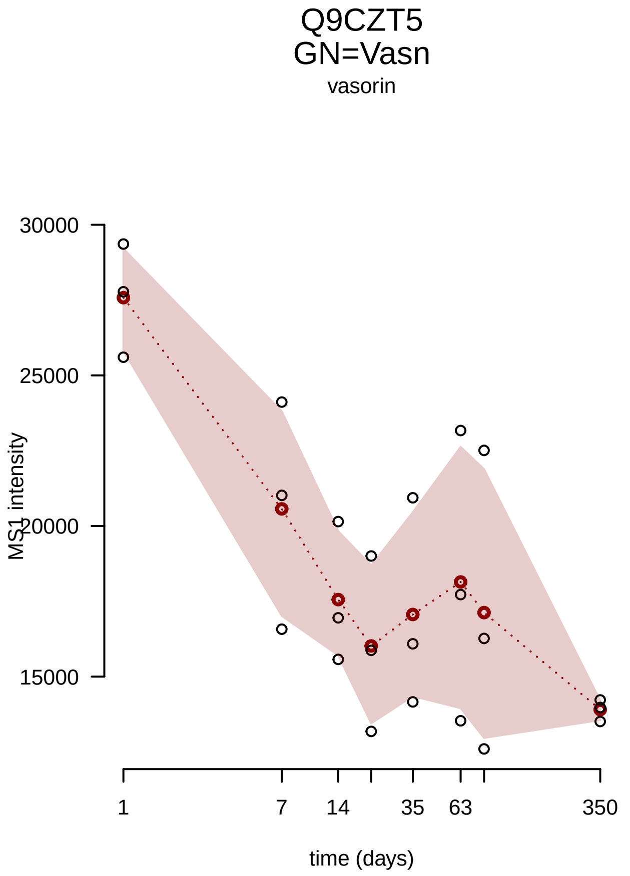
Q9CZT5
GN=Vasn
vasorin
30000
25000
20000
15000
MS1 intensity
1
7
14
35
63
350
time (days)

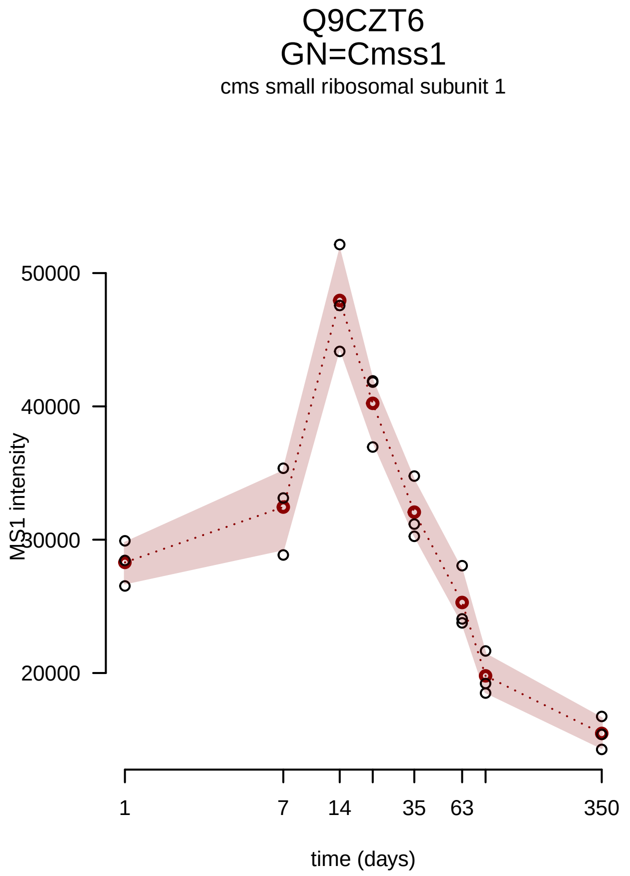
Q9CZT6
GN=Cmss1
cms small ribosomal subunit 1
50000
40000
30000
20000
MS1 intensity
1
7
14
35
63
350
time (days)

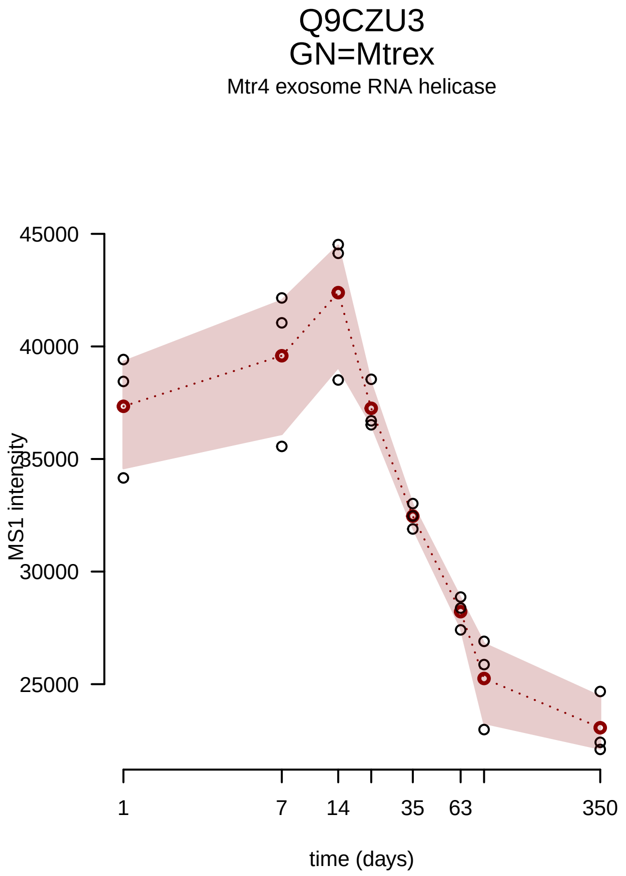
Q9CZU3
GN=Mtrex
Mtr4 exosome RNA helicase
45000
40000
35000
30000
25000
MS1 intensity
1
7
14
35
63
350
time (days)

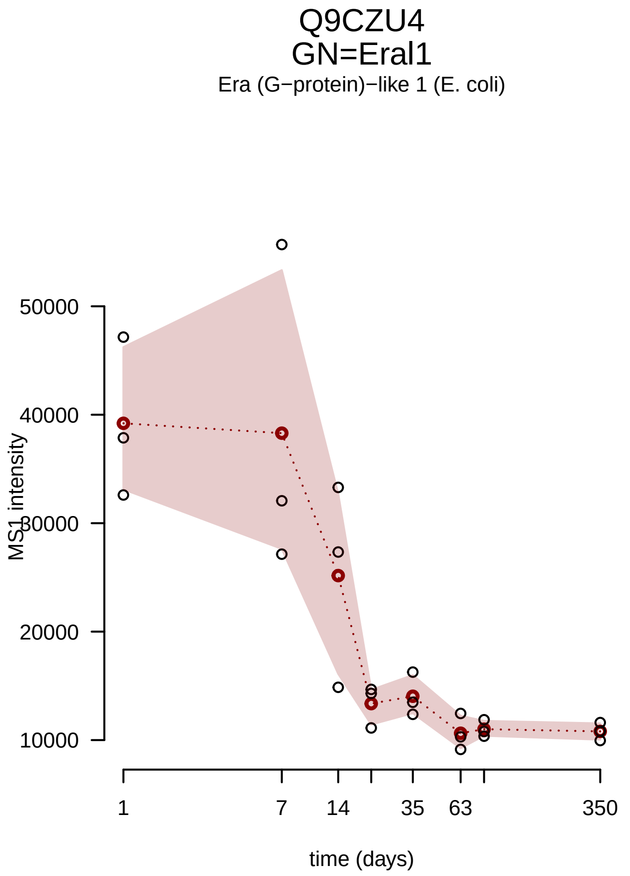
Q9CZU4
GN=Eral1
Era (G−protein)−like 1 (E. coli)
50000
40000
30000
20000
10000
MS1 intensity
1
7
14
35
63
350
time (days)

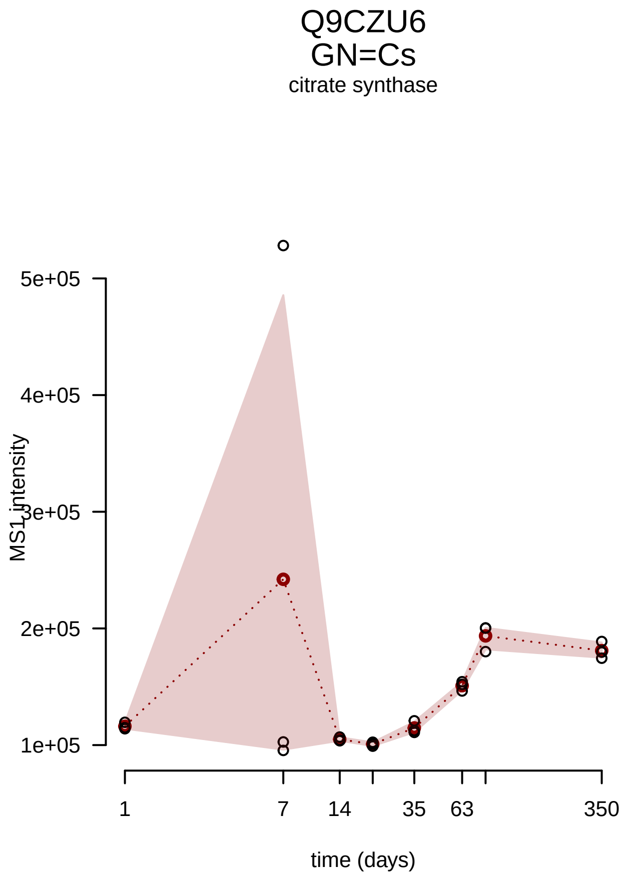
Q9CZU6
GN=Cs
citrate synthase
5e+05
4e+05
3e+05
2e+05
1e+05
MS1 intensity
1
7
14
35
63
350
time (days)

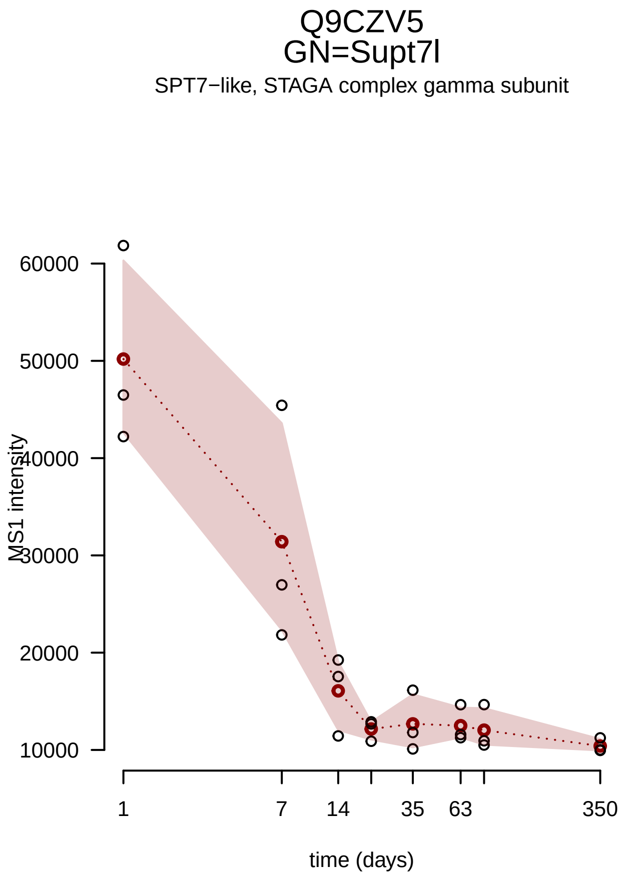
Q9CZV5
GN=Supt7l
SPT7−like, STAGA complex gamma subunit
60000
50000
40000
30000
20000
10000
MS1 intensity
1
7
14
35
63
350
time (days)

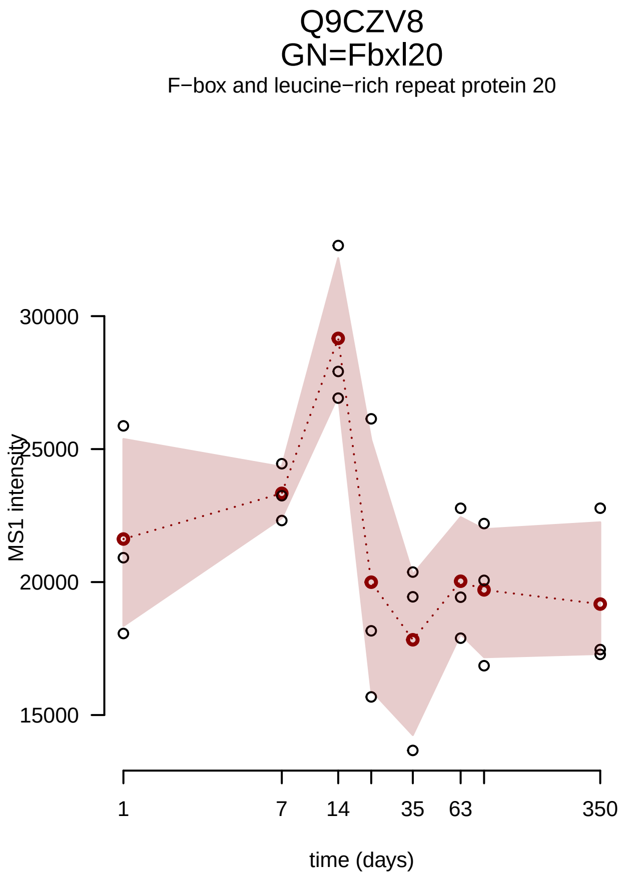
Q9CZV8
GN=Fbxl20
F−box and leucine−rich repeat protein 20
30000
25000
20000
15000
MS1 intensity
1
7
14
35
63
350
time (days)

Q9CZW2
GN=Cenpn
centromere protein N

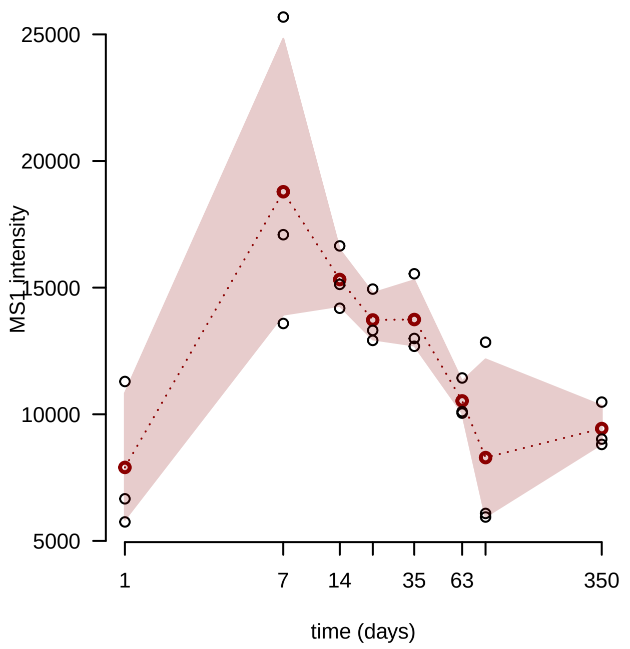

Q9CZW4
GN=Acsl3
acyl−CoA synthetase long−chain family me ...

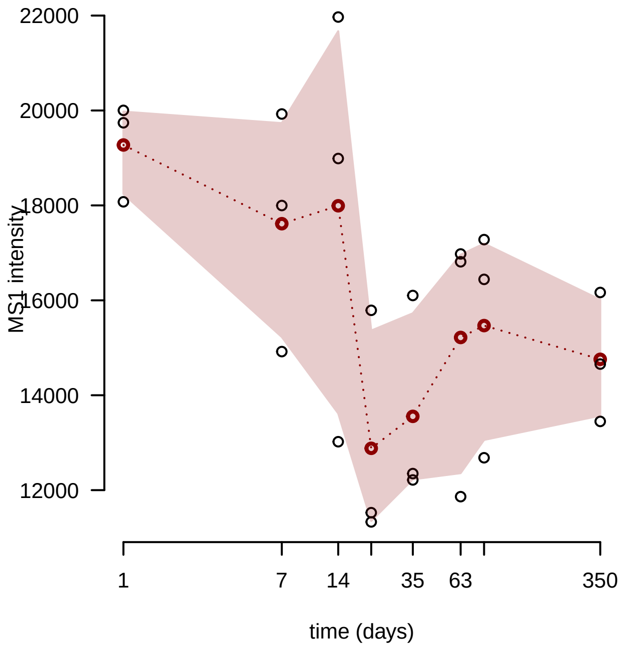

Q9CZW5
GN=Tomm70

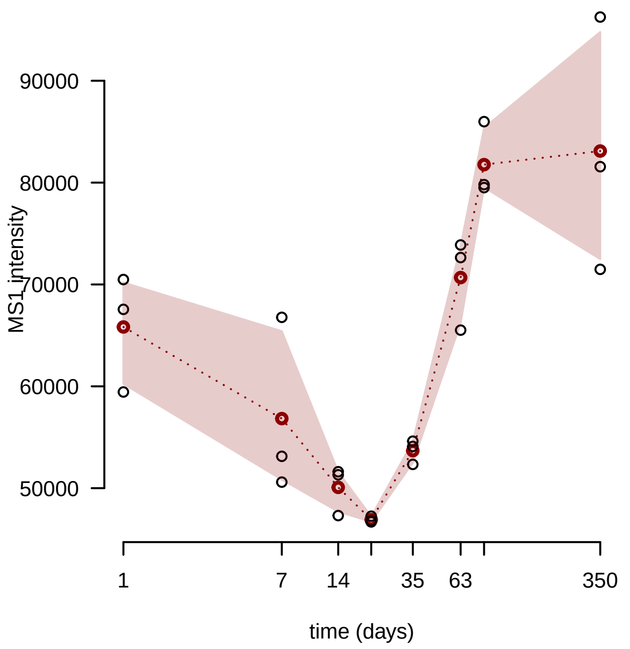

Q9CZX0
GN=Elp3
elongator acetyltransferase complex subu ...

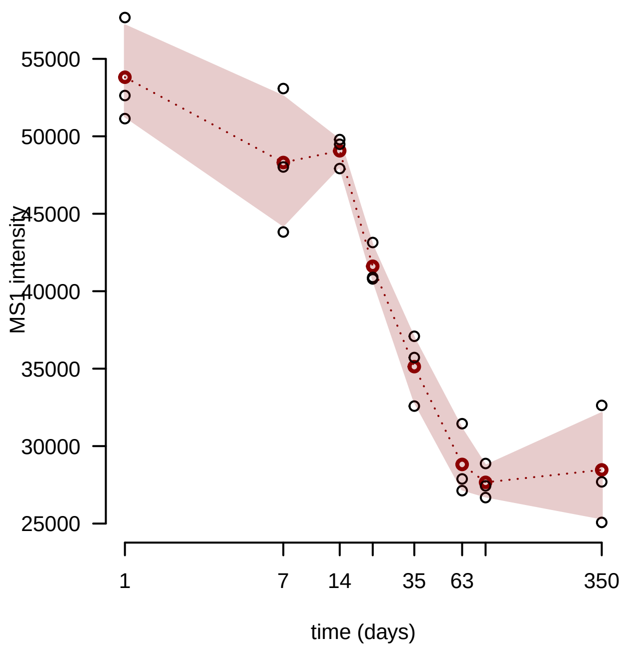

Q9CZX5
GN=Pinx1
PIN2/TERF1 interacting, telomerase inhib ...

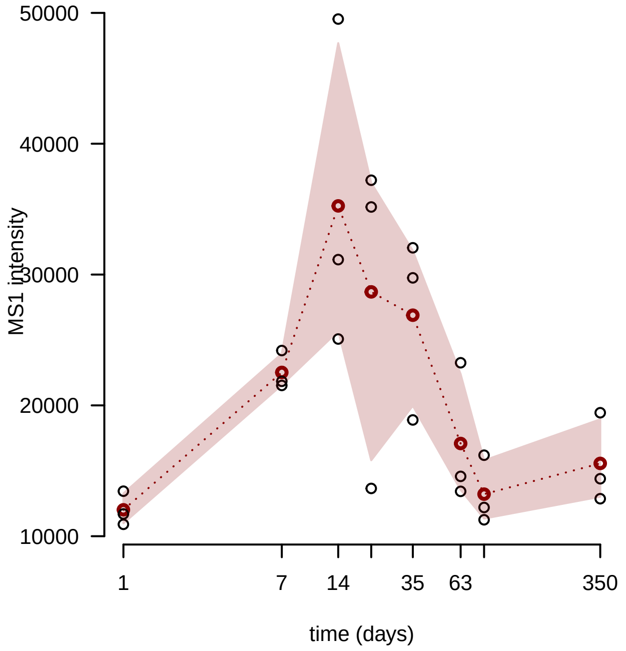

Q9CZX7
GN=Pip4p2
phosphatidylinositol−4,5−bisphosphate 4− ...

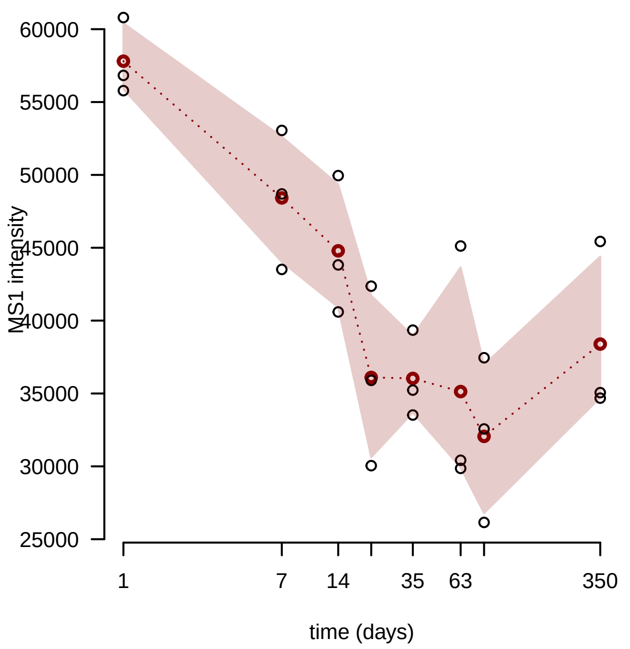

Q9CZX8
GN=Rps19
ribosomal protein S19

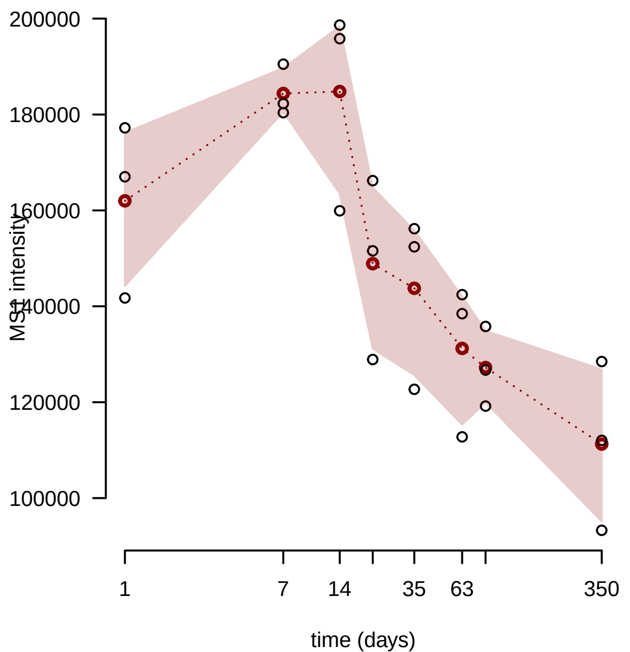

Q9CZX9
GN=Emc4
ER membrane protein complex subunit 4

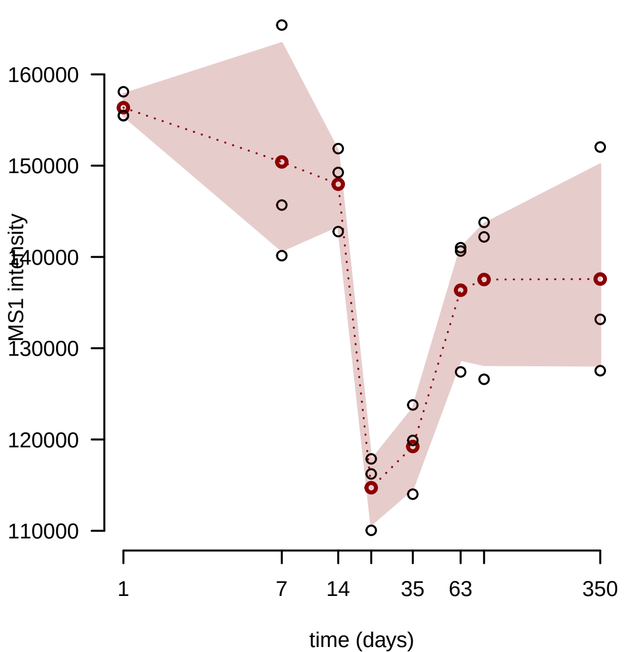

Q9CZY3
GN=Ube2v1
ubiquitin−conjugating enzyme E2 variant ...

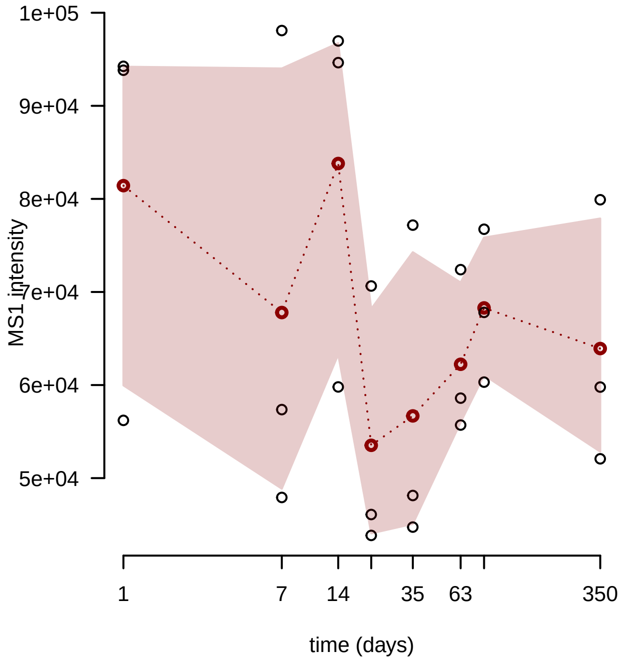

Q9D009
GN=Lipt2
lipoyl(octanoyl) transferase 2 (putative ...

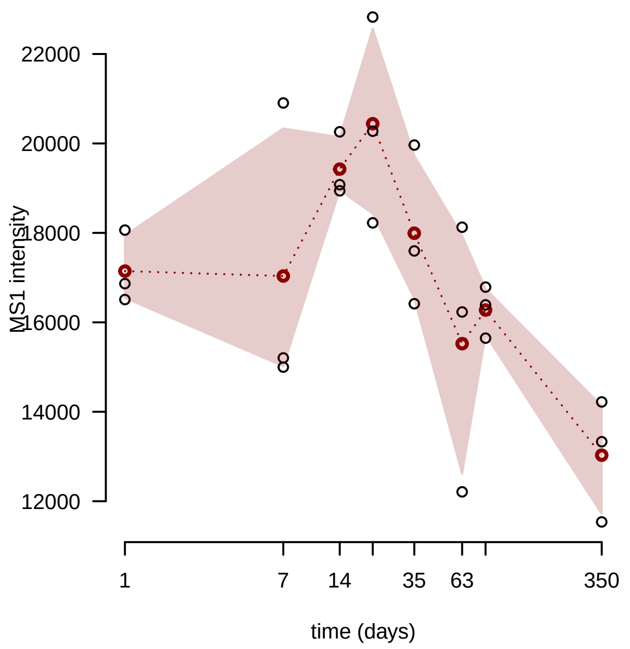

Q9D020
GN=Nt5c3a

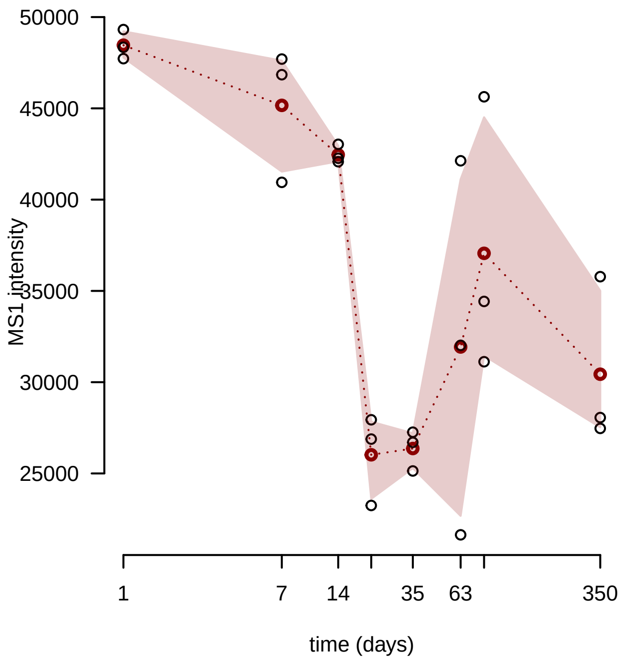

Q9D020
GN=Nt5c3a

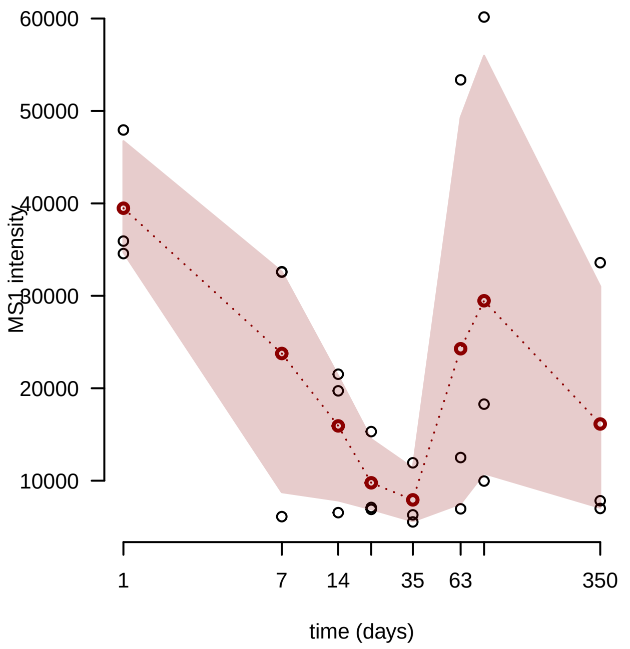

Q9D023
GN=Mpc2
mitochondrial pyruvate carrier 2

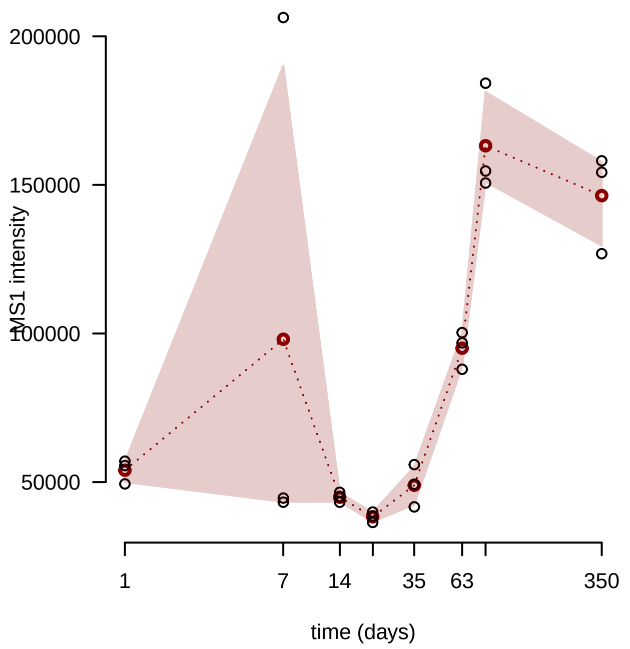

Q9D024
GN=Ccdc47
coiled−coil domain containing 47

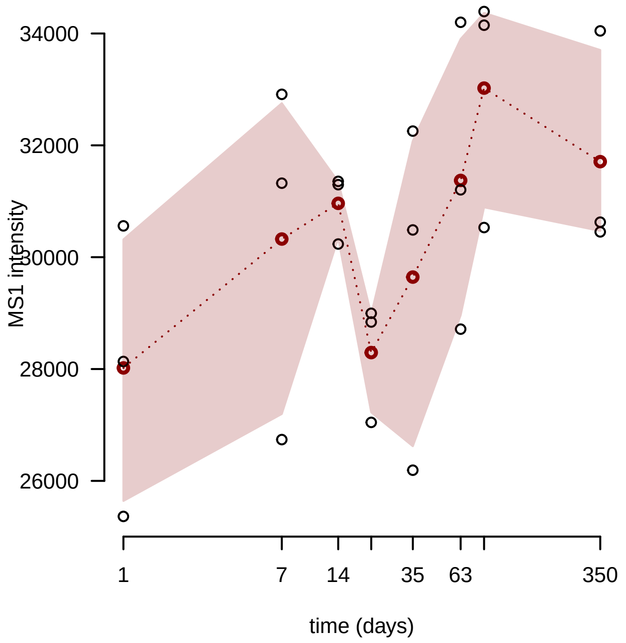

Q9D032
GN=Ssbp3
single−stranded DNA binding protein 3

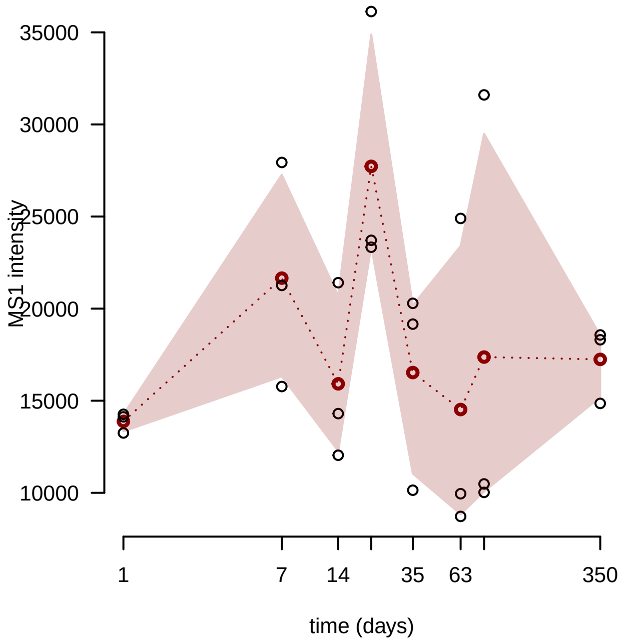

Q9D051
GN=Pdhb
pyruvate dehydrogenase (lipoamide) beta

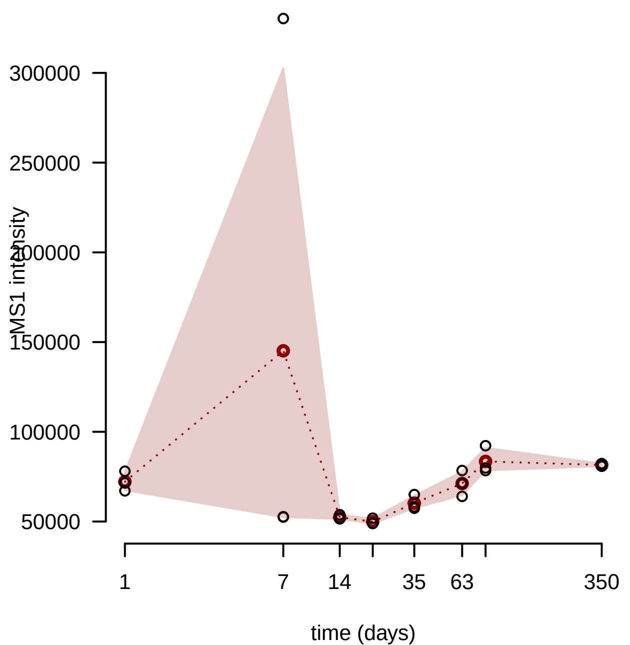

Q9D061
GN=Acbd6
acyl−Coenzyme A binding domain containin ...

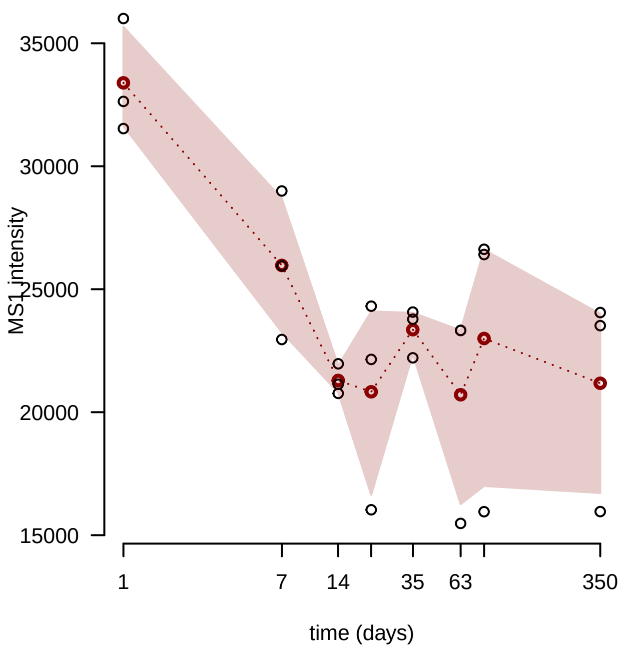

Q9D071
GN=Mms19
MMS19 cytosolic iron−sulfur assembly com ...

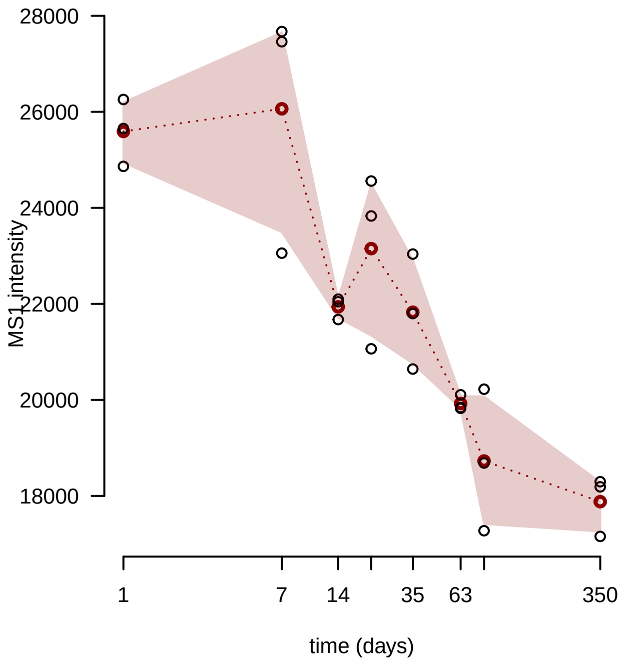

Q9D074
GN=Mgrn1
mahogunin, ring finger 1

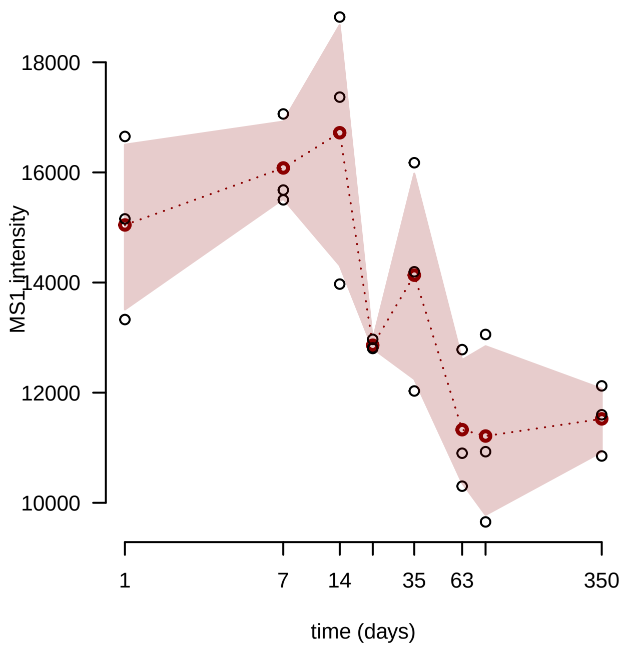

Q9D081
GN=Alg14
asparagine−linked glycosylation 14

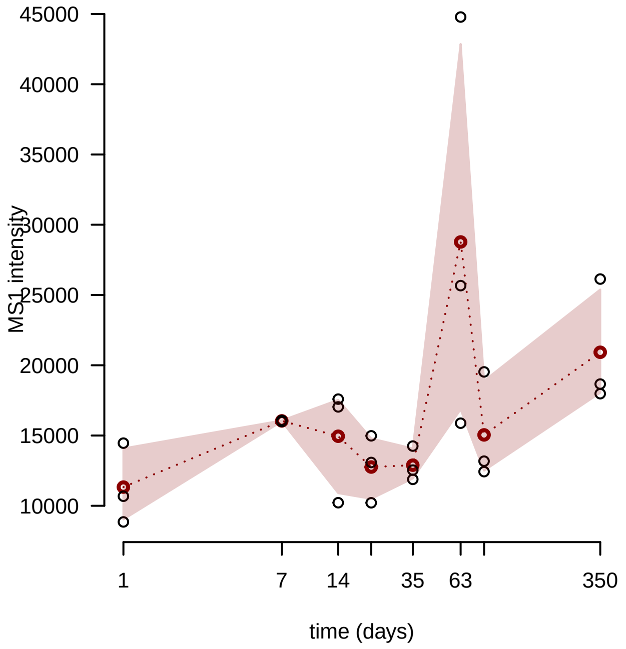

Q9D083
GN=Spc24
SPC24, NDC80 kinetochore complex compone ...

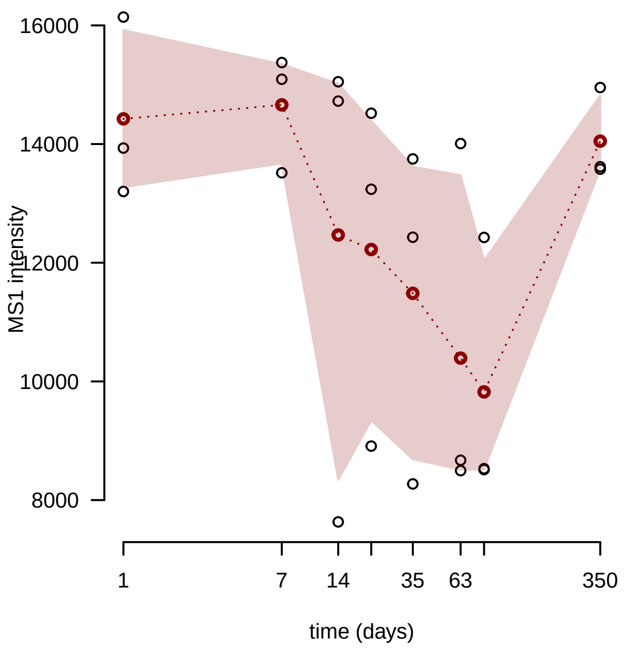

Q9D084
GN=Cenps
centromere protein S

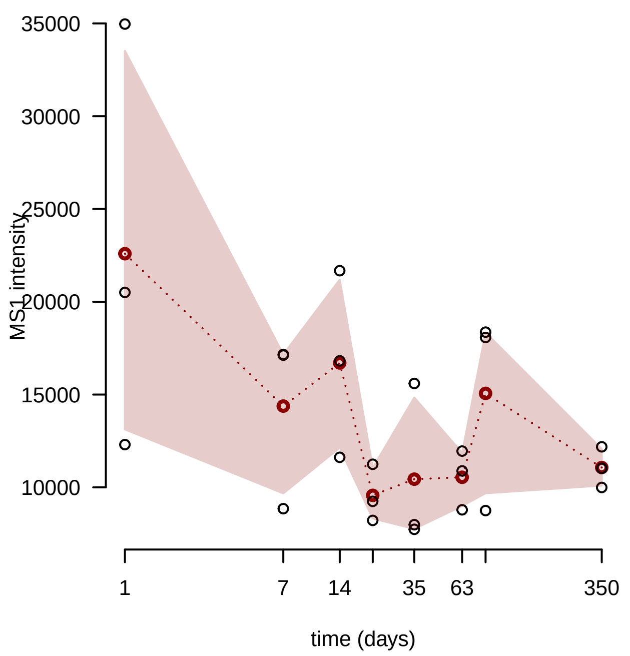

Q9D0A0
GN=Det1
de−etiolated homolog 1 (Arabidopsis)

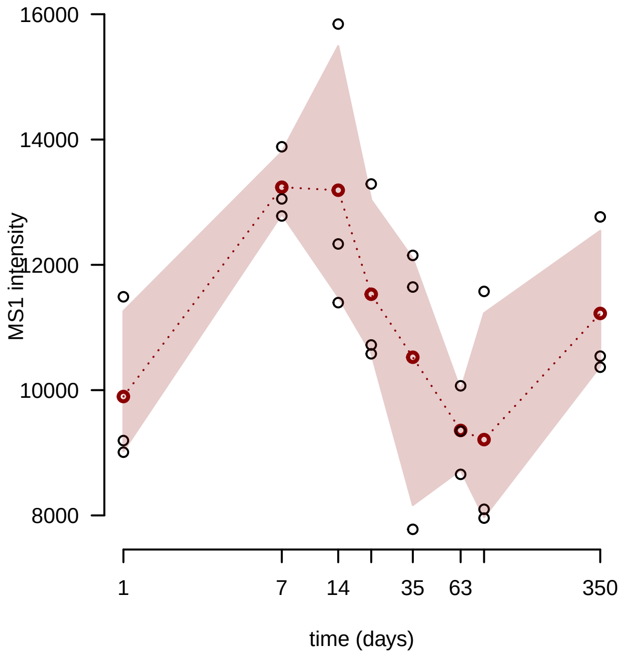

Q9D0A3
GN=Arpin
actin−related protein 2/3 complex inhibi ...

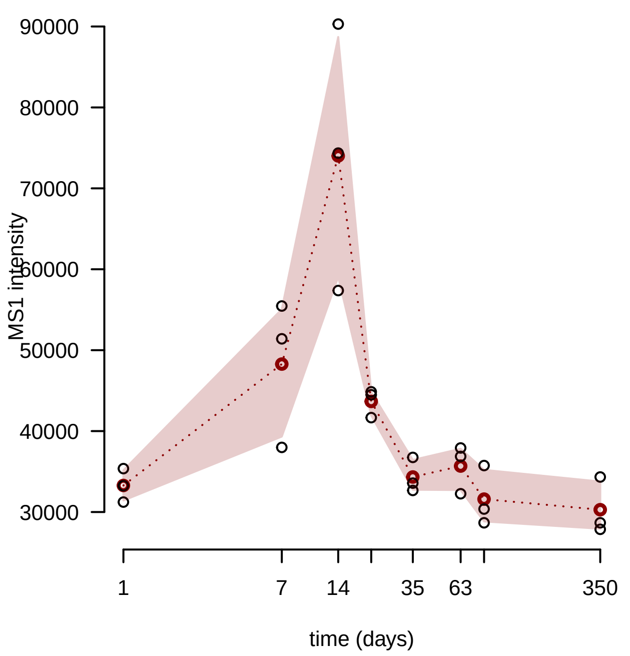

Q9D0B0
GN=Srsf9
serine and arginine−rich splicing factor ...

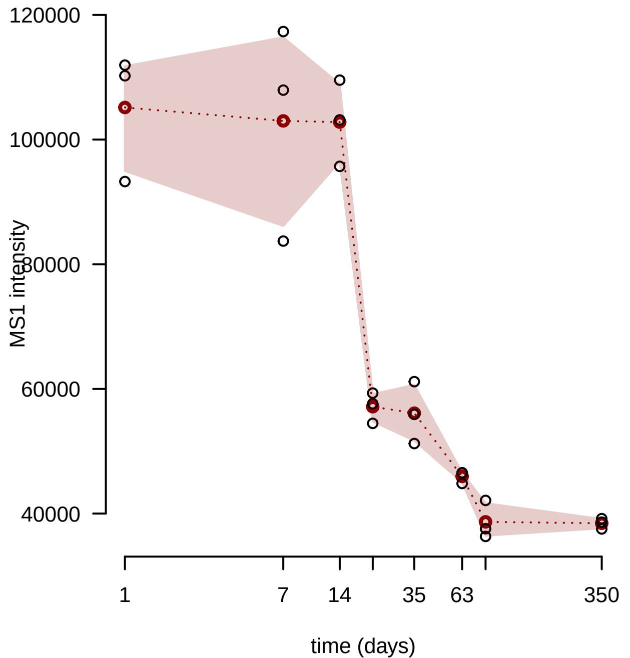

Q9D0B1
GN=Znf524

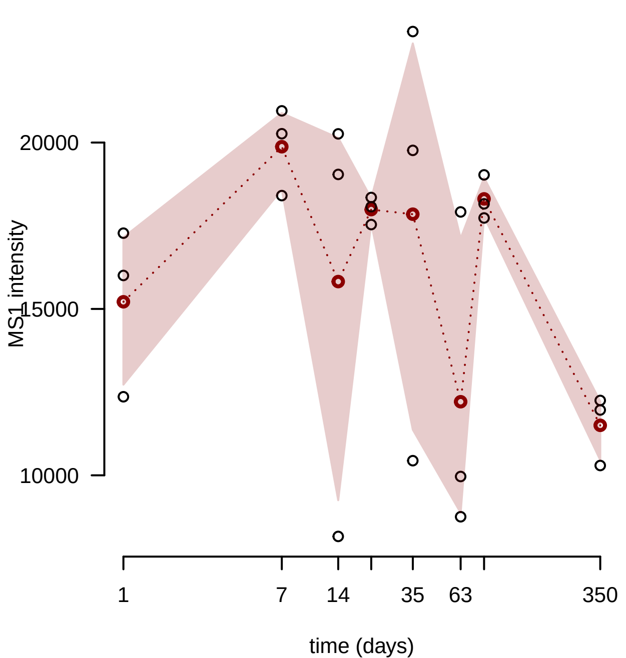

Q9D0B5
GN=Tstd3
thiosulfate sulfurtransferase (rhodanese ...

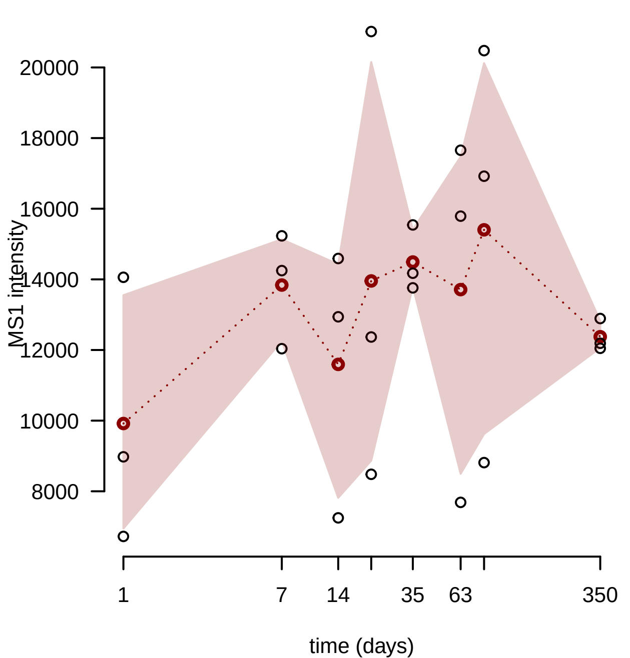

Q9D0B6
GN=Pbdc1
polysaccharide biosynthesis domain conta ...

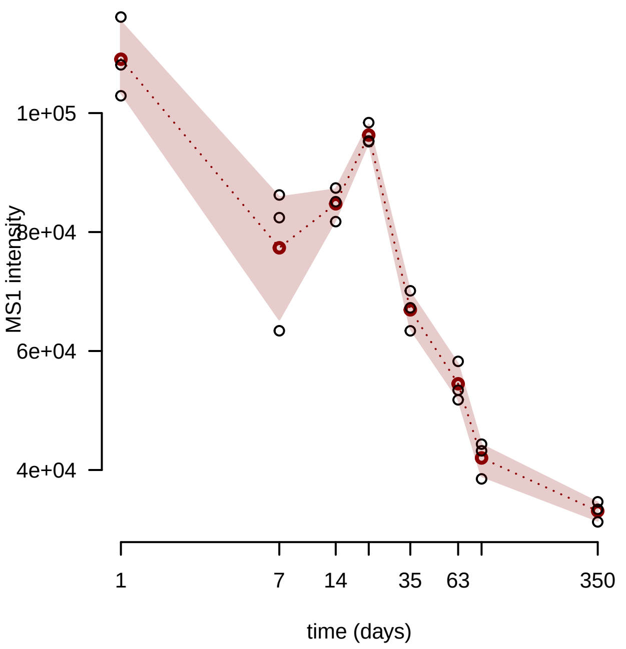

Q9D0C1
GN=Rnf115
ring finger protein 115

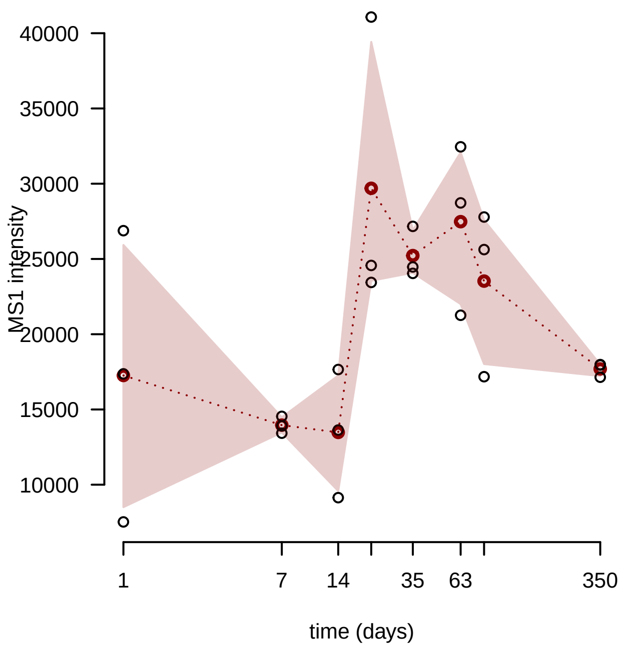

Q9D0C4
GN=Trmt5
TRM5 tRNA methyltransferase 5

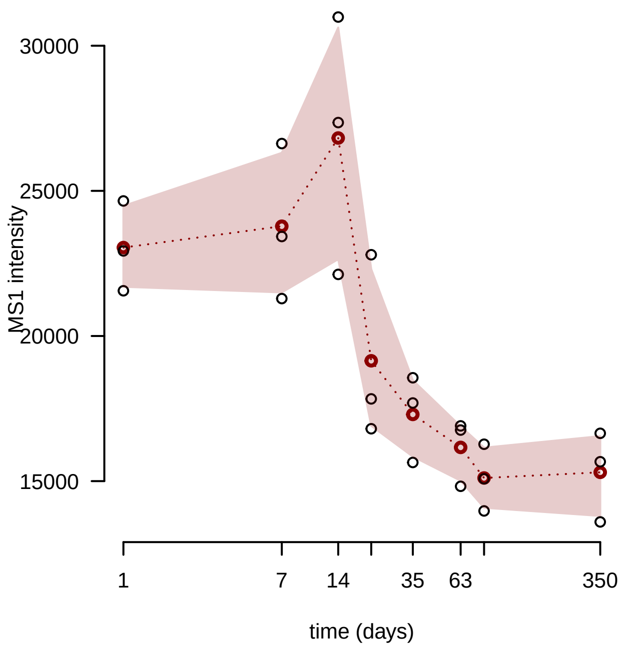

Q9D0D3
GN=Mtpap
mitochondrial poly(A) polymerase

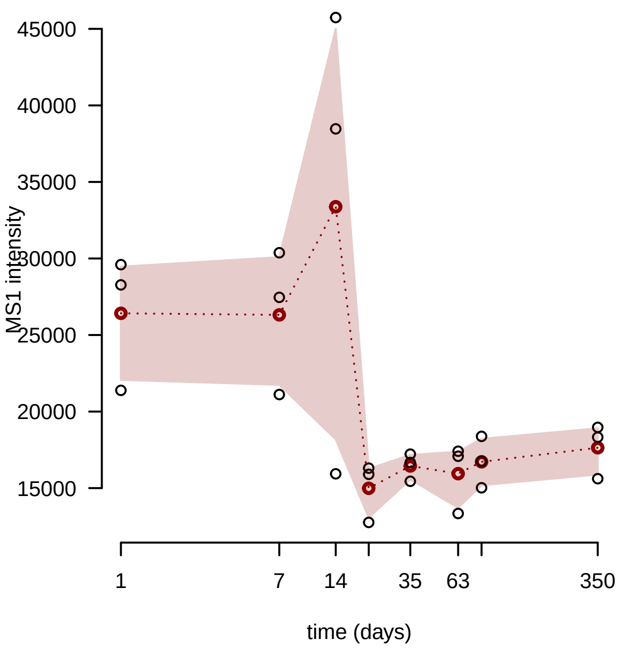

Q9D0D4
GN=Dimt1
DIM1 dimethyladenosine transferase 1−lik ...

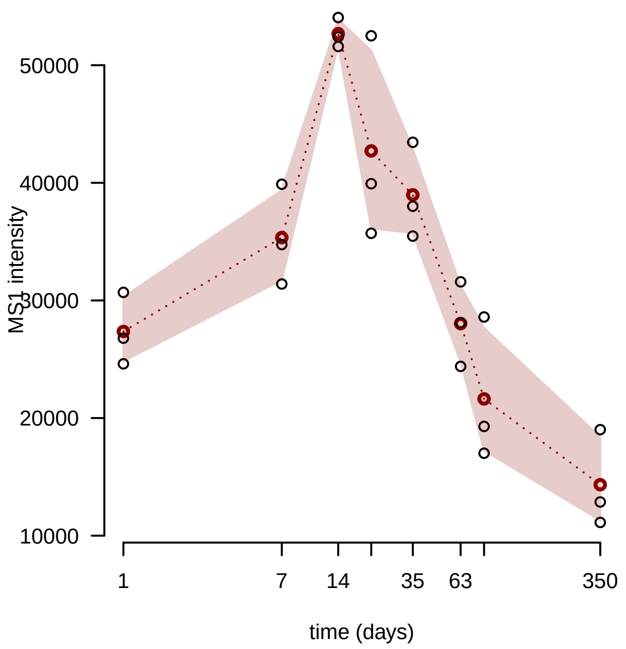

Q9D0D5
GN=Gtf2e1
general transcription factor II E, polyp ...

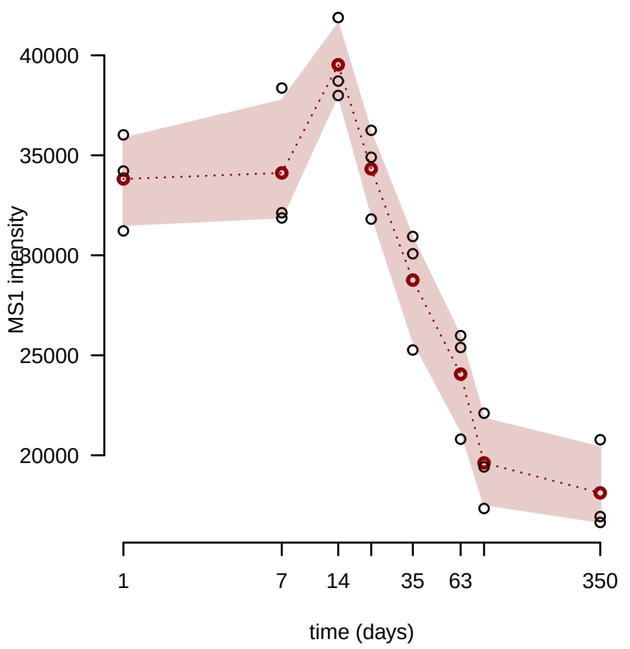

Q9D0E1
GN=Hnrnpm
heterogeneous nuclear ribonucleoprotein ...

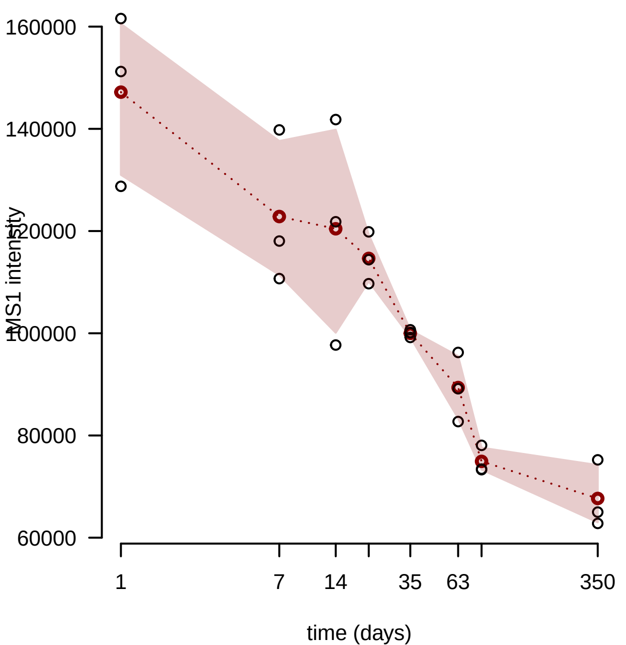

Q9D0E3
GN=Lysmd1
LysM, putative peptidoglycan−binding, do ...

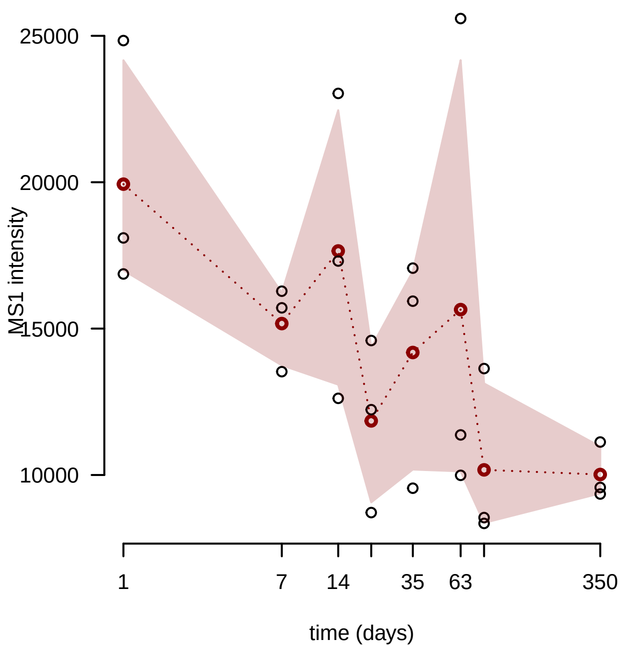

Q9D0F1
GN=Ndc80
NDC80 kinetochore complex component

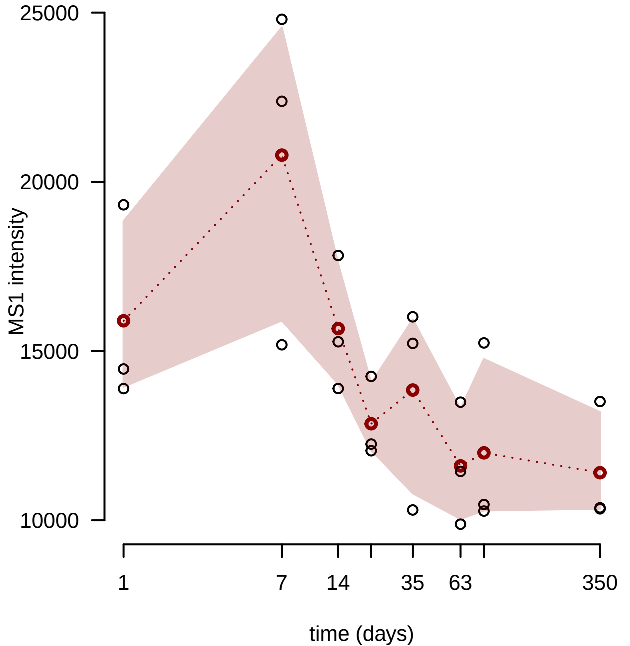

Q9D0F3
GN=Lman1
lectin, mannose−binding, 1

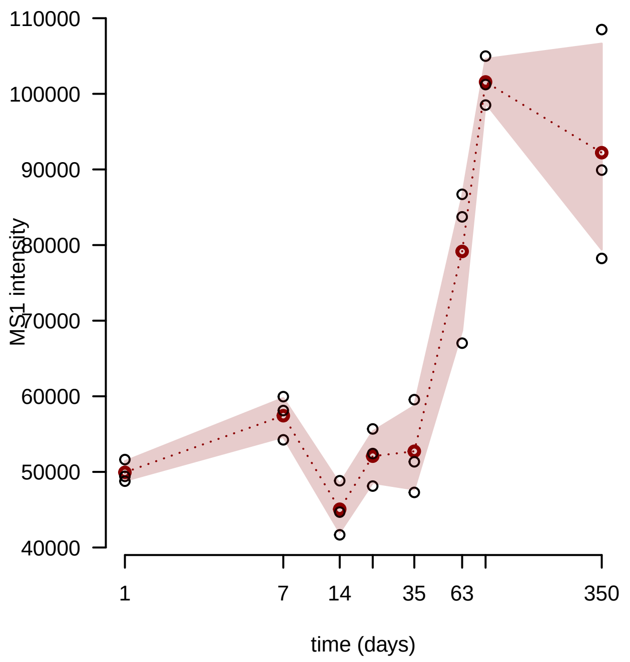

Q9D0F4
GN=Nkap
NFKB activating protein

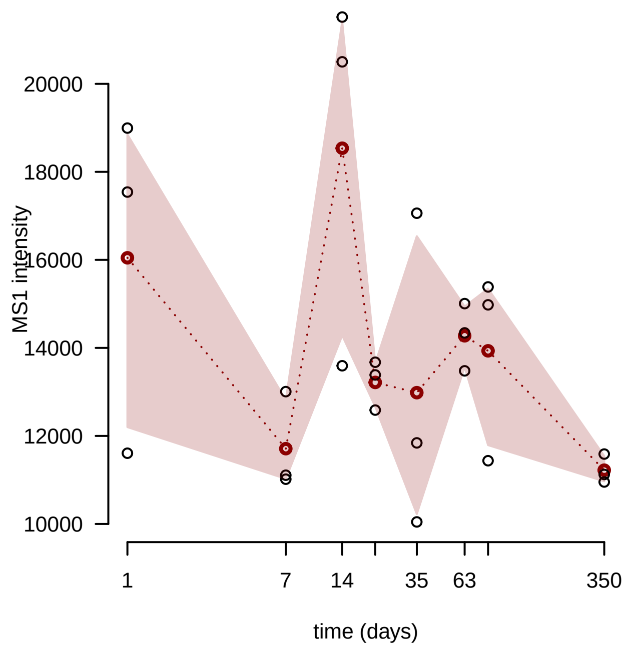

Q9D0F6
GN=Rfc5
replication factor C (activator 1) 5

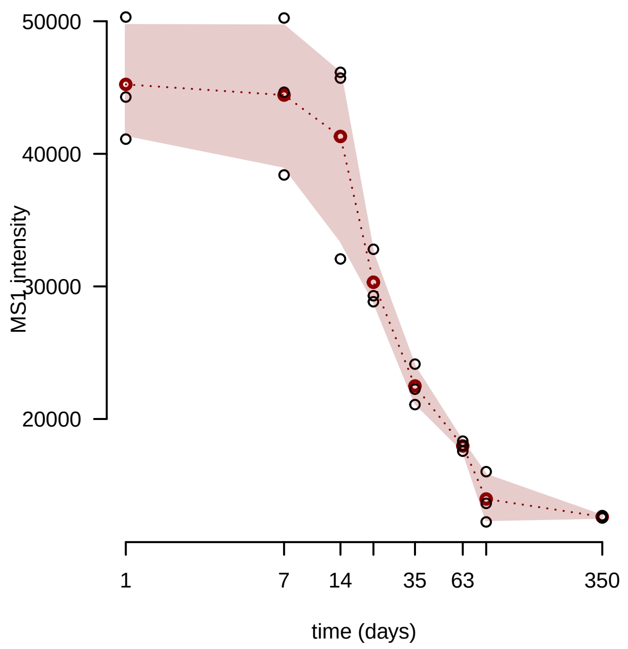

Q9D0F9
GN=Pgm1
phosphoglucomutase 1

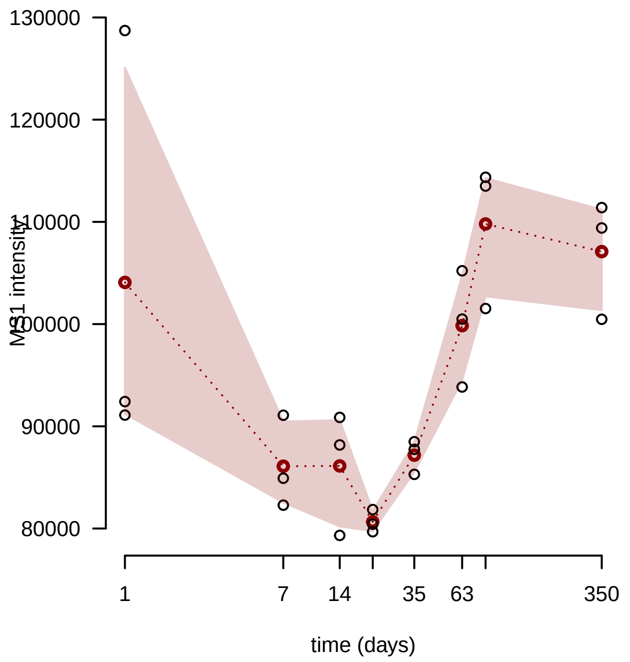

Q9D0G0
GN=Mrps30
mitochondrial ribosomal protein S30

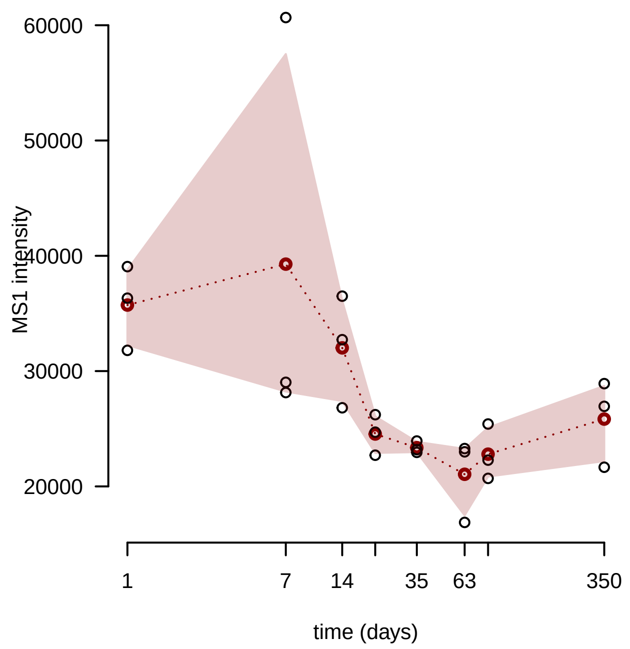

Q9D0I4
GN=Stx17
syntaxin 17

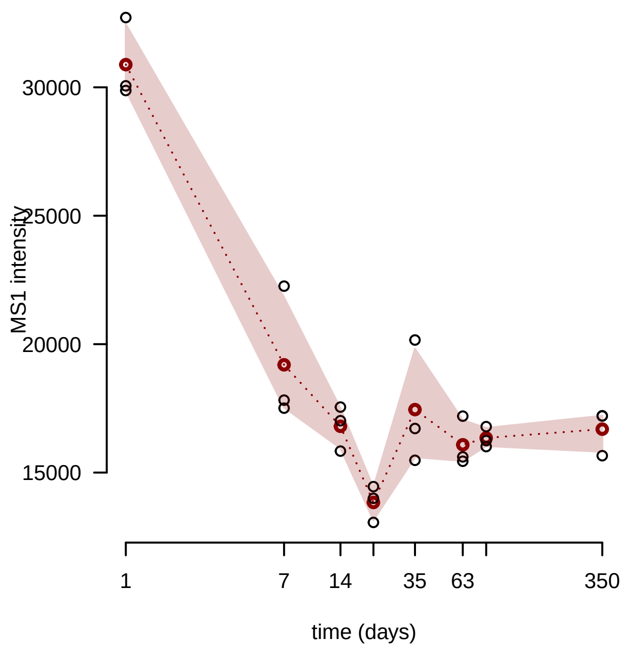

Q9D0I6
GN=Wdsub1
WD repeat, SAM and U−box domain containi ...

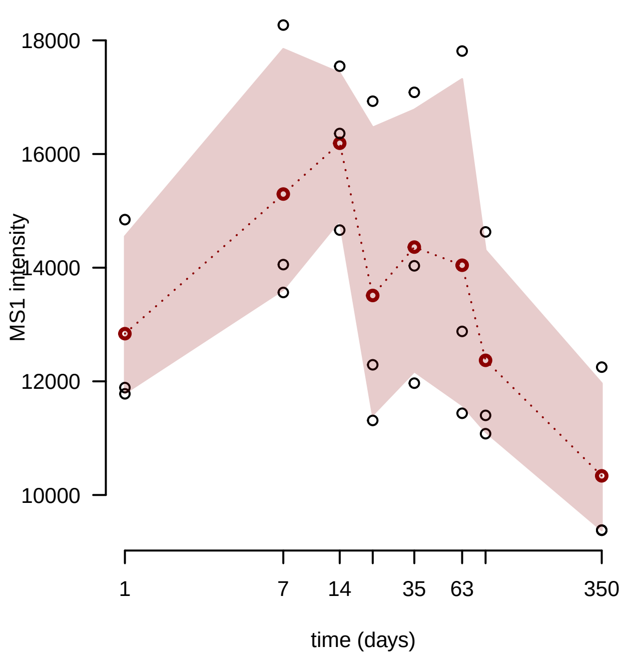

Q9D0I8
GN=Mrto4
mRNA turnover 4, ribosome maturation fac ...

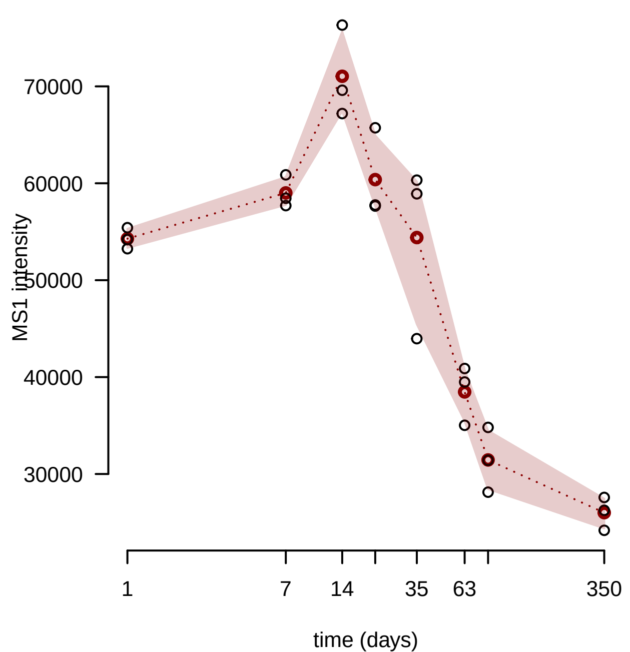

Q9D0I9
GN=Rars
arginyl−tRNA synthetase

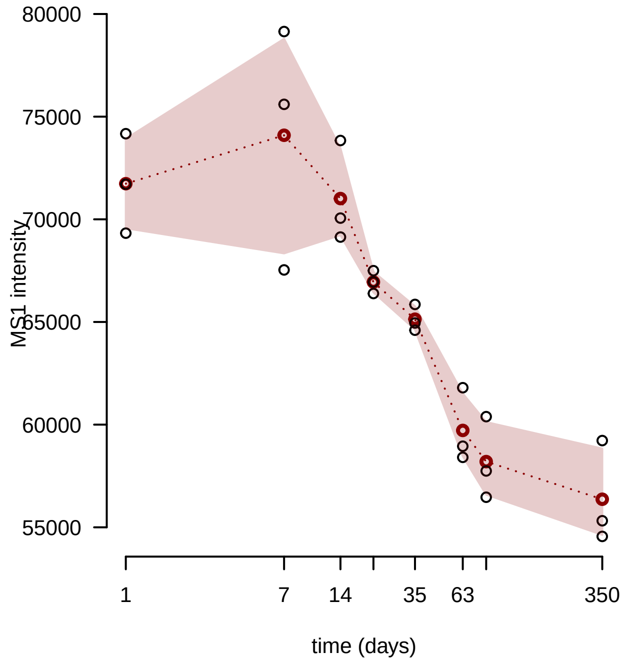

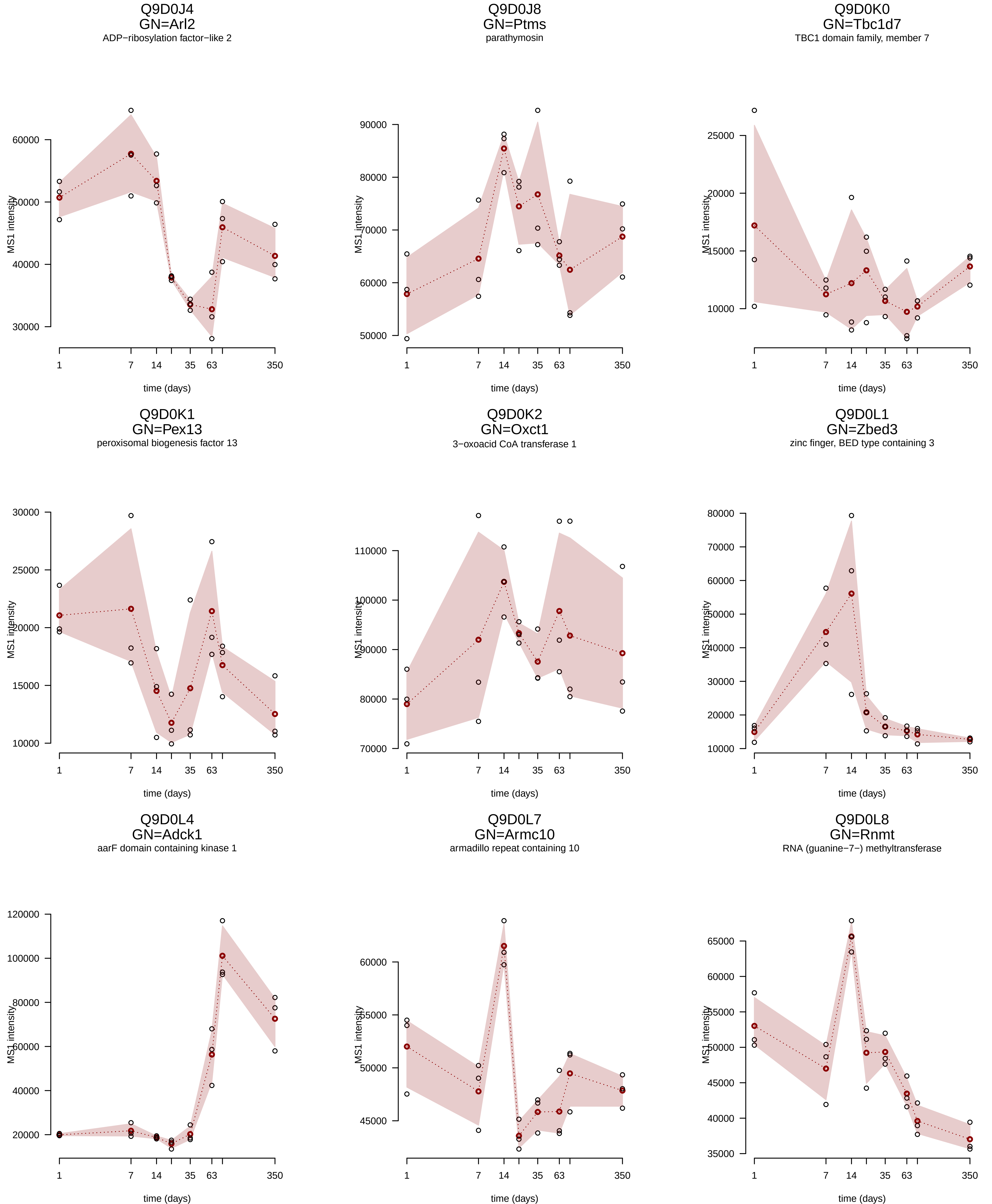

Q9D0J4
GN=Arl2
ADP−ribosylation factor−like 2
MS1 intensity
time (days)
Q9D0J8
GN=Ptms
parathymosin
MS1 intensity
time (days)
Q9D0K0
GN=Tbc1d7
TBC1 domain family, member 7
MS1 intensity
time (days)
Q9D0K1
GN=Pex13
peroxisomal biogenesis factor 13
MS1 intensity
time (days)
Q9D0K2
GN=Oxct1
3−oxoacid CoA transferase 1
MS1 intensity
time (days)
Q9D0L1
GN=Zbed3
zinc finger, BED type containing 3
MS1 intensity
time (days)
Q9D0L4
GN=Adck1
aarF domain containing kinase 1
MS1 intensity
time (days)
Q9D0L7
GN=Armc10
armadillo repeat containing 10
MS1 intensity
time (days)
Q9D0L8
GN=Rnmt
RNA (guanine−7−) methyltransferase
MS1 intensity
time (days)
1
7
14
35
63
350

Q9D0M0
GN=Exosc7
exosome component 7

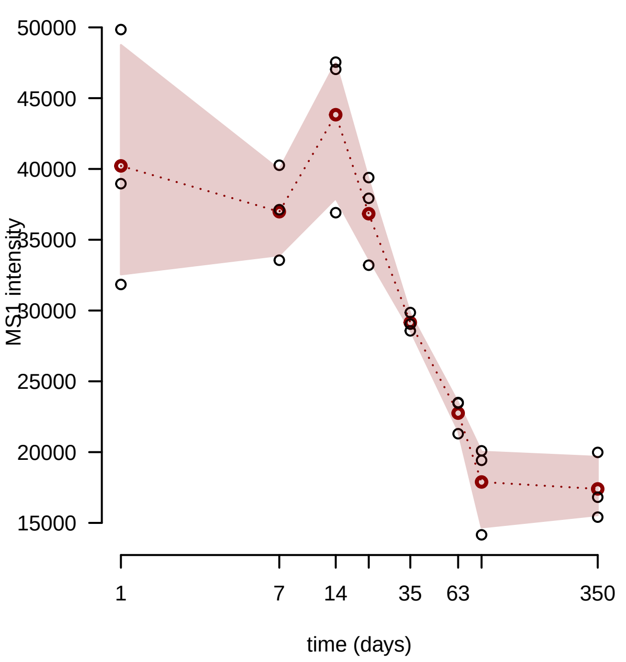

Q9D0M1
GN=Prpsap1
phosphoribosyl pyrophosphate synthetase− ...

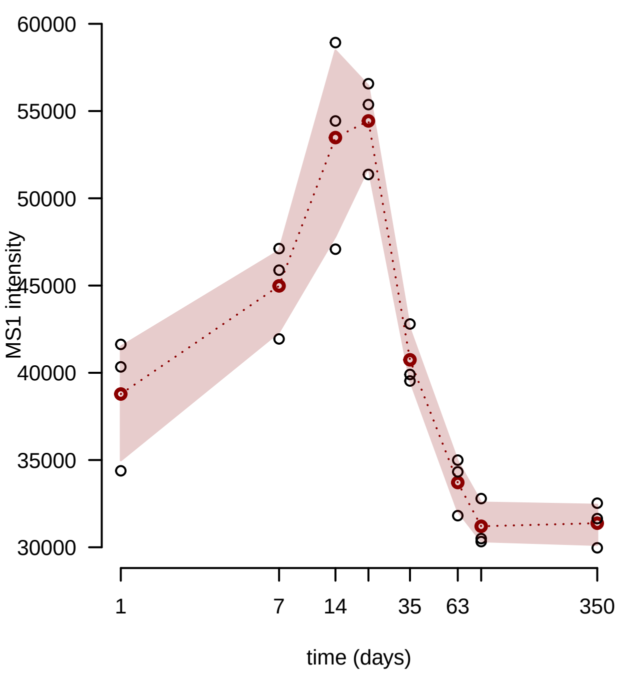

Q9D0M3
GN=Cyc1
cytochrome c−1

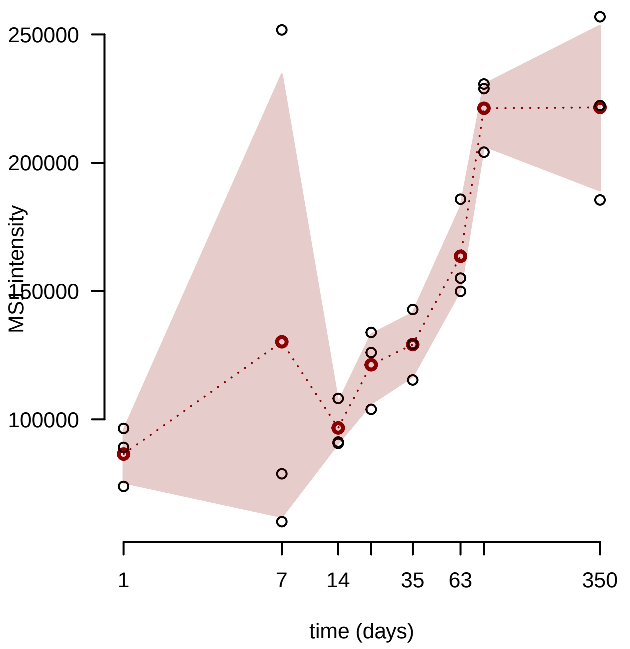

Q9D0M5
GN=Dynll2
dynein light chain LC8−type 2

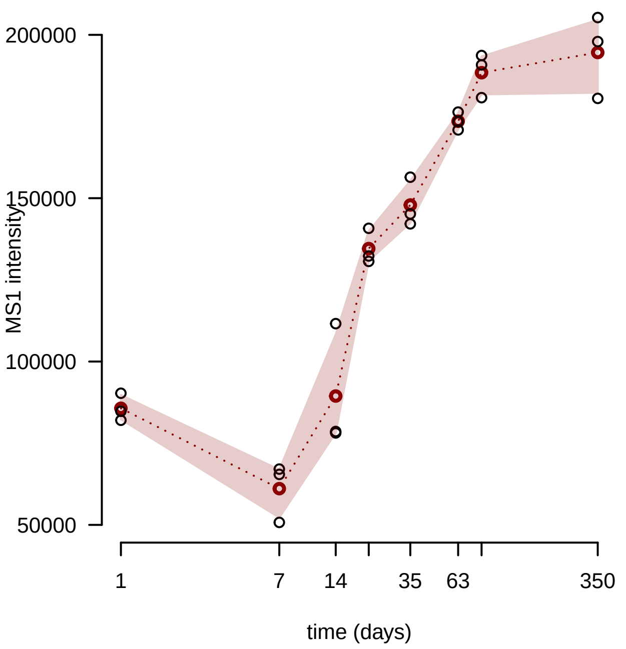

Q9D0N7
GN=Chaf1b
chromatin assembly factor 1, subunit B (...

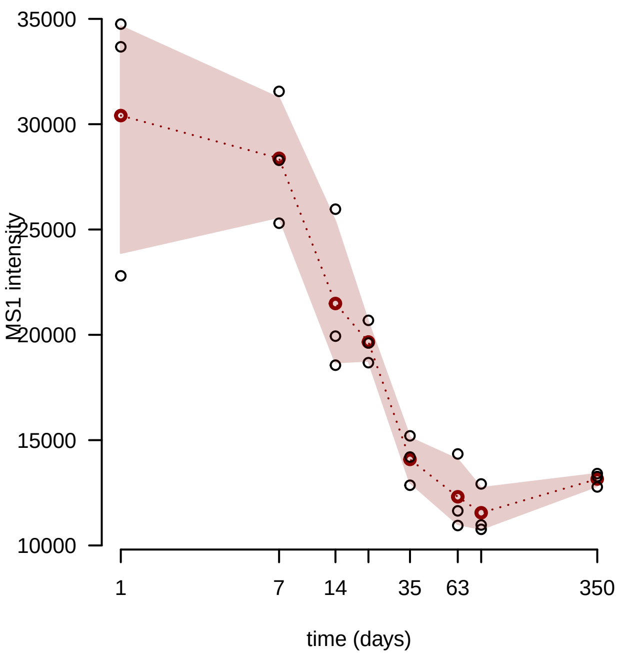

Q9D0P8
GN=Ift27
intraflagellar transport 27

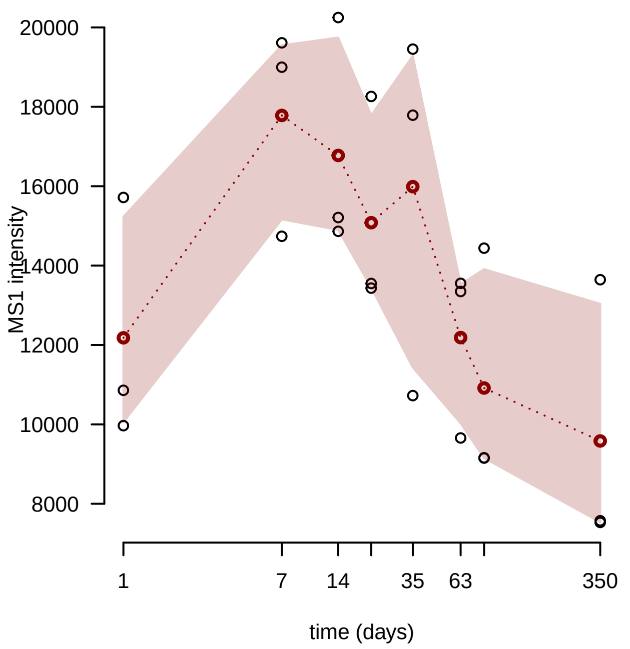

Q9D0Q7
GN=Mrpl45
mitochondrial ribosomal protein L45

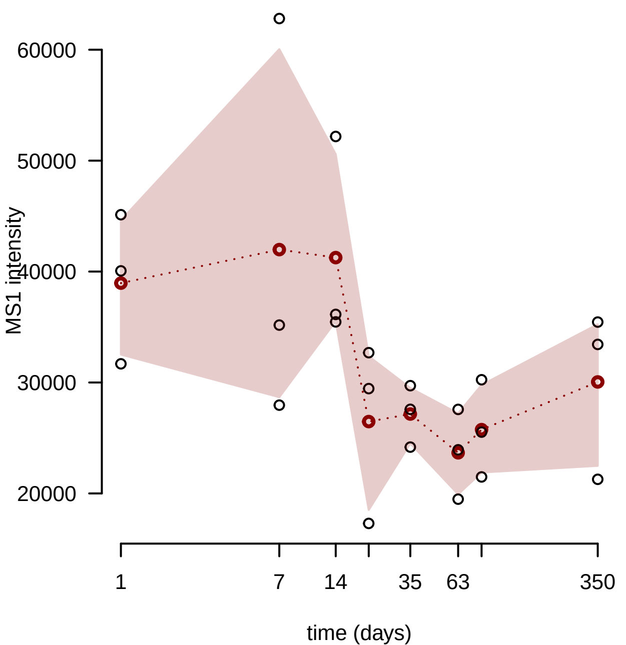

Q9D0R2
GN=Tars
threonyl−tRNA synthetase

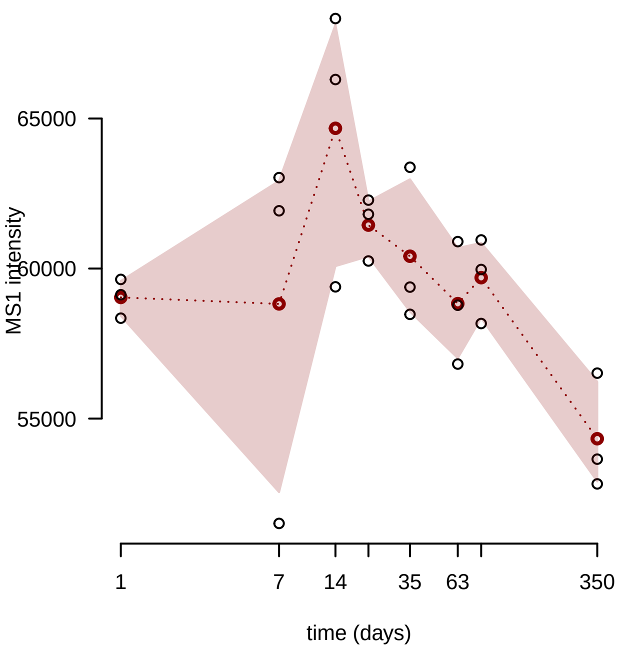

Q9D0R4
GN=Ddx56
DEAD box helicase 56

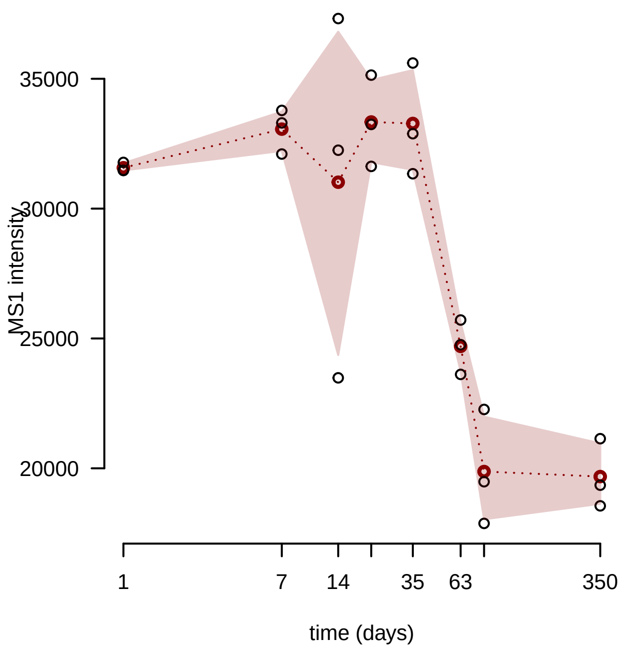

Q9D0R8
GN=Lsm12
LSM12 homolog

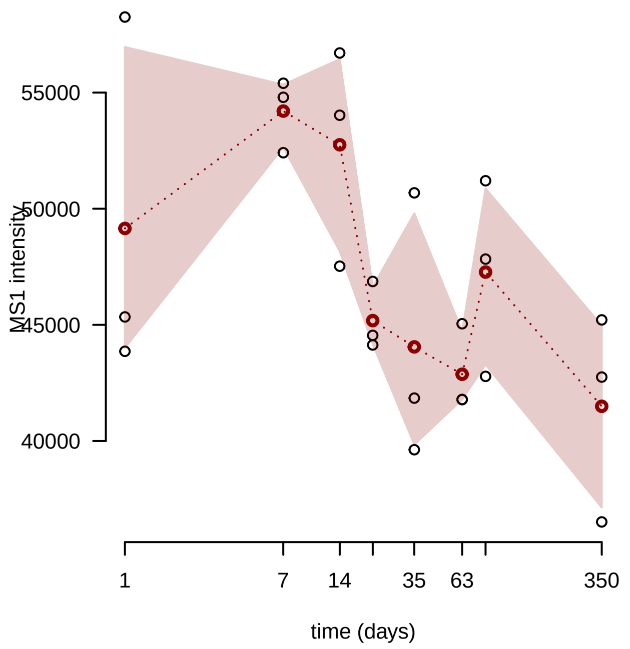

Q9D0R9
GN=Wdr89
WD repeat domain 89

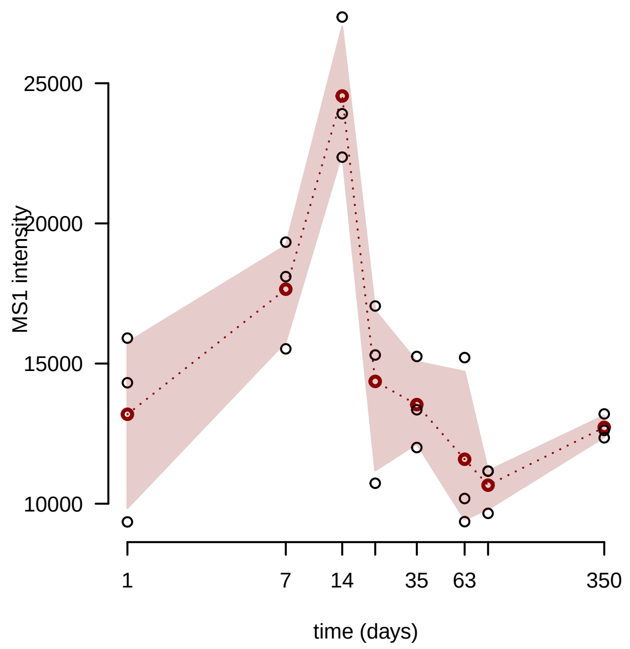

Q9D0S9
GN=Hint2
histidine triad nucleotide binding prote ...

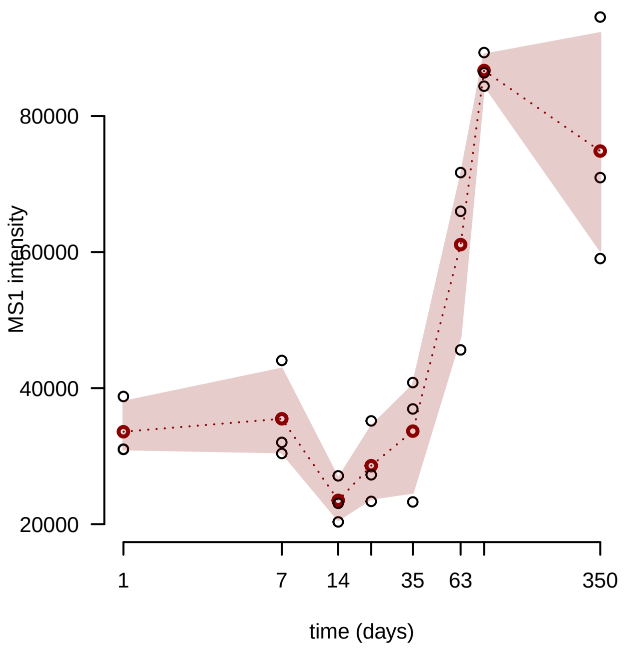

Q9D0T1
GN=Snu13
SNU13 homolog, small nuclear ribonucleop ...

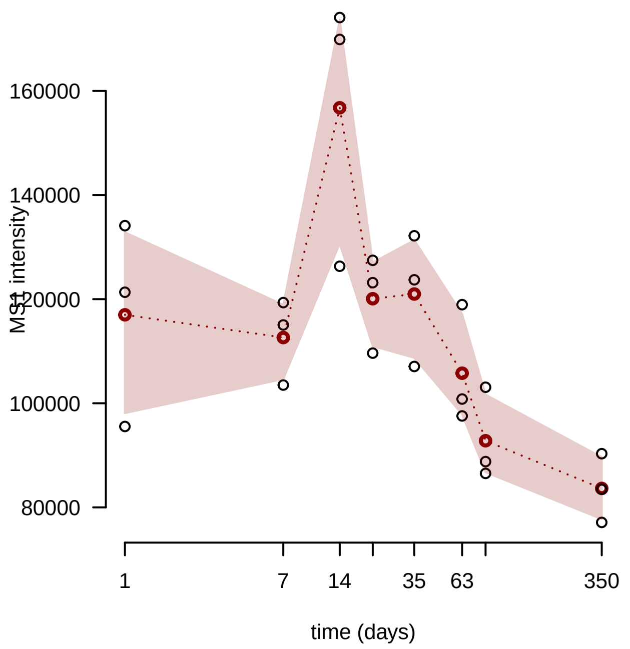

Q9D0T2
GN=Dusp12
dual specificity phosphatase 12

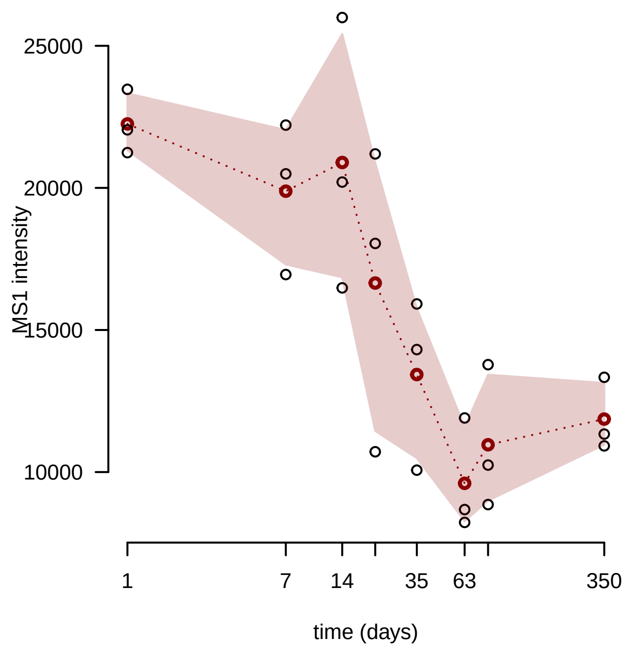

Q9D0V7
GN=Ebag9
estrogen receptor−binding fragment−assoc ...

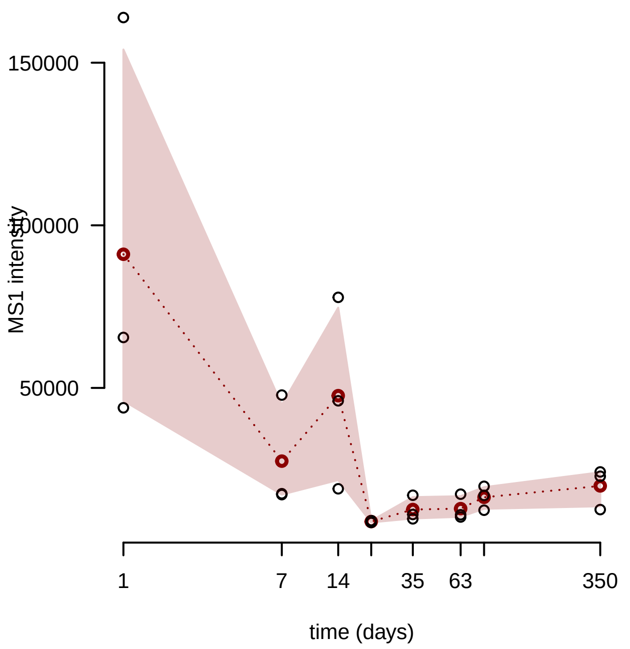

Q9D0W5
GN=Ppil1
peptidylprolyl isomerase (cyclophilin)−l ...

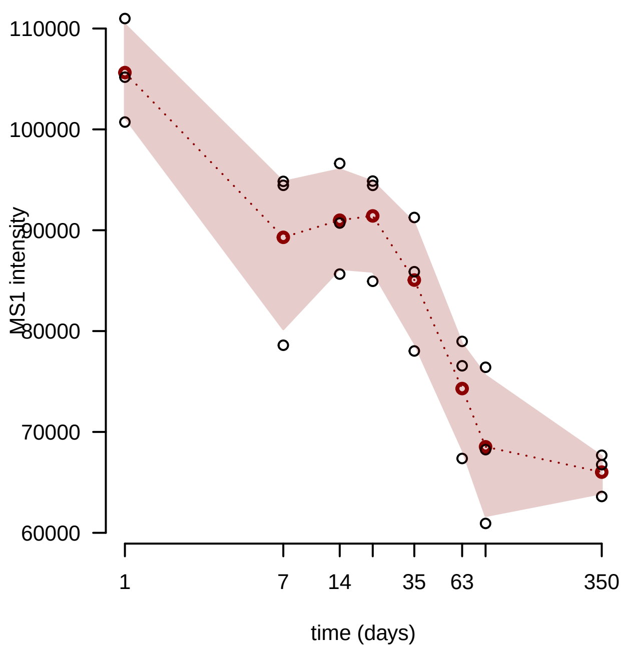

Q9D0Z3
GN=Tmem53
transmembrane protein 53

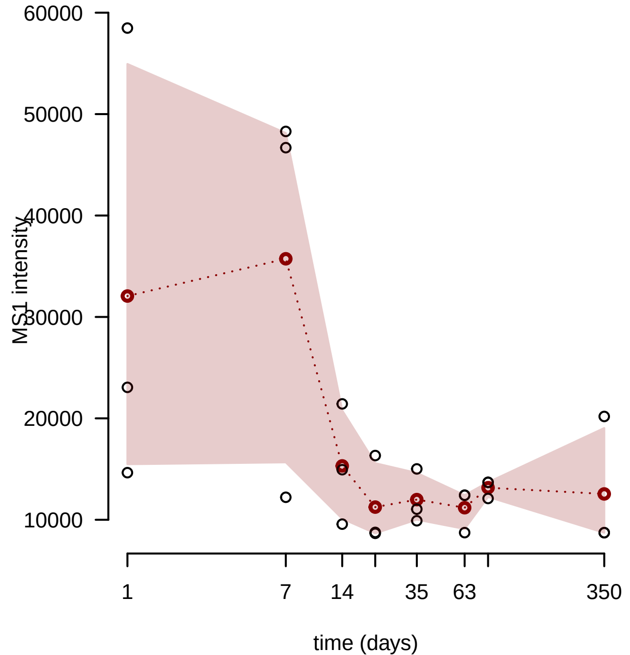

Q9D103
GN=Ifitm1
interferon induced transmembrane protein ...

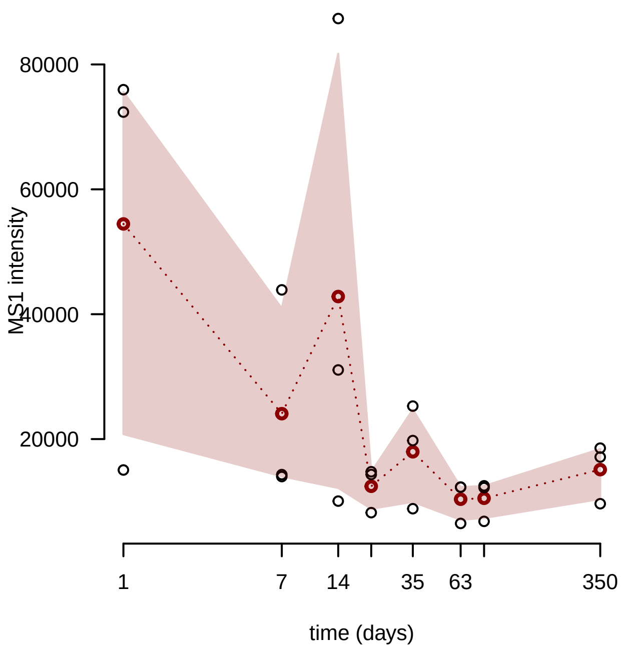

Q9D110
GN=Mthfs
5, 10−methenyltetrahydrofolate synthetas ...

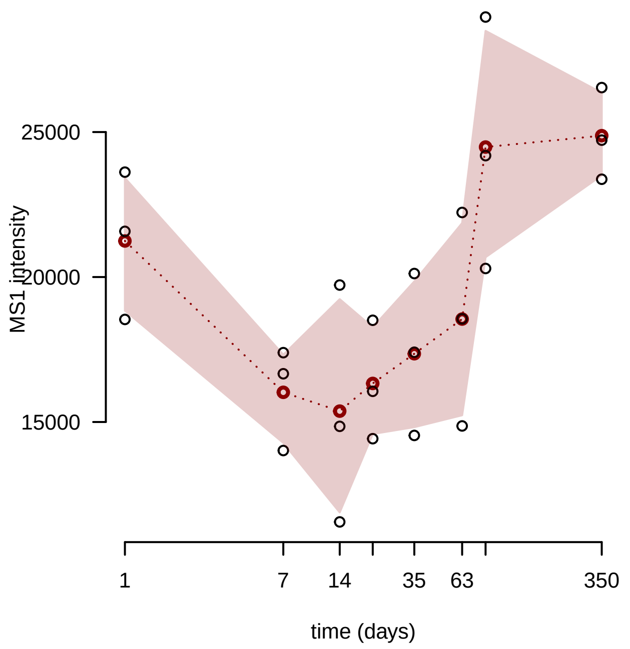

Q9D113
GN=Dnlz
DNL−type zinc finger

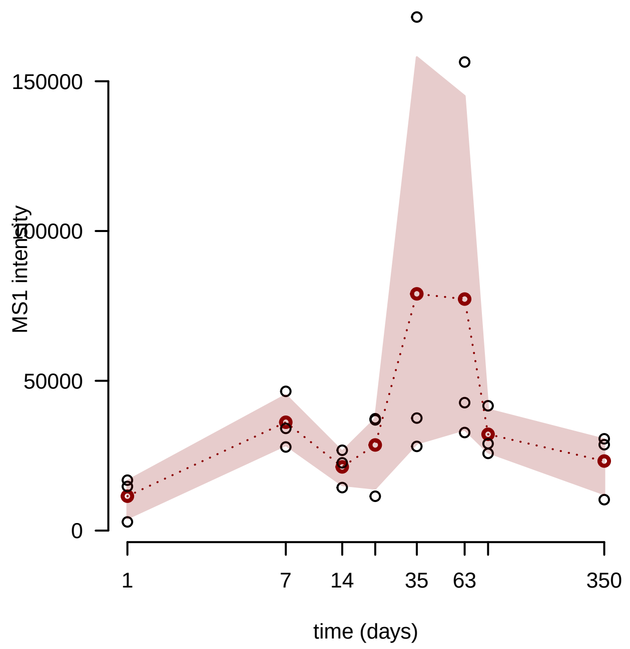

Q9D114
GN=Hddc3
HD domain containing 3

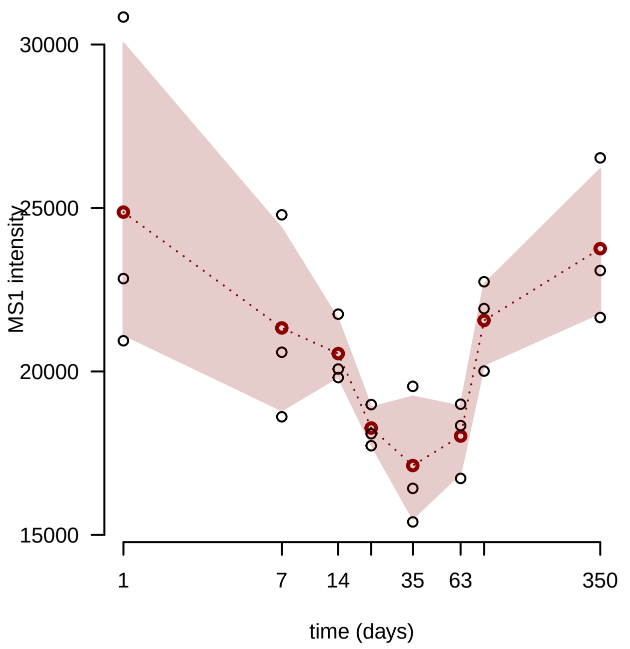

Q9D115
GN=Znf706

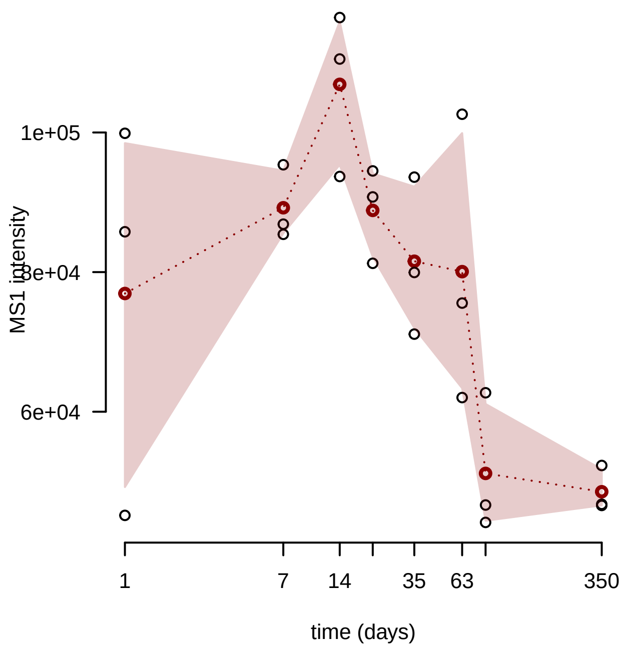

Q9D125
GN=Mrps25
mitochondrial ribosomal protein S25

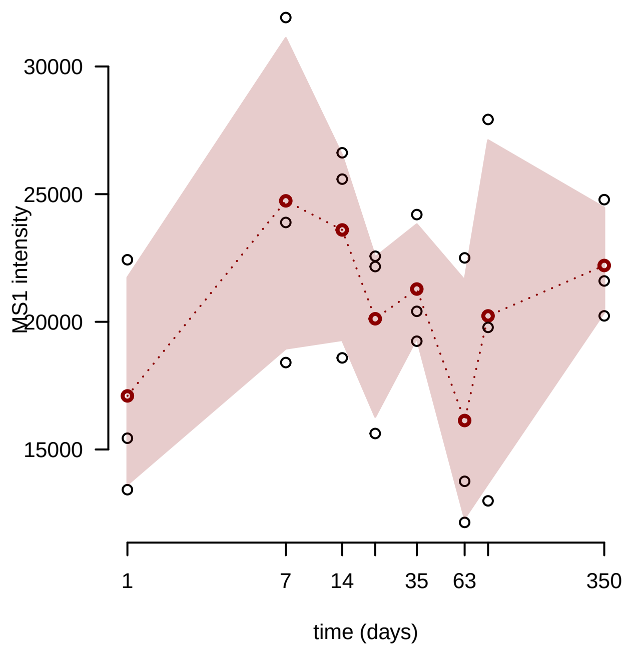

Q9D136
GN=Ogfod3
2−oxoglutarate and iron−dependent oxygen ...

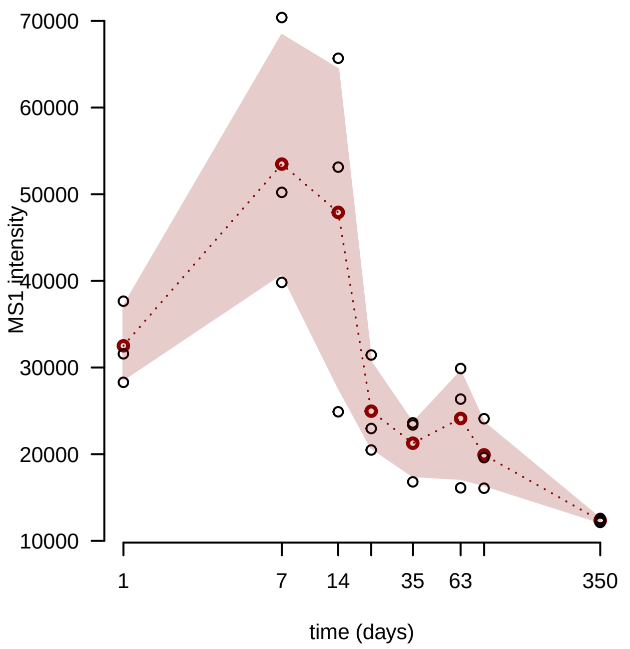

Q9D142
GN=Nudt14
nudix (nucleoside diphosphate linked moi ...

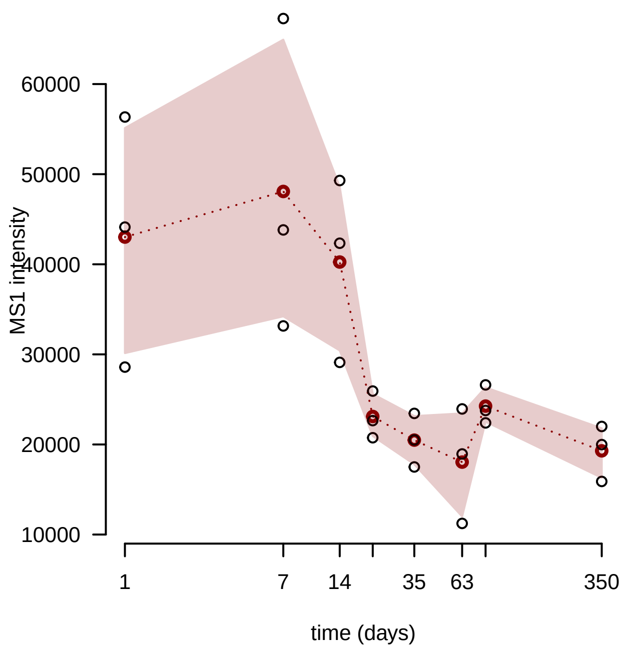

Q9D154
GN=Serpinb1a
serine (or cysteine) peptidase inhibitor ...

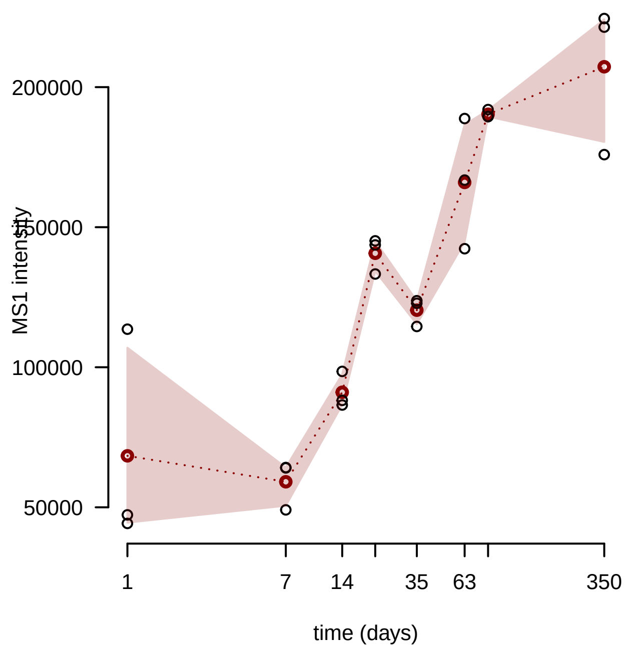

Q9D162
GN=Ccdc167
coiled−coil domain containing 167

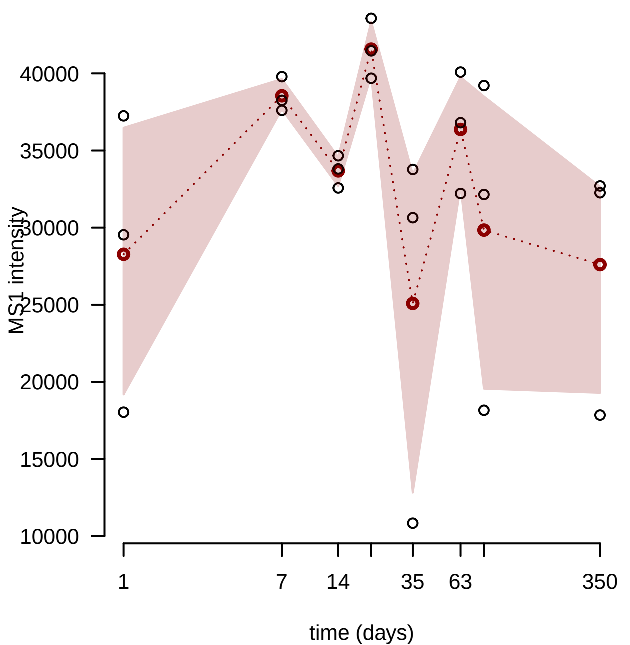

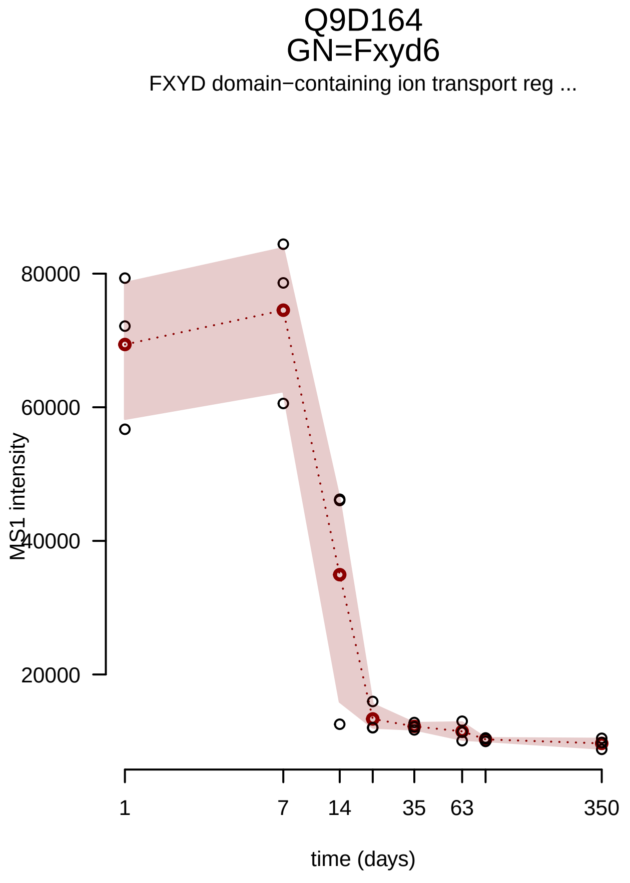
Q9D164
GN=Fxyd6
FXYD domain−containing ion transport reg ...
MS1 intensity
80000
60000
40000
20000
1
7
14
35
63
350
time (days)
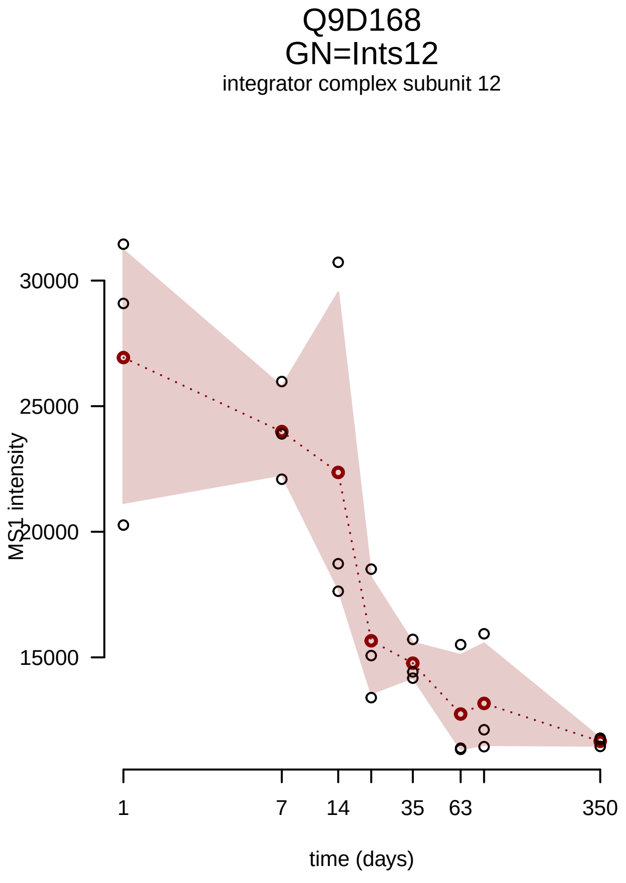
Q9D168
GN=Ints12
integrator complex subunit 12
MS1 intensity
30000
25000
20000
15000
1
7
14
35
63
350
time (days)
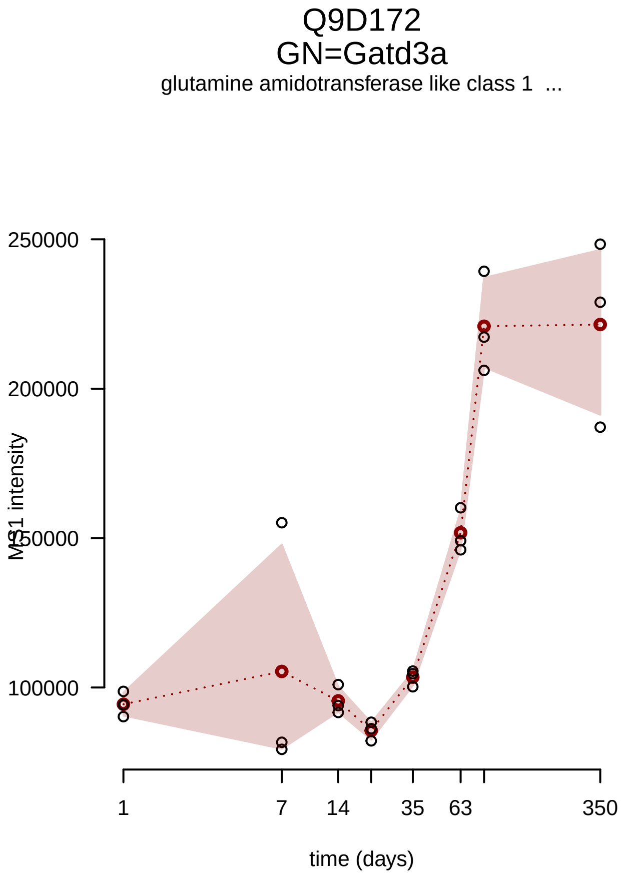
Q9D172
GN=Gatd3a
glutamine amidotransferase like class 1 ...
MS1 intensity
250000
200000
150000
100000
1
7
14
35
63
350
time (days)
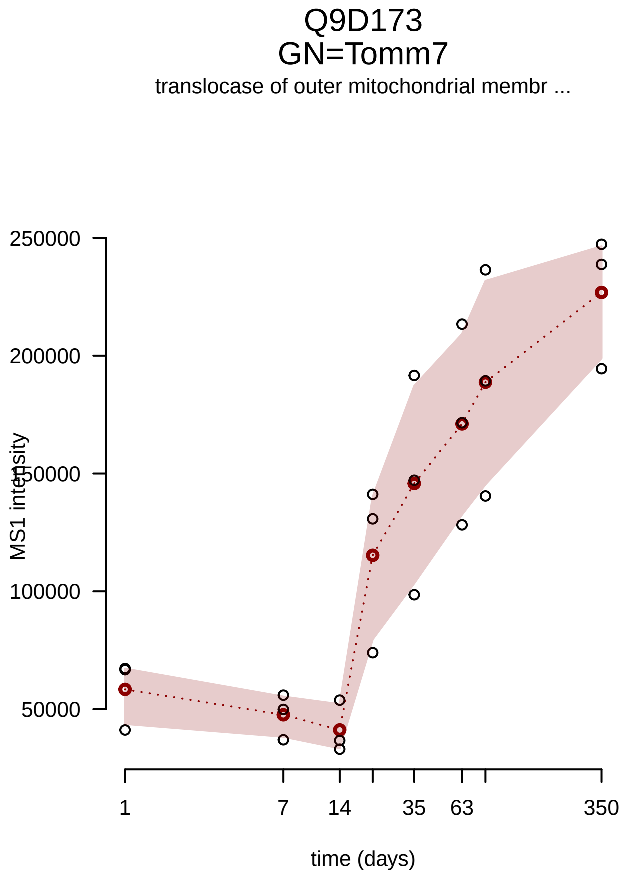
Q9D173
GN=Tomm7
translocase of outer mitochondrial membr ...
MS1 intensity
250000
200000
150000
100000
50000
1
7
14
35
63
350
time (days)
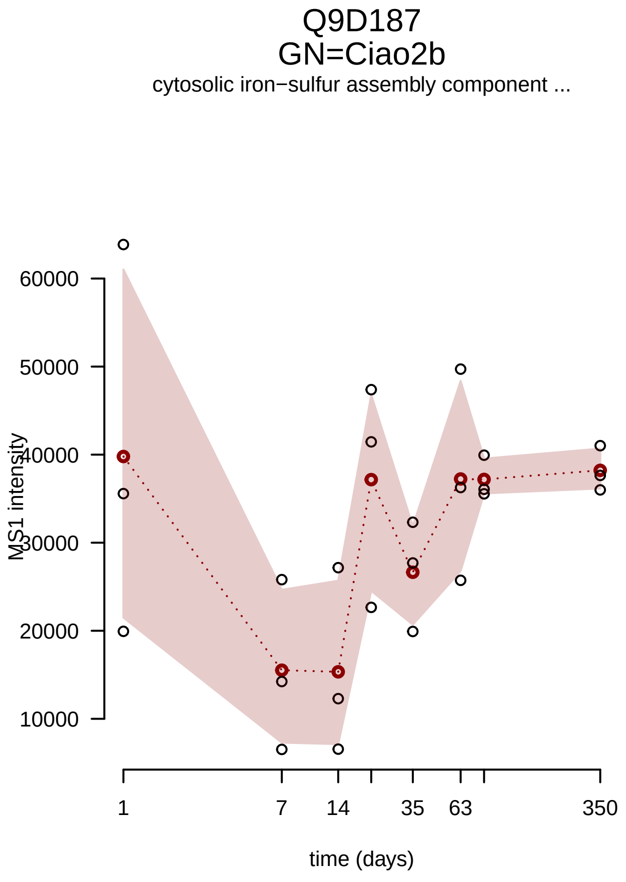
Q9D187
GN=Ciao2b
cytosolic iron−sulfur assembly component ...
MS1 intensity
60000
50000
40000
30000
20000
10000
1
7
14
35
63
350
time (days)
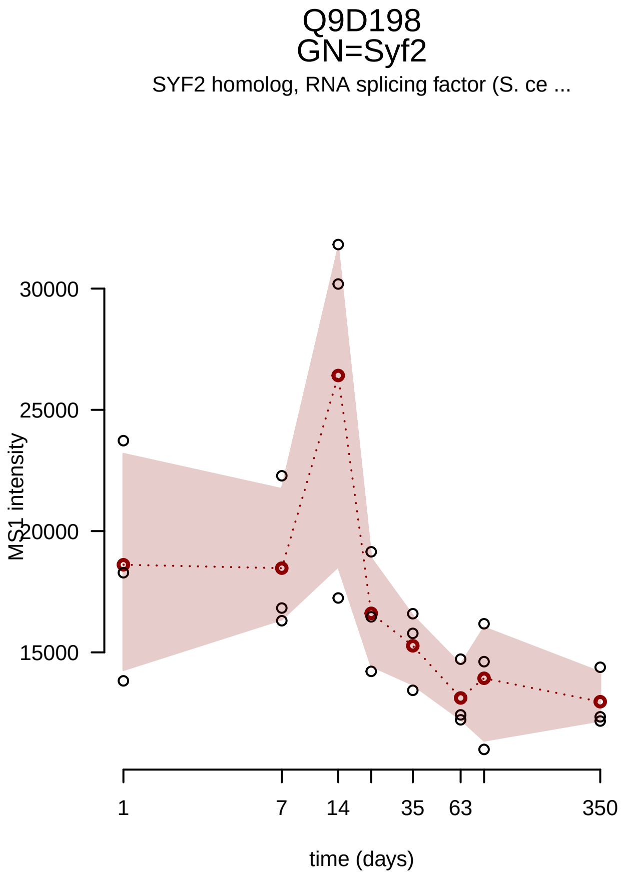
Q9D198
GN=Syf2
SYF2 homolog, RNA splicing factor (S. ce ...
MS1 intensity
30000
25000
20000
15000
1
7
14
35
63
350
time (days)
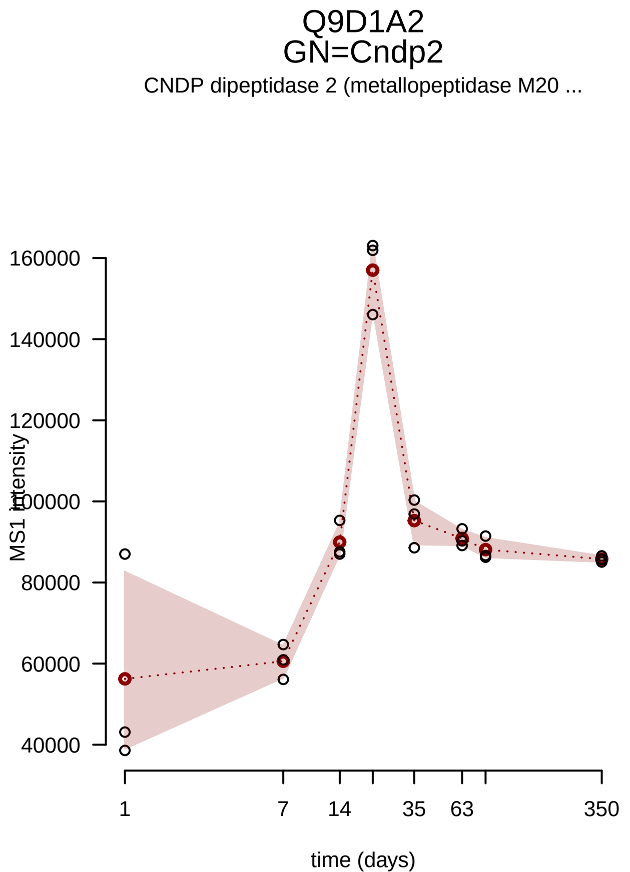
Q9D1A2
GN=Cndp2
CNDP dipeptidase 2 (metallopeptidase M20 ...
MS1 intensity
160000
140000
120000
100000
80000
60000
40000
1
7
14
35
63
350
time (days)
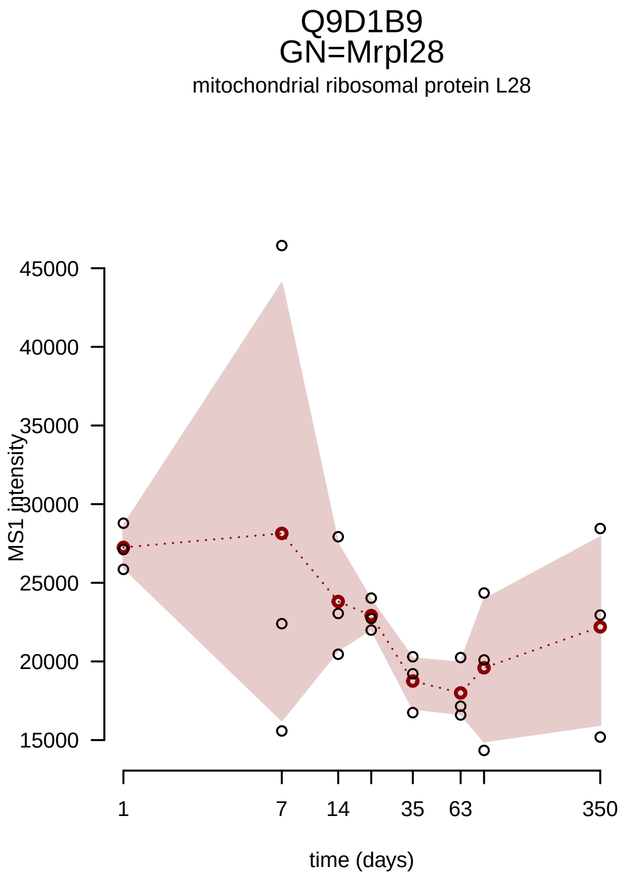
Q9D1B9
GN=Mrpl28
mitochondrial ribosomal protein L28
MS1 intensity
45000
40000
35000
30000
25000
20000
15000
1
7
14
35
63
350
time (days)
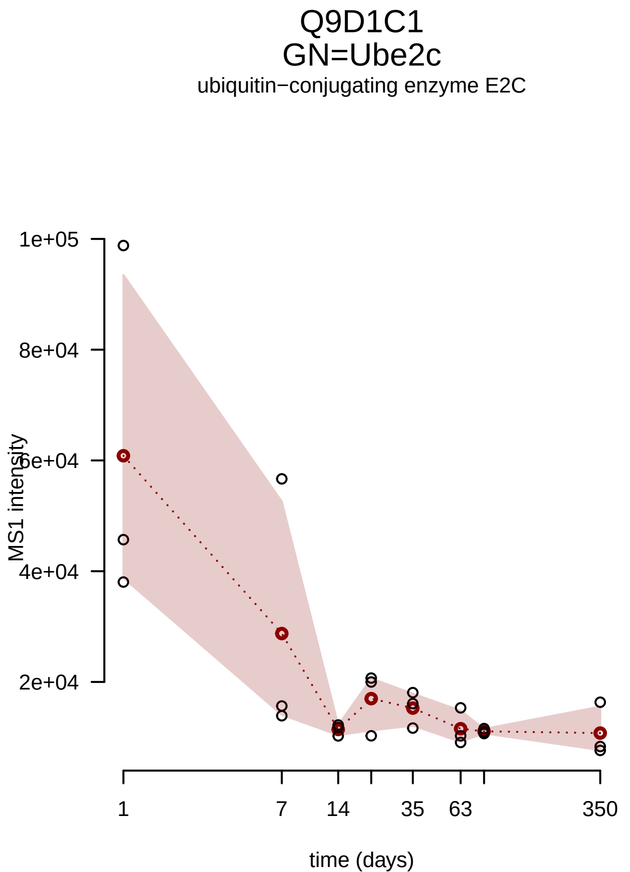
Q9D1C1
GN=Ube2c
ubiquitin−conjugating enzyme E2C
MS1 intensity
1e+05
8e+04
6e+04
4e+04
2e+04
1
7
14
35
63
350
time (days)

Q9D1C8
GN=Vps28
vacuolar protein sorting 28

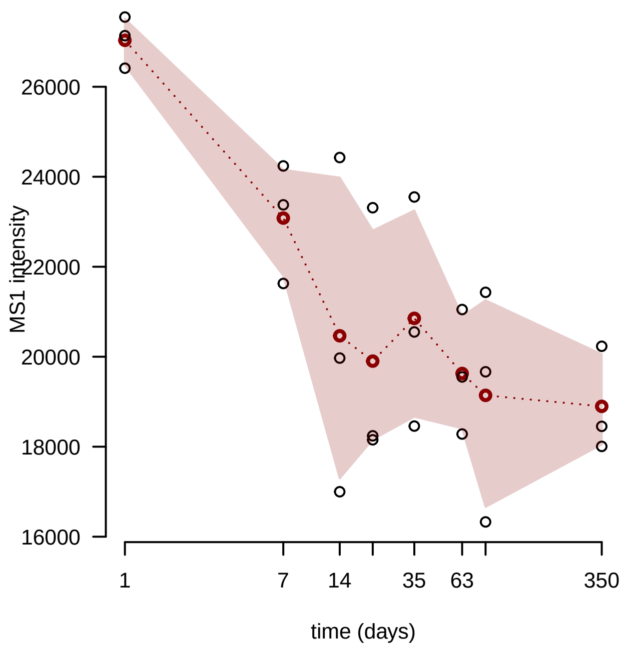

Q9D1C9
GN=Rrp7a
ribosomal RNA processing 7 homolog A

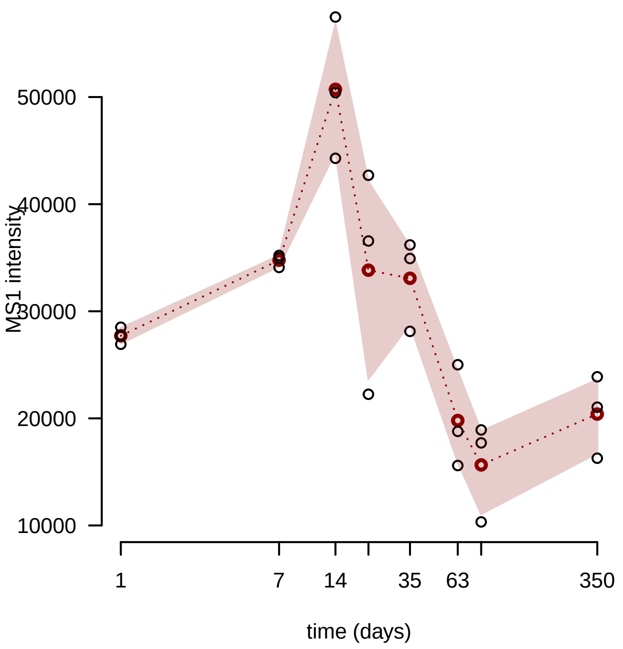

Q9D1D4
GN=Tmed10
transmembrane p24 trafficking protein 10

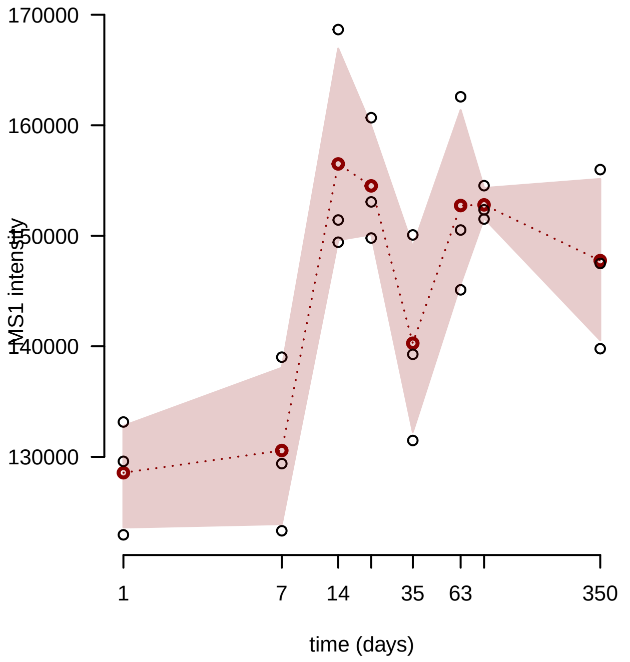

Q9D1D6
GN=Cthrc1
collagen triple helix repeat containing ...

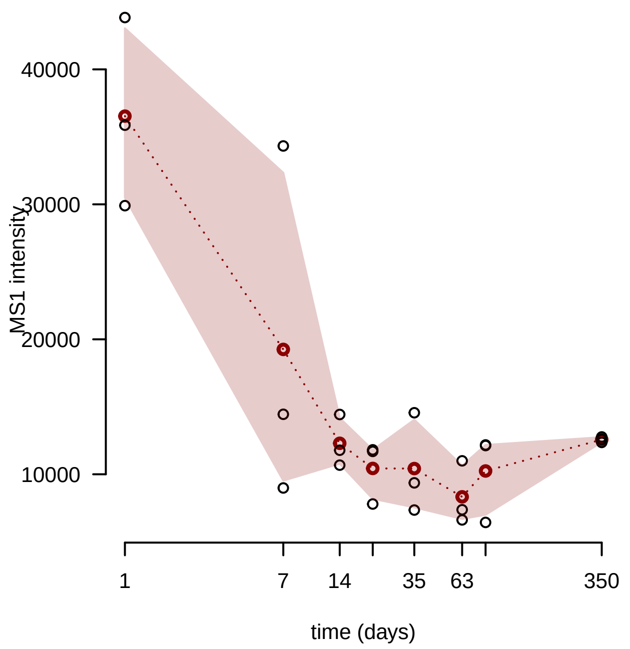

Q9D1E5
GN=Lmbr1l
limb region 1 like

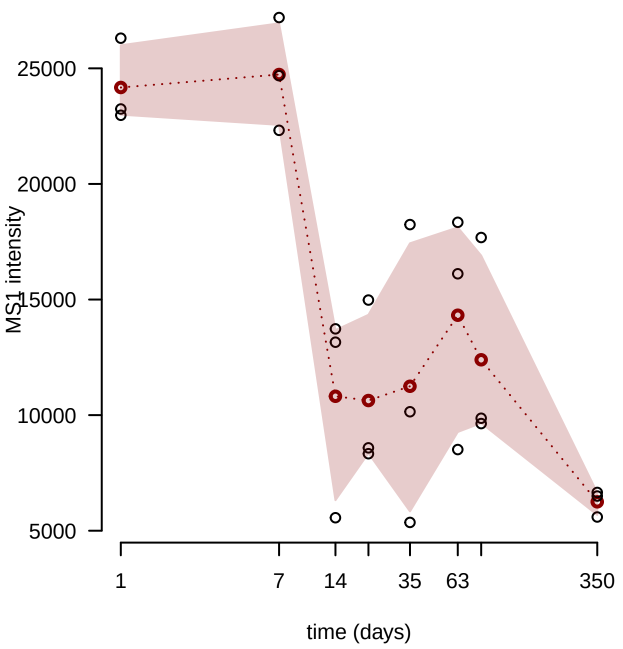

Q9D1E6
GN=Tbcb
tubulin folding cofactor B

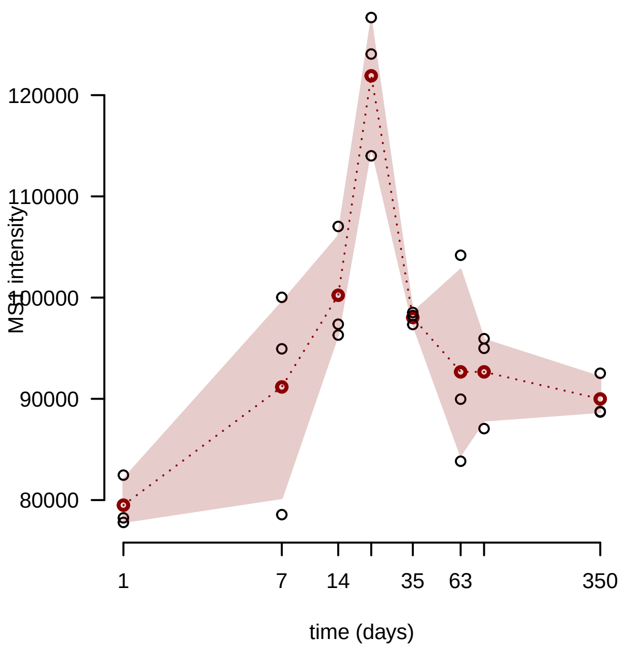

Q9D1E8
GN=Agpat5
1−acylglycerol−3−phosphate O−acyltransfe ...

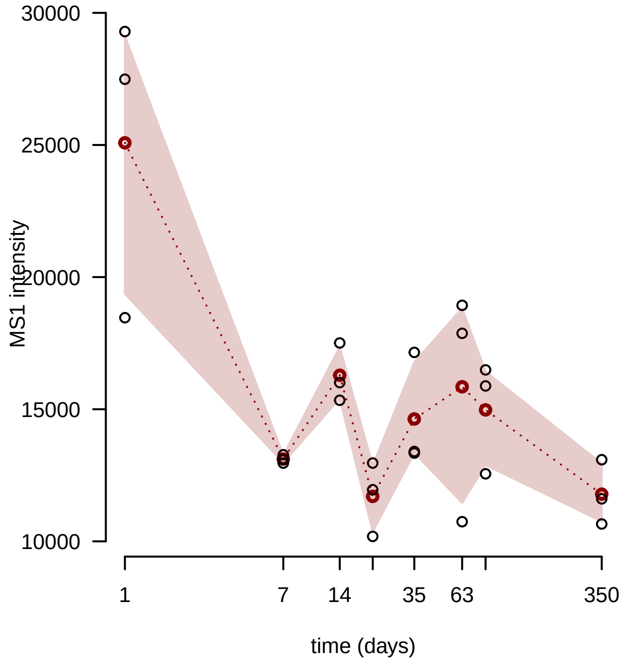

Q9D1F3
GN=

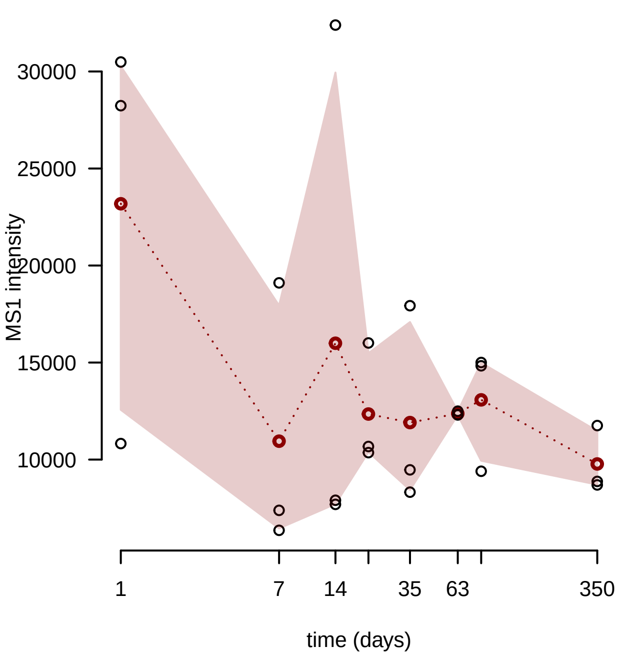

Q9D1F4
GN=Akt1s1
AKT1 substrate 1 (proline−rich)

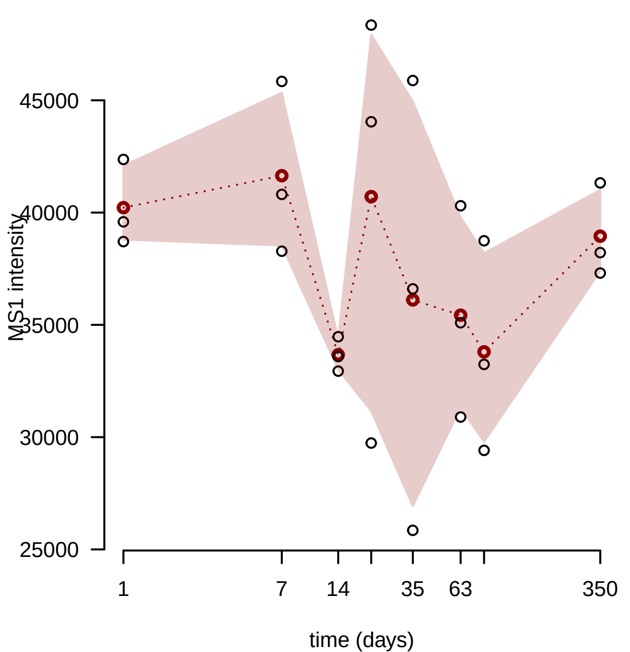

Q9D1G1
GN=Rab1b
RAB1B, member RAS oncogene family

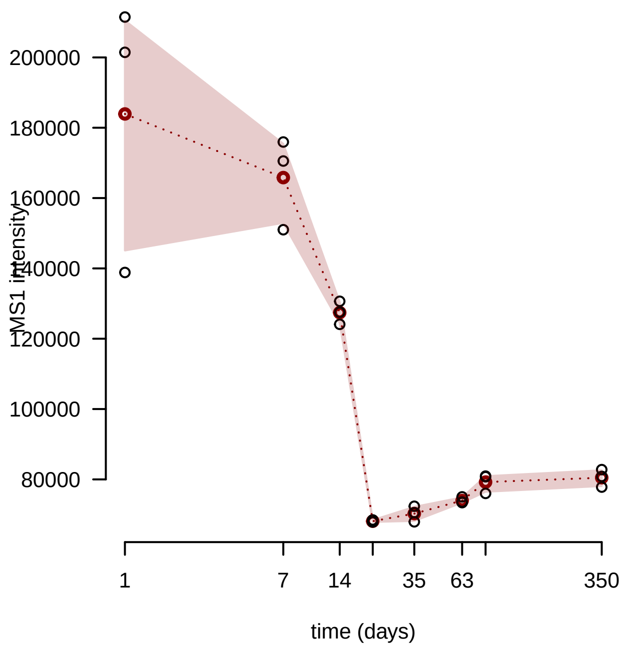

Q9D1G2
GN=Pmvk
phosphomevalonate kinase

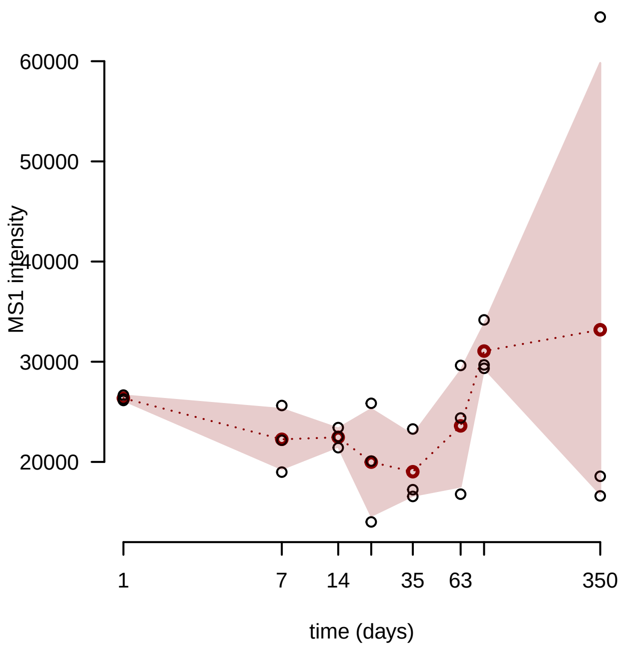

Q9D1G5
GN=Lrrc57
leucine rich repeat containing 57

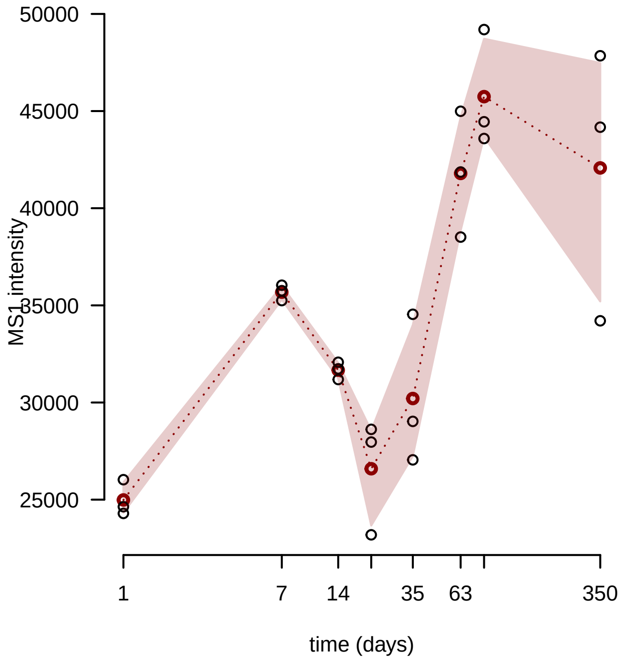

Q9D1H6
GN=Ndufaf4
NADH:ubiquinone oxidoreductase complex a ...

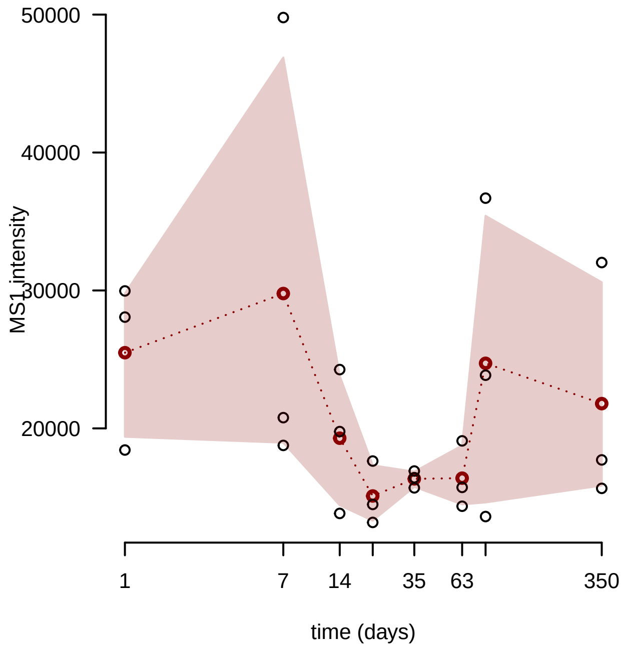

Q9D1H7
GN=Get4
golgi to ER traffic protein 4

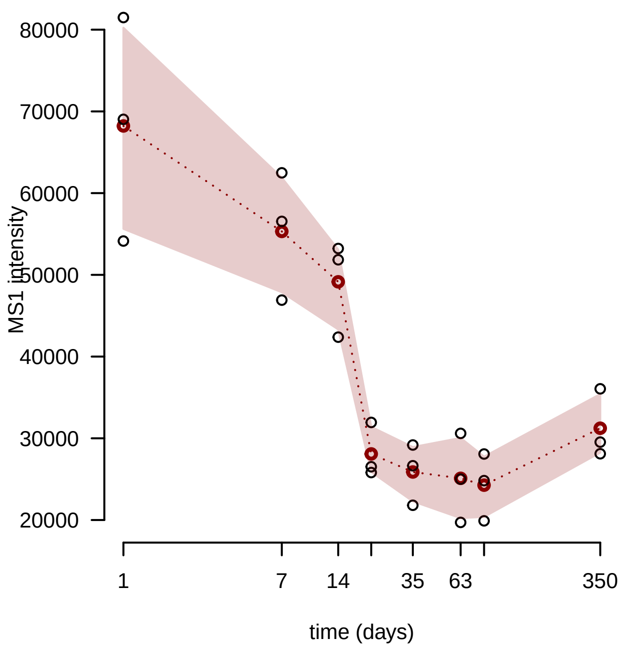

Q9D1H8
GN=Mrpl53
mitochondrial ribosomal protein L53

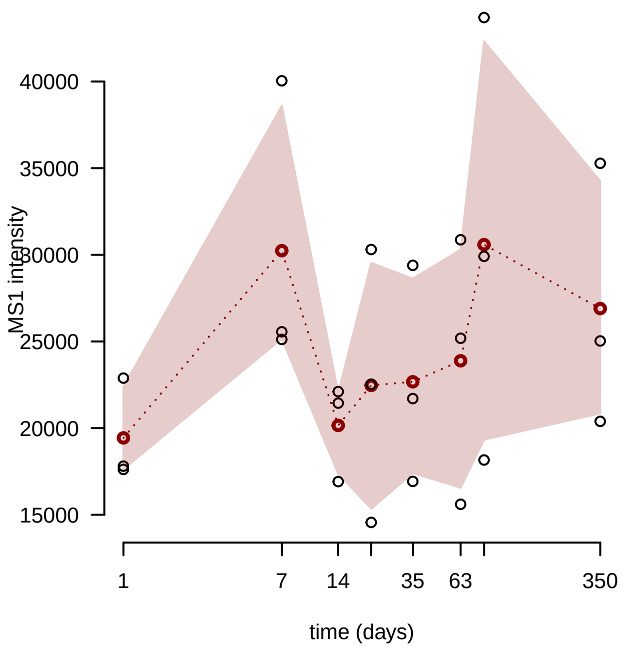

Q9D1H9
GN=Mfap4
microfibrillar−associated protein 4

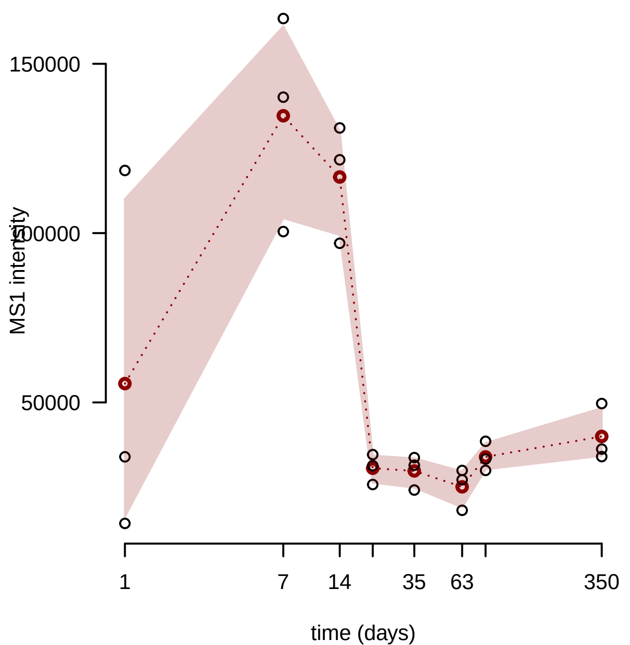

Q9D1I2
GN=Card19
caspase recruitment domain family, membe ...

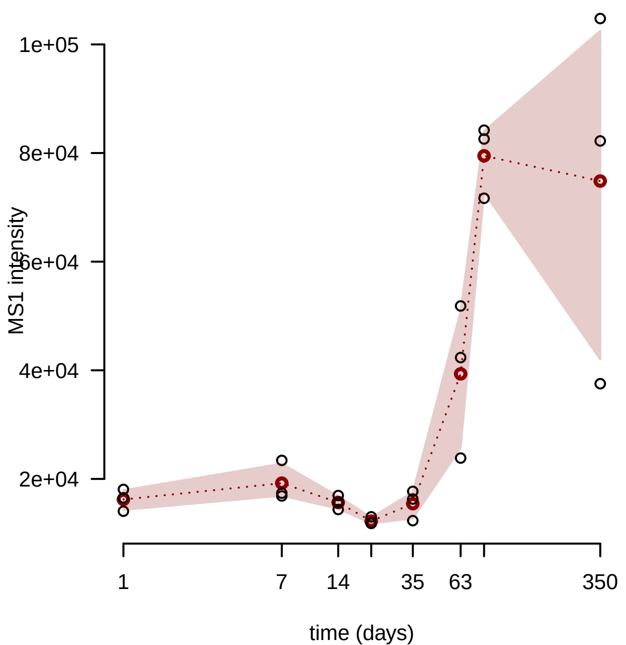

Q9D1I5
GN=Mcee
methylmalonyl CoA epimerase

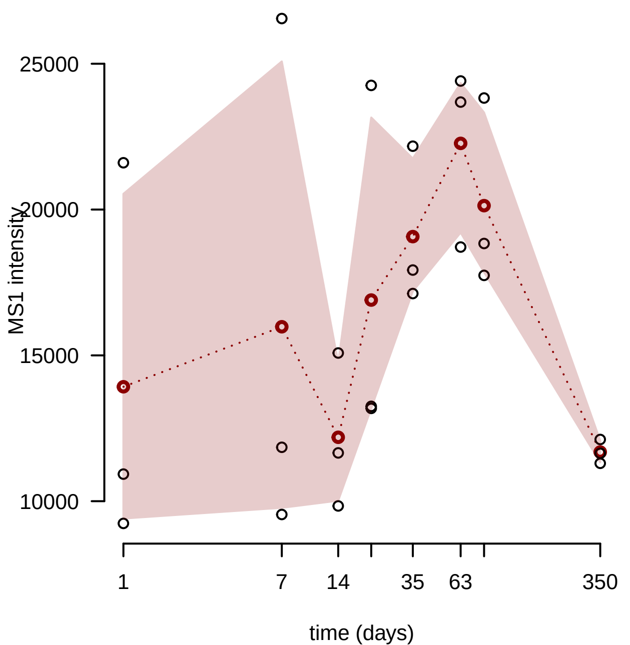

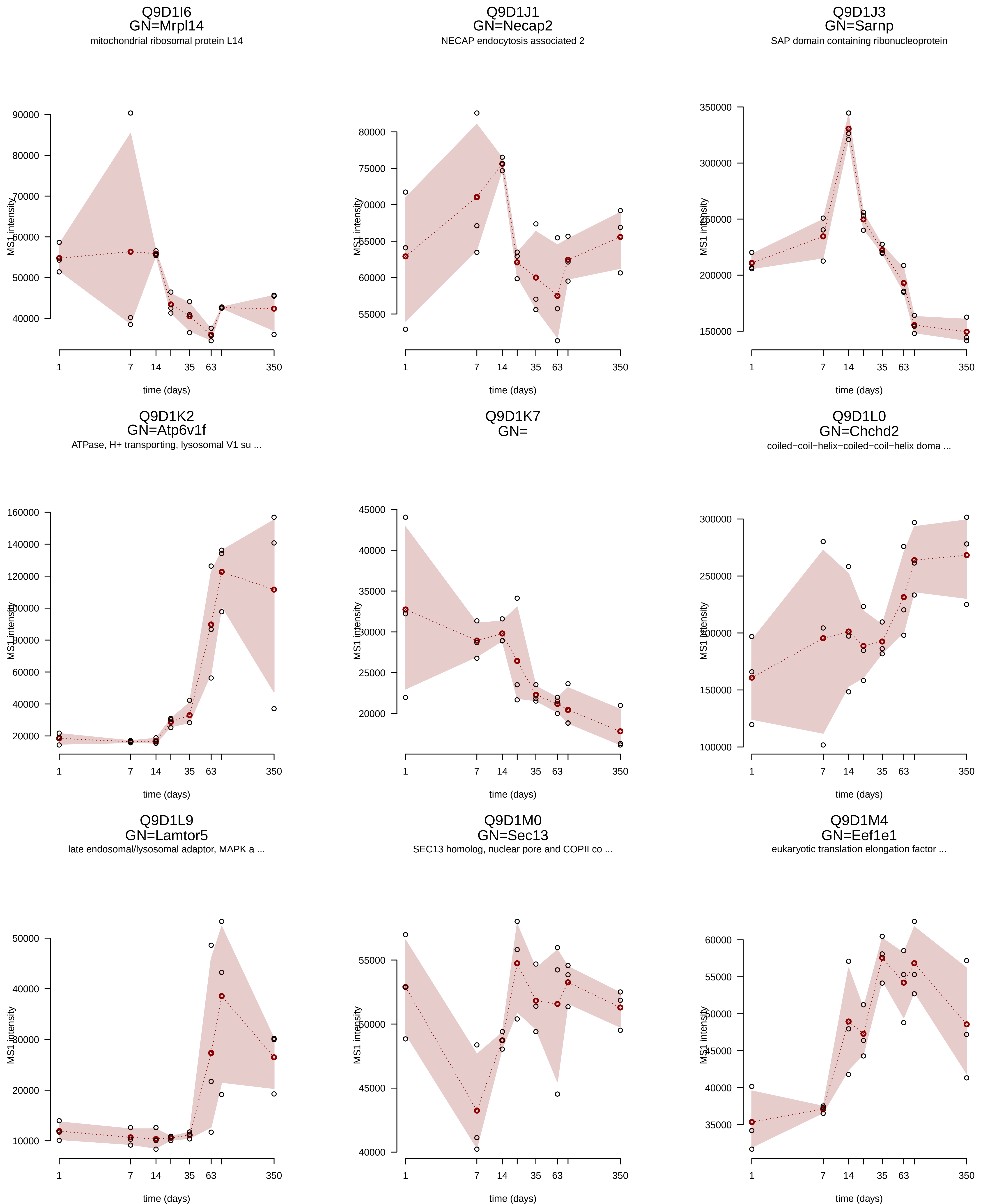
Q9D1I6
GN=Mrpl14
mitochondrial ribosomal protein L14
Q9D1J1
GN=Necap2
NECAP endocytosis associated 2
Q9D1J3
GN=Sarnp
SAP domain containing ribonucleoprotein
Q9D1K2
GN=Atp6v1f
ATPase, H+ transporting, lysosomal V1 su ...
Q9D1K7
GN=
Q9D1L0
GN=Chchd2
coiled−coil−helix−coiled−coil−helix doma ...
Q9D1L9
GN=Lamtor5
late endosomal/lysosomal adaptor, MAPK a ...
Q9D1M0
GN=Sec13
SEC13 homolog, nuclear pore and COPII co ...
Q9D1M4
GN=Eef1e1
eukaryotic translation elongation factor ...
MS1 intensity
time (days)
1
7
14
35
63
350

Q9D1M7
GN=Fkbp11
FK506 binding protein 11

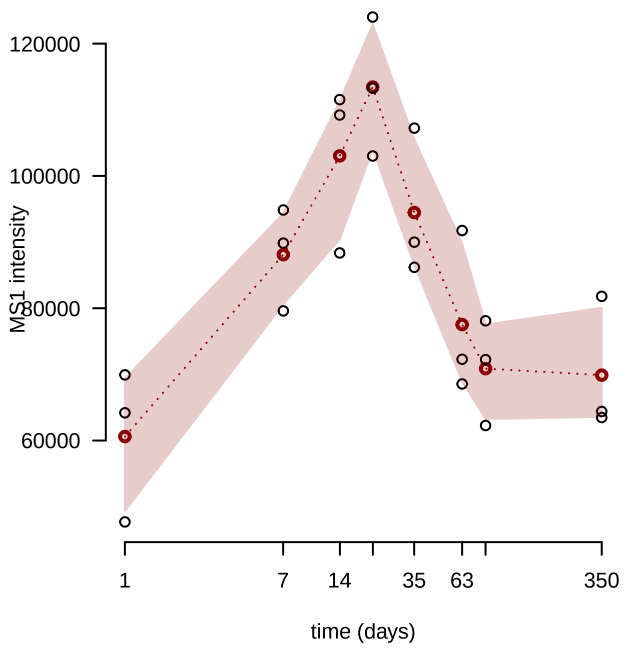

Q9D1N2
GN=Gpihbp1
GPI−anchored HDL−binding protein 1

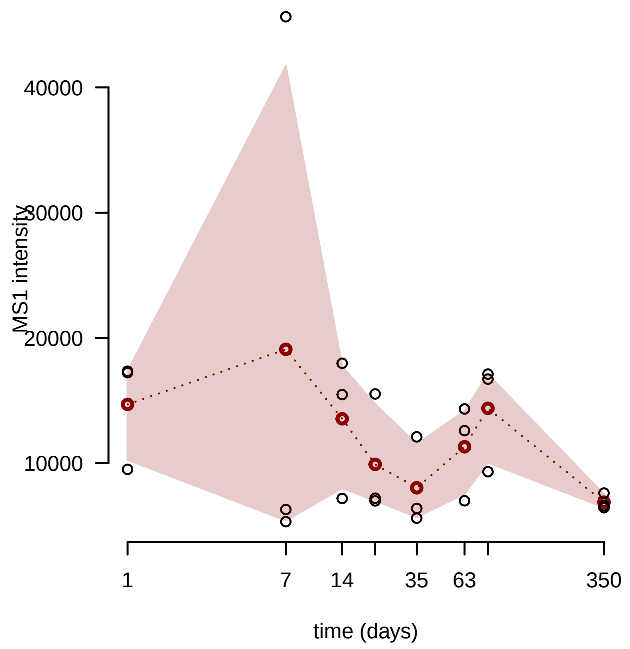

Q9D1N9
GN=Mrpl21
mitochondrial ribosomal protein L21

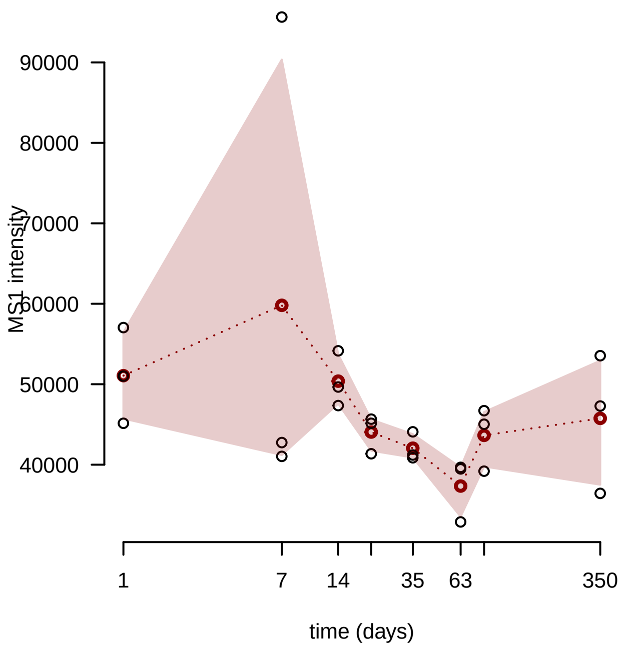

Q9D1P0
GN=Mrpl13
mitochondrial ribosomal protein L13

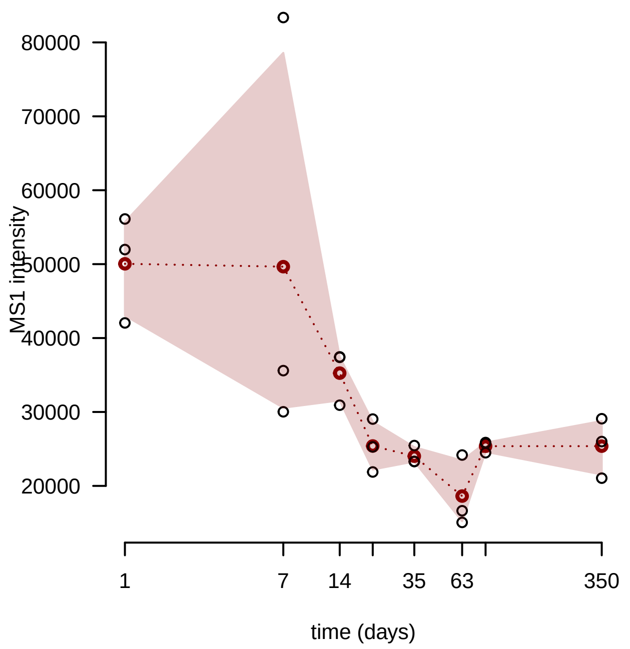

Q9D1P2
GN=Kat8
K(lysine) acetyltransferase 8

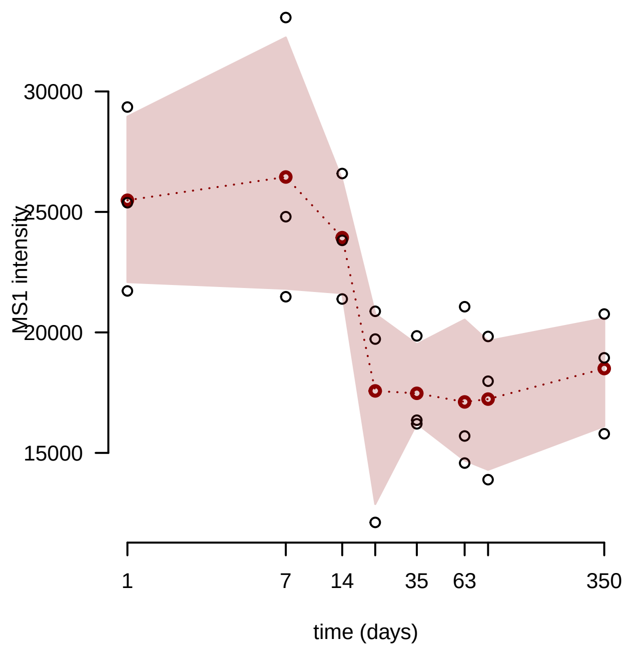

Q9D1P4
GN=Chordc1
cysteine and histidine−rich domain (CHOR ...

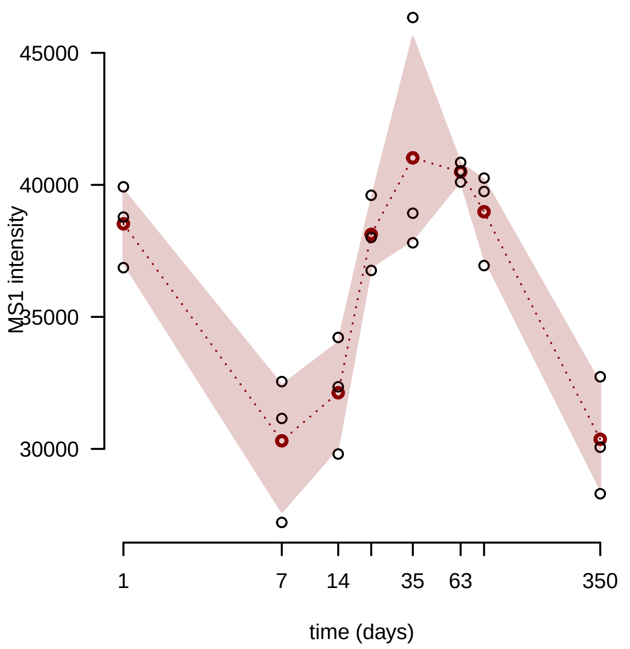

Q9D1Q1
GN=Mphosph6
M phase phosphoprotein 6

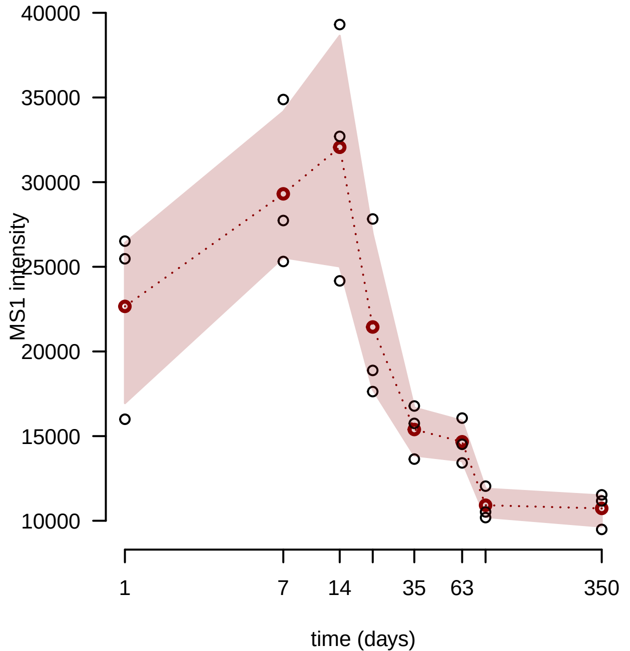

Q9D1Q4
GN=Dpm3
dolichyl−phosphate mannosyltransferase p ...

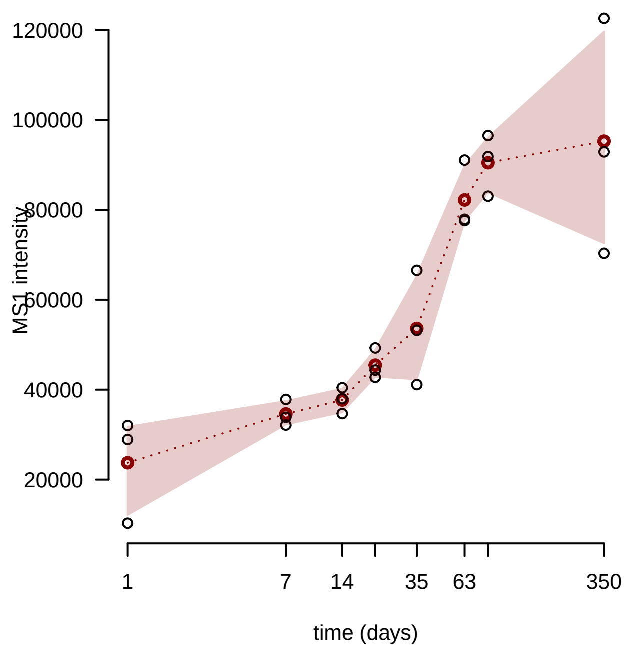

Q9D1Q6
GN=Erp44
endoplasmic reticulum protein 44

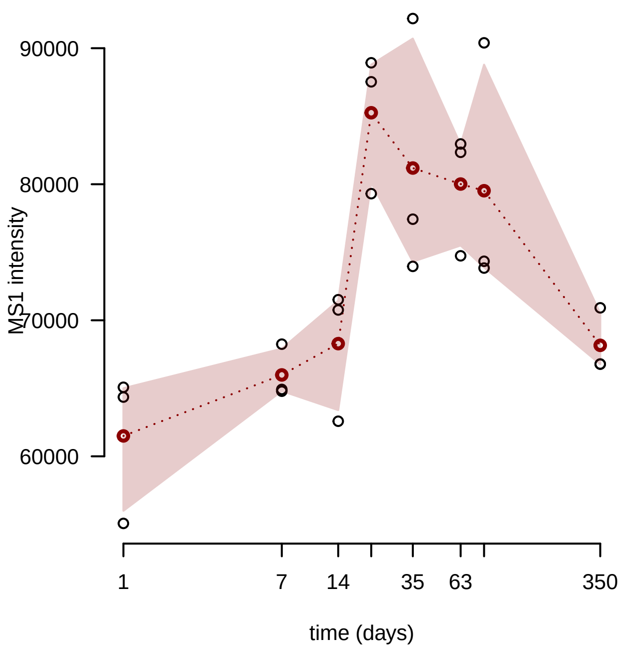

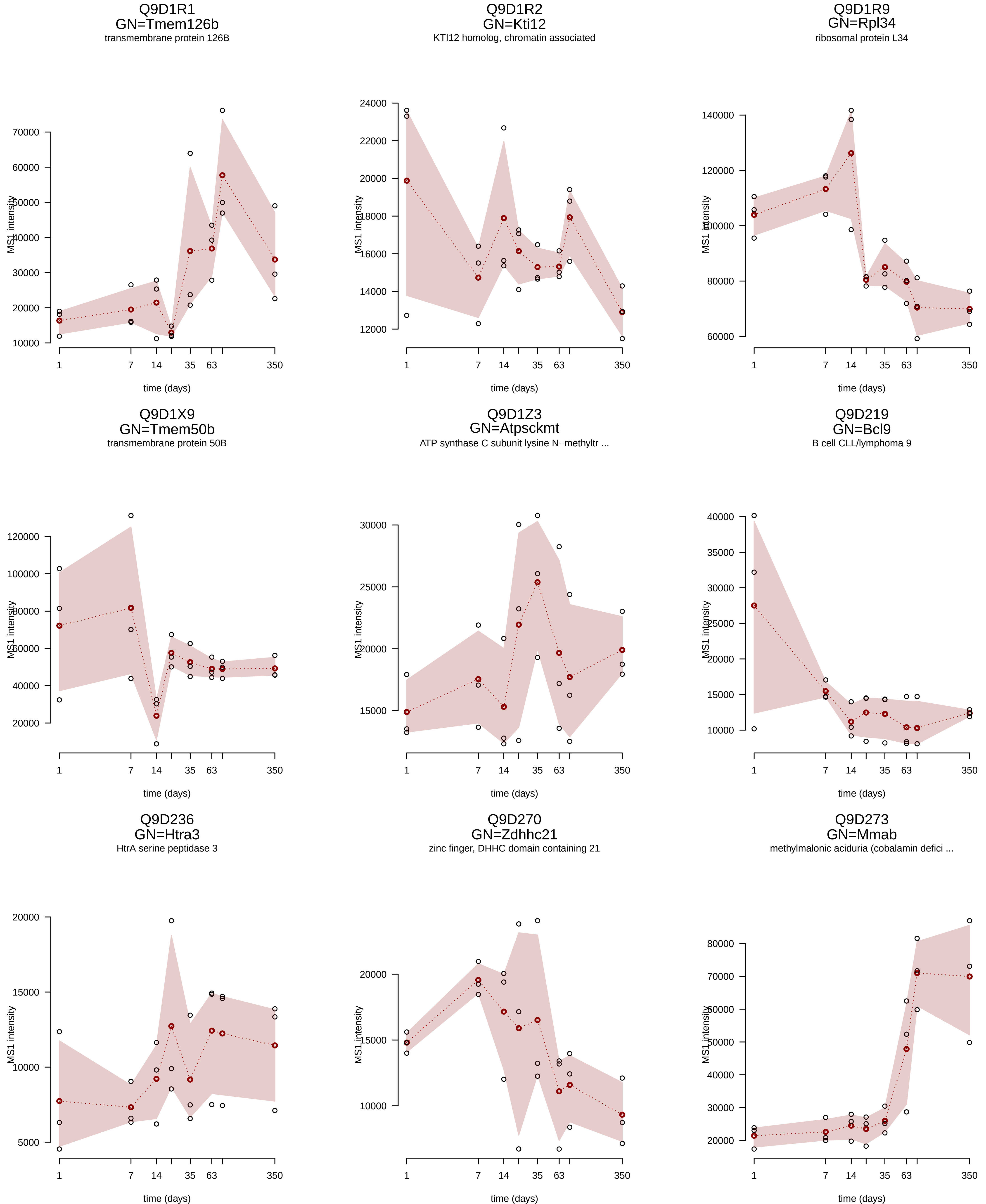
Q9D1R1
GN=Tmem126b
transmembrane protein 126B
Q9D1R2
GN=Kti12
KTI12 homolog, chromatin associated
Q9D1R9
GN=Rpl34
ribosomal protein L34
Q9D1X9
GN=Tmem50b
transmembrane protein 50B
Q9D1Z3
GN=Atpsckmt
ATP synthase C subunit lysine N−methyltr ...
Q9D219
GN=Bcl9
B cell CLL/lymphoma 9
Q9D236
GN=Htra3
HtrA serine peptidase 3
Q9D270
GN=Zdhhc21
zinc finger, DHHC domain containing 21
Q9D273
GN=Mmab
methylmalonic aciduria (cobalamin defici ...
MS1 intensity
time (days)
1
7
14
35
63
350

Q9D281
GN=Fam114a1
family with sequence similarity 114, mem ...

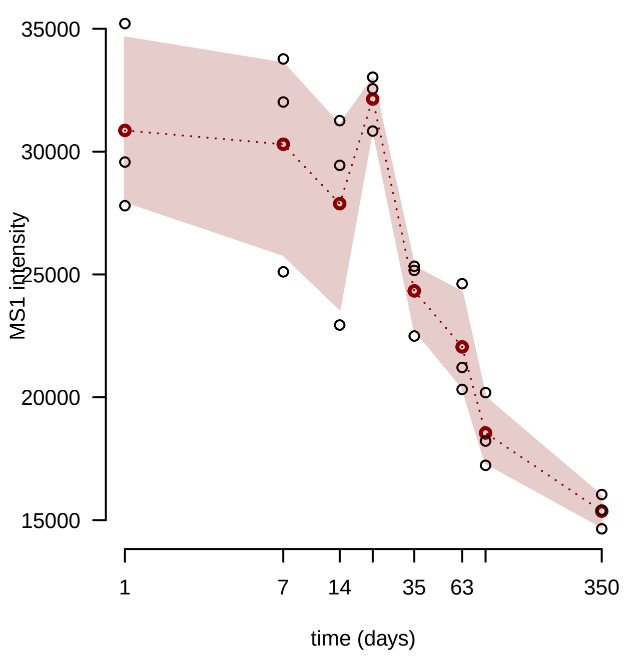

Q9D287
GN=Bcas2
BCAS2 pre−mRNA processing factor

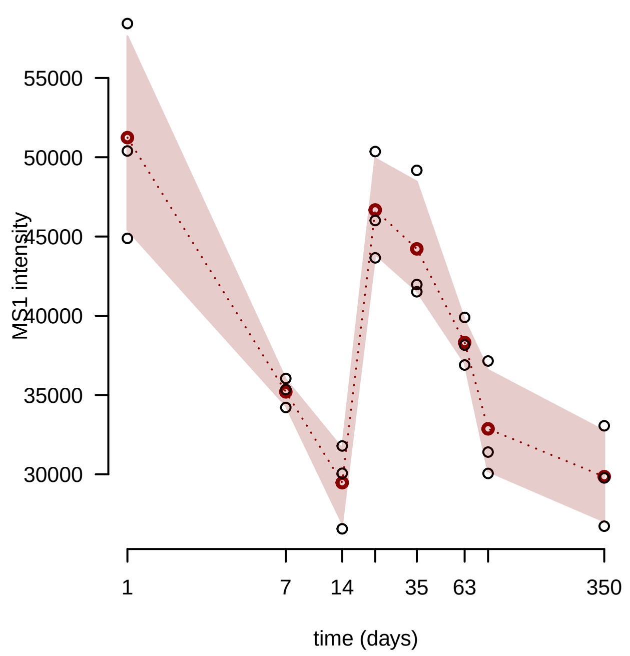

Q9D289
GN=Trappc6b
trafficking protein particle complex 6B

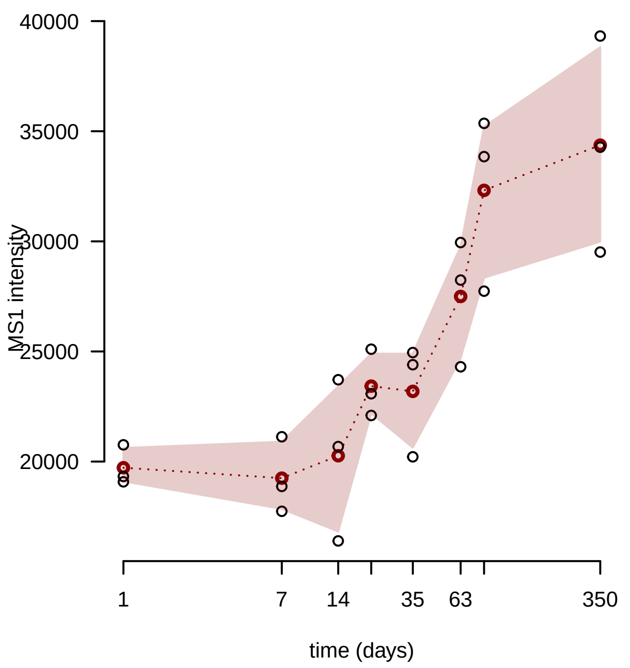

Q9D2C2
GN=Saal1
serum amyloid A−like 1

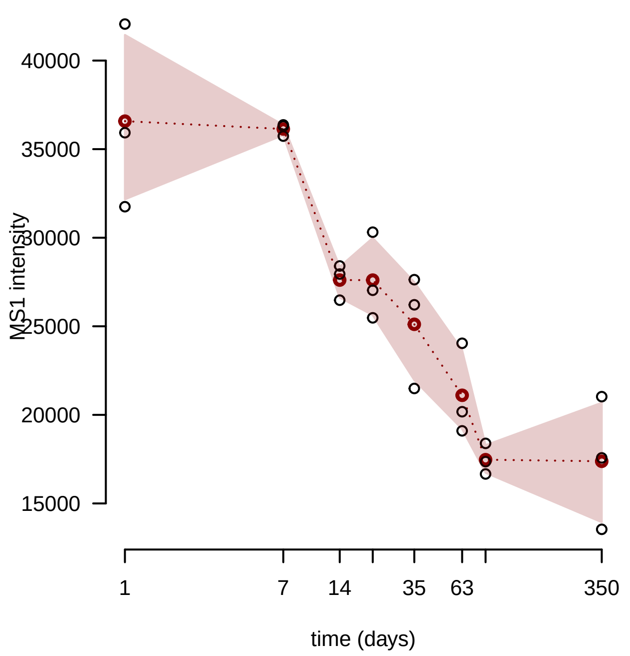

Q9D2C6
GN=Polr3h
polymerase (RNA) III (DNA directed) poly ...

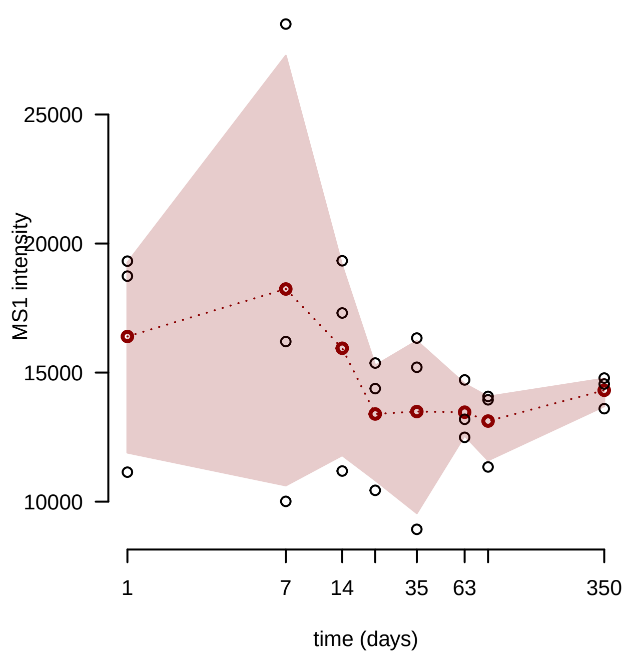

Q9D2C7
GN=Tmbim6
transmembrane BAX inhibitor motif contai ...

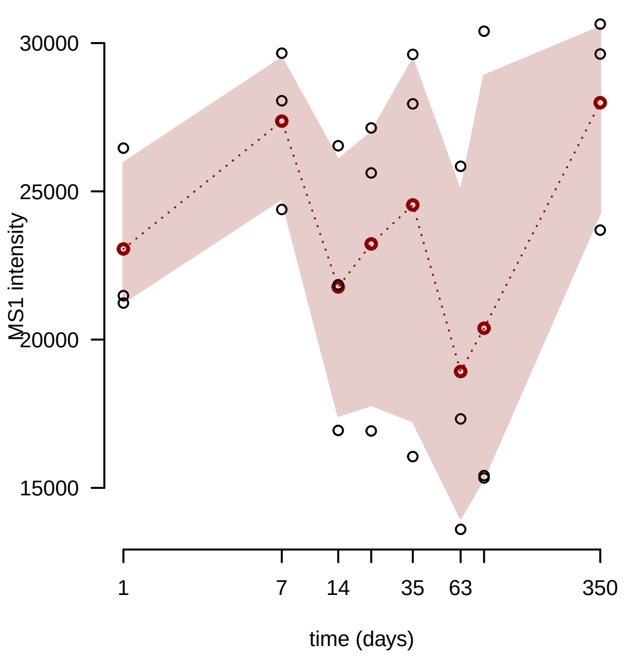

Q9D2D7
GN=Znf687

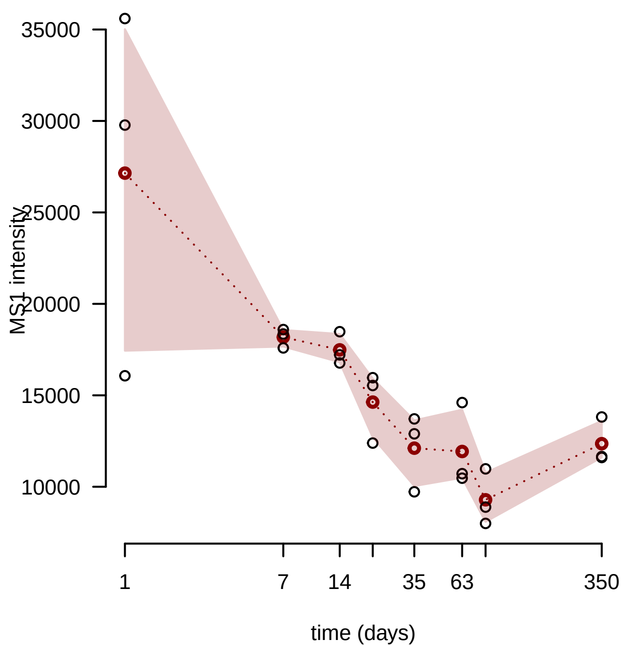

Q9D2D9
GN=Klhdc8b
kelch domain containing 8B

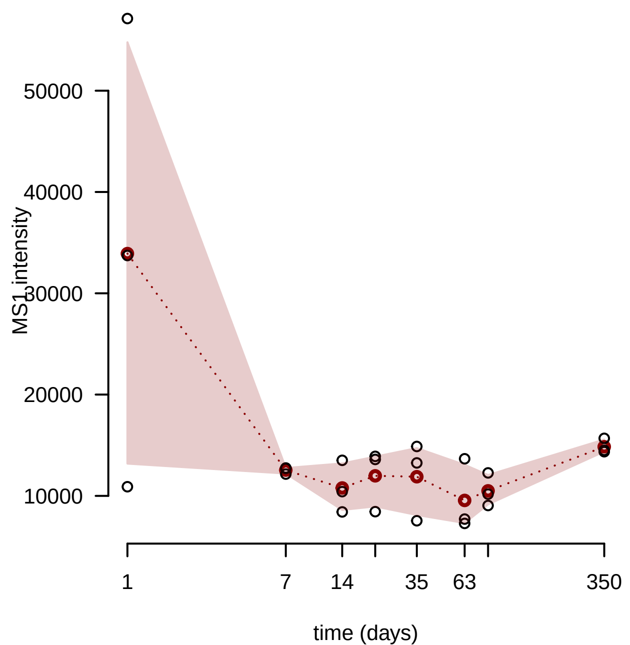

Q9D2E2
GN=Toe1
target of EGR1, member 1 (nuclear)

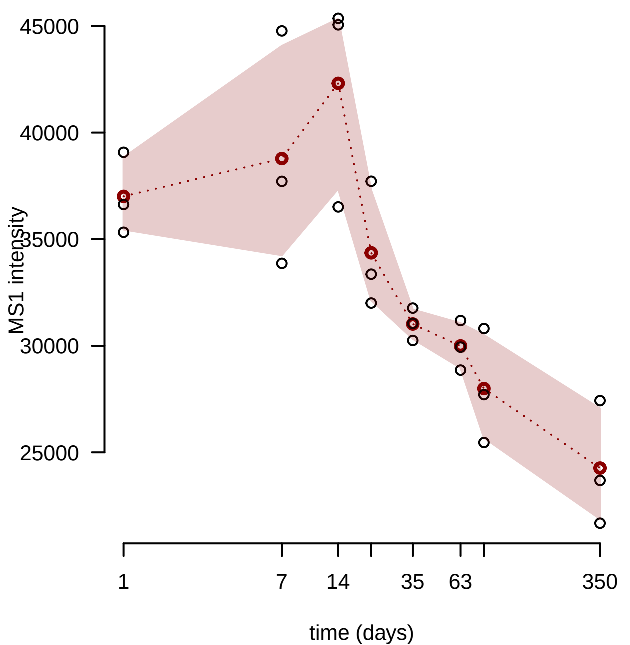

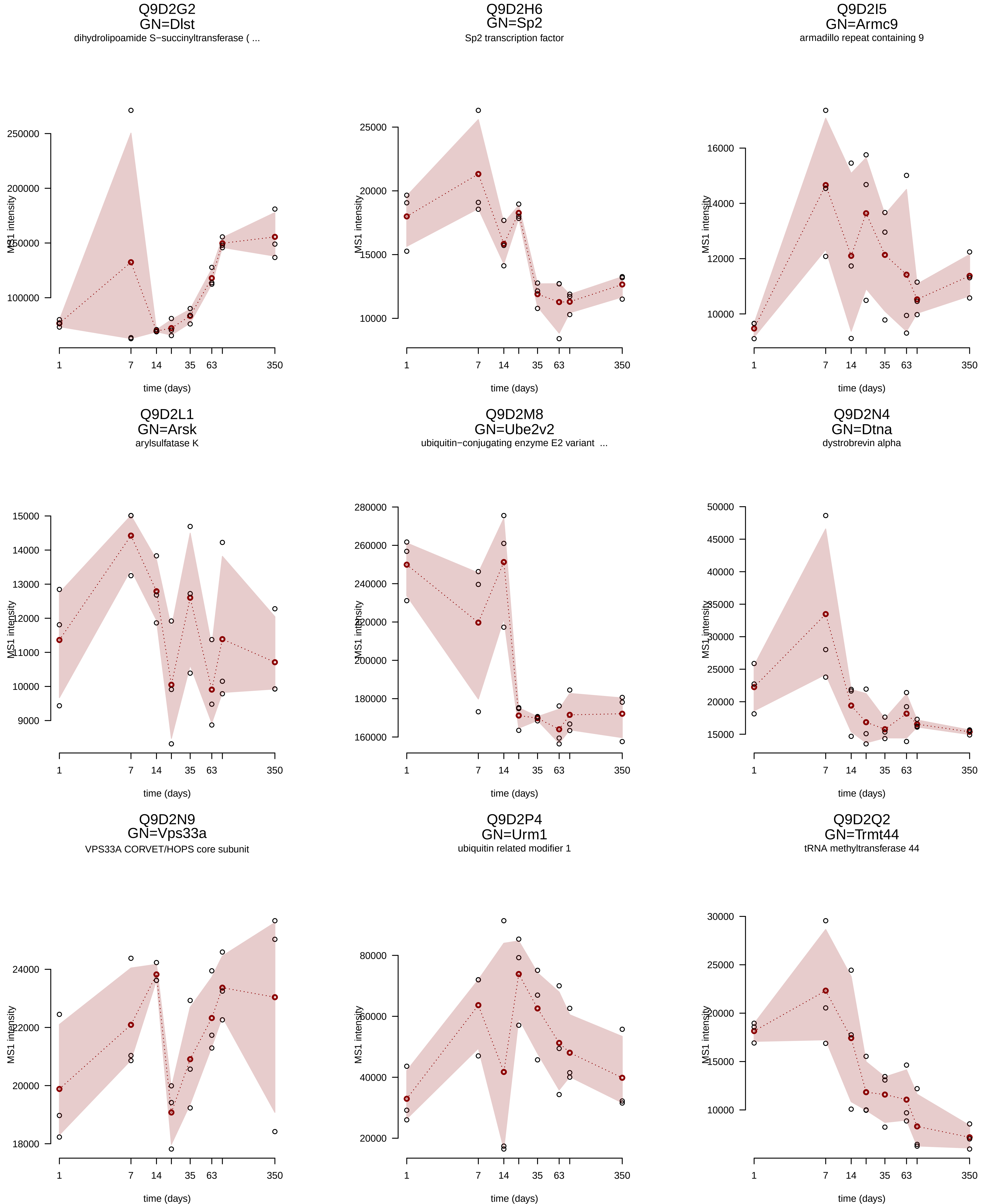

Q9D2G2
GN=Dlst
dihydrolipoamide S−succinyltransferase (...
Q9D2H6
GN=Sp2
Sp2 transcription factor
Q9D2I5
GN=Armc9
armadillo repeat containing 9
Q9D2L1
GN=Arsk
arylsulfatase K
Q9D2M8
GN=Ube2v2
ubiquitin−conjugating enzyme E2 variant ...
Q9D2N4
GN=Dtna
dystrobrevin alpha
Q9D2N9
GN=Vps33a
VPS33A CORVET/HOPS core subunit
Q9D2P4
GN=Urm1
ubiquitin related modifier 1
Q9D2Q2
GN=Trmt44
tRNA methyltransferase 44
MS1 intensity
time (days)
1
7
14
35
63
350

Q9D2Q3
GN=

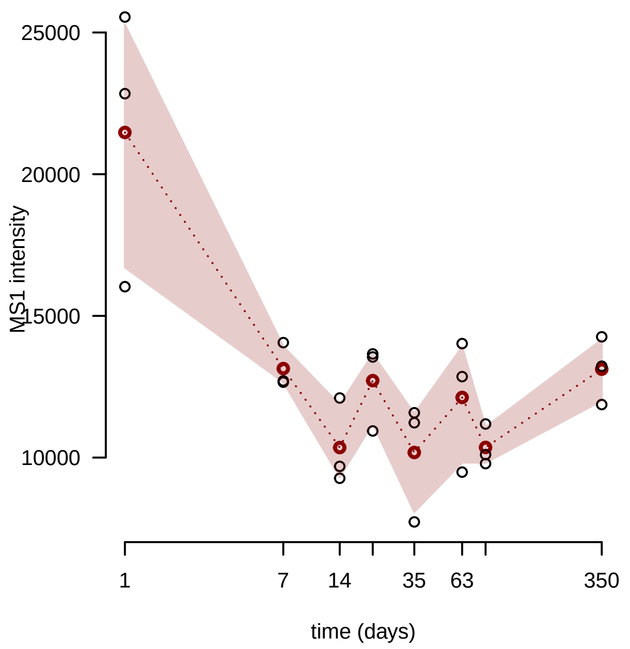

Q9D2Q8
GN=S100a14
S100 calcium binding protein A14

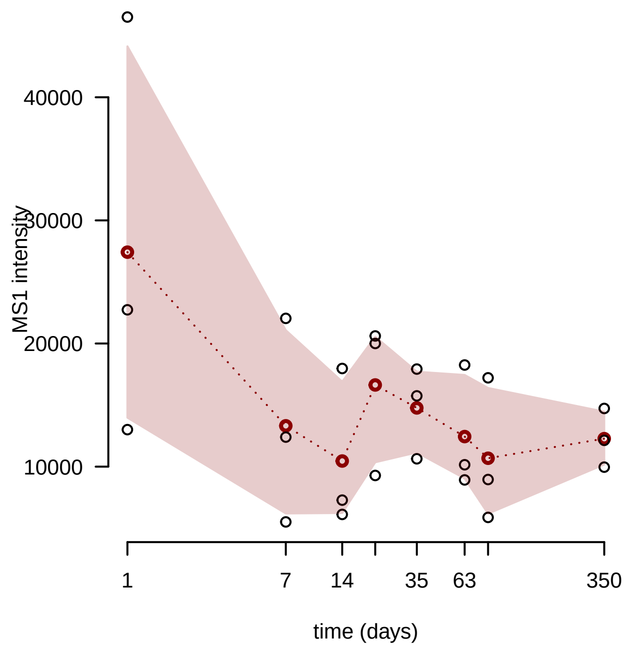

Q9D2R0
GN=Aacs
acetoacetyl−CoA synthetase

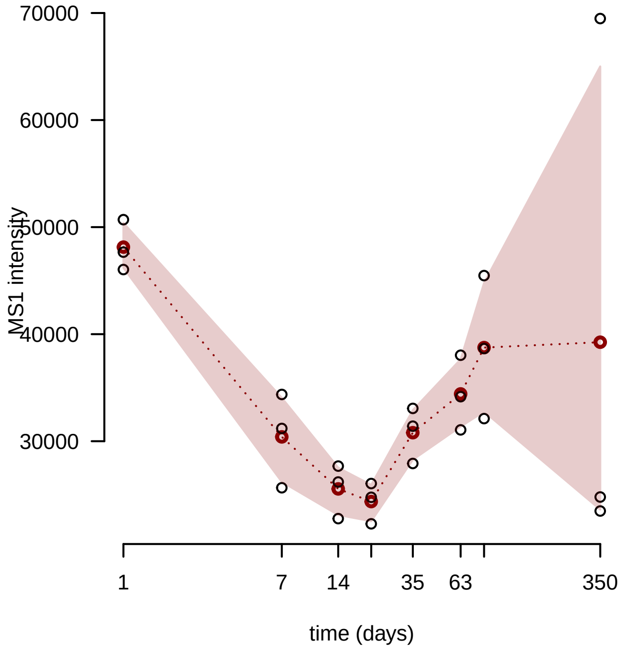

Q9D2R6
GN=Coa3
cytochrome C oxidase assembly factor 3

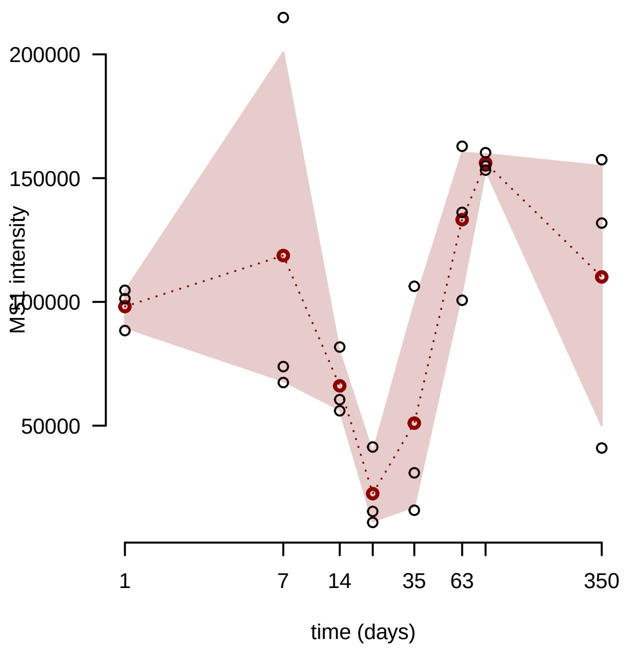

Q9D2R8
GN=Mrps33
mitochondrial ribosomal protein S33

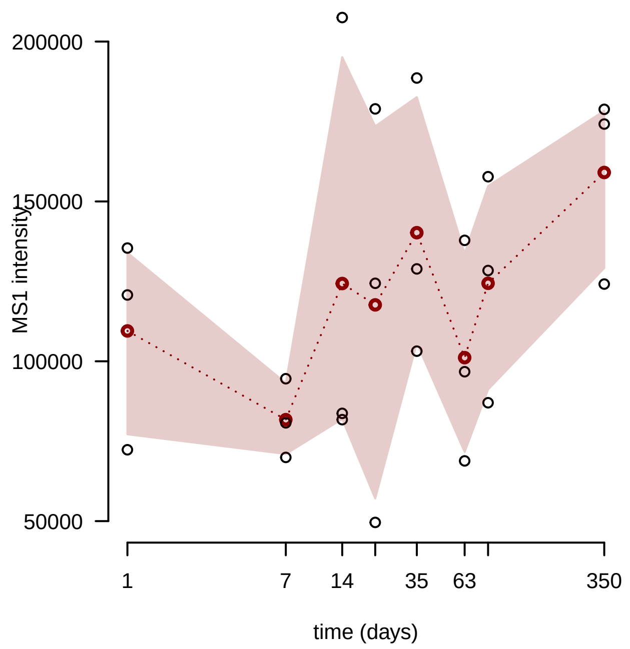

Q9D2U5
GN=Naa38
N(alpha)−acetyltransferase 38, NatC auxi ...

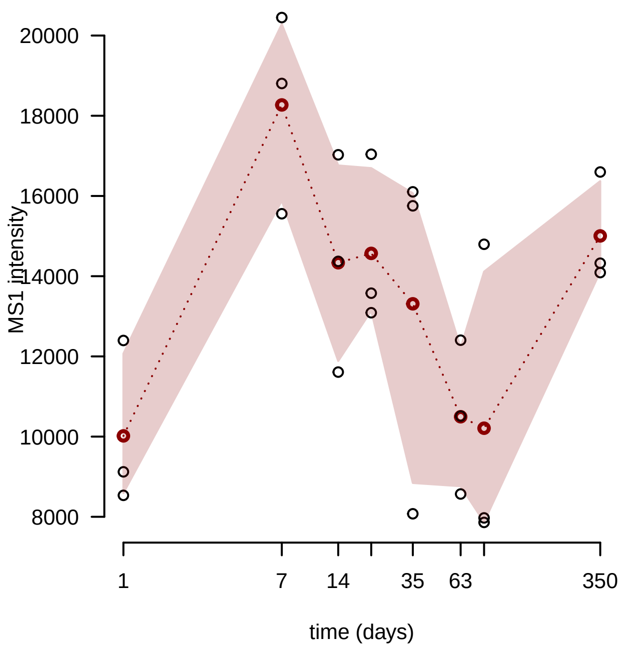

Q9D2V5
GN=Aar2
AAR2 splicing factor homolog

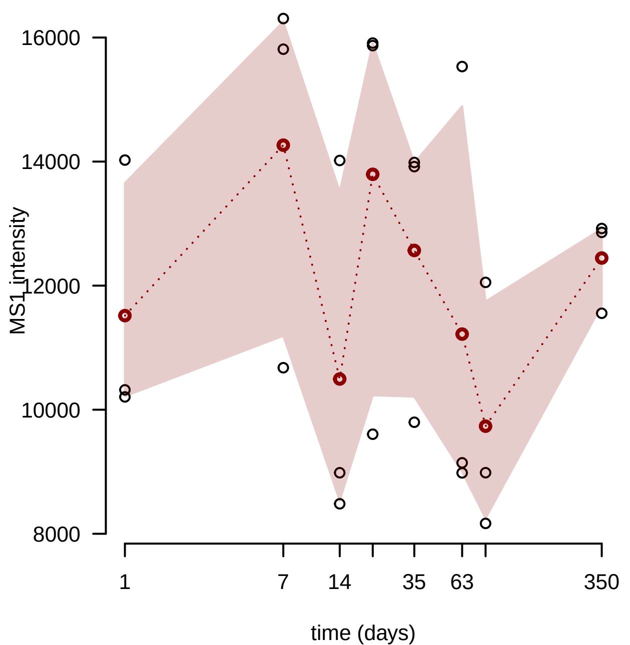

Q9D2V7
GN=Coro7
coronin 7

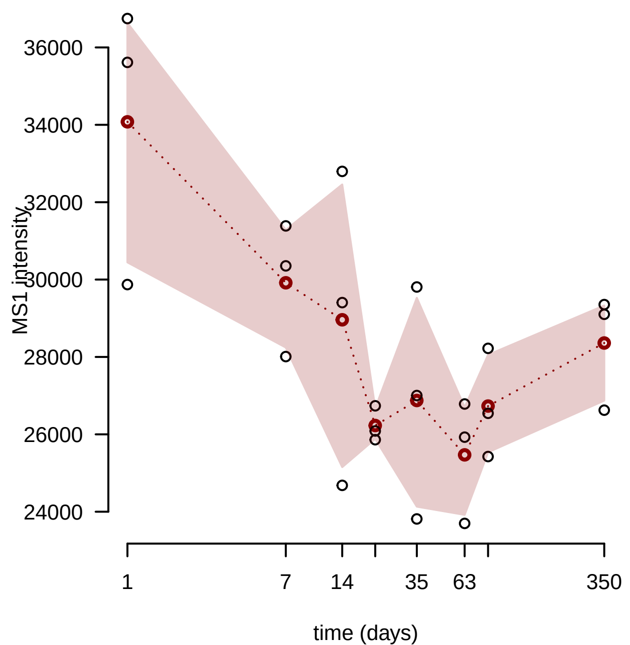

Q9D2V8
GN=Mfsd10
major facilitator superfamily domain con ...

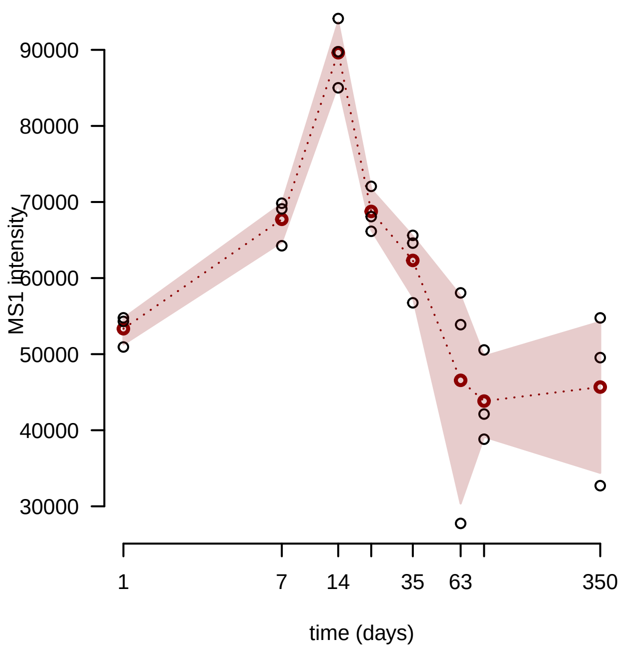

Q9D2X5
GN=Mau2
MAU2 sister chromatid cohesion factor

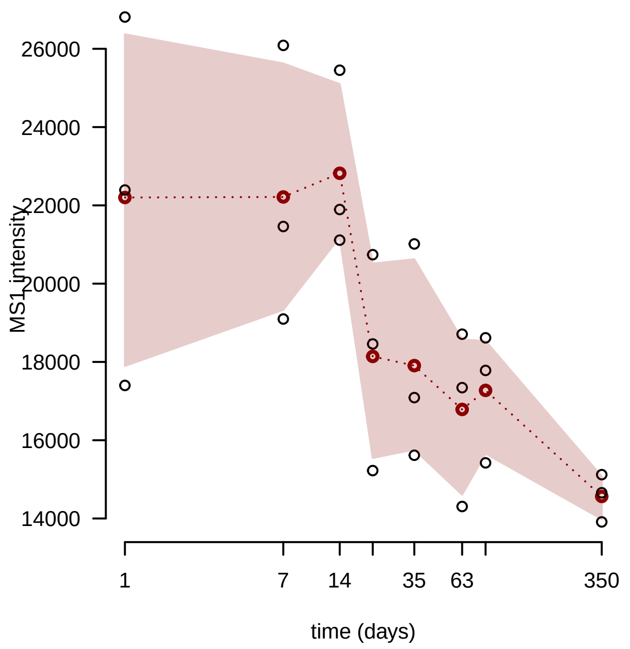

Q9D2X5
GN=Mau2
MAU2 sister chromatid cohesion factor

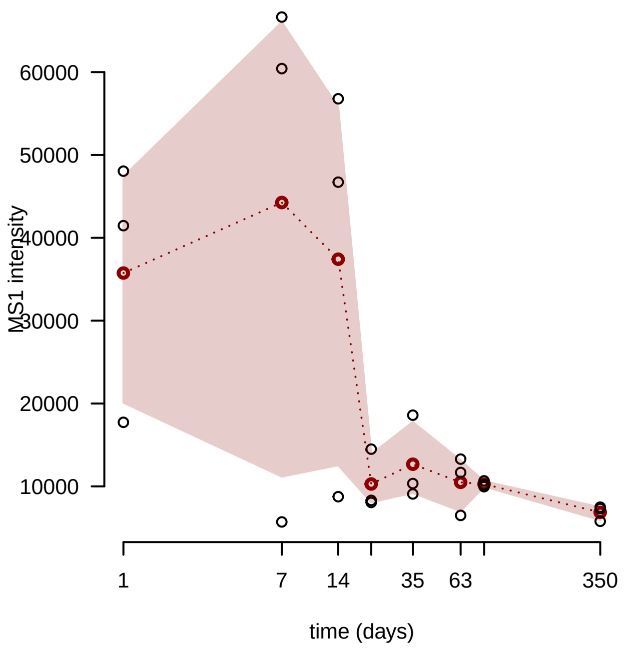

Q9D2Y4
GN=Mlkl
mixed lineage kinase domain−like

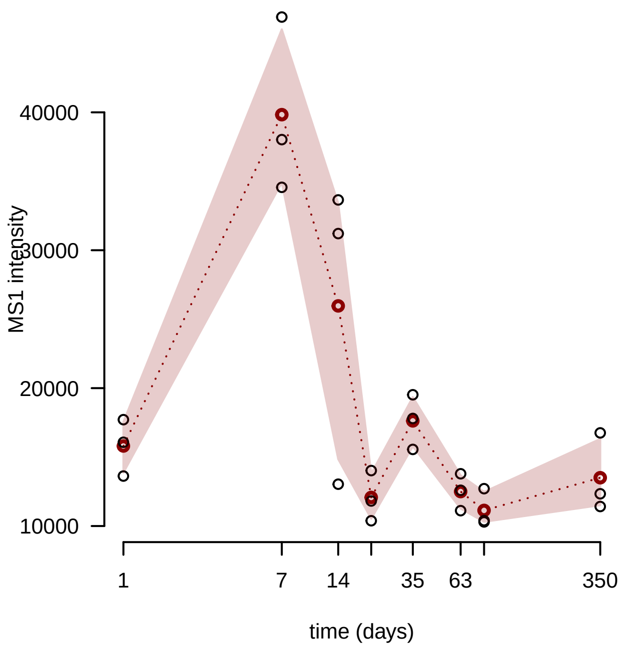

Q9D2Z4
GN=Senp8
SUMO peptidase family member, NEDD8 spec ...

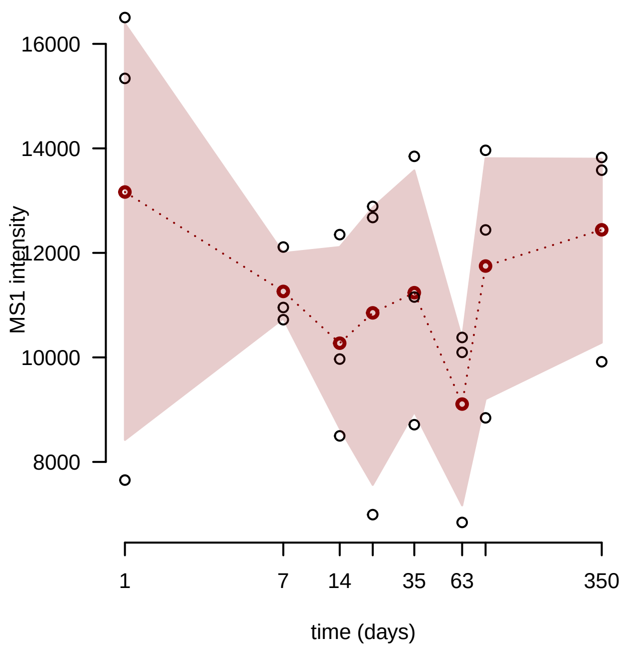

Q9D312
GN=Krt20
keratin 20

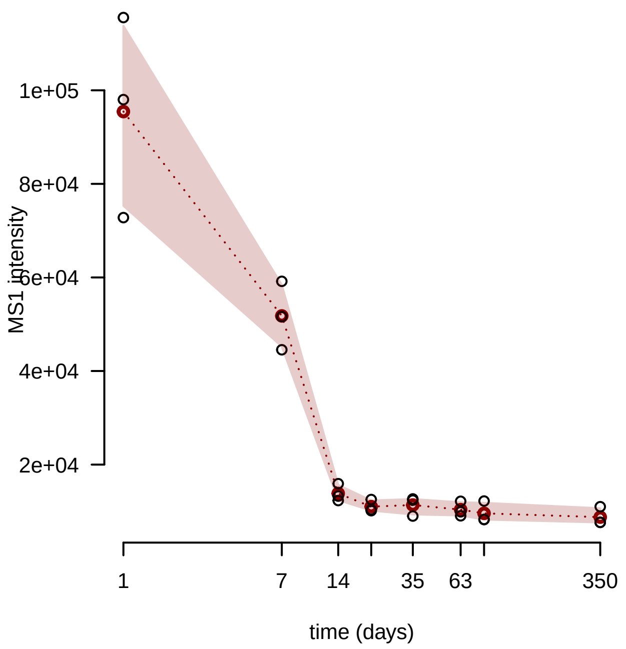

Q9D328
GN=Tmem35a
transmembrane protein 35A

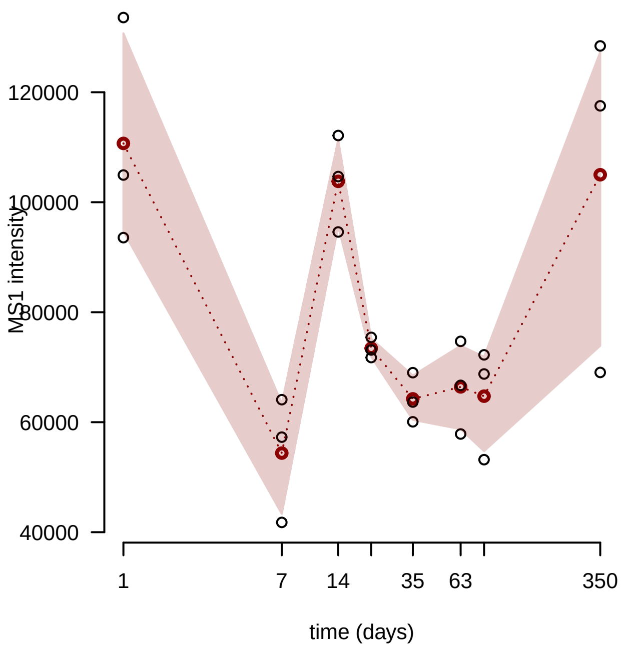

Q9D338
GN=Mrpl19
mitochondrial ribosomal protein L19

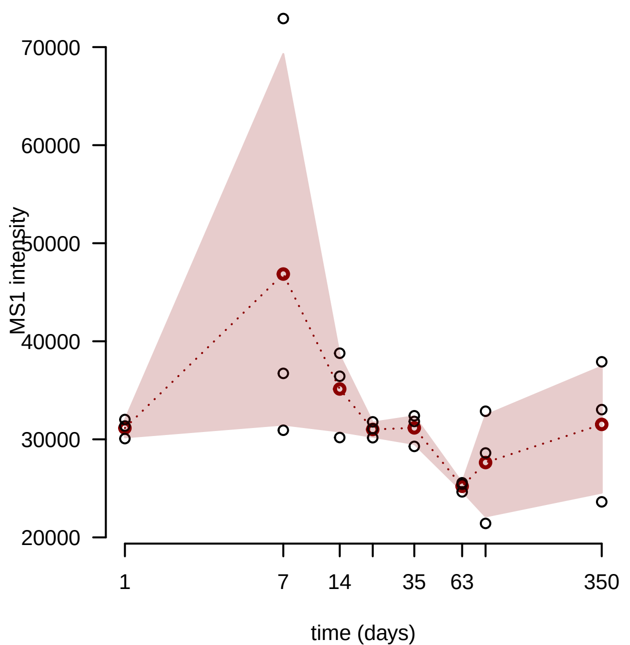

Q9D358
GN=Acp1
acid phosphatase 1, soluble

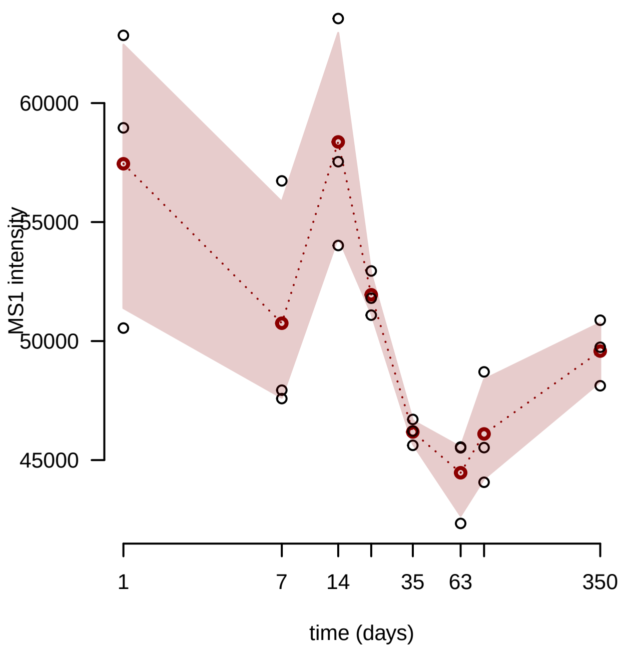

Q9D361
GN=Snrnp48
small nuclear ribonucleoprotein 48 (U11/ ...

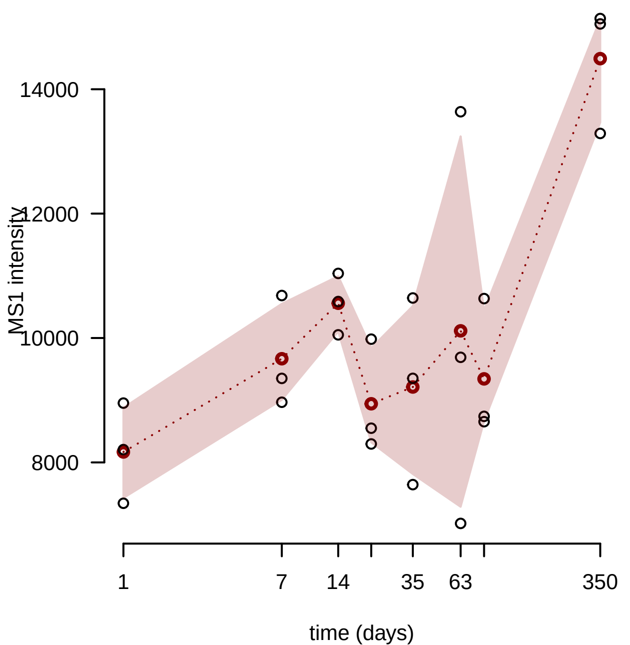

Q9D379
GN=Ephx1
epoxide hydrolase 1, microsomal

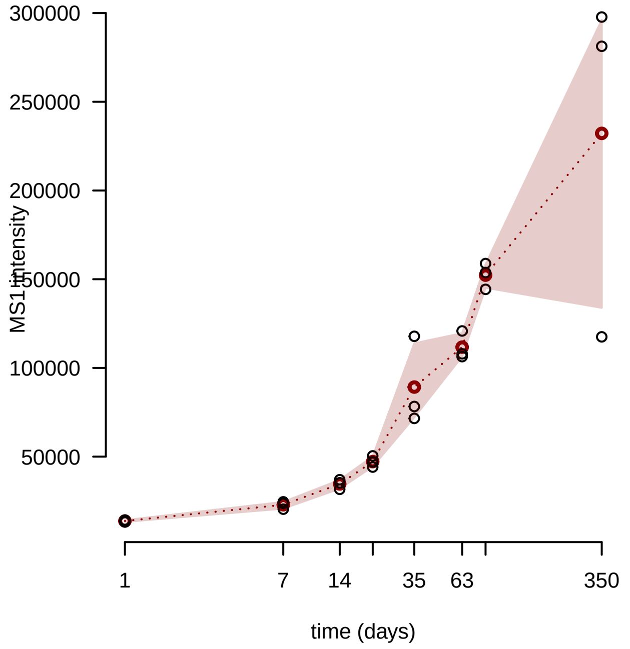

Q9D384
GN=Snrnp35
small nuclear ribonucleoprotein 35 (U11/ ...

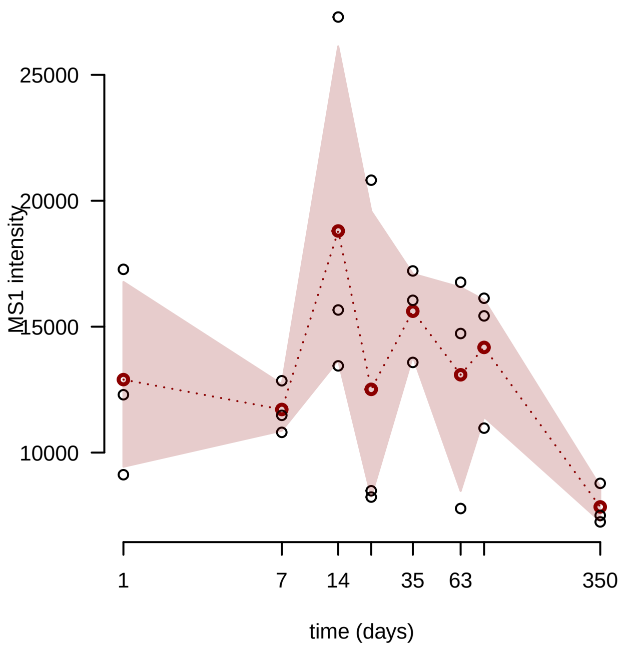

Q9D385
GN=Arl2bp
ADP−ribosylation factor−like 2 binding p ...

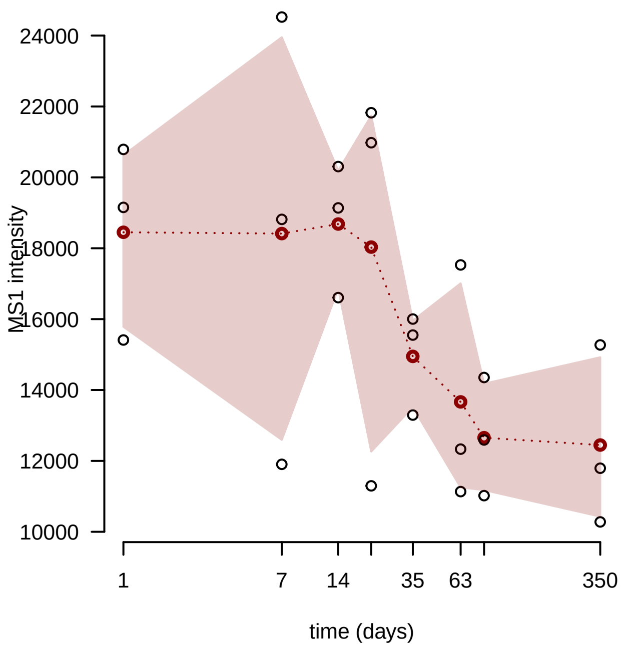

Q9D394
GN=Rufy3
RUN and FYVE domain containing 3

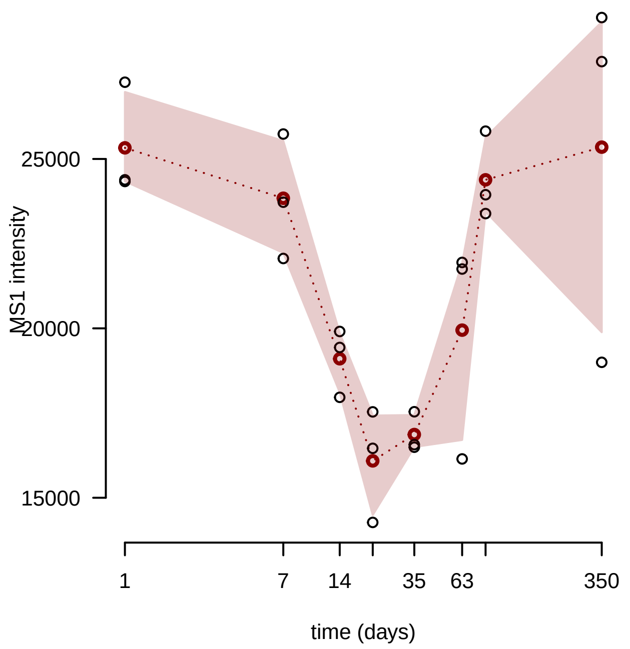

Q9D3A8
GN=Nos1ap
nitric oxide synthase 1 (neuronal) adapt ...

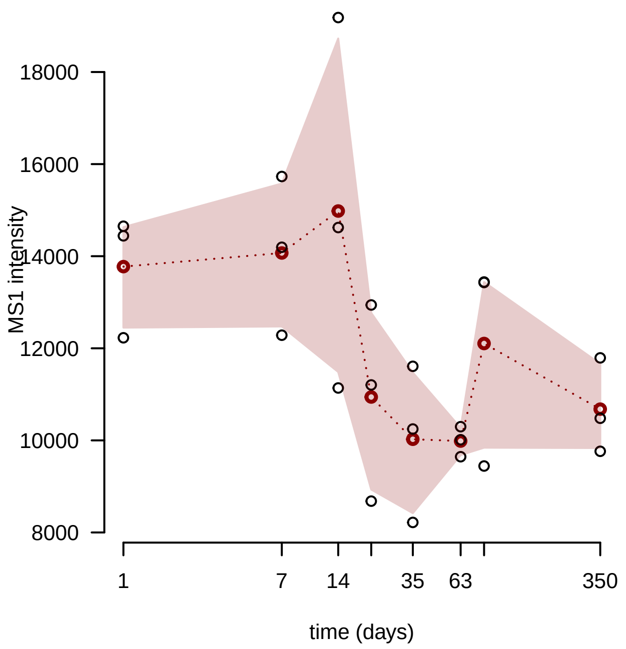

Q9D3B1
GN=Hacd2
3−hydroxyacyl−CoA dehydratase 2

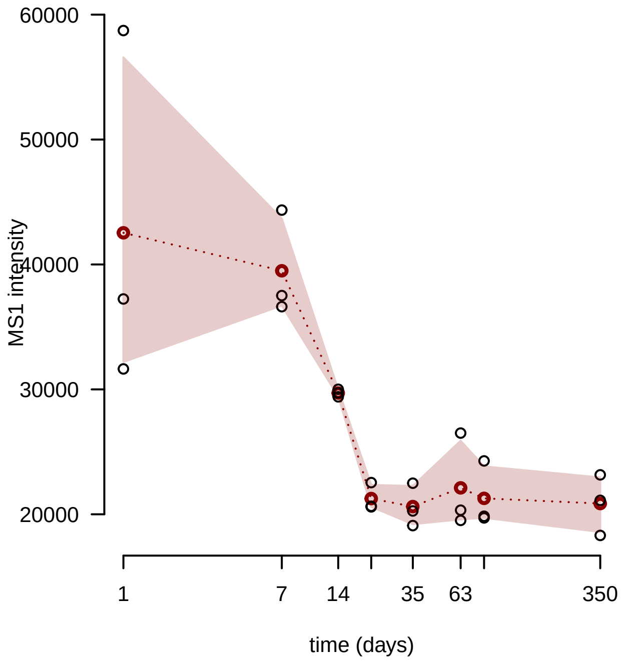

Q9D3D0
GN=Ttpal
tocopherol (alpha) transfer protein−like

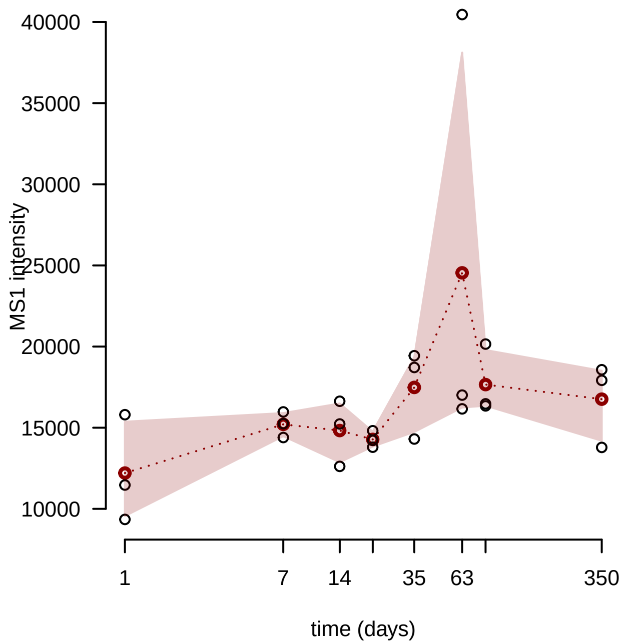

Q9D3D9
GN=Atp5f1d

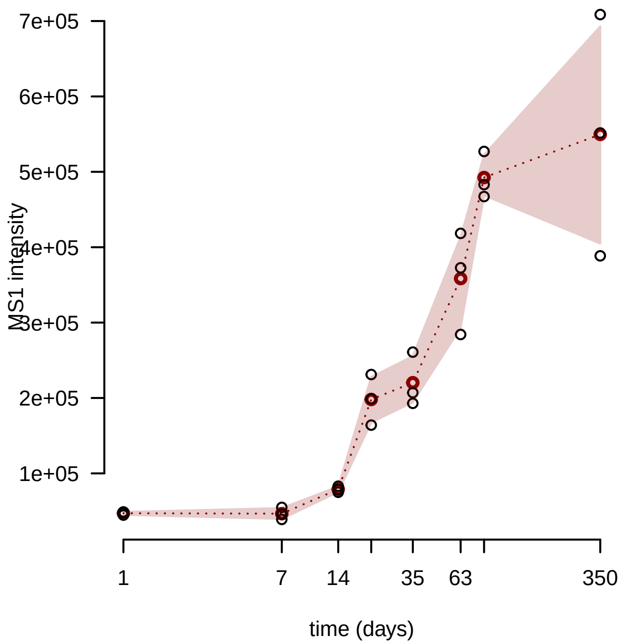

Q9D3E6
GN=Stag1
stromal antigen 1

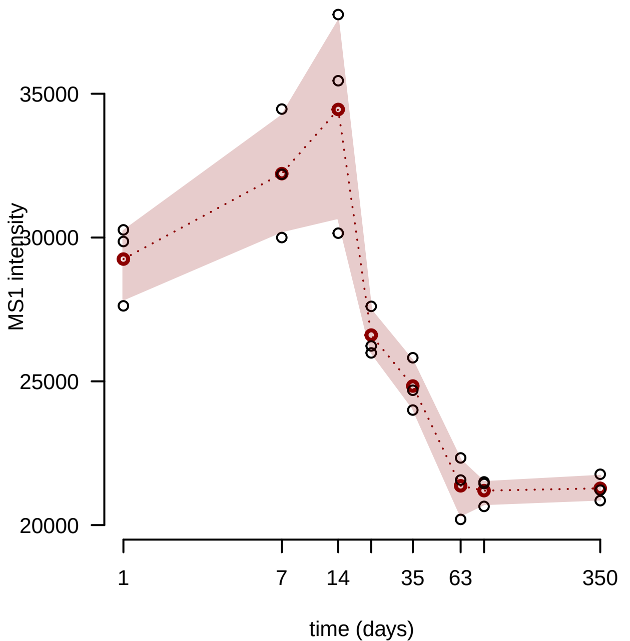

Q9D3G5
GN=Stx11
syntaxin 11

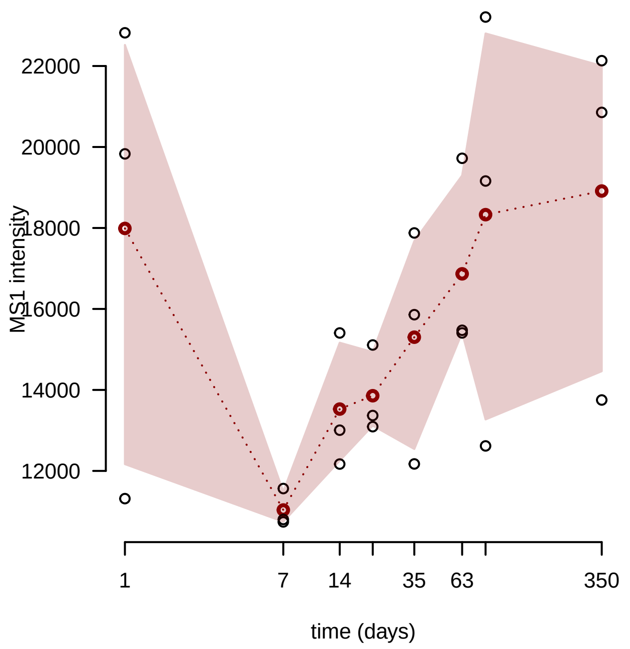

Q9D3L0
GN=Fam174a
family with sequence similarity 174, mem ...

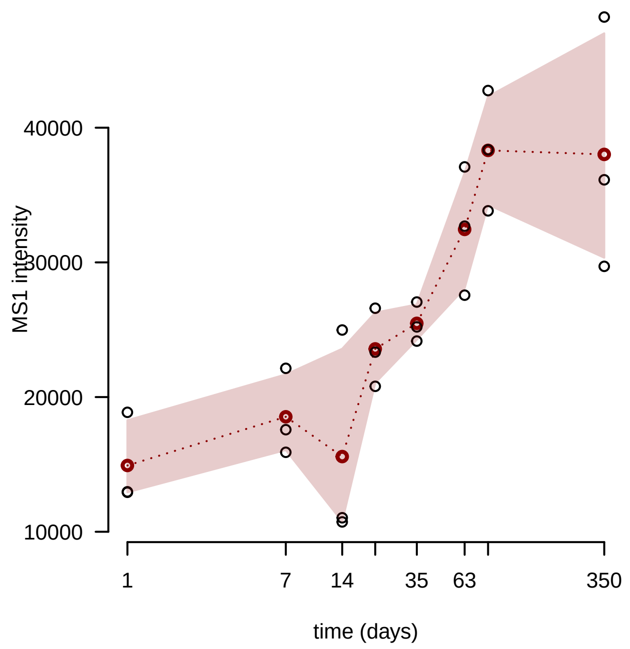

Q9D3P8
GN=Plgrkt
plasminogen receptor, C−terminal lysine ...

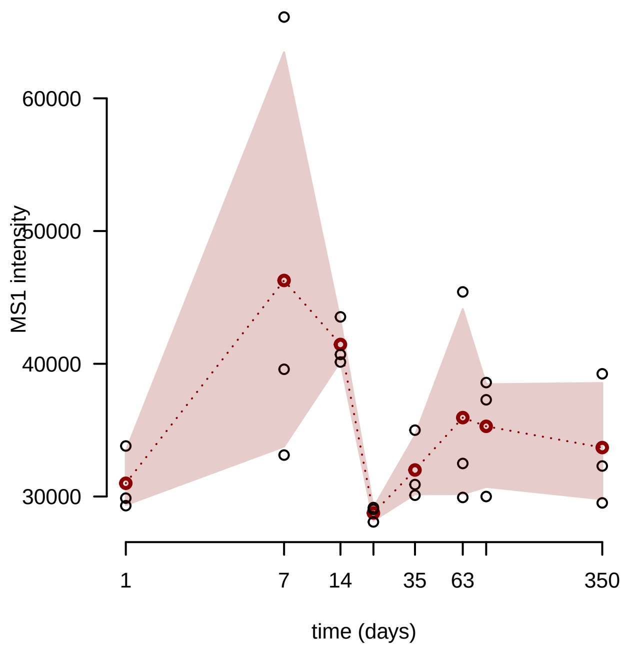

Q9D3R3
GN=Cep72
centrosomal protein 72

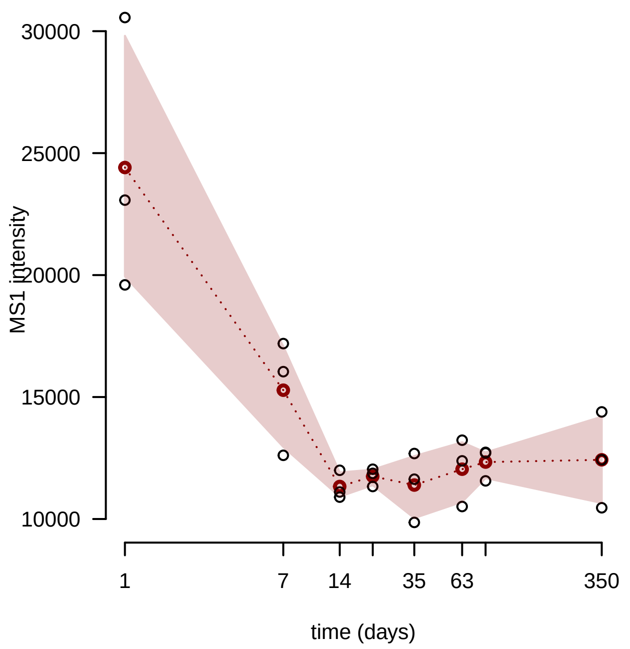

Q9D3R6
GN=Katnal2
katanin p60 subunit A−like 2

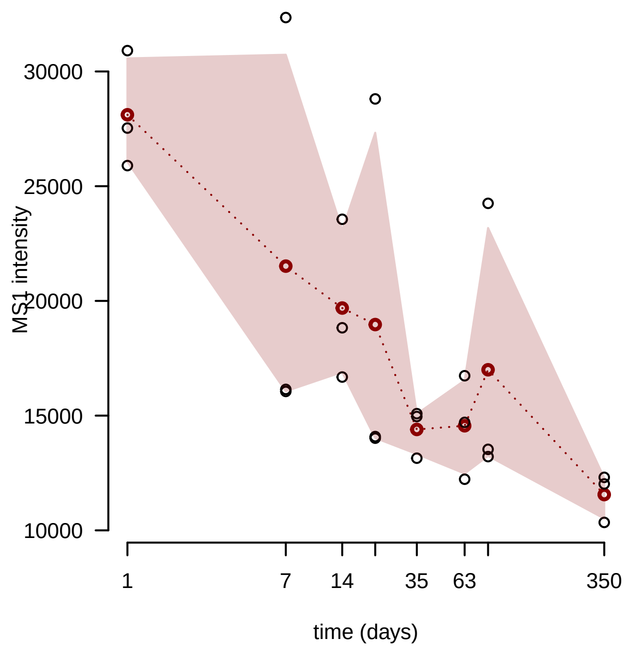

Q9D3S3
GN=Snx29
sorting nexin 29

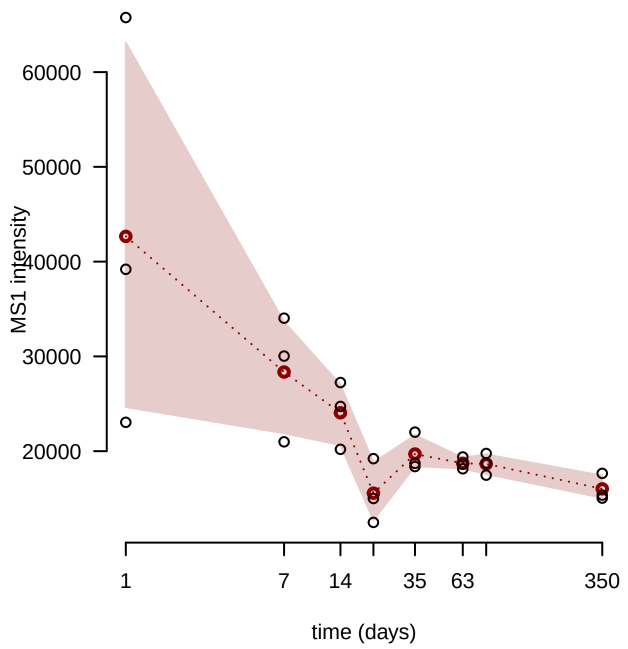

Q9D3U0
GN=Pus10
pseudouridylate synthase 10

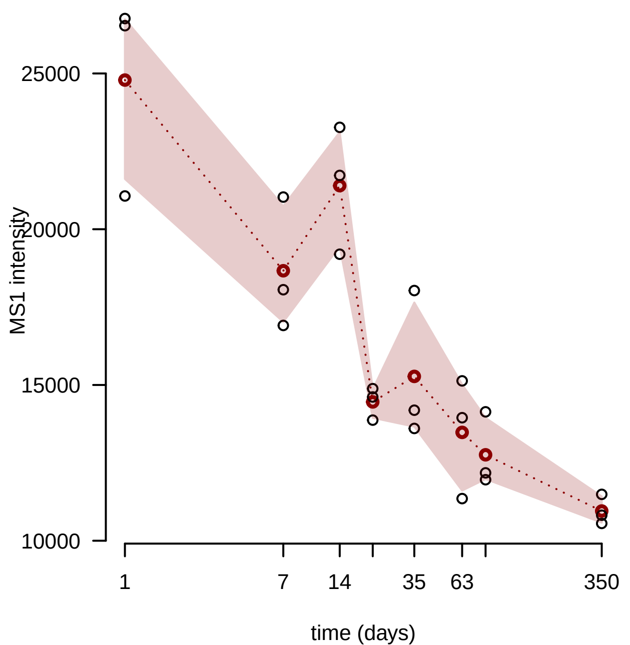

Q9D3W4
GN=Gpn3
GPN−loop GTPase 3

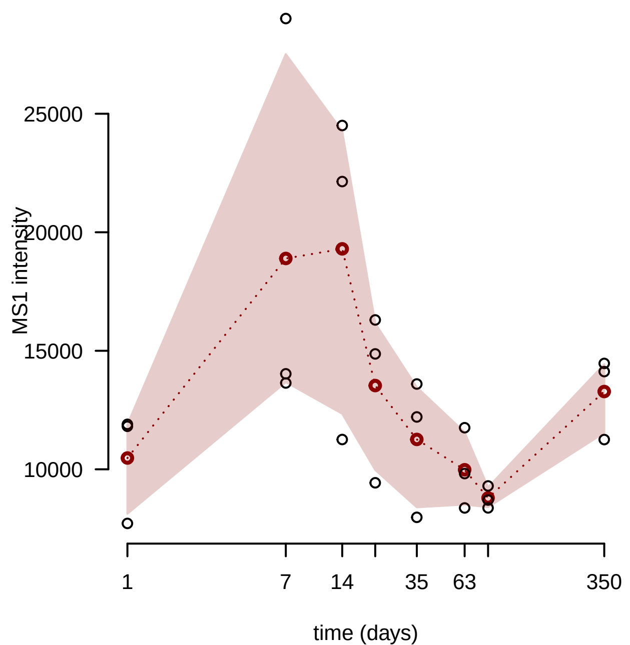

Q9D404
GN=Oxsm
3−oxoacyl−ACP synthase, mitochondrial

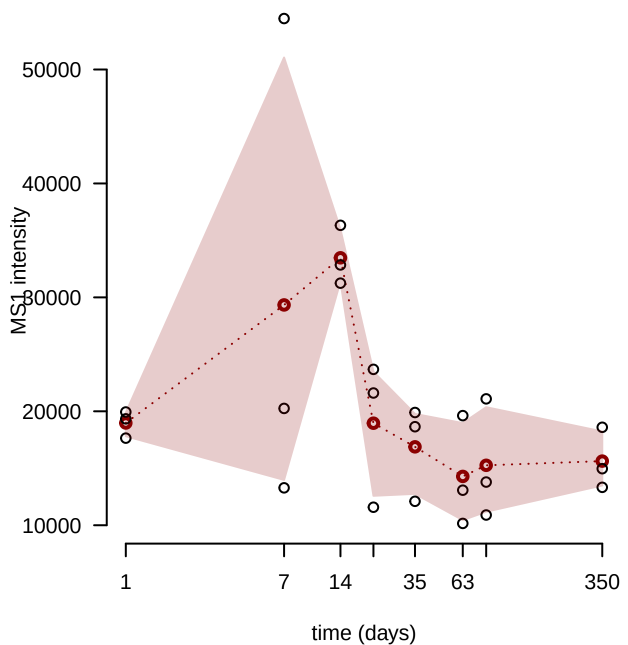

Q9D415
GN=Dlgap1
DLG associated protein 1

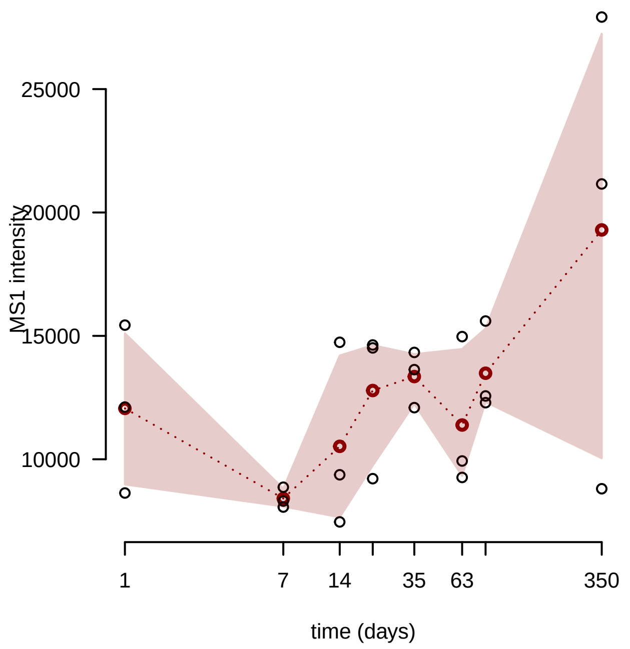

Q9D479
GN=Uvssa
UV stimulated scaffold protein A

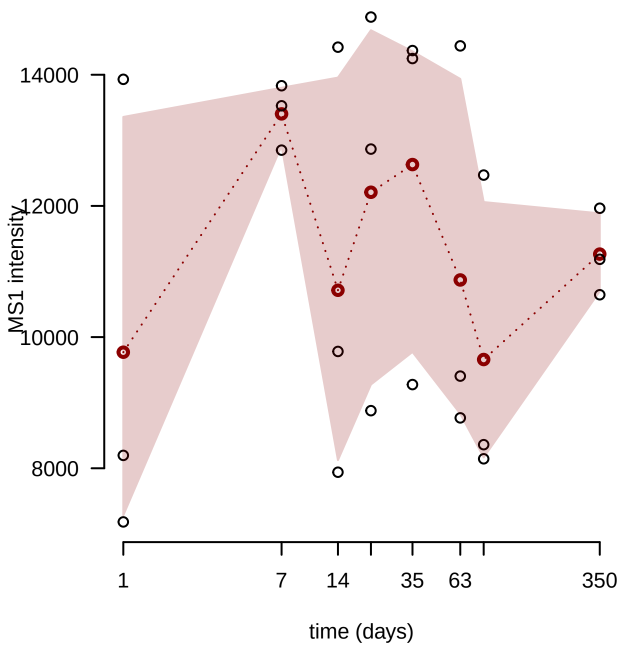

Q9D483
GN=Polr3c
polymerase (RNA) III (DNA directed) poly ...

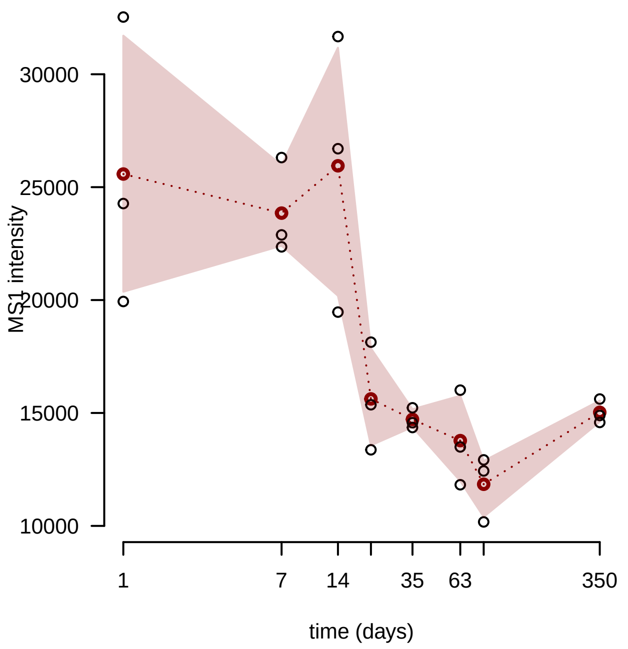

Q9D4D4
GN=Tktl2
transketolase−like 2

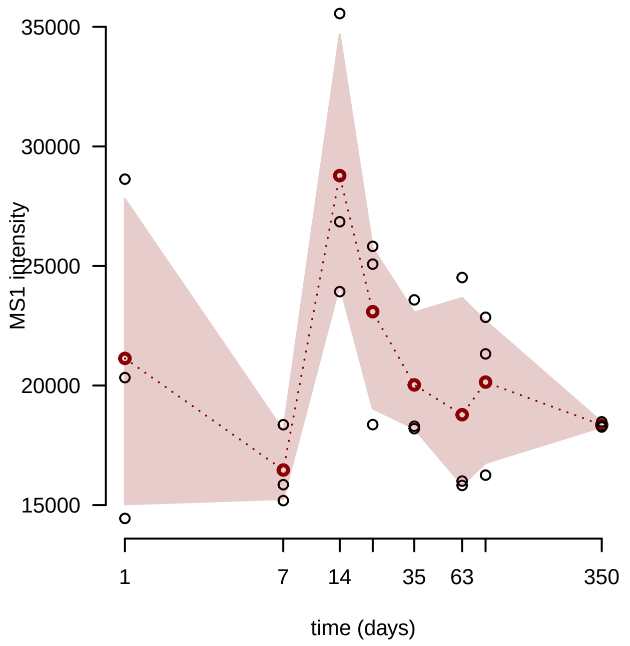

Q9D4F2
GN=Plpp6
phospholipid phosphatase 6

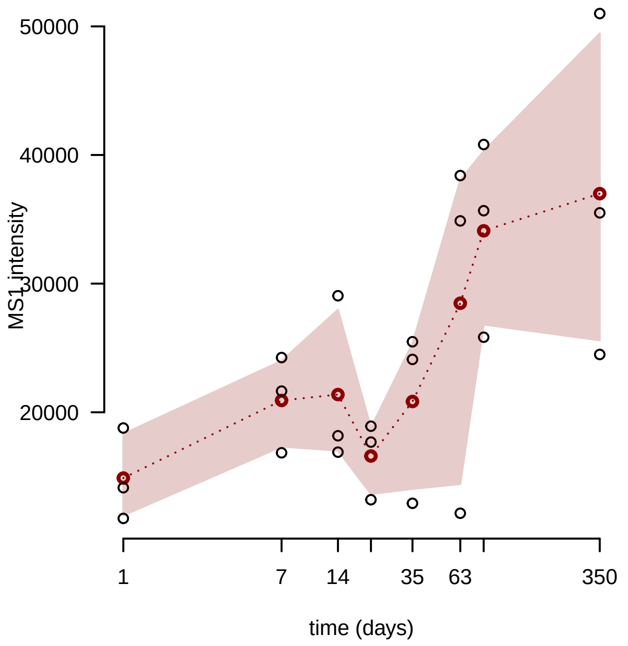

Q9D4F8
GN=Tubgcp4
tubulin, gamma complex associated protei ...

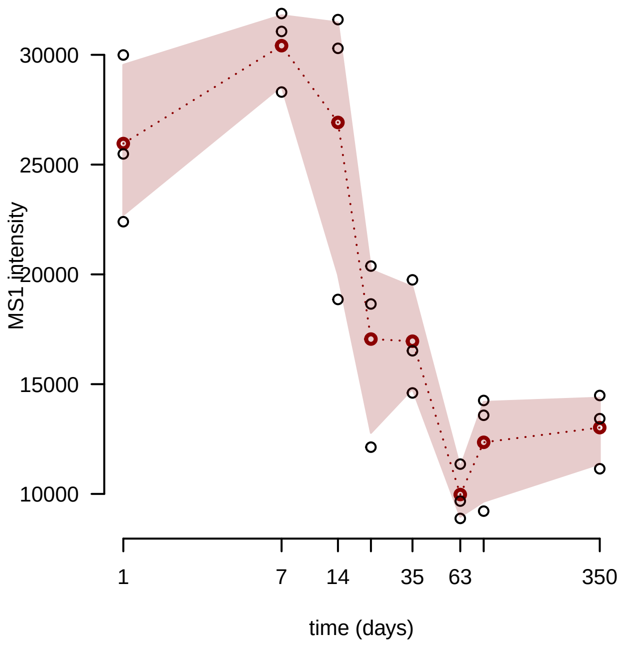

Q9D4H1
GN=Exoc2
exocyst complex component 2

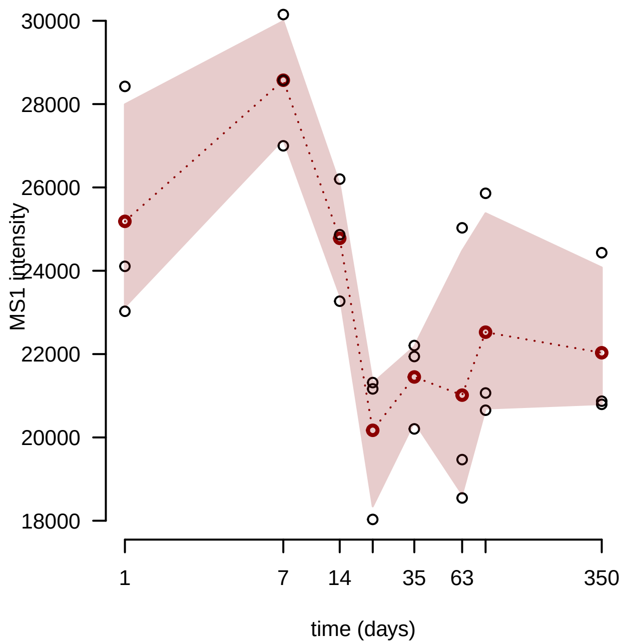

Q9D4H2
GN=Gcc1
golgi coiled coil 1

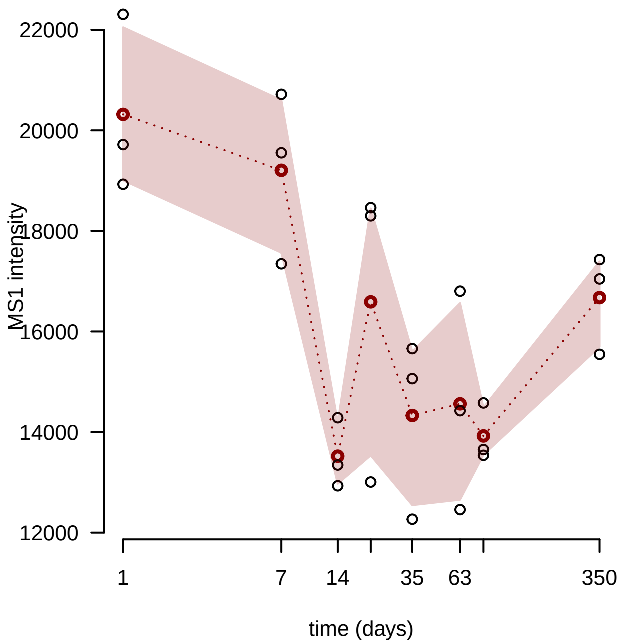

Q9D4H8
GN=Cul2
cullin 2

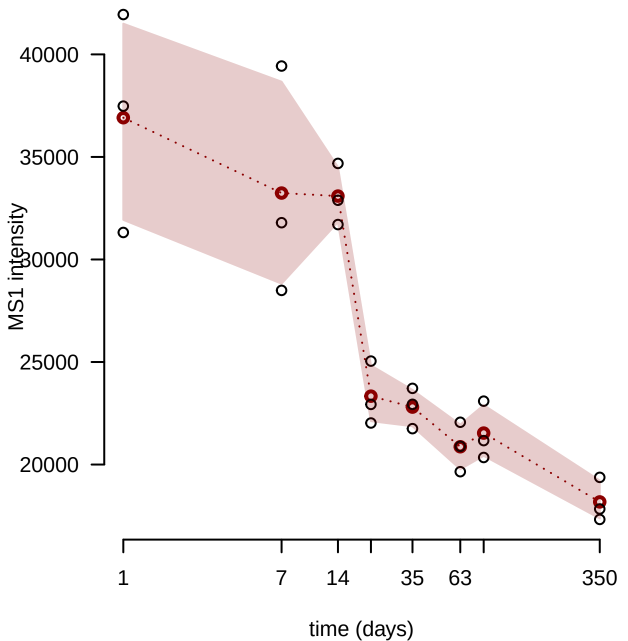

Q9D4H9
GN=Phf14
PHD finger protein 14

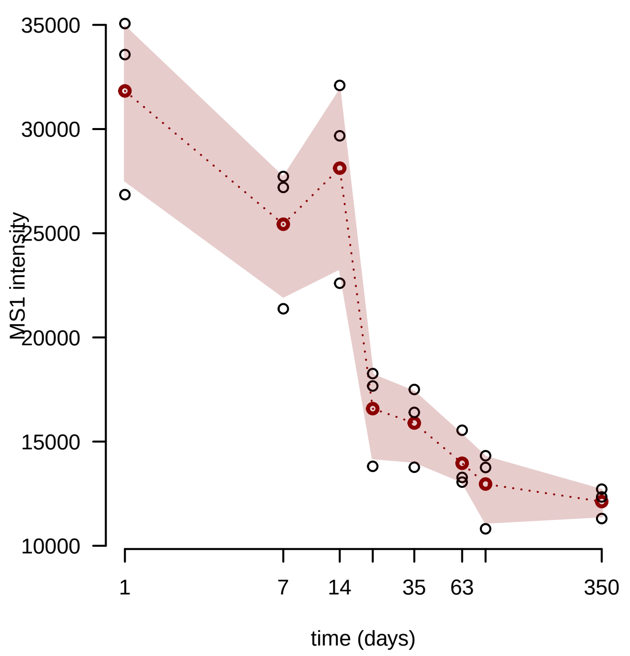

Q9D4J1
GN=Efhd1
EF hand domain containing 1

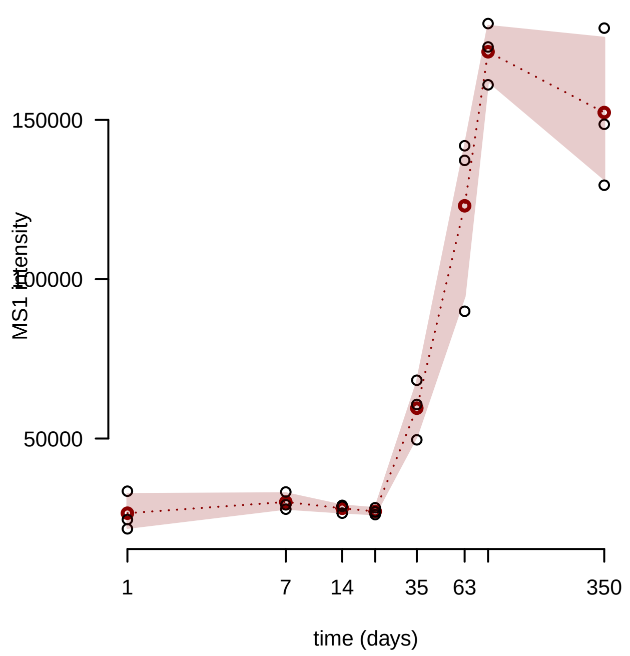

Q9D4J7
GN=Phf6
PHD finger protein 6

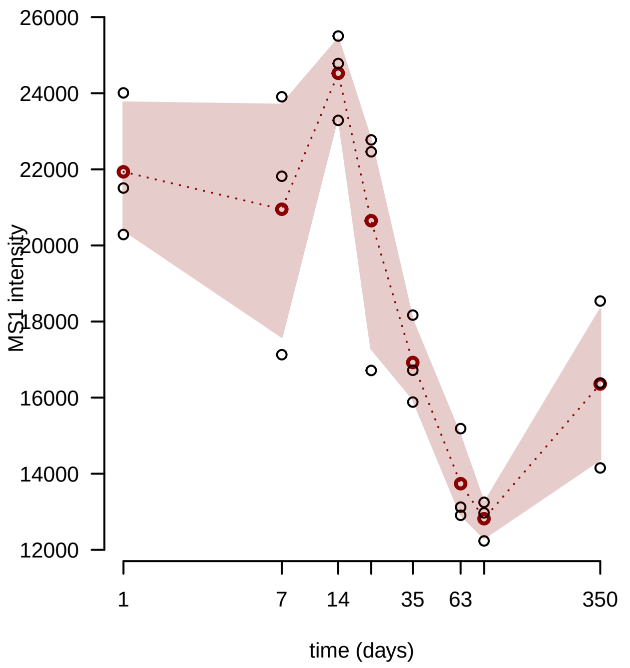

Q9D4P0
GN=Arl5b
ADP−ribosylation factor−like 5B

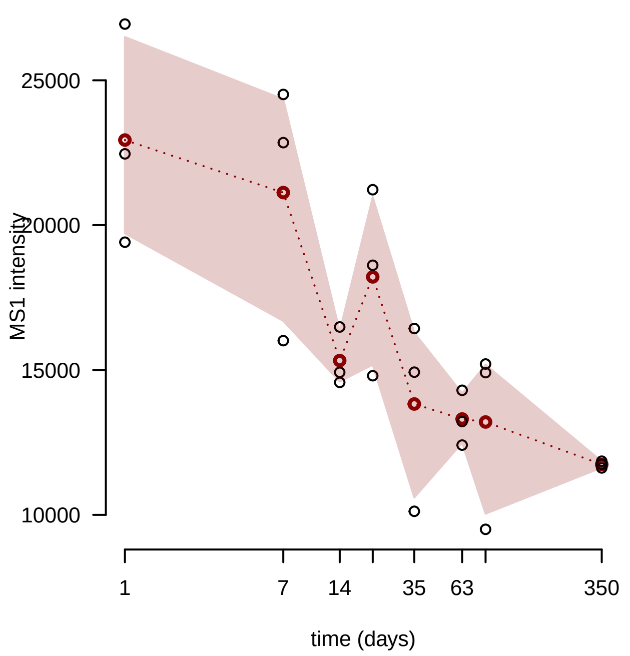

Q9D4V0
GN=Etnk1
ethanolamine kinase 1

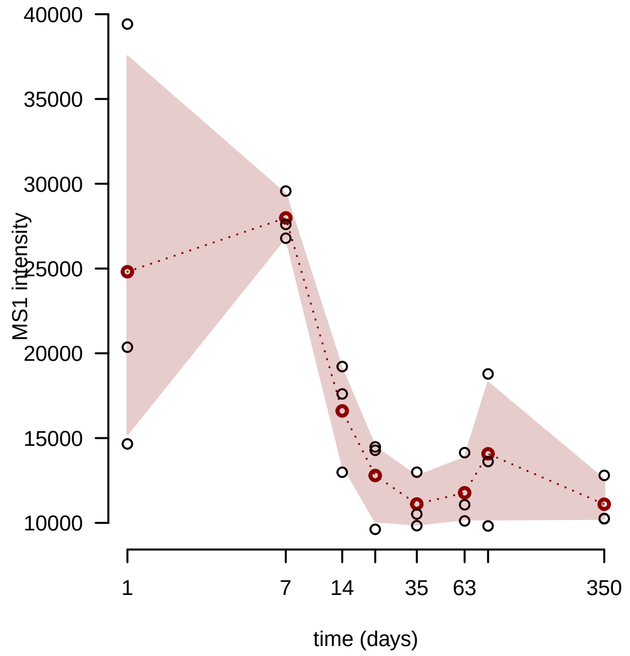

Q9D4V7
GN=Rabl3
RAB, member RAS oncogene family−like 3

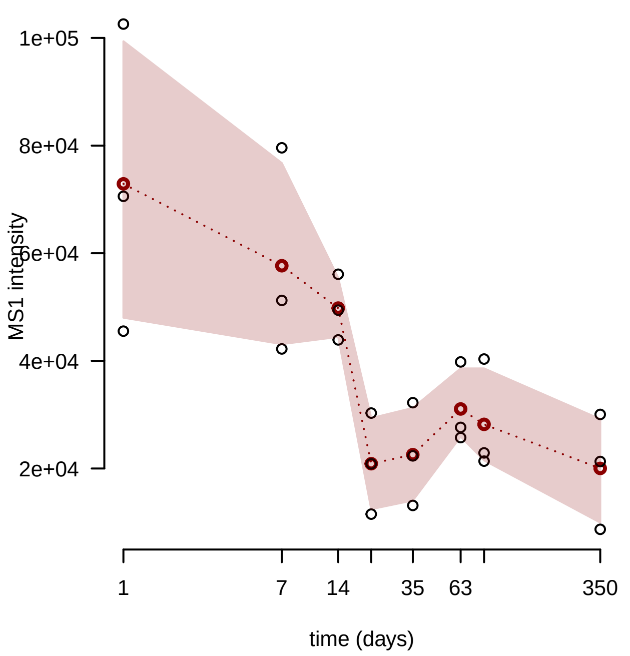

Q9D516
GN=Ccdc130
coiled−coil domain containing 130

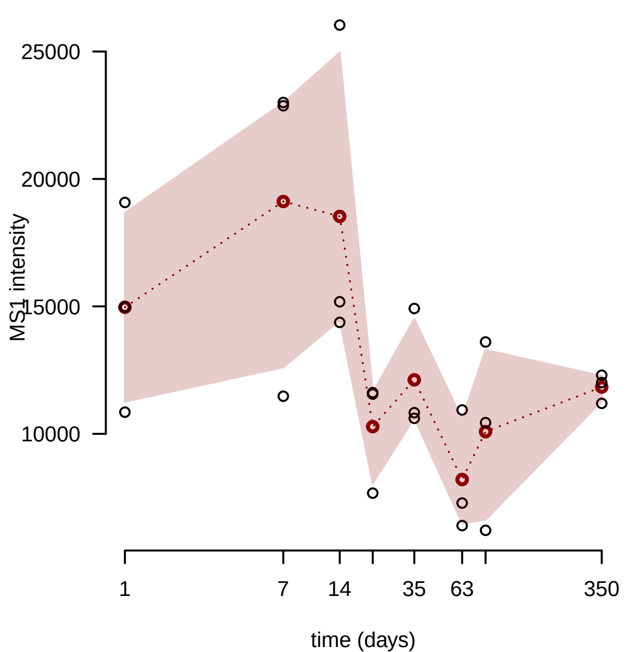

Q9D517
GN=Agpat3
1−acylglycerol−3−phosphate O−acyltransfe ...

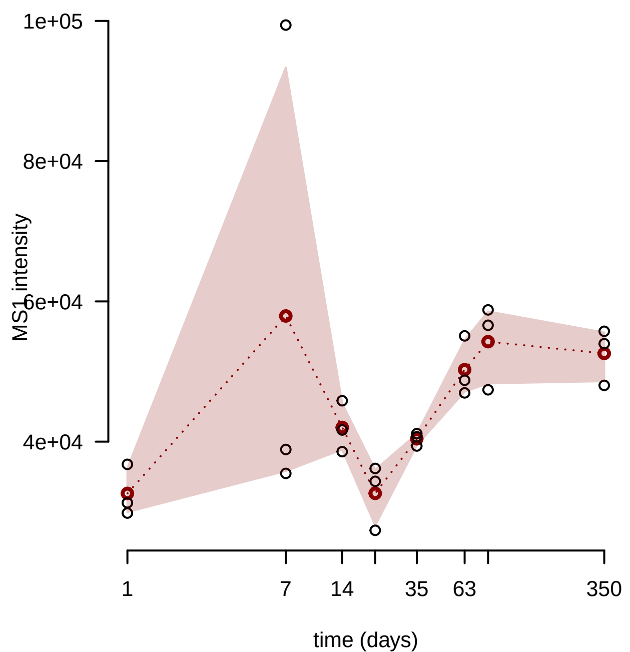

Q9D531
GN=Nxnl2
nucleoredoxin−like 2

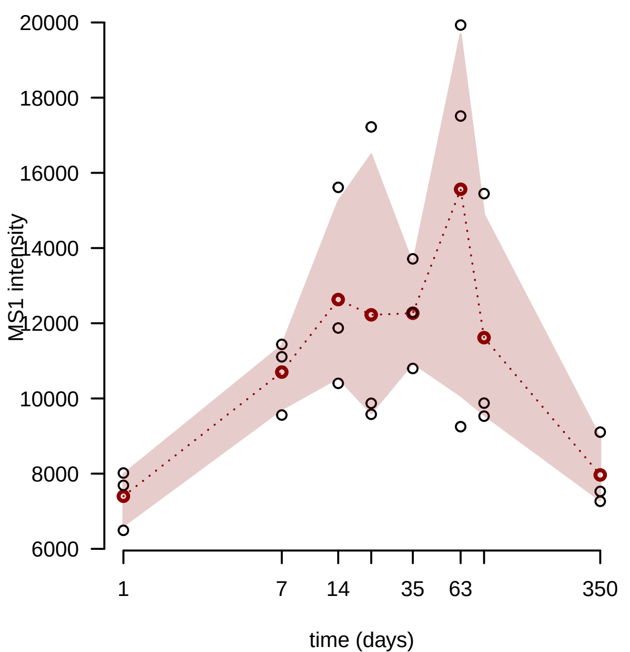

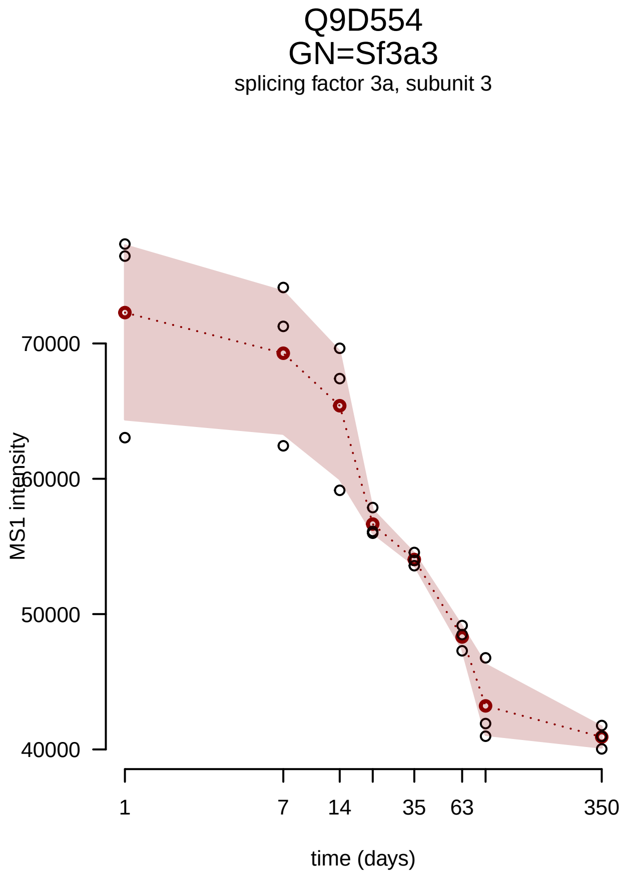
Q9D554
GN=Sf3a3
splicing factor 3a, subunit 3
MS1 intensity
40000 50000 60000 70000
1 7 14 35 63 350
time (days)

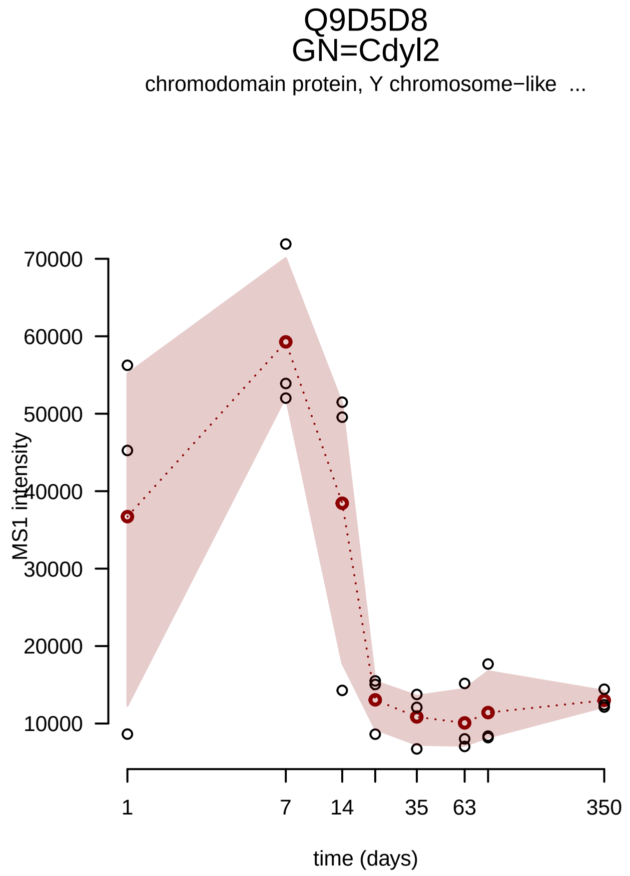
Q9D5D8
GN=Cdyl2
chromodomain protein, Y chromosome−like ...
MS1 intensity
10000 20000 30000 40000 50000 60000 70000
1 7 14 35 63 350
time (days)

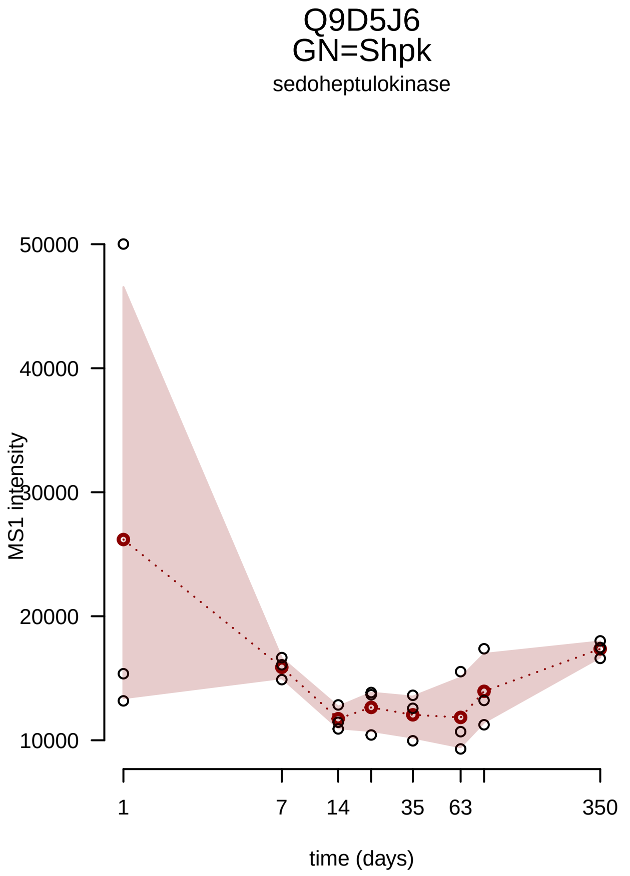
Q9D5J6
GN=Shpk
sedoheptulokinase
MS1 intensity
10000 20000 30000 40000 50000
1 7 14 35 63 350
time (days)

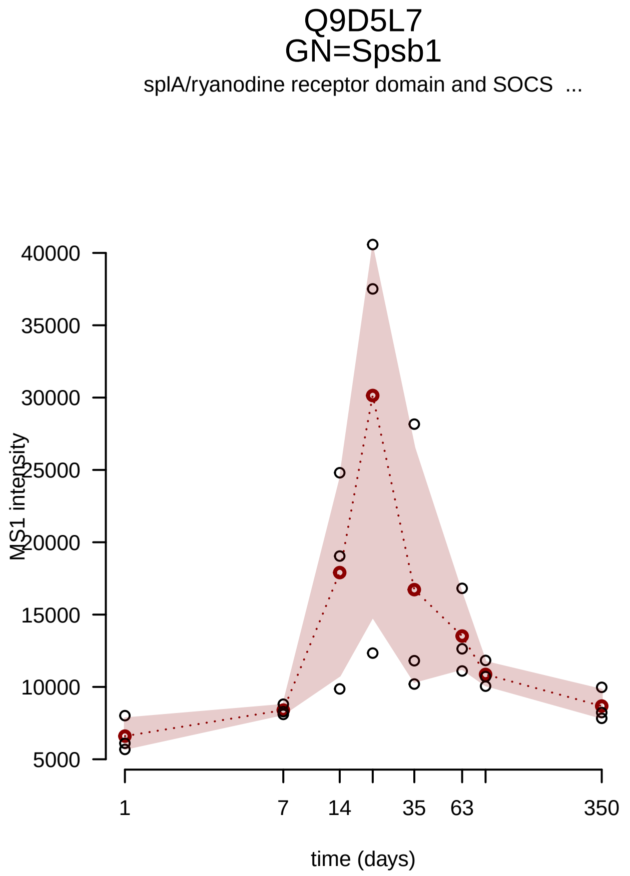
Q9D5L7
GN=Spsb1
splA/ryanodine receptor domain and SOCS ...
MS1 intensity
5000 10000 15000 20000 25000 30000 35000 40000
1 7 14 35 63 350
time (days)

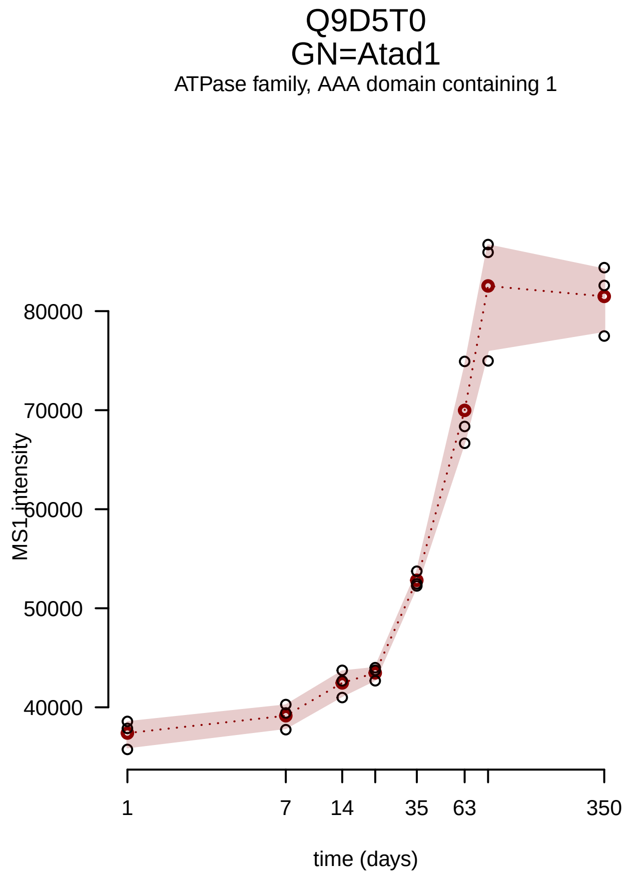
Q9D5T0
GN=Atad1
ATPase family, AAA domain containing 1
MS1 intensity
40000 50000 60000 70000 80000
1 7 14 35 63 350
time (days)

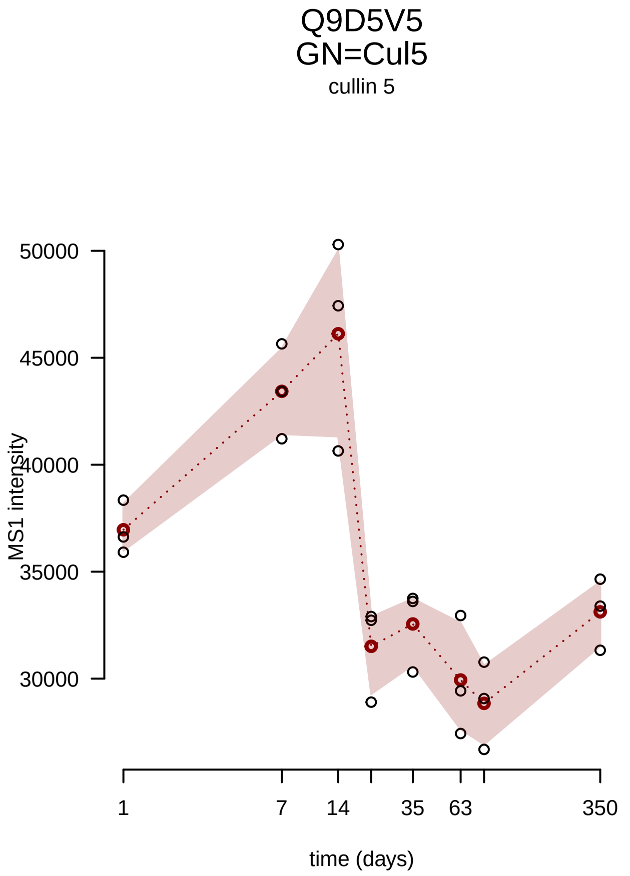
Q9D5V5
GN=Cul5
cullin 5
MS1 intensity
30000 35000 40000 45000 50000
1 7 14 35 63 350
time (days)

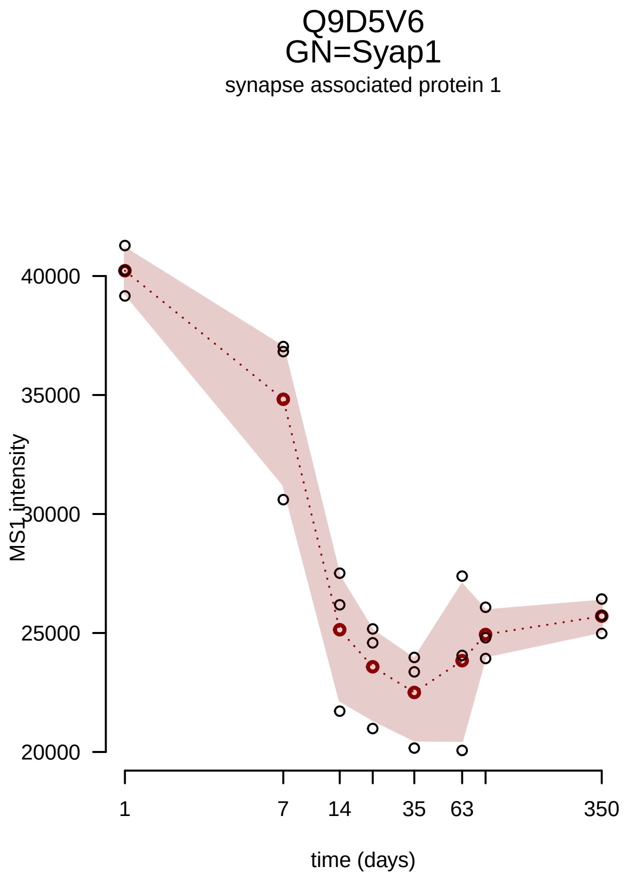
Q9D5V6
GN=Syap1
synapse associated protein 1
MS1 intensity
20000 25000 30000 35000 40000
1 7 14 35 63 350
time (days)

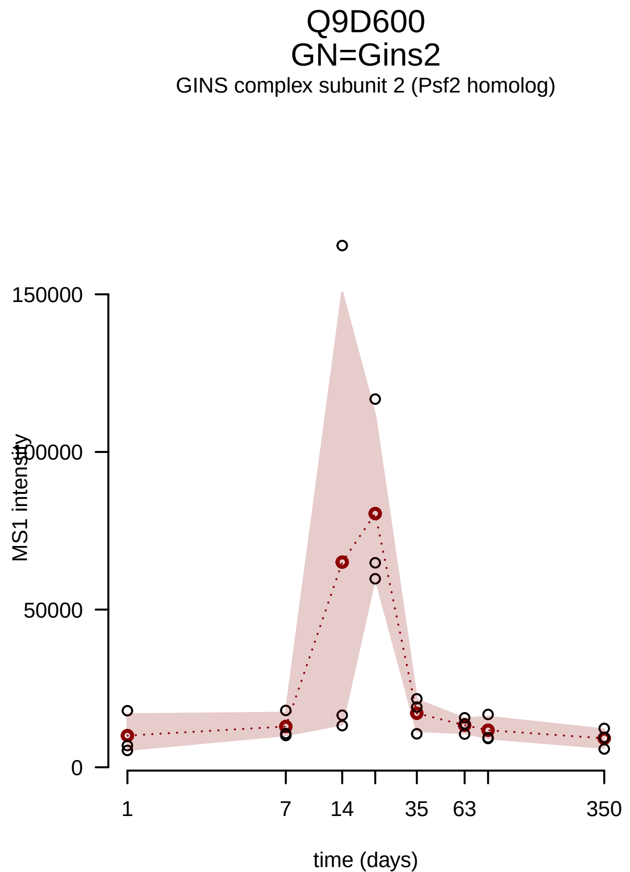
Q9D600
GN=Gins2
GINS complex subunit 2 (Psf2 homolog)
MS1 intensity
0 50000 100000 150000
1 7 14 35 63 350
time (days)

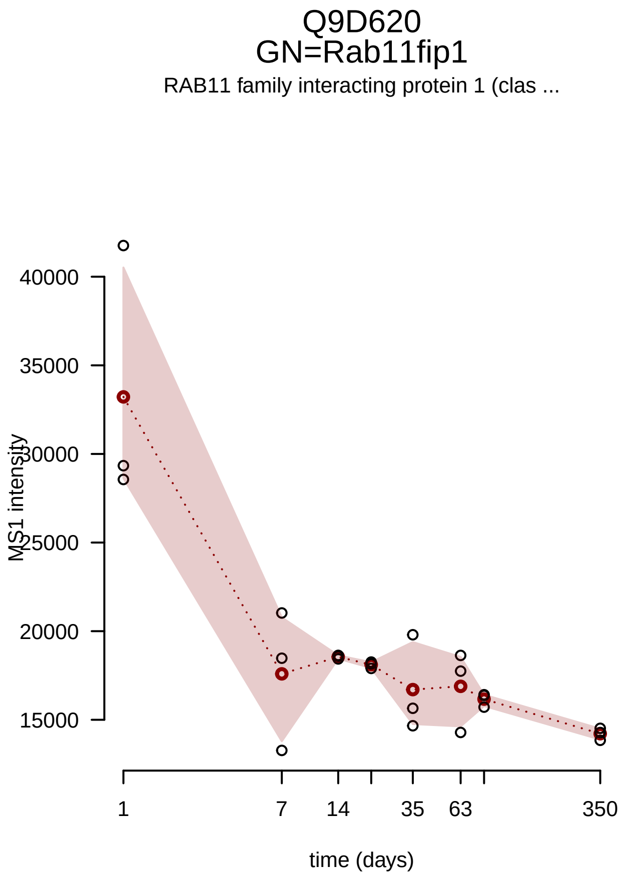
Q9D620
GN=Rab11fip1
RAB11 family interacting protein 1 (clas ...
MS1 intensity
15000 20000 25000 30000 35000 40000
1 7 14 35 63 350
time (days)

Q9D646
GN=Krt34
keratin 34

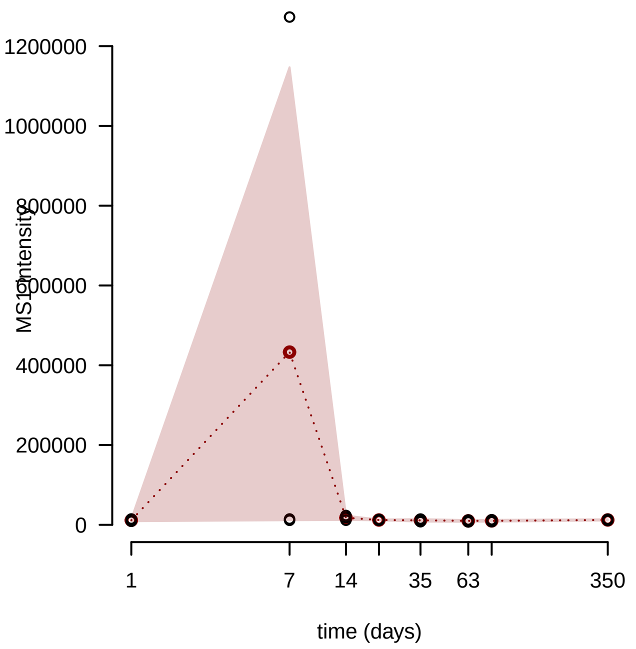

Q9D662
GN=Sec23b
SEC23 homolog B, COPII coat complex comp ...

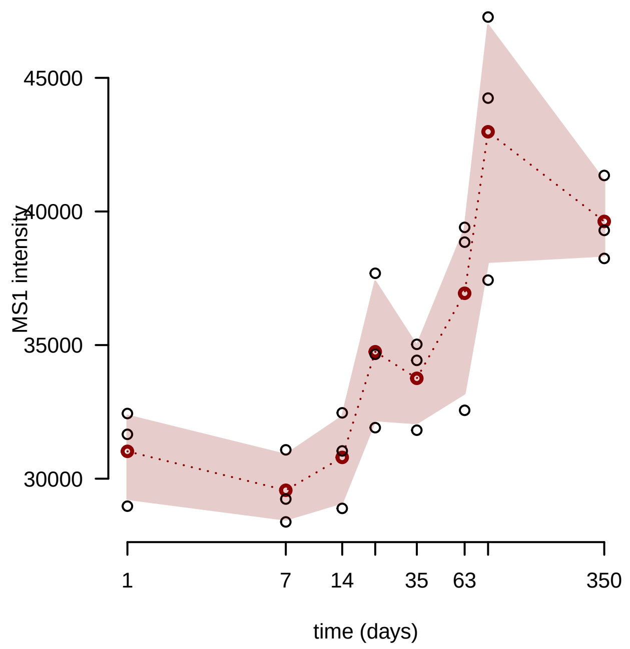

Q9D666
GN=Sun1
Sad1 and UNC84 domain containing 1

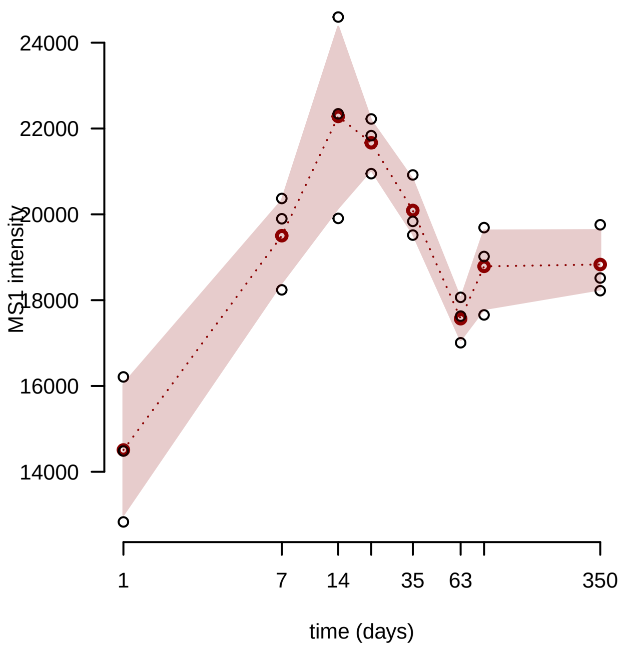

Q9D668
GN=Arrdc2
arrestin domain containing 2

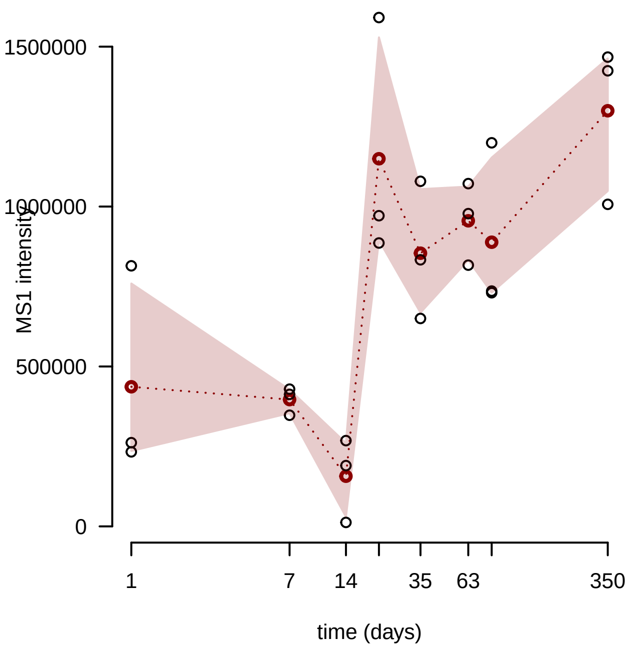

Q9D684
GN=Rin2
Ras and Rab interactor 2

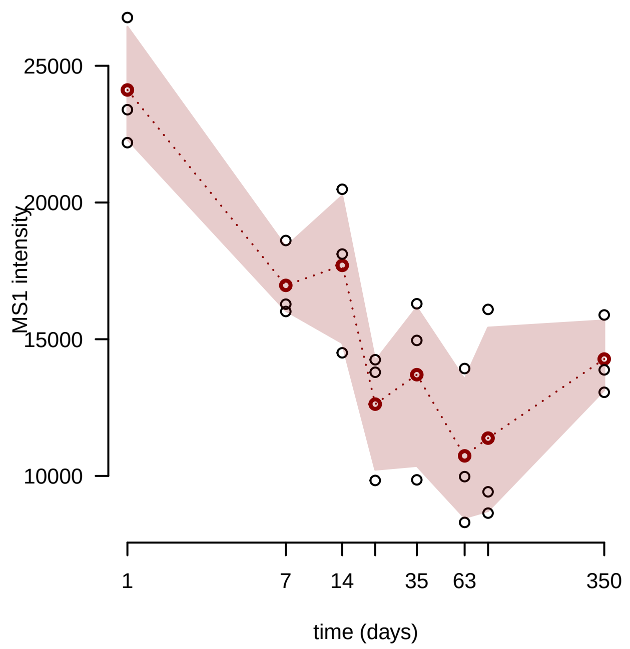

Q9D6F9
GN=Tubb4a
tubulin, beta 4A class IVA

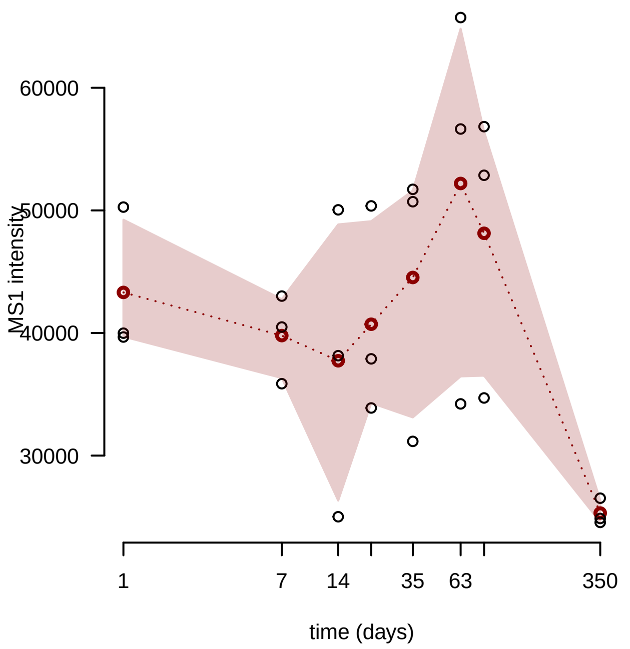

Q9D6H2
GN=Hspb11
heat shock protein family B (small), mem ...

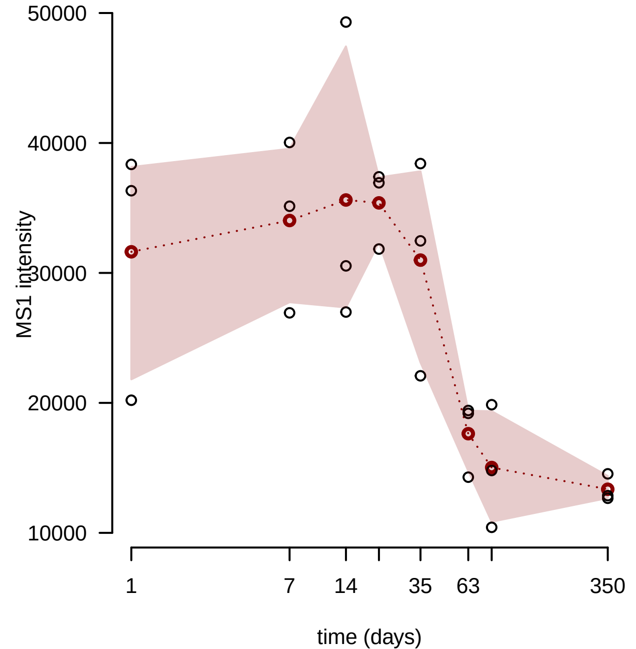

Q9D6I7
GN=Dipk1a
divergent protein kinase domain 1A

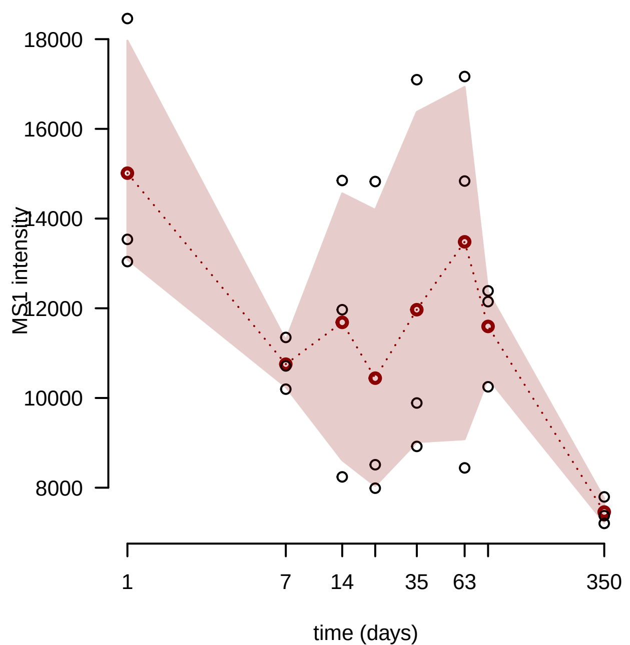

Q9D6J1
GN=Cers4
ceramide synthase 4

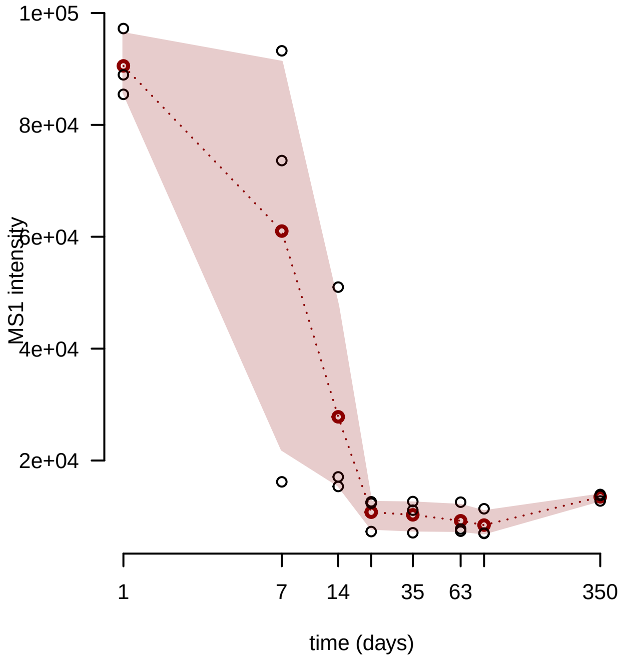

Q9D6J3
GN=Yju2
YJU2 splicing factor

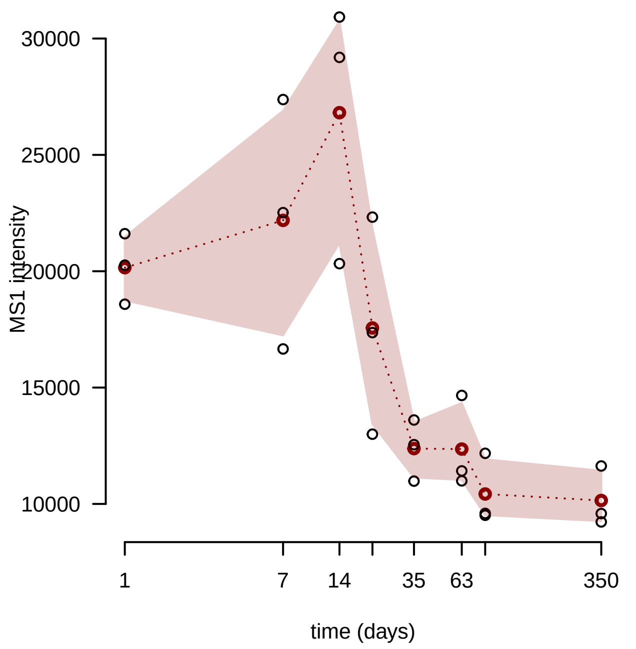

Q9D6J5
GN=Ndufb8
NADH:ubiquinone oxidoreductase subunit B ...

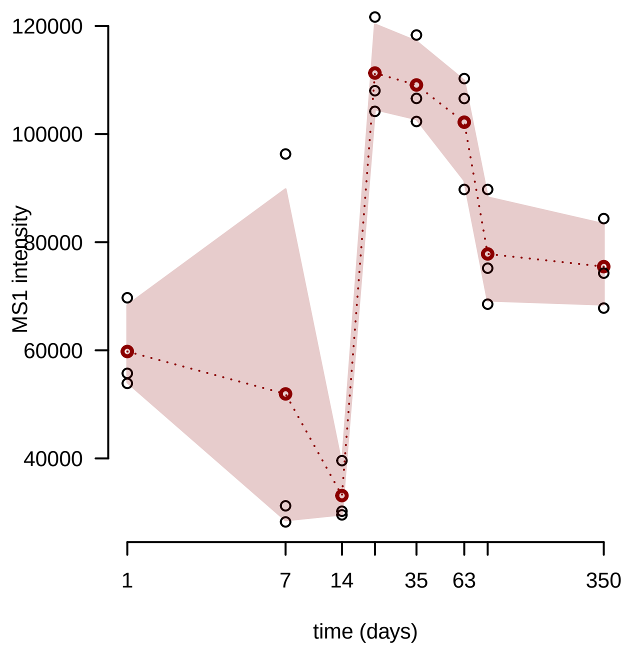

Q9D6J6
GN=Ndufv2
NADH:ubiquinone oxidoreductase core subu ...

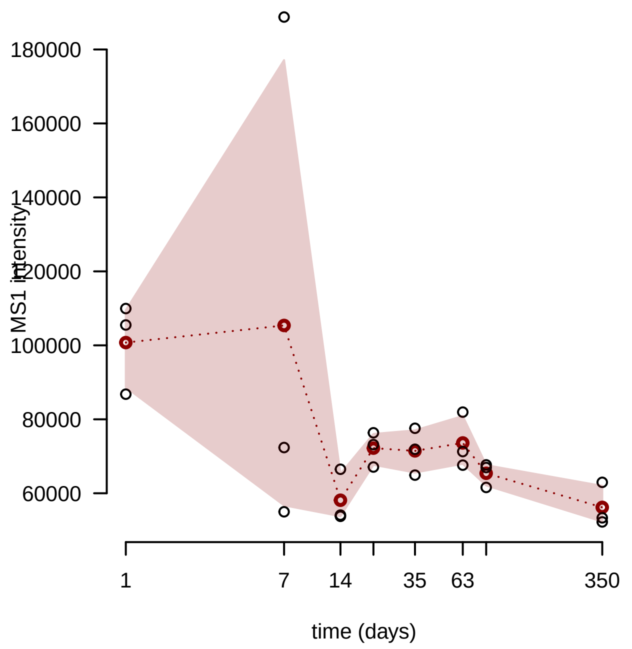

Q9D6K5
GN=Synj2bp
synaptojanin 2 binding protein

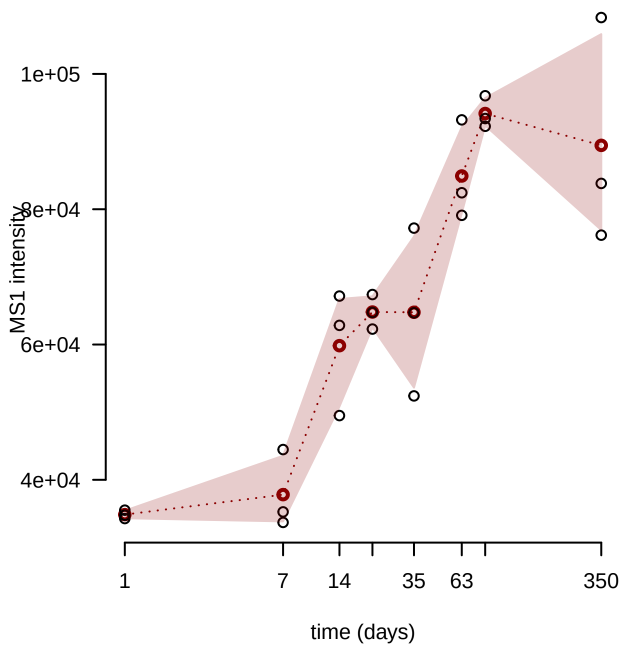

Q9D6K7
GN=Ttc33
tetratricopeptide repeat domain 33

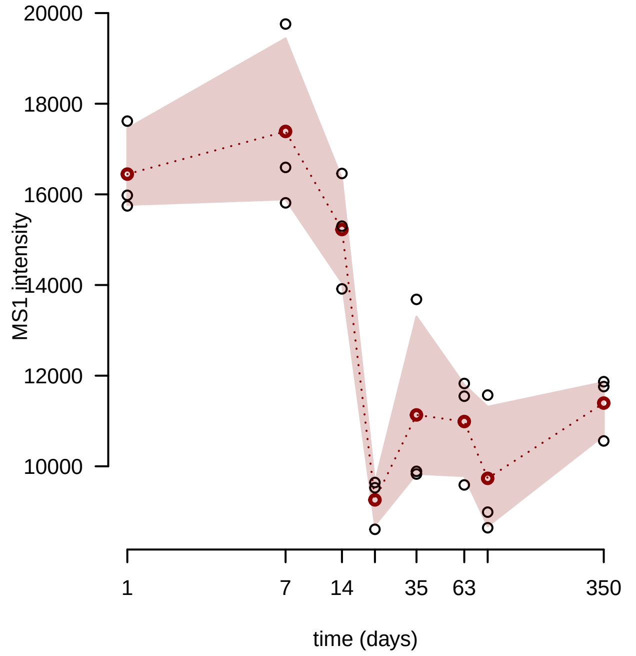

Q9D6K8
GN=Fundc2
FUN14 domain containing 2

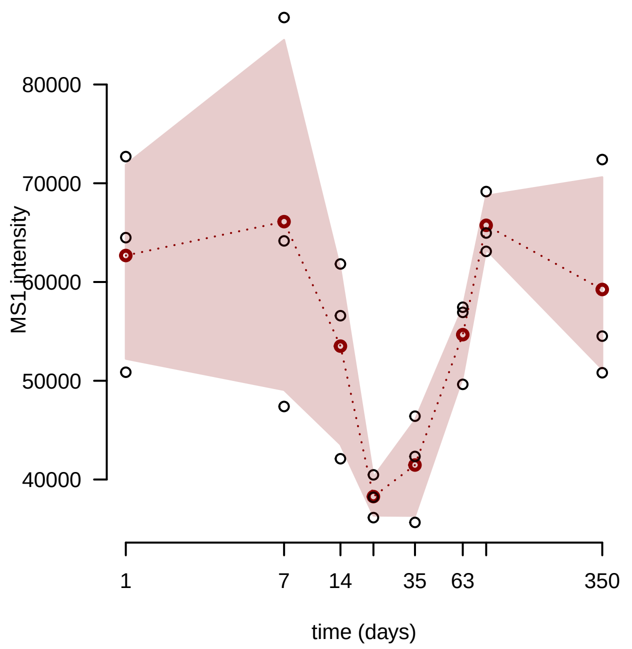

Q9D6K9
GN=Cers5
ceramide synthase 5

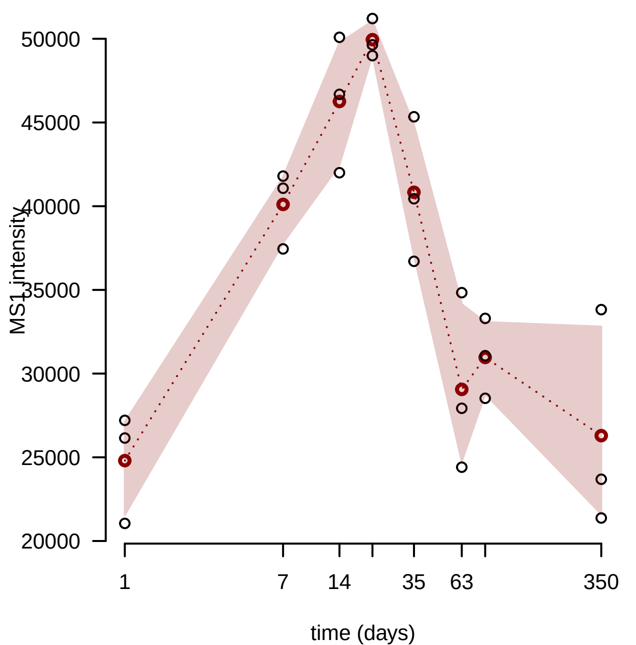

Q9D6L8
GN=Ppil3
peptidylprolyl isomerase (cyclophilin)−l ...

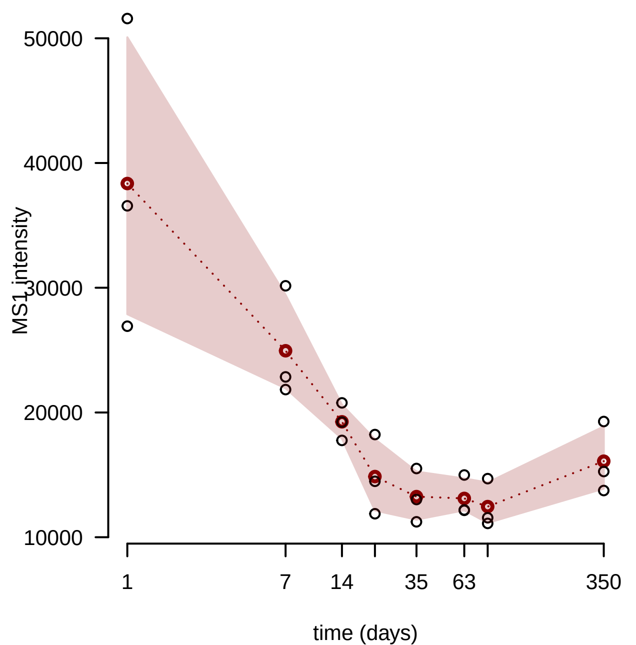

Q9D6M3
GN=Slc25a22
solute carrier family 25 (mitochondrial ...

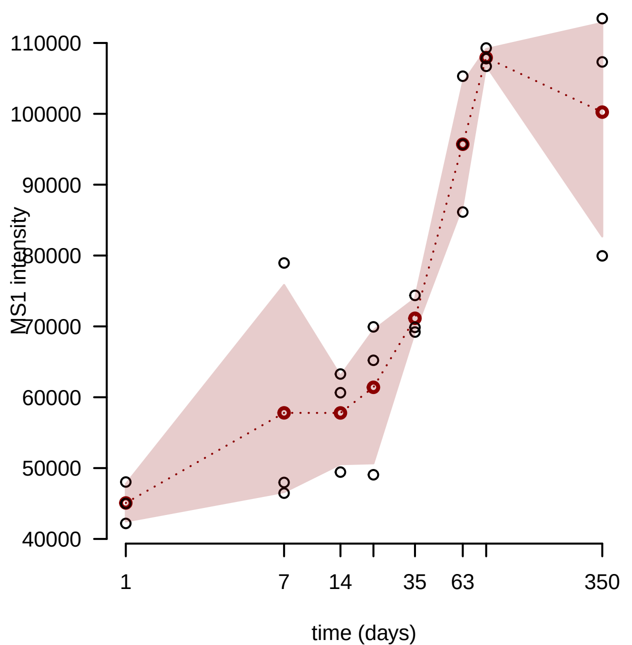

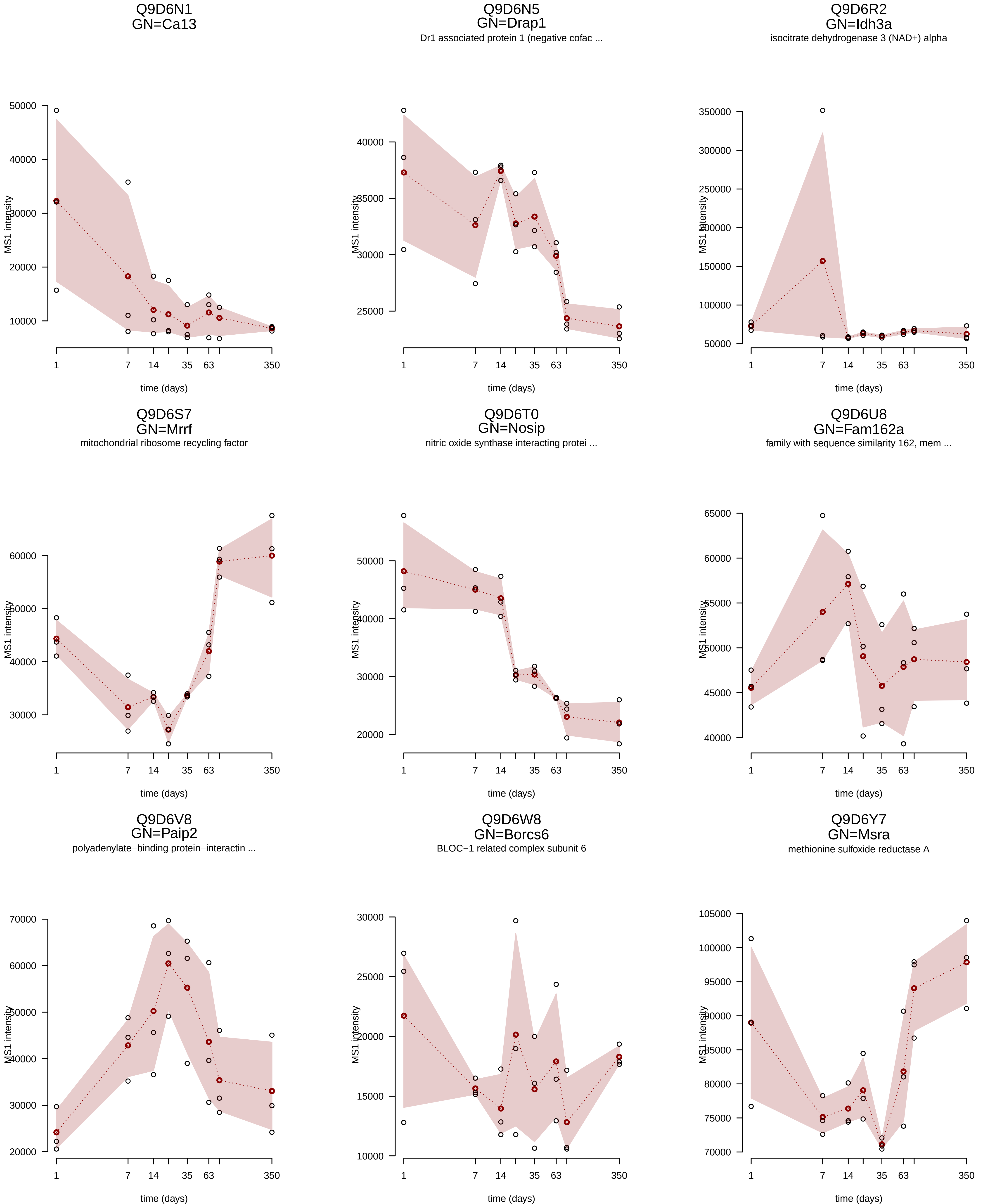

Q9D6N1
GN=Ca13
Q9D6N5
GN=Drap1
Dr1 associated protein 1 (negative cofac ...
Q9D6R2
GN=Idh3a
isocitrate dehydrogenase 3 (NAD+) alpha
Q9D6S7
GN=Mrrf
mitochondrial ribosome recycling factor
Q9D6T0
GN=Nosip
nitric oxide synthase interacting protei ...
Q9D6U8
GN=Fam162a
family with sequence similarity 162, mem ...
Q9D6V8
GN=Paip2
polyadenylate−binding protein−interactin ...
Q9D6W8
GN=Borcs6
BLOC−1 related complex subunit 6
Q9D6Y7
GN=Msra
methionine sulfoxide reductase A
MS1 intensity
time (days)
1
7
14
35
63
350

Q9D6Y9
GN=Gbe1
glucan (1,4−alpha−), branching enzyme 1

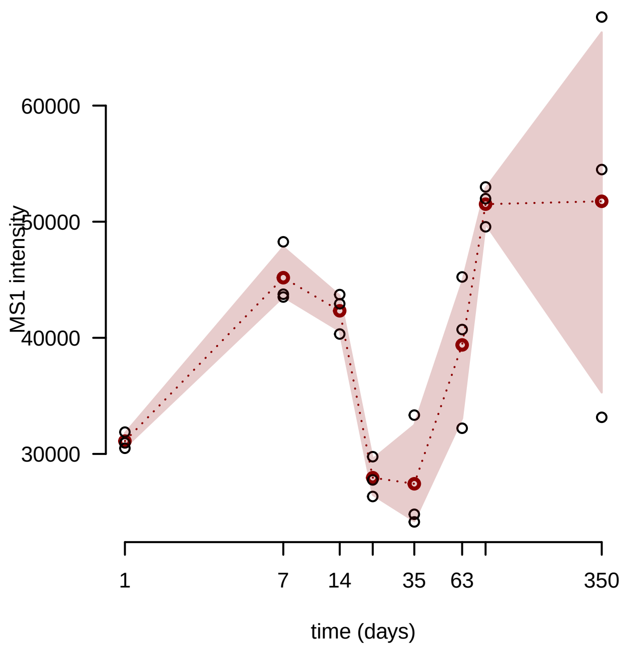

Q9D6Z0
GN=Alkbh7
alkB homolog 7

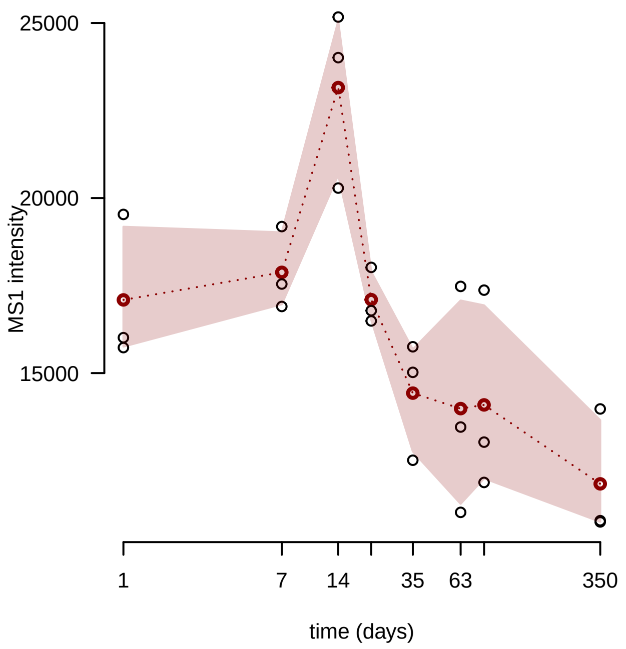

Q9D6Z1
GN=Nop56
NOP56 ribonucleoprotein

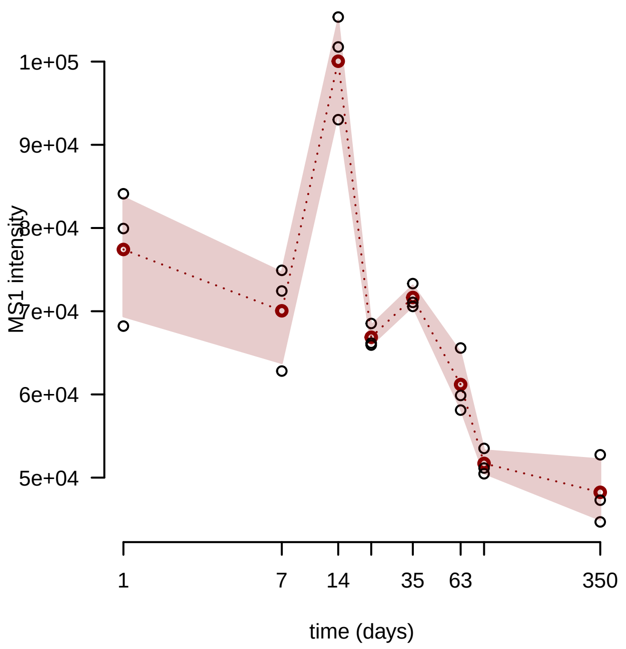

Q9D706
GN=Rpap3
RNA polymerase II associated protein 3

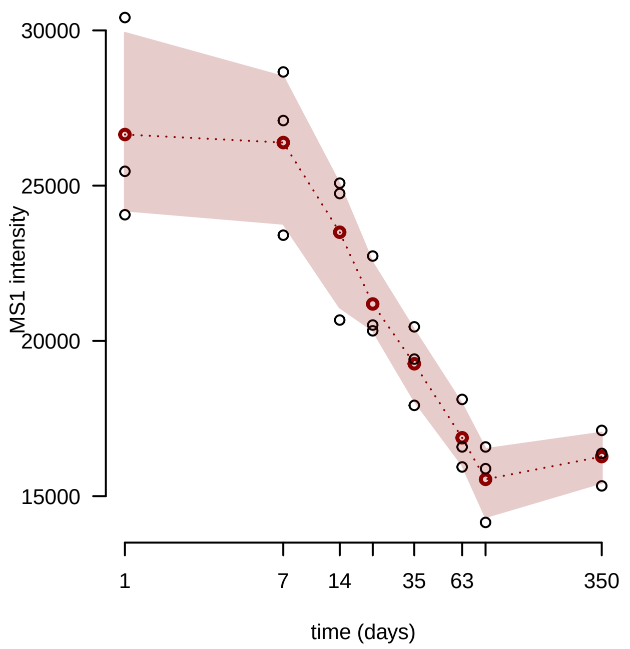

Q9D708
GN=S100a16
S100 calcium binding protein A16

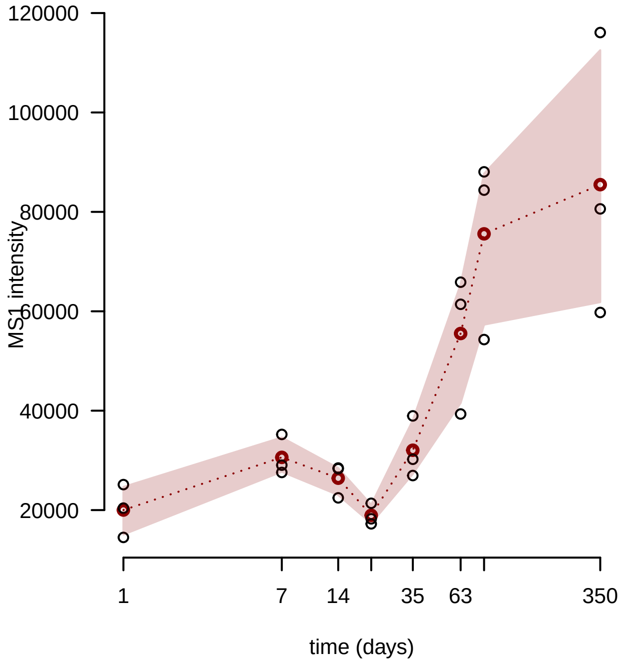

Q9D710
GN=Tmx2
thioredoxin−related transmembrane protei ...

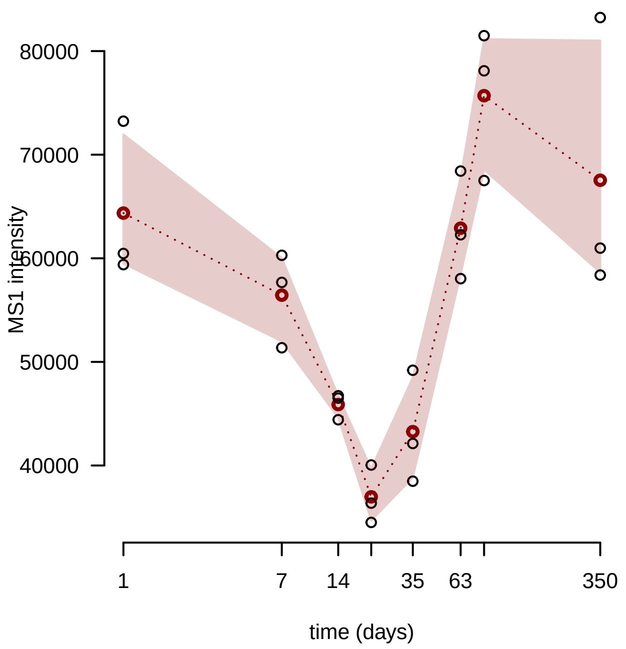

Q9D711
GN=Pir
pirin

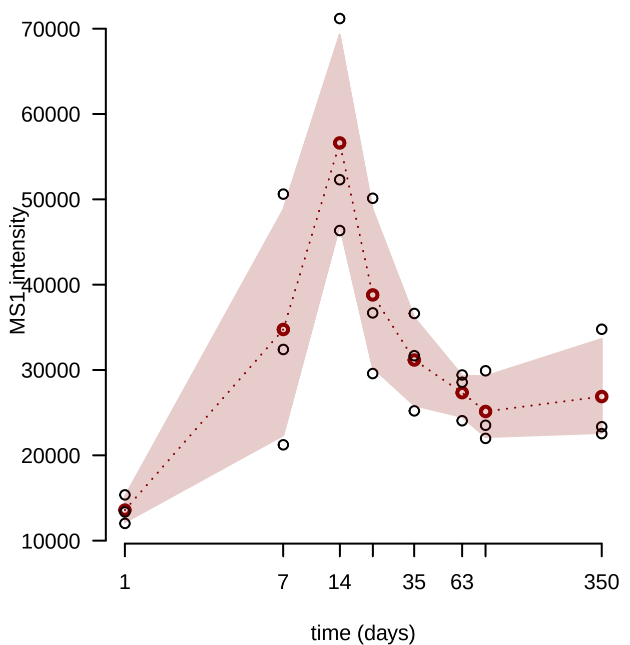

Q9D720
GN=Nsmce1
NSE1 homolog, SMC5−SMC6 complex componen ...

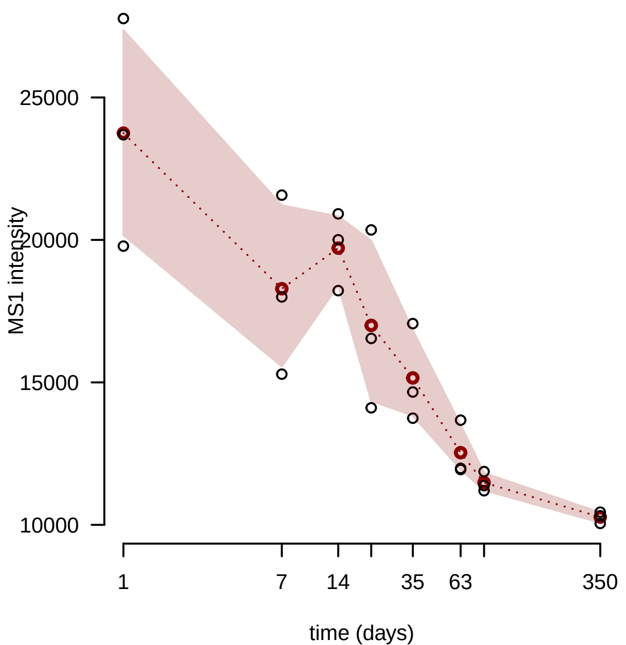

Q9D735
GN=Trir
telomerase RNA component interacting RNa ...

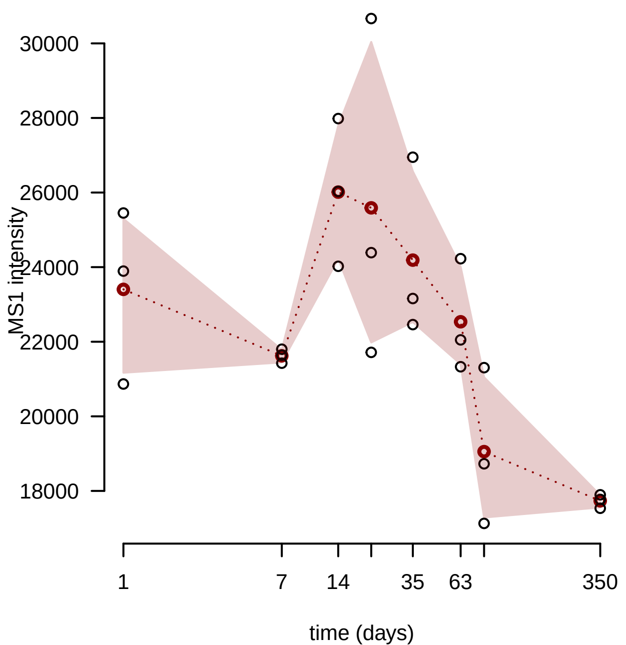

Q9D753
GN=Exosc8
exosome component 8

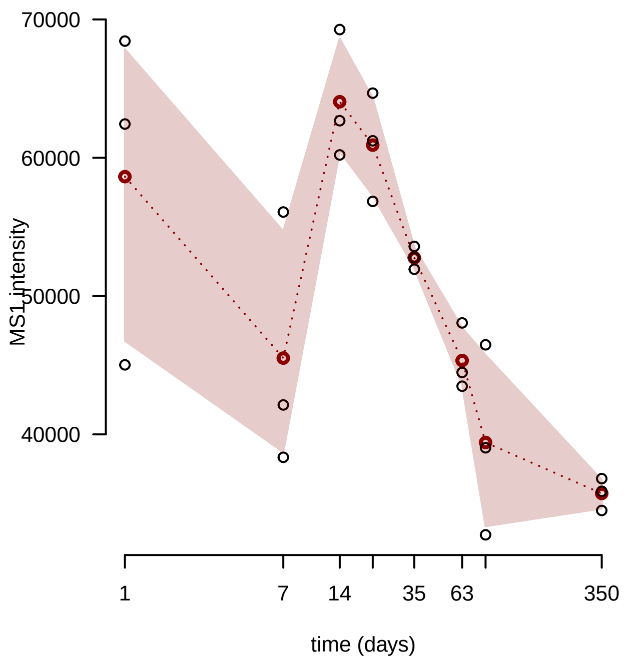

Q9D771
GN=Tmem206

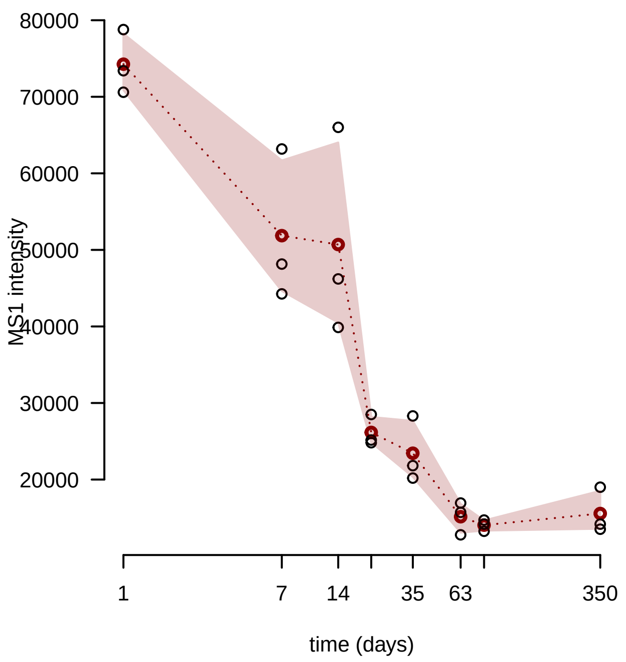

Q9D773
GN=Mrpl2
mitochondrial ribosomal protein L2

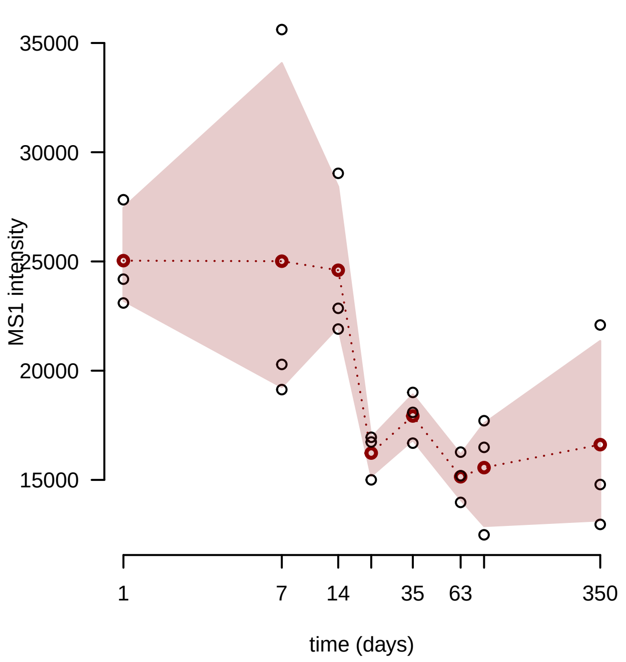

Q9D786
GN=Haus5
HAUS augmin−like complex, subunit 5

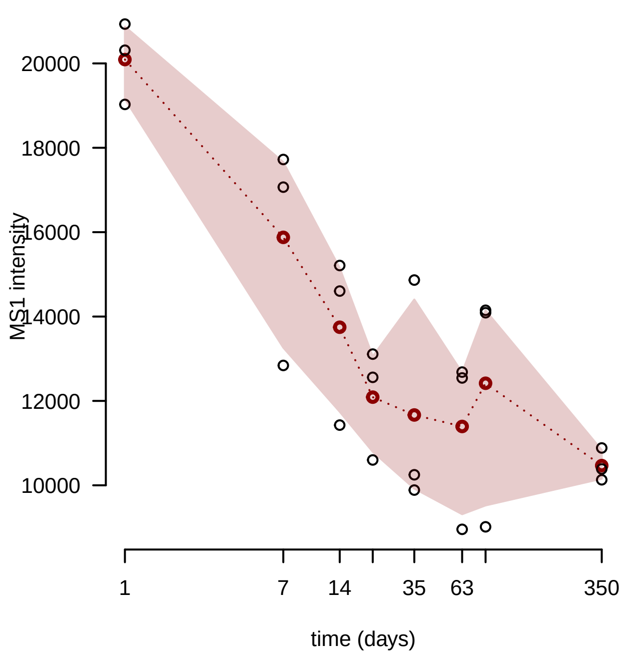

Q9D787
GN=Ppil2
peptidylprolyl isomerase (cyclophilin)−l ...

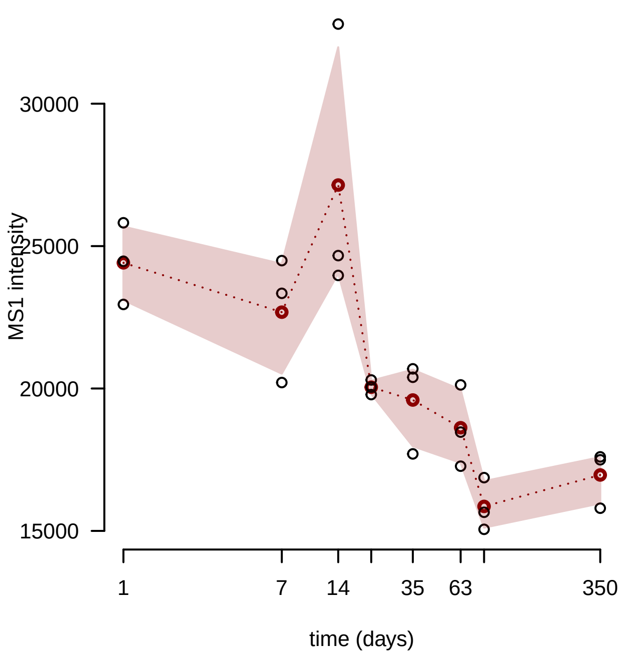

Q9D7A6
GN=Srp19
signal recognition particle 19

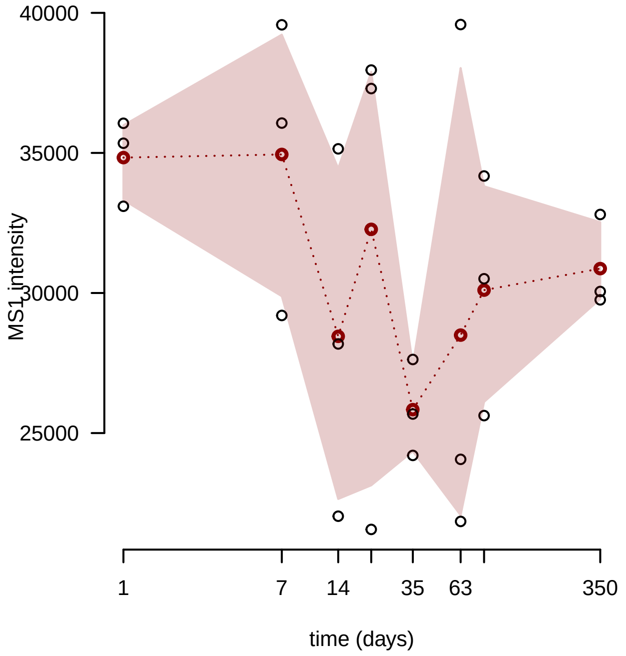

Q9D7A8
GN=Armc1
armadillo repeat containing 1

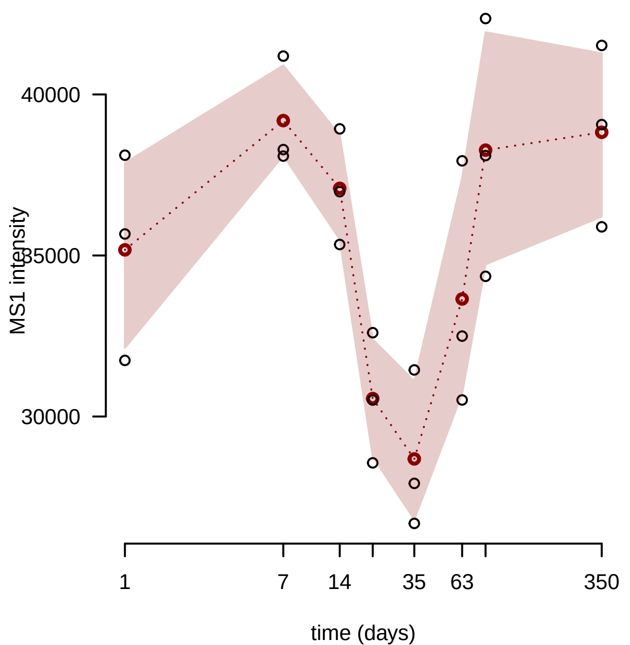

Q9D7B1
GN=Dus2
dihydrouridine synthase 2

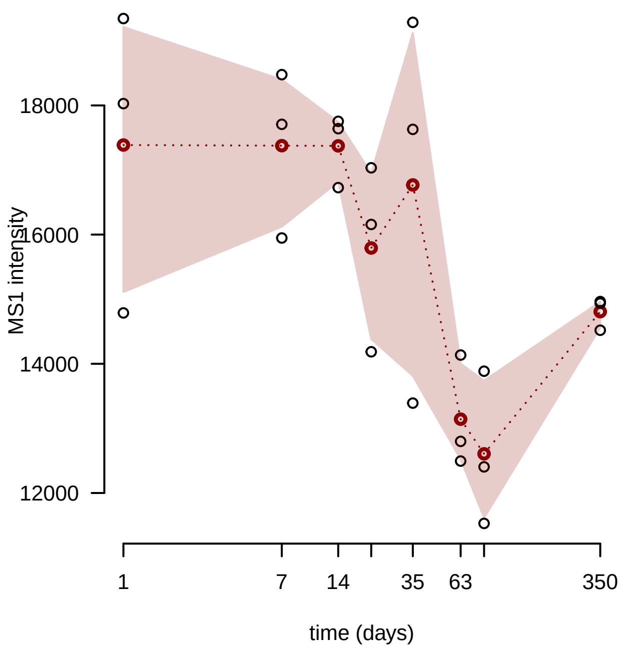

Q9D7B6
GN=Acad8
acyl−Coenzyme A dehydrogenase family, me ...

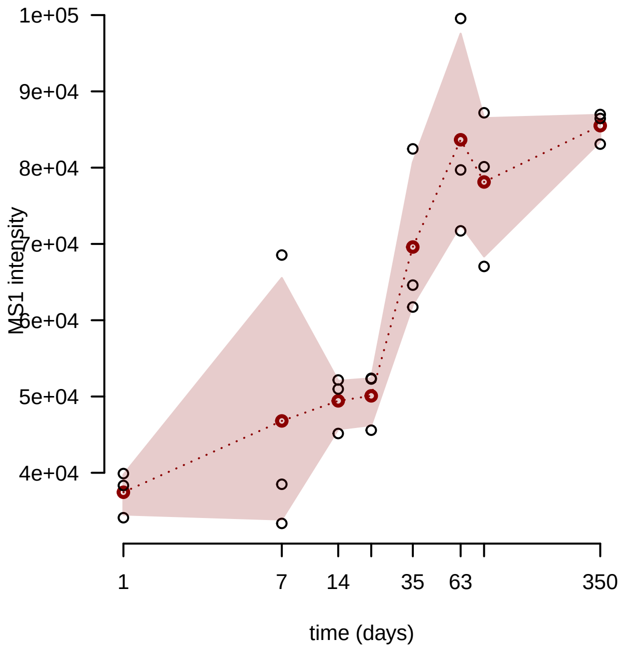

Q9D7B7
GN=Gpx8
glutathione peroxidase 8 (putative)

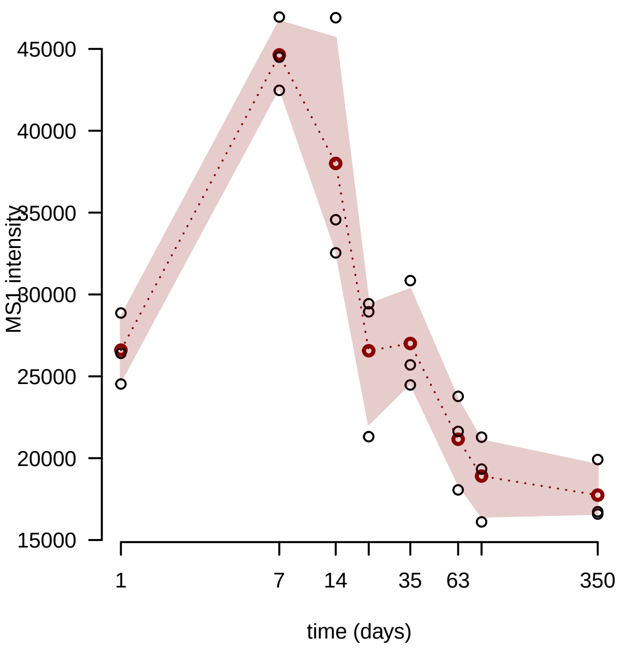

Q9D7E3
GN=Ovca2
candidate tumor suppressor in ovarian ca ...

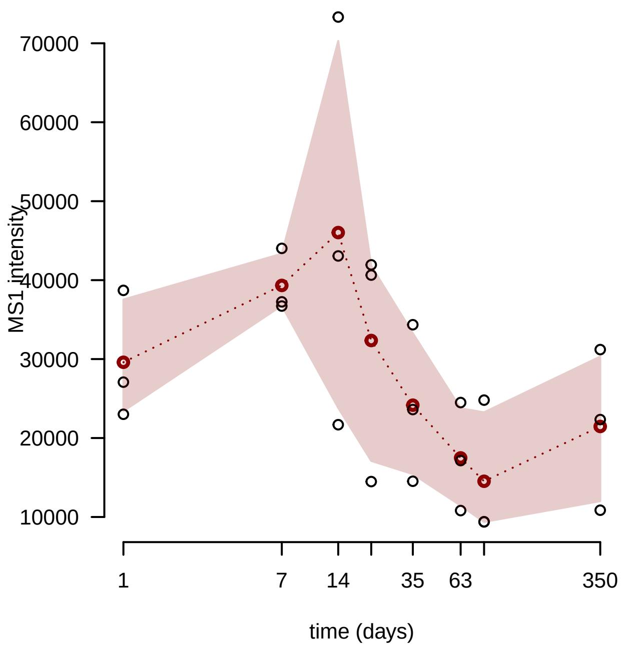

Q9D7E4
GN=

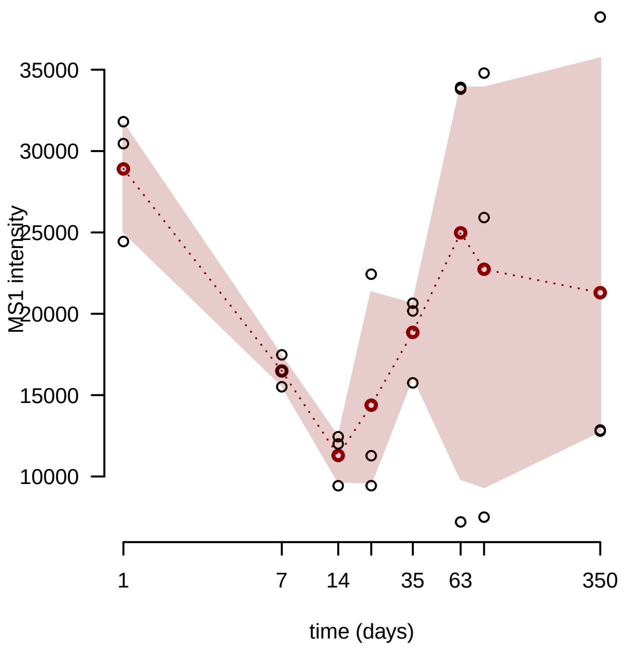

Q9D7G0
GN=Prps1
phosphoribosyl pyrophosphate synthetase ...

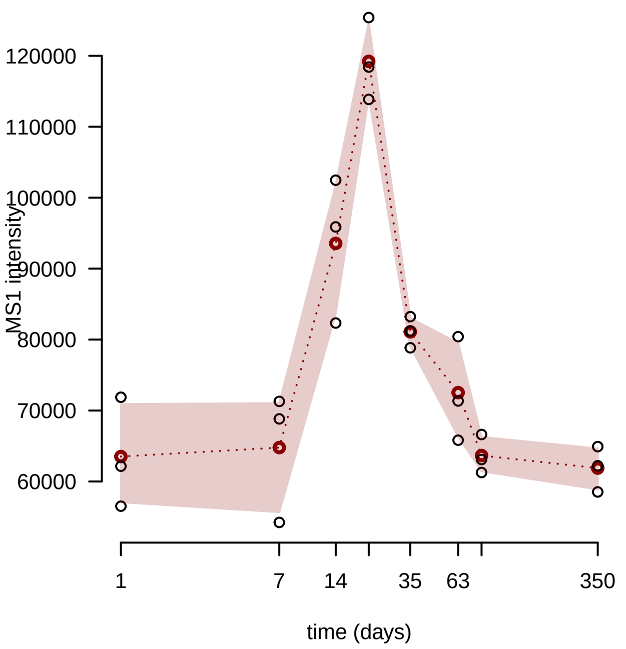

Q9D7H3
GN=RtcA

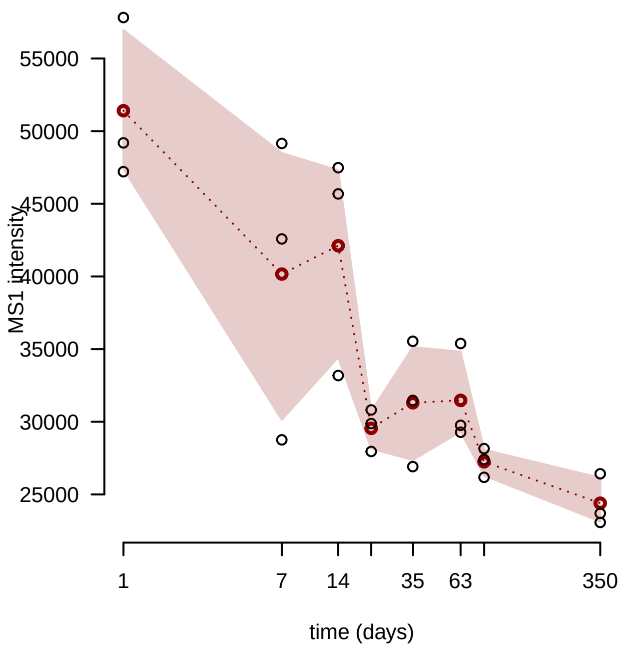

Q9D7I5
GN=Lhpp
phospholysine phosphohistidine inorganic ...

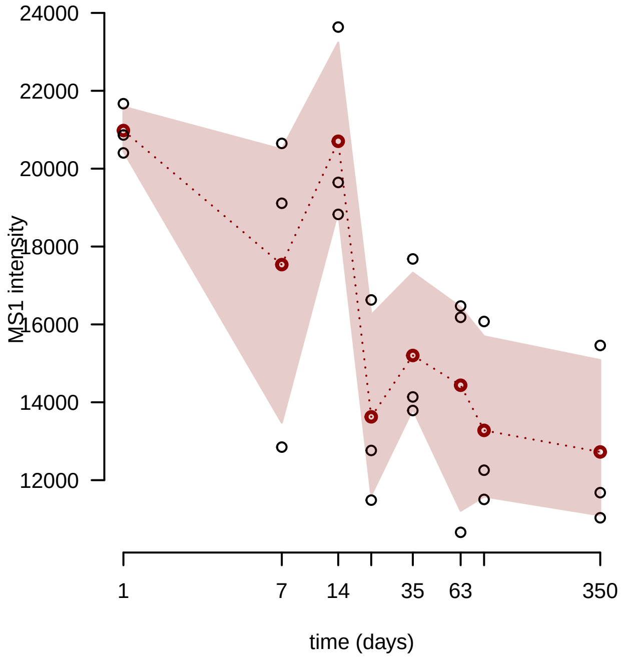

Q9D7J4
GN=Cox20
cytochrome c oxidase assembly protein 20

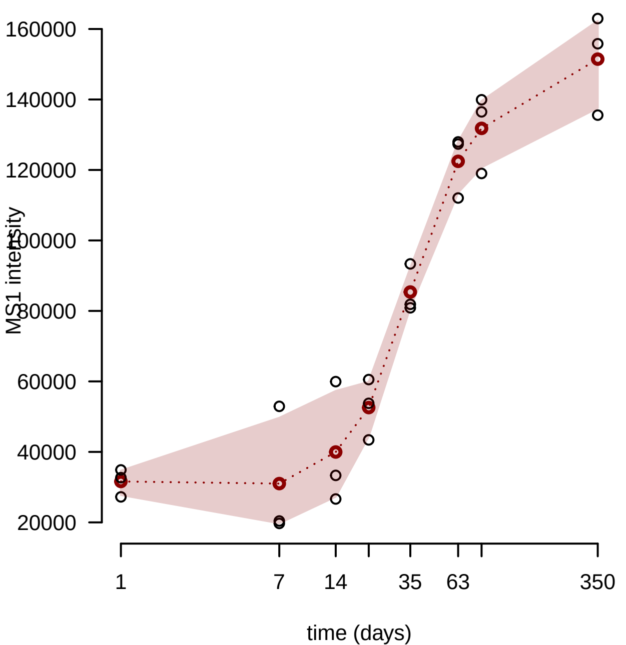

Q9D7J6
GN=Dnase1l1
deoxyribonuclease 1−like 1

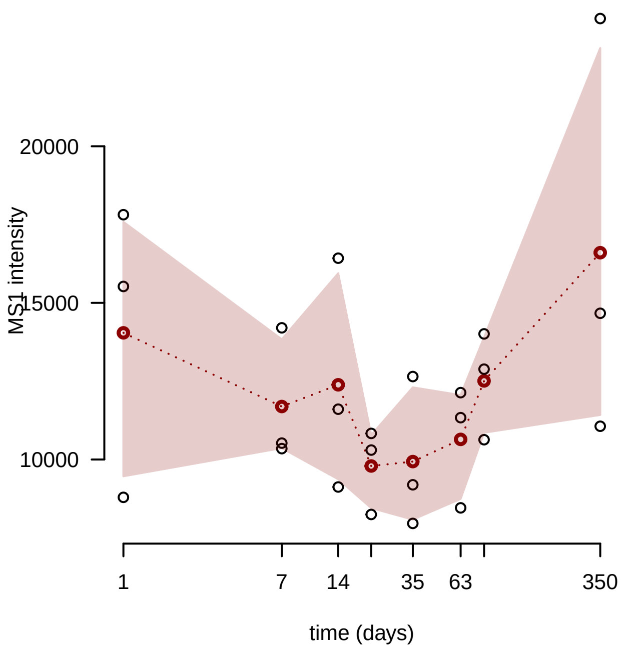

Q9D7J9
GN=Echdc3
enoyl Coenzyme A hydratase domain contai ...

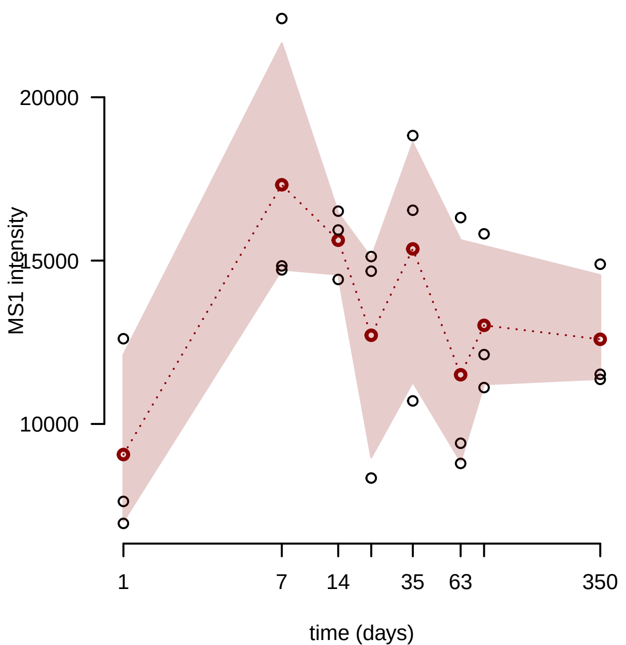

Q9D7K2
GN=Ten1
TEN1 telomerase capping complex subunit

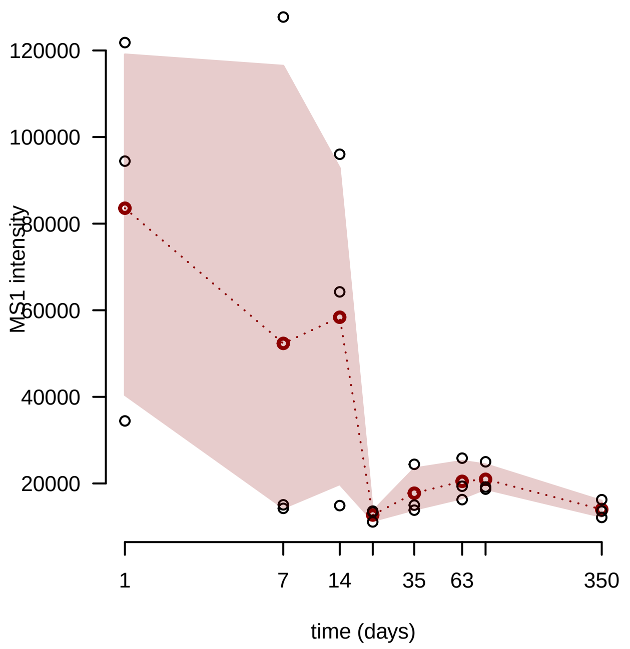

Q9D7K5
GN=Dmac2
distal membrane arm assembly complex 2

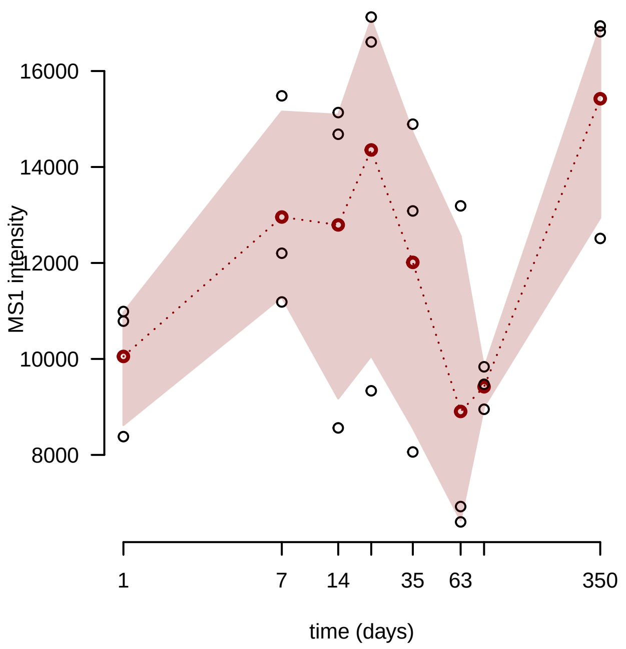

Q9D7M1
GN=Gid8
GID complex subunit 8

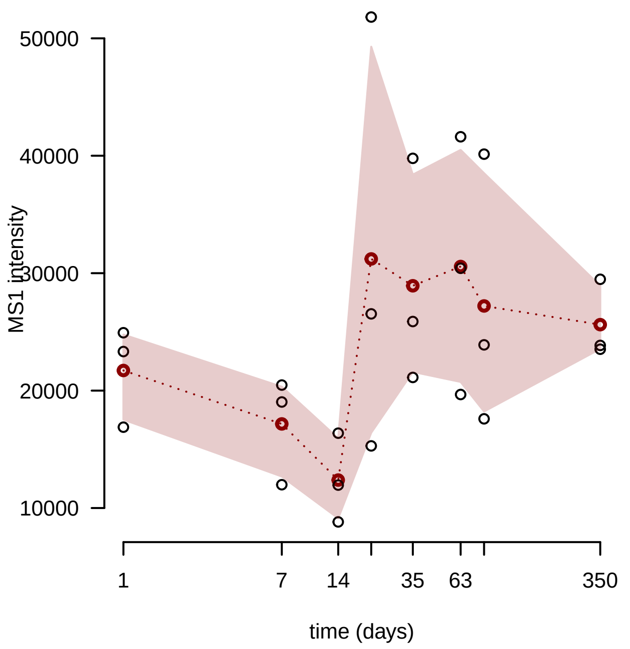

Q9D7M8
GN=Polr2d
polymerase (RNA) II (DNA directed) polyp ...

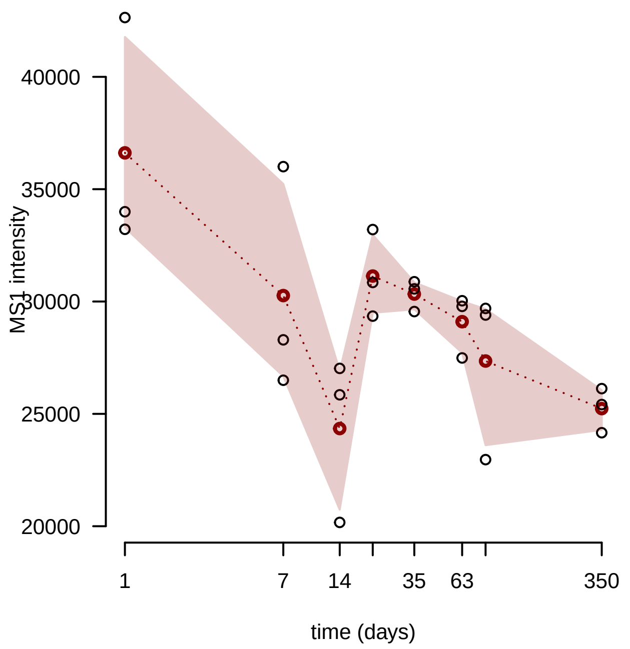

Q9D7N3
GN=Mrps9
mitochondrial ribosomal protein S9

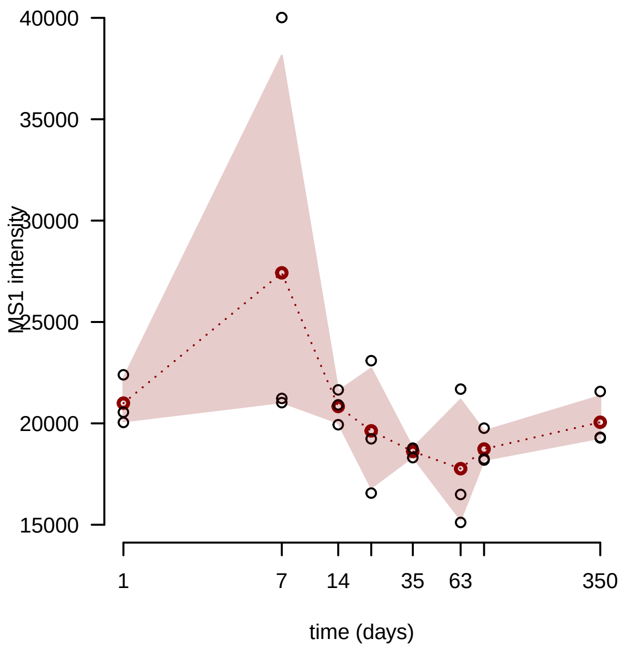

Q9D7N6
GN=Mrpl30
mitochondrial ribosomal protein L30

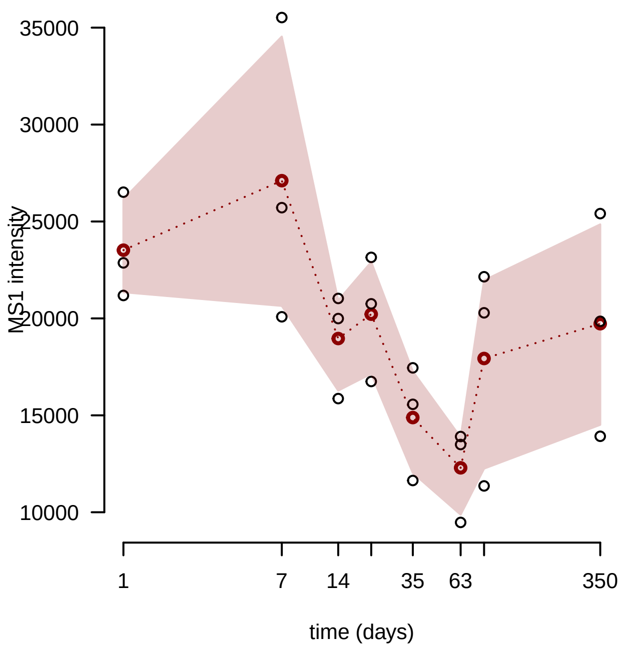

Q9D7N9
GN=Apmap
adipocyte plasma membrane associated pro ...

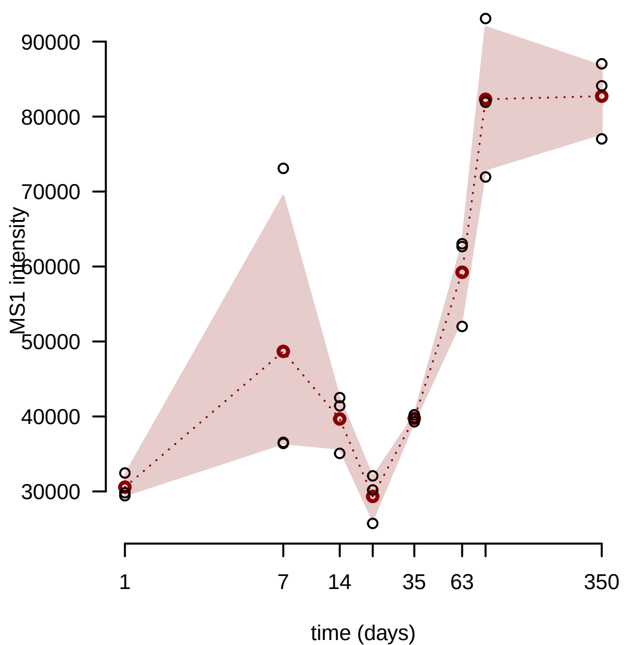

Q9D7P6
GN=Iscu
iron−sulfur cluster assembly enzyme

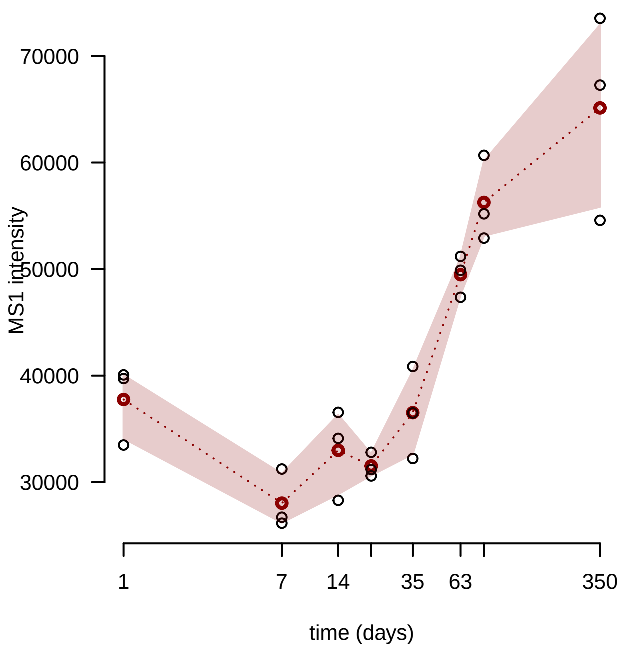

Q9D7S7
GN=Rpl22l1
ribosomal protein L22 like 1

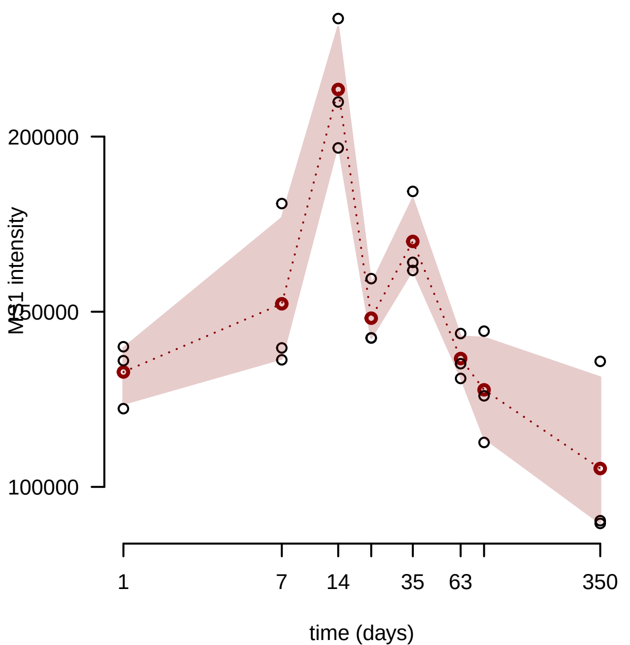

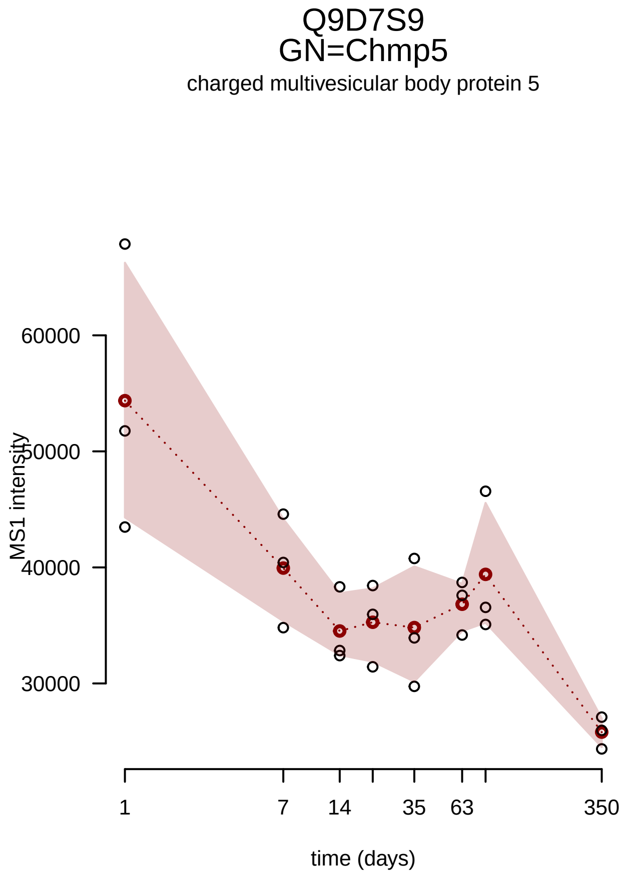
Q9D7S9
GN=Chmp5
charged multivesicular body protein 5
MS1 intensity
30000 40000 50000 60000
1 7 14 35 63 350
time (days)
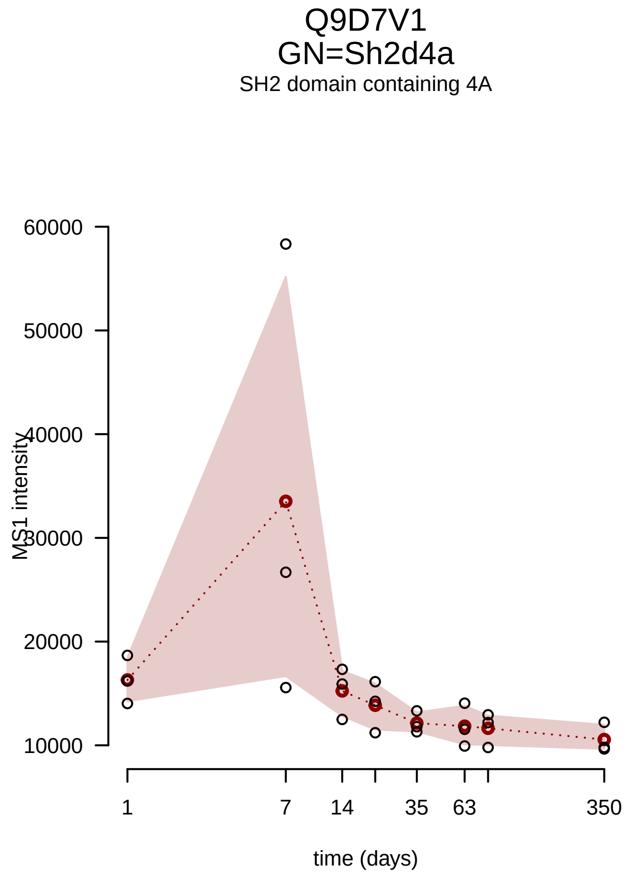
Q9D7V1
GN=Sh2d4a
SH2 domain containing 4A
MS1 intensity
10000 20000 30000 40000 50000 60000
1 7 14 35 63 350
time (days)
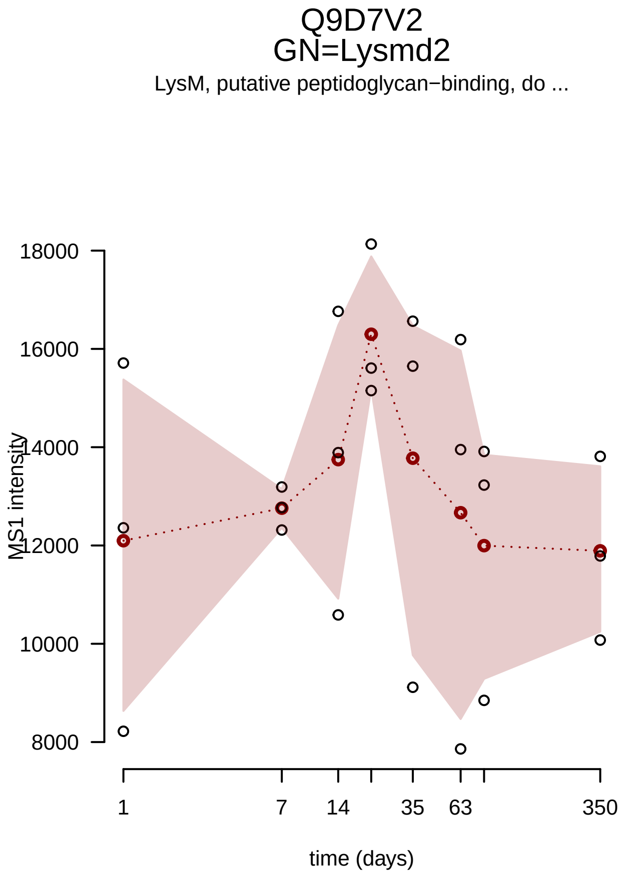
Q9D7V2
GN=Lysmd2
LysM, putative peptidoglycan−binding, do ...
MS1 intensity
8000 10000 12000 14000 16000 18000
1 7 14 35 63 350
time (days)
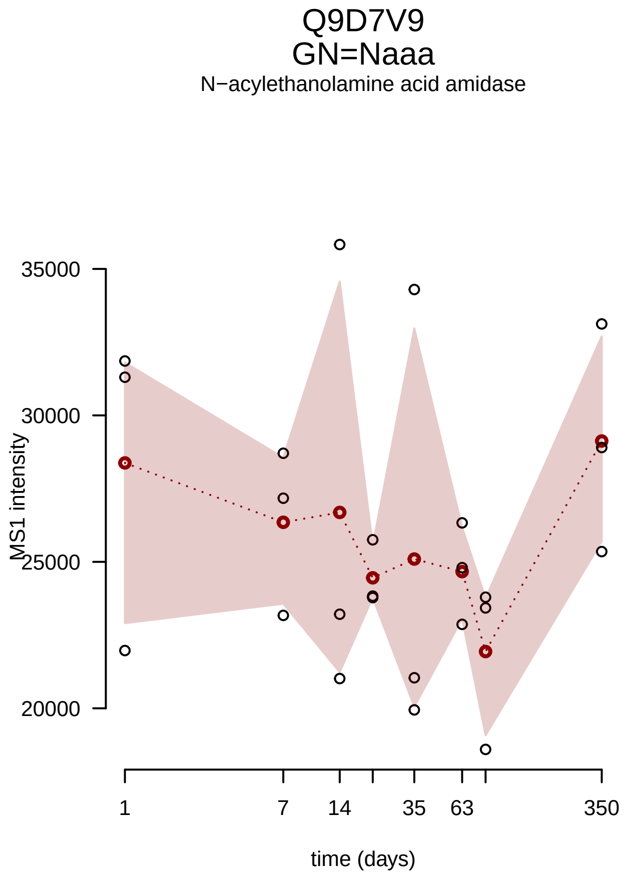
Q9D7V9
GN=Naaa
N−acylethanolamine acid amidase
MS1 intensity
20000 25000 30000 35000
1 7 14 35 63 350
time (days)
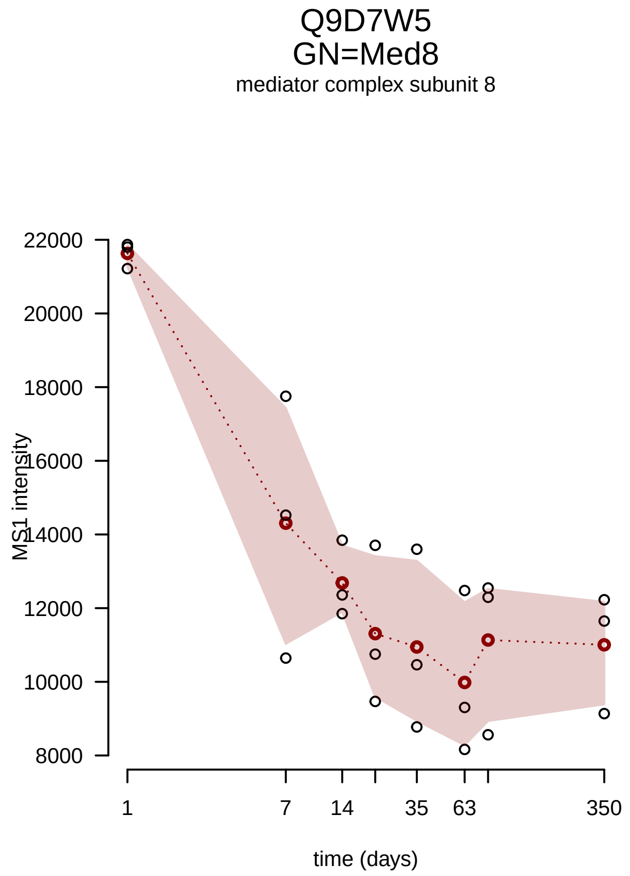
Q9D7W5
GN=Med8
mediator complex subunit 8
MS1 intensity
8000 10000 12000 14000 16000 18000 20000 22000
1 7 14 35 63 350
time (days)
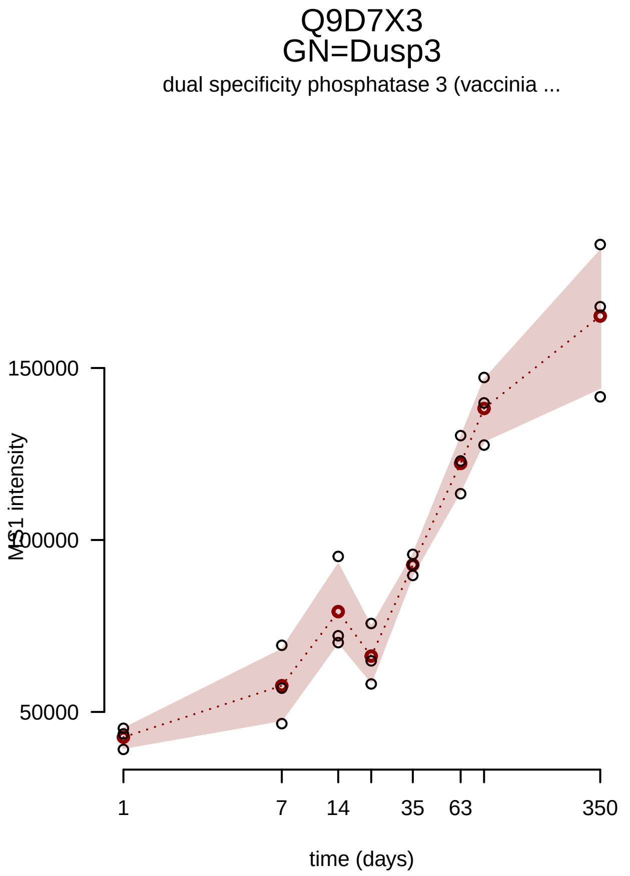
Q9D7X3
GN=Dusp3
dual specificity phosphatase 3 (vaccinia ...
MS1 intensity
50000 100000 150000
1 7 14 35 63 350
time (days)
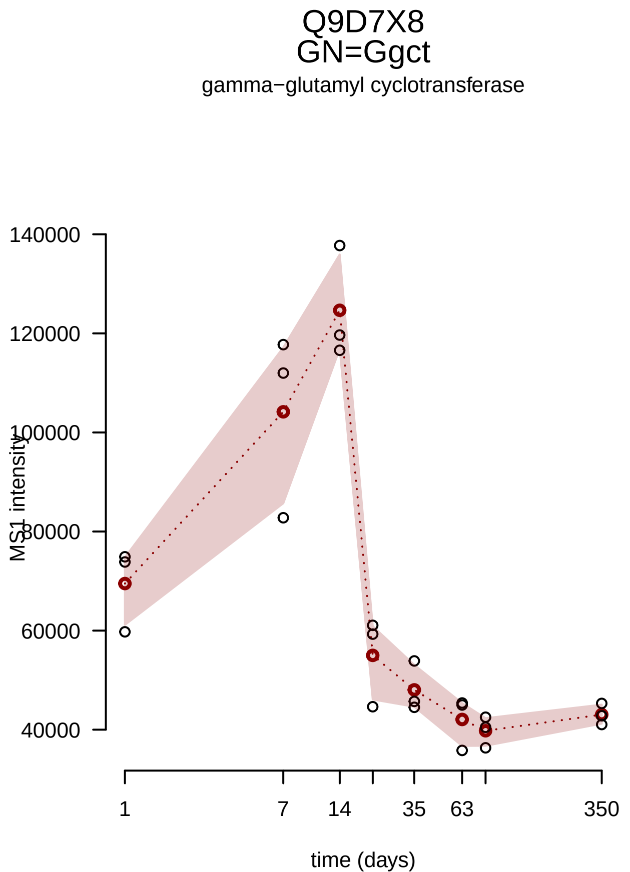
Q9D7X8
GN=Ggct
gamma−glutamyl cyclotransferase
MS1 intensity
40000 60000 80000 100000 120000 140000
1 7 14 35 63 350
time (days)
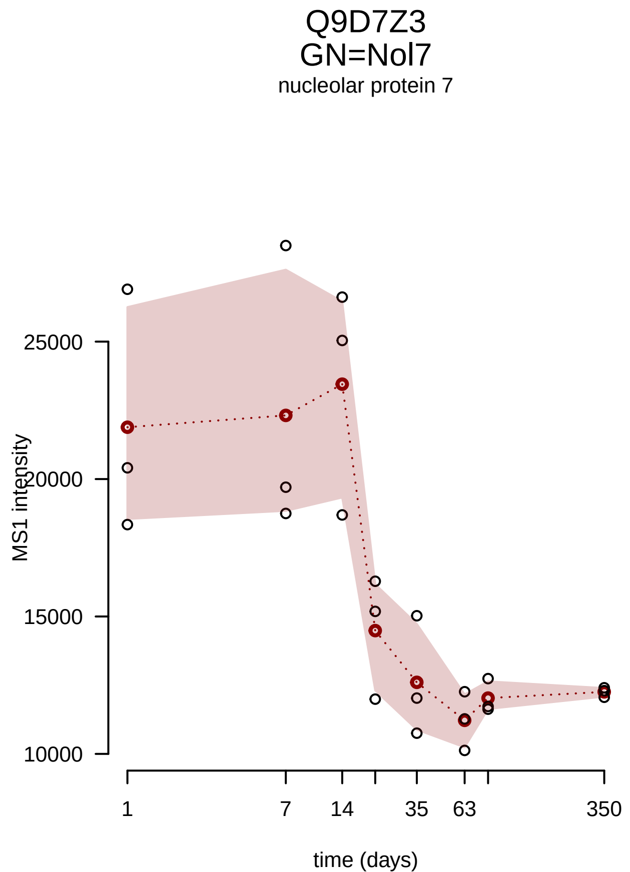
Q9D7Z3
GN=Nol7
nucleolar protein 7
MS1 intensity
10000 15000 20000 25000
1 7 14 35 63 350
time (days)
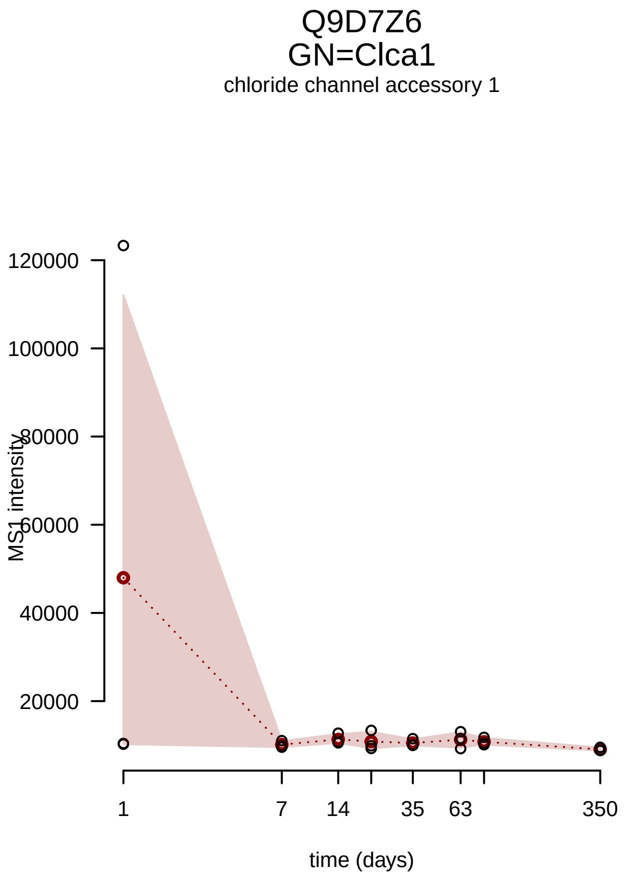
Q9D7Z6
GN=Clca1
chloride channel accessory 1
MS1 intensity
20000 40000 60000 80000 100000 120000
1 7 14 35 63 350
time (days)

Q9D819
GN=Ppa1
pyrophosphatase (inorganic) 1

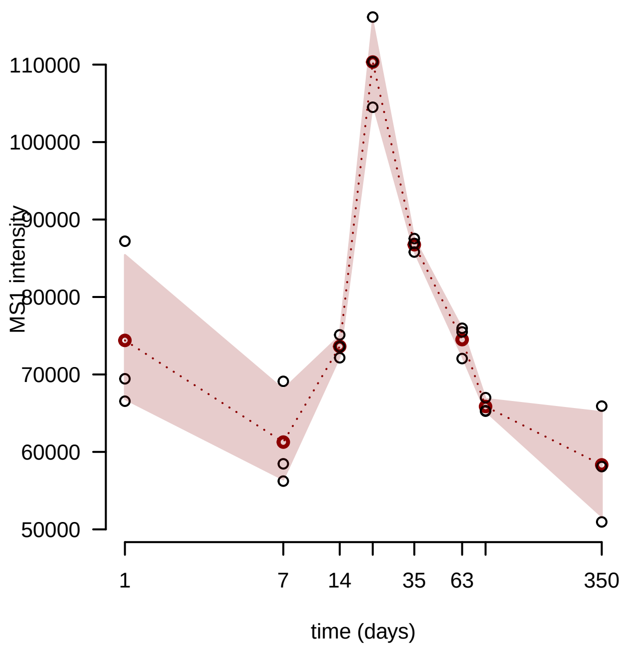

Q9D820
GN=Prorsd1
prolyl−tRNA synthetase domain containing ...

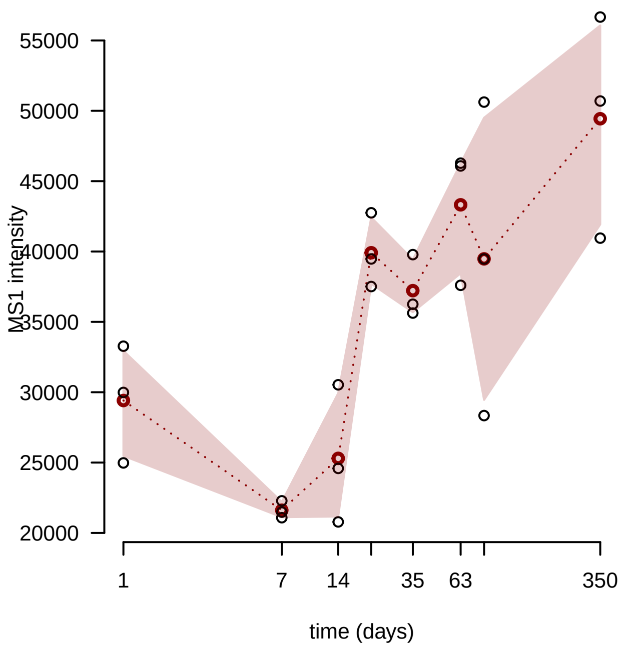

Q9D823
GN=Rpl37
ribosomal protein L37

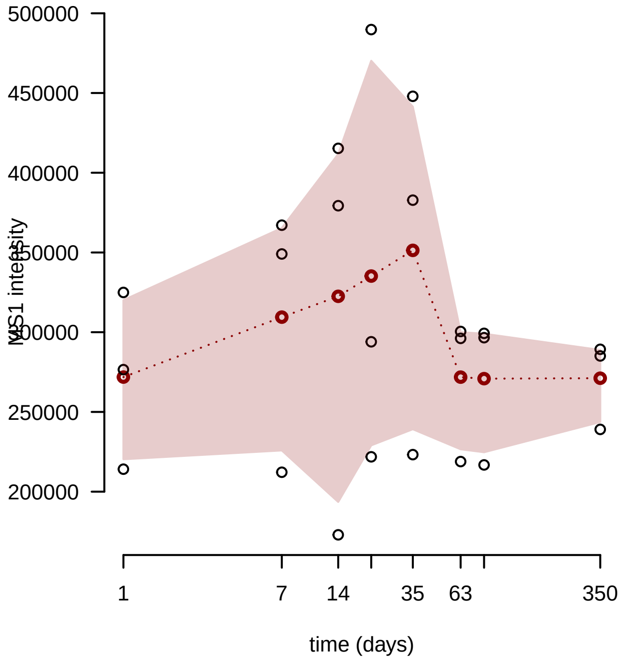

Q9D824
GN=Fip1l1
FIP1 like 1 (S. cerevisiae)

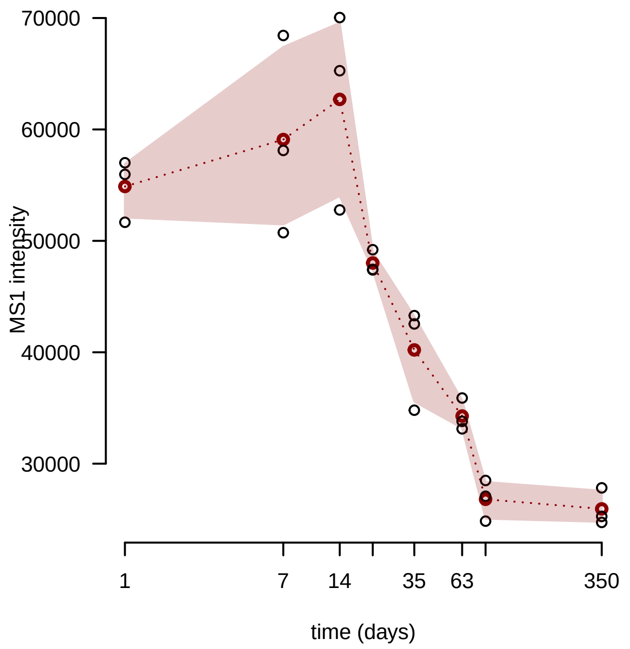

Q9D826
GN=Pipox
pipecolic acid oxidase

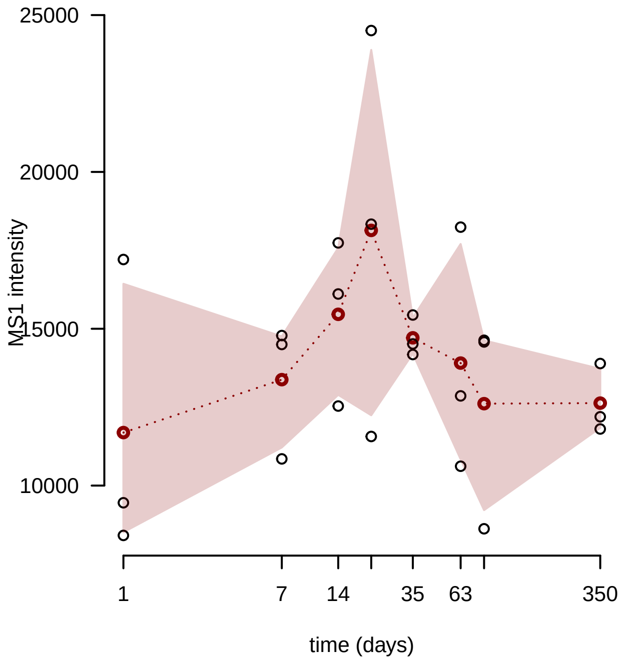

Q9D832
GN=Dnajb4
DnaJ heat shock protein family (Hsp40) m ...

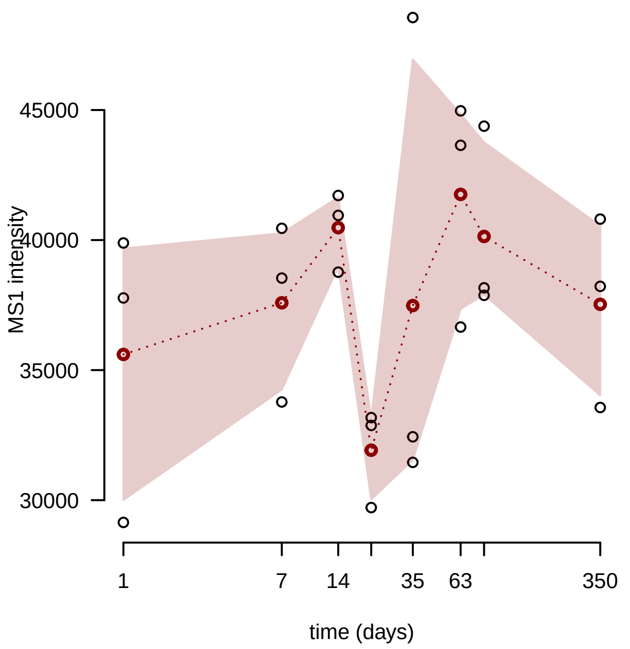

Q9D842
GN=Aplf
aprataxin and PNKP like factor

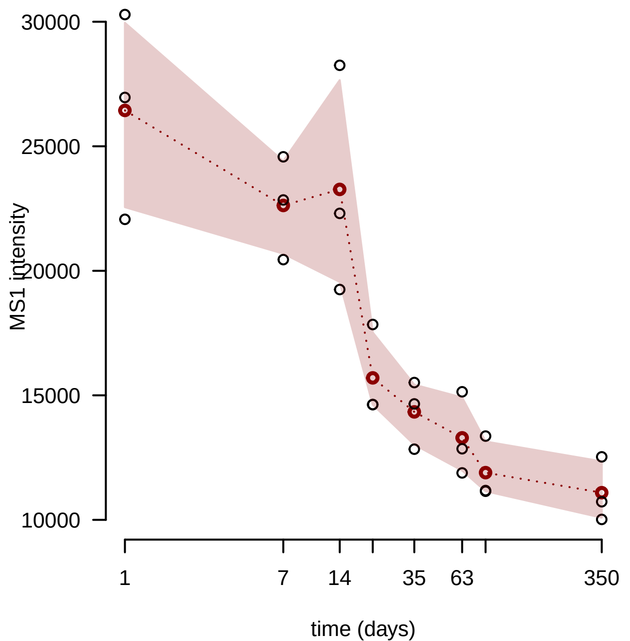

Q9D850
GN=Tmem68
transmembrane protein 68

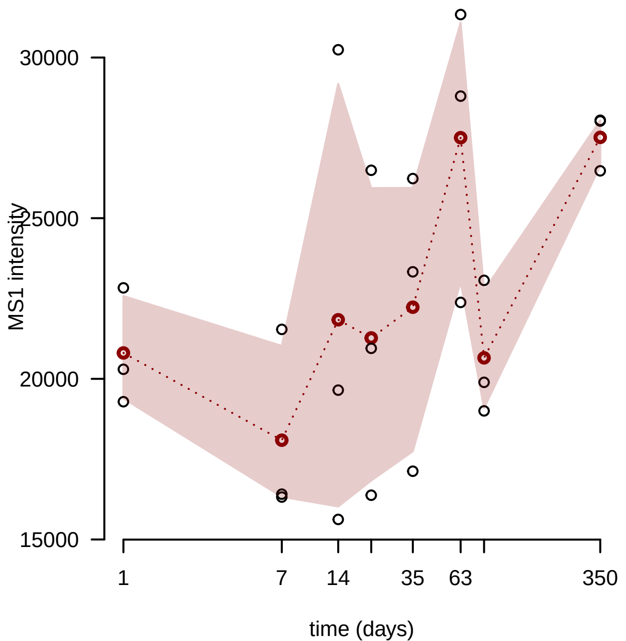

Q9D853
GN=Eef1akmt2
EEF1A lysine methyltransferase 2

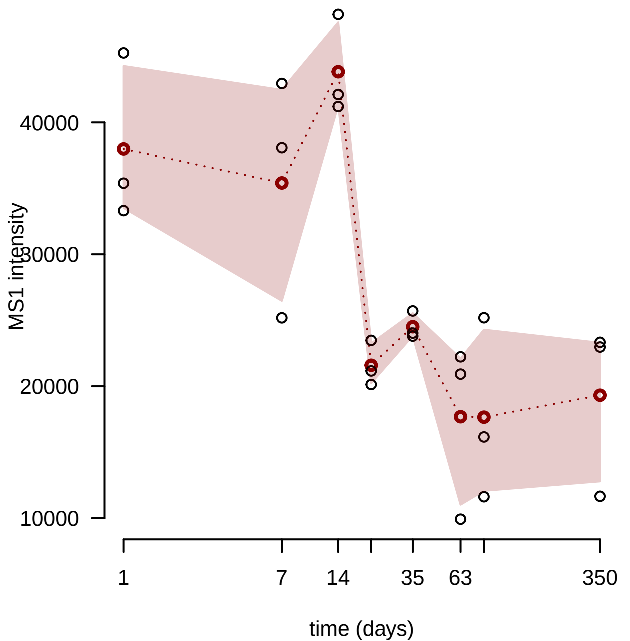

Q9D855
GN=Uqcrb
ubiquinol−cytochrome c reductase binding ...

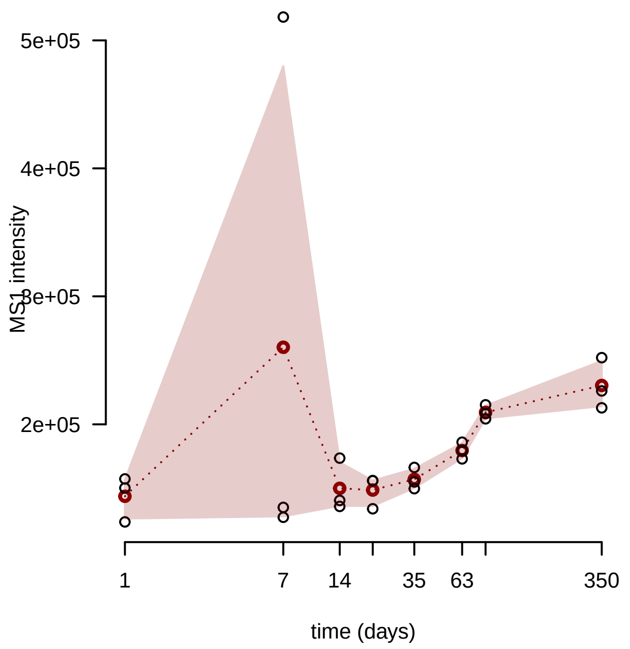

Q9D864
GN=Actr6
ARP6 actin−related protein 6

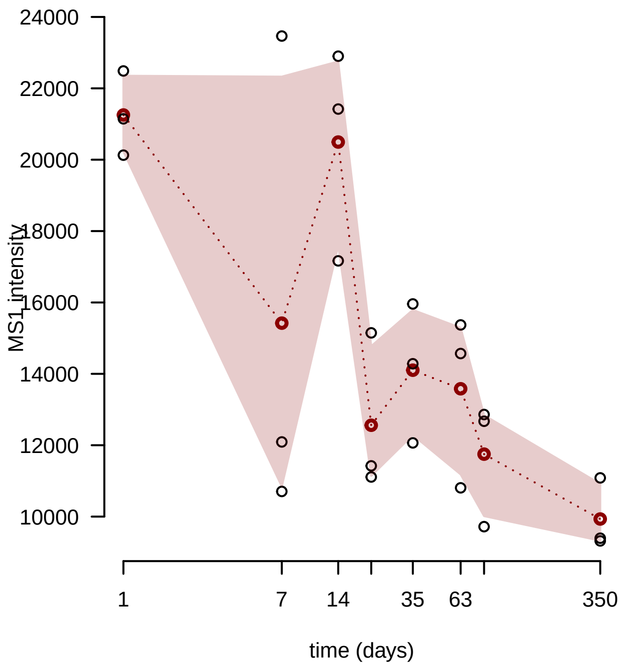

Q9D868
GN=Ppih
peptidyl prolyl isomerase H

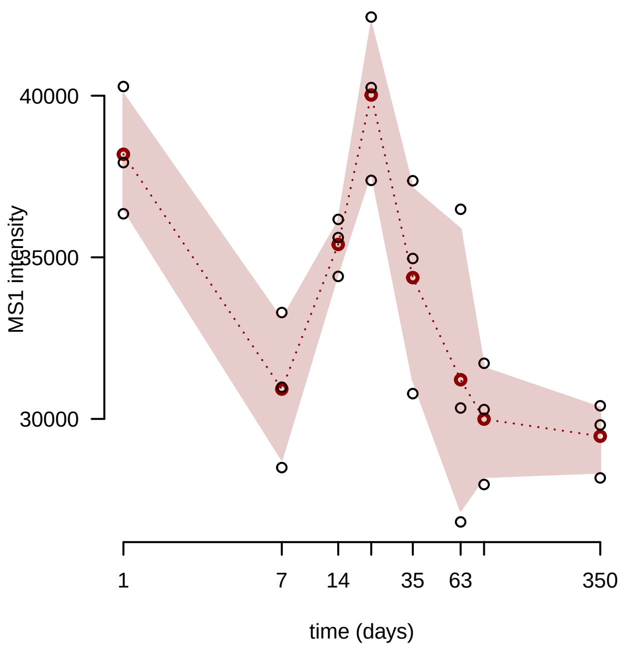

Q9D869
GN=Chp2
calcineurin−like EF hand protein 2

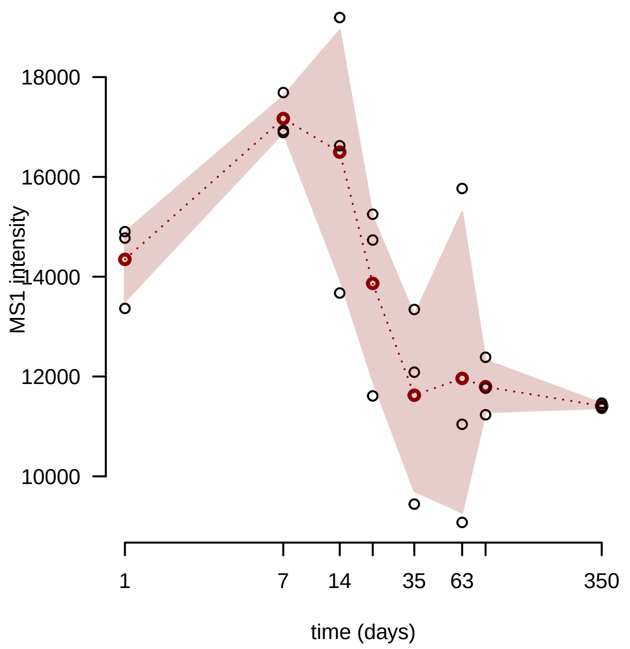

Q9D880
GN=Timm50
translocase of inner mitochondrial membr ...

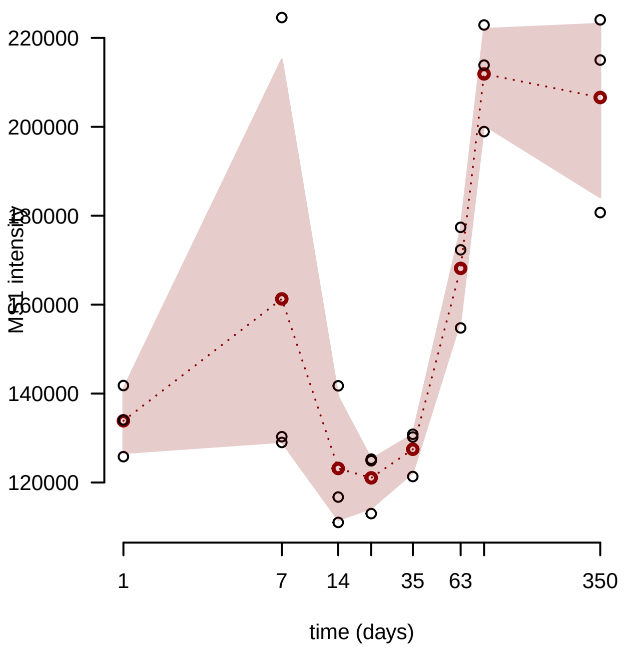

Q9D883
GN=U2af1
U2 small nuclear ribonucleoprotein auxil ...

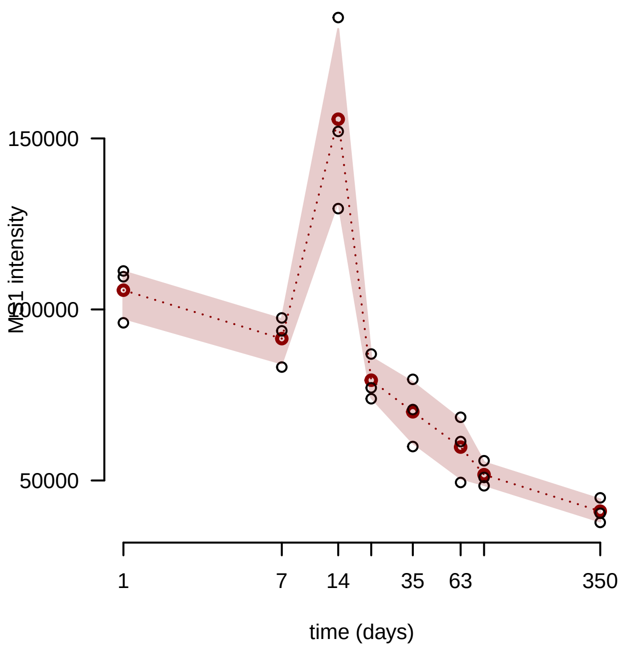

Q9D892
GN=Itpa
inosine triphosphatase (nucleoside triph ...

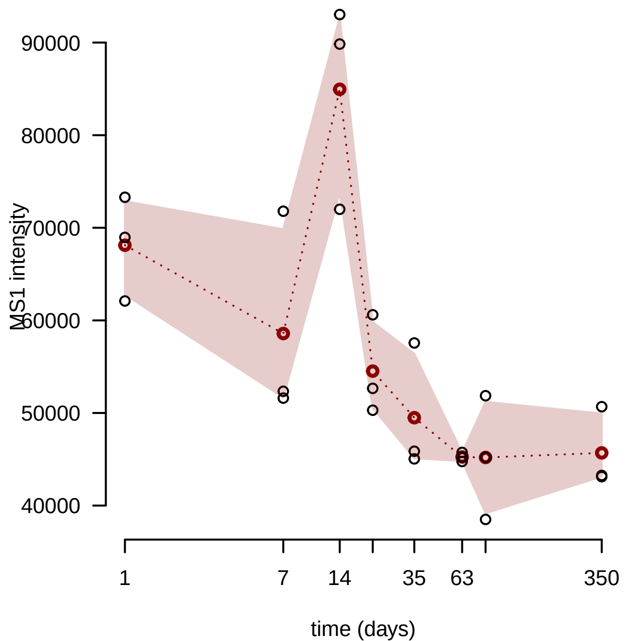

Q9D898
GN=Arpc5l
actin related protein 2/3 complex, subun ...

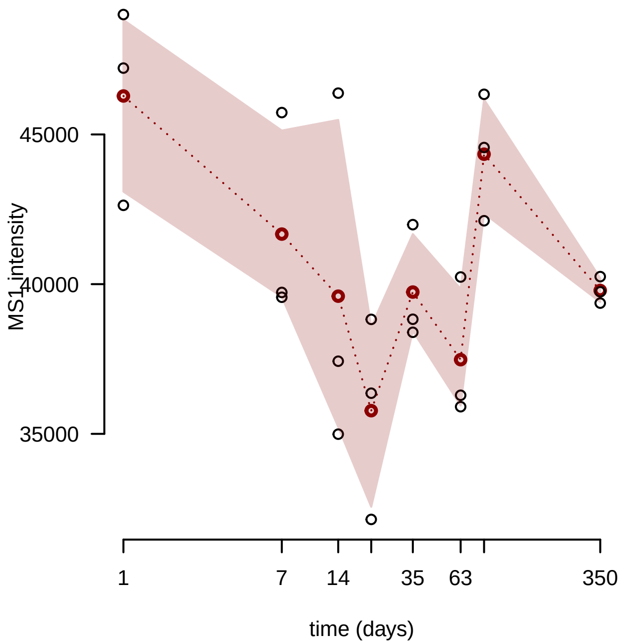

Q9D8B3
GN=Chmp4b
charged multivesicular body protein 4B

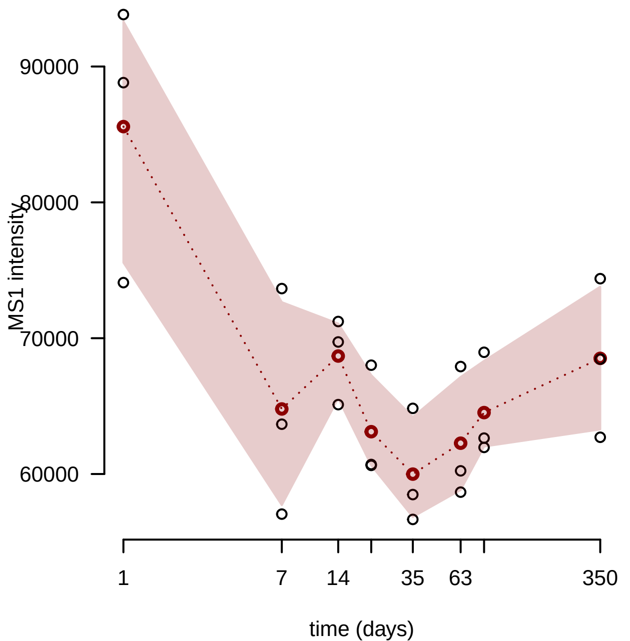

Q9D8B4
GN=Ndufa11
NADH:ubiquinone oxidoreductase subunit A ...

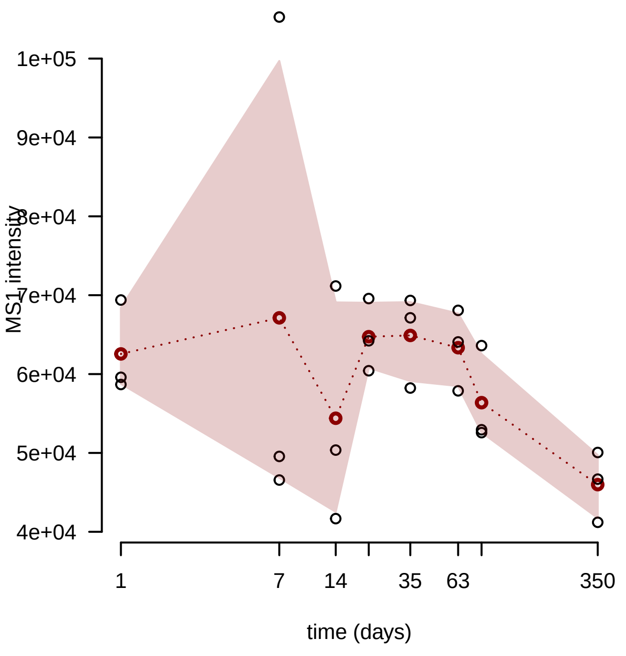

Q9D8B6
GN=Fam210b
family with sequence similarity 210, mem ...

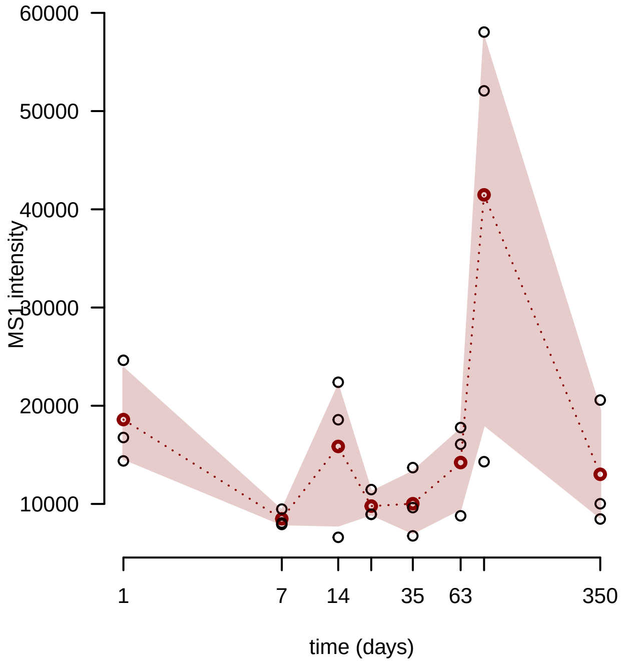

Q9D8B7
GN=Jam3
junction adhesion molecule 3

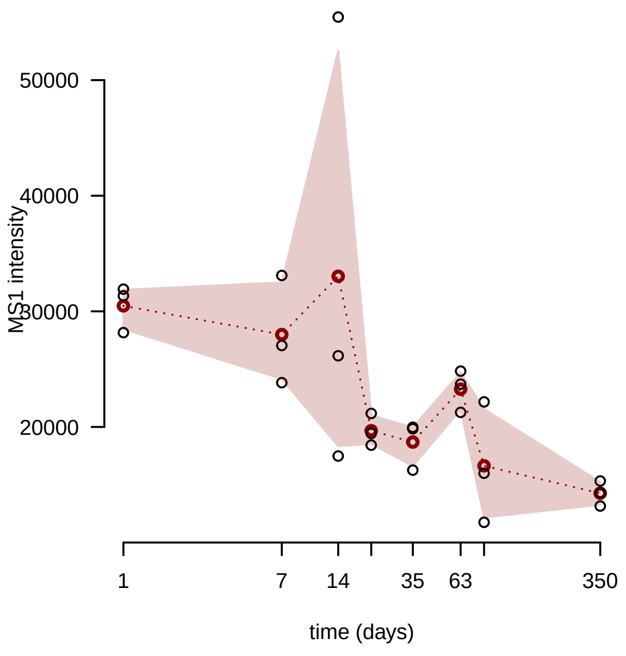

Q9D8C3
GN=Alg13
asparagine−linked glycosylation 13

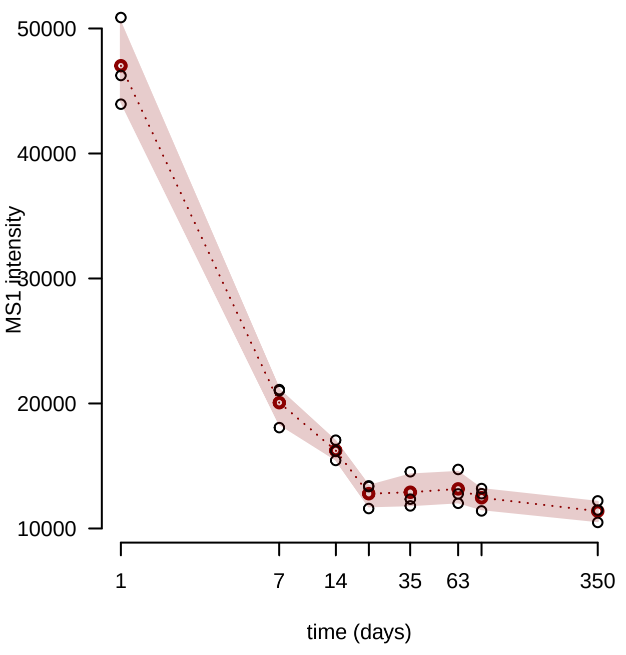

Q9D8C4
GN=Ifi35
interferon−induced protein 35

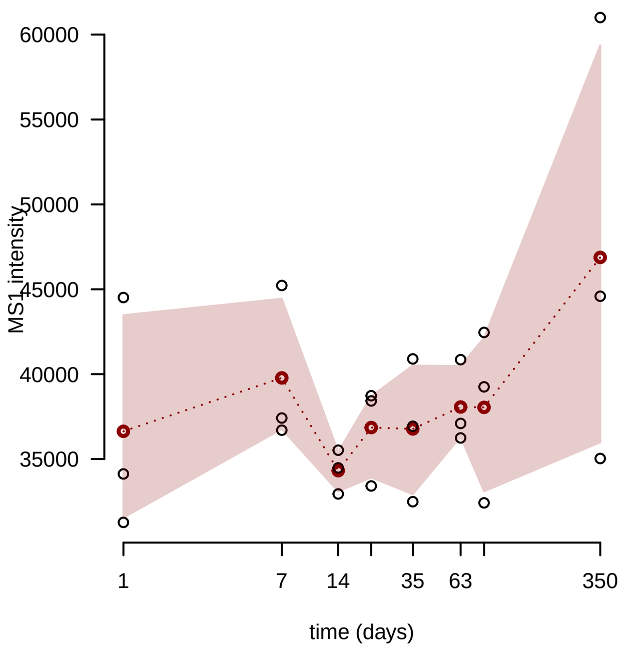

Q9D8E6
GN=Rpl4
ribosomal protein L4

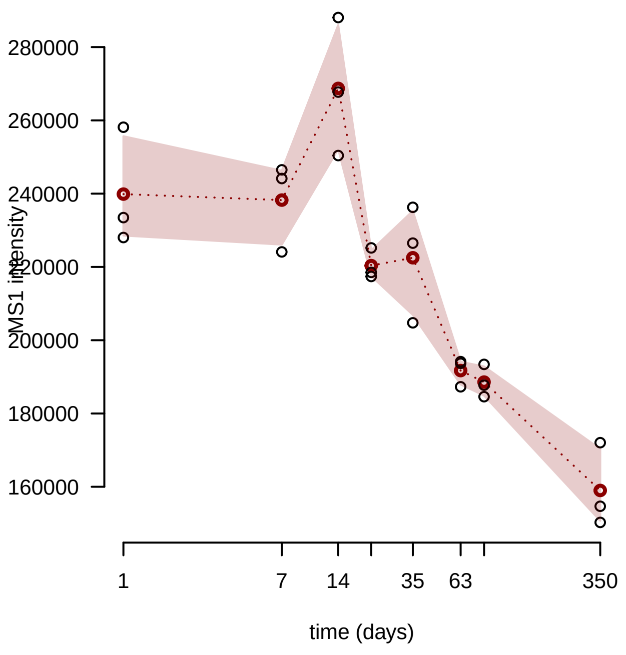

Q9D8F1
GN=Alkbh4
alkB homolog 4, lysine demethylase

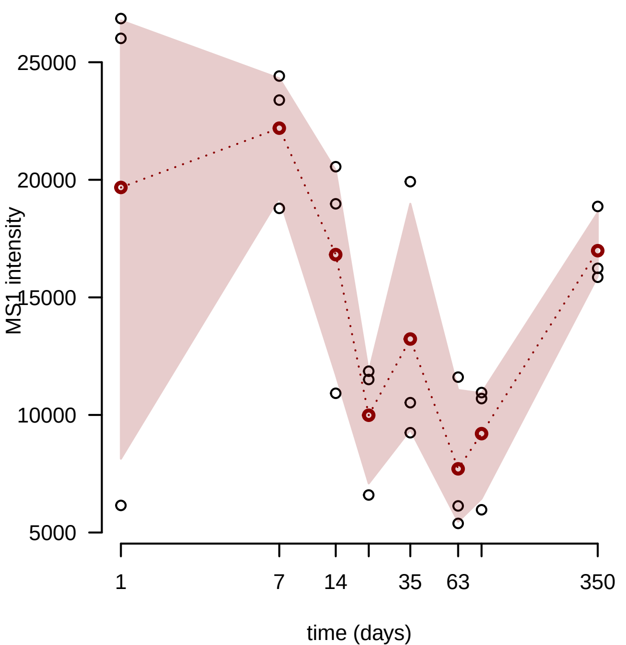

Q9D8H7
GN=Oma1
OMA1 zinc metallopeptidase

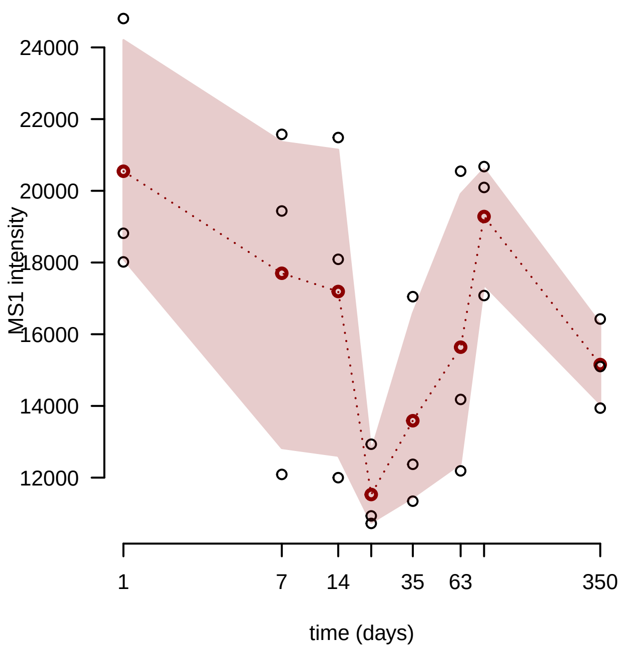

Q9D8K3
GN=Derl3
Der1−like domain family, member 3

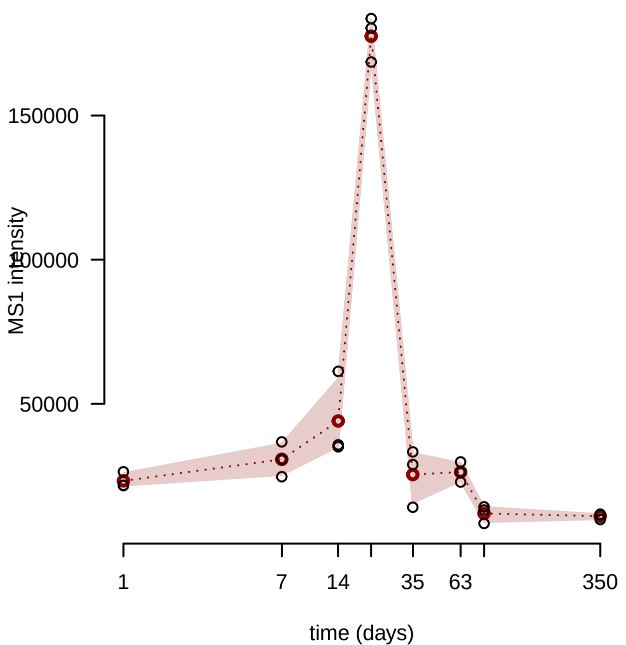

Q9D8L5
GN=Ccdc91
coiled−coil domain containing 91

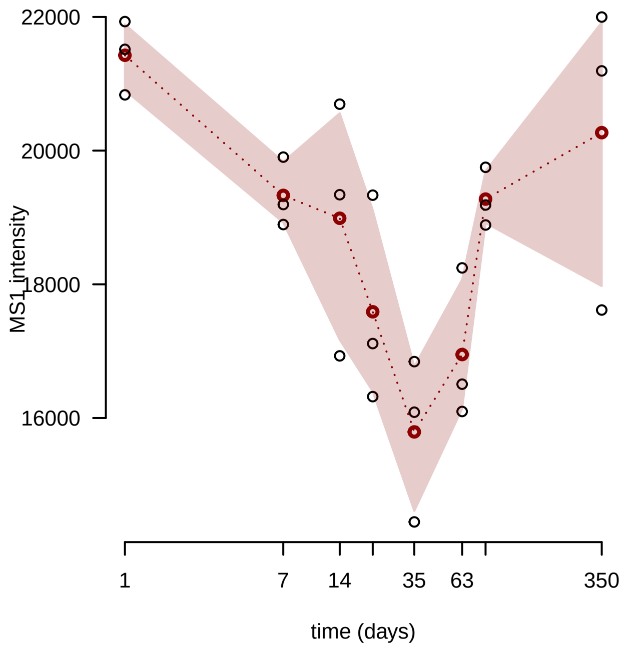

Q9D8M4
GN=Rpl7l1
ribosomal protein L7−like 1

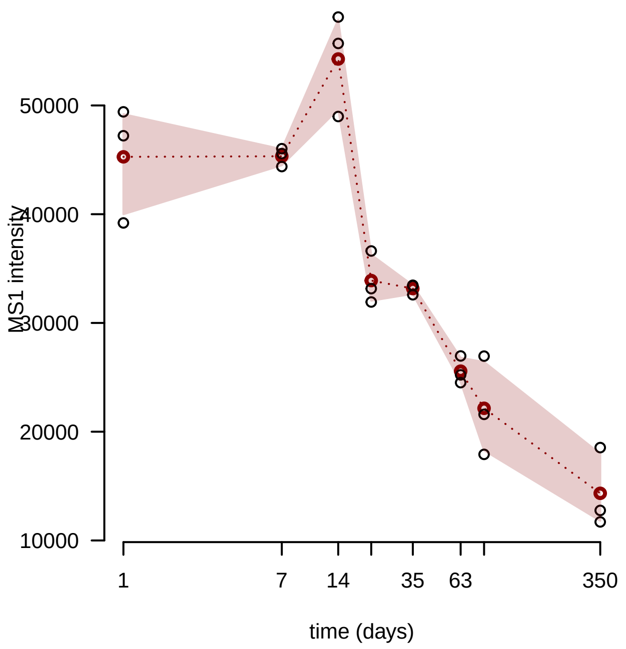

Q9D8M7
GN=Phf10
PHD finger protein 10

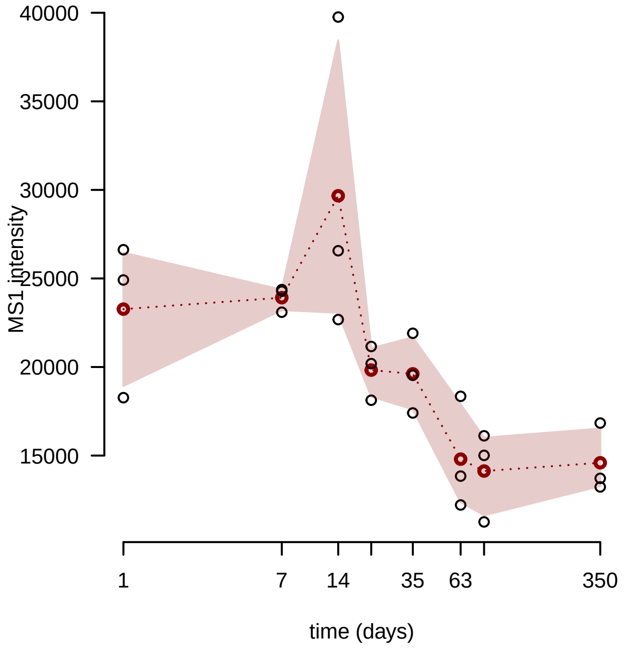

Q9D8N0
GN=Eef1g
eukaryotic translation elongation factor ...

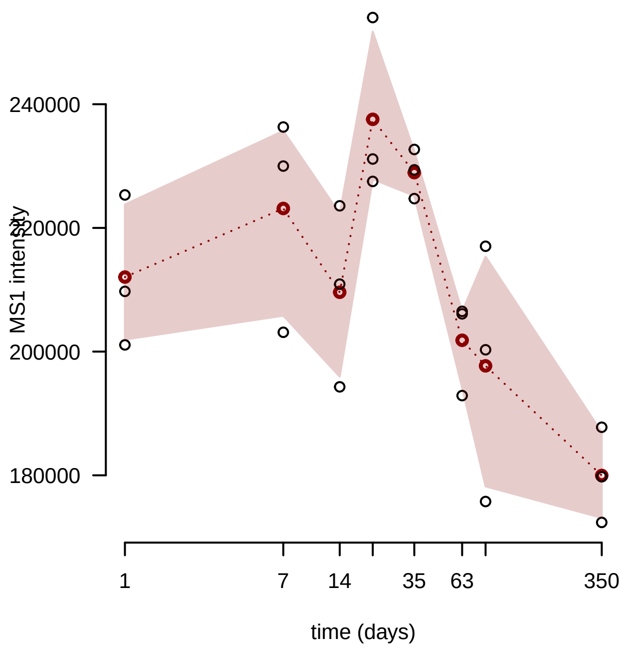

Q9D8N2
GN=Fam45a

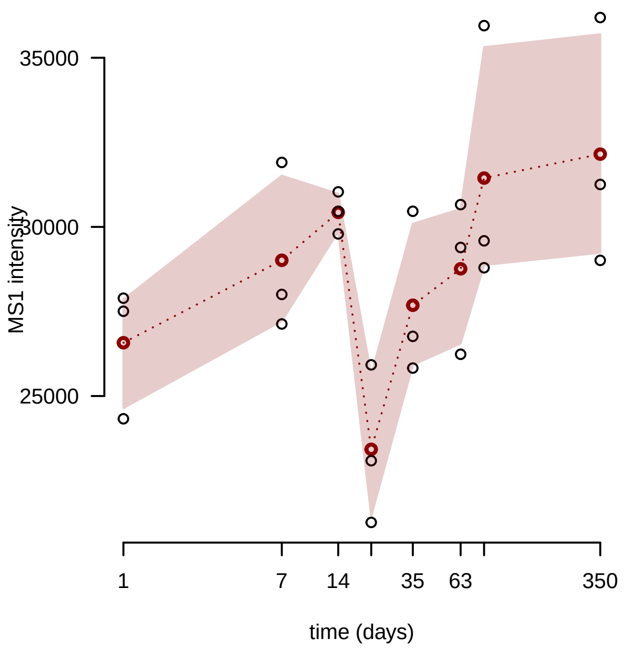

Q9D8N3
GN=Tmem86a
transmembrane protein 86A

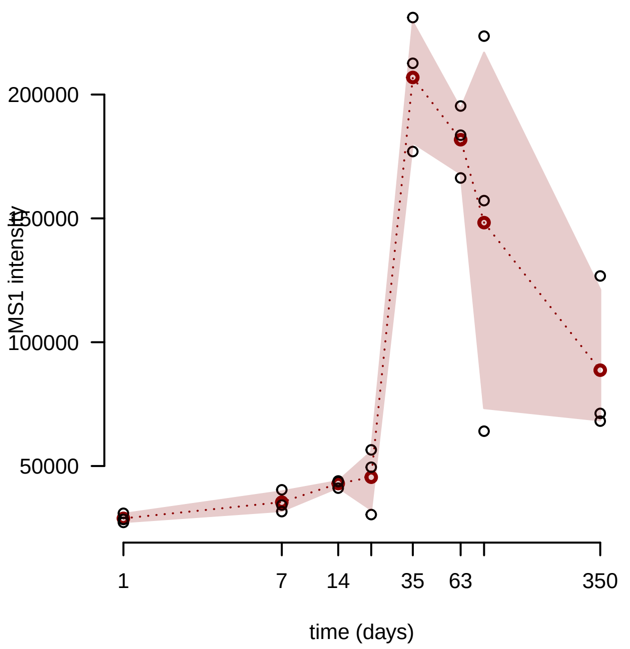

Q9D8P4
GN=Mrpl17
mitochondrial ribosomal protein L17

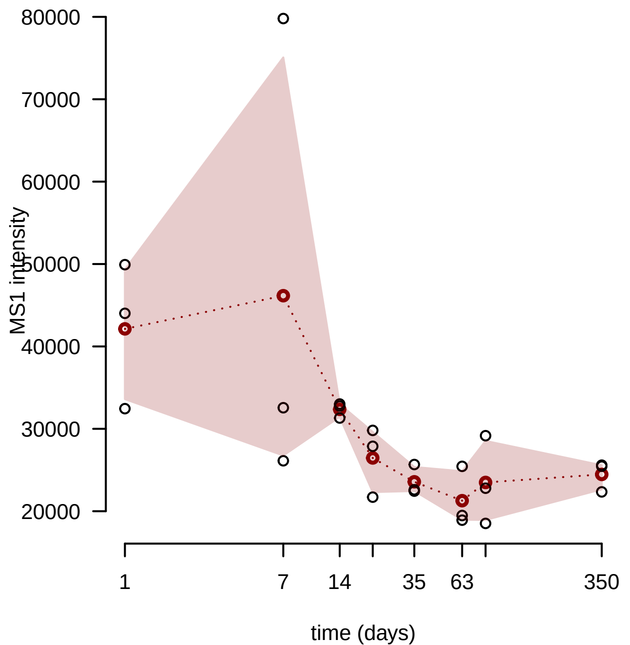

Q9D8S3
GN=Arfgap3
ADP−ribosylation factor GTPase activatin ...

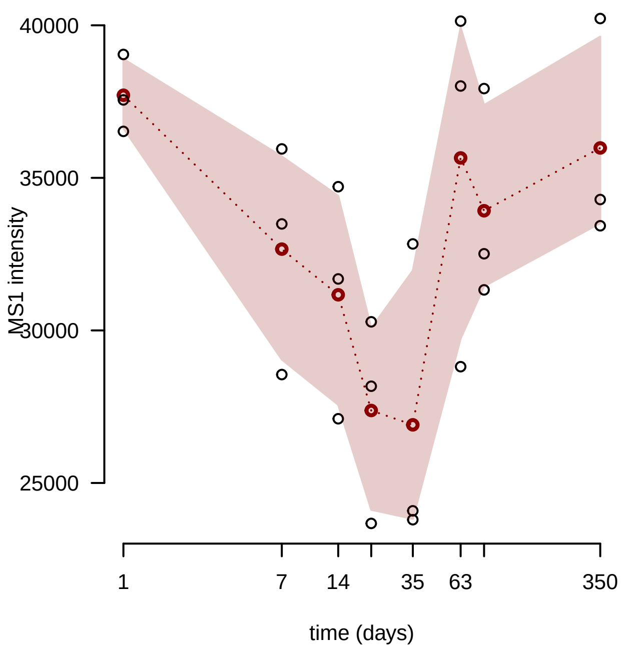

Q9D8S4
GN=Rexo2
RNA exonuclease 2

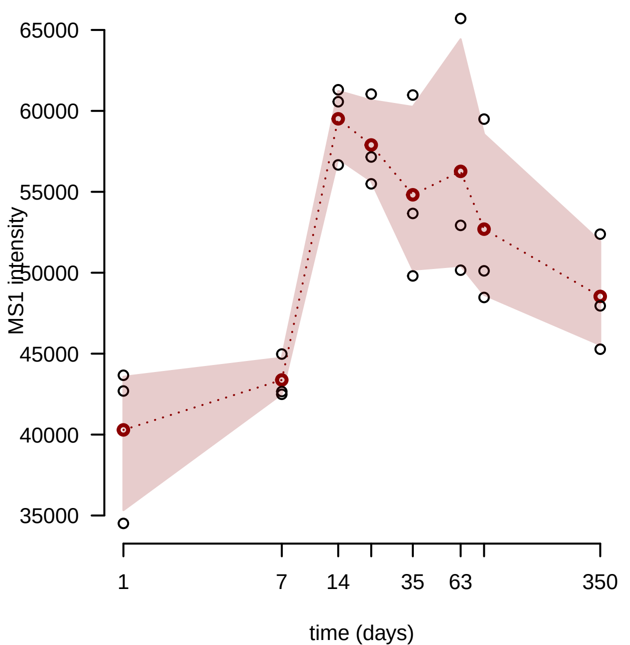

Q9D8S9
GN=Bola1
bolA−like 1 (E. coli)

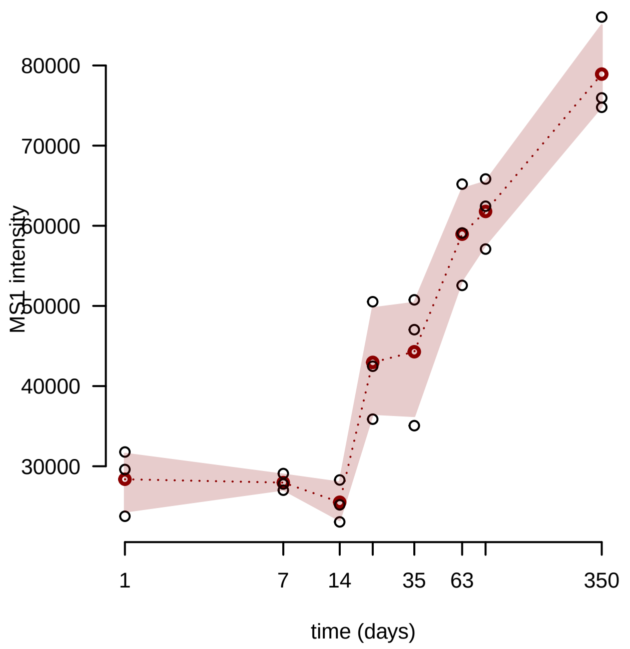

Q9D8T0
GN=Fam3a
FAM3 metabolism regulating signaling mol ...

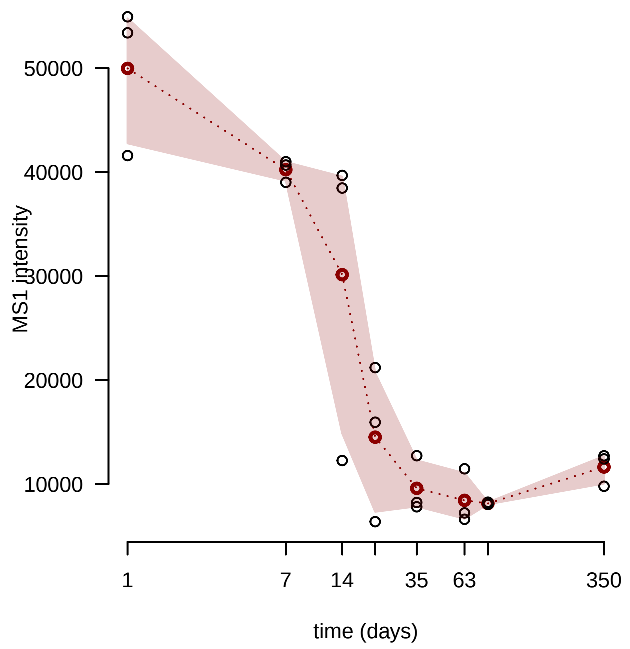

Q9D8T2
GN=Gsdmdc1

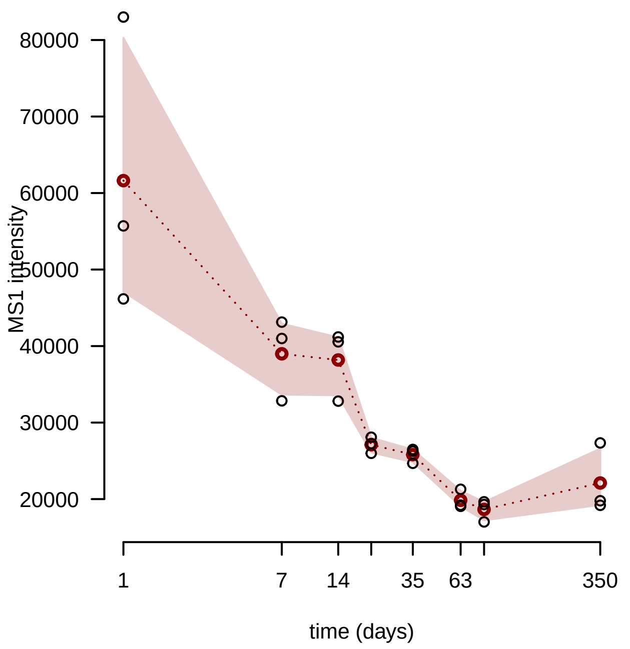

Q9D8T4
GN=Tvp23b
trans−golgi network vesicle protein 23B

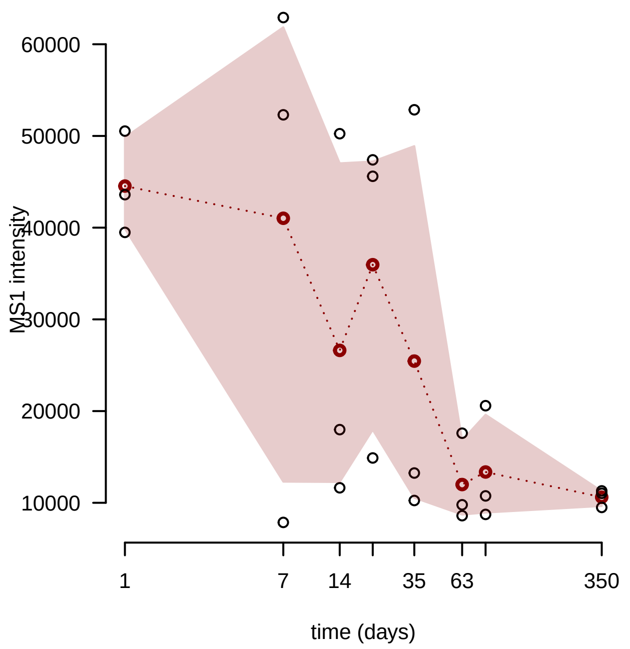

Q9D8T7
GN=Slirp
SRA stem−loop interacting RNA binding pr ...

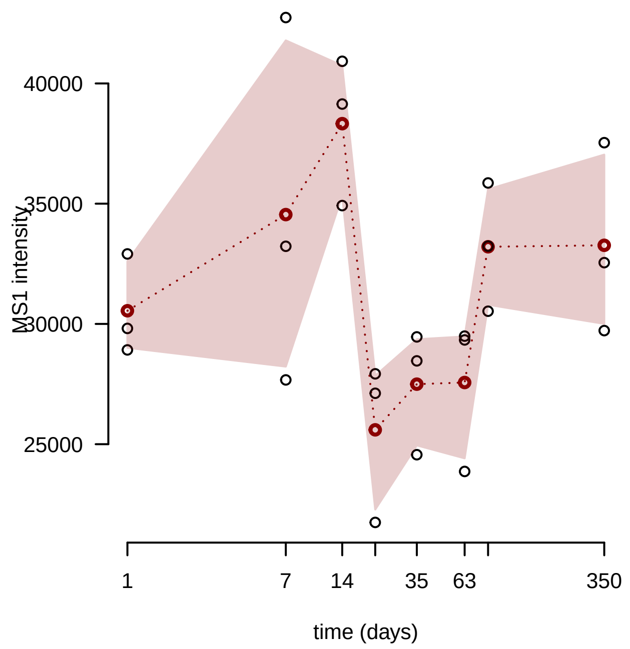

Q9D8U2
GN=Tmem41a
transmembrane protein 41a

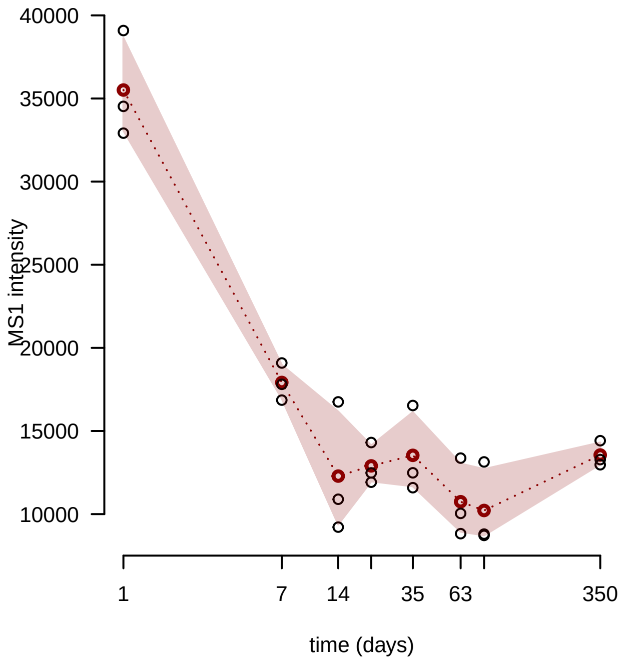

Q9D8U7
GN=Dtwd1
DTW domain containing 1

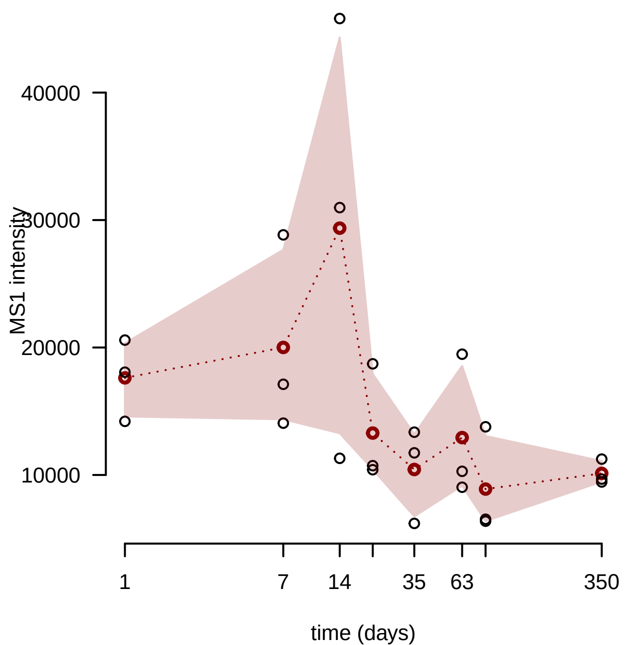

Q9D8U8
GN=Snx5
sorting nexin 5

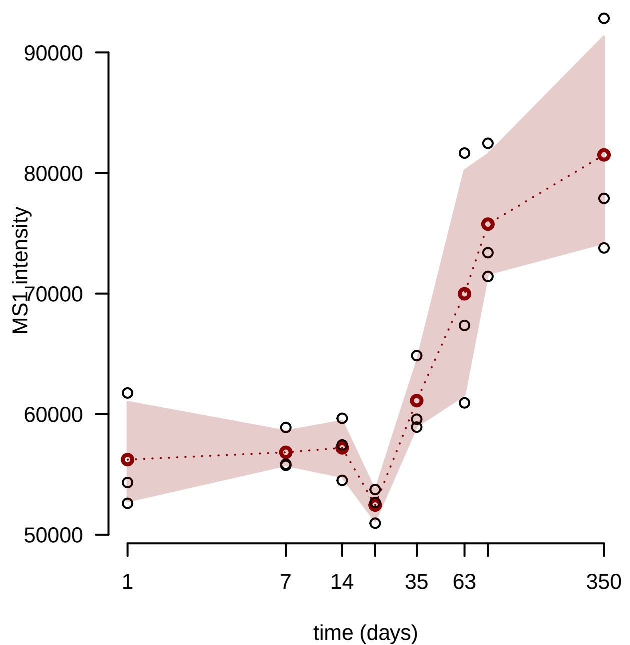

Q9D8V0
GN=Hm13

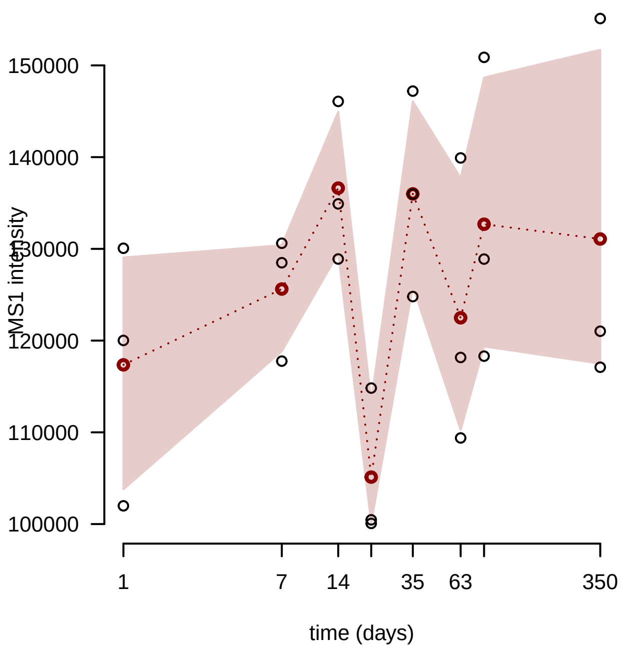

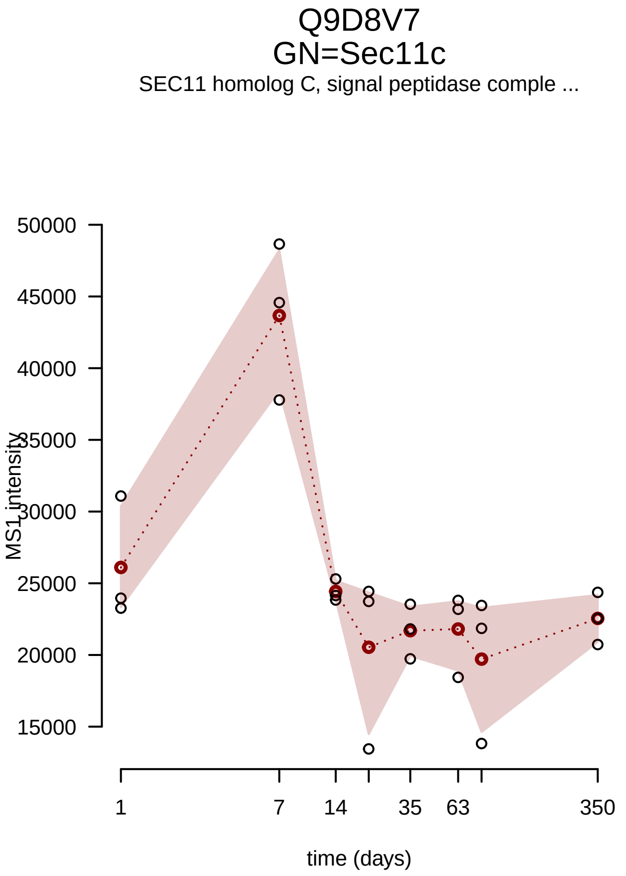
Q9D8V7
GN=Sec11c
SEC11 homolog C, signal peptidase comple ...
50000
45000
40000
35000
30000
25000
20000
15000
MS1 intensity
1
7
14
35
63
350
time (days)

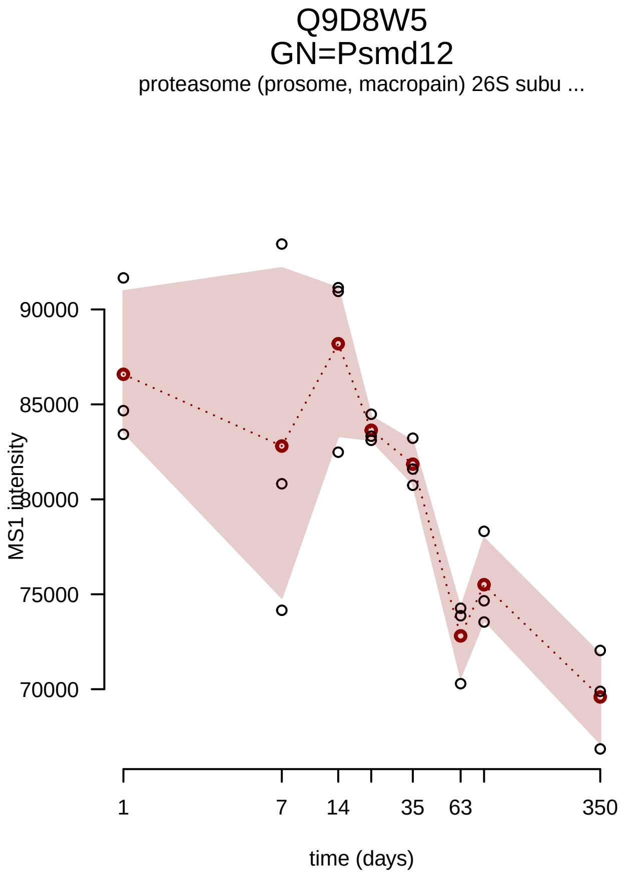
Q9D8W5
GN=Psmd12
proteasome (prosome, macropain) 26S subu ...
90000
85000
80000
75000
70000
MS1 intensity
1
7
14
35
63
350
time (days)

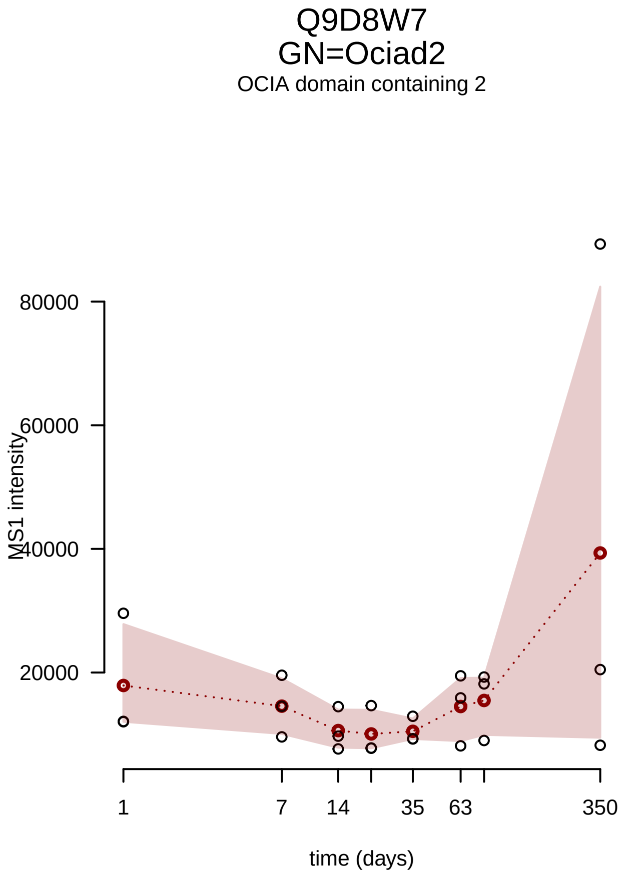
Q9D8W7
GN=Ociad2
OCIA domain containing 2
80000
60000
40000
20000
MS1 intensity
1
7
14
35
63
350
time (days)

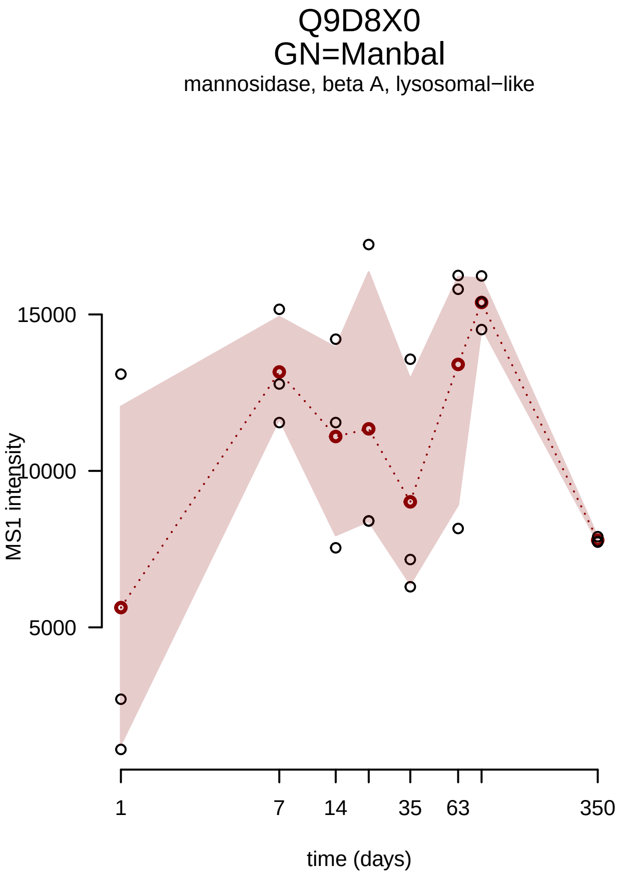
Q9D8X0
GN=Manbal
mannosidase, beta A, lysosomal−like
15000
10000
5000
MS1 intensity
1
7
14
35
63
350
time (days)

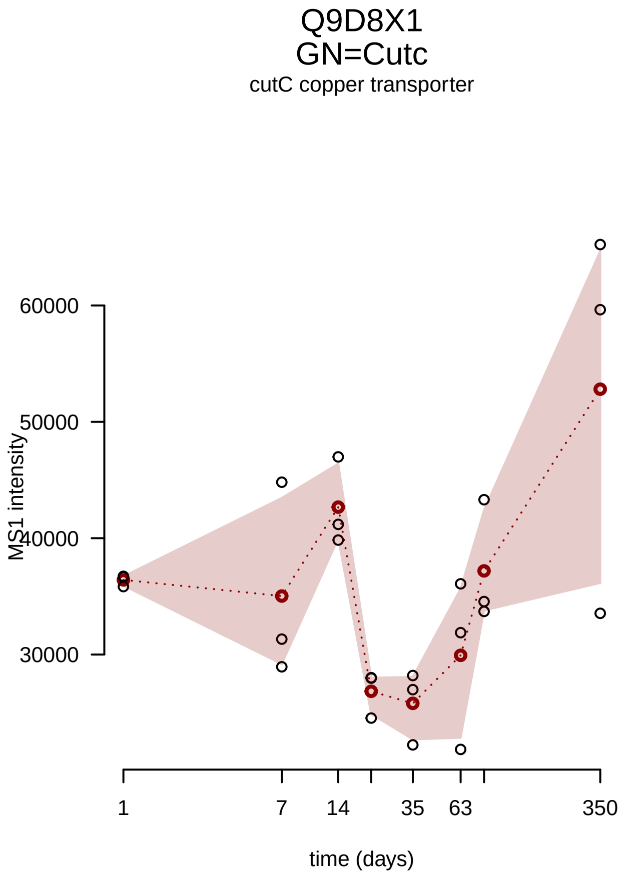
Q9D8X1
GN=Cutc
cutC copper transporter
60000
50000
40000
30000
MS1 intensity
1
7
14
35
63
350
time (days)

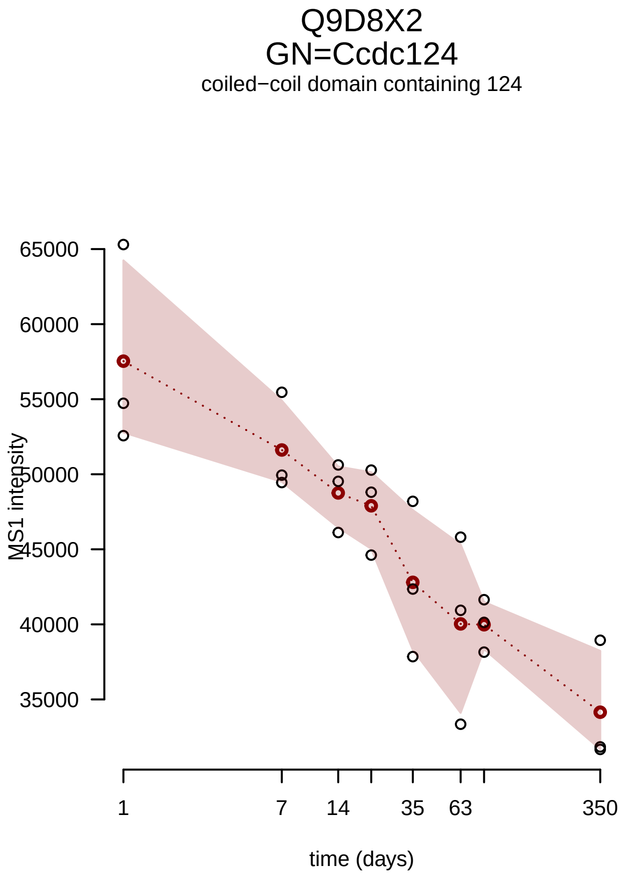
Q9D8X2
GN=Ccdc124
coiled−coil domain containing 124
65000
60000
55000
50000
45000
40000
35000
MS1 intensity
1
7
14
35
63
350
time (days)

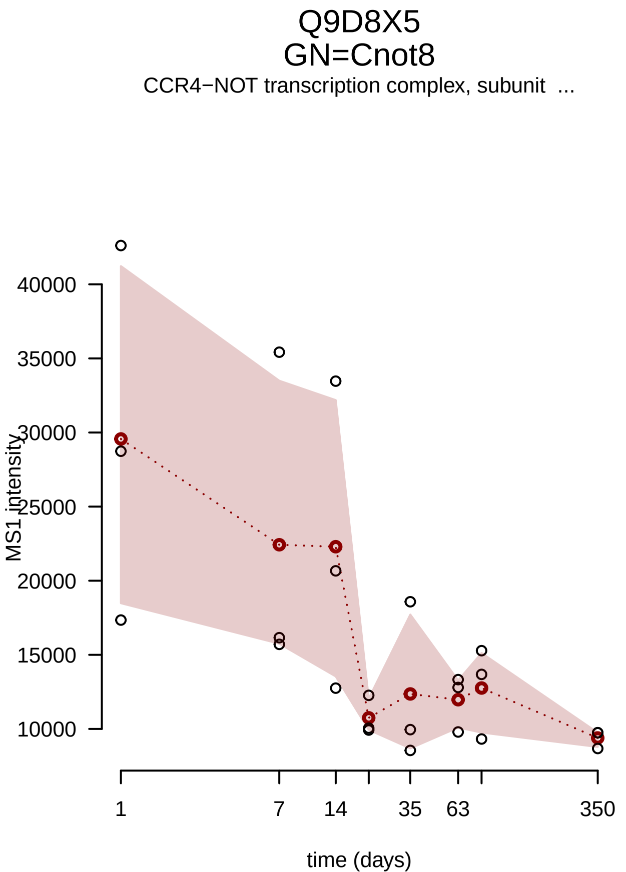
Q9D8X5
GN=Cnot8
CCR4−NOT transcription complex, subunit ...
40000
35000
30000
25000
20000
15000
10000
MS1 intensity
1
7
14
35
63
350
time (days)

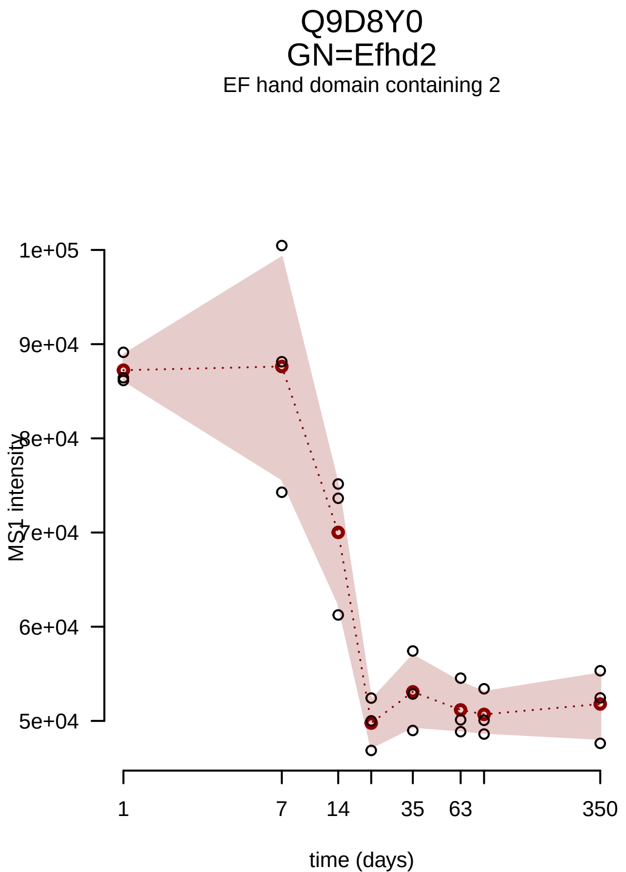
Q9D8Y0
GN=Efhd2
EF hand domain containing 2
1e+05
9e+04
8e+04
7e+04
6e+04
5e+04
MS1 intensity
1
7
14
35
63
350
time (days)

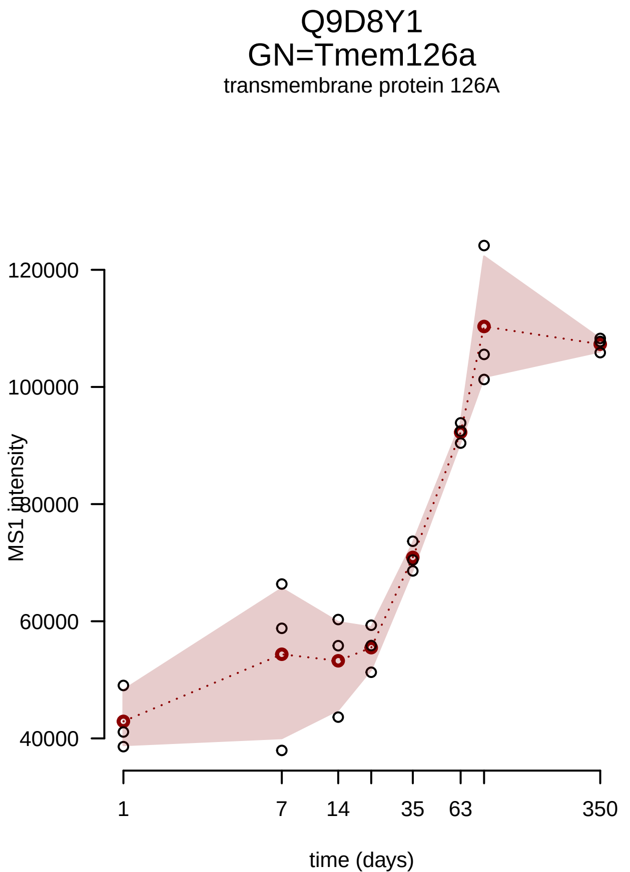
Q9D8Y1
GN=Tmem126a
transmembrane protein 126A
120000
100000
80000
60000
40000
MS1 intensity
1
7
14
35
63
350
time (days)

Q9D8Y8
GN=Ing5
inhibitor of growth family, member 5

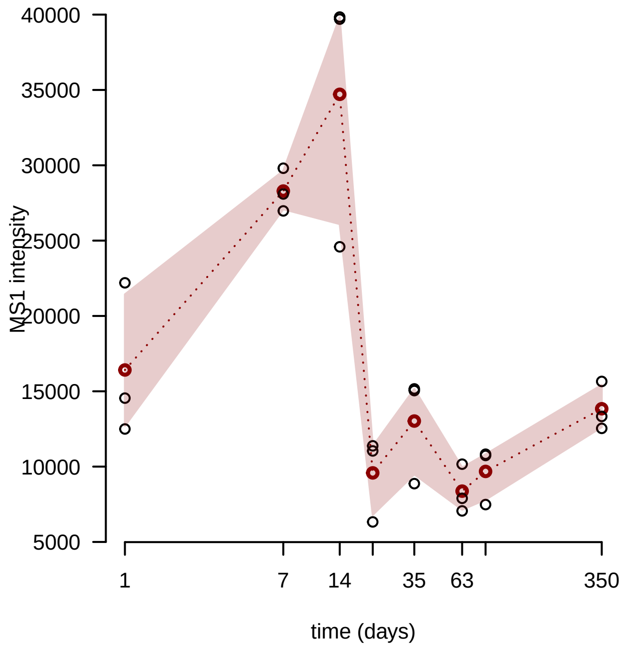

Q9D8Z1
GN=Ascc1
activating signal cointegrator 1 complex ...

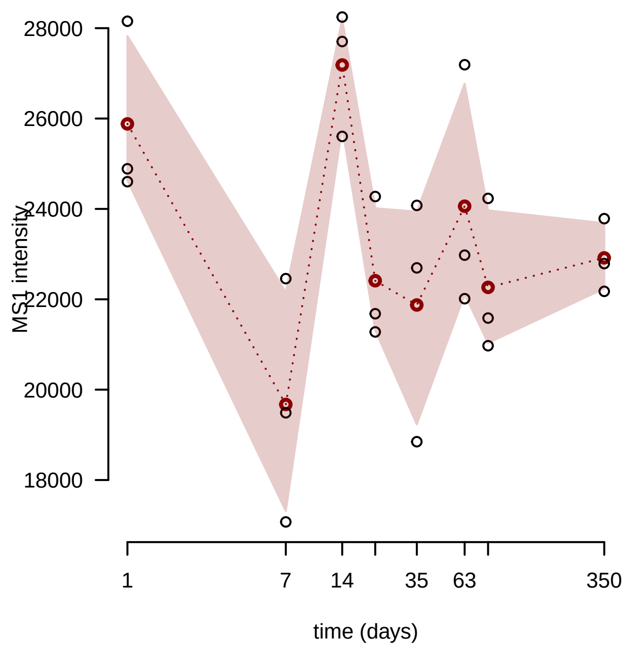

Q9D8Z6
GN=Atg101
autophagy related 101

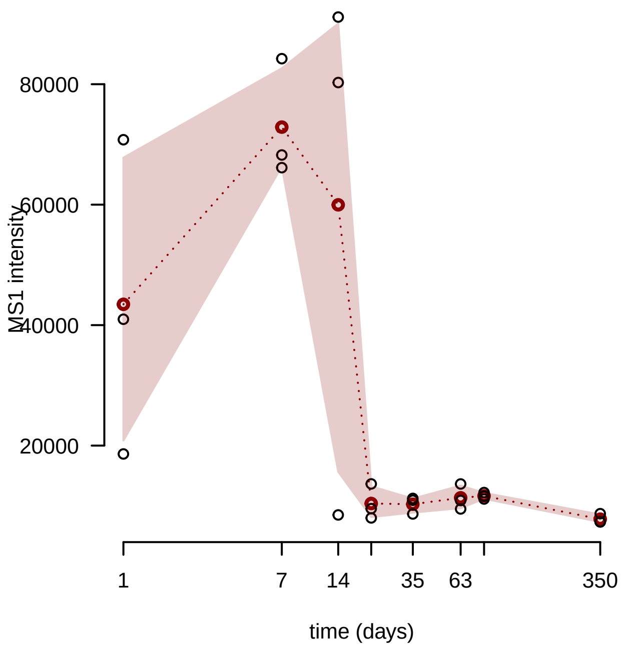

Q9D902
GN=Gtf2e2
general transcription factor II E, polyp ...

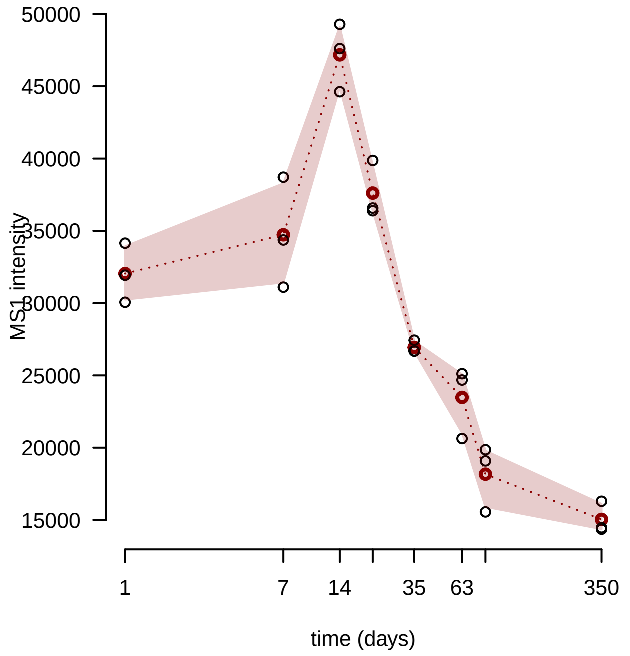

Q9D903
GN=Ebna1bp2
EBNA1 binding protein 2

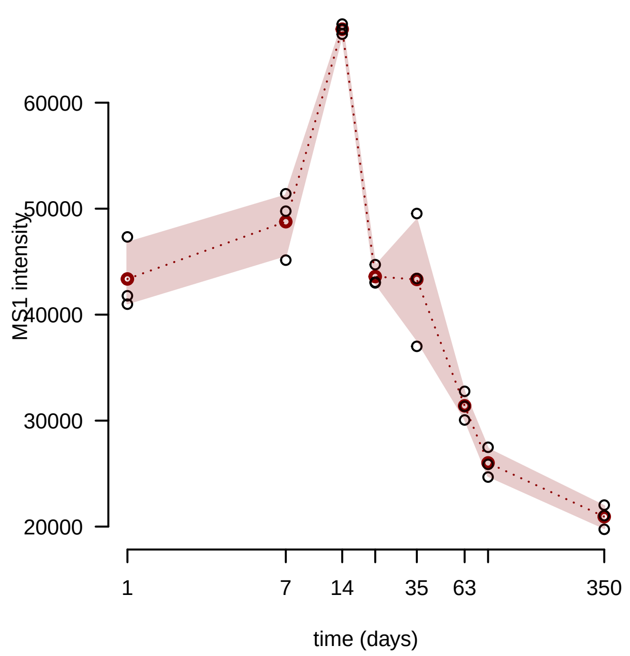

Q9D906
GN=Atg7
autophagy related 7

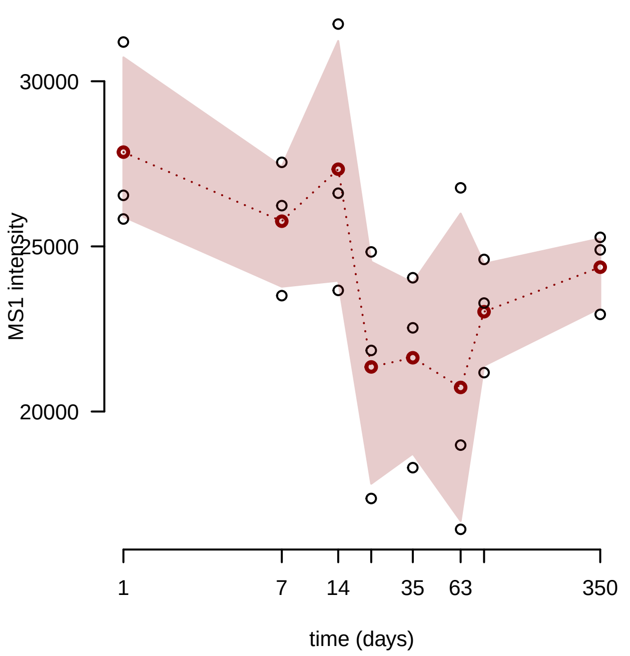

Q9D920
GN=Borcs5
BLOC−1 related complex subunit 5

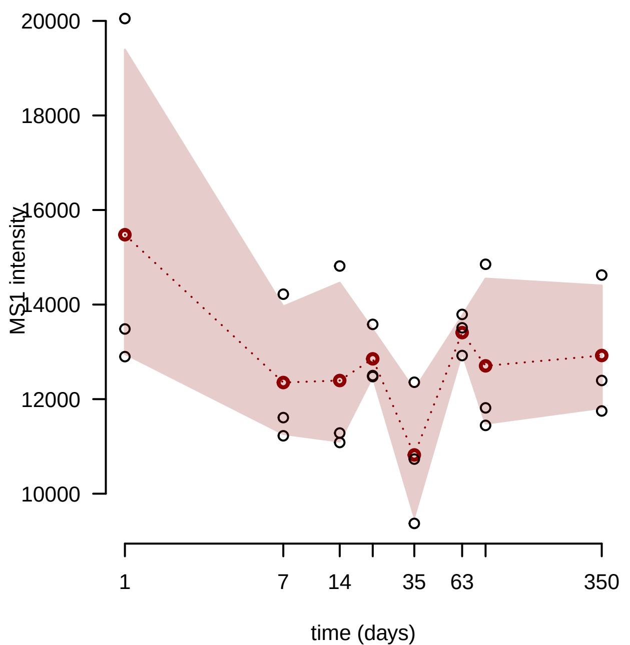

Q9D924
GN=Isca1
iron−sulfur cluster assembly 1

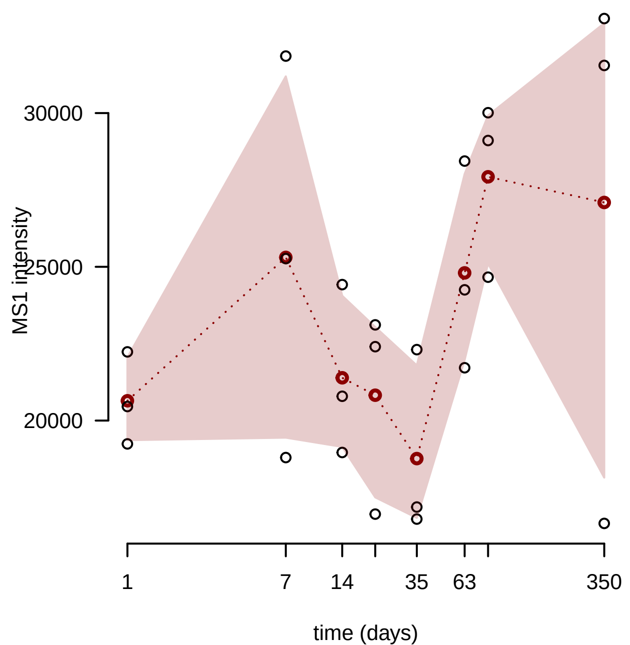

Q9D937
GN=

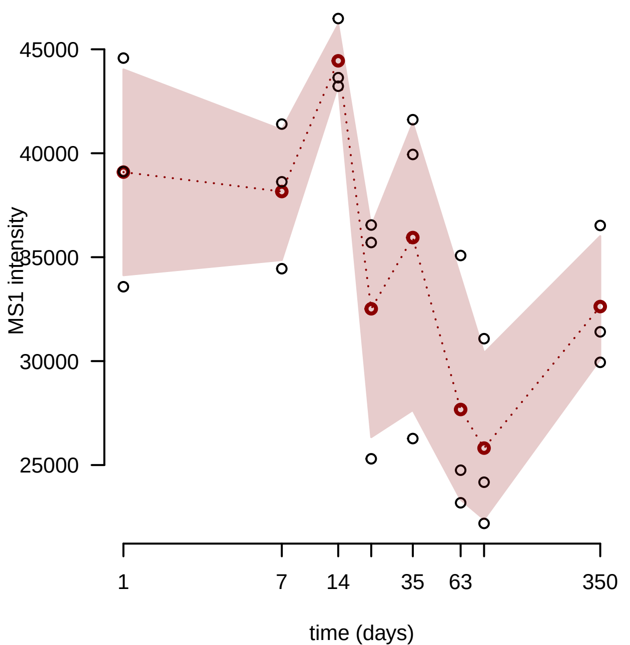

Q9D938
GN=Tmem160
transmembrane protein 160

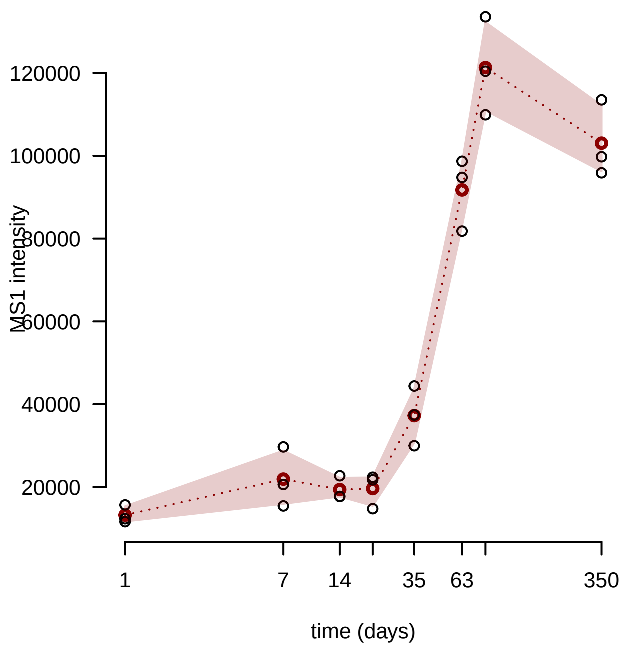

Q9D945
GN=Llph
LLP homolog, long−term synaptic facilita ...

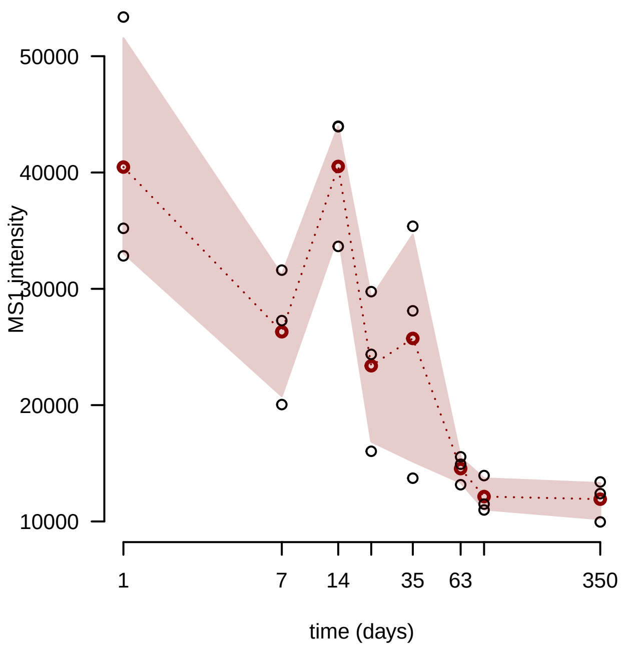

Q9D952
GN=Evpl
envoplakin

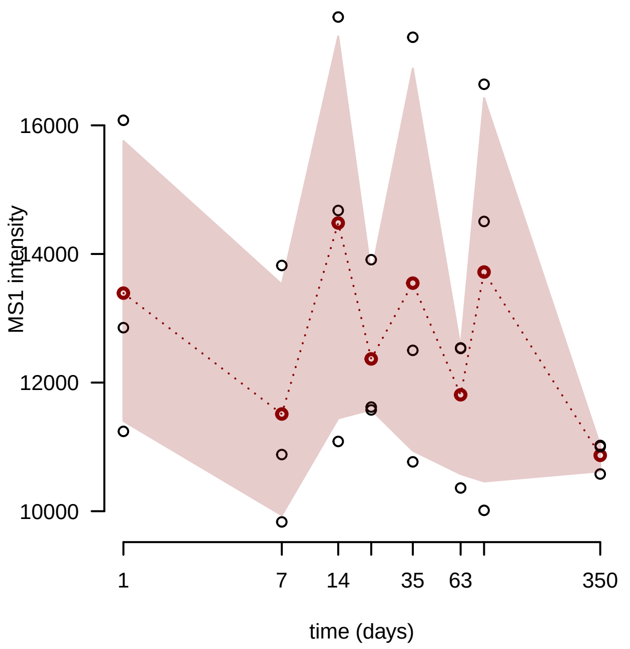

Q9D958
GN=Spcs1
signal peptidase complex subunit 1 homol ...

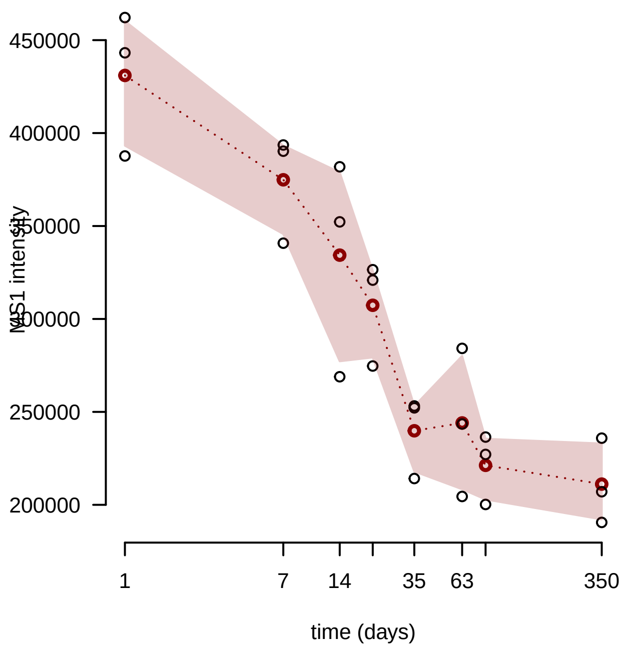

Q9D964
GN=Gatm
glycine amidinotransferase (L−arginine:g ...

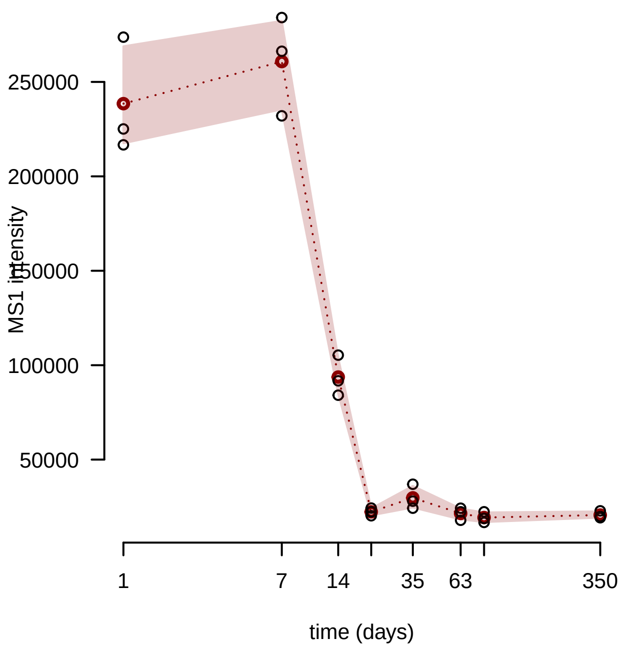

Q9D967
GN=Mdp1
magnesium−dependent phosphatase 1

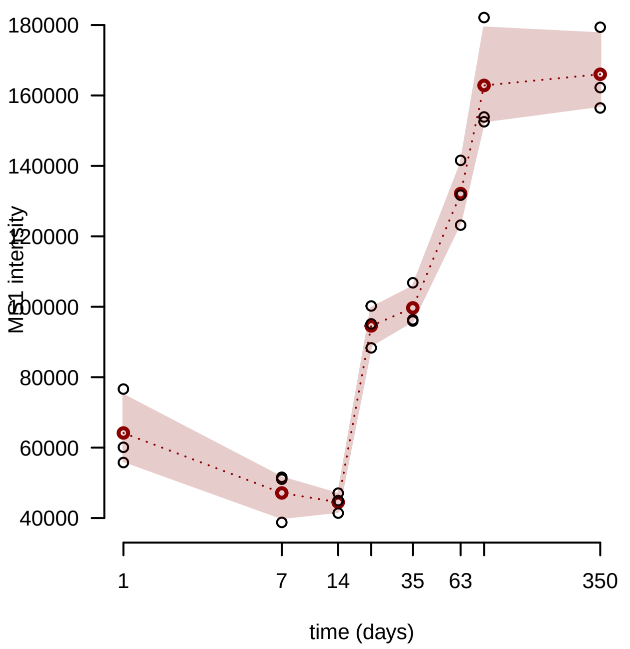

Q9D968
GN=Hcfc2
host cell factor C2

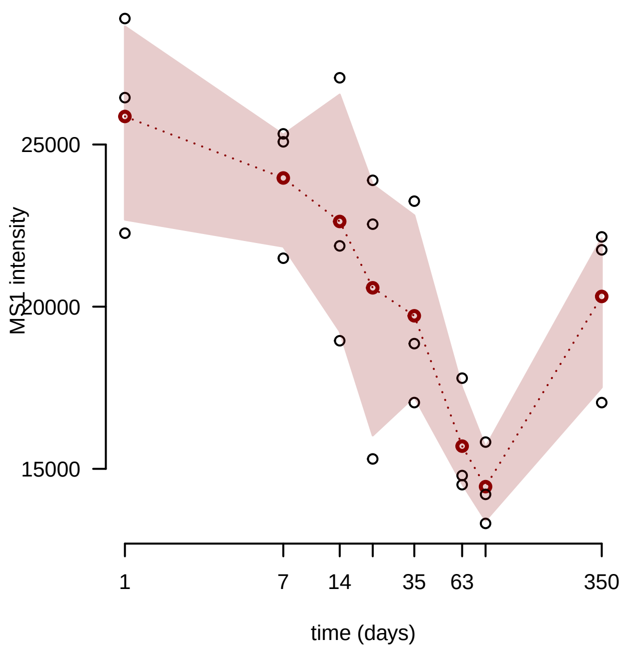

Q9D975
GN=Srxn1
sulfiredoxin 1 homolog (S. cerevisiae)

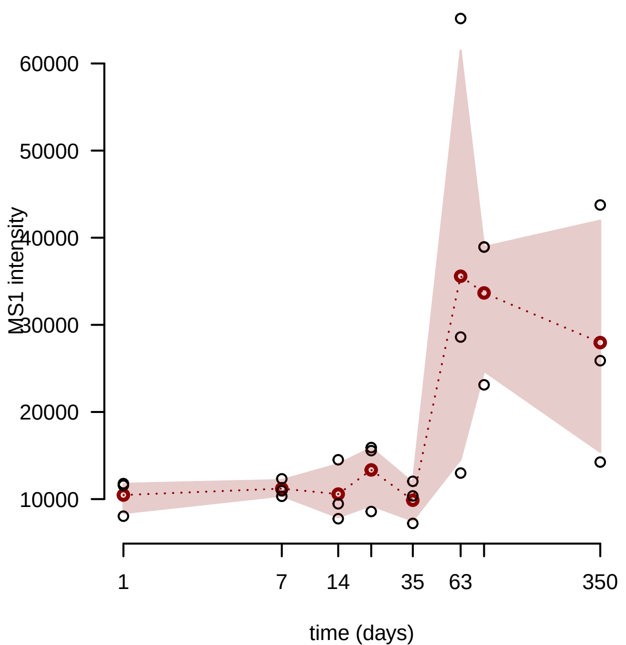

Q9D9B4
GN=Lrmda
leucine rich melanocyte differentiation ...

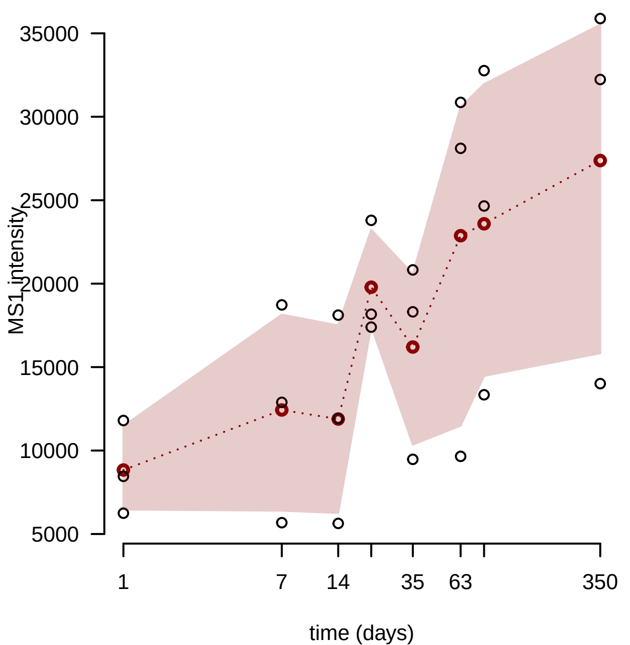

Q9D9E0
GN=Slc22a17
solute carrier family 22 (organic cation ...

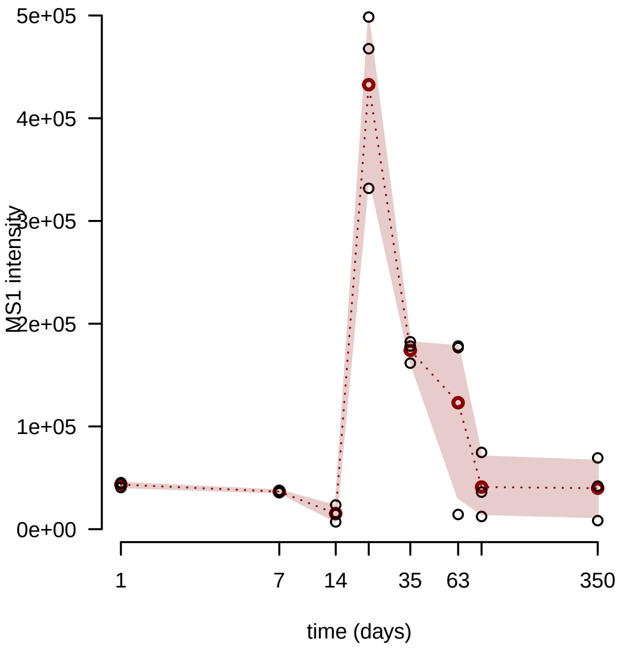

Q9D9G3
GN=Chic2
cysteine−rich hydrophobic domain 2

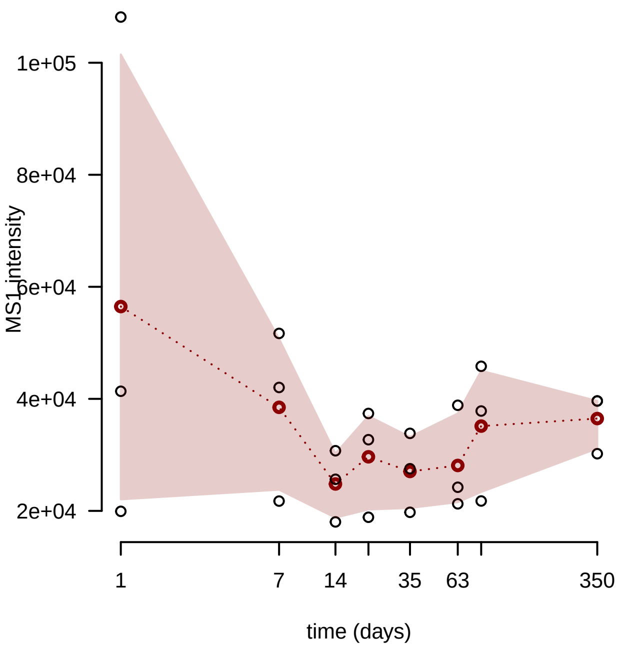

Q9D9H8
GN=

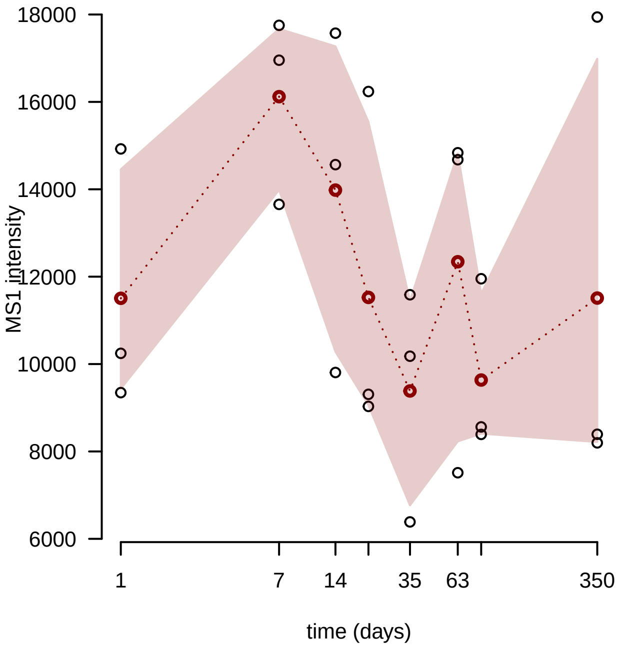

Q9D9I4
GN=Tbc1d20
TBC1 domain family, member 20

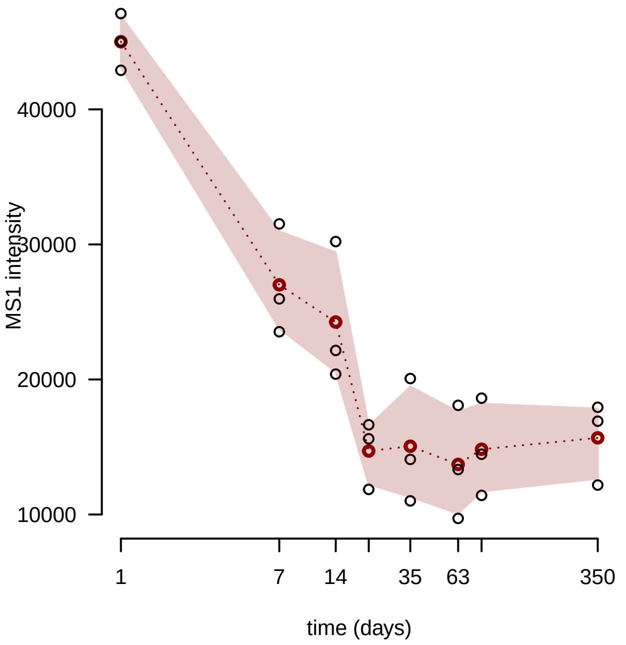

Q9D9K3
GN=Aven
apoptosis, caspase activation inhibitor

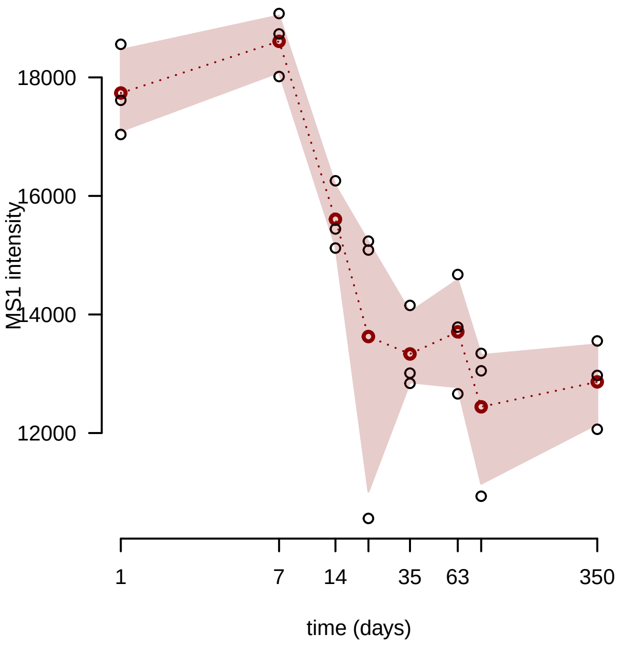

Q9D9V3
GN=Echdc1
enoyl Coenzyme A hydratase domain contai ...

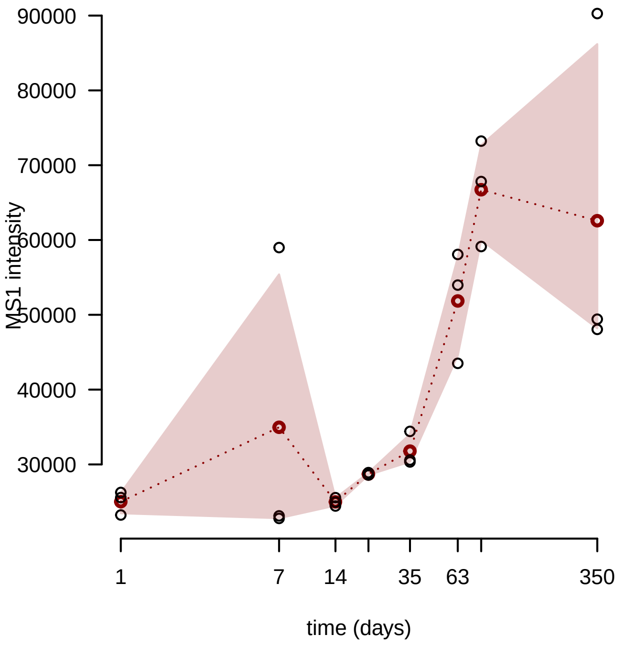

Q9D9Z5
GN=Dda1
DET1 and DDB1 associated 1

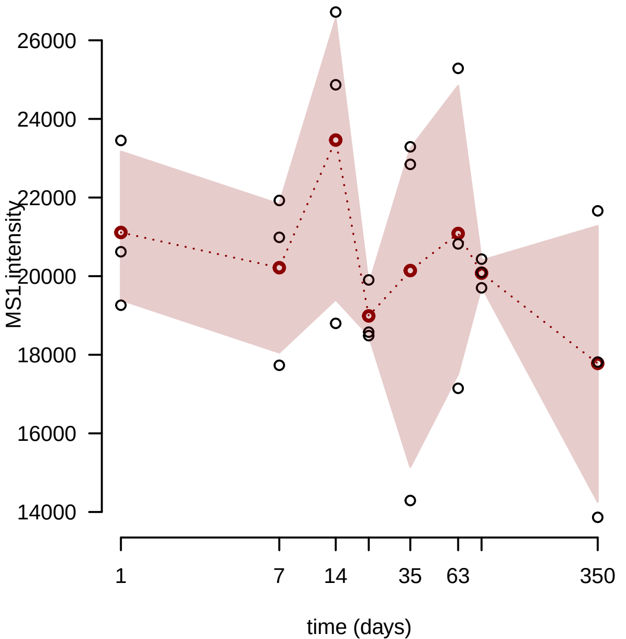

Q9DA08
GN=Sgf29
SAGA complex associated factor 29

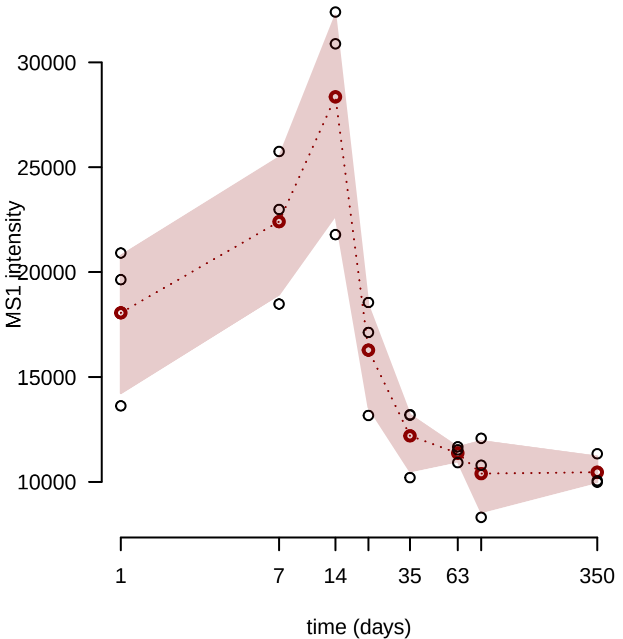

Q9DA19
GN=Cir1
corepressor interacting with RBPJ, 1

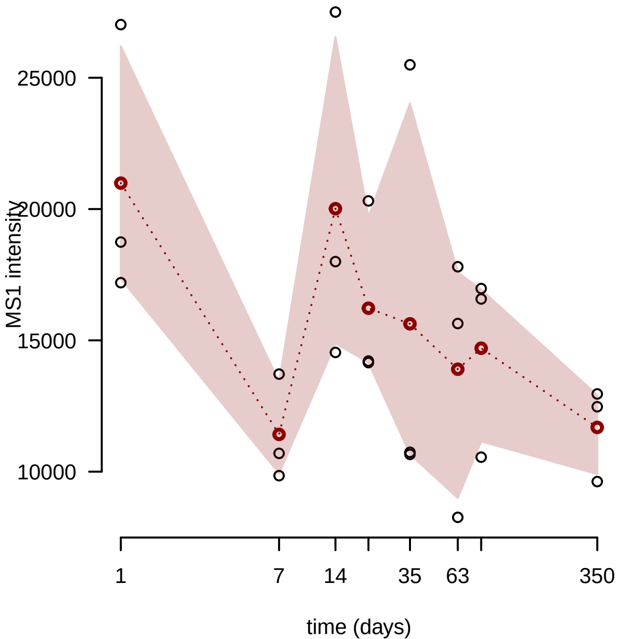

Q9DA69
GN=Ift43
intraflagellar transport 43

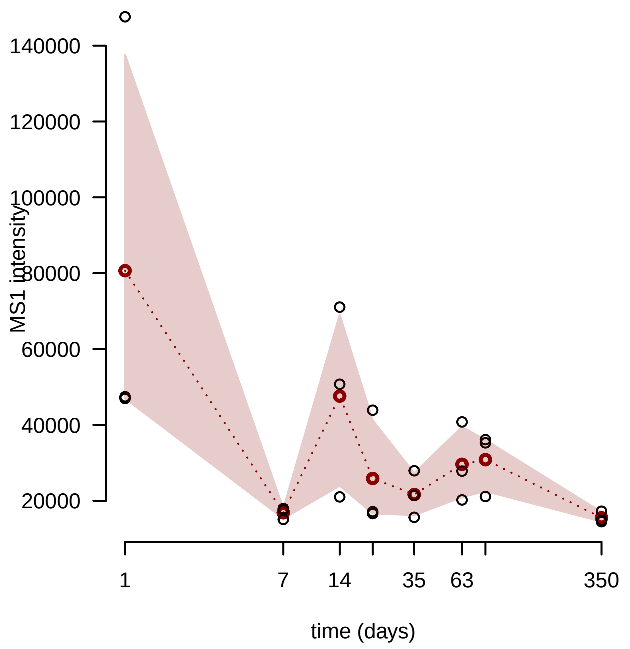

Q9DA79
GN=Dpep3
dipeptidase 3

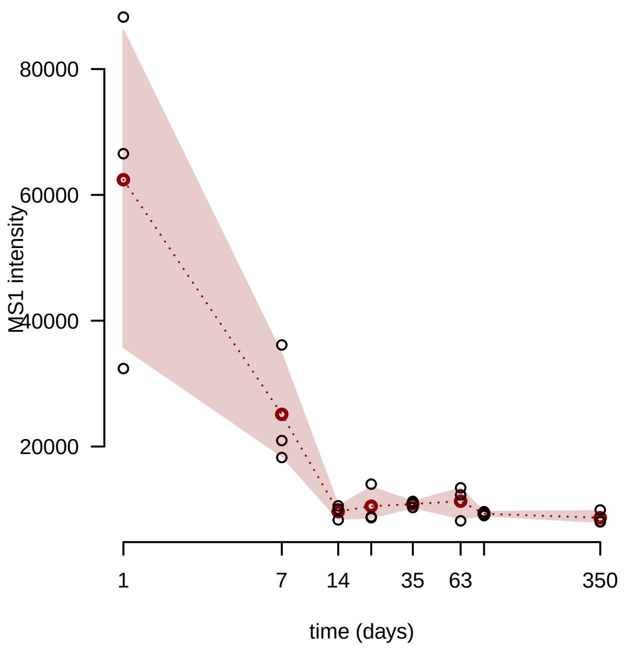

Q9DAA6
GN=Exosc1
exosome component 1

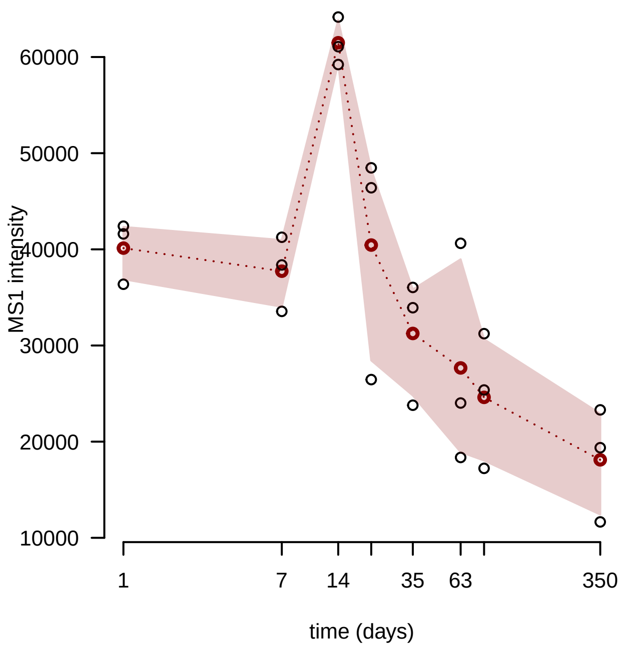

Q9DAD6
GN=Pfn3
profilin 3

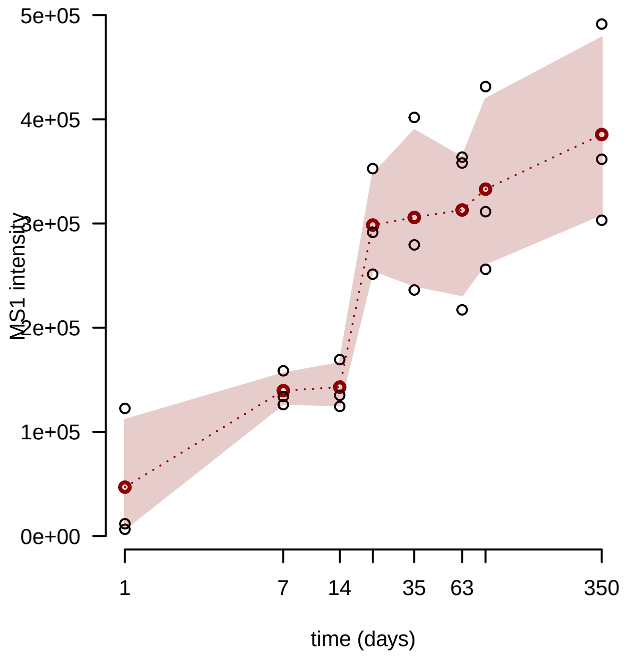

Q9DAI2
GN=Ift22
intraflagellar transport 22

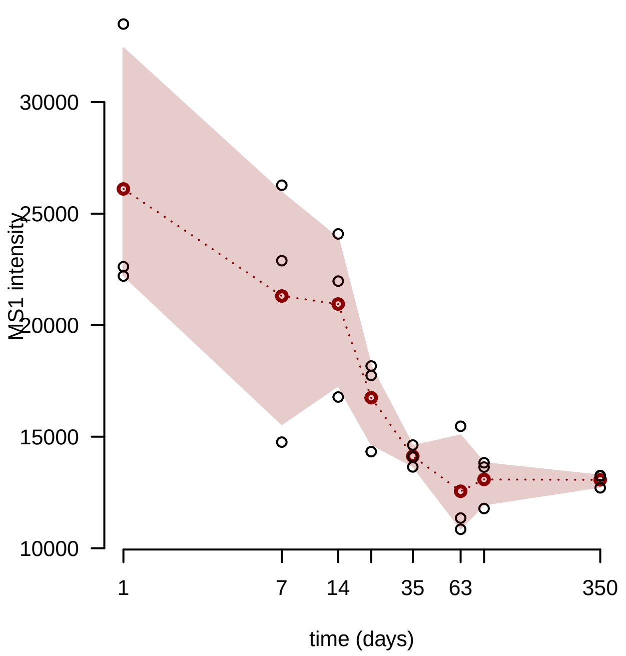

Q9DAJ4
GN=Wdr83
WD repeat domain containing 83

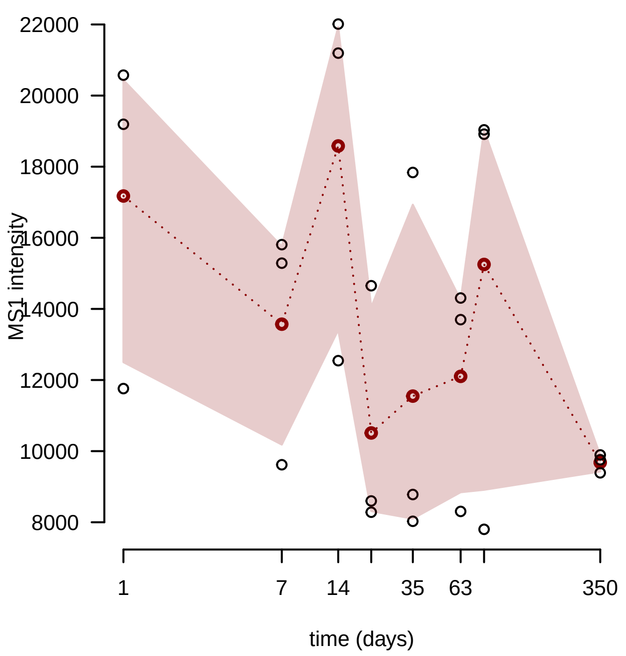

Q9DAK9
GN=Phpt1
phosphohistidine phosphatase 1

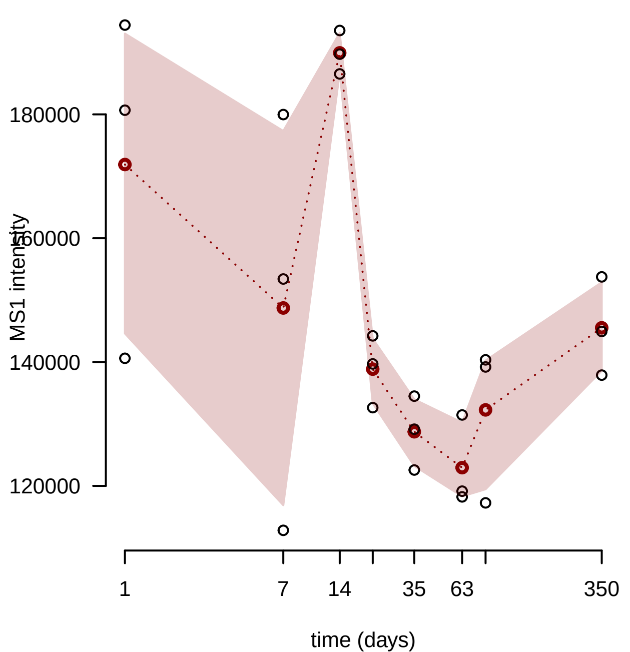

Q9DAM5
GN=Slc25a19
solute carrier family 25 (mitochondrial ...

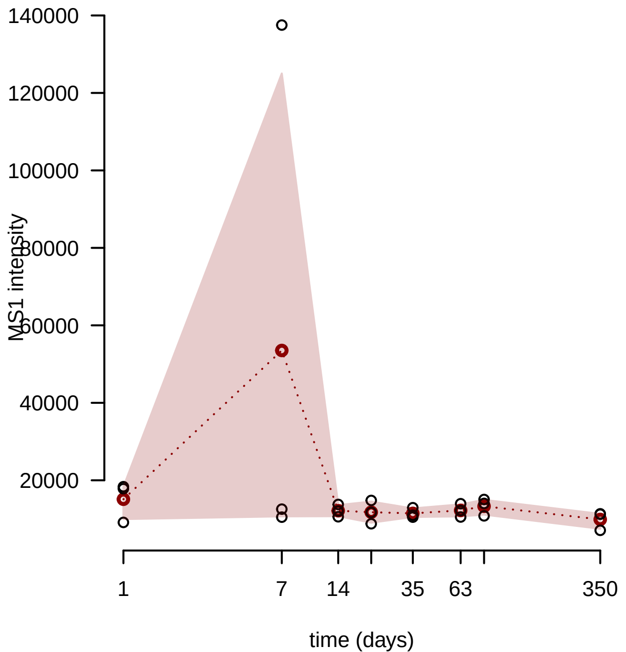

Q9DAM7
GN=Tmem263
transmembrane protein 263

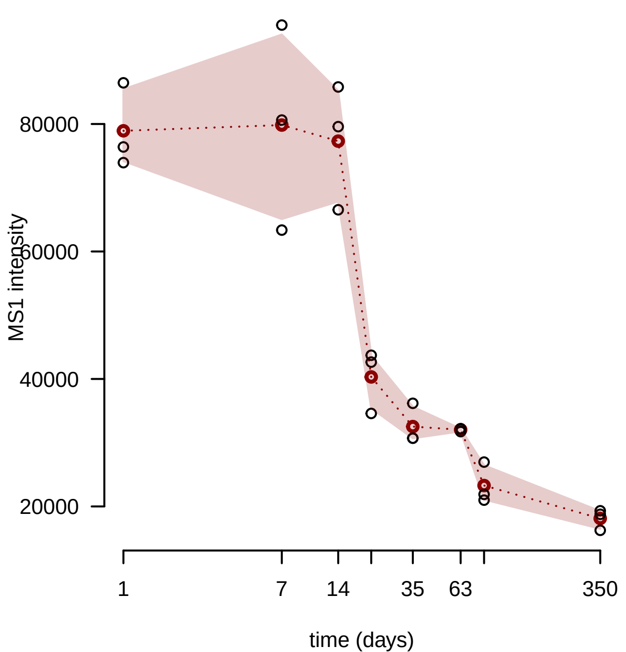

Q9DAN6
GN=Gtsf1
gametocyte specific factor 1

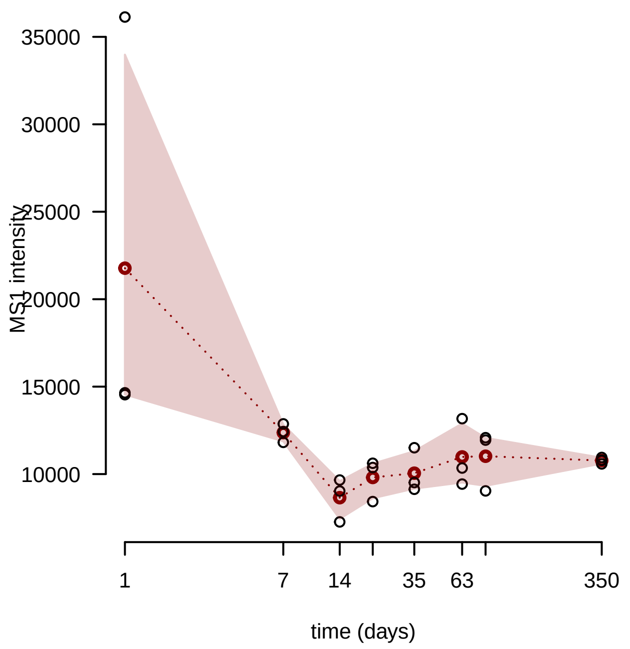

Q9DAN8
GN=Cst12
cystatin 12

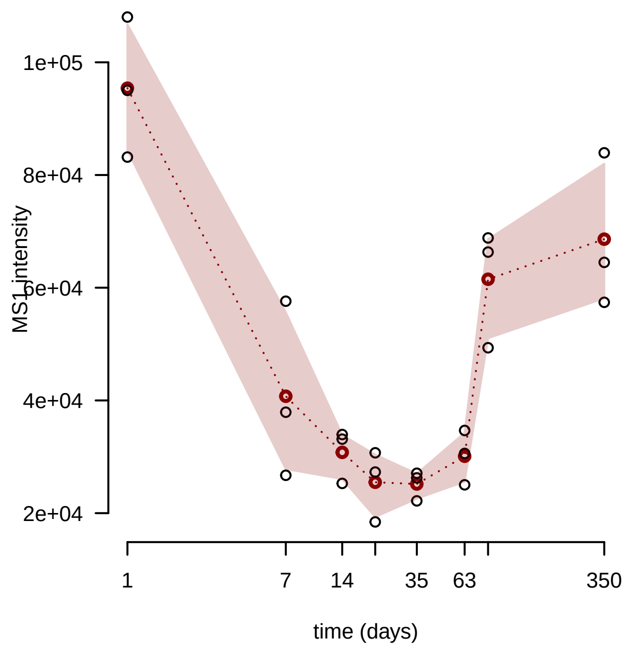

Q9DAR7
GN=Dcps
decapping enzyme, scavenger

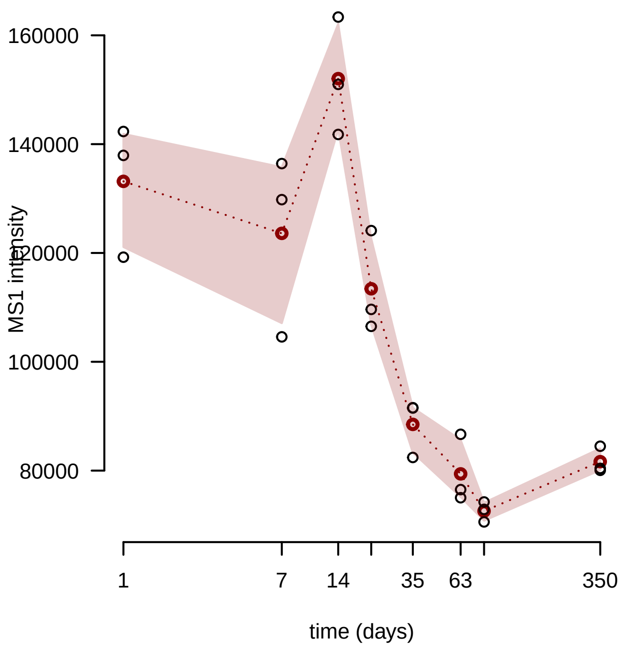

Q9DAS9
GN=Gng12
guanine nucleotide binding protein (G pr ...

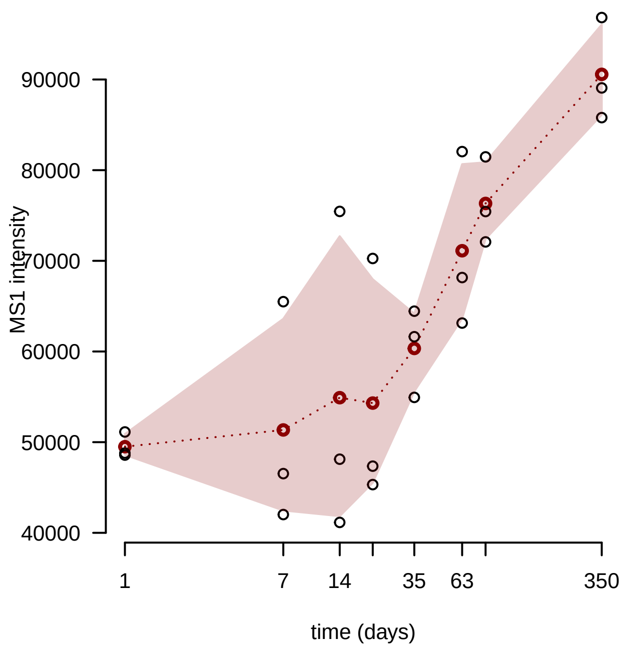

Q9DAT2
GN=Mrgbp
MRG/MORF4L binding protein

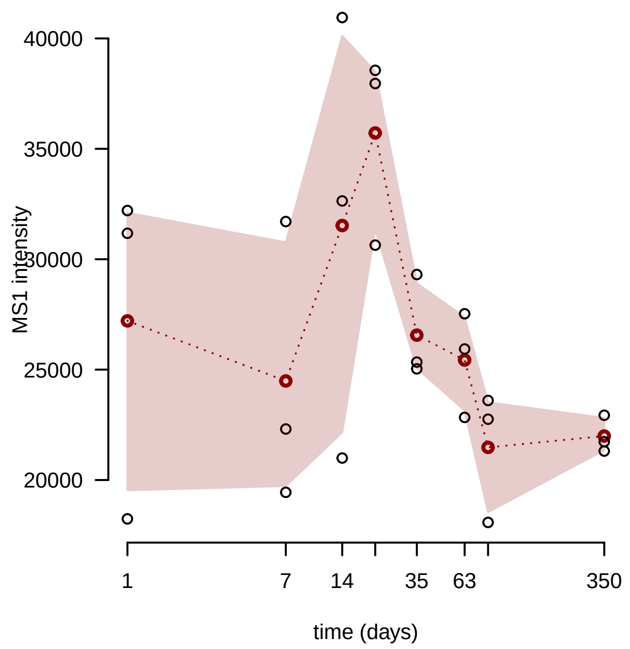

Q9DAT5
GN=Trmu
tRNA 5−methylaminomethyl−2−thiouridylate ...

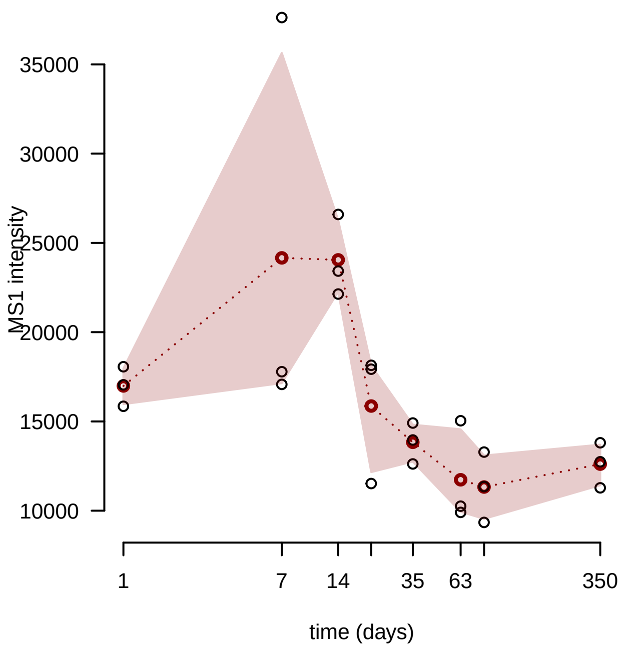

Q9DAU1
GN=Cnpy3
canopy FGF signaling regulator 3

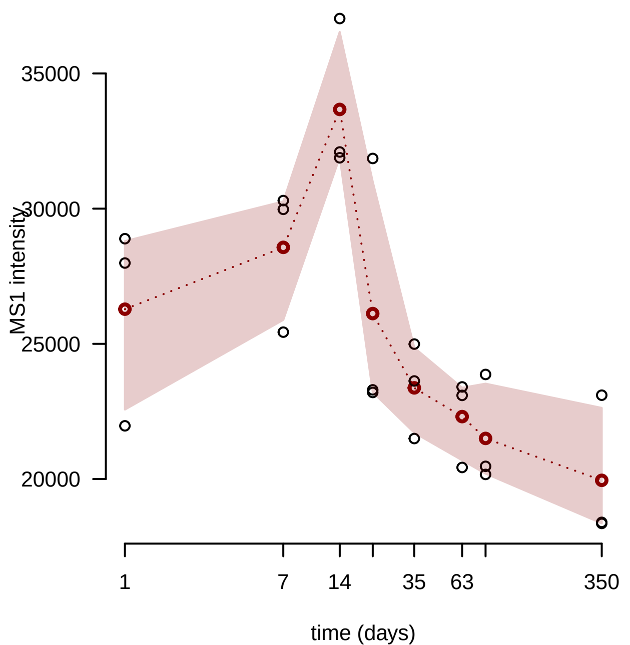

Q9DAU9
GN=Znf654

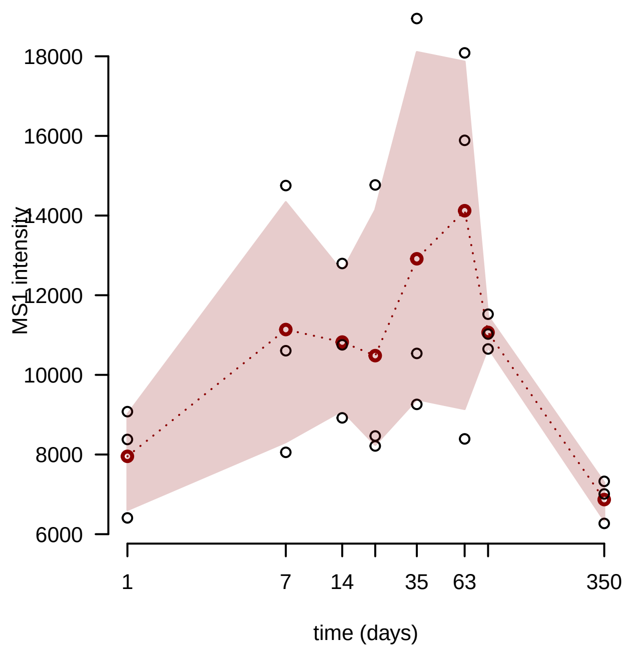

Q9DAV9
GN=Tmem38b
transmembrane protein 38B

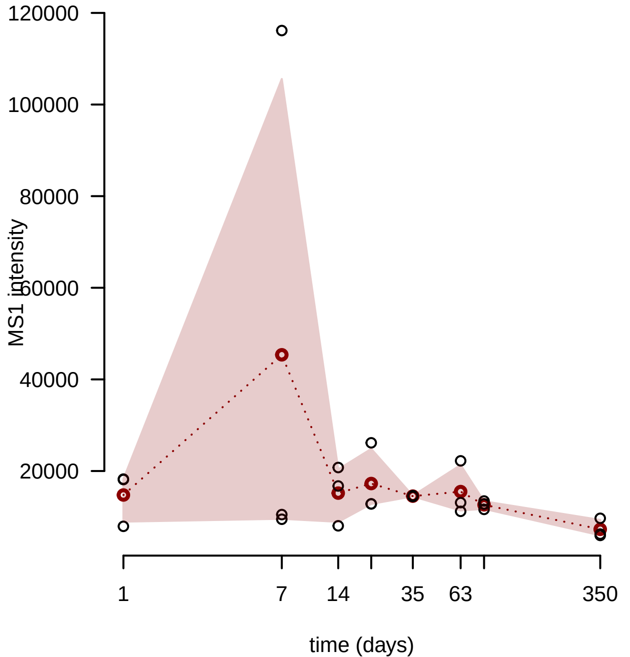

Q9DAW6
GN=Prpf4
pre−mRNA processing factor 4

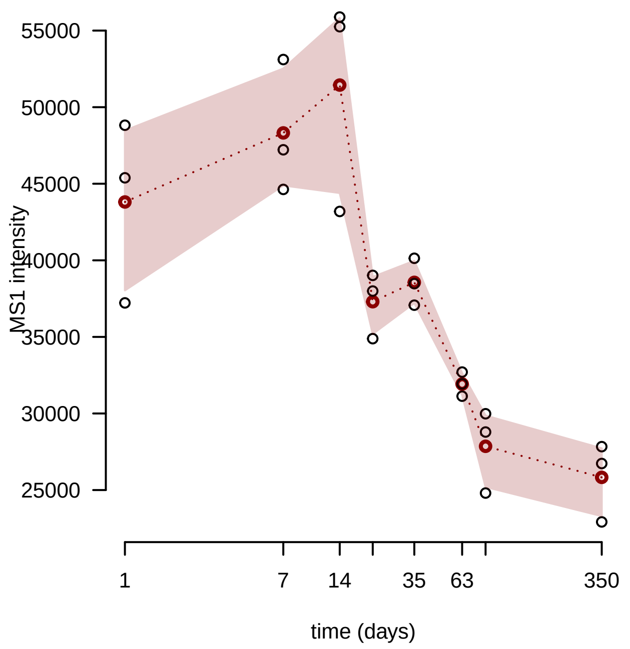

Q9DAW9
GN=Cnn3
calponin 3, acidic

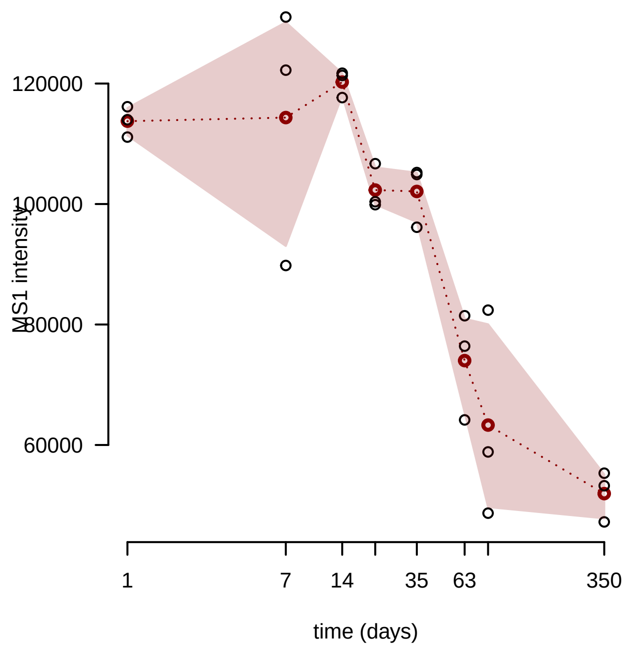

Q9DAX9
GN=Appbp2
amyloid beta precursor protein (cytoplas ...

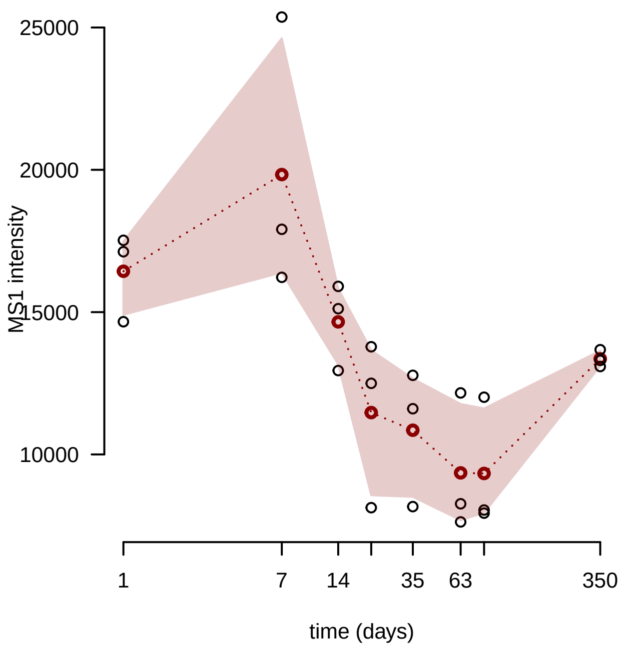

Q9DAZ9
GN=Zfyve19
zinc finger, FYVE domain containing 19

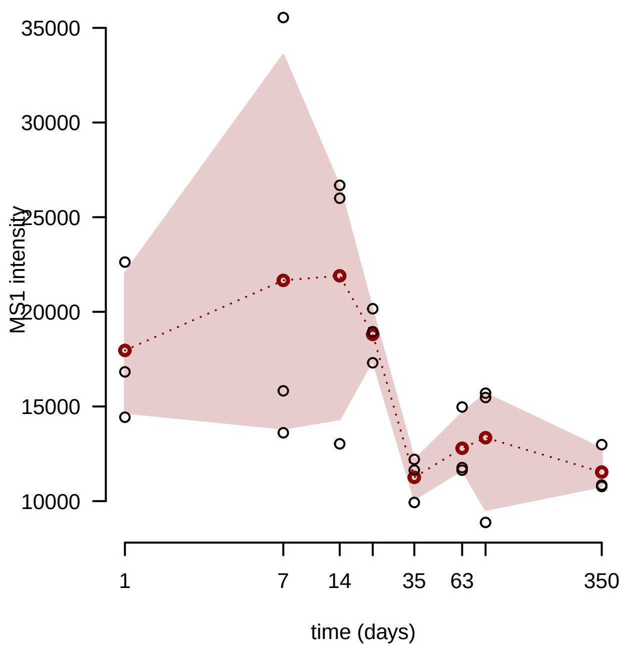

Q9DB00
GN=Gon4l
gon−4−like (C.elegans)

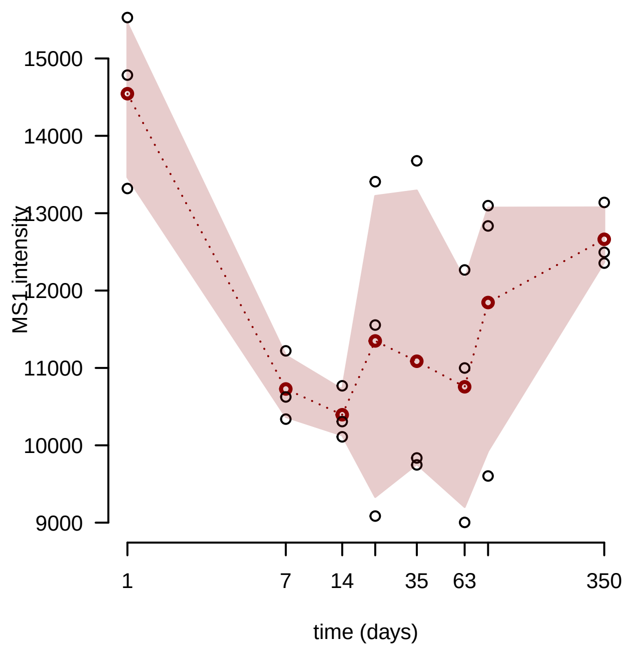

Q9DB05
GN=Napa
N−ethylmaleimide sensitive fusion protei ...

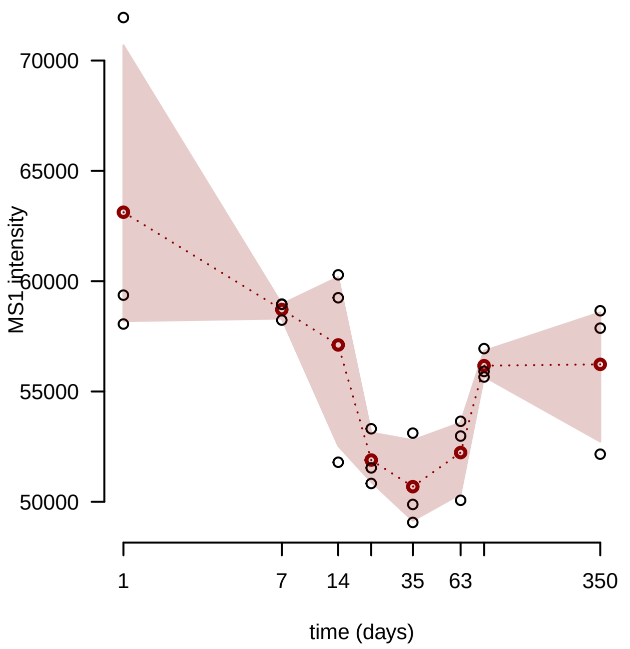

Q9DB07
GN=Ift46
intraflagellar transport 46

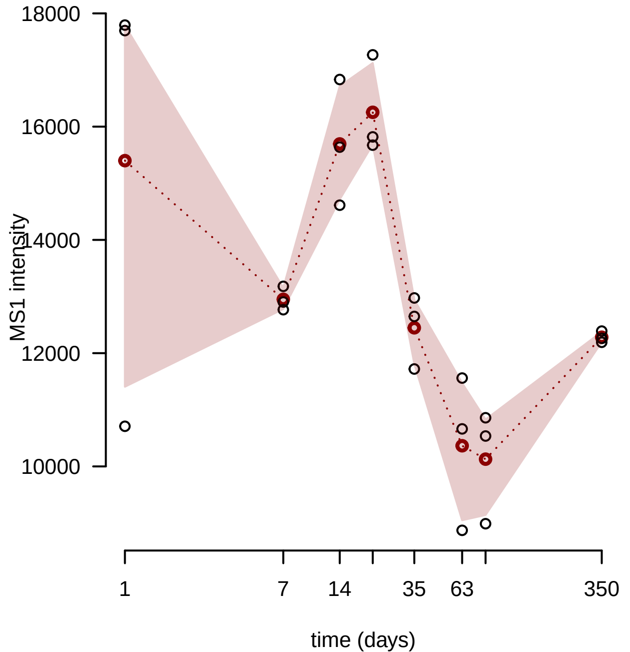

Q9DB10
GN=Smdt1
single−pass membrane protein with aspart ...

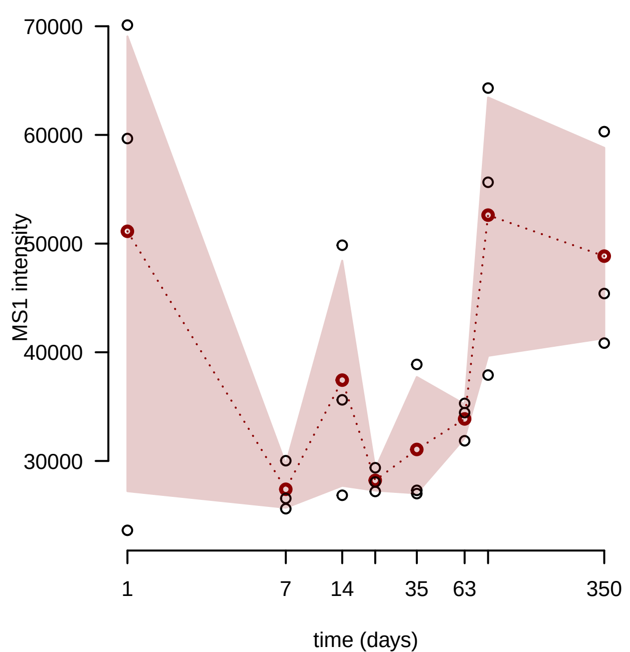

Q9DB15
GN=Mrpl12
mitochondrial ribosomal protein L12

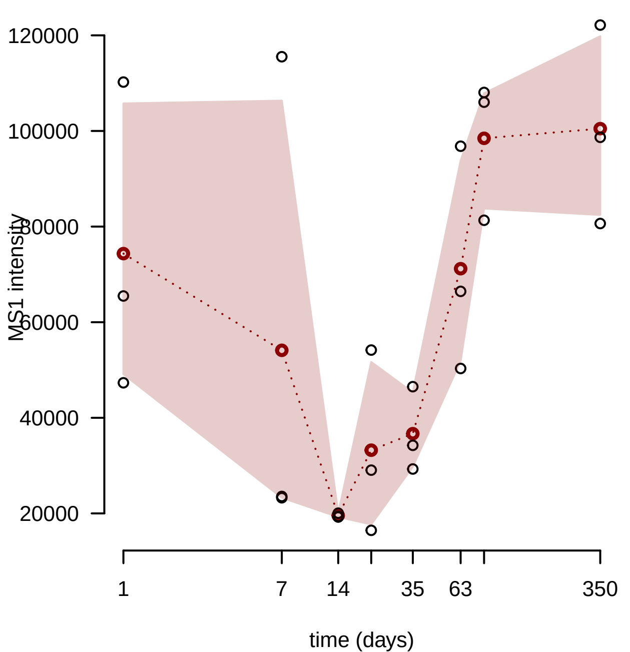

Q9DB16
GN=Cab39l
calcium binding protein 39−like

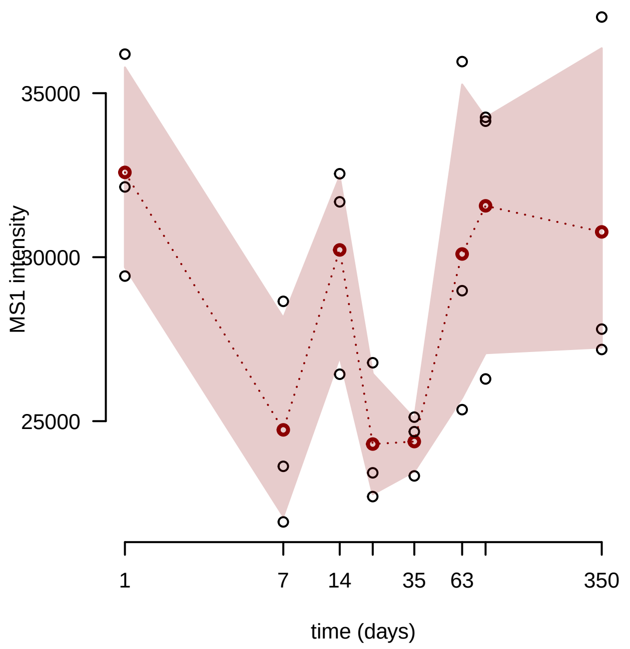

Q9DB20
GN=Atp5po

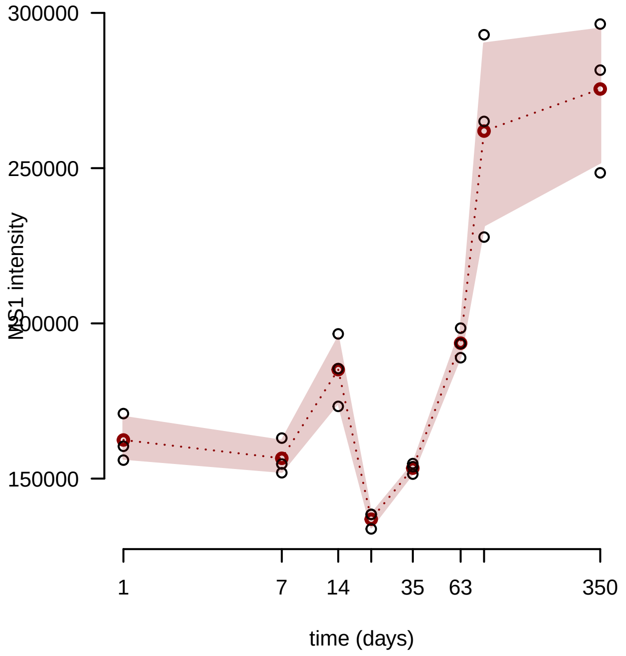

Q9DB25
GN=Alg5
asparagine−linked glycosylation 5 (dolic ...

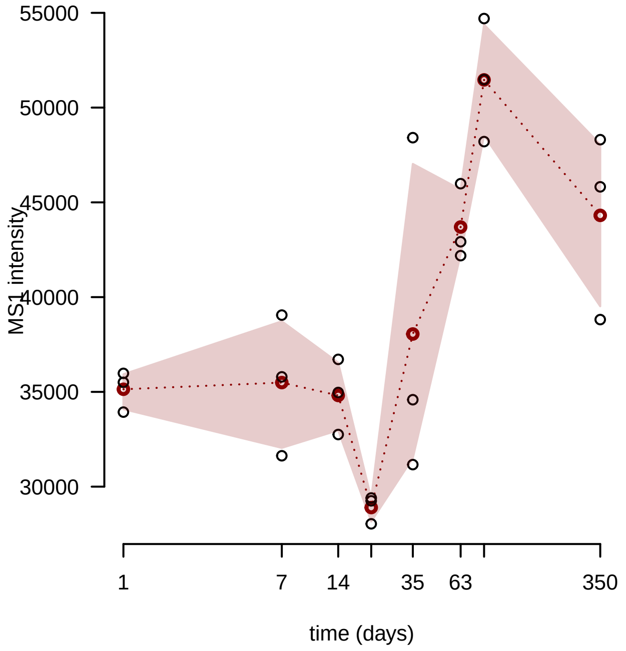

Q9DB26
GN=Phyhd1
phytanoyl−CoA dioxygenase domain contain ...

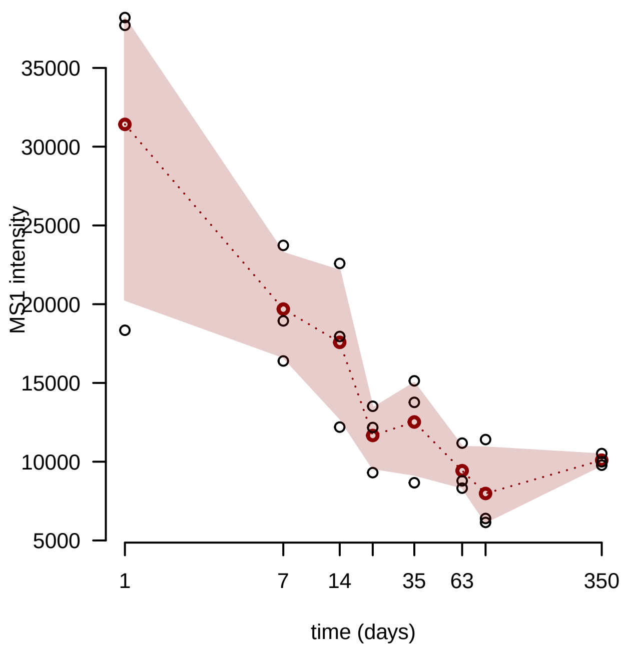

Q9DB27
GN=Mcts1
malignant T cell amplified sequence 1

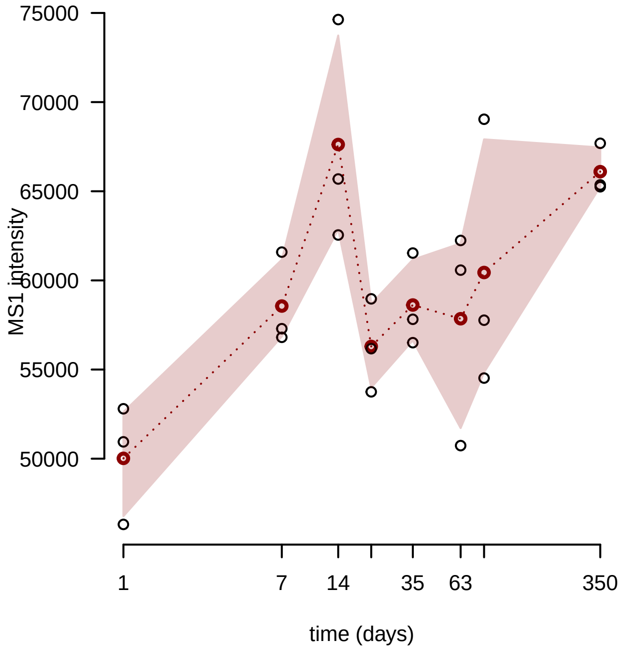

Q9DB28
GN=Pop5
processing of precursor 5, ribonuclease ...

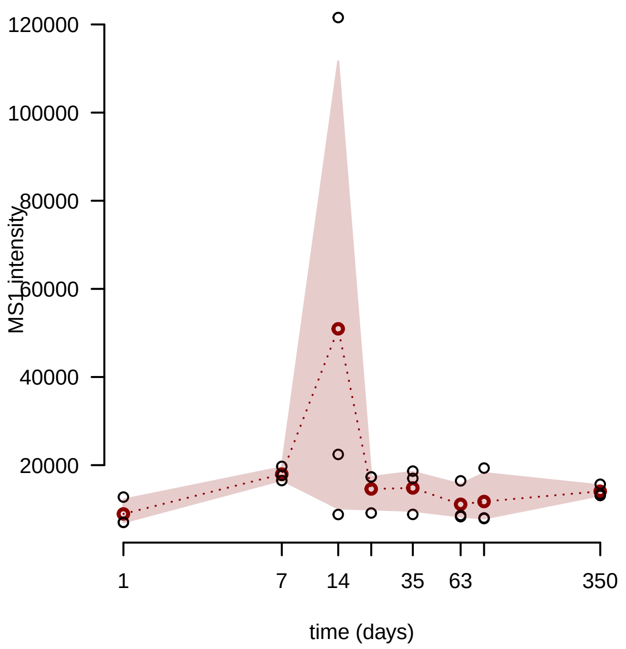

Q9DB29
GN=Iah1
isoamyl acetate−hydrolyzing esterase 1 h ...

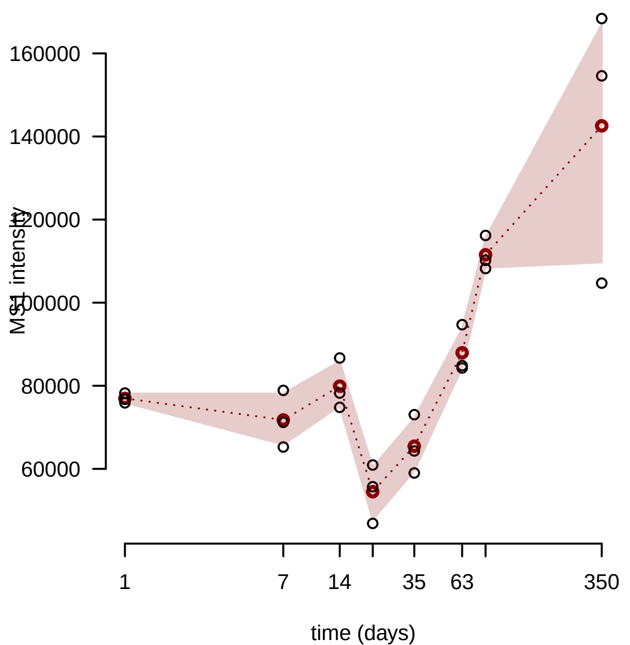

Q9DB30
GN=Phkg2
phosphorylase kinase, gamma 2 (testis)

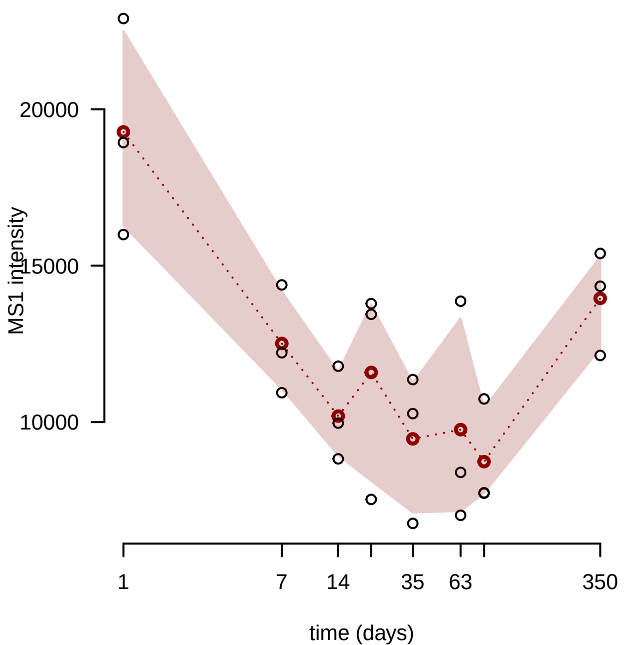

Q9DB32
GN=Haghl
hydroxyacylglutathione hydrolase−like

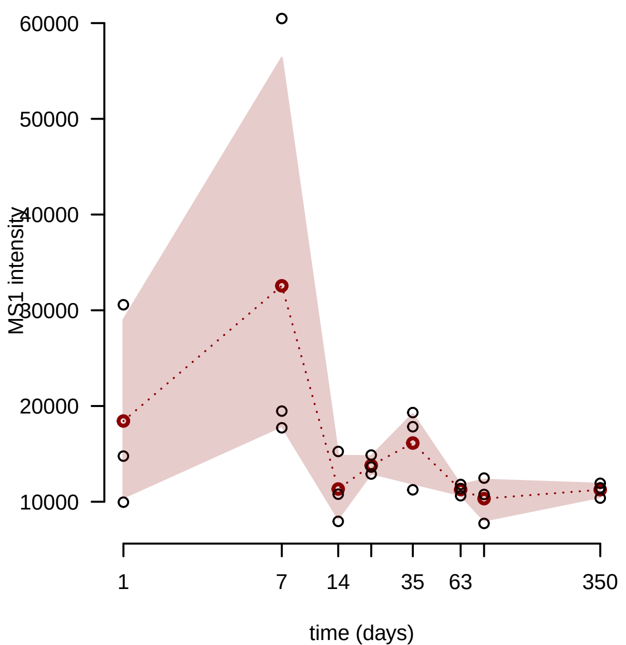

Q9DB34
GN=Chmp2a
charged multivesicular body protein 2A

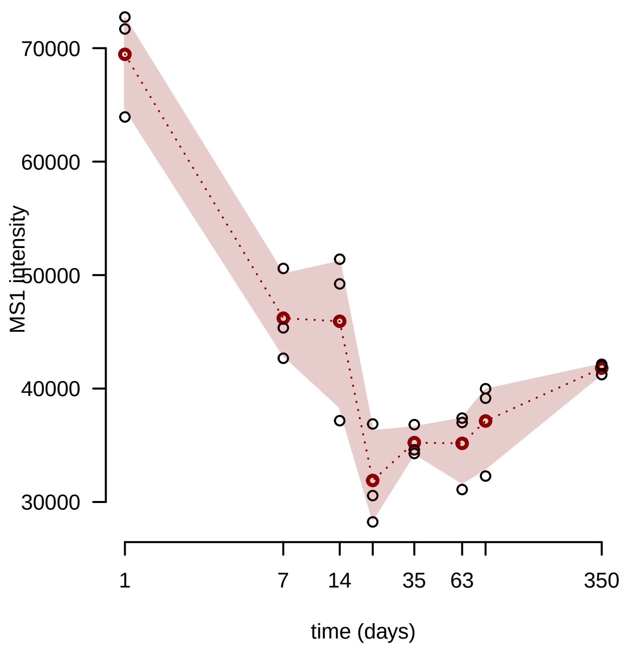

Q9DB38
GN=Znf580

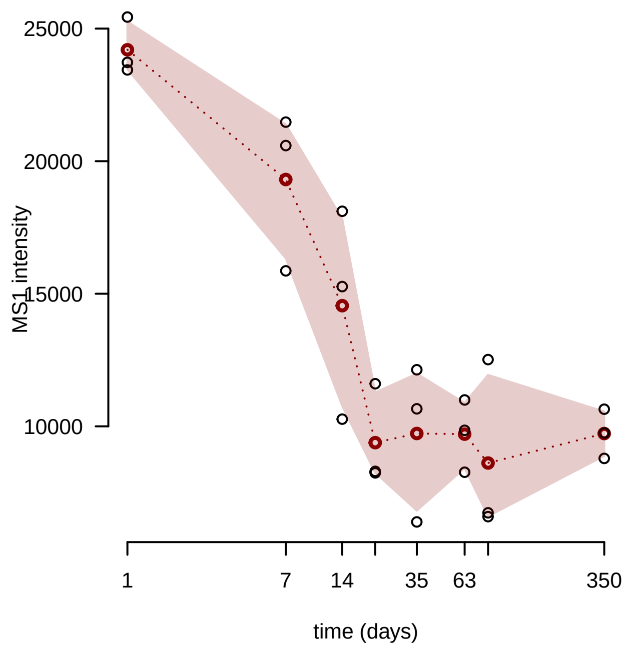

Q9DB40
GN=Med27
mediator complex subunit 27

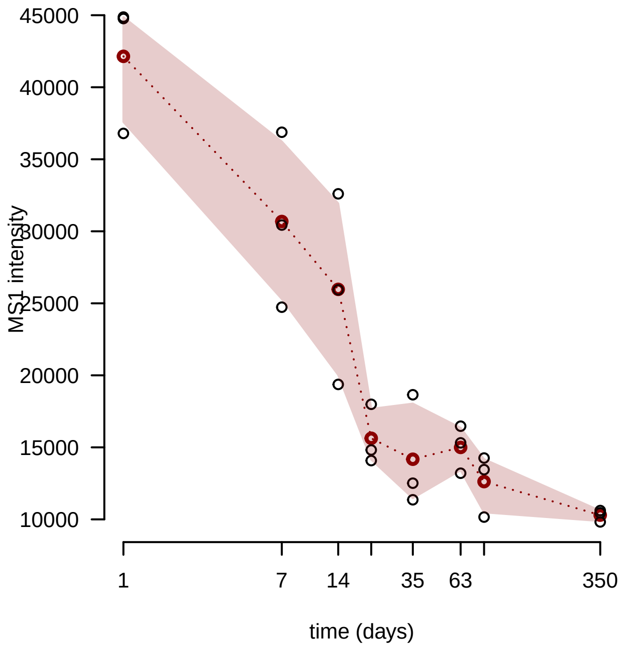

Q9DB41
GN=Slc25a18
solute carrier family 25 (mitochondrial ...

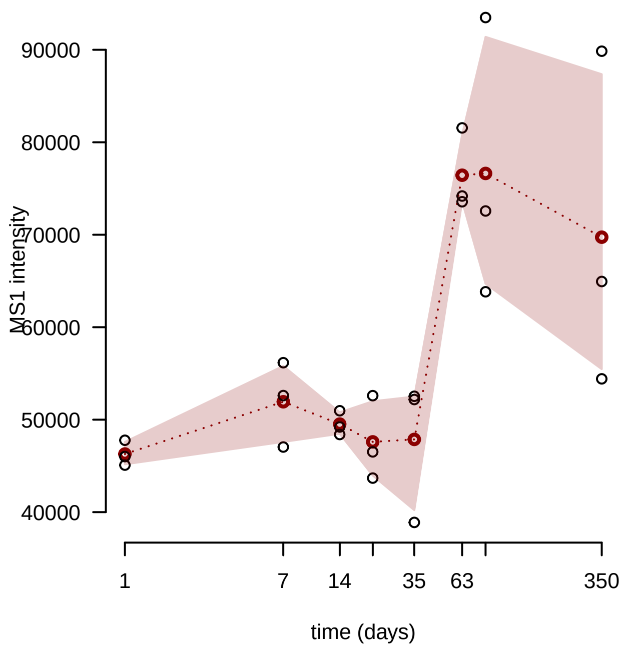

Q9DB42
GN=Znf593

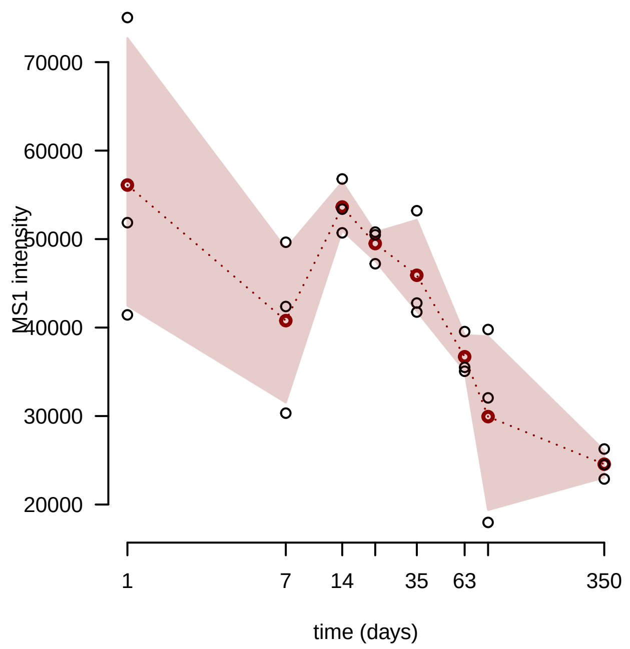

Q9DB43
GN=Zfpl1
zinc finger like protein 1

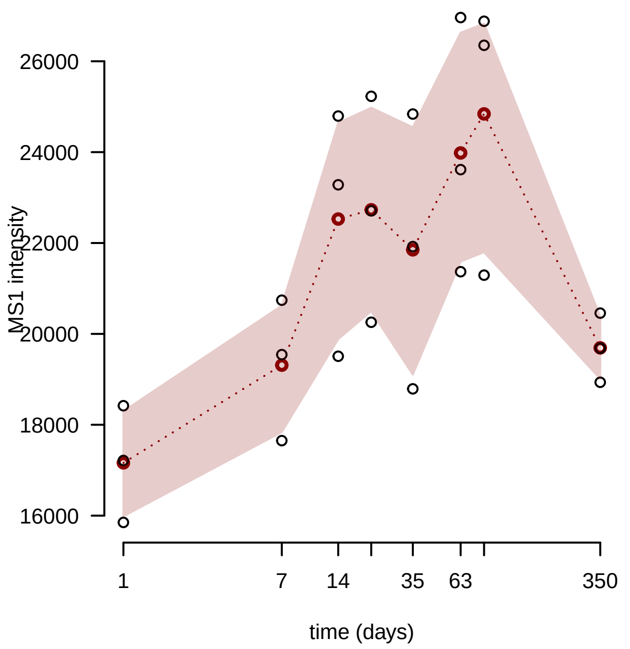

Q9DB50
GN=Ap1s2
adaptor−related protein complex 1, sigma ...

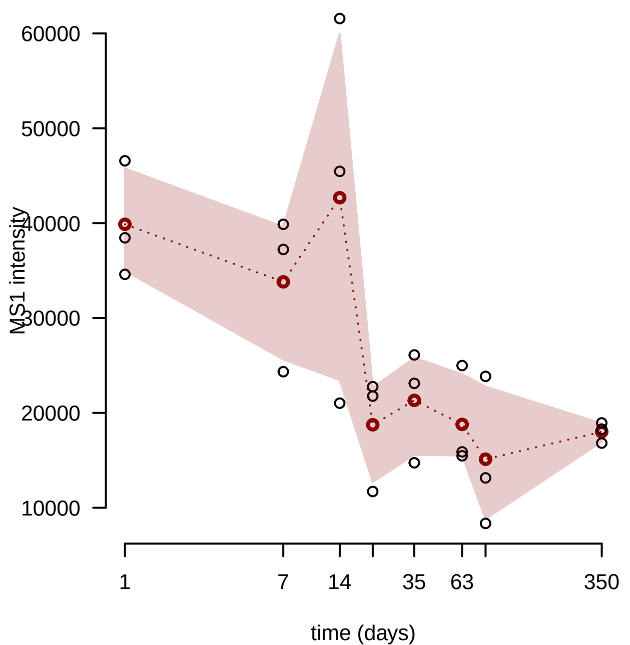

Q9DB52
GN=Fam122a
family with sequence similarity 122, mem ...

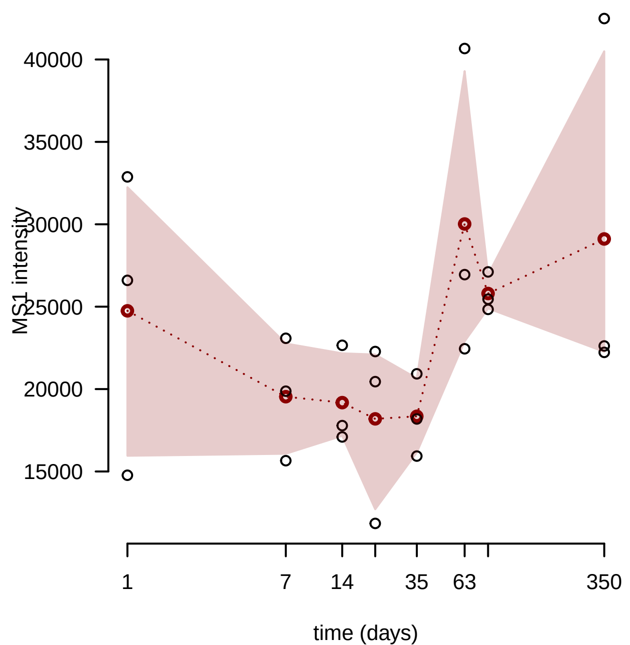

Q9DB60
GN=Prxl2b
peroxiredoxin like 2B

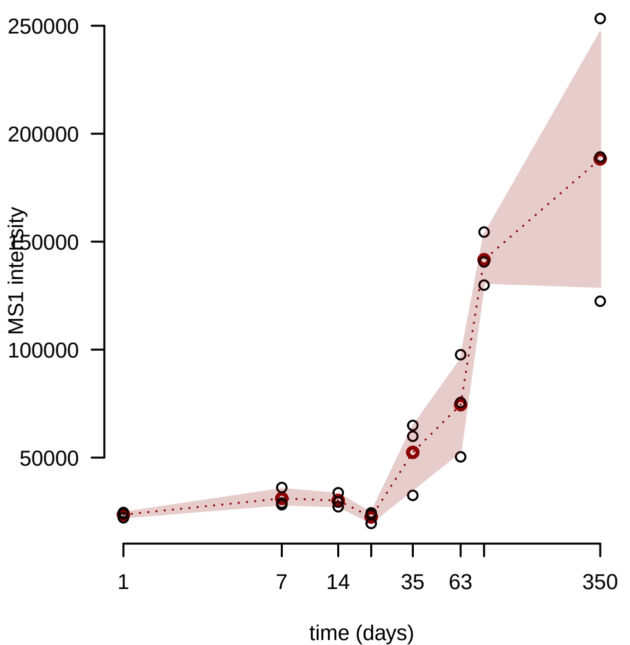

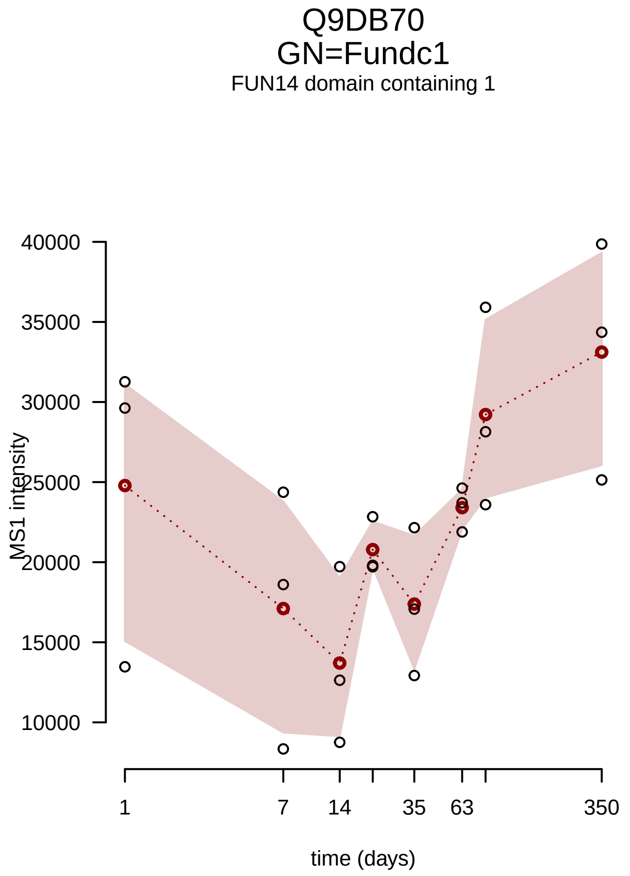
Q9DB70
GN=Fundc1
FUN14 domain containing 1
40000
35000
30000
25000
20000
15000
10000
MS1 intensity
1
7
14
35
63
350
time (days)

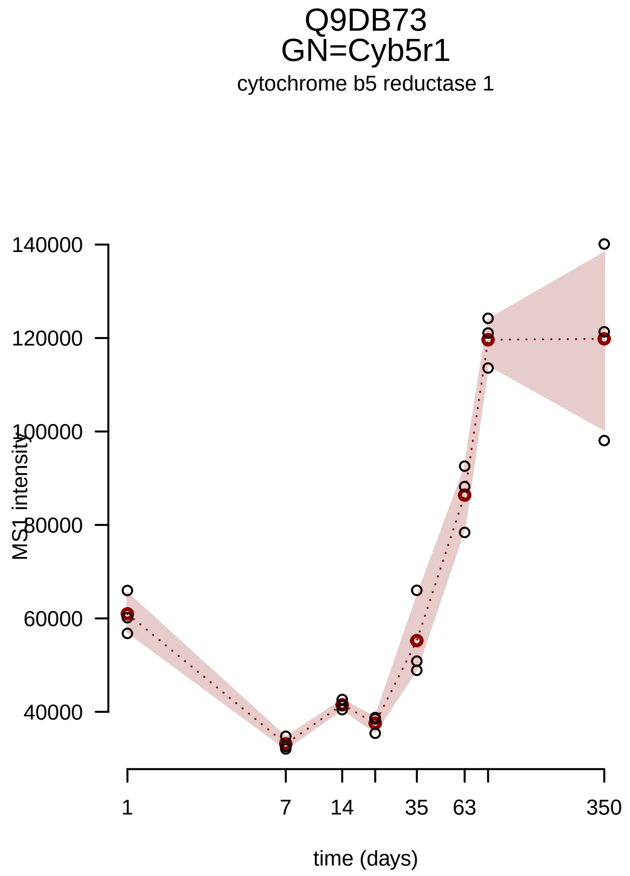
Q9DB73
GN=Cyb5r1
cytochrome b5 reductase 1
140000
120000
100000
80000
60000
40000
MS1 intensity
1
7
14
35
63
350
time (days)

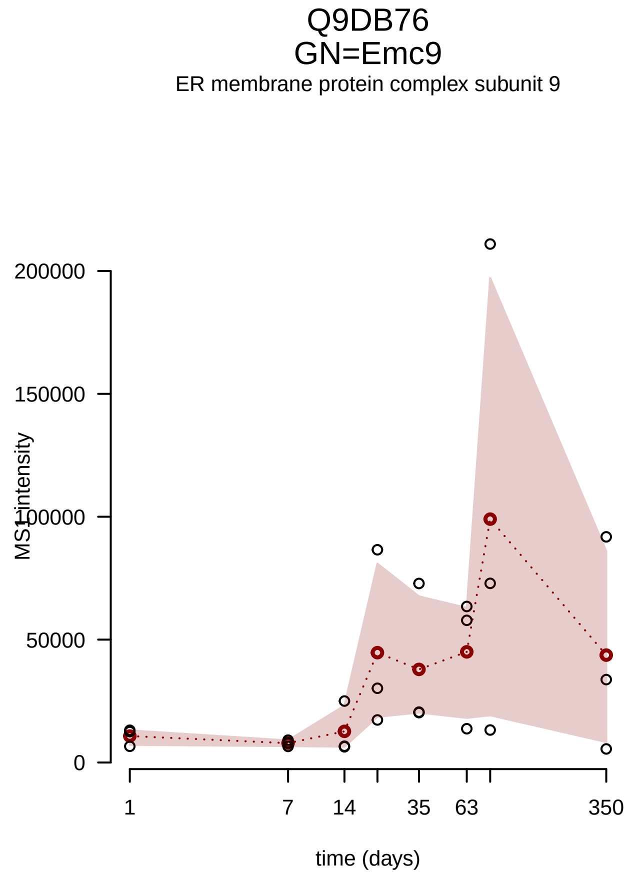
Q9DB76
GN=Emc9
ER membrane protein complex subunit 9
200000
150000
100000
50000
0
MS1 intensity
1
7
14
35
63
350
time (days)

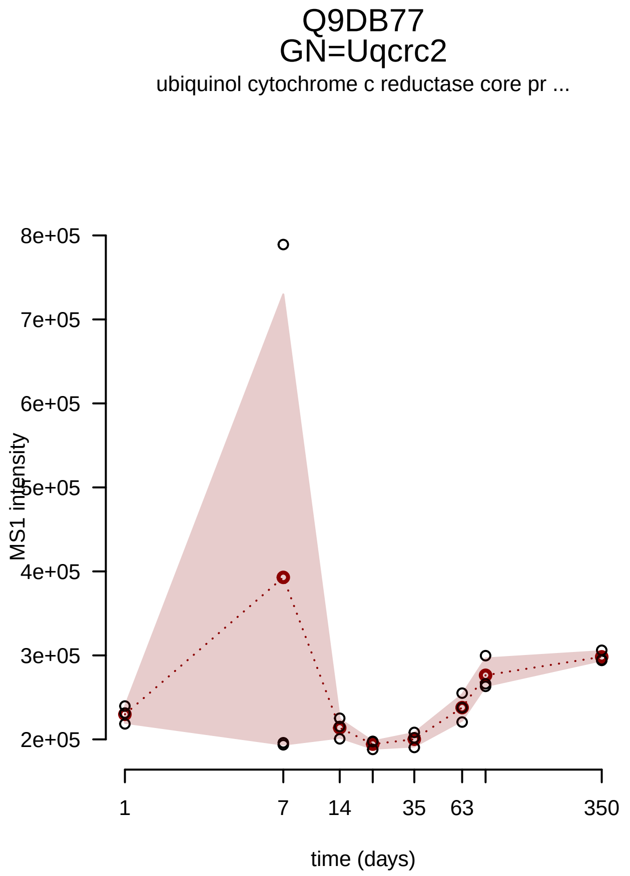
Q9DB77
GN=Uqcrc2
ubiquinol cytochrome c reductase core pr ...
8e+05
7e+05
6e+05
5e+05
4e+05
3e+05
2e+05
MS1 intensity
1
7
14
35
63
350
time (days)

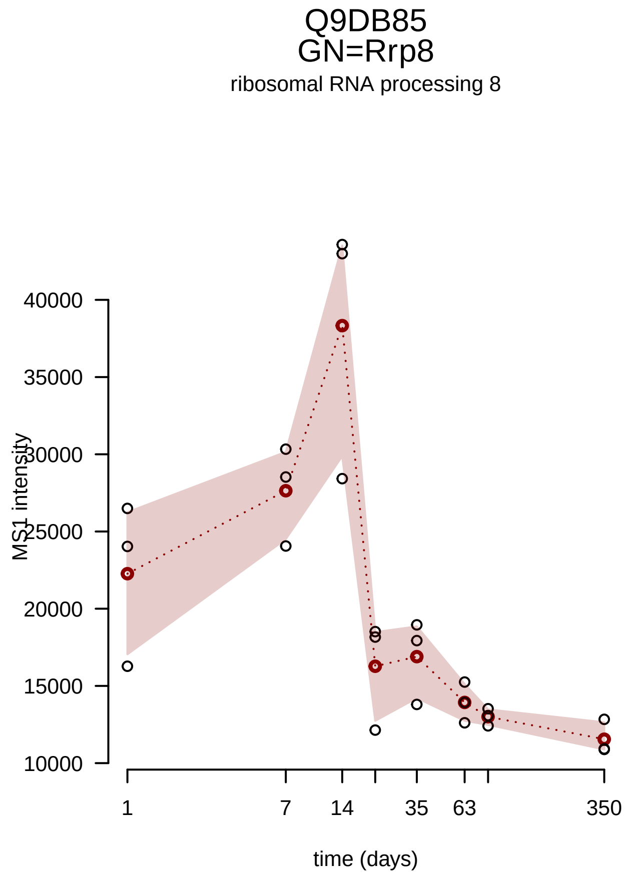
Q9DB85
GN=Rrp8
ribosomal RNA processing 8
40000
35000
30000
25000
20000
15000
10000
MS1 intensity
1
7
14
35
63
350
time (days)

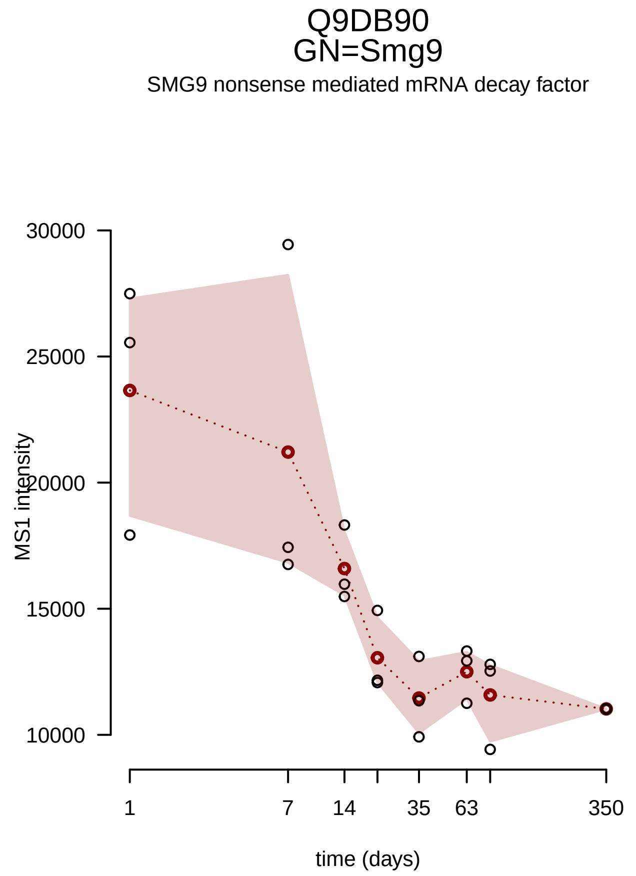
Q9DB90
GN=Smg9
SMG9 nonsense mediated mRNA decay factor
30000
25000
20000
15000
10000
MS1 intensity
1
7
14
35
63
350
time (days)

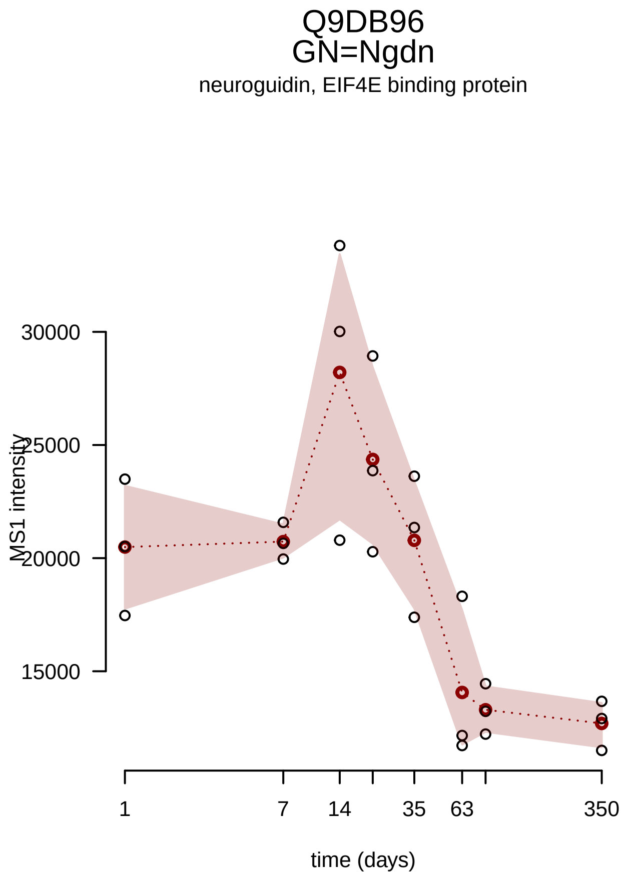
Q9DB96
GN=Ngdn
neuroguidin, EIF4E binding protein
30000
25000
20000
15000
MS1 intensity
1
7
14
35
63
350
time (days)

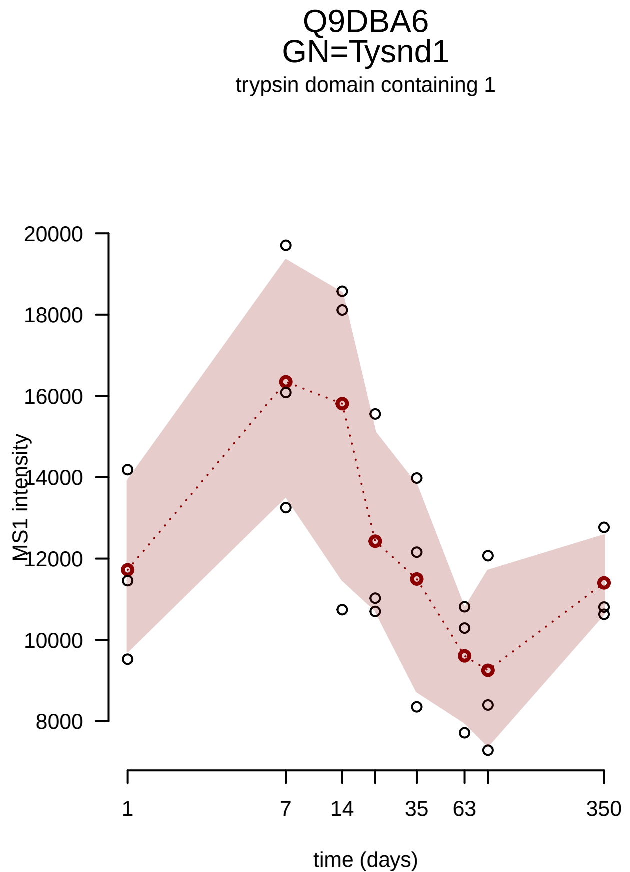
Q9DBA6
GN=Tysnd1
trypsin domain containing 1
20000
18000
16000
14000
12000
10000
8000
MS1 intensity
1
7
14
35
63
350
time (days)

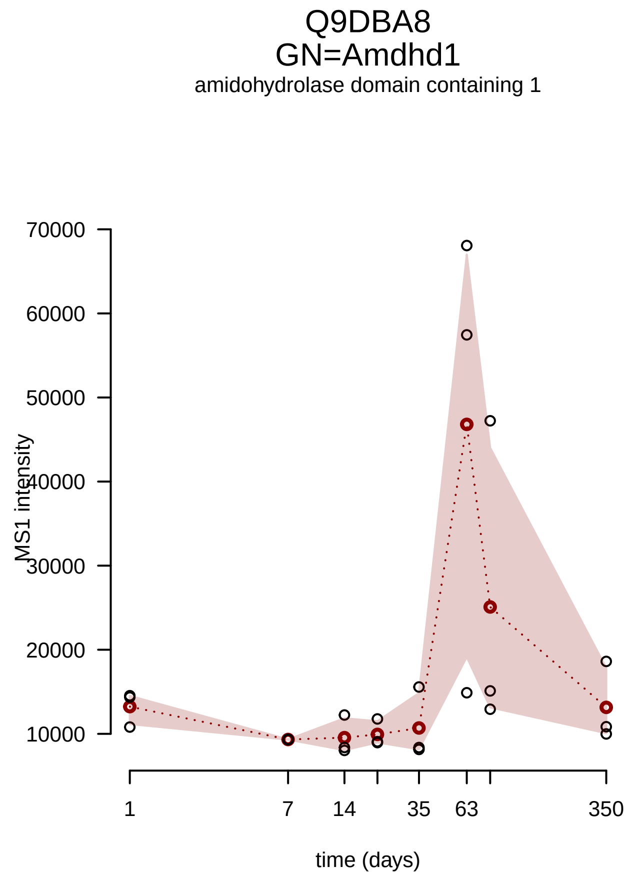
Q9DBA8
GN=Amdhd1
amidohydrolase domain containing 1
70000
60000
50000
40000
30000
20000
10000
MS1 intensity
1
7
14
35
63
350
time (days)

Q9DBA9
GN=Gtf2h1
general transcription factor II H, polyp ...

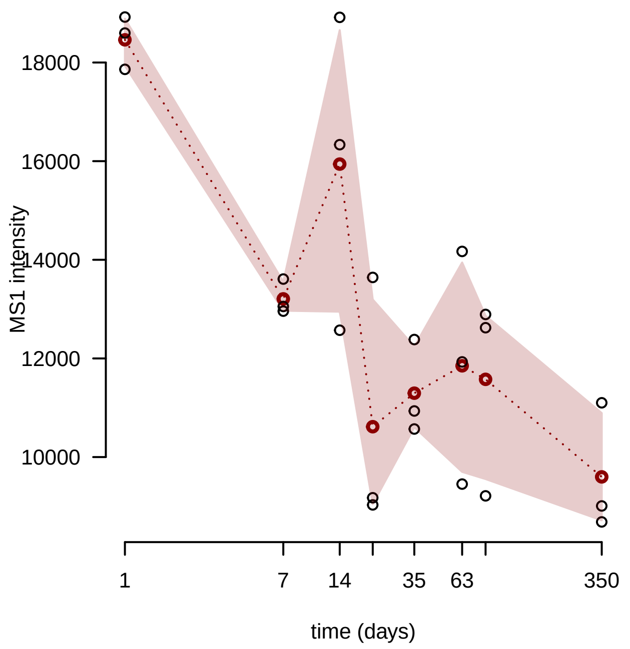

Q9DBB4
GN=Naa16
N(alpha)−acetyltransferase 16, NatA auxi ...

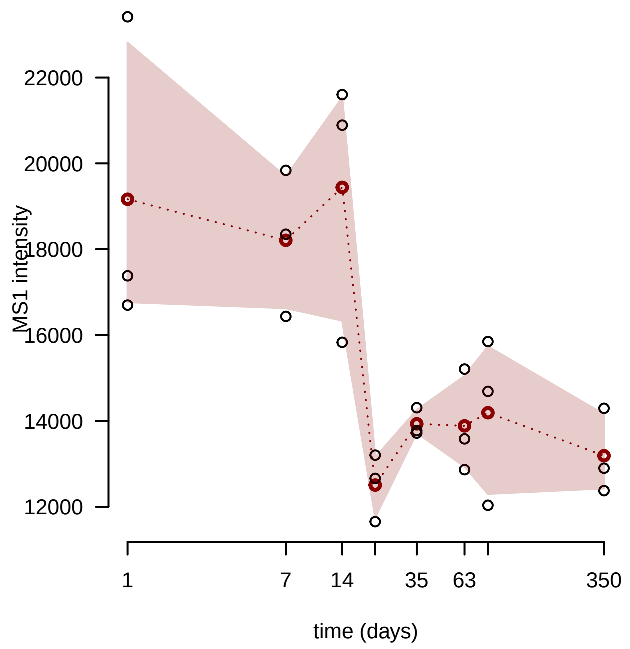

Q9DBB5
GN=Eif4e3
eukaryotic translation initiation factor ...

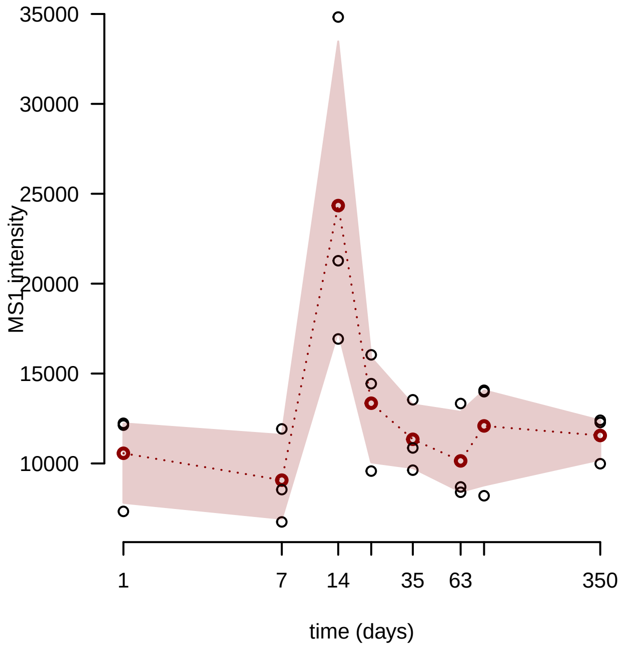

Q9DBB8
GN=Dhdh
dihydrodiol dehydrogenase (dimeric)

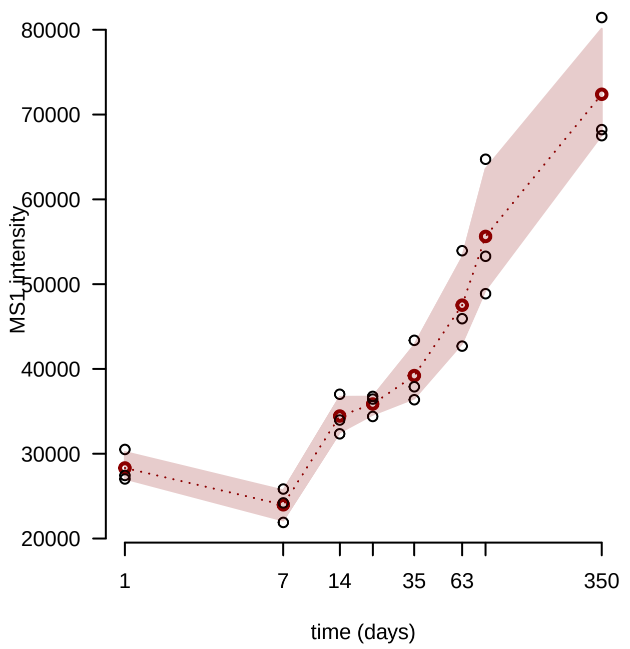

Q9DBB9
GN=Cpn2
carboxypeptidase N, polypeptide 2

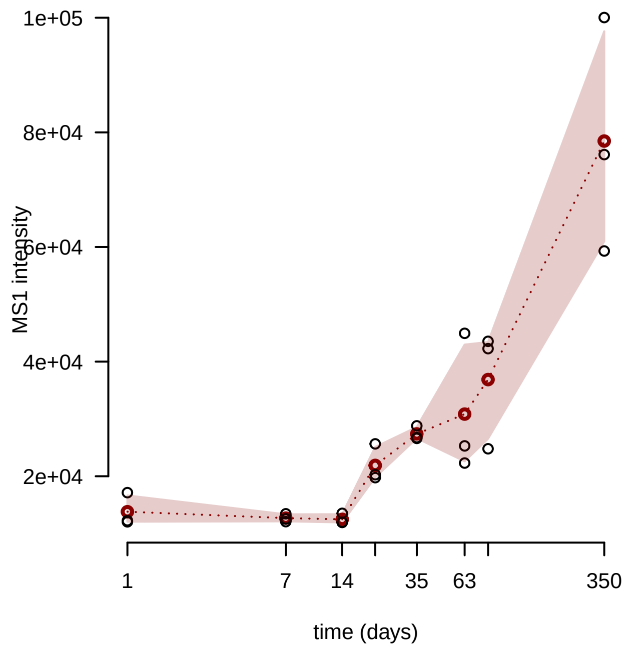

Q9DBC0
GN=Selenoo
selenoprotein O

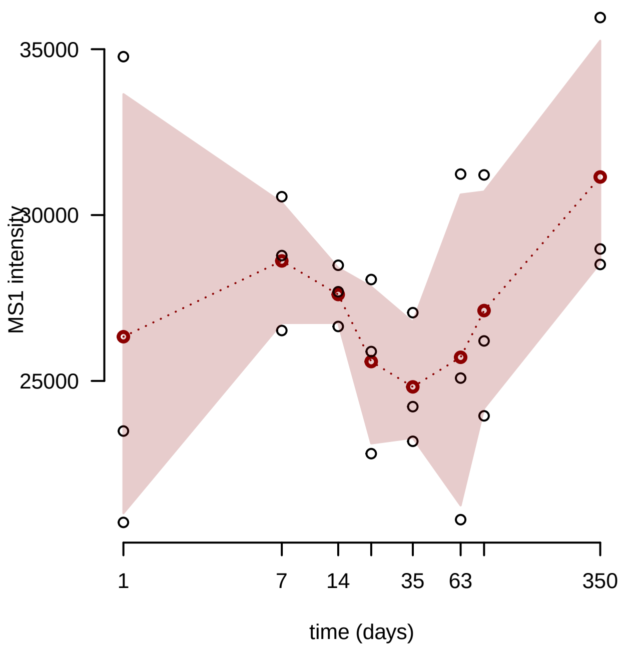

Q9DBC3
GN=Cmtr1
cap methyltransferase 1

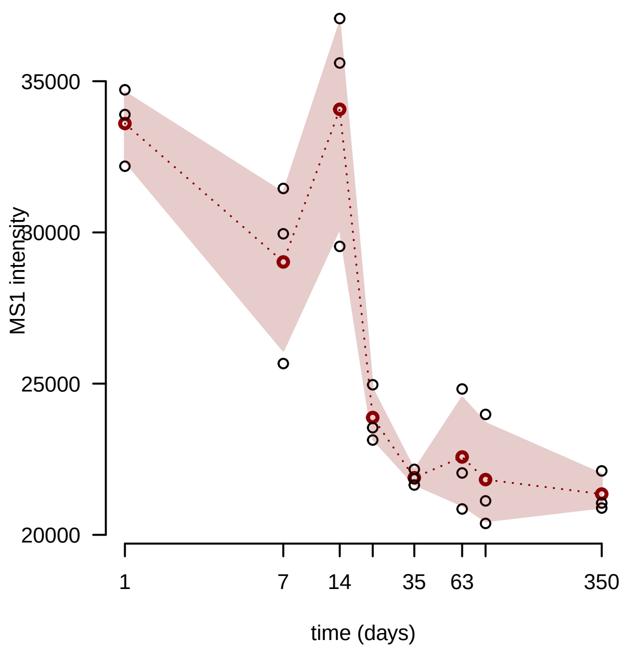

Q9DBC7
GN=Prkar1a
protein kinase, cAMP dependent regulator ...

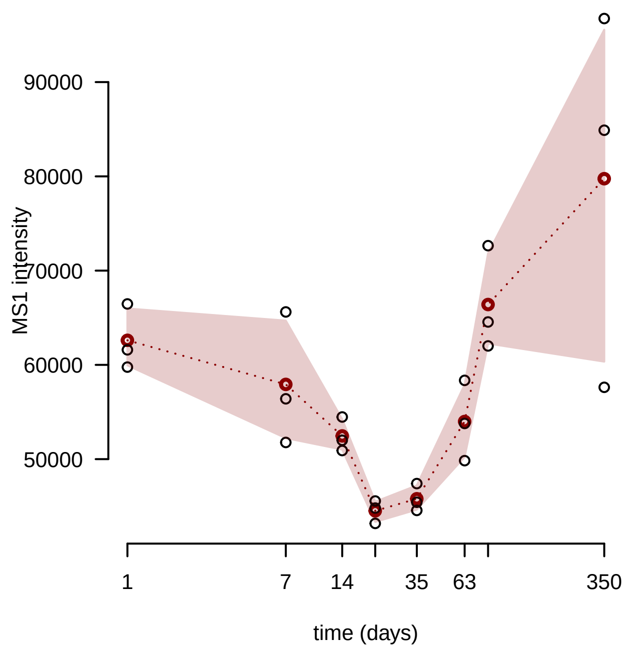

Q9DBD0
GN=Ica

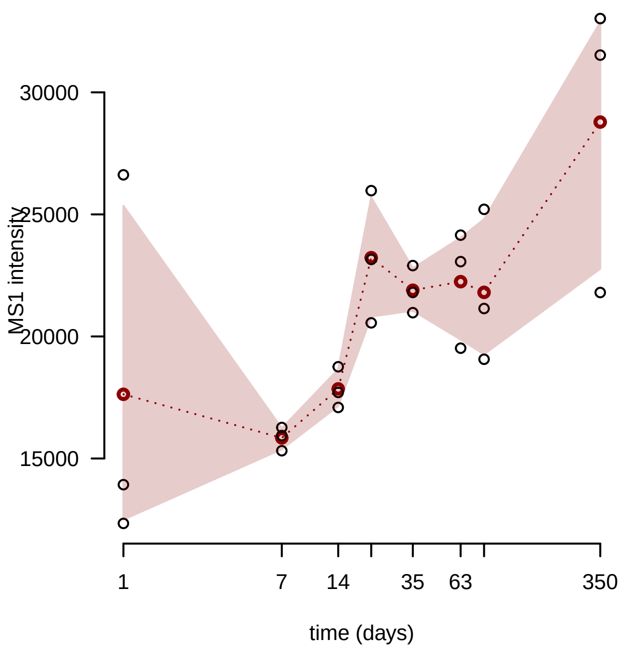

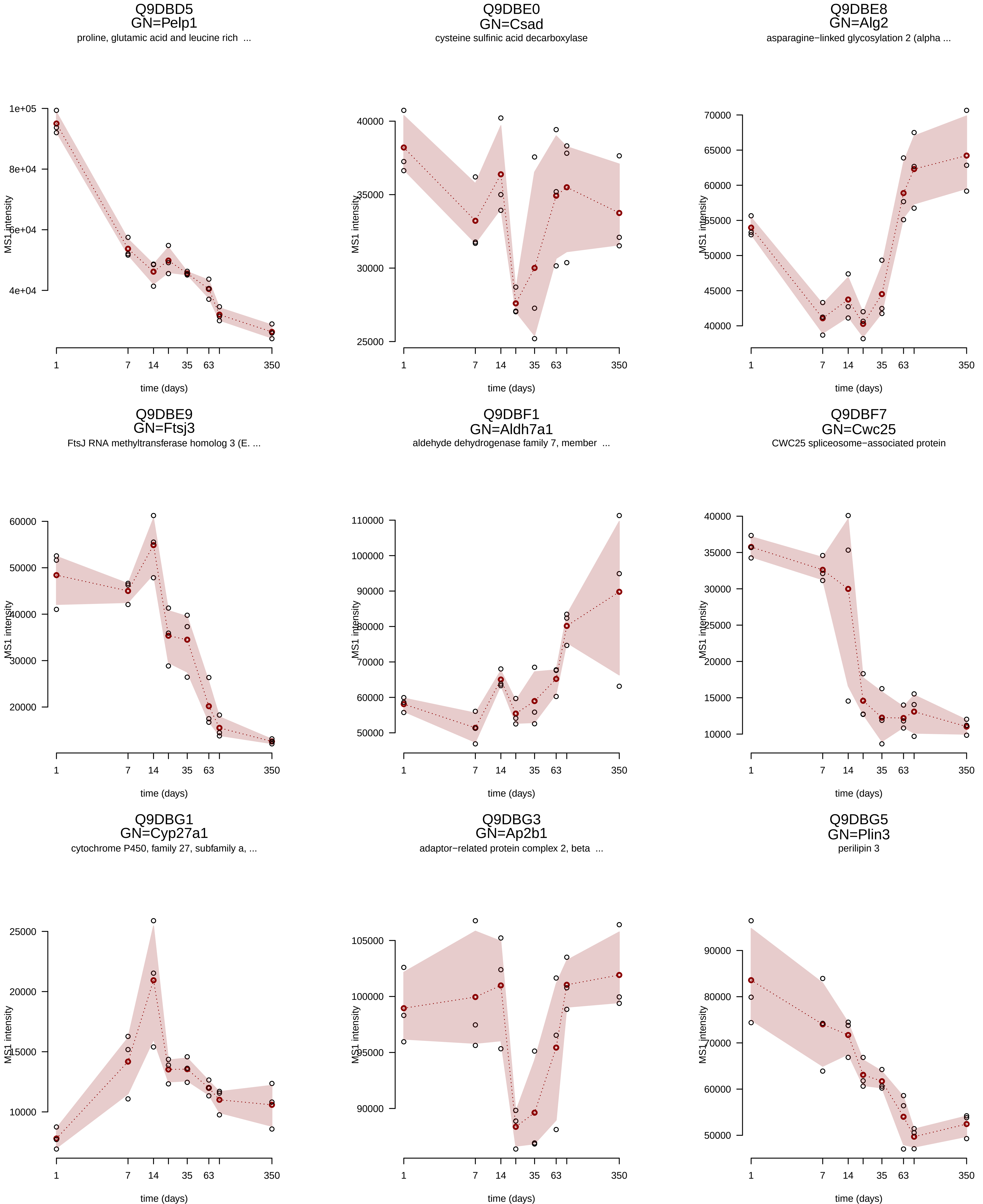
Q9DBD5
GN=Pelp1
proline, glutamic acid and leucine rich ...
1e+05
8e+04
6e+04
4e+04
MS1 intensity
1
7
14
35
63
350
time (days)
Q9DBE0
GN=Csad
cysteine sulfinic acid decarboxylase
40000
35000
30000
25000
MS1 intensity
1
7
14
35
63
350
time (days)
Q9DBE8
GN=Alg2
asparagine−linked glycosylation 2 (alpha ...
70000
65000
60000
55000
50000
45000
40000
MS1 intensity
1
7
14
35
63
350
time (days)
Q9DBE9
GN=Ftsj3
FtsJ RNA methyltransferase homolog 3 (E. ...
60000
50000
40000
30000
20000
MS1 intensity
1
7
14
35
63
350
time (days)
Q9DBF1
GN=Aldh7a1
aldehyde dehydrogenase family 7, member ...
110000
100000
90000
80000
70000
60000
50000
MS1 intensity
1
7
14
35
63
350
time (days)
Q9DBF7
GN=Cwc25
CWC25 spliceosome−associated protein
40000
35000
30000
25000
20000
15000
10000
MS1 intensity
1
7
14
35
63
350
time (days)
Q9DBG1
GN=Cyp27a1
cytochrome P450, family 27, subfamily a, ...
25000
20000
15000
10000
MS1 intensity
1
7
14
35
63
350
time (days)
Q9DBG3
GN=Ap2b1
adaptor−related protein complex 2, beta ...
105000
100000
95000
90000
MS1 intensity
1
7
14
35
63
350
time (days)
Q9DBG5
GN=Plin3
perilipin 3
90000
80000
70000
60000
50000
MS1 intensity
1
7
14
35
63
350
time (days)

Q9DBG6
GN=Rpn2
ribophorin II

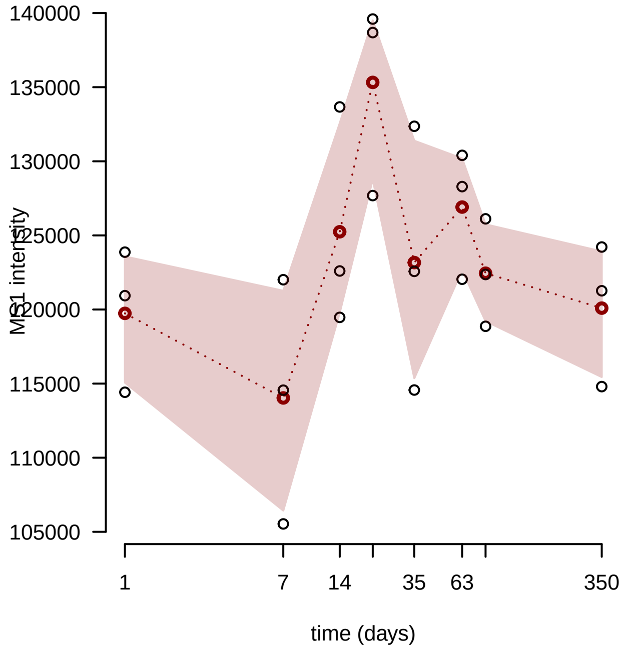

Q9DBG7
GN=Srpra

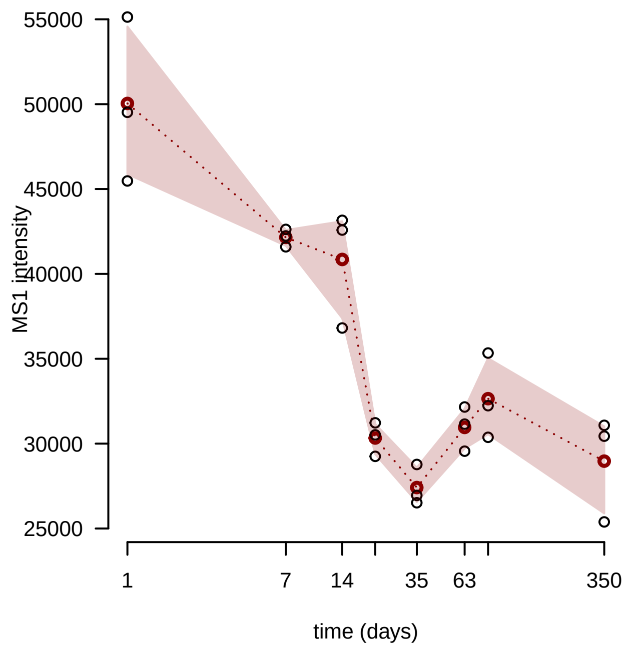

Q9DBG9
GN=Tax1bp3
Tax1 (human T cell leukemia virus type I ...

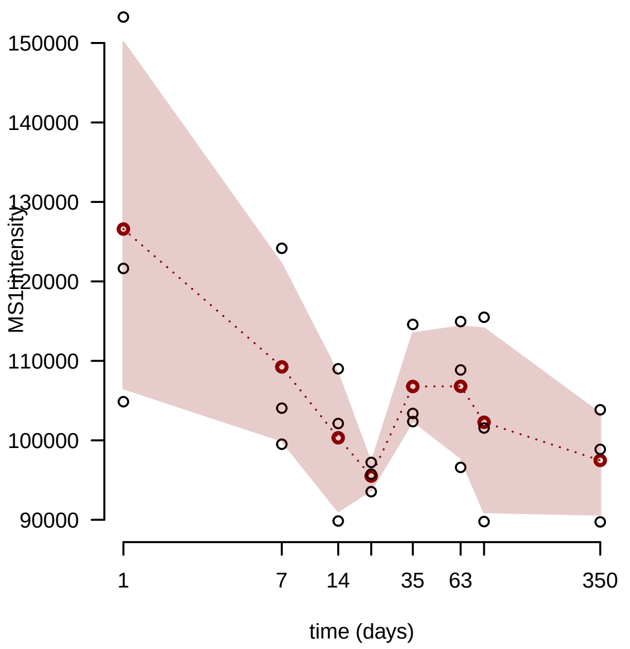

Q9DBH0
GN=Wwp2
WW domain containing E3 ubiquitin protei ...

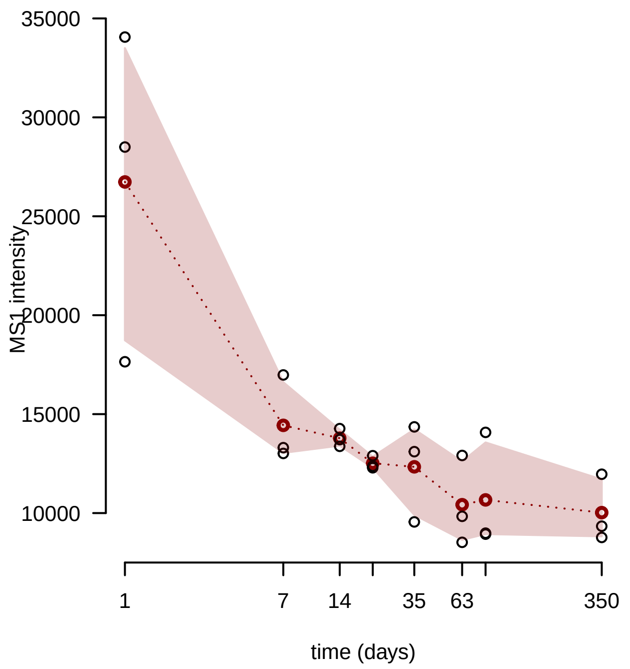

Q9DBH5
GN=Lman2
lectin, mannose−binding 2

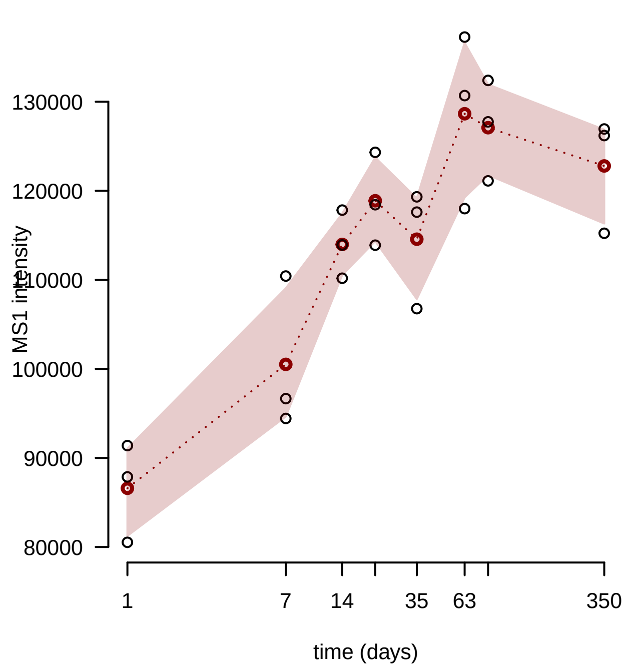

Q9DBJ1
GN=Pgam1
phosphoglycerate mutase 1

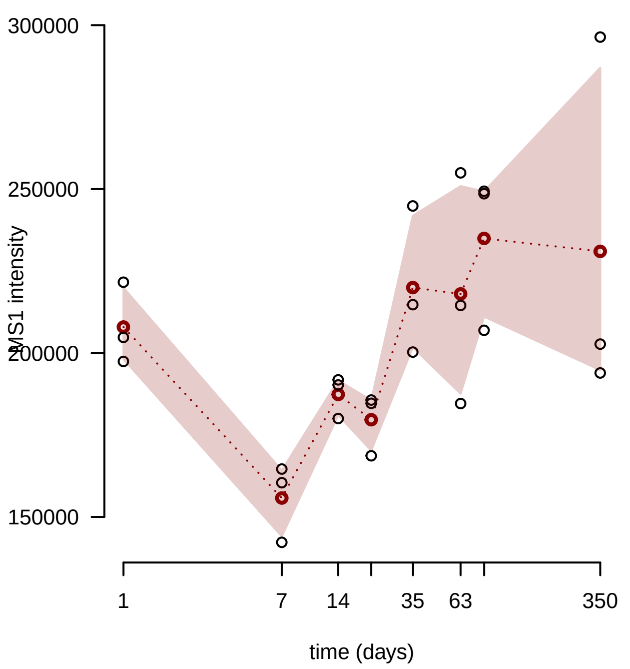

Q9DBJ3
GN=Baiap2l1
BAI1−associated protein 2−like 1

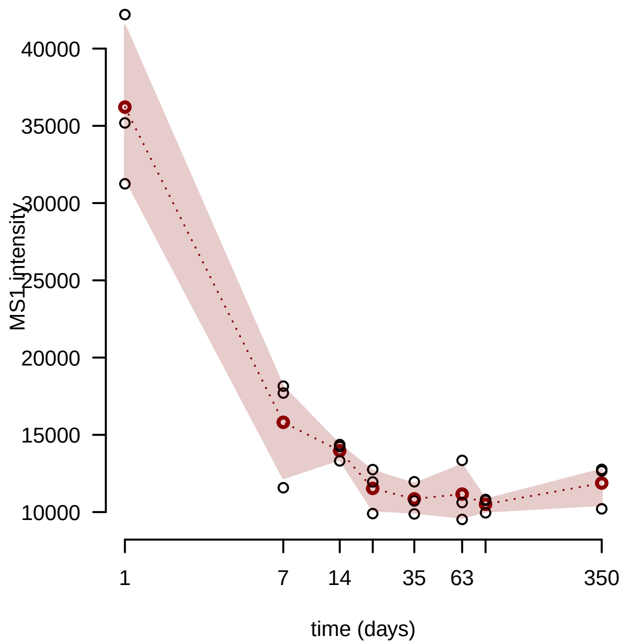

Q9DBK0
GN=Acot12
acyl−CoA thioesterase 12

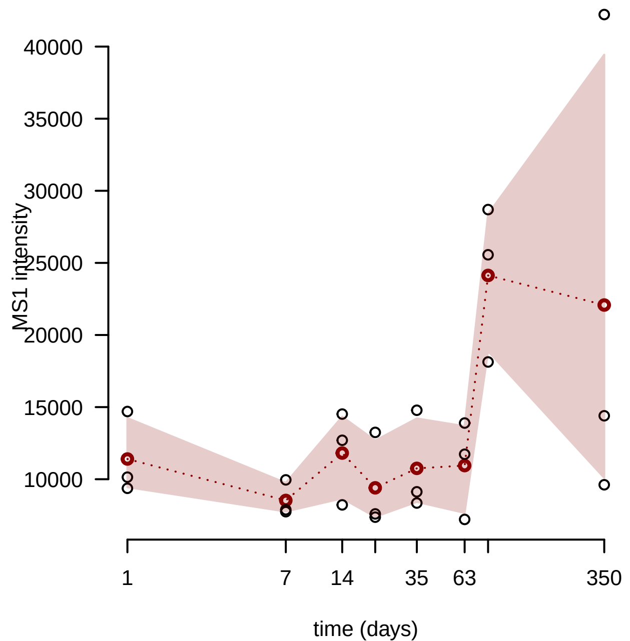

Q9DBL1
GN=Acadsb
acyl−Coenzyme A dehydrogenase, short/bra ...

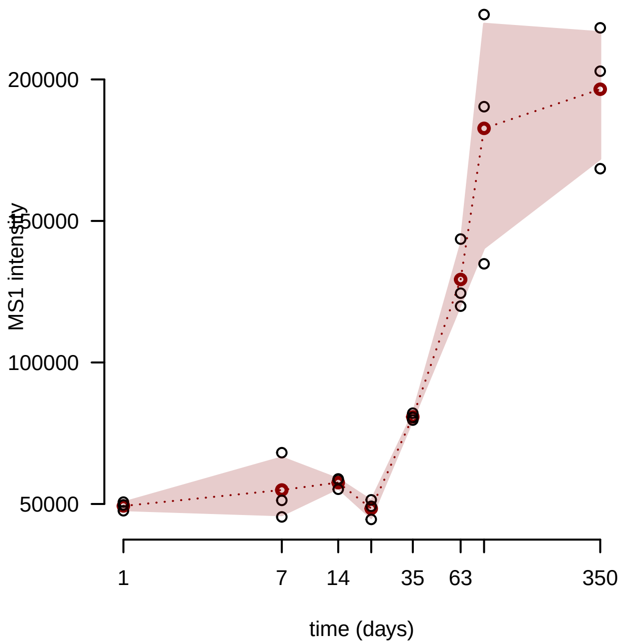

Q9DBL2
GN=Gdap2
ganglioside−induced differentiation−asso ...

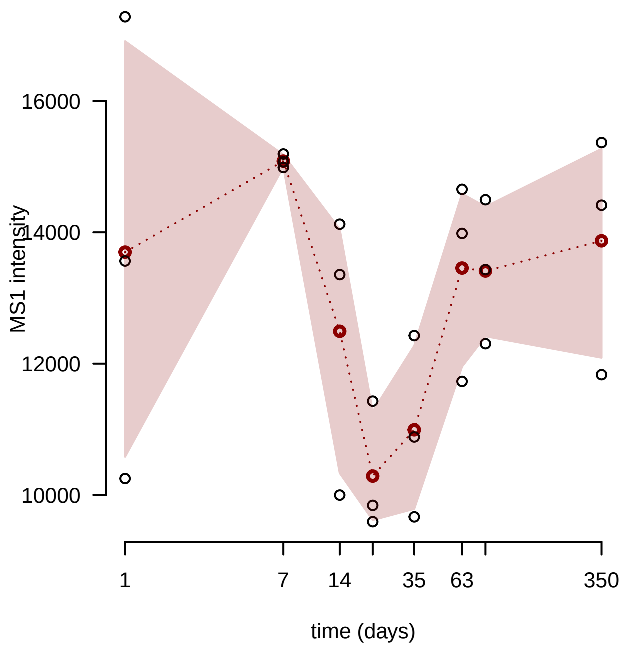

Q9DBL7
GN=Coasy
Coenzyme A synthase

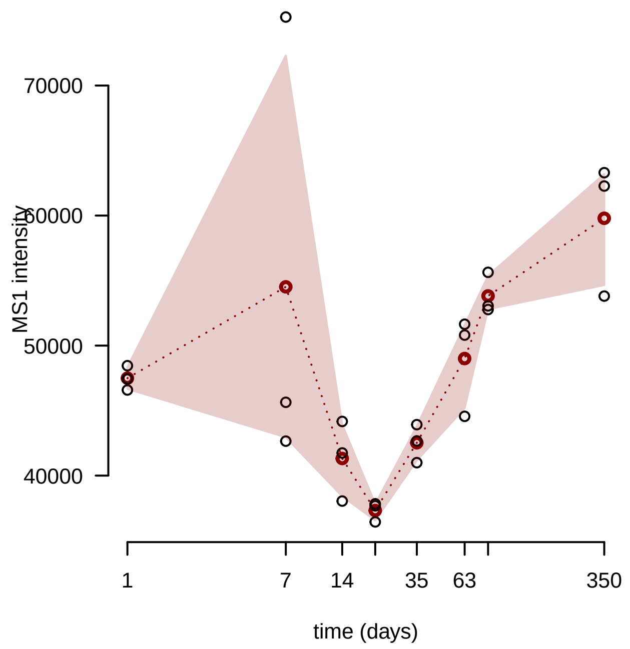

Q9DBL9
GN=Abhd5
abhydrolase domain containing 5

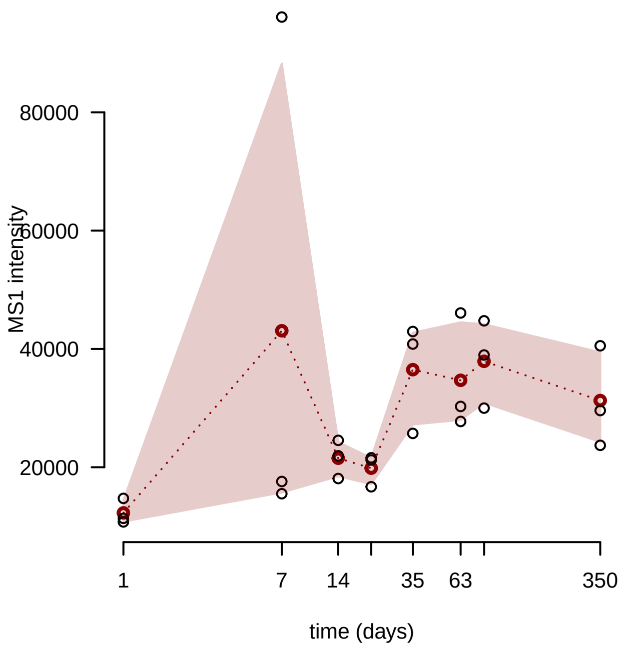

Q9DBM1
GN=Gpatch1
G patch domain containing 1

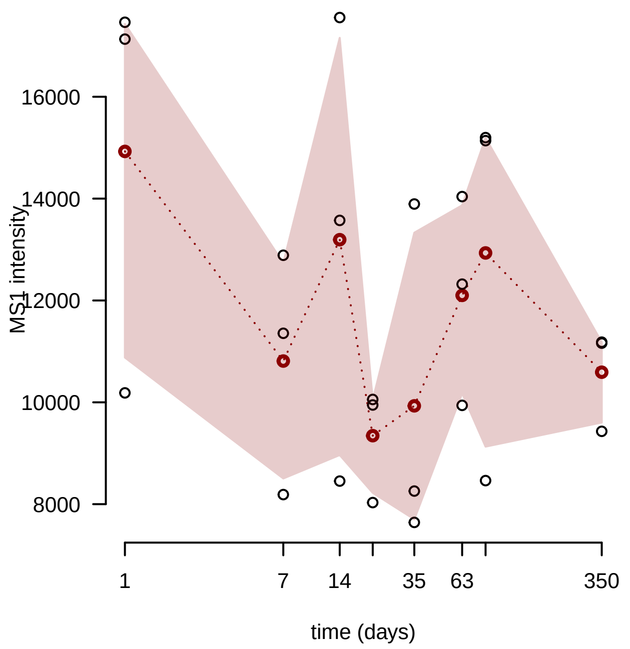

Q9DBM2
GN=Ehhadh
enoyl−Coenzyme A, hydratase/3−hydroxyacy ...

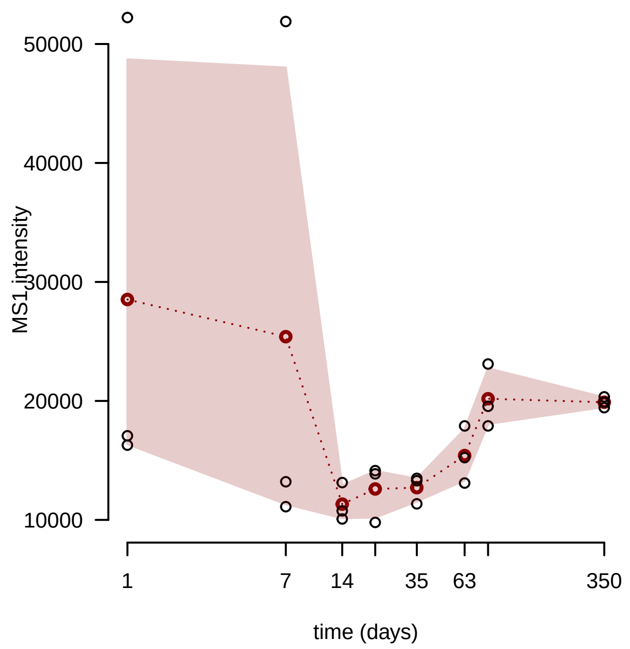

Q9DBN4
GN=P33monox

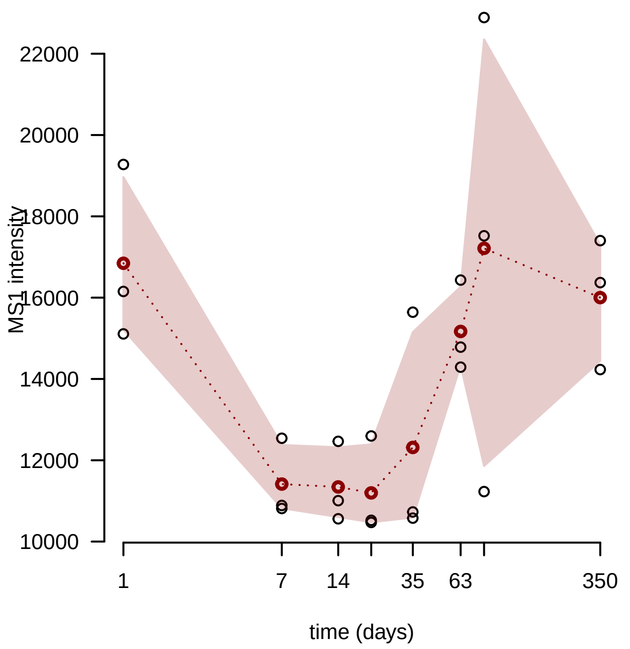

Q9DBN5
GN=Lonp2
lon peptidase 2, peroxisomal

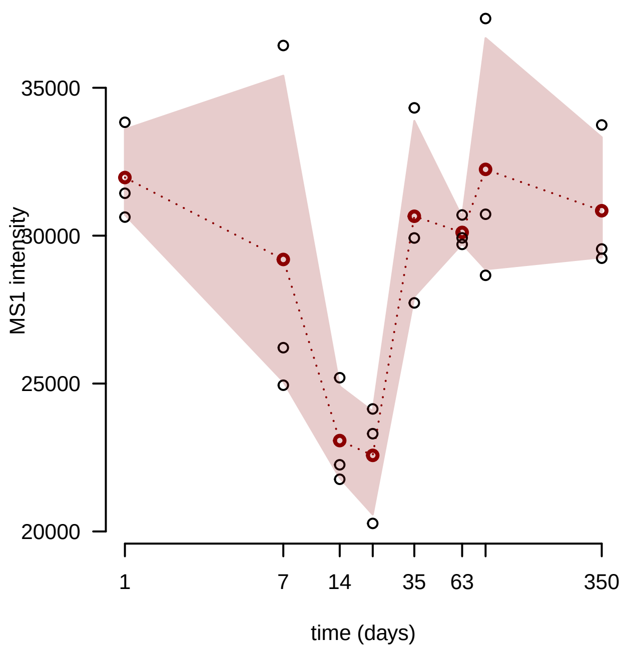

Q9DBN9
GN=Ddx59
DEAD box helicase 59

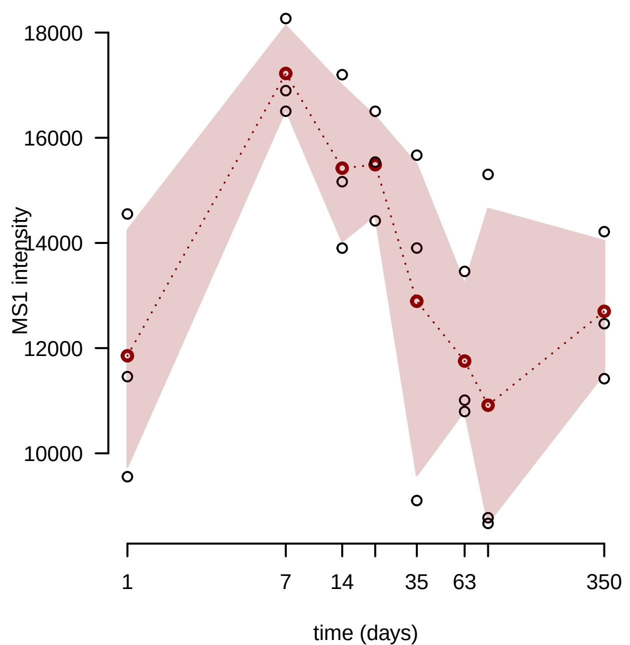

Q9DBP5
GN=Cmpk1
cytidine monophosphate (UMP−CMP) kinase ...

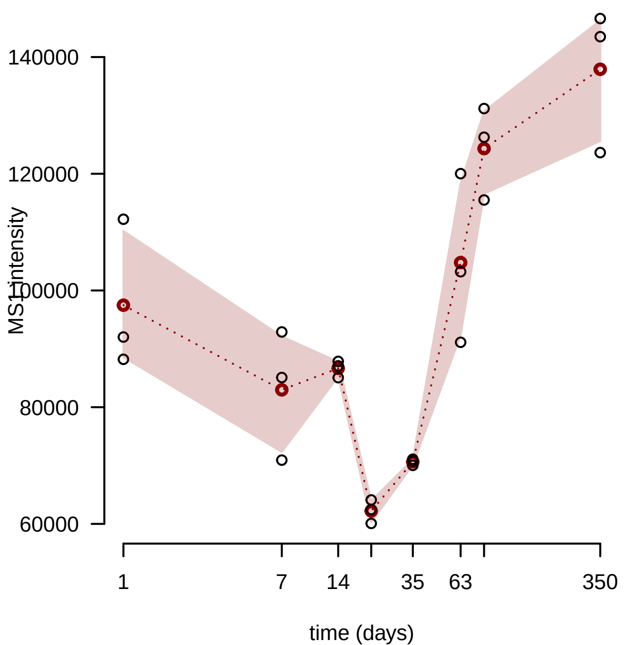

Q9DBQ7
GN=Scyl3
SCY1−like 3 (S. cerevisiae)

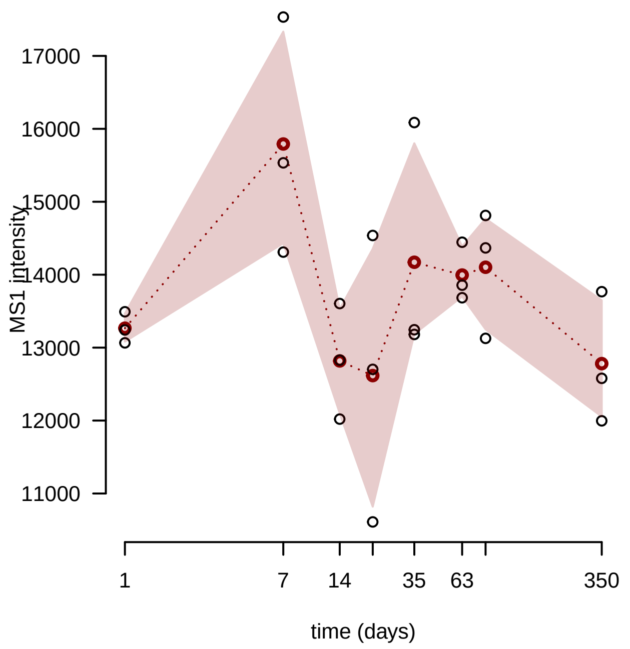

Q9DBR0
GN=Akap8
A kinase (PRKA) anchor protein 8

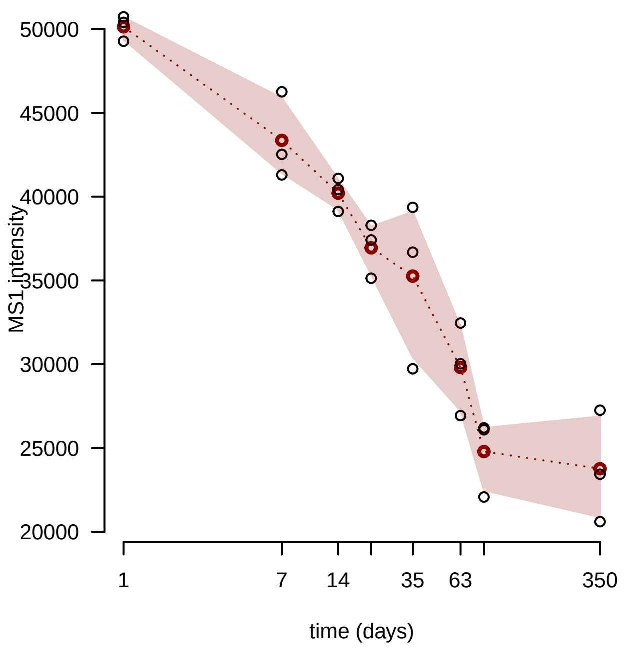

Q9DBR1
GN=Xrn2
5'−3' exoribonuclease 2

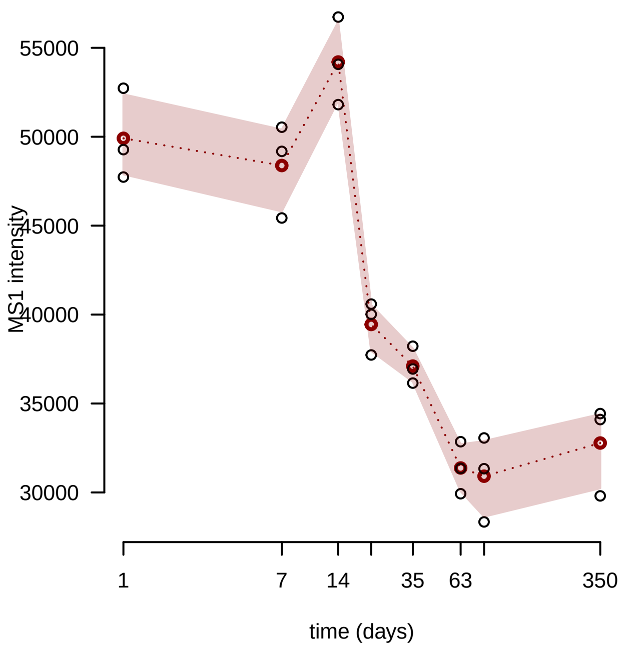

Q9DBR3
GN=Armc8
armadillo repeat containing 8

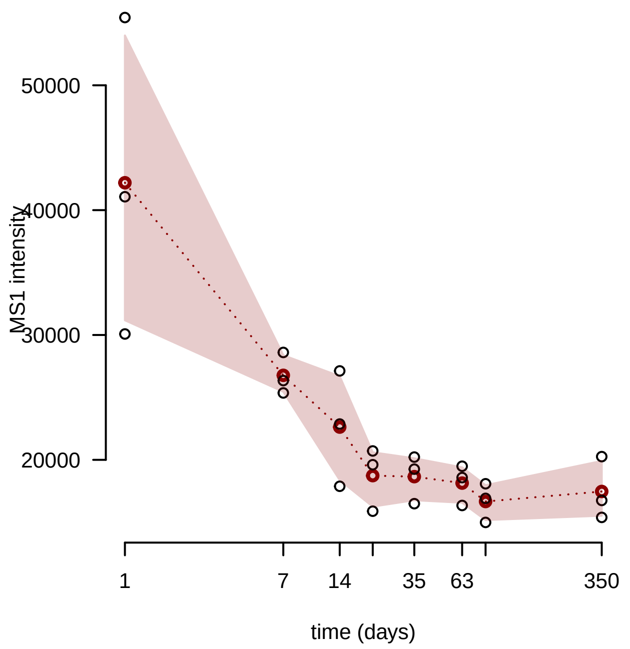

Q9DBR3
GN=Armc8
armadillo repeat containing 8

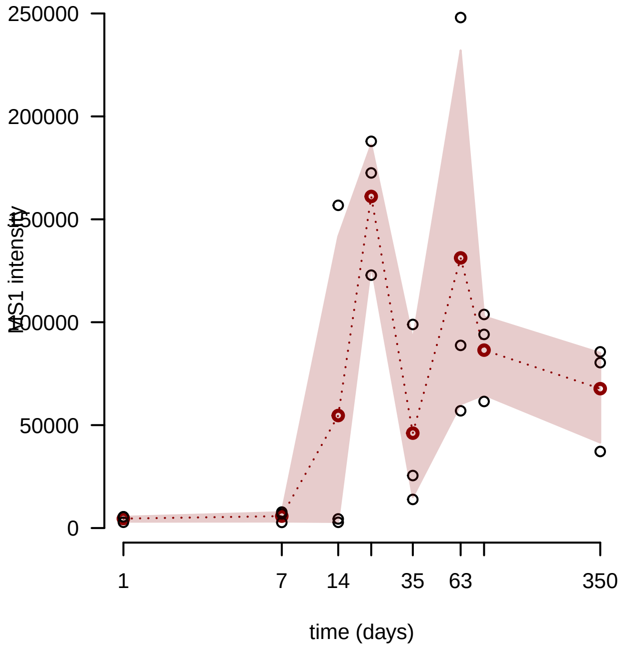

Q9DBR4
GN=Apbb2
amyloid beta (A4) precursor protein−bind ...

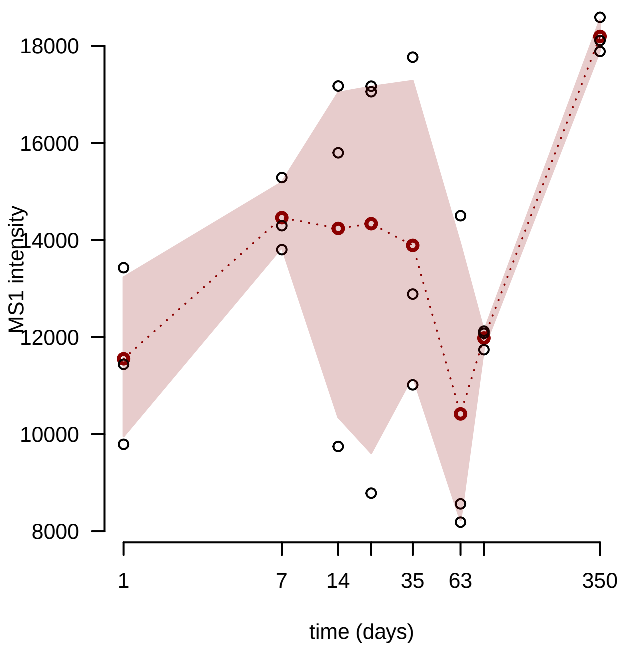

Q9DBR7
GN=Ppp1r12a
protein phosphatase 1, regulatory subuni ...

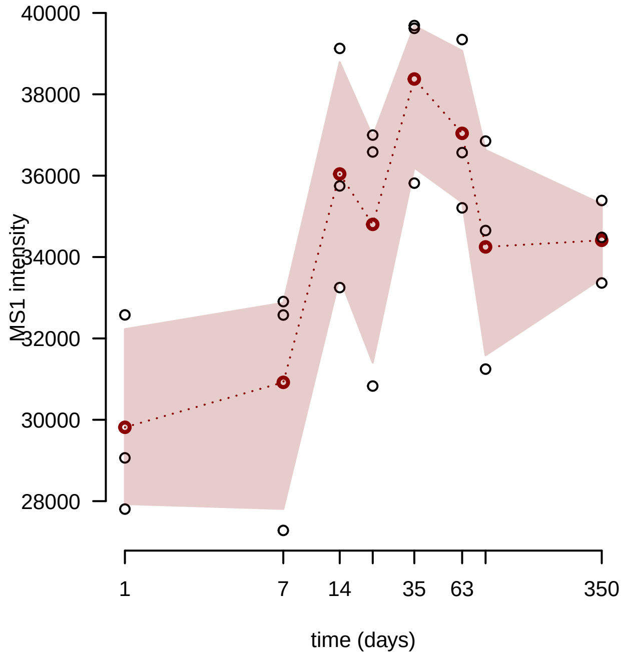

Q9DBS1
GN=Tmem43
transmembrane protein 43

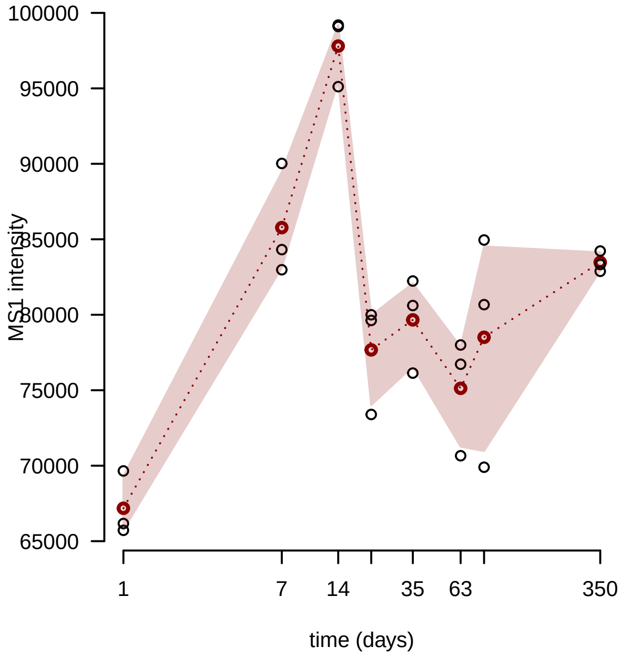

Q9DBS2
GN=Tprg1l

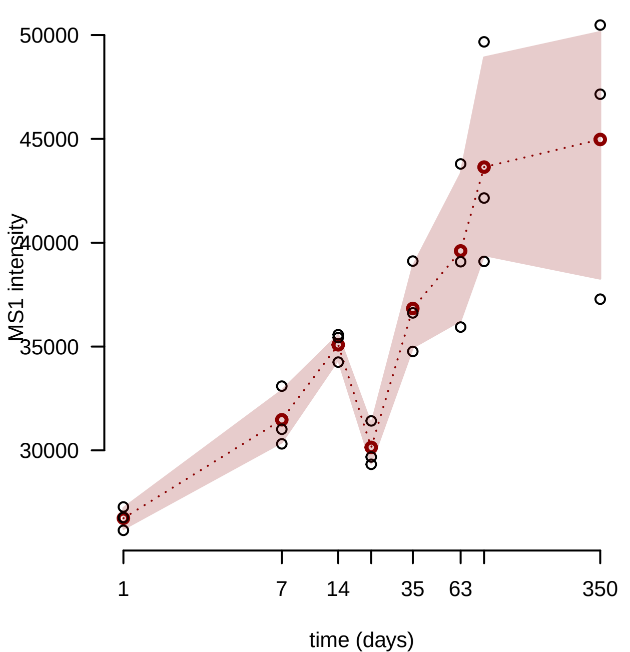

Q9DBS5
GN=Klc4
kinesin light chain 4

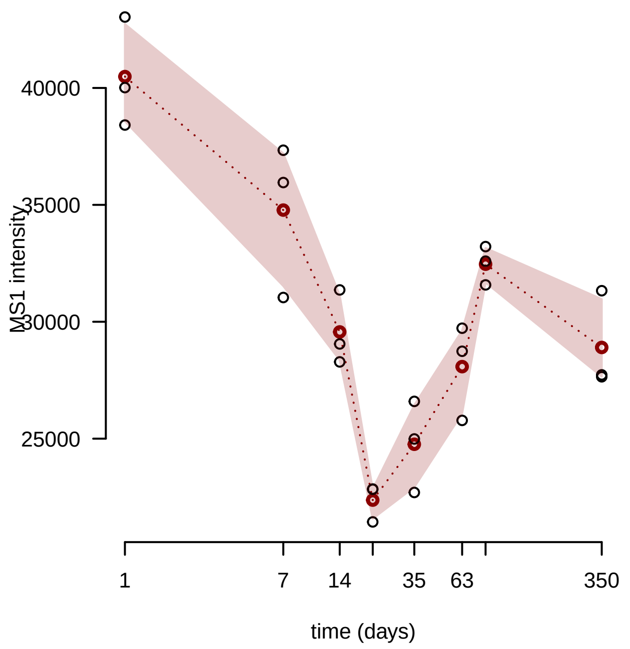

Q9DBS8
GN=Poc5
POC5 centriolar protein

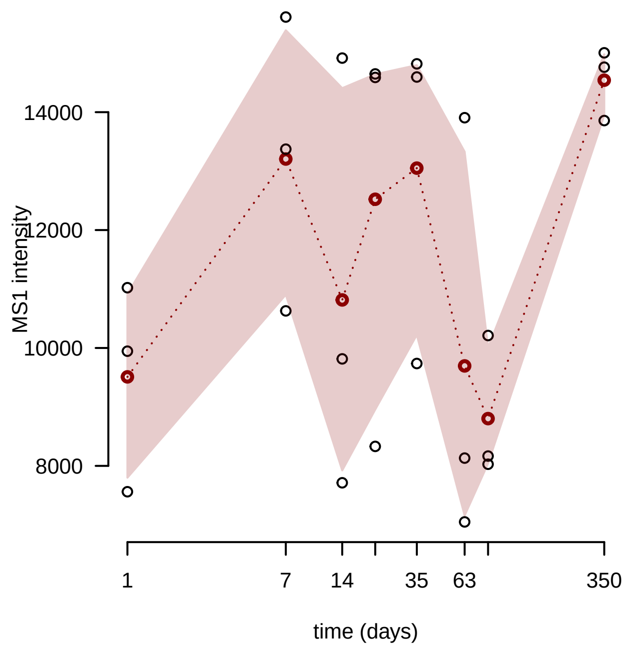

Q9DBS9
GN=Osbpl3
oxysterol binding protein−like 3

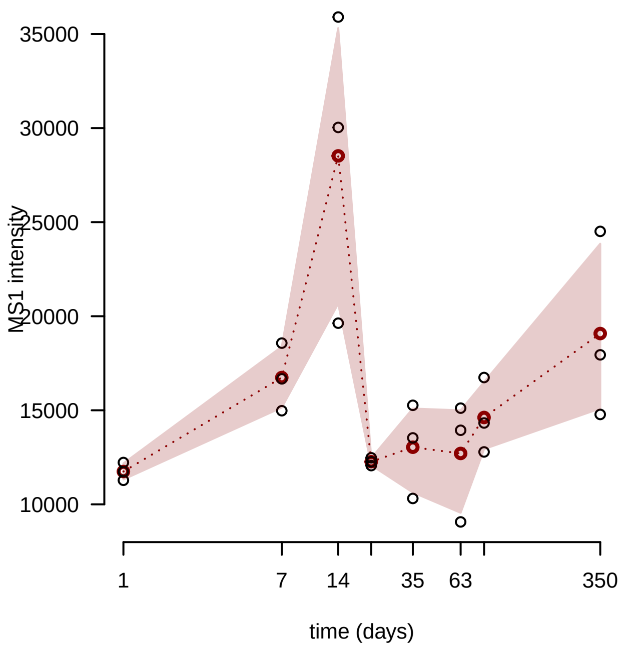

Q9DBT3
GN=Ccdc97
coiled−coil domain containing 97

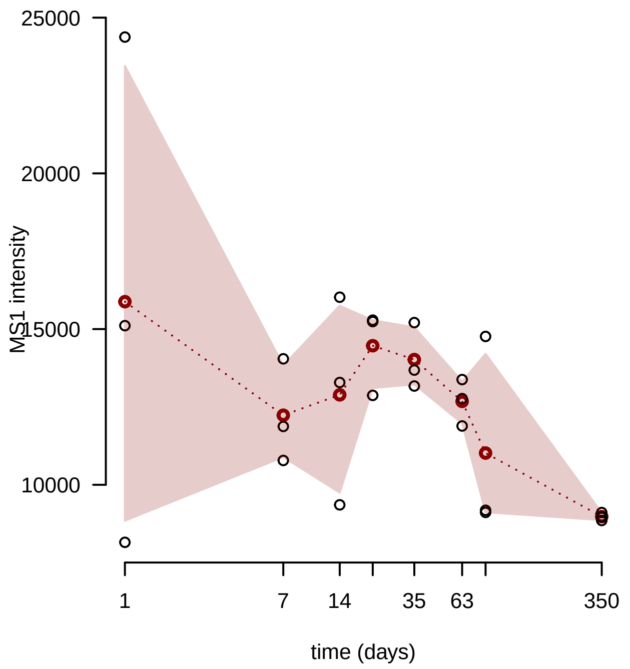

Q9DBT4
GN=Entpd4
ectonucleoside triphosphate diphosphohyd ...

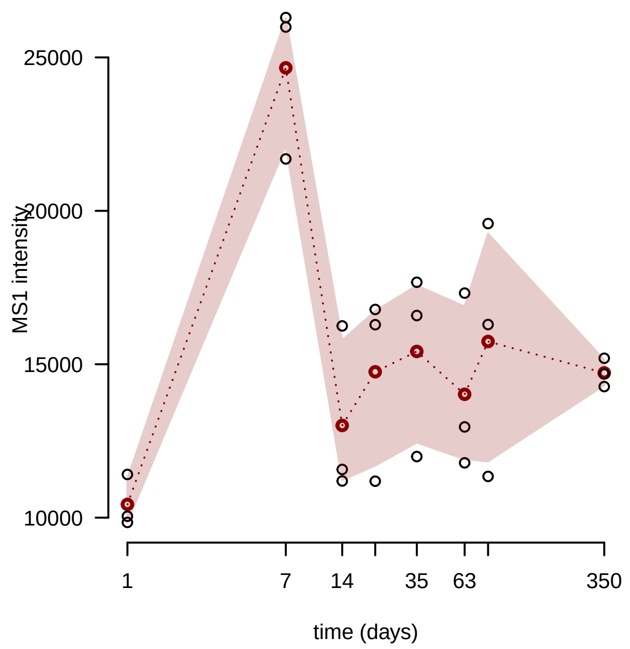

Q9DBT5
GN=Ampd2
adenosine monophosphate deaminase 2

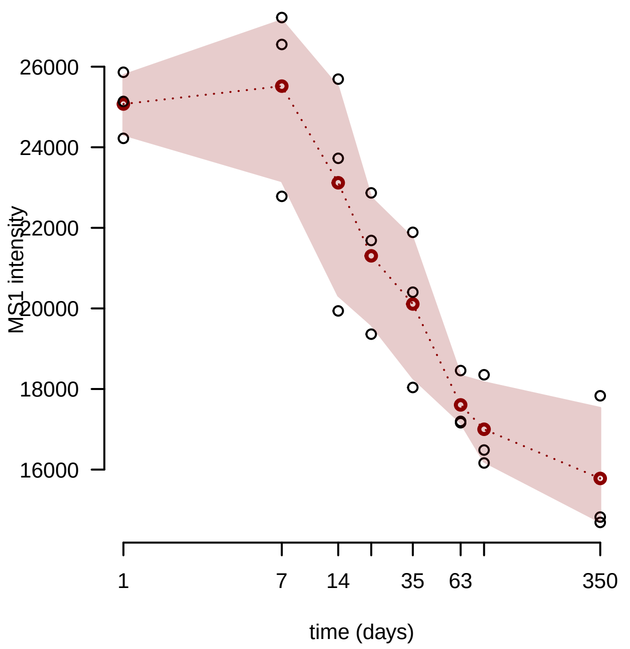

Q9DBU0
GN=Tm9sf1
transmembrane 9 superfamily member 1

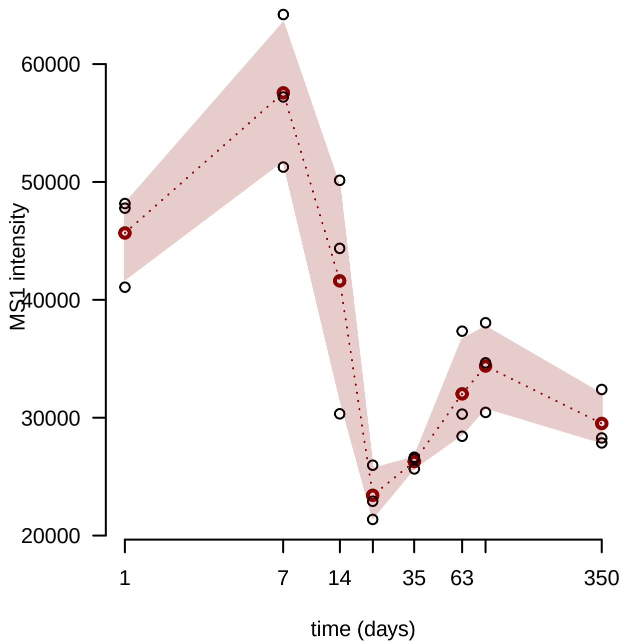

Q9DBU3
GN=Riok3
RIO kinase 3

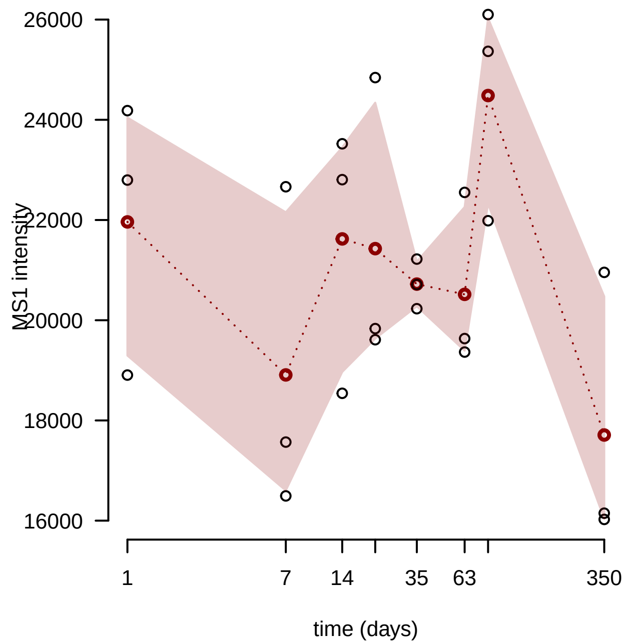

Q9DBU5
GN=Rnf6
ring finger protein (C3H2C3 type) 6

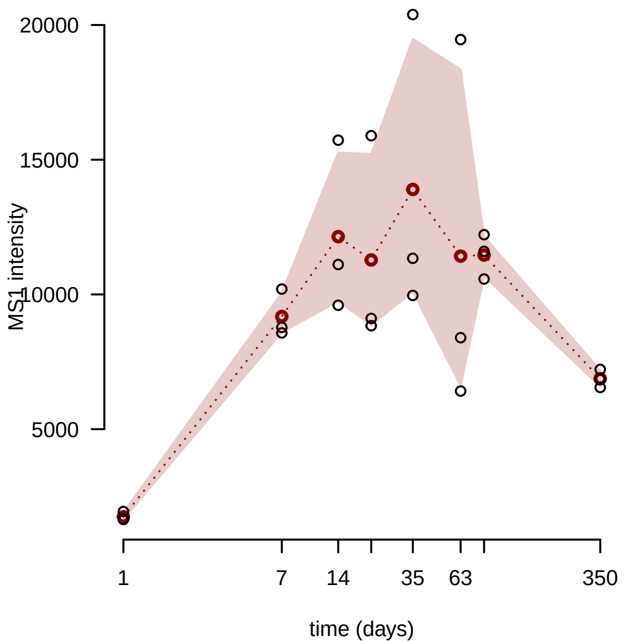

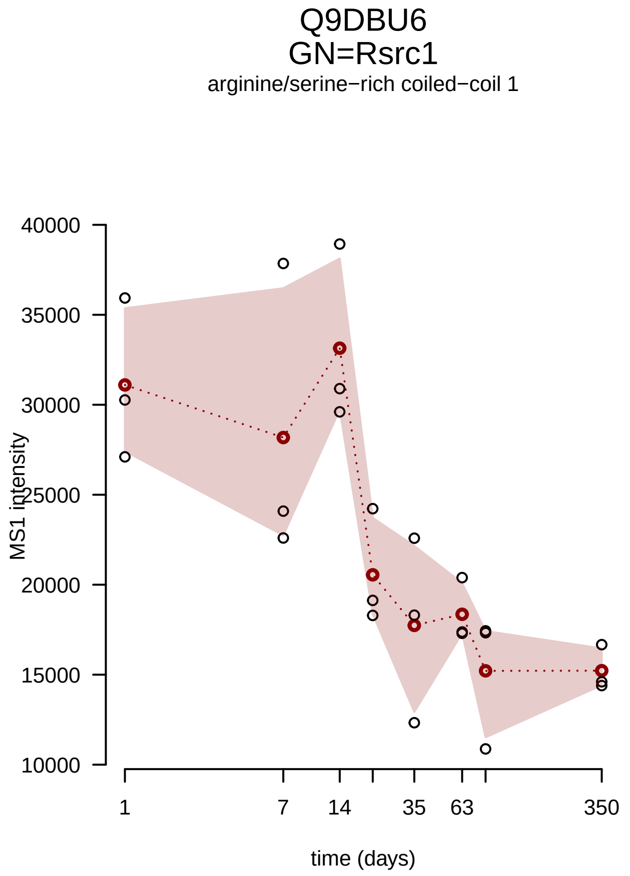
Q9DBU6
GN=Rsrc1
arginine/serine−rich coiled−coil 1
MS1 intensity
40000
35000
30000
25000
20000
15000
10000
1
7
14
35
63
350
time (days)

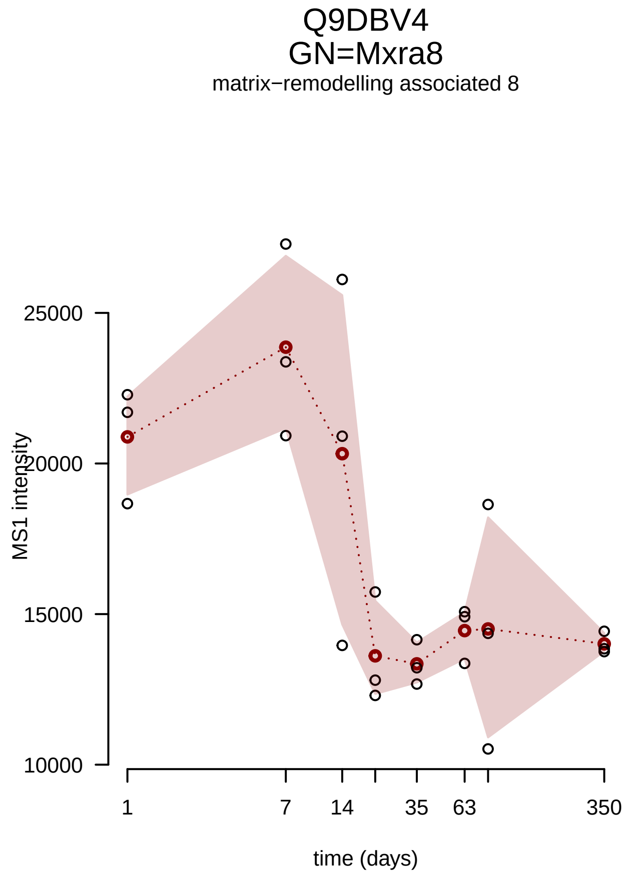
Q9DBV4
GN=Mxra8
matrix−remodelling associated 8
MS1 intensity
25000
20000
15000
10000
1
7
14
35
63
350
time (days)

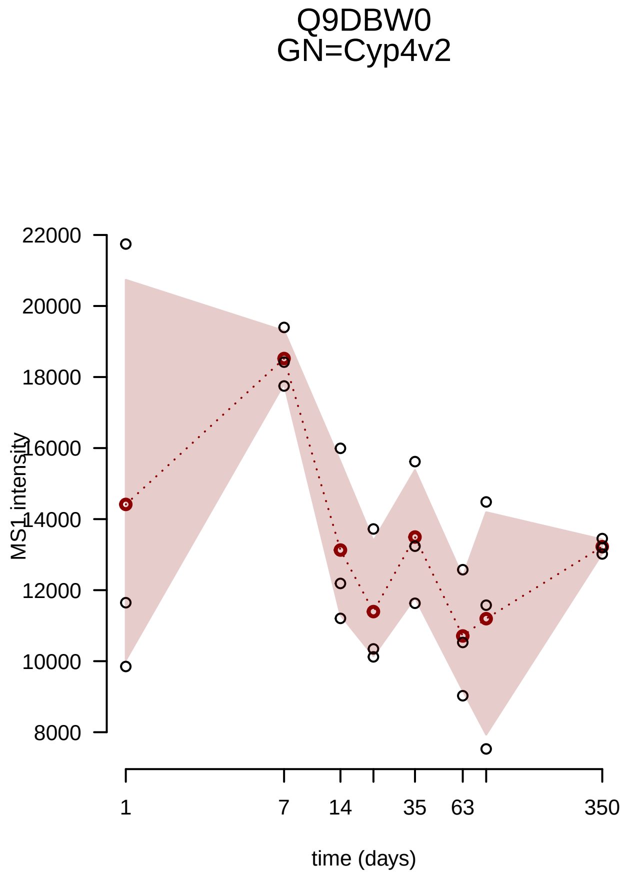
Q9DBW0
GN=Cyp4v2
MS1 intensity
22000
20000
18000
16000
14000
12000
10000
8000
1
7
14
35
63
350
time (days)

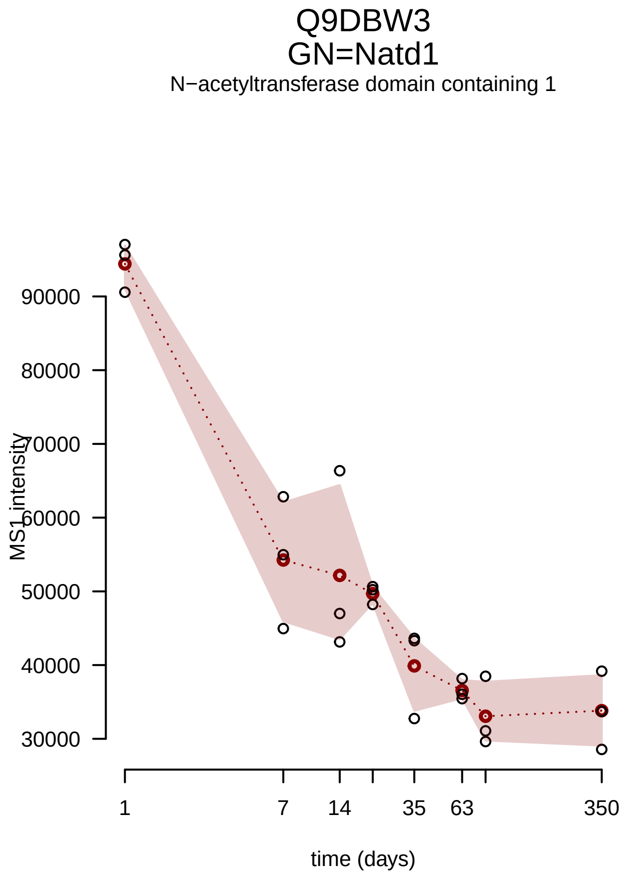
Q9DBW3
GN=Natd1
N−acetyltransferase domain containing 1
MS1 intensity
90000
80000
70000
60000
50000
40000
30000
1
7
14
35
63
350
time (days)

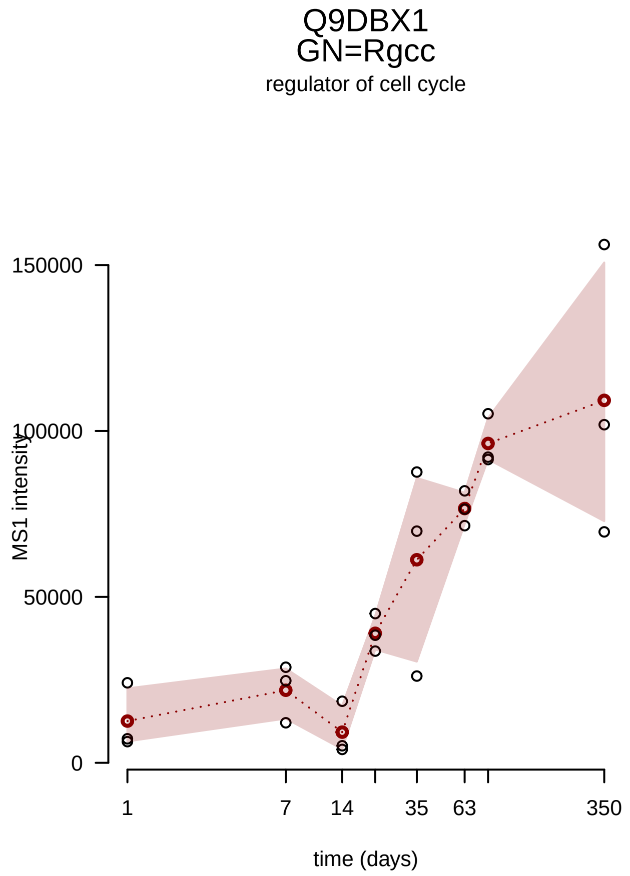
Q9DBX1
GN=Rgcc
regulator of cell cycle
MS1 intensity
150000
100000
50000
0
1
7
14
35
63
350
time (days)

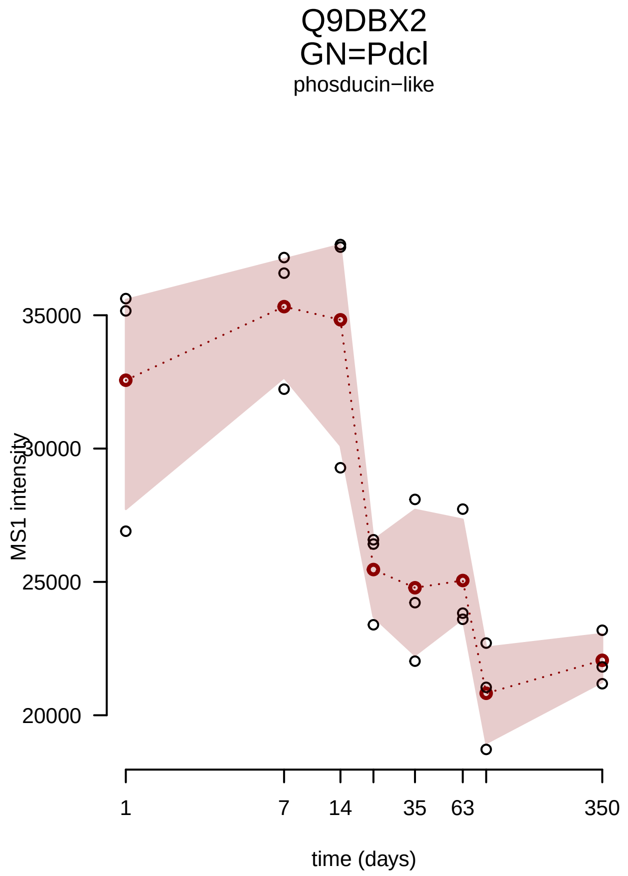
Q9DBX2
GN=Pdcl
phosducin−like
MS1 intensity
35000
30000
25000
20000
1
7
14
35
63
350
time (days)

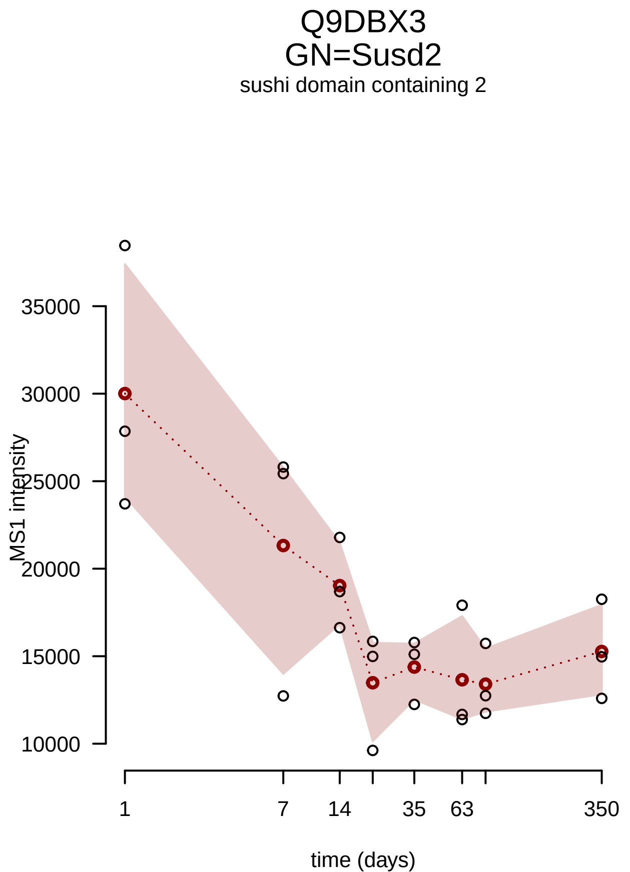
Q9DBX3
GN=Susd2
sushi domain containing 2
MS1 intensity
35000
30000
25000
20000
15000
10000
1
7
14
35
63
350
time (days)

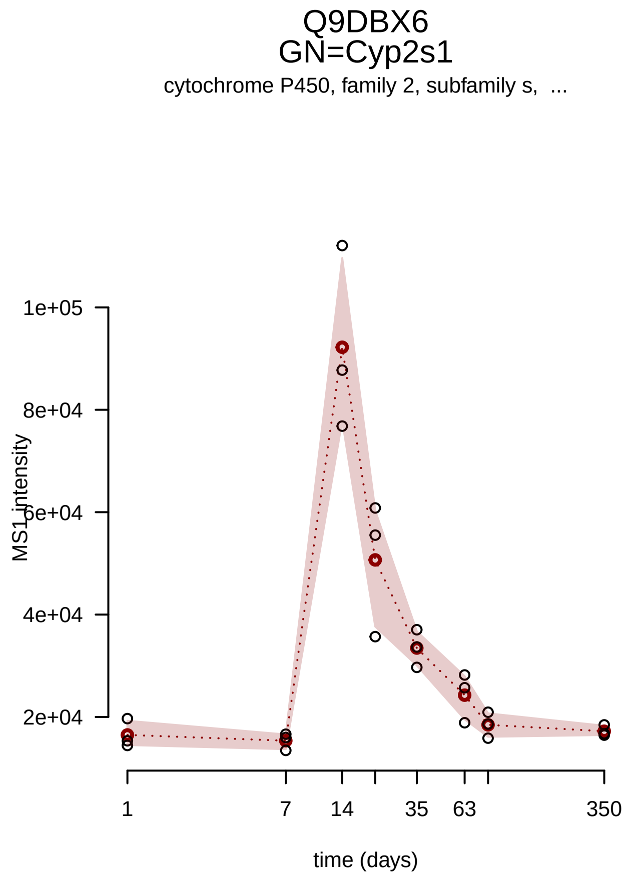
Q9DBX6
GN=Cyp2s1
cytochrome P450, family 2, subfamily s, ...
MS1 intensity
1e+05
8e+04
6e+04
4e+04
2e+04
1
7
14
35
63
350
time (days)

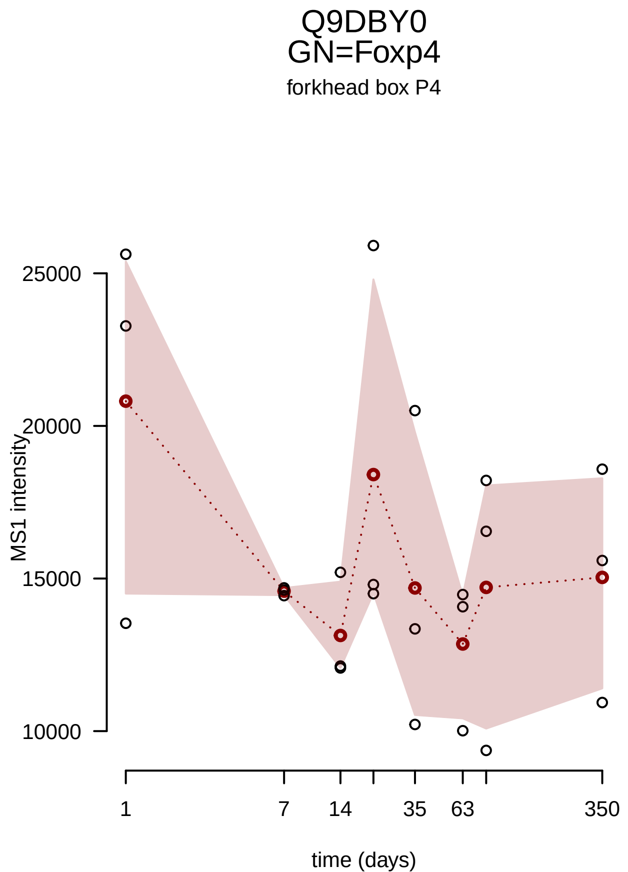
Q9DBY0
GN=Foxp4
forkhead box P4
MS1 intensity
25000
20000
15000
10000
1
7
14
35
63
350
time (days)

Q9DBY1
GN=Syvn1
synovial apoptosis inhibitor 1, synoviol ...

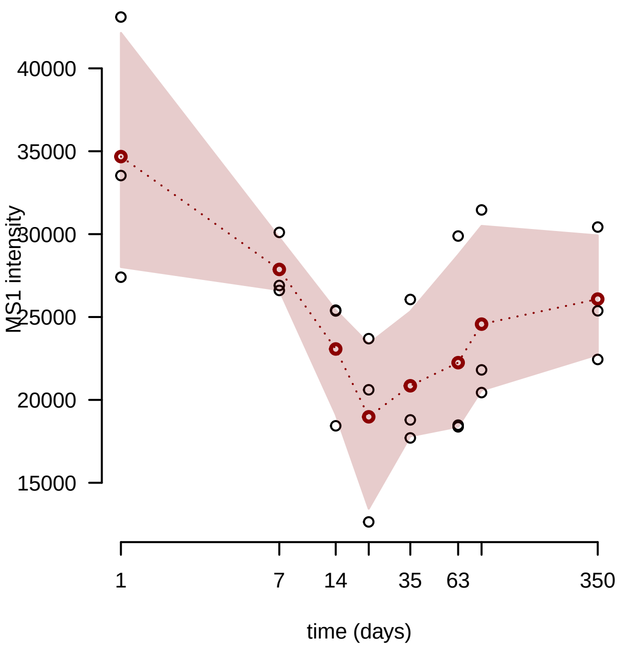

Q9DBY4
GN=Tril
TLR4 interactor with leucine−rich repeat ...

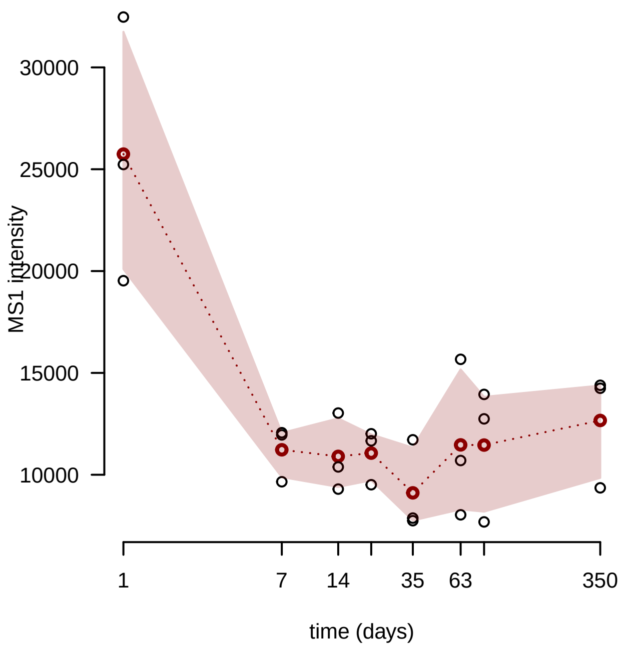

Q9DBY5
GN=Cbx6
chromobox 6

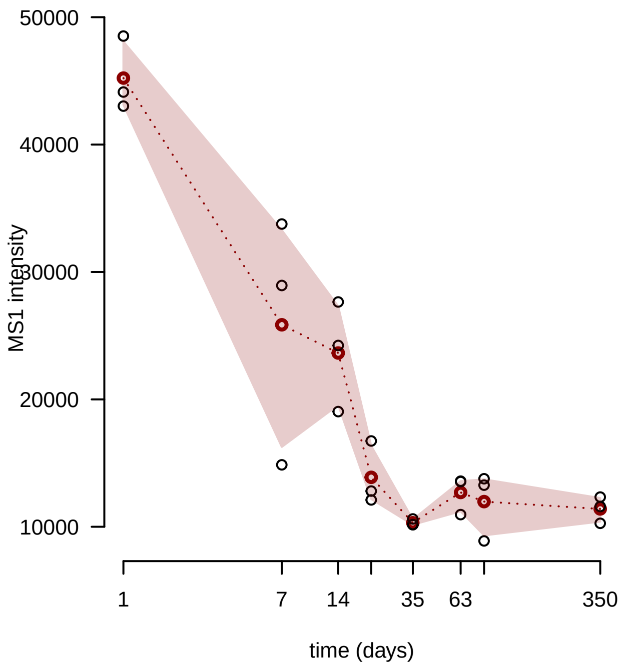

Q9DBY8
GN=Nvl
nuclear VCP−like

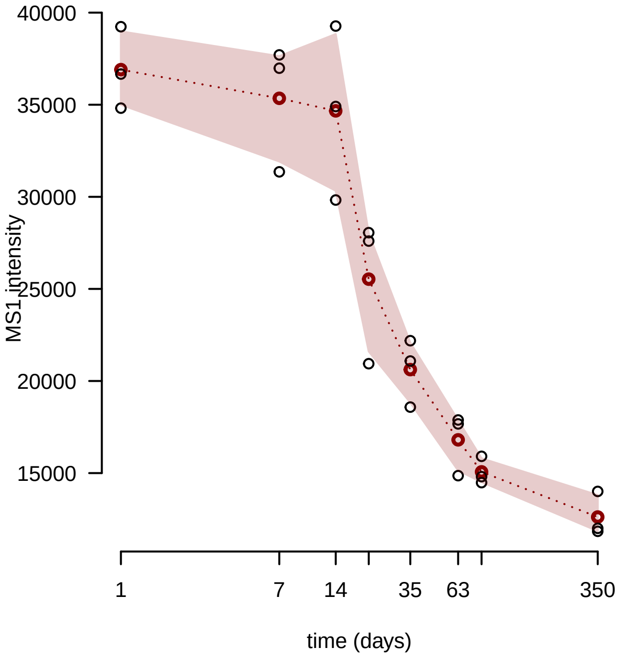

Q9DBZ1
GN=Ikbip
IKBKB interacting protein

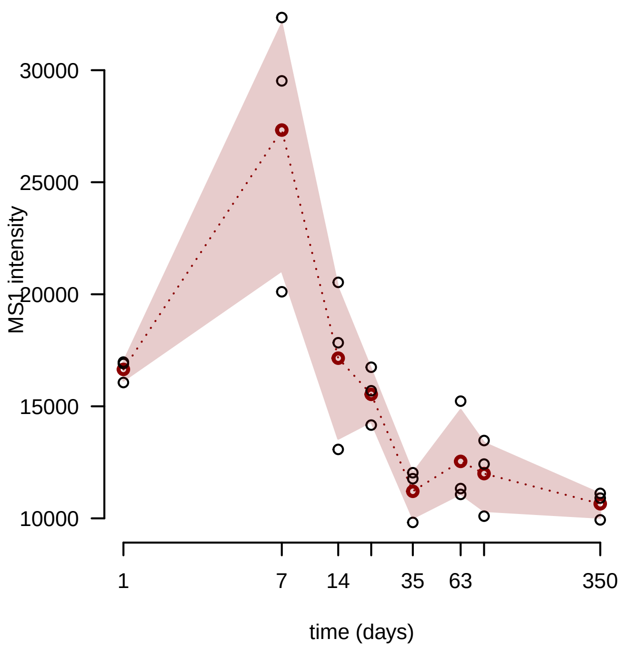

Q9DBZ1
GN=Ikbip
IKBKB interacting protein

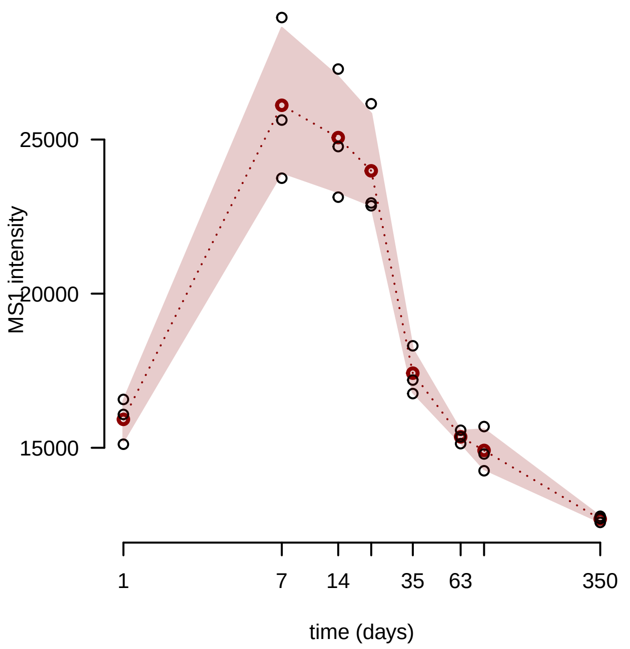

Q9DBZ5
GN=Eif3k
eukaryotic translation initiation factor ...

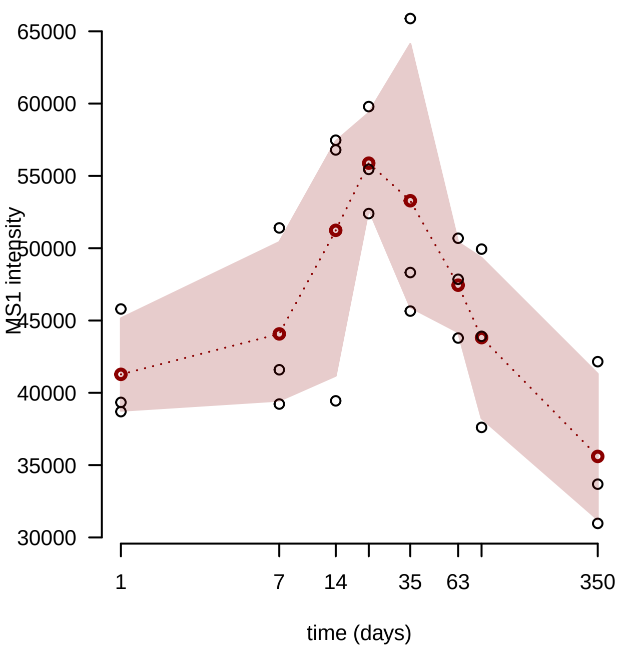

Q9DBZ9
GN=Syde1
synapse defective 1, Rho GTPase, homolog ...

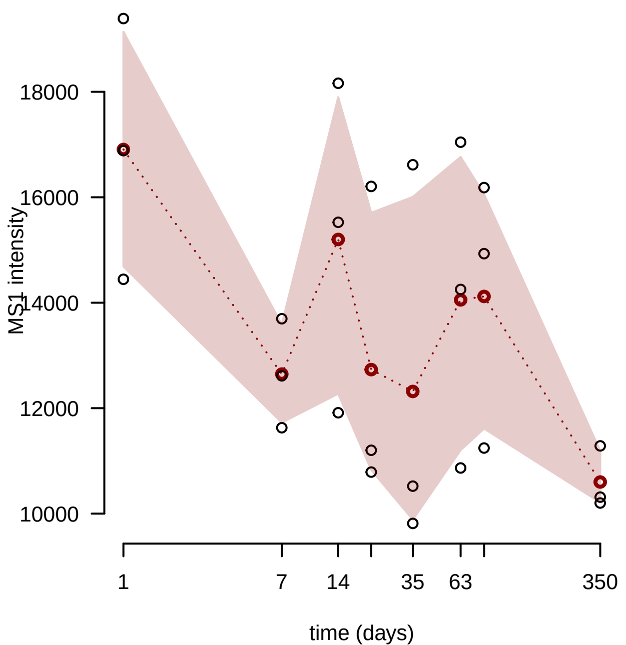

Q9DC04
GN=Rgs3
regulator of G−protein signaling 3

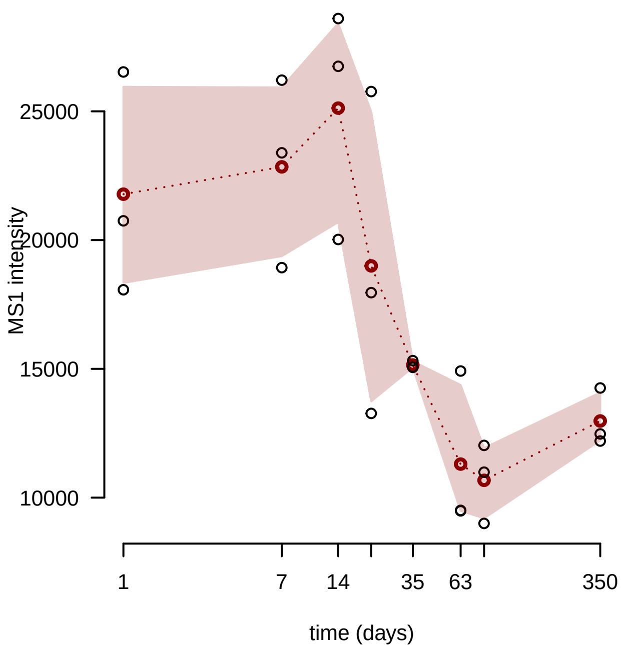

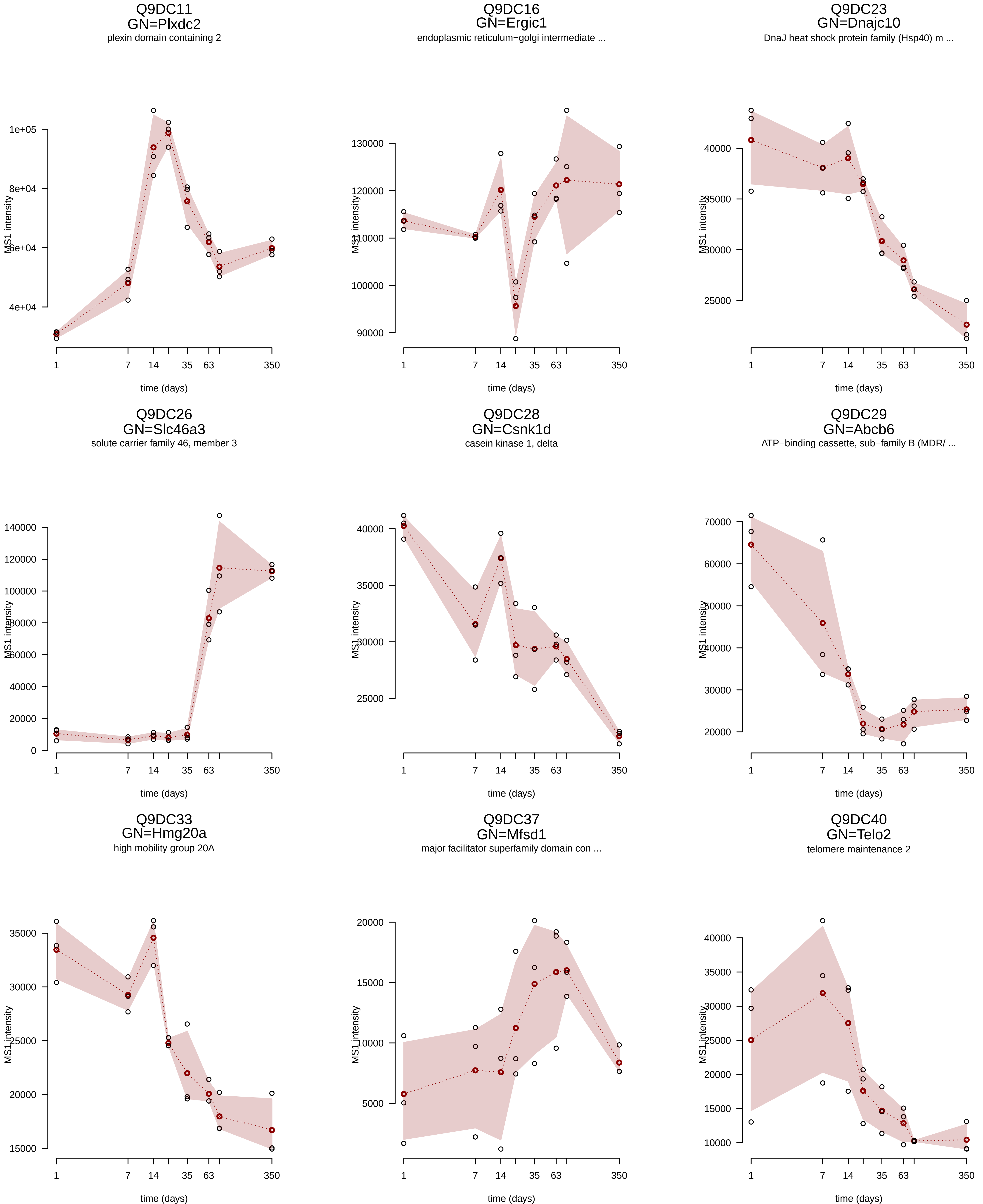
Q9DC11
GN=Plxdc2
plexin domain containing 2
1e+05
8e+04
6e+04
4e+04
MS1 intensity
1
7
14
35
63
350
time (days)
Q9DC16
GN=Ergic1
endoplasmic reticulum−golgi intermediate ...
130000
120000
110000
100000
90000
MS1 intensity
1
7
14
35
63
350
time (days)
Q9DC23
GN=Dnajc10
DnaJ heat shock protein family (Hsp40) m ...
40000
35000
30000
25000
MS1 intensity
1
7
14
35
63
350
time (days)
Q9DC26
GN=Slc46a3
solute carrier family 46, member 3
140000
120000
100000
80000
60000
40000
20000
0
MS1 intensity
1
7
14
35
63
350
time (days)
Q9DC28
GN=Csnk1d
casein kinase 1, delta
40000
35000
30000
25000
MS1 intensity
1
7
14
35
63
350
time (days)
Q9DC29
GN=Abcb6
ATP−binding cassette, sub−family B (MDR/ ...
70000
60000
50000
40000
30000
20000
MS1 intensity
1
7
14
35
63
350
time (days)
Q9DC33
GN=Hmg20a
high mobility group 20A
35000
30000
25000
20000
15000
MS1 intensity
1
7
14
35
63
350
time (days)
Q9DC37
GN=Mfsd1
major facilitator superfamily domain con ...
20000
15000
10000
5000
MS1 intensity
1
7
14
35
63
350
time (days)
Q9DC40
GN=Telo2
telomere maintenance 2
40000
35000
30000
25000
20000
15000
10000
MS1 intensity
1
7
14
35
63
350
time (days)

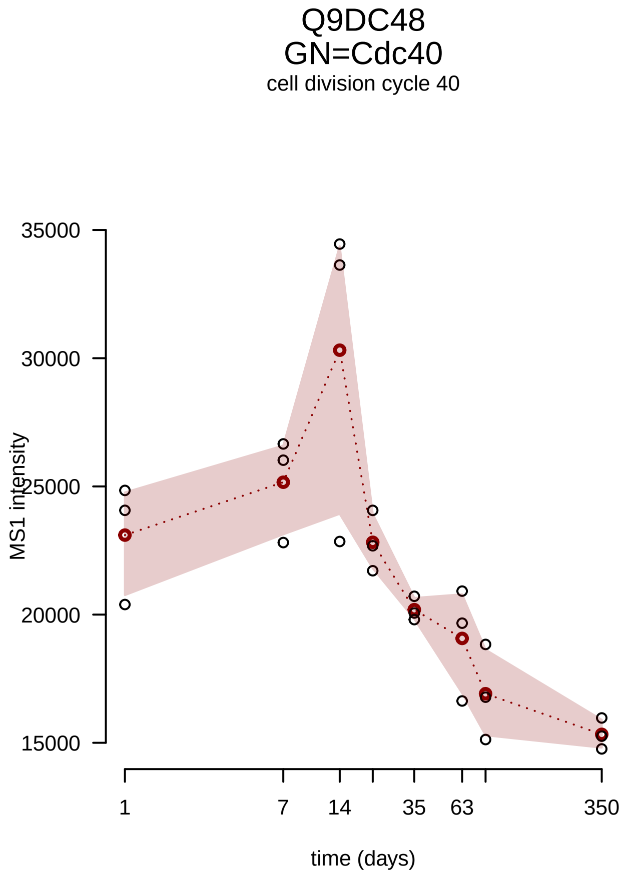
Q9DC48
GN=Cdc40
cell division cycle 40
35000
30000
25000
20000
15000
MS1 intensity
1
7
14
35
63
350
time (days)

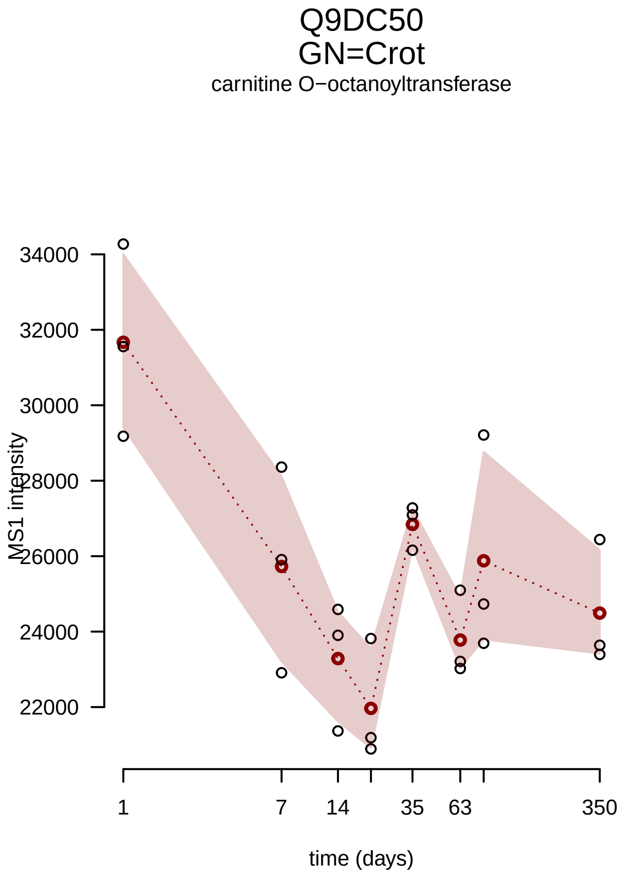
Q9DC50
GN=Crot
carnitine O−octanoyltransferase
34000
32000
30000
28000
26000
24000
22000
MS1 intensity
1
7
14
35
63
350
time (days)

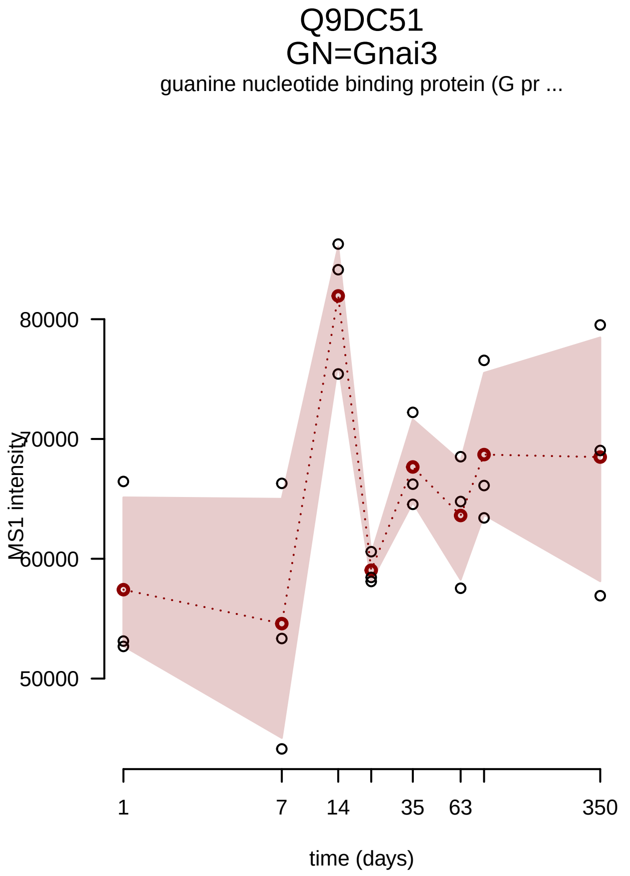
Q9DC51
GN=Gnai3
guanine nucleotide binding protein (G pr ...
80000
70000
60000
50000
MS1 intensity
1
7
14
35
63
350
time (days)

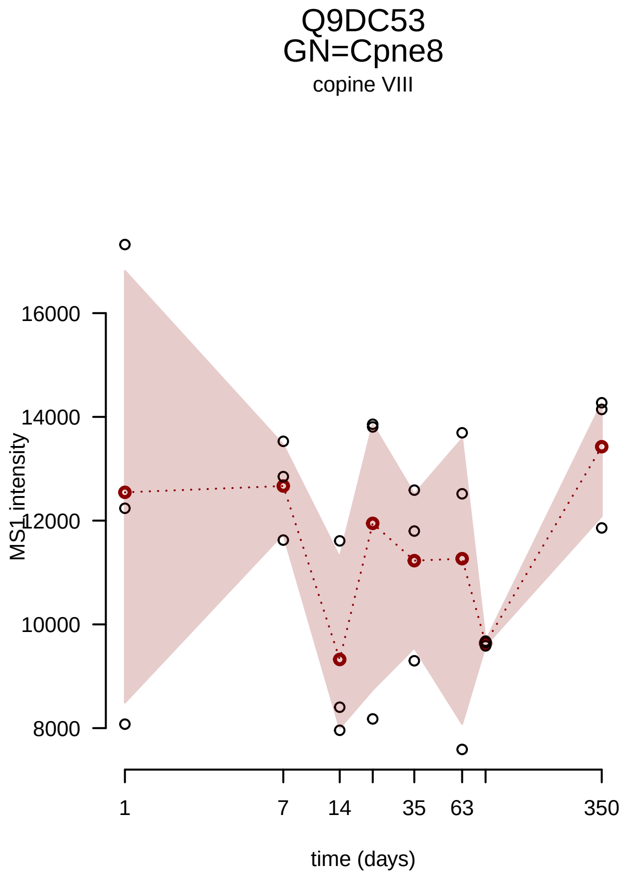
Q9DC53
GN=Cpne8
copine VIII
16000
14000
12000
10000
8000
MS1 intensity
1
7
14
35
63
350
time (days)

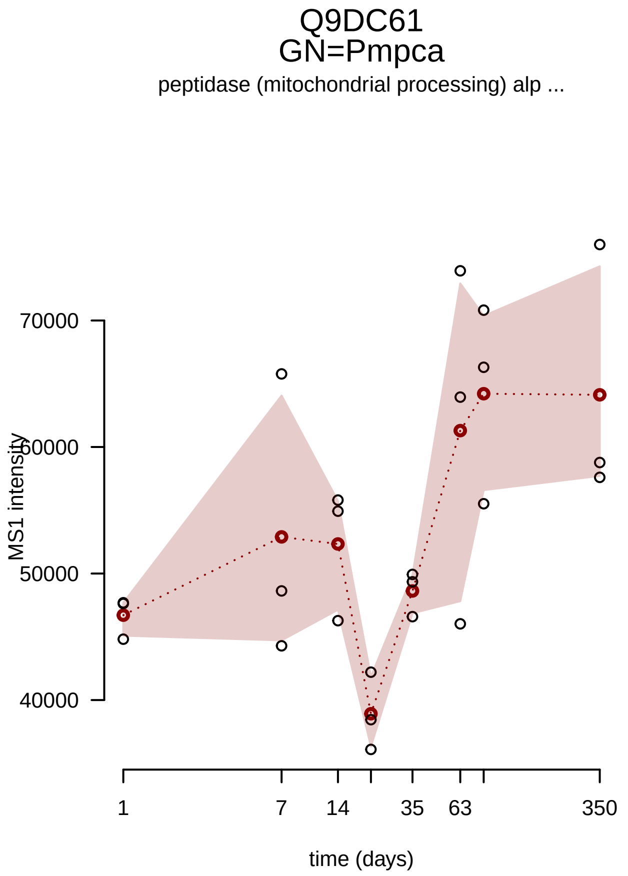
Q9DC61
GN=Pmpca
peptidase (mitochondrial processing) alp ...
70000
60000
50000
40000
MS1 intensity
1
7
14
35
63
350
time (days)

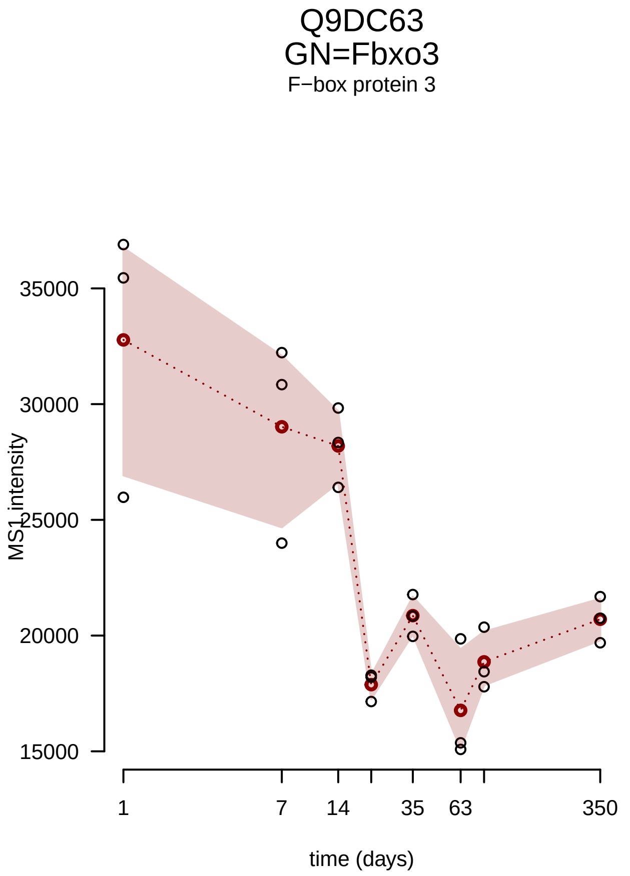
Q9DC63
GN=Fbxo3
F−box protein 3
35000
30000
25000
20000
15000
MS1 intensity
1
7
14
35
63
350
time (days)

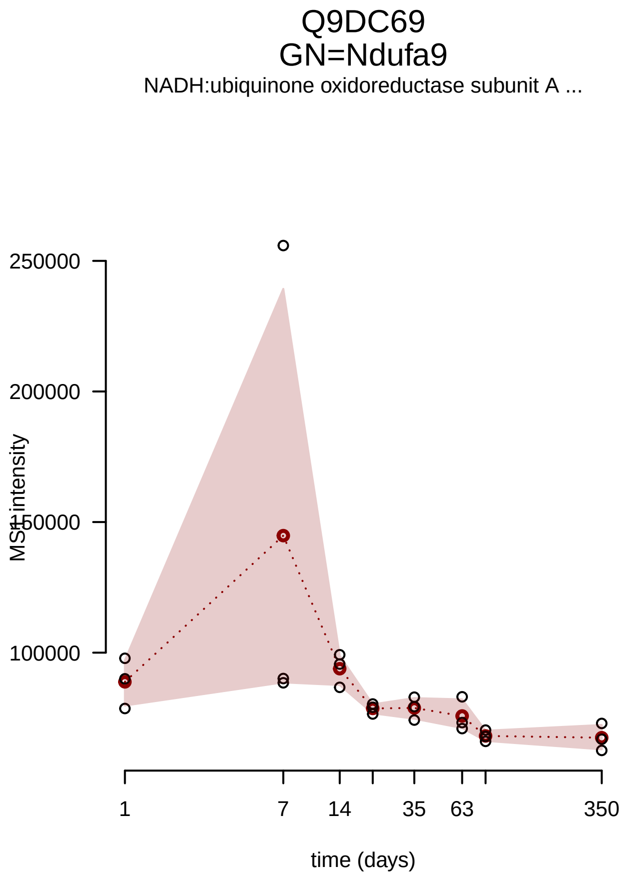
Q9DC69
GN=Ndufa9
NADH:ubiquinone oxidoreductase subunit A ...
250000
200000
150000
100000
MS1 intensity
1
7
14
35
63
350
time (days)

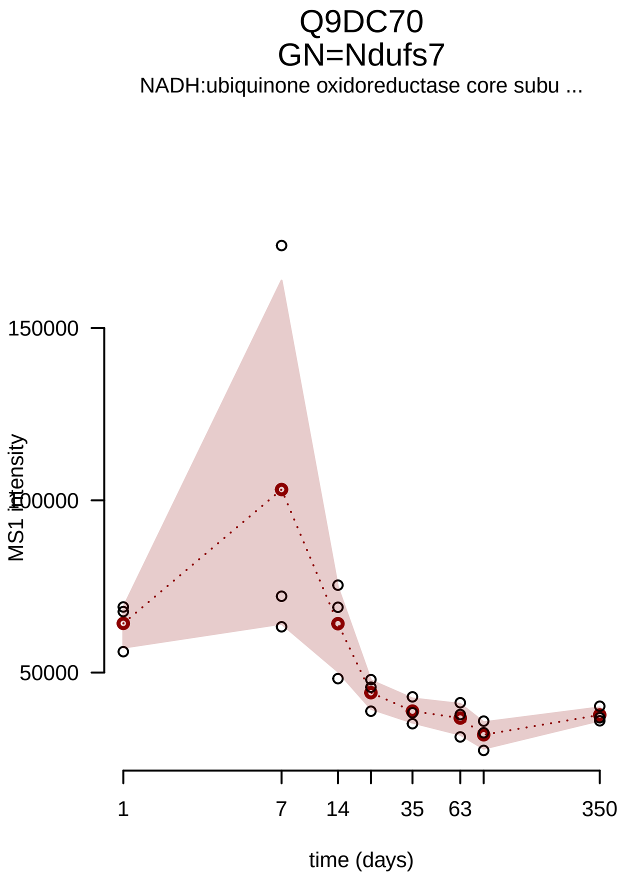
Q9DC70
GN=Ndufs7
NADH:ubiquinone oxidoreductase core subu ...
150000
100000
50000
MS1 intensity
1
7
14
35
63
350
time (days)

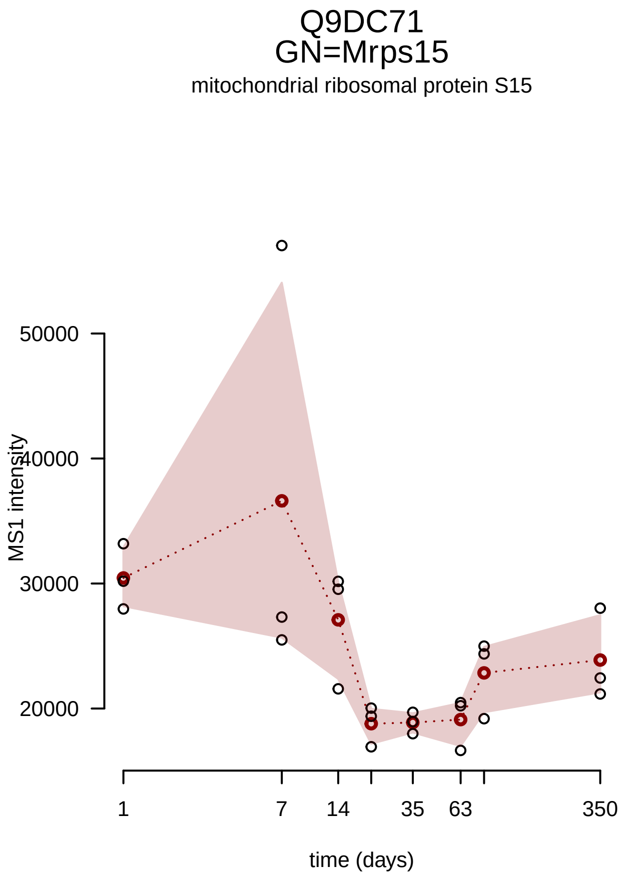
Q9DC71
GN=Mrps15
mitochondrial ribosomal protein S15
50000
40000
30000
20000
MS1 intensity
1
7
14
35
63
350
time (days)

Q9DC77
GN=Smpx
small muscle protein, X−linked

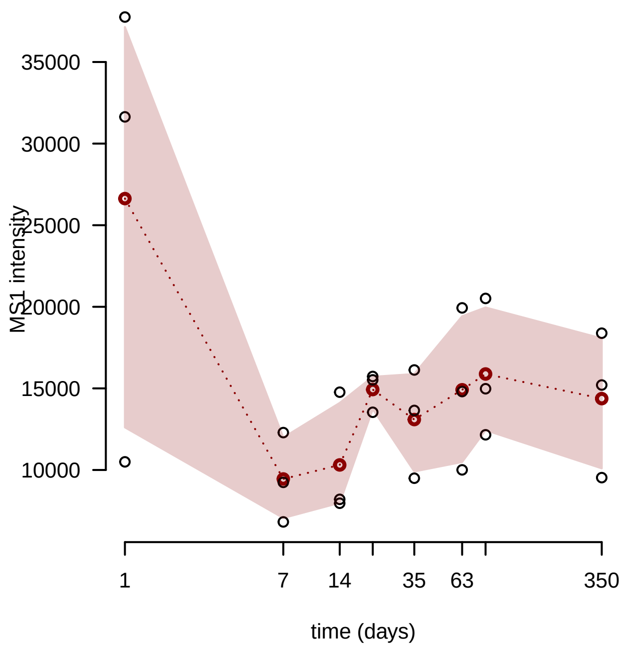

Q9DCA2
GN=Mrps11
mitochondrial ribosomal protein S11

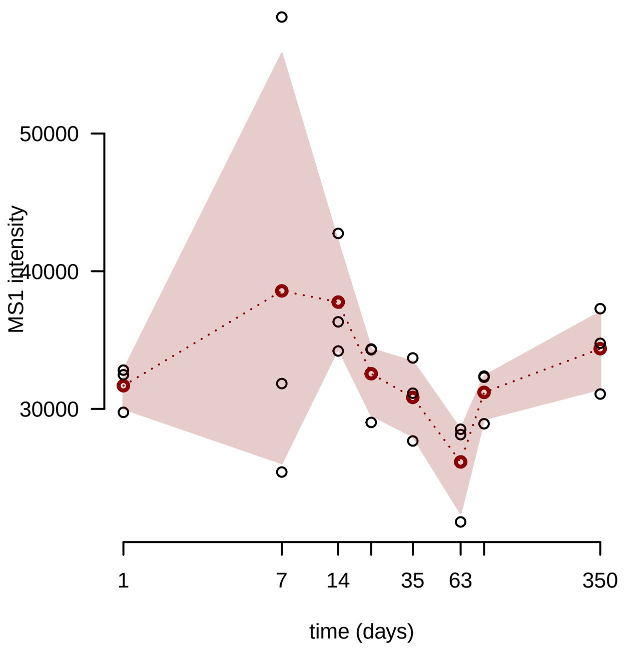

Q9DCA5
GN=Brix1
BRX1, biogenesis of ribosomes

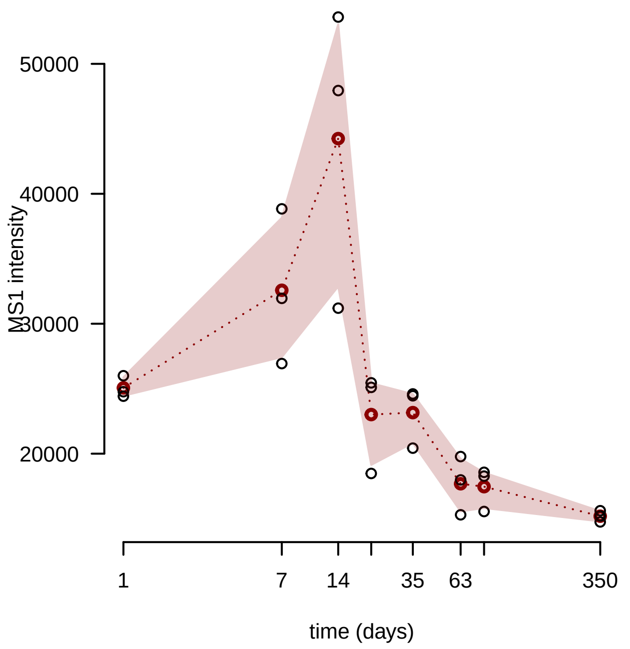

Q9DCB1
GN=Hmgn3
high mobility group nucleosomal binding ...

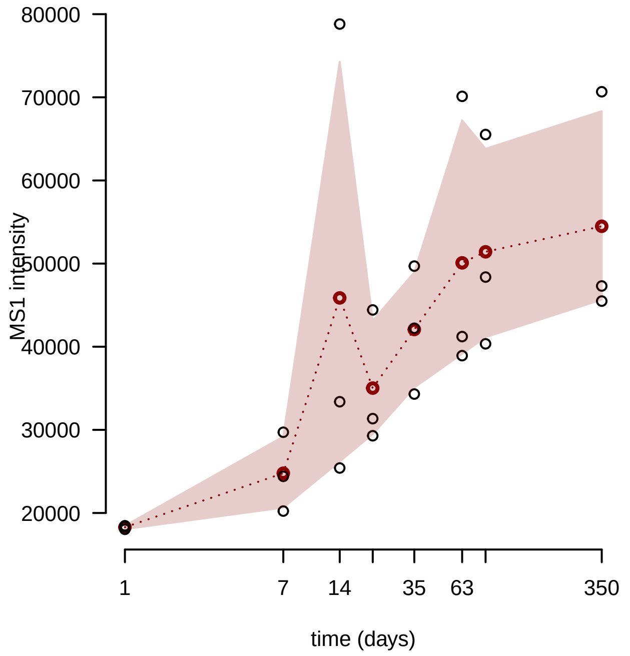

Q9DCB8
GN=Isca2
iron−sulfur cluster assembly 2

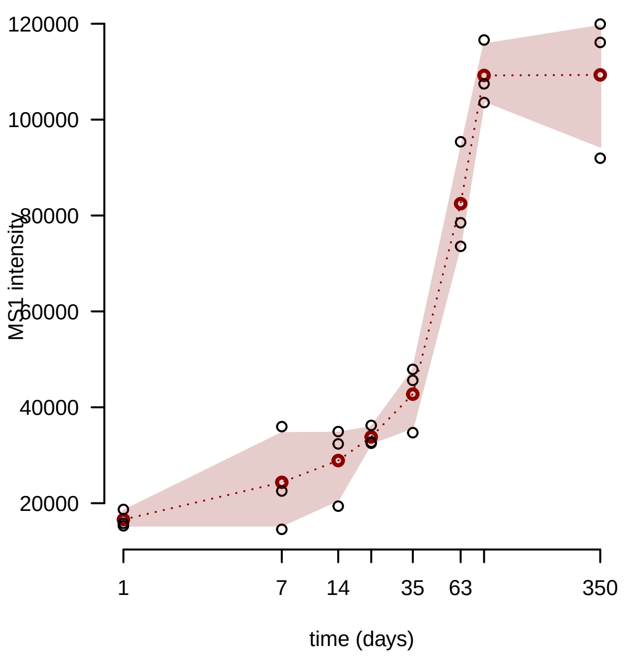

Q9DCC4
GN=Pycr3

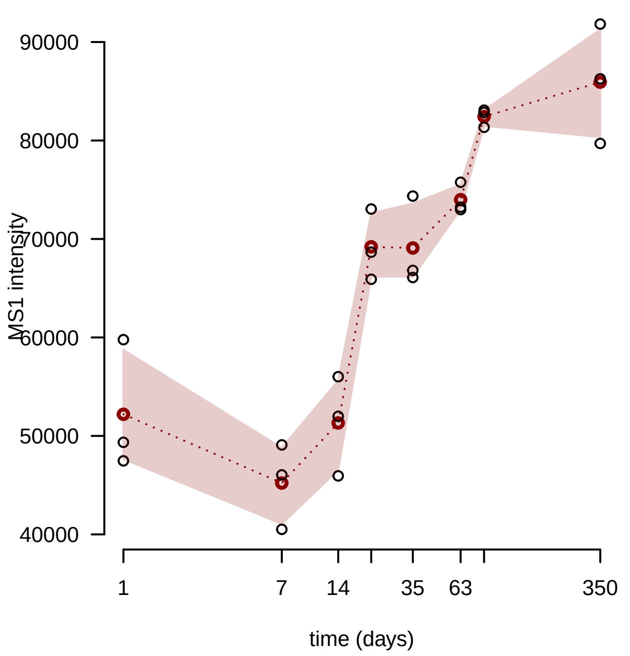

Q9DCC8
GN=Tomm20
translocase of outer mitochondrial membr ...

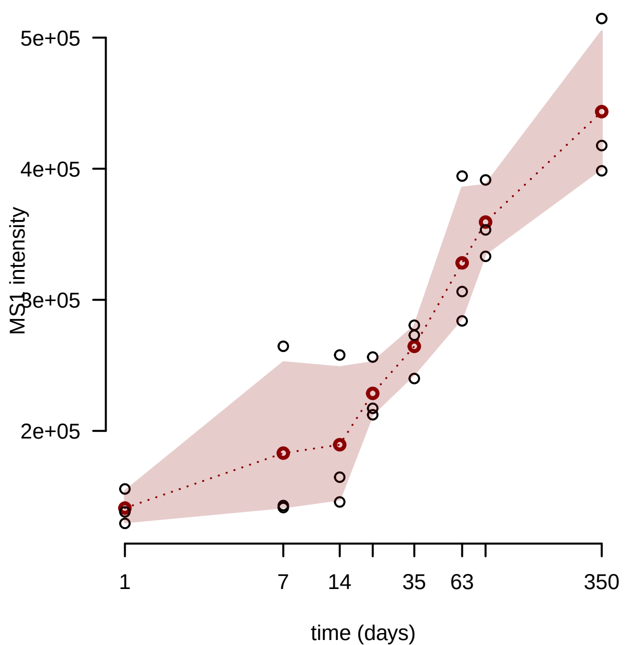

Q9DCD0
GN=Pgd
phosphogluconate dehydrogenase

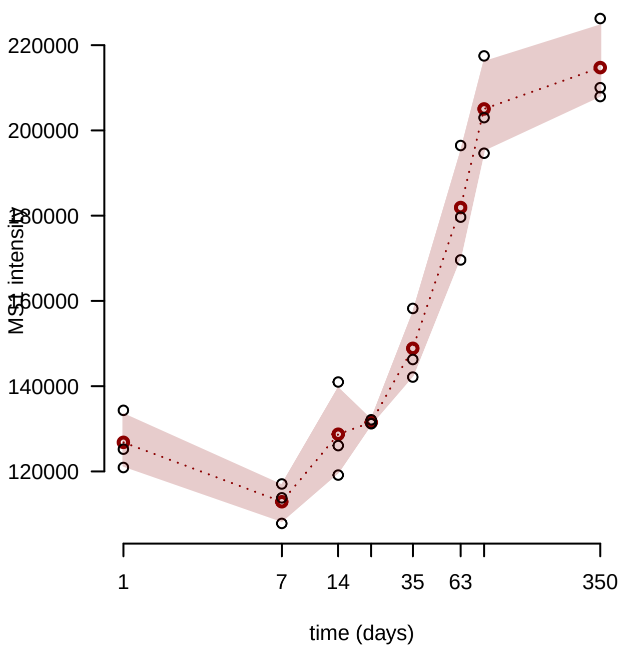

Q9DCD2
GN=Xab2
XPA binding protein 2

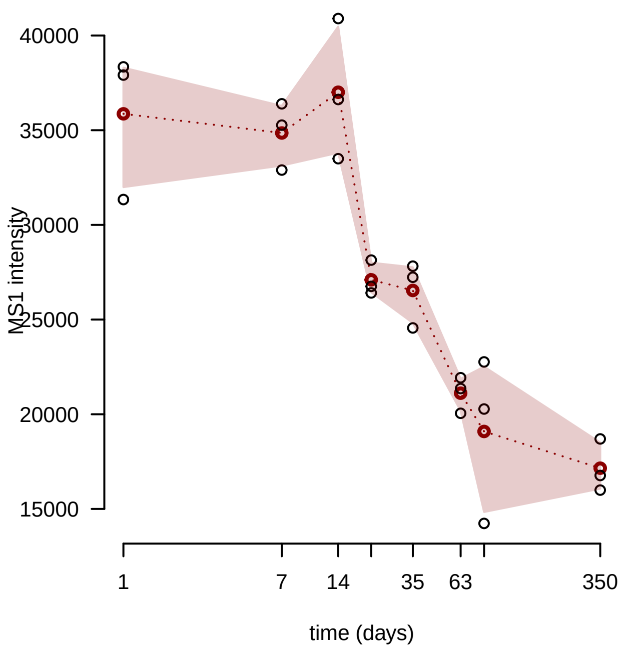

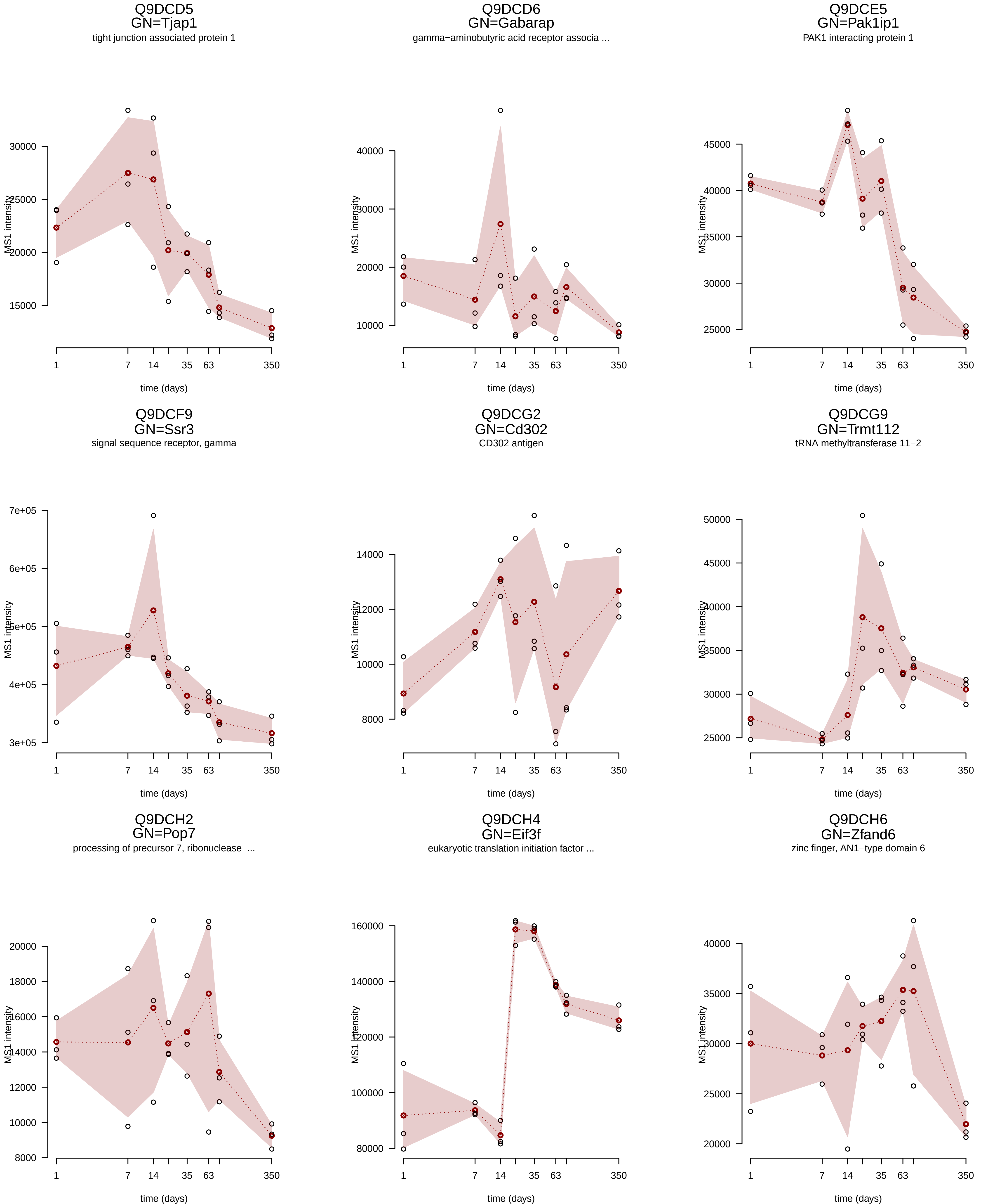
Q9DCD5
GN=Tjap1
tight junction associated protein 1
MS1 intensity
15000
20000
25000
30000
time (days)
1
7
14
35
63
350
Q9DCD6
GN=Gabarap
gamma−aminobutyric acid receptor associa ...
MS1 intensity
10000
20000
30000
40000
time (days)
Q9DCE5
GN=Pak1ip1
PAK1 interacting protein 1
MS1 intensity
25000
30000
35000
40000
45000
time (days)
Q9DCF9
GN=Ssr3
signal sequence receptor, gamma
MS1 intensity
3e+05
4e+05
5e+05
6e+05
7e+05
time (days)
Q9DCG2
GN=Cd302
CD302 antigen
MS1 intensity
8000
10000
12000
14000
time (days)
Q9DCG9
GN=Trmt112
tRNA methyltransferase 11−2
MS1 intensity
25000
30000
35000
40000
45000
50000
time (days)
Q9DCH2
GN=Pop7
processing of precursor 7, ribonuclease ...
MS1 intensity
8000
10000
12000
14000
16000
18000
20000
time (days)
Q9DCH4
GN=Eif3f
eukaryotic translation initiation factor ...
MS1 intensity
80000
100000
120000
140000
160000
time (days)
Q9DCH6
GN=Zfand6
zinc finger, AN1−type domain 6
MS1 intensity
20000
25000
30000
35000
40000
time (days)

Q9DCI3
GN=Stard3nl
STARD3 N−terminal like

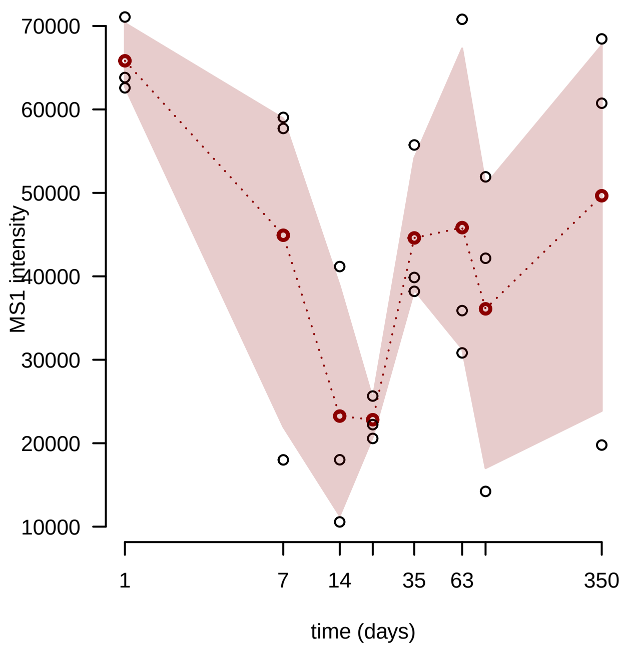

Q9DCI9
GN=Mrpl32
mitochondrial ribosomal protein L32

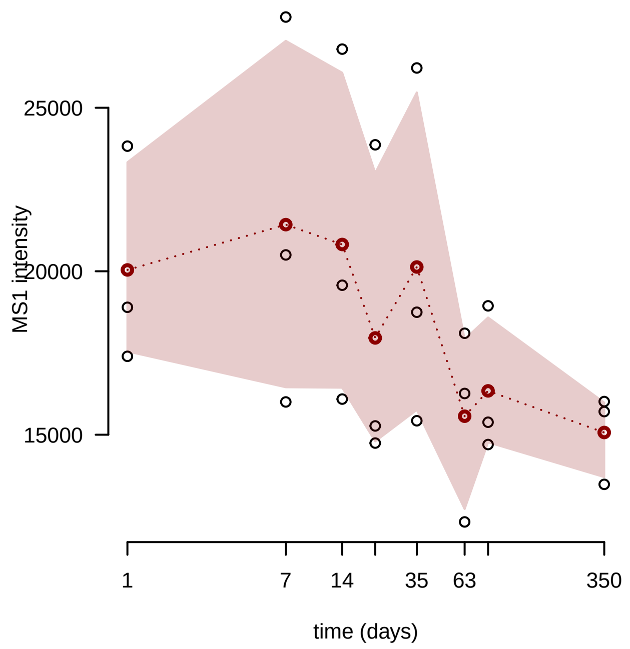

Q9DCJ1
GN=Mlst8
MTOR associated protein, LST8 homolog (S ...

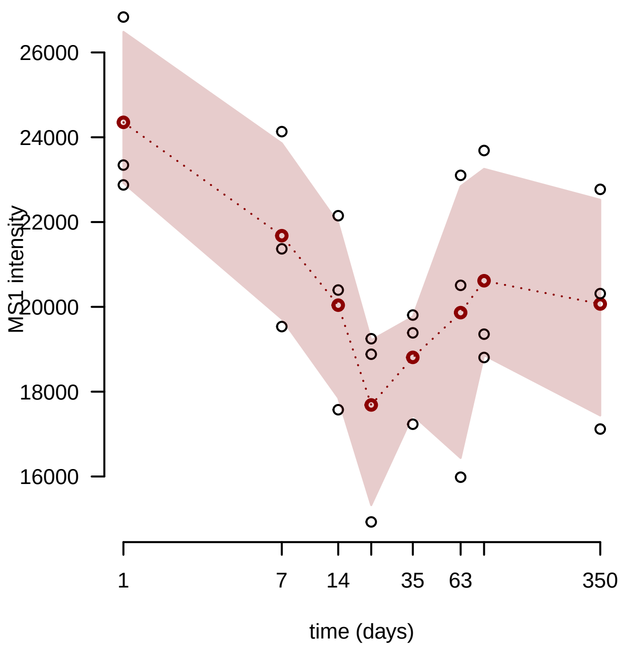

Q9DCJ5
GN=Ndufa8
NADH:ubiquinone oxidoreductase subunit A ...

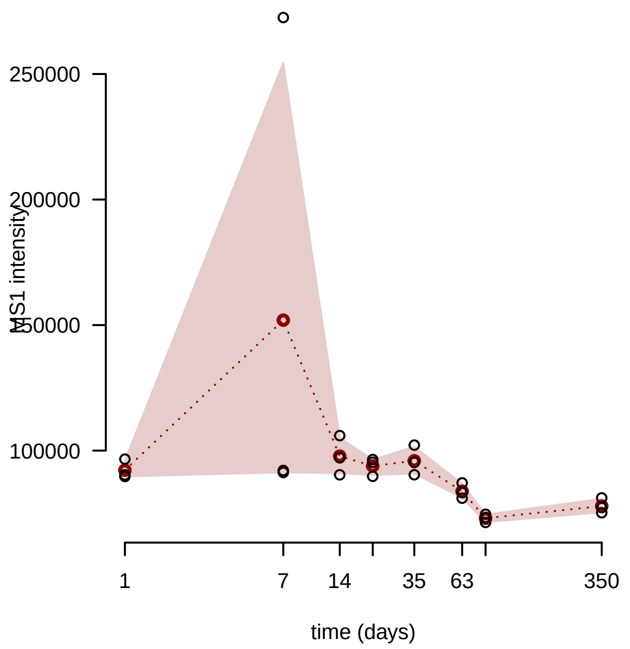

Q9DCJ7
GN=Aurkaip1
aurora kinase A interacting protein 1

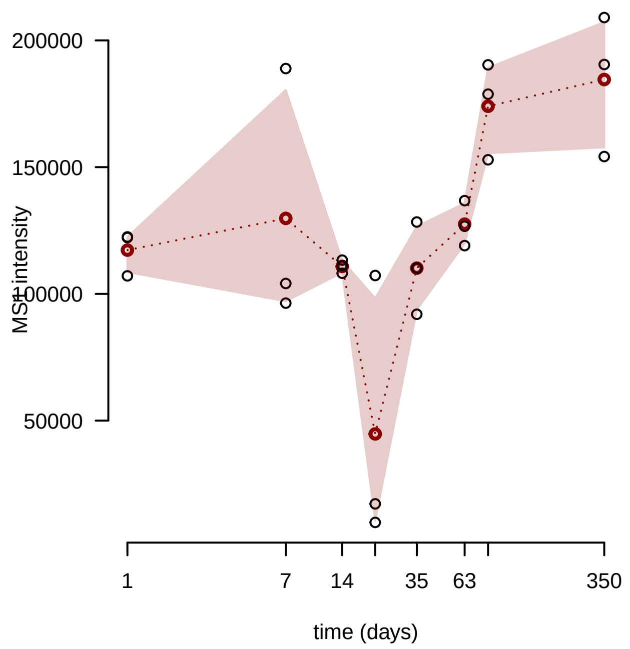

Q9DCJ9
GN=Npl
N−acetylneuraminate pyruvate lyase

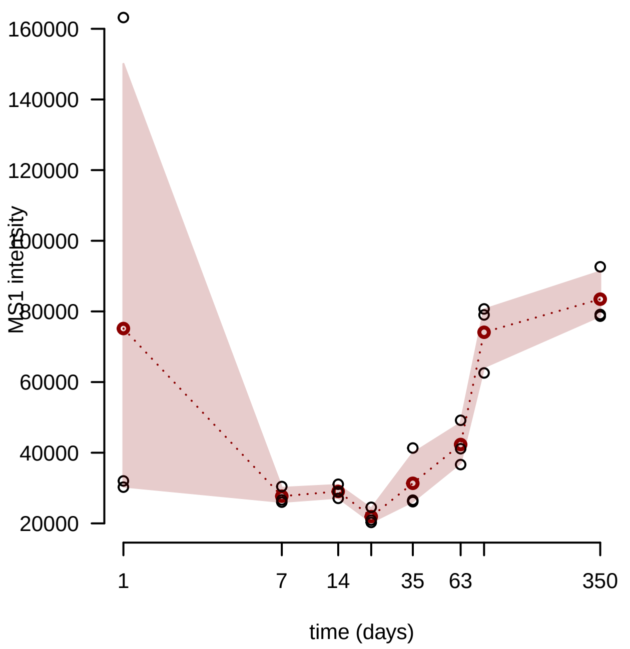

Q9DCK3
GN=Tspan4
tetraspanin 4

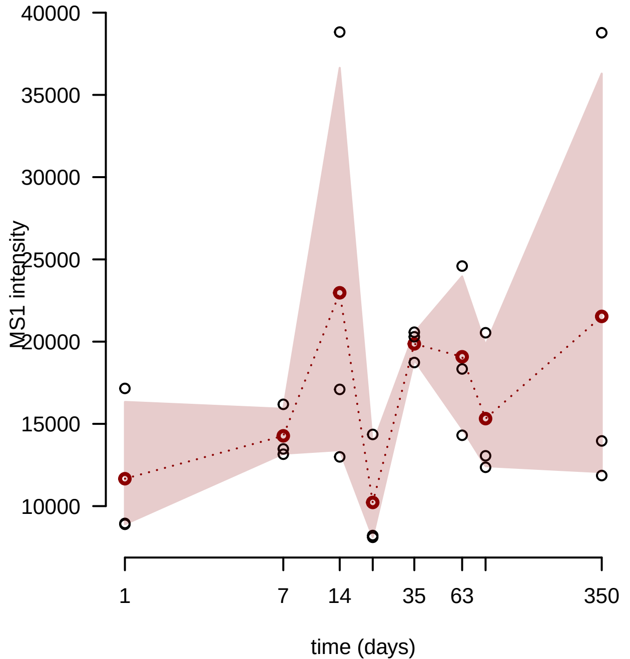

Q9DCL2
GN=Ciao2a
cytosolic iron−sulfur assembly component ...

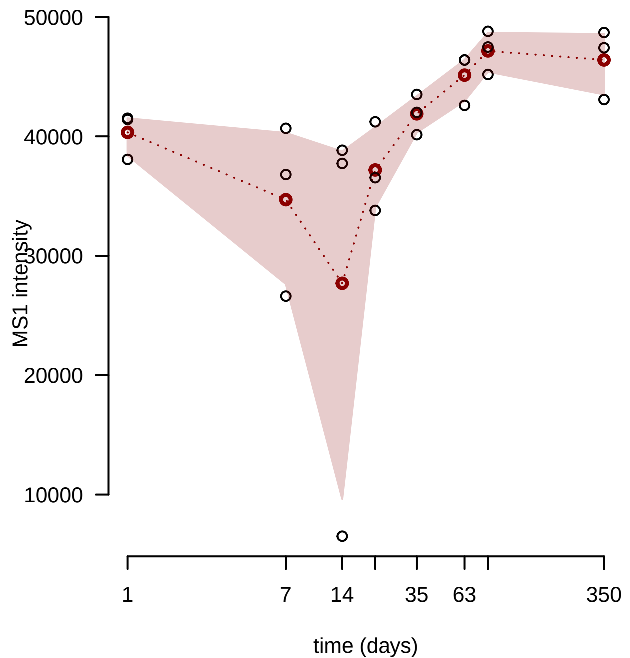

Q9DCL4
GN=Mettl15
methyltransferase like 15

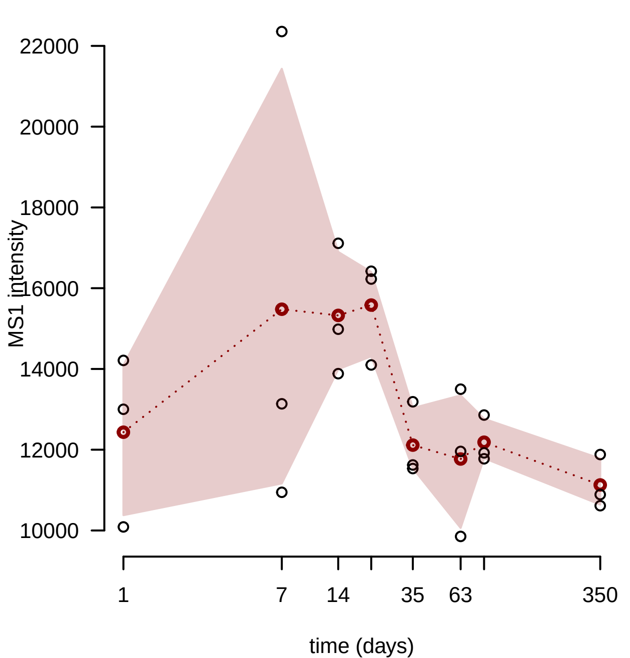

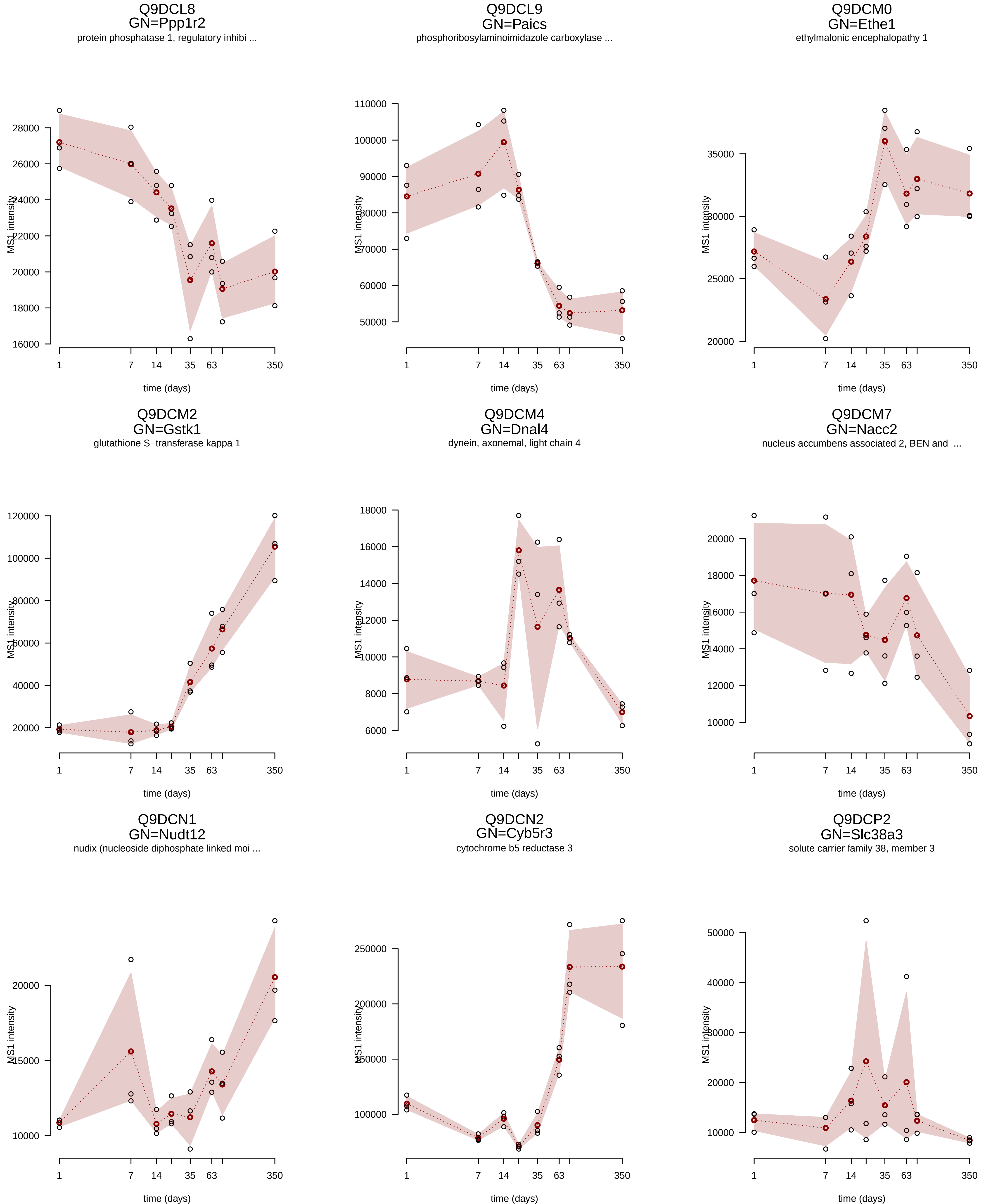
Q9DCL8
GN=Ppp1r2
protein phosphatase 1, regulatory inhibi ...
MS1 intensity
16000 18000 20000 22000 24000 26000 28000
time (days)
1 7 14 35 63 350
Q9DCL9
GN=Paics
phosphoribosylaminoimidazole carboxylase ...
MS1 intensity
50000 60000 70000 80000 90000 100000 110000
time (days)
1 7 14 35 63 350
Q9DCM0
GN=Ethe1
ethylmalonic encephalopathy 1
MS1 intensity
20000 25000 30000 35000
time (days)
1 7 14 35 63 350
Q9DCM2
GN=Gstk1
glutathione S−transferase kappa 1
MS1 intensity
20000 40000 60000 80000 100000 120000
time (days)
1 7 14 35 63 350
Q9DCM4
GN=Dnal4
dynein, axonemal, light chain 4
MS1 intensity
6000 8000 10000 12000 14000 16000 18000
time (days)
1 7 14 35 63 350
Q9DCM7
GN=Nacc2
nucleus accumbens associated 2, BEN and ...
MS1 intensity
10000 12000 14000 16000 18000 20000
time (days)
1 7 14 35 63 350
Q9DCN1
GN=Nudt12
nudix (nucleoside diphosphate linked moi ...
MS1 intensity
10000 15000 20000
time (days)
1 7 14 35 63 350
Q9DCN2
GN=Cyb5r3
cytochrome b5 reductase 3
MS1 intensity
100000 150000 200000 250000
time (days)
1 7 14 35 63 350
Q9DCP2
GN=Slc38a3
solute carrier family 38, member 3
MS1 intensity
10000 20000 30000 40000 50000
time (days)
1 7 14 35 63 350

Q9DCR2
GN=Ap3s1
adaptor−related protein complex 3, sigma ...

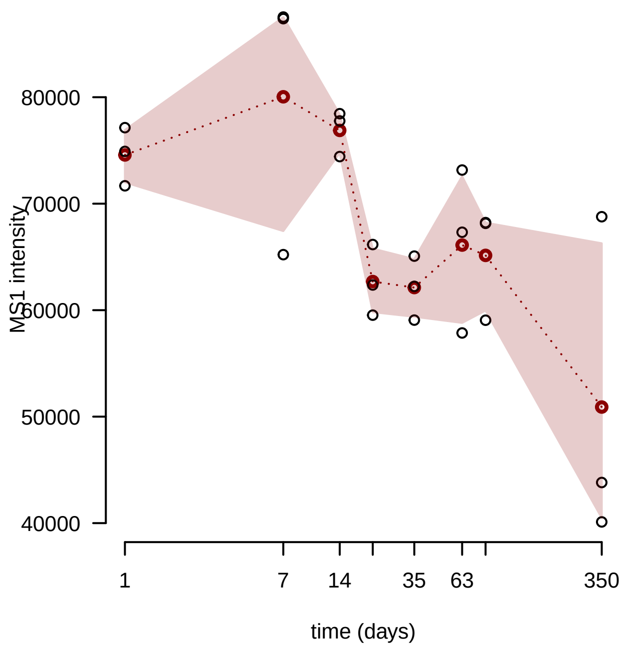

Q9DCS1
GN=Tmem176a
transmembrane protein 176A

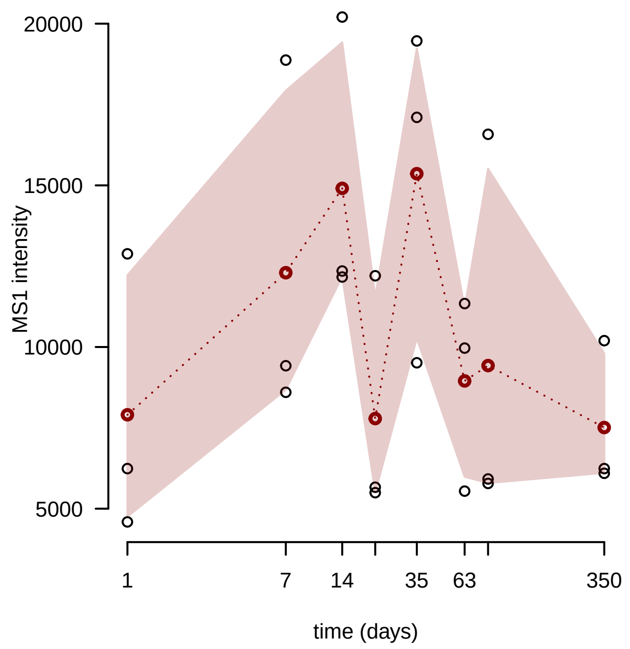

Q9DCS2
GN=Mettl26
methyltransferase like 26

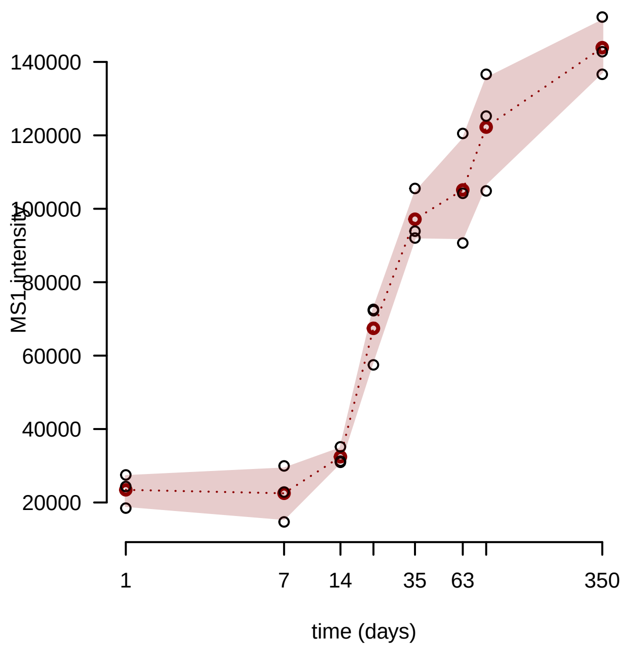

Q9DCS3
GN=Mecr
mitochondrial trans−2−enoyl−CoA reductas ...

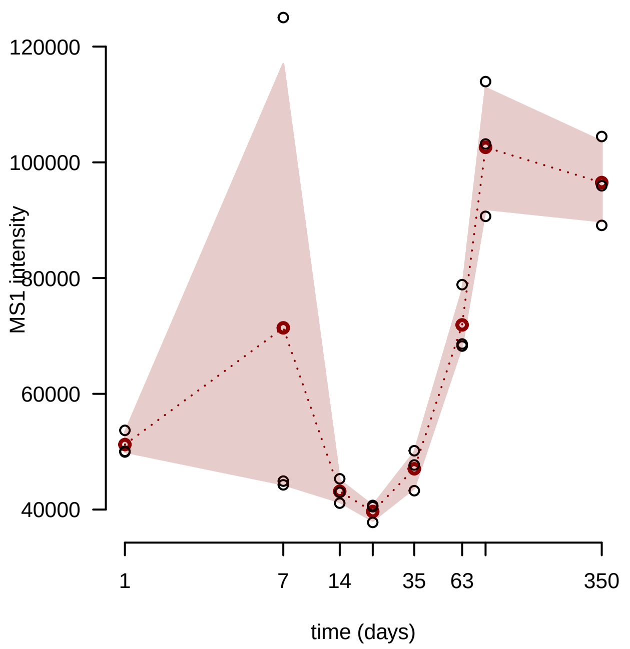

Q9DCS9
GN=Ndufb10
NADH:ubiquinone oxidoreductase subunit B ...

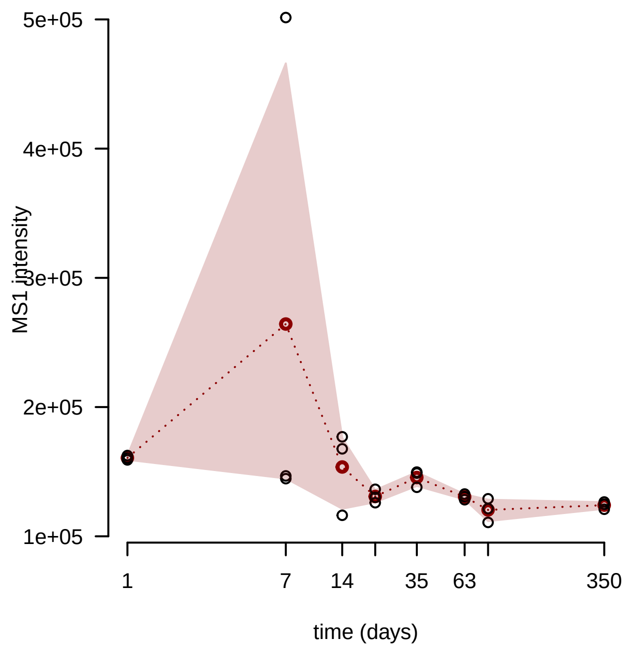

Q9DCT1
GN=Akr1e2

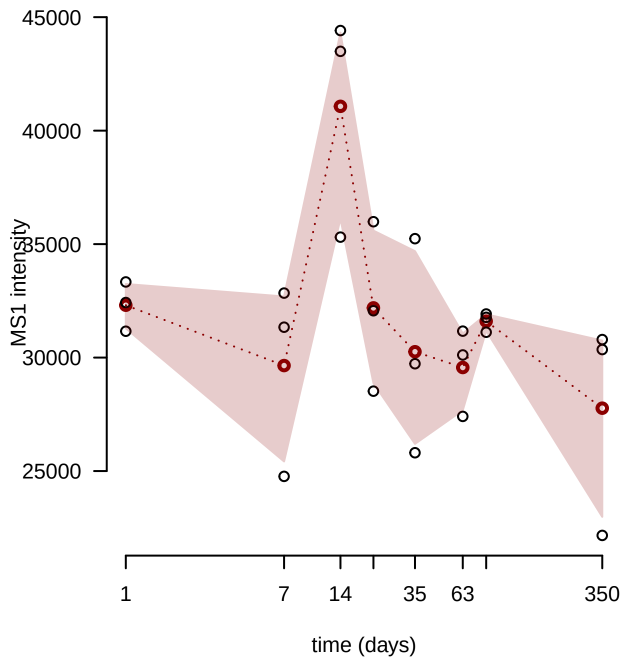

Q9DCT2
GN=Ndufs3
NADH:ubiquinone oxidoreductase core subu ...

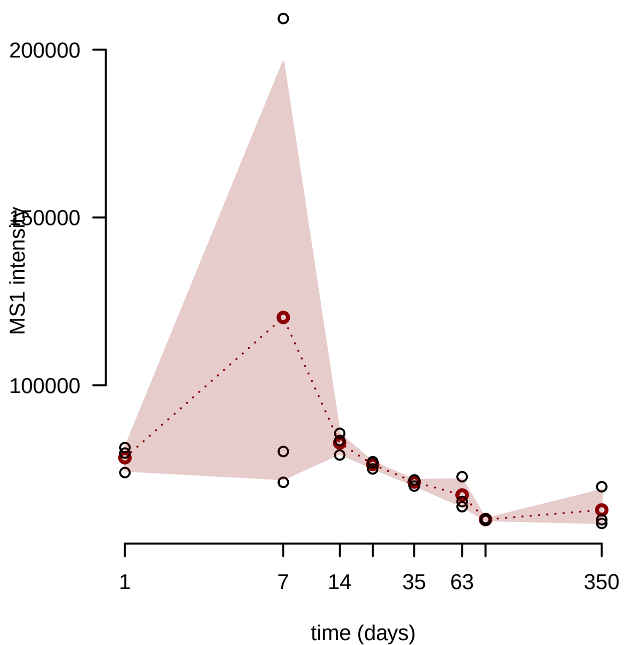

Q9DCT5
GN=Sdf2
stromal cell derived factor 2

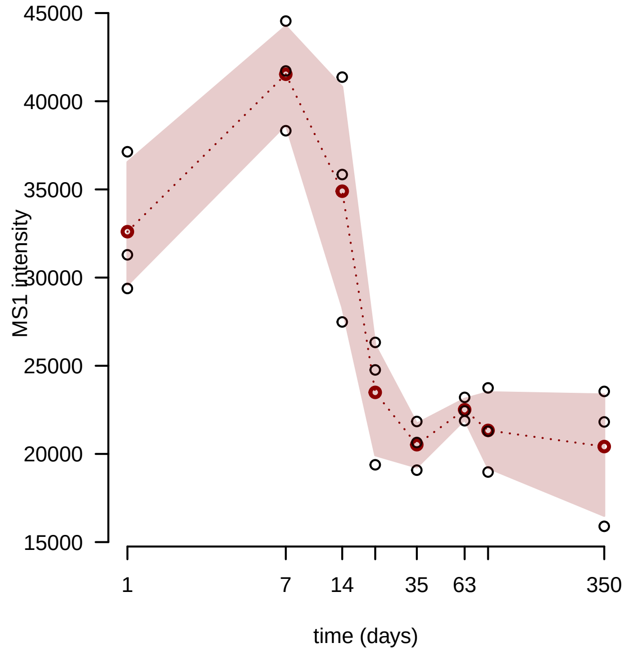

Q9DCT6
GN=Bap18

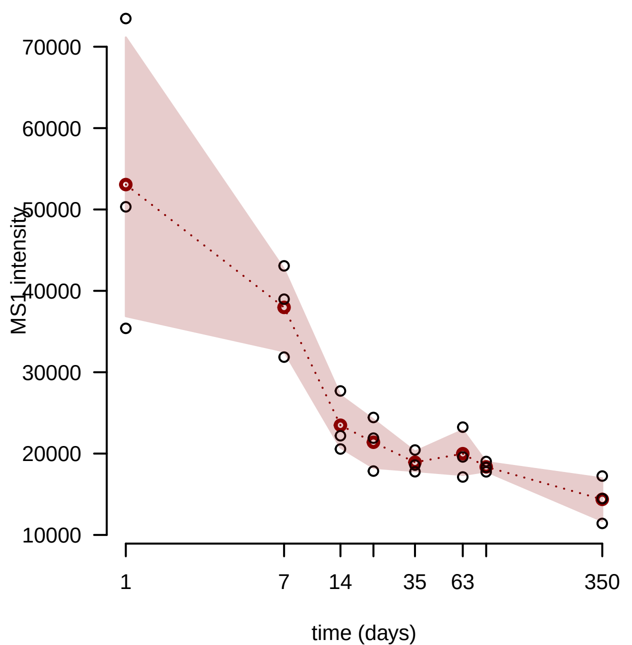

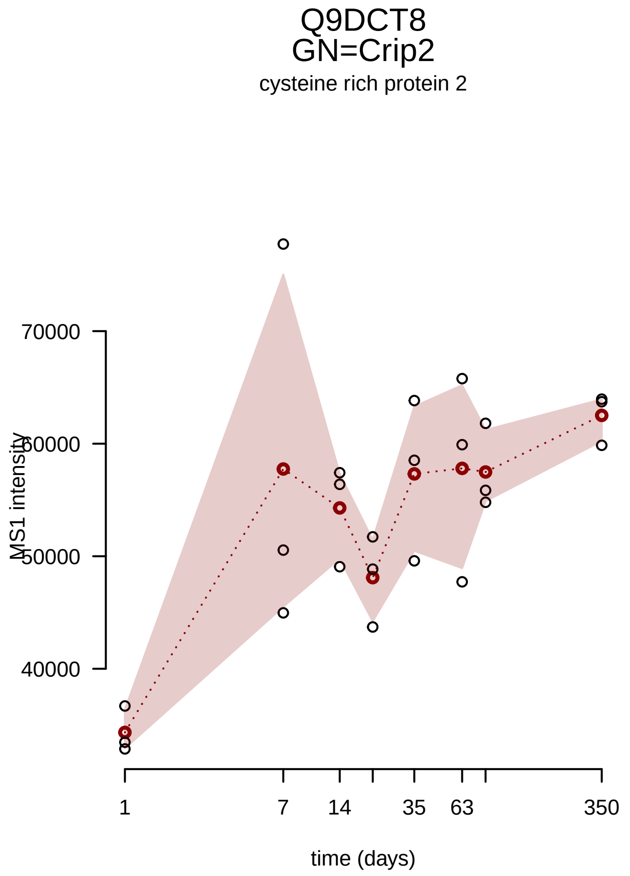
Q9DCT8
GN=Crip2
cysteine rich protein 2
MS1 intensity
70000
60000
50000
40000
1
7
14
35
63
350
time (days)

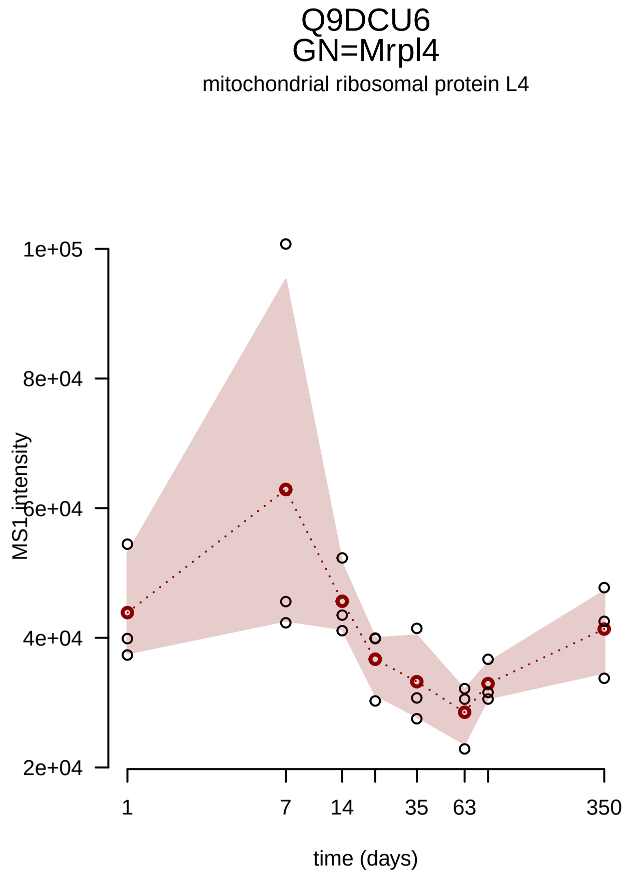
Q9DCU6
GN=Mrpl4
mitochondrial ribosomal protein L4
MS1 intensity
1e+05
8e+04
6e+04
4e+04
2e+04
1
7
14
35
63
350
time (days)

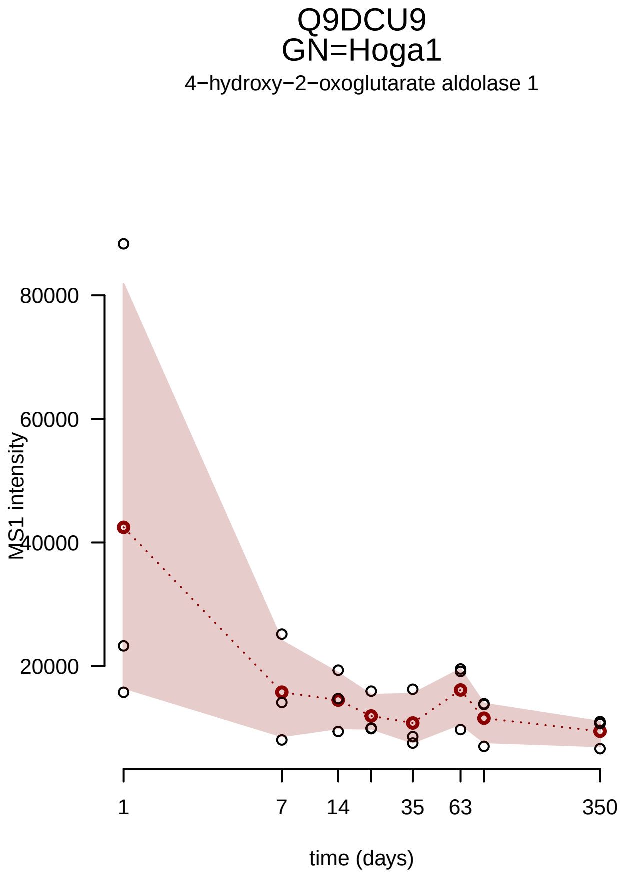
Q9DCU9
GN=Hoga1
4−hydroxy−2−oxoglutarate aldolase 1
MS1 intensity
80000
60000
40000
20000
1
7
14
35
63
350
time (days)

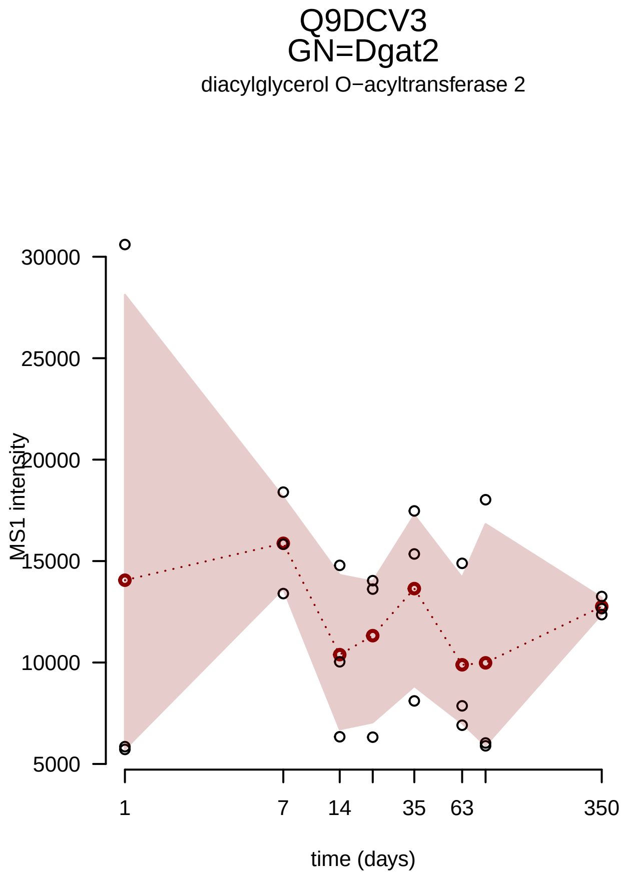
Q9DCV3
GN=Dgat2
diacylglycerol O−acyltransferase 2
MS1 intensity
30000
25000
20000
15000
10000
5000
1
7
14
35
63
350
time (days)

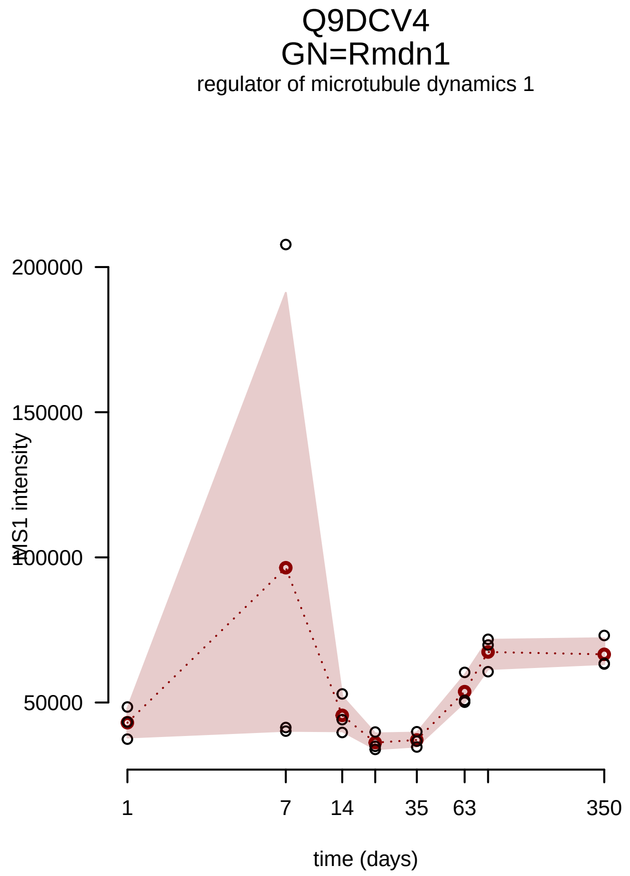
Q9DCV4
GN=Rmdn1
regulator of microtubule dynamics 1
MS1 intensity
200000
150000
100000
50000
1
7
14
35
63
350
time (days)

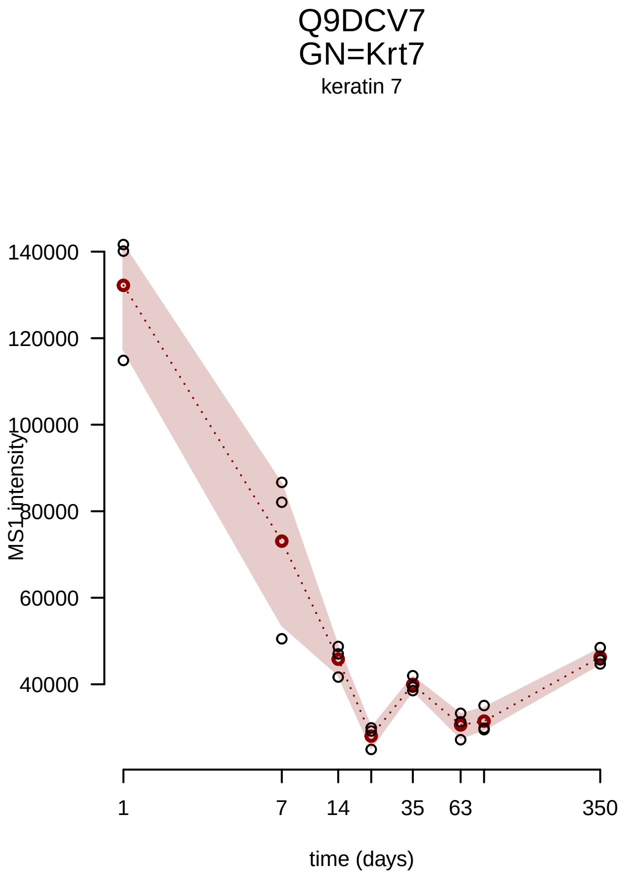
Q9DCV7
GN=Krt7
keratin 7
MS1 intensity
140000
120000
100000
80000
60000
40000
1
7
14
35
63
350
time (days)

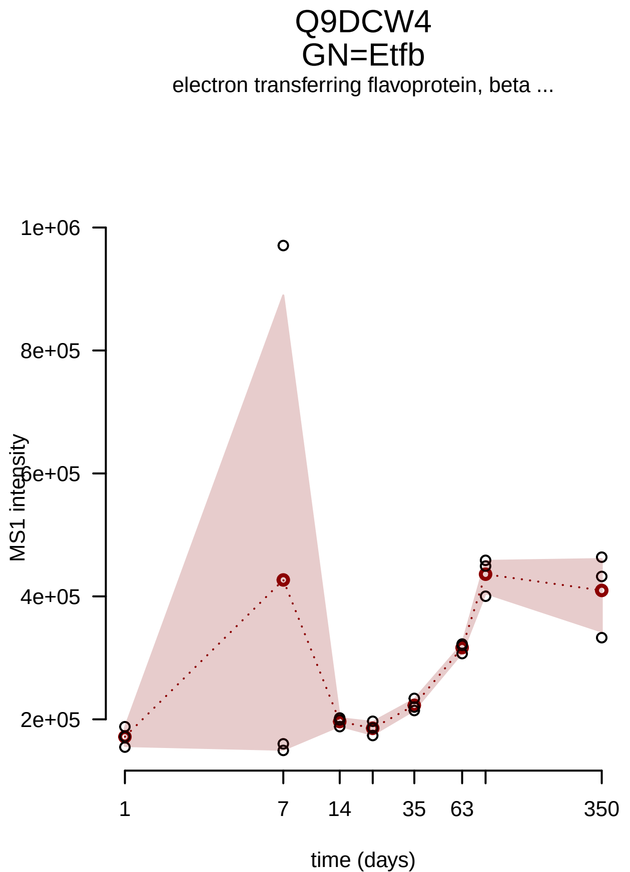
Q9DCW4
GN=Etfb
electron transferring flavoprotein, beta ...
MS1 intensity
1e+06
8e+05
6e+05
4e+05
2e+05
1
7
14
35
63
350
time (days)

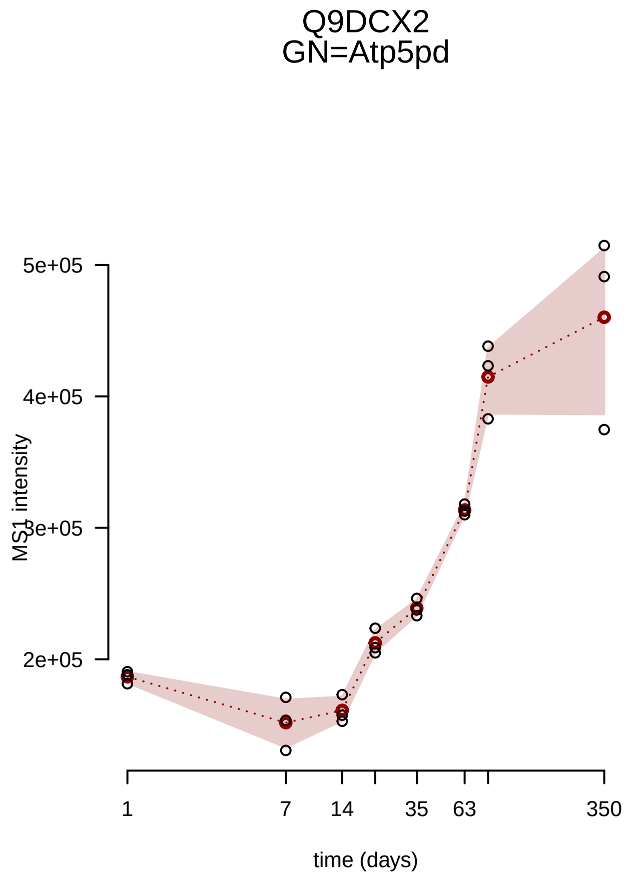
Q9DCX2
GN=Atp5pd
MS1 intensity
5e+05
4e+05
3e+05
2e+05
1
7
14
35
63
350
time (days)

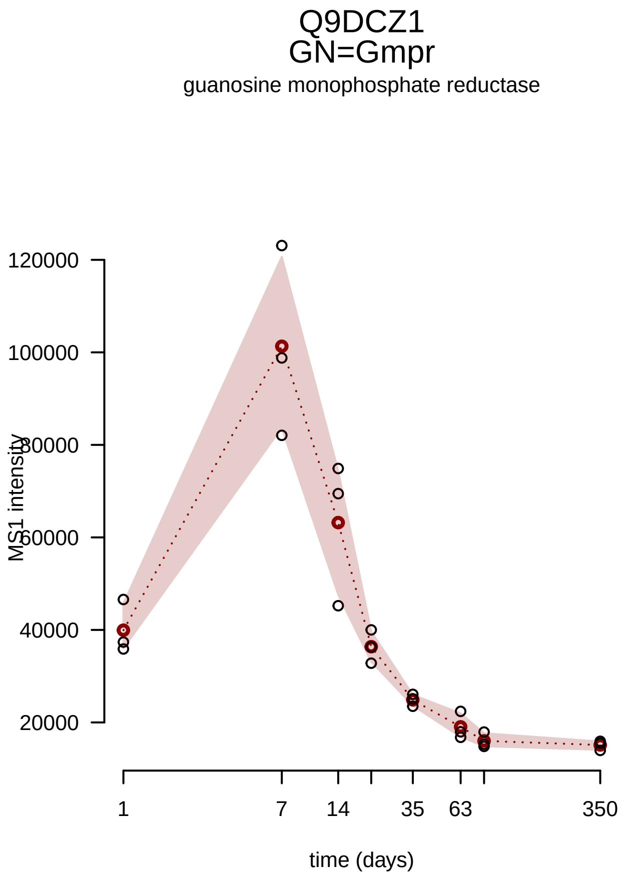
Q9DCZ1
GN=Gmpr
guanosine monophosphate reductase
MS1 intensity
120000
100000
80000
60000
40000
20000
1
7
14
35
63
350
time (days)

Q9DCZ4
GN=Apoo
apolipoprotein O

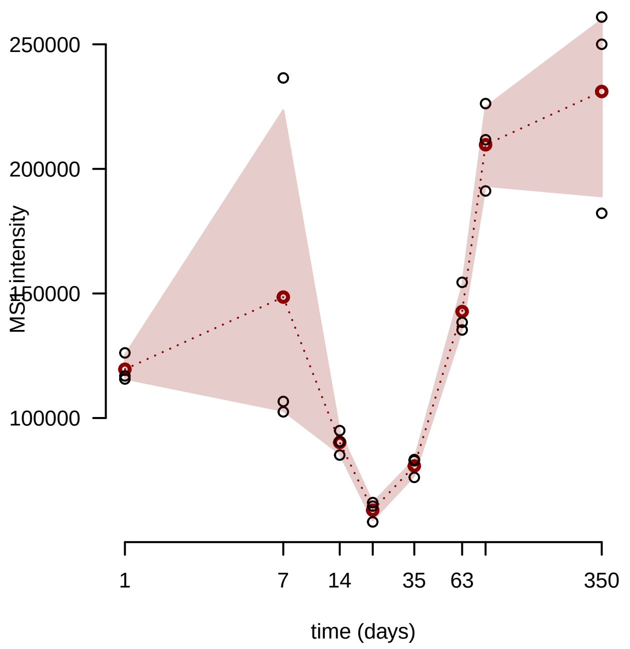

Q9DD02
GN=Hikeshi
heat shock protein nuclear import factor

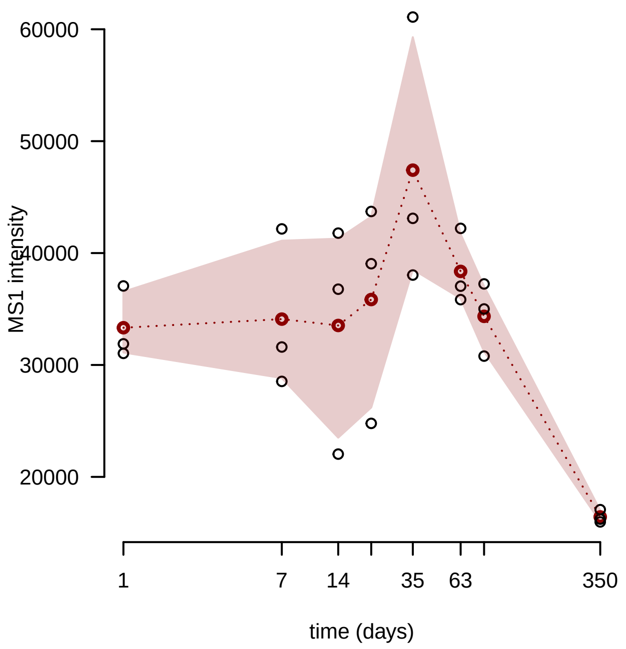

Q9DD03
GN=Rab13
RAB13, member RAS oncogene family

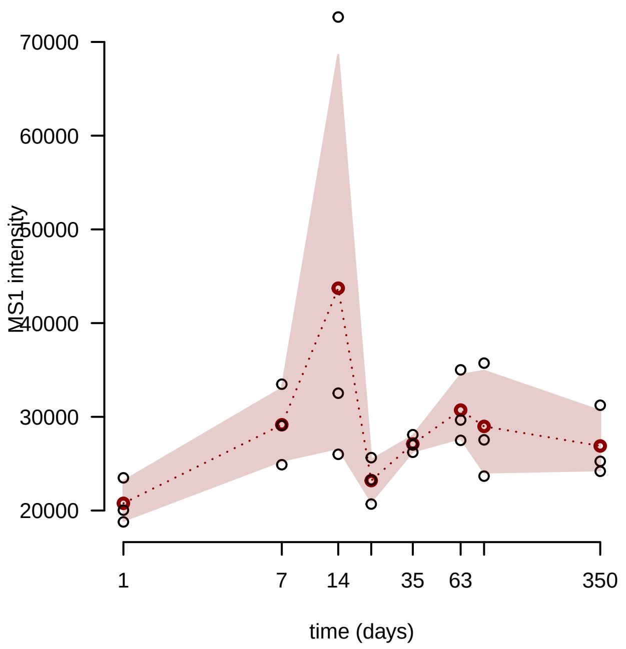

Q9DD06
GN=Rarres2
retinoic acid receptor responder (tazaro ...

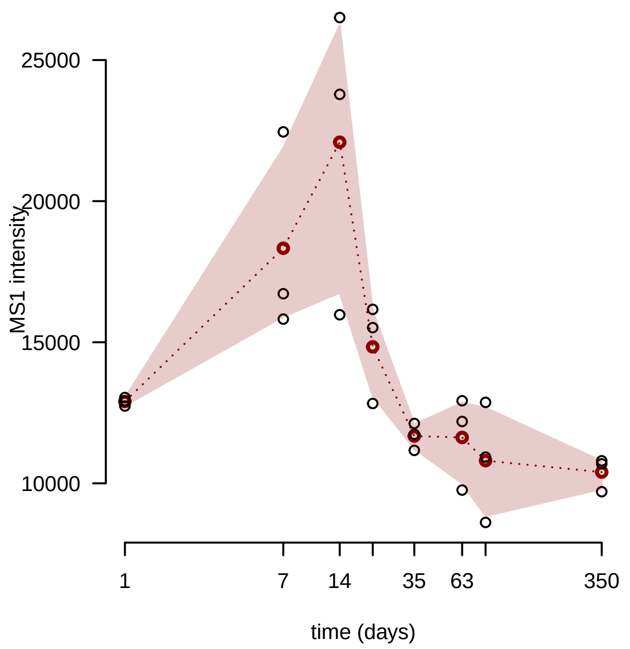

Q9DD18
GN=Dtd1
D−tyrosyl−tRNA deacylase 1

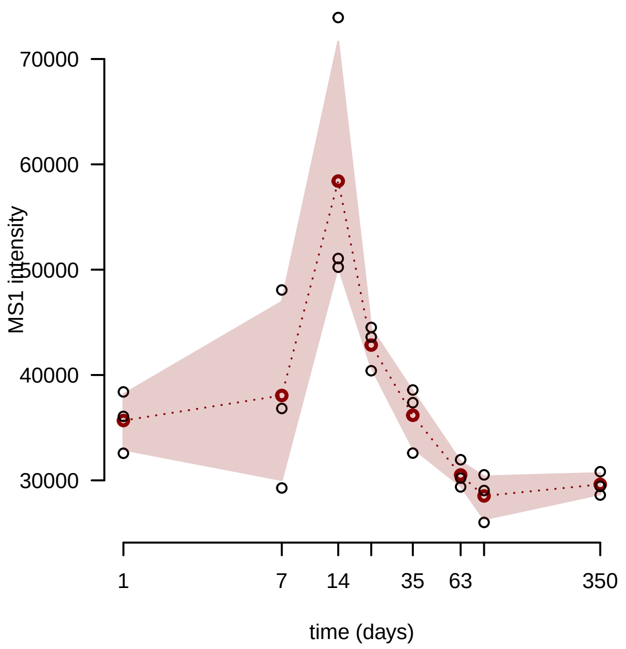

Q9DD24
GN=Tceal9
transcription elongation factor A like 9

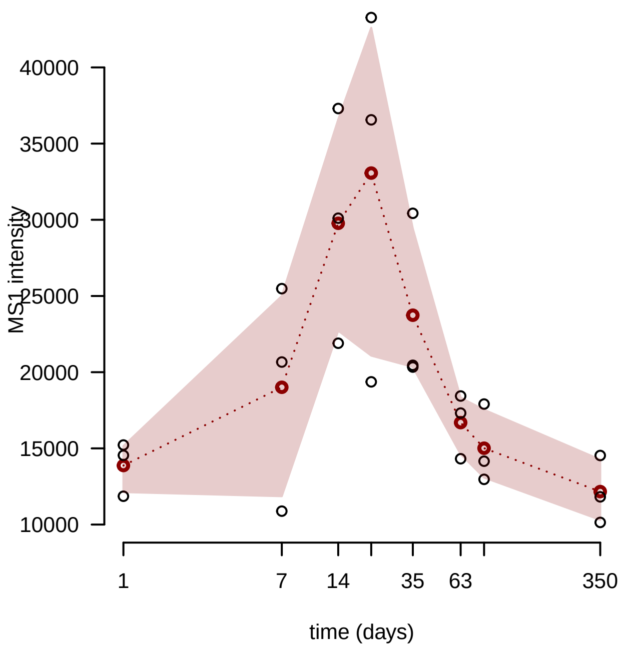

Q9EP52
GN=Twsg1
twisted gastrulation BMP signaling modul ...

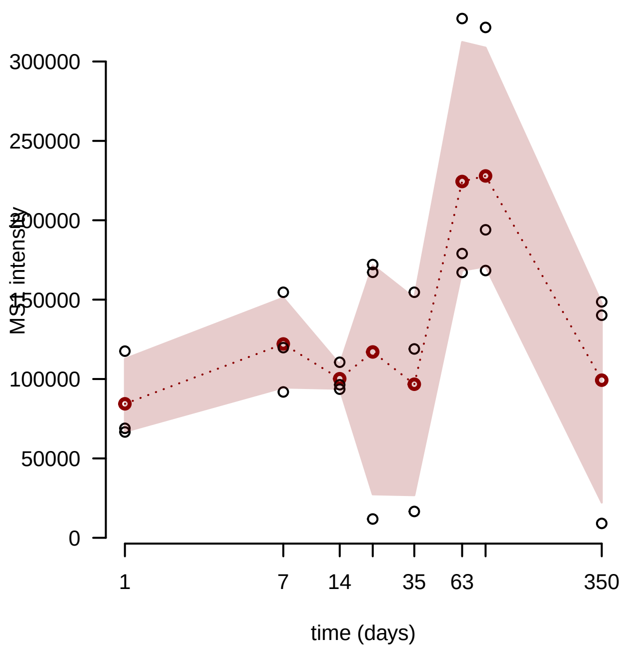

Q9EP53
GN=Tsc1
TSC complex subunit 1

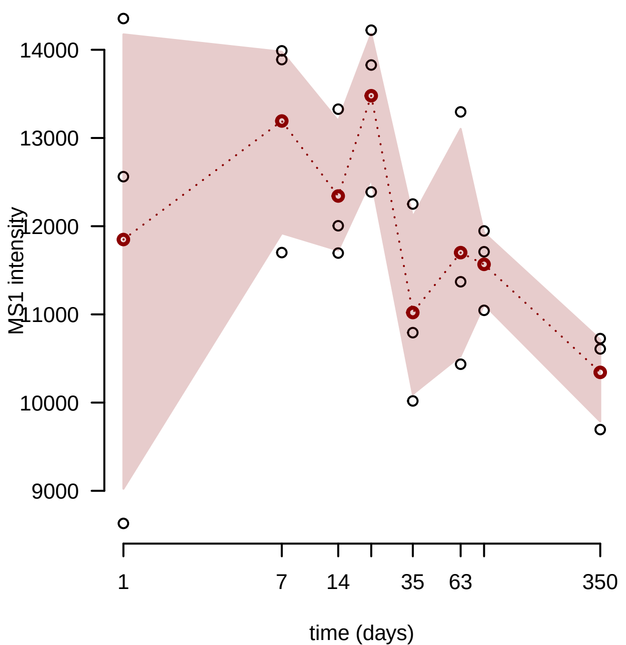

Q9EP69
GN=Sacm1l
SAC1 suppressor of actin mutations 1−lik ...

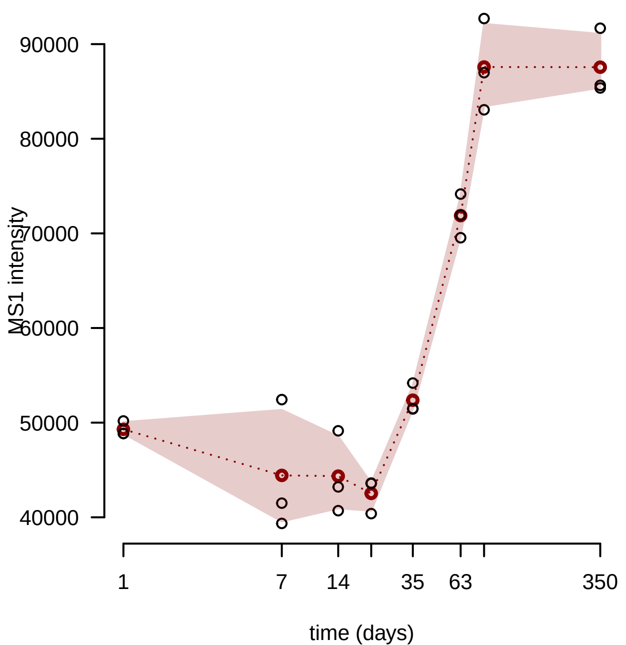

Q9EP71
GN=Rai14
retinoic acid induced 14

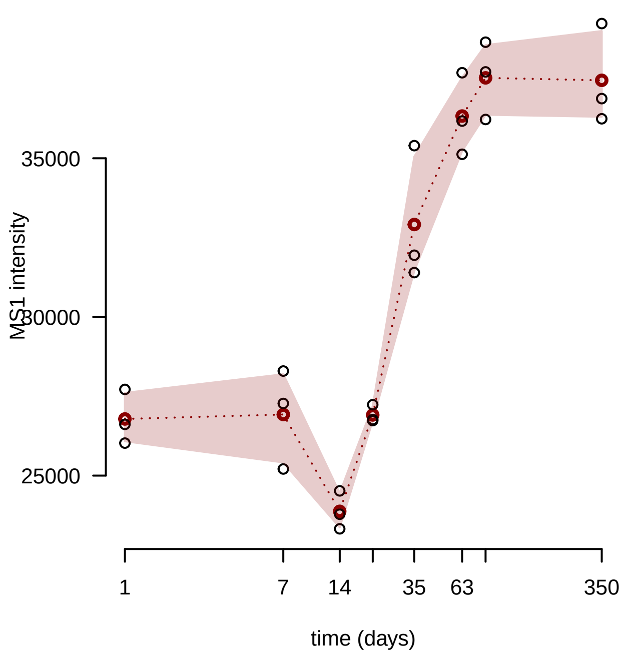

Q9EP72
GN=Emc7
ER membrane protein complex subunit 7

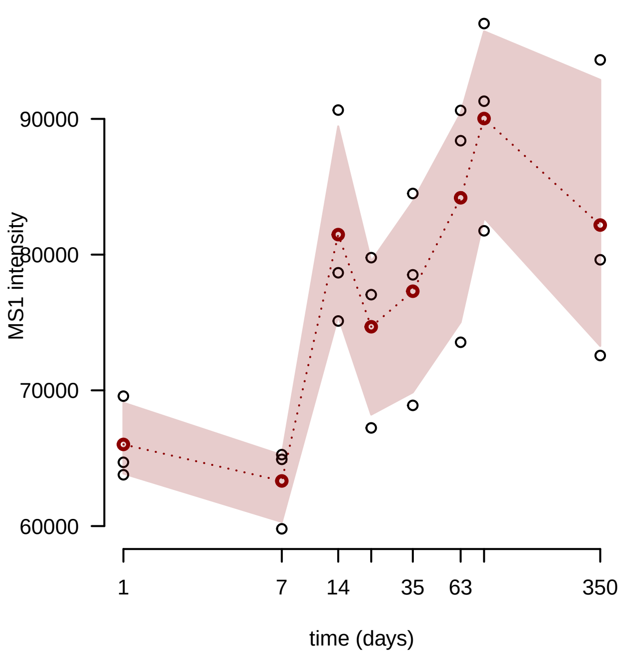

Q9EP75
GN=Cyp4f14
cytochrome P450, family 4, subfamily f, ...

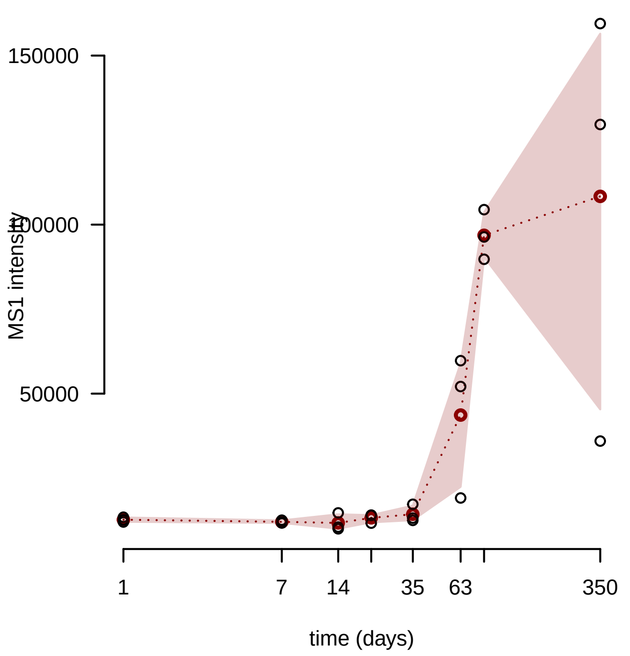

Q9EP82
GN=Wdr4
WD repeat domain 4

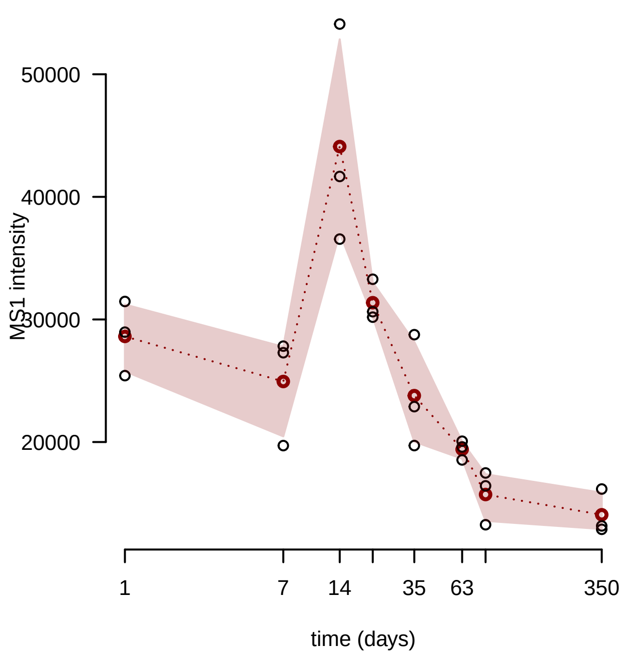

Q9EP89
GN=Lactb
lactamase, beta

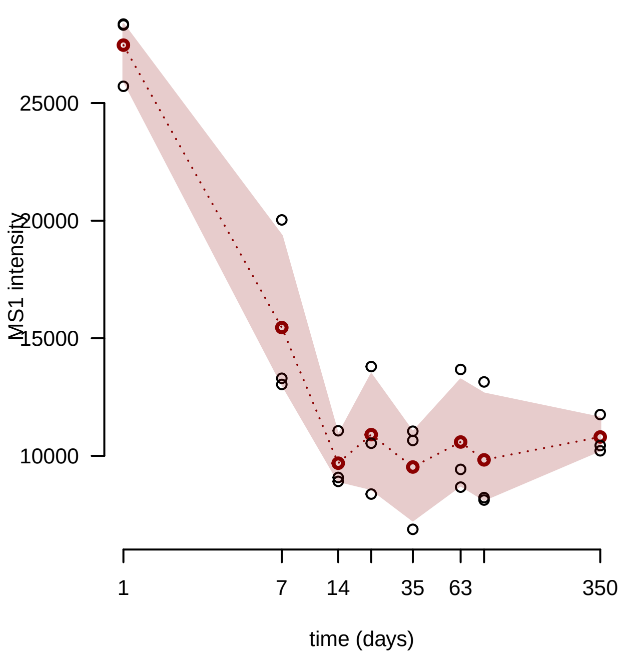

Q9EP97
GN=Senp3
SUMO/sentrin specific peptidase 3

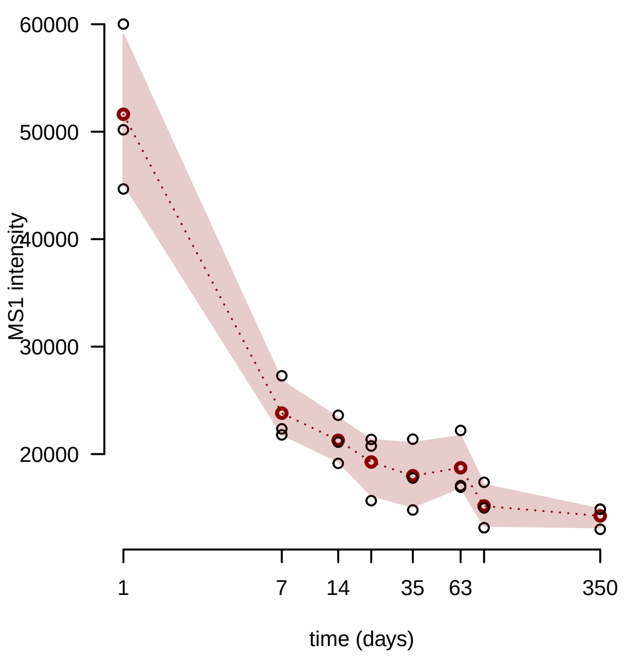

Q9EPA7
GN=Nmnat1
nicotinamide nucleotide adenylyltransfer ...

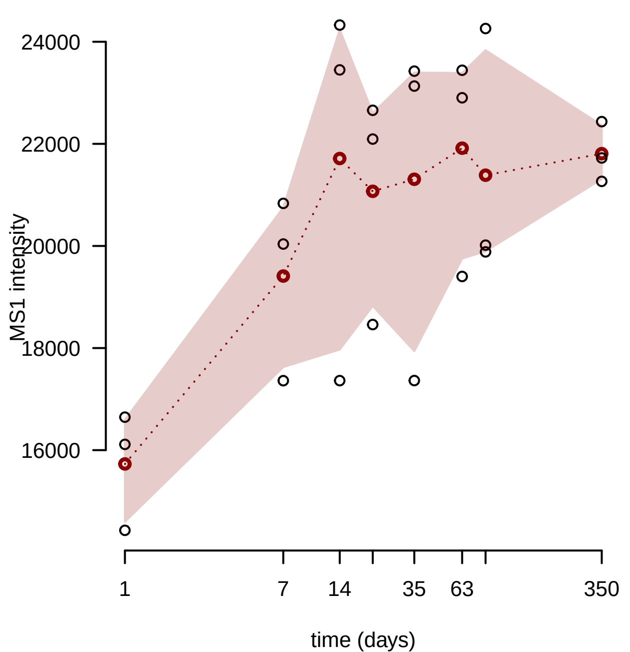

Q9EPB4
GN=Pycard
PYD and CARD domain containing

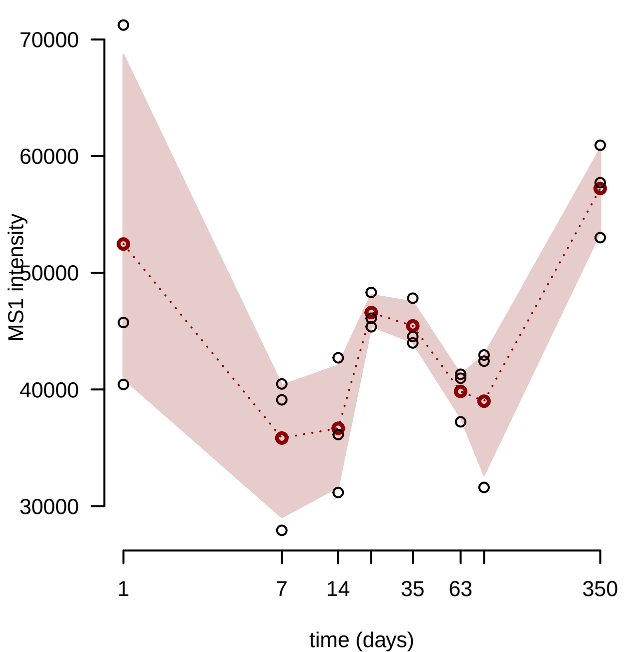

Q9EPB5
GN=Serhl
serine hydrolase−like

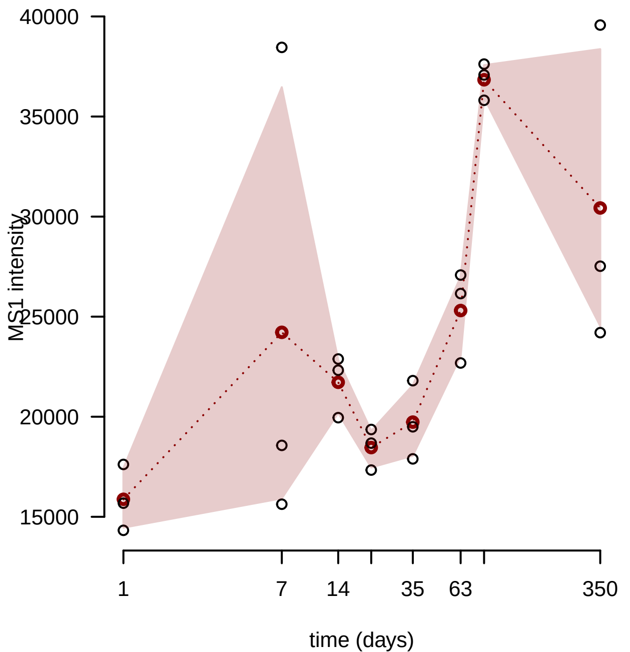

Q9EPC1
GN=Parva
parvin, alpha

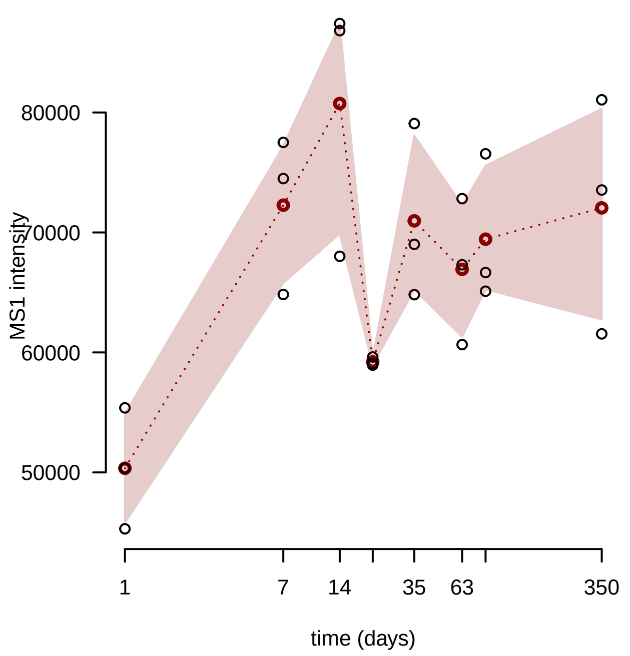

Q9EPC5
GN=Rbp7
retinol binding protein 7, cellular

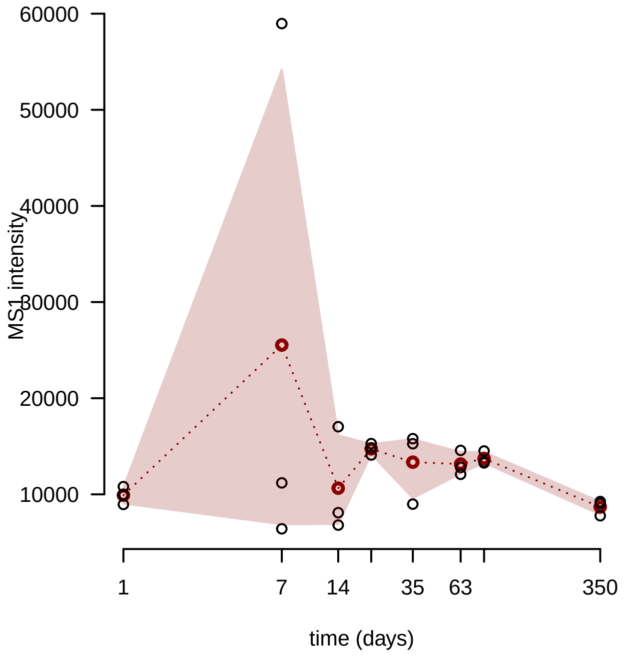

Q9EPE9
GN=Atp13a1
ATPase type 13A1

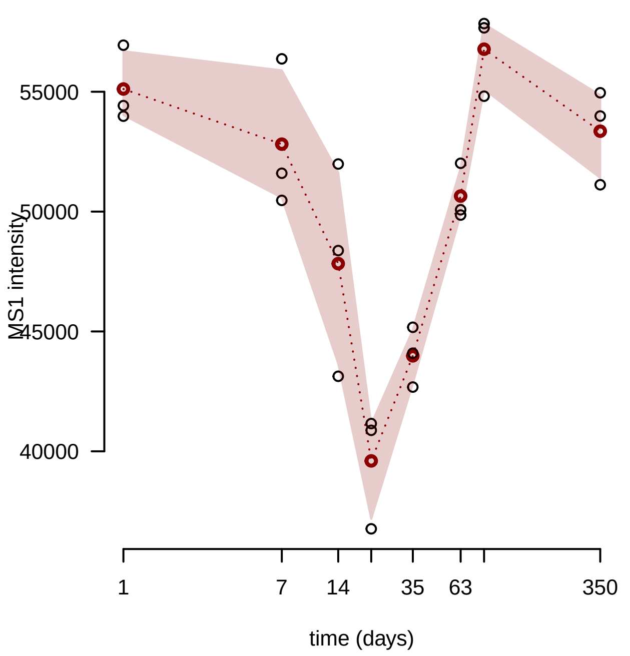

Q9EPJ9
GN=Arfgap1
ADP−ribosylation factor GTPase activatin ...

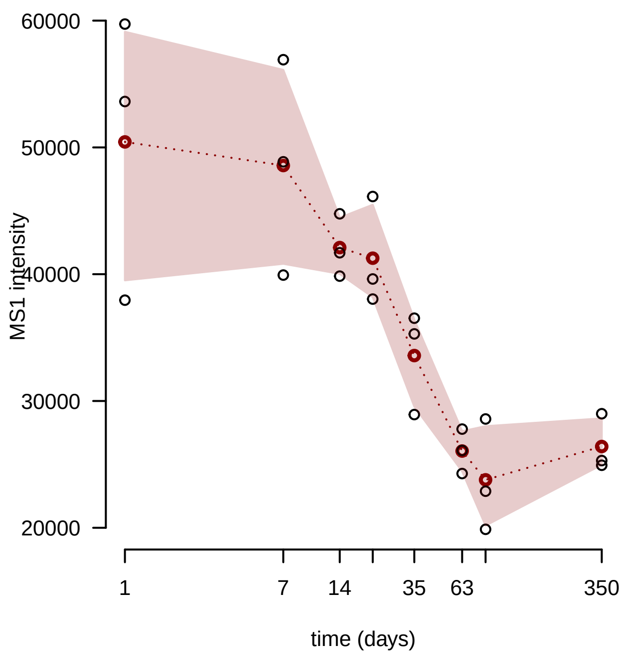

Q9EPK2
GN=Rp2
retinitis pigmentosa 2 homolog

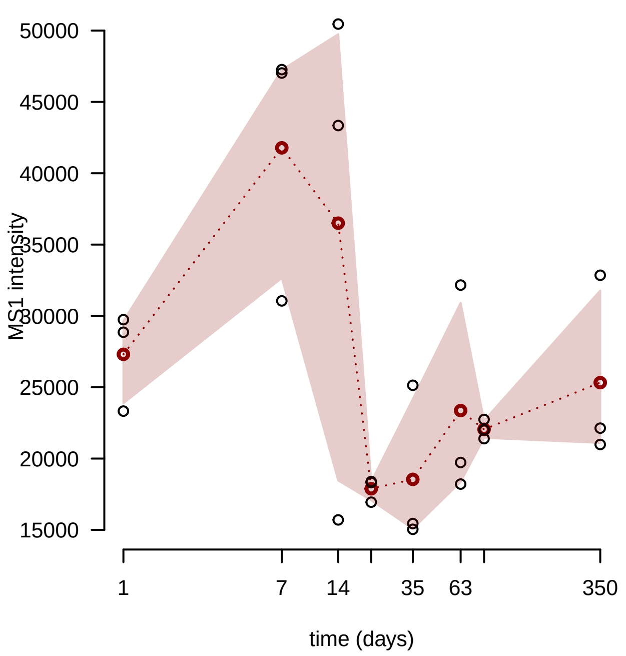

Q9EPK5
GN=Wwtr1
WW domain containing transcription regul ...

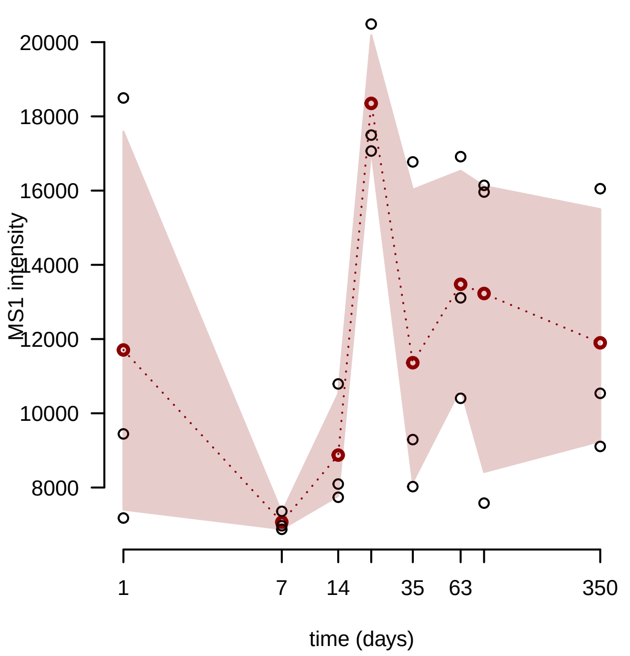

Q9EPK6
GN=Sil1
SIL1 nucleotide exchange factor

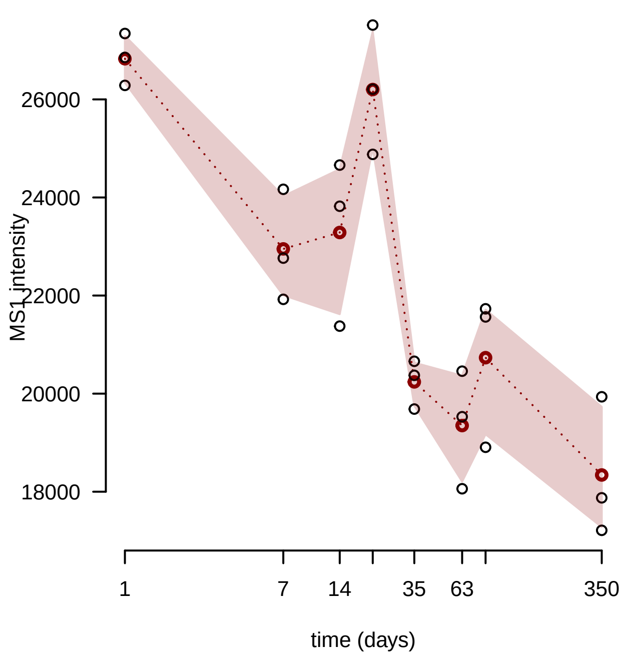

Q9EPK7
GN=Xpo7
exportin 7

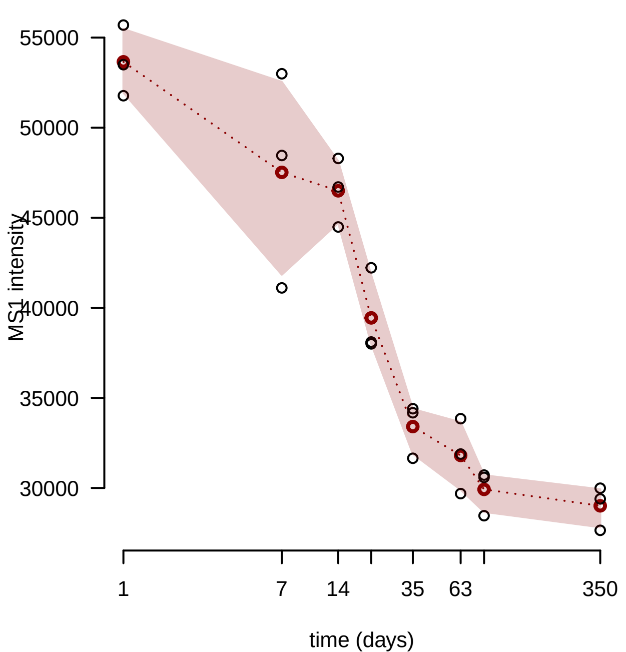

Q9EPL0
GN=Xylt2
xylosyltransferase II

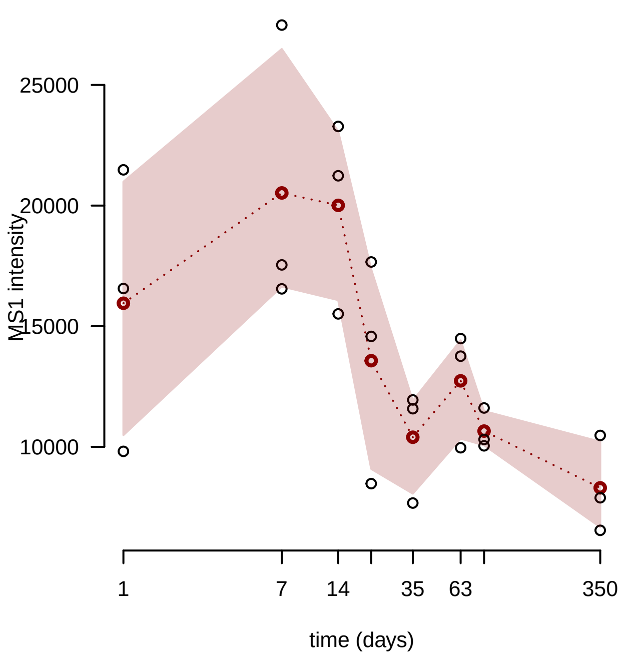

Q9EPL2
GN=Clstn1
calsyntenin 1

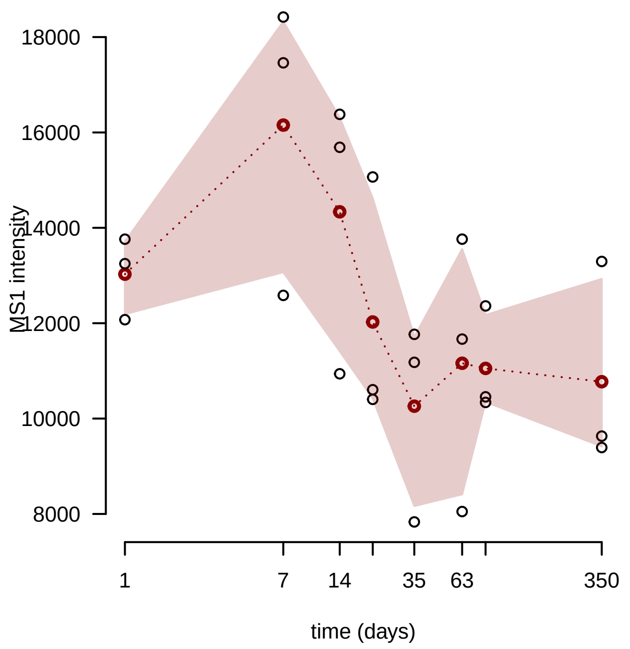

Q9EPL4
GN=Mettl9
methyltransferase like 9

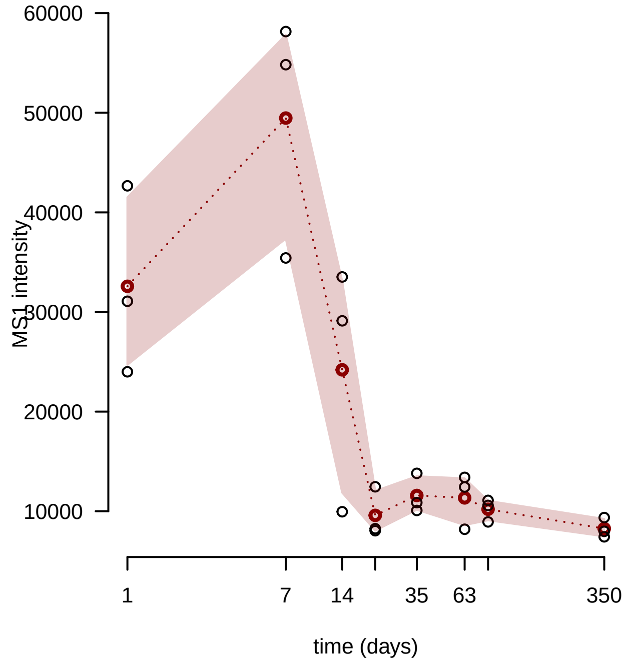

Q9EPL8
GN=Ipo7
importin 7

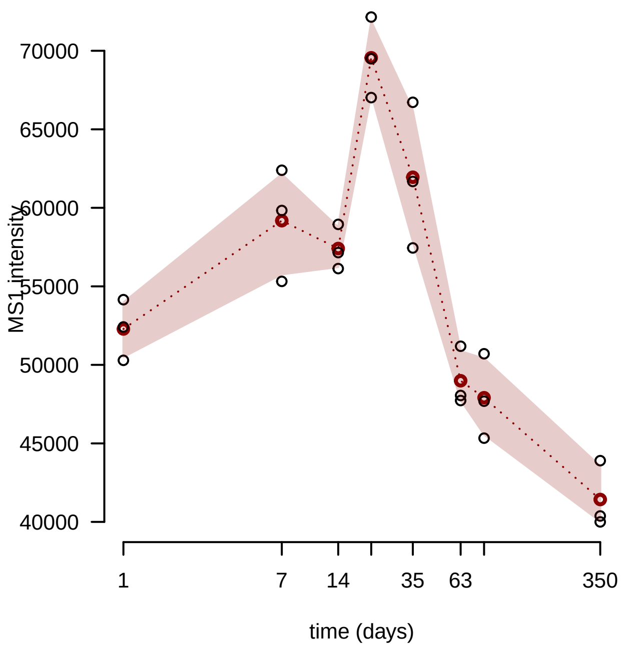

Q9EPL9
GN=Acox3
acyl−Coenzyme A oxidase 3, pristanoyl

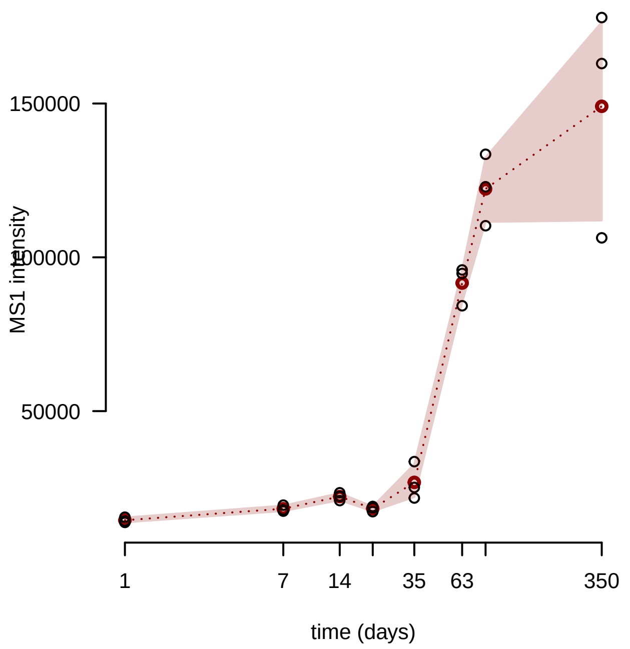

Q9EPM5
GN=Sync
syncoilin

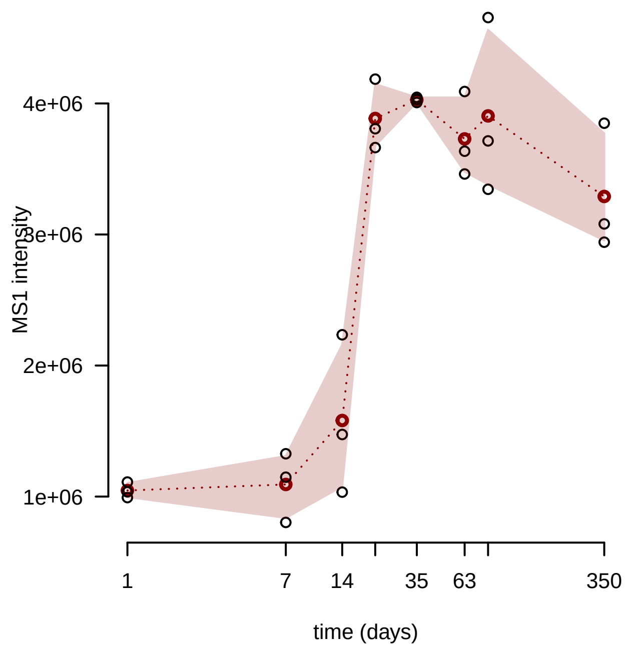

Q9EPN1
GN=Nbea
neurobeachin

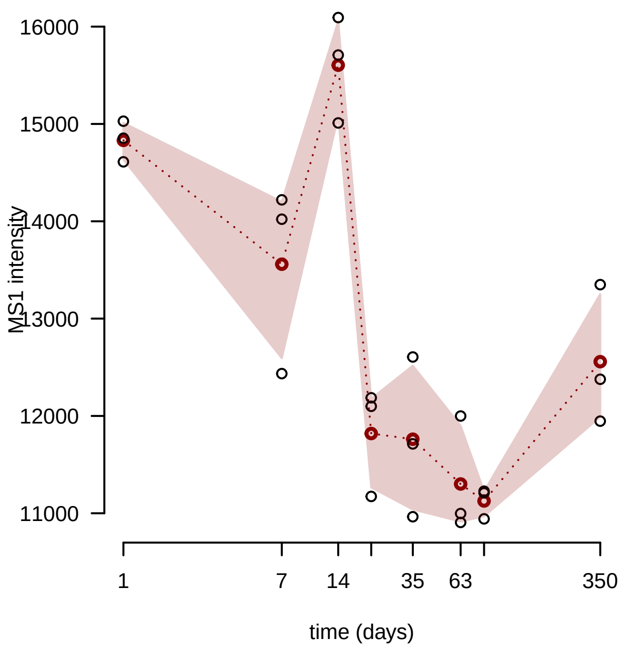

Q9EPQ7
GN=Stard5
StAR−related lipid transfer (START) doma ...

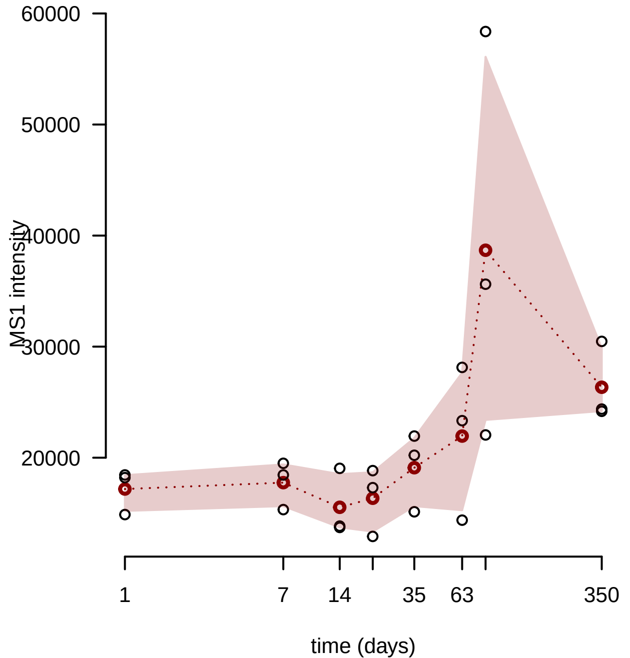

Q9EPQ8
GN=Tcf20
transcription factor 20

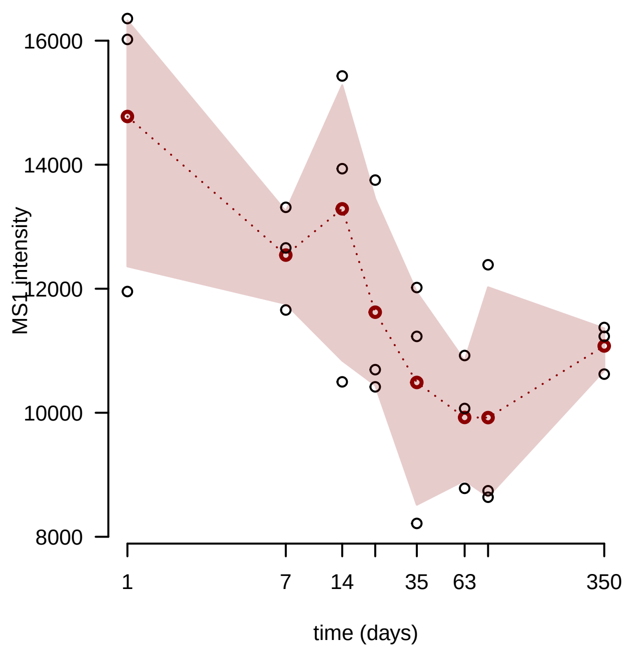

Q9EPR4
GN=Slc23a2
solute carrier family 23 (nucleobase tra ...

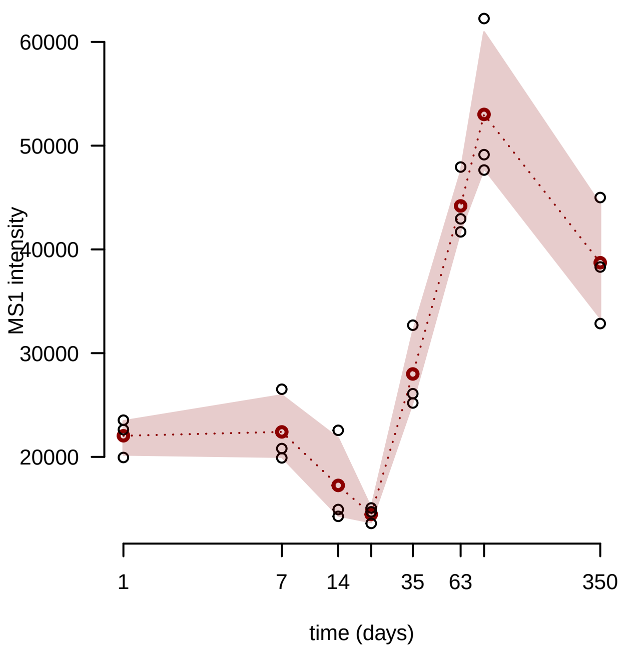

Q9EPR5
GN=Sorcs2
sortilin−related VPS10 domain containing ...

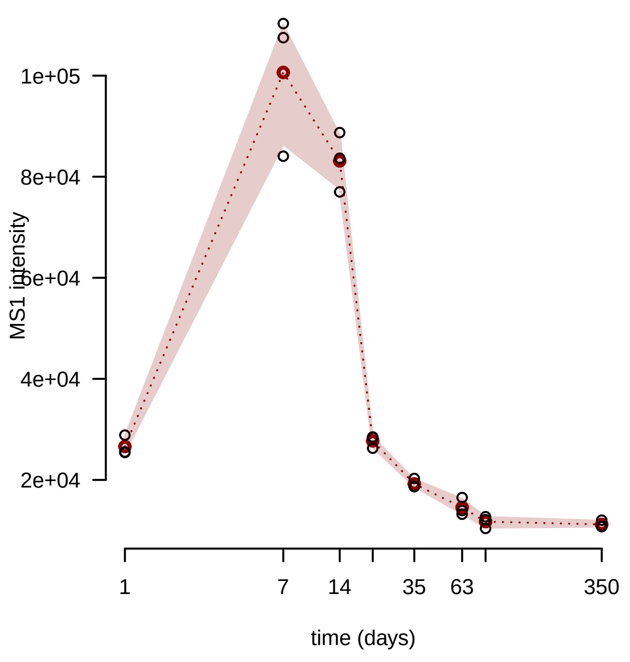

Q9EPS3
GN=Glce
glucuronyl C5−epimerase

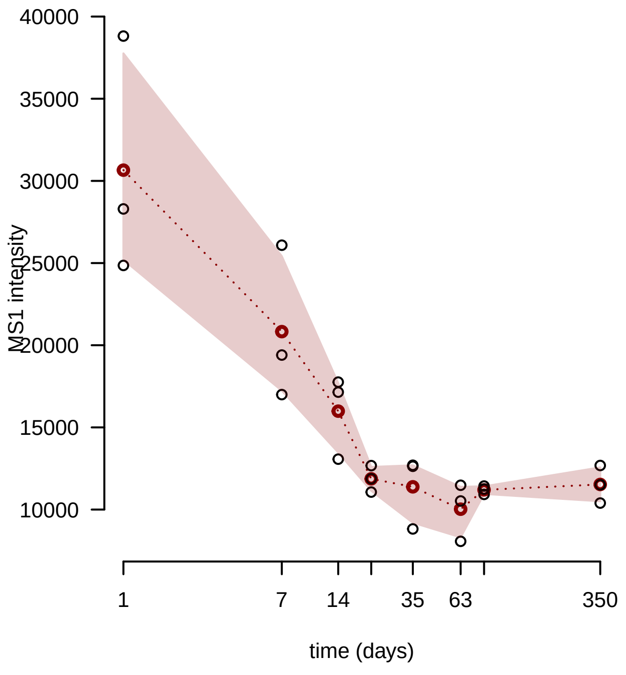

Q9EPT5
GN=Slco2a1
solute carrier organic anion transporter ...

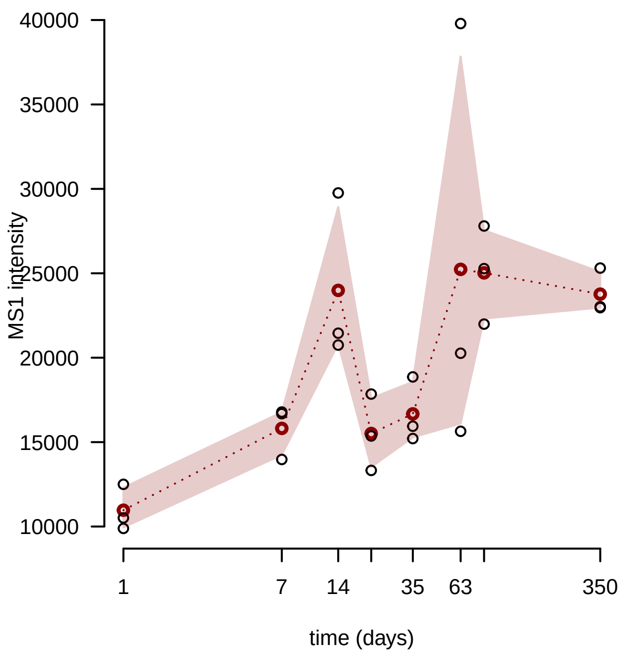

Q9EPU0
GN=Upf1
UPF1 regulator of nonsense transcripts h ...

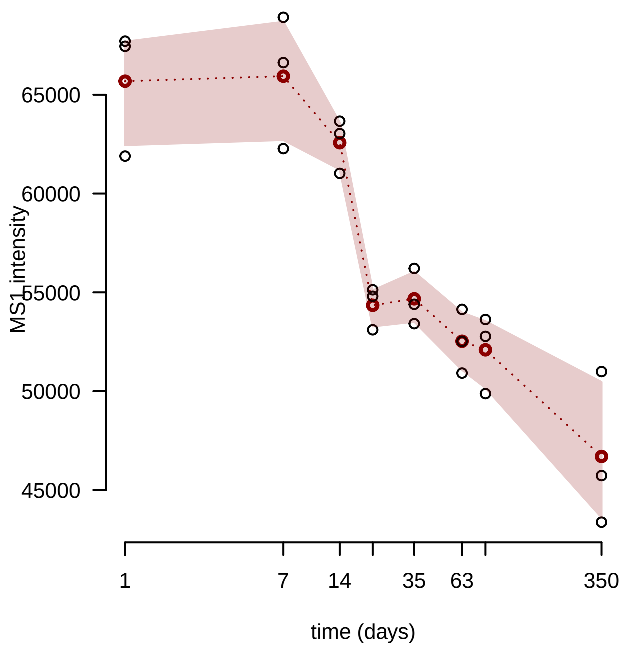

Q9EPU4
GN=Cpsf1
cleavage and polyadenylation specific fa ...

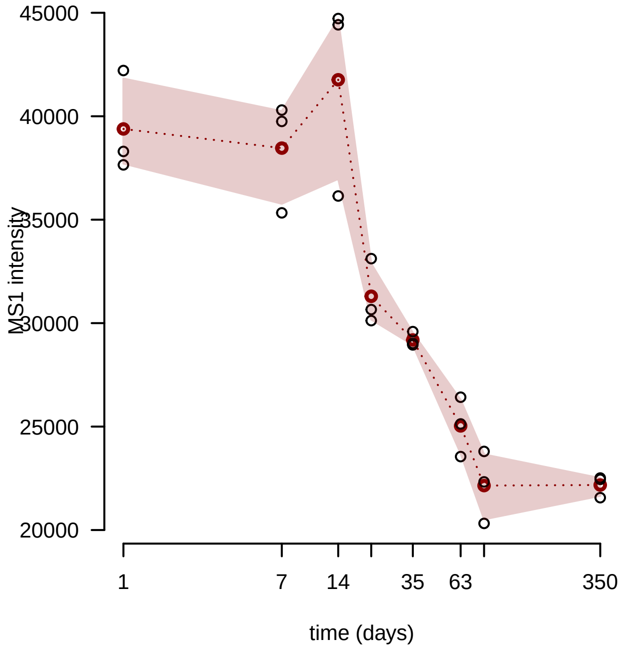

Q9EPV8
GN=Ubl5
ubiquitin−like 5

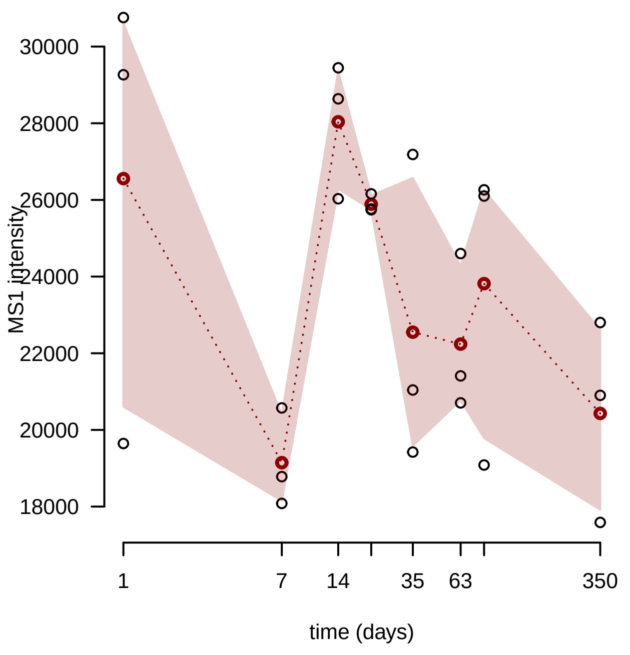

Q9EPW0
GN=Inpp4a
inositol polyphosphate−4−phosphatase, ty ...

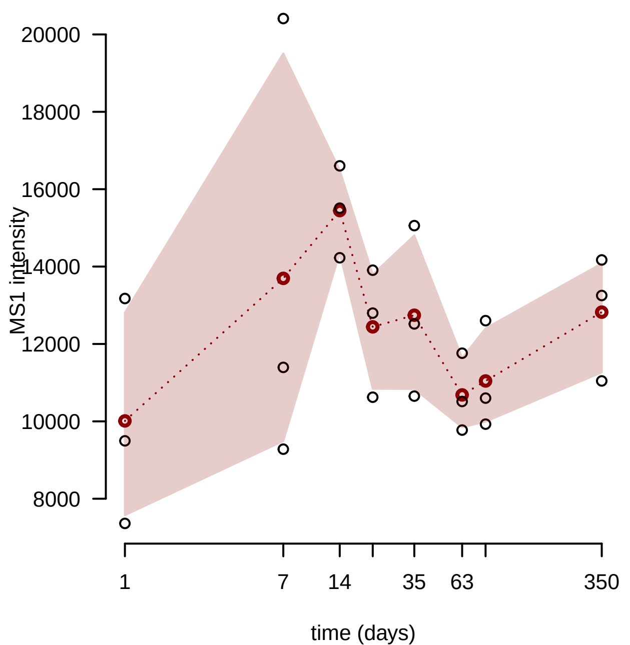

Q9EPX2
GN=Papln
papilin, proteoglycan−like sulfated glyc ...

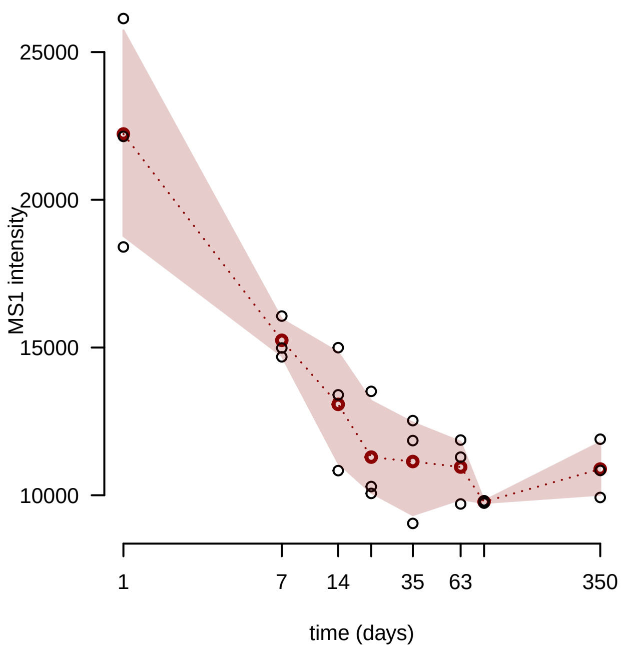

Q9EQ06
GN=Hsd17b11
hydroxysteroid (17−beta) dehydrogenase 1 ...

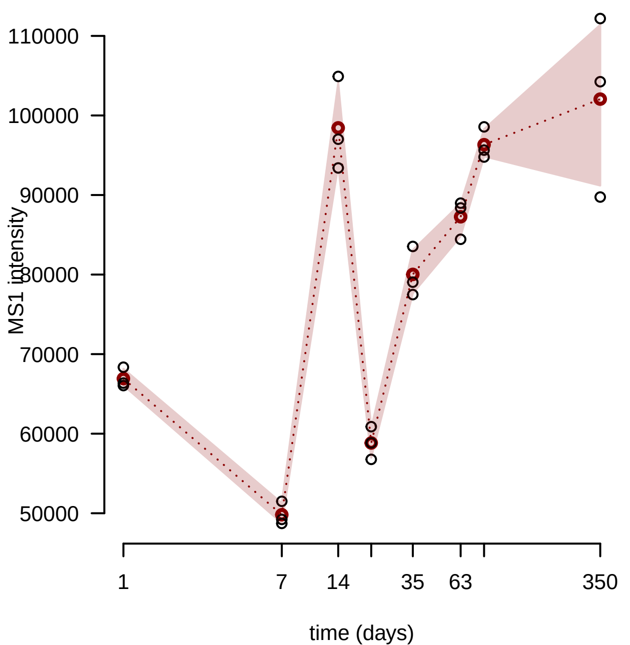

Q9EQ15
GN=Gnb1l
guanine nucleotide binding protein (G pr ...

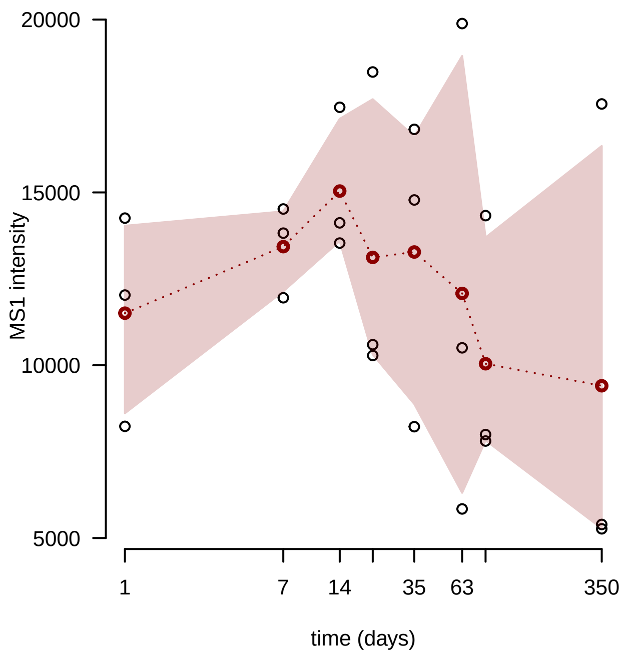

Q9EQ20
GN=Aldh6a1
aldehyde dehydrogenase family 6, subfami ...

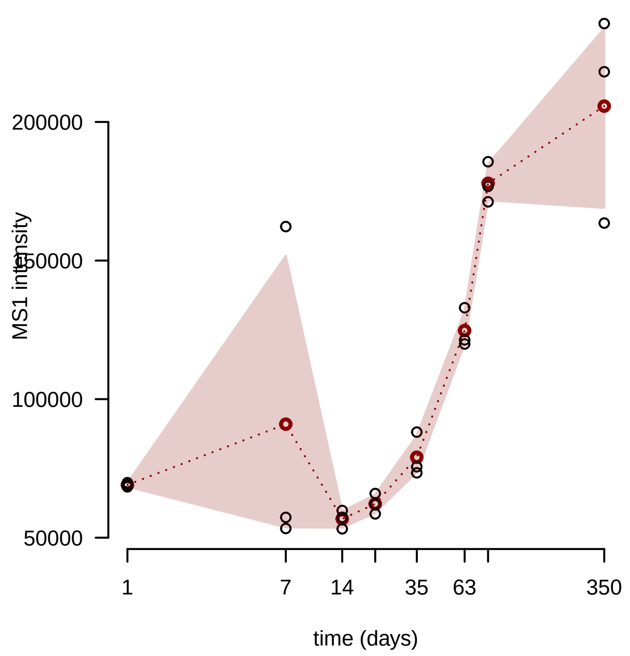

Q9EQ28
GN=Pold3
polymerase (DNA−directed), delta 3, acce ...

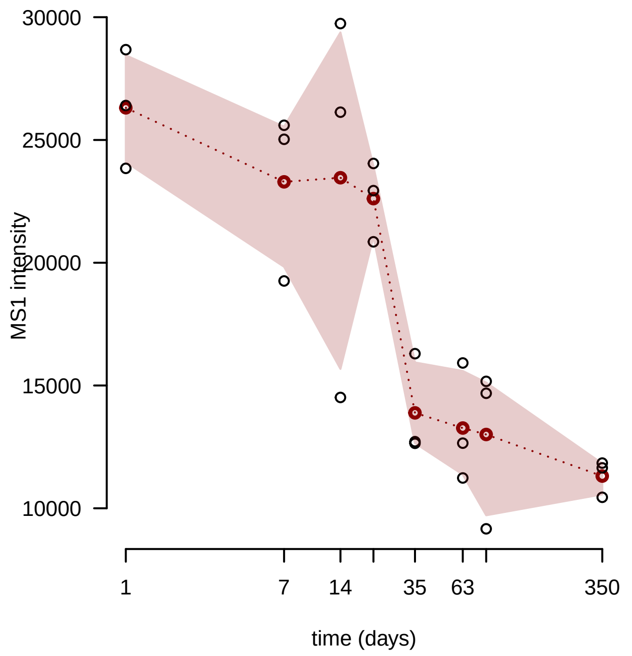

Q9EQ32
GN=Pik3ap1
phosphoinositide−3−kinase adaptor protei ...

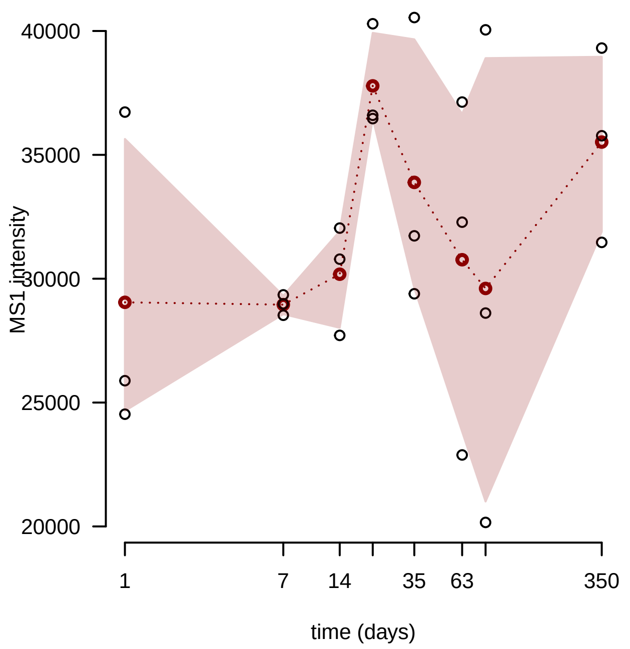

Q9EQ61
GN=Pes1
pescadillo ribosomal biogenesis factor 1

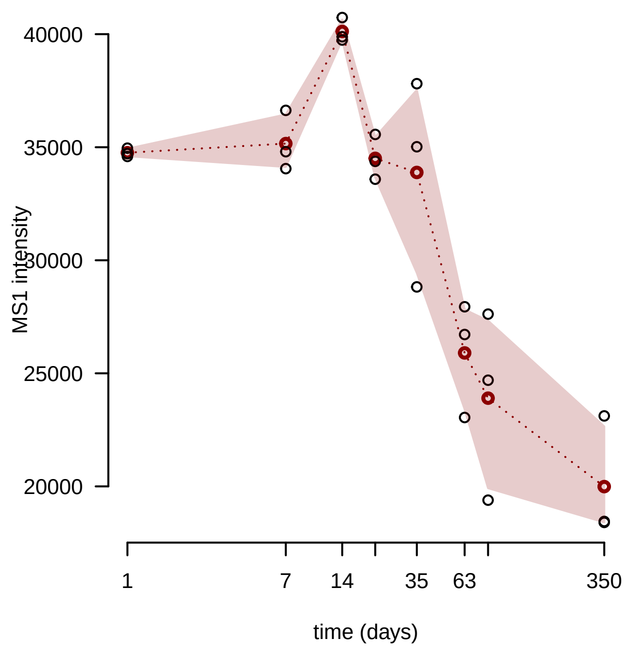

Q9EQ80
GN=Nif3l1
Ngg1 interacting factor 3−like 1 (S. pom ...

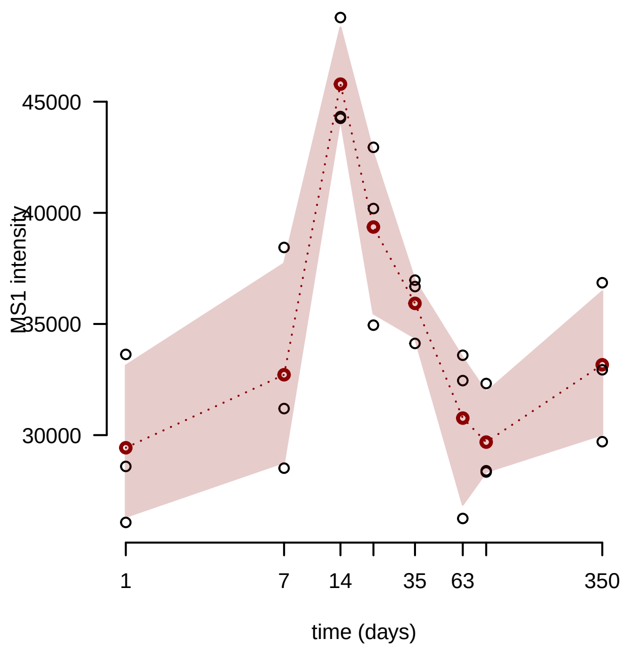

Q9EQC1
GN=Hsd3b7
hydroxy−delta−5−steroid dehydrogenase, 3 ...

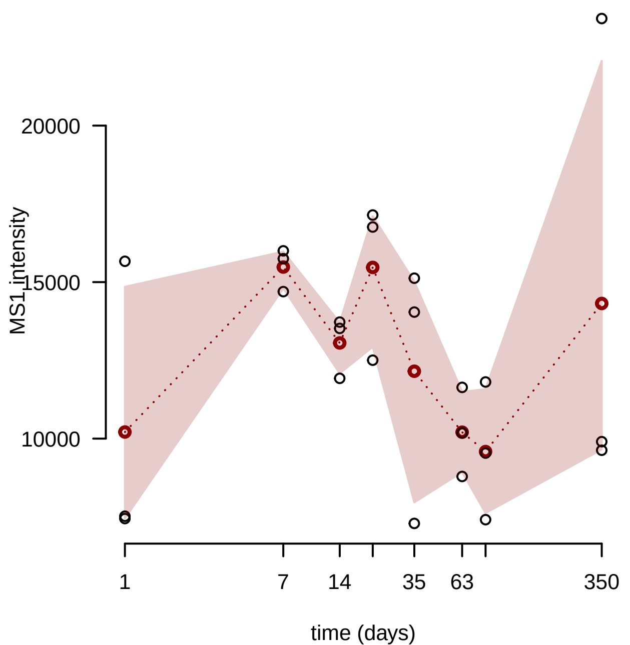

Q9EQC5
GN=Scyl1
SCY1−like 1 (S. cerevisiae)

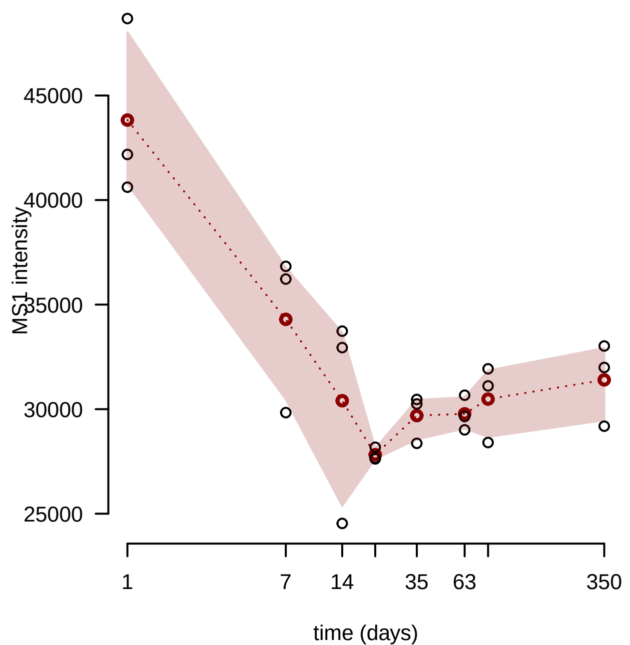

Q9EQF5
GN=Dpys
dihydropyrimidinase

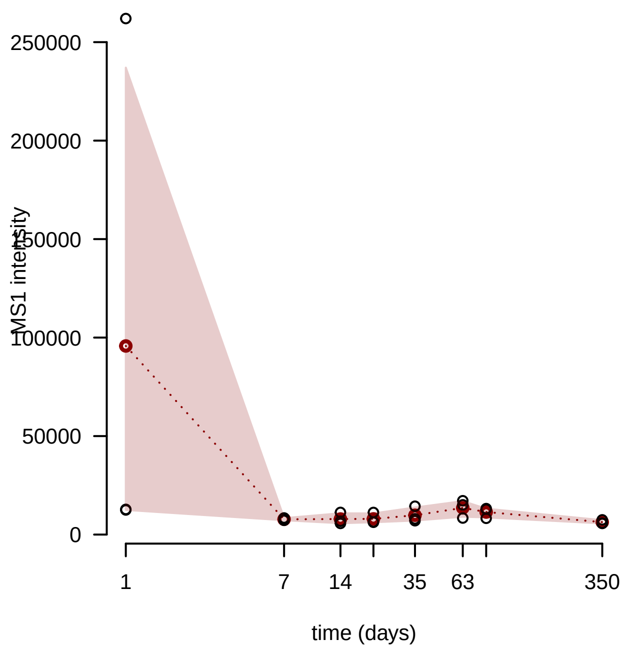

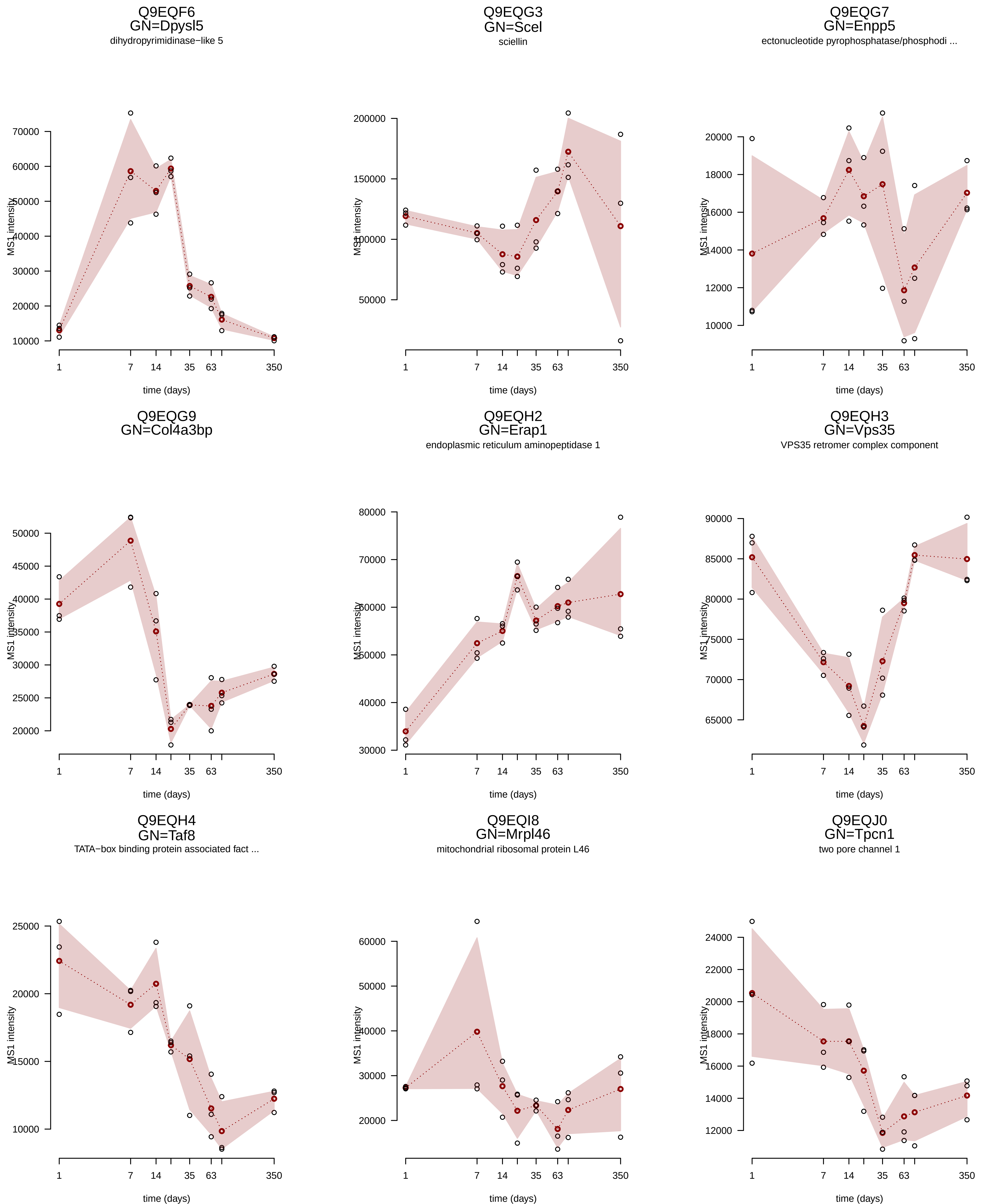
Q9EQF6
GN=Dpysl5
dihydropyrimidinase−like 5
Q9EQG3
GN=Scel
sciellin
Q9EQG7
GN=Enpp5
ectonucleotide pyrophosphatase/phosphodi ...
Q9EQG9
GN=Col4a3bp
Q9EQH2
GN=Erap1
endoplasmic reticulum aminopeptidase 1
Q9EQH3
GN=Vps35
VPS35 retromer complex component
Q9EQH4
GN=Taf8
TATA−box binding protein associated fact ...
Q9EQI8
GN=Mrpl46
mitochondrial ribosomal protein L46
Q9EQJ0
GN=Tpcn1
two pore channel 1
MS1 intensity
time (days)
1
7
14
35
63
350

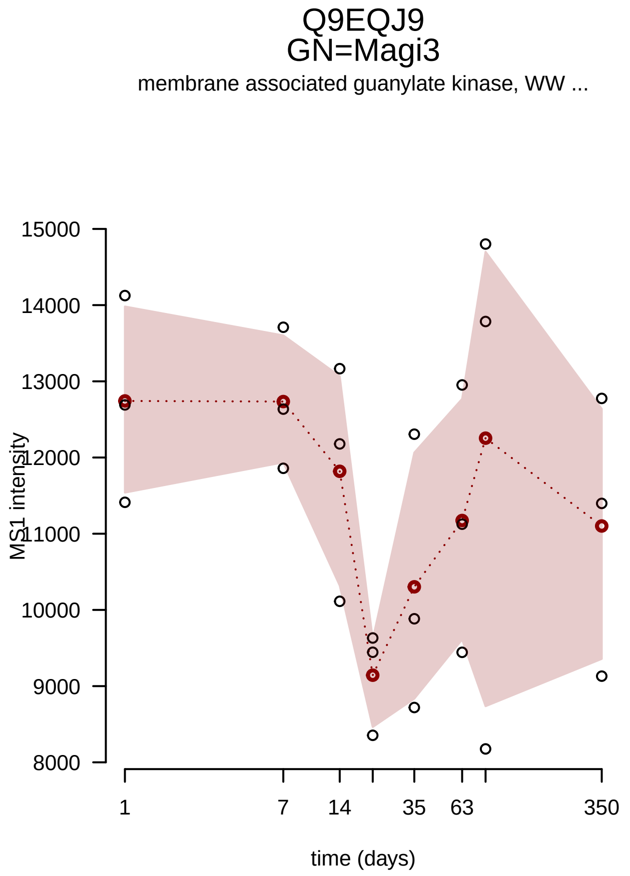
Q9EQJ9
GN=Magi3
membrane associated guanylate kinase, WW ...
MS1 intensity
15000
14000
13000
12000
11000
10000
9000
8000
1
7
14
35
63
350
time (days)

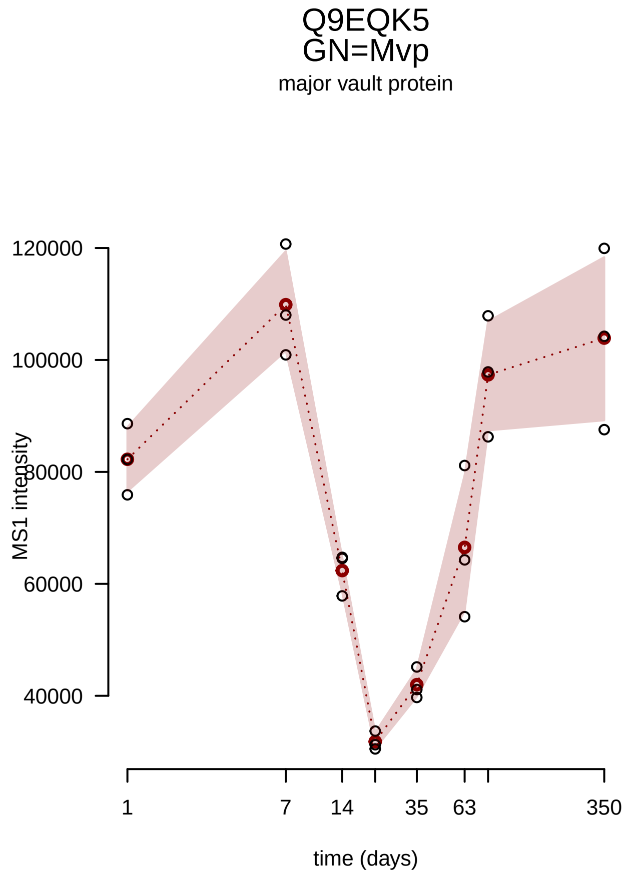
Q9EQK5
GN=Mvp
major vault protein
MS1 intensity
120000
100000
80000
60000
40000
1
7
14
35
63
350
time (days)

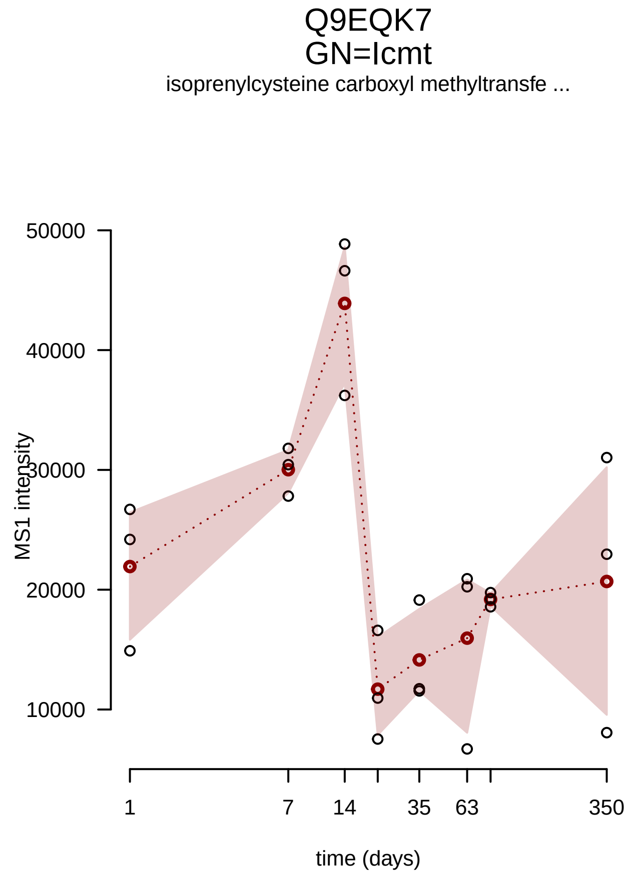
Q9EQK7
GN=Icmt
isoprenylcysteine carboxyl methyltransfe ...
MS1 intensity
50000
40000
30000
20000
10000
1
7
14
35
63
350
time (days)

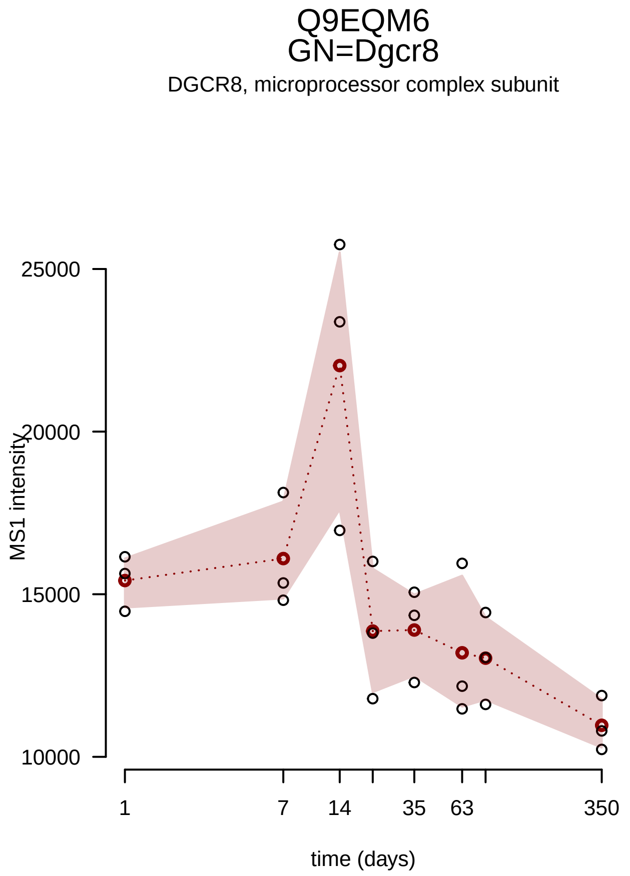
Q9EQM6
GN=Dgcr8
DGCR8, microprocessor complex subunit
MS1 intensity
25000
20000
15000
10000
1
7
14
35
63
350
time (days)

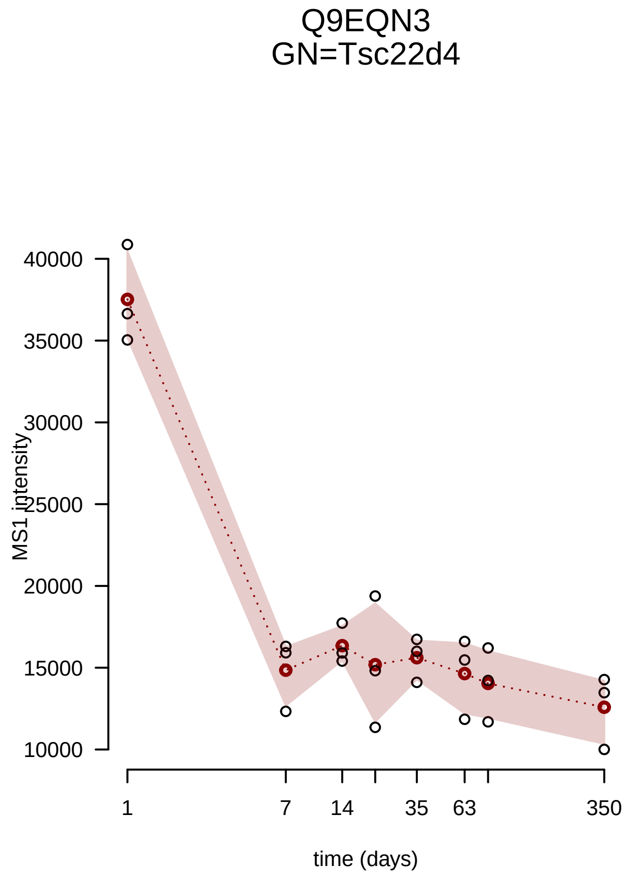
Q9EQN3
GN=Tsc22d4
MS1 intensity
40000
35000
30000
25000
20000
15000
10000
1
7
14
35
63
350
time (days)

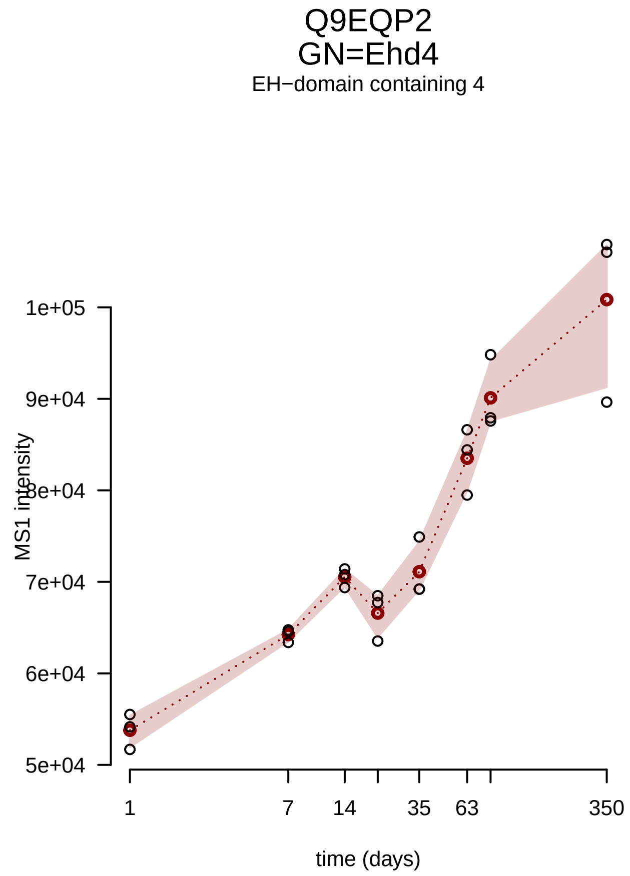
Q9EQP2
GN=Ehd4
EH−domain containing 4
MS1 intensity
1e+05
9e+04
8e+04
7e+04
6e+04
5e+04
1
7
14
35
63
350
time (days)

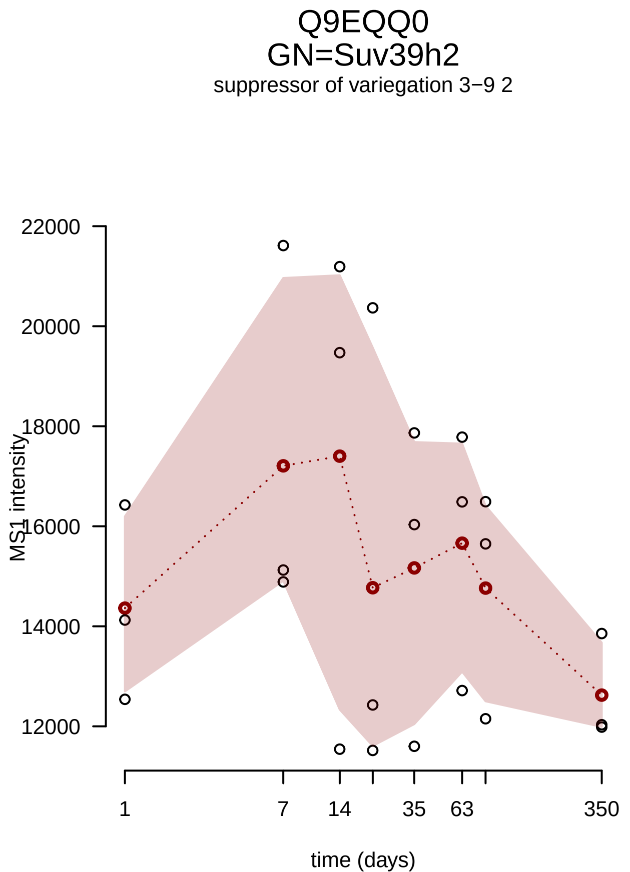
Q9EQQ0
GN=Suv39h2
suppressor of variegation 3−9 2
MS1 intensity
22000
20000
18000
16000
14000
12000
1
7
14
35
63
350
time (days)

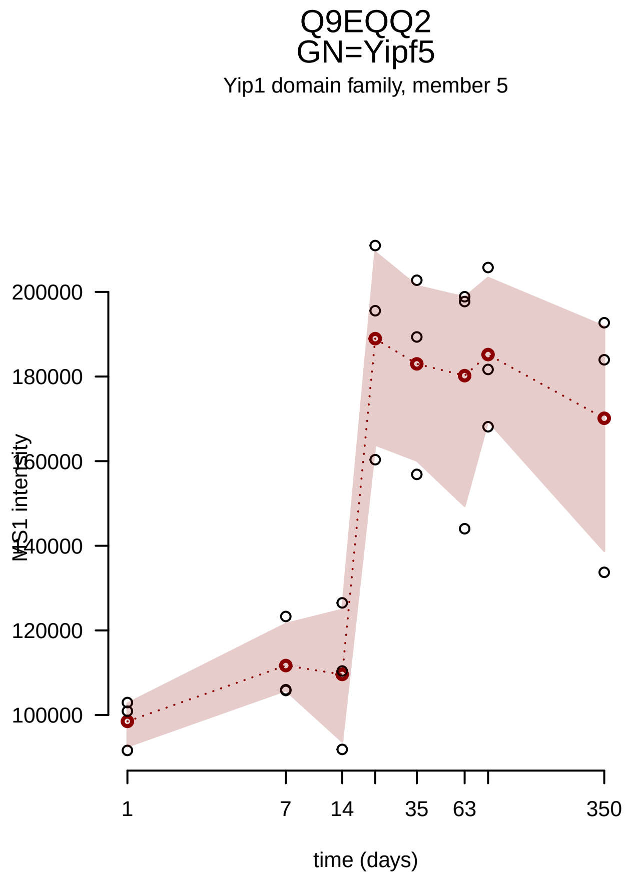
Q9EQQ2
GN=Yipf5
Yip1 domain family, member 5
MS1 intensity
200000
180000
160000
140000
120000
100000
1
7
14
35
63
350
time (days)

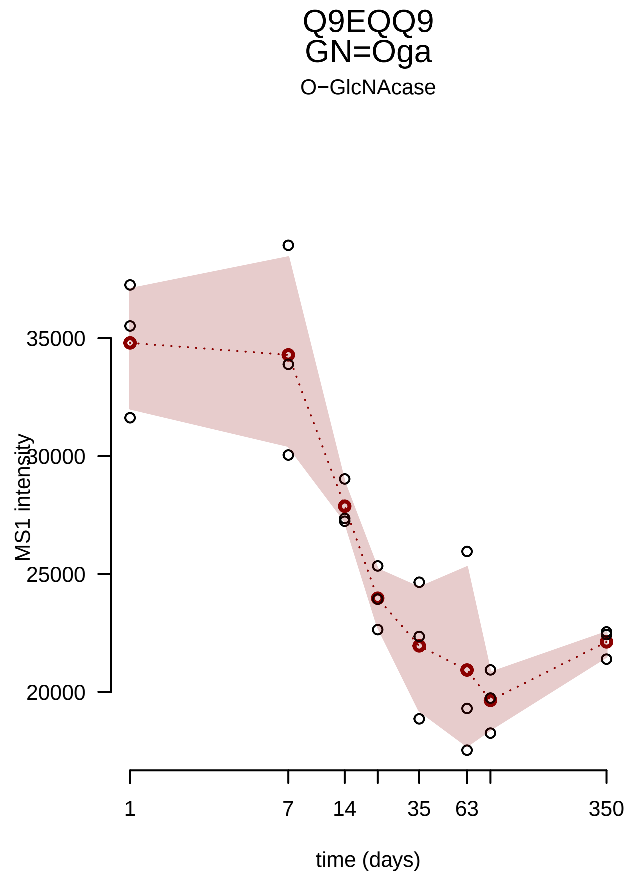
Q9EQQ9
GN=Oga
O−GlcNAcase
MS1 intensity
35000
30000
25000
20000
1
7
14
35
63
350
time (days)

Q9EQS3
GN=Mycbp
MYC binding protein

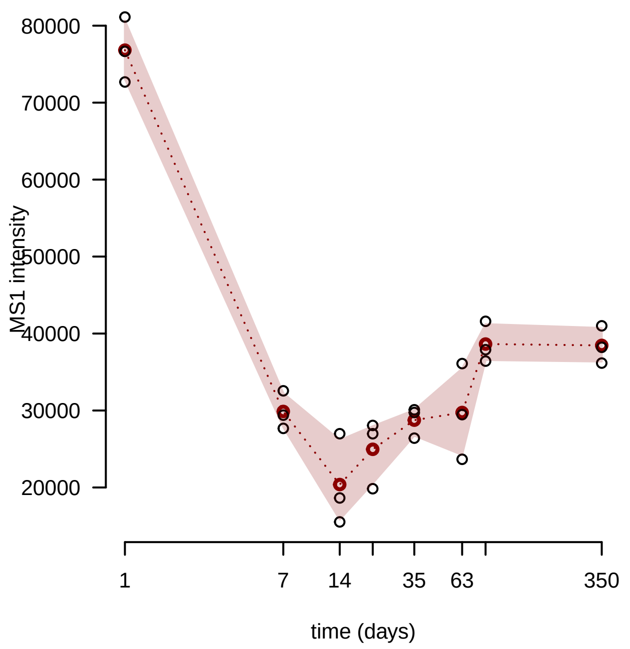

Q9EQS9
GN=Igdcc4
immunoglobulin superfamily, DCC subclass ...

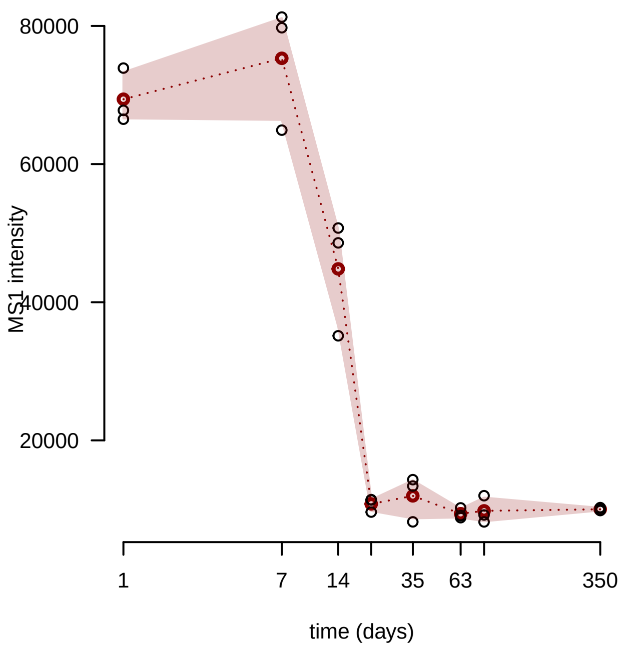

Q9EQU5
GN=Set
SET nuclear oncogene

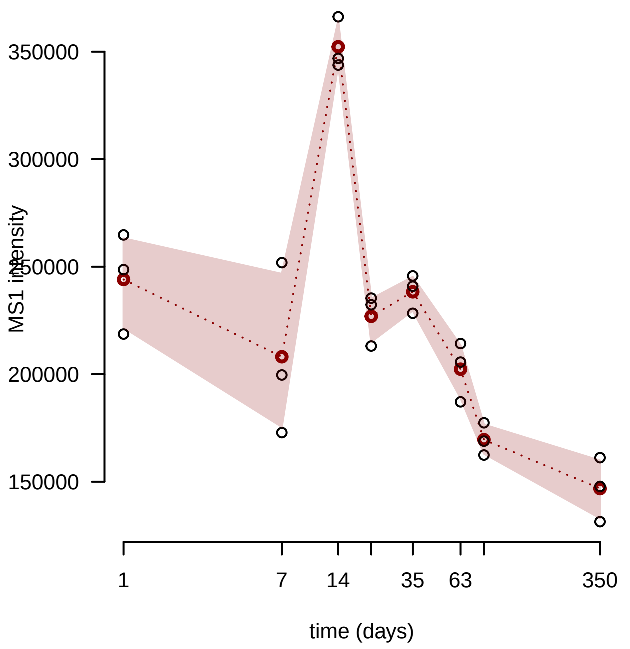

Q9EQU5
GN=Set
SET nuclear oncogene

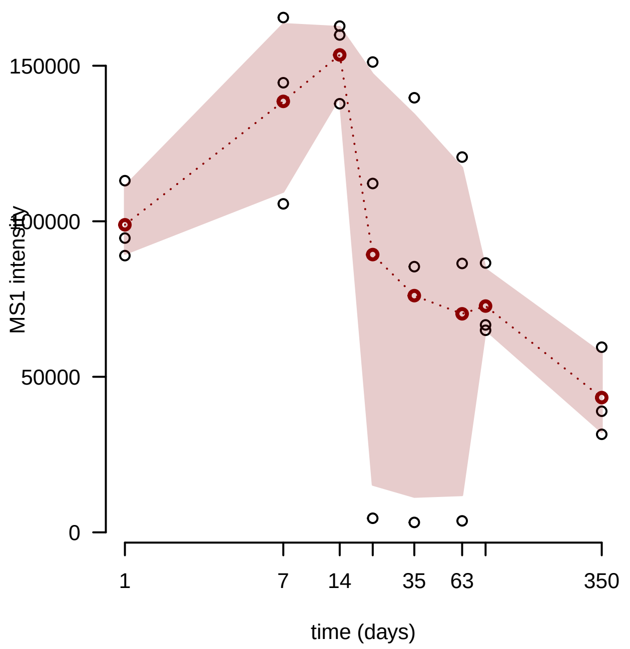

Q9EQW7
GN=Kif13a
kinesin family member 13A

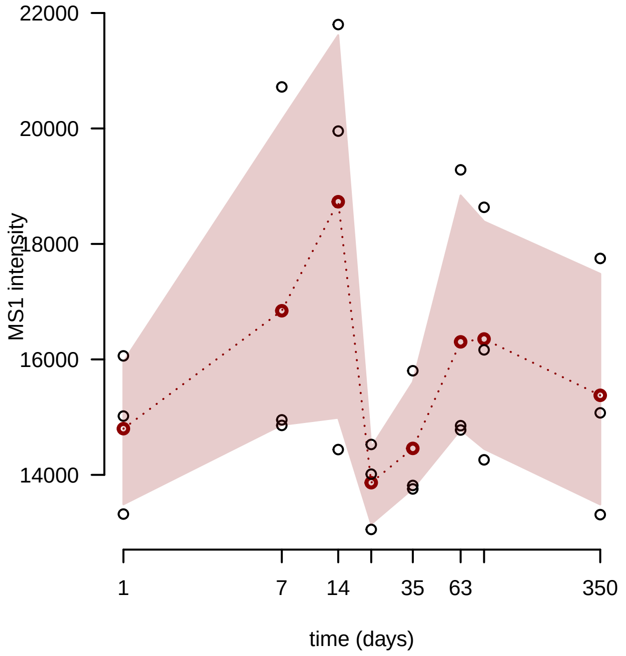

Q9EQX4
GN=Aif1l
allograft inflammatory factor 1−like

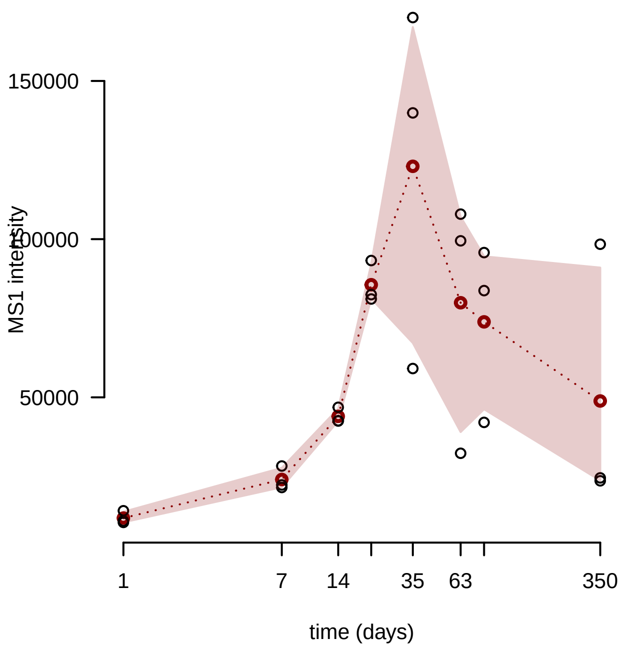

Q9EQY0
GN=Ern1
endoplasmic reticulum (ER) to nucleus si ...

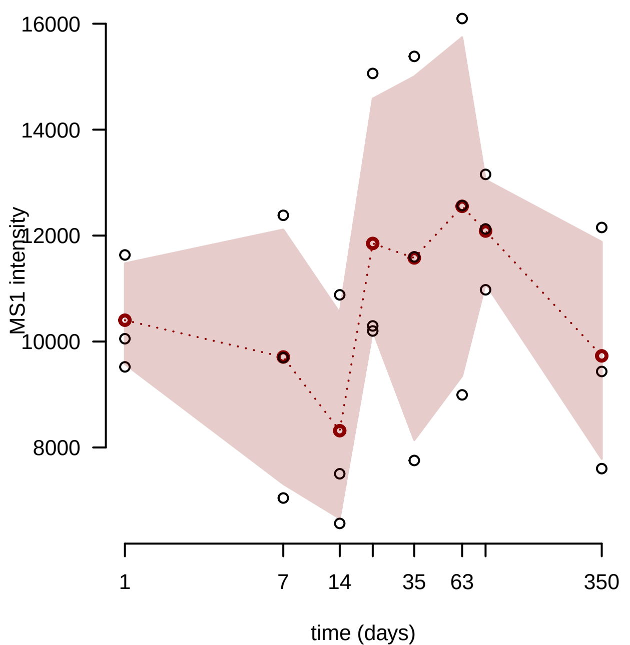

Q9ER00
GN=Stx12
syntaxin 12

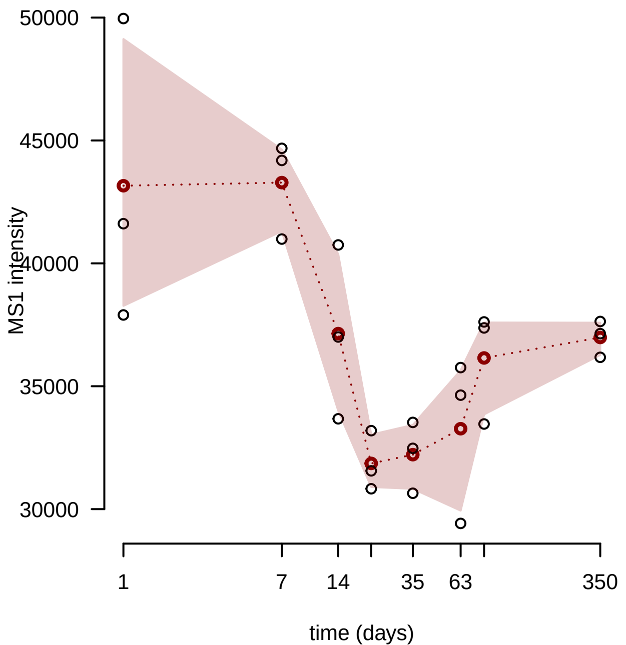

Q9ER38
GN=Tor3a
torsin family 3, member A

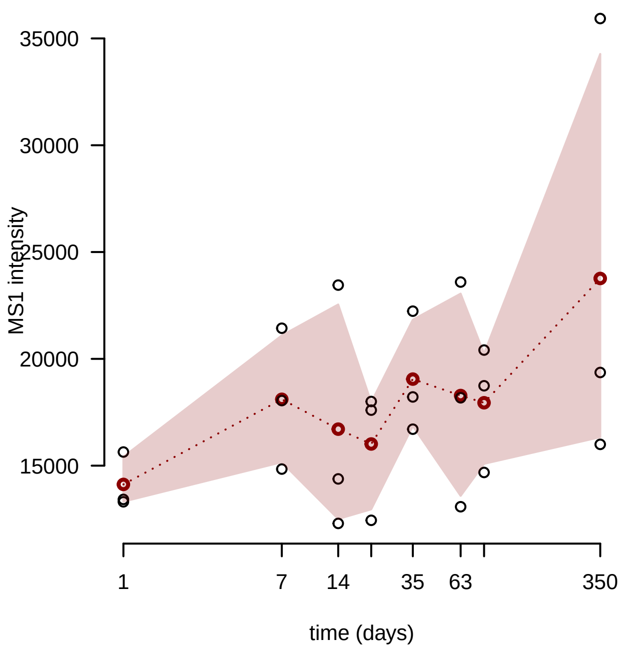

Q9ER39
GN=Tor1a
torsin family 1, member A (torsin A)

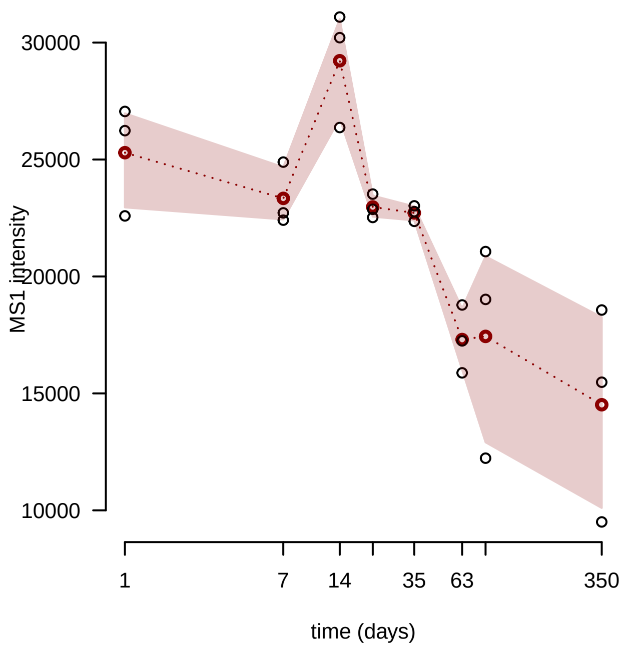

Q9ER41
GN=Tor1b
torsin family 1, member B

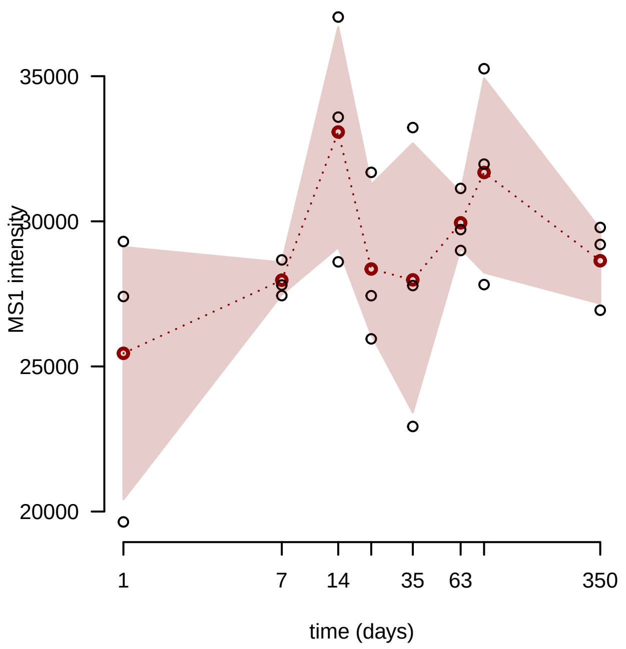

Q9ER58
GN=Spock2
sparc/osteonectin, cwcv and kazal−like d ...

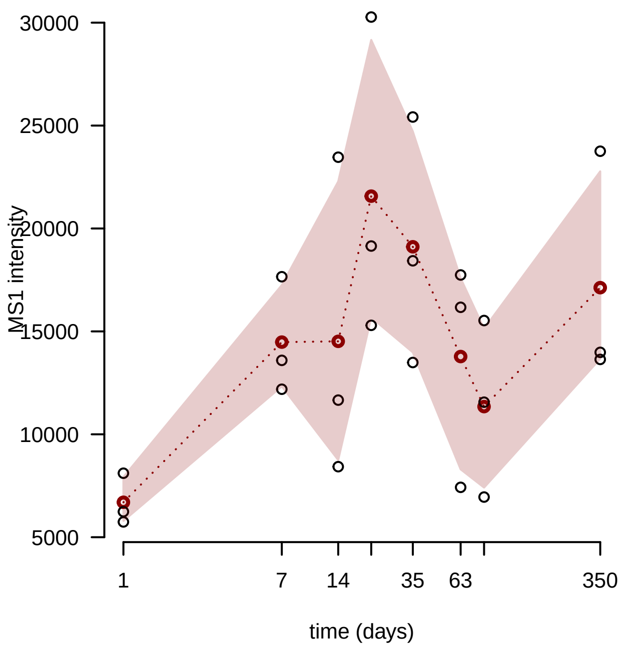

Q9ER64
GN=Osbpl5
oxysterol binding protein−like 5

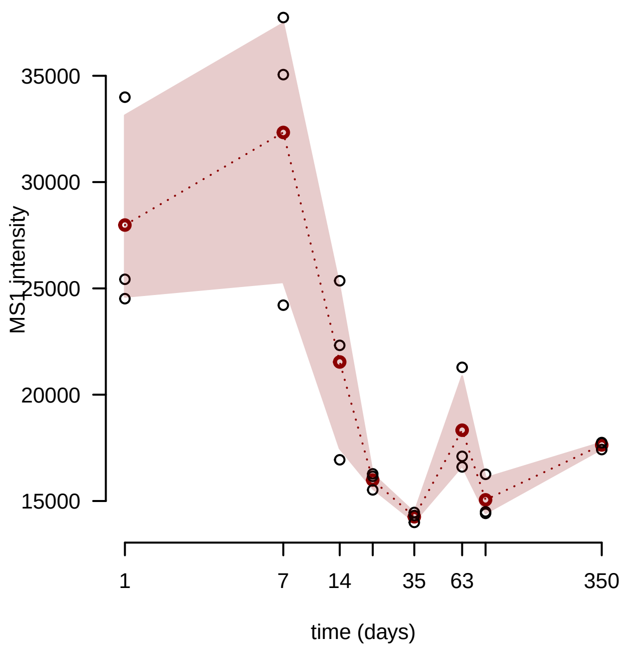

Q9ER69
GN=Wtap
WT1 associating protein

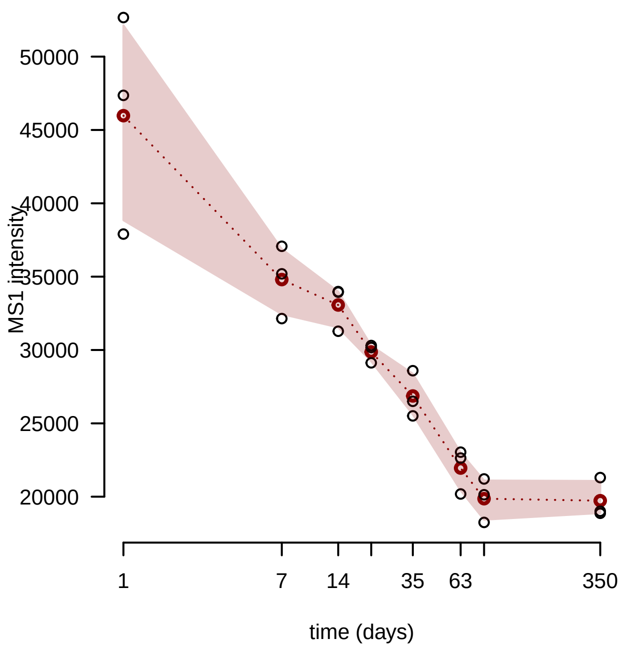

Q9ER71
GN=Rhoj
ras homolog family member J

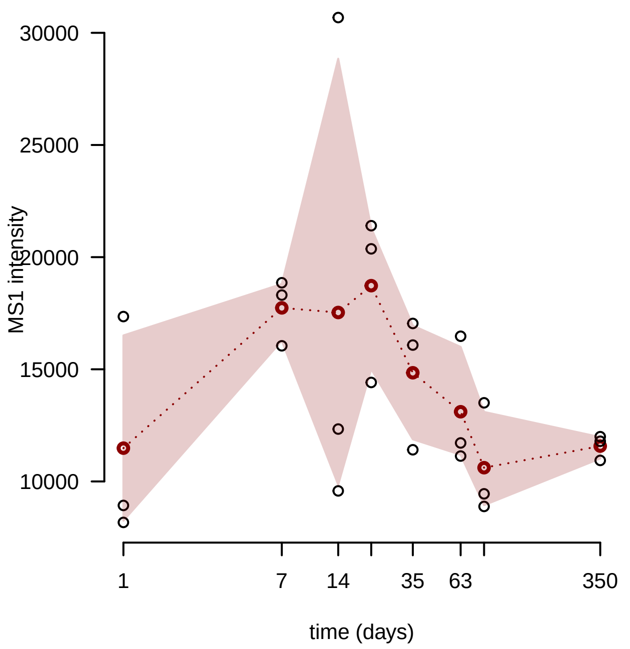

Q9ER72
GN=Cars
cysteinyl−tRNA synthetase

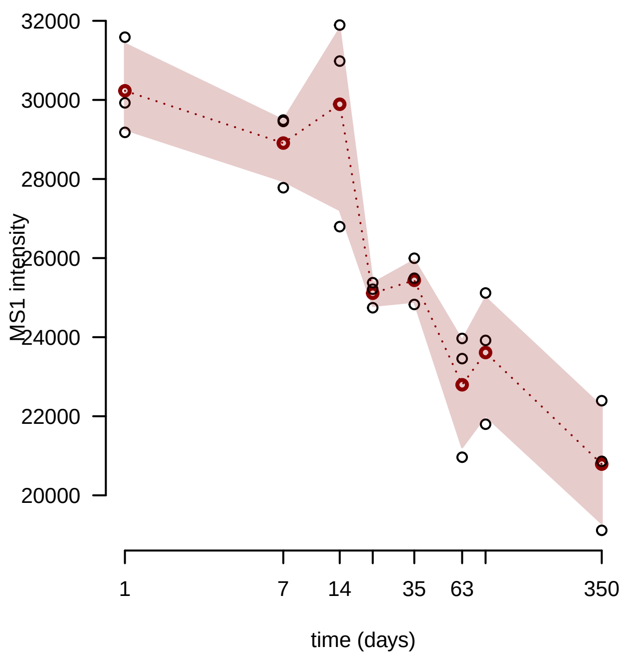

Q9ER73
GN=Elp4
elongator acetyltransferase complex subu ...

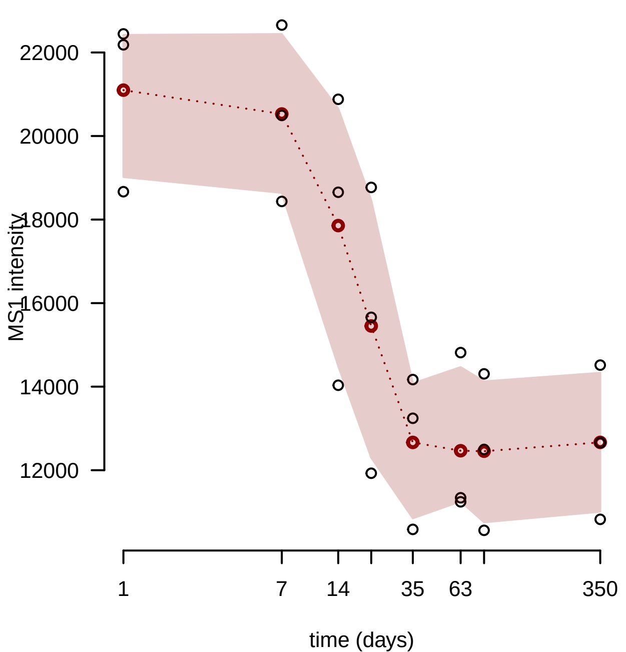

Q9ER81
GN=Tor1aip2

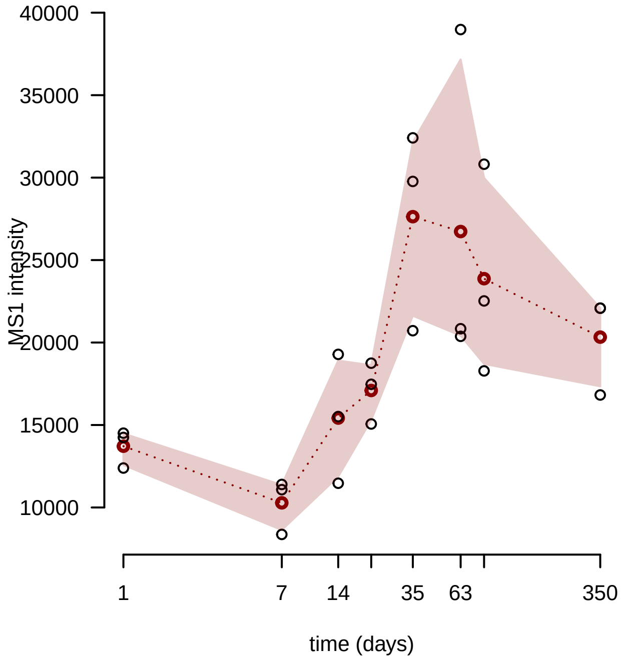

Q9ER88
GN=Dap3
death associated protein 3

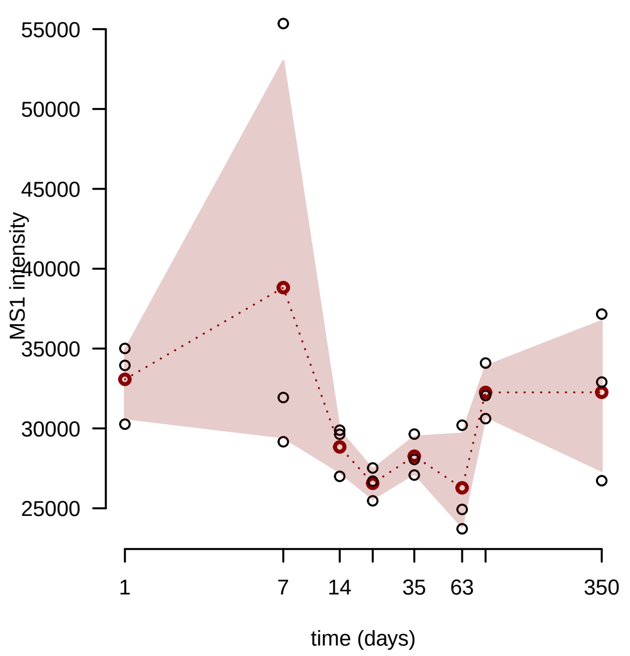

Q9ERA0
GN=Tfcp2
transcription factor CP2

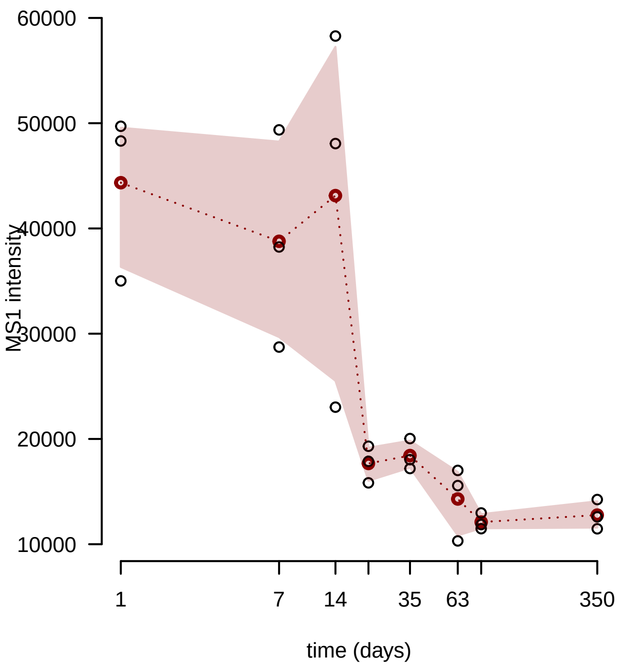

Q9ERA6
GN=Tfip11
tuftelin interacting protein 11

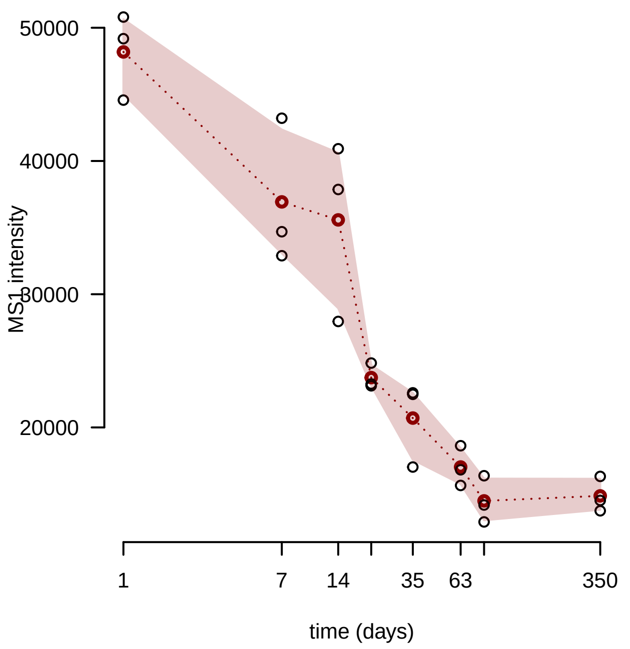

Q9ERB0
GN=Snap29
synaptosomal−associated protein 29

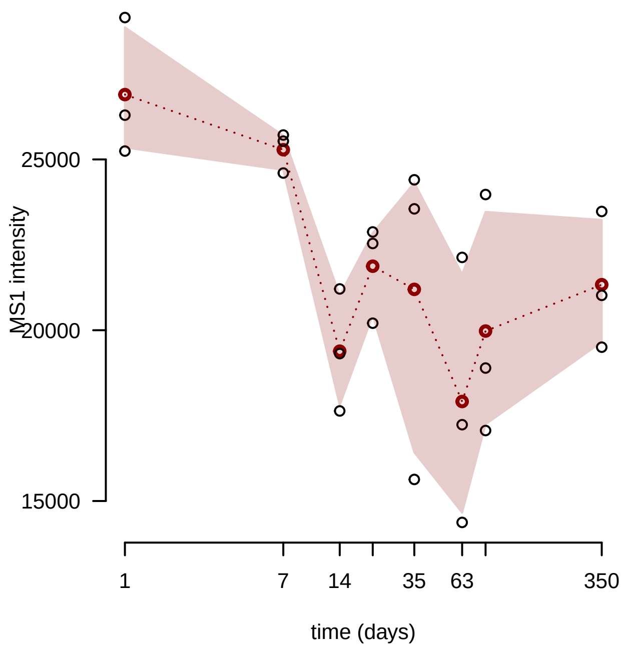

Q9ERD6
GN=Ralgps2
Ral GEF with PH domain and SH3 binding m ...

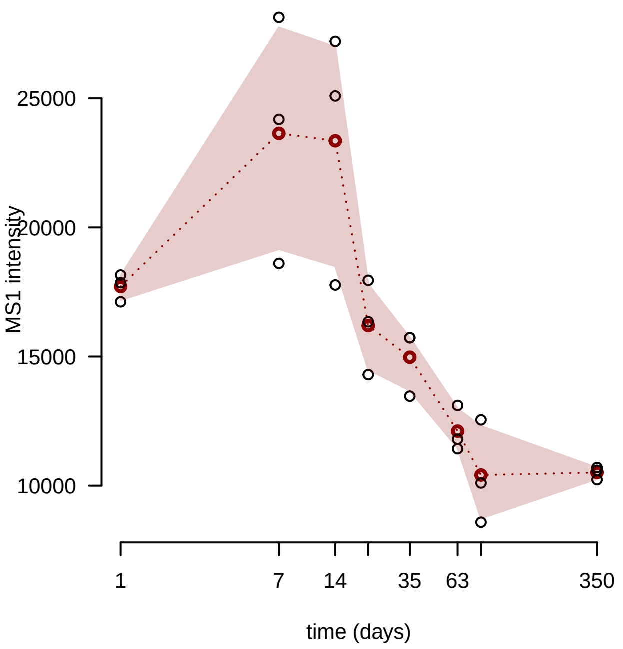

Q9ERD7
GN=Tubb3
tubulin, beta 3 class III

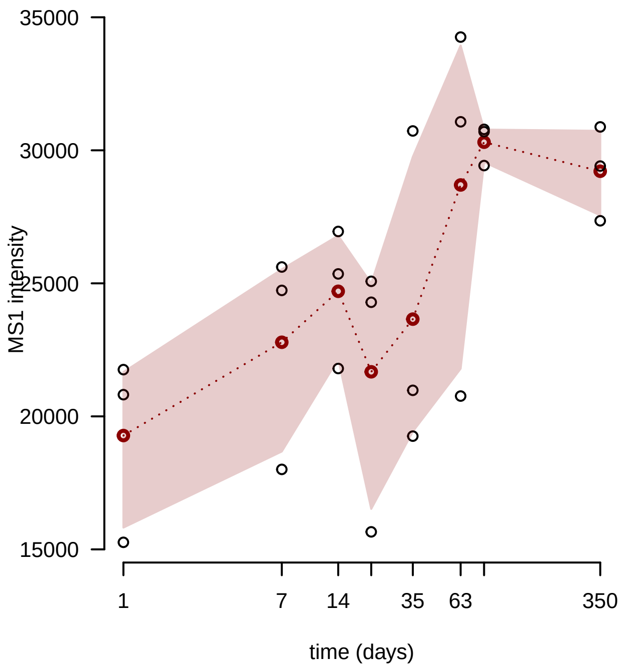

Q9ERE2
GN=Krt81
keratin 81

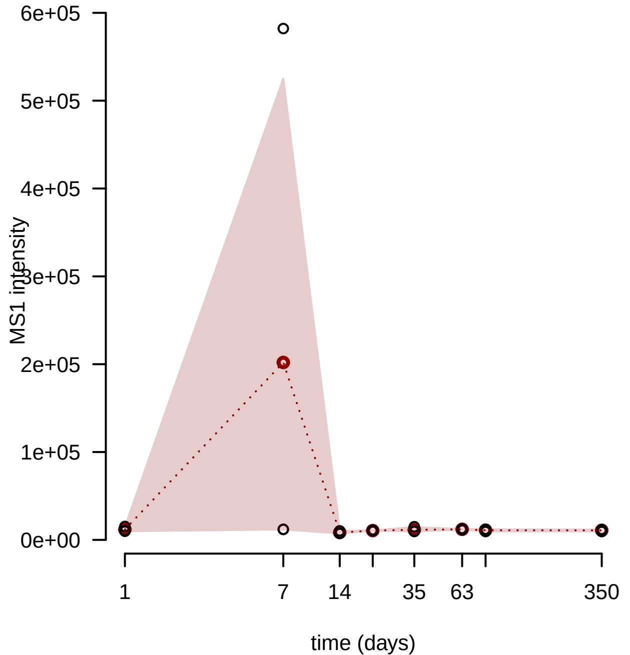

Q9ERE3
GN=Sgk3
serum/glucocorticoid regulated kinase 3

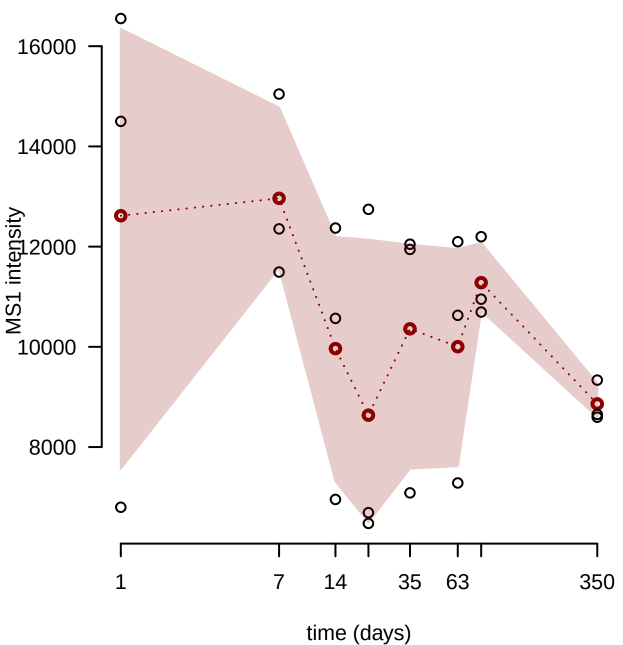

Q9ERE7
GN=Mesd
mesoderm development LRP chaperone

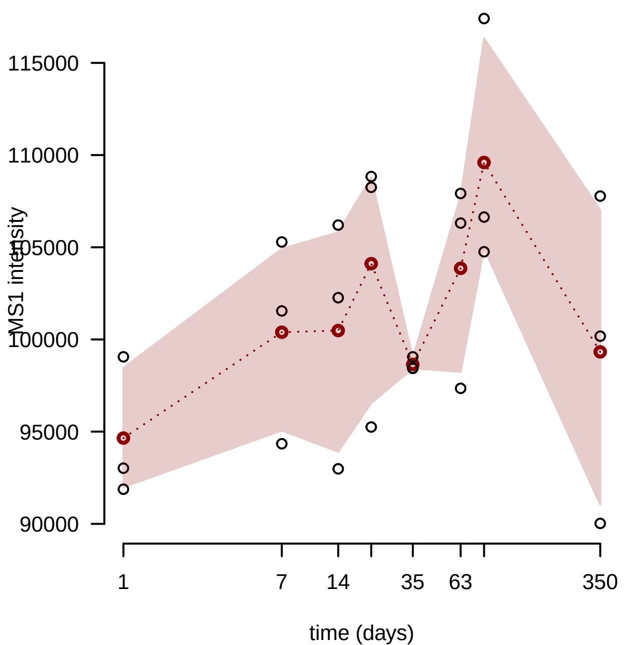

Q9ERF3
GN=Wdr61
WD repeat domain 61

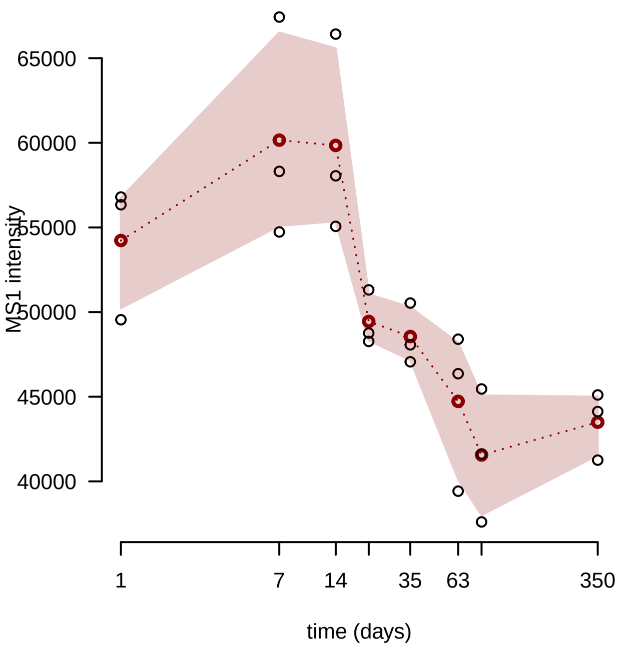

Q9ERG0
GN=Lima1
LIM domain and actin binding 1

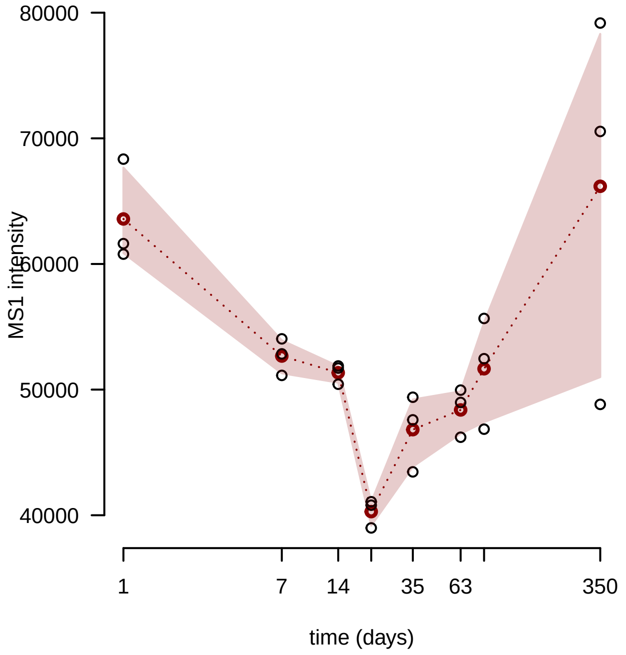

Q9ERG2
GN=Strn3
striatin, calmodulin binding protein 3

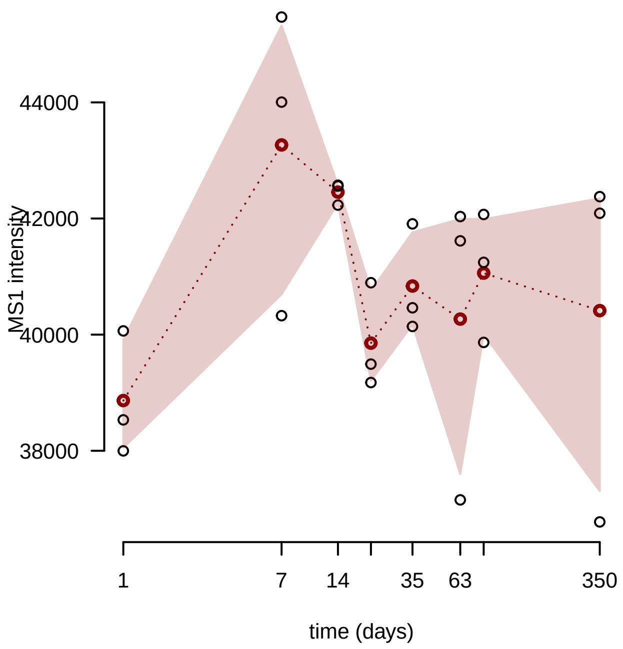

Q9ERH4
GN=Nusap1
nucleolar and spindle associated protein ...

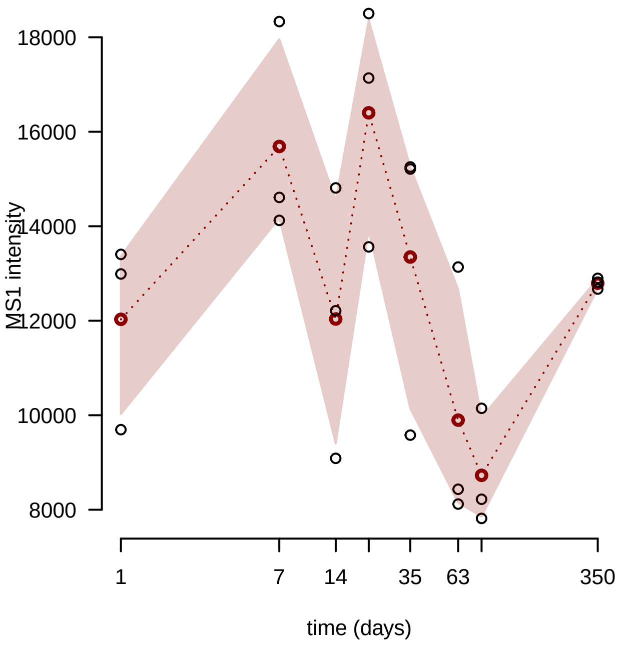

Q9ERI2
GN=Rab27a
RAB27A, member RAS oncogene family

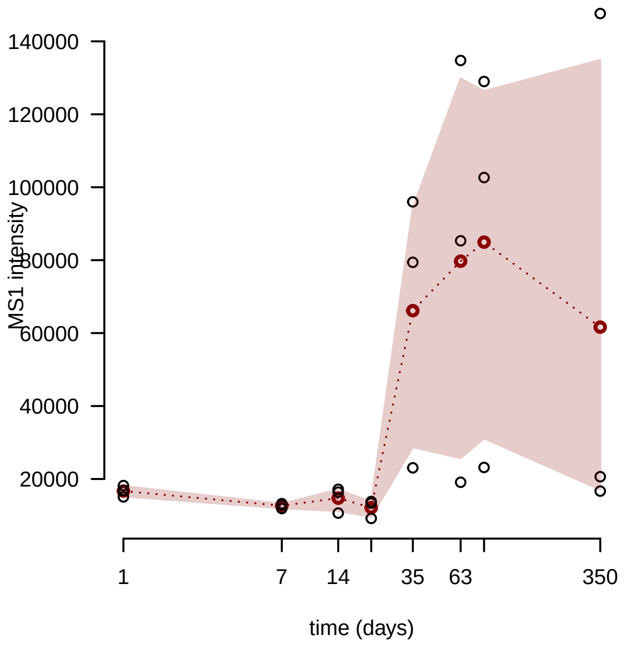

Q9ERI5
GN=Jmjd6
jumonji domain containing 6

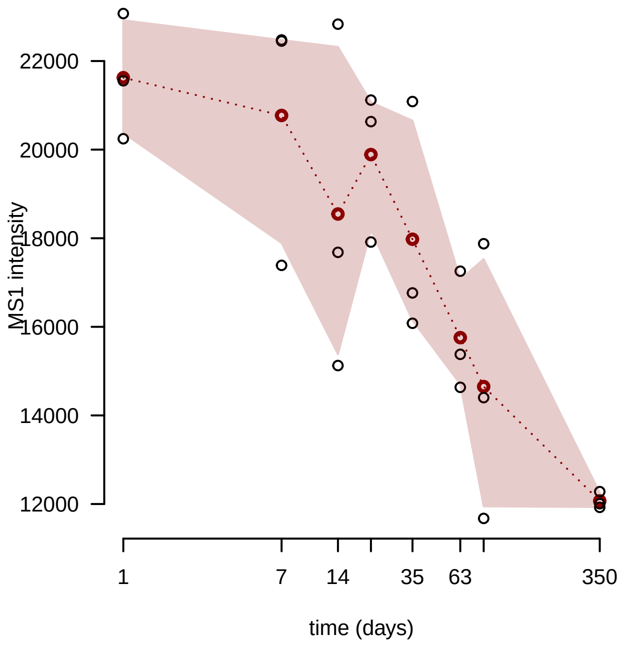

Q9ERI6
GN=Rdh14
retinol dehydrogenase 14 (all−trans and ...

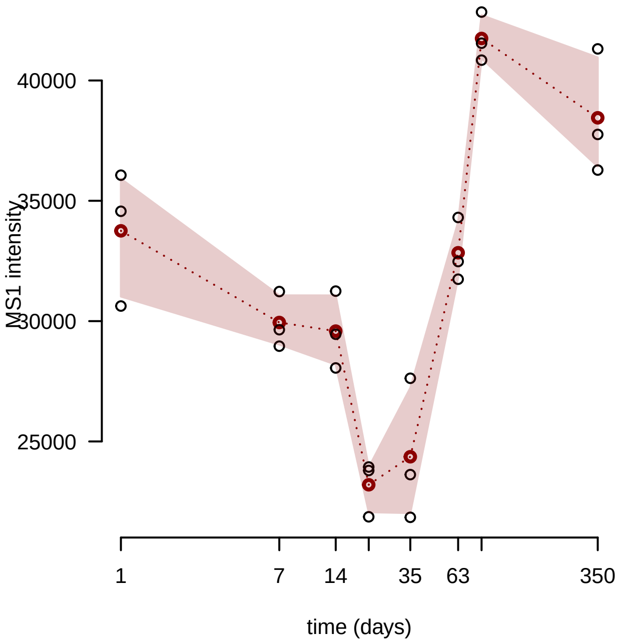

Q9ERK4
GN=Cse1l
chromosome segregation 1−like (S. cerevi ...

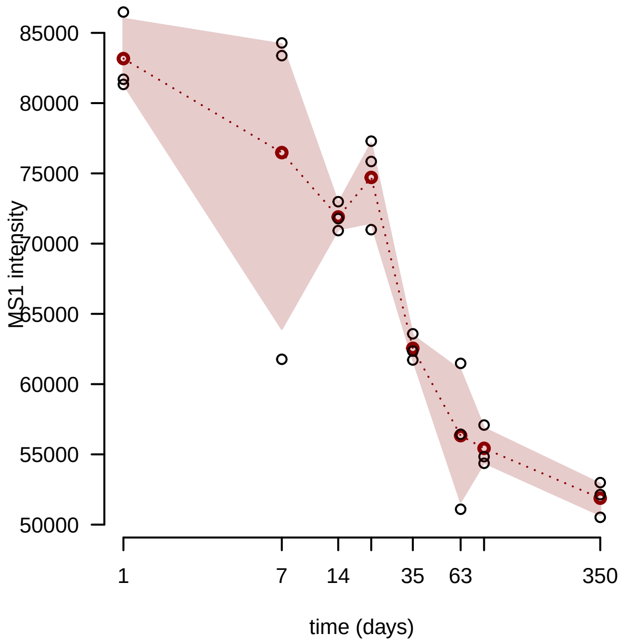

Q9ERL7
GN=Gmfg
glia maturation factor, gamma

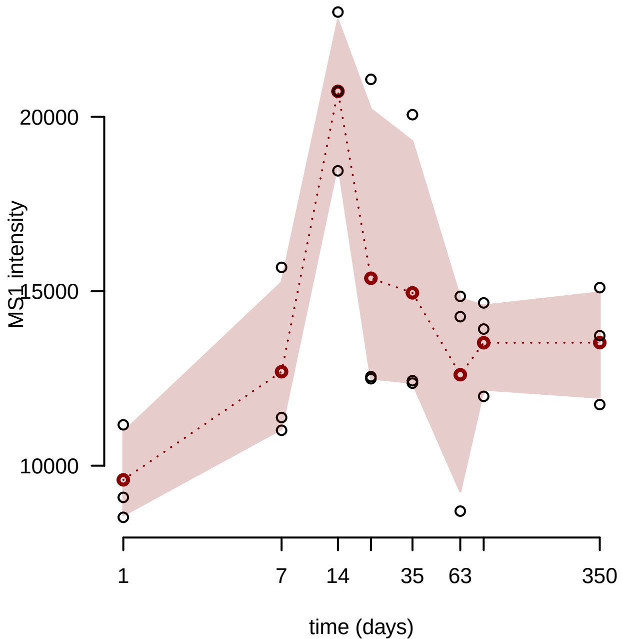

Q9ERL9
GN=Gucy1a1
guanylate cyclase 1, soluble, alpha 1

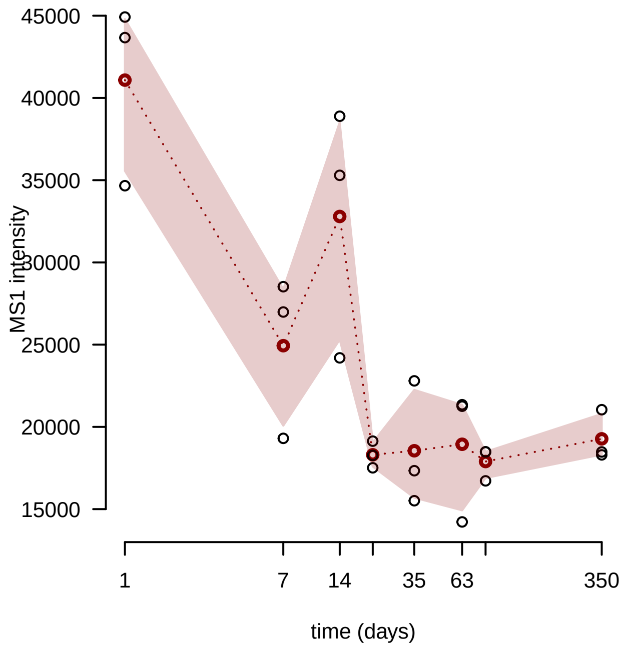

Q9ERN0
GN=Scamp2
secretory carrier membrane protein 2

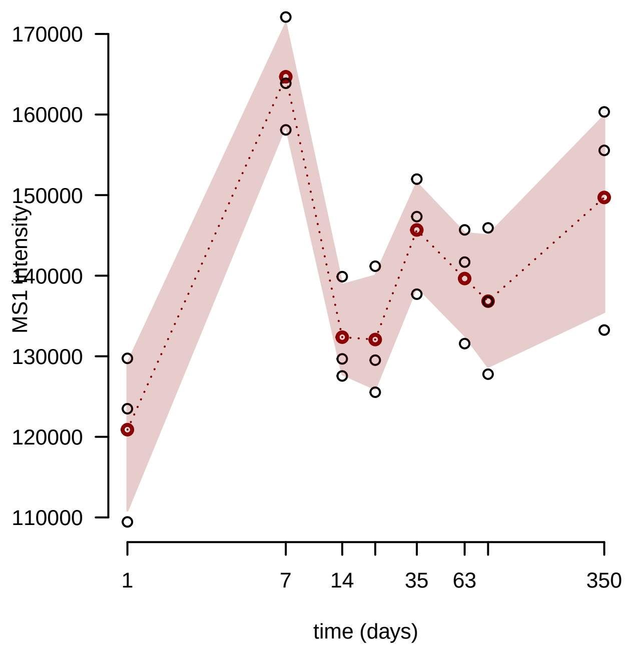

Q9ERQ3
GN=Znf704

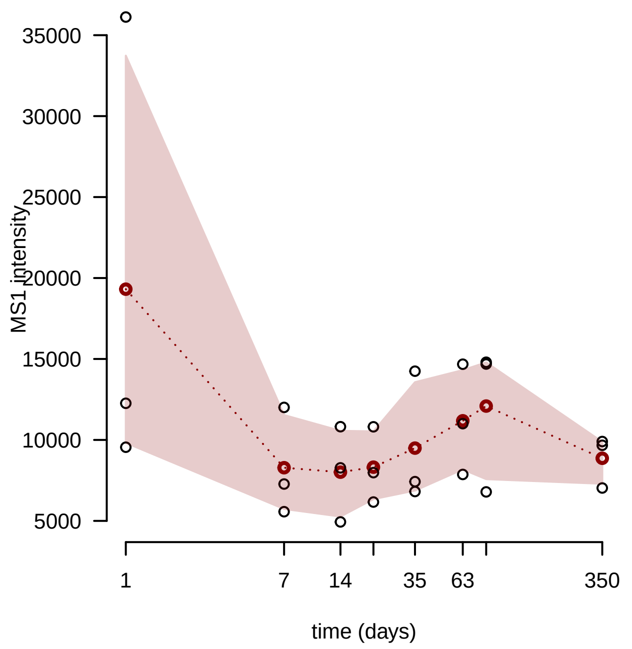

Q9ERR7
GN=Selenof
selenoprotein F

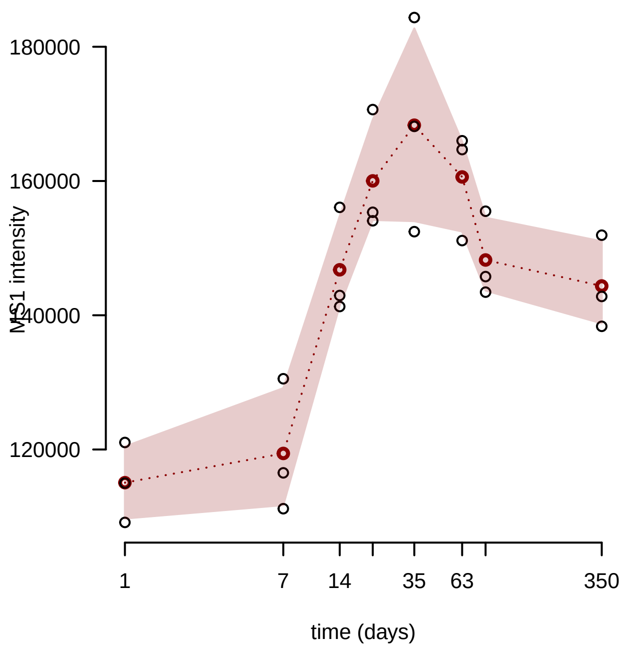

Q9ERR8
GN=Znf319

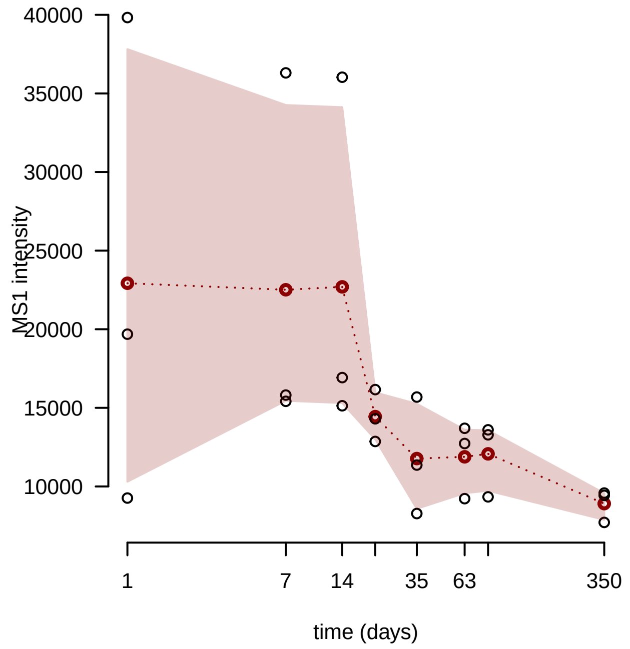

Q9ERS2
GN=Ndufa13
NADH:ubiquinone oxidoreductase subunit A ...

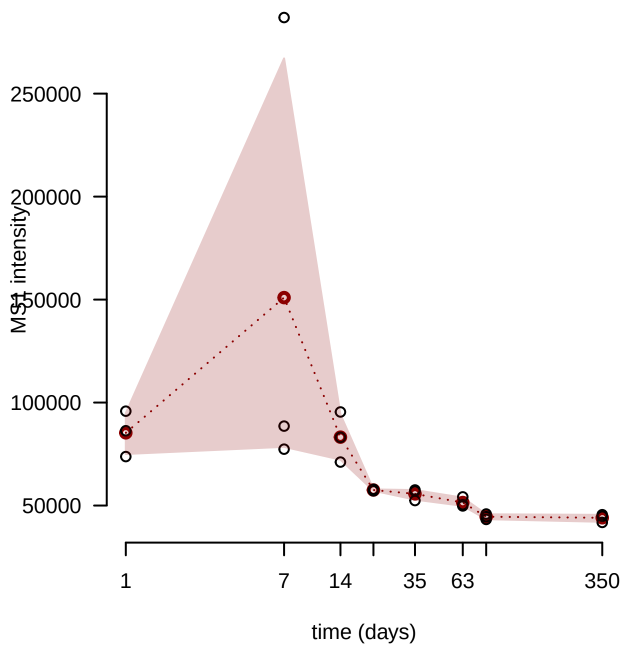

Q9ERS5
GN=Plekha2
pleckstrin homology domain−containing, f ...

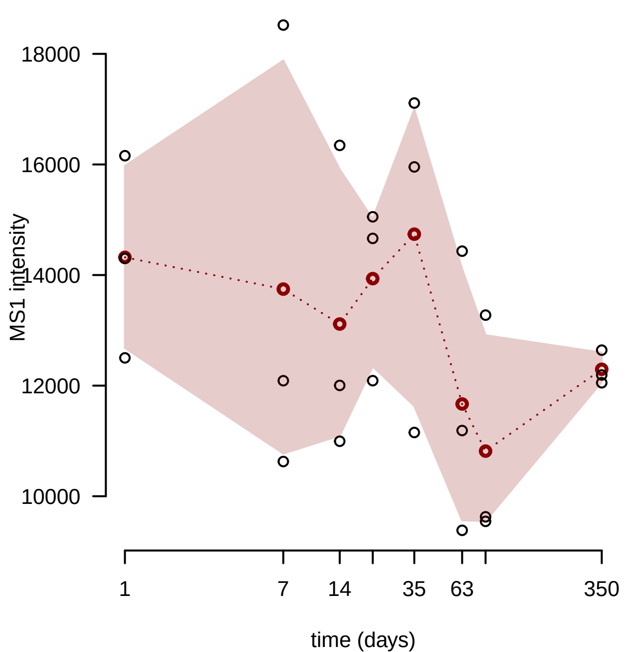

Q9ERU3
GN=Znf22

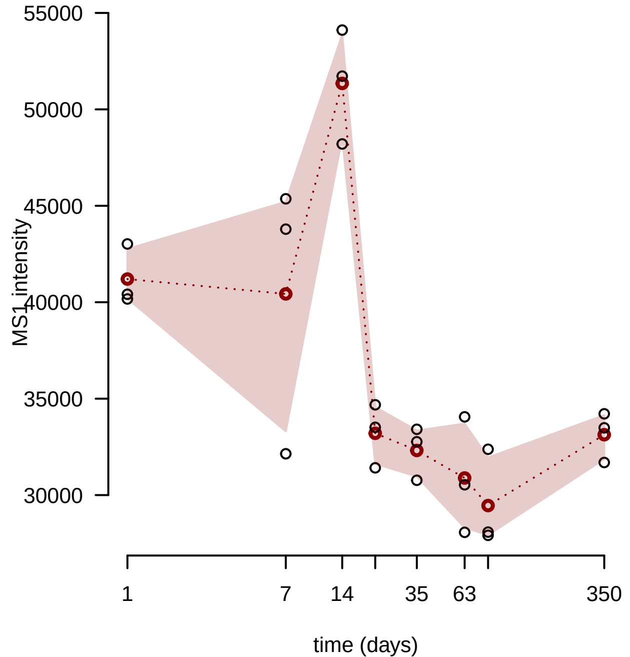

Q9ERU9
GN=Ranbp2
RAN binding protein 2

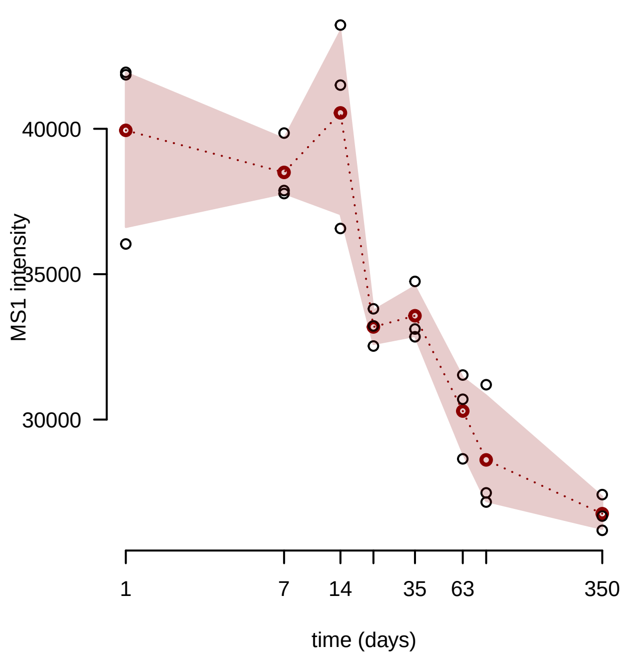

Q9ERV1
GN=Mkrn2
makorin, ring finger protein, 2

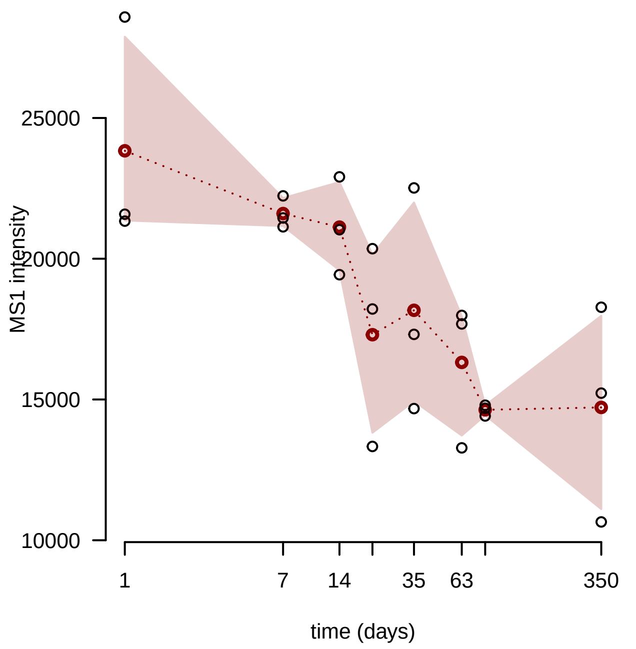

Q9ERY9
GN=Erg28
ergosterol biosynthesis 28

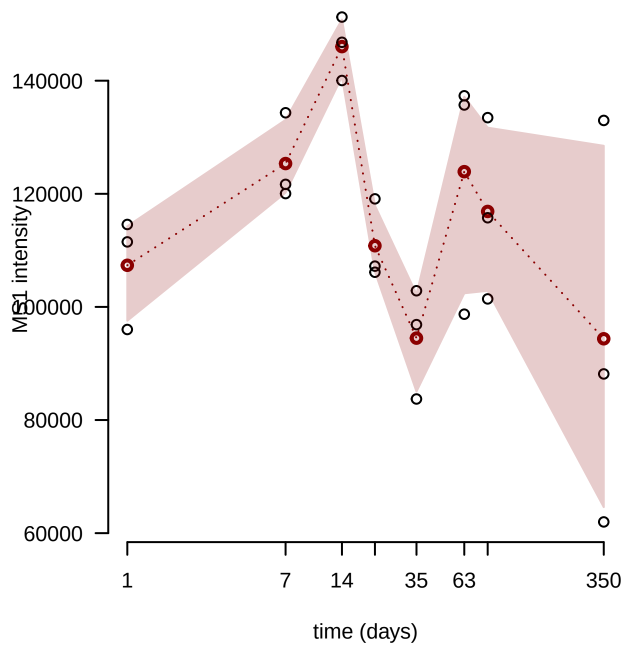

Q9ES00
GN=Ube4b
ubiquitination factor E4B

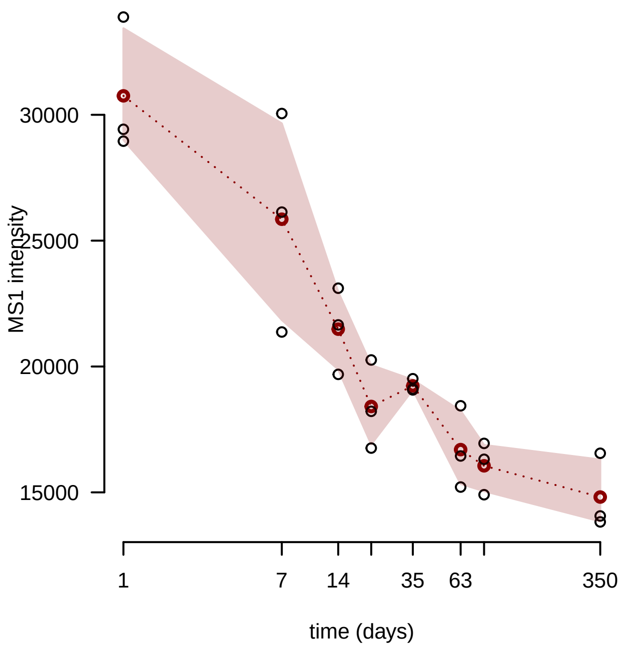

Q9ES18
GN=Foxj2
forkhead box J2

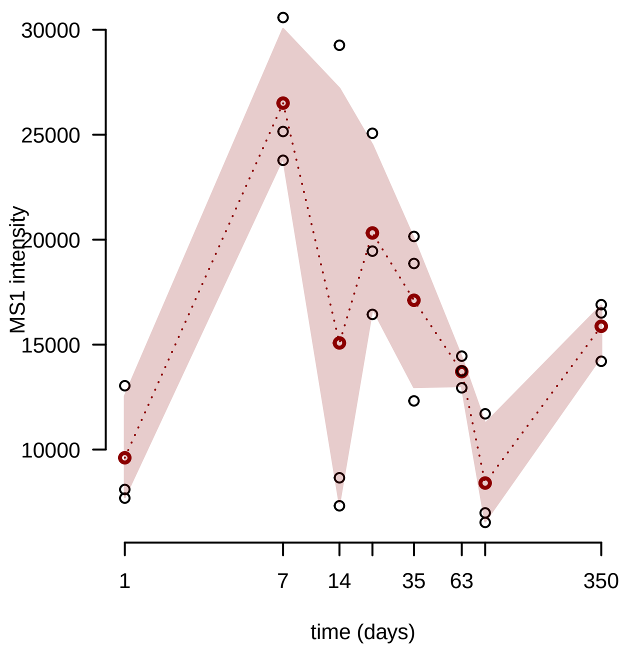

Q9ES28
GN=Arhgef7
Rho guanine nucleotide exchange factor (...

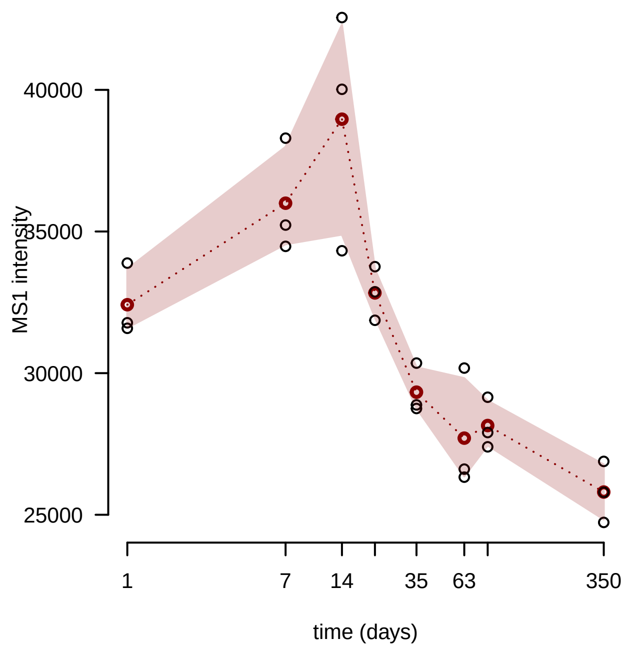

Q9ES28
GN=Arhgef7
Rho guanine nucleotide exchange factor (...

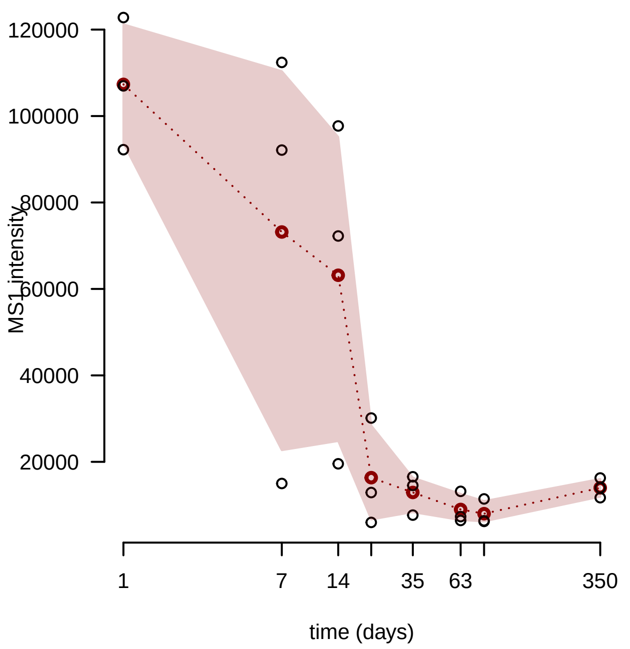

Q9ES34
GN=Ube3b
ubiquitin protein ligase E3B

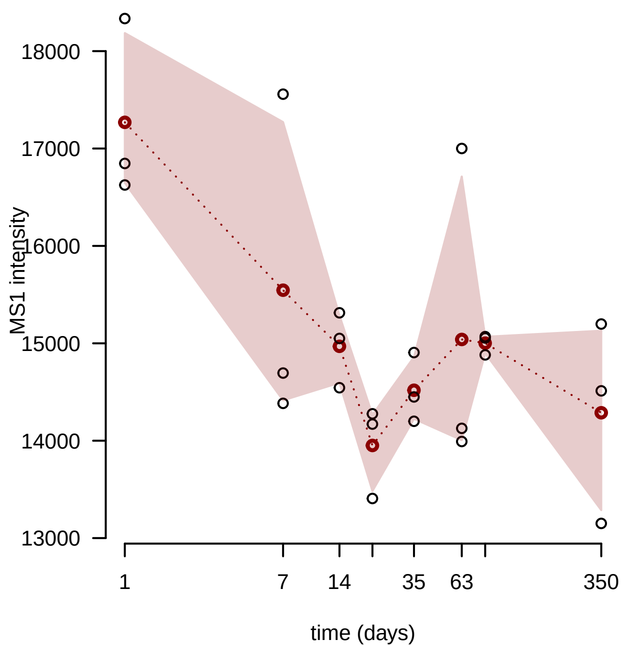

Q9ES46
GN=Parvb
parvin, beta

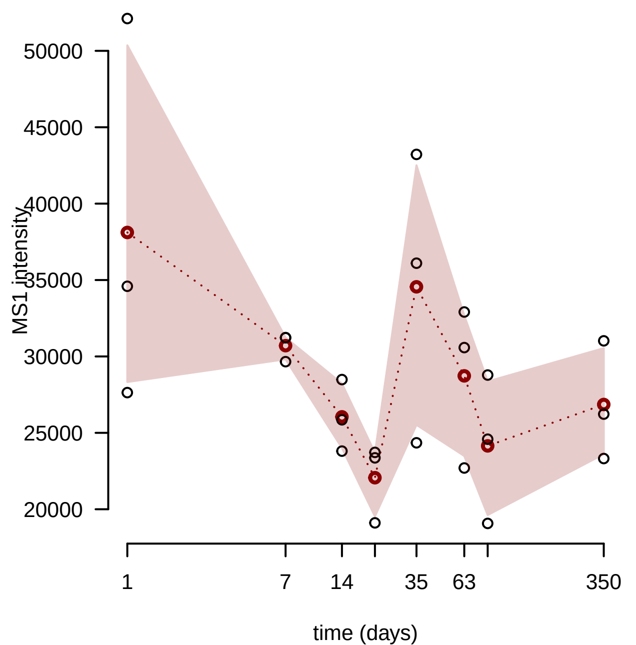

Q9ES52
GN=Inpp5d
inositol polyphosphate−5−phosphatase D

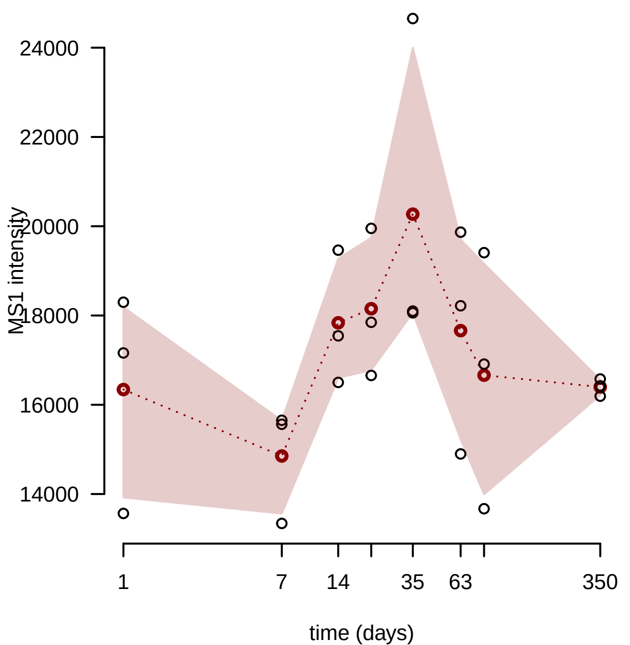

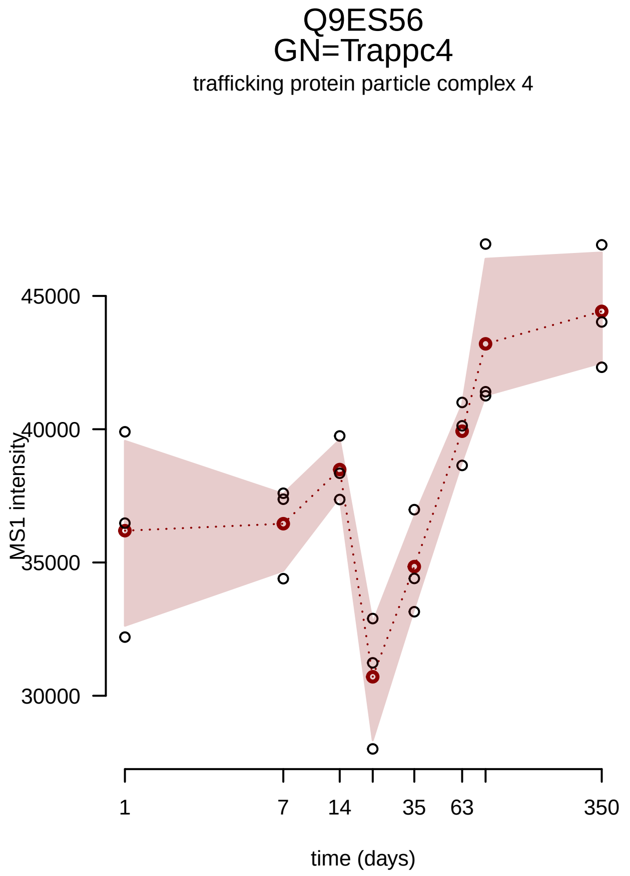
Q9ES56
GN=Trappc4
trafficking protein particle complex 4
MS1 intensity
30000
35000
40000
45000
1
7
14
35
63
350
time (days)

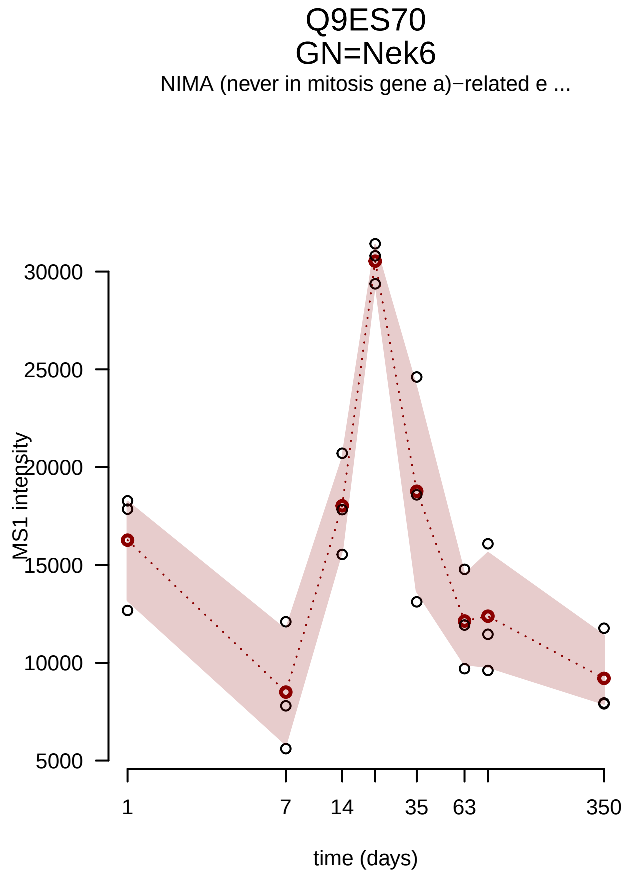
Q9ES70
GN=Nek6
NIMA (never in mitosis gene a)−related e ...
MS1 intensity
5000
10000
15000
20000
25000
30000
1
7
14
35
63
350
time (days)

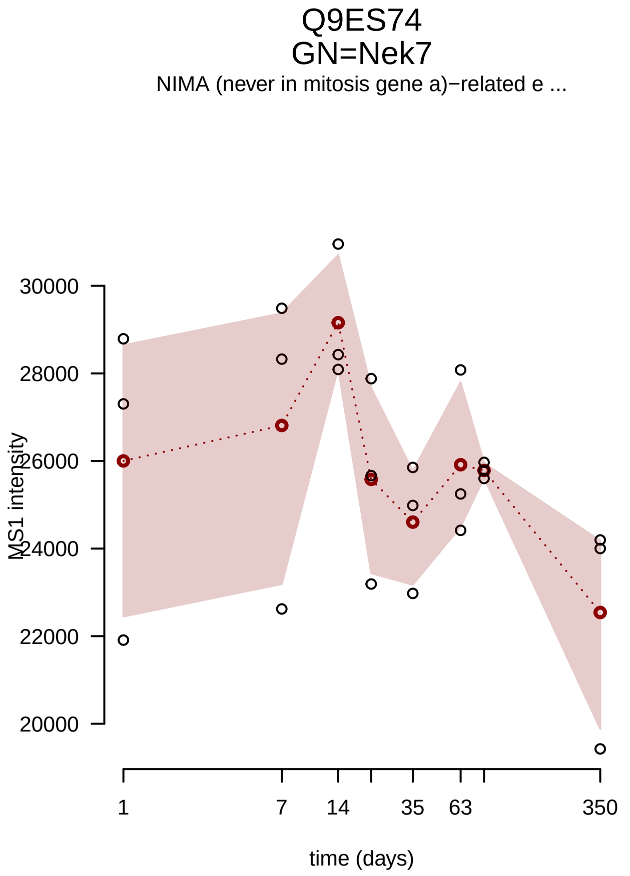
Q9ES74
GN=Nek7
NIMA (never in mitosis gene a)−related e ...
MS1 intensity
20000
22000
24000
26000
28000
30000
1
7
14
35
63
350
time (days)

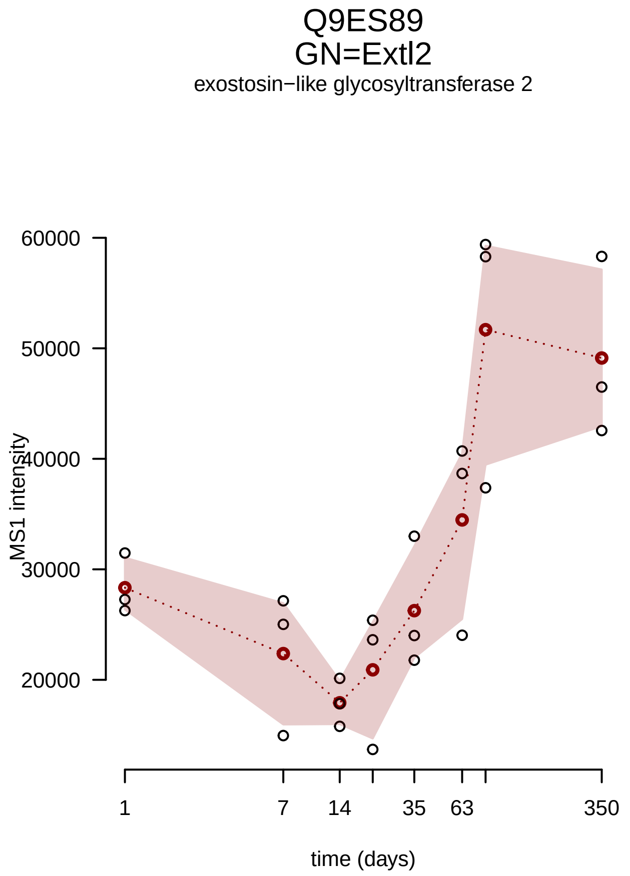
Q9ES89
GN=Extl2
exostosin−like glycosyltransferase 2
MS1 intensity
20000
30000
40000
50000
60000
1
7
14
35
63
350
time (days)

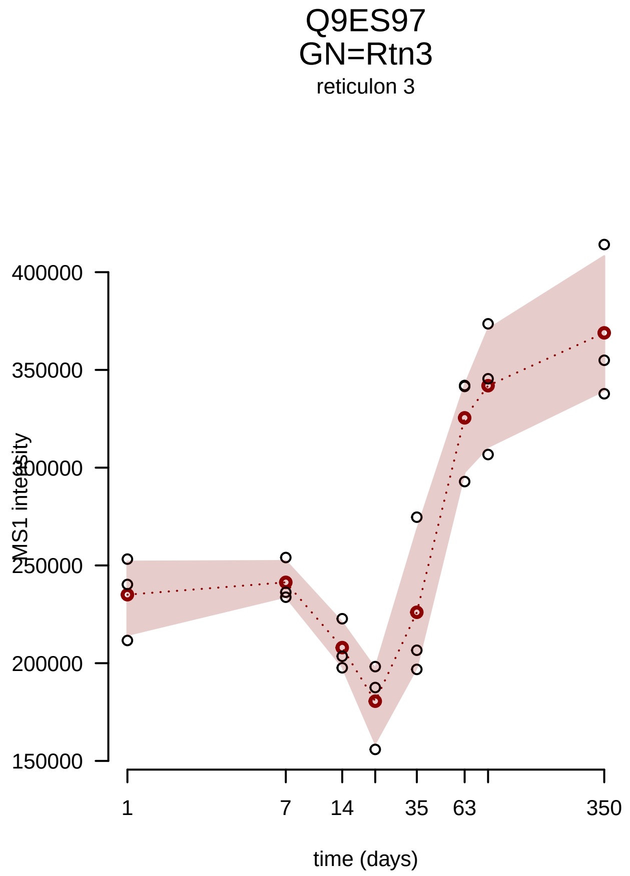
Q9ES97
GN=Rtn3
reticulon 3
MS1 intensity
150000
200000
250000
300000
350000
400000
1
7
14
35
63
350
time (days)

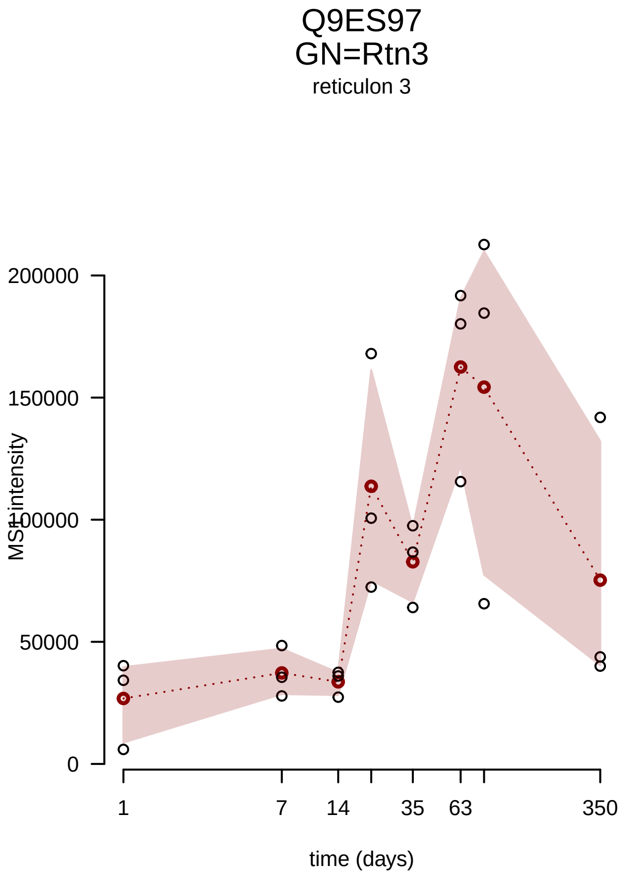
Q9ES97
GN=Rtn3
reticulon 3
MS1 intensity
0
50000
100000
150000
200000
1
7
14
35
63
350
time (days)

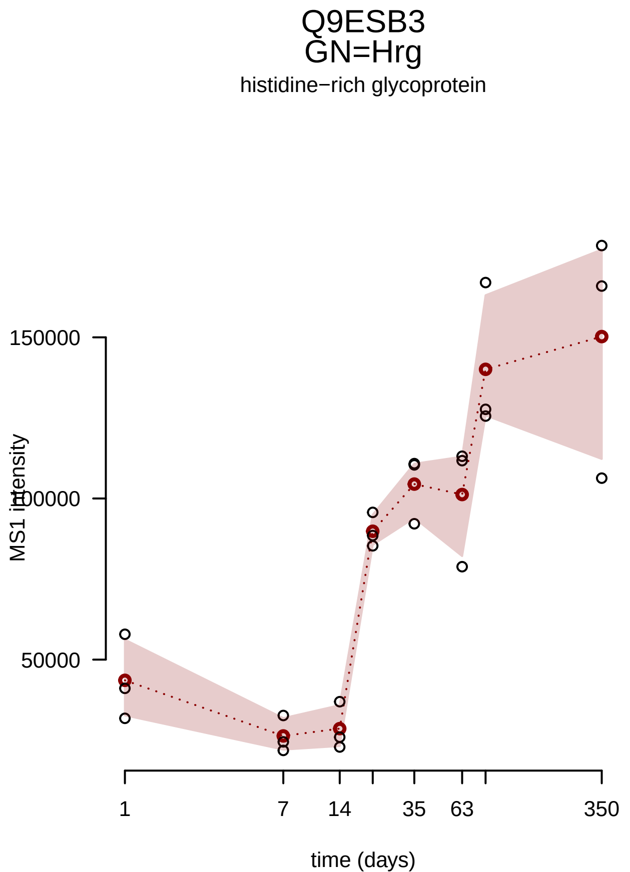
Q9ESB3
GN=Hrg
histidine−rich glycoprotein
MS1 intensity
50000
100000
150000
1
7
14
35
63
350
time (days)

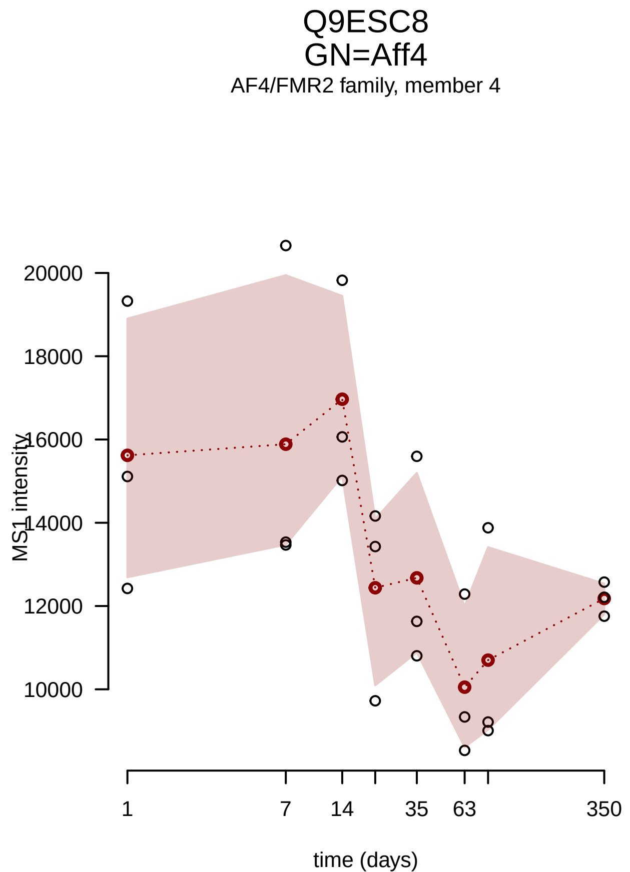
Q9ESC8
GN=Aff4
AF4/FMR2 family, member 4
MS1 intensity
10000
12000
14000
16000
18000
20000
1
7
14
35
63
350
time (days)

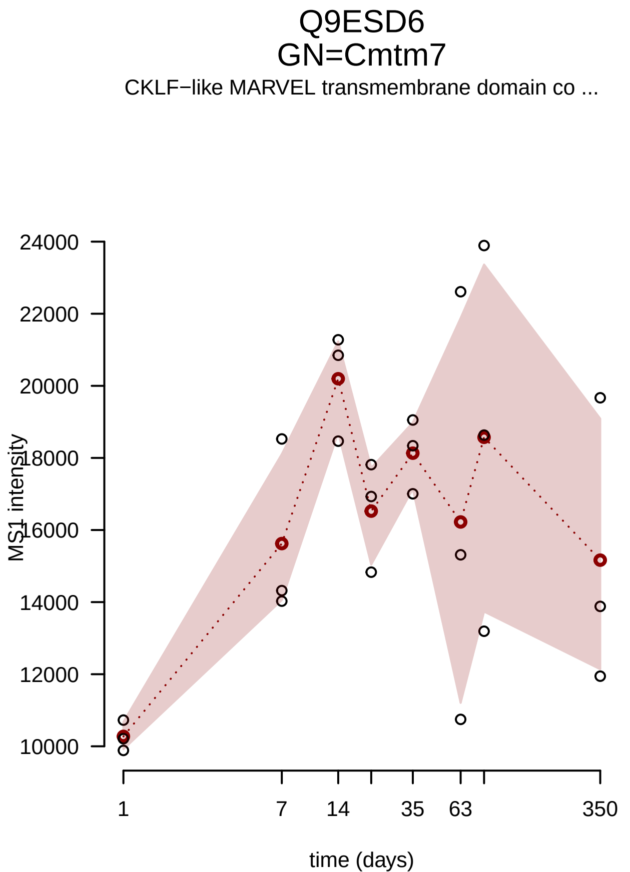
Q9ESD6
GN=Cmtm7
CKLF−like MARVEL transmembrane domain co ...
MS1 intensity
10000
12000
14000
16000
18000
20000
22000
24000
1
7
14
35
63
350
time (days)

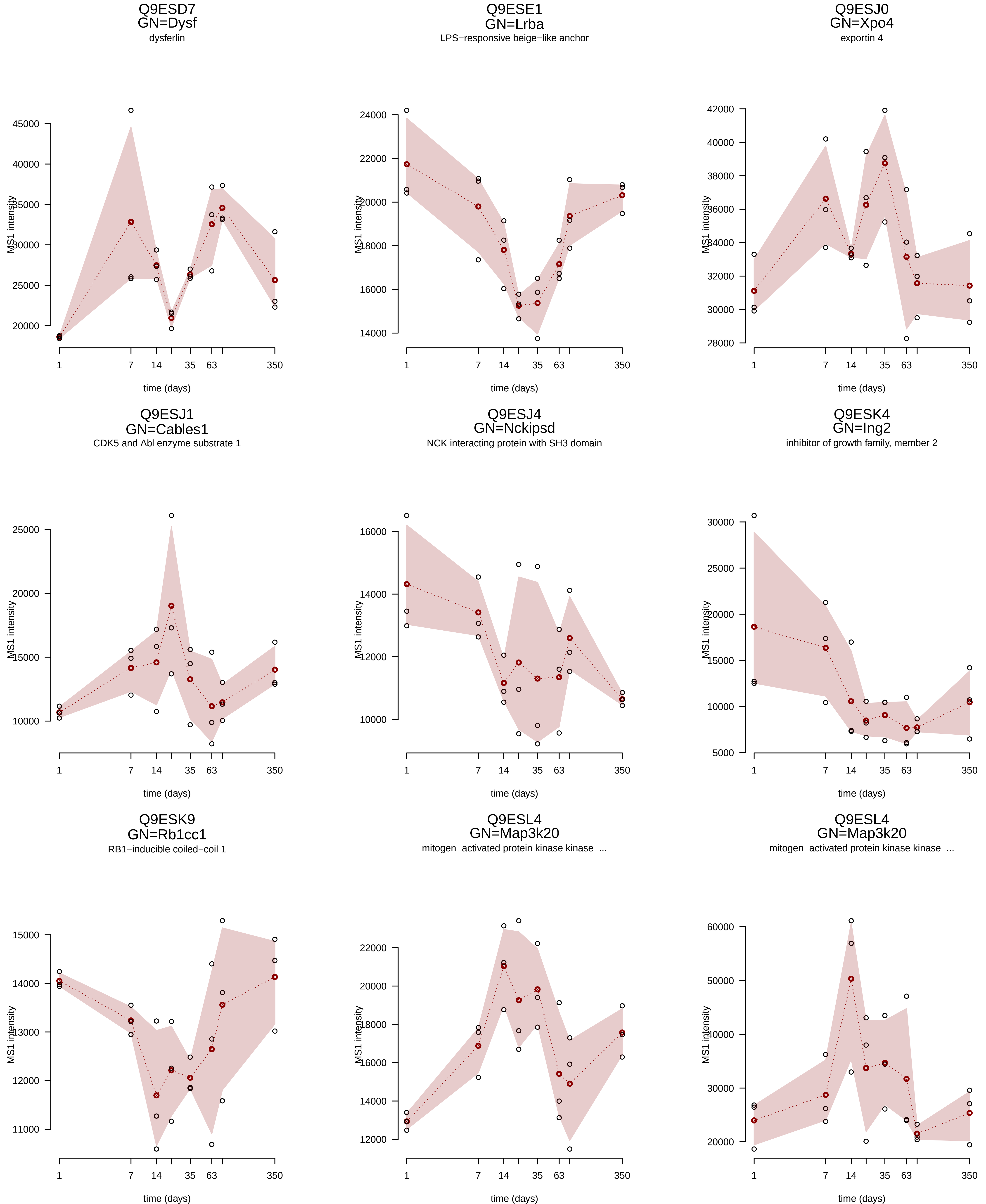

Q9ESD7
GN=Dysf
dysferlin
Q9ESE1
GN=Lrba
LPS−responsive beige−like anchor
Q9ESJ0
GN=Xpo4
exportin 4
Q9ESJ1
GN=Cables1
CDK5 and Abl enzyme substrate 1
Q9ESJ4
GN=Nckipsd
NCK interacting protein with SH3 domain
Q9ESK4
GN=Ing2
inhibitor of growth family, member 2
Q9ESK9
GN=Rb1cc1
RB1−inducible coiled−coil 1
Q9ESL4
GN=Map3k20
mitogen−activated protein kinase kinase ...
Q9ESL4
GN=Map3k20
mitogen−activated protein kinase kinase ...
MS1 intensity
time (days)
1
7
14
35
63
350

Q9ESM6
GN=Gdpd2
glycerophosphodiester phosphodiesterase ...

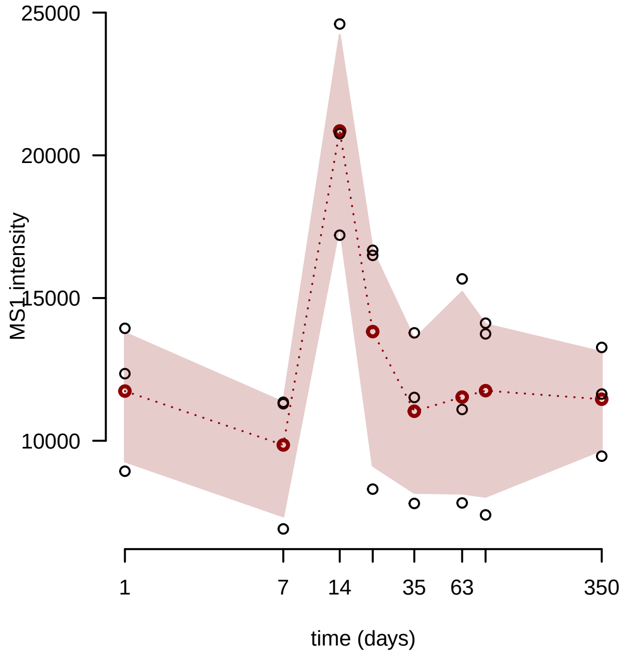

Q9ESN6
GN=Trim2
tripartite motif−containing 2

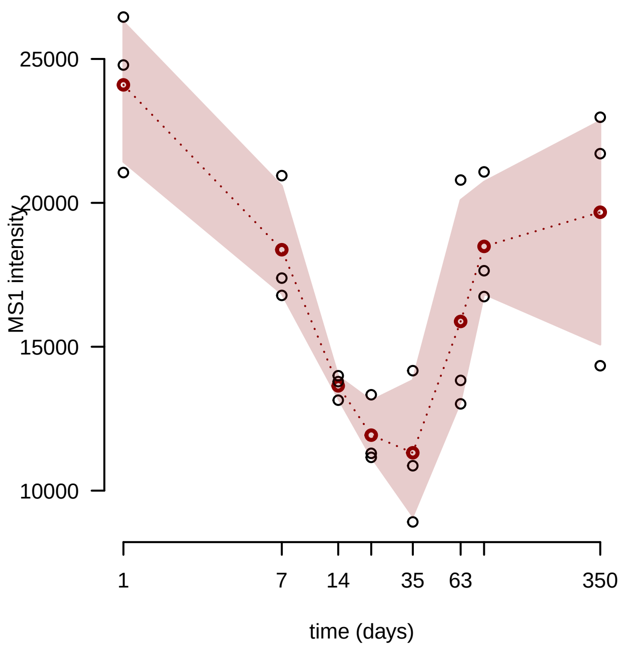

Q9ESN9
GN=Mapk8ip3
mitogen−activated protein kinase 8 inter ...

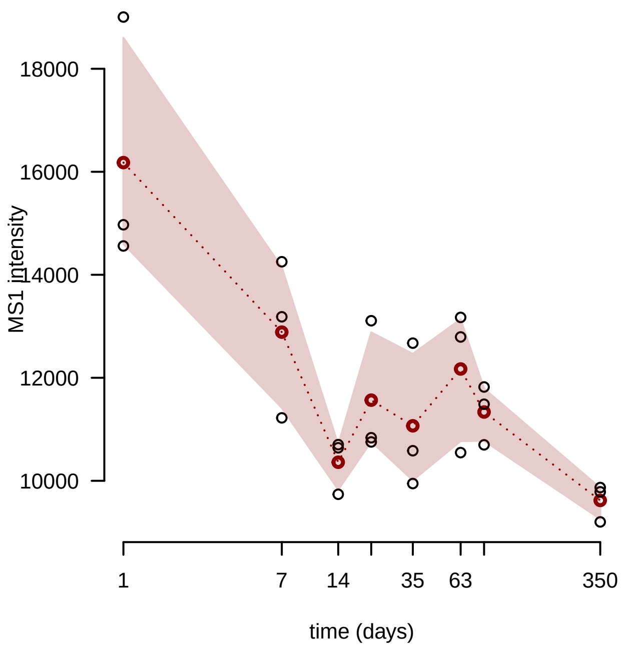

Q9ESP1
GN=Sdf2l1
stromal cell−derived factor 2−like 1

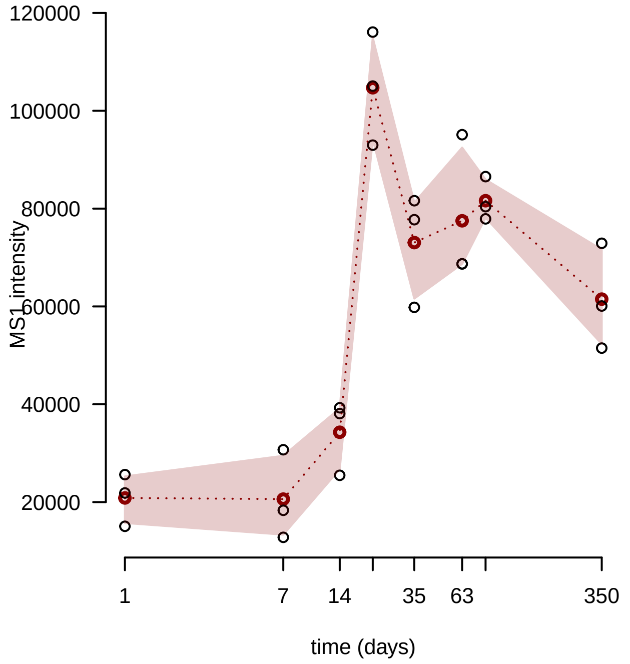

Q9EST1
GN=Gsdma
gasdermin A

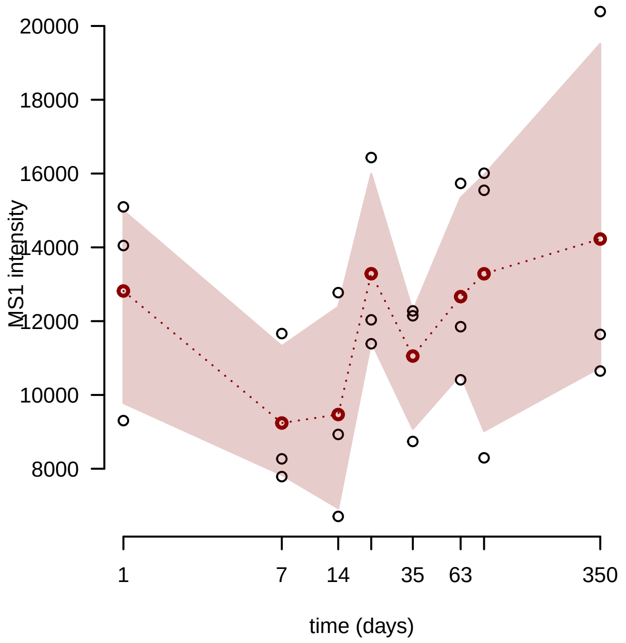

Q9EST3
GN=Eif4enif1
eukaryotic translation initiation factor ...

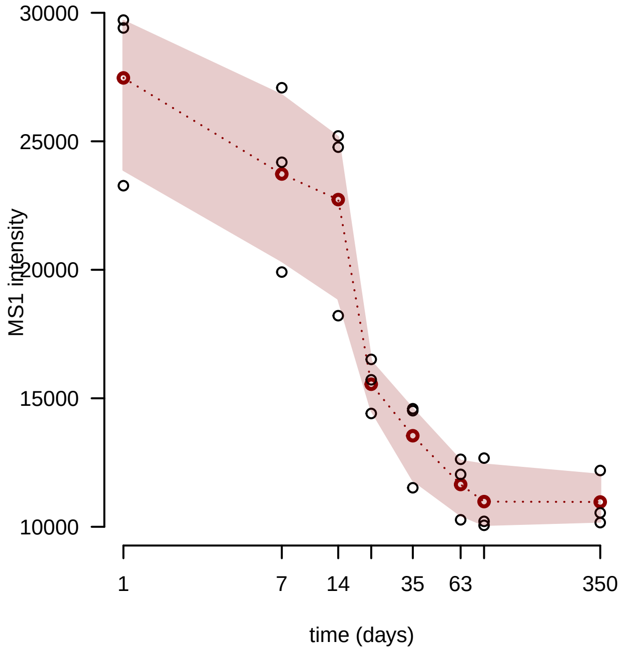

Q9EST3
GN=Eif4enif1
eukaryotic translation initiation factor ...

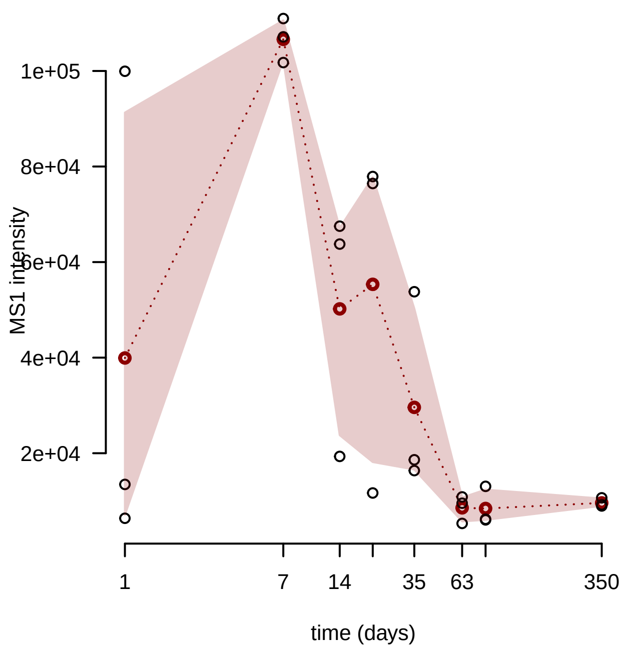

Q9EST4
GN=Psmg2
proteasome (prosome, macropain) assembly ...

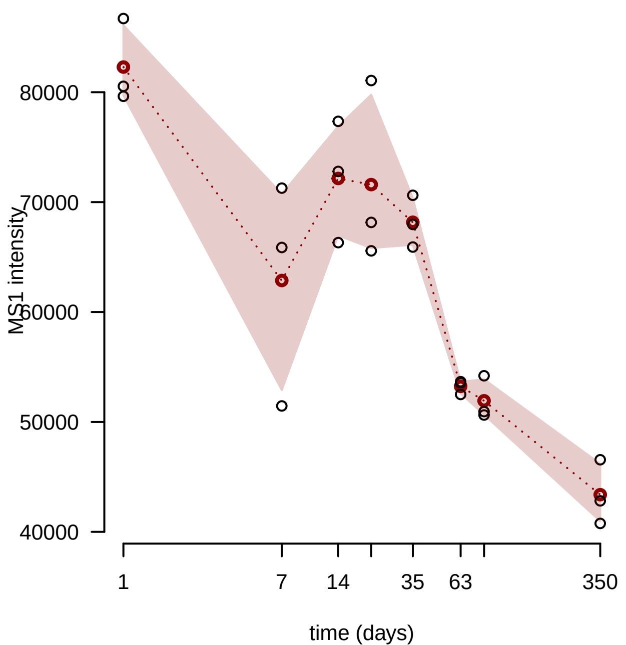

Q9EST5
GN=Anp32b
acidic (leucine−rich) nuclear phosphopro ...

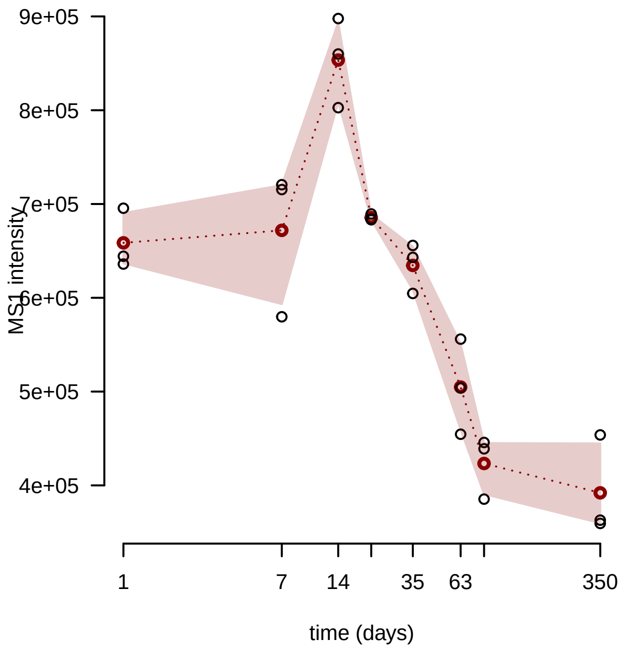

Q9ESU6
GN=Brd4
bromodomain containing 4

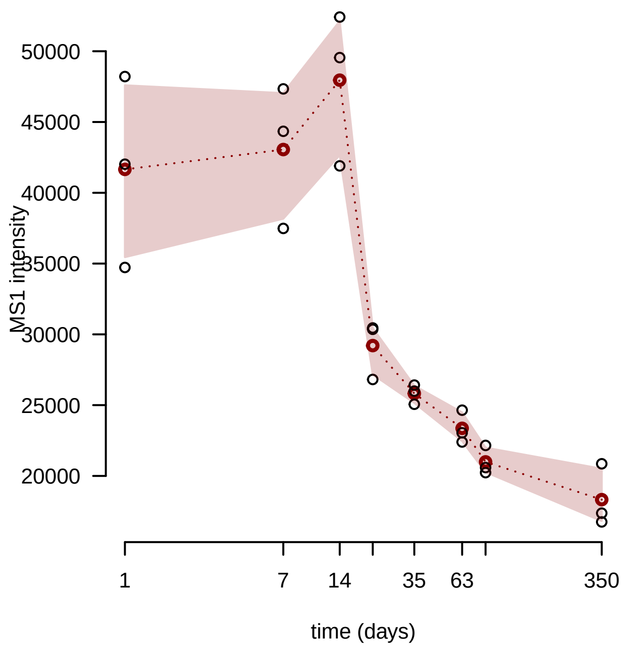

Q9ESV0
GN=Ddx24
DEAD box helicase 24

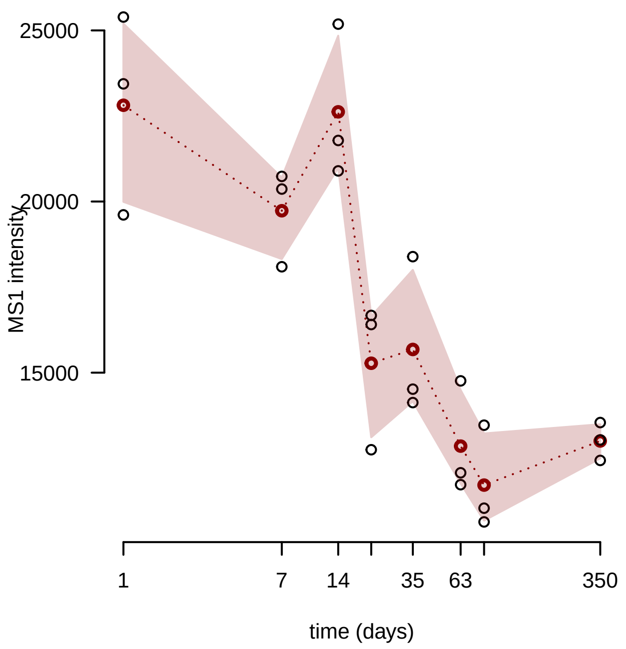

Q9ESW4
GN=Agk
acylglycerol kinase

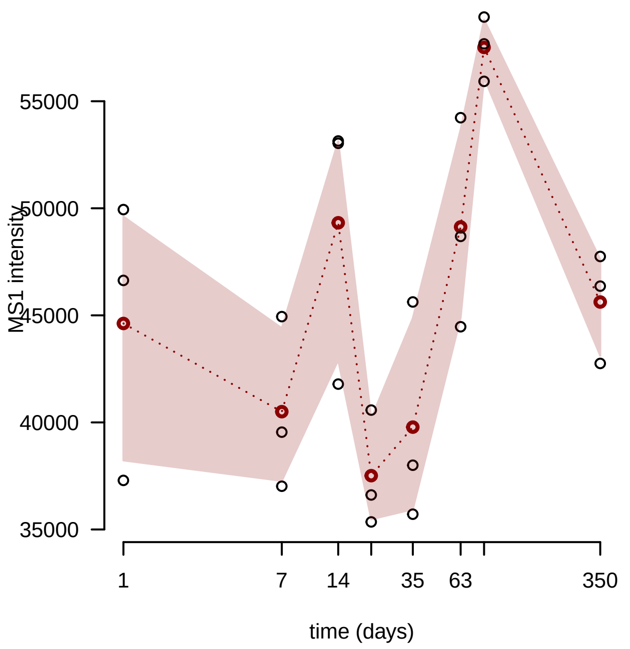

Q9ESW8
GN=Pgpep1
pyroglutamyl−peptidase I

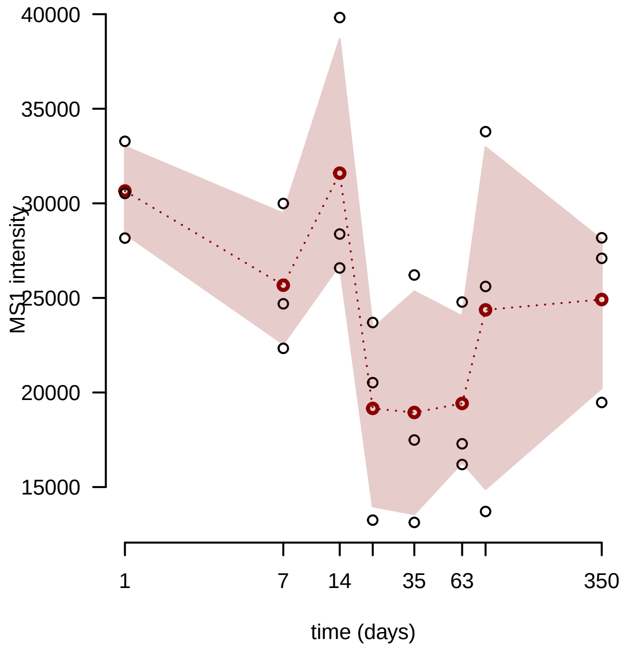

Q9ESX4
GN=Zcchc17
zinc finger, CCHC domain containing 17

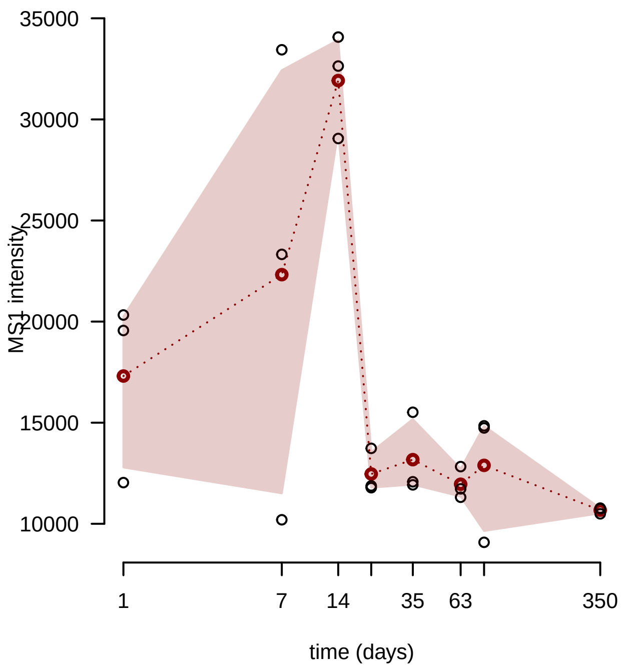

Q9ESX5
GN=Dkc1
dyskeratosis congenita 1, dyskerin

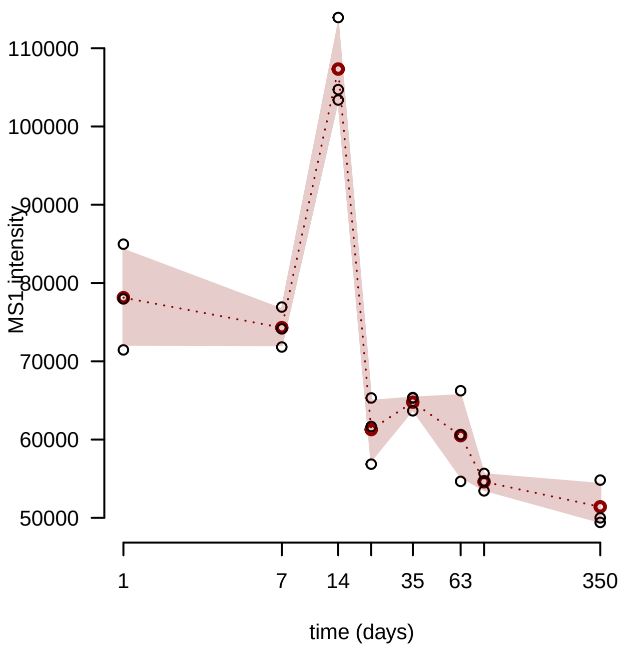

Q9ESZ8
GN=Gtf2i
general transcription factor II I

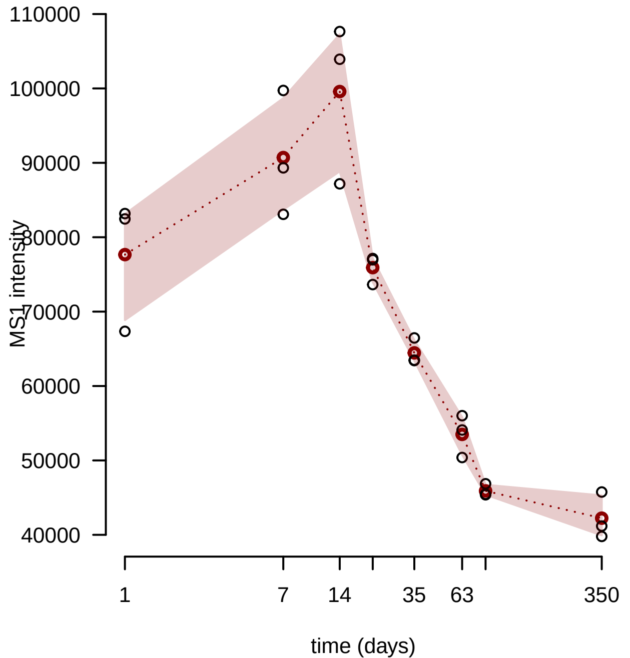

Q9ET01
GN=Pygl
liver glycogen phosphorylase

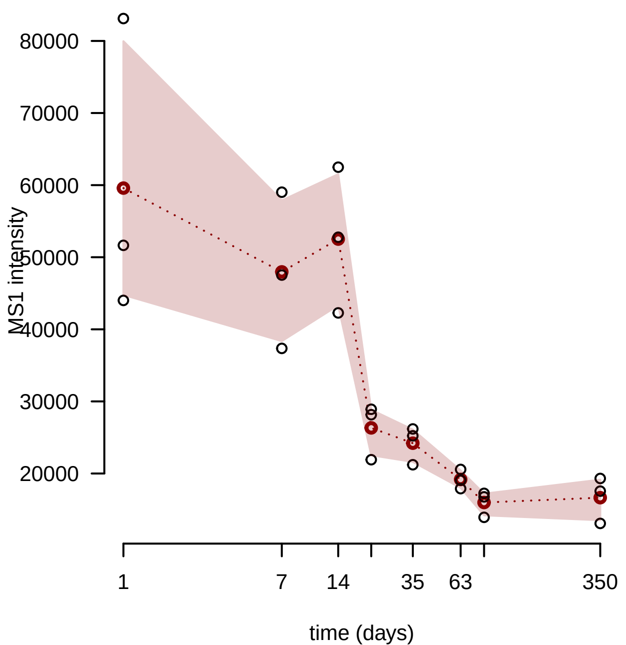

Q9ET22
GN=Dpp7
dipeptidylpeptidase 7

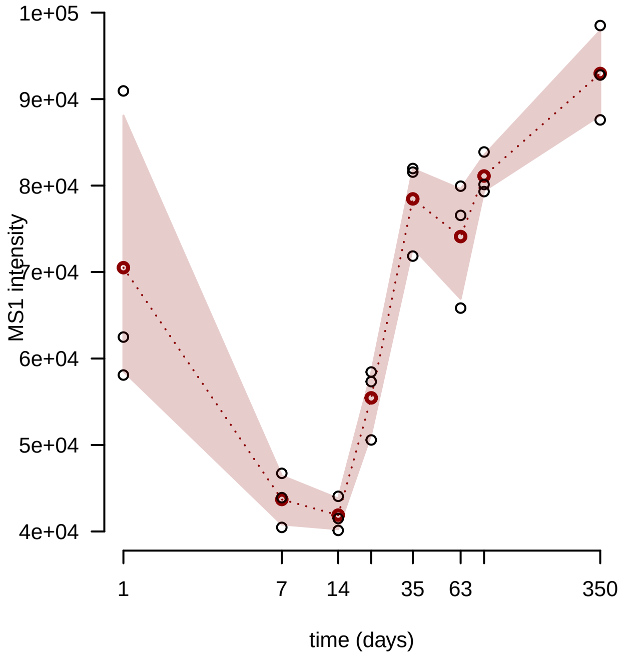

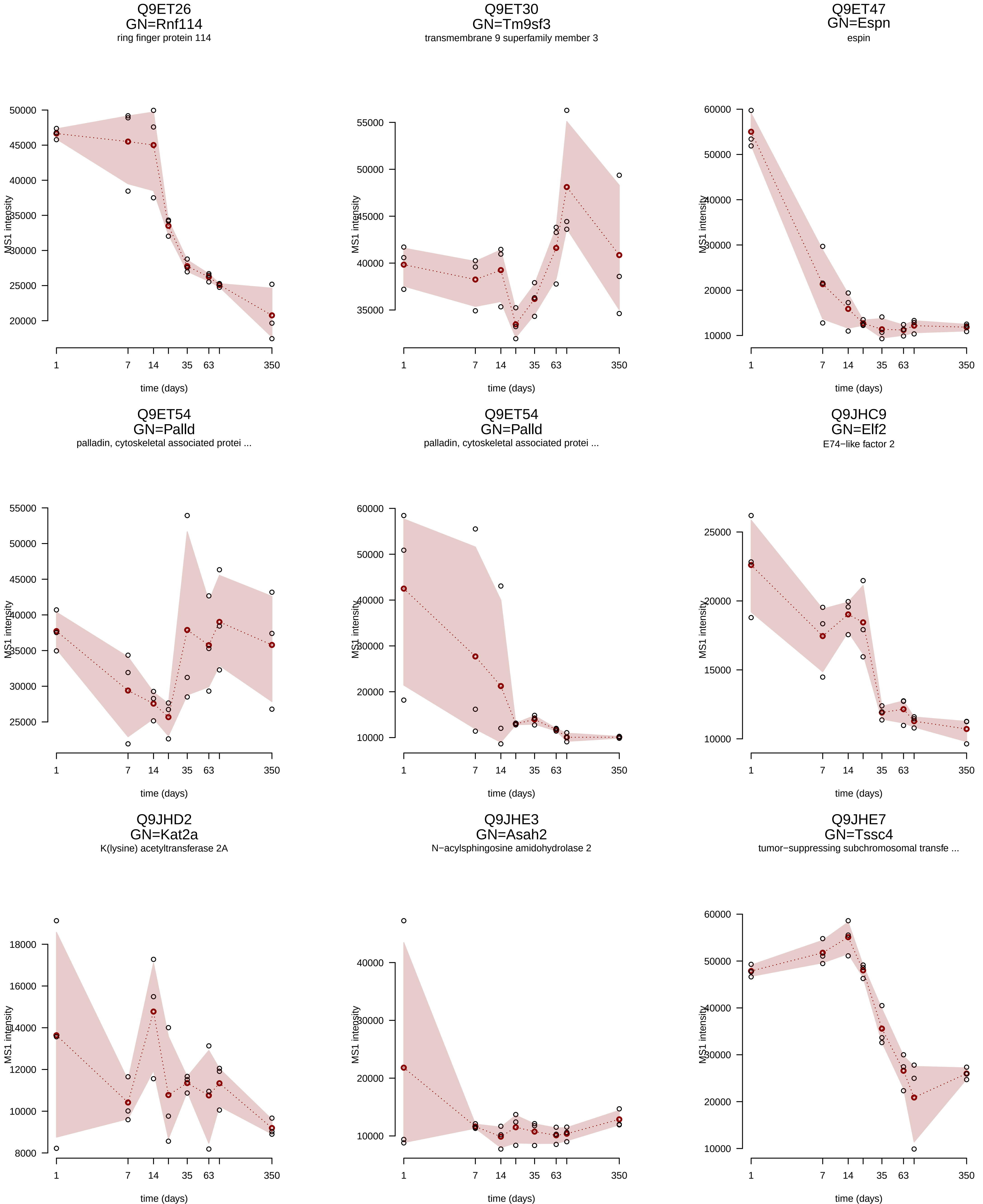
Q9ET26
GN=Rnf114
ring finger protein 114
MS1 intensity
50000
45000
40000
35000
30000
25000
20000
1
7
14
35
63
350
time (days)
Q9ET30
GN=Tm9sf3
transmembrane 9 superfamily member 3
MS1 intensity
55000
50000
45000
40000
35000
1
7
14
35
63
350
time (days)
Q9ET47
GN=Espn
espin
MS1 intensity
60000
50000
40000
30000
20000
10000
1
7
14
35
63
350
time (days)
Q9ET54
GN=Palld
palladin, cytoskeletal associated protei ...
MS1 intensity
55000
50000
45000
40000
35000
30000
25000
1
7
14
35
63
350
time (days)
Q9ET54
GN=Palld
palladin, cytoskeletal associated protei ...
MS1 intensity
60000
50000
40000
30000
20000
10000
1
7
14
35
63
350
time (days)
Q9JHC9
GN=Elf2
E74−like factor 2
MS1 intensity
25000
20000
15000
10000
1
7
14
35
63
350
time (days)
Q9JHD2
GN=Kat2a
K(lysine) acetyltransferase 2A
MS1 intensity
18000
16000
14000
12000
10000
8000
1
7
14
35
63
350
time (days)
Q9JHE3
GN=Asah2
N−acylsphingosine amidohydrolase 2
MS1 intensity
40000
30000
20000
10000
1
7
14
35
63
350
time (days)
Q9JHE7
GN=Tssc4
tumor−suppressing subchromosomal transfe ...
MS1 intensity
60000
50000
40000
30000
20000
10000
1
7
14
35
63
350
time (days)

Q9JHG1
GN=Pigp
phosphatidylinositol glycan anchor biosy ...

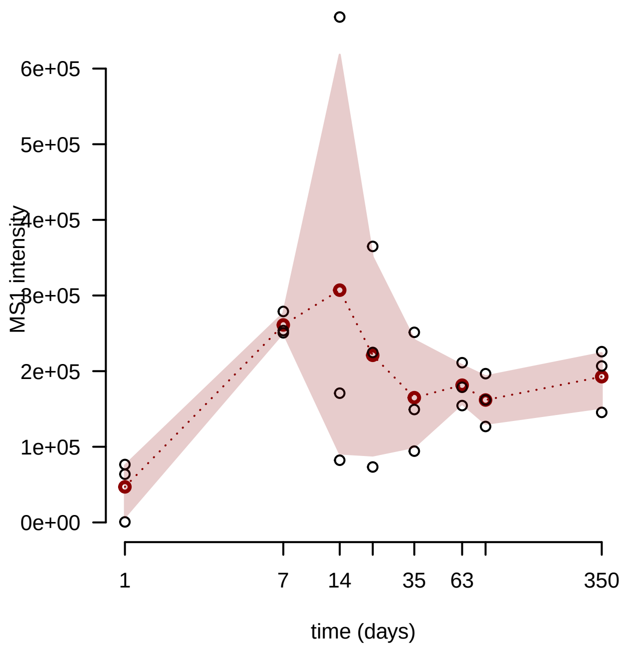

Q9JHG7
GN=Pik3cg
phosphatidylinositol−4,5−bisphosphate 3− ...

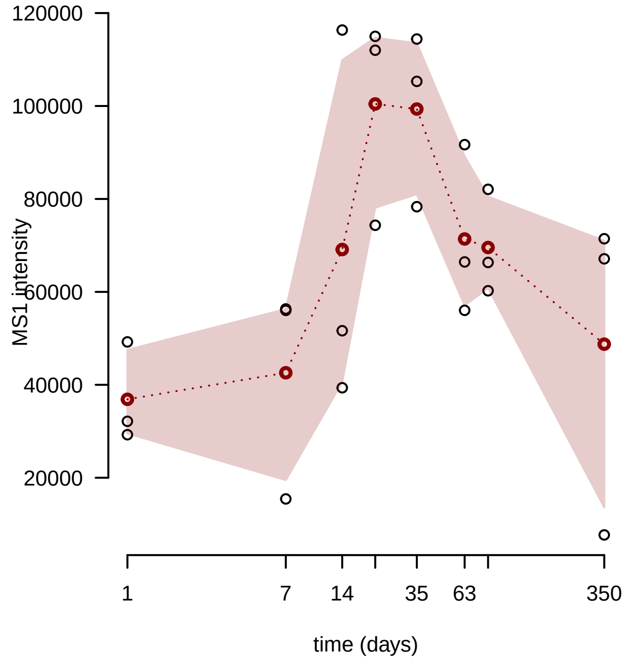

Q9JHH6
GN=Cpb2
carboxypeptidase B2 (plasma)

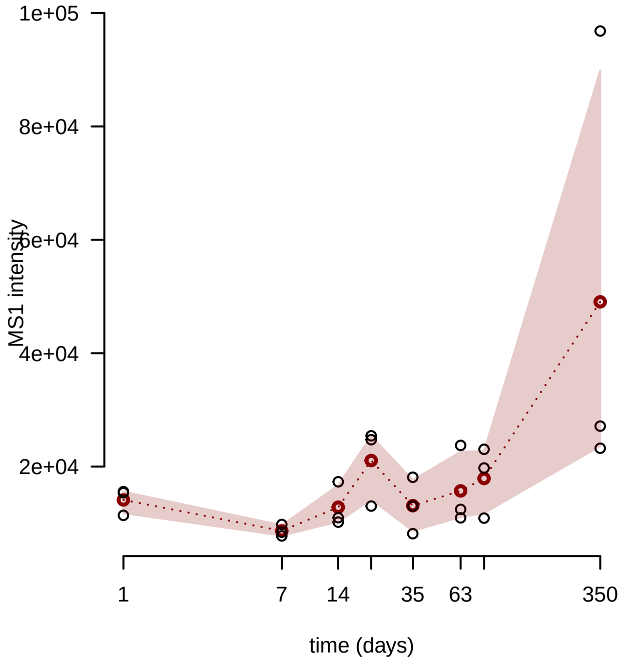

Q9JHH9
GN=Copz2
coatomer protein complex, subunit zeta 2

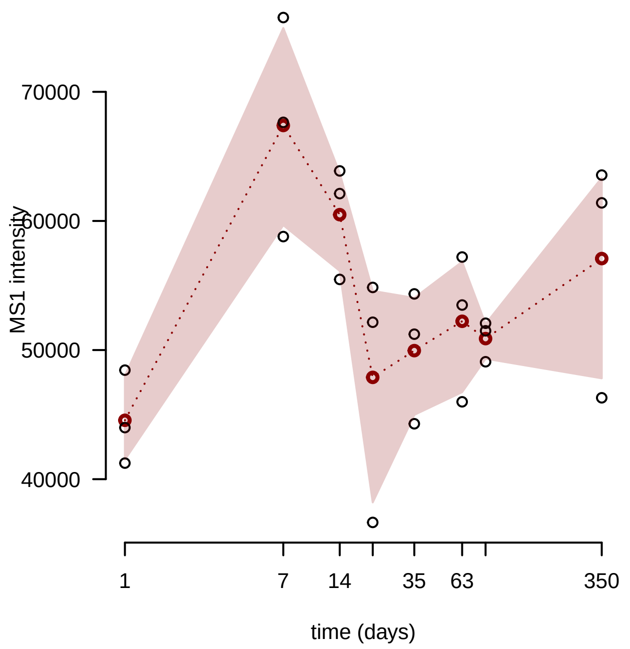

Q9JHI2
GN=Adat1
adenosine deaminase, tRNA−specific 1

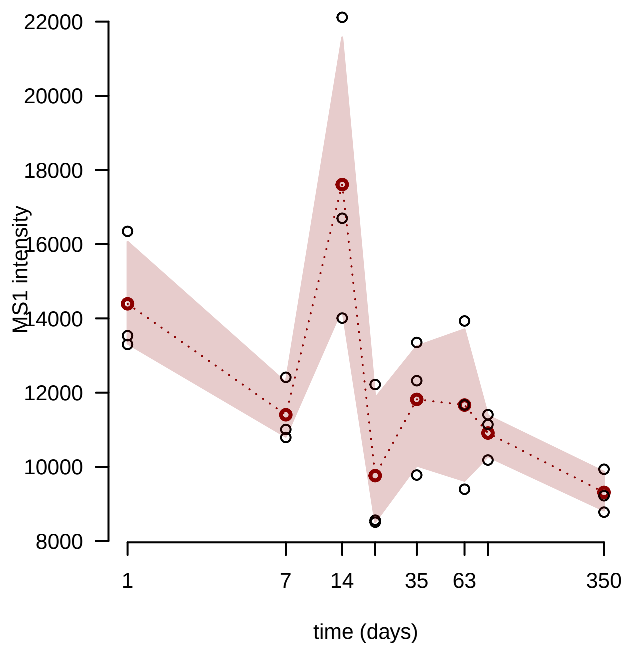

Q9JHI5
GN=Ivd
isovaleryl coenzyme A dehydrogenase

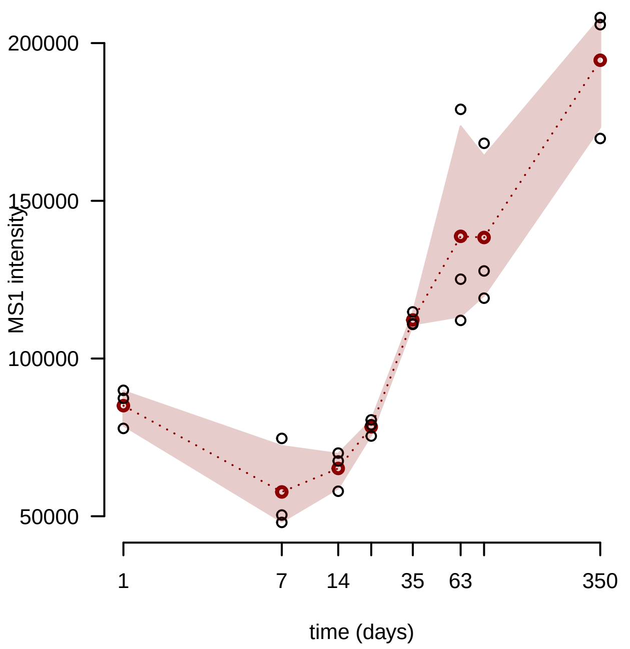

Q9JHI7
GN=Exosc9
exosome component 9

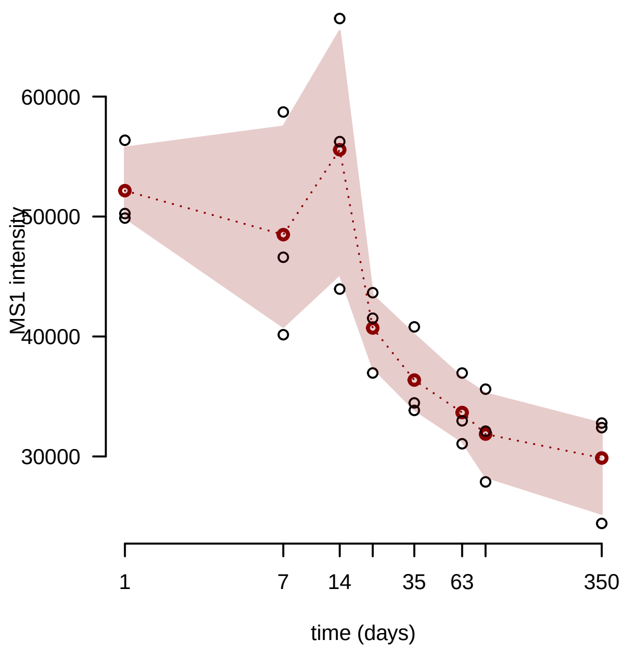

Q9JHJ0
GN=Tmod3
tropomodulin 3

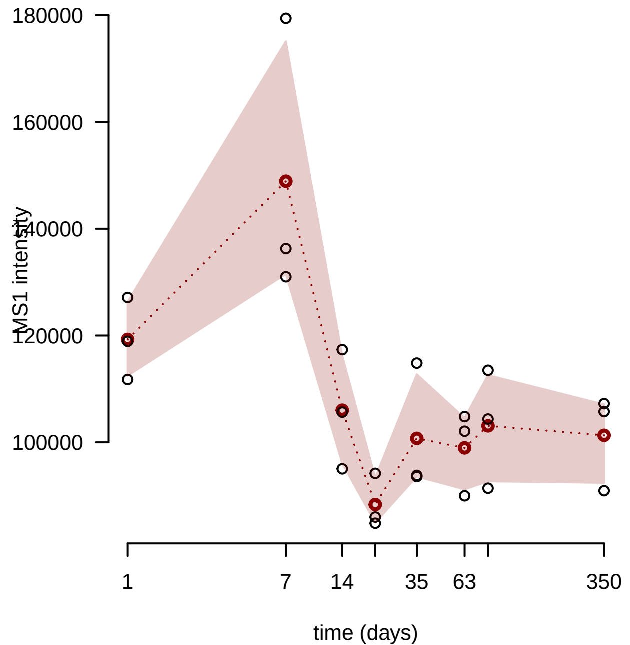

Q9JHJ3
GN=Glmp
glycosylated lysosomal membrane protein

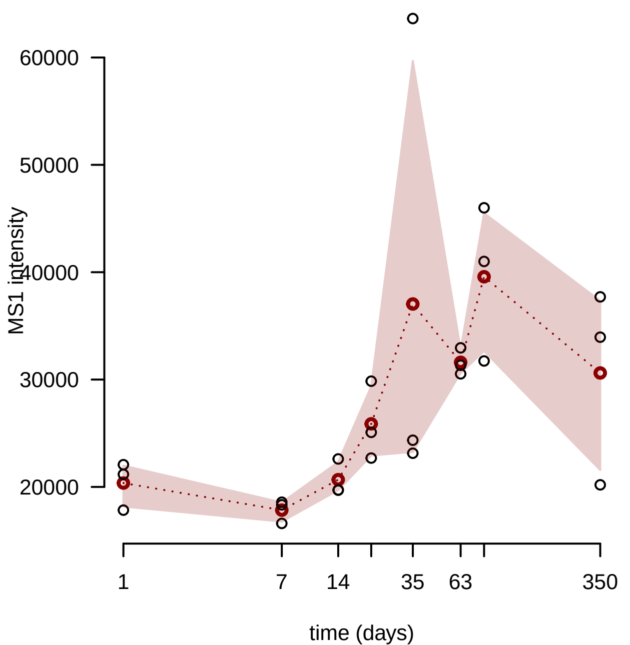

Q9JHJ8
GN=Icoslg

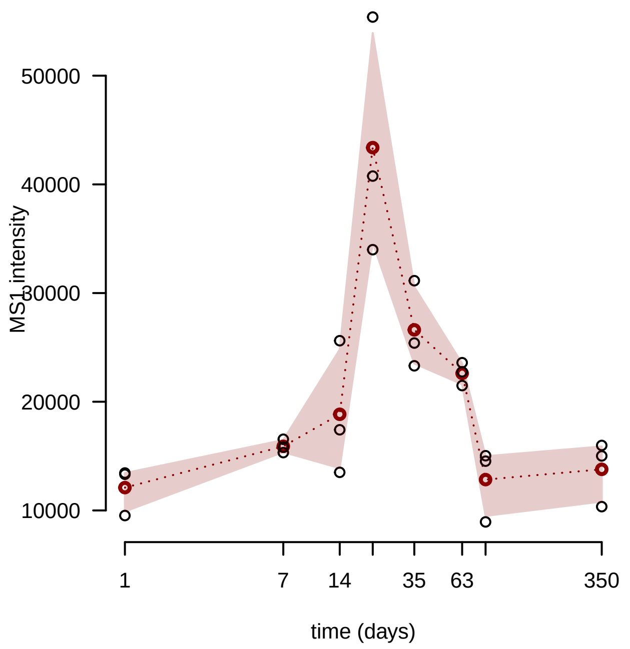

Q9JHK4
GN=Rabggta
Rab geranylgeranyl transferase, a subuni ...

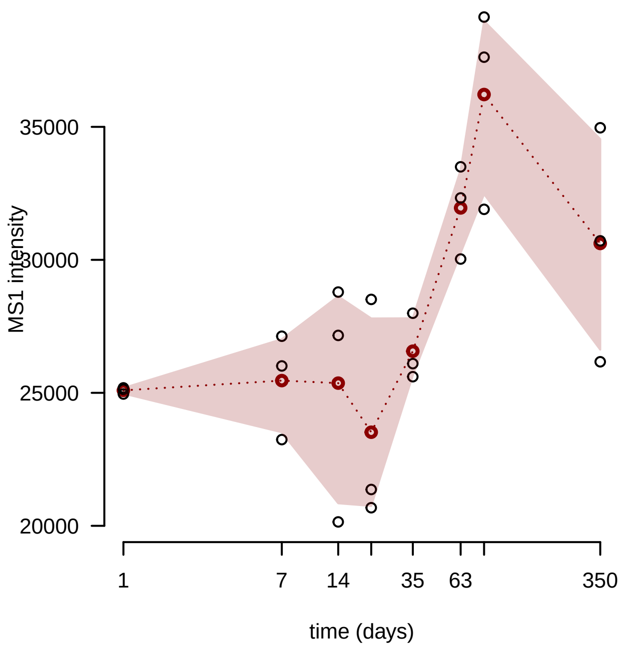

Q9JHK5
GN=Plek
pleckstrin

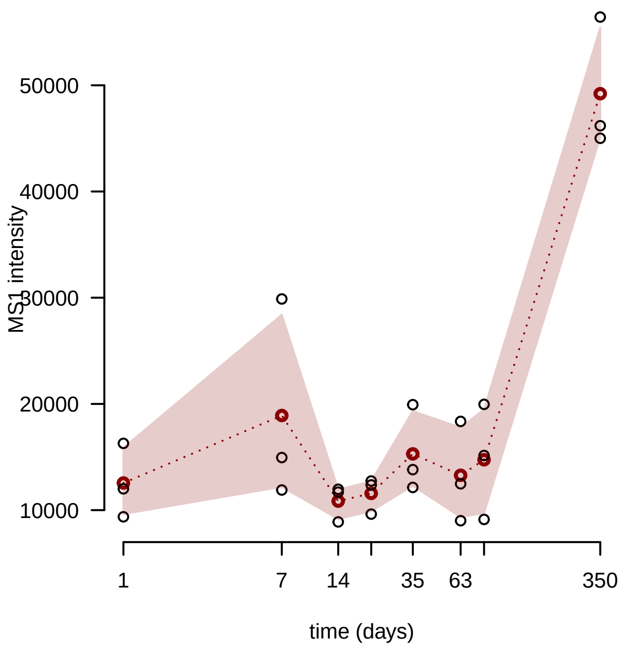

Q9JHL1
GN=Slc9a3r2
solute carrier family 9 (sodium/hydrogen ...

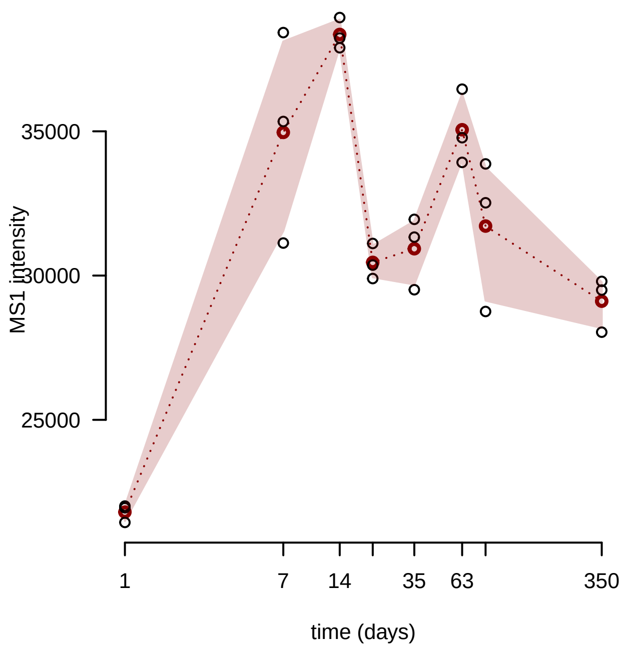

Q9JHP7
GN=Poglut2
protein O−glucosyltransferase 2

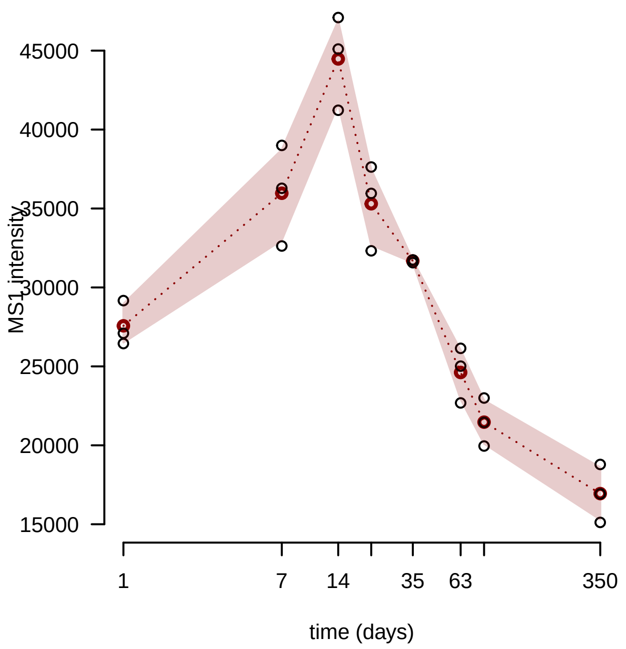

Q9JHQ5
GN=Lztfl1
leucine zipper transcription factor−like ...

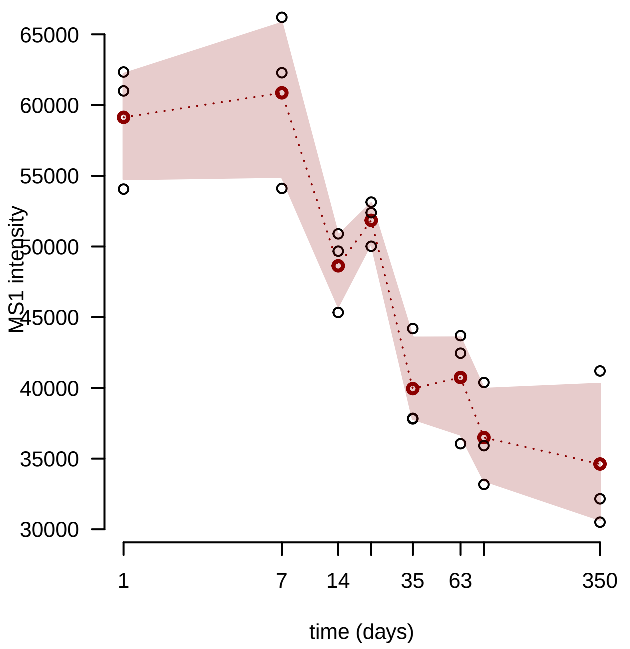

Q9JHR7
GN=Ide
insulin degrading enzyme

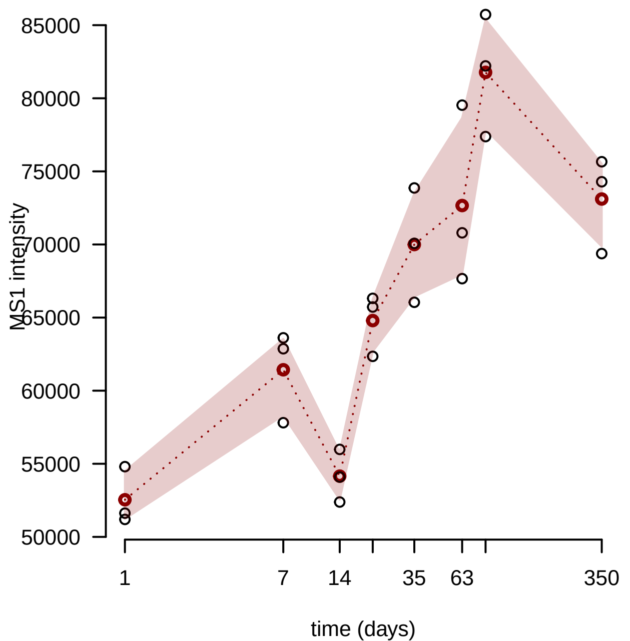

Q9JHR9
GN=Nrip2
nuclear receptor interacting protein 2

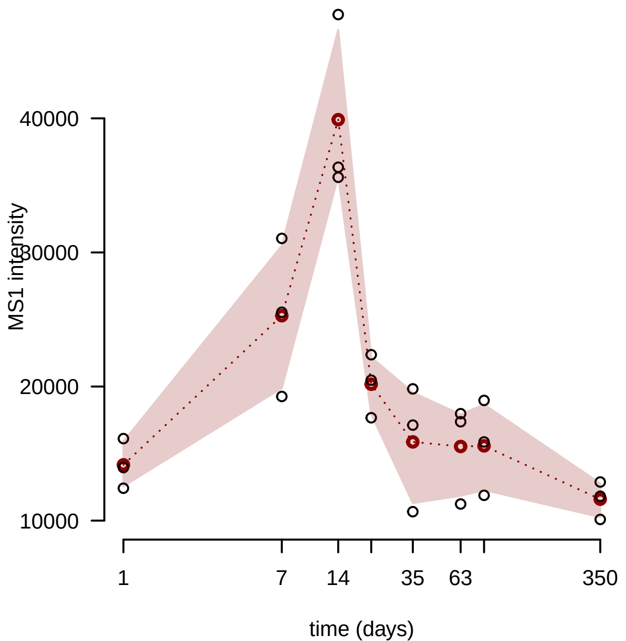

Q9JHS3
GN=Lamtor2
late endosomal/lysosomal adaptor, MAPK a ...

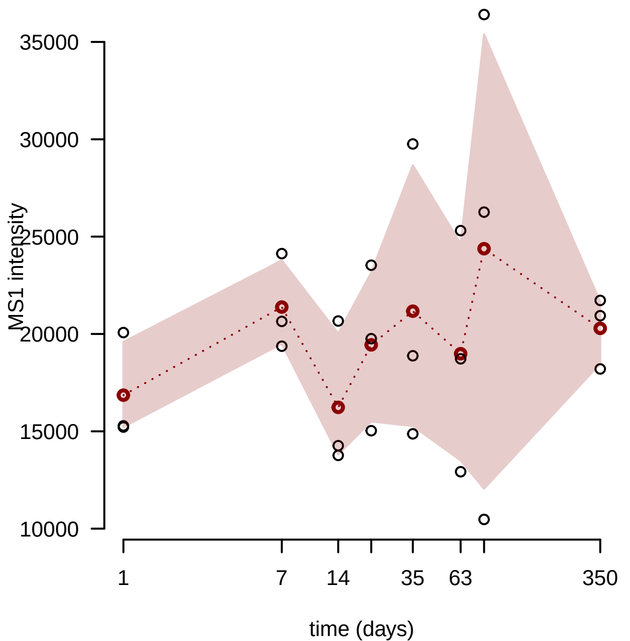

Q9JHS4
GN=Clpx
caseinolytic mitochondrial matrix peptid ...

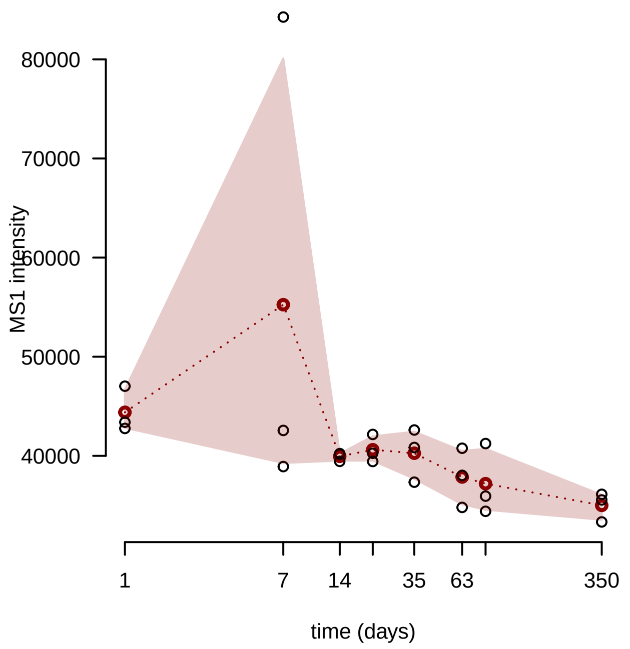

Q9JHS9
GN=Cwc15
CWC15 spliceosome−associated protein

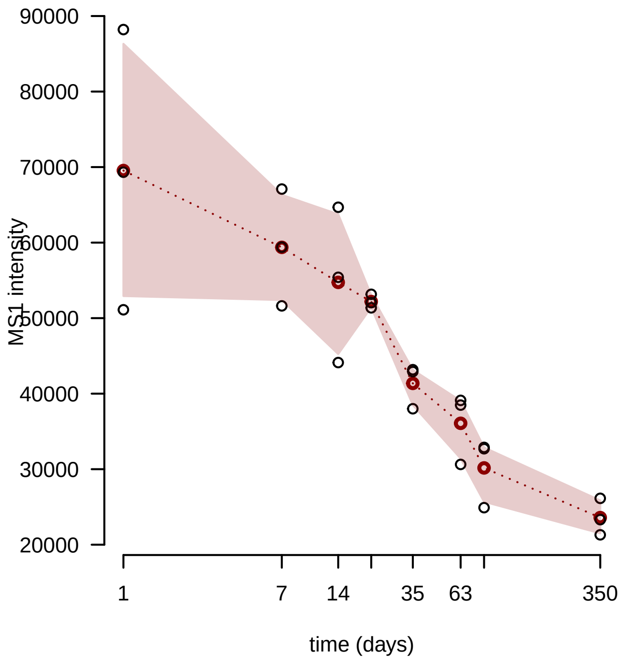

Q9JHT5
GN=Ammecr1
AMMECR nuclear protein 1

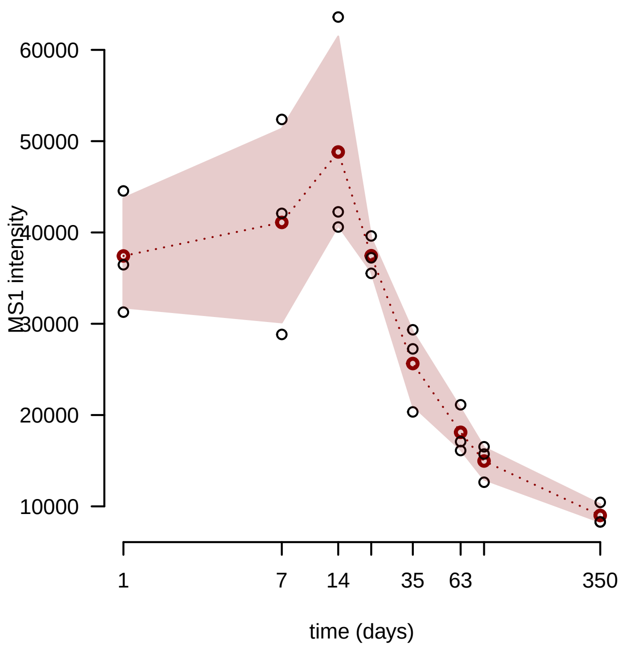

Q9JHU2
GN=Palmd
palmdelphin

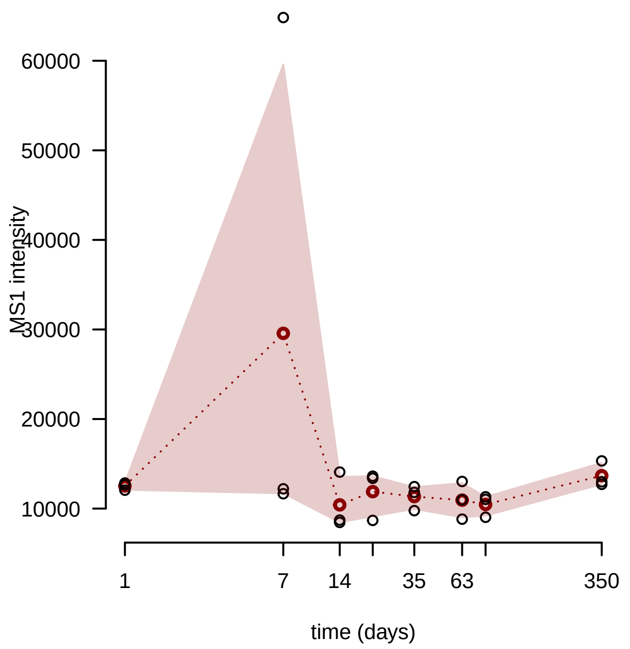

Q9JHU3
GN=Cdk20
cyclin−dependent kinase 20

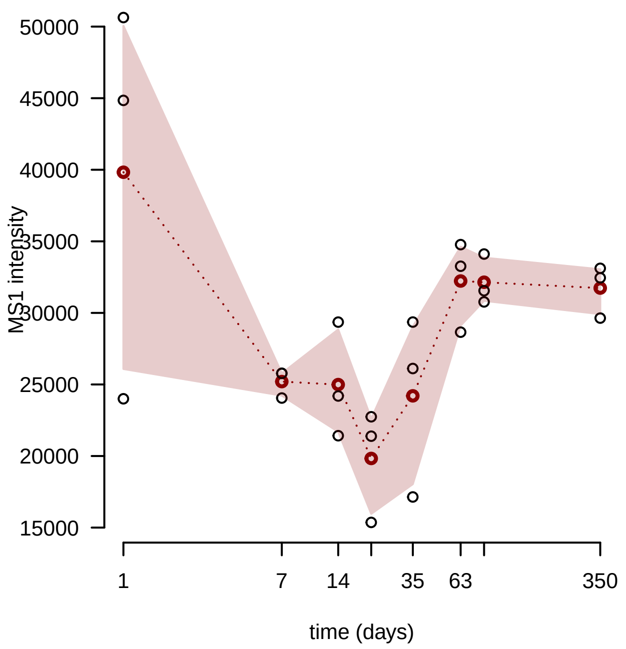

Q9JHU4
GN=Dync1h1
dynein cytoplasmic 1 heavy chain 1

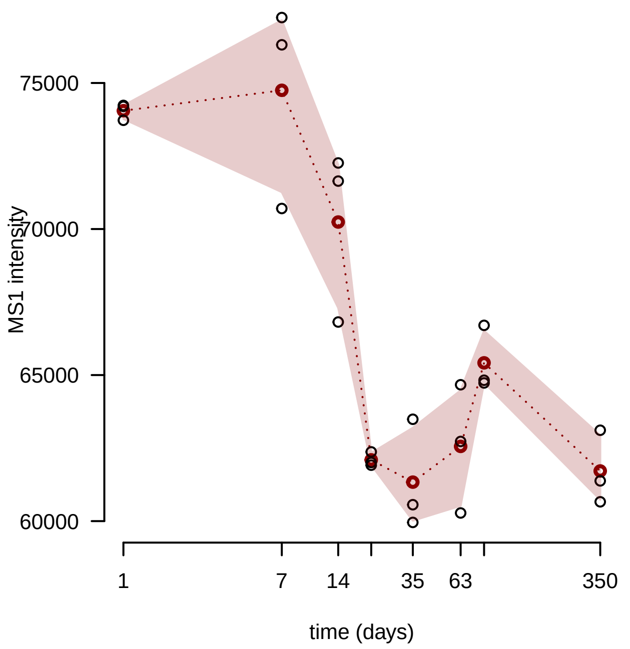

Q9JHU9
GN=Isyna1
myo−inositol 1−phosphate synthase A1

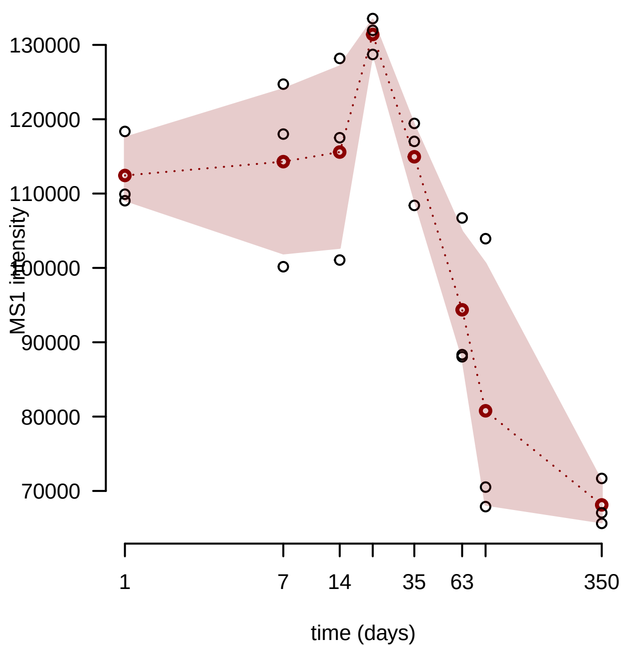

Q9JHW2
GN=Nit2
nitrilase family, member 2

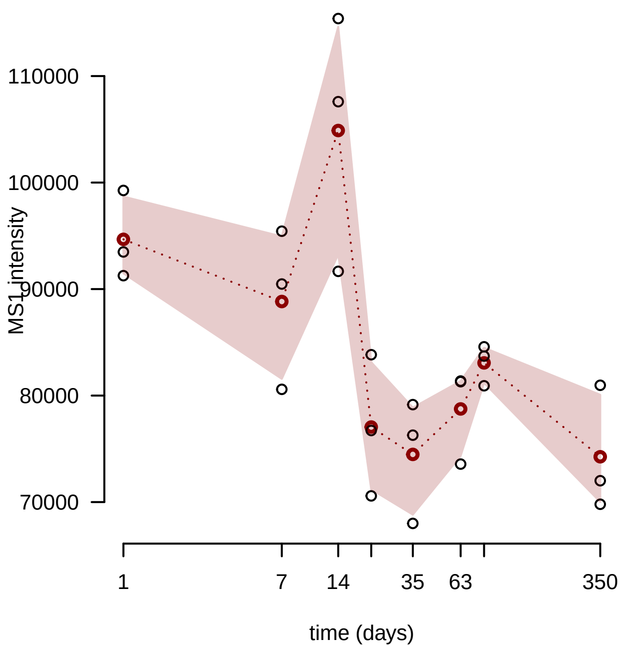

Q9JHW4
GN=Eefsec
eukaryotic elongation factor, selenocyst ...

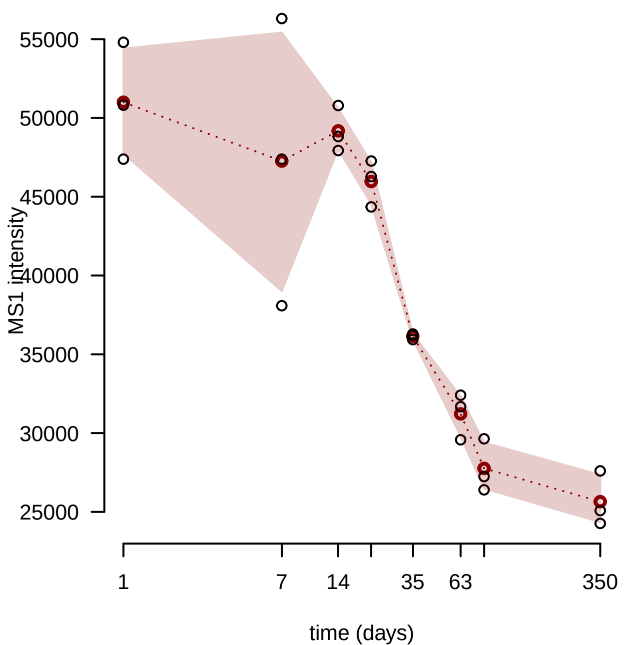

Q9JHW9
GN=Aldh1a3
aldehyde dehydrogenase family 1, subfami ...

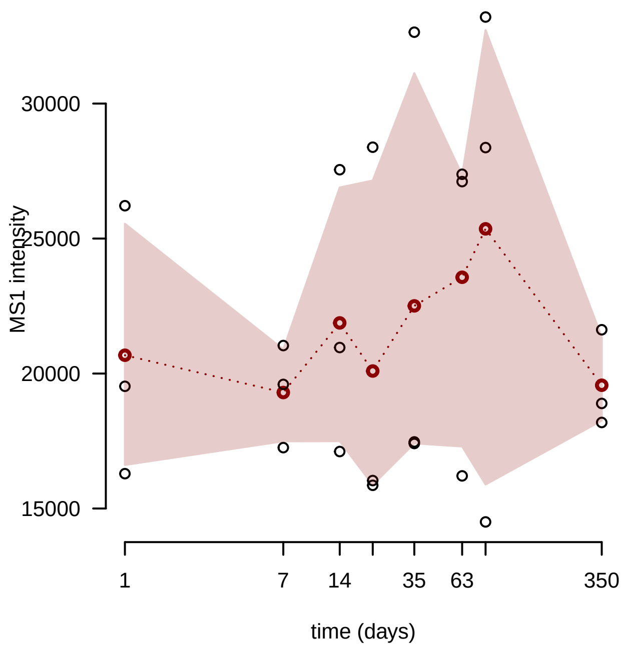

Q9JHZ2
GN=Ankh

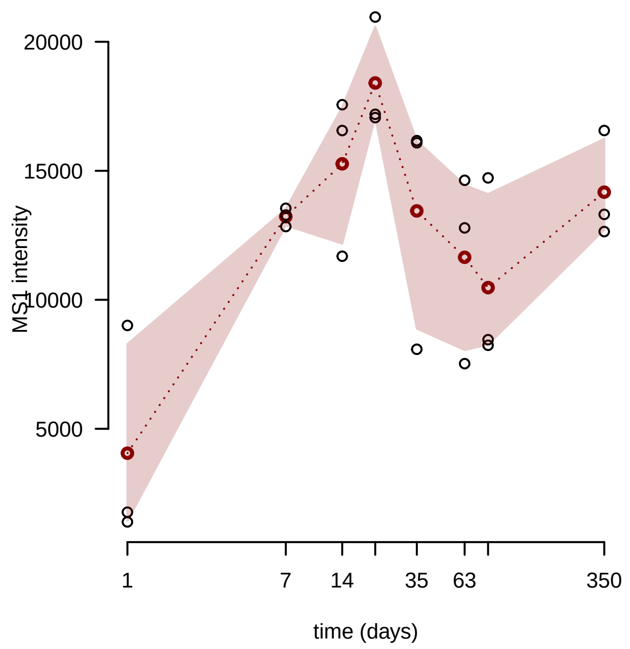

Q9JI10
GN=Stk3
serine/threonine kinase 3

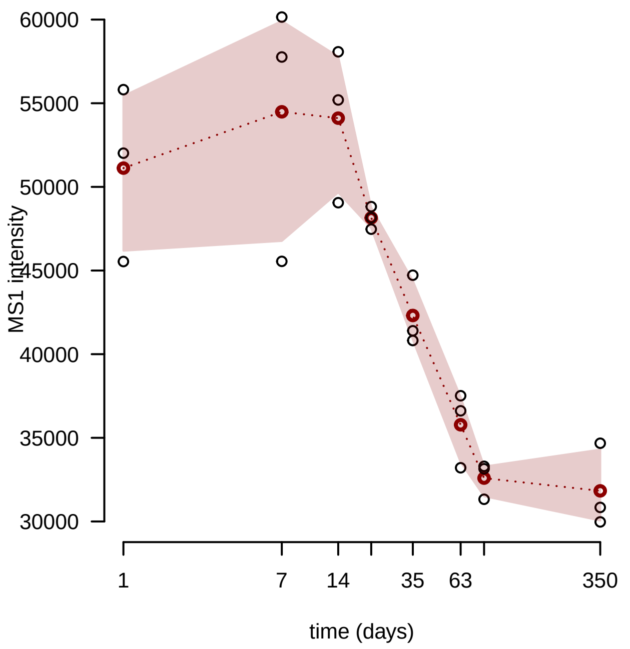

Q9JI11
GN=Stk4
serine/threonine kinase 4

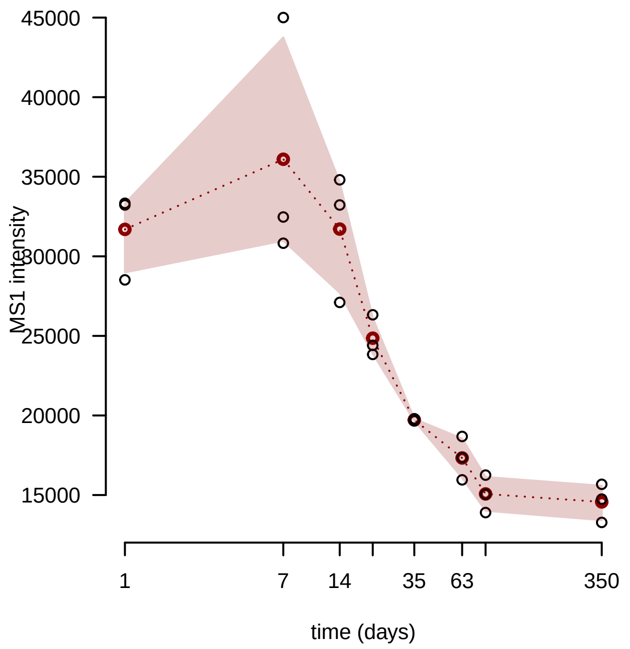

Q9JI13
GN=Utp3
UTP3 small subunit processome component

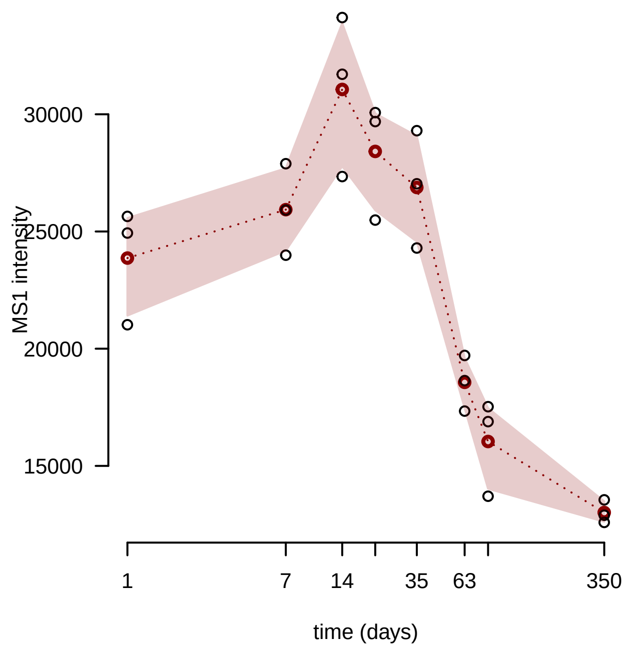

Q9JI19
GN=Fibp
fibroblast growth factor (acidic) intrac ...

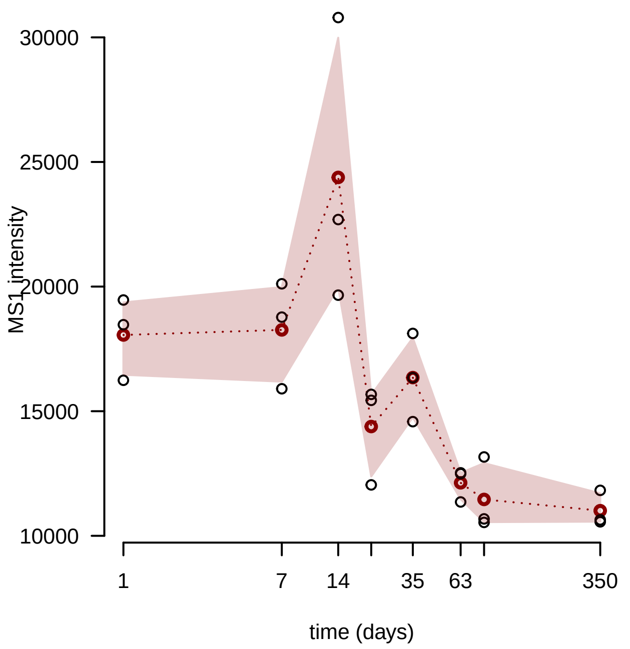

Q9JI39
GN=Abcb10
ATP−binding cassette, sub−family B (MDR/ ...

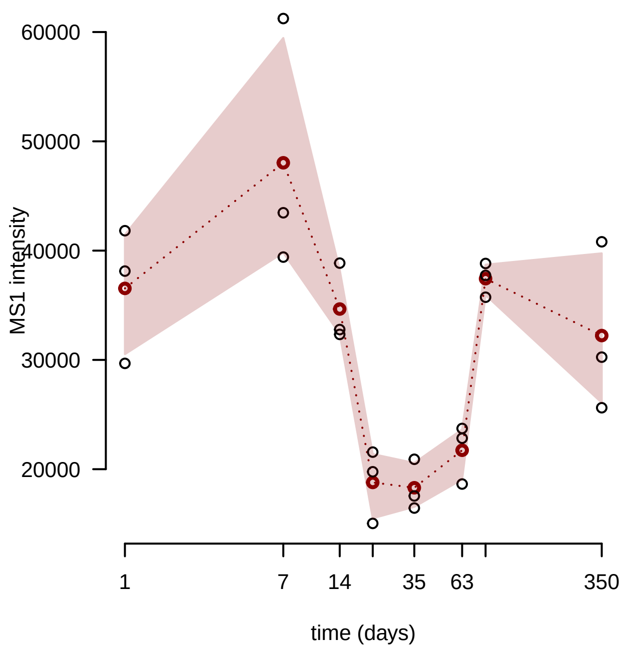

Q9JI44
GN=Dmap1
DNA methyltransferase 1−associated prote ...

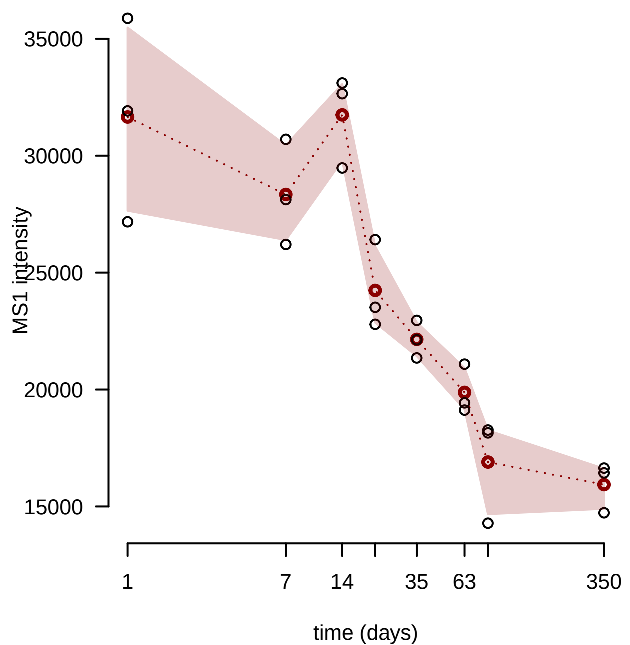

Q9JI46
GN=Nudt3
nudix (nucleotide diphosphate linked moi ...

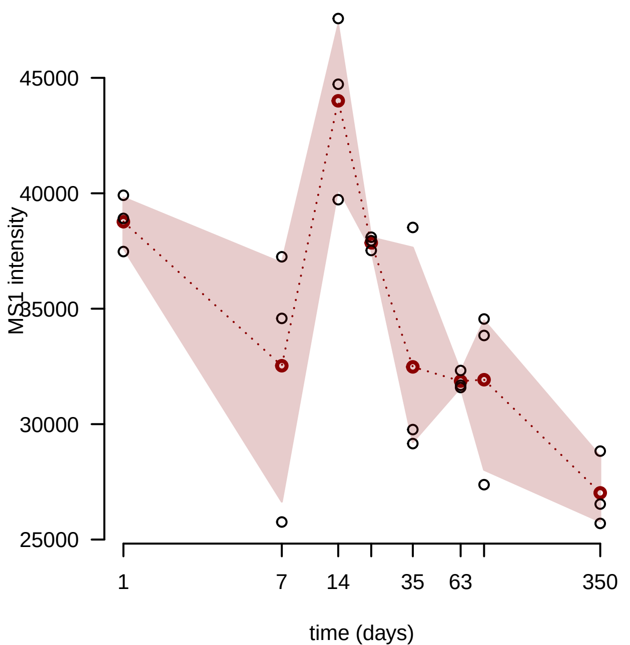

Q9JI48
GN=Plac8
placenta−specific 8

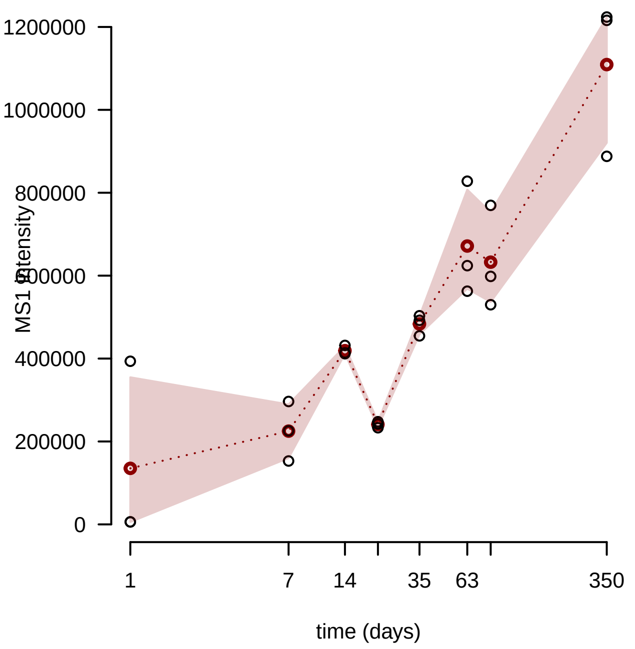

Q9JI57
GN=Gtf2ird1
general transcription factor II I repeat ...

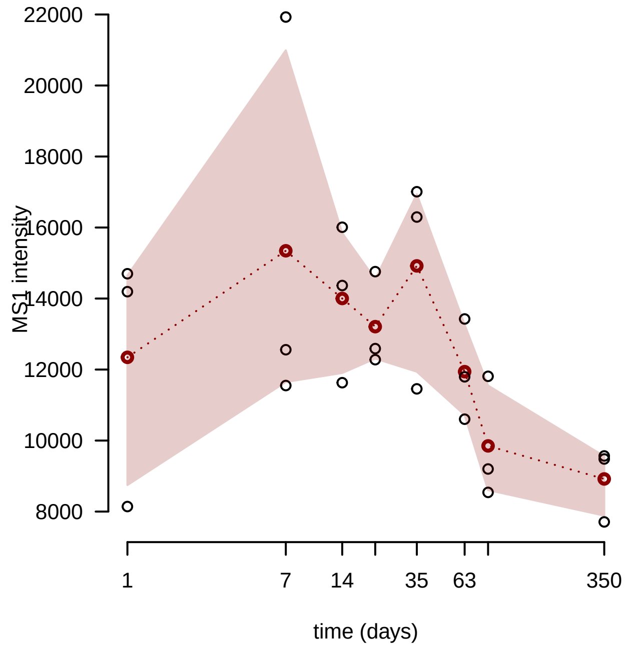

Q9JI75
GN=Nqo2
N−ribosyldihydronicotinamide quinone red ...

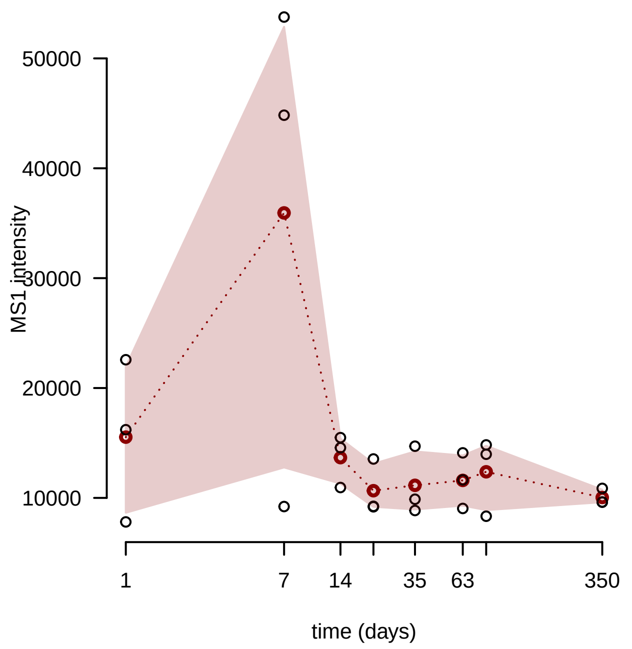

Q9JI78
GN=Ngly1
N−glycanase 1

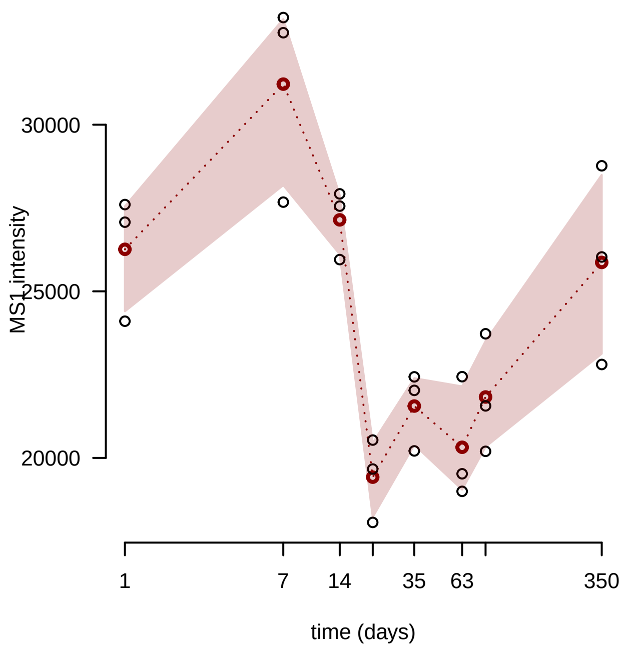

Q9JI90
GN=Rnf14
ring finger protein 14

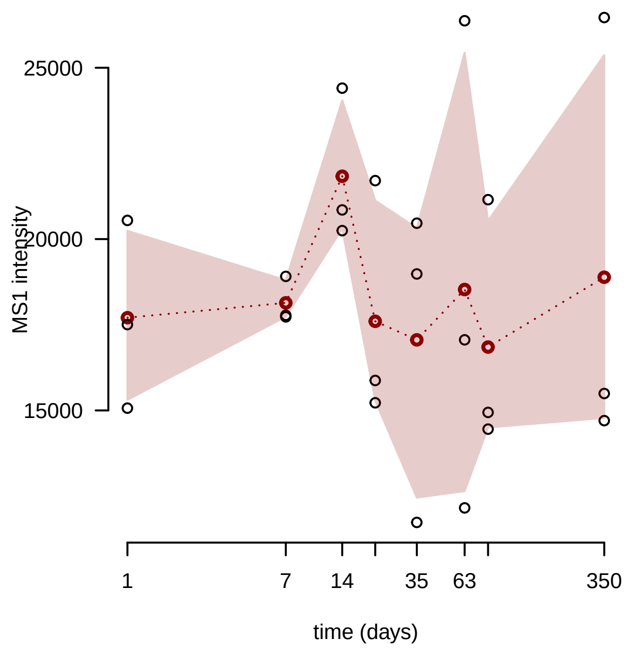

Q9JI91
GN=Actn2
actinin alpha 2

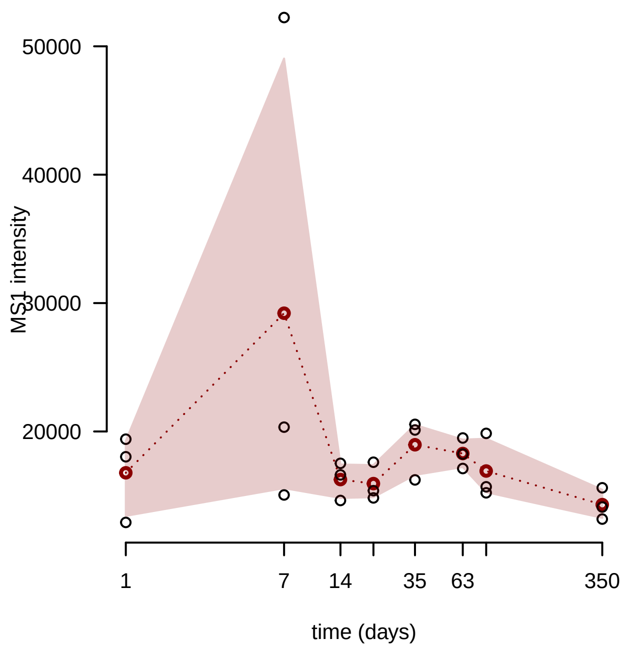

Q9JI99
GN=Sgpp1
sphingosine−1−phosphate phosphatase 1

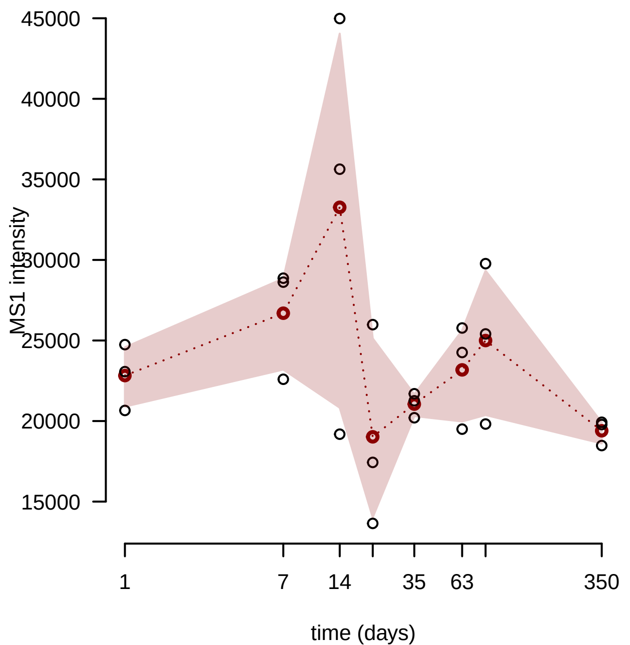

Q9JIA7
GN=Sphk2
sphingosine kinase 2

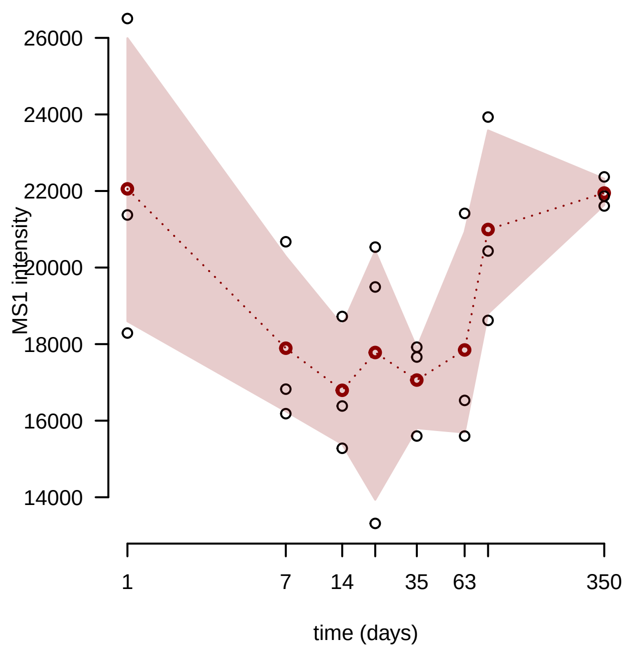

Q9JIB4
GN=Gtf2h2
general transcription factor II H, polyp ...

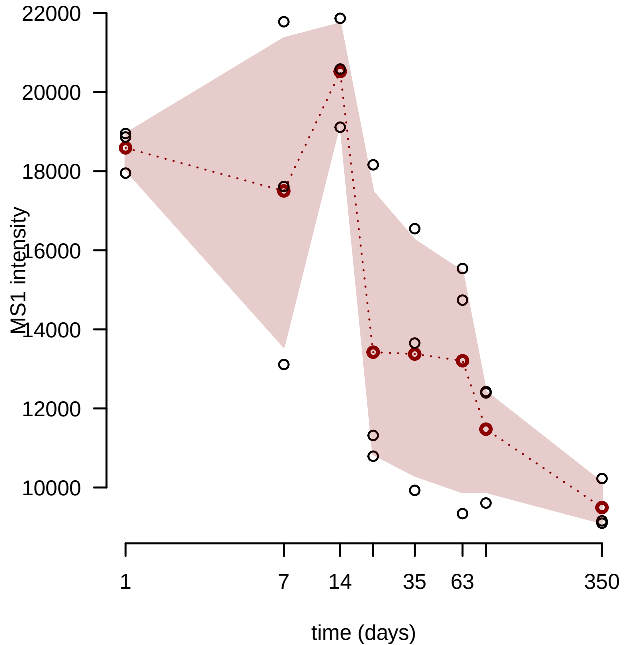

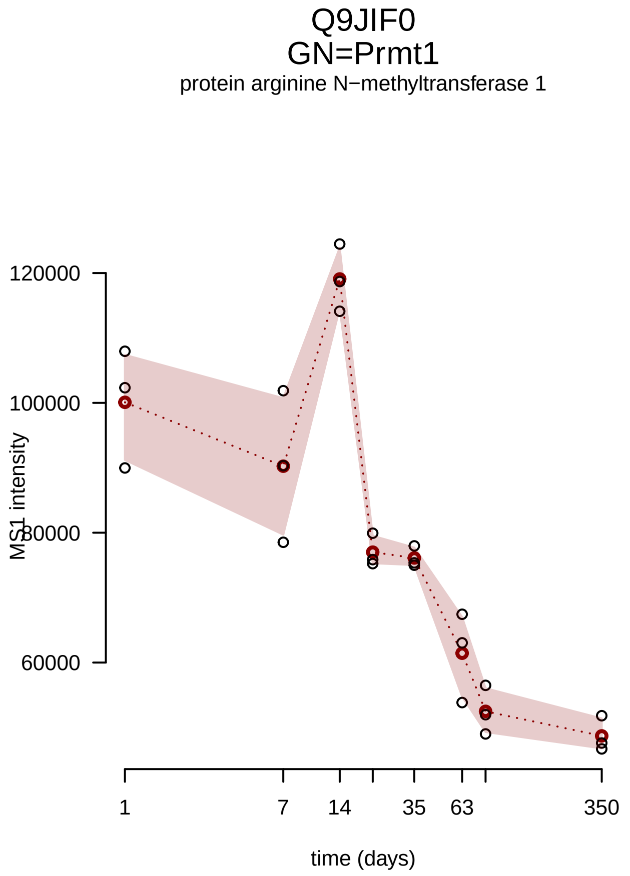
Q9JIF0
GN=Prmt1
protein arginine N−methyltransferase 1
120000
100000
80000
60000
MS1 intensity
1
7
14
35
63
350
time (days)

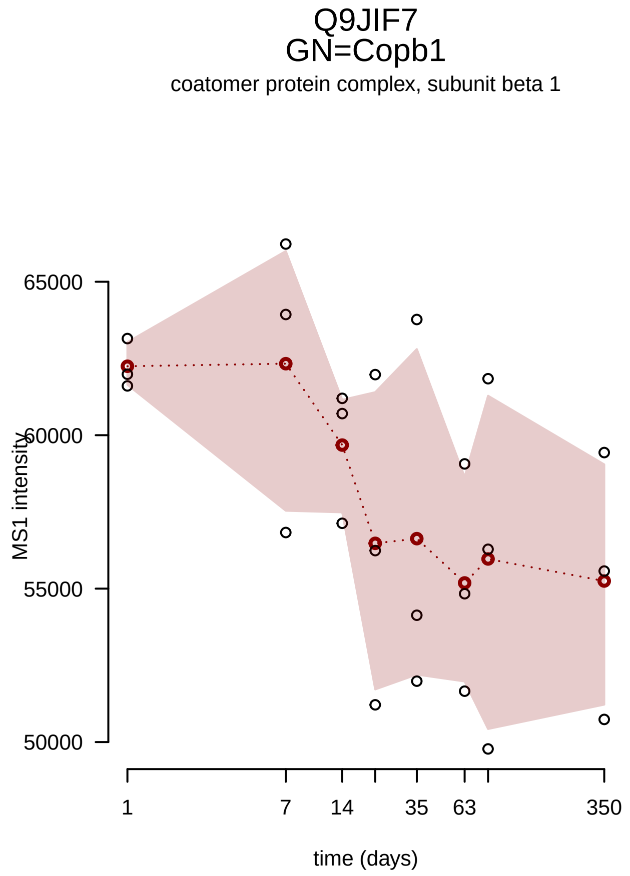
Q9JIF7
GN=Copb1
coatomer protein complex, subunit beta 1
65000
60000
55000
50000
MS1 intensity
1
7
14
35
63
350
time (days)

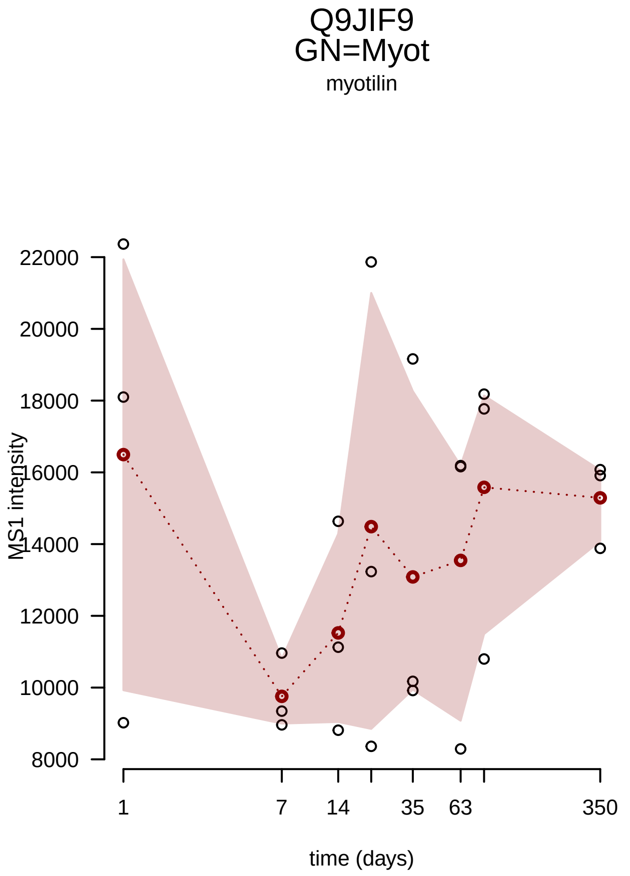
Q9JIF9
GN=Myot
myotilin
22000
20000
18000
16000
14000
12000
10000
8000
MS1 intensity
1
7
14
35
63
350
time (days)

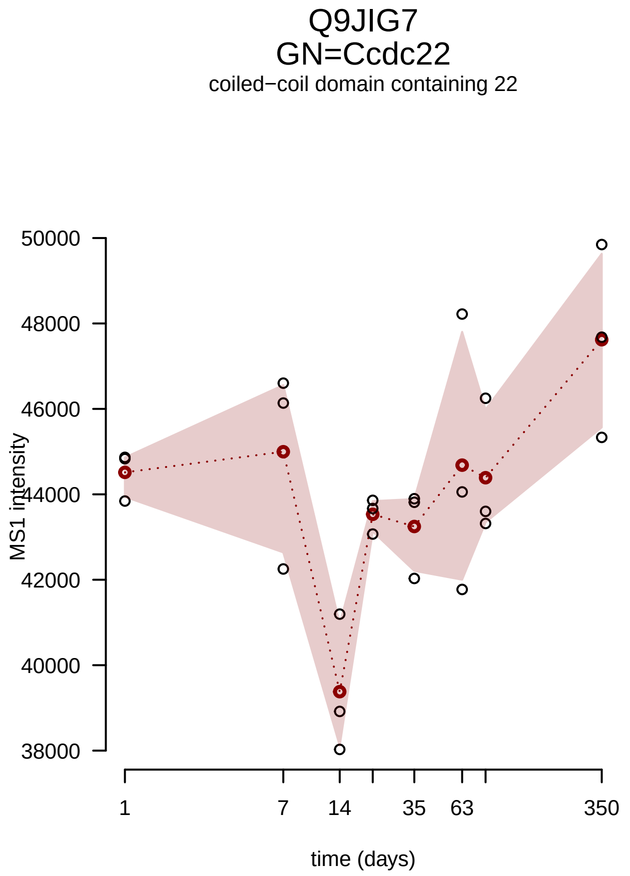
Q9JIG7
GN=Ccdc22
coiled−coil domain containing 22
50000
48000
46000
44000
42000
40000
38000
MS1 intensity
1
7
14
35
63
350
time (days)

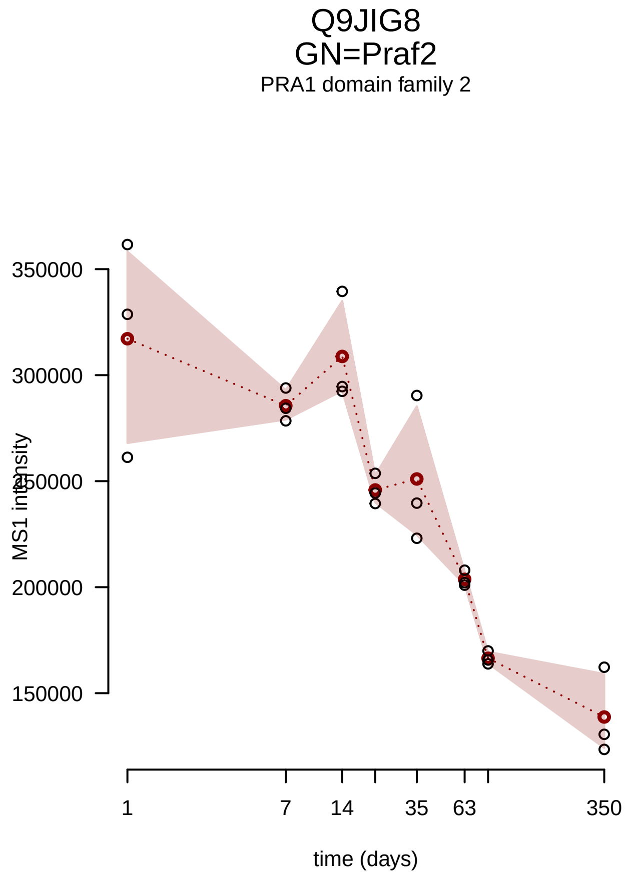
Q9JIG8
GN=Praf2
PRA1 domain family 2
350000
300000
250000
200000
150000
MS1 intensity
1
7
14
35
63
350
time (days)

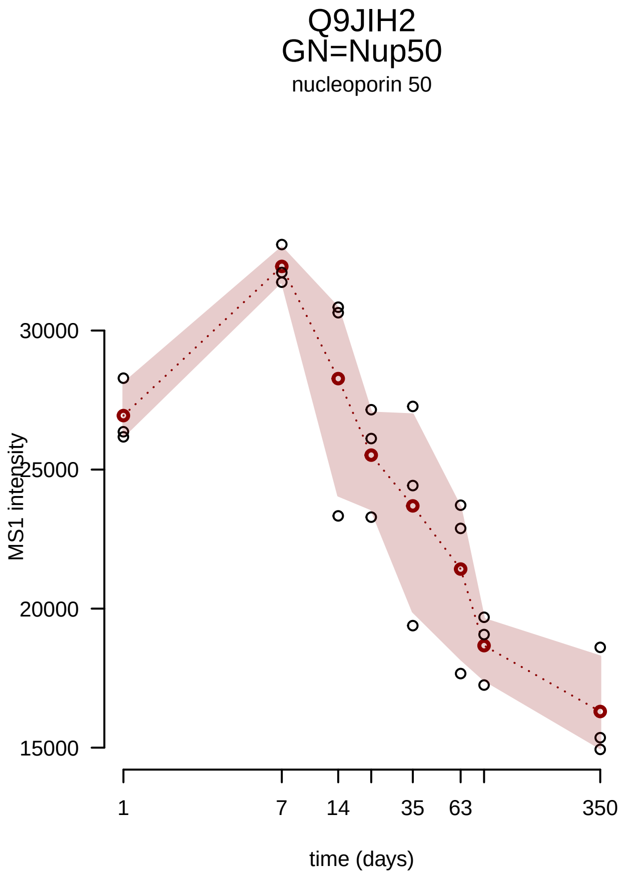
Q9JIH2
GN=Nup50
nucleoporin 50
30000
25000
20000
15000
MS1 intensity
1
7
14
35
63
350
time (days)

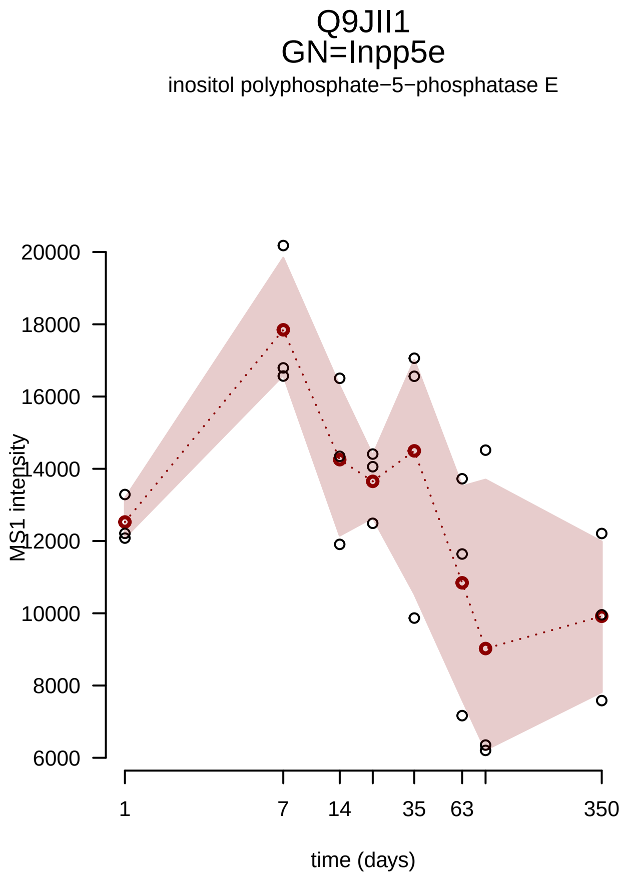
Q9JII1
GN=Inpp5e
inositol polyphosphate−5−phosphatase E
20000
18000
16000
14000
12000
10000
8000
6000
MS1 intensity
1
7
14
35
63
350
time (days)

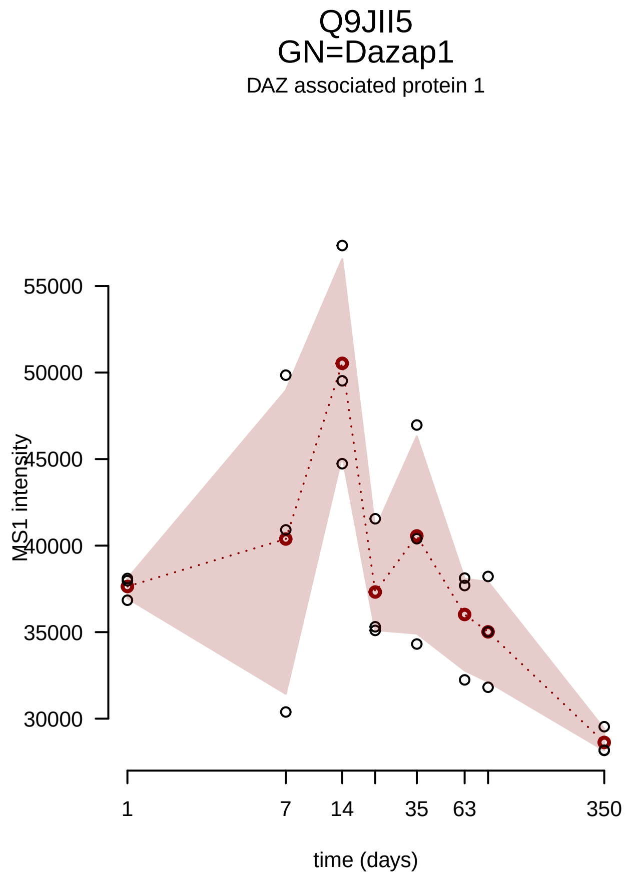
Q9JII5
GN=Dazap1
DAZ associated protein 1
55000
50000
45000
40000
35000
30000
MS1 intensity
1
7
14
35
63
350
time (days)

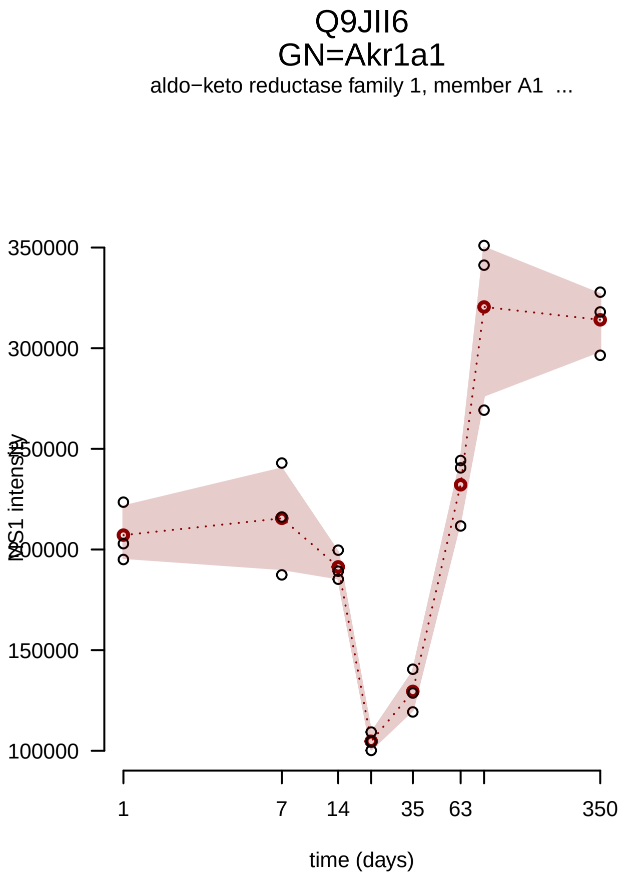
Q9JII6
GN=Akr1a1
aldo−keto reductase family 1, member A1 ...
350000
300000
250000
200000
150000
100000
MS1 intensity
1
7
14
35
63
350
time (days)

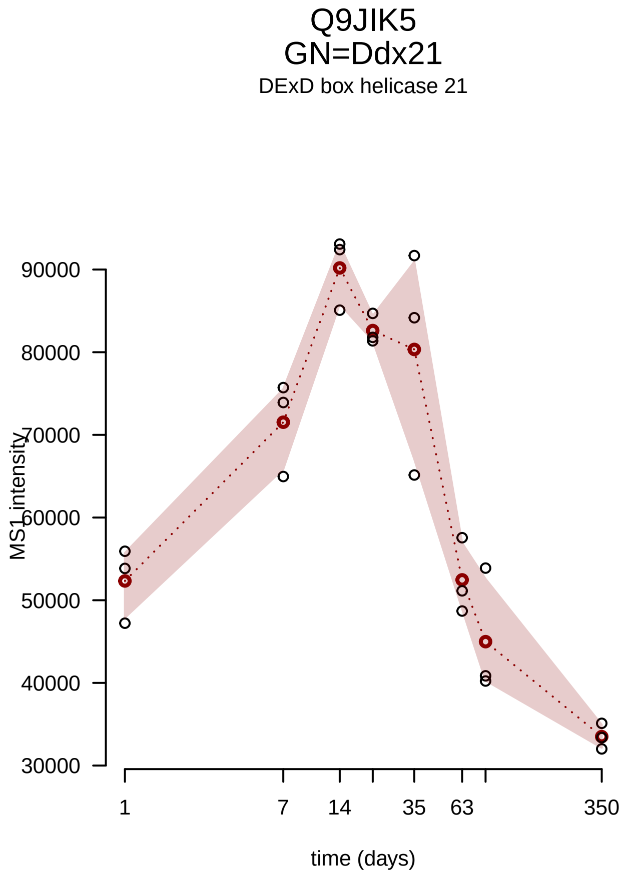
Q9JIK5
GN=Ddx21
DExD box helicase 21
MS1 intensity
30000
40000
50000
60000
70000
80000
90000
1
7
14
35
63
350
time (days)

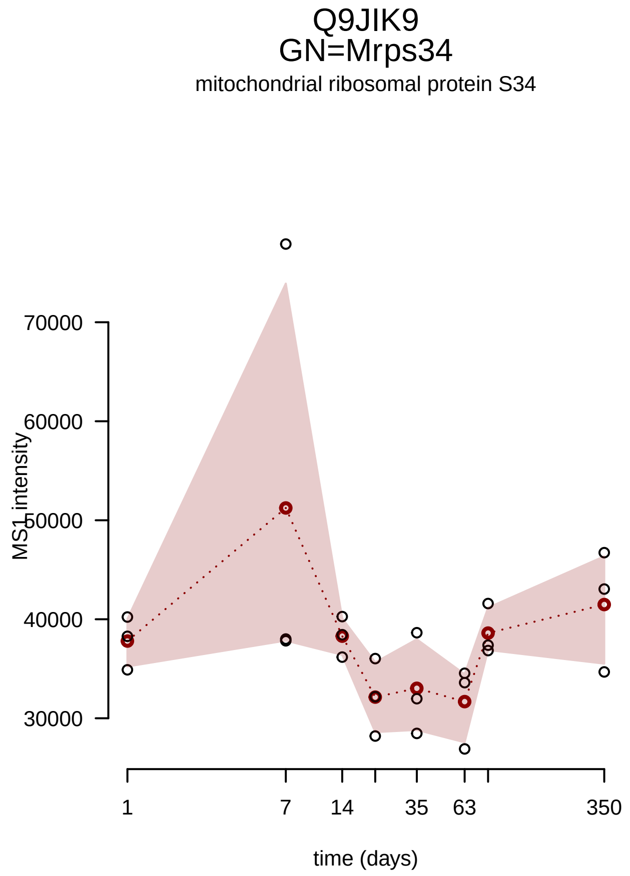
Q9JIK9
GN=Mrps34
mitochondrial ribosomal protein S34
MS1 intensity
30000
40000
50000
60000
70000
1
7
14
35
63
350
time (days)

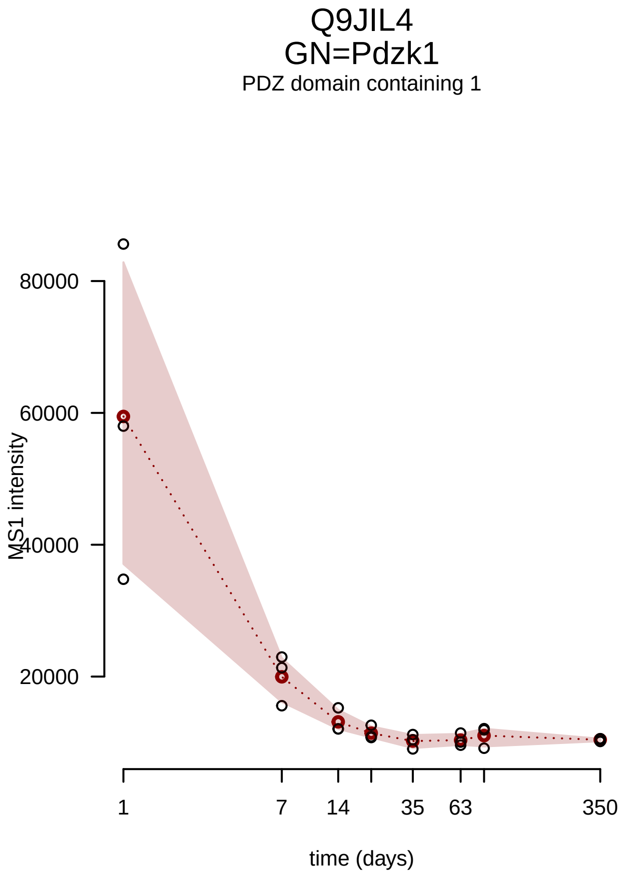
Q9JIL4
GN=Pdzk1
PDZ domain containing 1
MS1 intensity
20000
40000
60000
80000
1
7
14
35
63
350
time (days)

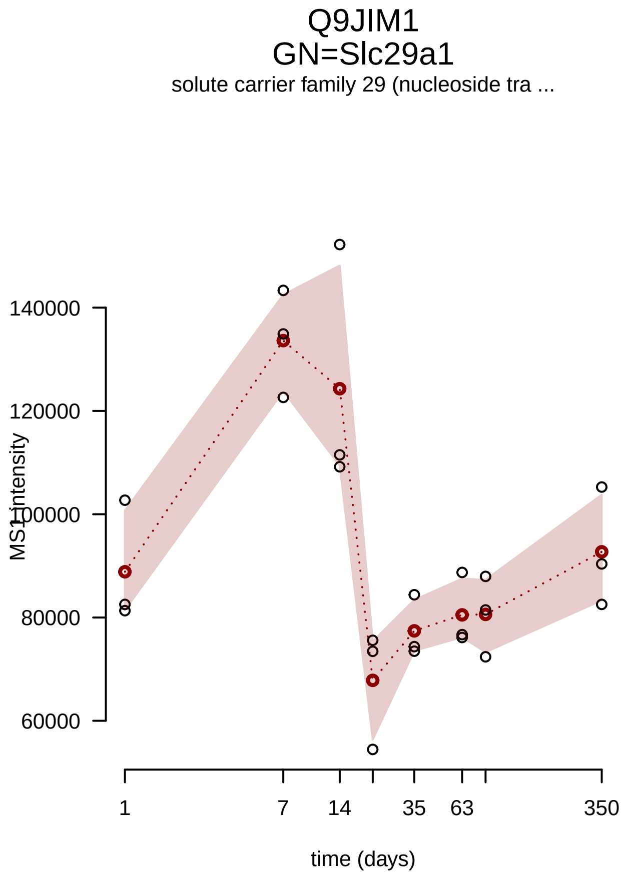
Q9JIM1
GN=Slc29a1
solute carrier family 29 (nucleoside tra ...
MS1 intensity
60000
80000
100000
120000
140000
1
7
14
35
63
350
time (days)

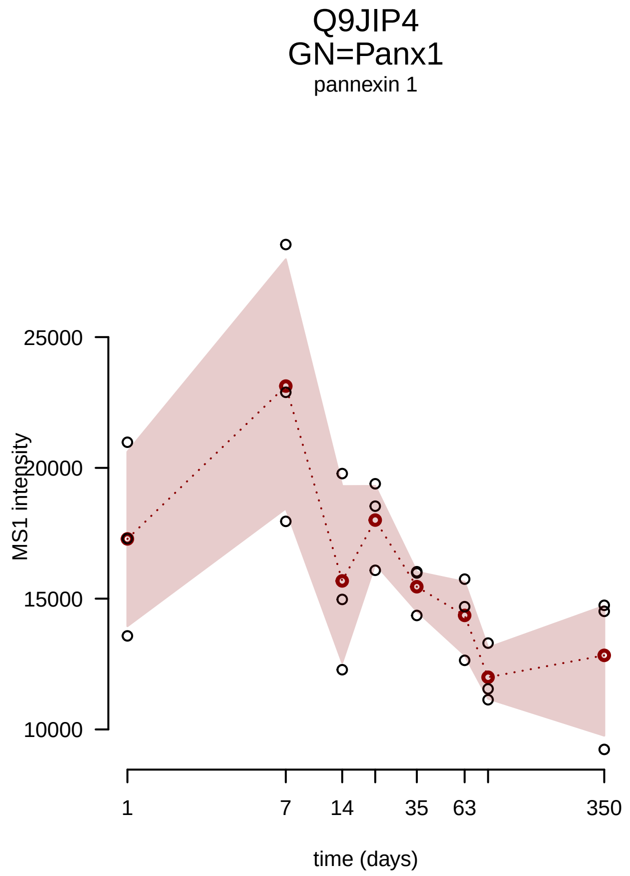
Q9JIP4
GN=Panx1
pannexin 1
MS1 intensity
10000
15000
20000
25000
1
7
14
35
63
350
time (days)

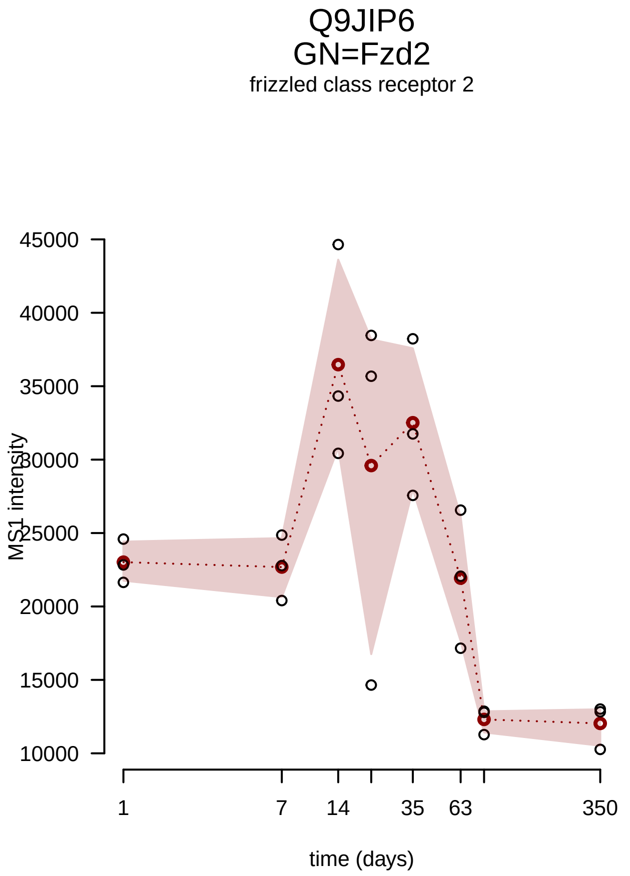
Q9JIP6
GN=Fzd2
frizzled class receptor 2
MS1 intensity
10000
15000
20000
25000
30000
35000
40000
45000
1
7
14
35
63
350
time (days)

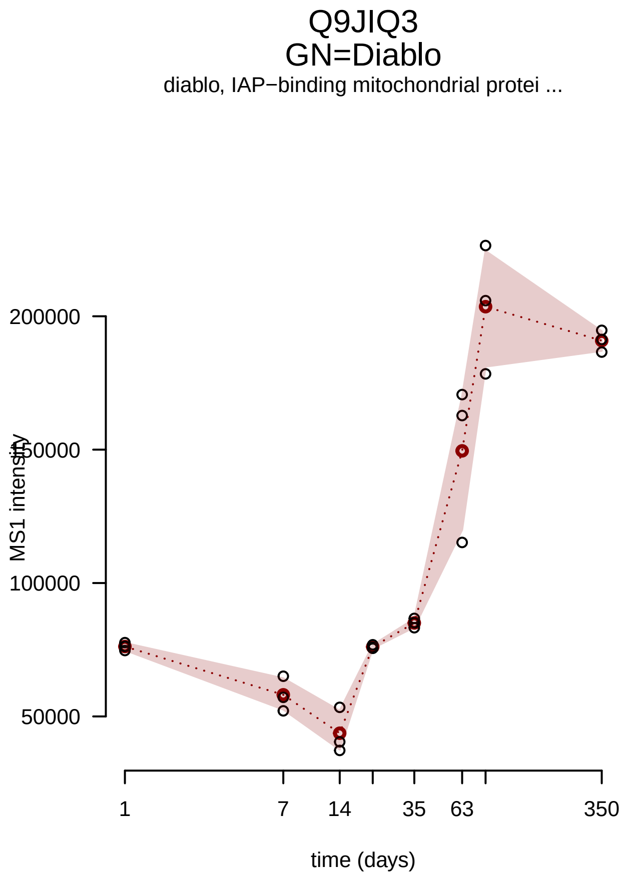
Q9JIQ3
GN=Diablo
diablo, IAP−binding mitochondrial protei ...
MS1 intensity
50000
100000
150000
200000
1
7
14
35
63
350
time (days)

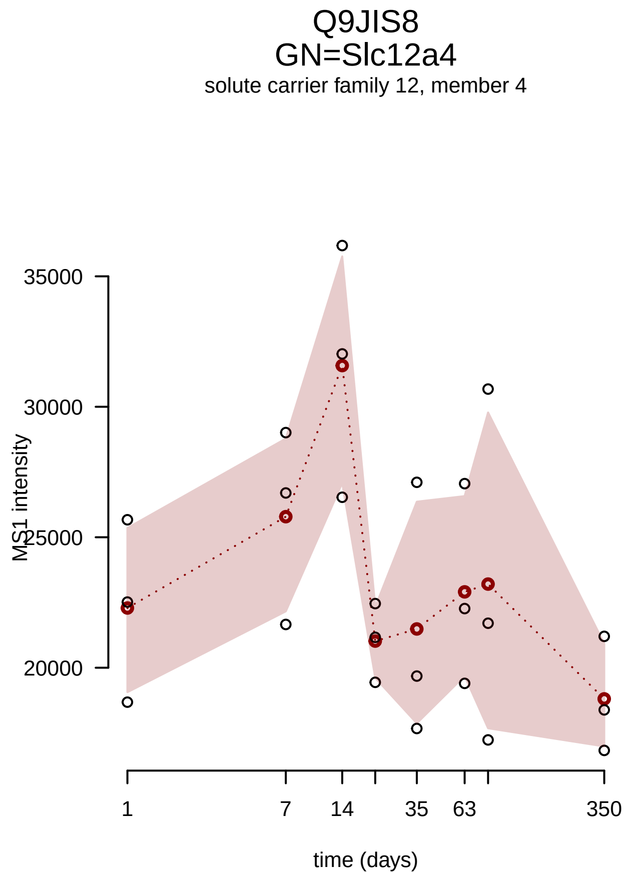
Q9JIS8
GN=Slc12a4
solute carrier family 12, member 4
MS1 intensity
20000
25000
30000
35000
1
7
14
35
63
350
time (days)

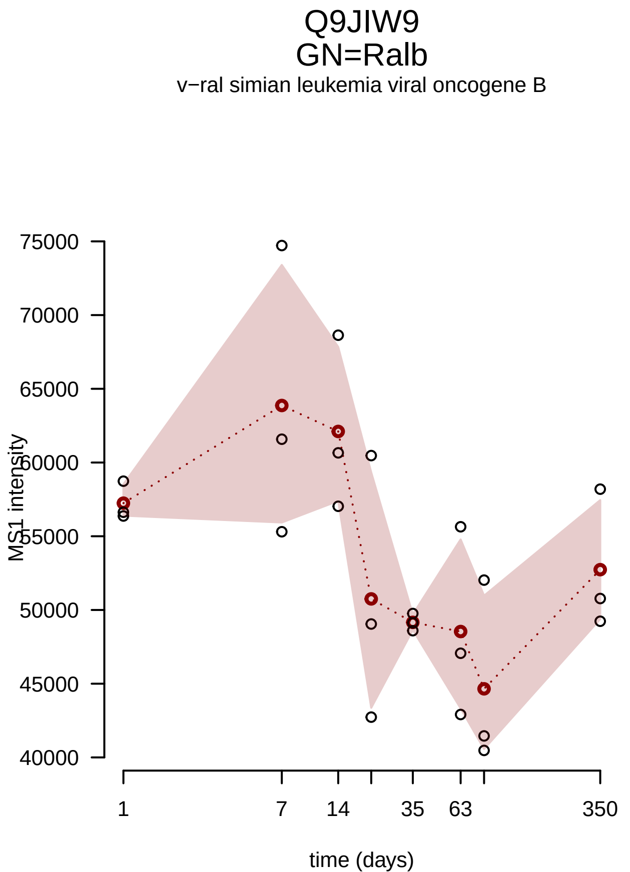
Q9JIW9
GN=Ralb
v−ral simian leukemia viral oncogene B
MS1 intensity
40000
45000
50000
55000
60000
65000
70000
75000
1
7
14
35
63
350
time (days)

Q9JIX0
GN=Eny2
ENY2 transcription and export complex 2 ...

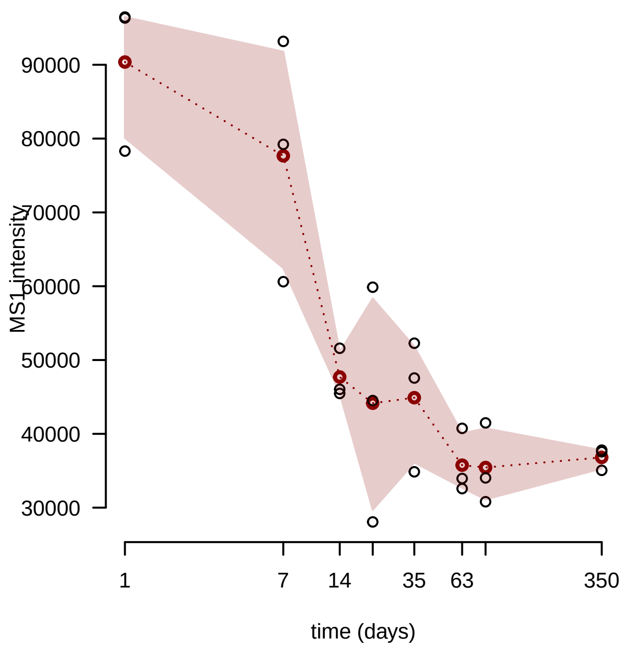

Q9JIX8
GN=Acin1
apoptotic chromatin condensation inducer ...

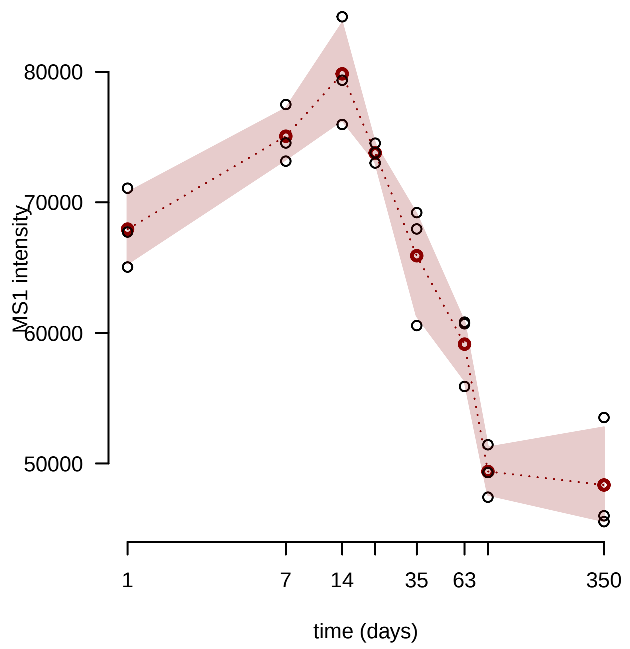

Q9JIX9
GN=Fastk
Fas−activated serine/threonine kinase

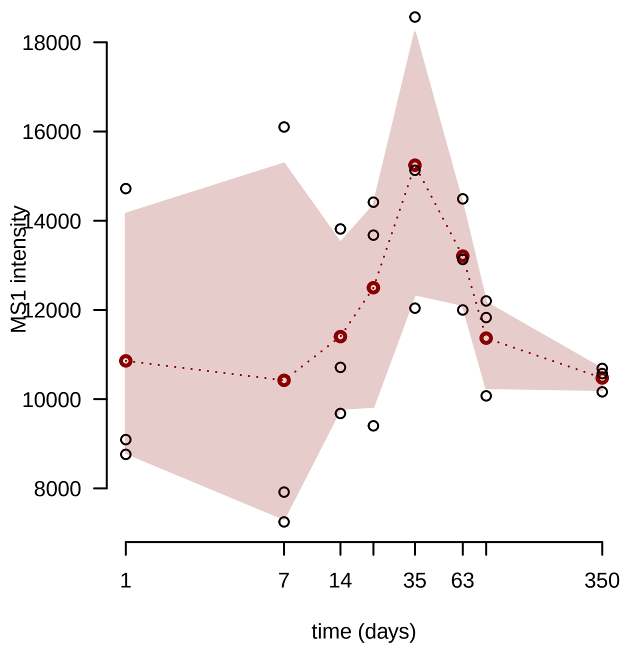

Q9JIY0
GN=Plekho1
pleckstrin homology domain containing, f ...

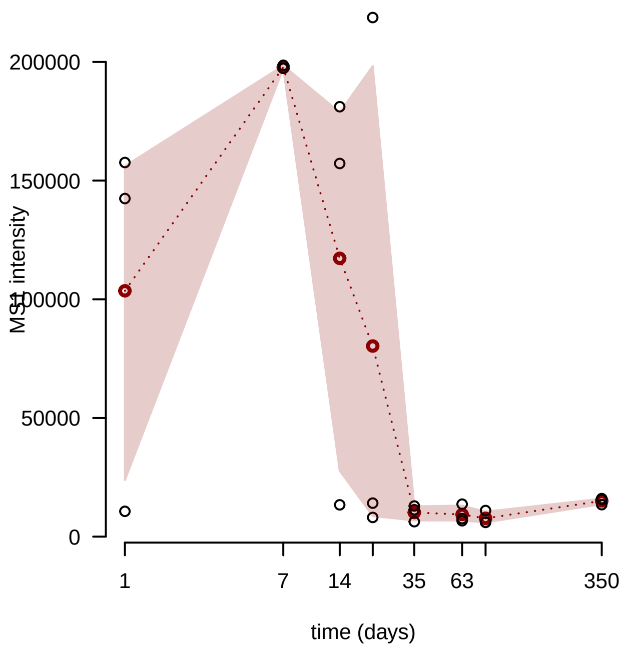

Q9JIY2
GN=Cbll1
Casitas B−lineage lymphoma−like 1

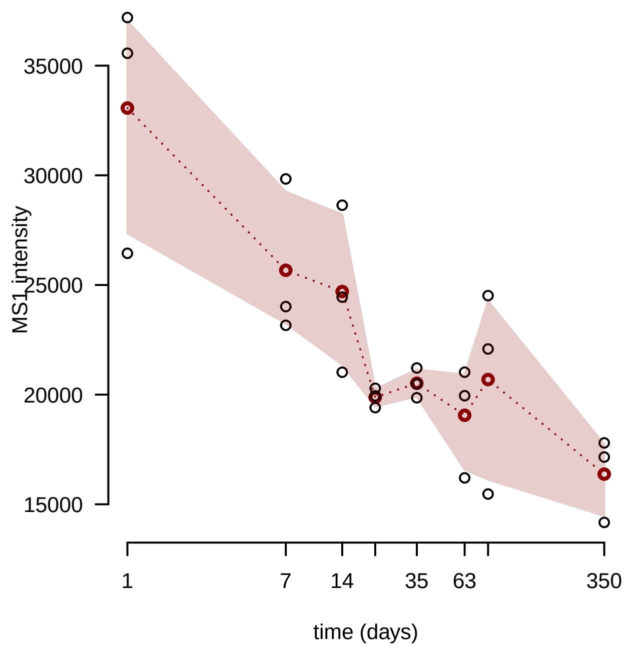

Q9JIY5
GN=Htra2
HtrA serine peptidase 2

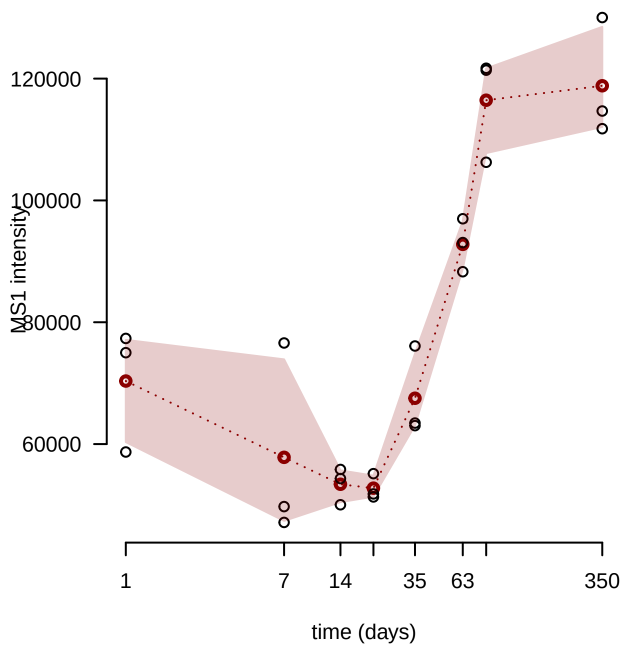

Q9JIZ9
GN=Plscr3
phospholipid scramblase 3

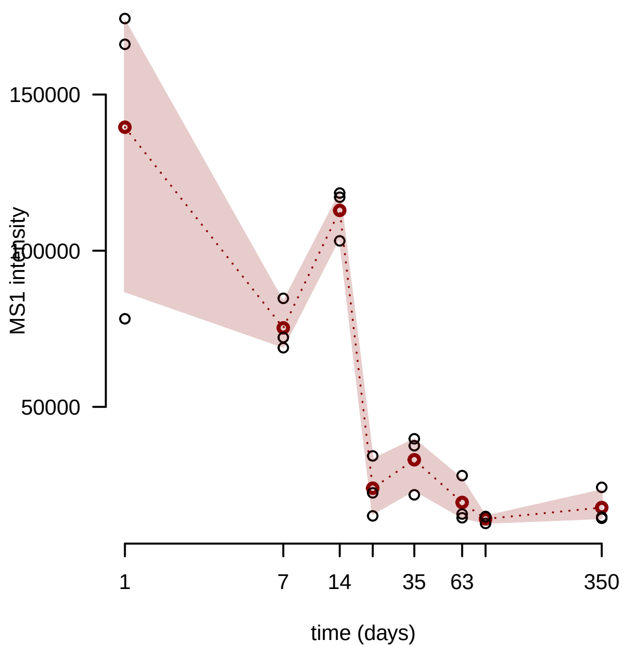

Q9JJ00
GN=Plscr1
phospholipid scramblase 1

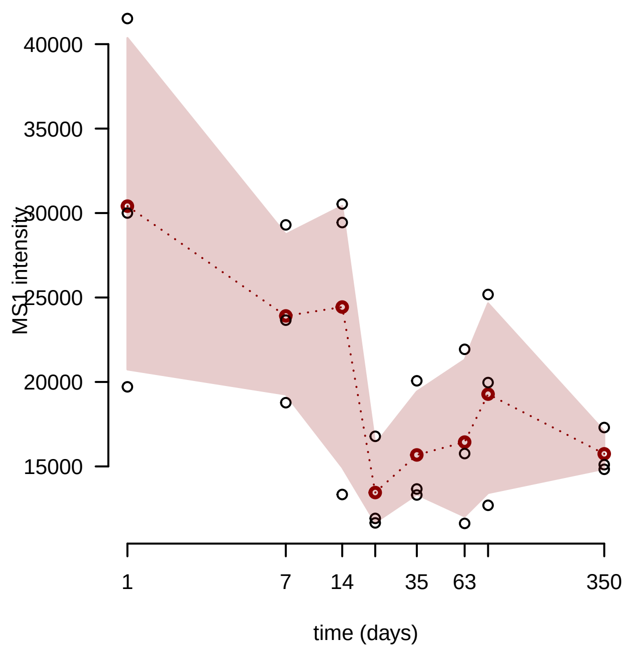

Q9JJ04
GN=B4galt4
UDP−Gal:betaGlcNAc beta 1,4−galactosyltr ...

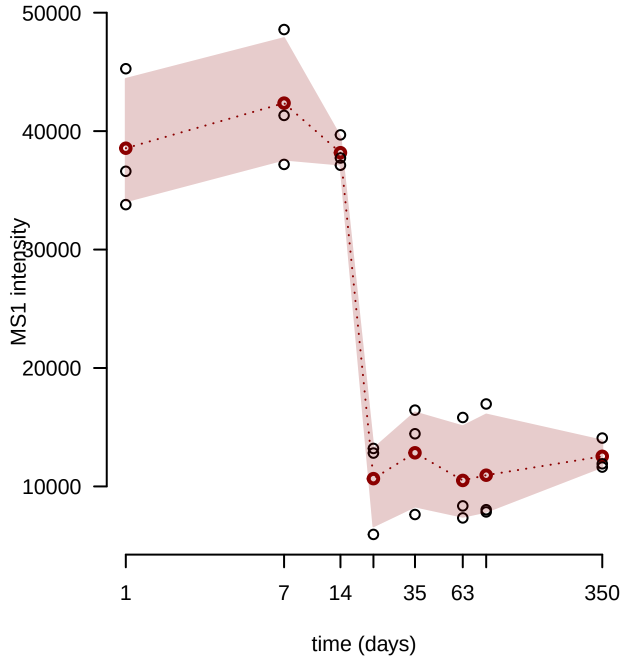

Q9JJ06
GN=C1galt1
core 1 synthase, glycoprotein−N−acetylga ...

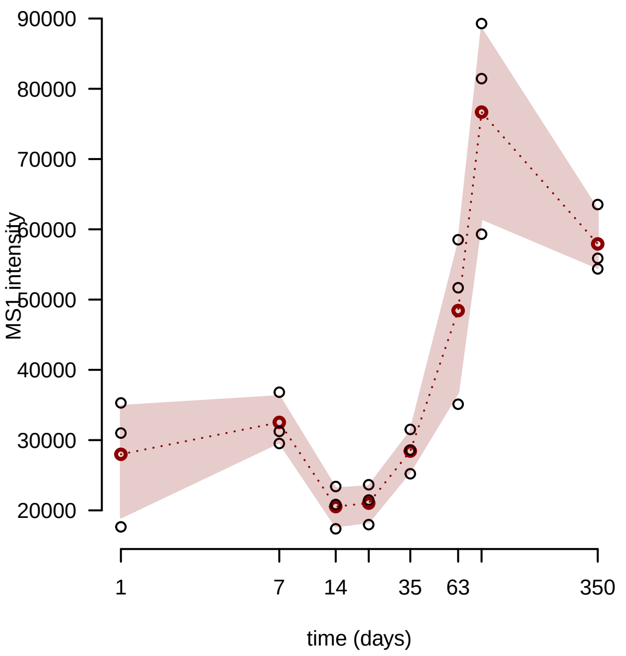

Q9JJ11
GN=Tacc3
transforming, acidic coiled−coil contain ...

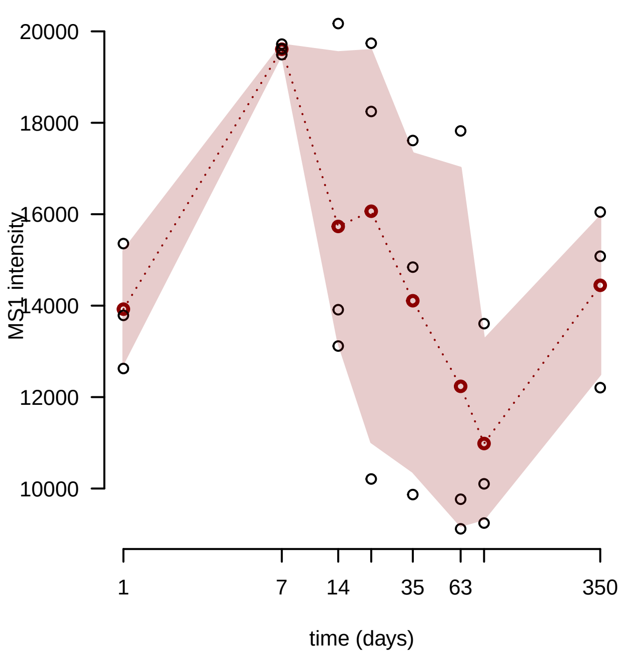

Q9JJ28
GN=Flii
flightless I actin binding protein

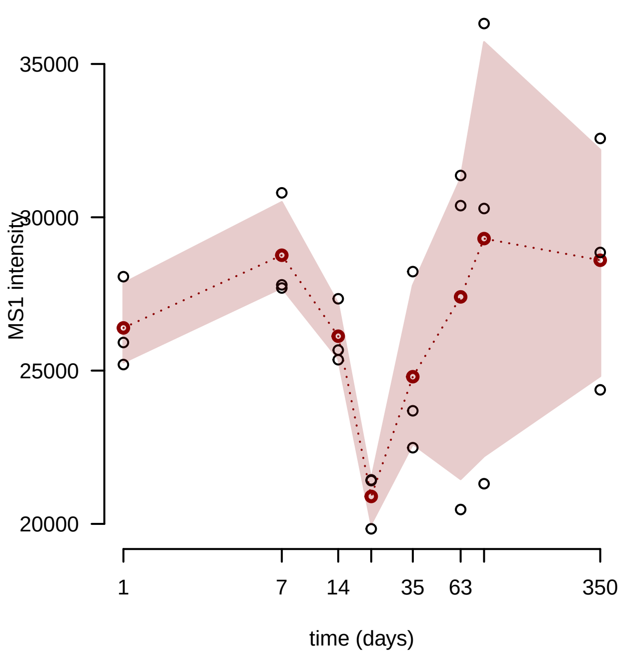

Q9JJ48
GN=Zc3h8
zinc finger CCCH type containing 8

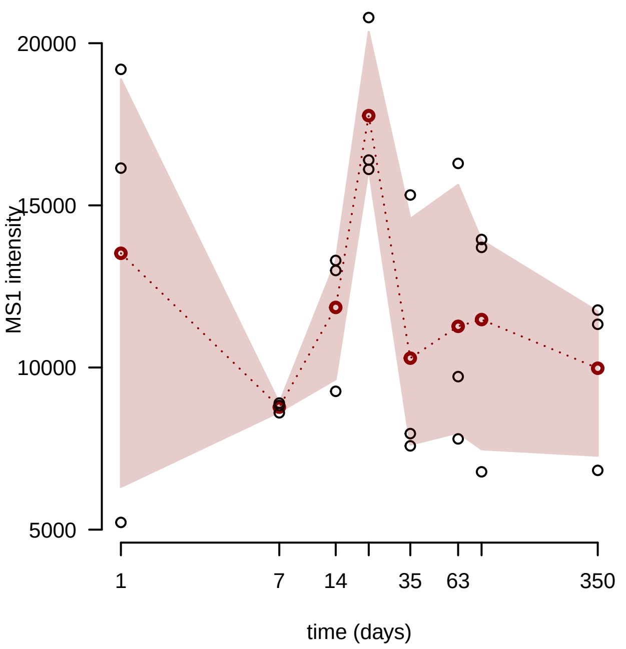

Q9JJ61
GN=Galnt16
polypeptide N−acetylgalactosaminyltransf ...

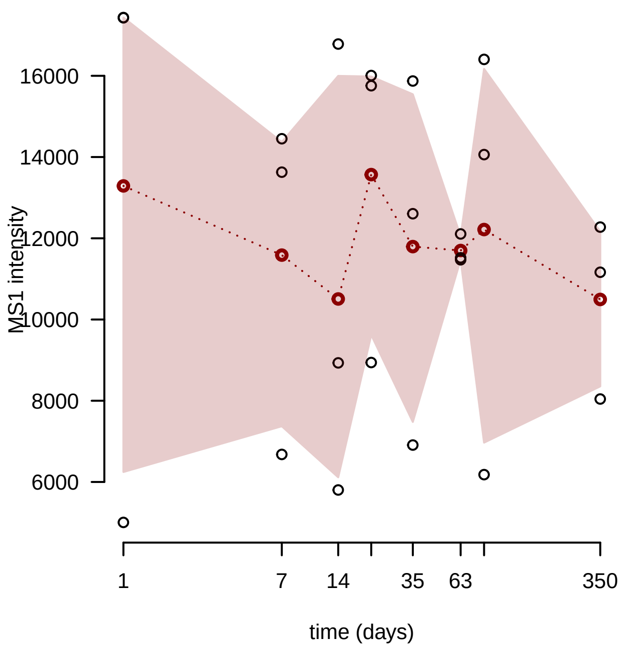

Q9JJ66
GN=Cdc20
cell division cycle 20

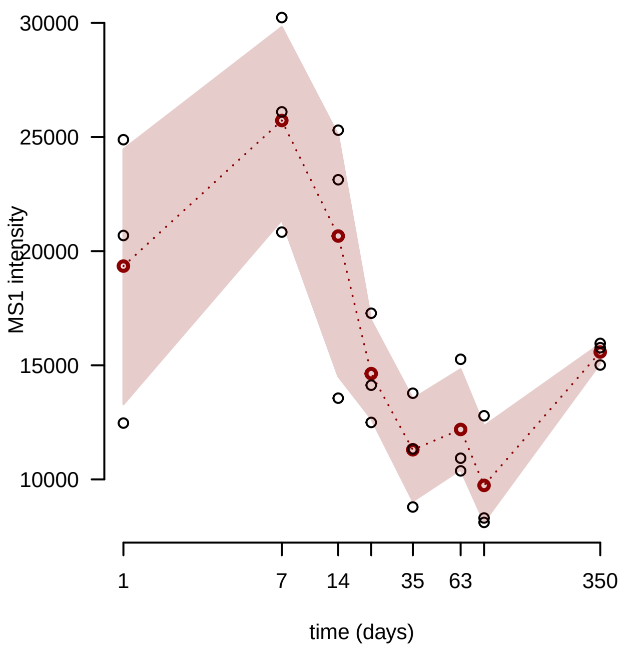

Q9JJ78
GN=Pbk
PDZ binding kinase

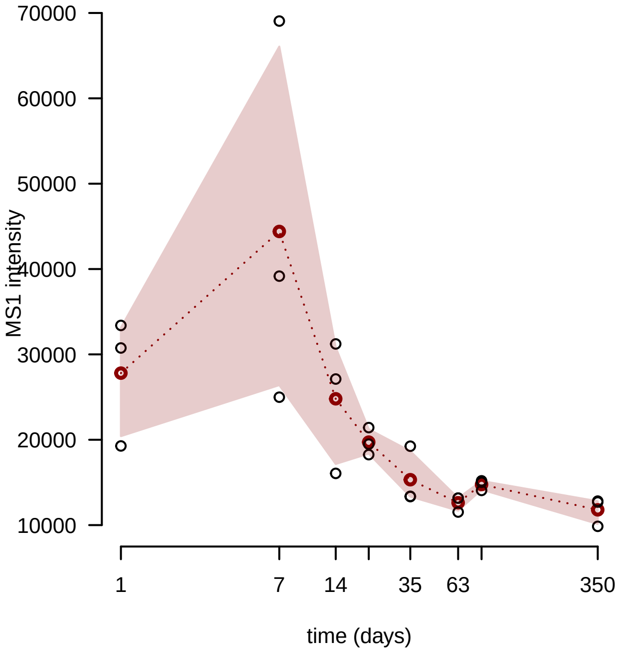

Q9JJ80
GN=Rpf2
ribosome production factor 2 homolog

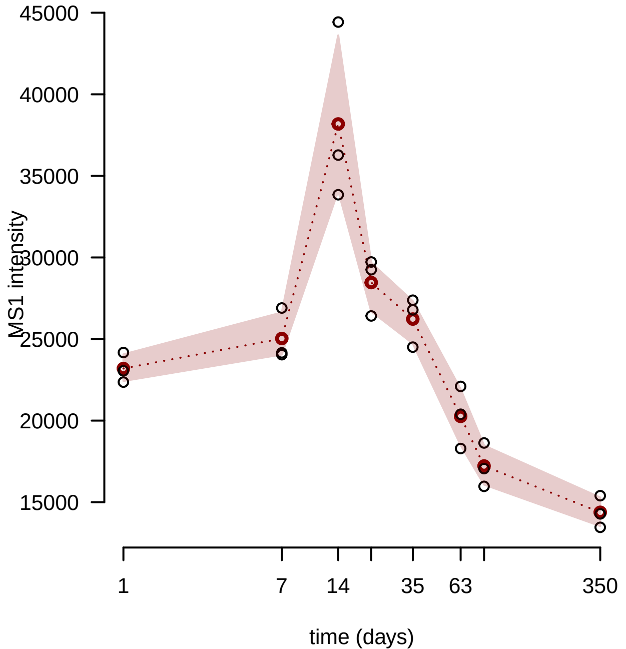

Q9JJ89
GN=Ccdc86
coiled−coil domain containing 86

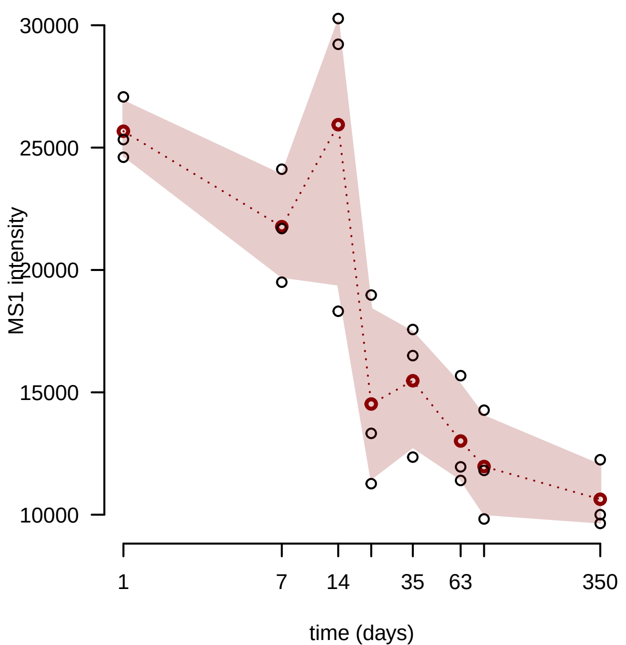

Q9JJA2
GN=Cog8
component of oligomeric golgi complex 8

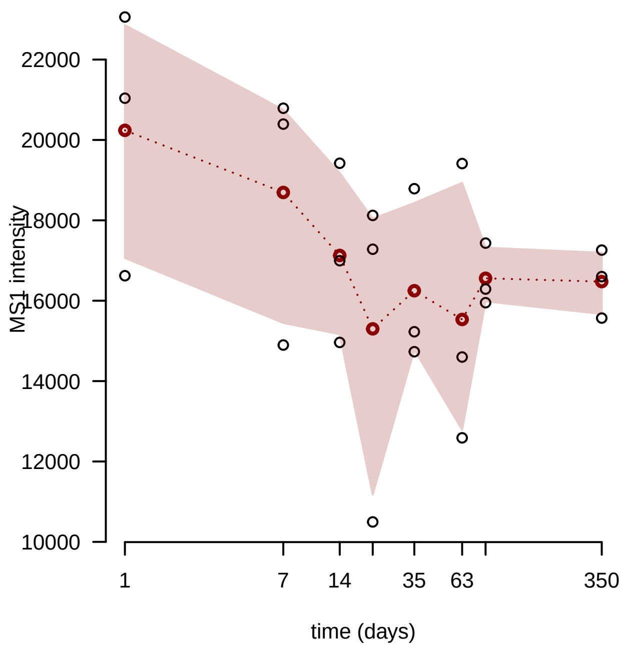

Q9JJA4
GN=Wdr12
WD repeat domain 12

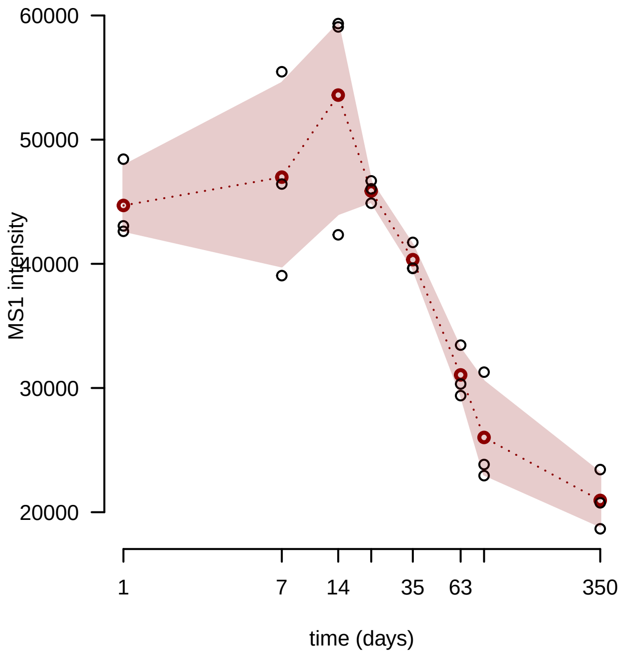

Q9JJA7
GN=Ccnl2
cyclin L2

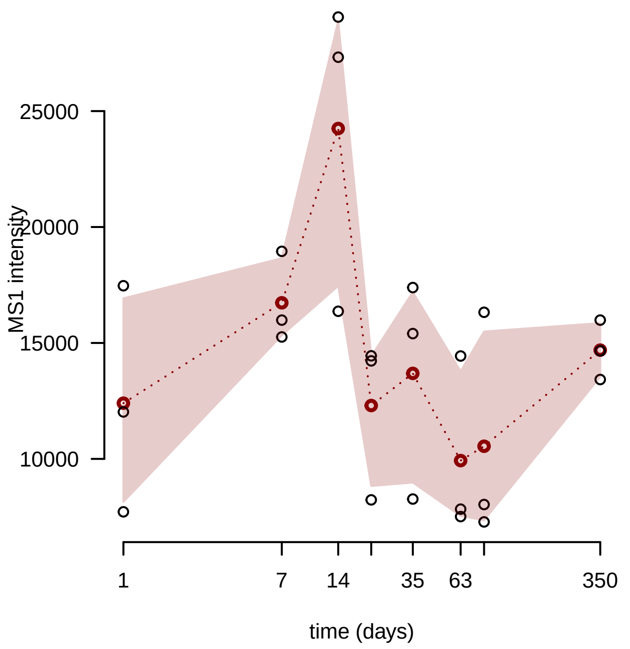

Q9JJC6
GN=Rilpl1
Rab interacting lysosomal protein−like 1

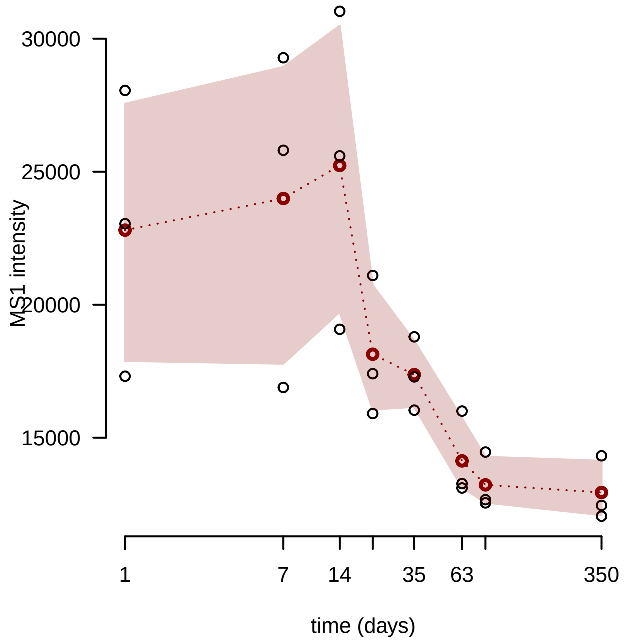

Q9JJC9
GN=Herpud2
HERPUD family member 2

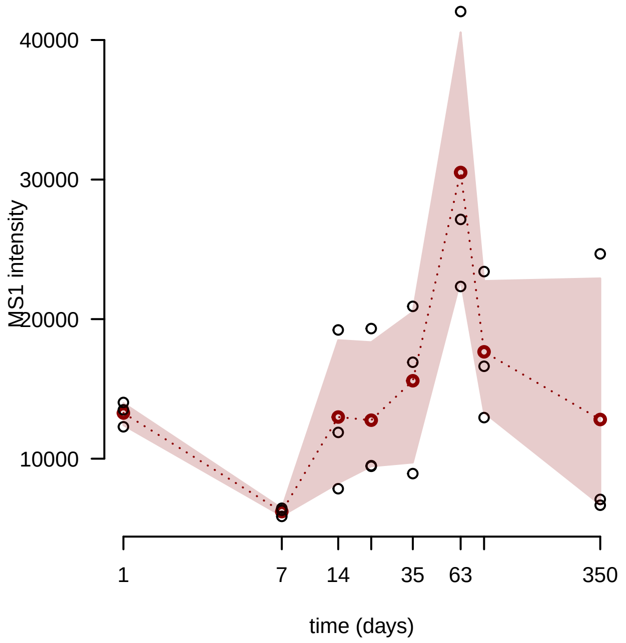

Q9JJD0
GN=Thap11
THAP domain containing 11

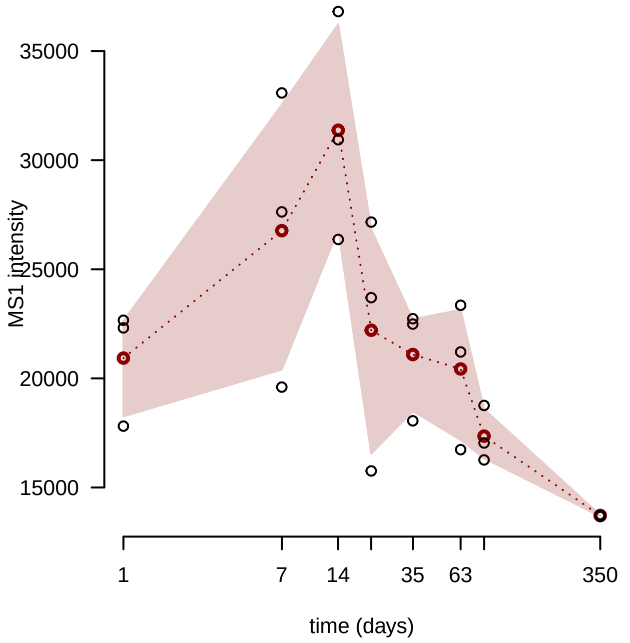

Q9JJE4
GN=Paqr4
progestin and adipoQ receptor family mem ...

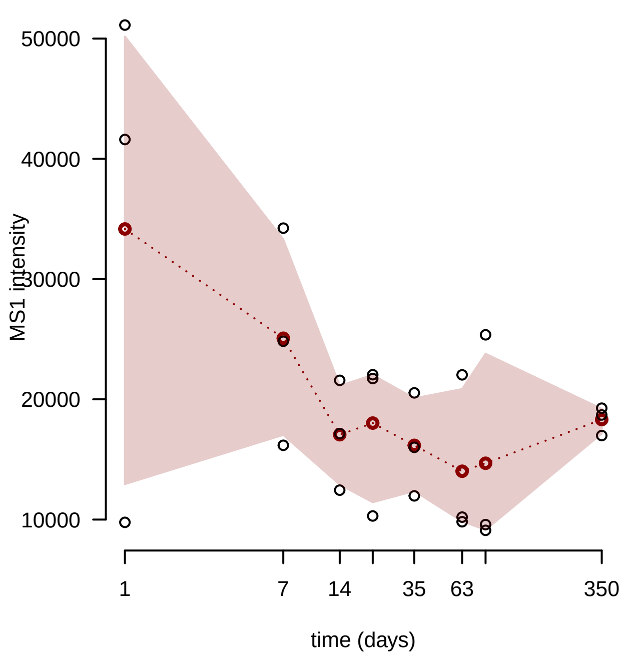

Q9JJE7
GN=Fads3
fatty acid desaturase 3

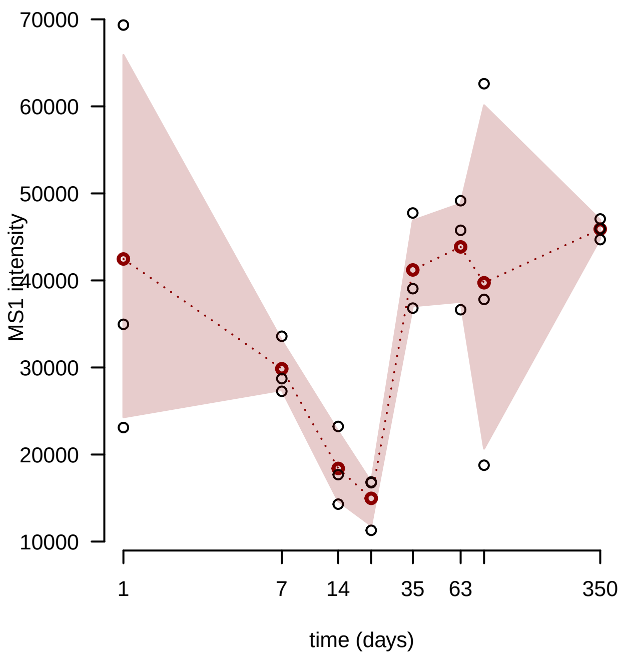

Q9JJF0
GN=Nap1l5
nucleosome assembly protein 1−like 5

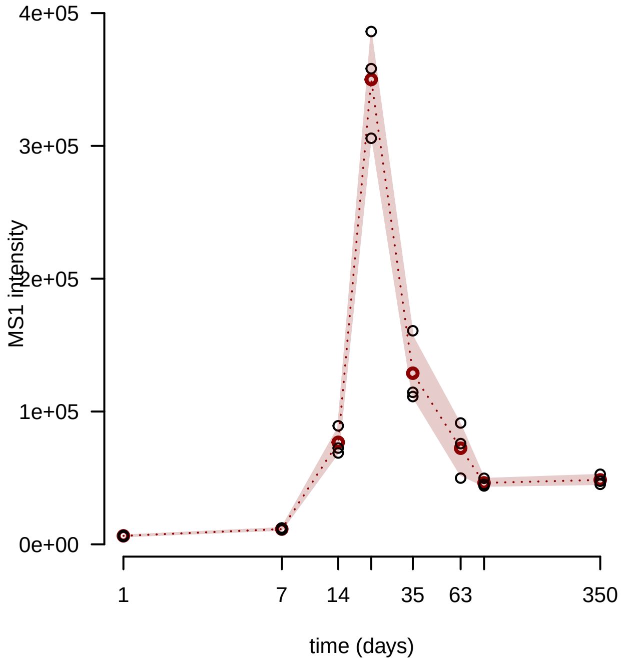

Q9JJF3
GN=Riox1
ribosomal oxygenase 1

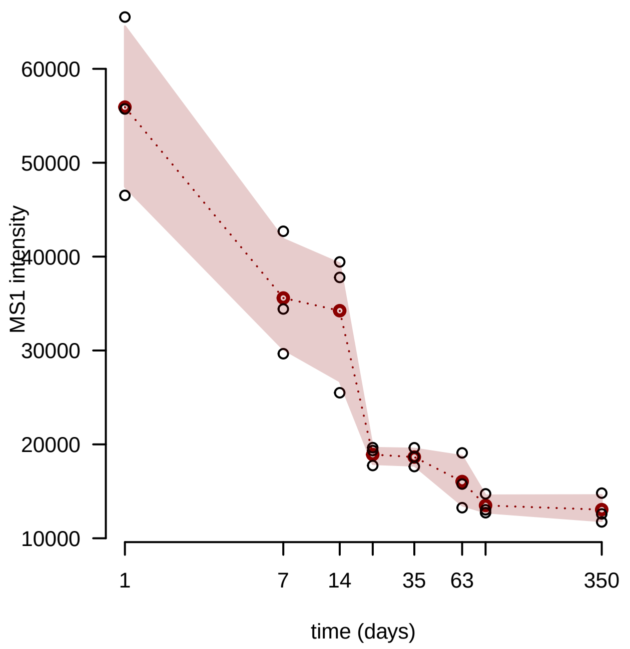

Q9JJF9
GN=Sppl2a
signal peptide peptidase like 2A

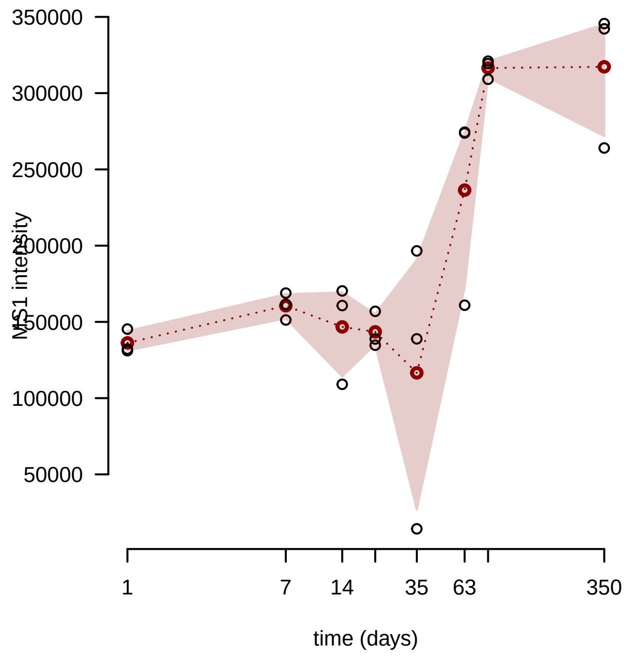

Q9JJG0
GN=Tacc2
transforming, acidic coiled−coil contain ...

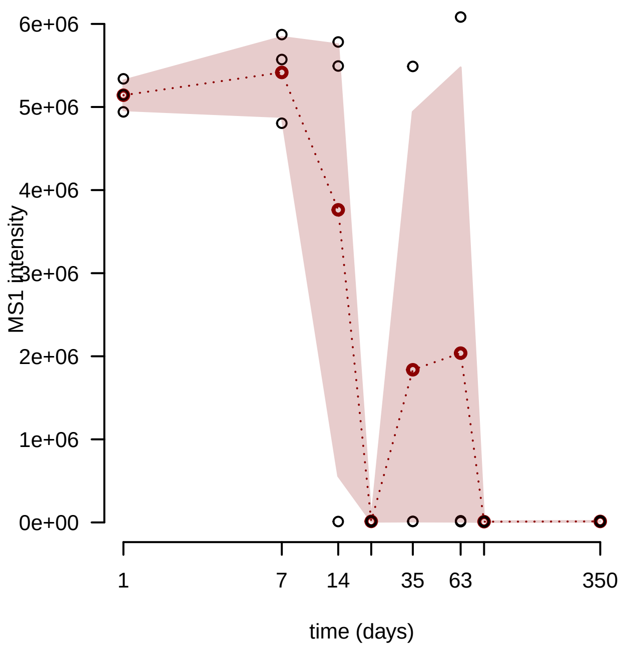

Q9JJG0
GN=Tacc2
transforming, acidic coiled−coil contain ...

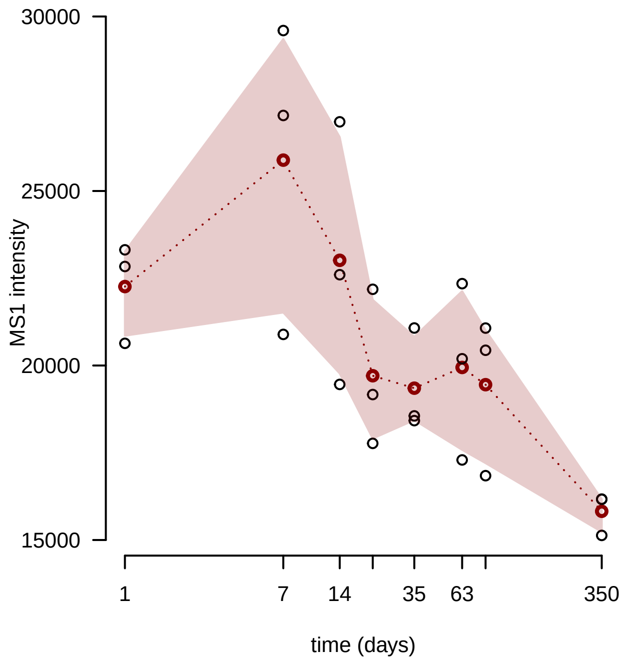

Q9JJG6
GN=Tmem47
transmembrane protein 47

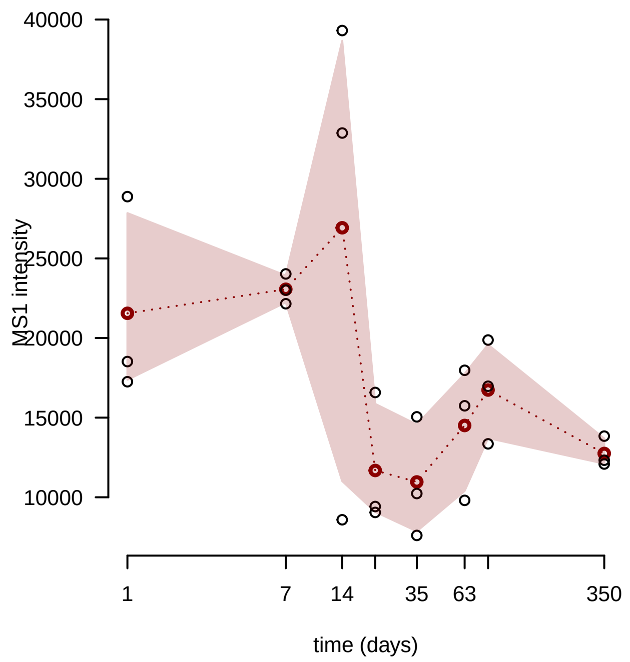

Q9JJG9
GN=Noa1
nitric oxide associated 1

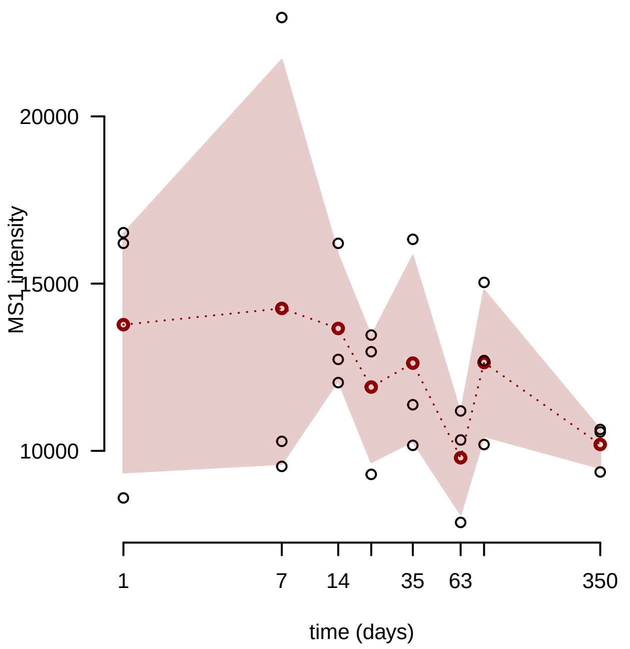

Q9JJH1
GN=Rnase4
ribonuclease, RNase A family 4

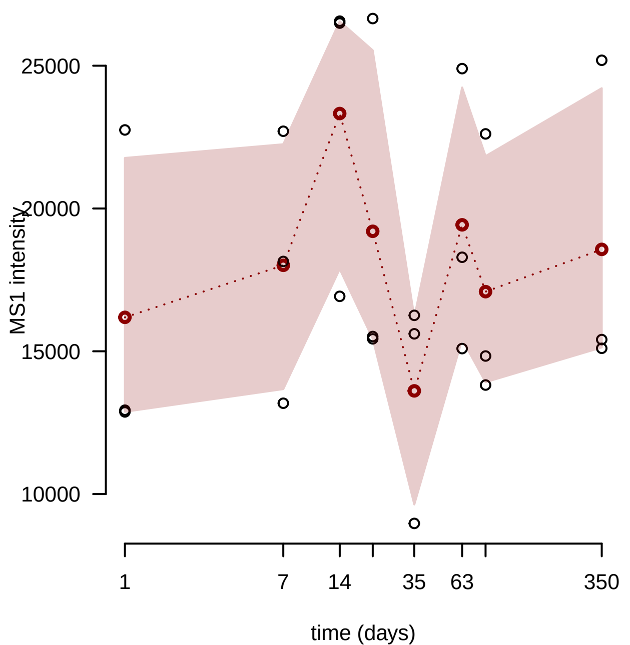

Q9JJI6
GN=Pigo
phosphatidylinositol glycan anchor biosy ...

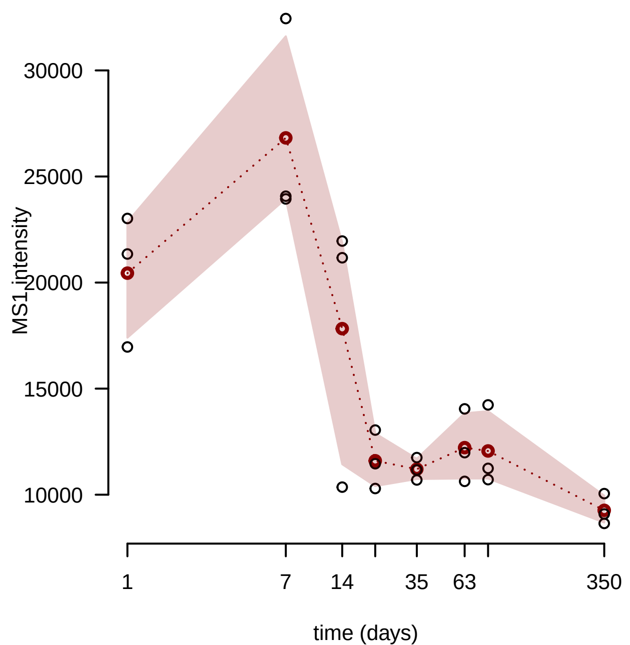

Q9JJI8
GN=Rpl38
ribosomal protein L38

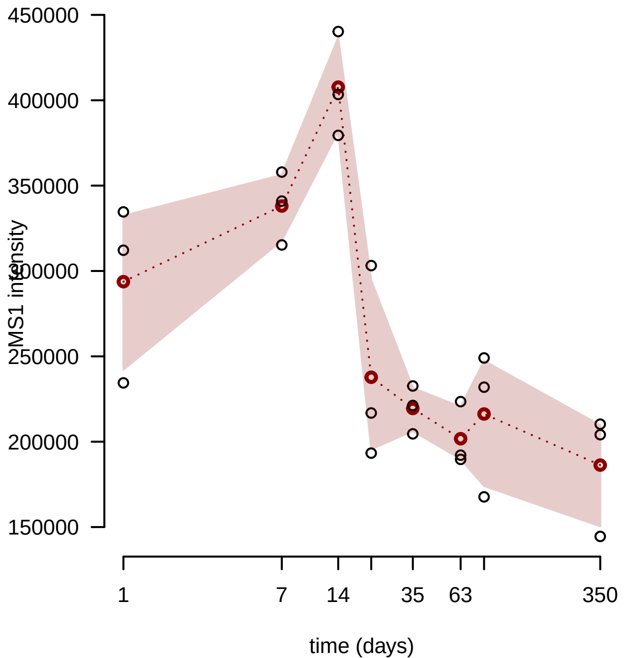

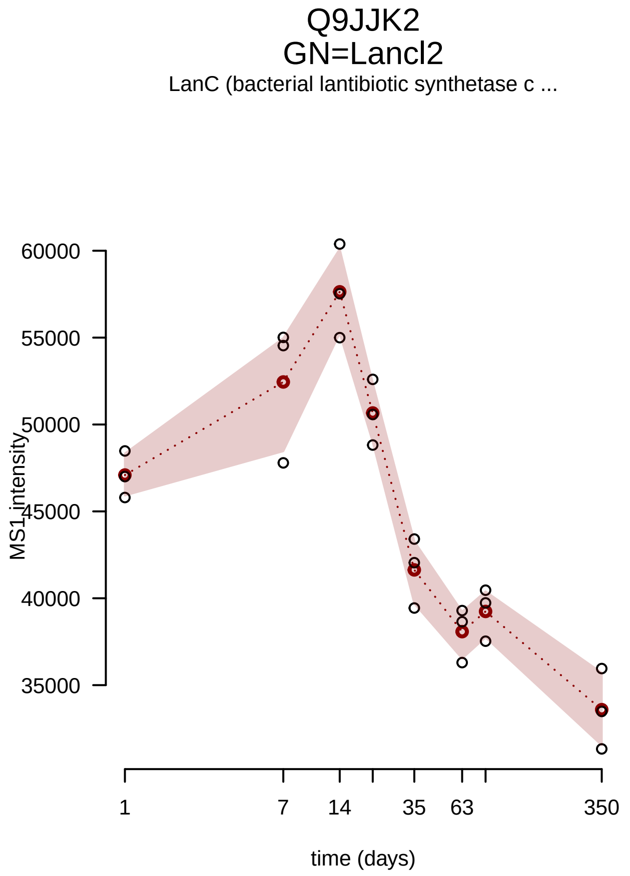
Q9JJK2
GN=Lancl2
LanC (bacterial lantibiotic synthetase c ...
MS1 intensity
60000
55000
50000
45000
40000
35000
1
7
14
35
63
350
time (days)
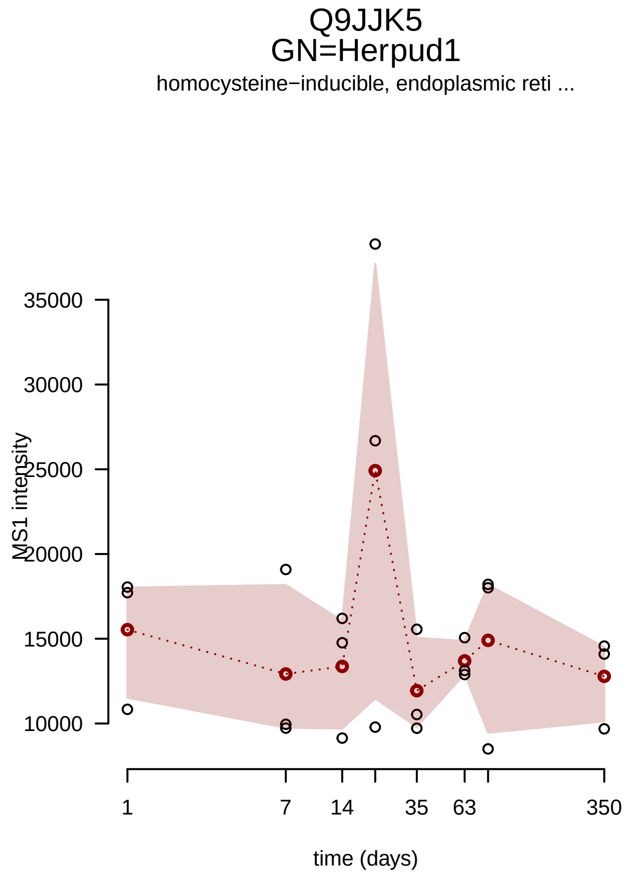
Q9JJK5
GN=Herpud1
homocysteine−inducible, endoplasmic reti ...
MS1 intensity
35000
30000
25000
20000
15000
10000
1
7
14
35
63
350
time (days)
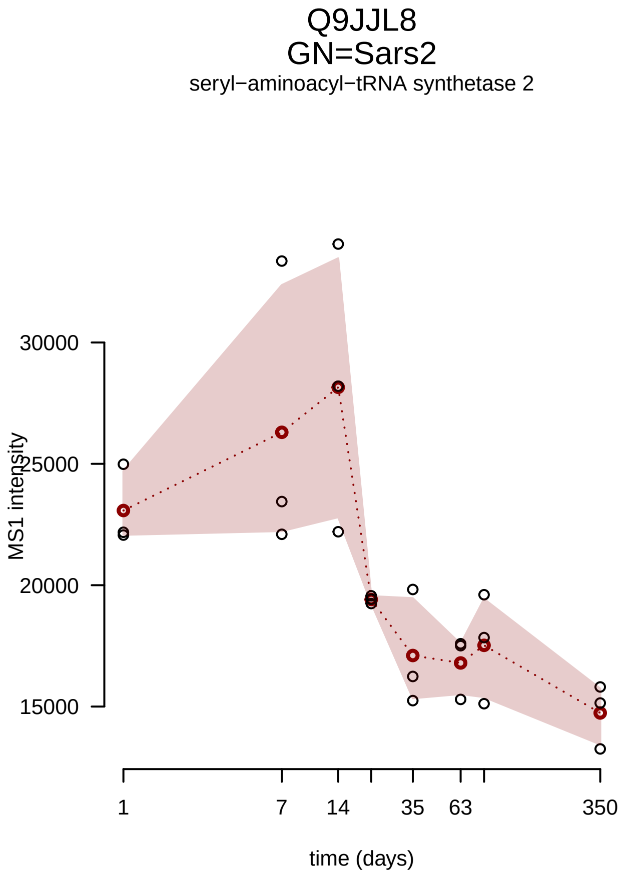
Q9JJL8
GN=Sars2
seryl−aminoacyl−tRNA synthetase 2
MS1 intensity
30000
25000
20000
15000
1
7
14
35
63
350
time (days)
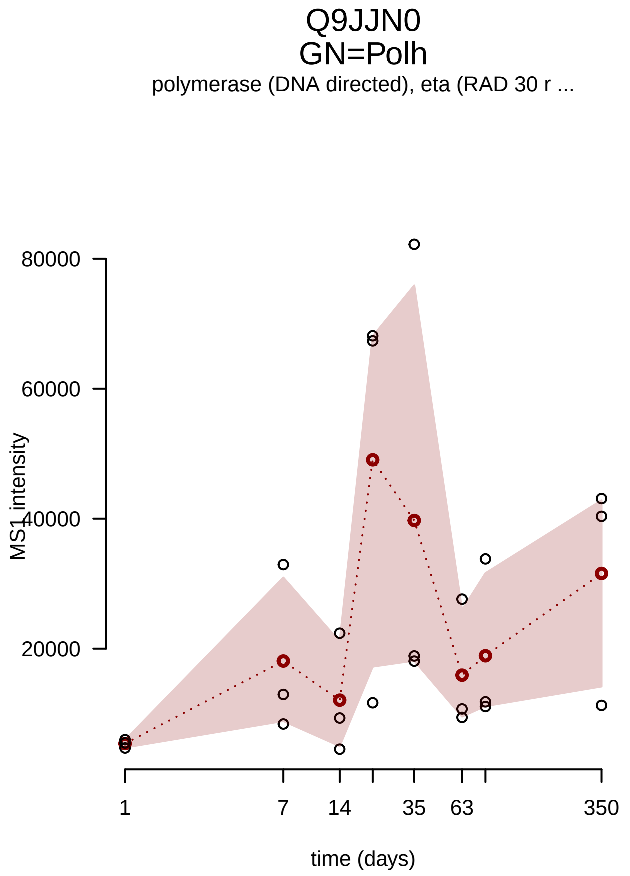
Q9JJN0
GN=Polh
polymerase (DNA directed), eta (RAD 30 r ...
MS1 intensity
80000
60000
40000
20000
1
7
14
35
63
350
time (days)
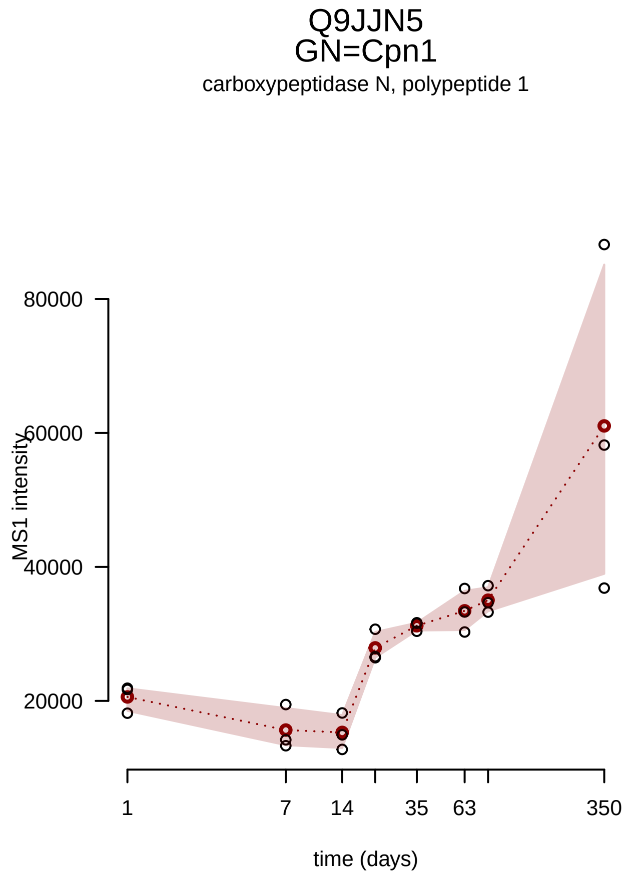
Q9JJN5
GN=Cpn1
carboxypeptidase N, polypeptide 1
MS1 intensity
80000
60000
40000
20000
1
7
14
35
63
350
time (days)
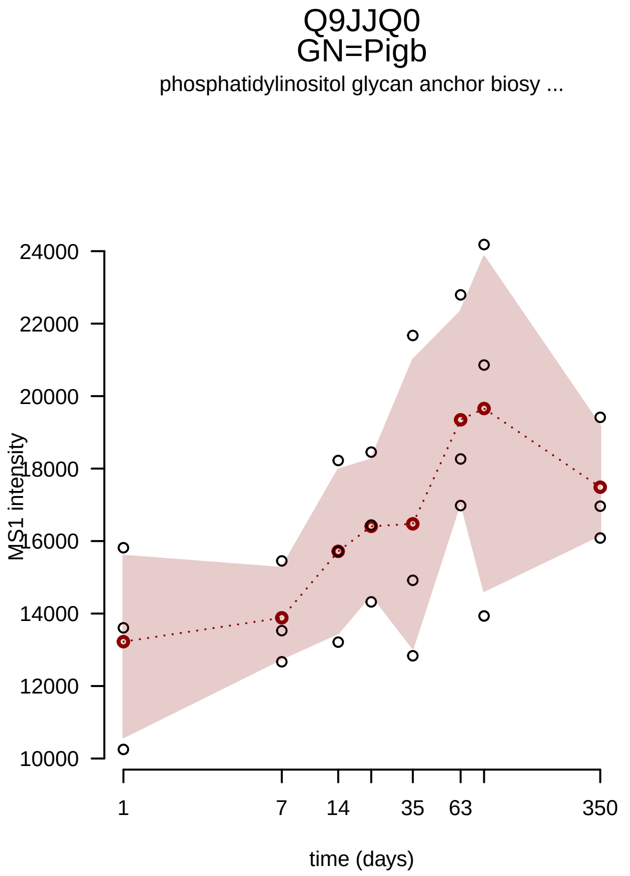
Q9JJQ0
GN=Pigb
phosphatidylinositol glycan anchor biosy ...
MS1 intensity
24000
22000
20000
18000
16000
14000
12000
10000
1
7
14
35
63
350
time (days)
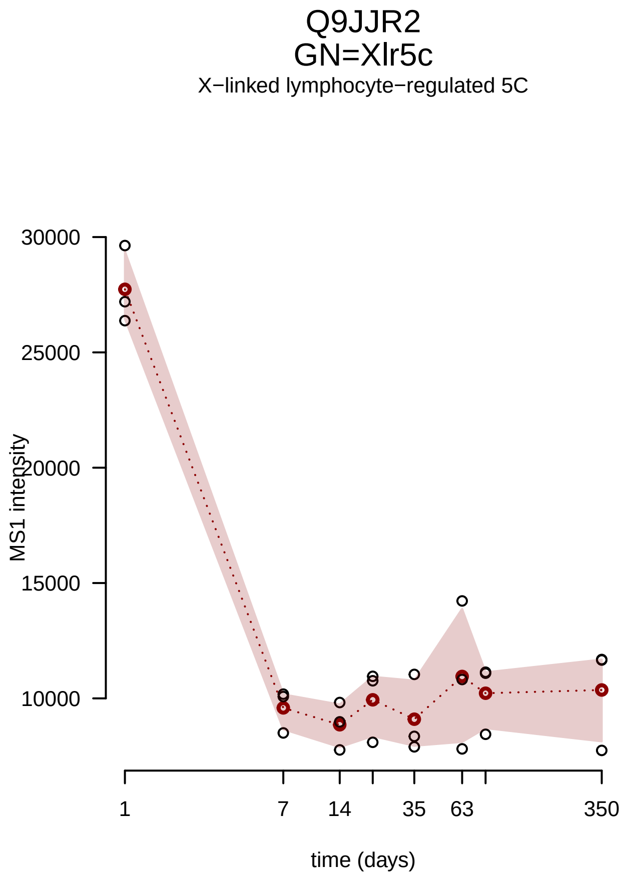
Q9JJR2
GN=Xlr5c
X−linked lymphocyte−regulated 5C
MS1 intensity
30000
25000
20000
15000
10000
1
7
14
35
63
350
time (days)
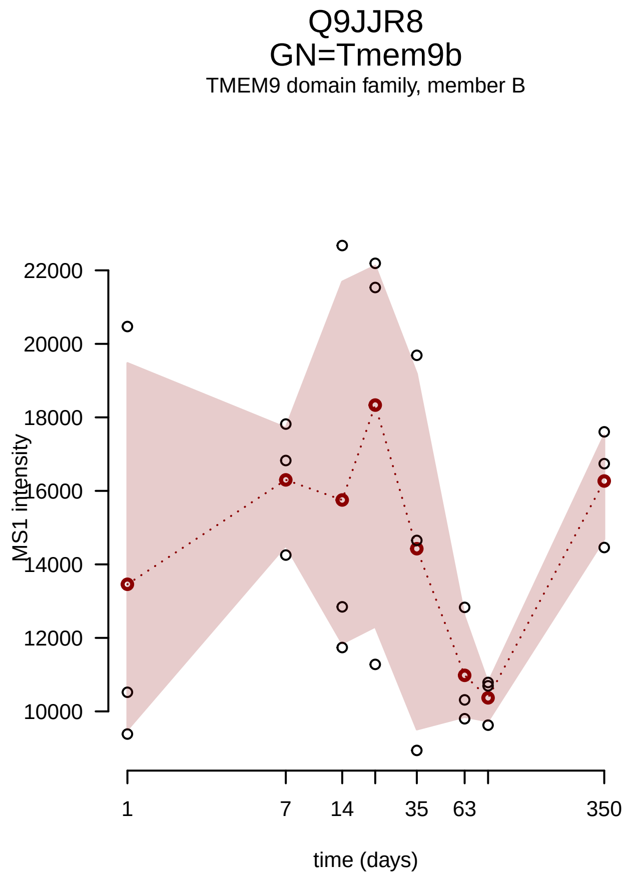
Q9JJR8
GN=Tmem9b
TMEM9 domain family, member B
MS1 intensity
22000
20000
18000
16000
14000
12000
10000
1
7
14
35
63
350
time (days)
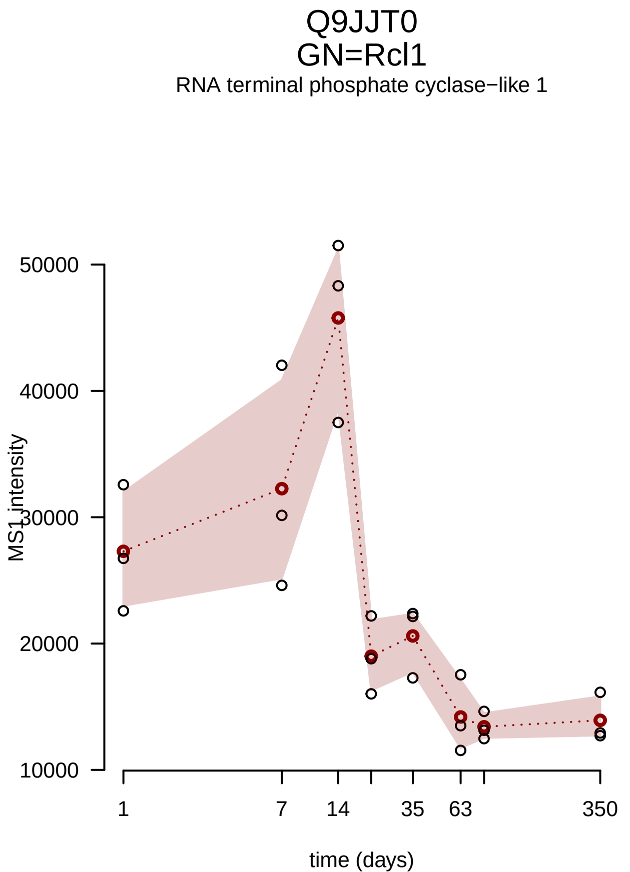
Q9JJT0
GN=Rcl1
RNA terminal phosphate cyclase−like 1
MS1 intensity
50000
40000
30000
20000
10000
1
7
14
35
63
350
time (days)

Q9JJT9
GN=Phax
phosphorylated adaptor for RNA export

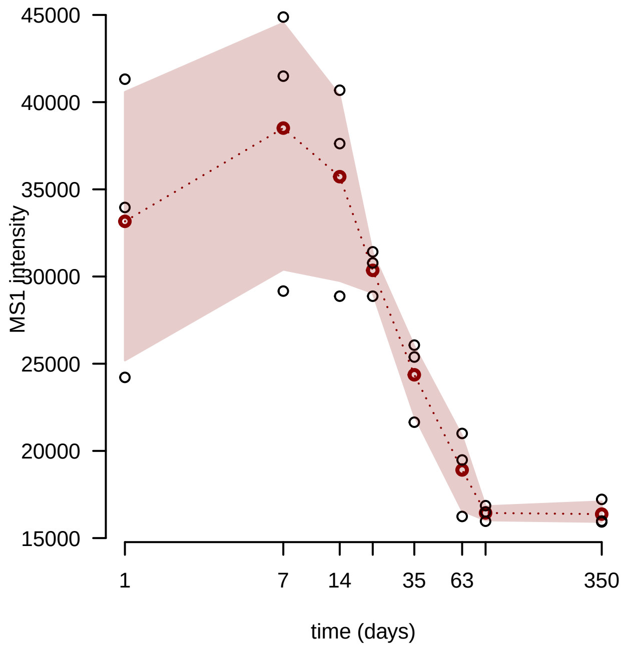

Q9JJU8
GN=Sh3bgrl
SH3−binding domain glutamic acid−rich pr ...

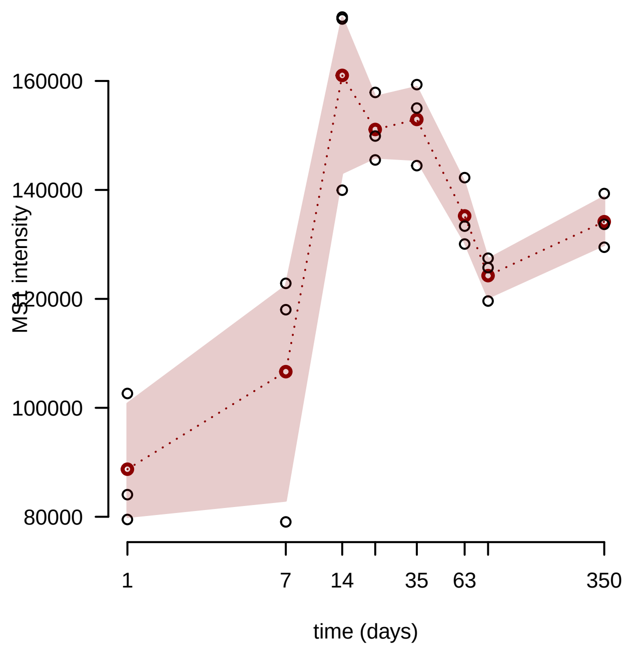

Q9JJV2
GN=Pfn2
profilin 2

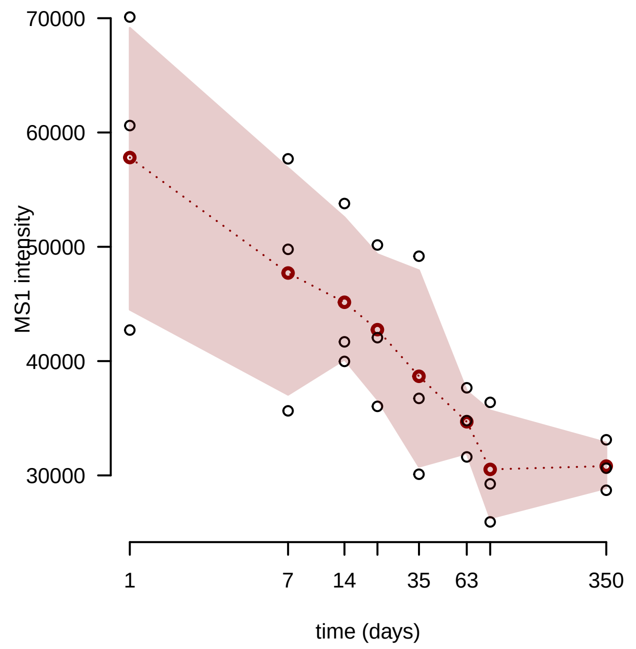

Q9JJW0
GN=Pxmp4
peroxisomal membrane protein 4

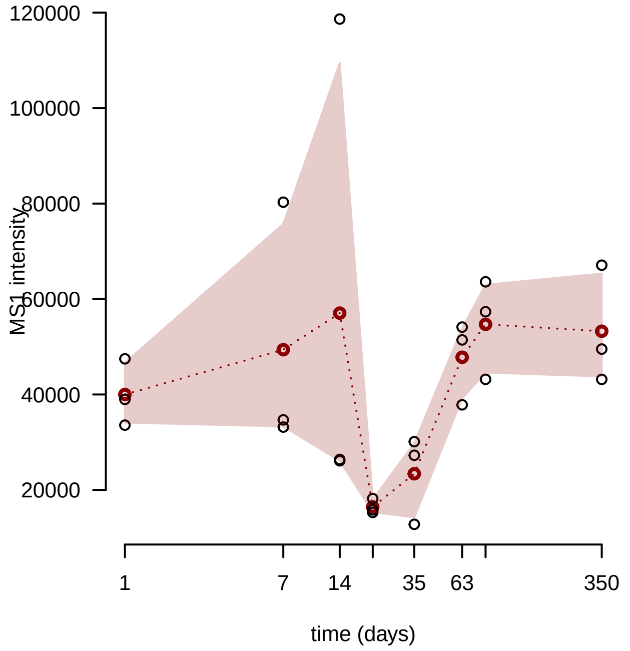

Q9JJX6
GN=P2rx4
purinergic receptor P2X, ligand−gated io ...

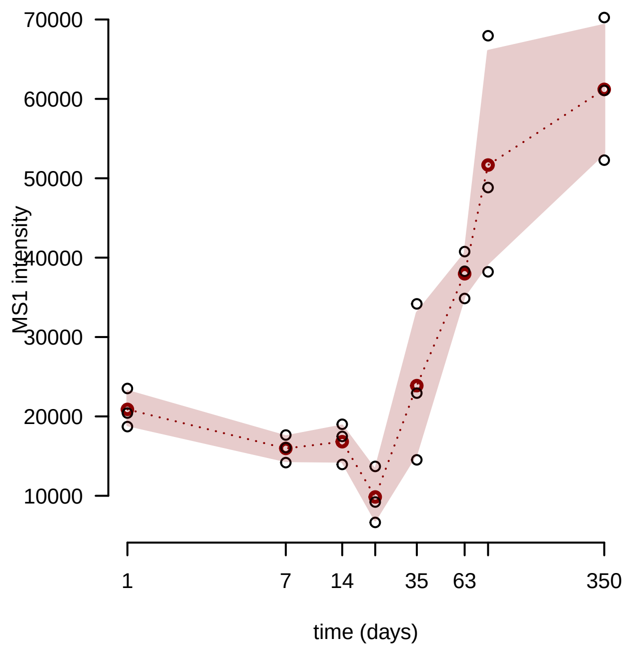

Q9JJX7
GN=Tdp2
tyrosyl−DNA phosphodiesterase 2

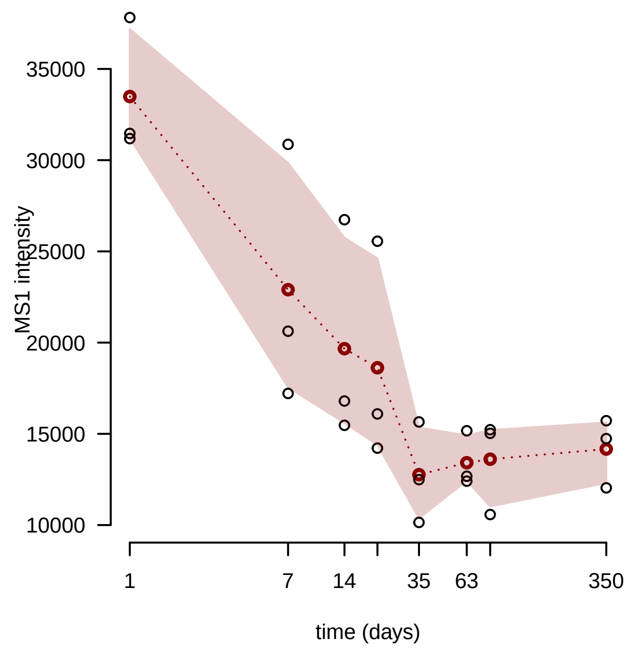

Q9JJY4
GN=Ddx20
DEAD box helicase 20

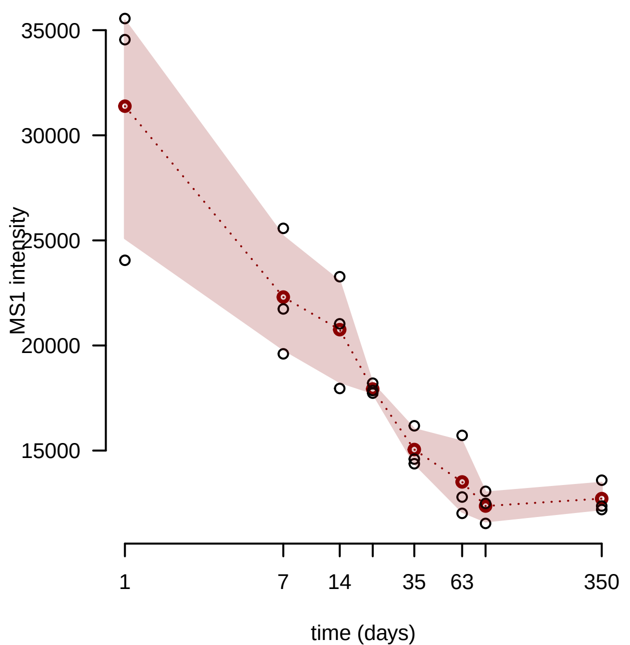

Q9JJZ2
GN=Tuba8
tubulin, alpha 8

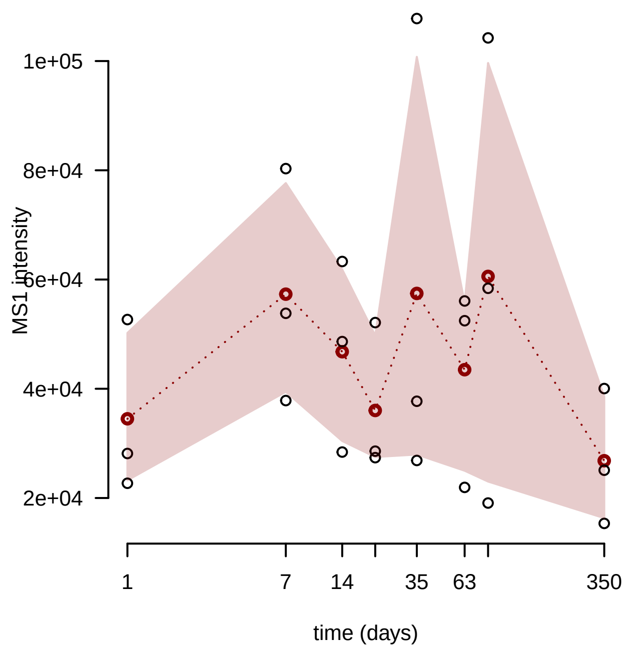

Q9JJZ4
GN=Ube2j1
ubiquitin−conjugating enzyme E2J 1

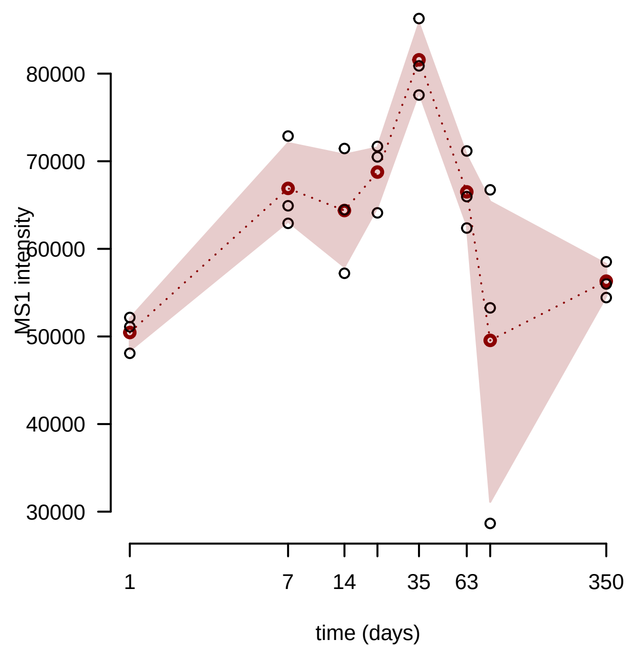

Q9JJZ5
GN=Egfl6
EGF−like−domain, multiple 6

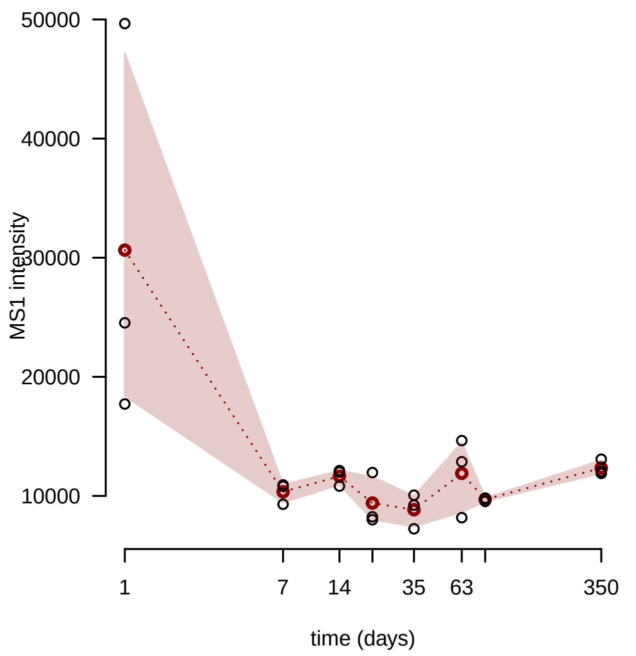

Q9JJZ6
GN=Klf13
Kruppel−like factor 13

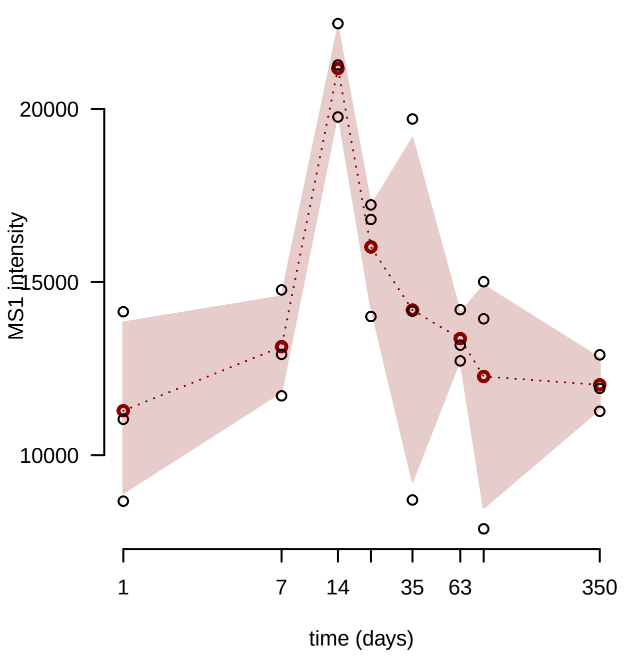

Q9JK23
GN=Psmg1
proteasome (prosome, macropain) assembly ...

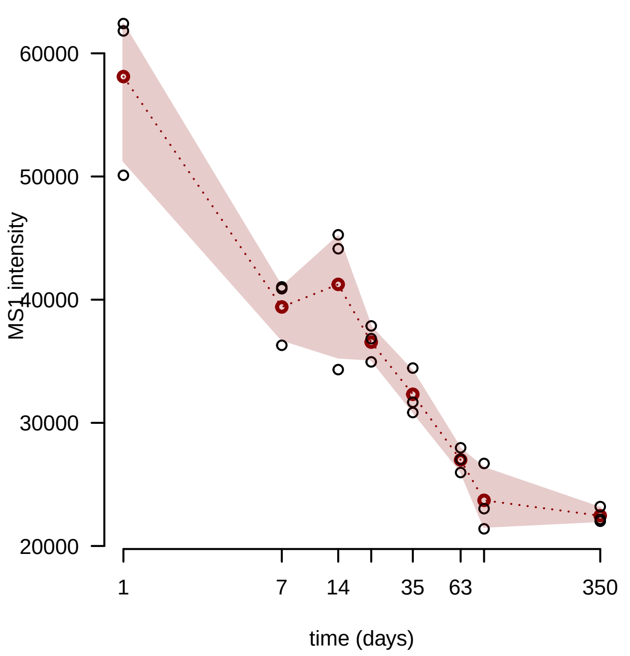

Q9JK30
GN=Orc3
origin recognition complex, subunit 3

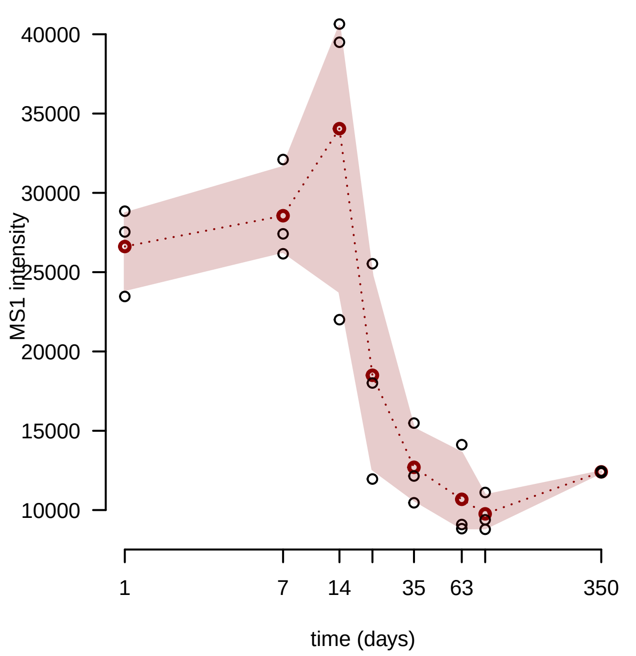

Q9JK38
GN=Gnpnat1
glucosamine−phosphate N−acetyltransferas ...

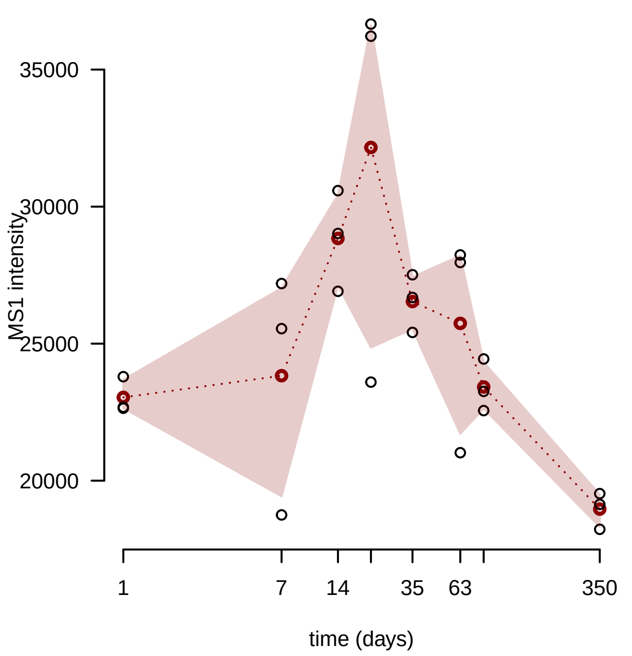

Q9JK42
GN=Pdk2
pyruvate dehydrogenase kinase, isoenzyme ...

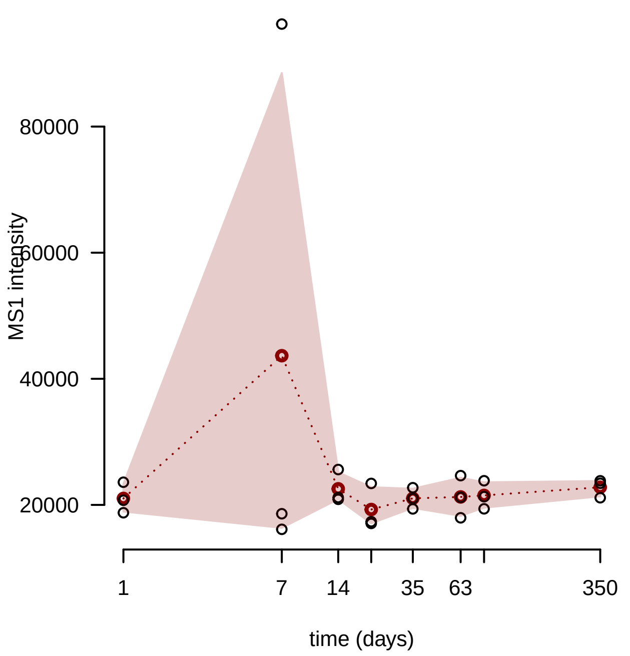

Q9JK48
GN=Sh3glb1
SH3−domain GRB2−like B1 (endophilin)

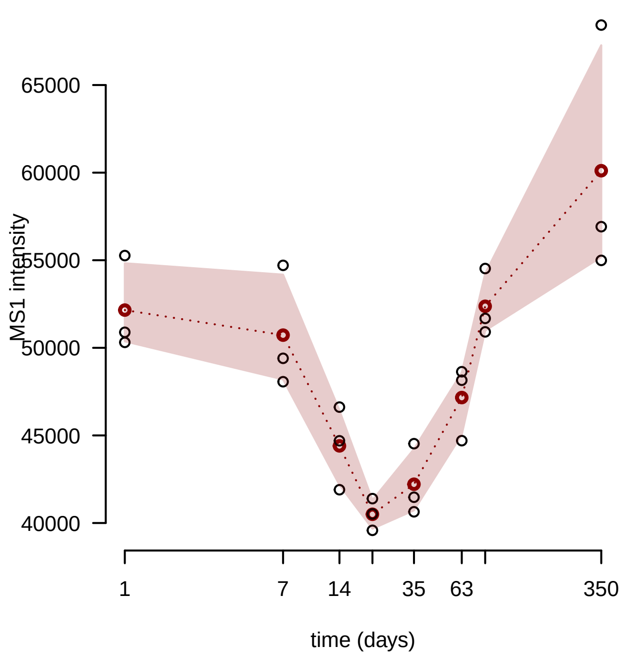

Q9JK53
GN=Prelp
proline arginine−rich end leucine−rich r ...

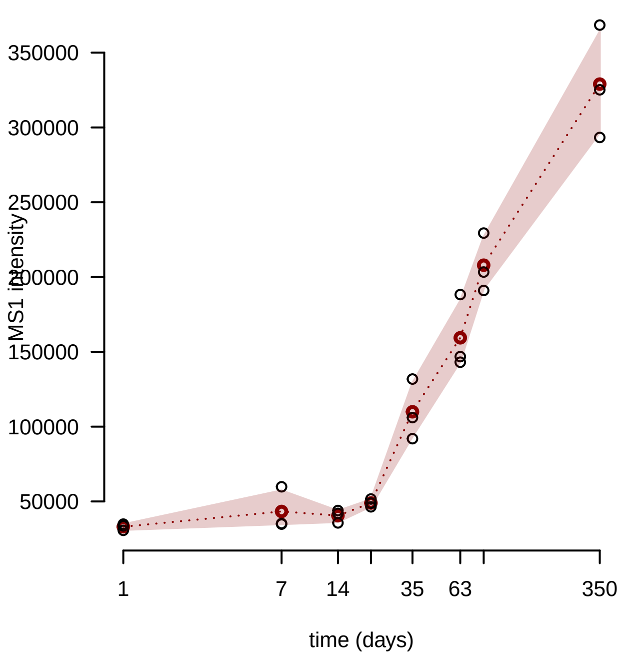

Q9JK81
GN=Myg1
melanocyte proliferating gene 1

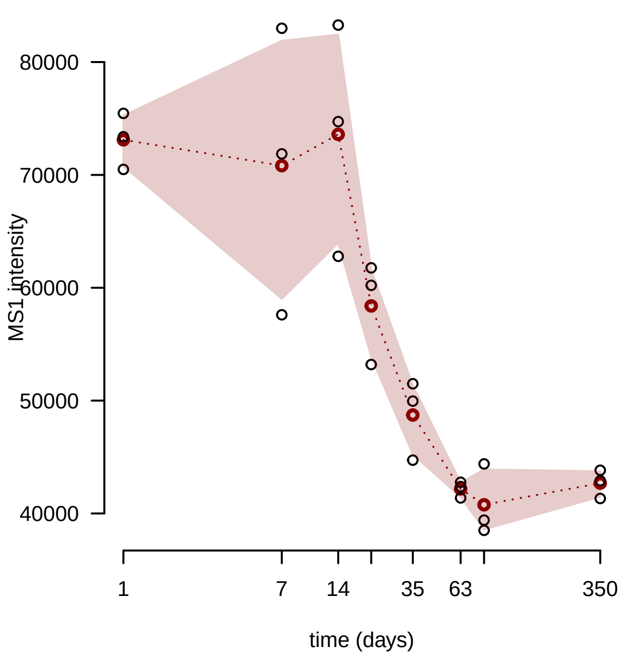

Q9JK83
GN=Pard6b
par−6 family cell polarity regulator bet ...

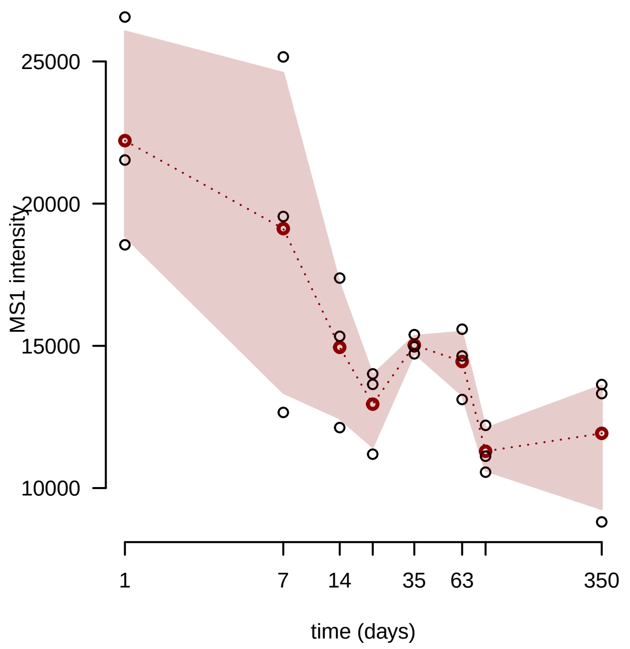

Q9JK84
GN=Pard6g
par−6 family cell polarity regulator gam ...

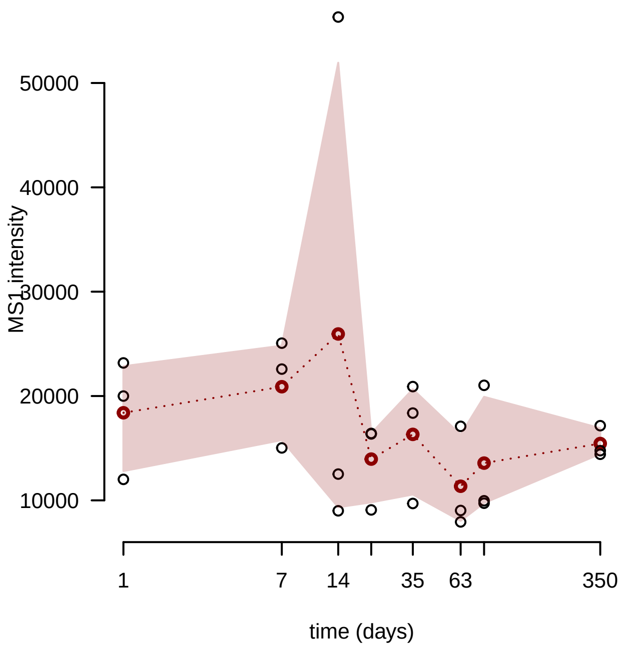

Q9JK91
GN=Mlh1
mutL homolog 1

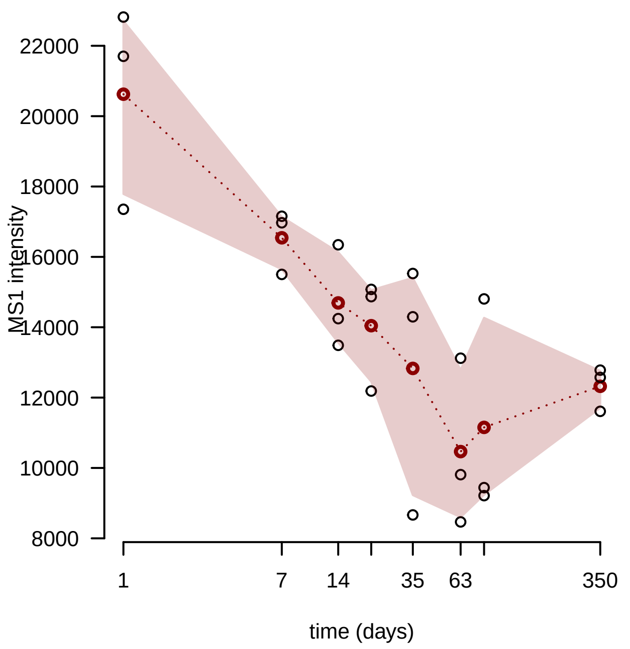

Q9JK92
GN=Hspb8
heat shock protein 8

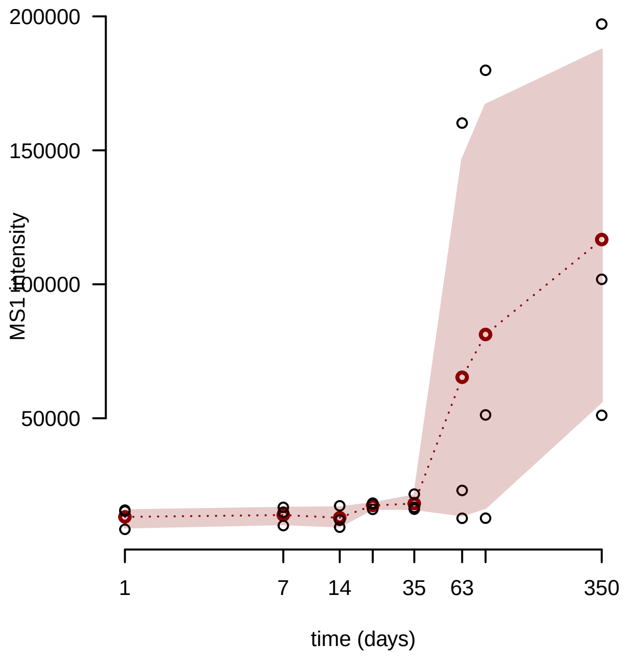

Q9JKB1
GN=Uchl3
ubiquitin carboxyl−terminal esterase L3 ...

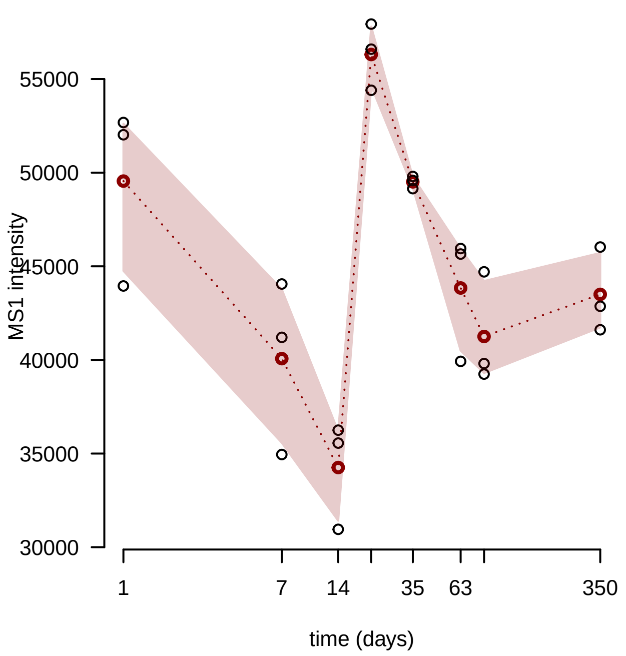

Q9JKB3
GN=Ybx3
Y box protein 3

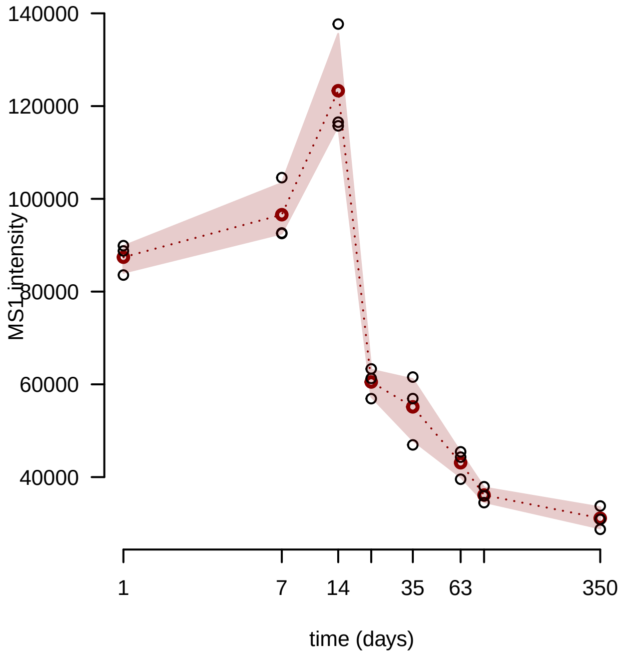

Q9JKB3
GN=Ybx3
Y box protein 3

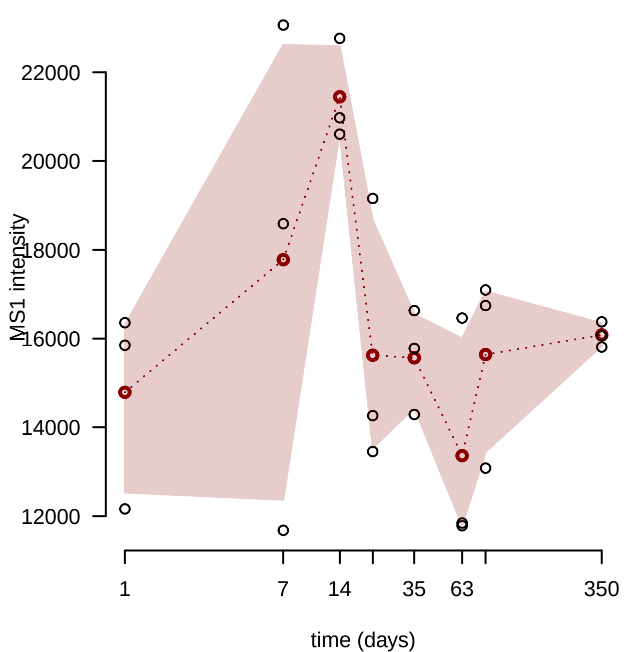

Q9JKC7
GN=Ap4m1
adaptor−related protein complex AP−4, mu ...

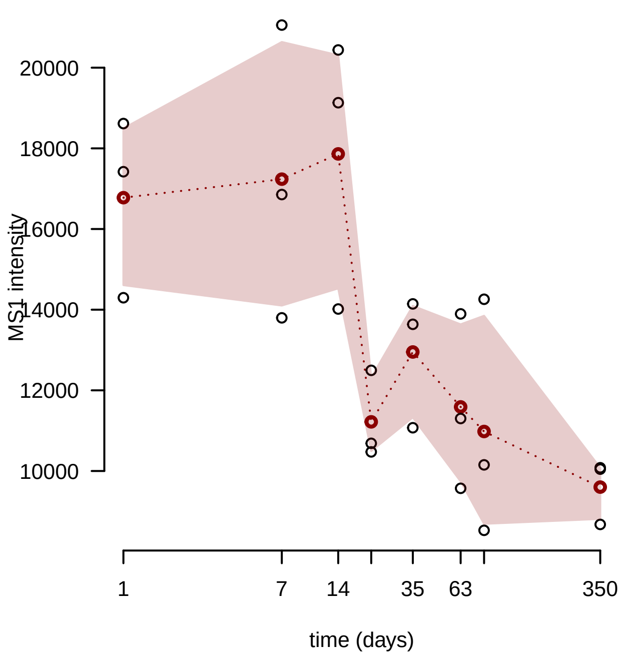

Q9JKC8
GN=Ap3m1
adaptor−related protein complex 3, mu 1 ...

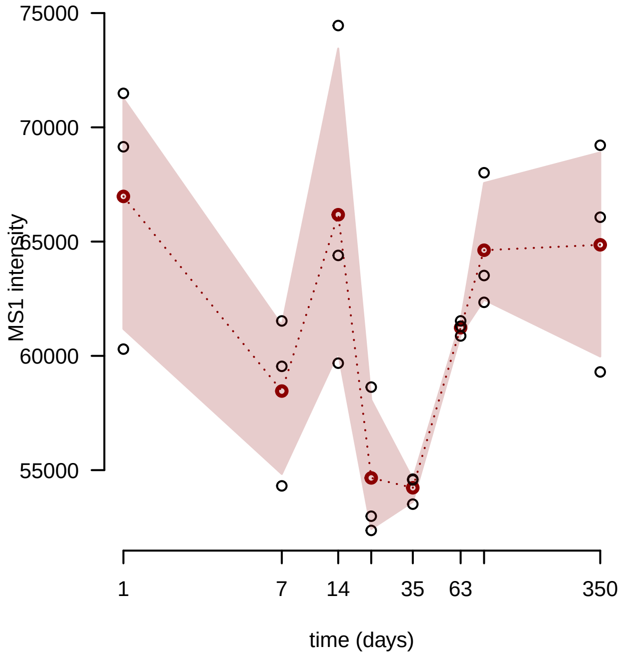

Q9JKD3
GN=Scamp5
secretory carrier membrane protein 5

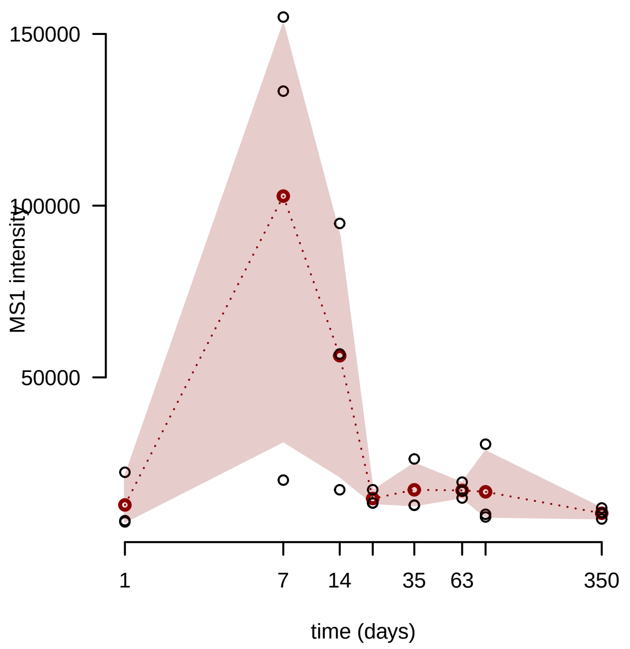

Q9JKF1
GN=Iqgap1
IQ motif containing GTPase activating pr ...

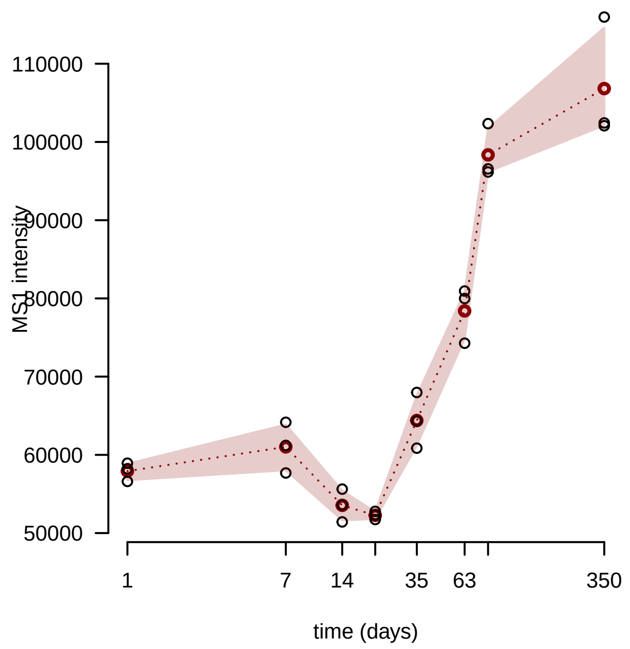

Q9JKF6
GN=Nectin1
nectin cell adhesion molecule 1

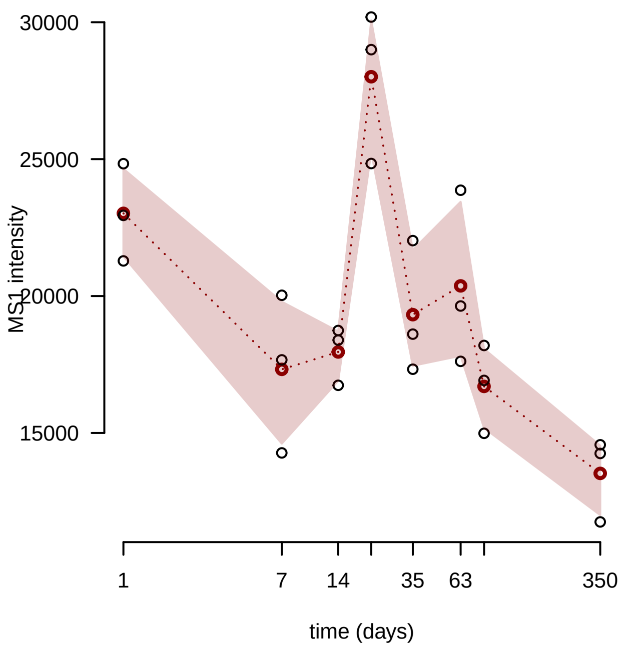

Q9JKF7
GN=Mrpl39
mitochondrial ribosomal protein L39

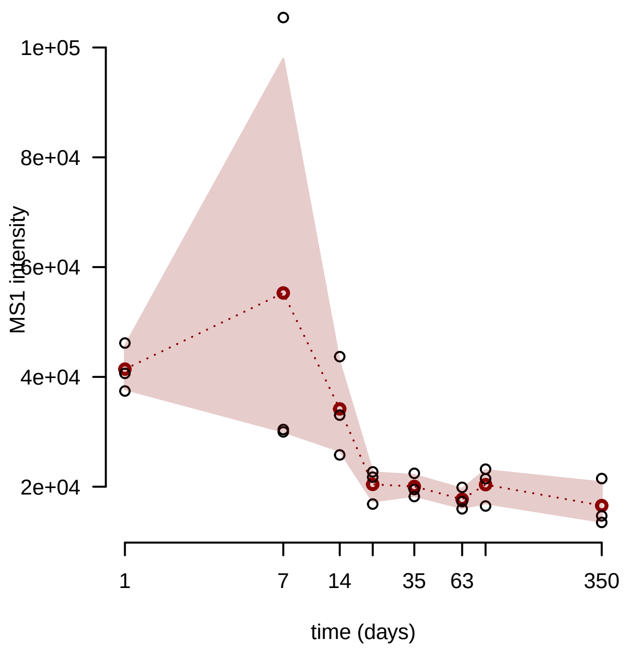

Q9JKK1
GN=Stx6
syntaxin 6

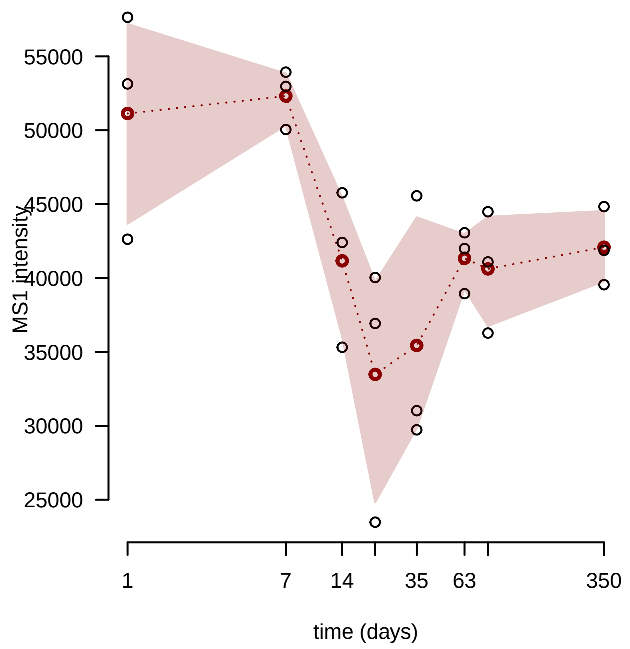

Q9JKK7
GN=Tmod2
tropomodulin 2

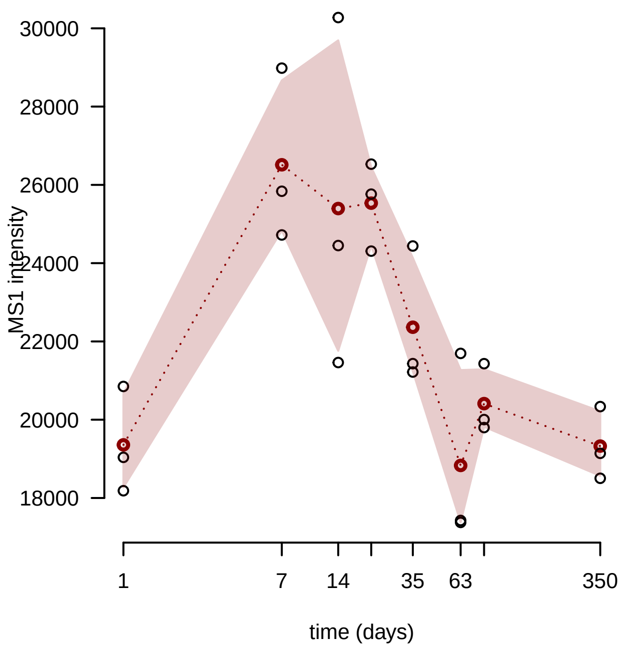

Q9JKK8
GN=Atr
ataxia telangiectasia and Rad3 related

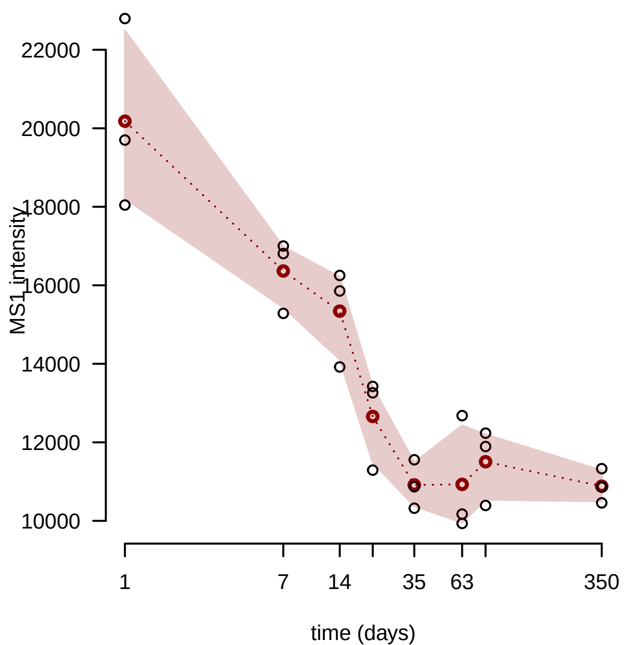

Q9JKL4
GN=Ndufaf3
NADH:ubiquinone oxidoreductase complex a ...

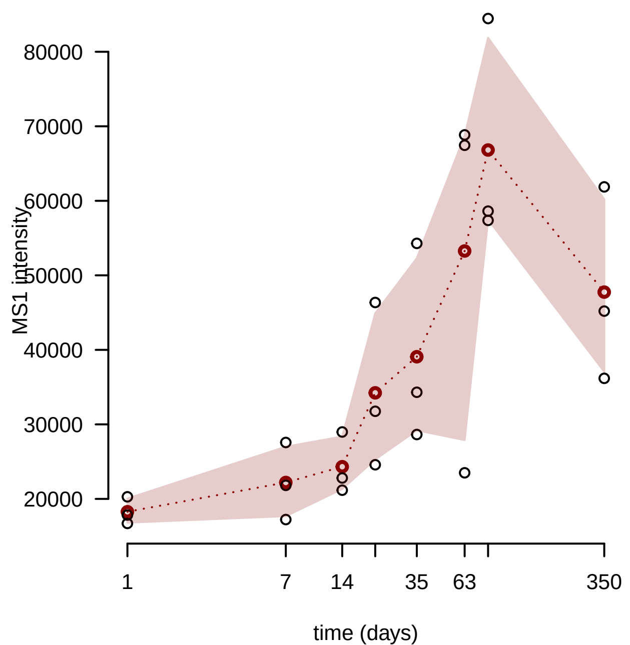

Q9JKN1
GN=Slc30a7
solute carrier family 30 (zinc transport ...

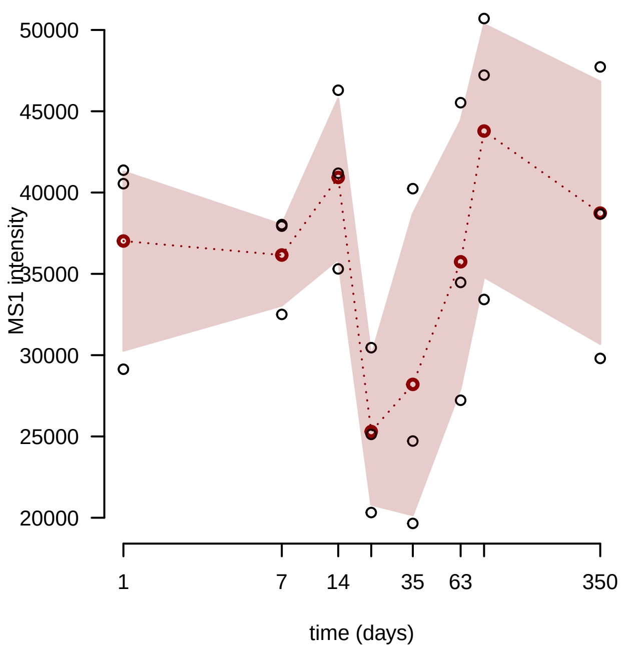

Q9JKN6
GN=Nova1
NOVA alternative splicing regulator 1

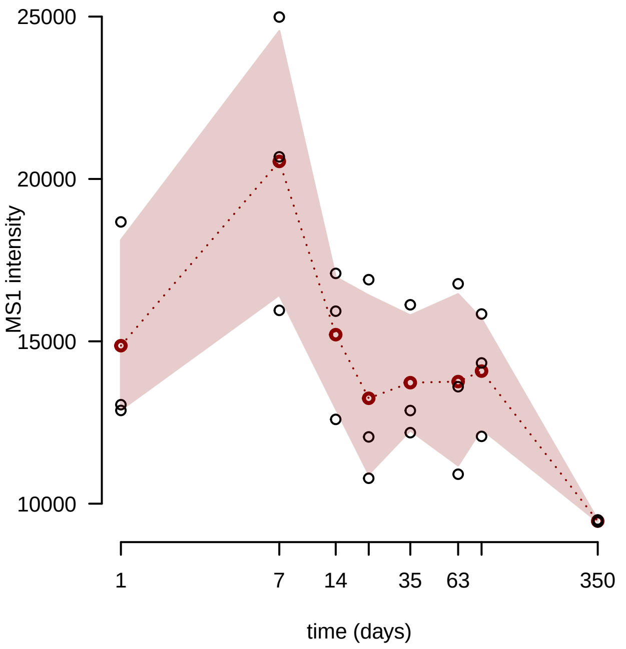

Q9JKP5
GN=Mbnl1
muscleblind like splicing factor 1

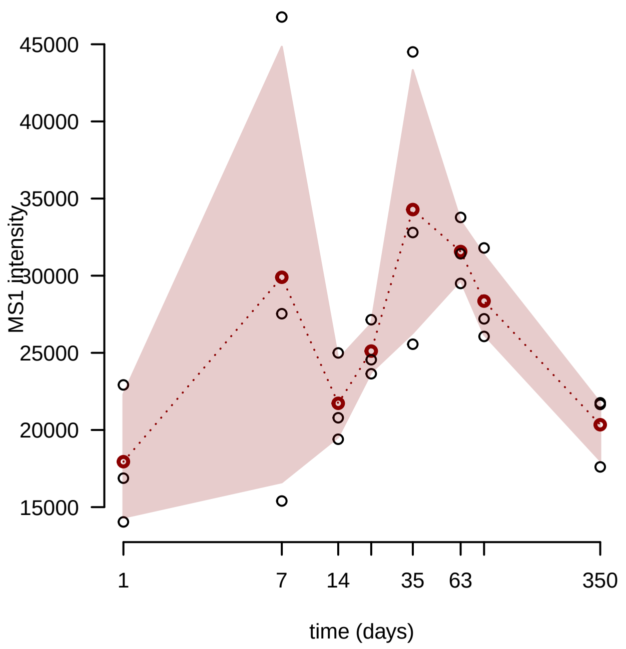

Q9JKP7
GN=Pole3
polymerase (DNA directed), epsilon 3 (p1 ...

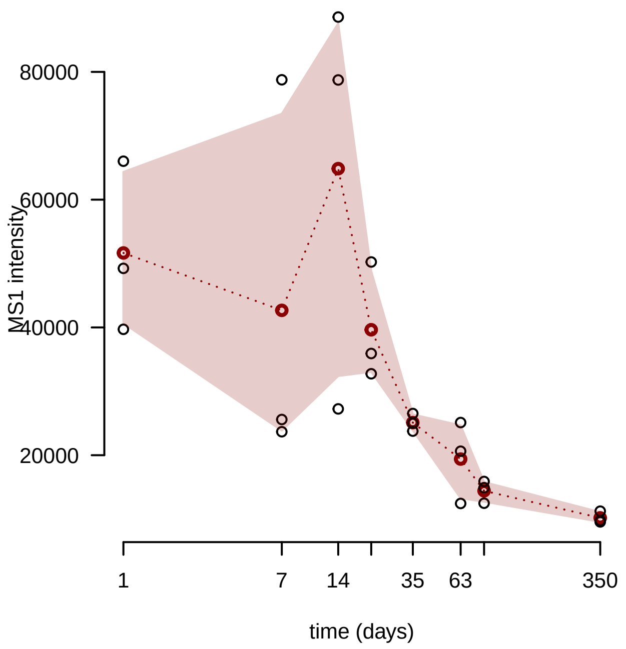

Q9JKP8
GN=Chrac1
chromatin accessibility complex 1

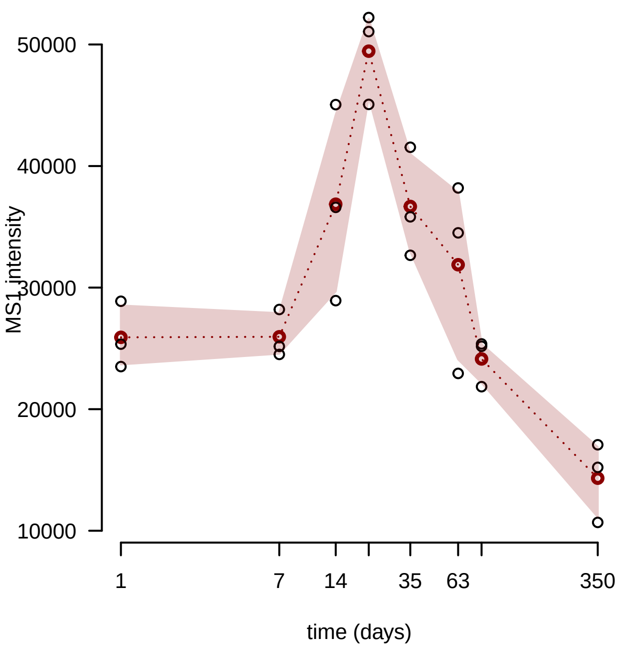

Q9JKR6
GN=Hyou1
hypoxia up−regulated 1

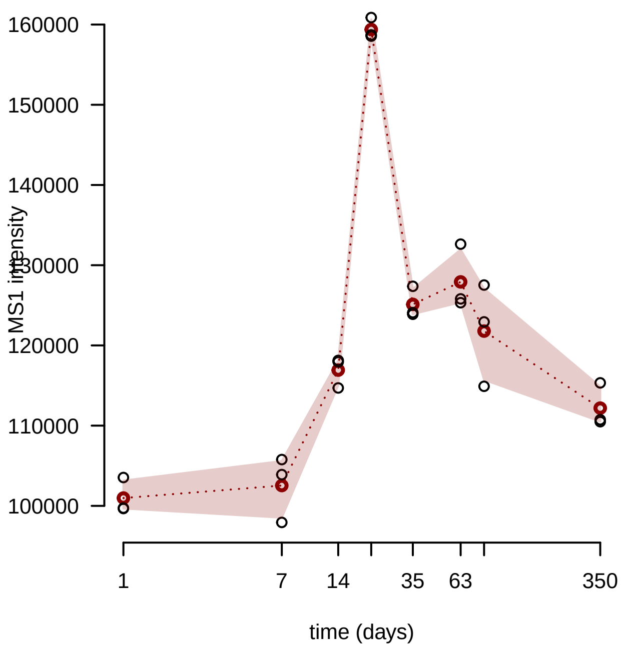

Q9JKS4
GN=Ldb3
LIM domain binding 3

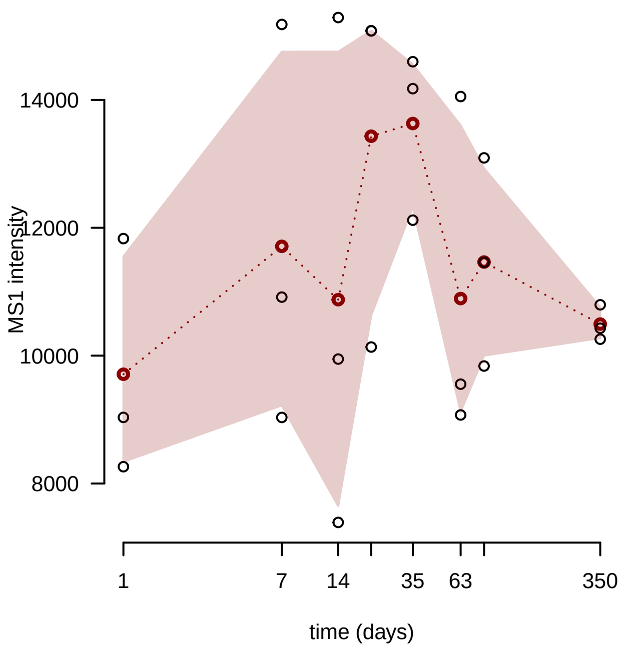

Q9JKS4
GN=Ldb3
LIM domain binding 3

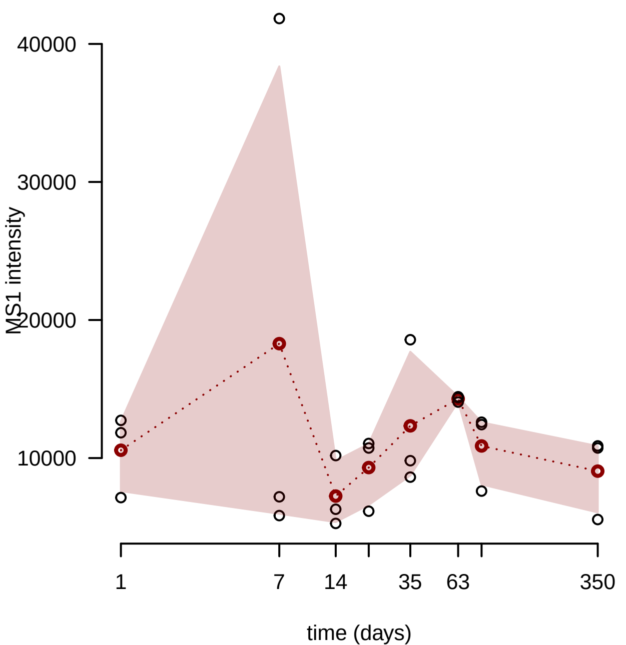

Q9JKS5
GN=Habp4
hyaluronic acid binding protein 4

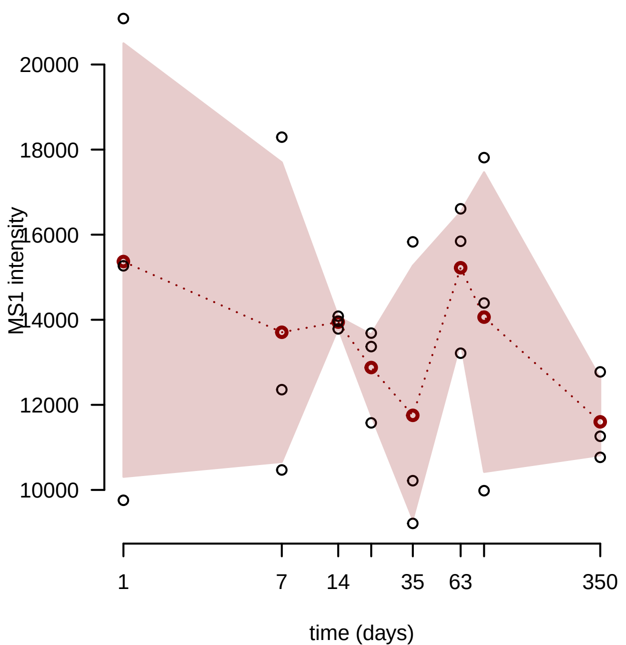

Q9JKV1
GN=Adrm1
adhesion regulating molecule 1 26S prote ...

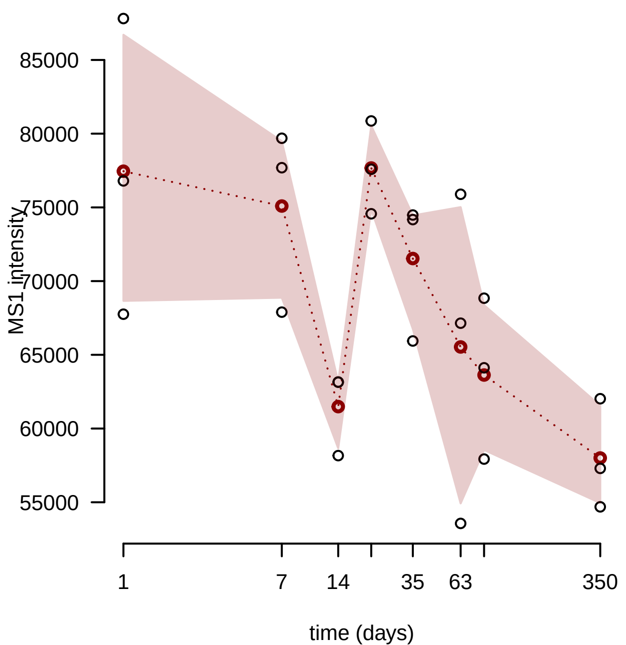

Q9JKV5
GN=Scamp4
secretory carrier membrane protein 4

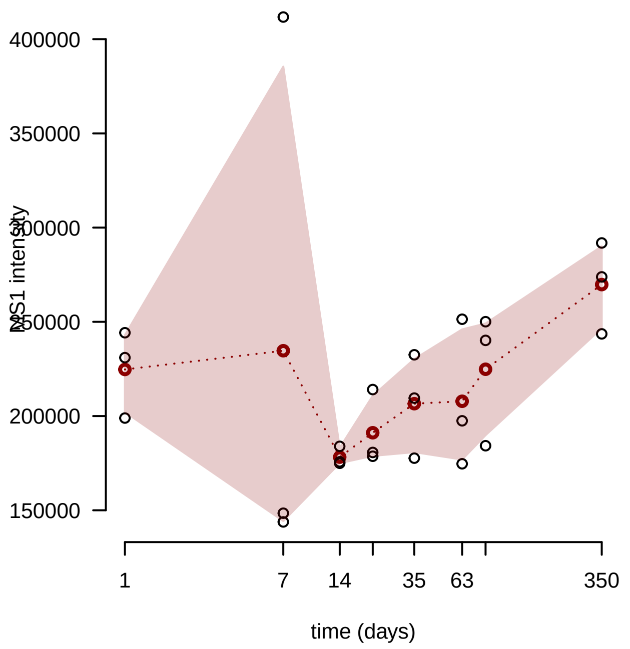

Q9JKW0
GN=Arl6ip1
ADP−ribosylation factor−like 6 interacti ...

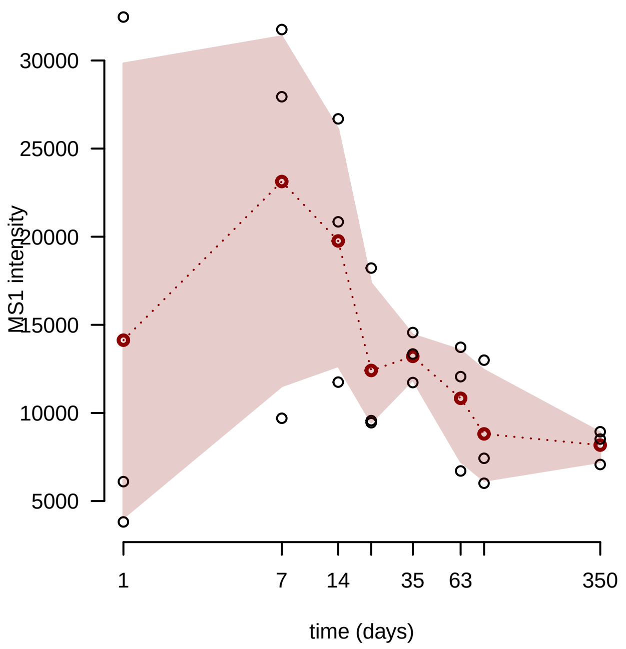

Q9JKX4
GN=Aatf
apoptosis antagonizing transcription fac ...

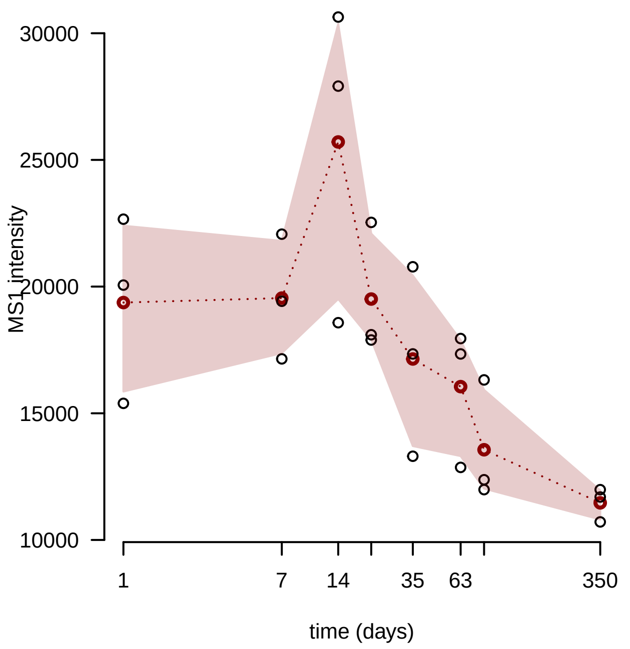

Q9JKX6
GN=Nudt5
nudix (nucleoside diphosphate linked moi ...

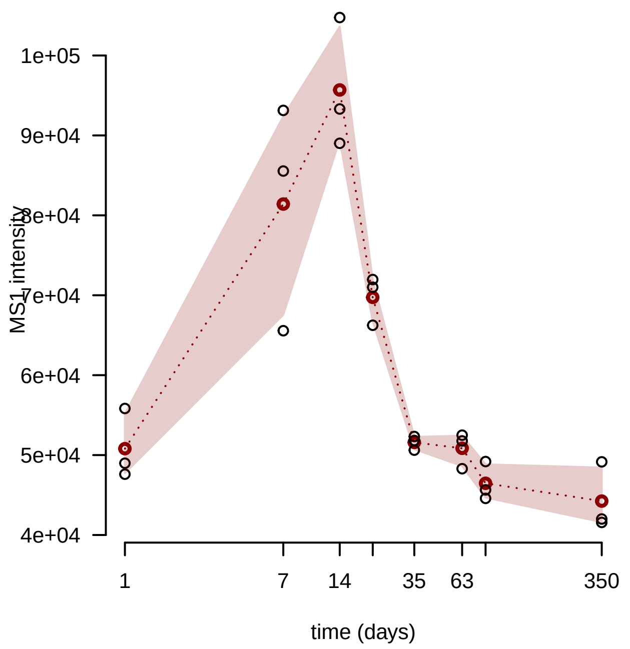

Q9JKY0
GN=Cnot9
CCR4−NOT transcription complex, subunit ...

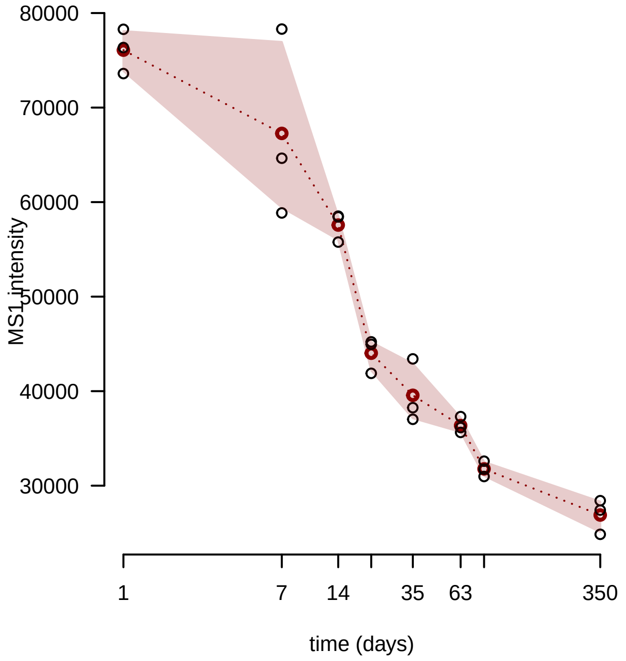

Q9JKY5
GN=Hip1r
huntingtin interacting protein 1 related

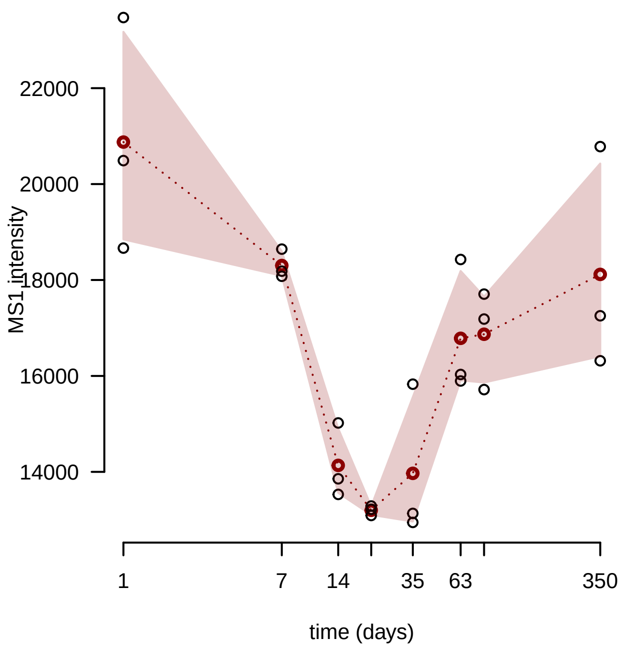

Q9JKZ2
GN=Slc5a3
solute carrier family 5 (inositol transp ...

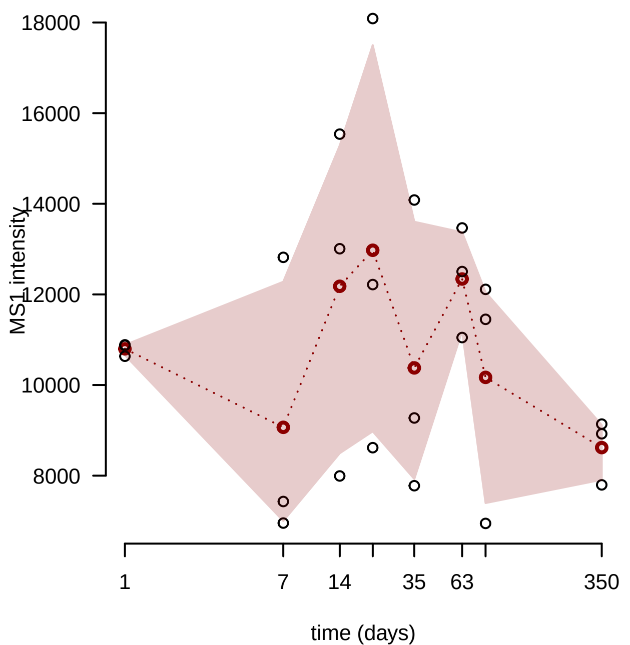

Q9JL06
GN=Lpar2
lysophosphatidic acid receptor 2

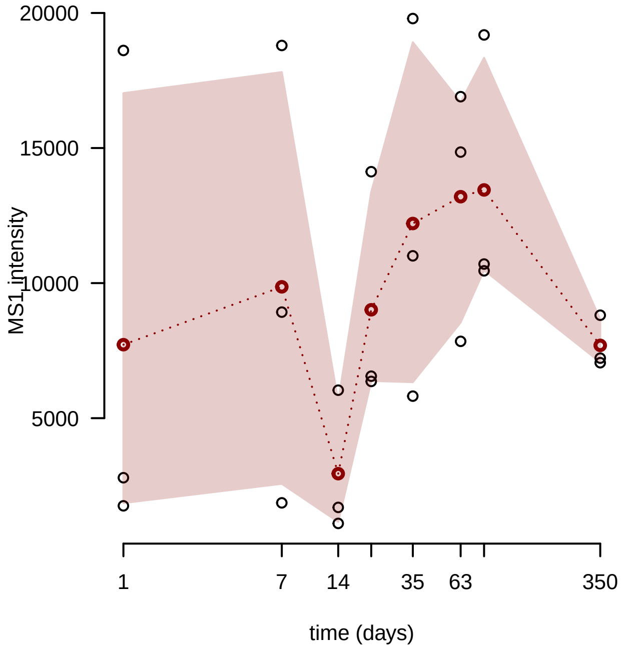

Q9JL15
GN=Lgals8
lectin, galactose binding, soluble 8

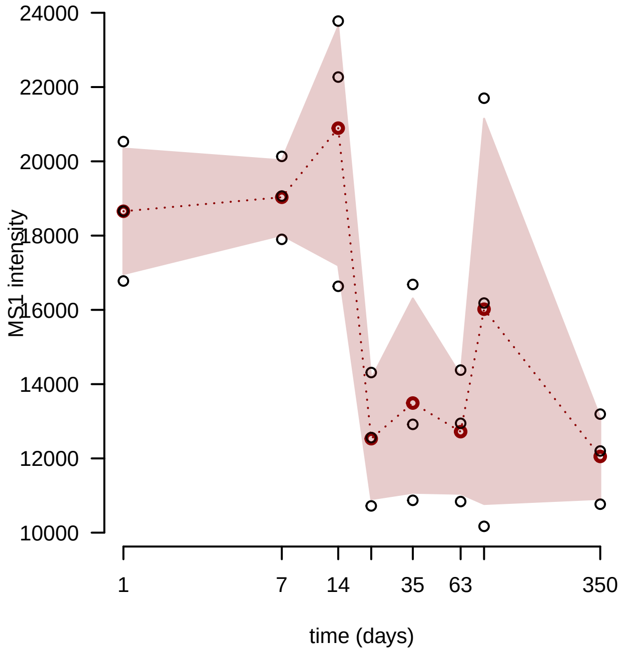

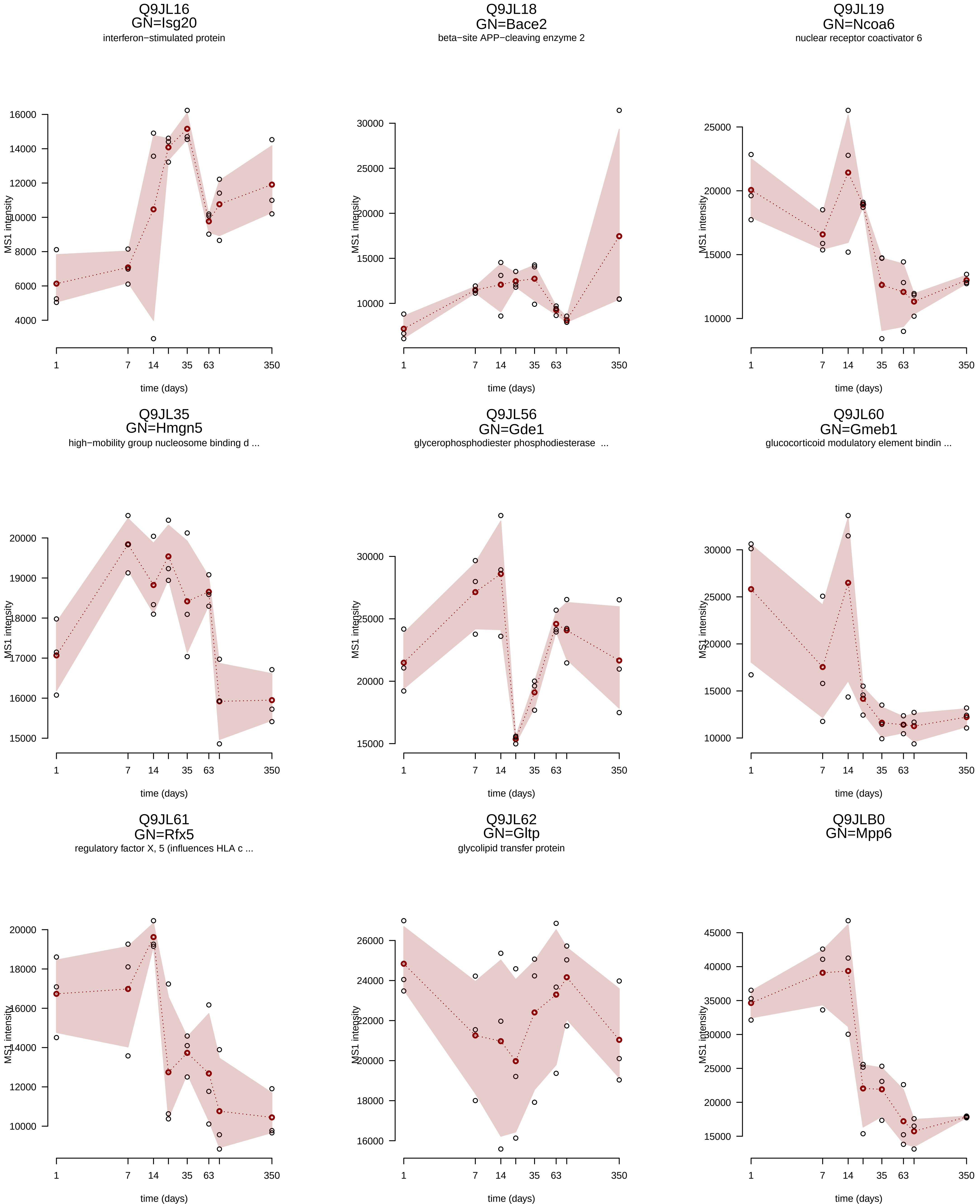
Q9JL16
GN=Isg20
interferon−stimulated protein
Q9JL18
GN=Bace2
beta−site APP−cleaving enzyme 2
Q9JL19
GN=Ncoa6
nuclear receptor coactivator 6
Q9JL35
GN=Hmgn5
high−mobility group nucleosome binding d ...
Q9JL56
GN=Gde1
glycerophosphodiester phosphodiesterase ...
Q9JL60
GN=Gmeb1
glucocorticoid modulatory element bindin ...
Q9JL61
GN=Rfx5
regulatory factor X, 5 (influences HLA c ...
Q9JL62
GN=Gltp
glycolipid transfer protein
Q9JLB0
GN=Mpp6
MS1 intensity
time (days)
1
7
14
35
63
350

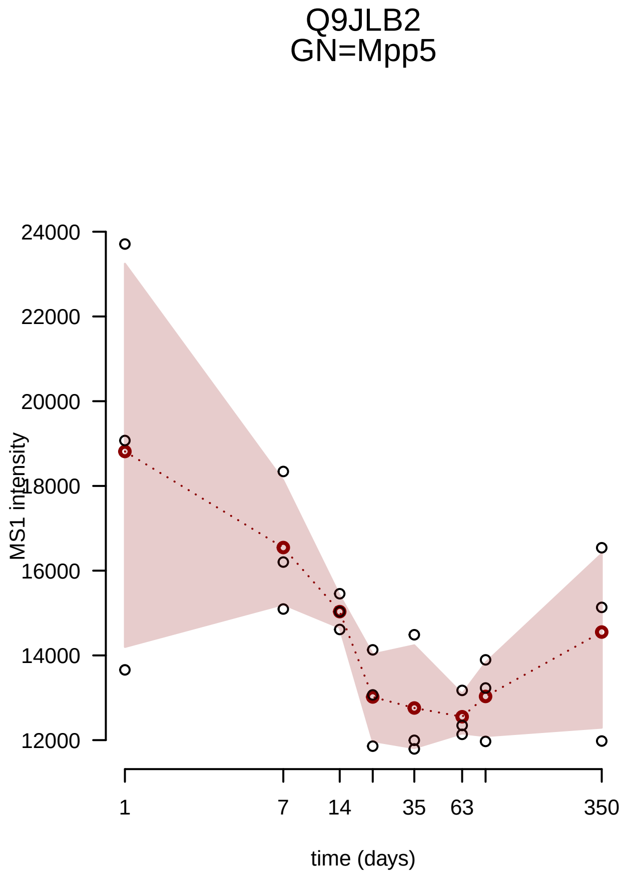
Q9JLB2
GN=Mpp5
MS1 intensity
time (days)
1
7
14
35
63
350
12000
14000
16000
18000
20000
22000
24000

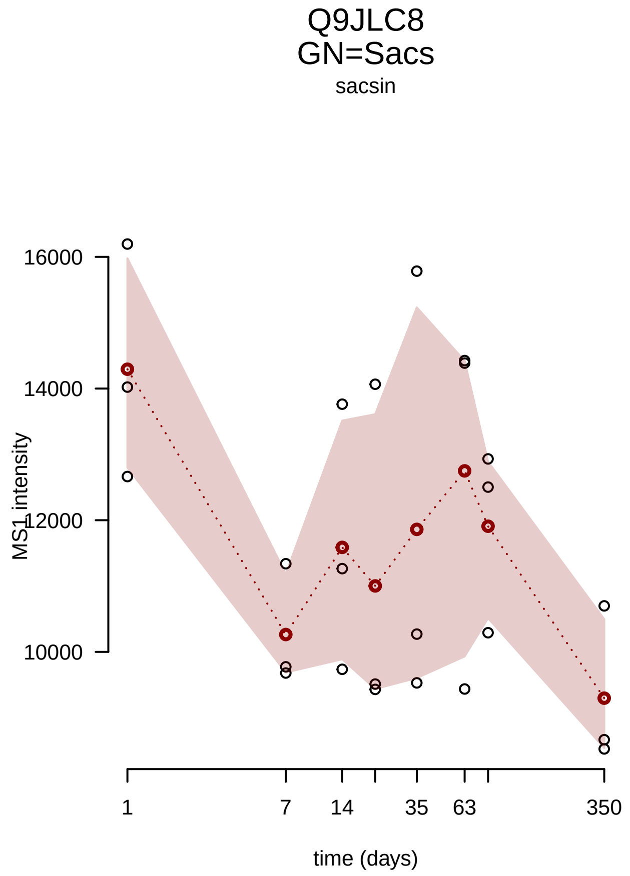
Q9JLC8
GN=Sacs
sacsin
MS1 intensity
time (days)
1
7
14
35
63
350
10000
12000
14000
16000

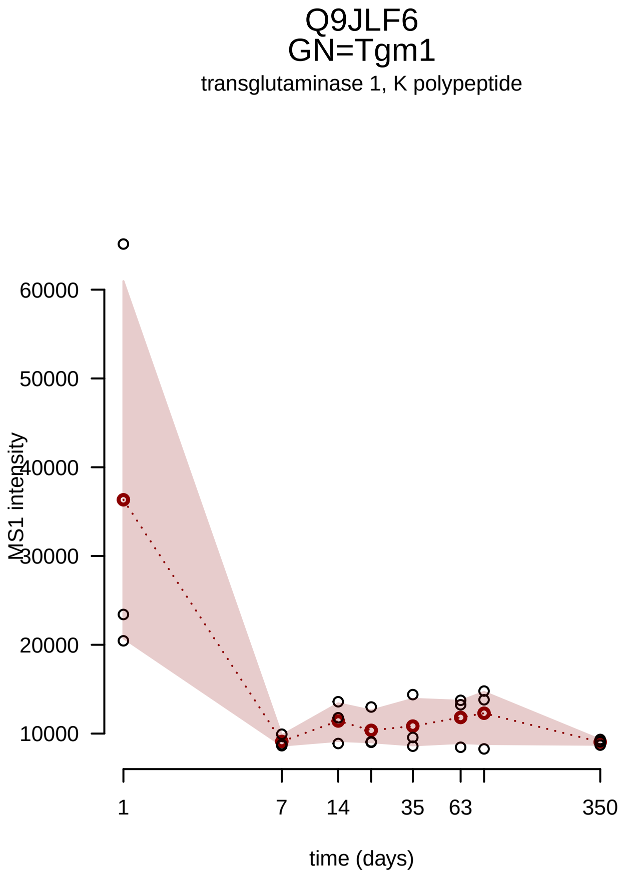
Q9JLF6
GN=Tgm1
transglutaminase 1, K polypeptide
MS1 intensity
time (days)
1
7
14
35
63
350
10000
20000
30000
40000
50000
60000

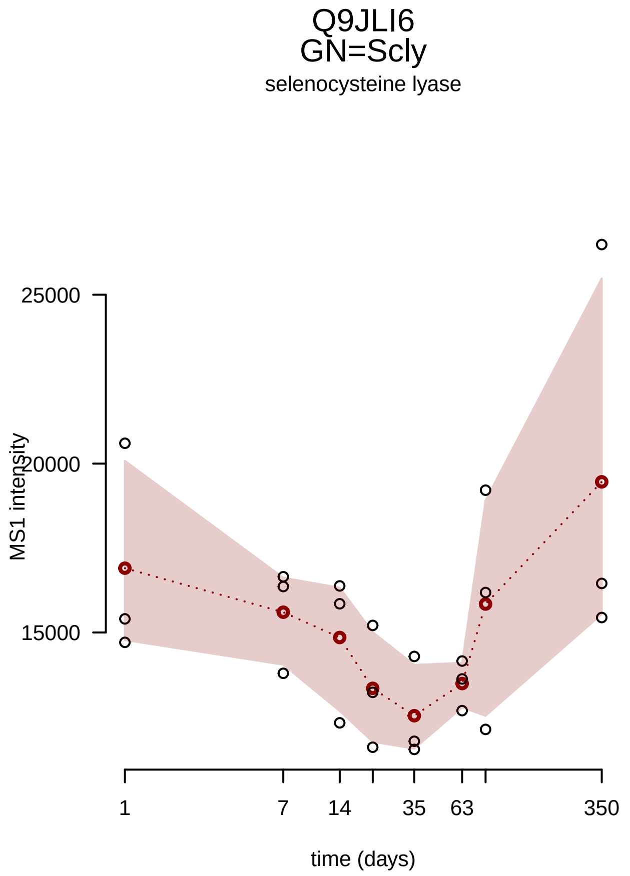
Q9JLI6
GN=Scly
selenocysteine lyase
MS1 intensity
time (days)
1
7
14
35
63
350
15000
20000
25000

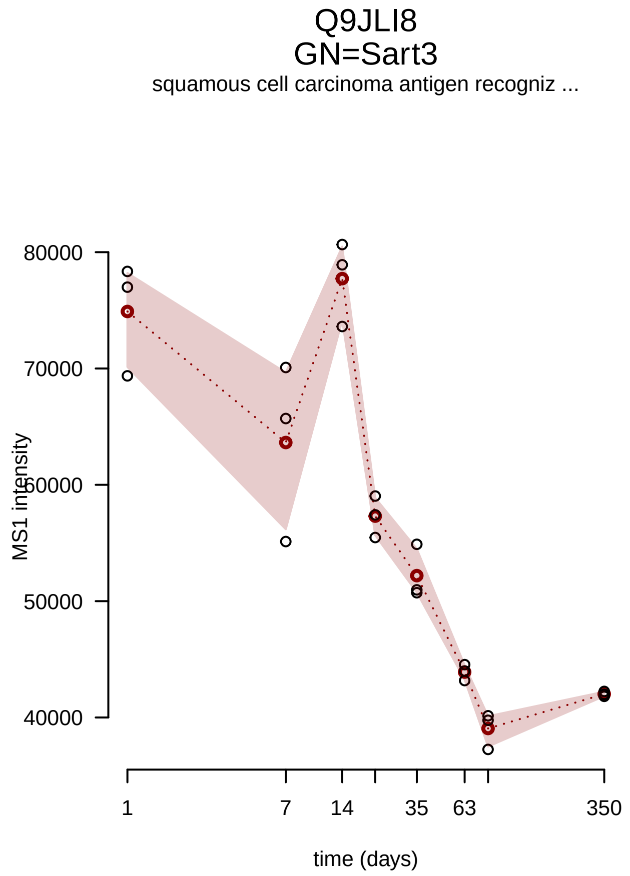
Q9JLI8
GN=Sart3
squamous cell carcinoma antigen recogniz ...
MS1 intensity
time (days)
1
7
14
35
63
350
40000
50000
60000
70000
80000

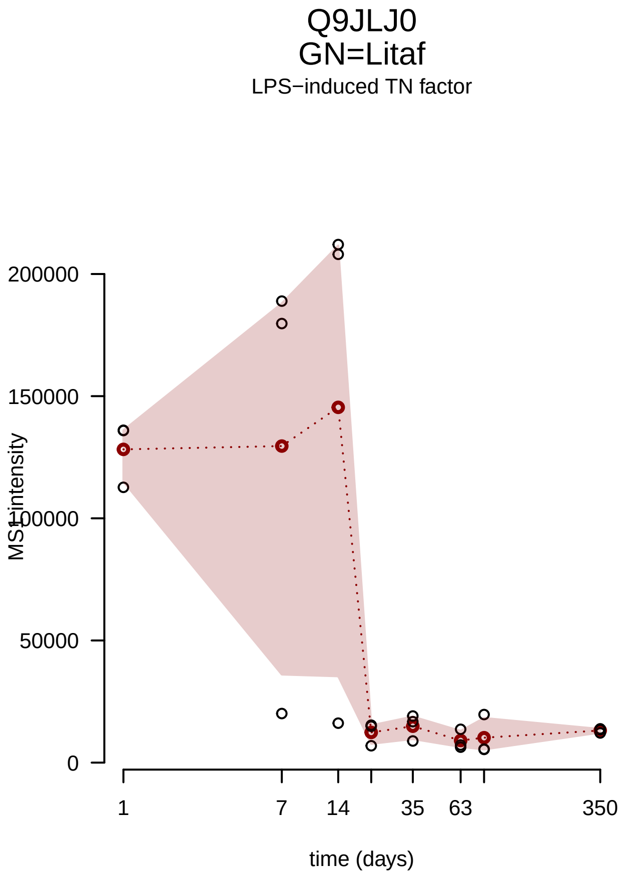
Q9JLJ0
GN=Litaf
LPS−induced TN factor
MS1 intensity
time (days)
1
7
14
35
63
350
0
50000
100000
150000
200000

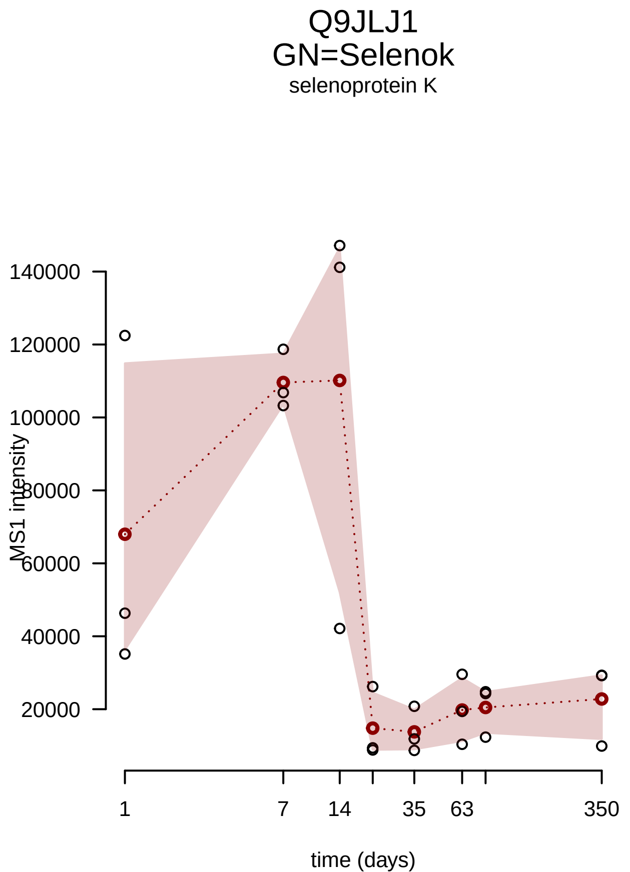
Q9JLJ1
GN=Selenok
selenoprotein K
MS1 intensity
time (days)
1
7
14
35
63
350
20000
40000
60000
80000
100000
120000
140000

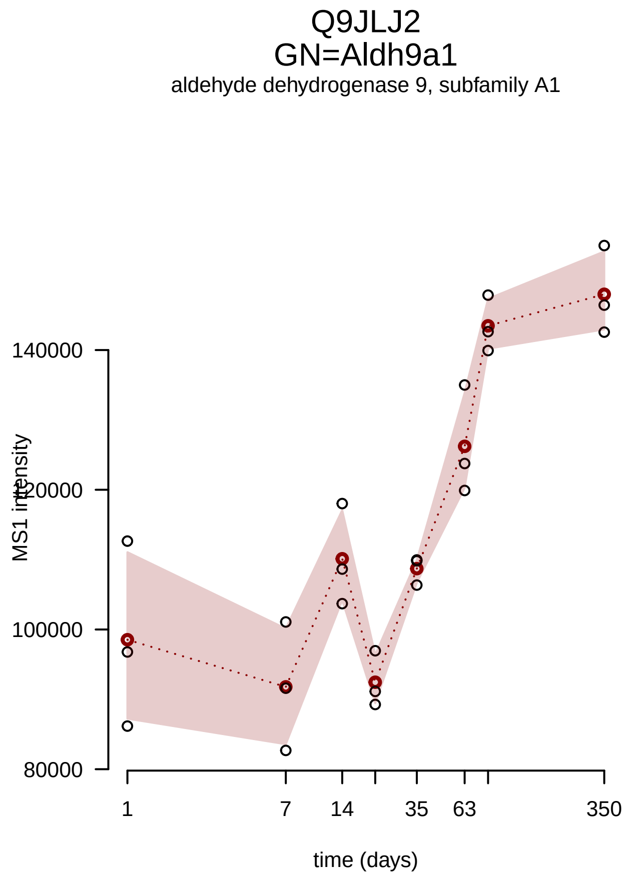
Q9JLJ2
GN=Aldh9a1
aldehyde dehydrogenase 9, subfamily A1
MS1 intensity
time (days)
1
7
14
35
63
350
80000
100000
120000
140000

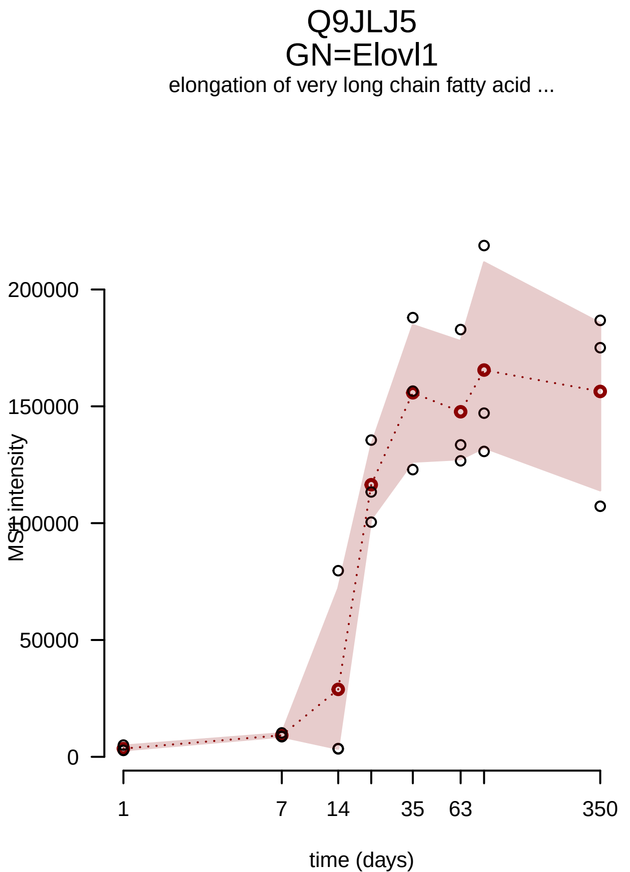
Q9JLJ5
GN=Elovl1
elongation of very long chain fatty acid ...
MS1 intensity
time (days)
1
7
14
35
63
350
0
50000
100000
150000
200000

Q9JLM4
GN=Zmym3
zinc finger, MYM−type 3

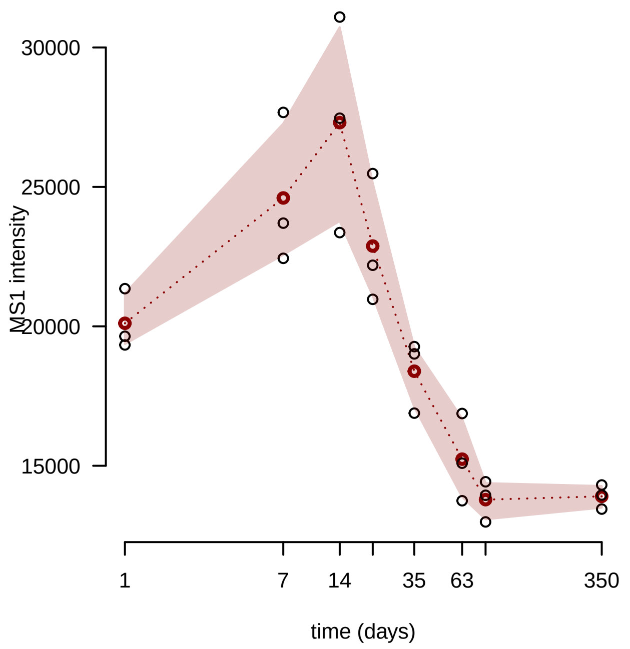

Q9JLM8
GN=Dclk1
doublecortin−like kinase 1

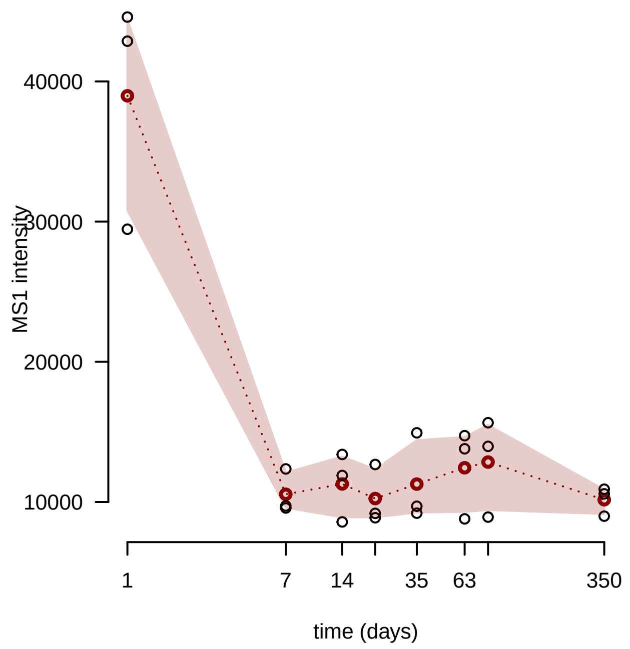

Q9JLM9
GN=Grb14
growth factor receptor bound protein 14

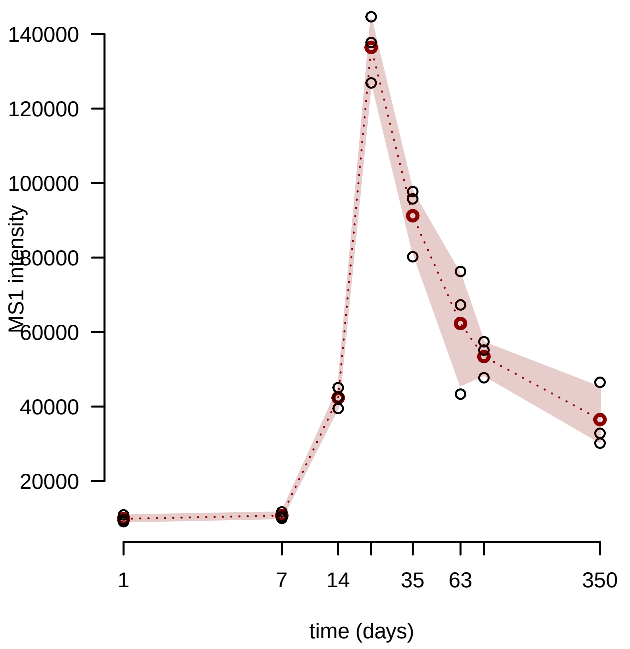

Q9JLN9
GN=Mtor
mechanistic target of rapamycin kinase

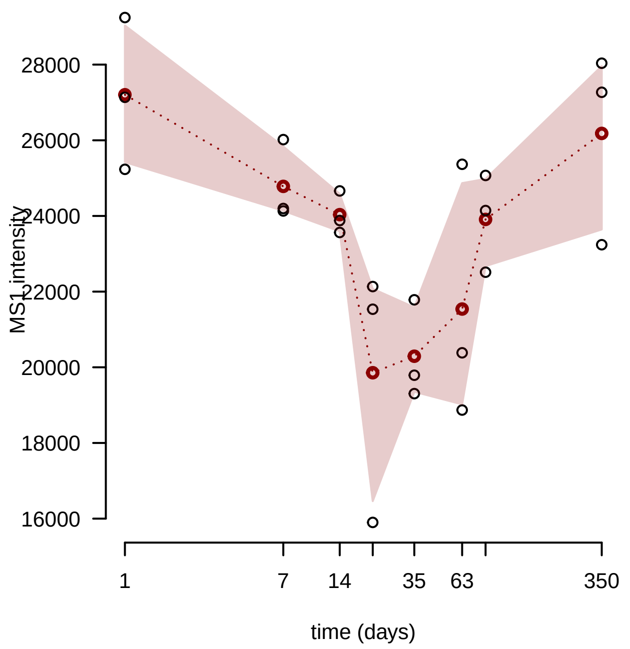

Q9JLQ0
GN=Cd2ap
CD2−associated protein

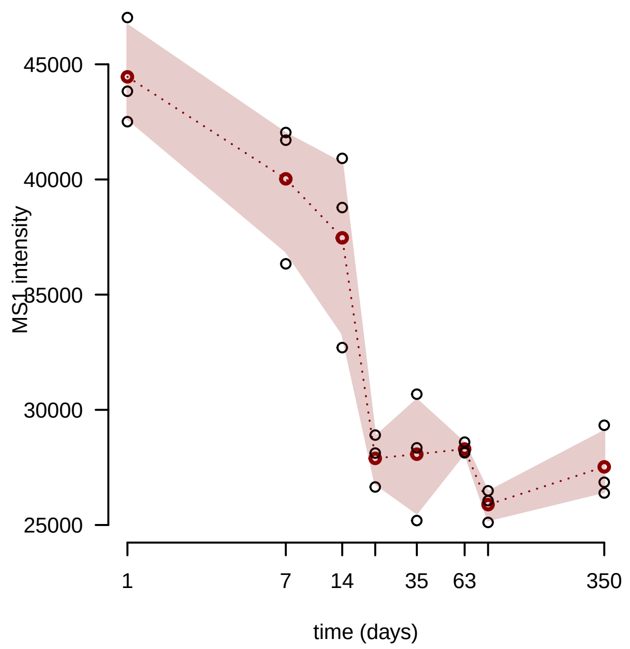

Q9JLQ2
GN=Git2
GIT ArfGAP 2

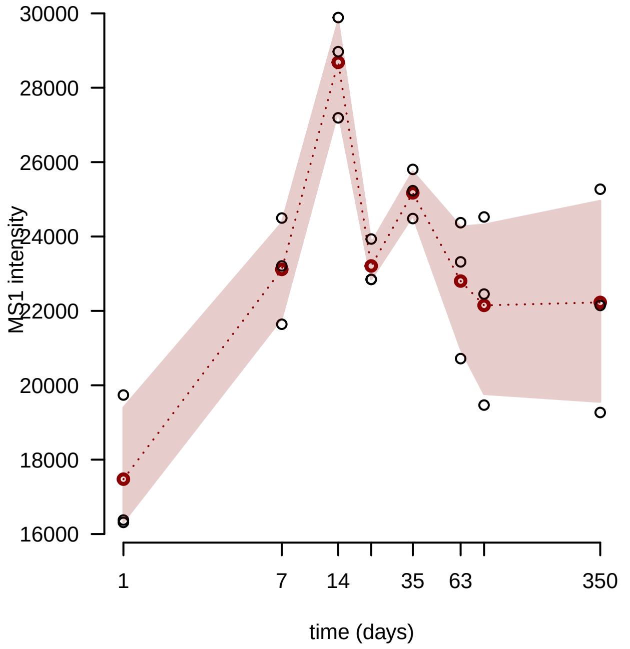

Q9JLR1
GN=Sec61a2
Sec61, alpha subunit 2 (S. cerevisiae)

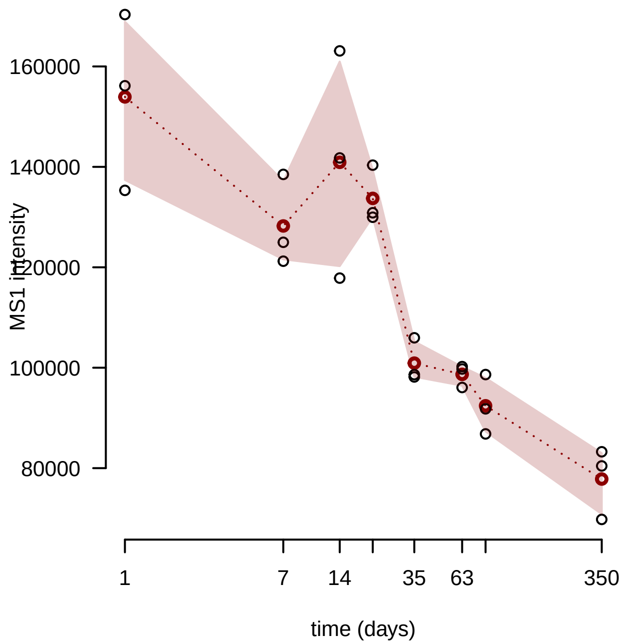

Q9JLR9
GN=Higd1a
HIG1 domain family, member 1A

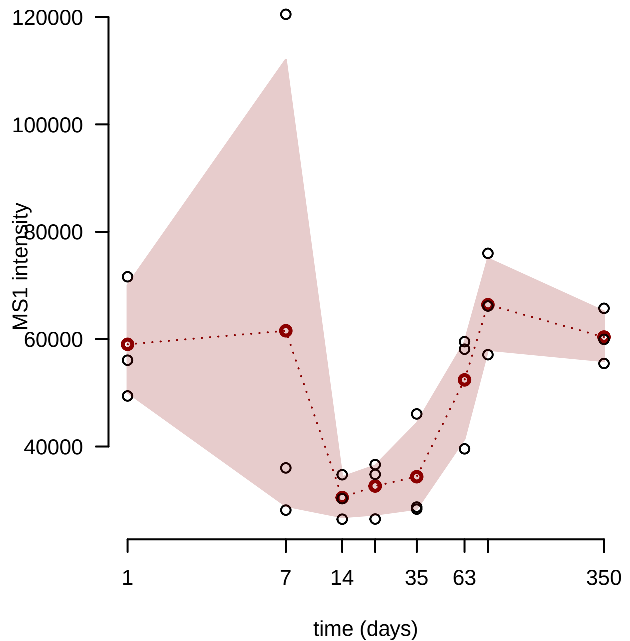

Q9JLT4
GN=Txnrd2
thioredoxin reductase 2

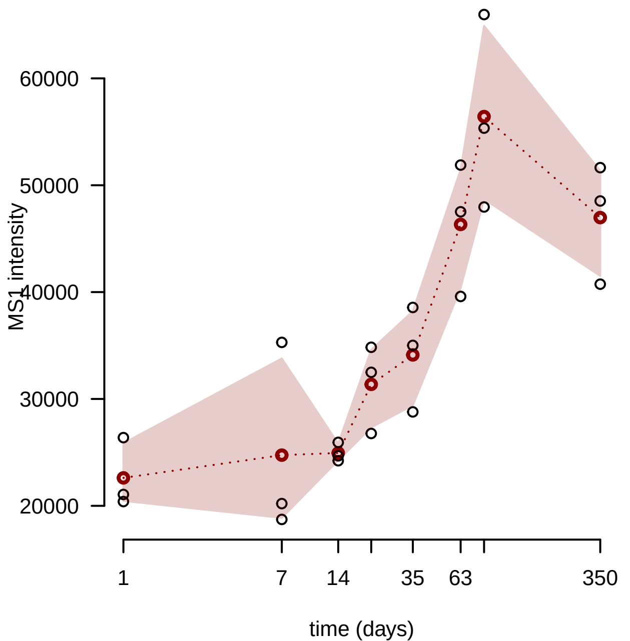

Q9JLV1
GN=Bag3
BCL2−associated athanogene 3

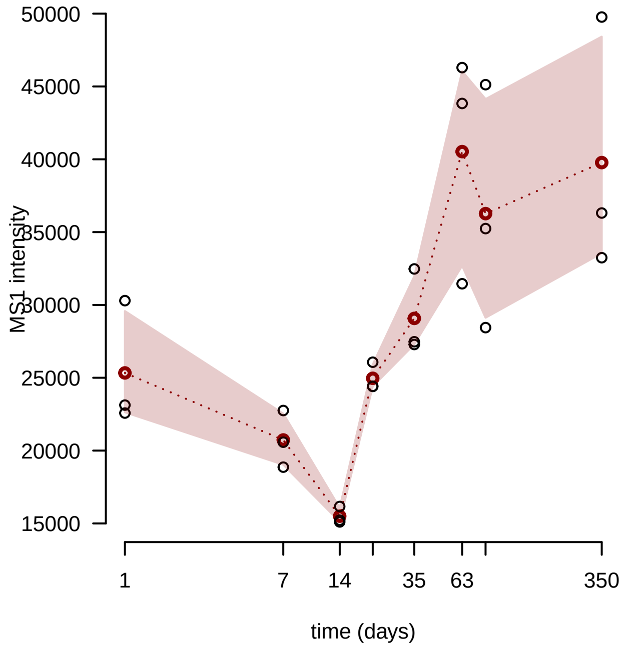

Q9JLV2
GN=Trpc4ap
transient receptor potential cation chan ...

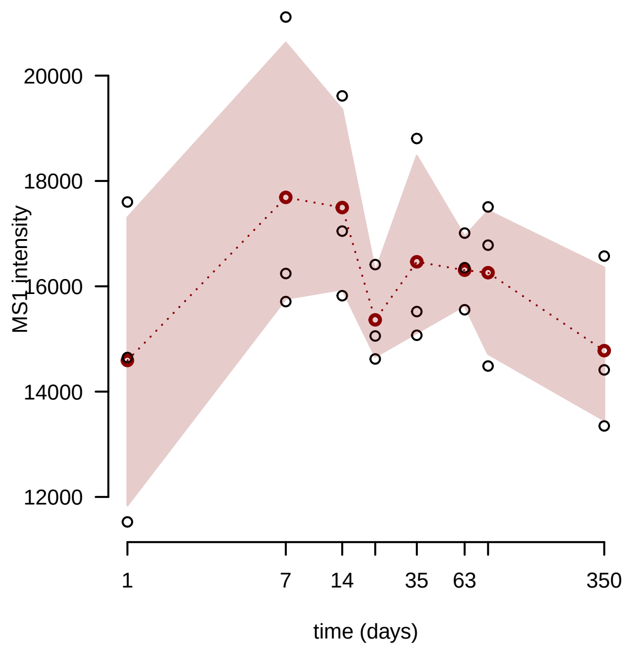

Q9JLV5
GN=Cul3
cullin 3

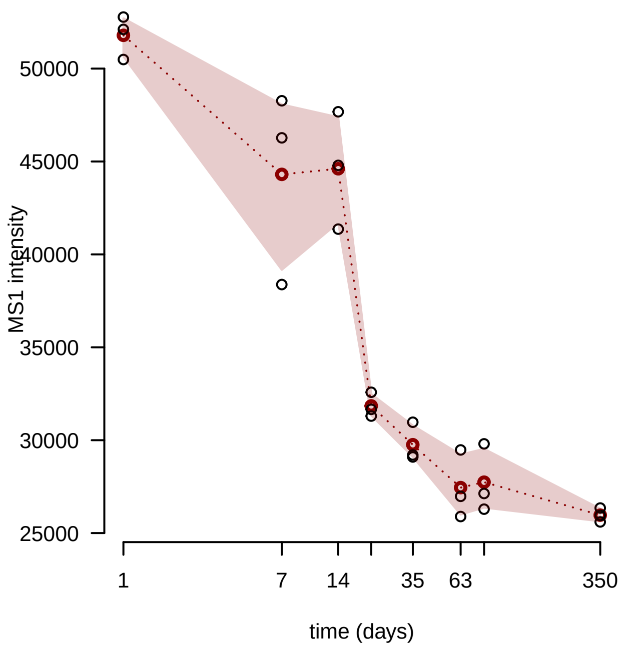

Q9JLV6
GN=Pnkp
polynucleotide kinase 3'− phosphatase

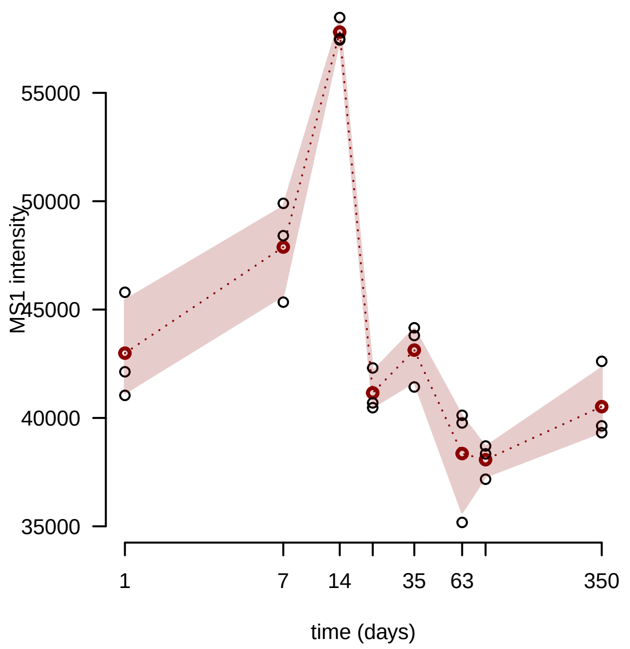

Q9JLZ3
GN=Auh
AU RNA binding protein/enoyl−coenzyme A ...

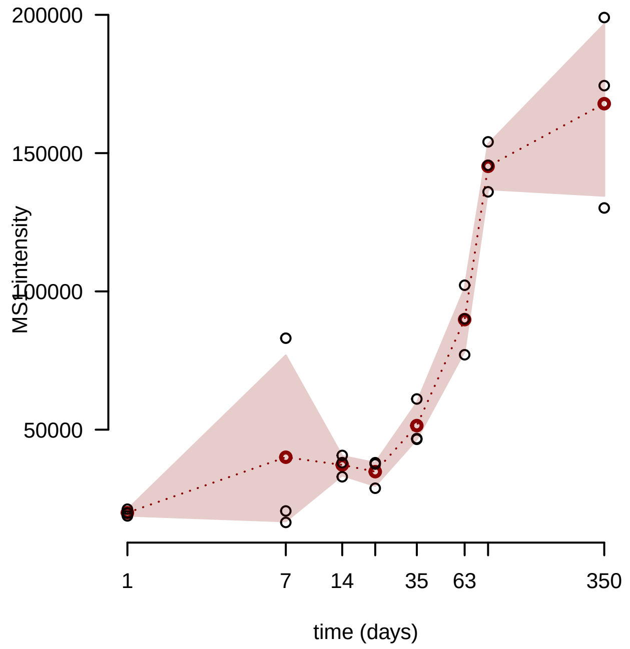

Q9JLZ6
GN=Hic2
hypermethylated in cancer 2

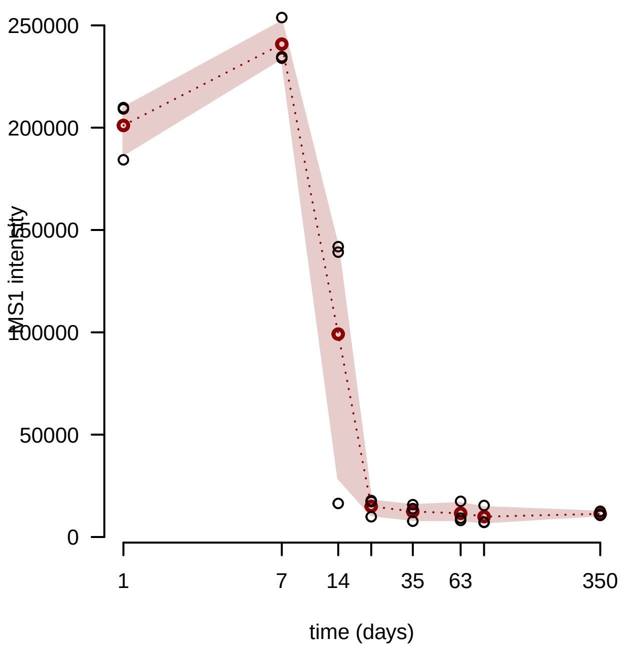

Q9JM05
GN=Pias4
protein inhibitor of activated STAT 4

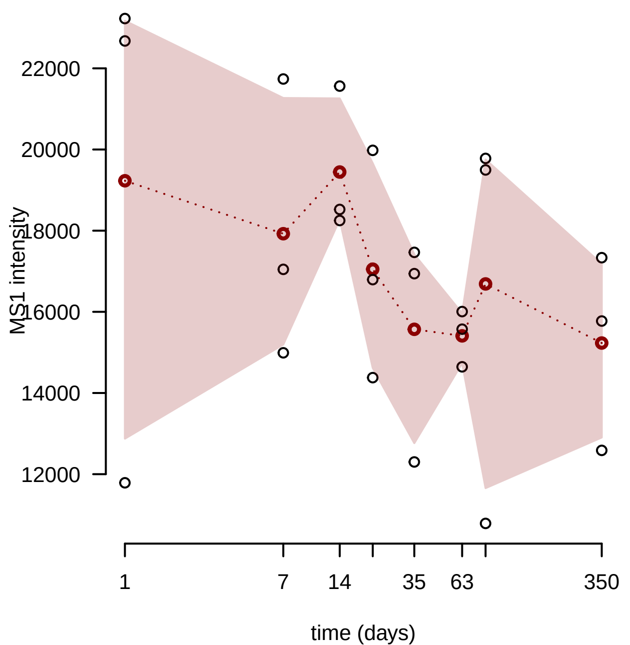

Q9JM13
GN=Rabgef1
RAB guanine nucleotide exchange factor (...

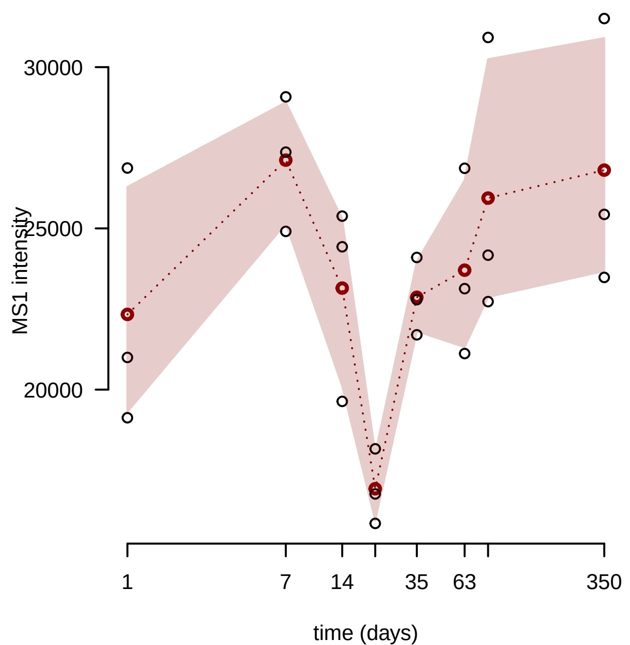

Q9JM14
GN=Nt5c
5',3'−nucleotidase, cytosolic

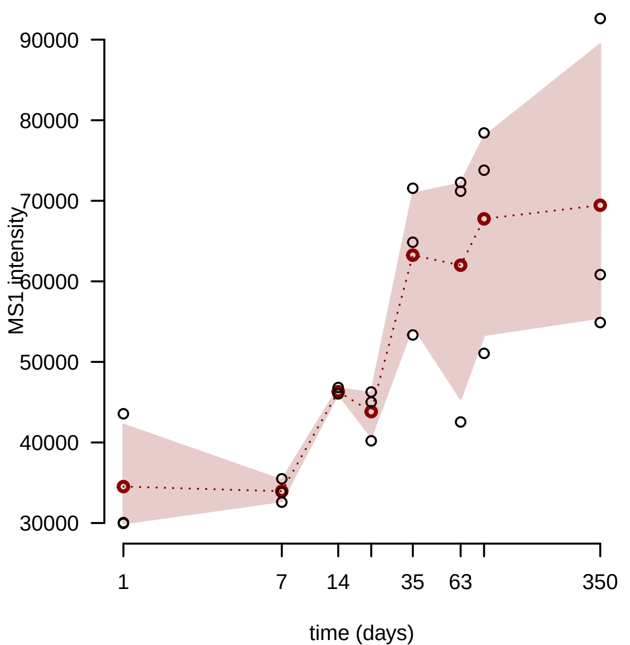

Q9JM51
GN=Ptges
prostaglandin E synthase

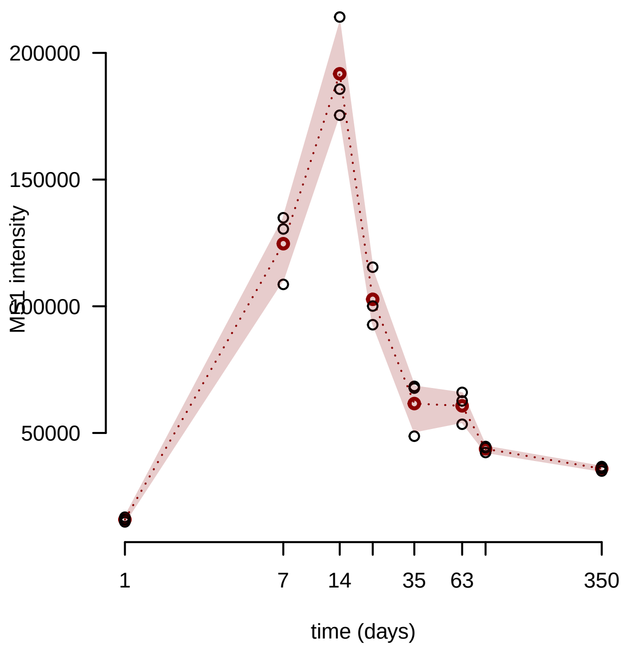

Q9JM52
GN=Mink1
misshapen−like kinase 1 (zebrafish)

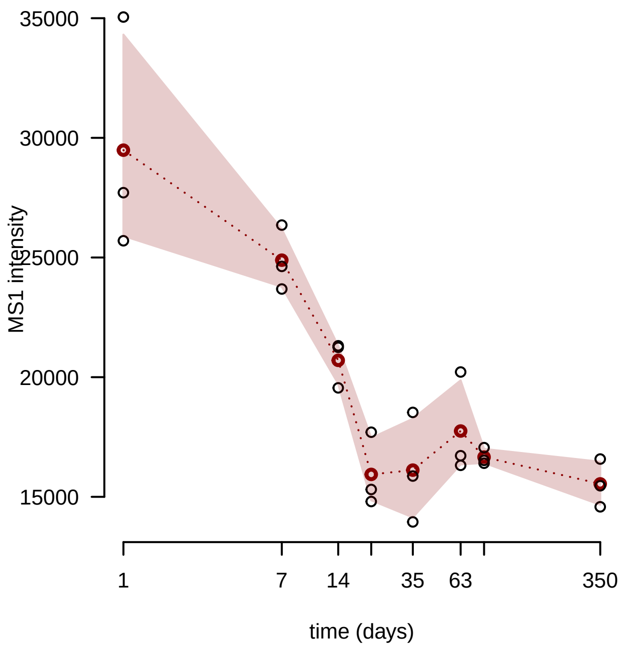

Q9JM62
GN=Reep6
receptor accessory protein 6

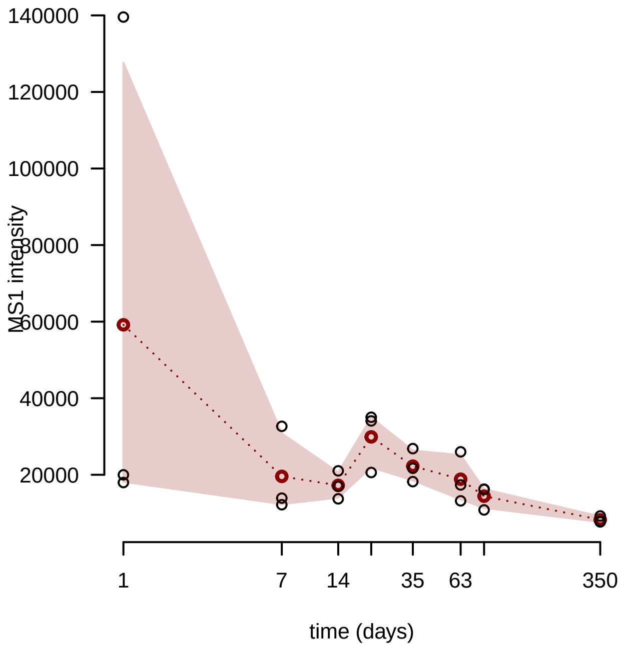

Q9JM76
GN=Arpc3
actin related protein 2/3 complex, subun ...

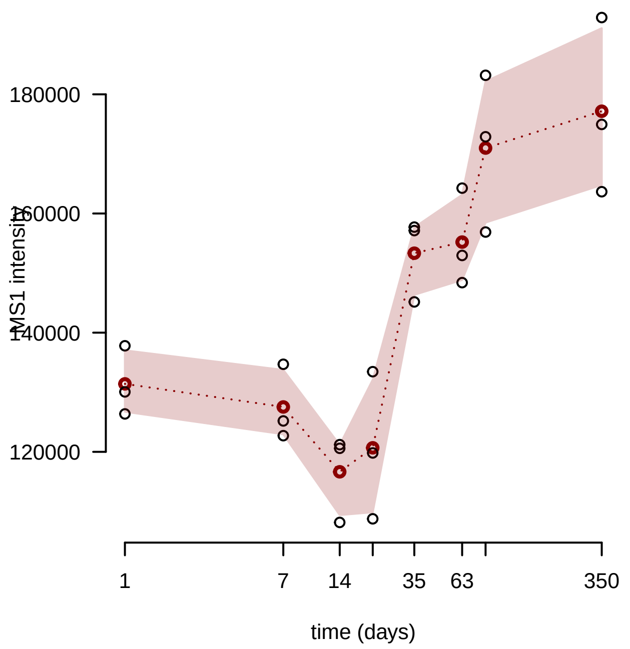

Q9JM93
GN=Arl6ip4
ADP−ribosylation factor−like 6 interacti ...

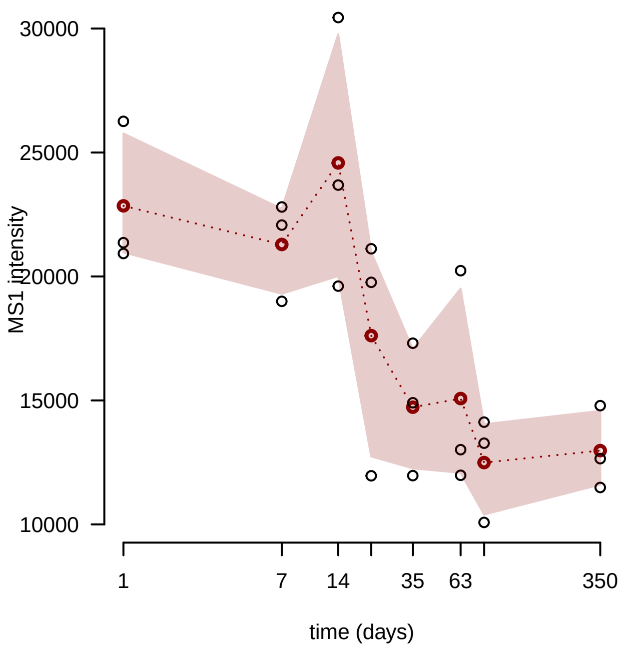

Q9JM96
GN=Cdc42ep4
CDC42 effector protein (Rho GTPase bindi ...

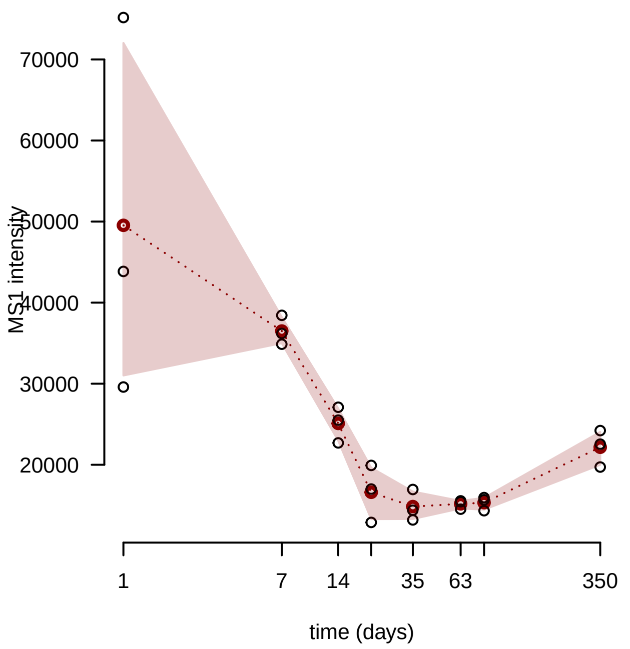

Q9JM98
GN=Wrap73
WD repeat containing, antisense to Trp73

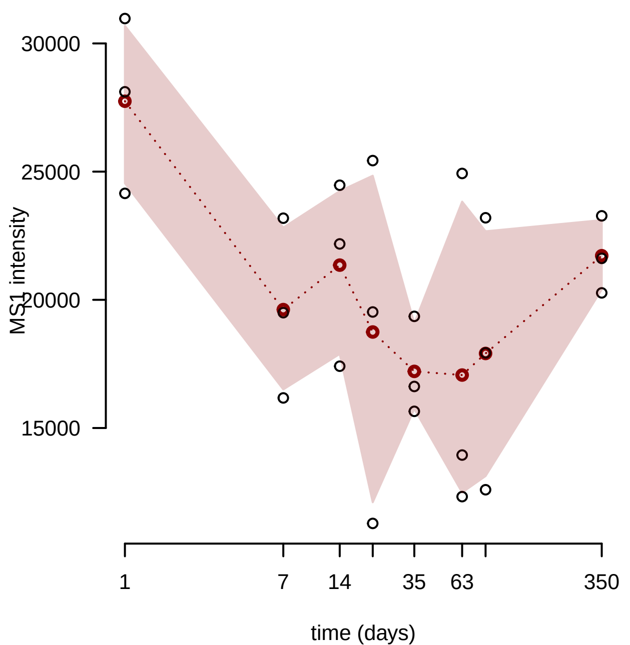

Q9JMA1
GN=Usp14
ubiquitin specific peptidase 14

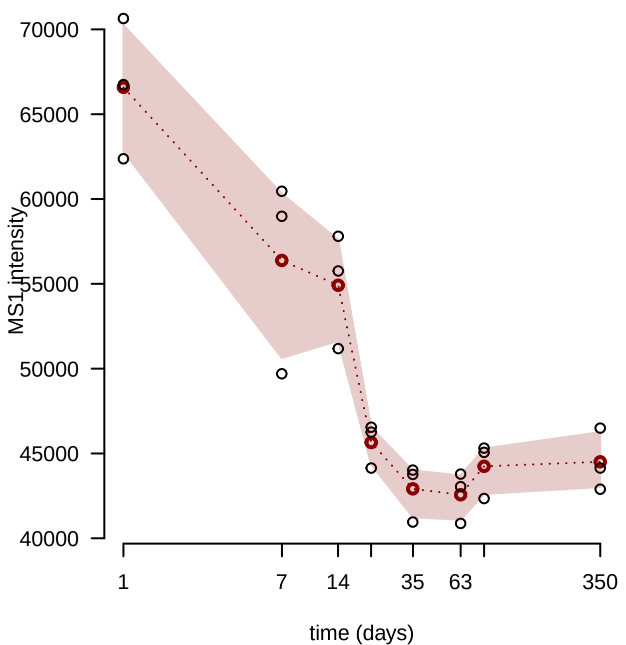

Q9JMA2
GN=Qtrt1
queuine tRNA−ribosyltransferase catalyti ...

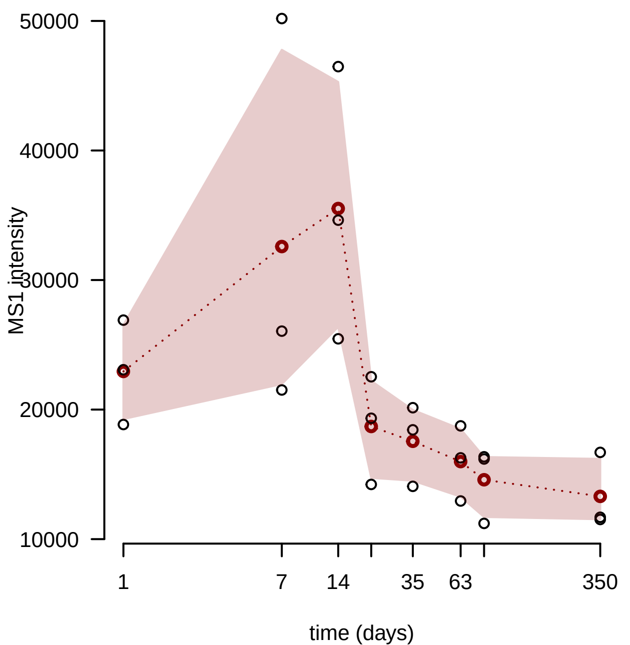

Q9JMB0
GN=Gkap1
G kinase anchoring protein 1

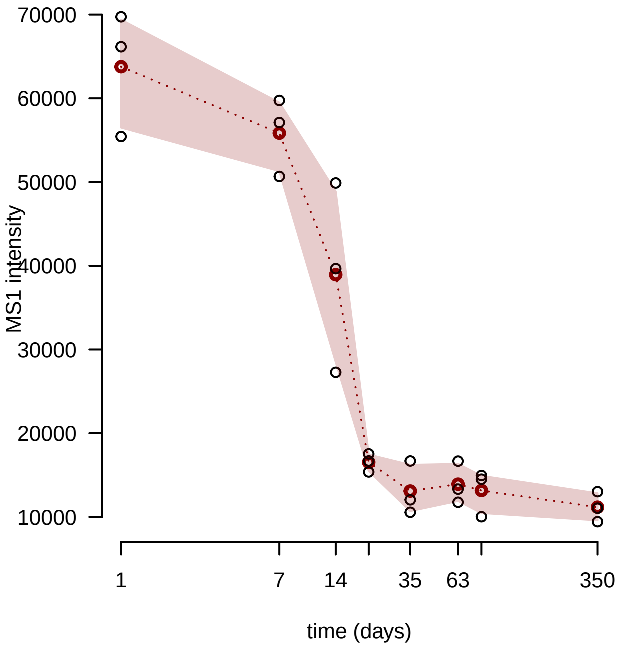

Q9JMC3
GN=Dnaja4
DnaJ heat shock protein family (Hsp40) m ...

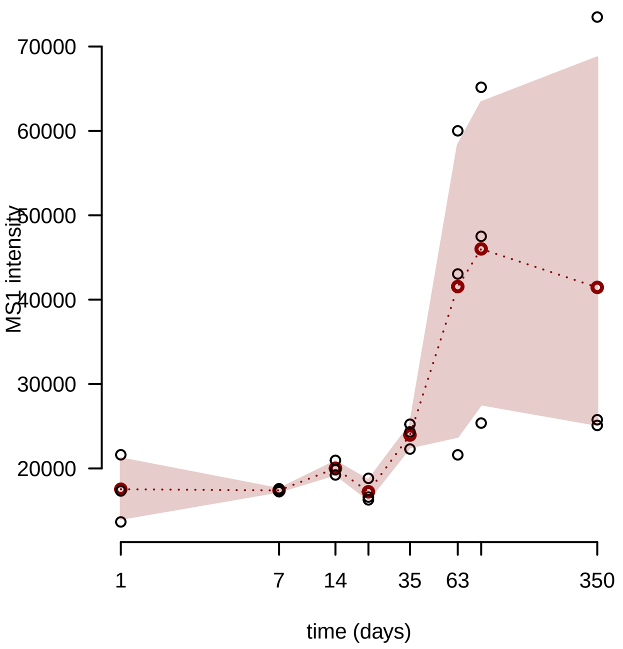

Q9JMC8
GN=Epb41l4b
erythrocyte membrane protein band 4.1 li ...

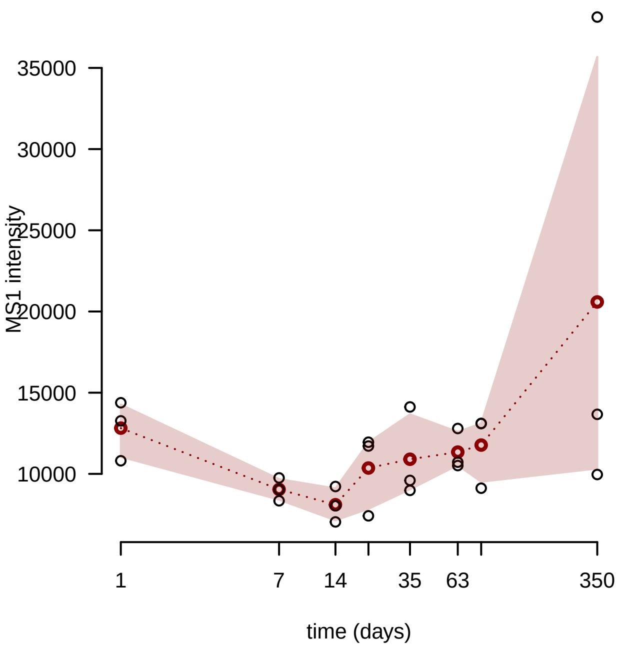

Q9JMD0
GN=Znf207

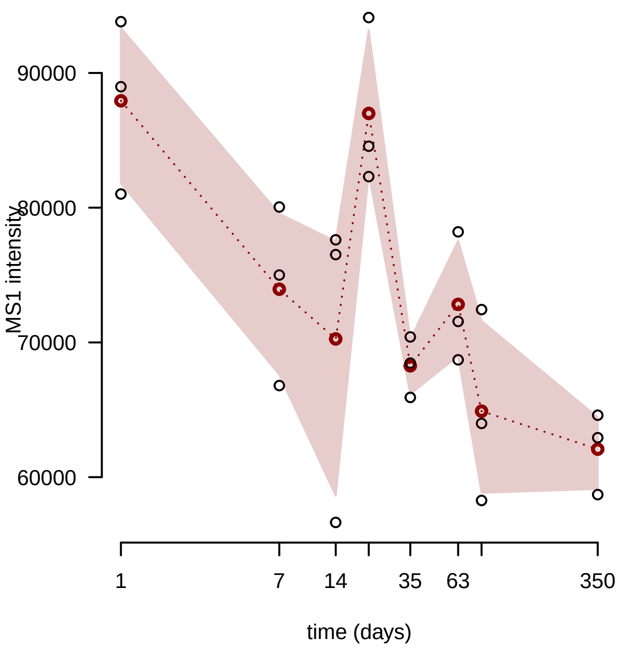

Q9JMD3
GN=Stard10
START domain containing 10

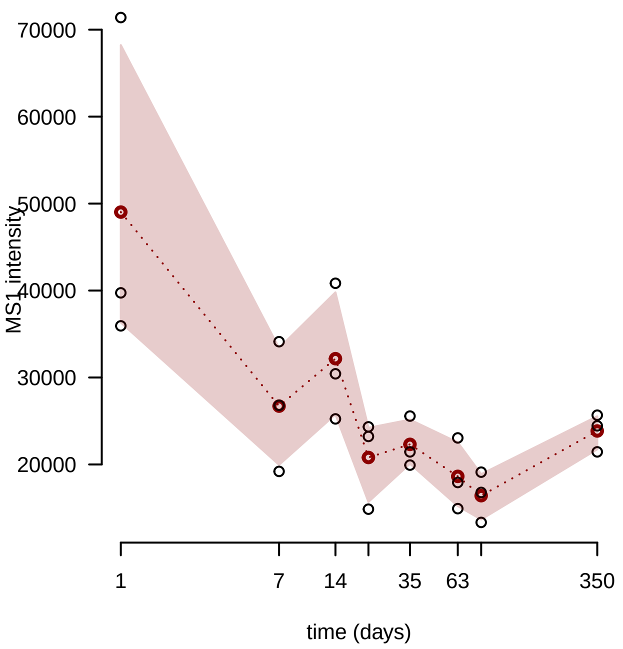

Q9JME2
GN=Chst11
carbohydrate sulfotransferase 11

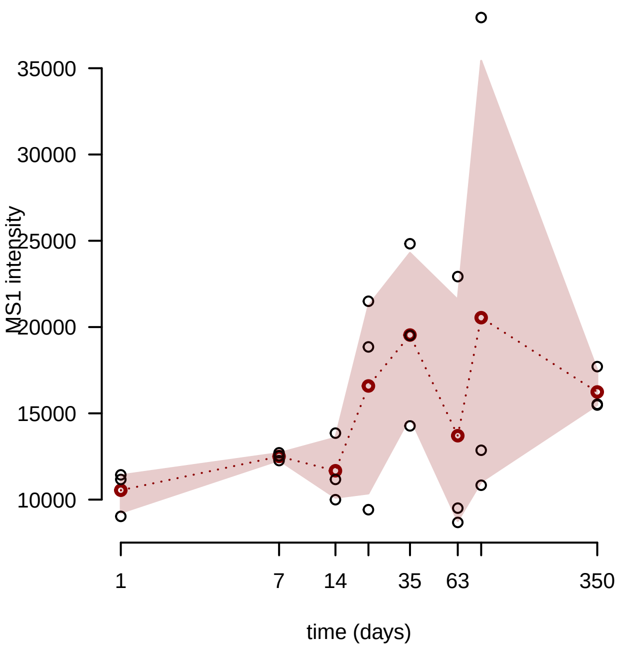

Q9JME7
GN=Trappc2l
trafficking protein particle complex 2L

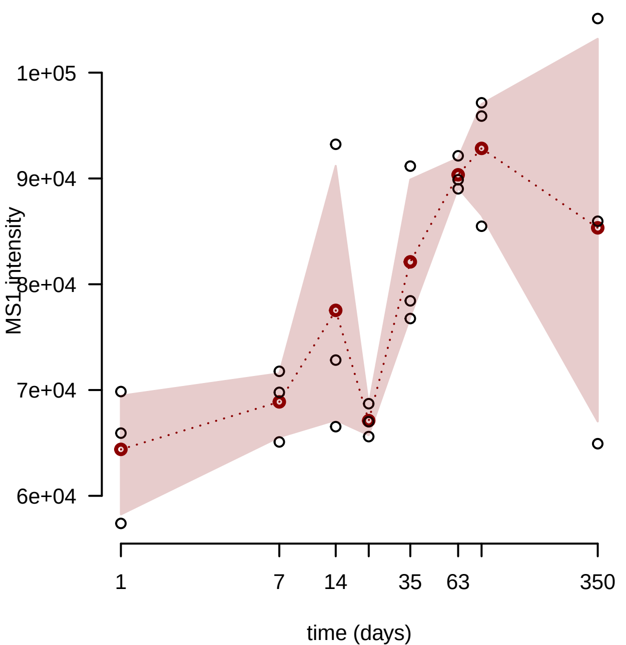

Q9JMF7
GN=Dolpp1
dolichyl pyrophosphate phosphatase 1

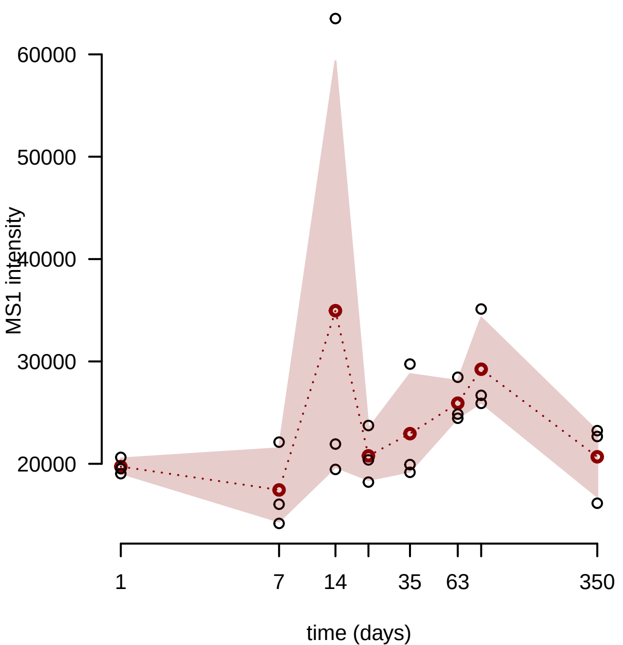

Q9JMG1
GN=Edf1
endothelial differentiation−related fact ...

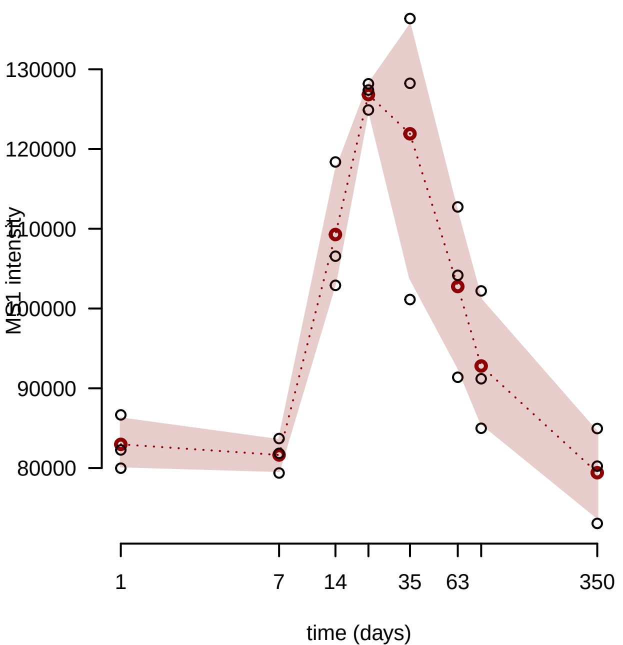

Q9JMG2
GN=C1galt1c1
C1GALT1−specific chaperone 1

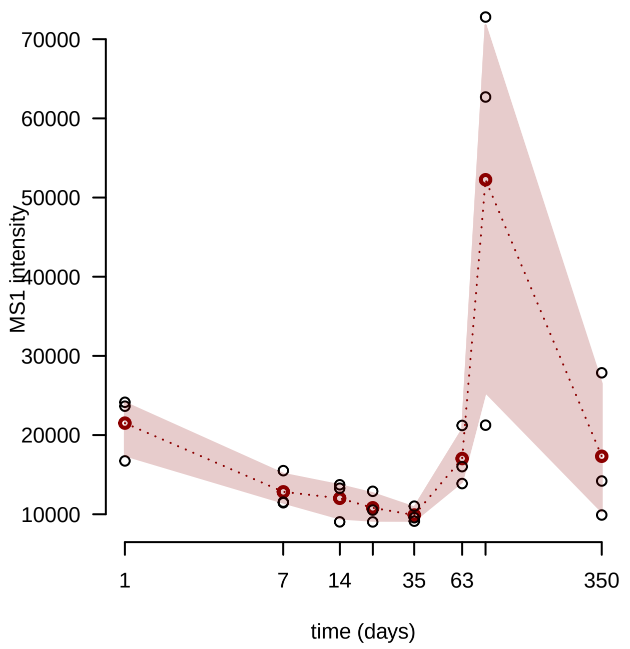

Q9JMG3
GN=Tmub1
transmembrane and ubiquitin−like domain ...

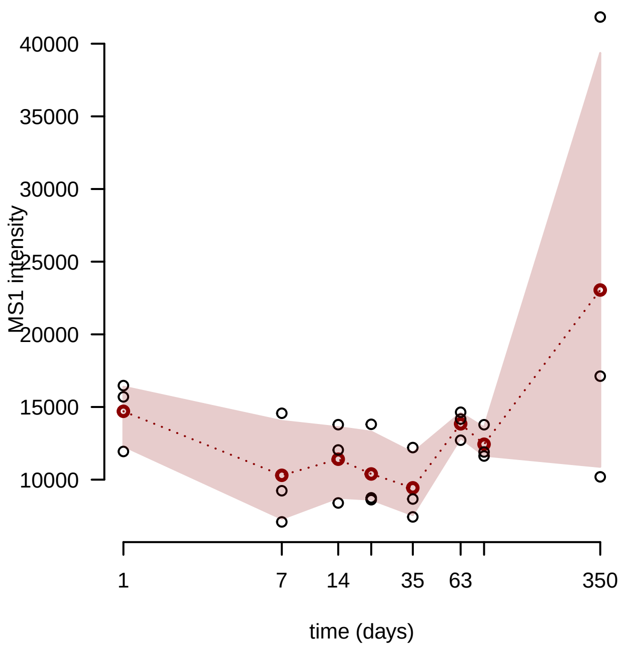

Q9JMG7
GN=Hdgfl3
HDGF like 3

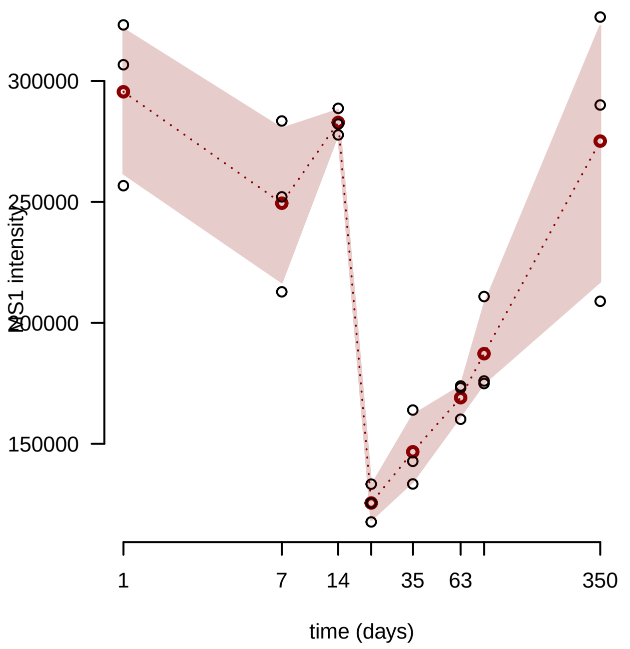

Q9JMH6
GN=Txnrd1
thioredoxin reductase 1

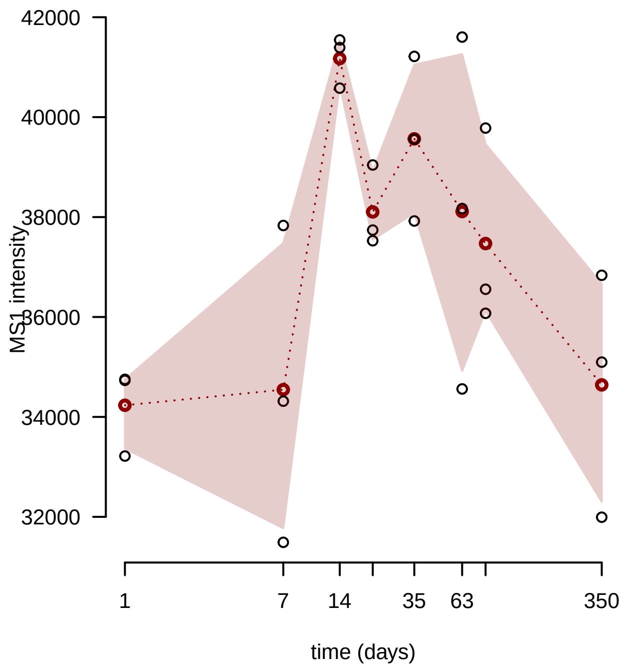

Q9JMH7
GN=Neu3
neuraminidase 3

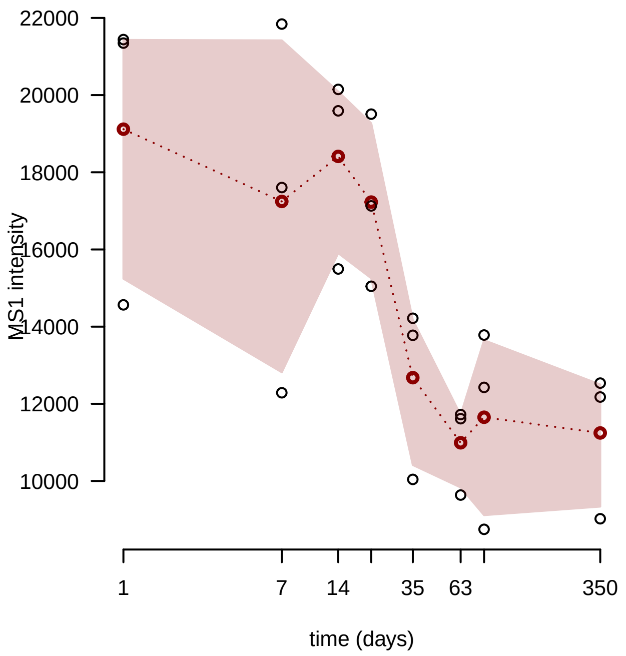

Q9JMH9
GN=Myo18a
myosin XVIIIA

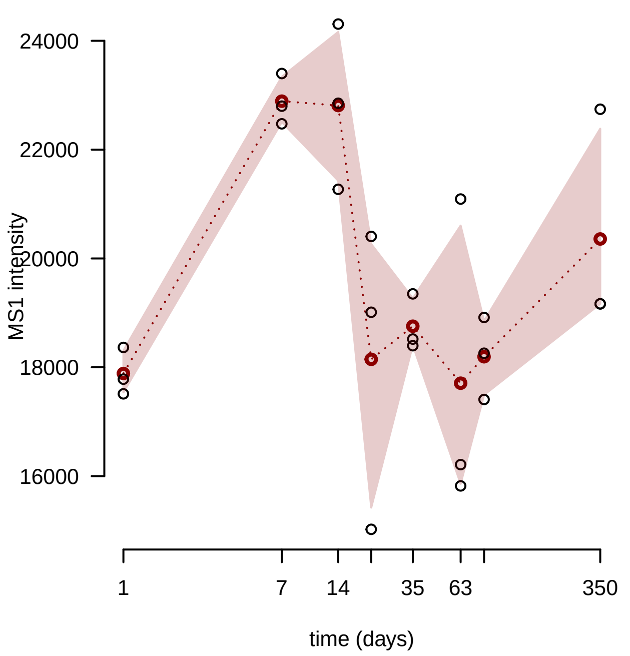

Q9JMI7
GN=Tex101
testis expressed gene 101

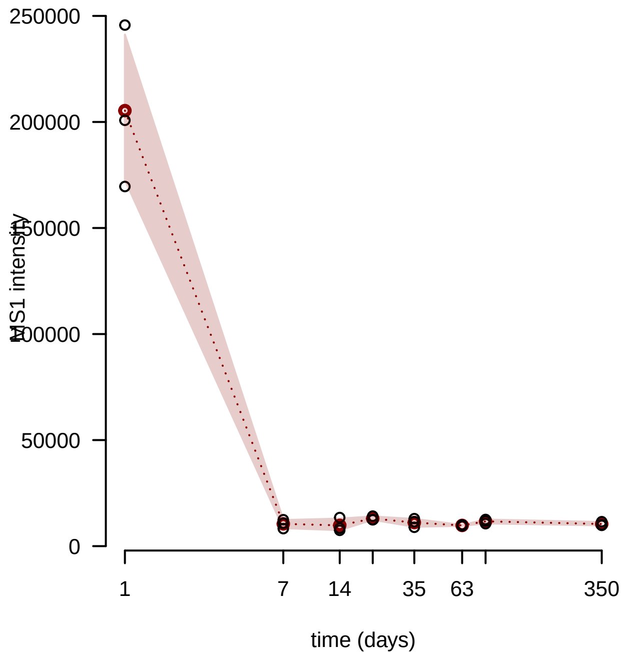

Q9JMK2
GN=Csnk1e
casein kinase 1, epsilon

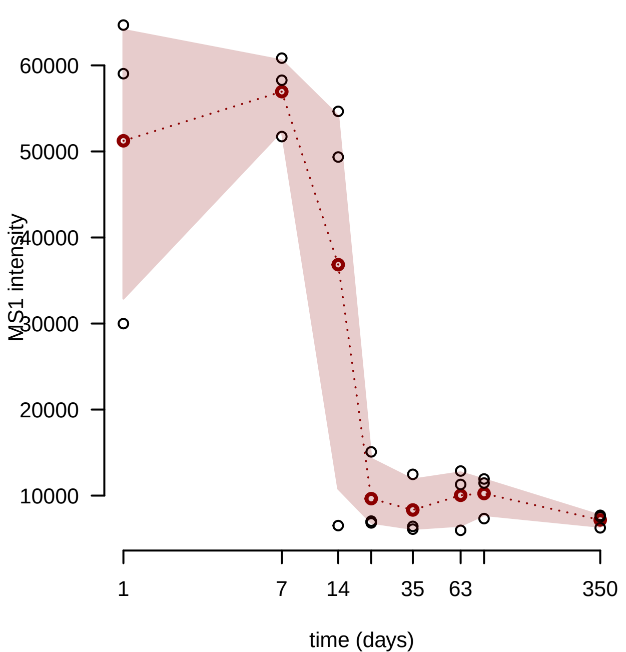

Q9NWG9
GN=Mageh1
MAGE family member H1

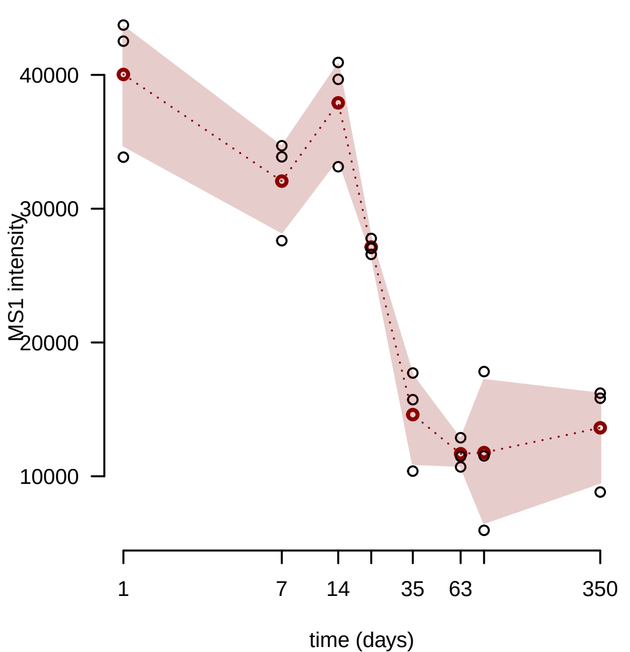

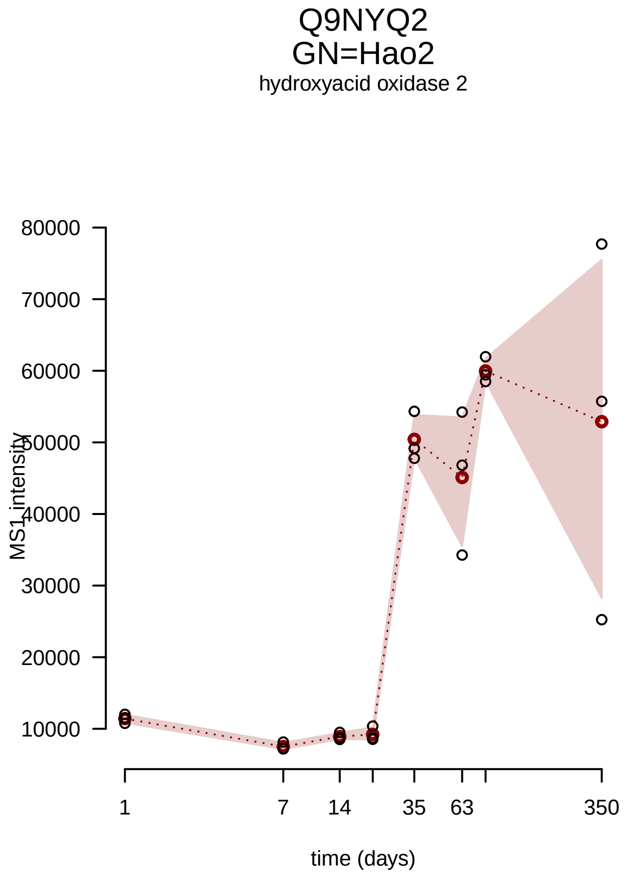
Q9NYQ2
GN=Hao2
hydroxyacid oxidase 2
MS1 intensity
10000
20000
30000
40000
50000
60000
70000
80000
1
7
14
35
63
350
time (days)

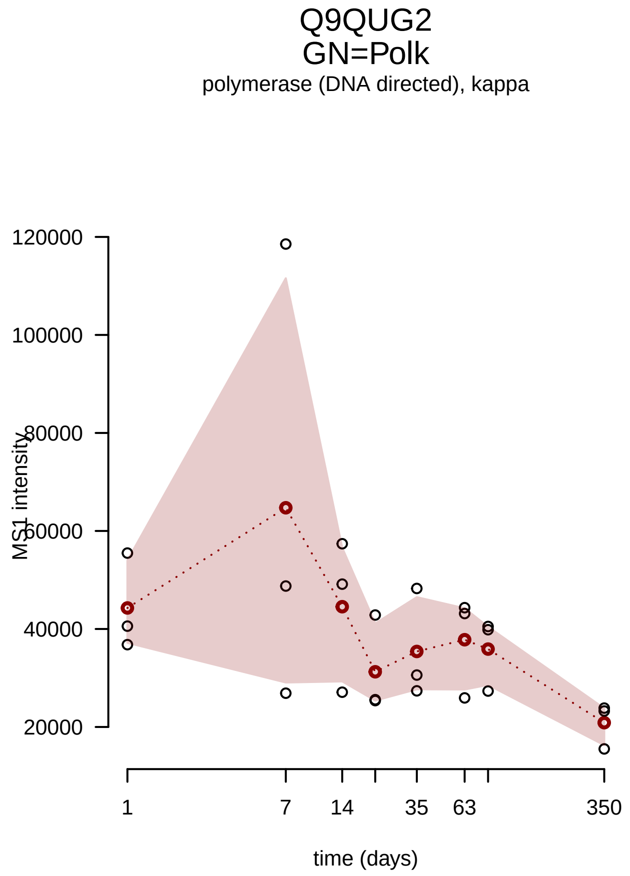
Q9QUG2
GN=Polk
polymerase (DNA directed), kappa
MS1 intensity
20000
40000
60000
80000
100000
120000
1
7
14
35
63
350
time (days)

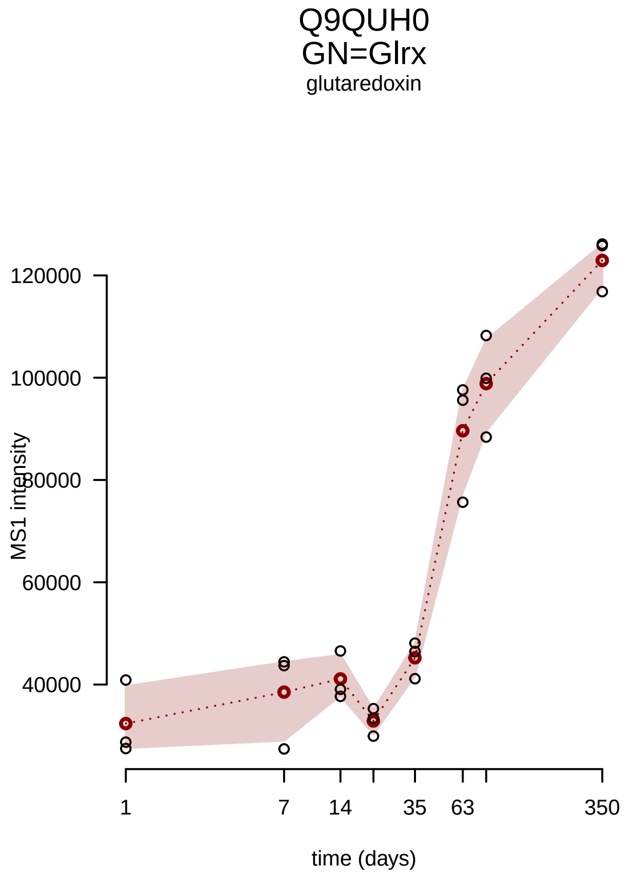
Q9QUH0
GN=Glrx
glutaredoxin
MS1 intensity
40000
60000
80000
100000
120000
1
7
14
35
63
350
time (days)

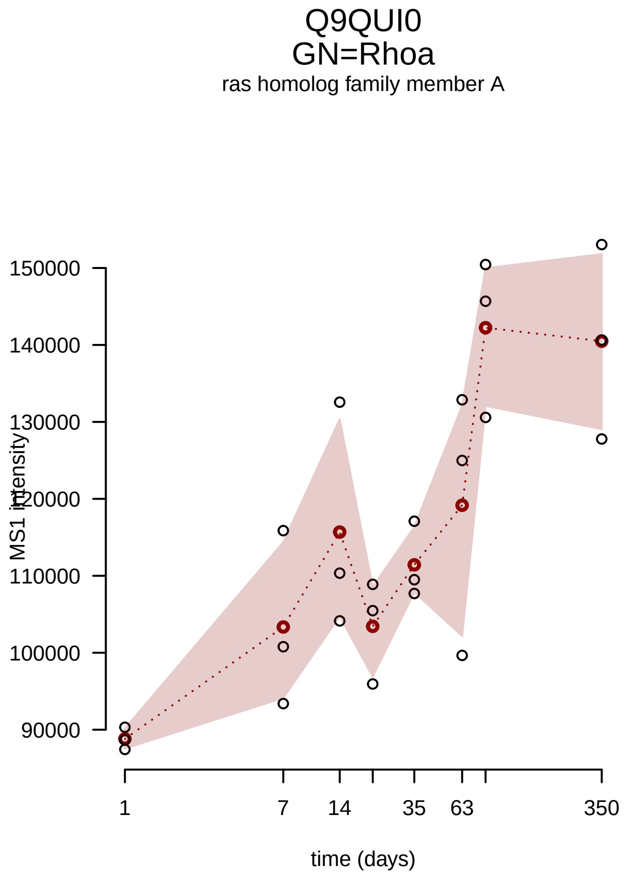
Q9QUI0
GN=Rhoa
ras homolog family member A
MS1 intensity
90000
100000
110000
120000
130000
140000
150000
1
7
14
35
63
350
time (days)

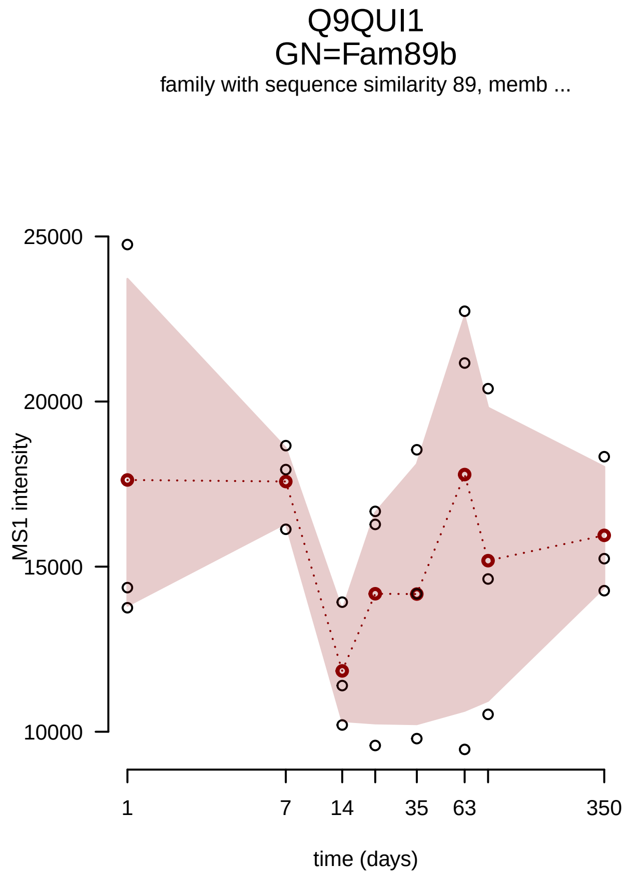
Q9QUI1
GN=Fam89b
family with sequence similarity 89, memb ...
MS1 intensity
10000
15000
20000
25000
1
7
14
35
63
350
time (days)

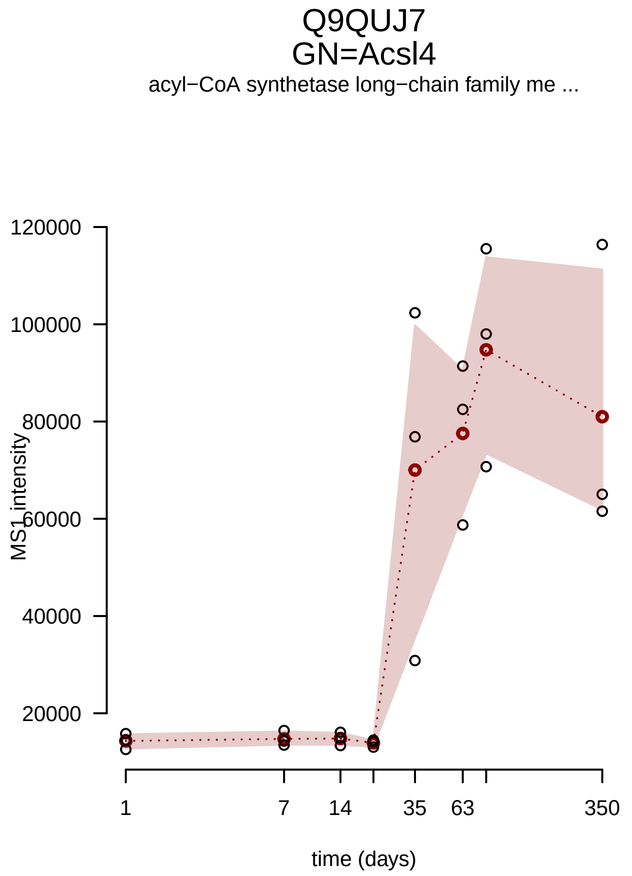
Q9QUJ7
GN=Acsl4
acyl−CoA synthetase long−chain family me ...
MS1 intensity
20000
40000
60000
80000
100000
120000
1
7
14
35
63
350
time (days)

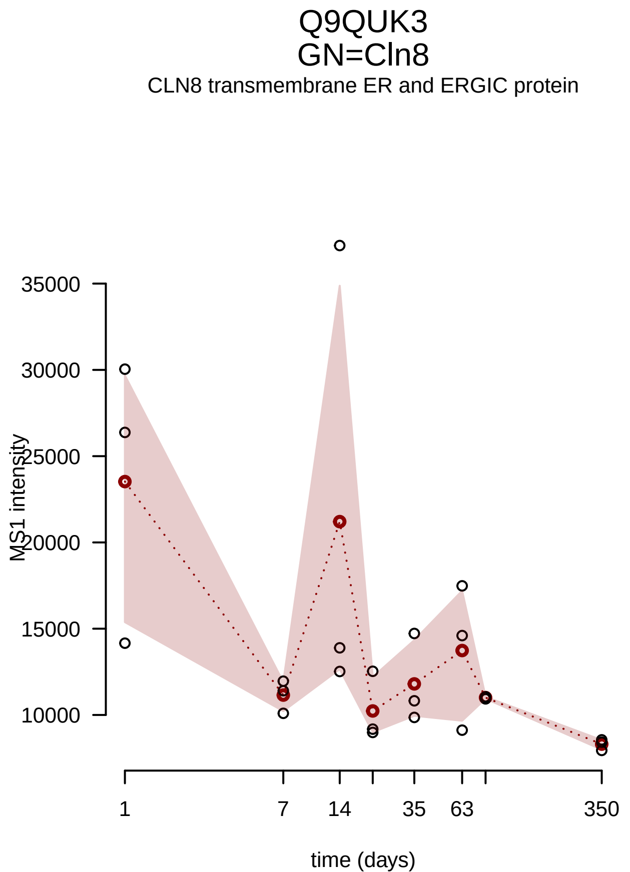
Q9QUK3
GN=Cln8
CLN8 transmembrane ER and ERGIC protein
MS1 intensity
10000
15000
20000
25000
30000
35000
1
7
14
35
63
350
time (days)

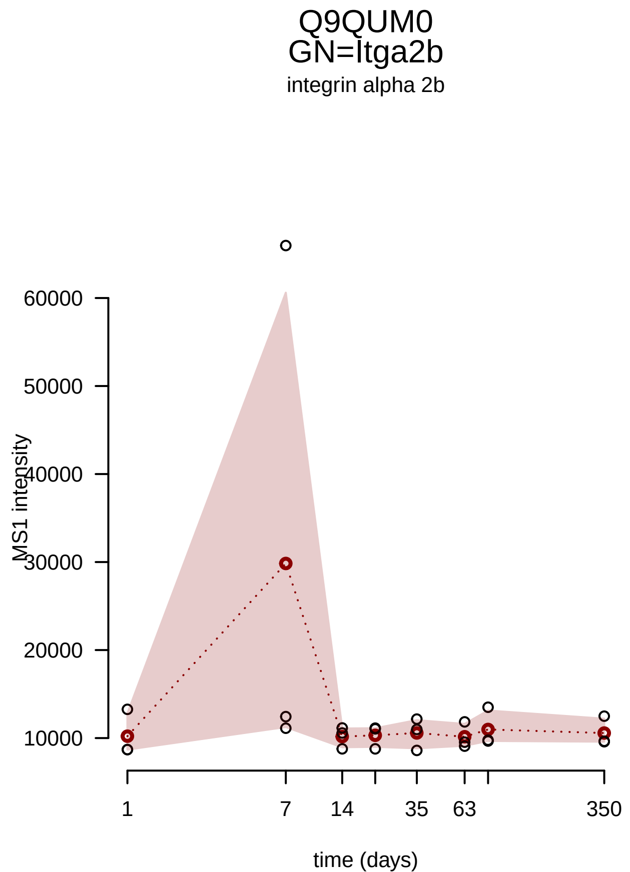
Q9QUM0
GN=Itga2b
integrin alpha 2b
MS1 intensity
10000
20000
30000
40000
50000
60000
1
7
14
35
63
350
time (days)

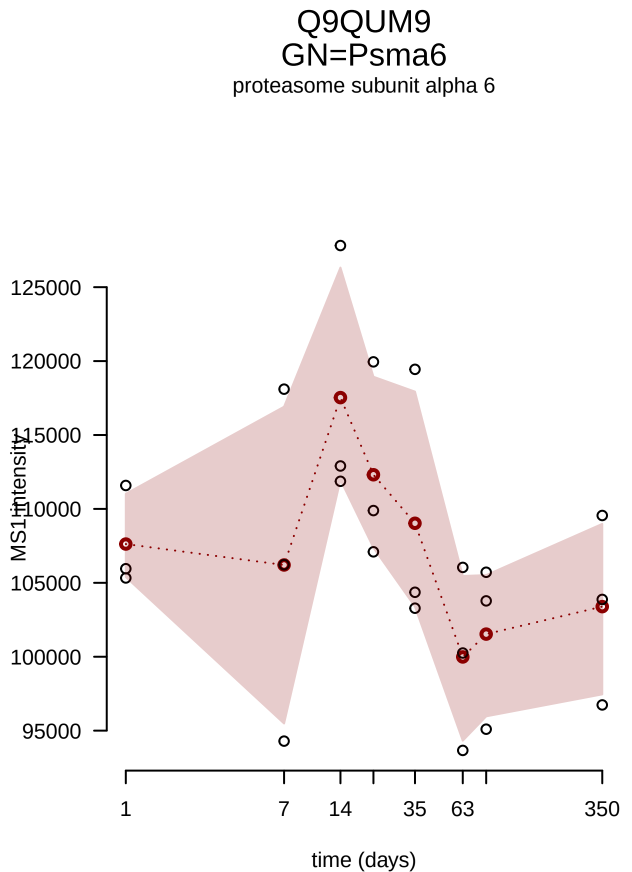
Q9QUM9
GN=Psma6
proteasome subunit alpha 6
MS1 intensity
95000
100000
105000
110000
115000
120000
125000
1
7
14
35
63
350
time (days)

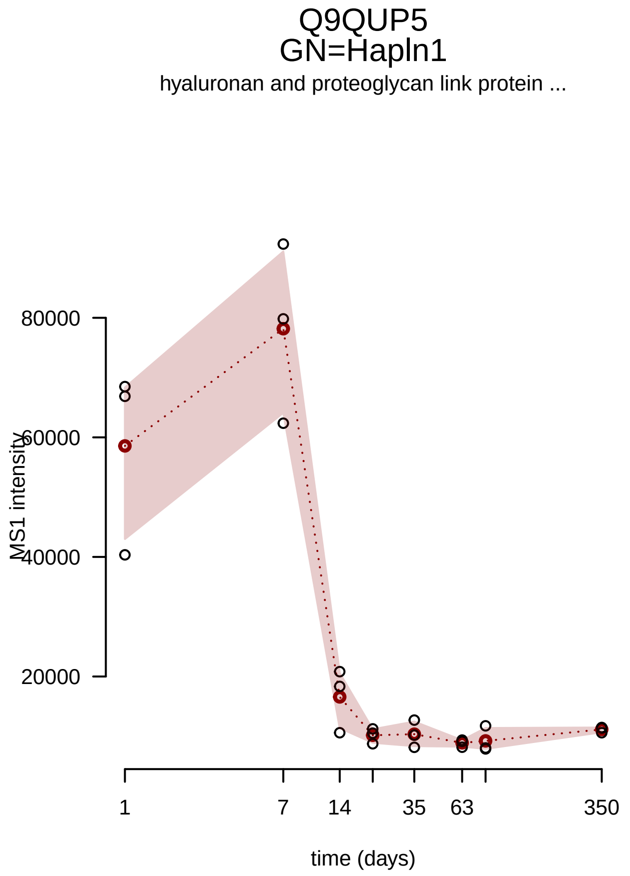
Q9QUP5
GN=Hapln1
hyaluronan and proteoglycan link protein ...
MS1 intensity
20000
40000
60000
80000
1
7
14
35
63
350
time (days)

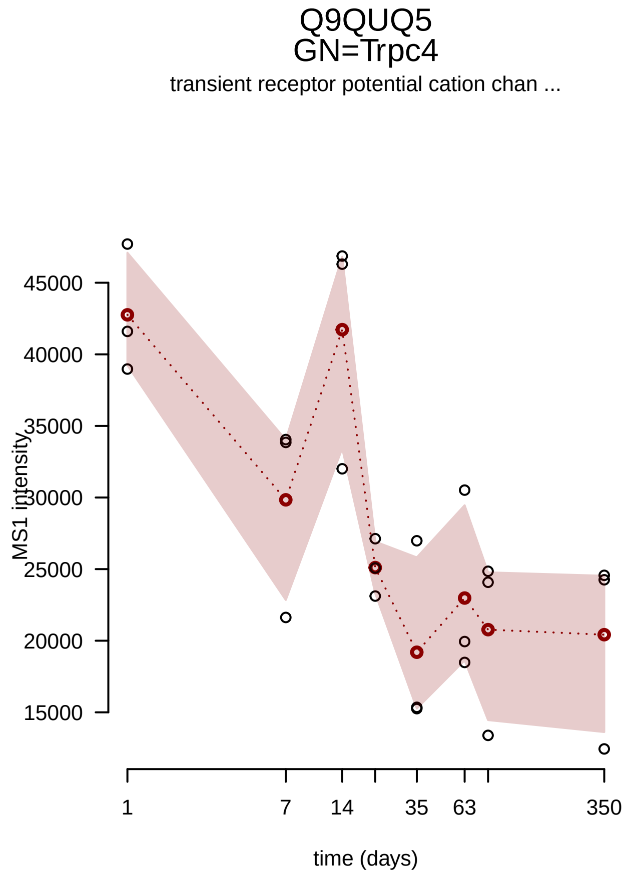
Q9QUQ5
GN=Trpc4
transient receptor potential cation chan ...
MS1 intensity
15000
20000
25000
30000
35000
40000
45000
1
7
14
35
63
350
time (days)

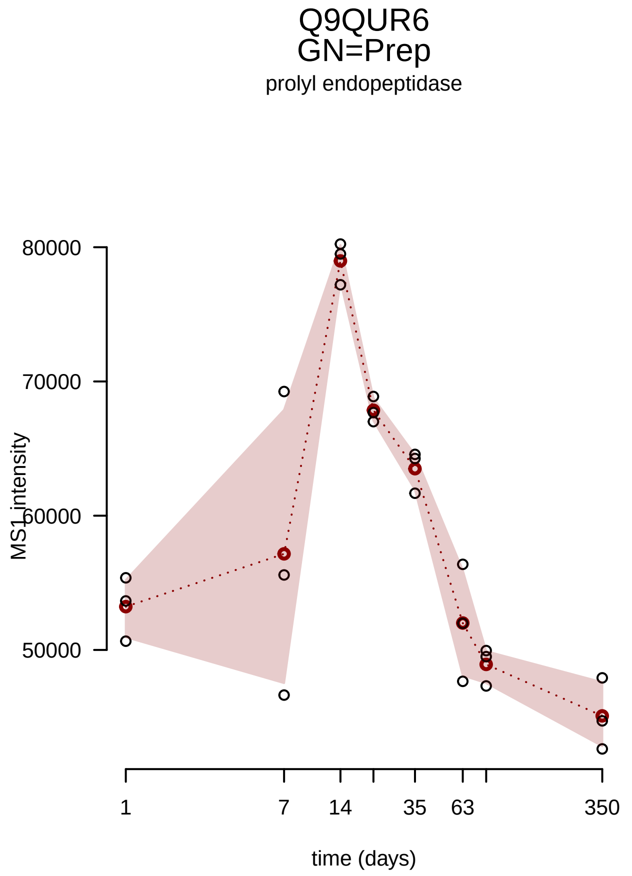
Q9QUR6
GN=Prep
prolyl endopeptidase
MS1 intensity
50000
60000
70000
80000
1
7
14
35
63
350
time (days)

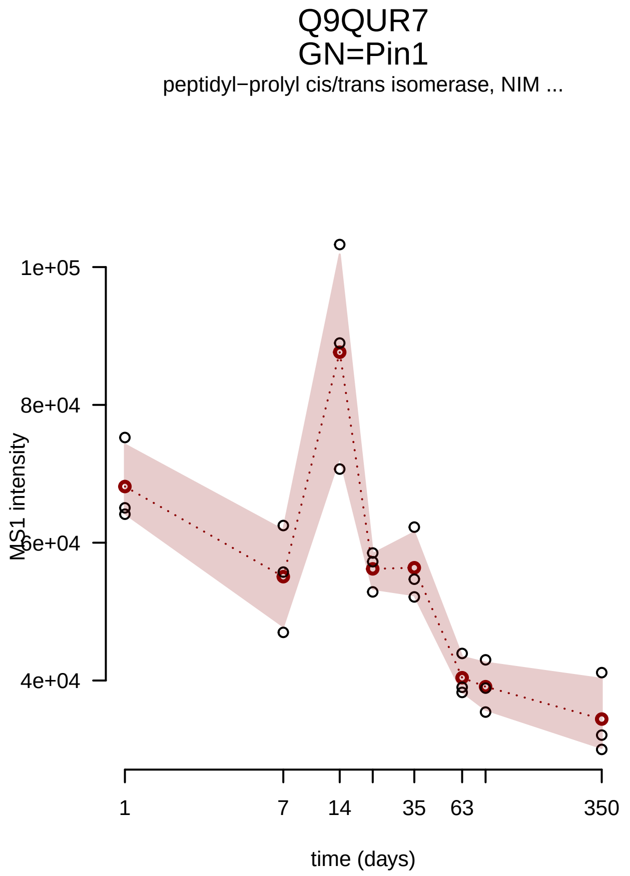
Q9QUR7
GN=Pin1
peptidyl−prolyl cis/trans isomerase, NIM ...
MS1 intensity
4e+04
6e+04
8e+04
1e+05
1
7
14
35
63
350
time (days)

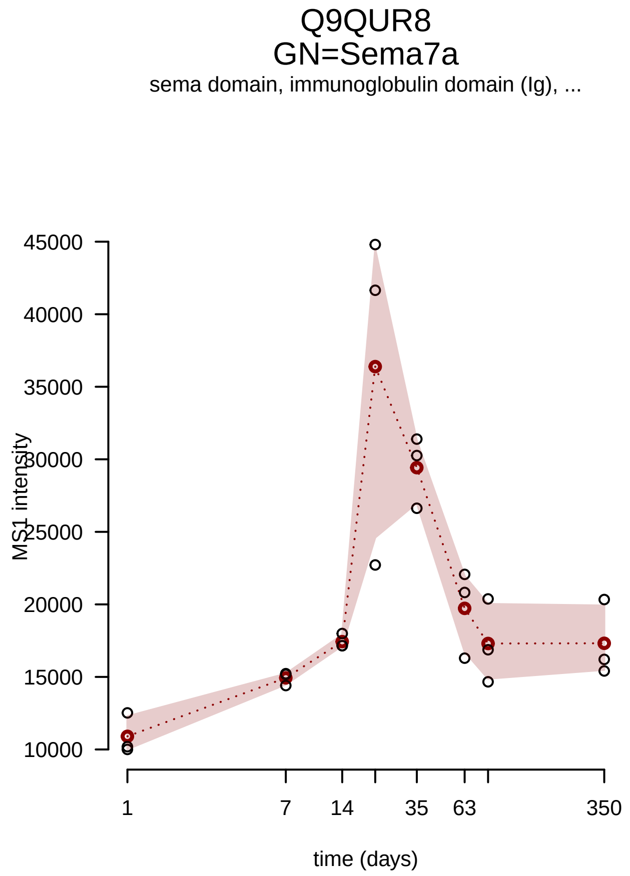
Q9QUR8
GN=Sema7a
sema domain, immunoglobulin domain (Ig), ...
MS1 intensity
10000
15000
20000
25000
30000
35000
40000
45000
1
7
14
35
63
350
time (days)

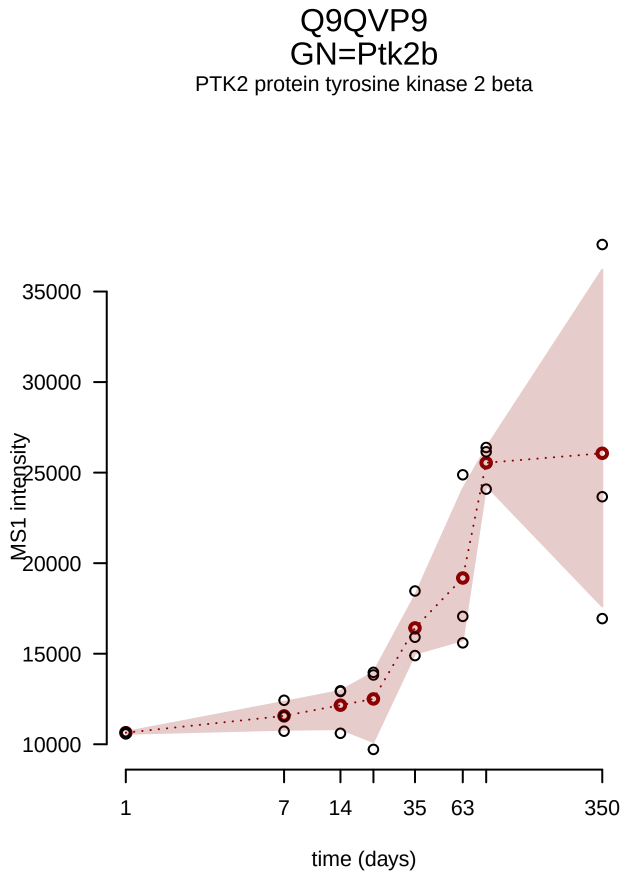
Q9QVP9
GN=Ptk2b
PTK2 protein tyrosine kinase 2 beta
MS1 intensity
10000
15000
20000
25000
30000
35000
1
7
14
35
63
350
time (days)

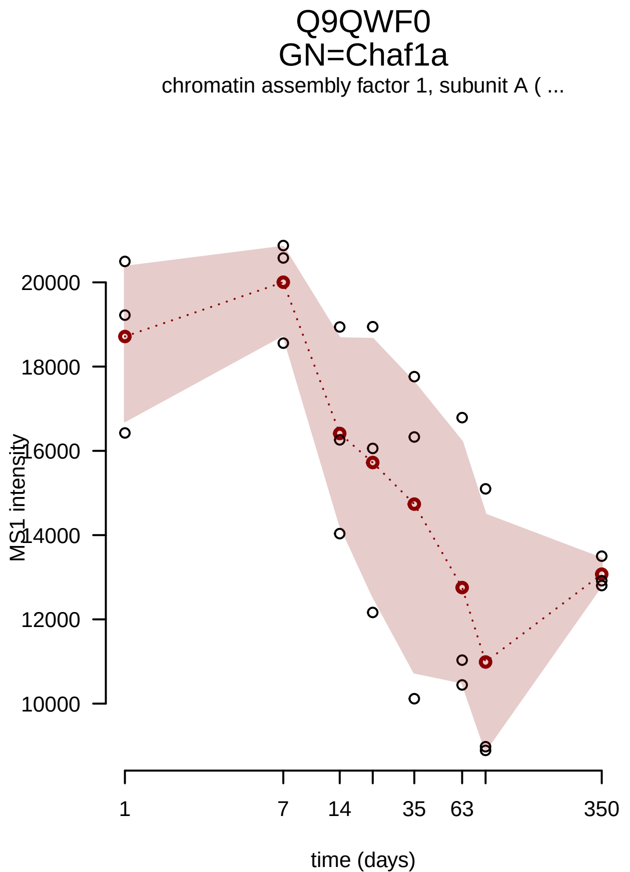
Q9QWF0
GN=Chaf1a
chromatin assembly factor 1, subunit A (...
MS1 intensity
10000
12000
14000
16000
18000
20000
1
7
14
35
63
350
time (days)

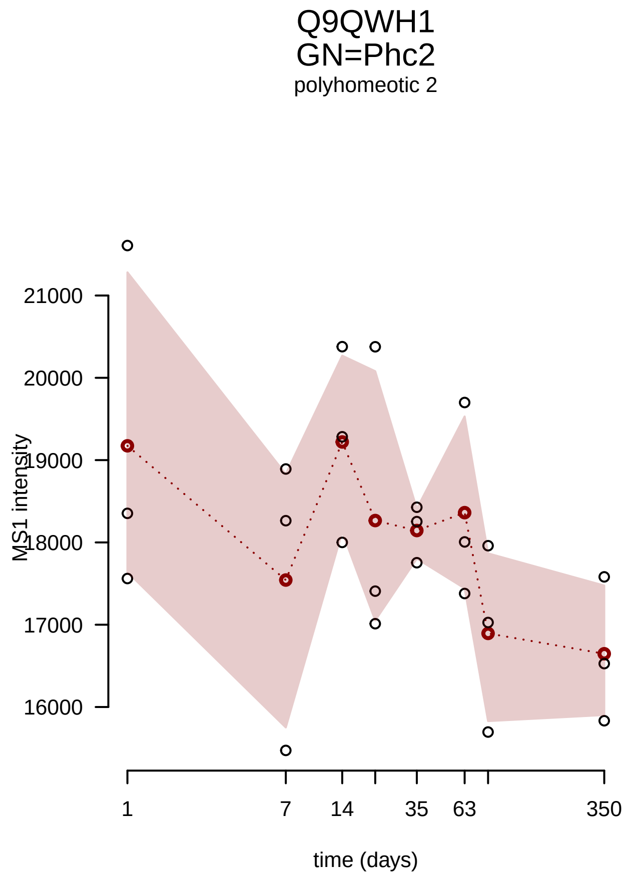
Q9QWH1
GN=Phc2
polyhomeotic 2
MS1 intensity
16000
17000
18000
19000
20000
21000
1
7
14
35
63
350
time (days)

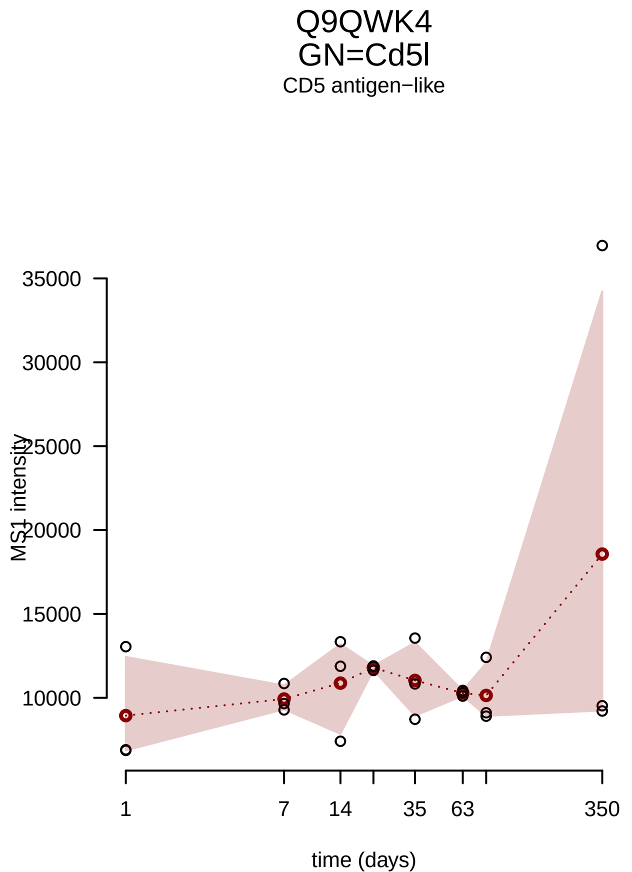
Q9QWK4
GN=Cd5l
CD5 antigen−like
MS1 intensity
10000
15000
20000
25000
30000
35000
1
7
14
35
63
350
time (days)

Q9QWL7
GN=Krt17
keratin 17

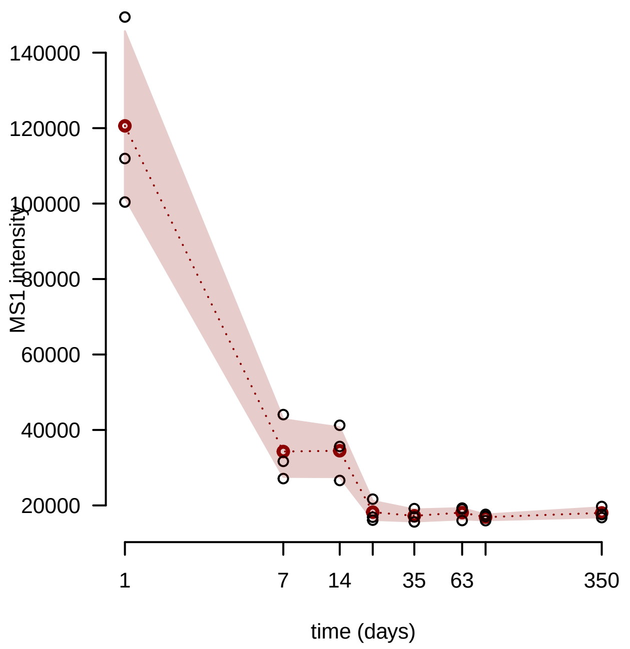

Q9QWR8
GN=Naga
N−acetyl galactosaminidase, alpha

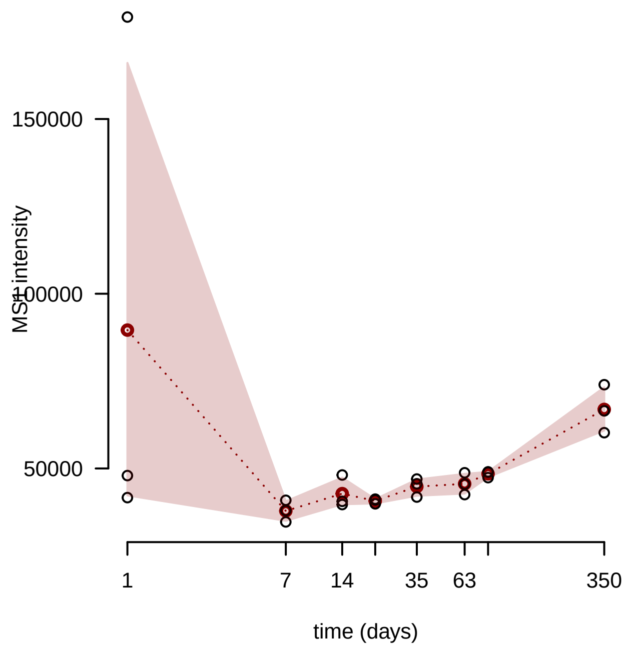

Q9QWT9
GN=Kifc1
kinesin family member C1

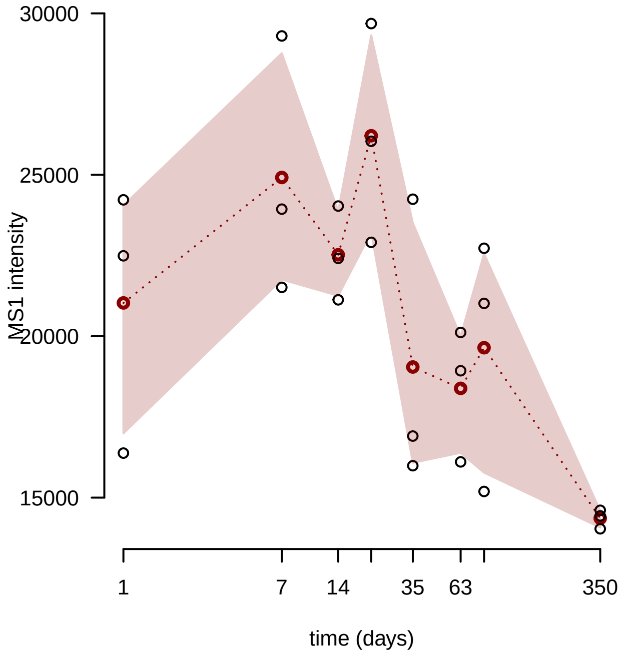

Q9QWV9
GN=Ccnt1
cyclin T1

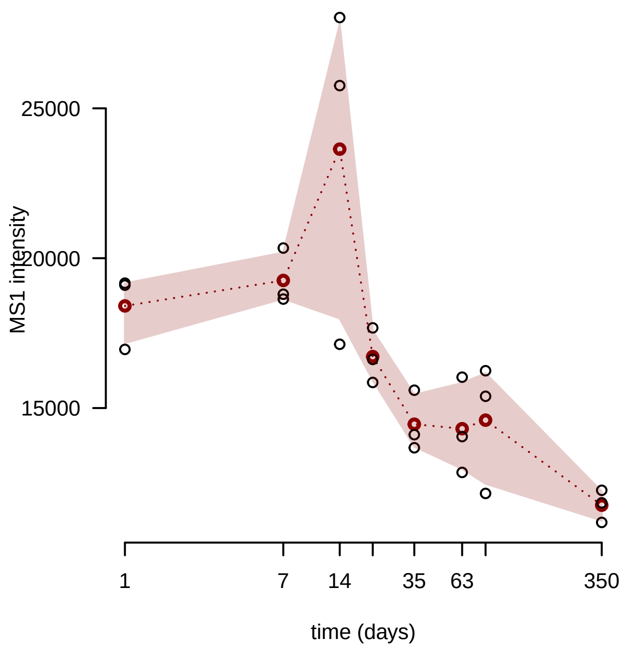

Q9QWW1
GN=Homer2
homer scaffolding protein 2

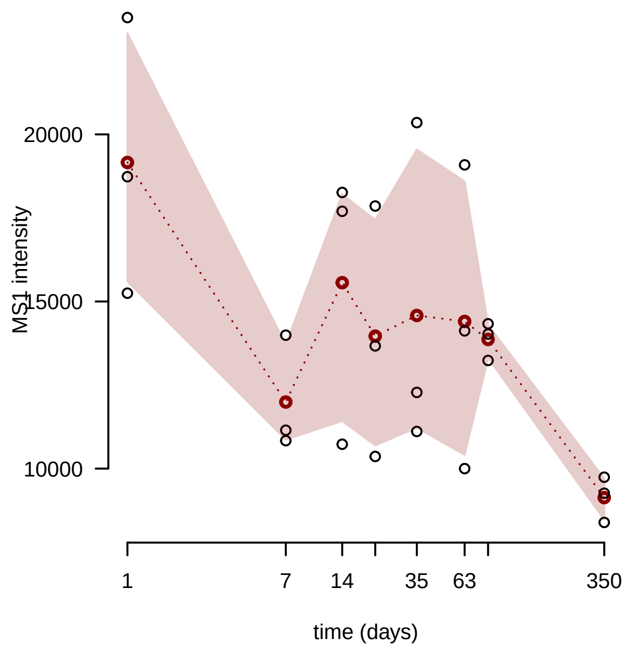

Q9QWY8
GN=Asap1
ArfGAP with SH3 domain, ankyrin repeat a ...

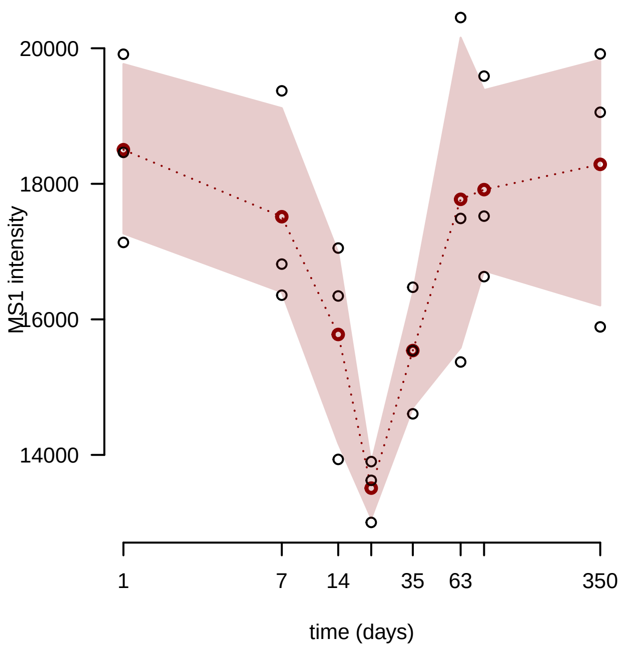

Q9QWZ1
GN=Rad1
RAD1 checkpoint DNA exonuclease

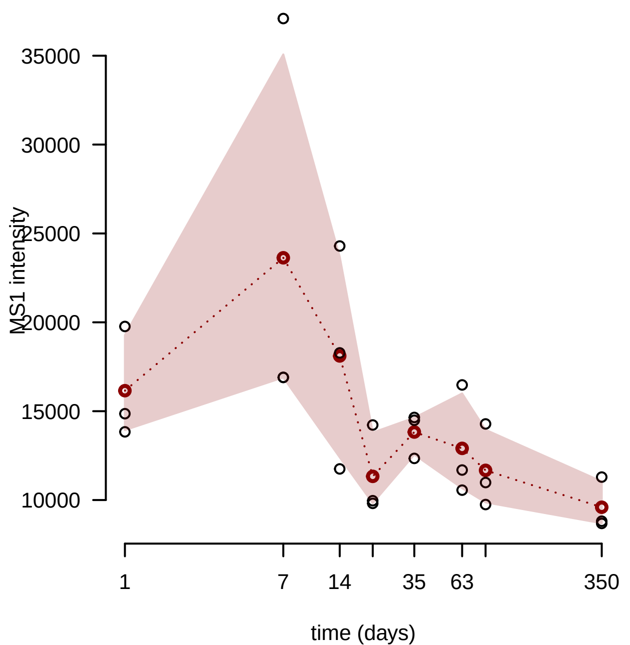

Q9QX11
GN=Cyth1
cytohesin 1

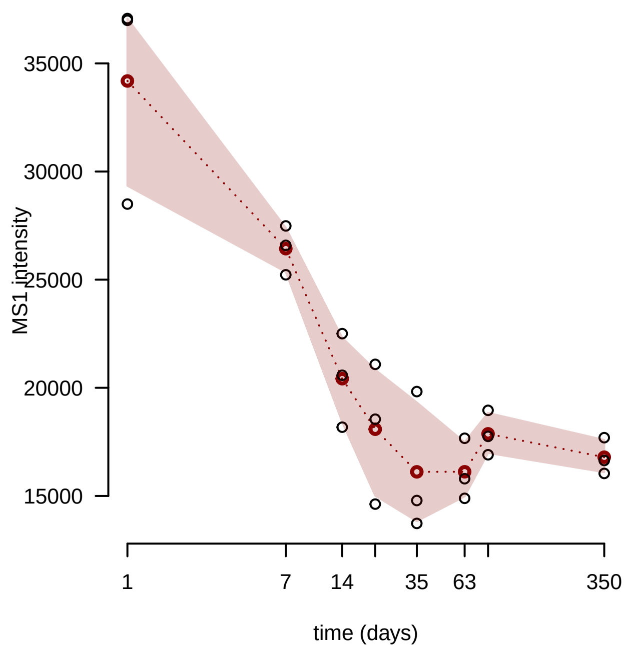

Q9QX15
GN=Clca3a1
chloride channel accessory 3A1

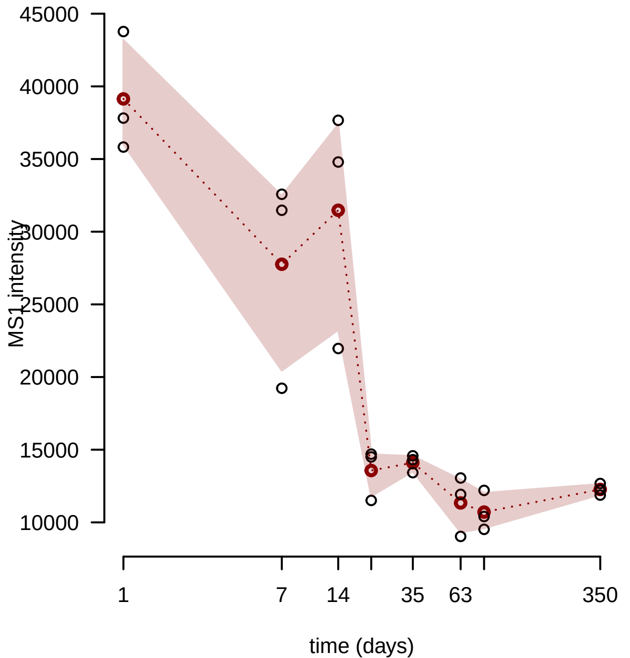

Q9QX47
GN=Son
Son DNA binding protein

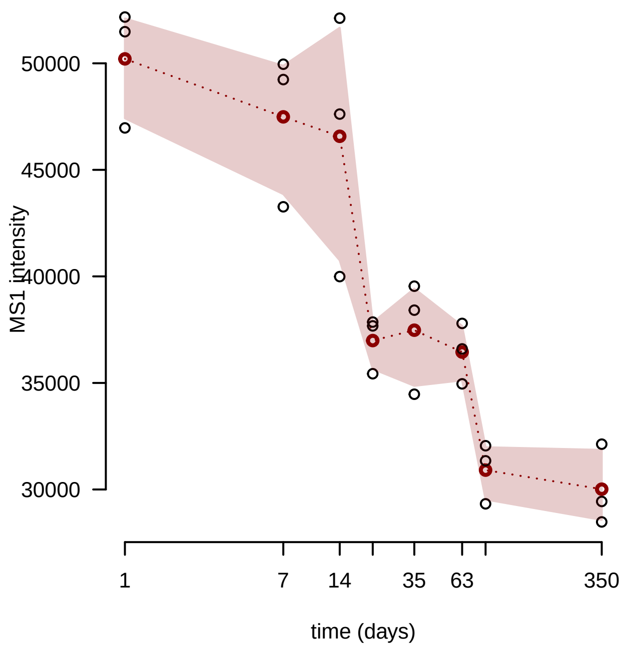

Q9QX60
GN=Dguok
deoxyguanosine kinase

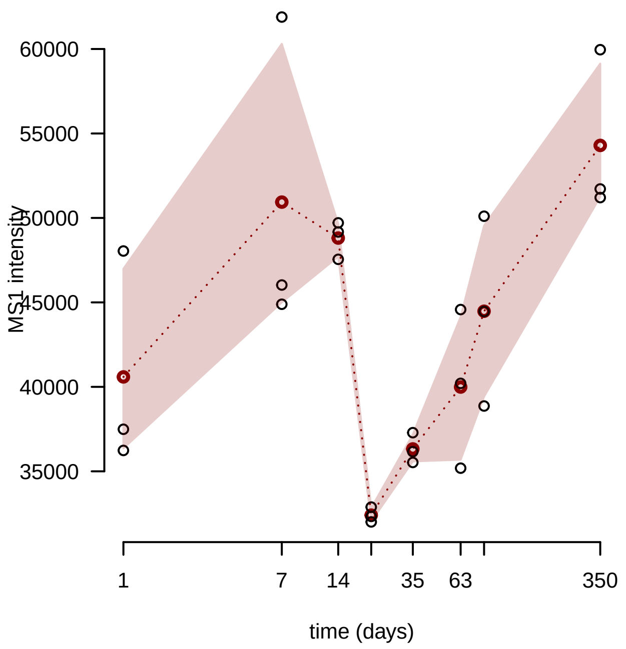

Q9QXA1
GN=Cyhr1
cysteine and histidine rich 1

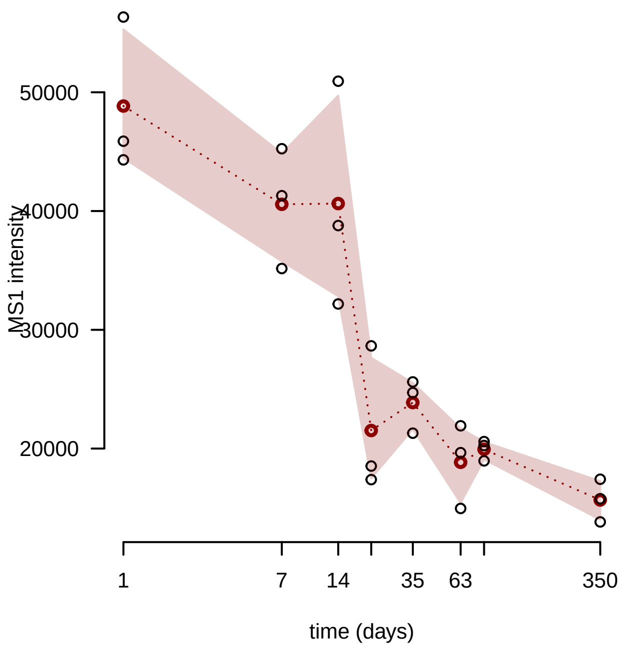

Q9QXA1
GN=Cyhr1
cysteine and histidine rich 1

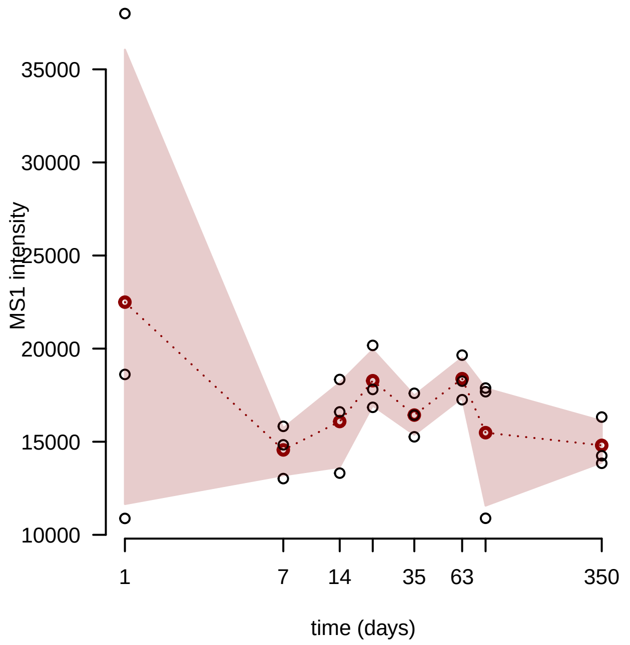

Q9QXA5
GN=Lsm4
LSM4 homolog, U6 small nuclear RNA and m ...

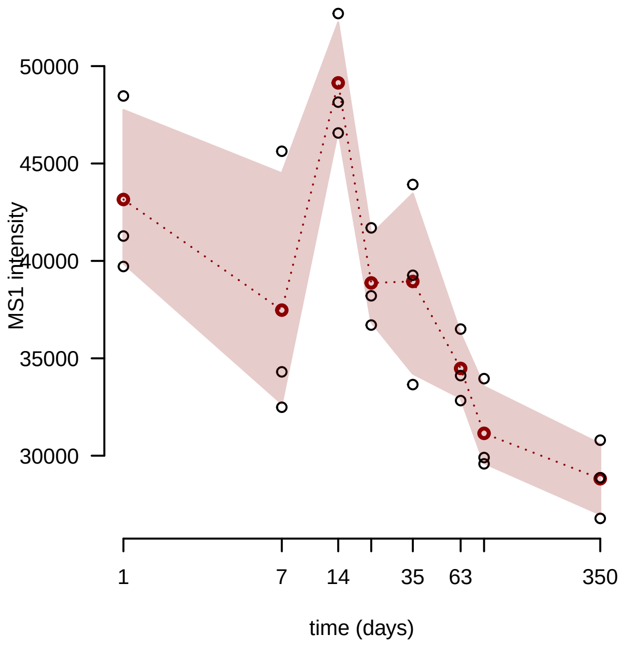

Q9QXA7
GN=Trim44
tripartite motif−containing 44

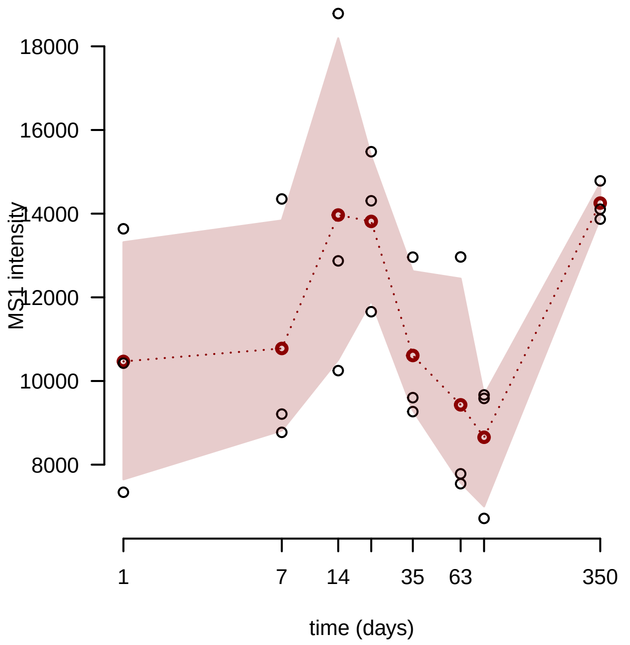

Q9QXB9
GN=Drg2
developmentally regulated GTP binding pr ...

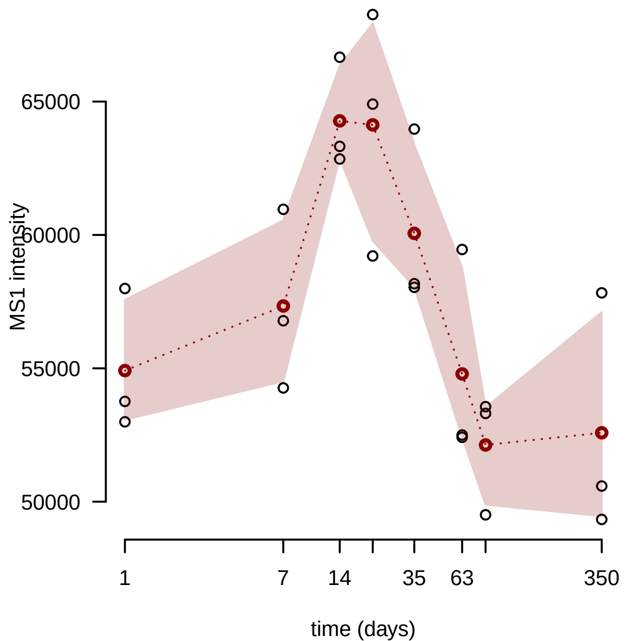

Q9QXC1
GN=Fetub
fetuin beta

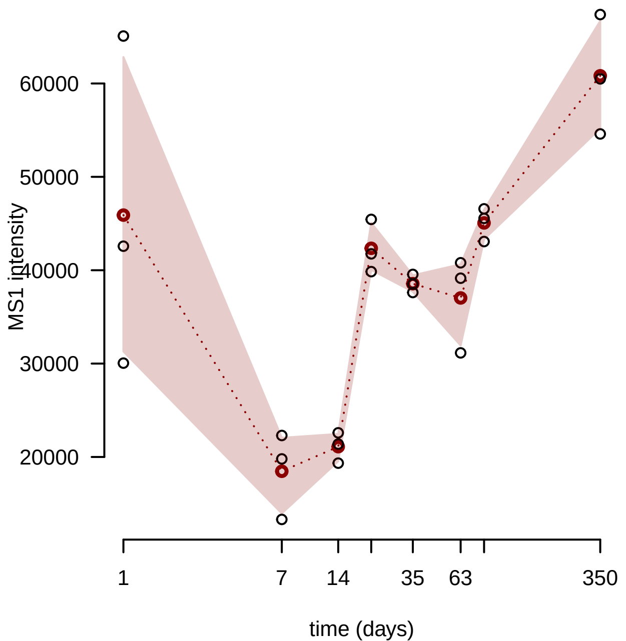

Q9QXD6
GN=Fbp1
fructose bisphosphatase 1

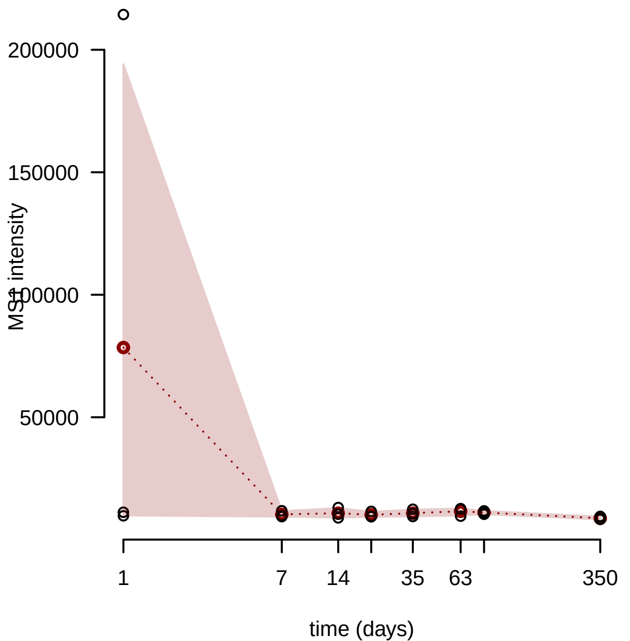

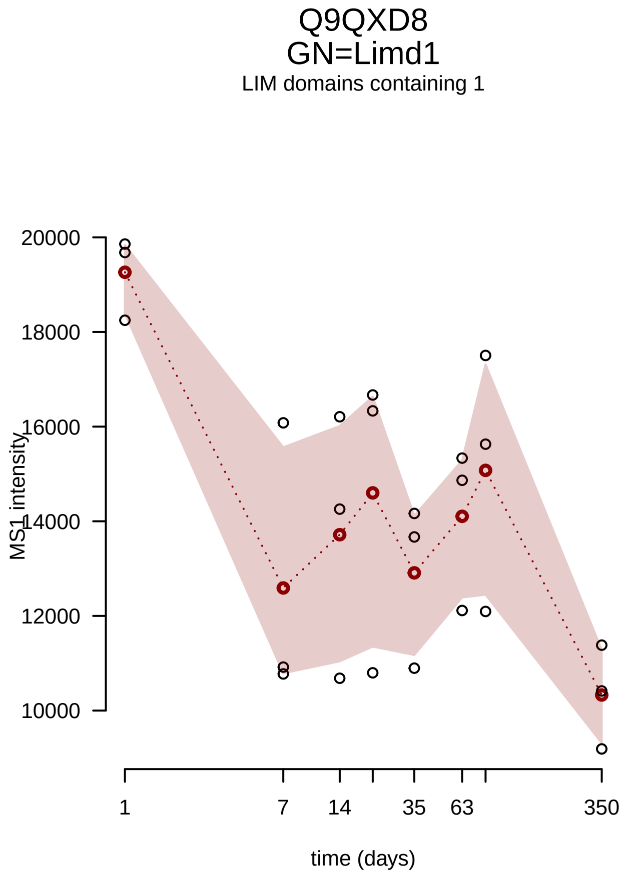
Q9QXD8
GN=Limd1
LIM domains containing 1
MS1 intensity
20000
18000
16000
14000
12000
10000
1
7
14
35
63
350
time (days)

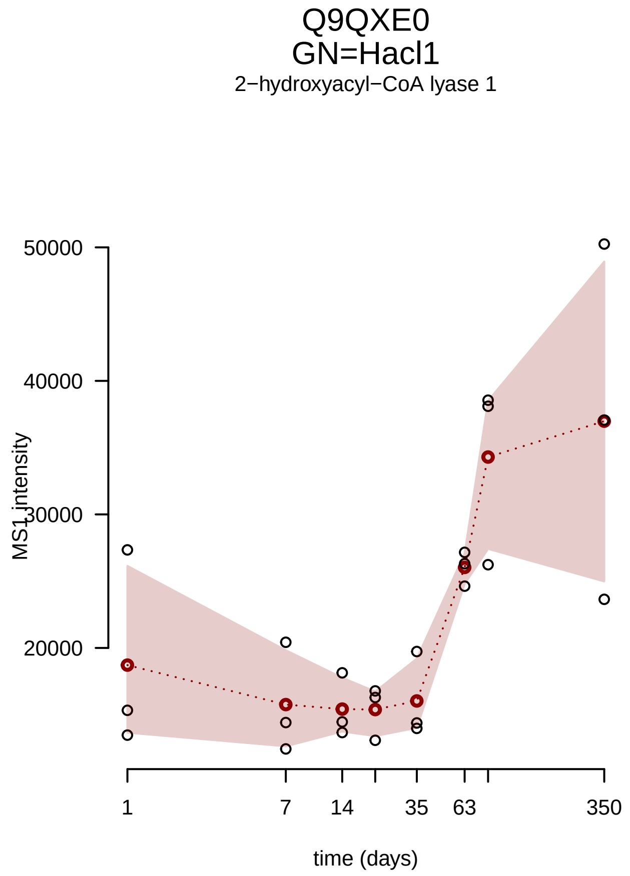
Q9QXE0
GN=Hacl1
2−hydroxyacyl−CoA lyase 1
MS1 intensity
50000
40000
30000
20000
1
7
14
35
63
350
time (days)

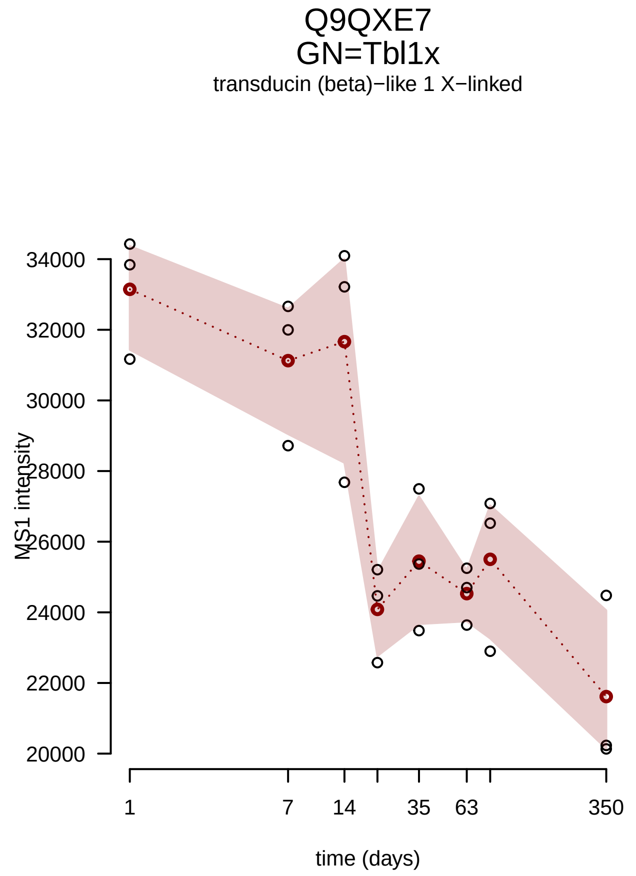
Q9QXE7
GN=Tbl1x
transducin (beta)−like 1 X−linked
MS1 intensity
34000
32000
30000
28000
26000
24000
22000
20000
1
7
14
35
63
350
time (days)

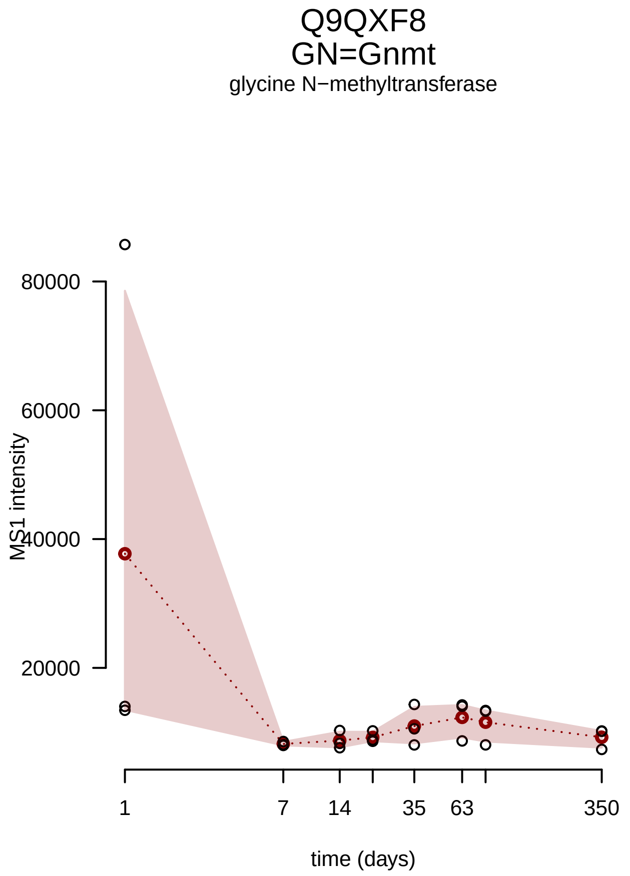
Q9QXF8
GN=Gnmt
glycine N−methyltransferase
MS1 intensity
80000
60000
40000
20000
1
7
14
35
63
350
time (days)

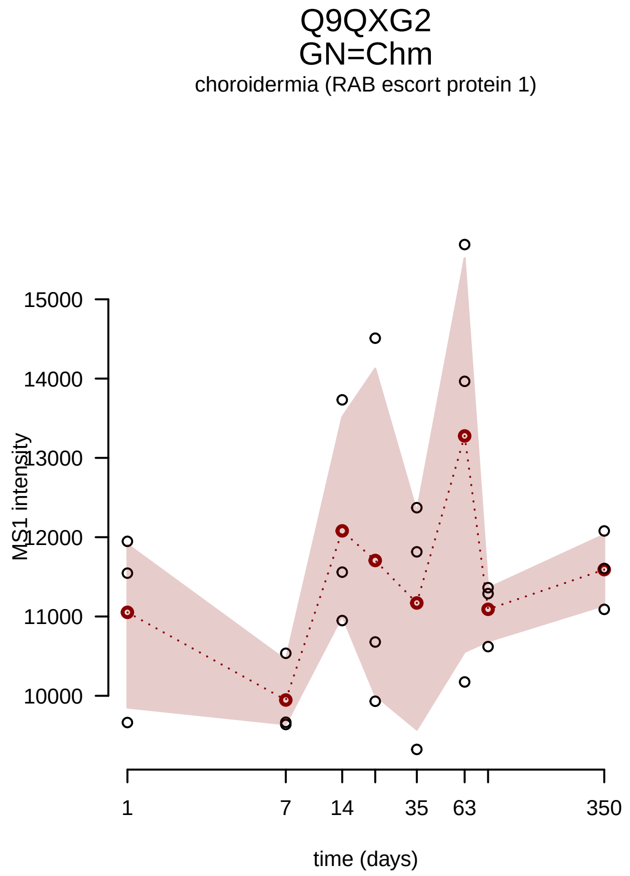
Q9QXG2
GN=Chm
choroidermia (RAB escort protein 1)
MS1 intensity
15000
14000
13000
12000
11000
10000
1
7
14
35
63
350
time (days)

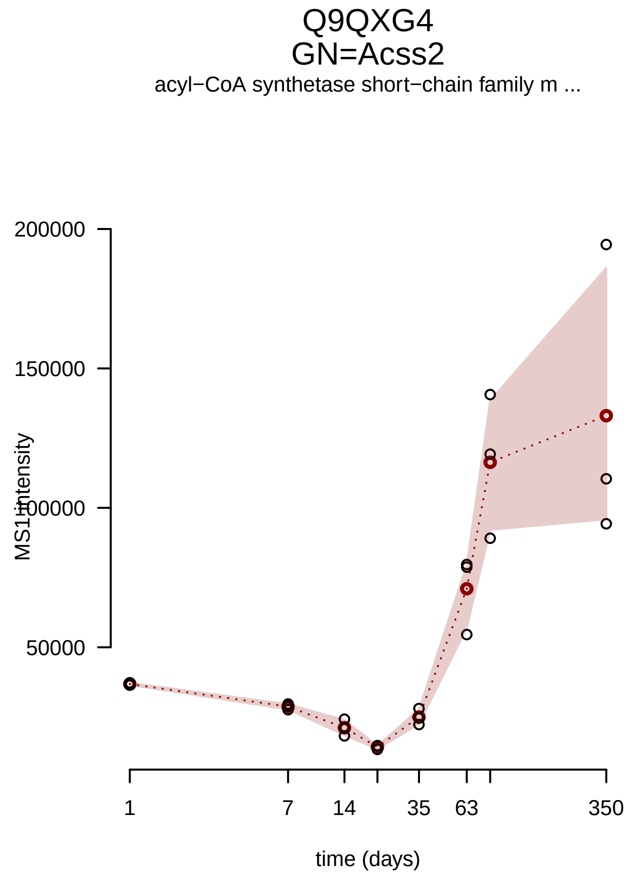
Q9QXG4
GN=Acss2
acyl−CoA synthetase short−chain family m ...
MS1 intensity
200000
150000
100000
50000
1
7
14
35
63
350
time (days)

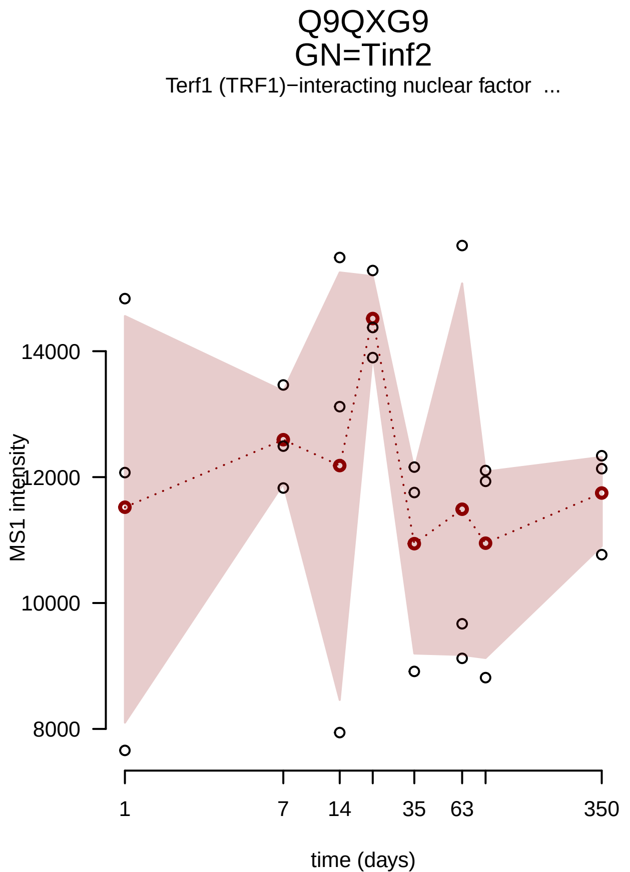
Q9QXG9
GN=Tinf2
Terf1 (TRF1)−interacting nuclear factor ...
MS1 intensity
14000
12000
10000
8000
1
7
14
35
63
350
time (days)

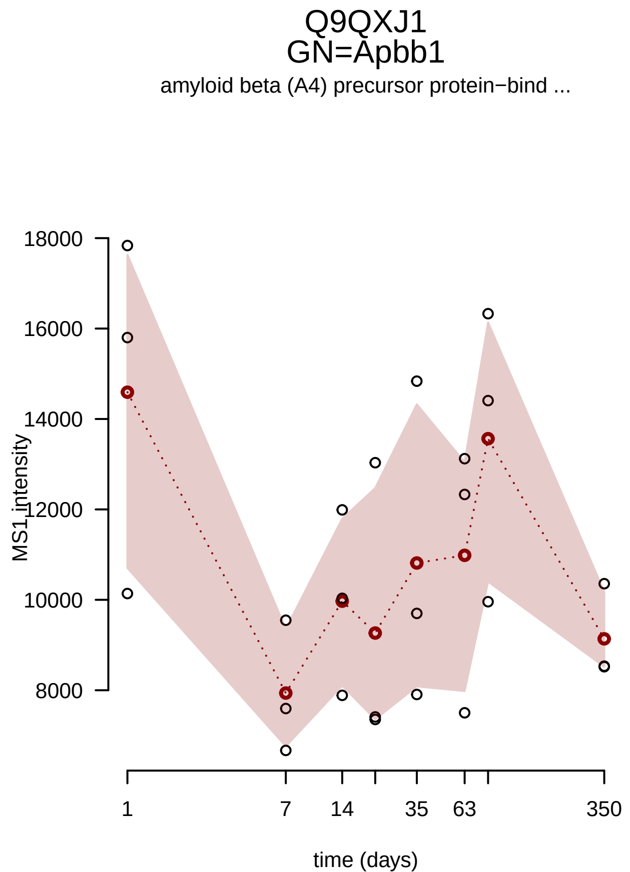
Q9QXJ1
GN=Apbb1
amyloid beta (A4) precursor protein−bind ...
MS1 intensity
18000
16000
14000
12000
10000
8000
1
7
14
35
63
350
time (days)

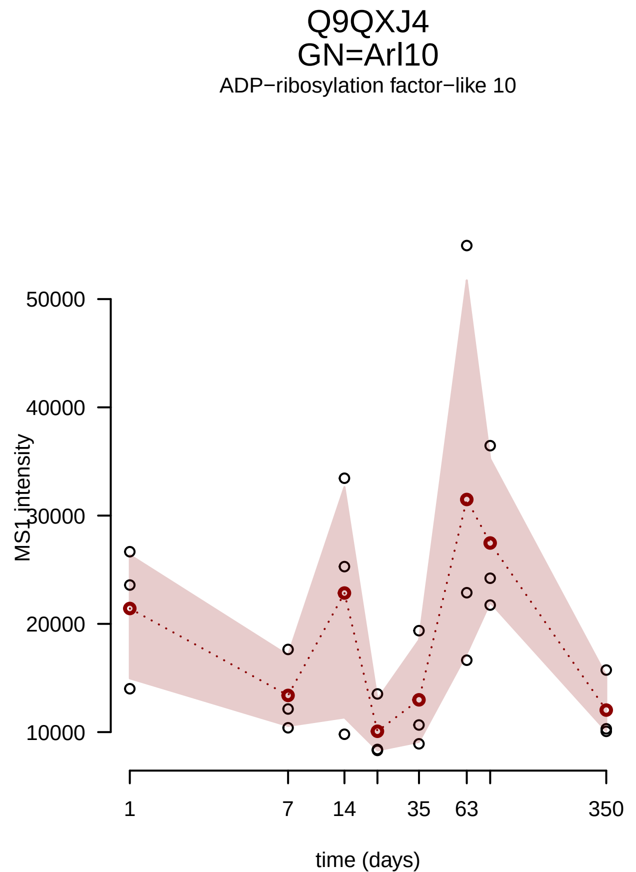
Q9QXJ4
GN=Arl10
ADP−ribosylation factor−like 10
MS1 intensity
50000
40000
30000
20000
10000
1
7
14
35
63
350
time (days)

Q9QXK2
GN=Rad18
RAD18 E3 ubiquitin protein ligase

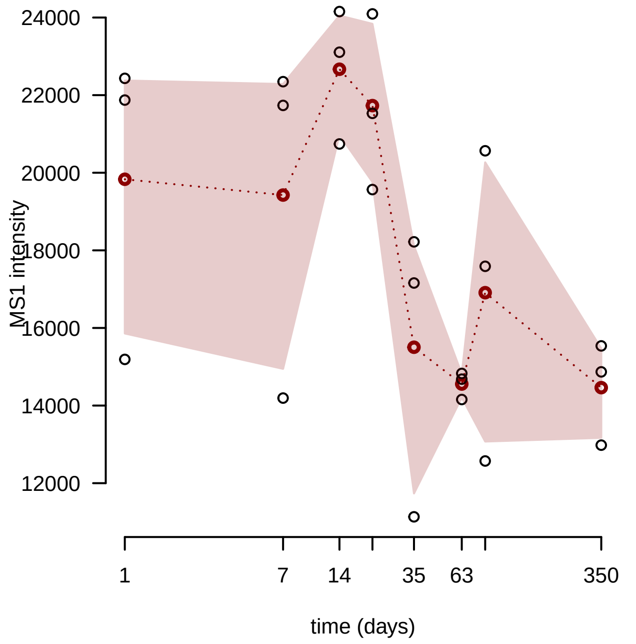

Q9QXK3
GN=Copg2
coatomer protein complex, subunit gamma ...

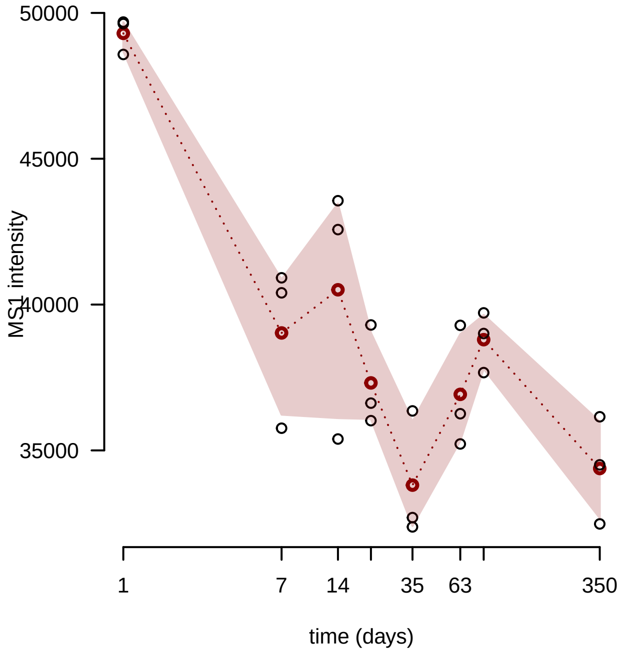

Q9QXK7
GN=Cpsf3
cleavage and polyadenylation specificity ...

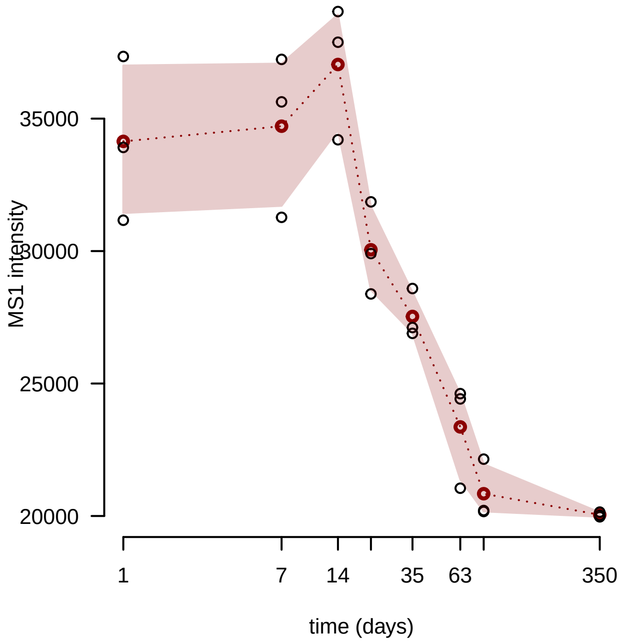

Q9QXL1
GN=Kif21b
kinesin family member 21B

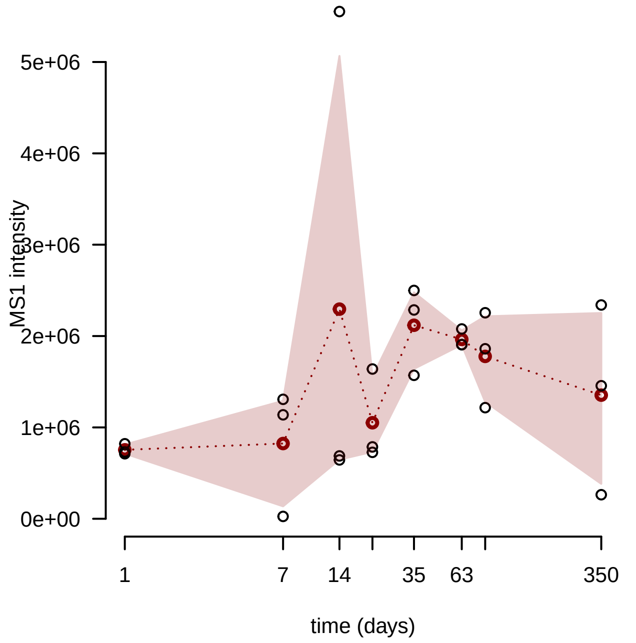

Q9QXL2
GN=Kif21a
kinesin family member 21A

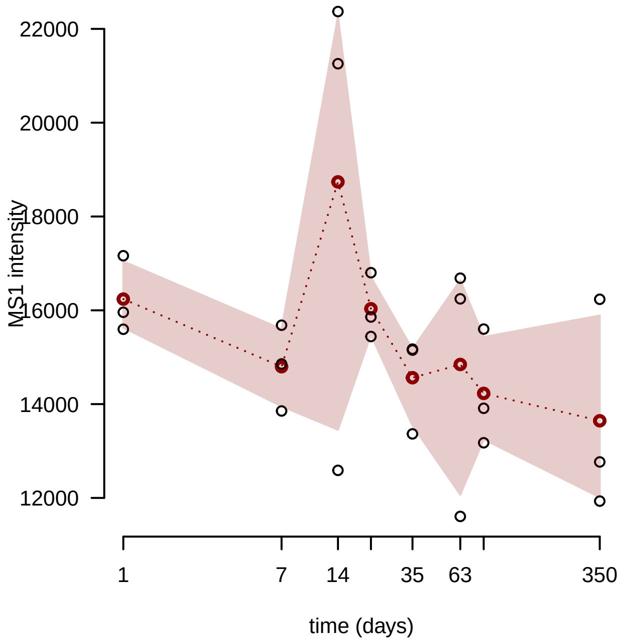

Q9QXL8
GN=Nme7
NME/NM23 family member 7

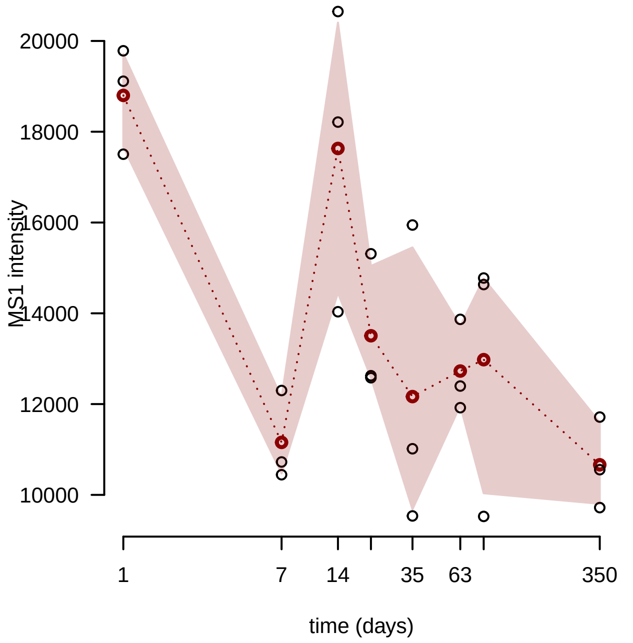

Q9QXM0
GN=Abhd2
abhydrolase domain containing 2

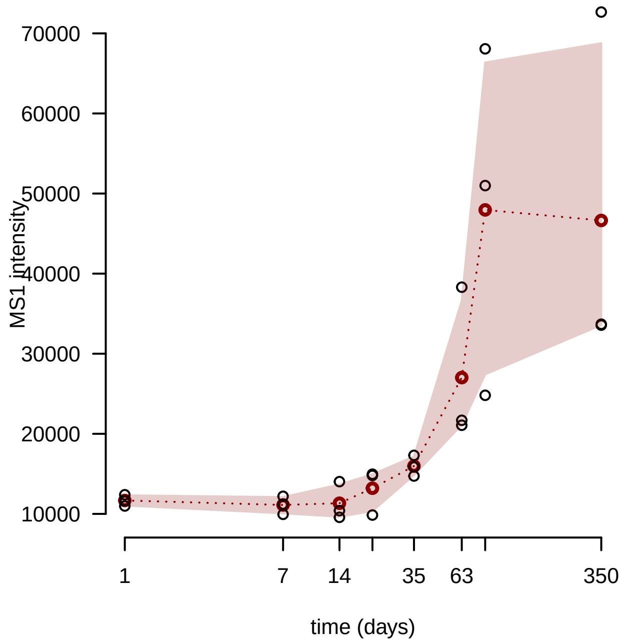

Q9QXM1
GN=Jmy
junction−mediating and regulatory protei ...

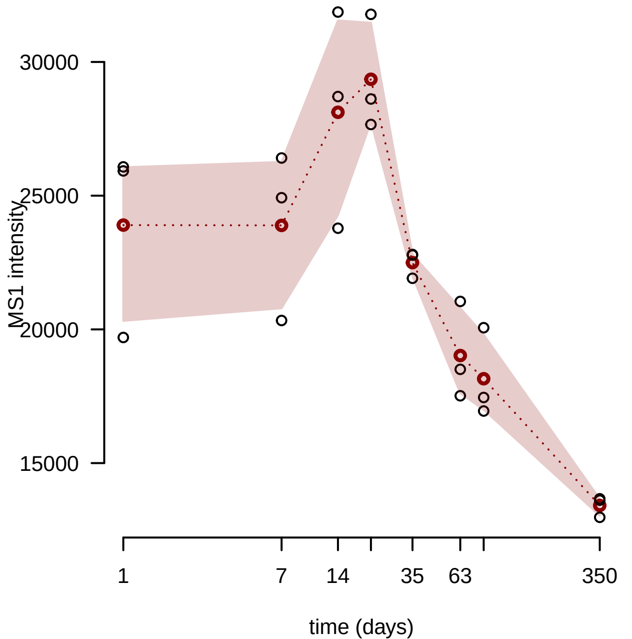

Q9QXN0
GN=Shroom3
shroom family member 3

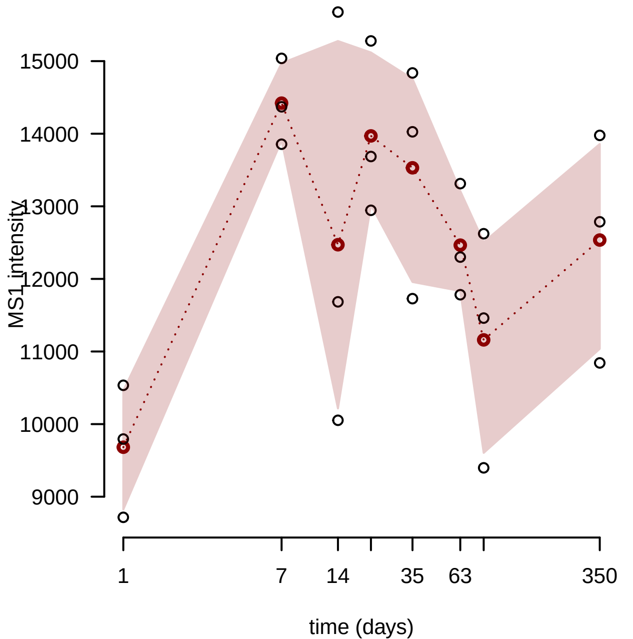

Q9QXN3
GN=Trip4
thyroid hormone receptor interactor 4

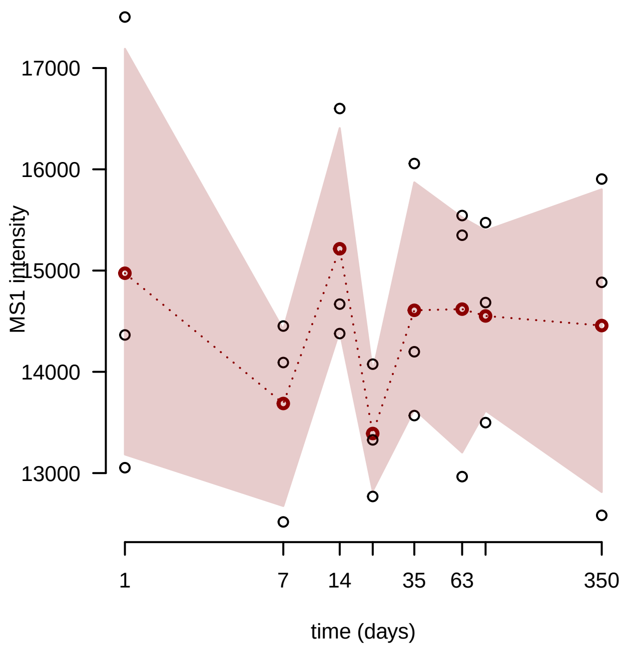

Q9QXN5
GN=Miox
myo−inositol oxygenase

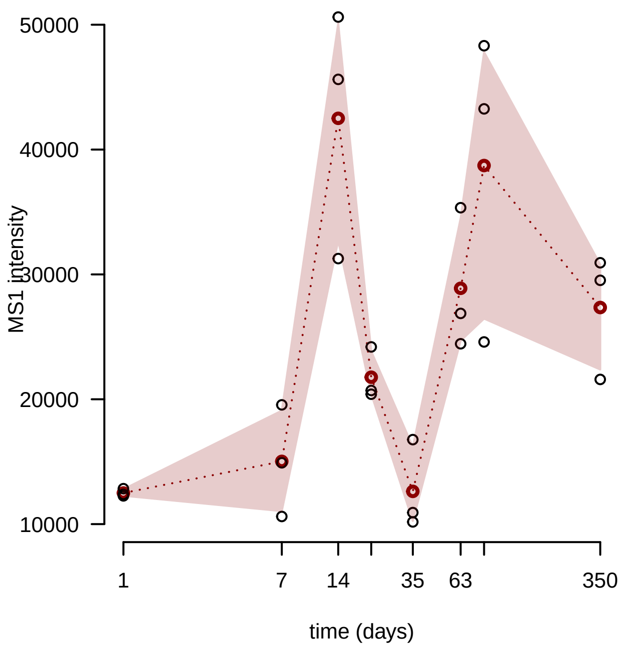

Q9QXP7
GN=C1qtnf1
C1q and tumor necrosis factor related pr ...

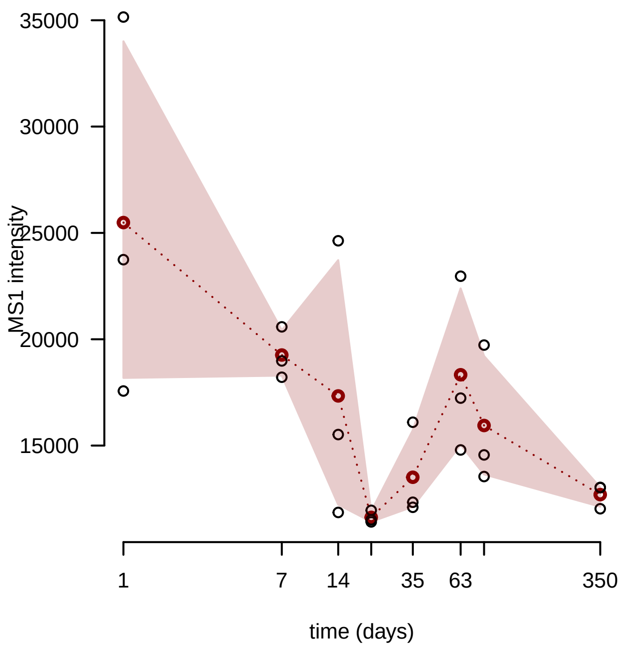

Q9QXS1
GN=Plec
plectin

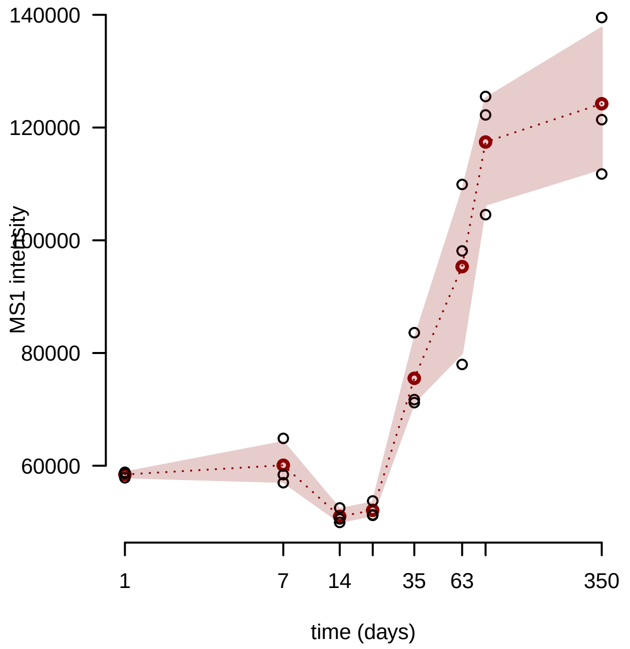

Q9QXS1
GN=Plec
plectin

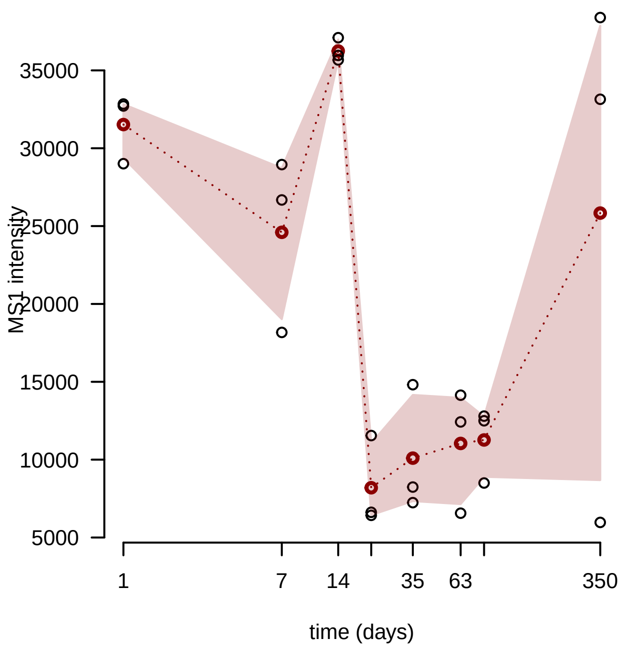

Q9QXS1
GN=Plec
plectin

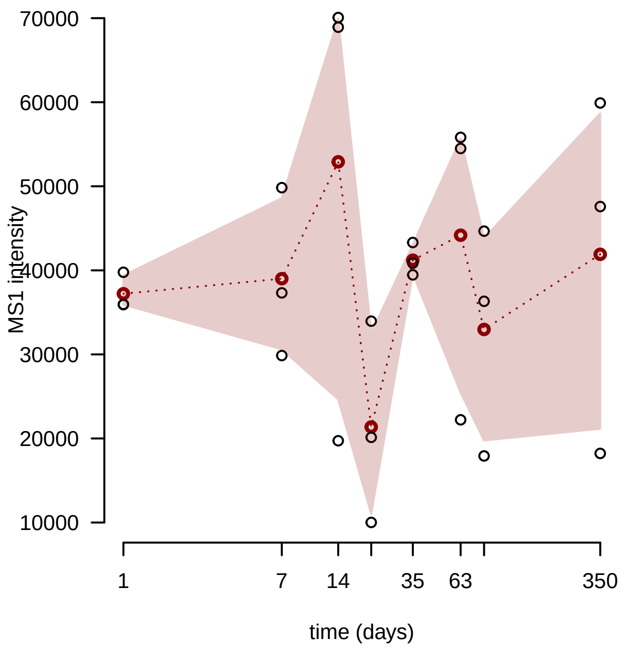

Q9QXS6
GN=Dbn1
drebrin 1

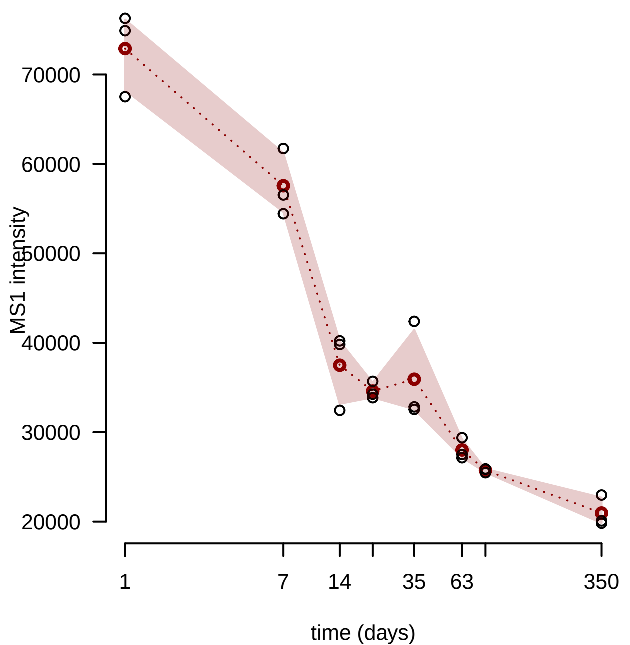

Q9QXT0
GN=Cnpy2
canopy FGF signaling regulator 2

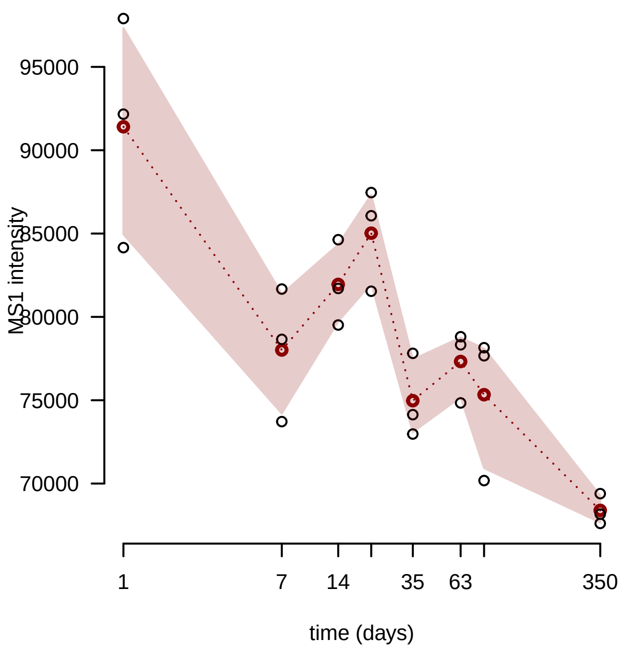

Q9QXT5
GN=Egfl7
EGF−like domain 7

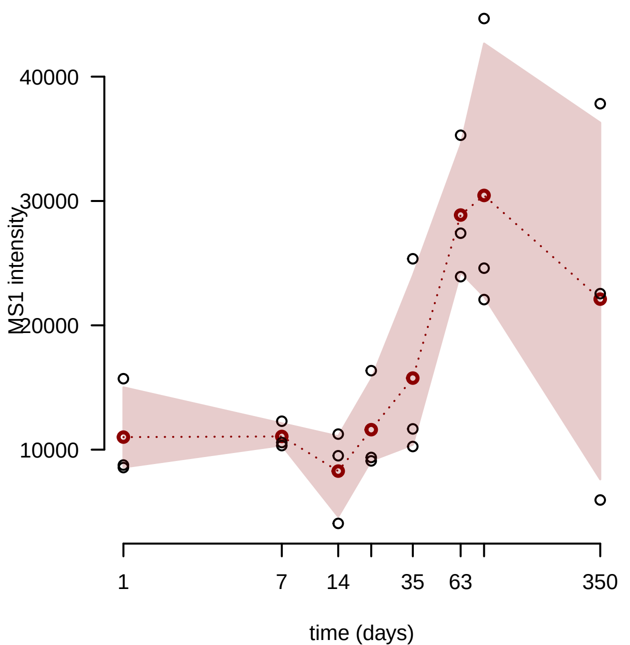

Q9QXT8
GN=Kcnip3

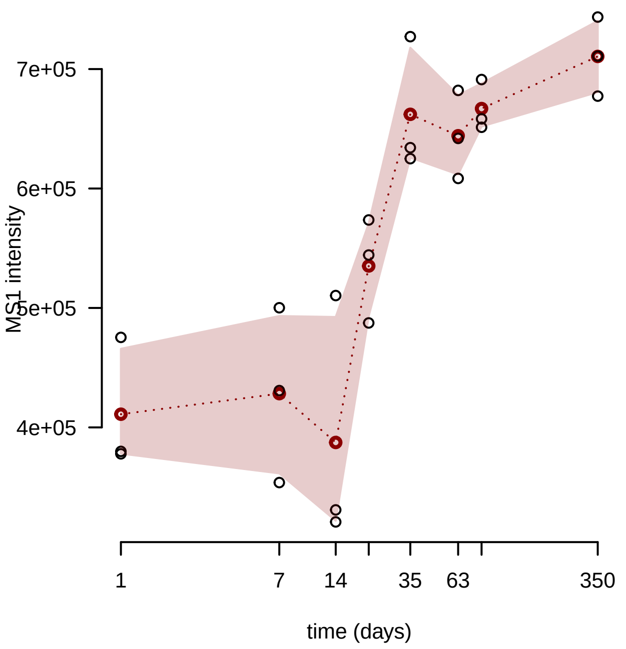

Q9QXV1
GN=Cbx8
chromobox 8

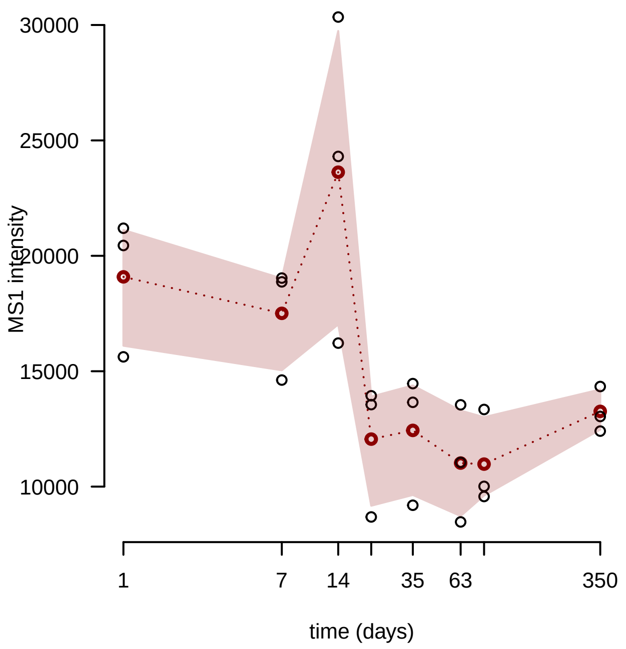

Q9QXV3
GN=Ing1
inhibitor of growth family, member 1

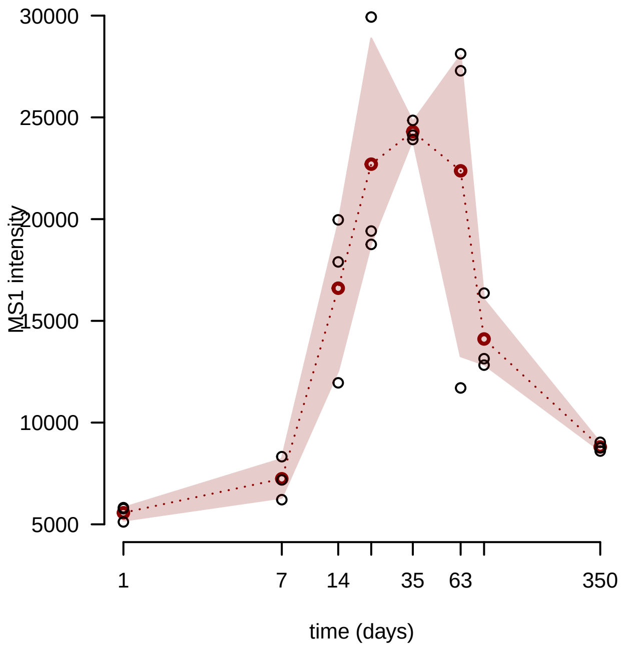

Q9QXW9
GN=Slc7a8
solute carrier family 7 (cationic amino ...

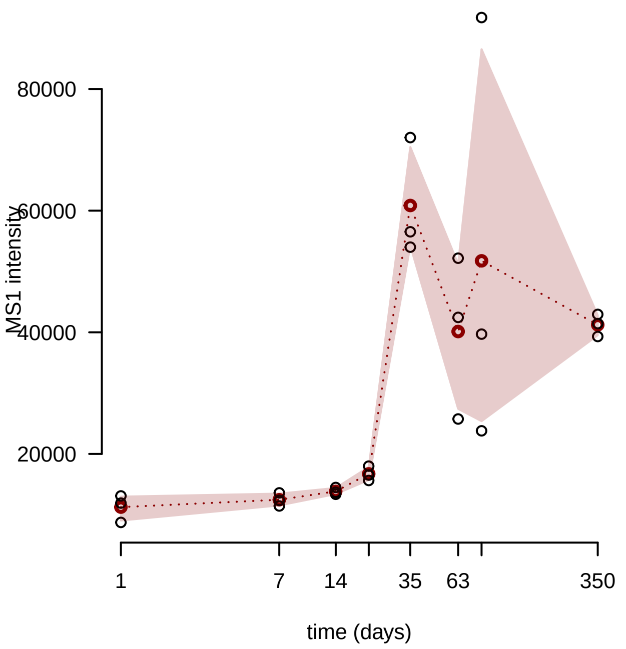

Q9QXX0
GN=Jag1
jagged 1

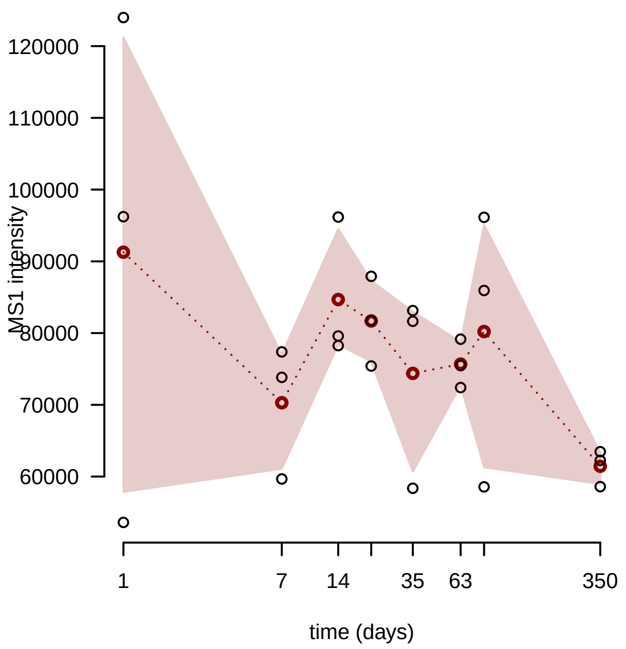

Q9QXX4
GN=Slc25a13
solute carrier family 25 (mitochondrial ...

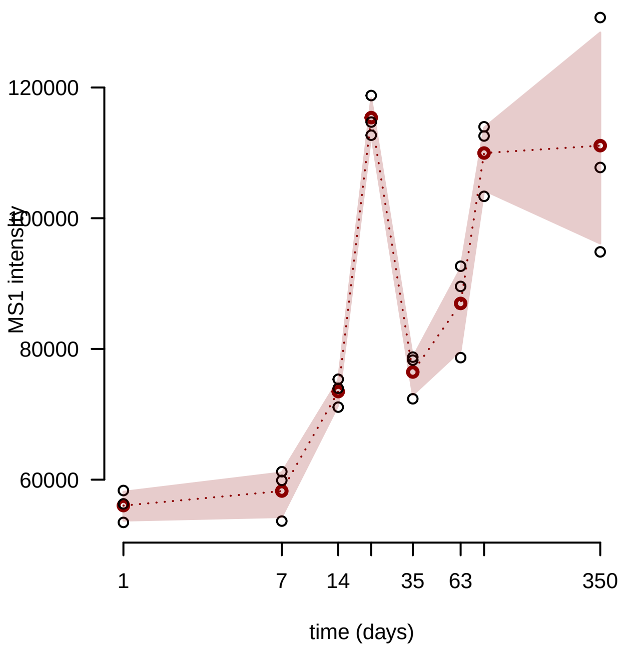

Q9QXY6
GN=Ehd3
EH−domain containing 3

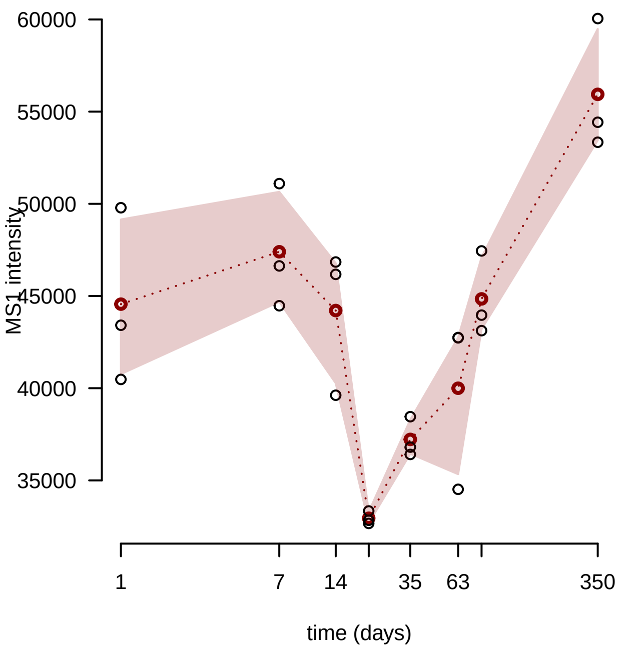

Q9QXY9
GN=Pex3
peroxisomal biogenesis factor 3

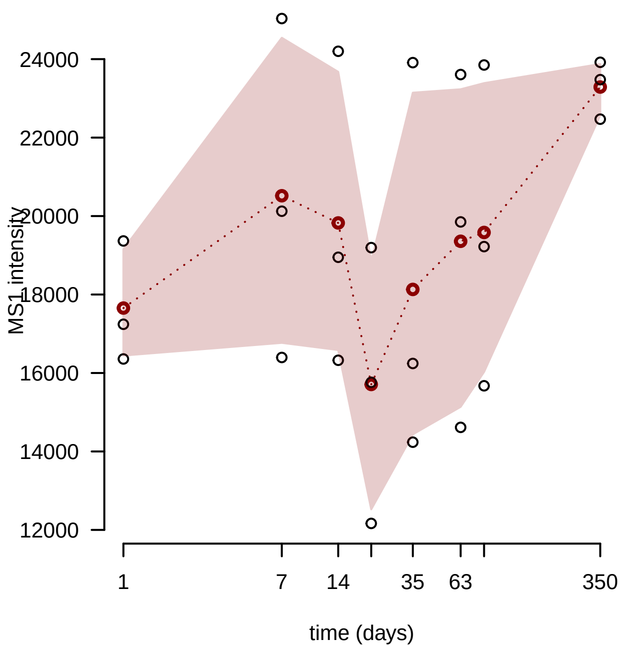

Q9QXZ0
GN=Macf1

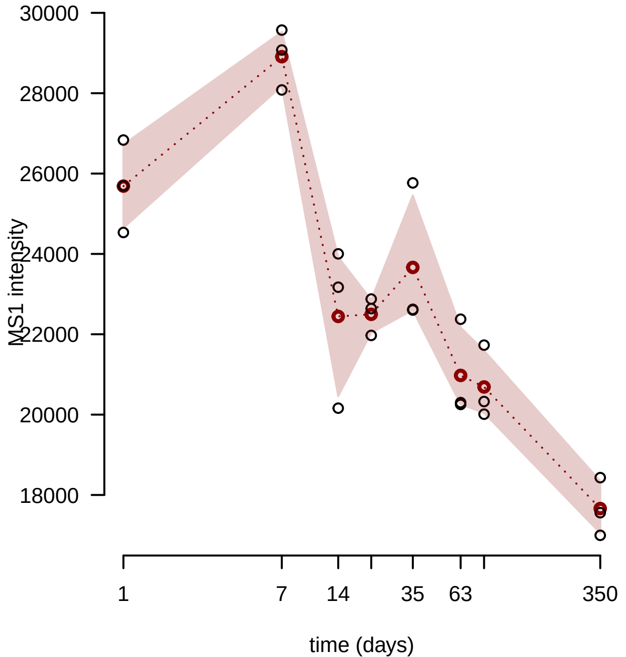

Q9QXZ0
GN=Macf1

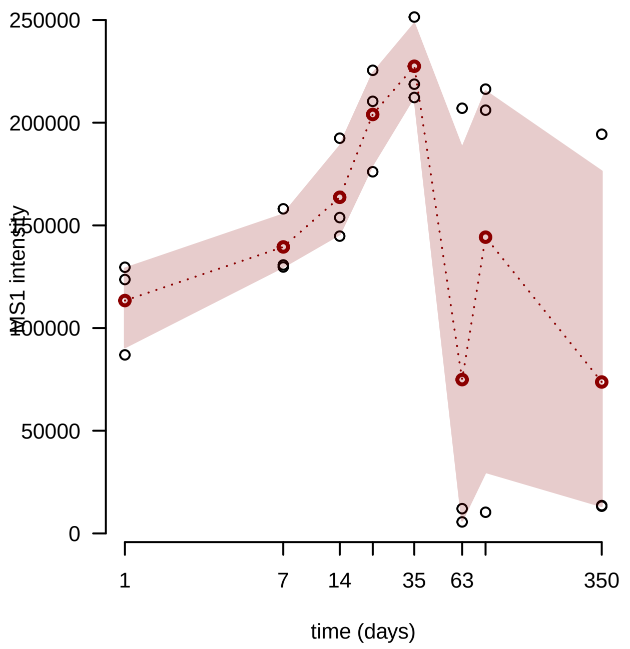

Q9QY06
GN=Myo9b
myosin IXb

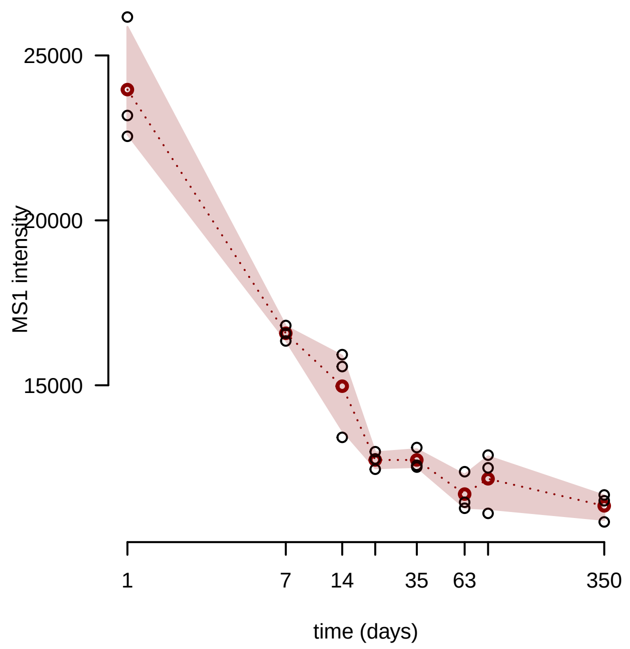

Q9QY15
GN=Ddx25
DEAD box helicase 25

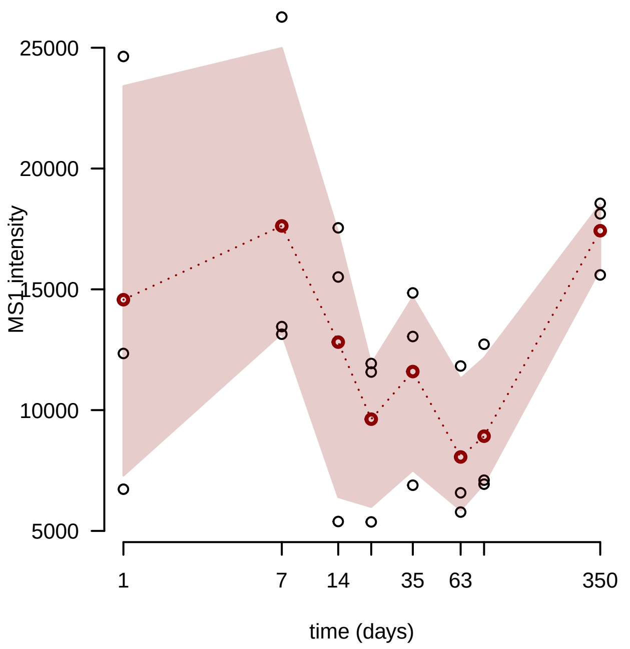

Q9QY23
GN=Pkp3
plakophilin 3

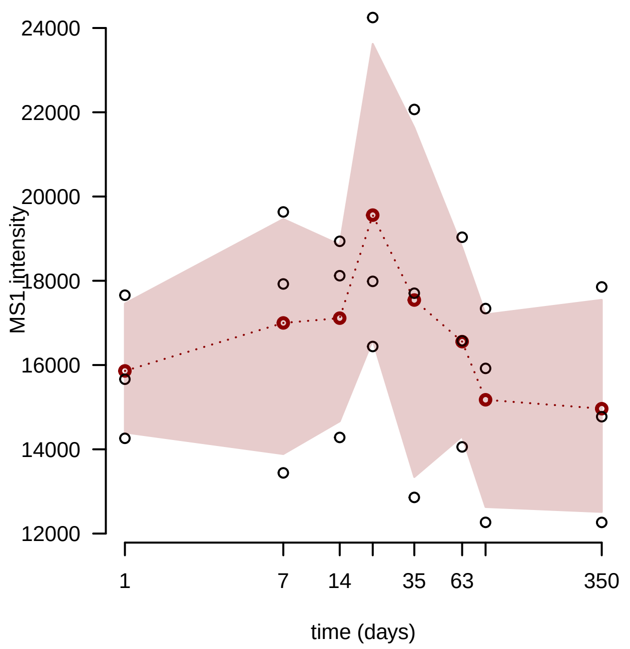

Q9QY30
GN=Abcb11
ATP−binding cassette, sub−family B (MDR/ ...

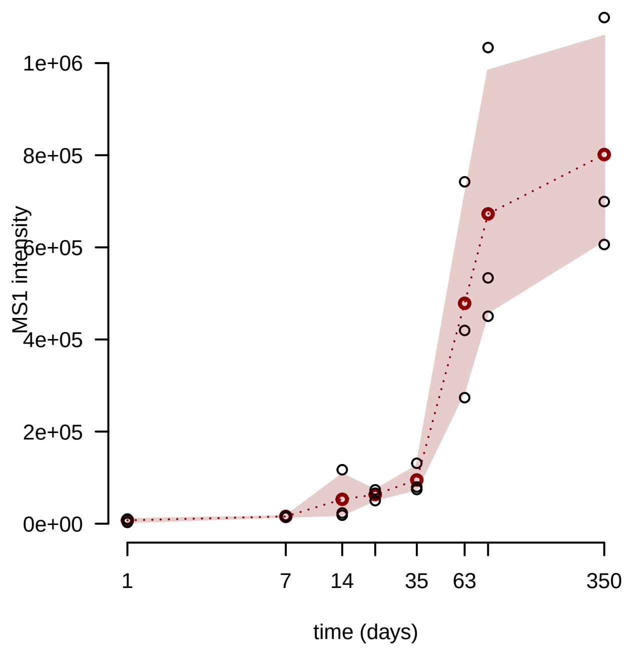

Q9QY36
GN=Naa10
N(alpha)−acetyltransferase 10, NatA cata ...

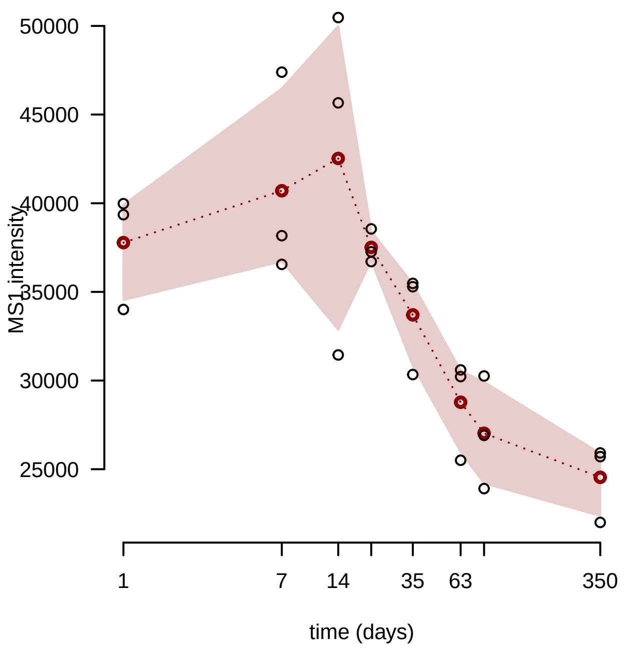

Q9QY40
GN=Plxnb3
plexin B3

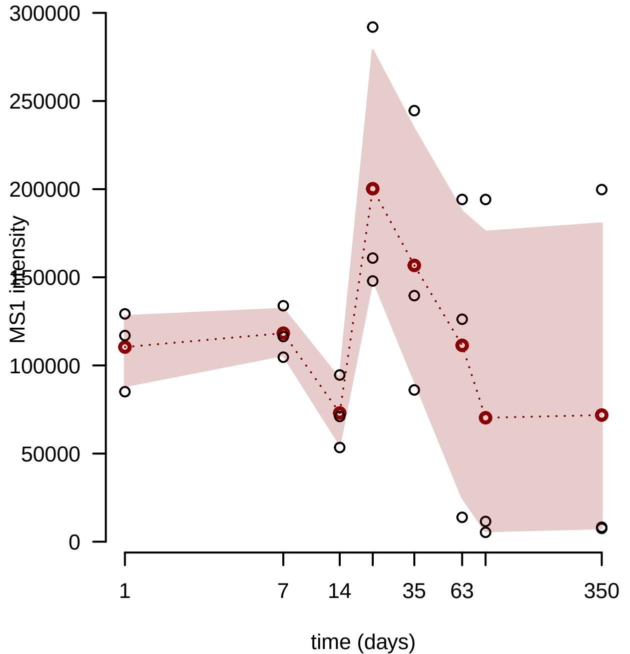

Q9QY53
GN=Nphp1
nephronophthisis 1 (juvenile) homolog (h ...

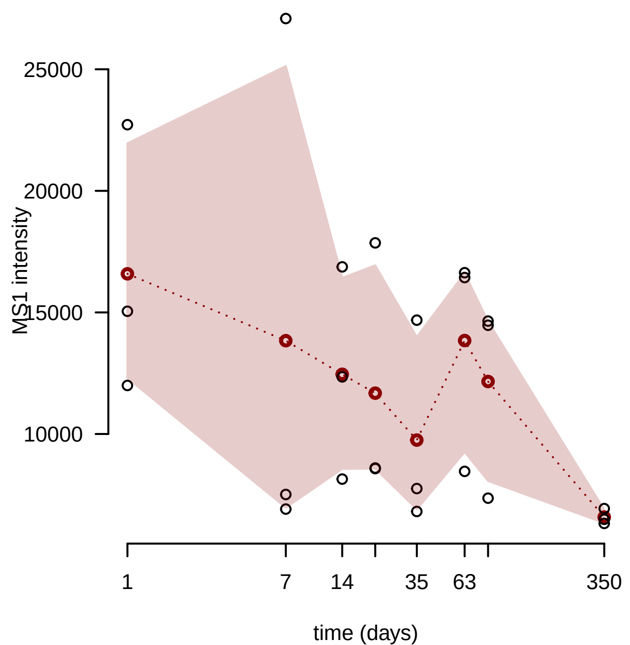

Q9QY66
GN=Znhit2
zinc finger, HIT domain containing 2

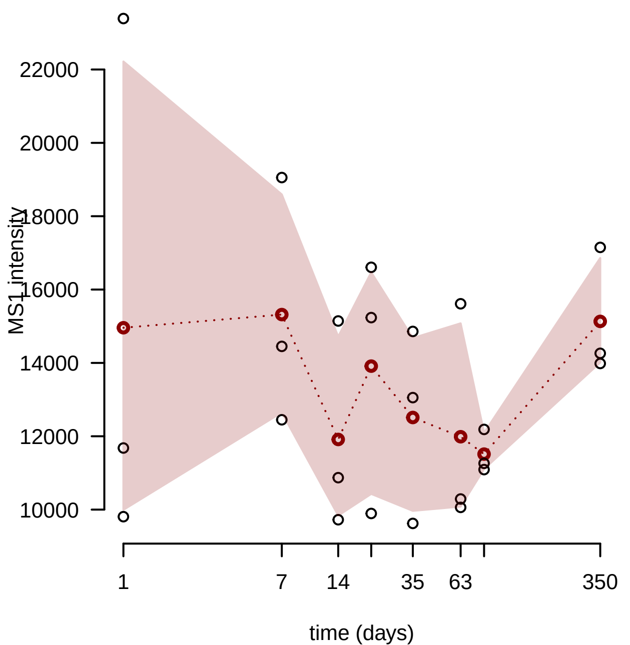

Q9QY73
GN=Tmem59
transmembrane protein 59

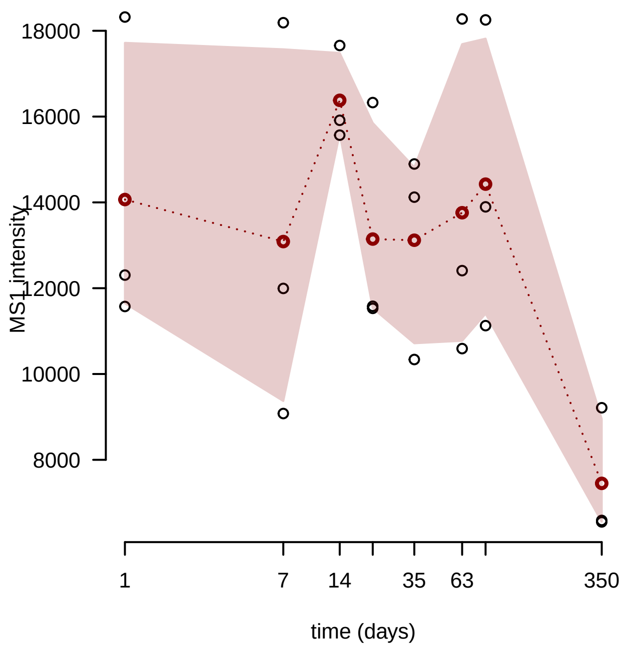

Q9QY76
GN=Vapb
vesicle−associated membrane protein, ass ...

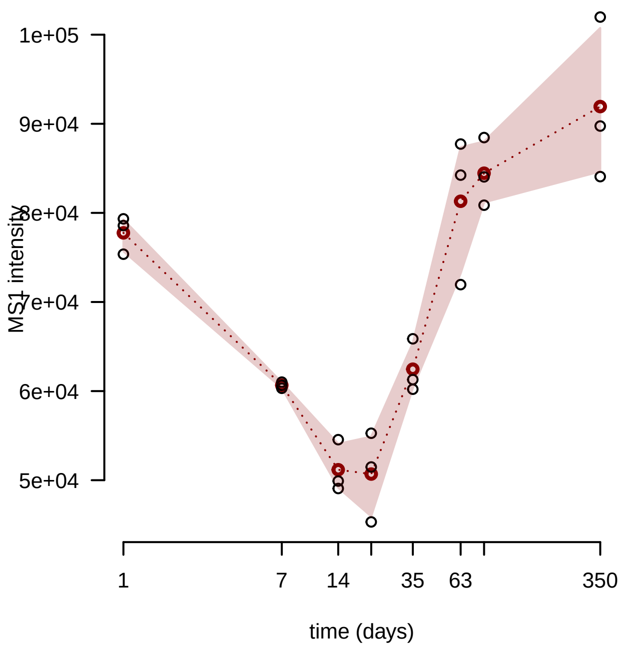

Q9QY81
GN=Nup210
nucleoporin 210

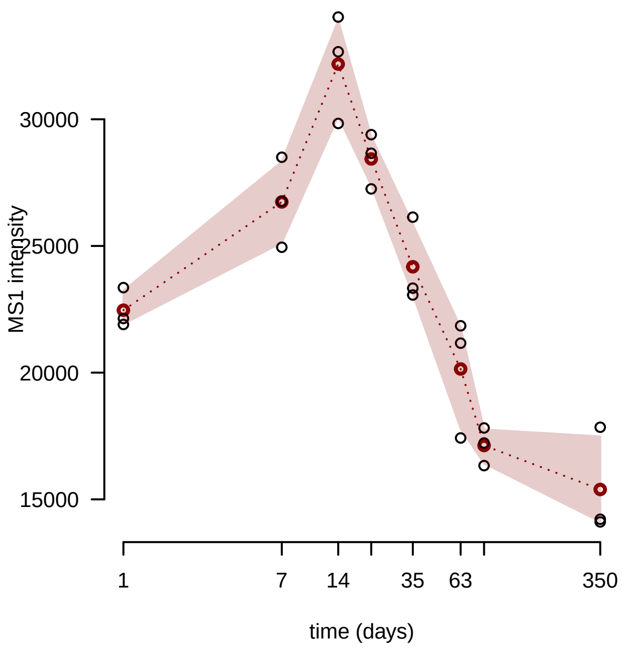

Q9QY93
GN=Dctpp1
dCTP pyrophosphatase 1

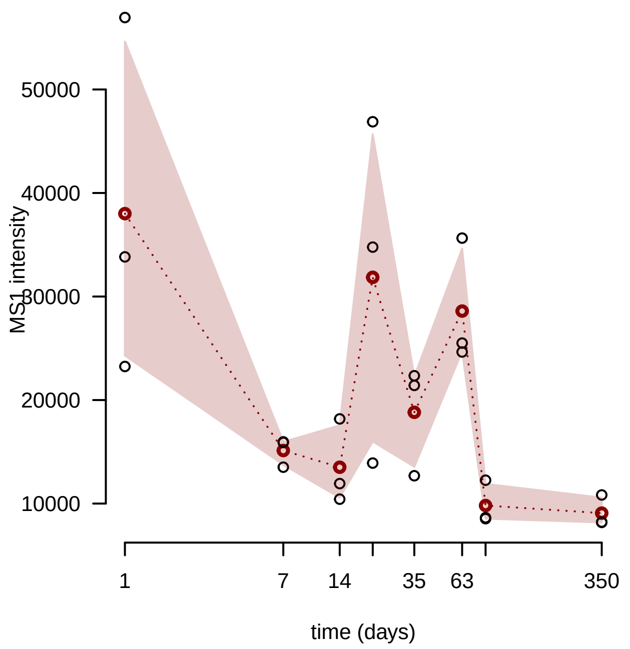

Q9QYA2
GN=Tomm40
translocase of outer mitochondrial membr ...

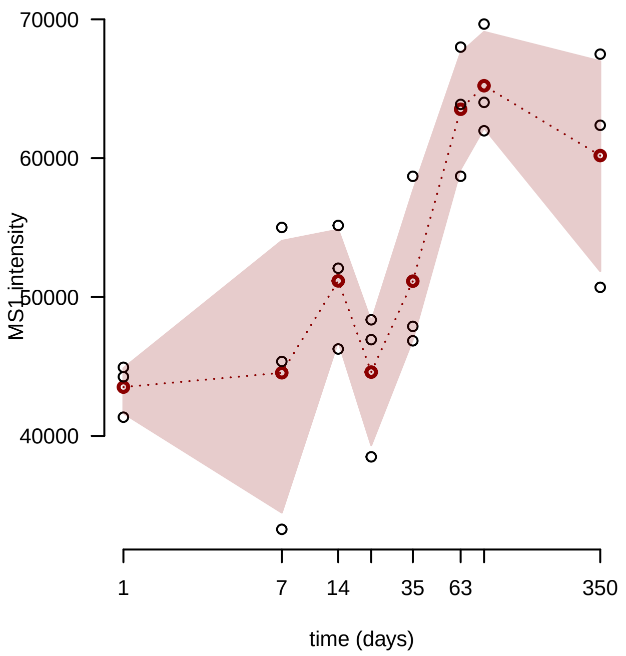

Q9QYB1
GN=Clic4
chloride intracellular channel 4 (mitoch ...

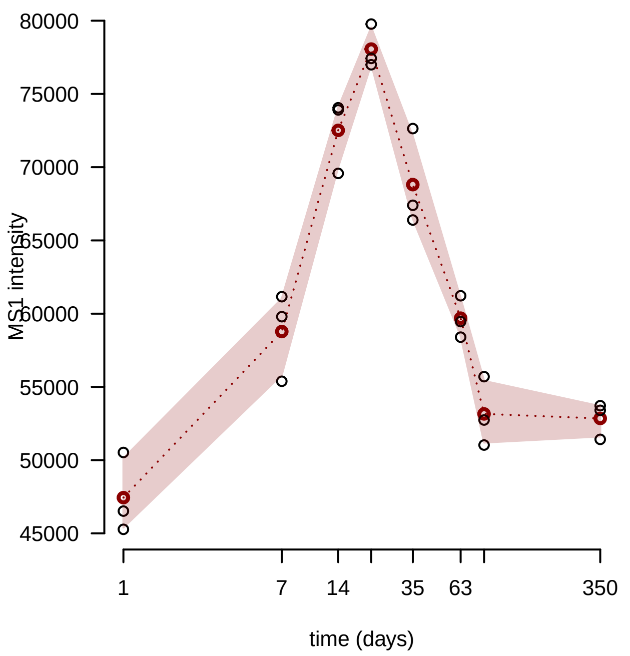

Q9QYB2
GN=Dach1
dachshund family transcription factor 1

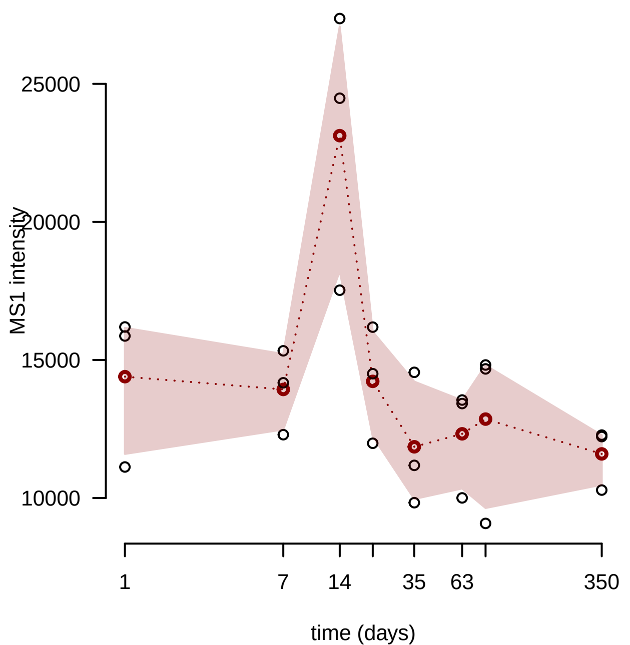

Q9QYB5
GN=Add3
adducin 3 (gamma)

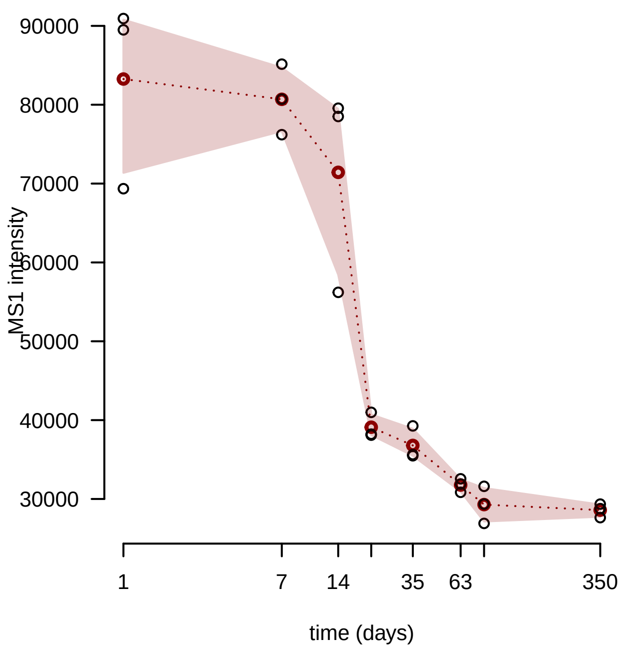

Q9QYB5
GN=Add3
adducin 3 (gamma)

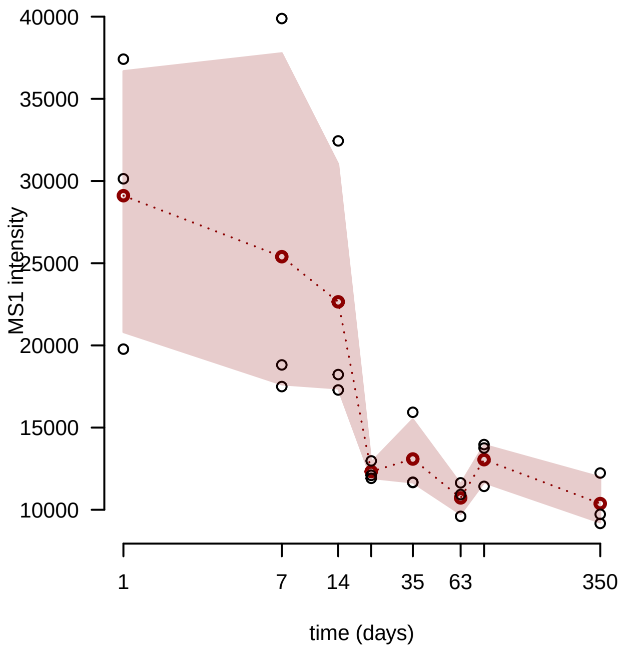

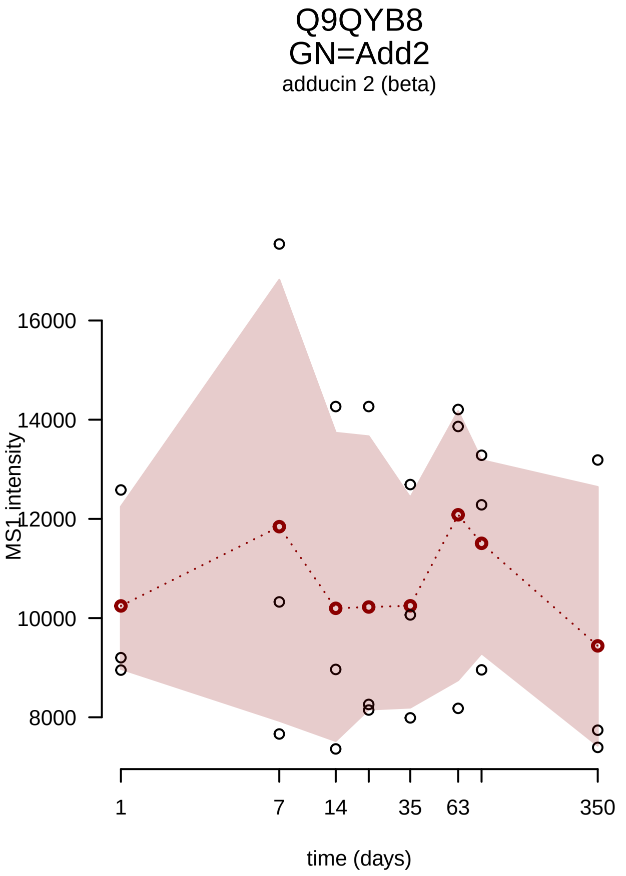
Q9QYB8
GN=Add2
adducin 2 (beta)
MS1 intensity
8000
10000
12000
14000
16000
1
7
14
35
63
350
time (days)
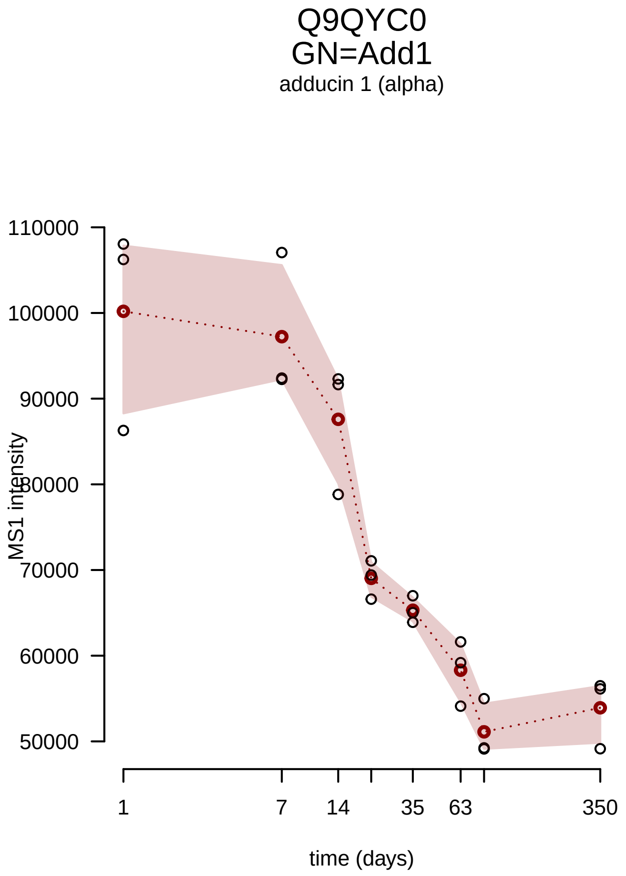
Q9QYC0
GN=Add1
adducin 1 (alpha)
MS1 intensity
50000
60000
70000
80000
90000
100000
110000
1
7
14
35
63
350
time (days)
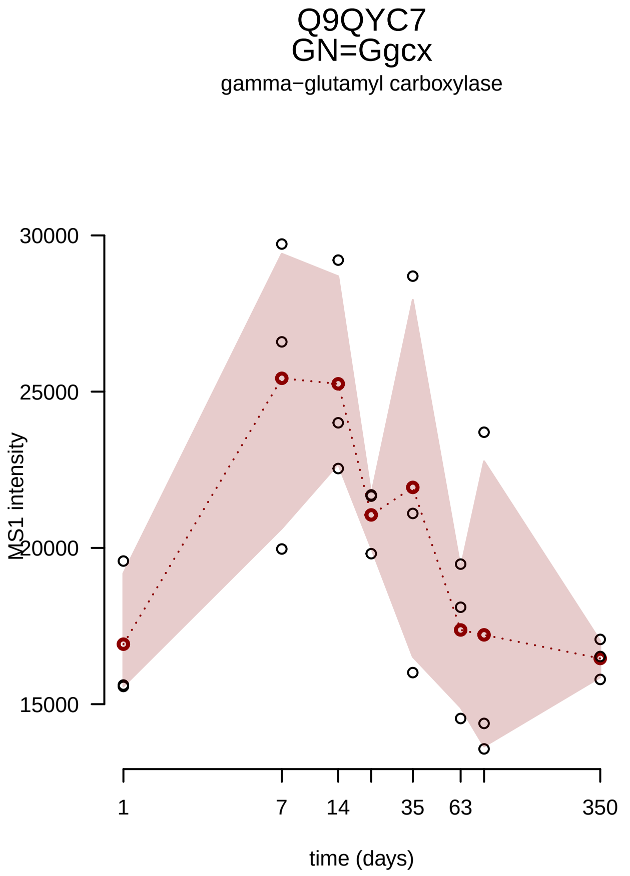
Q9QYC7
GN=Ggcx
gamma−glutamyl carboxylase
MS1 intensity
15000
20000
25000
30000
1
7
14
35
63
350
time (days)
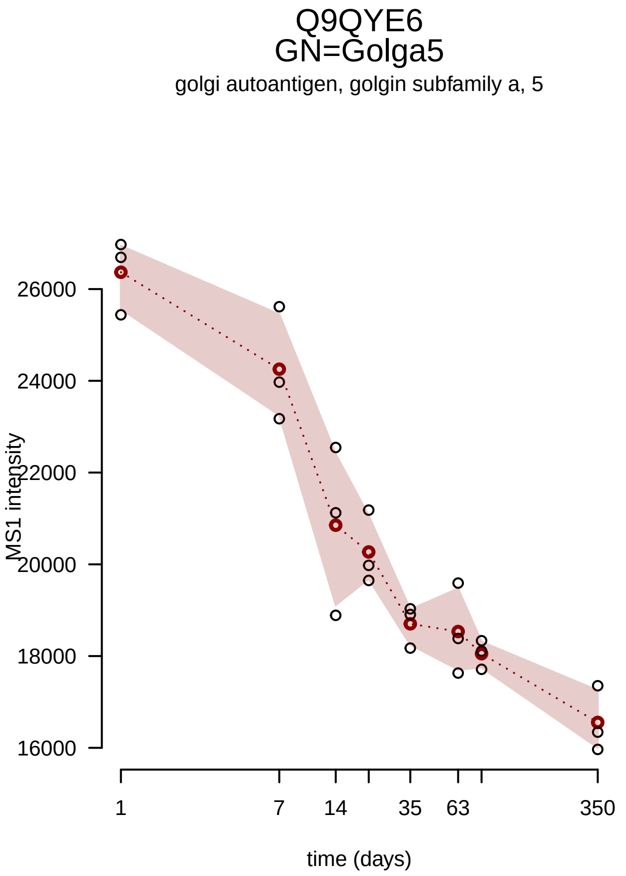
Q9QYE6
GN=Golga5
golgi autoantigen, golgin subfamily a, 5
MS1 intensity
16000
18000
20000
22000
24000
26000
1
7
14
35
63
350
time (days)
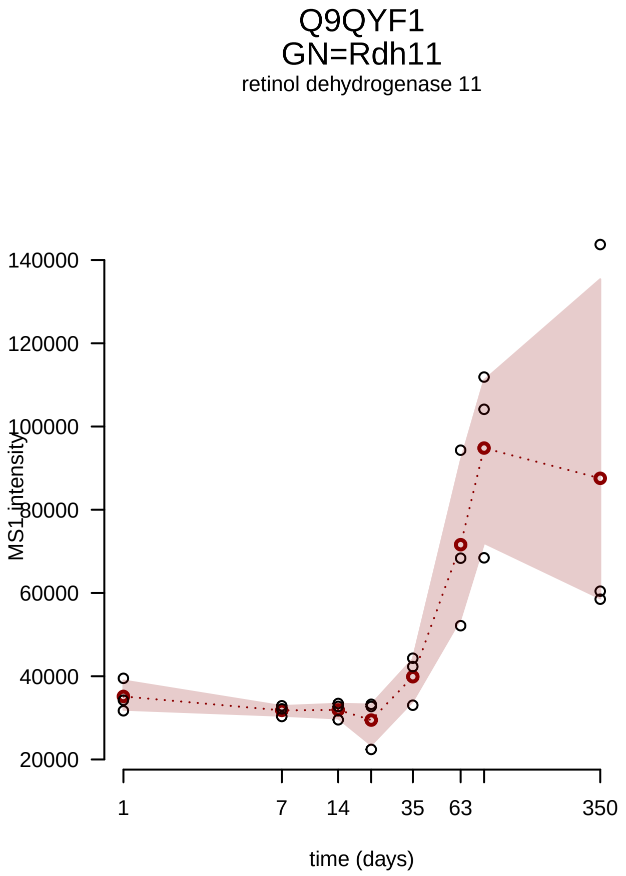
Q9QYF1
GN=Rdh11
retinol dehydrogenase 11
MS1 intensity
20000
40000
60000
80000
100000
120000
140000
1
7
14
35
63
350
time (days)
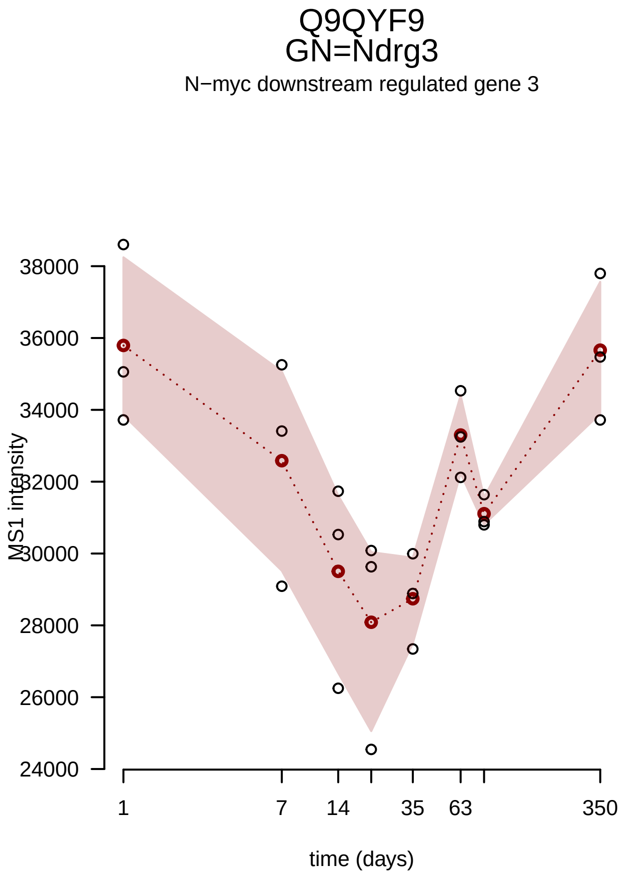
Q9QYF9
GN=Ndrg3
N−myc downstream regulated gene 3
MS1 intensity
24000
26000
28000
30000
32000
34000
36000
38000
1
7
14
35
63
350
time (days)
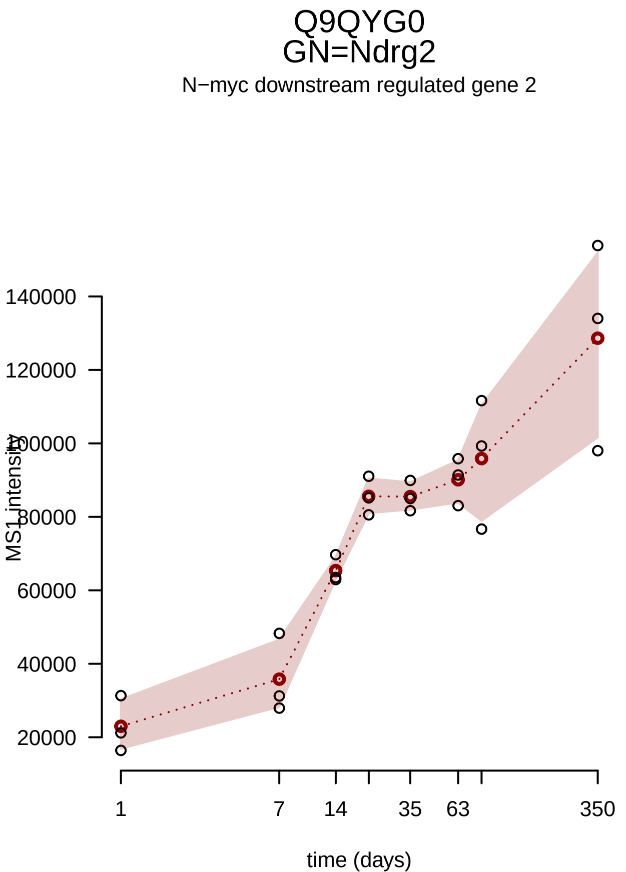
Q9QYG0
GN=Ndrg2
N−myc downstream regulated gene 2
MS1 intensity
20000
40000
60000
80000
100000
120000
140000
1
7
14
35
63
350
time (days)
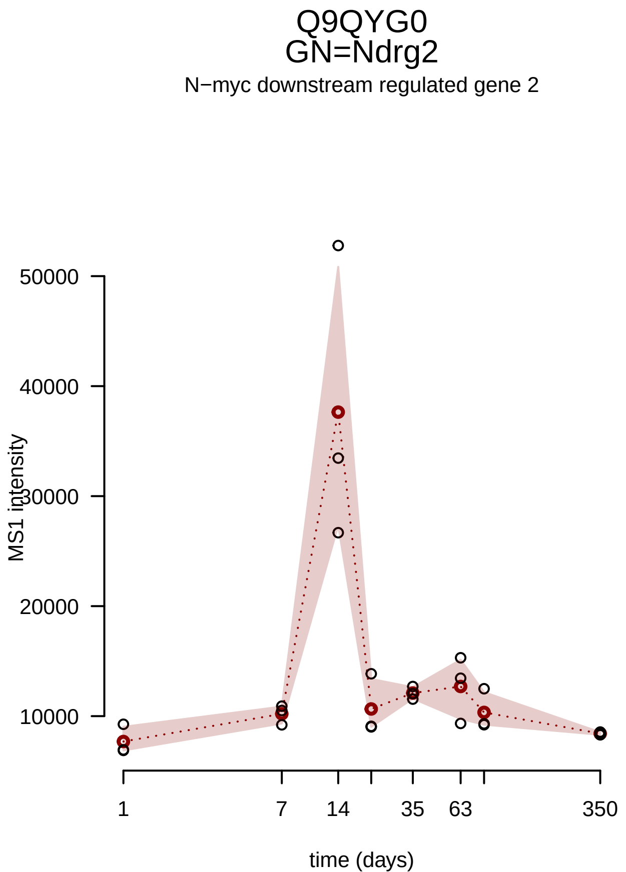
Q9QYG0
GN=Ndrg2
N−myc downstream regulated gene 2
MS1 intensity
10000
20000
30000
40000
50000
1
7
14
35
63
350
time (days)
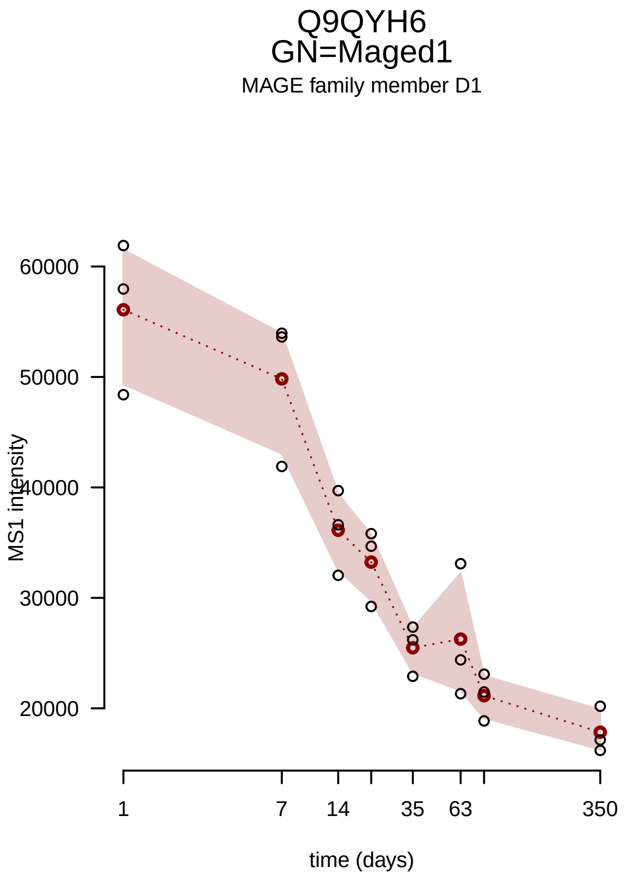
Q9QYH6
GN=Maged1
MAGE family member D1
MS1 intensity
20000
30000
40000
50000
60000
1
7
14
35
63
350
time (days)

Q9QYI3
GN=Dnajc7
DnaJ heat shock protein family (Hsp40) m ...

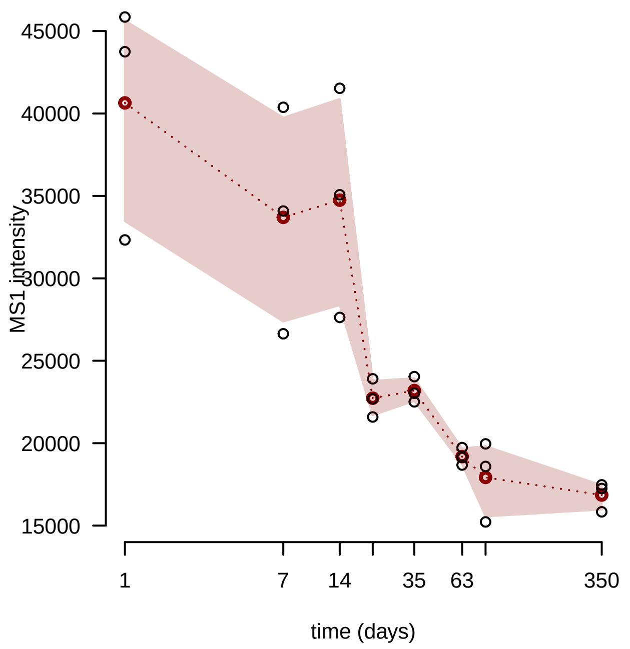

Q9QYI4
GN=Dnajb12
DnaJ heat shock protein family (Hsp40) m ...

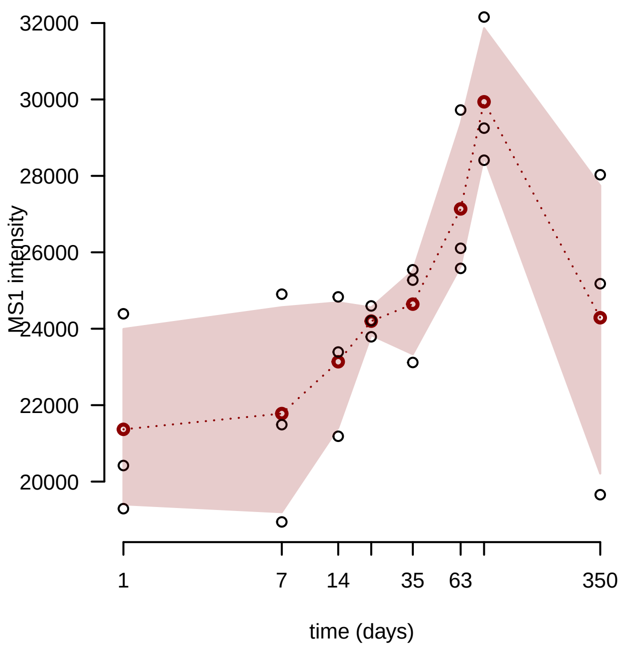

Q9QYI5
GN=Dnajb2
DnaJ heat shock protein family (Hsp40) m ...

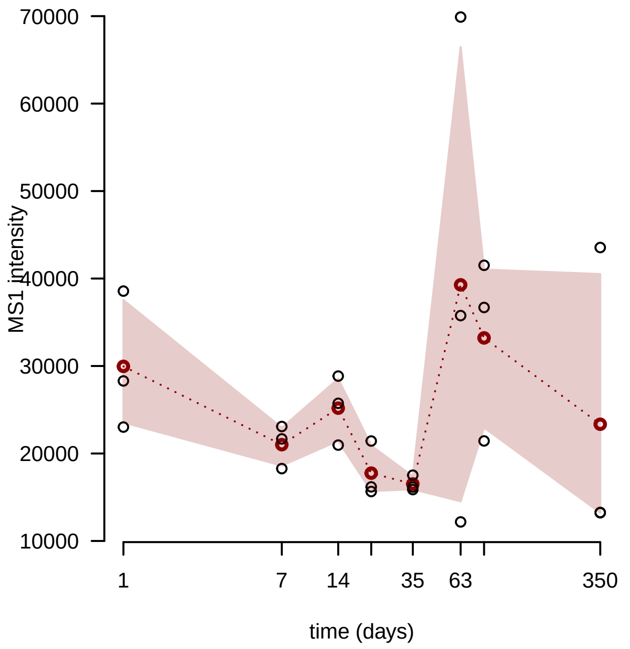

Q9QYI6
GN=Dnajb9
DnaJ heat shock protein family (Hsp40) m ...

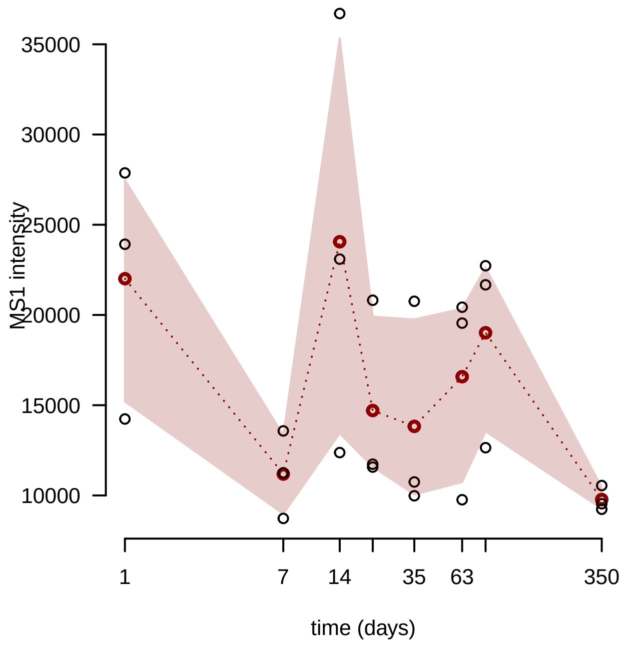

Q9QYJ0
GN=Dnaja2
DnaJ heat shock protein family (Hsp40) m ...

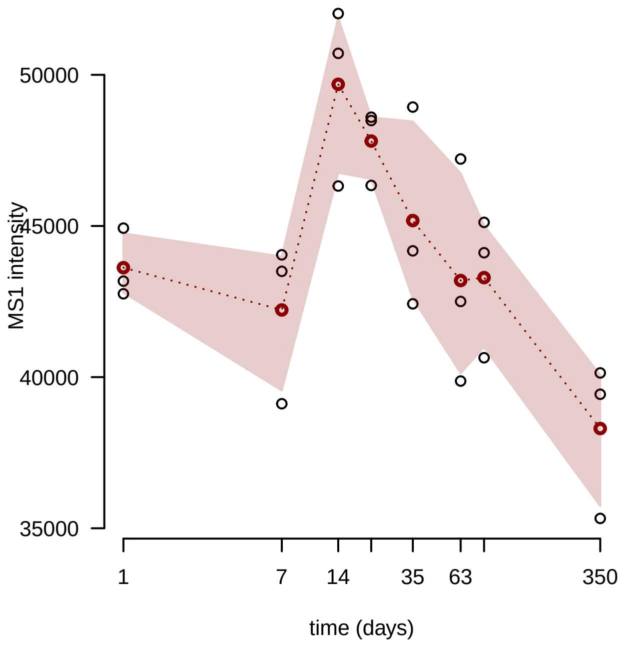

Q9QYJ3
GN=Dnajb1
DnaJ heat shock protein family (Hsp40) m ...

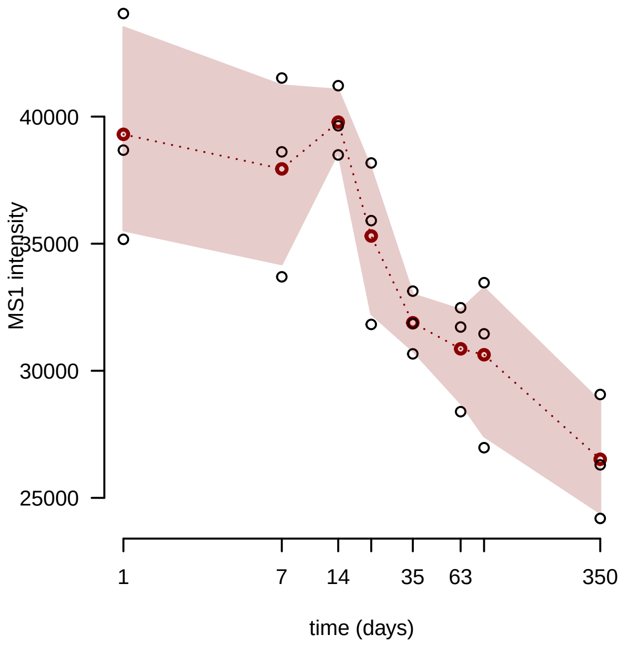

Q9QYL7
GN=Abt1
activator of basal transcription 1

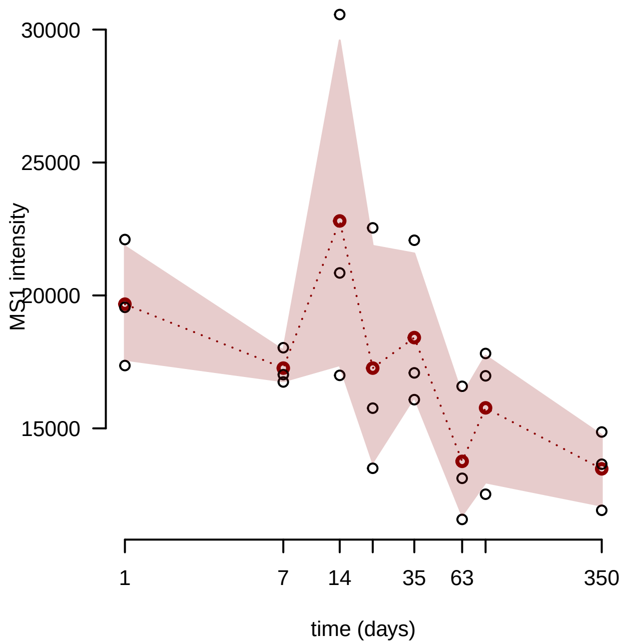

Q9QYM5
GN=Rnd2
Rho family GTPase 2

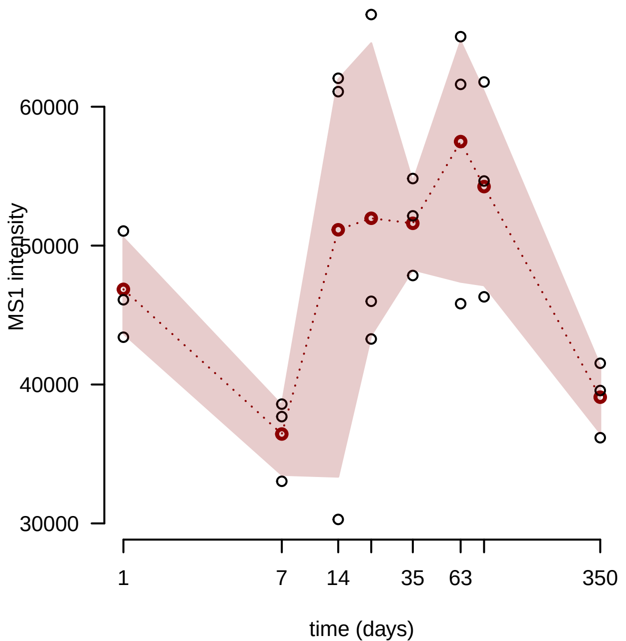

Q9QYM8
GN=Cenph
centromere protein H

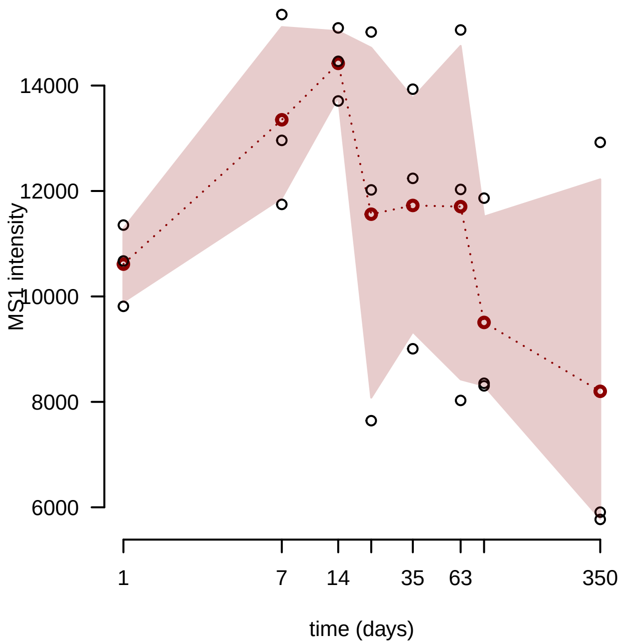

Q9QYP6
GN=Azi2
5−azacytidine induced gene 2

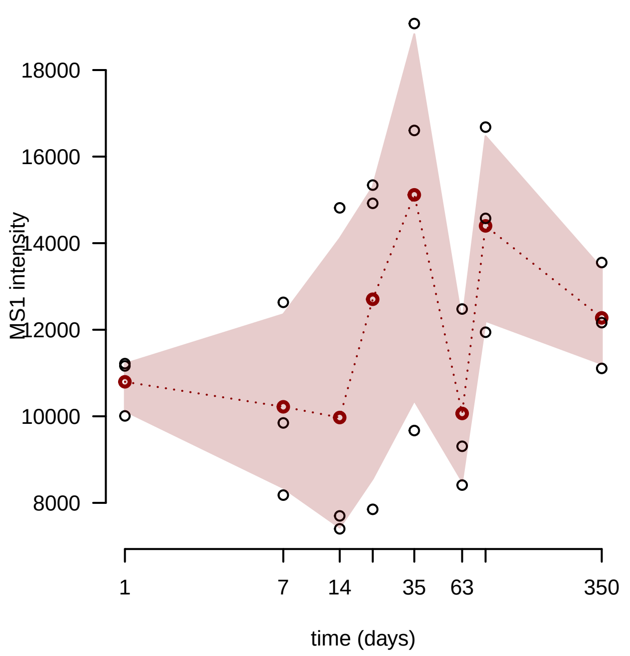

Q9QYR6
GN=Map1a
microtubule−associated protein 1 A

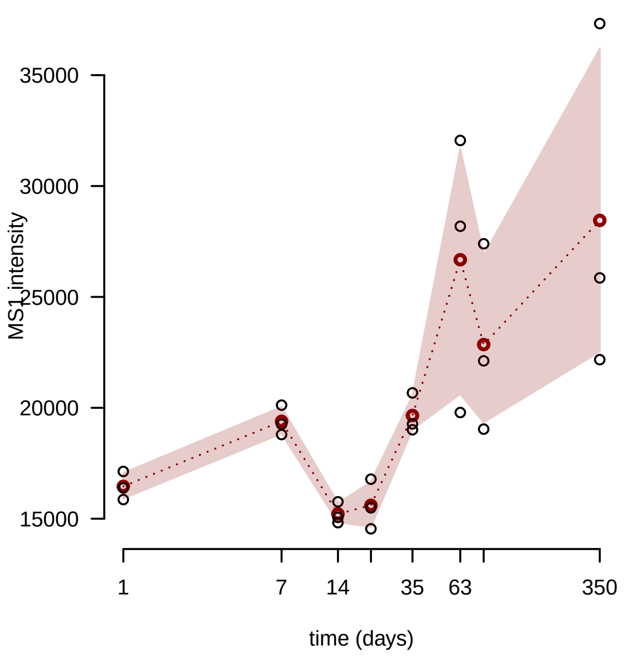

Q9QYR9
GN=Acot2
acyl−CoA thioesterase 2

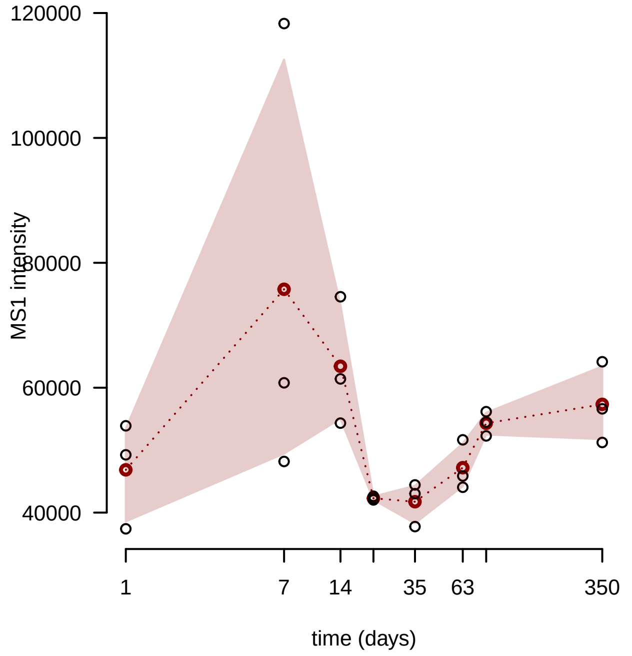

Q9QYS9
GN=Qki
quaking, KH domain containing RNA bindin ...

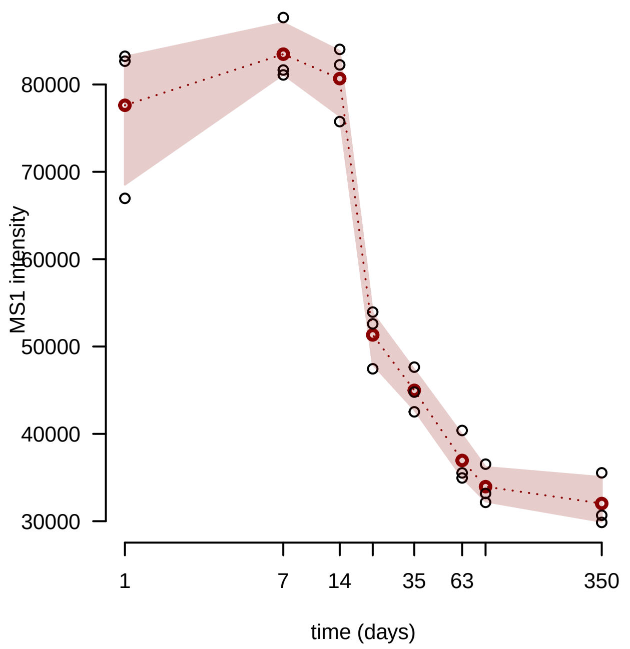

Q9QYT7
GN=Pigq
phosphatidylinositol glycan anchor biosy ...

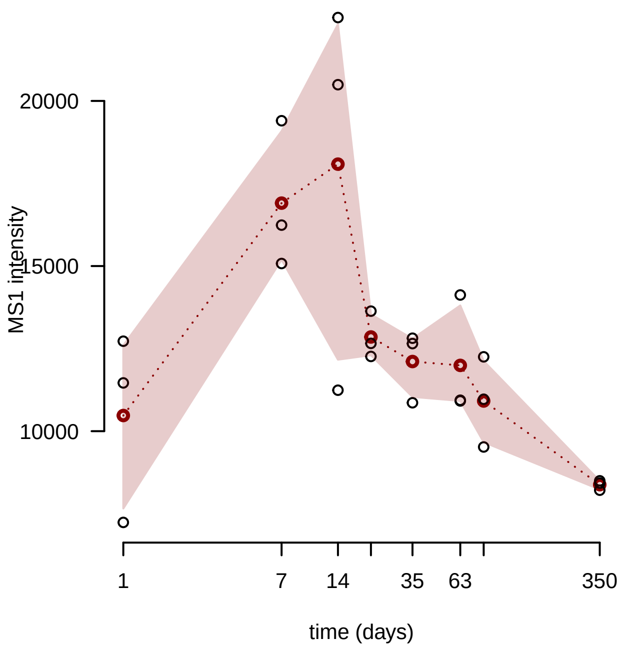

Q9QYY0
GN=Gab1
growth factor receptor bound protein 2−a ...

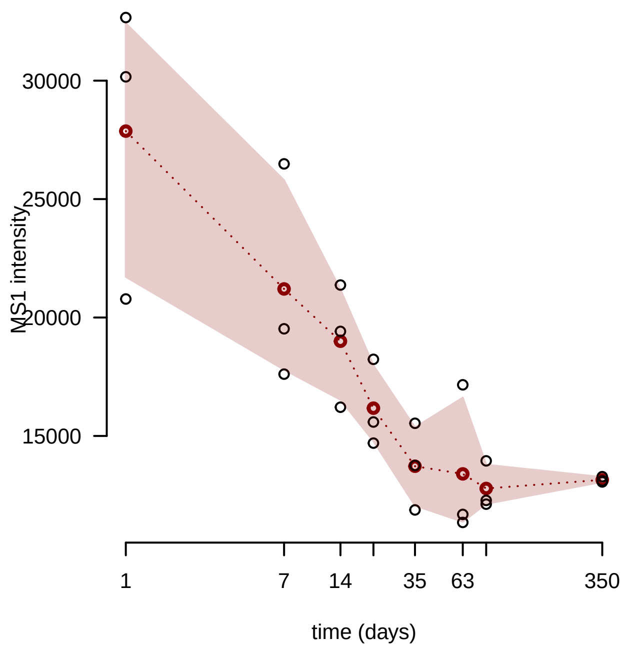

Q9QYY8
GN=Spast
spastin

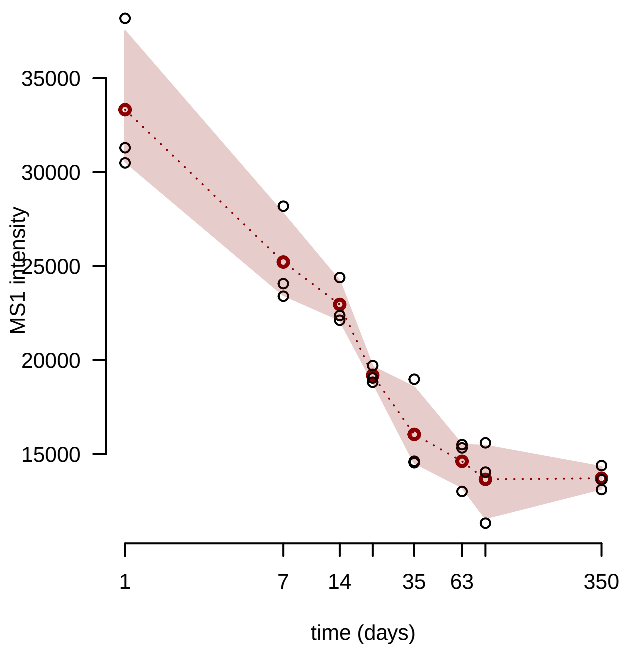

Q9QZ03
GN=Slc39a1
solute carrier family 39 (zinc transport ...

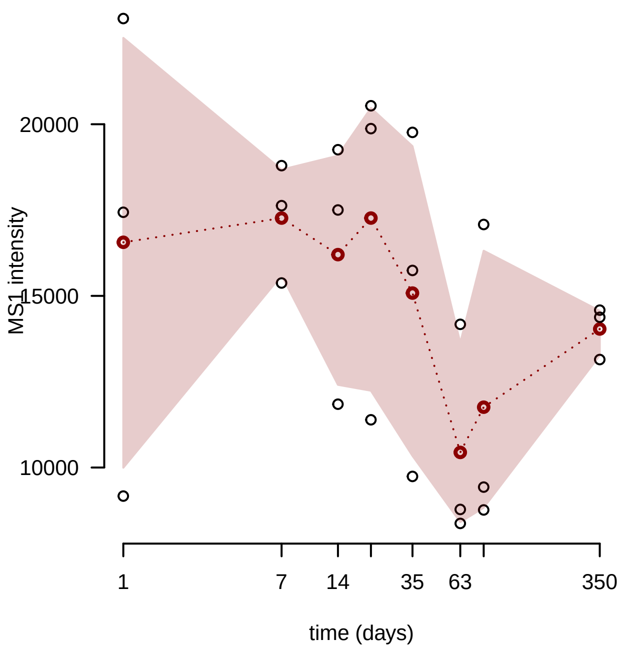

Q9QZ05
GN=Eif2ak4
eukaryotic translation initiation factor ...

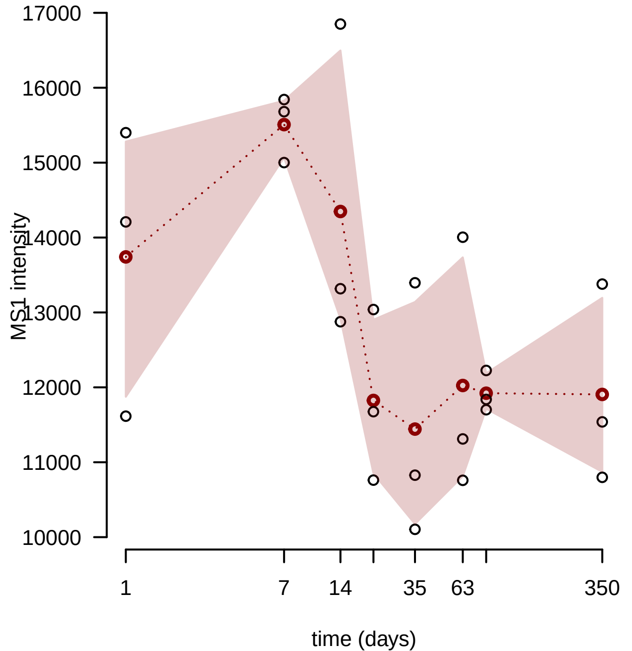

Q9QZ06
GN=Tollip
toll interacting protein

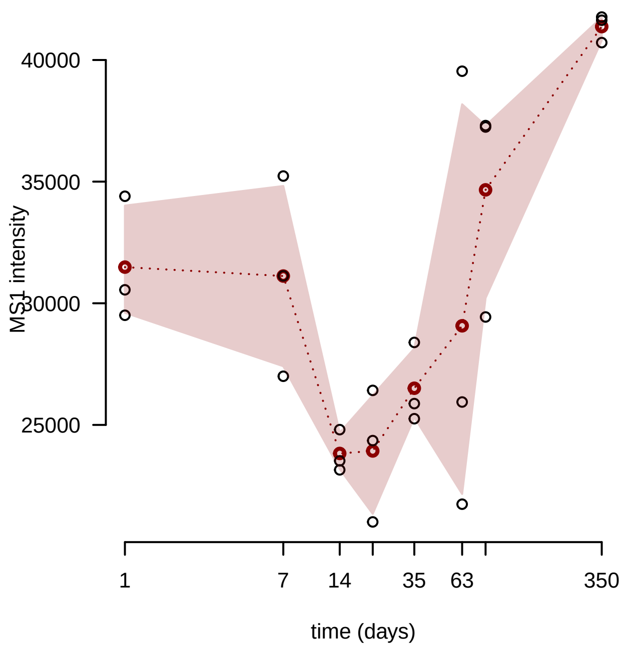

Q9QZ08
GN=Nagk
N−acetylglucosamine kinase

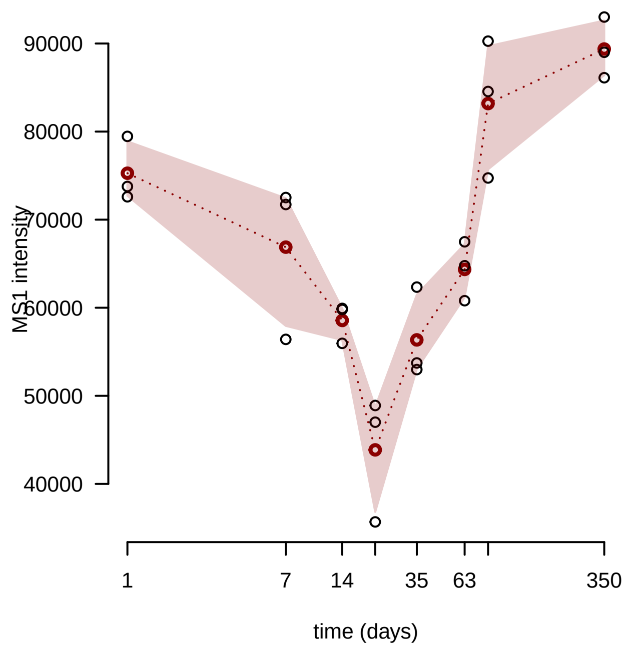

Q9QZ23
GN=Nfu1
NFU1 iron−sulfur cluster scaffold

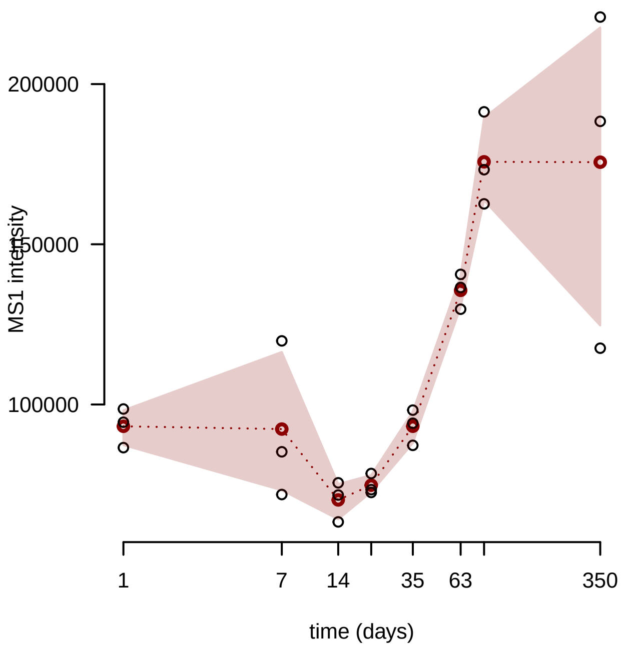

Q9QZ47
GN=Tnnt3
troponin T3, skeletal, fast

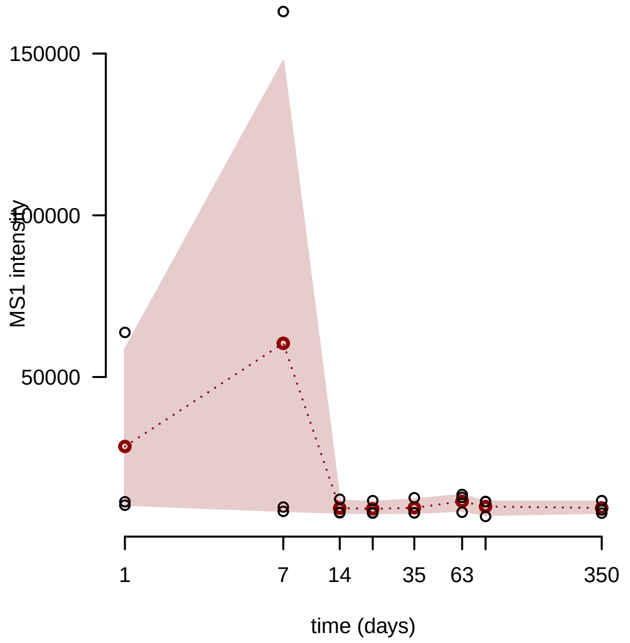

Q9QZ49
GN=Ubxn8
UBX domain protein 8

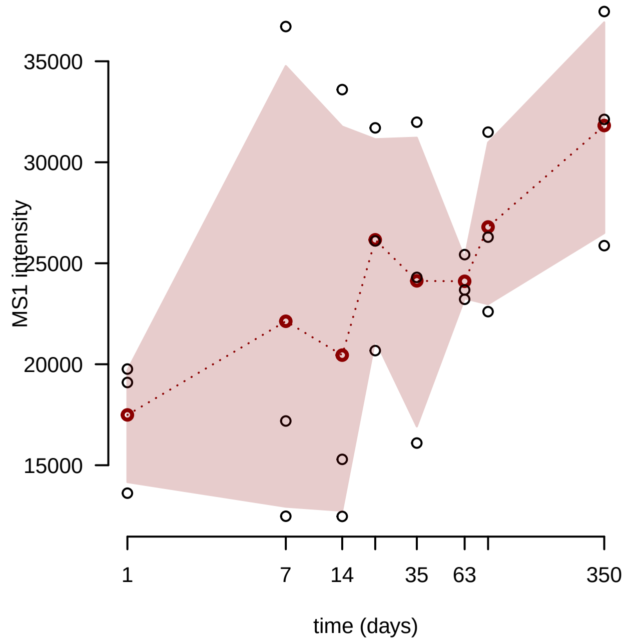

Q9QZ67
GN=Ppm1d
protein phosphatase 1D magnesium−depende ...

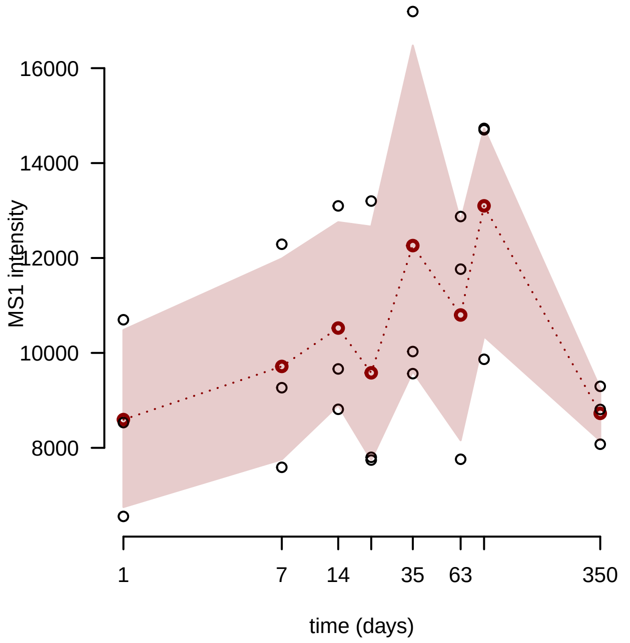

Q9QZ73
GN=Dcun1d1
DCN1, defective in cullin neddylation 1, ...

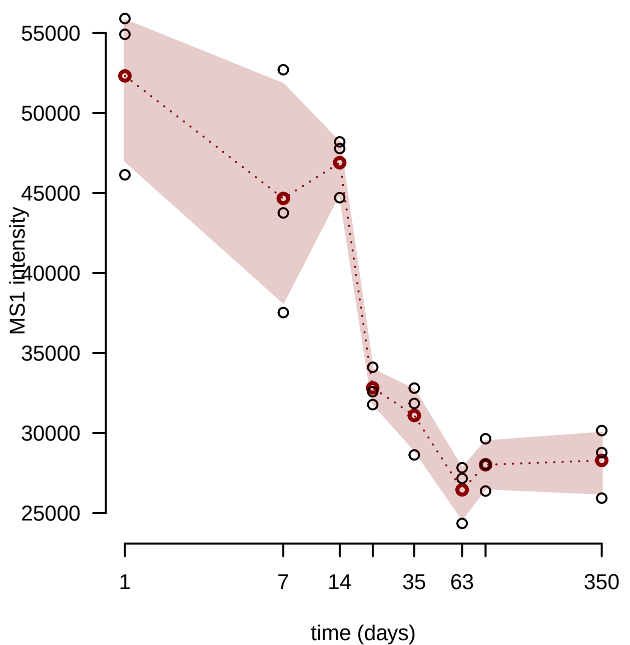

Q9QZ82
GN=Cyp11a1
cytochrome P450, family 11, subfamily a, ...

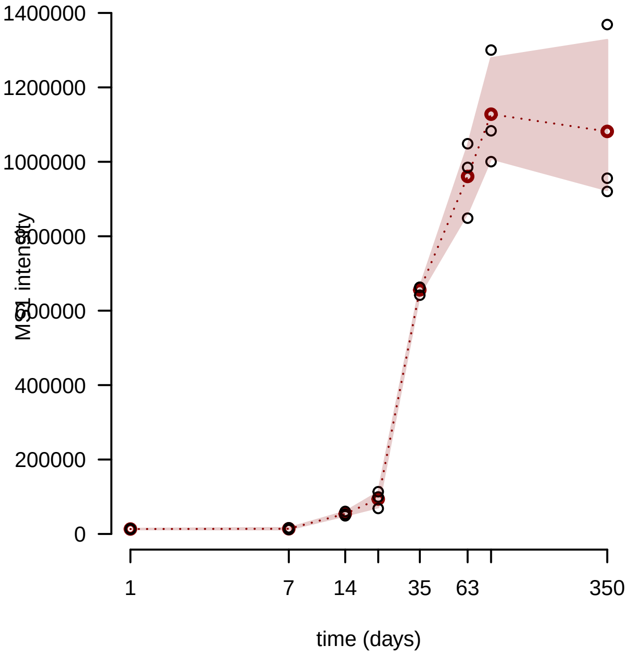

Q9QZ85
GN=Iigp1
interferon inducible GTPase 1

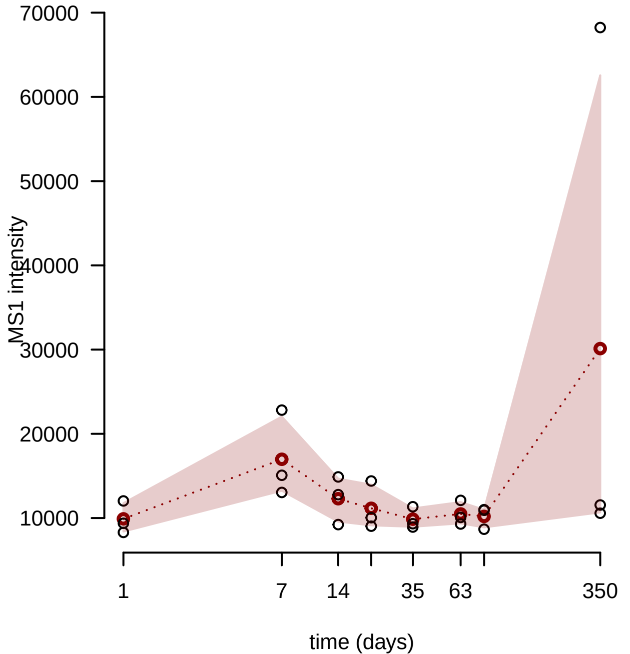

Q9QZ88
GN=Vps29
VPS29 retromer complex component

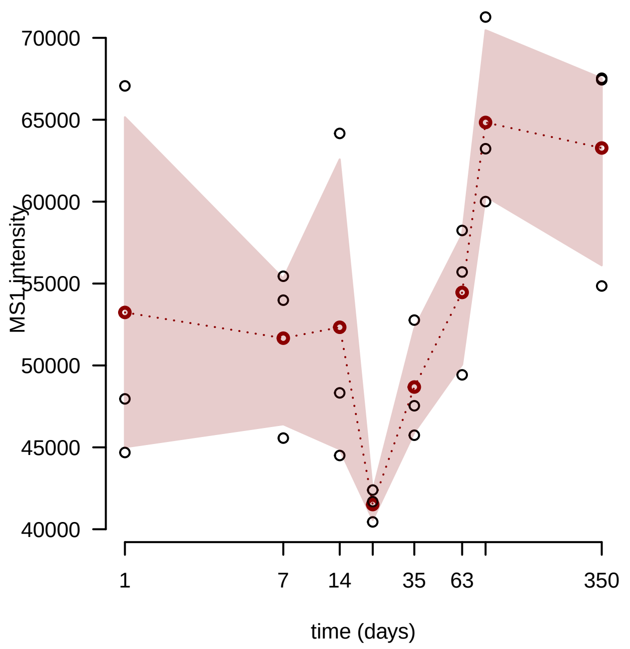

Q9QZA0
GN=Ca5b

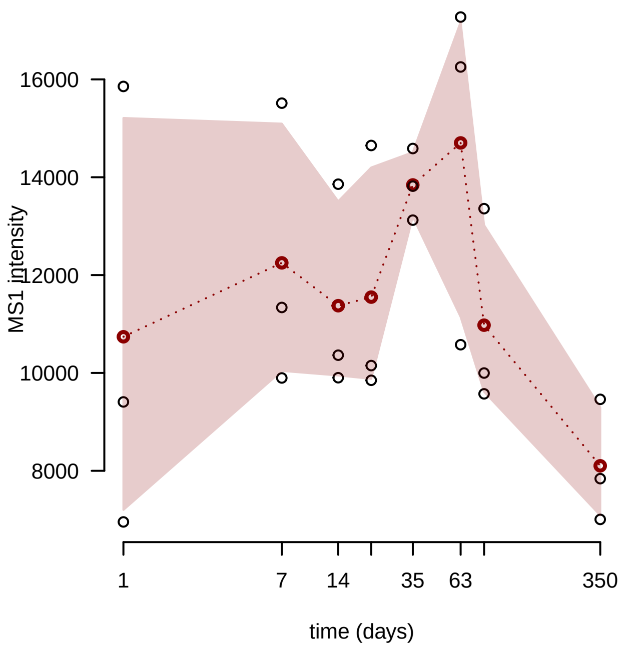

Q9QZB7
GN=Actr10
ARP10 actin−related protein 10

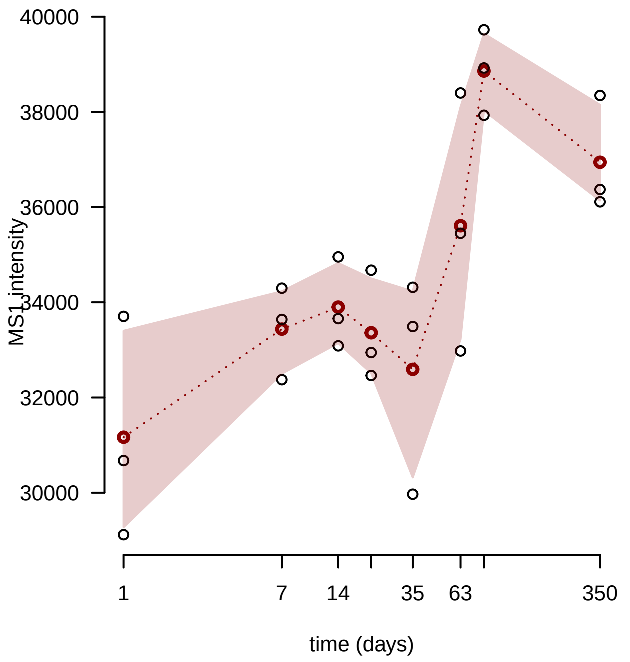

Q9QZB9
GN=Dctn5
dynactin 5

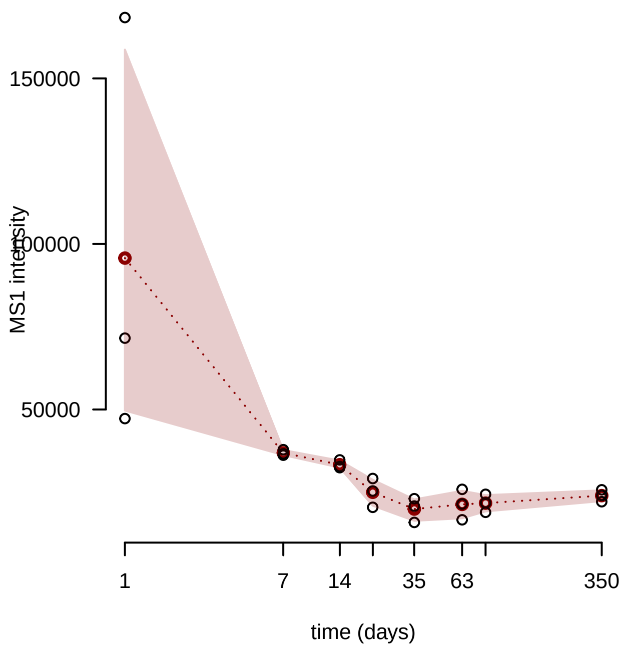

Q9QZC2
GN=Plxnc1
plexin C1

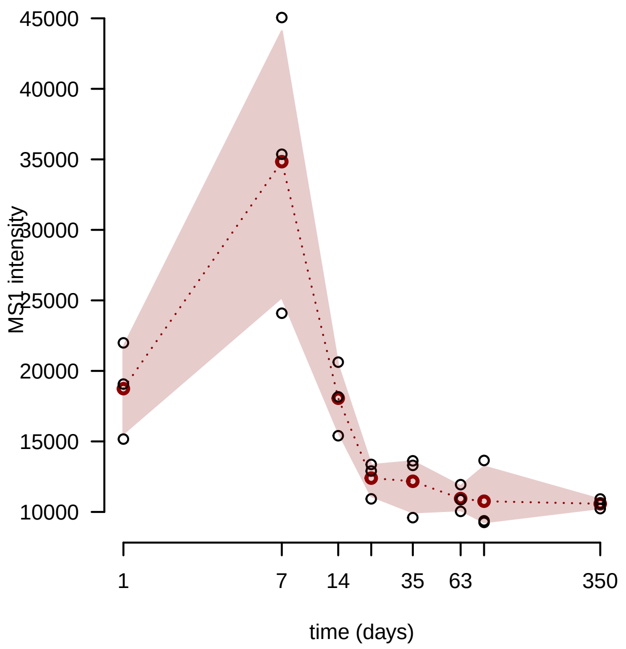

Q9QZC8
GN=Abhd1
abhydrolase domain containing 1

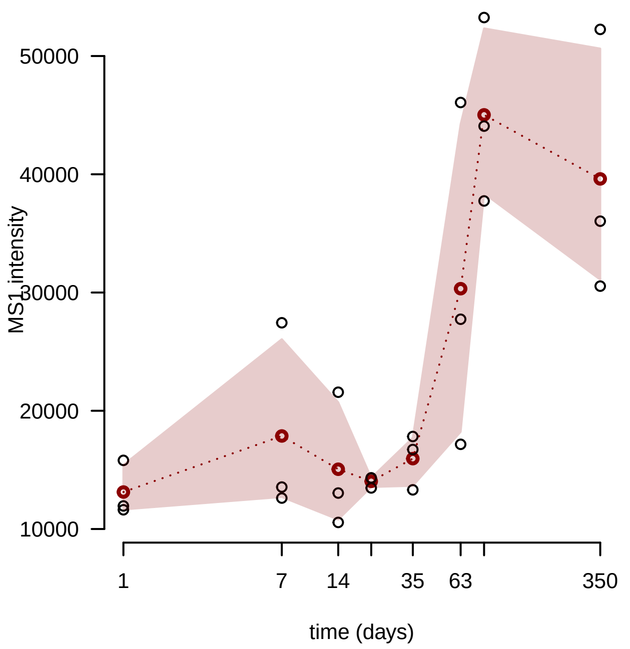

Q9QZD4
GN=Ercc4
excision repair cross−complementing rode ...

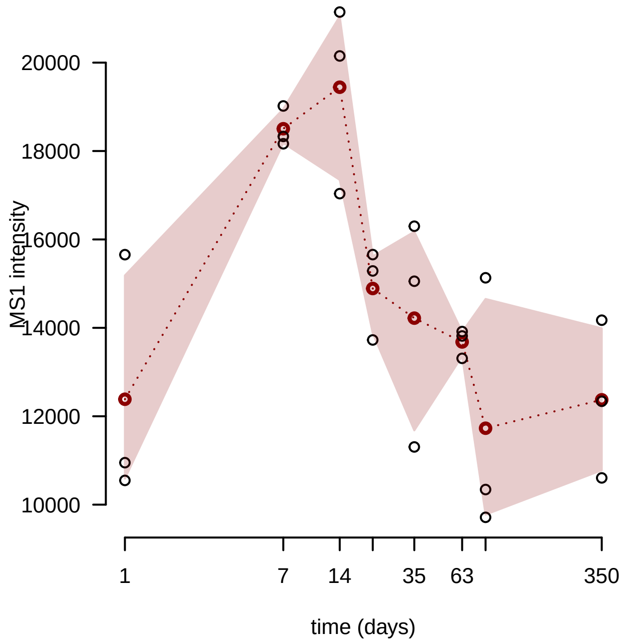

Q9QZD8
GN=Slc25a10
solute carrier family 25 (mitochondrial ...

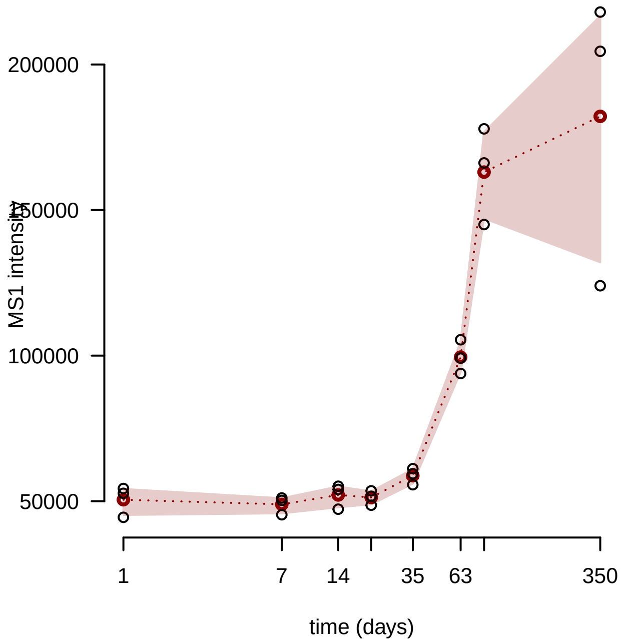

Q9QZD9
GN=Eif3i
eukaryotic translation initiation factor ...

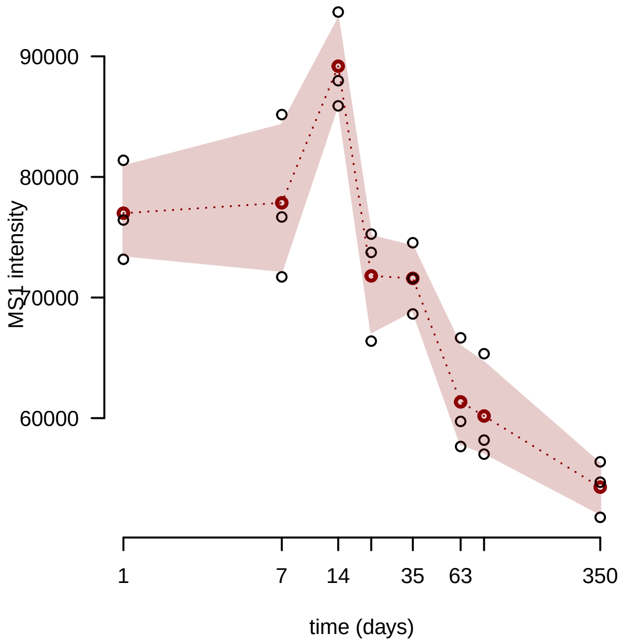

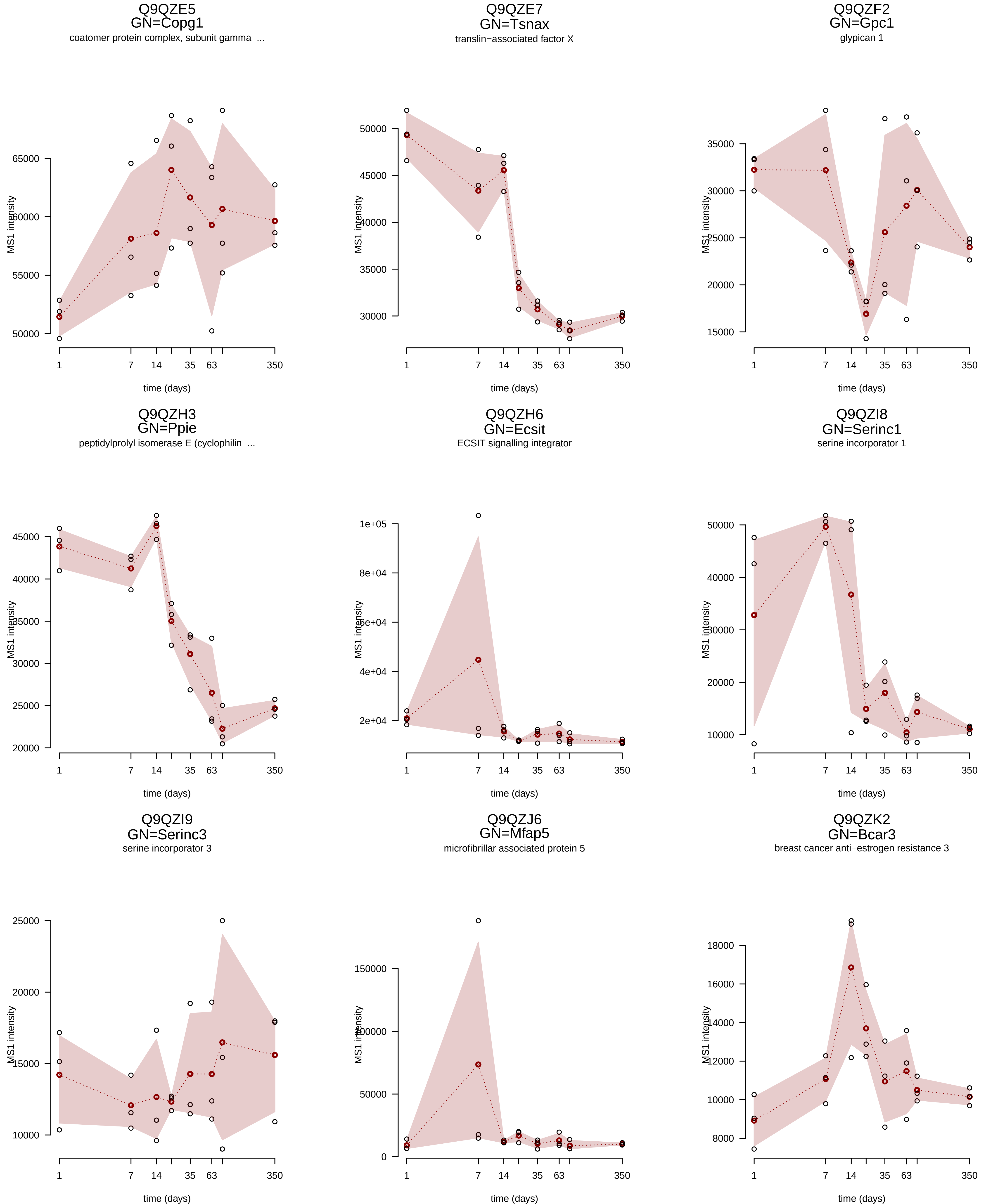
Q9QZE5
GN=Copg1
coatomer protein complex, subunit gamma ...
MS1 intensity
50000 55000 60000 65000
1 7 14 35 63 350
time (days)
Q9QZE7
GN=Tsnax
translin−associated factor X
MS1 intensity
30000 35000 40000 45000 50000
1 7 14 35 63 350
time (days)
Q9QZF2
GN=Gpc1
glypican 1
MS1 intensity
15000 20000 25000 30000 35000
1 7 14 35 63 350
time (days)
Q9QZH3
GN=Ppie
peptidylprolyl isomerase E (cyclophilin ...
MS1 intensity
20000 25000 30000 35000 40000 45000
1 7 14 35 63 350
time (days)
Q9QZH6
GN=Ecsit
ECSIT signalling integrator
MS1 intensity
2e+04 4e+04 6e+04 8e+04 1e+05
1 7 14 35 63 350
time (days)
Q9QZI8
GN=Serinc1
serine incorporator 1
MS1 intensity
10000 20000 30000 40000 50000
1 7 14 35 63 350
time (days)
Q9QZI9
GN=Serinc3
serine incorporator 3
MS1 intensity
10000 15000 20000 25000
1 7 14 35 63 350
time (days)
Q9QZJ6
GN=Mfap5
microfibrillar associated protein 5
MS1 intensity
0 50000 100000 150000
1 7 14 35 63 350
time (days)
Q9QZK2
GN=Bcar3
breast cancer anti−estrogen resistance 3
MS1 intensity
8000 10000 12000 14000 16000 18000
1 7 14 35 63 350
time (days)

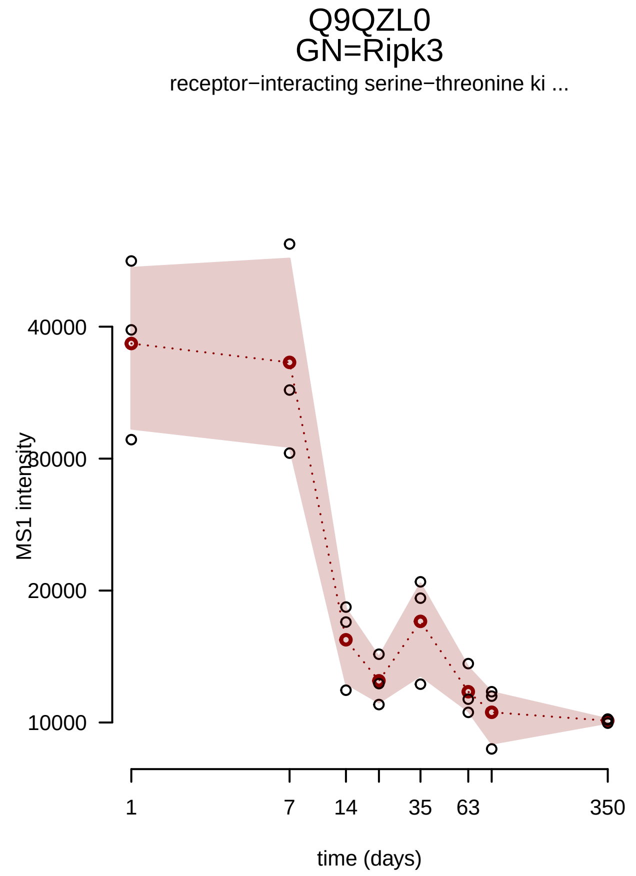
Q9QZL0
GN=Ripk3
receptor−interacting serine−threonine ki ...
MS1 intensity
40000
30000
20000
10000
1
7
14
35
63
350
time (days)

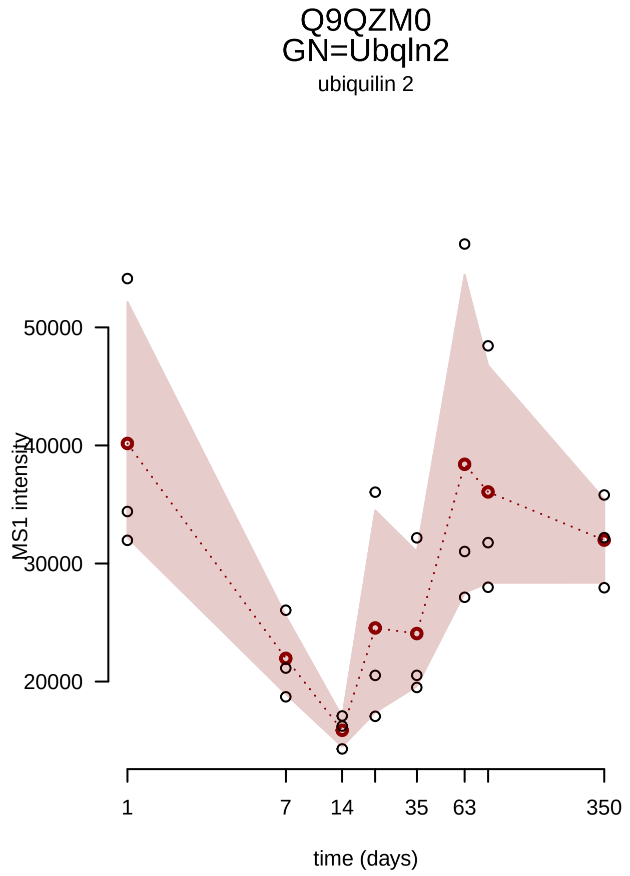
Q9QZM0
GN=Ubqln2
ubiquilin 2
MS1 intensity
50000
40000
30000
20000
1
7
14
35
63
350
time (days)

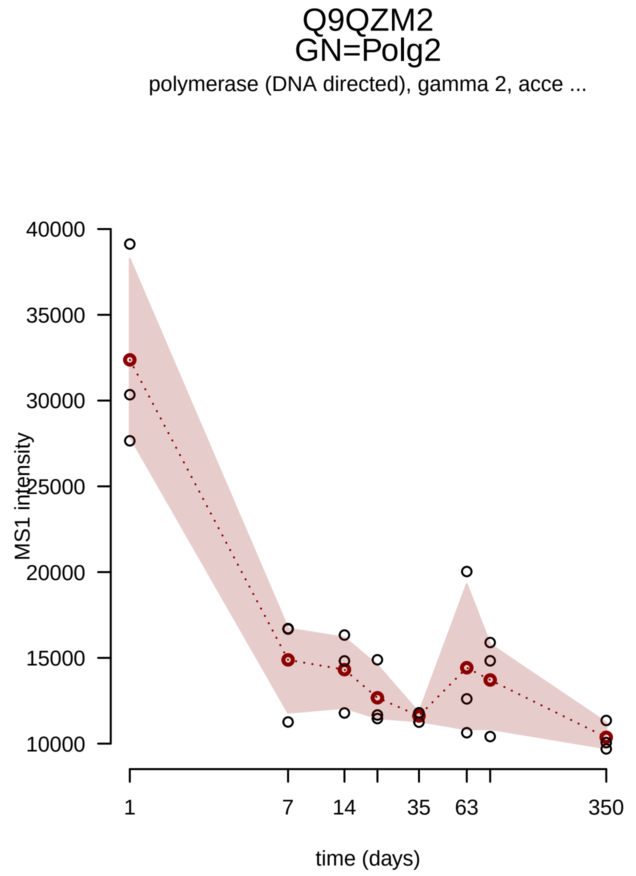
Q9QZM2
GN=Polg2
polymerase (DNA directed), gamma 2, acce ...
MS1 intensity
40000
35000
30000
25000
20000
15000
10000
1
7
14
35
63
350
time (days)

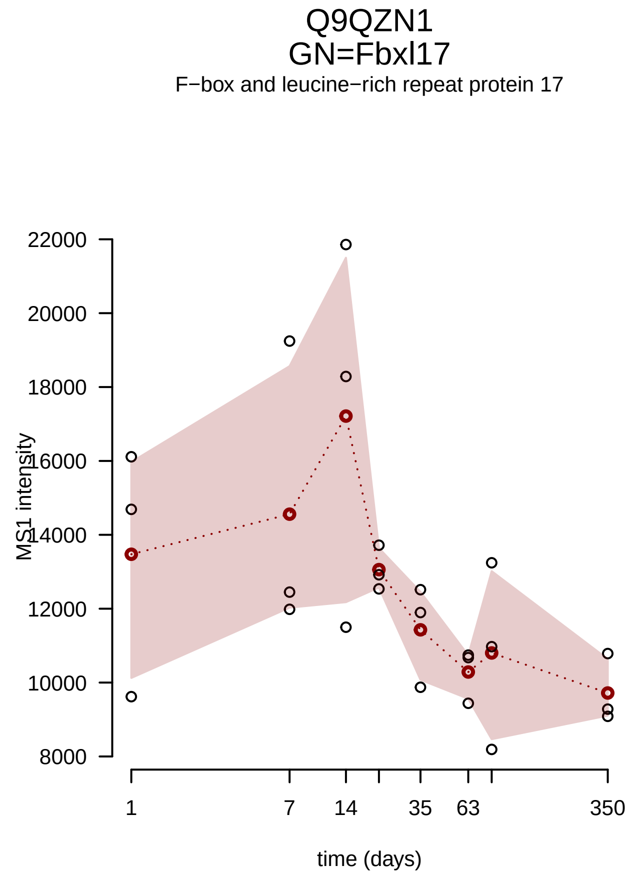
Q9QZN1
GN=Fbxl17
F−box and leucine−rich repeat protein 17
MS1 intensity
22000
20000
18000
16000
14000
12000
10000
8000
1
7
14
35
63
350
time (days)

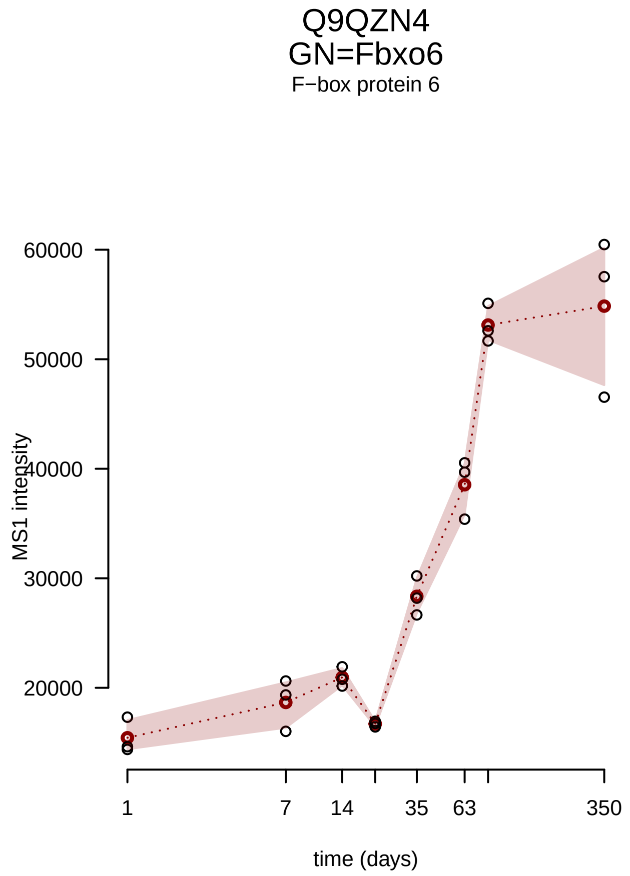
Q9QZN4
GN=Fbxo6
F−box protein 6
MS1 intensity
60000
50000
40000
30000
20000
1
7
14
35
63
350
time (days)

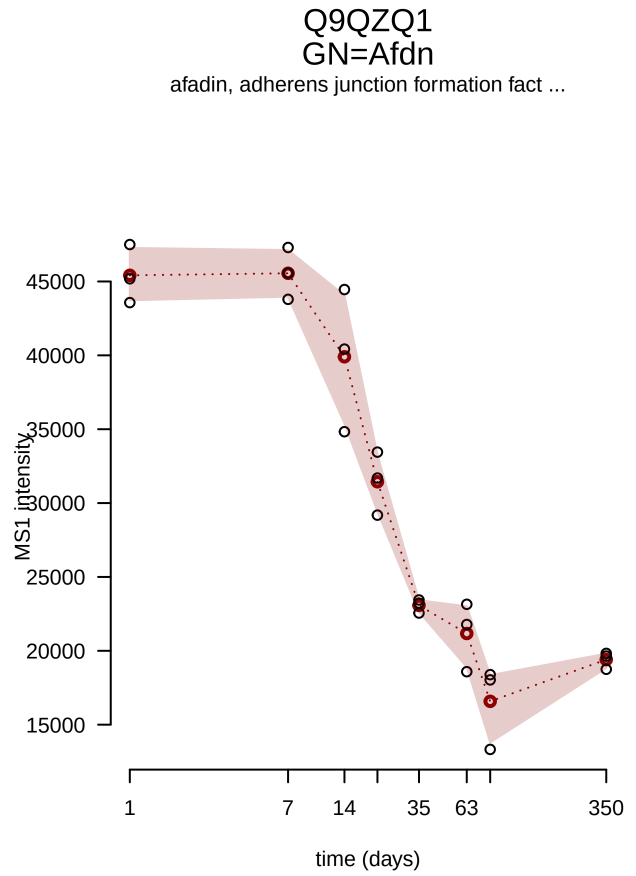
Q9QZQ1
GN=Afdn
afadin, adherens junction formation fact ...
MS1 intensity
45000
40000
35000
30000
25000
20000
15000
1
7
14
35
63
350
time (days)

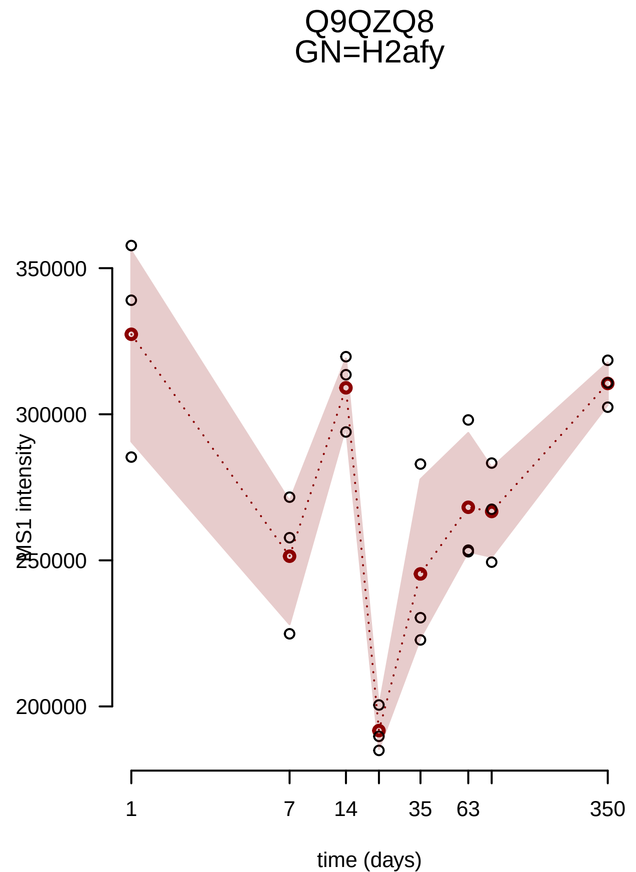
Q9QZQ8
GN=H2afy
MS1 intensity
350000
300000
250000
200000
1
7
14
35
63
350
time (days)

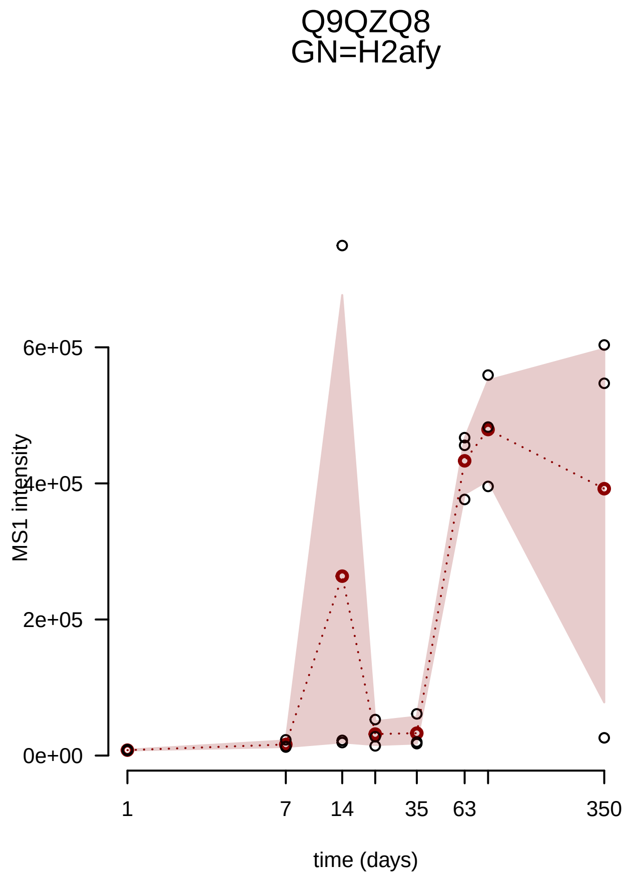
Q9QZQ8
GN=H2afy
MS1 intensity
6e+05
4e+05
2e+05
0e+00
1
7
14
35
63
350
time (days)

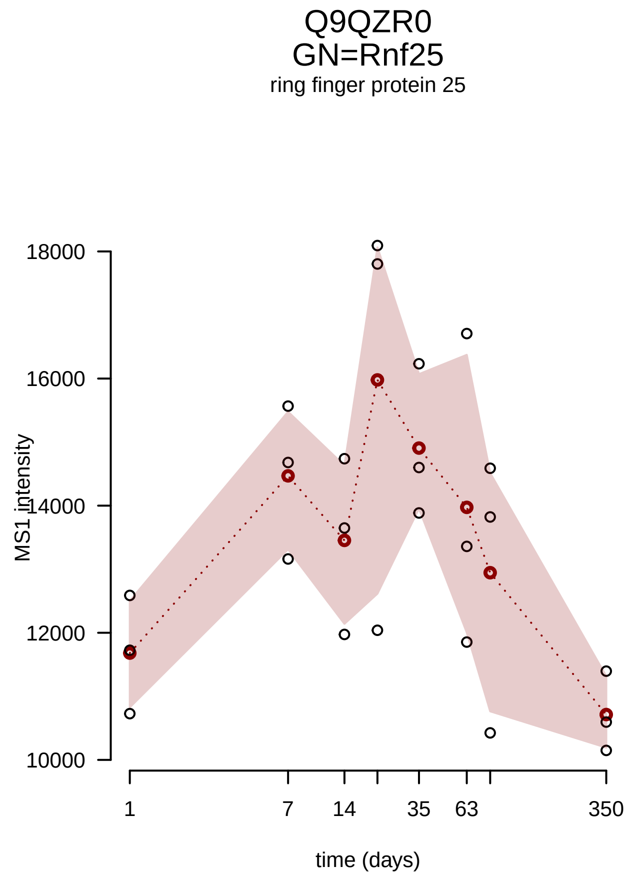
Q9QZR0
GN=Rnf25
ring finger protein 25
MS1 intensity
18000
16000
14000
12000
10000
1
7
14
35
63
350
time (days)

Q9QZR9
GN=Col4a4
collagen, type IV, alpha 4

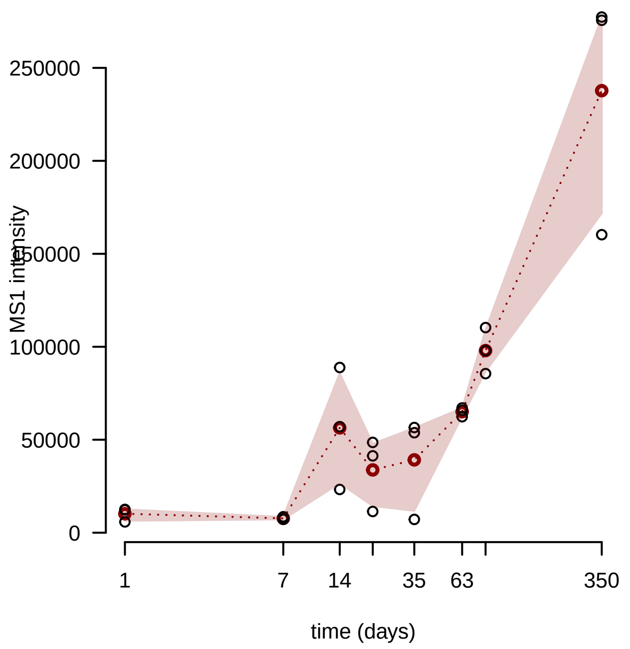

Q9QZS0
GN=Col4a3
collagen, type IV, alpha 3

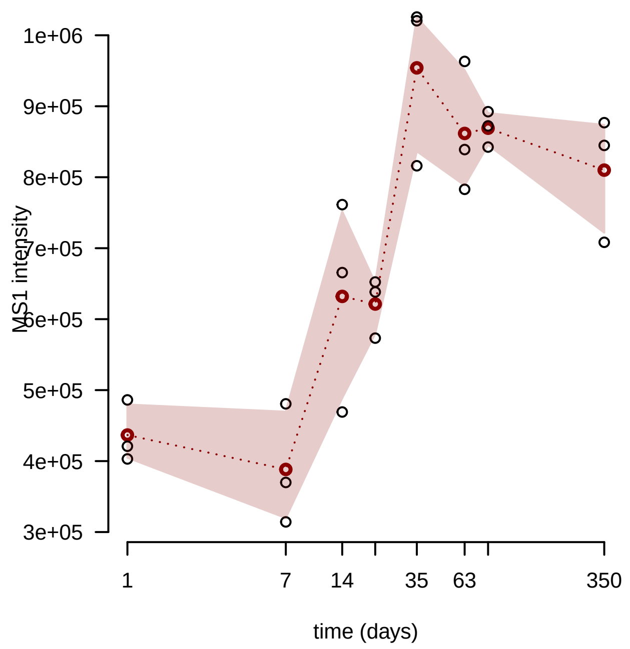

Q9QZS3
GN=Numb
NUMB endocytic adaptor protein

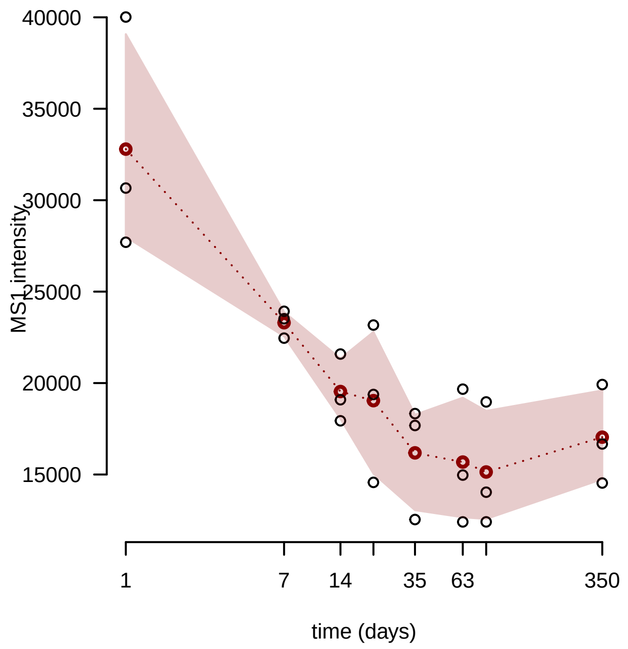

Q9QZU9
GN=Ube2l6
ubiquitin−conjugating enzyme E2L 6

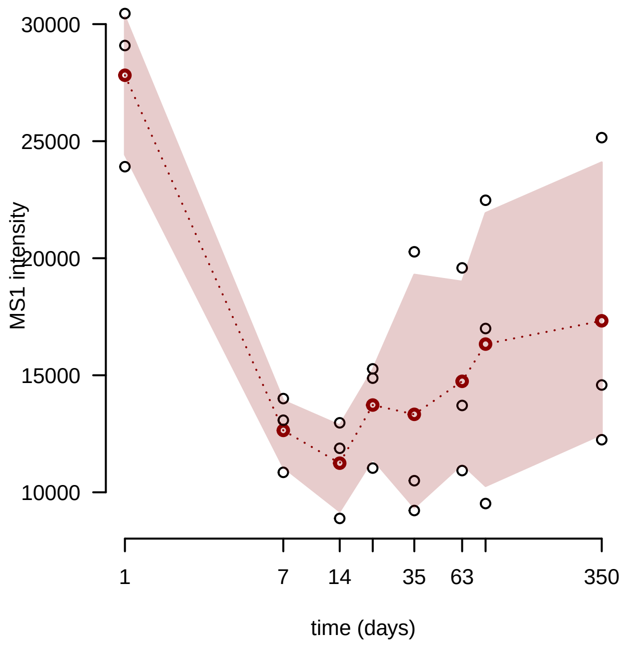

Q9QZV9
GN=Nxt1
NTF2−related export protein 1

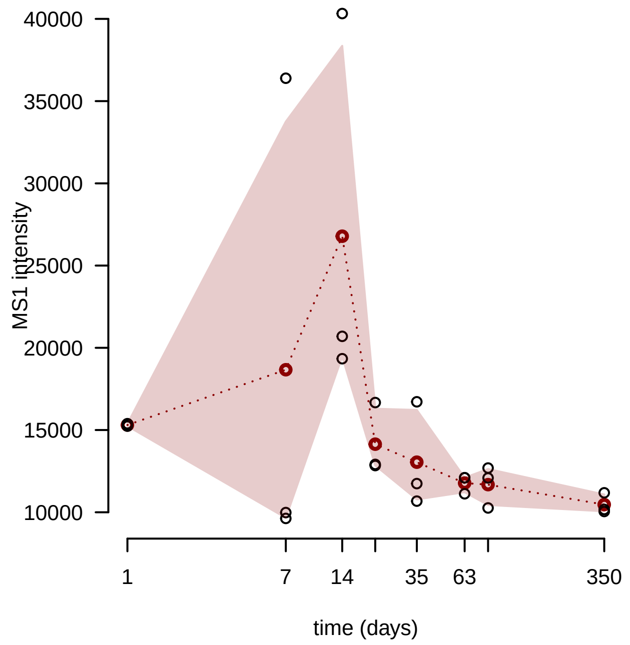

Q9QZW0
GN=Atp11c
ATPase, class VI, type 11C

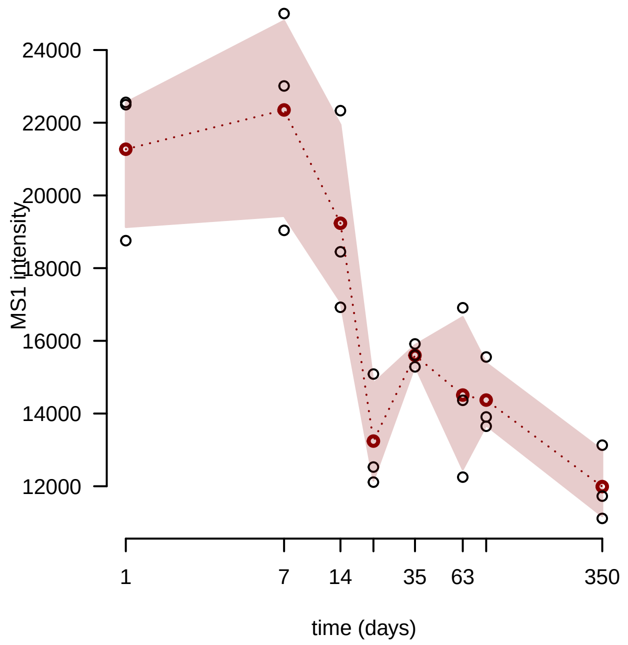

Q9QZX7
GN=Srr
serine racemase

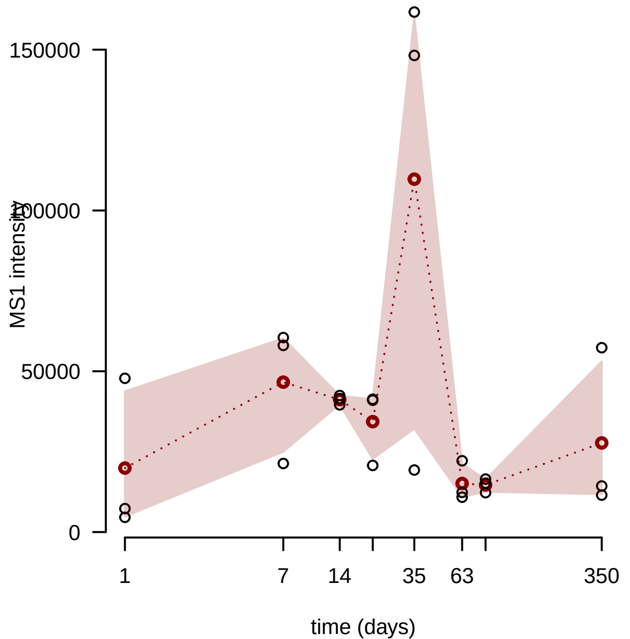

Q9QZZ4
GN=Myo15a
myosin XVA

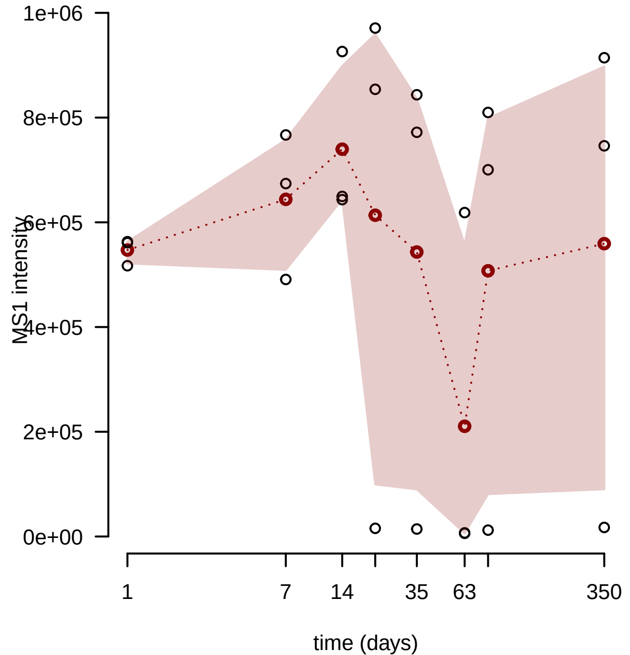

Q9QZZ6
GN=Dpt
dermatopontin

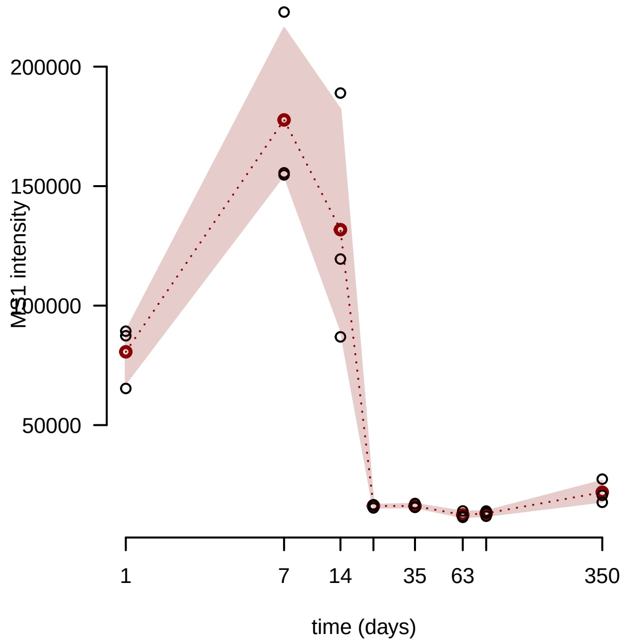

Q9R002
GN=Ifi202

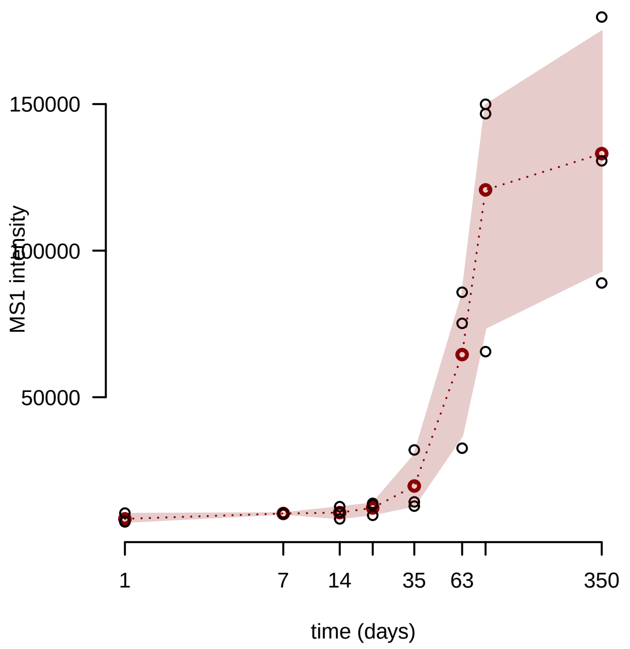

Q9R008
GN=Mvk
mevalonate kinase

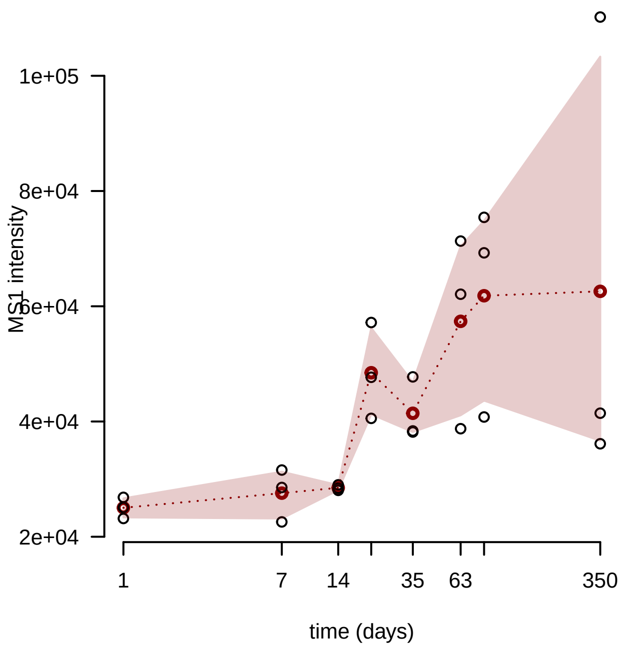

Q9R013
GN=Ctsf
cathepsin F

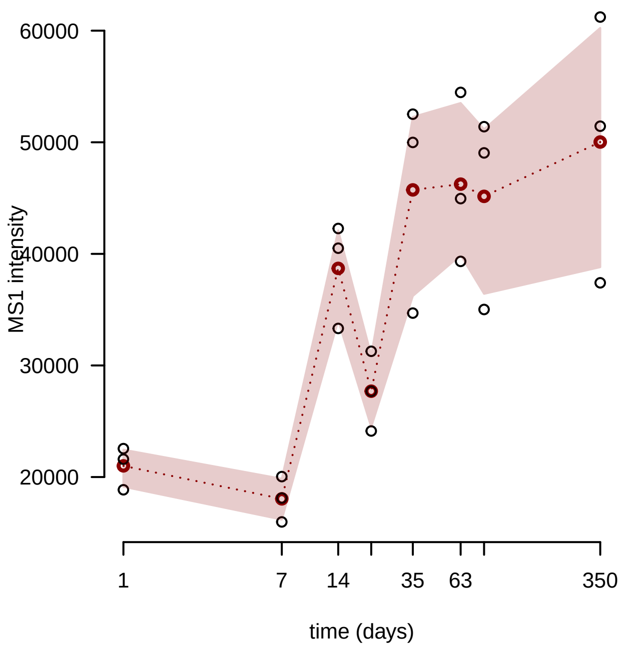

Q9R020
GN=Zranb2
zinc finger, RAN−binding domain containi ...

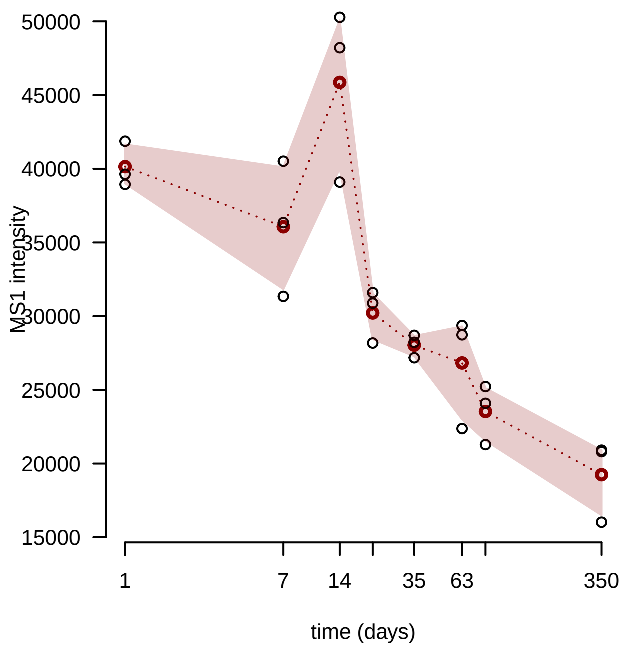

Q9R045
GN=Angptl2
angiopoietin−like 2

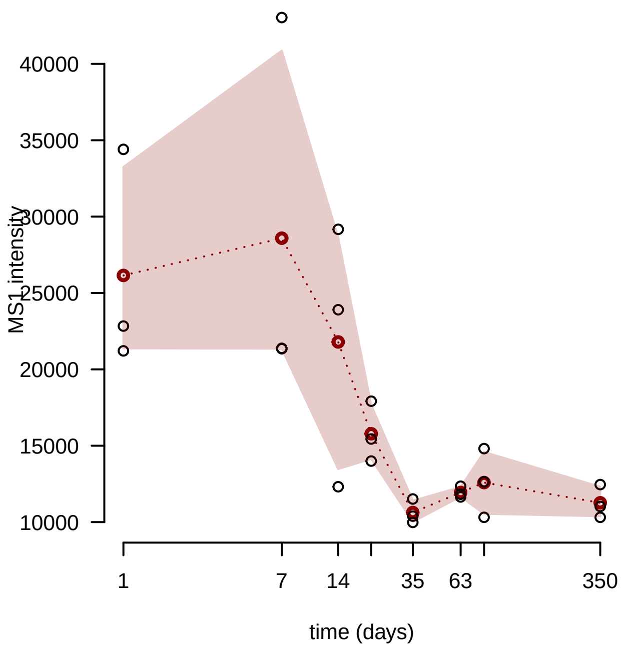

Q9R049
GN=Amfr
autocrine motility factor receptor

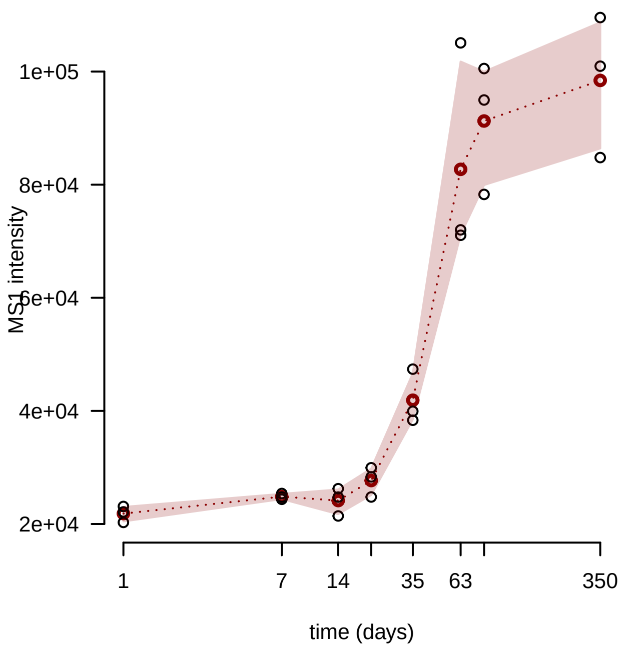

Q9R059
GN=Fhl3
four and a half LIM domains 3

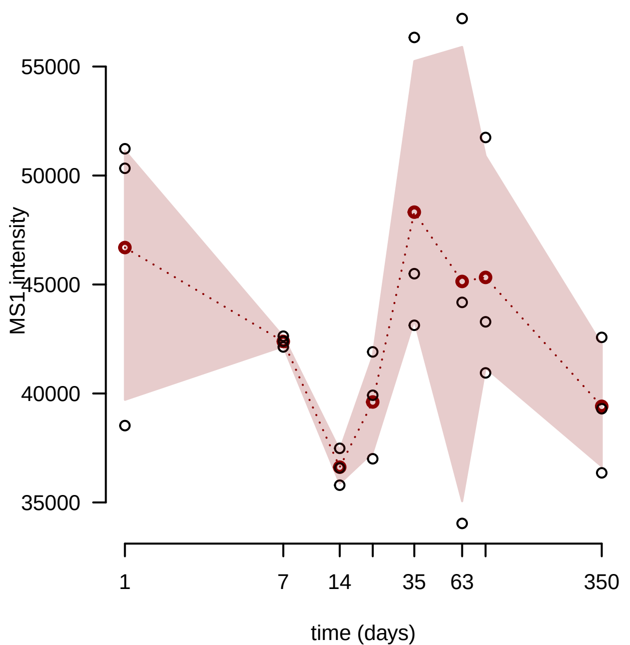

Q9R060
GN=Nubp1
nucleotide binding protein 1

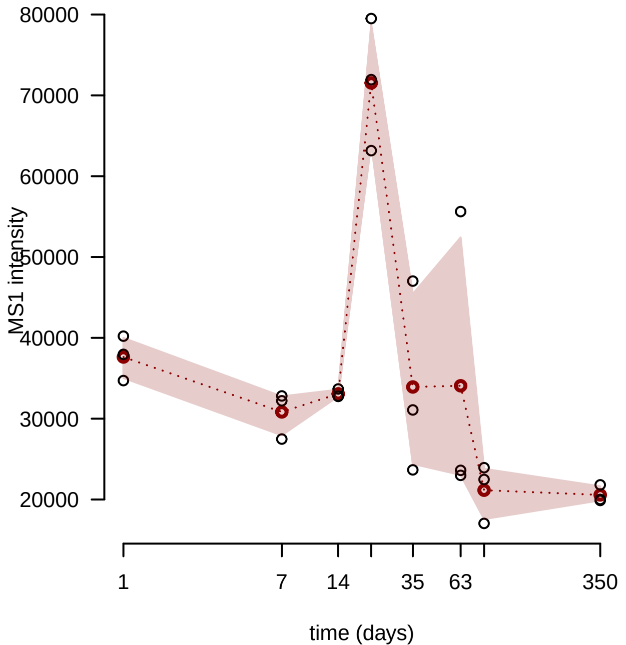

Q9R061
GN=Nubp2
nucleotide binding protein 2

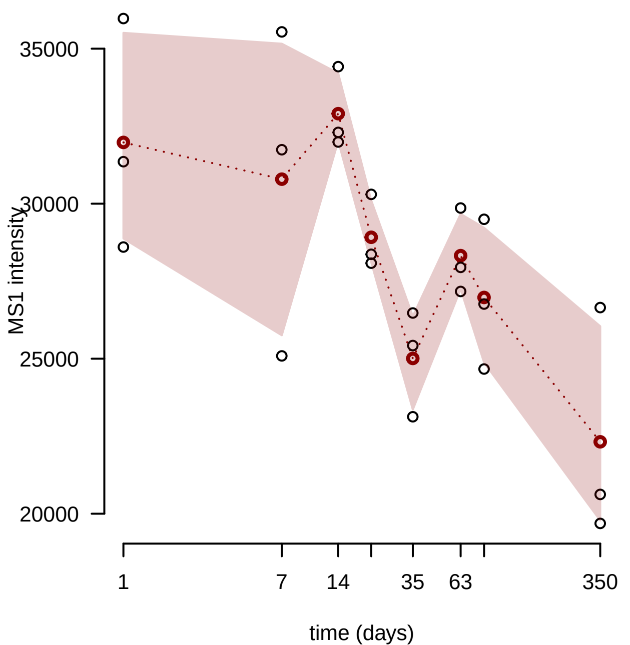

Q9R062
GN=Gyg1

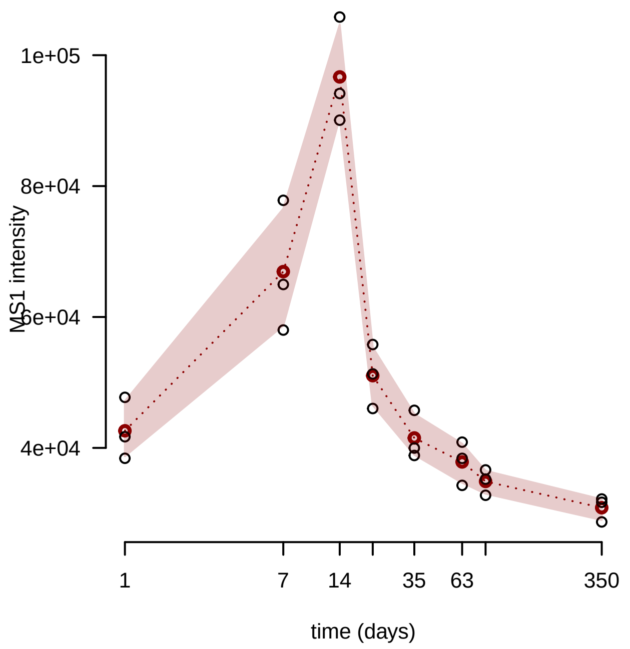

Q9R069
GN=Bcam
basal cell adhesion molecule

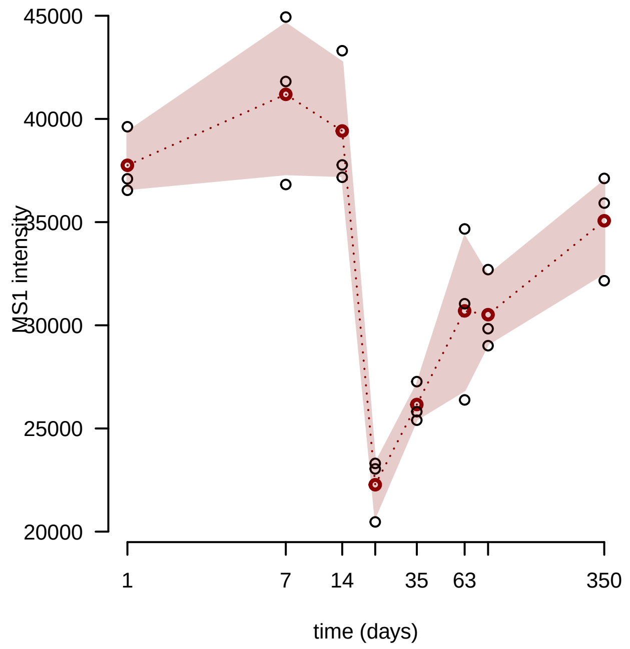

Q9R078
GN=Prkab1
protein kinase, AMP−activated, beta 1 no ...

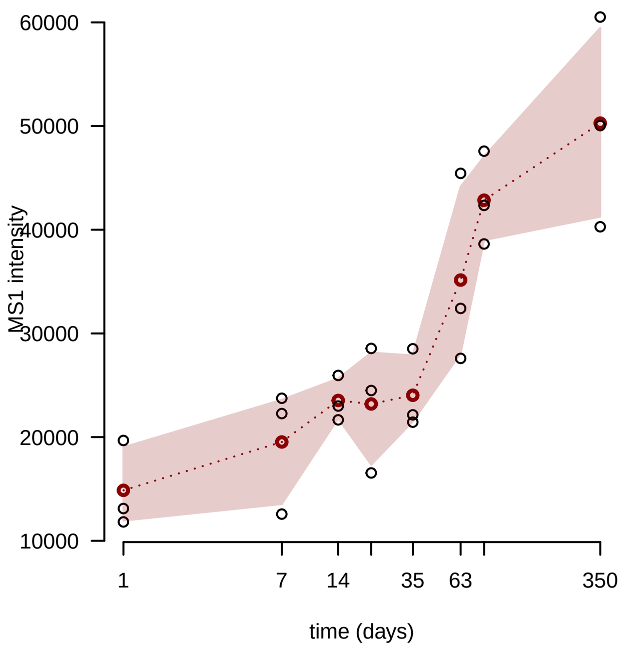

Q9R087
GN=Gpc6
glypican 6

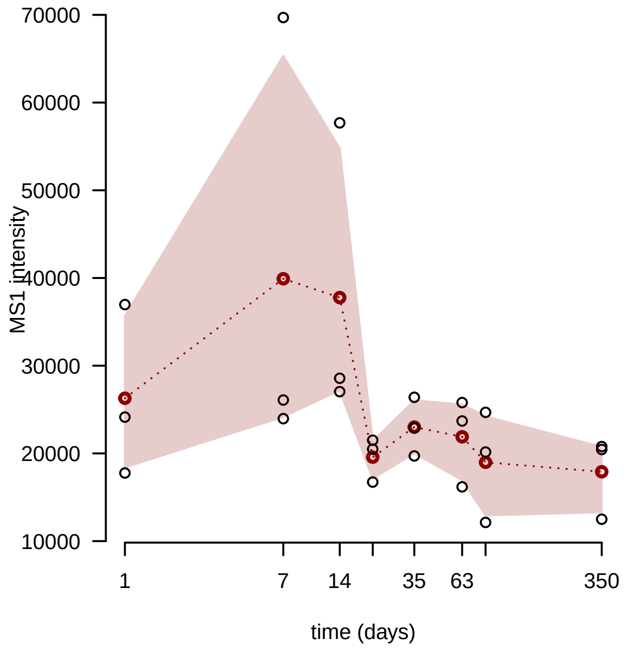

Q9R088
GN=Tk2
thymidine kinase 2, mitochondrial

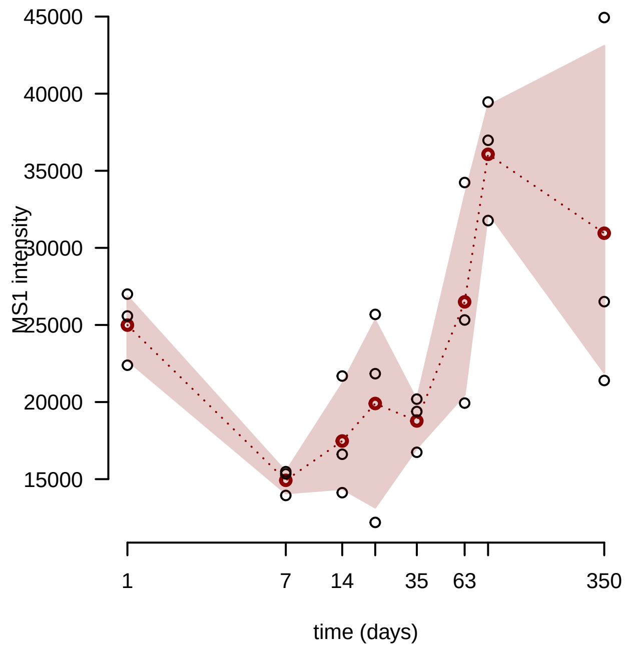

Q9R098
GN=Hgfac
hepatocyte growth factor activator

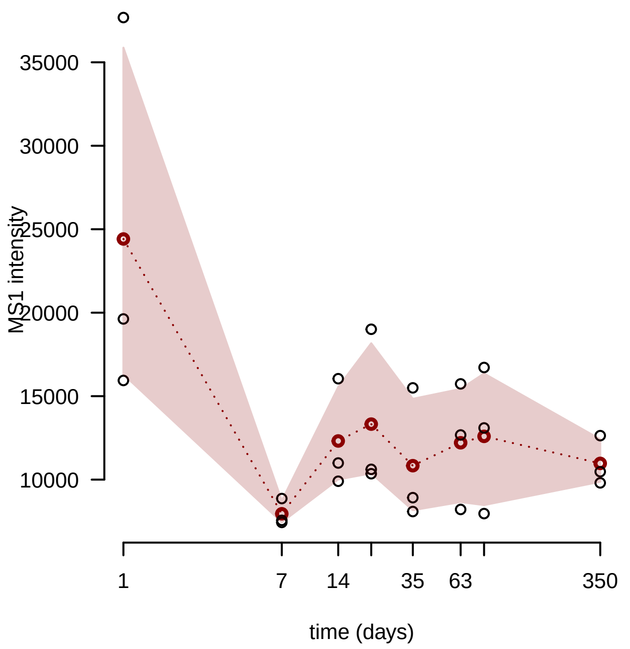

Q9R099
GN=Tbl2
transducin (beta)−like 2

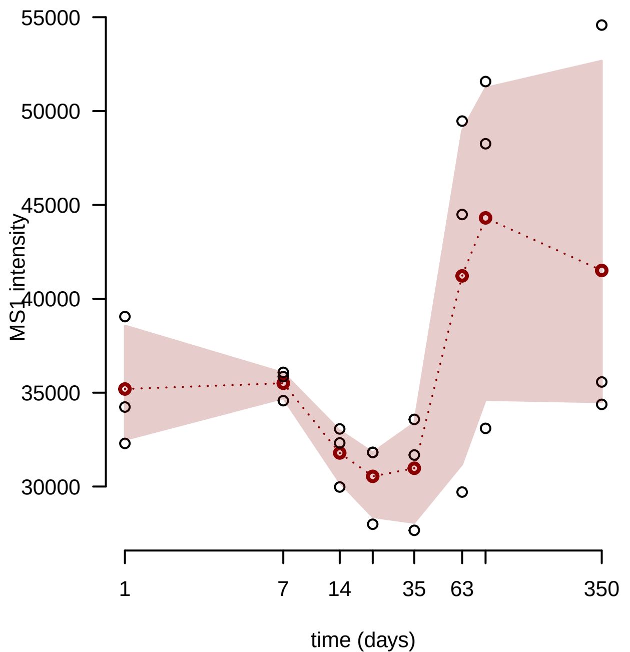

Q9R0A0
GN=Pex14
peroxisomal biogenesis factor 14

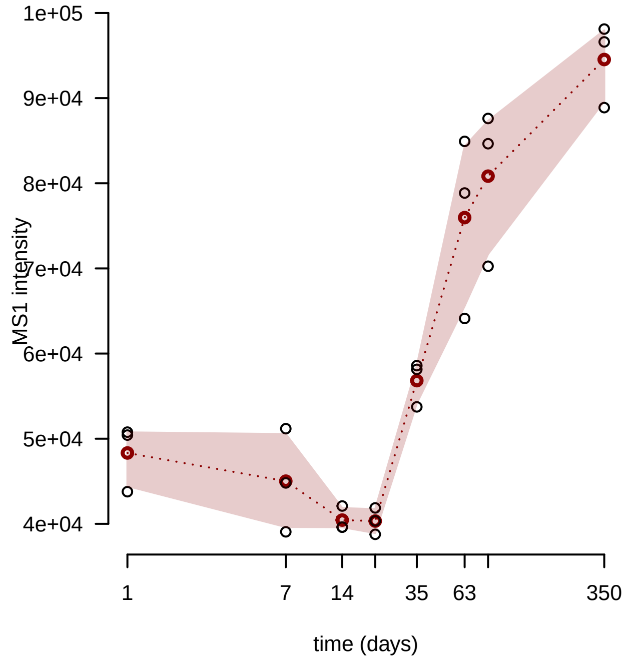

Q9R0A1
GN=Clcn2
chloride channel, voltage−sensitive 2

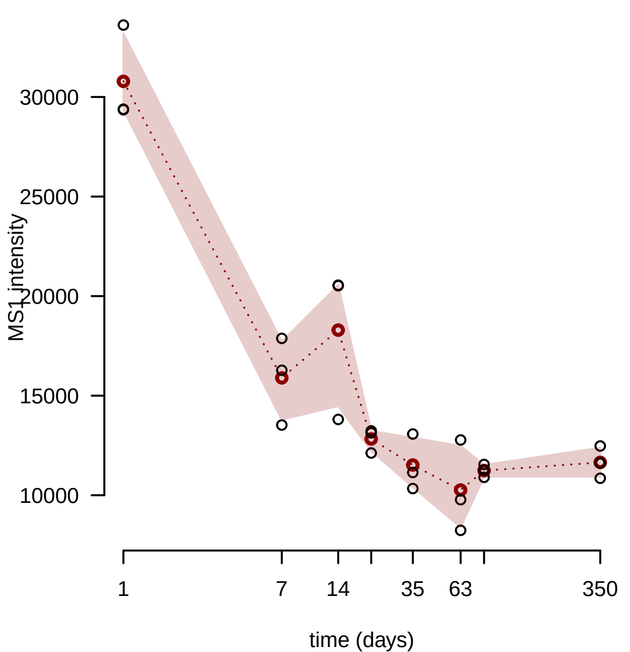

Q9R0A5
GN=Nek3
NIMA (never in mitosis gene a)−related e ...

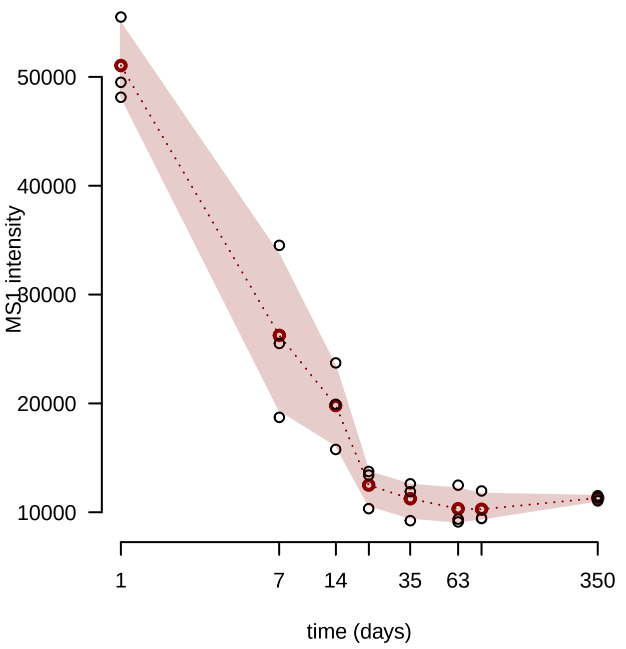

Q9R0B6
GN=Lamc3
laminin gamma 3

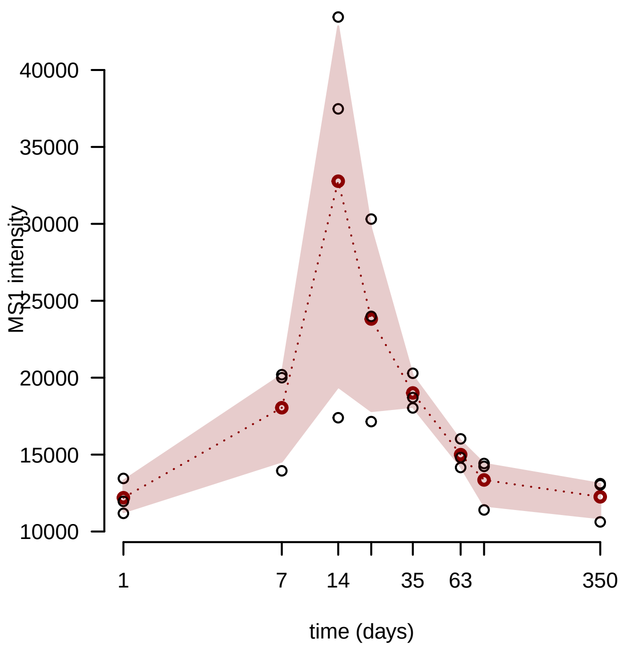

Q9R0B7
GN=Znf346

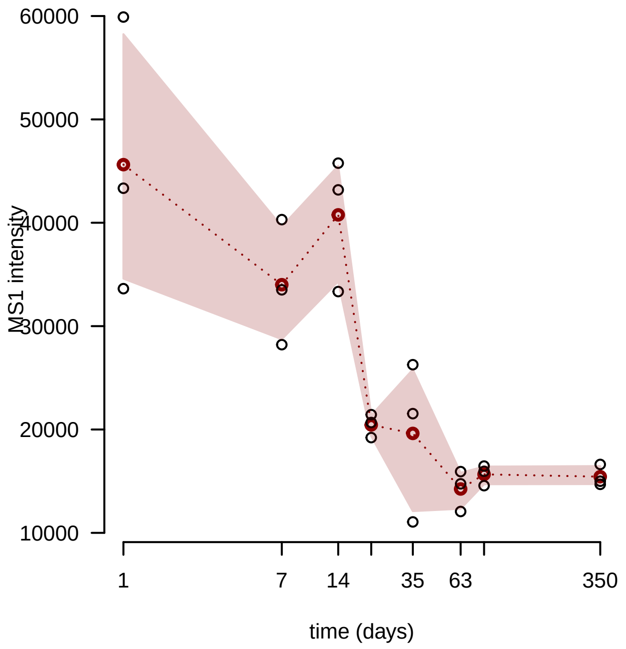

Q9R0B9
GN=Plod2
procollagen lysine, 2−oxoglutarate 5−dio ...

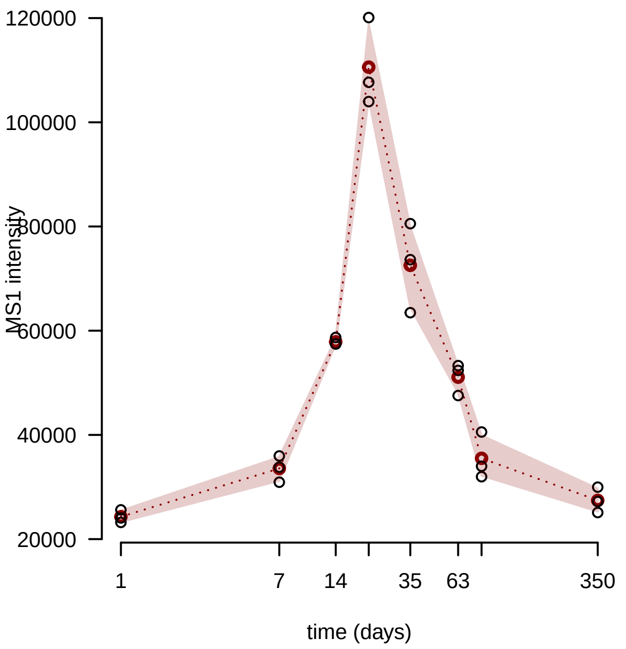

Q9R0D8
GN=Wdr54
WD repeat domain 54

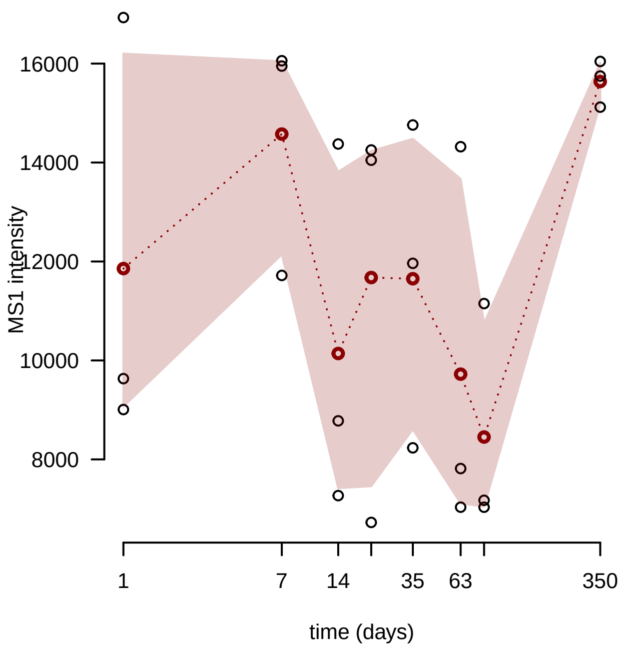

Q9R0E1
GN=Plod3
procollagen−lysine, 2−oxoglutarate 5−dio ...

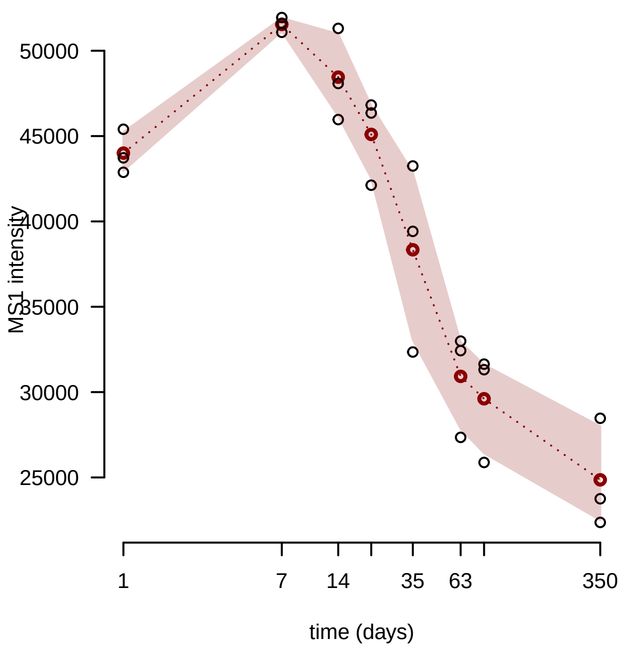

Q9R0E2
GN=Plod1
procollagen−lysine, 2−oxoglutarate 5−dio ...

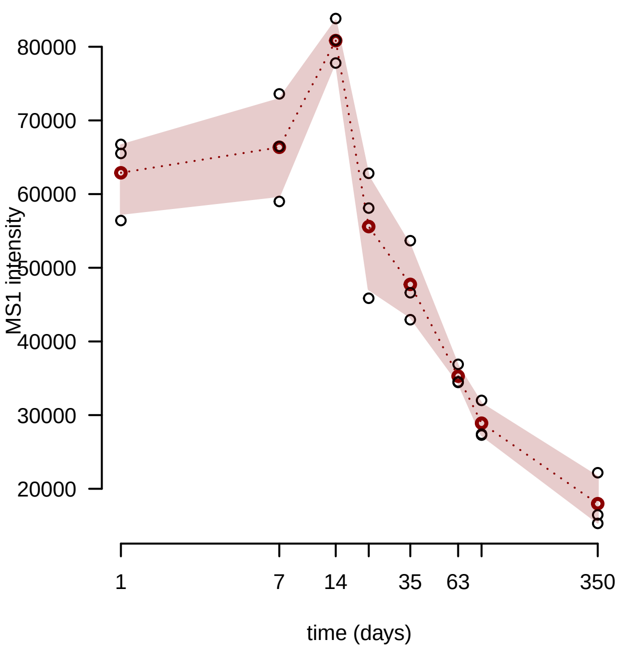

Q9R0G7
GN=Zeb2
zinc finger E−box binding homeobox 2

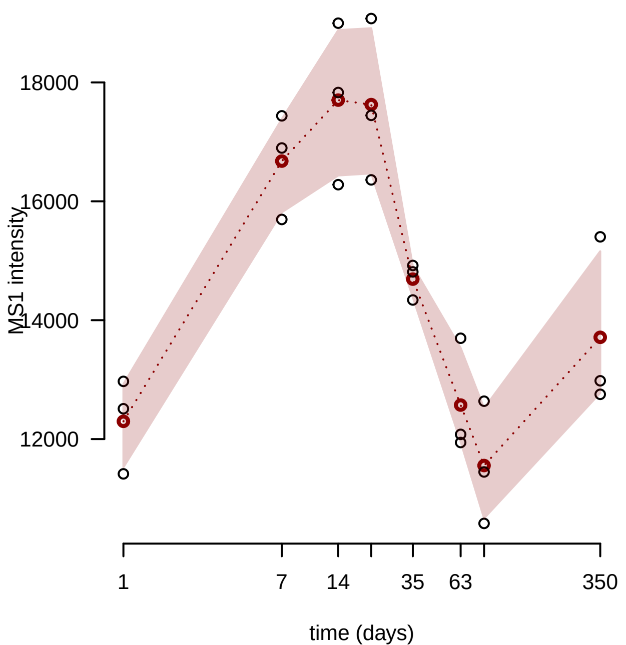

Q9R0G8
GN=Nrk
Nik related kinase

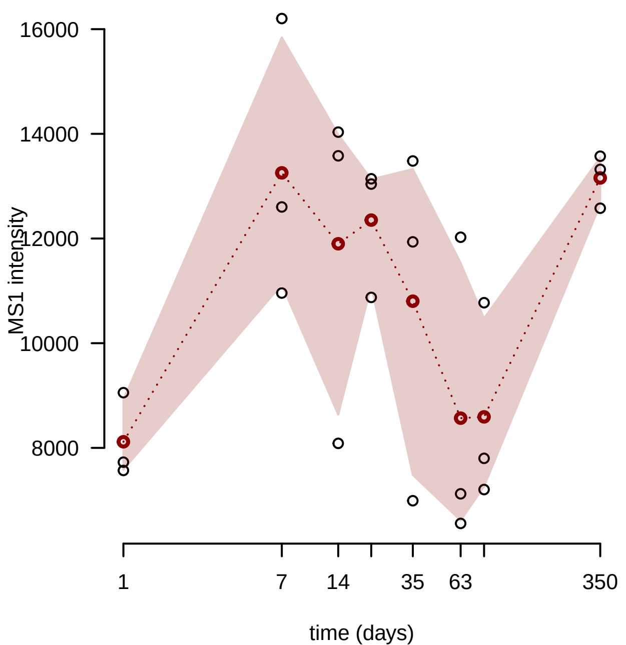

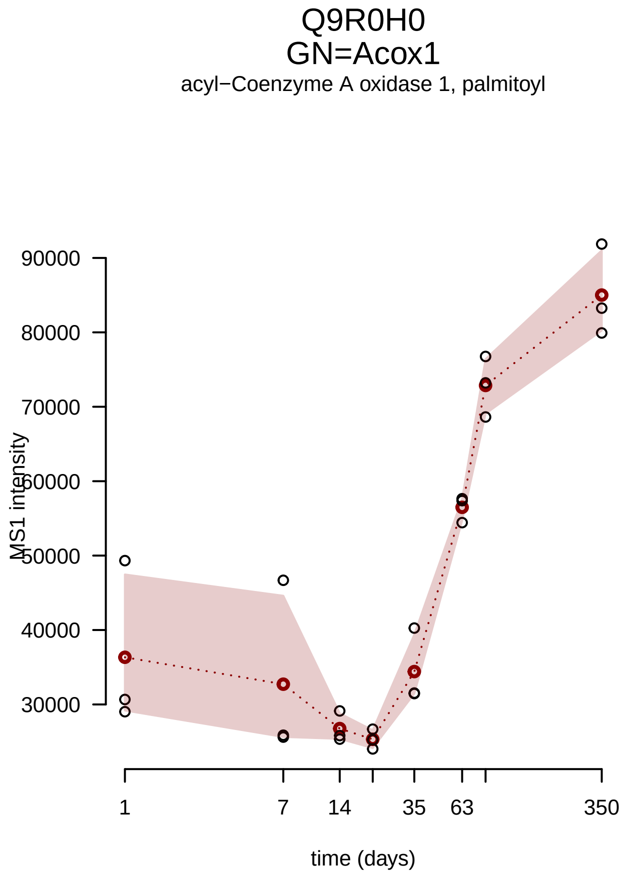
Q9R0H0
GN=Acox1
acyl−Coenzyme A oxidase 1, palmitoyl
MS1 intensity
90000
80000
70000
60000
50000
40000
30000
1
7
14
35
63
350
time (days)

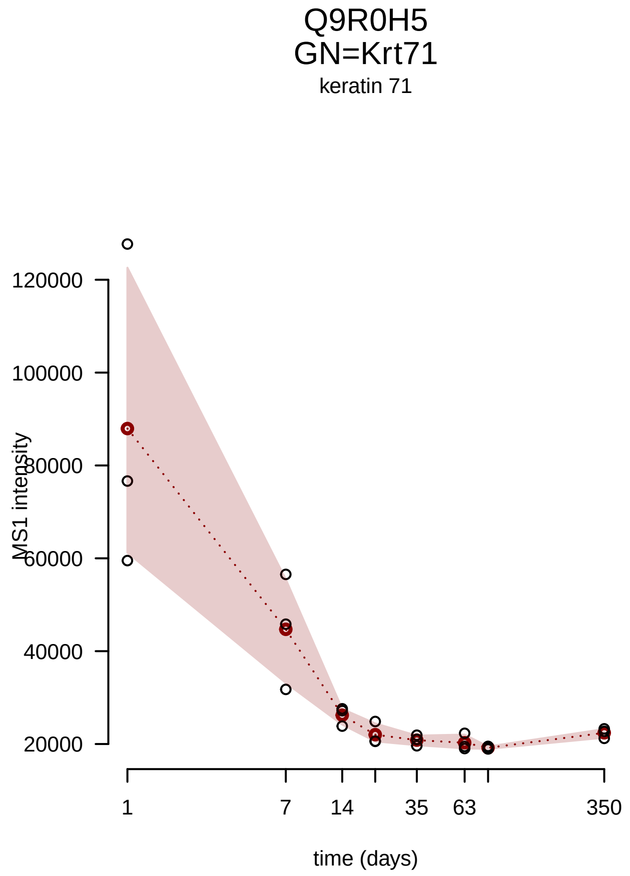
Q9R0H5
GN=Krt71
keratin 71
MS1 intensity
120000
100000
80000
60000
40000
20000
1
7
14
35
63
350
time (days)

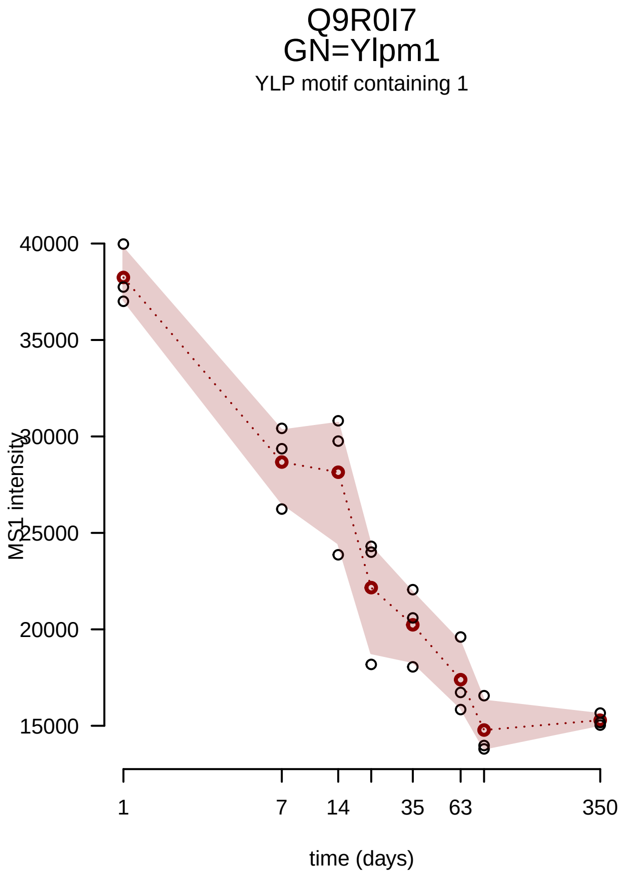
Q9R0I7
GN=Ylpm1
YLP motif containing 1
MS1 intensity
40000
35000
30000
25000
20000
15000
1
7
14
35
63
350
time (days)

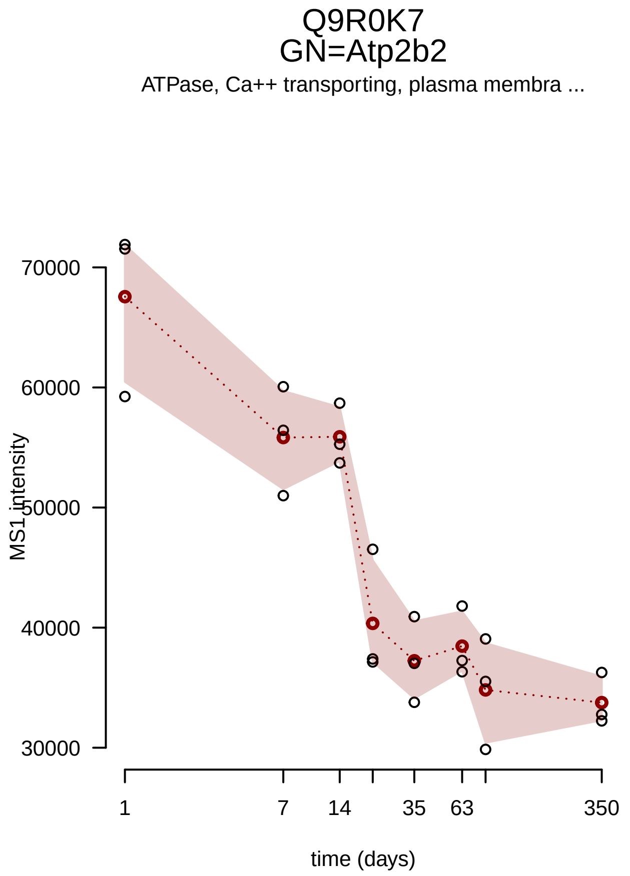
Q9R0K7
GN=Atp2b2
ATPase, Ca++ transporting, plasma membra ...
MS1 intensity
70000
60000
50000
40000
30000
1
7
14
35
63
350
time (days)

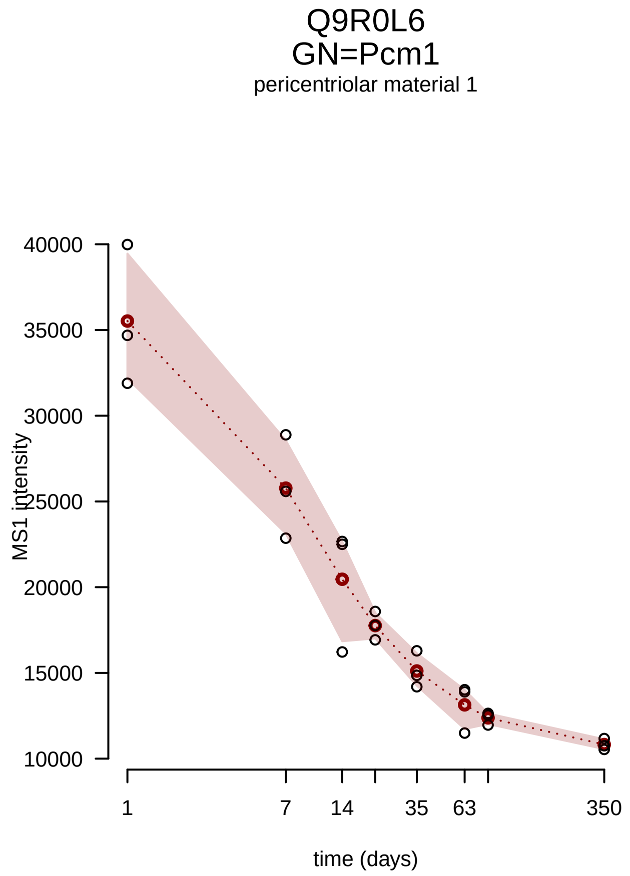
Q9R0L6
GN=Pcm1
pericentriolar material 1
MS1 intensity
40000
35000
30000
25000
20000
15000
10000
1
7
14
35
63
350
time (days)

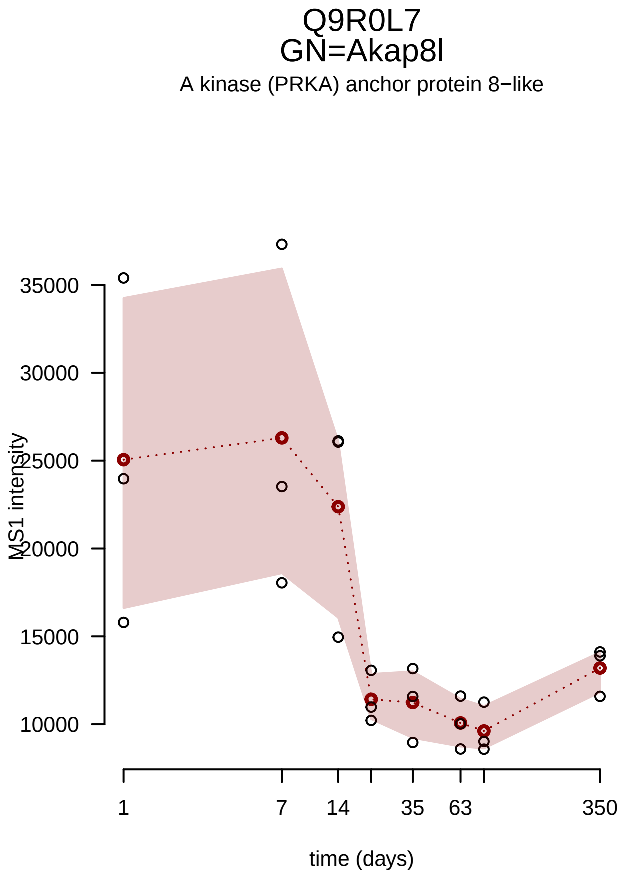
Q9R0L7
GN=Akap8l
A kinase (PRKA) anchor protein 8−like
MS1 intensity
35000
30000
25000
20000
15000
10000
1
7
14
35
63
350
time (days)

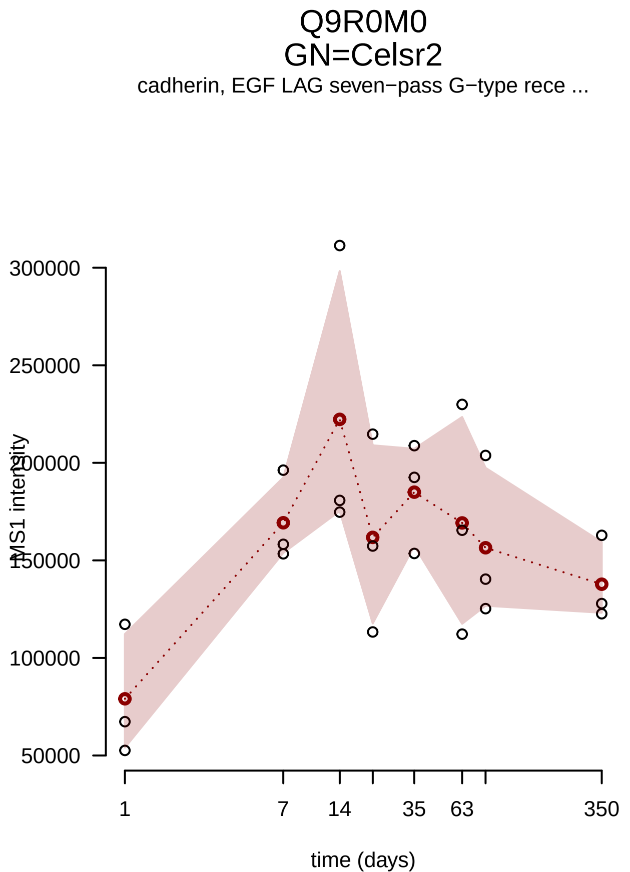
Q9R0M0
GN=Celsr2
cadherin, EGF LAG seven−pass G−type rece ...
MS1 intensity
300000
250000
200000
150000
100000
50000
1
7
14
35
63
350
time (days)

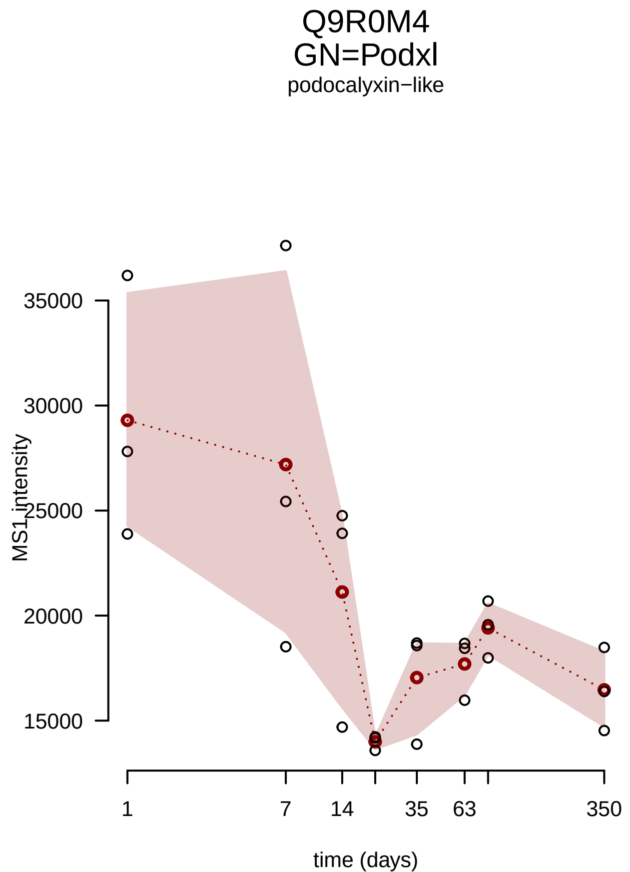
Q9R0M4
GN=Podxl
podocalyxin−like
MS1 intensity
35000
30000
25000
20000
15000
1
7
14
35
63
350
time (days)

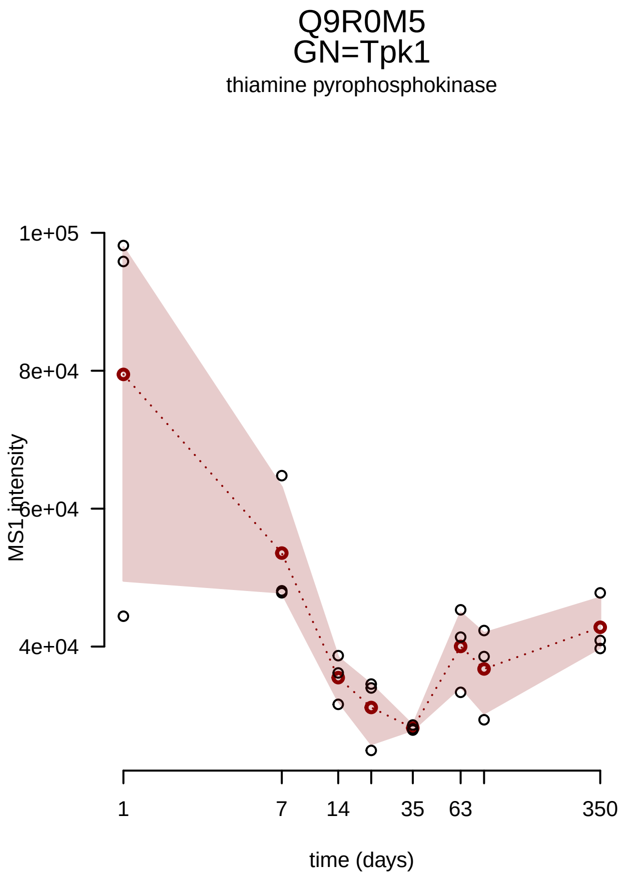
Q9R0M5
GN=Tpk1
thiamine pyrophosphokinase
MS1 intensity
1e+05
8e+04
6e+04
4e+04
1
7
14
35
63
350
time (days)

Q9R0M6
GN=Rab9a

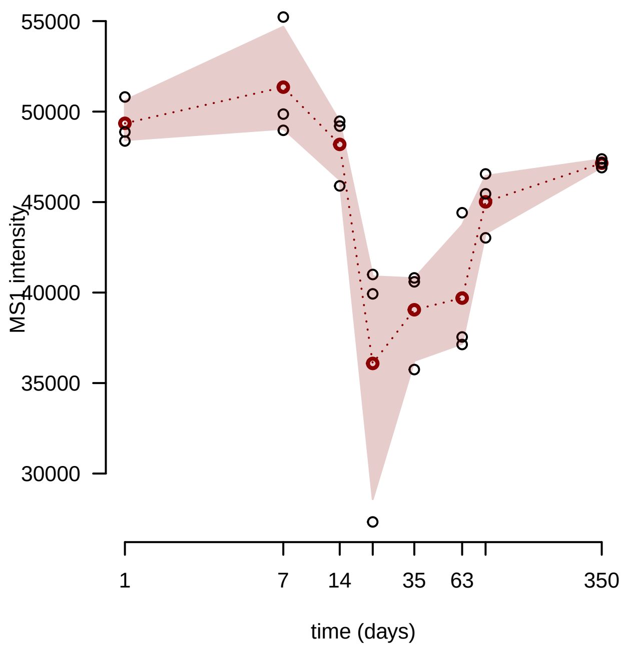

Q9R0M8
GN=Slc35a2
solute carrier family 35 (UDP−galactose ...

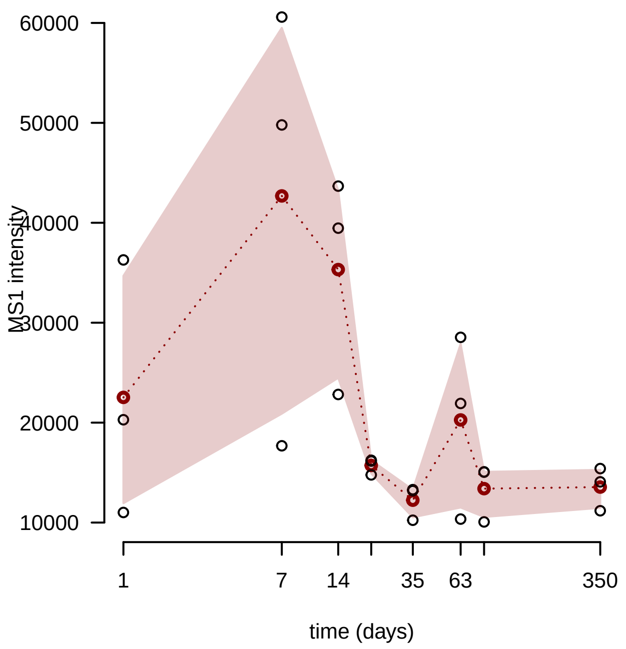

Q9R0N0
GN=Galk1
galactokinase 1

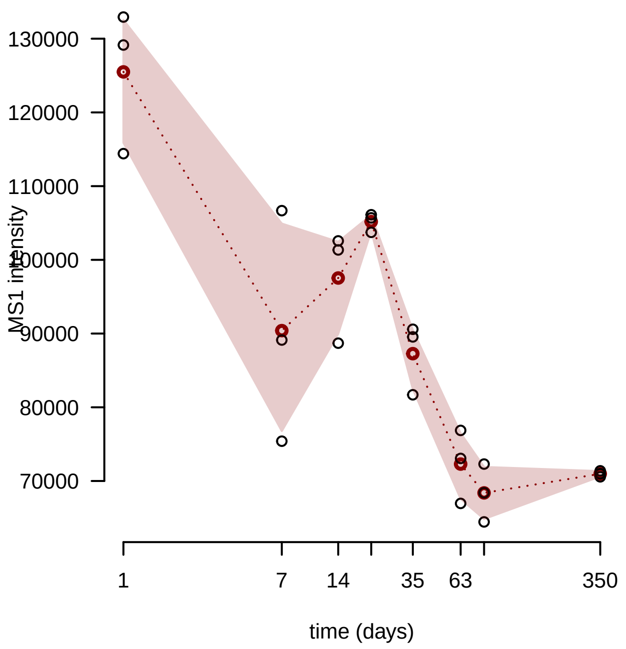

Q9R0N7
GN=Syt7
synaptotagmin VII

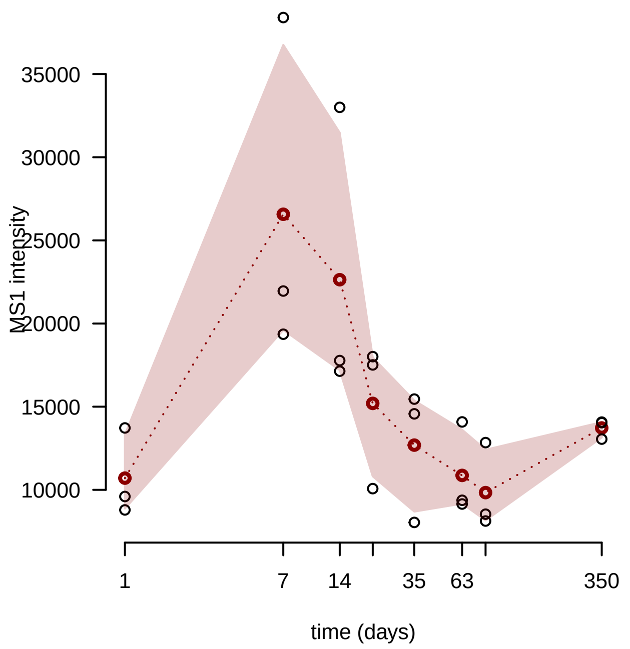

Q9R0P3
GN=Esd
esterase D/formylglutathione hydrolase

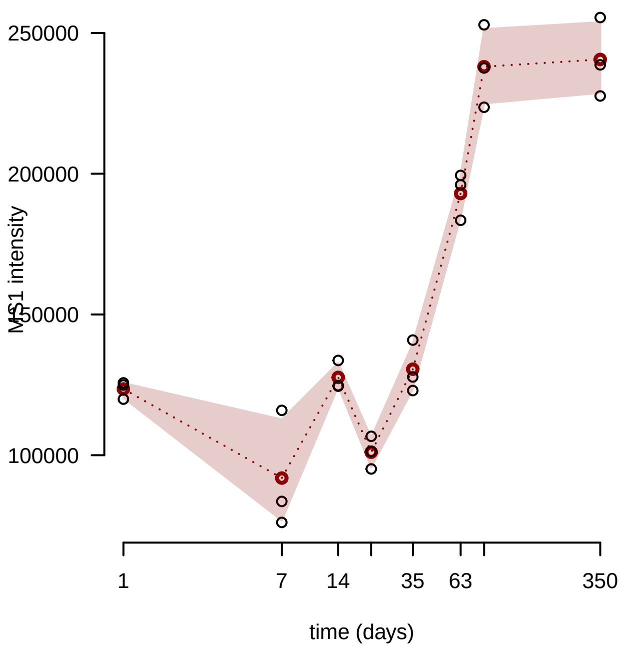

Q9R0P4
GN=Smap

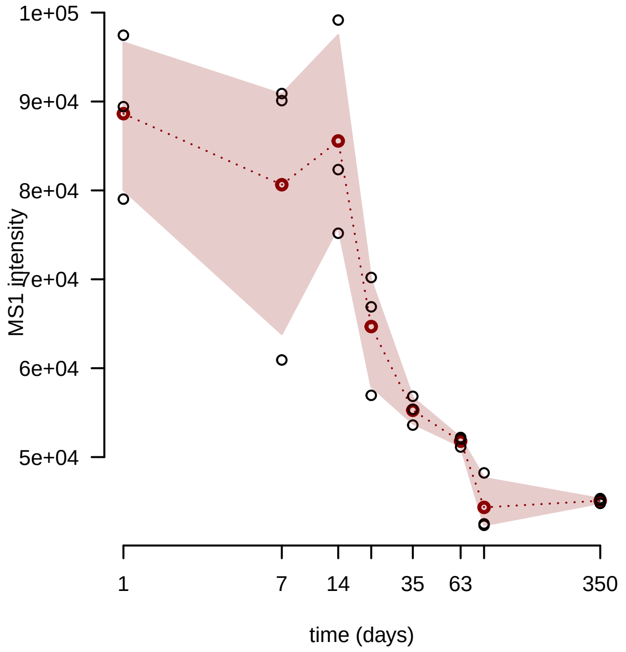

Q9R0P5
GN=Dstn
destrin

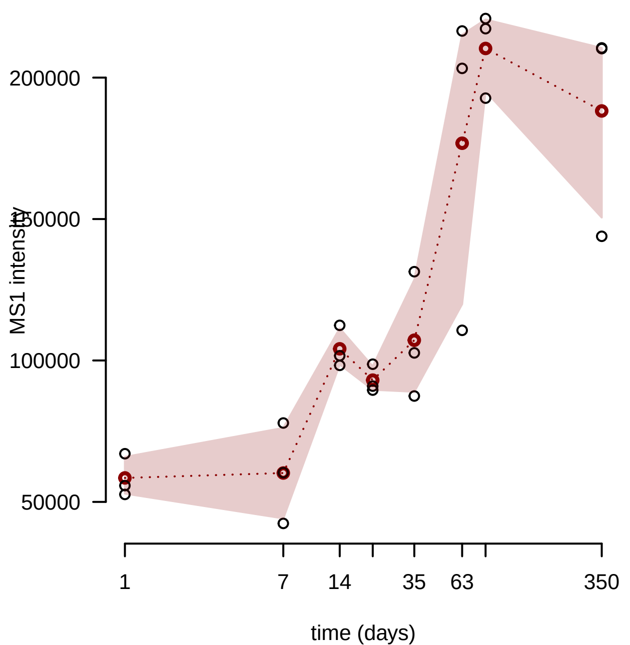

Q9R0P6
GN=Sec11a
SEC11 homolog A, signal peptidase comple ...

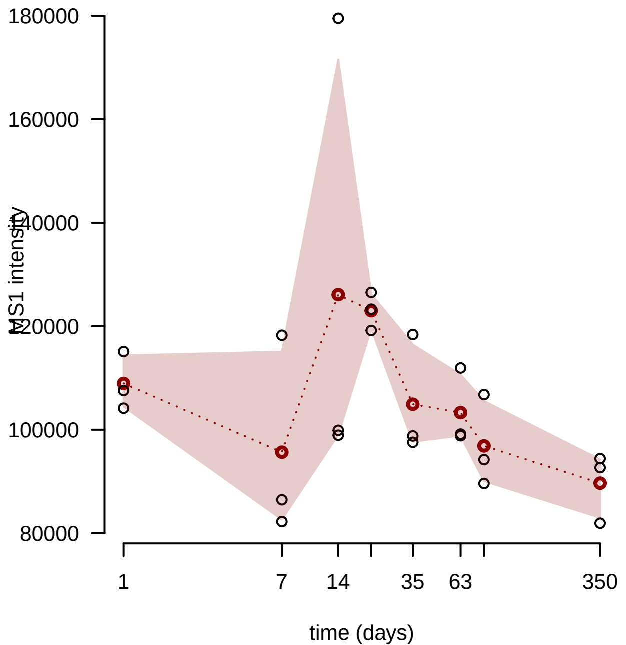

Q9R0P9
GN=Uchl1
ubiquitin carboxy−terminal hydrolase L1

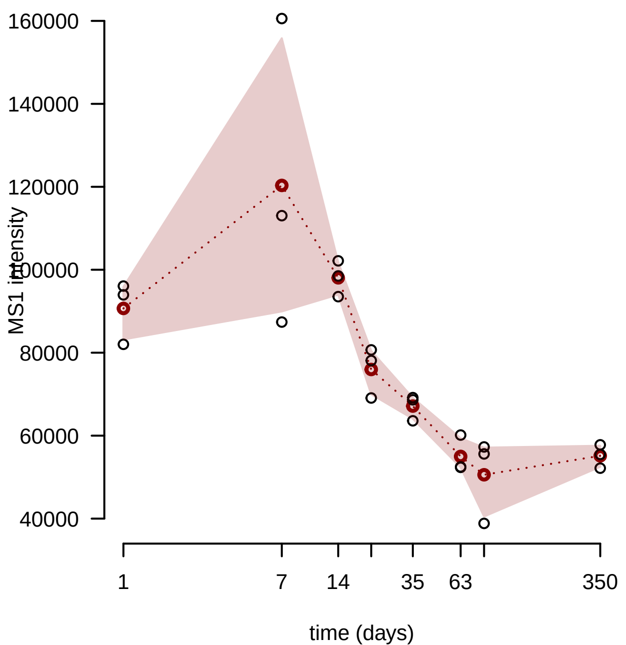

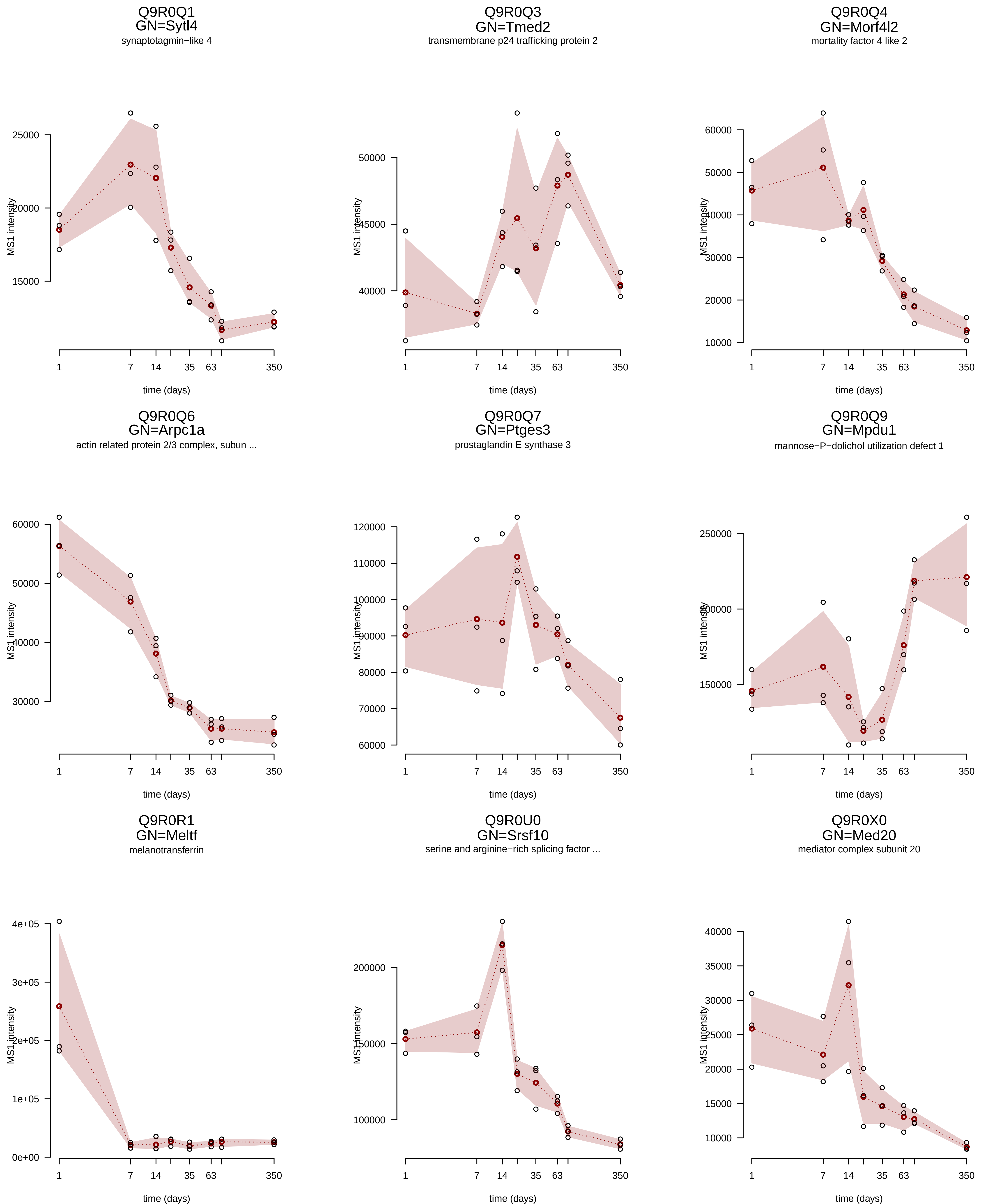
Q9R0Q1
GN=Sytl4
synaptotagmin−like 4
Q9R0Q3
GN=Tmed2
transmembrane p24 trafficking protein 2
Q9R0Q4
GN=Morf4l2
mortality factor 4 like 2
Q9R0Q6
GN=Arpc1a
actin related protein 2/3 complex, subun ...
Q9R0Q7
GN=Ptges3
prostaglandin E synthase 3
Q9R0Q9
GN=Mpdu1
mannose−P−dolichol utilization defect 1
Q9R0R1
GN=Meltf
melanotransferrin
Q9R0U0
GN=Srsf10
serine and arginine−rich splicing factor ...
Q9R0X0
GN=Med20
mediator complex subunit 20
MS1 intensity
time (days)
1
7
14
35
63
350
25000
20000
15000
50000
45000
40000
60000
30000
10000
120000
110000
100000
90000
80000
70000
250000
200000
150000
4e+05
3e+05
2e+05
1e+05
0e+00
35000

Q9R0X4
GN=Acot9
acyl−CoA thioesterase 9

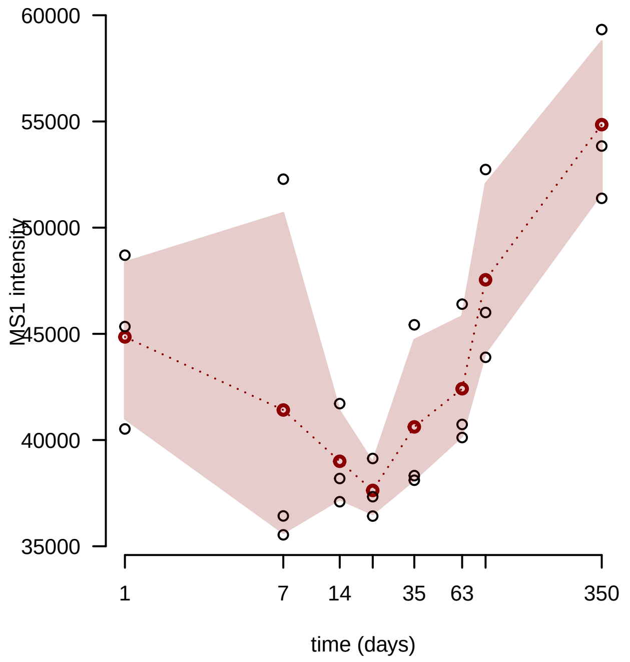

Q9R0X5
GN=Rpgr
retinitis pigmentosa GTPase regulator

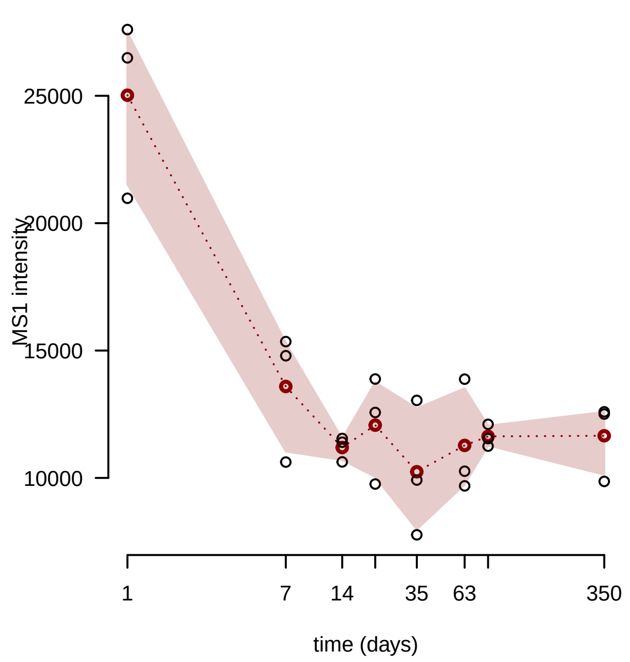

Q9R0Y5
GN=Ak1
adenylate kinase 1

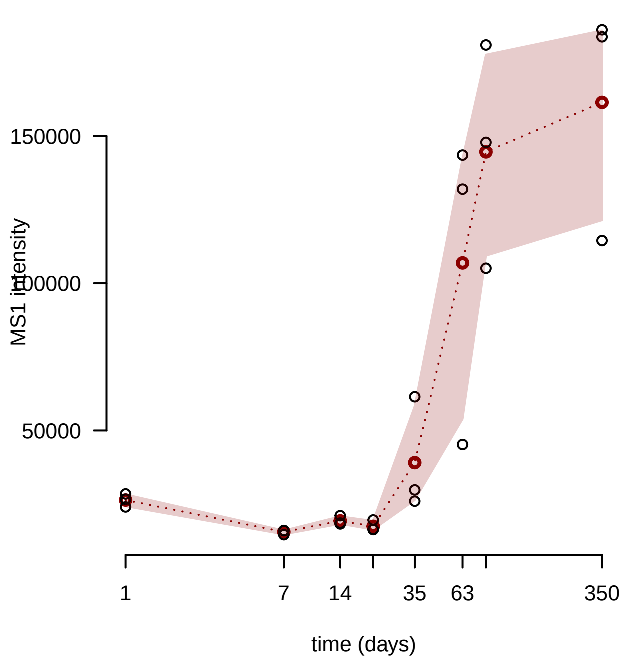

Q9R0Z9
GN=Dlc1
deleted in liver cancer 1

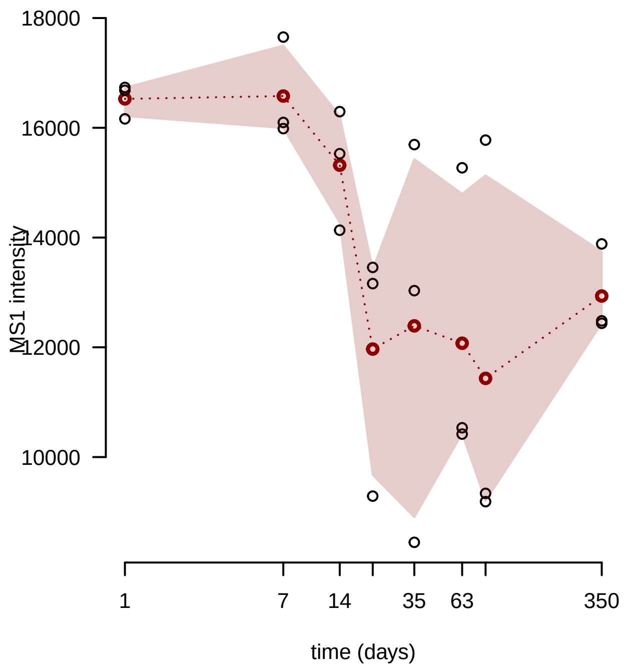

Q9R100
GN=Cdh17
cadherin 17

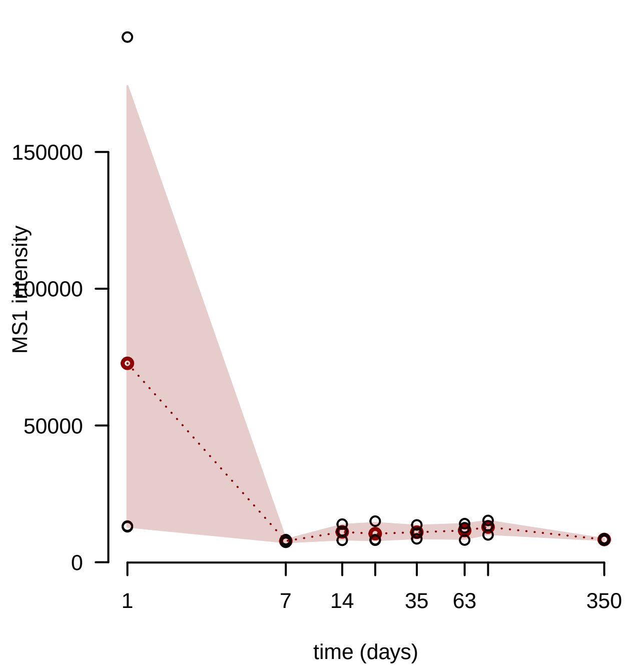

Q9R111
GN=Gda
guanine deaminase

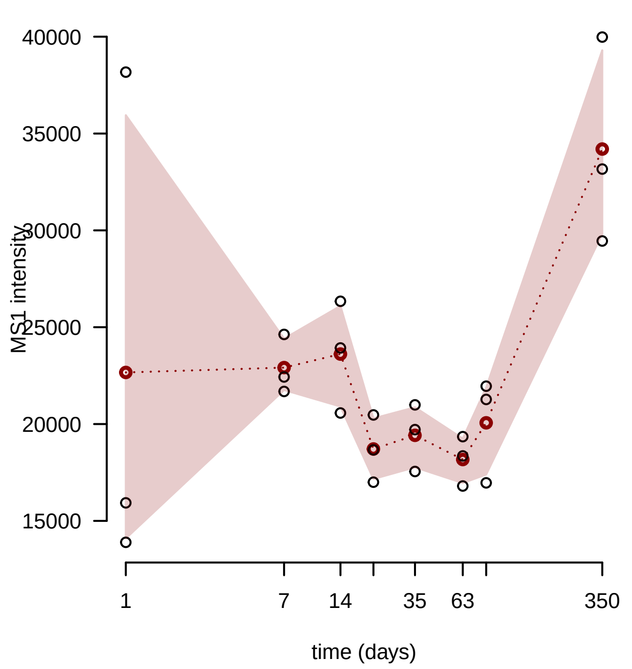

Q9R112
GN=Sqor
sulfide quinone oxidoreductase

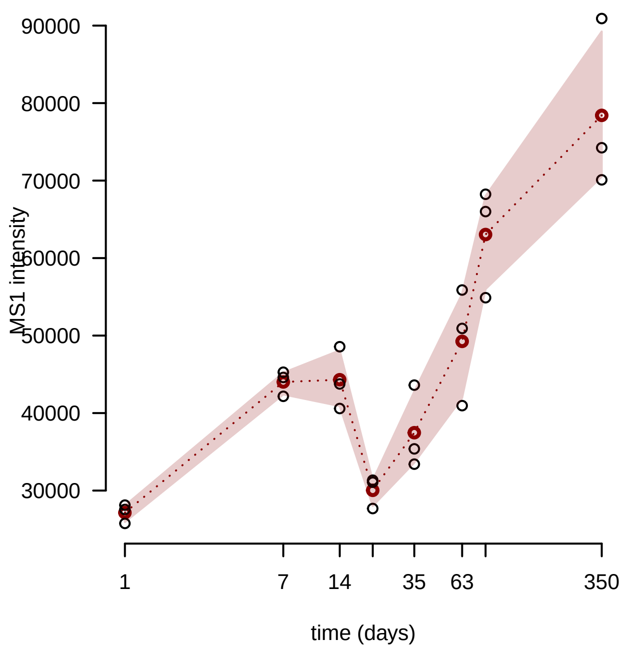

Q9R118
GN=Htra1
HtrA serine peptidase 1

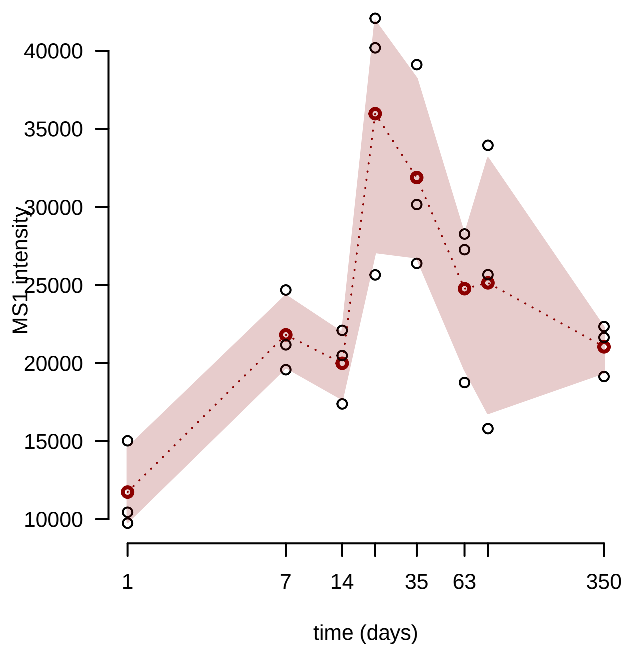

Q9R190
GN=Mta2
metastasis−associated gene family, membe ...

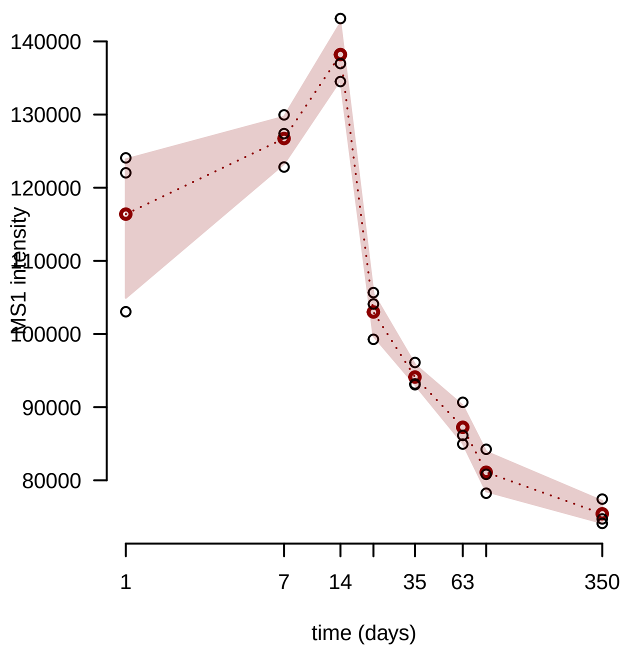

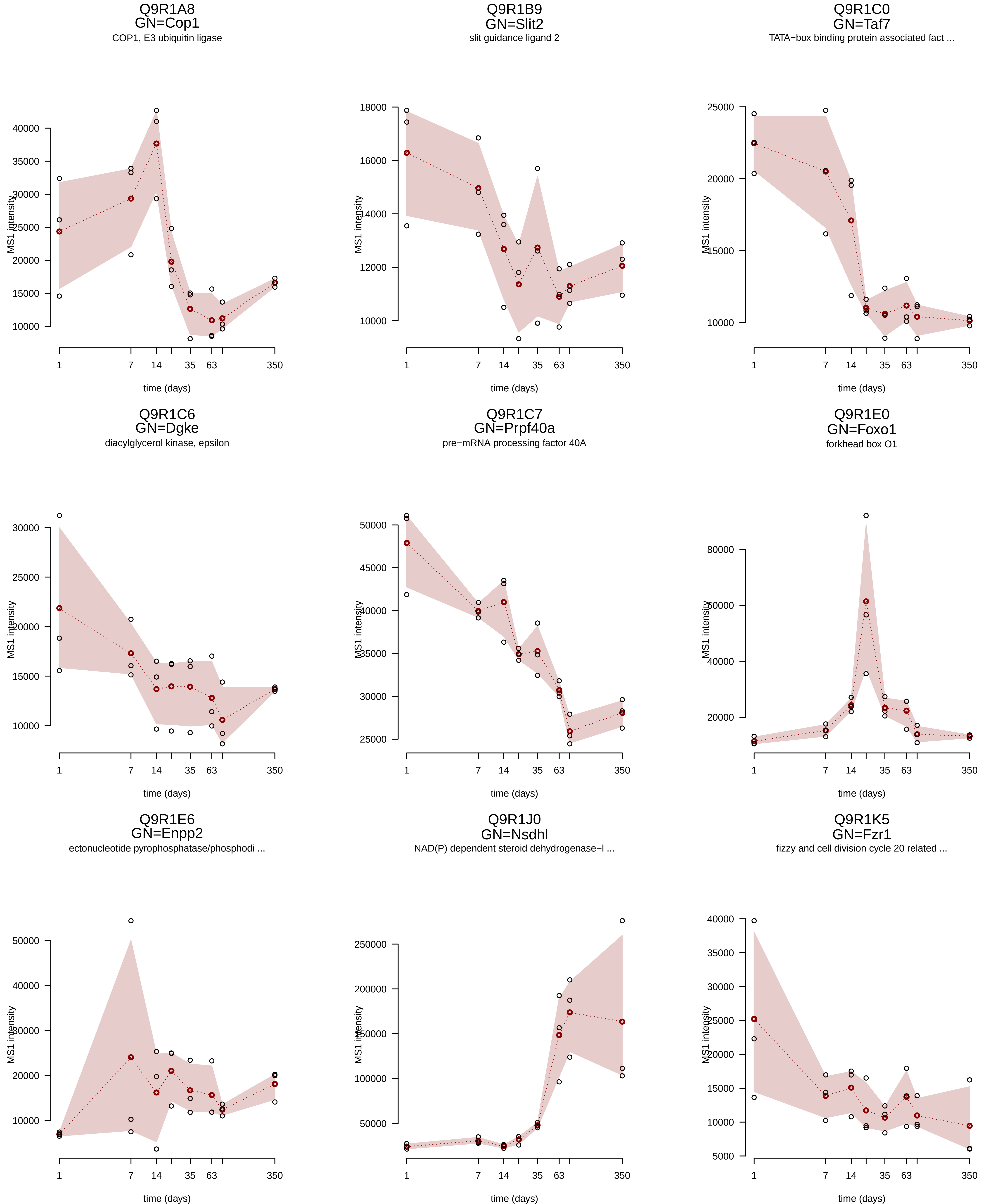
Q9R1A8
GN=Cop1
COP1, E3 ubiquitin ligase
Q9R1B9
GN=Slit2
slit guidance ligand 2
Q9R1C0
GN=Taf7
TATA−box binding protein associated fact ...
Q9R1C6
GN=Dgke
diacylglycerol kinase, epsilon
Q9R1C7
GN=Prpf40a
pre−mRNA processing factor 40A
Q9R1E0
GN=Foxo1
forkhead box O1
Q9R1E6
GN=Enpp2
ectonucleotide pyrophosphatase/phosphodi ...
Q9R1J0
GN=Nsdhl
NAD(P) dependent steroid dehydrogenase−l ...
Q9R1K5
GN=Fzr1
fizzy and cell division cycle 20 related ...
MS1 intensity
time (days)
1
7
14
35
63
350

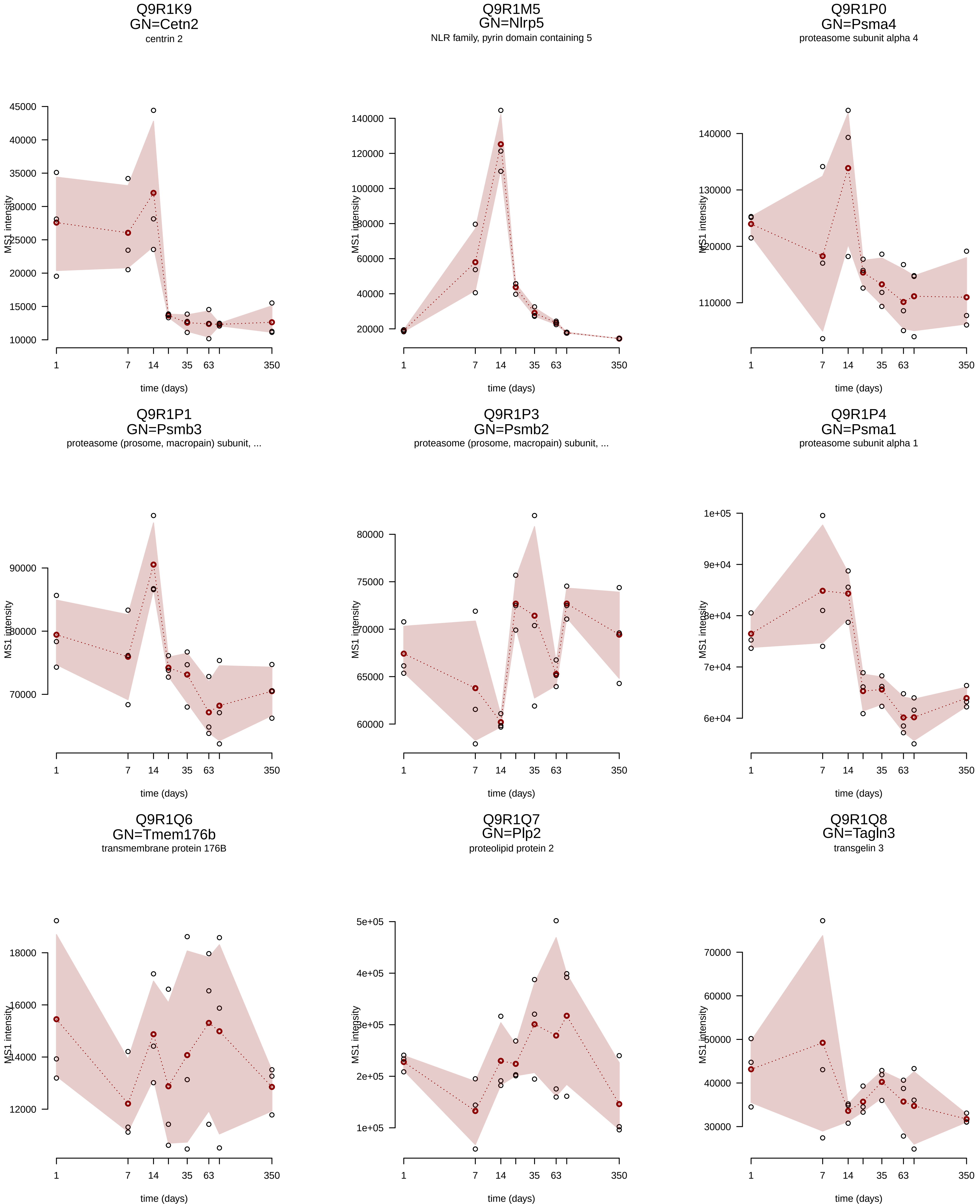
Q9R1K9
GN=Cetn2
centrin 2
Q9R1M5
GN=Nlrp5
NLR family, pyrin domain containing 5
Q9R1P0
GN=Psma4
proteasome subunit alpha 4
Q9R1P1
GN=Psmb3
proteasome (prosome, macropain) subunit, ...
Q9R1P3
GN=Psmb2
proteasome (prosome, macropain) subunit, ...
Q9R1P4
GN=Psma1
proteasome subunit alpha 1
Q9R1Q6
GN=Tmem176b
transmembrane protein 176B
Q9R1Q7
GN=Plp2
proteolipid protein 2
Q9R1Q8
GN=Tagln3
transgelin 3
MS1 intensity
time (days)
1
7
14
35
63
350

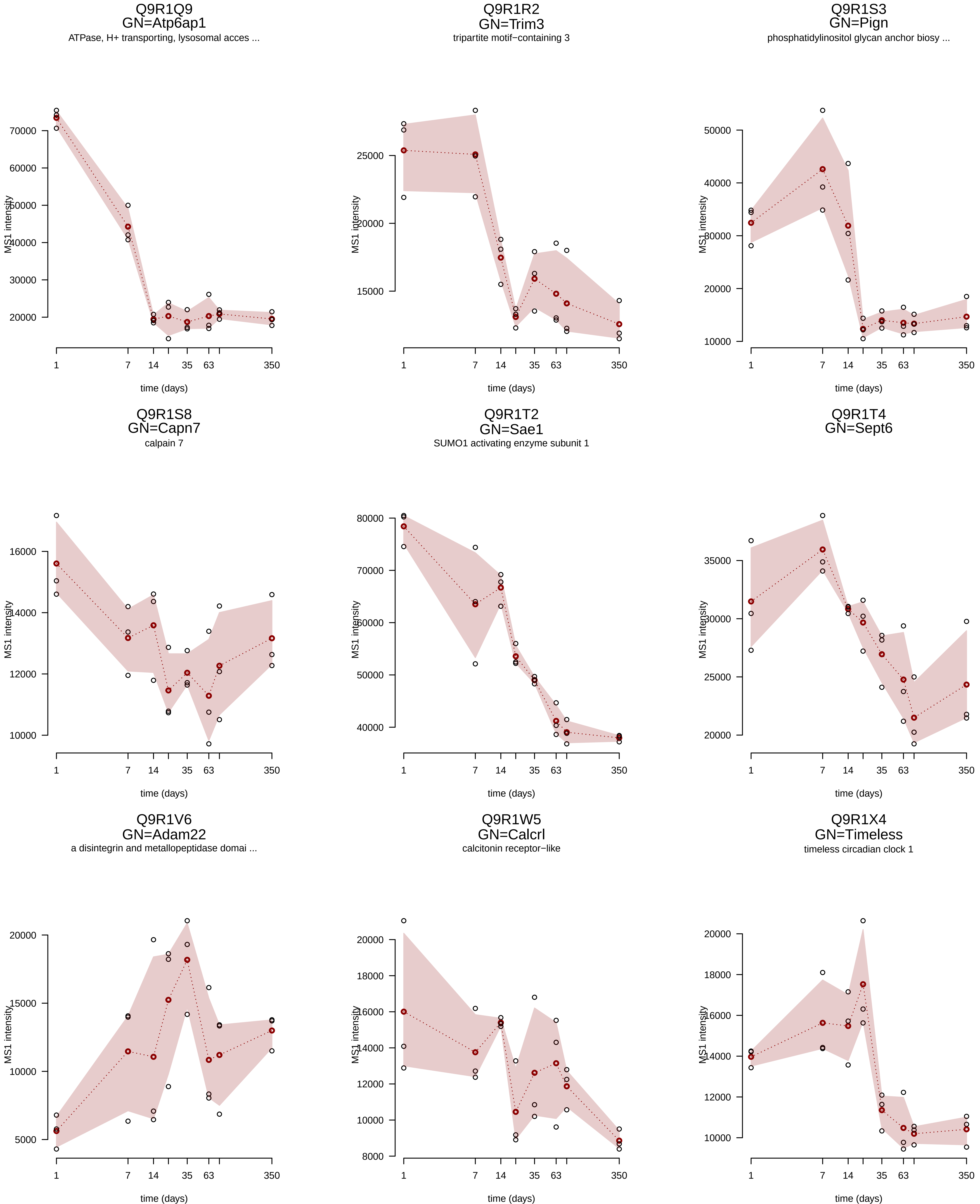
Q9R1Q9
GN=Atp6ap1
ATPase, H+ transporting, lysosomal acces ...
MS1 intensity
70000
60000
50000
40000
30000
20000
1
7
14
35
63
350
time (days)
Q9R1R2
GN=Trim3
tripartite motif−containing 3
MS1 intensity
25000
20000
15000
1
7
14
35
63
350
time (days)
Q9R1S3
GN=Pign
phosphatidylinositol glycan anchor biosy ...
MS1 intensity
50000
40000
30000
20000
10000
1
7
14
35
63
350
time (days)
Q9R1S8
GN=Capn7
calpain 7
MS1 intensity
16000
14000
12000
10000
1
7
14
35
63
350
time (days)
Q9R1T2
GN=Sae1
SUMO1 activating enzyme subunit 1
MS1 intensity
80000
70000
60000
50000
40000
1
7
14
35
63
350
time (days)
Q9R1T4
GN=Sept6
MS1 intensity
35000
30000
25000
20000
1
7
14
35
63
350
time (days)
Q9R1V6
GN=Adam22
a disintegrin and metallopeptidase domai ...
MS1 intensity
20000
15000
10000
5000
1
7
14
35
63
350
time (days)
Q9R1W5
GN=Calcrl
calcitonin receptor−like
MS1 intensity
20000
18000
16000
14000
12000
10000
8000
1
7
14
35
63
350
time (days)
Q9R1X4
GN=Timeless
timeless circadian clock 1
MS1 intensity
20000
18000
16000
14000
12000
10000
1
7
14
35
63
350
time (days)

Q9R1Y5
GN=Hic1
hypermethylated in cancer 1

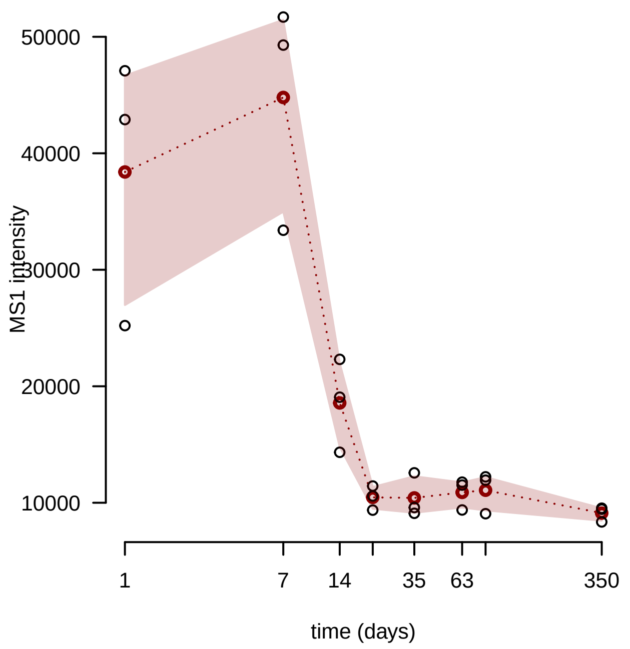

Q9R1Z7
GN=Pts
6−pyruvoyl−tetrahydropterin synthase

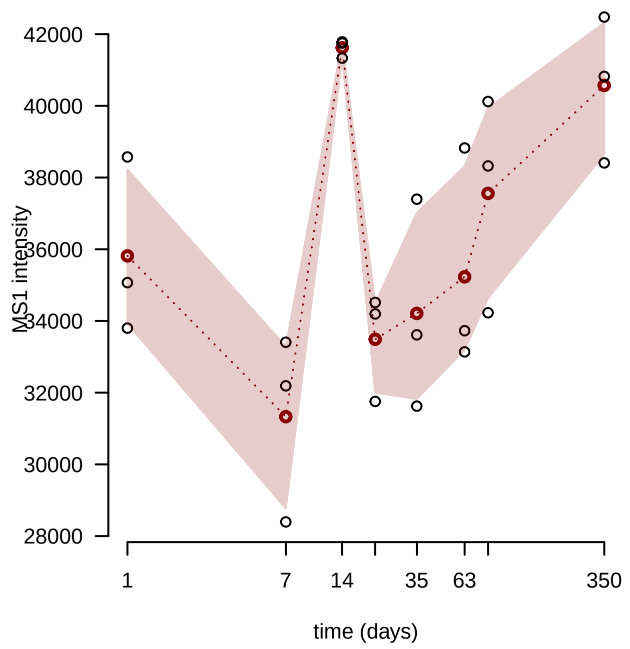

Q9R1Z8
GN=Sorbs3
sorbin and SH3 domain containing 3

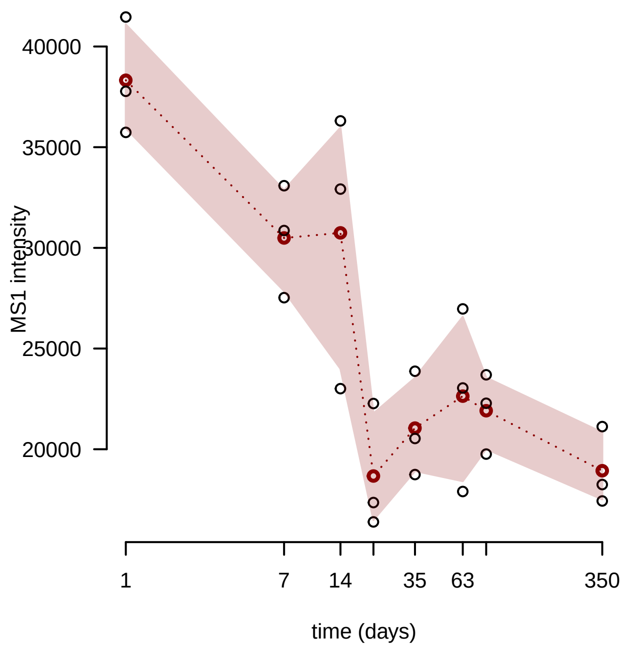

Q9R207
GN=Nbn
nibrin

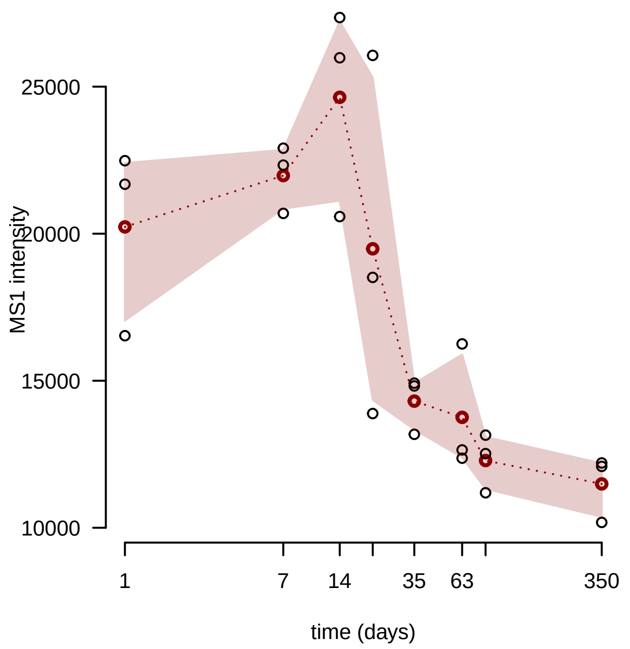

Q9R210
GN=Tfeb
transcription factor EB

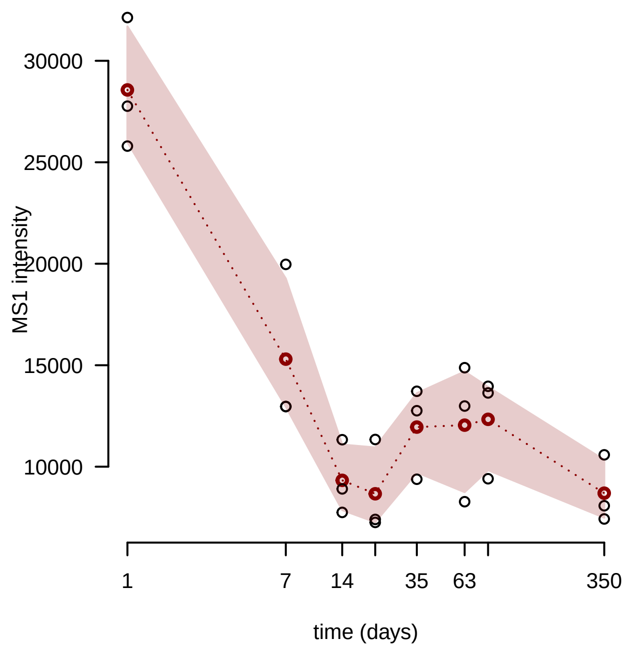

Q9R226
GN=Khdrbs3
KH domain containing, RNA binding, signa ...

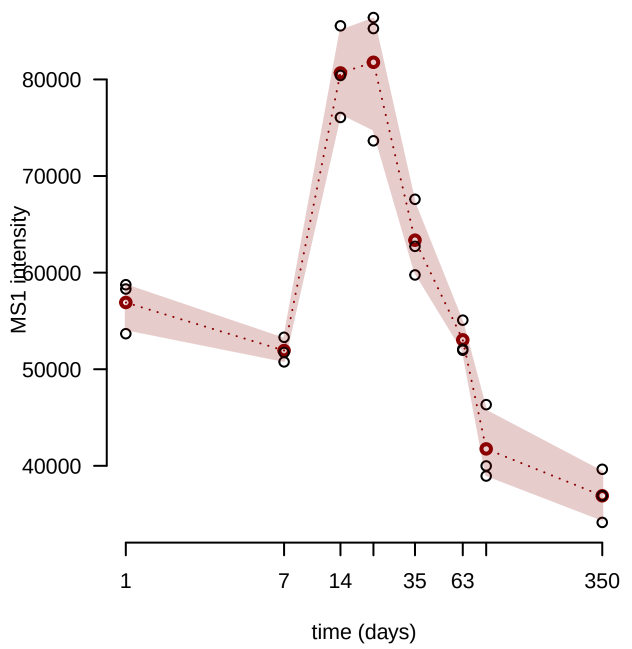

Q9R233
GN=Tapbp
TAP binding protein

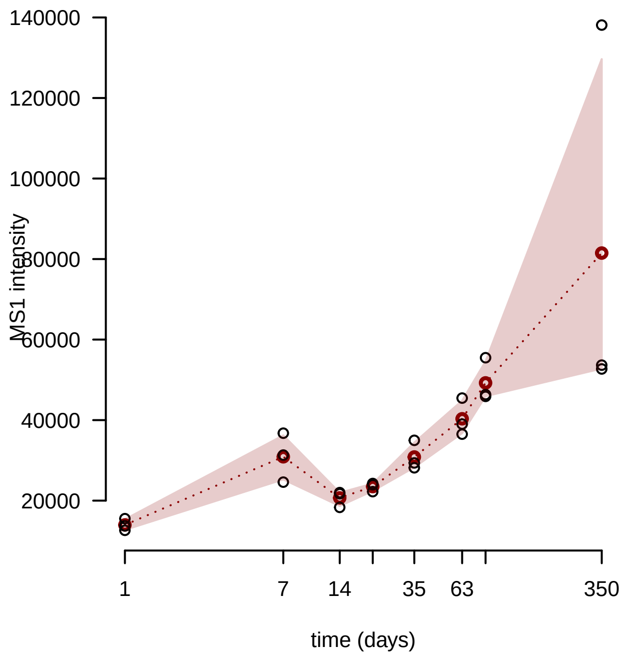

Q9R257
GN=Hebp1
heme binding protein 1

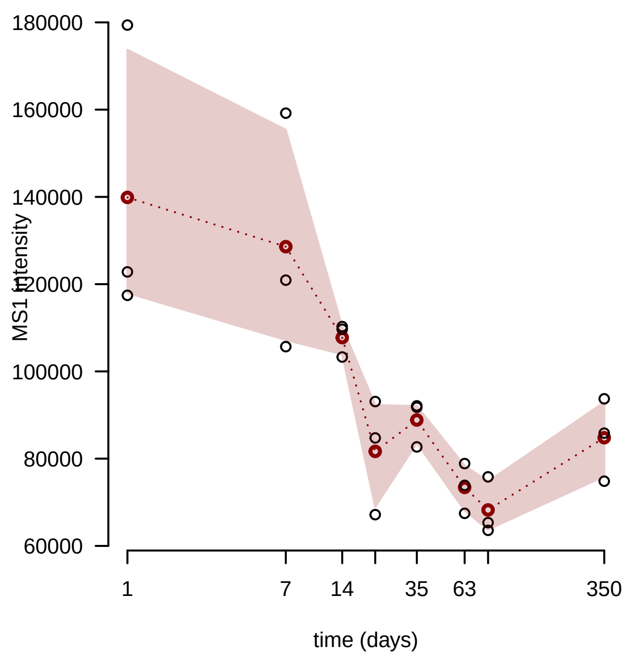

Q9R269
GN=Ppl
periplakin

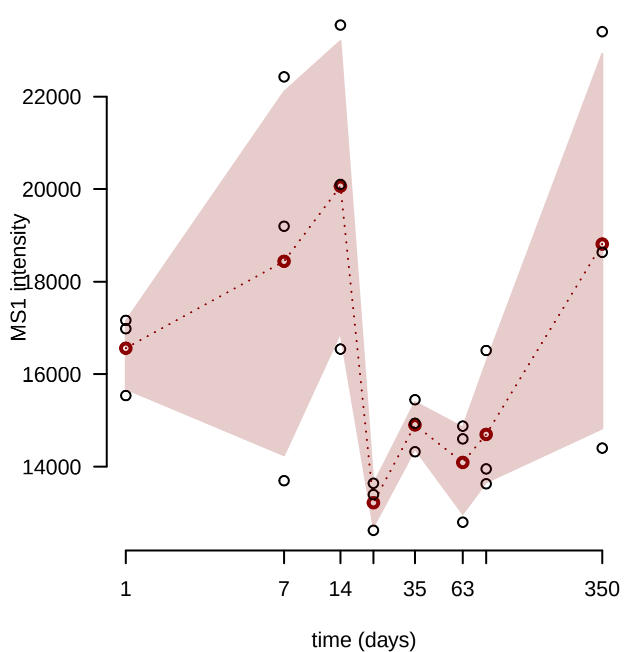

Q9R2B6
GN=St6galnac4
ST6 (alpha−N−acetyl−neuraminyl−2,3−beta− ...

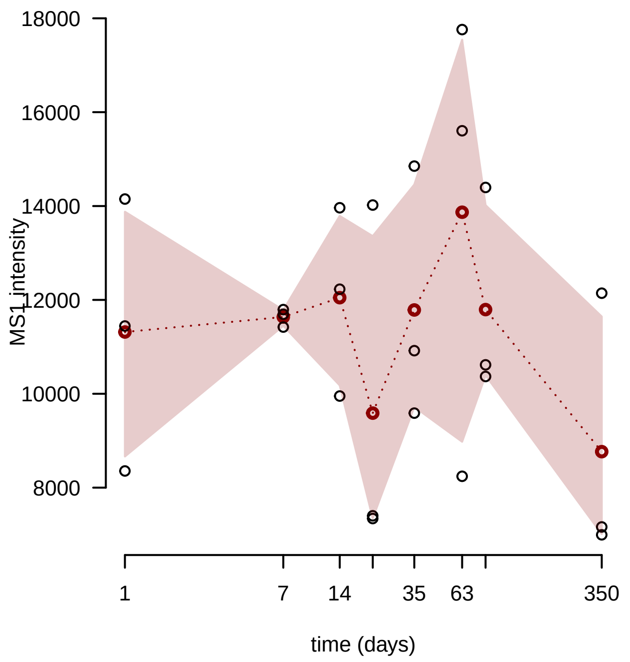

Q9WTI7
GN=Myo1c
myosin IC

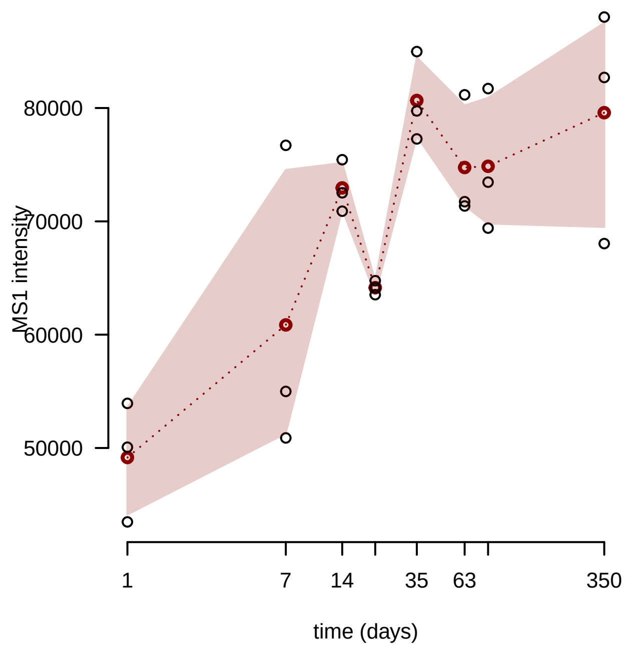

Q9WTI7
GN=Myo1c
myosin IC

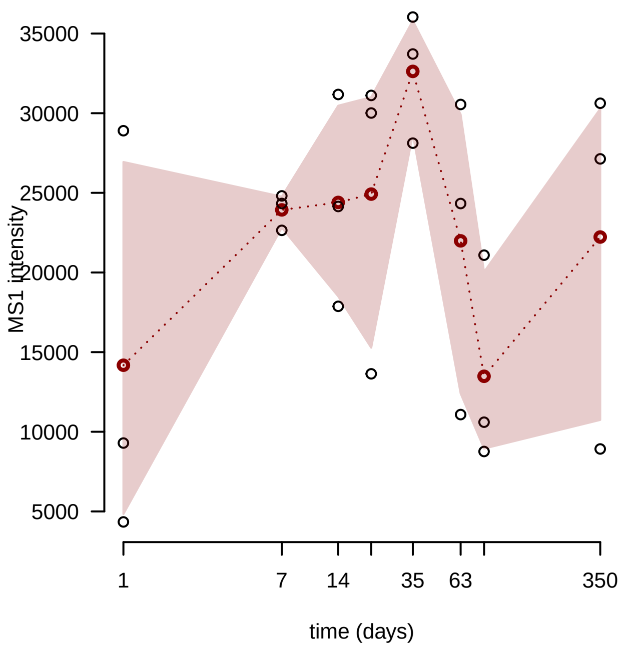

Q9WTI7
GN=Myo1c
myosin IC

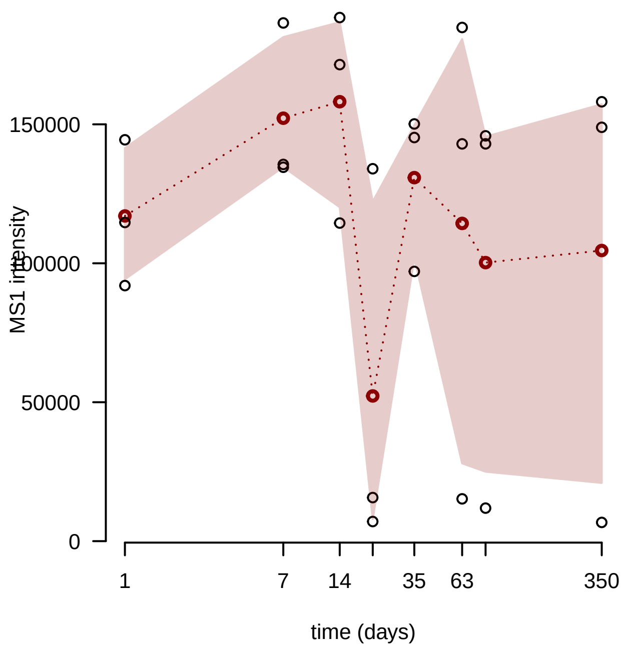

Q9WTJ4
GN=Fiz1
Flt3 interacting zinc finger protein 1

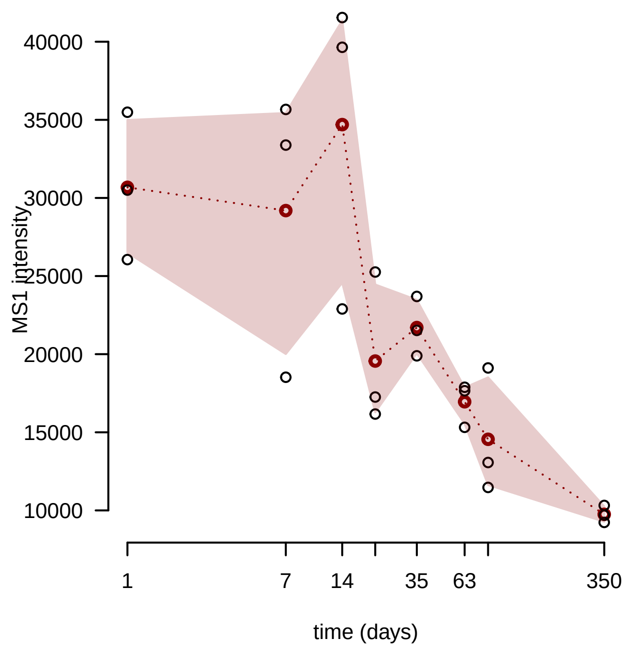

Q9WTK2
GN=Cdyl
chromodomain protein, Y chromosome−like

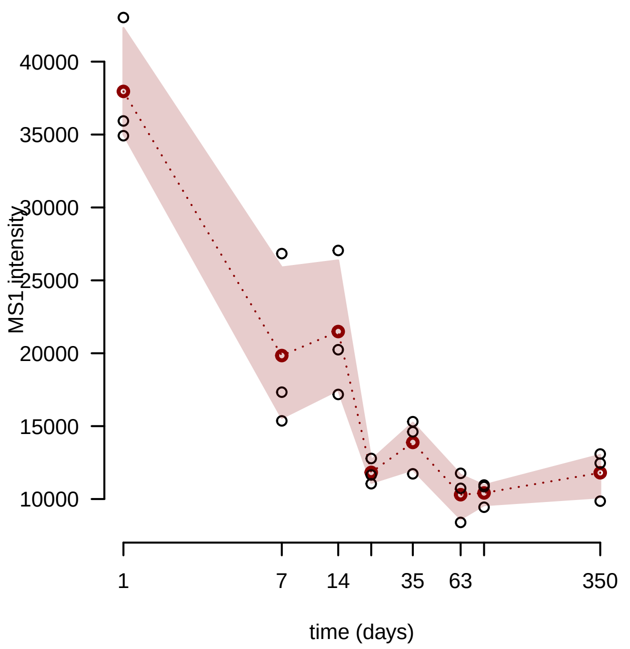

Q9WTK3
GN=Gpaa1
GPI anchor attachment protein 1

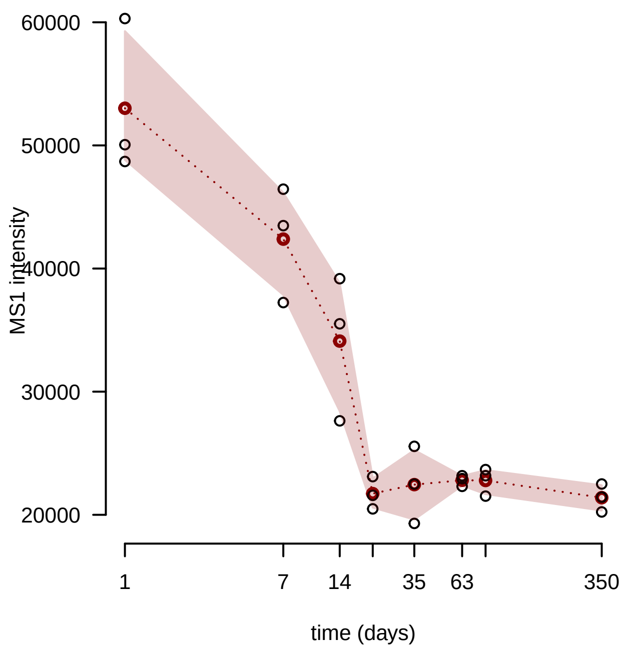

Q9WTK5
GN=Nfkb2
nuclear factor of kappa light polypeptid ...

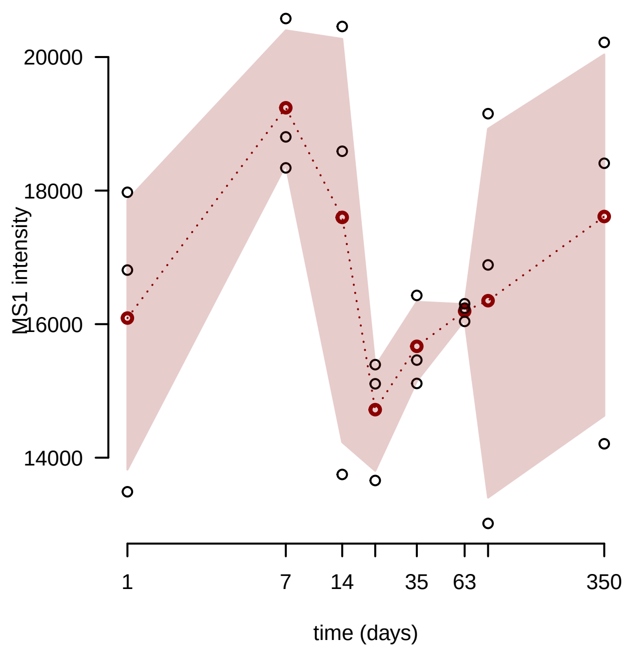

Q9WTK7
GN=Stk11
serine/threonine kinase 11

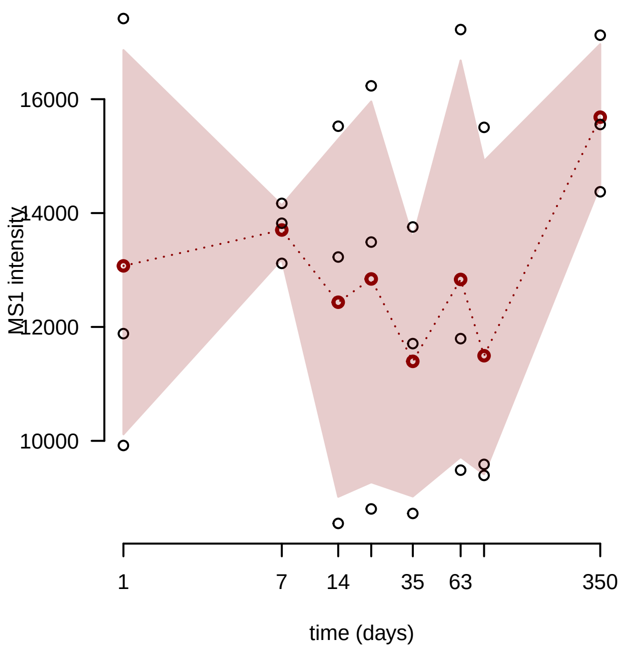

Q9WTL4
GN=Insrr
insulin receptor−related receptor

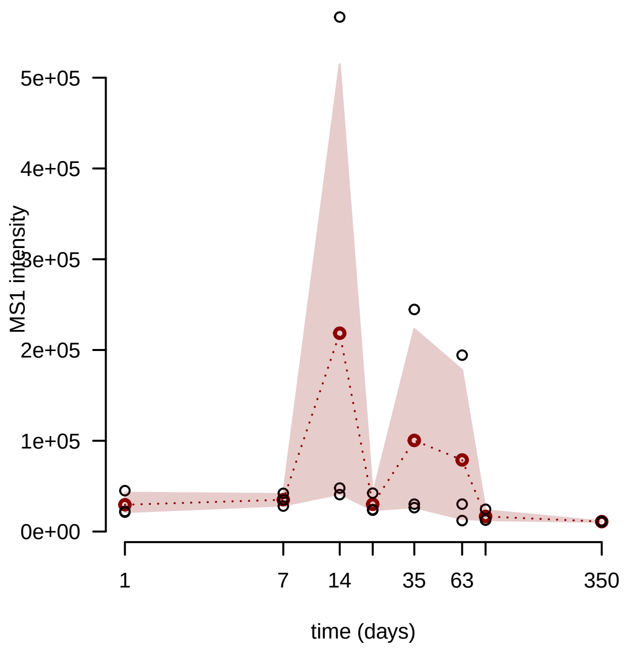

Q9WTL7
GN=Lypla2
lysophospholipase 2

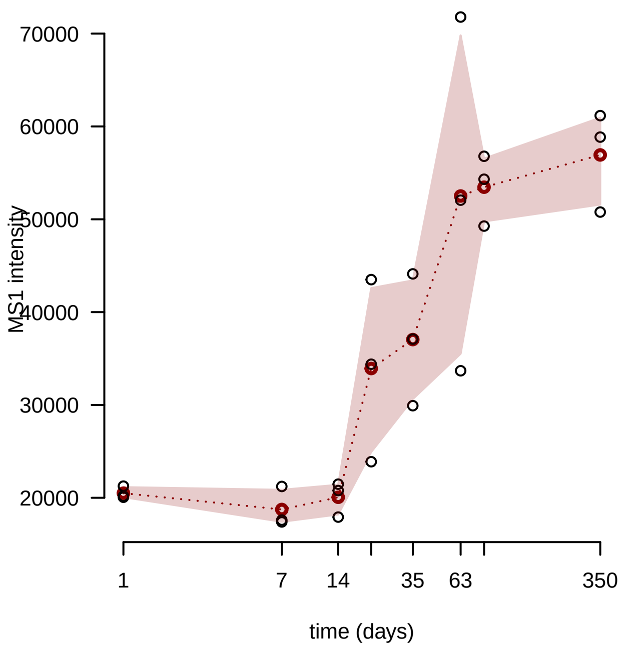

Q9WTM5
GN=Ruvbl2
RuvB−like protein 2

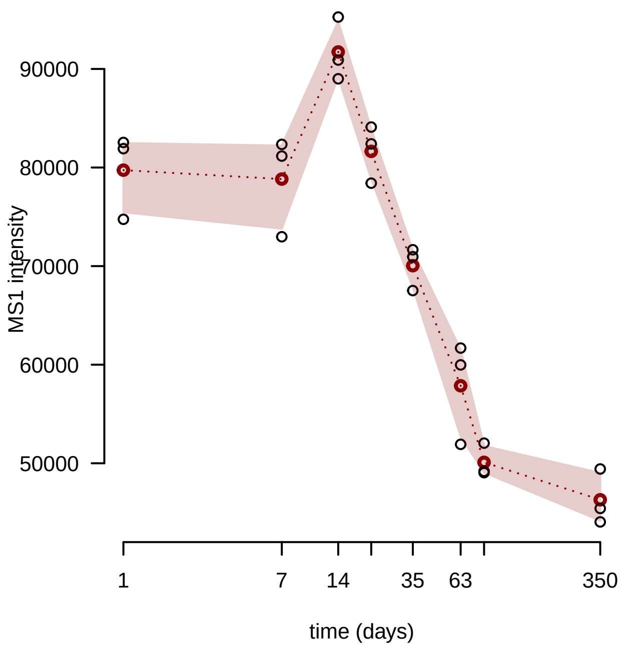

Q9WTN0
GN=Ggps1
geranylgeranyl diphosphate synthase 1

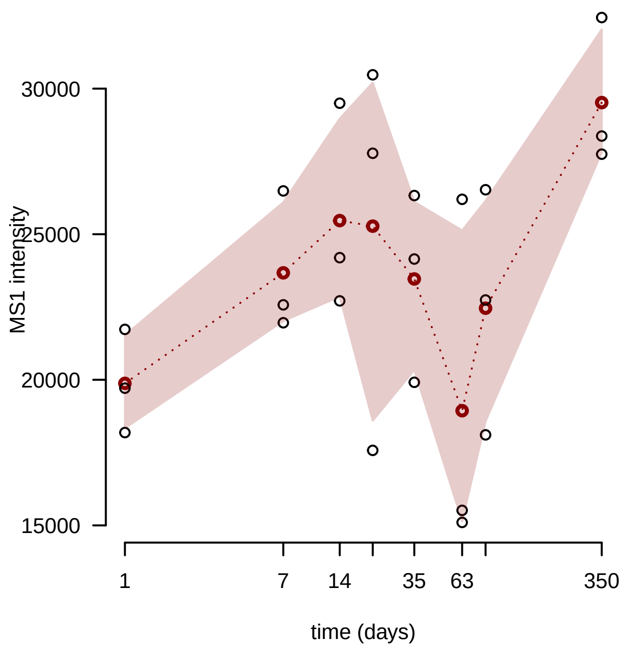

Q9WTN3
GN=Srebf1
sterol regulatory element binding transc ...

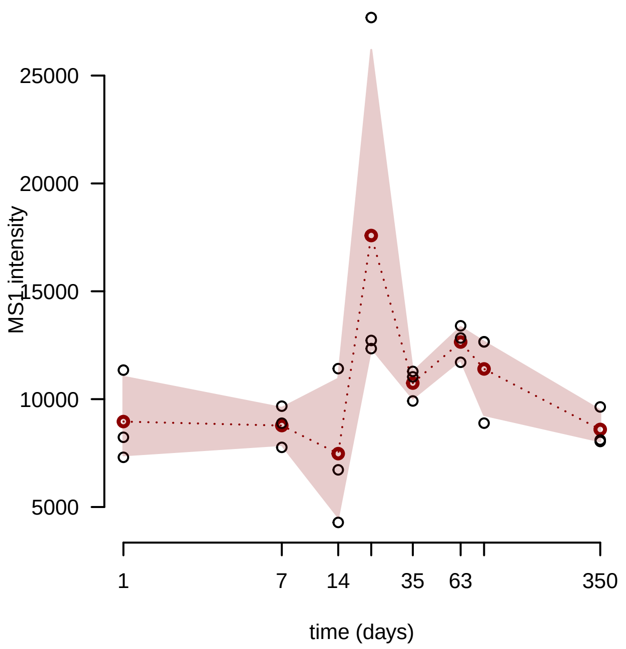

Q9WTP6
GN=Ak2
adenylate kinase 2

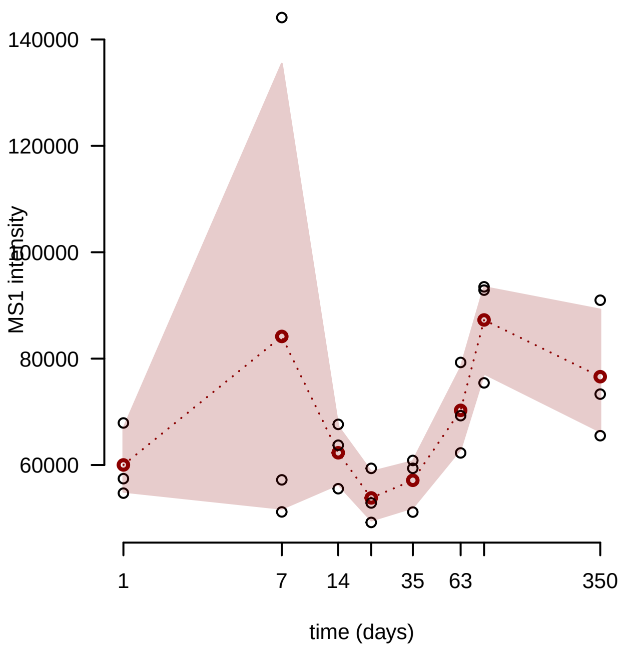

Q9WTP7
GN=Ak3
adenylate kinase 3

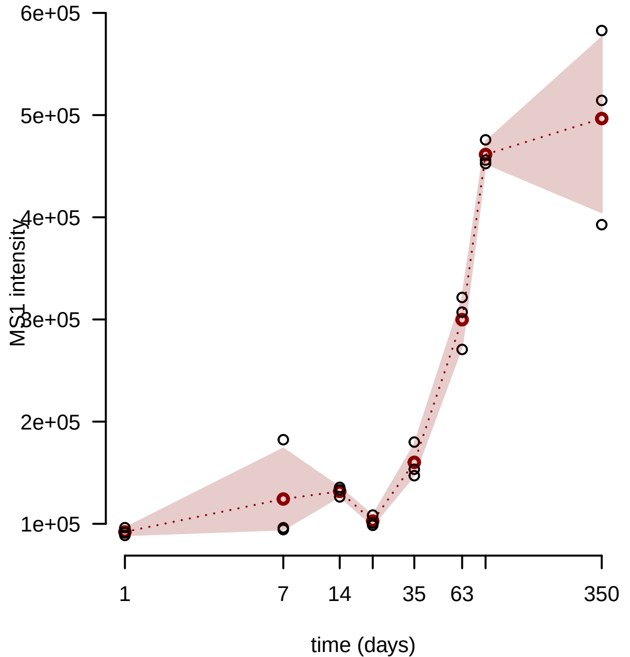

Q9WTQ5
GN=Akap12
A kinase (PRKA) anchor protein (gravin) ...

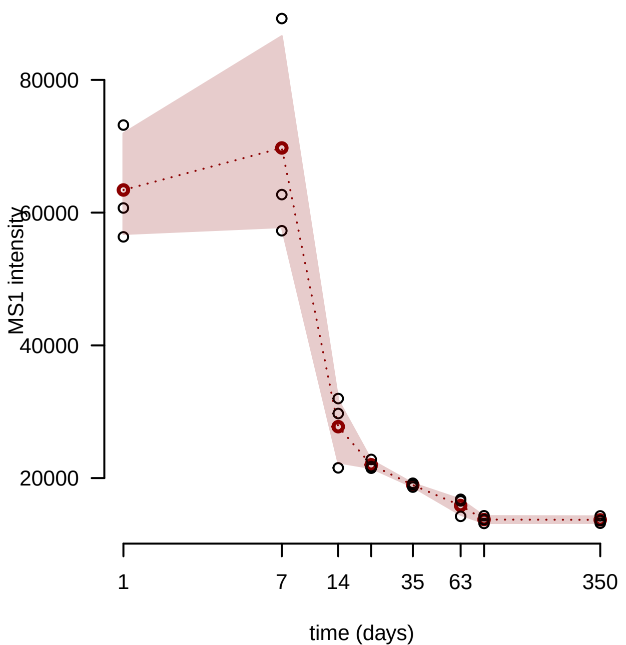

Q9WTQ8
GN=Timm23
translocase of inner mitochondrial membr ...

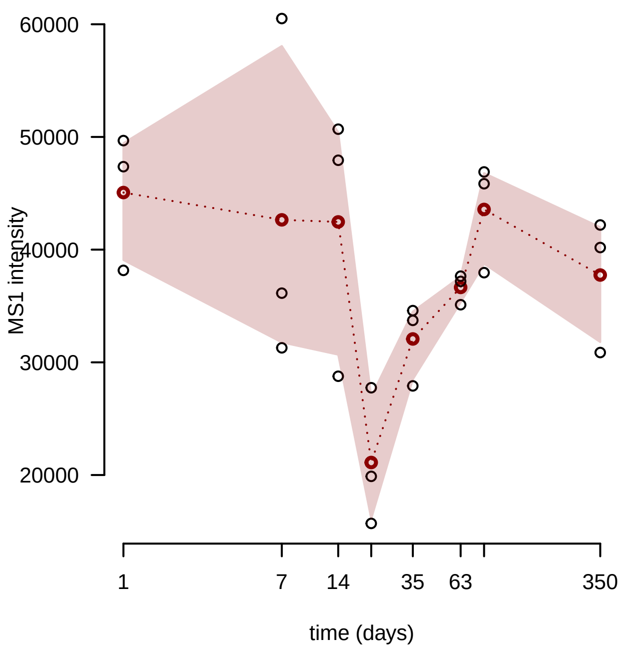

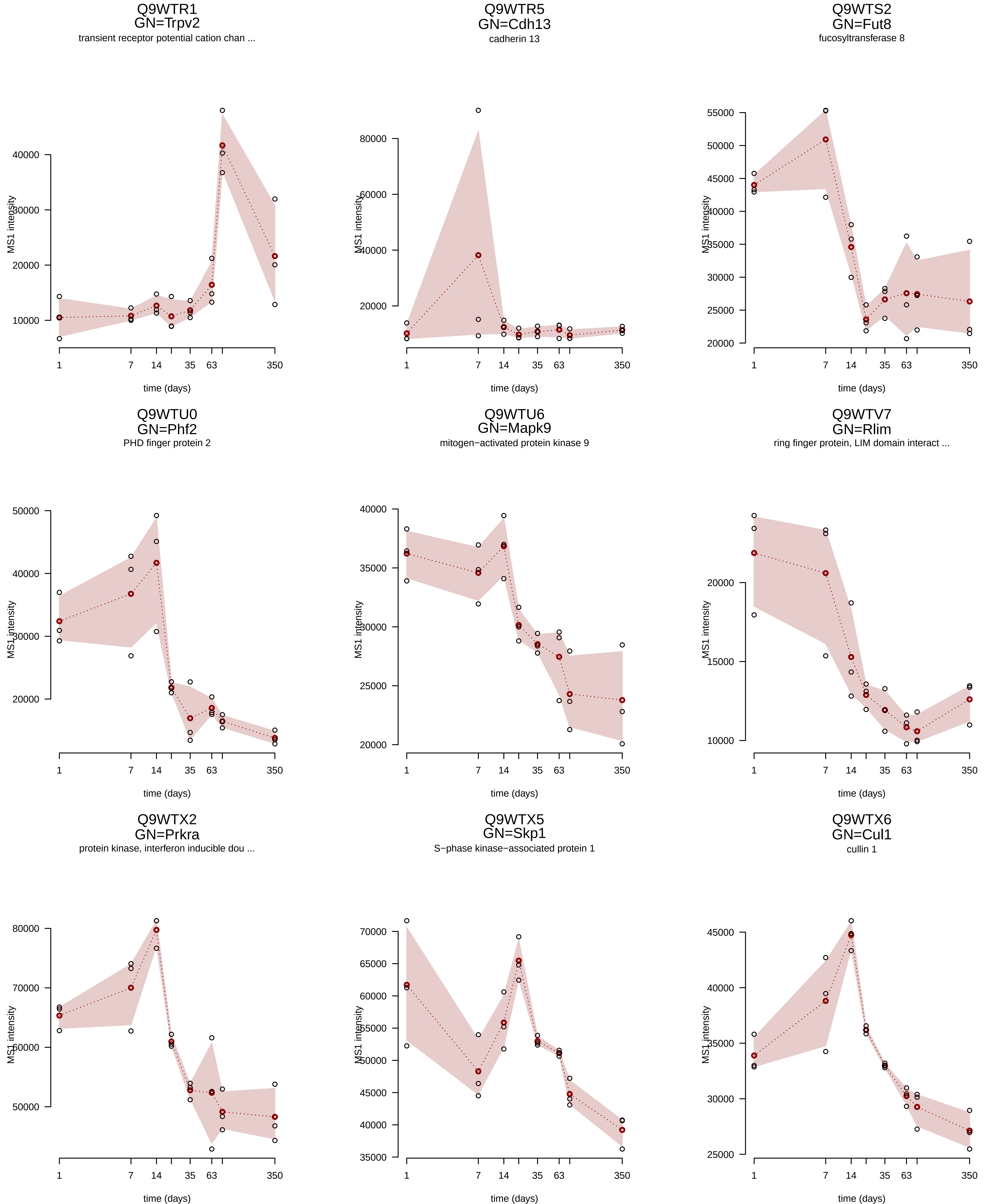
Q9WTR1
GN=Trpv2
transient receptor potential cation chan ...
MS1 intensity
10000
20000
30000
40000
1
7
14
35
63
350
time (days)
Q9WTR5
GN=Cdh13
cadherin 13
MS1 intensity
20000
40000
60000
80000
1
7
14
35
63
350
time (days)
Q9WTS2
GN=Fut8
fucosyltransferase 8
MS1 intensity
20000
25000
30000
35000
40000
45000
50000
55000
1
7
14
35
63
350
time (days)
Q9WTU0
GN=Phf2
PHD finger protein 2
MS1 intensity
20000
30000
40000
50000
1
7
14
35
63
350
time (days)
Q9WTU6
GN=Mapk9
mitogen−activated protein kinase 9
MS1 intensity
20000
25000
30000
35000
40000
1
7
14
35
63
350
time (days)
Q9WTV7
GN=Rlim
ring finger protein, LIM domain interact ...
MS1 intensity
10000
15000
20000
1
7
14
35
63
350
time (days)
Q9WTX2
GN=Prkra
protein kinase, interferon inducible dou ...
MS1 intensity
50000
60000
70000
80000
1
7
14
35
63
350
time (days)
Q9WTX5
GN=Skp1
S−phase kinase−associated protein 1
MS1 intensity
35000
40000
45000
50000
55000
60000
65000
70000
1
7
14
35
63
350
time (days)
Q9WTX6
GN=Cul1
cullin 1
MS1 intensity
25000
30000
35000
40000
45000
1
7
14
35
63
350
time (days)

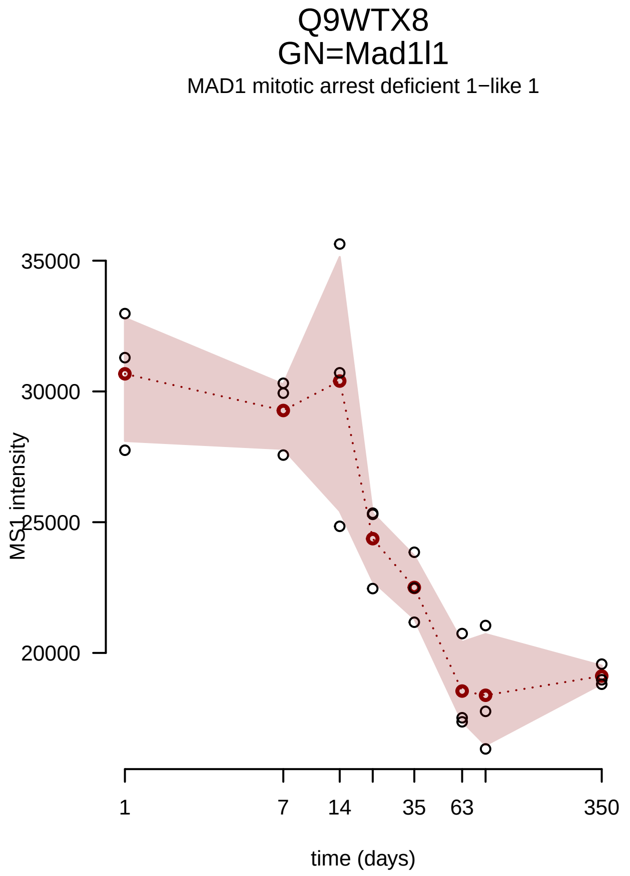
Q9WTX8
GN=Mad1l1
MAD1 mitotic arrest deficient 1−like 1
35000
30000
25000
20000
MS1 intensity
1
7
14
35
63
350
time (days)

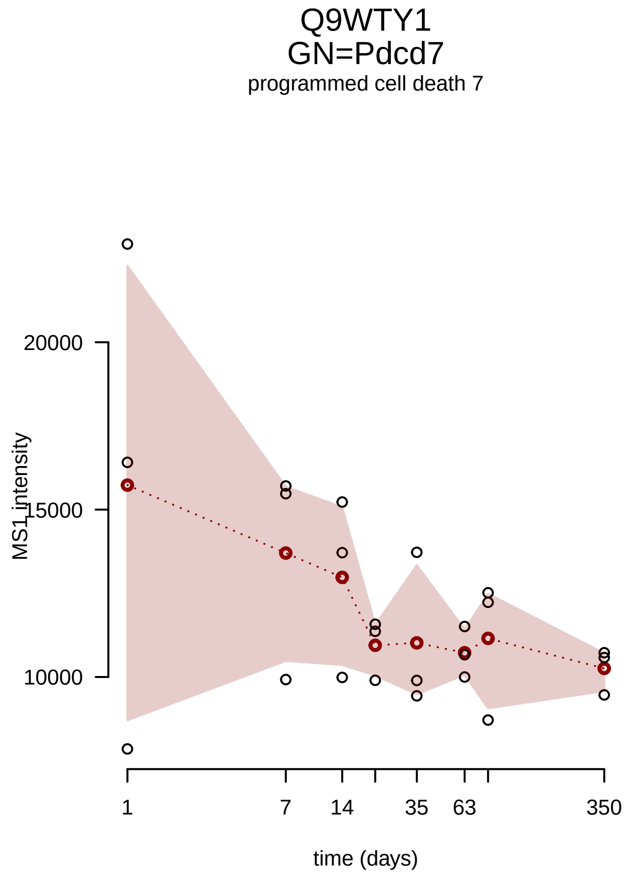
Q9WTY1
GN=Pdcd7
programmed cell death 7
20000
15000
10000
MS1 intensity
1
7
14
35
63
350
time (days)

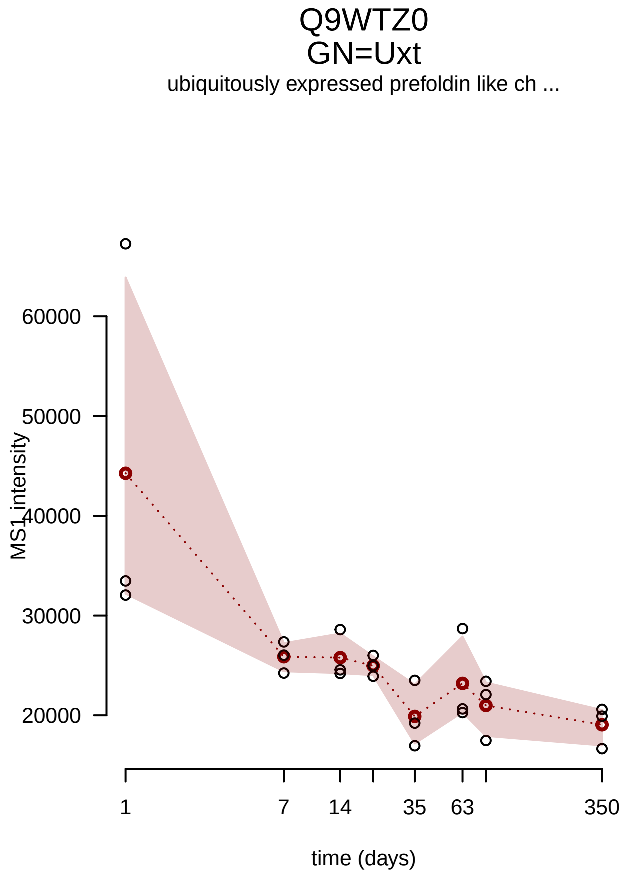
Q9WTZ0
GN=Uxt
ubiquitously expressed prefoldin like ch ...
60000
50000
40000
30000
20000
MS1 intensity
1
7
14
35
63
350
time (days)

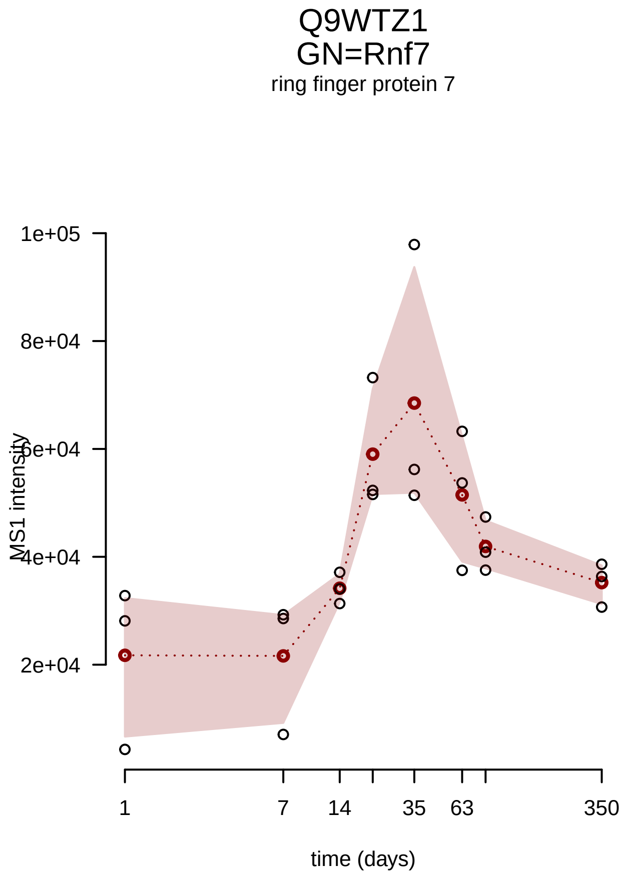
Q9WTZ1
GN=Rnf7
ring finger protein 7
1e+05
8e+04
6e+04
4e+04
2e+04
MS1 intensity
1
7
14
35
63
350
time (days)

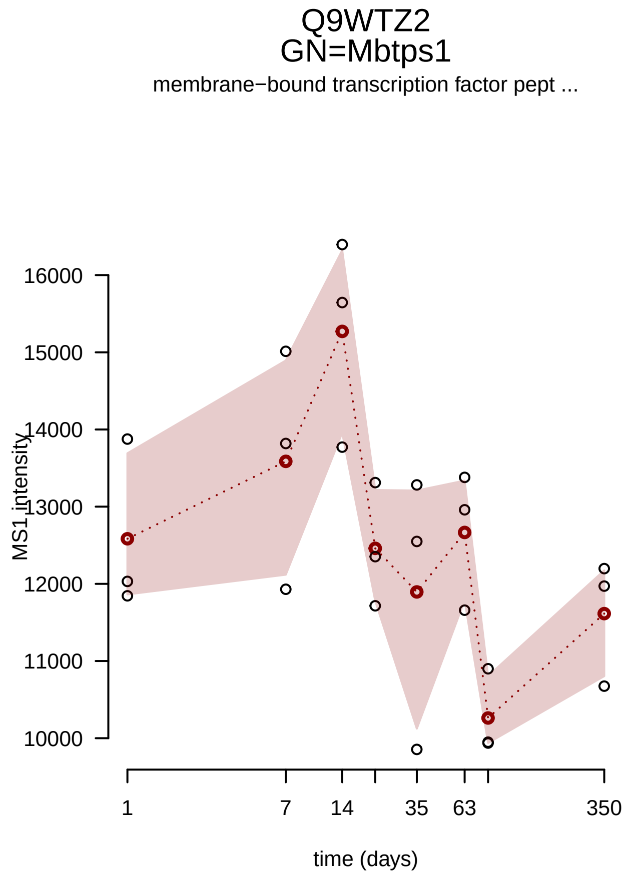
Q9WTZ2
GN=Mbtps1
membrane−bound transcription factor pept ...
16000
15000
14000
13000
12000
11000
10000
MS1 intensity
1
7
14
35
63
350
time (days)

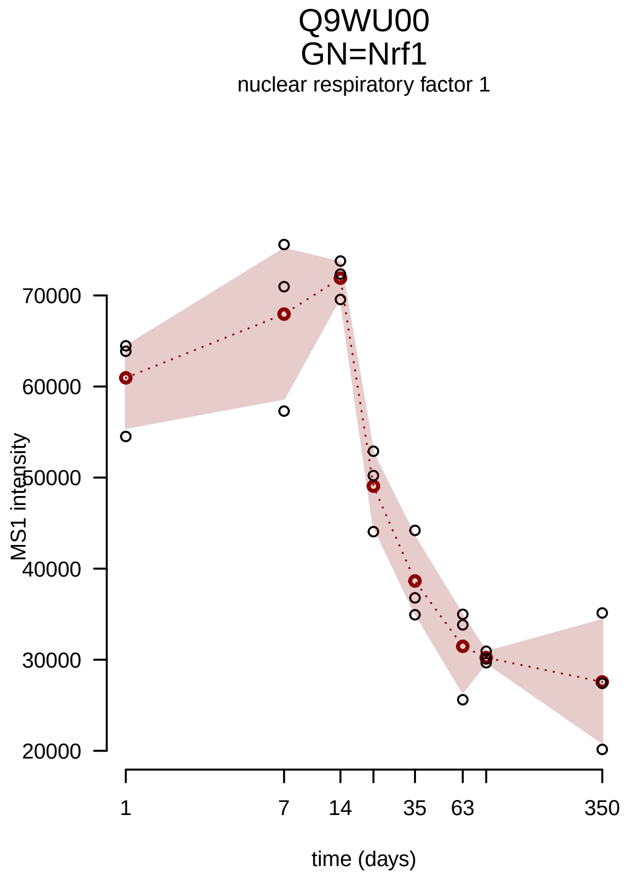
Q9WU00
GN=Nrf1
nuclear respiratory factor 1
70000
60000
50000
40000
30000
20000
MS1 intensity
1
7
14
35
63
350
time (days)

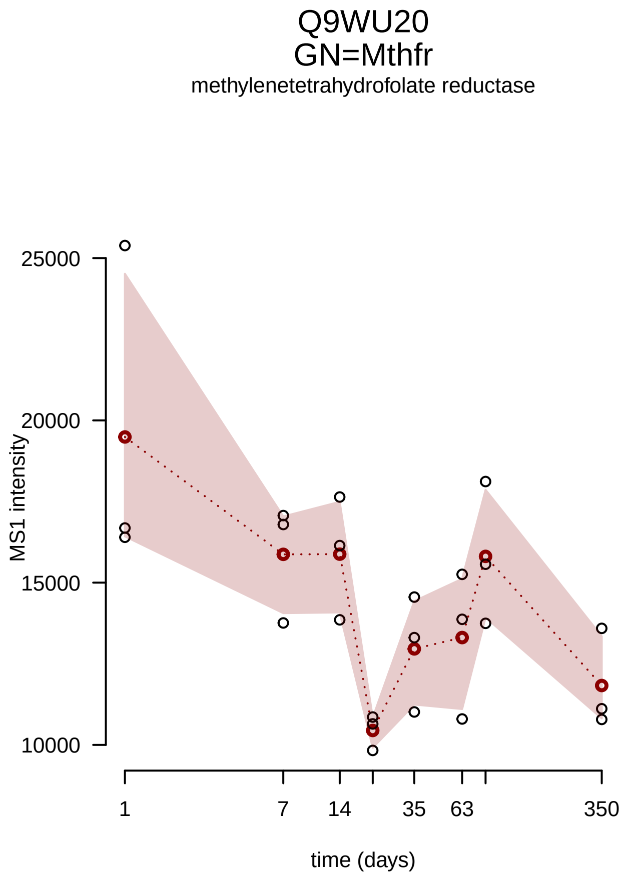
Q9WU20
GN=Mthfr
methylenetetrahydrofolate reductase
25000
20000
15000
10000
MS1 intensity
1
7
14
35
63
350
time (days)

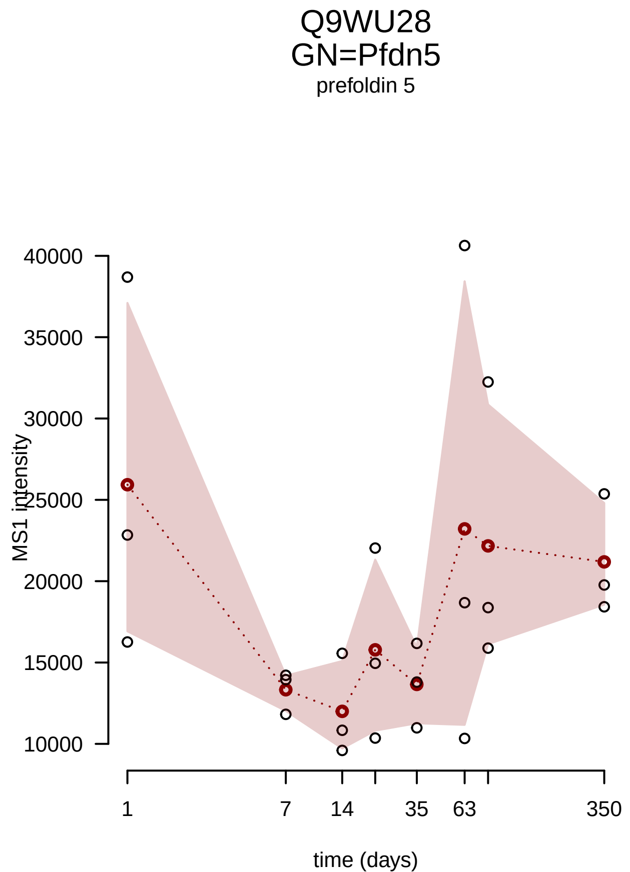
Q9WU28
GN=Pfdn5
prefoldin 5
40000
35000
30000
25000
20000
15000
10000
MS1 intensity
1
7
14
35
63
350
time (days)

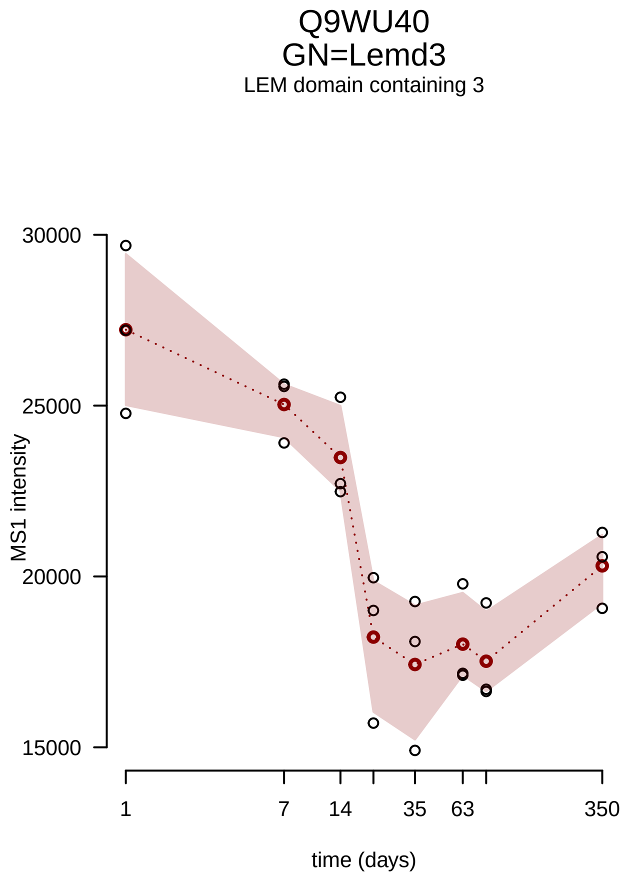
Q9WU40
GN=Lemd3
LEM domain containing 3
30000
25000
20000
15000
MS1 intensity
1
7
14
35
63
350
time (days)

Q9WU42
GN=Ncor2
nuclear receptor co−repressor 2

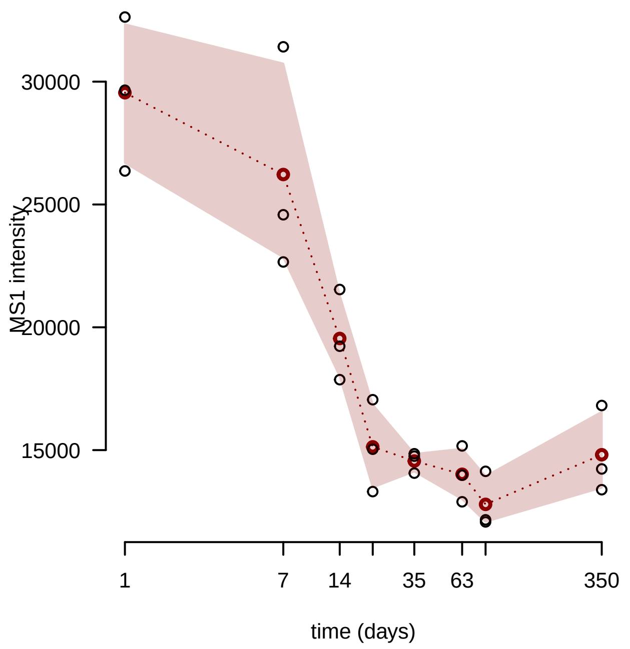

Q9WU56
GN=Pus1
pseudouridine synthase 1

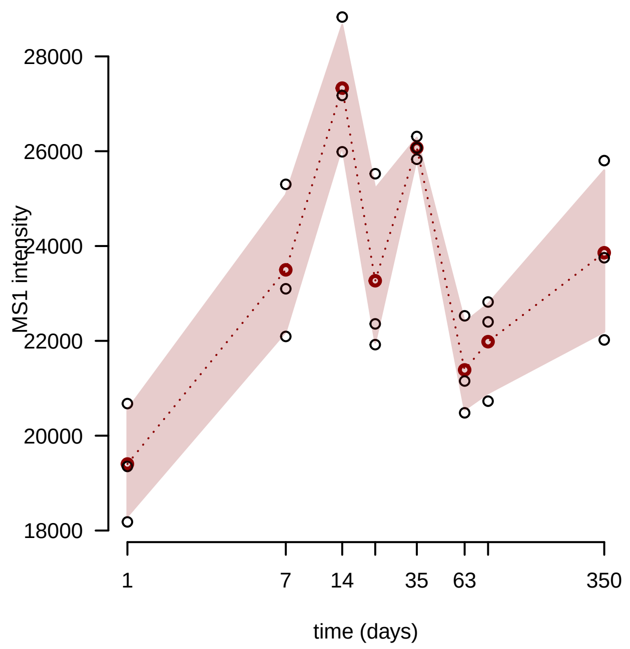

Q9WU60
GN=Atrn
attractin

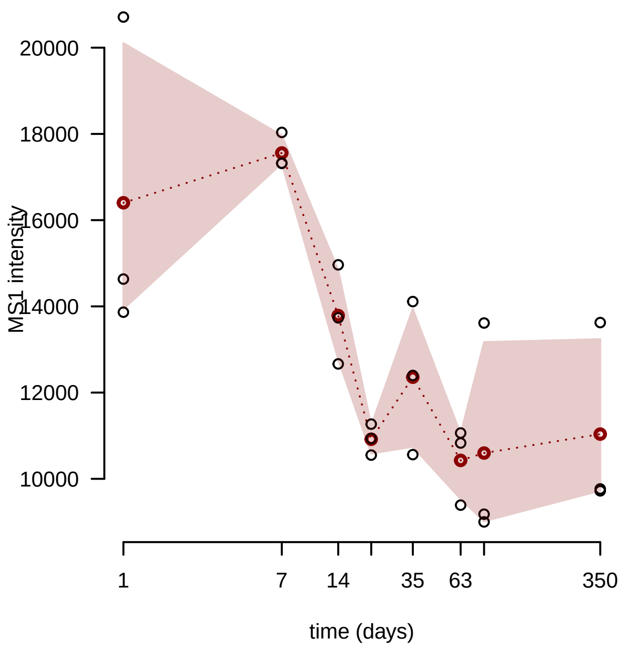

Q9WU62
GN=Incenp
inner centromere protein

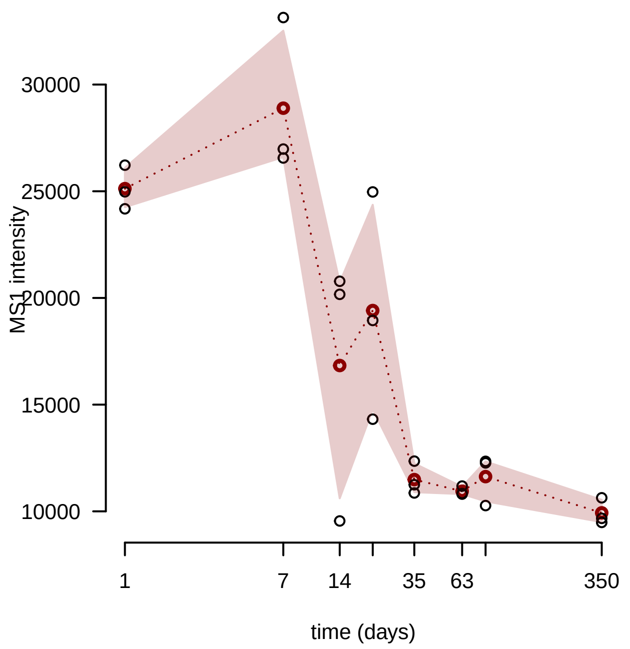

Q9WU63
GN=Hebp2
heme binding protein 2

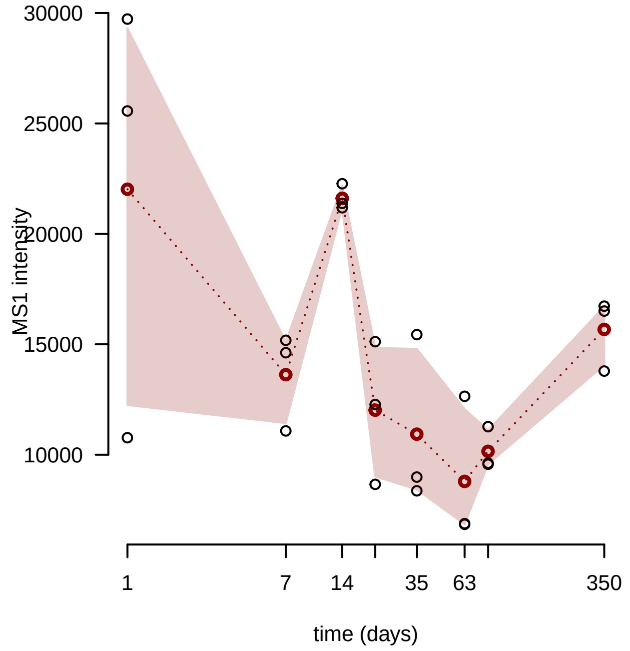

Q9WU78
GN=Pdcd6ip
programmed cell death 6 interacting prot ...

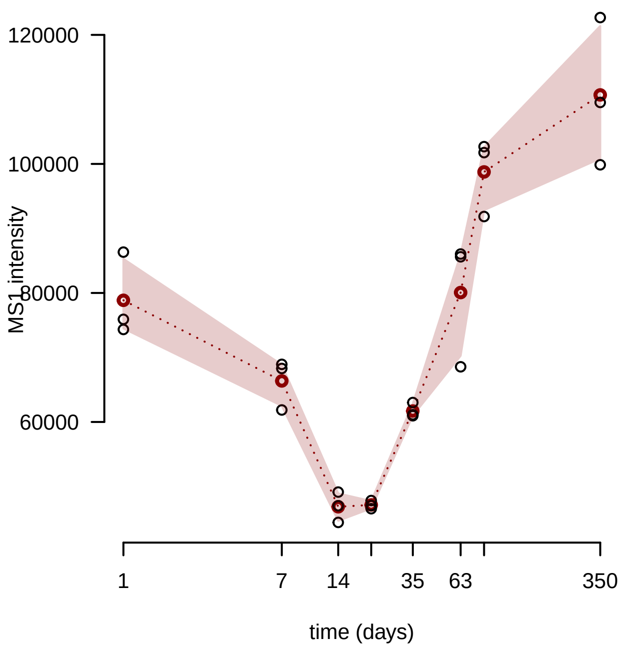

Q9WU79
GN=Prodh
proline dehydrogenase

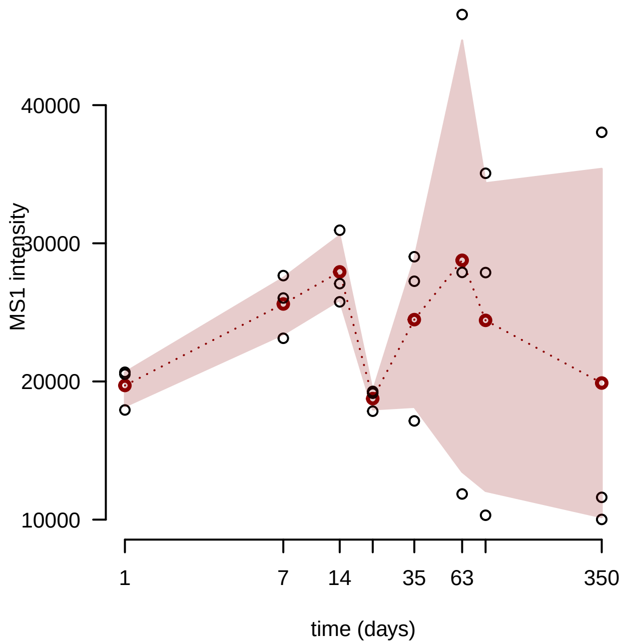

Q9WU81
GN=Slc37a2
solute carrier family 37 (glycerol−3−pho ...

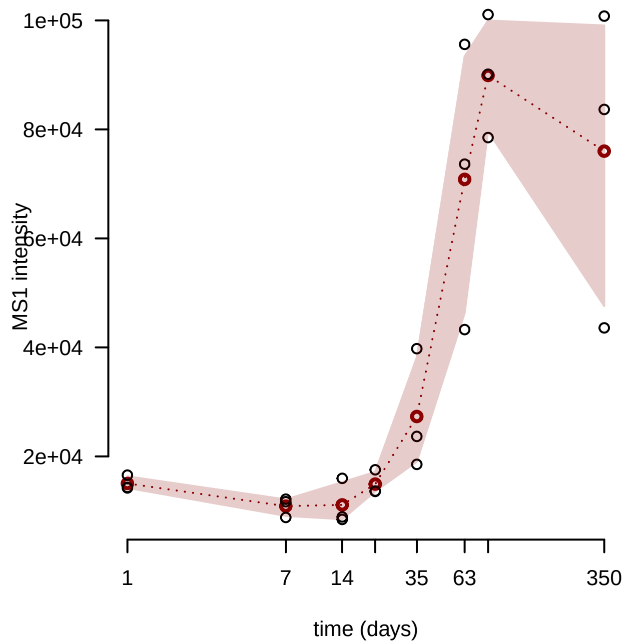

Q9WU84
GN=Ccs
copper chaperone for superoxide dismutas ...

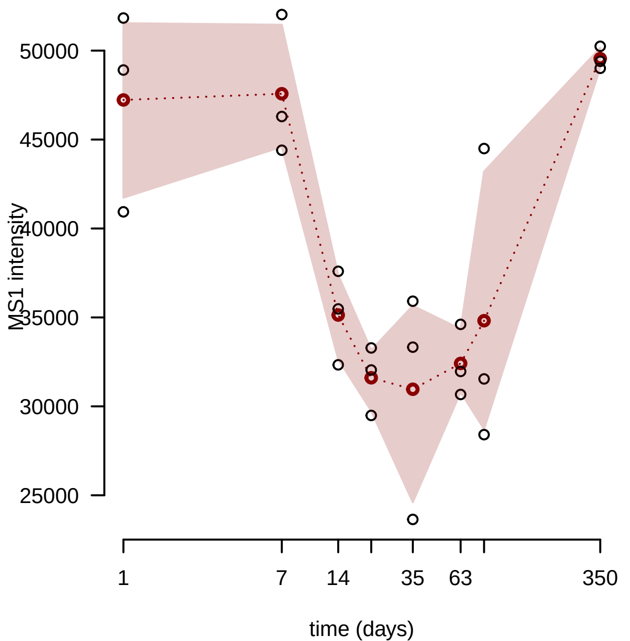

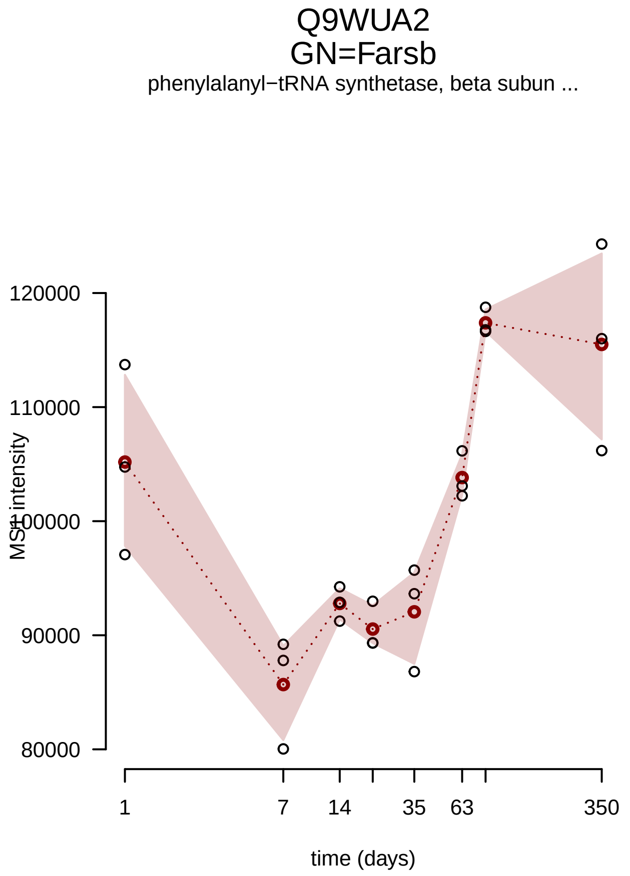
Q9WUA2
GN=Farsb
phenylalanyl−tRNA synthetase, beta subun ...
MS1 intensity
80000
90000
100000
110000
120000
1
7
14
35
63
350
time (days)
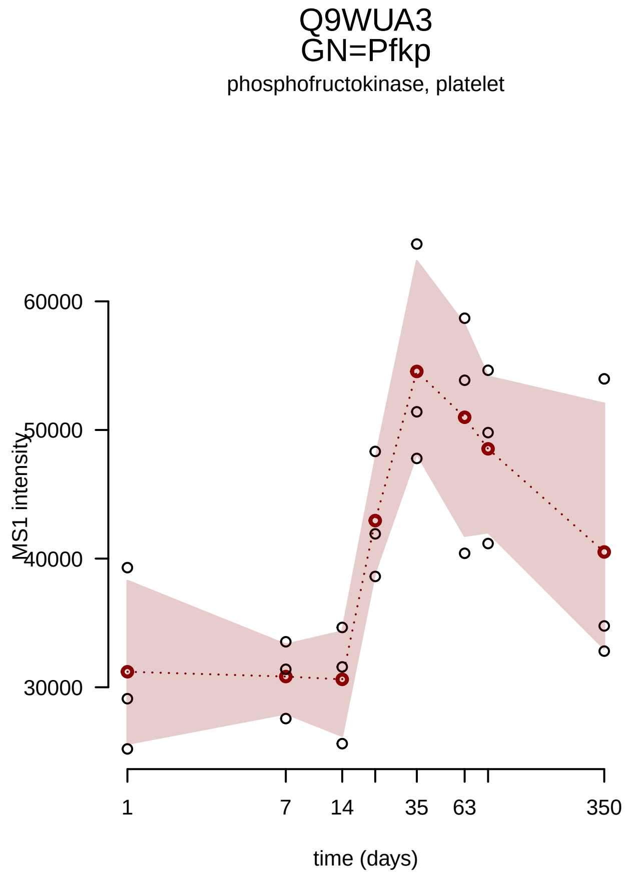
Q9WUA3
GN=Pfkp
phosphofructokinase, platelet
MS1 intensity
30000
40000
50000
60000
1
7
14
35
63
350
time (days)
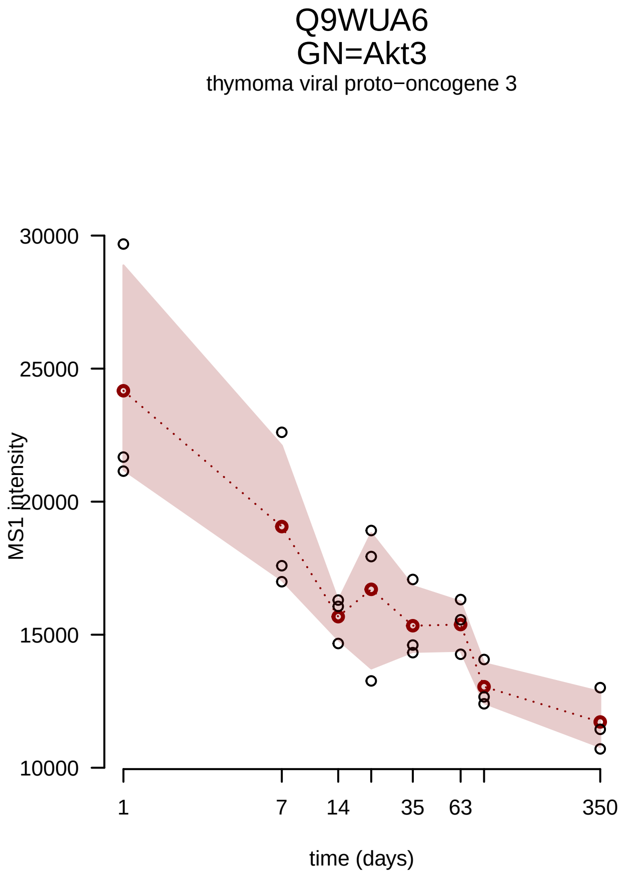
Q9WUA6
GN=Akt3
thymoma viral proto−oncogene 3
MS1 intensity
10000
15000
20000
25000
30000
1
7
14
35
63
350
time (days)
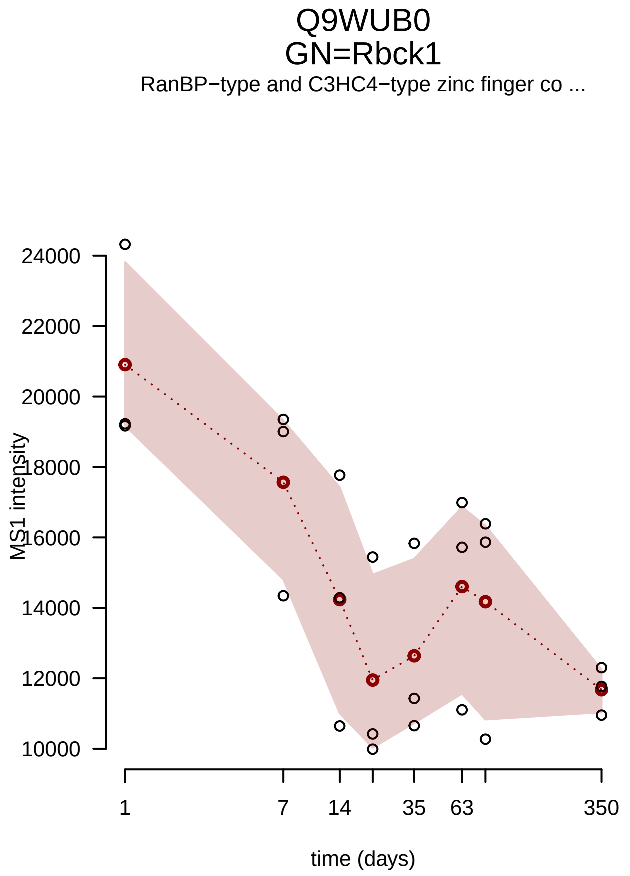
Q9WUB0
GN=Rbck1
RanBP−type and C3HC4−type zinc finger co ...
MS1 intensity
10000
12000
14000
16000
18000
20000
22000
24000
1
7
14
35
63
350
time (days)
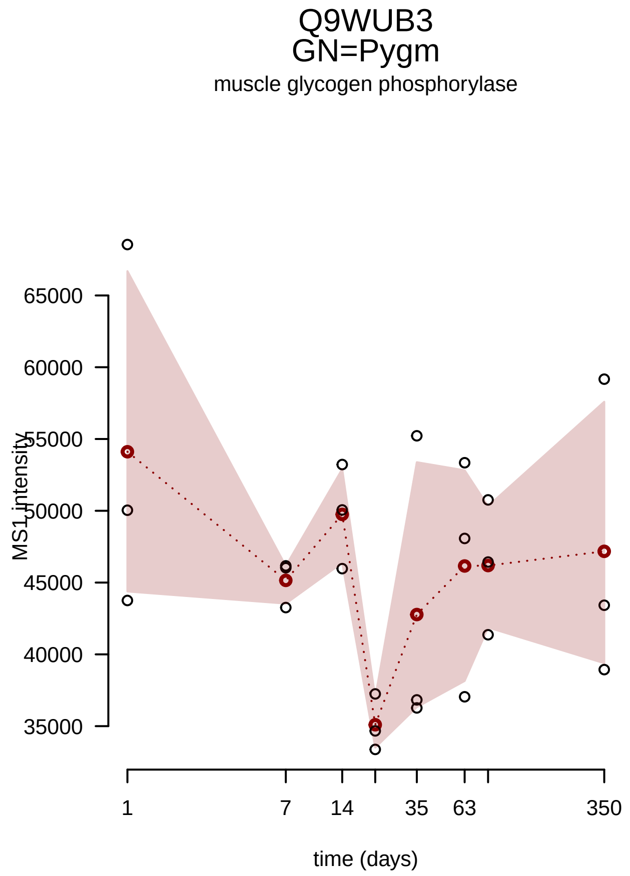
Q9WUB3
GN=Pygm
muscle glycogen phosphorylase
MS1 intensity
35000
40000
45000
50000
55000
60000
65000
1
7
14
35
63
350
time (days)
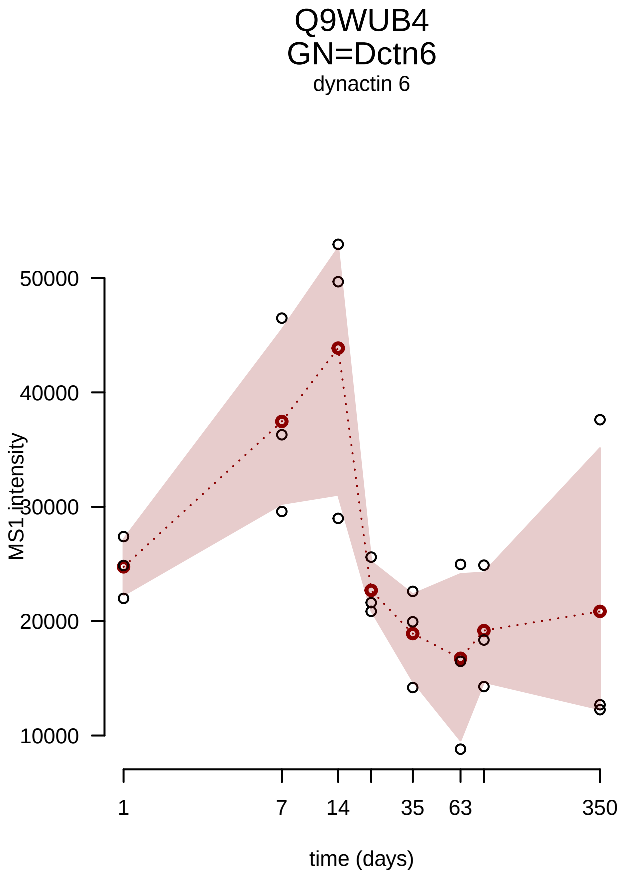
Q9WUB4
GN=Dctn6
dynactin 6
MS1 intensity
10000
20000
30000
40000
50000
1
7
14
35
63
350
time (days)
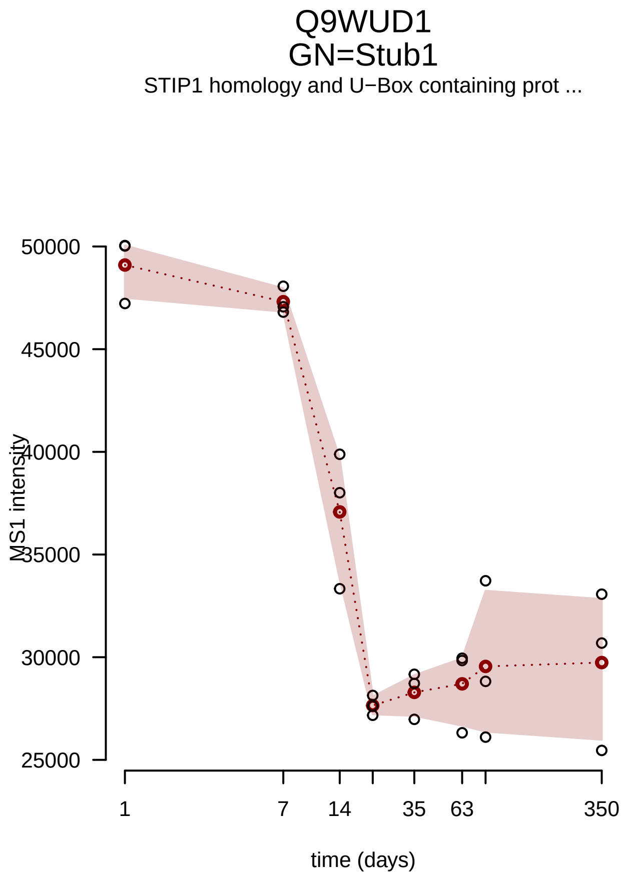
Q9WUD1
GN=Stub1
STIP1 homology and U−Box containing prot ...
MS1 intensity
25000
30000
35000
40000
45000
50000
1
7
14
35
63
350
time (days)
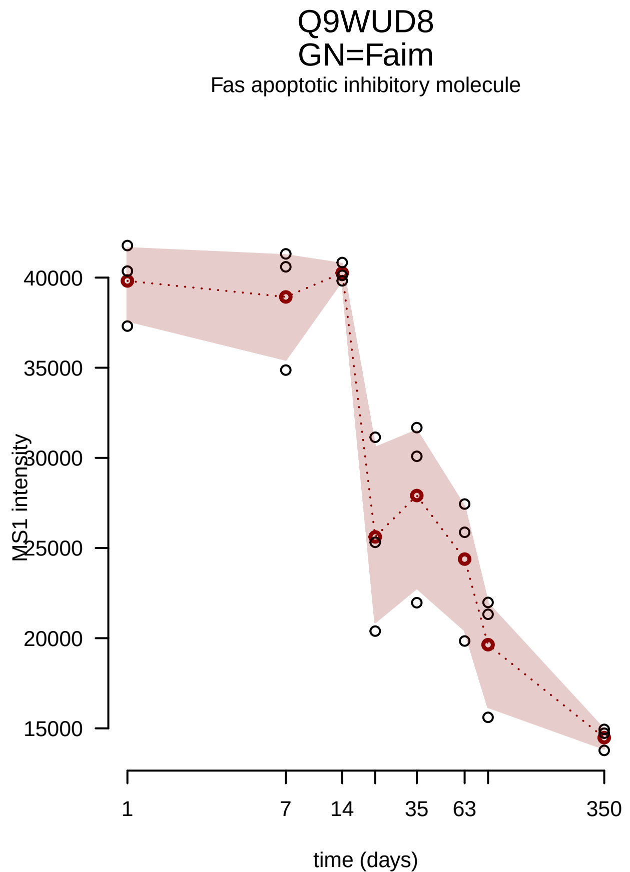
Q9WUD8
GN=Faim
Fas apoptotic inhibitory molecule
MS1 intensity
15000
20000
25000
30000
35000
40000
1
7
14
35
63
350
time (days)
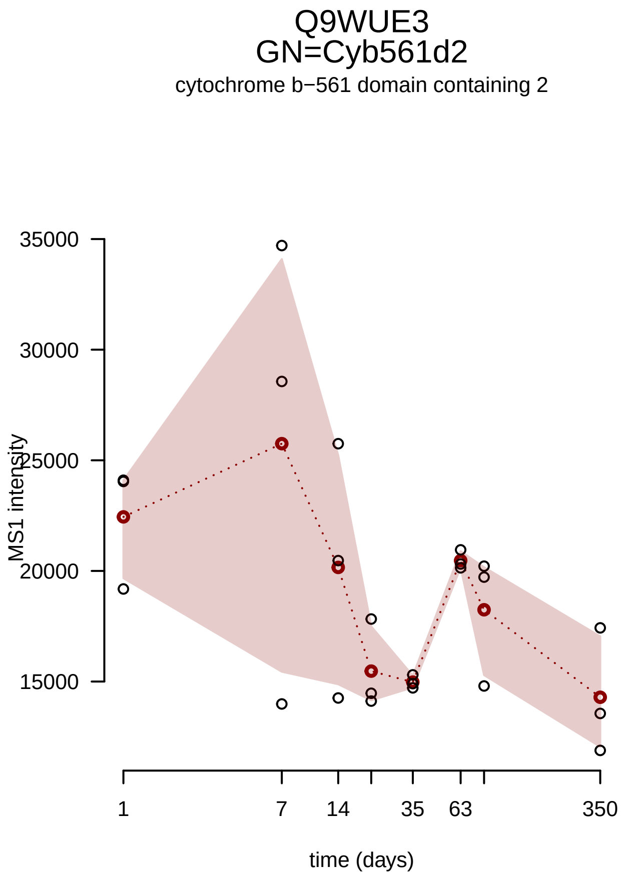
Q9WUE3
GN=Cyb561d2
cytochrome b−561 domain containing 2
MS1 intensity
15000
20000
25000
30000
35000
1
7
14
35
63
350
time (days)

Q9WUE4
GN=Nprl2
NPR2 like, GATOR1 complex subunit

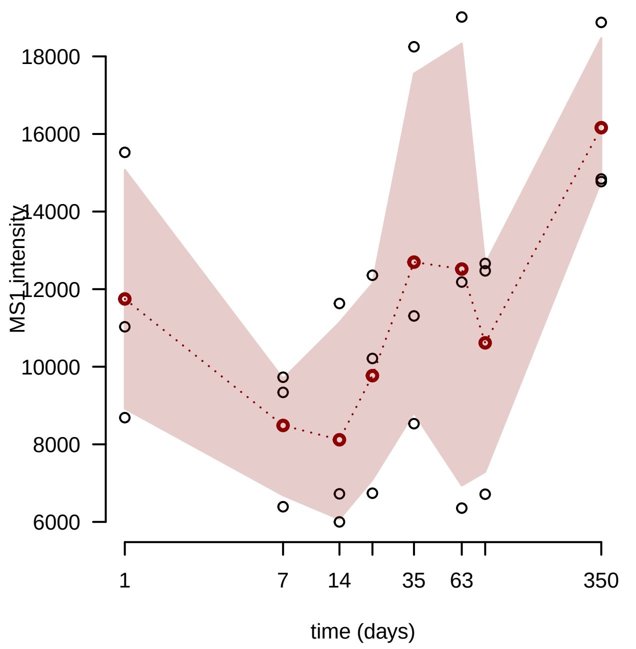

Q9WUF3
GN=Casp8ap2
caspase 8 associated protein 2

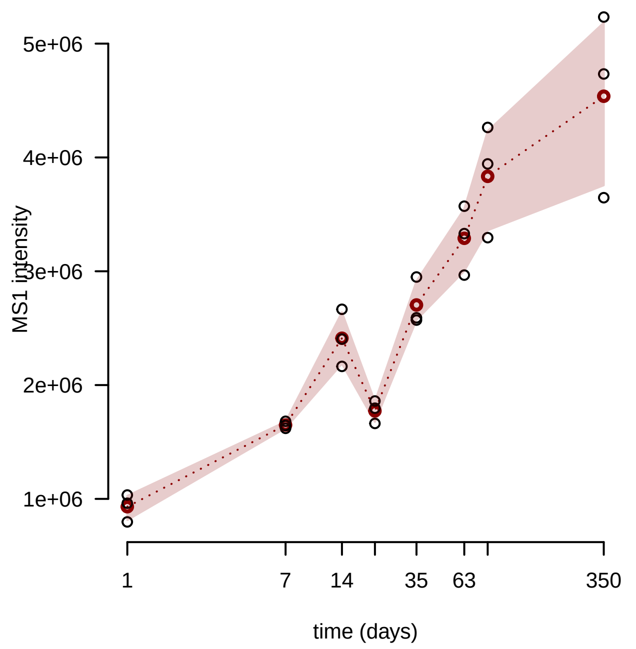

Q9WUH1
GN=Tmem115
transmembrane protein 115

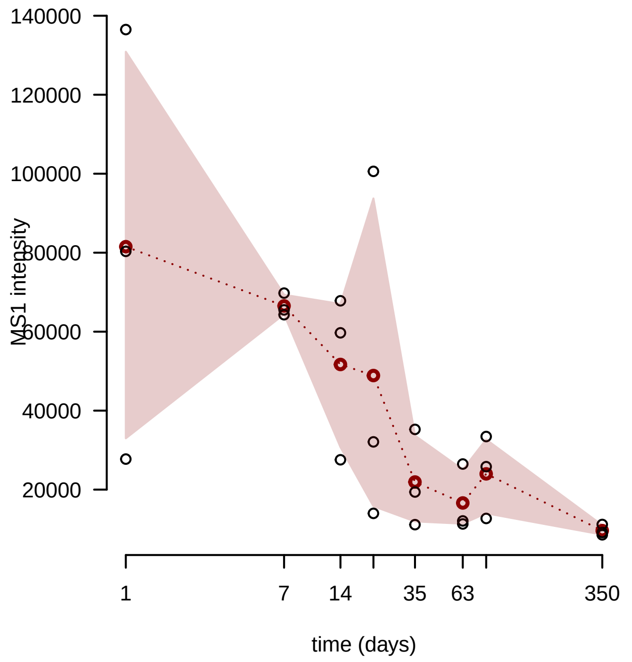

Q9WUJ8
GN=Orc6
origin recognition complex, subunit 6

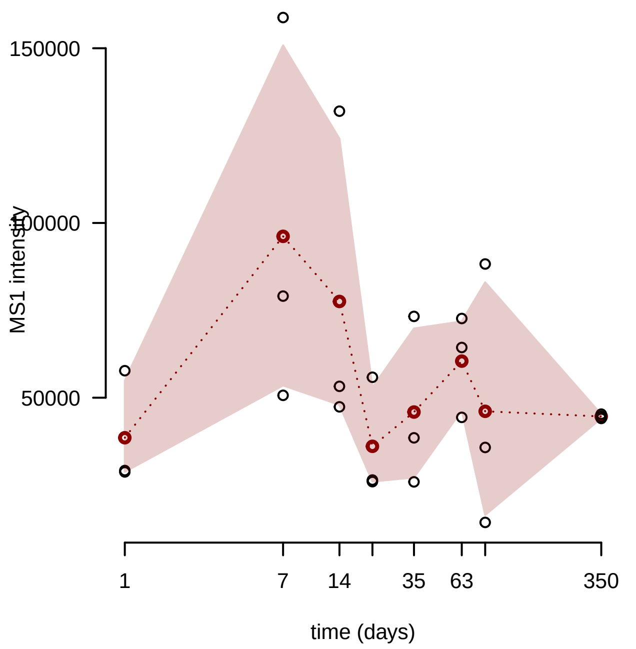

Q9WUK2
GN=Eif4h
eukaryotic translation initiation factor ...

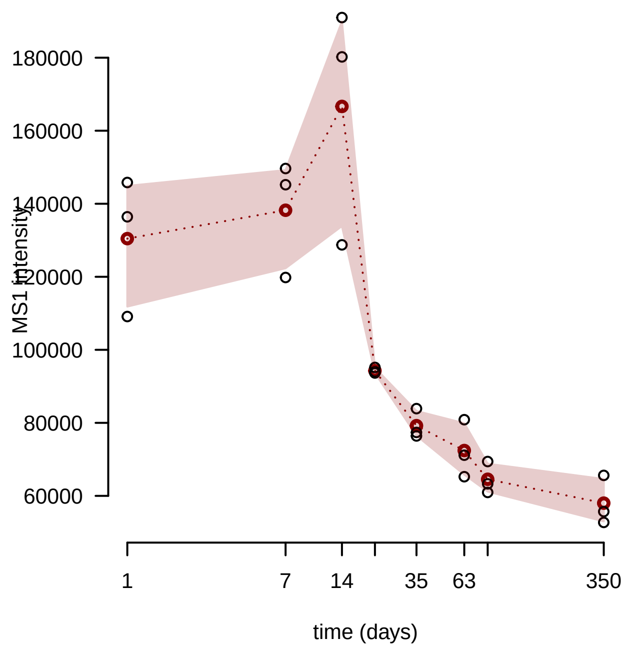

Q9WUK4
GN=Rfc2
replication factor C (activator 1) 2

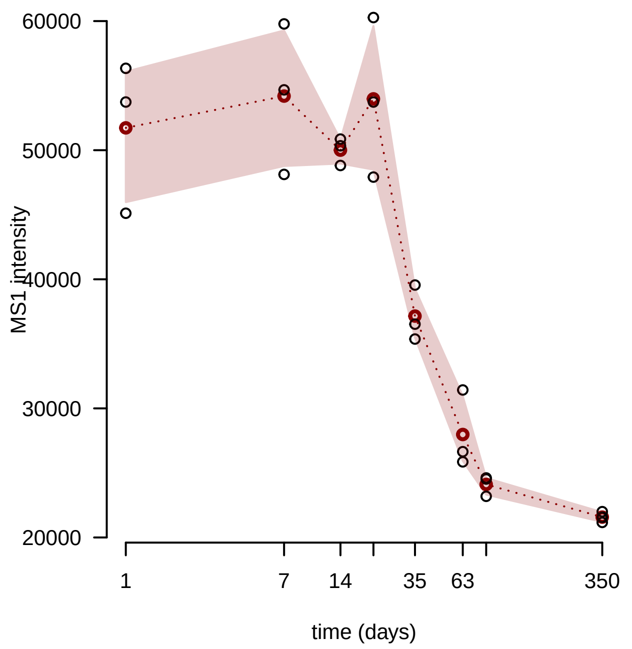

Q9WUL7
GN=Arl3
ADP−ribosylation factor−like 3

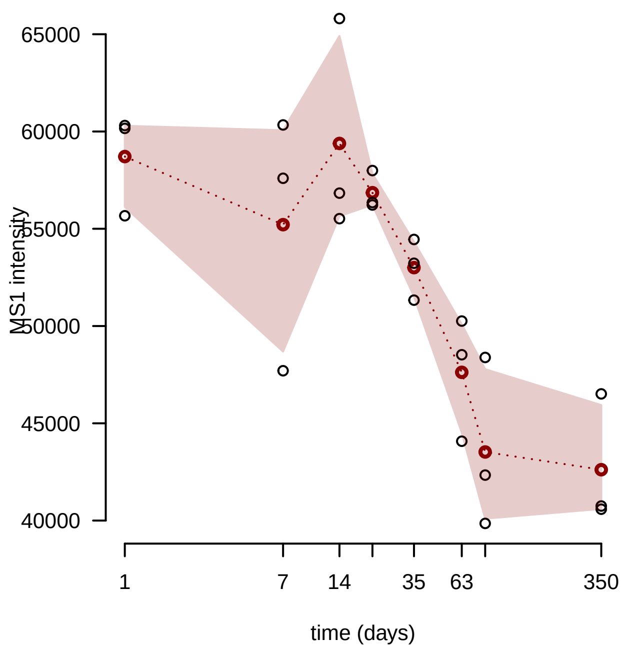

Q9WUM3
GN=Coro1b
coronin, actin binding protein 1B

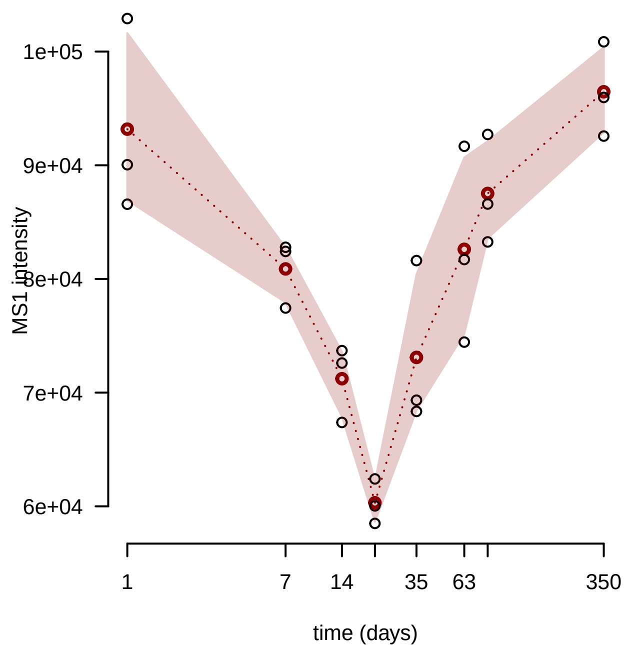

Q9WUM4
GN=Coro1c
coronin, actin binding protein 1C

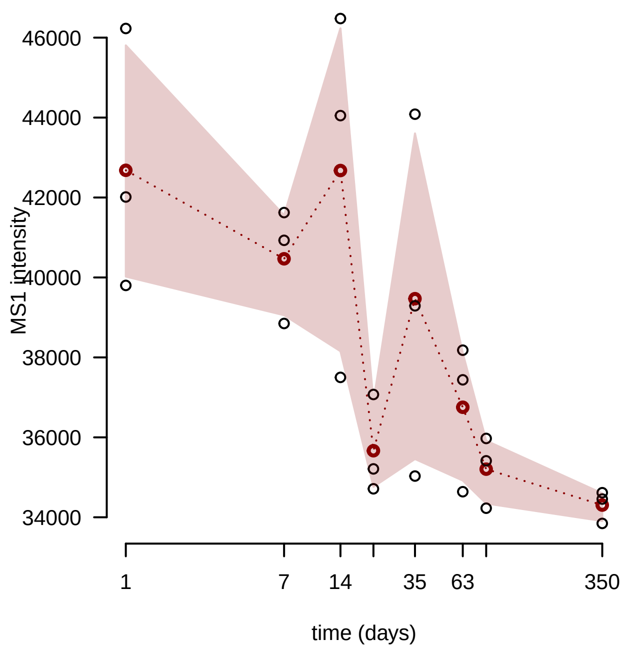

Q9WUM5
GN=Suclg1
succinate−CoA ligase, GDP−forming, alpha ...

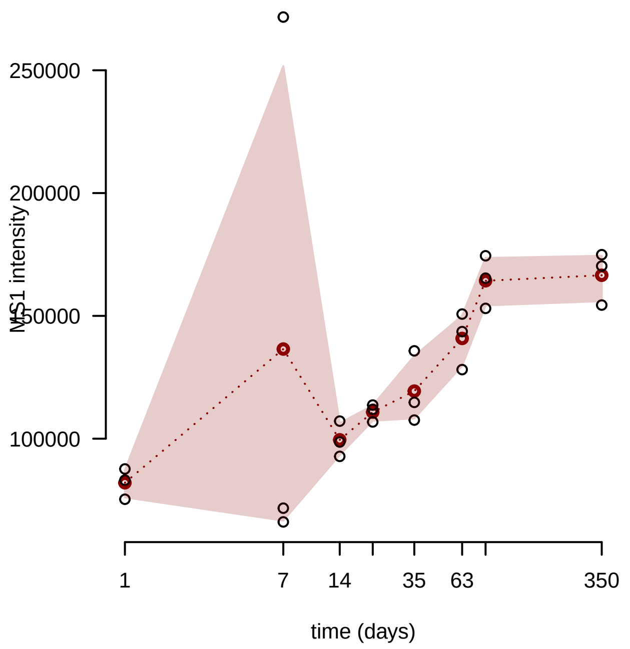

Q9WUN2
GN=Tbk1
TANK−binding kinase 1

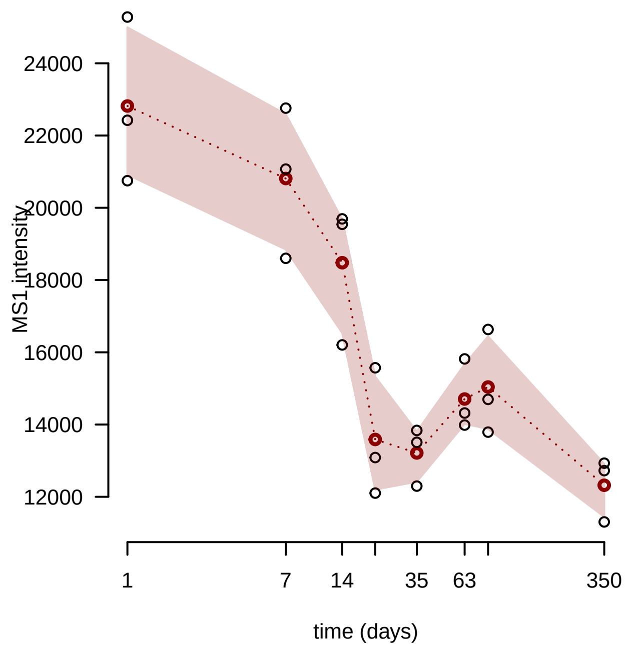

Q9WUP4
GN=Srd5a3
steroid 5 alpha−reductase 3

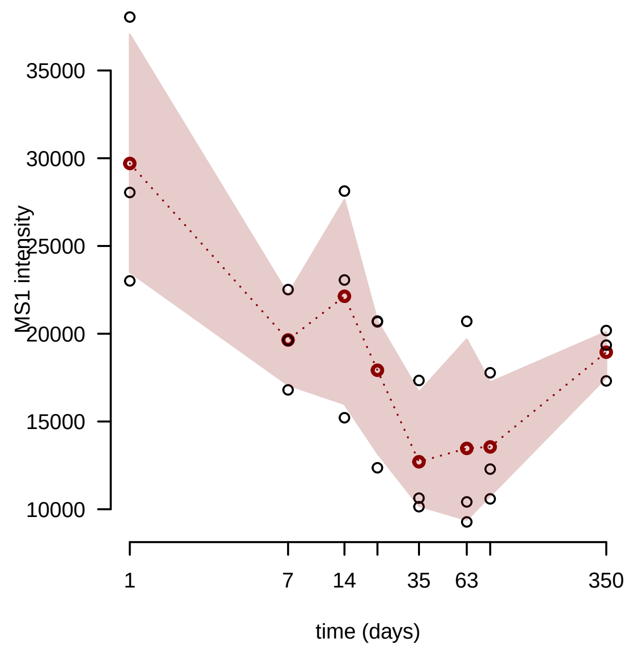

Q9WUP7
GN=Uchl5
ubiquitin carboxyl−terminal esterase L5

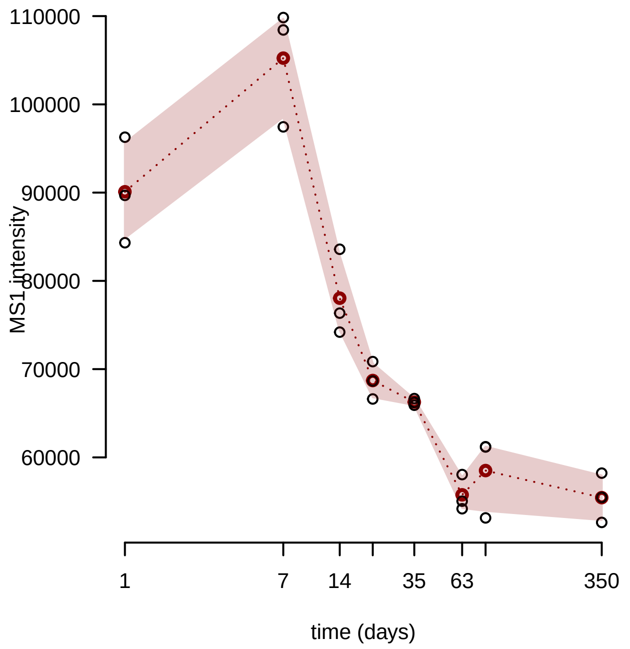

Q9WUQ2
GN=Preb
prolactin regulatory element binding

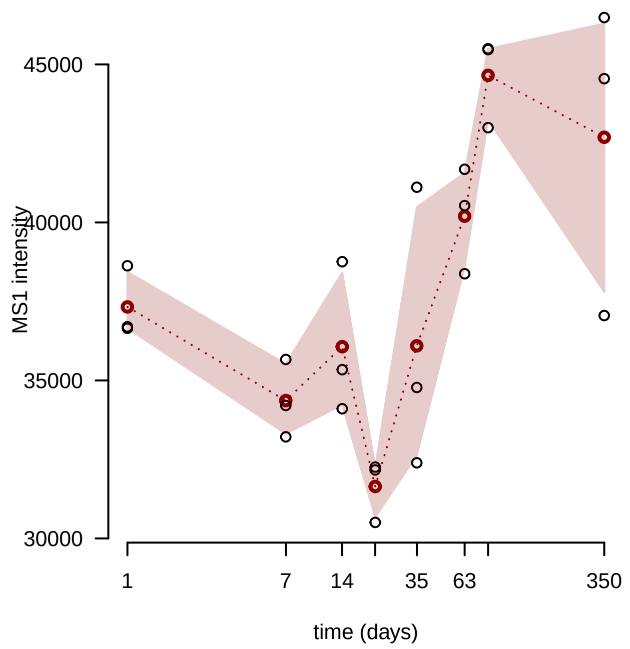

Q9WUR2
GN=Eci2
enoyl−Coenzyme A delta isomerase 2

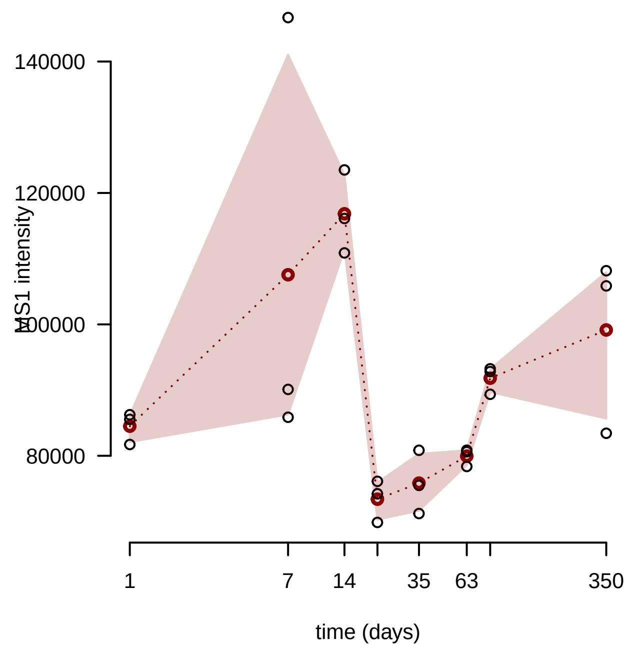

Q9WUR9
GN=Ak4
adenylate kinase 4

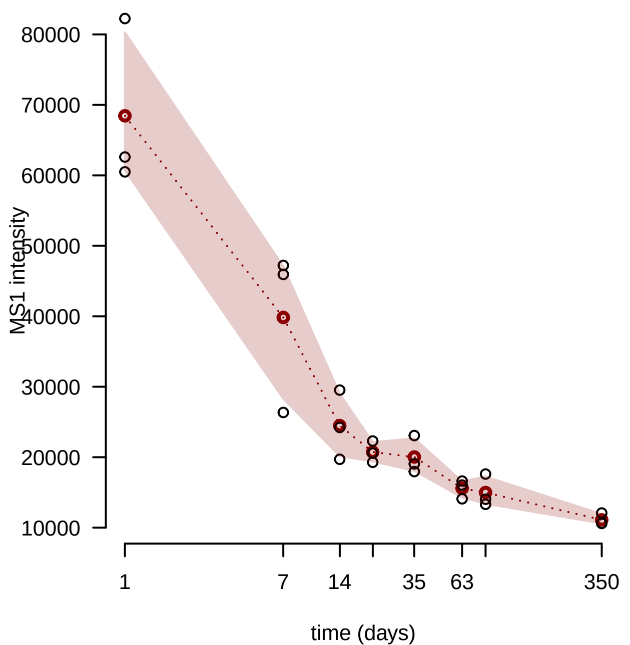

Q9WUU7
GN=Ctsz
cathepsin Z

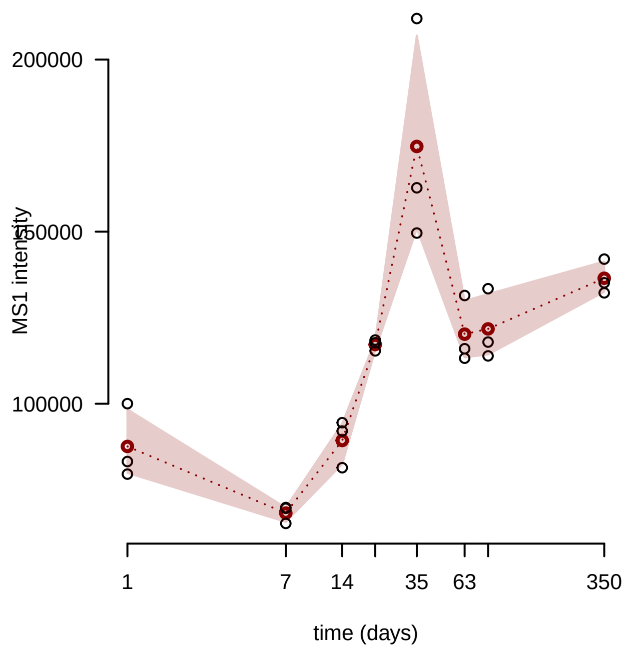

Q9WUU8
GN=Tnip1
TNFAIP3 interacting protein 1

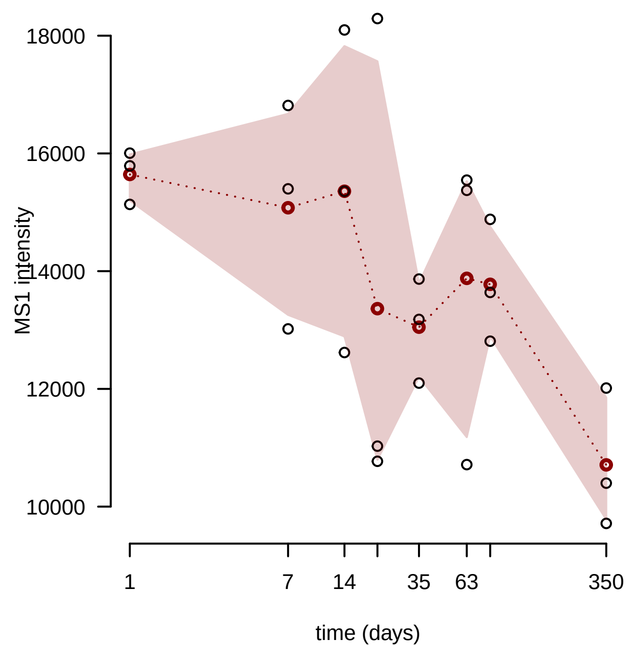

Q9WUU9
GN=Mcm3ap
minichromosome maintenance complex compo ...

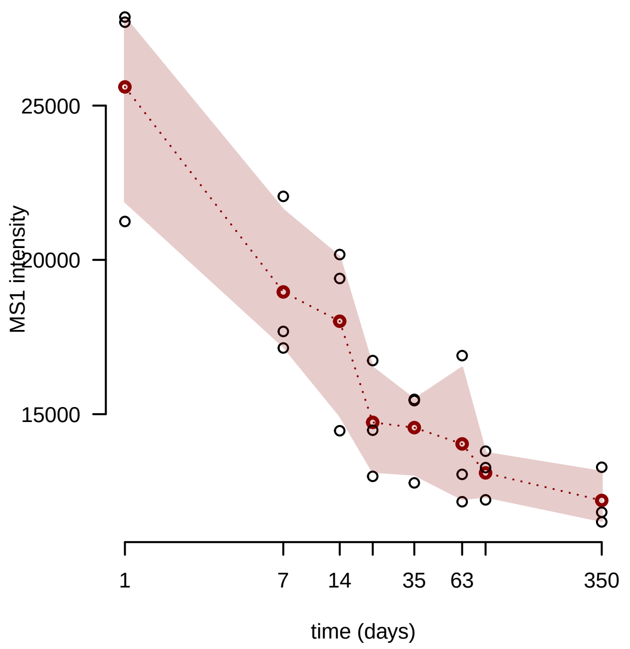

Q9WUV0
GN=Orc5
origin recognition complex, subunit 5

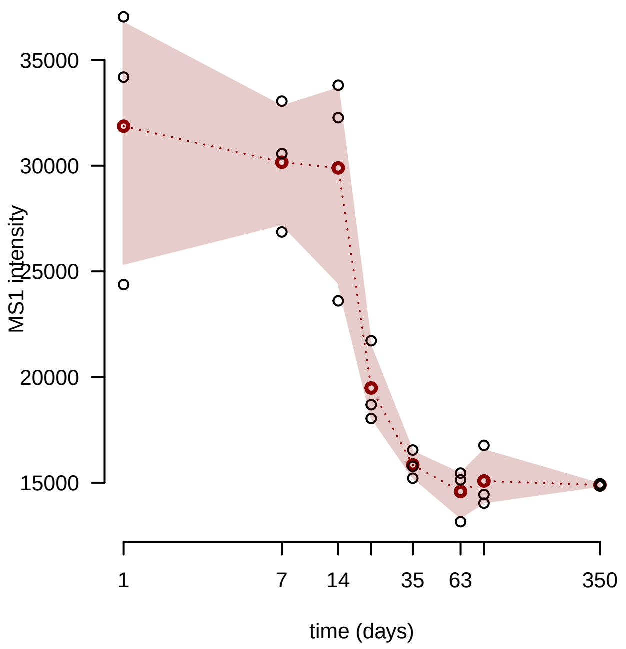

Q9WUV2
GN=St6galnac3
ST6 (alpha−N−acetyl−neuraminyl−2,3−beta− ...

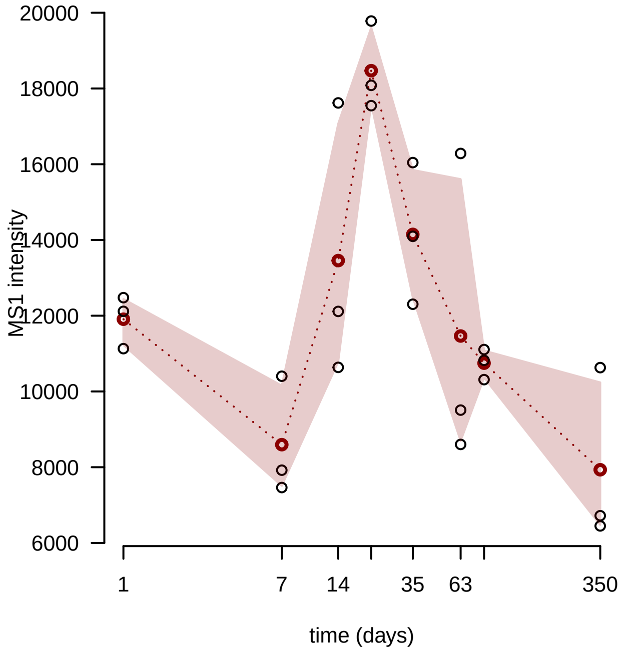

Q9WUX5
GN=Mrvi1

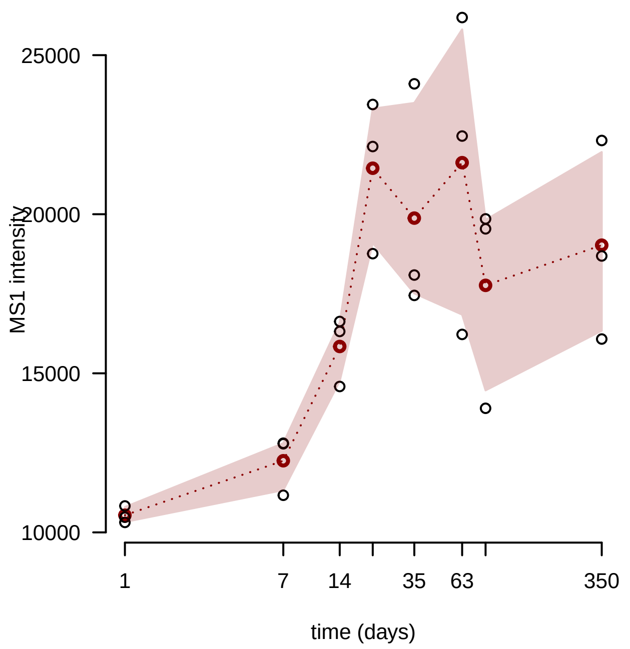

Q9WUZ7
GN=Sh3bgr
SH3−binding domain glutamic acid−rich pr ...

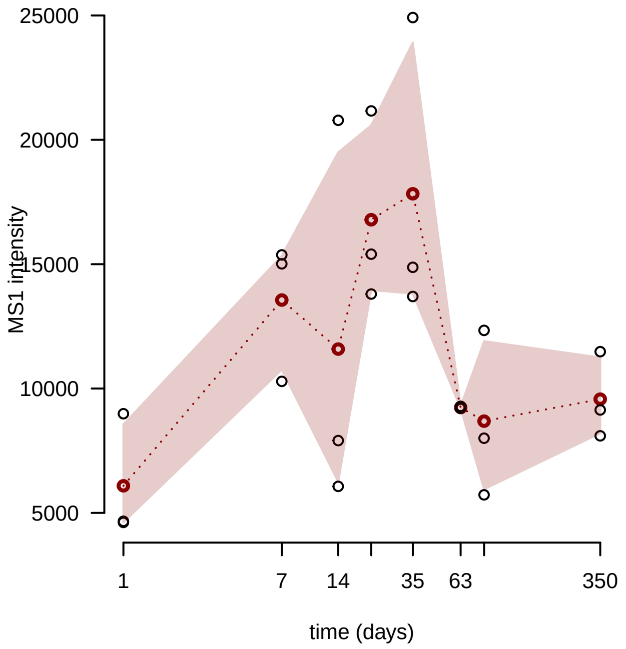

Q9WUZ9
GN=Entpd5
ectonucleoside triphosphate diphosphohyd ...

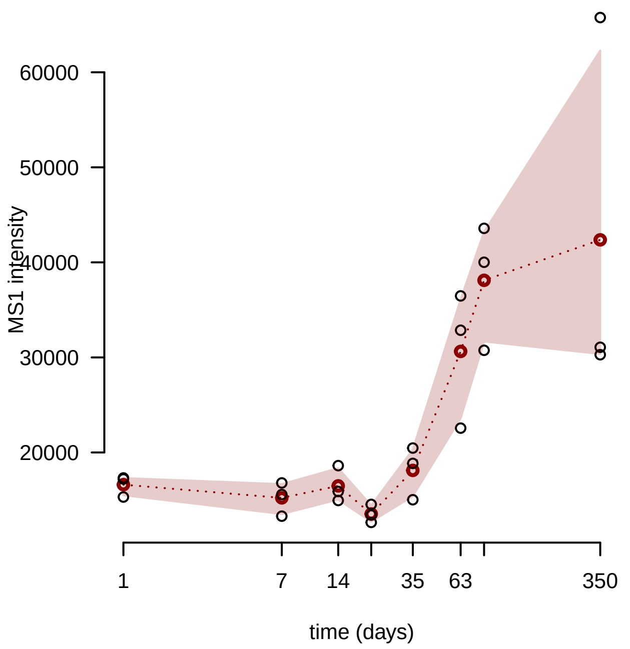

Q9WV02
GN=Rbmx
RNA binding motif protein, X chromosome

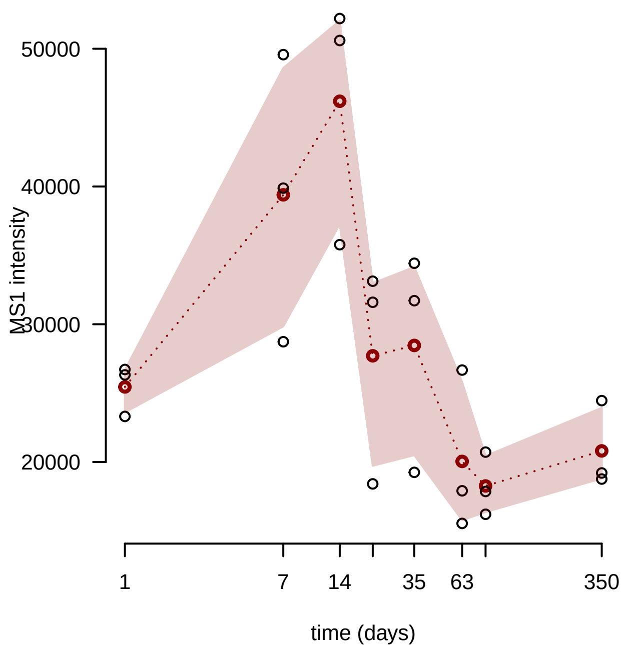

Q9WV03
GN=Fam50a
family with sequence similarity 50, memb ...

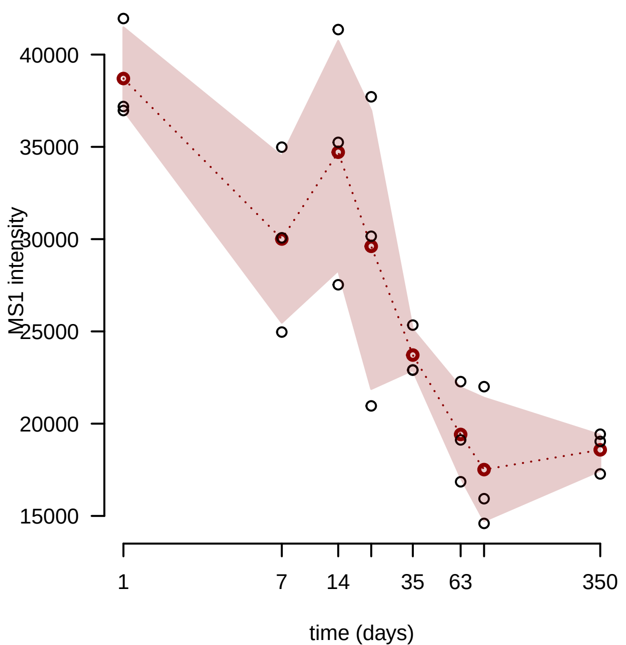

Q9WV30
GN=Nfat5
nuclear factor of activated T cells 5

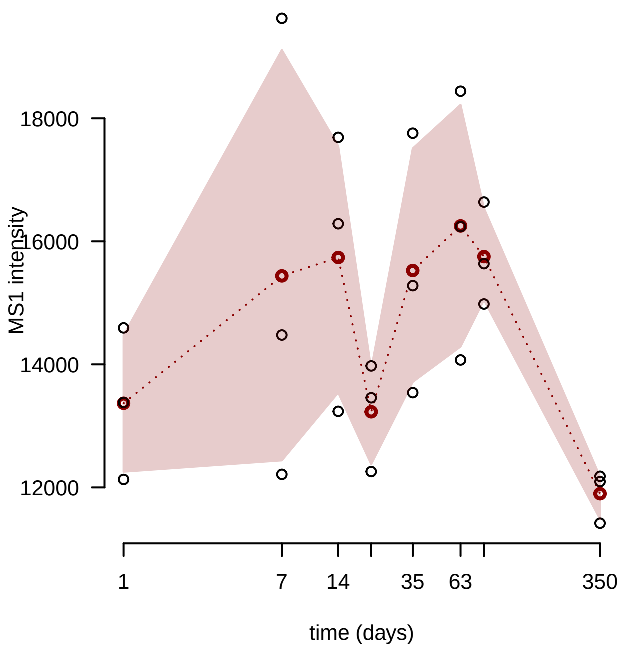

Q9WV32
GN=Arpc1b
actin related protein 2/3 complex, subun ...

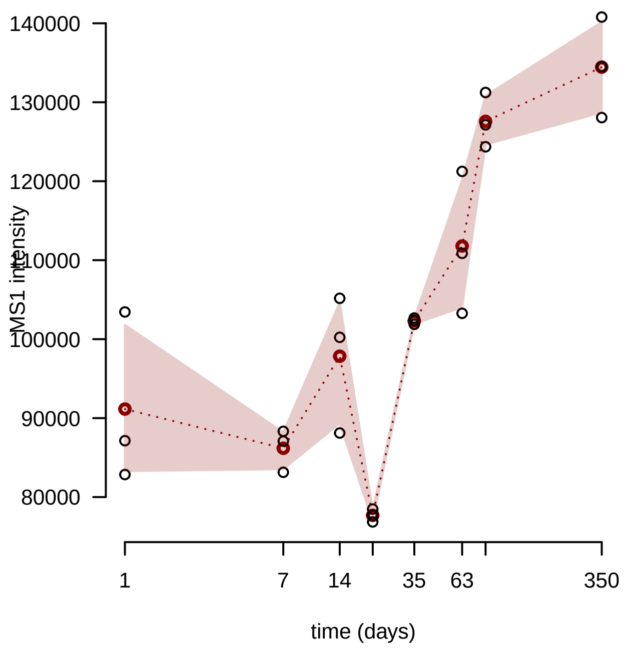

Q9WV34
GN=Mpp2
membrane protein, palmitoylated 2 (MAGUK ...

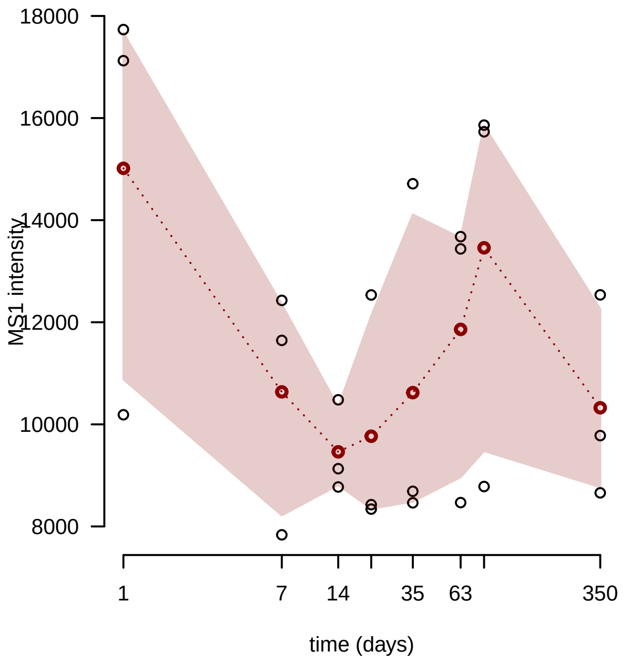

Q9WV35
GN=Apobec2
apolipoprotein B mRNA editing enzyme, ca ...

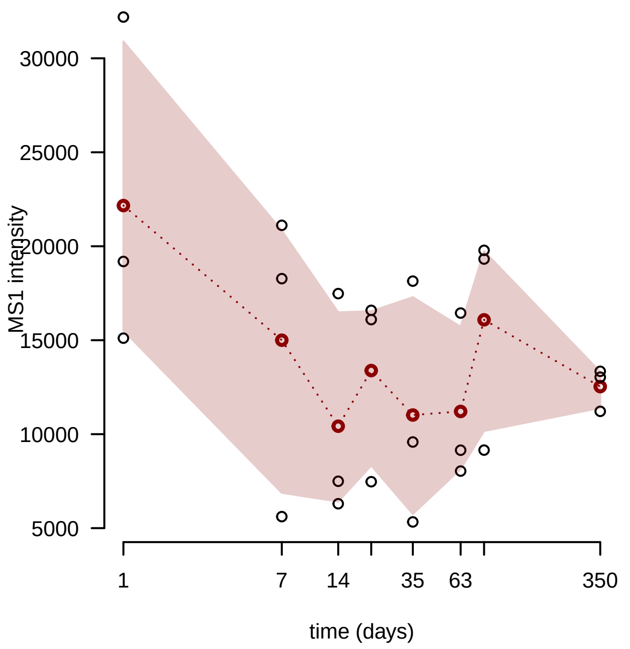

Q9WV54
GN=Asah1
N−acylsphingosine amidohydrolase 1

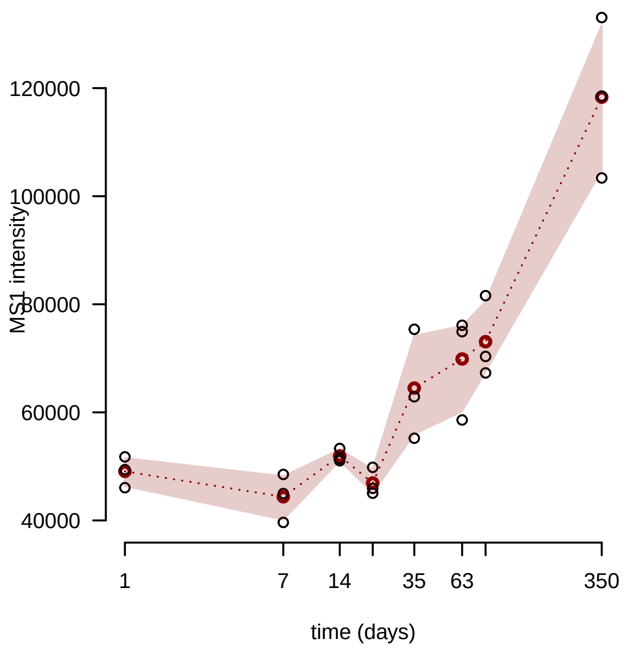

Q9WV55
GN=Vapa
vesicle−associated membrane protein, ass ...

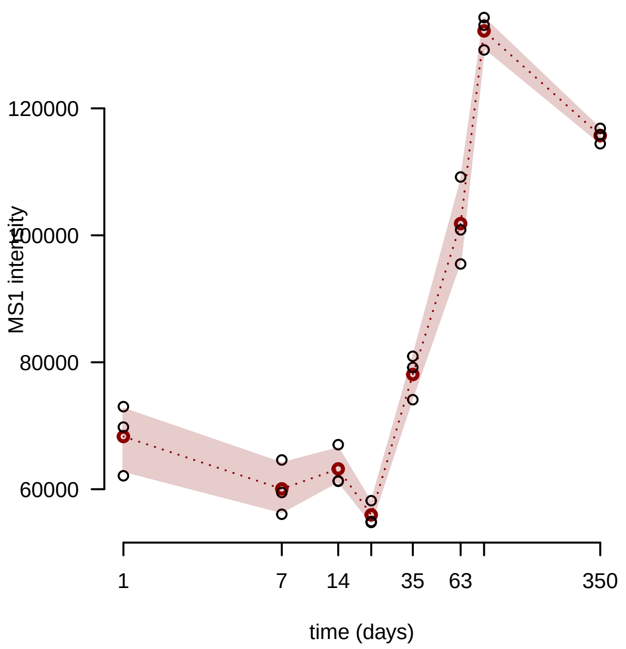

Q9WV60
GN=Gsk3b
glycogen synthase kinase 3 beta

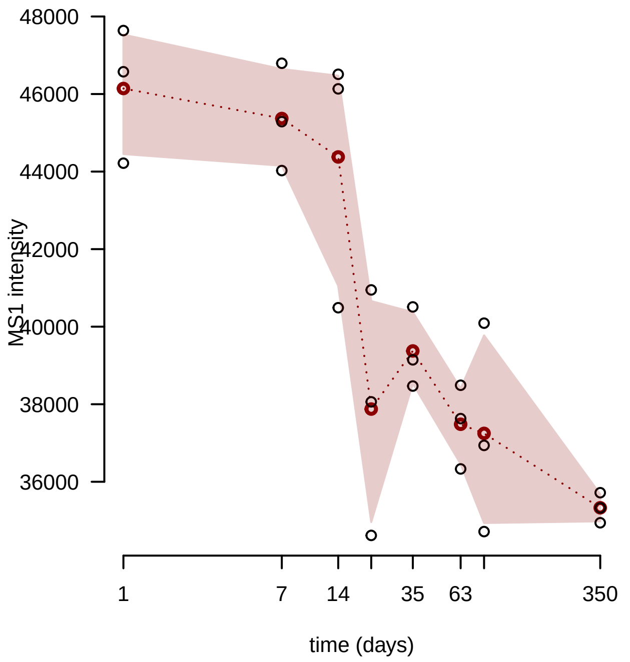

Q9WV68
GN=Decr2
2−4−dienoyl−Coenzyme A reductase 2, pero ...

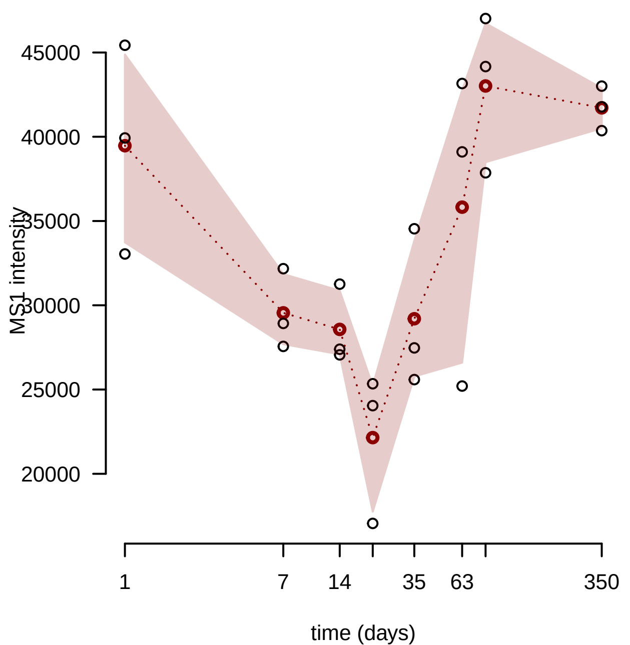

Q9WV69
GN=Dmtn
dematin actin binding protein

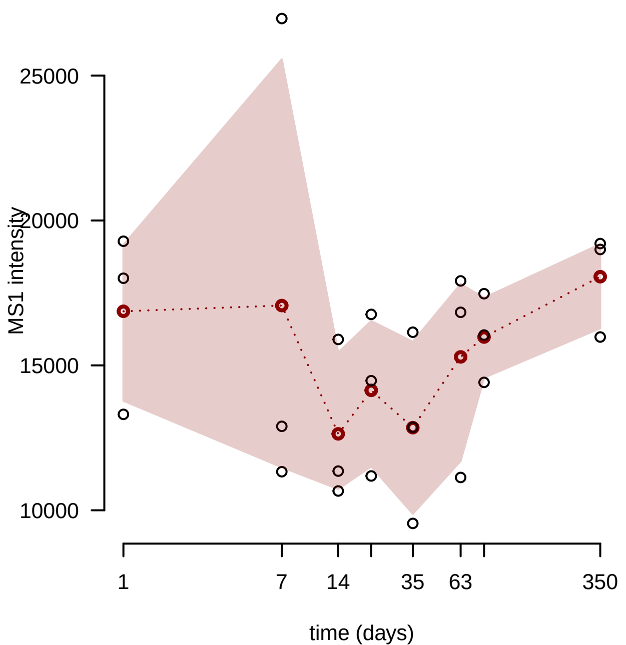

Q9WV70
GN=Noc2l
NOC2 like nucleolar associated transcrip ...

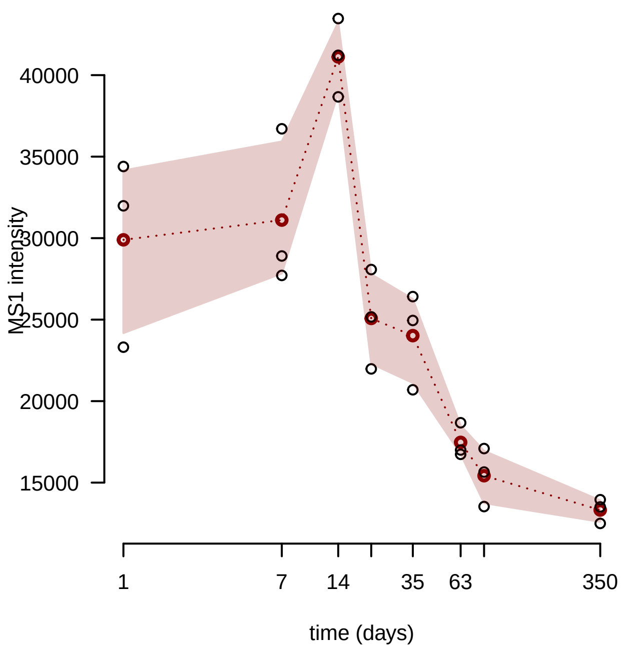

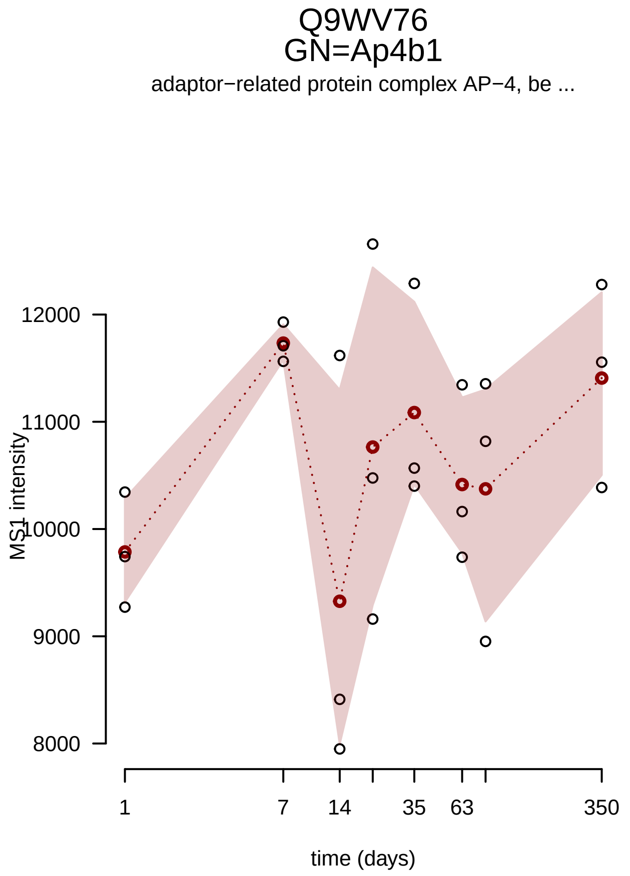
Q9WV76
GN=Ap4b1
adaptor−related protein complex AP−4, be ...
MS1 intensity
8000
9000
10000
11000
12000
1
7
14
35
63
350
time (days)

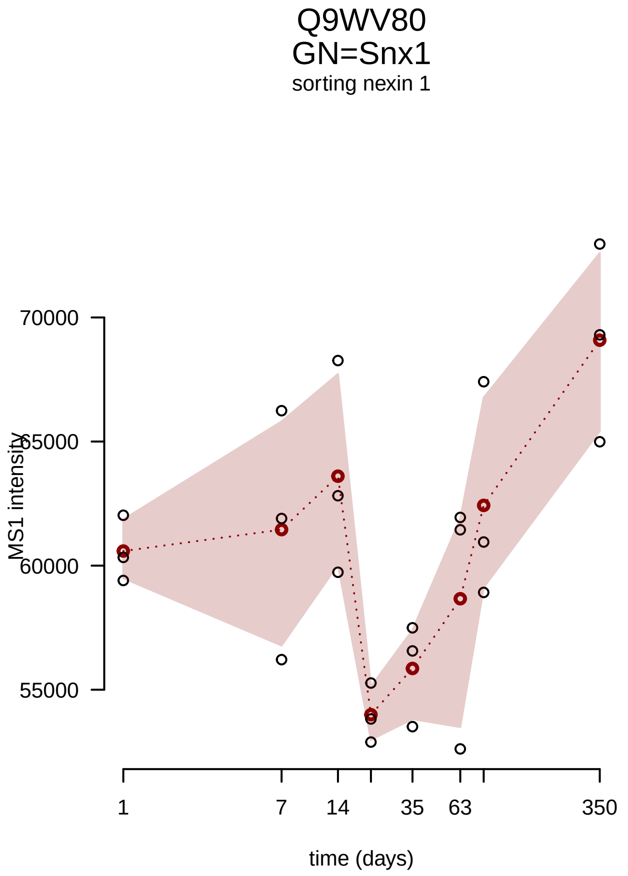
Q9WV80
GN=Snx1
sorting nexin 1
MS1 intensity
55000
60000
65000
70000
1
7
14
35
63
350
time (days)

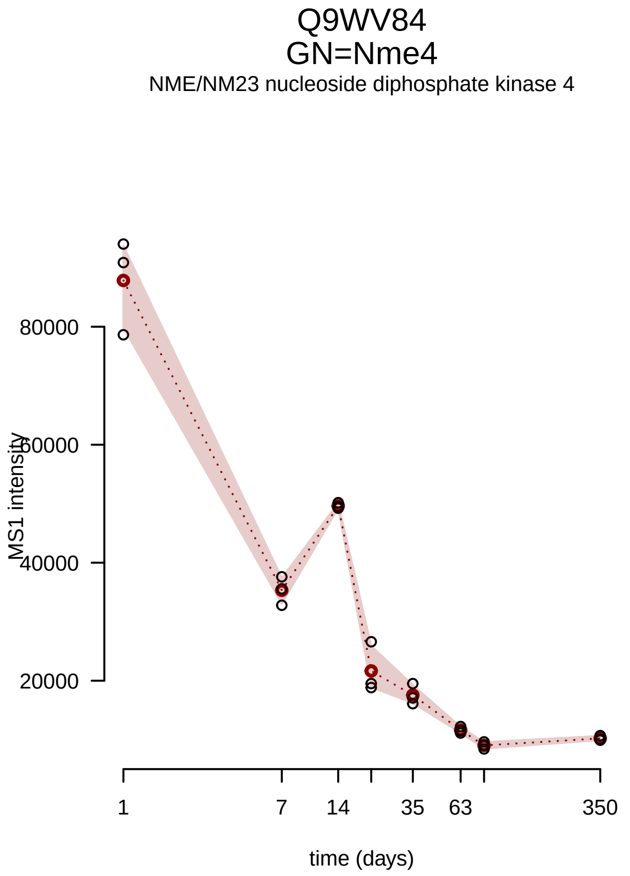
Q9WV84
GN=Nme4
NME/NM23 nucleoside diphosphate kinase 4
MS1 intensity
20000
40000
60000
80000
1
7
14
35
63
350
time (days)

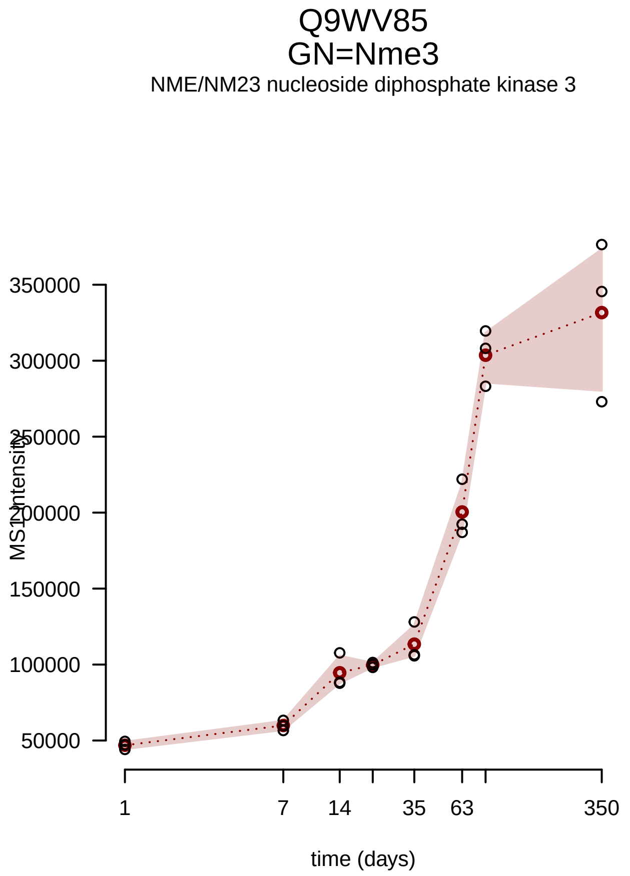
Q9WV85
GN=Nme3
NME/NM23 nucleoside diphosphate kinase 3
MS1 intensity
50000
100000
150000
200000
250000
300000
350000
1
7
14
35
63
350
time (days)

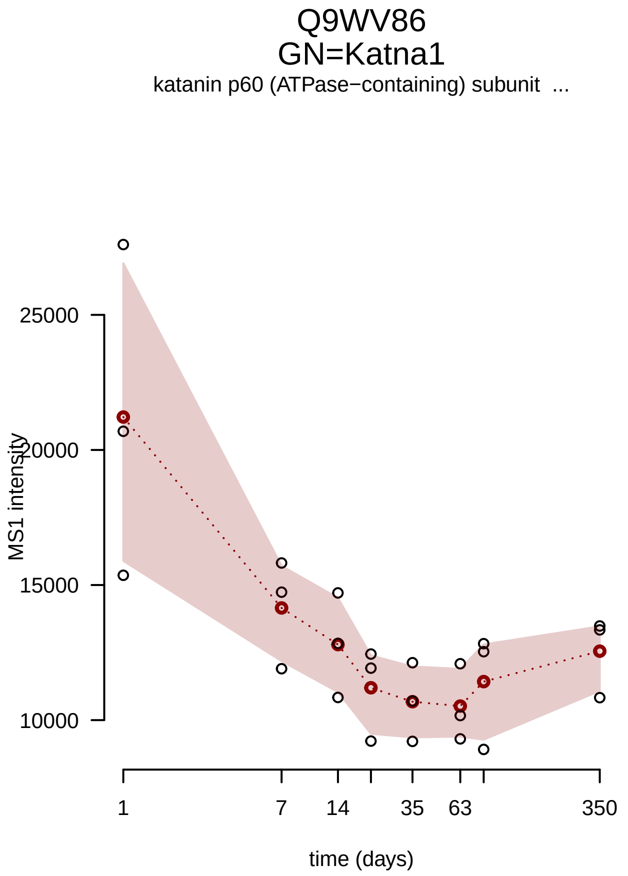
Q9WV86
GN=Katna1
katanin p60 (ATPase−containing) subunit ...
MS1 intensity
10000
15000
20000
25000
1
7
14
35
63
350
time (days)

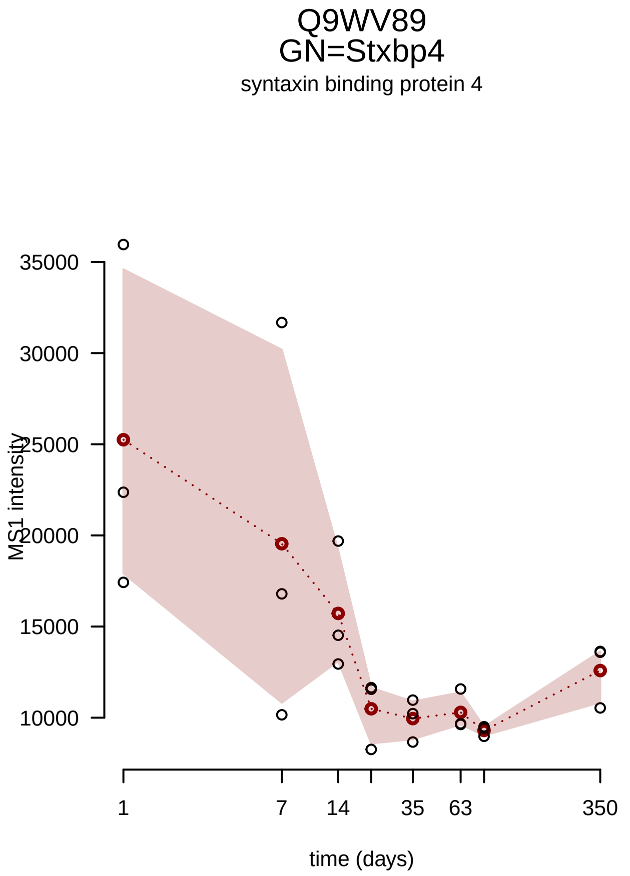
Q9WV89
GN=Stxbp4
syntaxin binding protein 4
MS1 intensity
10000
15000
20000
25000
30000
35000
1
7
14
35
63
350
time (days)

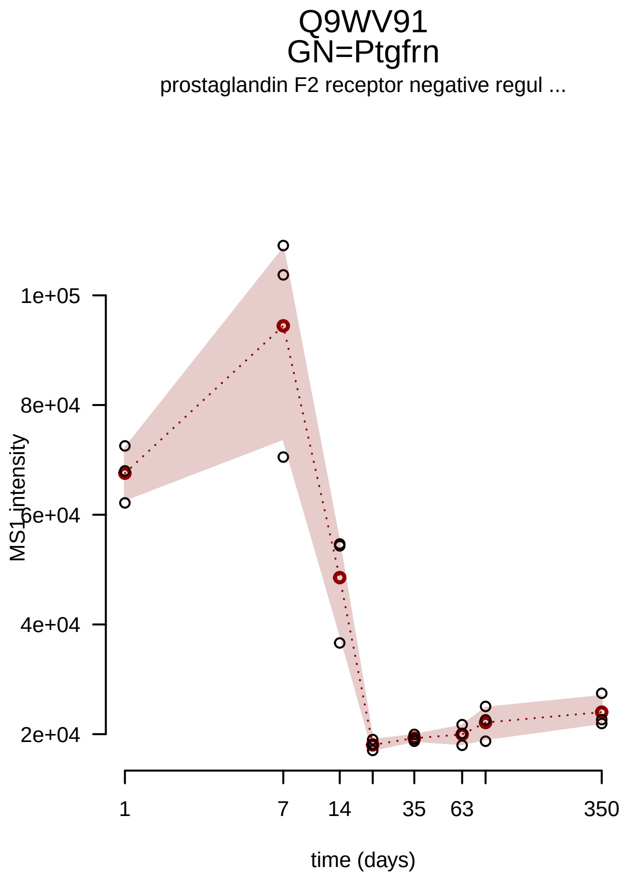
Q9WV91
GN=Ptgfrn
prostaglandin F2 receptor negative regul ...
MS1 intensity
2e+04
4e+04
6e+04
8e+04
1e+05
1
7
14
35
63
350
time (days)

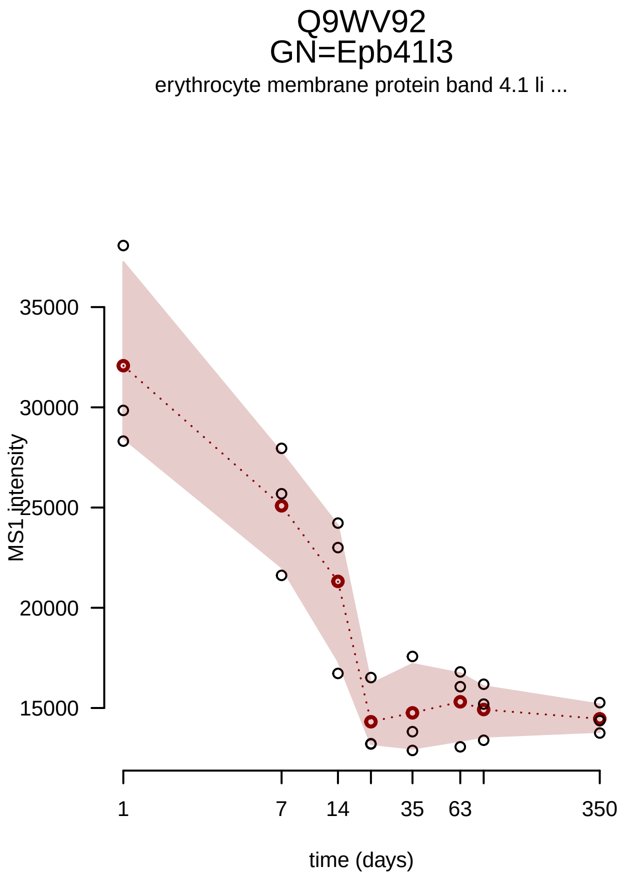
Q9WV92
GN=Epb41l3
erythrocyte membrane protein band 4.1 li ...
MS1 intensity
15000
20000
25000
30000
35000
1
7
14
35
63
350
time (days)

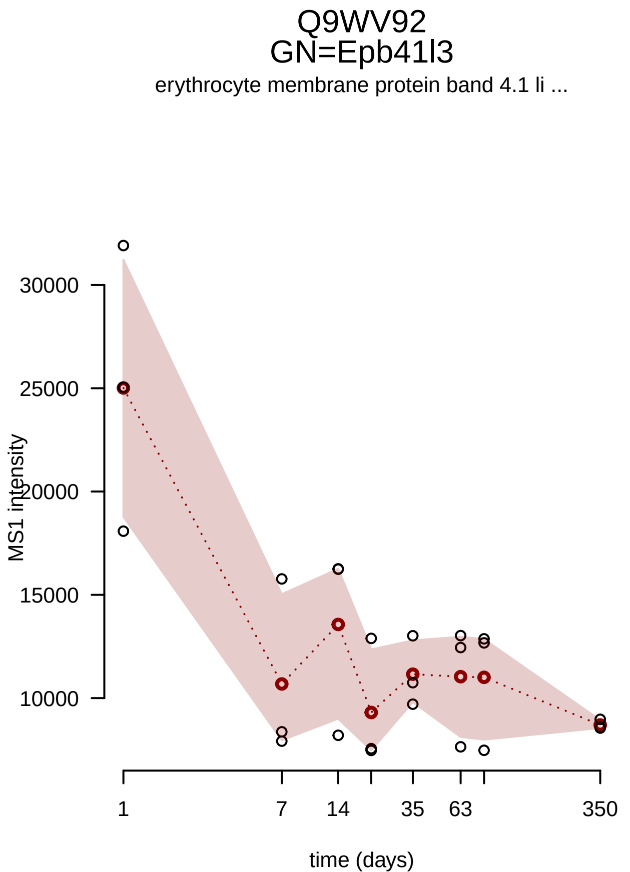
Q9WV92
GN=Epb41l3
erythrocyte membrane protein band 4.1 li ...
MS1 intensity
10000
15000
20000
25000
30000
1
7
14
35
63
350
time (days)

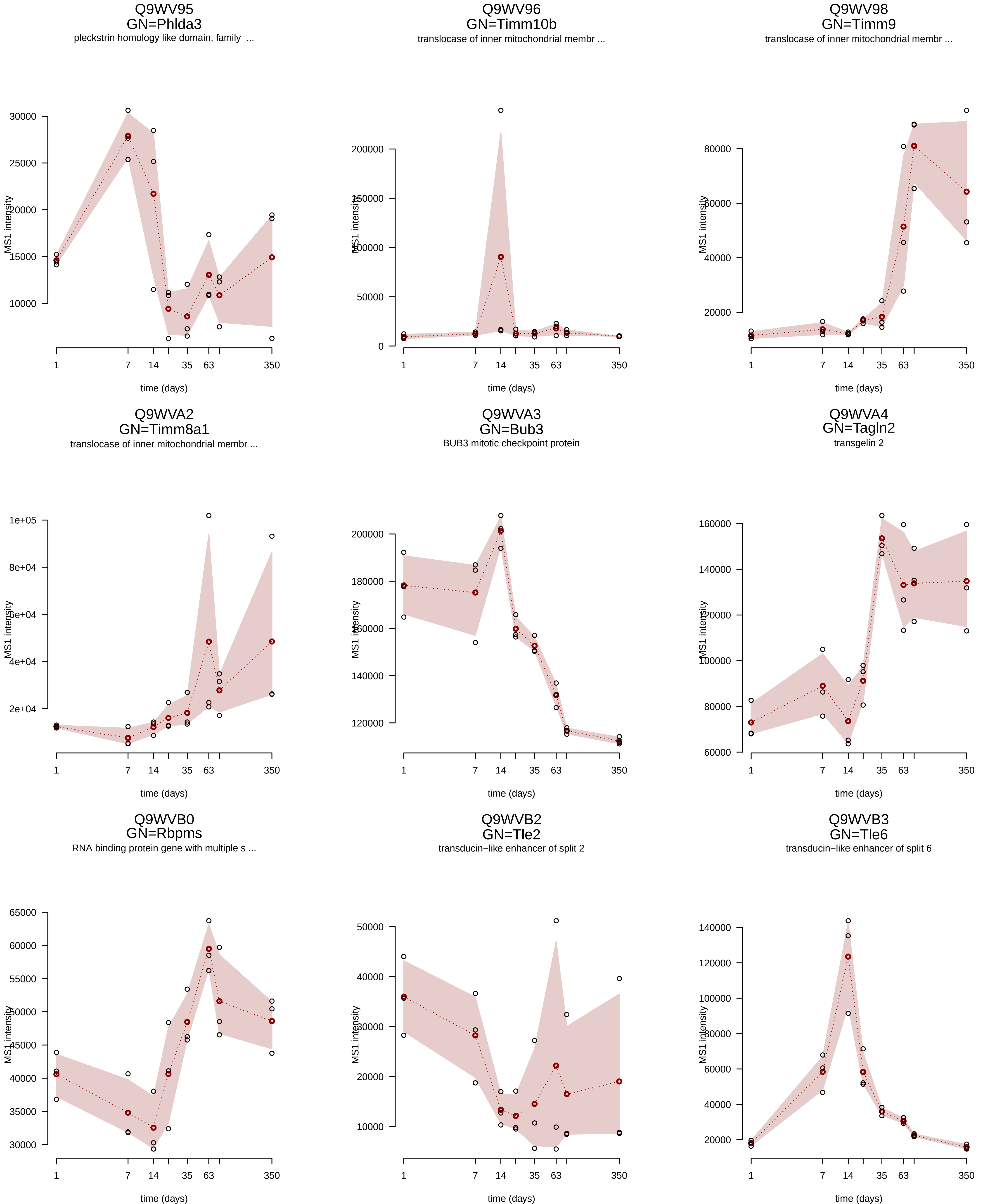
Q9WV95
GN=Phlda3
pleckstrin homology like domain, family ...
MS1 intensity
10000
15000
20000
25000
30000
1
7
14
35
63
350
time (days)
Q9WV96
GN=Timm10b
translocase of inner mitochondrial membr ...
MS1 intensity
0
50000
100000
150000
200000
1
7
14
35
63
350
time (days)
Q9WV98
GN=Timm9
translocase of inner mitochondrial membr ...
MS1 intensity
20000
40000
60000
80000
1
7
14
35
63
350
time (days)
Q9WVA2
GN=Timm8a1
translocase of inner mitochondrial membr ...
MS1 intensity
2e+04
4e+04
6e+04
8e+04
1e+05
1
7
14
35
63
350
time (days)
Q9WVA3
GN=Bub3
BUB3 mitotic checkpoint protein
MS1 intensity
120000
140000
160000
180000
200000
1
7
14
35
63
350
time (days)
Q9WVA4
GN=Tagln2
transgelin 2
MS1 intensity
60000
80000
100000
120000
140000
160000
1
7
14
35
63
350
time (days)
Q9WVB0
GN=Rbpms
RNA binding protein gene with multiple s ...
MS1 intensity
30000
35000
40000
45000
50000
55000
60000
65000
1
7
14
35
63
350
time (days)
Q9WVB2
GN=Tle2
transducin−like enhancer of split 2
MS1 intensity
10000
20000
30000
40000
50000
1
7
14
35
63
350
time (days)
Q9WVB3
GN=Tle6
transducin−like enhancer of split 6
MS1 intensity
20000
40000
60000
80000
100000
120000
140000
1
7
14
35
63
350
time (days)

Q9WVB4
GN=Slit3
slit guidance ligand 3

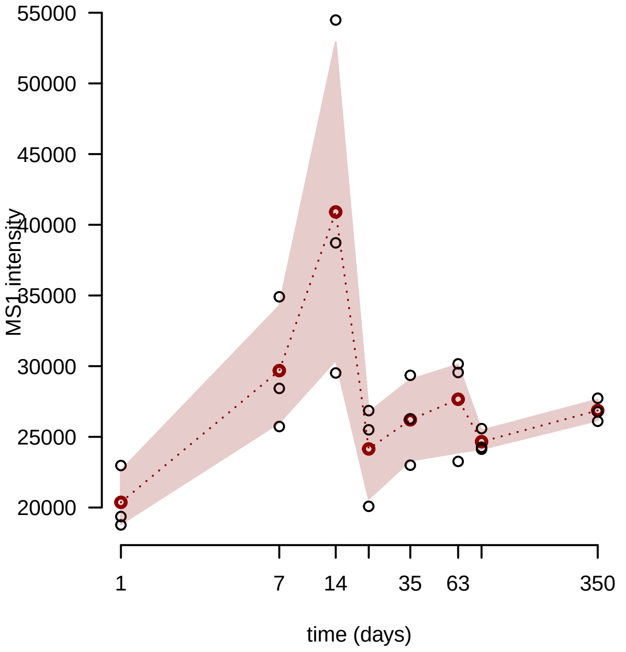

Q9WVC3
GN=Cav2
caveolin 2

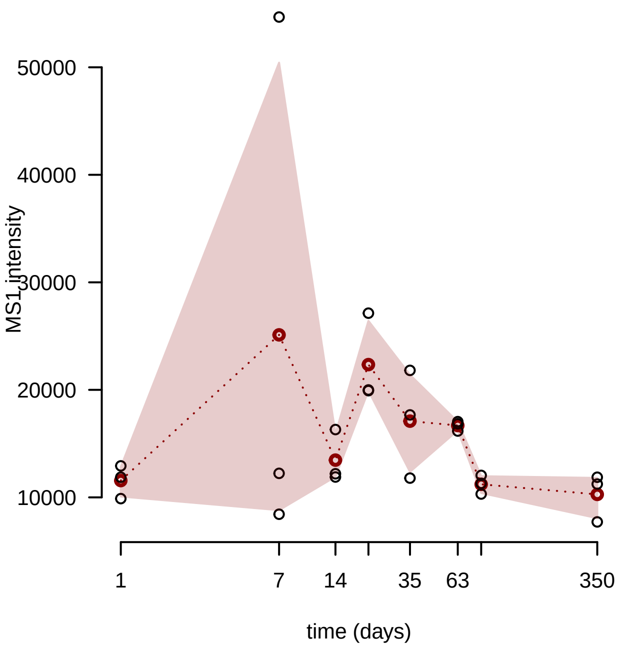

Q9WVC6
GN=Sgk1
serum/glucocorticoid regulated kinase 1

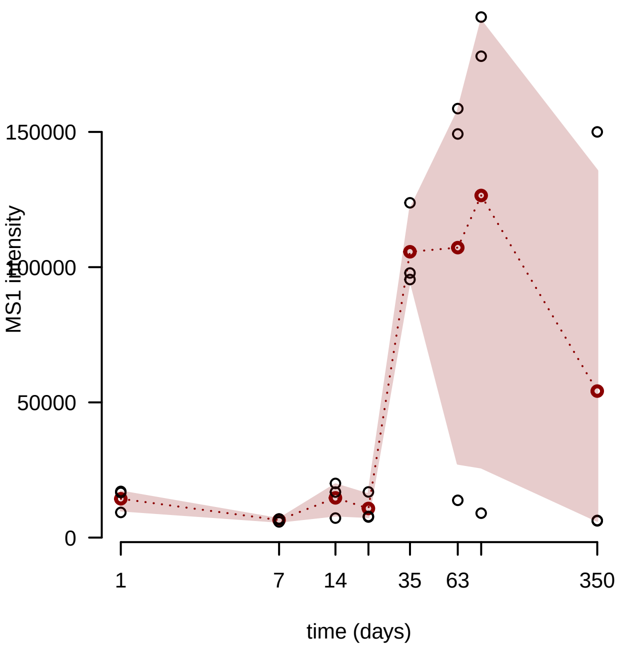

Q9WVD4
GN=Clcn5
chloride channel, voltage−sensitive 5

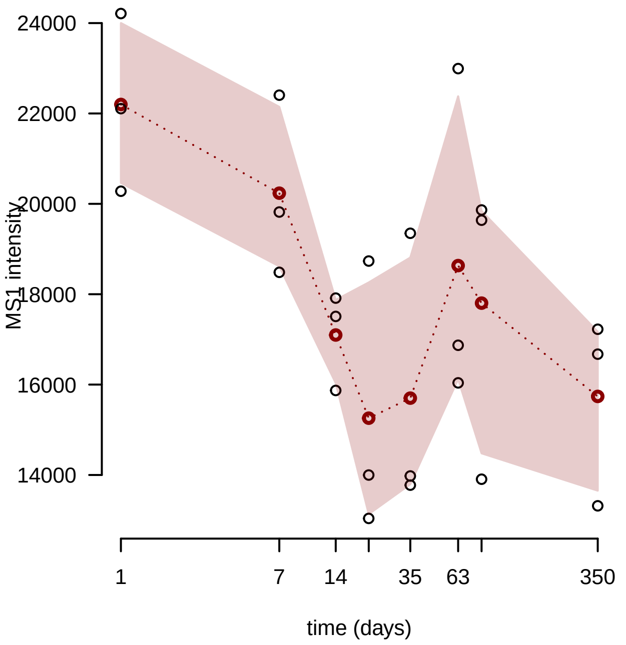

Q9WVD5
GN=Slc25a15
solute carrier family 25 (mitochondrial ...

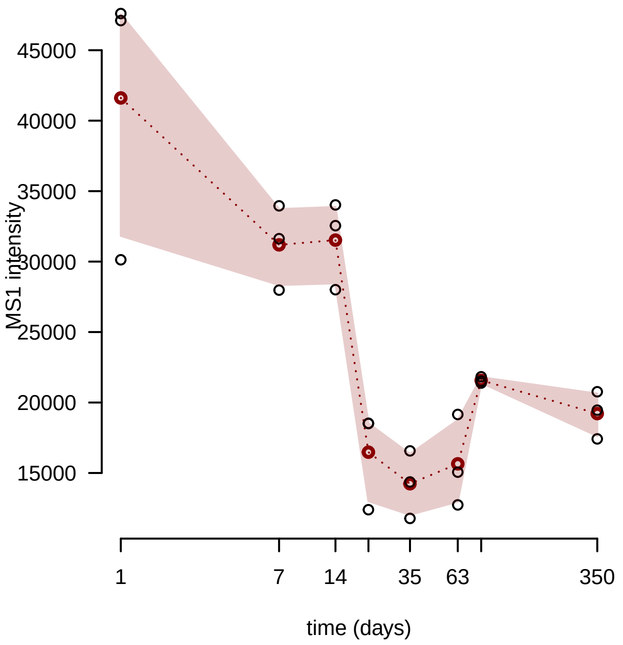

Q9WVE8
GN=Pacsin2
protein kinase C and casein kinase subst ...

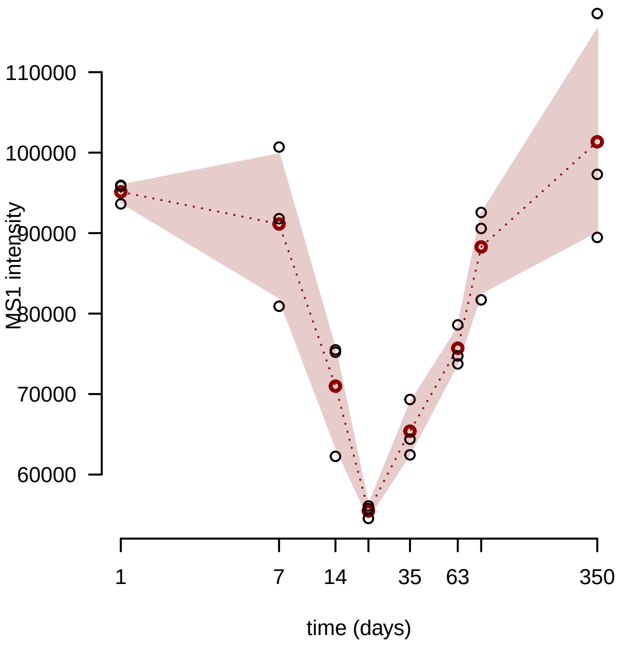

Q9WVF7
GN=Pole
polymerase (DNA directed), epsilon

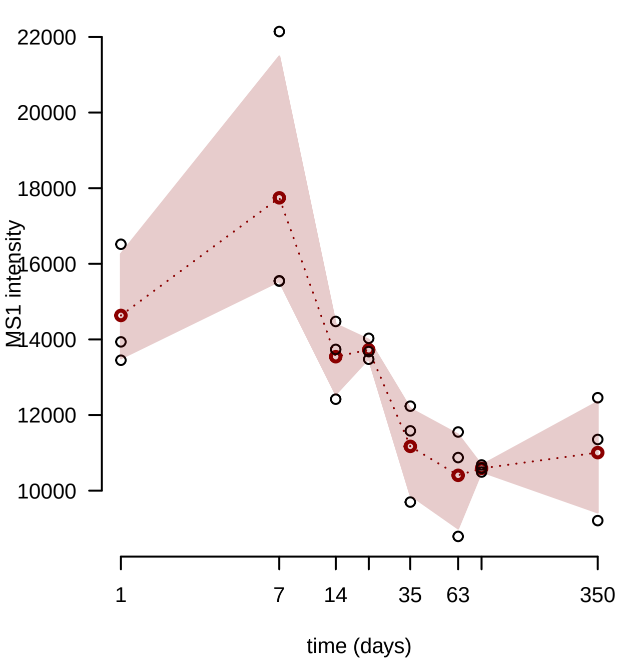

Q9WVF8
GN=Tusc2
tumor suppressor 2, mitochondrial calciu ...

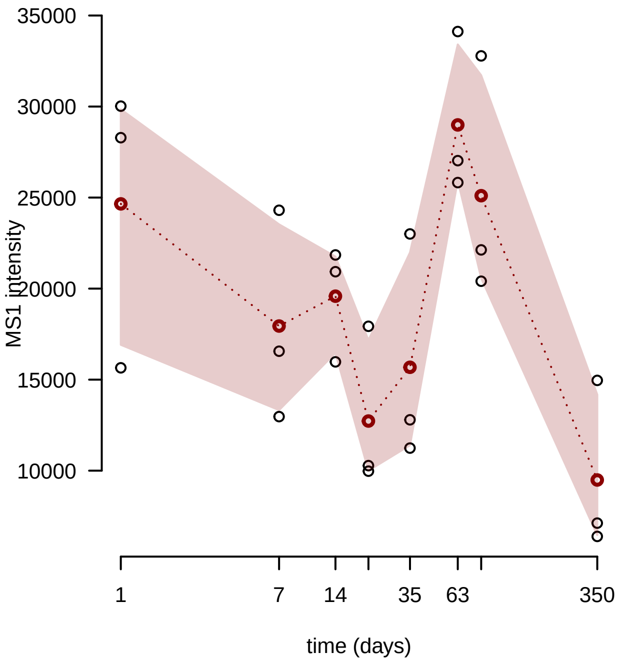

Q9WVG5
GN=Lipg
lipase, endothelial

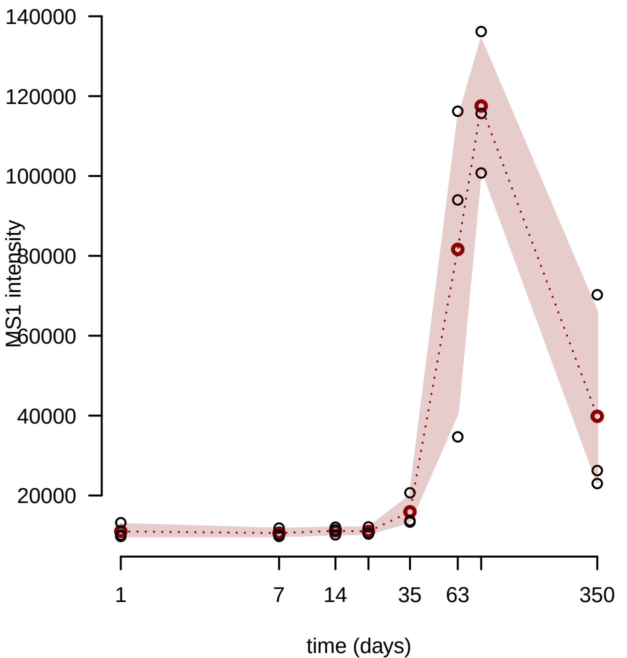

Q9WVG6
GN=Carm1
coactivator−associated arginine methyltr ...

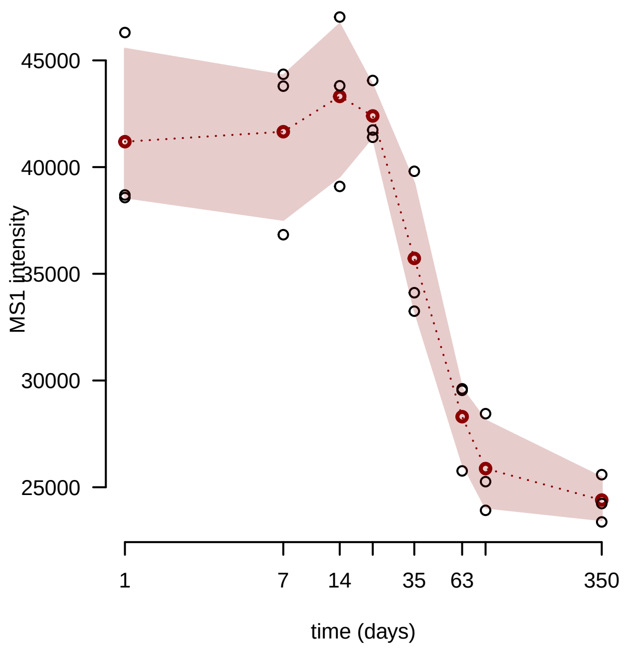

Q9WVG9
GN=Msl3
MSL complex subunit 3

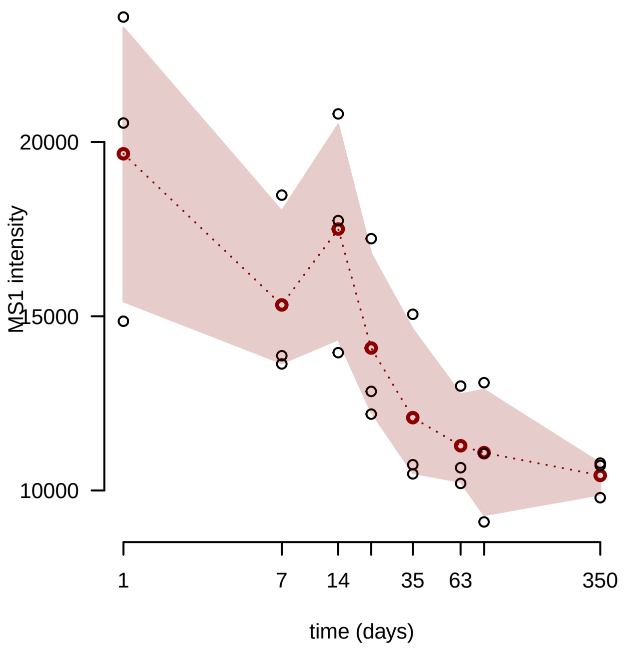

Q9WVH4
GN=Foxo3
forkhead box O3

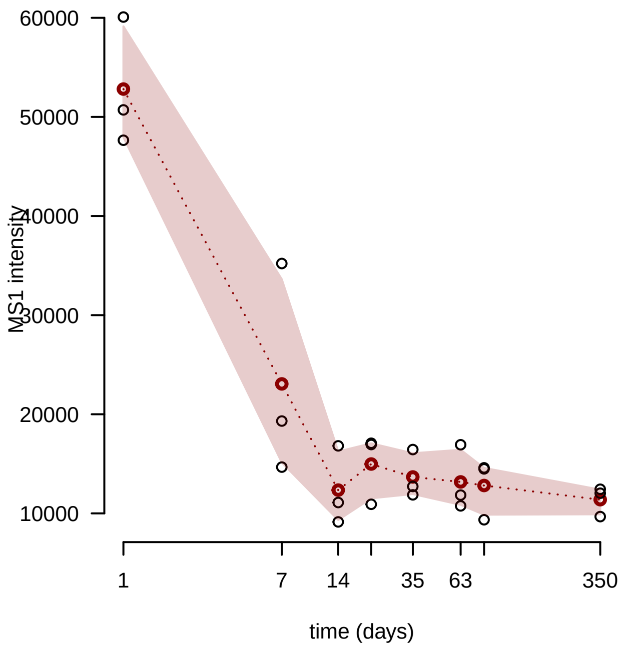

Q9WVH9
GN=Fbln5
fibulin 5

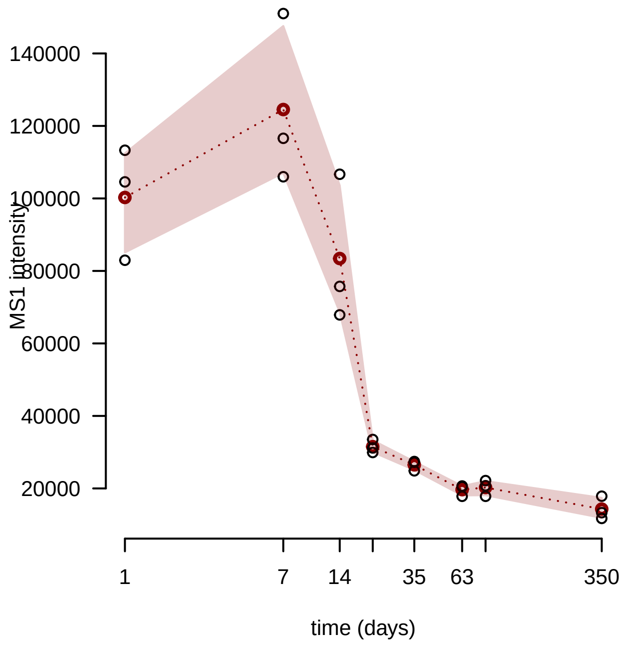

Q9WVJ2
GN=Psmd13
proteasome (prosome, macropain) 26S subu ...

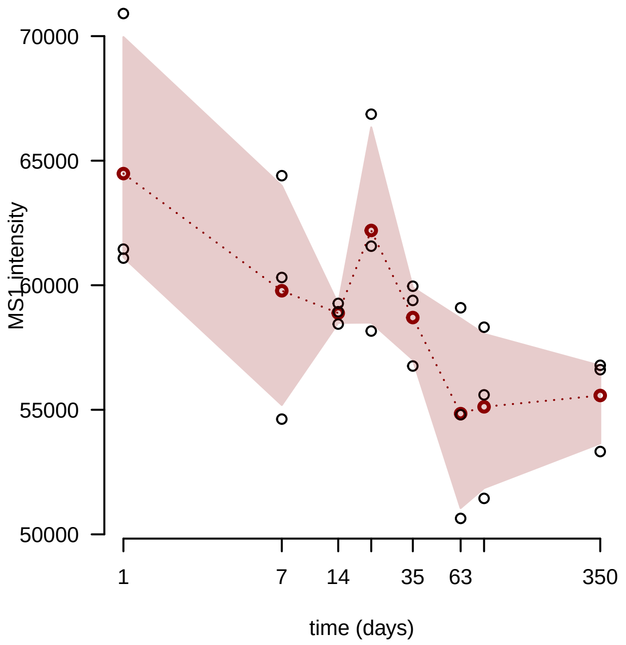

Q9WVJ3
GN=Cpq
carboxypeptidase Q

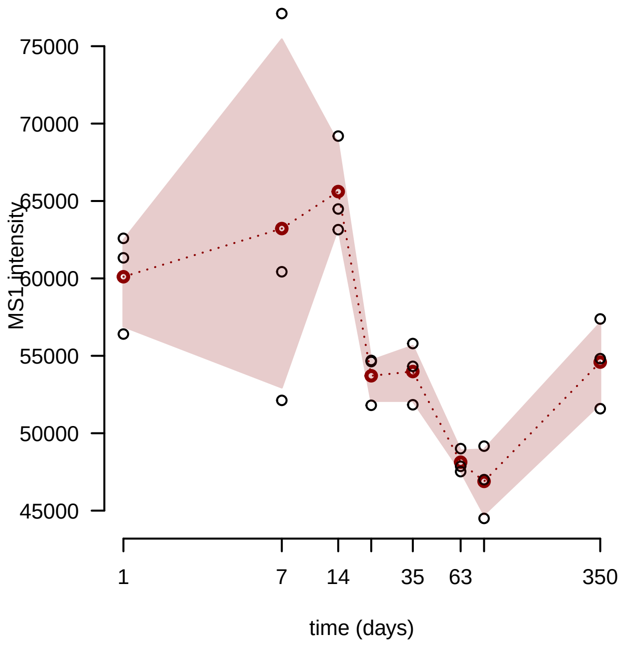

Q9WVJ5
GN=Crybb1
crystallin, beta B1

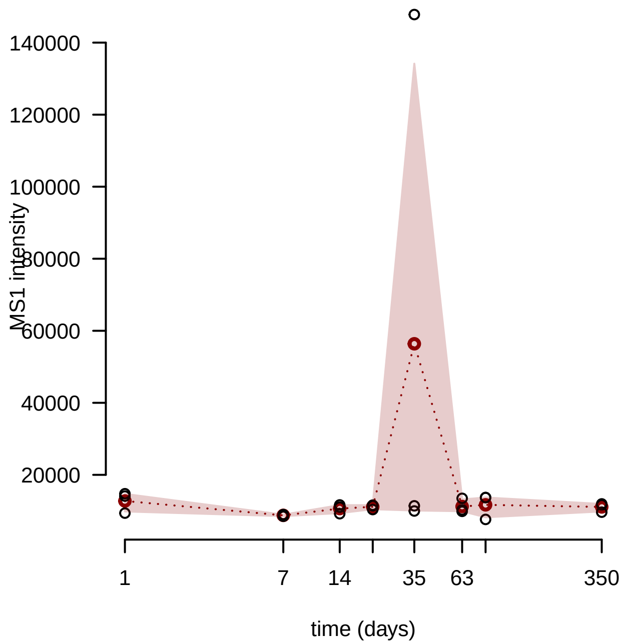

Q9WVJ9
GN=Efemp2
epidermal growth factor−containing fibul ...

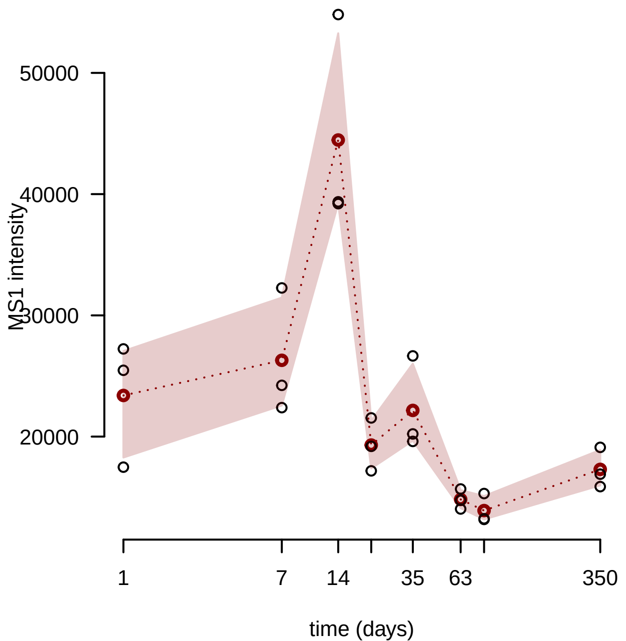

Q9WVK0
GN=Agtrap
angiotensin II, type I receptor−associat ...

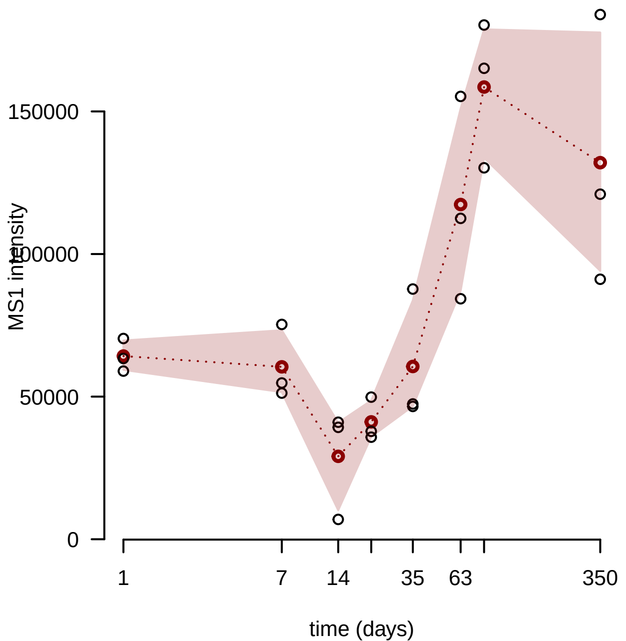

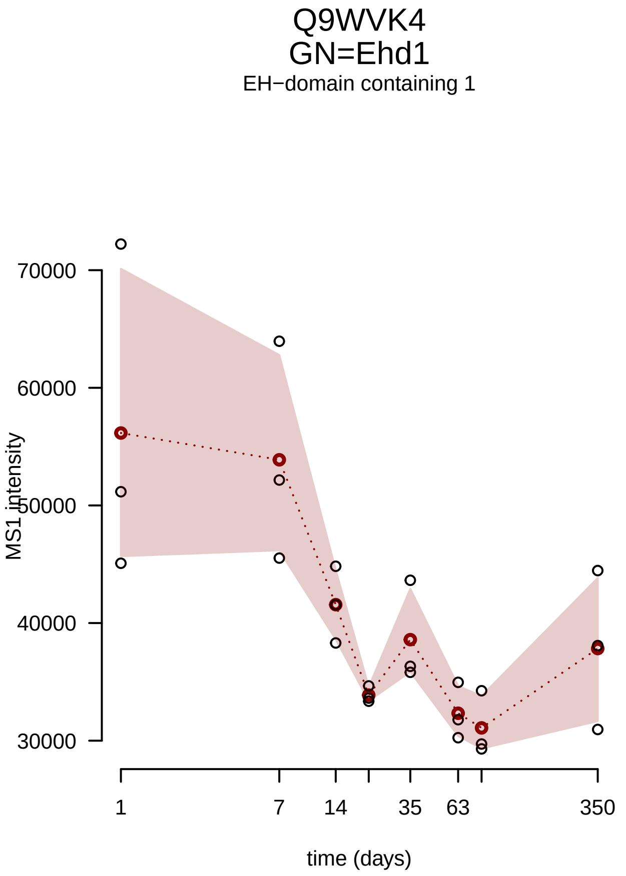
Q9WVK4
GN=Ehd1
EH−domain containing 1
MS1 intensity
70000
60000
50000
40000
30000
1
7
14
35
63
350
time (days)

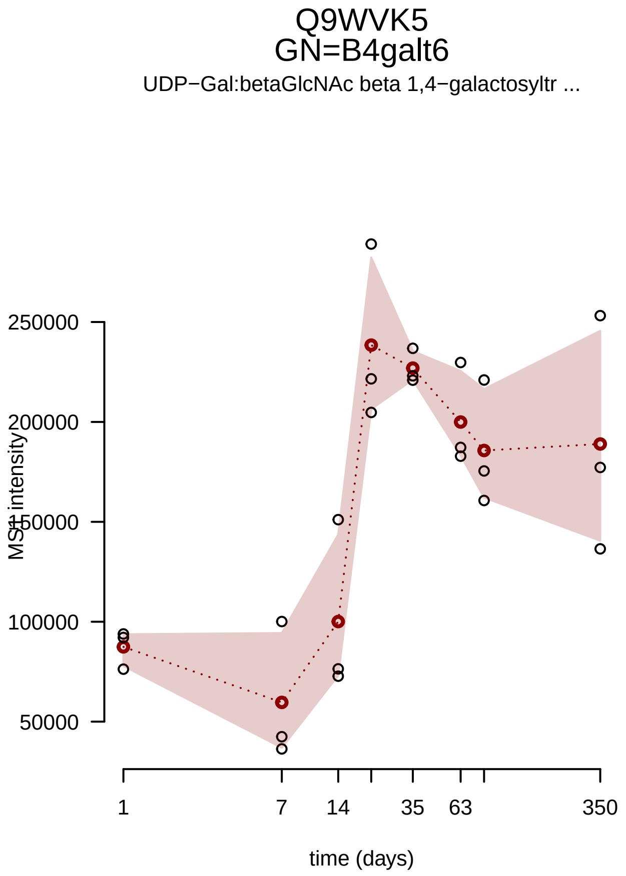
Q9WVK5
GN=B4galt6
UDP−Gal:betaGlcNAc beta 1,4−galactosyltr ...
MS1 intensity
250000
200000
150000
100000
50000
1
7
14
35
63
350
time (days)

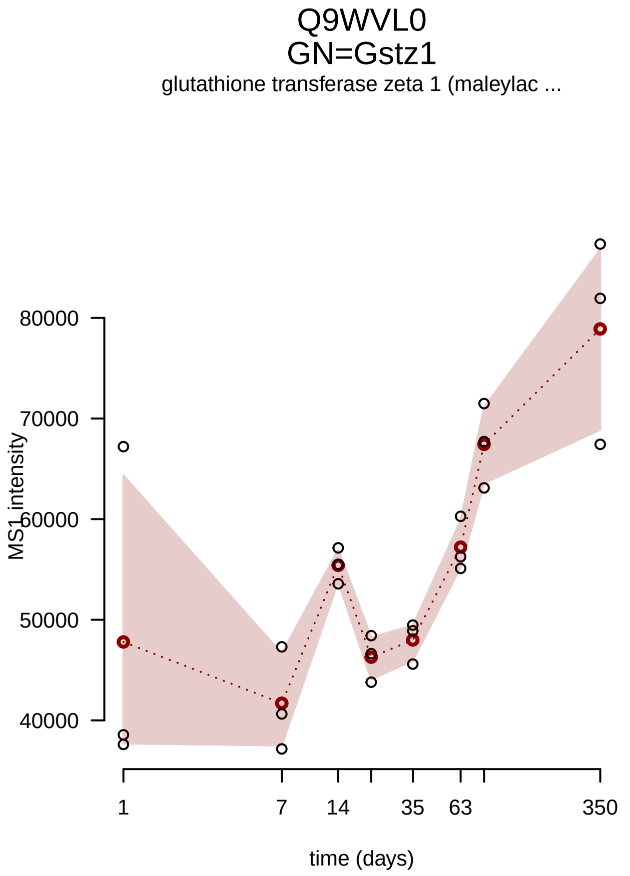
Q9WVL0
GN=Gstz1
glutathione transferase zeta 1 (maleylac ...
MS1 intensity
80000
70000
60000
50000
40000
1
7
14
35
63
350
time (days)

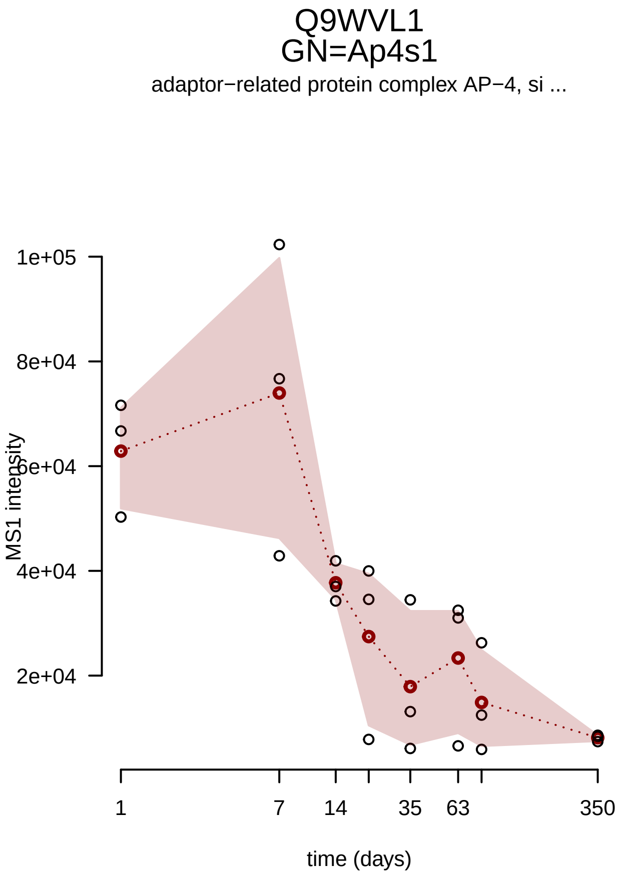
Q9WVL1
GN=Ap4s1
adaptor−related protein complex AP−4, si ...
MS1 intensity
1e+05
8e+04
6e+04
4e+04
2e+04
1
7
14
35
63
350
time (days)

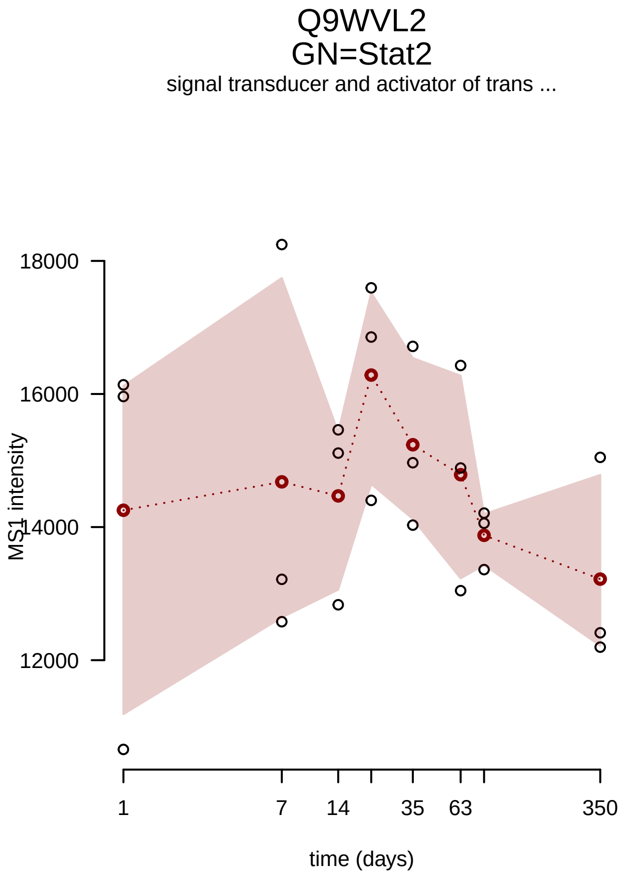
Q9WVL2
GN=Stat2
signal transducer and activator of trans ...
MS1 intensity
18000
16000
14000
12000
1
7
14
35
63
350
time (days)

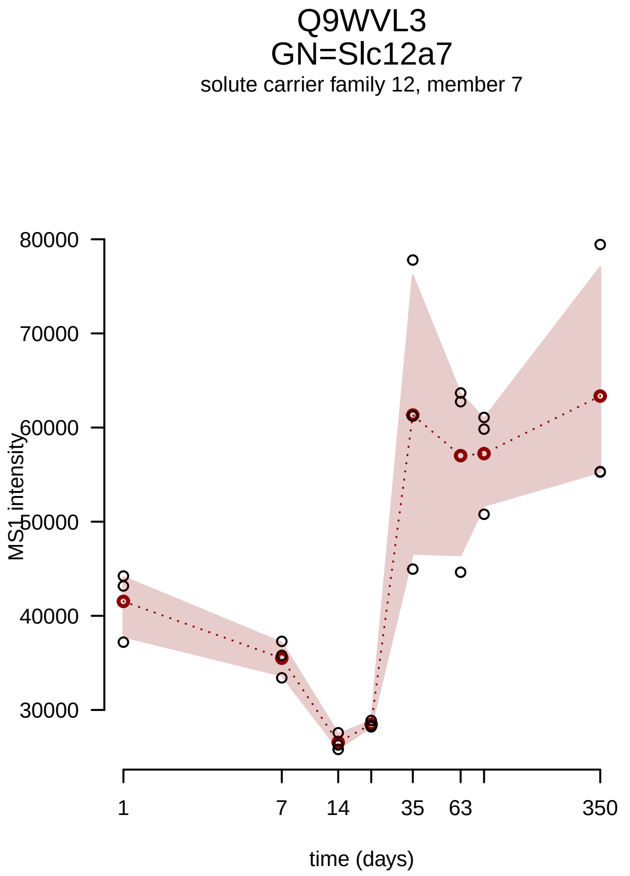
Q9WVL3
GN=Slc12a7
solute carrier family 12, member 7
MS1 intensity
80000
70000
60000
50000
40000
30000
1
7
14
35
63
350
time (days)

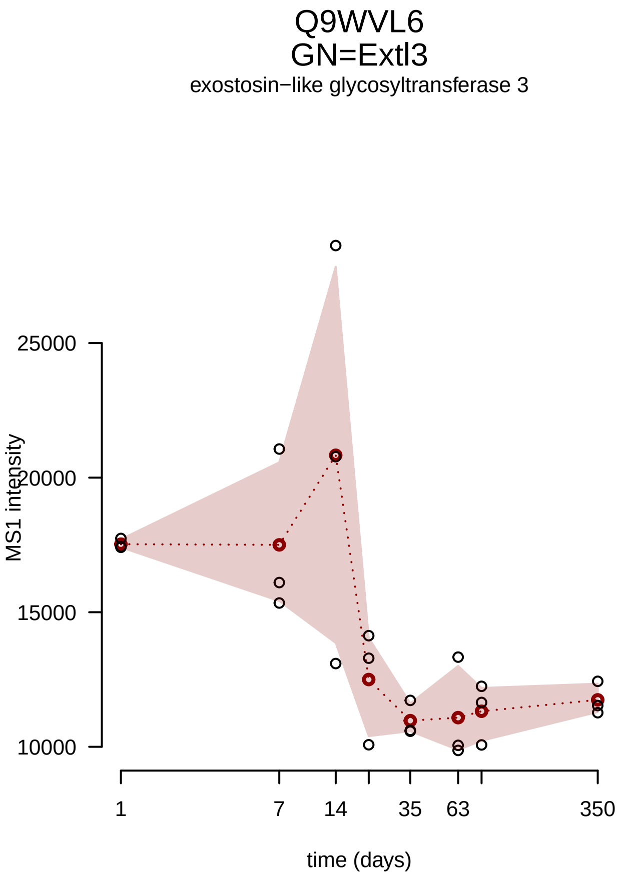
Q9WVL6
GN=Extl3
exostosin−like glycosyltransferase 3
MS1 intensity
25000
20000
15000
10000
1
7
14
35
63
350
time (days)

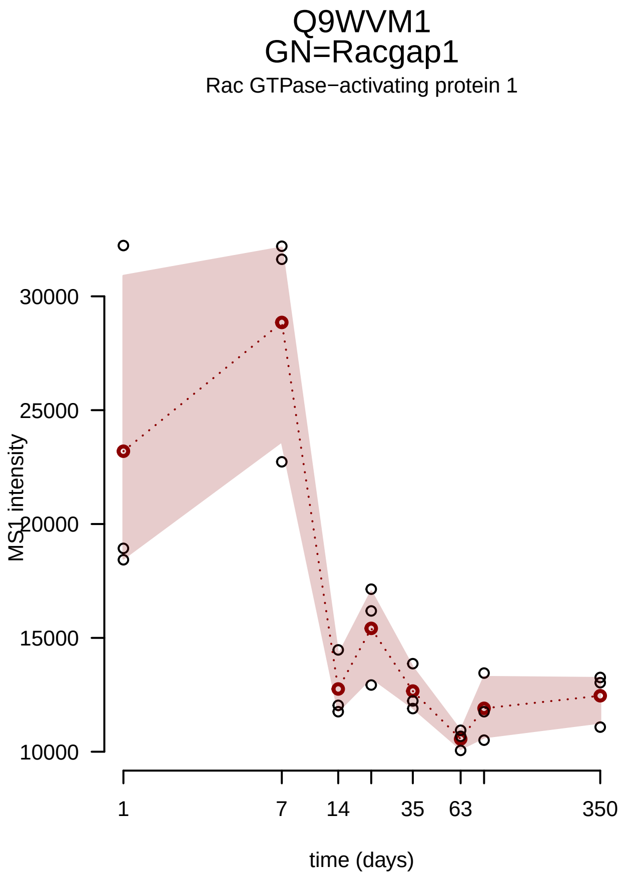
Q9WVM1
GN=Racgap1
Rac GTPase−activating protein 1
MS1 intensity
30000
25000
20000
15000
10000
1
7
14
35
63
350
time (days)

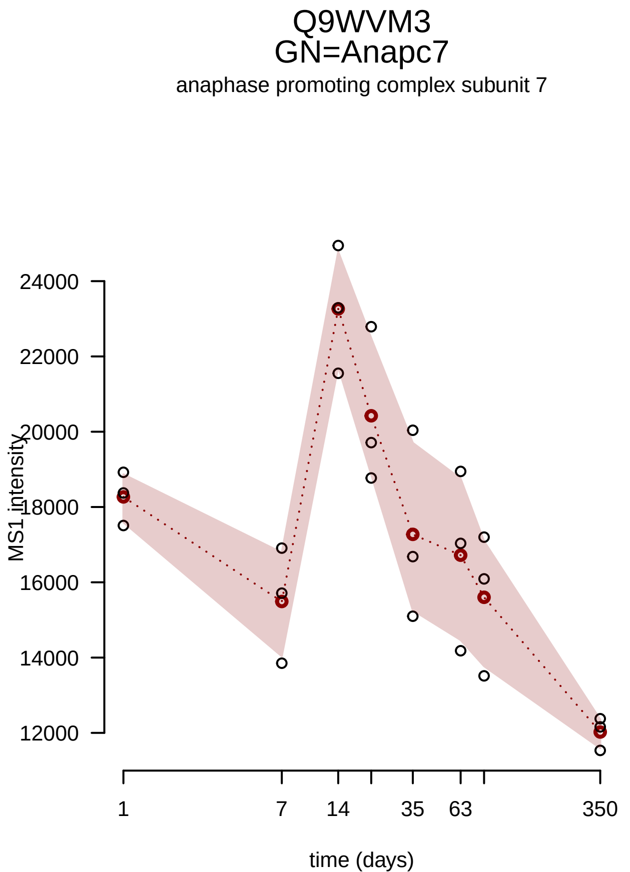
Q9WVM3
GN=Anapc7
anaphase promoting complex subunit 7
MS1 intensity
24000
22000
20000
18000
16000
14000
12000
1
7
14
35
63
350
time (days)

Q9WVP1
GN=Ap1m2
adaptor protein complex AP−1, mu 2 subun ...

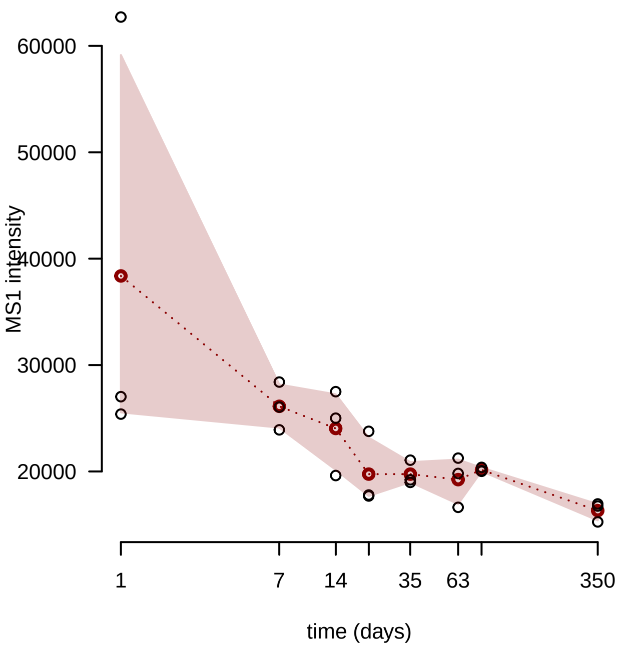

Q9WVP6
GN=Papolb
poly (A) polymerase beta (testis specifi ...

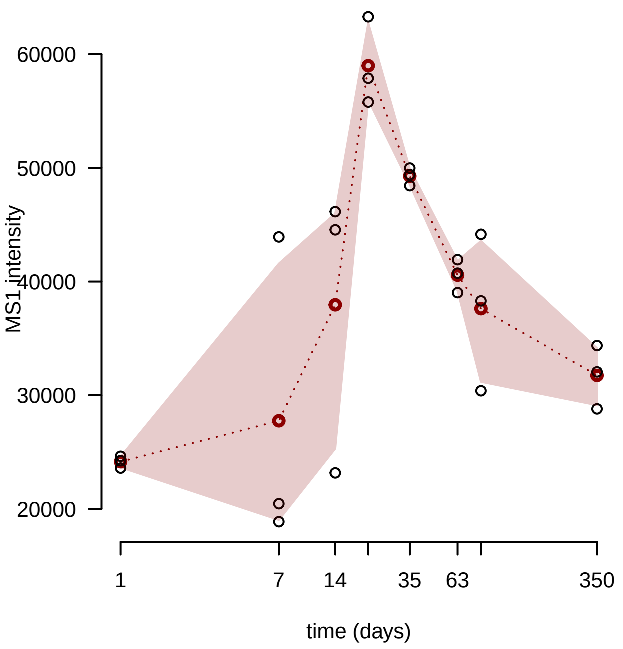

Q9WVQ1
GN=Magi2
membrane associated guanylate kinase, WW ...

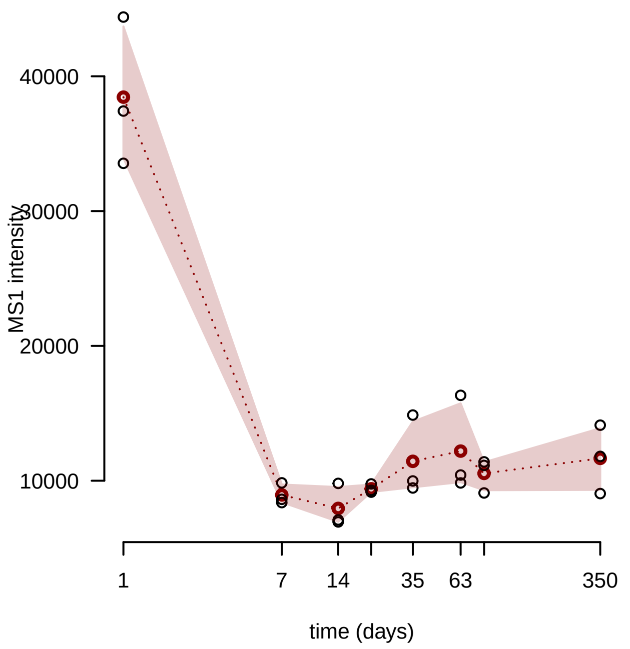

Q9WVQ5
GN=Apip
APAF1 interacting protein

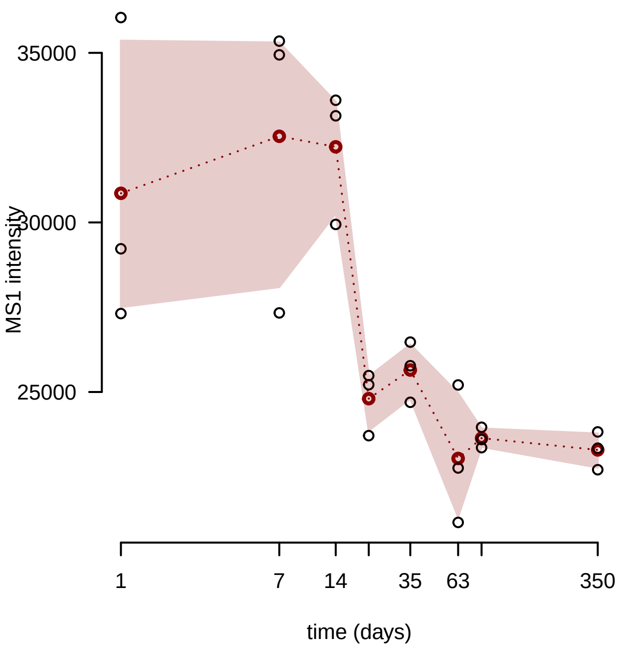

Q9WVR4
GN=Fxr2
FMR1 autosomal homolog 2

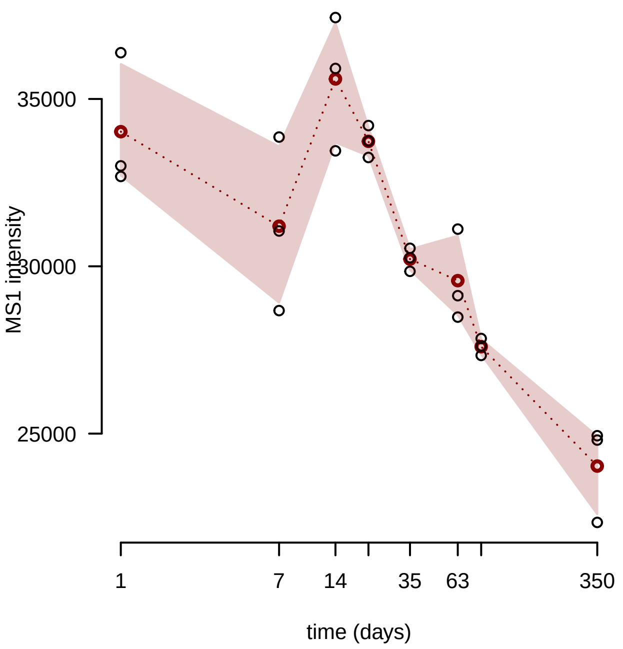

Q9WVS8
GN=Mapk7
mitogen−activated protein kinase 7

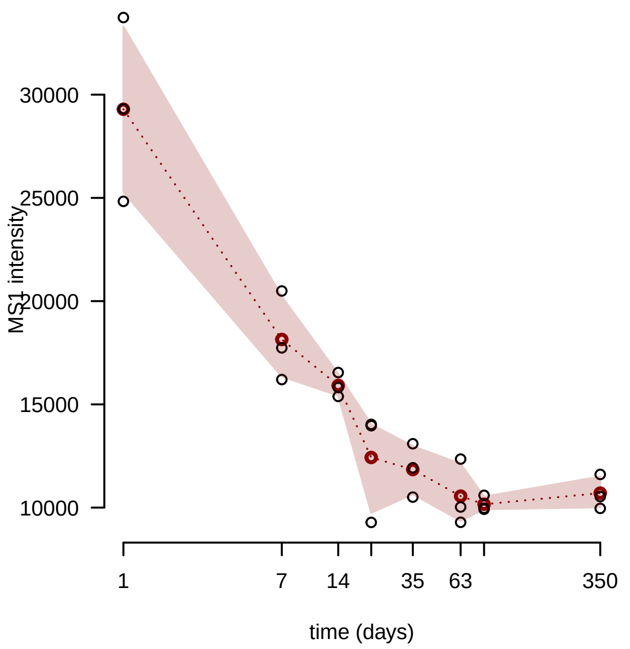

Q9WVT6
GN=Ca14

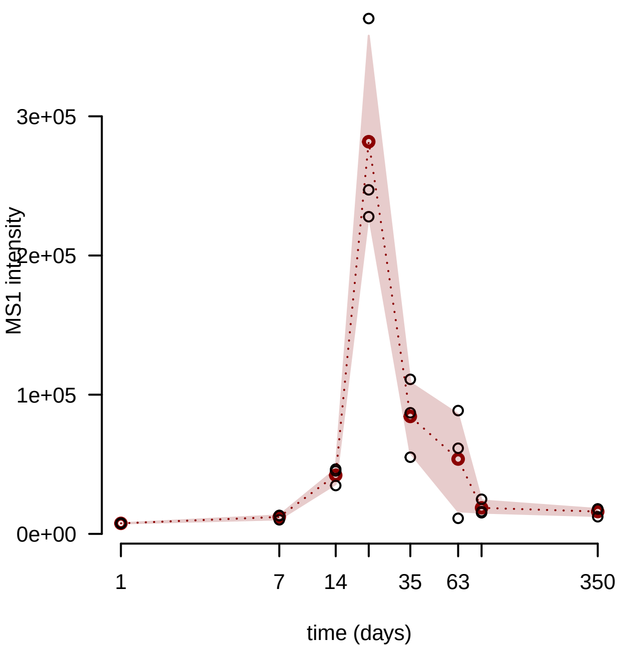

Q9Z0E0
GN=Ncdn
neurochondrin

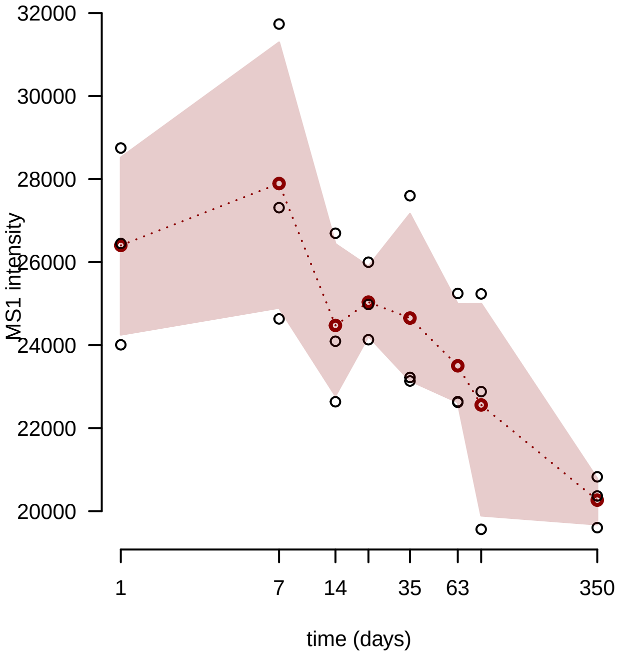

Q9Z0E6
GN=Gbp2
guanylate binding protein 2

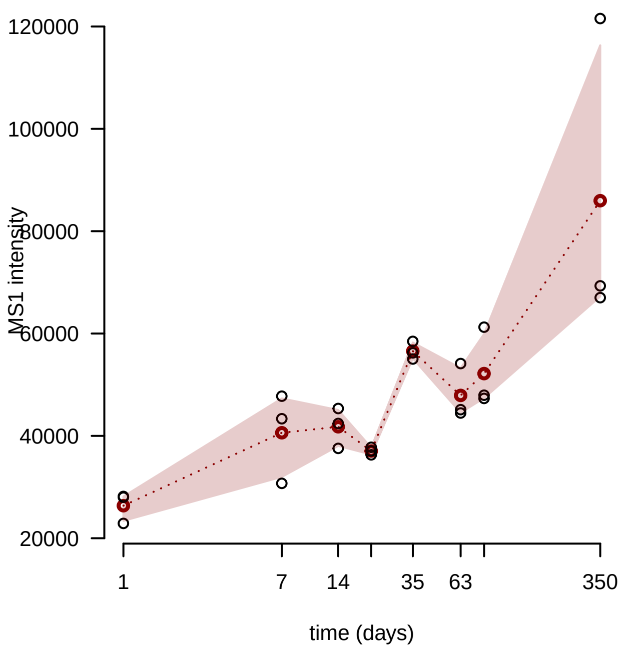

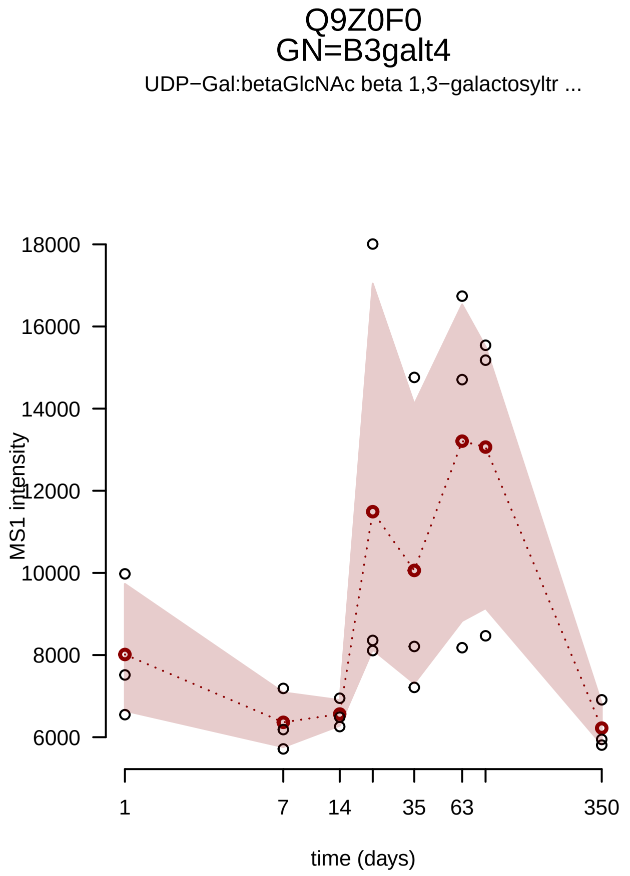
Q9Z0F0
GN=B3galt4
UDP−Gal:betaGlcNAc beta 1,3−galactosyltr ...
MS1 intensity
18000
16000
14000
12000
10000
8000
6000
1
7
14
35
63
350
time (days)

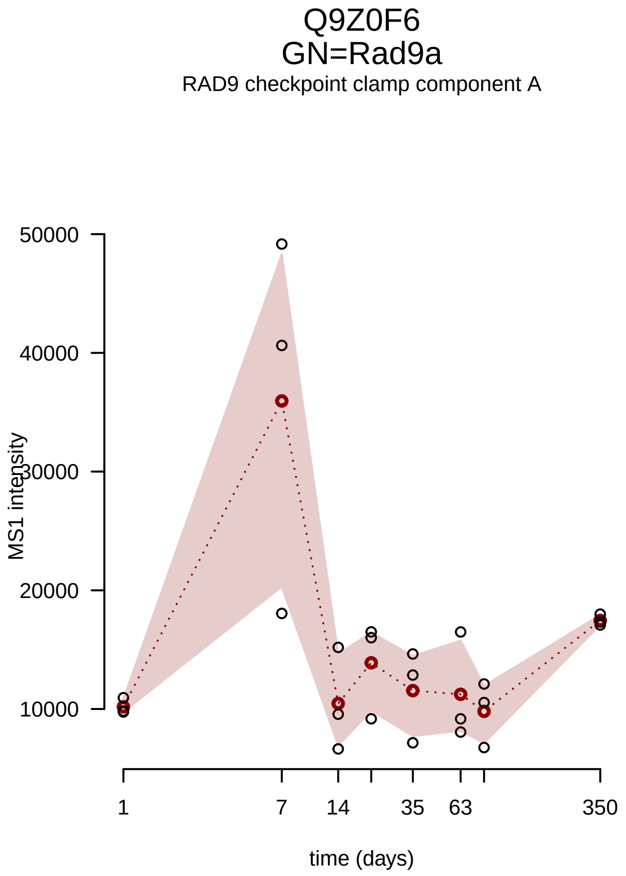
Q9Z0F6
GN=Rad9a
RAD9 checkpoint clamp component A
MS1 intensity
50000
40000
30000
20000
10000
1
7
14
35
63
350
time (days)

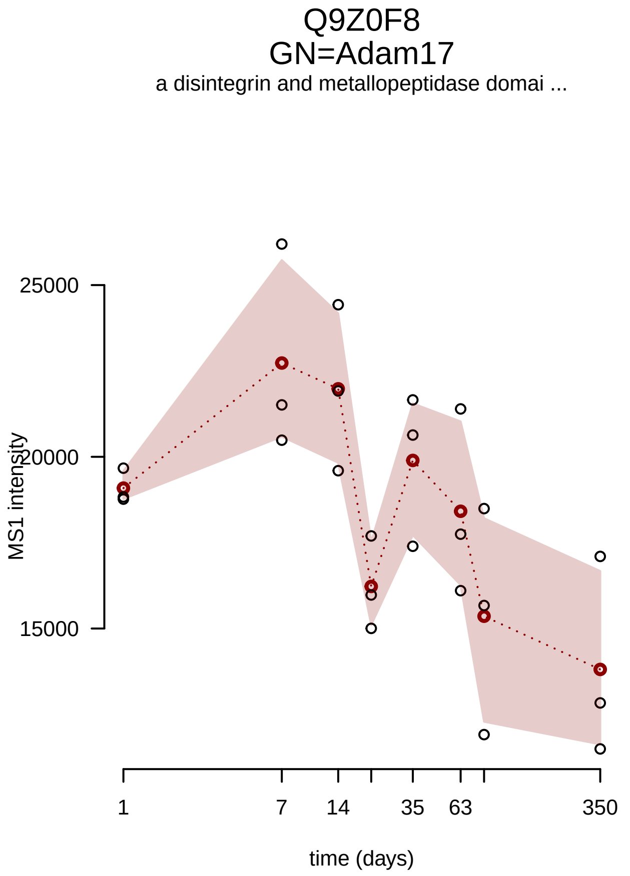
Q9Z0F8
GN=Adam17
a disintegrin and metallopeptidase domai ...
MS1 intensity
25000
20000
15000
1
7
14
35
63
350
time (days)

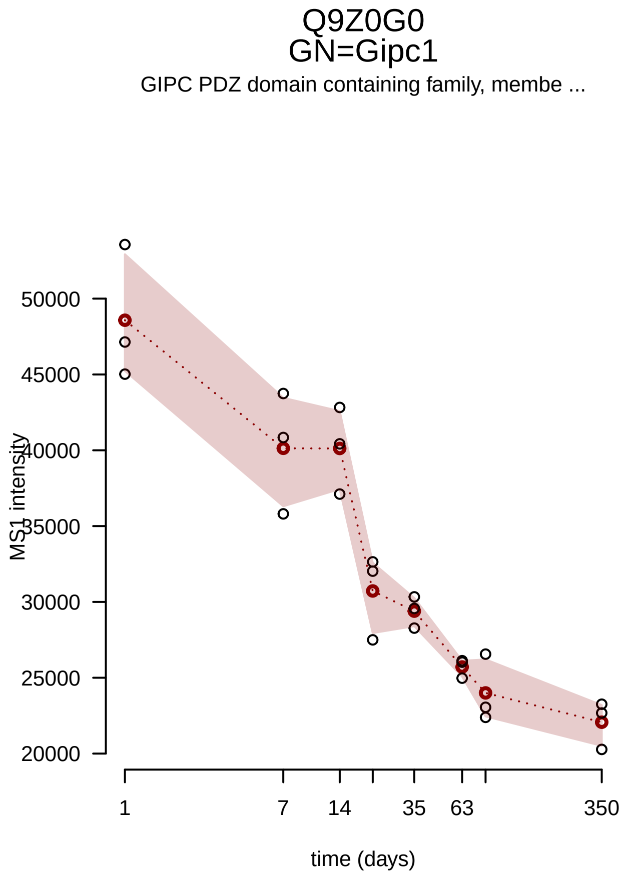
Q9Z0G0
GN=Gipc1
GIPC PDZ domain containing family, membe ...
MS1 intensity
50000
45000
40000
35000
30000
25000
20000
1
7
14
35
63
350
time (days)

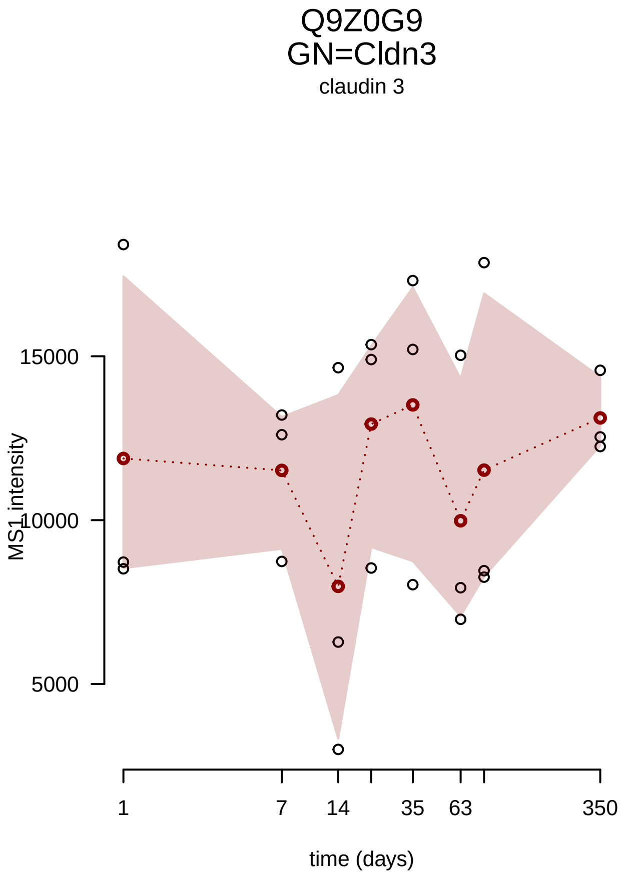
Q9Z0G9
GN=Cldn3
claudin 3
MS1 intensity
15000
10000
5000
1
7
14
35
63
350
time (days)

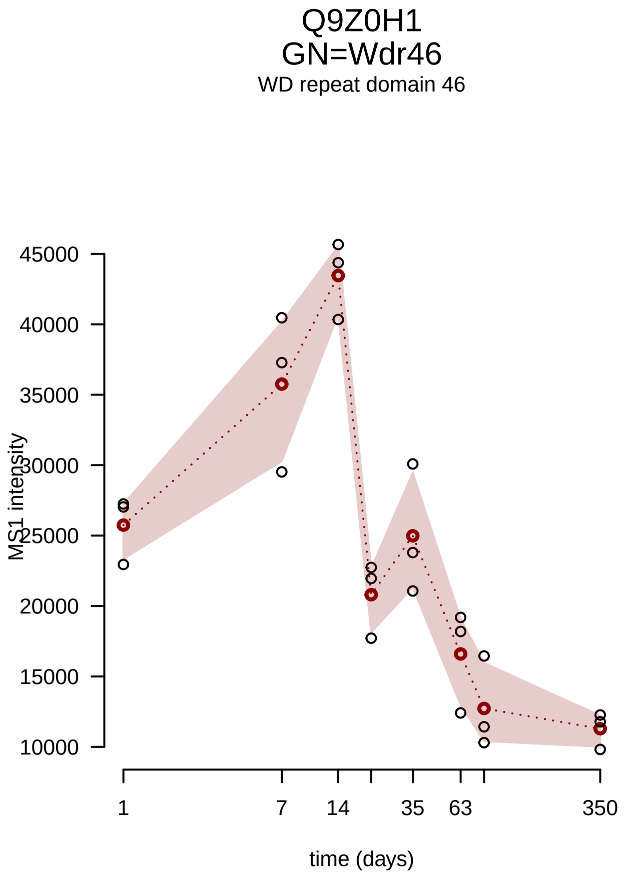
Q9Z0H1
GN=Wdr46
WD repeat domain 46
MS1 intensity
45000
40000
35000
30000
25000
20000
15000
10000
1
7
14
35
63
350
time (days)

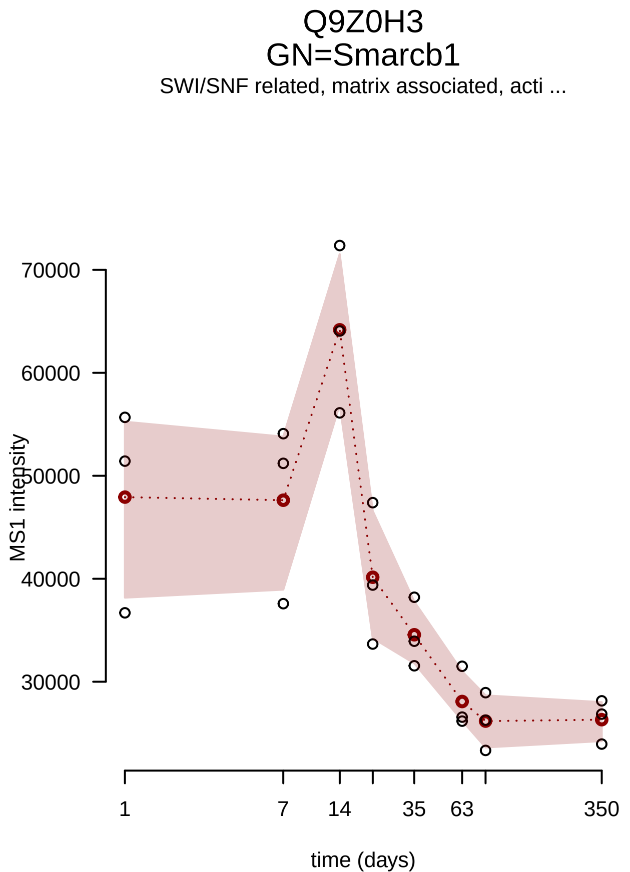
Q9Z0H3
GN=Smarcb1
SWI/SNF related, matrix associated, acti ...
MS1 intensity
70000
60000
50000
40000
30000
1
7
14
35
63
350
time (days)

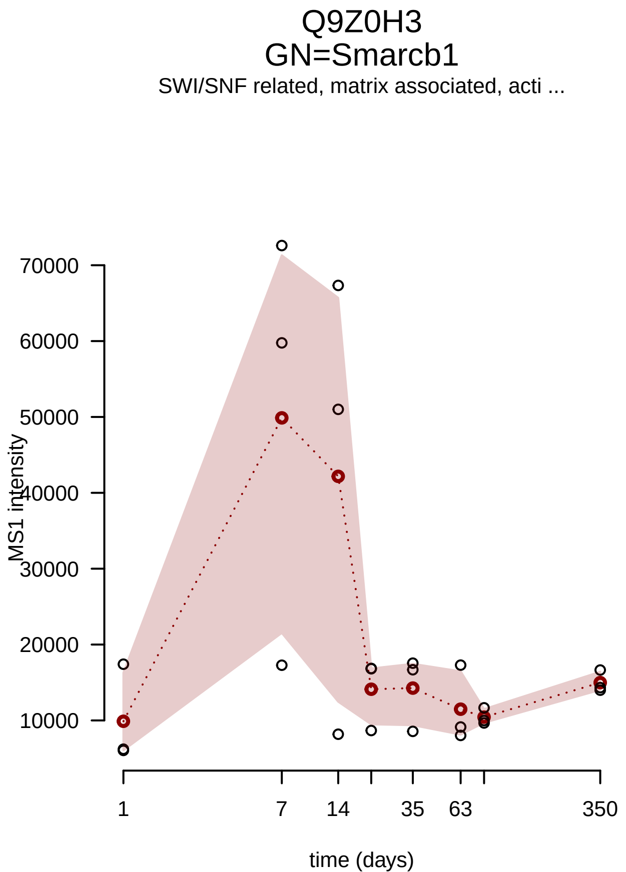
Q9Z0H3
GN=Smarcb1
SWI/SNF related, matrix associated, acti ...
MS1 intensity
70000
60000
50000
40000
30000
20000
10000
1
7
14
35
63
350
time (days)

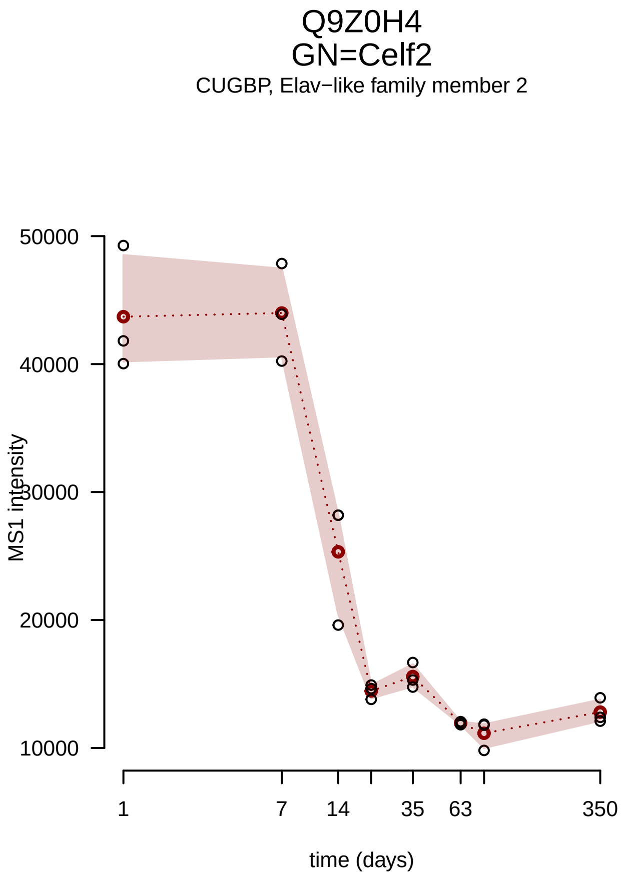
Q9Z0H4
GN=Celf2
CUGBP, Elav−like family member 2
MS1 intensity
50000
40000
30000
20000
10000
1
7
14
35
63
350
time (days)

Q9Z0H7
GN=Bcl10
B cell leukemia/lymphoma 10

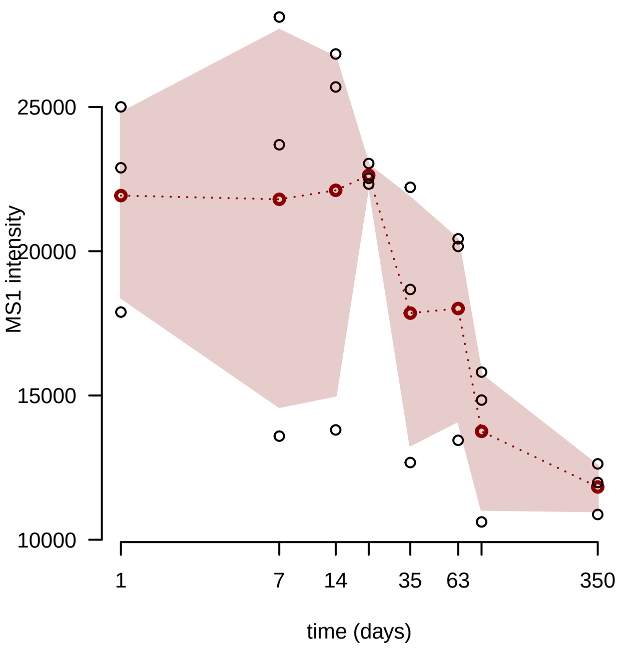

Q9Z0H8
GN=Clip2
CAP−GLY domain containing linker protein ...

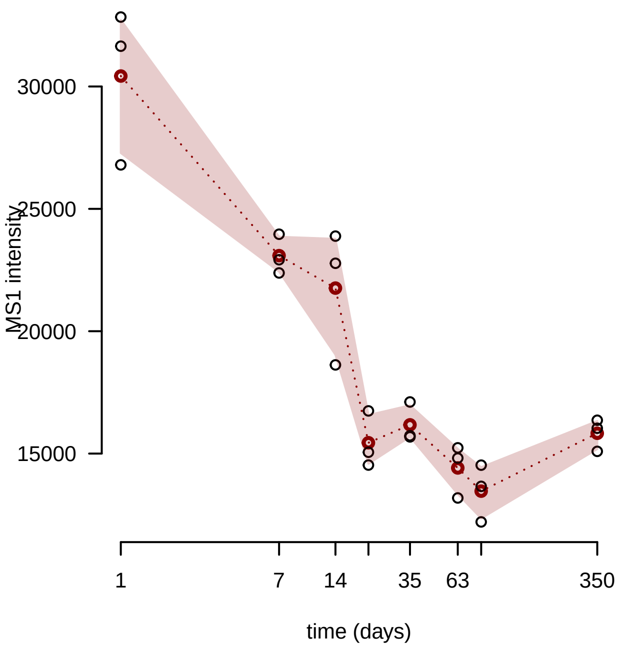

Q9Z0J0
GN=Npc2
NPC intracellular cholesterol transporte ...

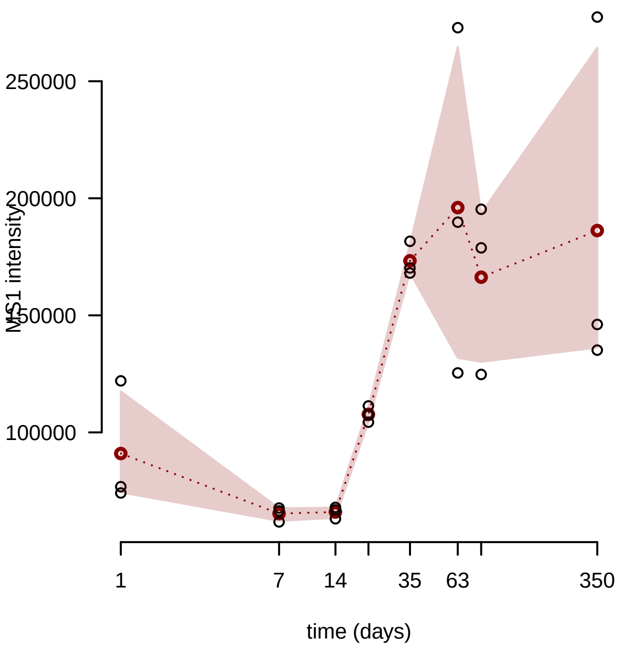

Q9Z0J1
GN=Reck
reversion−inducing−cysteine−rich protein ...

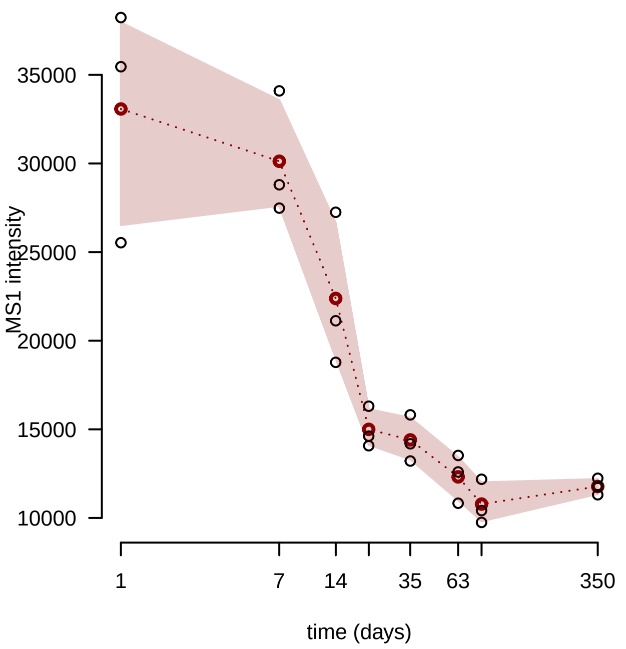

Q9Z0J4
GN=Nos1
nitric oxide synthase 1, neuronal

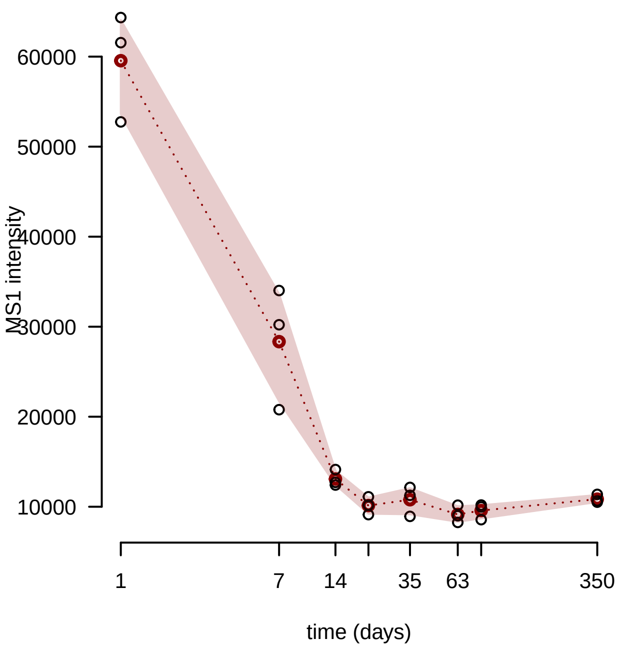

Q9Z0K8
GN=Vnn1
vanin 1

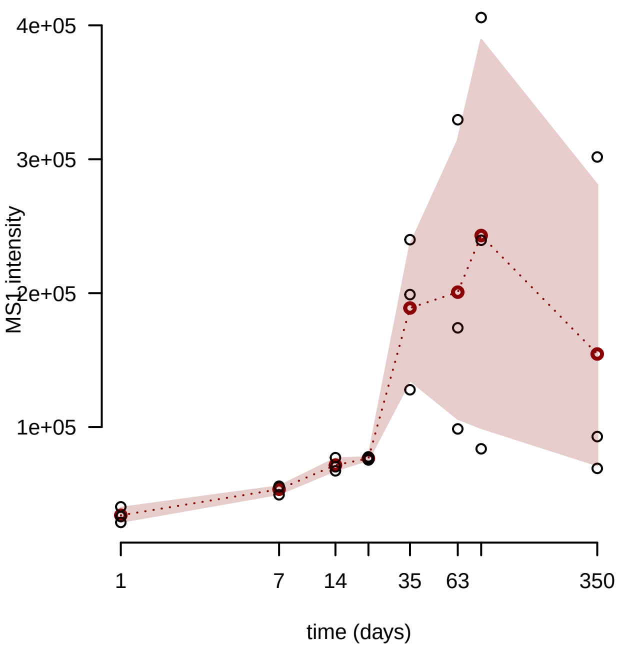

Q9Z0L0
GN=Tpbg
trophoblast glycoprotein

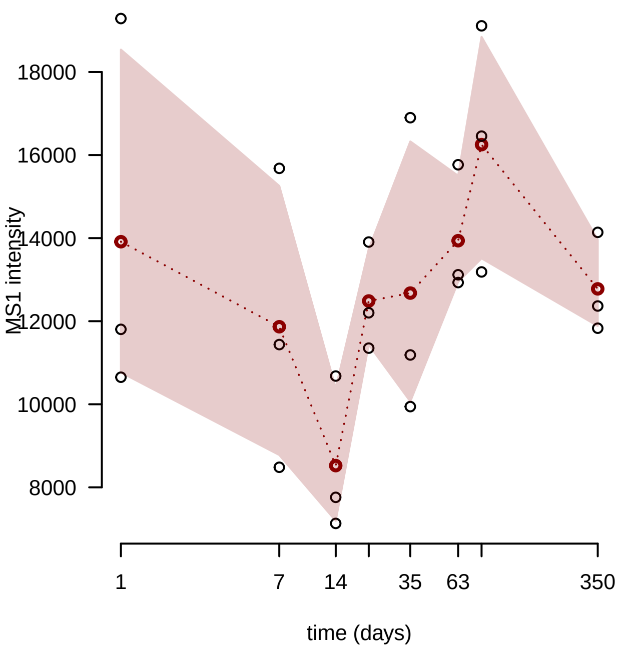

Q9Z0L8
GN=Ggh
gamma−glutamyl hydrolase

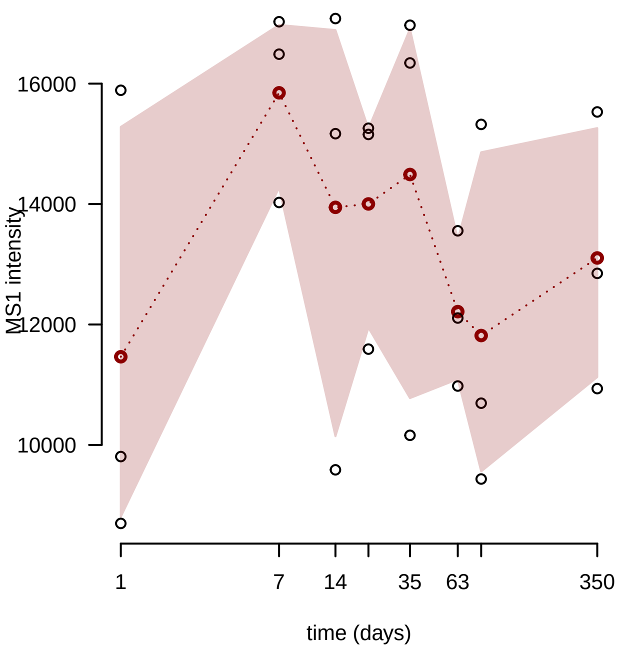

Q9Z0M5
GN=Lipa
lysosomal acid lipase A

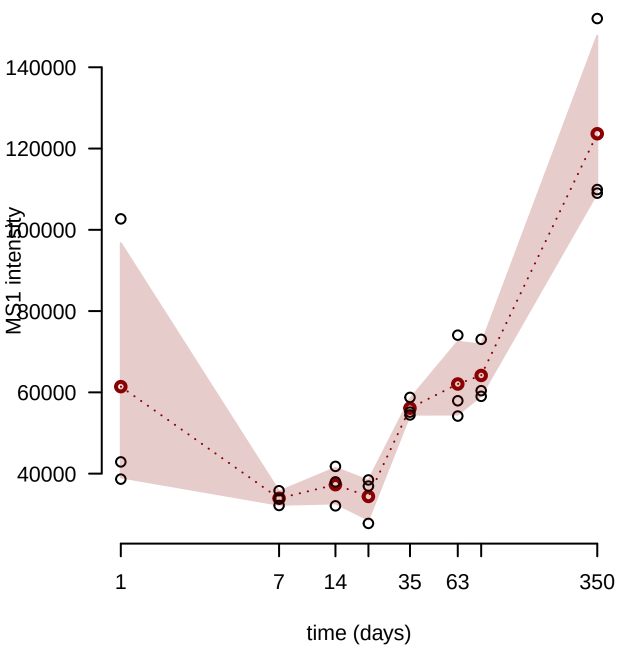

Q9Z0M6
GN=Cd97

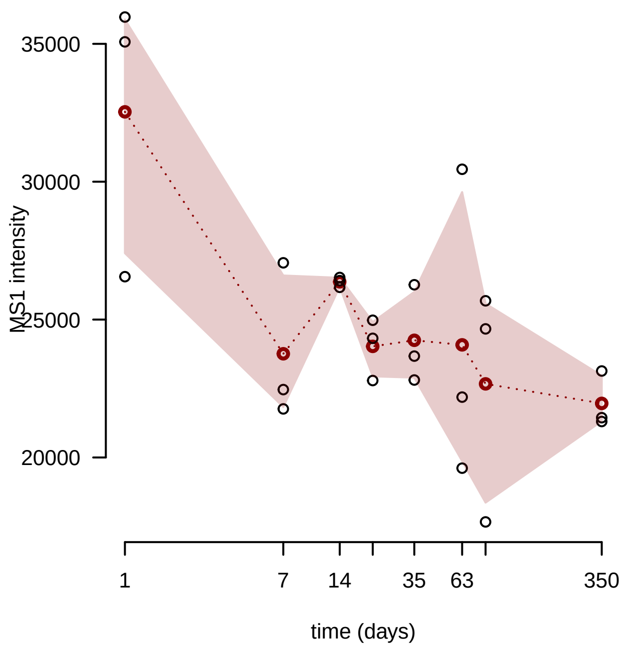

Q9Z0N1
GN=Eif2s3x
eukaryotic translation initiation factor ...

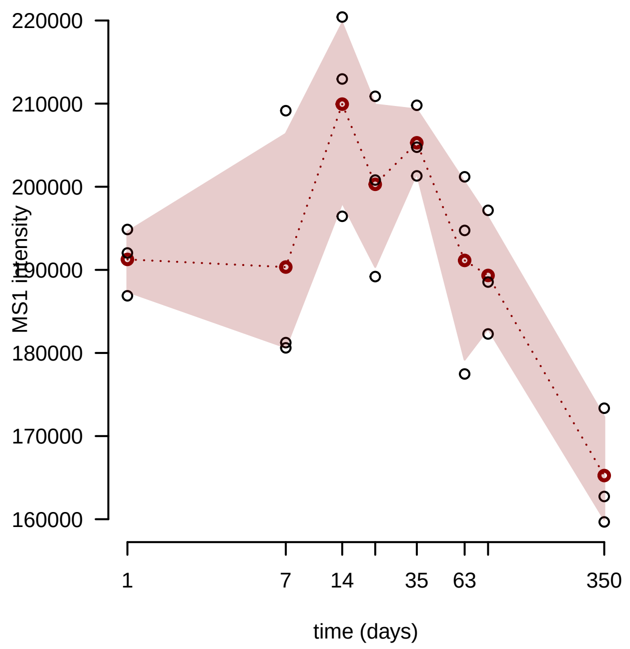

Q9Z0N2
GN=Eif2s3y
eukaryotic translation initiation factor ...

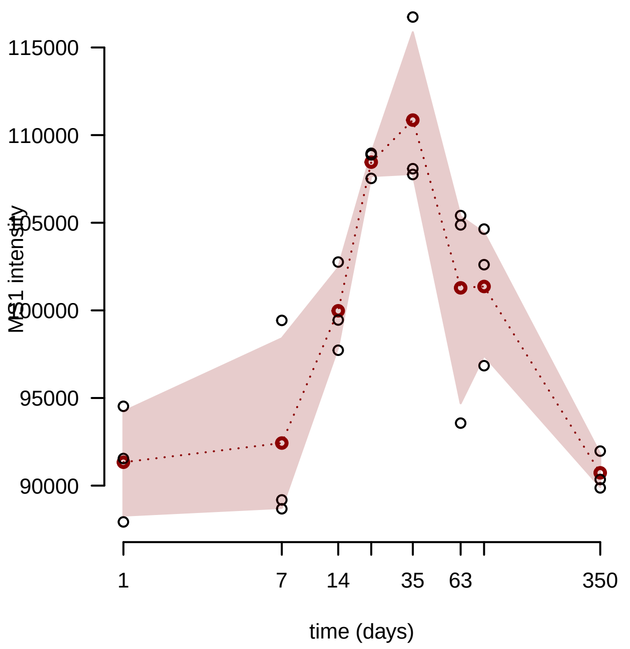

Q9Z0P4
GN=Palm
paralemmin

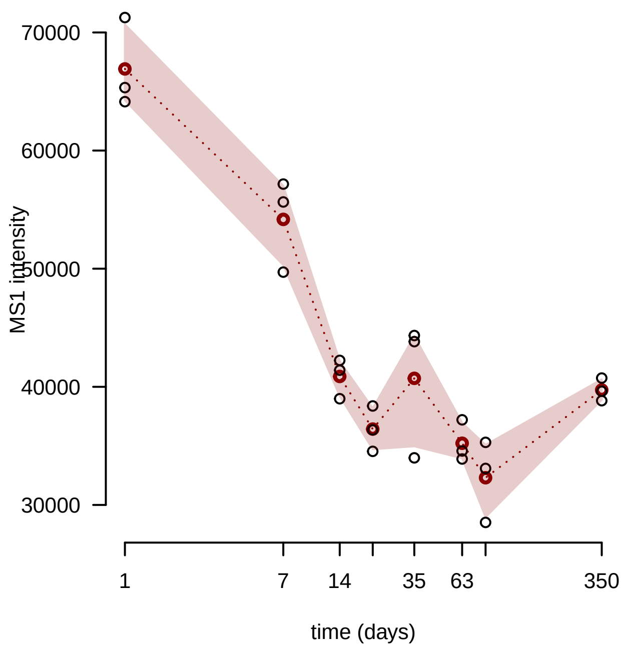

Q9Z0P5
GN=Twf2
twinfilin actin binding protein 2

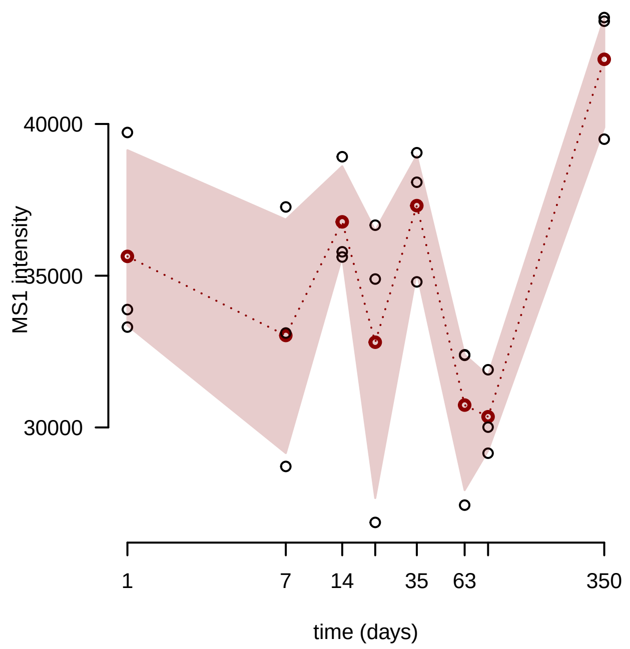

Q9Z0P7
GN=Sufu
SUFU negative regulator of hedgehog sign ...

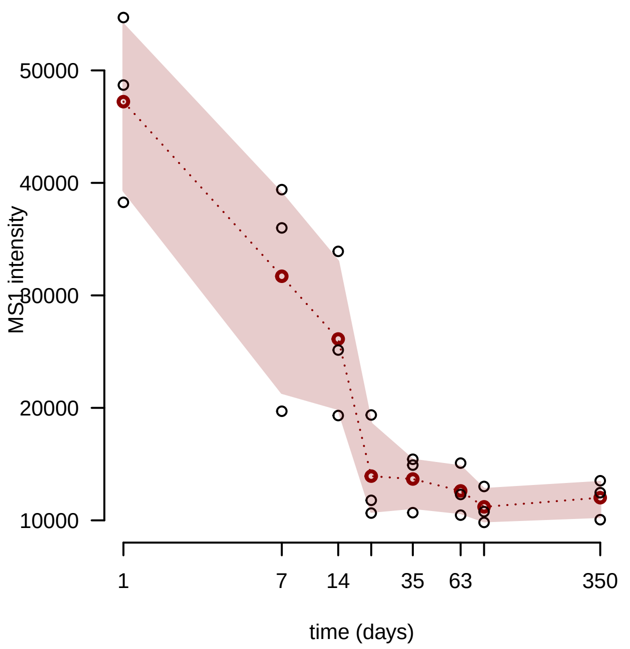

Q9Z0R4
GN=Itsn1
intersectin 1 (SH3 domain protein 1A)

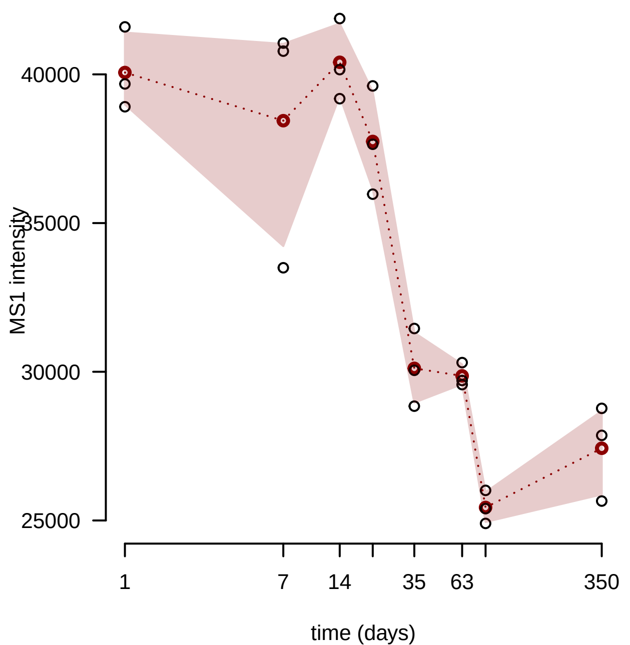

Q9Z0R6
GN=Itsn2
intersectin 2

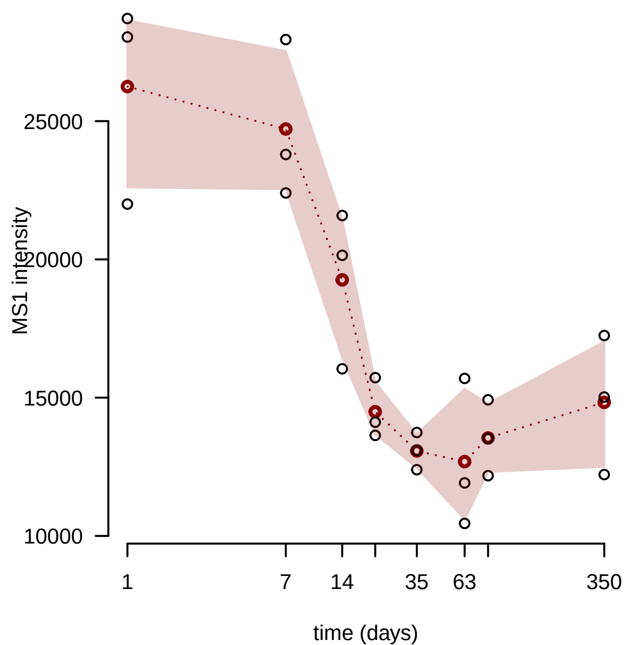

Q9Z0R9
GN=Fads2
fatty acid desaturase 2

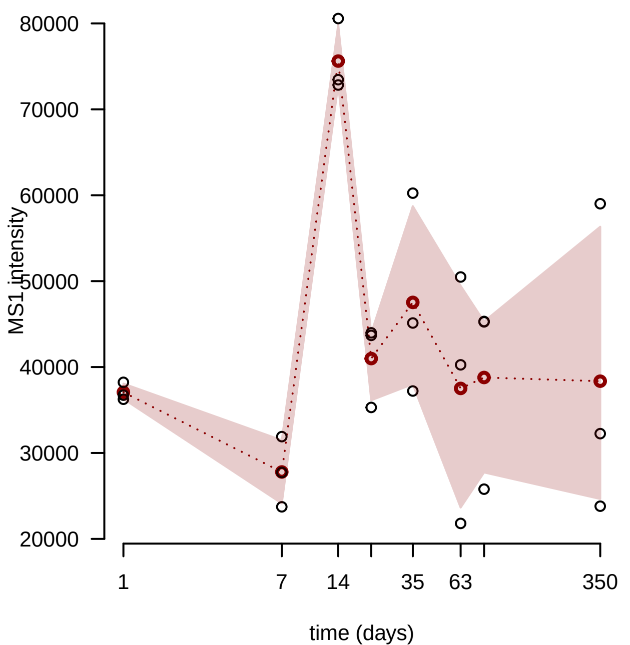

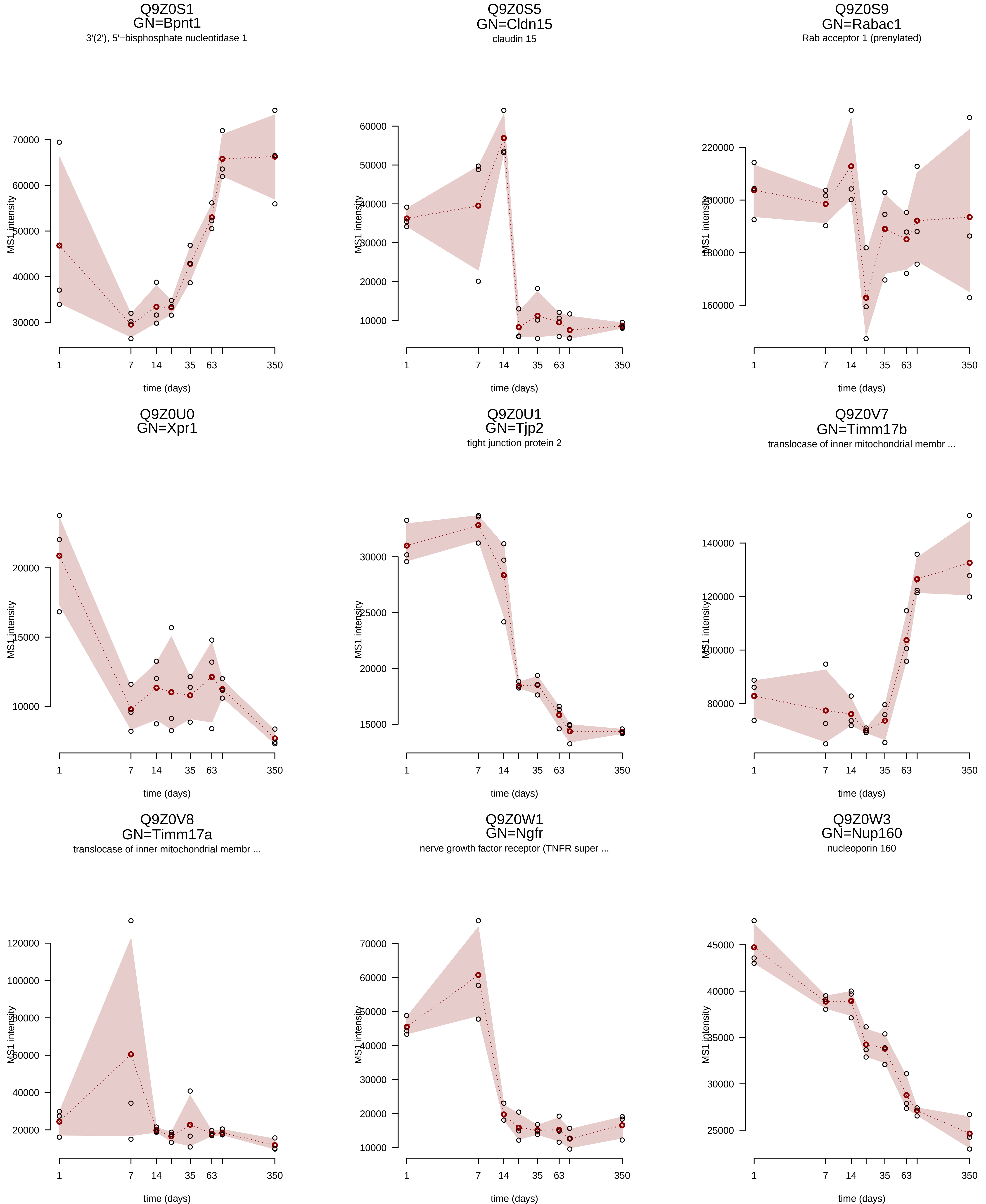
Q9Z0S1
GN=Bpnt1
3'(2'), 5'−bisphosphate nucleotidase 1
MS1 intensity
time (days)
Q9Z0S5
GN=Cldn15
claudin 15
MS1 intensity
time (days)
Q9Z0S9
GN=Rabac1
Rab acceptor 1 (prenylated)
MS1 intensity
time (days)
Q9Z0U0
GN=Xpr1
MS1 intensity
time (days)
Q9Z0U1
GN=Tjp2
tight junction protein 2
MS1 intensity
time (days)
Q9Z0V7
GN=Timm17b
translocase of inner mitochondrial membr ...
MS1 intensity
time (days)
Q9Z0V8
GN=Timm17a
translocase of inner mitochondrial membr ...
MS1 intensity
time (days)
Q9Z0W1
GN=Ngfr
nerve growth factor receptor (TNFR super ...
MS1 intensity
time (days)
Q9Z0W3
GN=Nup160
nucleoporin 160
MS1 intensity
time (days)

Q9Z0X0
GN=Cdc42ep5
CDC42 effector protein (Rho GTPase bindi ...

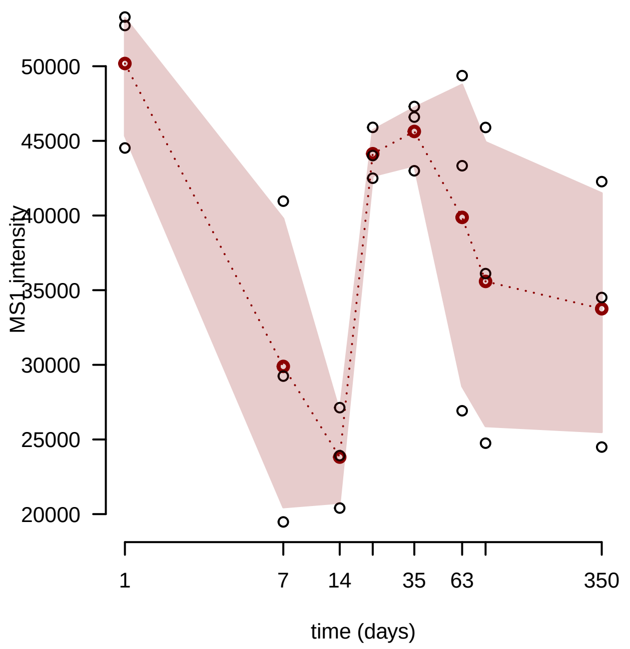

Q9Z0X1
GN=Aifm1
apoptosis−inducing factor, mitochondrion ...

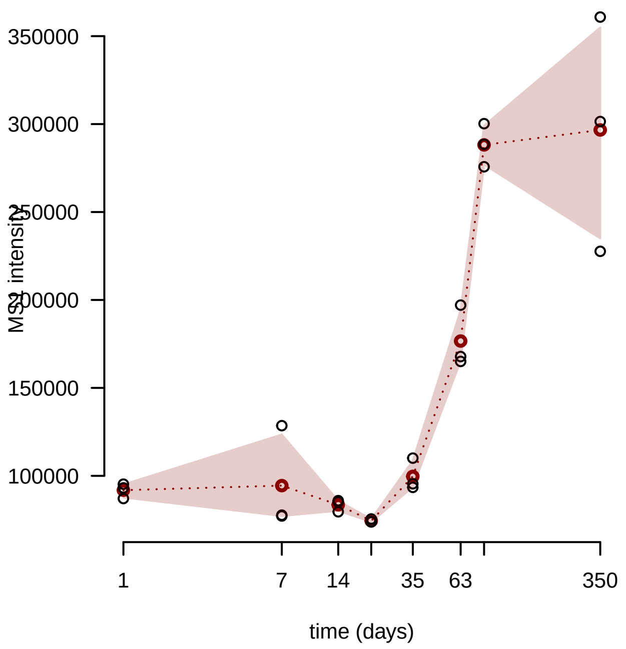

Q9Z0X4
GN=Pde3a
phosphodiesterase 3A, cGMP inhibited

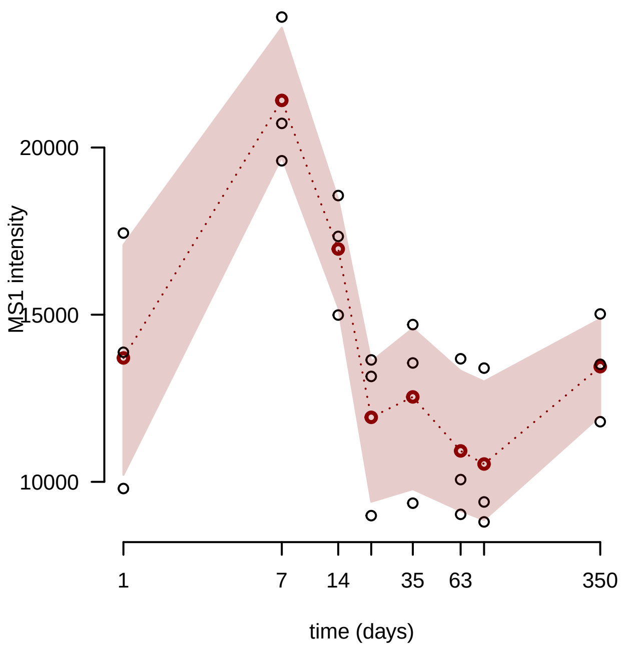

Q9Z0Y1
GN=Dctn3
dynactin 3

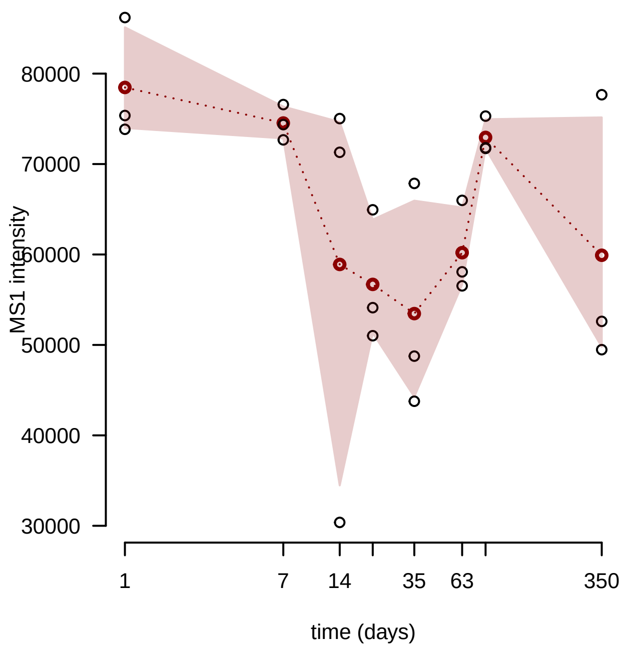

Q9Z0Z4
GN=Heph
hephaestin

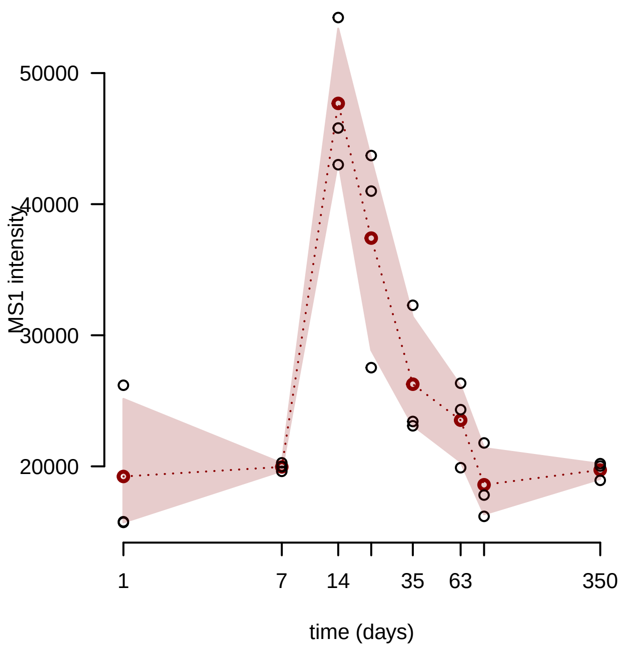

Q9Z100
GN=Cpxm1
carboxypeptidase X 1 (M14 family)

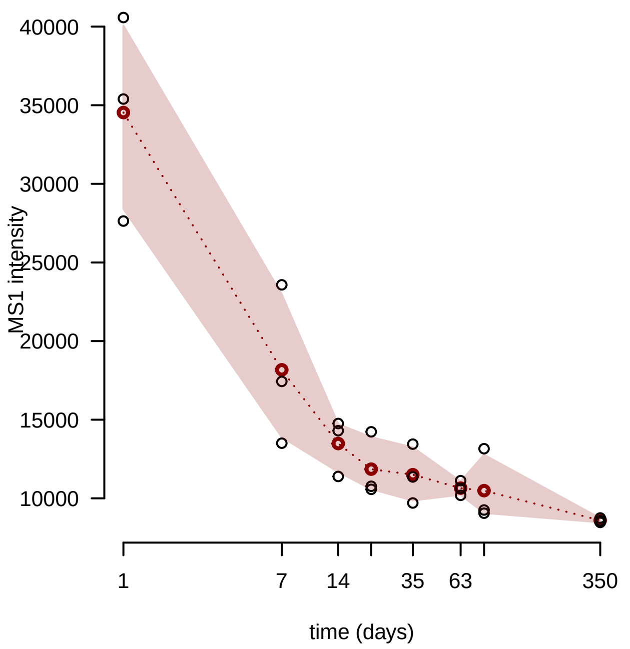

Q9Z103
GN=Adnp
activity−dependent neuroprotective prote ...

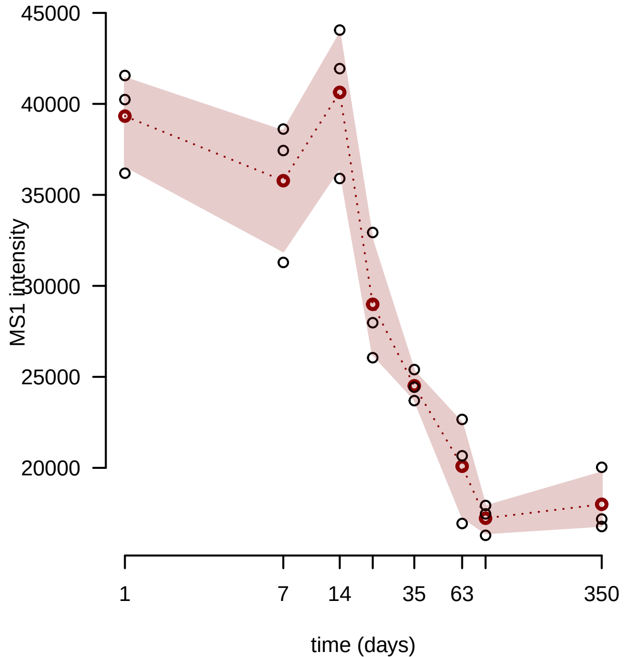

Q9Z104
GN=Hmg20b
high mobility group 20B

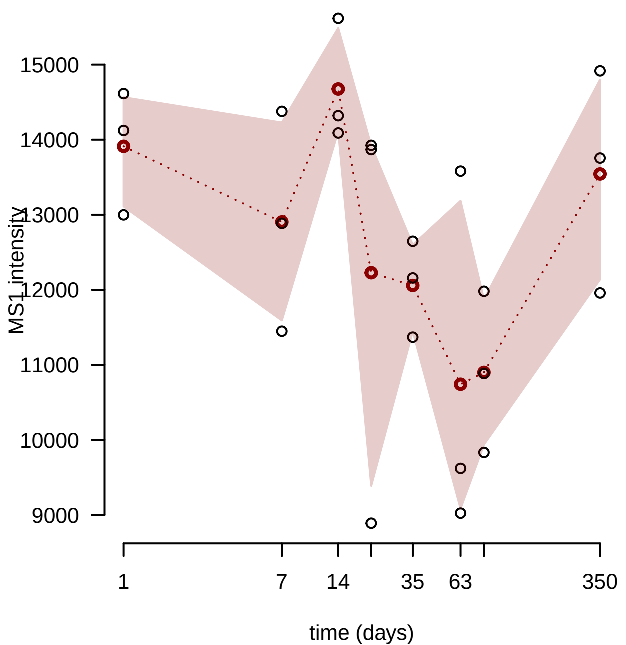

Q9Z108
GN=Stau1
staufen double−stranded RNA binding prot ...

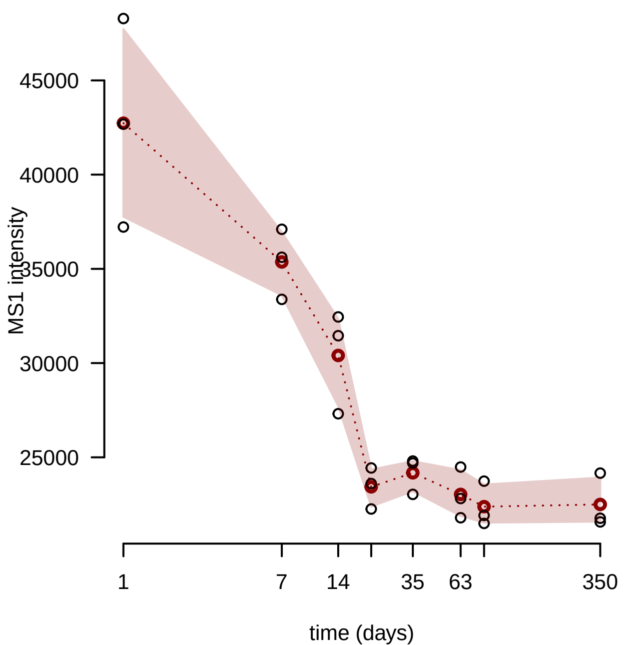

Q9Z110
GN=Aldh18a1
aldehyde dehydrogenase 18 family, member ...

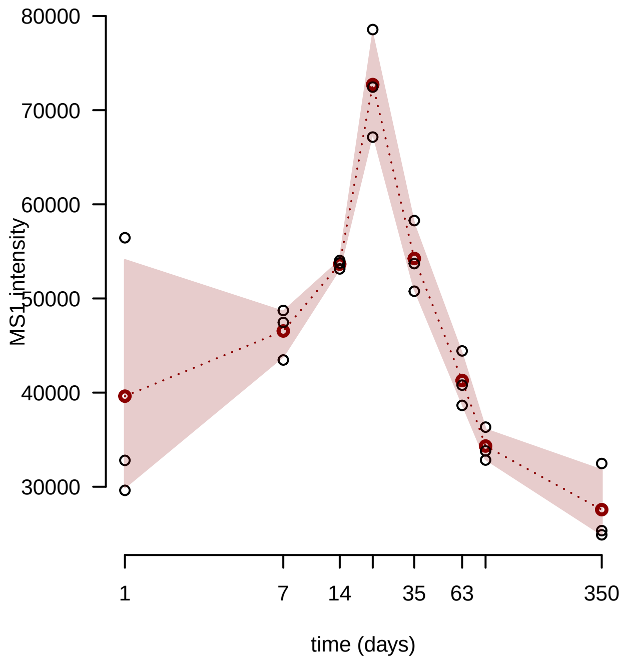

Q9Z120
GN=Mettl1
methyltransferase like 1

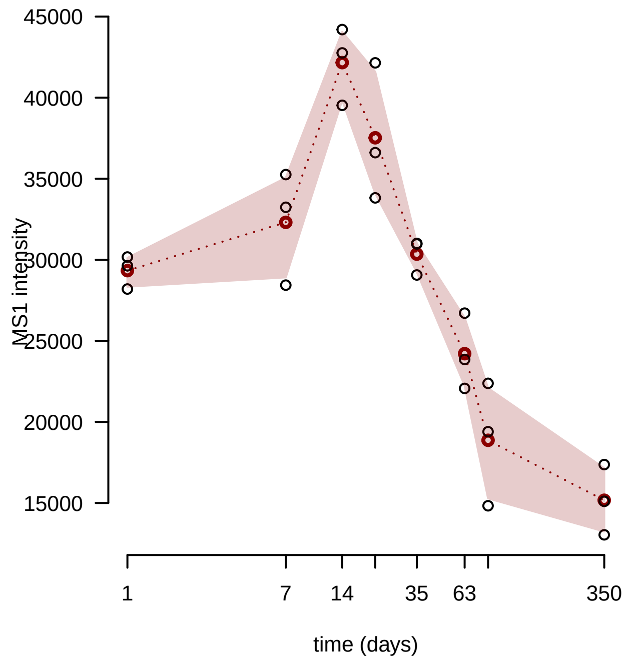

Q9Z126
GN=Pf4
platelet factor 4

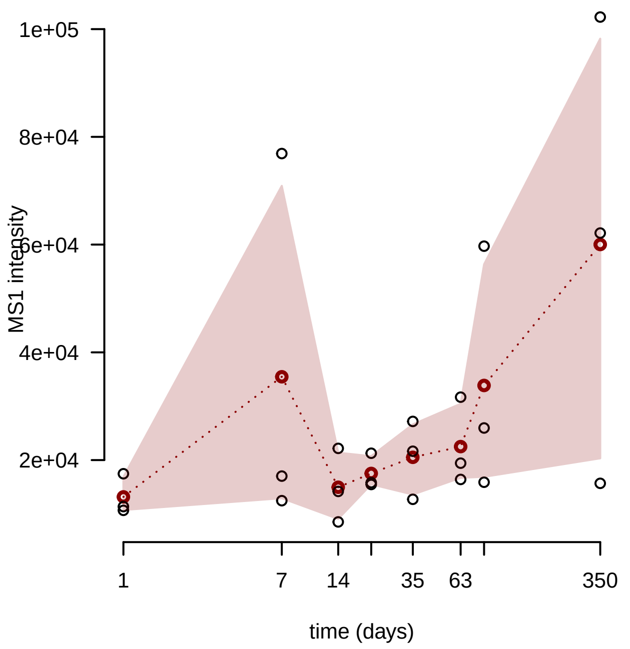

Q9Z127
GN=Slc7a5
solute carrier family 7 (cationic amino ...

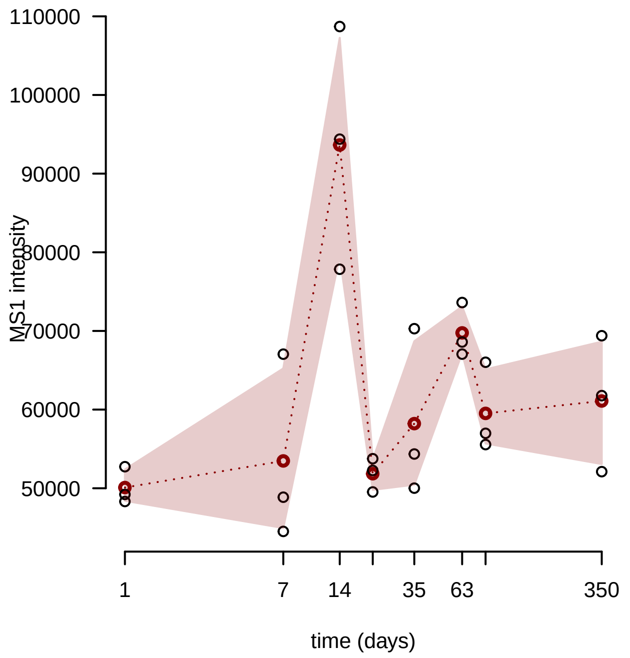

Q9Z129
GN=Recql
RecQ protein−like

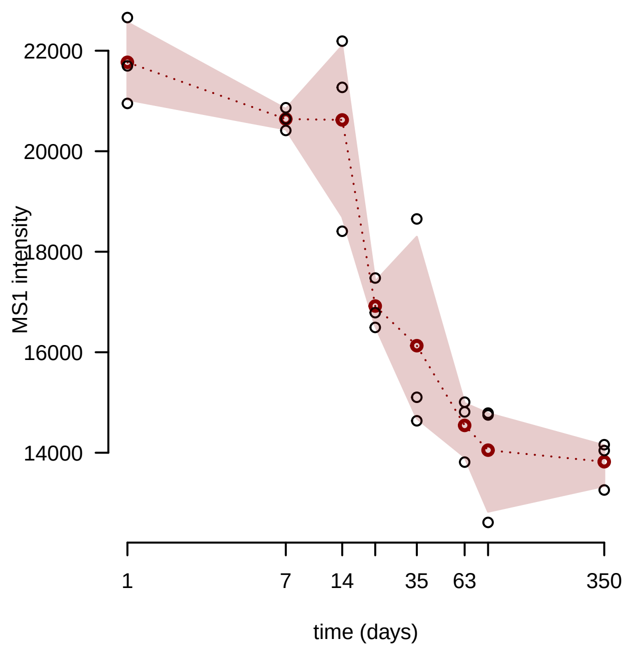

Q9Z130
GN=Hnrnpdl
heterogeneous nuclear ribonucleoprotein ...

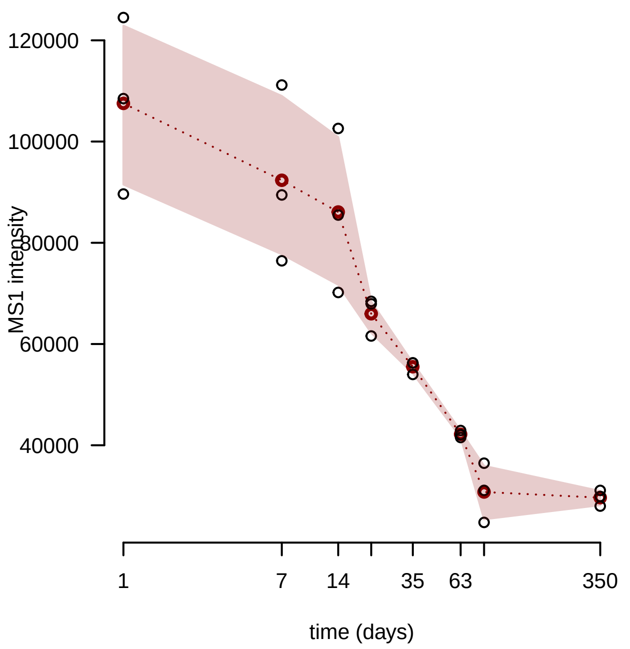

Q9Z138
GN=Ror2
receptor tyrosine kinase−like orphan rec ...

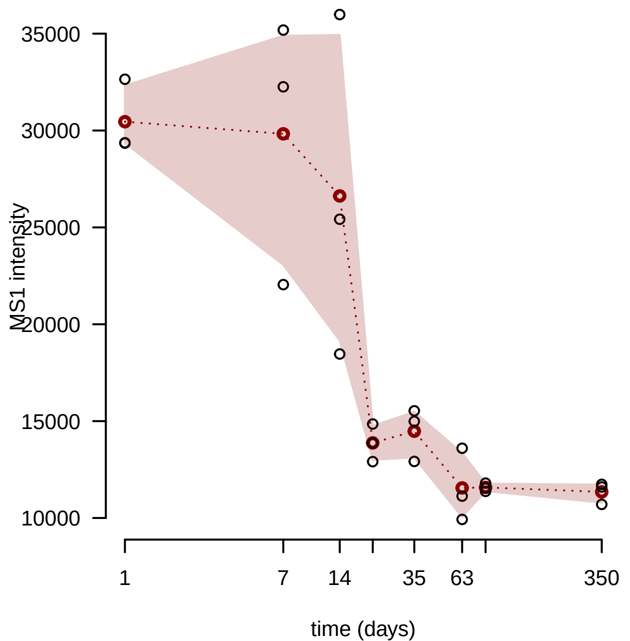

Q9Z148
GN=Ehmt2
euchromatic histone lysine N−methyltrans ...

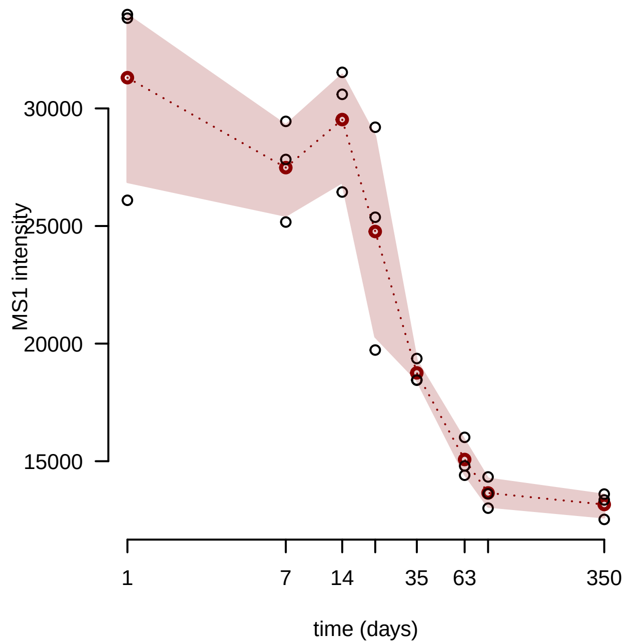

Q9Z160
GN=Cog1
component of oligomeric golgi complex 1

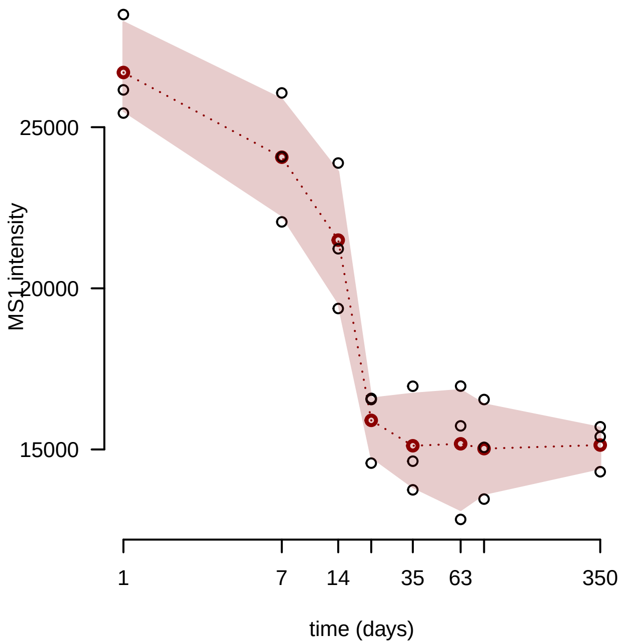

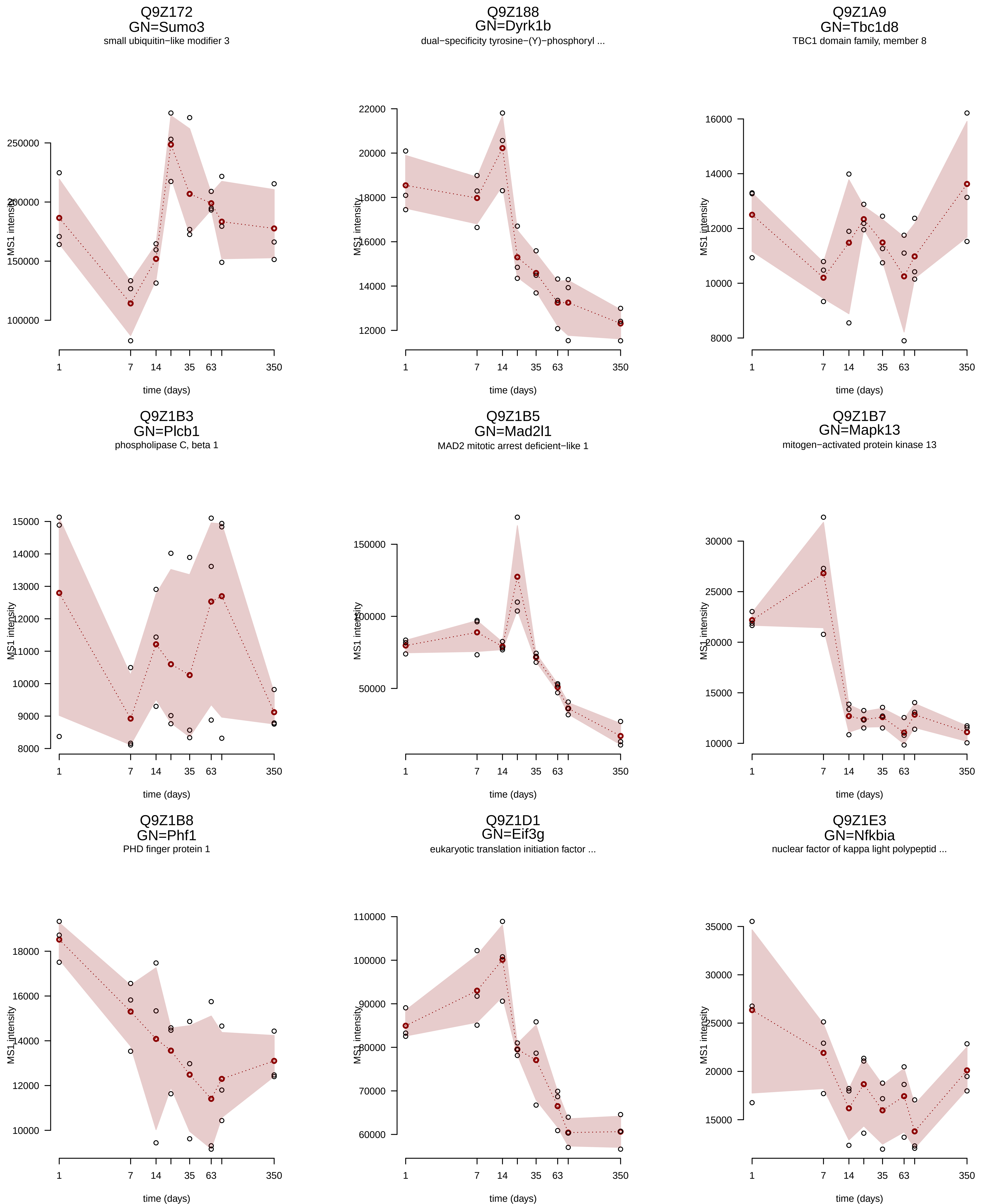
Q9Z172
GN=Sumo3
small ubiquitin−like modifier 3
Q9Z188
GN=Dyrk1b
dual−specificity tyrosine−(Y)−phosphoryl ...
Q9Z1A9
GN=Tbc1d8
TBC1 domain family, member 8
Q9Z1B3
GN=Plcb1
phospholipase C, beta 1
Q9Z1B5
GN=Mad2l1
MAD2 mitotic arrest deficient−like 1
Q9Z1B7
GN=Mapk13
mitogen−activated protein kinase 13
Q9Z1B8
GN=Phf1
PHD finger protein 1
Q9Z1D1
GN=Eif3g
eukaryotic translation initiation factor ...
Q9Z1E3
GN=Nfkbia
nuclear factor of kappa light polypeptid ...
MS1 intensity
time (days)
1
7
14
35
63
350

Q9Z1E4
GN=Gys1
glycogen synthase 1, muscle

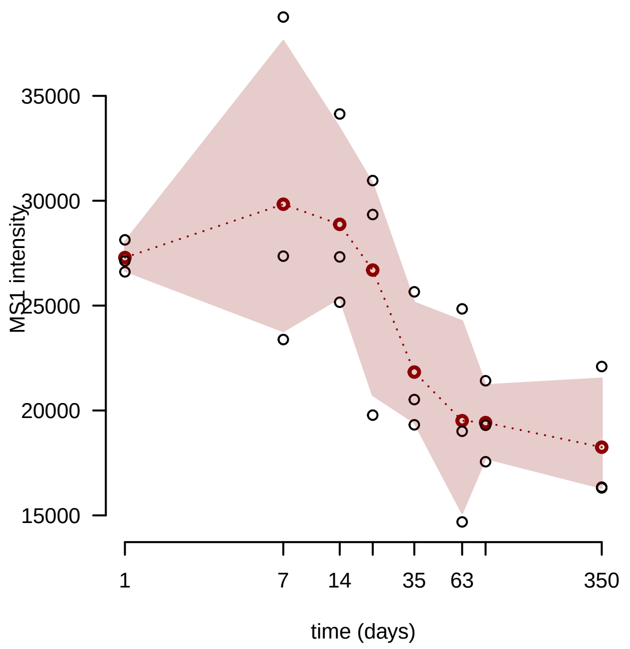

Q9Z1F9
GN=Uba2
ubiquitin−like modifier activating enzym ...

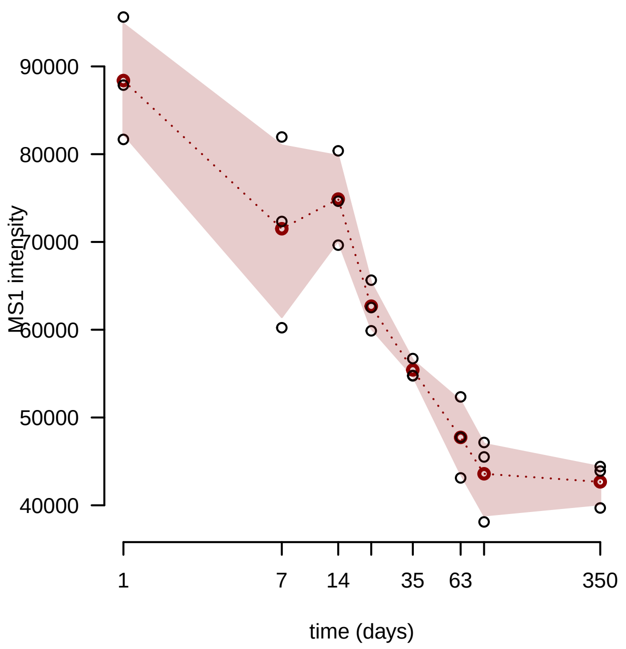

Q9Z1G3
GN=Atp6v1c1
ATPase, H+ transporting, lysosomal V1 su ...

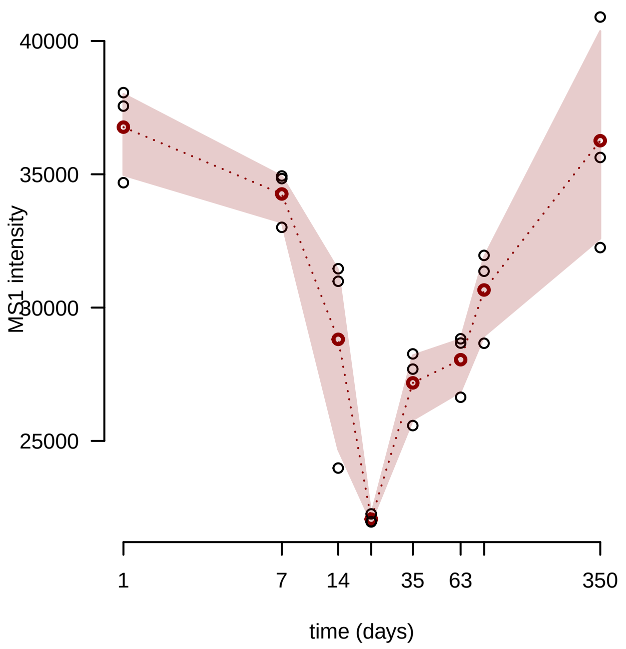

Q9Z1G4
GN=Atp6v0a1
ATPase, H+ transporting, lysosomal V0 su ...

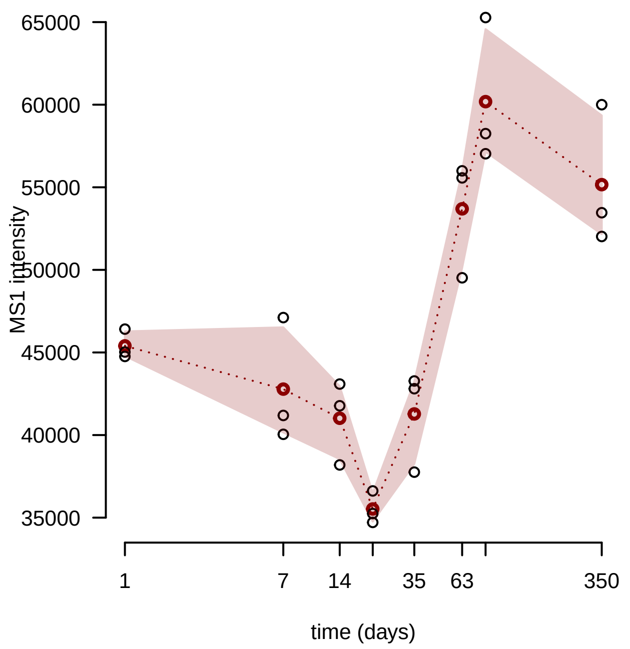

Q9Z1J1
GN=Tcf7l1
transcription factor 7 like 1 (T cell sp ...

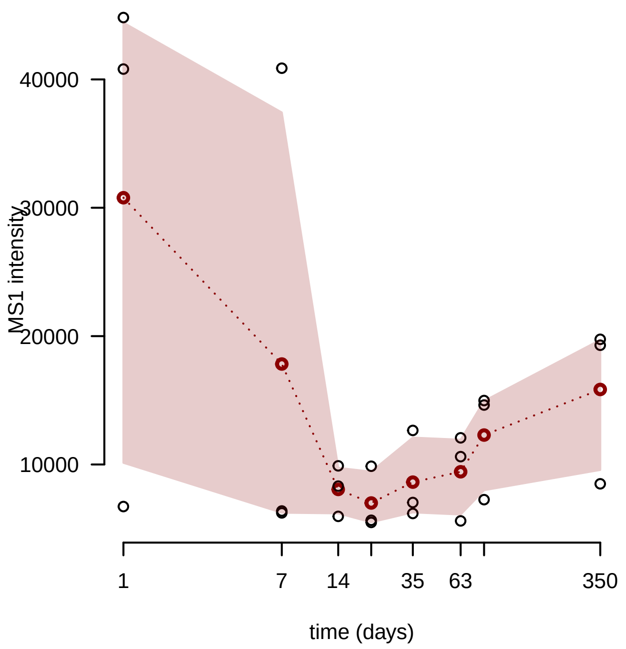

Q9Z1J2
GN=Nek4
NIMA (never in mitosis gene a)−related e ...

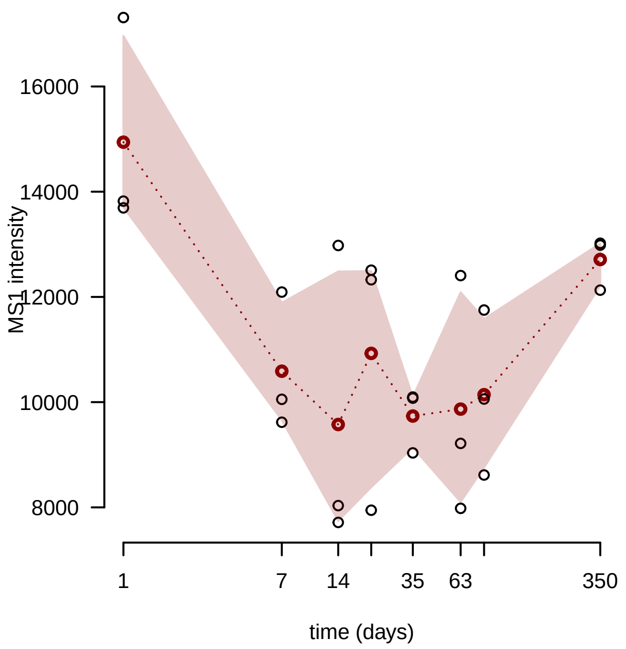

Q9Z1J3
GN=Nfs1
nitrogen fixation gene 1 (S. cerevisiae)

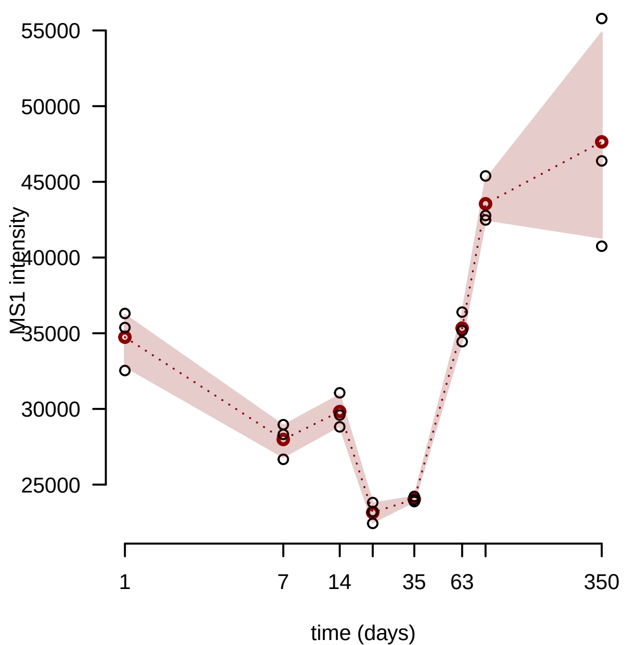

Q9Z1K5
GN=Arih1
ariadne RBR E3 ubiquitin protein ligase ...

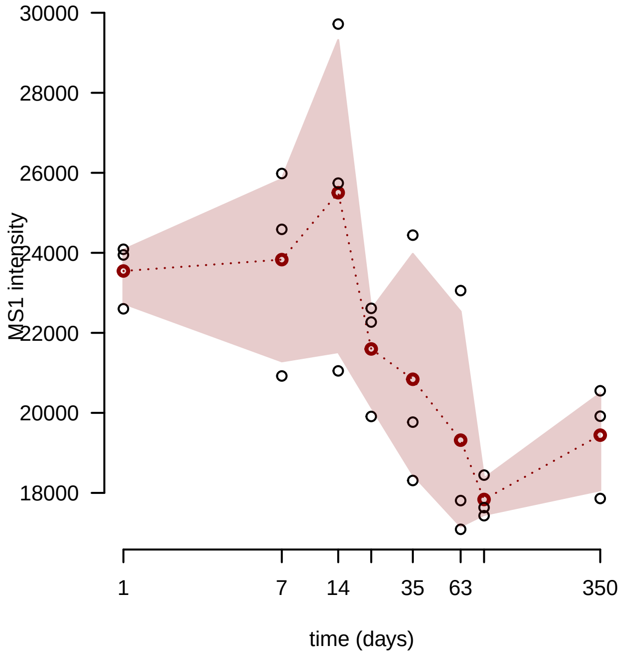

Q9Z1K6
GN=Arih2
ariadne RBR E3 ubiquitin protein ligase ...

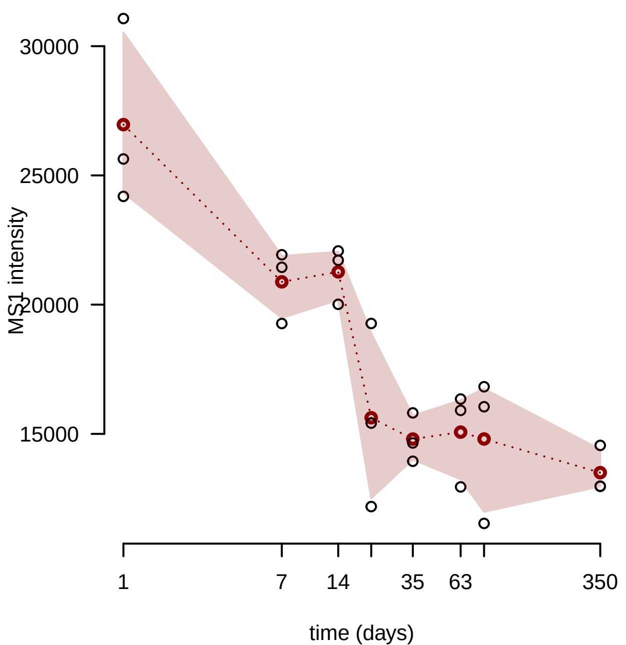

Q9Z1K8
GN=Slc7a7
solute carrier family 7 (cationic amino ...

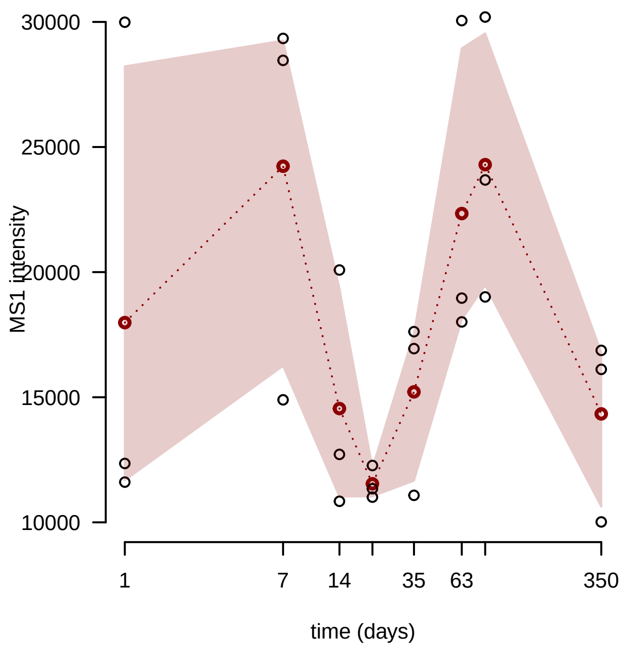

Q9Z1L3
GN=Dedd
death effector domain−containing

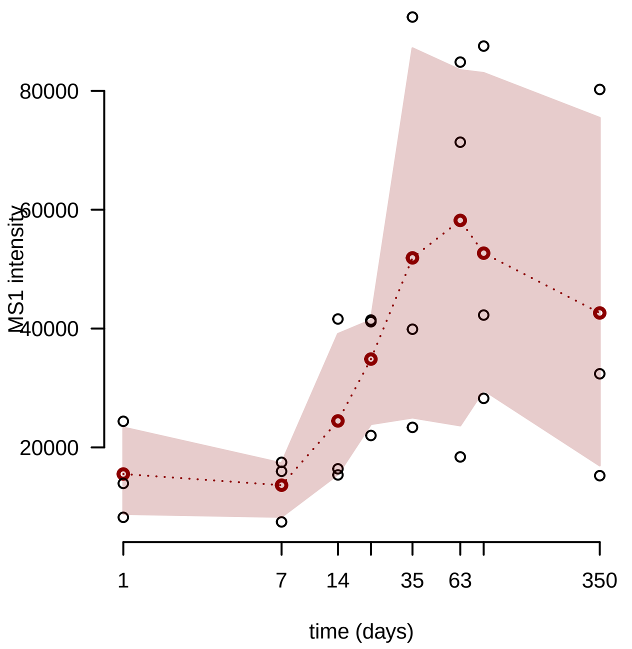

Q9Z1M4
GN=Rps6kb2
ribosomal protein S6 kinase, polypeptide ...

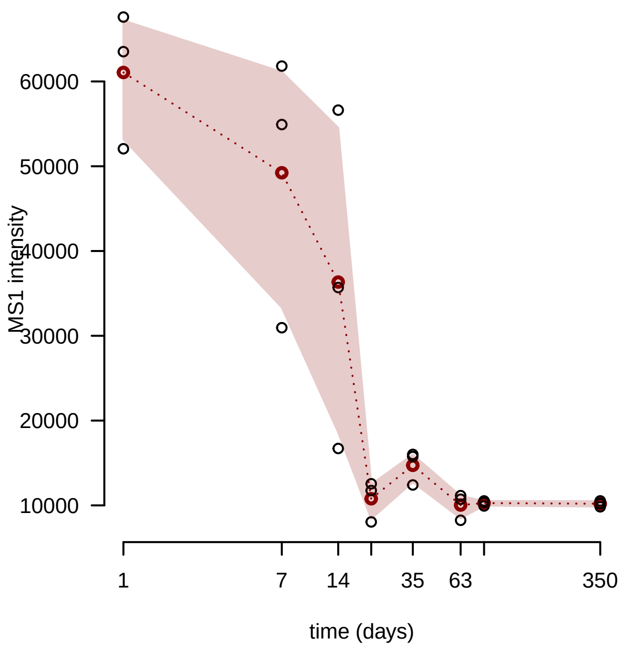

Q9Z1M7
GN=Large1
LARGE xylosyl− and glucuronyltransferase ...

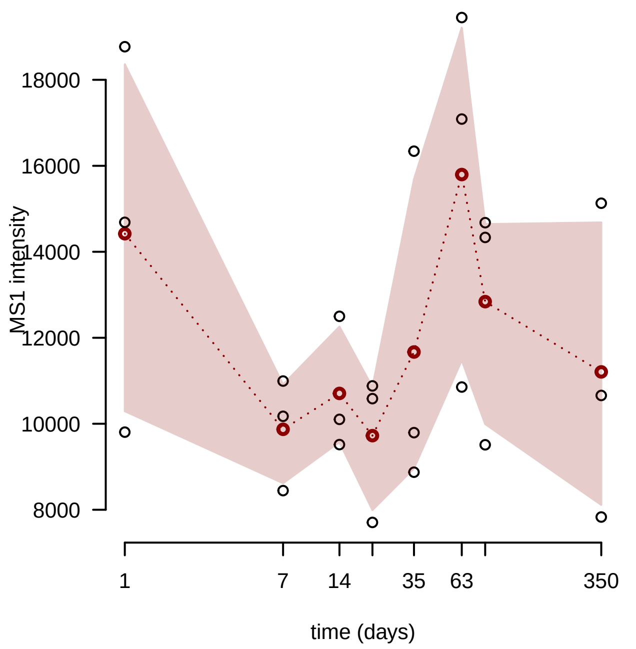

Q9Z1M8
GN=Ik
IK cytokine

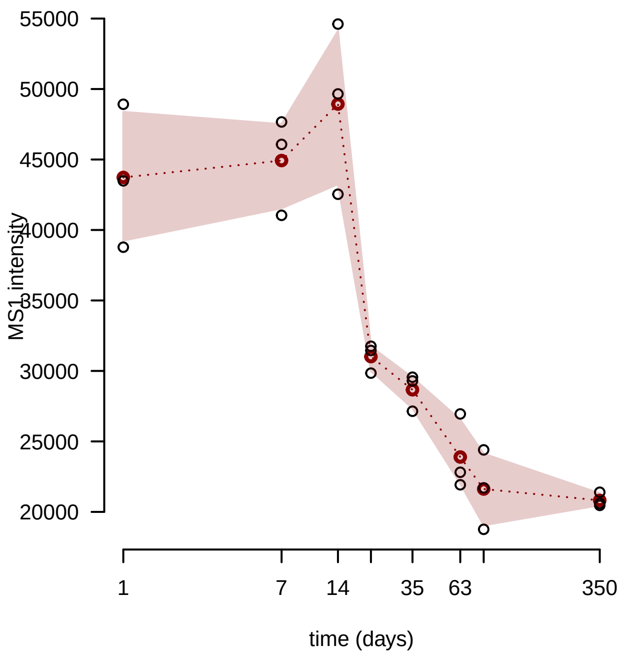

Q9Z1N2
GN=Orc1
origin recognition complex, subunit 1

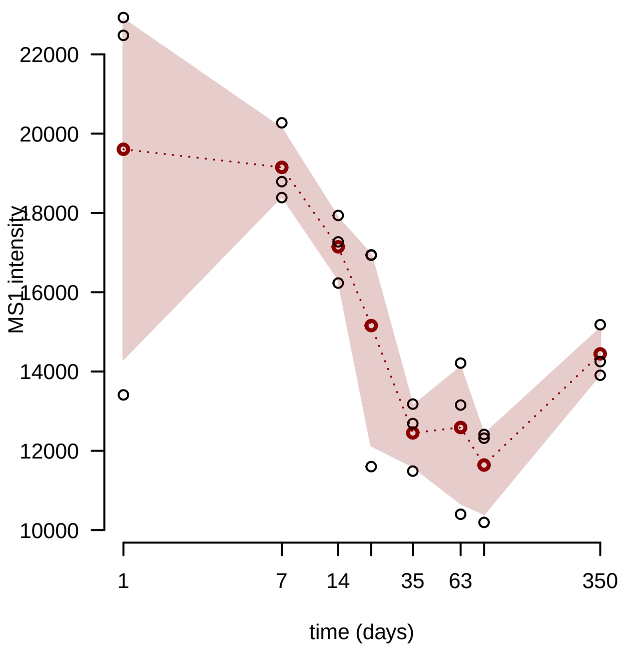

Q9Z1N5
GN=Ddx39b
DEAD box helicase 39b

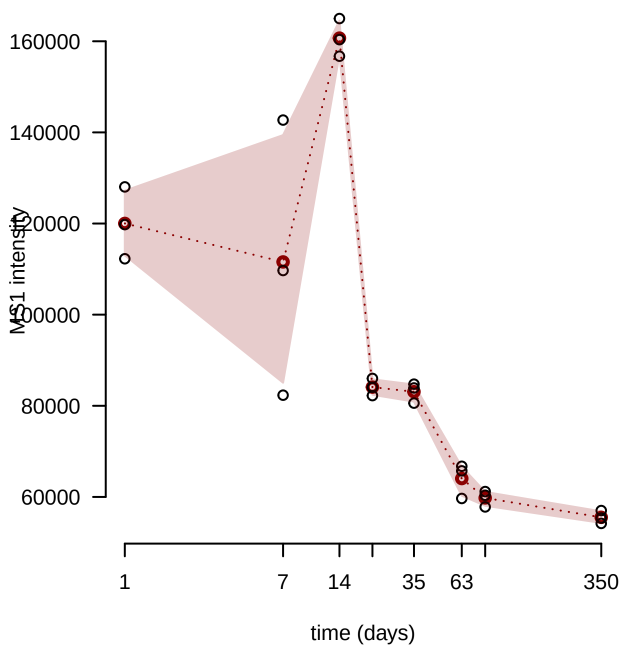

Q9Z1N6
GN=Sfrp4
secreted frizzled−related protein 4

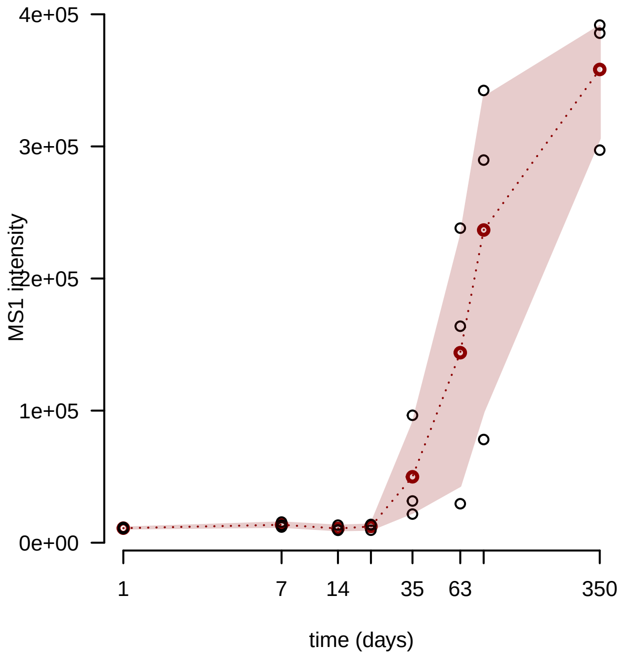

Q9Z1P6
GN=Ndufa7
NADH:ubiquinone oxidoreductase subunit A ...

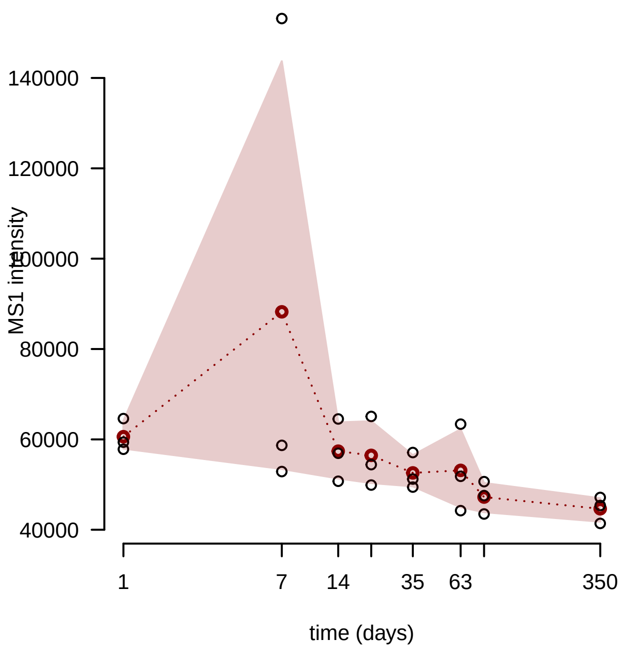

Q9Z1P7
GN=Kank3
KN motif and ankyrin repeat domains 3

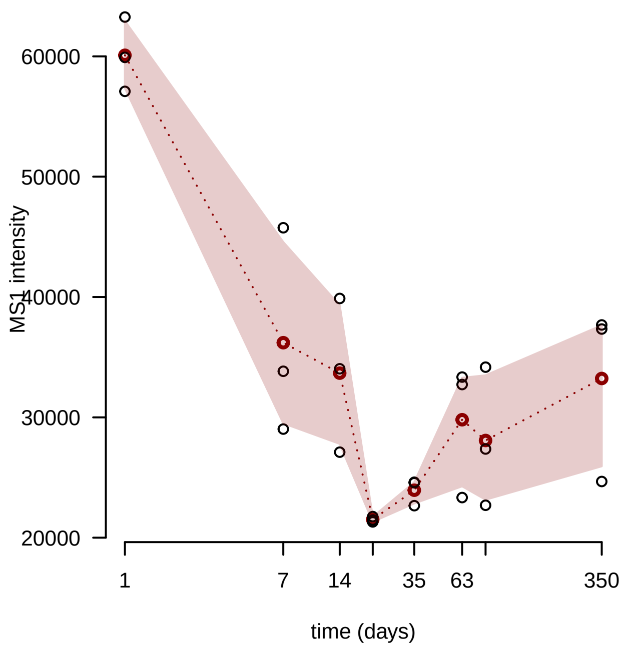

Q9Z1Q2
GN=Abhd16a
abhydrolase domain containing 16A

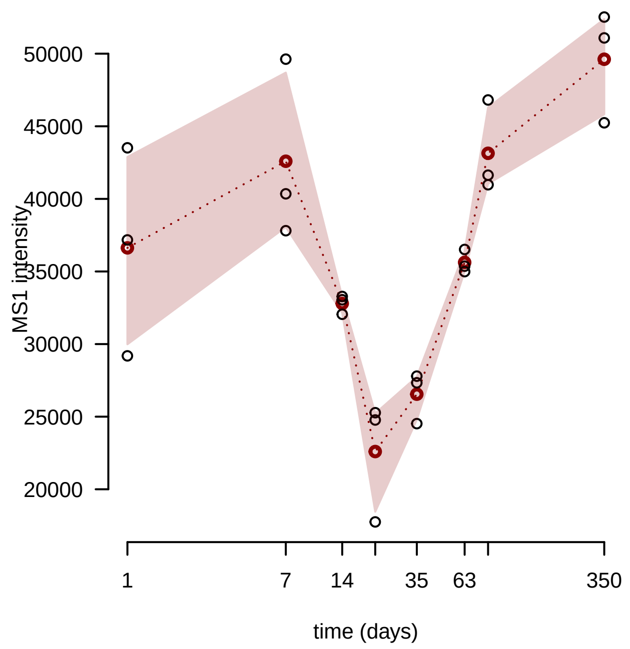

Q9Z1Q5
GN=Clic1
chloride intracellular channel 1

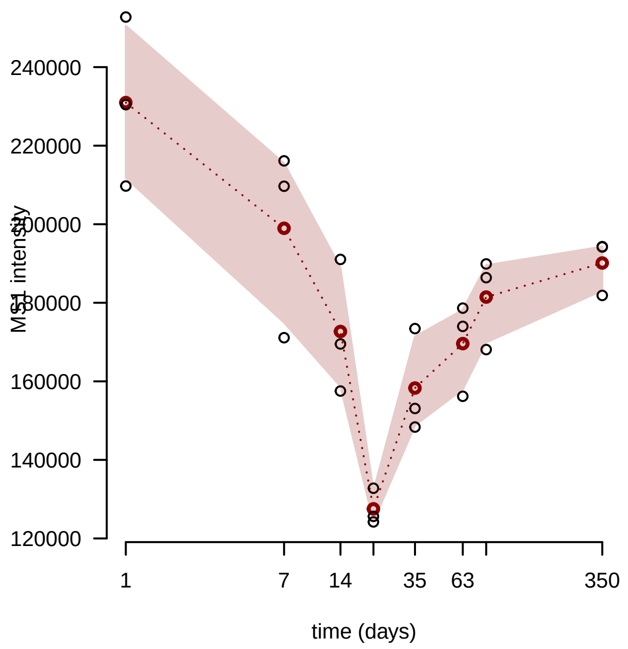

Q9Z1Q9
GN=Vars
valyl−tRNA synthetase

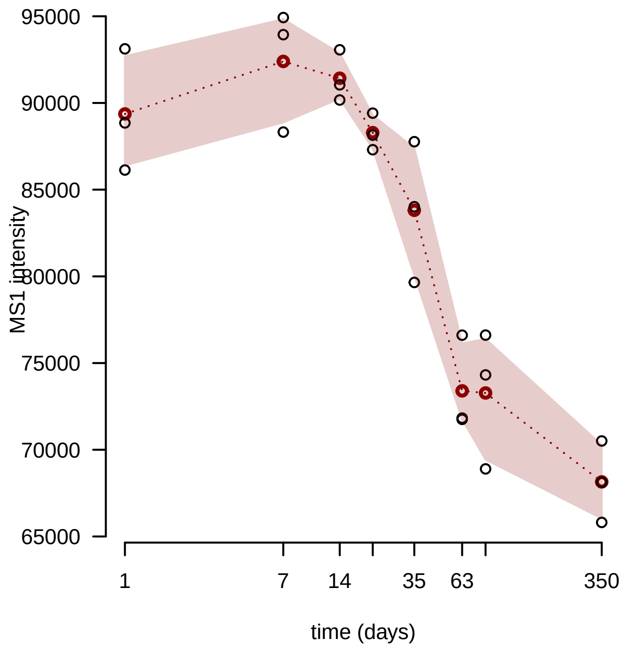

Q9Z1R2
GN=Bag6
BCL2−associated athanogene 6

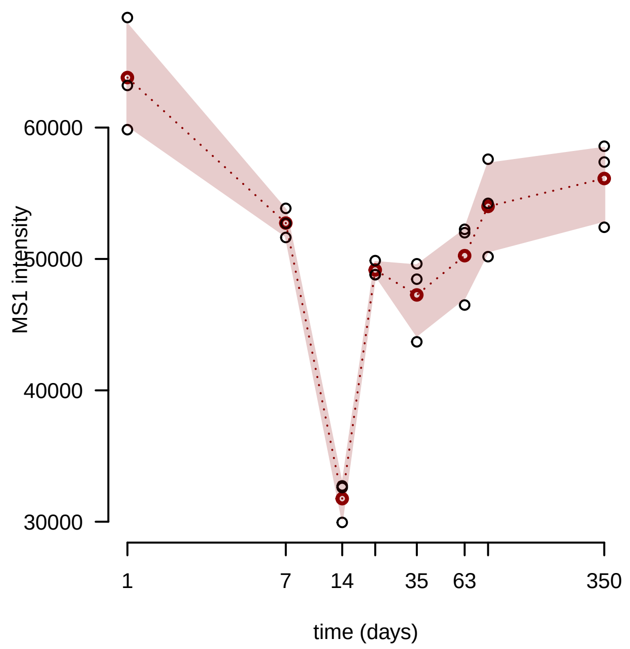

Q9Z1R3
GN=Apom
apolipoprotein M

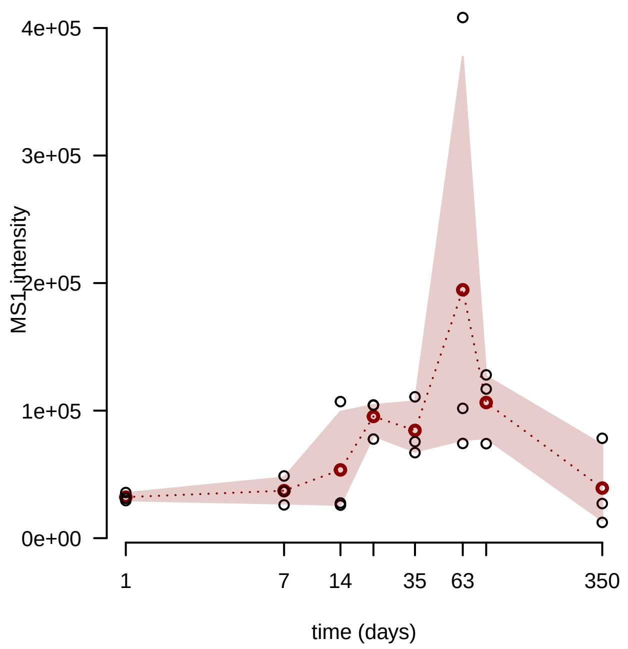

Q9Z1R4
GN=D17h6s53e

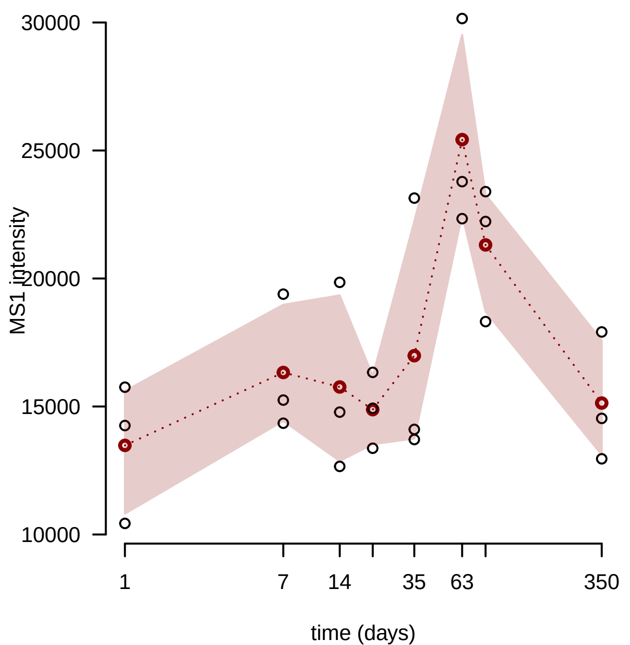

Q9Z1S0
GN=Bub1b
BUB1B, mitotic checkpoint serine/threoni ...

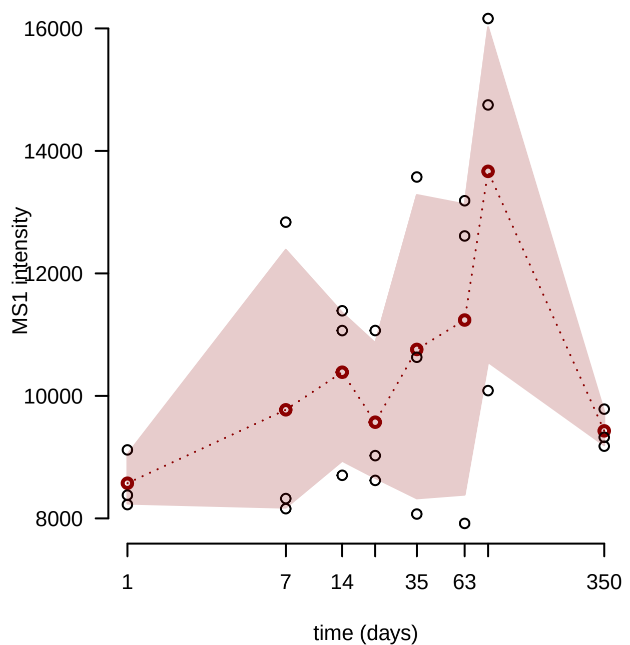

Q9Z1S5
GN=Sept3

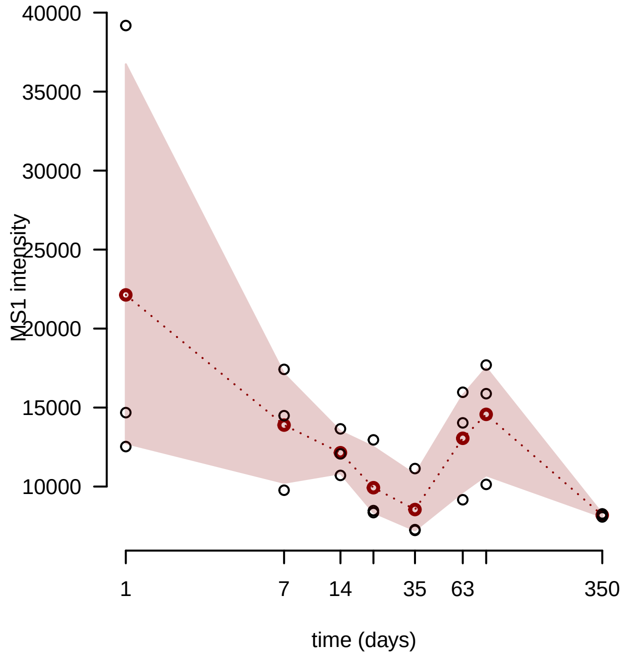

Q9Z1S8
GN=Gab2
growth factor receptor bound protein 2−a ...

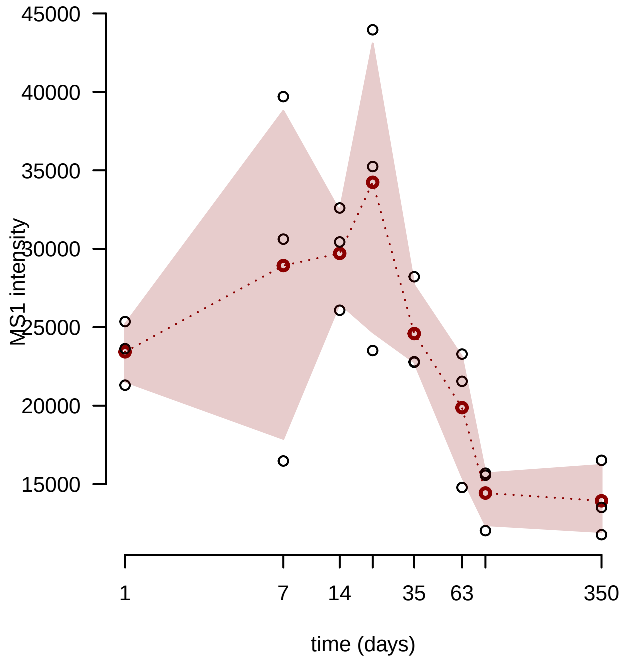

Q9Z1T1
GN=Ap3b1
adaptor−related protein complex 3, beta ...

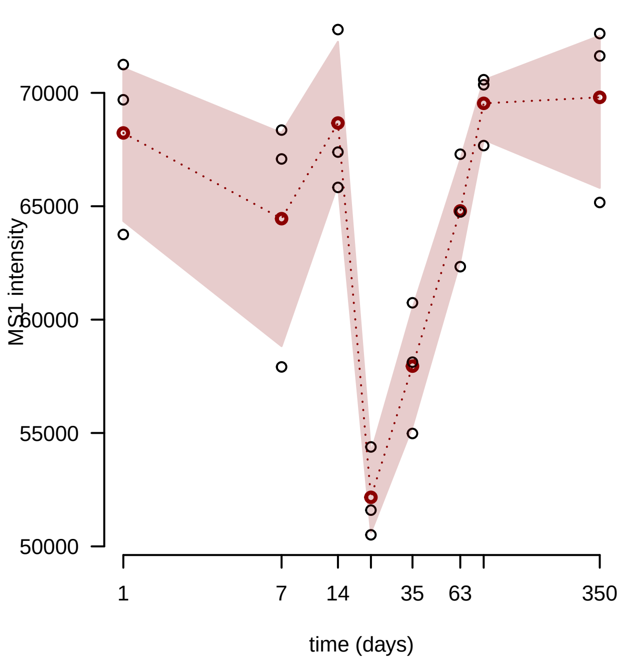

Q9Z1T2
GN=Thbs4
thrombospondin 4

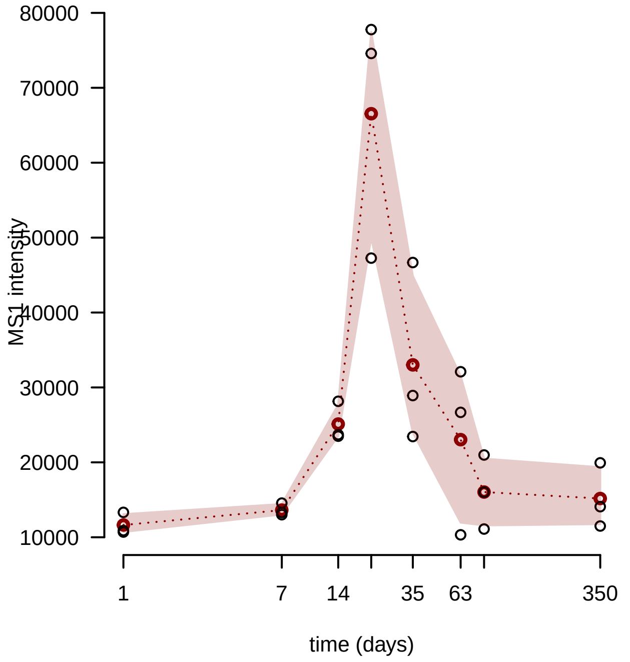

Q9Z1T6
GN=Pikfyve
phosphoinositide kinase, FYVE type zinc ...

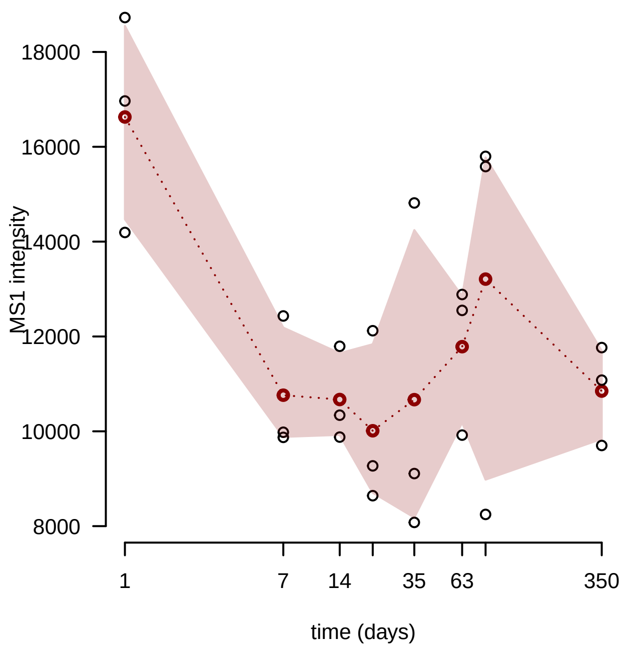

Q9Z1W9
GN=Stk39
serine/threonine kinase 39

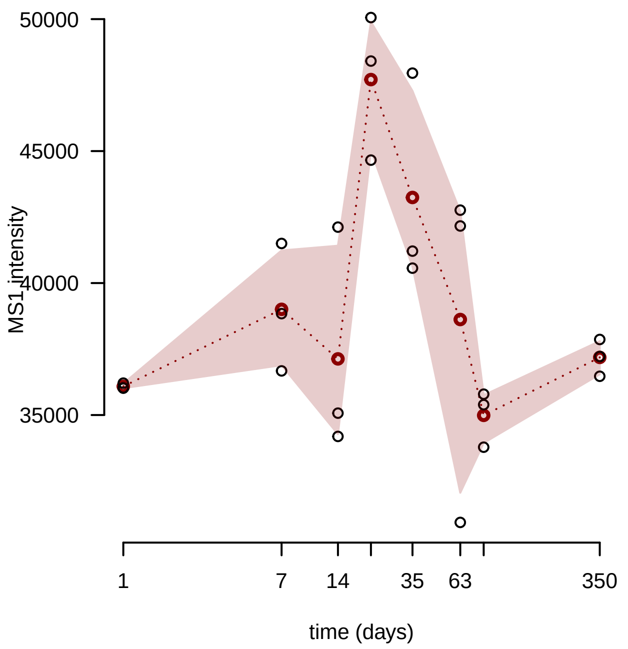

Q9Z1X2
GN=Ptdss2
phosphatidylserine synthase 2

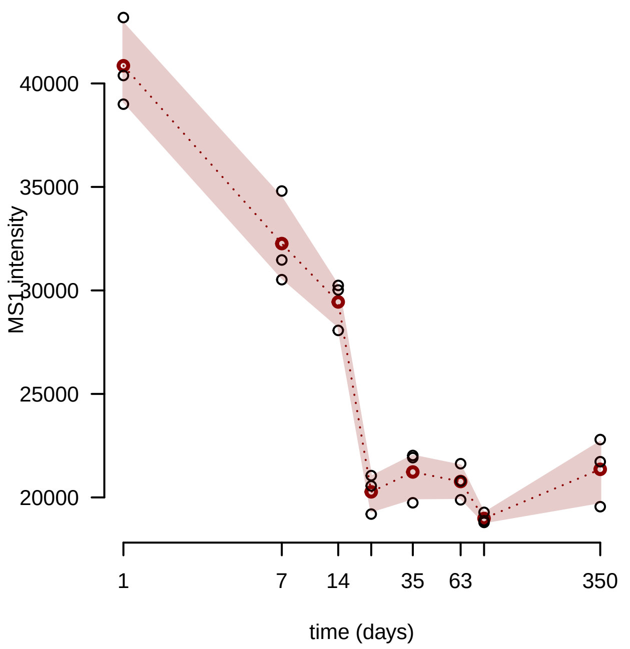

Q9Z1X4
GN=Ilf3
interleukin enhancer binding factor 3

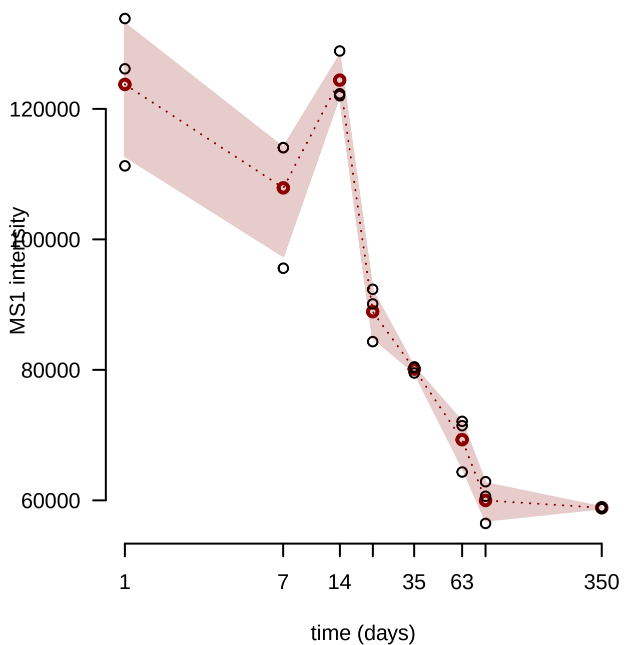

Q9Z1Y4
GN=Trip6
thyroid hormone receptor interactor 6

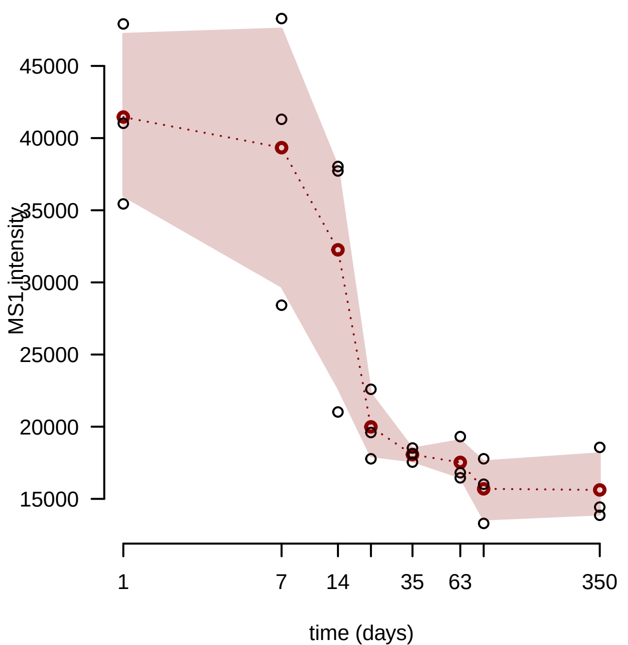

Q9Z1Z0
GN=Uso1
USO1 vesicle docking factor

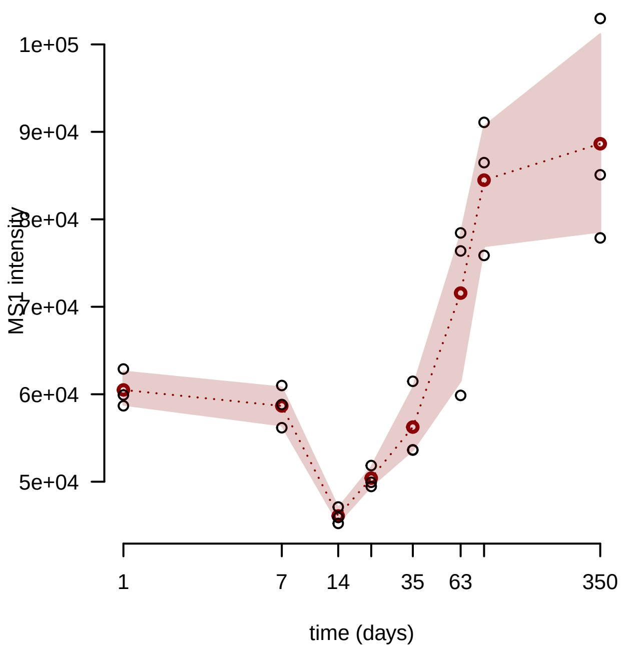

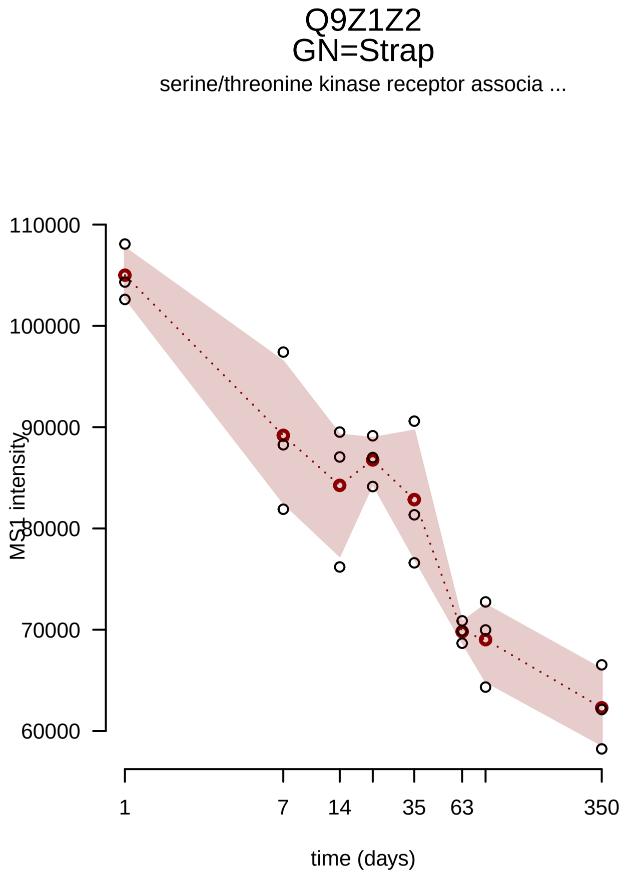
Q9Z1Z2
GN=Strap
serine/threonine kinase receptor associa ...
MS1 intensity
110000
100000
90000
80000
70000
60000
1
7
14
35
63
350
time (days)

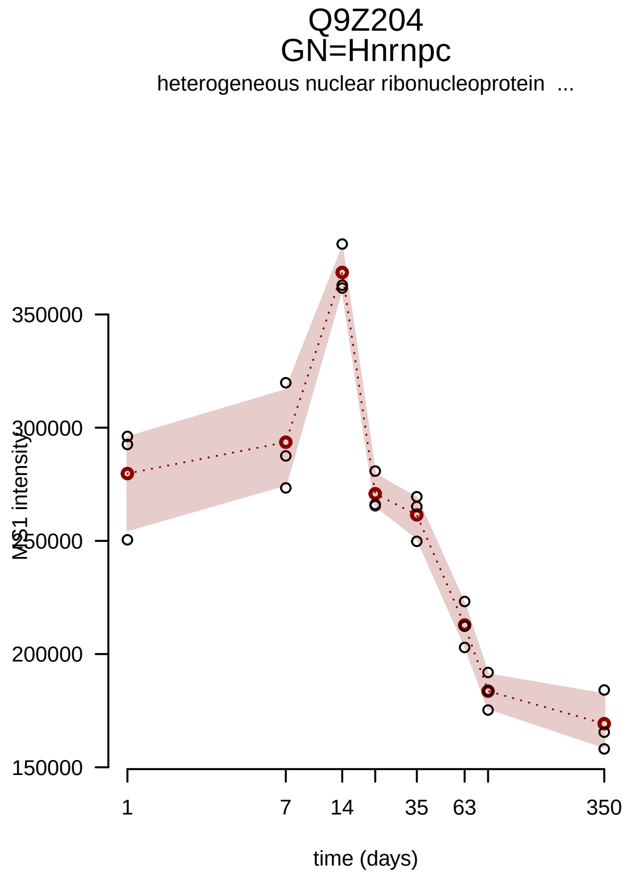
Q9Z204
GN=Hnrnpc
heterogeneous nuclear ribonucleoprotein ...
MS1 intensity
350000
300000
250000
200000
150000
1
7
14
35
63
350
time (days)

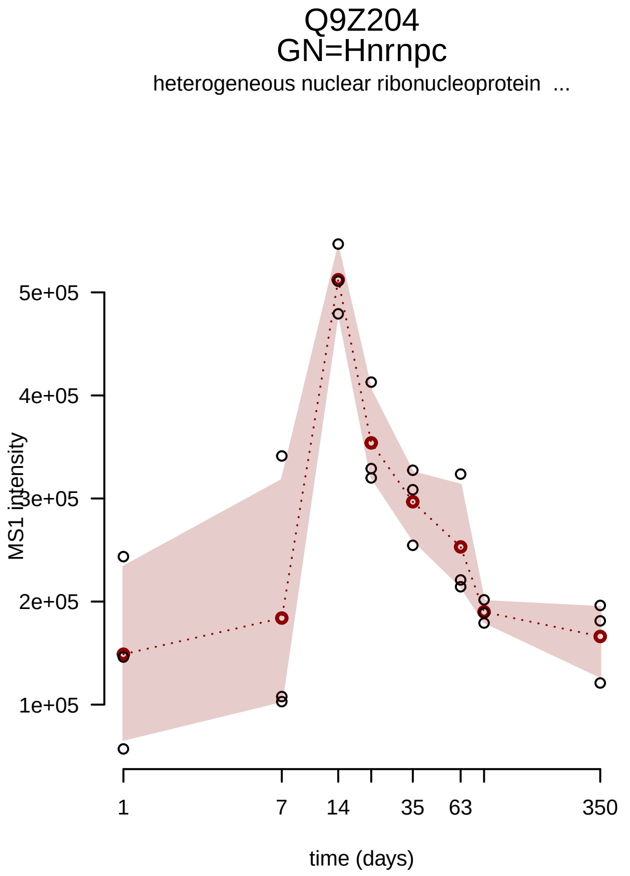
Q9Z204
GN=Hnrnpc
heterogeneous nuclear ribonucleoprotein ...
MS1 intensity
5e+05
4e+05
3e+05
2e+05
1e+05
1
7
14
35
63
350
time (days)

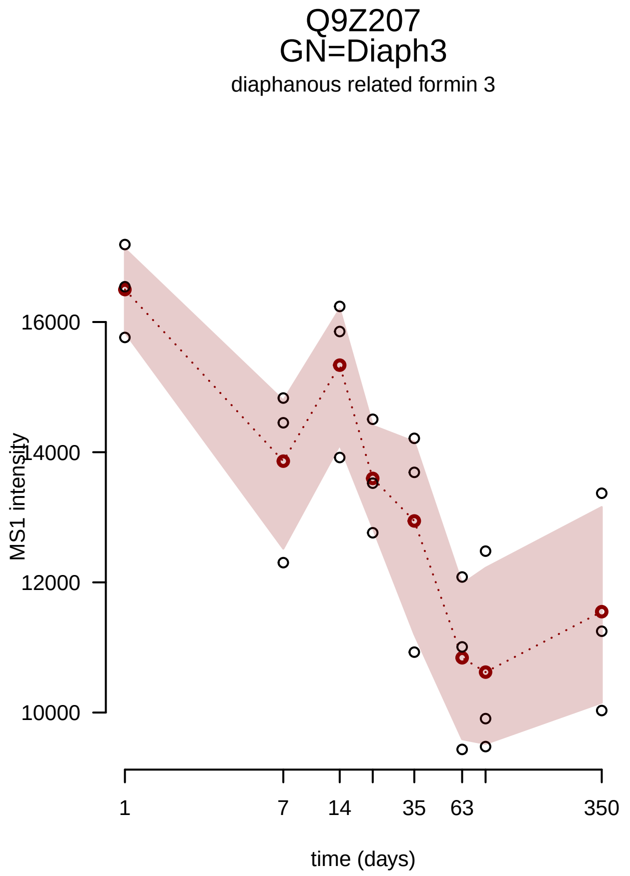
Q9Z207
GN=Diaph3
diaphanous related formin 3
MS1 intensity
16000
14000
12000
10000
1
7
14
35
63
350
time (days)

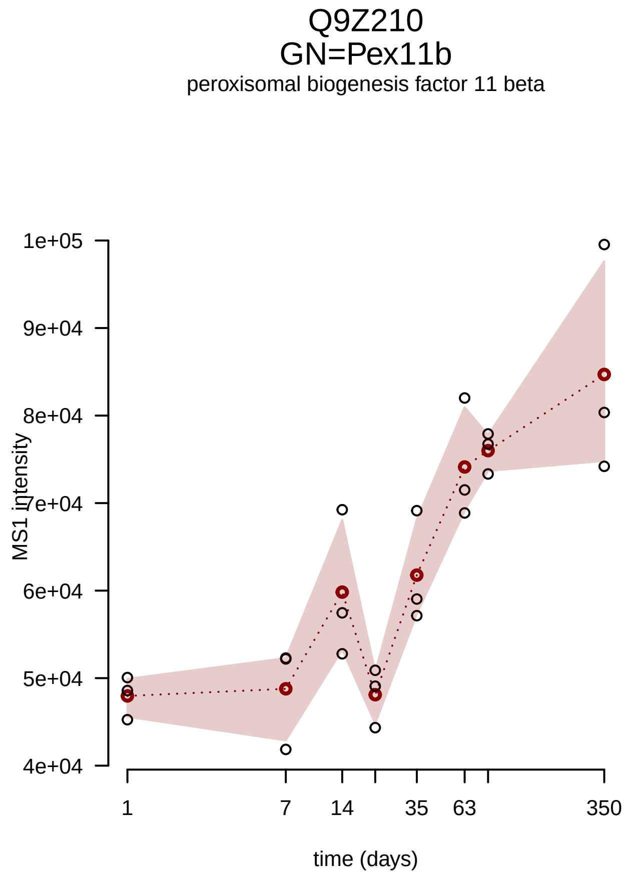
Q9Z210
GN=Pex11b
peroxisomal biogenesis factor 11 beta
MS1 intensity
1e+05
9e+04
8e+04
7e+04
6e+04
5e+04
4e+04
1
7
14
35
63
350
time (days)

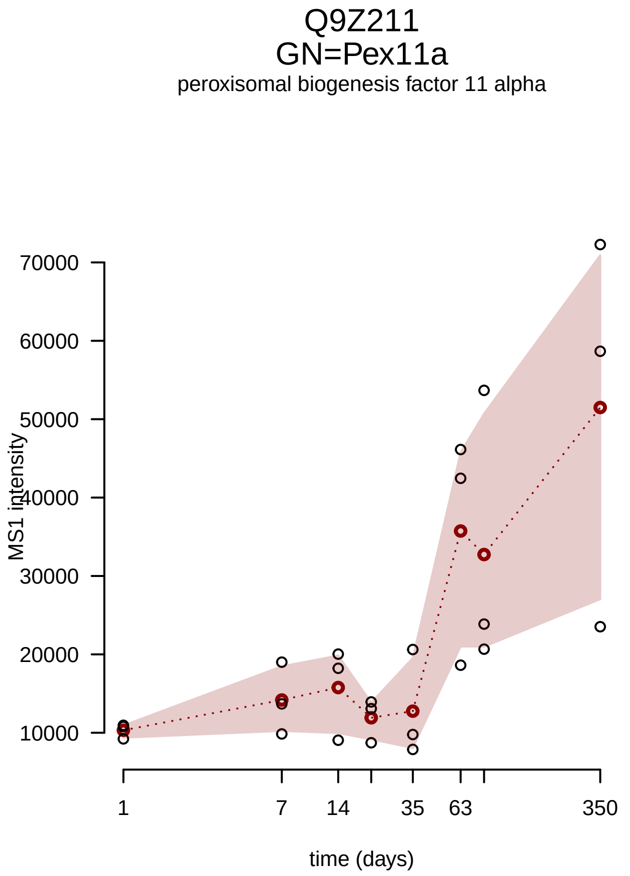
Q9Z211
GN=Pex11a
peroxisomal biogenesis factor 11 alpha
MS1 intensity
70000
60000
50000
40000
30000
20000
10000
1
7
14
35
63
350
time (days)

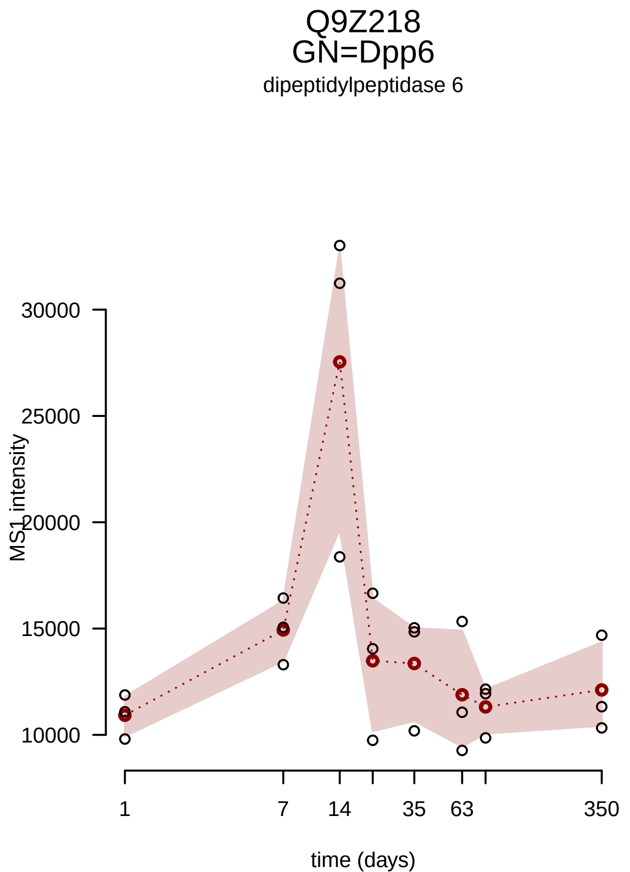
Q9Z218
GN=Dpp6
dipeptidylpeptidase 6
MS1 intensity
30000
25000
20000
15000
10000
1
7
14
35
63
350
time (days)

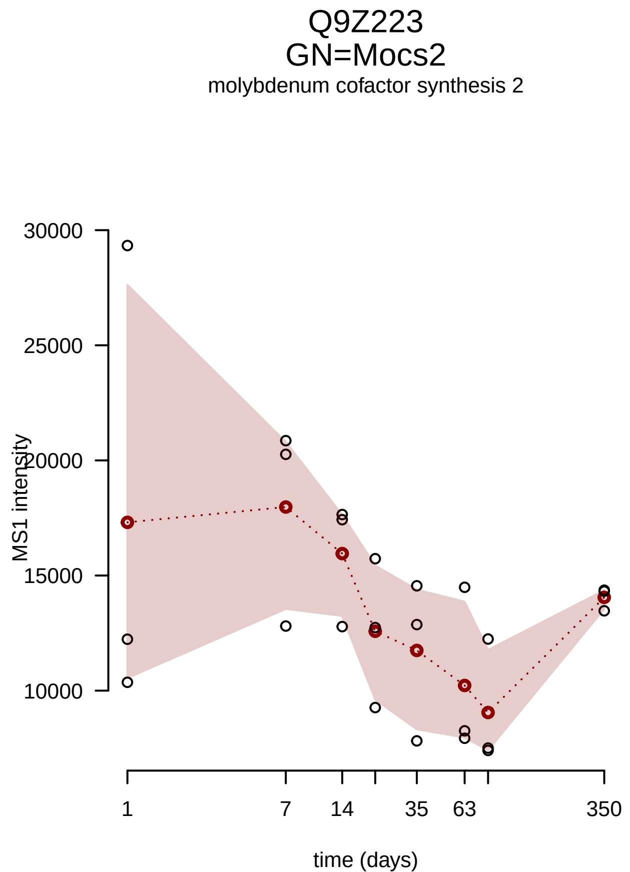
Q9Z223
GN=Mocs2
molybdenum cofactor synthesis 2
MS1 intensity
30000
25000
20000
15000
10000
1
7
14
35
63
350
time (days)

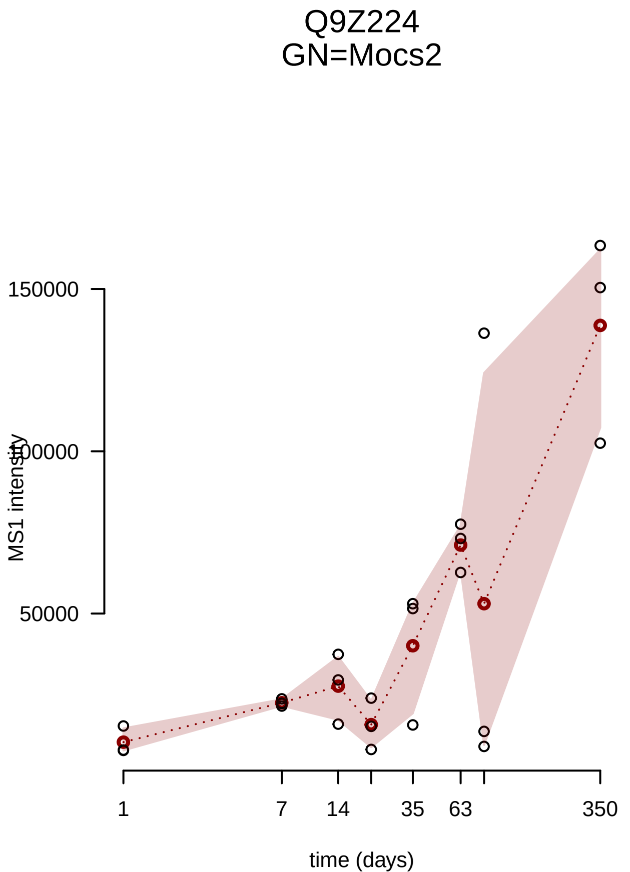
Q9Z224
GN=Mocs2
MS1 intensity
150000
100000
50000
1
7
14
35
63
350
time (days)

Q9Z239
GN=Fxyd1
FXYD domain−containing ion transport reg ...

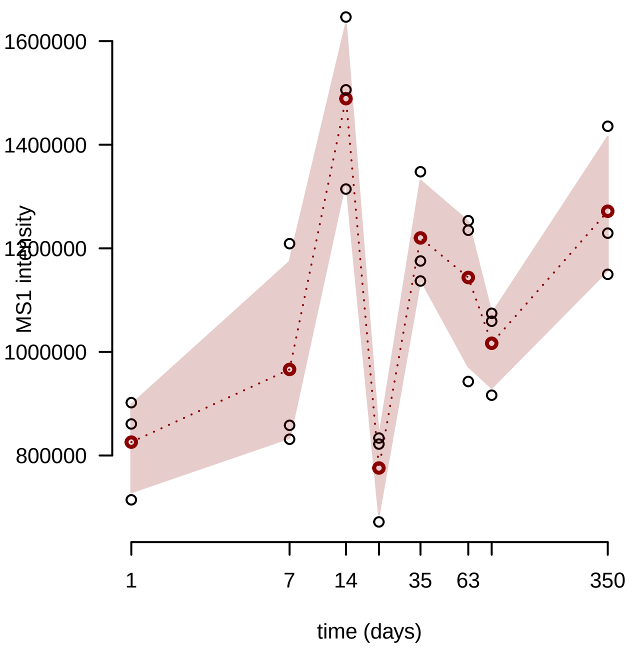

Q9Z247
GN=Fkbp9
FK506 binding protein 9

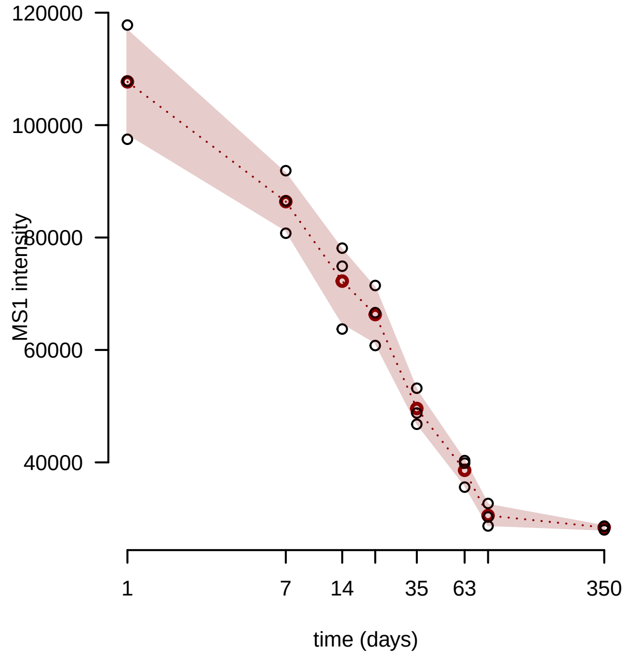

Q9Z248
GN=Aebp2
AE binding protein 2

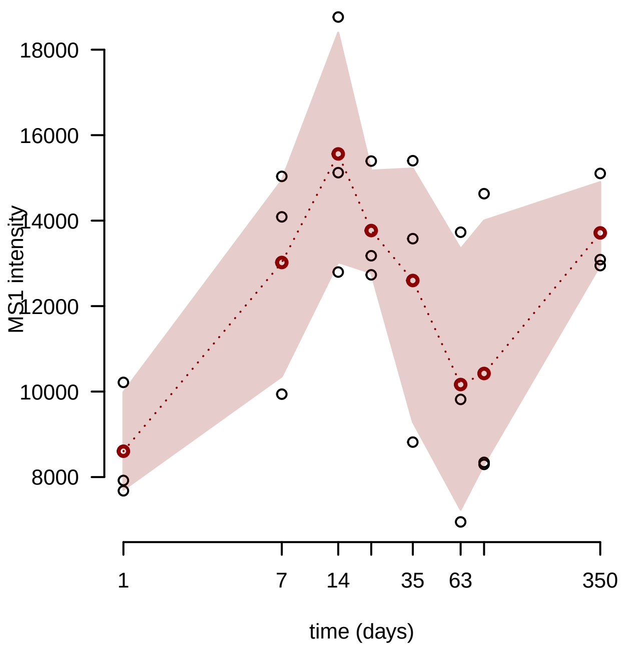

Q9Z255
GN=Ube2a
ubiquitin−conjugating enzyme E2A

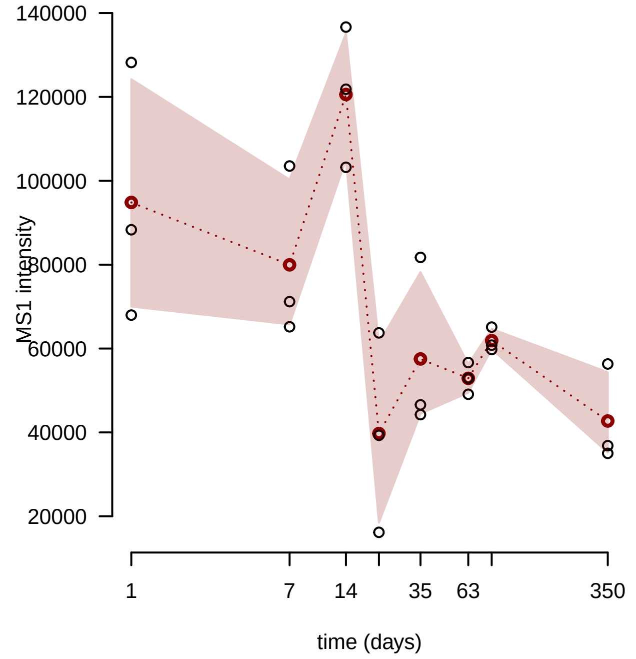

Q9Z266
GN=Snapin
SNAP−associated protein

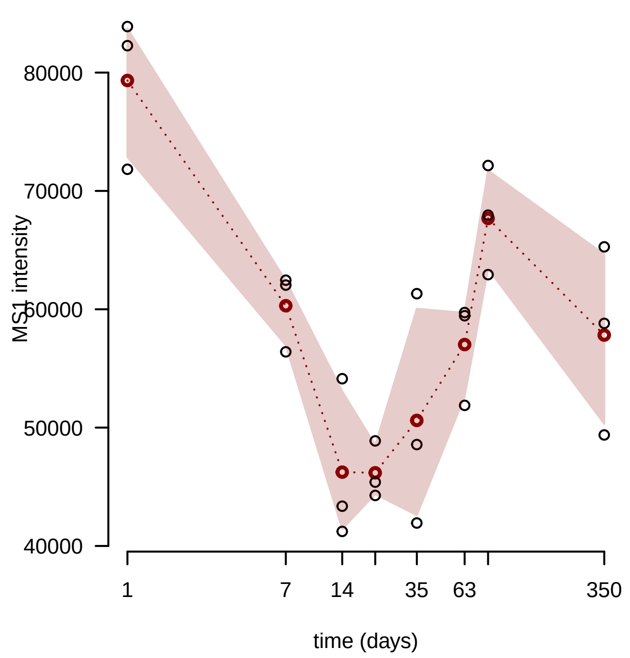

Q9Z268
GN=Rasal1
RAS protein activator like 1 (GAP1 like)

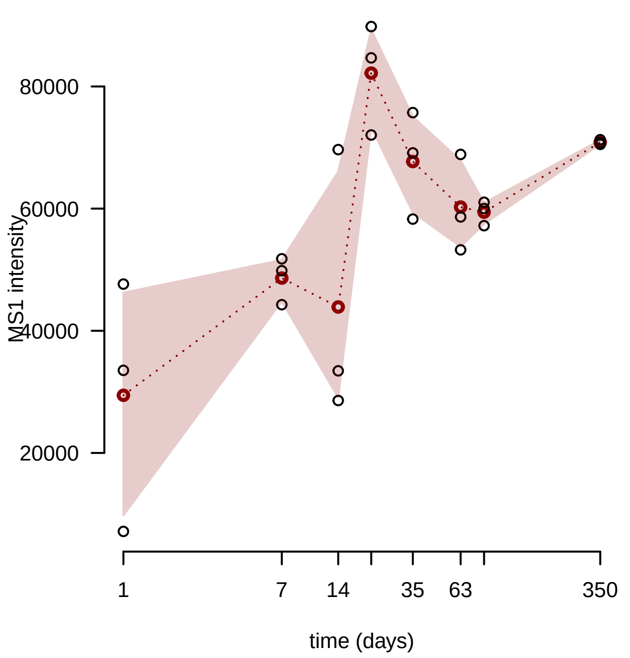

Q9Z277
GN=Baz1b
bromodomain adjacent to zinc finger doma ...

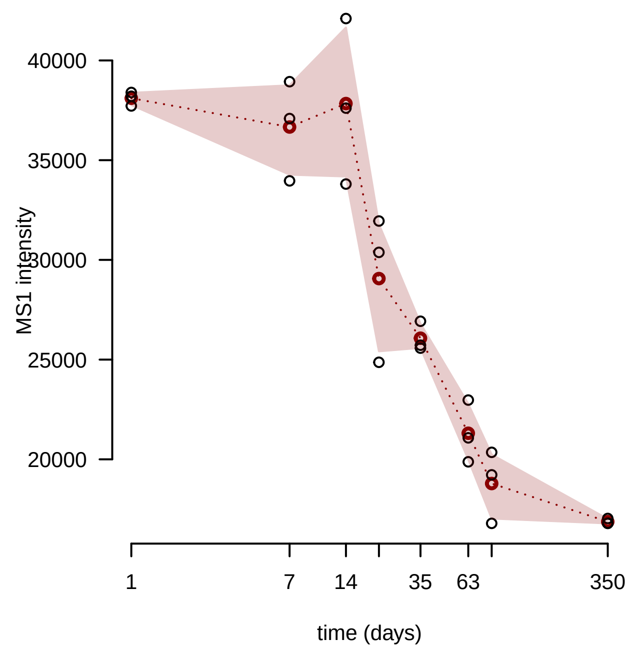

Q9Z280
GN=Pld1
phospholipase D1

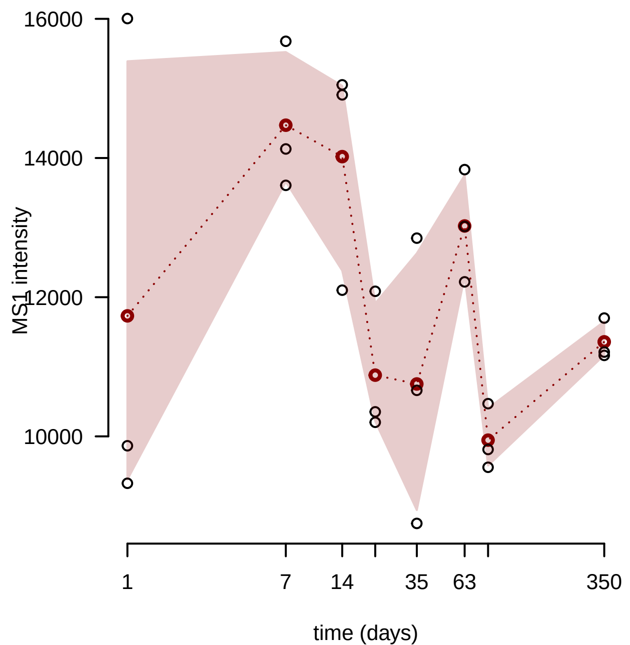

Q9Z2A0
GN=Pdpk1
3−phosphoinositide dependent protein kin ...

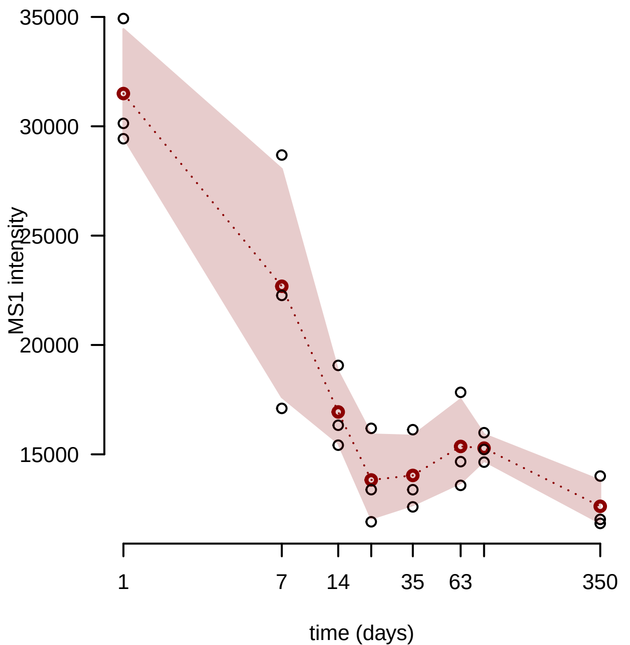

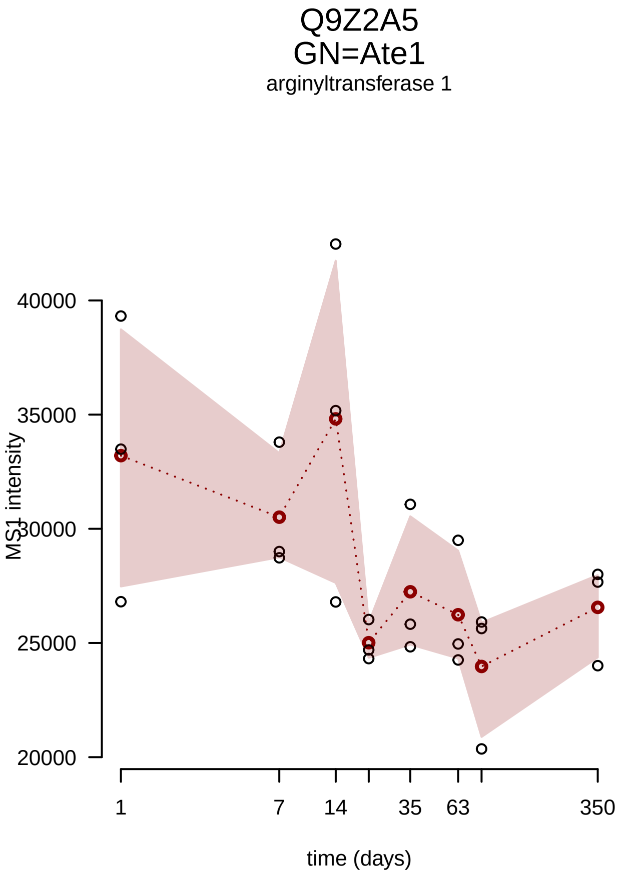
Q9Z2A5
GN=Ate1
arginyltransferase 1
MS1 intensity
20000
25000
30000
35000
40000
1
7
14
35
63
350
time (days)

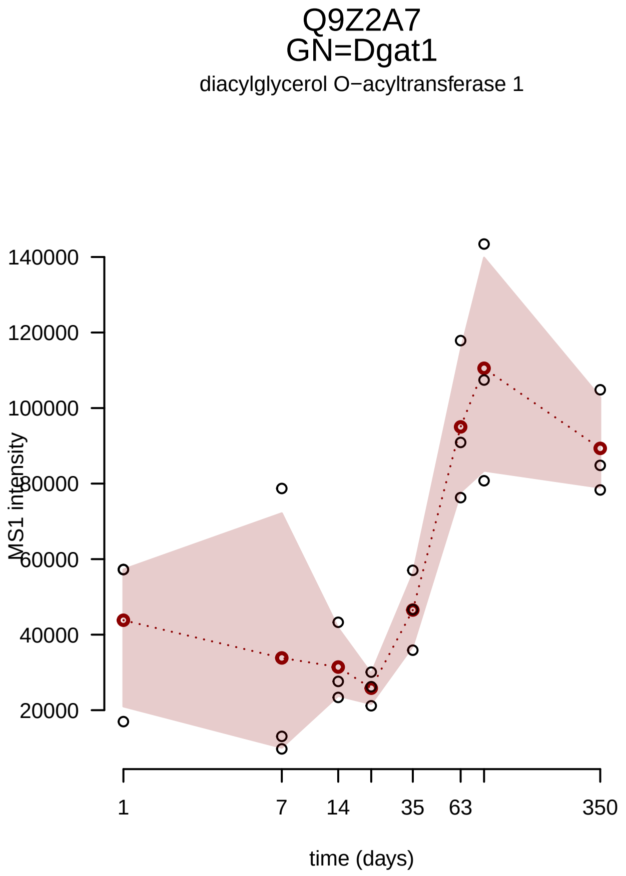
Q9Z2A7
GN=Dgat1
diacylglycerol O−acyltransferase 1
MS1 intensity
20000
40000
60000
80000
100000
120000
140000
1
7
14
35
63
350
time (days)

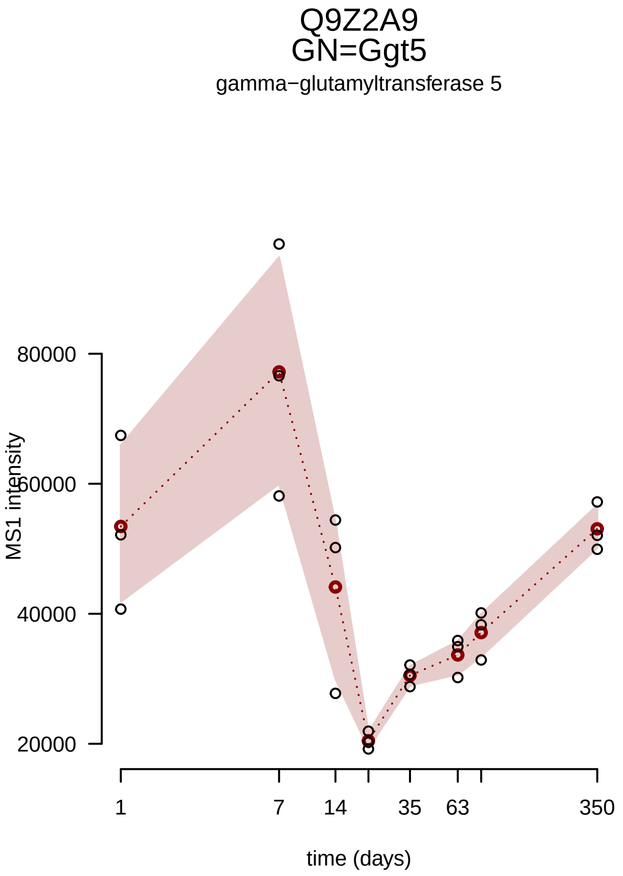
Q9Z2A9
GN=Ggt5
gamma−glutamyltransferase 5
MS1 intensity
20000
40000
60000
80000
1
7
14
35
63
350
time (days)

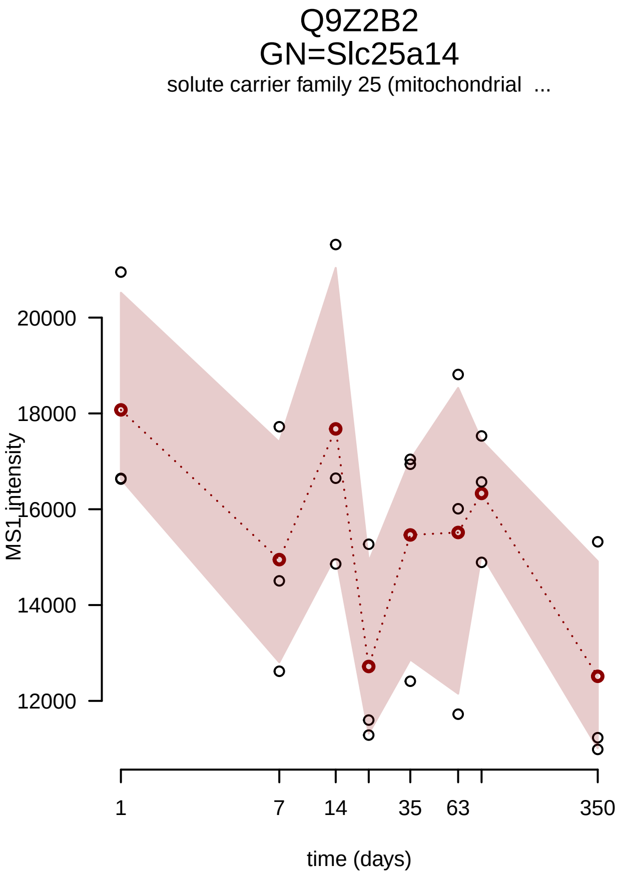
Q9Z2B2
GN=Slc25a14
solute carrier family 25 (mitochondrial ...
MS1 intensity
12000
14000
16000
18000
20000
1
7
14
35
63
350
time (days)

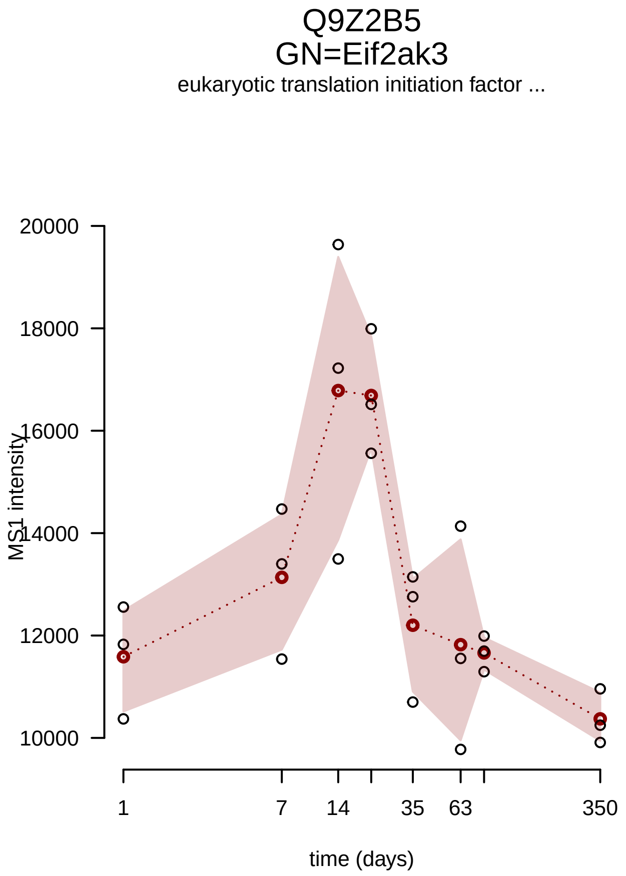
Q9Z2B5
GN=Eif2ak3
eukaryotic translation initiation factor ...
MS1 intensity
10000
12000
14000
16000
18000
20000
1
7
14
35
63
350
time (days)

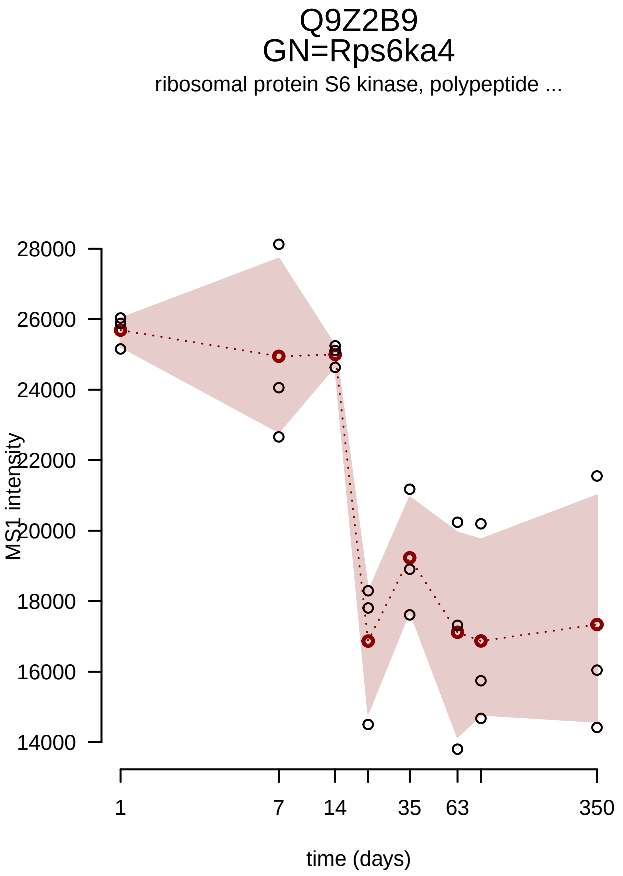
Q9Z2B9
GN=Rps6ka4
ribosomal protein S6 kinase, polypeptide ...
MS1 intensity
14000
16000
18000
20000
22000
24000
26000
28000
1
7
14
35
63
350
time (days)

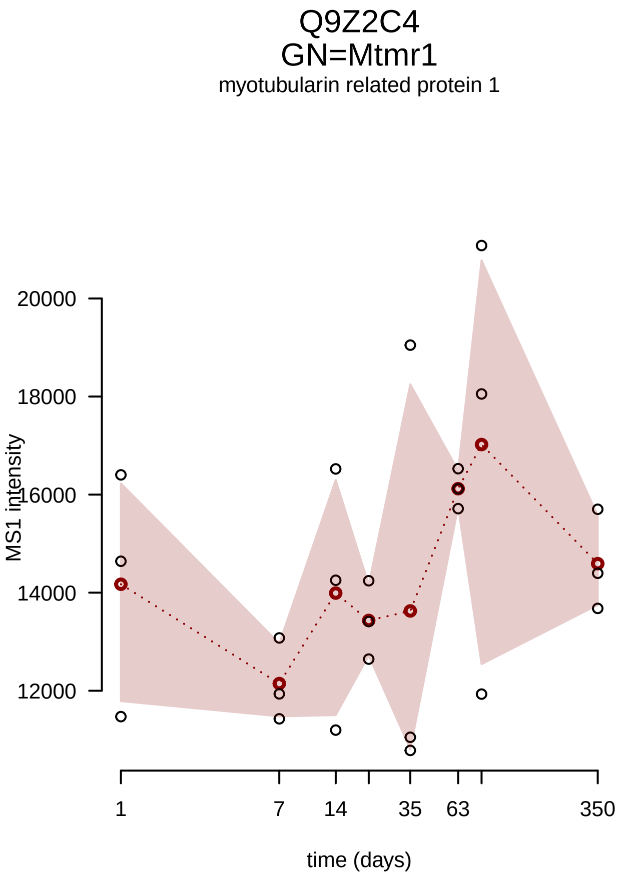
Q9Z2C4
GN=Mtmr1
myotubularin related protein 1
MS1 intensity
12000
14000
16000
18000
20000
1
7
14
35
63
350
time (days)

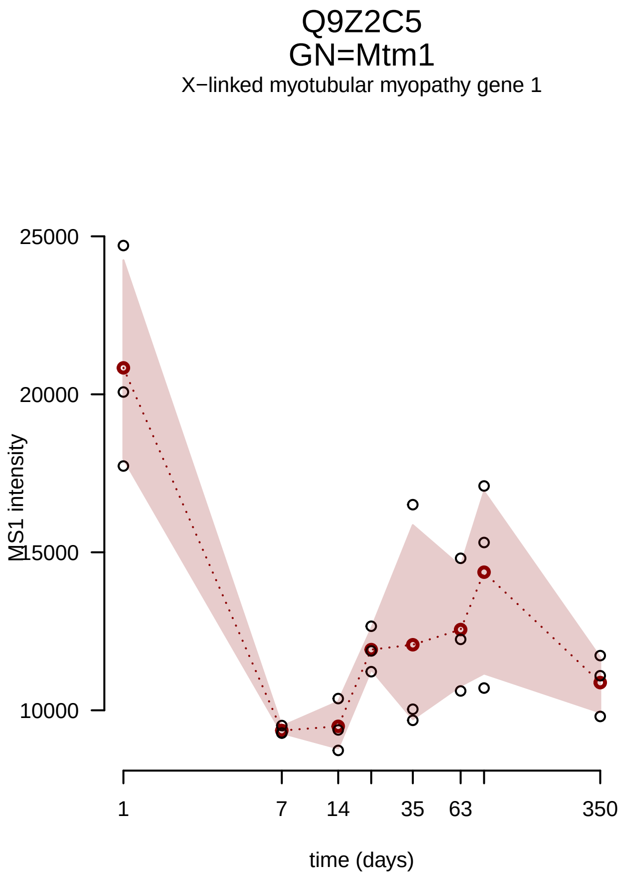
Q9Z2C5
GN=Mtm1
X−linked myotubular myopathy gene 1
MS1 intensity
10000
15000
20000
25000
1
7
14
35
63
350
time (days)

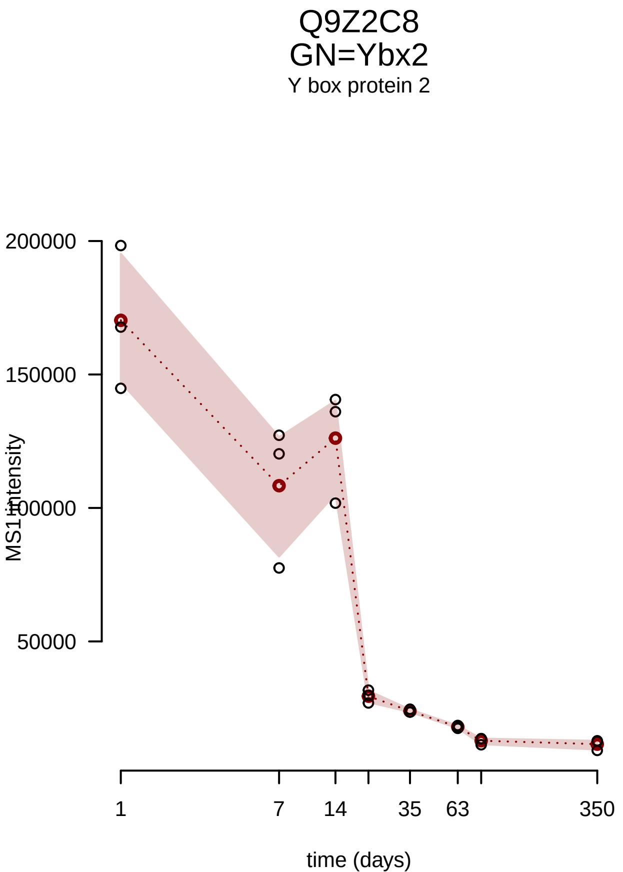
Q9Z2C8
GN=Ybx2
Y box protein 2
MS1 intensity
50000
100000
150000
200000
1
7
14
35
63
350
time (days)

Q9Z2C9
GN=Mtmr7
myotubularin related protein 7

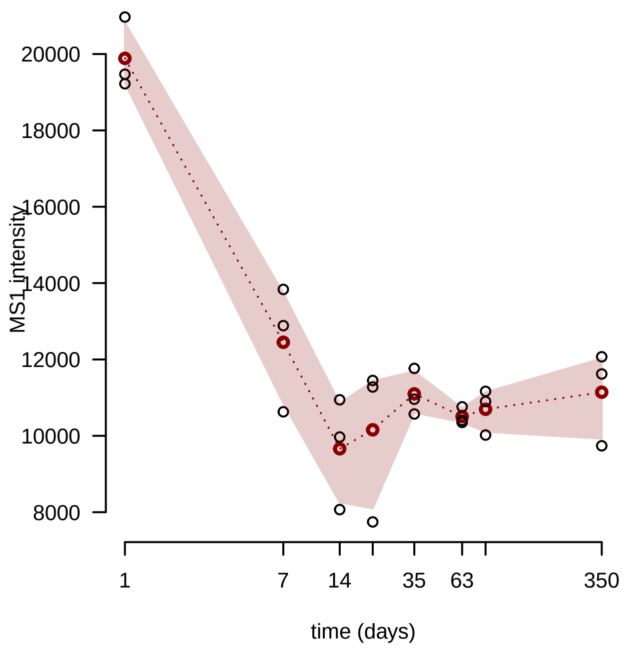

Q9Z2D0
GN=Mtmr9
myotubularin related protein 9

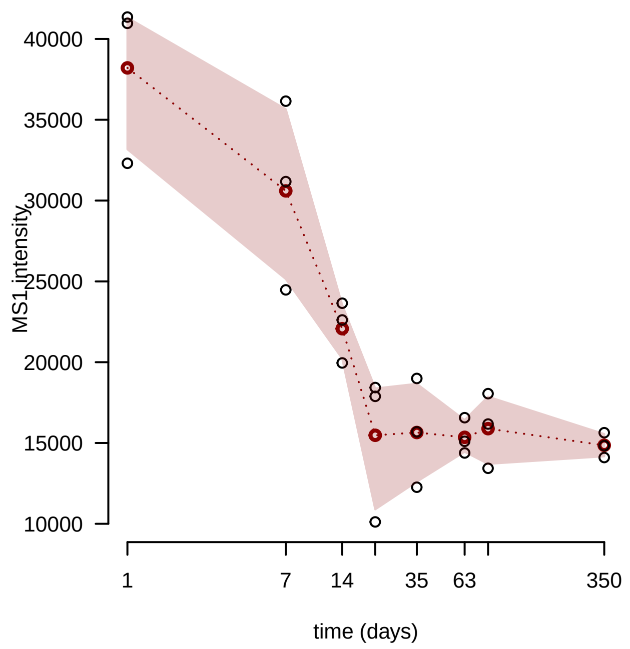

Q9Z2D1
GN=Mtmr2
myotubularin related protein 2

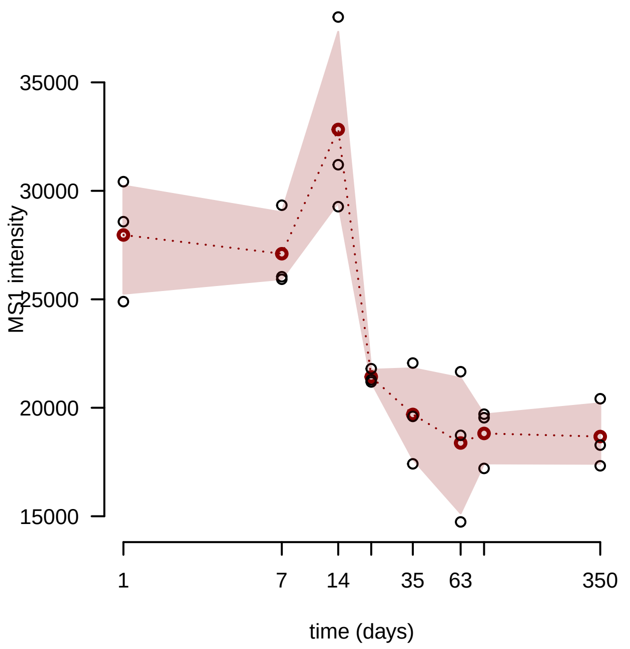

Q9Z2D6
GN=Mecp2
methyl CpG binding protein 2

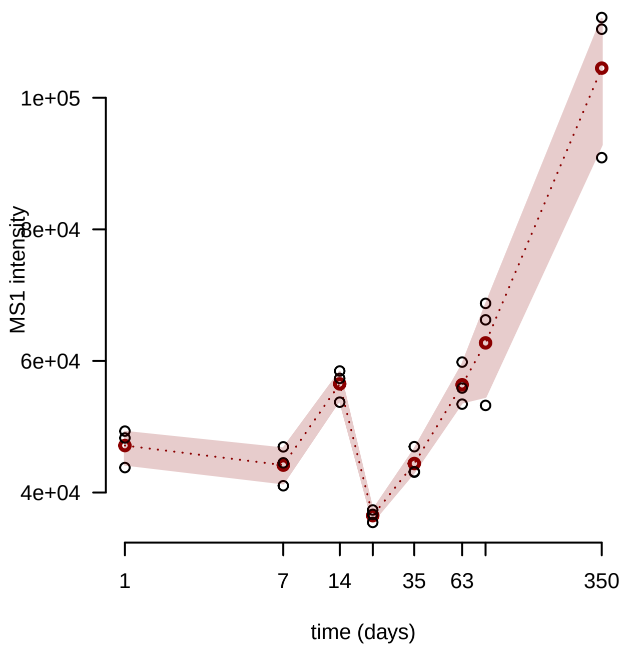

Q9Z2D8
GN=Mbd3
methyl−CpG binding domain protein 3

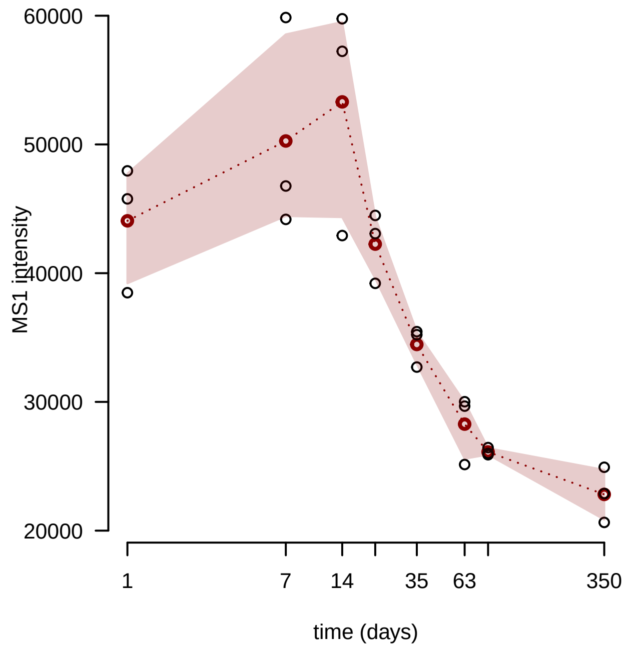

Q9Z2E1
GN=Mbd2
methyl−CpG binding domain protein 2

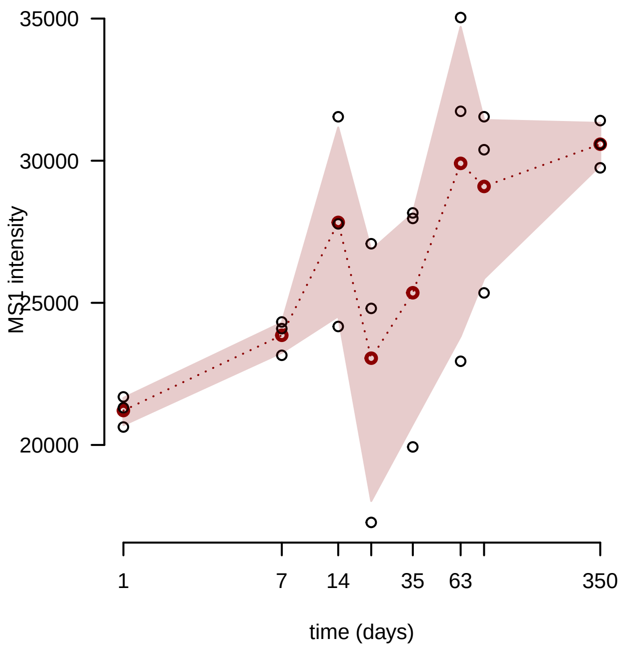

Q9Z2E2
GN=Mbd1
methyl−CpG binding domain protein 1

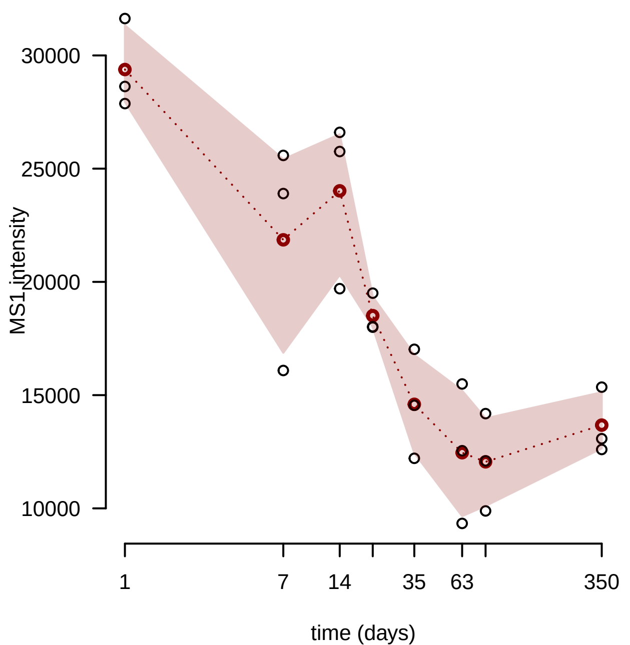

Q9Z2E9
GN=Bscl2
Berardinelli−Seip congenital lipodystrop ...

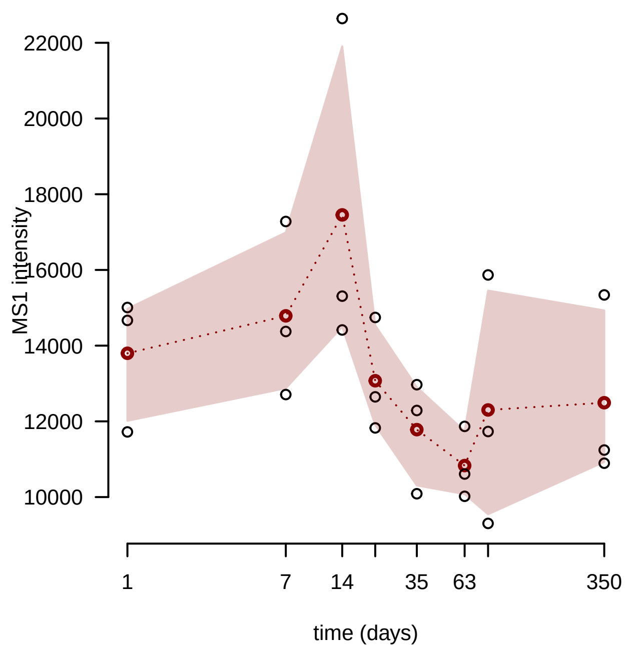

Q9Z2F2
GN=Oasl2
2'−5' oligoadenylate synthetase−like 2

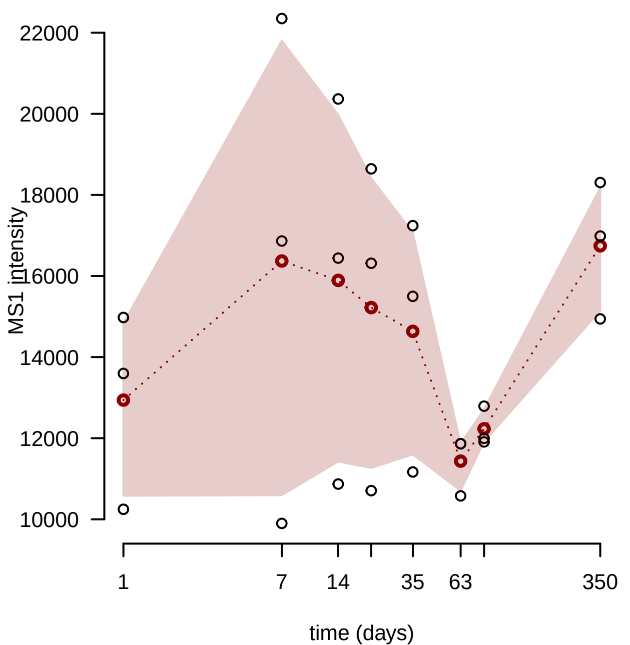

Q9Z2F7
GN=Bnip3l
BCL2/adenovirus E1B interacting protein ...

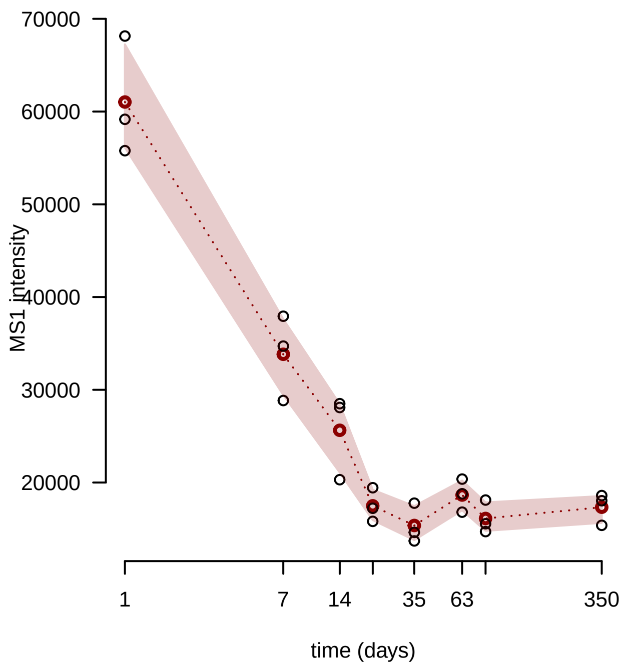

Q9Z2G0
GN=Fem1b
fem 1 homolog b

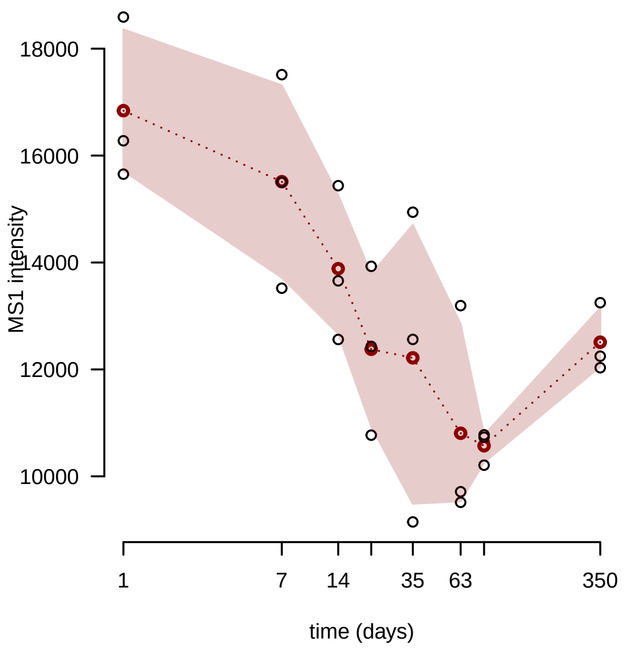

Q9Z2G6
GN=Sel1l
sel−1 suppressor of lin−12−like (C. eleg ...

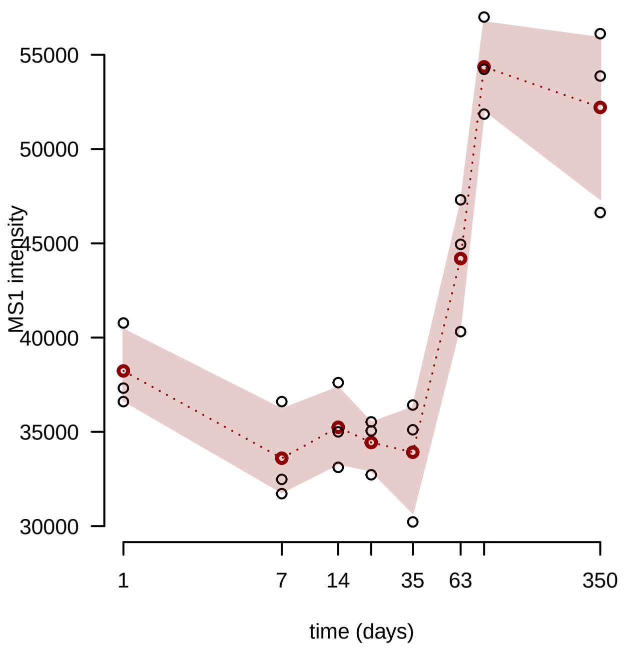

Q9Z2G9
GN=Htatip2
HIV−1 Tat interactive protein 2

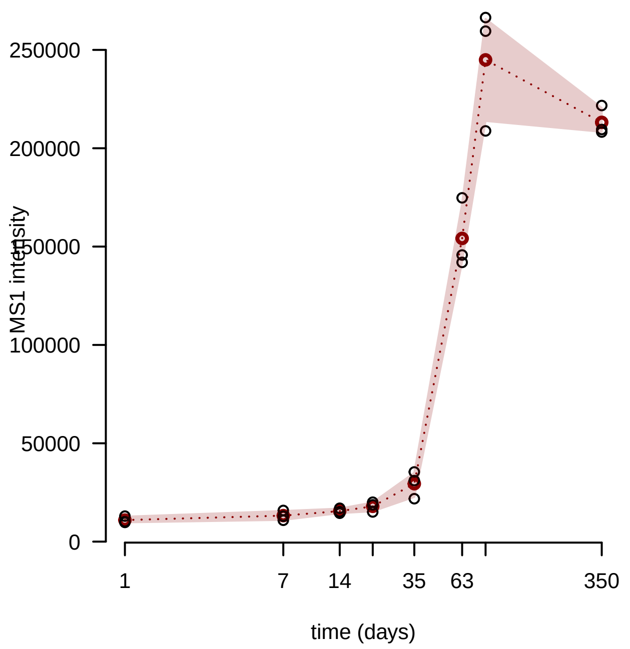

Q9Z2H5
GN=Epb41l1
erythrocyte membrane protein band 4.1 li ...

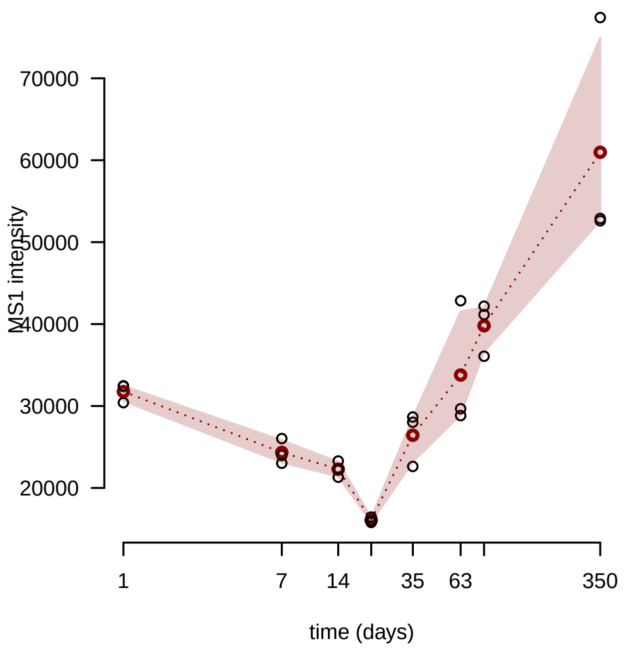

Q9Z2I0
GN=Letm1
leucine zipper−EF−hand containing transm ...

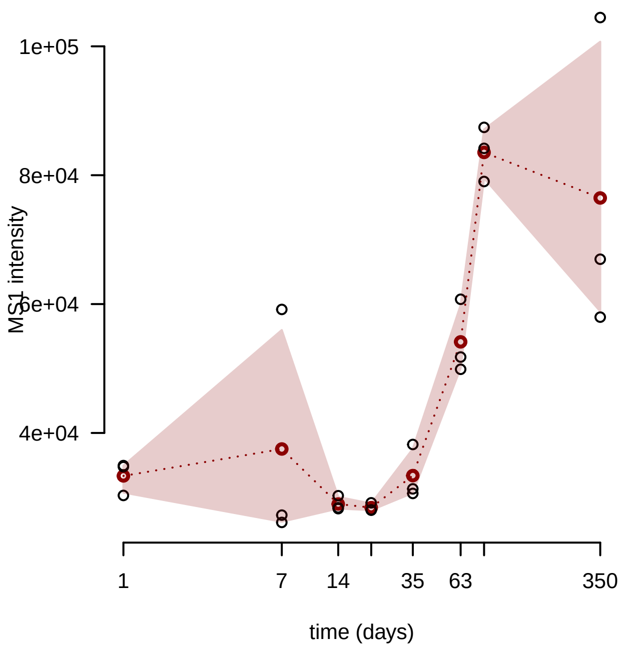

Q9Z2I8
GN=Suclg2
succinate−Coenzyme A ligase, GDP−forming ...

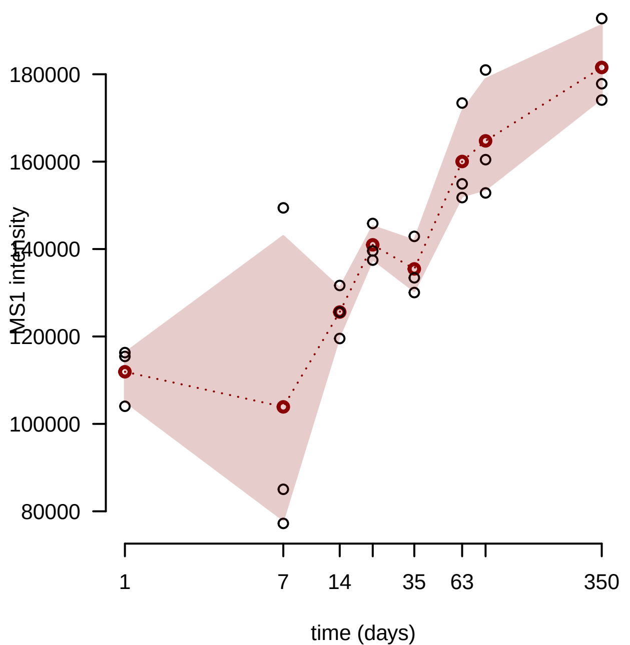

Q9Z2I8
GN=Suclg2
succinate−Coenzyme A ligase, GDP−forming ...

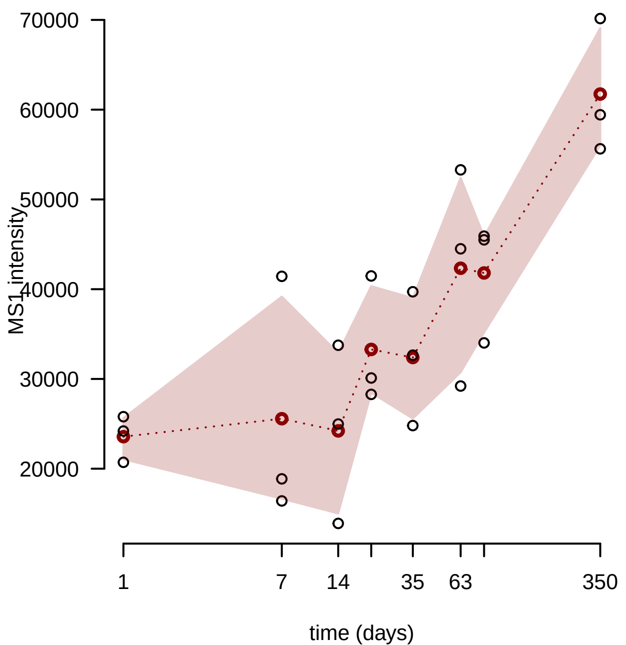

Q9Z2I9
GN=Sucla2
succinate−Coenzyme A ligase, ADP−forming ...

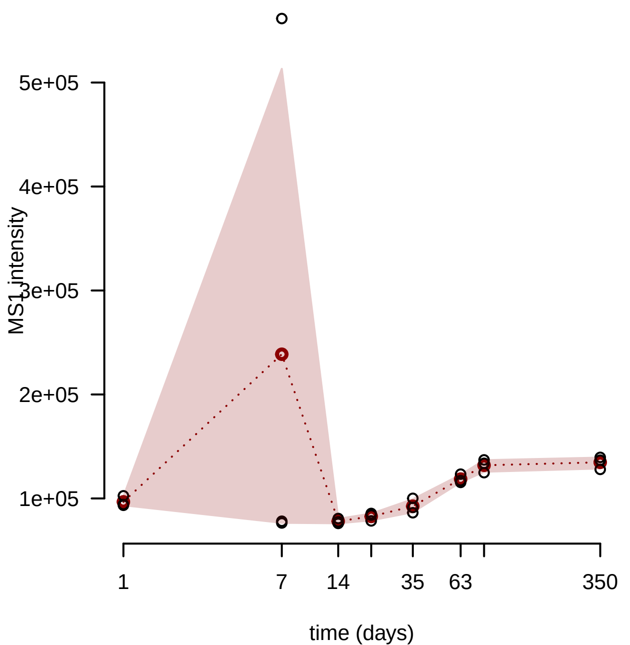

Q9Z2K1
GN=Krt16
keratin 16

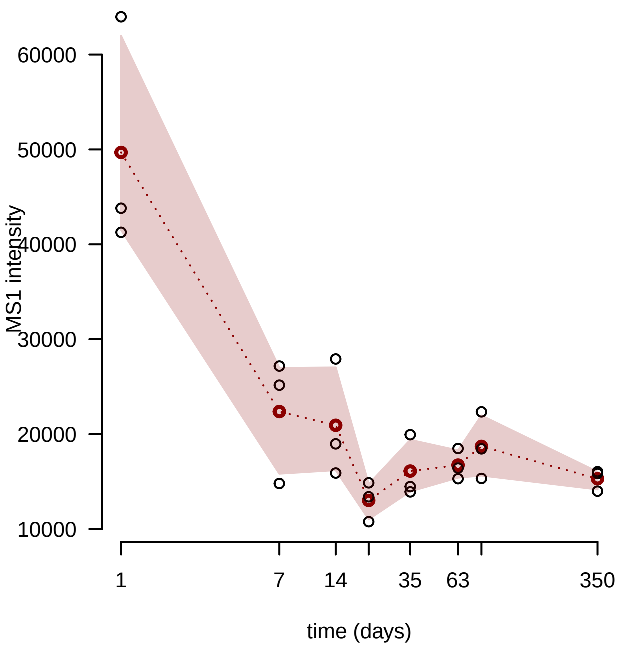

Q9Z2L6
GN=Minpp1
multiple inositol polyphosphate histidin ...

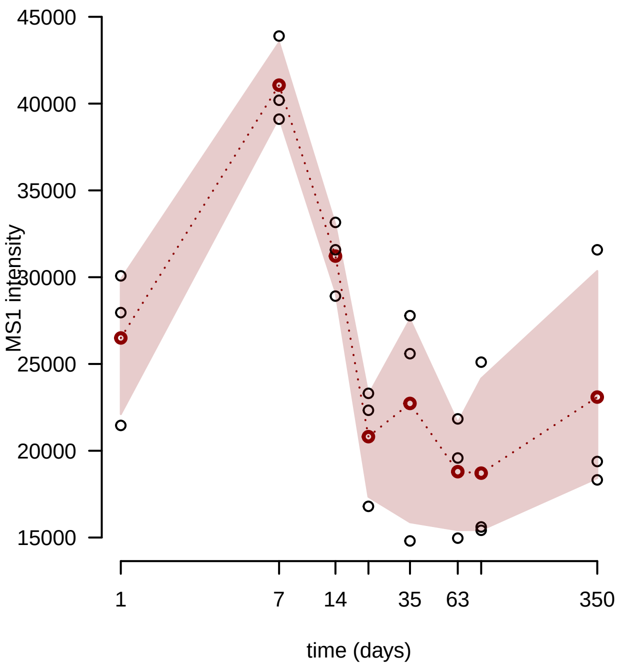

Q9Z2L7
GN=Crlf3
cytokine receptor−like factor 3

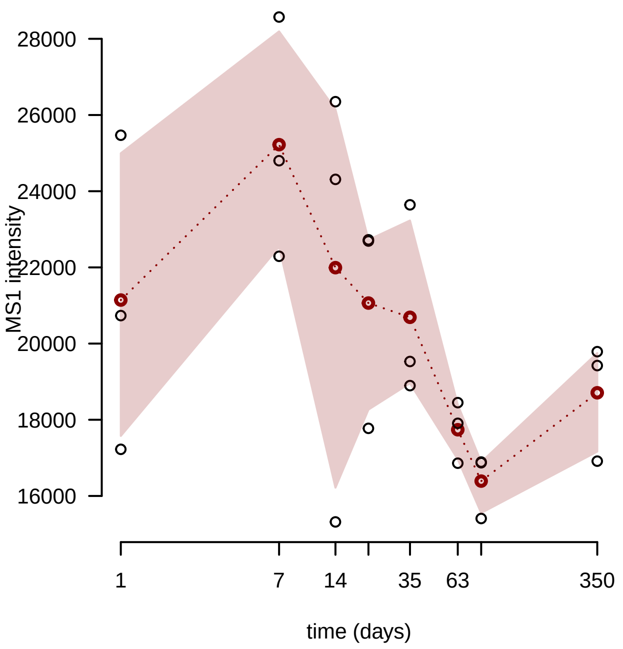

Q9Z2M6
GN=Ubl3
ubiquitin−like 3

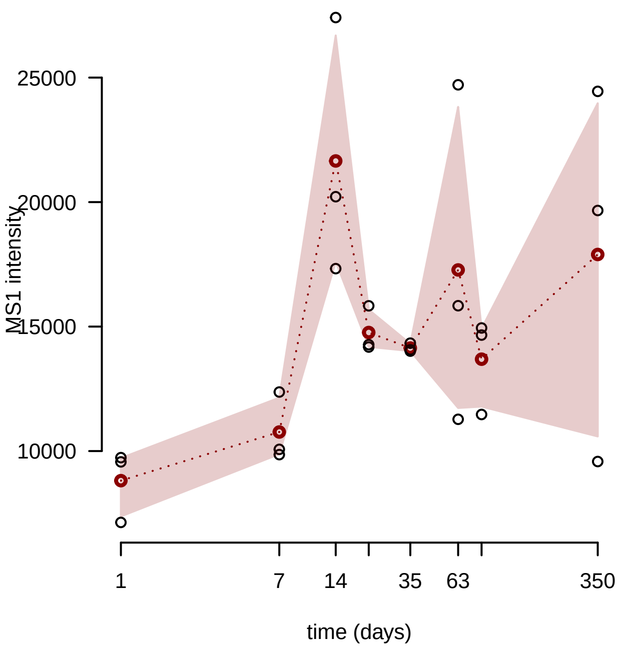

Q9Z2M7
GN=Pmm2
phosphomannomutase 2

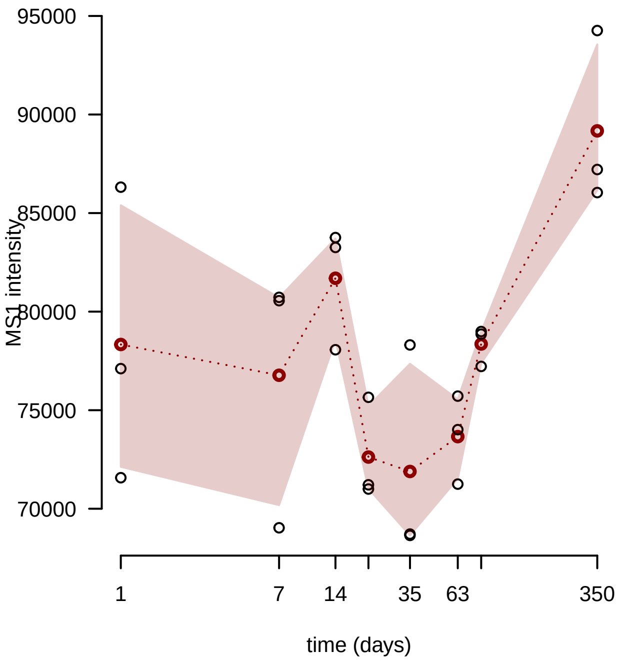

Q9Z2N8
GN=Actl6a
actin−like 6A

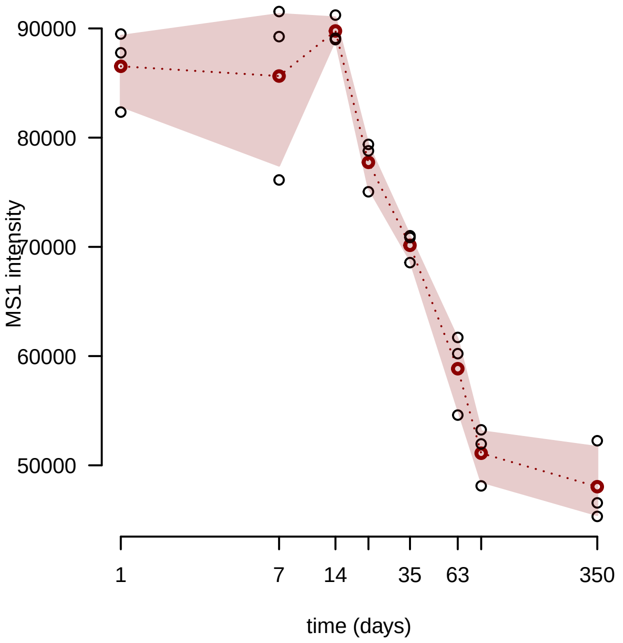

Q9Z2P8
GN=Vamp5
vesicle−associated membrane protein 5

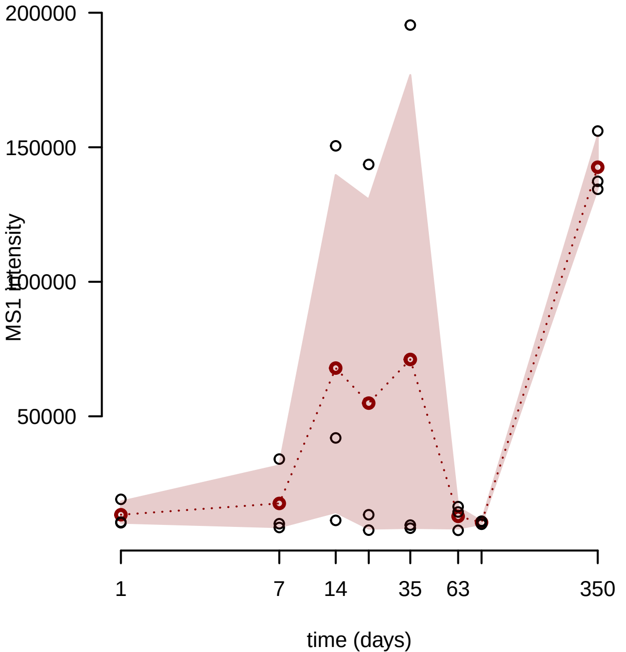

Q9Z2Q2
GN=Knop1
lysine rich nucleolar protein 1

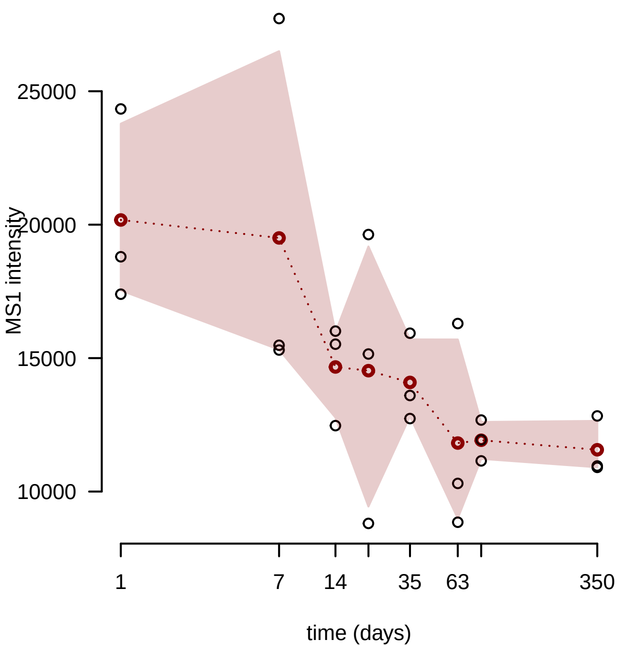

Q9Z2Q5
GN=Mrpl40
mitochondrial ribosomal protein L40

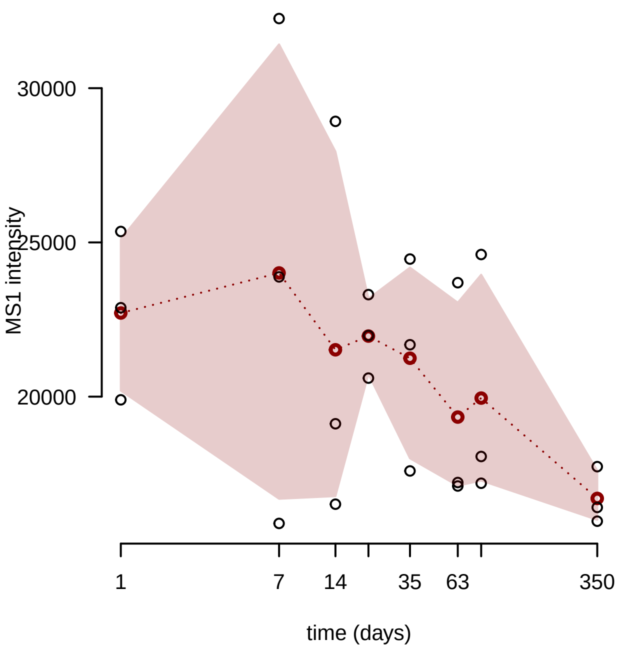

Q9Z2Q6
GN=Sept5

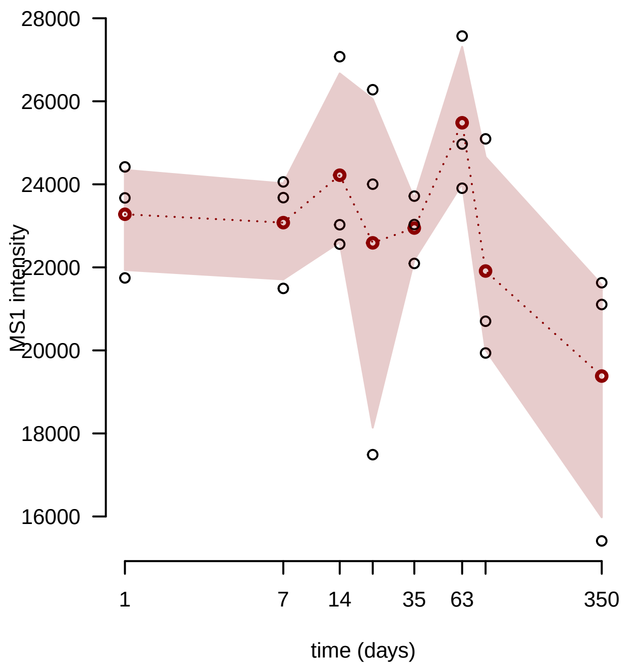

Q9Z2R6
GN=Unc119
unc−119 lipid binding chaperone

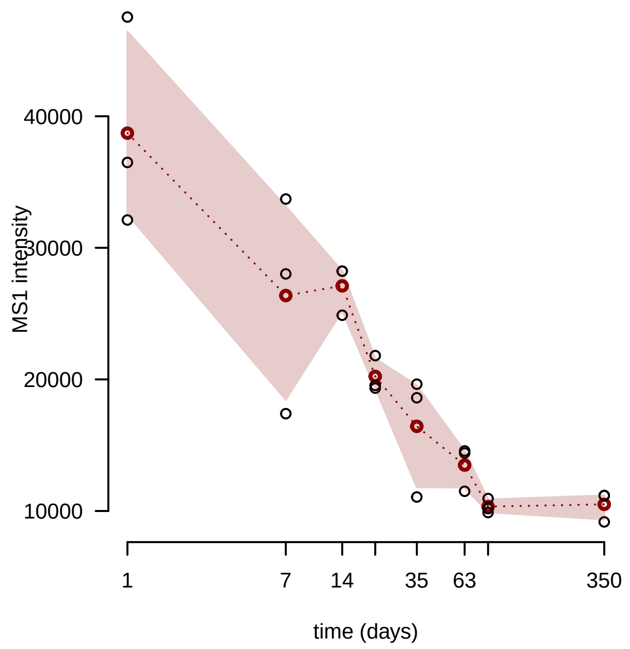

Q9Z2T6
GN=Krt85

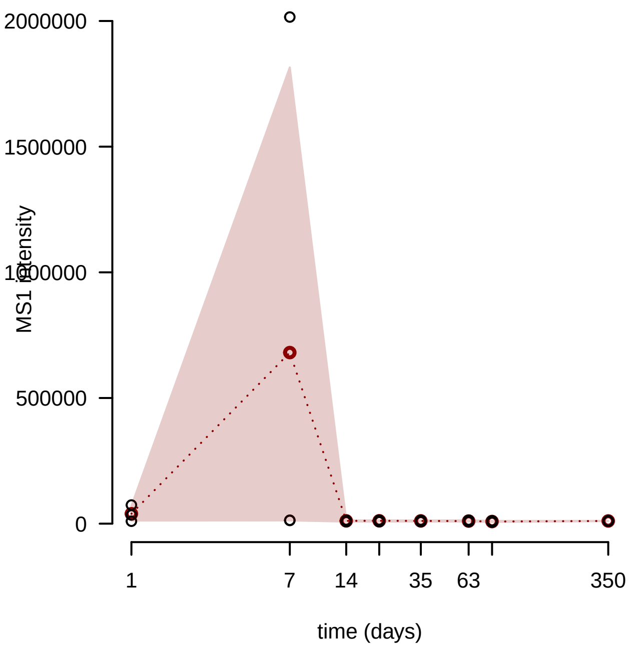

Q9Z2U0
GN=Psma7
proteasome subunit alpha 7

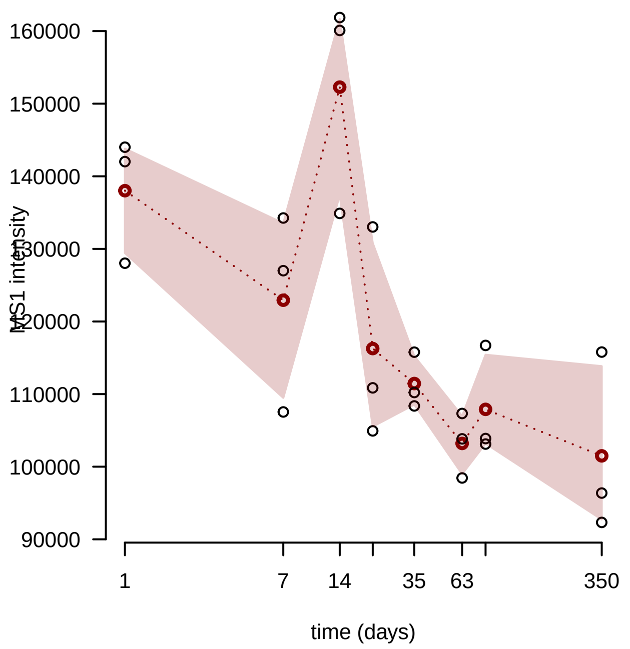

Q9Z2U1
GN=Psma5
proteasome subunit alpha 5

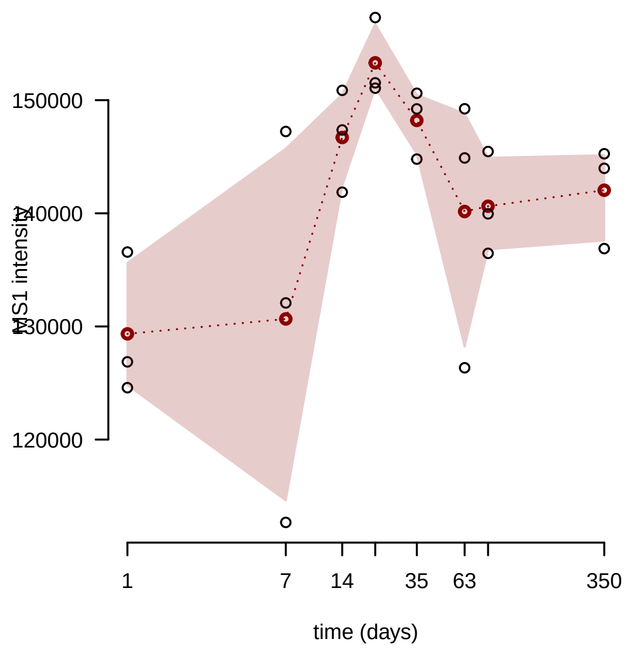

Q9Z2U2
GN=Zfp292
zinc finger protein 292

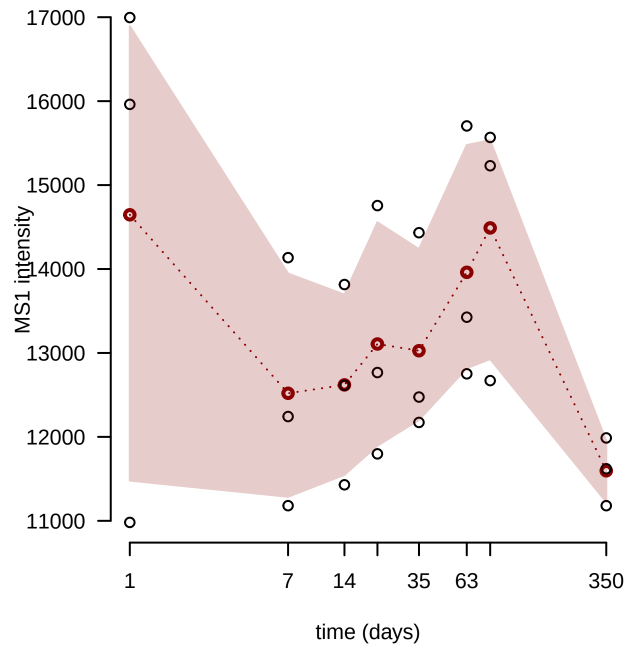

Q9Z2V4
GN=Pck1
phosphoenolpyruvate carboxykinase 1, cyt ...

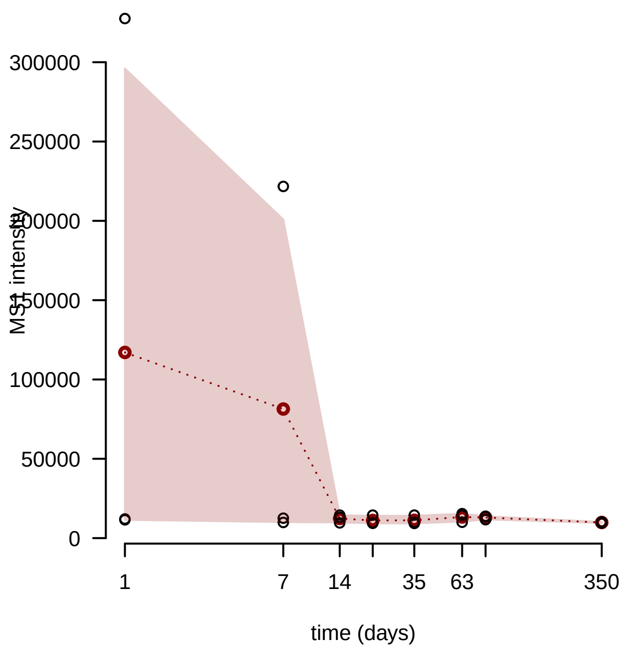

Q9Z2V5
GN=Hdac6
histone deacetylase 6

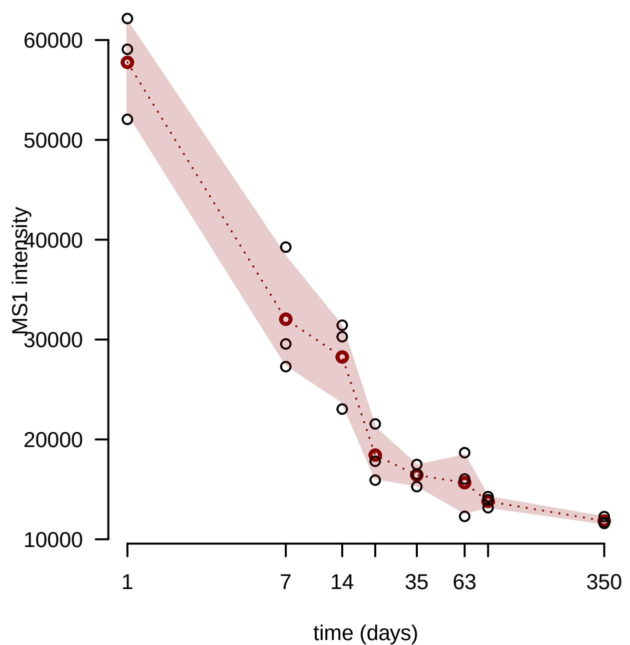

Q9Z2V6
GN=Hdac5
histone deacetylase 5

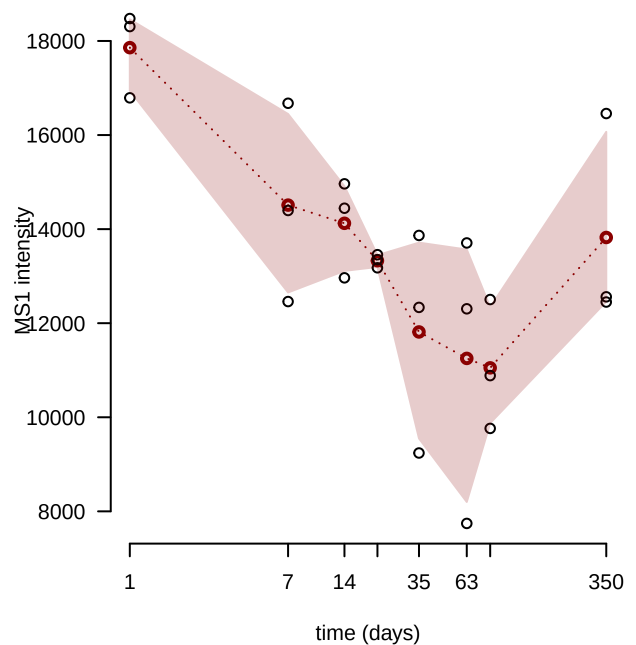

Q9Z2W0
GN=Dnpep
aspartyl aminopeptidase

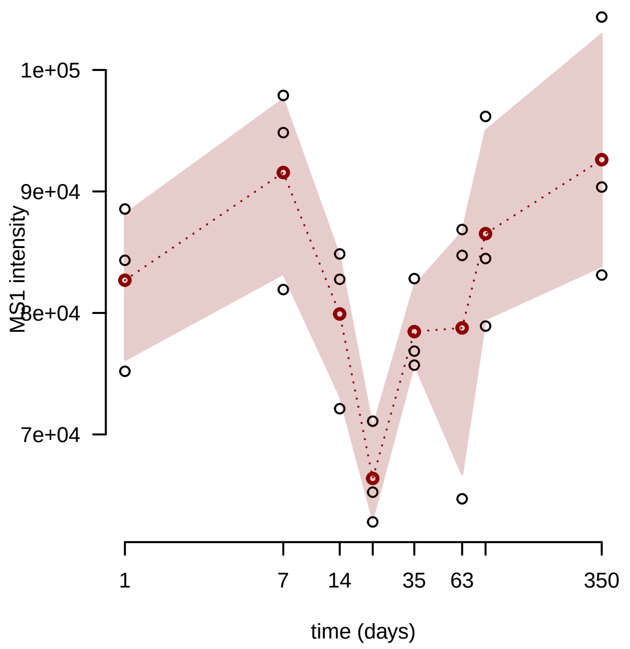

Q9Z2W1
GN=Stk25
serine/threonine kinase 25 (yeast)

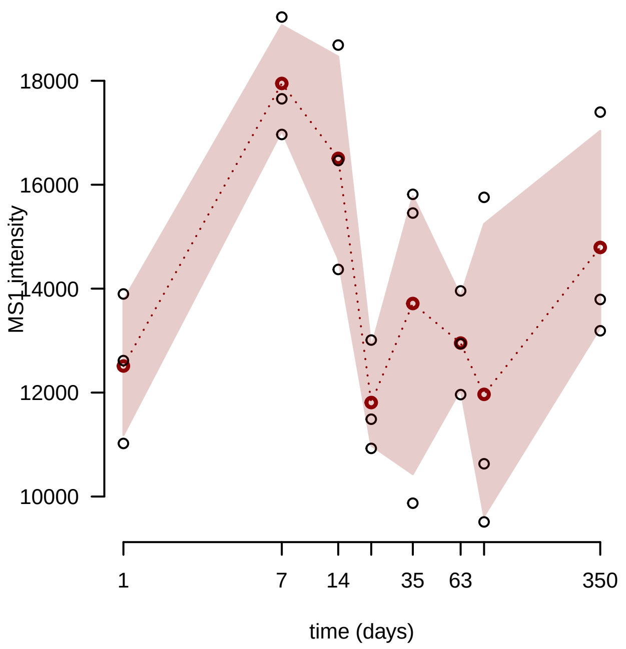

Q9Z2X1
GN=Hnrnpf
heterogeneous nuclear ribonucleoprotein ...

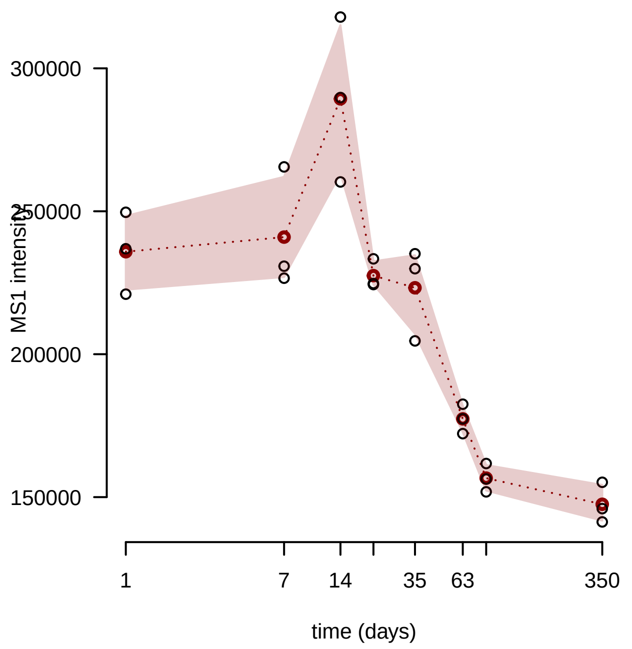

Q9Z2X2
GN=Psmd10
proteasome (prosome, macropain) 26S subu ...

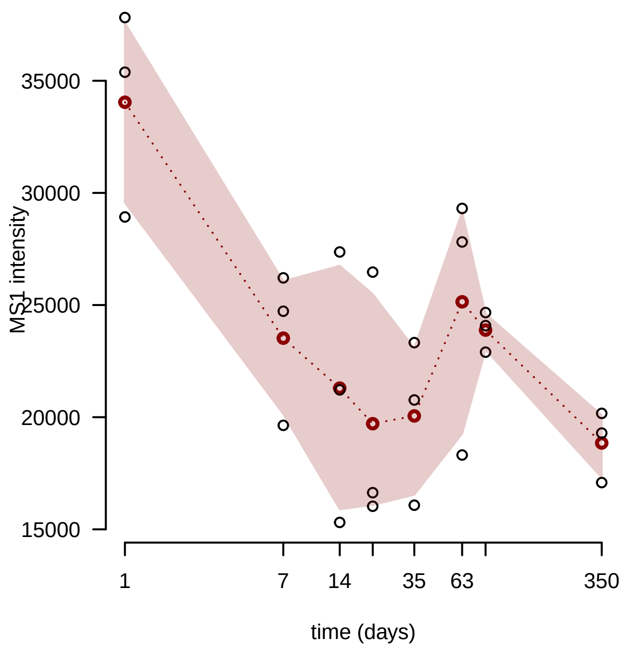

Q9Z2X8
GN=Keap1
kelch−like ECH−associated protein 1

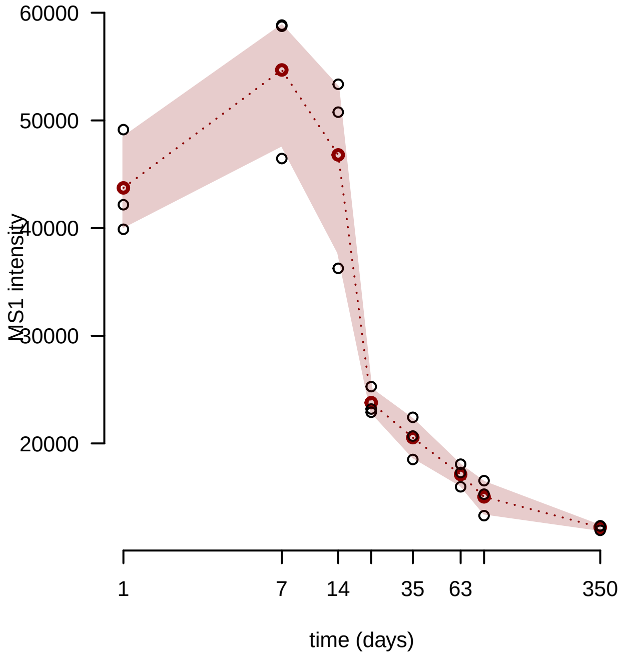

Q9Z2Y2
GN=B4galt2
UDP−Gal:betaGlcNAc beta 1,4− galactosylt ...

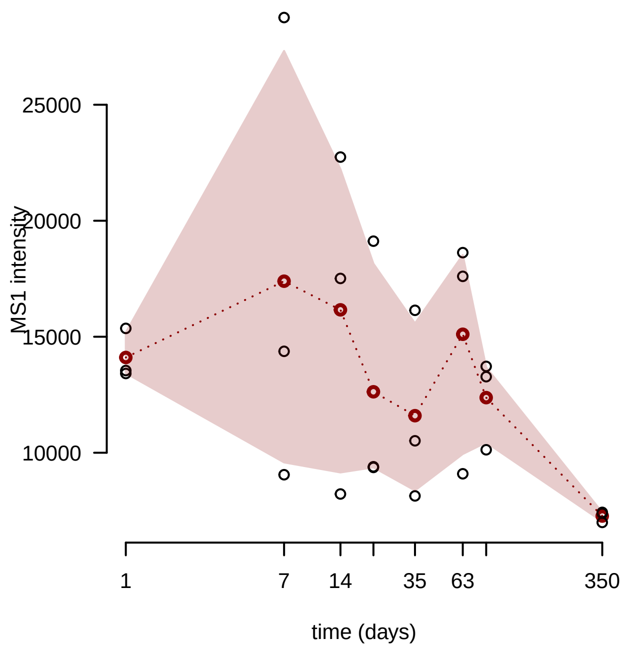

Q9Z2Y3
GN=Homer1
homer scaffolding protein 1

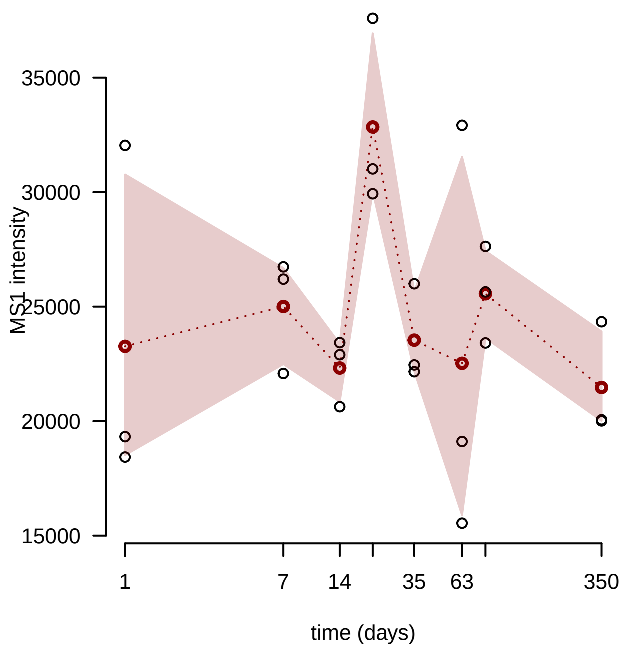

Q9Z2Y8
GN=Plpbp
pyridoxal phosphate binding protein

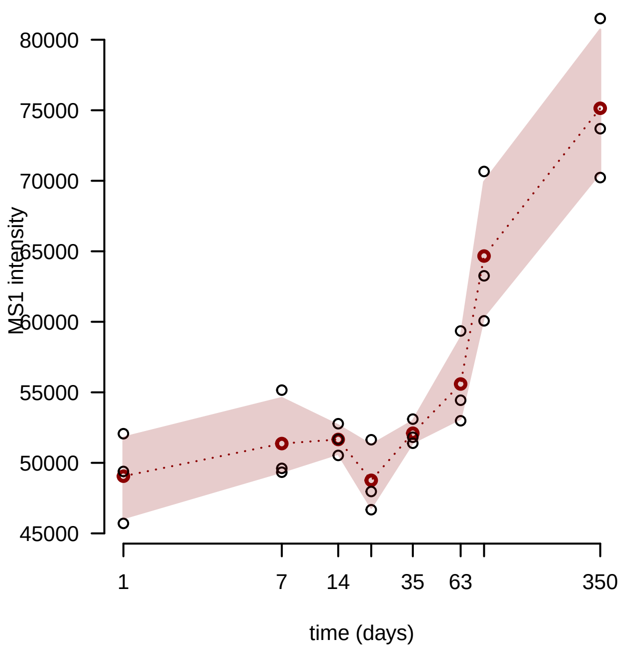

Q9Z2Z6
GN=Slc25a20
solute carrier family 25 (mitochondrial ...

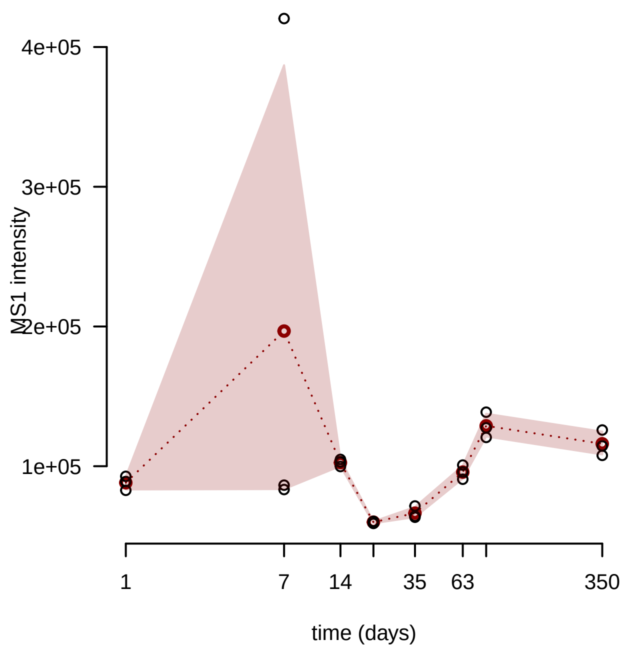

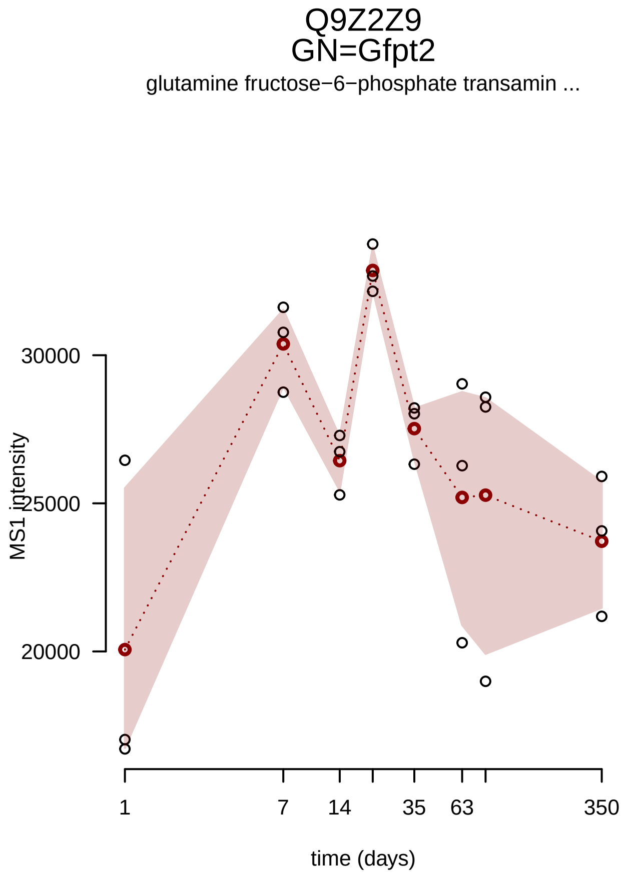
Q9Z2Z9
GN=Gfpt2
glutamine fructose−6−phosphate transamin ...
MS1 intensity
20000
25000
30000
1
7
14
35
63
350
time (days)

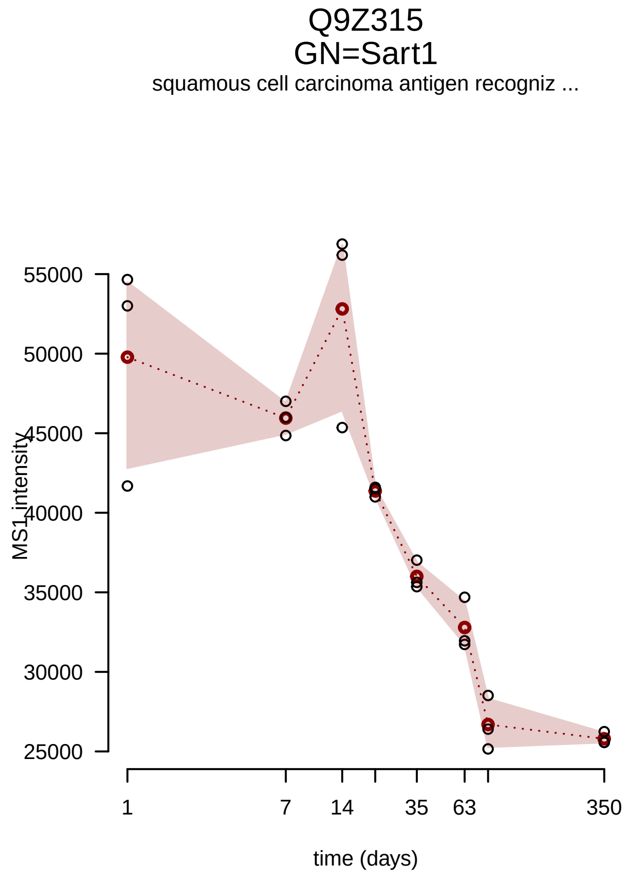
Q9Z315
GN=Sart1
squamous cell carcinoma antigen recogniz ...
MS1 intensity
25000
30000
35000
40000
45000
50000
55000
1
7
14
35
63
350
time (days)

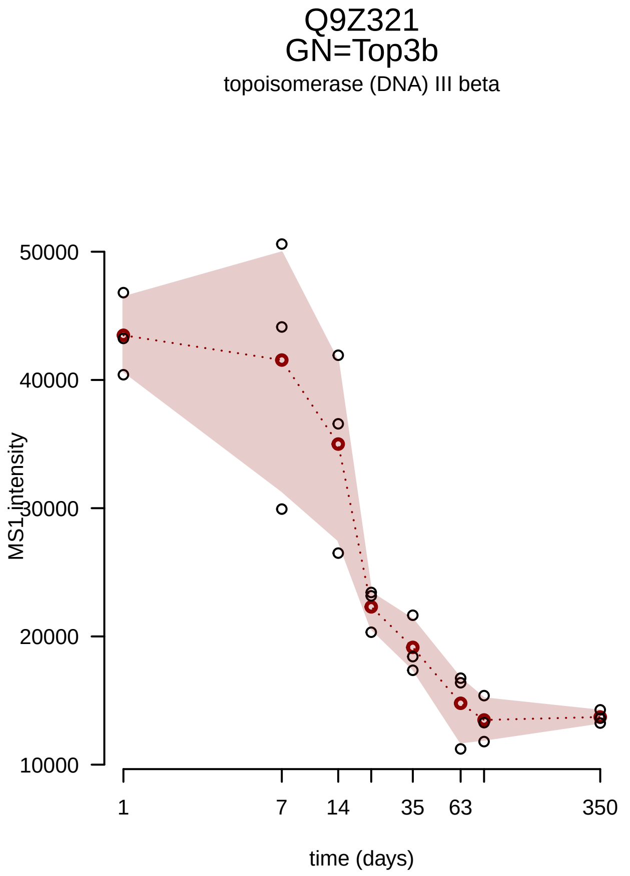
Q9Z321
GN=Top3b
topoisomerase (DNA) III beta
MS1 intensity
10000
20000
30000
40000
50000
1
7
14
35
63
350
time (days)

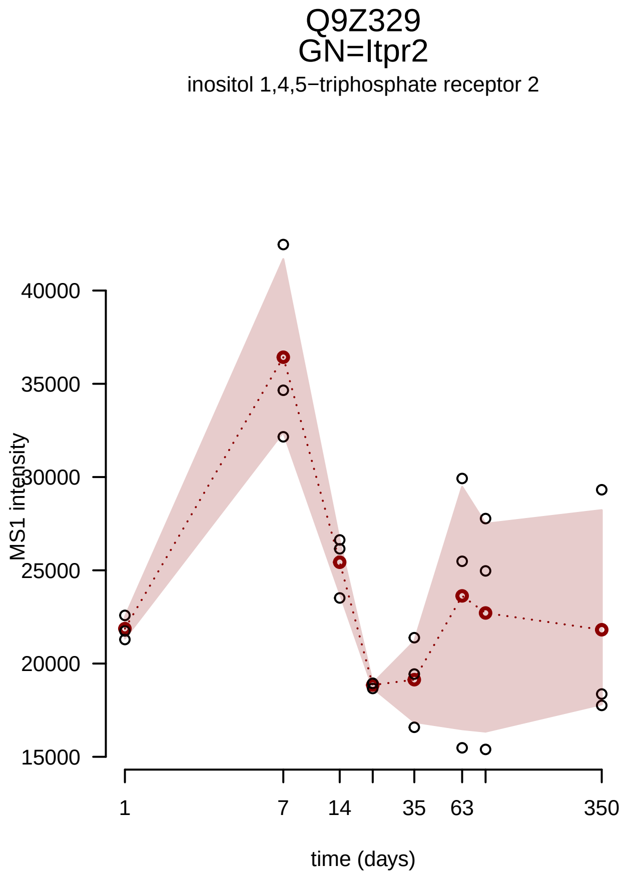
Q9Z329
GN=Itpr2
inositol 1,4,5−triphosphate receptor 2
MS1 intensity
15000
20000
25000
30000
35000
40000
1
7
14
35
63
350
time (days)

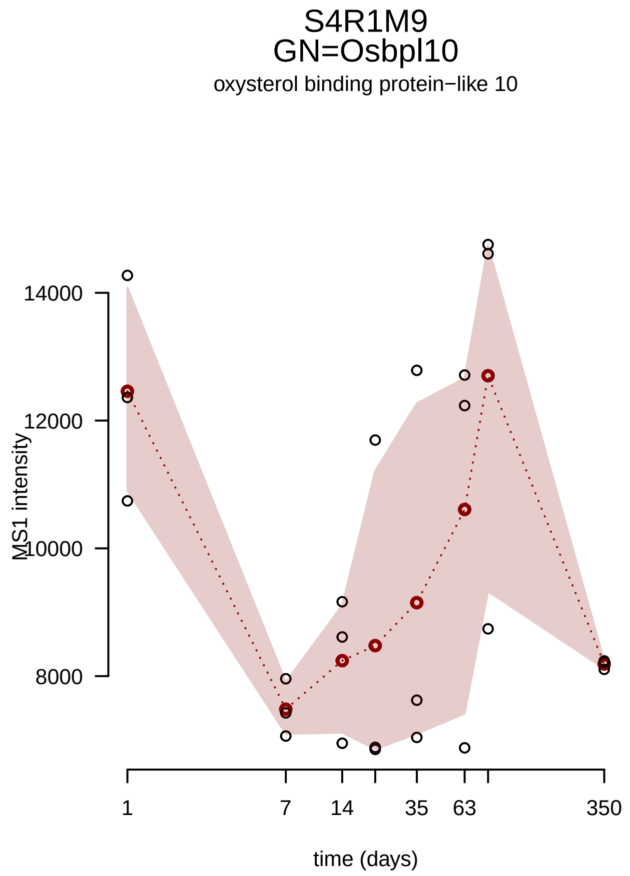
S4R1M9
GN=Osbpl10
oxysterol binding protein−like 10
MS1 intensity
8000
10000
12000
14000
1
7
14
35
63
350
time (days)

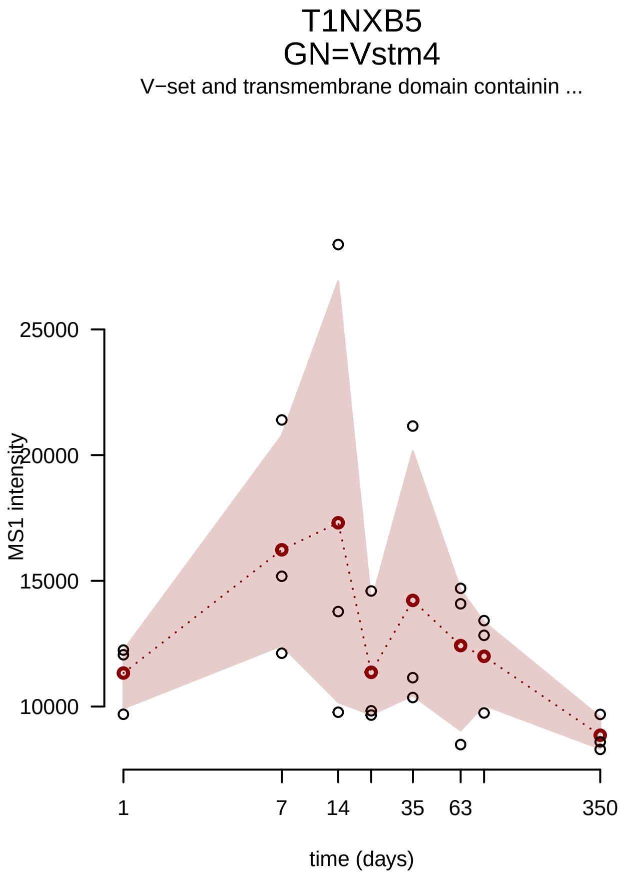
T1NXB5
GN=Vstm4
V−set and transmembrane domain containin ...
MS1 intensity
10000
15000
20000
25000
1
7
14
35
63
350
time (days)

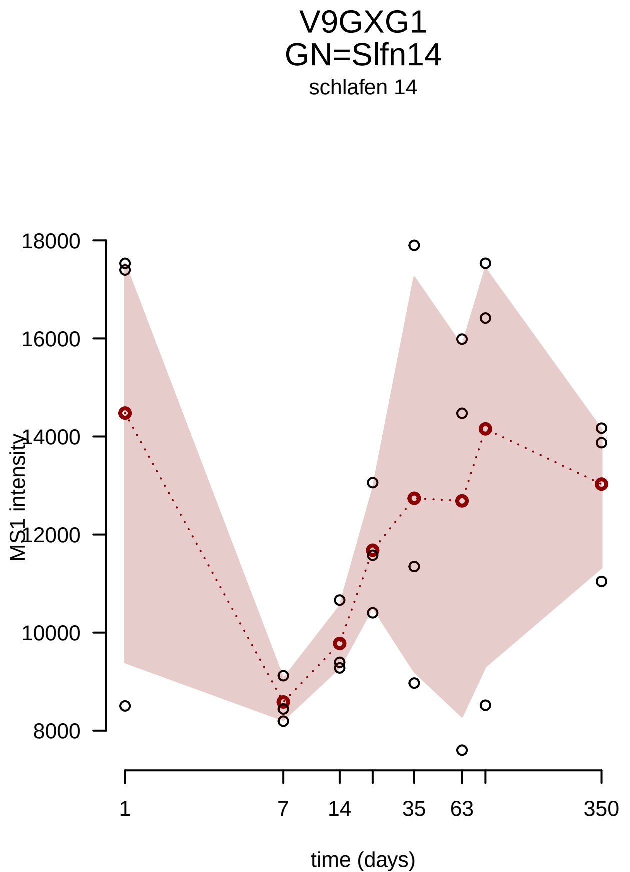
V9GXG1
GN=Slfn14
schlafen 14
MS1 intensity
8000
10000
12000
14000
16000
18000
1
7
14
35
63
350
time (days)
