# Supplementary material for: The maintenance of oocytes in the mammalian ovary involves extreme protein longevity
Source: Nat Cell Biol. 2024 Jun 20;26(7):1124–38. doi: 10.1038/s41556-024-01442-7 (PMC11252011; doi:10.1038/s41556-024-01442-7)

P62806

>sp|P62806|H4_MOUSE

Histone H4

GN=H4c1

Med H1/2: 253.8 days

Q5   H1/2: 165.3 days

Q95 H1/2: 465 days

reliable model fit: yes

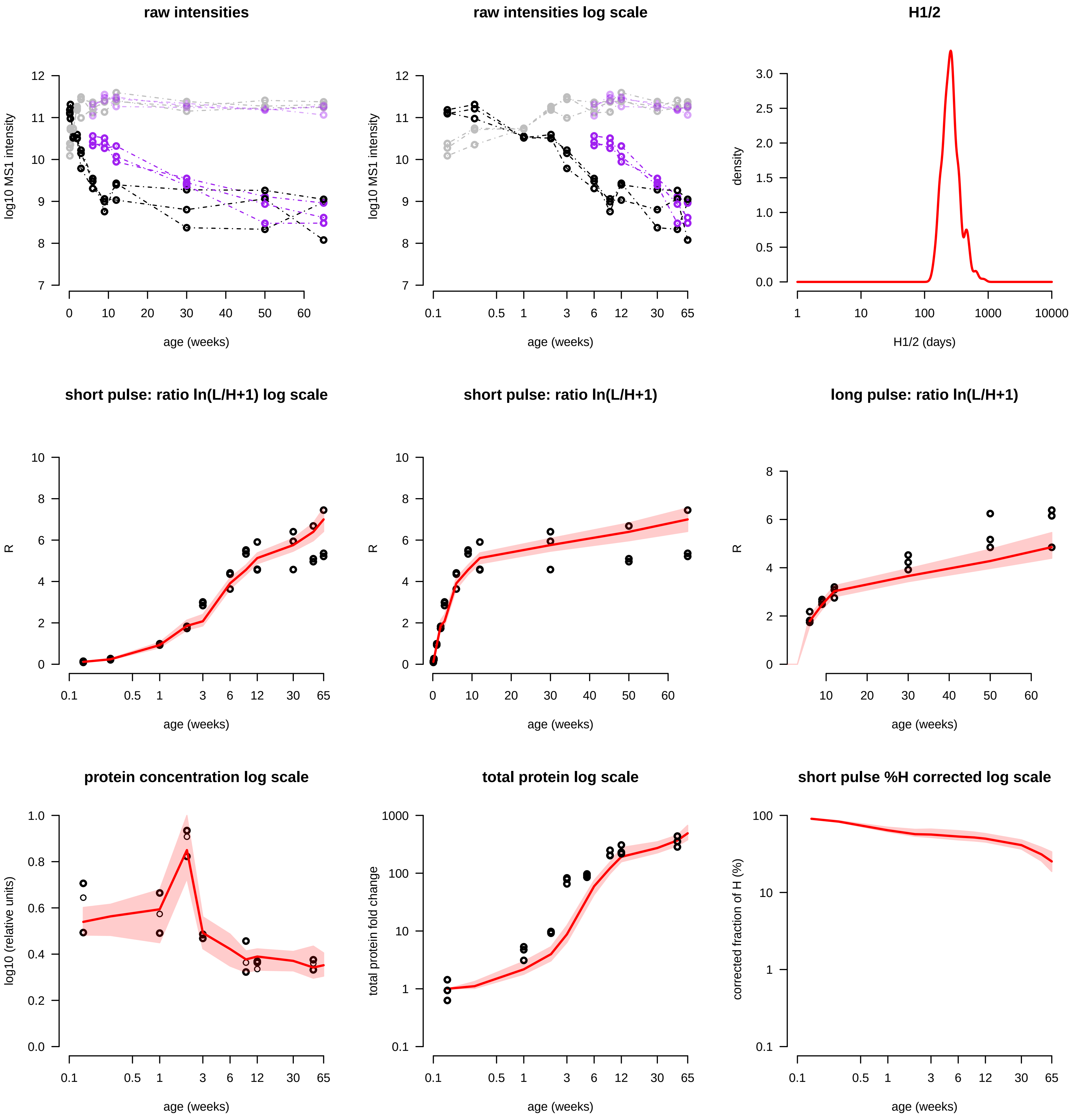

Q8K023;Q8K023−2

>sp|Q8K023|AKC1H_MOUSE

Aldo−keto reductase family 1 member C18

GN=Akr1c18

Med H1/2: 40.7 days

Q5 H1/2: 26.4 days

Q95 H1/2: 115.2 days

reliable model fit: yes

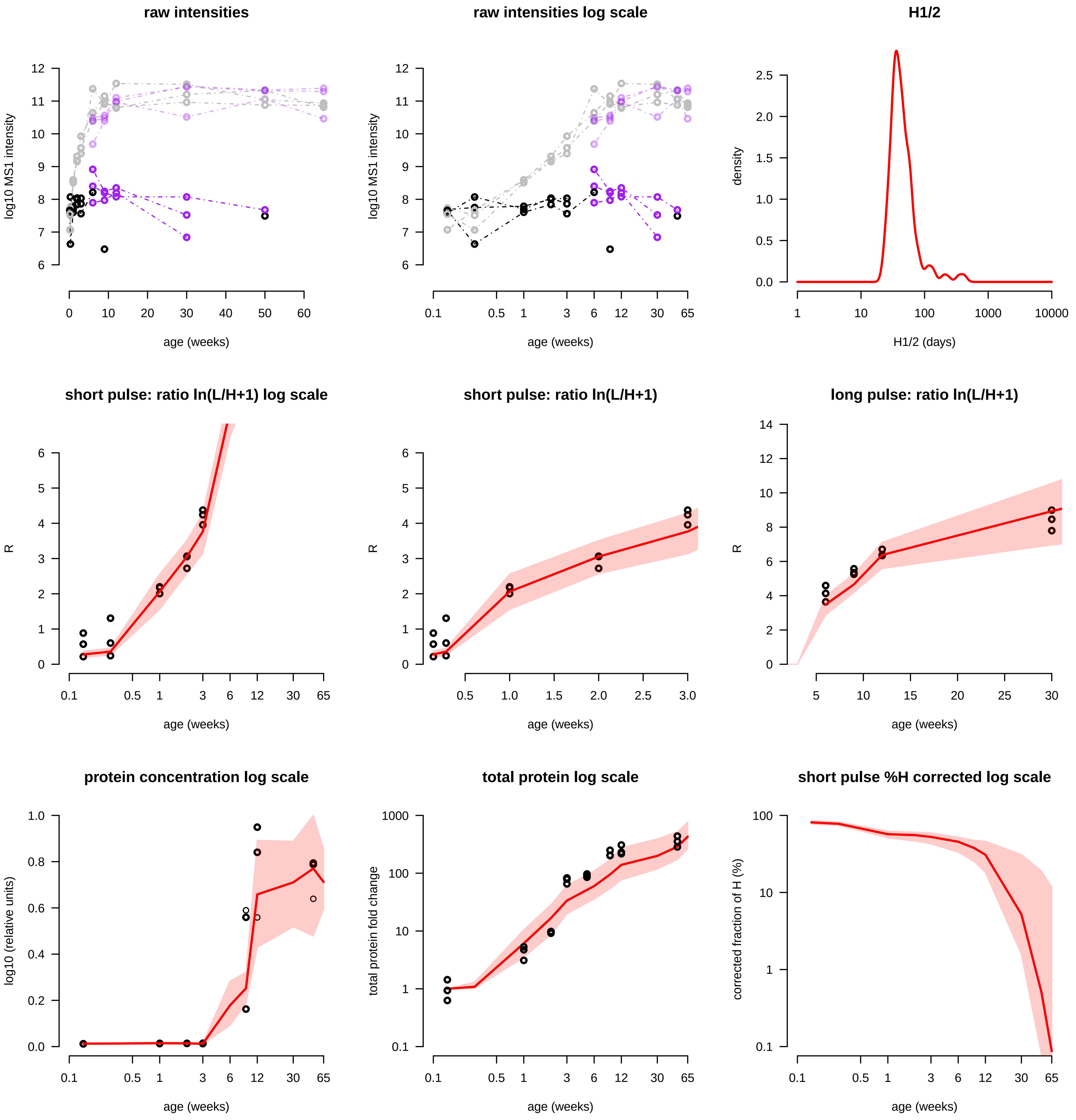

Q6ZWY9;Q64478

>sp|Q6ZWY9|H2B1C_MOUSE

Histone H2B type 1−C/E/G

GN=H2bc4

Med H1/2: 190.7 days

Q5 H1/2: 131.9 days

Q95 H1/2: 306.8 days

reliable model fit: yes

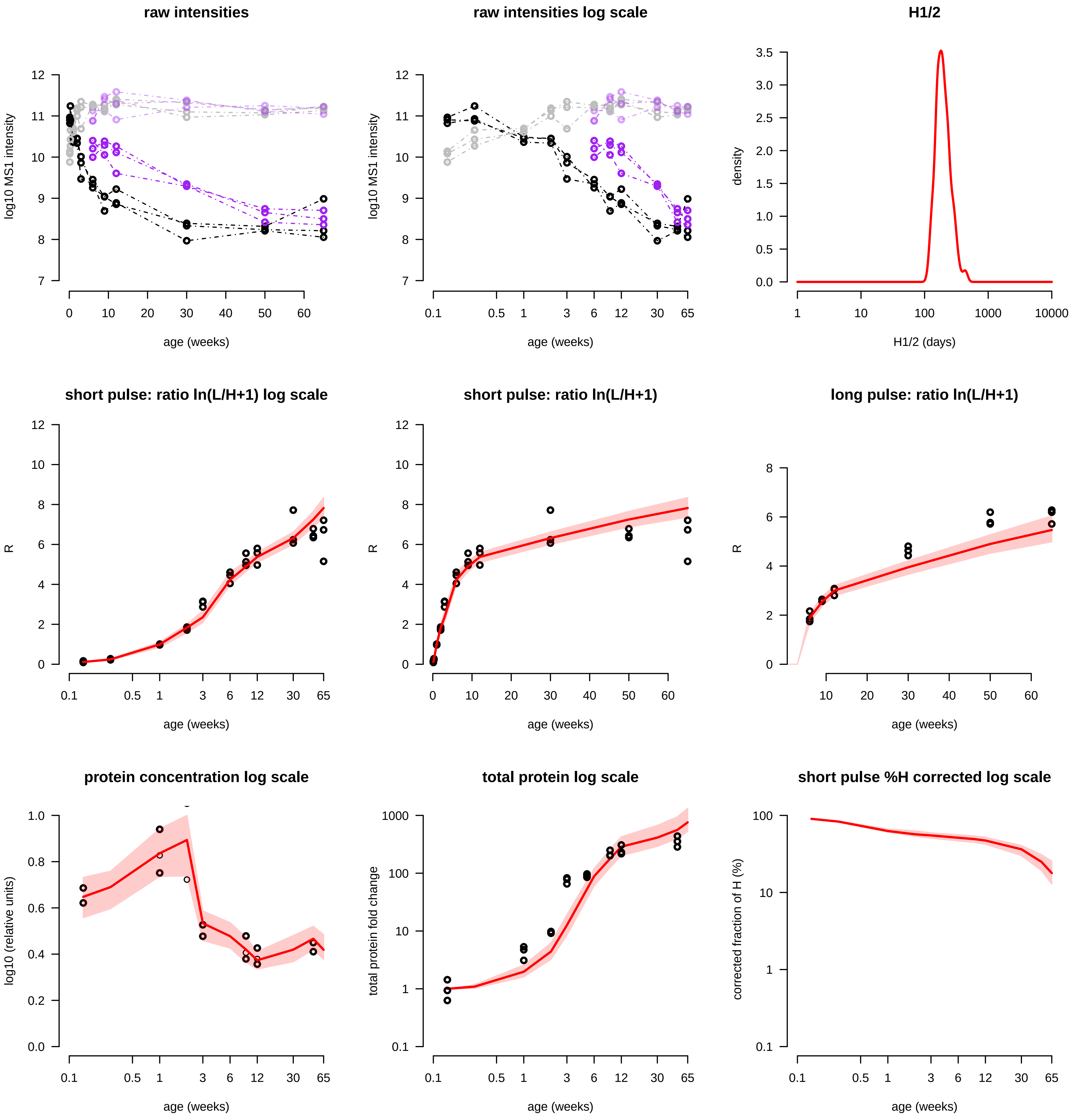

P63260

>sp|P63260|ACTG_MOUSE

Actin, cytoplasmic 2

GN=Actg1

Med H1/2: 143.4 days

Q5 H1/2: 81 days

Q95 H1/2: 435.7 days

reliable model fit: yes

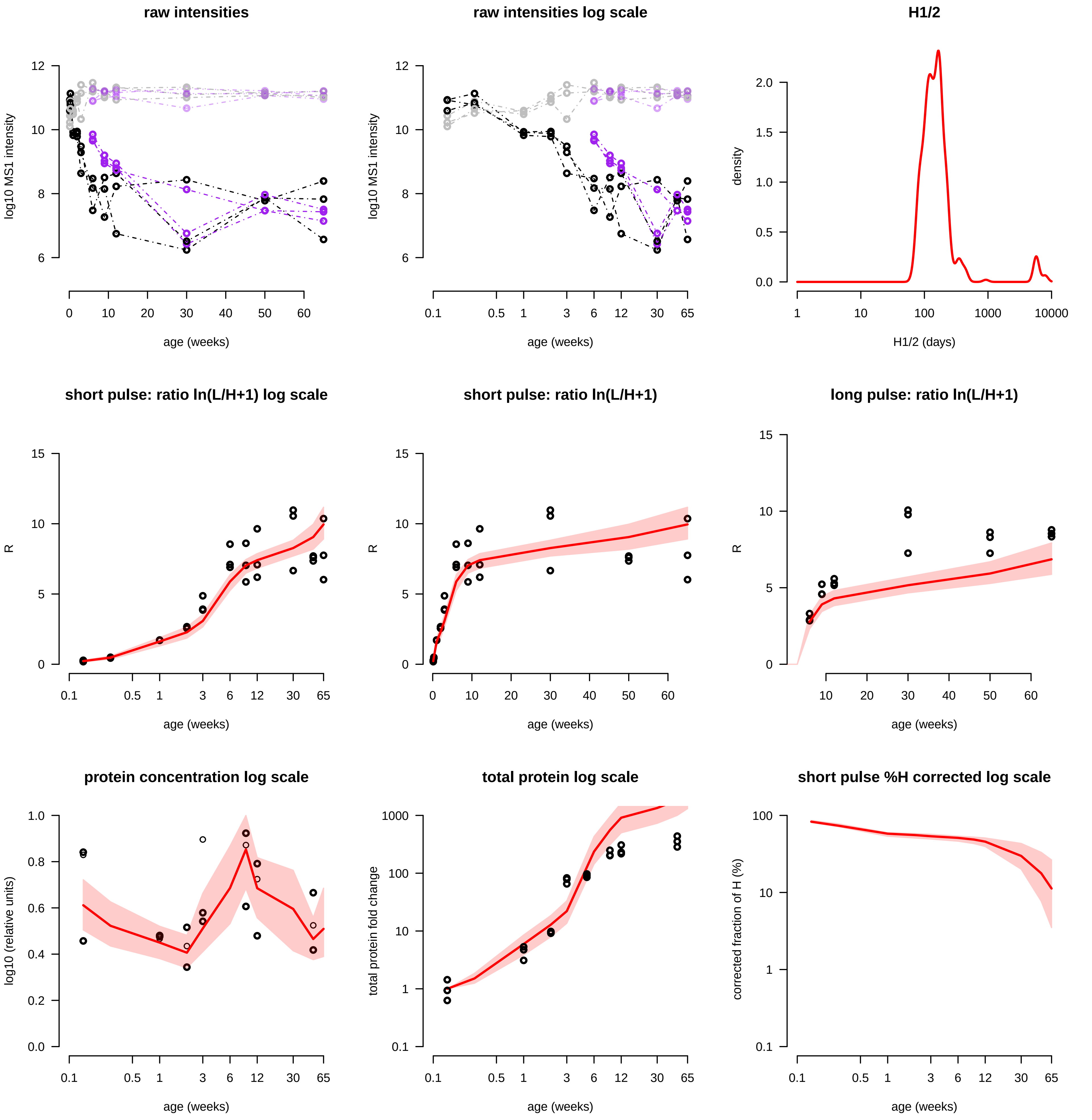

P34914

>sp|P34914|HYES_MOUSE

Bifunctional epoxide hydrolase 2

GN=Ephx2

Med H1/2: 80.6 days

Q5 H1/2: 57.7 days

Q95 H1/2: 146.2 days

reliable model fit: yes

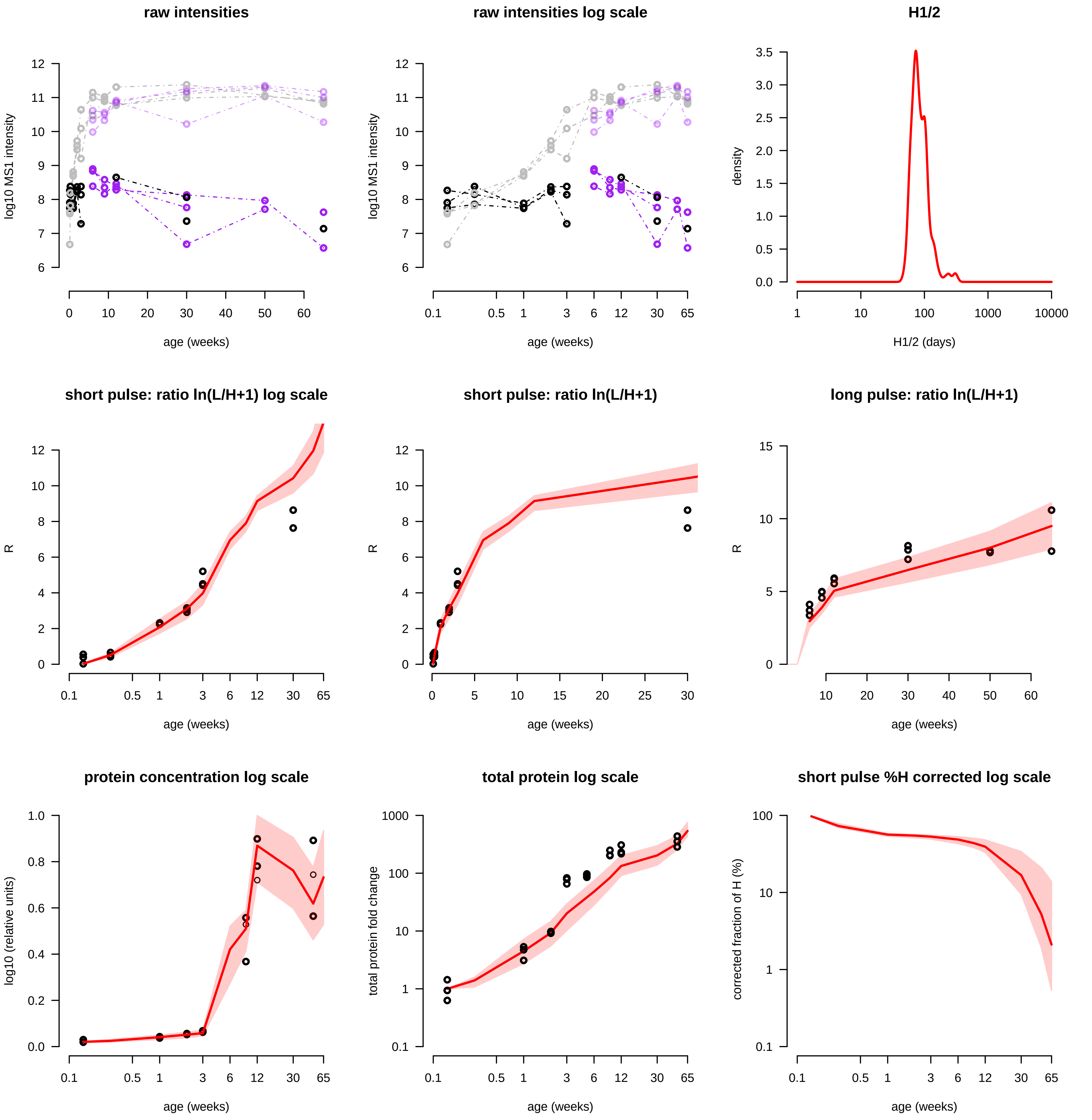

P24815

>sp|P24815|3BHS1_MOUSE

3 beta−hydroxysteroid dehydrogenase/Delta 5−−>4−isomerase type 1

GN=Hsd3b1

Med H1/2: 91.9 days

Q5 H1/2: 60.6 days

Q95 H1/2: 216.2 days

reliable model fit: yes

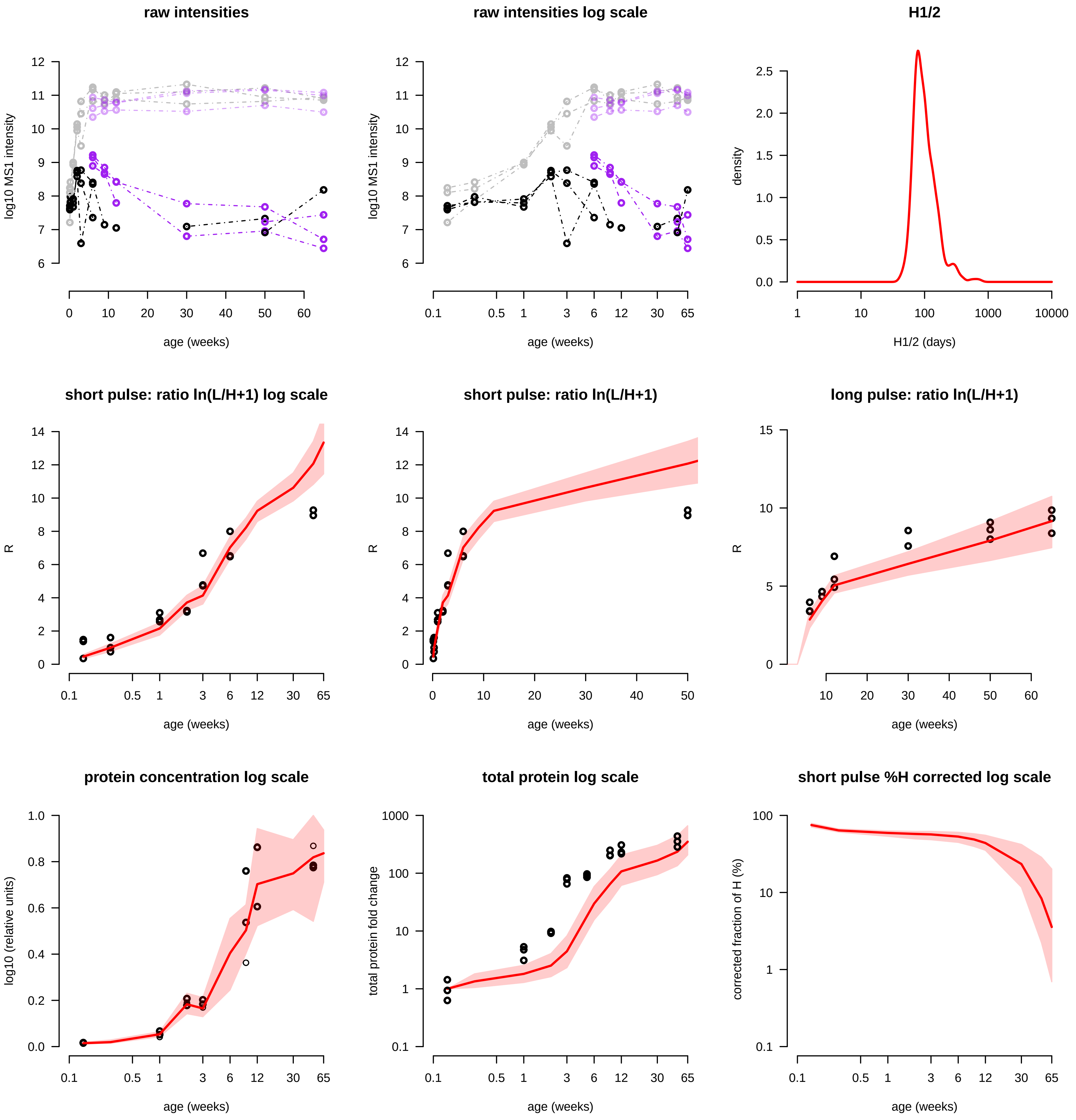

P63038

>sp|P63038|CH60_MOUSE

60 kDa heat shock protein, mitochondrial

GN=Hspd1

Med H1/2: 88 days

Q5 H1/2: 58.5 days

Q95 H1/2: 121.2 days

reliable model fit: yes

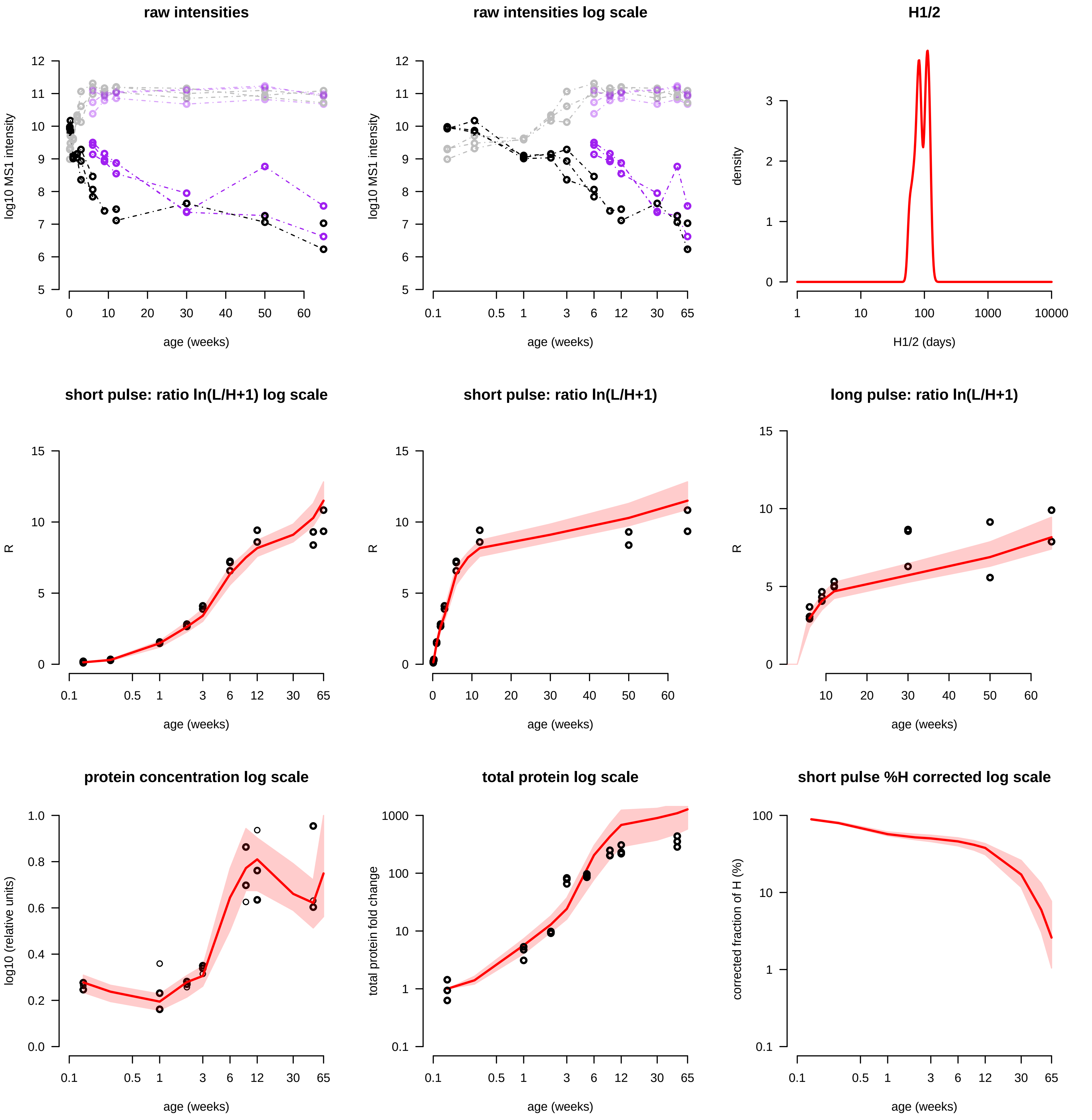

O88844

>sp|O88844|IDHC_MOUSE

Isocitrate dehydrogenase [NADP] cytoplasmic

GN=Idh1

Med H1/2: 94.1 days

Q5 H1/2: 69.8 days

Q95 H1/2: 138.1 days

reliable model fit: yes

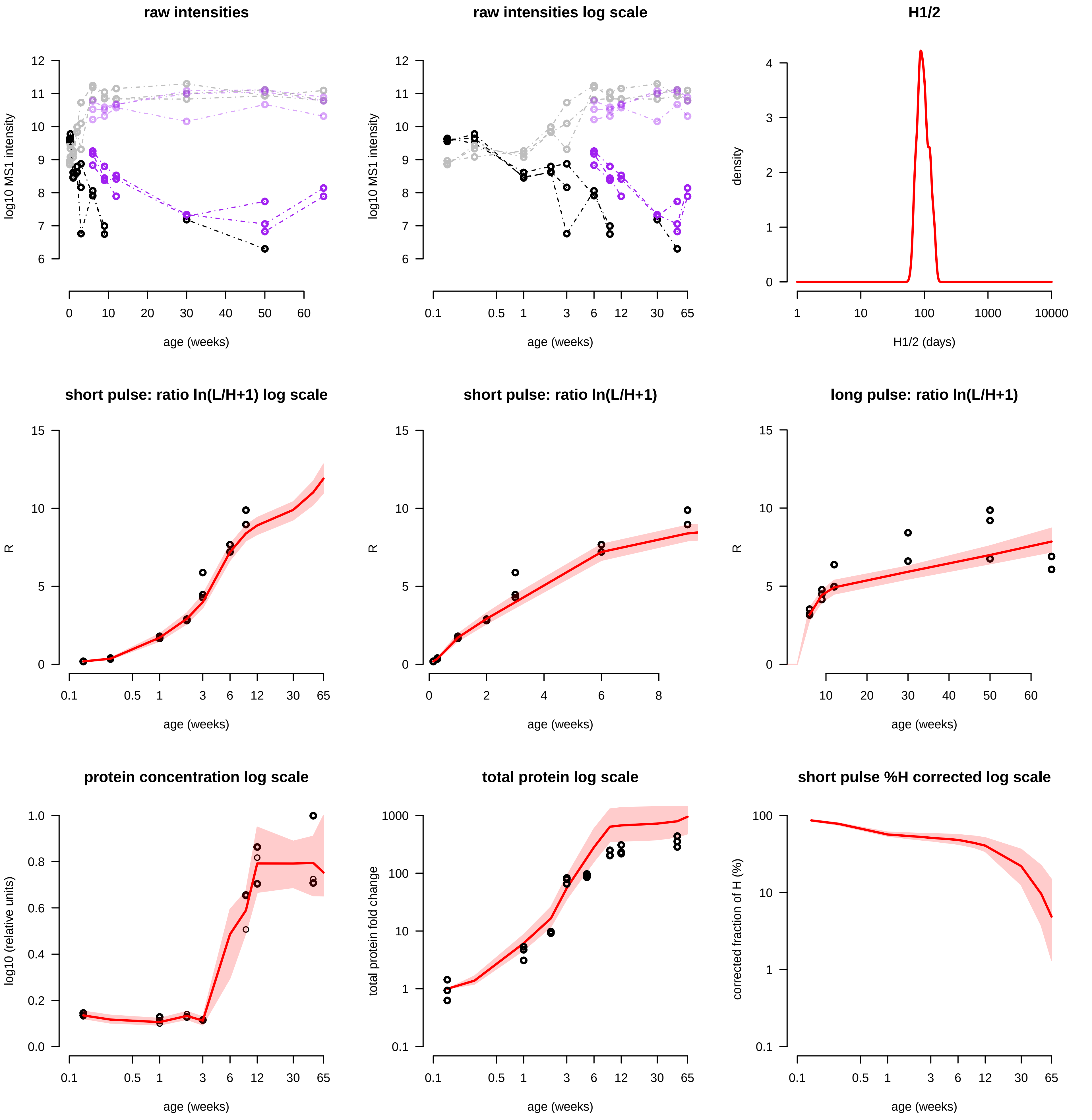

P20152

>sp|P20152|VIME_MOUSE

Vimentin

GN=Vim

Med H1/2: 97.3 days

Q5 H1/2: 59.9 days

Q95 H1/2: 301.4 days

reliable model fit: yes

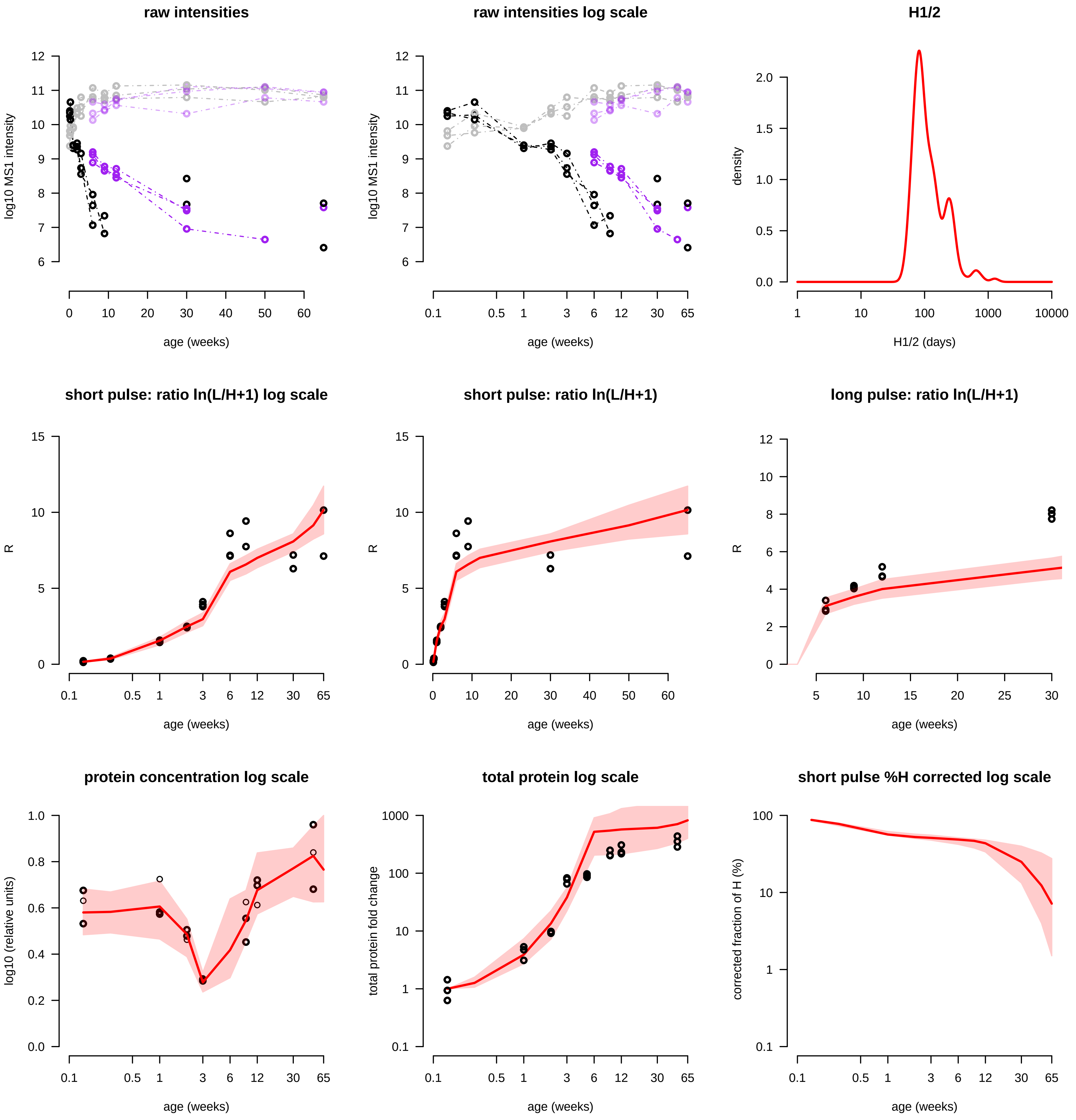

P10126

>sp|P10126|EF1A1_MOUSE

Elongation factor 1−alpha 1

GN=Eef1a1

Med H1/2: 605.5 days

Q5 H1/2: 217.8 days

Q95 H1/2: 6942.2 days

reliable model fit: yes

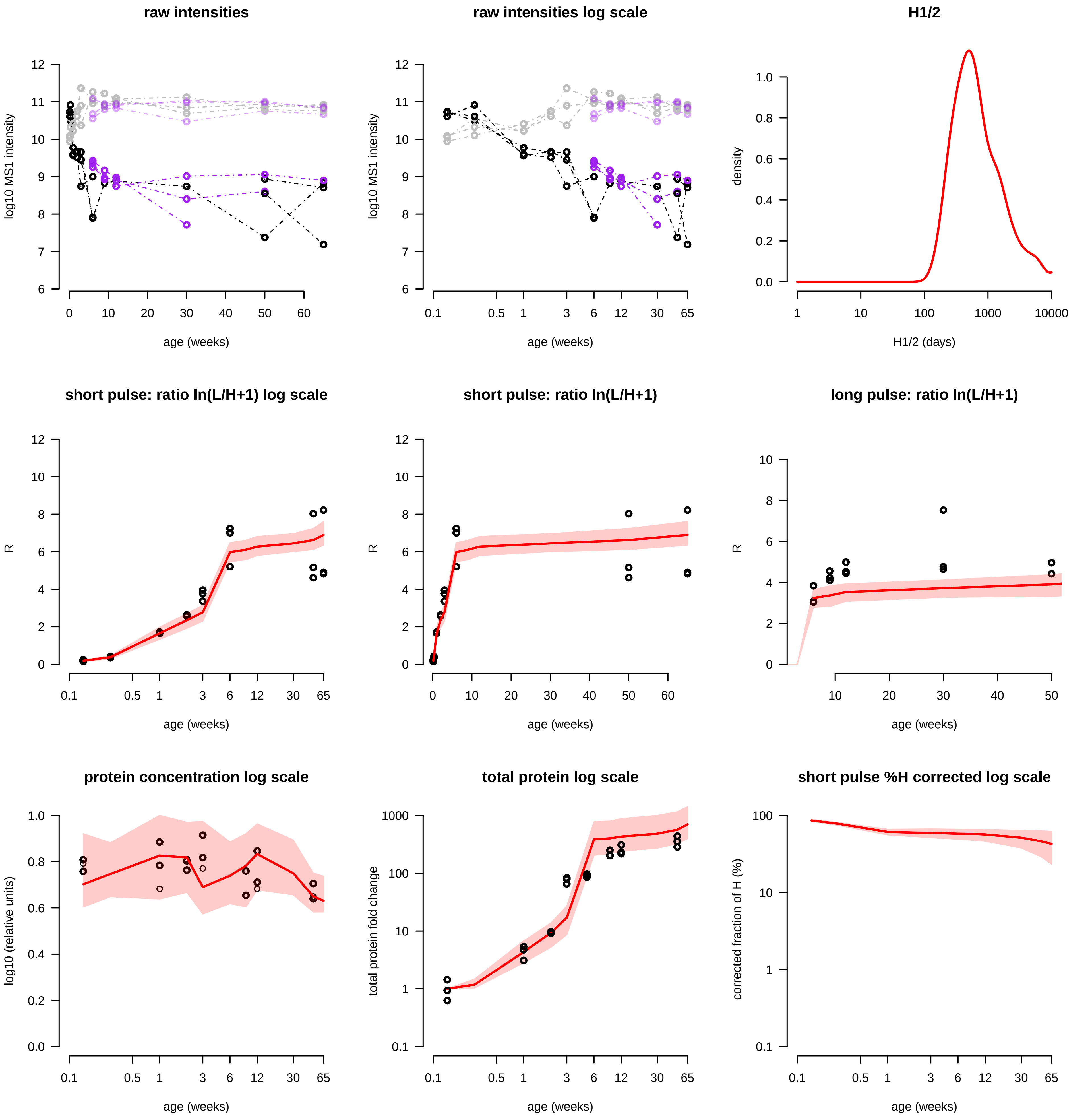

P16858

>sp|P16858|G3P_MOUSE

Glyceraldehyde−3−phosphate dehydrogenase

GN=Gapdh

Med H1/2: 18.1 days

Q5 H1/2: 15 days

Q95 H1/2: 20.3 days

reliable model fit: yes

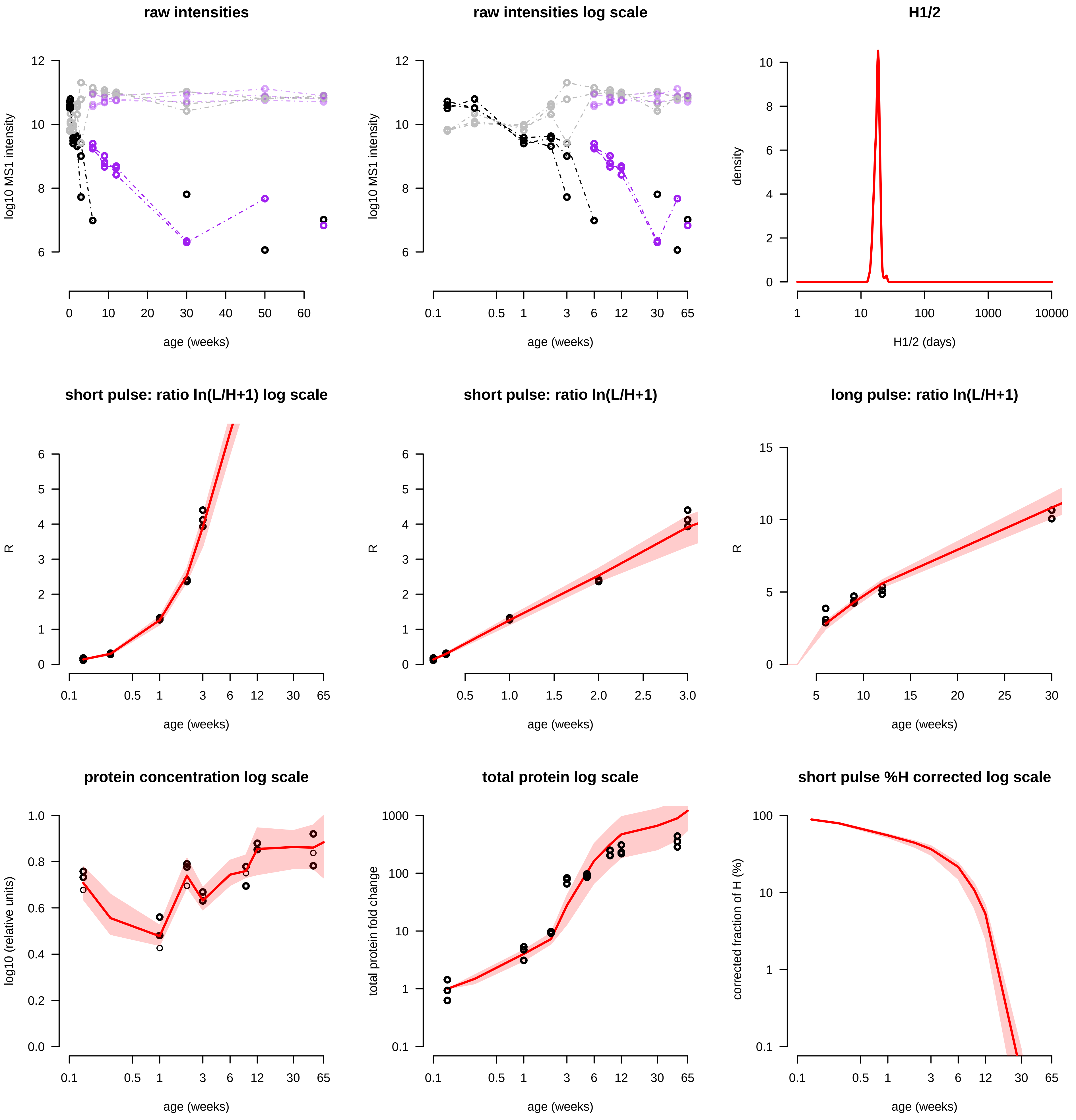

P06801

>sp|P06801|MAOX_MOUSE

NADP−dependent malic enzyme

GN=Me1

Med H1/2: 598.6 days

Q5 H1/2: 218.2 days

Q95 H1/2: 6671.1 days

reliable model fit: no

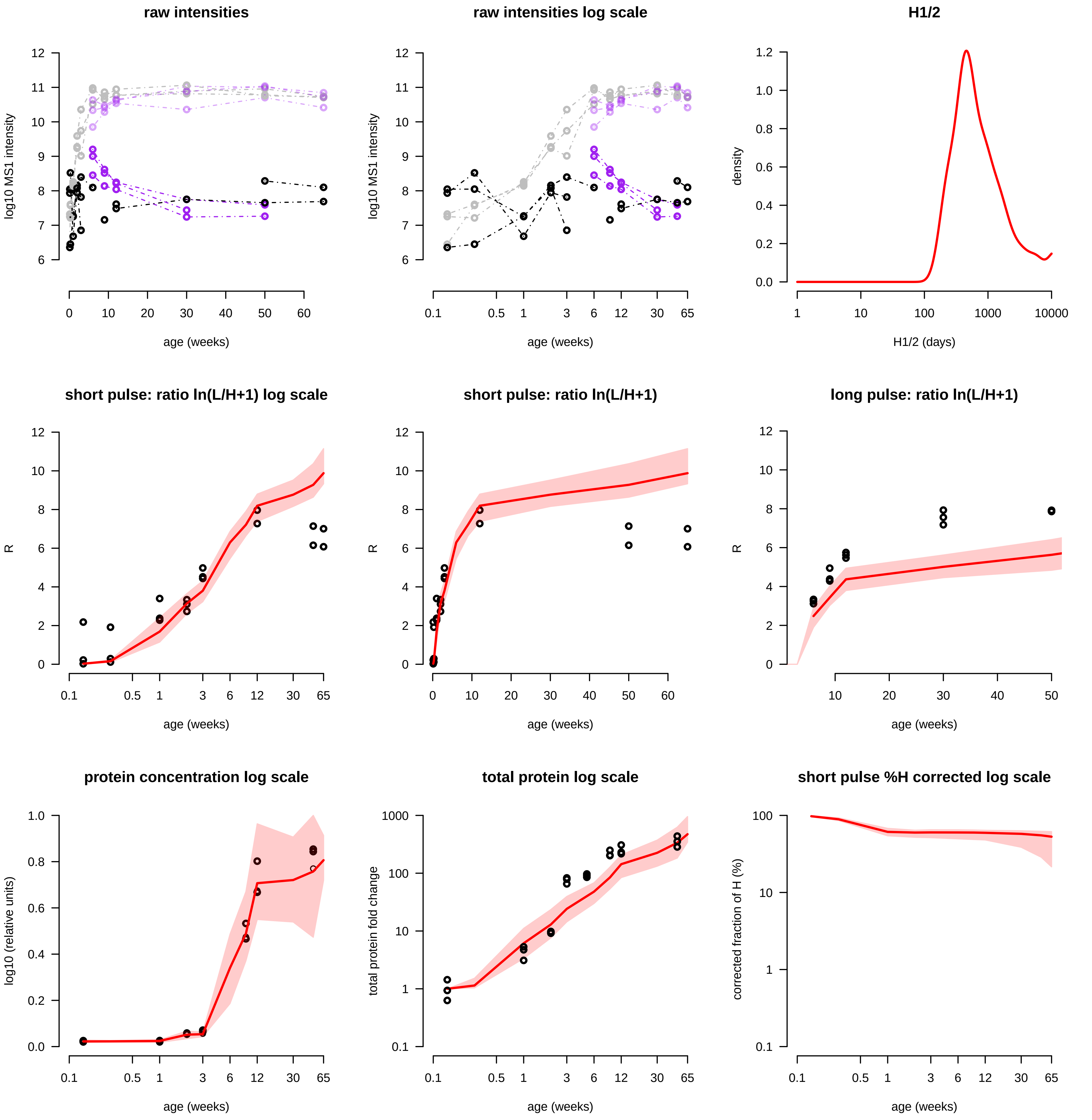

P68369

>sp|P68369|TBA1A_MOUSE

Tubulin alpha−1A chain

GN=Tuba1a

Med H1/2: 59.6 days

Q5 H1/2: 43.2 days

Q95 H1/2: 93.3 days

reliable model fit: yes

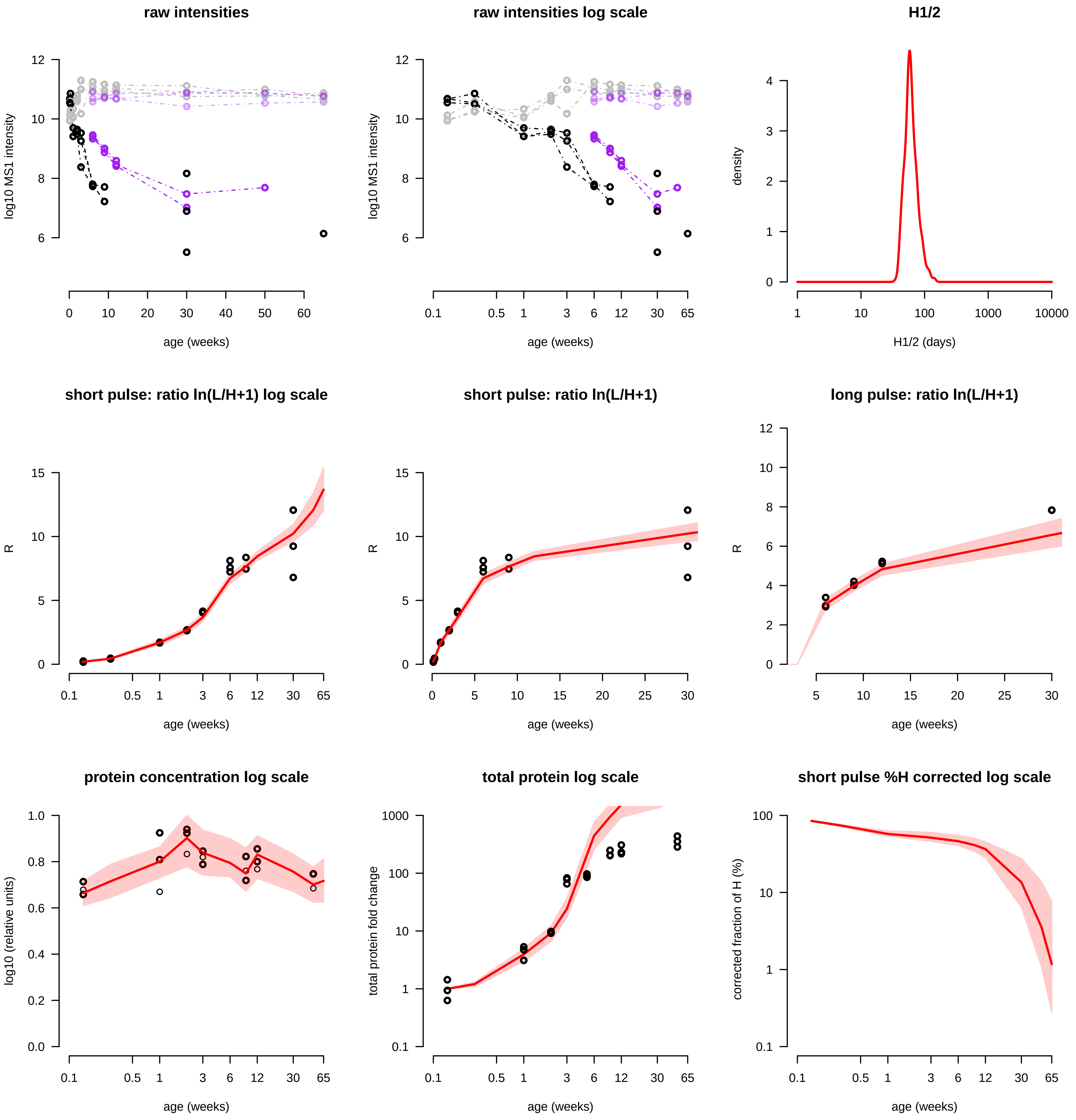

Q9QZ82

>sp|Q9QZ82|CP11A_MOUSE

Cholesterol side−chain cleavage enzyme, mitochondrial

GN=Cyp11a1

Med H1/2: 645.7 days

Q5 H1/2: 215.3 days

Q95 H1/2: 7159.5 days

reliable model fit: yes

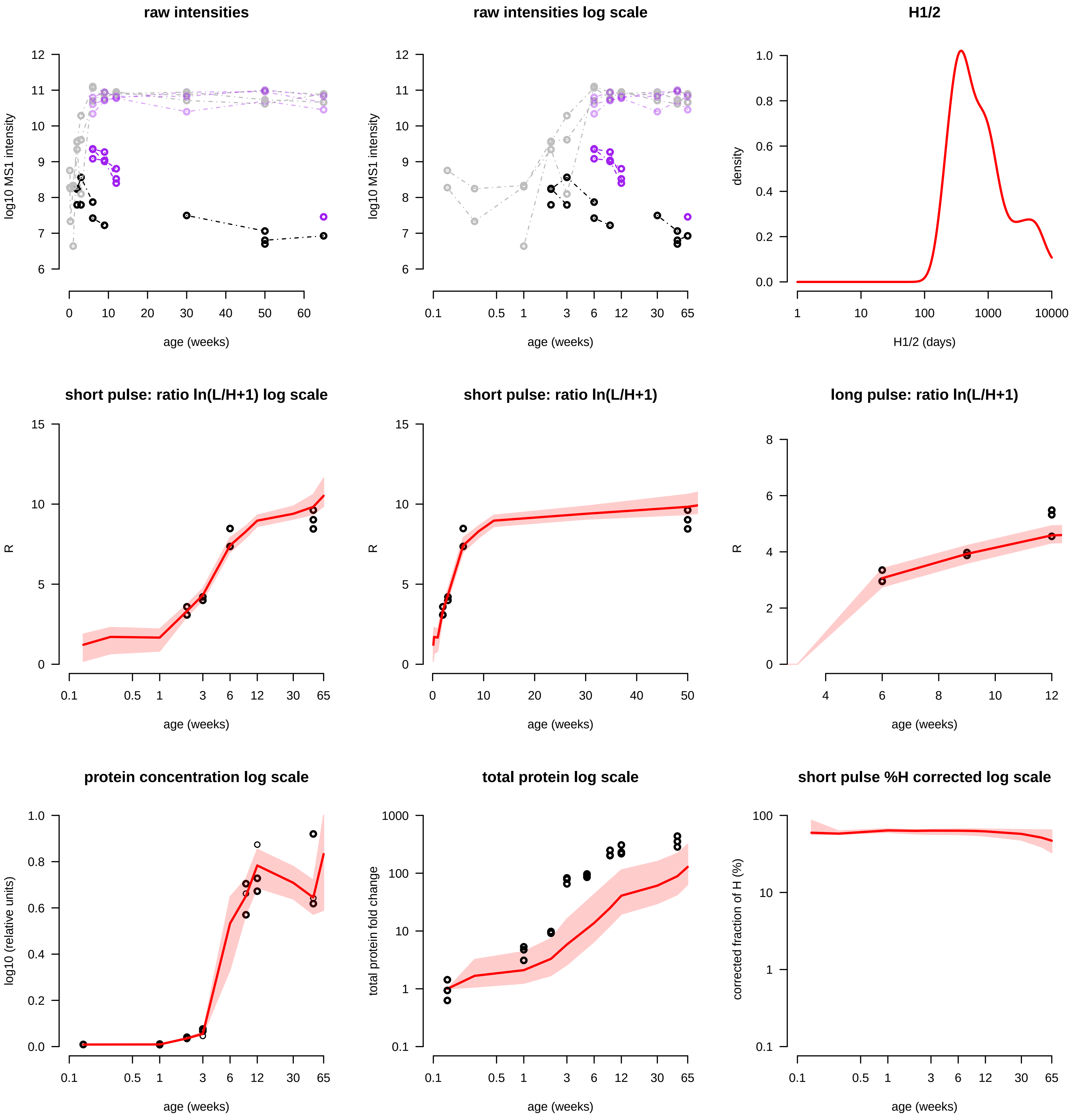

P15864

>sp|P15864|H12_MOUSE

Histone H1.2

GN=H1−2

Med H1/2: 94.6 days

Q5 H1/2: 78.2 days

Q95 H1/2: 120.4 days

reliable model fit: yes

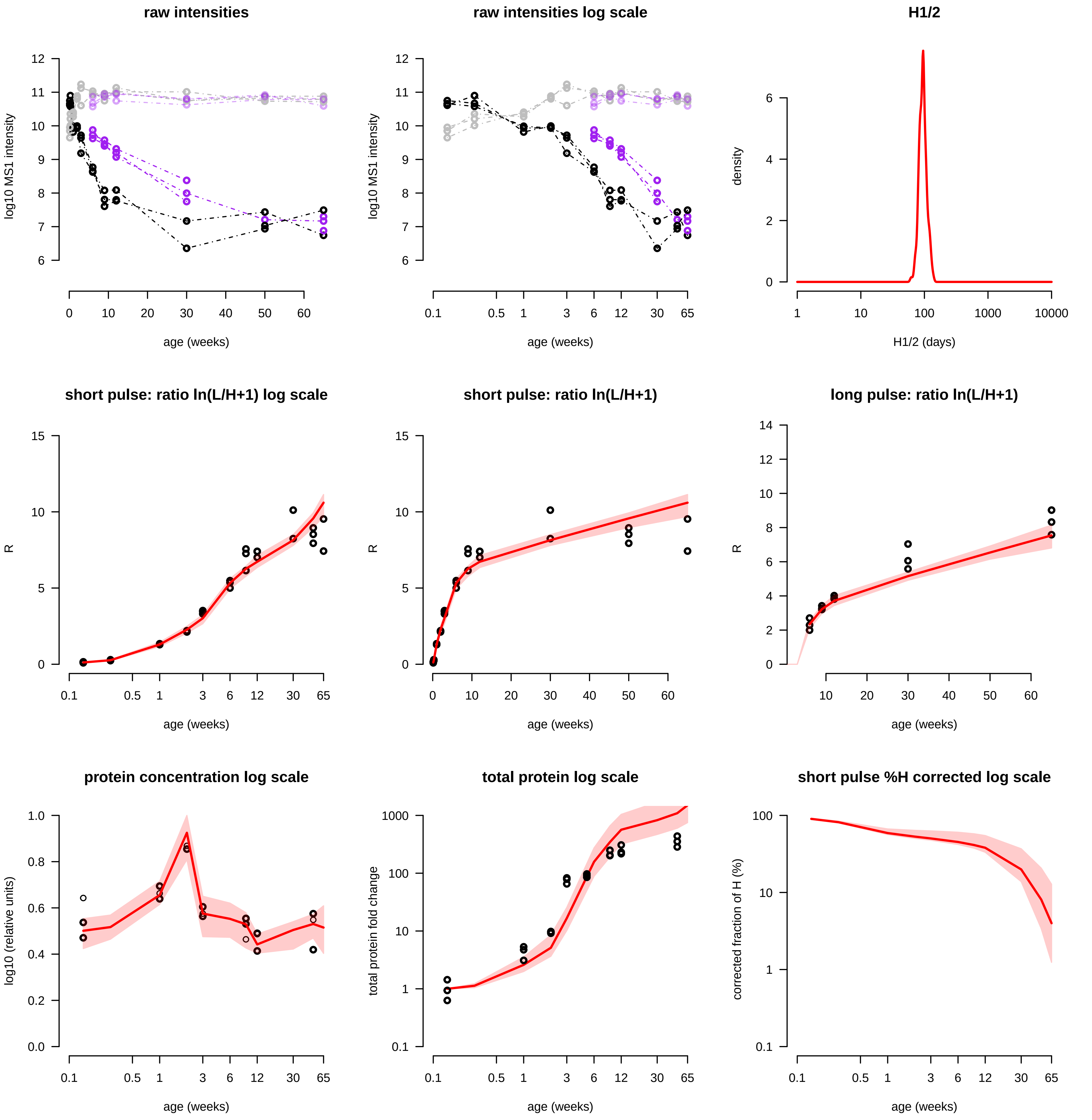

P35700

>sp|P35700|PRDX1_MOUSE

Peroxiredoxin−1

GN=Prdx1

Med H1/2: 149.1 days

Q5 H1/2: 101.4 days

Q95 H1/2: 266.8 days

reliable model fit: yes

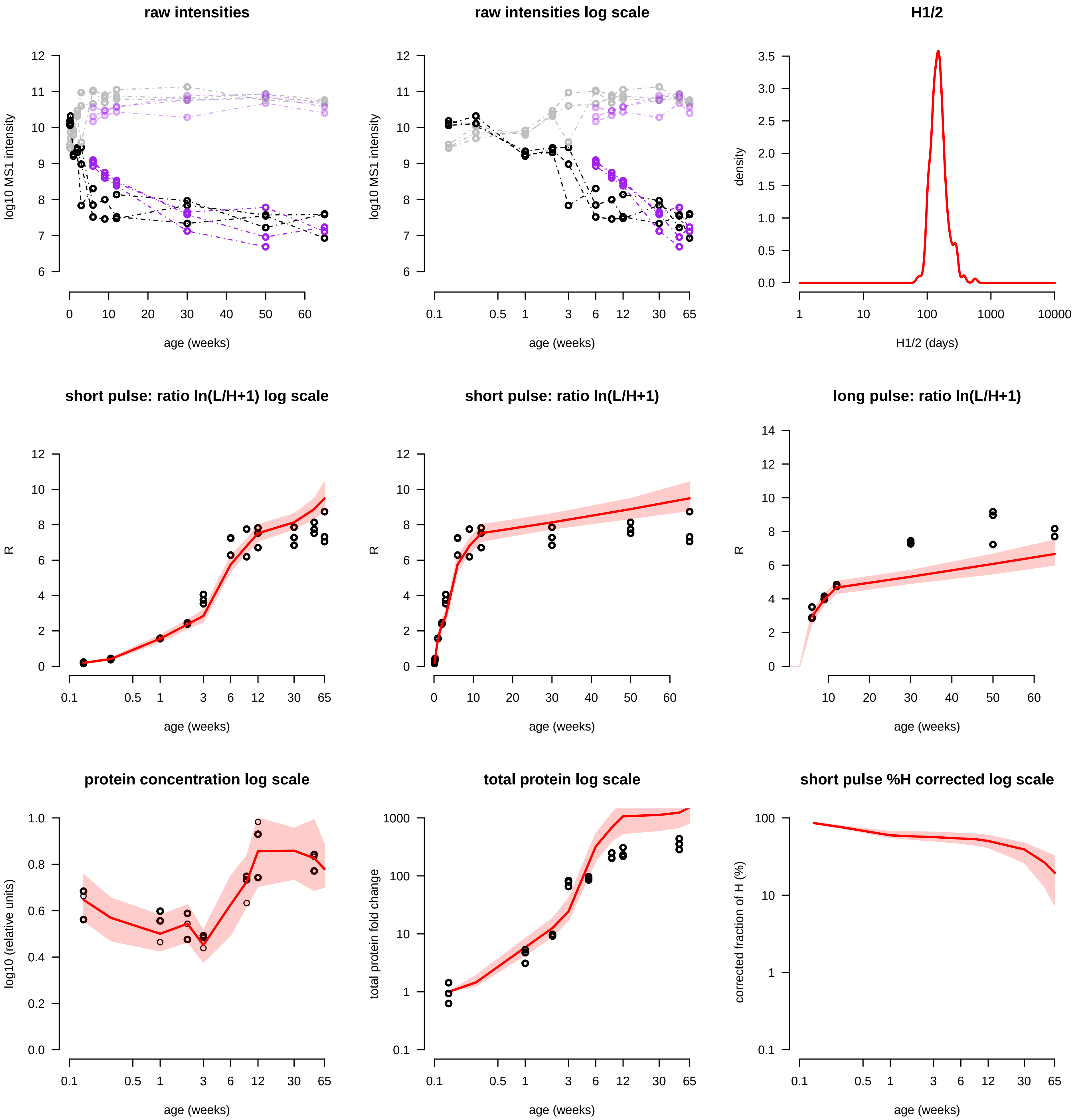

P63017

>sp|P63017|HSP7C_MOUSE

Heat shock cognate 71 kDa protein

GN=Hspa8

Med H1/2: 459.9 days

Q5 H1/2: 150.9 days

Q95 H1/2: 4435.5 days

reliable model fit: yes

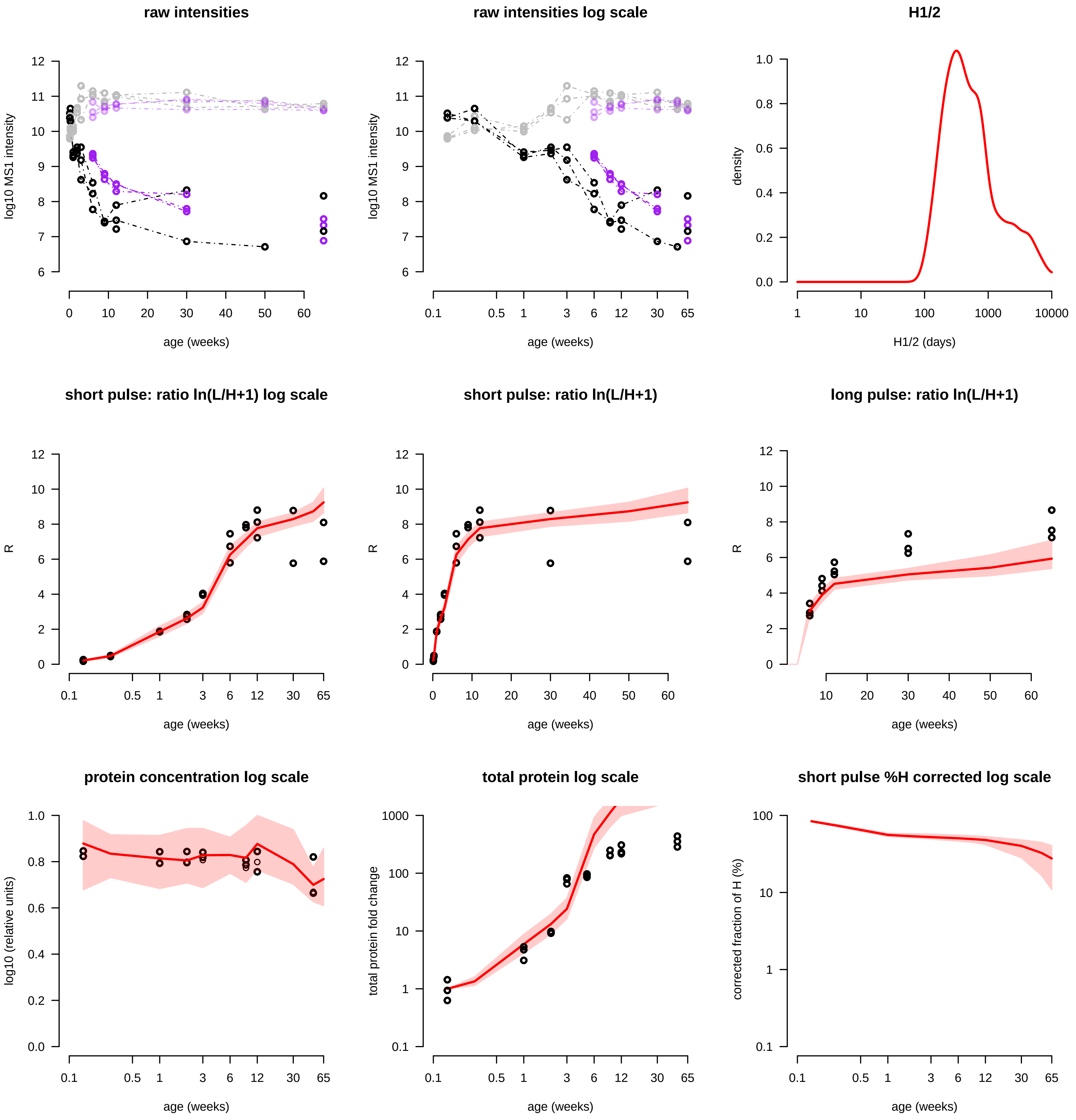

Q03265

>sp|Q03265|ATPA_MOUSE

ATP synthase subunit alpha, mitochondrial

GN=Atp5f1a

Med H1/2: 26.8 days

Q5 H1/2: 18.4 days

Q95 H1/2: 41.1 days

reliable model fit: yes

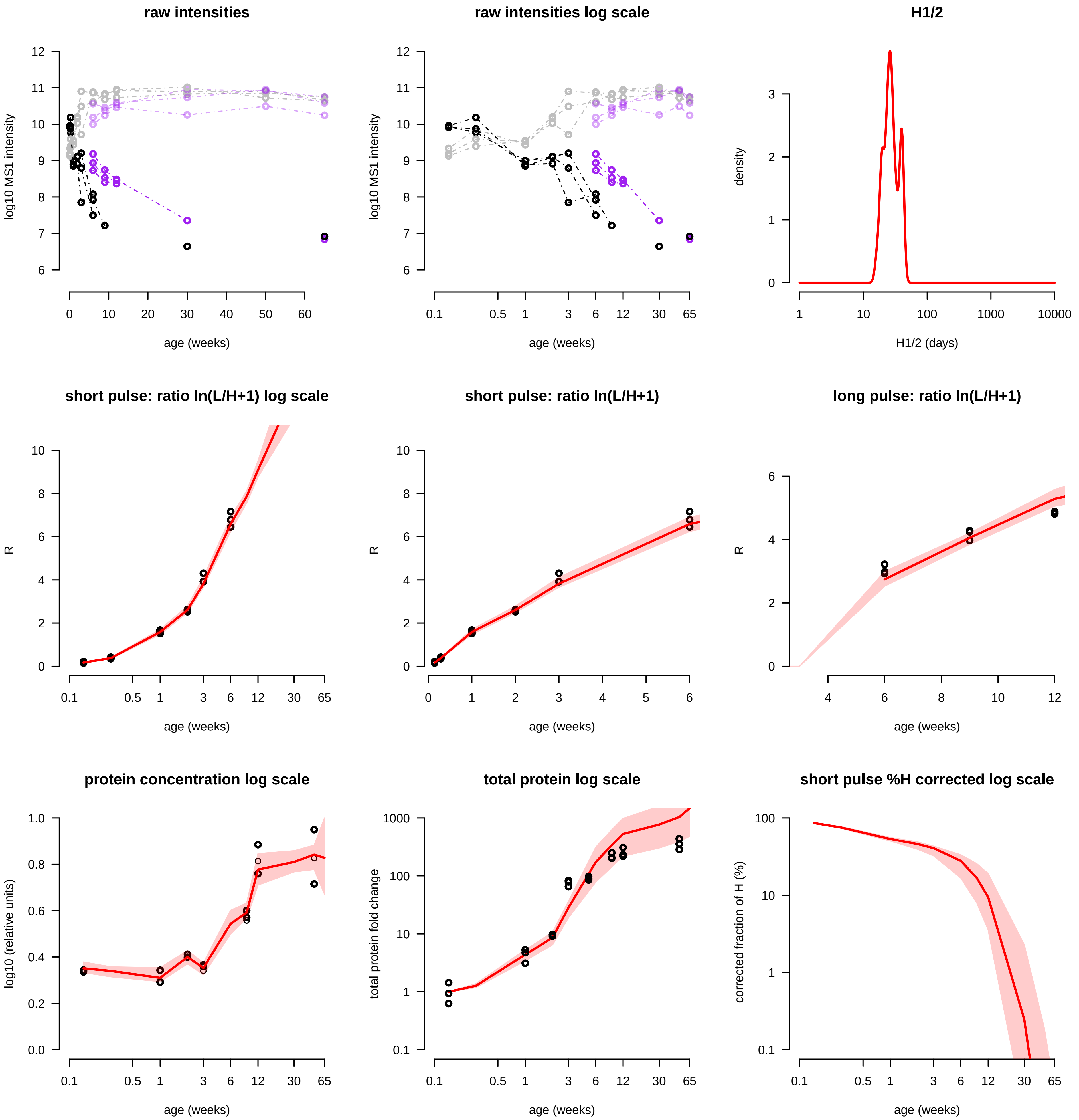

P20029

>sp|P20029|BIP_MOUSE

Endoplasmic reticulum chaperone BiP

GN=Hspa5

Med H1/2: 53.3 days

Q5 H1/2: 40.4 days

Q95 H1/2: 73.6 days

reliable model fit: yes

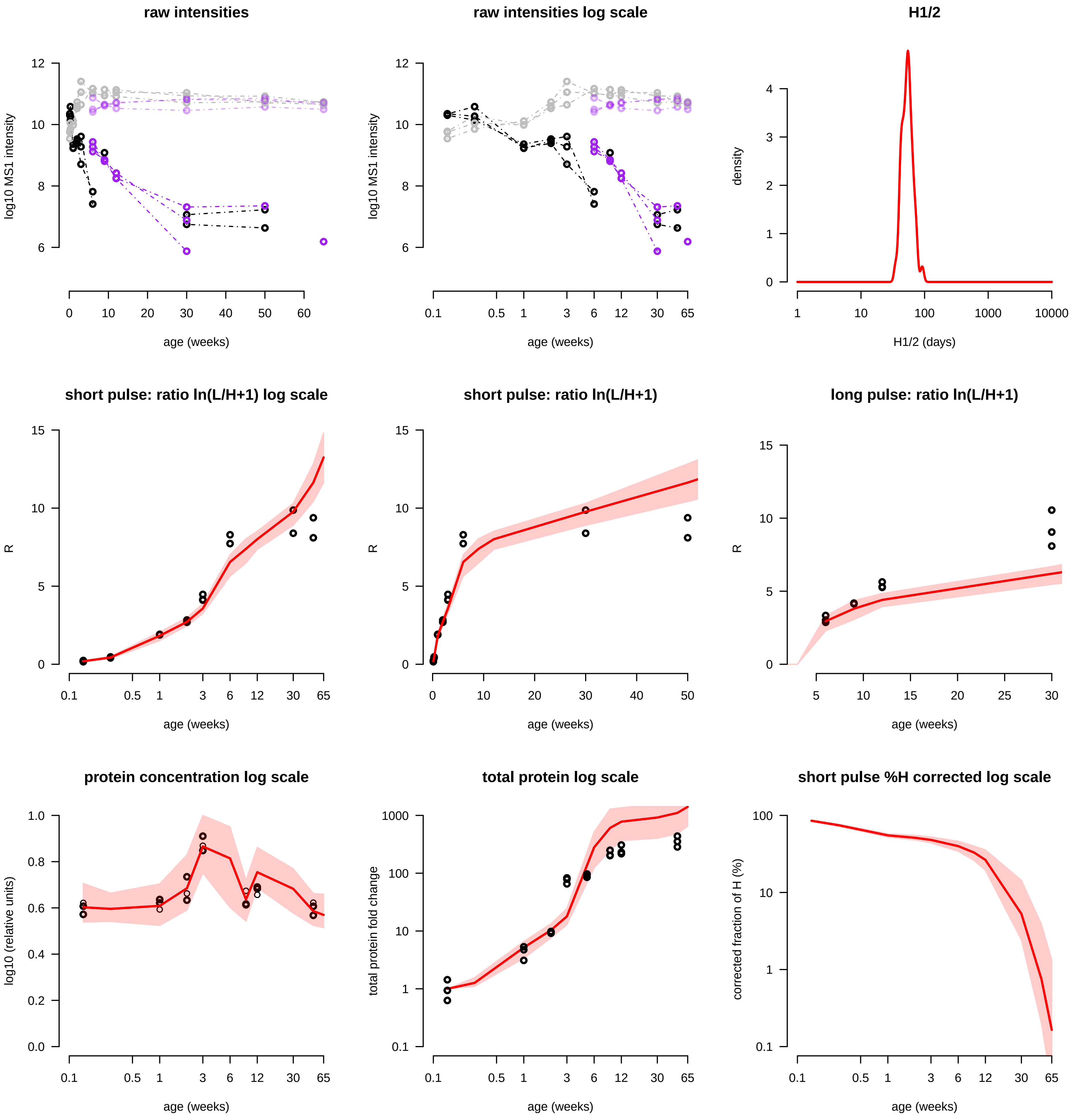

P45376

>sp|P45376|ALDR_MOUSE

Aldo−keto reductase family 1 member B1

GN=Akr1b1

Med H1/2: 50.5 days

Q5 H1/2: 39 days

Q95 H1/2: 68.1 days

reliable model fit: yes

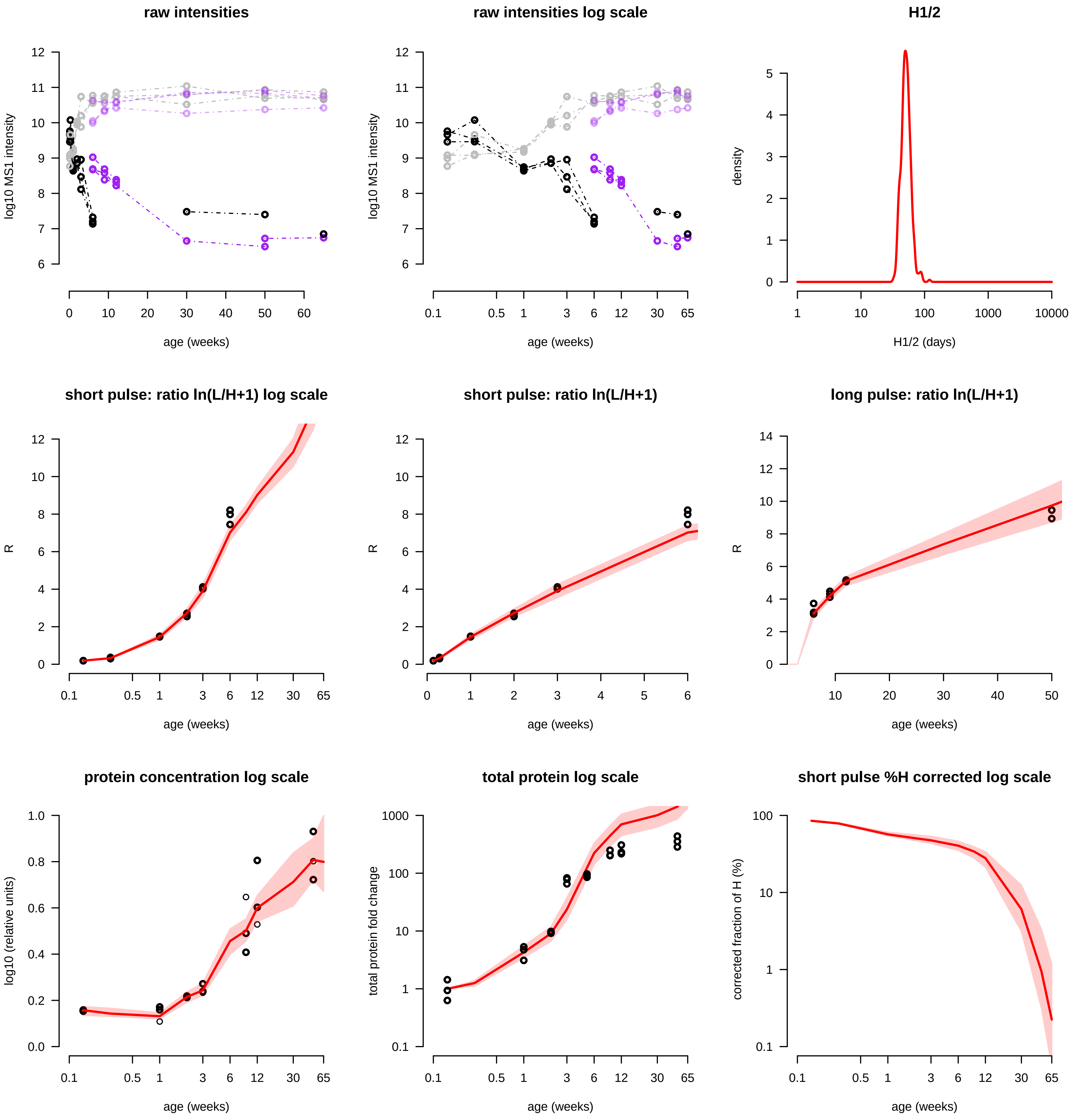

P56480

>sp|P56480|ATPB_MOUSE

ATP synthase subunit beta, mitochondrial

GN=Atp5f1b

Med H1/2: 63.5 days

Q5 H1/2: 49.6 days

Q95 H1/2: 82.2 days

reliable model fit: yes

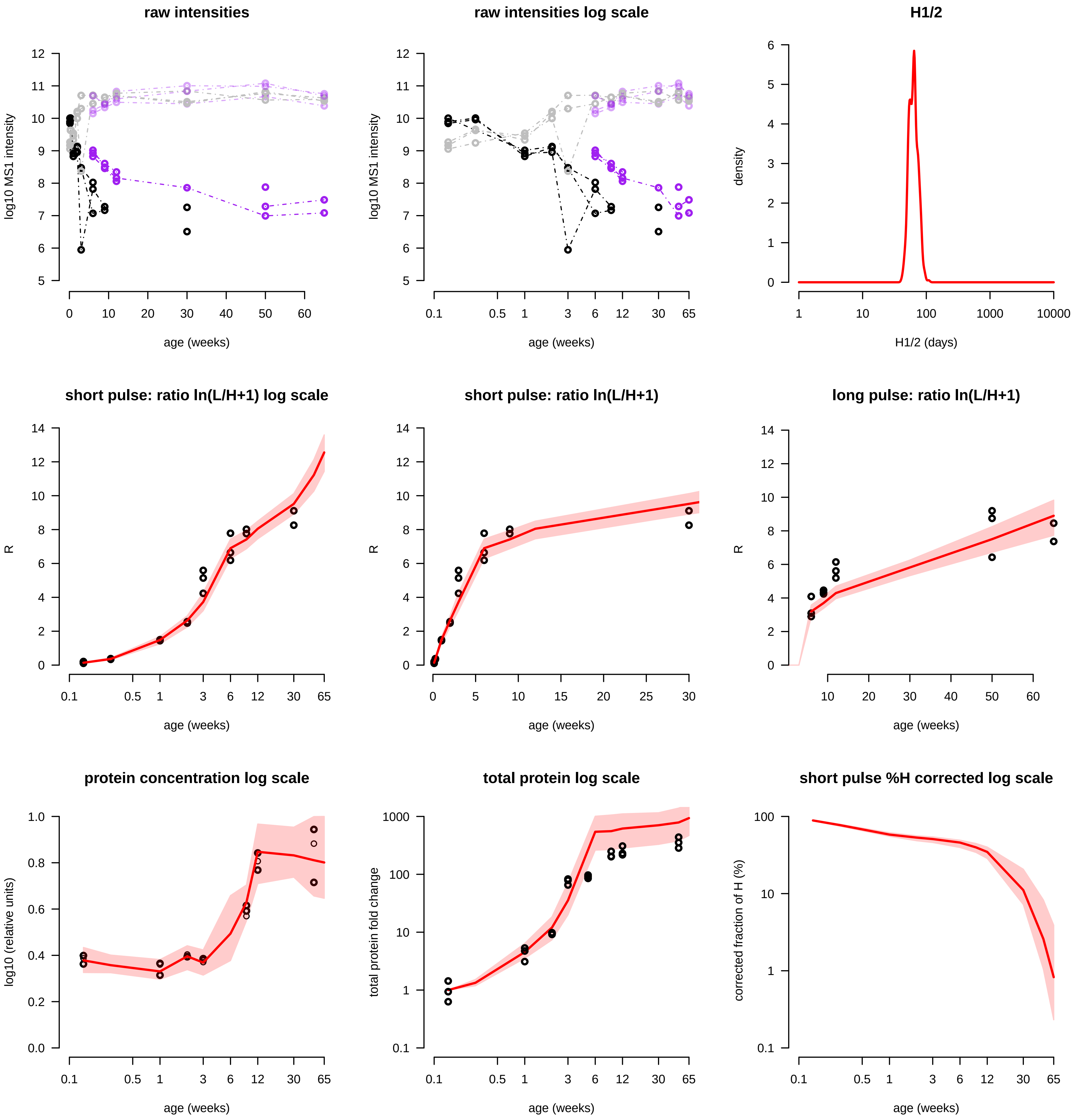

P62737

>sp|P62737|ACTA_MOUSE

Actin, aortic smooth muscle

GN=Acta2

Med H1/2: 8.2 days

Q5 H1/2: 6.8 days

Q95 H1/2: 10.4 days

reliable model fit: yes

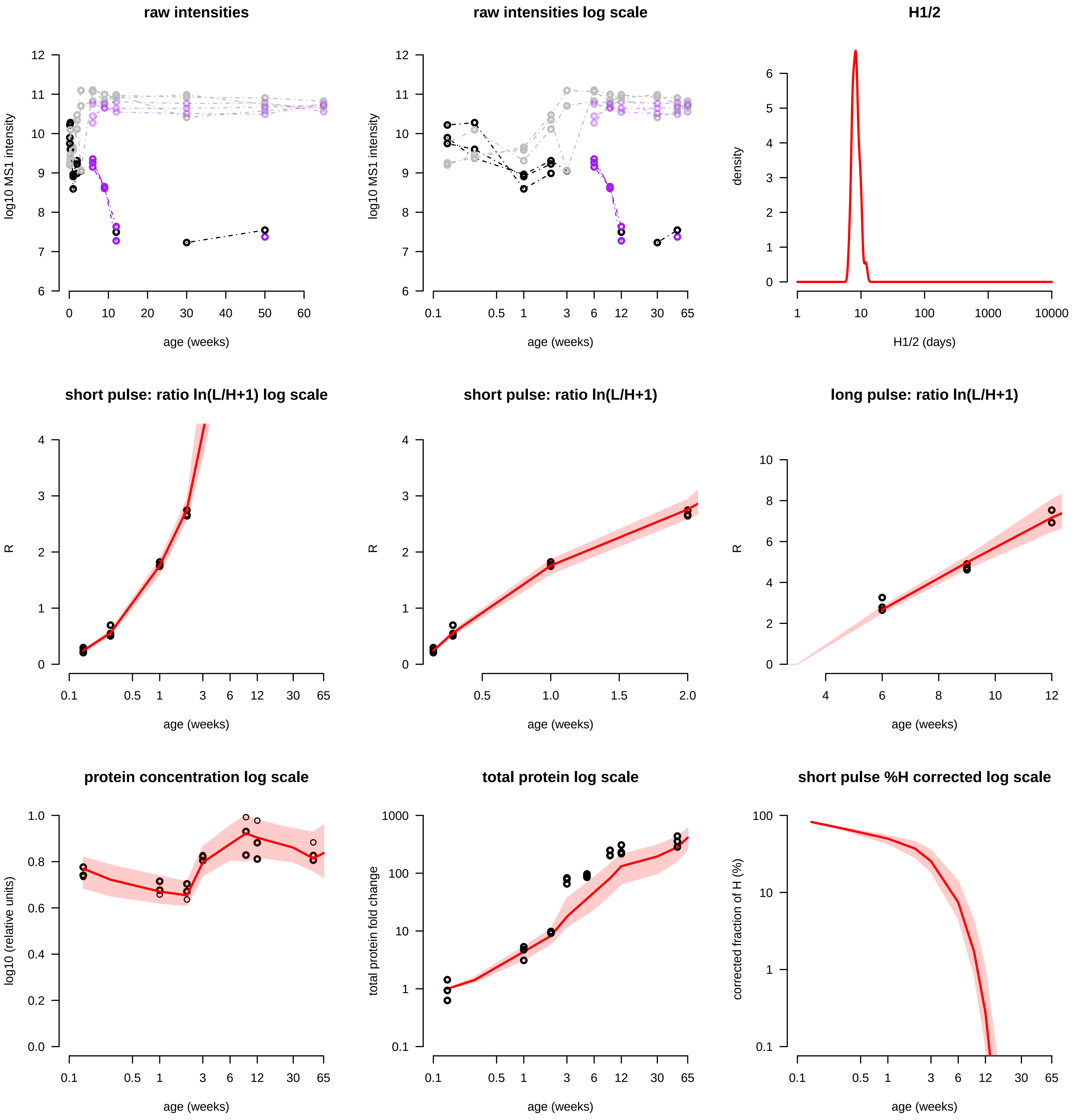

P15626

>sp|P15626|GSTM2_MOUSE

Glutathione S−transferase Mu 2

GN=Gstm2

Med H1/2: 156.8 days

Q5 H1/2: 95.2 days

Q95 H1/2: 284.8 days

reliable model fit: yes

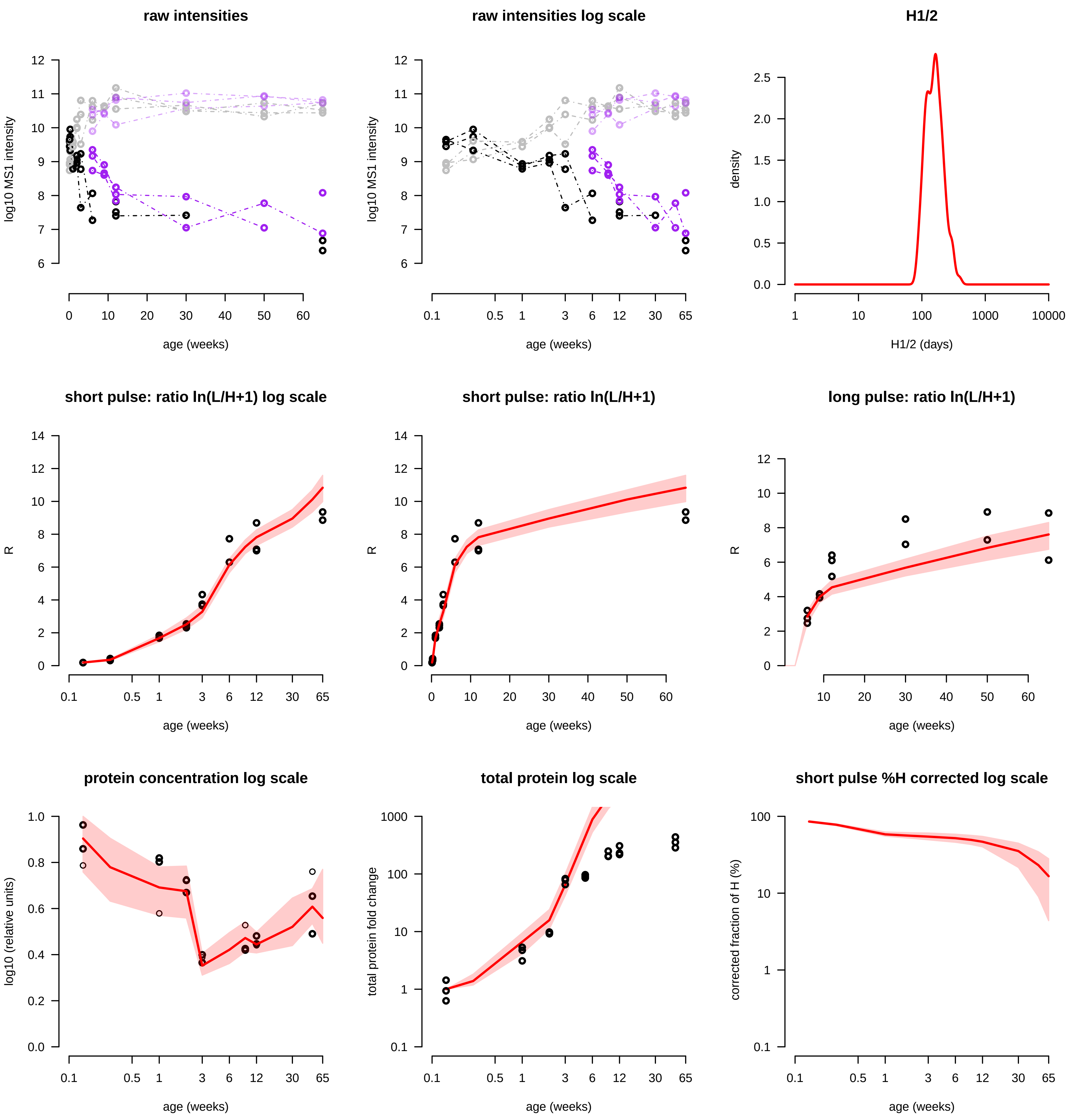

Q921I1

>sp|Q921I1|TRFE_MOUSE

Serotransferrin

GN=Tf

Med H1/2: 76.3 days

Q5 H1/2: 47.3 days

Q95 H1/2: 3091.3 days

reliable model fit: yes

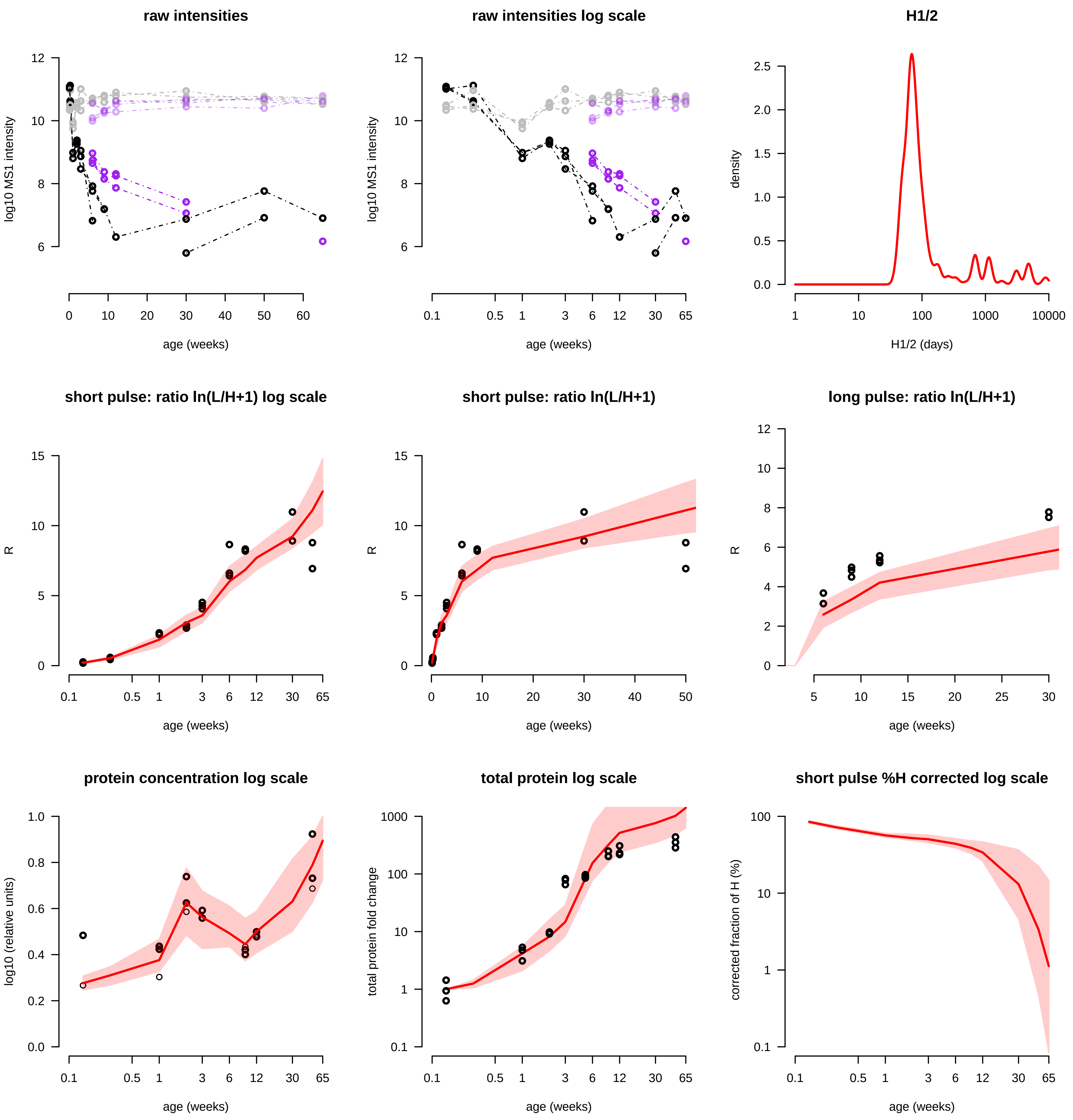

P14211

>sp|P14211|CALR_MOUSE

Calreticulin

GN=Calr

Med H1/2: 604.3 days

Q5 H1/2: 192.1 days

Q95 H1/2: 8660.1 days

reliable model fit: yes

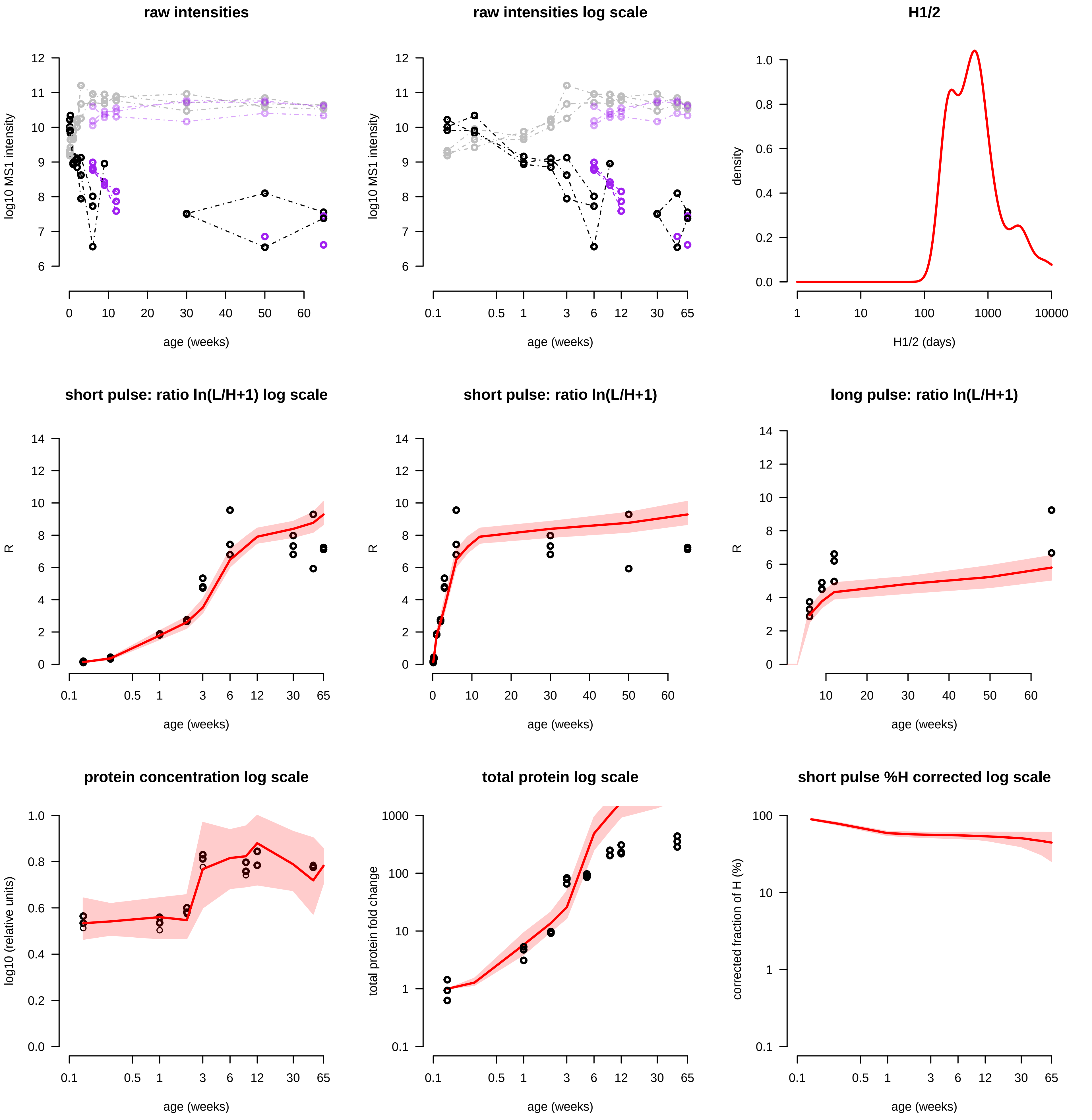

Q60932−2;Q60932

>sp|Q60932−2|VDAC1_MOUSE

Isoform Mt−VDAC1 of Voltage−dependent anion−selective channel protein 1

GN=Vdac1

Med H1/2: 25.5 days

Q5 H1/2: 19.5 days

Q95 H1/2: 34.2 days

reliable model fit: yes

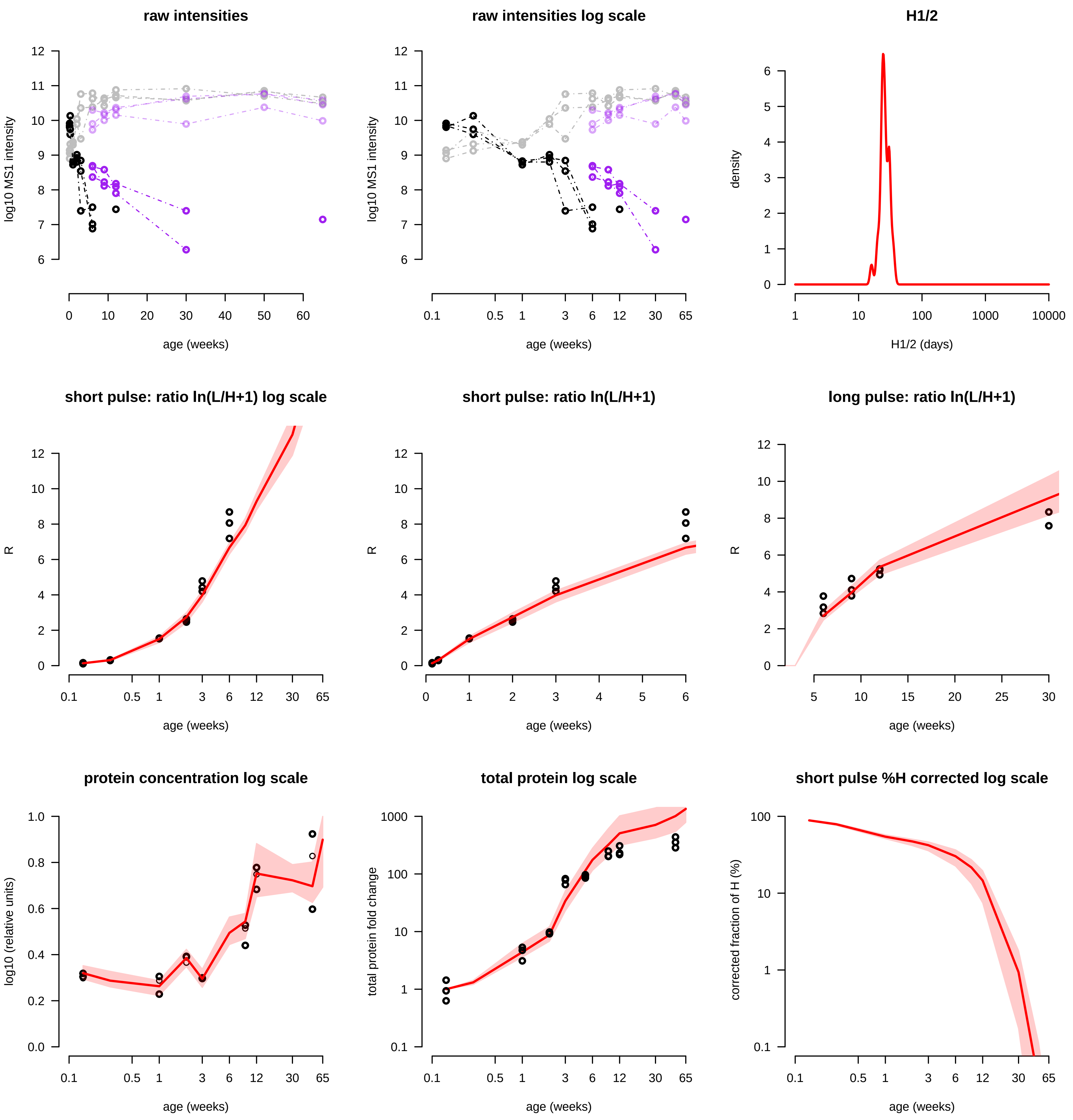

P17742

>sp|P17742|PPIA_MOUSE

Peptidyl−prolyl cis−trans isomerase A

GN=Ppia

Med H1/2: 186.9 days

Q5 H1/2: 113.5 days

Q95 H1/2: 512.4 days

reliable model fit: yes

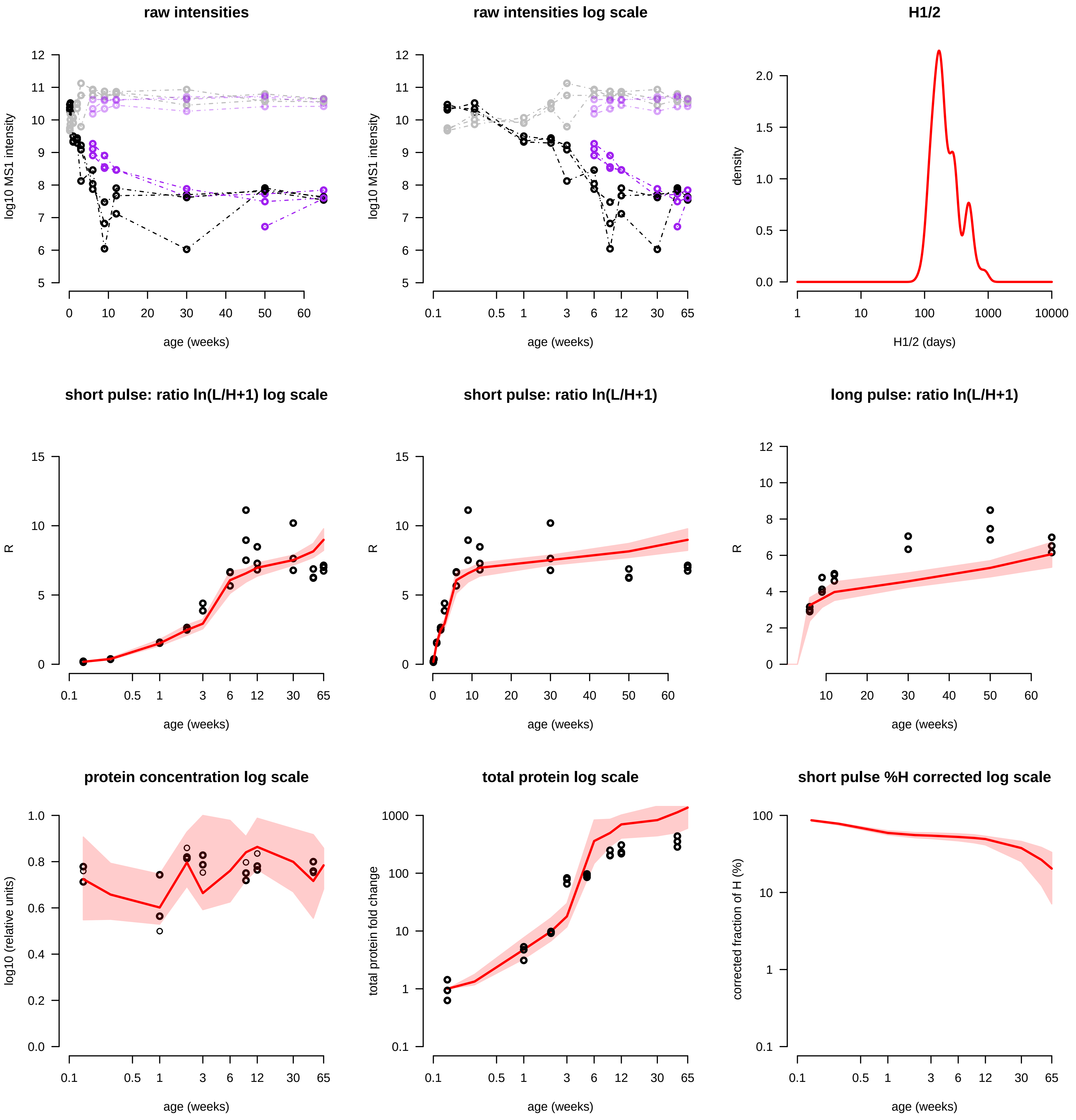

P84228

>sp|P84228|H32_MOUSE

Histone H3.2

GN=H3c2

Med H1/2: 786 days

Q5 H1/2: 308.9 days

Q95 H1/2: 16727.6 days

reliable model fit: yes

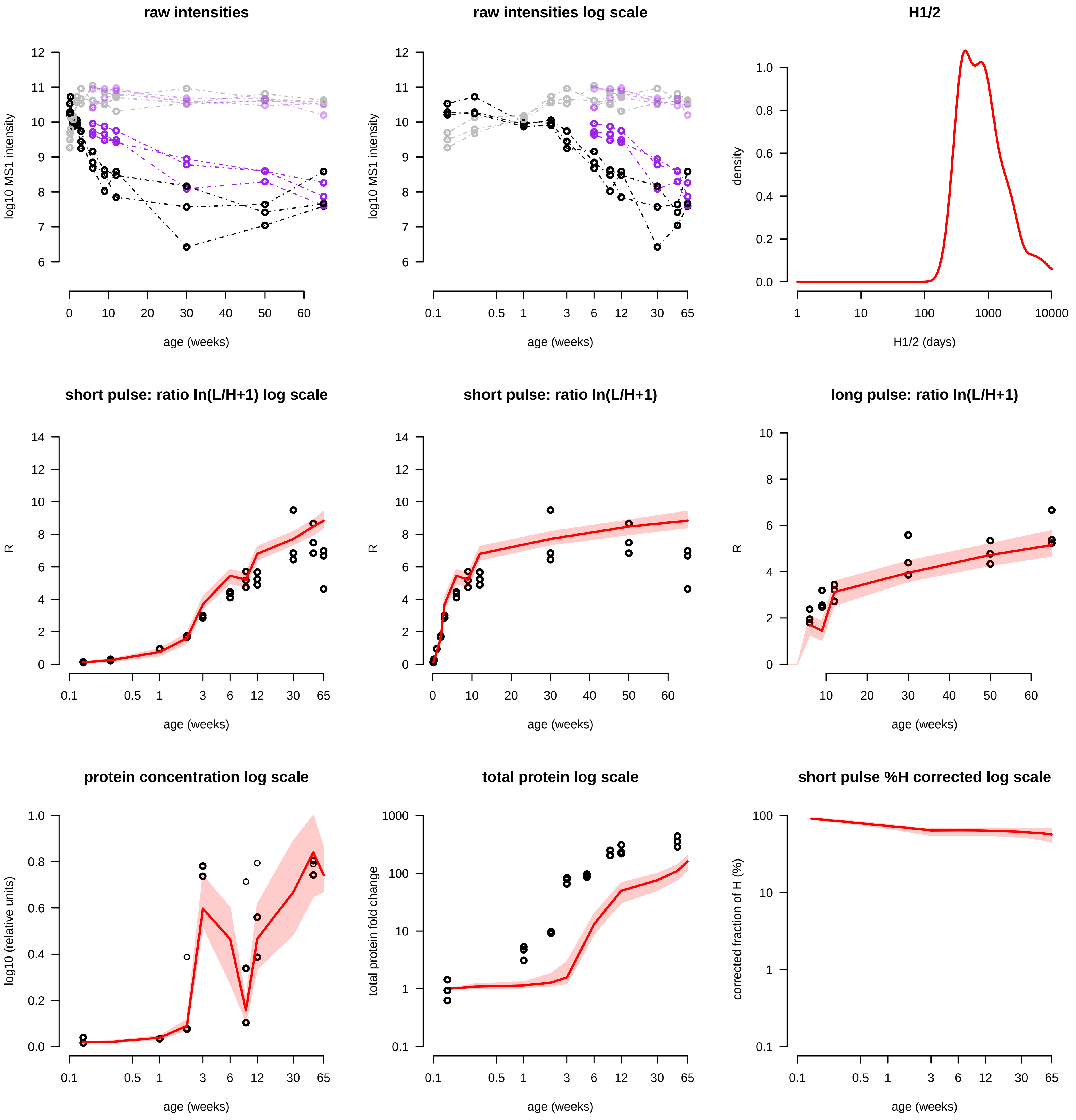

P07356

>sp|P07356|ANXA2_MOUSE

Annexin A2

GN=Anxa2

Med H1/2: 342.7 days

Q5 H1/2: 147.9 days

Q95 H1/2: 1968.4 days

reliable model fit: yes

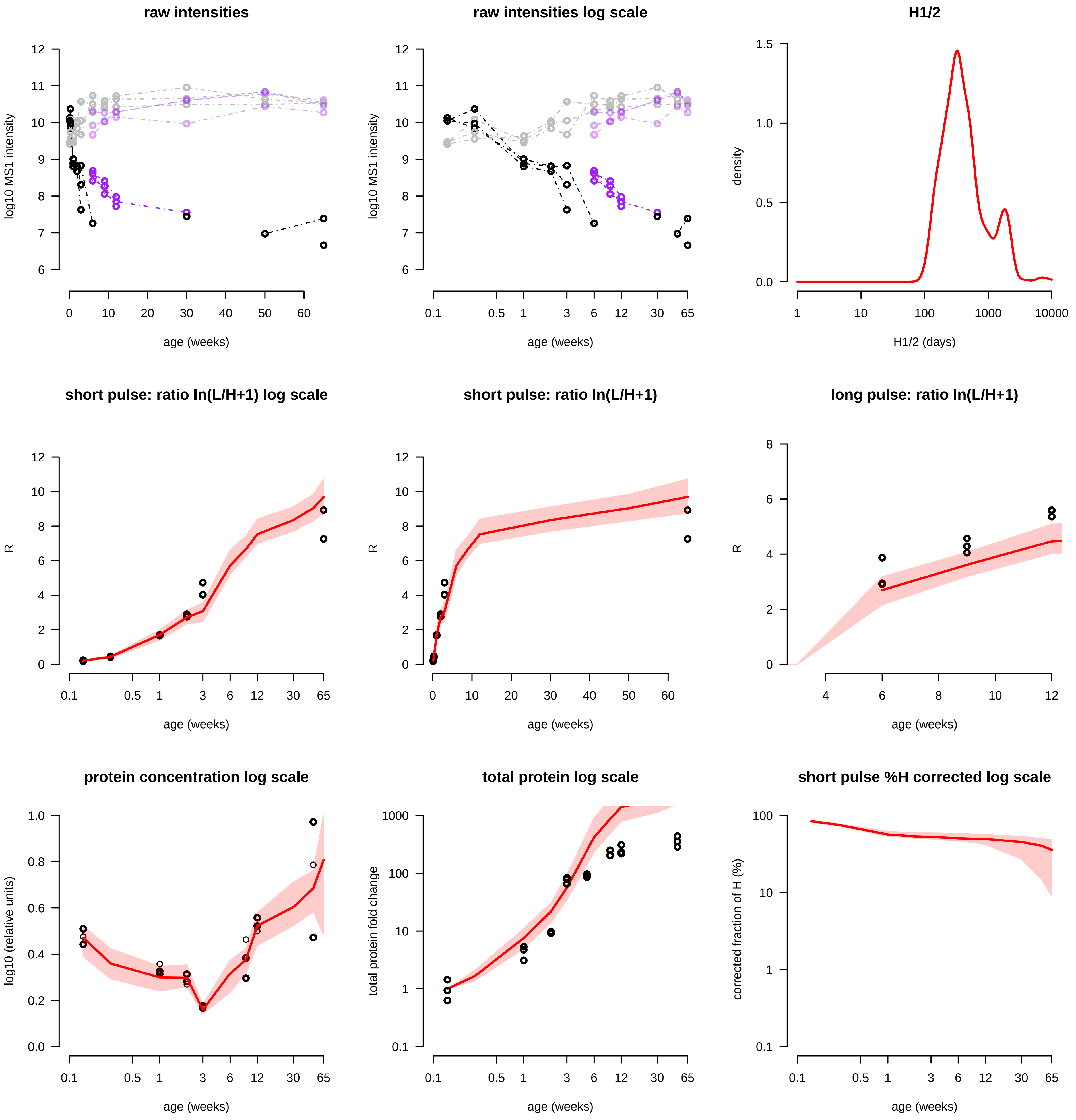

Q8VDD5

>sp|Q8VDD5|MYH9_MOUSE

Myosin−9

GN=Myh9

Med H1/2: 494.5 days

Q5 H1/2: 185.2 days

Q95 H1/2: 13449 days

reliable model fit: yes

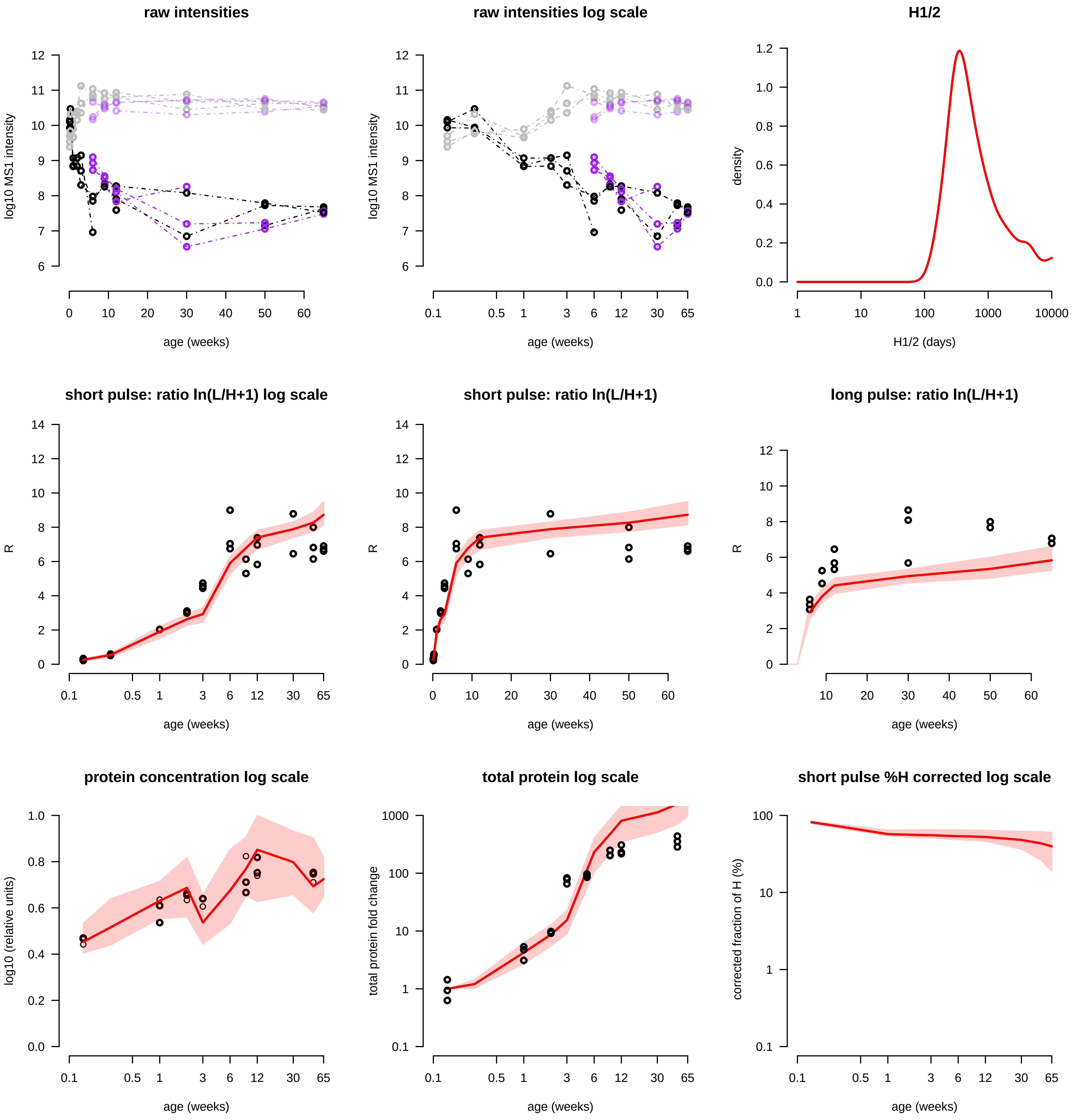

P27773

>sp|P27773|PDIA3_MOUSE

Protein disulfide−isomerase A3

GN=Pdia3

Med H1/2: 261.3 days

Q5 H1/2: 148.5 days

Q95 H1/2: 13115.2 days

reliable model fit: yes

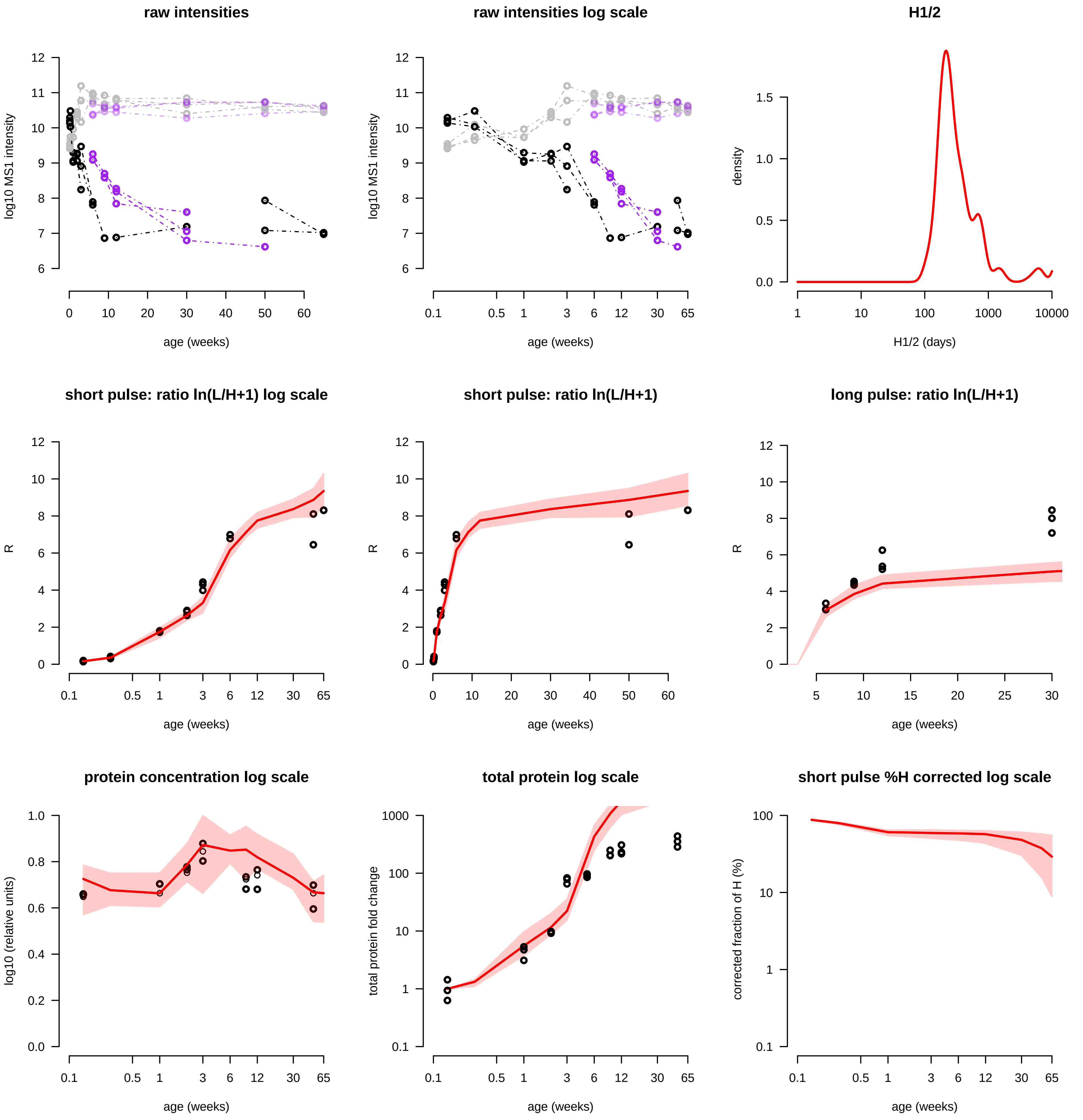

Q64433

>sp|Q64433|CH10_MOUSE

10 kDa heat shock protein, mitochondrial

GN=Hspe1

Med H1/2: 81.4 days

Q5 H1/2: 49.5 days

Q95 H1/2: 153.3 days

reliable model fit: yes

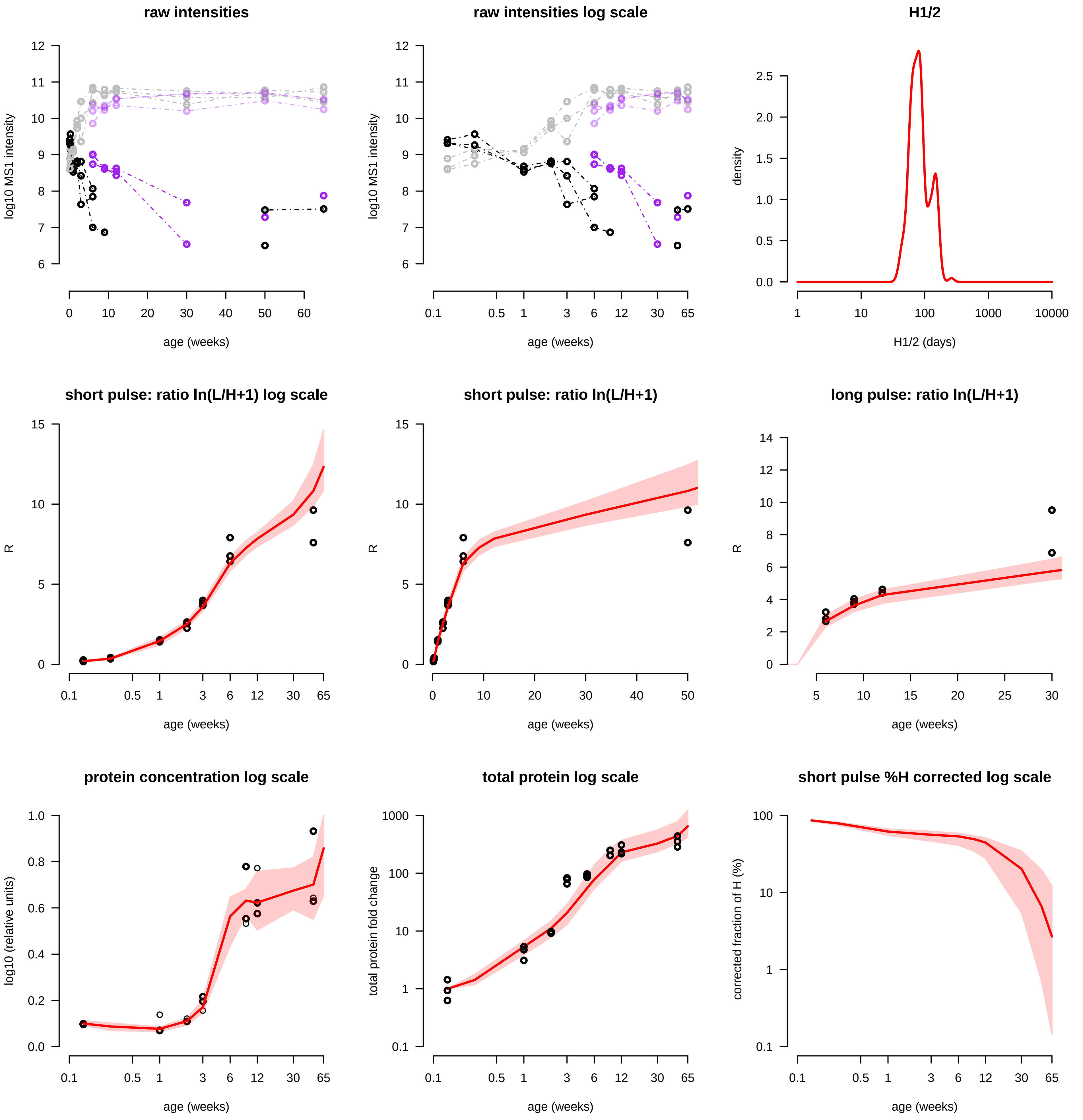

P09103

>sp|P09103|PDIA1_MOUSE

Protein disulfide−isomerase

GN=P4hb

Med H1/2: 2839.5 days

Q5 H1/2: 673.5 days

Q95 H1/2: 91291.4 days

reliable model fit: yes

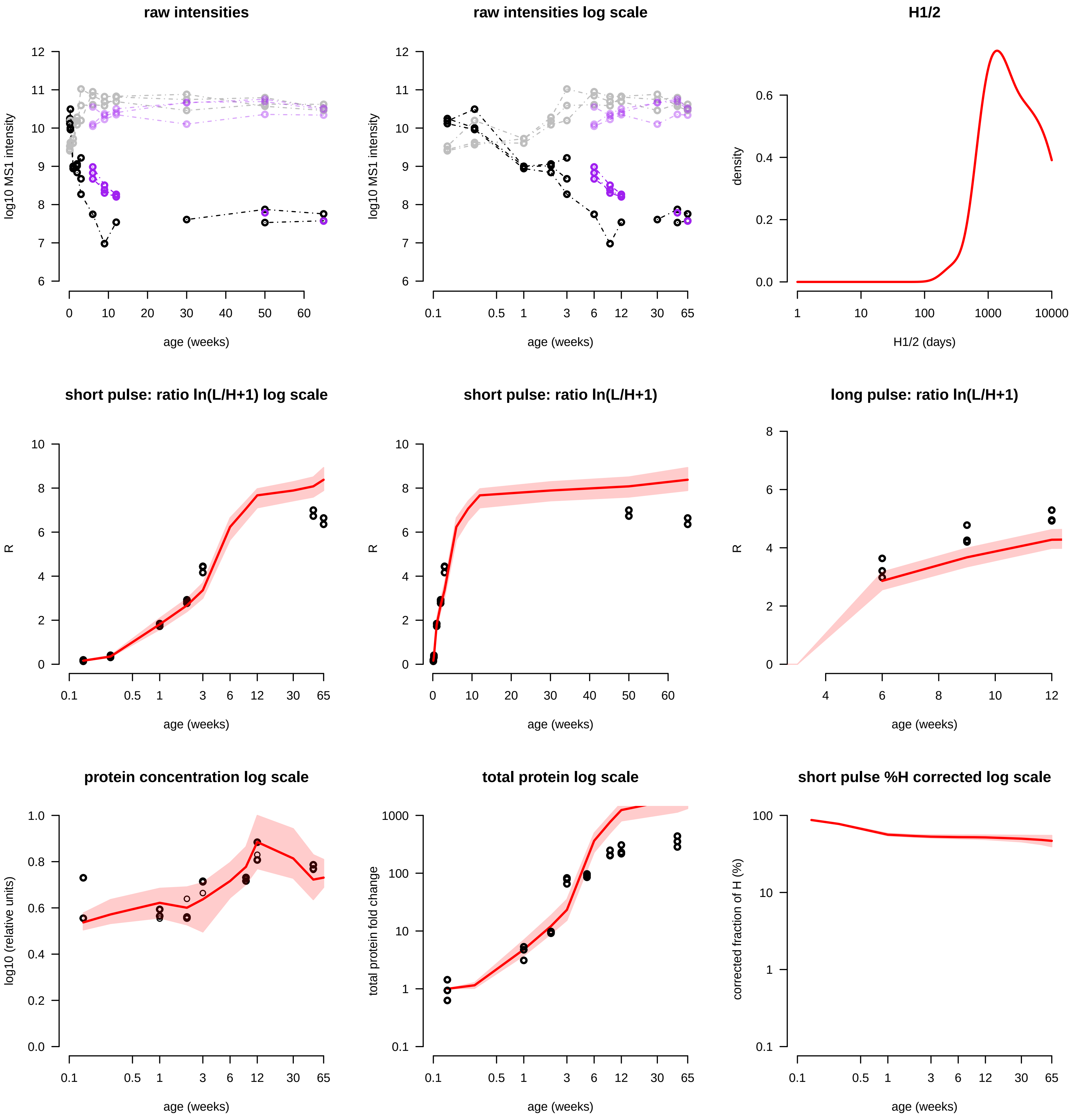

P29391;P49945

>sp|P29391|FRIL1_MOUSE

Ferritin light chain 1

GN=Ftl1

Med H1/2: 87 days

Q5 H1/2: 51.4 days

Q95 H1/2: 232.2 days

reliable model fit: yes

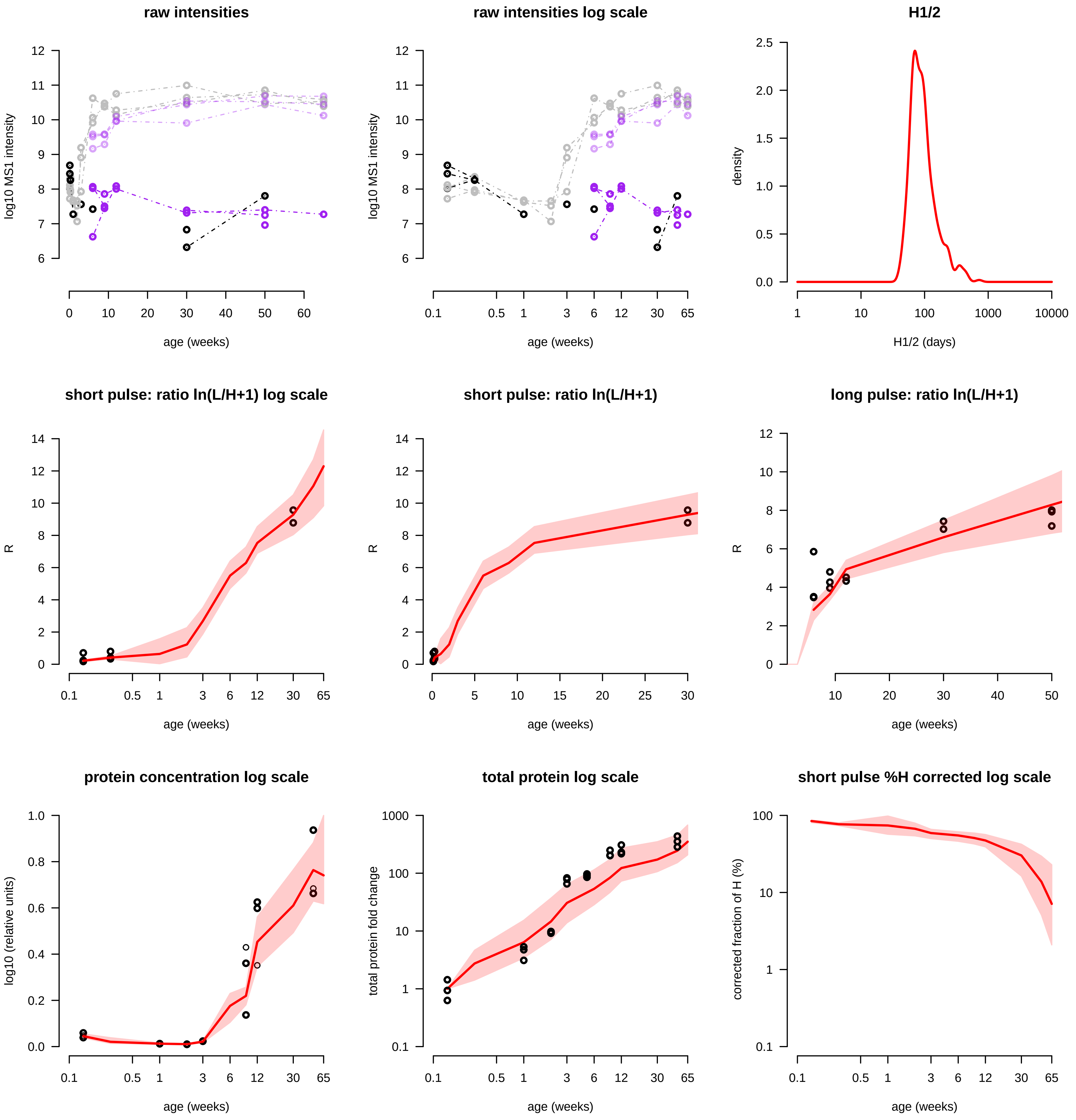

P52480

>sp|P52480|KPYM_MOUSE

Pyruvate kinase PKM

GN=Pkm

Med H1/2: 363.3 days

Q5 H1/2: 123.5 days

Q95 H1/2: 3479.3 days

reliable model fit: yes

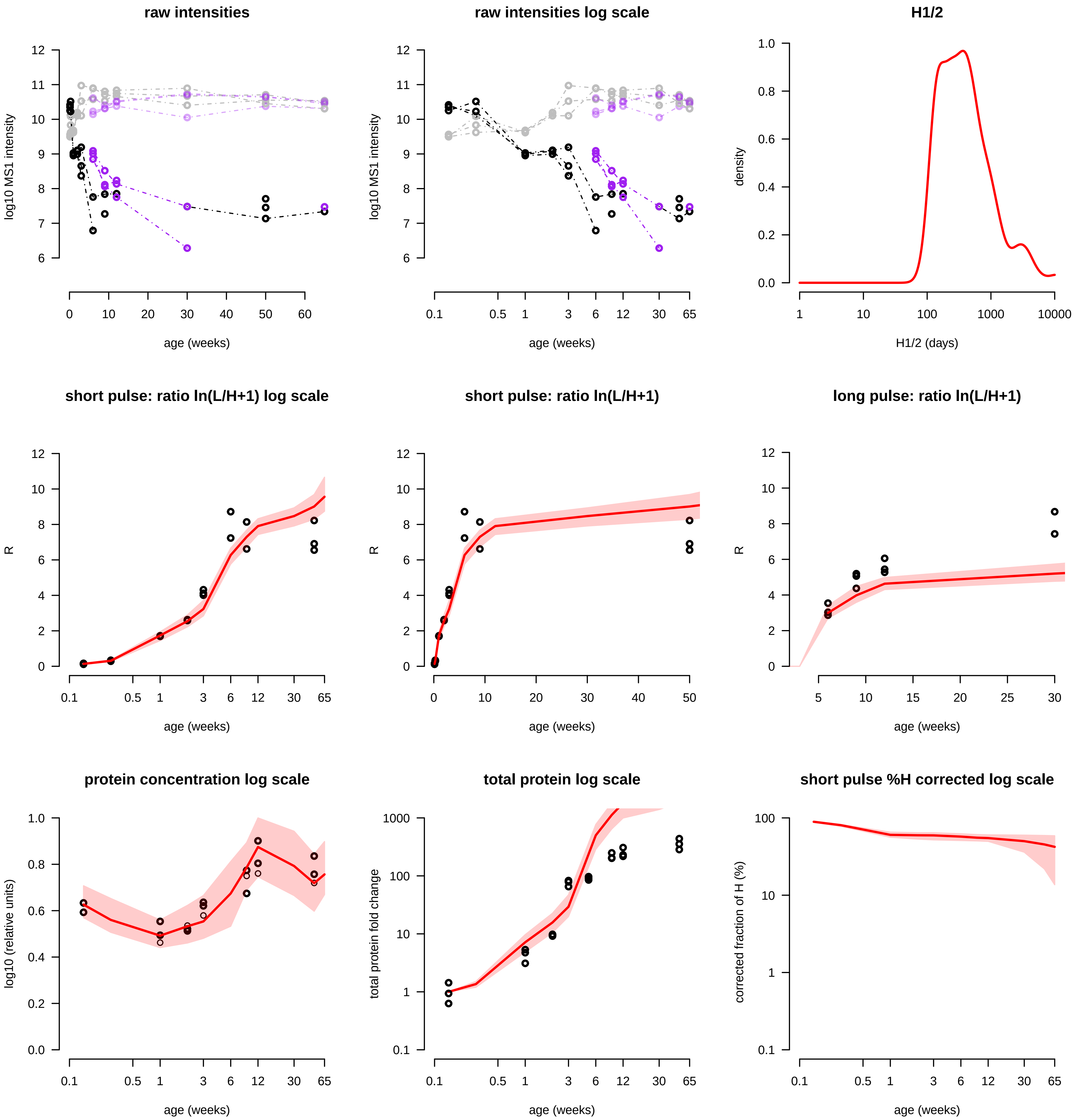

P38647

>sp|P38647|GRP75_MOUSE

Stress−70 protein, mitochondrial

GN=Hspa9

Med H1/2: 436.8 days

Q5 H1/2: 159.1 days

Q95 H1/2: 3334.3 days

reliable model fit: yes

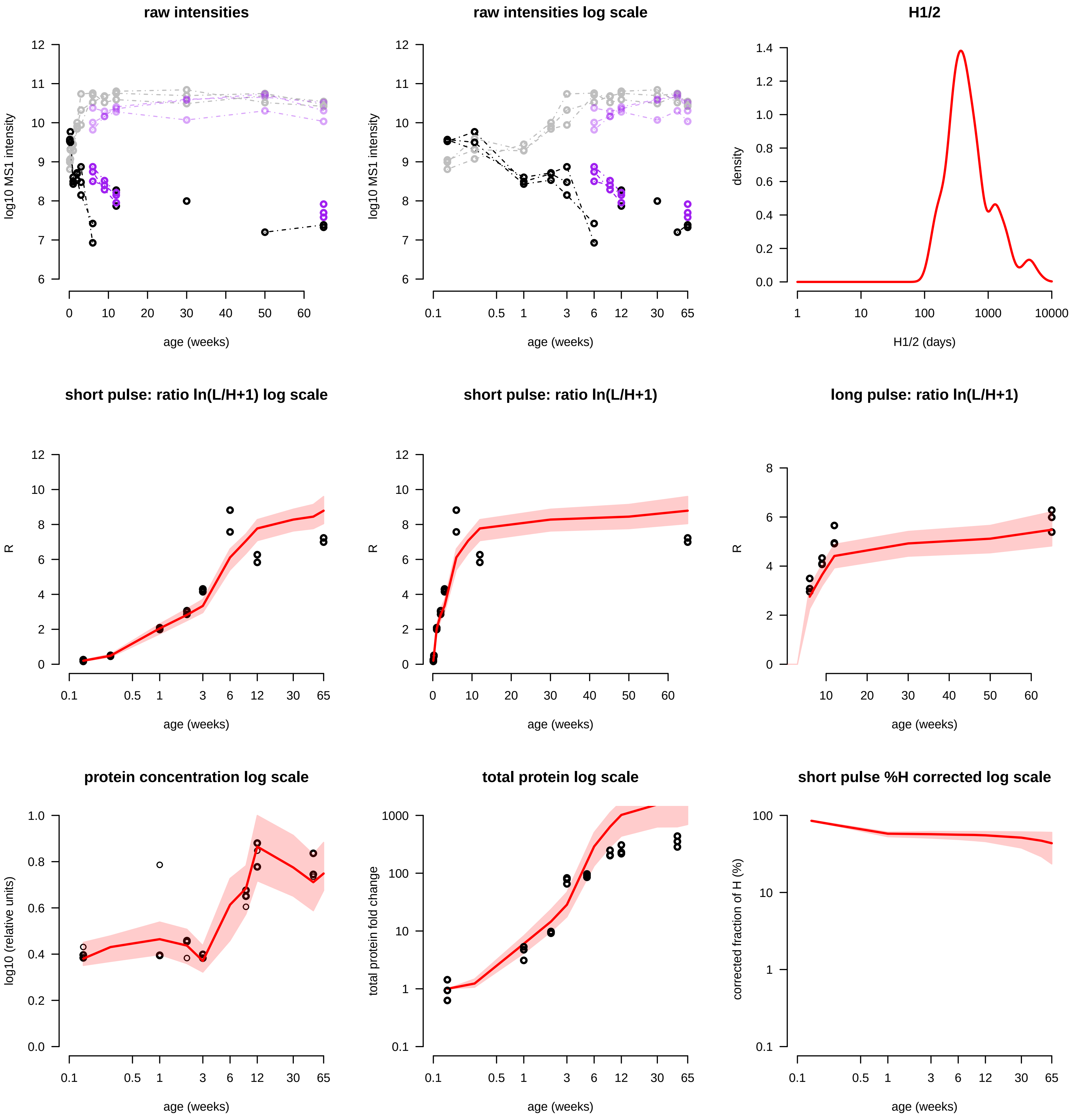

Q8BTM8

>sp|Q8BTM8|FLNA_MOUSE

Filamin−A

GN=Flna

Med H1/2: 209.8 days

Q5 H1/2: 89.7 days

Q95 H1/2: 1342 days

reliable model fit: no

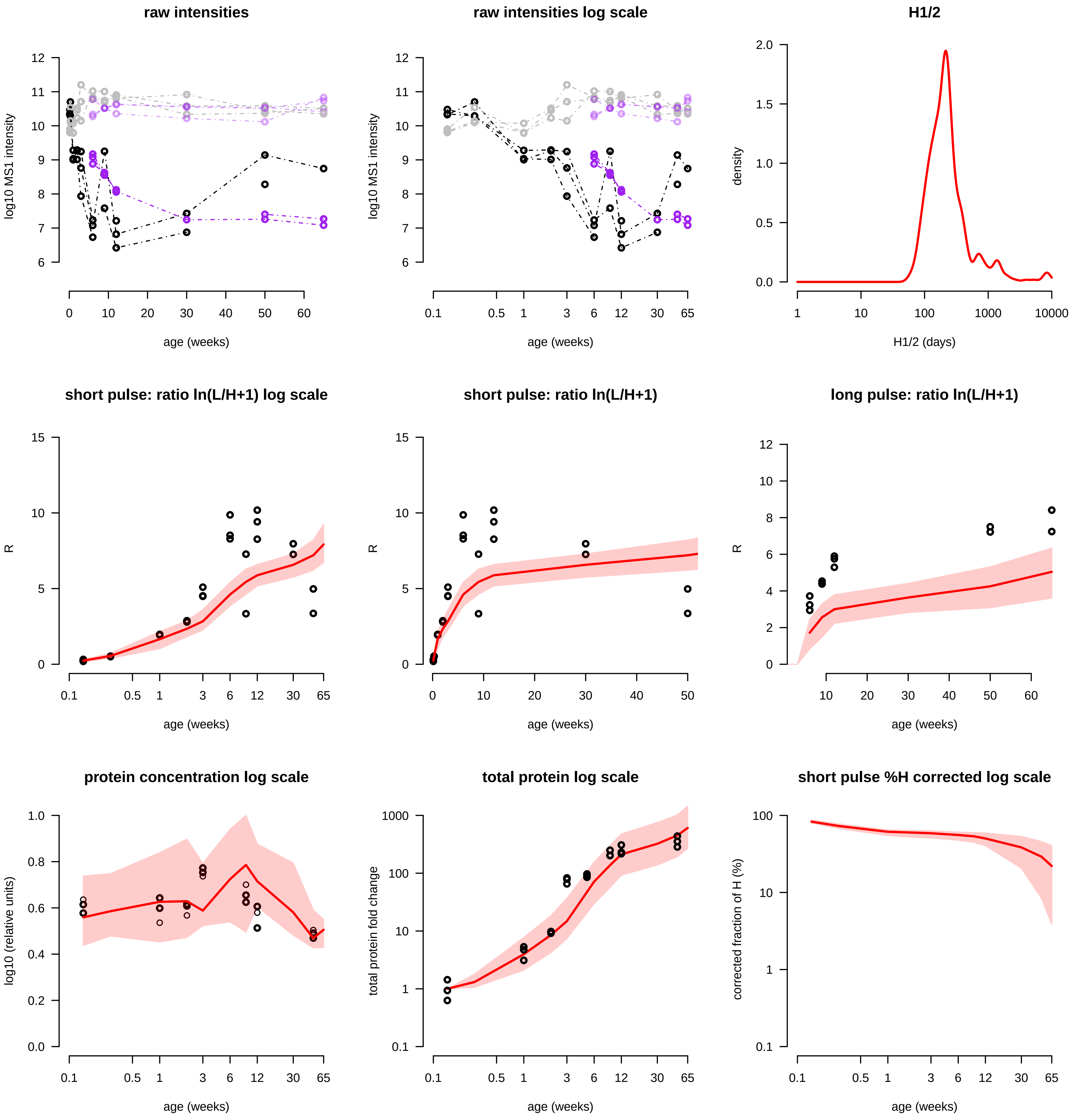

P17182

>sp|P17182|ENOA_MOUSE

Alpha−enolase

GN=Eno1

Med H1/2: 270.1 days

Q5 H1/2: 126.1 days

Q95 H1/2: 941.5 days

reliable model fit: yes

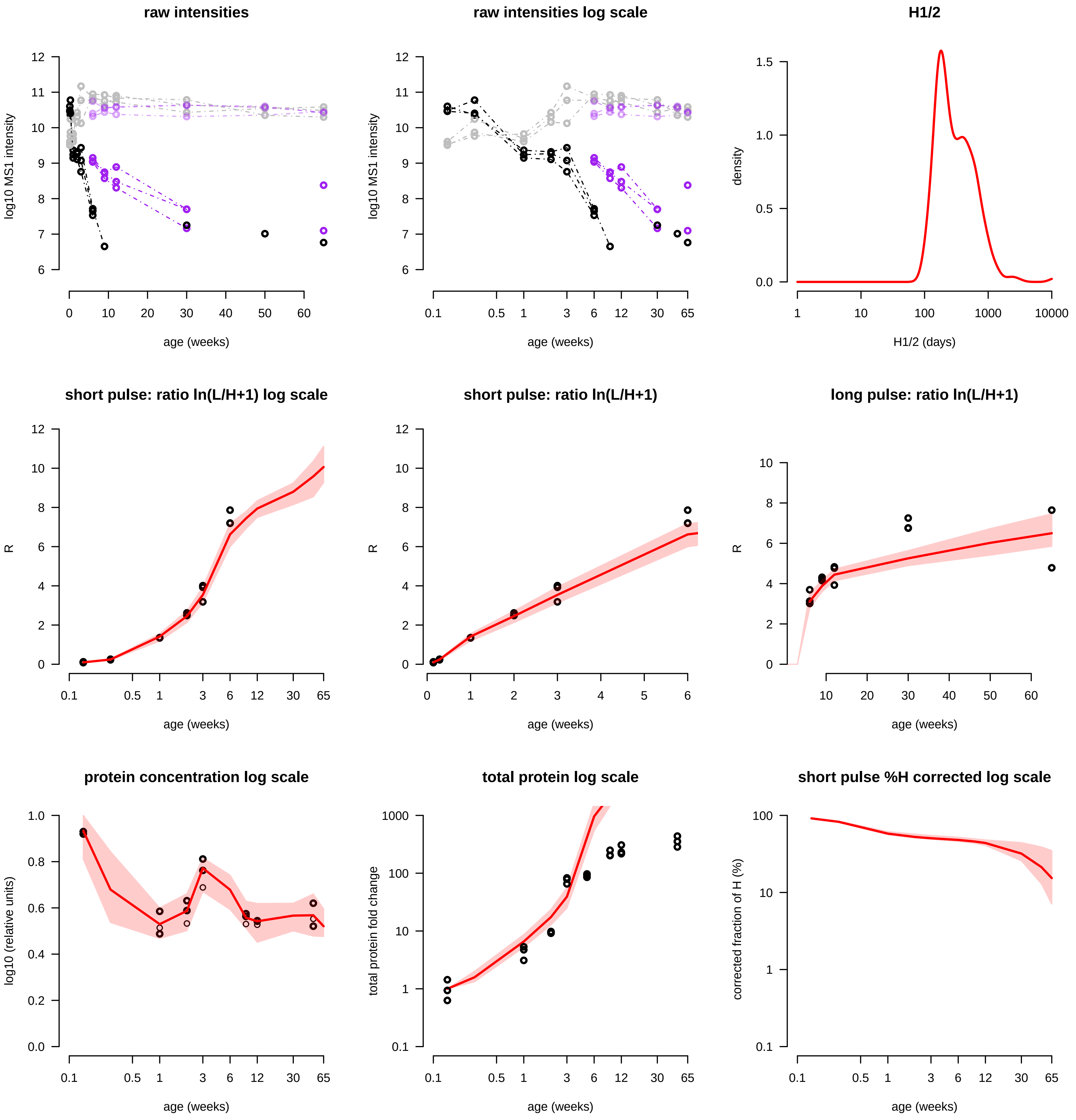

P08249

>sp|P08249|MDHM_MOUSE

Malate dehydrogenase, mitochondrial

GN=Mdh2

Med H1/2: 208.4 days

Q5 H1/2: 89.1 days

Q95 H1/2: 640.9 days

reliable model fit: yes

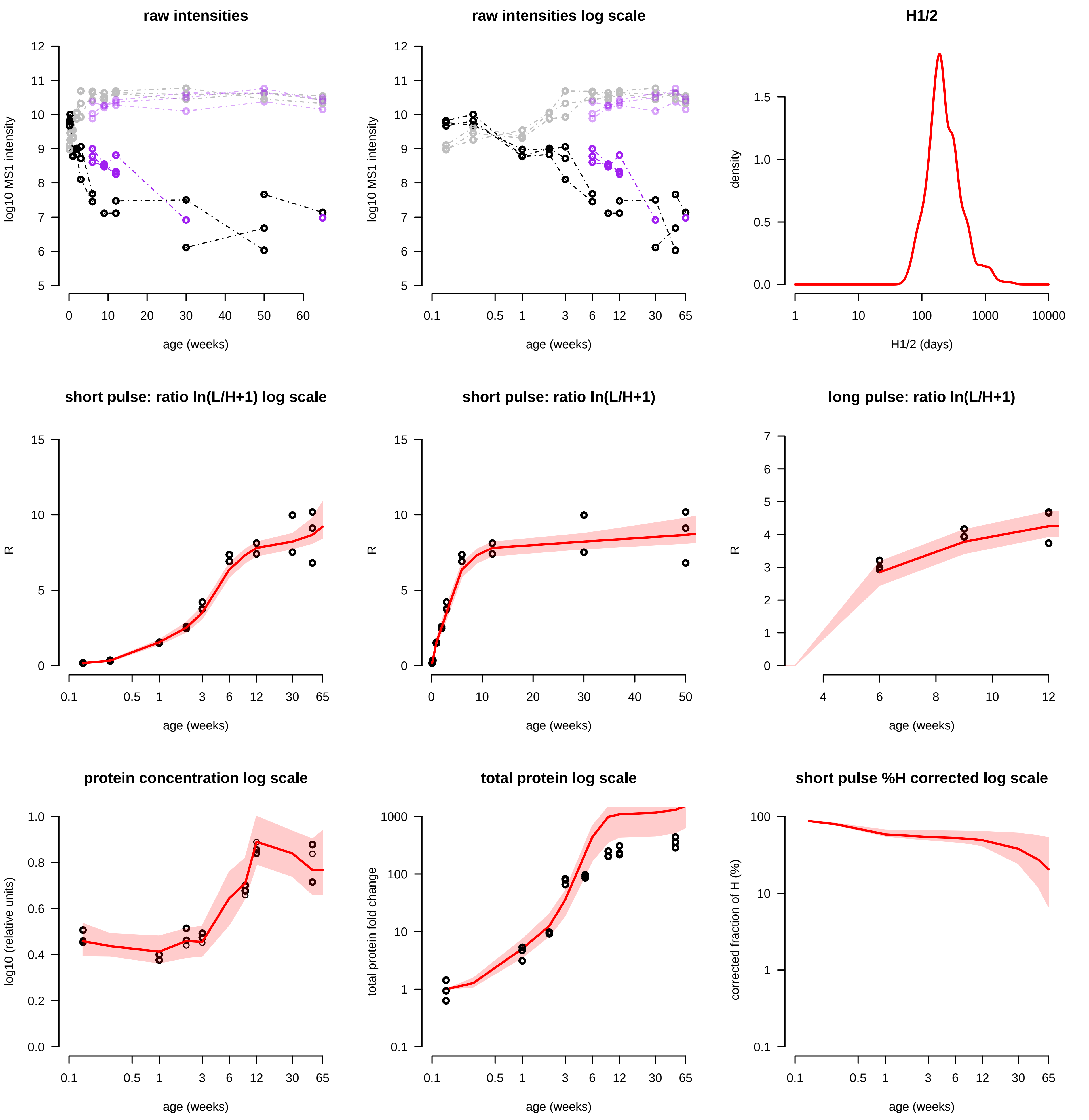

P24549

>sp|P24549|AL1A1_MOUSE

Retinal dehydrogenase 1

GN=Aldh1a1

Med H1/2: 84.8 days

Q5 H1/2: 56.7 days

Q95 H1/2: 176 days

reliable model fit: yes

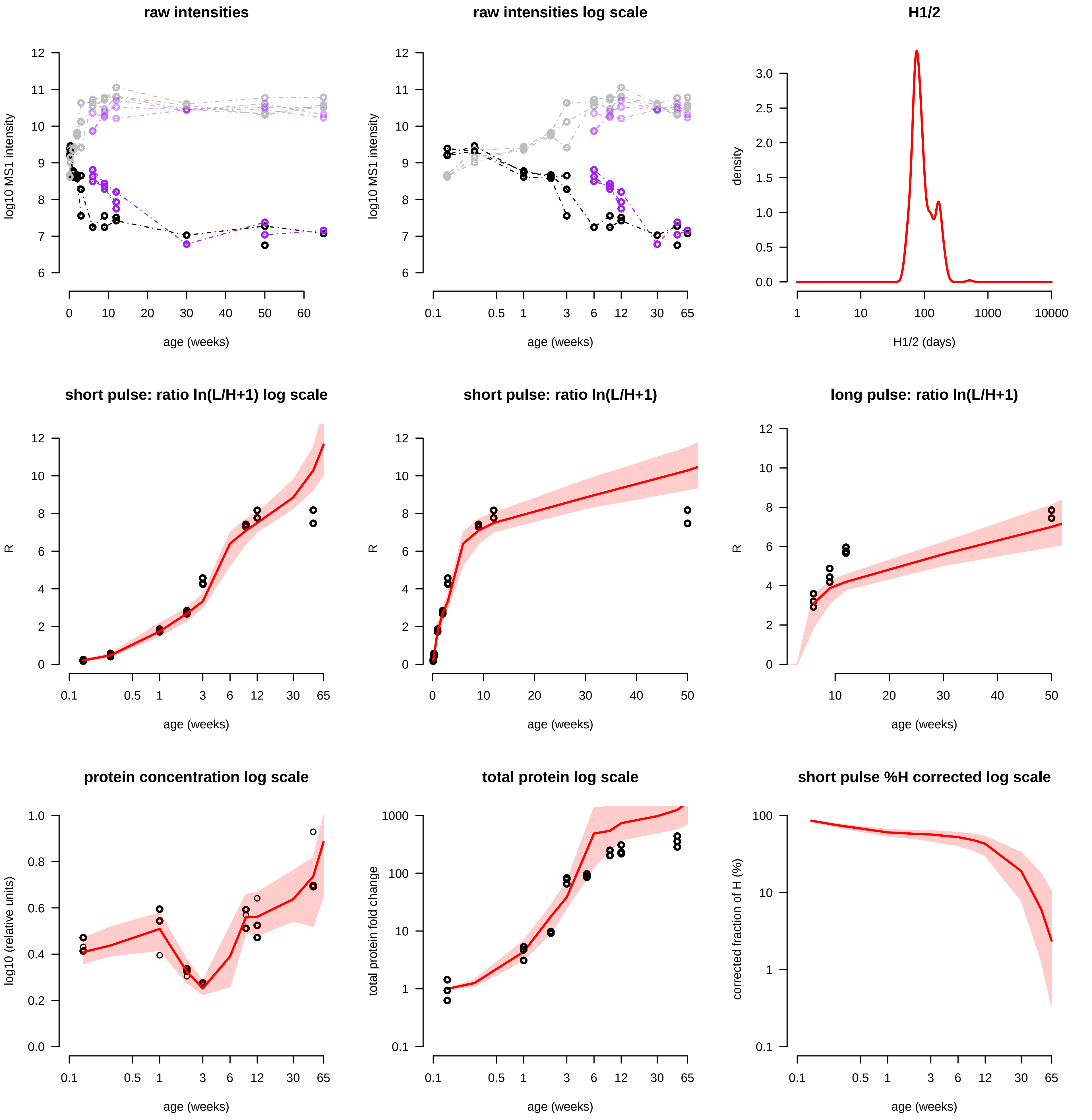

P08113

>sp|P08113|ENPL_MOUSE

Endoplasmin

GN=Hsp90b1

Med H1/2: 1166.3 days

Q5 H1/2: 307 days

Q95 H1/2: 14128.1 days

reliable model fit: yes

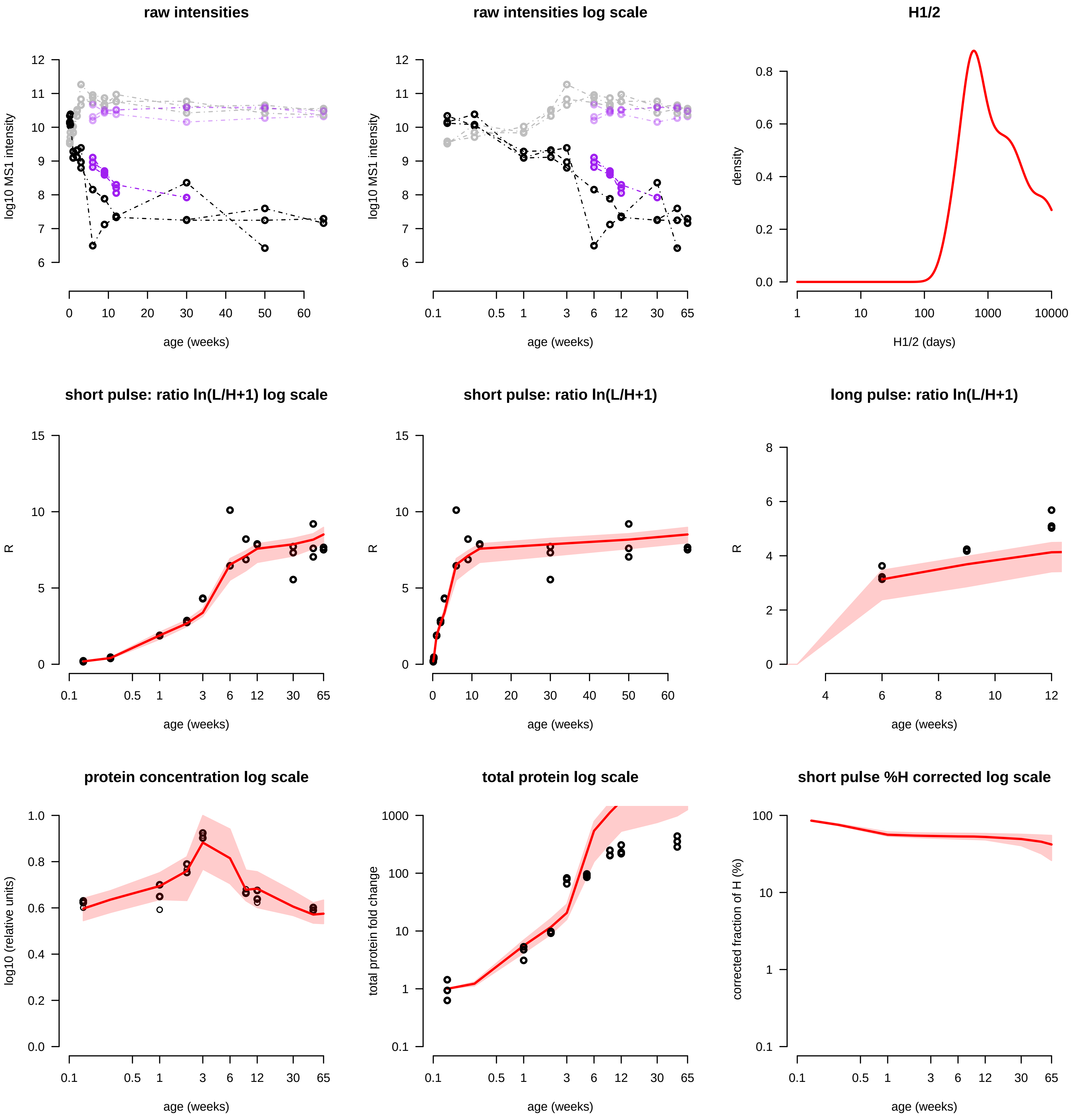

P07901

>sp|P07901|HS90A_MOUSE

Heat shock protein HSP 90−alpha

GN=Hsp90aa1

Med H1/2: 13.2 days

Q5 H1/2: 10.2 days

Q95 H1/2: 17.7 days

reliable model fit: yes

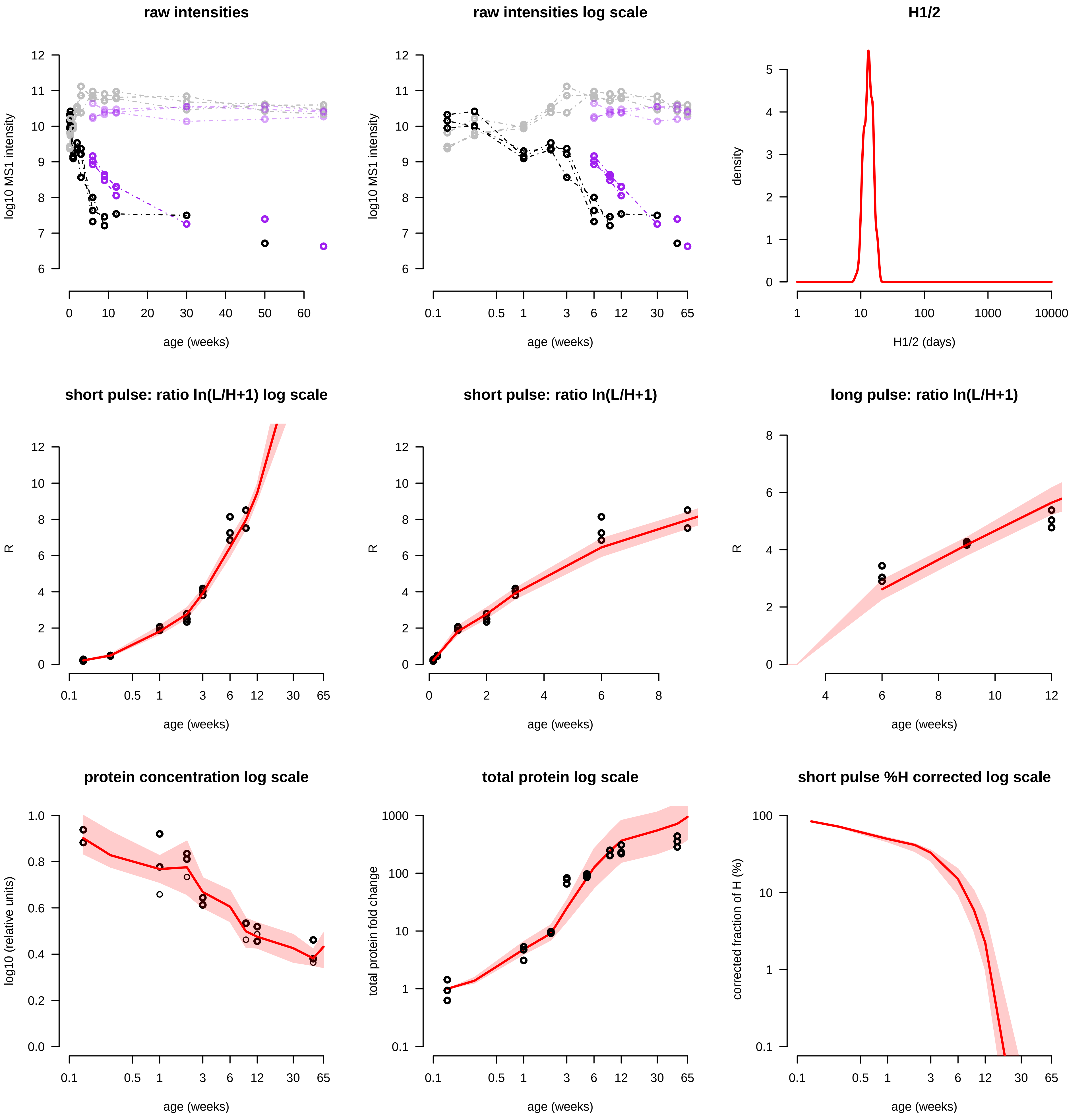

P40142

>sp|P40142|TKT_MOUSE

Transketolase

GN=Tkt

Med H1/2: 13.1 days

Q5 H1/2: 11 days

Q95 H1/2: 16.8 days

reliable model fit: yes

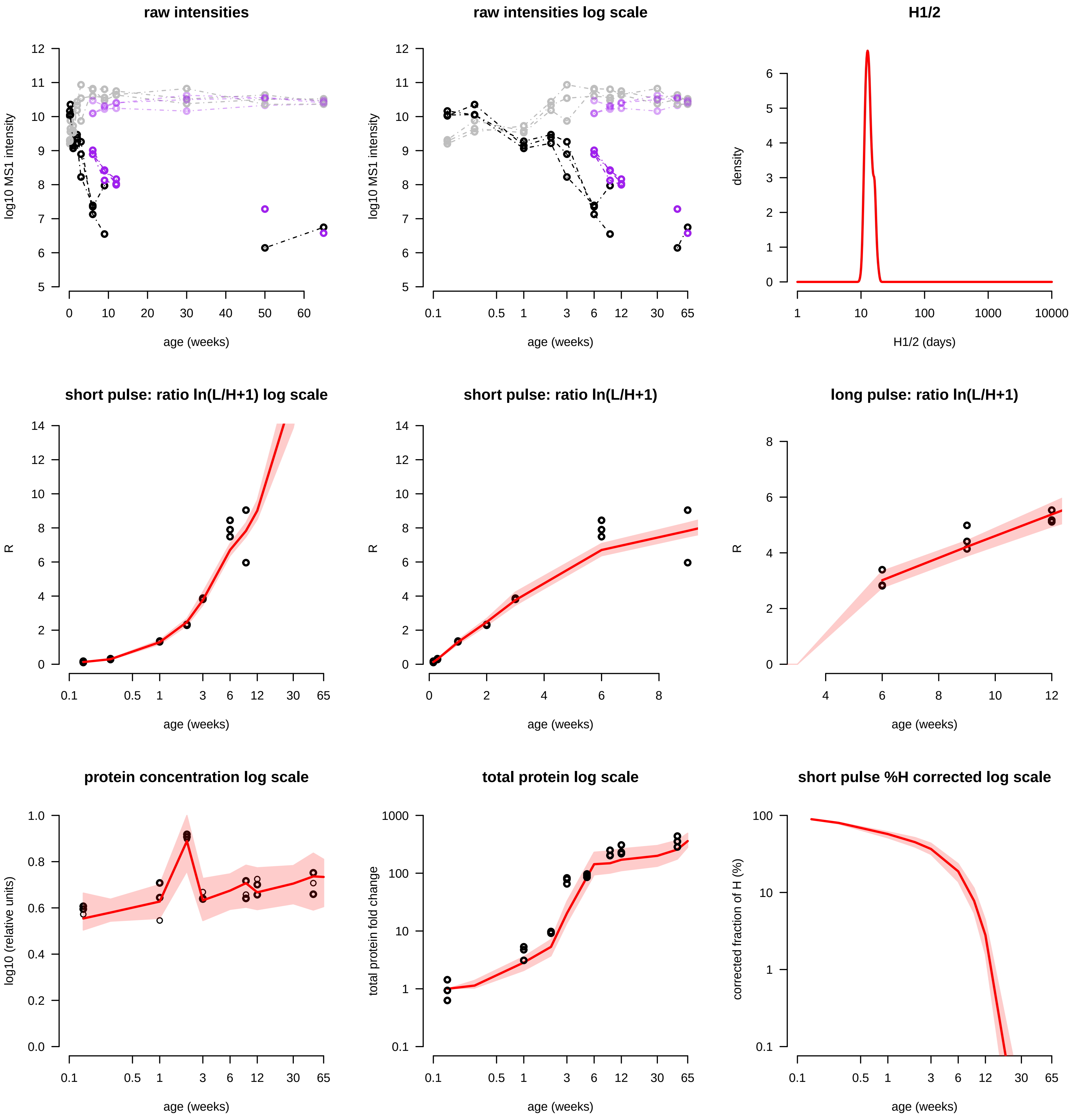

Q99KI0

>sp|Q99KI0|ACON_MOUSE

Aconitate hydratase, mitochondrial

GN=Aco2

Med H1/2: 72.8 days

Q5 H1/2: 42.1 days

Q95 H1/2: 133.3 days

reliable model fit: yes

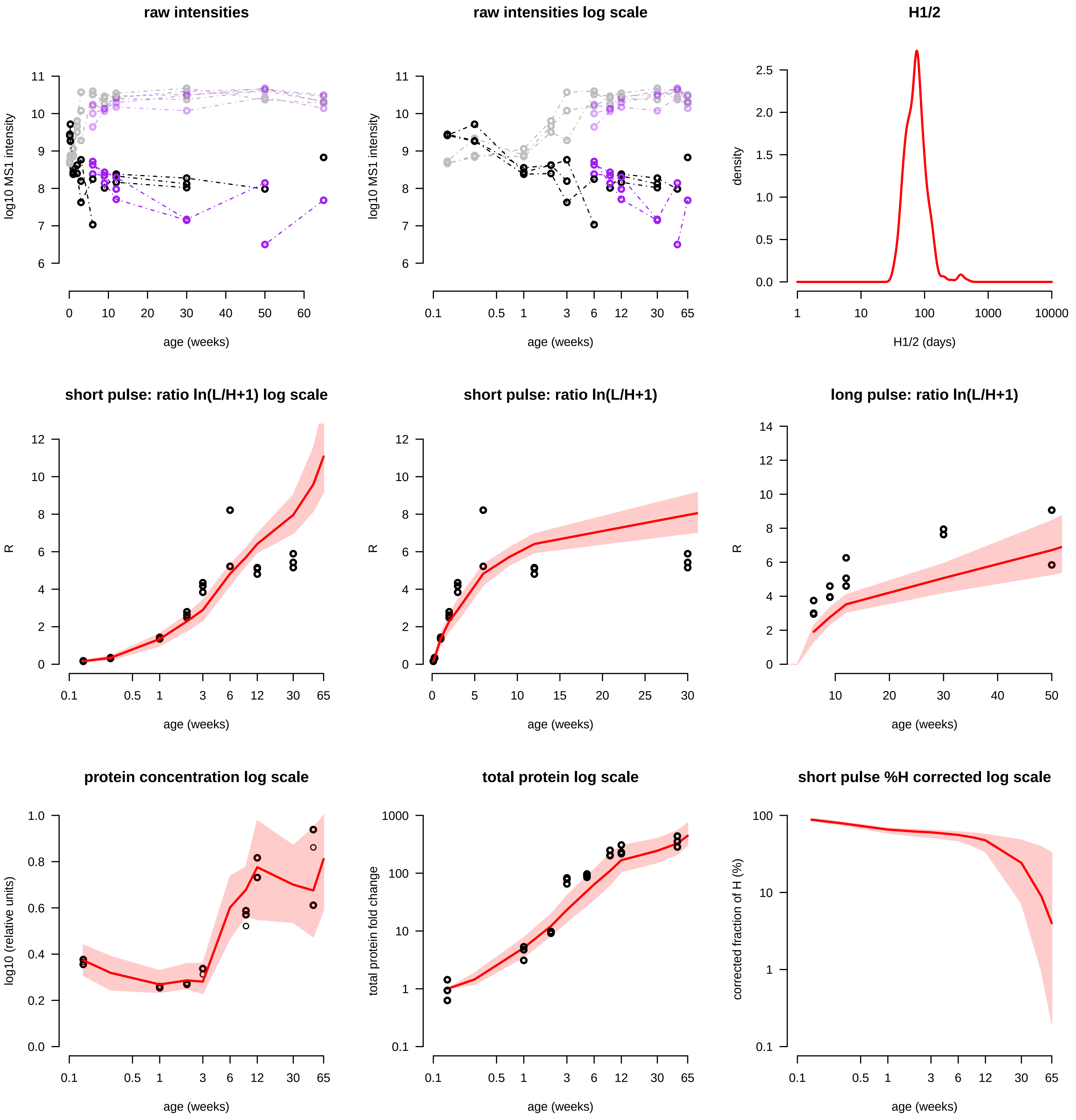

P11499

>sp|P11499|HS90B_MOUSE

Heat shock protein HSP 90−beta

GN=Hsp90ab1

Med H1/2: 12.5 days

Q5 H1/2: 9.4 days

Q95 H1/2: 17.5 days

reliable model fit: yes

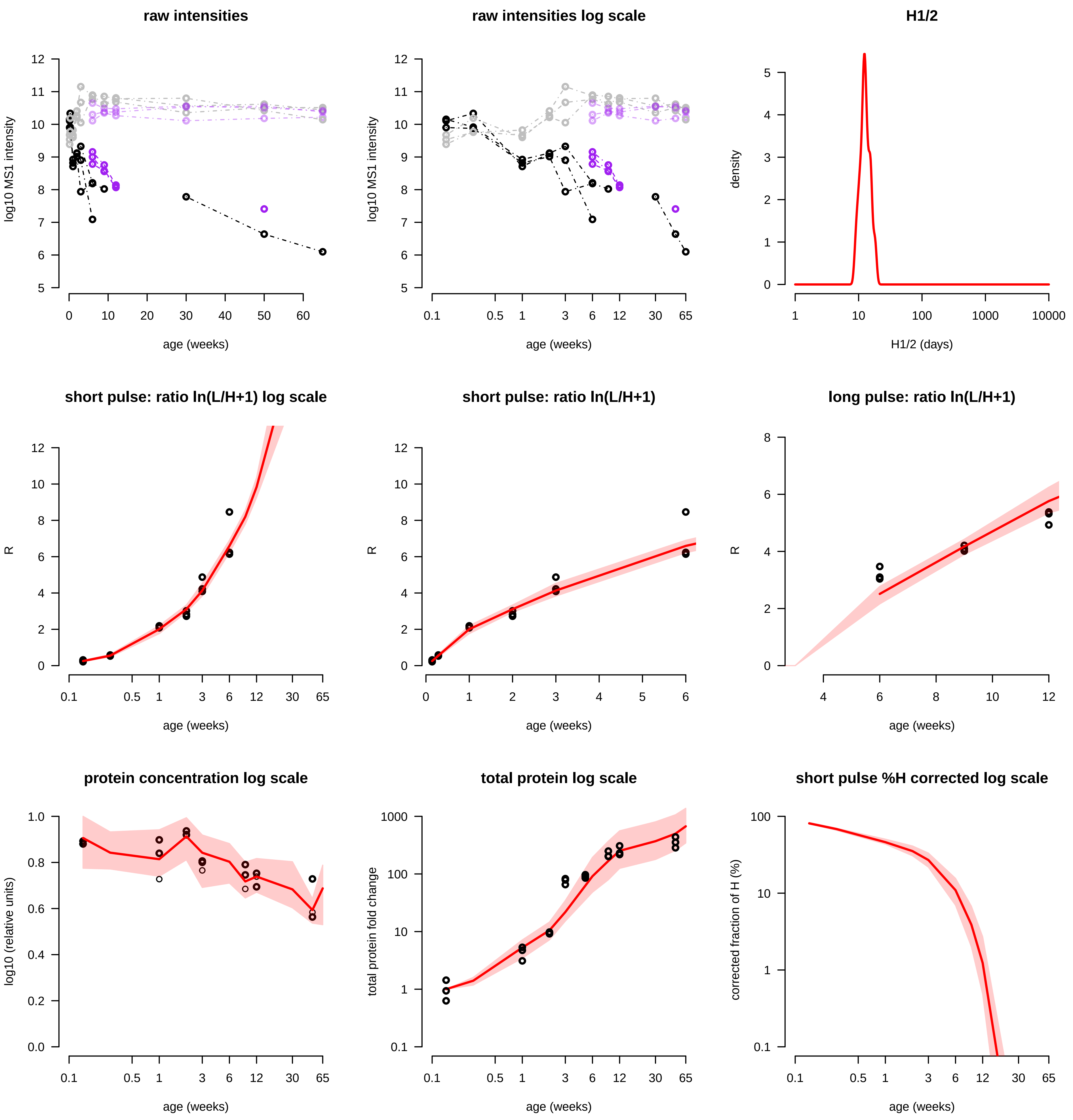

P68372

>sp|P68372|TBB4B_MOUSE

Tubulin beta−4B chain

GN=Tubb4b

Med H1/2: 68.5 days

Q5 H1/2: 41.4 days

Q95 H1/2: 123.9 days

reliable model fit: yes

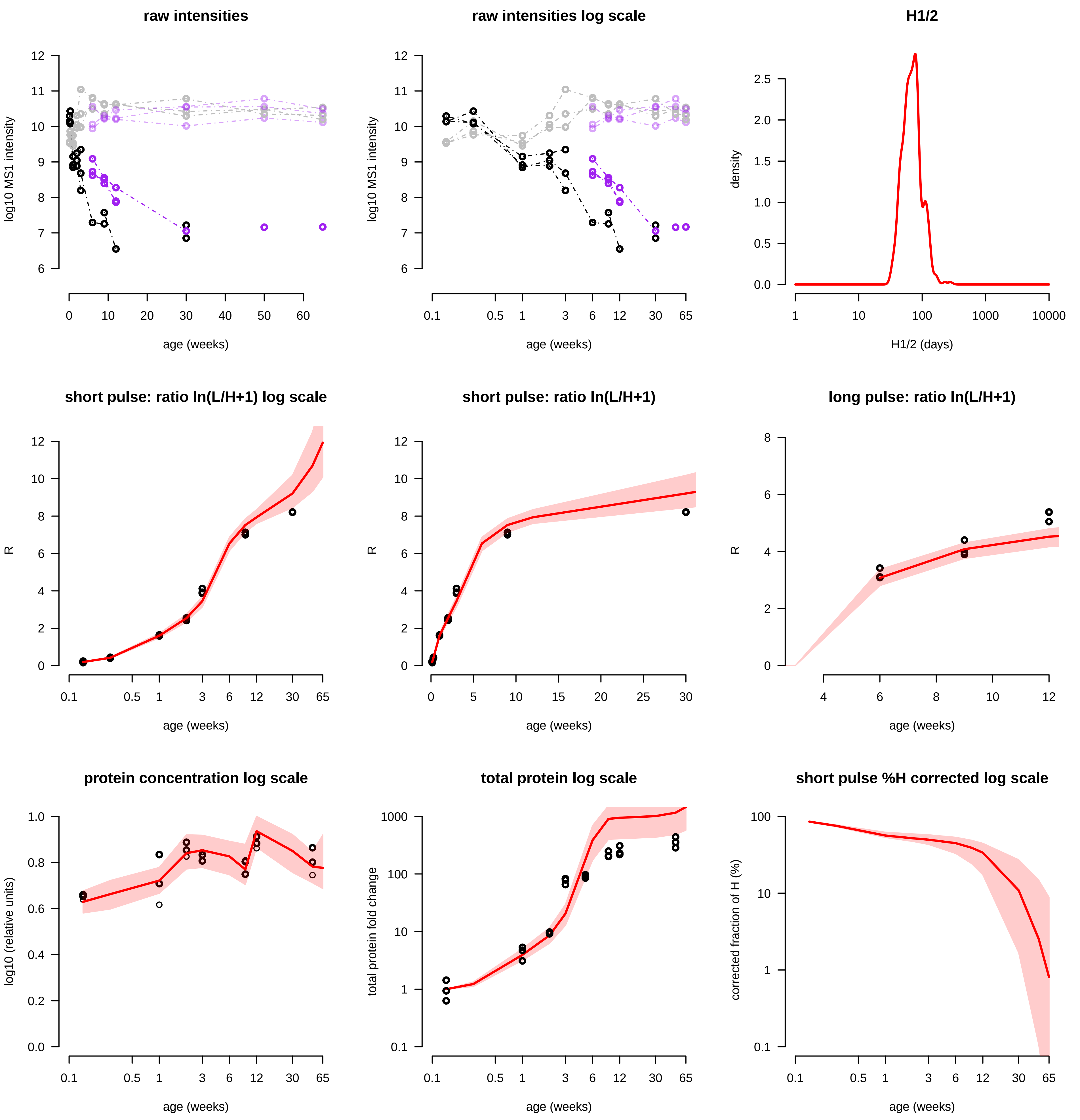

P58252

>sp|P58252|EF2_MOUSE

Elongation factor 2

GN=Eef2

Med H1/2: 483.9 days

Q5 H1/2: 203.6 days

Q95 H1/2: 3516.3 days

reliable model fit: yes

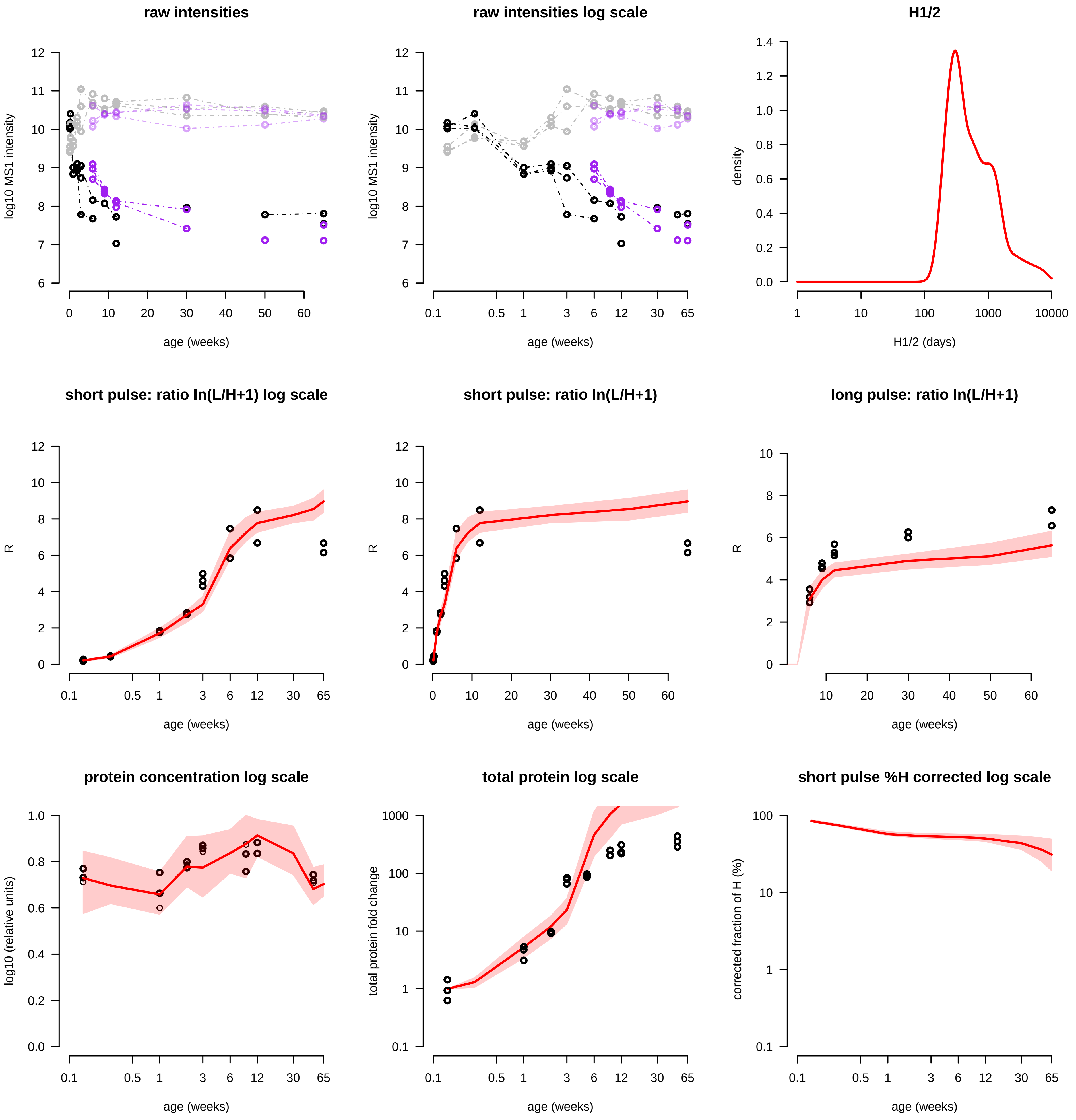

Q01853

>sp|Q01853|TERA_MOUSE

Transitional endoplasmic reticulum ATPase

GN=Vcp

Med H1/2: 172.4 days

Q5 H1/2: 106.7 days

Q95 H1/2: 344.5 days

reliable model fit: yes

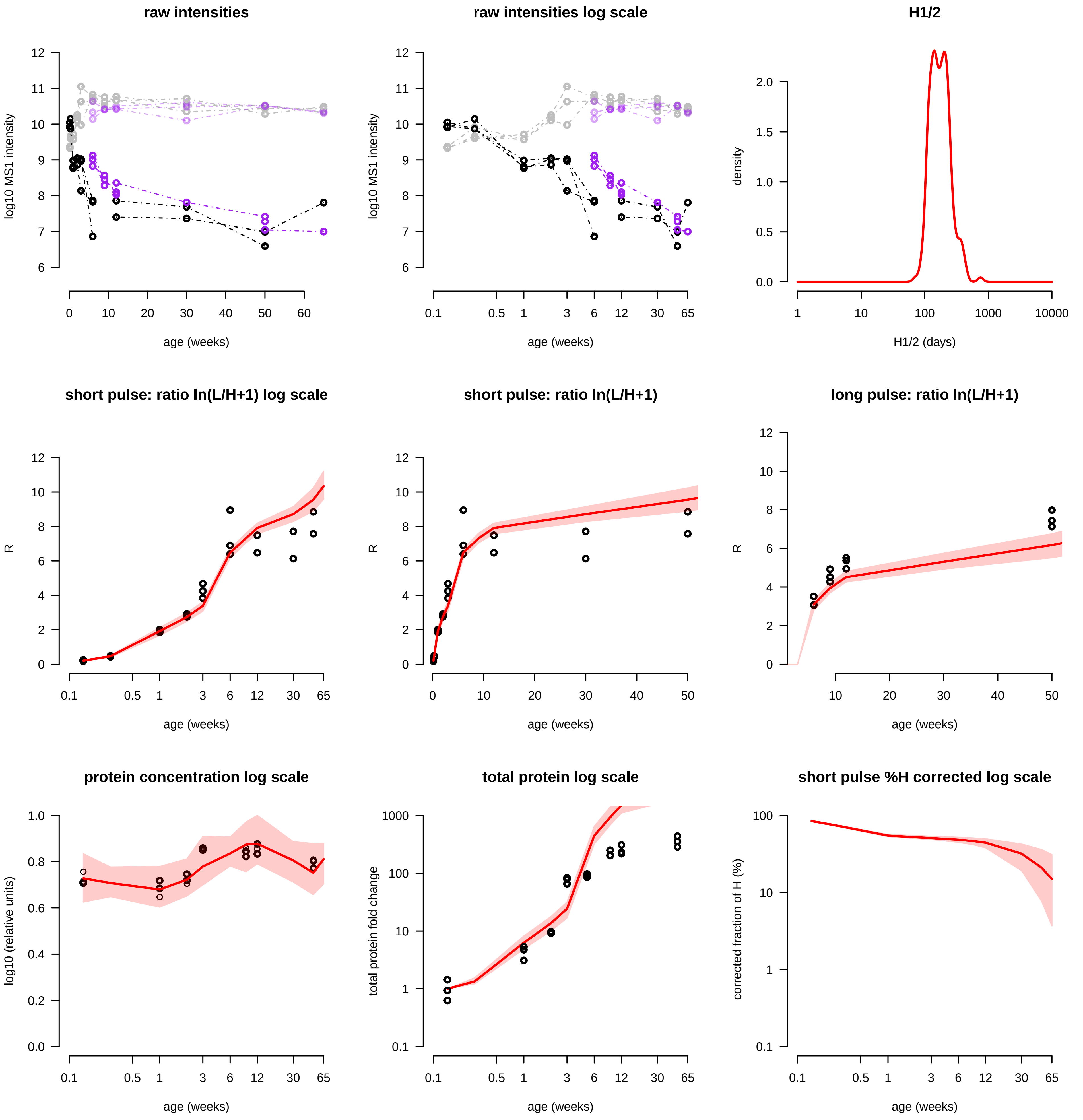

Q61578

>sp|Q61578|ADRO_MOUSE

NADPH:adrenodoxin oxidoreductase, mitochondrial

GN=Fdxr

Med H1/2: 35.5 days

Q5 H1/2: 27.5 days

Q95 H1/2: 53.3 days

reliable model fit: yes

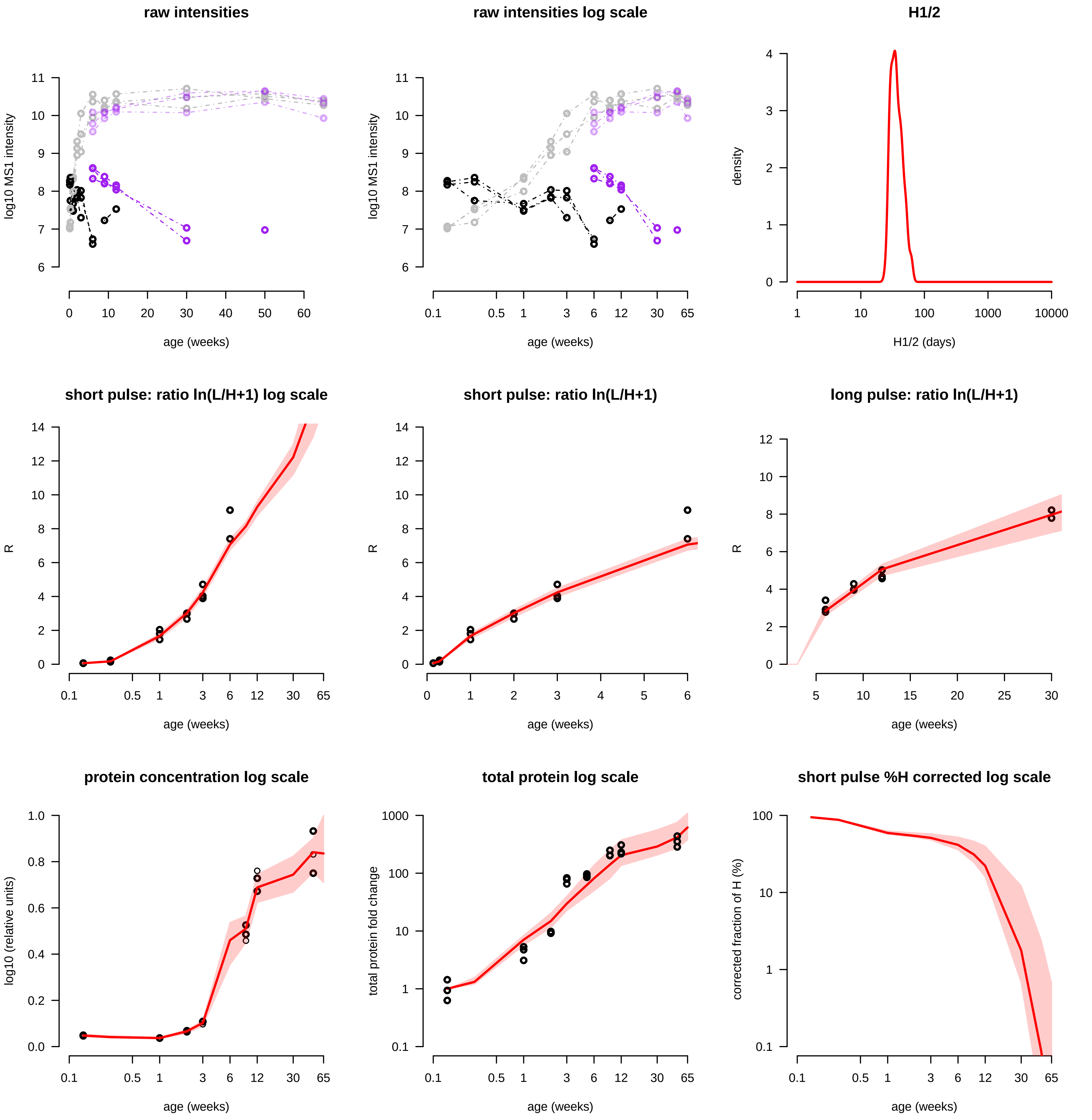

P05064

>sp|P05064|ALDOA_MOUSE

Fructose−bisphosphate aldolase A

GN=Aldoa

Med H1/2: 46.4 days

Q5 H1/2: 34.8 days

Q95 H1/2: 59.5 days

reliable model fit: yes

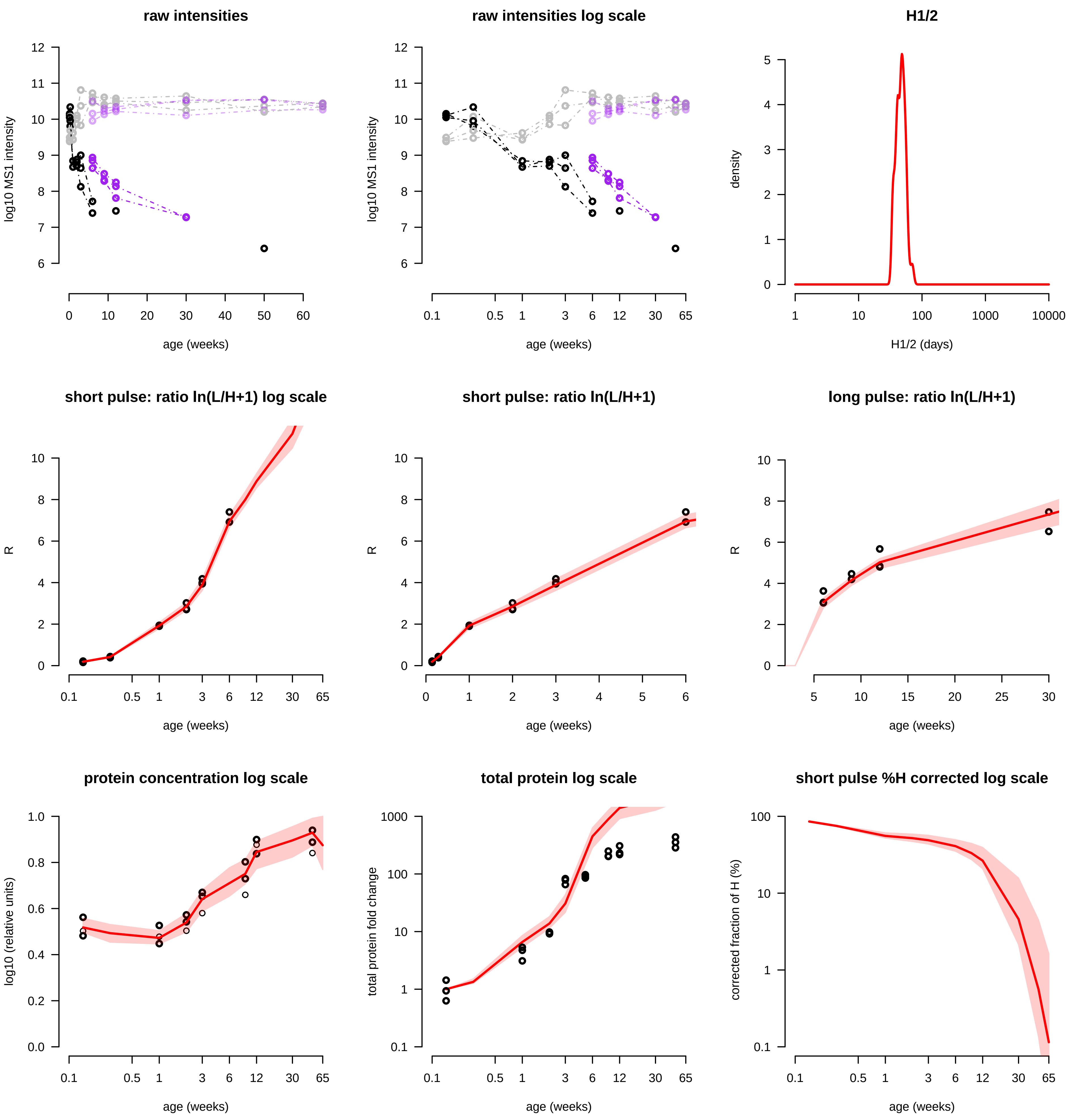

P43274

>sp|P43274|H14_MOUSE

Histone H1.4

GN=H1−4

Med H1/2: 33.8 days

Q5 H1/2: 27.9 days

Q95 H1/2: 58.7 days

reliable model fit: yes

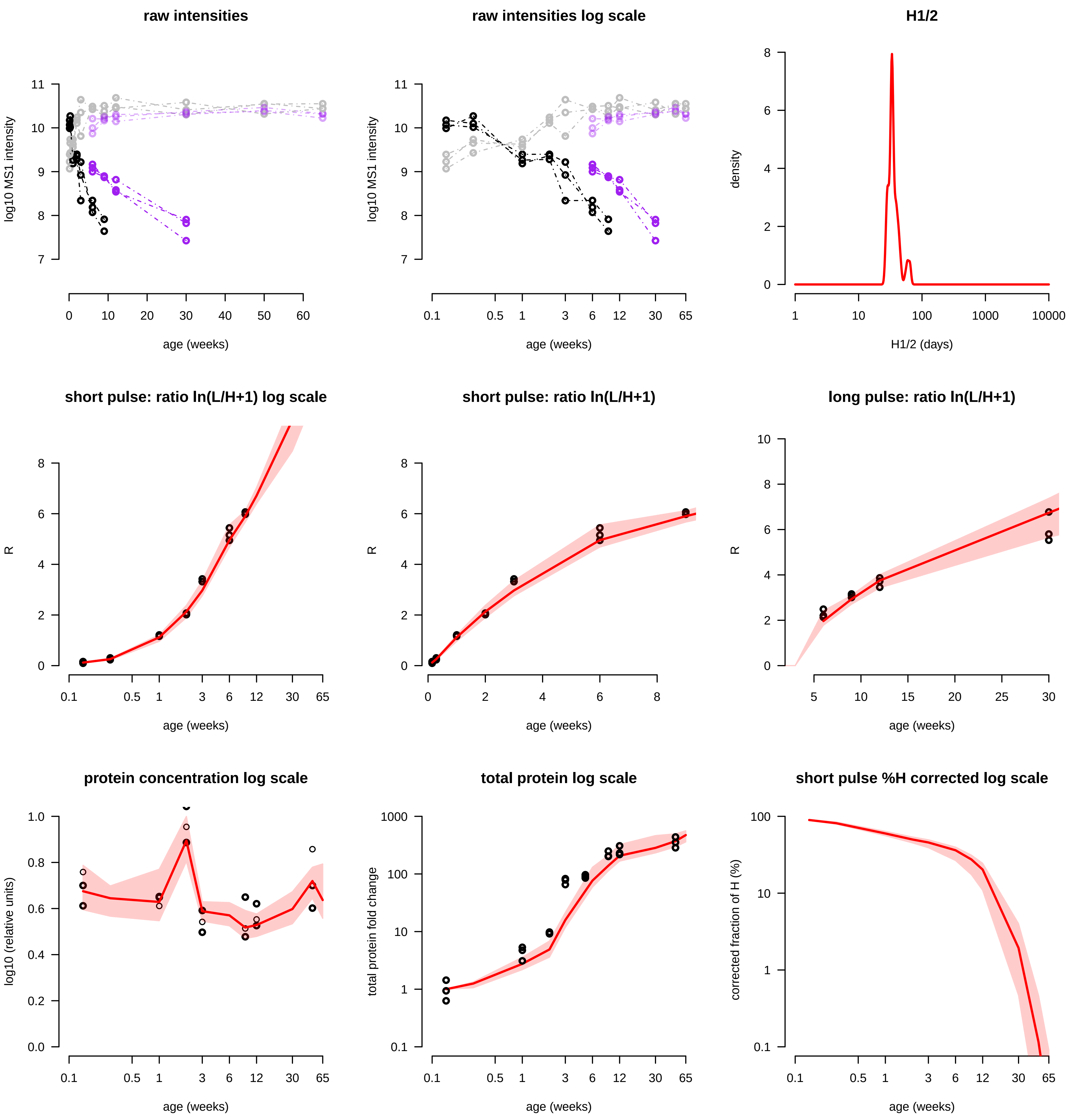

Q00896

>sp|Q00896|A1AT3_MOUSE

Alpha−1−antitrypsin 1−3

GN=Serpina1c

Med H1/2: 68.6 days

Q5 H1/2: 37.5 days

Q95 H1/2: 101.9 days

reliable model fit: yes

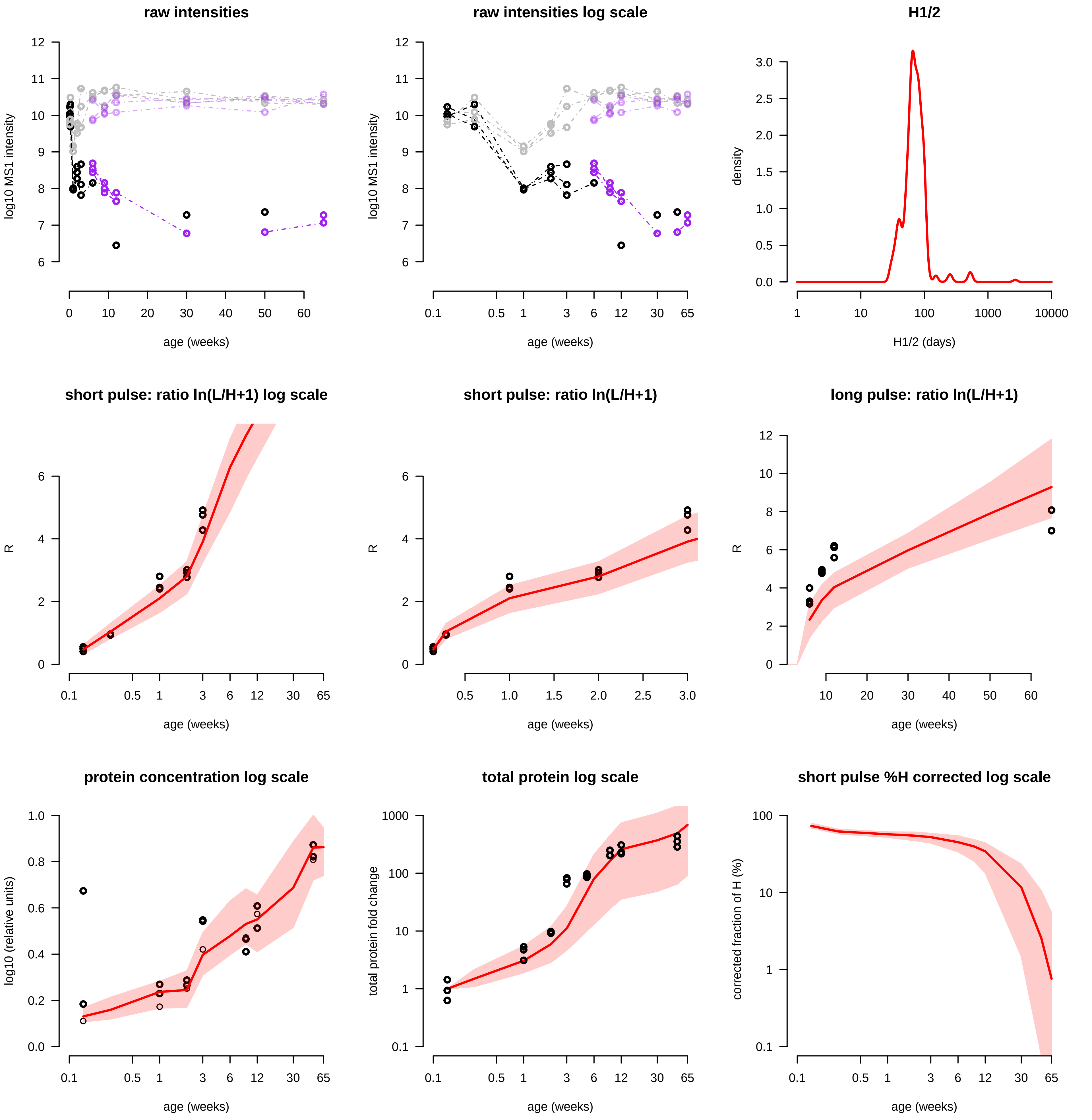

Q8BW75

>sp|Q8BW75|AOFB_MOUSE

Amine oxidase [flavin−containing] B

GN=Maob

Med H1/2: 110.3 days

Q5 H1/2: 56.4 days

Q95 H1/2: 523.3 days

reliable model fit: no

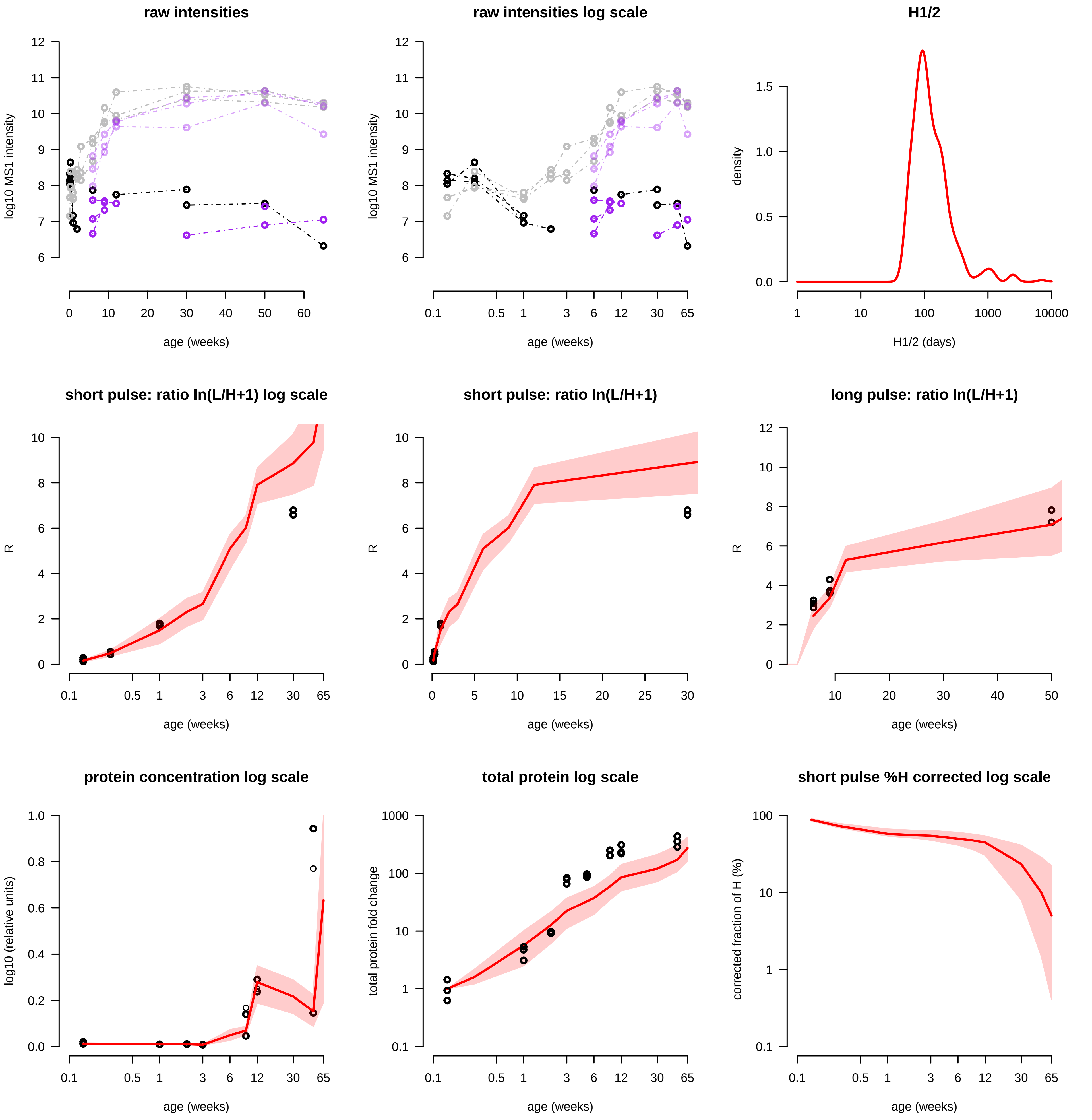

Q61838

>sp|Q61838|PZP_MOUSE

Pregnancy zone protein

GN=Pzp

Med H1/2: 129.7 days

Q5 H1/2: 72.2 days

Q95 H1/2: 1039.4 days

reliable model fit: yes

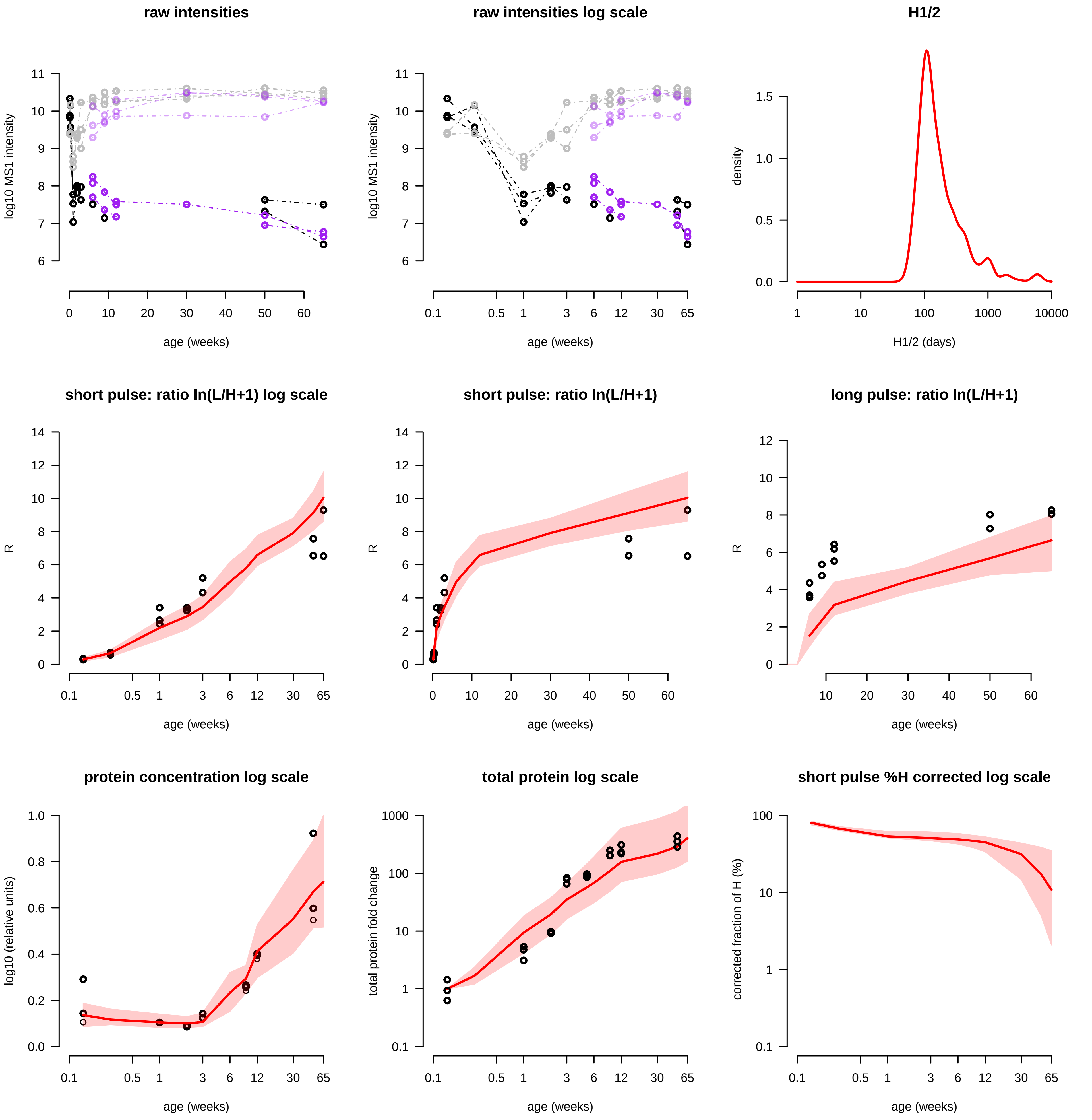

P47738

>sp|P47738|ALDH2_MOUSE

Aldehyde dehydrogenase, mitochondrial

GN=Aldh2

Med H1/2: 337.4 days

Q5 H1/2: 119.1 days

Q95 H1/2: 3592.8 days

reliable model fit: yes

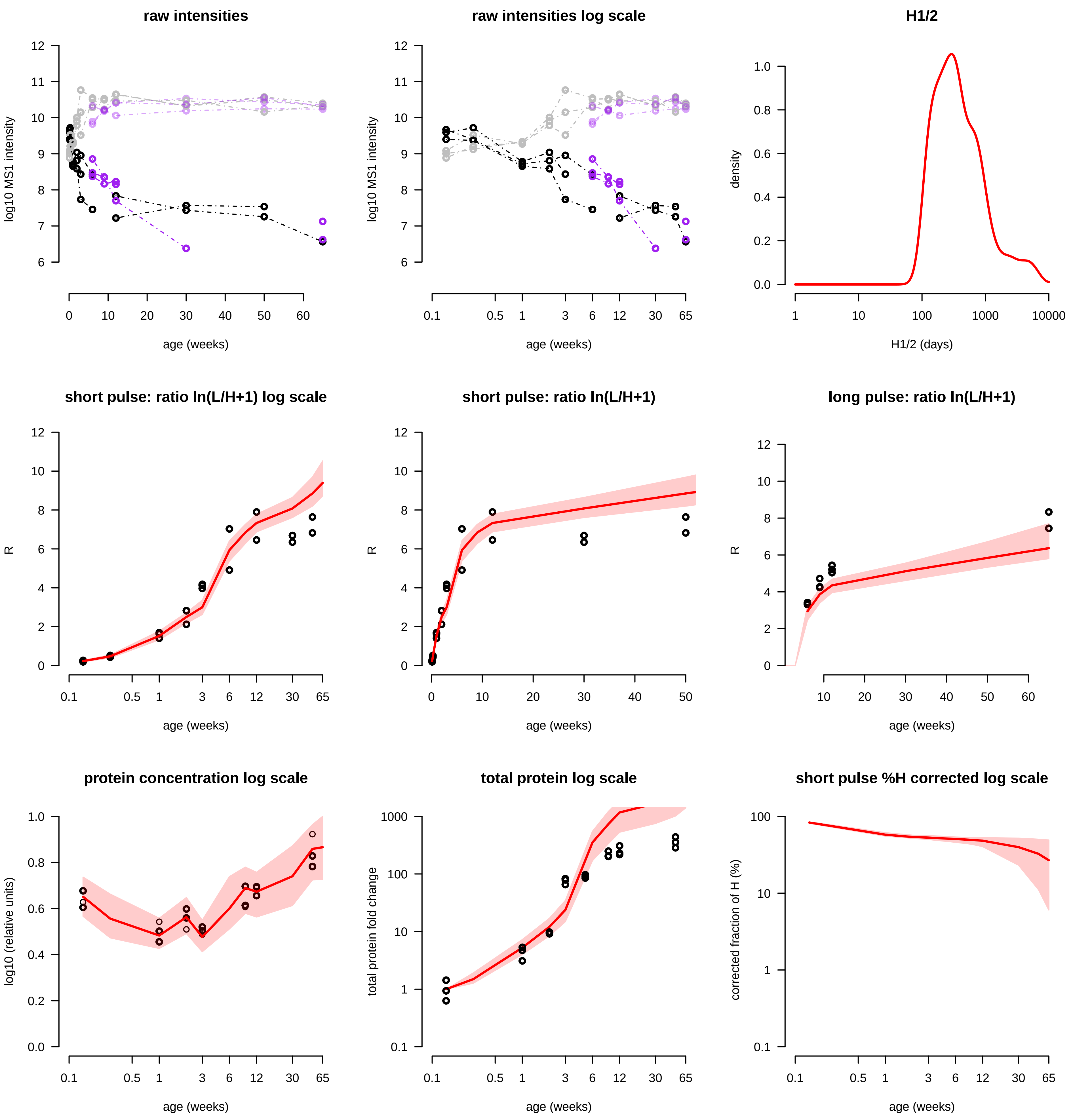

P20108

>sp|P20108|PRDX3_MOUSE

Thioredoxin−dependent peroxide reductase, mitochondrial

GN=Prdx3

Med H1/2: 234.3 days

Q5 H1/2: 129.3 days

Q95 H1/2: 595 days

reliable model fit: yes

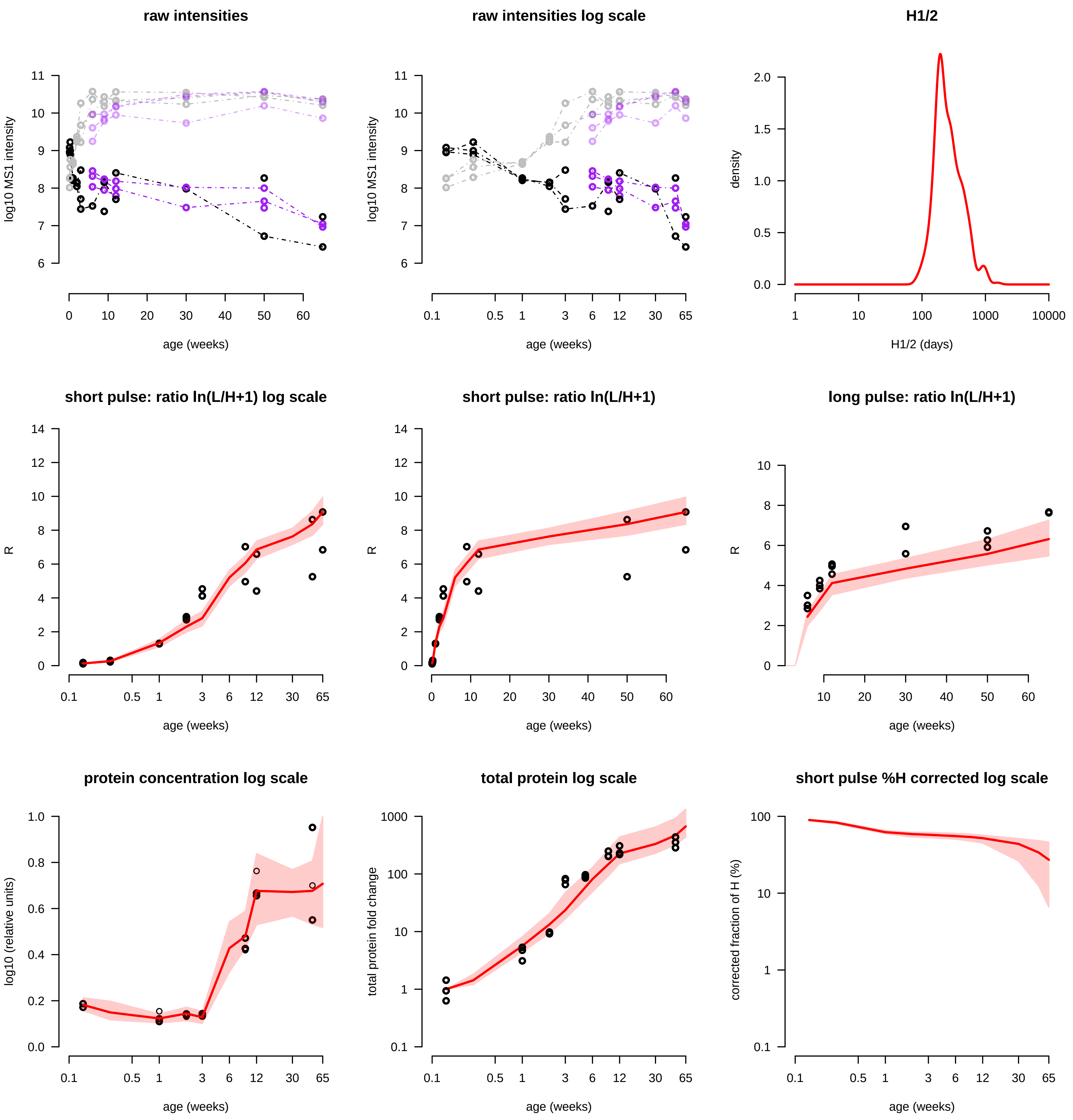

P48962

>sp|P48962|ADT1_MOUSE

ADP/ATP translocase 1

GN=Slc25a4

Med H1/2: 39.9 days

Q5 H1/2: 30.5 days

Q95 H1/2: 47.6 days

reliable model fit: yes

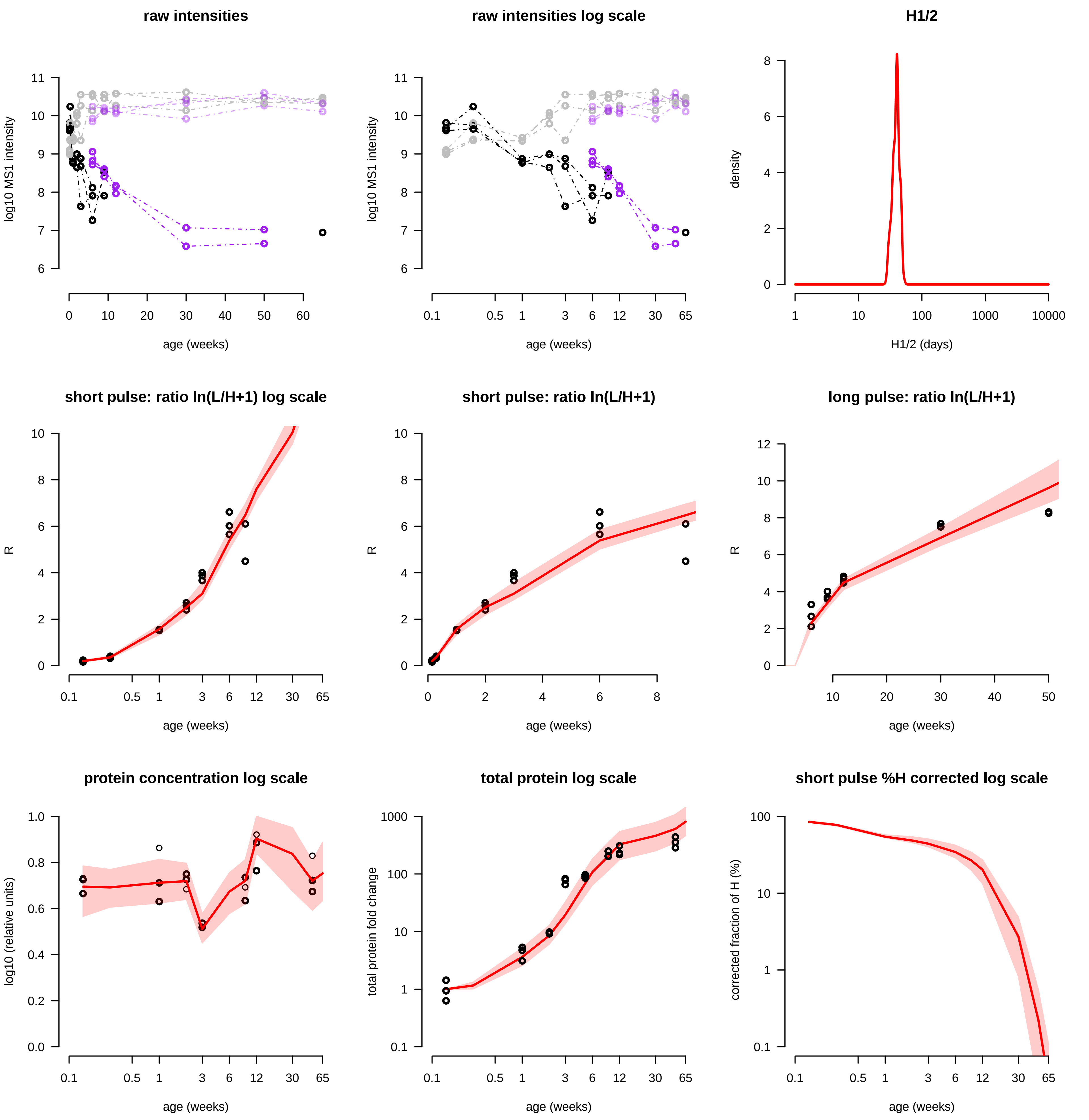

O08638

>sp|O08638|MYH11_MOUSE

Myosin−11

GN=Myh11

Med H1/2: 447.8 days

Q5 H1/2: 162 days

Q95 H1/2: 3605.8 days

reliable model fit: yes

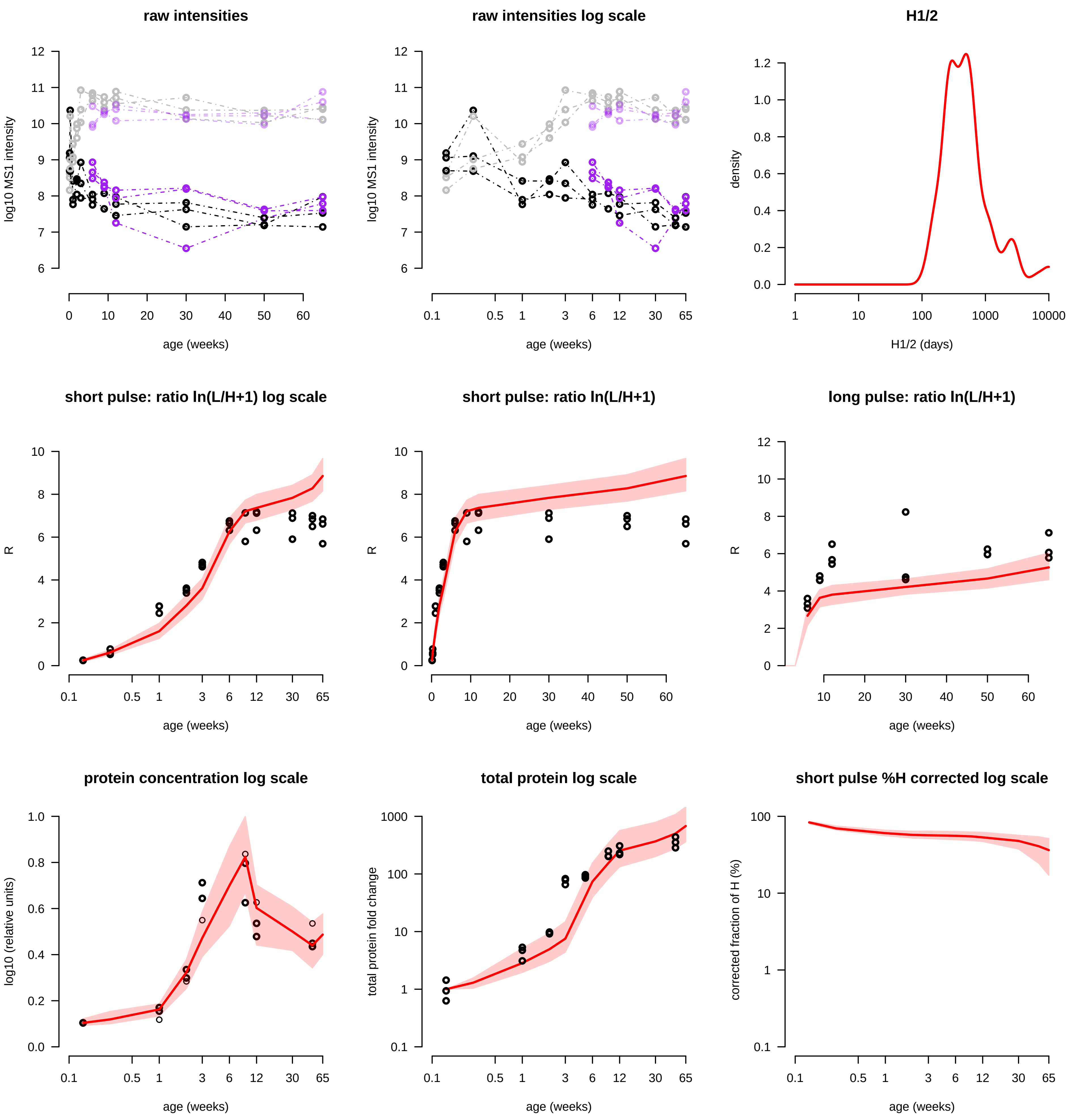

Q61171

>sp|Q61171|PRDX2_MOUSE

Peroxiredoxin−2

GN=Prdx2

Med H1/2: 27 days

Q5 H1/2: 23.5 days

Q95 H1/2: 32.9 days

reliable model fit: yes

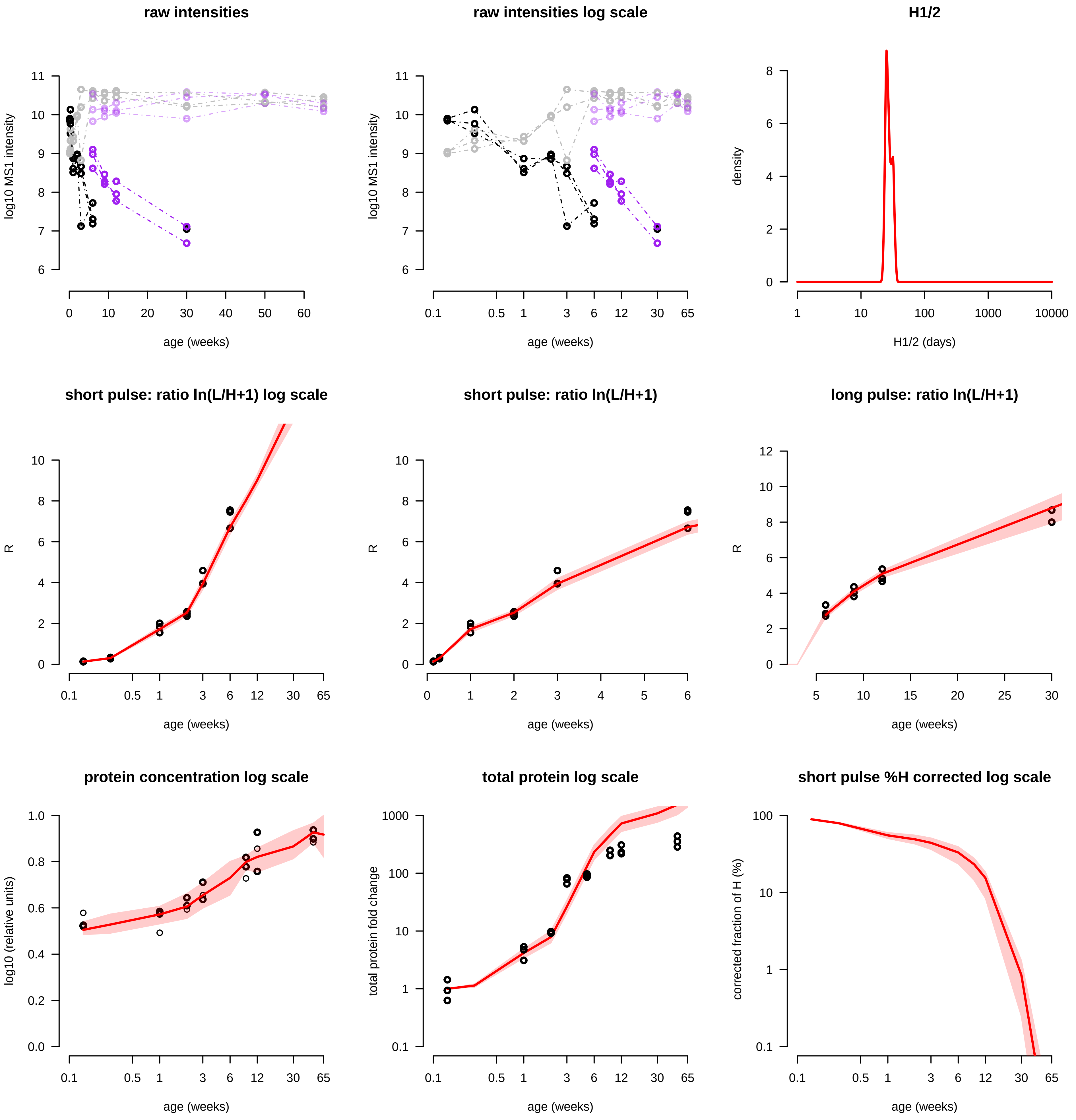

P10922

>sp|P10922|H10_MOUSE

Histone H1.0

GN=H1−0

Med H1/2: 49.6 days

Q5 H1/2: 41.6 days

Q95 H1/2: 65.6 days

reliable model fit: yes

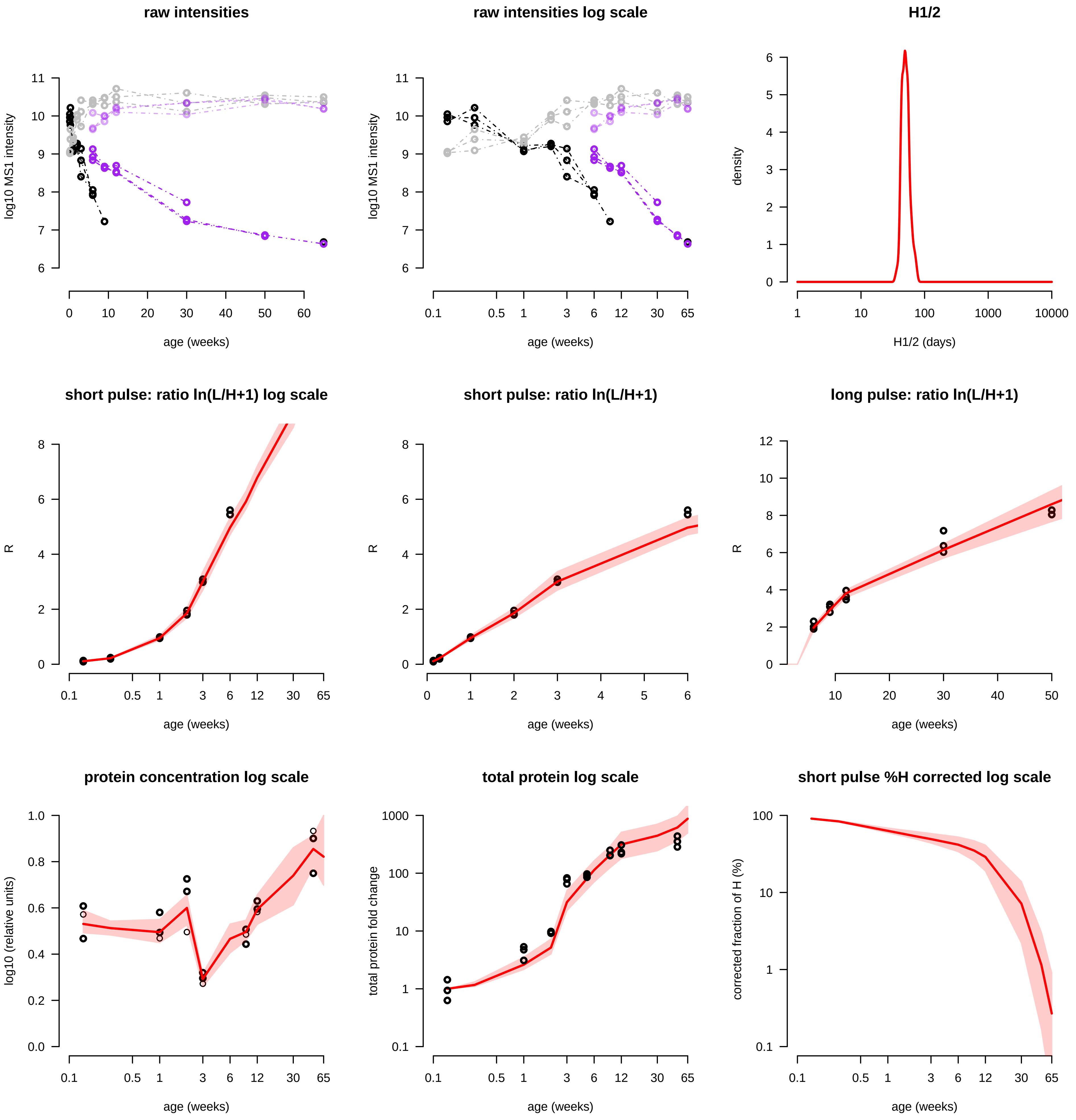

Q8VEM8

>sp|Q8VEM8|MPCP_MOUSE

Phosphate carrier protein, mitochondrial

GN=Slc25a3

Med H1/2: 1339.1 days

Q5 H1/2: 390.5 days

Q95 H1/2: 16873.4 days

reliable model fit: yes

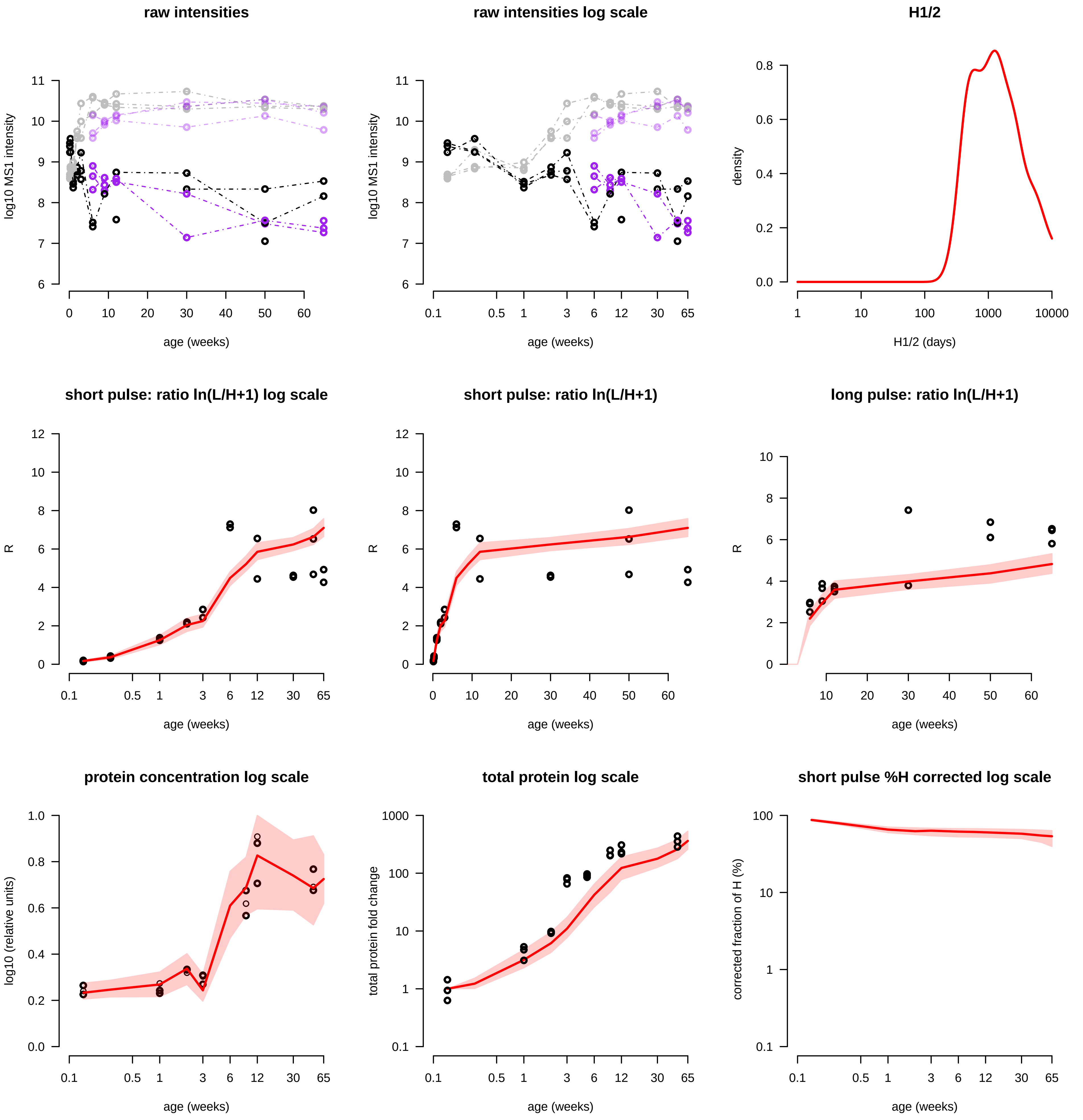

P63101

>sp|P63101|1433Z_MOUSE

14−3−3 protein zeta/delta

GN=Ywhaz

Med H1/2: 21.1 days

Q5 H1/2: 15.6 days

Q95 H1/2: 25.3 days

reliable model fit: yes

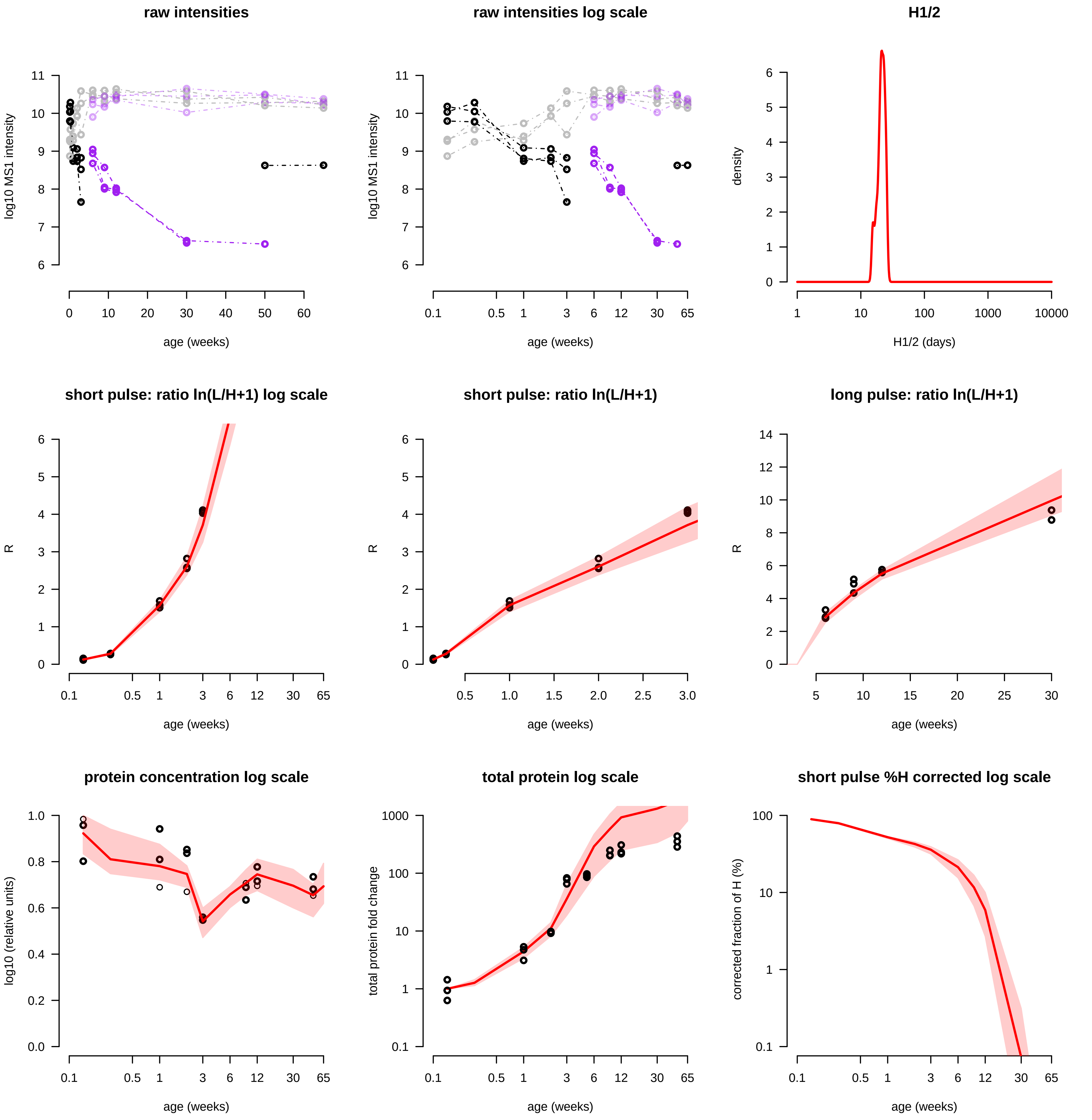

Q68FD5

>sp|Q68FD5|CLH1_MOUSE

Clathrin heavy chain 1

GN=Cltc

Med H1/2: 297.6 days

Q5 H1/2: 159.7 days

Q95 H1/2: 1829.8 days

reliable model fit: yes

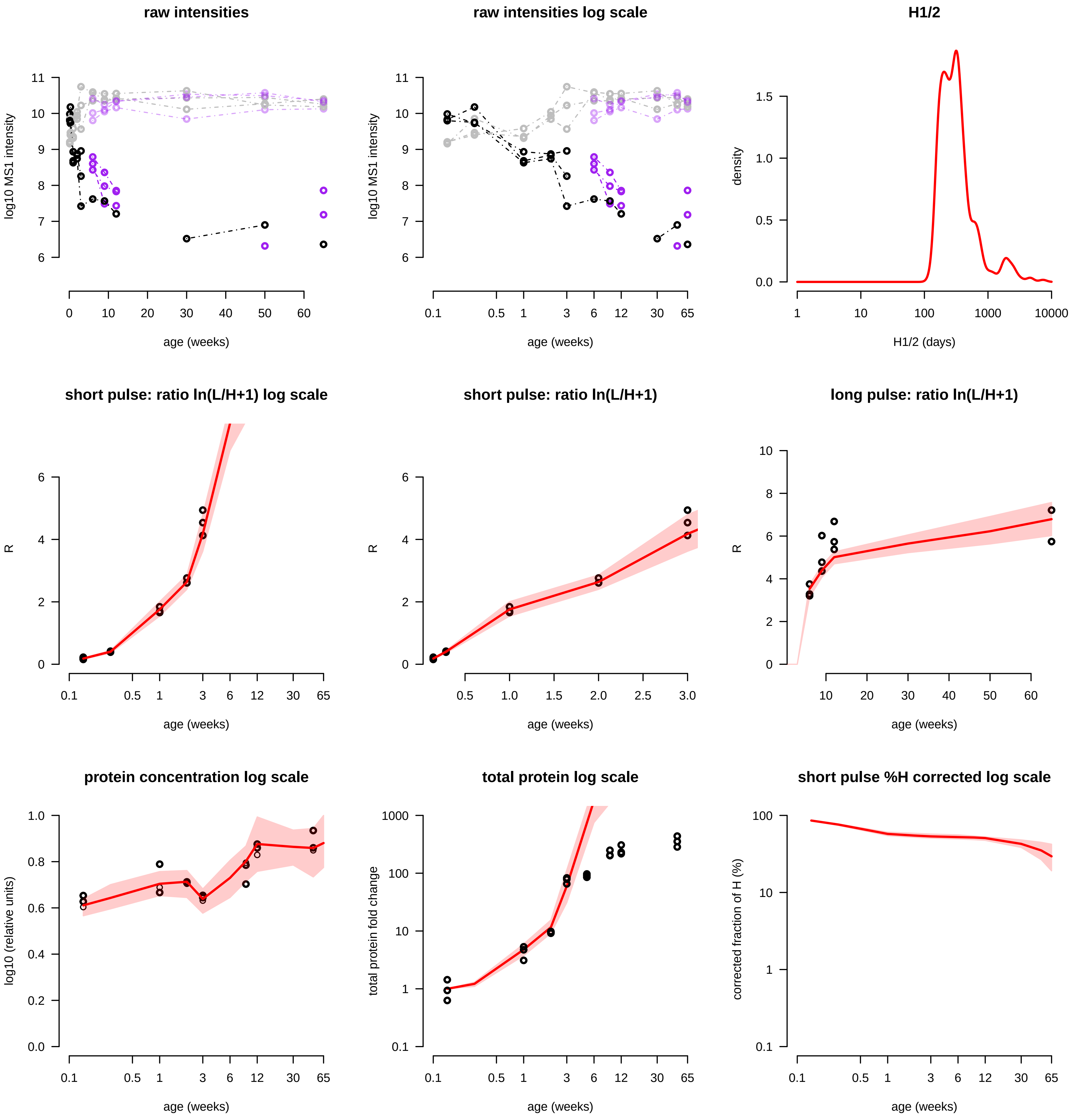

P18760

>sp|P18760|COF1_MOUSE

Cofilin−1

GN=Cfl1

Med H1/2: 9.7 days

Q5 H1/2: 7.9 days

Q95 H1/2: 13.6 days

reliable model fit: yes

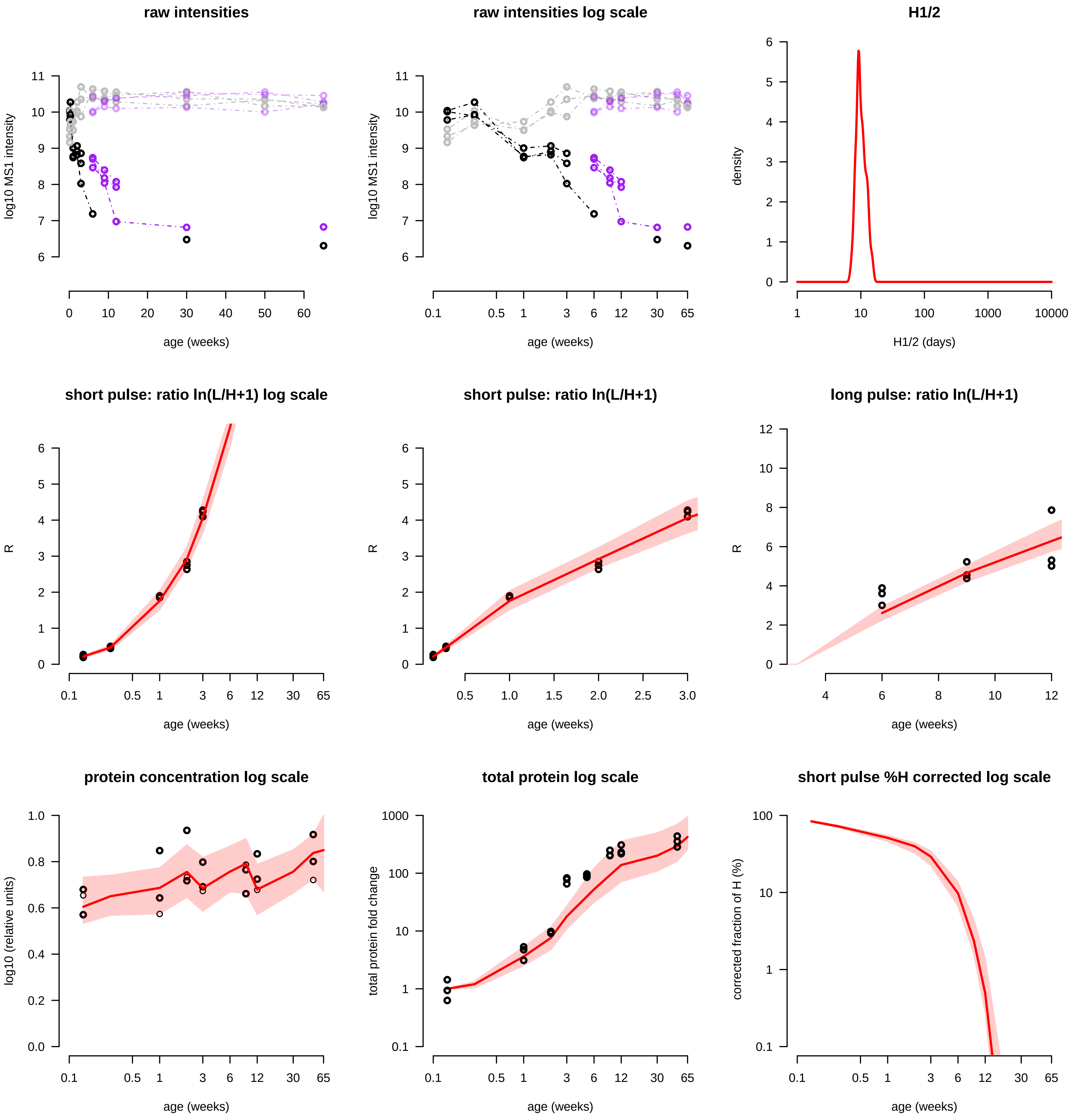

Q9CQF9

>sp|Q9CQF9|PCYOX_MOUSE

Prenylcysteine oxidase

GN=Pcyox1

Med H1/2: 10.7 days

Q5 H1/2: 9 days

Q95 H1/2: 12.4 days

reliable model fit: yes

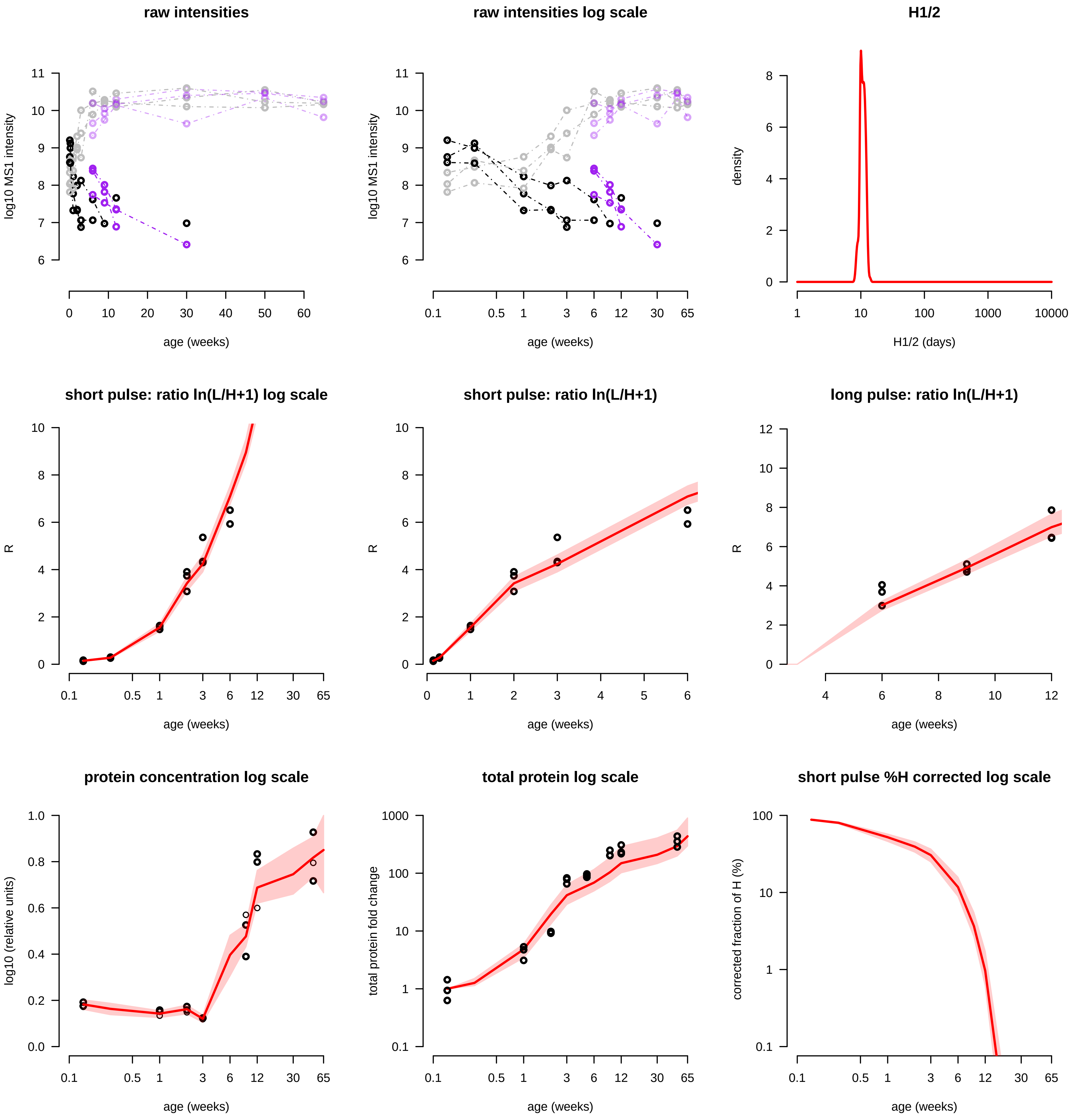

Q99LC5

>sp|Q99LC5|ETFA_MOUSE

Electron transfer flavoprotein subunit alpha, mitochondrial

GN=Etfa

Med H1/2: 38.9 days

Q5 H1/2: 27.3 days

Q95 H1/2: 61.2 days

reliable model fit: yes

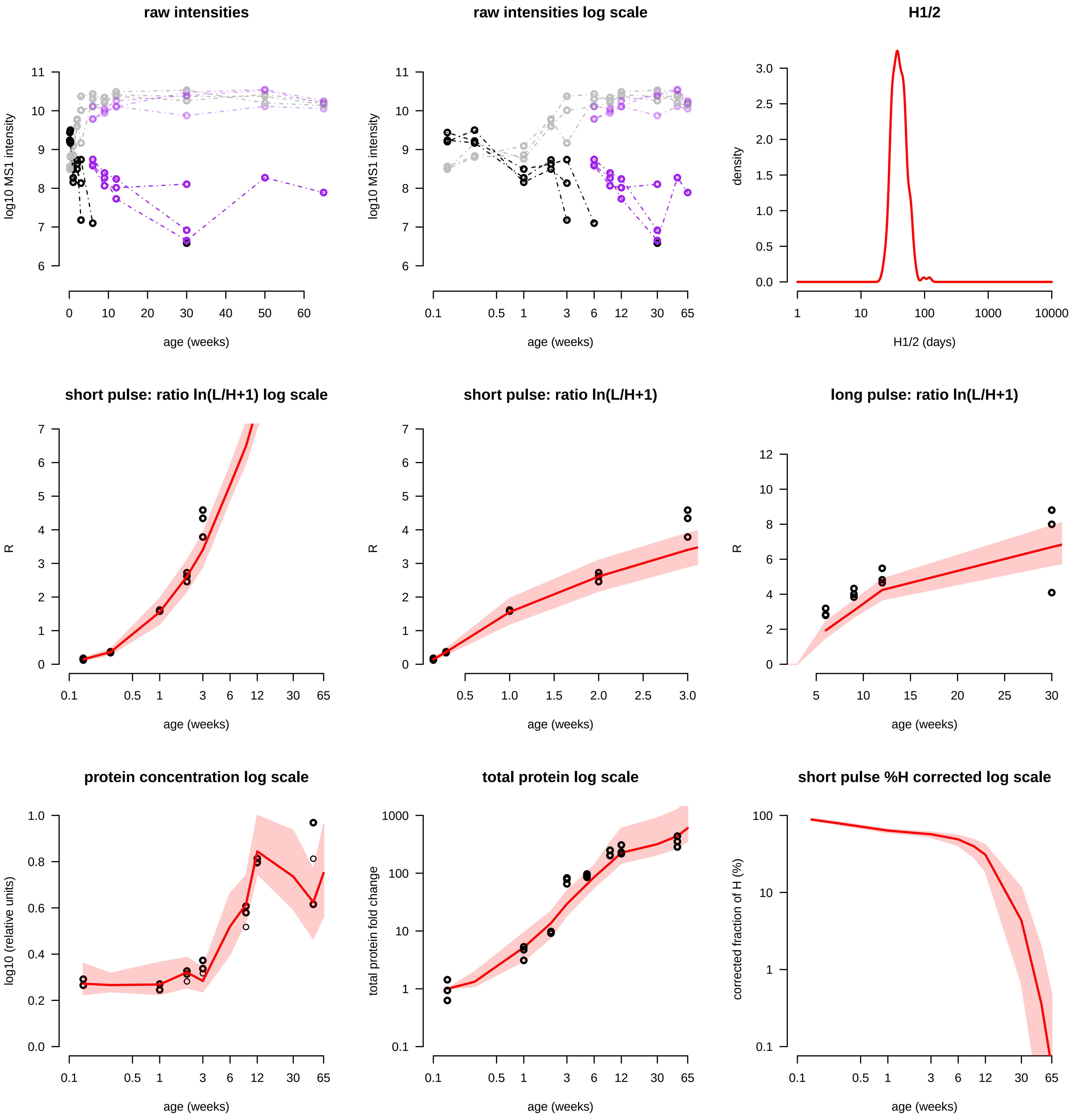

P06795

>sp|P06795|MDR1B_MOUSE

ATP−dependent translocase ABCB1

GN=Abcb1b

Med H1/2: 34.2 days

Q5 H1/2: 19.2 days

Q95 H1/2: 123.8 days

reliable model fit: yes

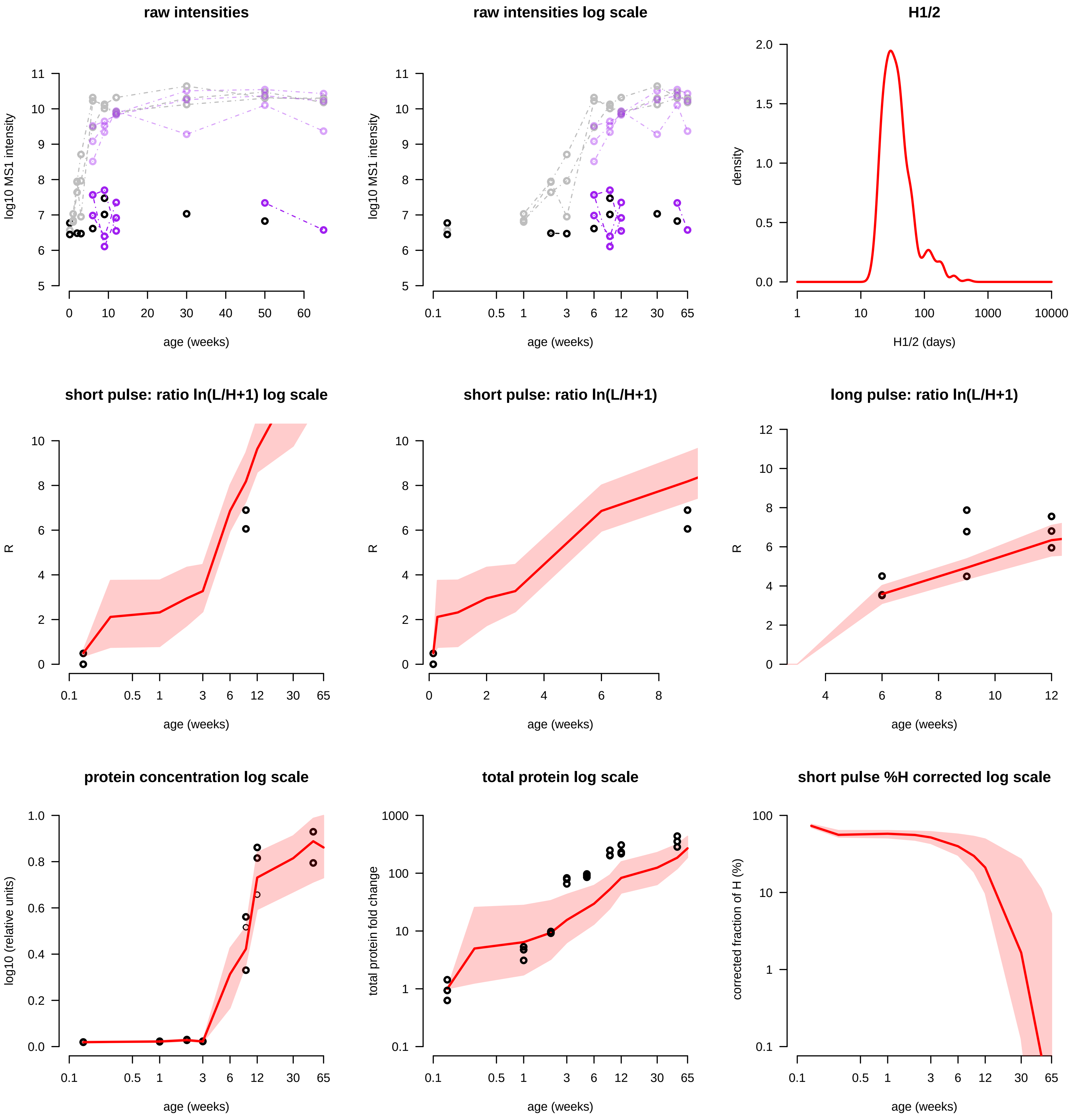

P63158

>sp|P63158|HMGB1_MOUSE

High mobility group protein B1

GN=Hmgb1

Med H1/2: 44.1 days

Q5 H1/2: 29.7 days

Q95 H1/2: 149.5 days

reliable model fit: no

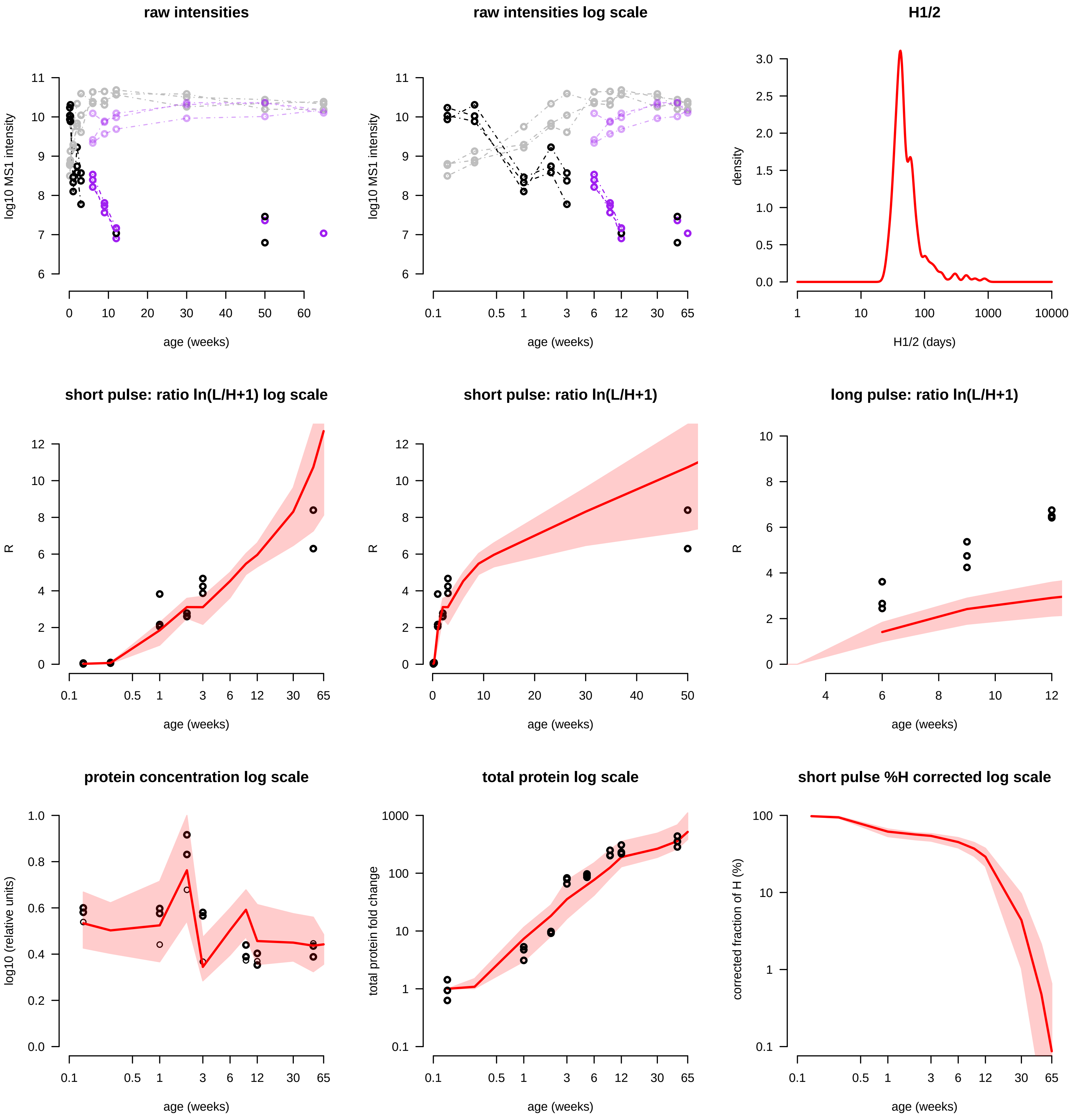

P15532

>sp|P15532|NDKA_MOUSE

Nucleoside diphosphate kinase A

GN=Nme1

Med H1/2: 16.2 days

Q5 H1/2: 12.5 days

Q95 H1/2: 21.8 days

reliable model fit: yes

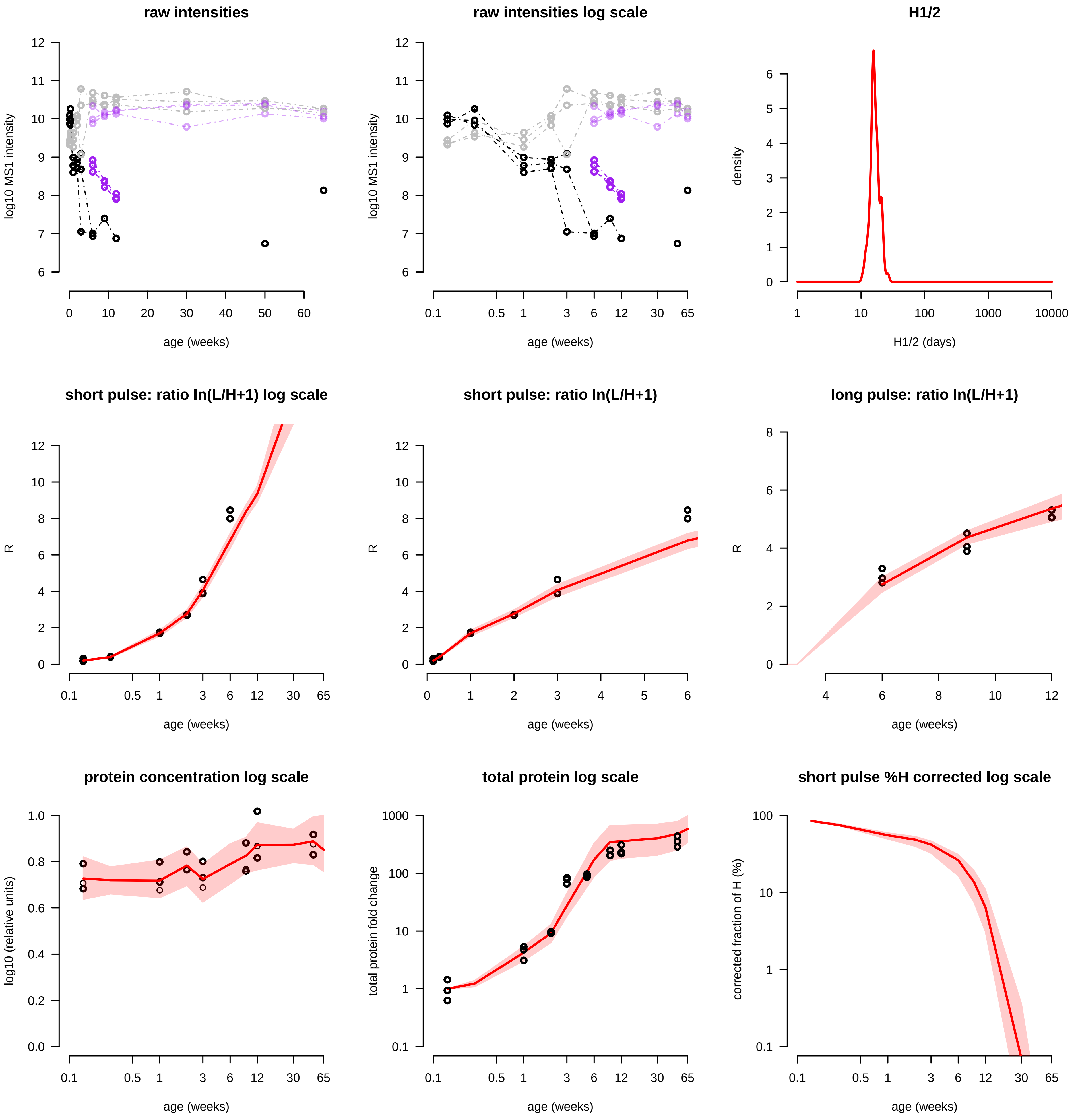

Q8BMS1

>sp|Q8BMS1|ECHA_MOUSE

Trifunctional enzyme subunit alpha, mitochondrial

GN=Hadha

Med H1/2: 31.7 days

Q5 H1/2: 19.7 days

Q95 H1/2: 57 days

reliable model fit: yes

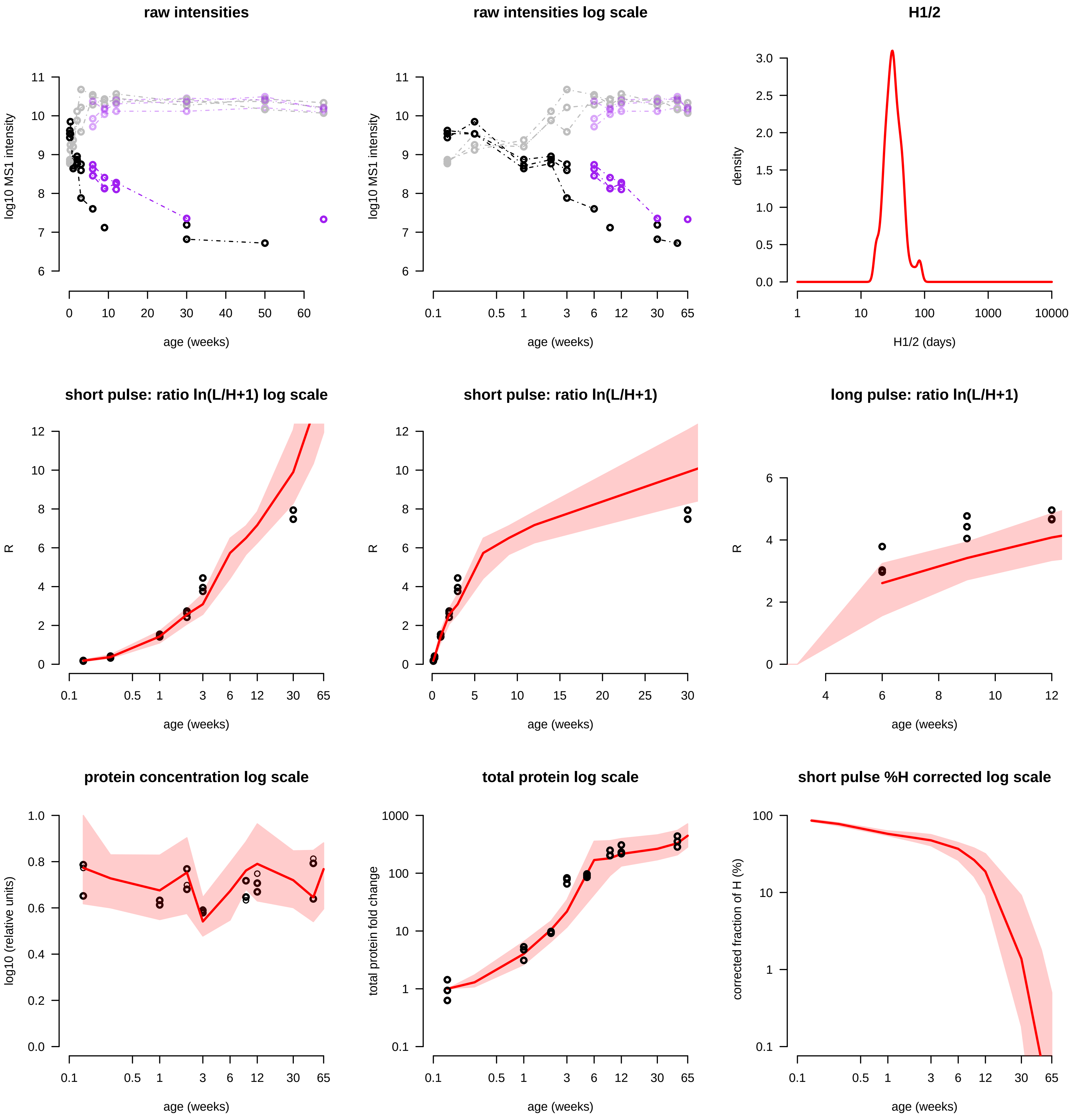

Q8CGK3

>sp|Q8CGK3|LONM_MOUSE

Lon protease homolog, mitochondrial

GN=Lonp1

Med H1/2: 11.5 days

Q5 H1/2: 8.6 days

Q95 H1/2: 16 days

reliable model fit: yes

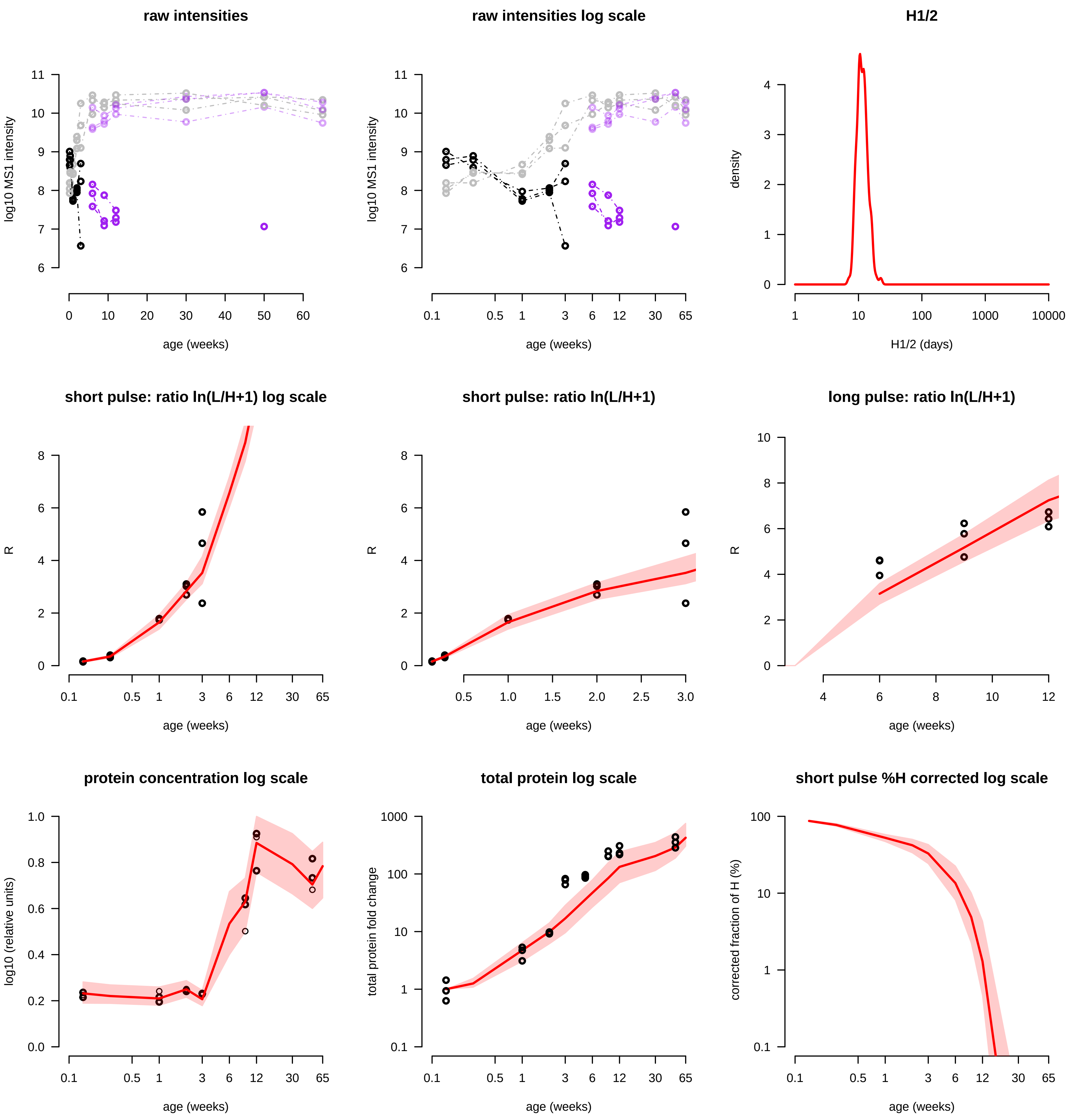

Q60930

>sp|Q60930|VDAC2_MOUSE

Voltage−dependent anion−selective channel protein 2

GN=Vdac2

Med H1/2: 13.3 days

Q5 H1/2: 10.6 days

Q95 H1/2: 17.2 days

reliable model fit: yes

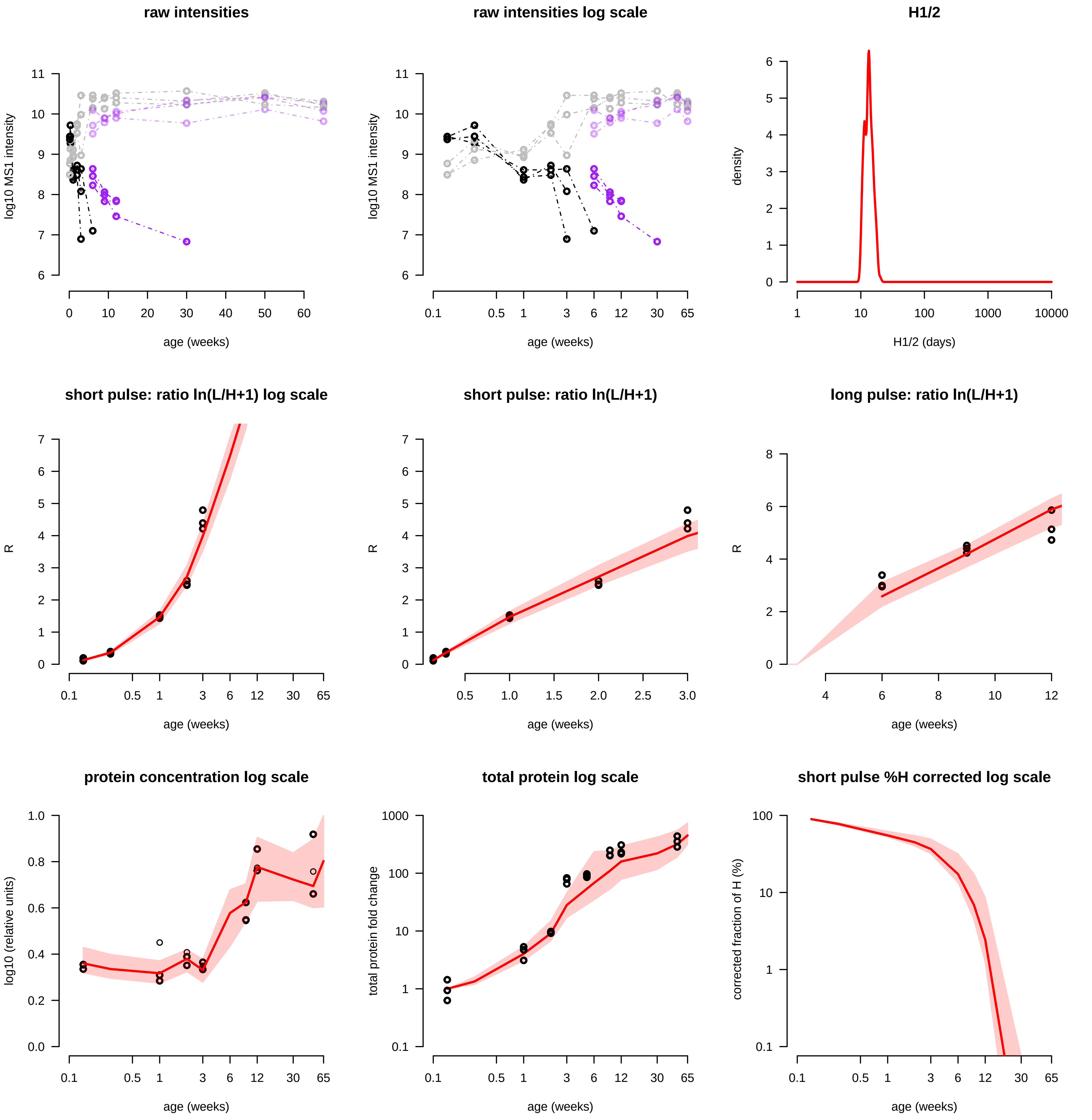

P62259

>sp|P62259|1433E_MOUSE

14−3−3 protein epsilon

GN=Ywhae

Med H1/2: 10.7 days

Q5 H1/2: 8.6 days

Q95 H1/2: 15.2 days

reliable model fit: yes

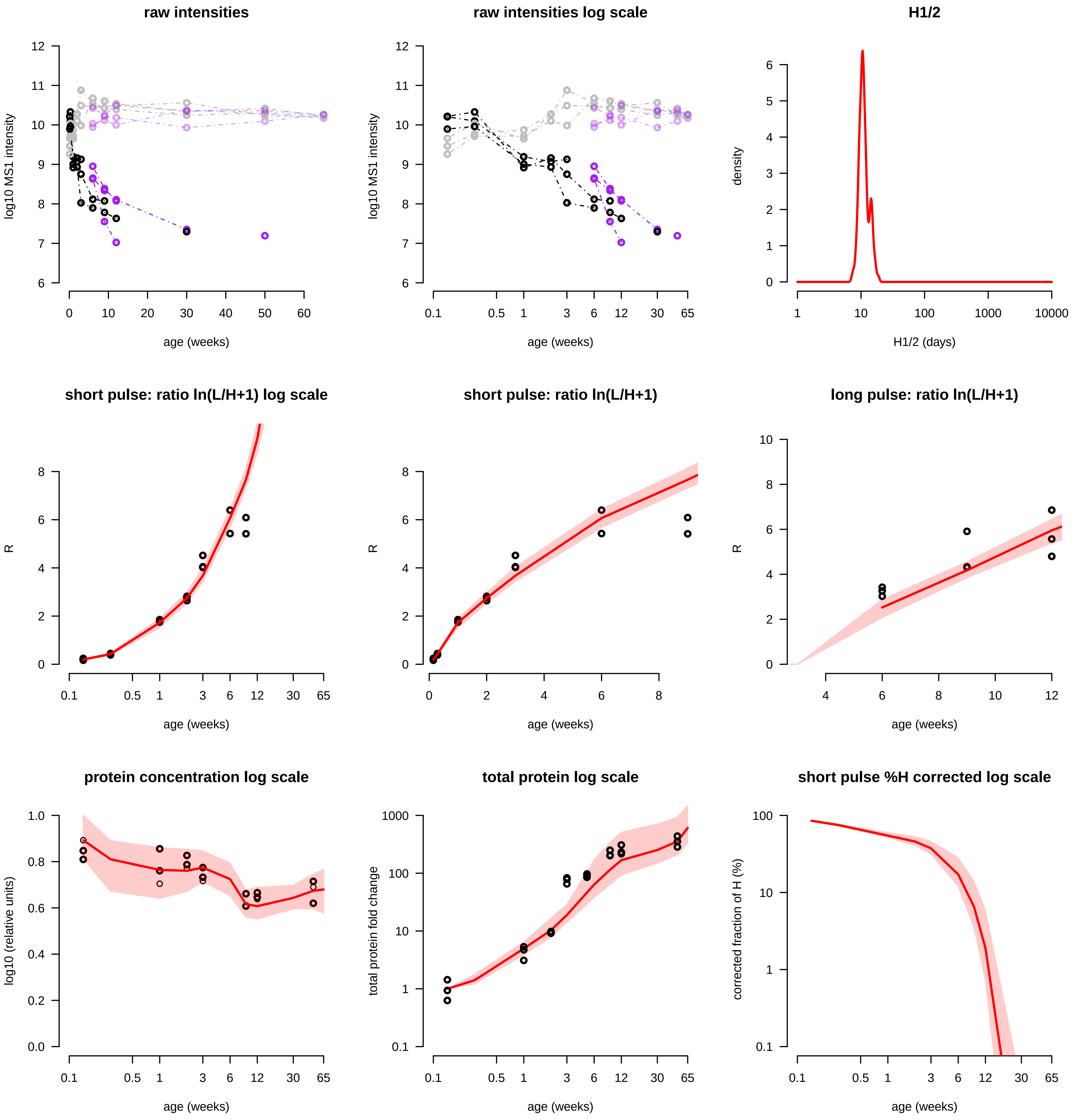

Q64133

>sp|Q64133|AOFA_MOUSE

Amine oxidase [flavin−containing] A

GN=Maoa

Med H1/2: 21.5 days

Q5 H1/2: 15.8 days

Q95 H1/2: 43.8 days

reliable model fit: yes

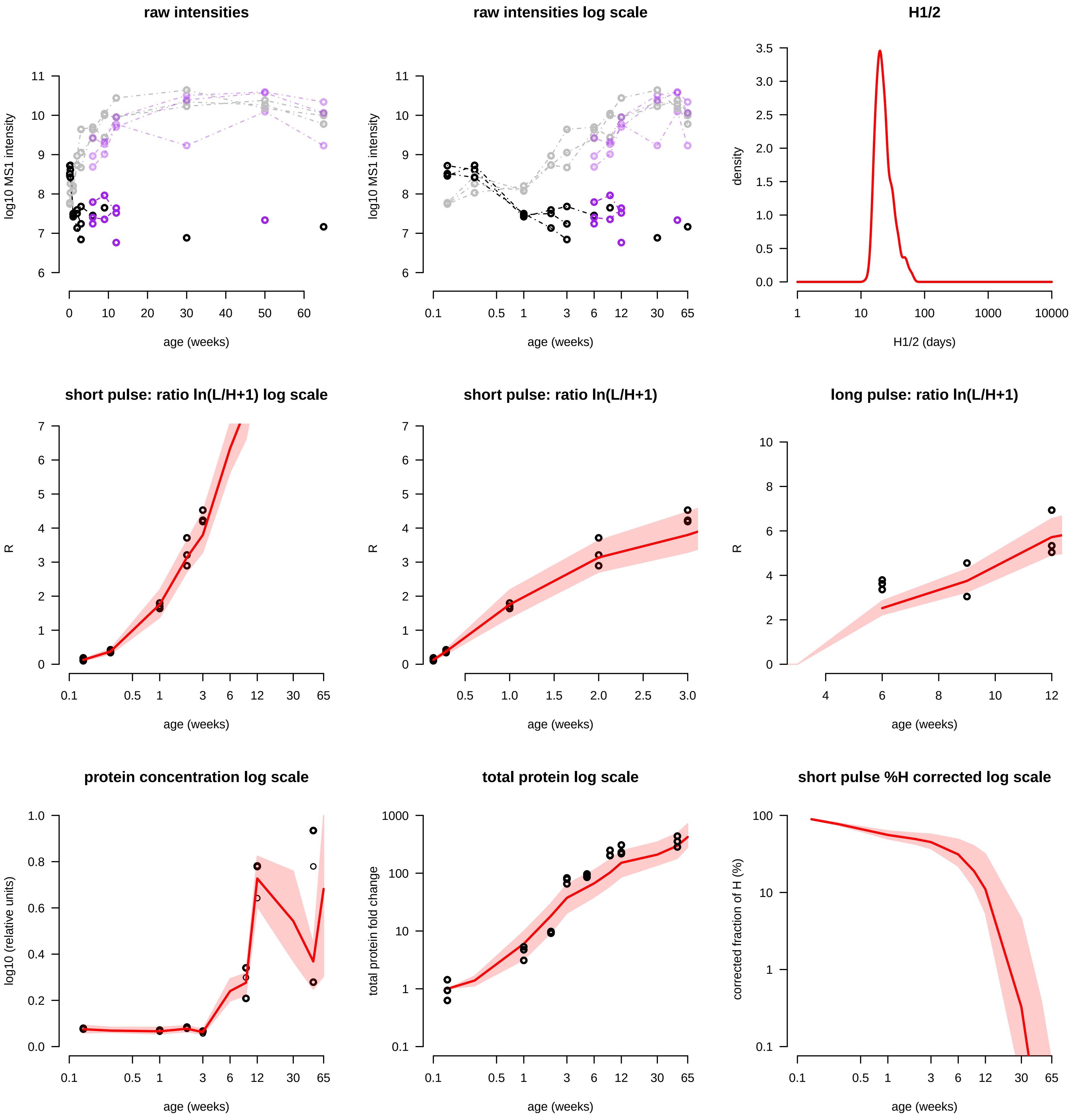

P57780

>sp|P57780|ACTN4_MOUSE

Alpha−actinin−4

GN=Actn4

Med H1/2: 100.2 days

Q5 H1/2: 44 days

Q95 H1/2: 953.6 days

reliable model fit: yes

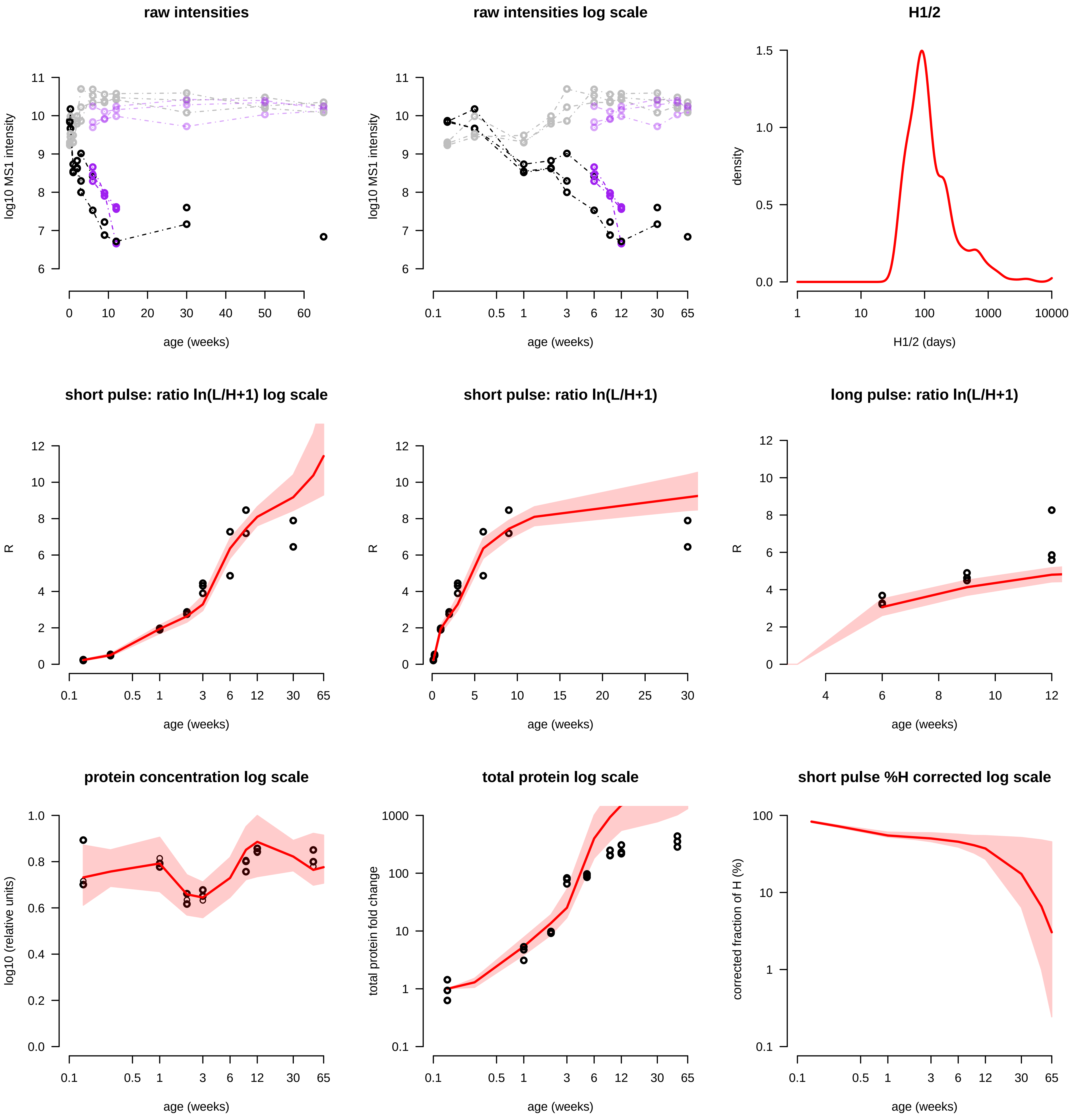

P09411

>sp|P09411|PGK1_MOUSE

Phosphoglycerate kinase 1

GN=Pgk1

Med H1/2: 1065.4 days

Q5 H1/2: 406.8 days

Q95 H1/2: 9164.9 days

reliable model fit: yes

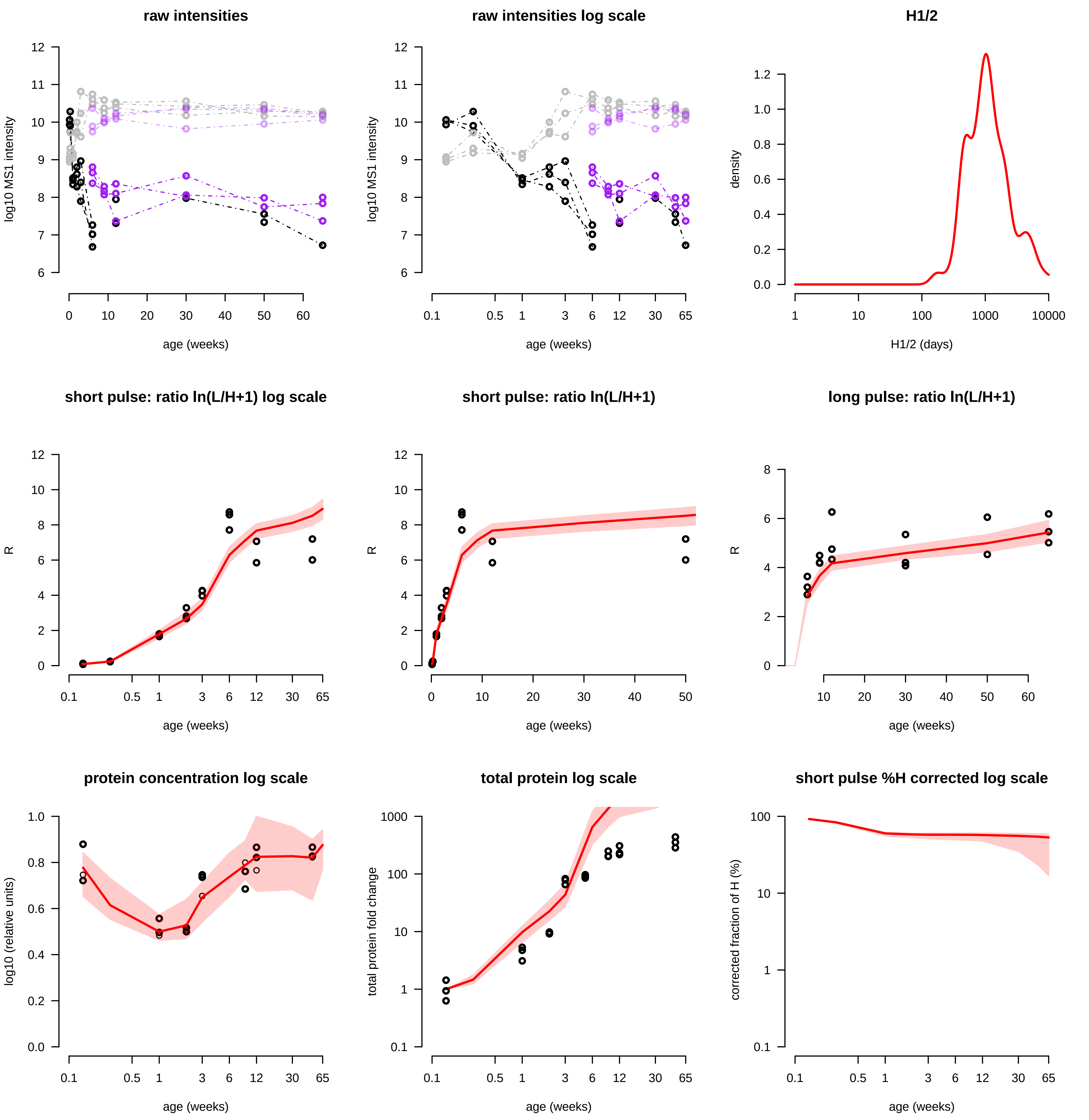

Q93092

>sp|Q93092|TALDO_MOUSE

Transaldolase

GN=Taldo1

Med H1/2: 262 days

Q5 H1/2: 146.9 days

Q95 H1/2: 941.5 days

reliable model fit: yes

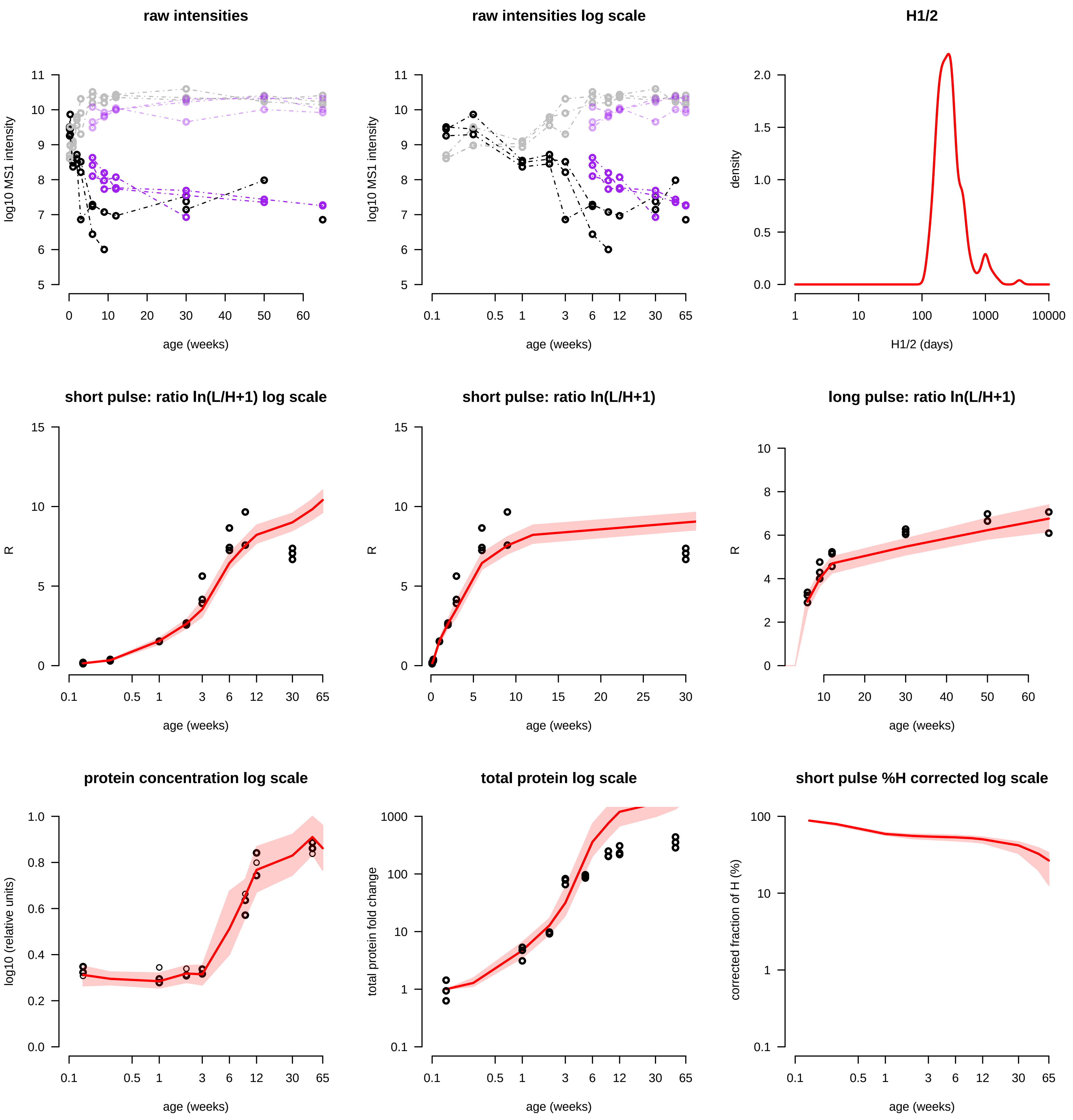

Q91V92

>sp|Q91V92|ACLY_MOUSE

ATP−citrate synthase

GN=Acly

Med H1/2: 225.6 days

Q5 H1/2: 64.5 days

Q95 H1/2: 2035.3 days

reliable model fit: yes

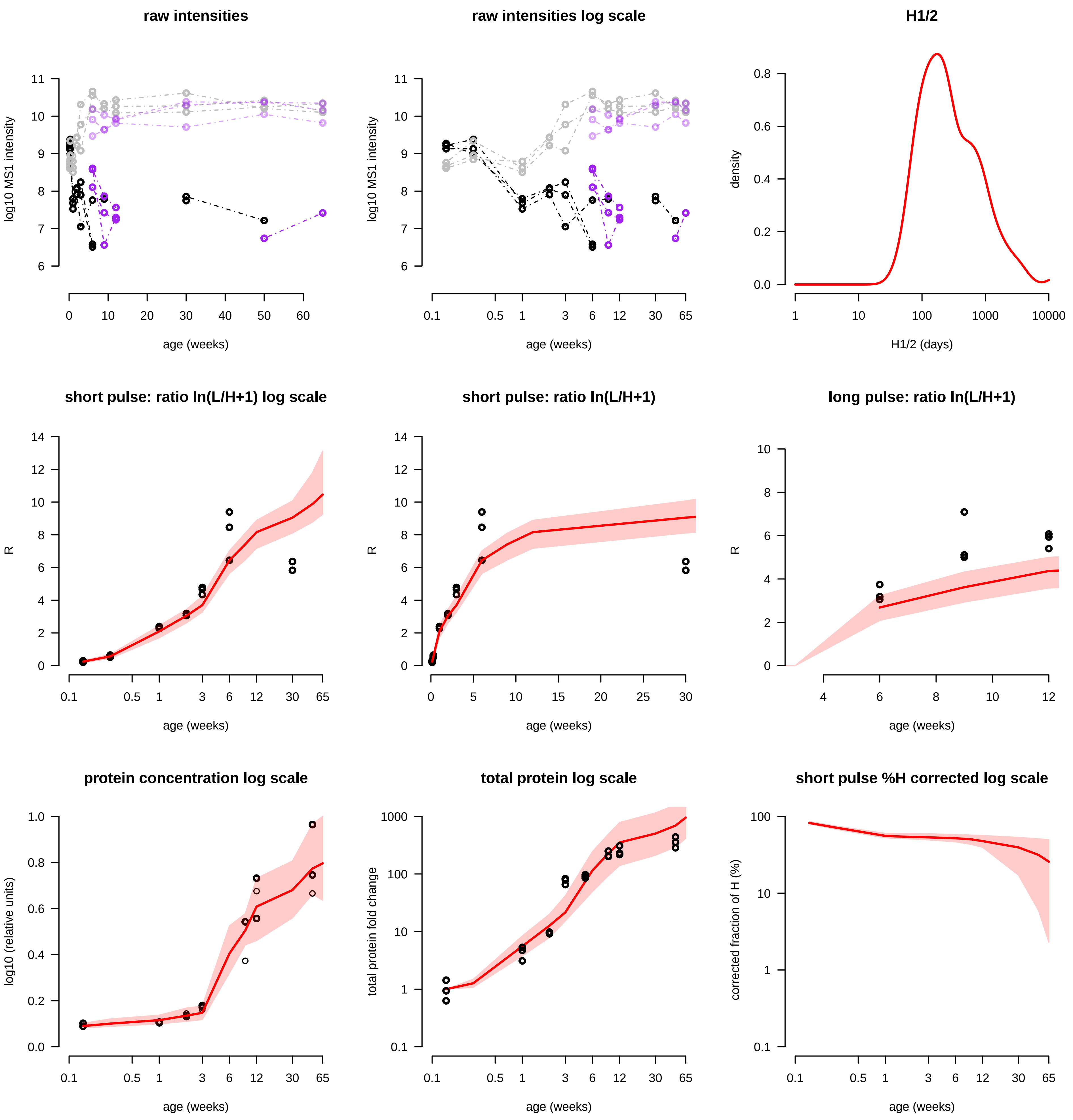

P48678;P48678−2;P48678−3

>sp|P48678|LMNA_MOUSE

Prelamin−A/C

GN=Lmna

Med H1/2: 17.6 days

Q5 H1/2: 13.5 days

Q95 H1/2: 27.2 days

reliable model fit: yes

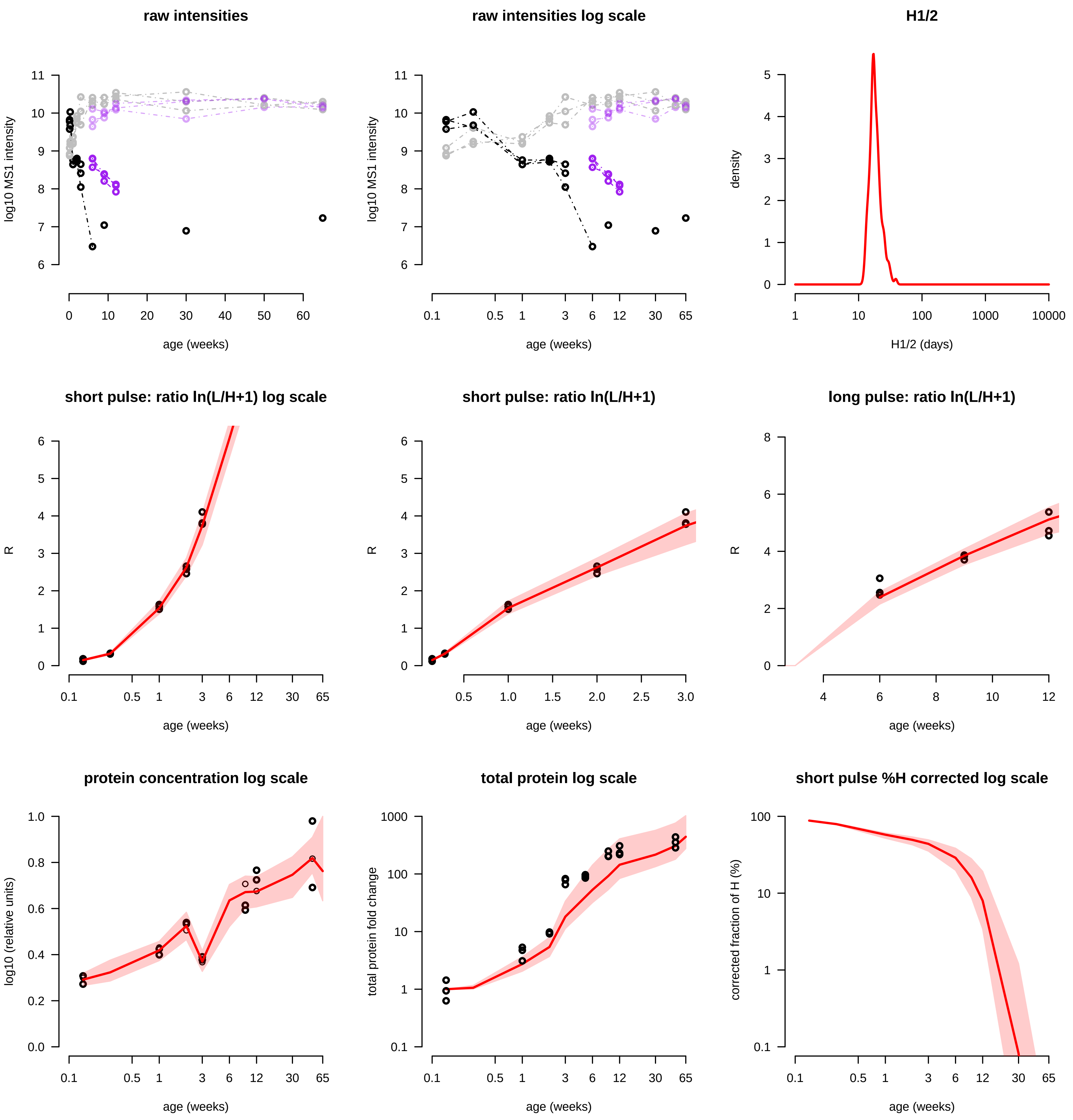

Q8BG05

>sp|Q8BG05|ROA3_MOUSE

Heterogeneous nuclear ribonucleoprotein A3

GN=Hnrnpa3

Med H1/2: 11.4 days

Q5 H1/2: 9.9 days

Q95 H1/2: 14.7 days

reliable model fit: yes

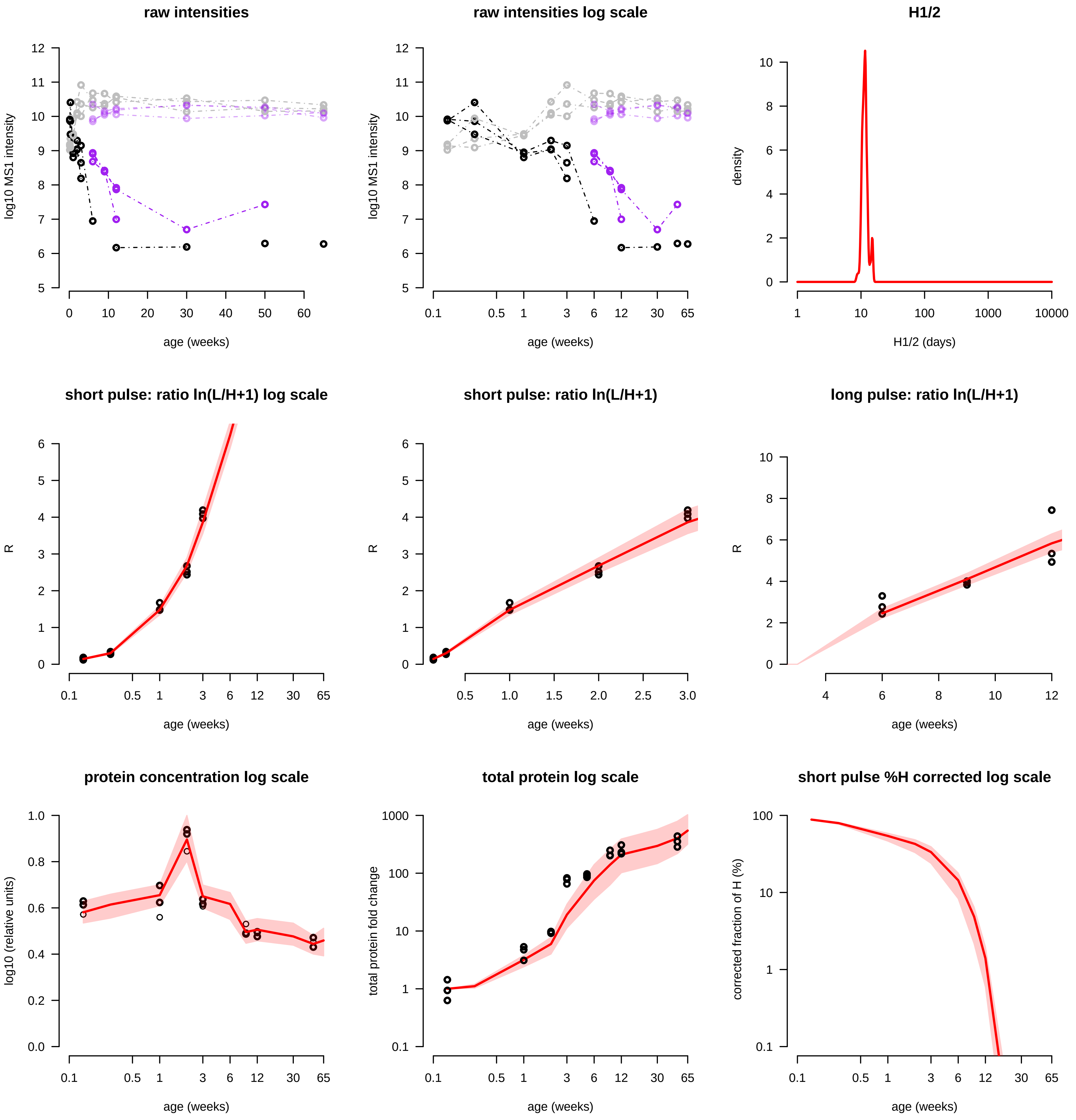

P26039

>sp|P26039|TLN1_MOUSE

Talin−1

GN=Tln1

Med H1/2: 981.7 days

Q5 H1/2: 301.6 days

Q95 H1/2: 7292.5 days

reliable model fit: yes

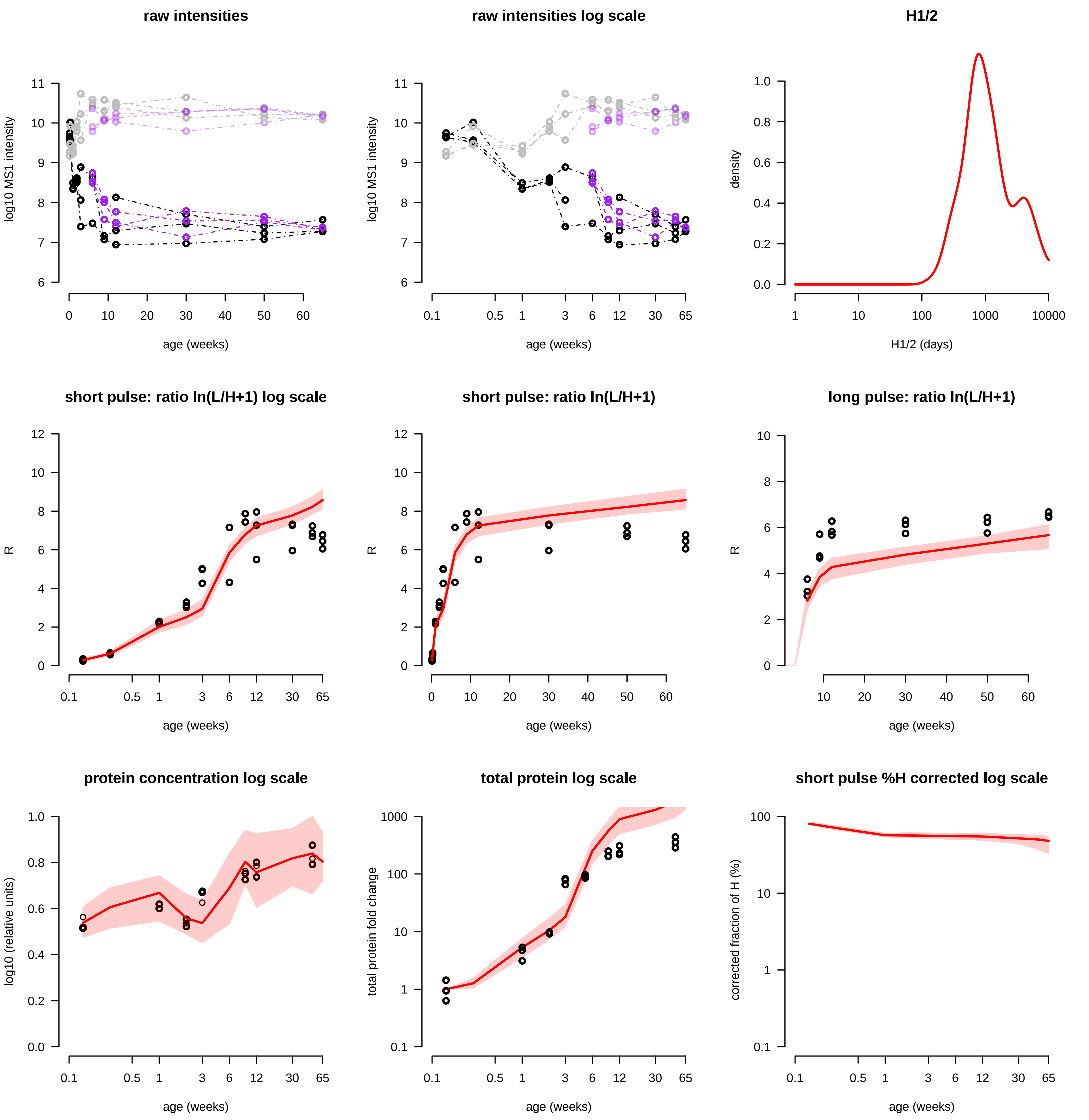

P17751−2;P17751

>sp|P17751−2|TPIS_MOUSE

Isoform 2 of Triosephosphate isomerase

GN=Tpi1

Med H1/2: 104.2 days

Q5 H1/2: 47.6 days

Q95 H1/2: 405.2 days

reliable model fit: yes

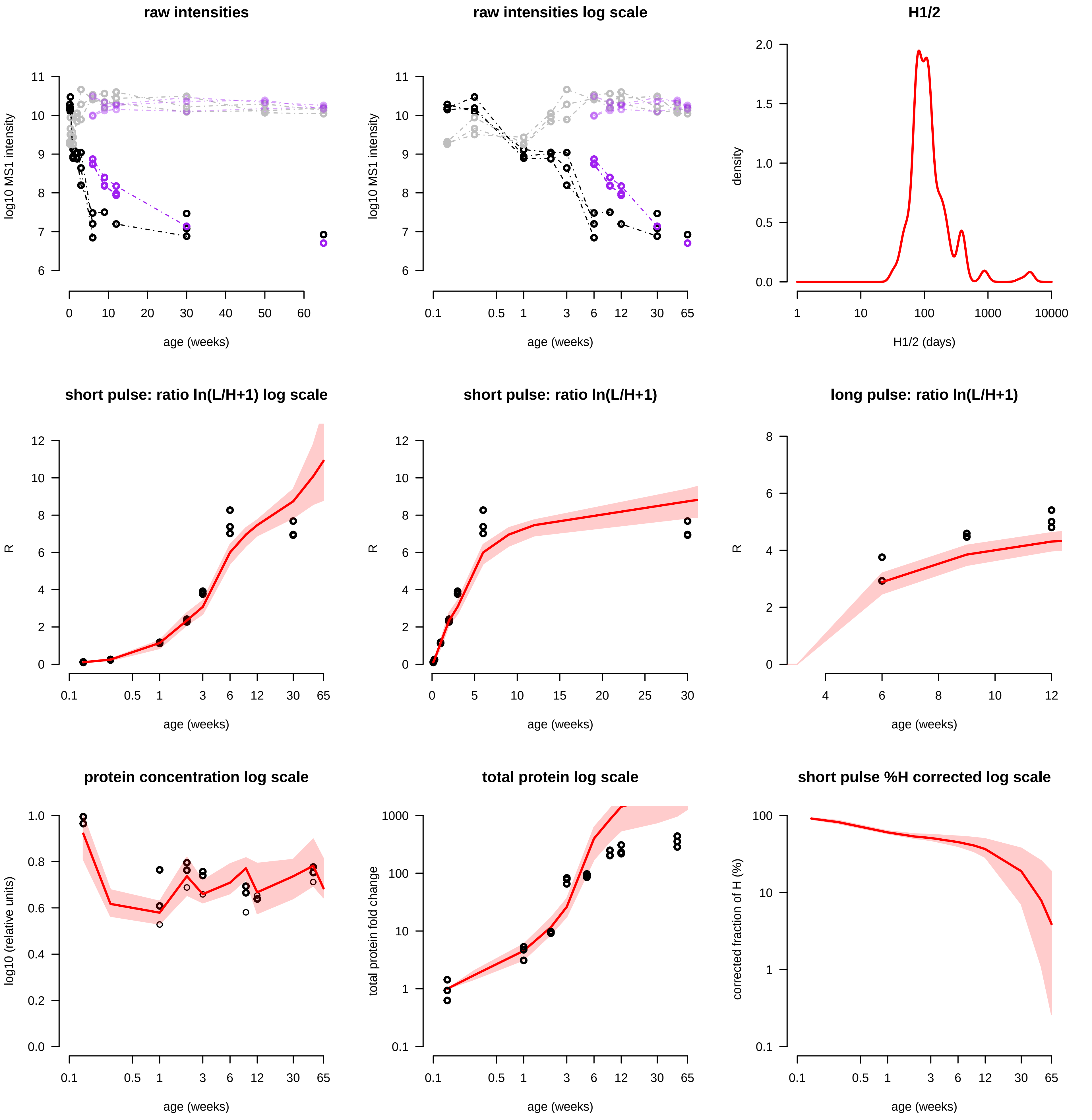

P07759

>sp|P07759|SPA3K_MOUSE

Serine protease inhibitor A3K

GN=Serpina3k

Med H1/2: 7.1 days

Q5 H1/2: 5.4 days

Q95 H1/2: 10.2 days

reliable model fit: yes

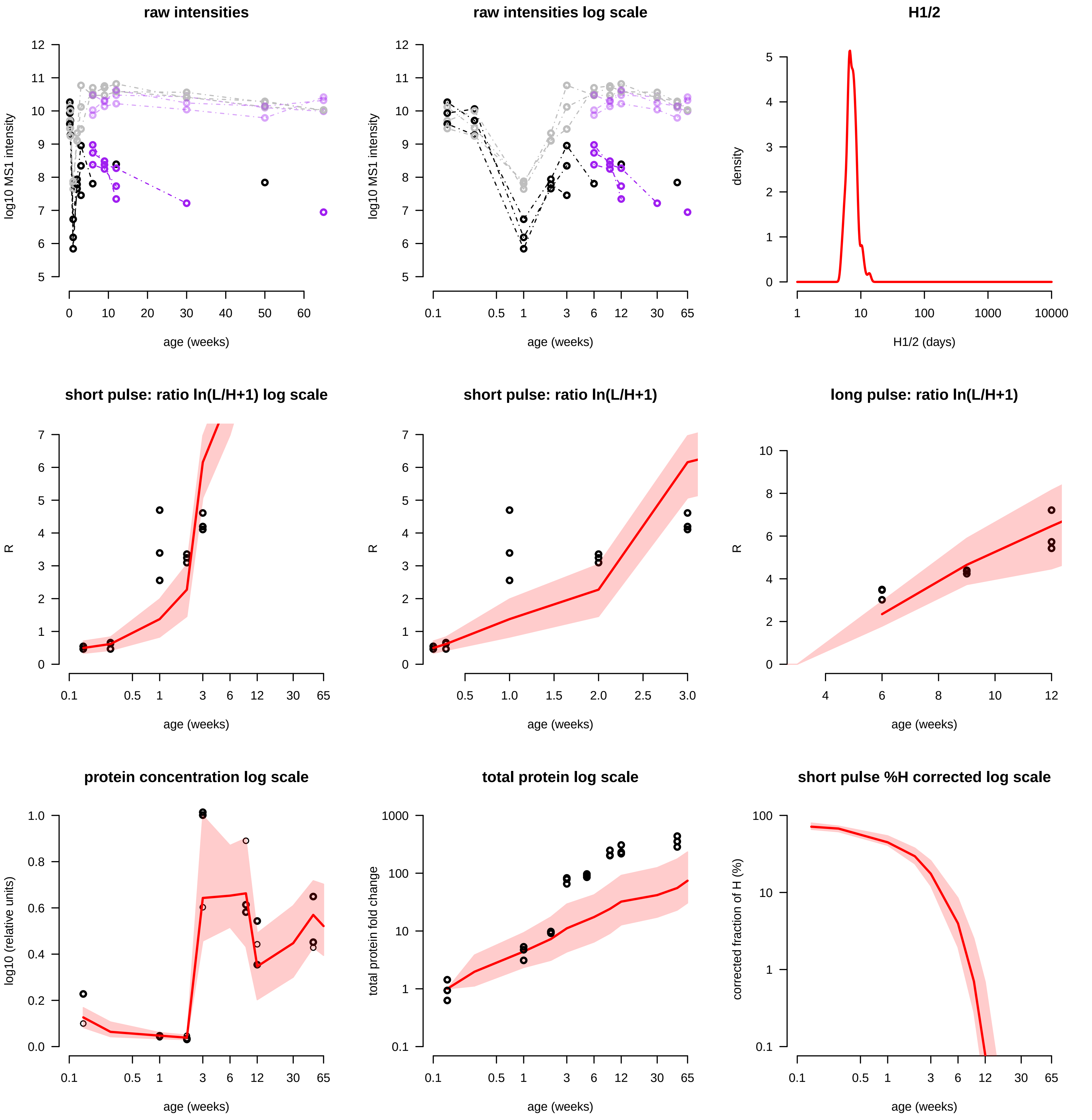

Q8BWT1

>sp|Q8BWT1|THIM_MOUSE

3−ketoacyl−CoA thiolase, mitochondrial

GN=Acaa2

Med H1/2: 472.2 days

Q5 H1/2: 169.4 days

Q95 H1/2: 3097.3 days

reliable model fit: yes

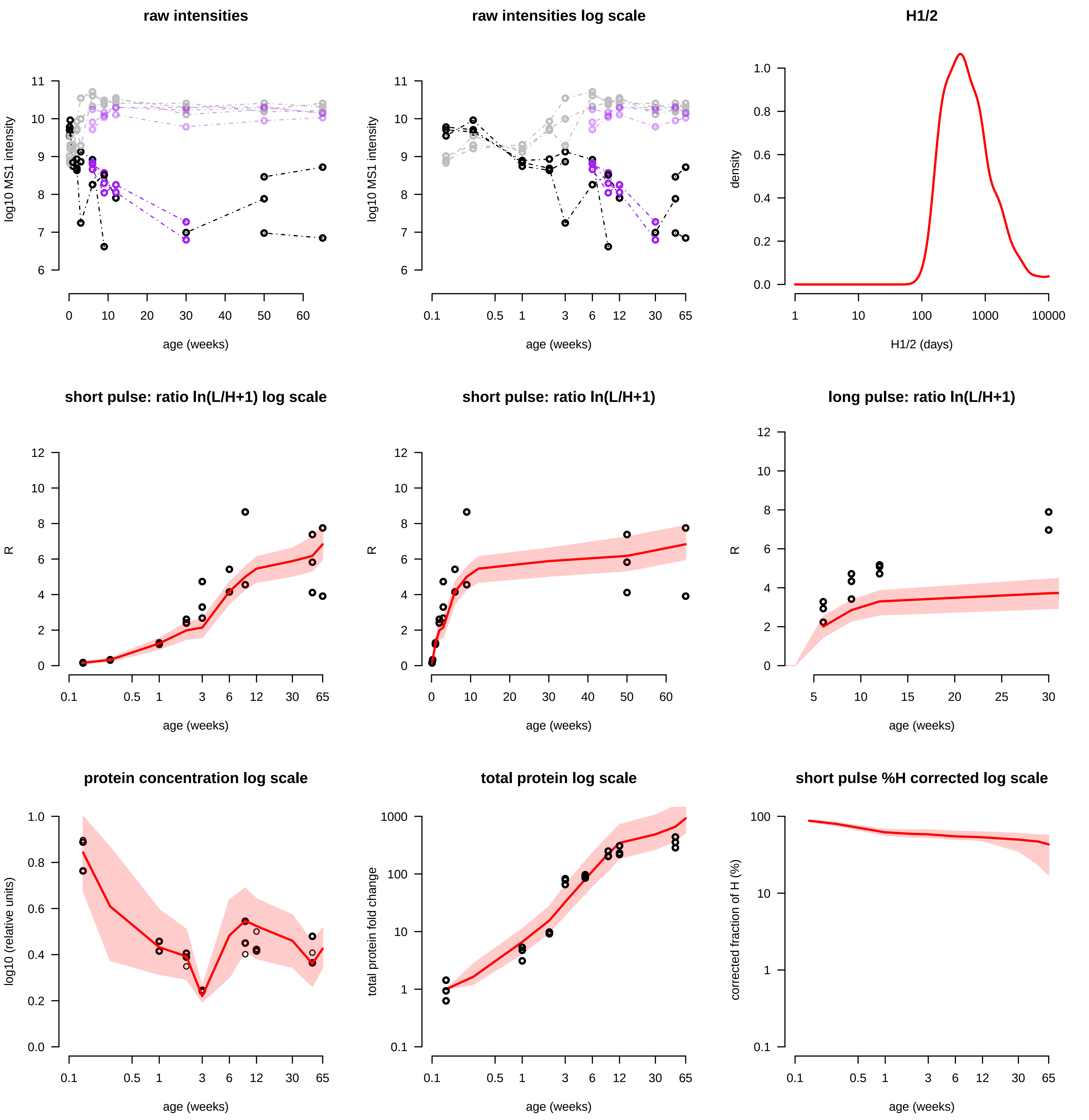

Q9JHU4

>sp|Q9JHU4|DYHC1_MOUSE

Cytoplasmic dynein 1 heavy chain 1

GN=Dync1h1

Med H1/2: 330.1 days

Q5 H1/2: 149 days

Q95 H1/2: 1500.2 days

reliable model fit: yes

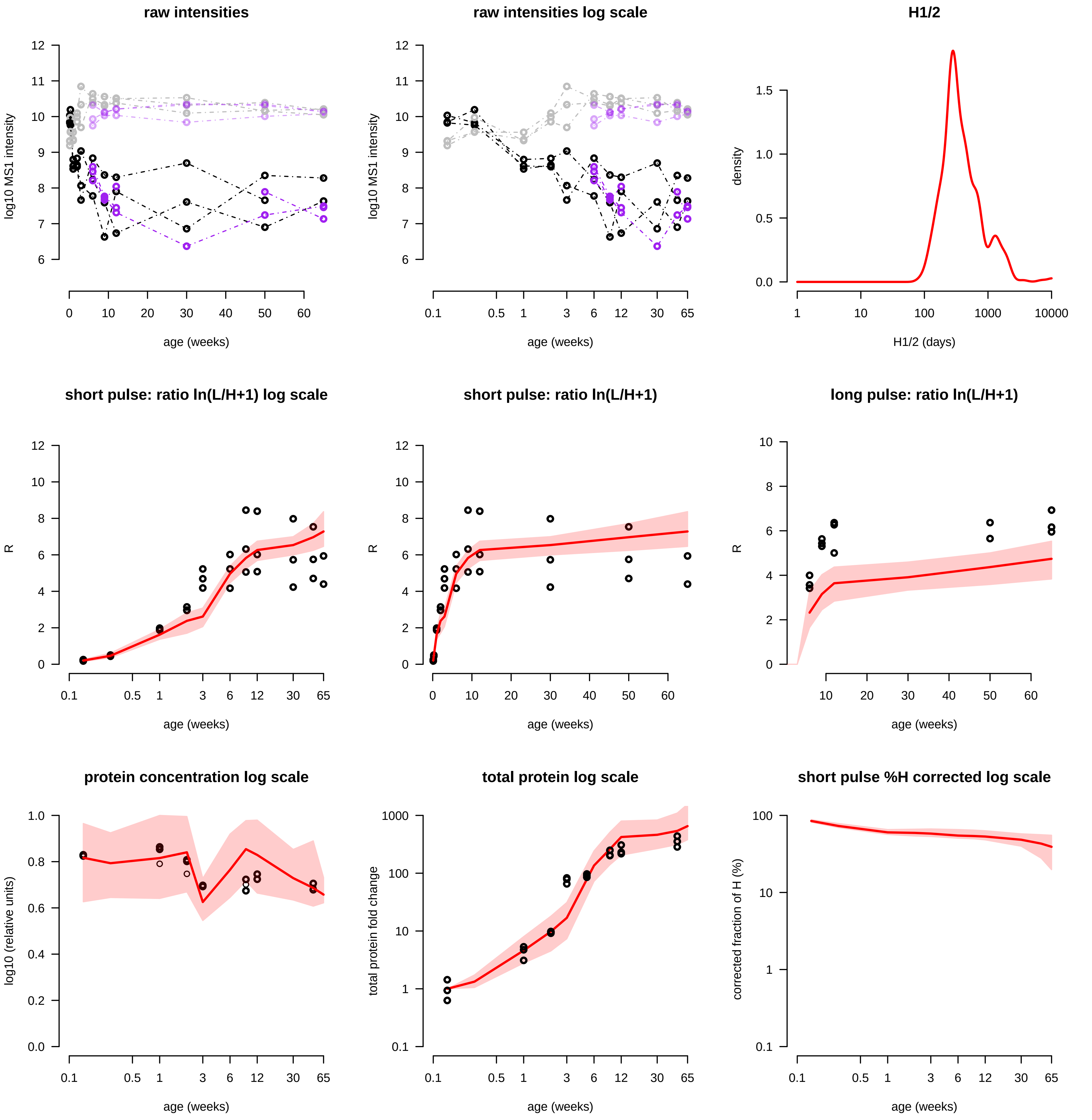

P51881

>sp|P51881|ADT2_MOUSE

ADP/ATP translocase 2

GN=Slc25a5

Med H1/2: 38.5 days

Q5 H1/2: 29.7 days

Q95 H1/2: 50.7 days

reliable model fit: yes

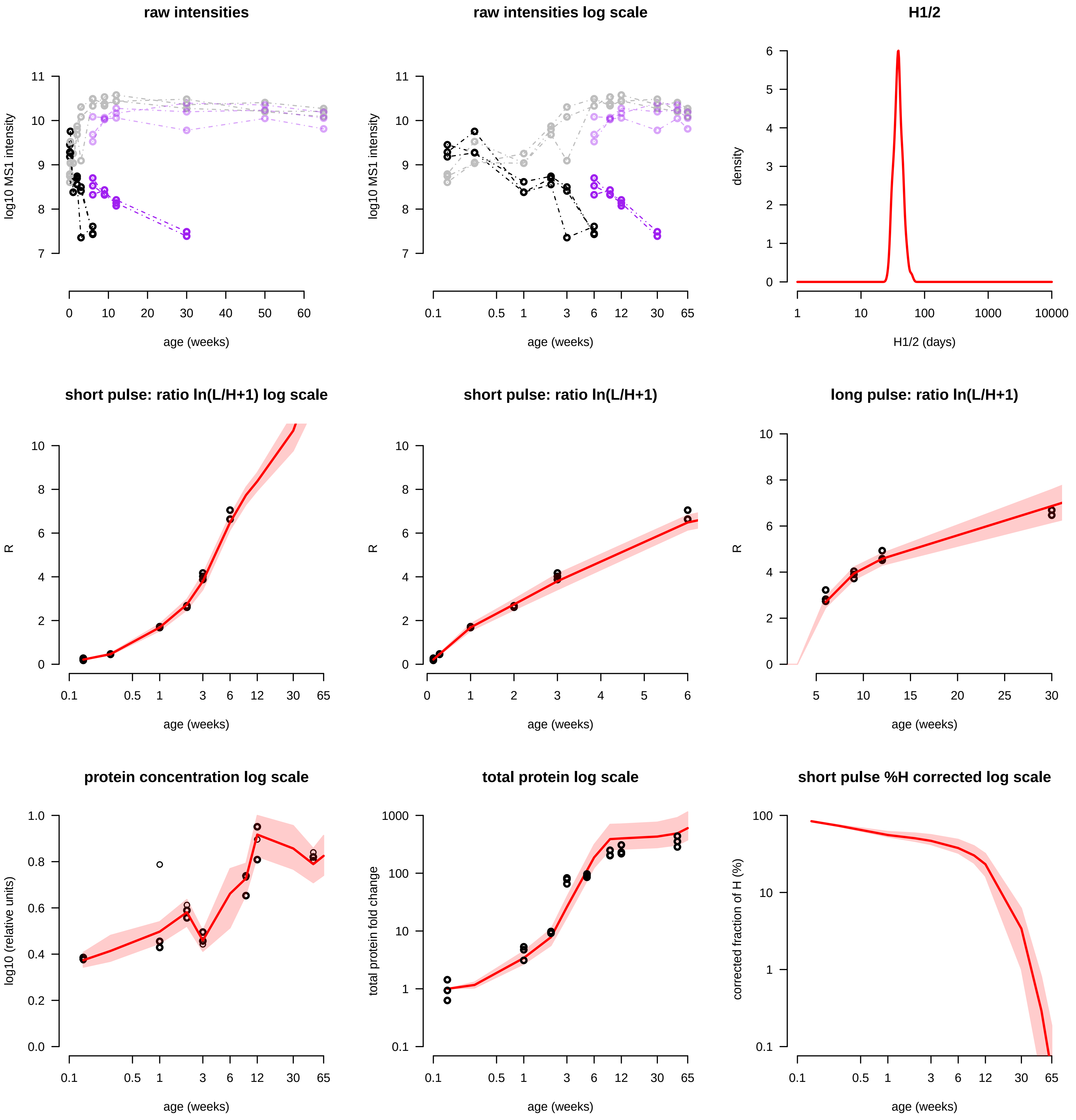

Q05920

>sp|Q05920|PYC_MOUSE

Pyruvate carboxylase, mitochondrial

GN=Pc

Med H1/2: 17.3 days

Q5 H1/2: 10.9 days

Q95 H1/2: 24.2 days

reliable model fit: yes

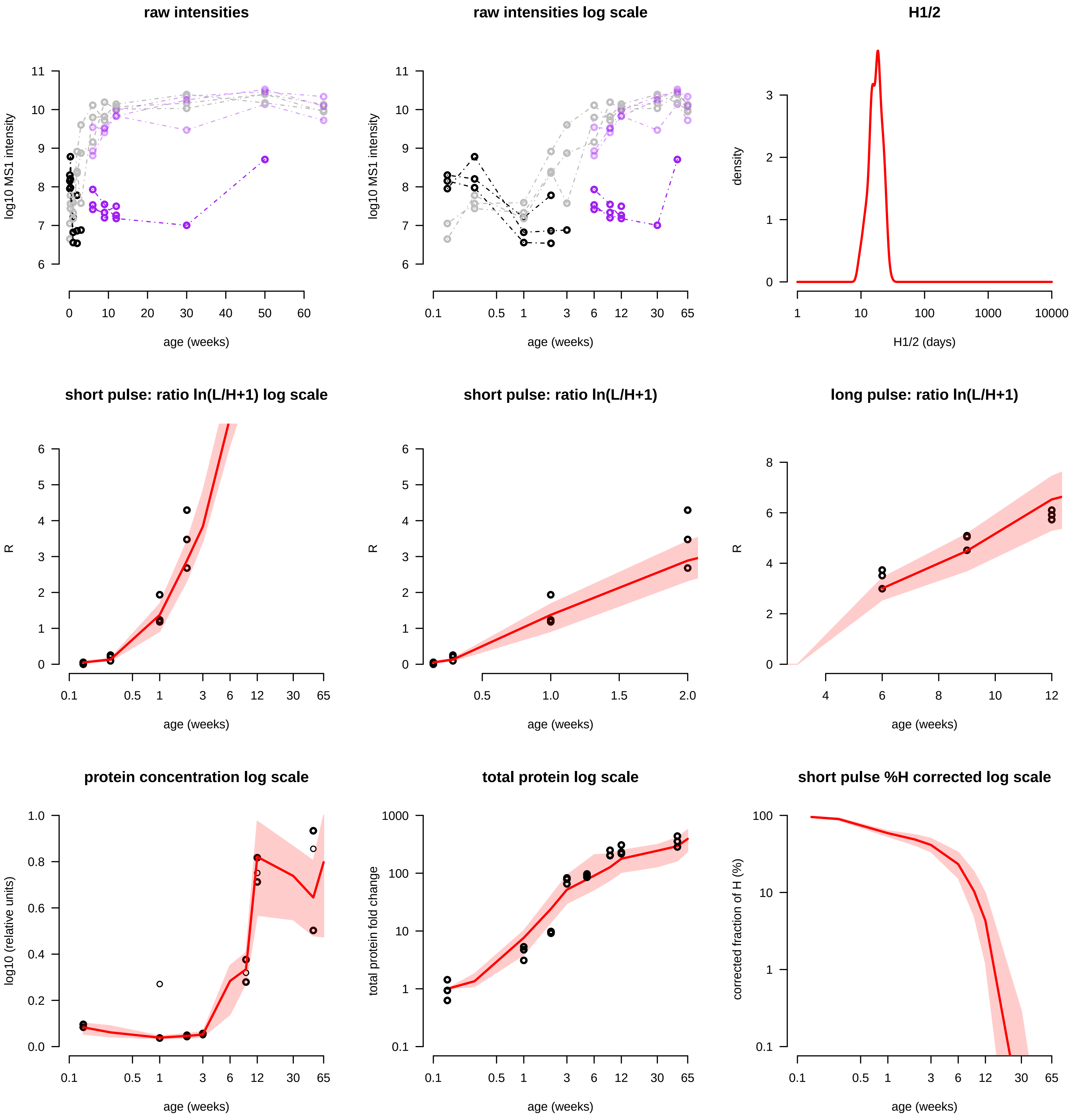

P09528

>sp|P09528|FRIH_MOUSE

Ferritin heavy chain

GN=Fth1

Med H1/2: 16.7 days

Q5 H1/2: 13.4 days

Q95 H1/2: 23.9 days

reliable model fit: yes

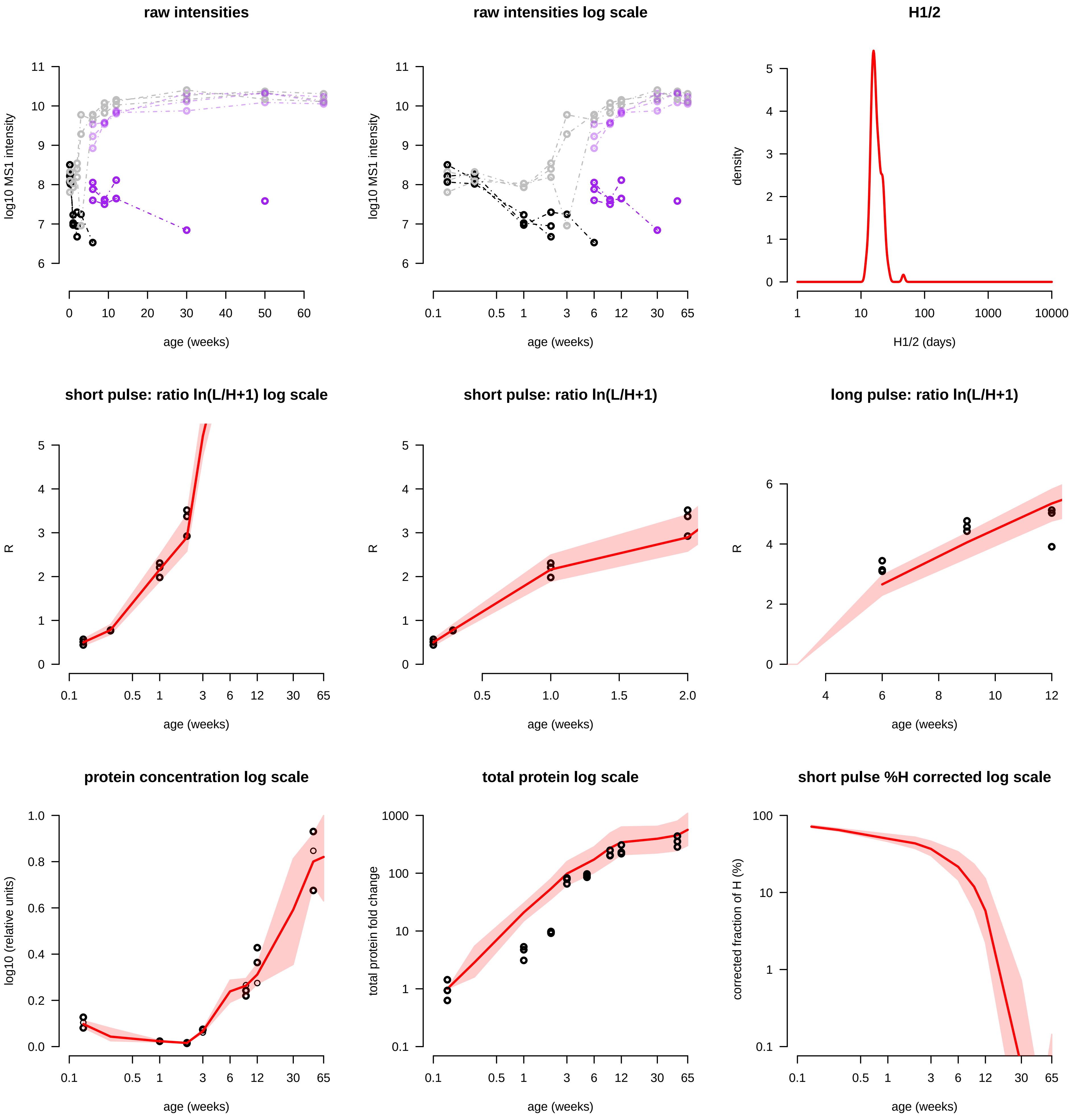

Q9Z0X1

>sp|Q9Z0X1|AIFM1_MOUSE

Apoptosis−inducing factor 1, mitochondrial

GN=Aifm1

Med H1/2: 262.5 days

Q5 H1/2: 71.7 days

Q95 H1/2: 3387.7 days

reliable model fit: no

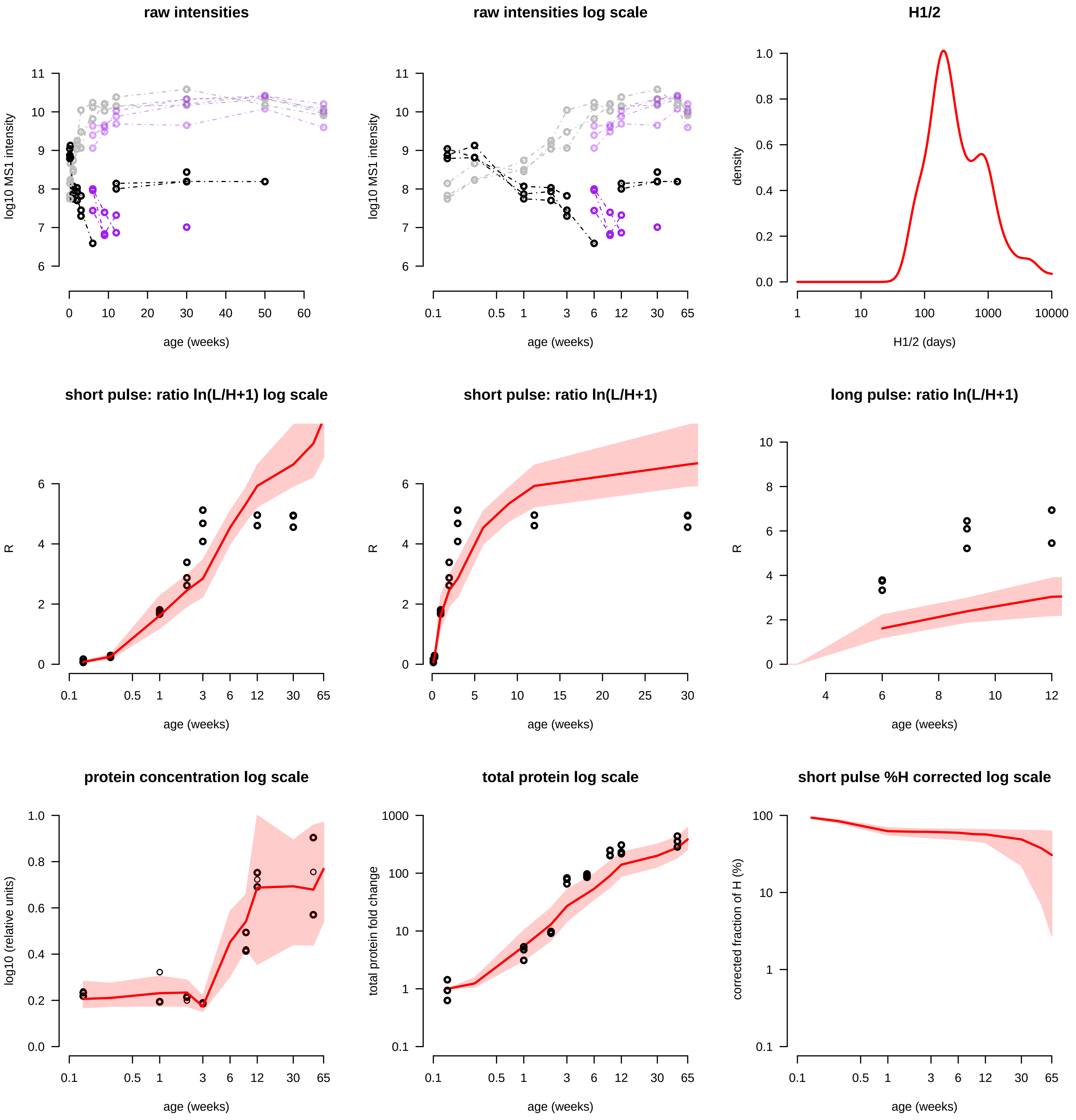

Q9DCX2

>sp|Q9DCX2|ATP5H_MOUSE

ATP synthase subunit d, mitochondrial

GN=Atp5pd

Med H1/2: 113.1 days

Q5 H1/2: 67.4 days

Q95 H1/2: 574.6 days

reliable model fit: no

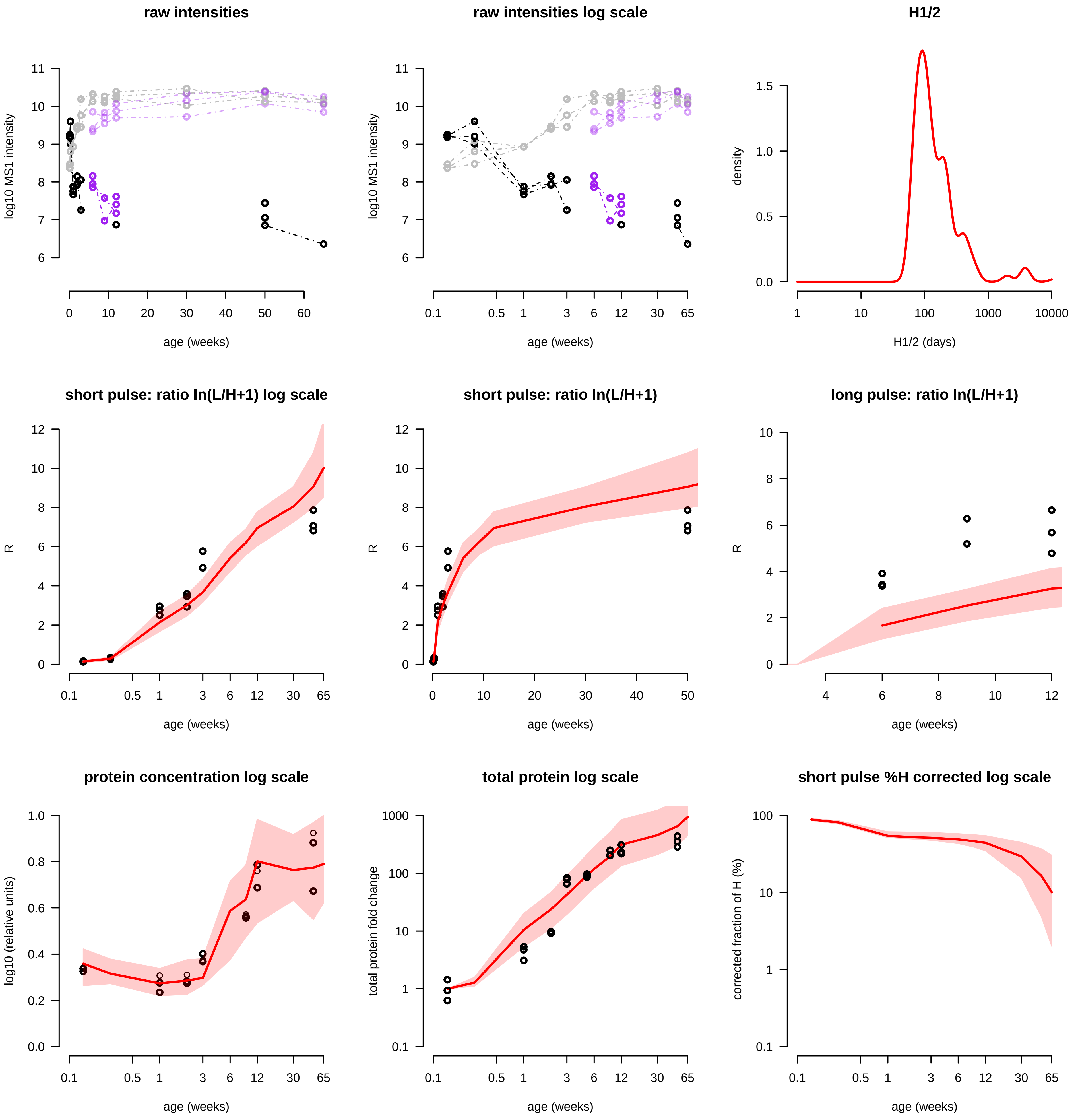

P35564

>sp|P35564|CALX_MOUSE

Calnexin

GN=Canx

Med H1/2: 551.2 days

Q5 H1/2: 164.2 days

Q95 H1/2: 2630.6 days

reliable model fit: yes

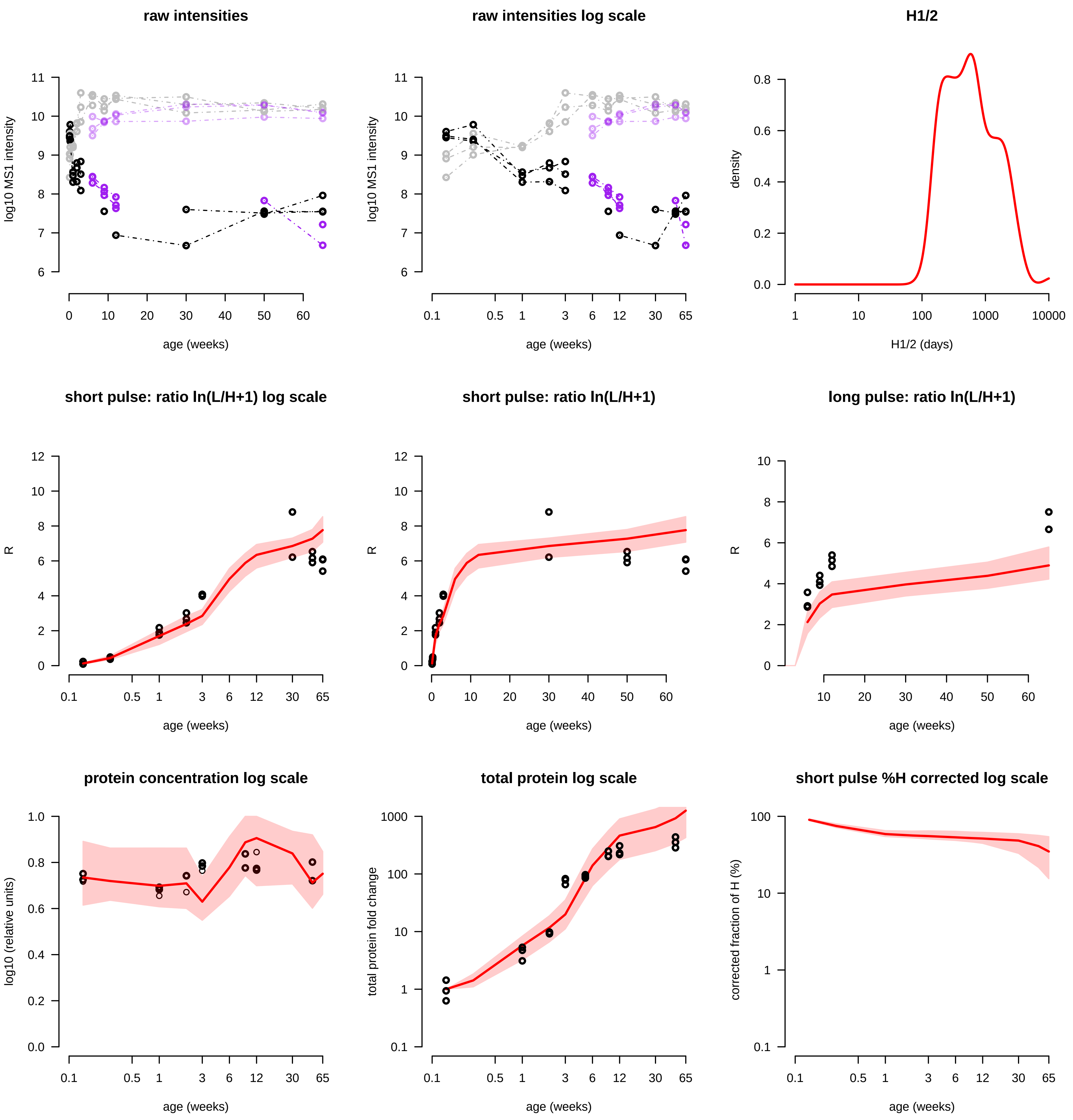

P18242

>sp|P18242|CATD_MOUSE

Cathepsin D

GN=Ctsd

Med H1/2: 14.4 days

Q5 H1/2: 11 days

Q95 H1/2: 19.3 days

reliable model fit: yes

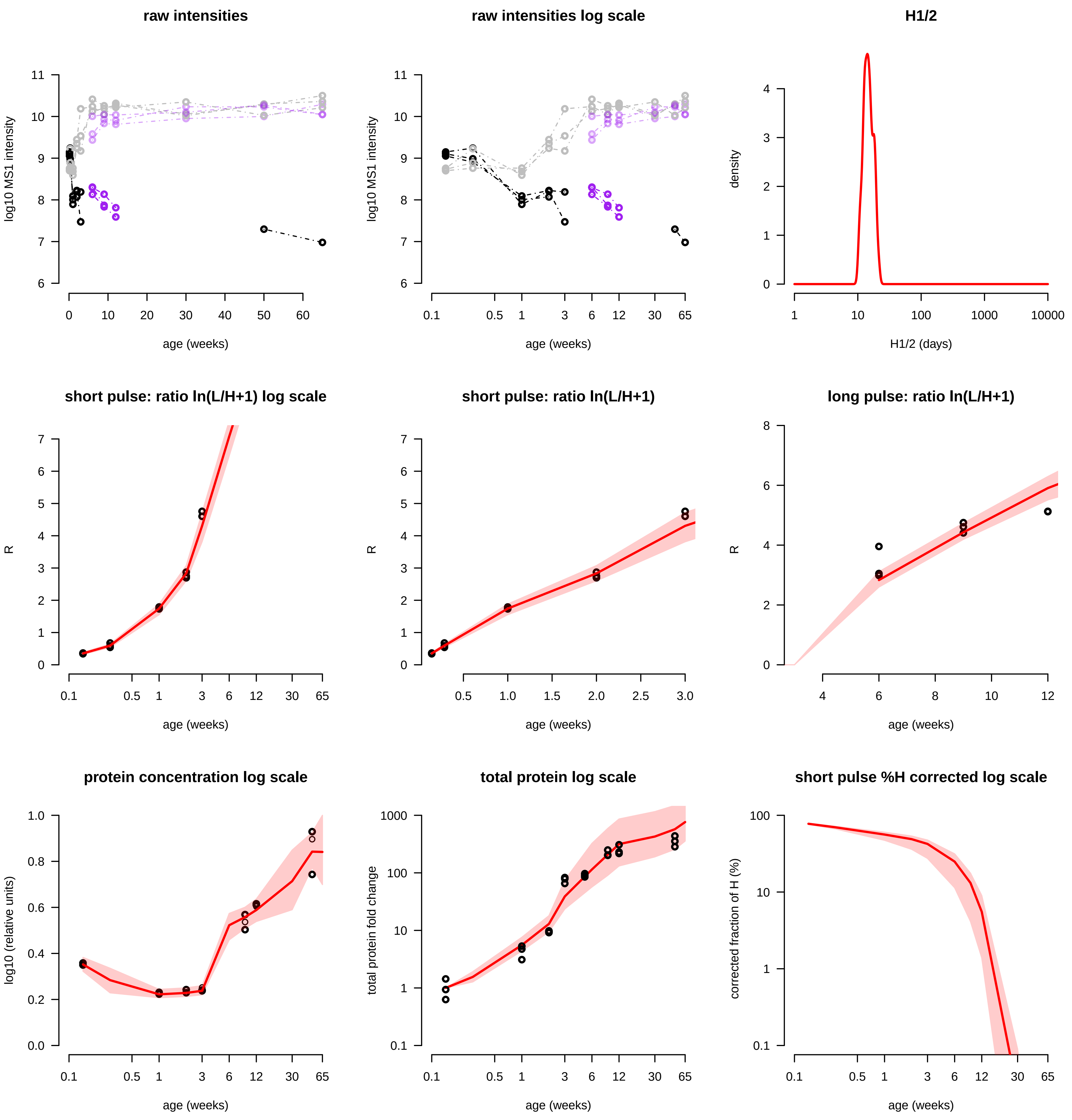

Q60854

>sp|Q60854|SPB6_MOUSE

Serpin B6

GN=Serpinb6

Med H1/2: 79.4 days

Q5 H1/2: 56.2 days

Q95 H1/2: 166.4 days

reliable model fit: yes

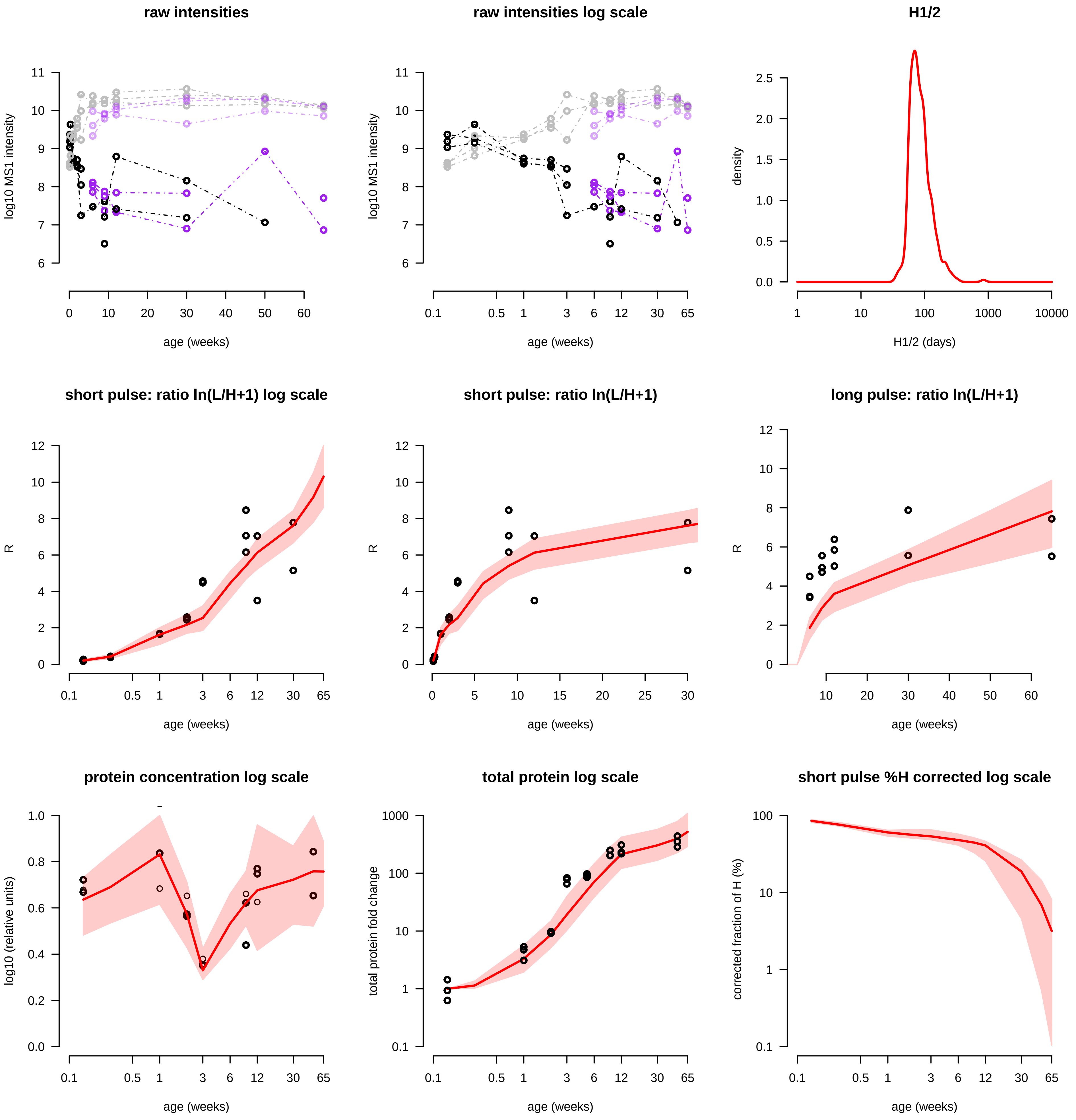

P16045

>sp|P16045|LEG1_MOUSE

Galectin−1

GN=Lgals1

Med H1/2: 14.1 days

Q5 H1/2: 10.1 days

Q95 H1/2: 22.4 days

reliable model fit: yes

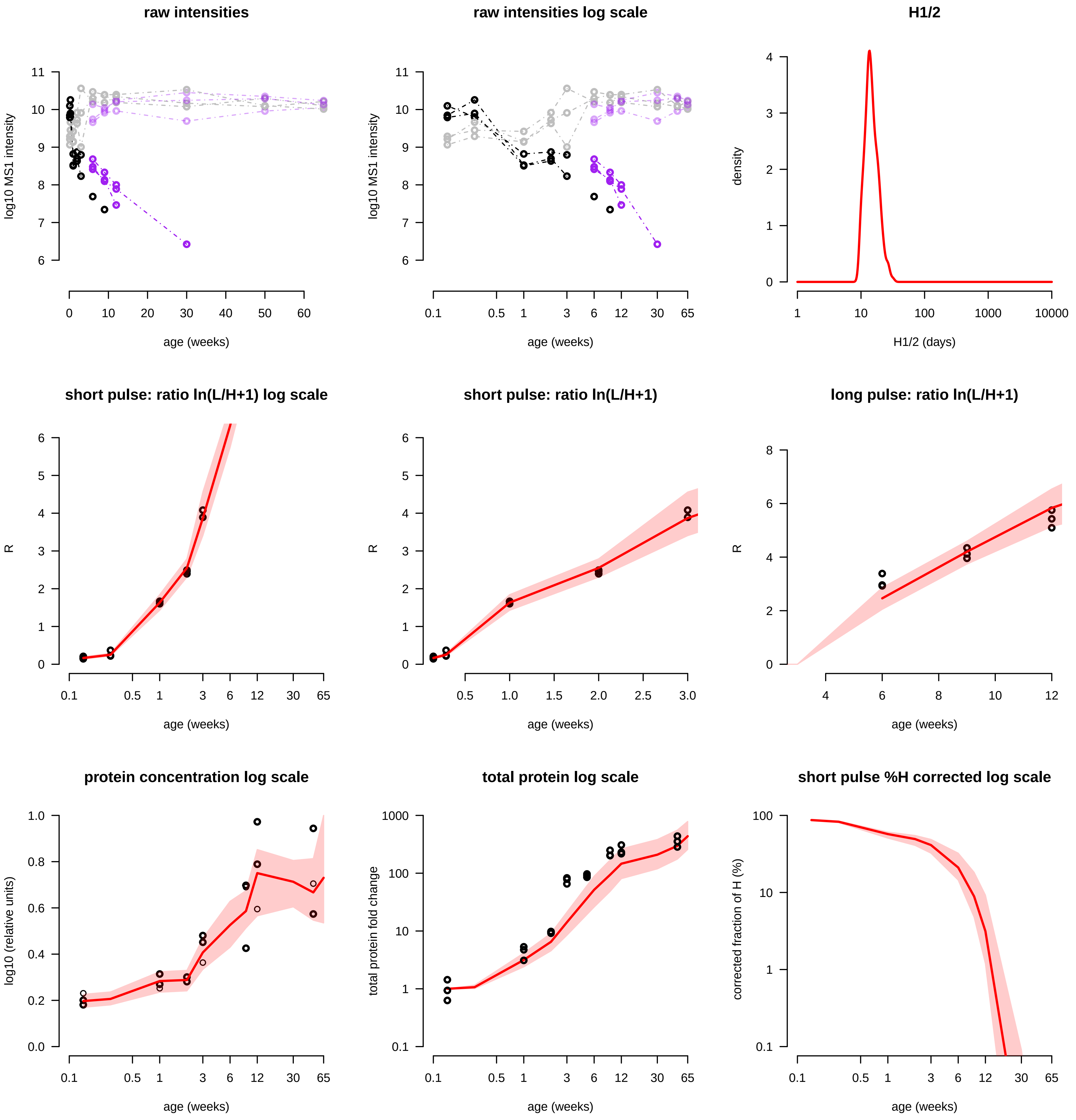

Q64523;Q6GSS7

>sp|Q64523|H2A2C_MOUSE

Histone H2A type 2−C

GN=H2ac20

Med H1/2: 18.2 days

Q5 H1/2: 7.7 days

Q95 H1/2: 93.3 days

reliable model fit: no

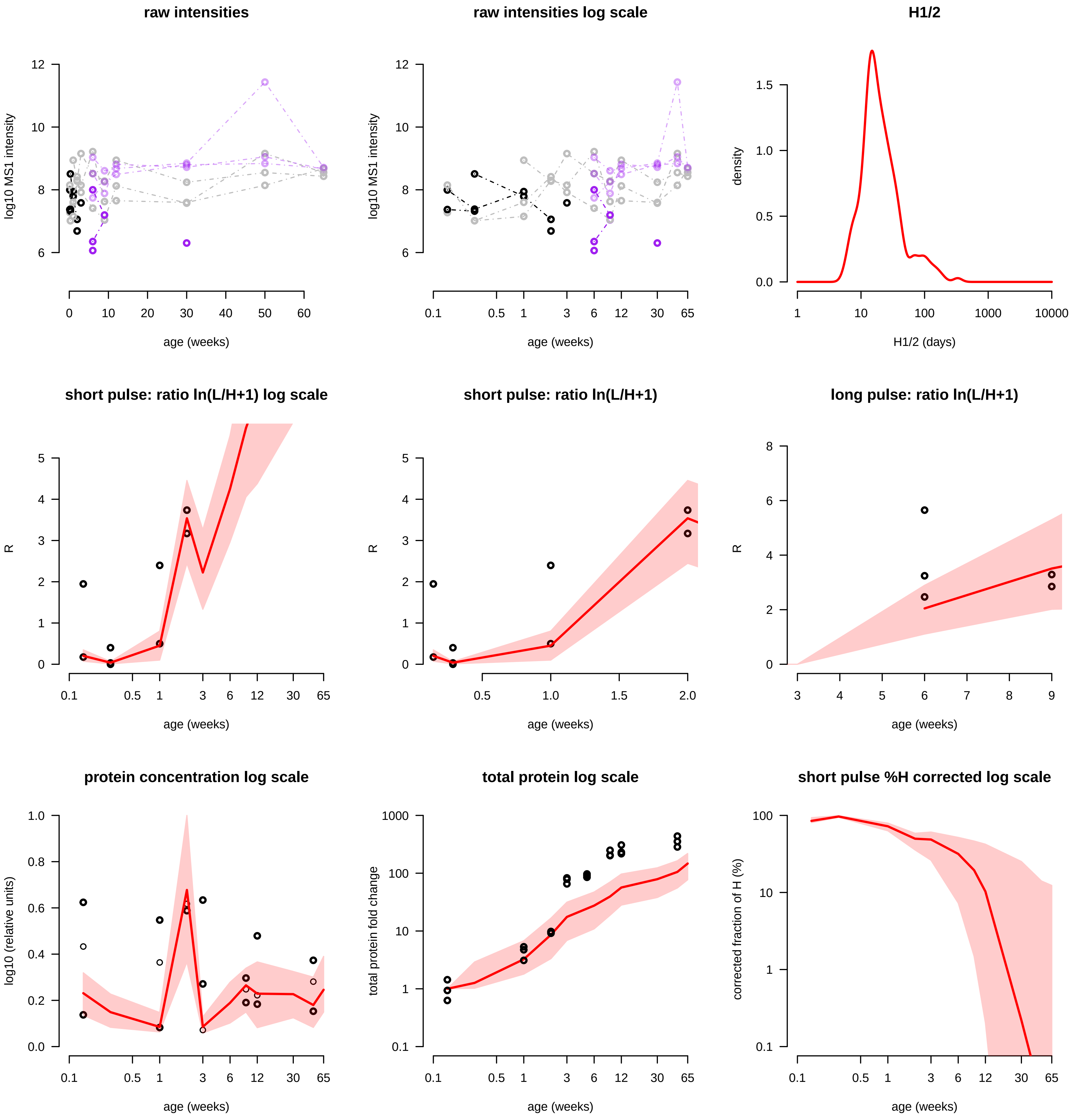

P01027

>sp|P01027|CO3_MOUSE

Complement C3

GN=C3

Med H1/2: 2721.4 days

Q5 H1/2: 743 days

Q95 H1/2: 35777.7 days

reliable model fit: yes

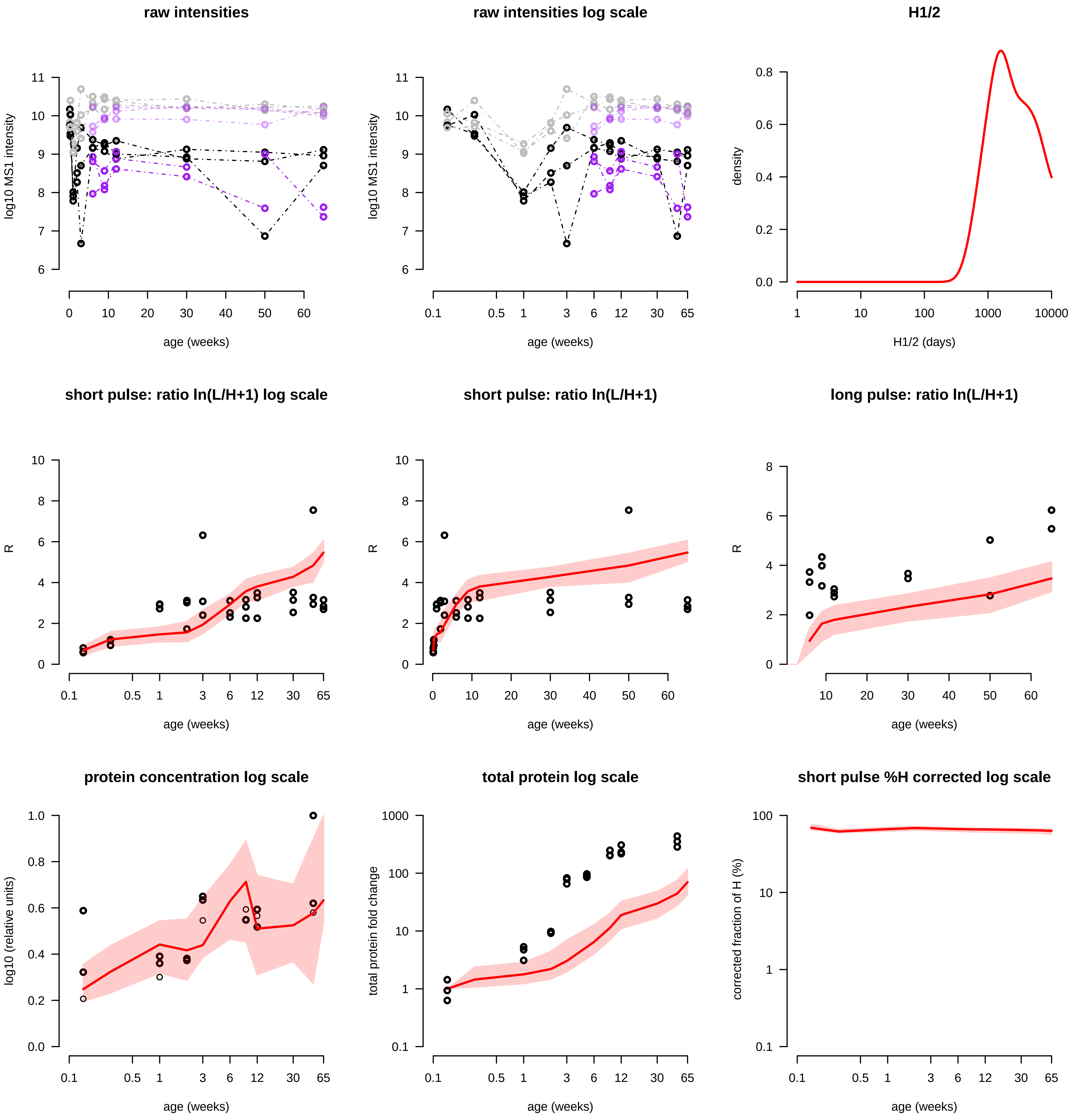

Q9DBJ1

>sp|Q9DBJ1|PGAM1_MOUSE

Phosphoglycerate mutase 1

GN=Pgam1

Med H1/2: 66.2 days

Q5 H1/2: 46 days

Q95 H1/2: 94.4 days

reliable model fit: yes

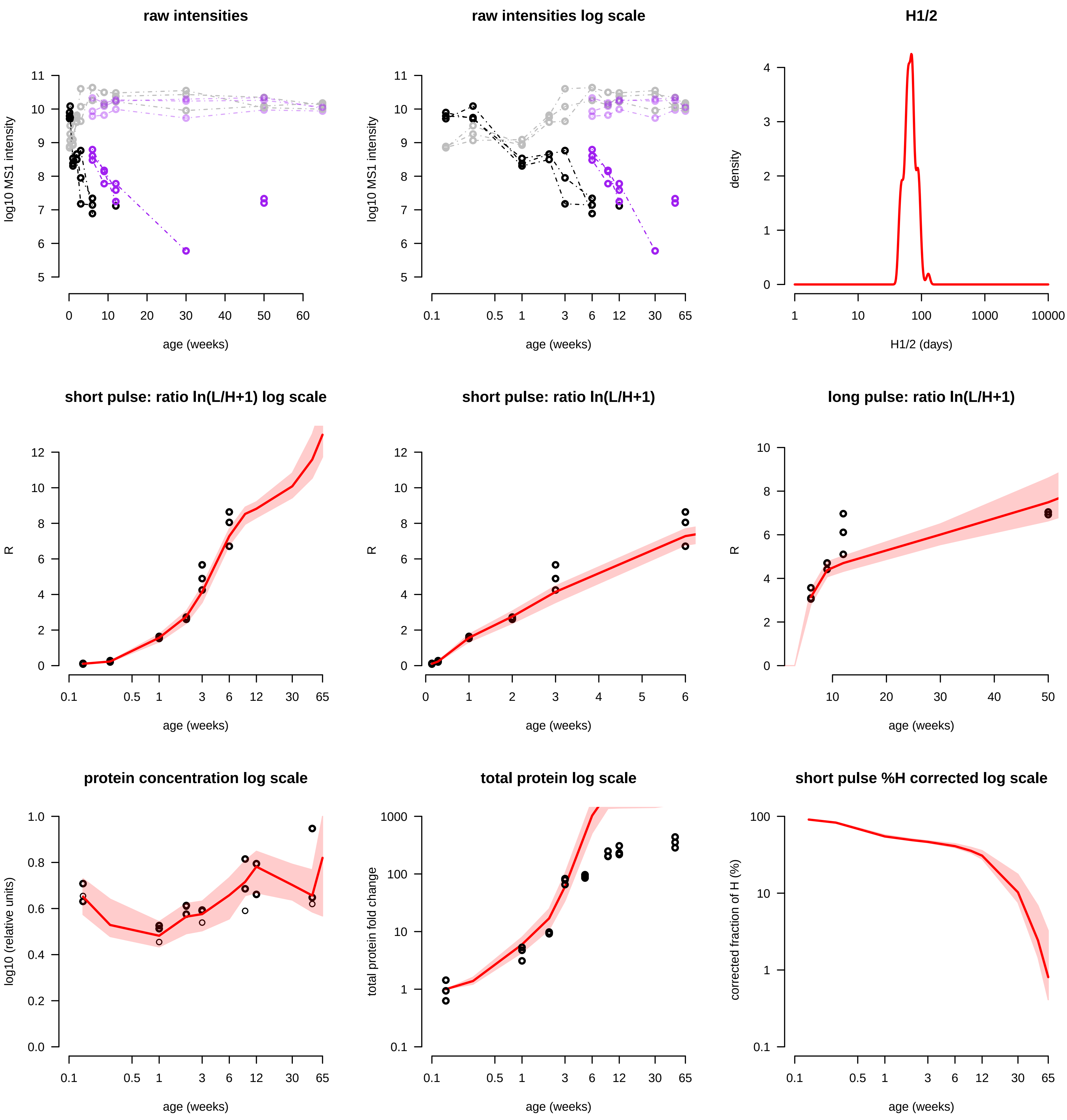

P32261

>sp|P32261|ANT3_MOUSE

Antithrombin−III

GN=Serpinc1

Med H1/2: 10.1 days

Q5 H1/2: 8.5 days

Q95 H1/2: 12.2 days

reliable model fit: yes

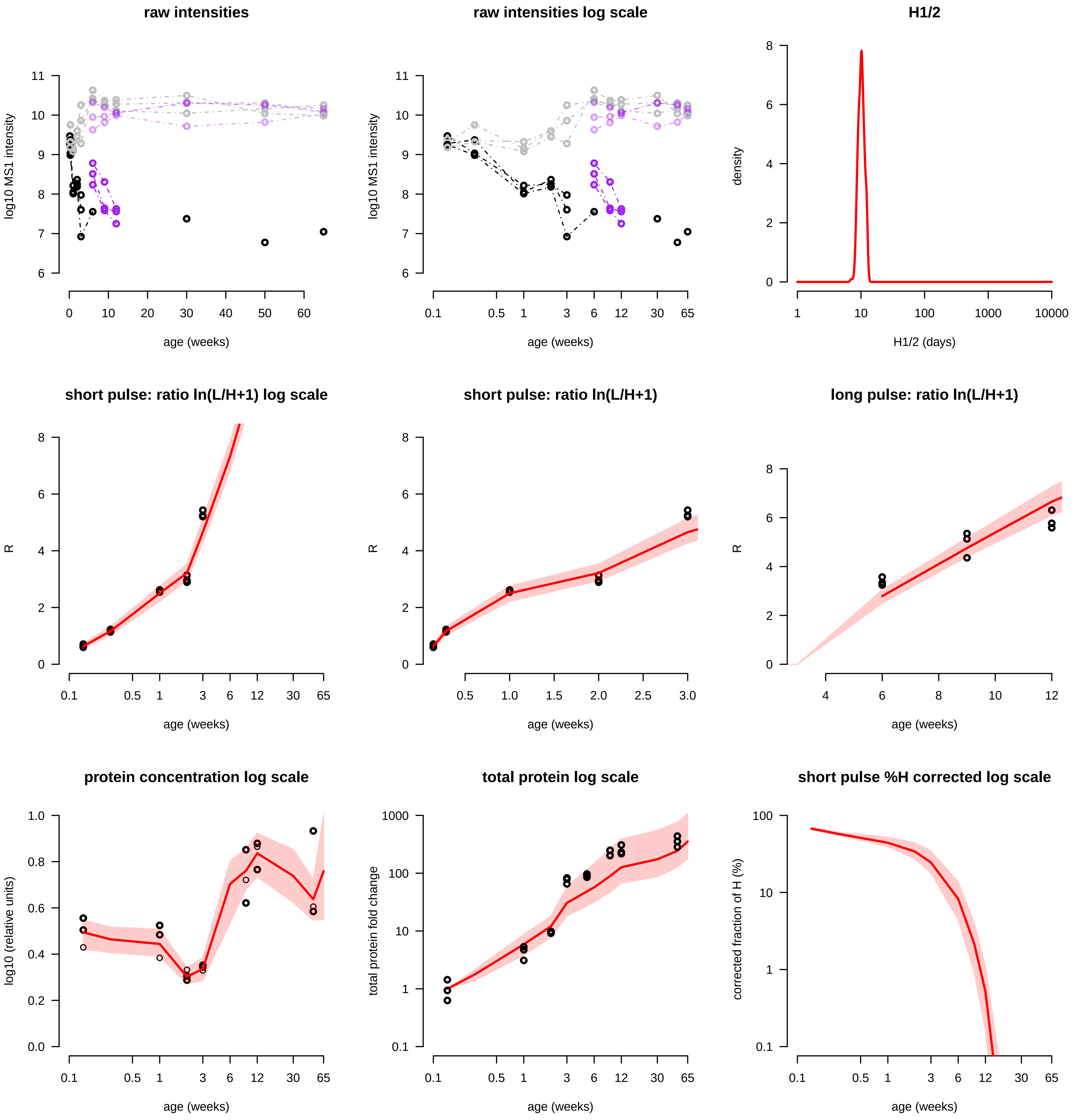

P16546−2

>sp|P16546−2|SPTN1_MOUSE

Isoform 2 of Spectrin alpha chain, non−erythrocytic 1

GN=Sptan1

Med H1/2: 161.9 days

Q5 H1/2: 81.7 days

Q95 H1/2: 600.5 days

reliable model fit: yes

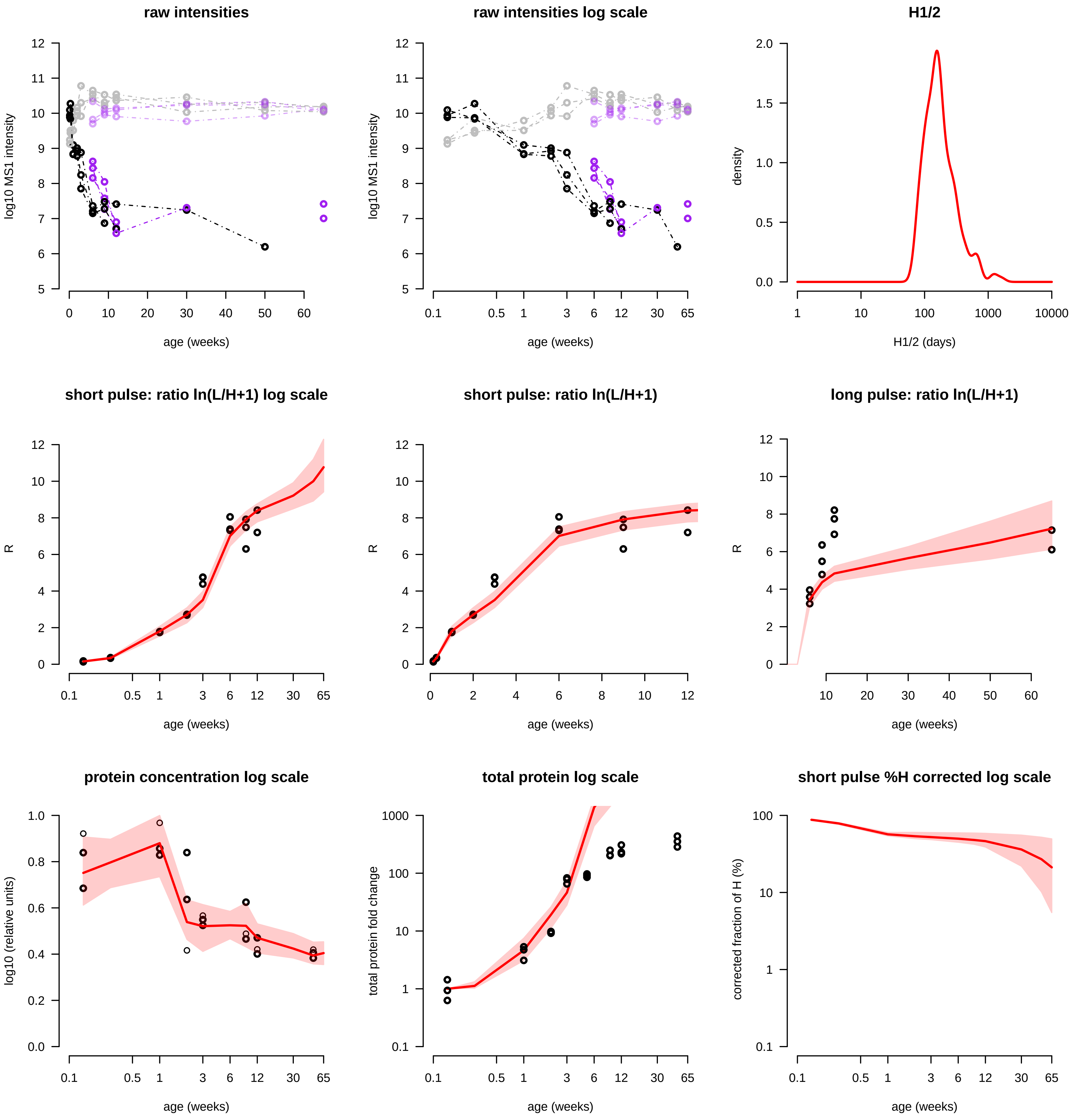

Q00623

>sp|Q00623|APOA1_MOUSE

Apolipoprotein A−I

GN=Apoa1

Med H1/2: 172.4 days

Q5 H1/2: 82.8 days

Q95 H1/2: 627.6 days

reliable model fit: yes

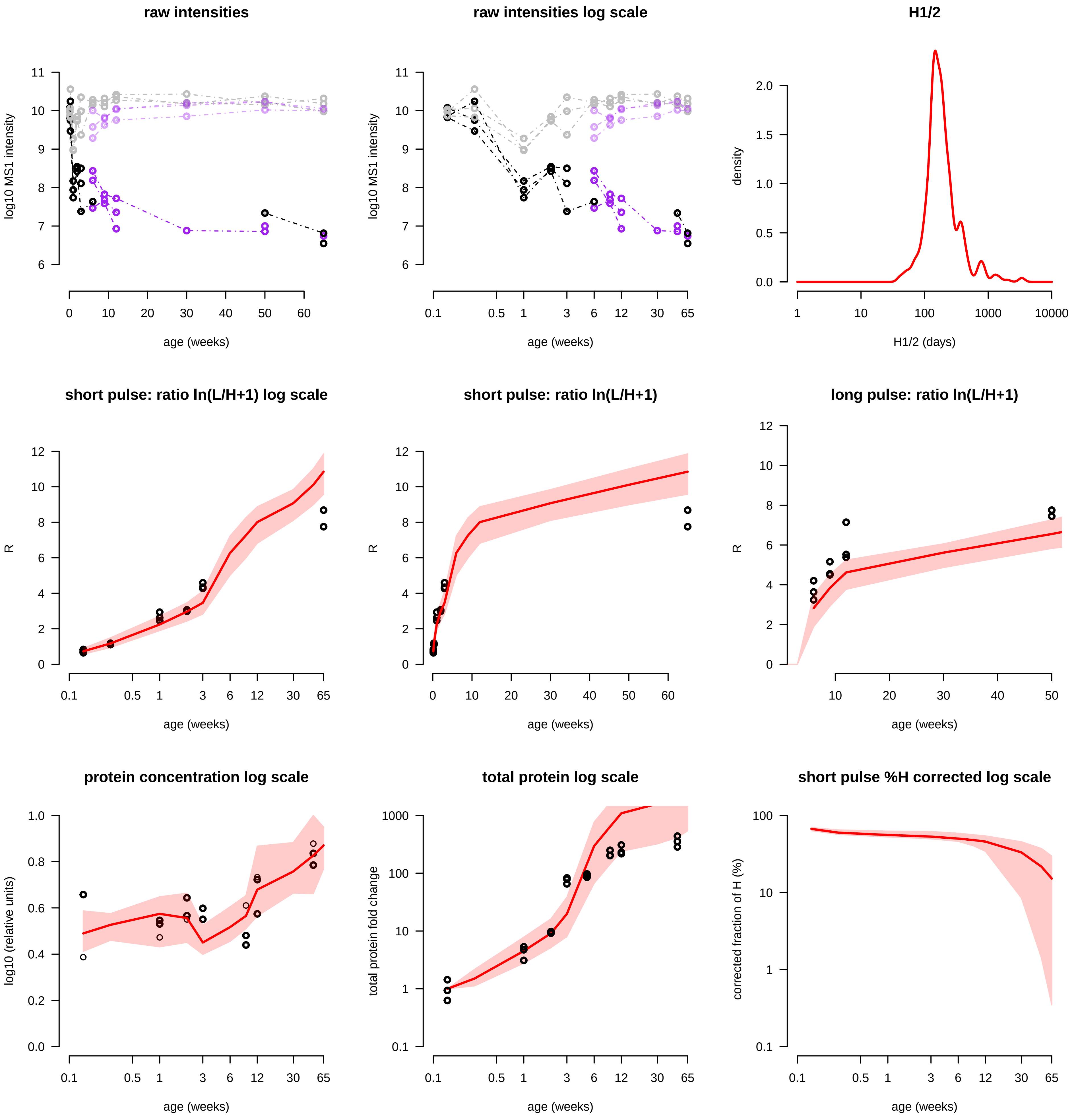

P62983

>sp|P62983|RS27A_MOUSE

Ubiquitin−40S ribosomal protein S27a

GN=Rps27a

Med H1/2: 9.3 days

Q5 H1/2: 7.4 days

Q95 H1/2: 13.3 days

reliable model fit: yes

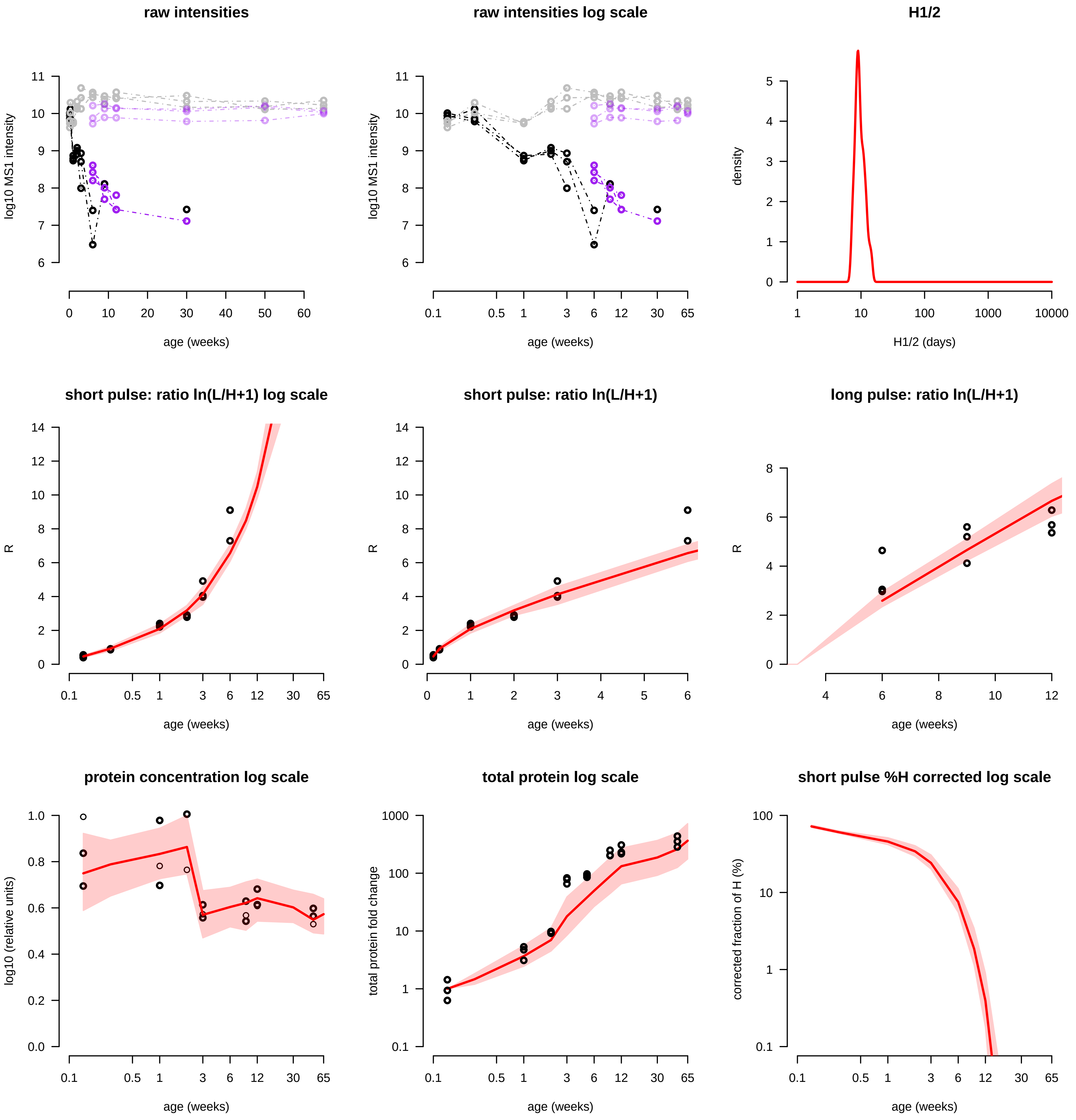

P09405

>sp|P09405|NUCL_MOUSE

Nucleolin

GN=Ncl

Med H1/2: 131.4 days

Q5 H1/2: 99 days

Q95 H1/2: 193.5 days

reliable model fit: yes

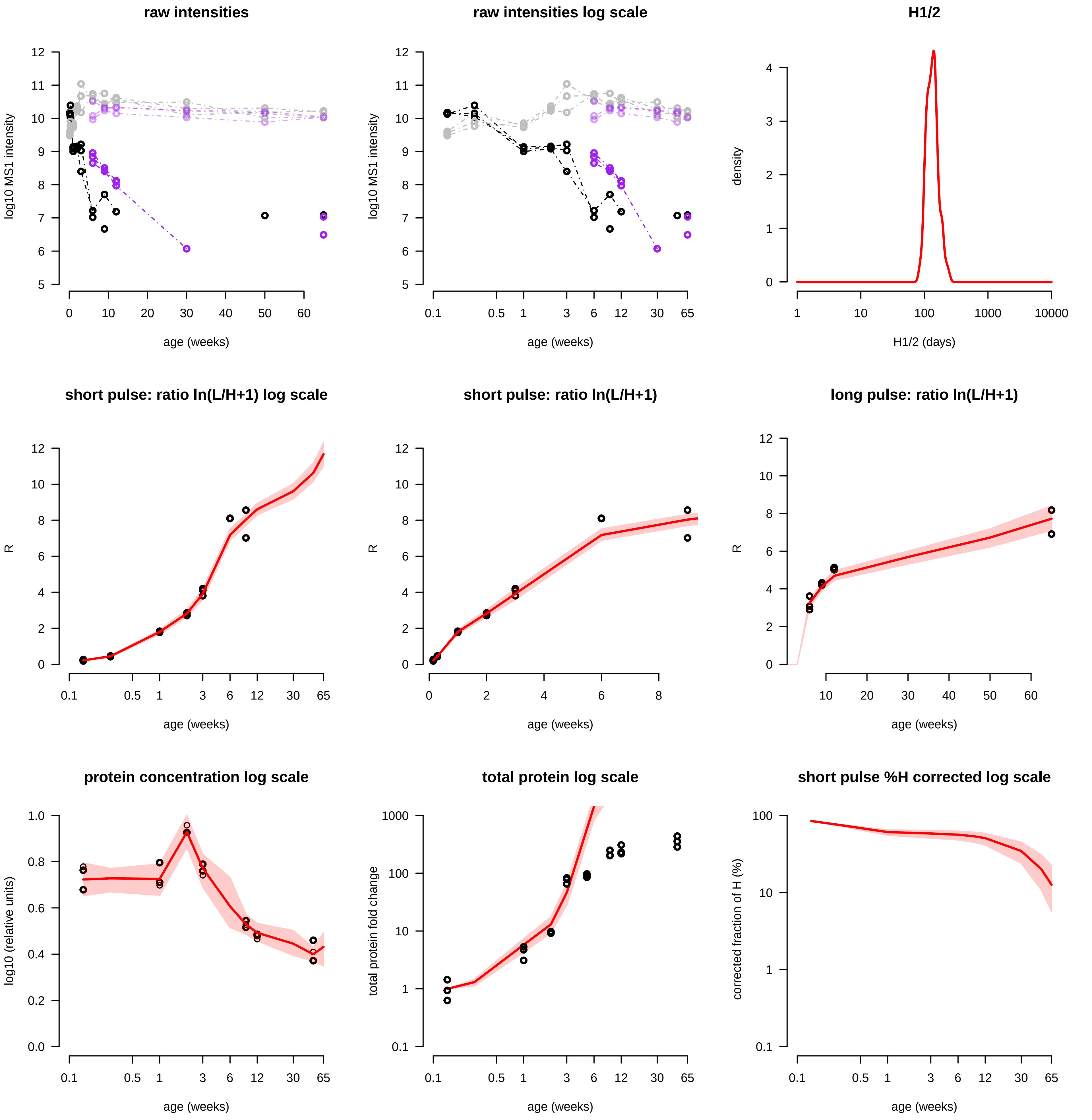

O88569

>sp|O88569|ROA2_MOUSE

Heterogeneous nuclear ribonucleoproteins A2/B1

GN=Hnrnpa2b1

Med H1/2: 18.8 days

Q5 H1/2: 14.3 days

Q95 H1/2: 22.9 days

reliable model fit: yes

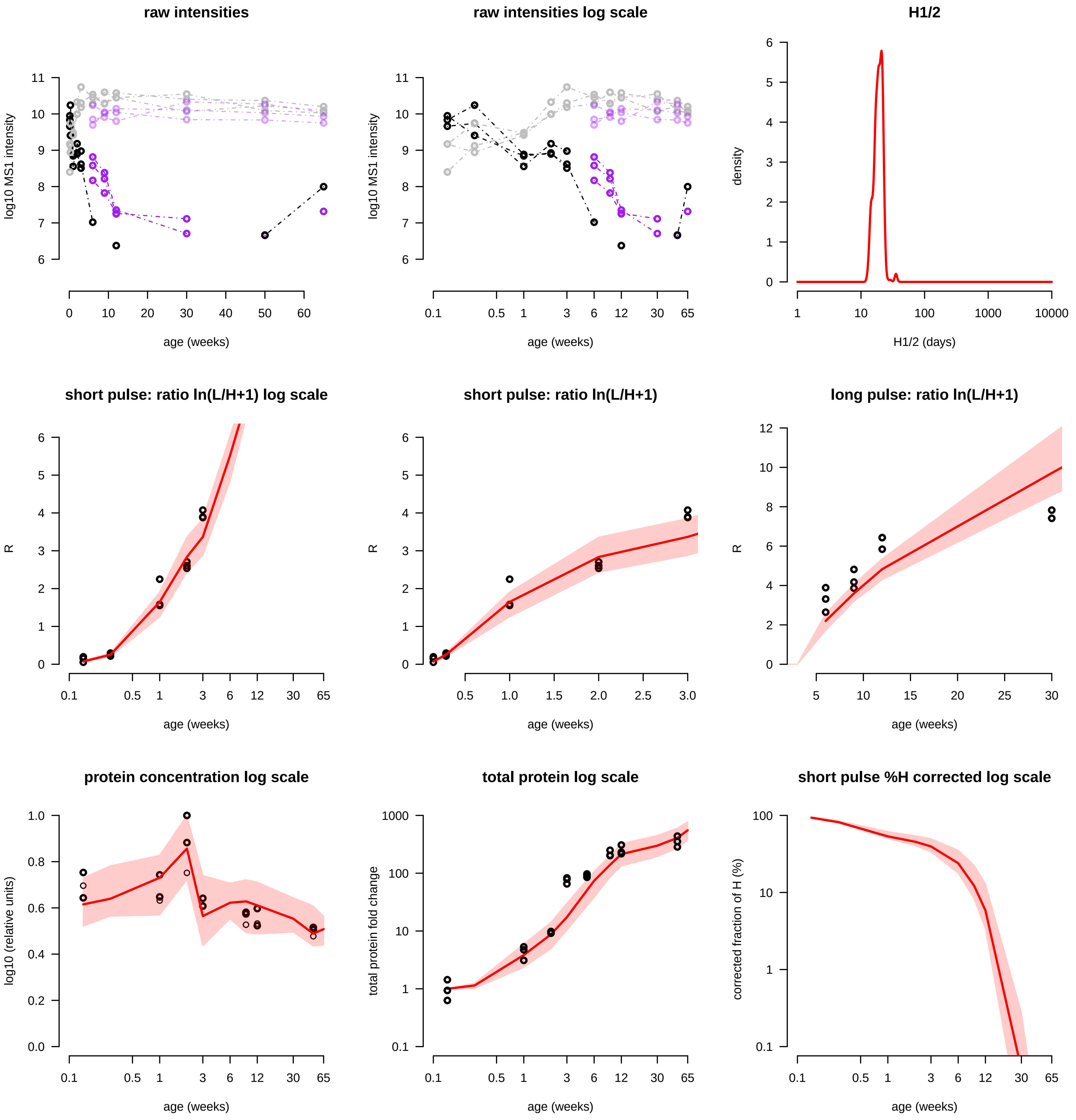

Q920E5

>sp|Q920E5|FPPS_MOUSE

Farnesyl pyrophosphate synthase

GN=Fdps

Med H1/2: 20 days

Q5 H1/2: 13.1 days

Q95 H1/2: 41.6 days

reliable model fit: yes

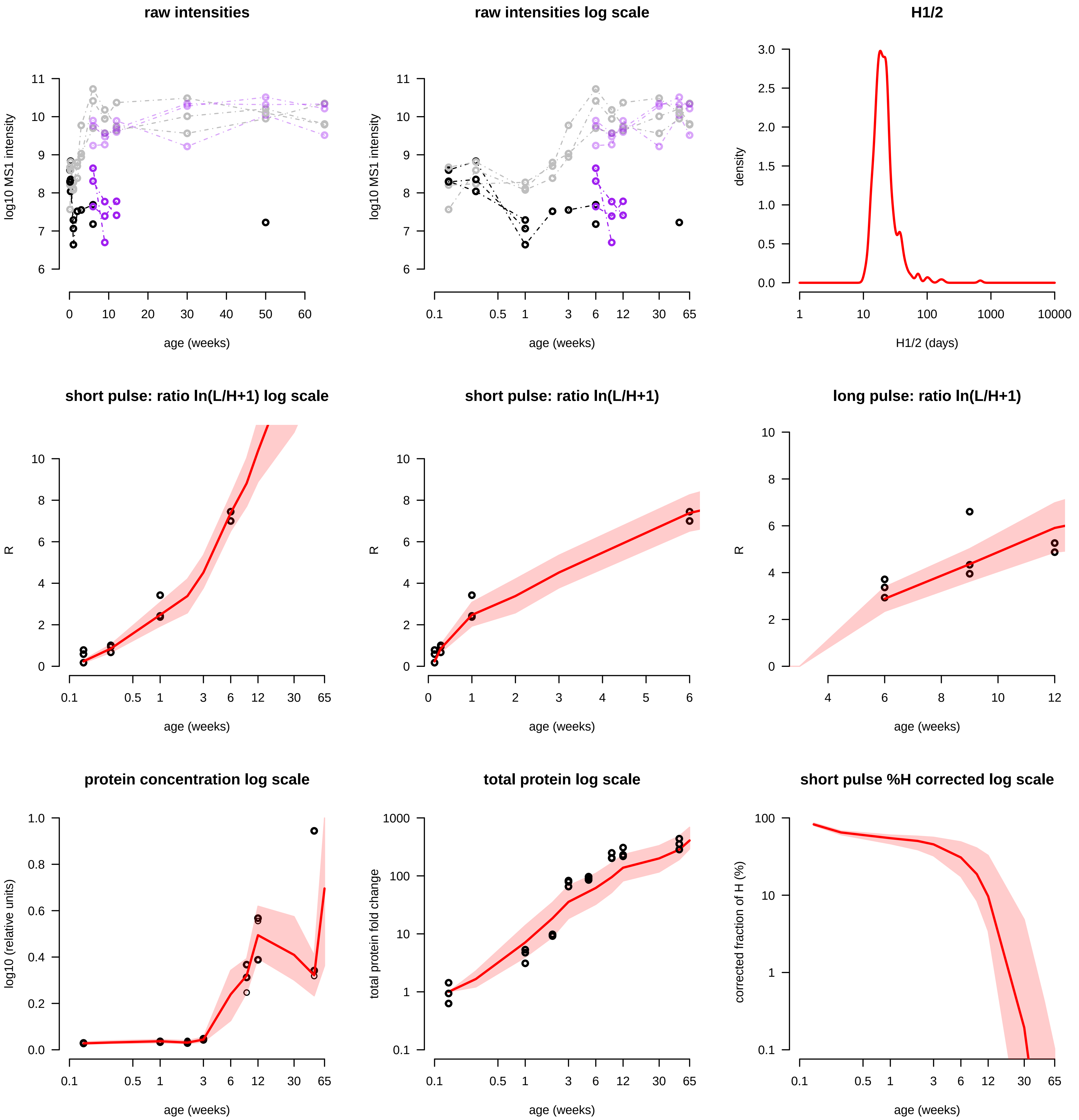

)XS1−10;Q9QXS1−9;Q9QXS1−5;Q9QXS1−16;Q9QXS1−4;

>sp|Q9QXS1−2|PLEC_MOUSE

Isoform PLEC−1 of Plectin

GN=Plec

Med H1/2: 180.4 days

Q5 H1/2: 104.2 days

Q95 H1/2: 1076.2 days

reliable model fit: yes

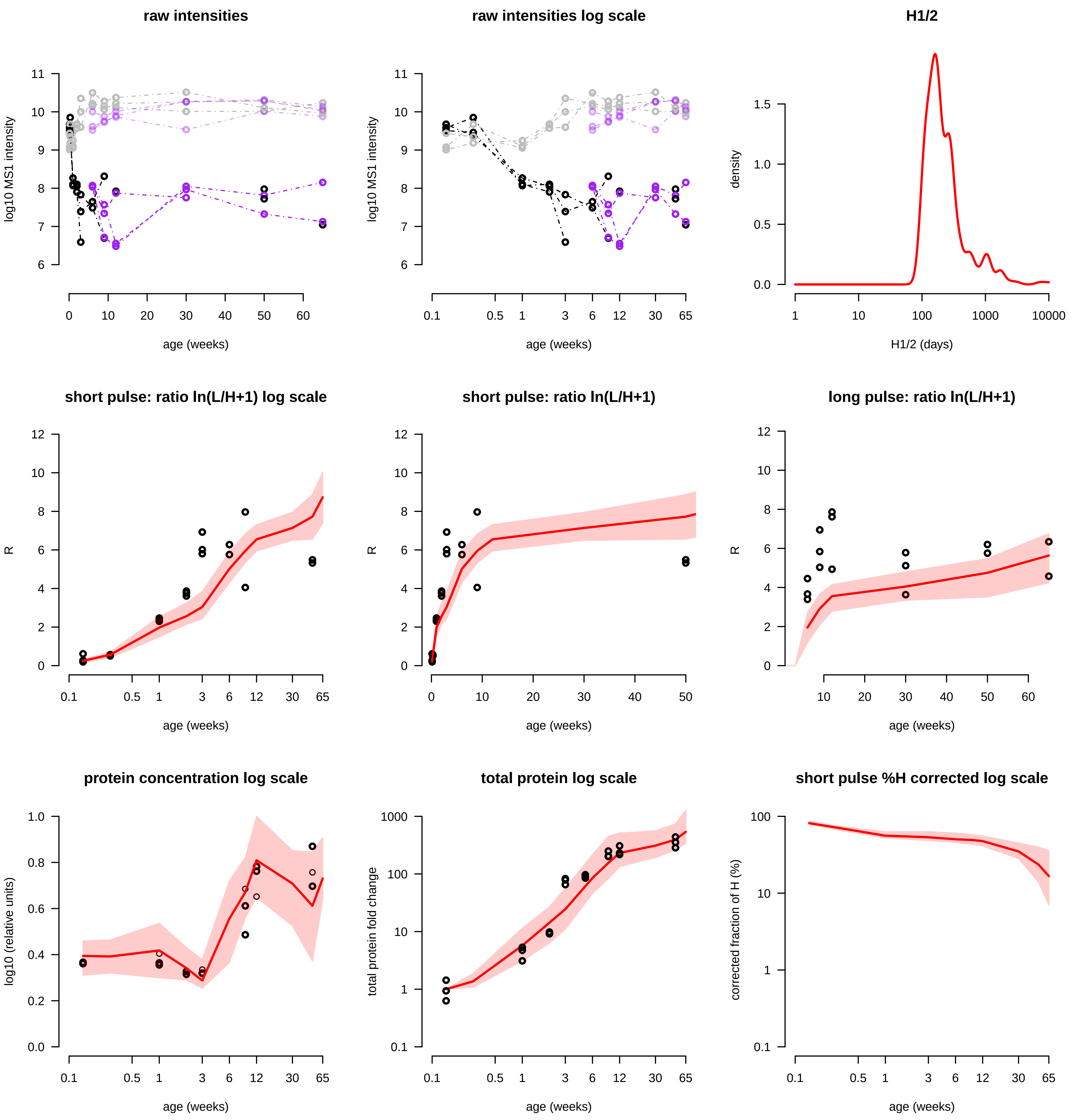

P61979−2;P61979

>sp|P61979−2|HNRPK_MOUSE

Isoform 2 of Heterogeneous nuclear ribonucleoprotein K

GN=Hnrnpk

Med H1/2: 152.8 days

Q5 H1/2: 109.1 days

Q95 H1/2: 383.6 days

reliable model fit: yes

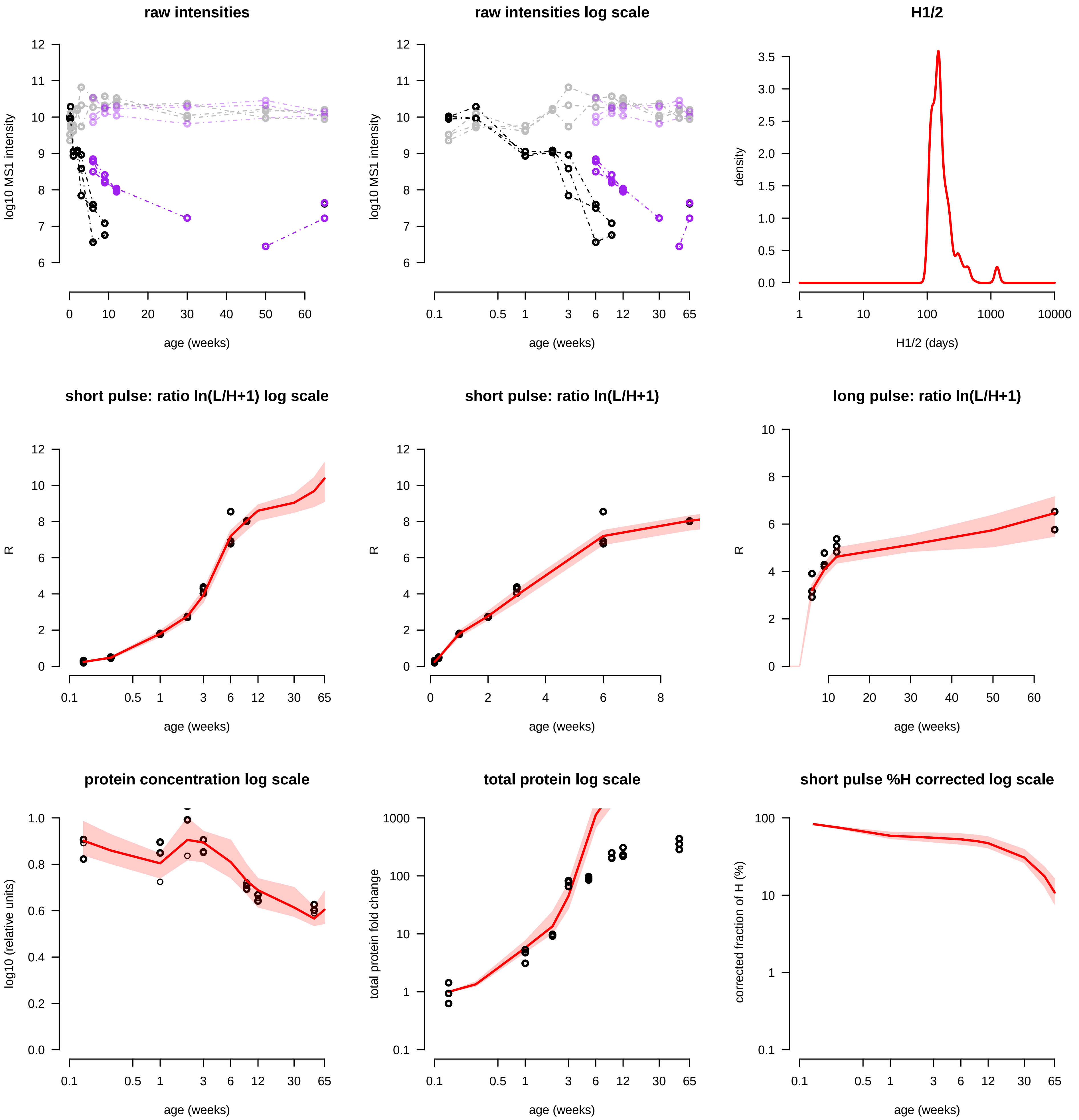

P10649

>sp|P10649|GSTM1_MOUSE

Glutathione S−transferase Mu 1

GN=Gstm1

Med H1/2: 13.6 days

Q5 H1/2: 10.8 days

Q95 H1/2: 19 days

reliable model fit: yes

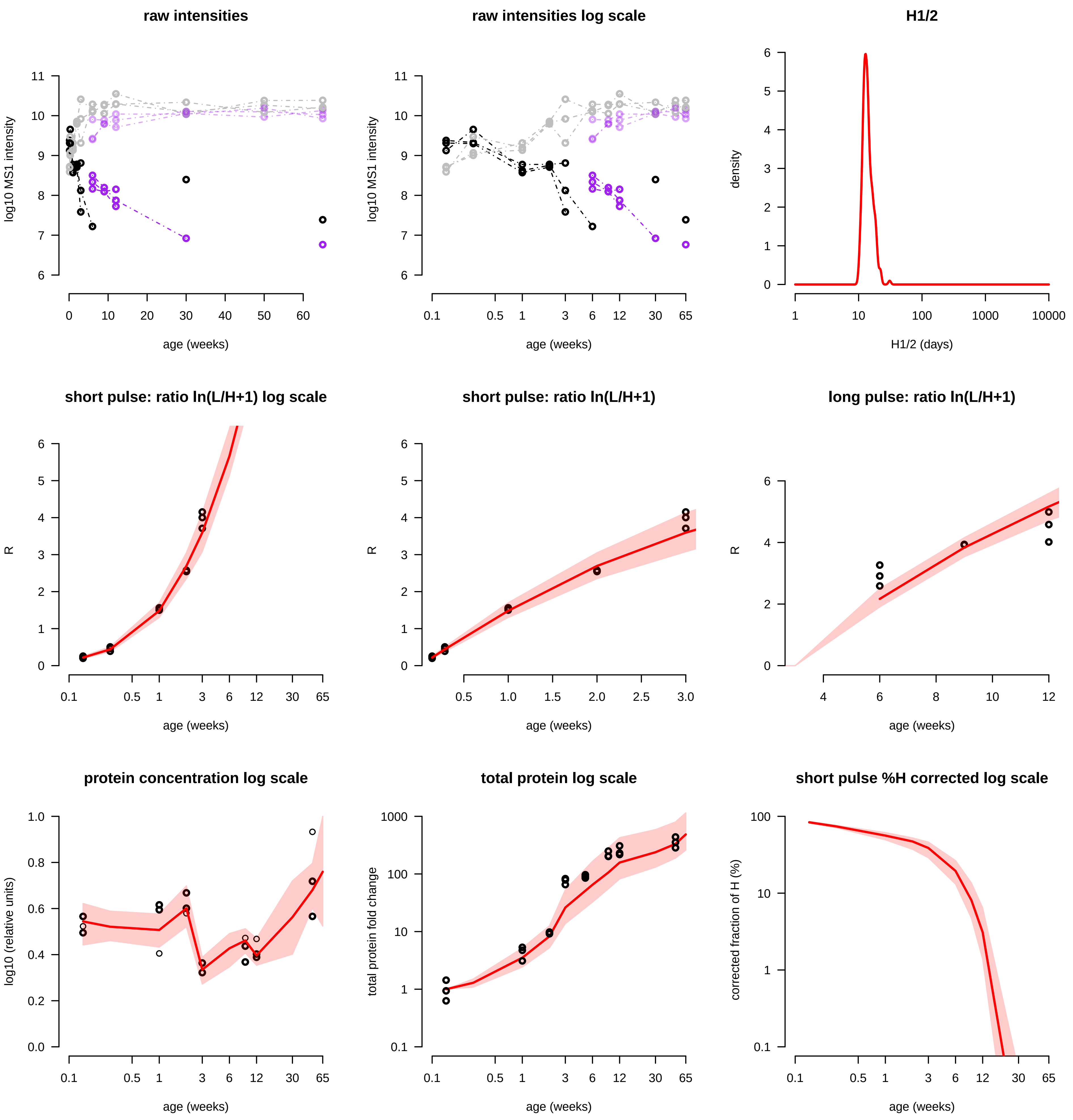

P14152

>sp|P14152|MDHC_MOUSE

Malate dehydrogenase, cytoplasmic

GN=Mdh1

Med H1/2: 11 days

Q5 H1/2: 9.4 days

Q95 H1/2: 15 days

reliable model fit: yes

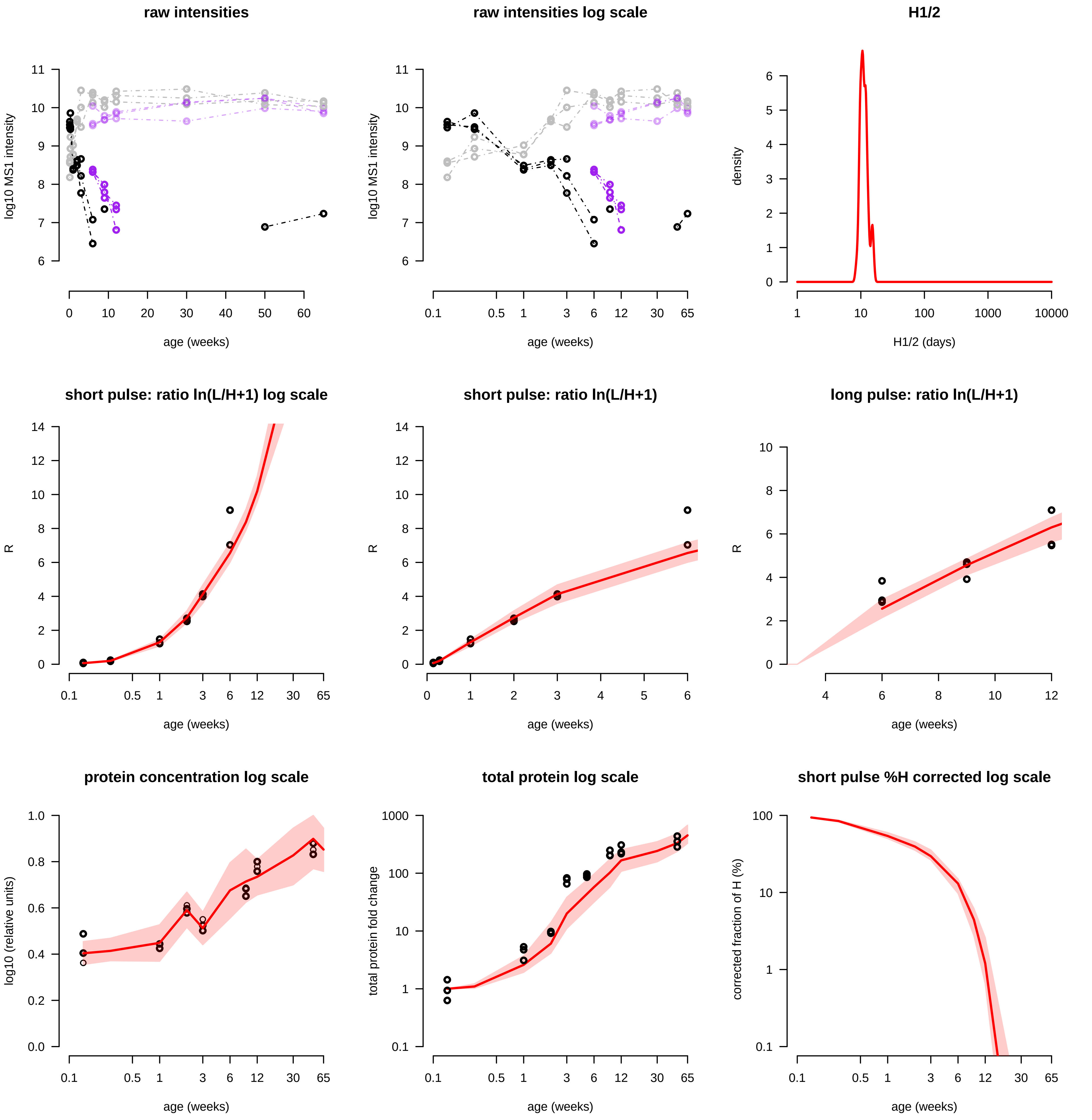

P97742

>sp|P97742|CPT1A_MOUSE

Carnitine O−palmitoyltransferase 1, liver isoform

GN=Cpt1a

Med H1/2: 21.2 days

Q5 H1/2: 15.4 days

Q95 H1/2: 32.2 days

reliable model fit: yes

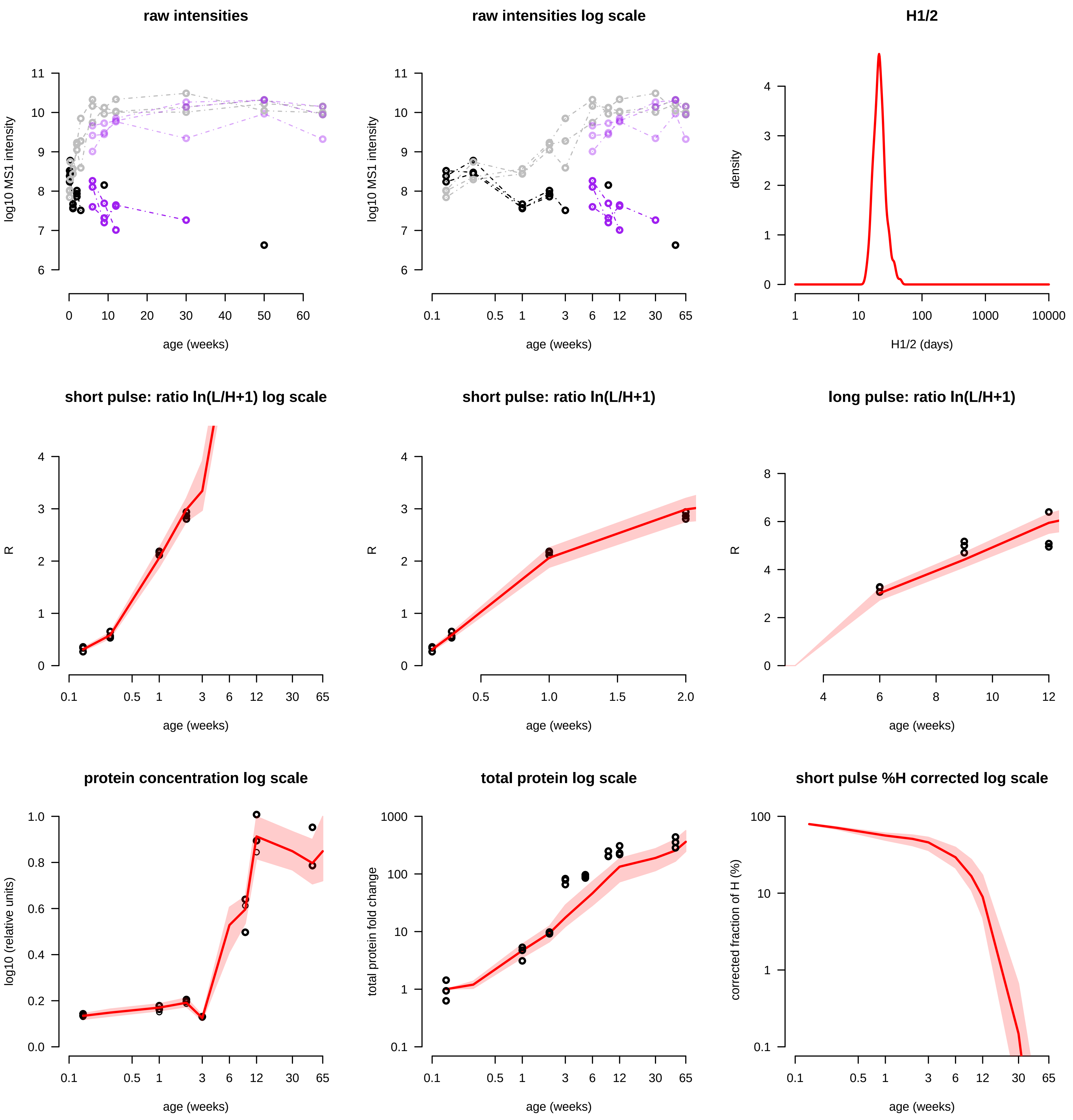

P16125

>sp|P16125|LDHB_MOUSE

L−lactate dehydrogenase B chain

GN=Ldhb

Med H1/2: 12.8 days

Q5 H1/2: 10.2 days

Q95 H1/2: 15.9 days

reliable model fit: yes

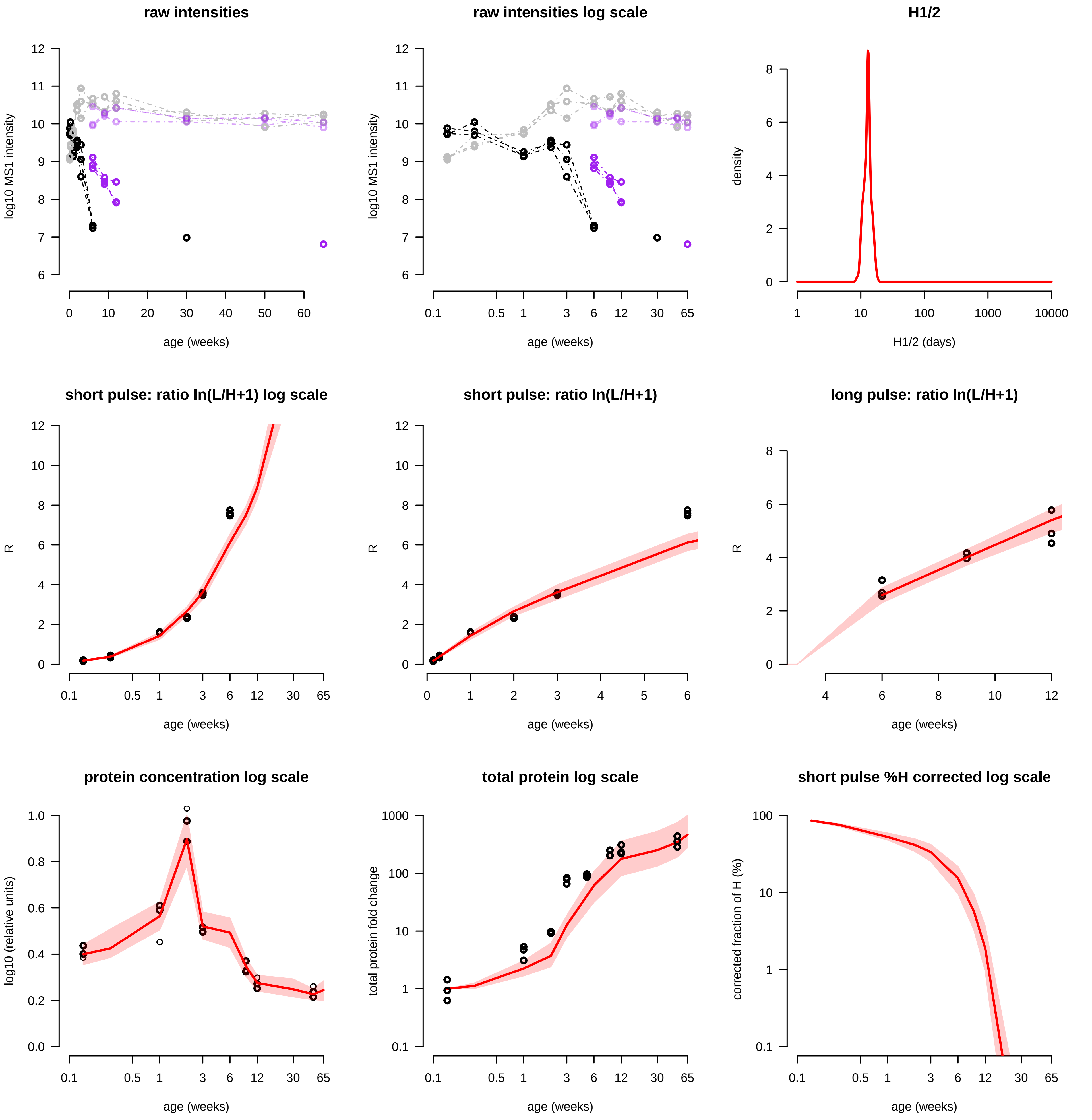

Q62261

>sp|Q62261|SPTB2_MOUSE

Spectrin beta chain, non−erythrocytic 1

GN=Sptbn1

Med H1/2: 165.9 days

Q5 H1/2: 75.2 days

Q95 H1/2: 460.3 days

reliable model fit: yes

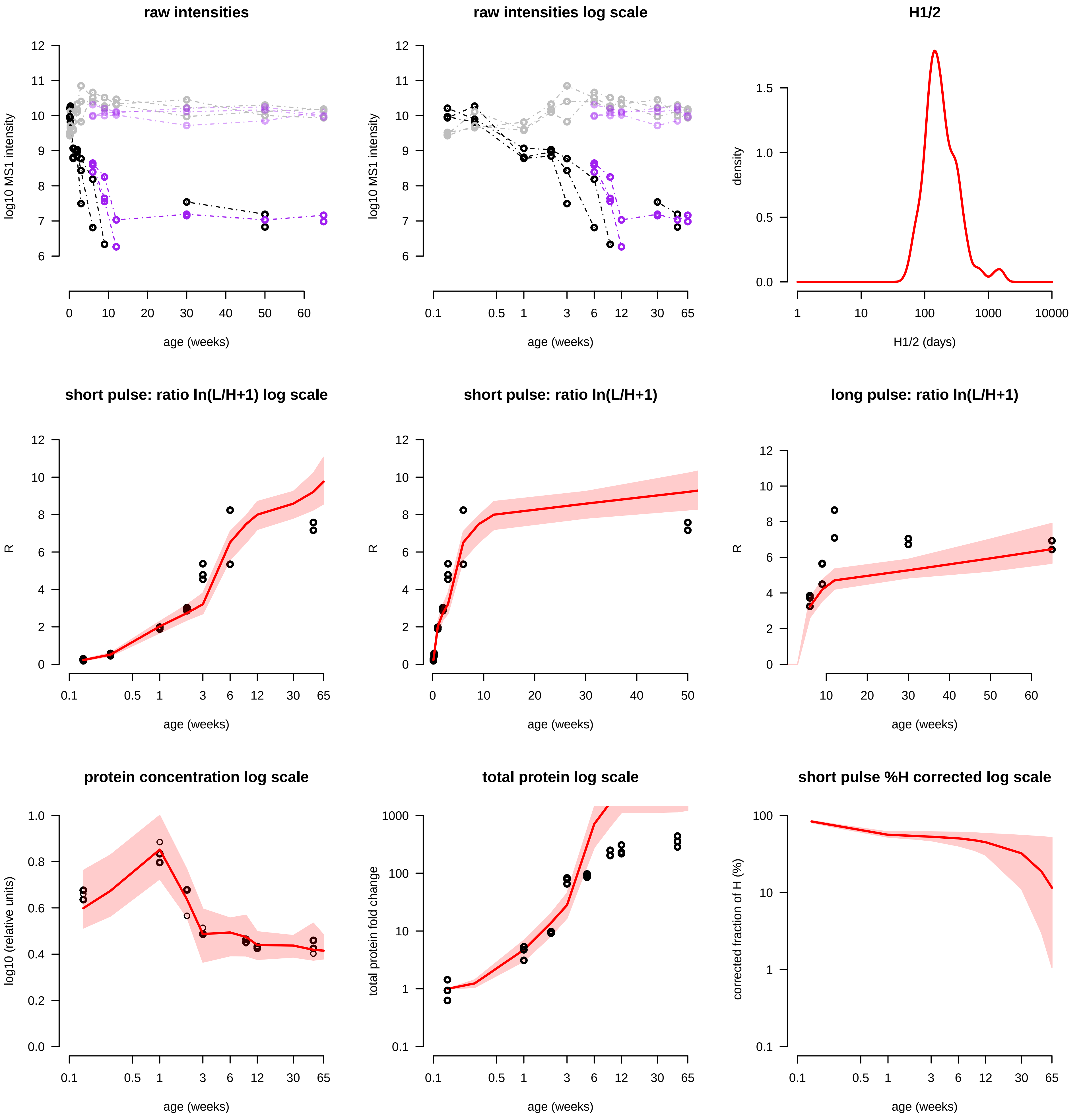

Q9R0P5

>sp|Q9R0P5|DEST_MOUSE

Destrin

GN=Dstn

Med H1/2: 382.4 days

Q5 H1/2: 154.2 days

Q95 H1/2: 3587.2 days

reliable model fit: yes

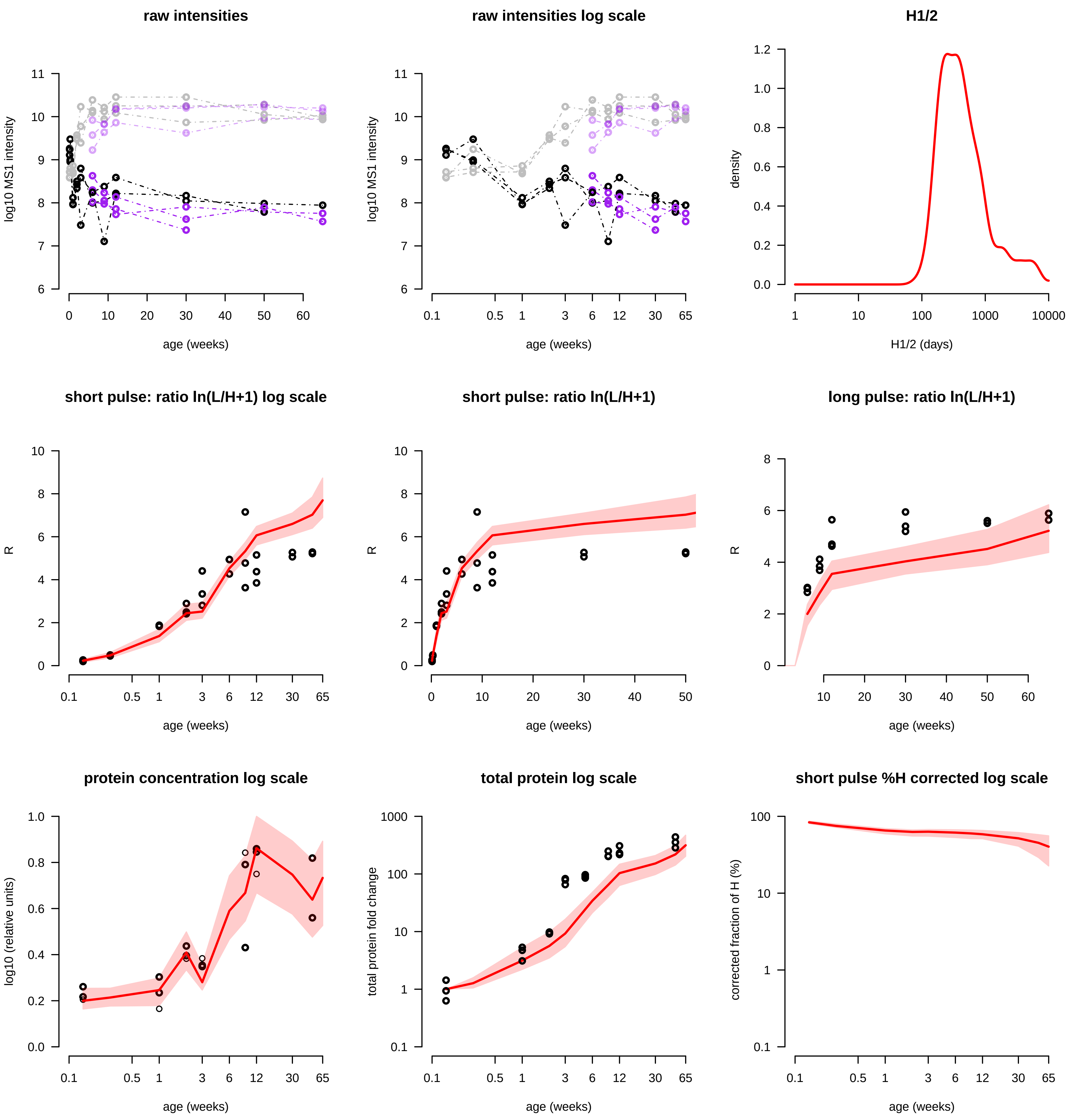

P54227

>sp|P54227|STMN1_MOUSE

Stathmin

GN=Stmn1

Med H1/2: 16.6 days

Q5 H1/2: 13.2 days

Q95 H1/2: 23.6 days

reliable model fit: yes

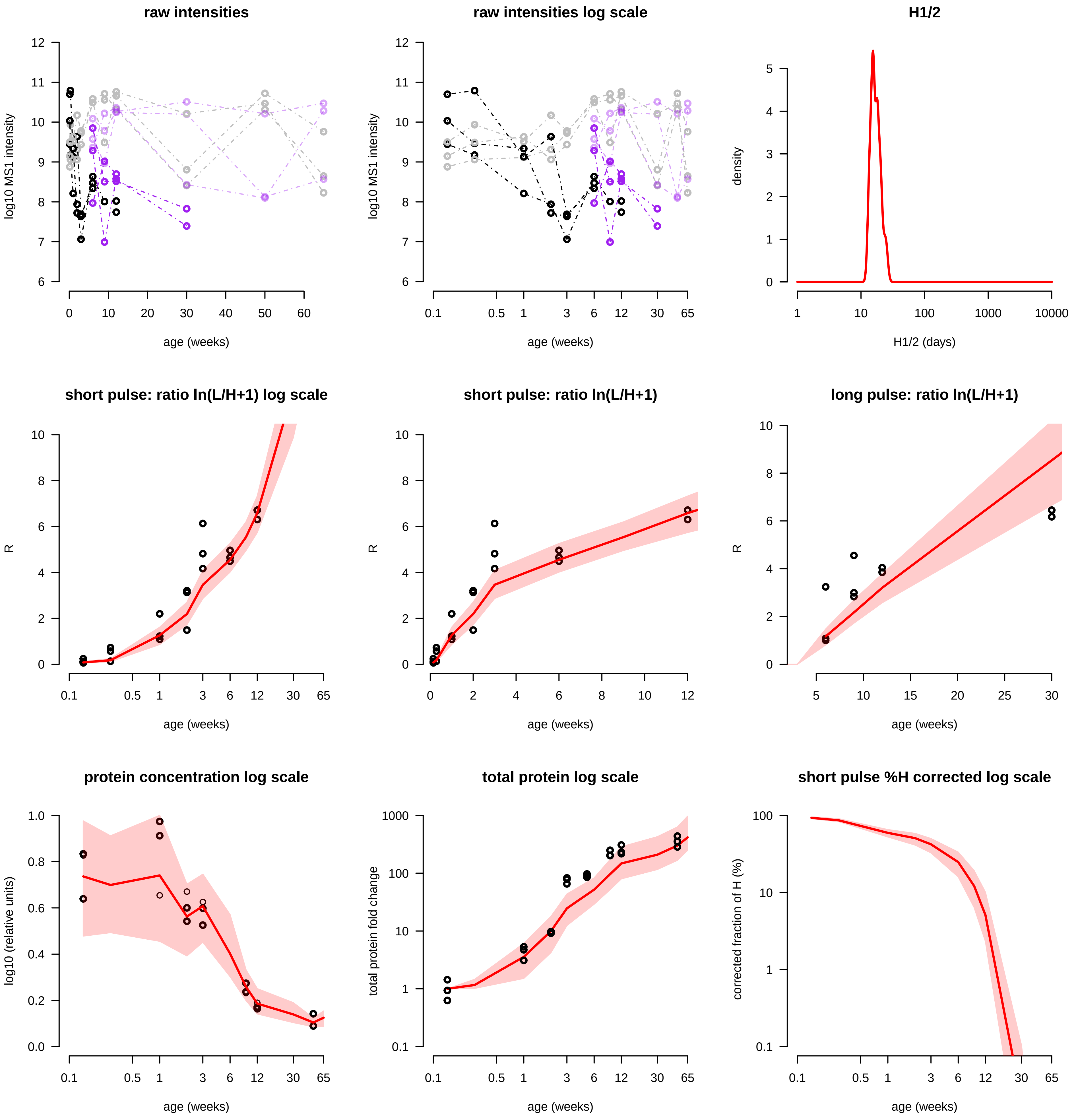

Q64727

>sp|Q64727|VINC_MOUSE

Vinculin

GN=Vcl

Med H1/2: 8.7 days

Q5 H1/2: 7.3 days

Q95 H1/2: 10.5 days

reliable model fit: yes

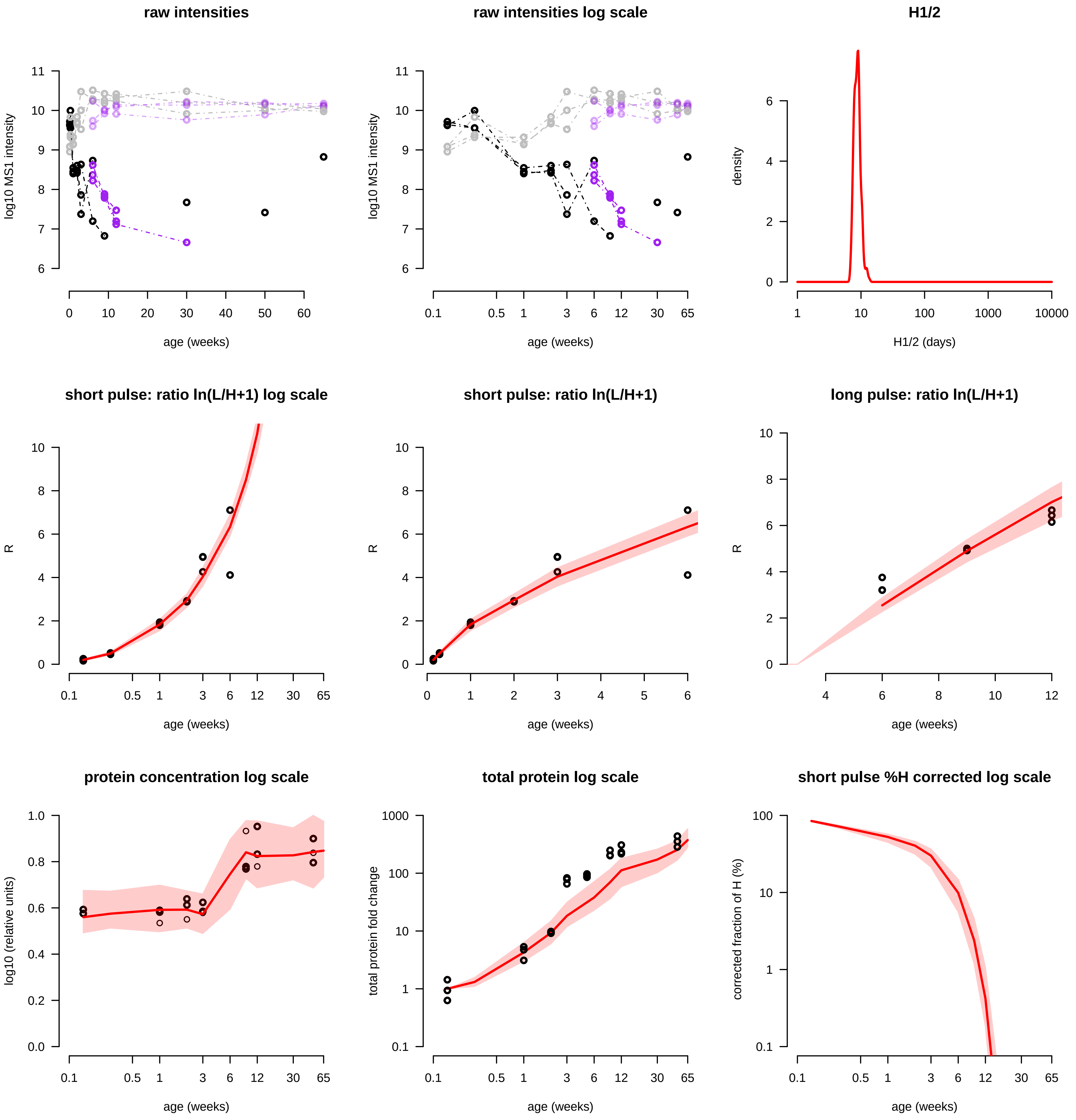

Q91VR2

>sp|Q91VR2|ATPG_MOUSE

ATP synthase subunit gamma, mitochondrial

GN=Atp5f1c

Med H1/2: 892.4 days

Q5 H1/2: 303.8 days

Q95 H1/2: 5051.2 days

reliable model fit: yes

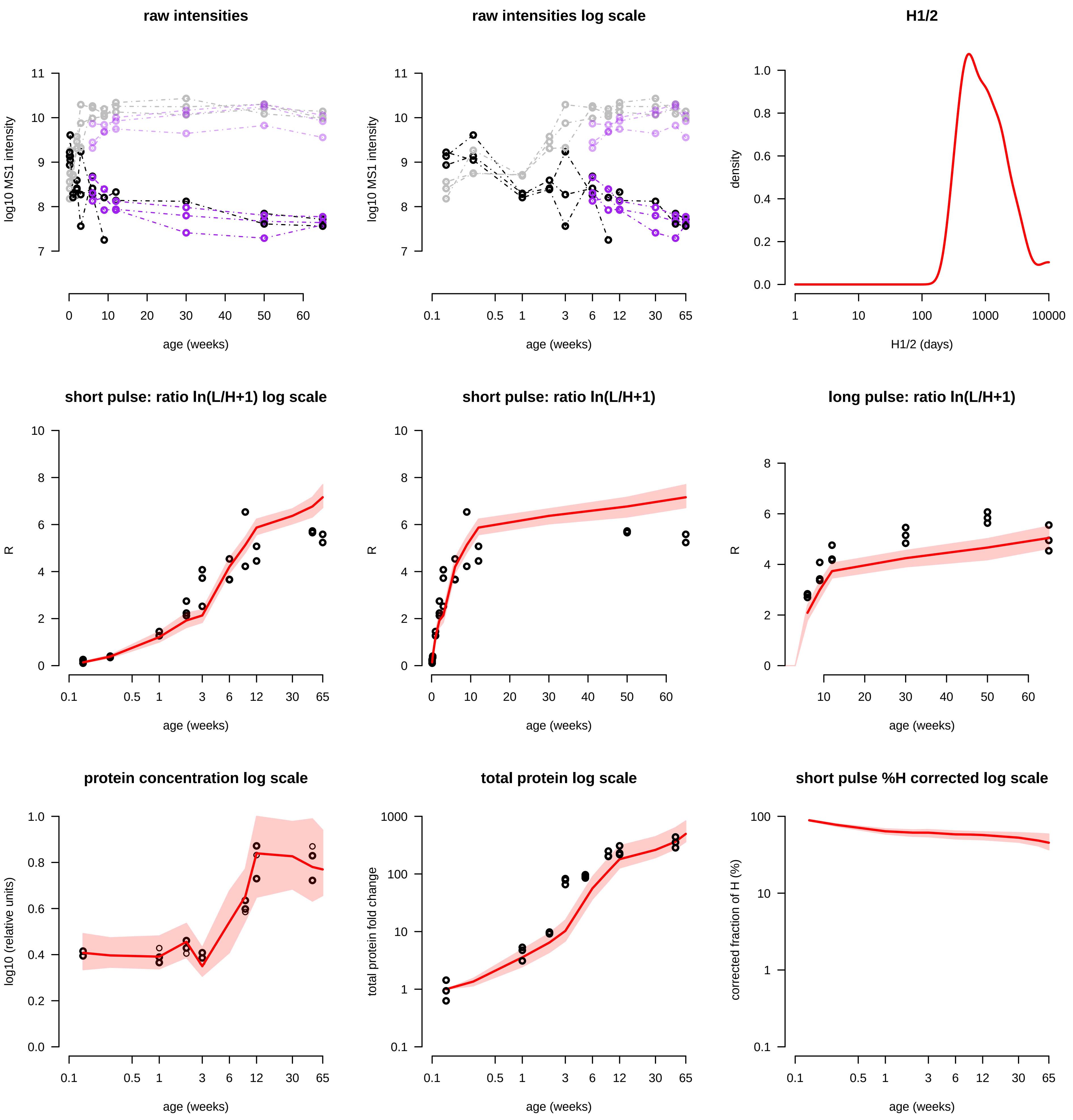

P62962

>sp|P62962|PROF1_MOUSE

Profilin−1

GN=Pfn1

Med H1/2: 14.4 days

Q5 H1/2: 10.2 days

Q95 H1/2: 21 days

reliable model fit: yes

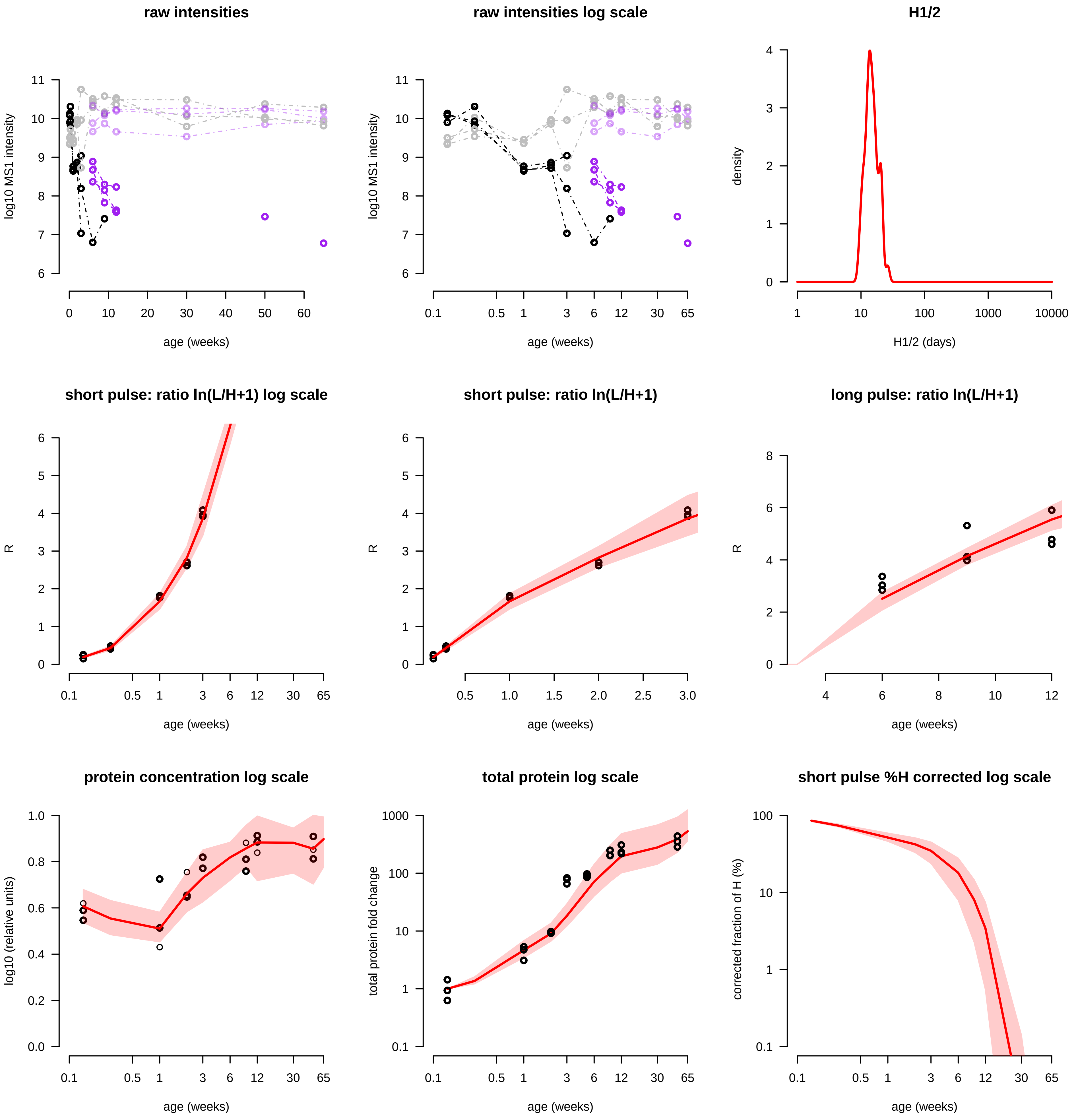

Q02053

>sp|Q02053|UBA1_MOUSE

Ubiquitin−like modifier−activating enzyme 1

GN=Uba1

Med H1/2: 460.8 days

Q5 H1/2: 168.7 days

Q95 H1/2: 5613.3 days

reliable model fit: no

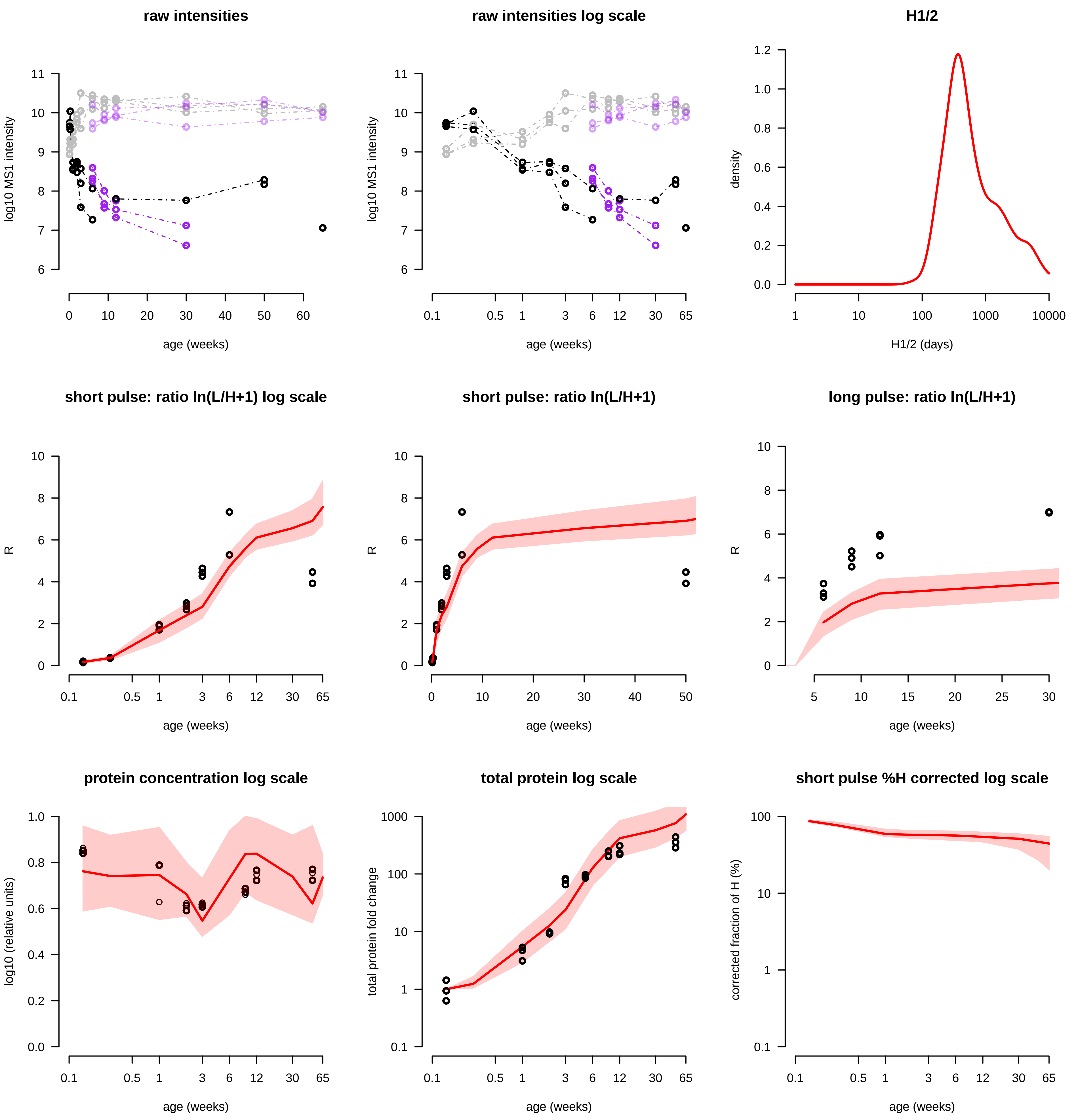

P14733

>sp|P14733|LMNB1_MOUSE

Lamin−B1

GN=Lmnb1

Med H1/2: 23.5 days

Q5 H1/2: 19.3 days

Q95 H1/2: 28.5 days

reliable model fit: yes

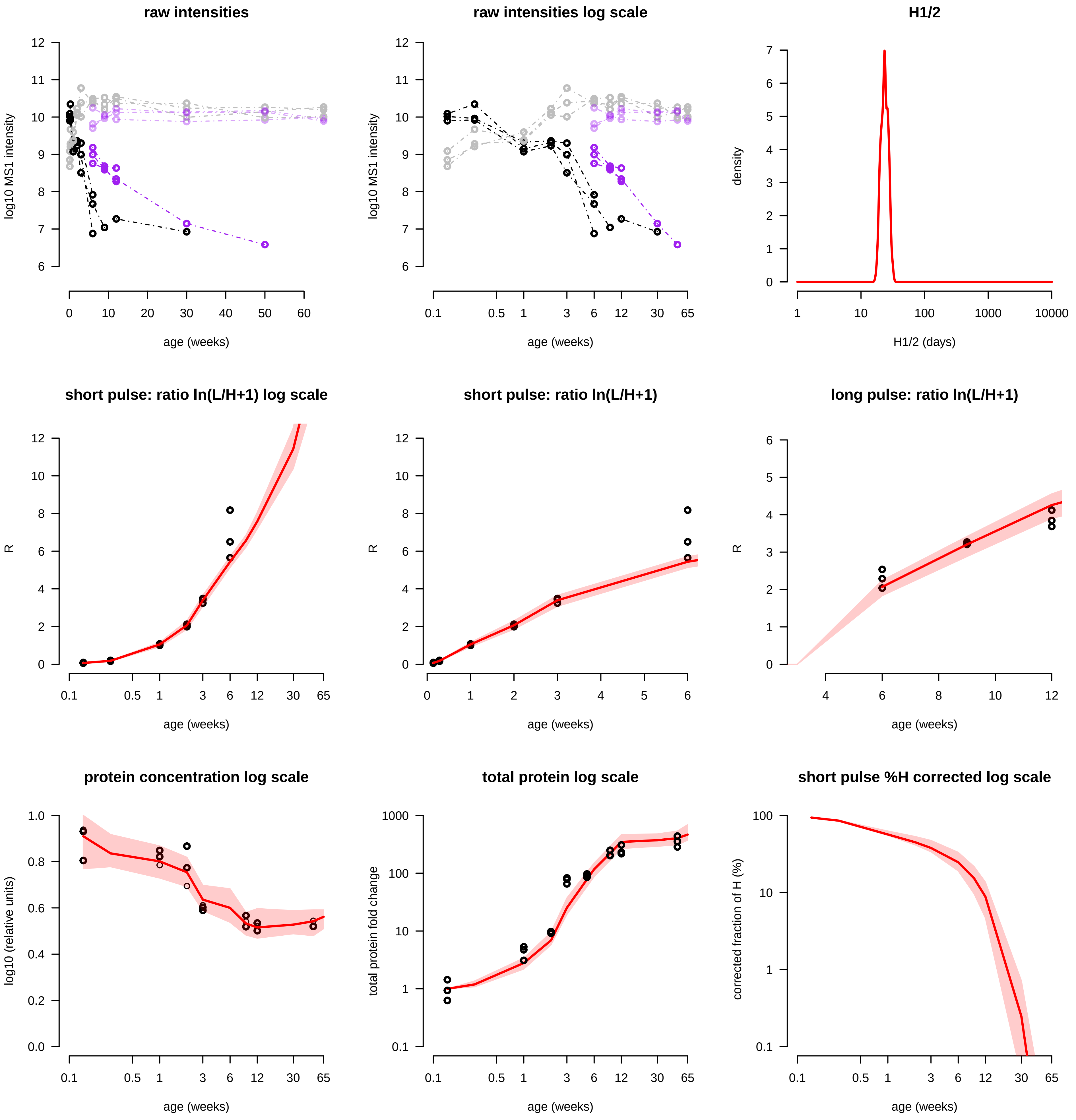

P99027

>sp|P99027|RLA2_MOUSE

60S acidic ribosomal protein P2

GN=Rplp2

Med H1/2: 8.5 days

Q5 H1/2: 7.3 days

Q95 H1/2: 9.9 days

reliable model fit: yes

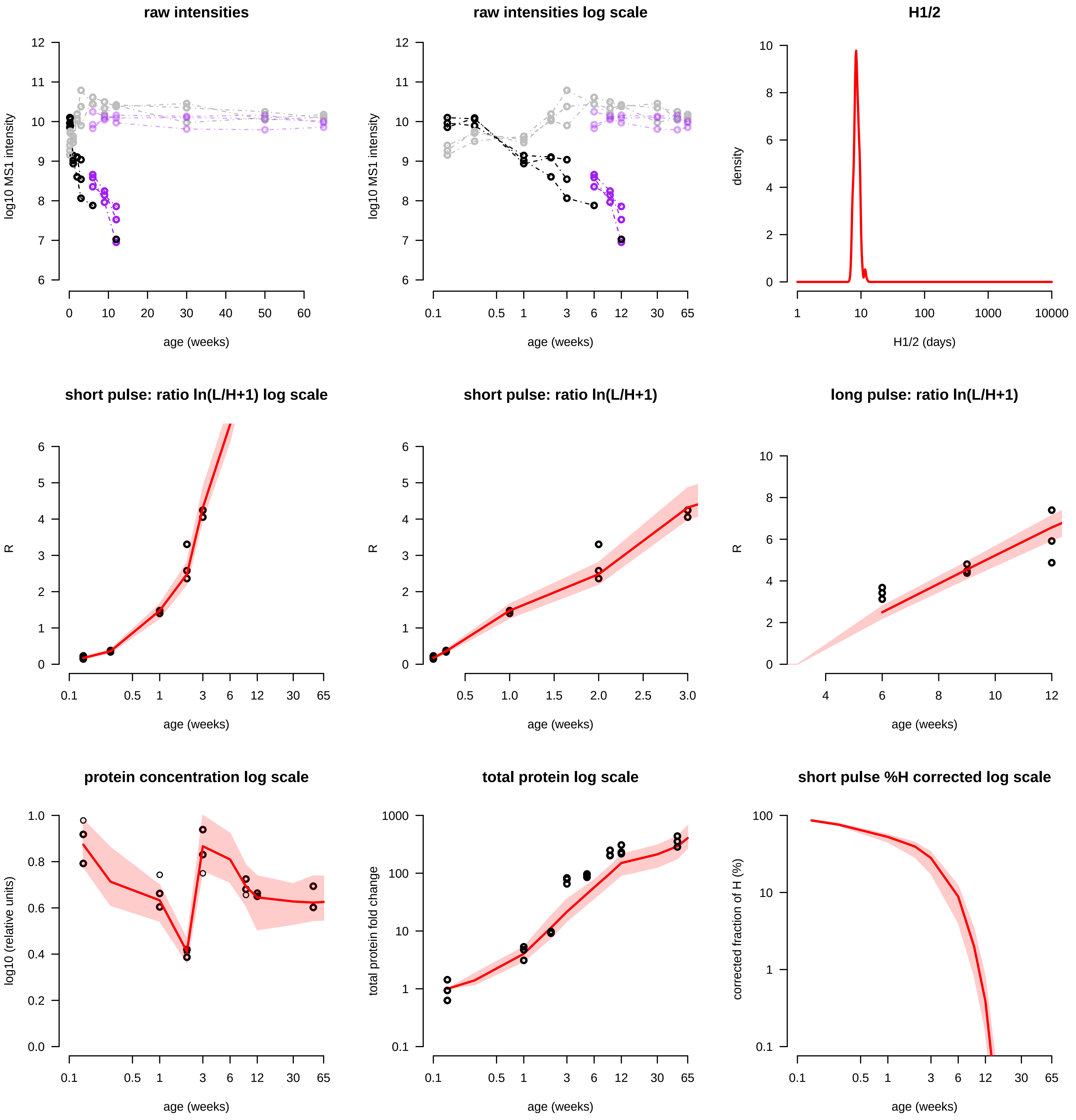

P08003

>sp|P08003|PDIA4_MOUSE

Protein disulfide−isomerase A4

GN=Pdia4

Med H1/2: 181.1 days

Q5 H1/2: 116.7 days

Q95 H1/2: 435.2 days

reliable model fit: yes

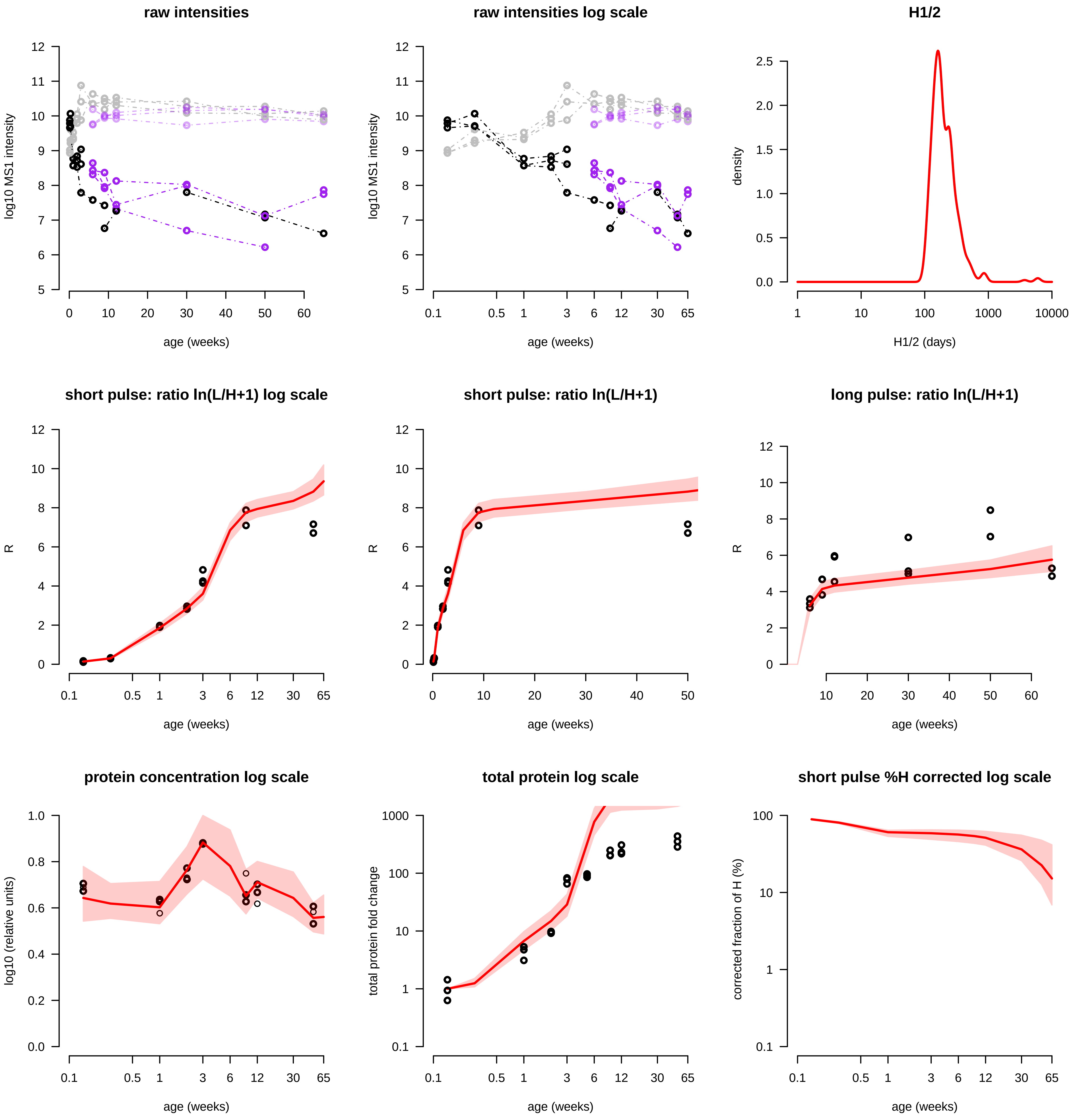

Q60597;Q60597−2

>sp|Q60597|ODO1_MOUSE

2−oxoglutarate dehydrogenase, mitochondrial

GN=Ogdh

Med H1/2: 226.4 days

Q5 H1/2: 82.7 days

Q95 H1/2: 2208.5 days

reliable model fit: no

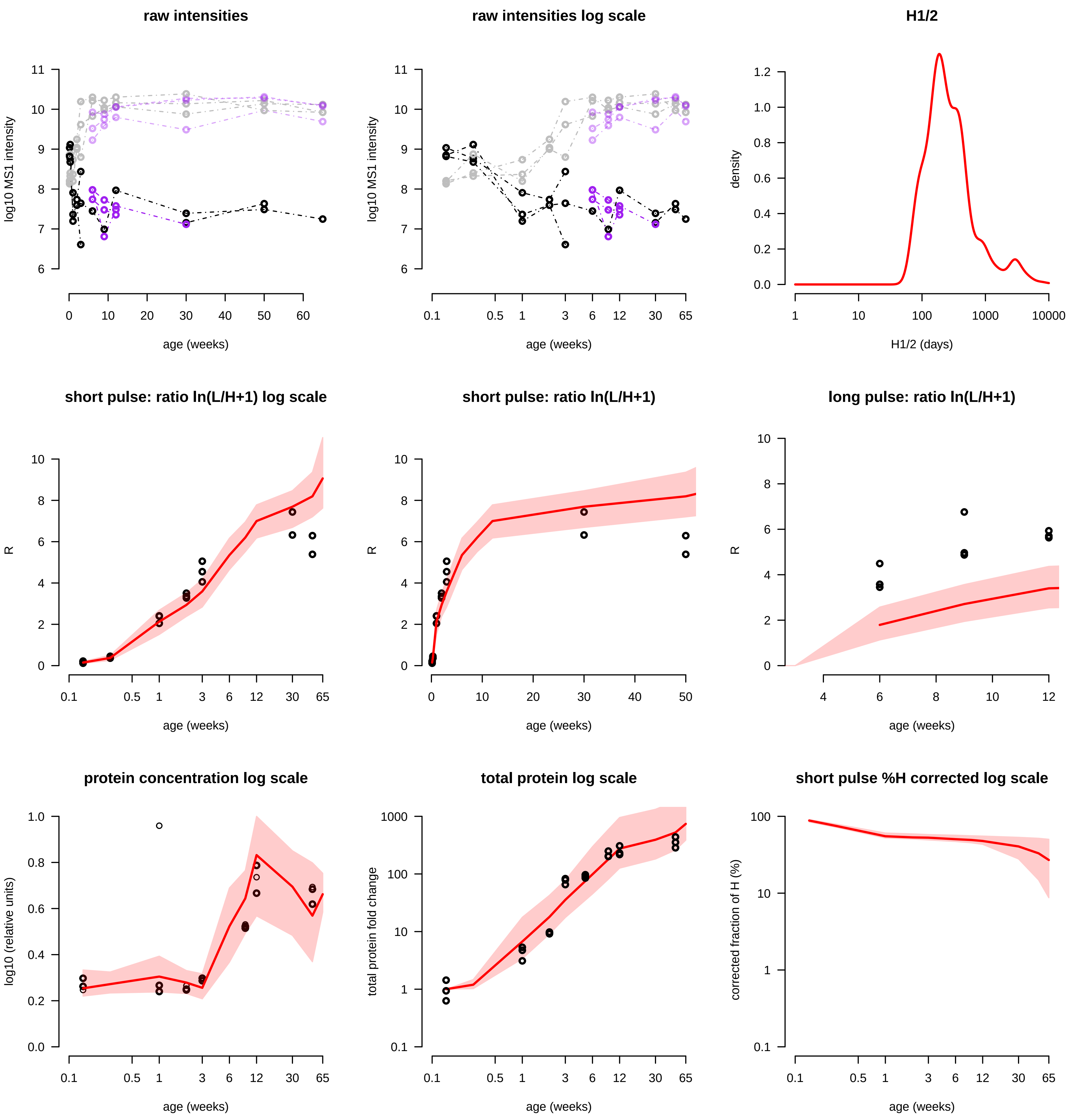

P54869

>sp|P54869|HMCS2_MOUSE

Hydroxymethylglutaryl−CoA synthase, mitochondrial

GN=Hmgcs2

Med H1/2: 132 days

Q5 H1/2: 79.4 days

Q95 H1/2: 492.2 days

reliable model fit: yes

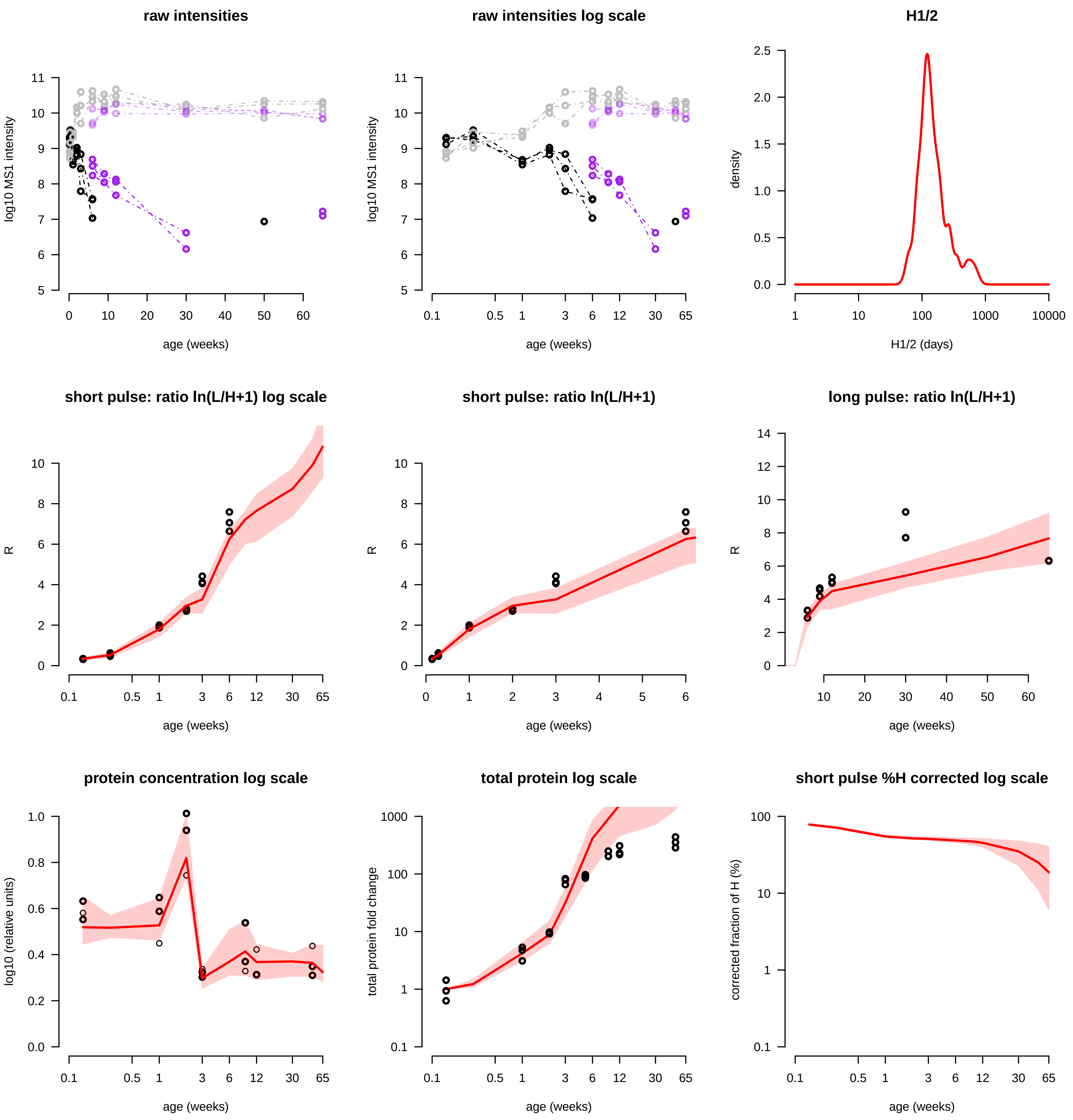

Q9DCN2−2;Q9DCN2

>sp|Q9DCN2−2|NB5R3_MOUSE

Isoform 2 of NADH−cytochrome b5 reductase 3

GN=Cyb5r3

Med H1/2: 11.7 days

Q5 H1/2: 9.3 days

Q95 H1/2: 15.6 days

reliable model fit: yes

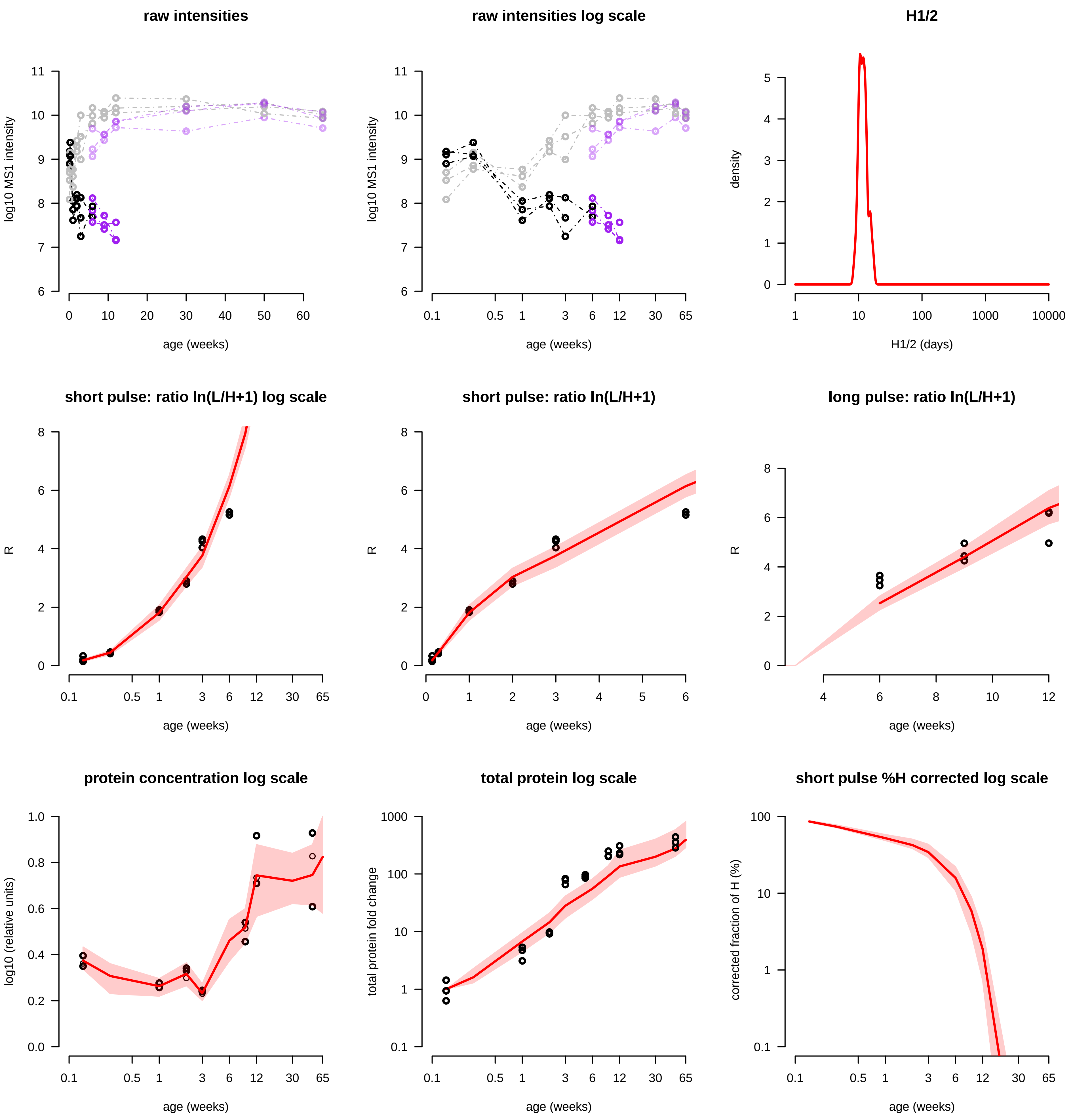

P50247

>sp|P50247|SAHH_MOUSE

Adenosylhomocysteinase

GN=Ahcy

Med H1/2: 103.7 days

Q5 H1/2: 78.4 days

Q95 H1/2: 148.6 days

reliable model fit: yes

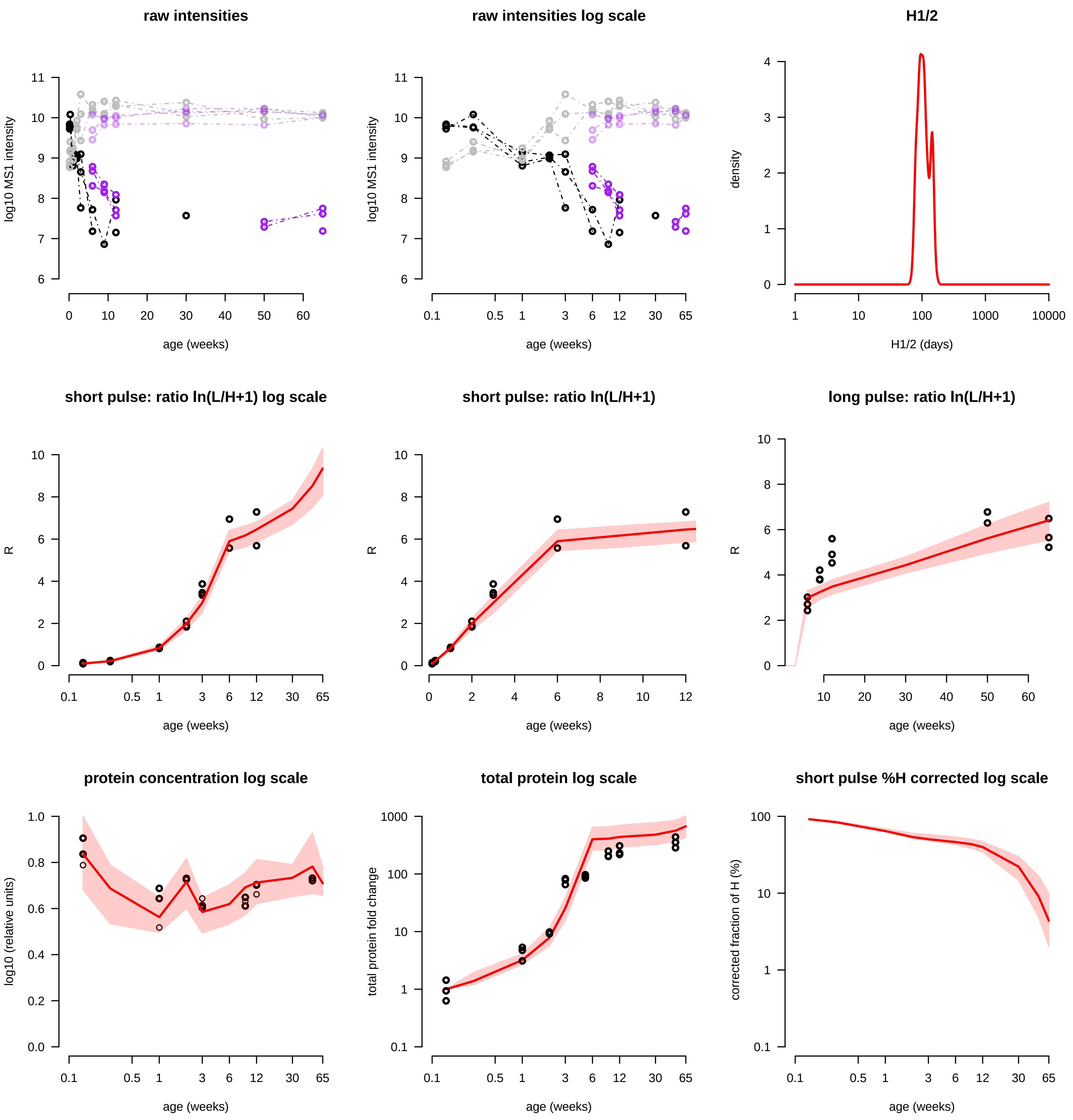

P21107−2

>sp|P21107−2|TPM3_MOUSE

Isoform 2 of Tropomyosin alpha−3 chain

GN=Tpm3

Med H1/2: 40.1 days

Q5 H1/2: 31.1 days

Q95 H1/2: 54.2 days

reliable model fit: yes

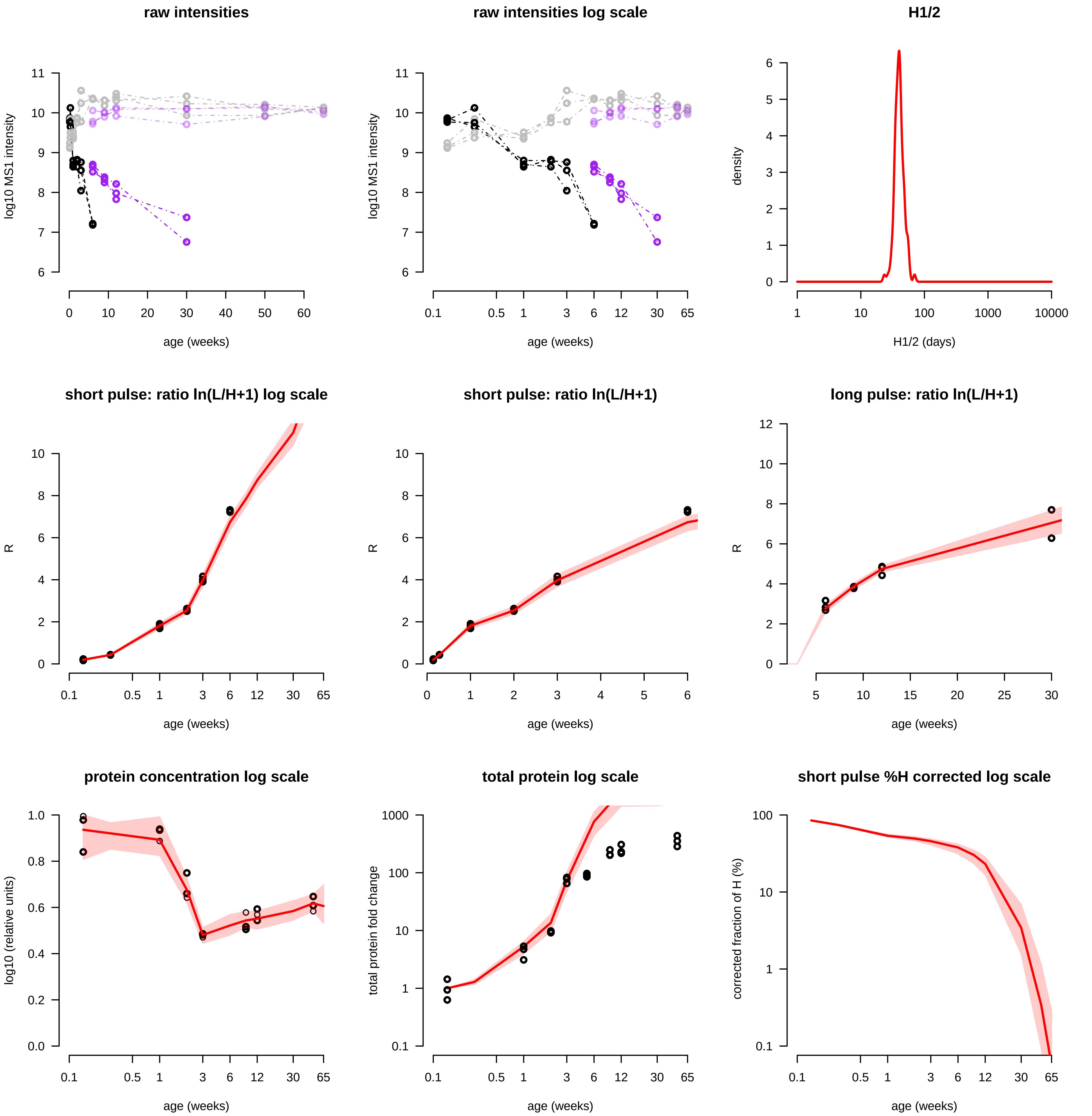

Q7TPR4

>sp|Q7TPR4|ACTN1_MOUSE

Alpha−actinin−1

GN=Actn1

Med H1/2: 616.1 days

Q5 H1/2: 168 days

Q95 H1/2: 4433.3 days

reliable model fit: no

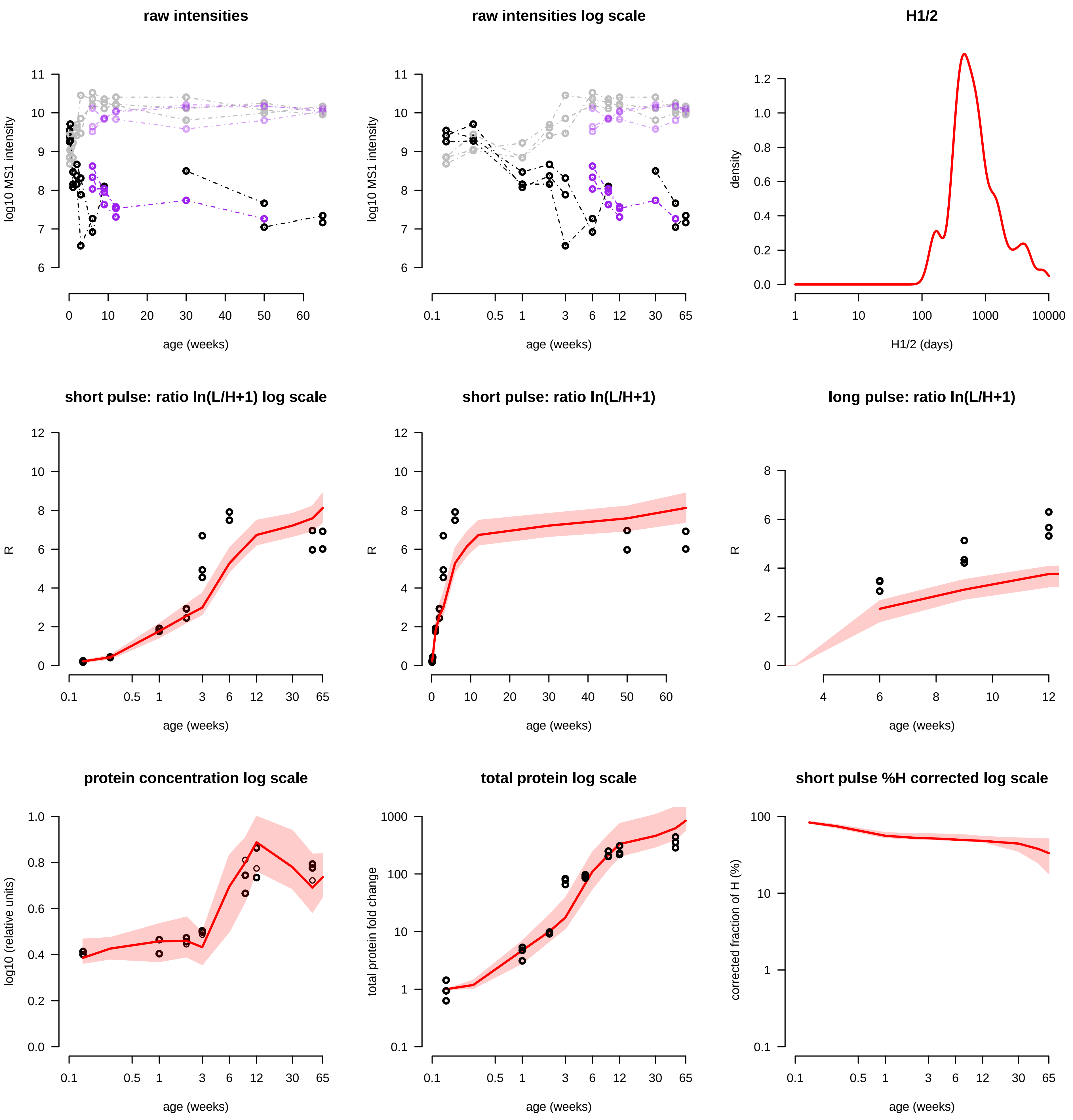

Q61425

>sp|Q61425|HCDH_MOUSE

Hydroxyacyl−coenzyme A dehydrogenase, mitochondrial

GN=Hadh

Med H1/2: 12.7 days

Q5 H1/2: 9.5 days

Q95 H1/2: 20.6 days

reliable model fit: yes

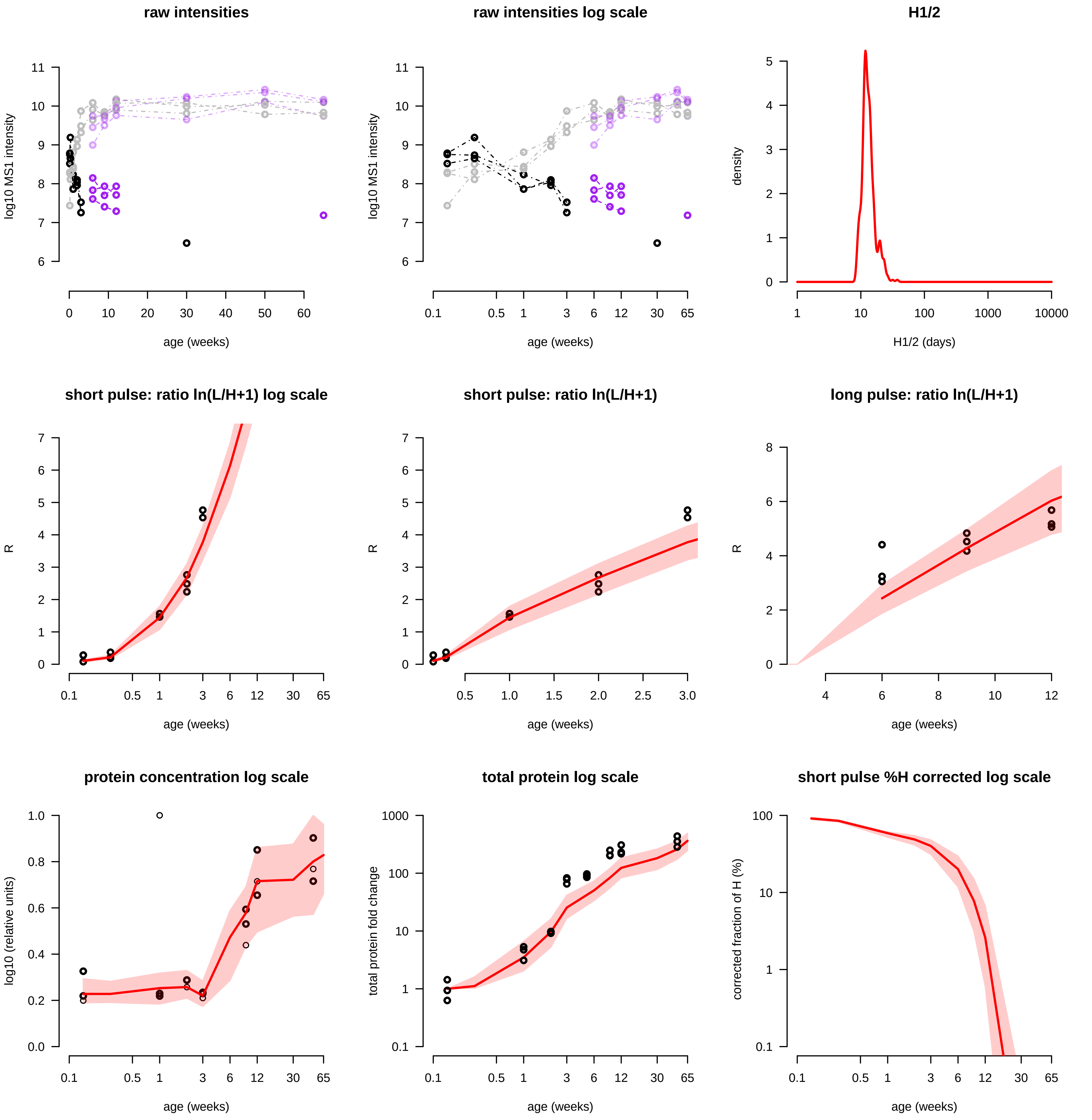

Q9WV55

>sp|Q9WV55|VAPA_MOUSE

Vesicle−associated membrane protein−associated protein A

GN=Vapa

Med H1/2: 11 days

Q5 H1/2: 9.2 days

Q95 H1/2: 13.7 days

reliable model fit: yes

raw intensities

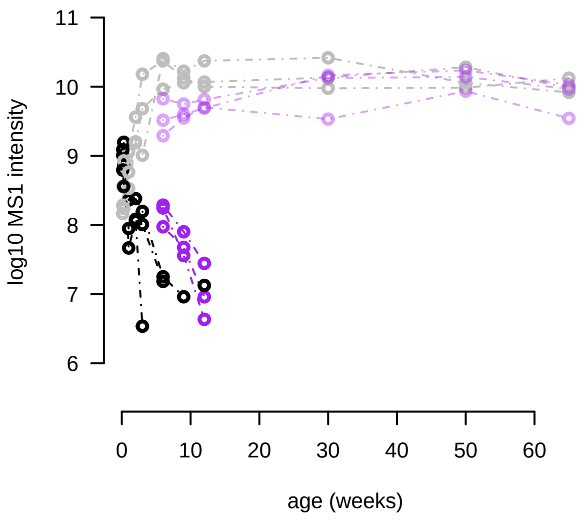

raw intensities log scale

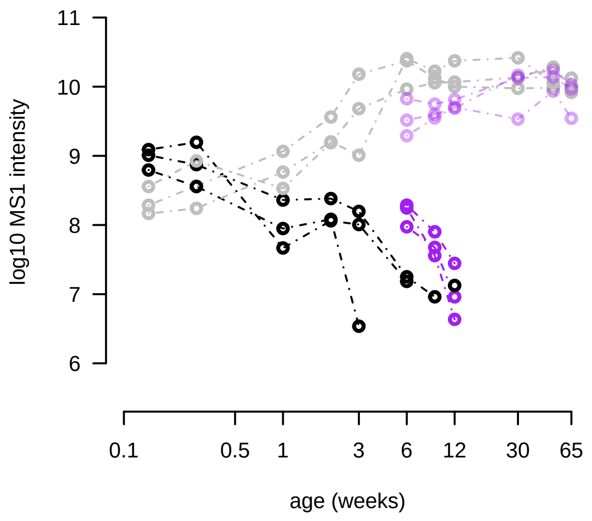

H1/2

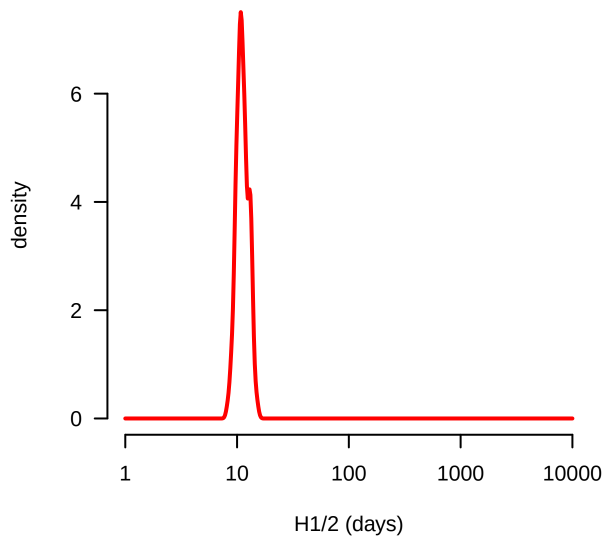

short pulse: ratio ln(L/H+1) log scale

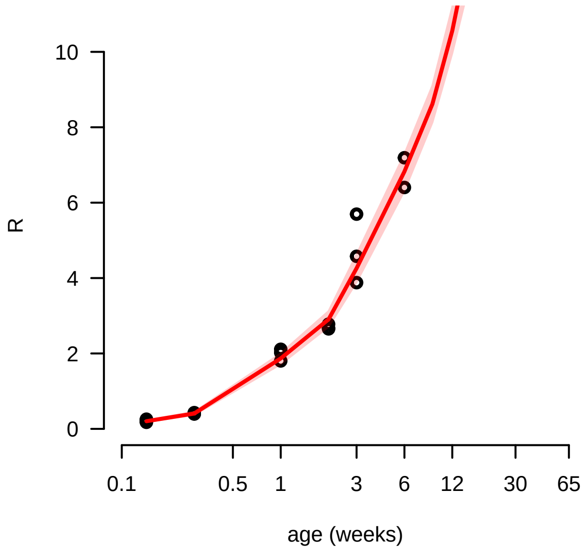

short pulse: ratio ln(L/H+1)

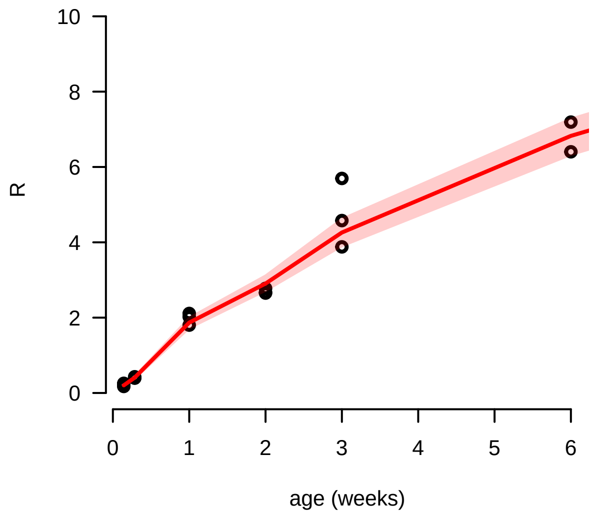

long pulse: ratio ln(L/H+1)

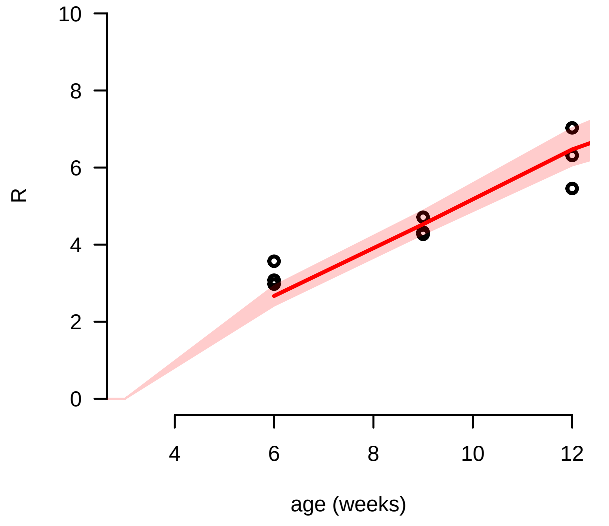

protein concentration log scale

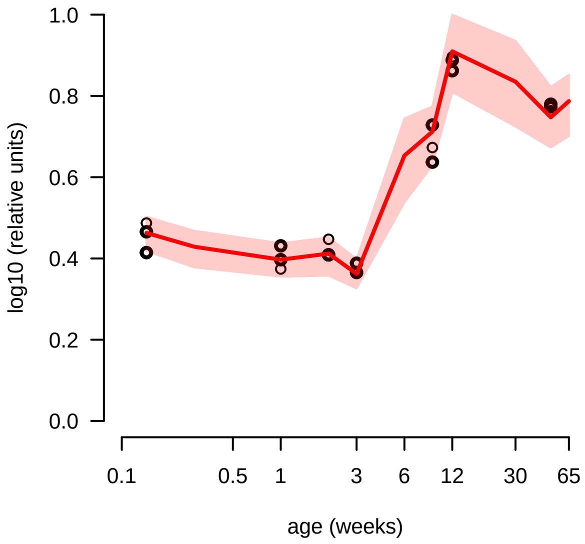

total protein log scale

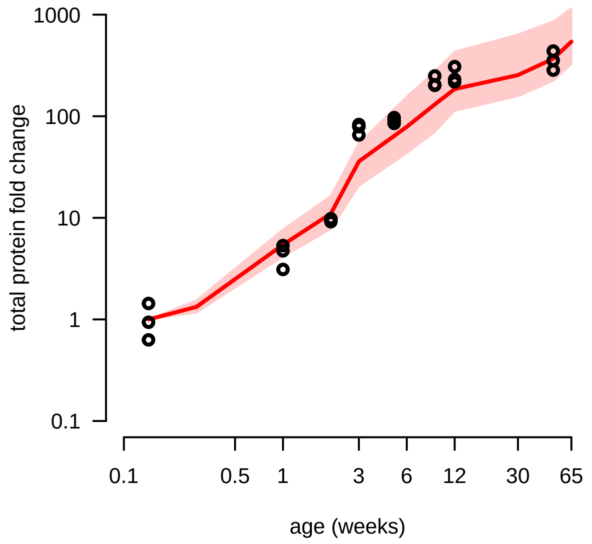

short pulse %H corrected log scale

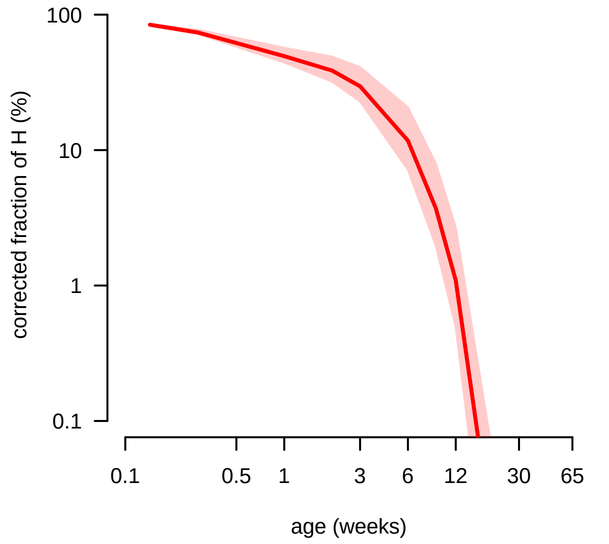

Q9ERK4

>sp|Q9ERK4|XPO2_MOUSE

Exportin−2

GN=Cse1l

Med H1/2: 1079.5 days

Q5 H1/2: 358.3 days

Q95 H1/2: 9882.4 days

reliable model fit: yes

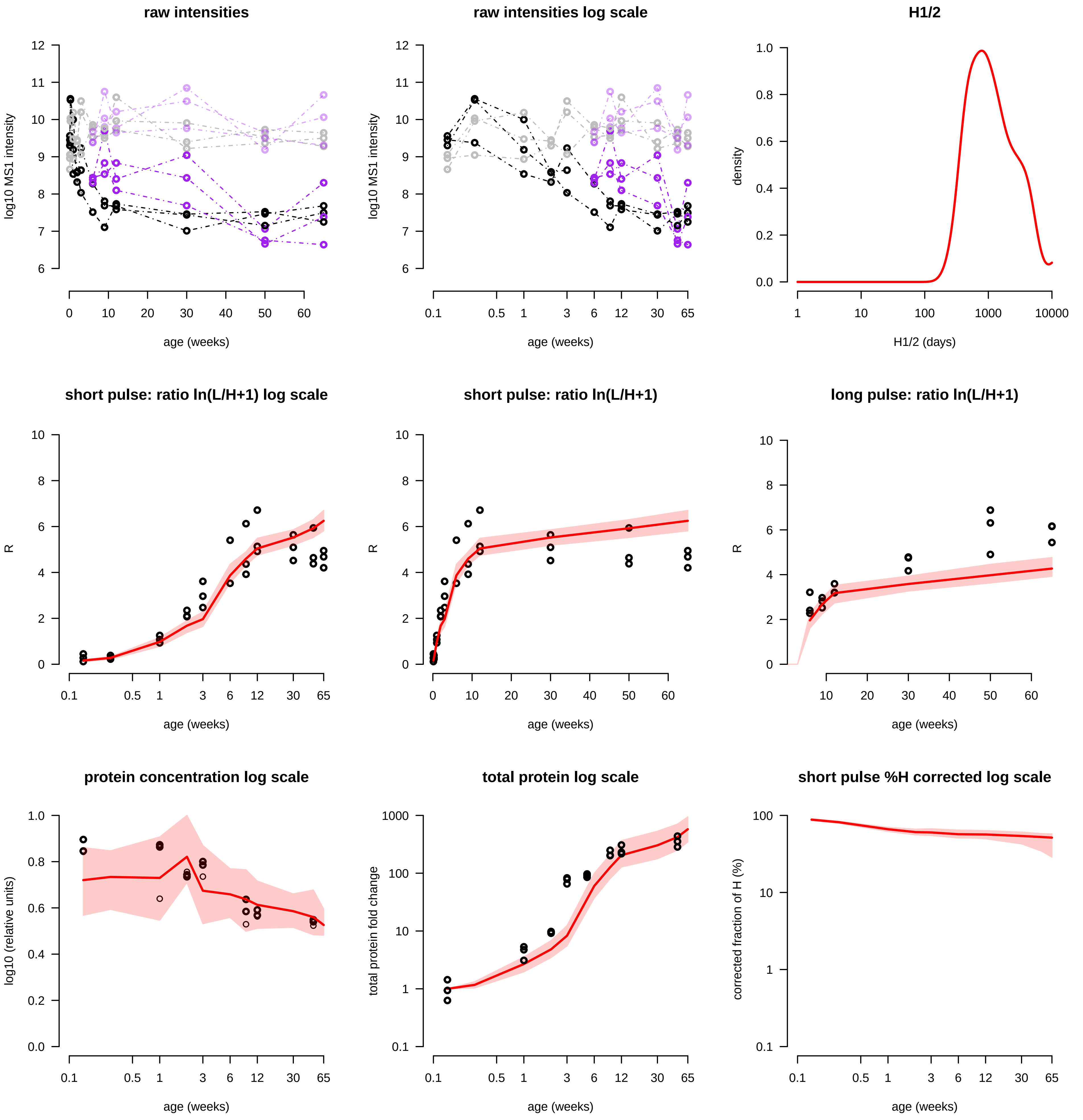

Q99PT1

>sp|Q99PT1|GDIR1_MOUSE

Rho GDP−dissociation inhibitor 1

GN=Arhgdia

Med H1/2: 6.5 days

Q5 H1/2: 5.4 days

Q95 H1/2: 8.6 days

reliable model fit: yes

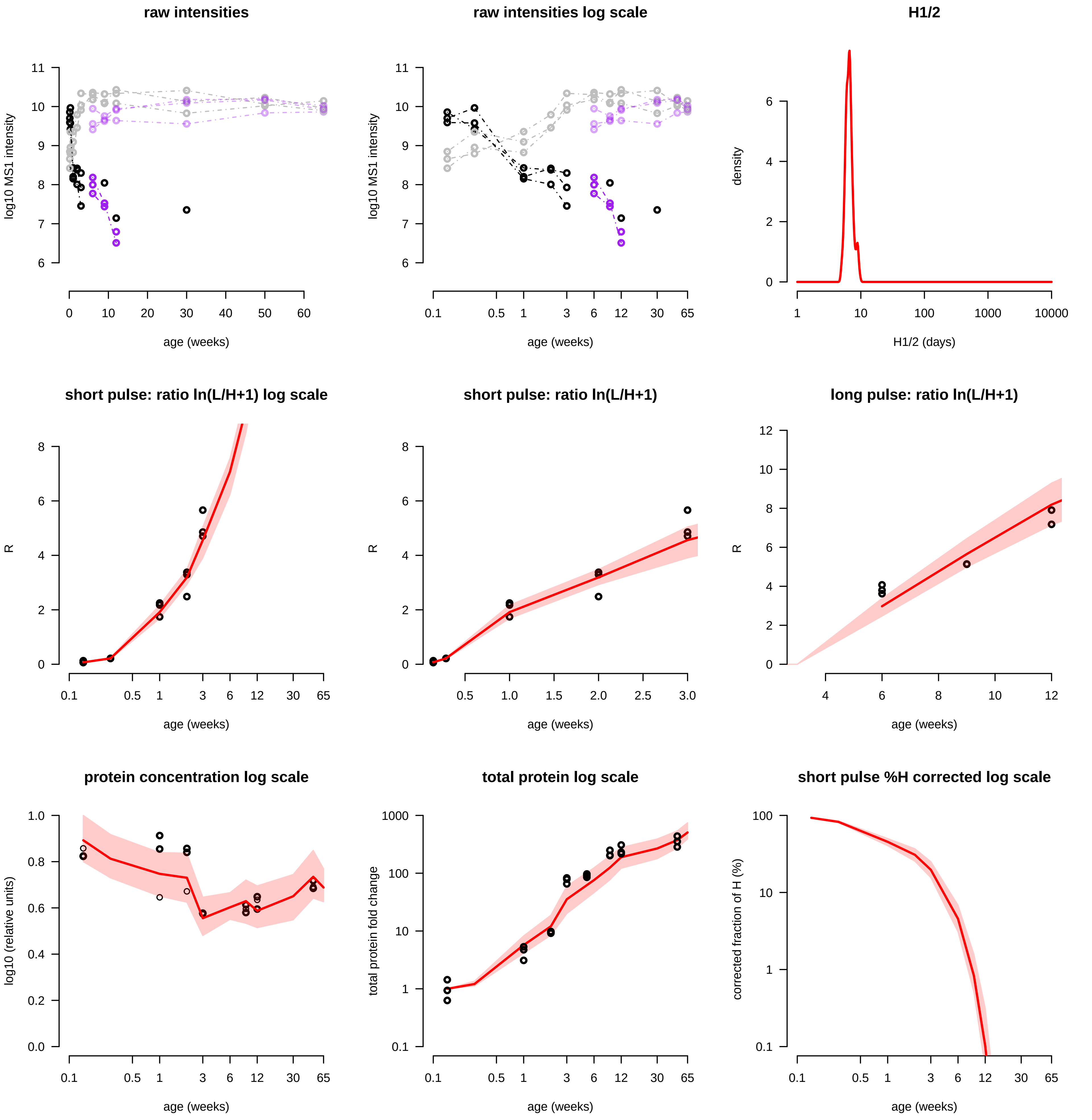

P24472

>sp|P24472|GSTA4_MOUSE

Glutathione S−transferase A4

GN=Gsta4

Med H1/2: 17.7 days

Q5 H1/2: 12.3 days

Q95 H1/2: 34.8 days

reliable model fit: yes

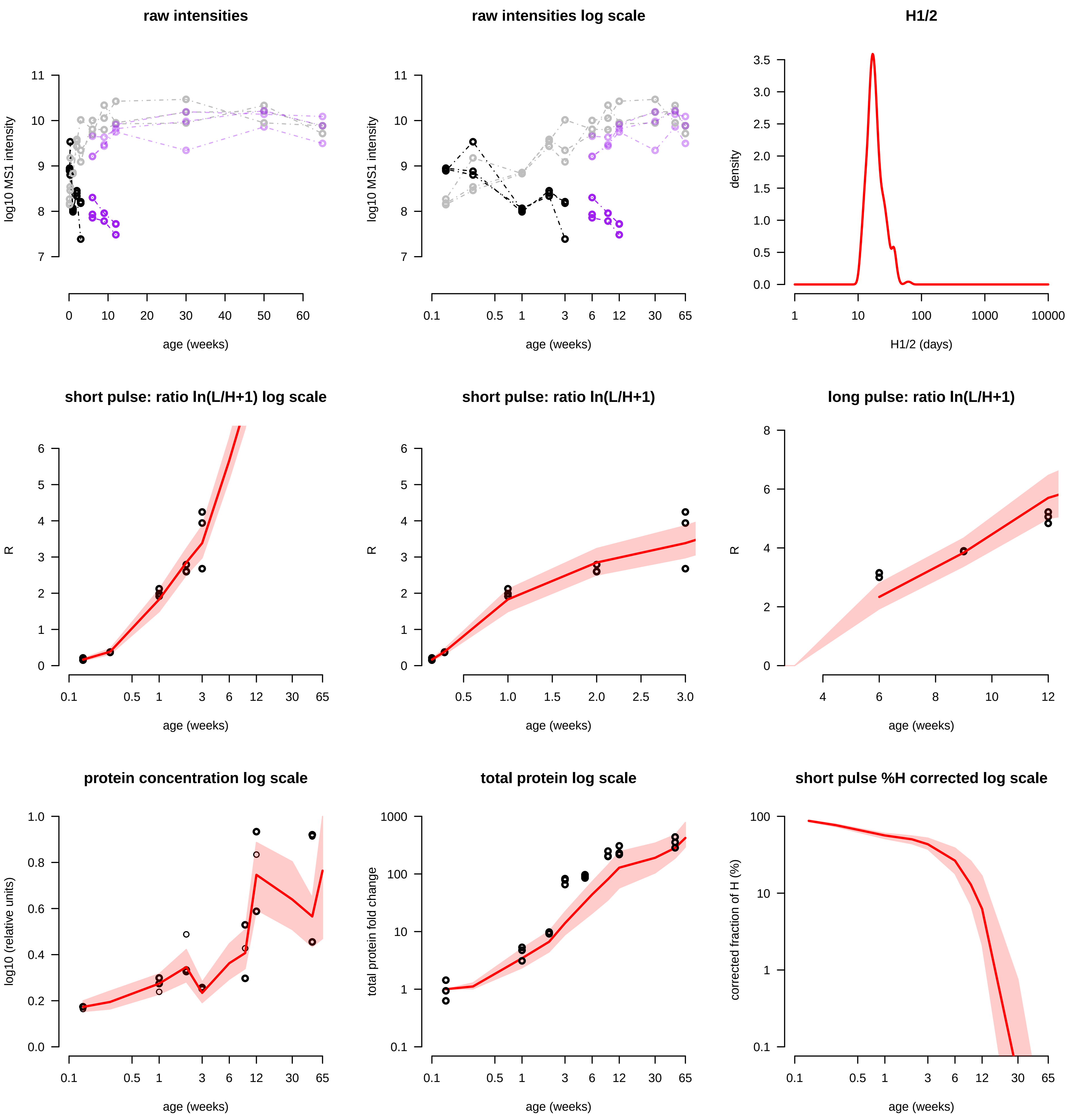

Q8VEK3;Q8VEK3−2

>sp|Q8VEK3|HNRPU_MOUSE

Heterogeneous nuclear ribonucleoprotein U

GN=Hnrnpu

Med H1/2: 13 days

Q5 H1/2: 10.5 days

Q95 H1/2: 17.9 days

reliable model fit: yes

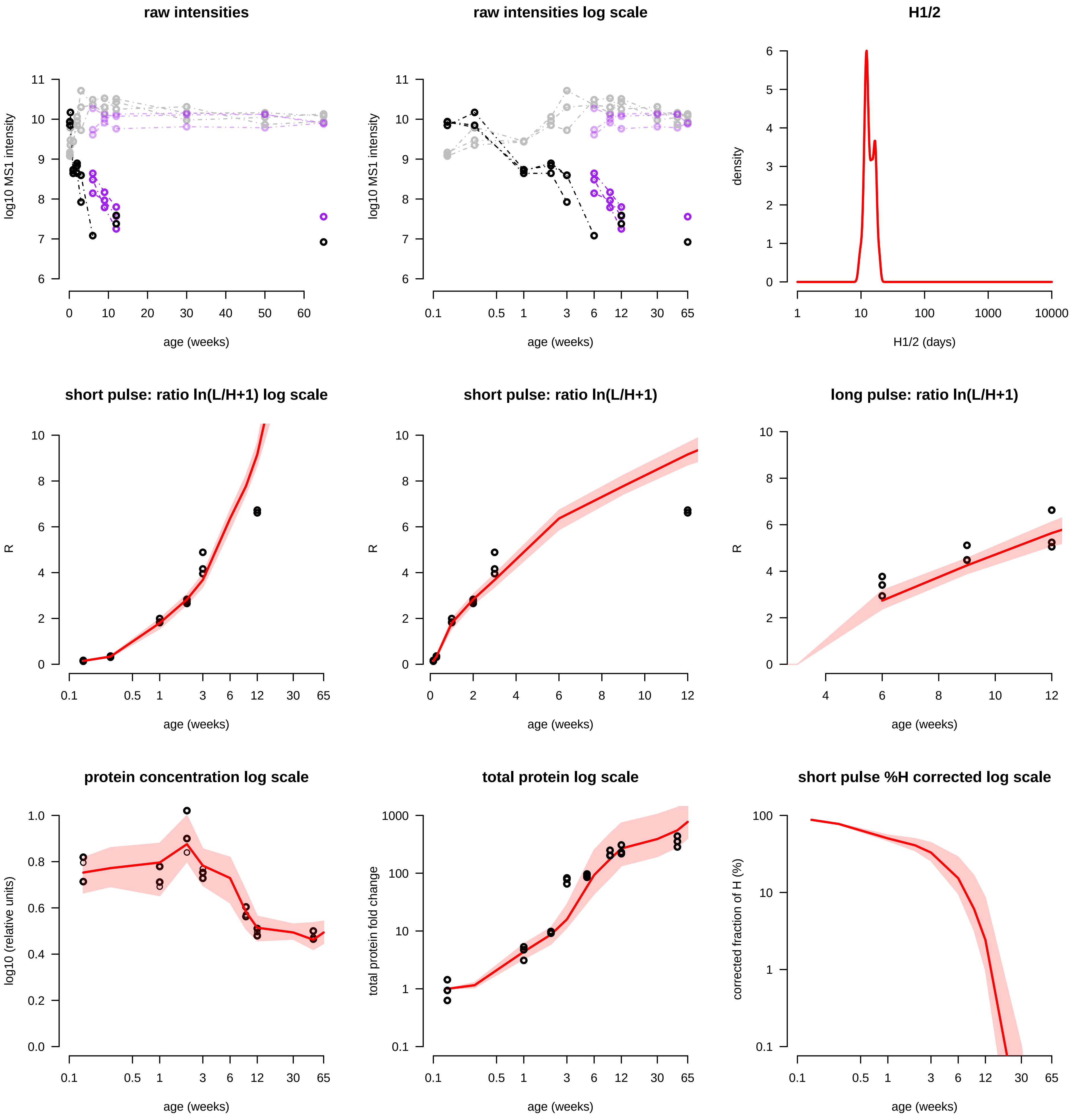

P14824

>sp|P14824|ANXA6_MOUSE

Annexin A6

GN=Anxa6

Med H1/2: 813 days

Q5 H1/2: 253 days

Q95 H1/2: 14020.3 days

reliable model fit: yes

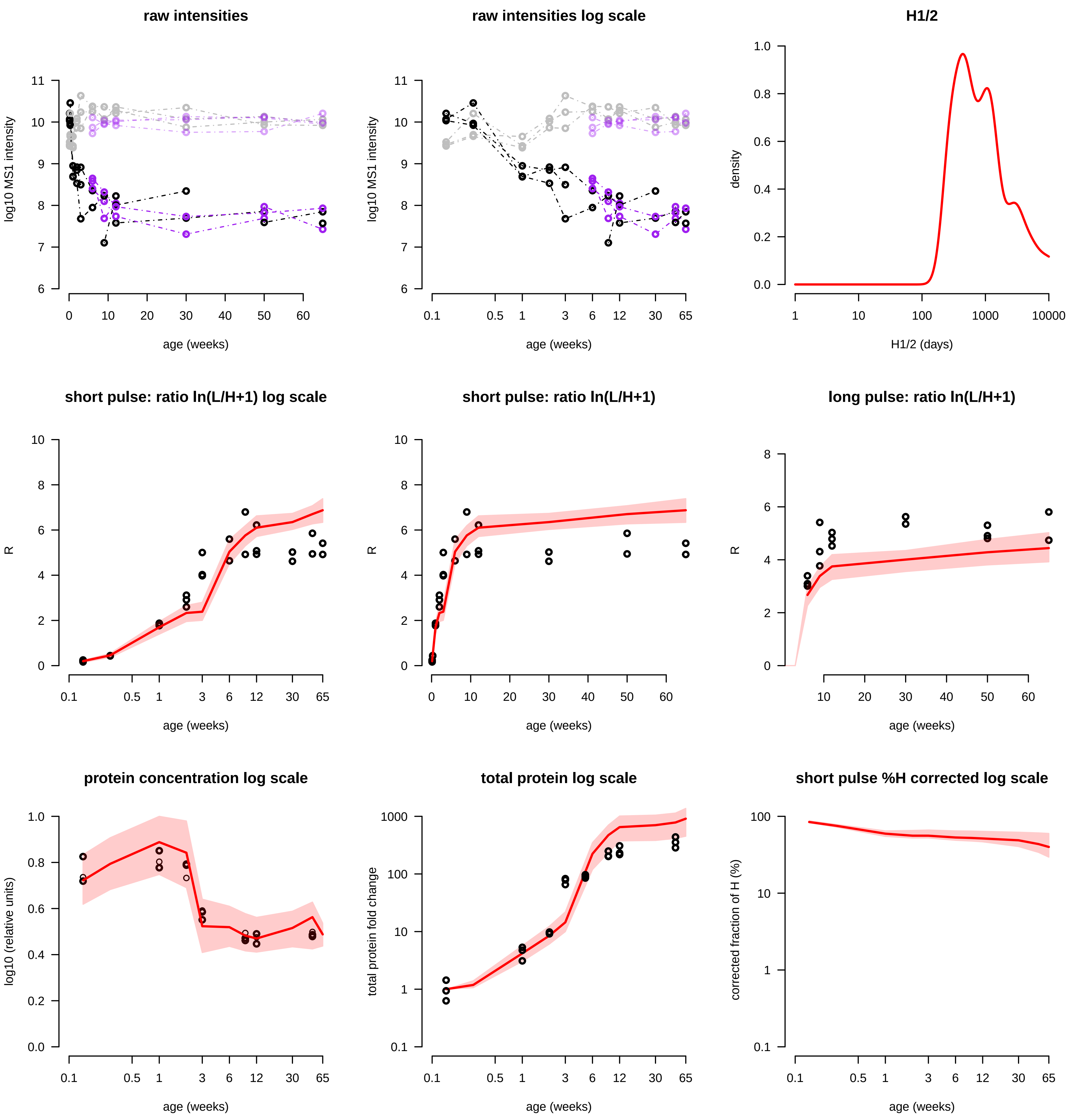

P62908

>sp|P62908|RS3_MOUSE

40S ribosomal protein S3

GN=Rps3

Med H1/2: 1320 days

Q5 H1/2: 359.2 days

Q95 H1/2: 17320.2 days

reliable model fit: no

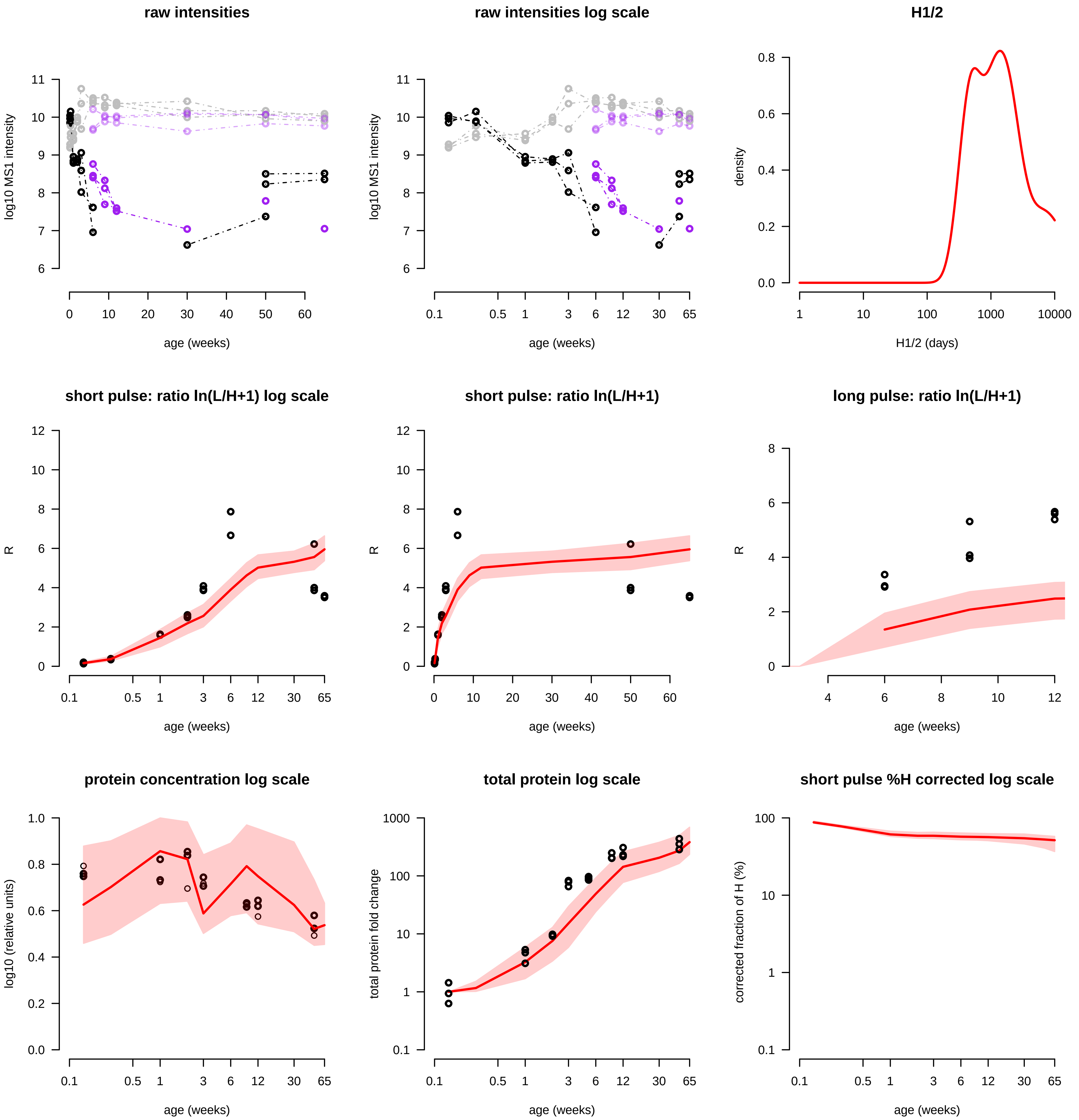

P70296

>sp|P70296|PEBP1_MOUSE

Phosphatidylethanolamine−binding protein 1

GN=Pebp1

Med H1/2: 11.5 days

Q5 H1/2: 9 days

Q95 H1/2: 15.9 days

reliable model fit: yes

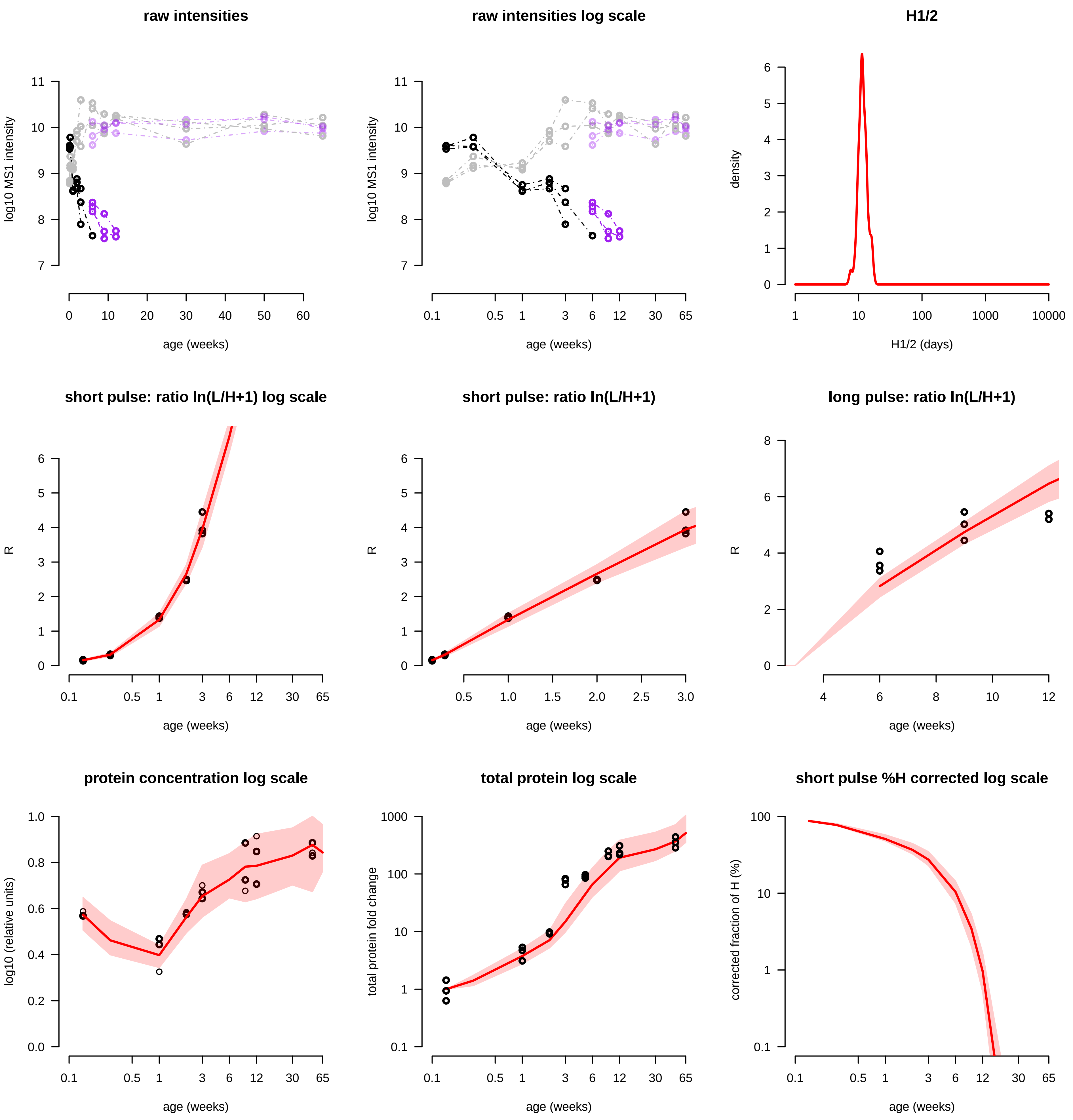

P32020;P32020−2

>sp|P32020|SCP2_MOUSE

Sterol carrier protein 2

GN=Scp2

Med H1/2: 106.5 days

Q5 H1/2: 61.2 days

Q95 H1/2: 255.2 days

reliable model fit: yes

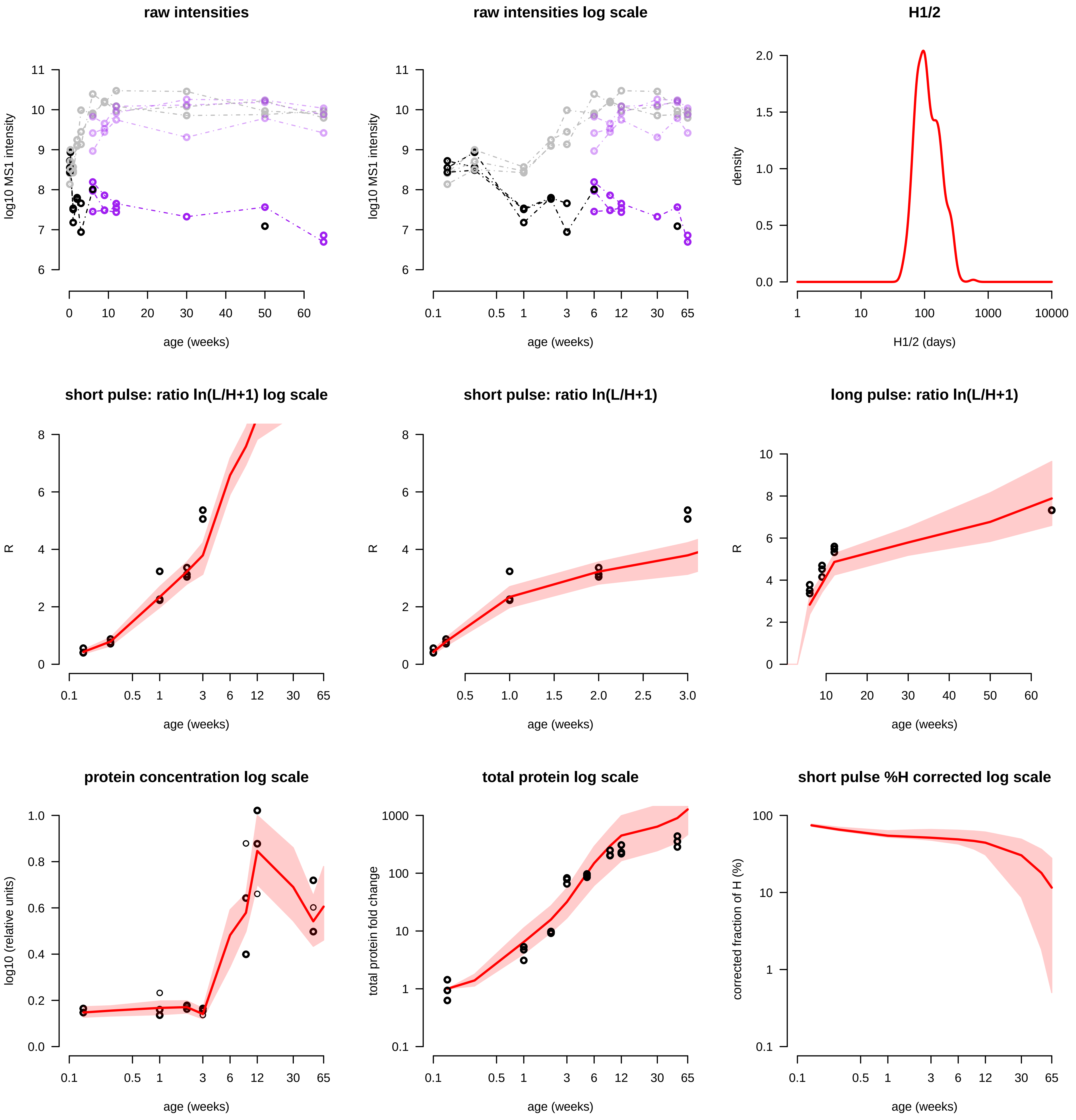

P25444

>sp|P25444|RS2_MOUSE

40S ribosomal protein S2

GN=Rps2

Med H1/2: 181.6 days

Q5 H1/2: 113.2 days

Q95 H1/2: 361.6 days

reliable model fit: yes

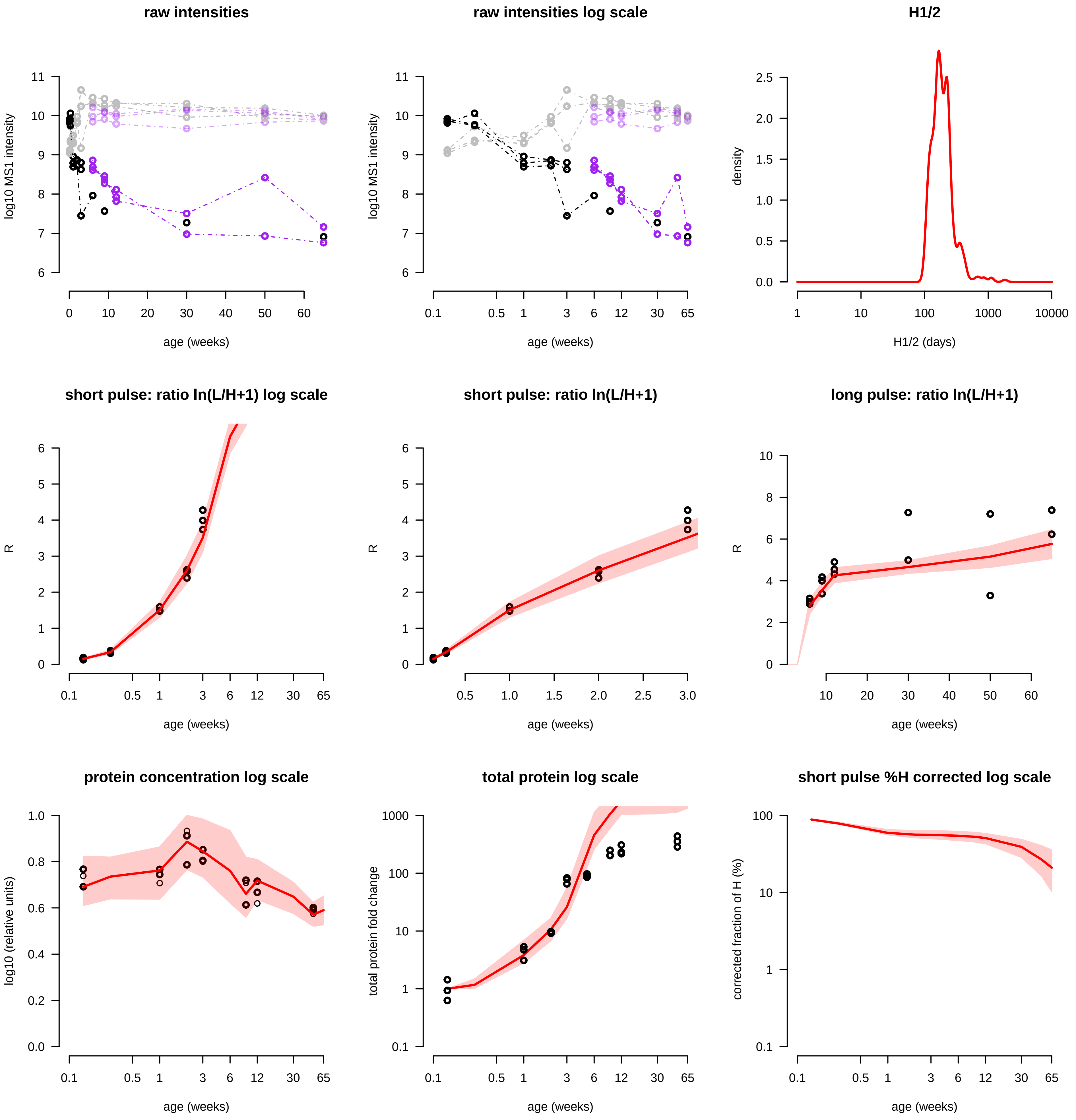

P14873

>sp|P14873|MAP1B_MOUSE

Microtubule−associated protein 1B

GN=Map1b

Med H1/2: 106.9 days

Q5 H1/2: 63.8 days

Q95 H1/2: 239.4 days

reliable model fit: yes

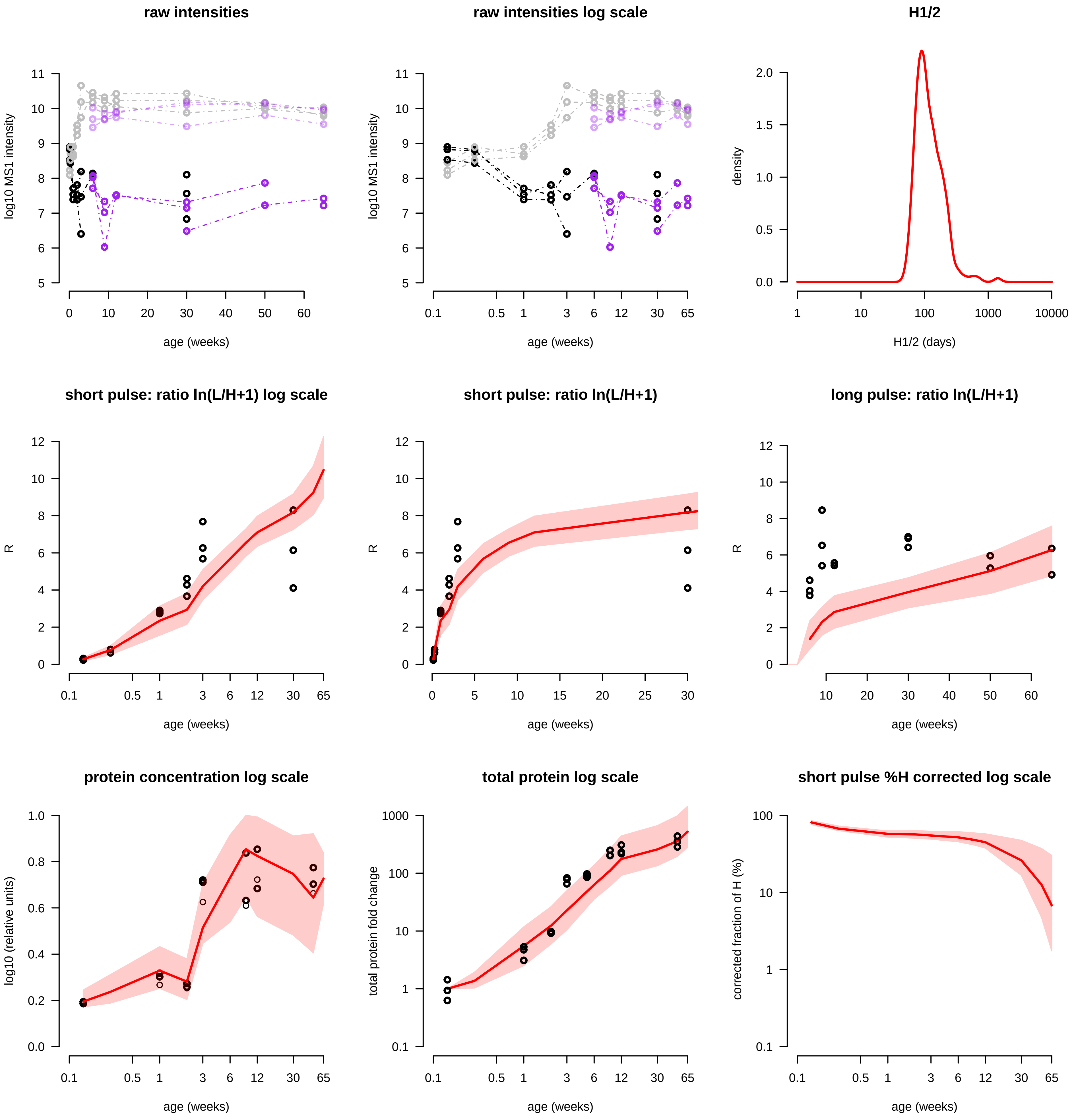

P67778

>sp|P67778|PHB_MOUSE

Prohibitin

GN=Phb

Med H1/2: 17.3 days

Q5 H1/2: 13.1 days

Q95 H1/2: 27 days

reliable model fit: yes

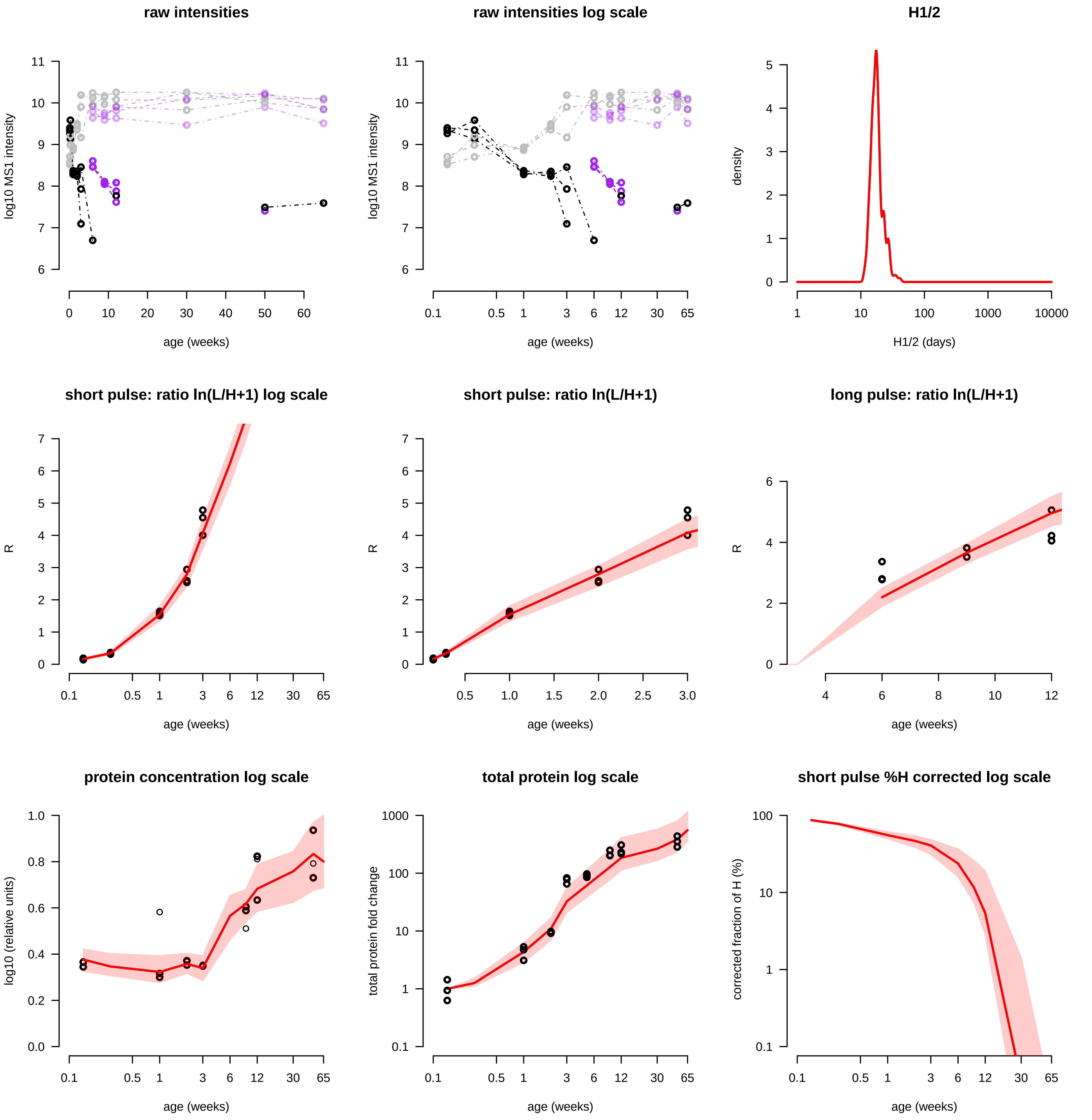

P08228

>sp|P08228|SODC_MOUSE

Superoxide dismutase [Cu−Zn]

GN=Sod1

Med H1/2: 16.8 days

Q5 H1/2: 13.5 days

Q95 H1/2: 24.6 days

reliable model fit: yes

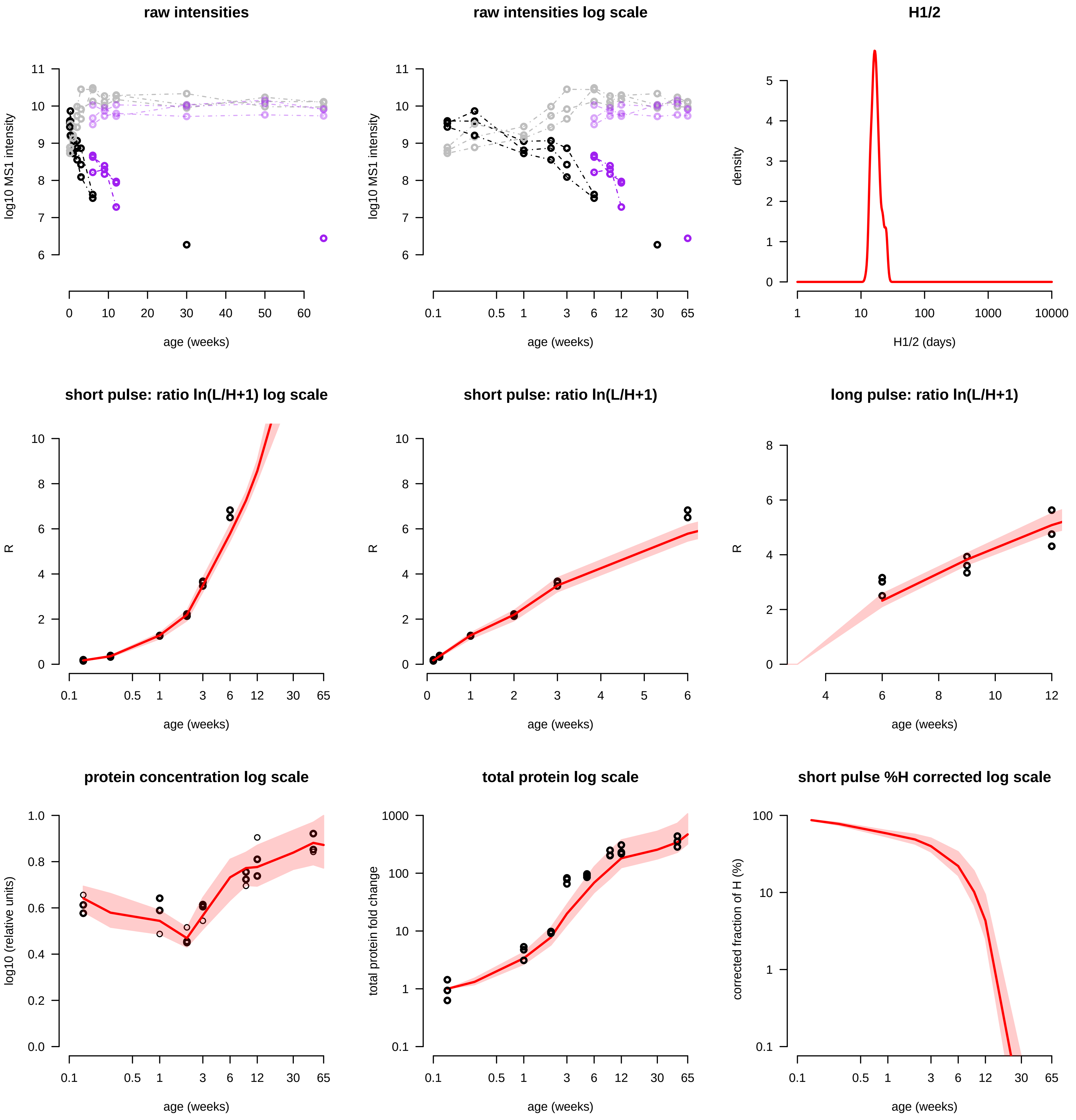

P10107

>sp|P10107|ANXA1_MOUSE

Annexin A1

GN=Anxa1

Med H1/2: 13 days

Q5 H1/2: 10.1 days

Q95 H1/2: 17.8 days

reliable model fit: yes

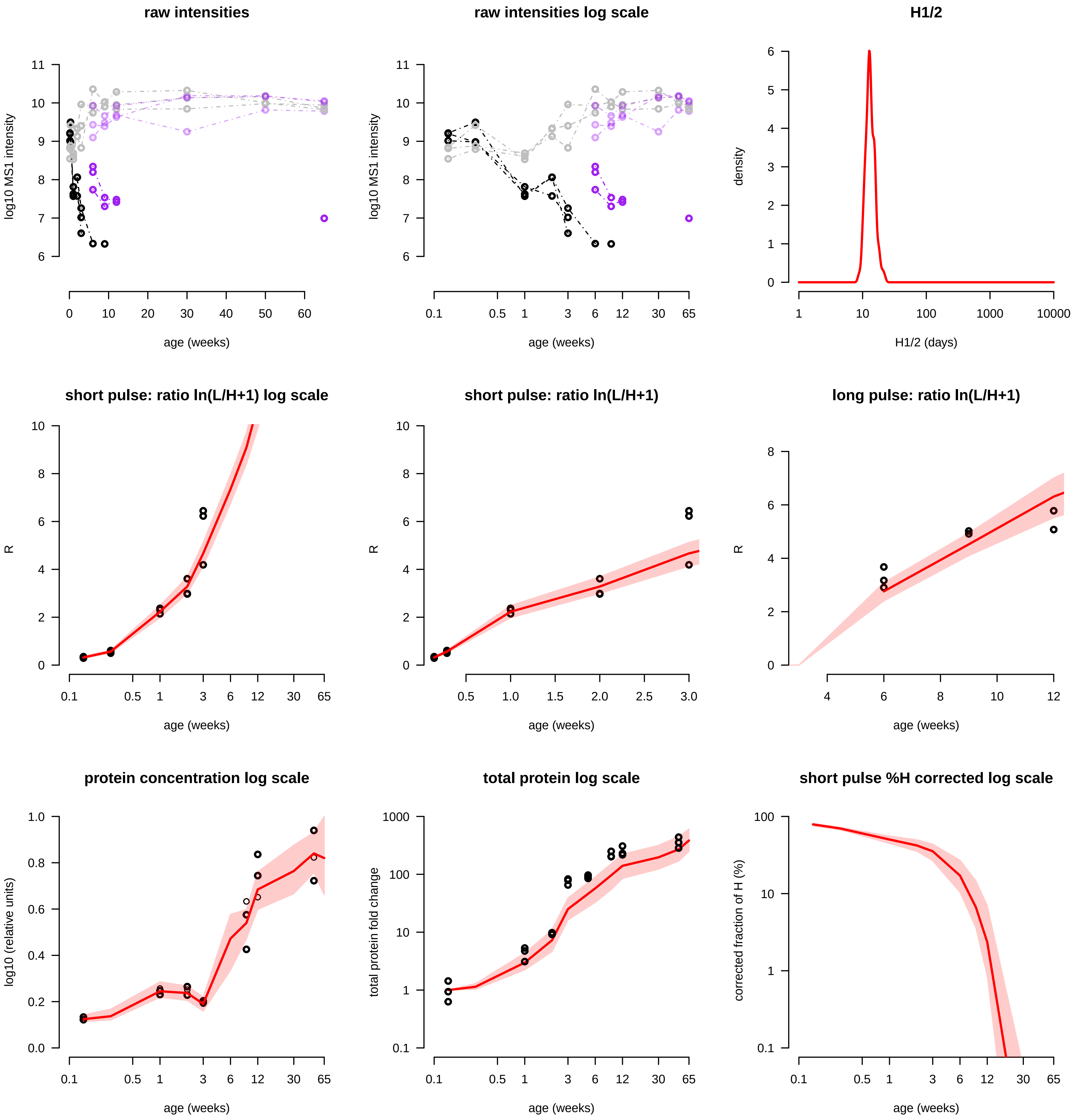

P84078;P61205

>sp|P84078|ARF1_MOUSE

ADP−ribosylation factor 1

GN=Arf1

Med H1/2: 13.8 days

Q5 H1/2: 9.4 days

Q95 H1/2: 19.2 days

reliable model fit: yes

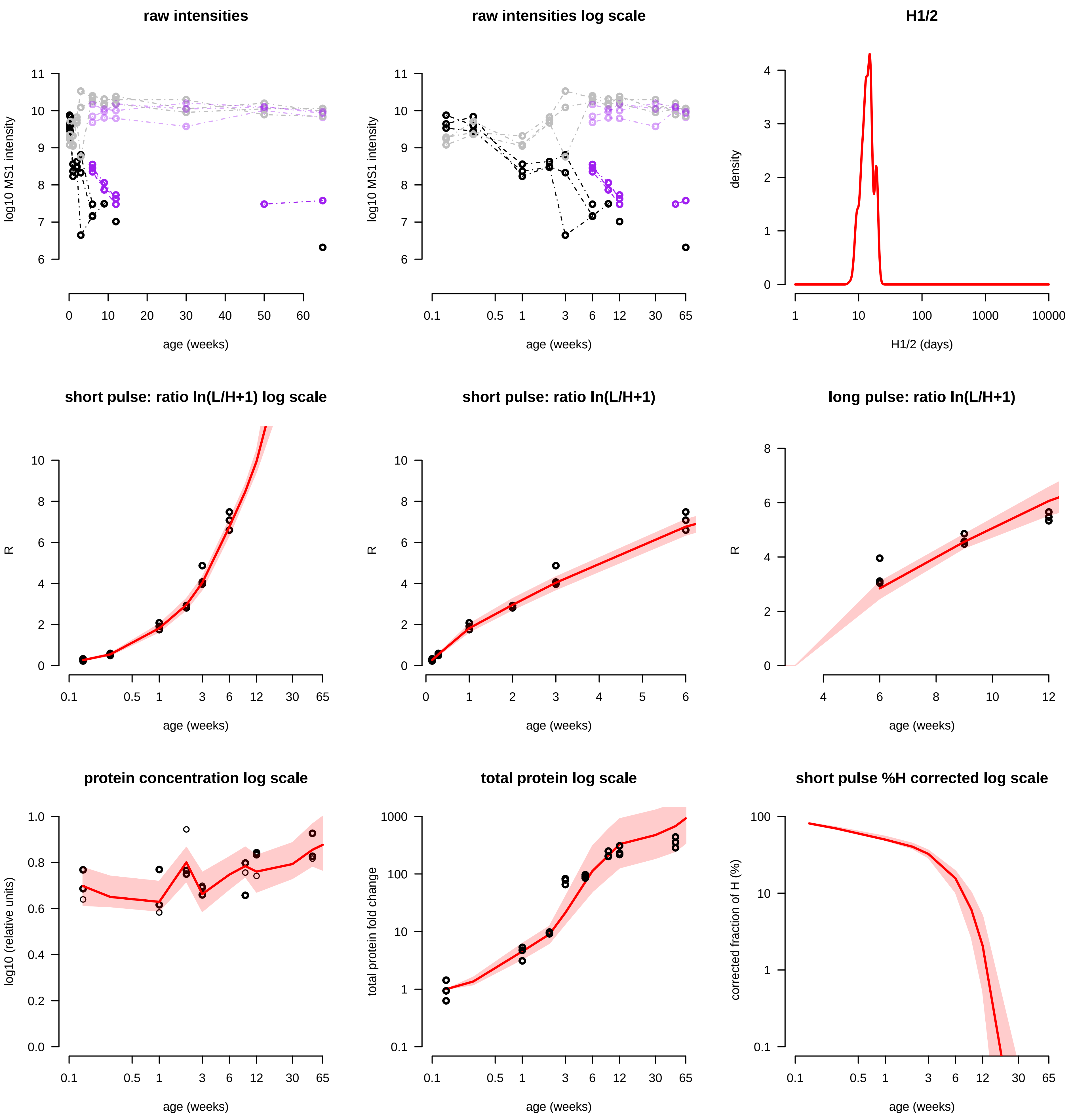

Q9JII6

>sp|Q9JII6|AK1A1_MOUSE

Aldo−keto reductase family 1 member A1

GN=Akr1a1

Med H1/2: 28.8 days

Q5 H1/2: 21.7 days

Q95 H1/2: 42.5 days

reliable model fit: yes

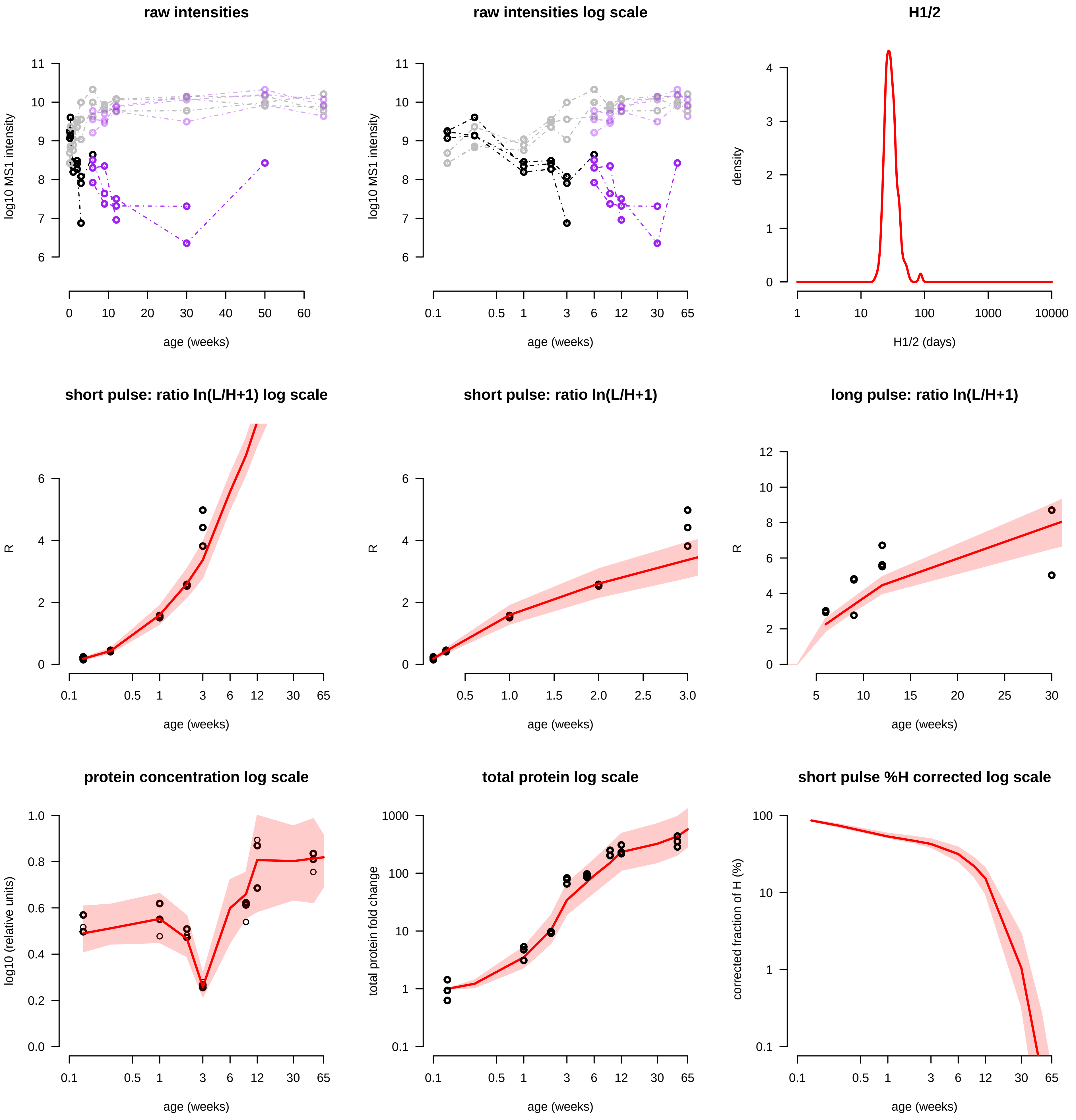

Q61941

>sp|Q61941|NNTM_MOUSE

NAD(P) transhydrogenase, mitochondrial

GN=Nnt

Med H1/2: 12.8 days

Q5 H1/2: 9.5 days

Q95 H1/2: 17.6 days

reliable model fit: yes

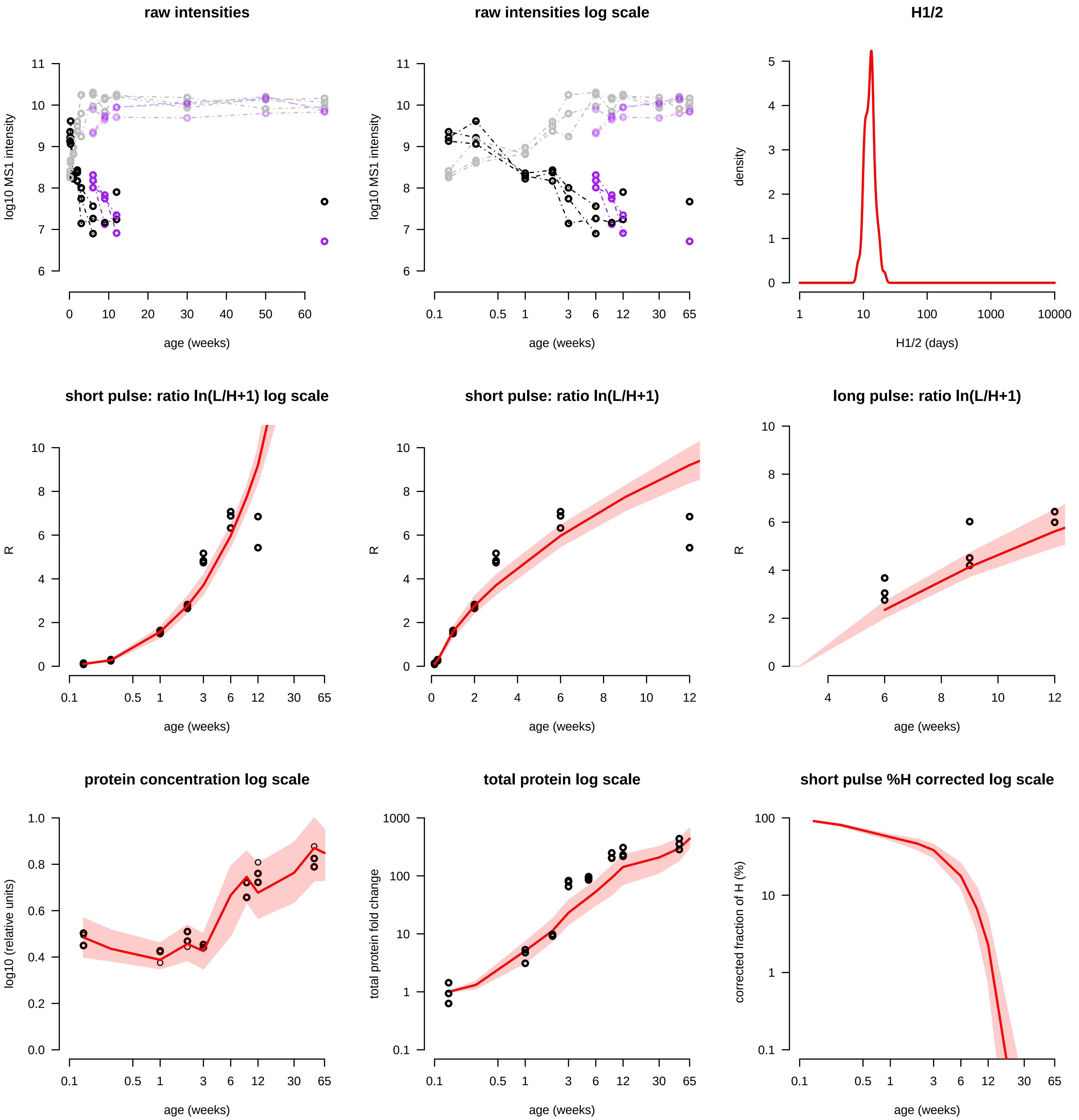

Q9JKF1

>sp|Q9JKF1|IQGA1_MOUSE

Ras GTPase−activating−like protein IQGAP1

GN=Iqgap1

Med H1/2: 7 days

Q5 H1/2: 6 days

Q95 H1/2: 8.2 days

reliable model fit: yes

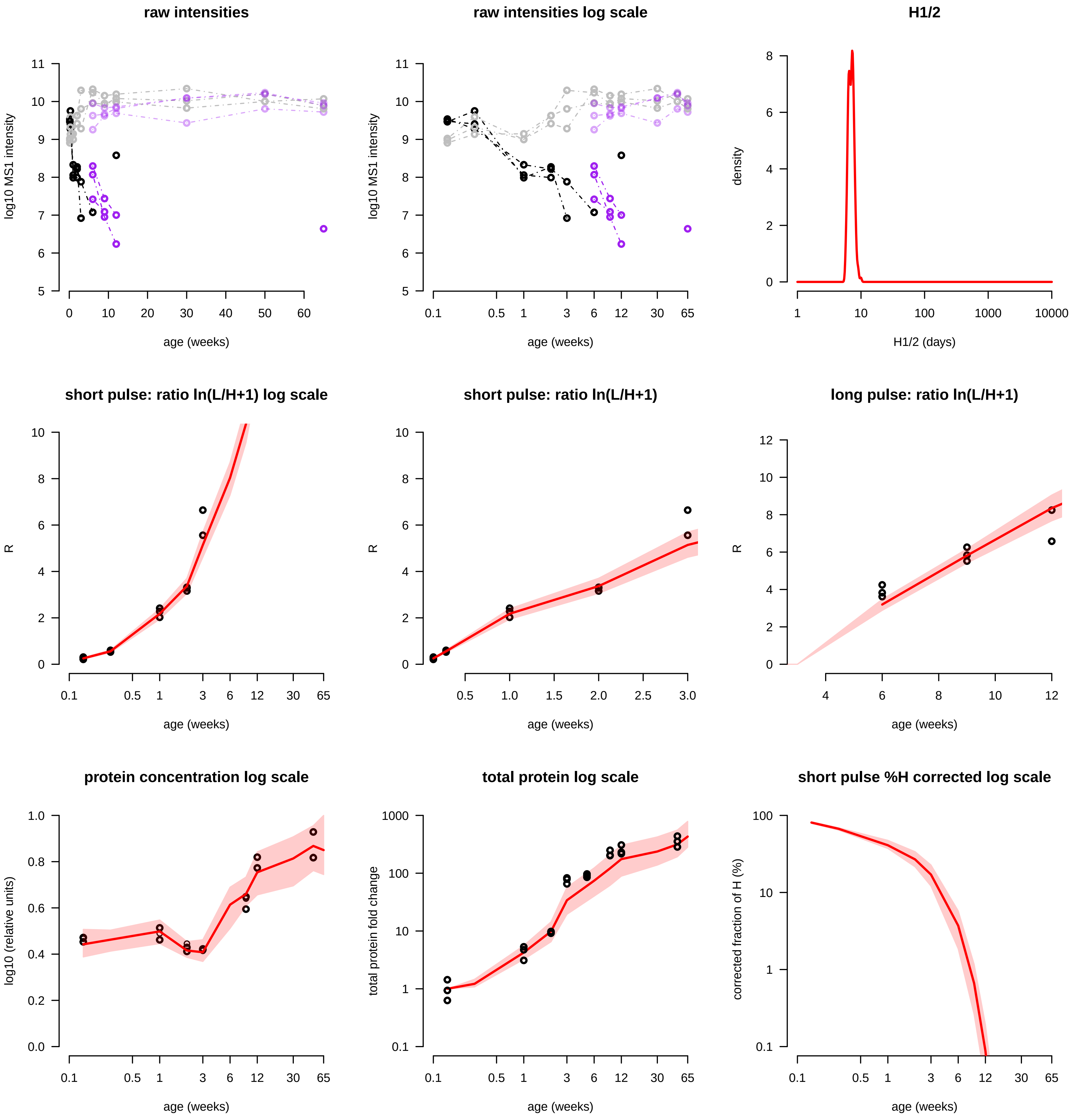

P14869

>sp|P14869|RLA0_MOUSE

60S acidic ribosomal protein P0

GN=Rplp0

Med H1/2: 11.3 days

Q5 H1/2: 9.6 days

Q95 H1/2: 13.8 days

reliable model fit: yes

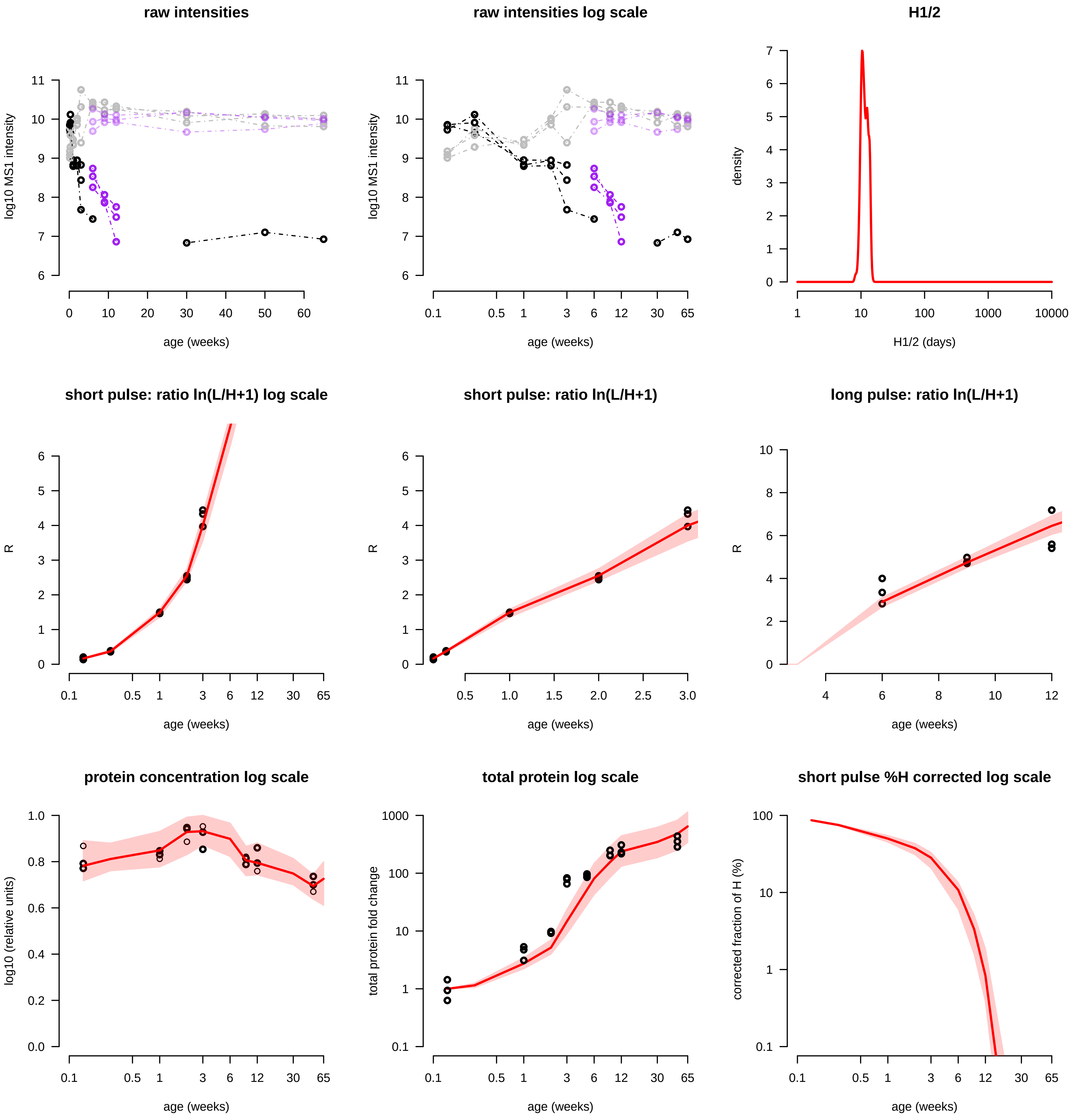

Q6IRU2

>sp|Q6IRU2|TPM4_MOUSE

Tropomyosin alpha−4 chain

GN=Tpm4

Med H1/2: 11 days

Q5 H1/2: 9.3 days

Q95 H1/2: 13.6 days

reliable model fit: yes

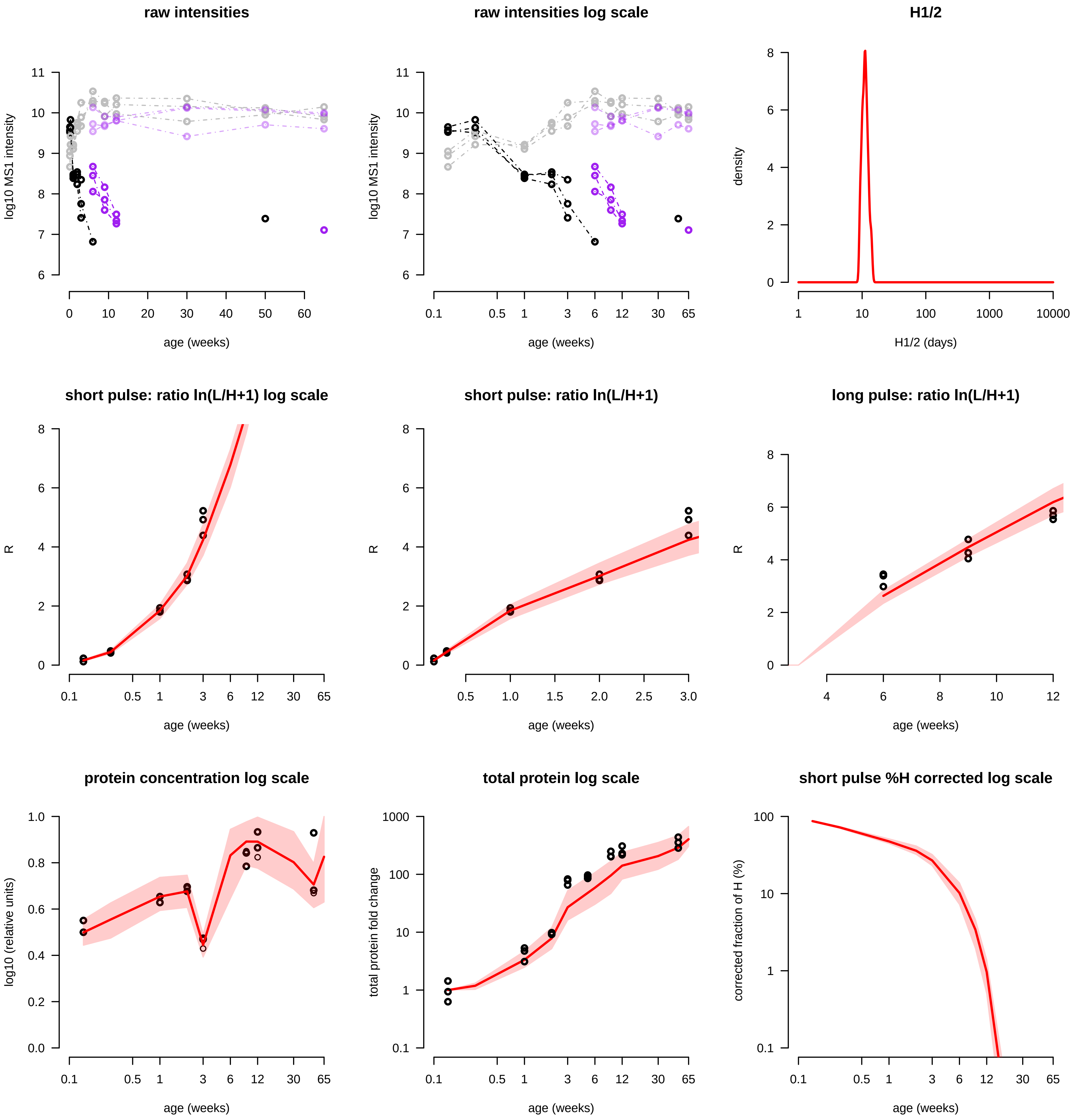

O88342

>sp|O88342|WDR1_MOUSE

WD repeat−containing protein 1

GN=Wdr1

Med H1/2: 7.1 days

Q5 H1/2: 6.2 days

Q95 H1/2: 9.1 days

reliable model fit: yes

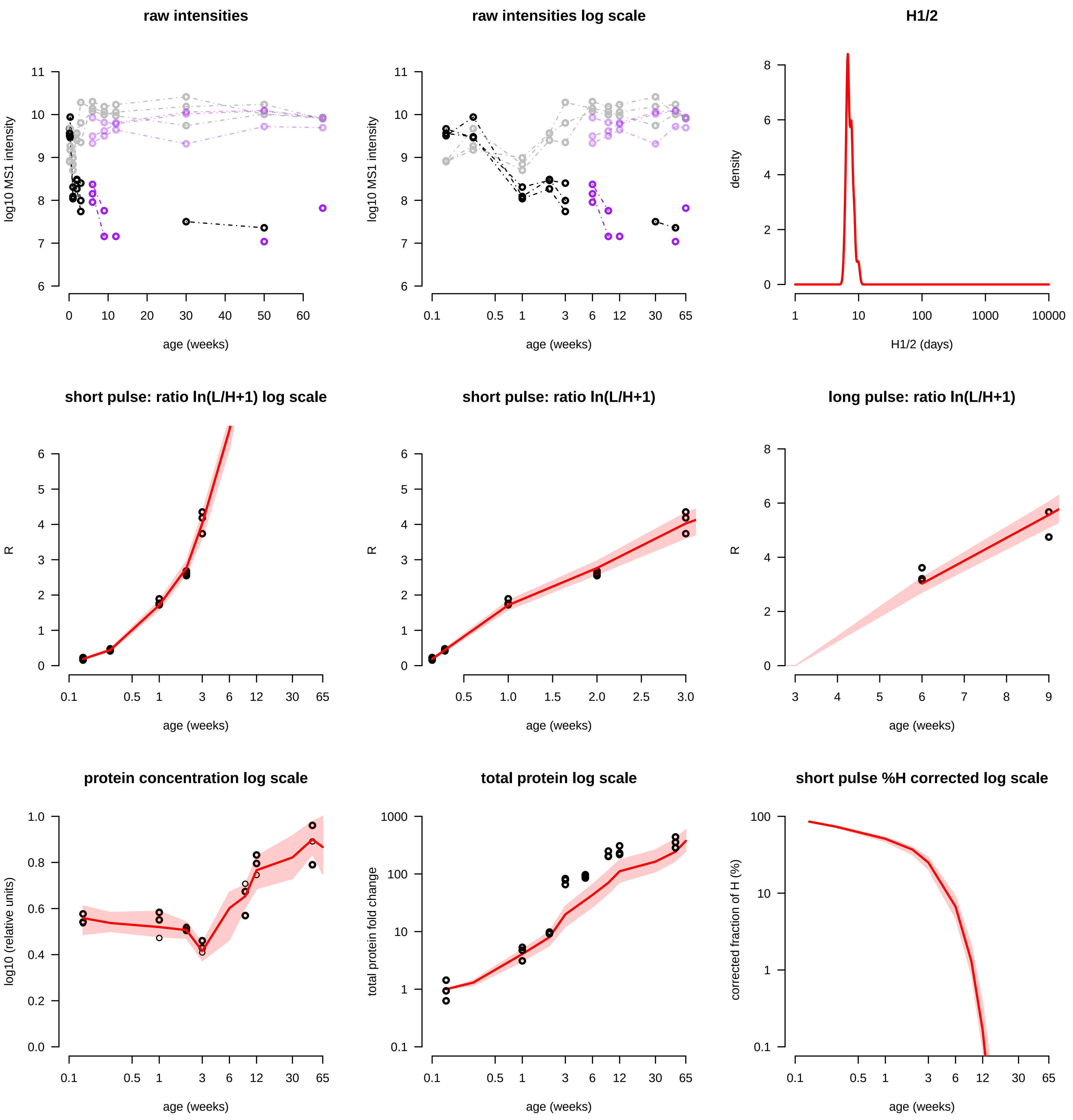

P12970

>sp|P12970|RL7A_MOUSE

60S ribosomal protein L7a

GN=Rpl7a

Med H1/2: 10.1 days

Q5 H1/2: 8.4 days

Q95 H1/2: 12.8 days

reliable model fit: yes

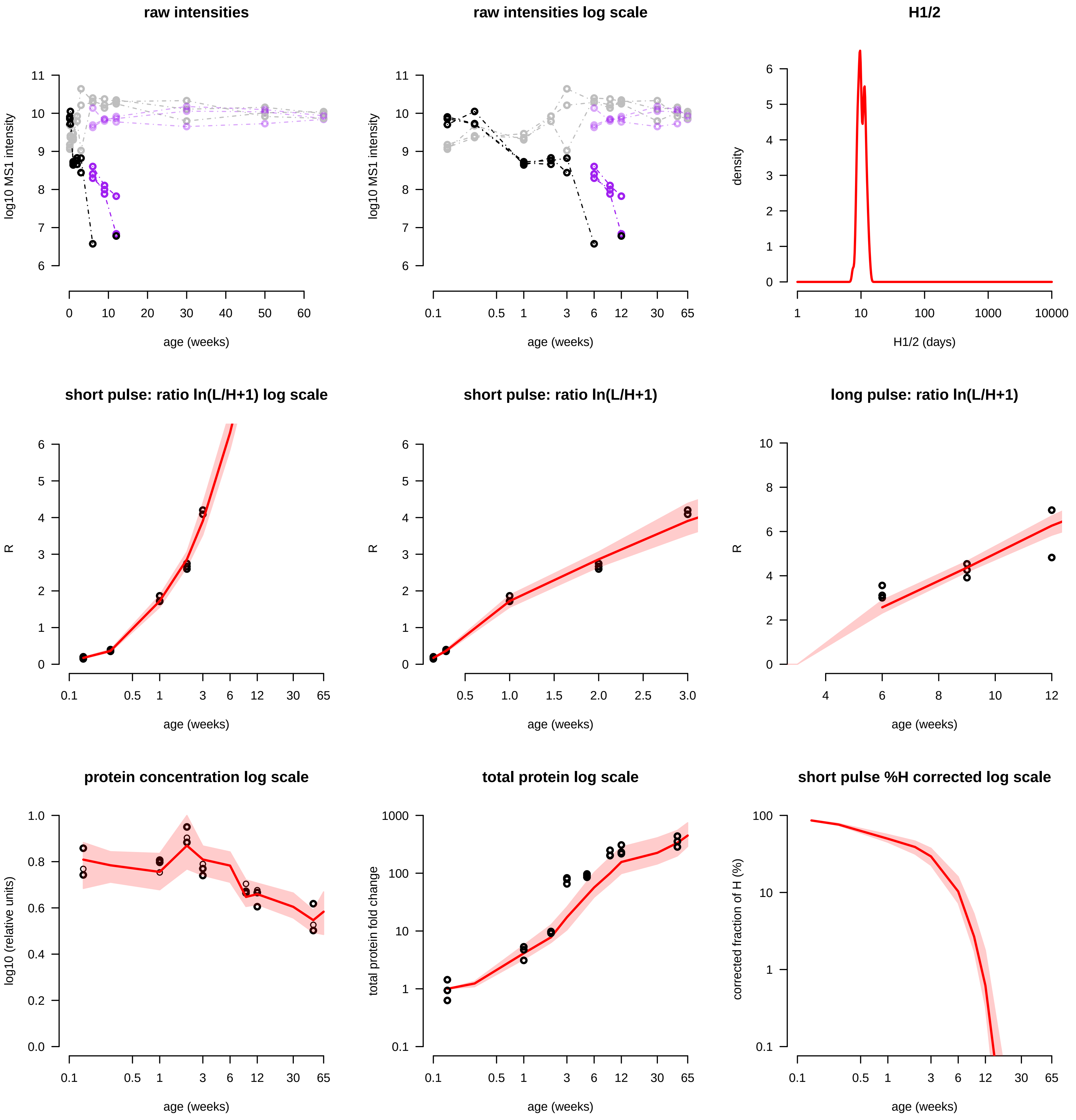

Q61598;Q61598−2

>sp|Q61598|GDIB_MOUSE

Rab GDP dissociation inhibitor beta

GN=Gdi2

Med H1/2: 236.4 days

Q5 H1/2: 93.5 days

Q95 H1/2: 1592.5 days

reliable model fit: yes

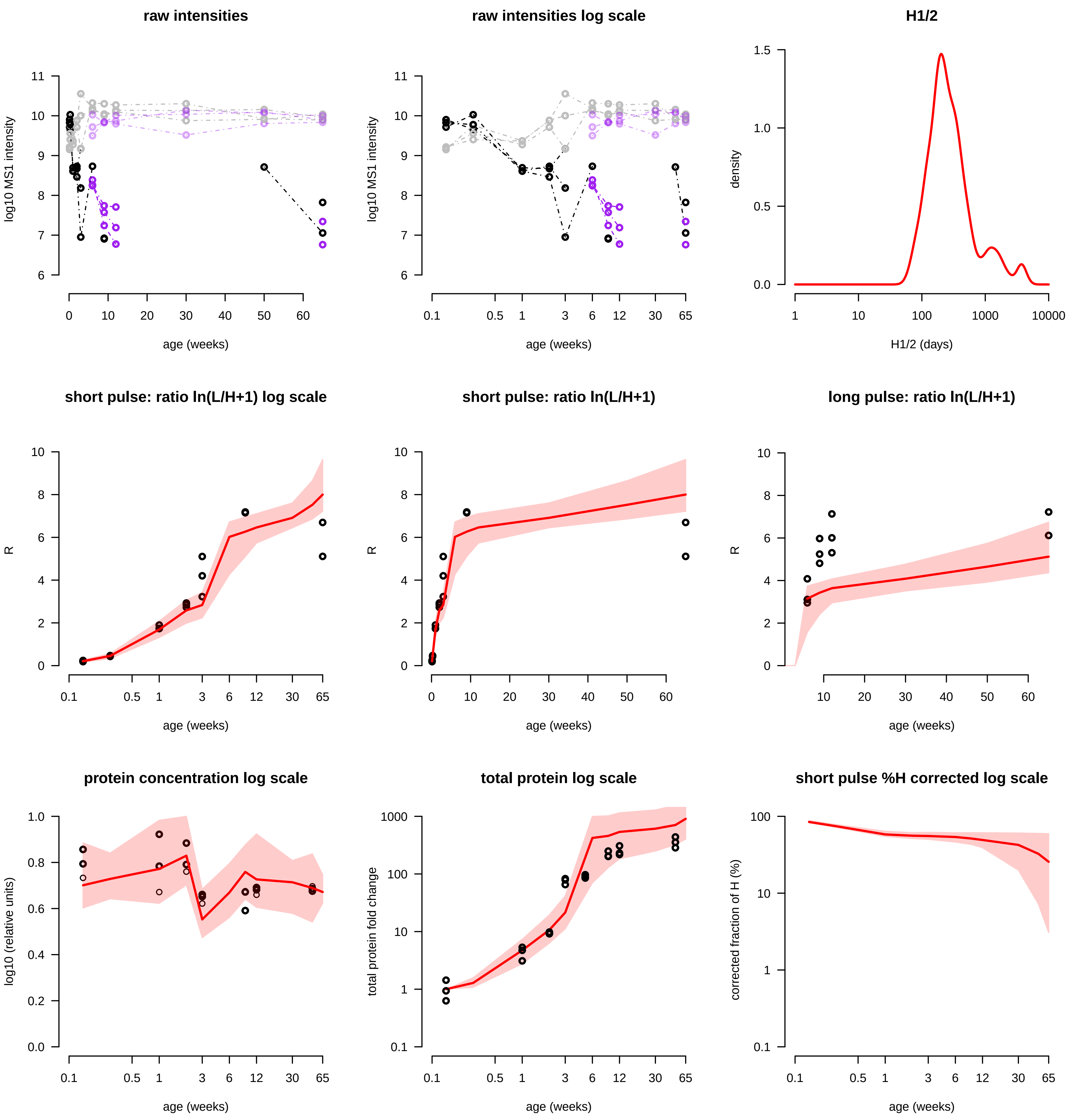

Q99N16

>sp|Q99N16|CP4F3_MOUSE

Cytochrome P450 4F3

GN=Cyp4f3

Med H1/2: 23.2 days

Q5 H1/2: 12.5 days

Q95 H1/2: 72.4 days

reliable model fit: yes

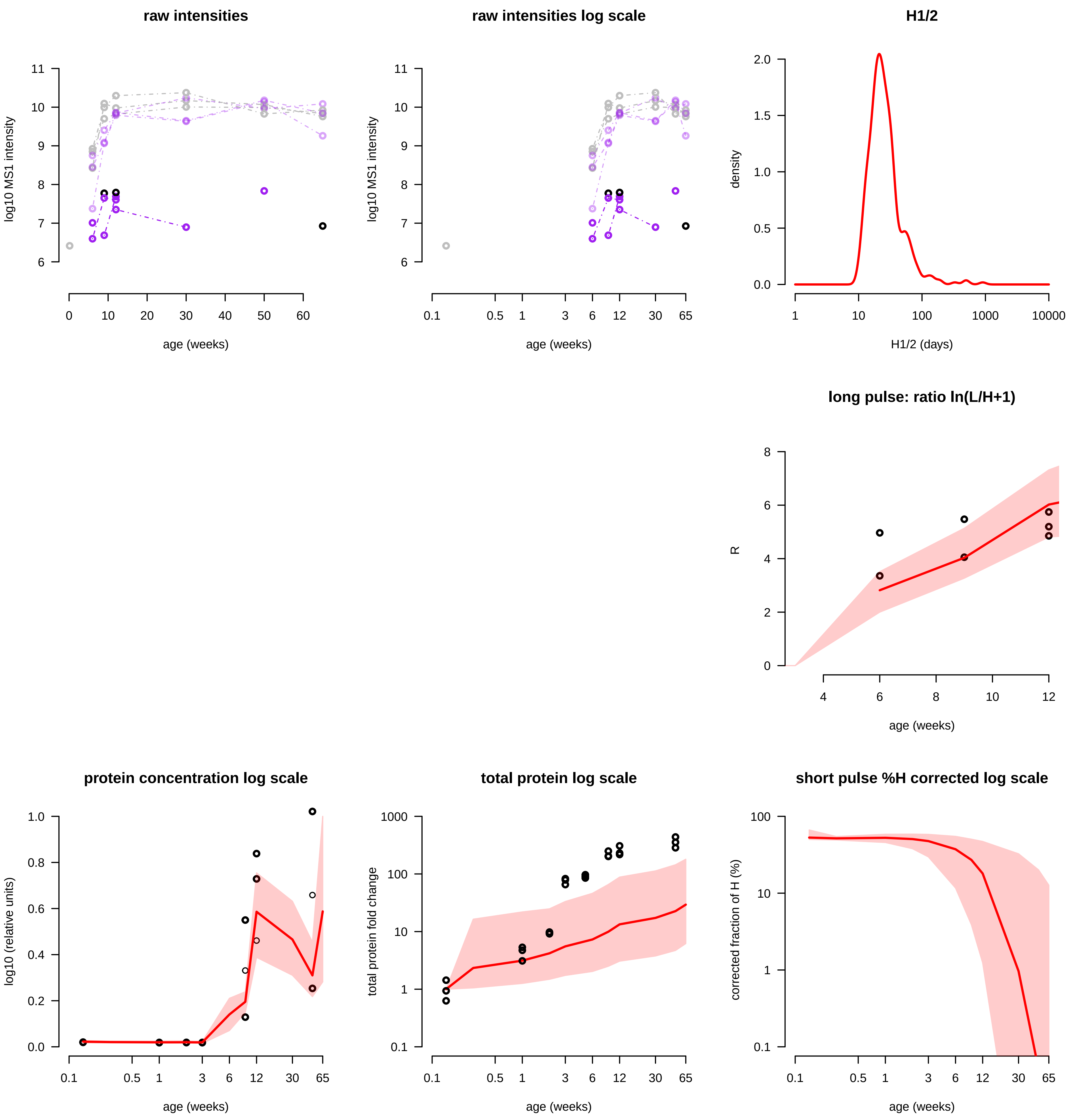

P00329

>sp|P00329|ADH1_MOUSE

Alcohol dehydrogenase 1

GN=Adh1

Med H1/2: 11.1 days

Q5 H1/2: 8 days

Q95 H1/2: 15 days

reliable model fit: yes

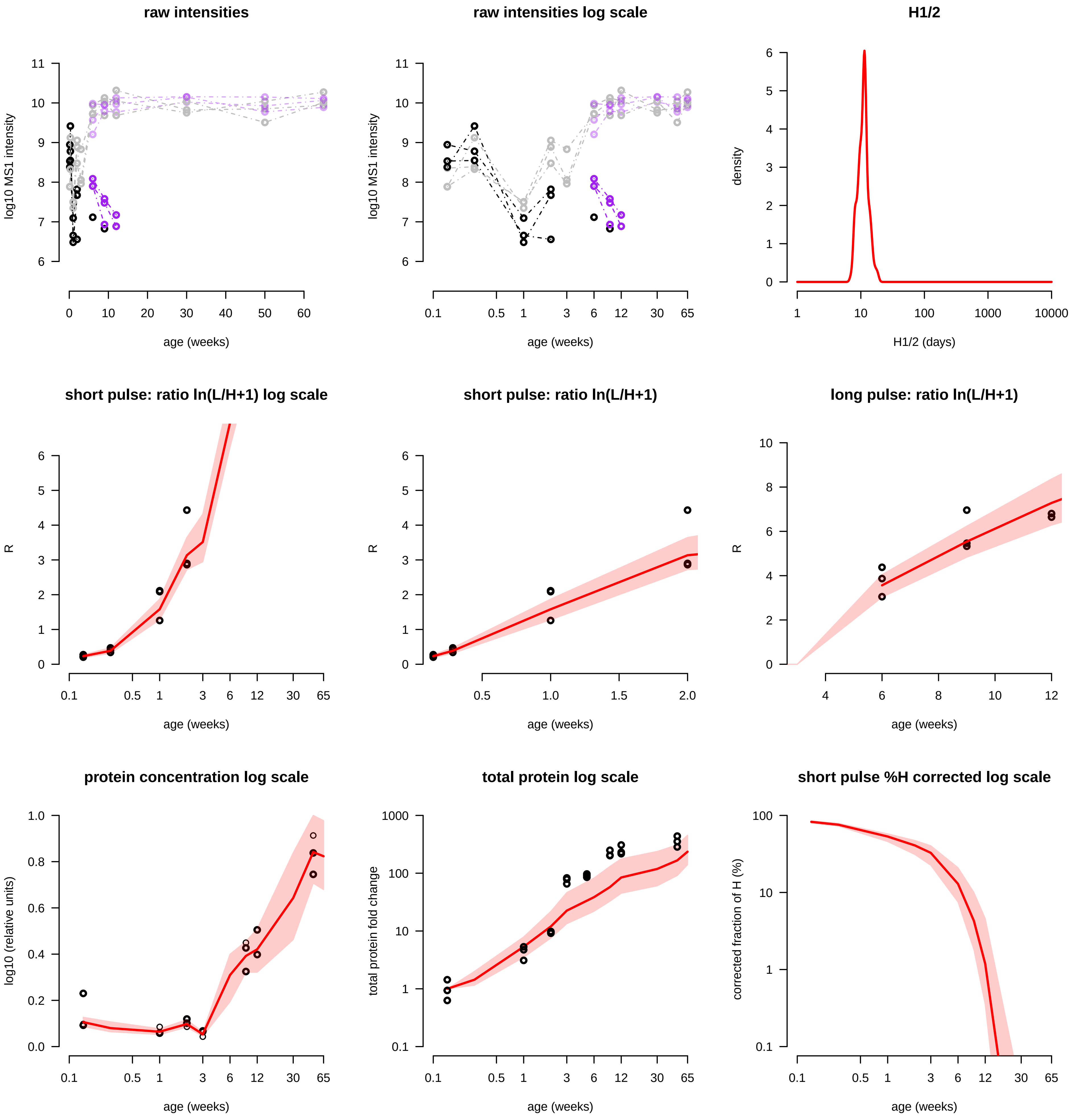

Q19LI2

>sp|Q19LI2|A1BG_MOUSE

Alpha−1B−glycoprotein

GN=A1bg

Med H1/2: 1772.5 days

Q5 H1/2: 519.6 days

Q95 H1/2: 14871.7 days

reliable model fit: no

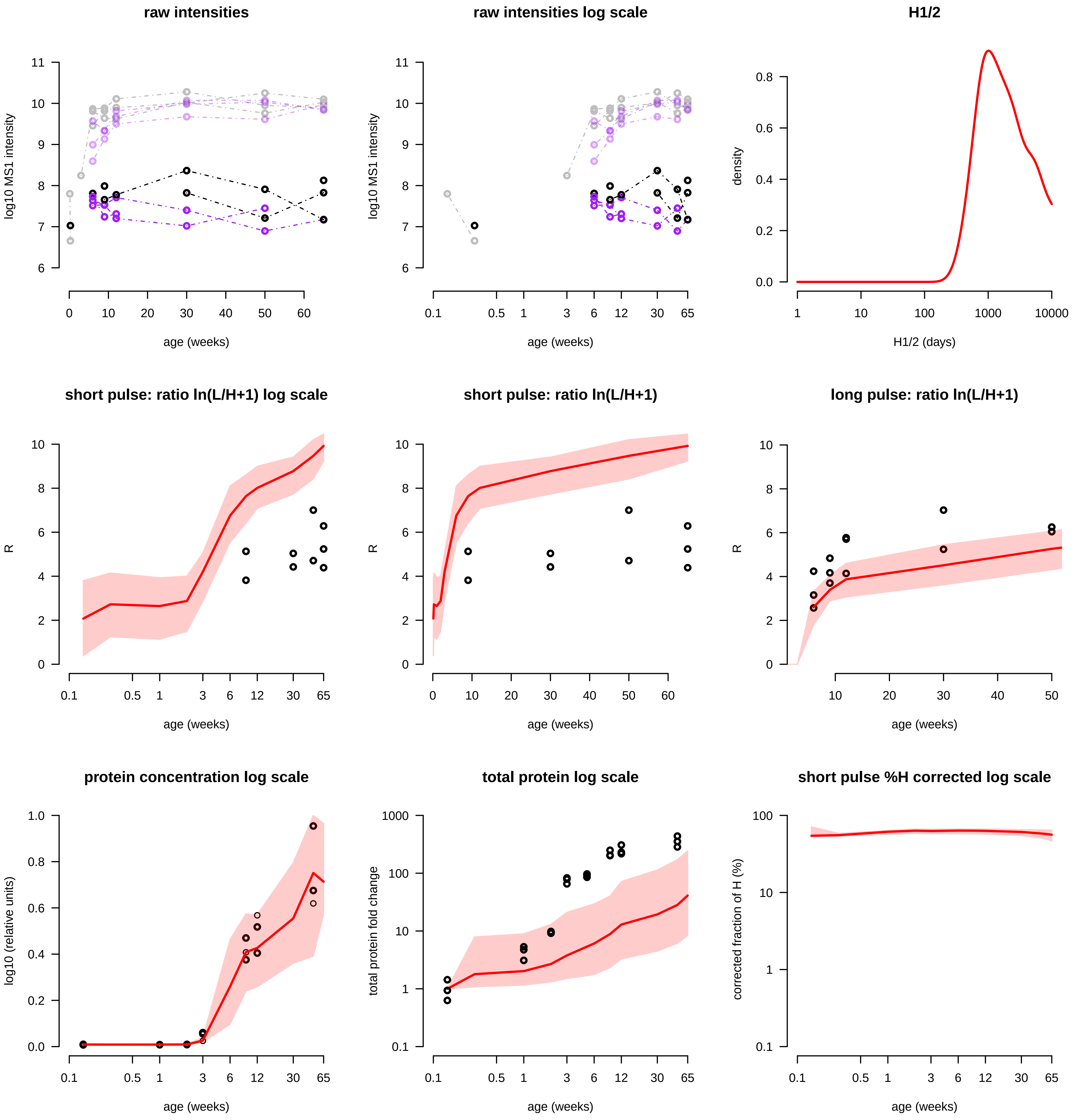

Q9D172

>sp|Q9D172|GAL3A_MOUSE

ıtamine amidotransferase−like class 1 domain−containing protein 3A, mitochond

GN=Gatd3a

Med H1/2: 11.5 days

Q5 H1/2: 9.2 days

Q95 H1/2: 14.5 days

reliable model fit: yes

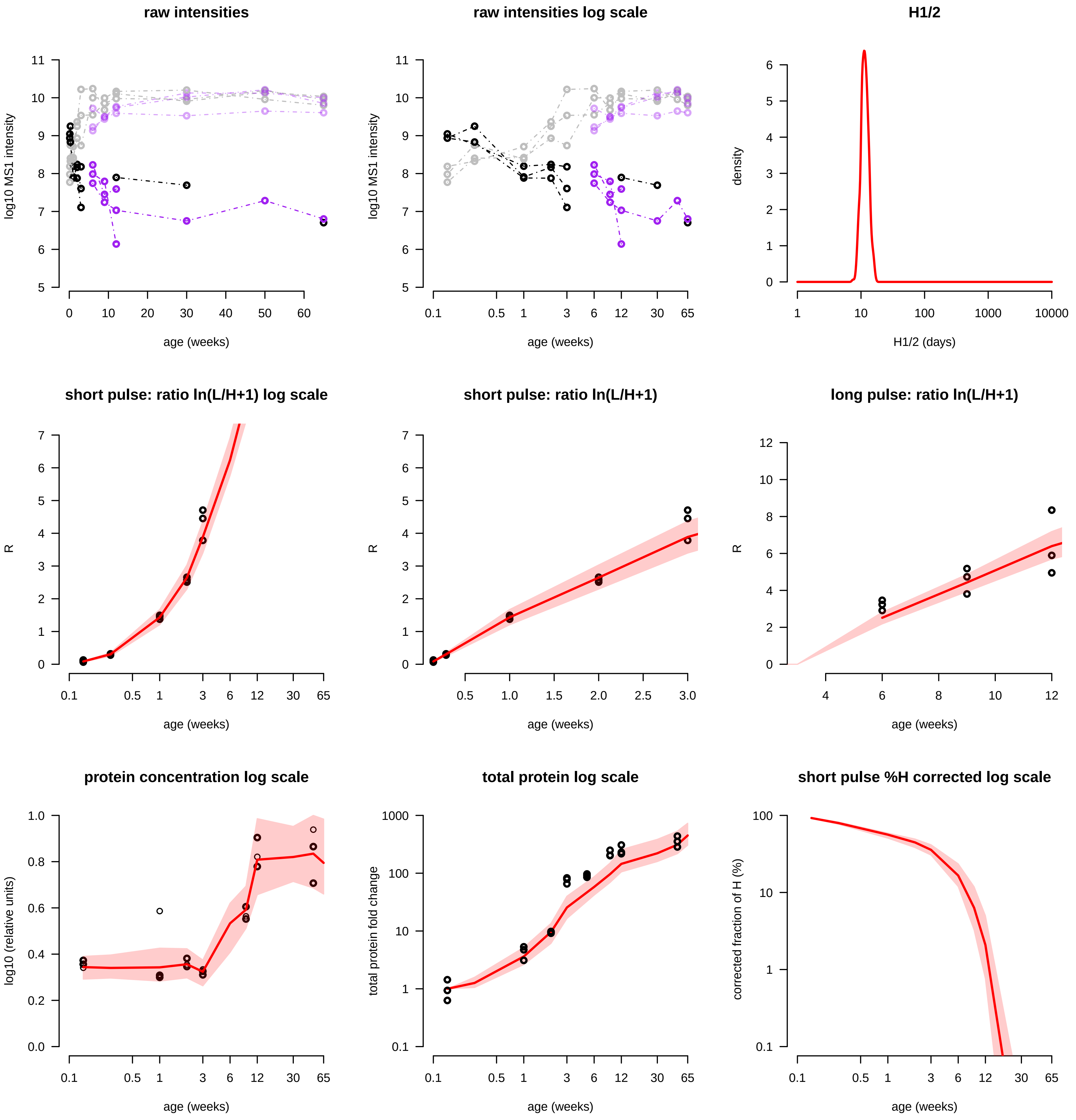

P99024

>sp|P99024|TBB5_MOUSE

Tubulin beta−5 chain

GN=Tubb5

Med H1/2: 15.5 days

Q5 H1/2: 10.5 days

Q95 H1/2: 22.9 days

reliable model fit: yes

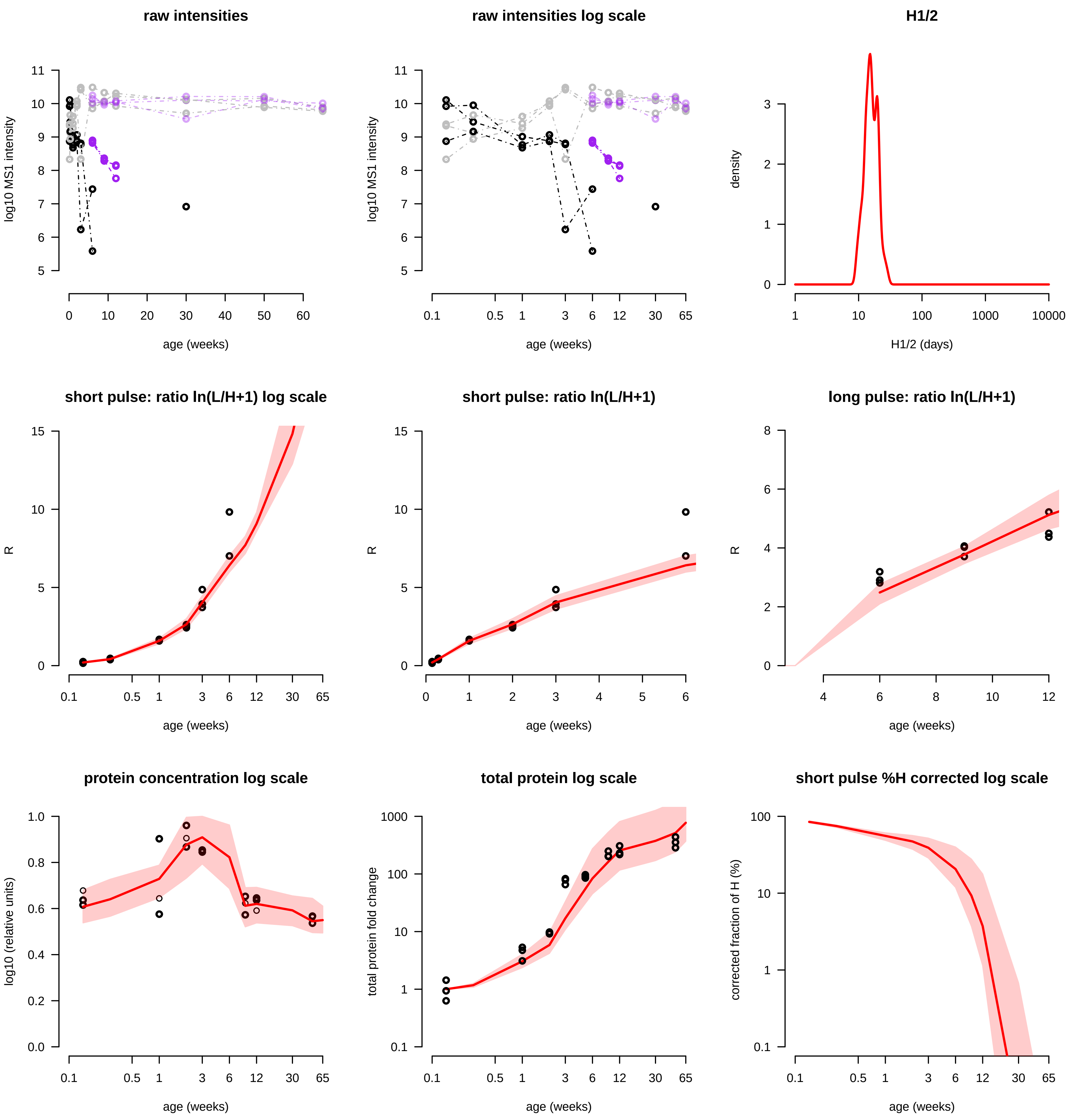

Q9DB20

>sp|Q9DB20|ATPO_MOUSE

ATP synthase subunit O, mitochondrial

GN=Atp5po

Med H1/2: 14.5 days

Q5 H1/2: 11.3 days

Q95 H1/2: 17.6 days

reliable model fit: yes

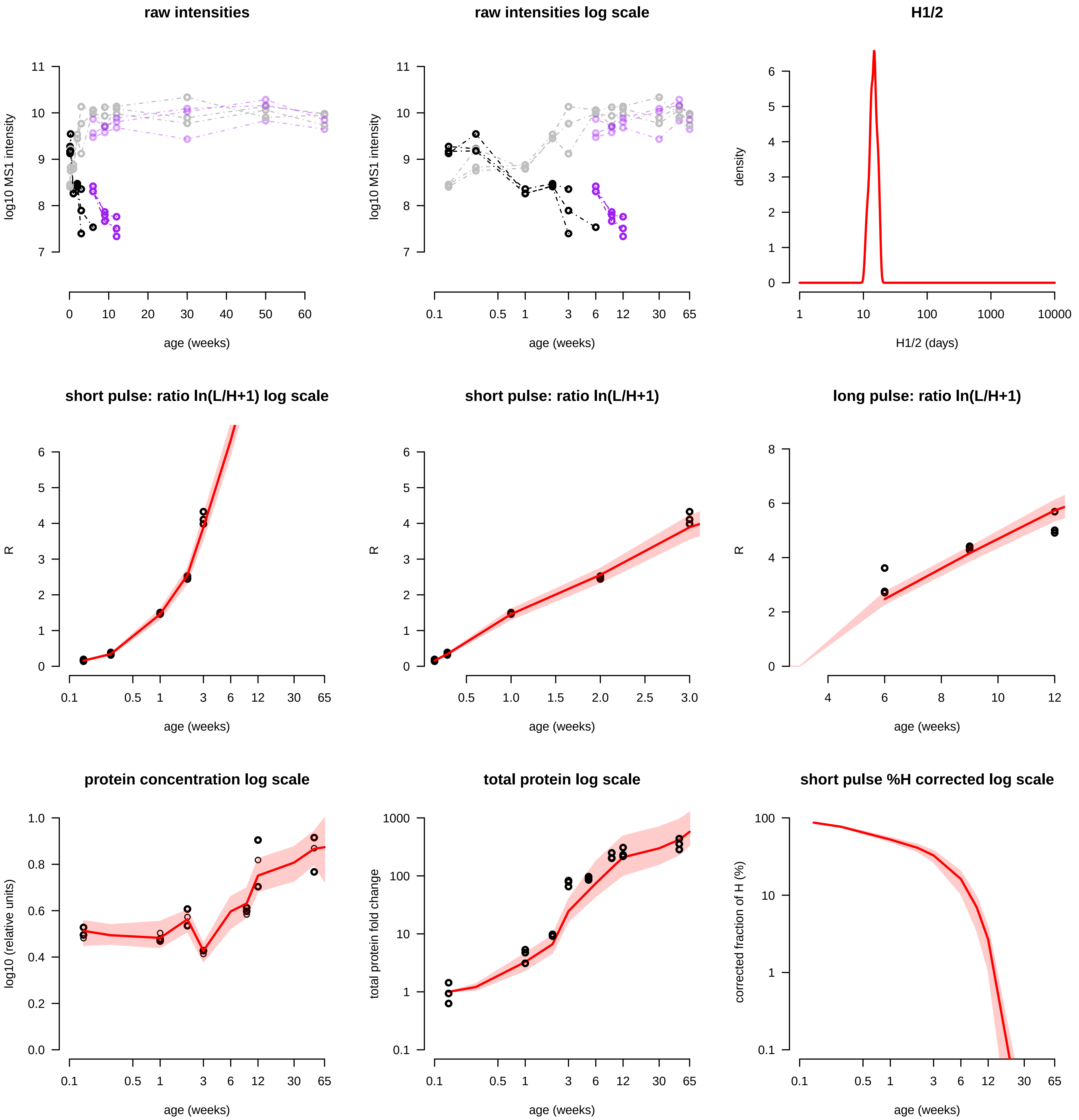

Q60931

>sp|Q60931|VDAC3_MOUSE

Voltage−dependent anion−selective channel protein 3

GN=Vdac3

Med H1/2: 11.4 days

Q5 H1/2: 10 days

Q95 H1/2: 14.8 days

reliable model fit: yes

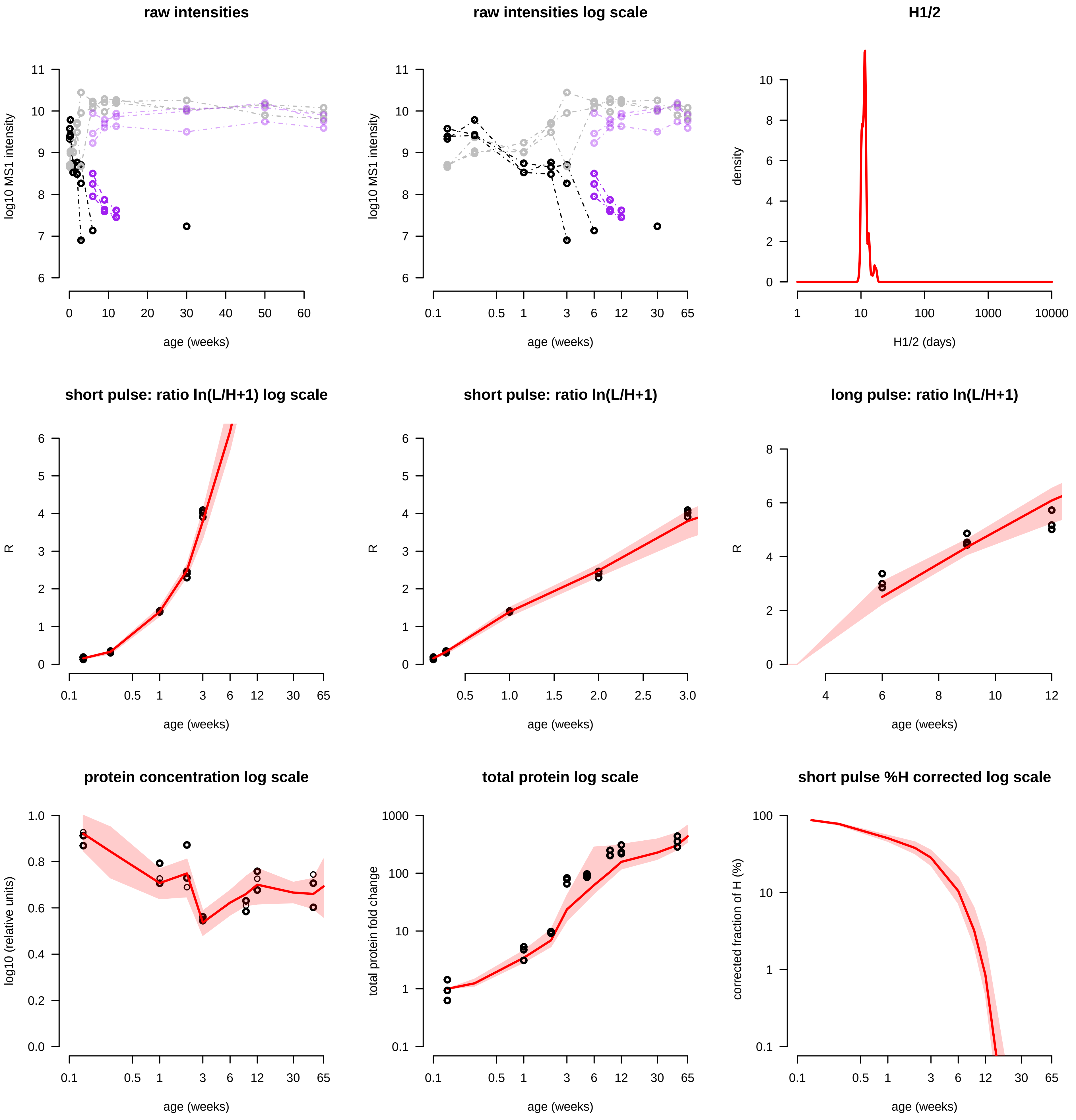

Q8K2B3

>sp|Q8K2B3|SDHA_MOUSE

Succinate dehydrogenase [ubiquinone] flavoprotein subunit, mitochondrial

GN=Sdha

Med H1/2: 10.2 days

Q5 H1/2: 8.1 days

Q95 H1/2: 13.3 days

reliable model fit: yes

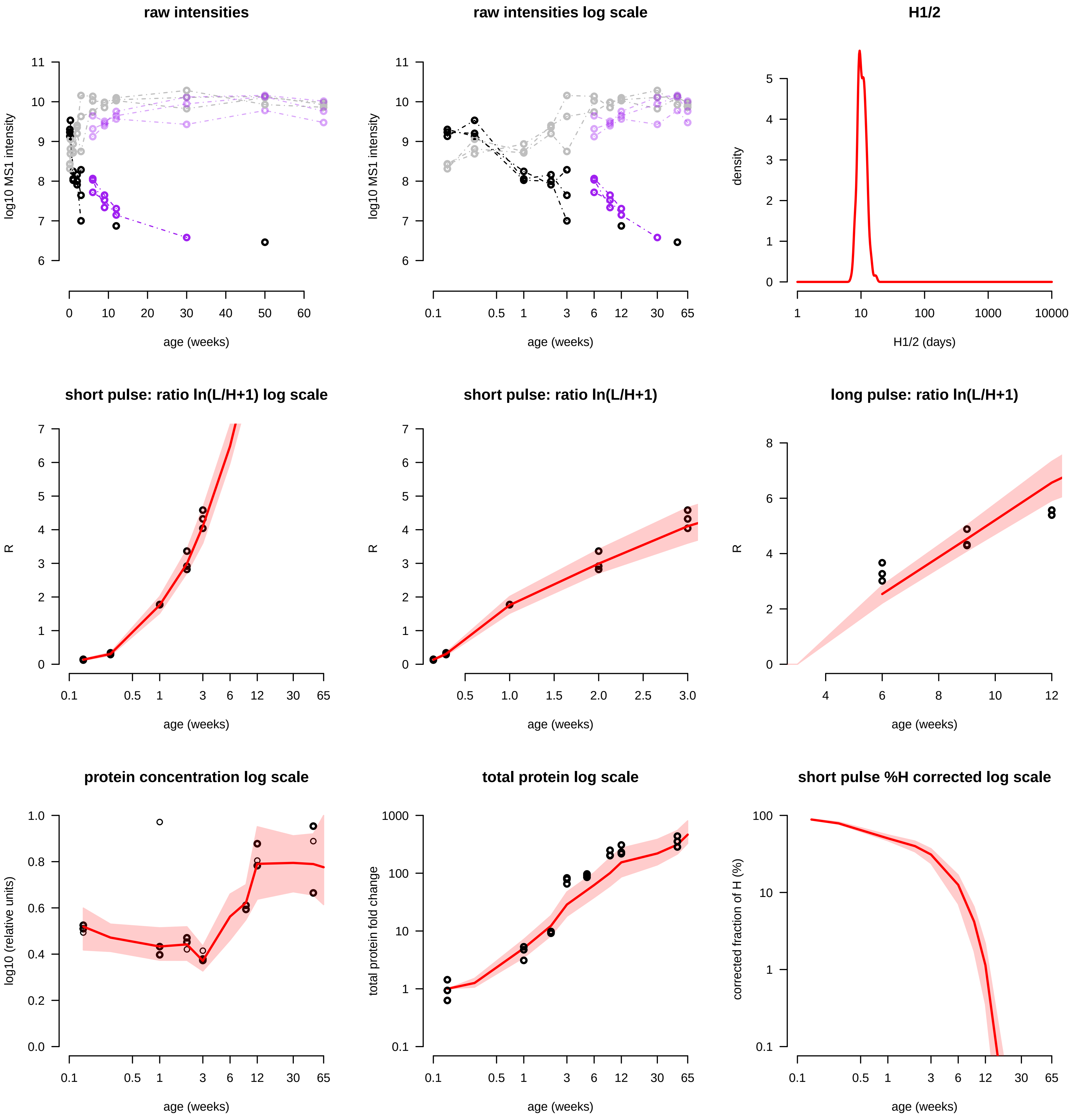

Q9D8E6

>sp|Q9D8E6|RL4_MOUSE

60S ribosomal protein L4

GN=Rpl4

Med H1/2: 15.2 days

Q5 H1/2: 11.3 days

Q95 H1/2: 21.5 days

reliable model fit: yes

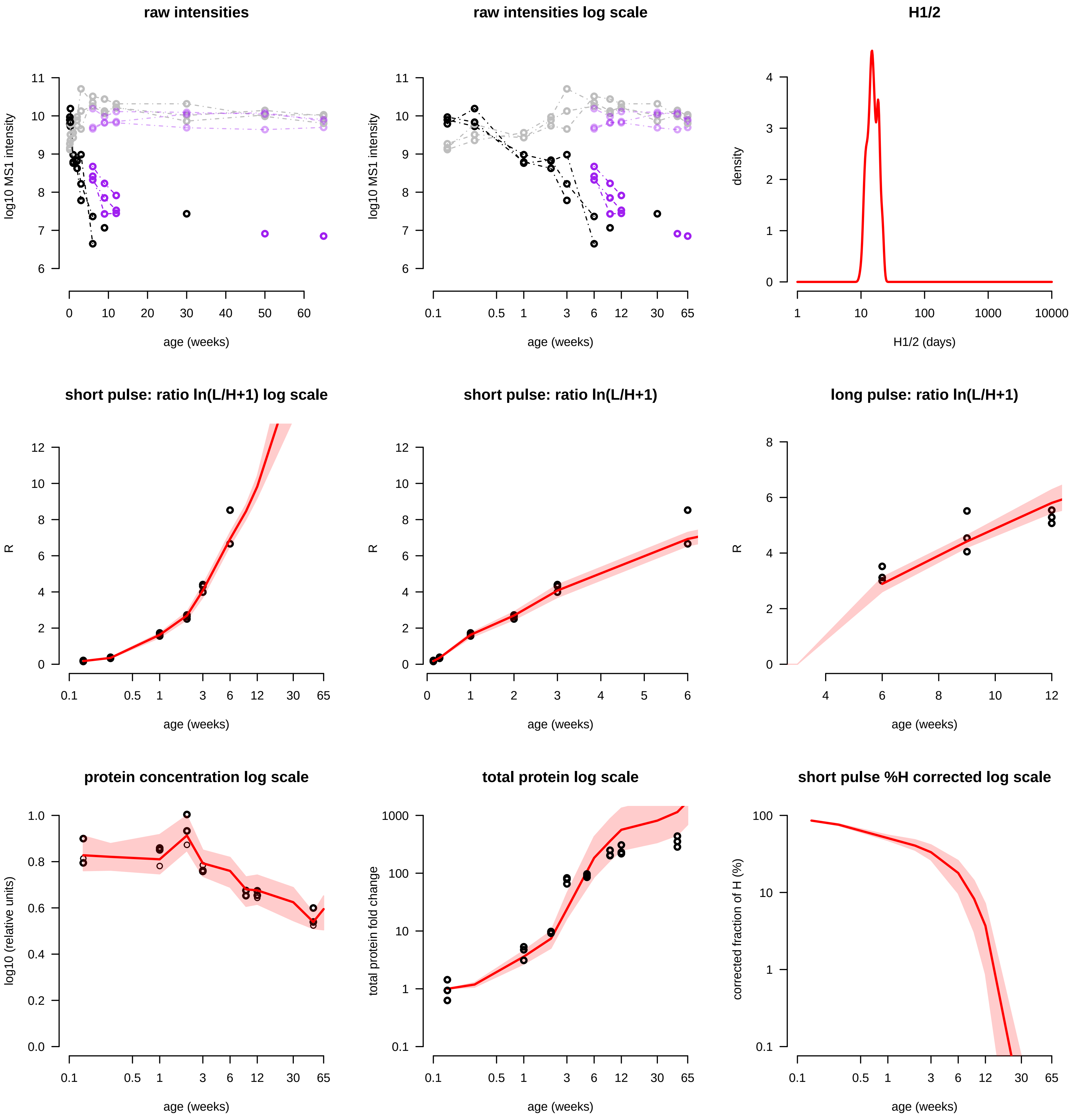

Q8BFR5;Q8BFR5−2

>sp|Q8BFR5|EFTU_MOUSE

Elongation factor Tu, mitochondrial

GN=Tufm

Med H1/2: 588.3 days

Q5 H1/2: 214.5 days

Q95 H1/2: 5642.9 days

reliable model fit: yes

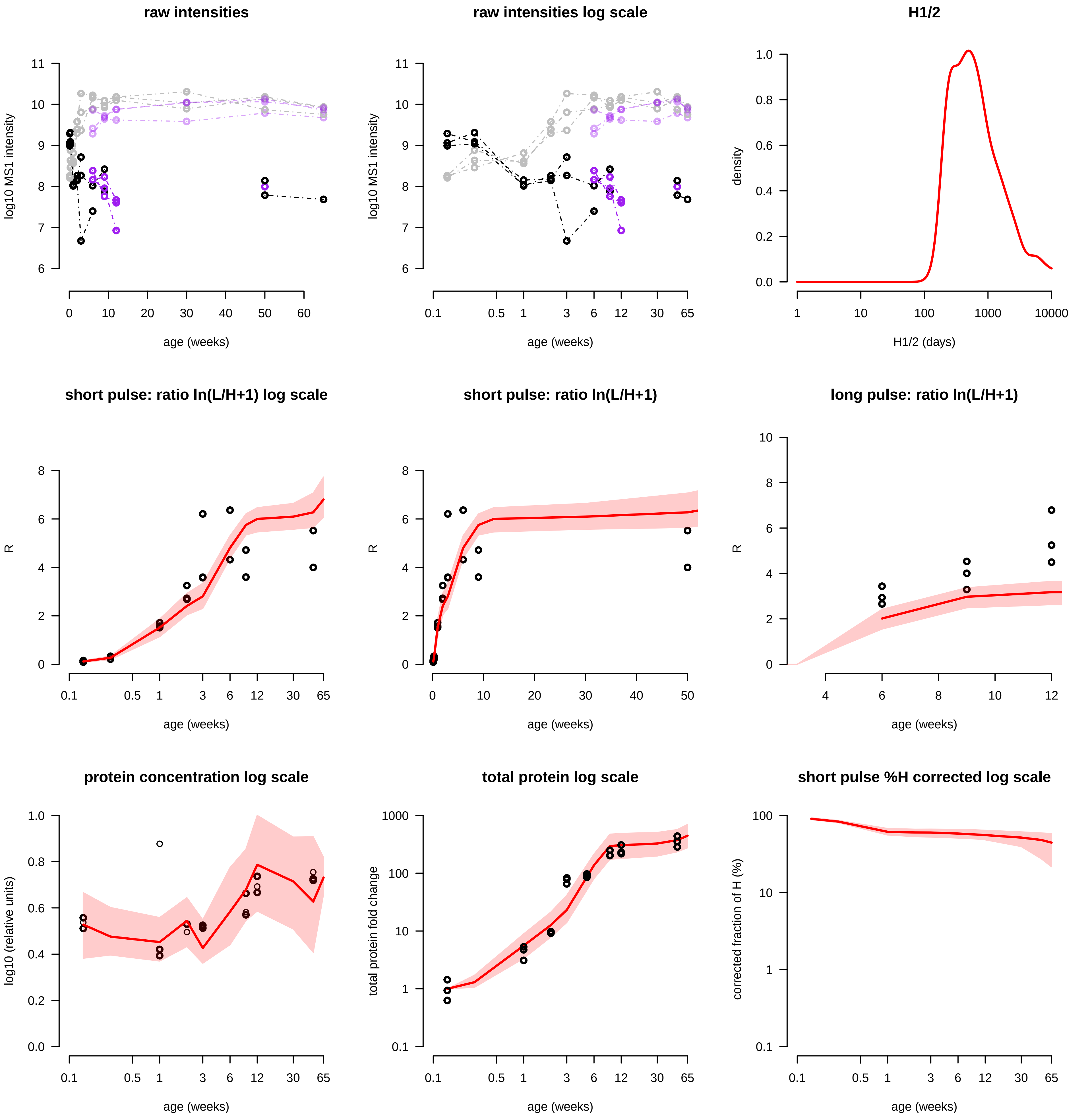

Q01768

>sp|Q01768|NDKB_MOUSE

Nucleoside diphosphate kinase B

GN=Nme2

Med H1/2: 15.3 days

Q5 H1/2: 11.1 days

Q95 H1/2: 20.1 days

reliable model fit: yes

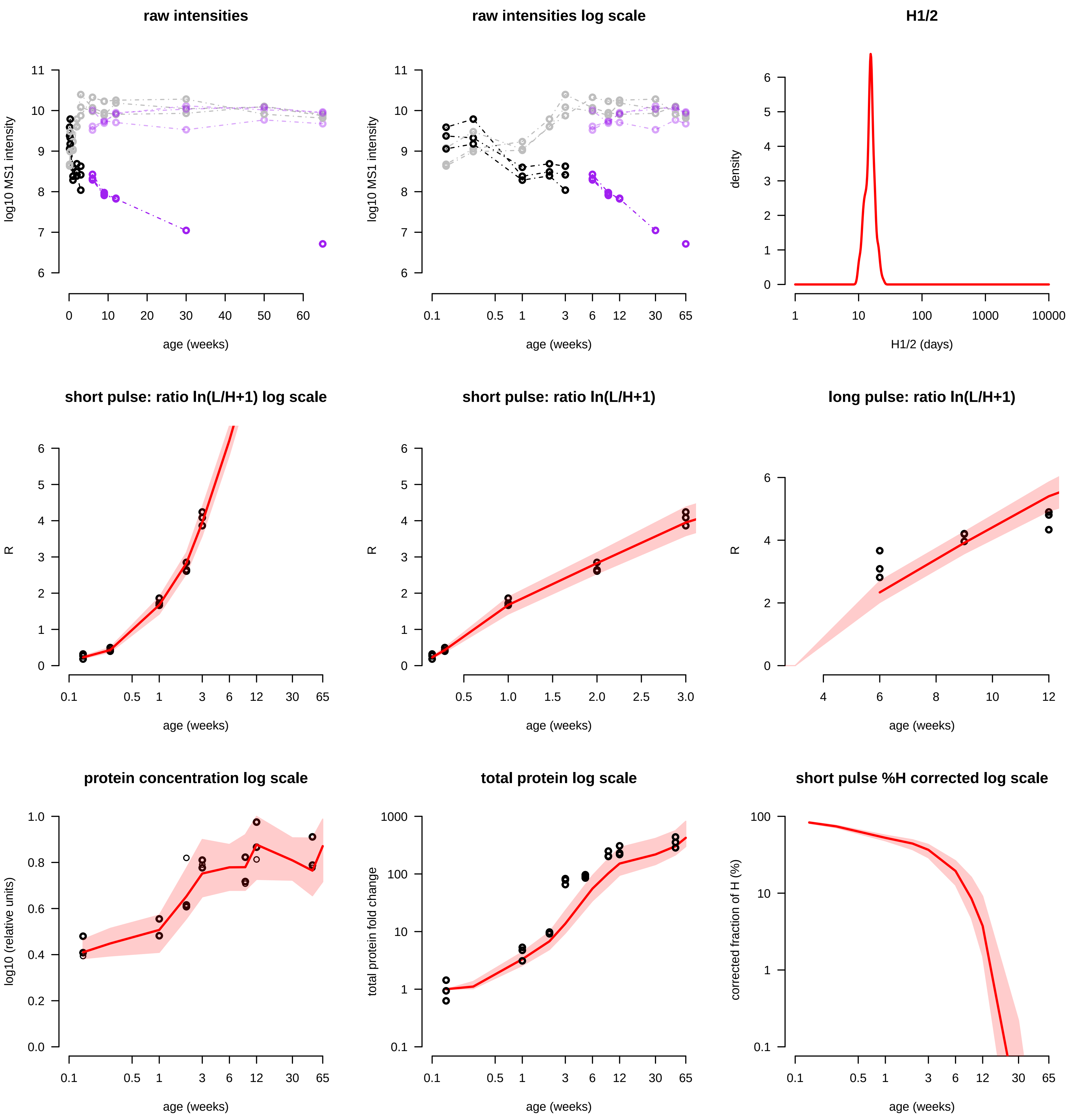

P0DP28;P0DP27;P0DP26

>sp|P0DP28|CALM3_MOUSE

Calmodulin−3

GN=Calm3

Med H1/2: 34 days

Q5 H1/2: 23.4 days

Q95 H1/2: 54.2 days

reliable model fit: yes

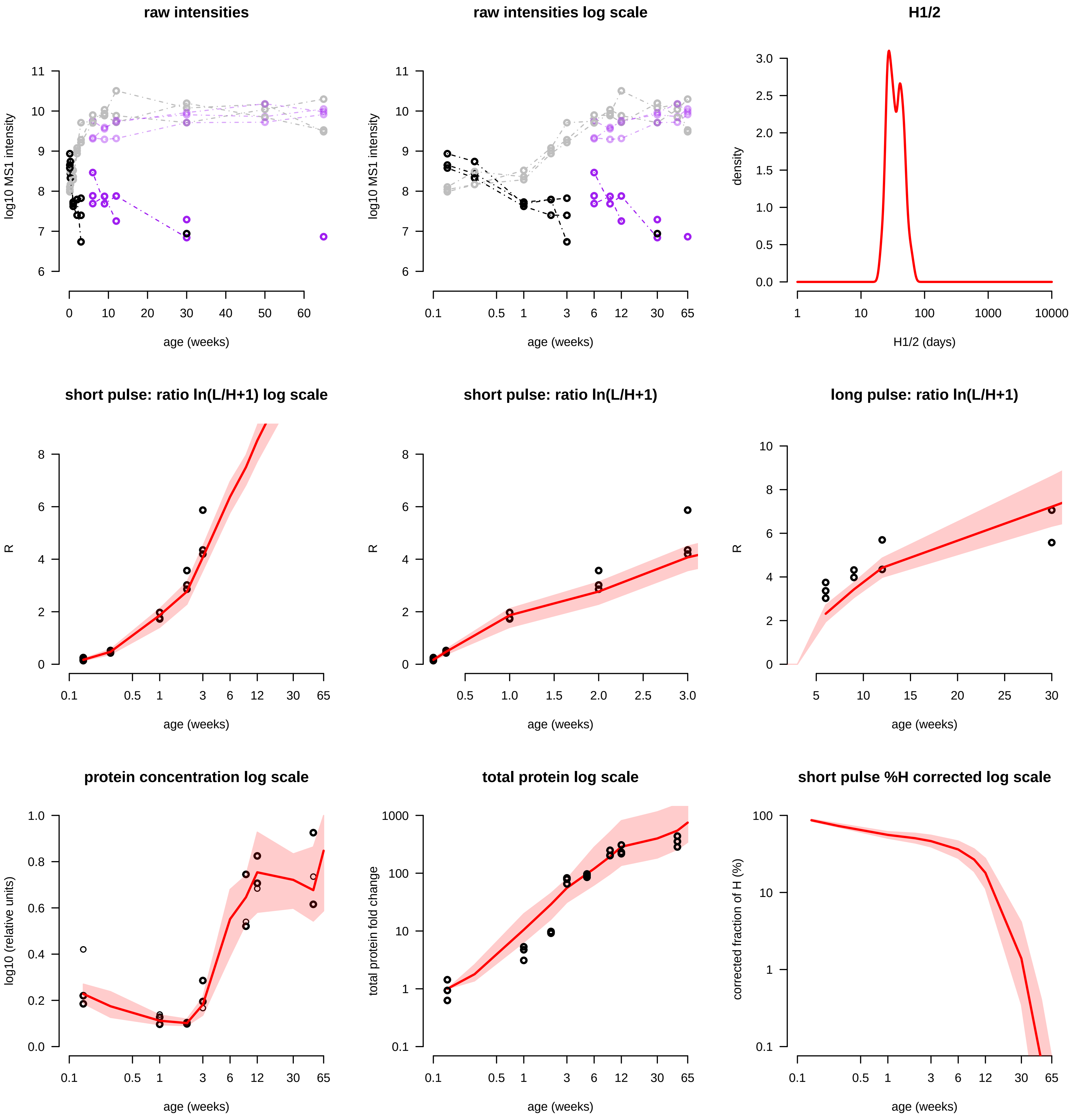

Q9QZQ8

>sp|Q9QZQ8|H2AY_MOUSE

Core histone macro−H2A.1

GN=Macroh2a1

Med H1/2: 13.8 days

Q5 H1/2: 11.5 days

Q95 H1/2: 17.7 days

reliable model fit: yes

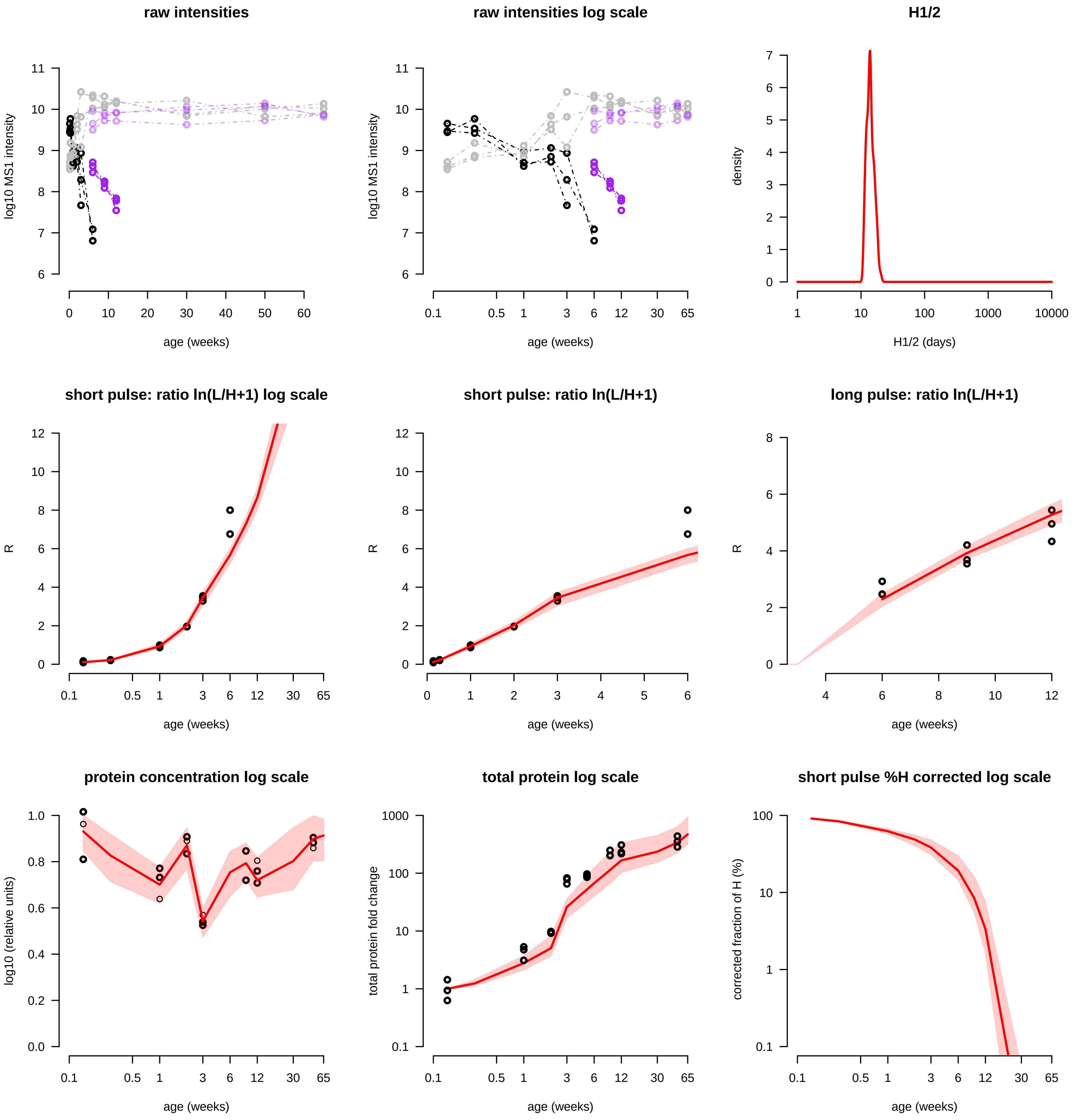

P62897

>sp|P62897|CYC_MOUSE

Cytochrome c, somatic

GN=Cycs

Med H1/2: 147.2 days

Q5 H1/2: 59.5 days

Q95 H1/2: 1352.6 days

reliable model fit: yes

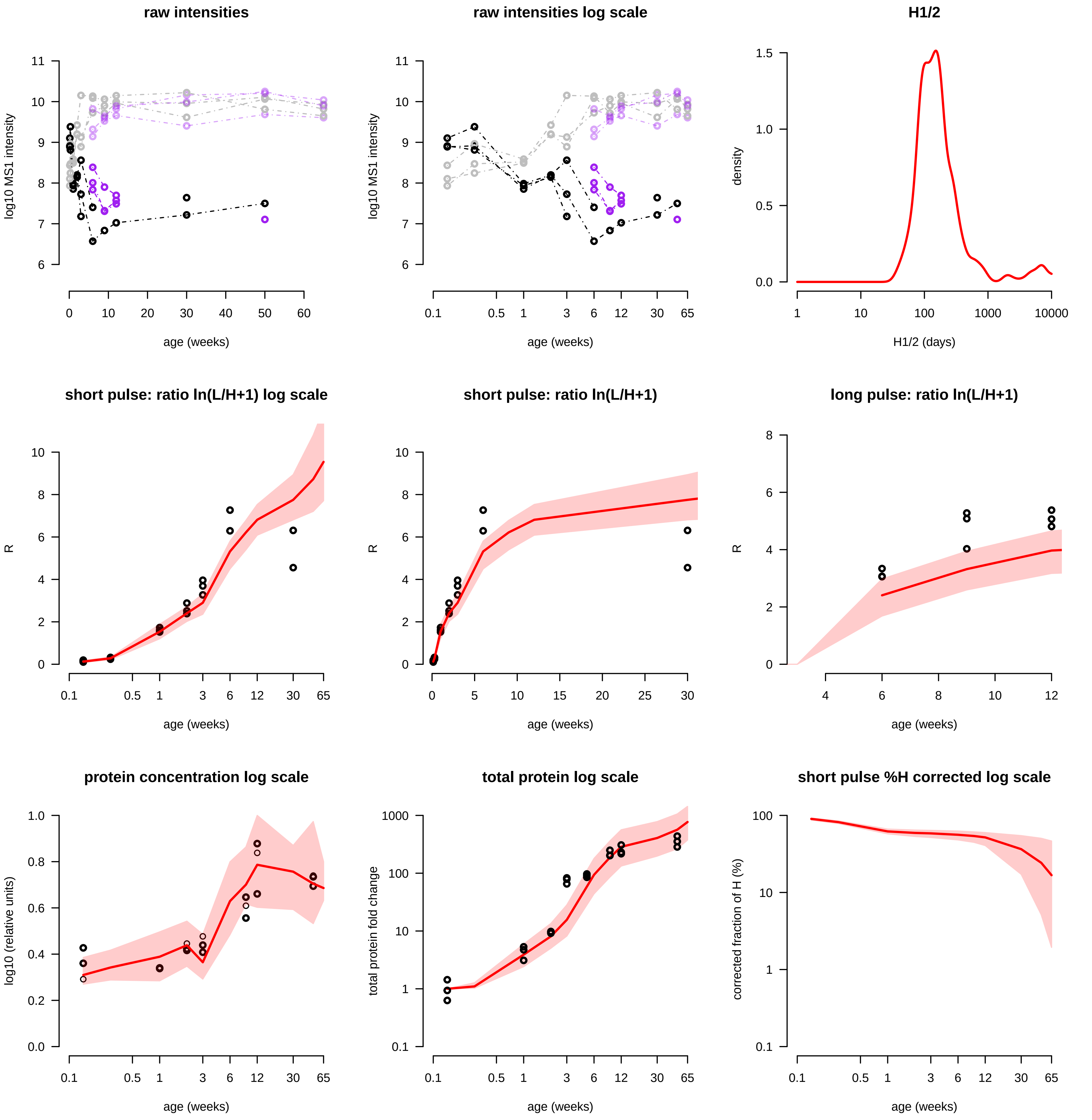

P26043

>sp|P26043|RADI_MOUSE

Radixin

GN=Rdx

Med H1/2: 9.1 days

Q5 H1/2: 7.1 days

Q95 H1/2: 11.8 days

reliable model fit: yes

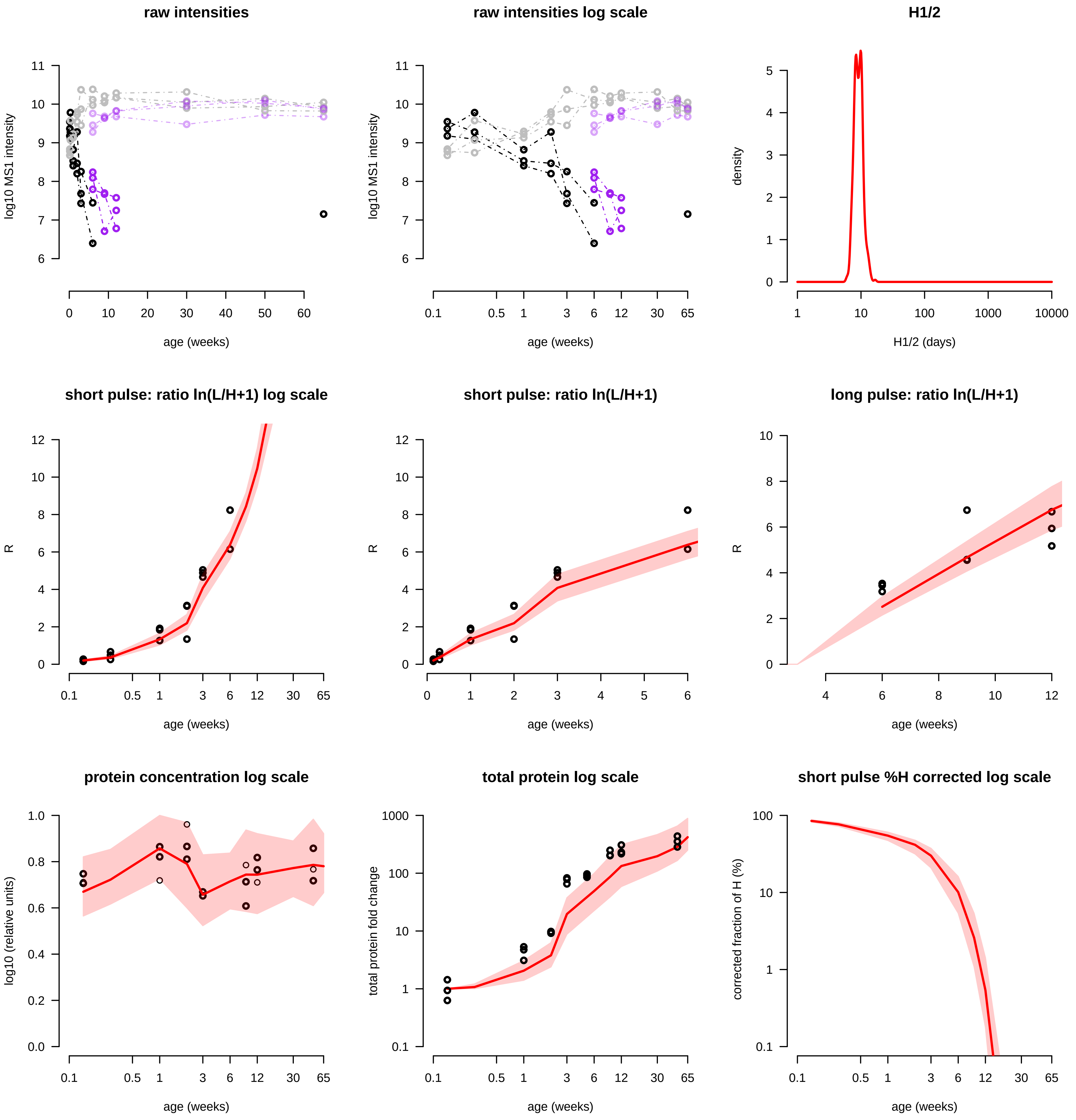

P13020−2

>sp|P13020−2|GELS_MOUSE

Isoform 2 of Gelsolin

GN=Gsn

Med H1/2: 182.4 days

Q5 H1/2: 56.6 days

Q95 H1/2: 1053.8 days

reliable model fit: yes

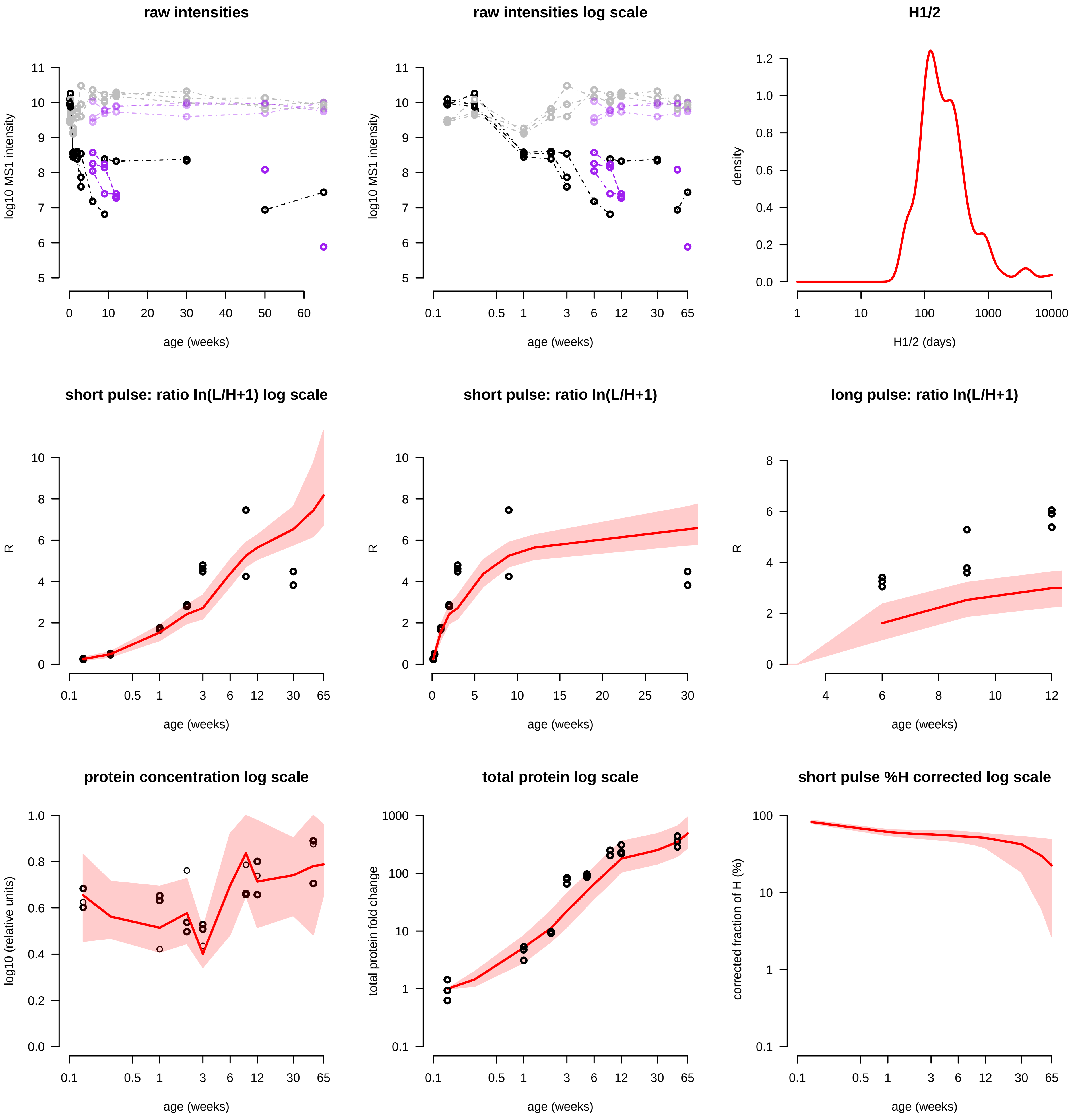

P42125

>sp|P42125|ECI1_MOUSE

Enoyl−CoA delta isomerase 1, mitochondrial

GN=Eci1

Med H1/2: 15.3 days

Q5 H1/2: 11.1 days

Q95 H1/2: 21.8 days

reliable model fit: yes

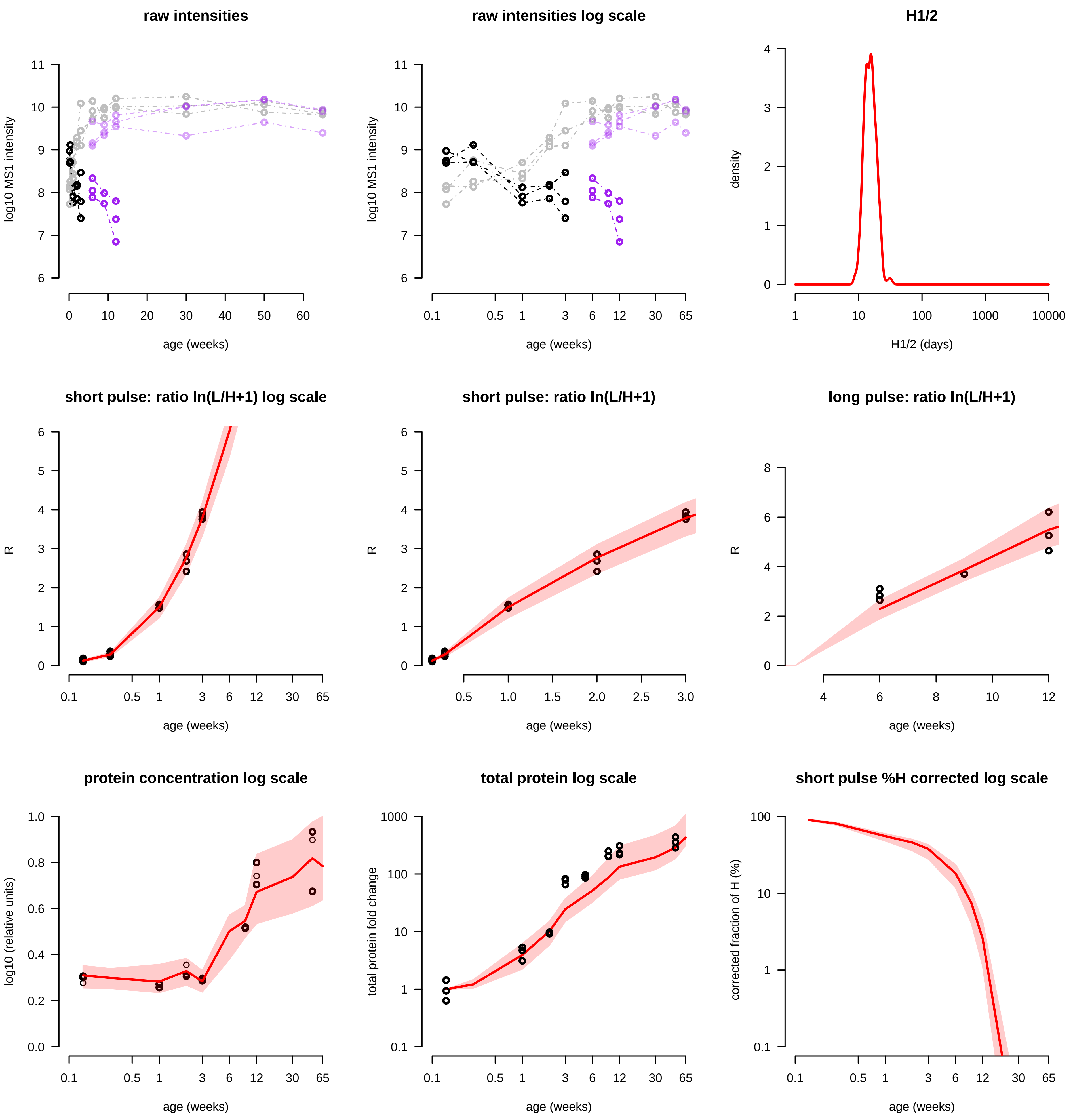

Q9CZU6

>sp|Q9CZU6|CISY_MOUSE

Citrate synthase, mitochondrial

GN=Cs

Med H1/2: 15.4 days

Q5 H1/2: 11.9 days

Q95 H1/2: 20.9 days

reliable model fit: yes

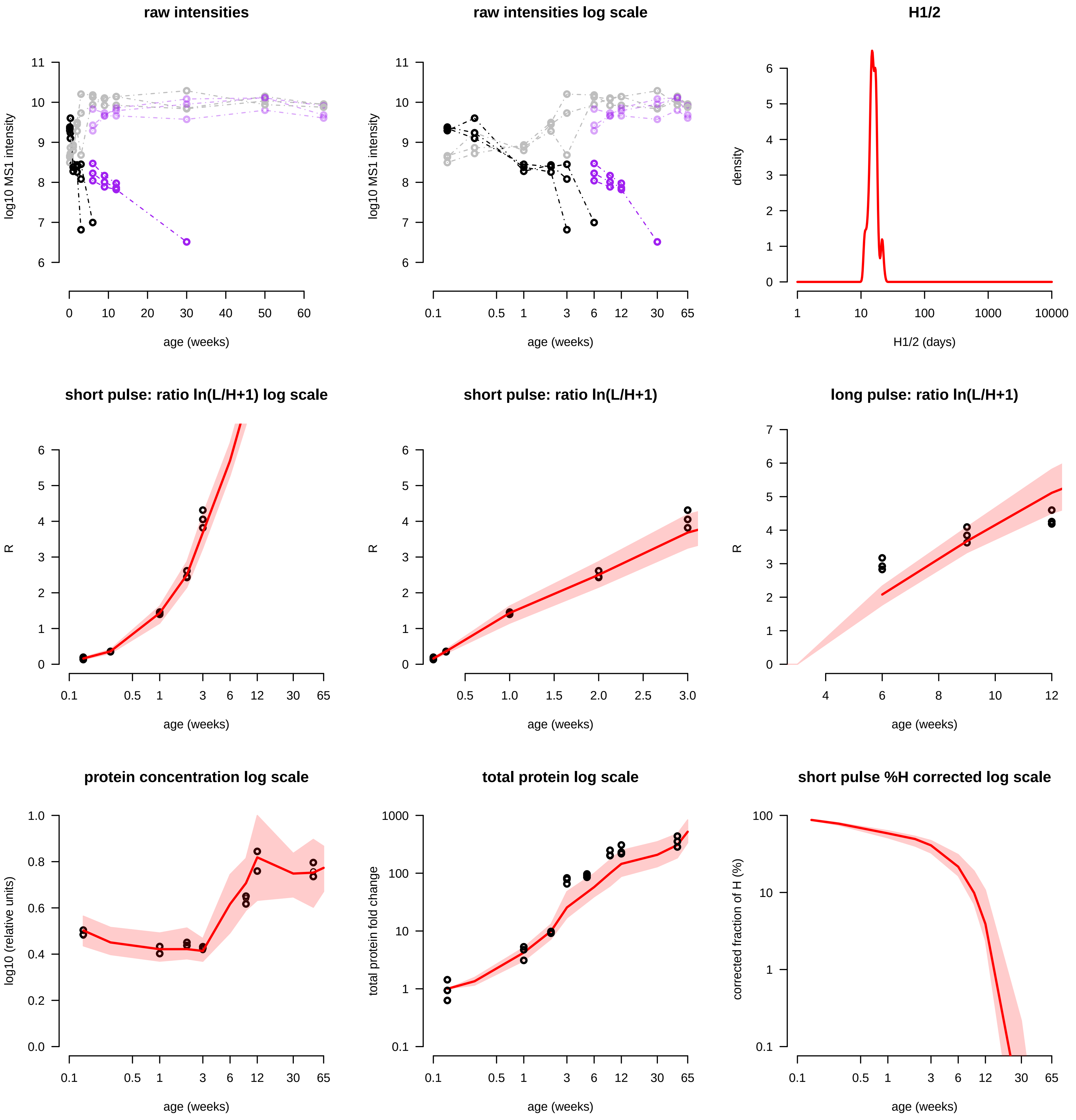

P06745

>sp|P06745|G6PI_MOUSE

Glucose−6−phosphate isomerase

GN=Gpi

Med H1/2: 1372.7 days

Q5 H1/2: 383.1 days

Q95 H1/2: 13633.1 days

reliable model fit: yes

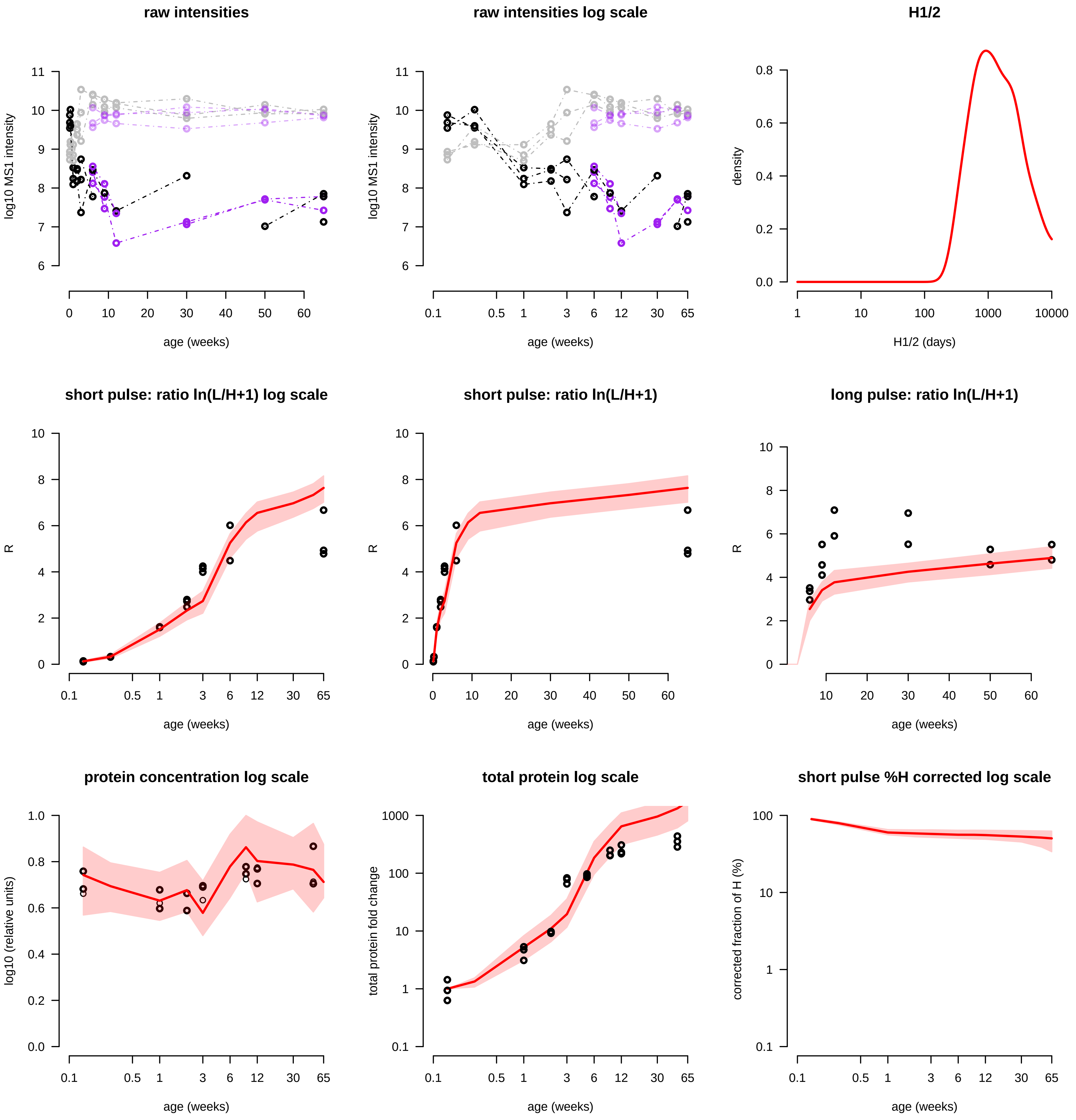

Q61316

>sp|Q61316|HSP74_MOUSE

Heat shock 70 kDa protein 4

GN=Hspa4

Med H1/2: 207.8 days

Q5 H1/2: 101 days

Q95 H1/2: 1565.2 days

reliable model fit: yes

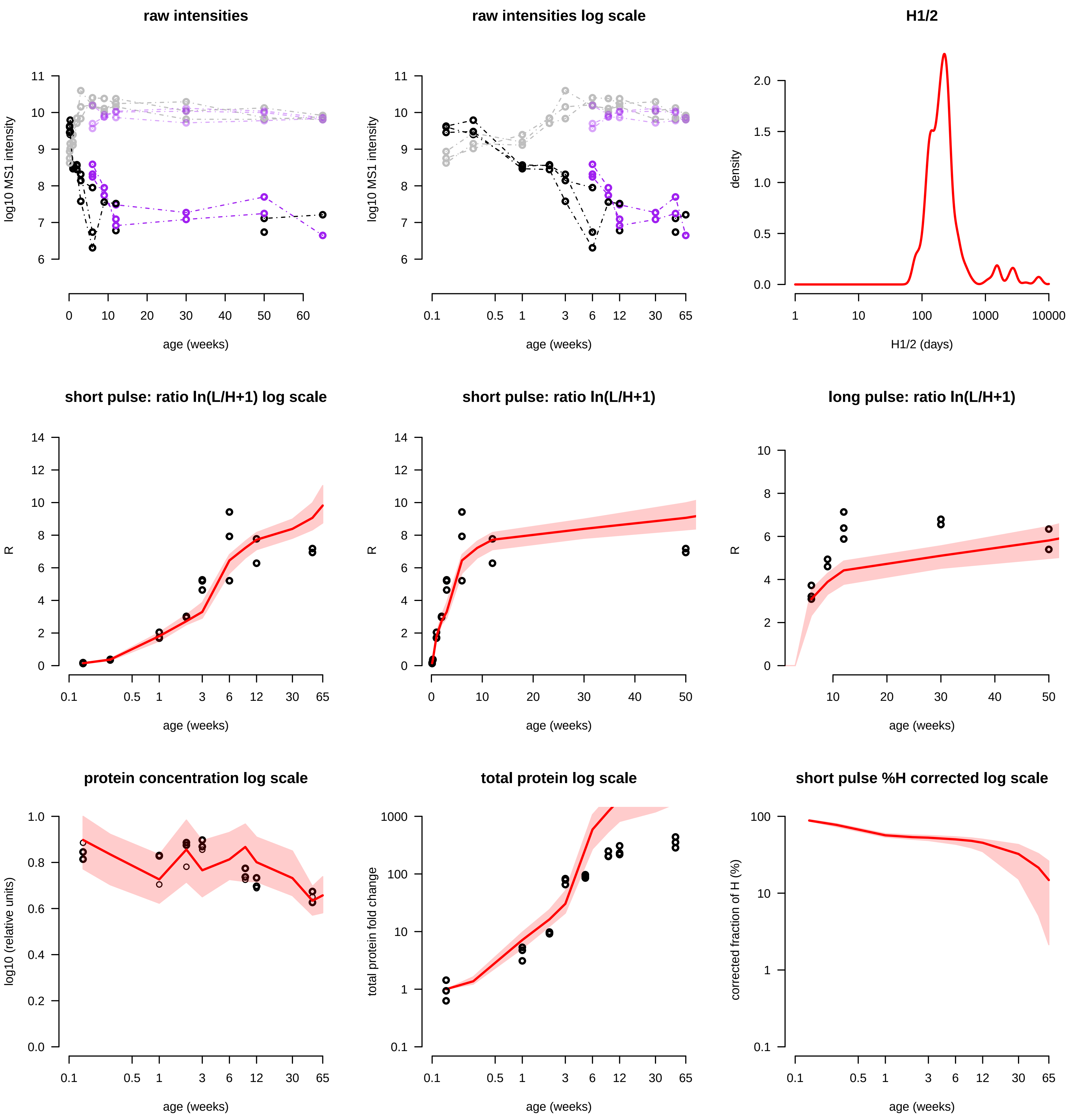

Q8BFZ3

>sp|Q8BFZ3|ACTBL_MOUSE

Beta−actin−like protein 2

GN=Actbl2

Med H1/2: 1219 days

Q5 H1/2: 379 days

Q95 H1/2: 11305 days

reliable model fit: yes

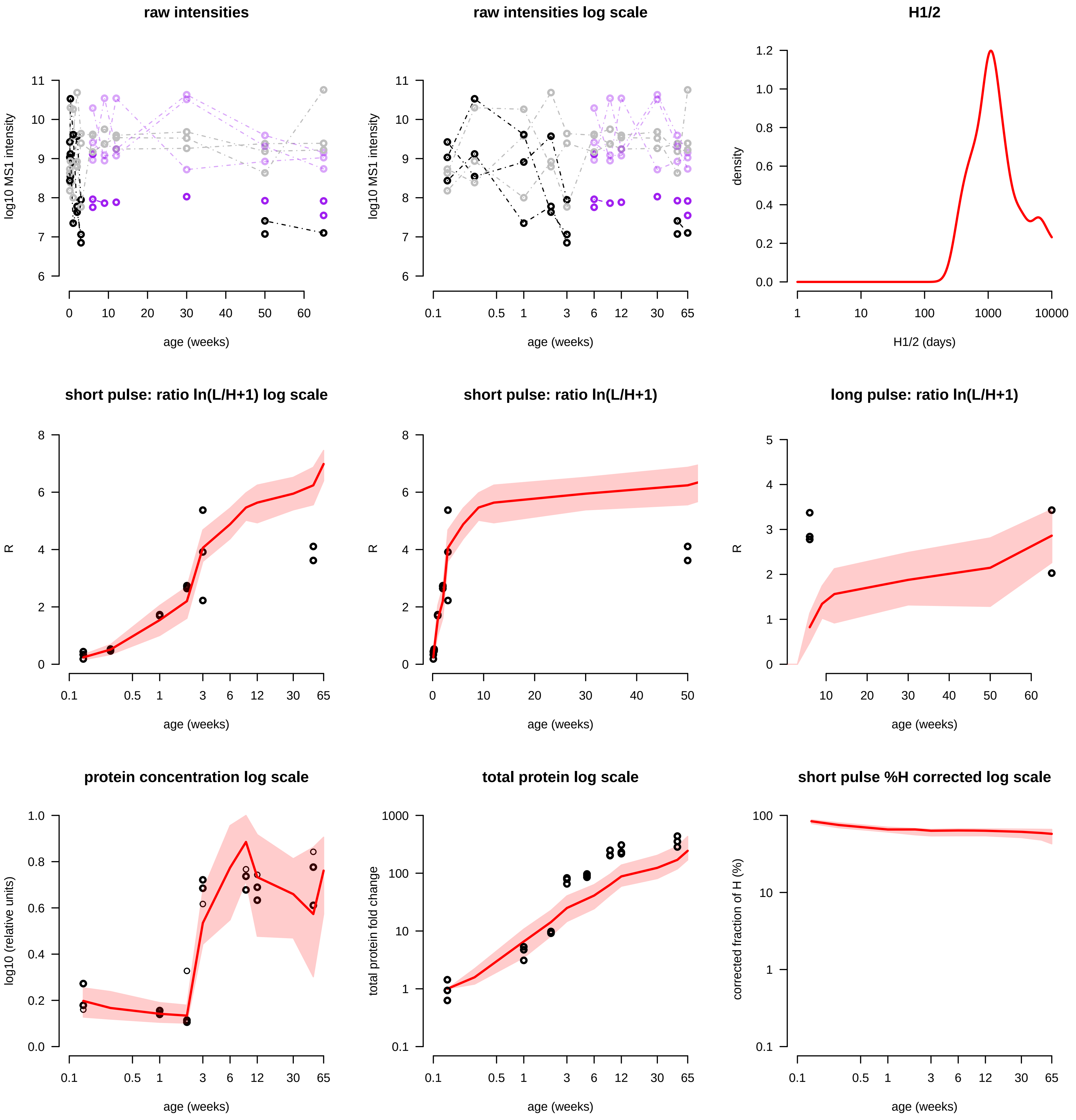

P10639

>sp|P10639|THIO_MOUSE

Thioredoxin

GN=Txn

Med H1/2: 13.6 days

Q5 H1/2: 10 days

Q95 H1/2: 19.4 days

reliable model fit: yes

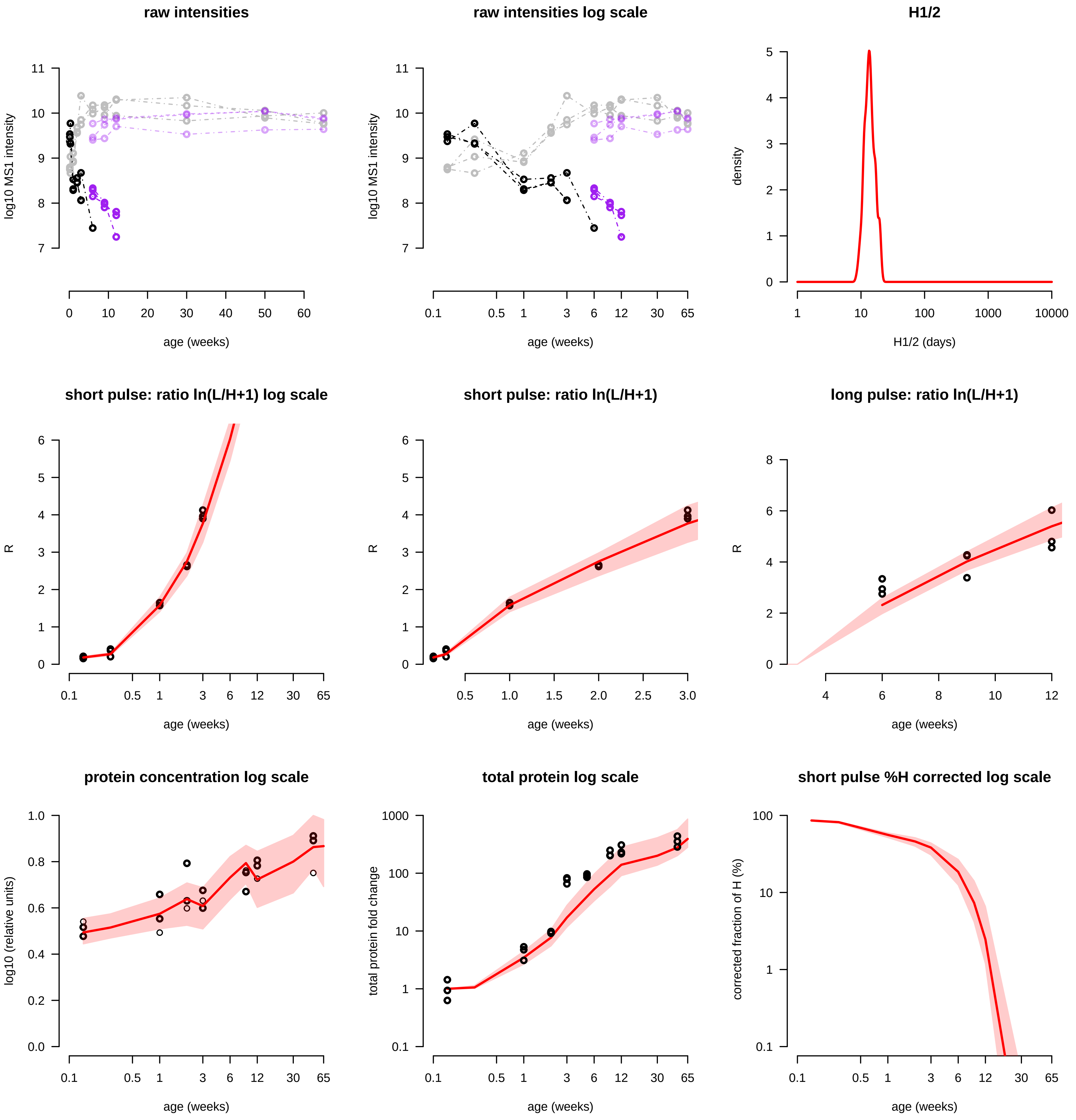

P62821

>sp|P62821|RAB1A_MOUSE

Ras−related protein Rab−1A

GN=Rab1A

Med H1/2: 9.9 days

Q5 H1/2: 8.3 days

Q95 H1/2: 11.7 days

reliable model fit: yes

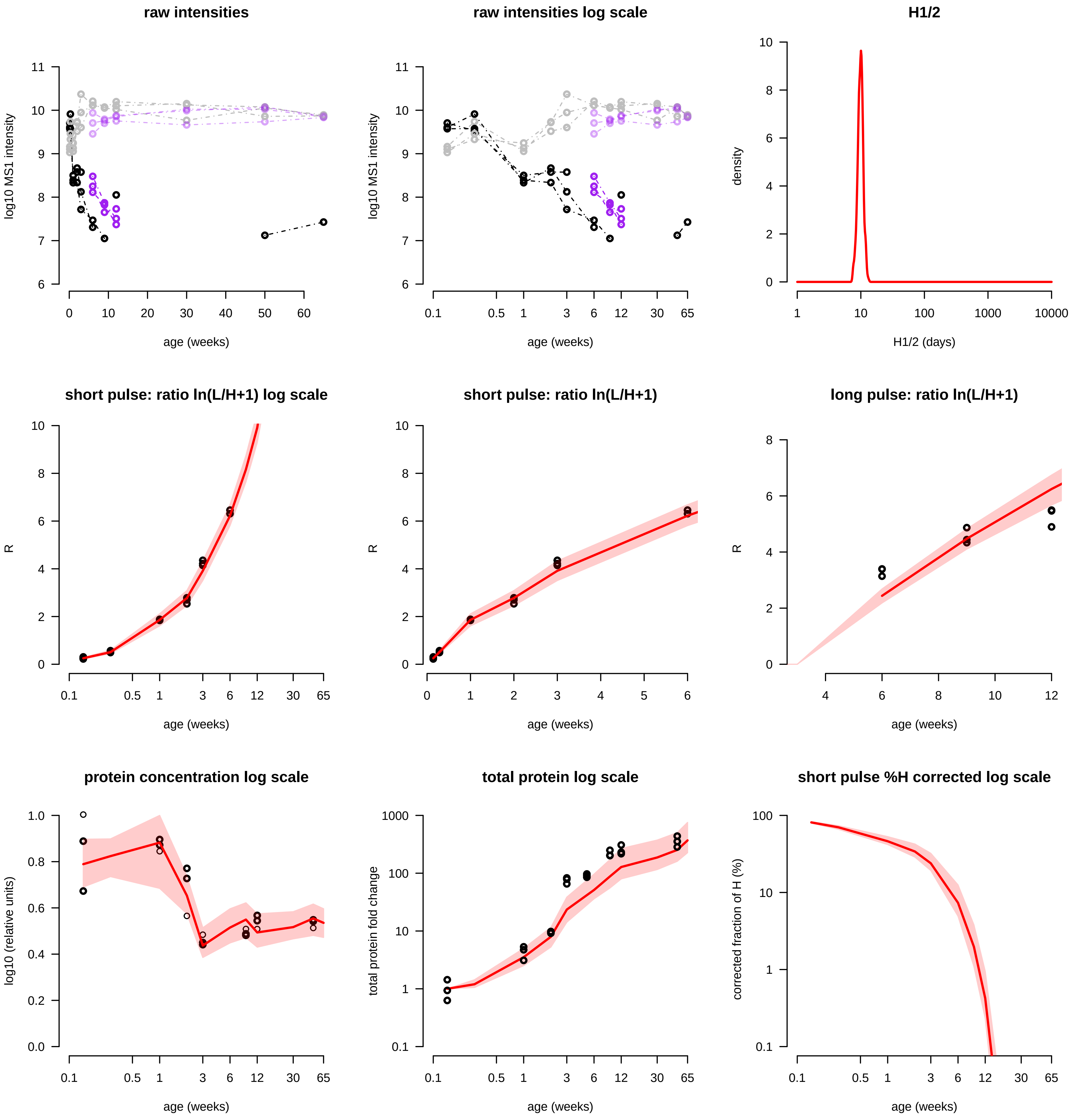

Q3UW53

>sp|Q3UW53|NIBA1_MOUSE

Protein Niban 1

GN=Niban1

Med H1/2: 5.8 days

Q5 H1/2: 4.1 days

Q95 H1/2: 9.3 days

reliable model fit: yes

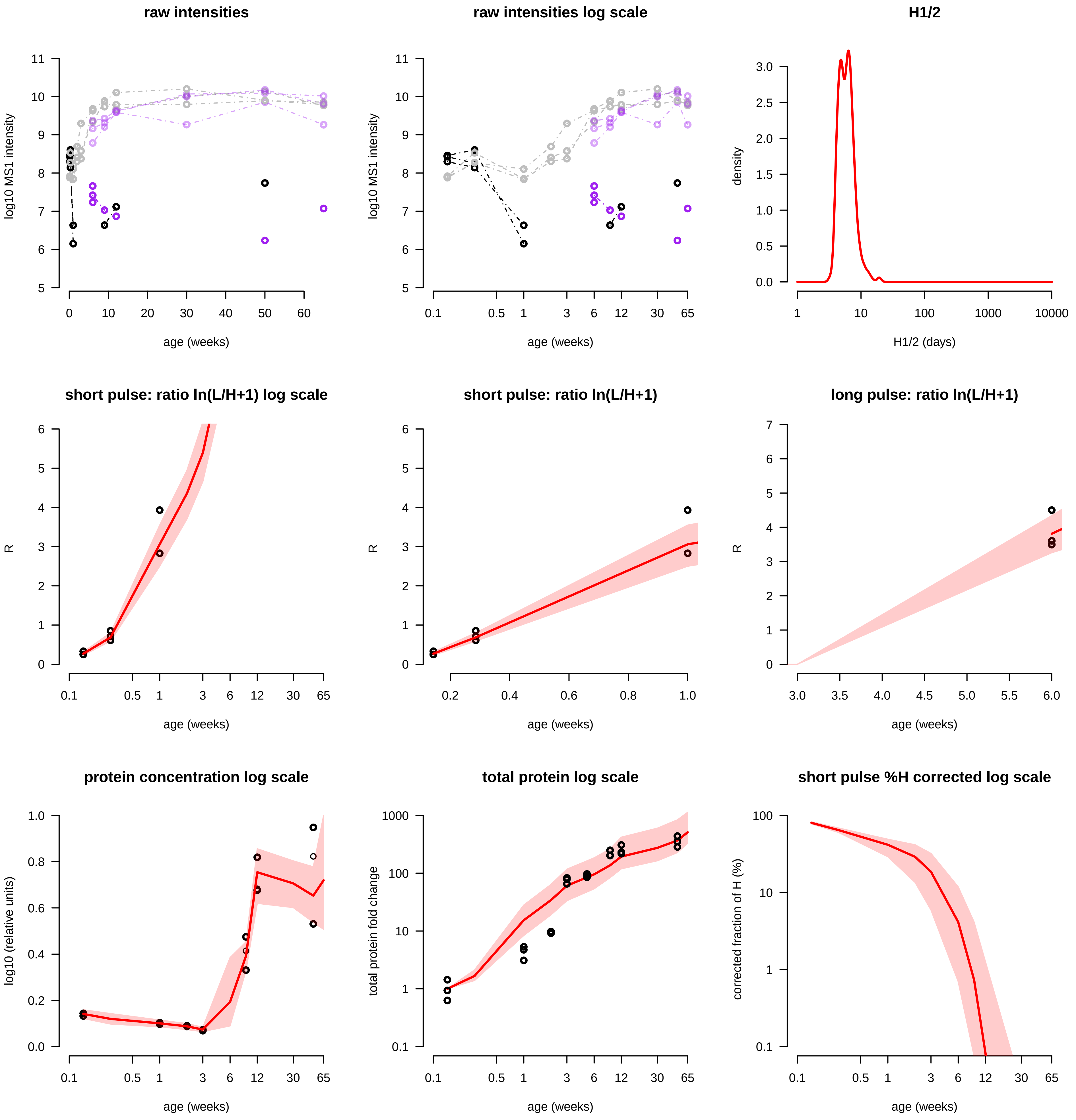

P37804

>sp|P37804|TAGL_MOUSE

Transgelin

GN=Tagln

Med H1/2: 293.2 days

Q5 H1/2: 120.3 days

Q95 H1/2: 4192.3 days

reliable model fit: yes

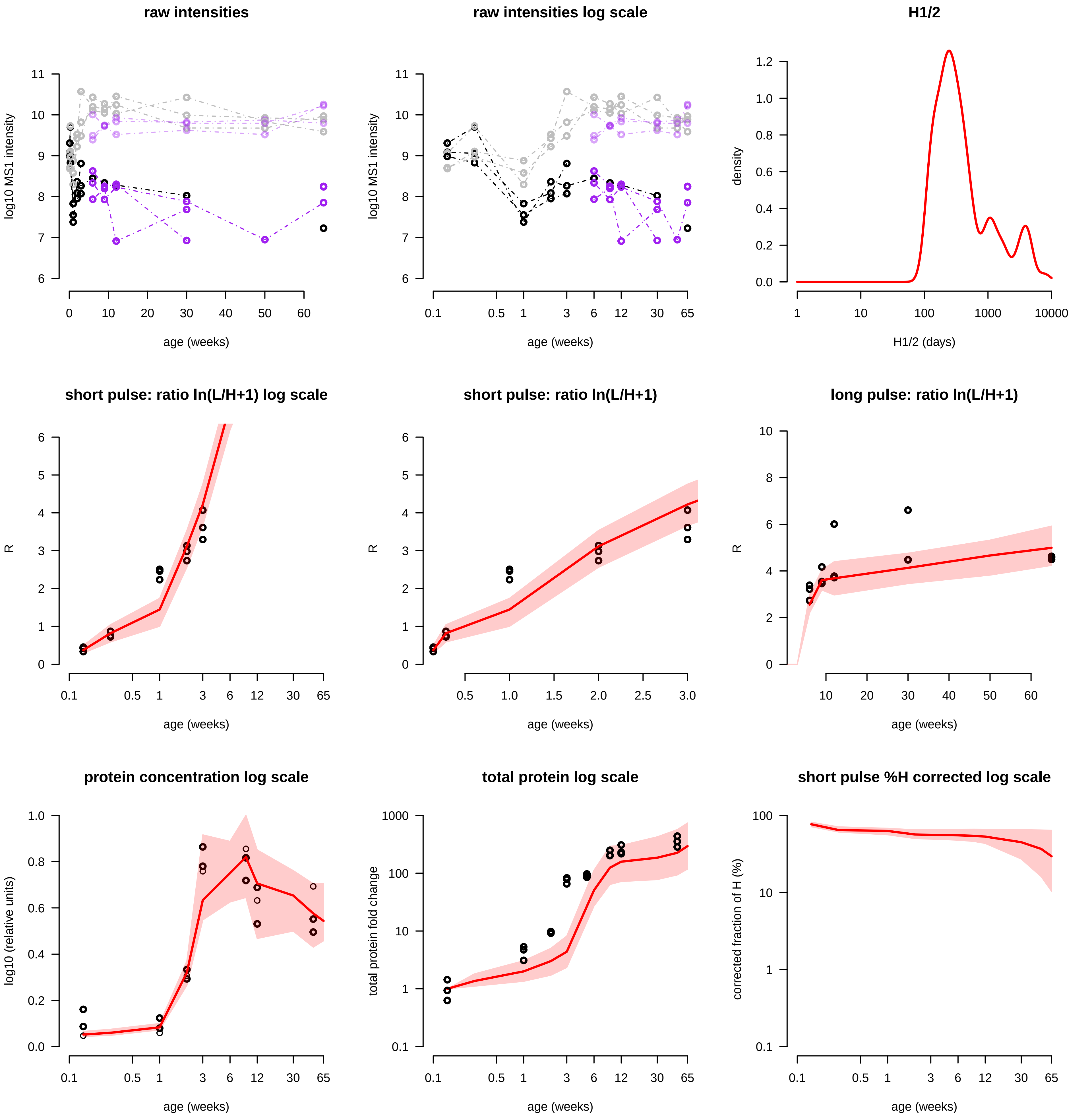

Q8R081

>sp|Q8R081|HNRPL_MOUSE

Heterogeneous nuclear ribonucleoprotein L

GN=Hnrnpl

Med H1/2: 917.6 days

Q5 H1/2: 274.9 days

Q95 H1/2: 6349.4 days

reliable model fit: yes

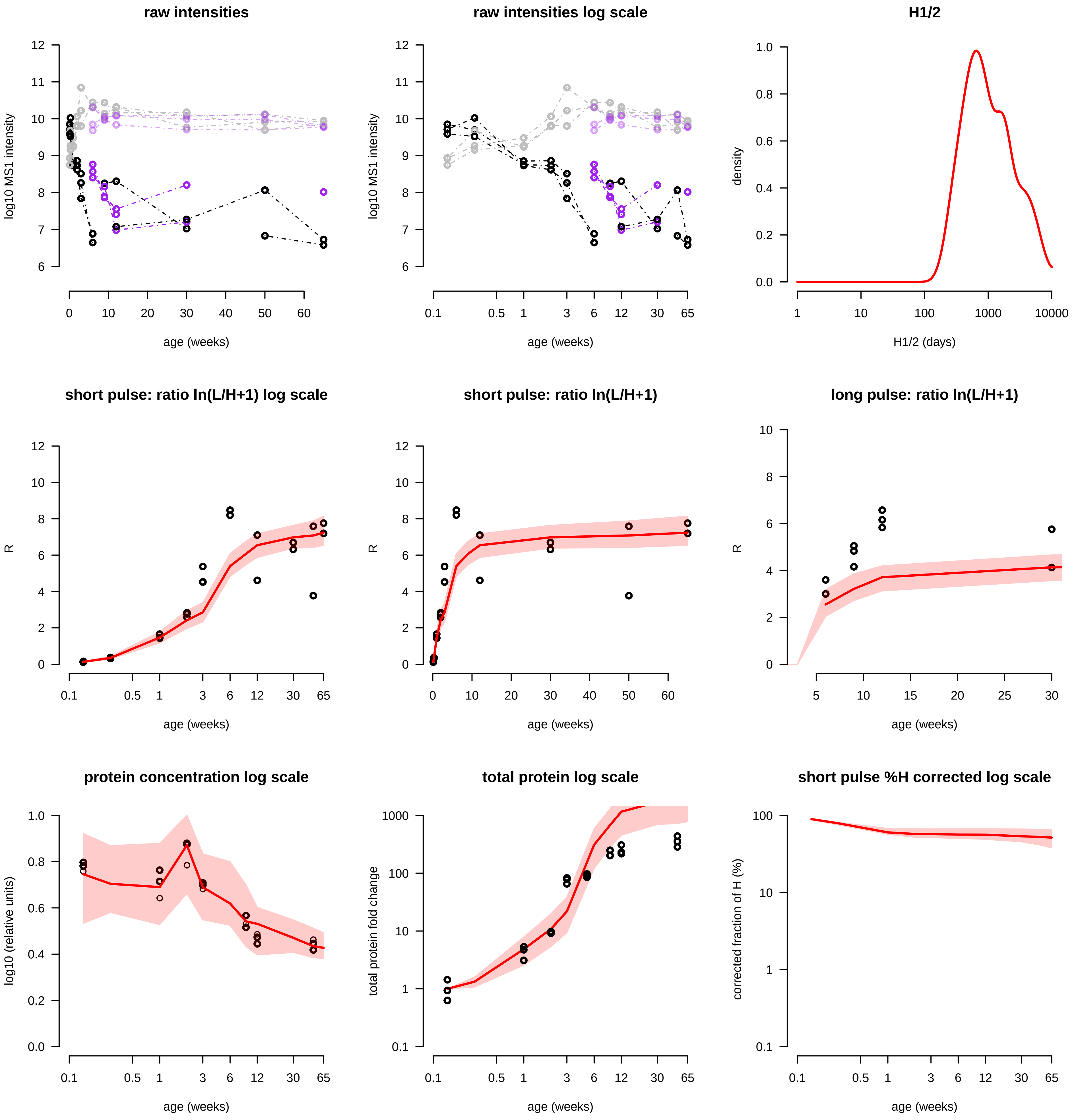

P40124

>sp|P40124|CAP1_MOUSE

Adenylyl cyclase−associated protein 1

GN=Cap1

Med H1/2: 6.8 days

Q5 H1/2: 5.8 days

Q95 H1/2: 7.8 days

reliable model fit: yes

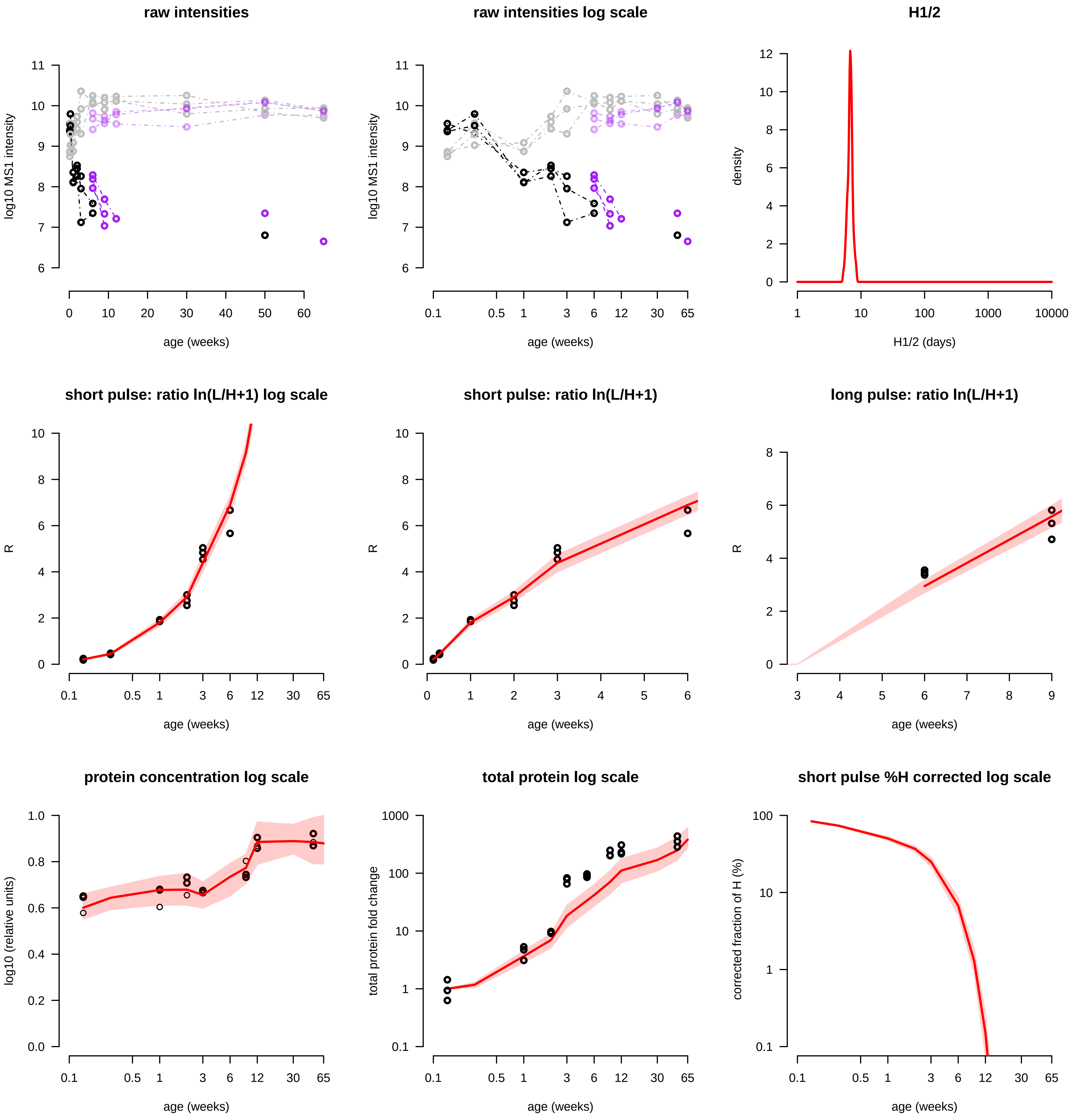

P27659

>sp|P27659|RL3_MOUSE

60S ribosomal protein L3

GN=Rpl3

Med H1/2: 9.8 days

Q5 H1/2: 8.2 days

Q95 H1/2: 11.7 days

reliable model fit: yes

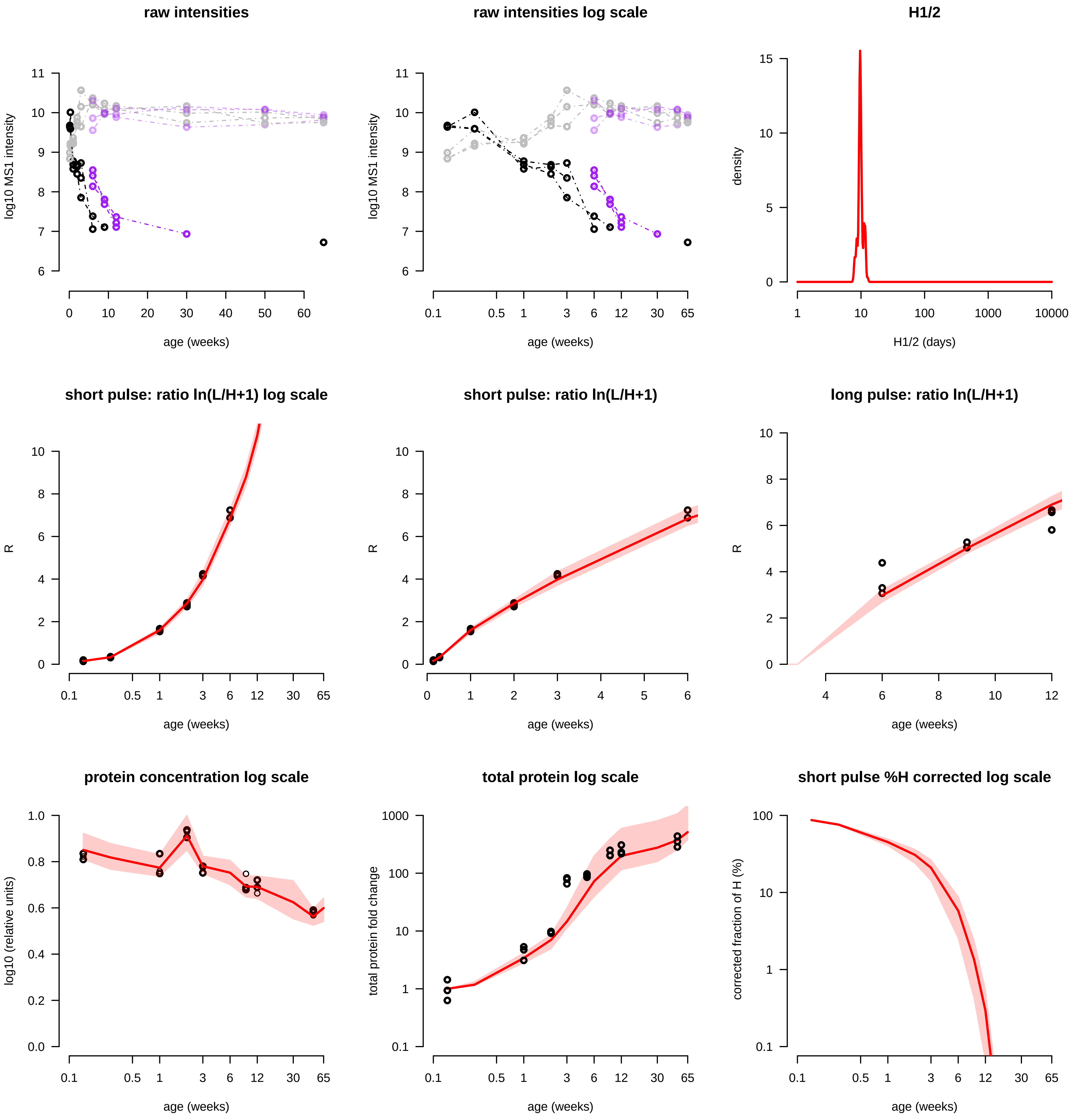

P43276

>sp|P43276|H15_MOUSE

Histone H1.5

GN=H1−5

Med H1/2: 39.6 days

Q5 H1/2: 34.9 days

Q95 H1/2: 62.9 days

reliable model fit: yes

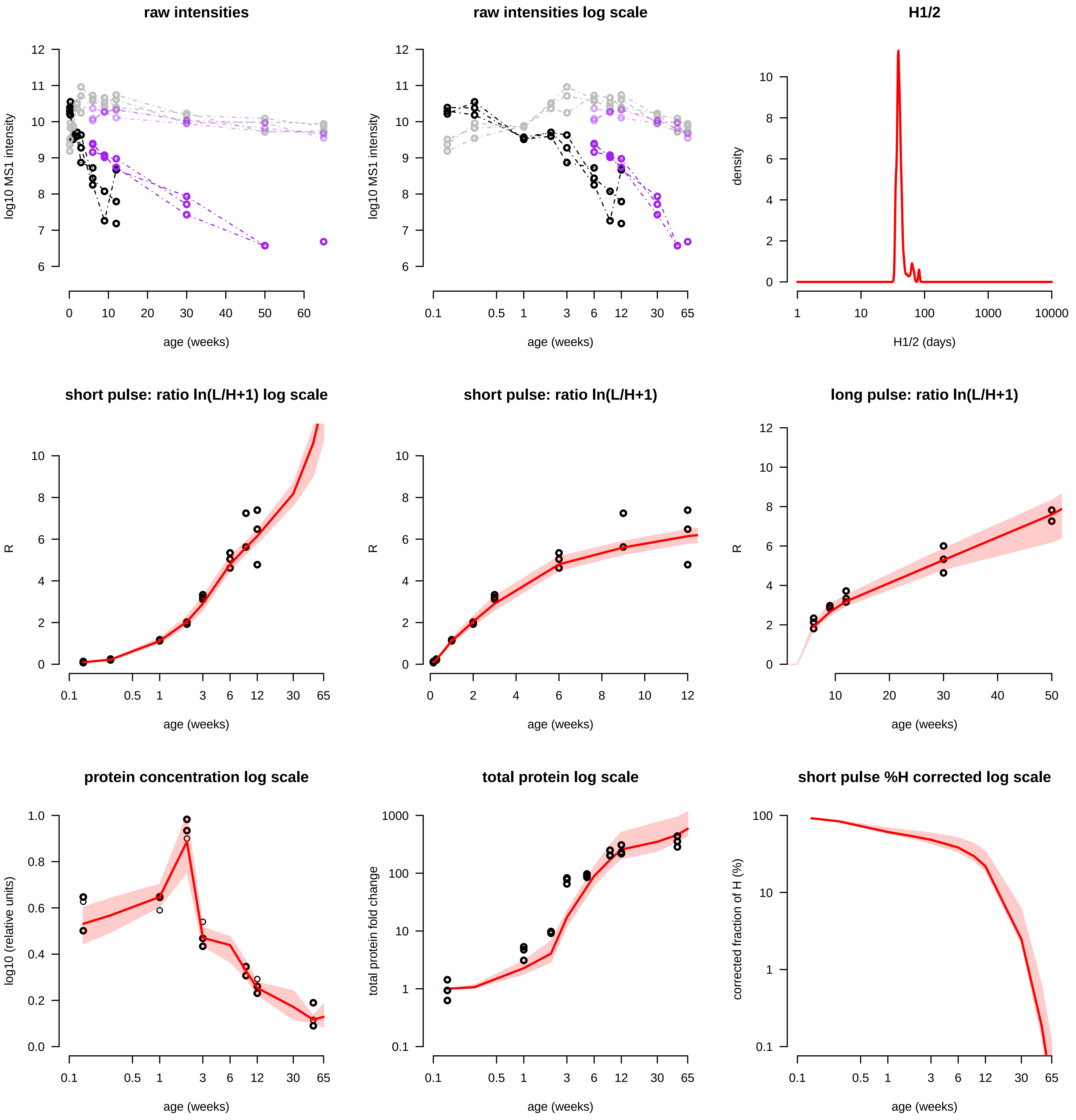

Q99PU5

>sp|Q99PU5|ACBG1_MOUSE

Long−chain−fatty−acid−−CoA ligase ACSBG1

GN=Acsbg1

Med H1/2: 15.6 days

Q5 H1/2: 11.1 days

Q95 H1/2: 25.6 days

reliable model fit: yes

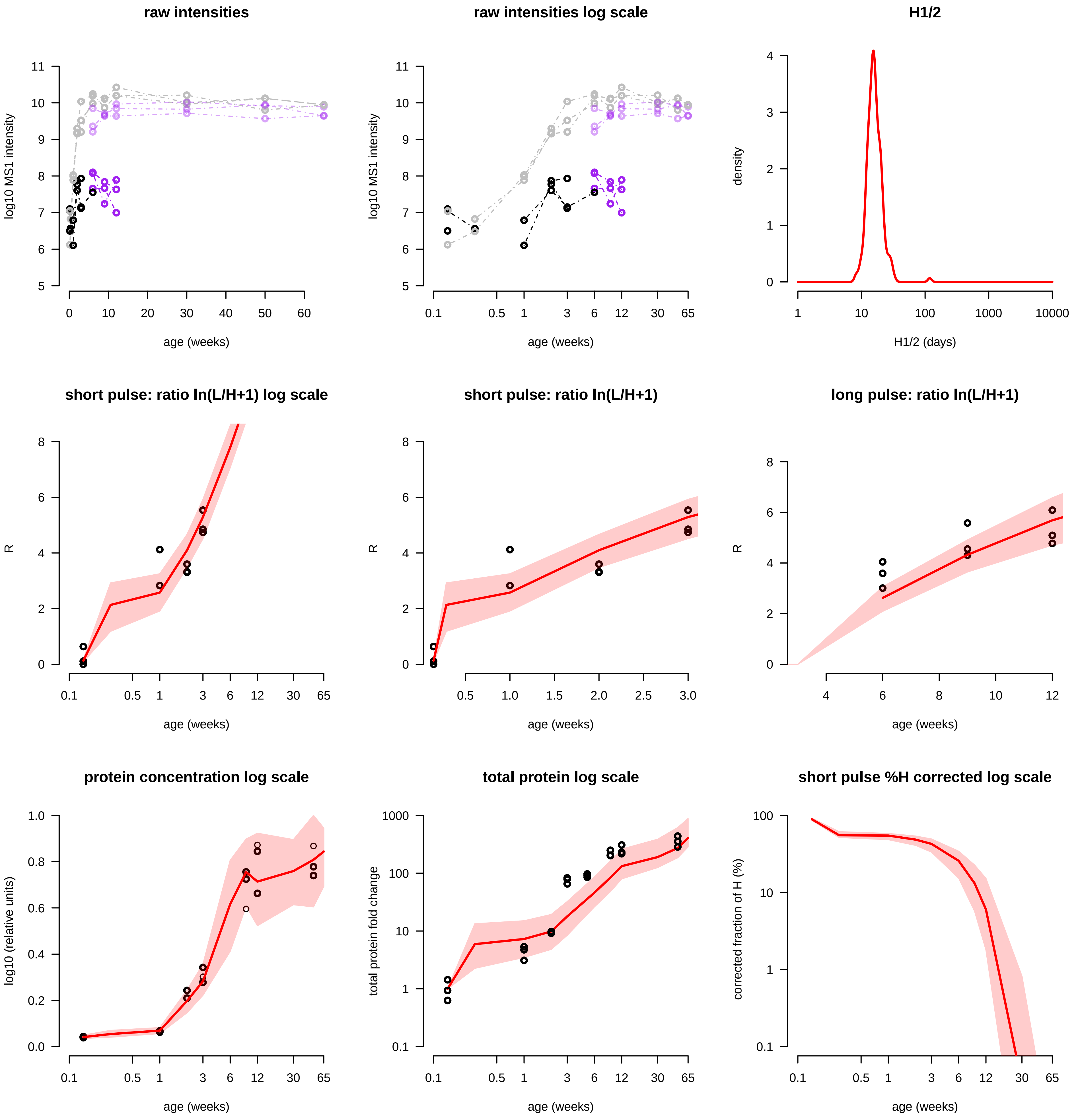

O08709

>sp|O08709|PRDX6_MOUSE

Peroxiredoxin−6

GN=Prdx6

Med H1/2: 9.8 days

Q5 H1/2: 7.9 days

Q95 H1/2: 14.2 days

reliable model fit: yes

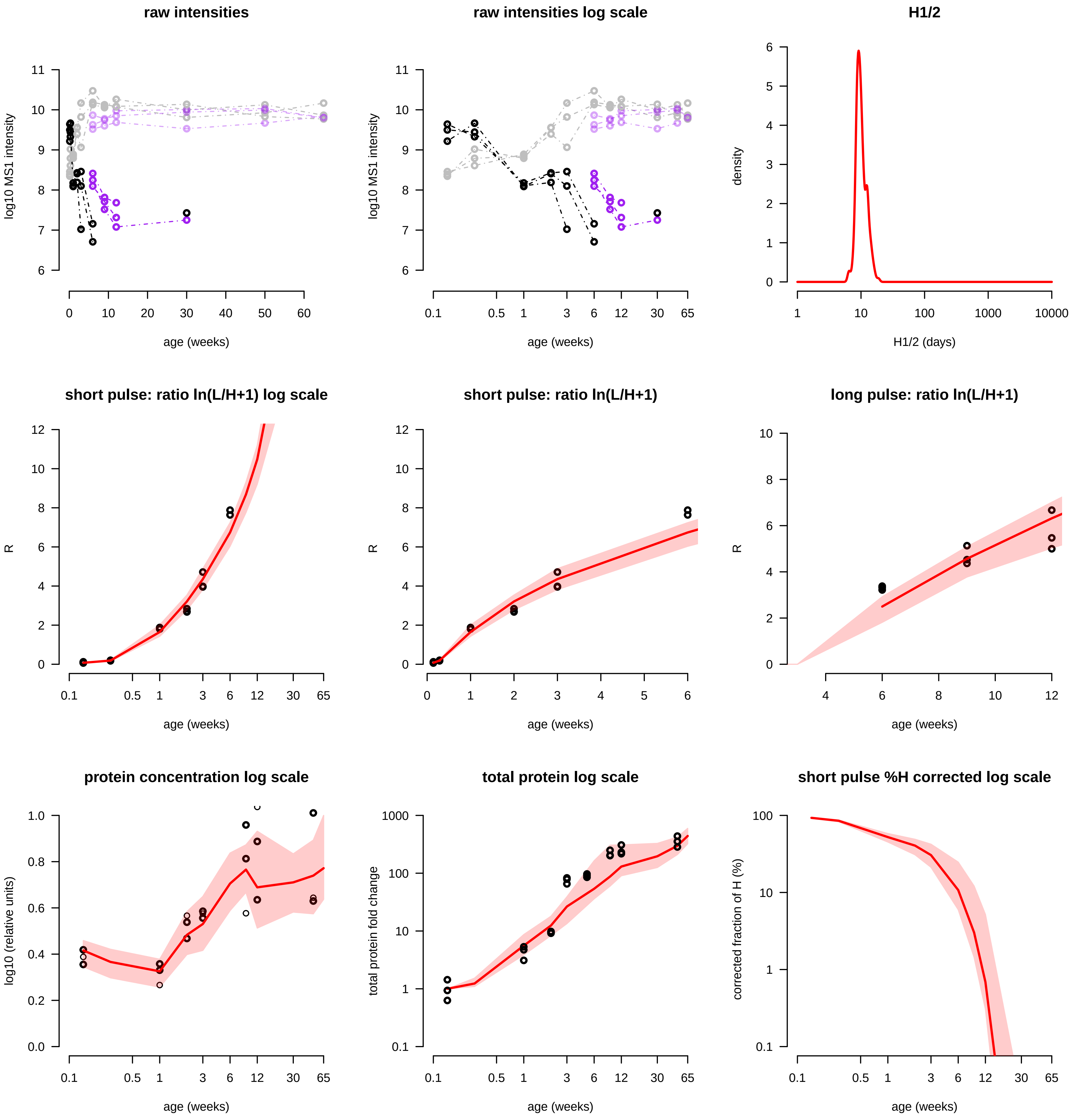

P06151

>sp|P06151|LDHA_MOUSE

L−lactate dehydrogenase A chain

GN=Ldha

Med H1/2: 31.9 days

Q5 H1/2: 25.2 days

Q95 H1/2: 38.3 days

reliable model fit: yes

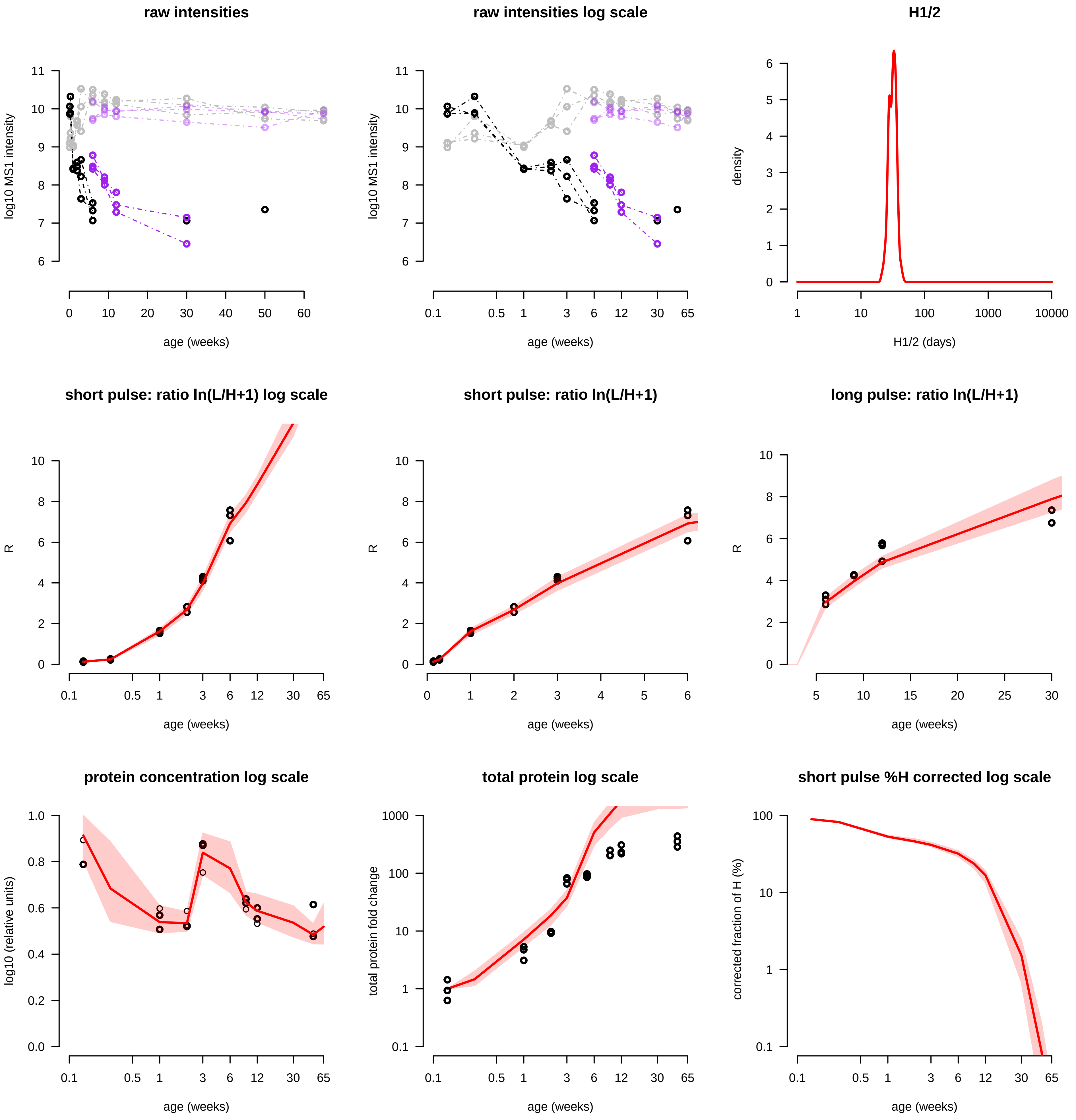

P19324

>sp|P19324|SERPH_MOUSE

Serpin H1

GN=Serpinh1

Med H1/2: 241.2 days

Q5 H1/2: 110.7 days

Q95 H1/2: 2144.1 days

reliable model fit: yes

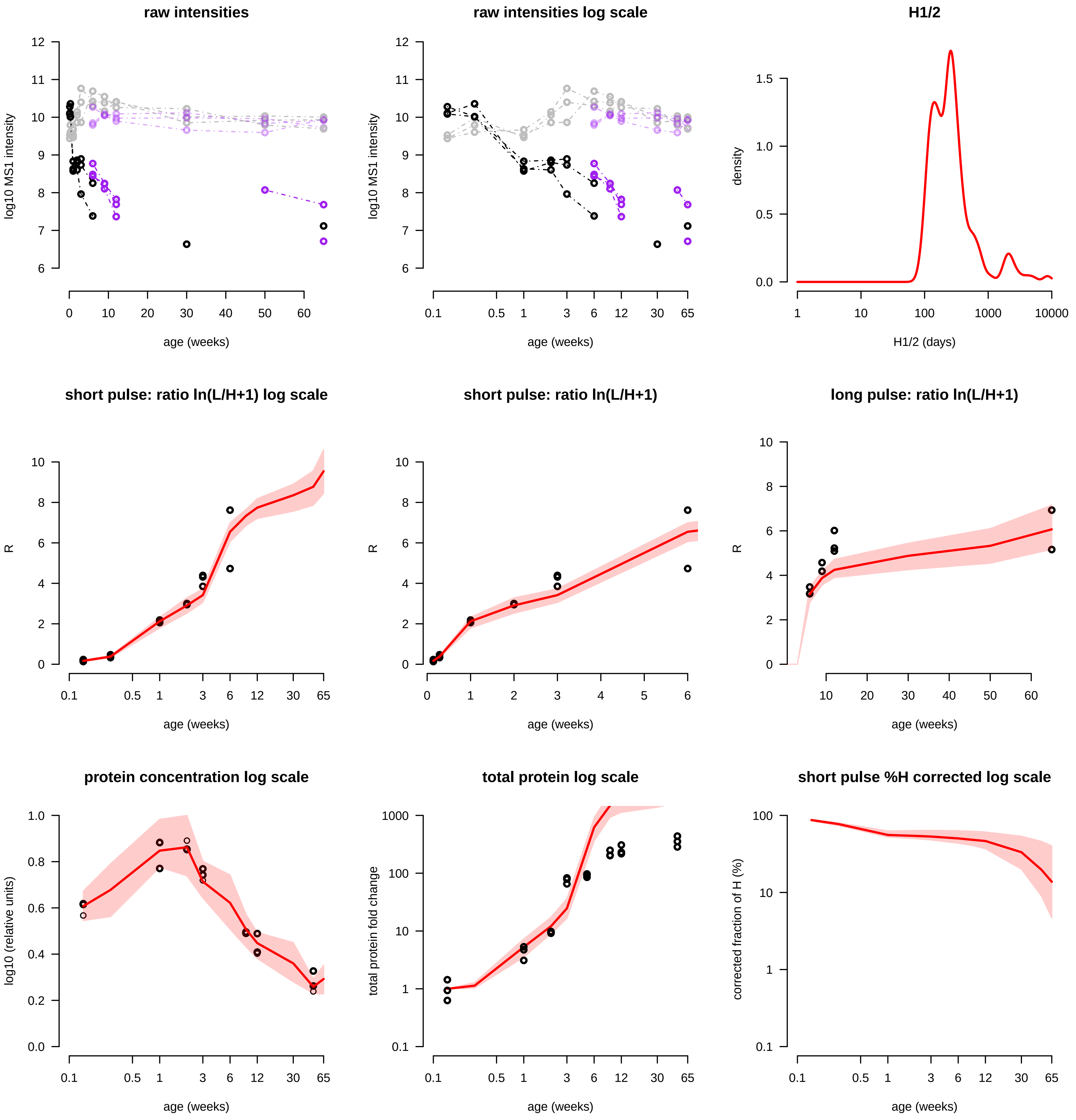

P24369

>sp|P24369|PPIB_MOUSE

Peptidyl−prolyl cis−trans isomerase B

GN=Ppib

Med H1/2: 11.5 days

Q5 H1/2: 9.6 days

Q95 H1/2: 14 days

reliable model fit: yes

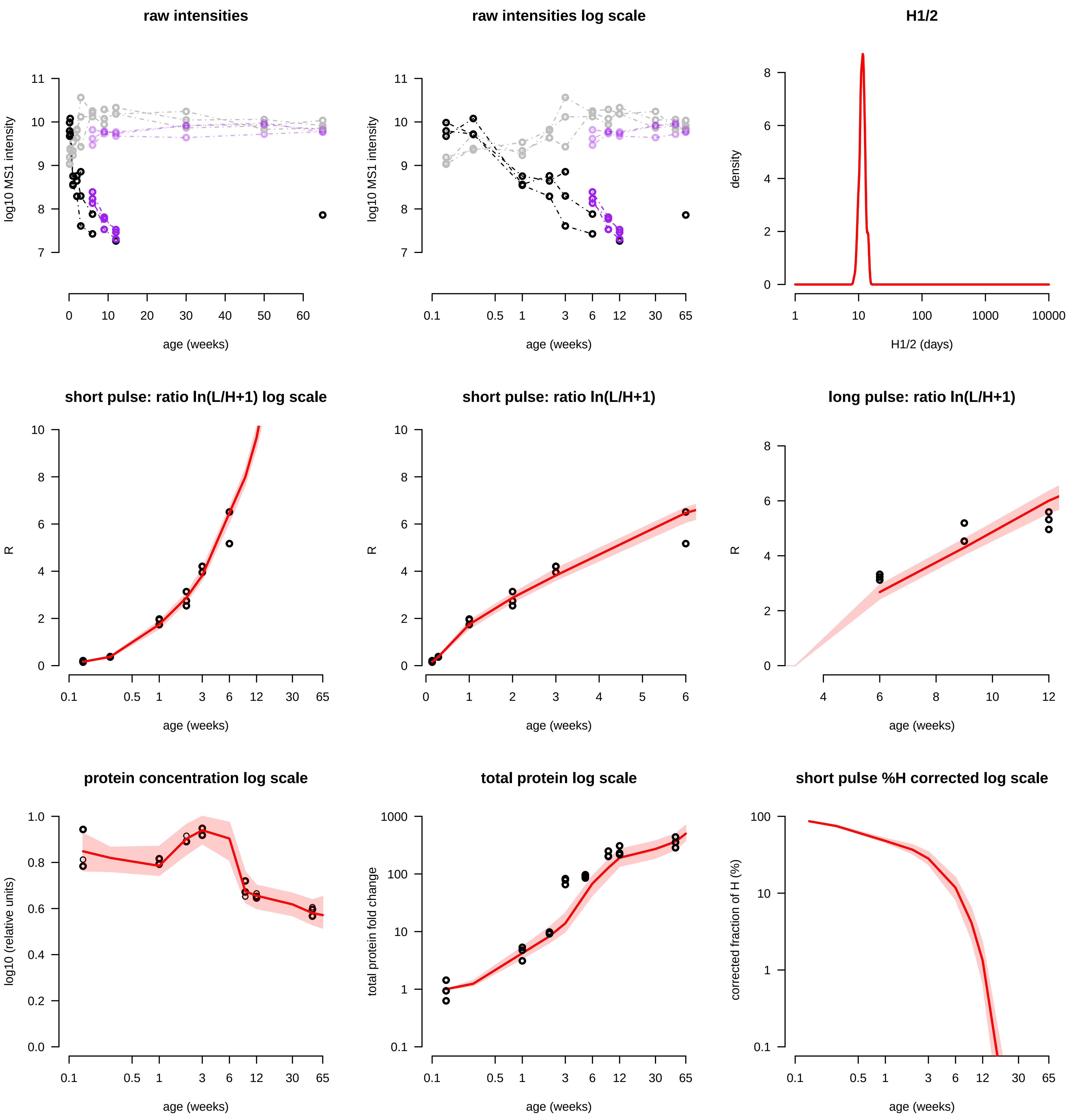

P01869;P01868

>sp|P01869|IGH1M_MOUSE

Ig gamma−1 chain C region, membrane−bound form

GN=Ighg1

Med H1/2: 291.6 days

Q5 H1/2: 119.7 days

Q95 H1/2: 1920.5 days

reliable model fit: yes

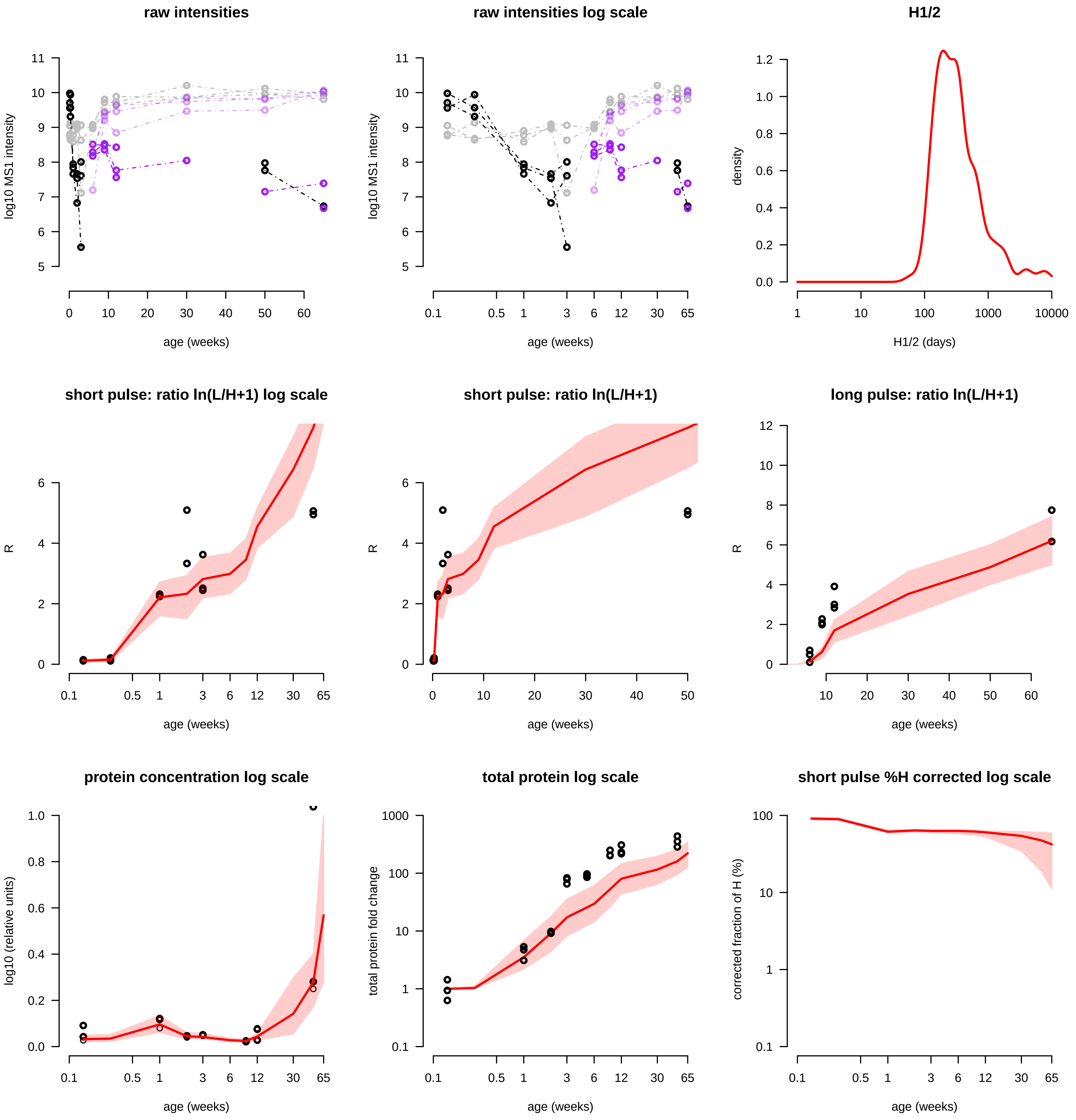

Q9DCW4

>sp|Q9DCW4|ETFB_MOUSE

Electron transfer flavoprotein subunit beta

GN=Etfb

Med H1/2: 291 days

Q5 H1/2: 129.9 days

Q95 H1/2: 11988.5 days

reliable model fit: yes

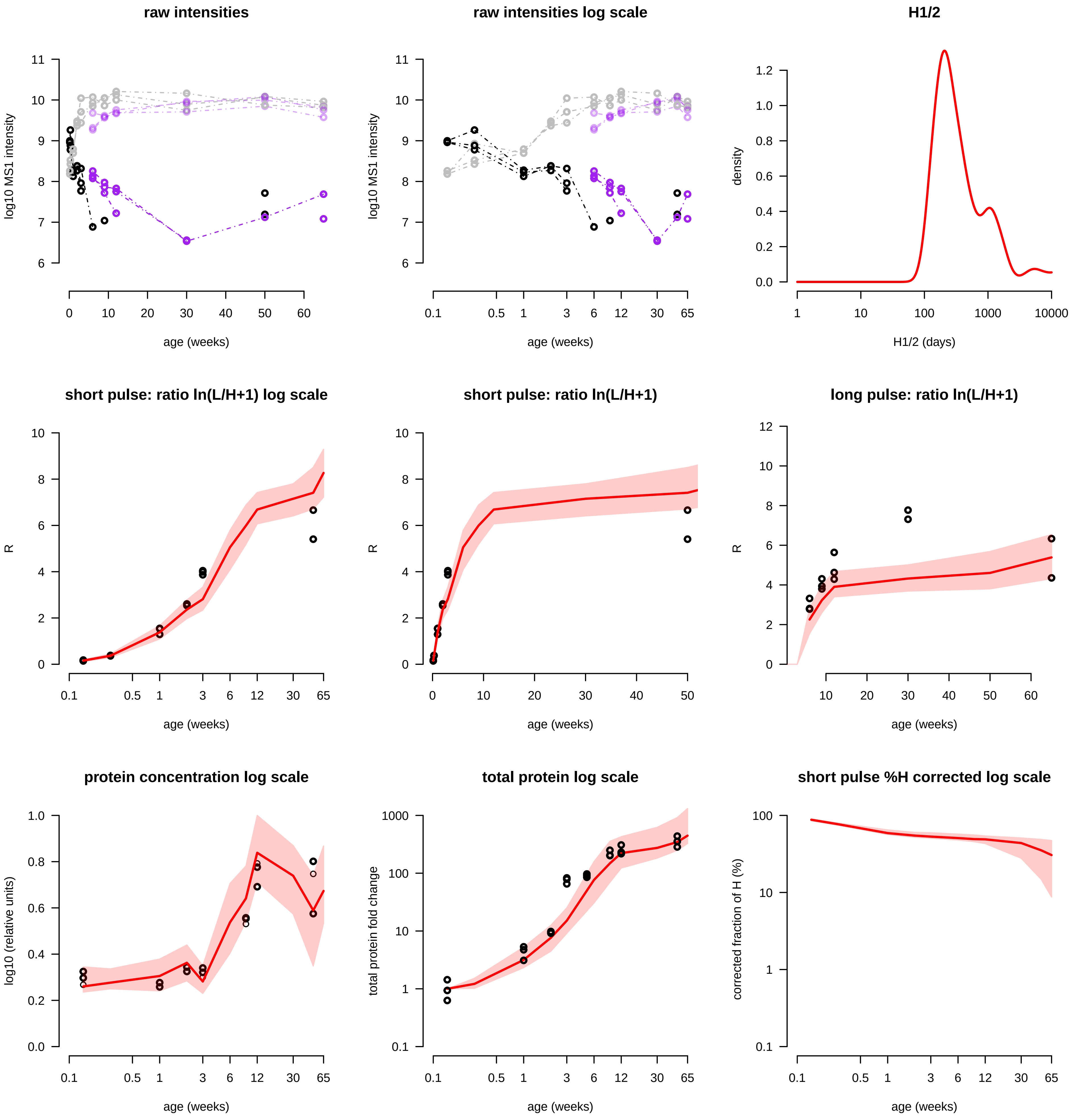

P48036

>sp|P48036|ANXA5_MOUSE

Annexin A5

GN=Anxa5

Med H1/2: 12.9 days

Q5 H1/2: 10 days

Q95 H1/2: 18.4 days

reliable model fit: yes

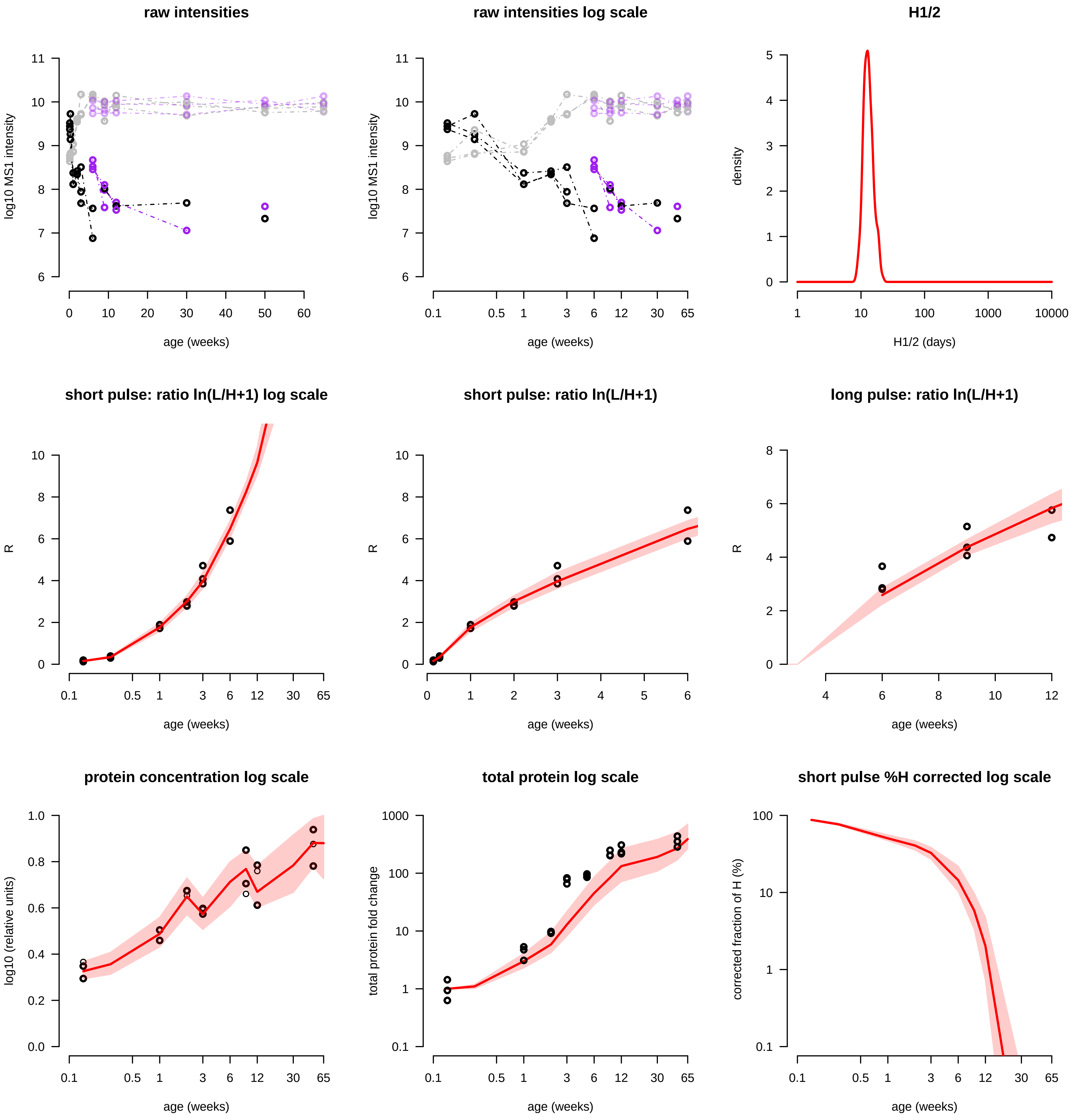

O35129

>sp|O35129|PHB2_MOUSE

Prohibitin−2

GN=Phb2

Med H1/2: 936.6 days

Q5 H1/2: 296.5 days

Q95 H1/2: 11698.3 days

reliable model fit: yes

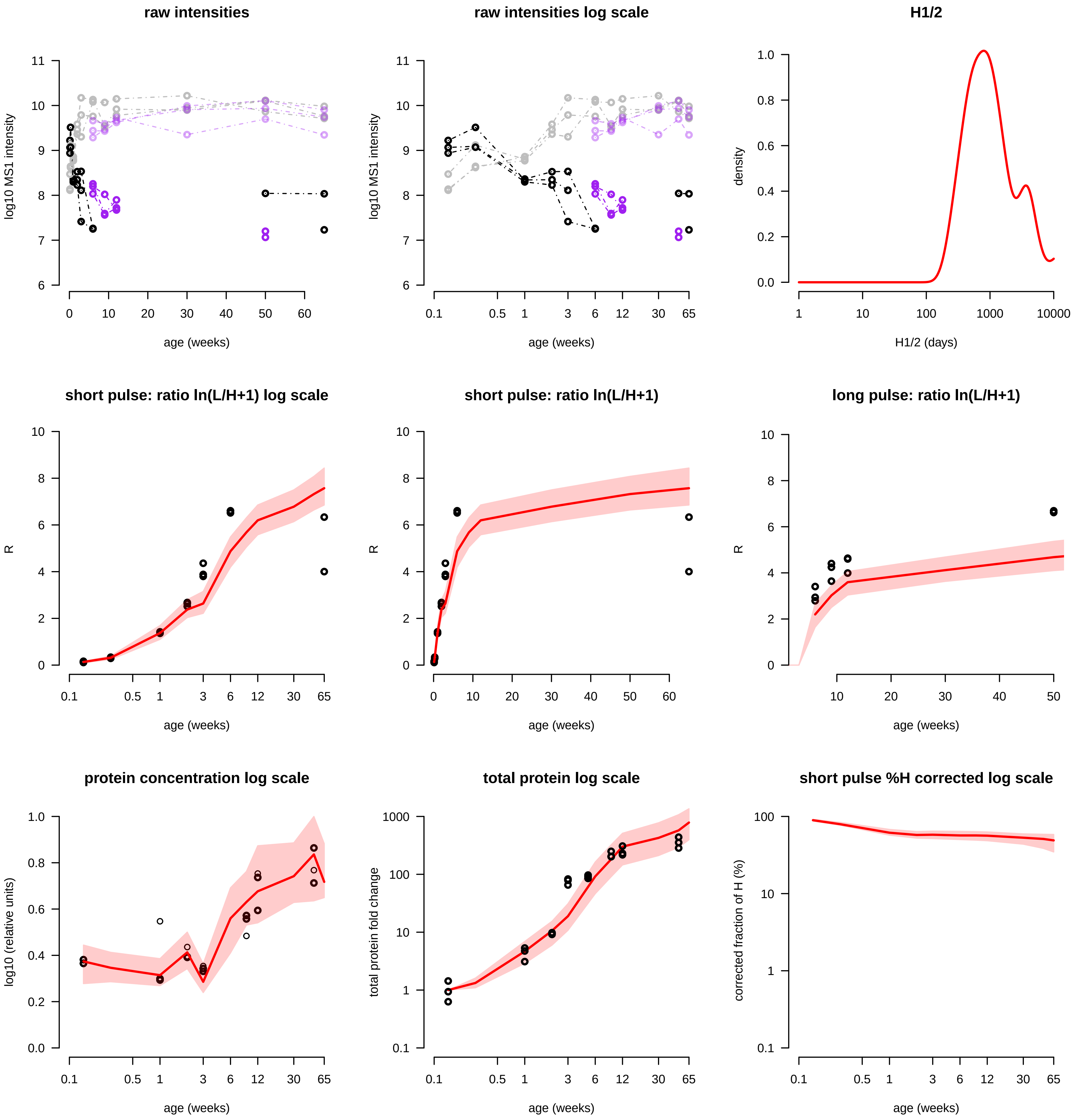

Q9CX86

>sp|Q9CX86|ROA0_MOUSE

Heterogeneous nuclear ribonucleoprotein A0

GN=Hnrnpa0

Med H1/2: 10.8 days

Q5 H1/2: 9 days

Q95 H1/2: 13.8 days

reliable model fit: yes

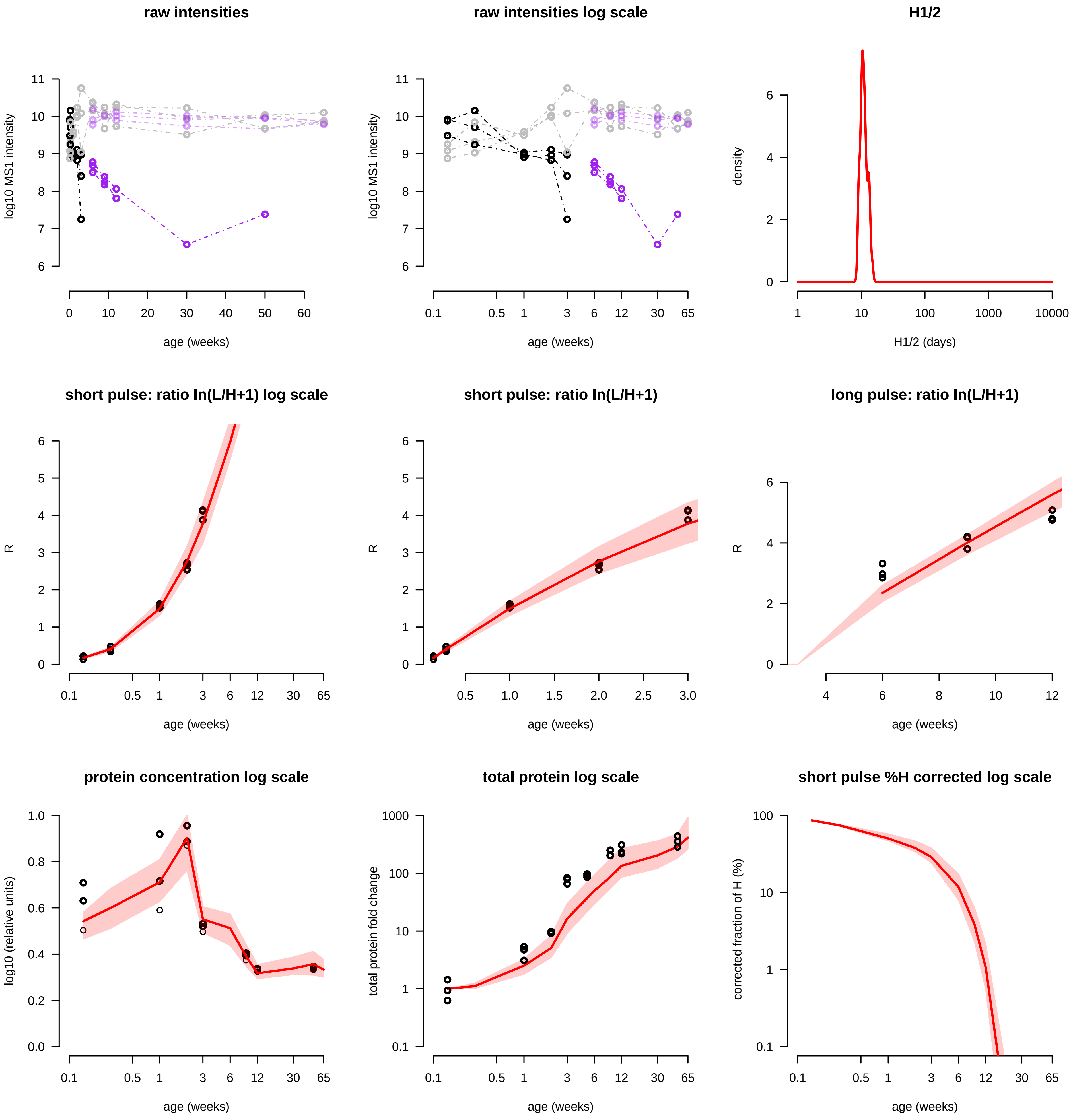

P97807−2;P97807

>sp|P97807−2|FUMH_MOUSE

Isoform Cytoplasmic of Fumarate hydratase, mitochondrial

GN=Fh

Med H1/2: 122.4 days

Q5 H1/2: 69.2 days

Q95 H1/2: 395.5 days

reliable model fit: yes

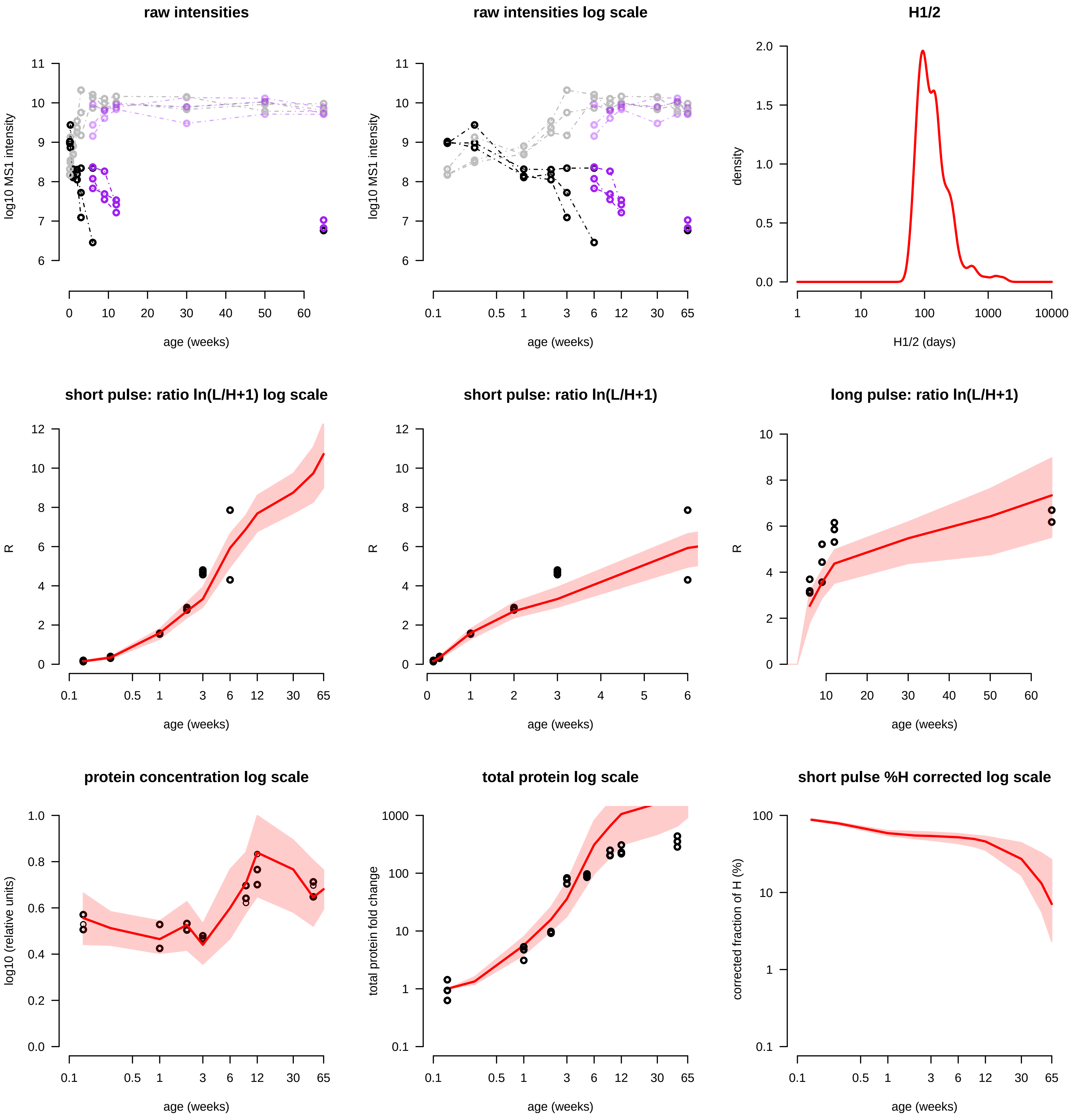

P07091

>sp|P07091|S10A4_MOUSE

Protein S100−A4

GN=S100a4

Med H1/2: 72.5 days

Q5 H1/2: 29.8 days

Q95 H1/2: 1683.8 days

reliable model fit: yes

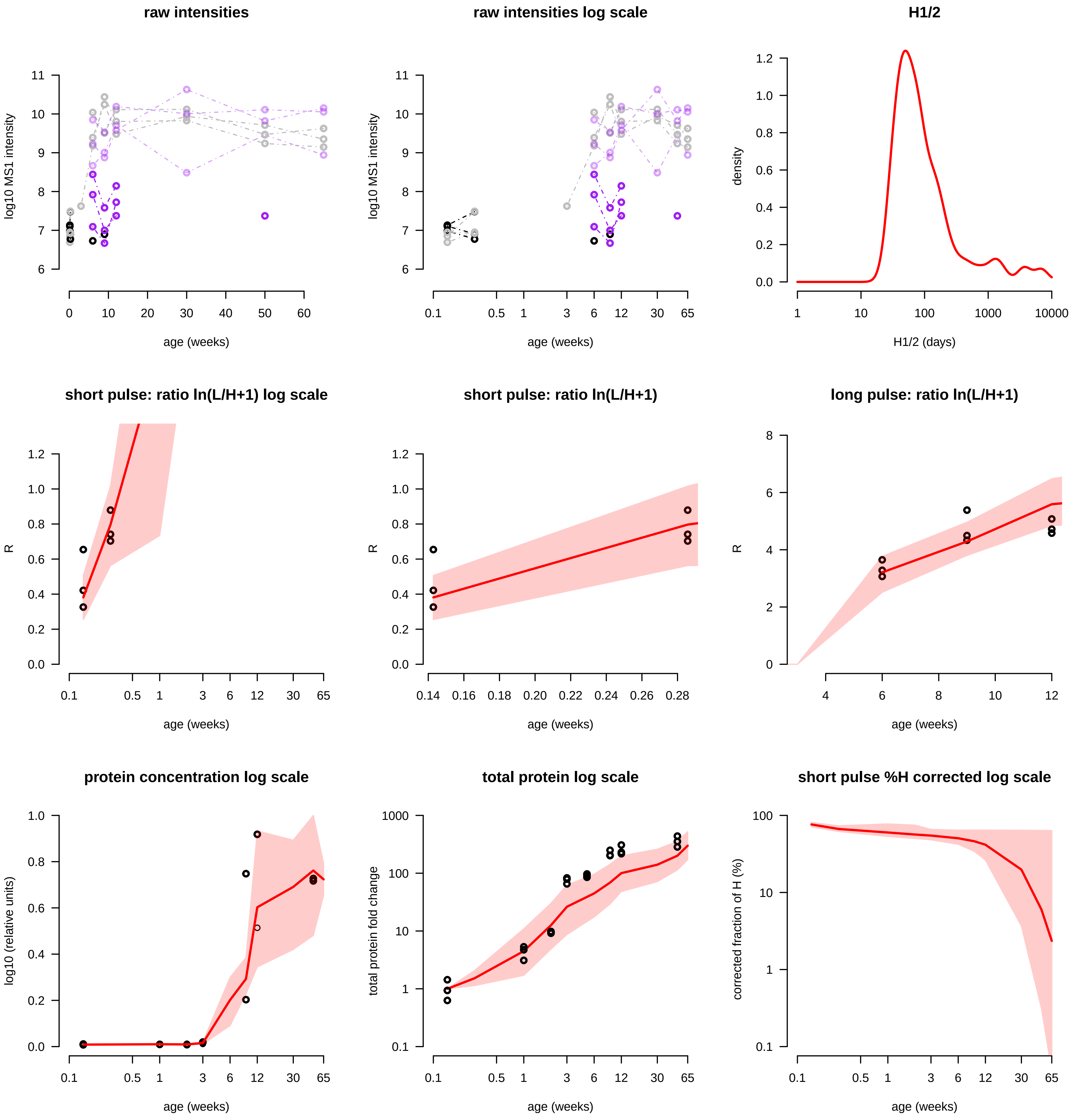

Q8QZT1

>sp|Q8QZT1|THIL_MOUSE

Acetyl−CoA acetyltransferase, mitochondrial

GN=Acat1

Med H1/2: 9.8 days

Q5 H1/2: 8.1 days

Q95 H1/2: 11.5 days

reliable model fit: yes

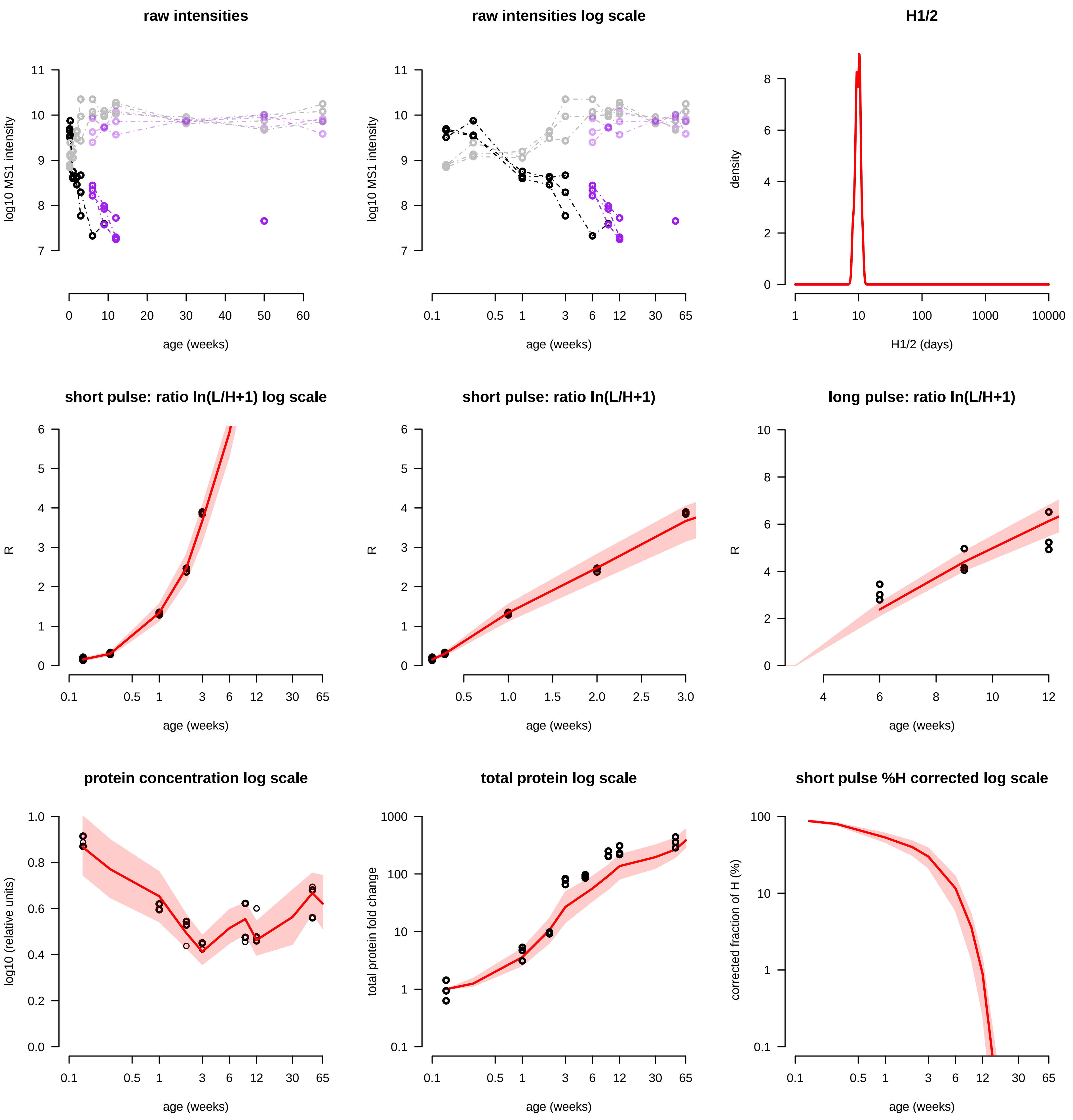

Q80X90

>sp|Q80X90|FLNB_MOUSE

Filamin−B

GN=Flnb

Med H1/2: 717.8 days

Q5 H1/2: 254.7 days

Q95 H1/2: 9519.2 days

reliable model fit: yes

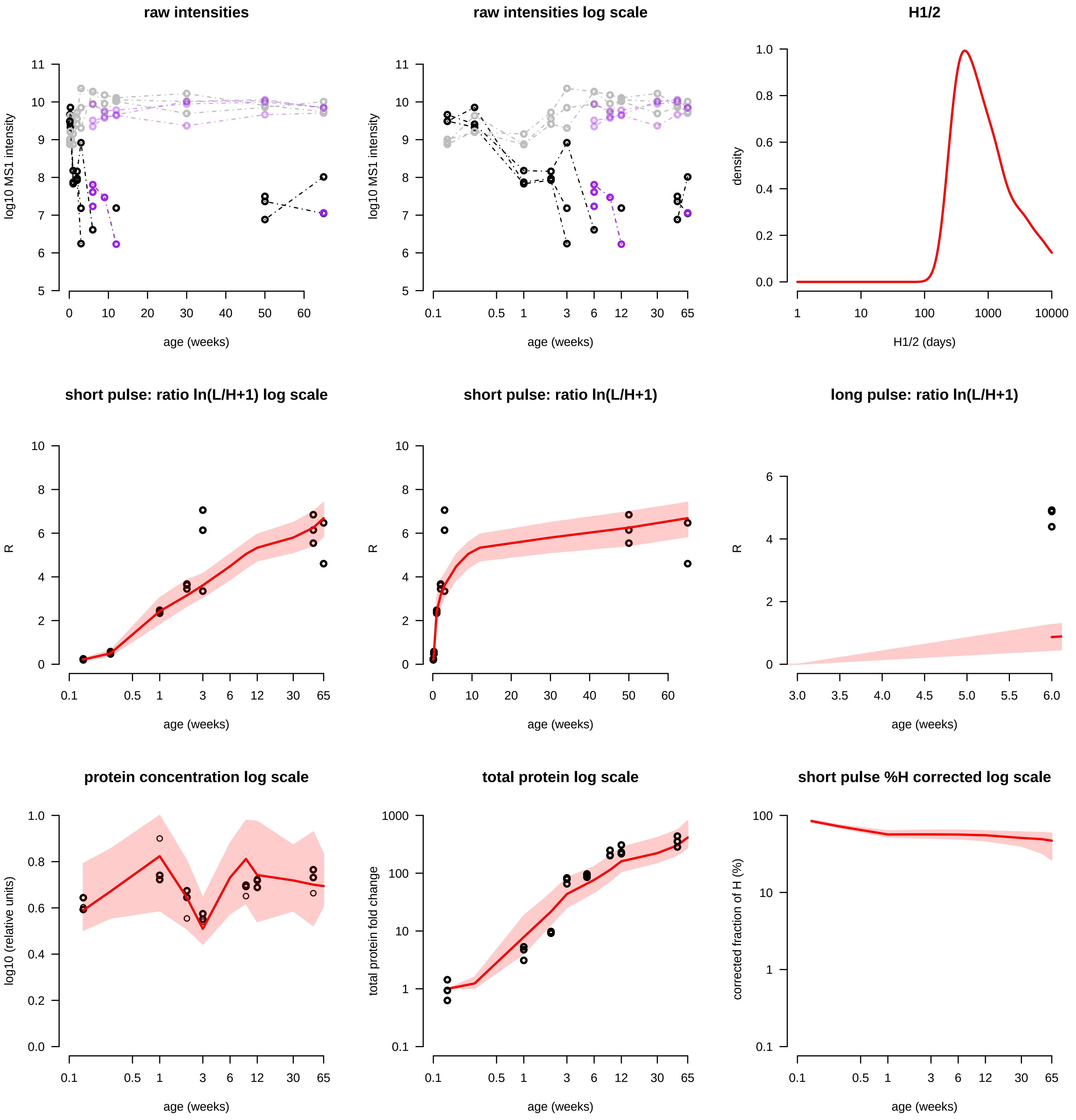

Q9DB77

>sp|Q9DB77|QCR2_MOUSE

Cytochrome b−c1 complex subunit 2, mitochondrial

GN=Uqcrc2

Med H1/2: 11.9 days

Q5 H1/2: 9.2 days

Q95 H1/2: 17.2 days

reliable model fit: yes

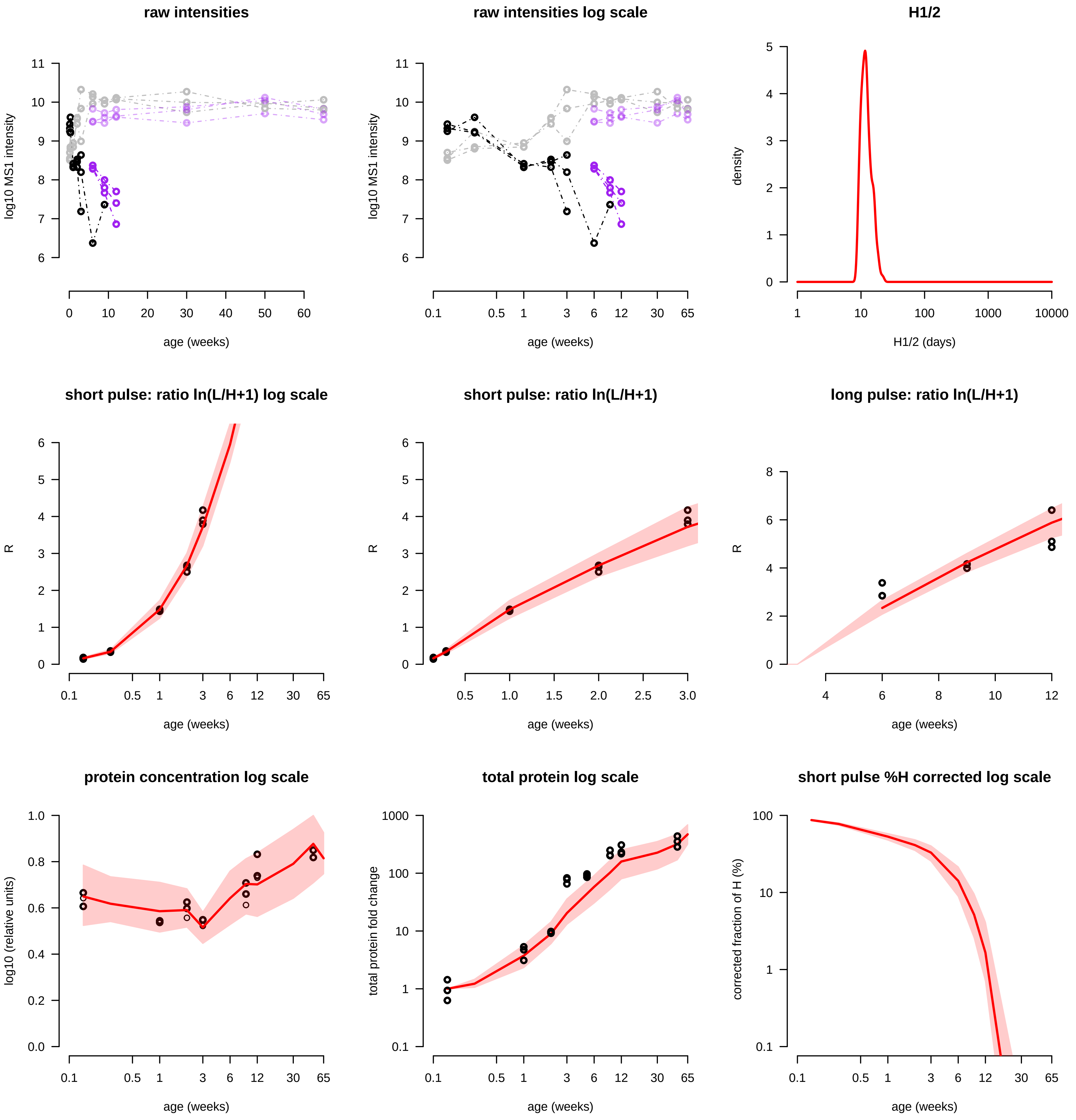

Q8VDN2

>sp|Q8VDN2|AT1A1_MOUSE

Sodium/potassium−transporting ATPase subunit alpha−1

GN=Atp1a1

Med H1/2: 44.7 days

Q5 H1/2: 32.6 days

Q95 H1/2: 62 days

reliable model fit: yes

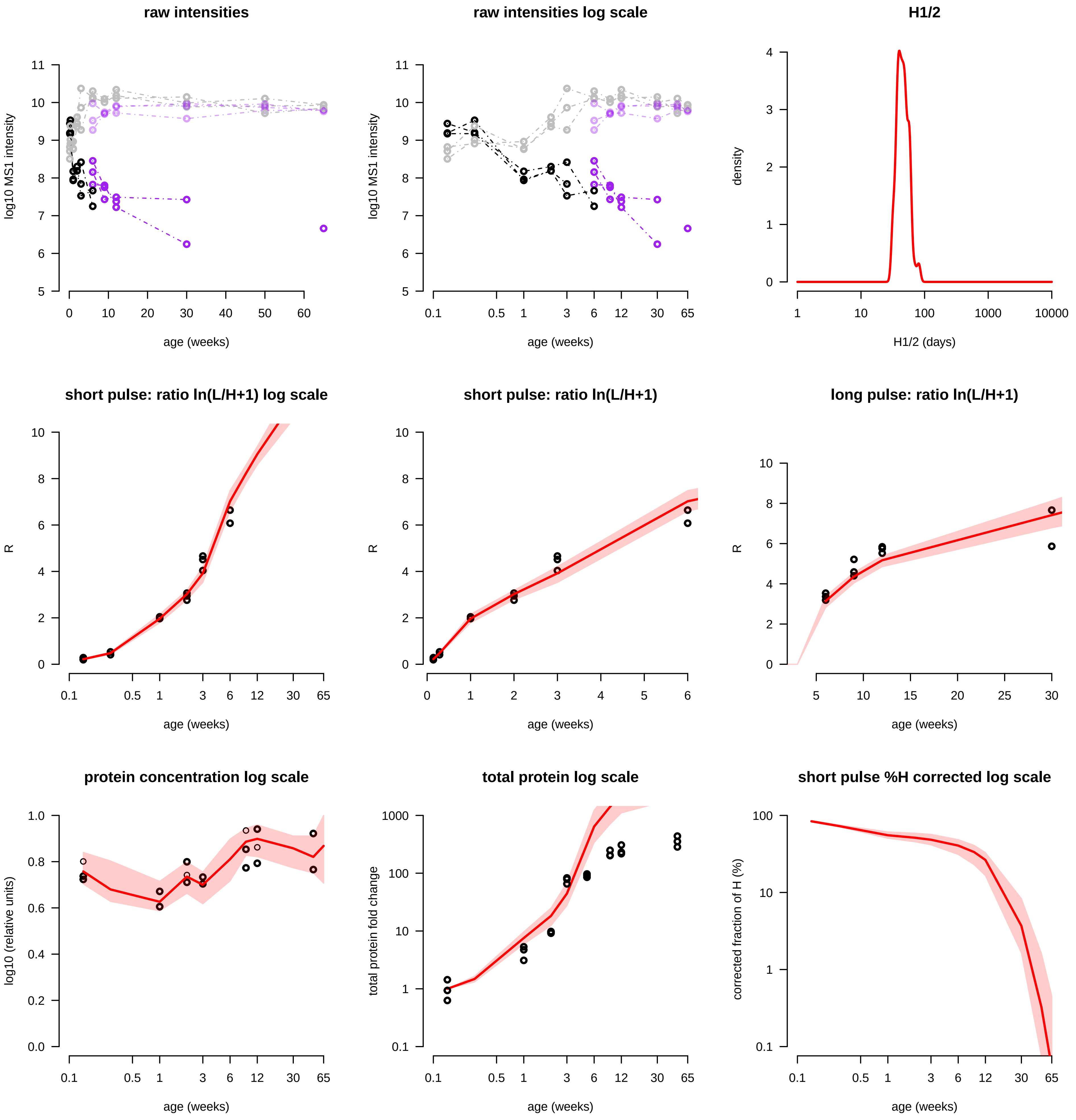

P31786

>sp|P31786|ACBP_MOUSE

Acyl−CoA−binding protein

GN=Dbi

Med H1/2: 10.2 days

Q5 H1/2: 7.7 days

Q95 H1/2: 16.4 days

reliable model fit: yes

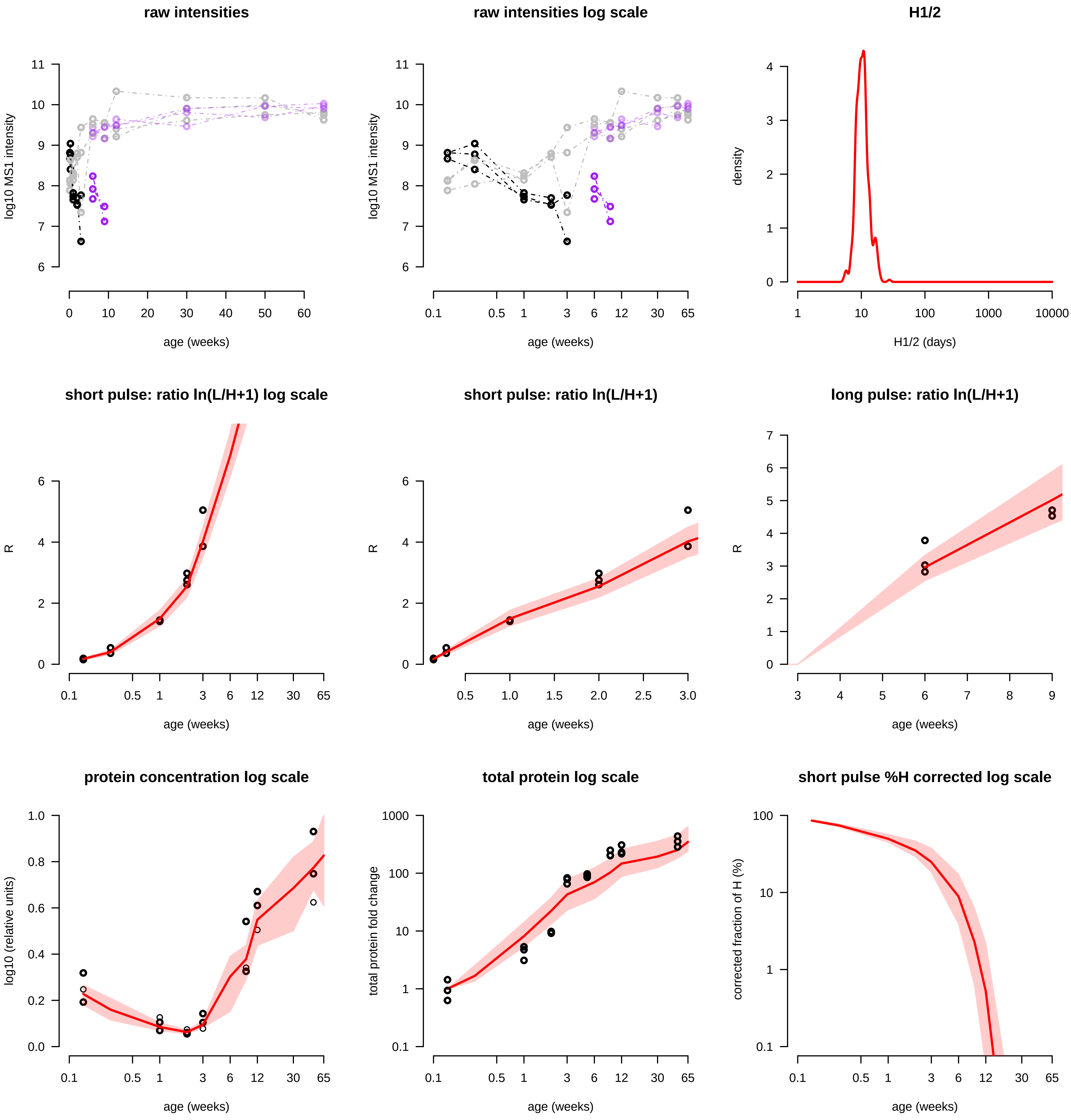

P16015

>sp|P16015|CAH3_MOUSE

Carbonic anhydrase 3

GN=Ca3

Med H1/2: 3.8 days

Q5 H1/2: 2.5 days

Q95 H1/2: 21.5 days

reliable model fit: yes

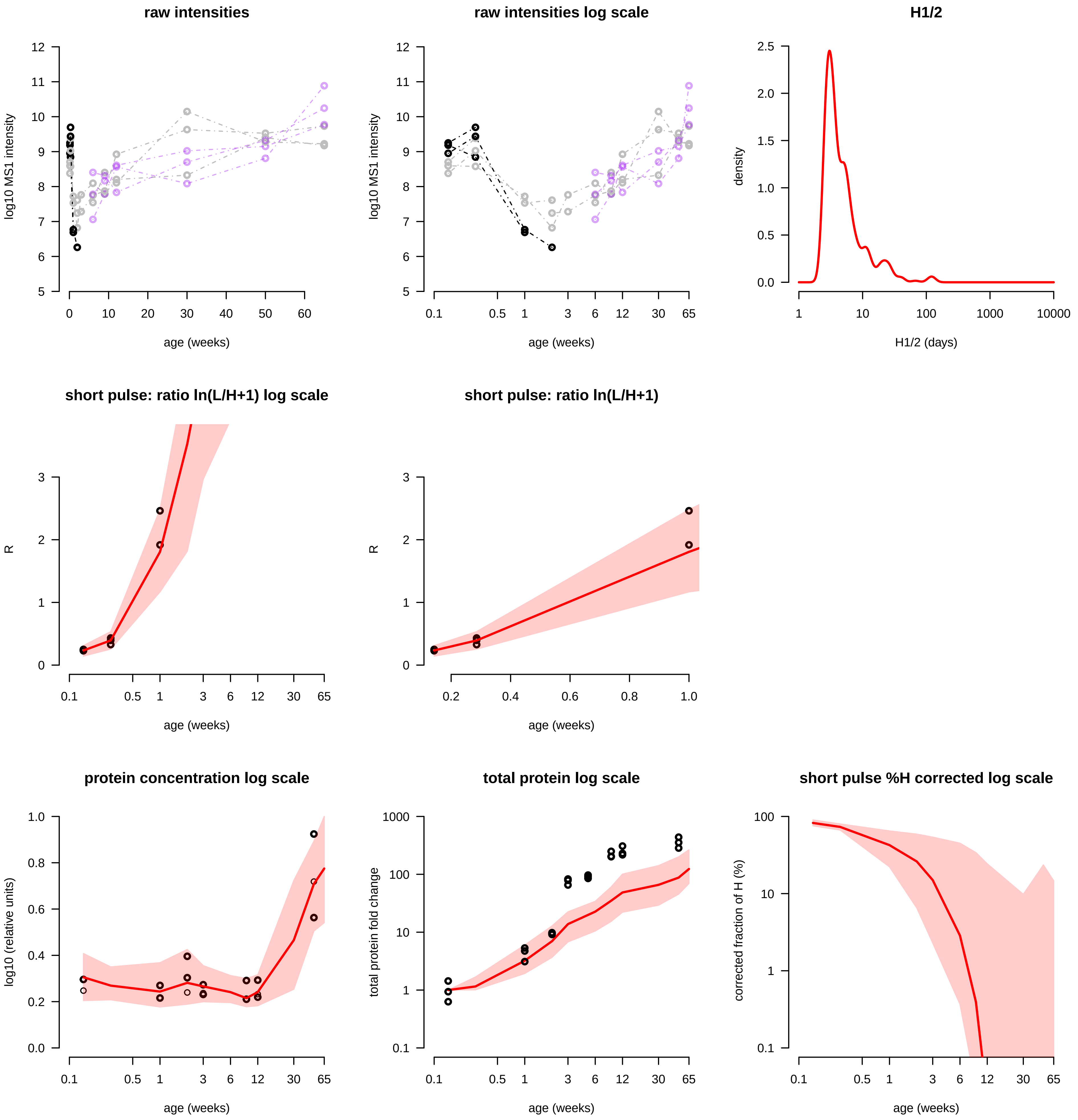

P47911

>sp|P47911|RL6_MOUSE

60S ribosomal protein L6

GN=Rpl6

Med H1/2: 24.5 days

Q5 H1/2: 18.5 days

Q95 H1/2: 32.4 days

reliable model fit: yes

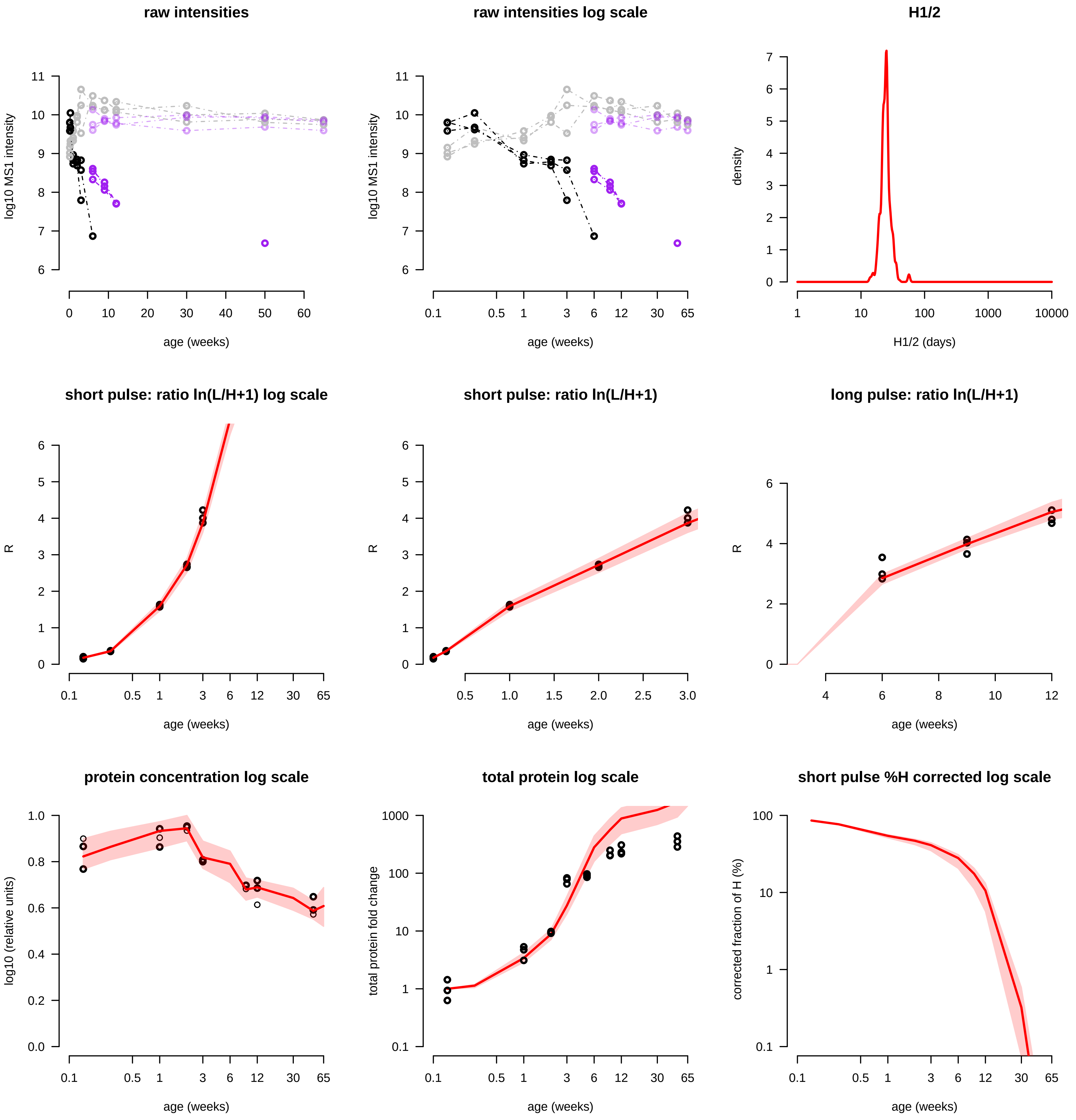

Q99KQ4

>sp|Q99KQ4|NAMPT_MOUSE

Nicotinamide phosphoribosyltransferase

GN=Nampt

Med H1/2: 41.6 days

Q5 H1/2: 27.7 days

Q95 H1/2: 189.6 days

reliable model fit: yes

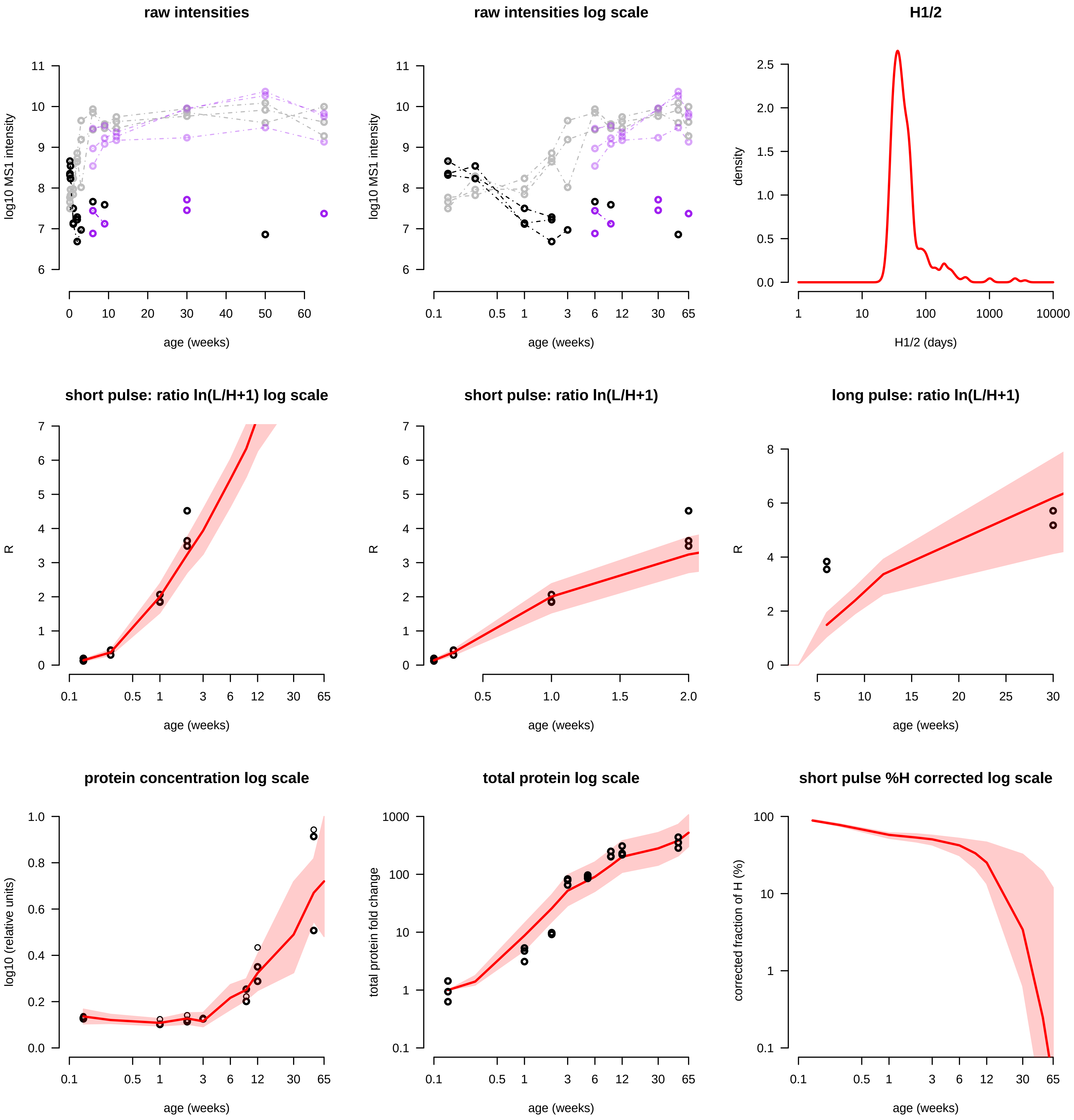

Q9CQQ7

>sp|Q9CQQ7|AT5F1_MOUSE

ATP synthase F(0) complex subunit B1, mitochondrial

GN=Atp5pb

Med H1/2: 11.8 days

Q5 H1/2: 9.8 days

Q95 H1/2: 15.4 days

reliable model fit: yes

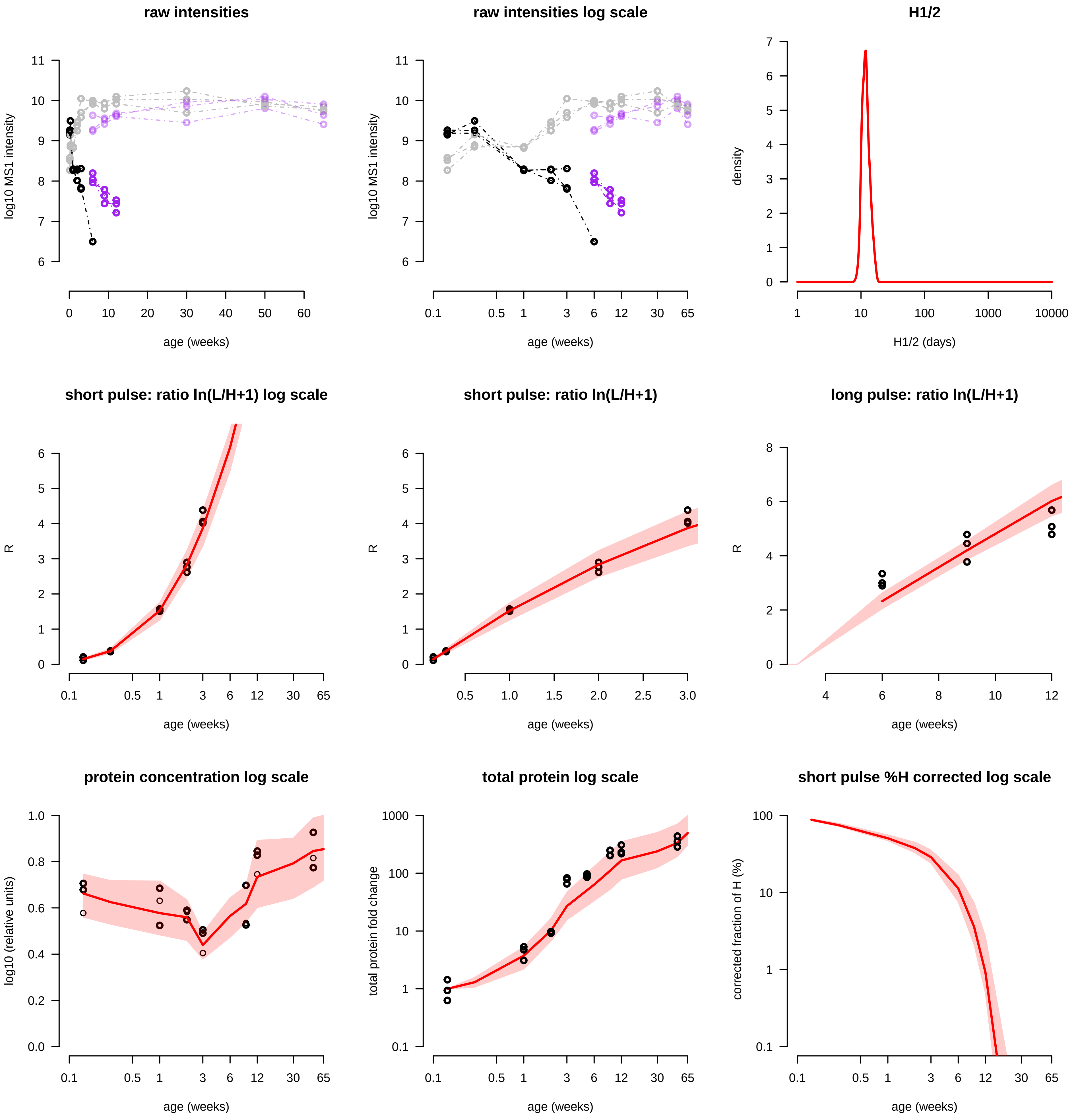

P99029−2;P99029

>sp|P99029−2|PRDX5_MOUSE

Isoform Cytoplasmic+peroxisomal of Peroxiredoxin−5, mitochondrial

GN=Prdx5

Med H1/2: 548.4 days

Q5 H1/2: 149.2 days

Q95 H1/2: 4402.3 days

reliable model fit: yes

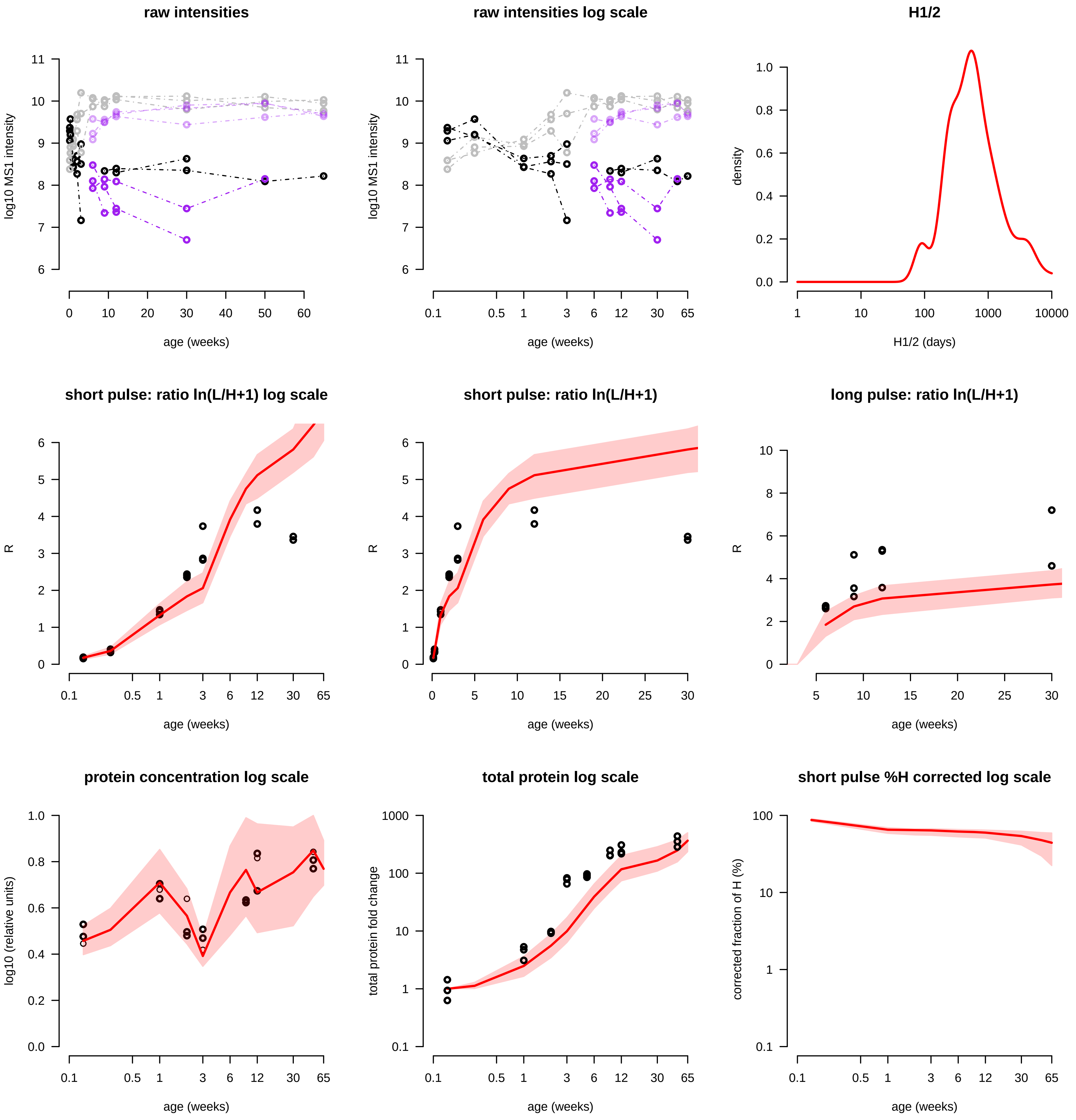

Q9D379

>sp|Q9D379|HYEP_MOUSE

Epoxide hydrolase 1

GN=Ephx1

Med H1/2: 9.5 days

Q5 H1/2: 7.2 days

Q95 H1/2: 12.5 days

reliable model fit: yes

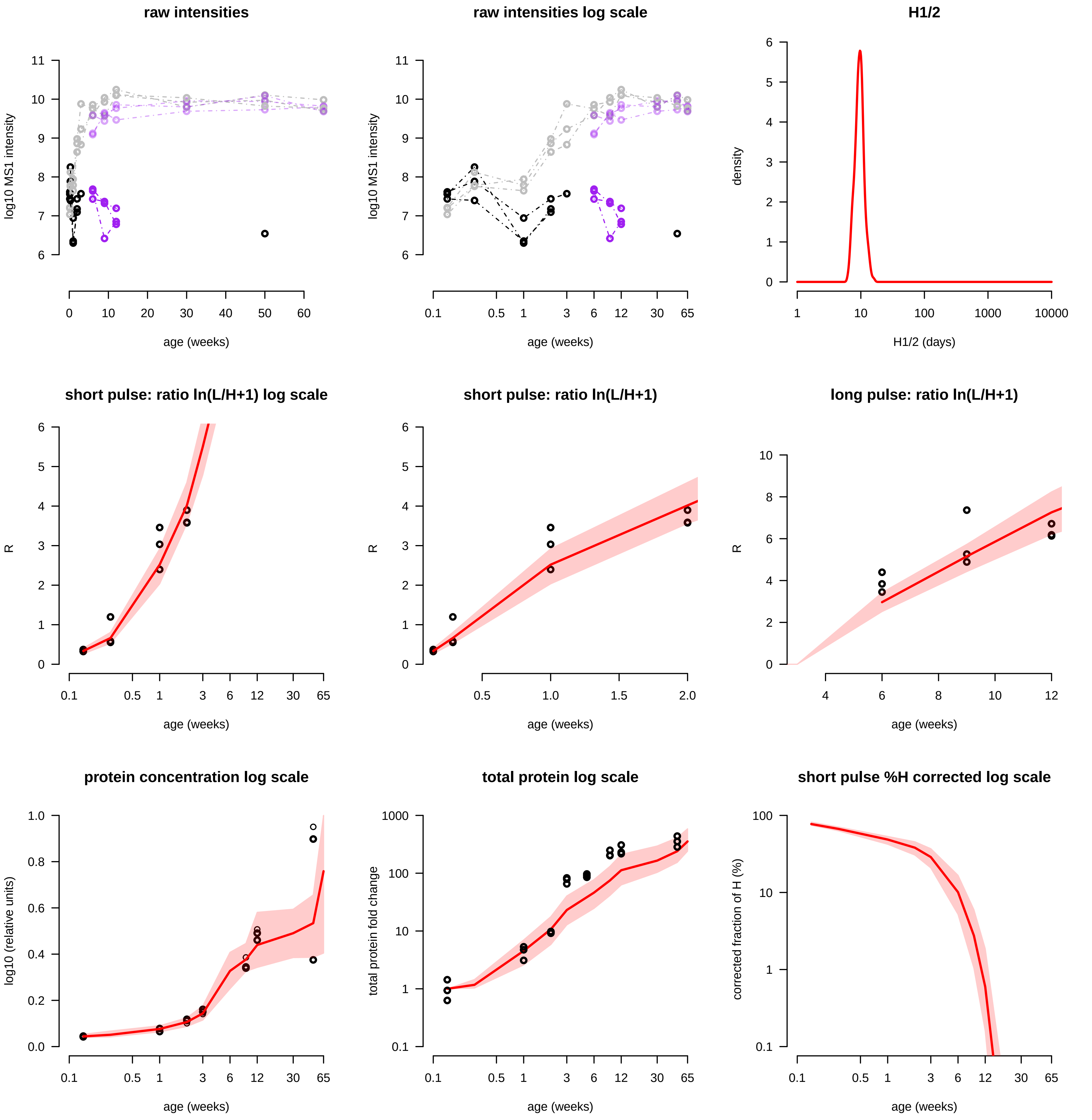

Q91YQ5

>sp|Q91YQ5|RPN1_MOUSE

Dolichyl−diphosphooligosaccharide−−protein glycosyltransferase subunit 1

GN=Rpn1

Med H1/2: 28 days

Q5 H1/2: 23 days

Q95 H1/2: 34.1 days

reliable model fit: yes

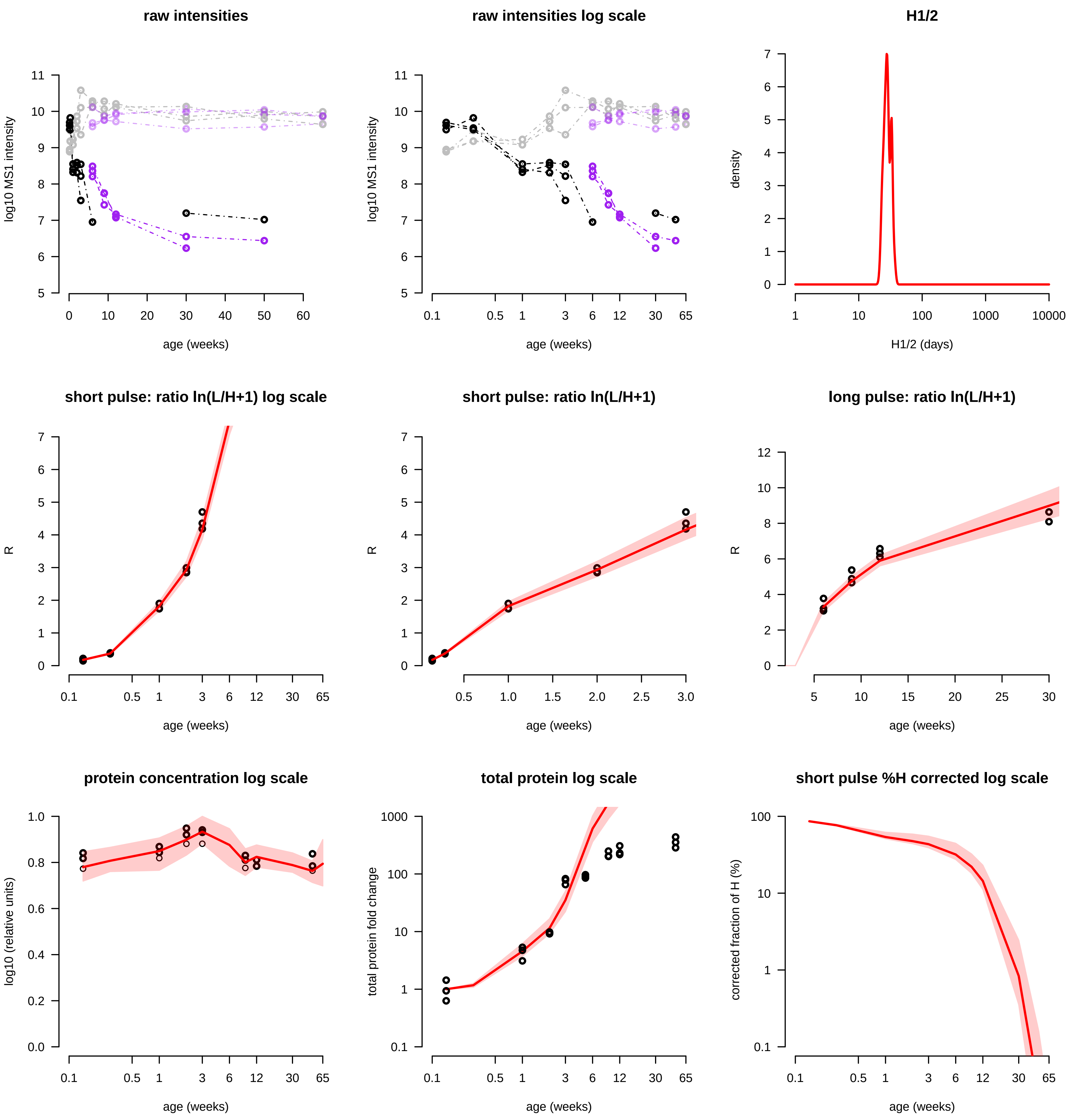

P14069

>sp|P14069|S10A6_MOUSE

Protein S100−A6

GN=S100a6

Med H1/2: 226.7 days

Q5 H1/2: 59.2 days

Q95 H1/2: 2988.4 days

reliable model fit: yes

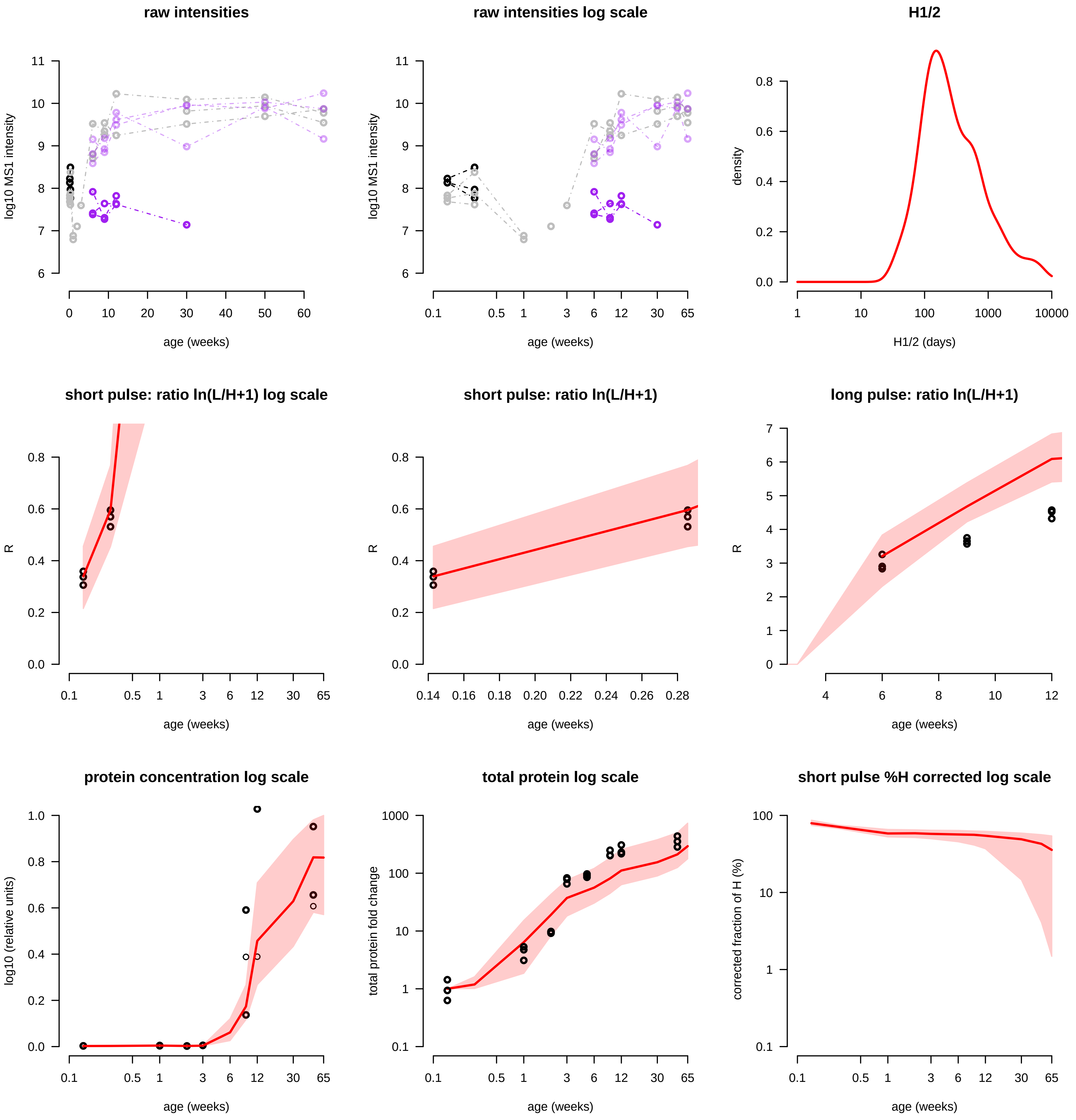

O08749

>sp|O08749|DLDH_MOUSE

Dihydrolipoyl dehydrogenase, mitochondrial

GN=Dld

Med H1/2: 532.9 days

Q5 H1/2: 231.1 days

Q95 H1/2: 7472.2 days

reliable model fit: yes

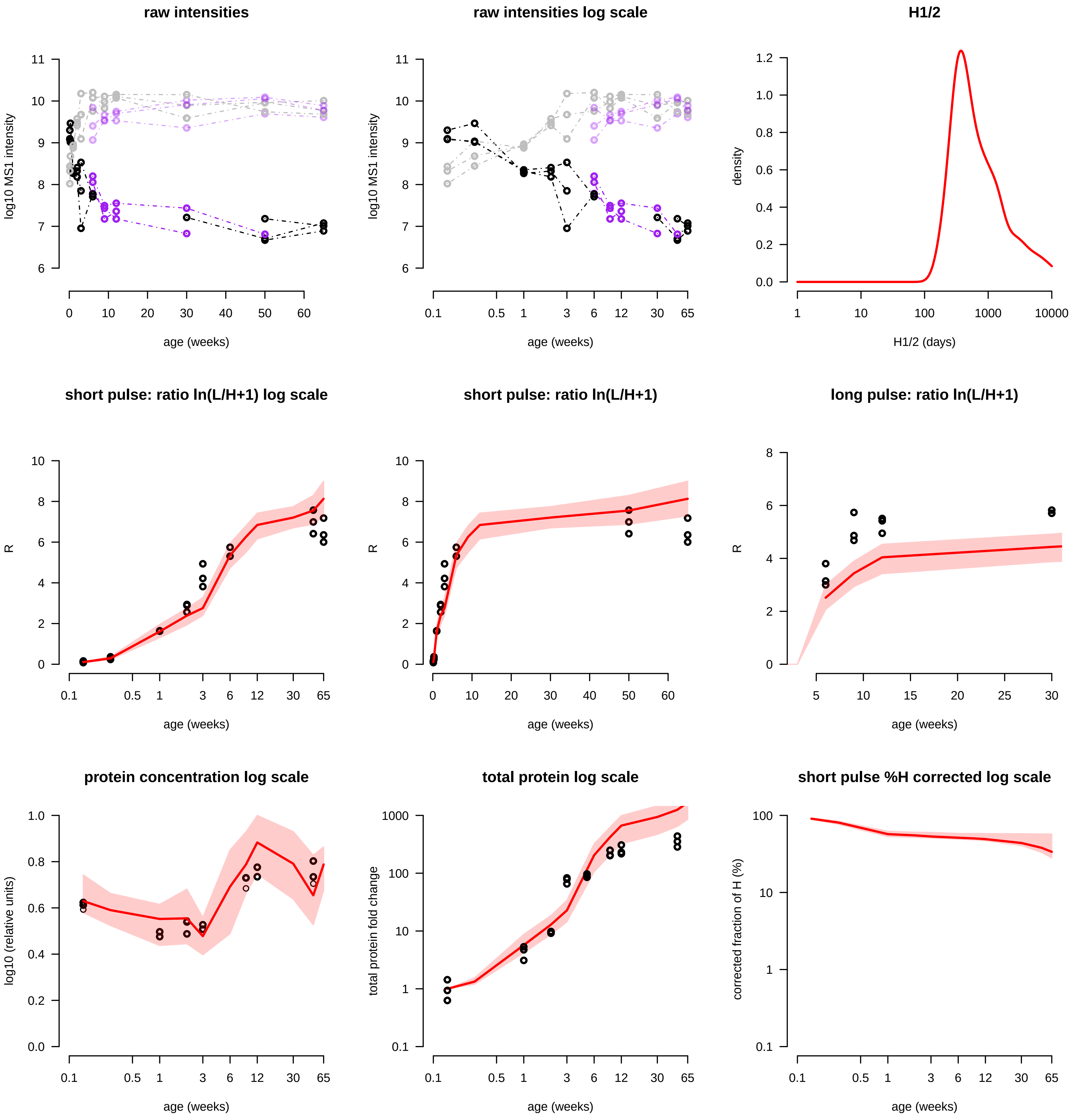

Q8VIJ6

>sp|Q8VIJ6|SFPQ_MOUSE

Splicing factor, proline− and glutamine−rich

GN=Sfpq

Med H1/2: 439.3 days

Q5 H1/2: 205.2 days

Q95 H1/2: 3447.1 days

reliable model fit: yes

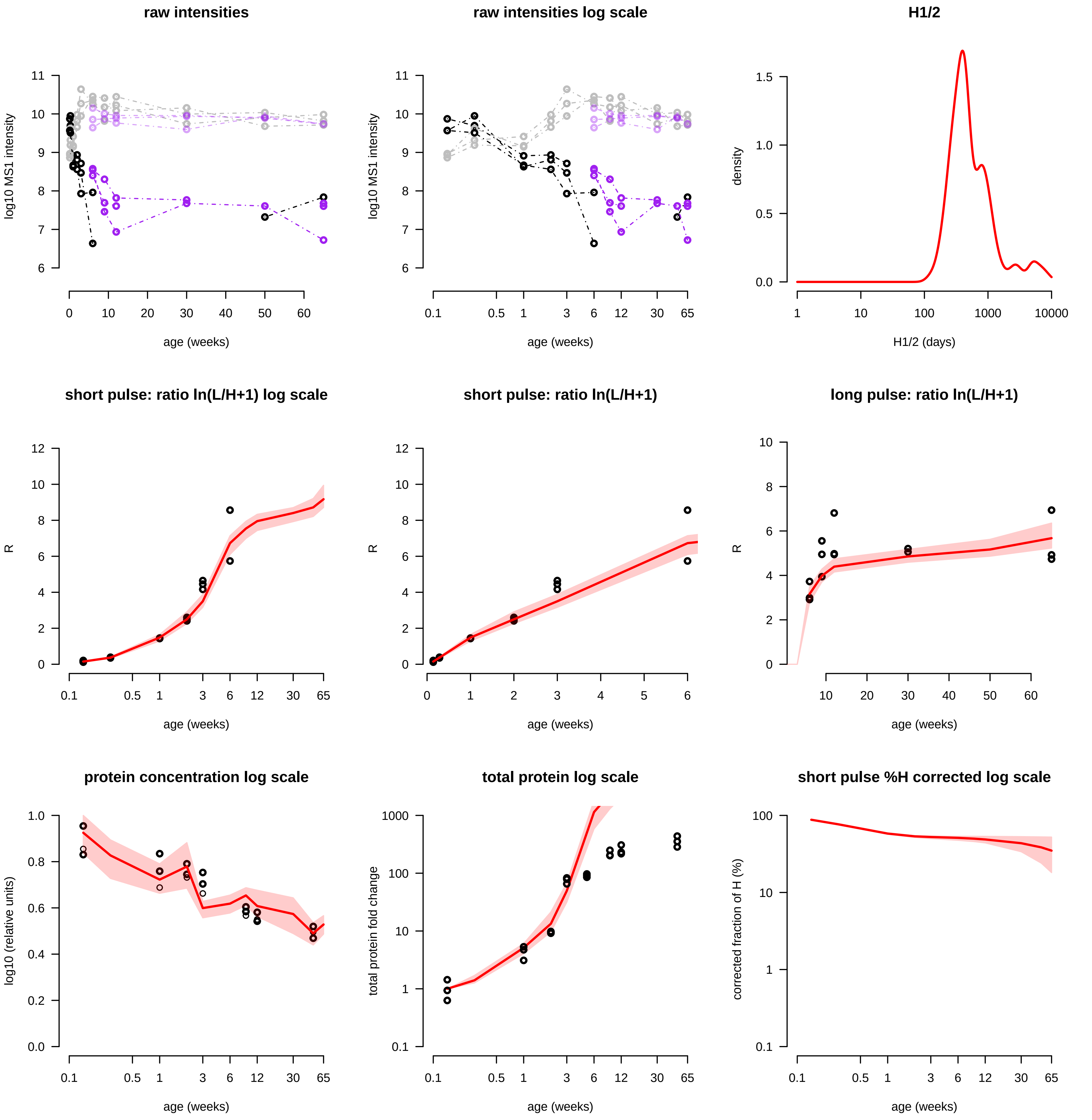

P60843

>sp|P60843|IF4A1_MOUSE

Eukaryotic initiation factor 4A−I

GN=Eif4a1

Med H1/2: 11.3 days

Q5 H1/2: 9.4 days

Q95 H1/2: 13.7 days

reliable model fit: yes

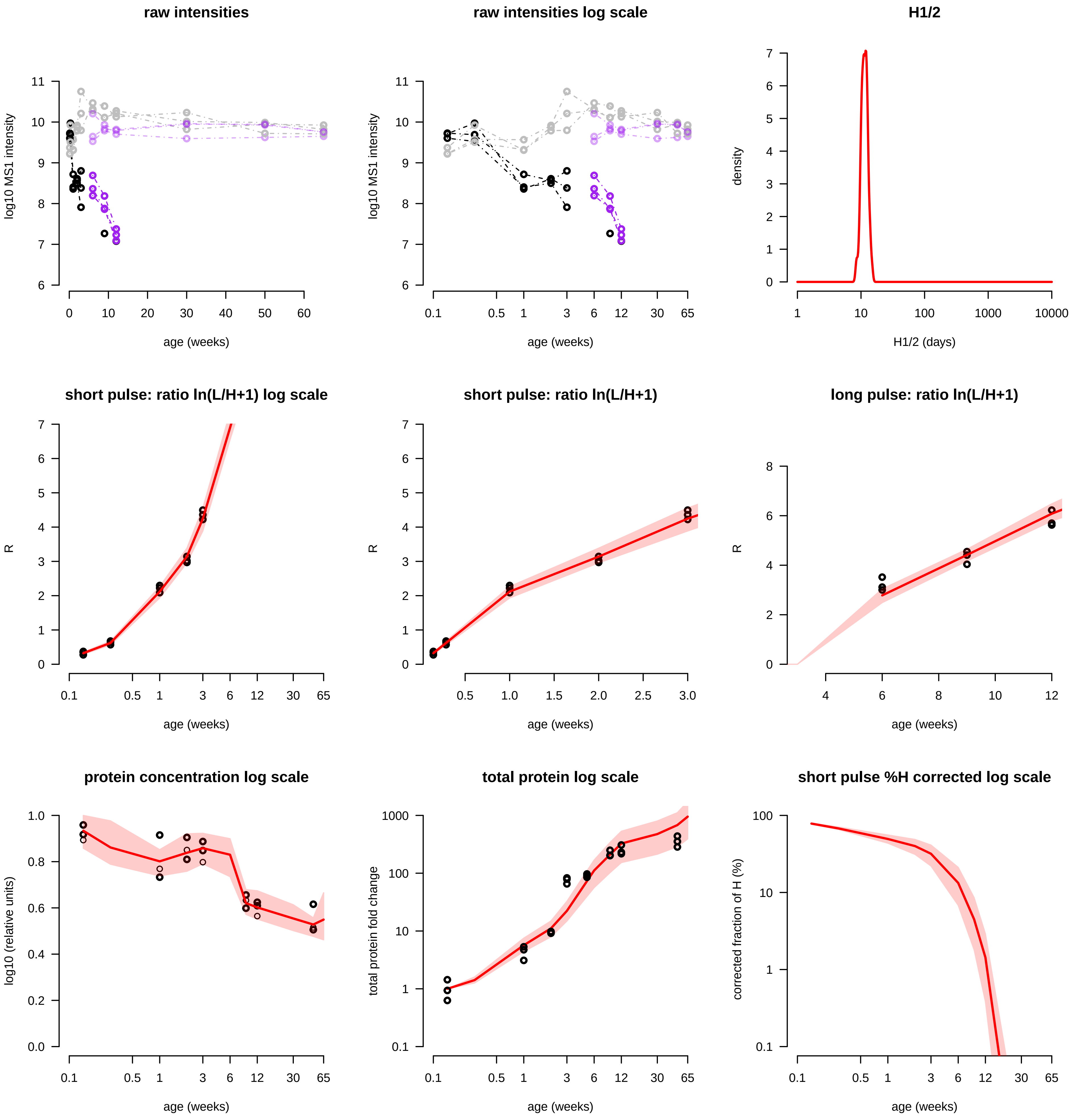

P62827

>sp|P62827|RAN_MOUSE

GTP−binding nuclear protein Ran

GN=Ran

Med H1/2: 710.4 days

Q5 H1/2: 211.3 days

Q95 H1/2: 5171.1 days

reliable model fit: yes

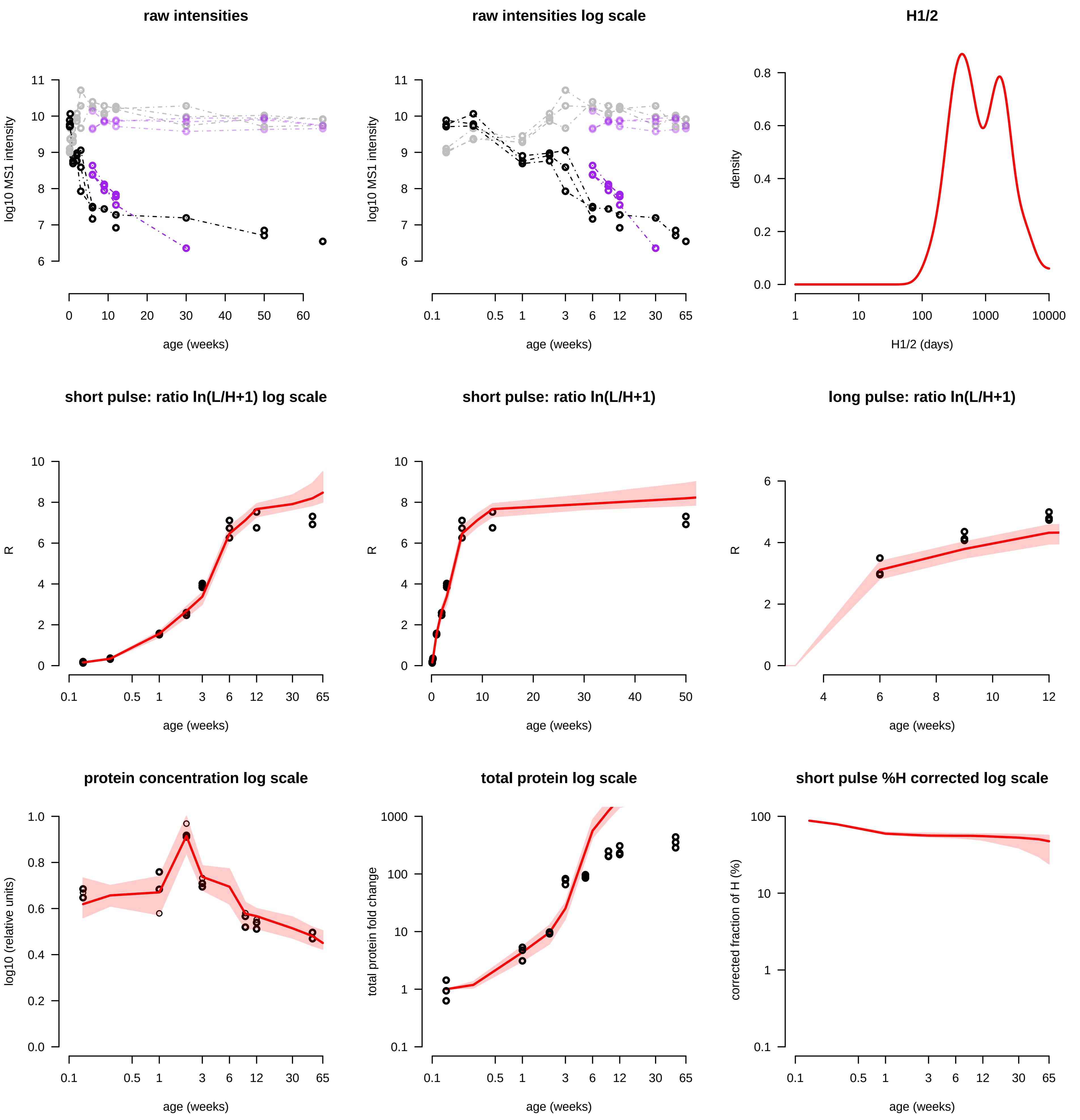

P53026

>sp|P53026|RL10A_MOUSE

60S ribosomal protein L10a

GN=Rpl10a

Med H1/2: 685.8 days

Q5 H1/2: 253.2 days

Q95 H1/2: 3805.3 days

reliable model fit: yes

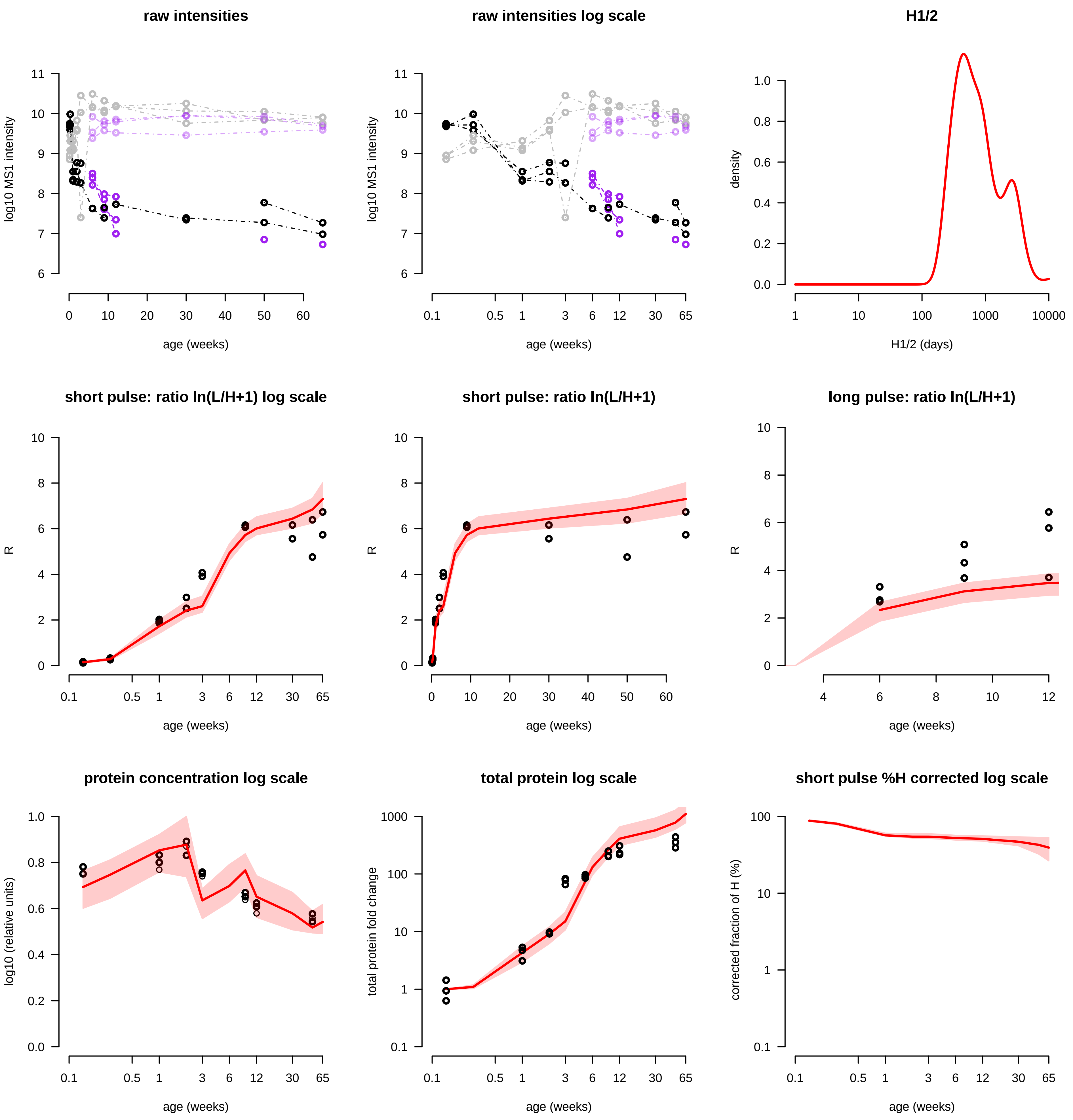

Q61879

>sp|Q61879|MYH10_MOUSE

Myosin−10

GN=Myh10

Med H1/2: 314.2 days

Q5 H1/2: 138.4 days

Q95 H1/2: 1150.5 days

reliable model fit: yes

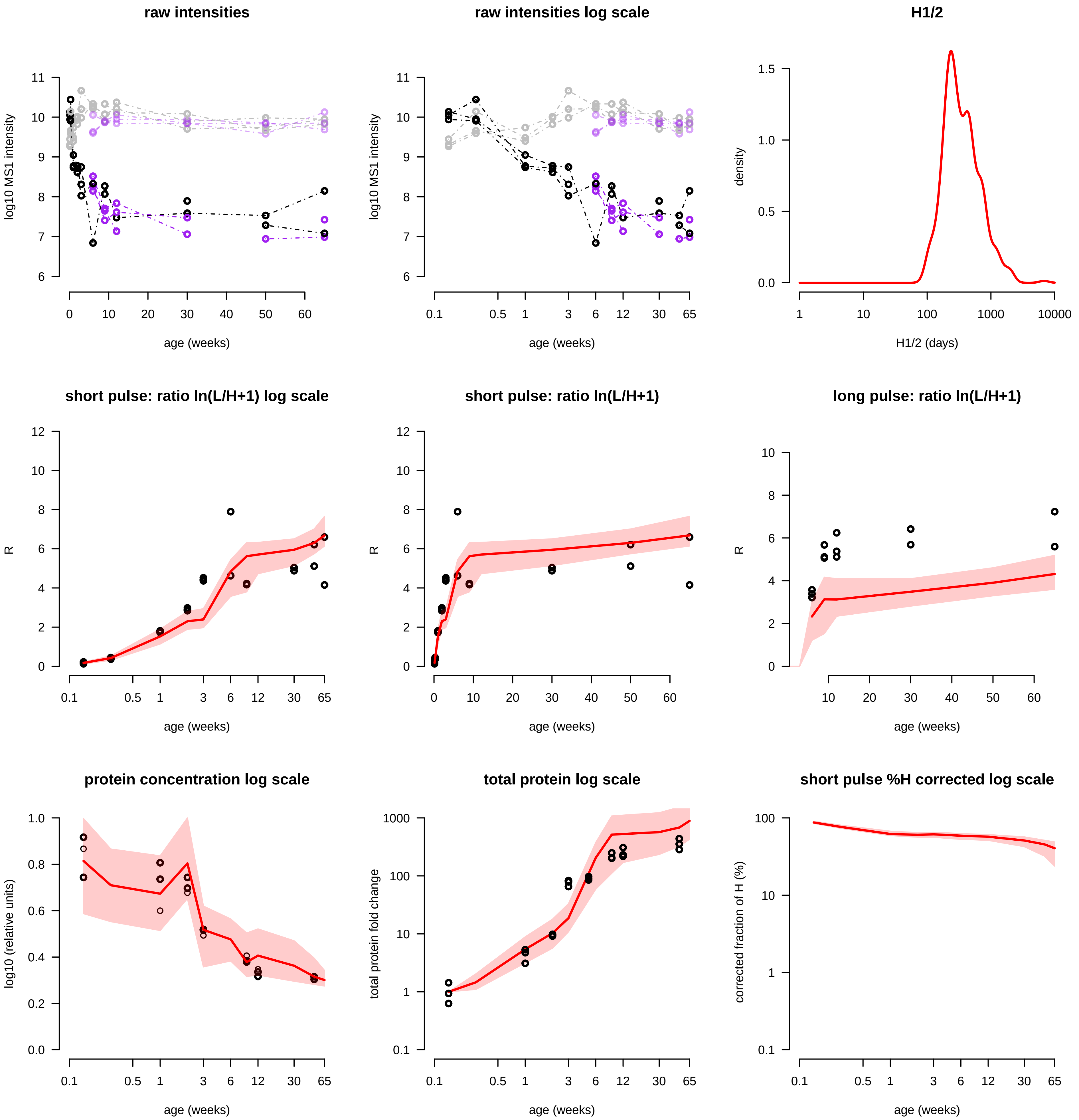

P05202

>sp|P05202|AATM_MOUSE

Aspartate aminotransferase, mitochondrial

GN=Got2

Med H1/2: 12 days

Q5 H1/2: 9.5 days

Q95 H1/2: 16.3 days

reliable model fit: yes

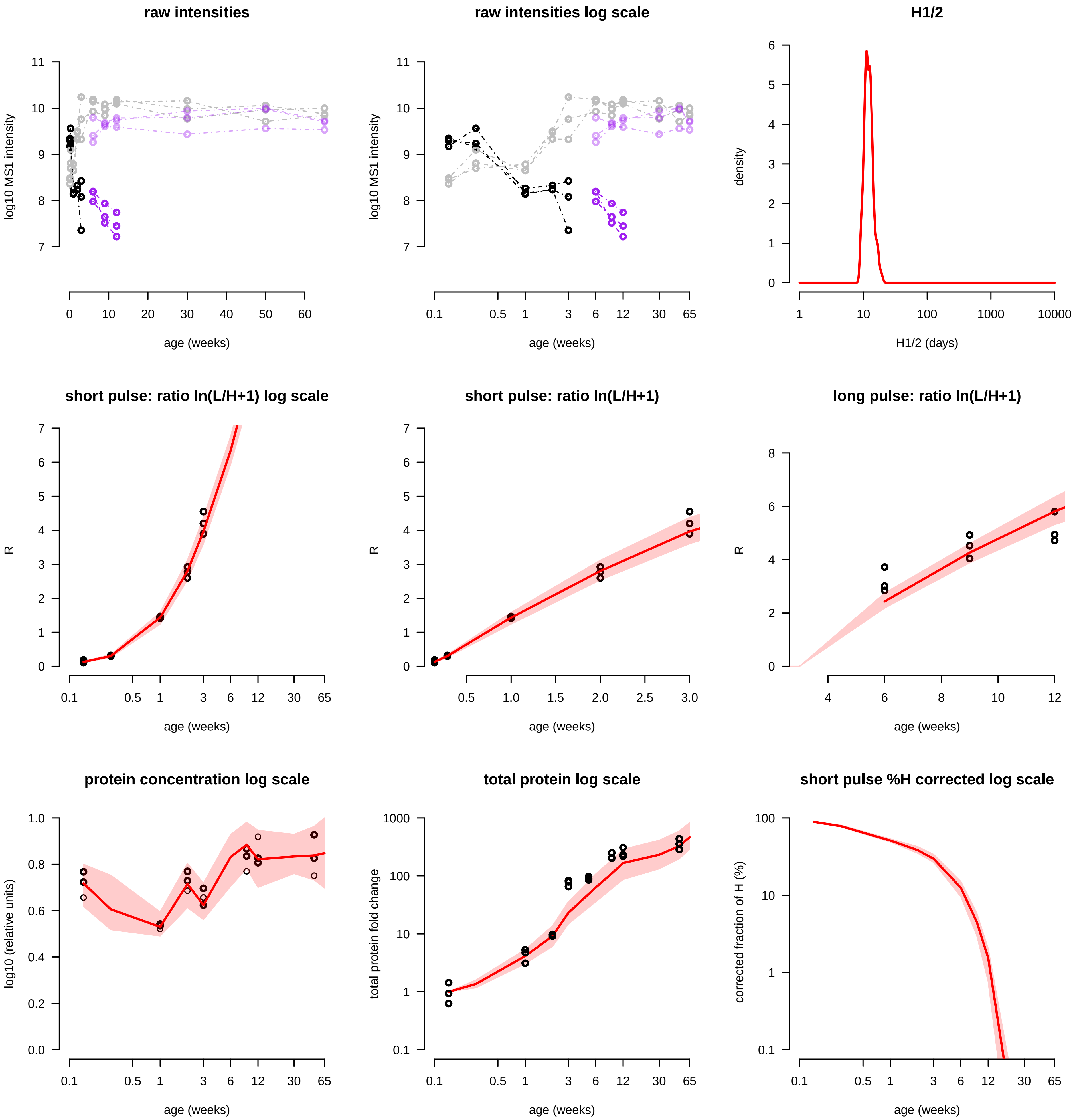

P19157

>sp|P19157|GSTP1_MOUSE

Glutathione S−transferase P 1

GN=Gstp1

Med H1/2: 7.4 days

Q5 H1/2: 5.8 days

Q95 H1/2: 11.9 days

reliable model fit: yes

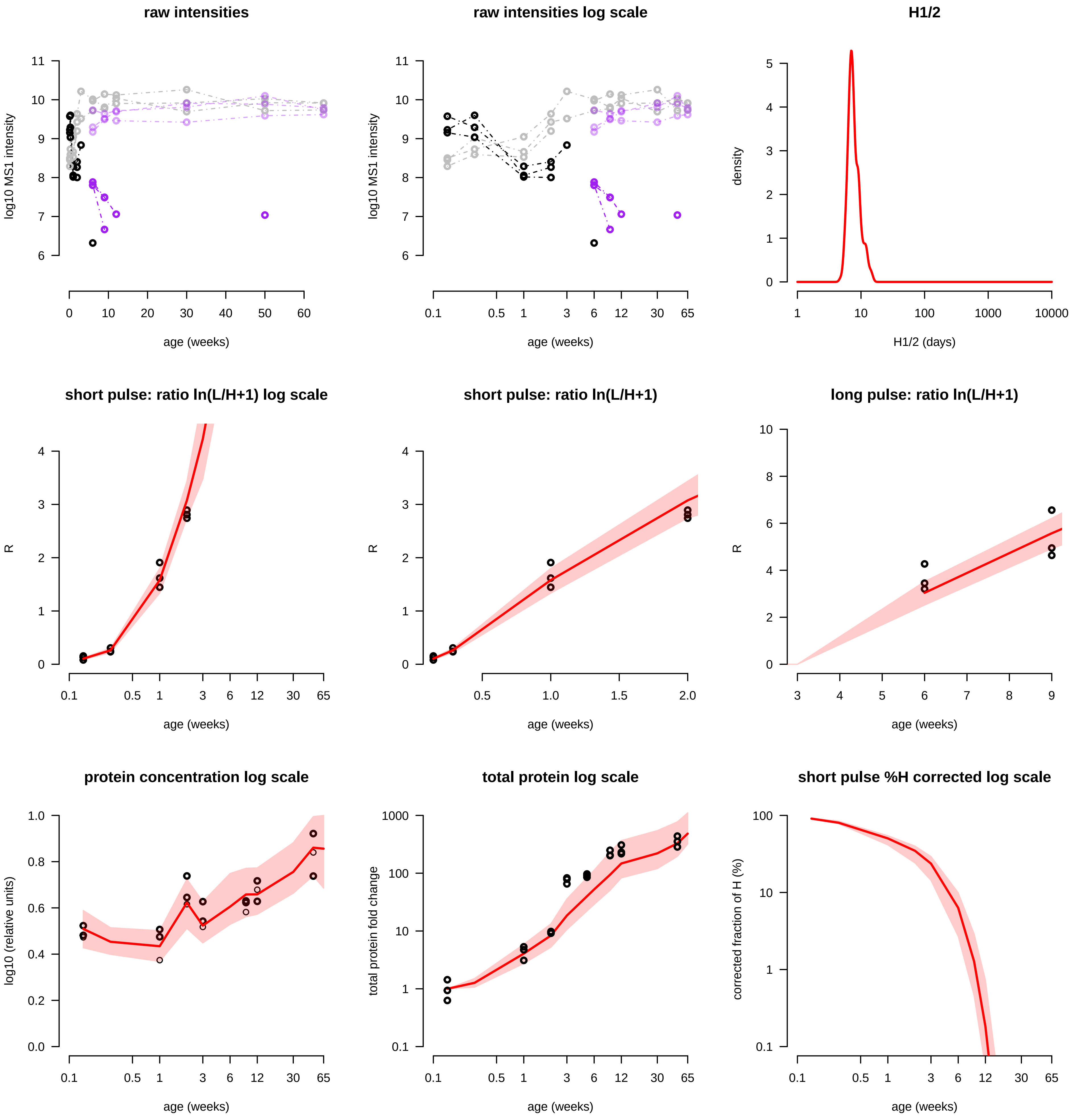

P49312;P49312−2

>sp|P49312|ROA1_MOUSE

Heterogeneous nuclear ribonucleoprotein A1

GN=Hnrnpa1

Med H1/2: 11.7 days

Q5 H1/2: 9.4 days

Q95 H1/2: 13.5 days

reliable model fit: yes

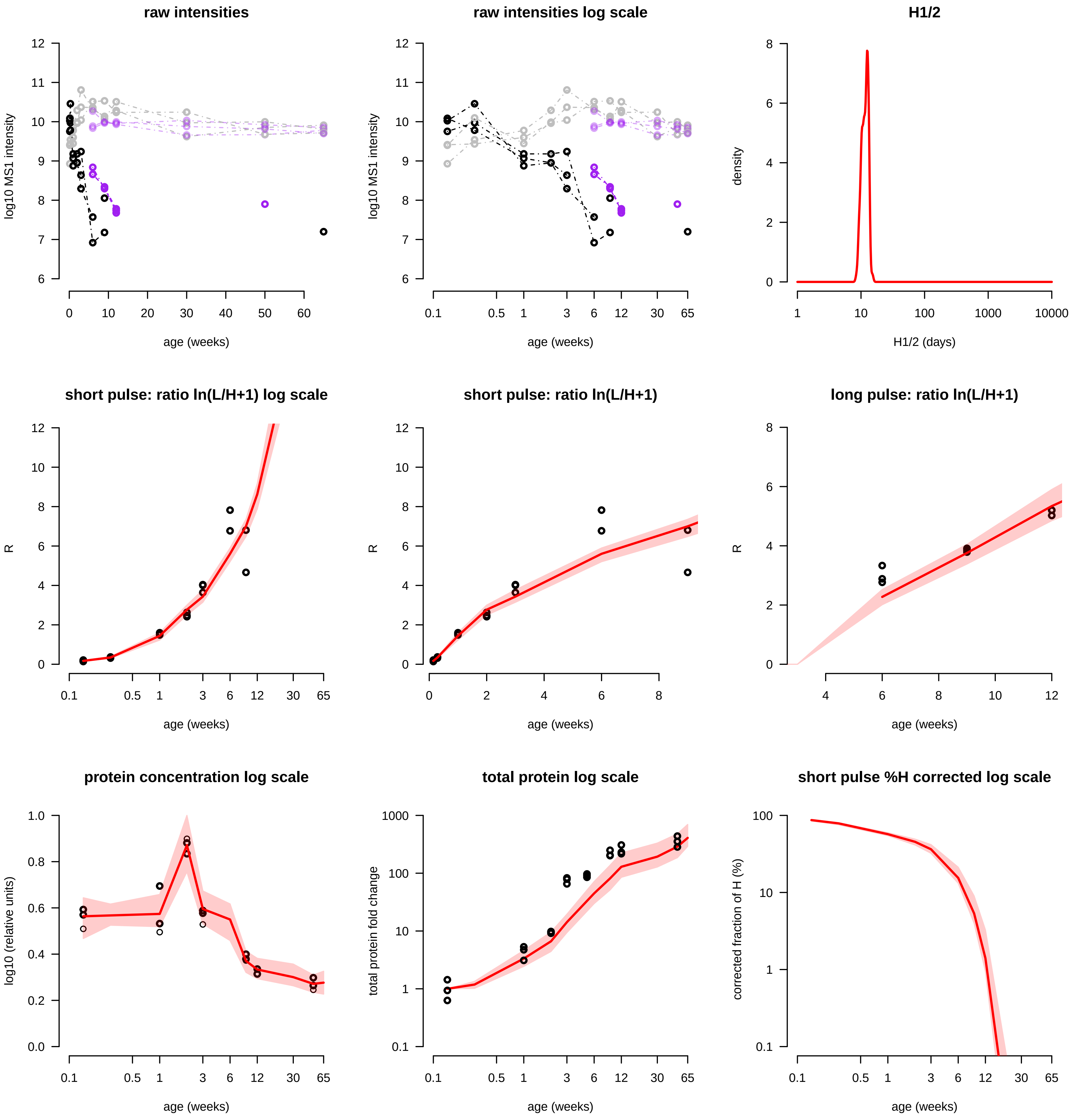

Q61937

>sp|Q61937|NPM_MOUSE

Nucleophosmin

GN=Npm1

Med H1/2: 10 days

Q5 H1/2: 8.4 days

Q95 H1/2: 13.1 days

reliable model fit: yes

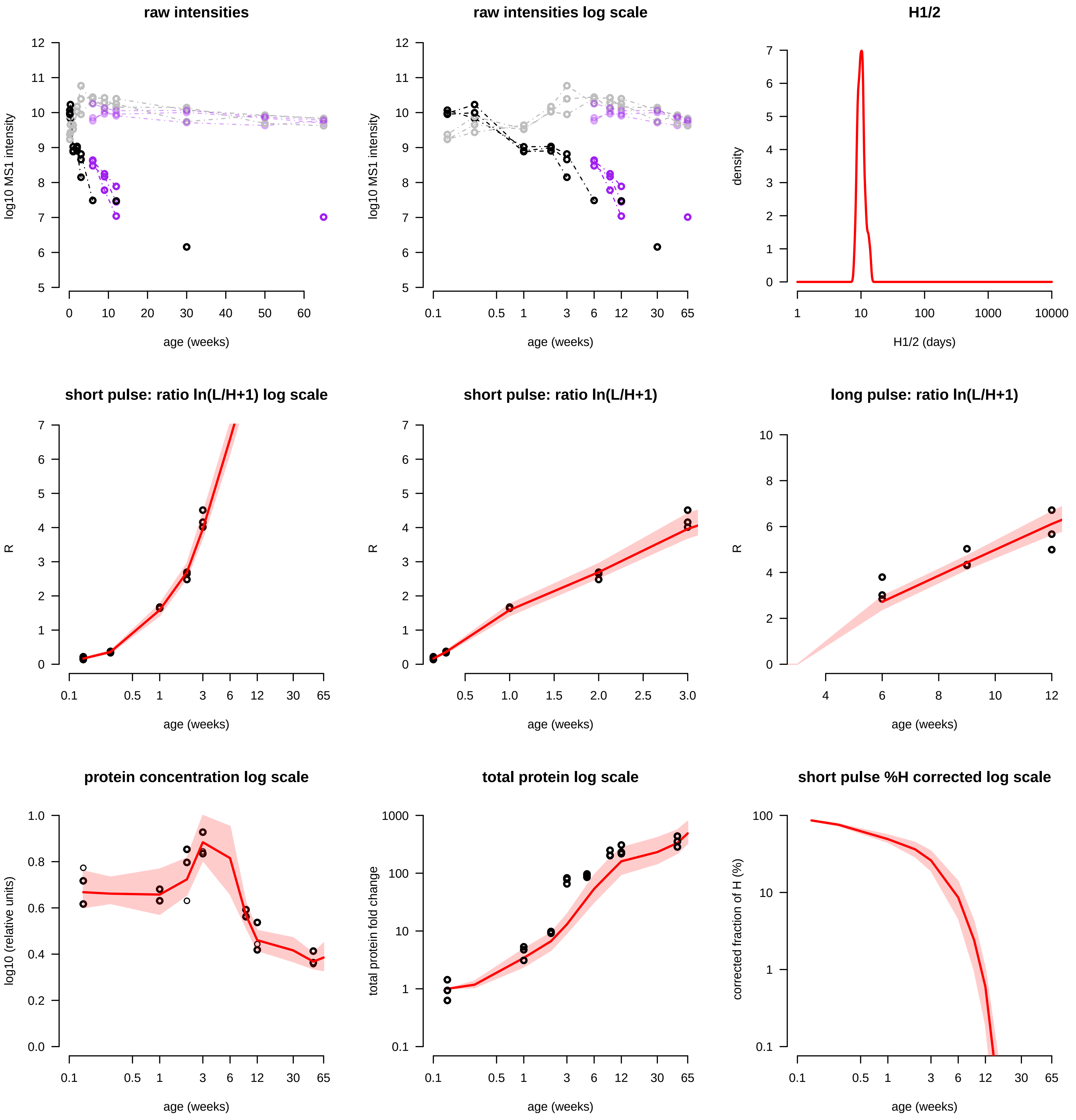

Q99JY0

>sp|Q99JY0|ECHB_MOUSE

Trifunctional enzyme subunit beta, mitochondrial

GN=Hadhb

Med H1/2: 11.5 days

Q5 H1/2: 9.3 days

Q95 H1/2: 14.3 days

reliable model fit: yes

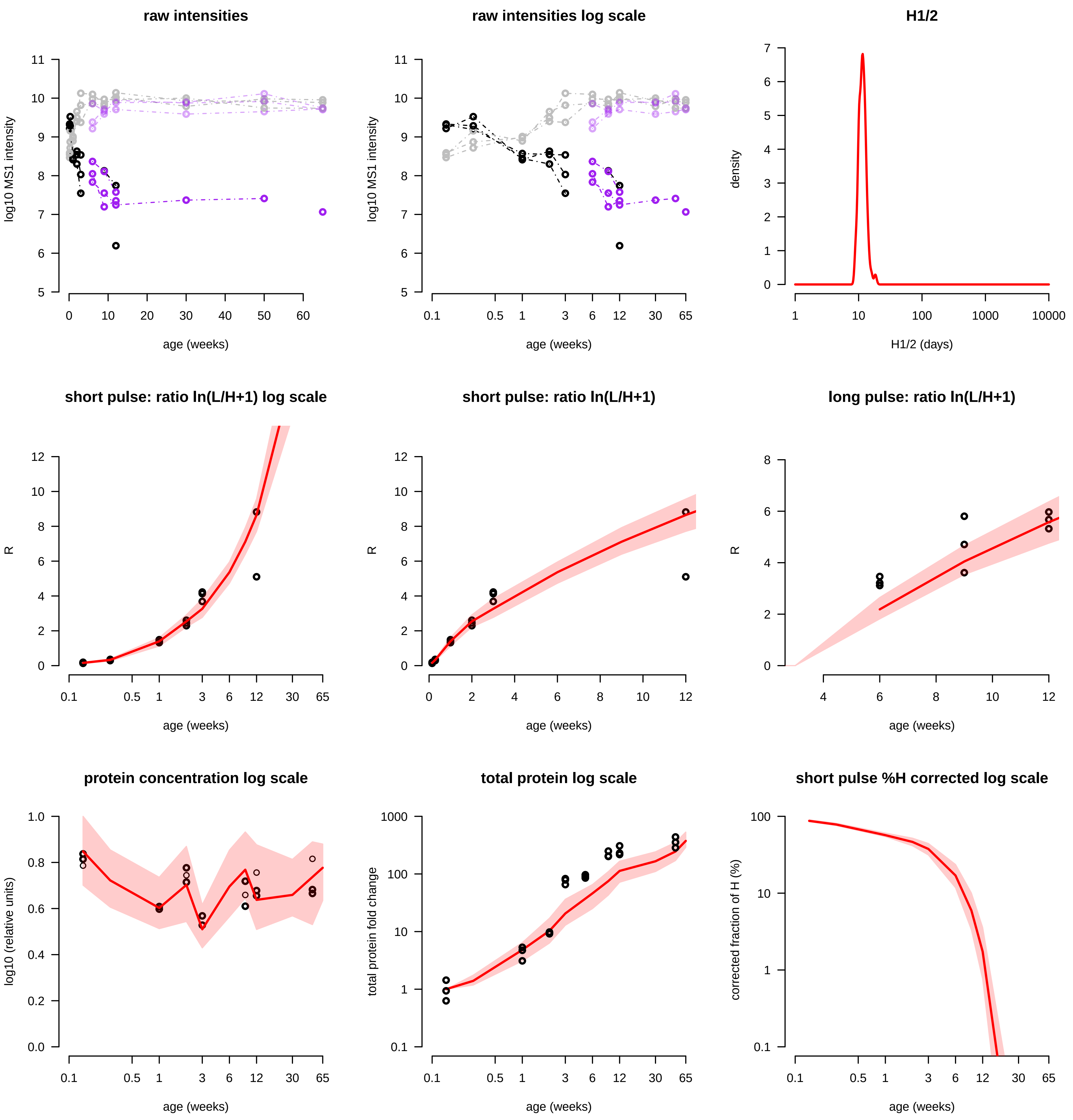

Q9WTP7

>sp|Q9WTP7|KAD3_MOUSE

GTP:AMP phosphotransferase AK3, mitochondrial

GN=Ak3

Med H1/2: 695.3 days

Q5 H1/2: 265.3 days

Q95 H1/2: 8953.9 days

reliable model fit: yes

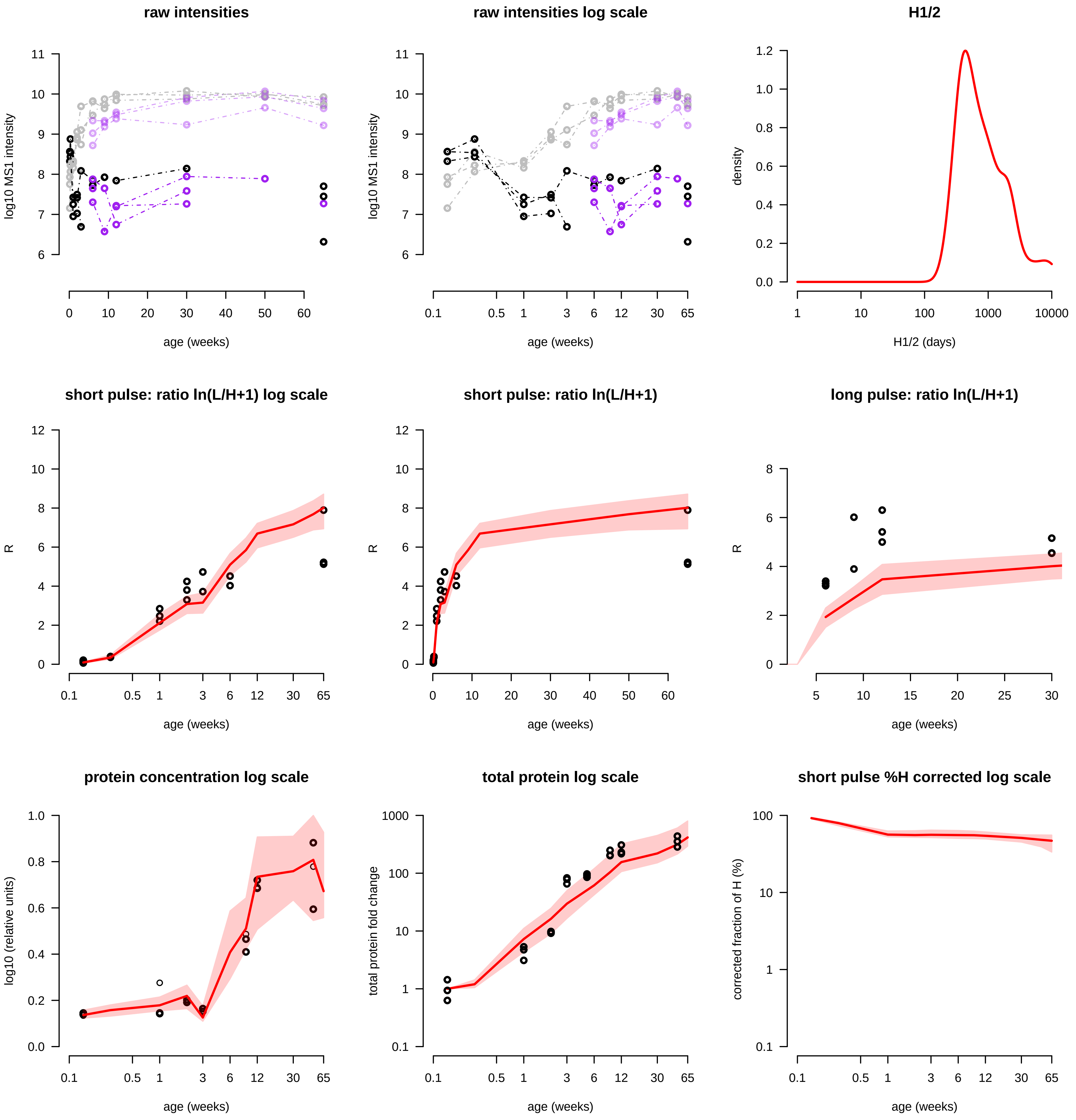

Q9JKR6

>sp|Q9JKR6|HYOU1_MOUSE

Hypoxia up−regulated protein 1

GN=Hyou1

Med H1/2: 197.1 days

Q5 H1/2: 138.5 days

Q95 H1/2: 412.3 days

reliable model fit: yes

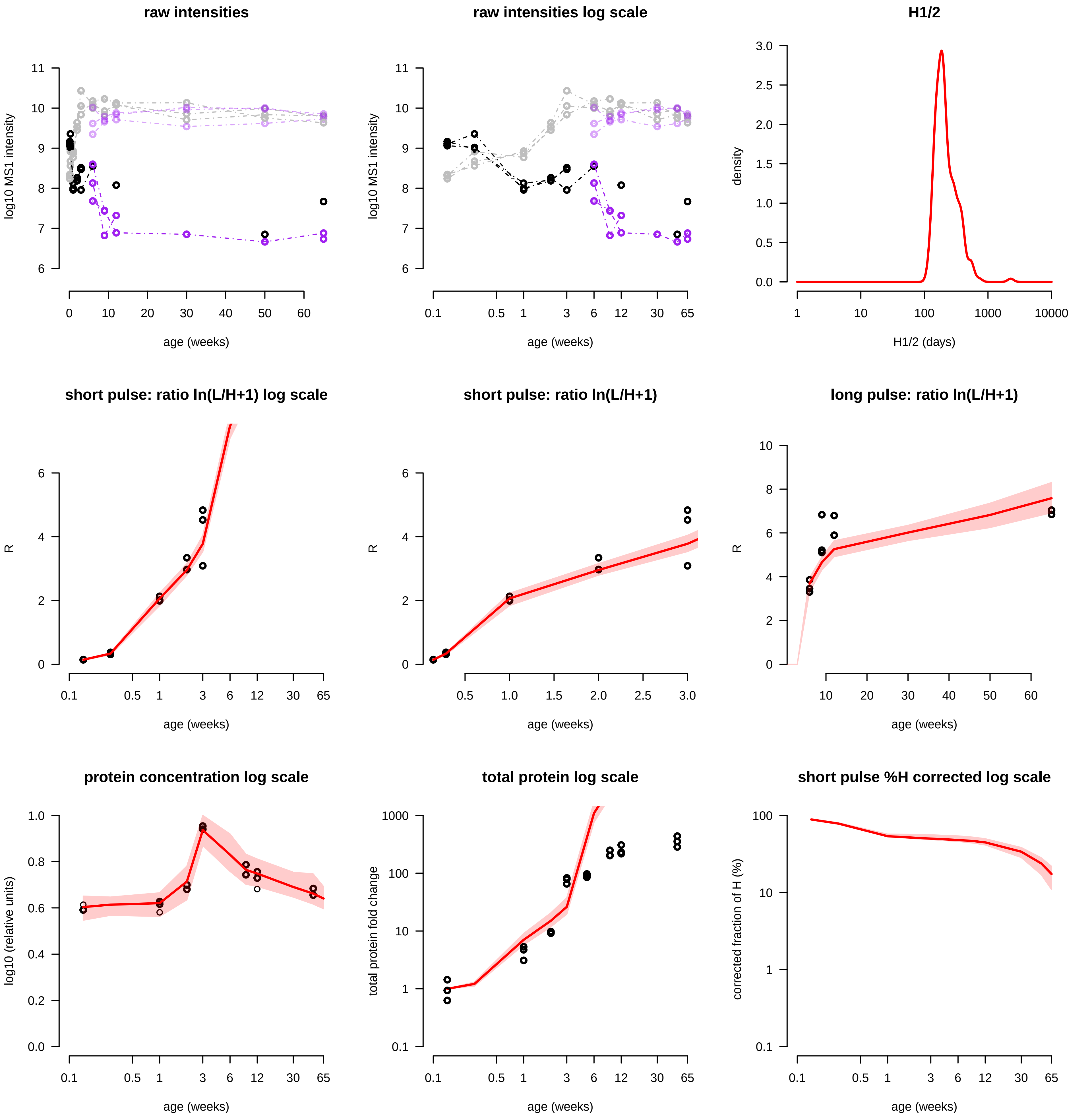

P26350

>sp|P26350|PTMA_MOUSE

Prothymosin alpha

GN=Ptma

Med H1/2: 7.9 days

Q5 H1/2: 6.8 days

Q95 H1/2: 10.3 days

reliable model fit: yes

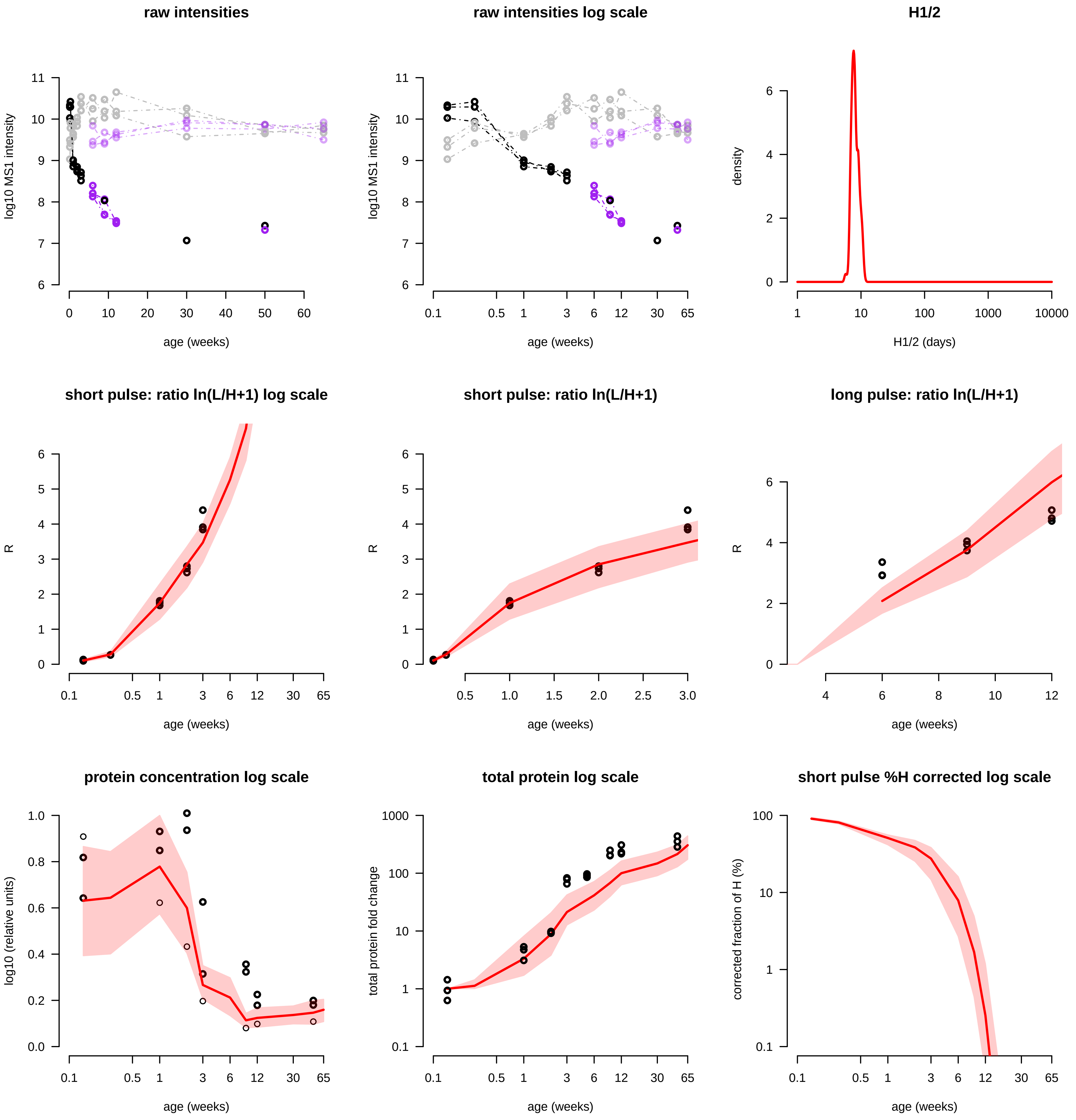

P58771−2

>sp|P58771−2|TPM1_MOUSE

Isoform 2 of Tropomyosin alpha−1 chain

GN=Tpm1

Med H1/2: 15.6 days

Q5 H1/2: 10.3 days

Q95 H1/2: 27.7 days

reliable model fit: yes

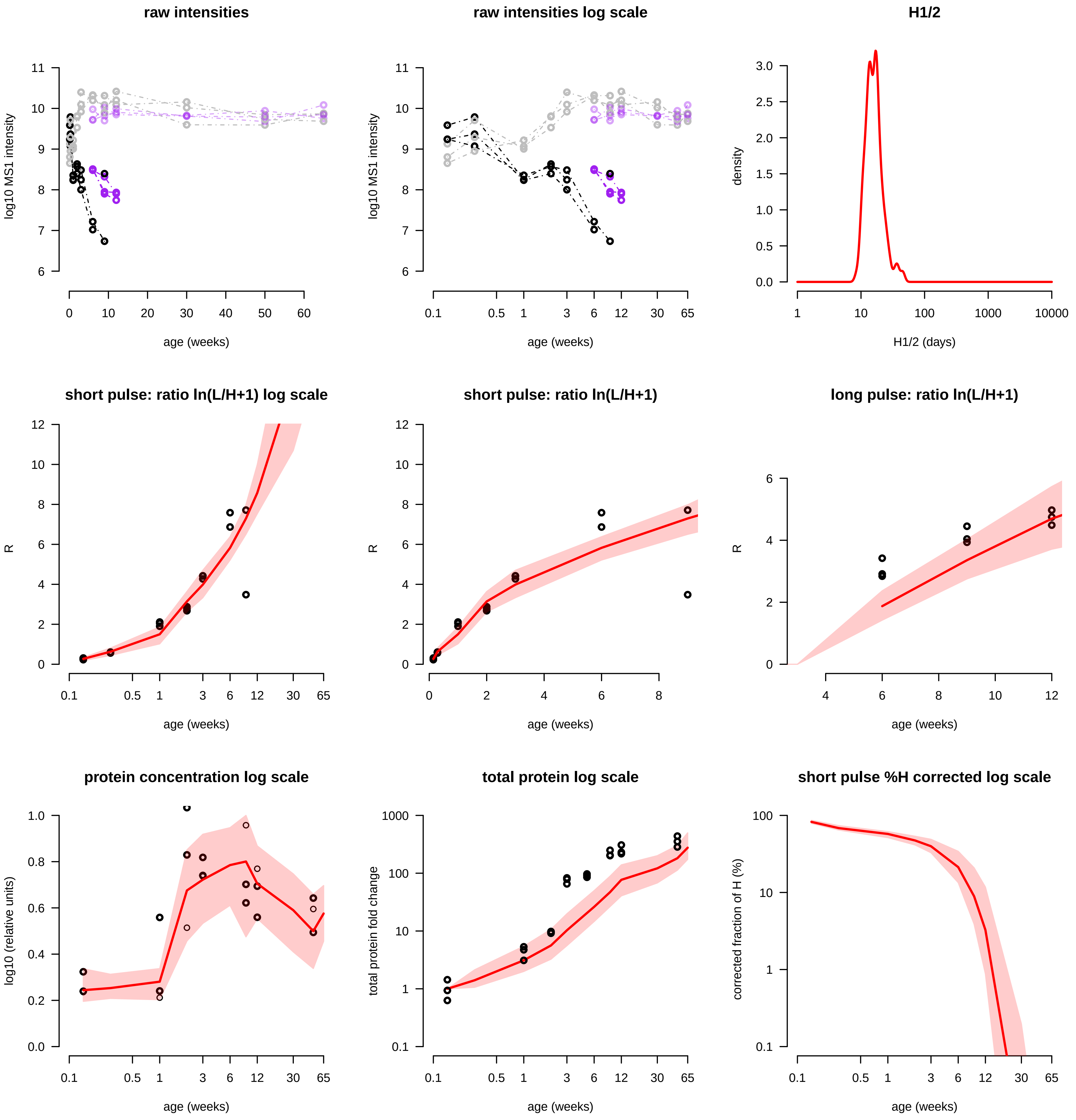

P19096

>sp|P19096|FAS_MOUSE

Fatty acid synthase

GN=Fasn

Med H1/2: 6.6 days

Q5 H1/2: 5.5 days

Q95 H1/2: 8.3 days

reliable model fit: yes

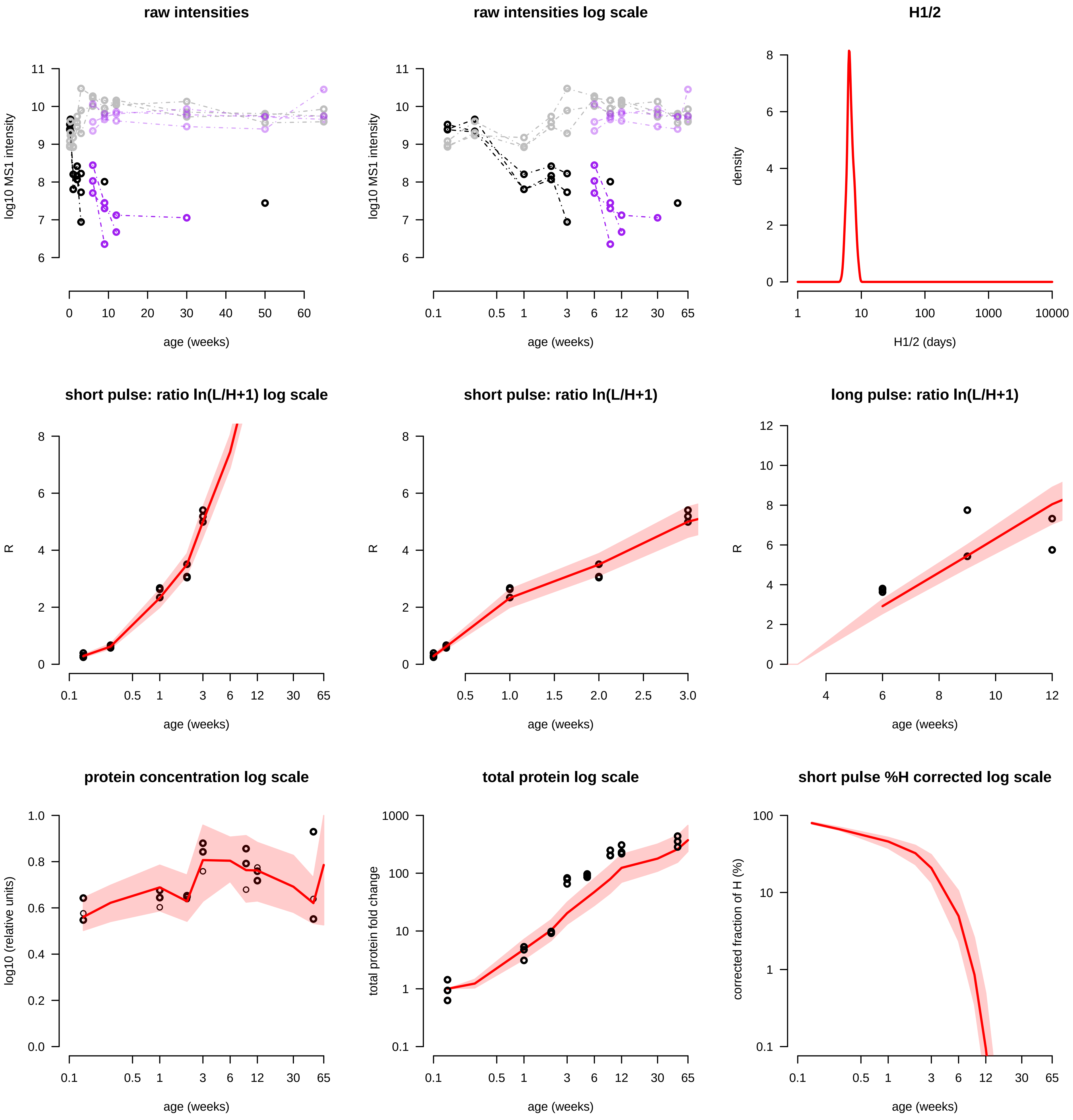

Q80YX1;Q80YX1−3;Q80YX1−4;Q80YX1−5

>sp|Q80YX1|TENA_MOUSE

Tenascin

GN=Tnc

Med H1/2: 89.5 days

Q5 H1/2: 47 days

Q95 H1/2: 645.6 days

reliable model fit: yes

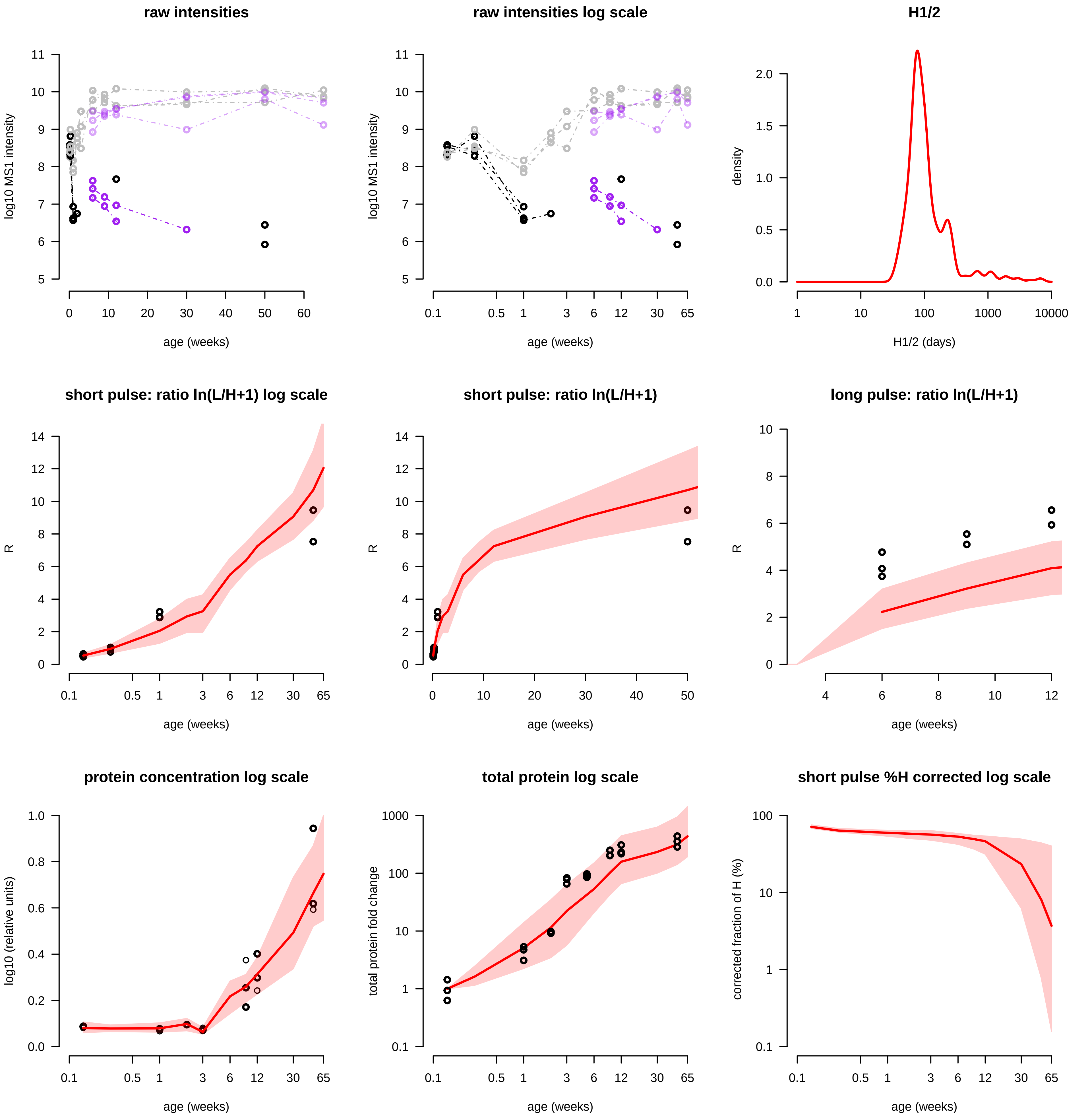

Q91ZA3

>sp|Q91ZA3|PCCA_MOUSE

Propionyl−CoA carboxylase alpha chain, mitochondrial

GN=Pcca

Med H1/2: 233.3 days

Q5 H1/2: 105.2 days

Q95 H1/2: 1922.2 days

reliable model fit: no

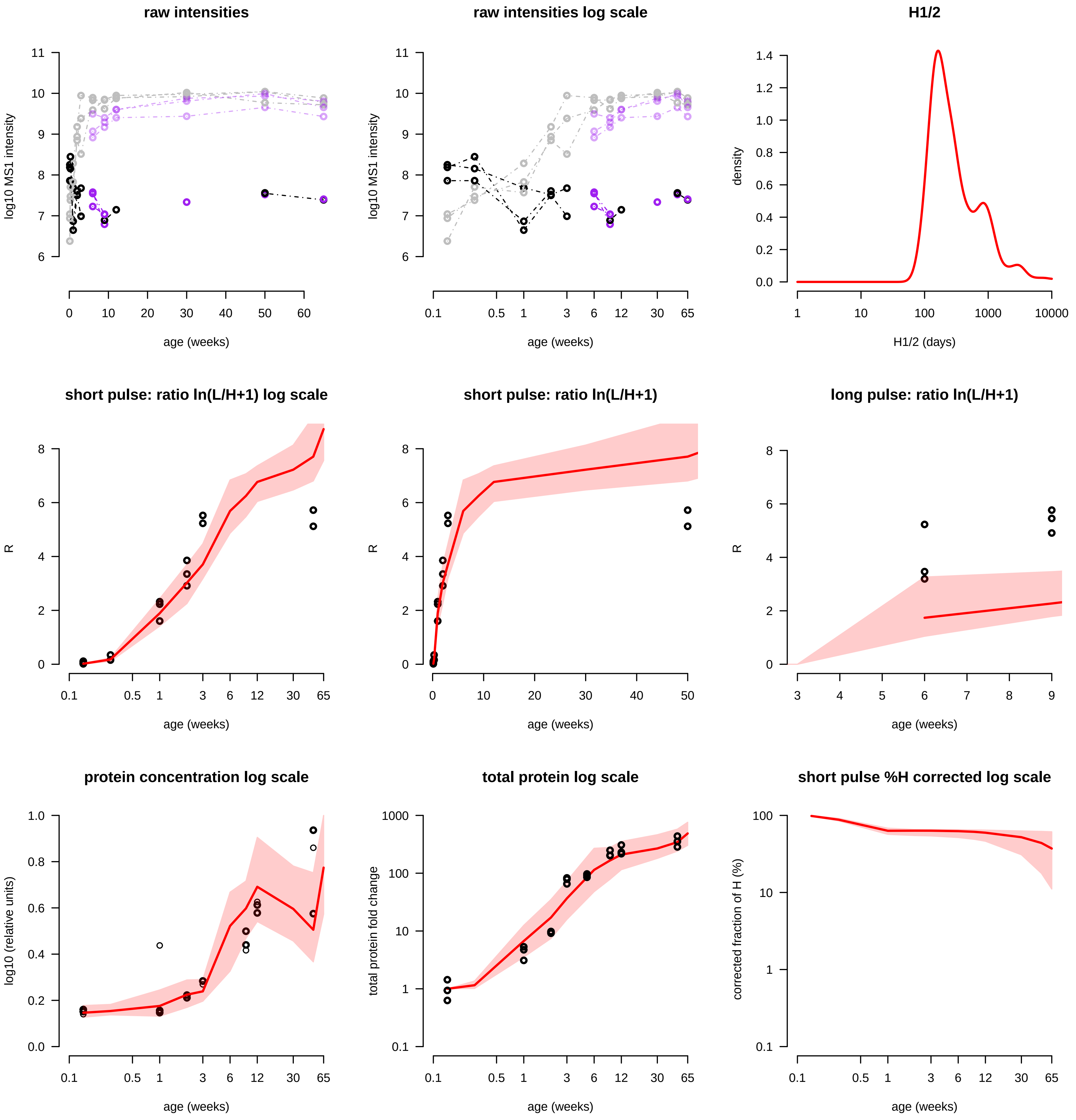

Q8BMD8

>sp|Q8BMD8|SCMC1_MOUSE

Calcium−binding mitochondrial carrier protein SCaMC−1

GN=Slc25a24

Med H1/2: 14.2 days

Q5 H1/2: 9.7 days

Q95 H1/2: 26.9 days

reliable model fit: yes

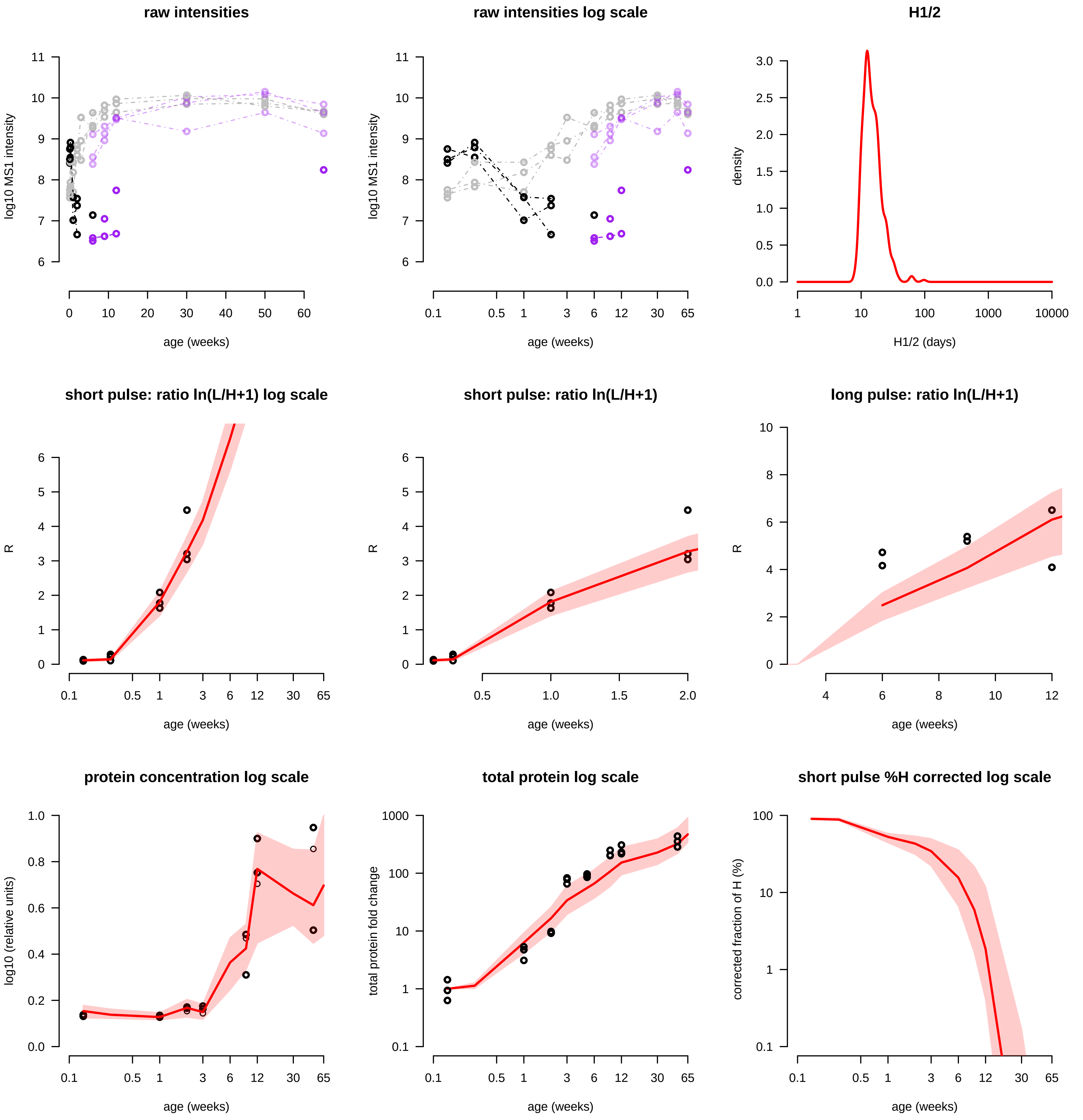

P52196

>sp|P52196|THTR_MOUSE

Thiosulfate sulfurtransferase

GN=Tst

Med H1/2: 10.8 days

Q5 H1/2: 9.2 days

Q95 H1/2: 15.7 days

reliable model fit: yes

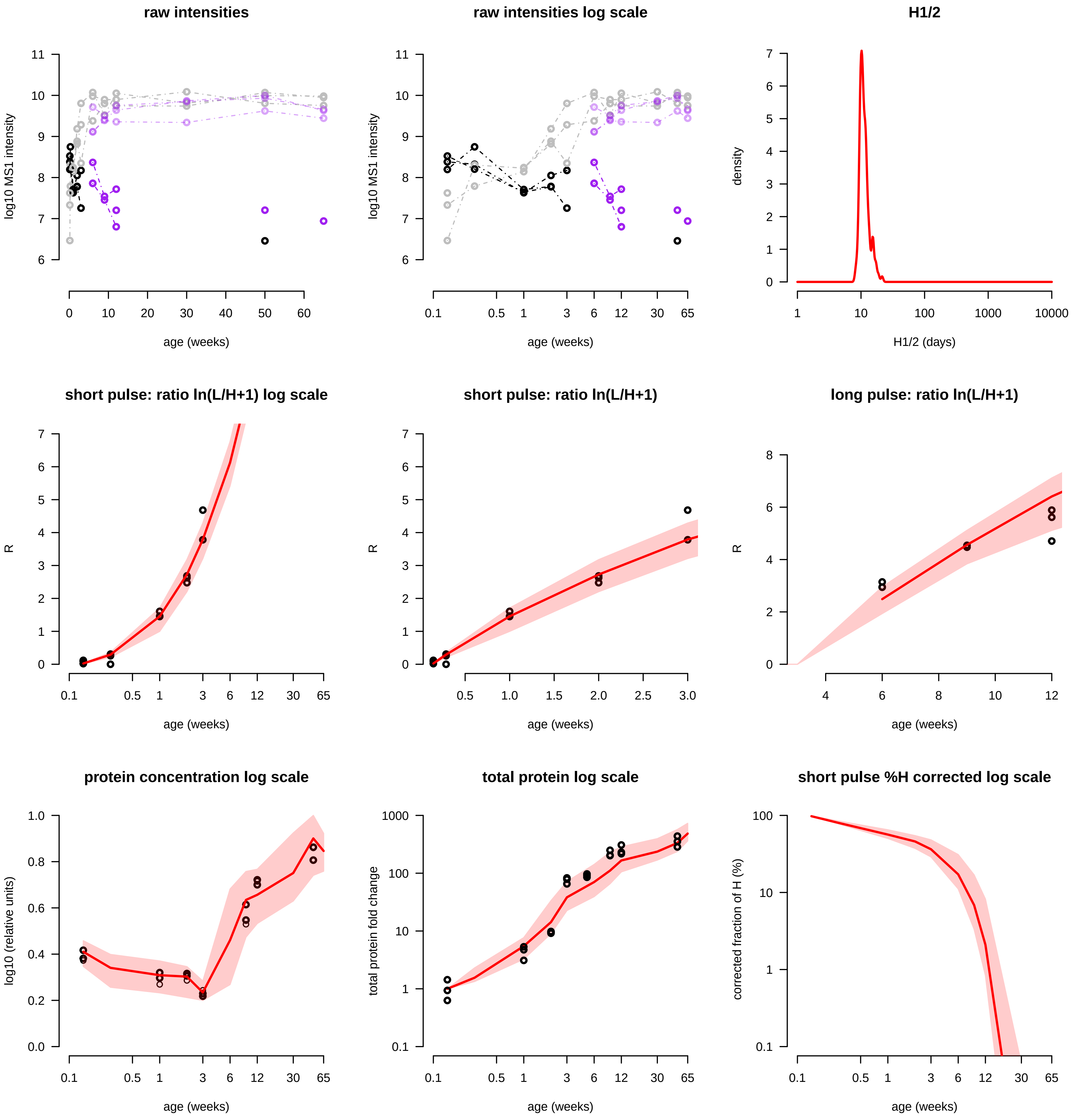

P63242

>sp|P63242|IF5A1_MOUSE

Eukaryotic translation initiation factor 5A−1

GN=Eif5a

Med H1/2: 10.4 days

Q5 H1/2: 9.1 days

Q95 H1/2: 14 days

reliable model fit: yes

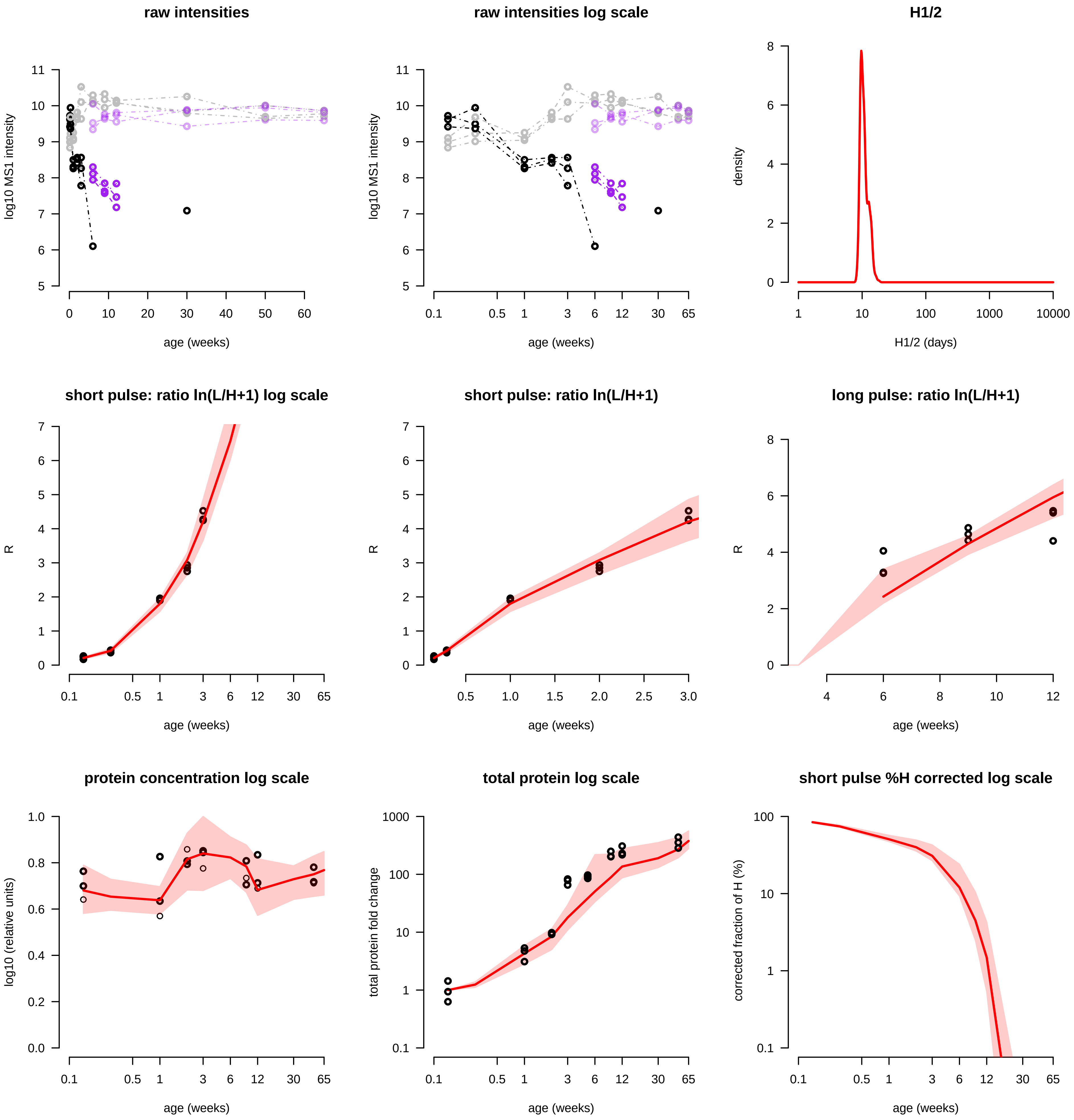

Q3THW5;P0C0S6

>sp|Q3THW5|H2AV_MOUSE

Histone H2A.V

GN=H2az2

Med H1/2: 18.2 days

Q5 H1/2: 14 days

Q95 H1/2: 24.4 days

reliable model fit: yes

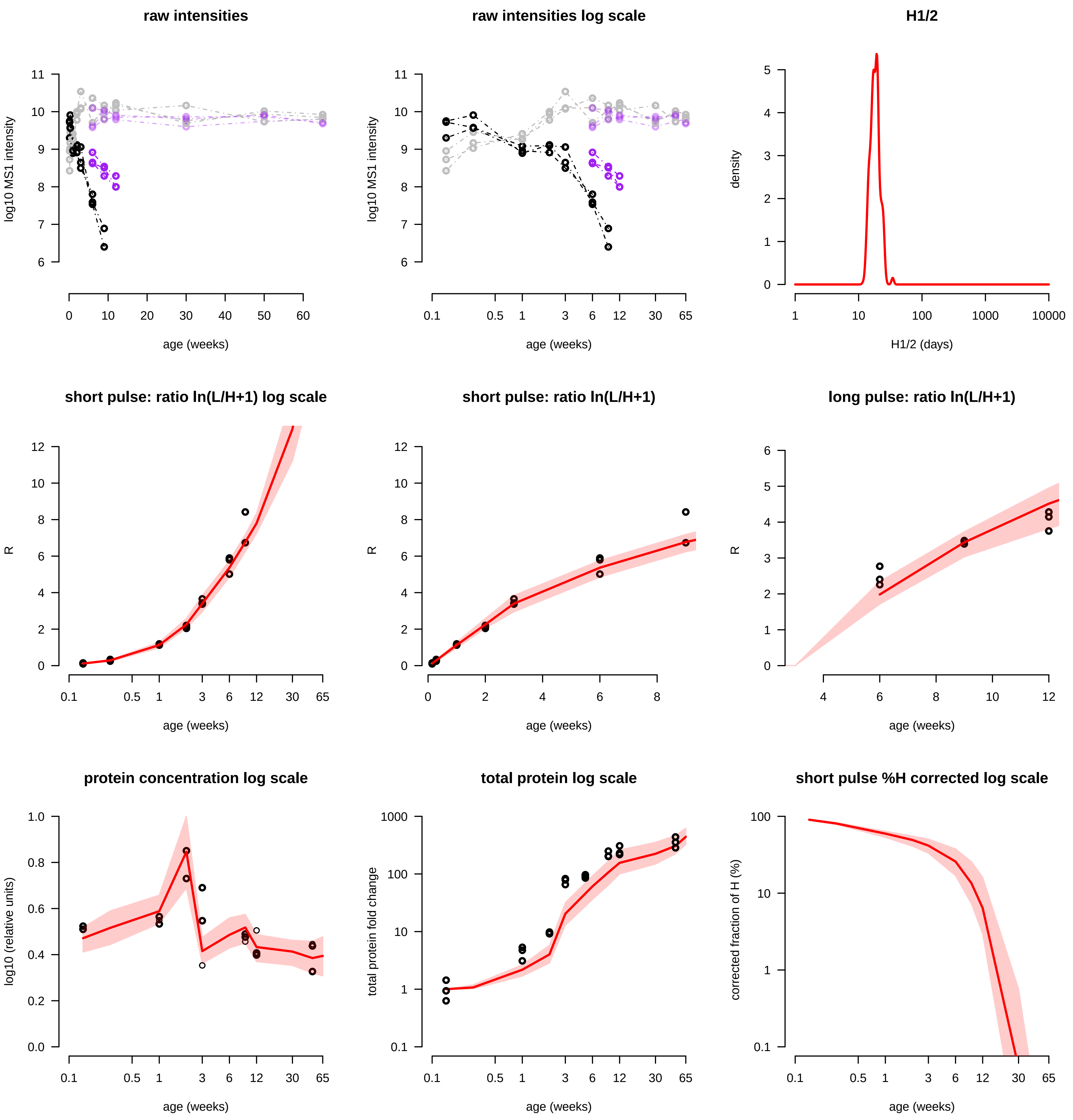

P42932

>sp|P42932|TCPQ_MOUSE

T−complex protein 1 subunit theta

GN=Cct8

Med H1/2: 1044 days

Q5 H1/2: 235.1 days

Q95 H1/2: 11313.2 days

reliable model fit: yes

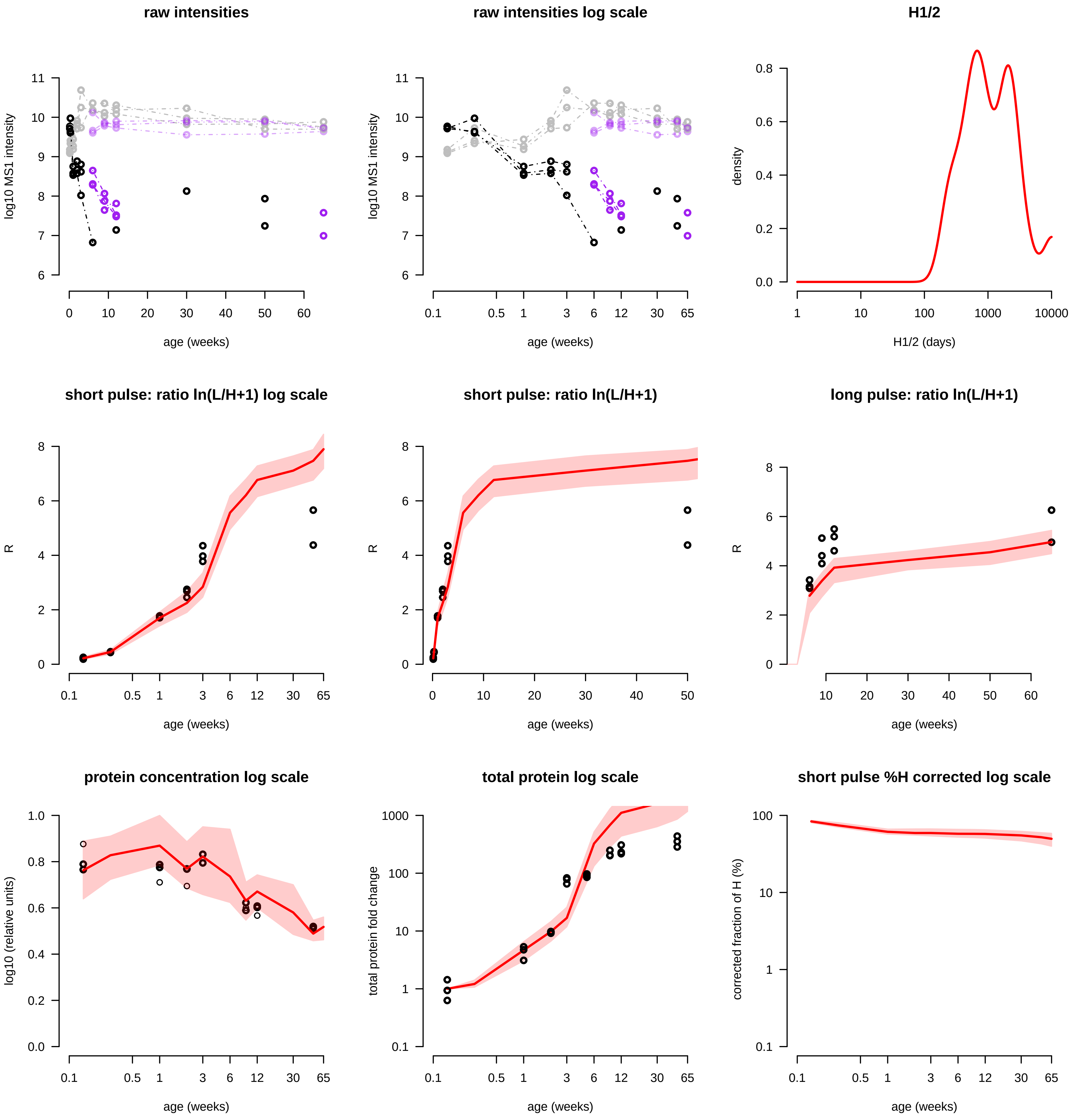

P62082

>sp|P62082|RS7_MOUSE

40S ribosomal protein S7

GN=Rps7

Med H1/2: 6.4 days

Q5 H1/2: 5.3 days

Q95 H1/2: 8 days

reliable model fit: yes

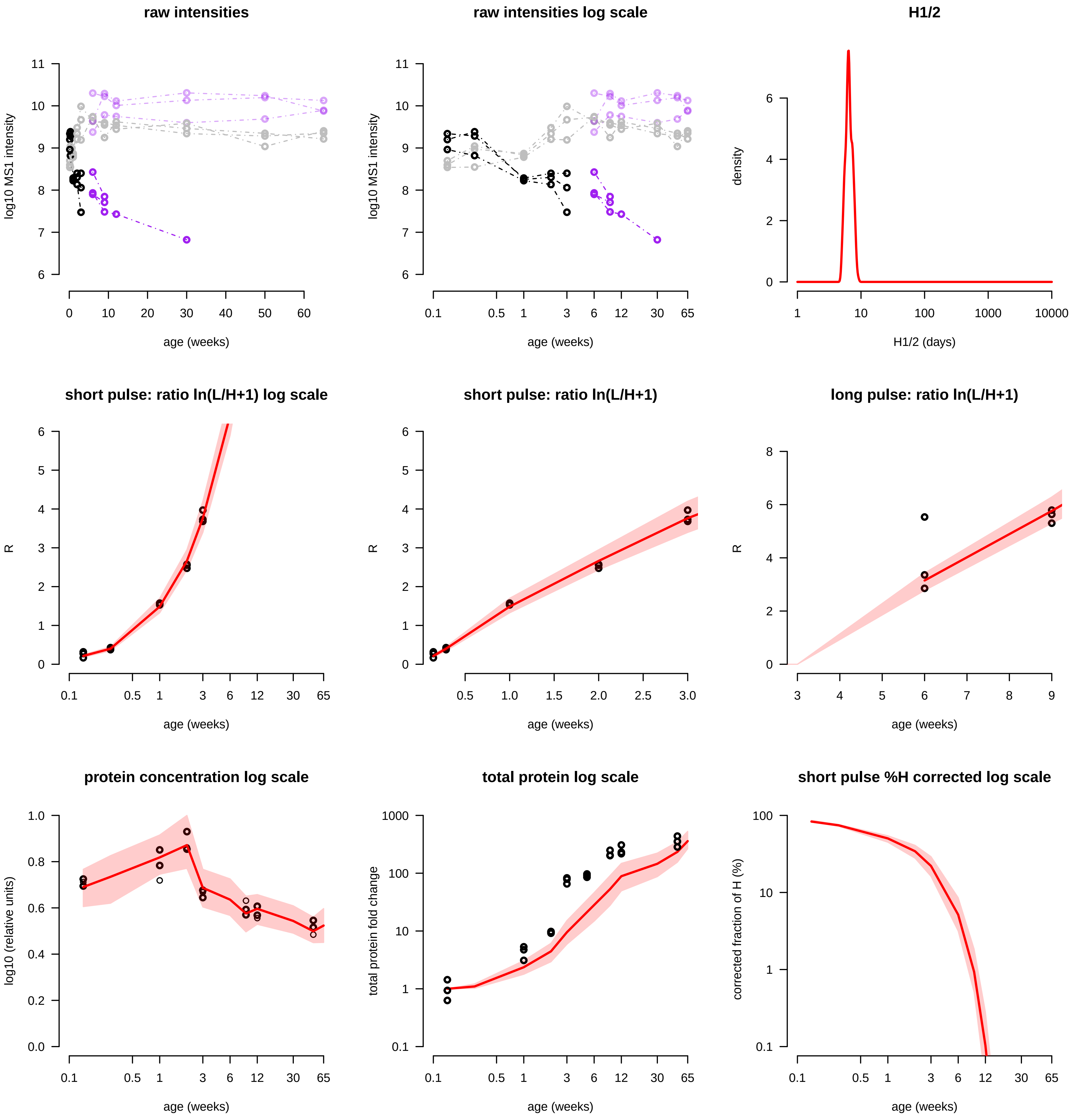

P50544

>sp|P50544|ACADV_MOUSE

Very long−chain specific acyl−CoA dehydrogenase, mitochondrial

GN=Acadvl

Med H1/2: 13.9 days

Q5 H1/2: 10.5 days

Q95 H1/2: 21.6 days

reliable model fit: yes

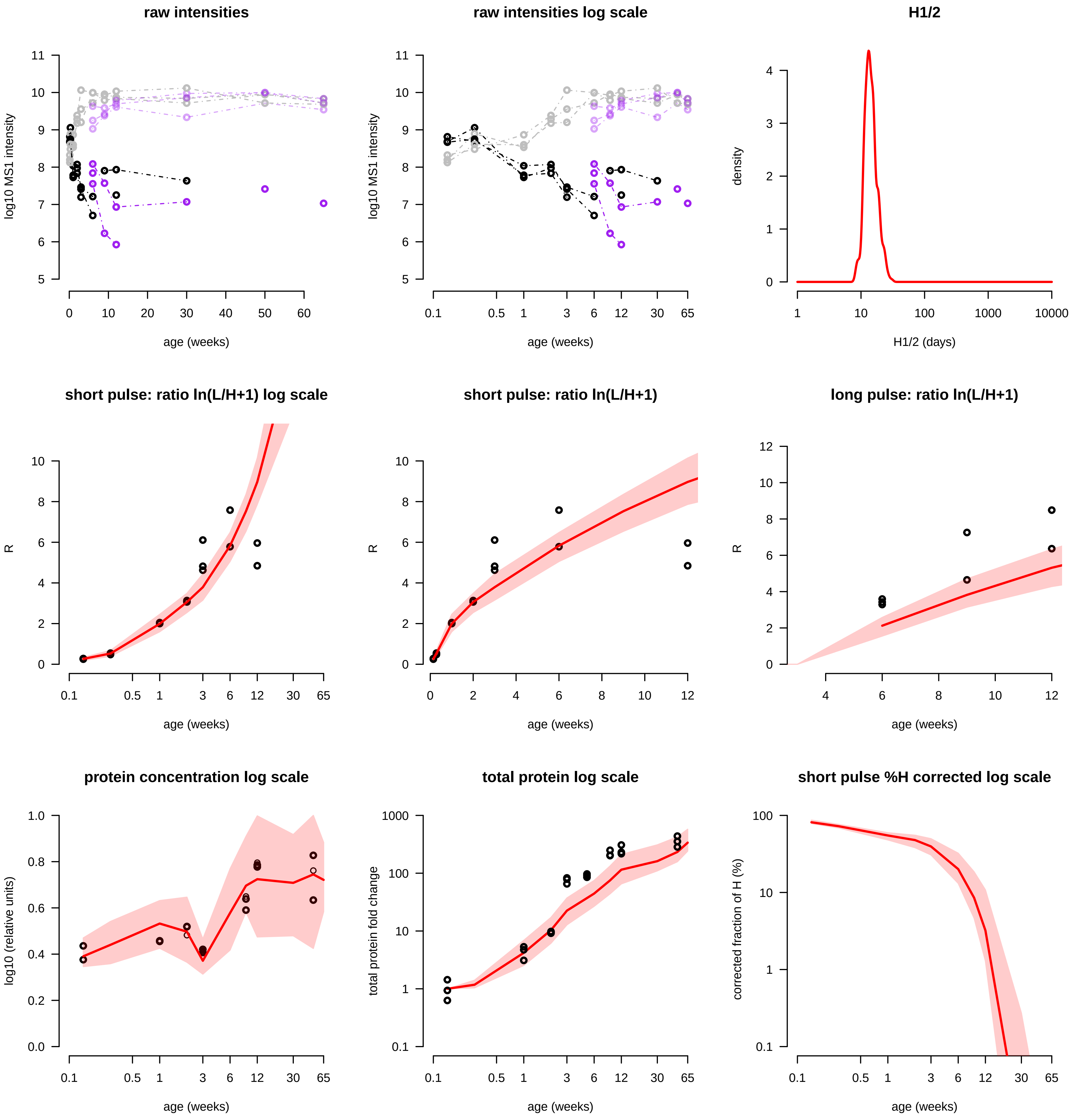

Q9CPR4

>sp|Q9CPR4|RL17_MOUSE

60S ribosomal protein L17

GN=Rpl17

Med H1/2: 7.8 days

Q5 H1/2: 6.4 days

Q95 H1/2: 10.6 days

reliable model fit: yes

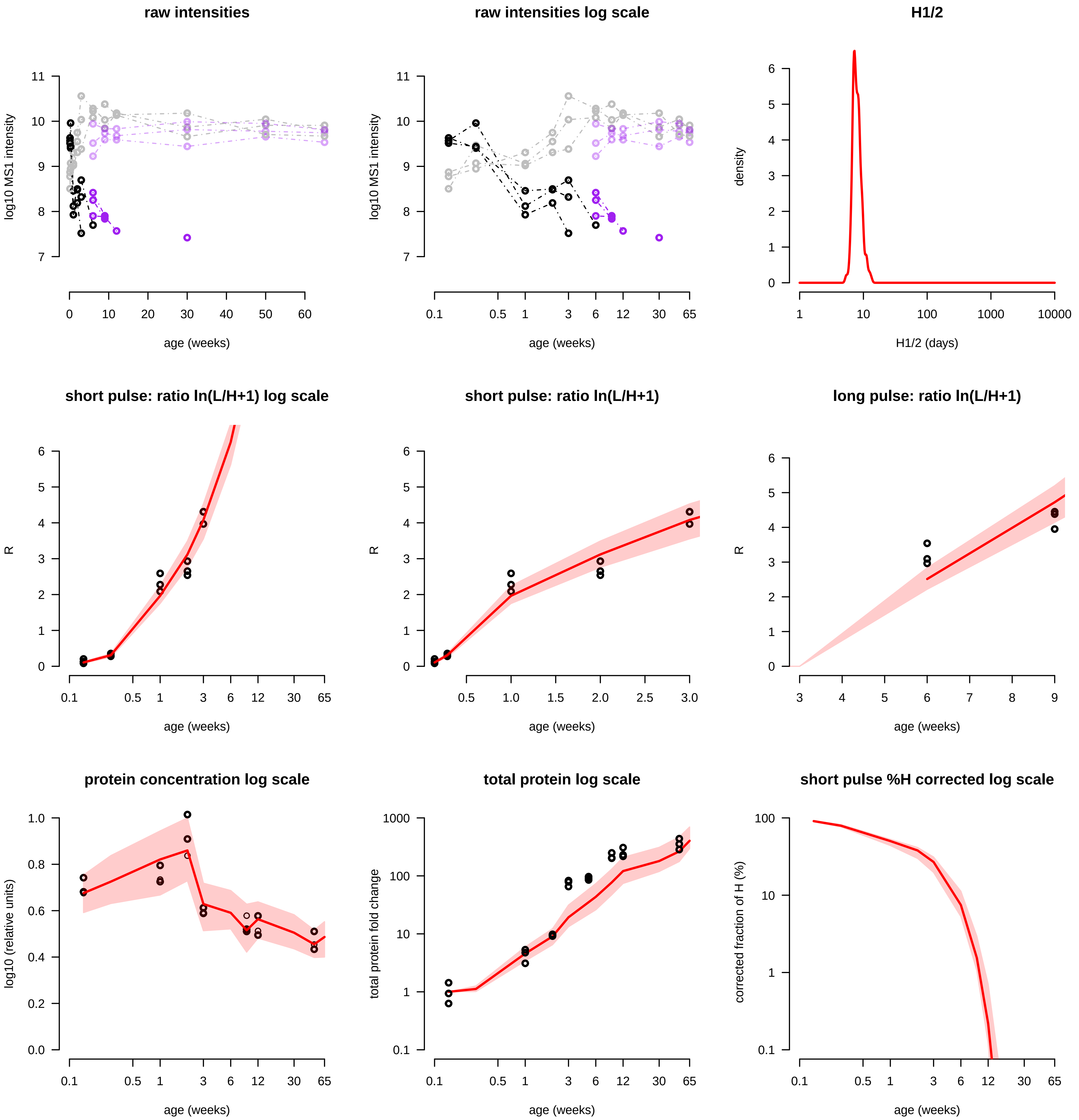

P08207

>sp|P08207|S10AA_MOUSE

Protein S100−A10

GN=S100a10

Med H1/2: 12.4 days

Q5 H1/2: 4.4 days

Q95 H1/2: 125 days

reliable model fit: yes

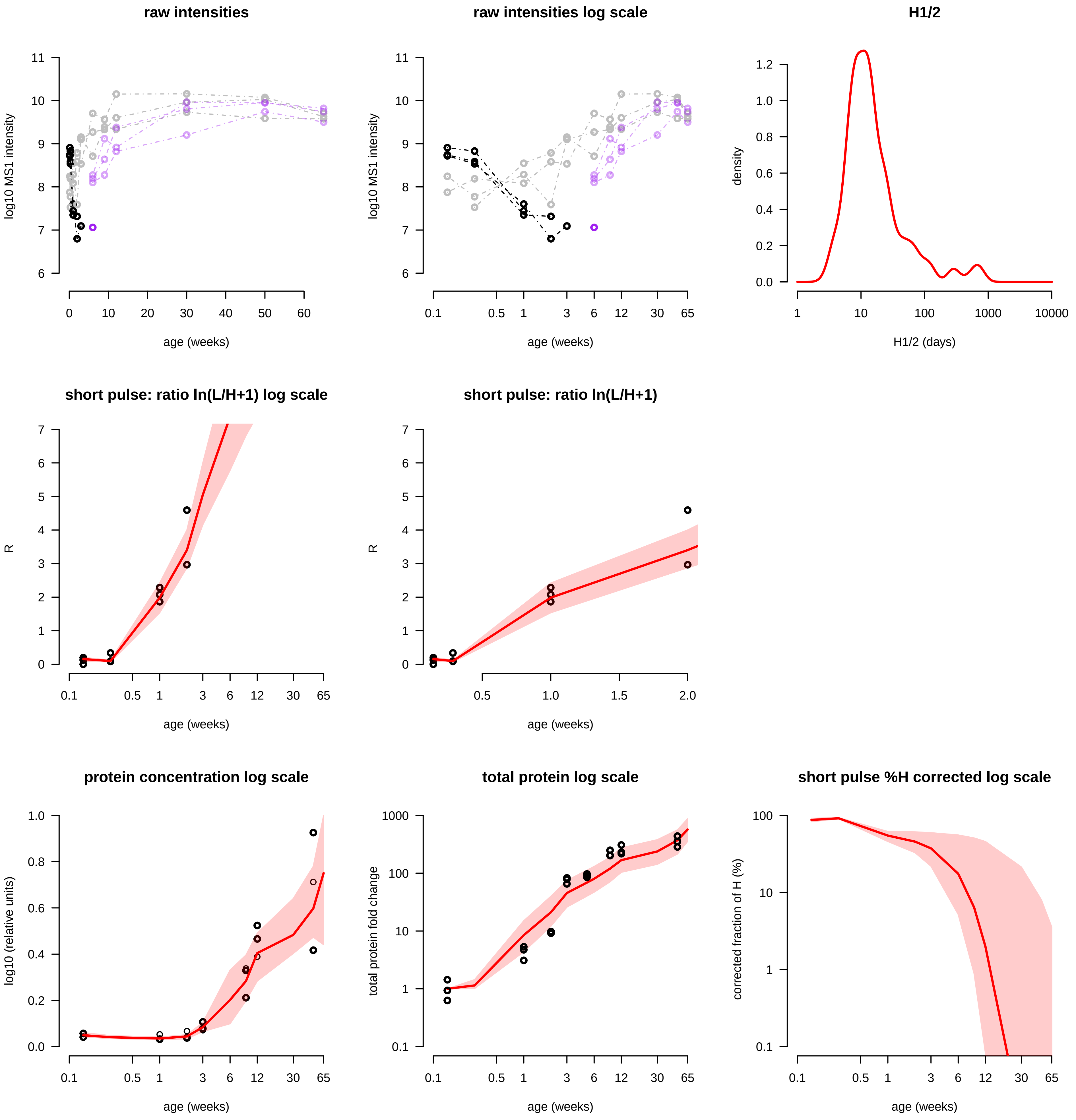

Q6ZWQ0

>sp|Q6ZWQ0|SYNE2_MOUSE

Nesprin−2

GN=Syne2

Med H1/2: 504.6 days

Q5 H1/2: 213.9 days

Q95 H1/2: 3530.5 days

reliable model fit: yes

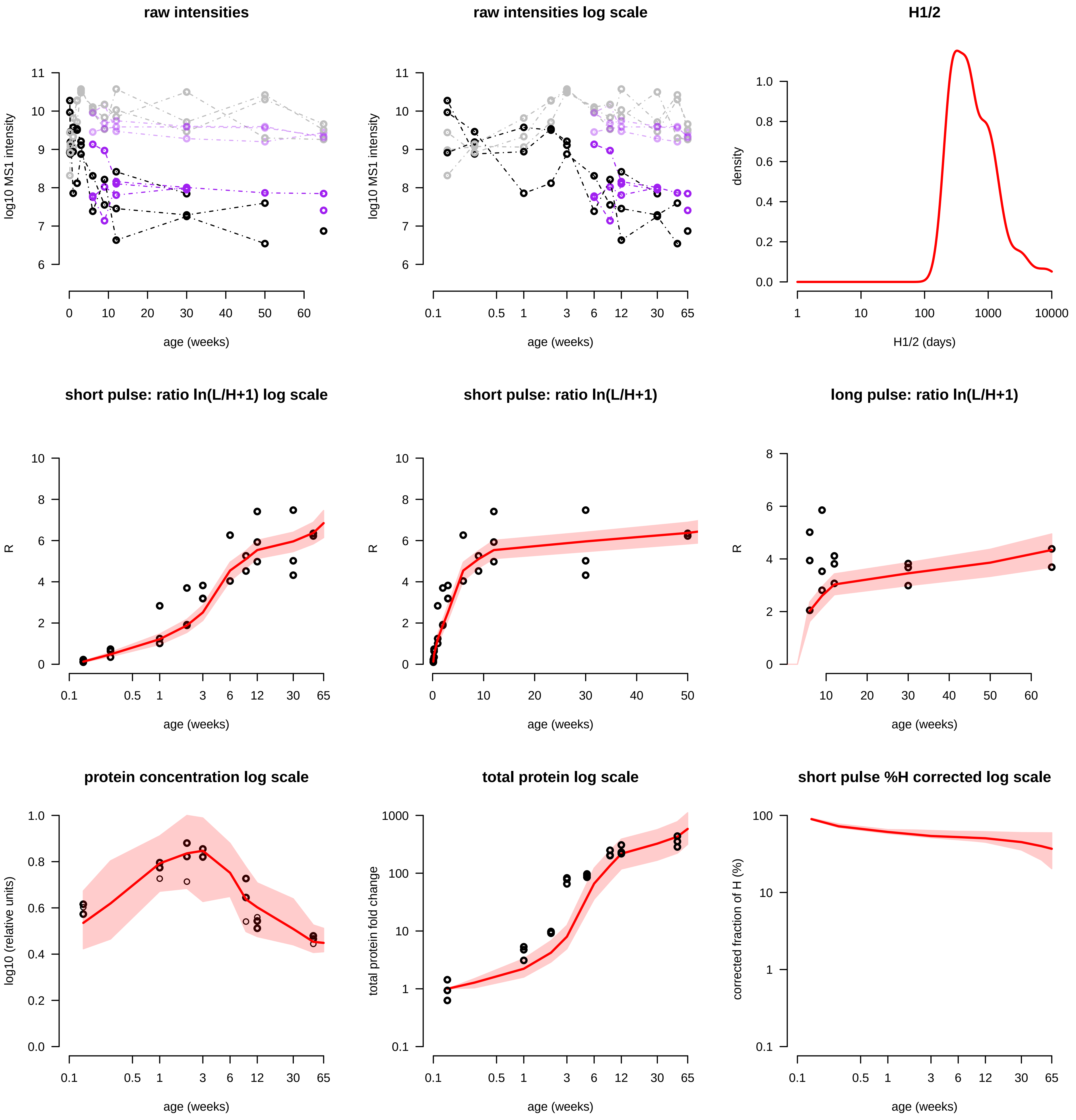

P14148

>sp|P14148|RL7_MOUSE

60S ribosomal protein L7

GN=Rpl7

Med H1/2: 87.7 days

Q5 H1/2: 56.5 days

Q95 H1/2: 132.9 days

reliable model fit: yes

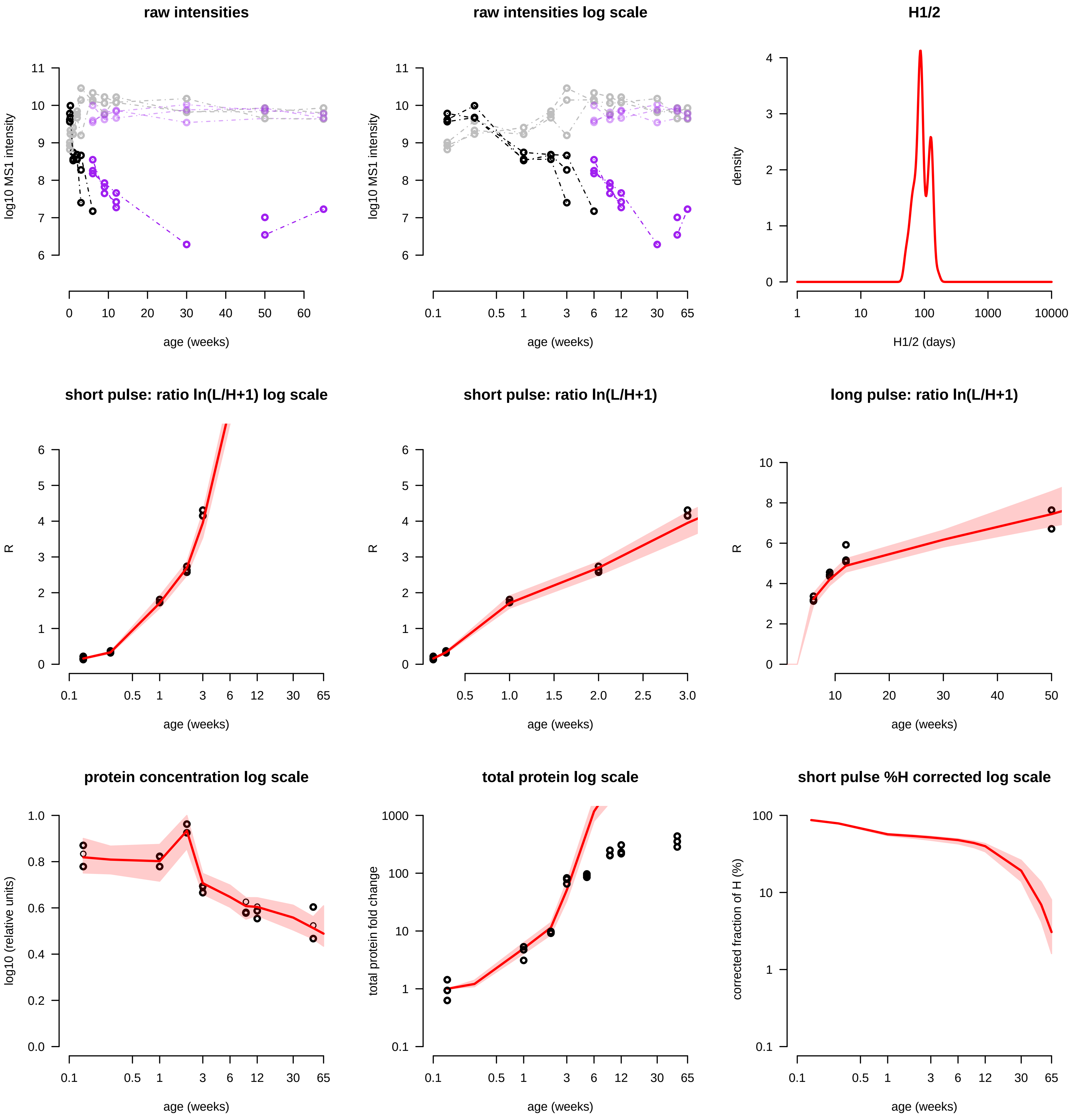

Q99PL5

>sp|Q99PL5|RRBP1_MOUSE

Ribosome−binding protein 1

GN=Rrbp1

Med H1/2: 187.7 days

Q5 H1/2: 56.6 days

Q95 H1/2: 1279.6 days

reliable model fit: yes

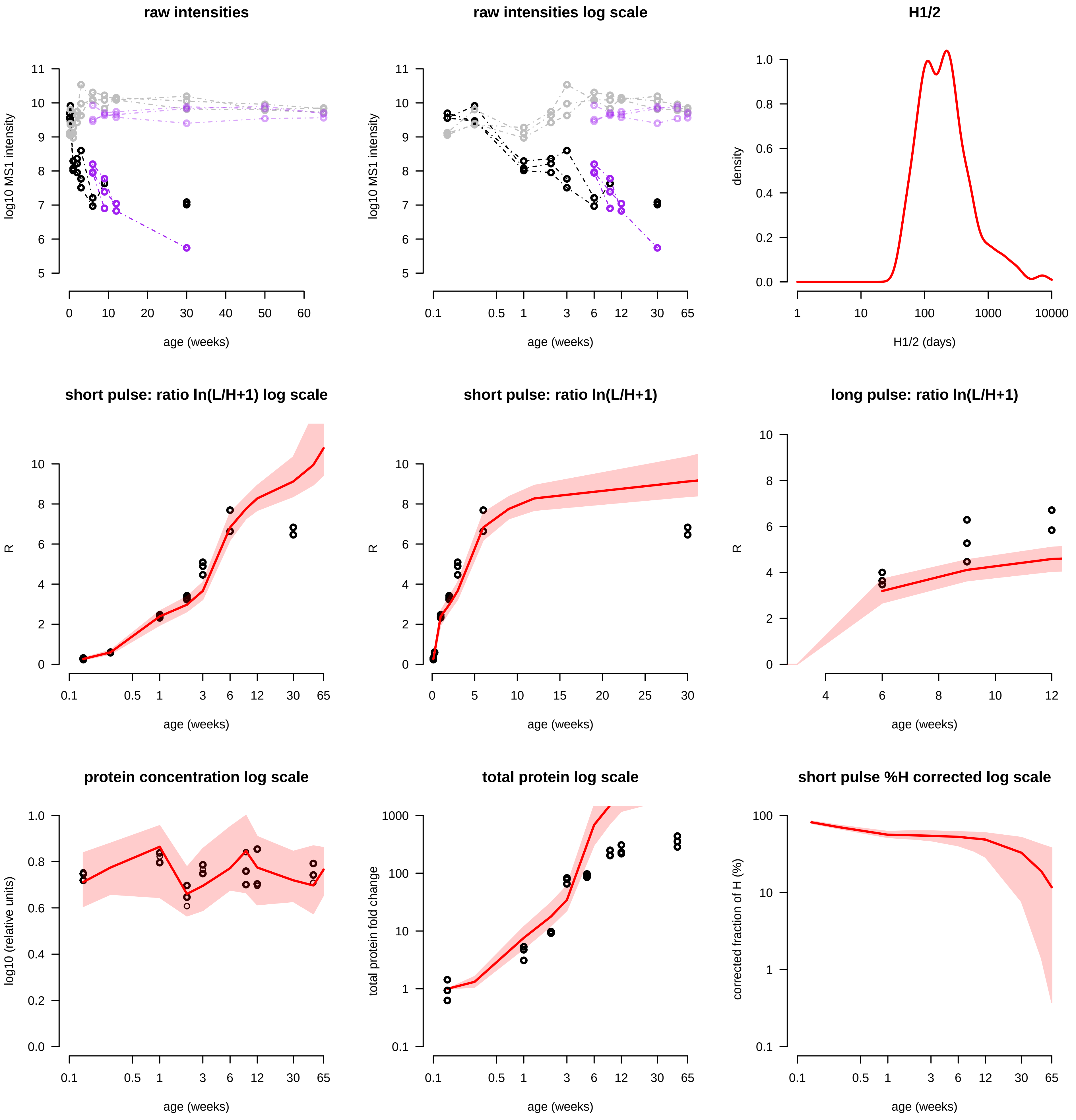

Q9D8N0

>sp|Q9D8N0|EF1G_MOUSE

Elongation factor 1−gamma

GN=Eef1g

Med H1/2: 1775.9 days

Q5 H1/2: 181 days

Q95 H1/2: 22386.5 days

reliable model fit: yes

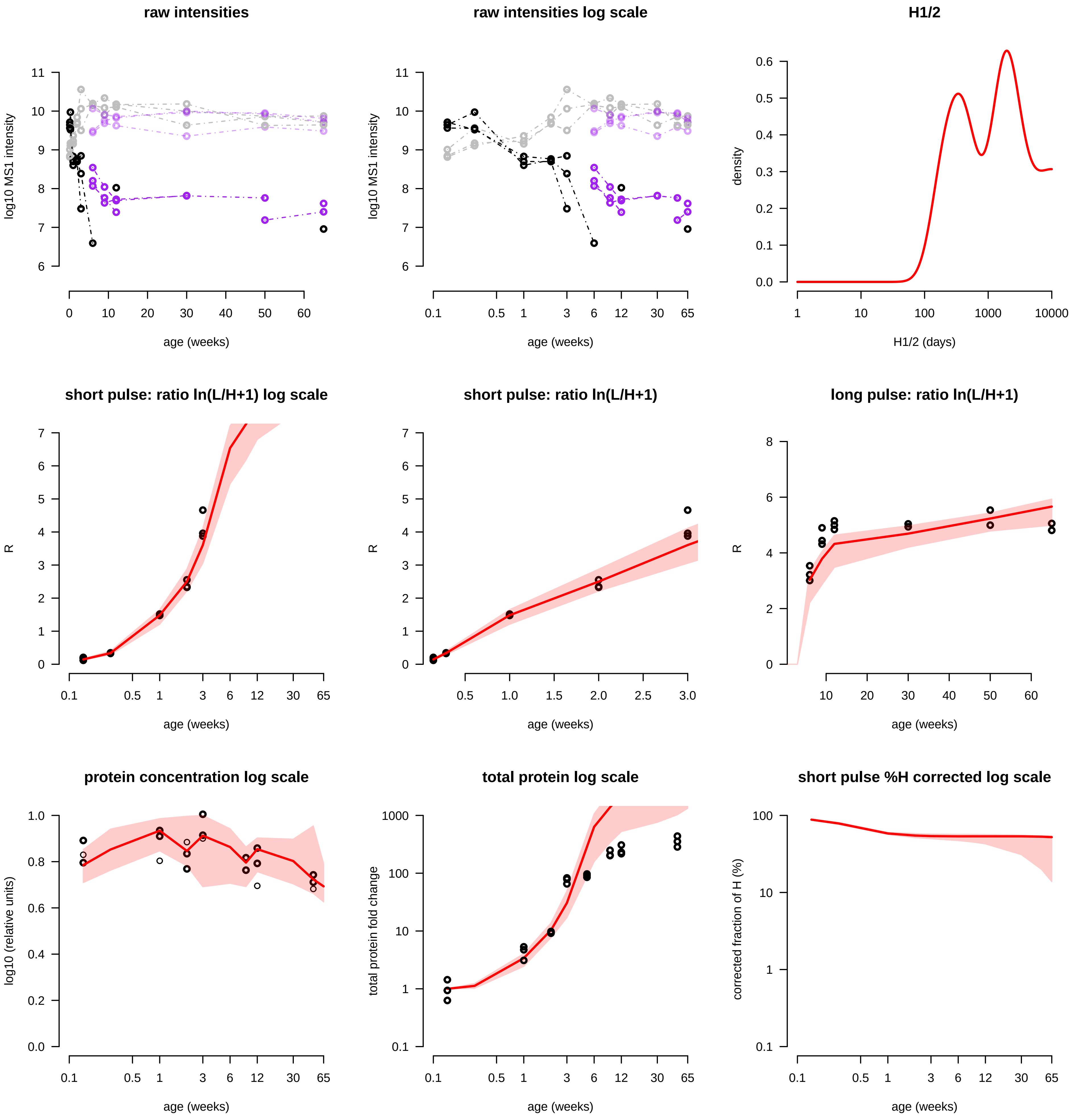

Q99LX0

>sp|Q99LX0|PARK7_MOUSE

Parkinson disease protein 7 homolog

GN=Park7

Med H1/2: 11.6 days

Q5 H1/2: 9 days

Q95 H1/2: 15 days

reliable model fit: yes

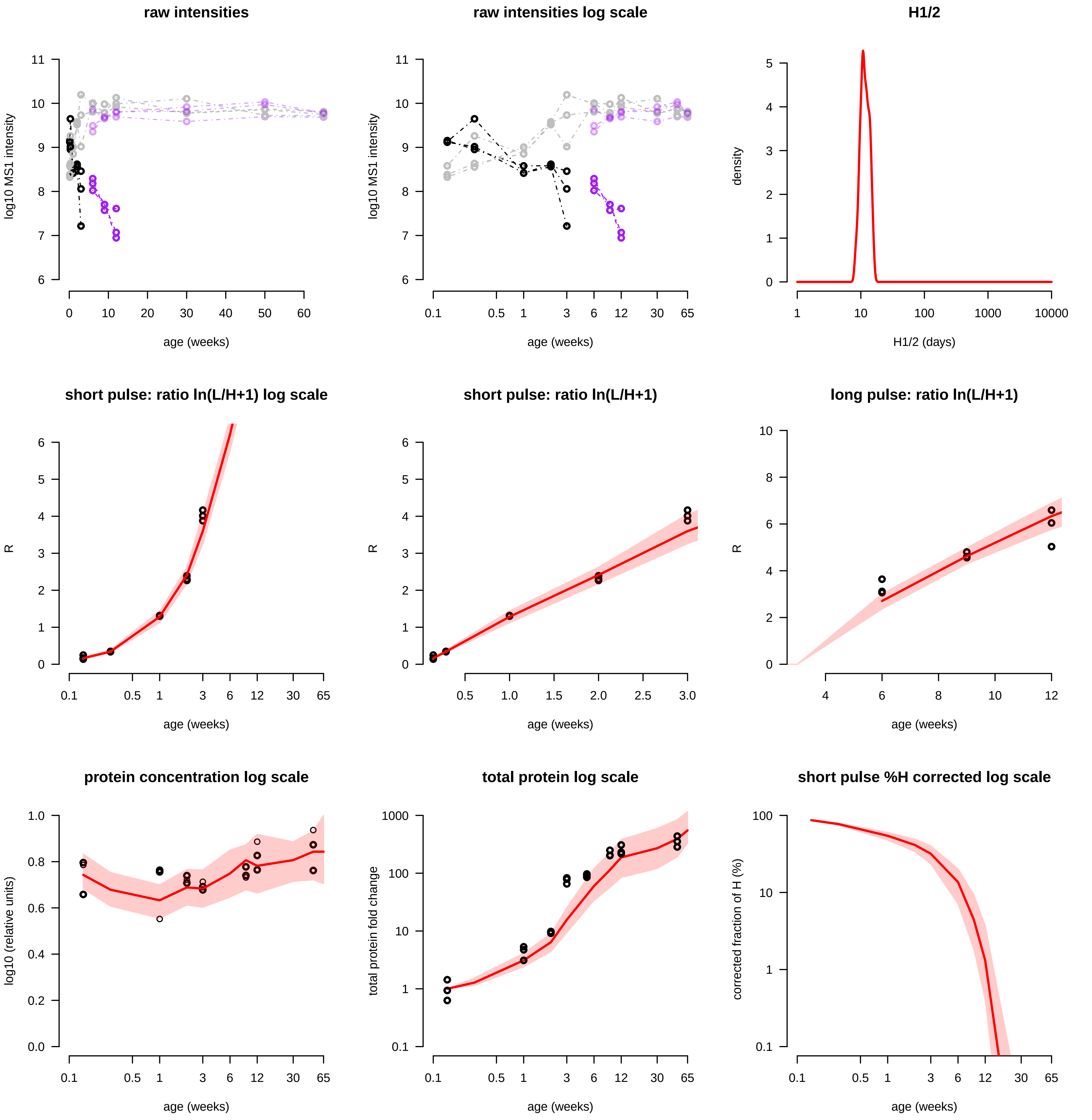

P43277

>sp|P43277|H13_MOUSE

Histone H1.3

GN=H1−3

Med H1/2: 21 days

Q5 H1/2: 16.6 days

Q95 H1/2: 30.9 days

reliable model fit: yes

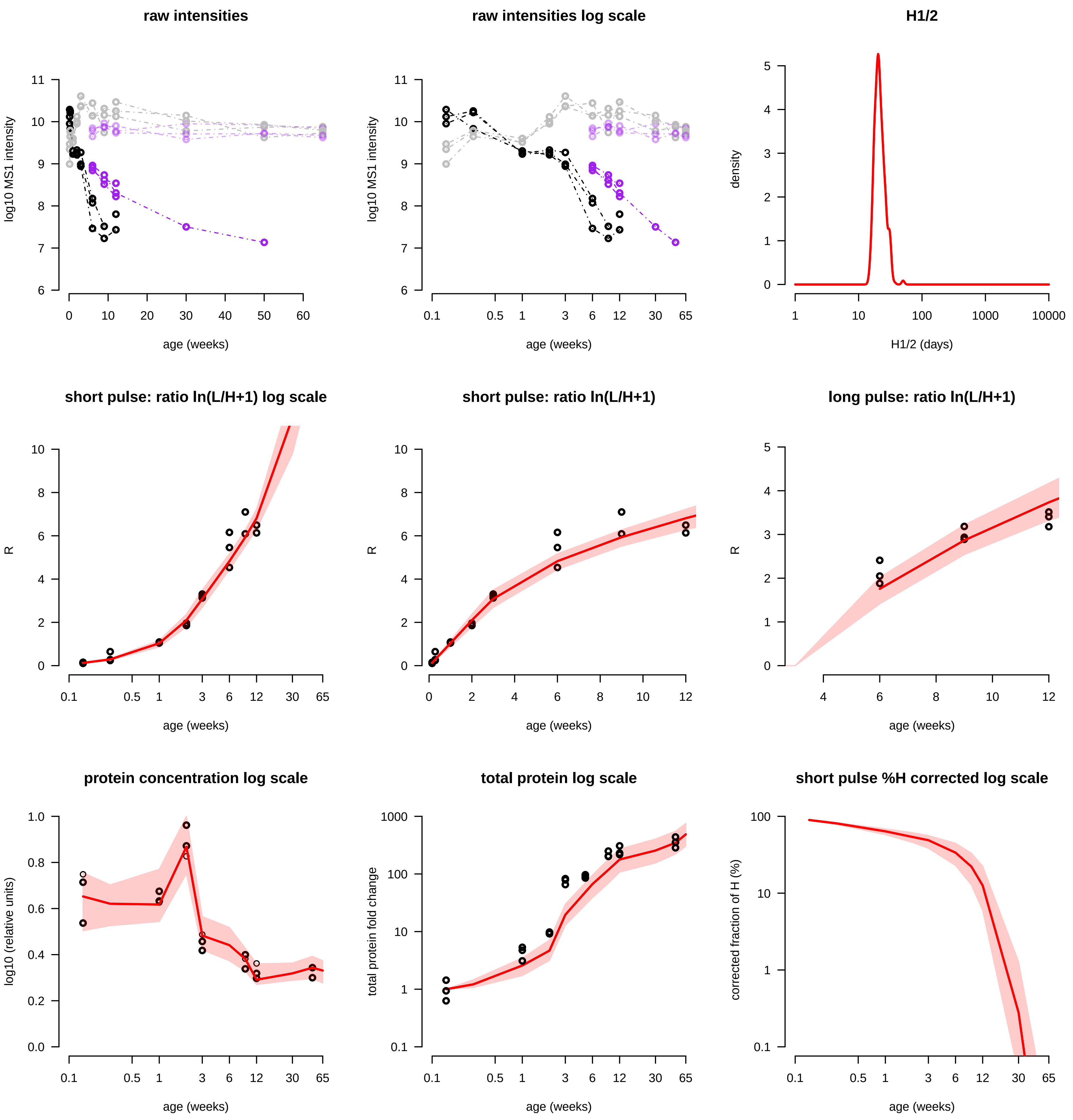

P62751

>sp|P62751|RL23A_MOUSE

60S ribosomal protein L23a

GN=Rpl23a

Med H1/2: 331 days

Q5 H1/2: 70.9 days

Q95 H1/2: 7066.7 days

reliable model fit: yes

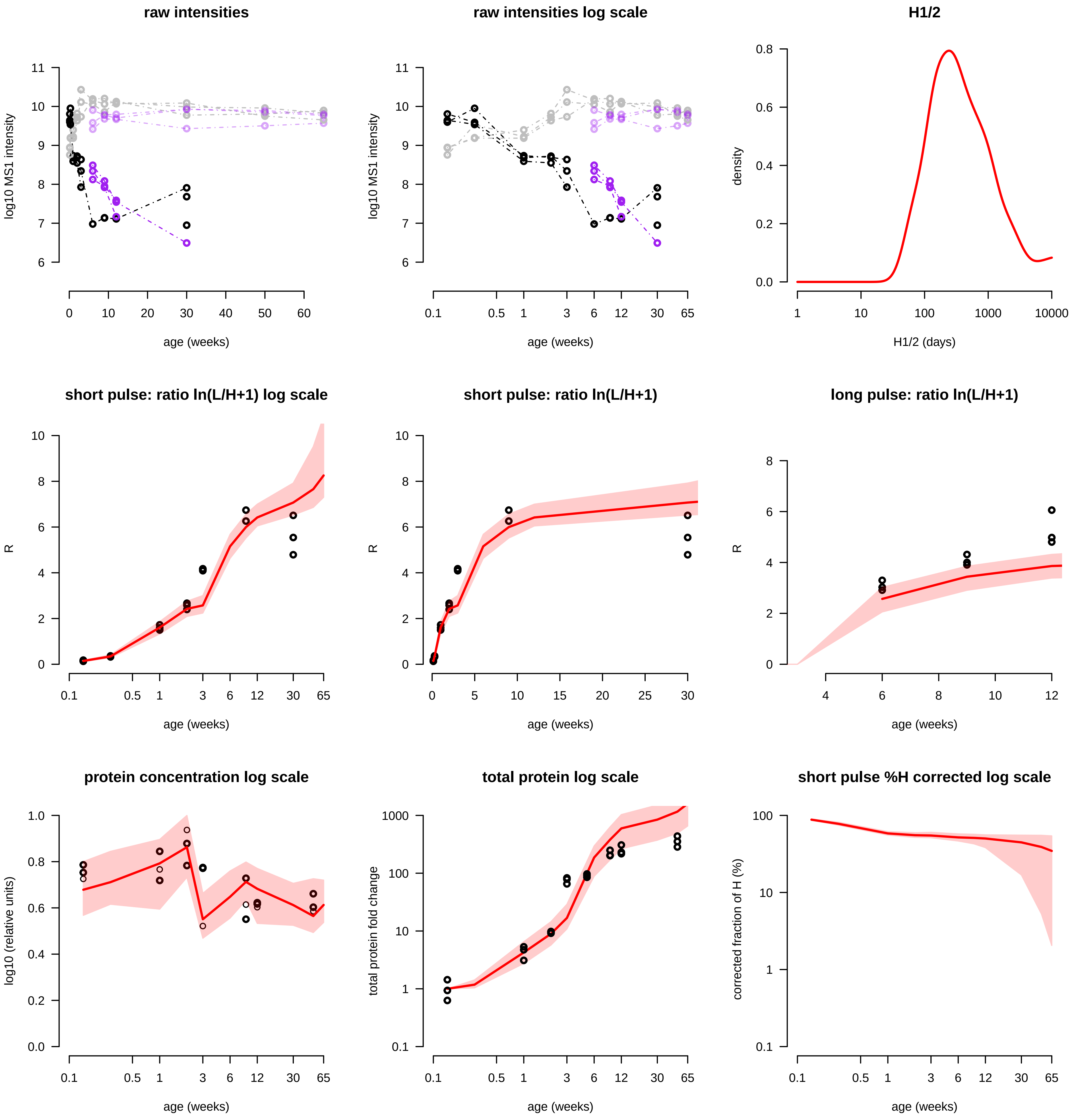

Q64435

>sp|Q64435|UD16_MOUSE

UDP−glucuronosyltransferase 1−6

GN=Ugt1a6

Med H1/2: 42.4 days

Q5 H1/2: 18.8 days

Q95 H1/2: 241 days

reliable model fit: yes

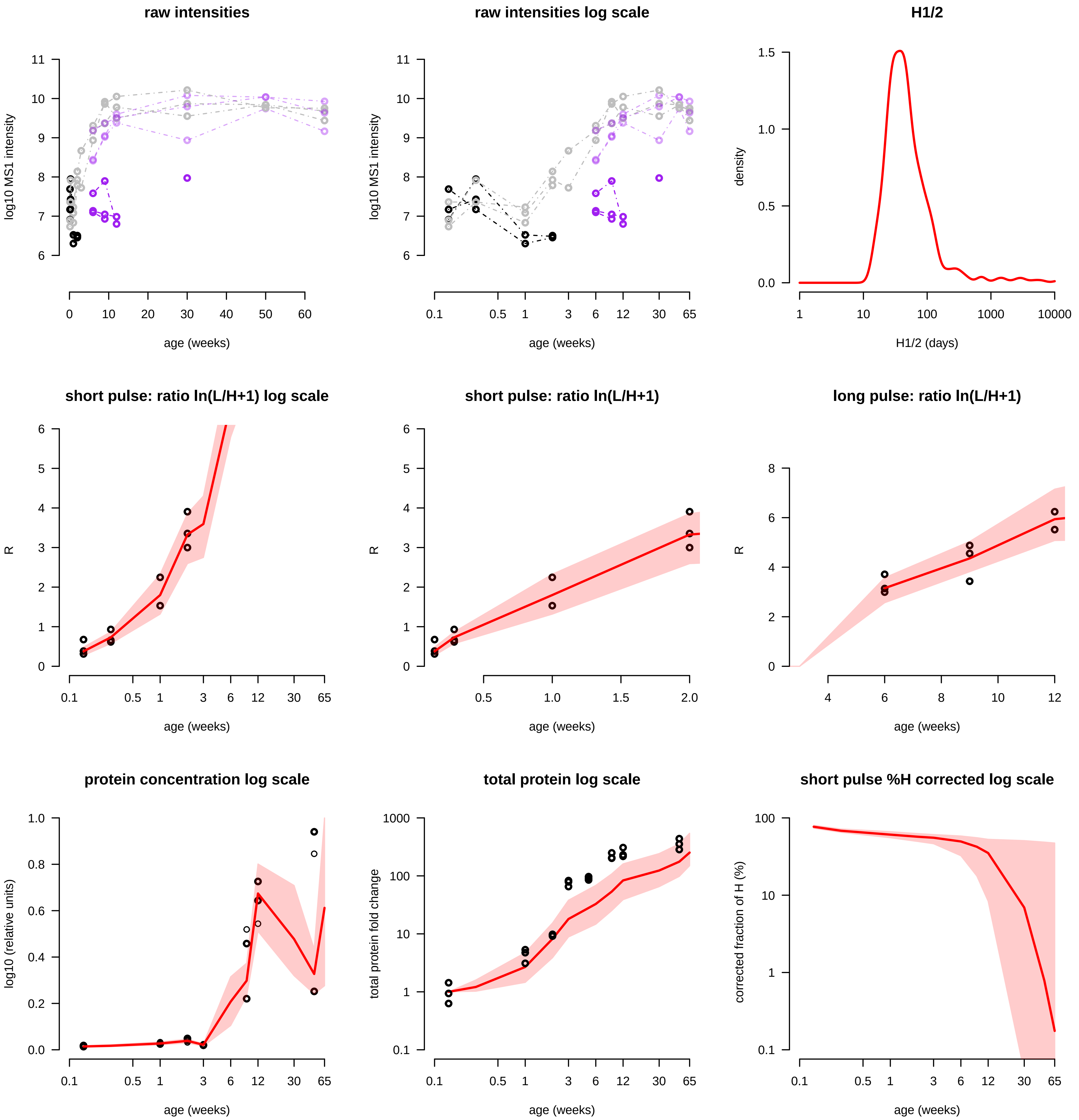

Q60605−2

>sp|Q60605−2|MYL6_MOUSE

Isoform Smooth muscle of Myosin light polypeptide 6

GN=Myl6

Med H1/2: 15.5 days

Q5 H1/2: 11.7 days

Q95 H1/2: 22.3 days

reliable model fit: yes

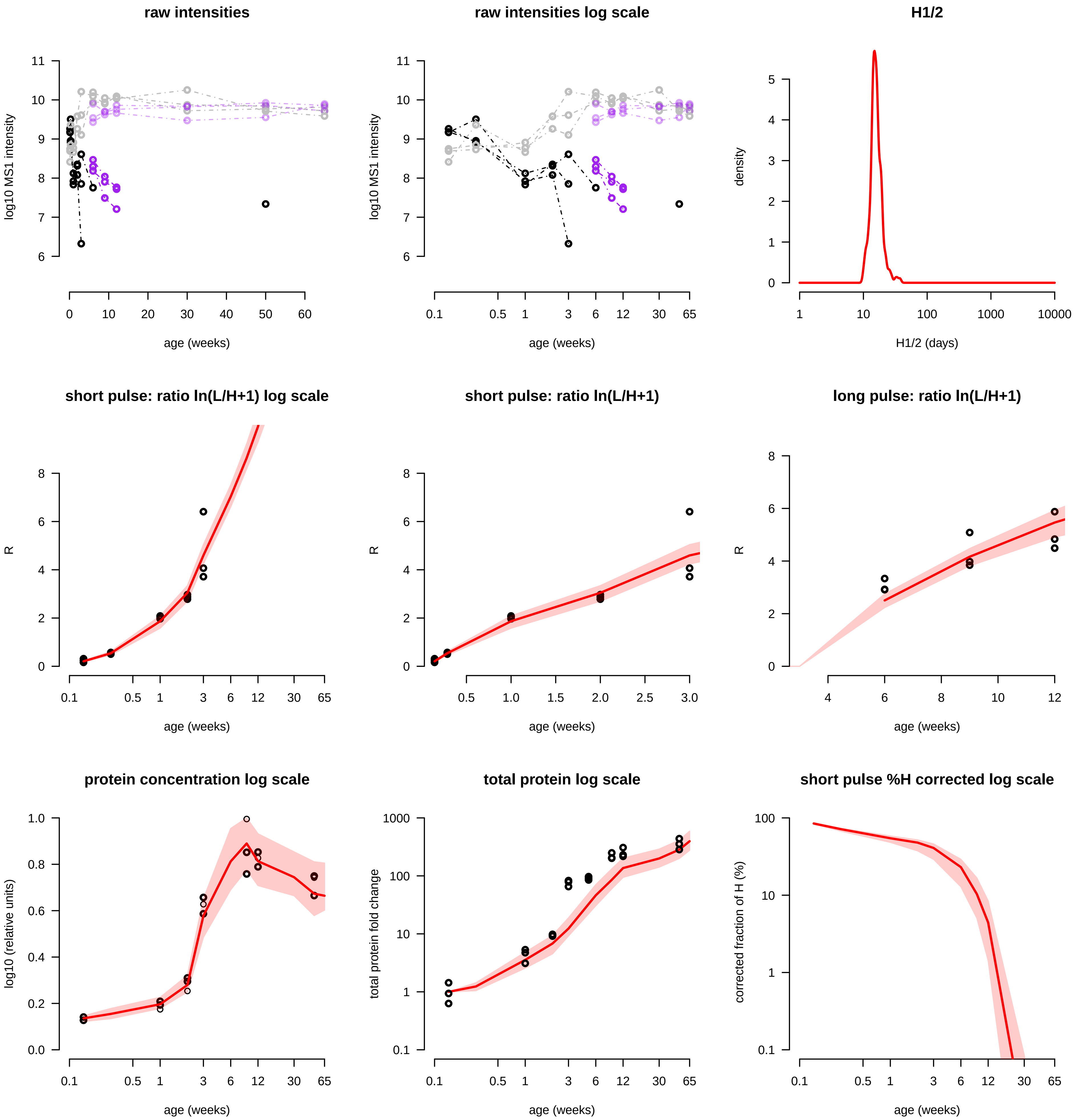

P46656

>sp|P46656|ADX_MOUSE

Adrenodoxin, mitochondrial

GN=Fdx1

Med H1/2: 42.6 days

Q5 H1/2: 28.6 days

Q95 H1/2: 155.3 days

reliable model fit: yes

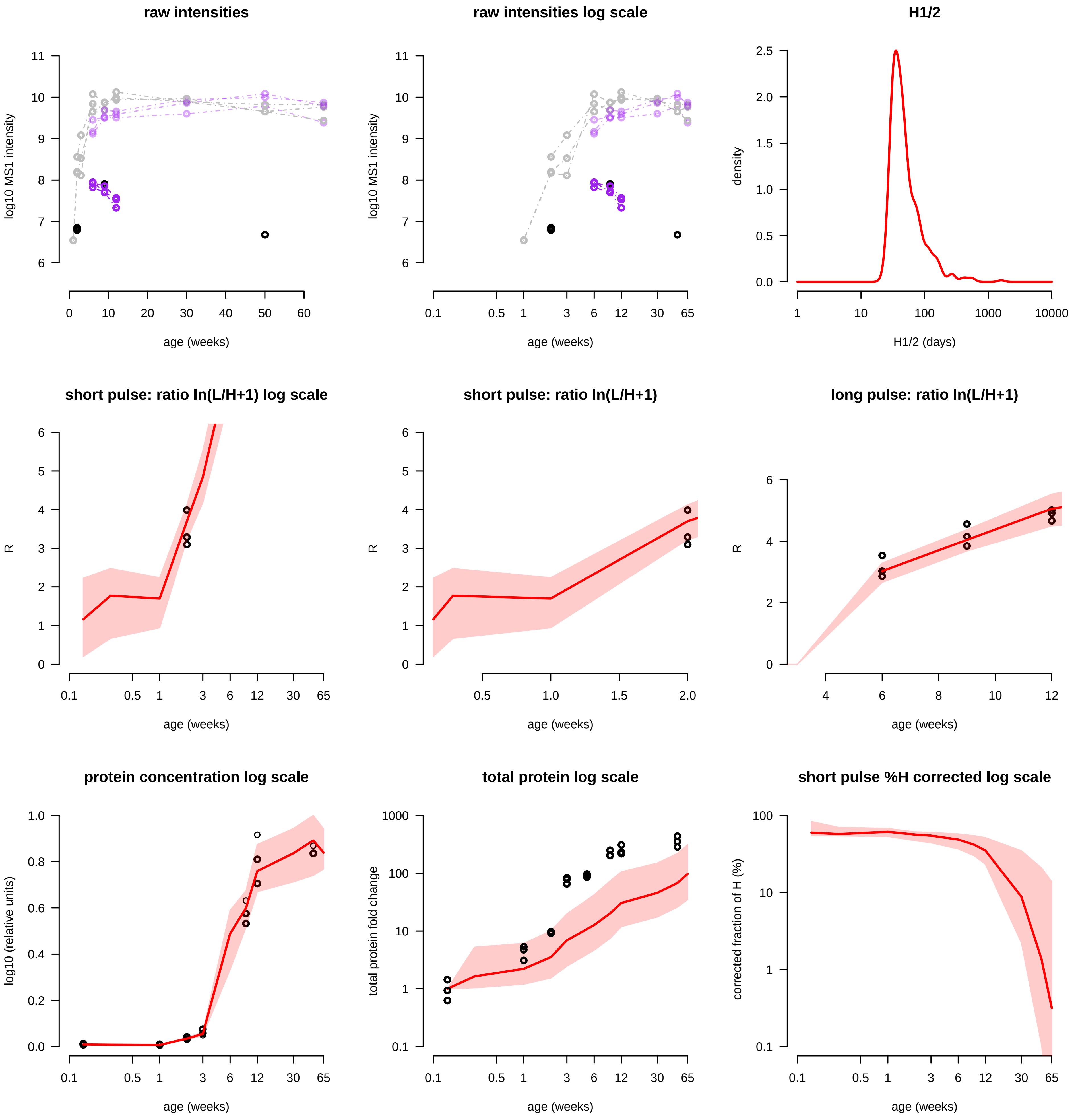

Q61263

>sp|Q61263|SOAT1_MOUSE

Sterol O−acyltransferase 1

GN=Soat1

Med H1/2: 576.1 days

Q5 H1/2: 224.2 days

Q95 H1/2: 7959 days

reliable model fit: yes

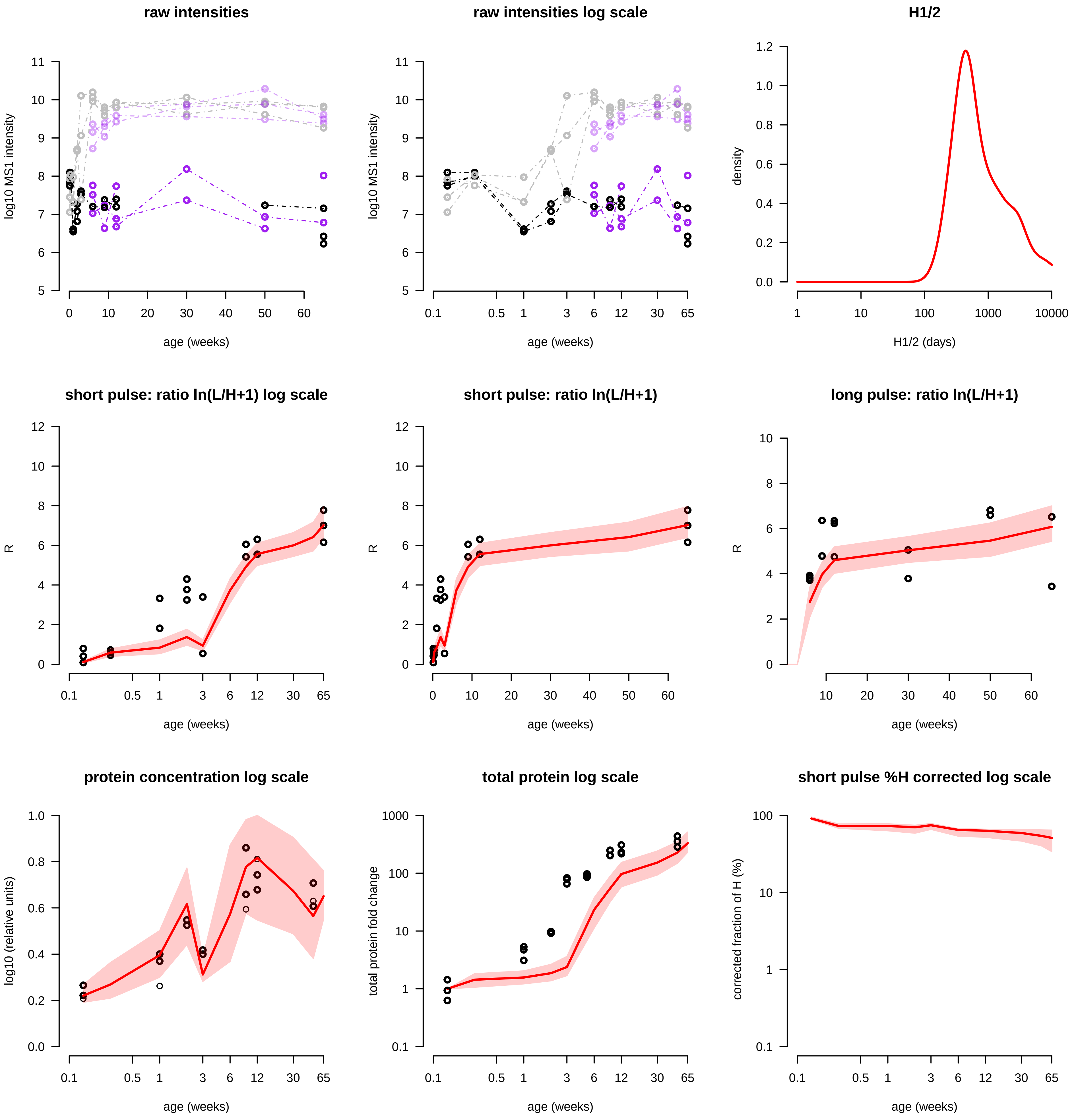

Q6ZQ38

>sp|Q6ZQ38|CAND1_MOUSE

Cullin−associated NEDD8−dissociated protein 1

GN=Cand1

Med H1/2: 9.1 days

Q5 H1/2: 7.7 days

Q95 H1/2: 11 days

reliable model fit: yes

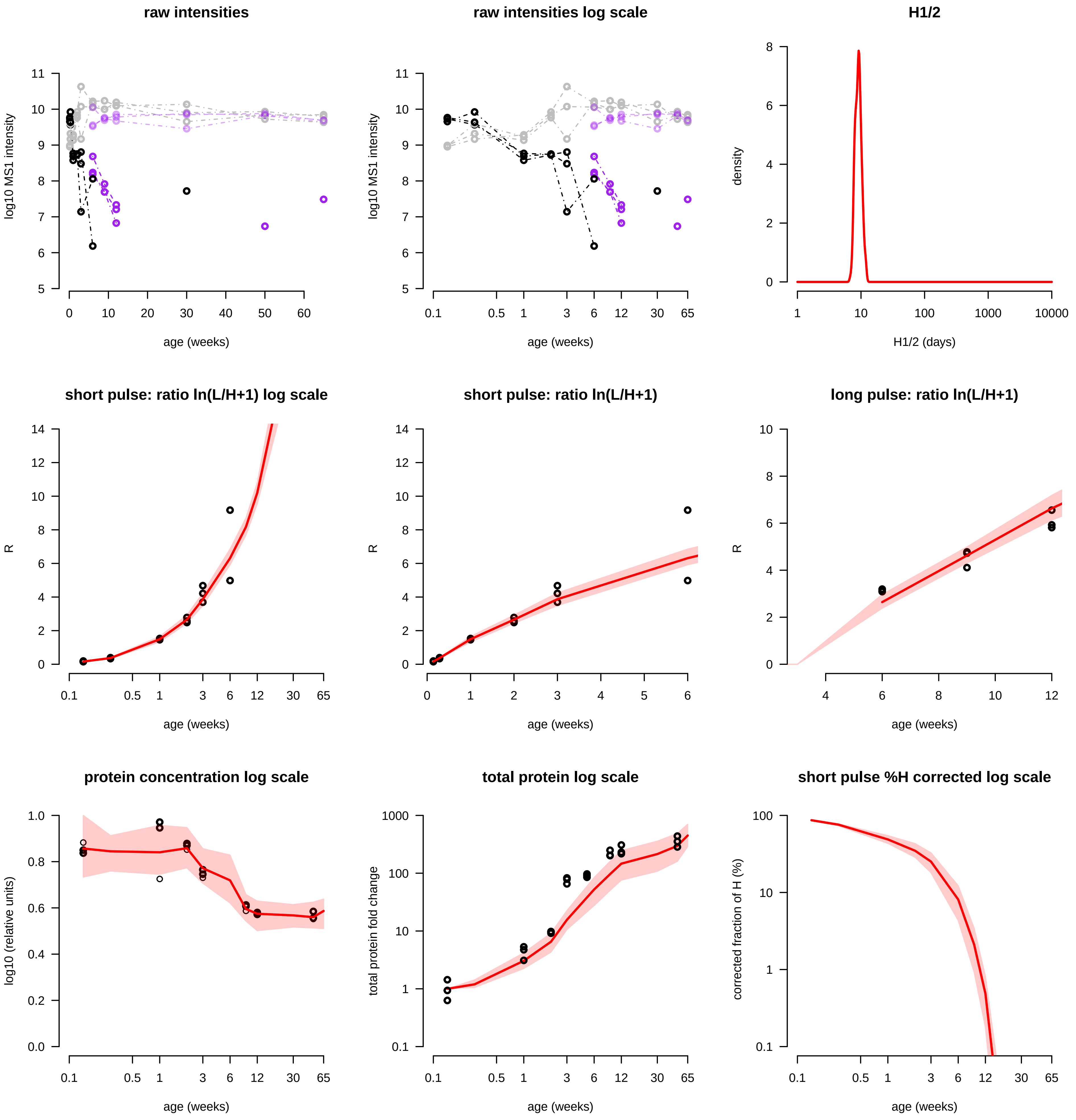

Q04857

>sp|Q04857|CO6A1_MOUSE

Collagen alpha−1(VI) chain

GN=Col6a1

Med H1/2: 8.4 days

Q5 H1/2: 7 days

Q95 H1/2: 10.1 days

reliable model fit: yes

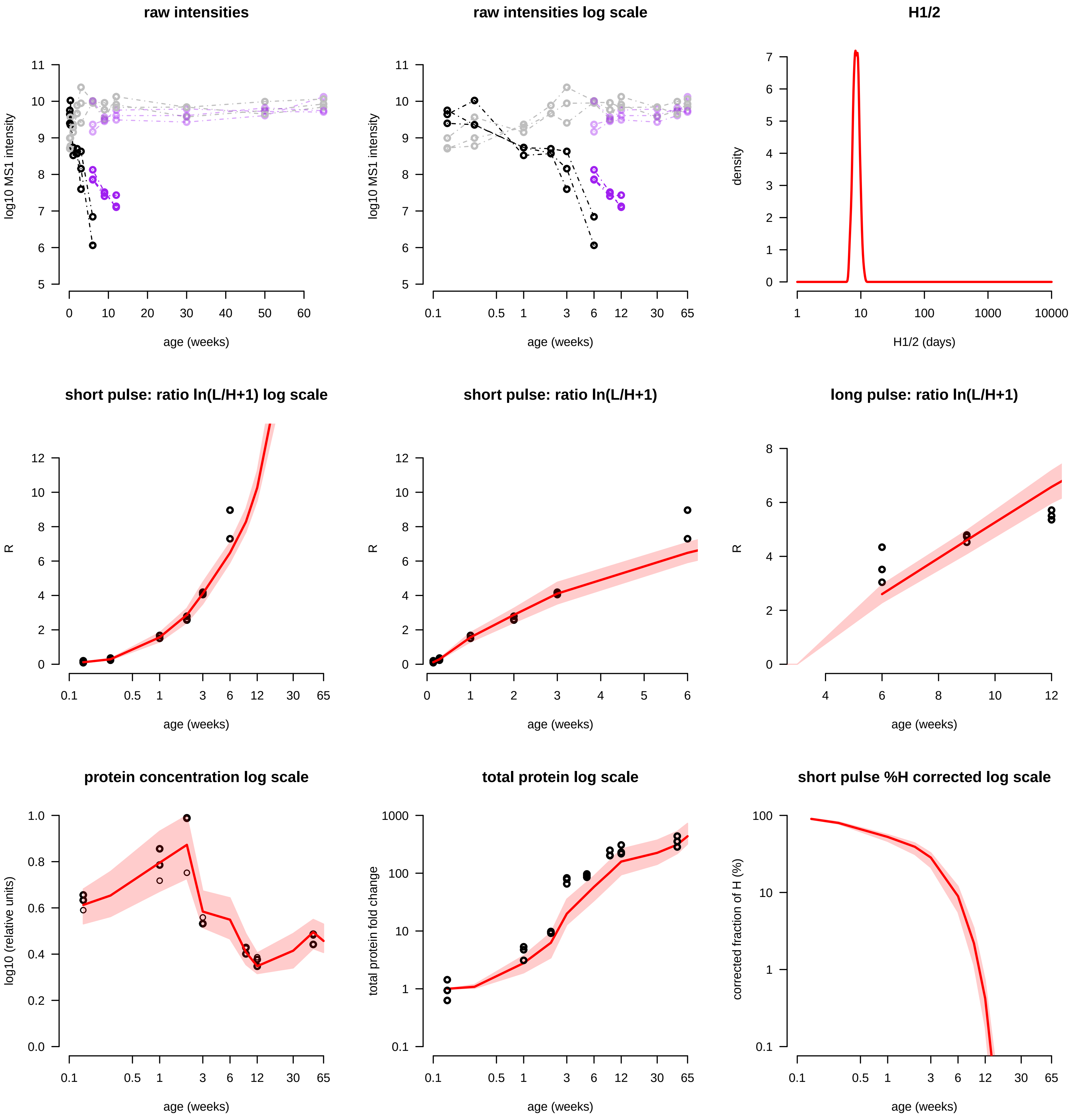

P97371

>sp|P97371|PSME1_MOUSE

Proteasome activator complex subunit 1

GN=Psme1

Med H1/2: 10.1 days

Q5 H1/2: 8.2 days

Q95 H1/2: 12.4 days

reliable model fit: yes

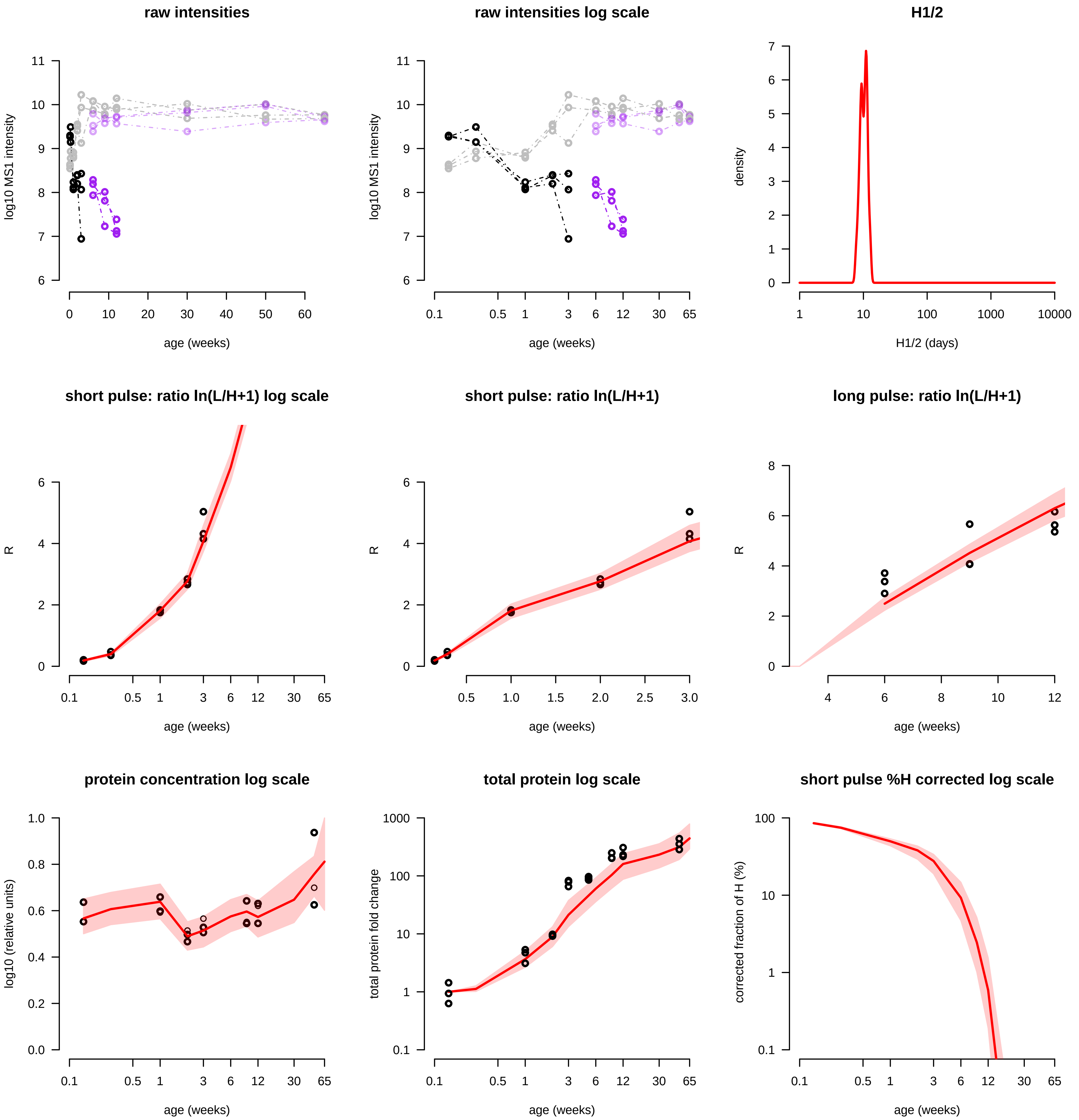

P60710

>sp|P60710|ACTB_MOUSE

Actin, cytoplasmic 1

GN=Actb

Med H1/2: 5.1 days

Q5 H1/2: 4.1 days

Q95 H1/2: 6.3 days

reliable model fit: yes

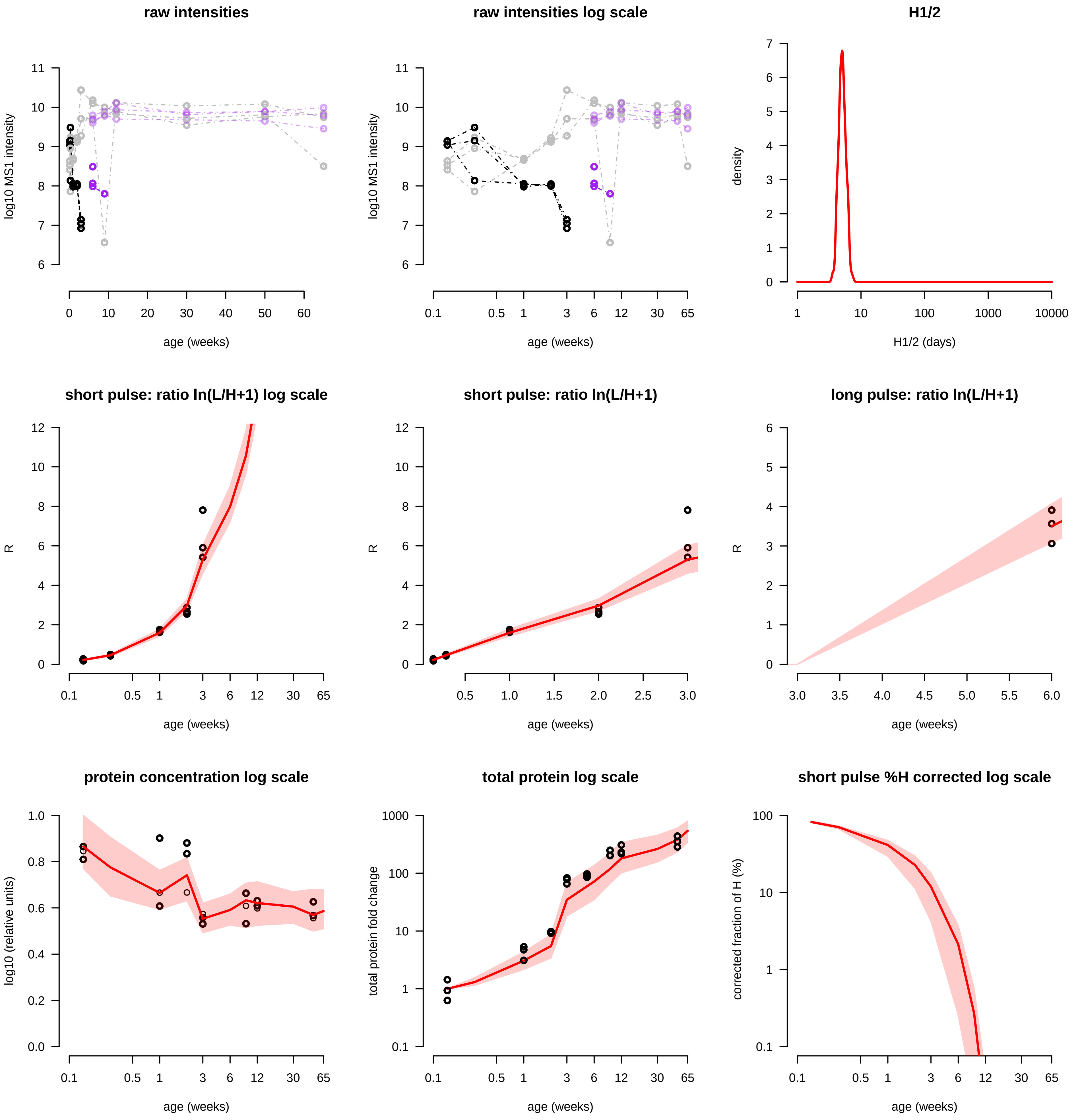

P62830

>sp|P62830|RL23_MOUSE

60S ribosomal protein L23

GN=Rpl23

Med H1/2: 225.7 days

Q5 H1/2: 141.1 days

Q95 H1/2: 787.9 days

reliable model fit: yes

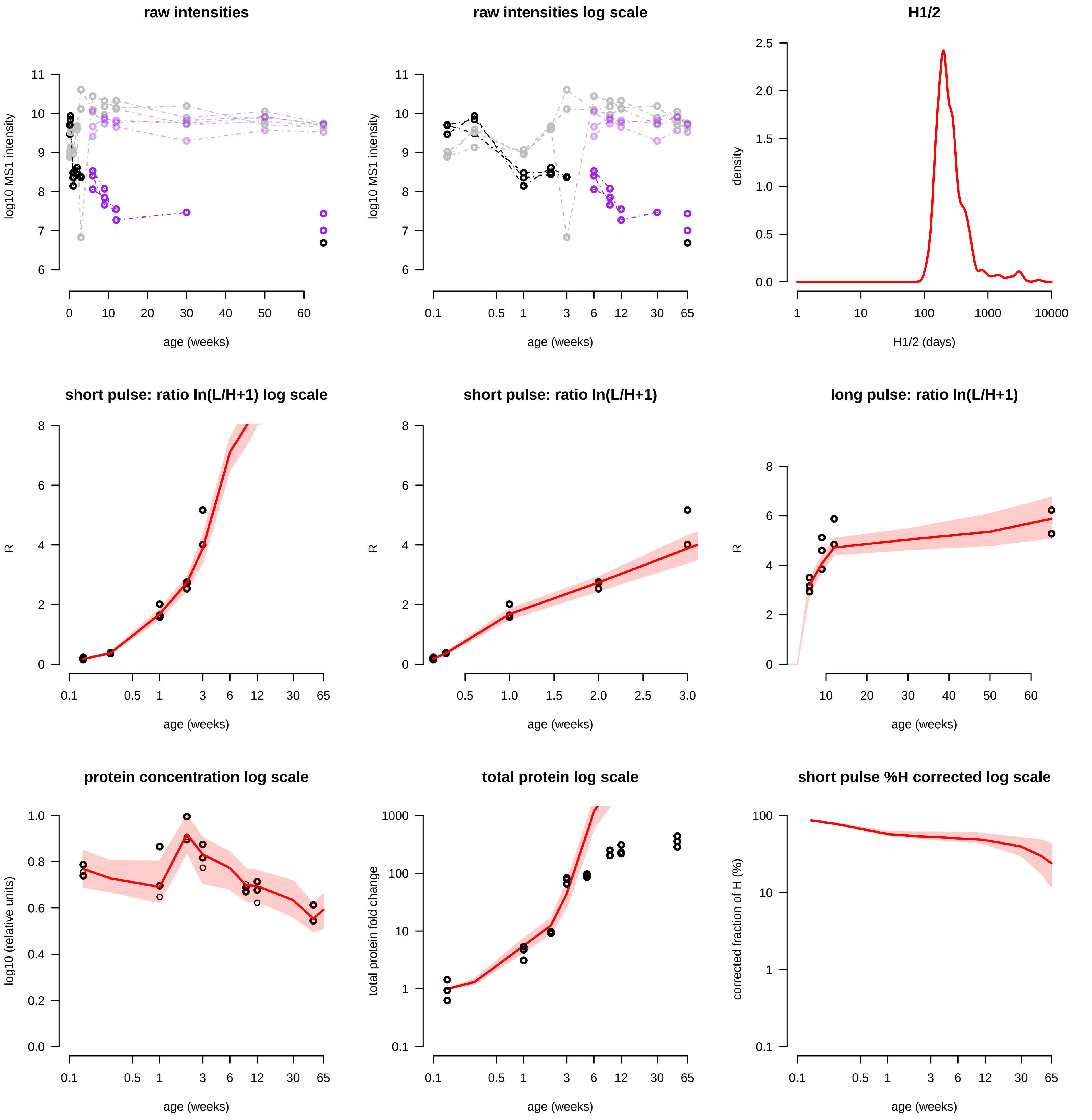

Q60864

>sp|Q60864|STIP1_MOUSE

Stress−induced−phosphoprotein 1

GN=Stip1

Med H1/2: 174.1 days

Q5 H1/2: 92.9 days

Q95 H1/2: 438.6 days

reliable model fit: yes

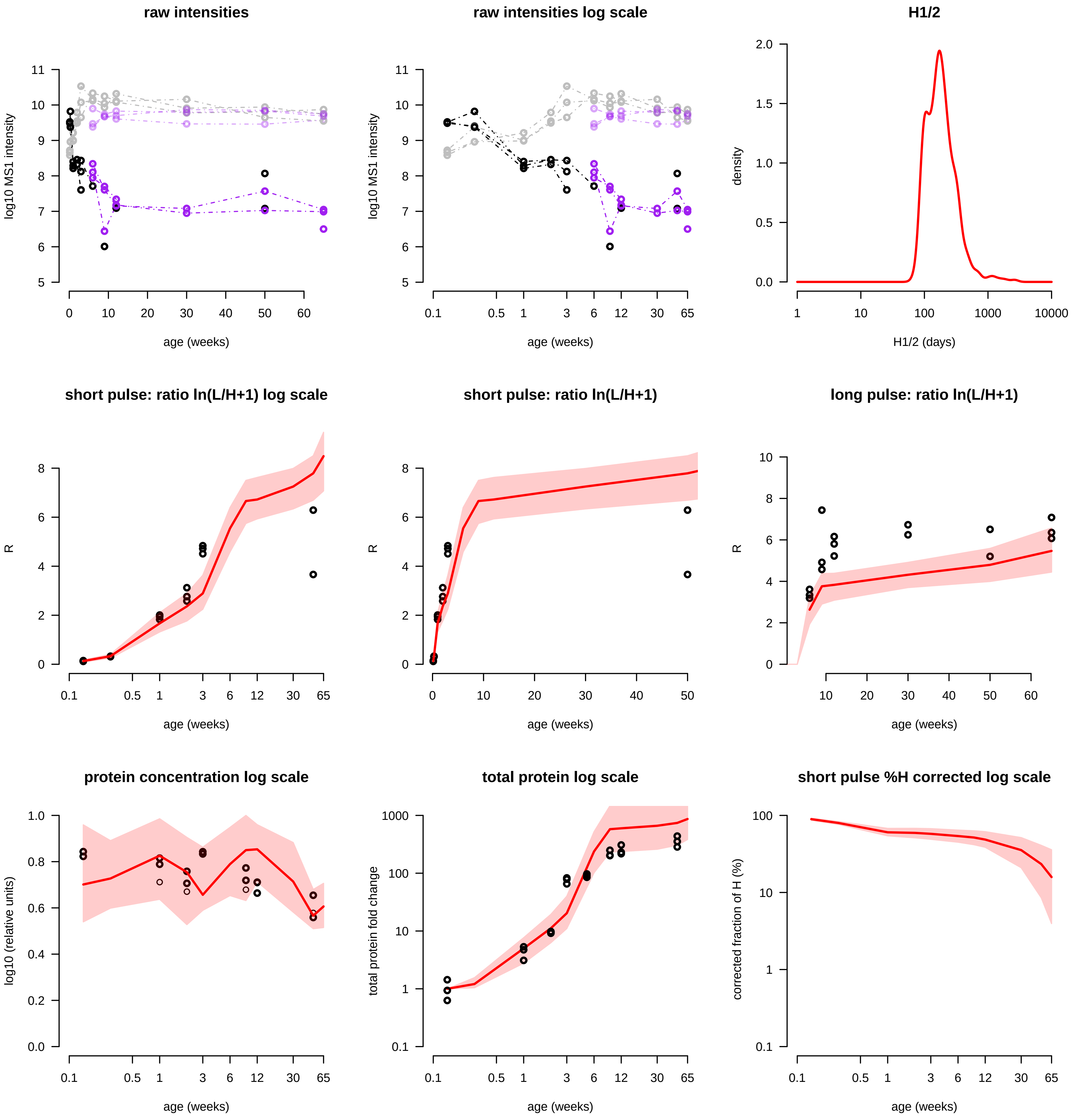

P47962

>sp|P47962|RL5_MOUSE

60S ribosomal protein L5

GN=Rpl5

Med H1/2: 11.7 days

Q5 H1/2: 8.9 days

Q95 H1/2: 14.9 days

reliable model fit: yes

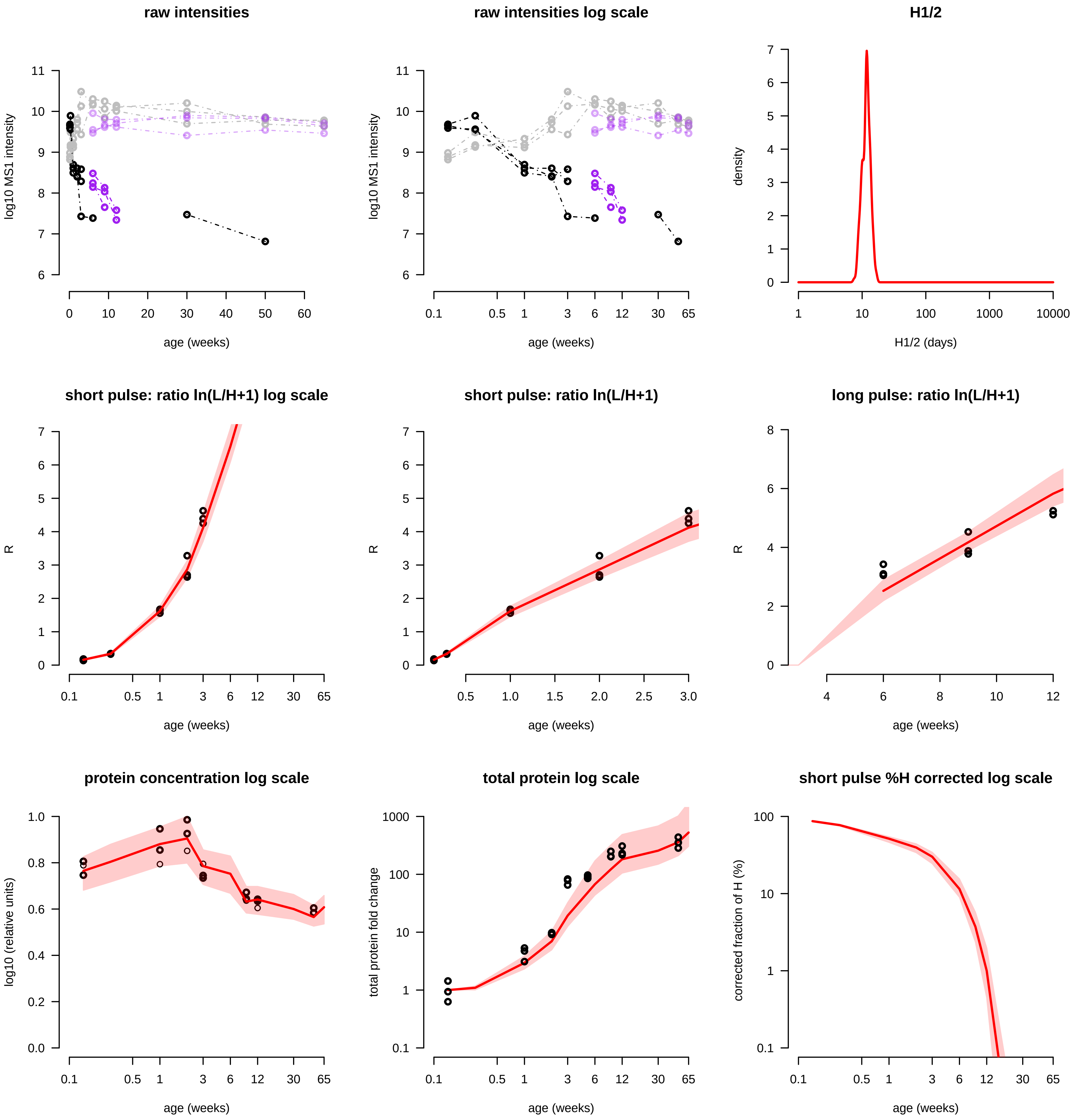

Q9DCD0

>sp|Q9DCD0|6PGD_MOUSE

6−phosphogluconate dehydrogenase, decarboxylating

GN=Pgd

Med H1/2: 242.9 days

Q5 H1/2: 114.1 days

Q95 H1/2: 3958.3 days

reliable model fit: yes

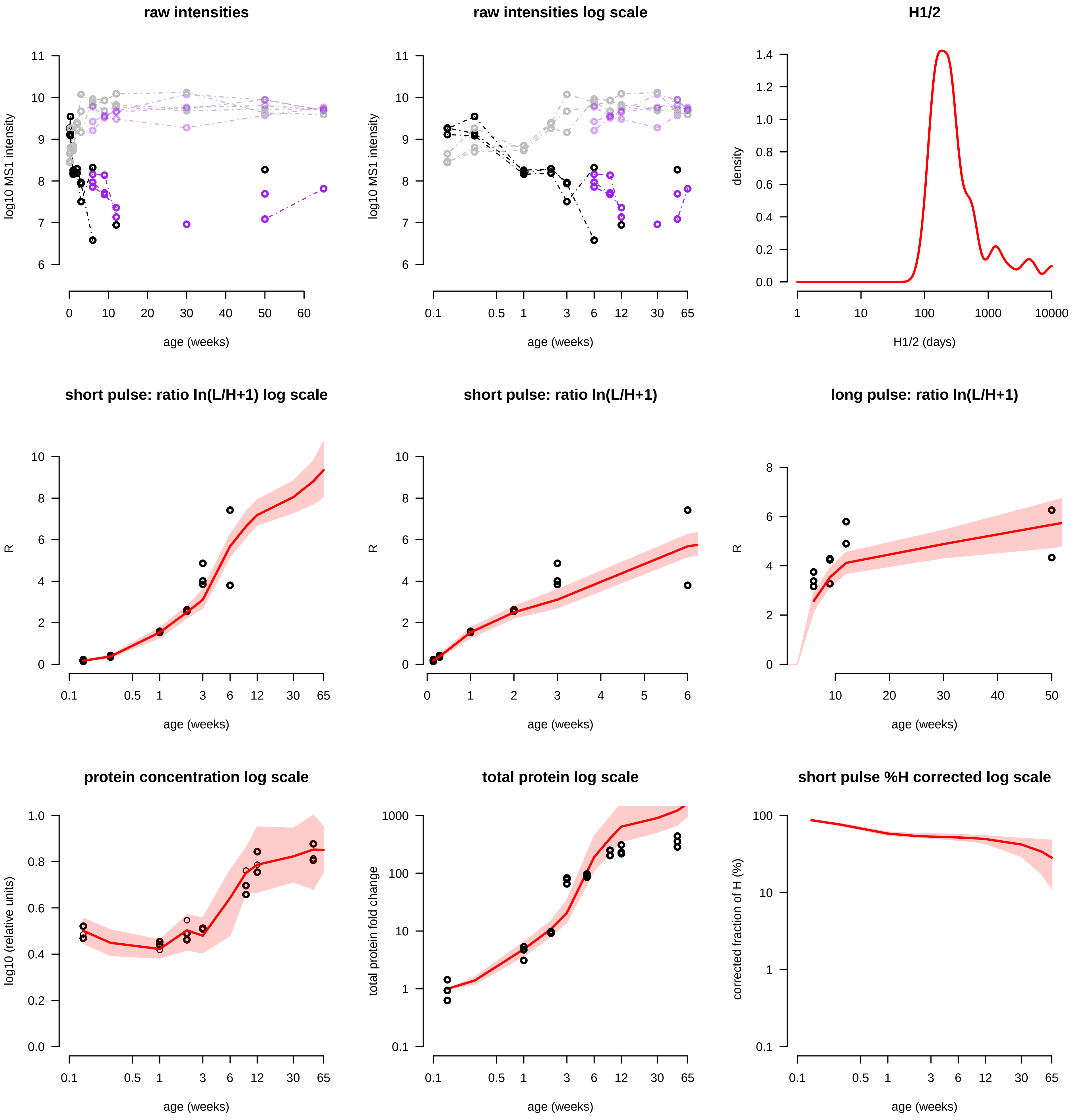

Q921G7

>sp|Q921G7|ETFD_MOUSE

Electron transfer flavoprotein−ubiquinone oxidoreductase, mitochondrial

GN=Etfdh

Med H1/2: 2051.1 days

Q5 H1/2: 472.3 days

Q95 H1/2: 22352.6 days

reliable model fit: yes

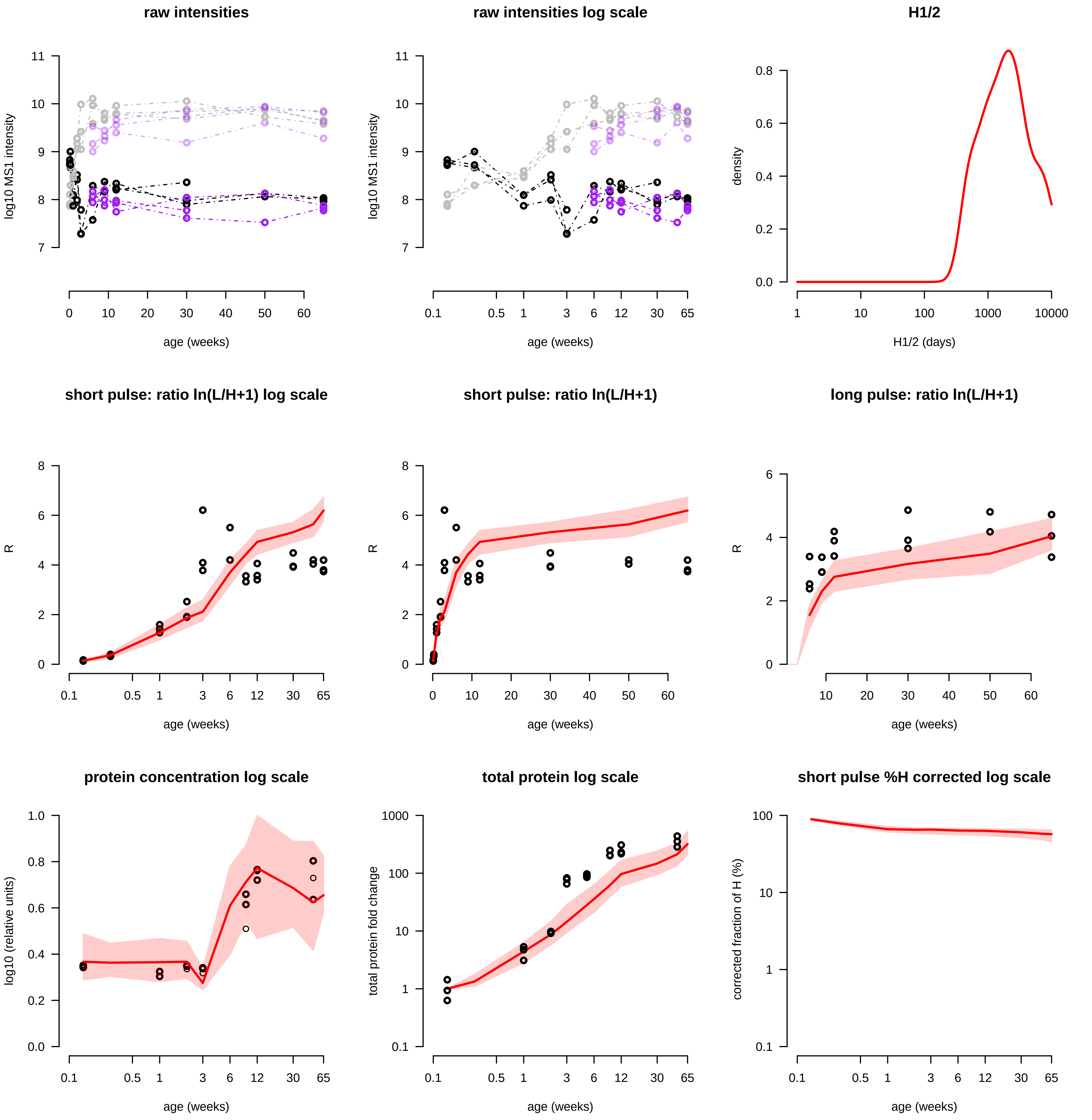

Q91Z53

>sp|Q91Z53|GRHPR_MOUSE

Glyoxylate reductase/hydroxypyruvate reductase

GN=Grhpr

Med H1/2: 9.8 days

Q5 H1/2: 8.4 days

Q95 H1/2: 11.8 days

reliable model fit: yes

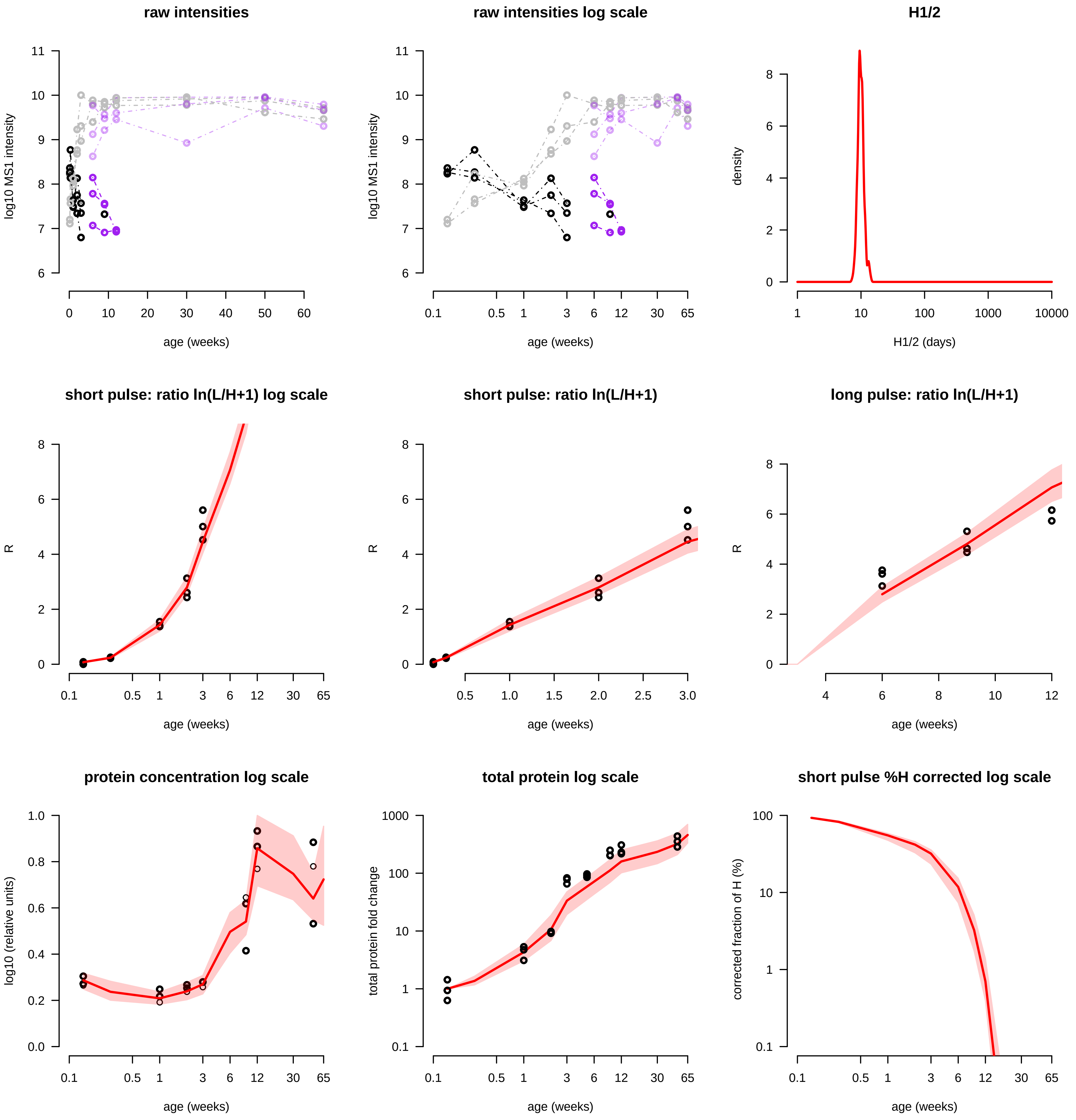

P62702

>sp|P62702|RS4X_MOUSE

40S ribosomal protein S4, X isoform

GN=Rps4x

Med H1/2: 223.3 days

Q5 H1/2: 85 days

Q95 H1/2: 934.2 days

reliable model fit: yes

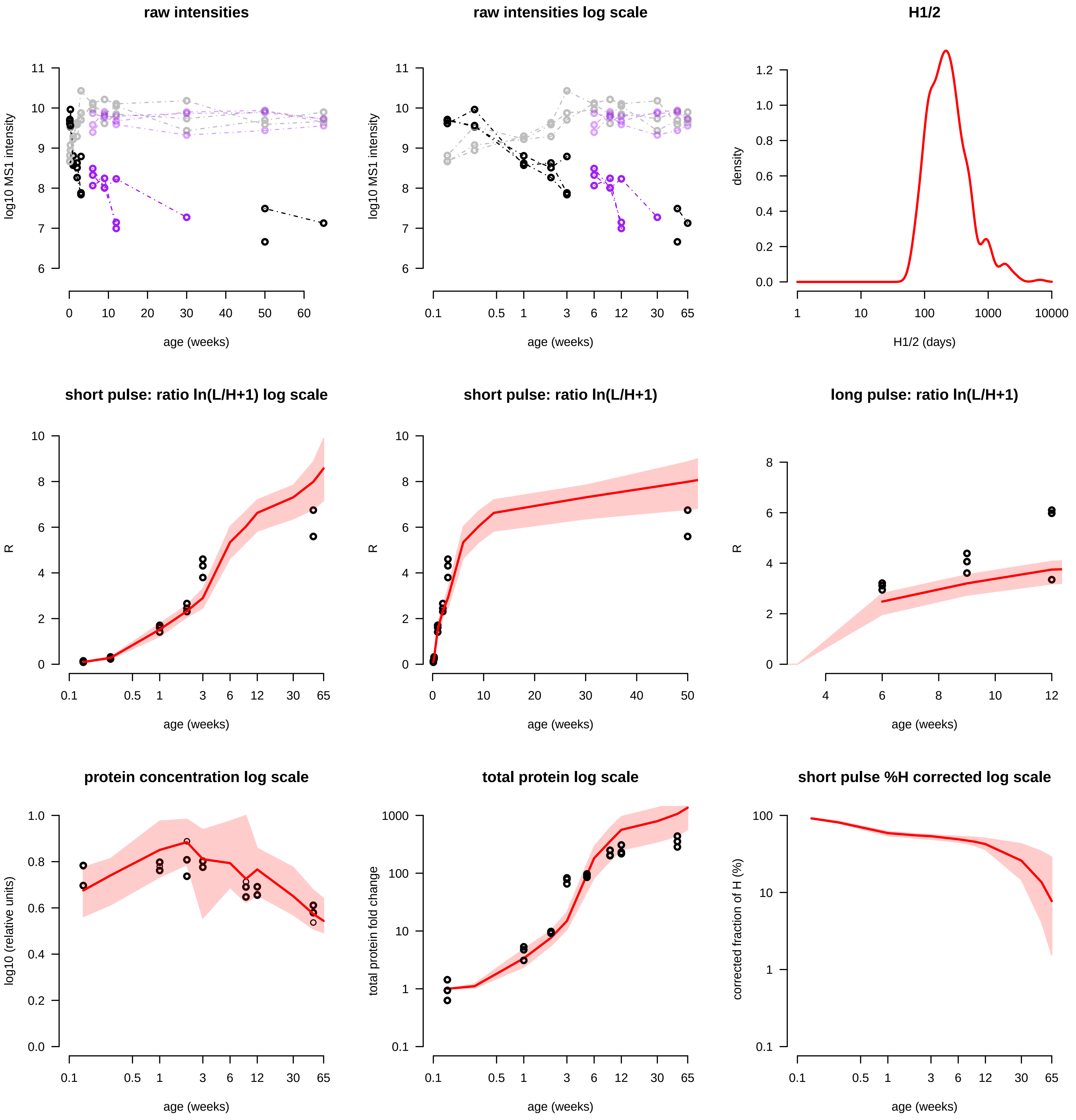

Q6NS45;Q6NS45−2;Q6NS45−3

>sp|Q6NS45|CCD66_MOUSE

Coiled−coil domain−containing protein 66

GN=Ccdc66

Med H1/2: 31.8 days

Q5 H1/2: 21.2 days

Q95 H1/2: 67.8 days

reliable model fit: yes

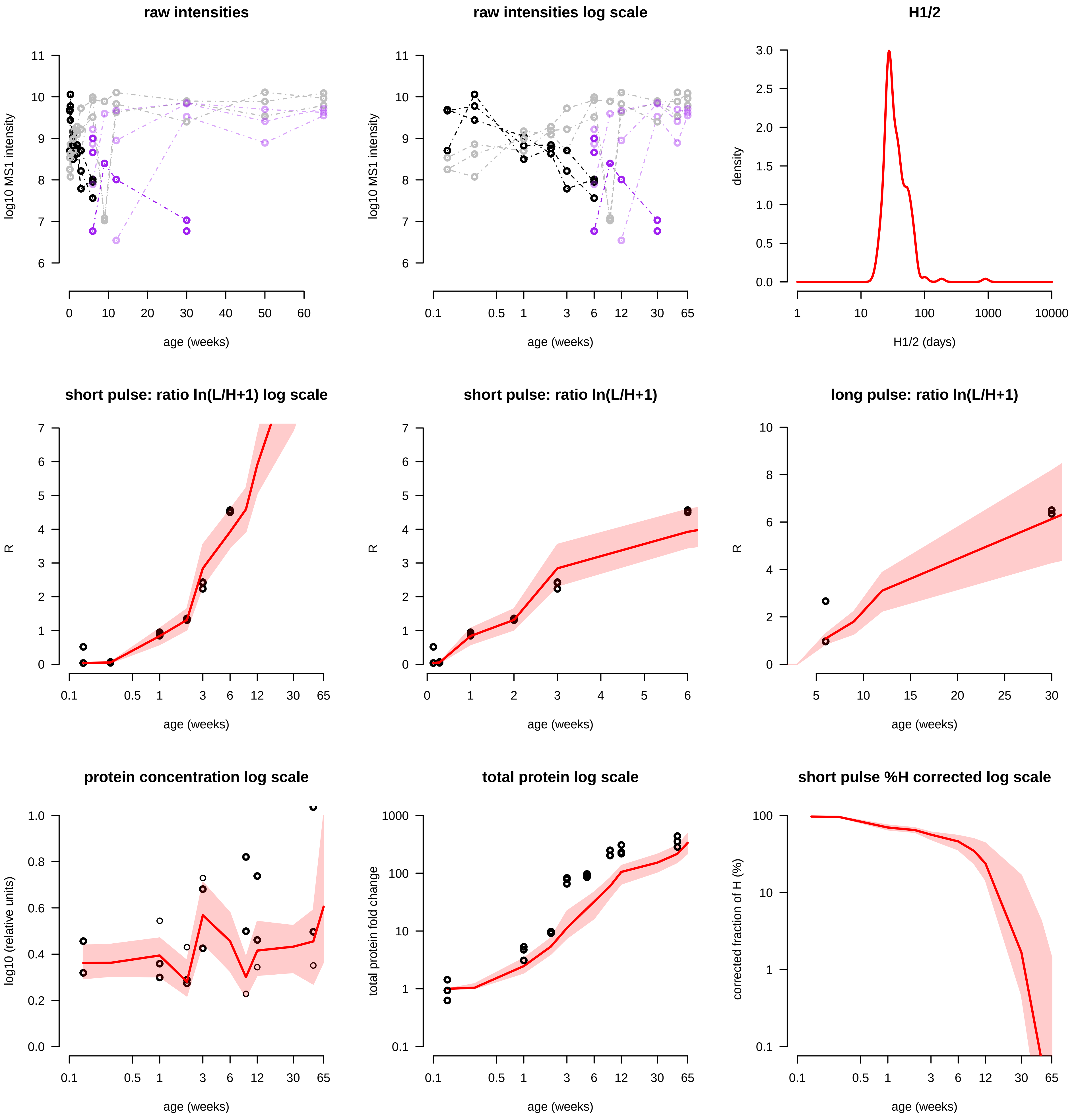

P51859

>sp|P51859|HDGF_MOUSE

Hepatoma−derived growth factor

GN=Hdgf

Med H1/2: 7.5 days

Q5 H1/2: 6 days

Q95 H1/2: 8.9 days

reliable model fit: yes

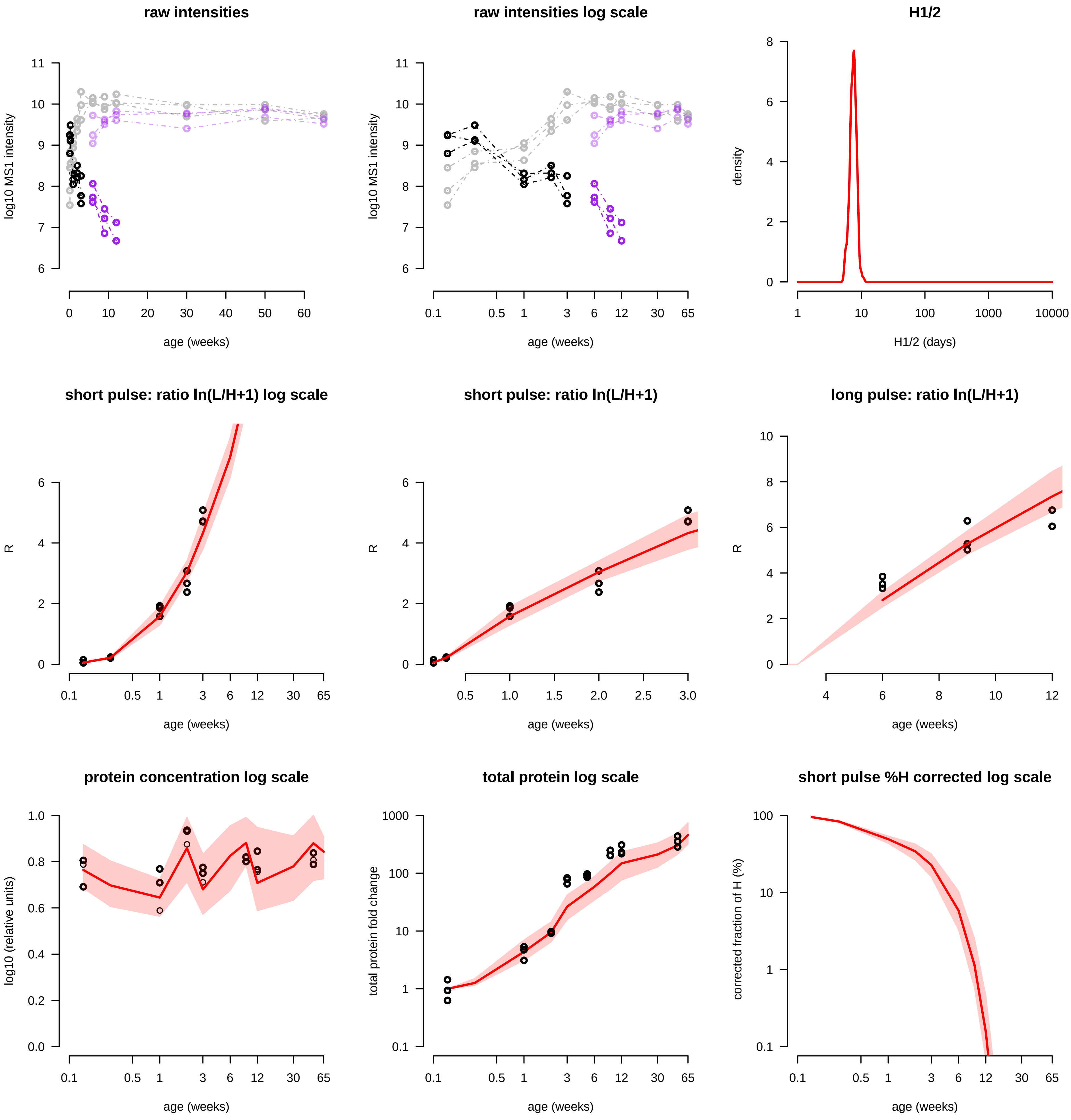

P17225

>sp|P17225|PTBP1_MOUSE

Polypyrimidine tract−binding protein 1

GN=Ptbp1

Med H1/2: 6.8 days

Q5 H1/2: 5.7 days

Q95 H1/2: 8.5 days

reliable model fit: yes

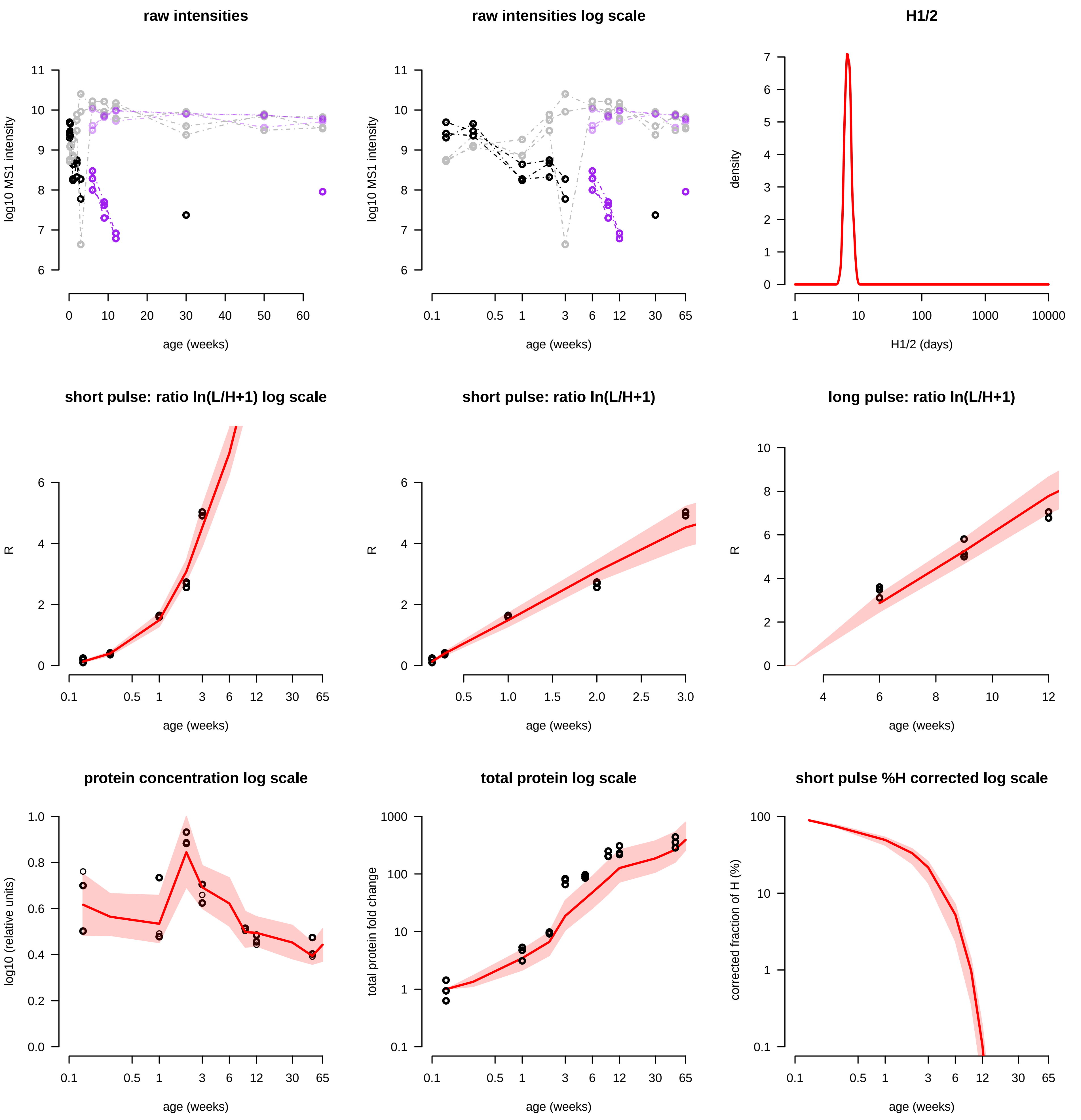

P80317

>sp|P80317|TCPZ_MOUSE

T−complex protein 1 subunit zeta

GN=Cct6a

Med H1/2: 9.9 days

Q5 H1/2: 8.5 days

Q95 H1/2: 11 days

reliable model fit: yes

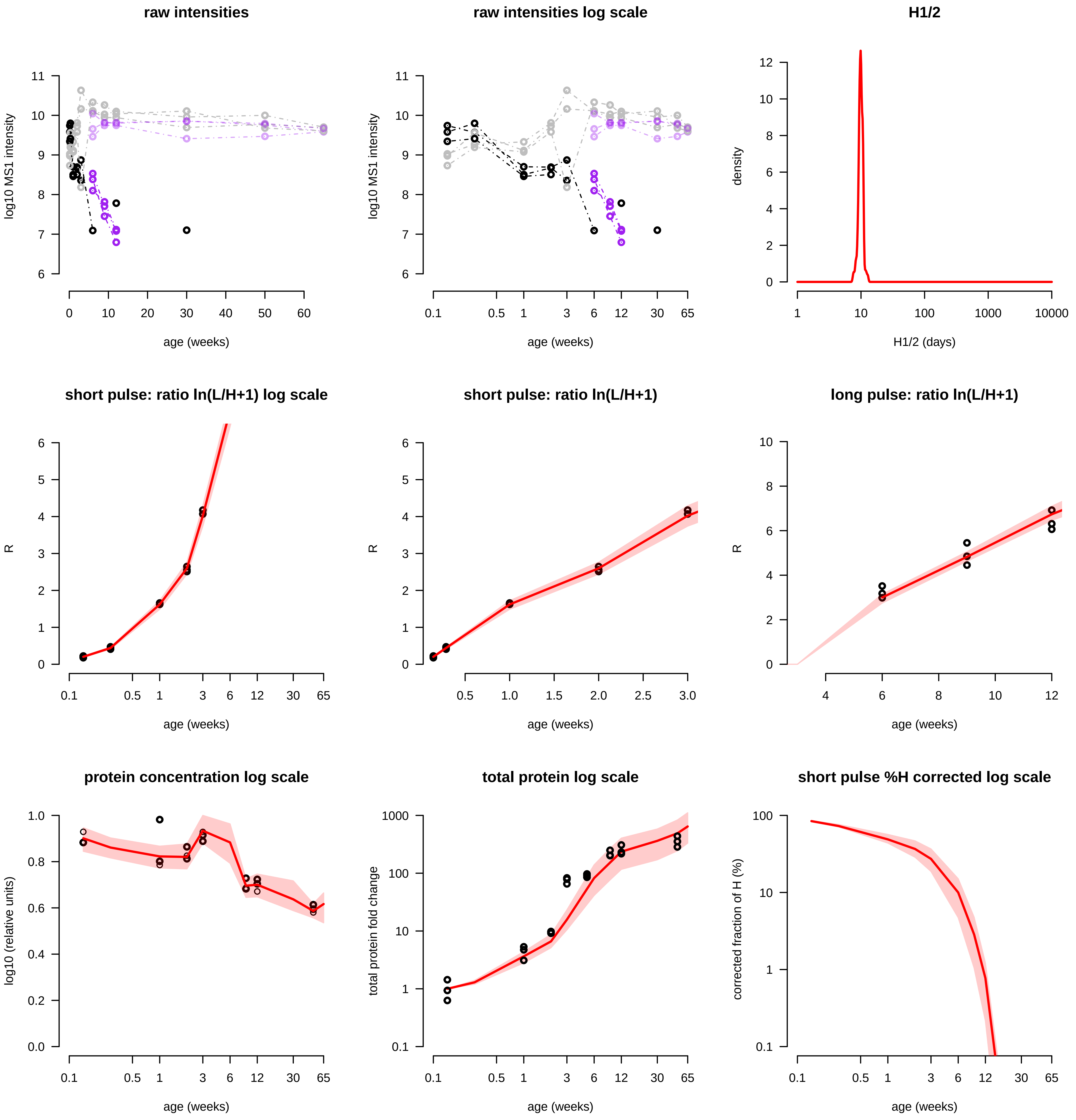

P23953

>sp|P23953|EST1C_MOUSE

Carboxylesterase 1C

GN=Ces1c

Med H1/2: 298.5 days

Q5 H1/2: 108.7 days

Q95 H1/2: 2417.6 days

reliable model fit: yes

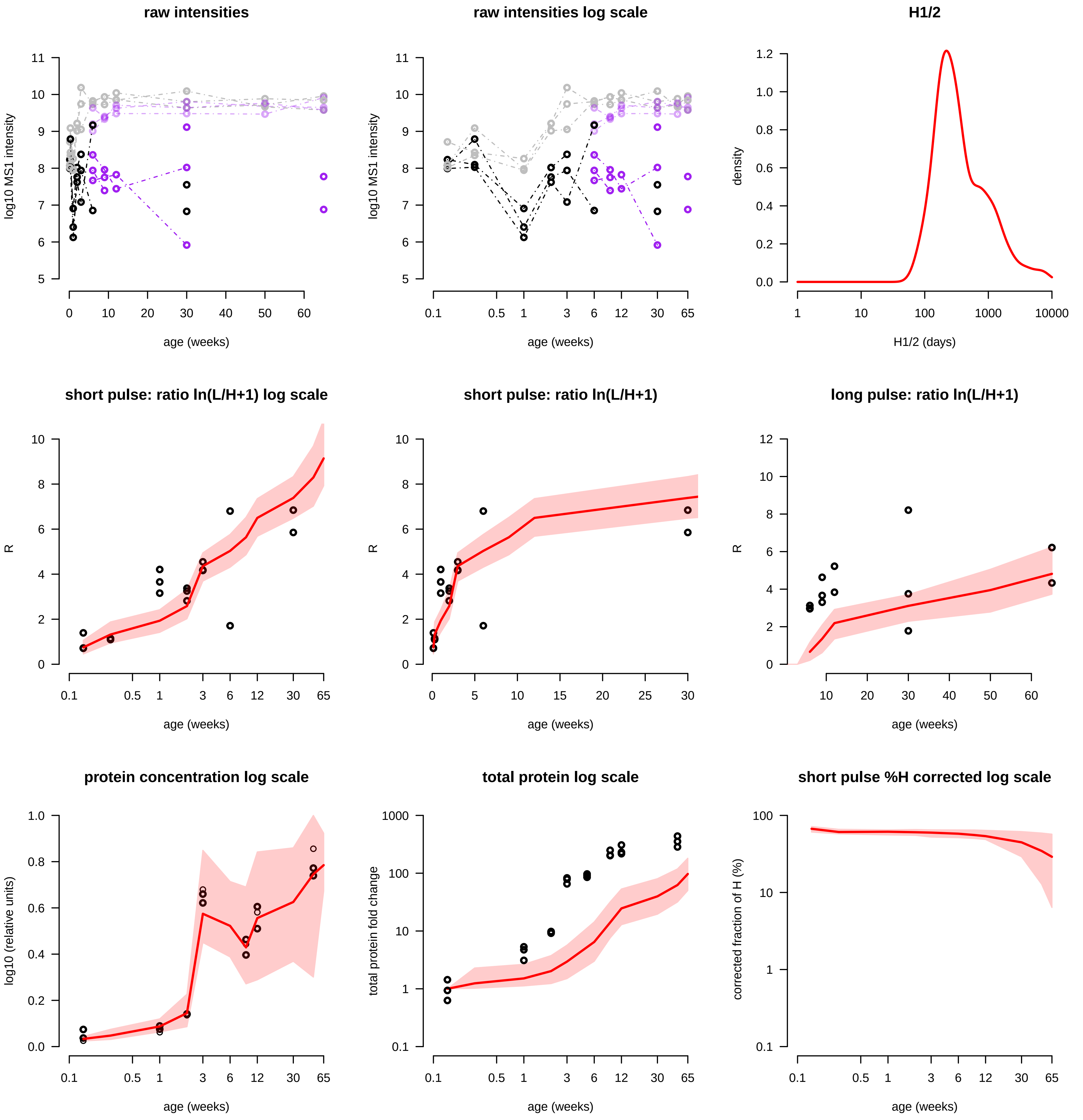

P68040

>sp|P68040|RACK1_MOUSE

Receptor of activated protein C kinase 1

GN=Rack1

Med H1/2: 8.2 days

Q5 H1/2: 7.2 days

Q95 H1/2: 9.7 days

reliable model fit: yes

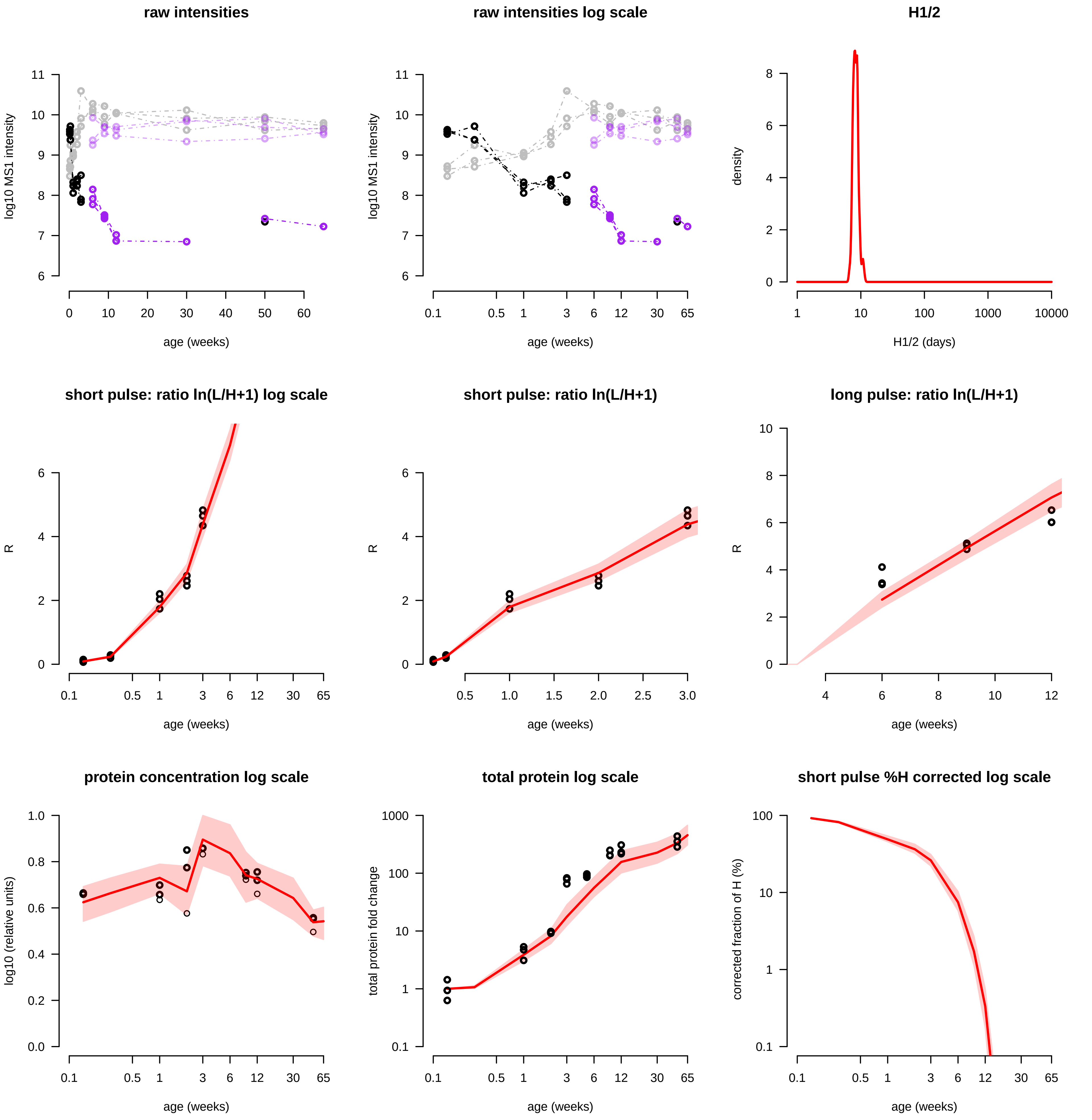

Q9Z2I0

>sp|Q9Z2I0|LETM1_MOUSE

Mitochondrial proton/calcium exchanger protein

GN=Letm1

Med H1/2: 4.9 days

Q5 H1/2: 3.7 days

Q95 H1/2: 7.2 days

reliable model fit: yes

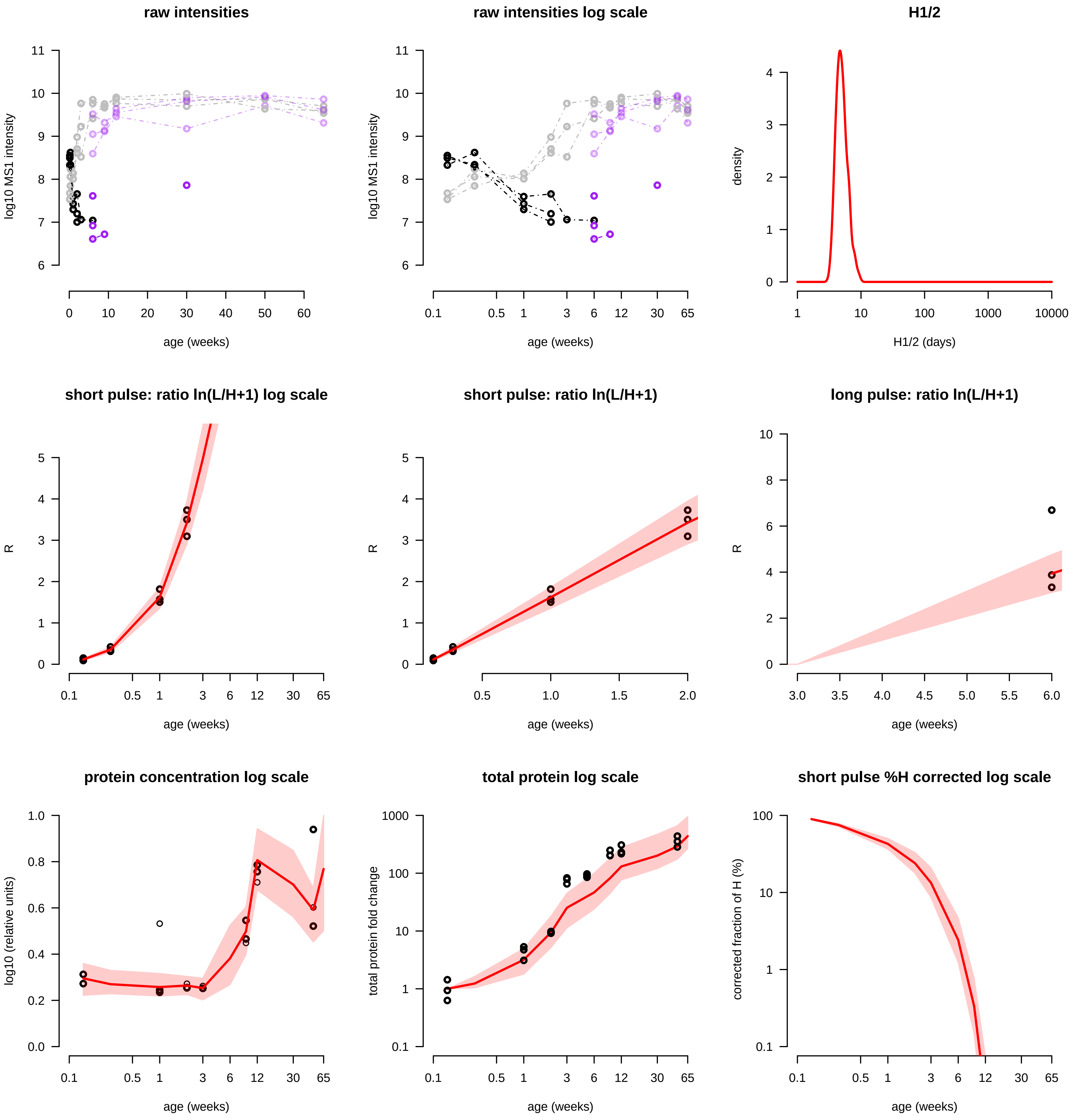

Q9EQU5;Q9EQU5−3

>sp|Q9EQU5|SET_MOUSE

Protein SET

GN=Set

Med H1/2: 31 days

Q5 H1/2: 23.7 days

Q95 H1/2: 40.5 days

reliable model fit: yes

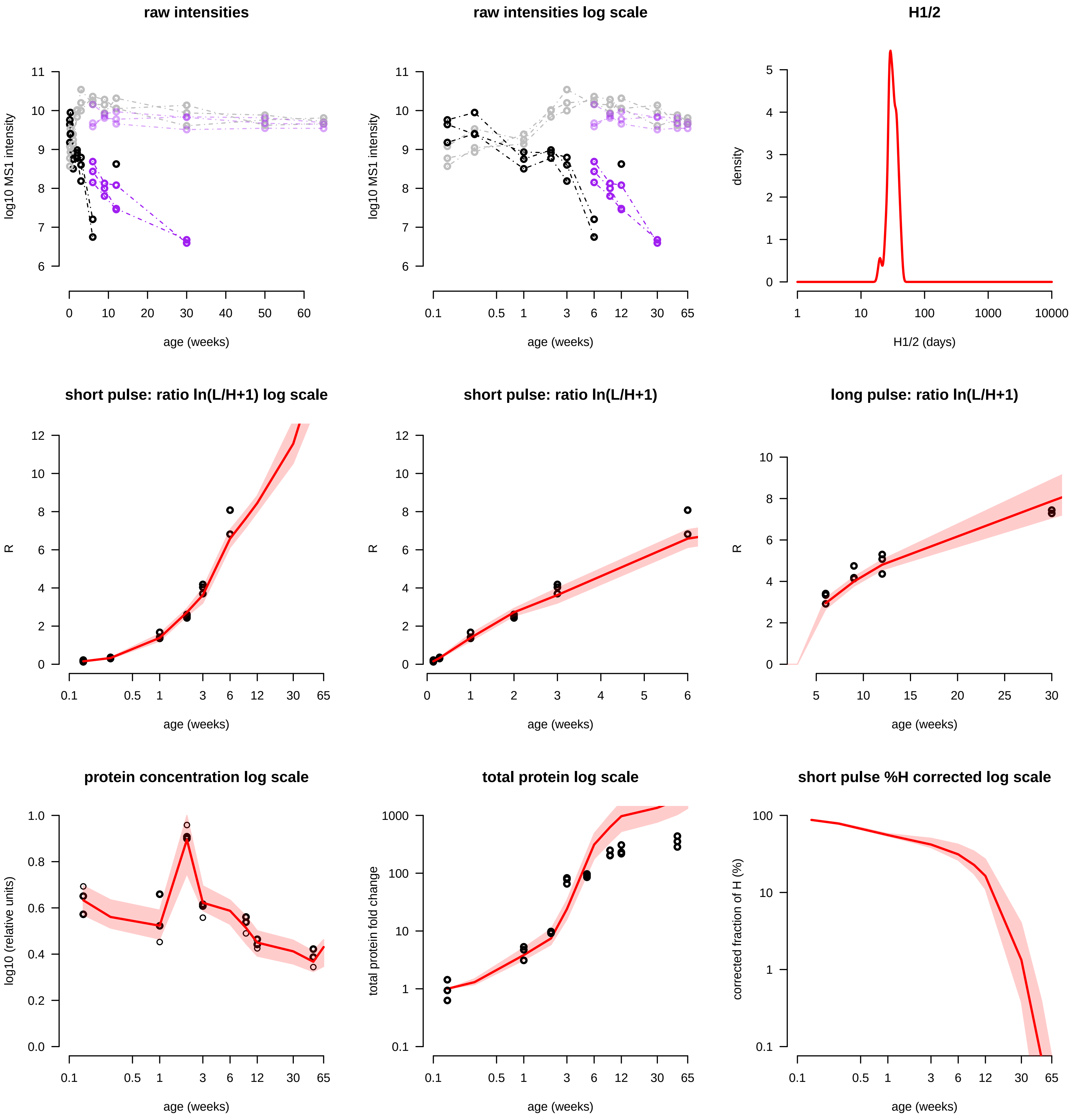

Q9CPV4;Q9CPV4−3;Q9CPV4−2

>sp|Q9CPV4|GLOD4_MOUSE

Glyoxalase domain−containing protein 4

GN=Glod4

Med H1/2: 133.8 days

Q5 H1/2: 85.6 days

Q95 H1/2: 307.8 days

reliable model fit: yes

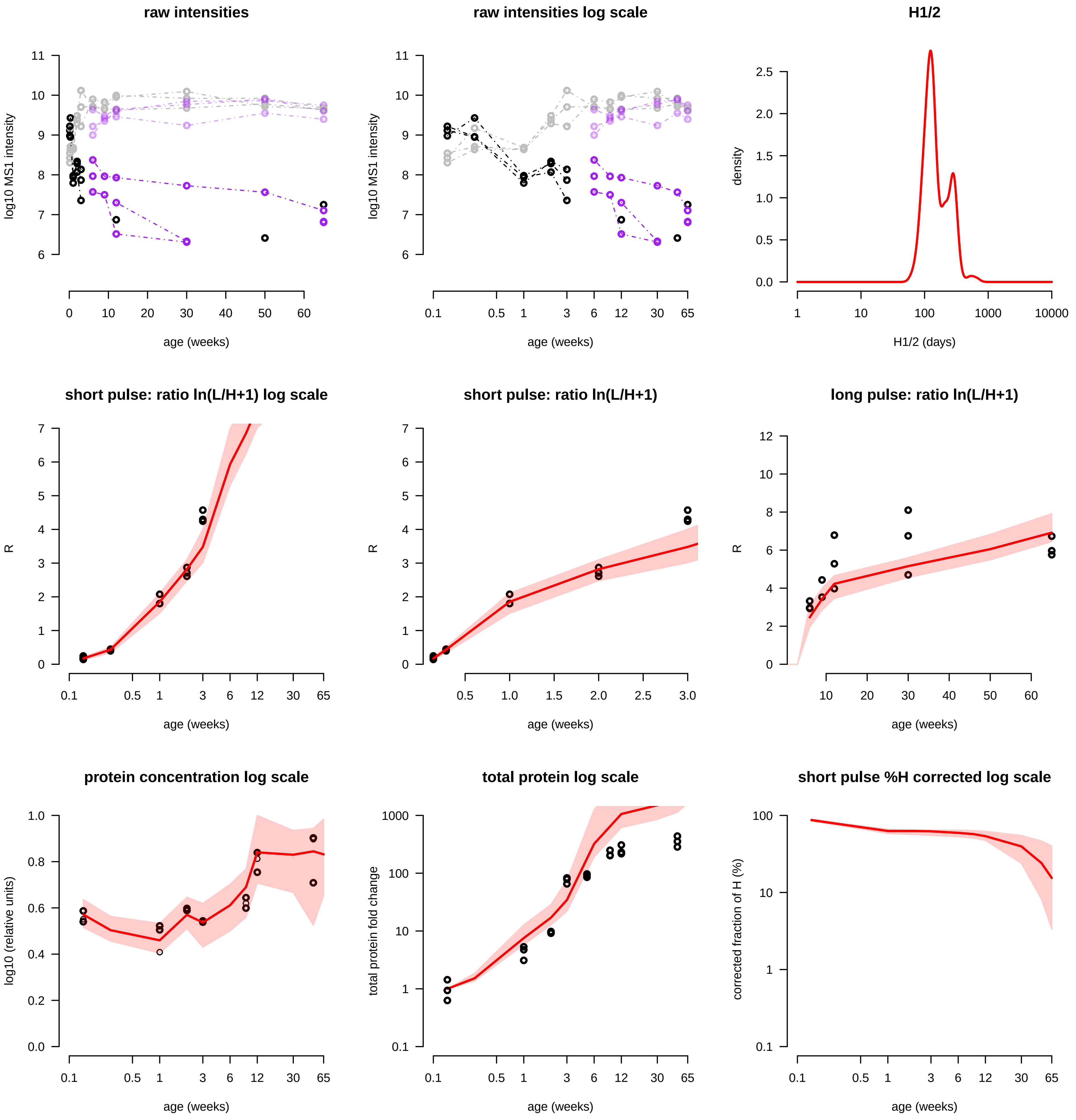

Q9D0E1−2;Q9D0E1

>sp|Q9D0E1−2|HNRPM_MOUSE

Isoform 2 of Heterogeneous nuclear ribonucleoprotein M

GN=Hnrnpm

Med H1/2: 7.6 days

Q5 H1/2: 6.4 days

Q95 H1/2: 9.8 days

reliable model fit: yes

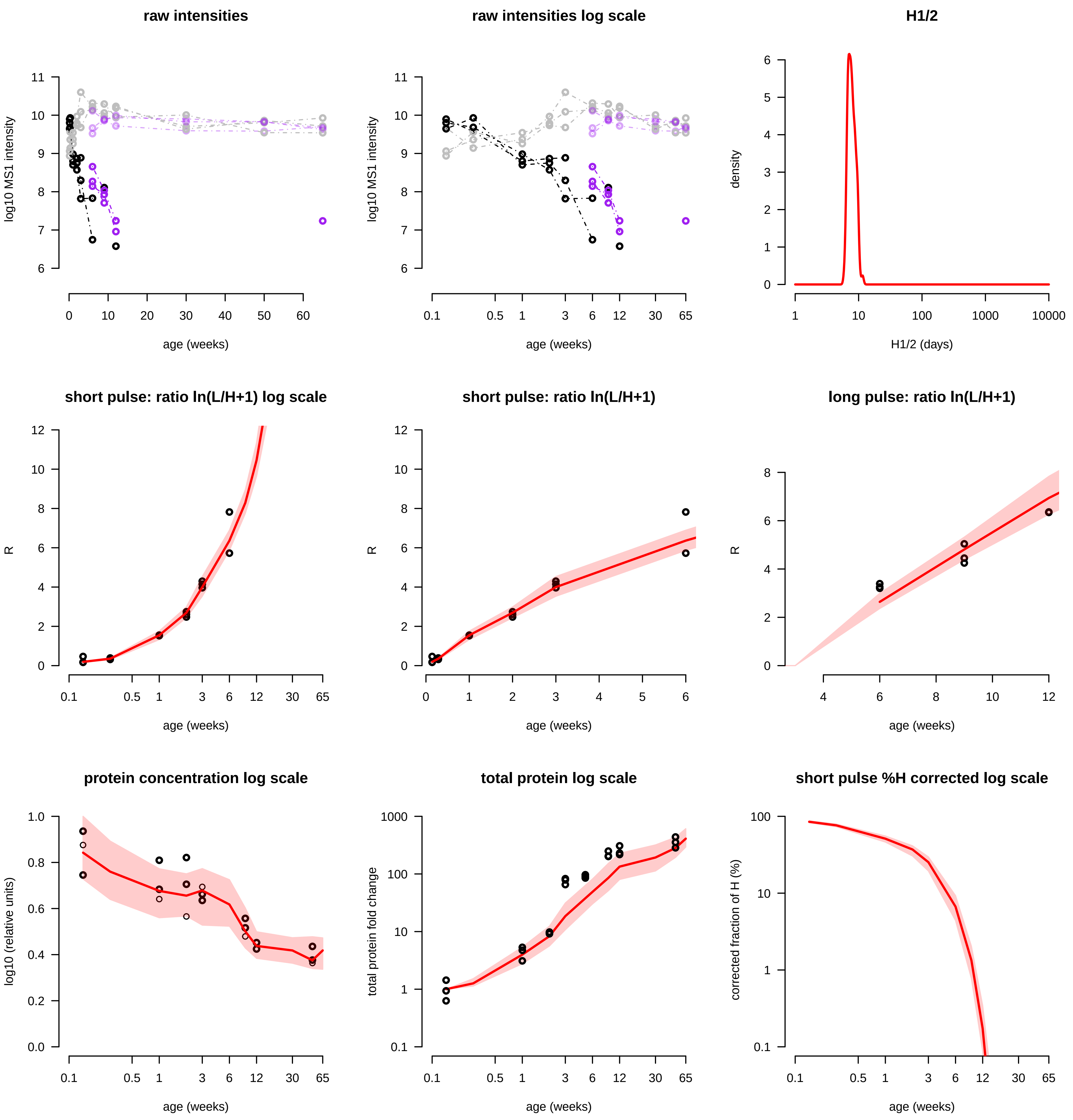

Q99P72;Q99P72−3

>sp|Q99P72|RTN4_MOUSE

Reticulon−4

GN=Rtn4

Med H1/2: 6.3 days

Q5 H1/2: 5.6 days

Q95 H1/2: 7.7 days

reliable model fit: yes

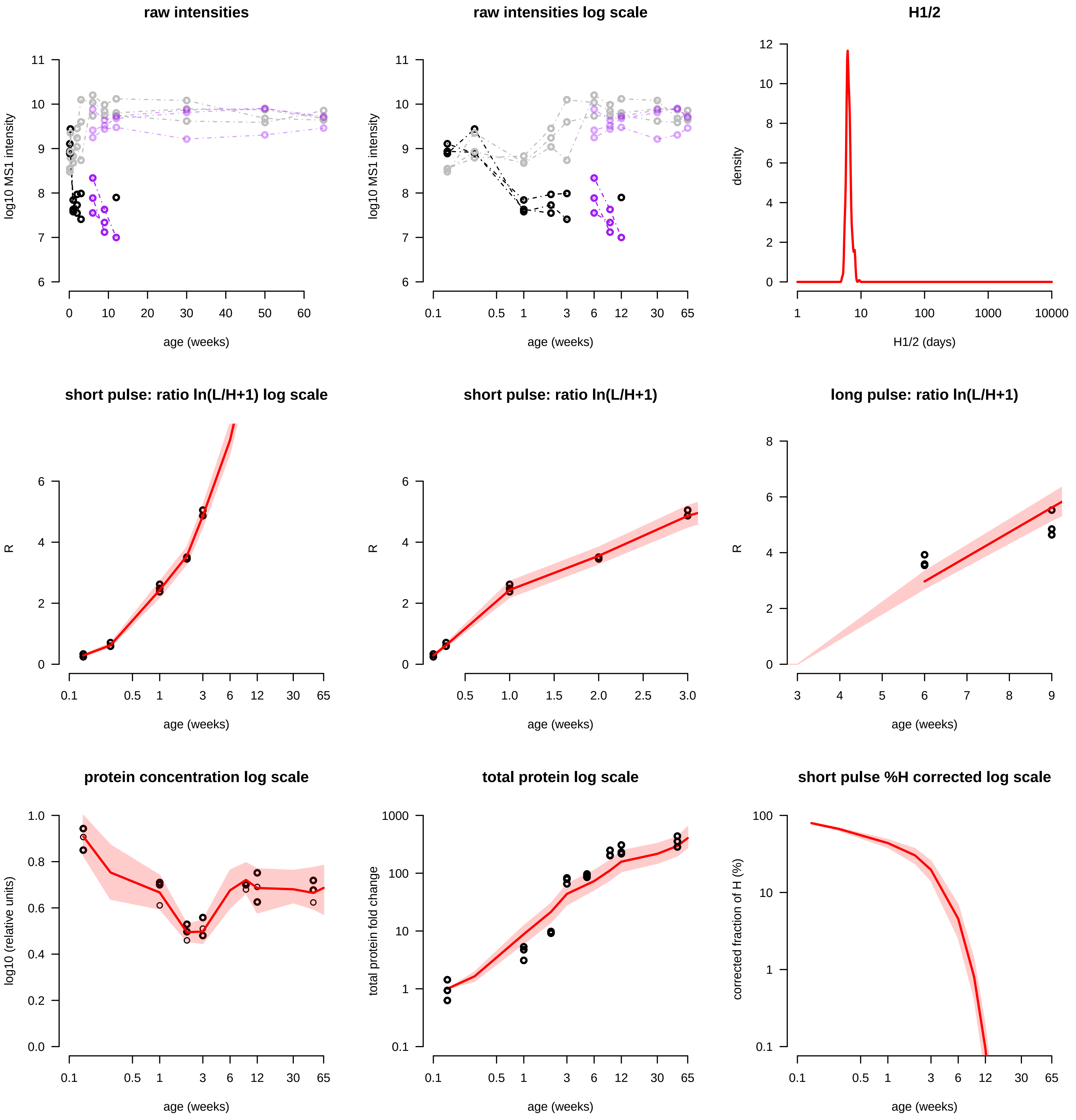

Q8CIE6

>sp|Q8CIE6|COPA_MOUSE

Coatomer subunit alpha

GN=Copa

Med H1/2: 18.9 days

Q5 H1/2: 12.8 days

Q95 H1/2: 33.2 days

reliable model fit: yes

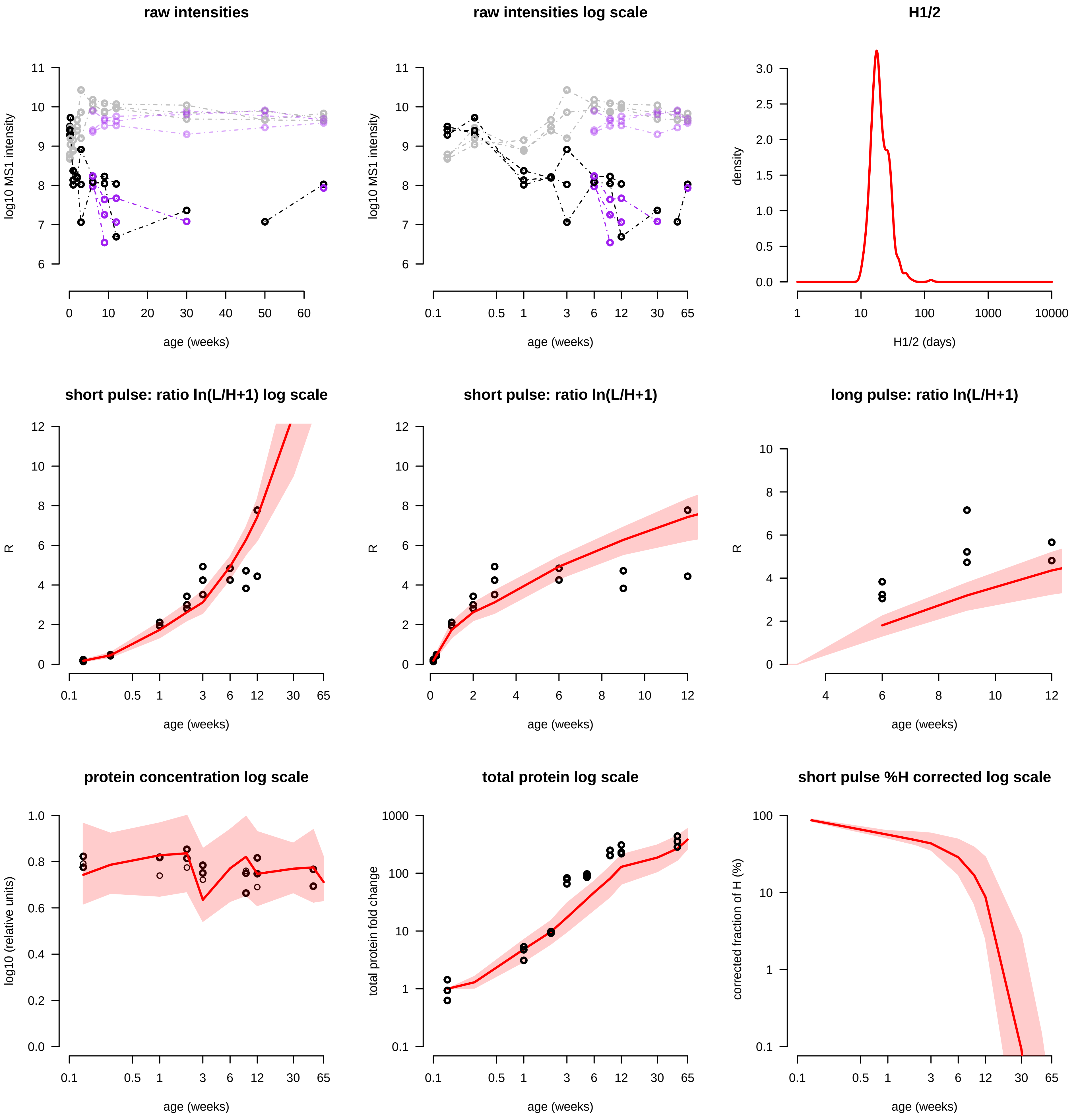

P80314

>sp|P80314|TCPB_MOUSE

T−complex protein 1 subunit beta

GN=Cct2

Med H1/2: 8.9 days

Q5 H1/2: 6.9 days

Q95 H1/2: 11.1 days

reliable model fit: yes

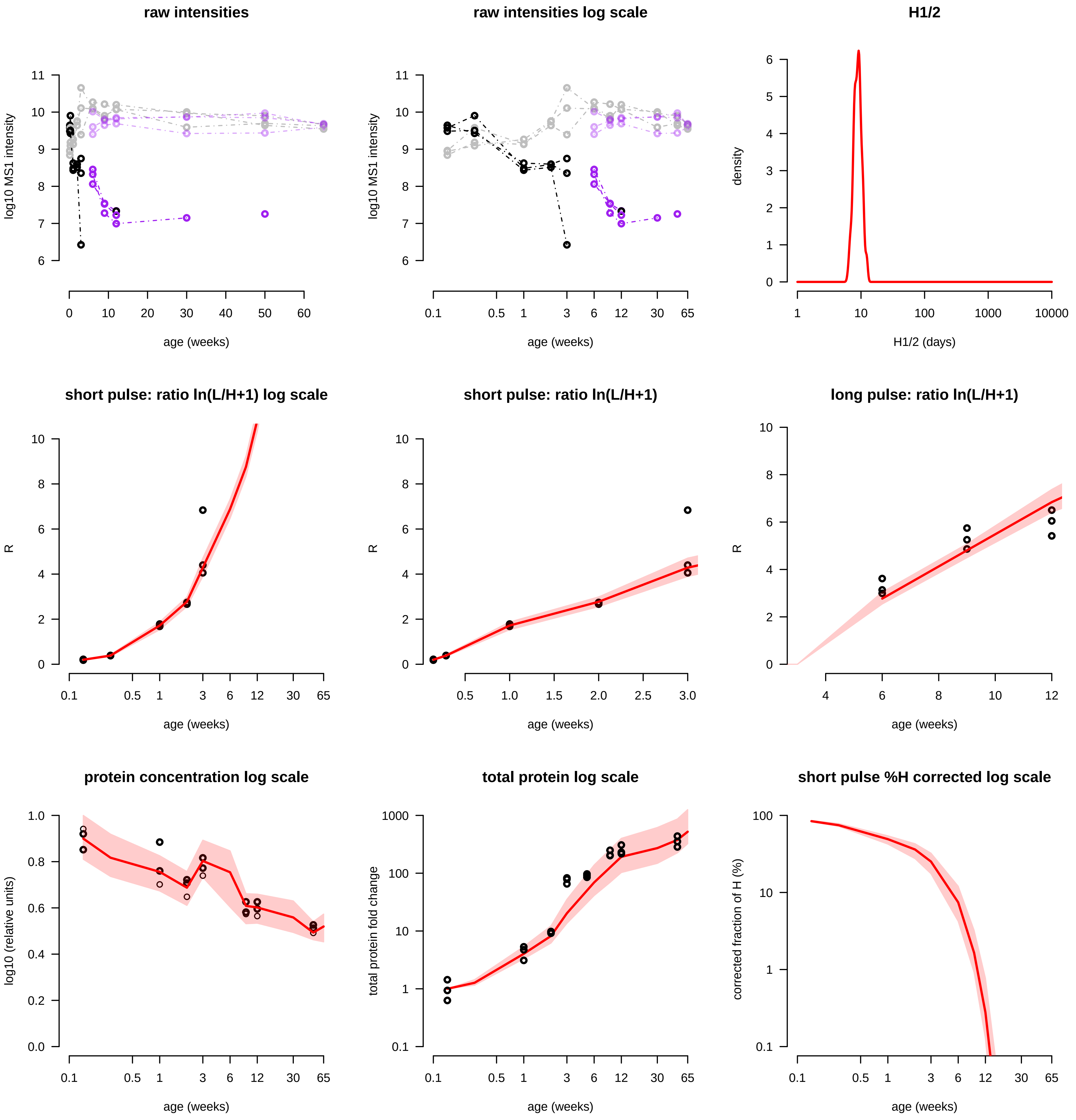

P06728

>sp|P06728|APOA4_MOUSE

Apolipoprotein A−IV

GN=Apoa4

Med H1/2: 49.6 days

Q5 H1/2: 29.6 days

Q95 H1/2: 85.9 days

reliable model fit: no

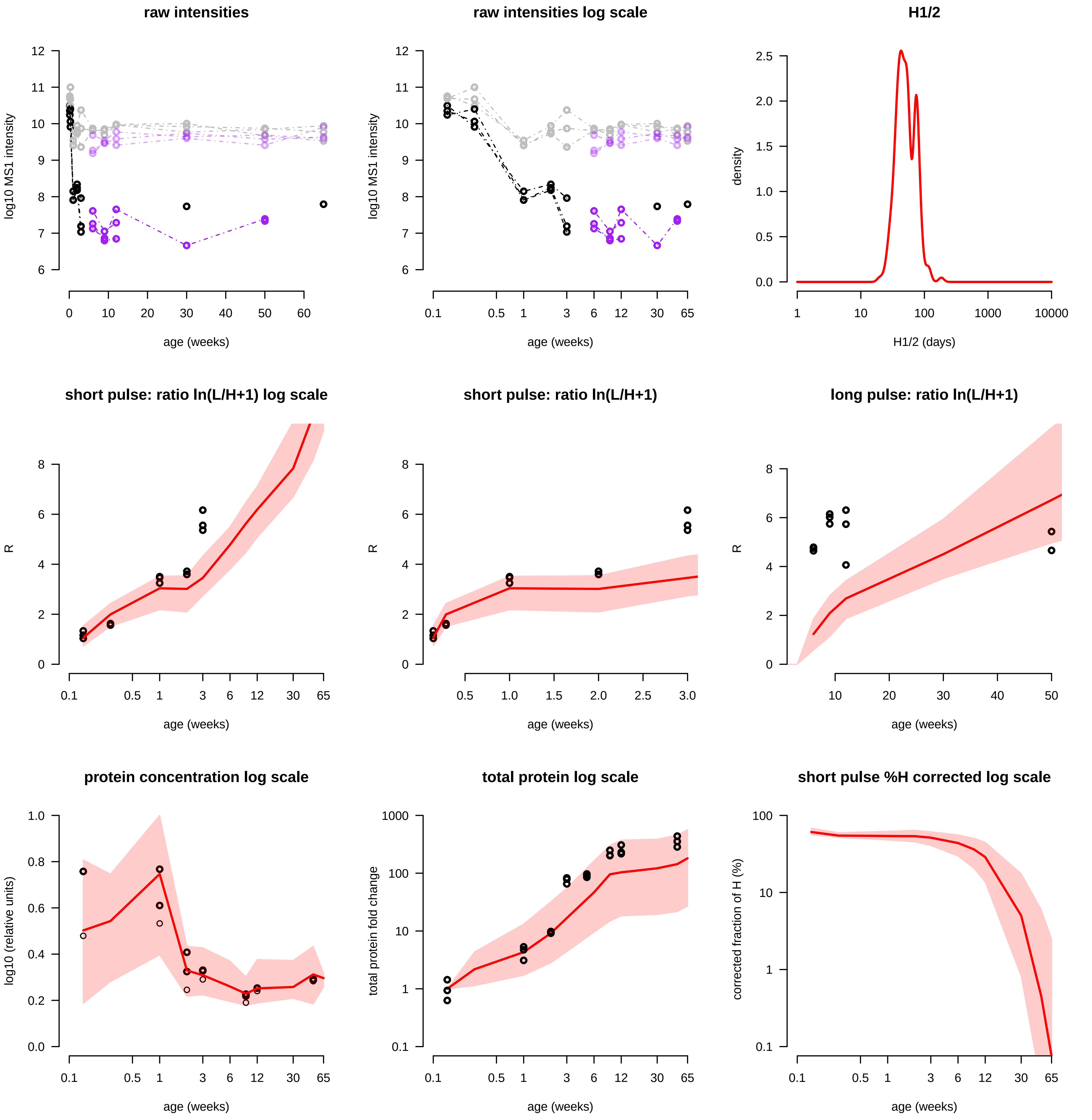

P11276

>sp|P11276|FINC_MOUSE

Fibronectin

GN=Fn1

Med H1/2: 43.3 days

Q5 H1/2: 28.2 days

Q95 H1/2: 83.9 days

reliable model fit: yes

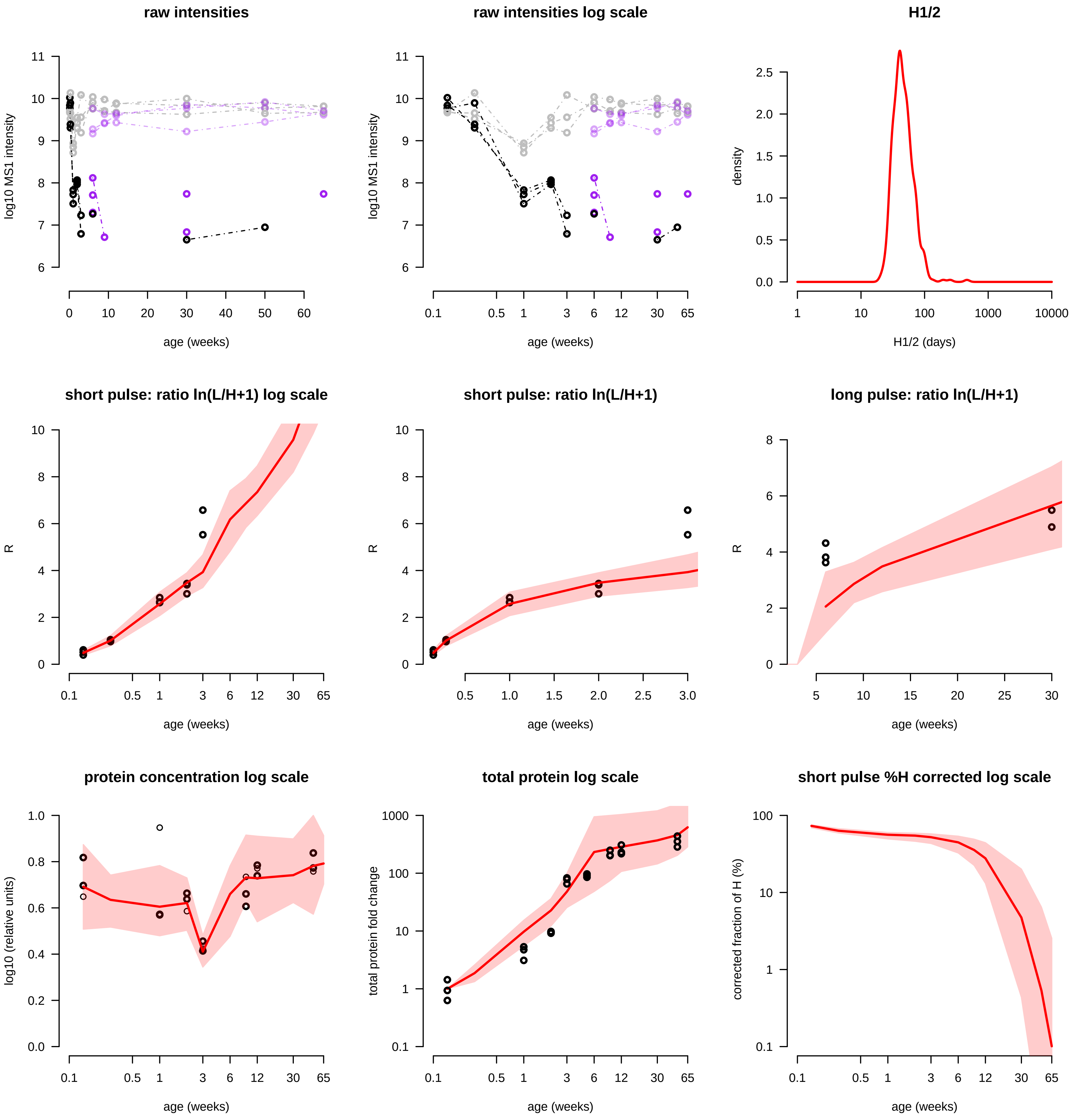

P97351

>sp|P97351|RS3A_MOUSE

40S ribosomal protein S3a

GN=Rps3a

Med H1/2: 14.2 days

Q5 H1/2: 10.5 days

Q95 H1/2: 19.9 days

reliable model fit: yes

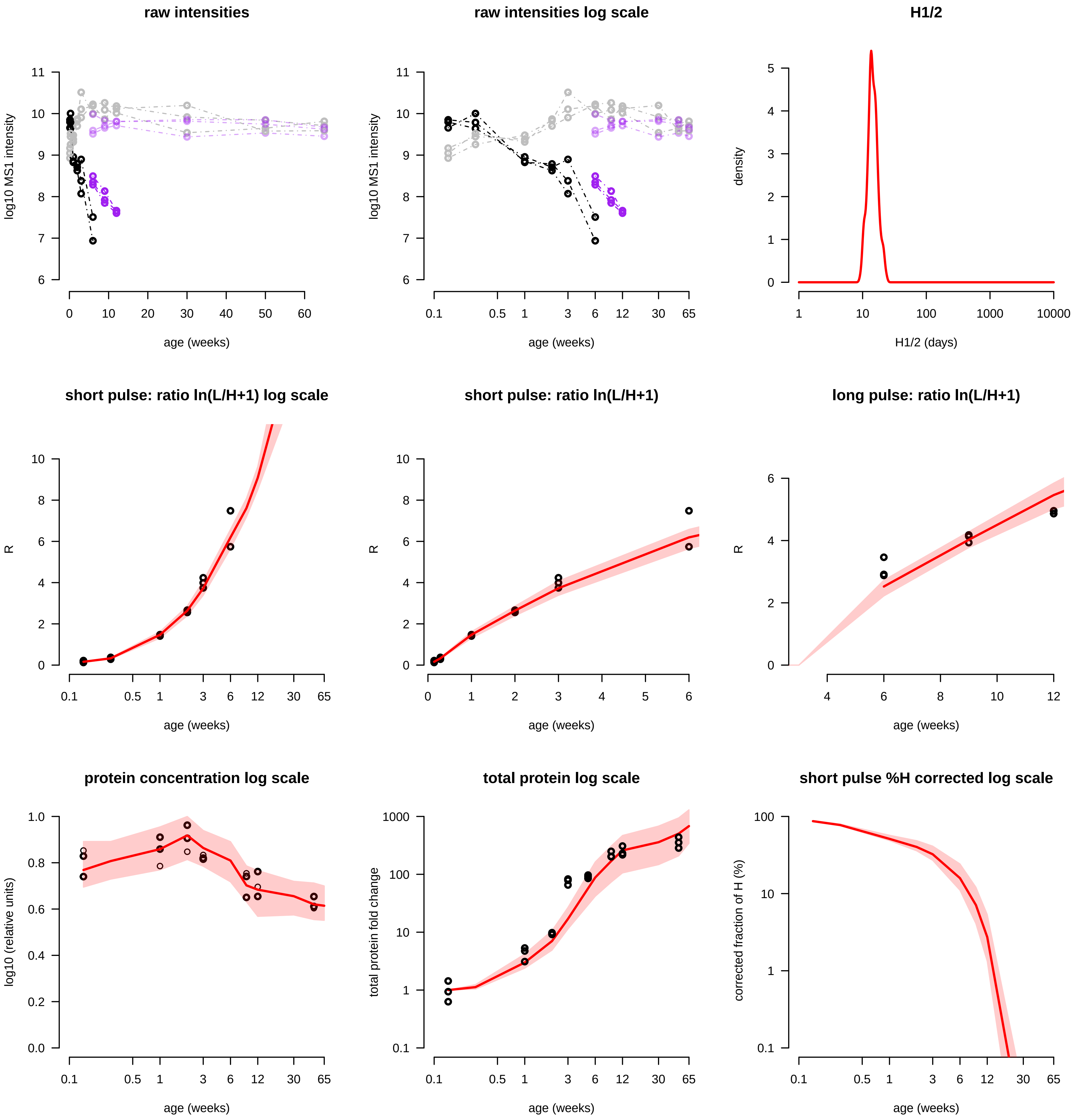

E9Q7G0

>sp|E9Q7G0|NUMA1_MOUSE

Nuclear mitotic apparatus protein 1

GN=Numa1

Med H1/2: 26.8 days

Q5 H1/2: 19.7 days

Q95 H1/2: 40.6 days

reliable model fit: no

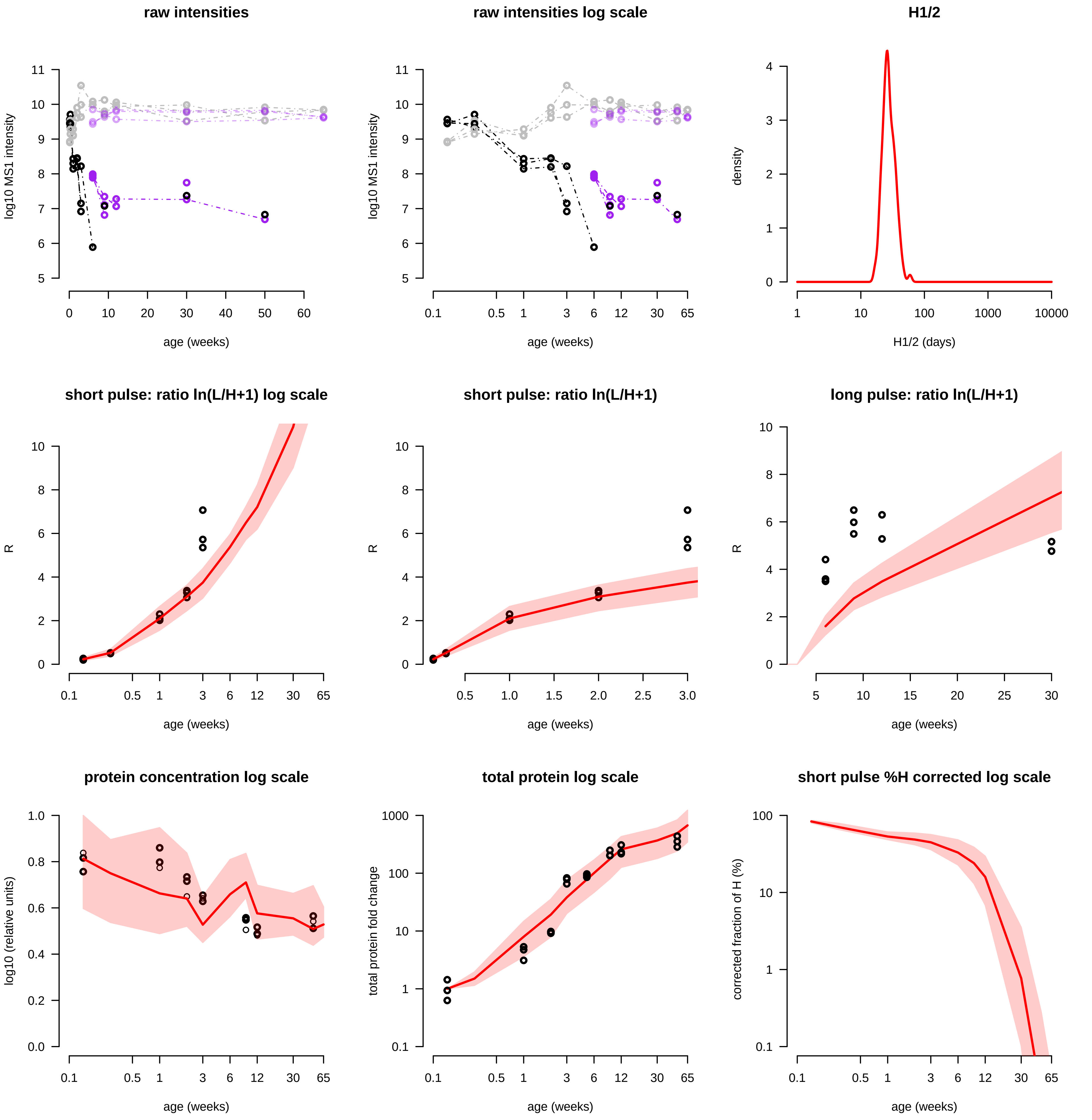

P80316

>sp|P80316|TCPE_MOUSE

T−complex protein 1 subunit epsilon

GN=Cct5

Med H1/2: 317.6 days

Q5 H1/2: 123.9 days

Q95 H1/2: 792.6 days

reliable model fit: yes

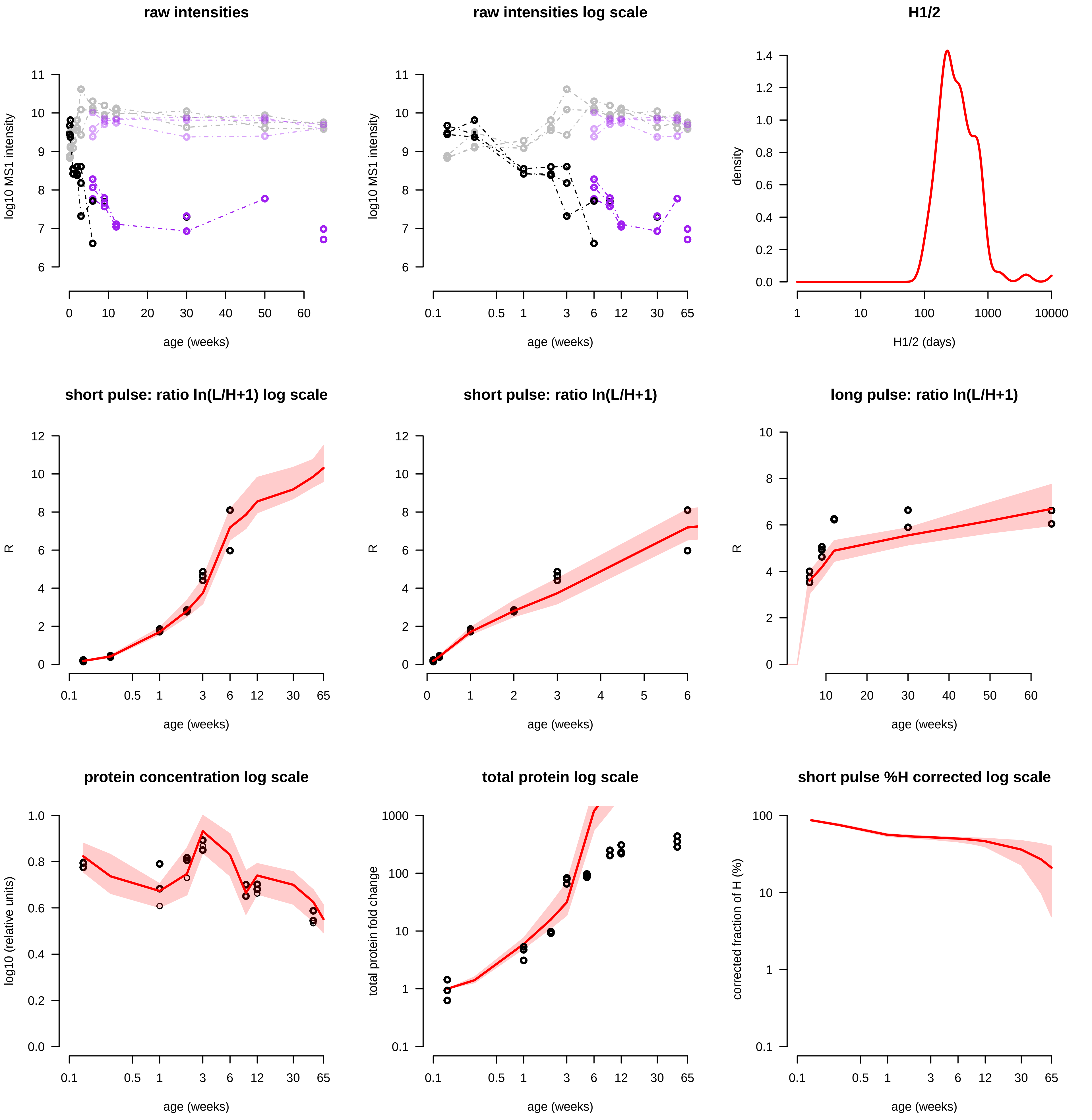

O55143

>sp|O55143|AT2A2_MOUSE

Sarcoplasmic/endoplasmic reticulum calcium ATPase 2

GN=Atp2a2

Med H1/2: 1021.4 days

Q5 H1/2: 278.3 days

Q95 H1/2: 15718.4 days

reliable model fit: yes

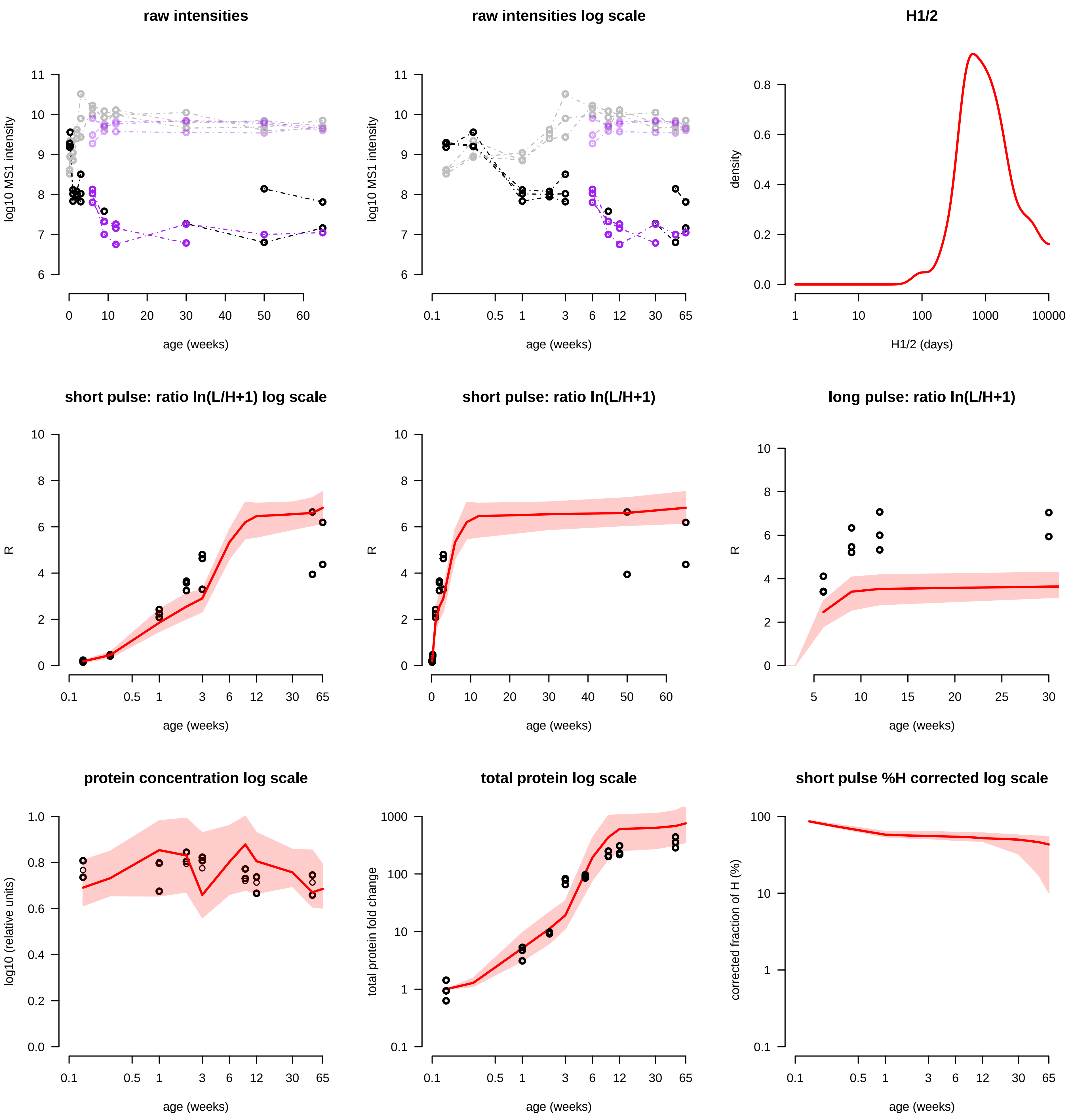

P80318

>sp|P80318|TCPG_MOUSE

T−complex protein 1 subunit gamma

GN=Cct3

Med H1/2: 13.1 days

Q5 H1/2: 9.9 days

Q95 H1/2: 20 days

reliable model fit: no

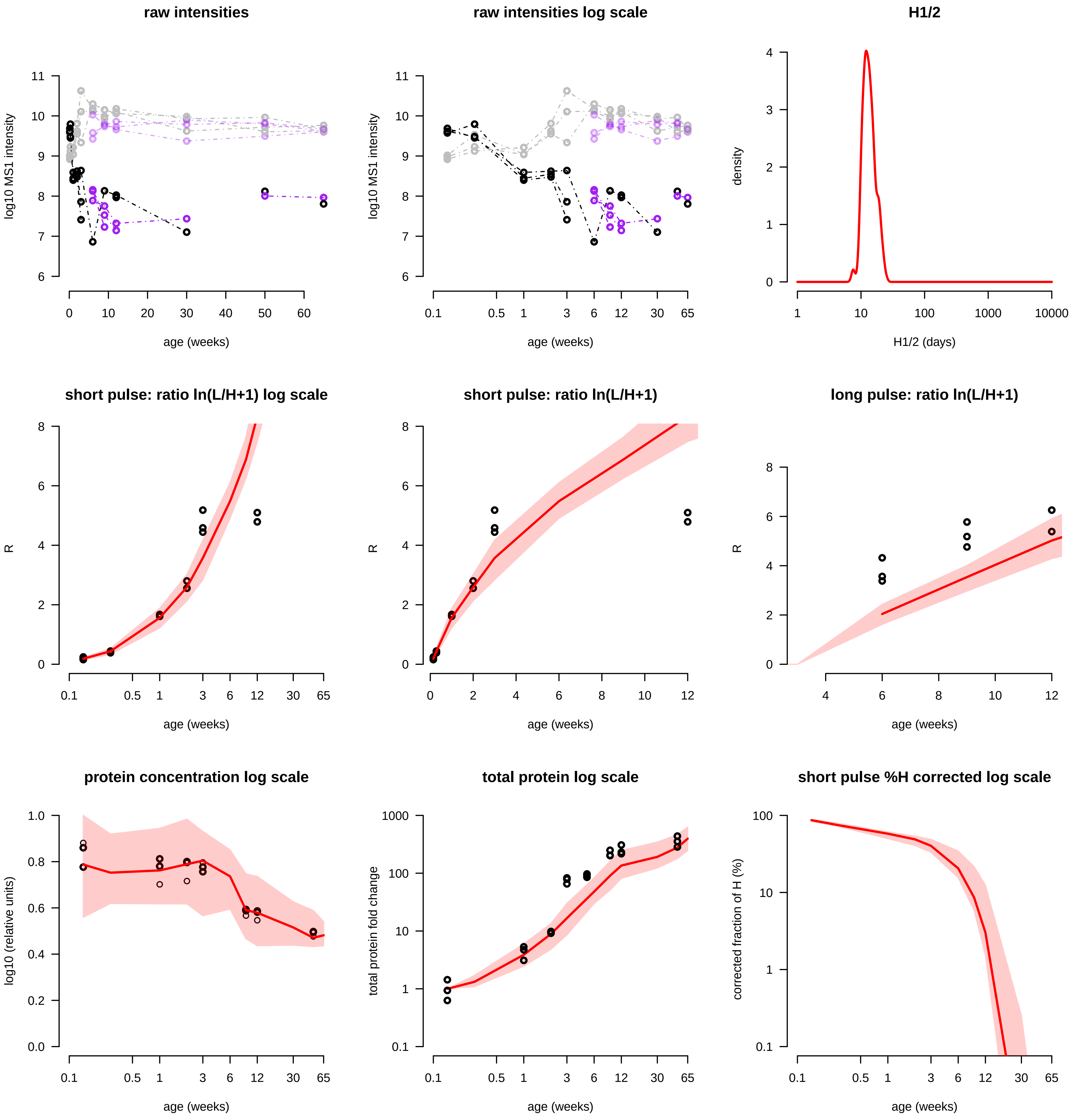

Q9JIF7

>sp|Q9JIF7|COPB_MOUSE

Coatomer subunit beta

GN=Copb1

Med H1/2: 7.8 days

Q5 H1/2: 6.6 days

Q95 H1/2: 9.4 days

reliable model fit: yes

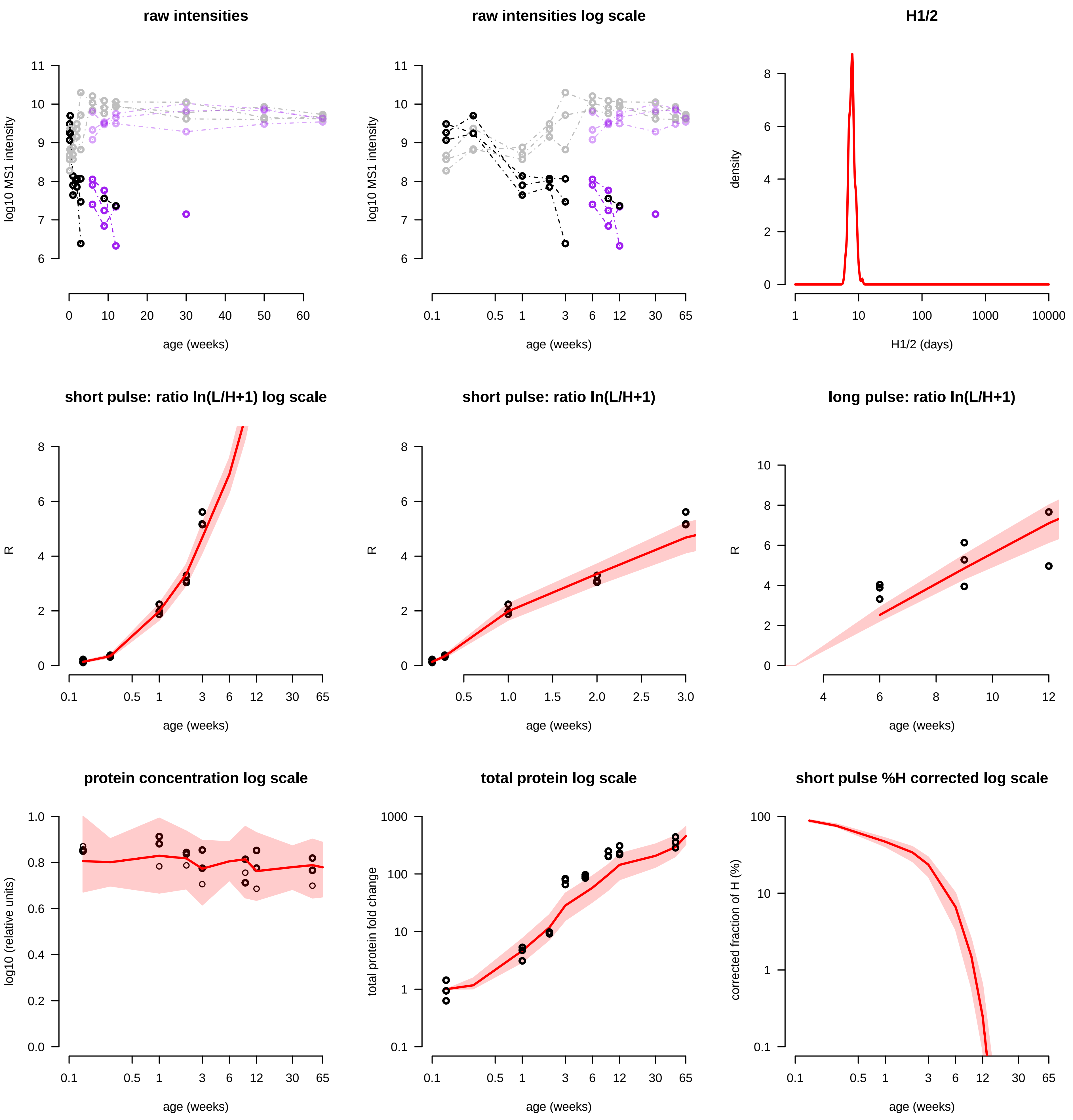

Q9CZS1

>sp|Q9CZS1|AL1B1_MOUSE

Aldehyde dehydrogenase X, mitochondrial

GN=Aldh1b1

Med H1/2: 8.6 days

Q5 H1/2: 7.5 days

Q95 H1/2: 9.8 days

reliable model fit: yes

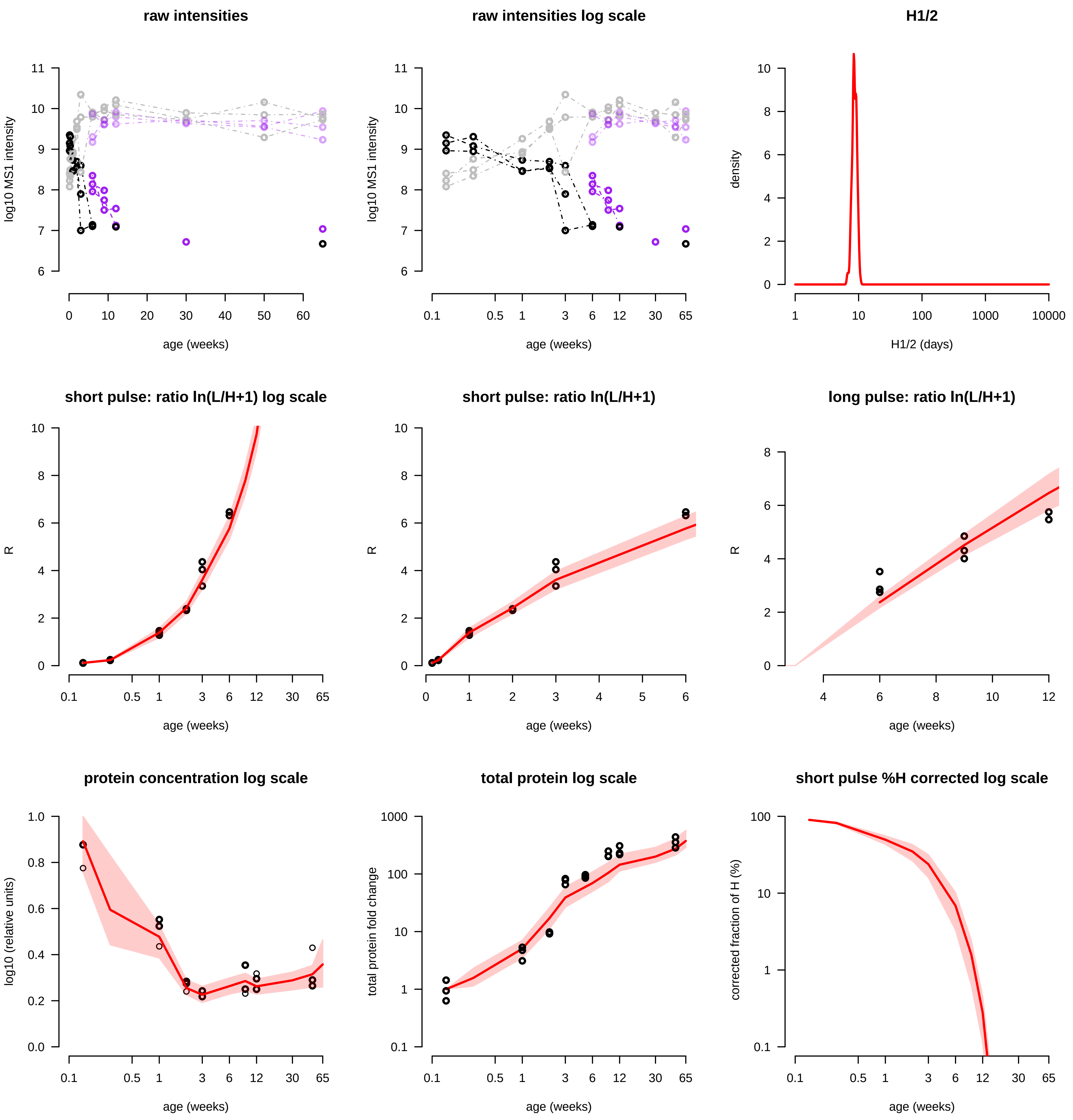

P24270

>sp|P24270|CATA_MOUSE

Catalase

GN=Cat

Med H1/2: 12 days

Q5 H1/2: 10 days

Q95 H1/2: 16.3 days

reliable model fit: yes

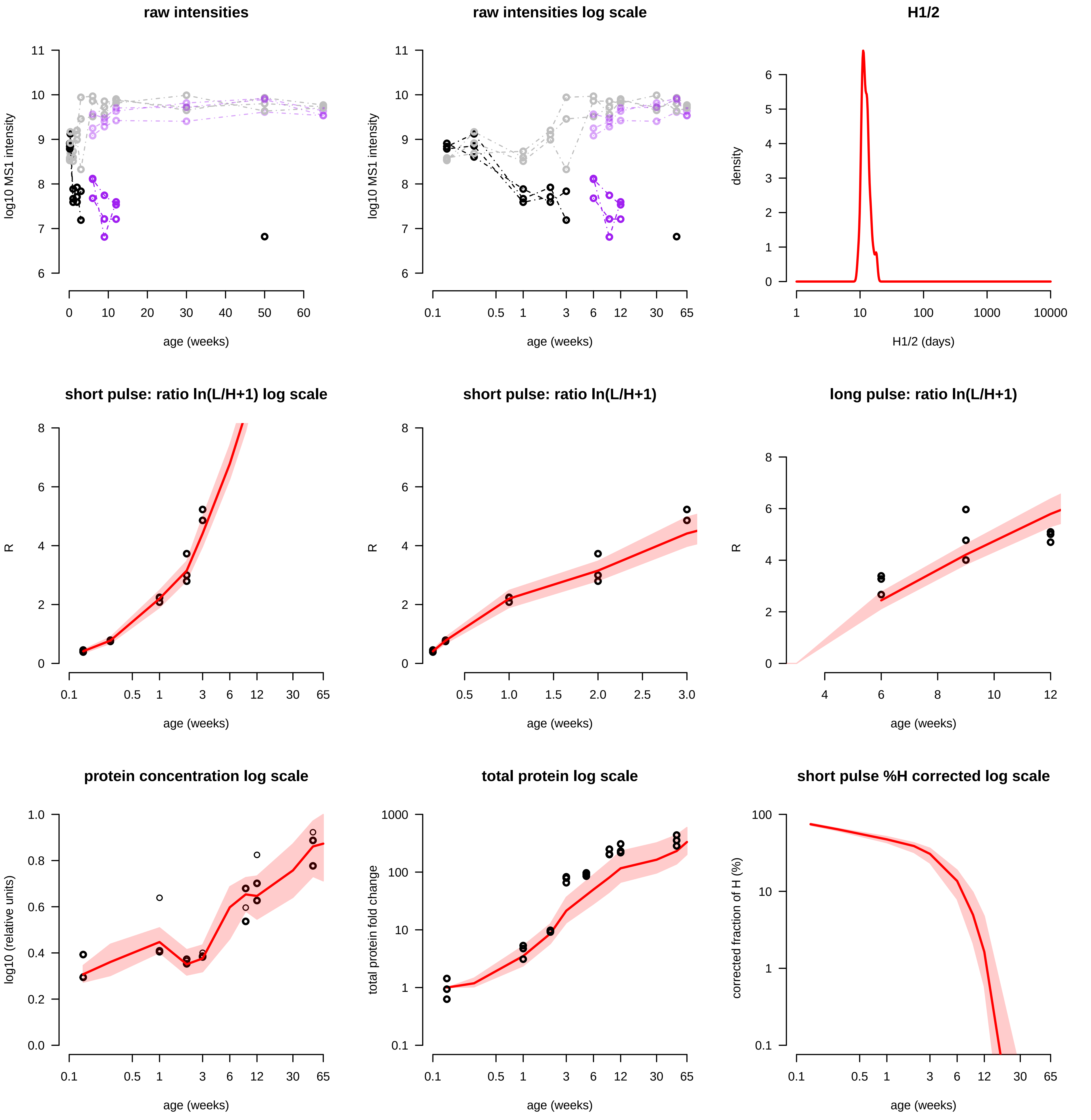

Q9CZX8

>sp|Q9CZX8|RS19_MOUSE

40S ribosomal protein S19

GN=Rps19

Med H1/2: 222.7 days

Q5 H1/2: 100.6 days

Q95 H1/2: 628.9 days

reliable model fit: yes

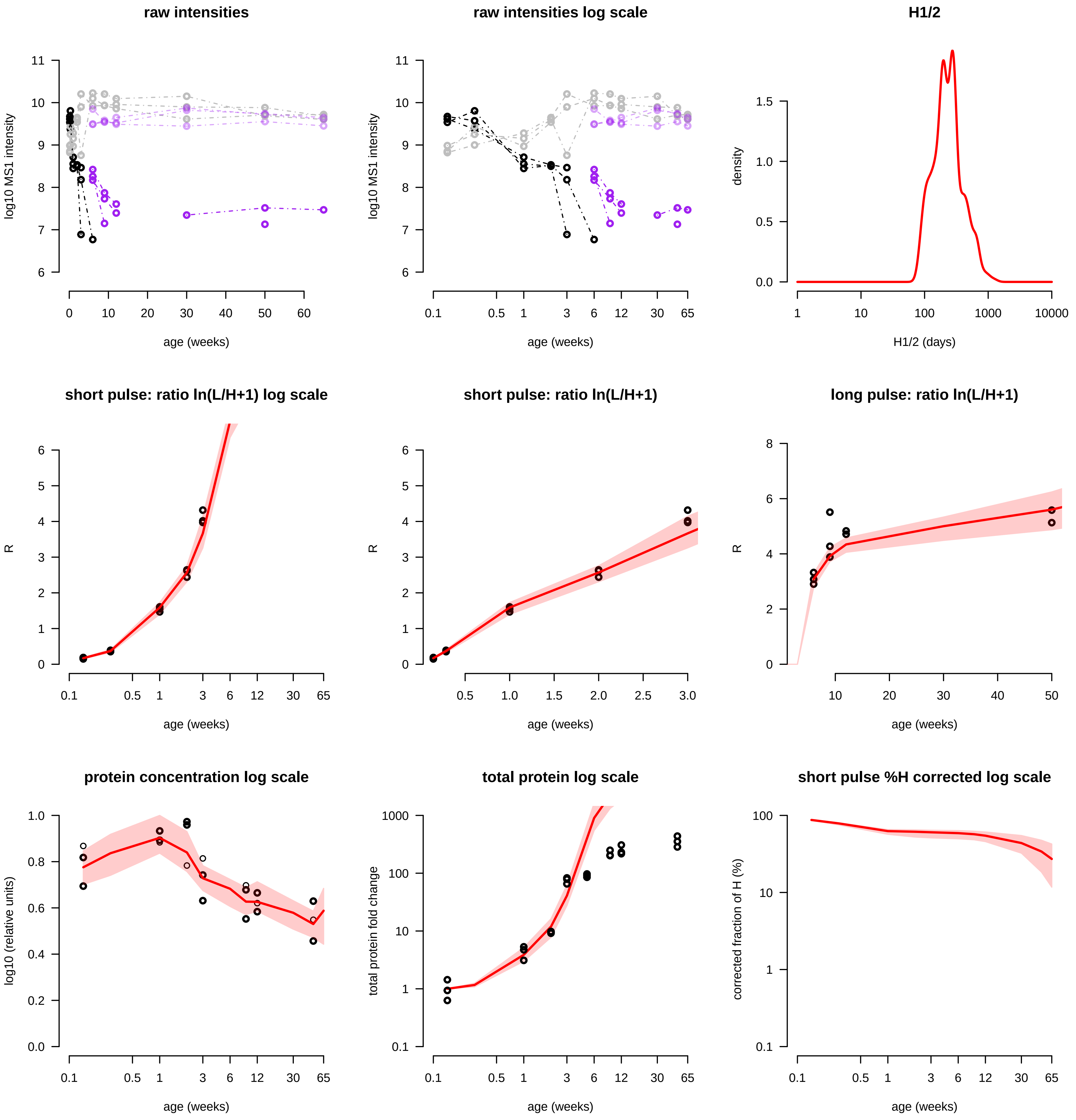

Q99M71

>sp|Q99M71|EPDR1_MOUSE

Mammalian ependymin−related protein 1

GN=Epdr1

Med H1/2: 6.7 days

Q5 H1/2: 4.2 days

Q95 H1/2: 11.1 days

reliable model fit: yes

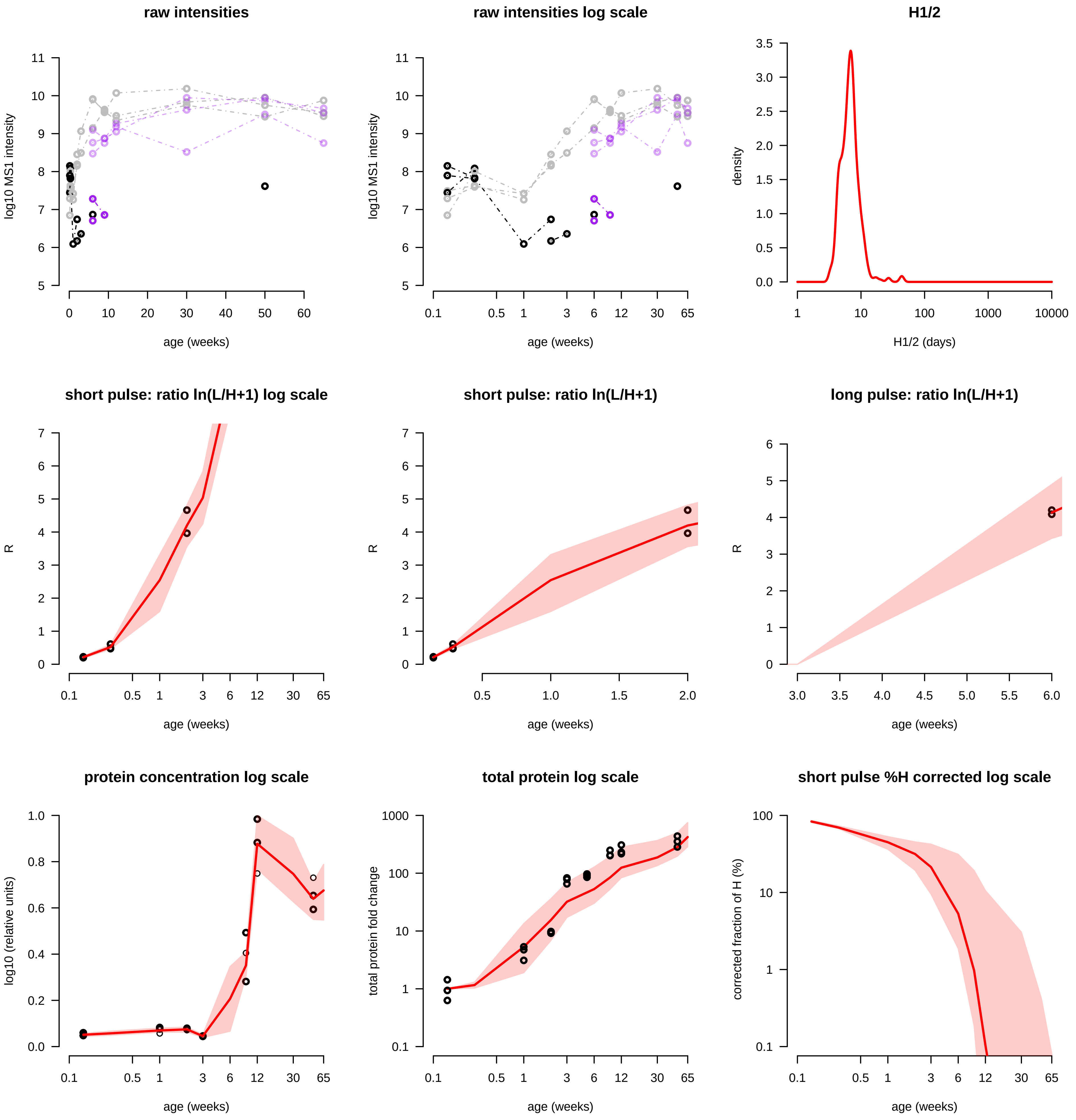

P51885

>sp|P51885|LUM_MOUSE

Lumican

GN=Lum

Med H1/2: 15.5 days

Q5 H1/2: 12.7 days

Q95 H1/2: 19.3 days

reliable model fit: yes

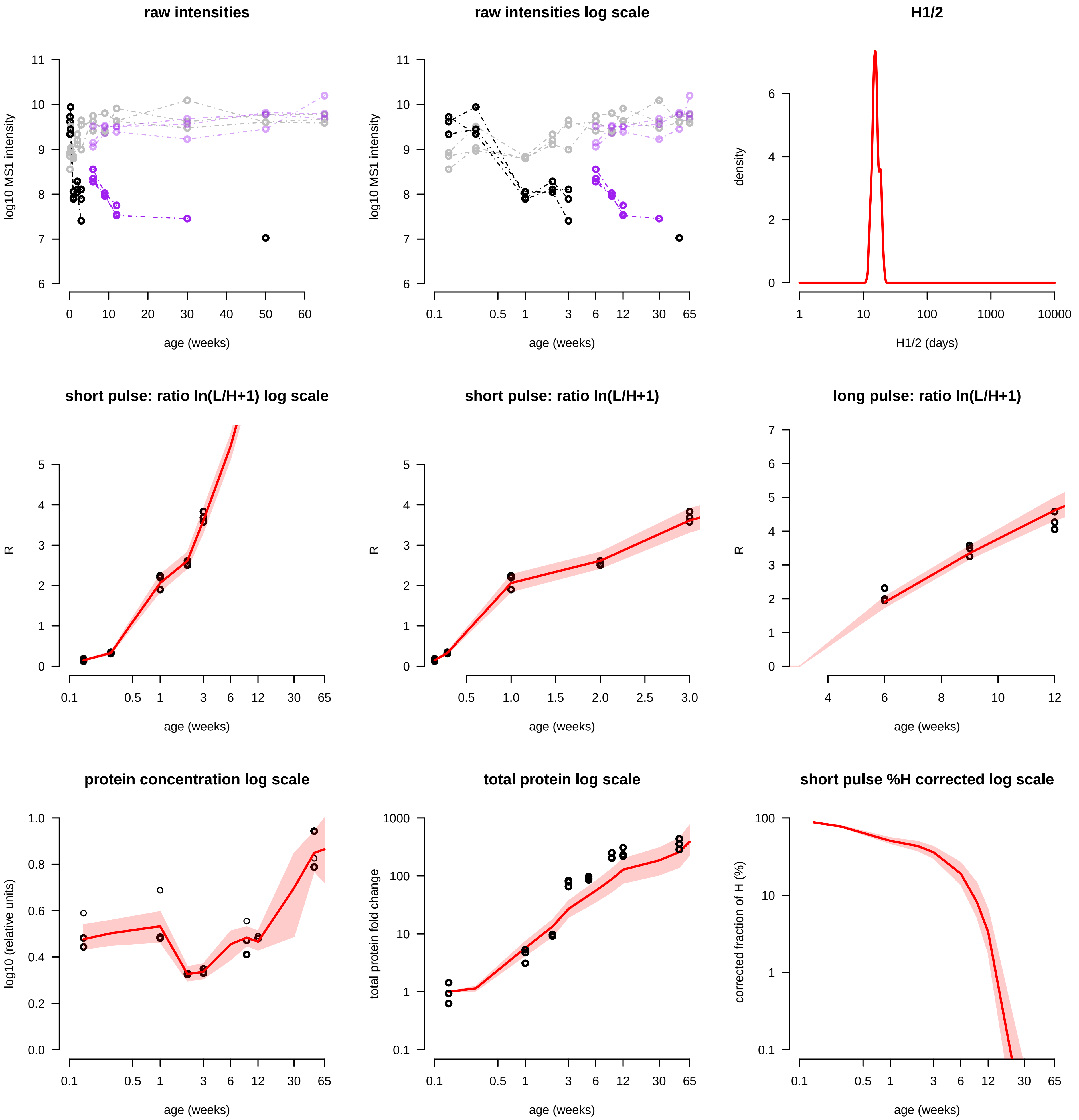

P54071

>sp|P54071|IDHP_MOUSE

Isocitrate dehydrogenase [NADP], mitochondrial

GN=Idh2

Med H1/2: 12.3 days

Q5 H1/2: 9.6 days

Q95 H1/2: 17.6 days

reliable model fit: yes

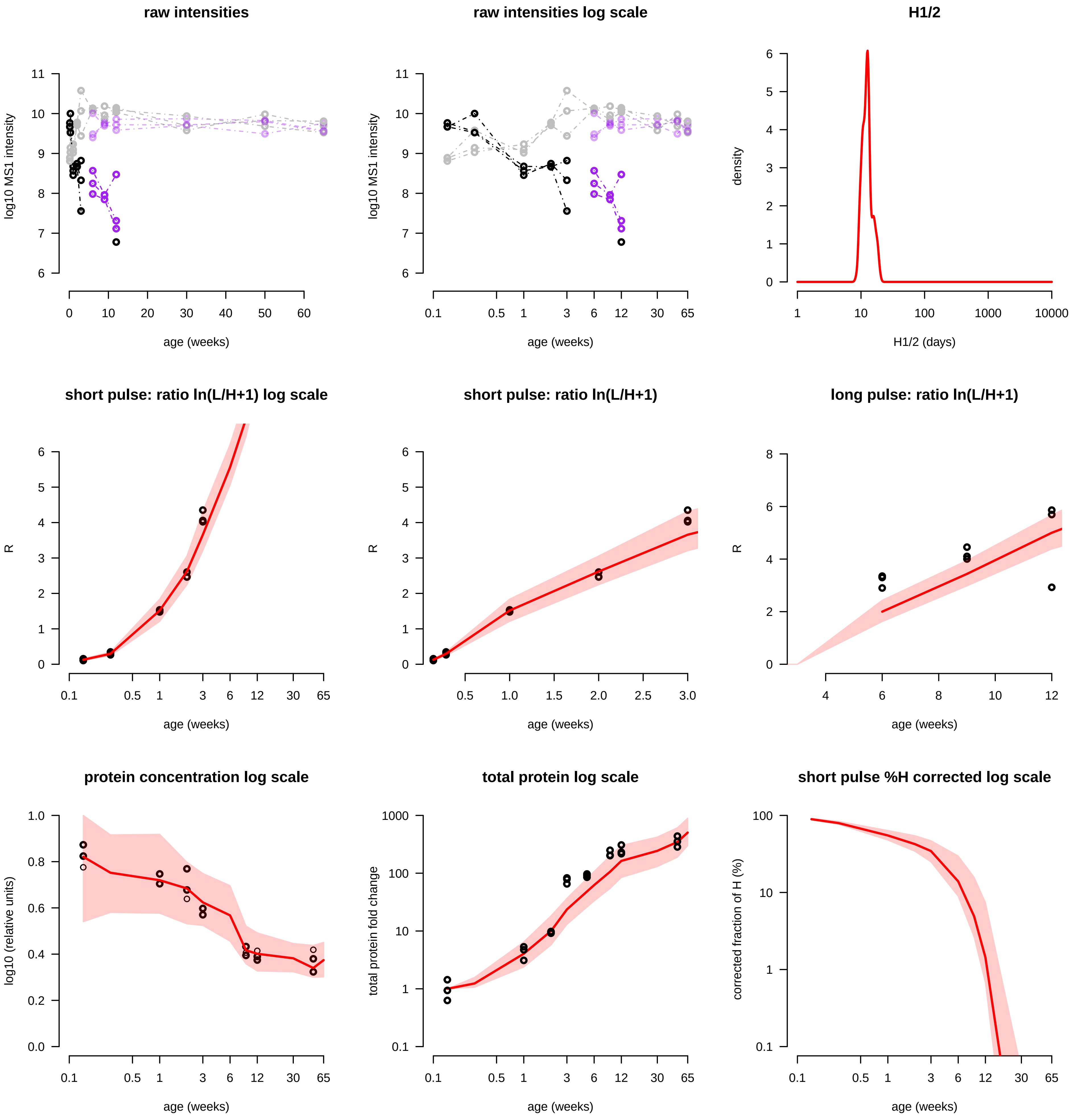

Q9R0P9

>sp|Q9R0P9|UCHL1_MOUSE

Ubiquitin carboxyl−terminal hydrolase isozyme L1

GN=Uchl1

Med H1/2: 1441.6 days

Q5 H1/2: 521.5 days

Q95 H1/2: 40829.5 days

reliable model fit: yes

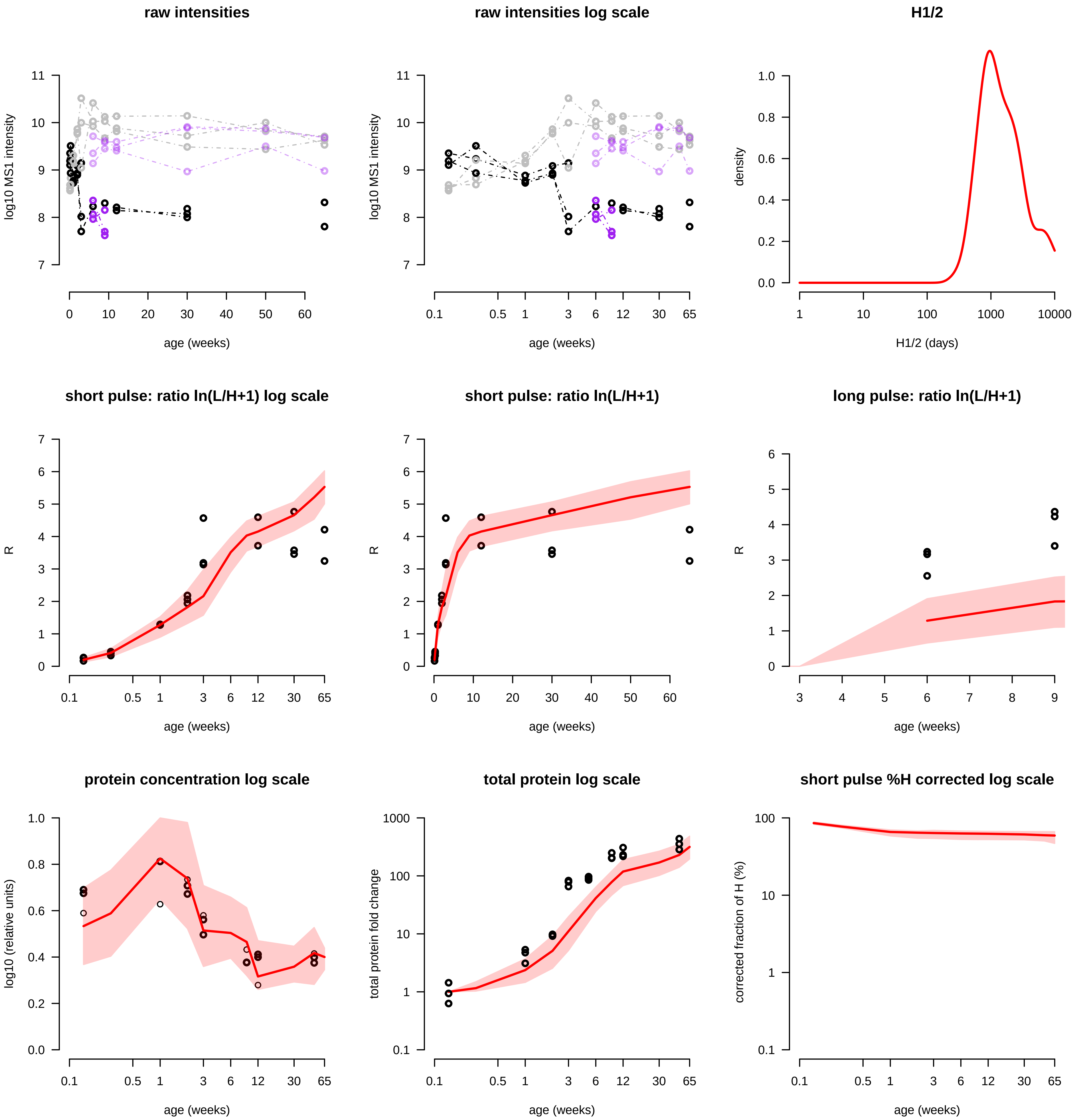

Q76MZ3

>sp|Q76MZ3|2AAA_MOUSE

rine/threonine−protein phosphatase 2A 65 kDa regulatory subunit A alpha isofo

GN=Ppp2r1a

Med H1/2: 1004.8 days

Q5 H1/2: 217.3 days

Q95 H1/2: 16873.6 days

reliable model fit: no

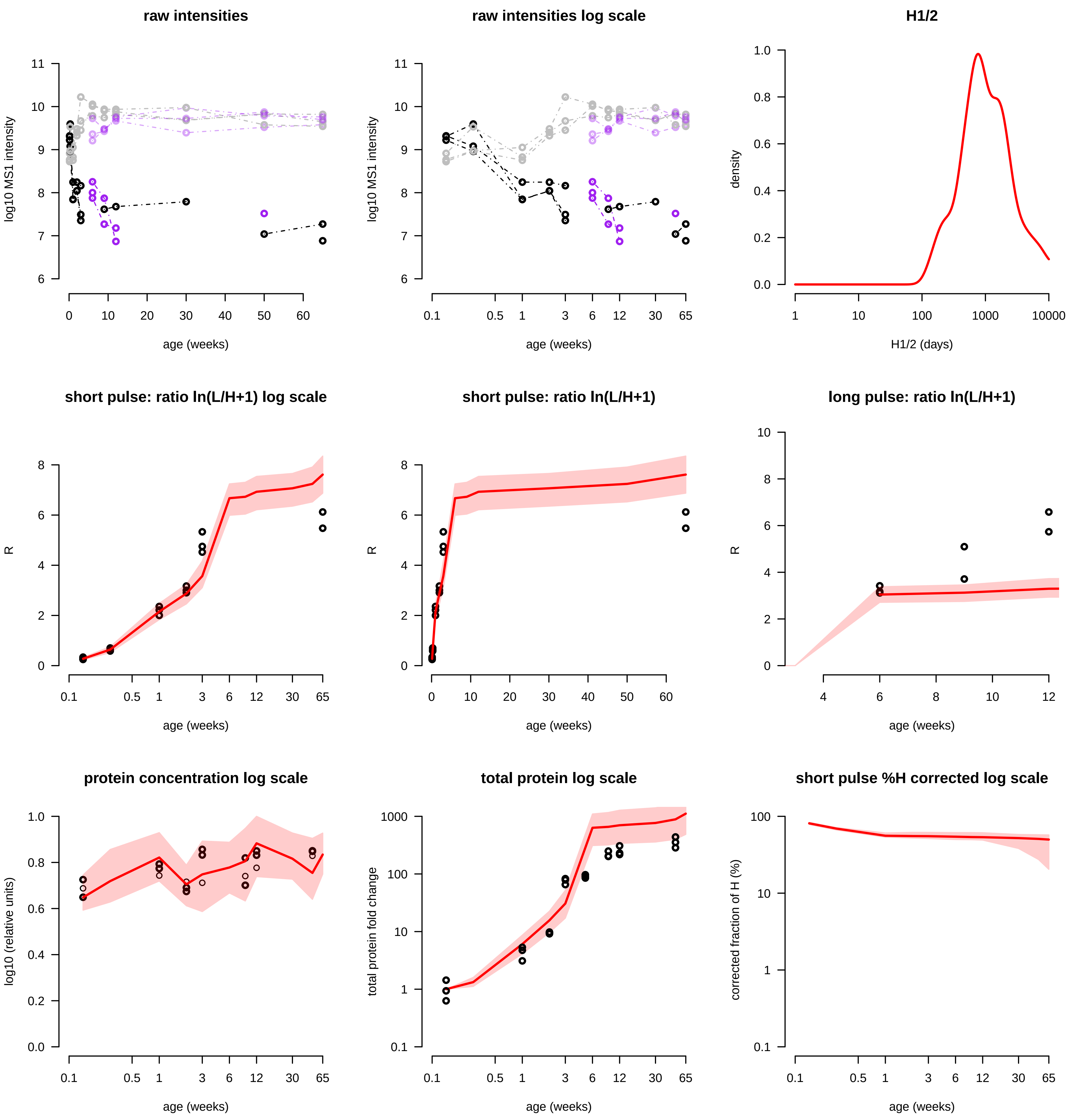

Q6ZWN5

>sp|Q6ZWN5|RS9_MOUSE

40S ribosomal protein S9

GN=Rps9

Med H1/2: 12.4 days

Q5 H1/2: 9.8 days

Q95 H1/2: 17.4 days

reliable model fit: yes

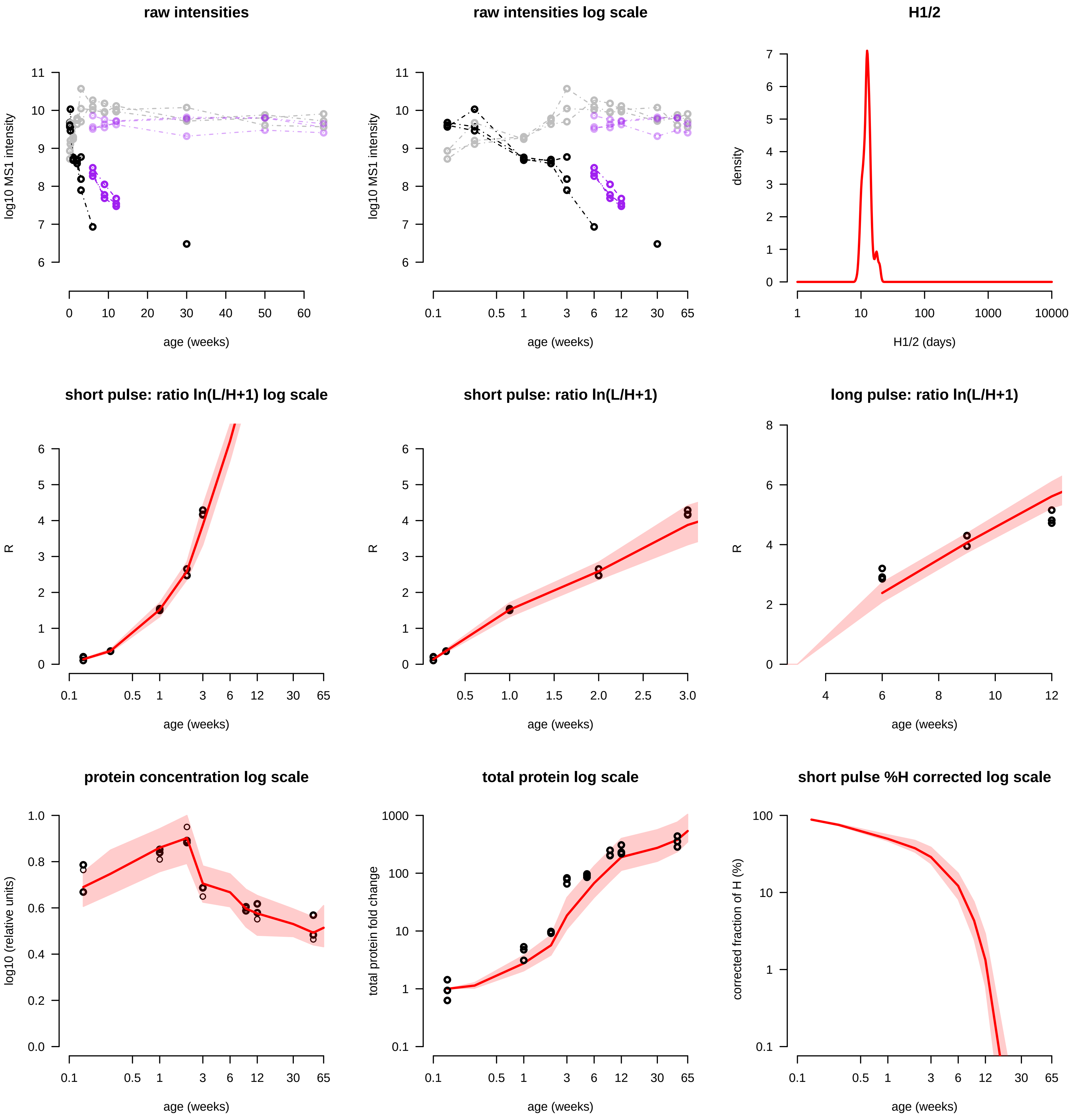

P05622;P05622−2

>sp|P05622|PGFRB_MOUSE

Platelet−derived growth factor receptor beta

GN=Pdgfrb

Med H1/2: 607.9 days

Q5 H1/2: 124.8 days

Q95 H1/2: 8735.3 days

reliable model fit: yes

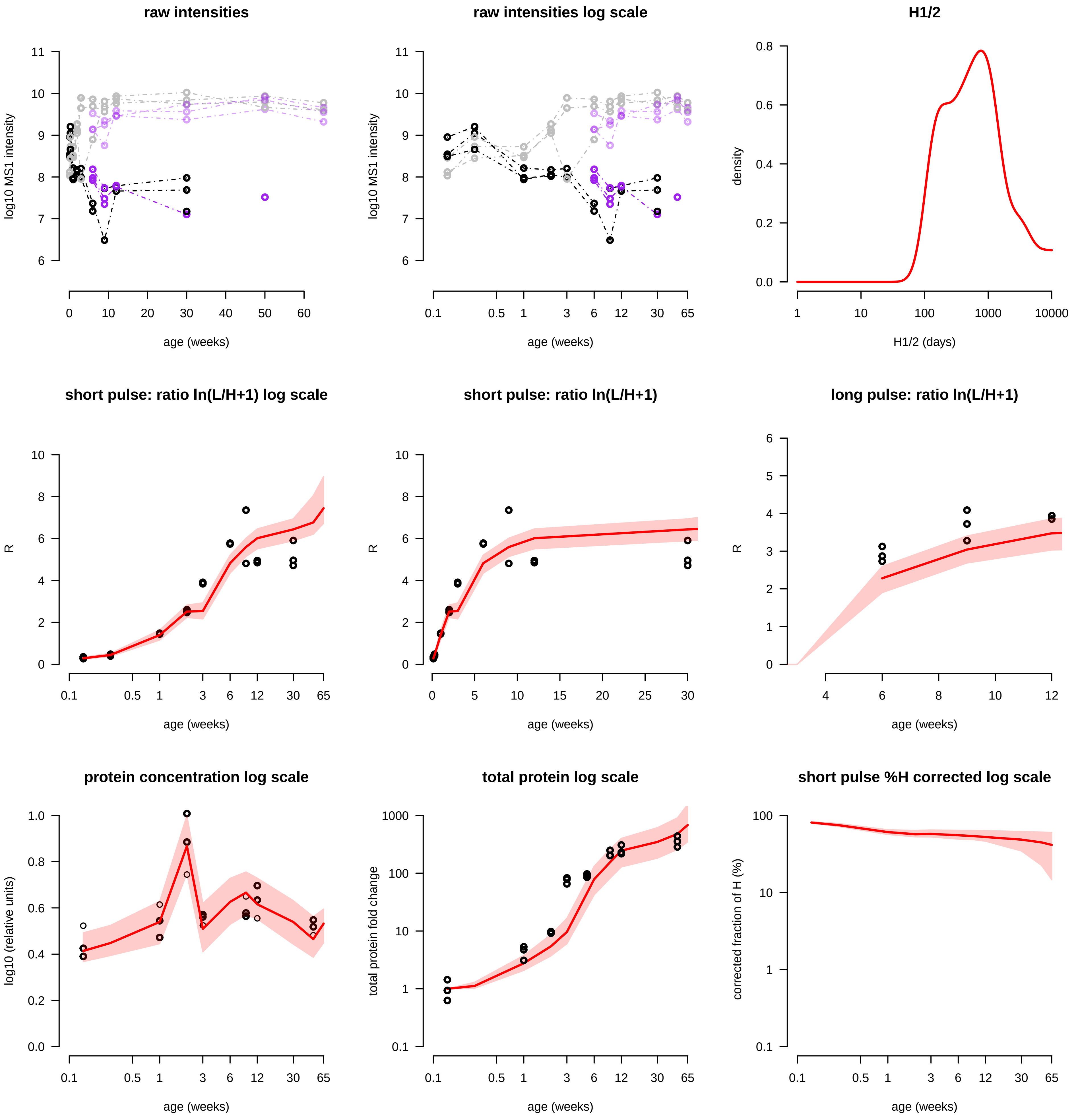

P07309

>sp|P07309|TTHY_MOUSE

Transthyretin

GN=Ttr

Med H1/2: 9.2 days

Q5 H1/2: 7 days

Q95 H1/2: 13.4 days

reliable model fit: yes

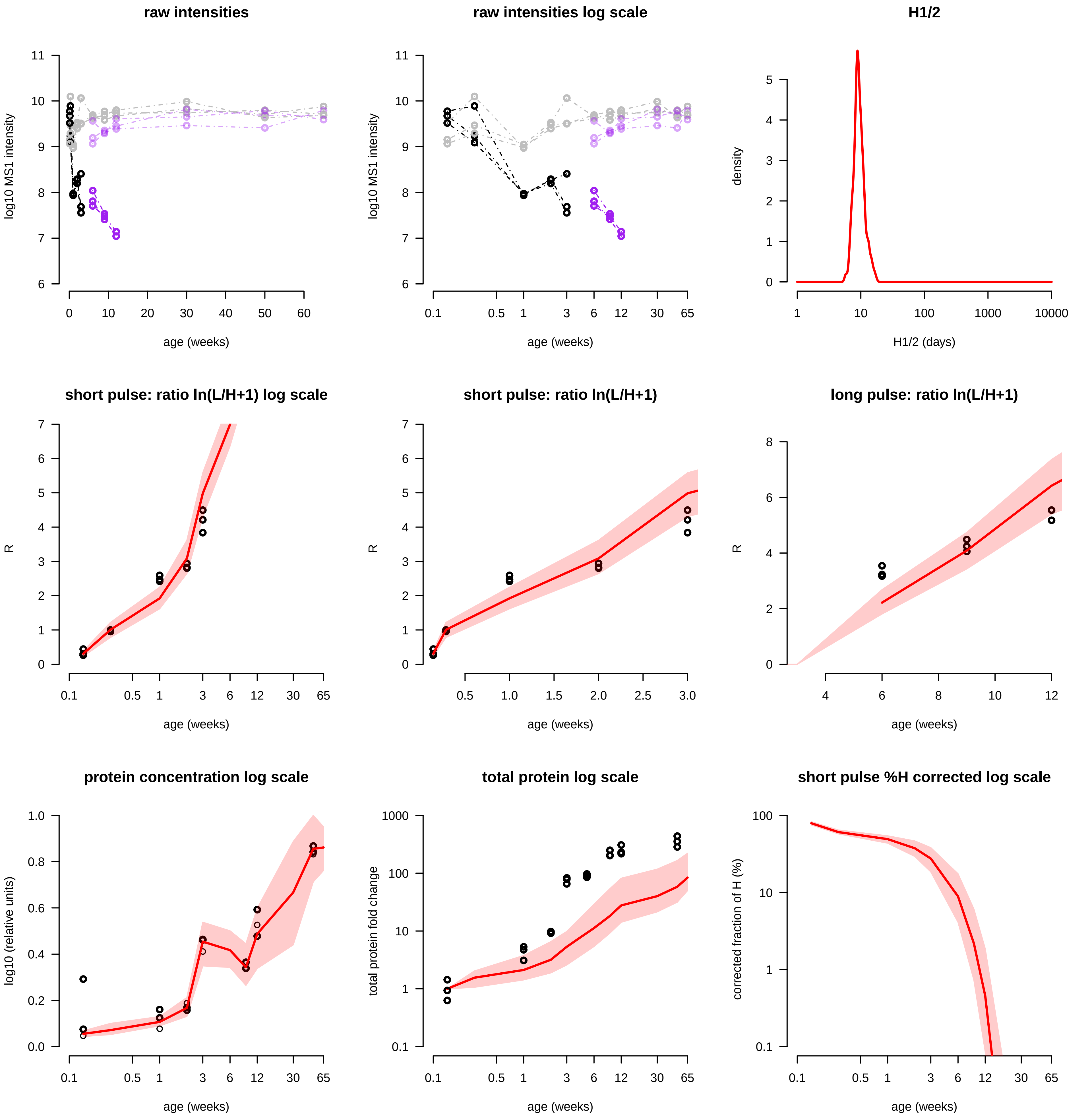

Q6PB66

>sp|Q6PB66|LPPRC_MOUSE

Leucine−rich PPR motif−containing protein, mitochondrial

GN=Lrpprc

Med H1/2: 8.4 days

Q5 H1/2: 6.9 days

Q95 H1/2: 10.9 days

reliable model fit: yes

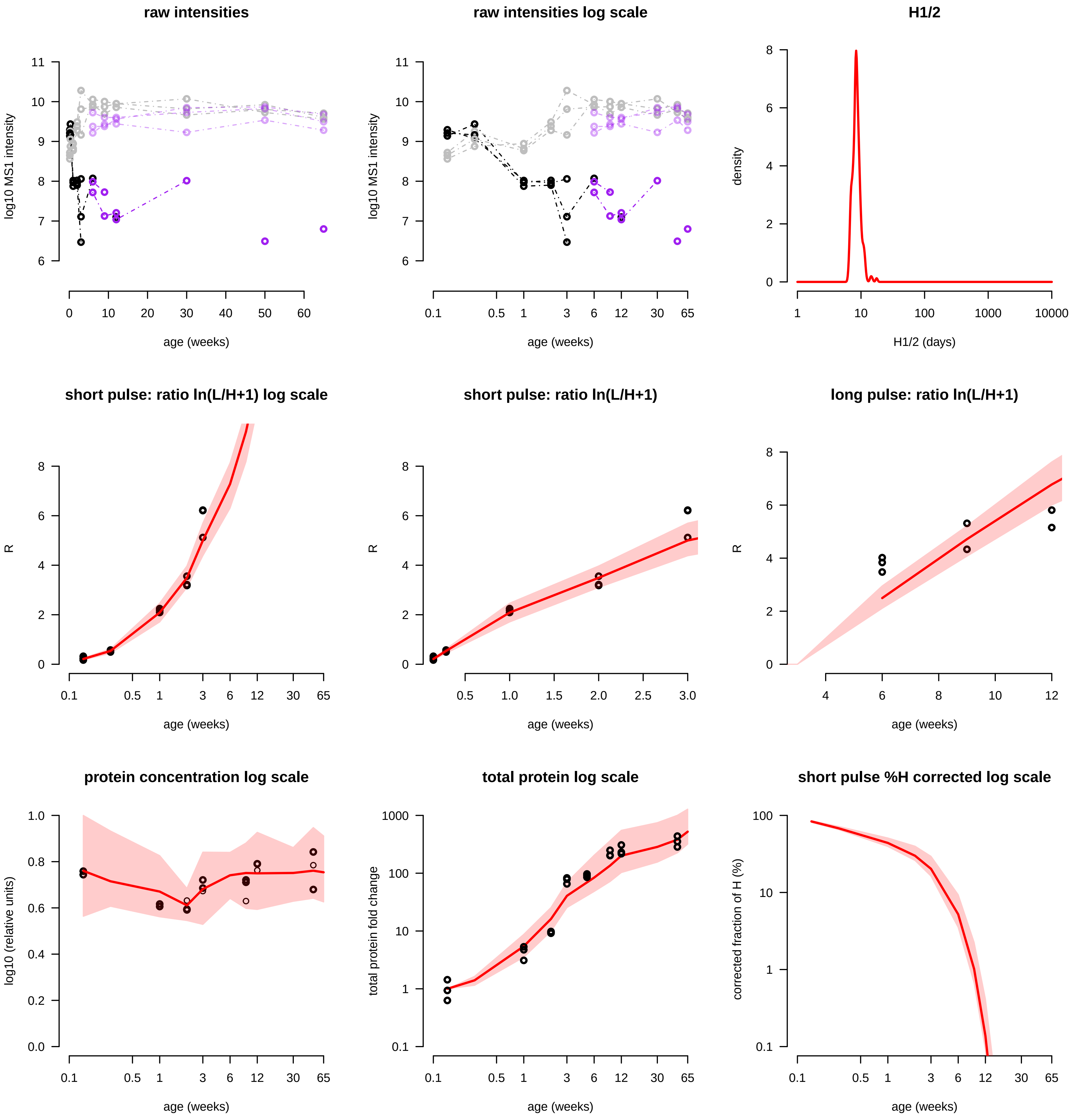

Q91X72

>sp|Q91X72|HEMO_MOUSE

Hemopexin

GN=Hpx

Med H1/2: 14 days

Q5 H1/2: 10 days

Q95 H1/2: 27.4 days

reliable model fit: yes

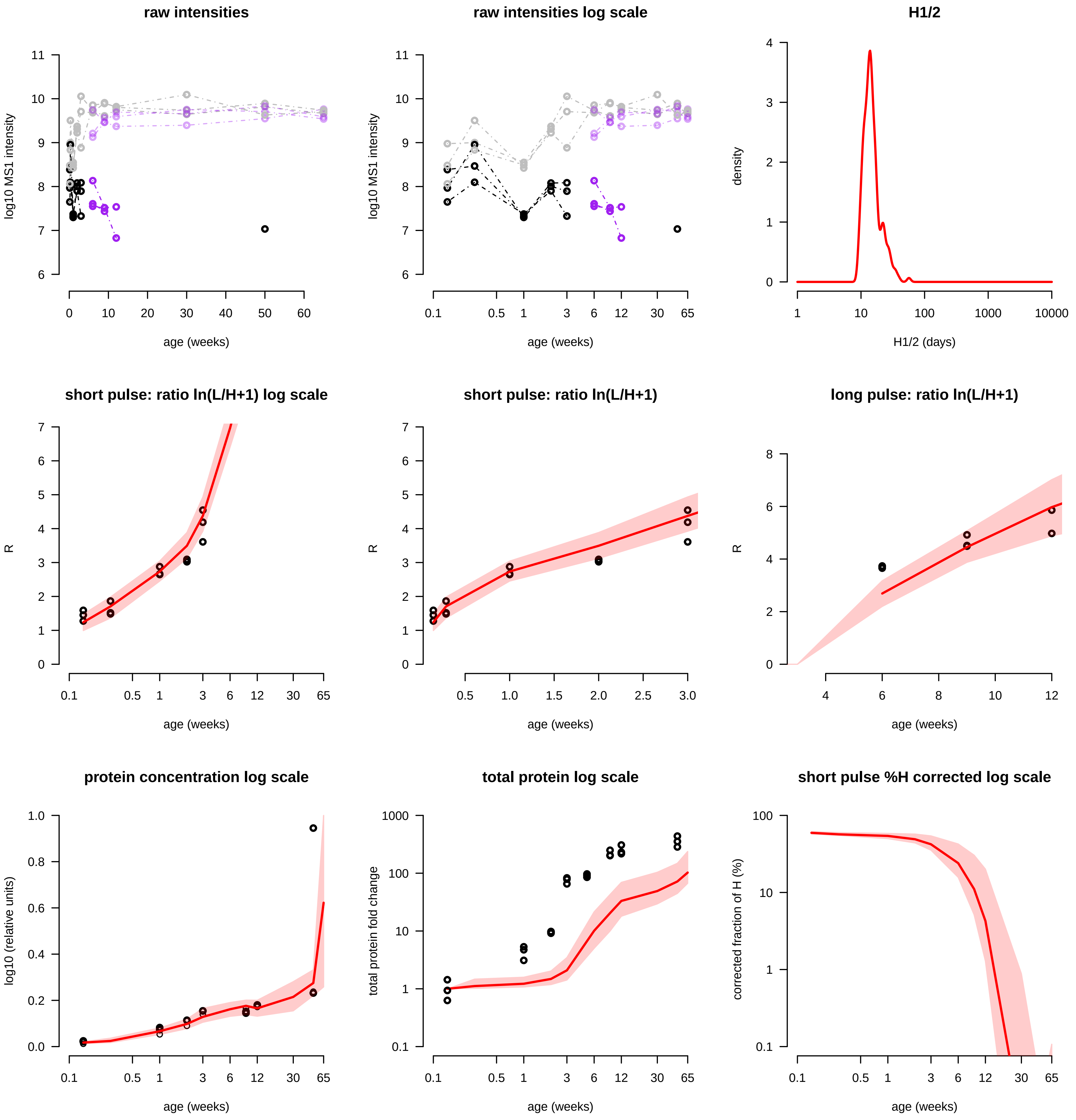

Q8BH95

>sp|Q8BH95|ECHM_MOUSE

Enoyl−CoA hydratase, mitochondrial

GN=Echs1

Med H1/2: 17 days

Q5 H1/2: 12.3 days

Q95 H1/2: 32.8 days

reliable model fit: yes

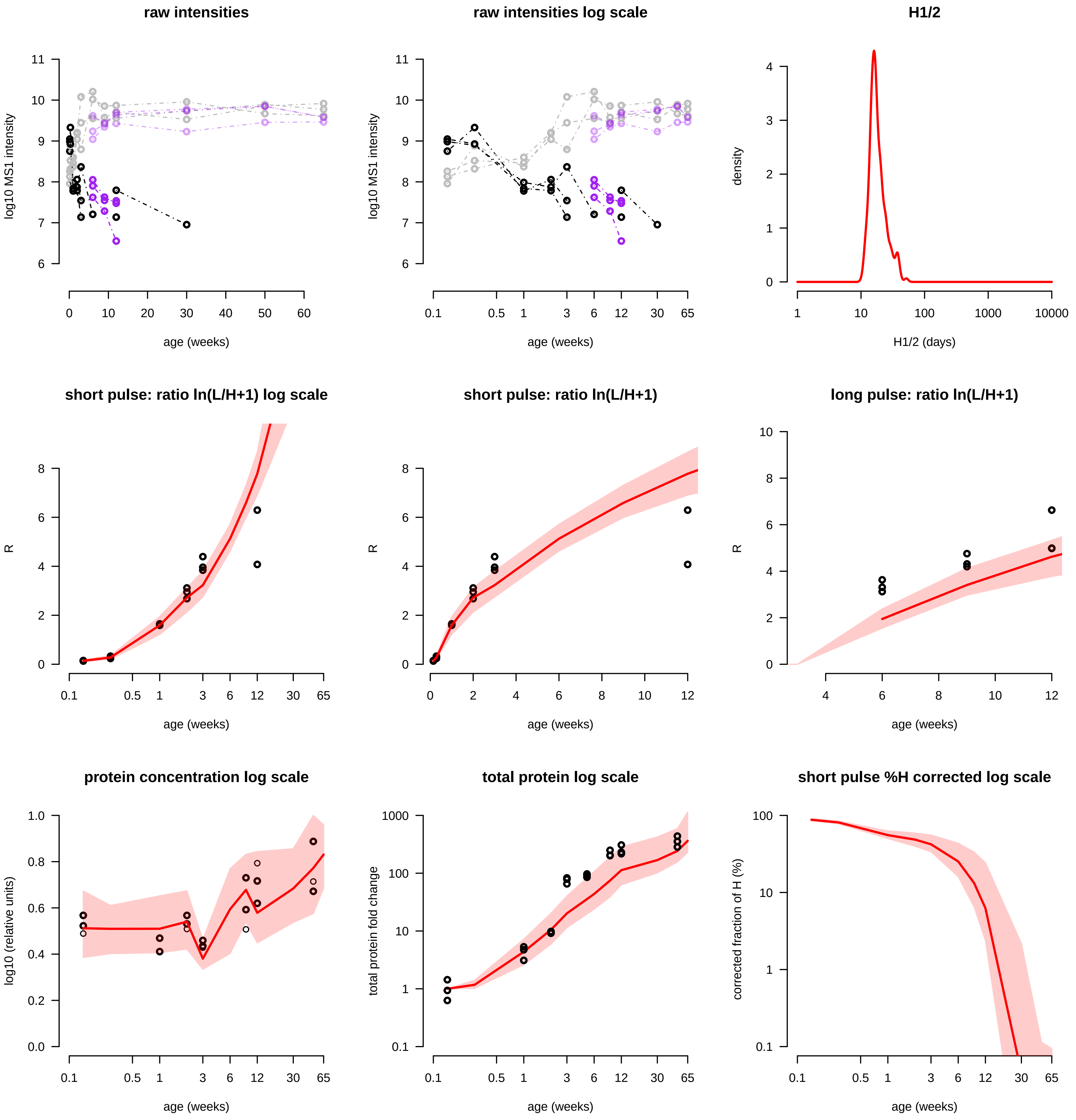

P45952

>sp|P45952|ACADM_MOUSE

Medium−chain specific acyl−CoA dehydrogenase, mitochondrial

GN=Acadm

Med H1/2: 9.9 days

Q5 H1/2: 7.9 days

Q95 H1/2: 12.7 days

reliable model fit: yes

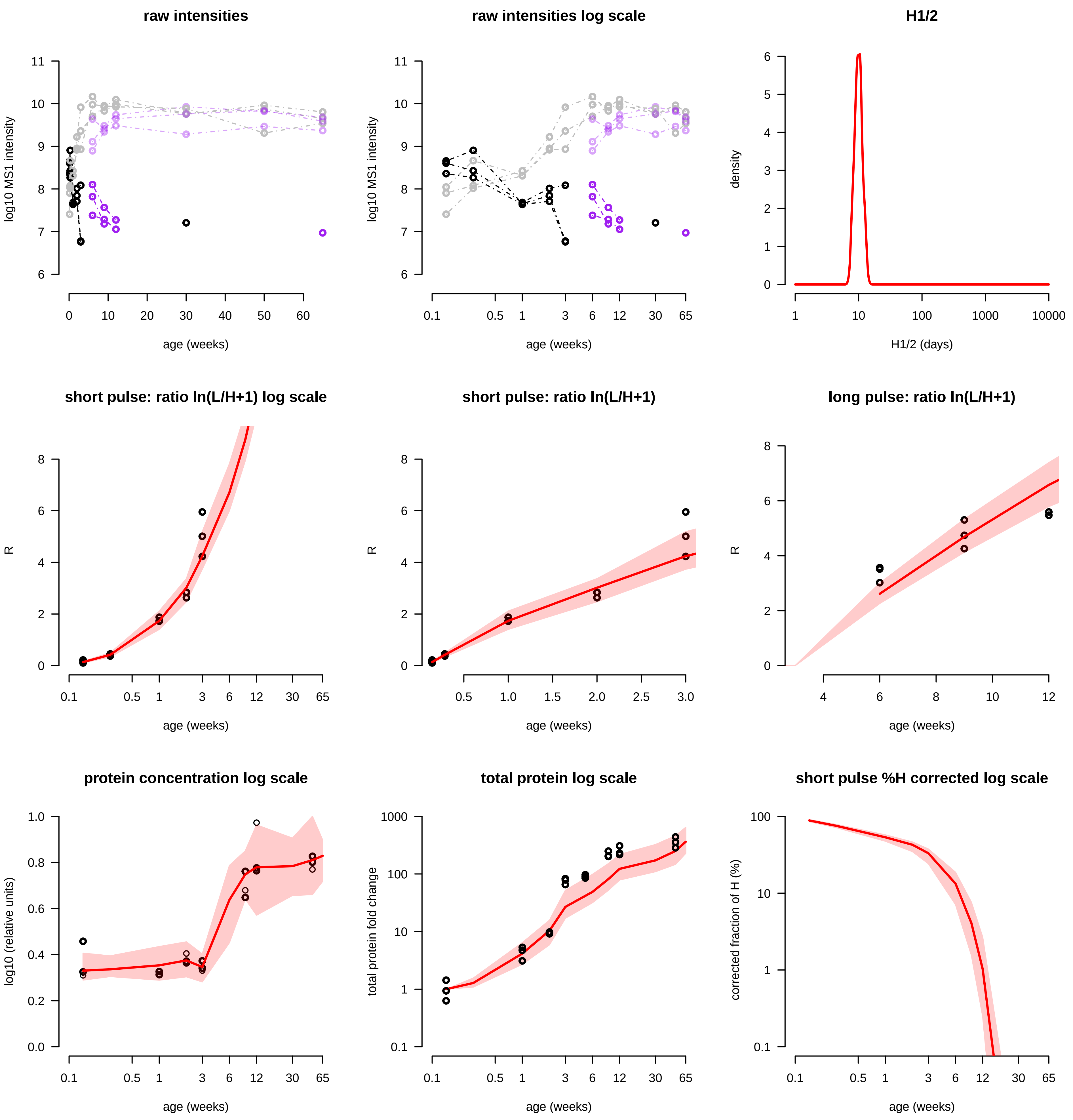

P19253

>sp|P19253|RL13A_MOUSE

60S ribosomal protein L13a

GN=Rpl13a

Med H1/2: 431.3 days

Q5 H1/2: 140.1 days

Q95 H1/2: 3812.8 days

reliable model fit: yes

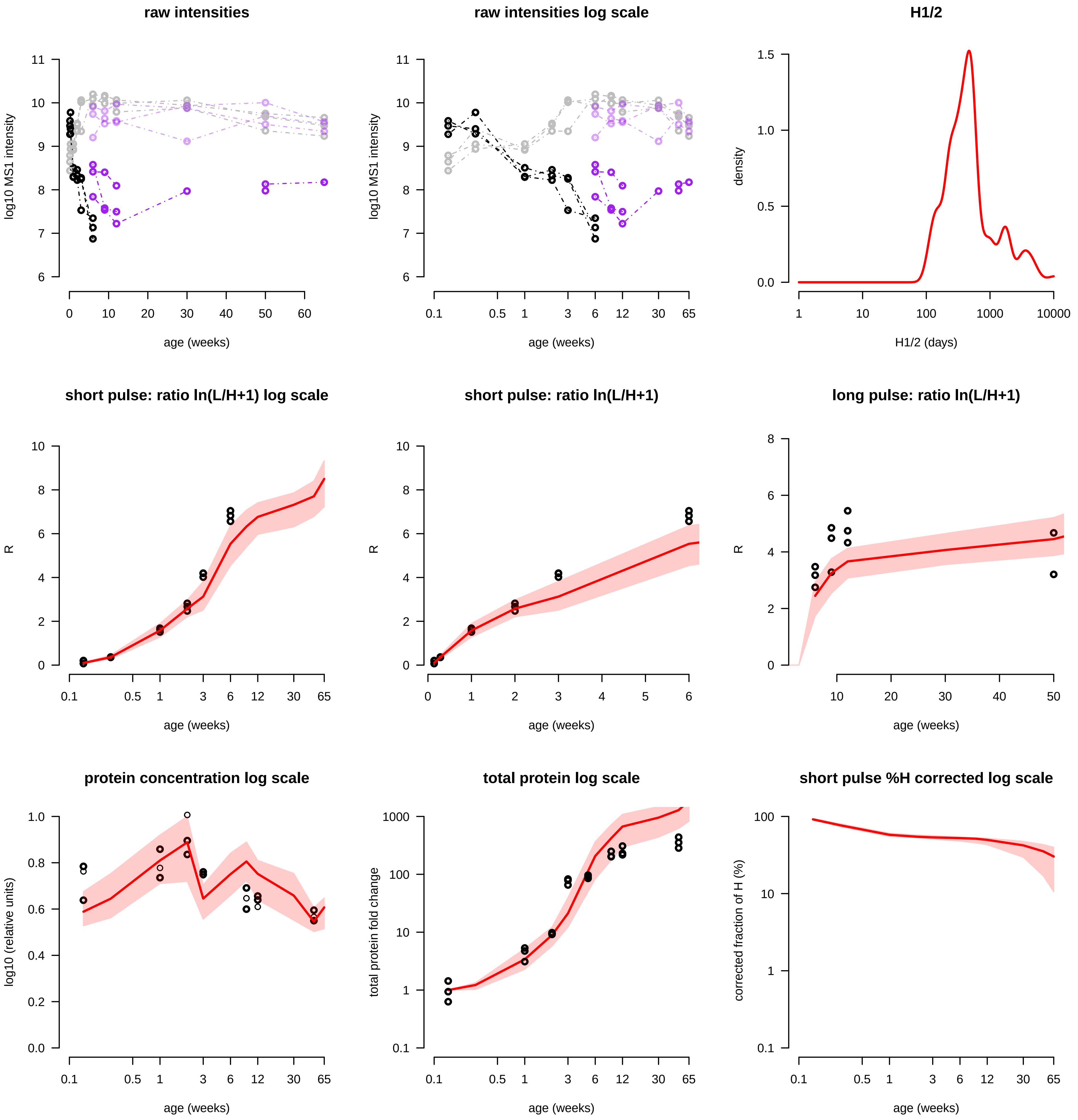

Q9R0P3

>sp|Q9R0P3|ESTD_MOUSE

S−formylglutathione hydrolase

GN=Esd

Med H1/2: 2245 days

Q5 H1/2: 623.4 days

Q95 H1/2: 26132.8 days

reliable model fit: no

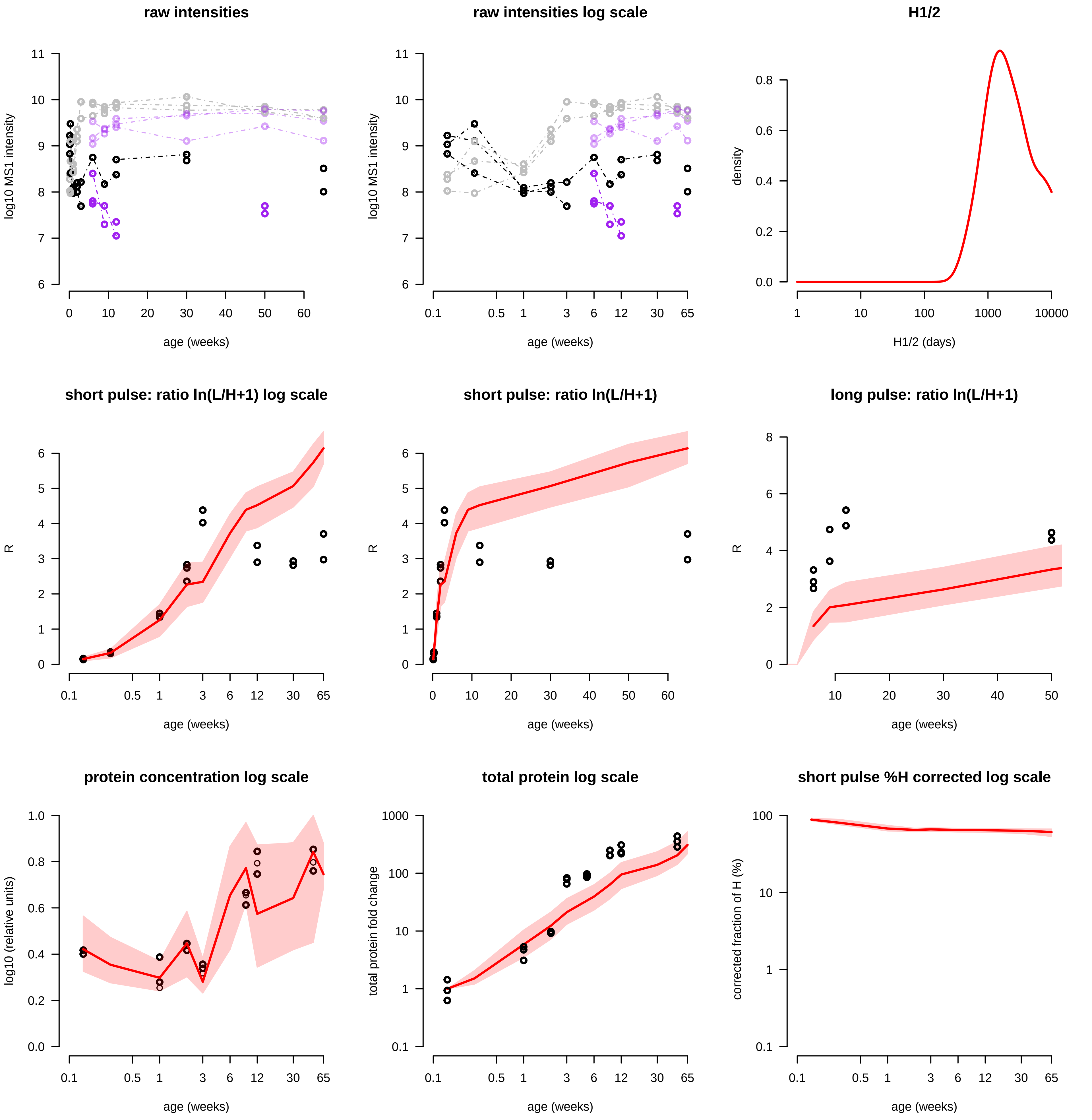

Q9EQ20

>sp|Q9EQ20|MMSA_MOUSE

Methylmalonate−semialdehyde dehydrogenase [acylating], mitochondrial

GN=Aldh6a1

Med H1/2: 105.7 days

Q5 H1/2: 46 days

Q95 H1/2: 249.4 days

reliable model fit: yes

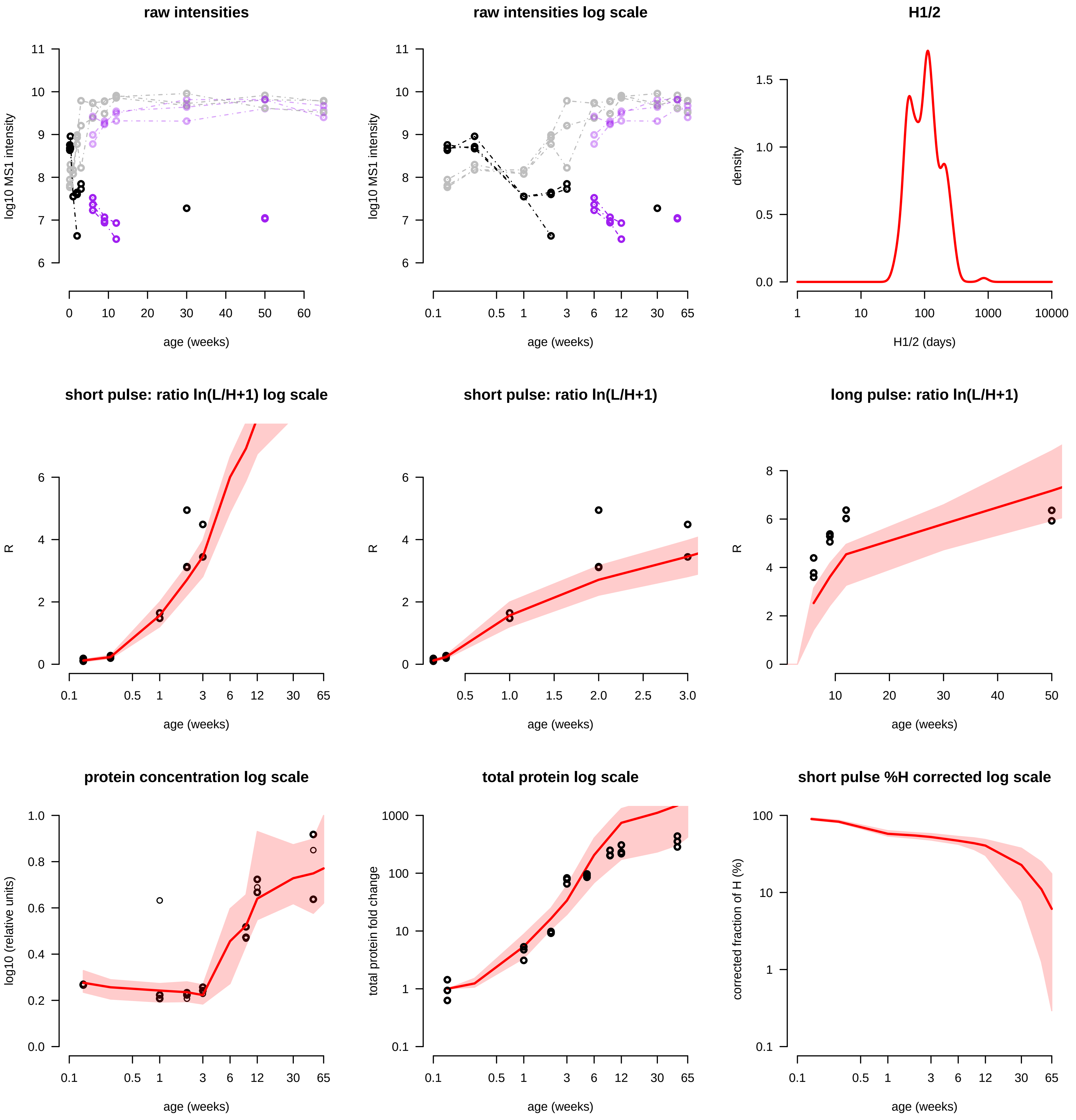

P51174

>sp|P51174|ACADL_MOUSE

Long−chain specific acyl−CoA dehydrogenase, mitochondrial

GN=Acadl

Med H1/2: 495.5 days

Q5 H1/2: 161 days

Q95 H1/2: 3264.8 days

reliable model fit: no

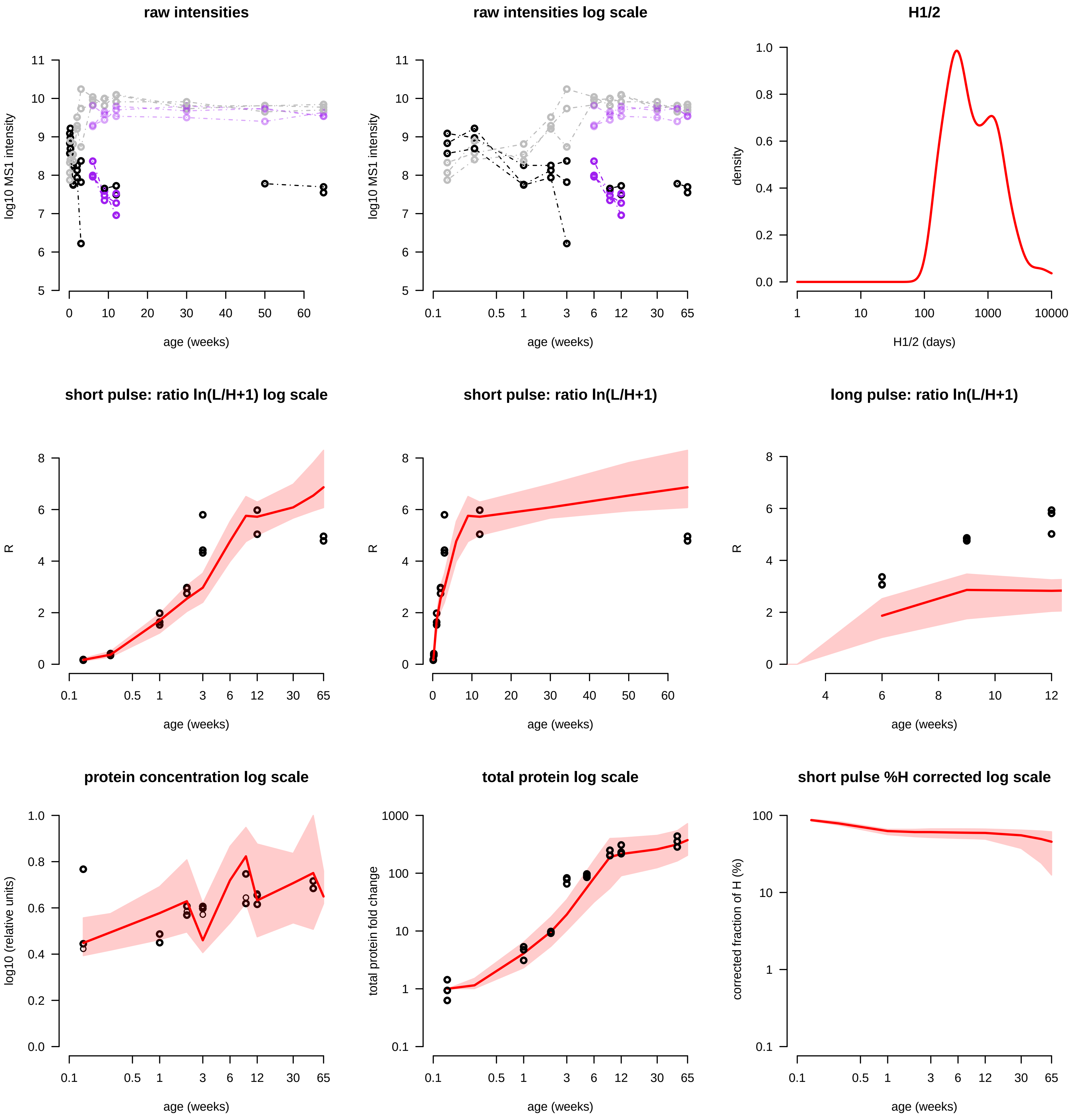

P09671

>sp|P09671|SODM_MOUSE

Superoxide dismutase [Mn], mitochondrial

GN=Sod2

Med H1/2: 75.2 days

Q5 H1/2: 47.8 days

Q95 H1/2: 130.3 days

reliable model fit: yes

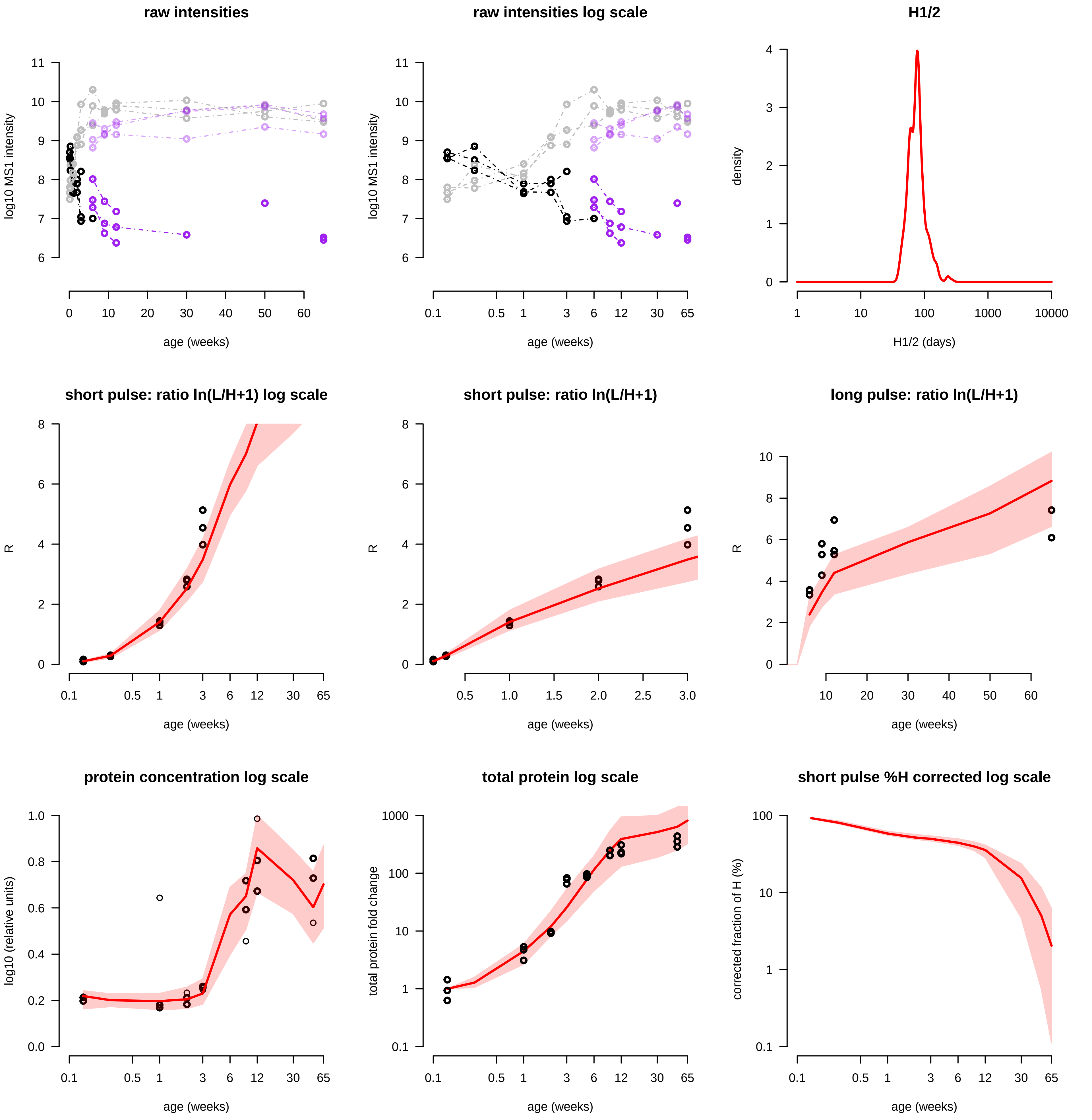

P61982

>sp|P61982|1433G_MOUSE

14−3−3 protein gamma

GN=Ywhag

Med H1/2: 12.5 days

Q5 H1/2: 10.2 days

Q95 H1/2: 16.7 days

reliable model fit: yes

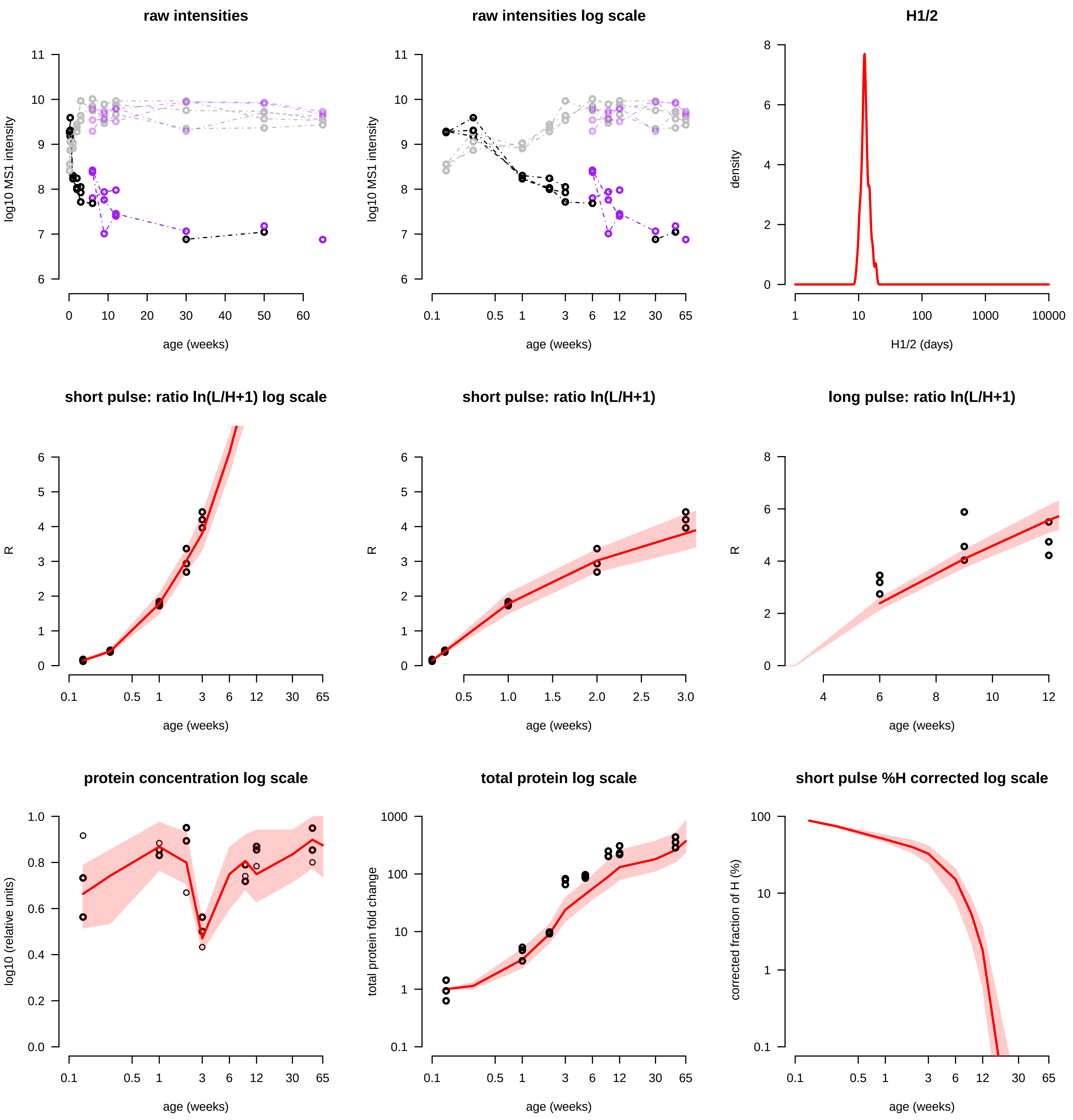

Q9WU78

>sp|Q9WU78|PDC6I_MOUSE

Programmed cell death 6−interacting protein

GN=Pdcd6ip

Med H1/2: 8.9 days

Q5 H1/2: 7.2 days

Q95 H1/2: 11.4 days

reliable model fit: yes

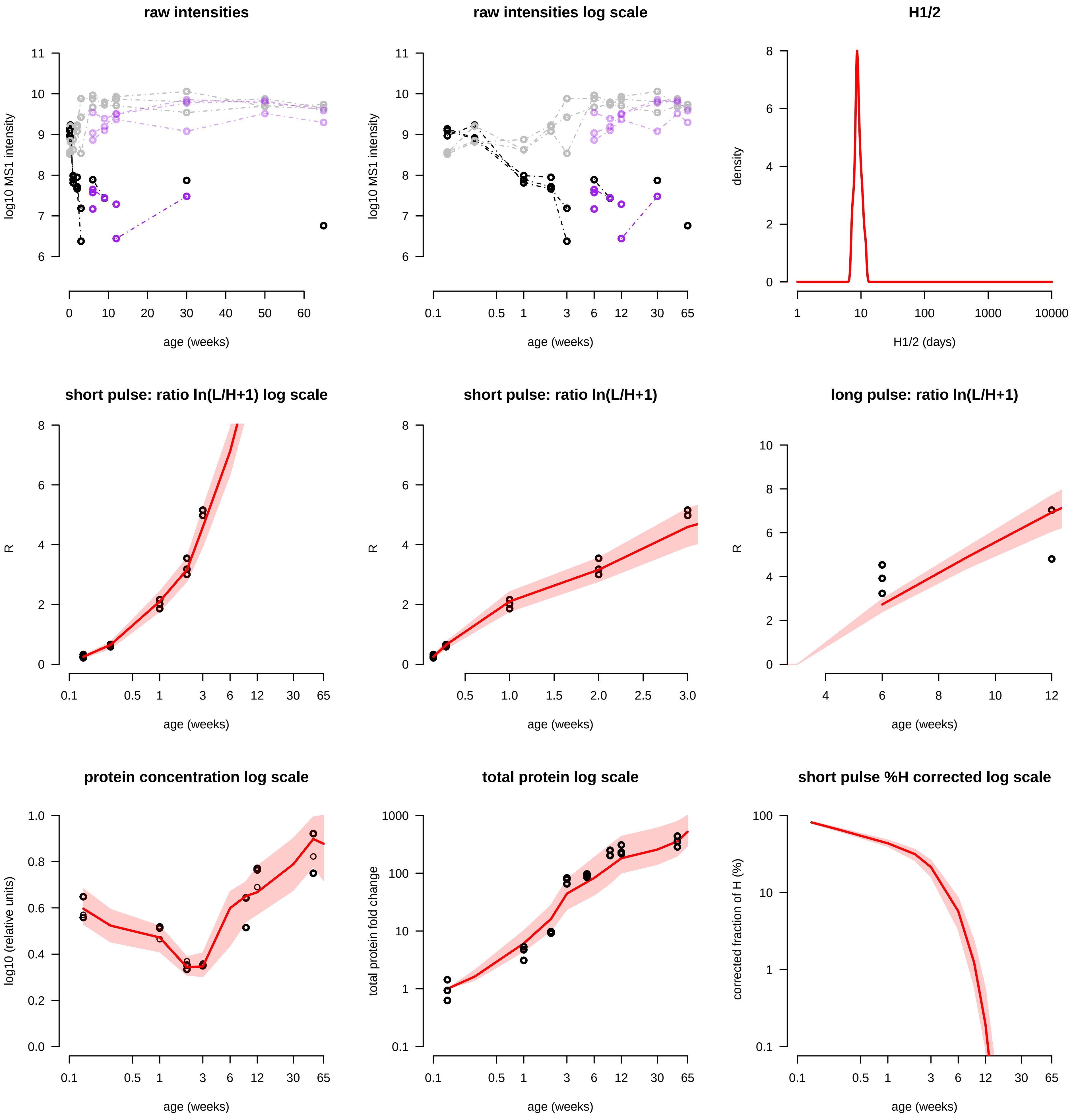

P24527

>sp|P24527|LKHA4_MOUSE

Leukotriene A−4 hydrolase

GN=Lta4h

Med H1/2: 10.6 days

Q5 H1/2: 8.4 days

Q95 H1/2: 15.1 days

reliable model fit: yes

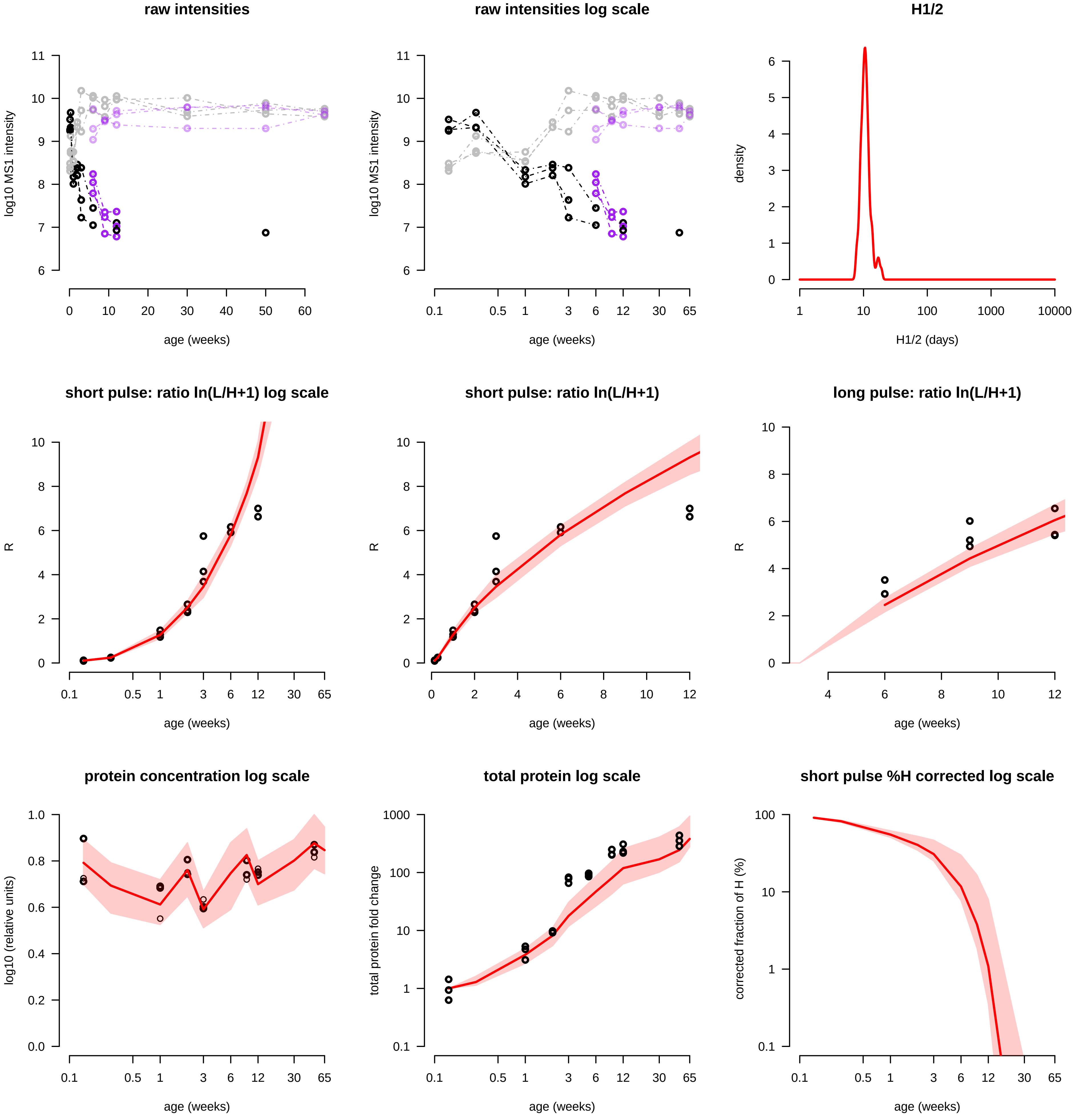

Q8BIJ6

>sp|Q8BIJ6|SYIM_MOUSE

Isoleucine−−tRNA ligase, mitochondrial

GN=Iars2

Med H1/2: 364.8 days

Q5 H1/2: 110.7 days

Q95 H1/2: 5568.9 days

reliable model fit: yes

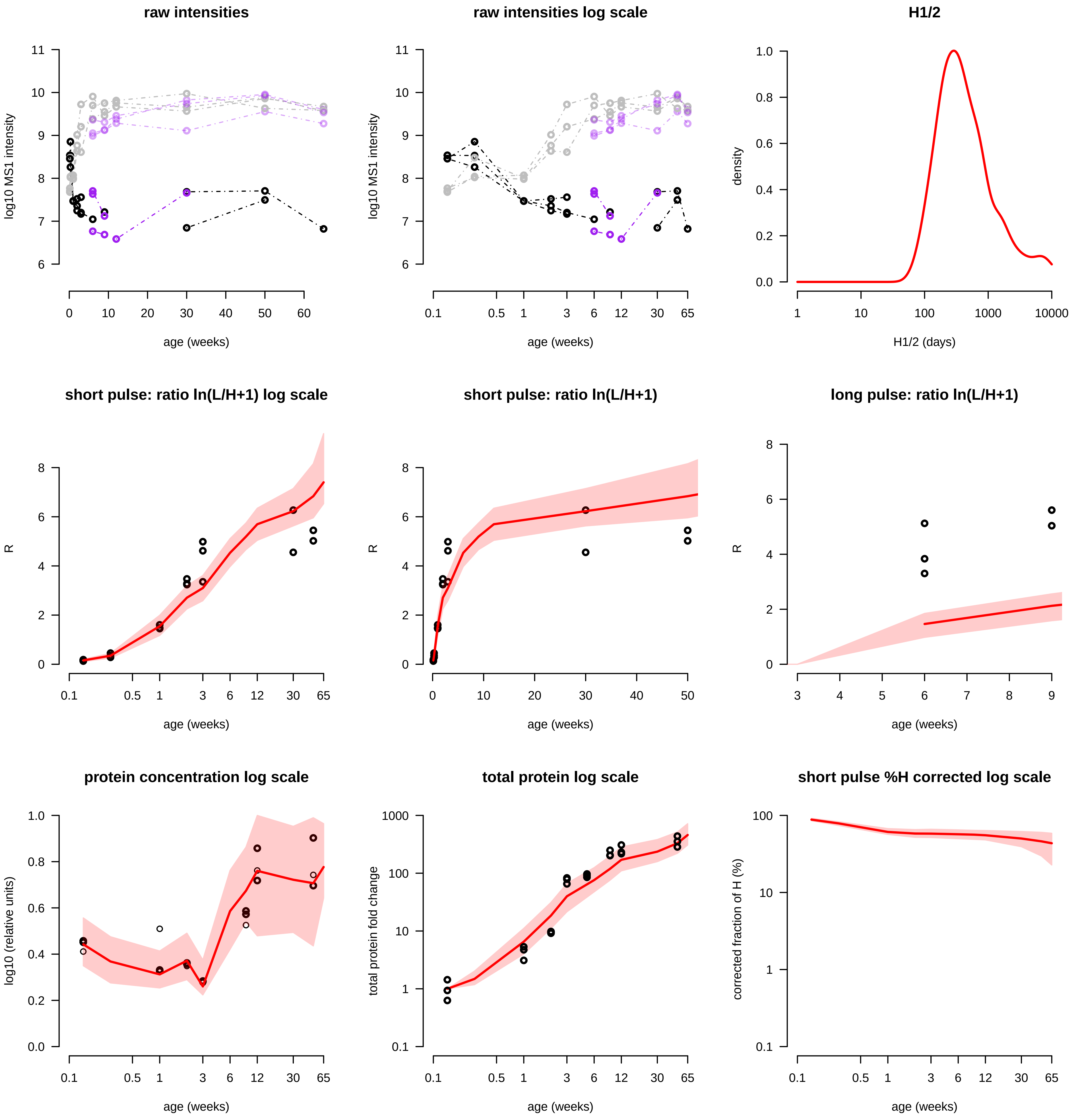

O70318

>sp|O70318|E41L2_MOUSE

Band 4.1−like protein 2

GN=Epb41l2

Med H1/2: 108.1 days

Q5 H1/2: 71 days

Q95 H1/2: 246.8 days

reliable model fit: no

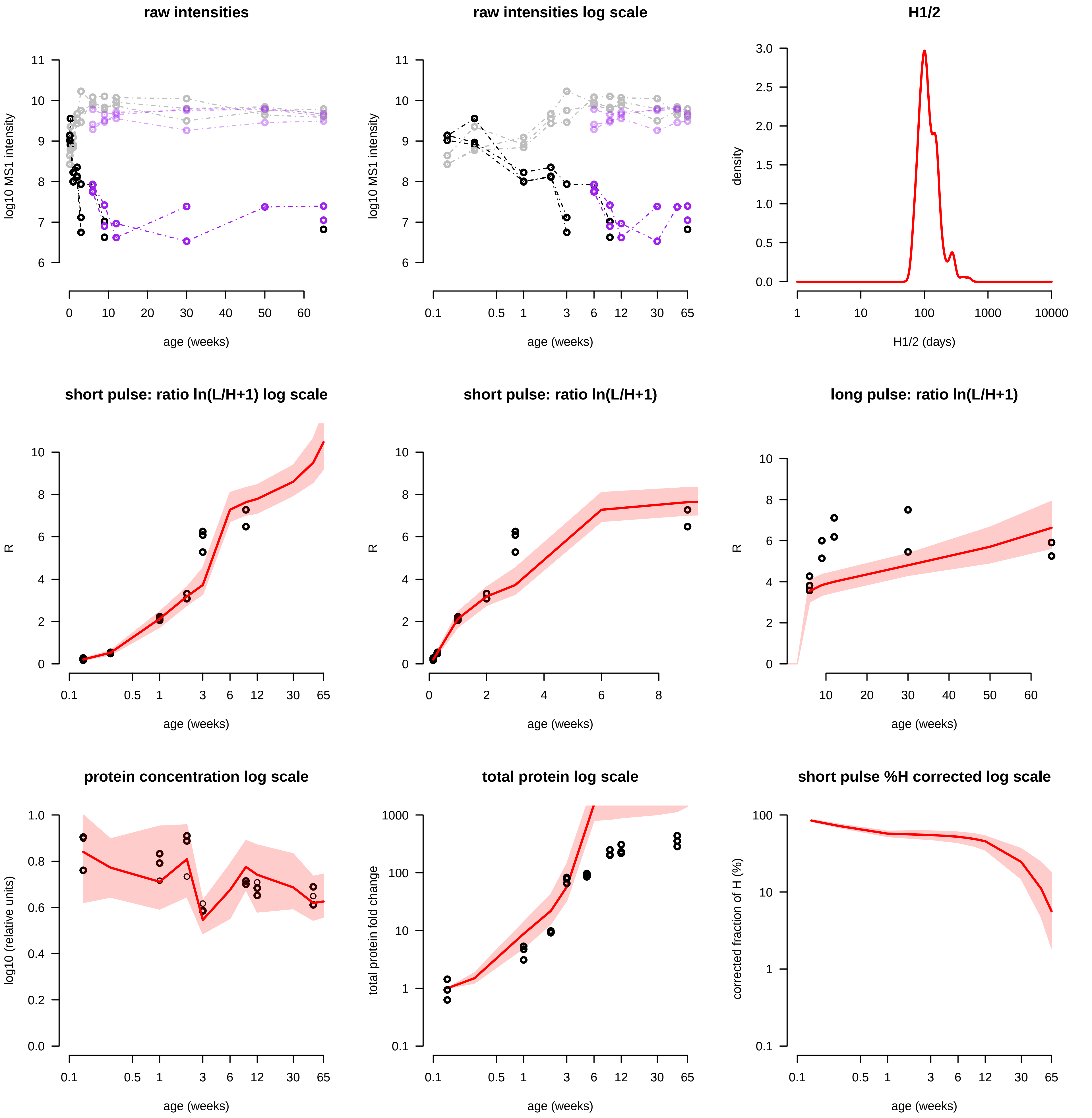

P19783

>sp|P19783|COX41_MOUSE

Cytochrome c oxidase subunit 4 isoform 1, mitochondrial

GN=Cox4i1

Med H1/2: 7.9 days

Q5 H1/2: 6.4 days

Q95 H1/2: 11.6 days

reliable model fit: yes

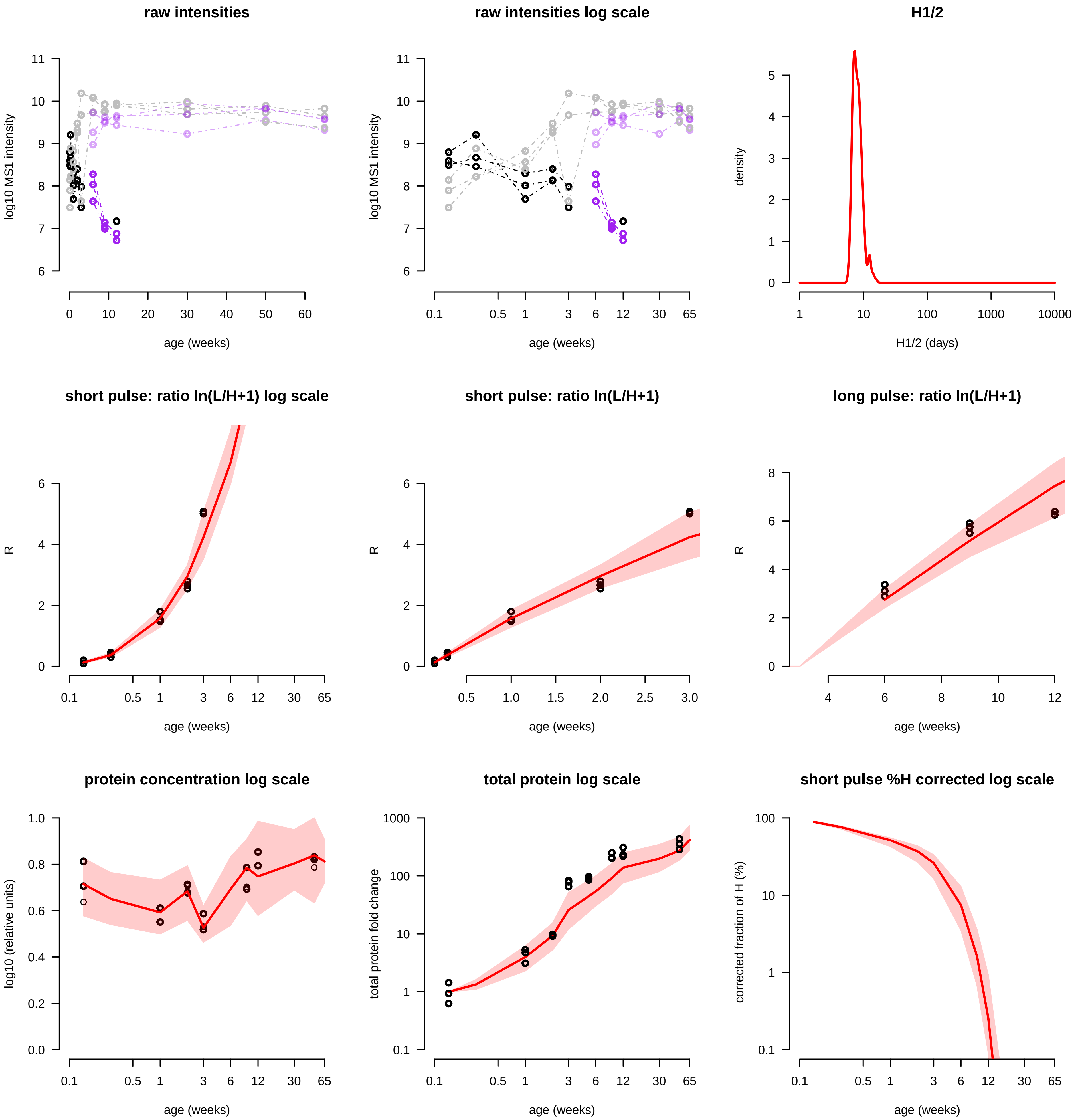

O35381

>sp|O35381|AN32A_MOUSE

Acidic leucine−rich nuclear phosphoprotein 32 family member A

GN=Anp32a

Med H1/2: 10.1 days

Q5 H1/2: 8 days

Q95 H1/2: 13.4 days

reliable model fit: yes

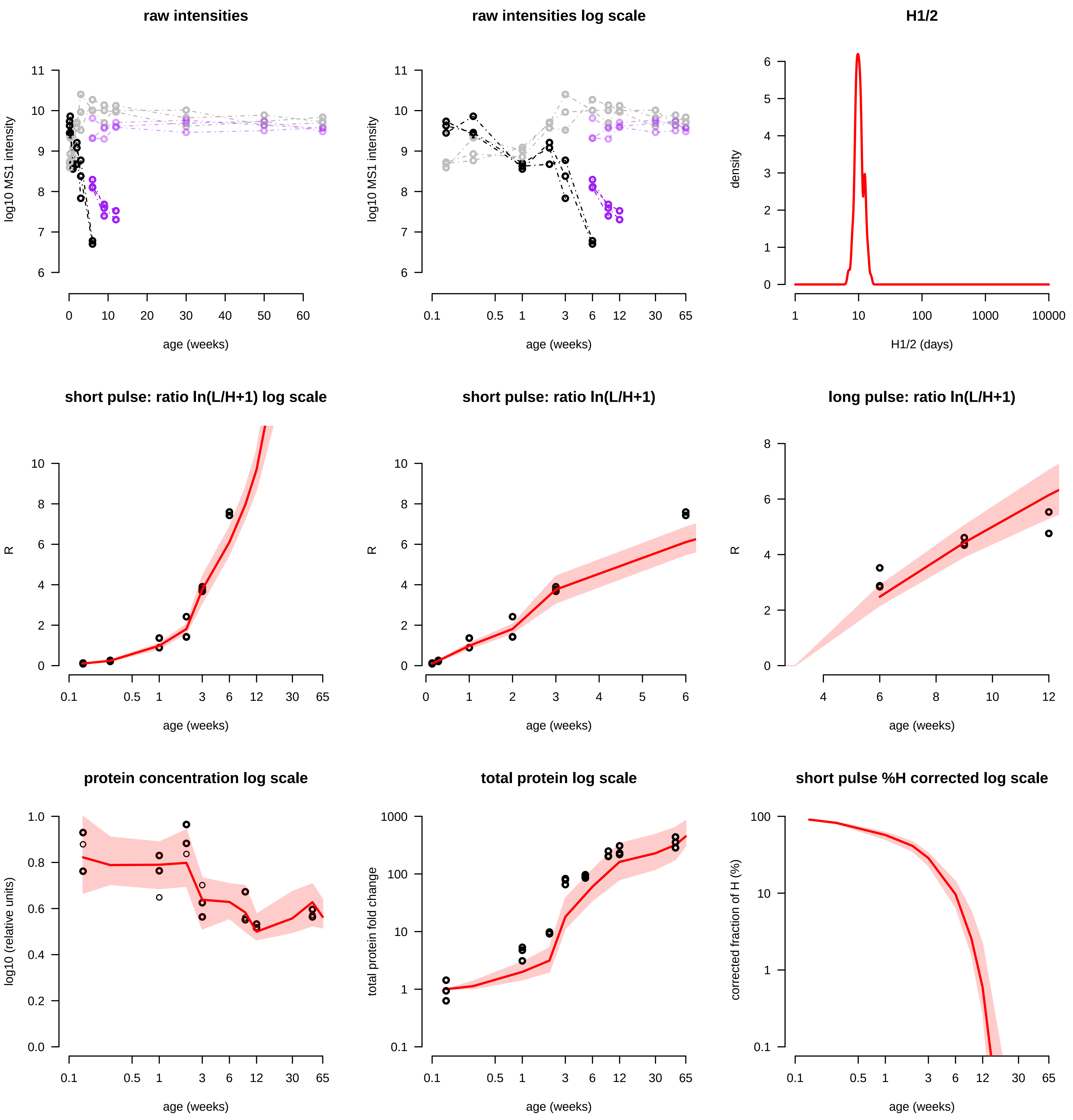

P80313

>sp|P80313|TCPH_MOUSE

T−complex protein 1 subunit eta

GN=Cct7

Med H1/2: 11.4 days

Q5 H1/2: 9.4 days

Q95 H1/2: 18.7 days

reliable model fit: no

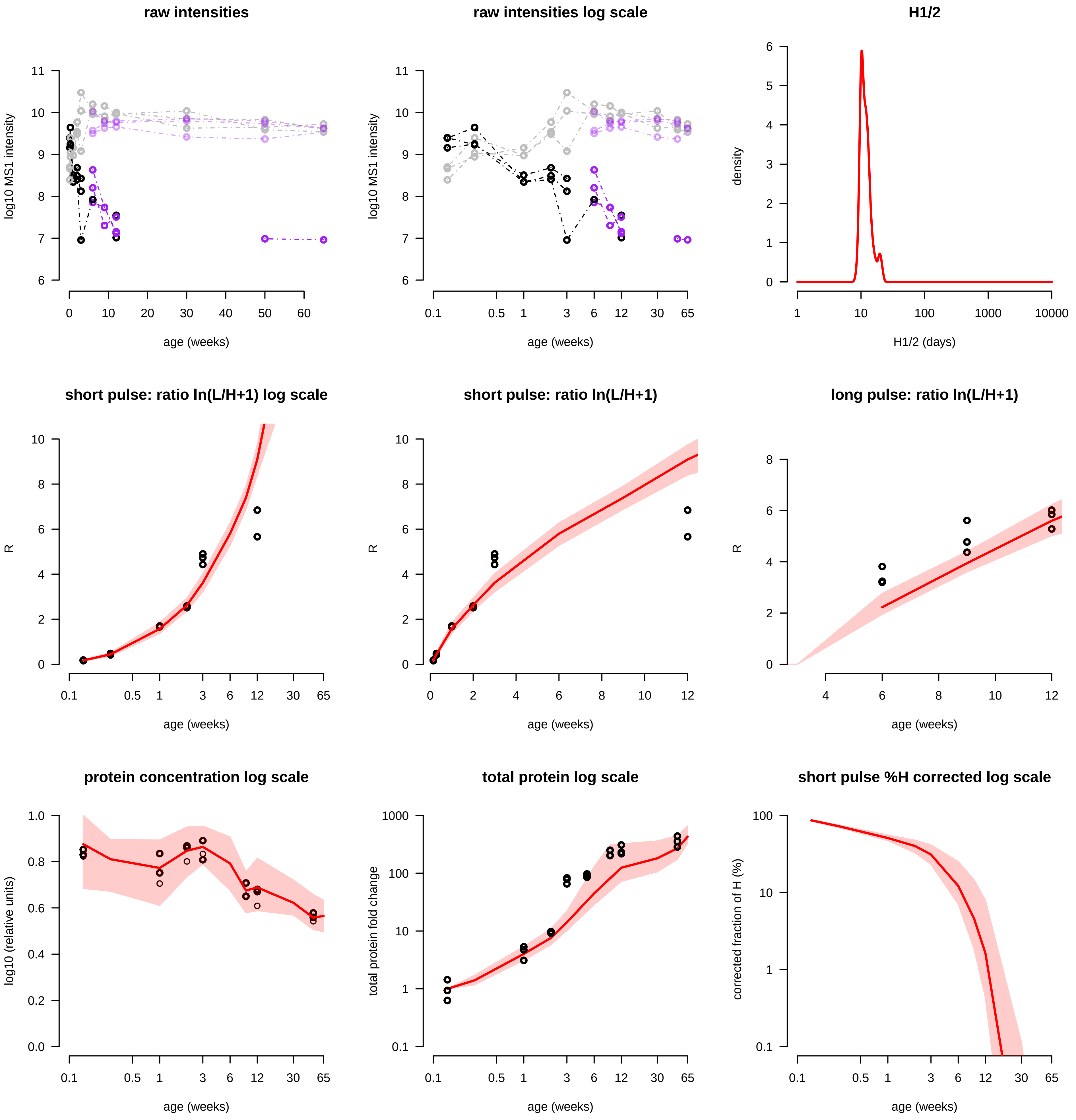

Q921H8

>sp|Q921H8|THIKA_MOUSE

3−ketoacyl−CoA thiolase A, peroxisomal

GN=Acaa1a

Med H1/2: 6.8 days

Q5 H1/2: 5.6 days

Q95 H1/2: 8.5 days

reliable model fit: yes

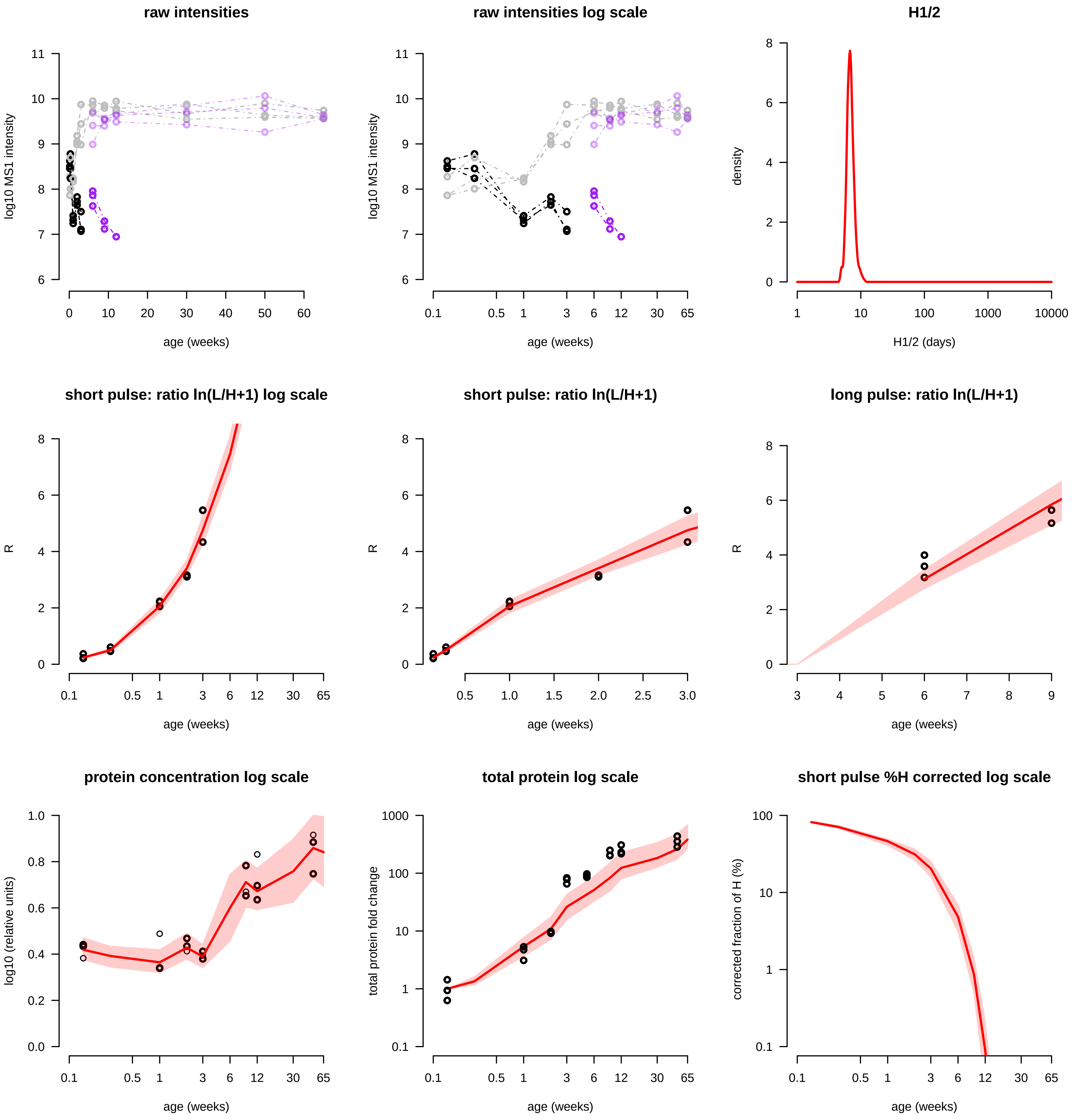

P26443

>sp|P26443|DHE3_MOUSE

Glutamate dehydrogenase 1, mitochondrial

GN=Glud1

Med H1/2: 2290.1 days

Q5 H1/2: 589.9 days

Q95 H1/2: 18404.3 days

reliable model fit: yes

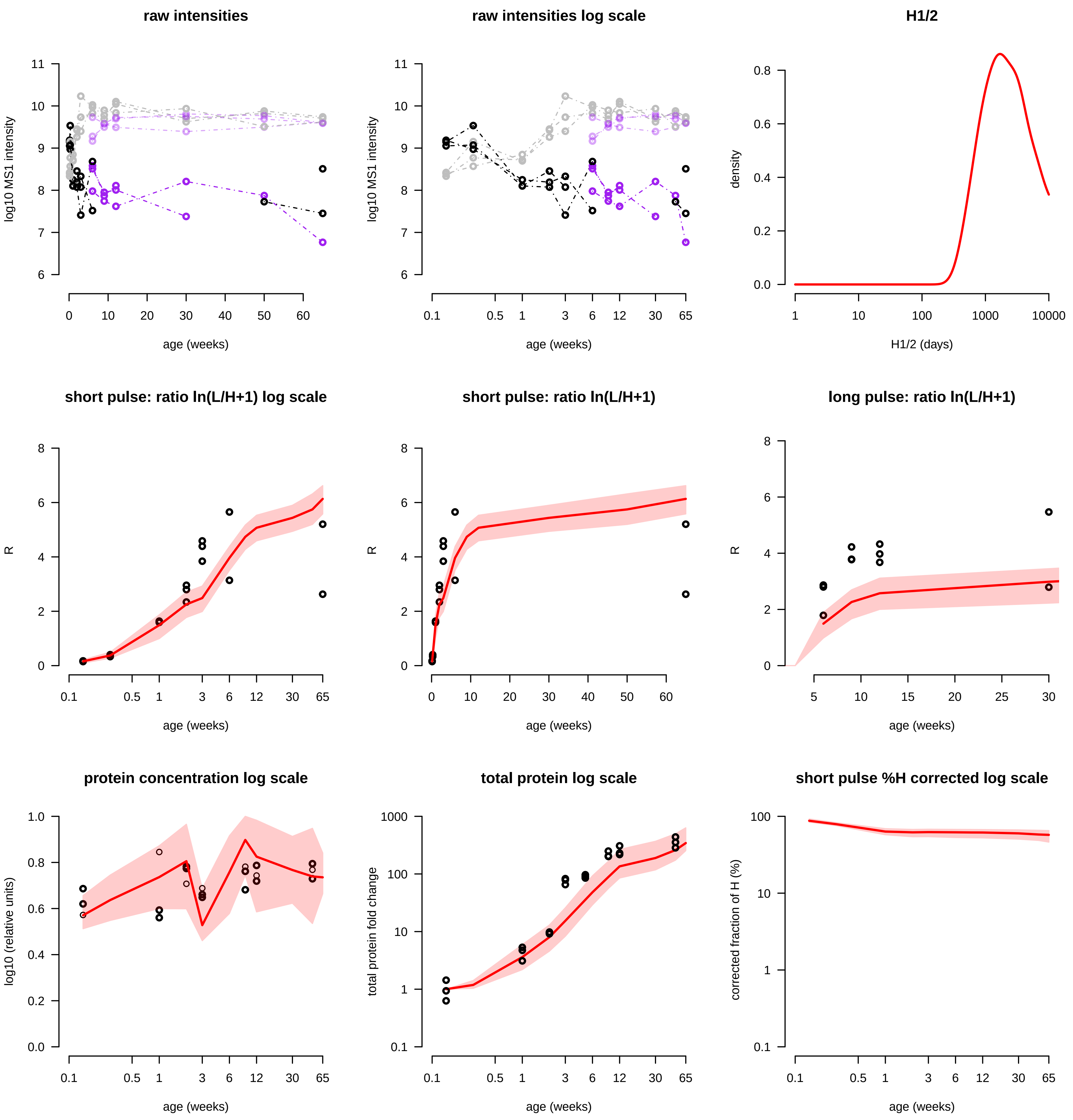

Q62425

>sp|Q62425|NDUA4_MOUSE

Cytochrome c oxidase subunit NDUFA4

GN=Ndufa4

Med H1/2: 582.3 days

Q5 H1/2: 171.5 days

Q95 H1/2: 7702 days

reliable model fit: no

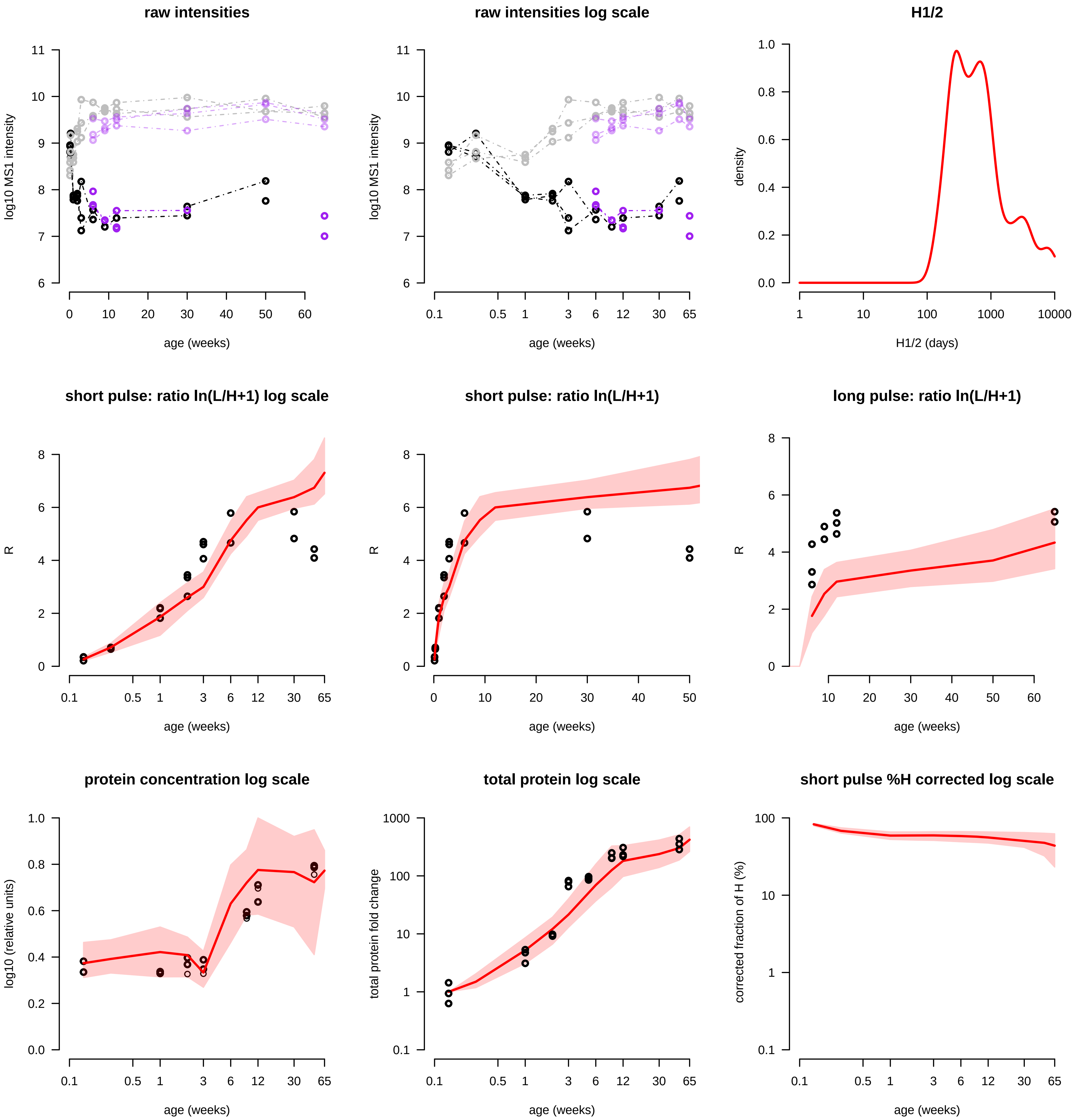

Q99KC8

>sp|Q99KC8|VMA5A_MOUSE

von Willebrand factor A domain−containing protein 5A

GN=Vwa5a

Med H1/2: 591.4 days

Q5 H1/2: 239.7 days

Q95 H1/2: 8792.7 days

reliable model fit: yes

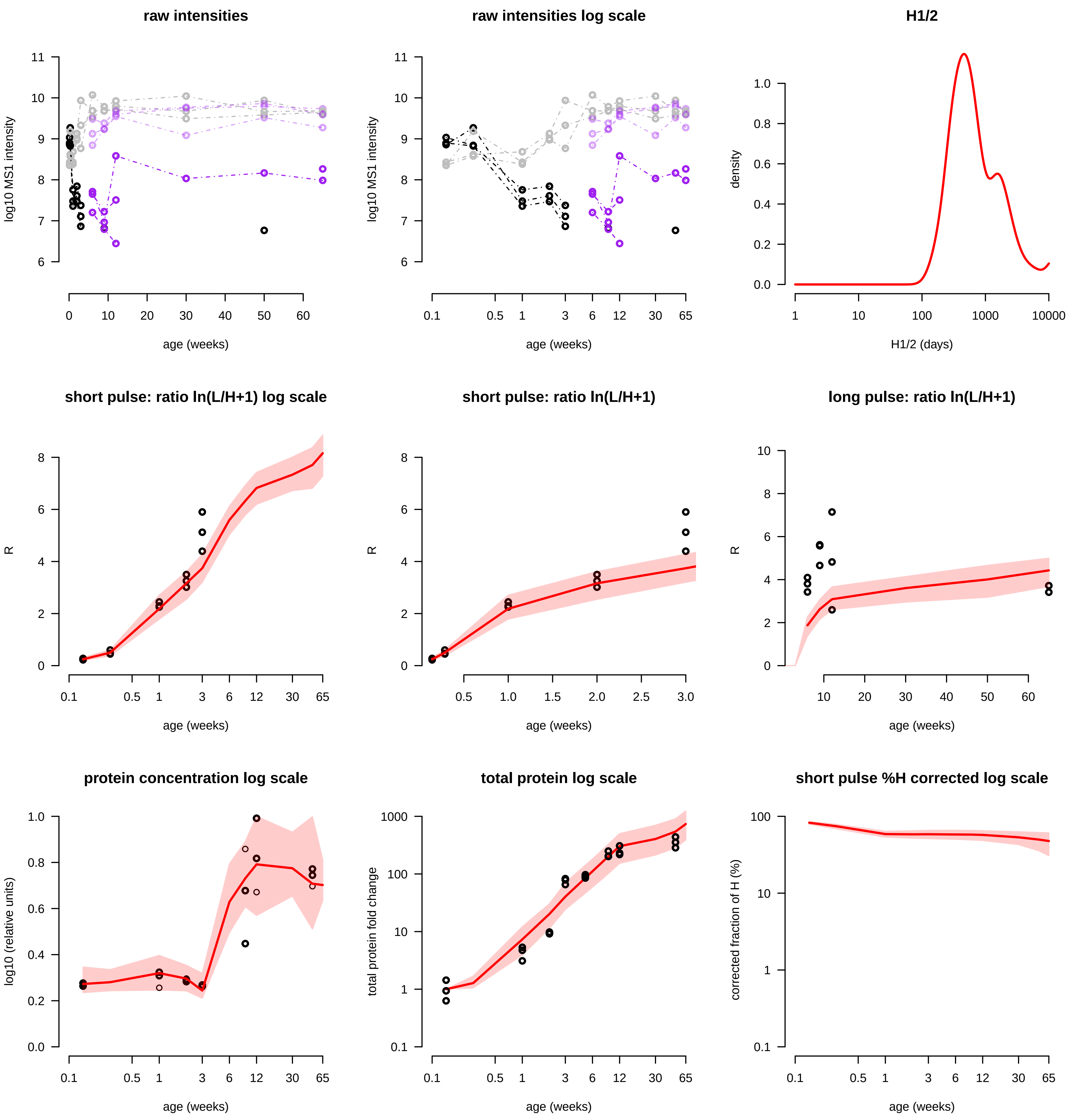

P26041

>sp|P26041|MOES_MOUSE

Moesin

GN=Msn

Med H1/2: 870.3 days

Q5 H1/2: 240.8 days

Q95 H1/2: 11346.1 days

reliable model fit: yes

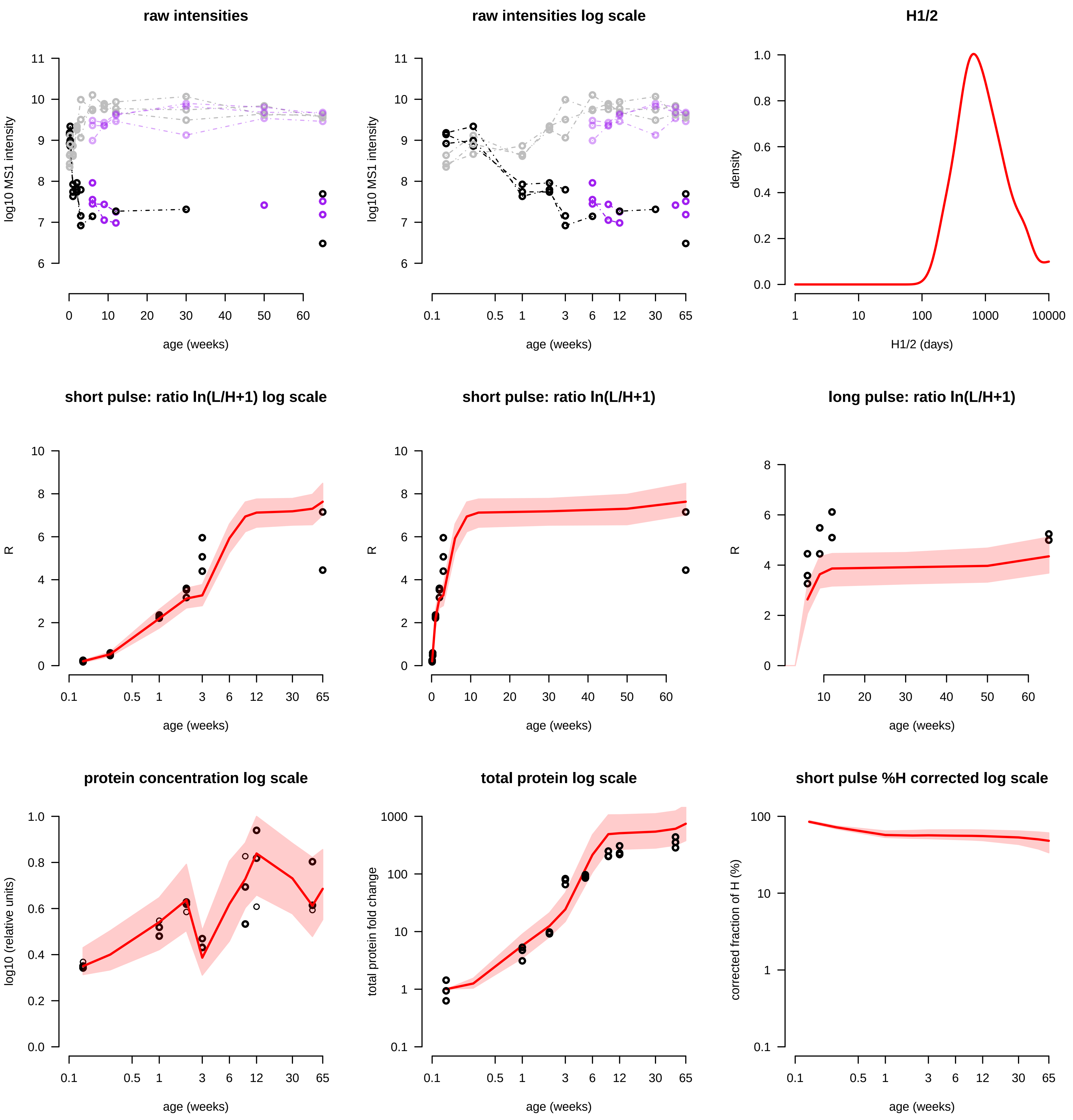

Q60847;Q60847−2;Q60847−5;Q60847−3;Q60847−4

>sp|Q60847|COCA1_MOUSE

Collagen alpha−1(XII) chain

GN=Col12a1

Med H1/2: 9.1 days

Q5 H1/2: 7.2 days

Q95 H1/2: 13.2 days

reliable model fit: yes

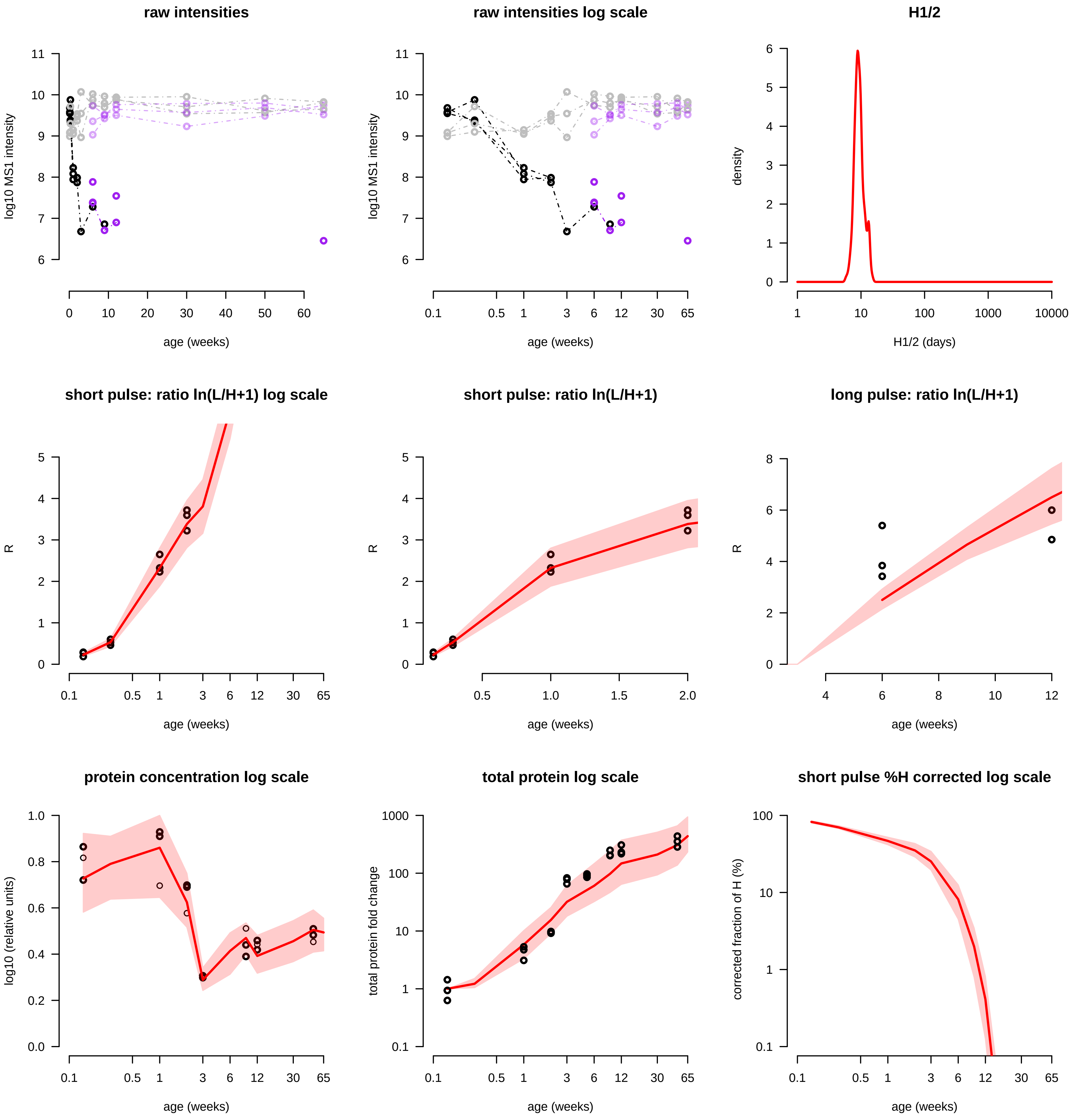

Q8BSE0

>sp|Q8BSE0|RMD2_MOUSE

Regulator of microtubule dynamics protein 2

GN=Rmdn2

Med H1/2: 842.7 days

Q5 H1/2: 258.8 days

Q95 H1/2: 7573.8 days

reliable model fit: yes

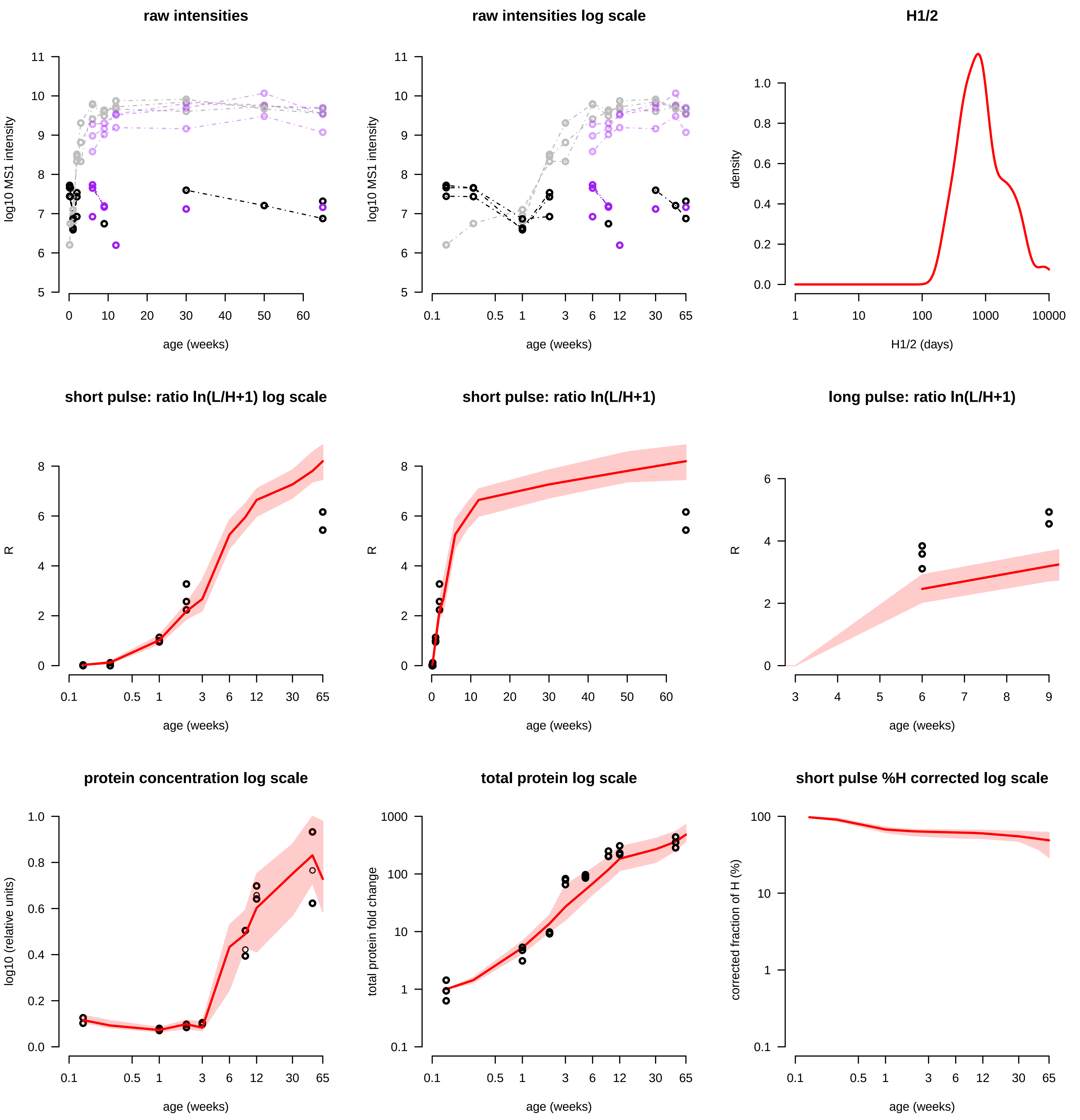

P62281

>sp|P62281|RS11_MOUSE

40S ribosomal protein S11

GN=Rps11

Med H1/2: 13.1 days

Q5 H1/2: 10.2 days

Q95 H1/2: 18.5 days

reliable model fit: yes

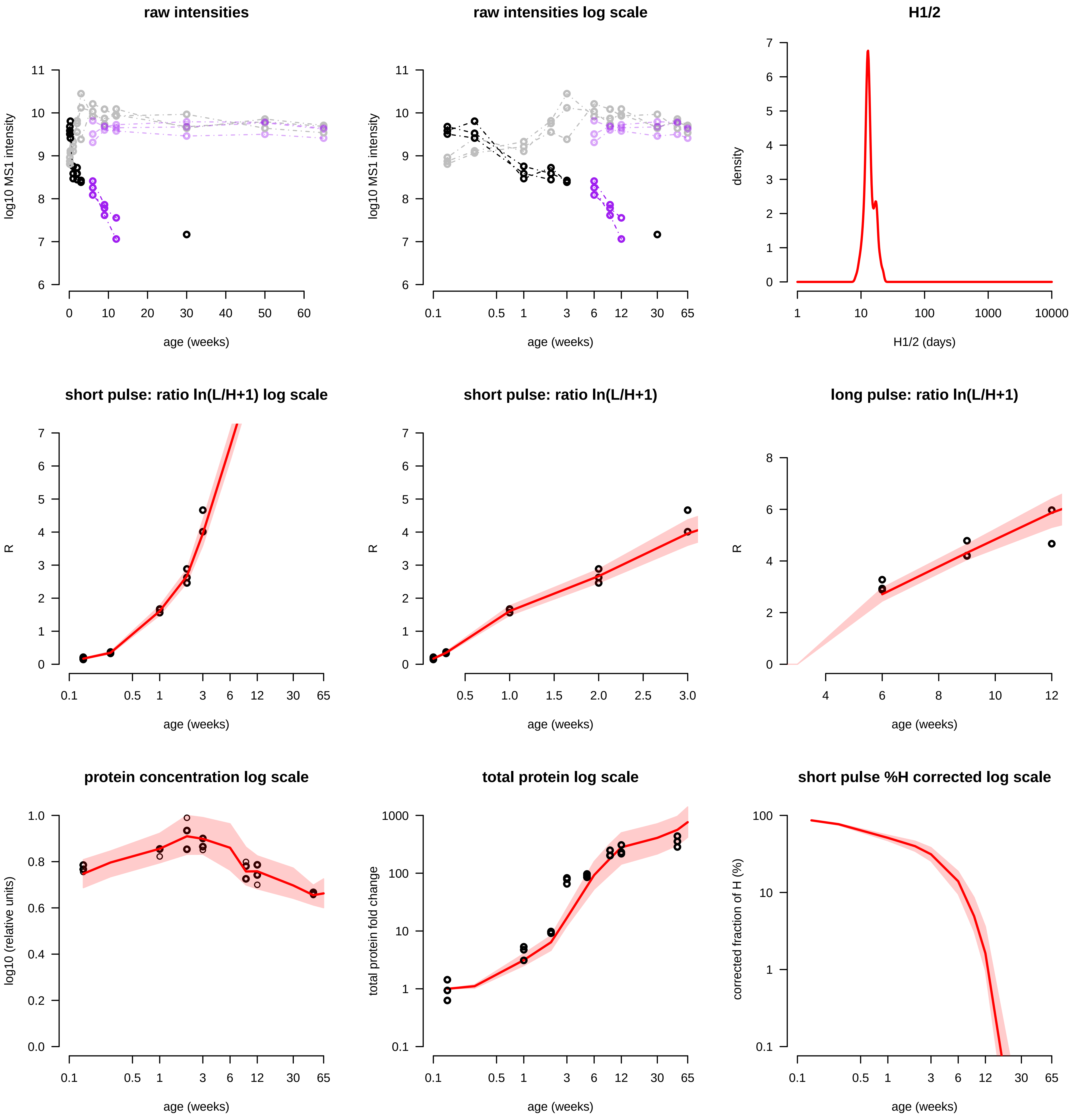

O70456

>sp|O70456|1433S_MOUSE

14−3−3 protein sigma

GN=Sfn

Med H1/2: 9.9 days

Q5 H1/2: 8.2 days

Q95 H1/2: 12.6 days

reliable model fit: yes

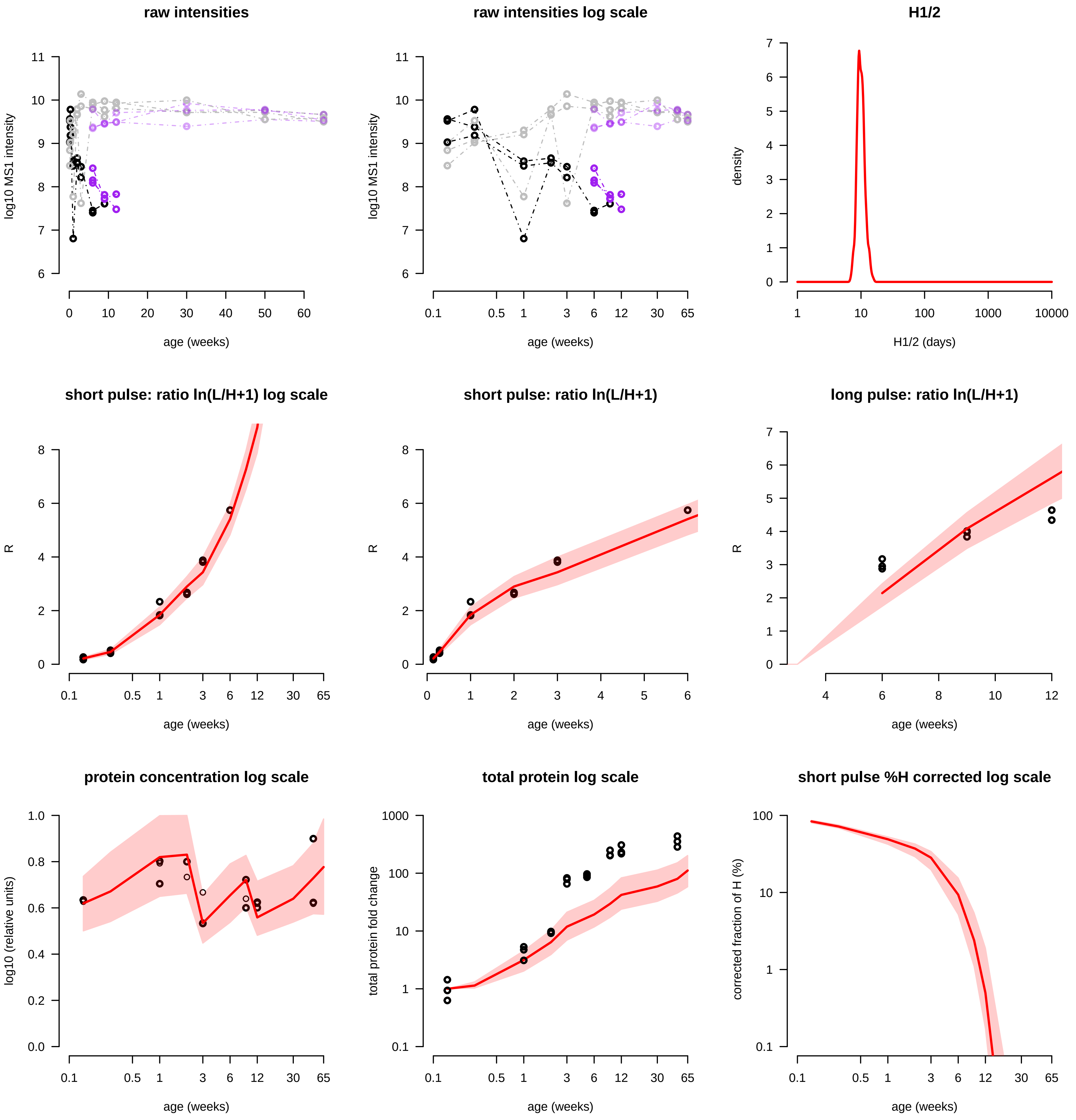

Q9Z204−2;Q9Z204−4;Q9Z204−3

>sp|Q9Z204−2|HNRPC_MOUSE

Isoform C1 of Heterogeneous nuclear ribonucleoproteins C1/C2

GN=Hnrnpc

Med H1/2: 6.8 days

Q5 H1/2: 5.5 days

Q95 H1/2: 9.1 days

reliable model fit: yes

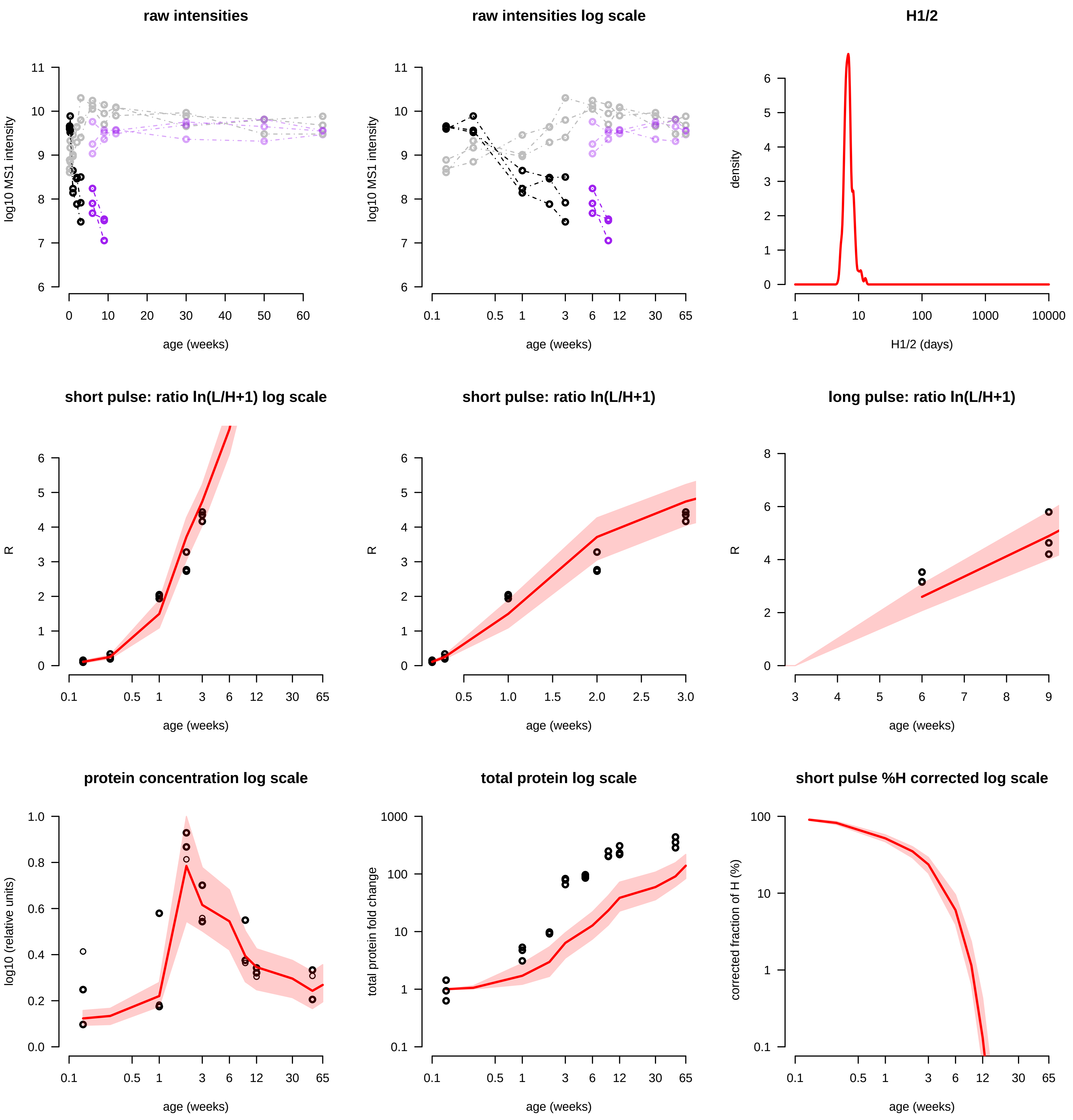

P29699

>sp|P29699|FETUA_MOUSE

Alpha−2−HS−glycoprotein

GN=Ahsg

Med H1/2: 7.4 days

Q5 H1/2: 6.1 days

Q95 H1/2: 9.5 days

reliable model fit: yes

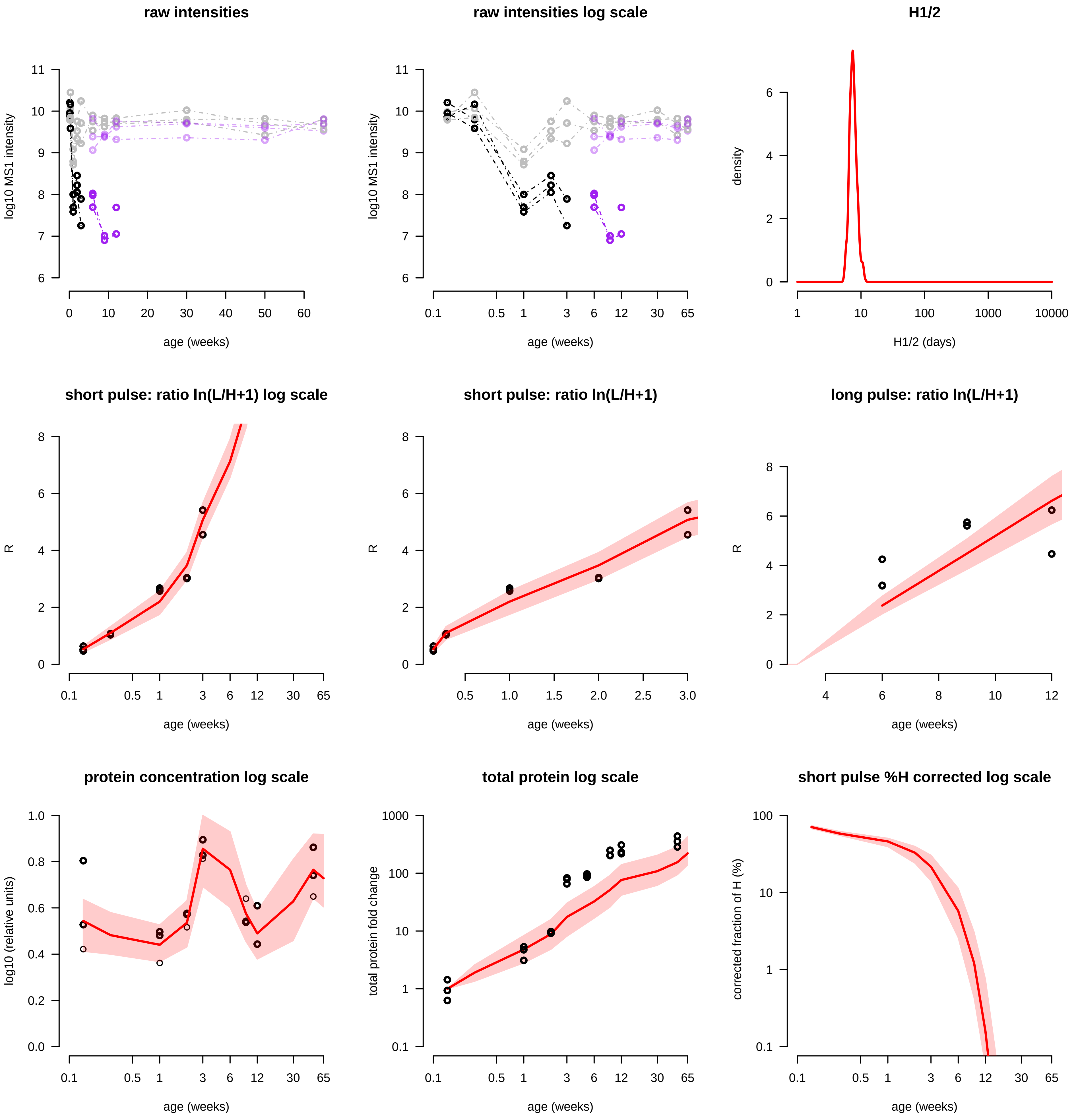

P63028

>sp|P63028|TCTP_MOUSE

Translationally−controlled tumor protein

GN=Tpt1

Med H1/2: 654.5 days

Q5 H1/2: 240.8 days

Q95 H1/2: 6415.5 days

reliable model fit: no

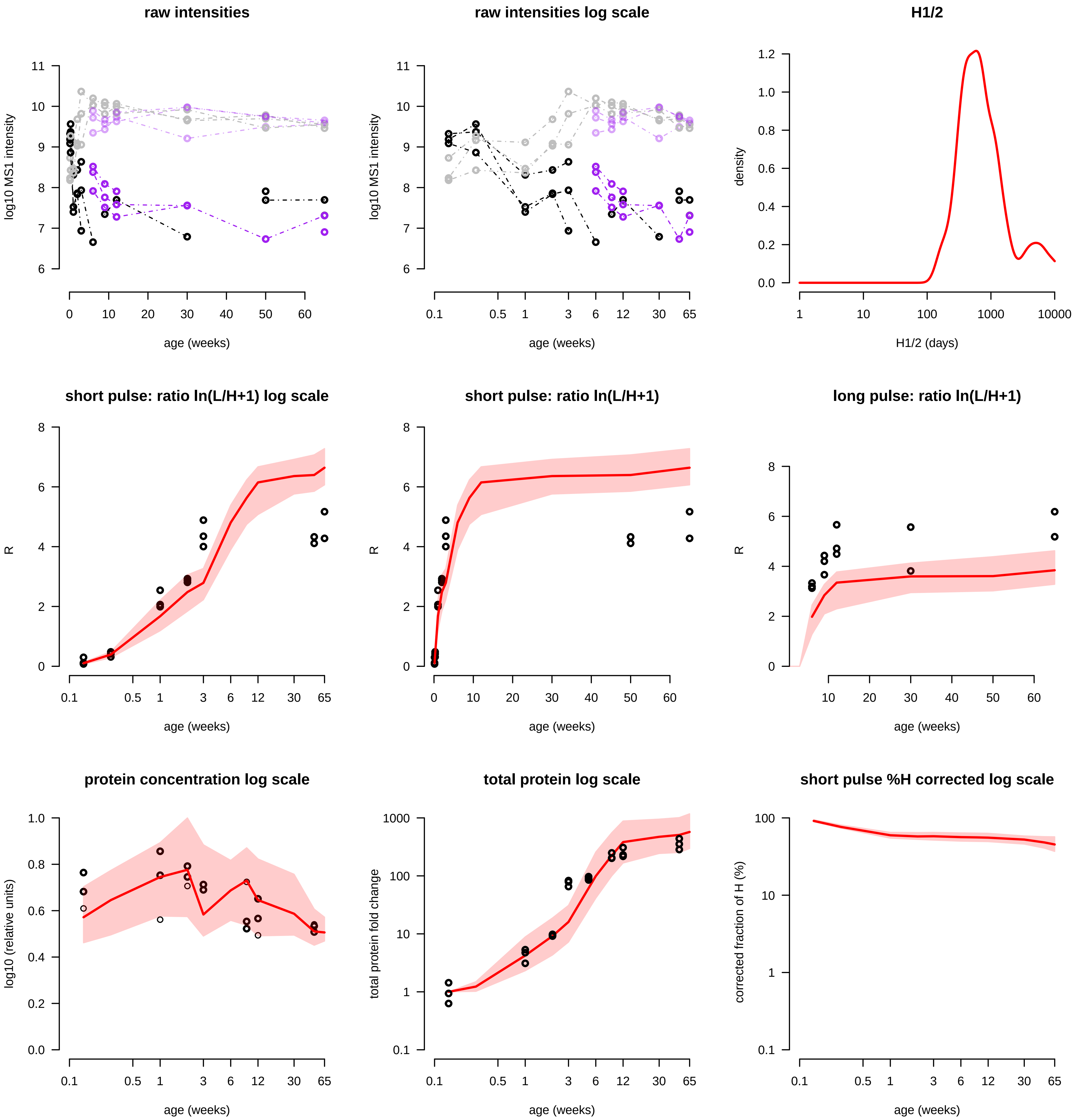

Q9CQV8;Q9CQV8−2

>sp|Q9CQV8|1433B_MOUSE

14−3−3 protein beta/alpha

GN=Ywhab

Med H1/2: 12.2 days

Q5 H1/2: 9.6 days

Q95 H1/2: 16.2 days

reliable model fit: yes

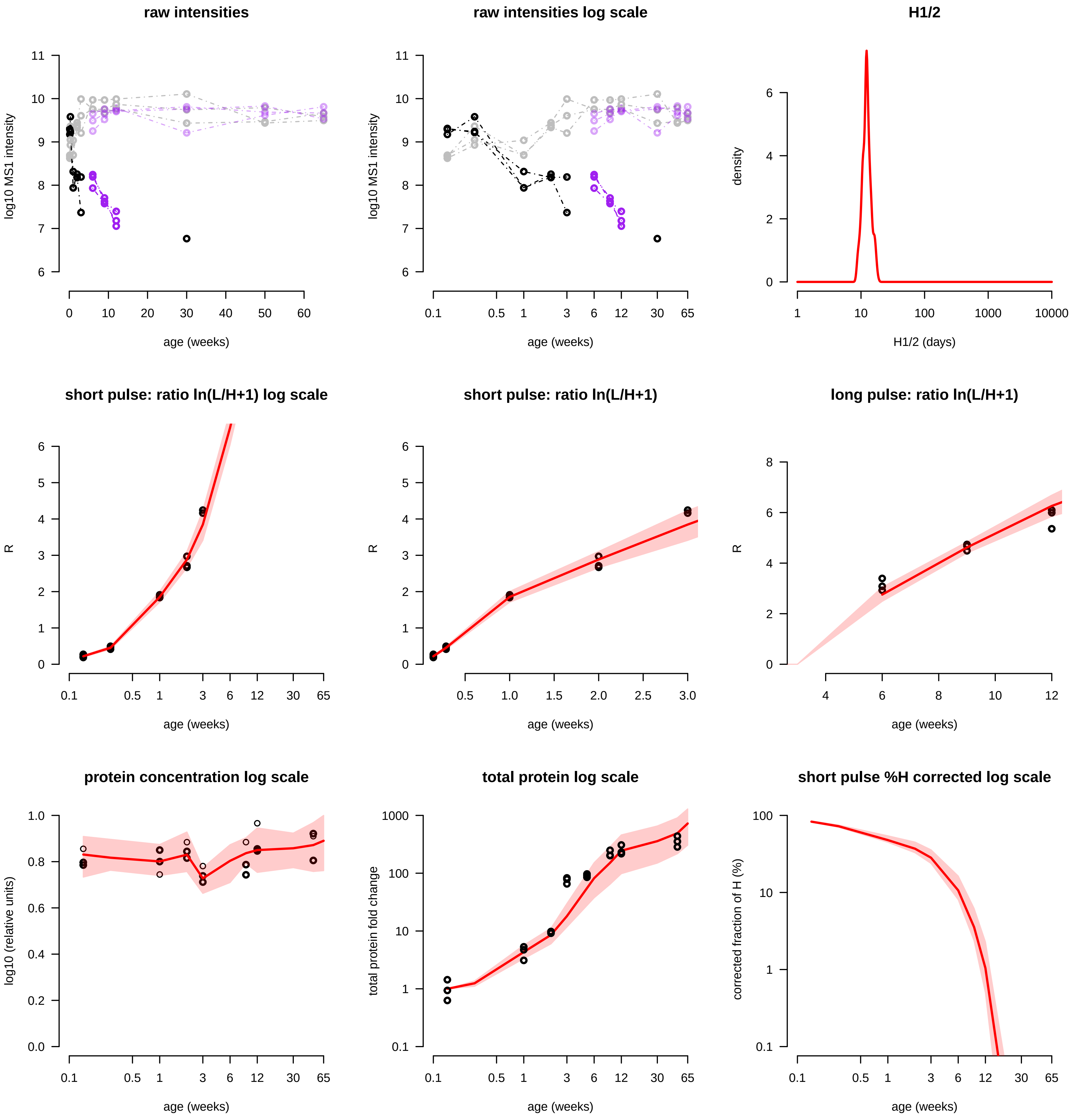

P97315

>sp|P97315|CSRP1_MOUSE

Cysteine and glycine−rich protein 1

GN=Csrp1

Med H1/2: 7.7 days

Q5 H1/2: 6.7 days

Q95 H1/2: 9.7 days

reliable model fit: yes

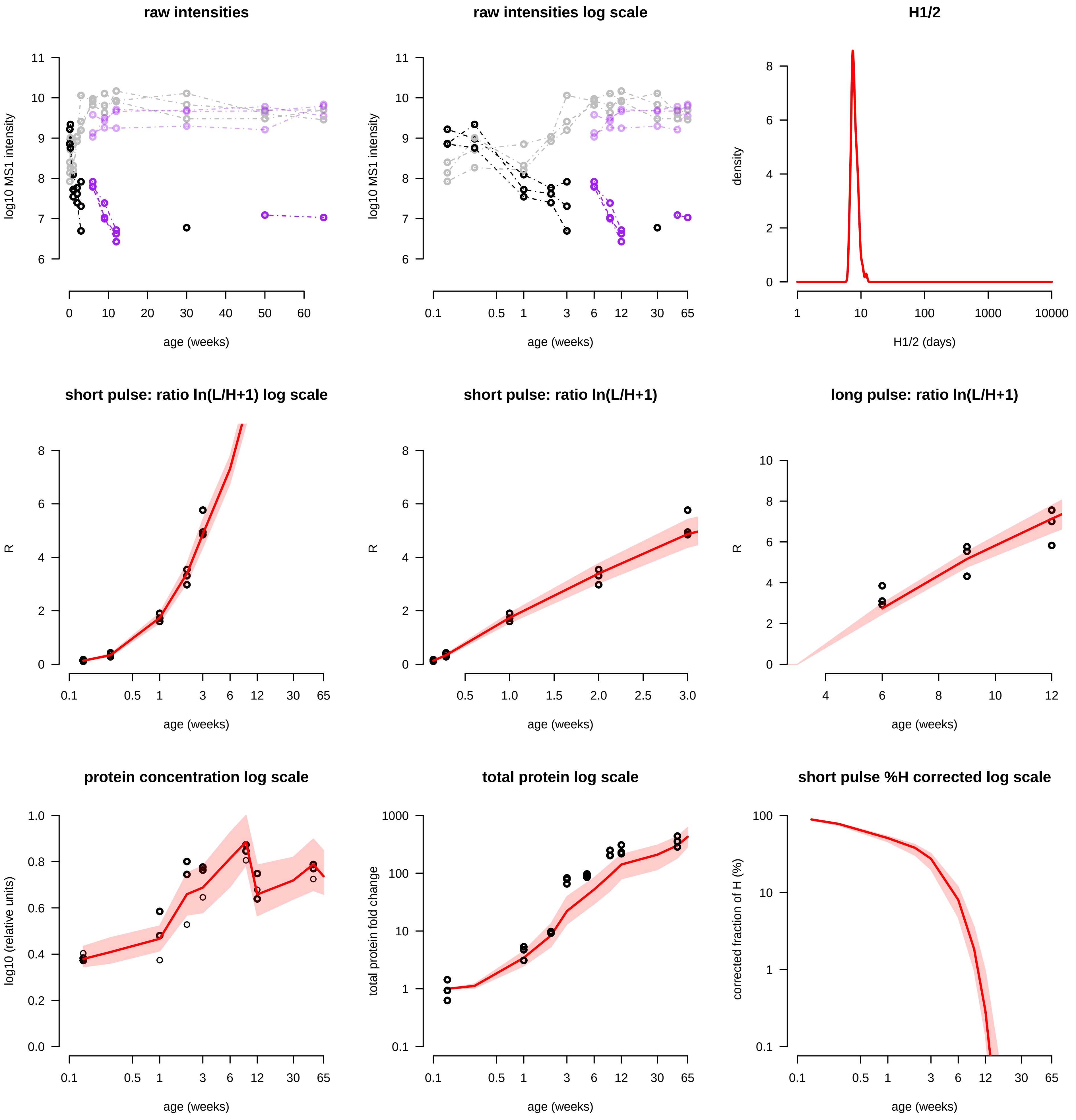

Q8BHN3−2

>sp|Q8BHN3−2|GANAB_MOUSE

Isoform 2 of Neutral alpha−glucosidase AB

GN=Ganab

Med H1/2: 43.7 days

Q5 H1/2: 31.7 days

Q95 H1/2: 76.2 days

reliable model fit: yes

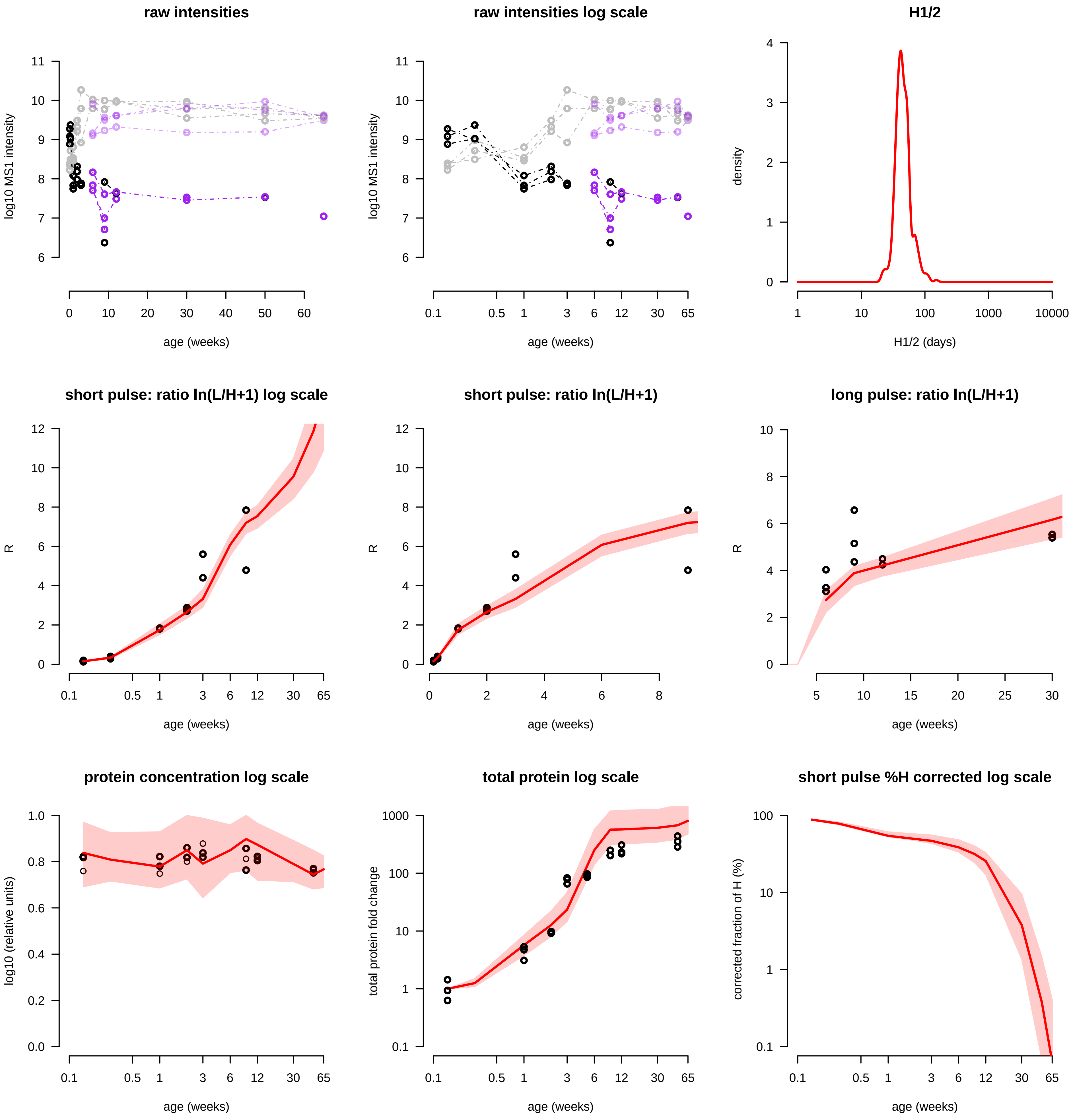

Q9CZ13

>sp|Q9CZ13|QCR1_MOUSE

Cytochrome b−c1 complex subunit 1, mitochondrial

GN=Uqcrc1

Med H1/2: 7.6 days

Q5 H1/2: 6.3 days

Q95 H1/2: 9.8 days

reliable model fit: yes

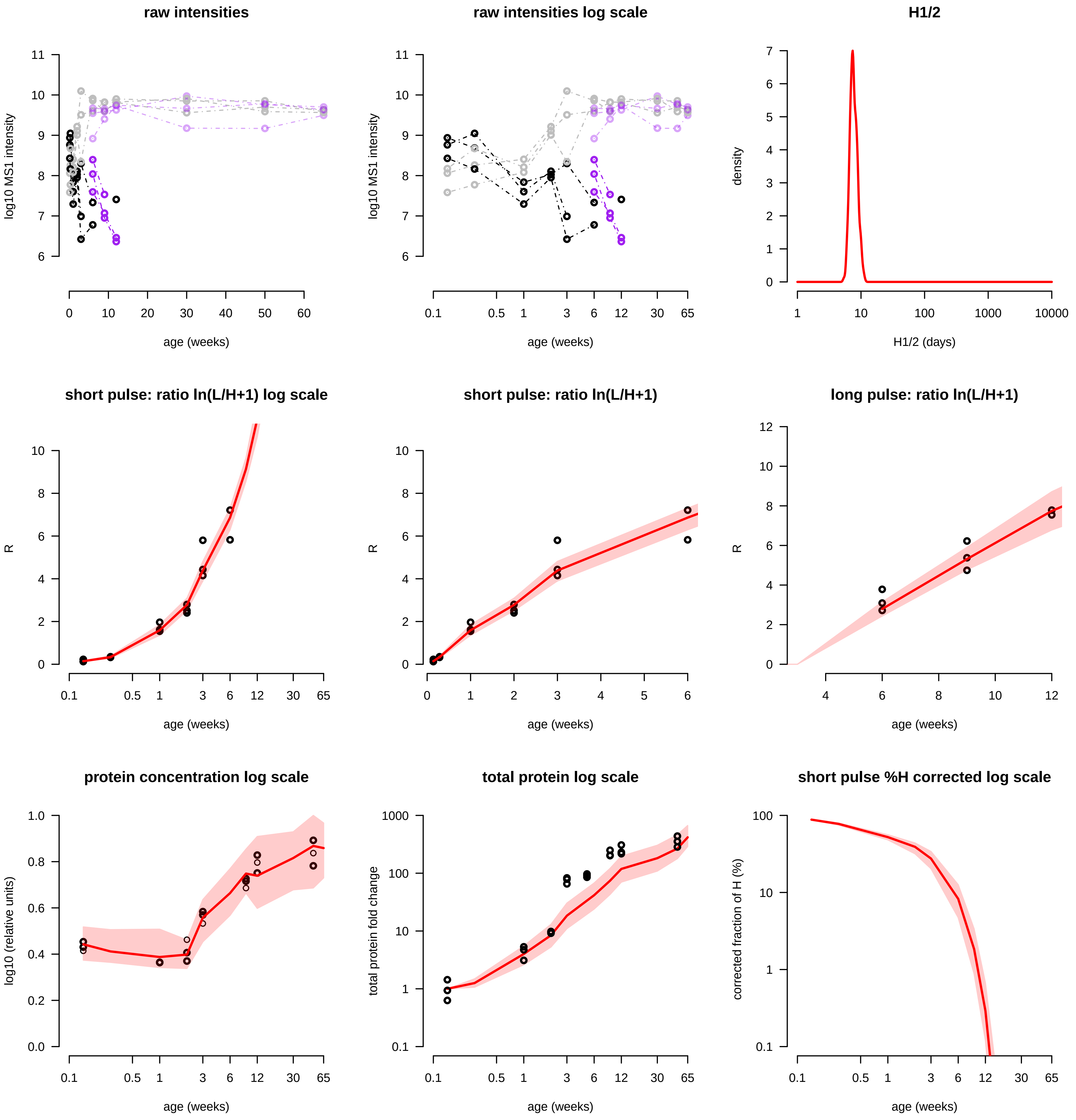

Q9JHI5

>sp|Q9JHI5|IVD_MOUSE

Isovaleryl−CoA dehydrogenase, mitochondrial

GN=Ivd

Med H1/2: 10 days

Q5 H1/2: 8.1 days

Q95 H1/2: 14.9 days

reliable model fit: yes

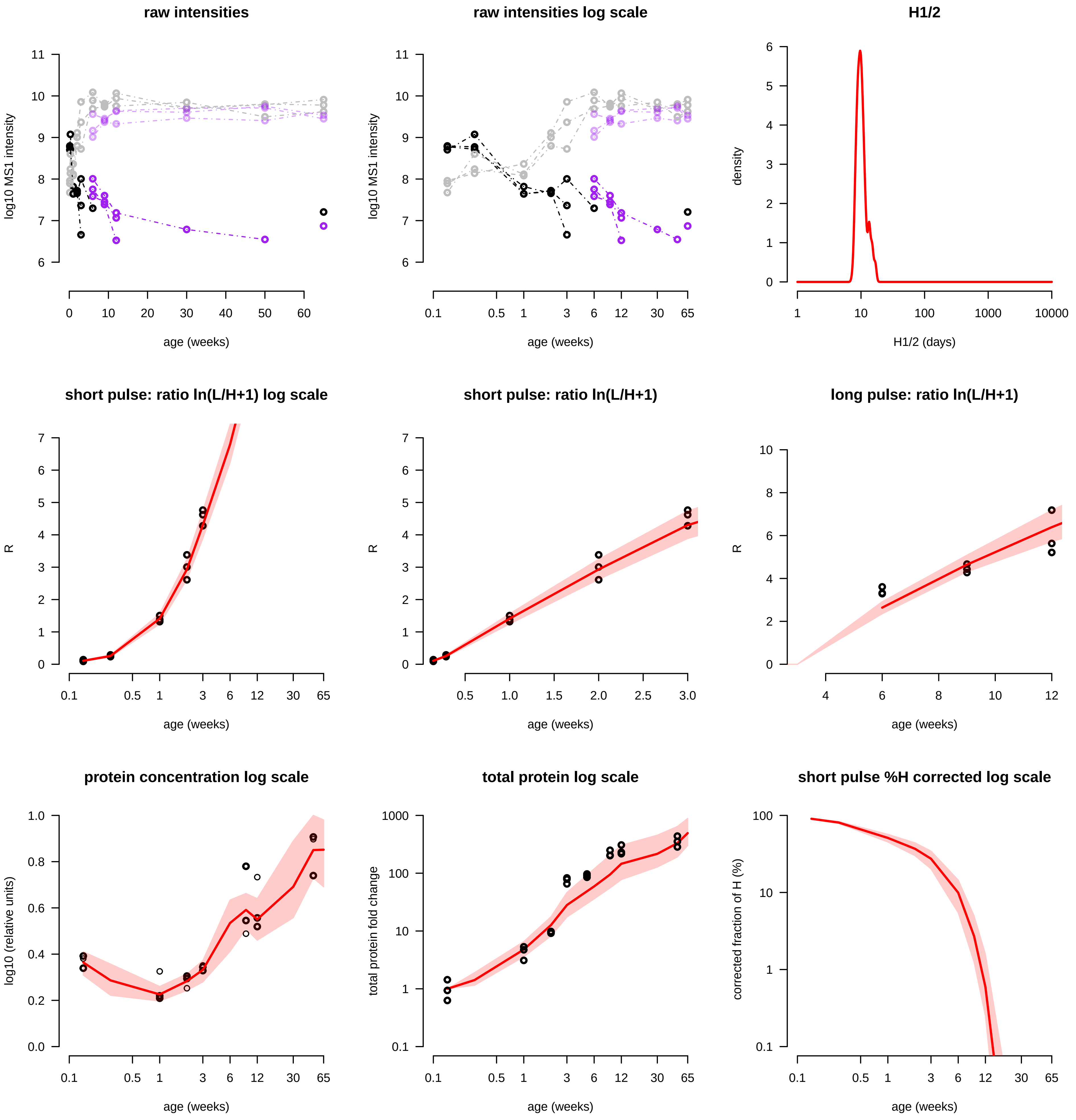

Q9CXW4

>sp|Q9CXW4|RL11_MOUSE

60S ribosomal protein L11

GN=Rpl11

Med H1/2: 13.5 days

Q5 H1/2: 10.7 days

Q95 H1/2: 18.4 days

reliable model fit: yes

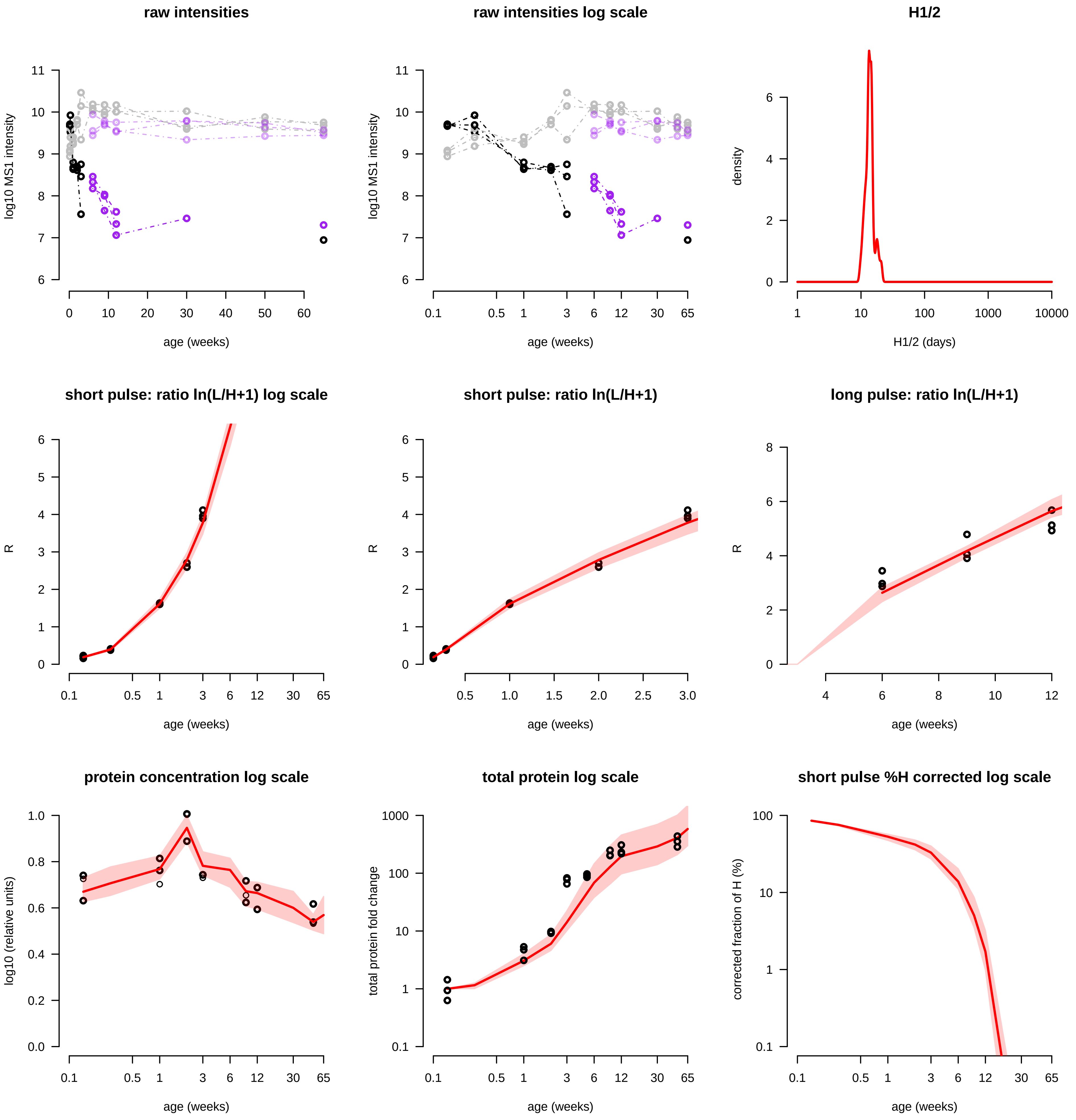

O35887

>sp|O35887|CALU_MOUSE

Calumenin

GN=Calu

Med H1/2: 7.2 days

Q5 H1/2: 6.3 days

Q95 H1/2: 8.7 days

reliable model fit: yes

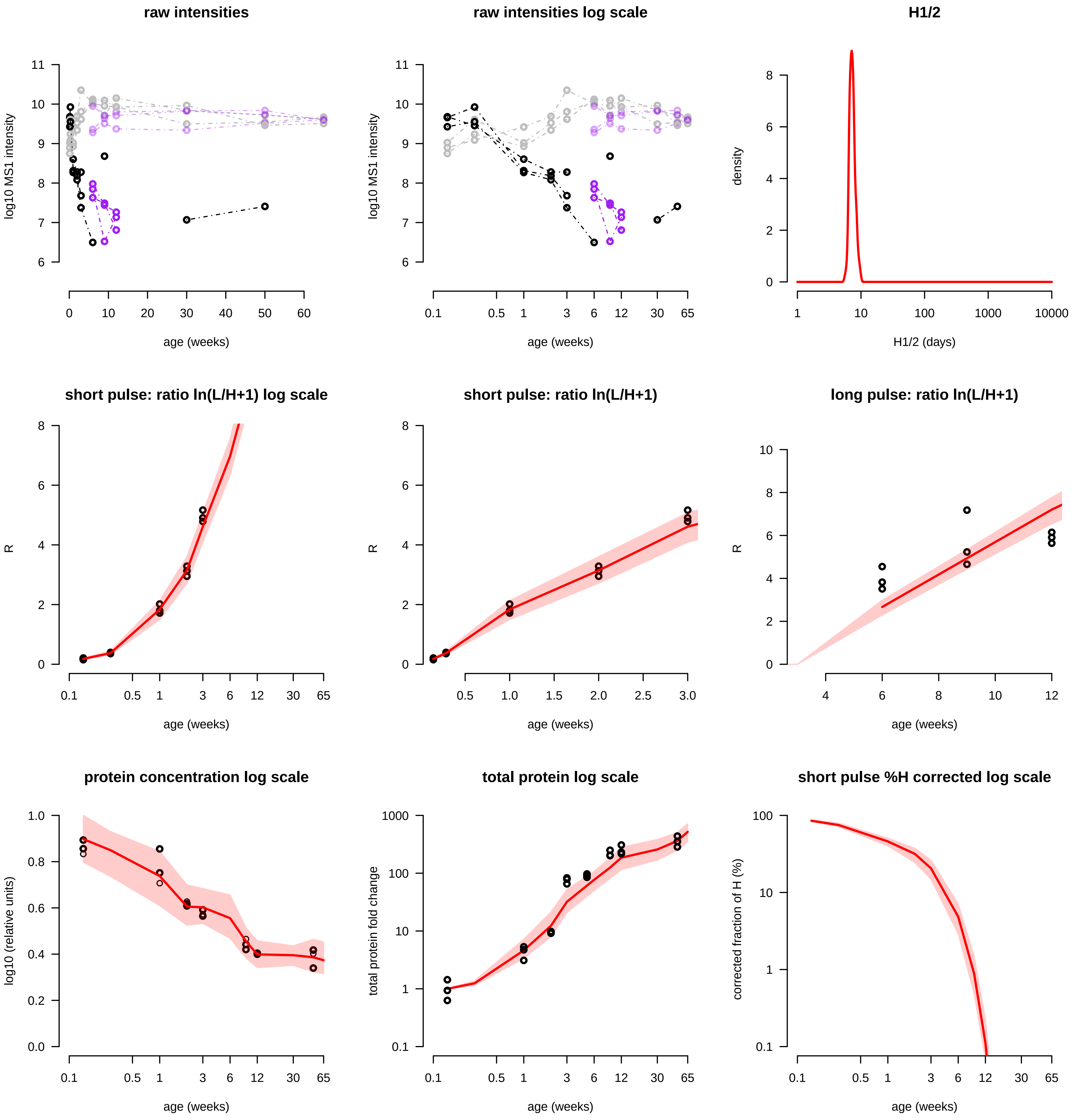

Q9Z2I8

>sp|Q9Z2I8|SUCB2_MOUSE

Succinate−−CoA ligase [GDP−forming] subunit beta, mitochondrial

GN=Suclg2

Med H1/2: 34.4 days

Q5 H1/2: 24.1 days

Q95 H1/2: 54.9 days

reliable model fit: yes

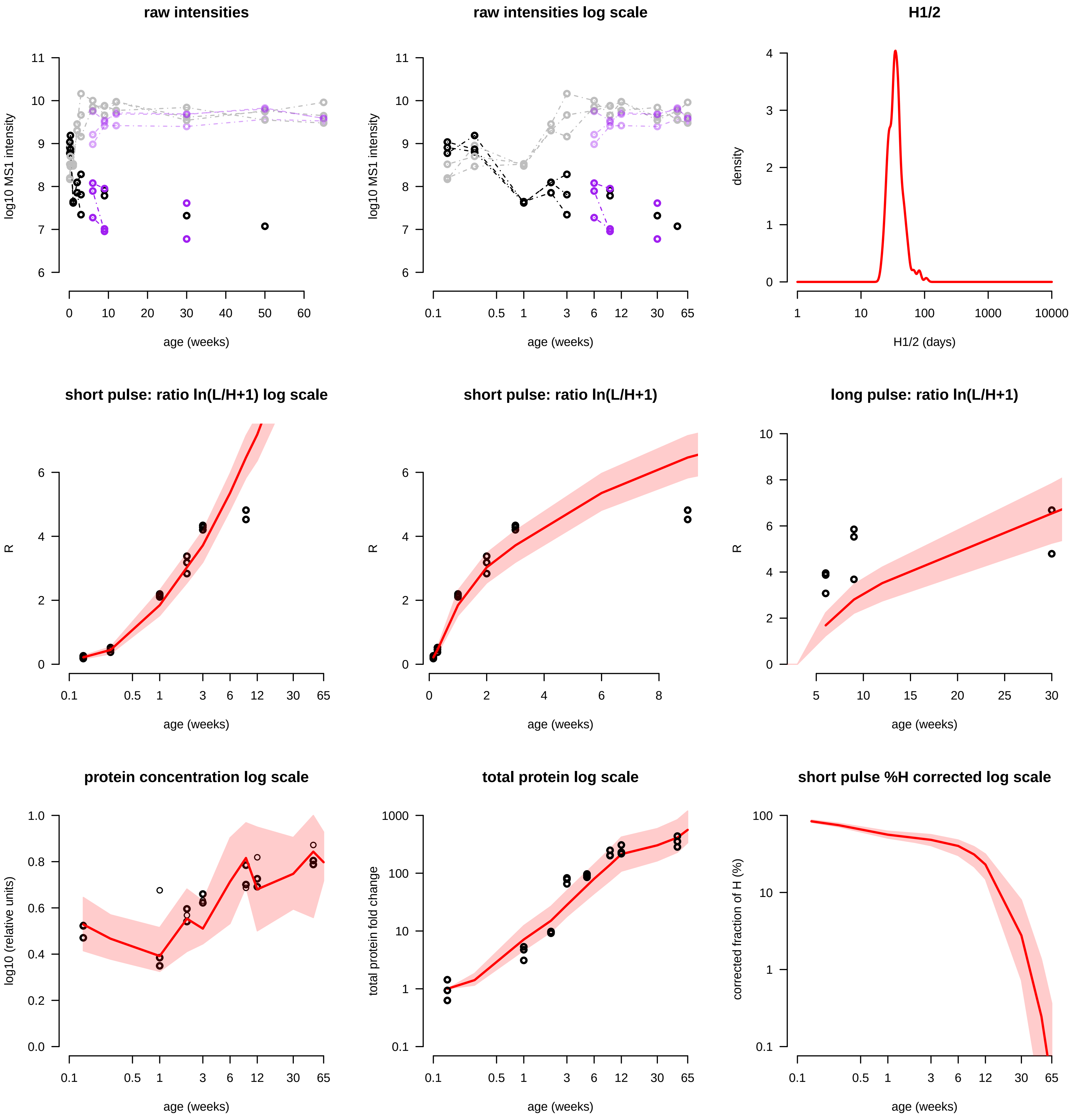

O35658

>sp|O35658|C1QBP_MOUSE

Complement component 1 Q subcomponent−binding protein, mitochondrial

GN=C1qbp

Med H1/2: 7 days

Q5 H1/2: 4.2 days

Q95 H1/2: 10.5 days

reliable model fit: yes

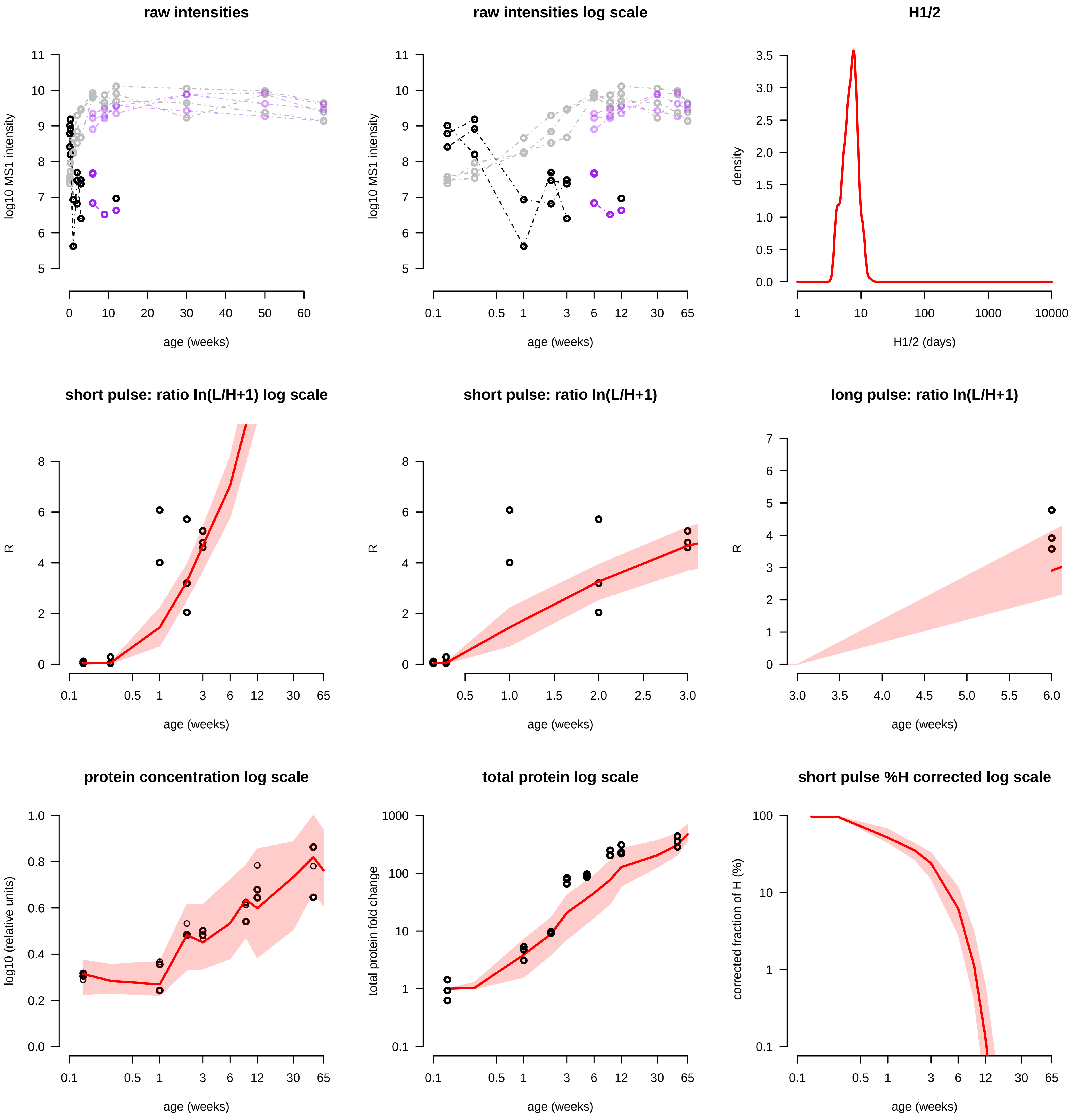

Q9CQN1

>sp|Q9CQN1|TRAP1_MOUSE

Heat shock protein 75 kDa, mitochondrial

GN=Trap1

Med H1/2: 6.7 days

Q5 H1/2: 5 days

Q95 H1/2: 9.6 days

reliable model fit: yes

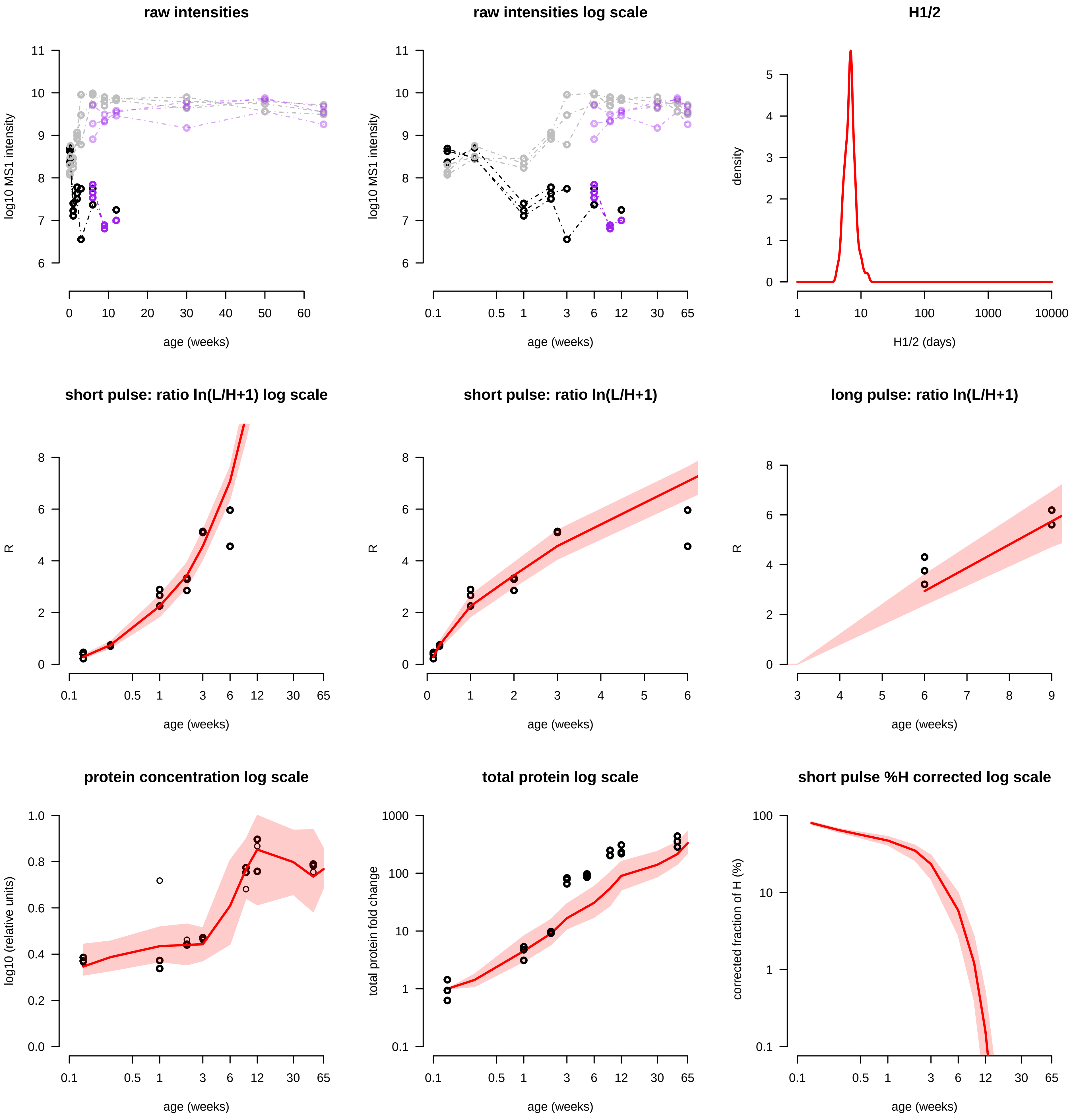

Q3TEA8;Q3TEA8−3

>sp|Q3TEA8|HP1B3_MOUSE

Heterochromatin protein 1−binding protein 3

GN=Hp1bp3

Med H1/2: 12.2 days

Q5 H1/2: 9.4 days

Q95 H1/2: 16.3 days

reliable model fit: yes

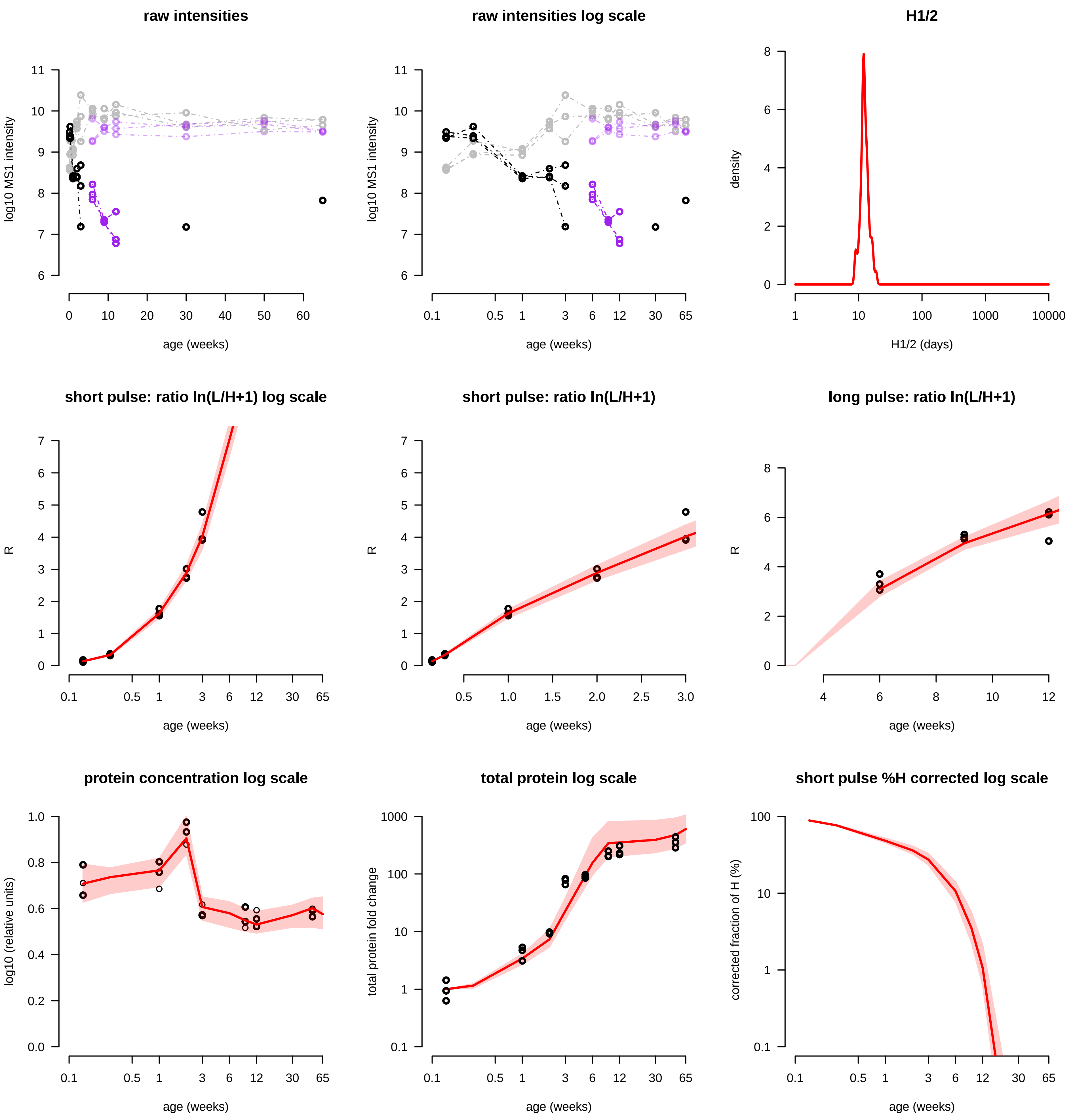

P68254;P68254−2

>sp|P68254|1433T_MOUSE

14−3−3 protein theta

GN=Ywhaq

Med H1/2: 8.7 days

Q5 H1/2: 7.3 days

Q95 H1/2: 10.2 days

reliable model fit: yes

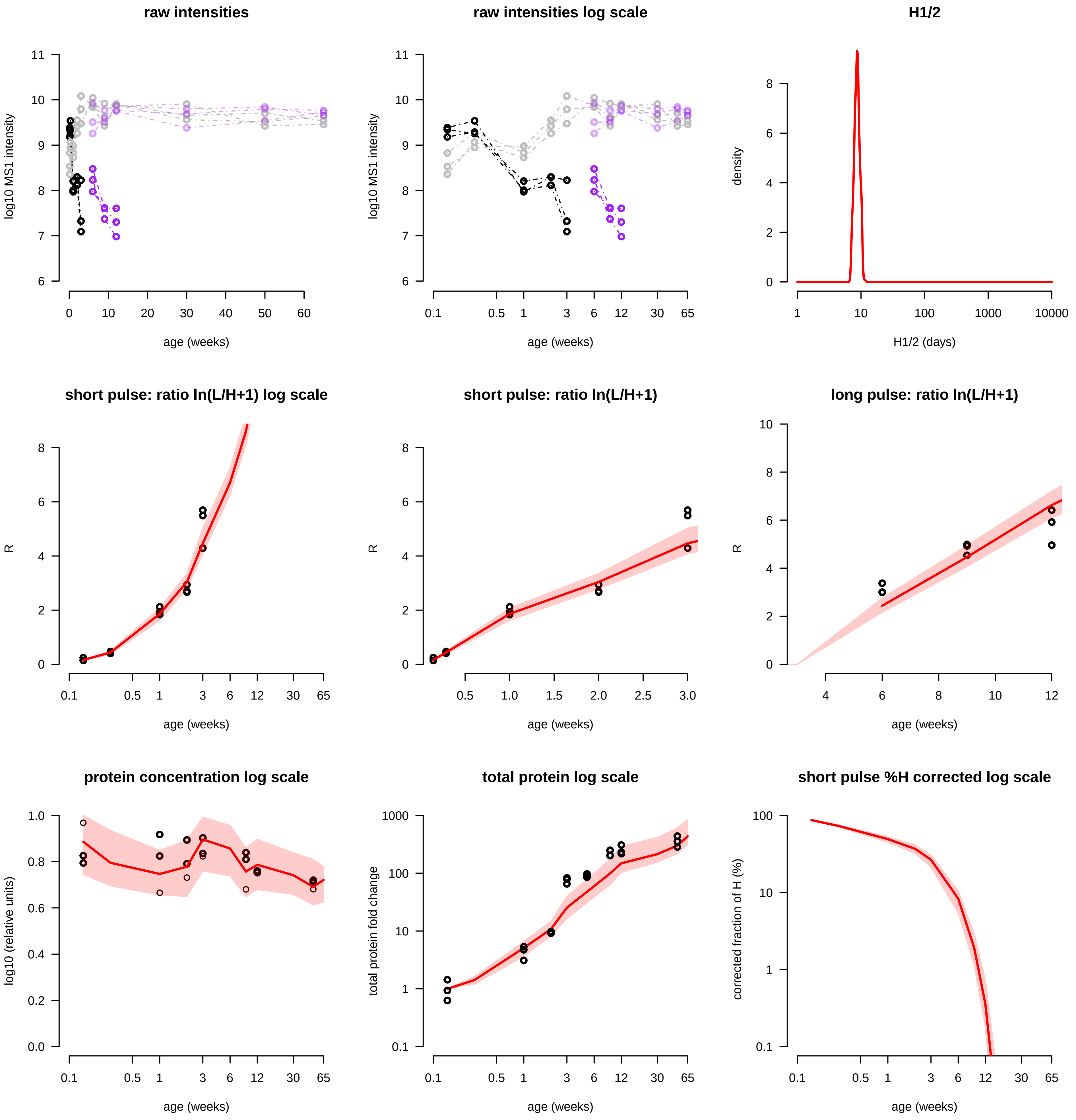

Q8VDJ3

>sp|Q8VDJ3|VIGLN_MOUSE

Vigilin

GN=Hdlbp

Med H1/2: 125.6 days

Q5 H1/2: 49.6 days

Q95 H1/2: 1158.7 days

reliable model fit: no

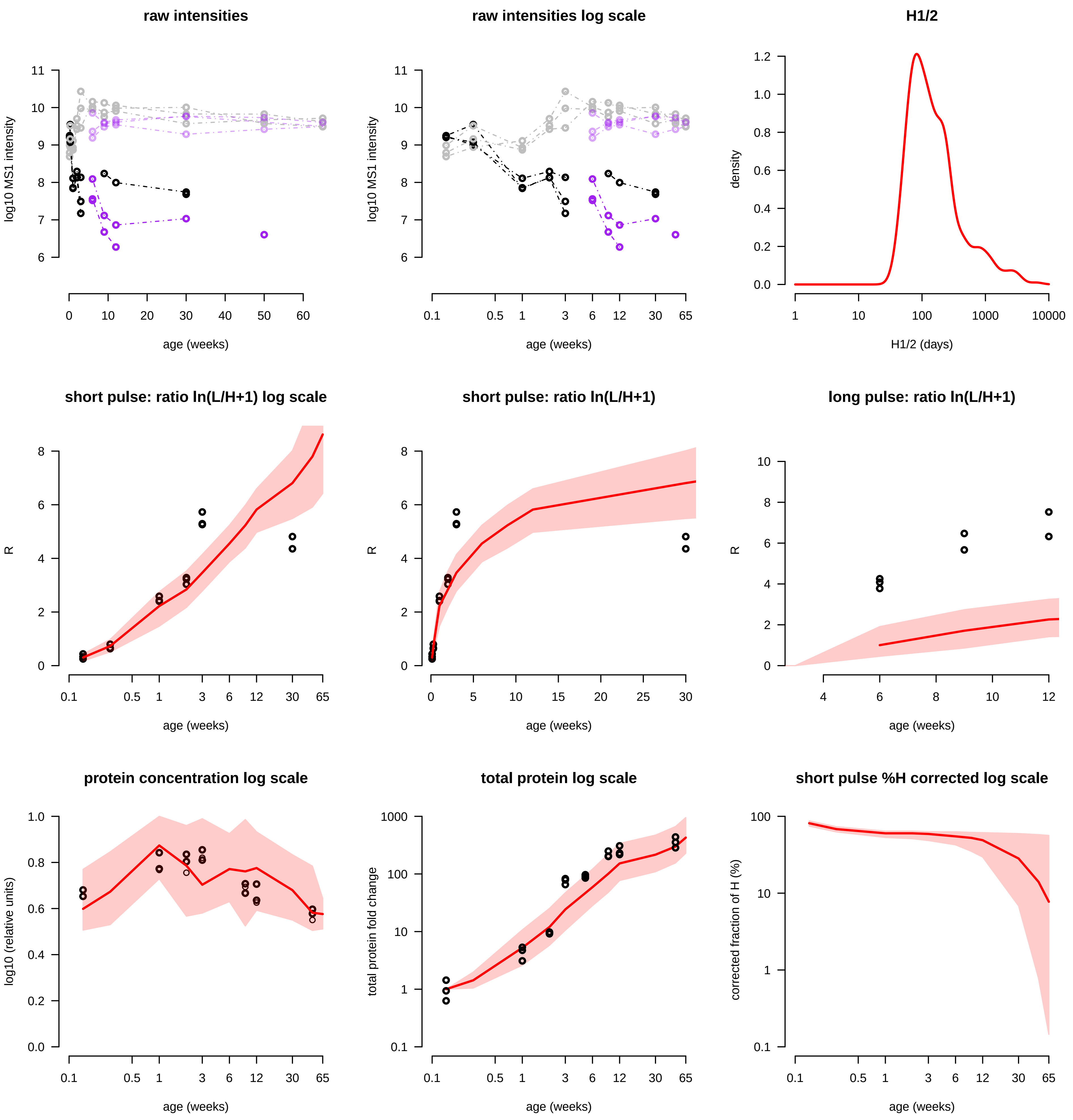

P29758

>sp|P29758|OAT_MOUSE

Ornithine aminotransferase, mitochondrial

GN=Oat

Med H1/2: 5.7 days

Q5 H1/2: 4.7 days

Q95 H1/2: 6.9 days

reliable model fit: yes

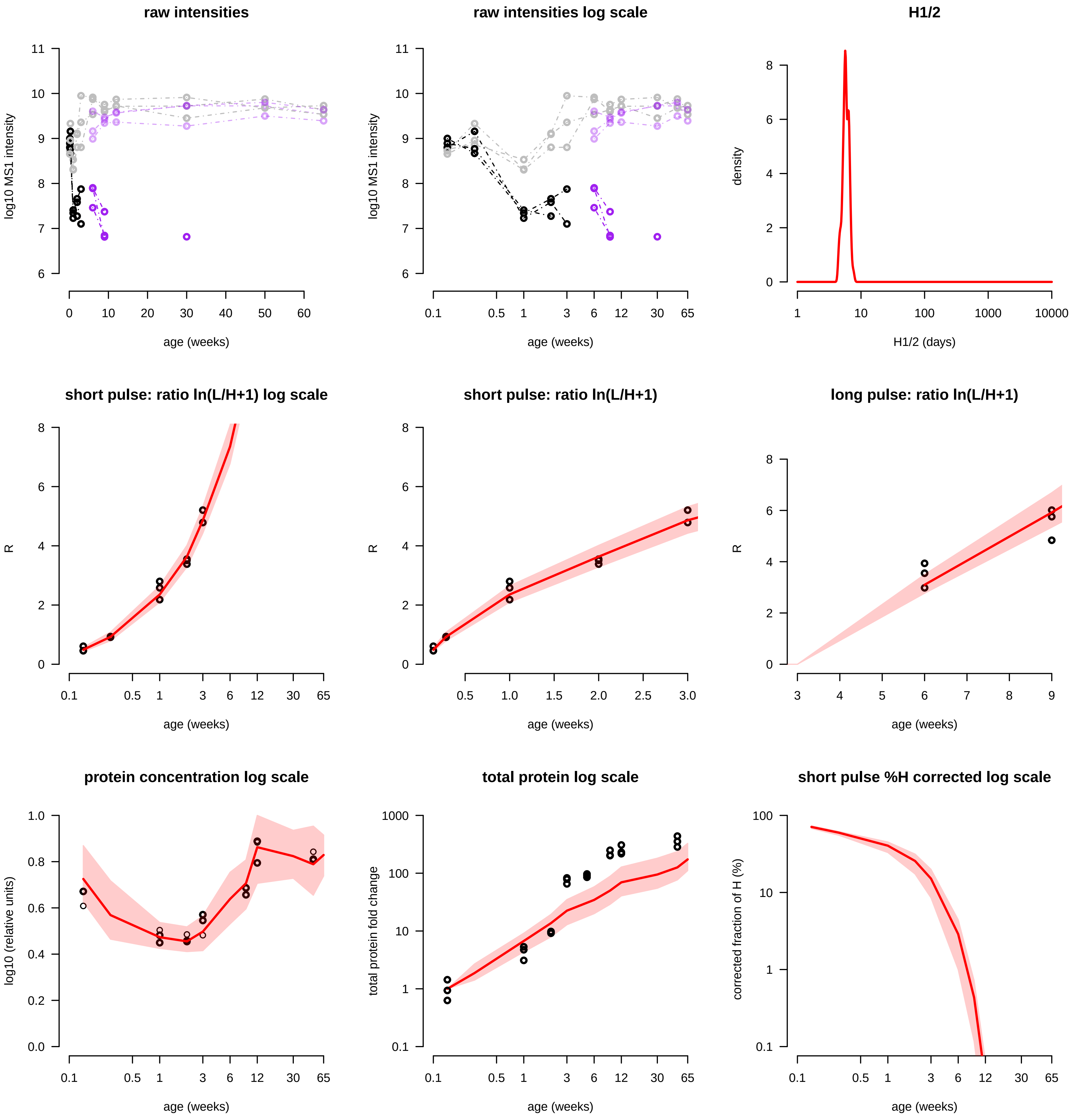

P62242

>sp|P62242|RS8_MOUSE

40S ribosomal protein S8

GN=Rps8

Med H1/2: 13.1 days

Q5 H1/2: 10.5 days

Q95 H1/2: 16.6 days

reliable model fit: yes

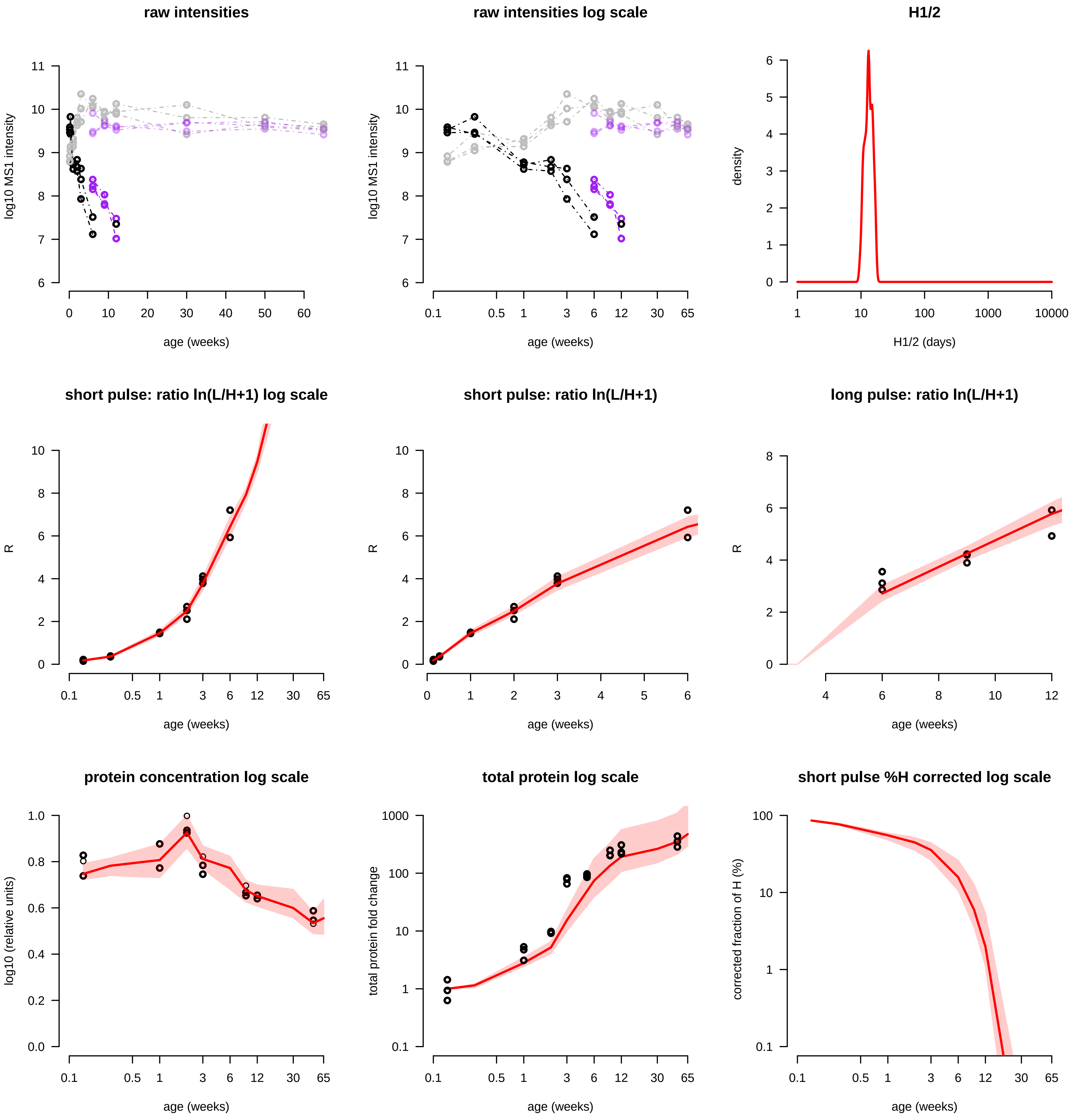

Q9Z1N5

>sp|Q9Z1N5|DX39B_MOUSE

Spliceosome RNA helicase Ddx39b

GN=Ddx39b

Med H1/2: 13.7 days

Q5 H1/2: 10 days

Q95 H1/2: 19.4 days

reliable model fit: yes

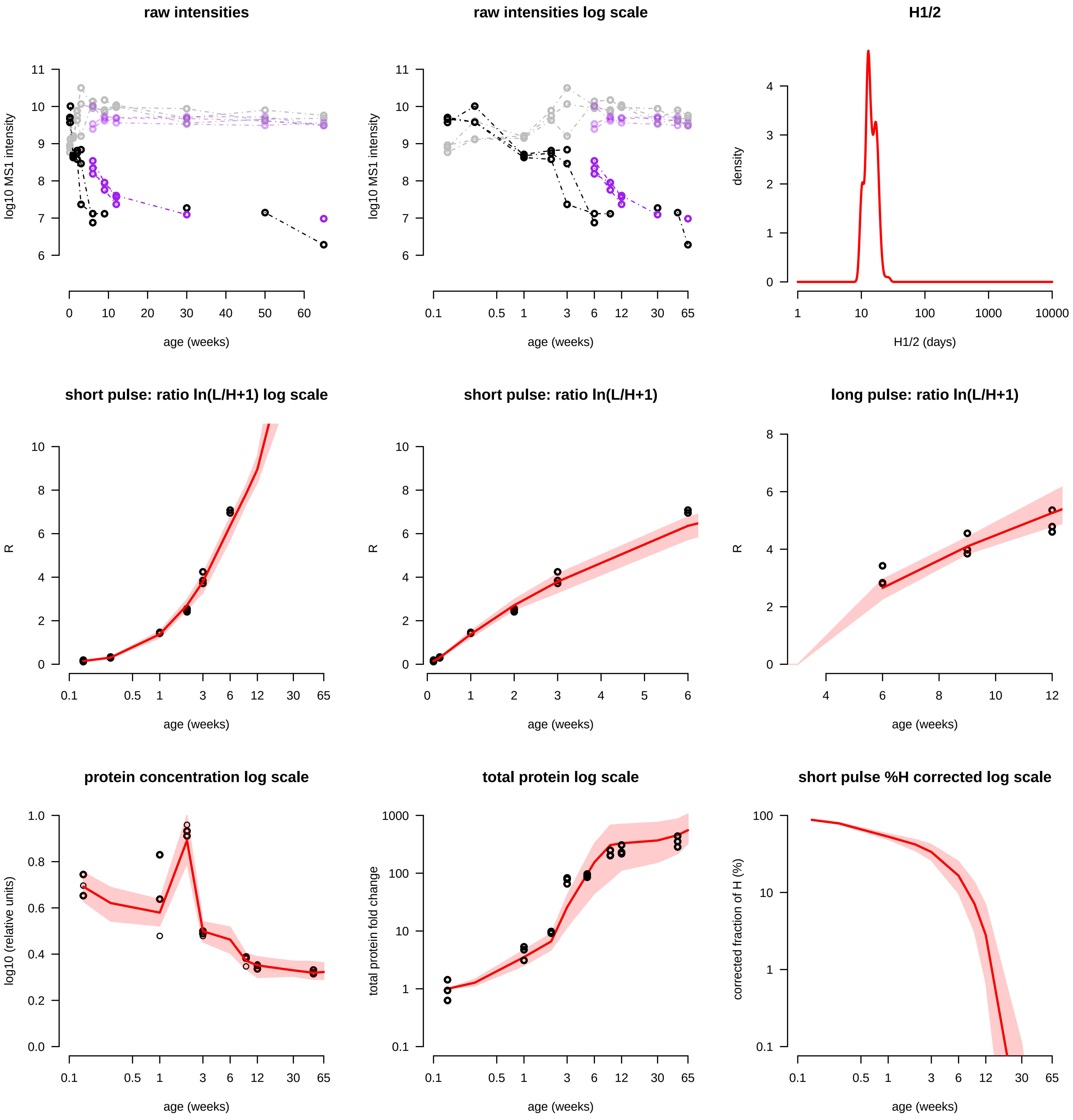

Q8CGC7

>sp|Q8CGC7|SYEP_MOUSE

Bifunctional glutamate/proline−−tRNA ligase

GN=Eprs1

Med H1/2: 4.6 days

Q5 H1/2: 4.1 days

Q95 H1/2: 5.4 days

reliable model fit: yes

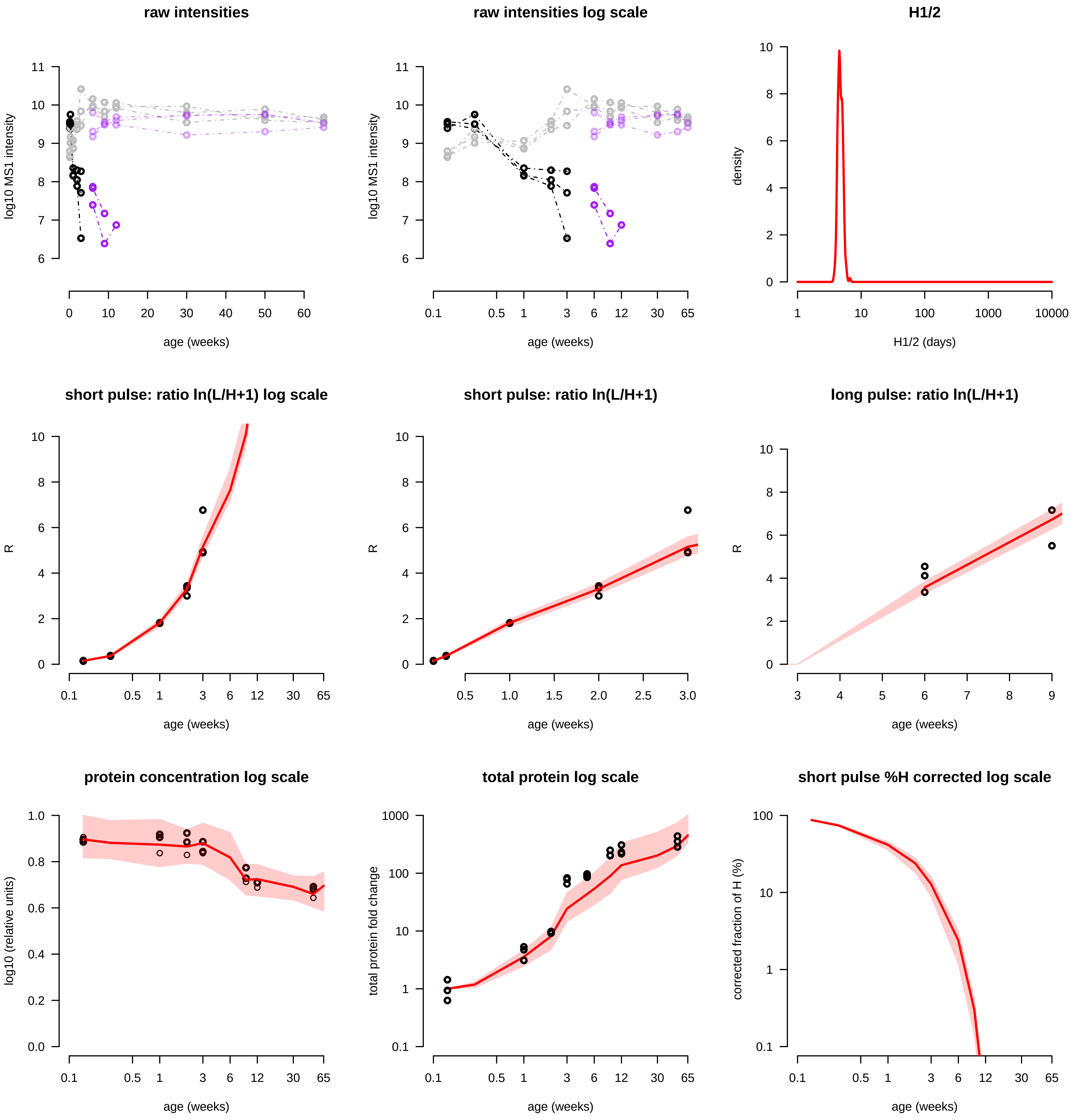

Q9JLJ2

>sp|Q9JLJ2|AL9A1_MOUSE

4−trimethylaminobutyraldehyde dehydrogenase

GN=Aldh9a1

Med H1/2: 10.8 days

Q5 H1/2: 8.9 days

Q95 H1/2: 13.1 days

reliable model fit: yes

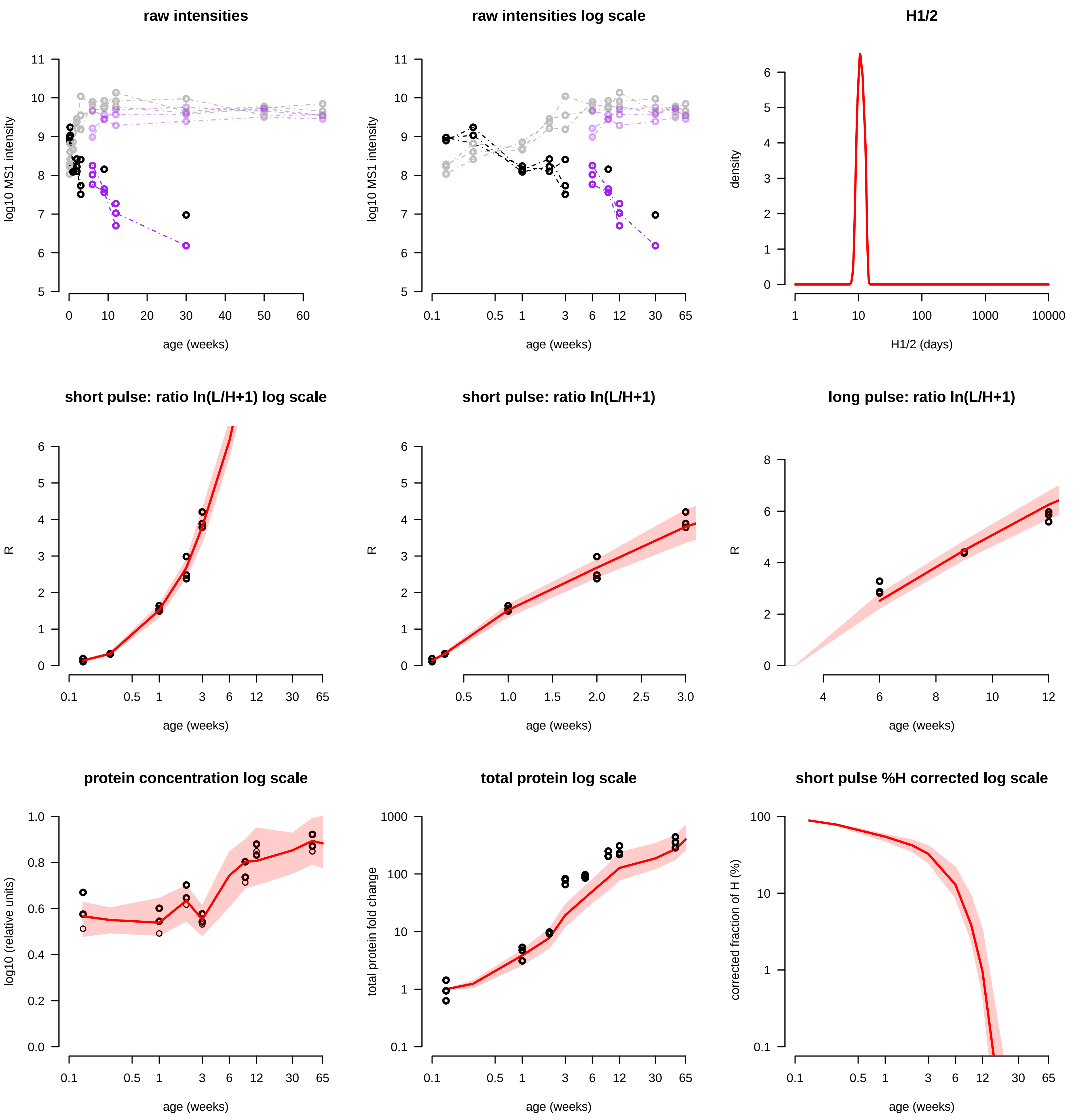

P14206

>sp|P14206|RSSA_MOUSE

40S ribosomal protein SA

GN=Rpsa

Med H1/2: 257.2 days

Q5 H1/2: 119.9 days

Q95 H1/2: 2479.4 days

reliable model fit: yes

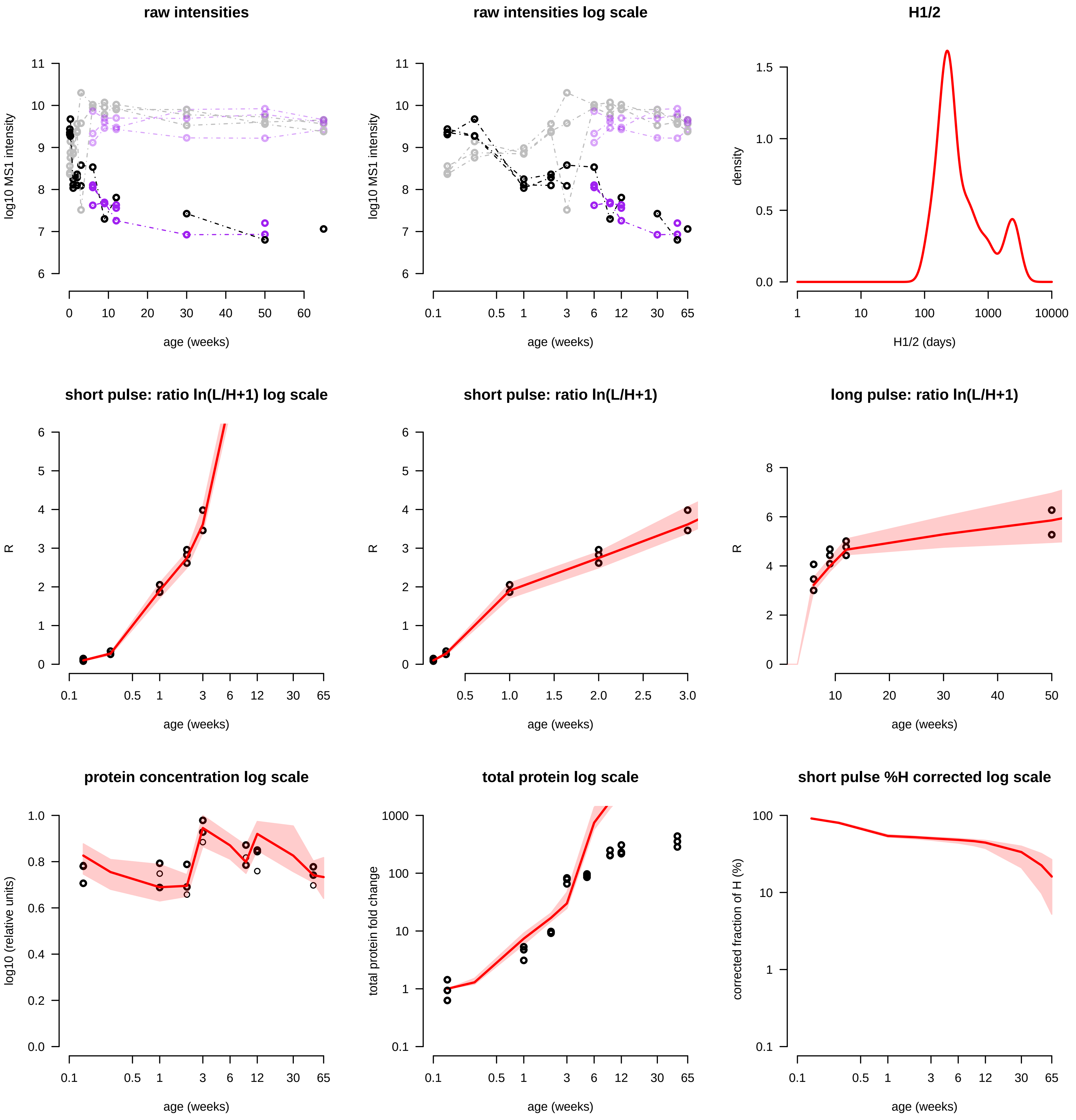

Q06185

>sp|Q06185|ATP5I_MOUSE

ATP synthase subunit e, mitochondrial

GN=Atp5me

Med H1/2: 13.6 days

Q5 H1/2: 10.8 days

Q95 H1/2: 18.1 days

reliable model fit: yes

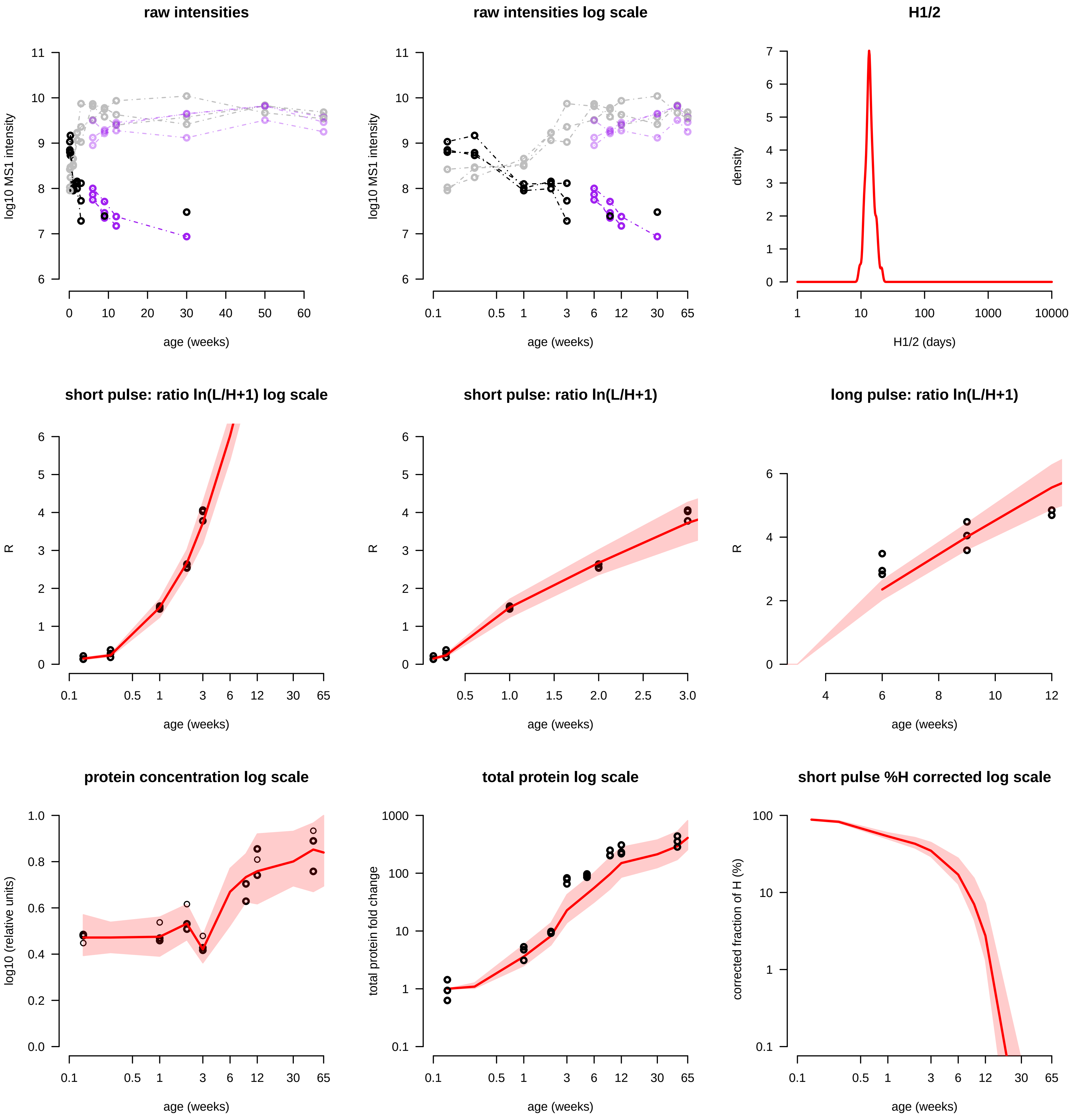

Q9WTP6−2;Q9WTP6

>sp|Q9WTP6−2|KAD2_MOUSE

Isoform 2 of Adenylate kinase 2, mitochondrial

GN=Ak2

Med H1/2: 62.5 days

Q5 H1/2: 30.8 days

Q95 H1/2: 185 days

reliable model fit: yes

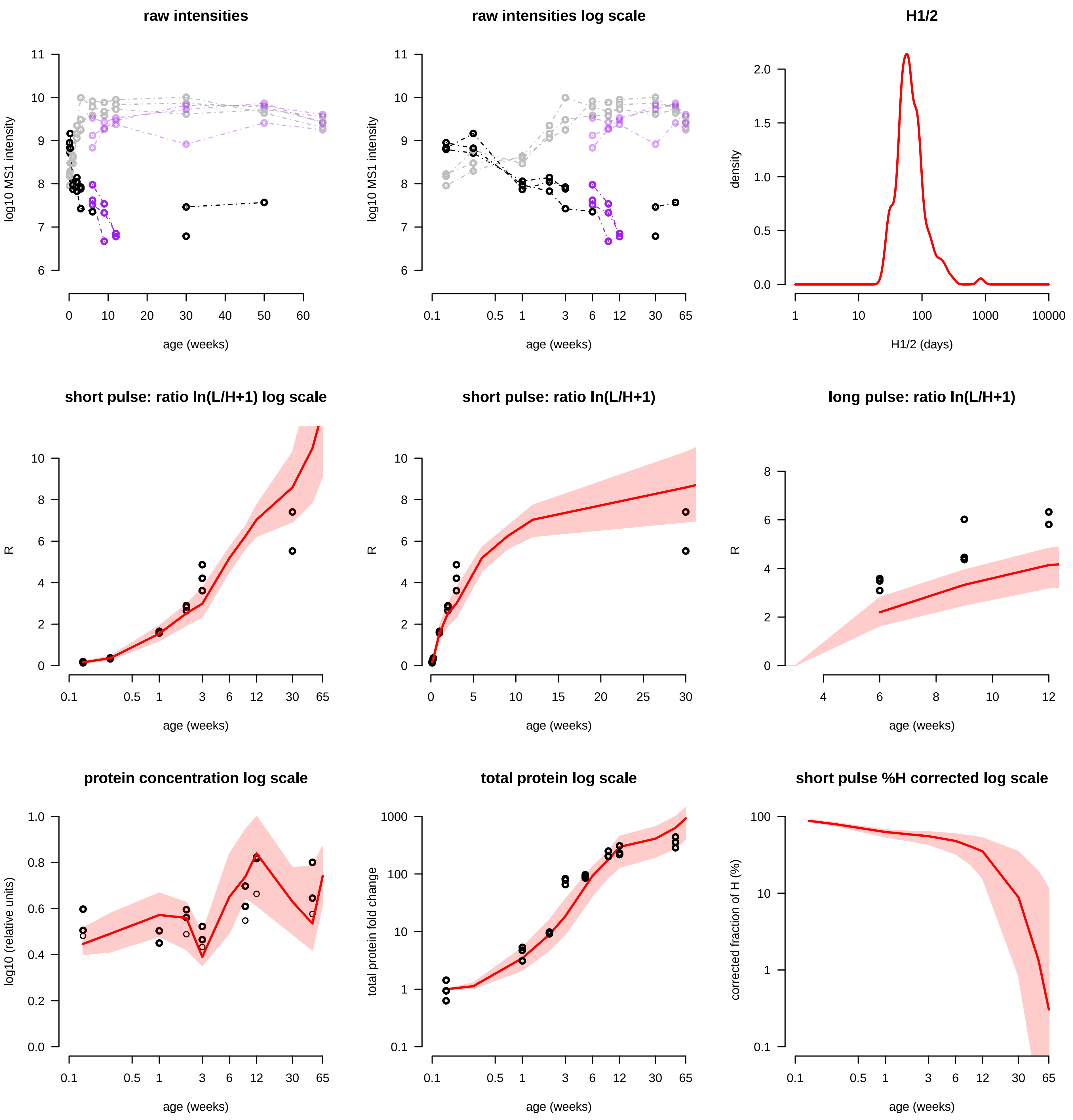

P37040

>sp|P37040|NCPR_MOUSE

NADPH−−cytochrome P450 reductase

GN=Por

Med H1/2: 6.3 days

Q5 H1/2: 4.8 days

Q95 H1/2: 8.3 days

reliable model fit: yes

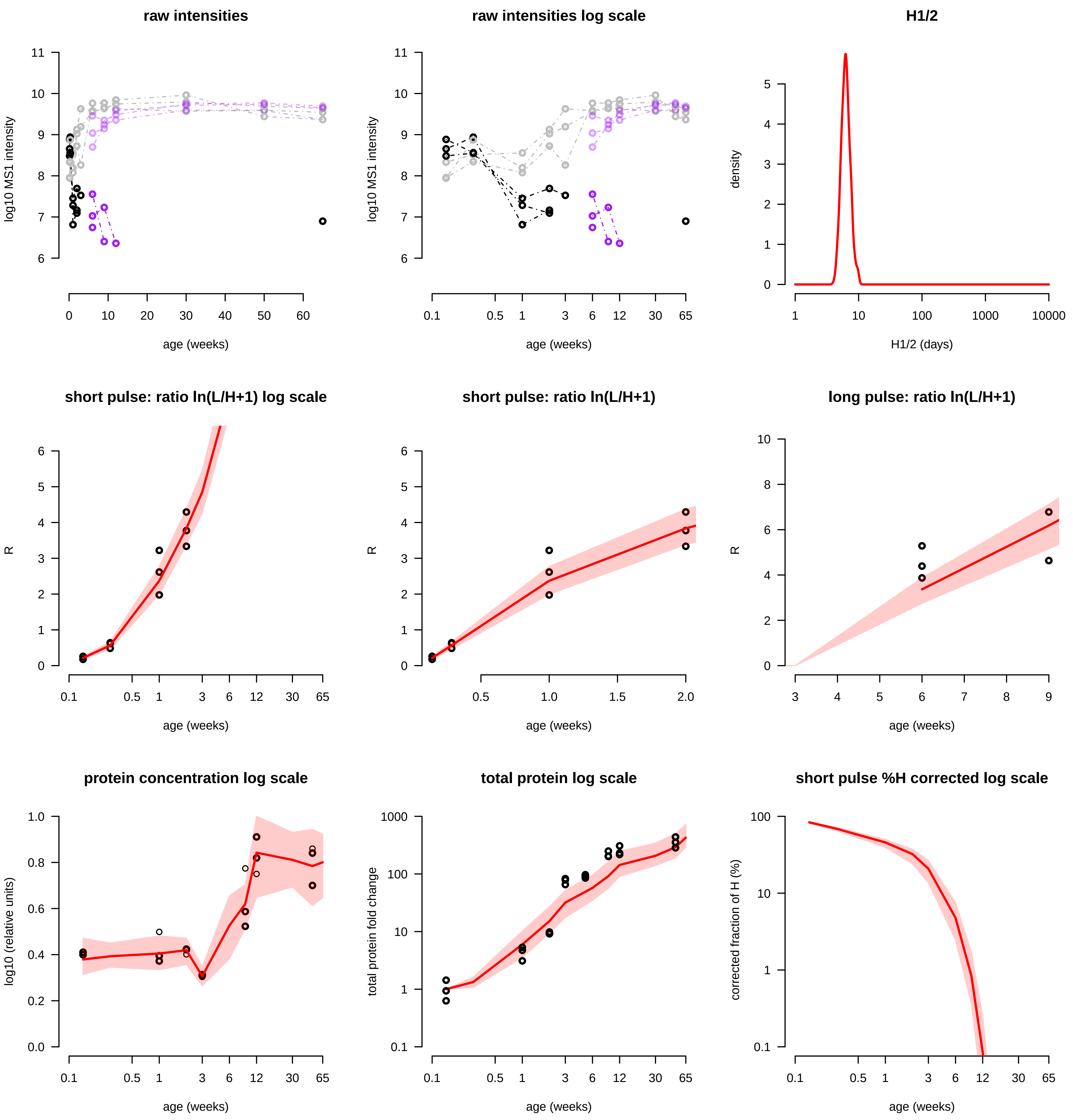

P46638

>sp|P46638|RB11B_MOUSE

Ras−related protein Rab−11B

GN=Rab11b

Med H1/2: 12.9 days

Q5   H1/2: 10 days

Q95 H1/2: 20 days

reliable model fit: yes

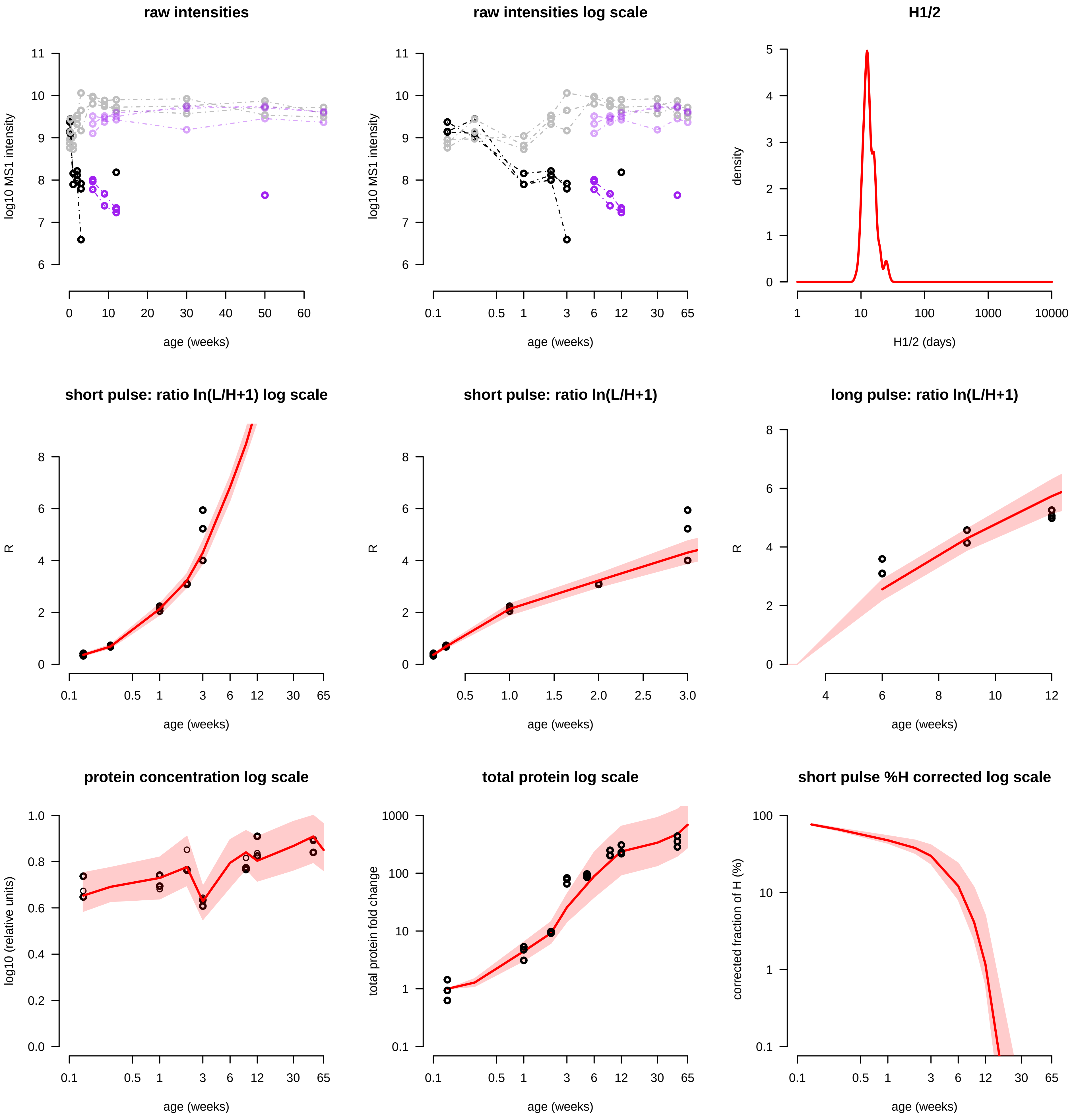

A6H584

>sp|A6H584|CO6A5_MOUSE

Collagen alpha−5(VI) chain

GN=Col6a5

Med H1/2: 569.3 days

Q5 H1/2: 166.9 days

Q95 H1/2: 8091.5 days

reliable model fit: no

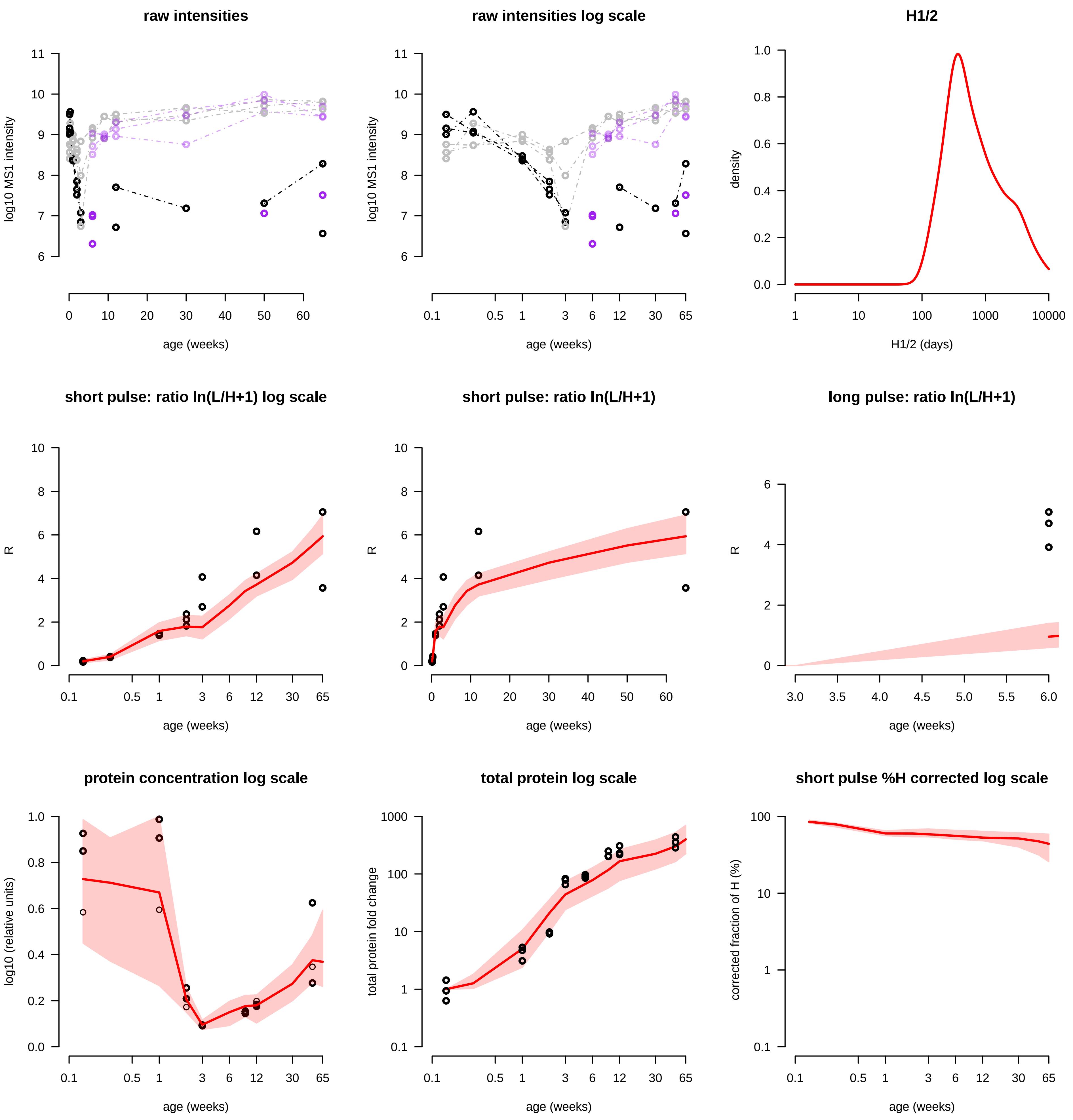

Q9CQA3

>sp|Q9CQA3|SDHB_MOUSE

Succinate dehydrogenase [ubiquinone] iron−sulfur subunit, mitochondrial

GN=Sdhb

Med H1/2: 10.3 days

Q5 H1/2: 8.3 days

Q95 H1/2: 13.3 days

reliable model fit: yes

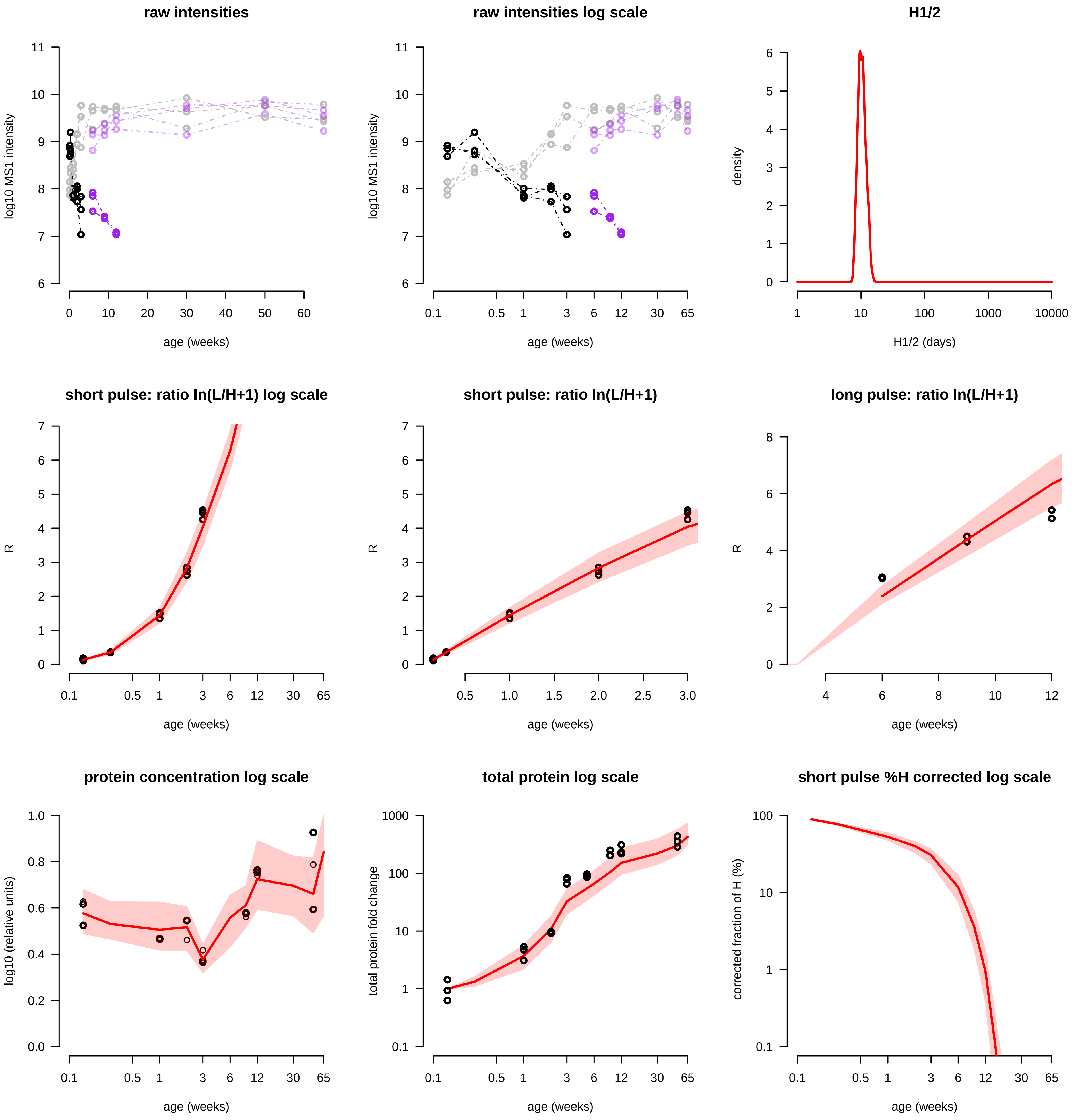

Q9Z2I9

>sp|Q9Z2I9|SUCB1_MOUSE

Succinate−−CoA ligase [ADP−forming] subunit beta, mitochondrial

GN=Sucla2

Med H1/2: 487 days

Q5 H1/2: 135.8 days

Q95 H1/2: 3596.2 days

reliable model fit: yes

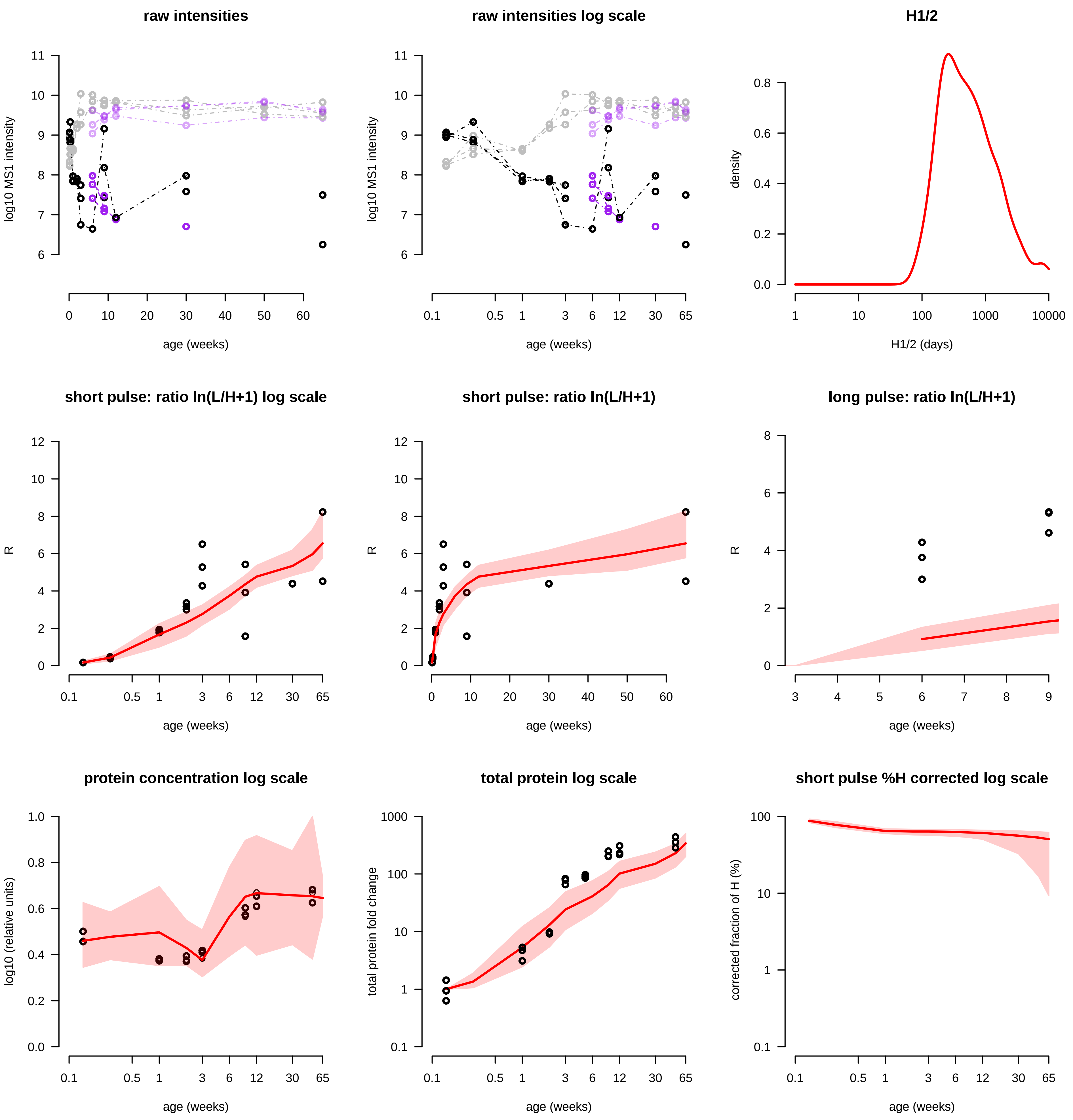

Q9DBG6

>sp|Q9DBG6|RPN2_MOUSE

Dolichyl−diphosphooligosaccharide−−protein glycosyltransferase subunit 2

GN=Rpn2

Med H1/2: 8.6 days

Q5 H1/2: 7.1 days

Q95 H1/2: 11 days

reliable model fit: yes

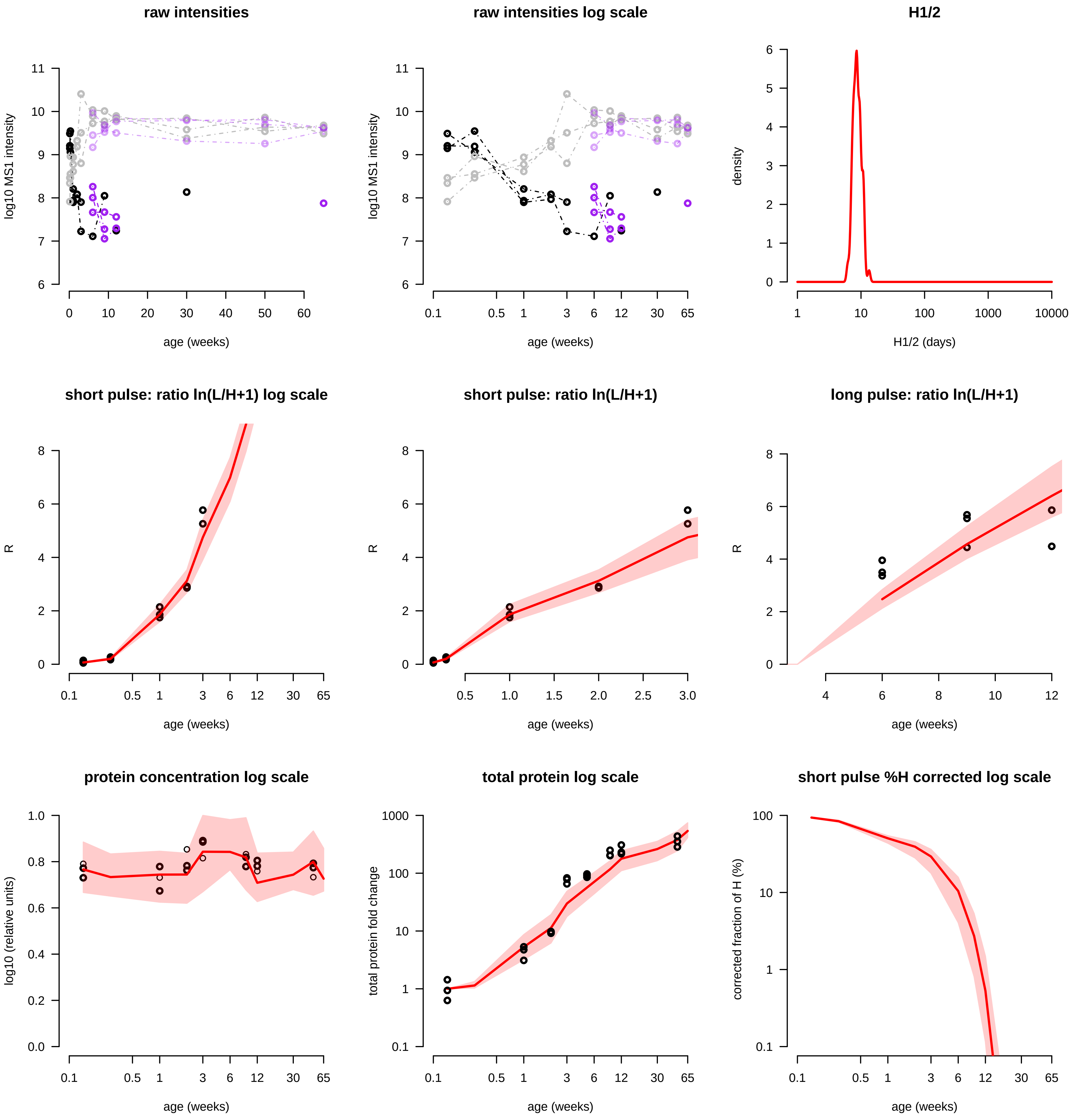

Q8CCK0

>sp|Q8CCK0|H2AW_MOUSE

Core histone macro−H2A.2

GN=Macroh2a2

Med H1/2: 13.9 days

Q5 H1/2: 11.3 days

Q95 H1/2: 17.1 days

reliable model fit: yes

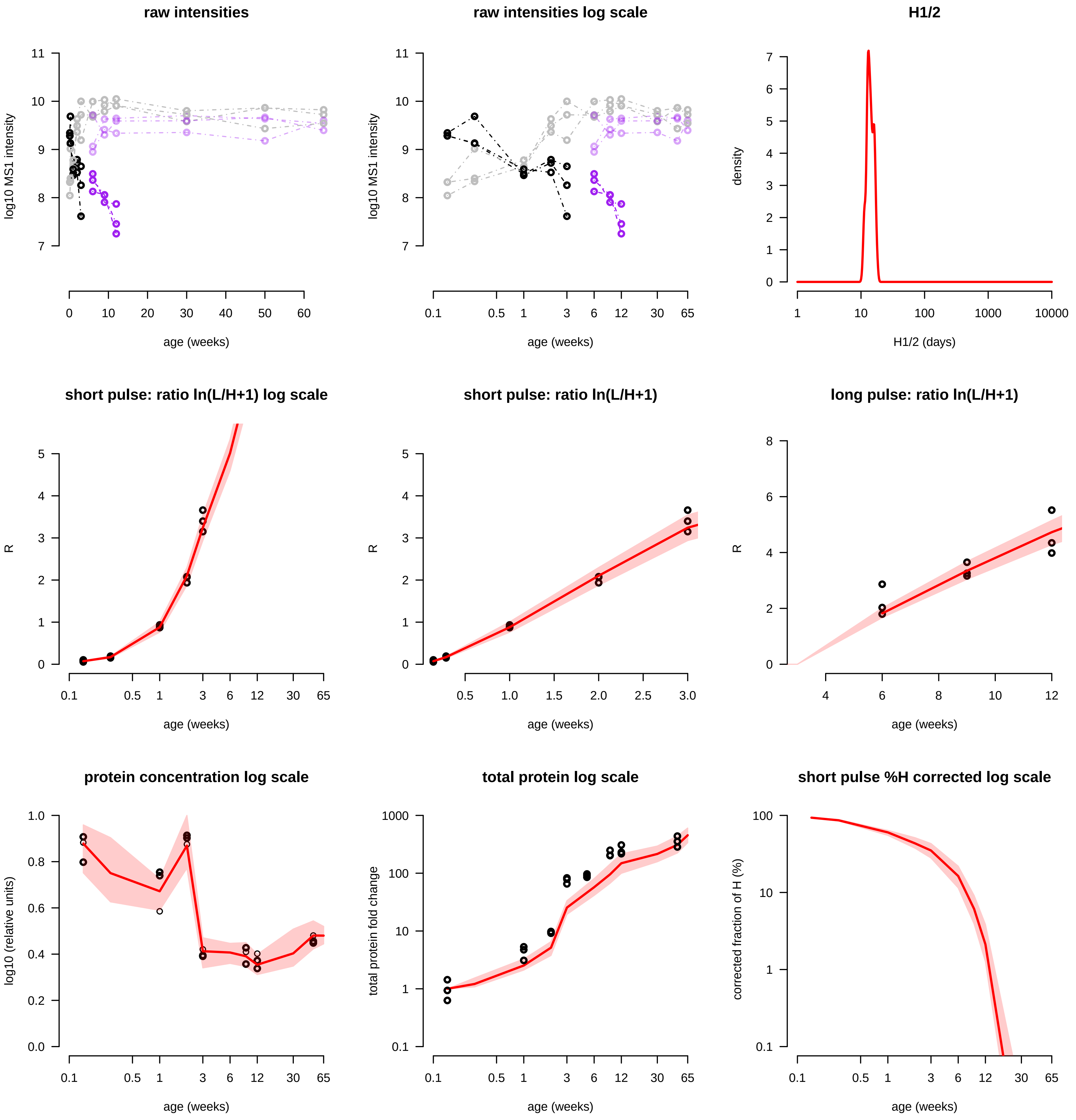

O35643

>sp|O35643|AP1B1_MOUSE

AP−1 complex subunit beta−1

GN=Ap1b1

Med H1/2: 5.5 days

Q5 H1/2: 4.6 days

Q95 H1/2: 6.7 days

reliable model fit: yes

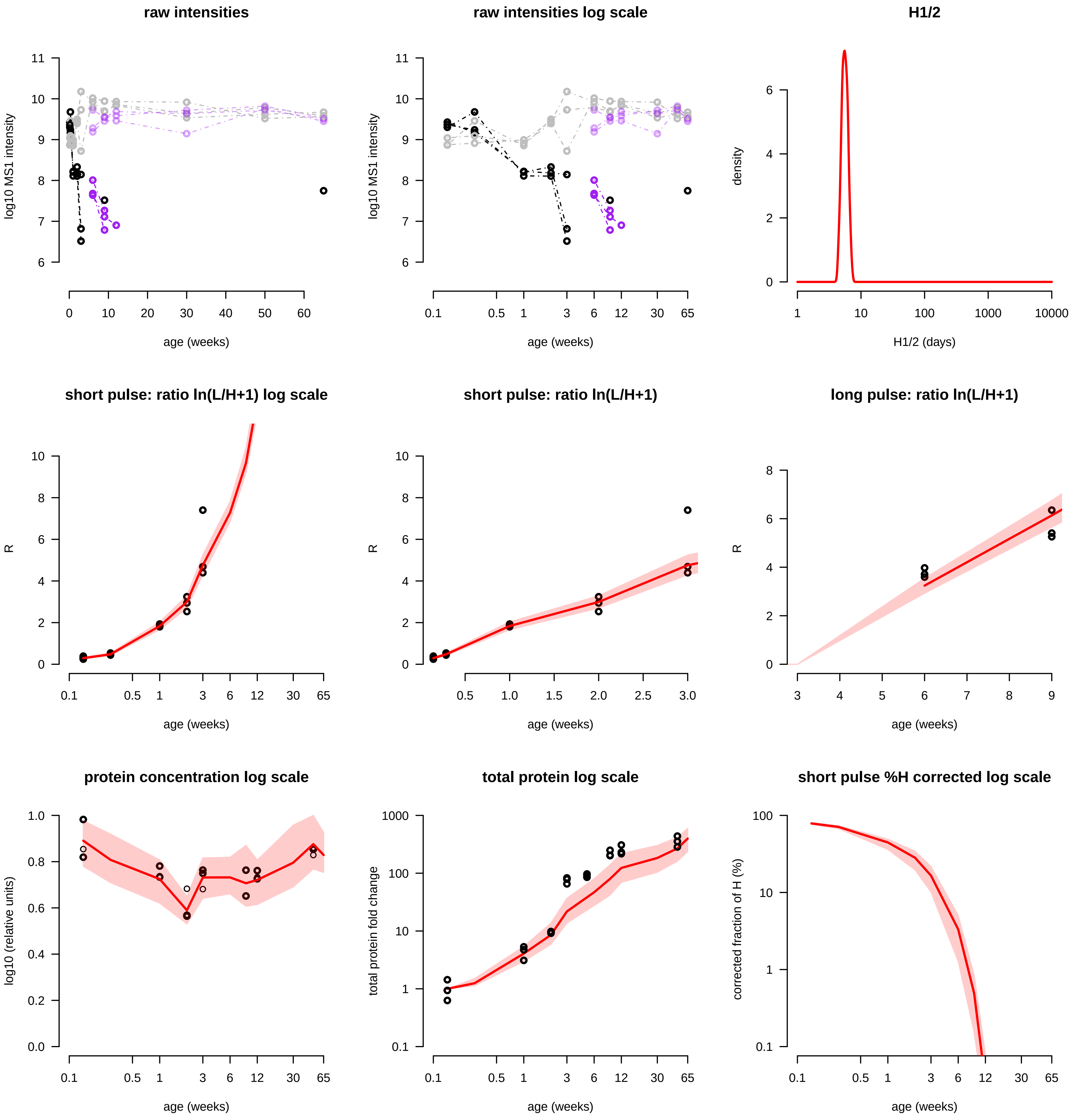

P11352

>sp|P11352|GPX1_MOUSE

Glutathione peroxidase 1

GN=Gpx1

Med H1/2: 12.2 days

Q5 H1/2: 8.8 days

Q95 H1/2: 21.4 days

reliable model fit: yes

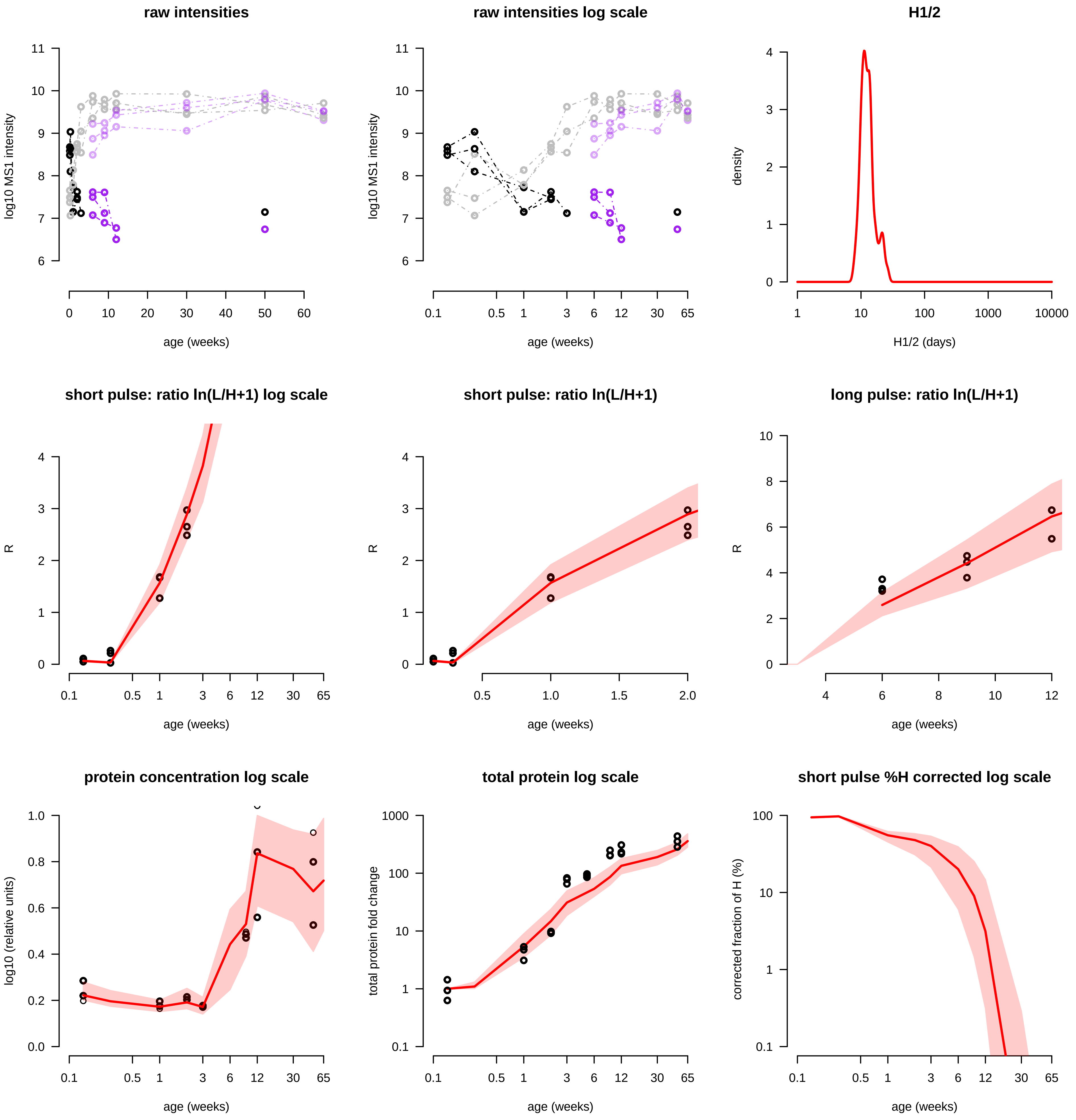

Q7TMK9

>sp|Q7TMK9|HNRPQ_MOUSE

Heterogeneous nuclear ribonucleoprotein Q

GN=Syncrip

Med H1/2: 7.4 days

Q5 H1/2: 6.2 days

Q95 H1/2: 8.8 days

reliable model fit: yes

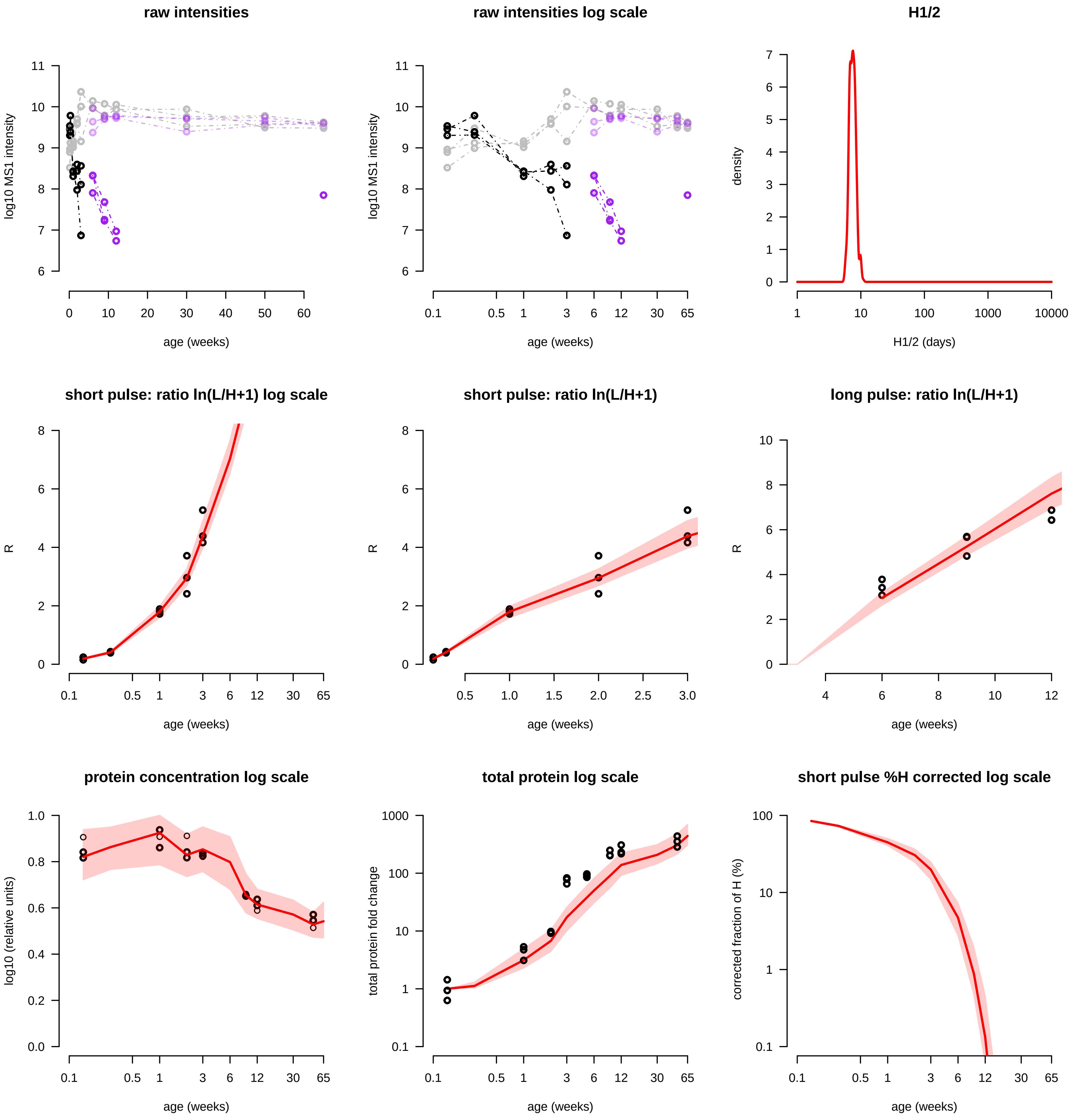

P21981

>sp|P21981|TGM2_MOUSE

Protein−glutamine gamma−glutamyltransferase 2

GN=Tgm2

Med H1/2: 12.1 days

Q5 H1/2: 8.9 days

Q95 H1/2: 15.9 days

reliable model fit: yes

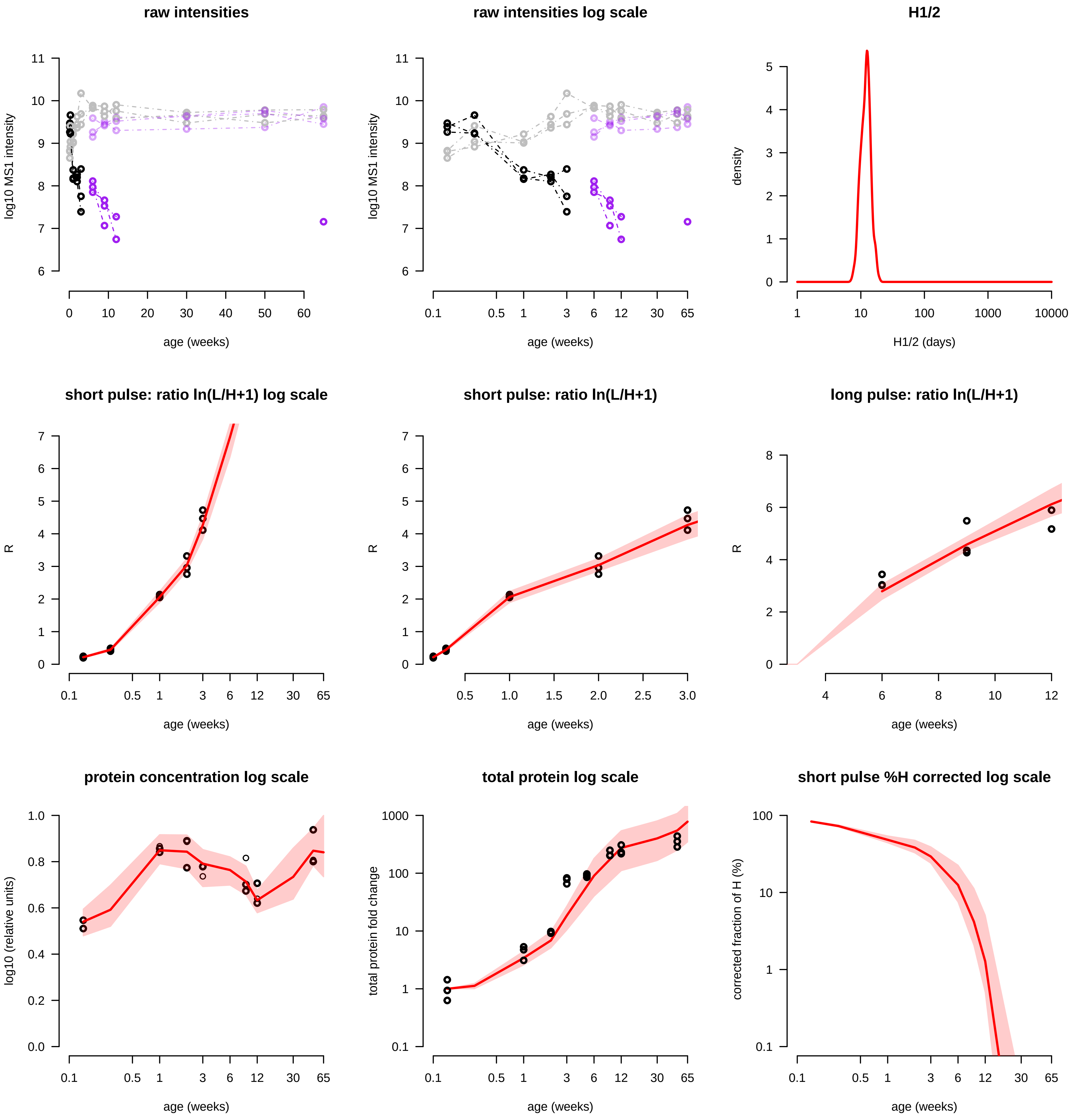

Q60668−3;Q60668;Q60668−4;Q60668−2

>sp|Q60668−3|HNRPD_MOUSE

Isoform 3 of Heterogeneous nuclear ribonucleoprotein D0

GN=Hnrnpd

Med H1/2: 1206 days

Q5 H1/2: 395.3 days

Q95 H1/2: 14668 days

reliable model fit: no

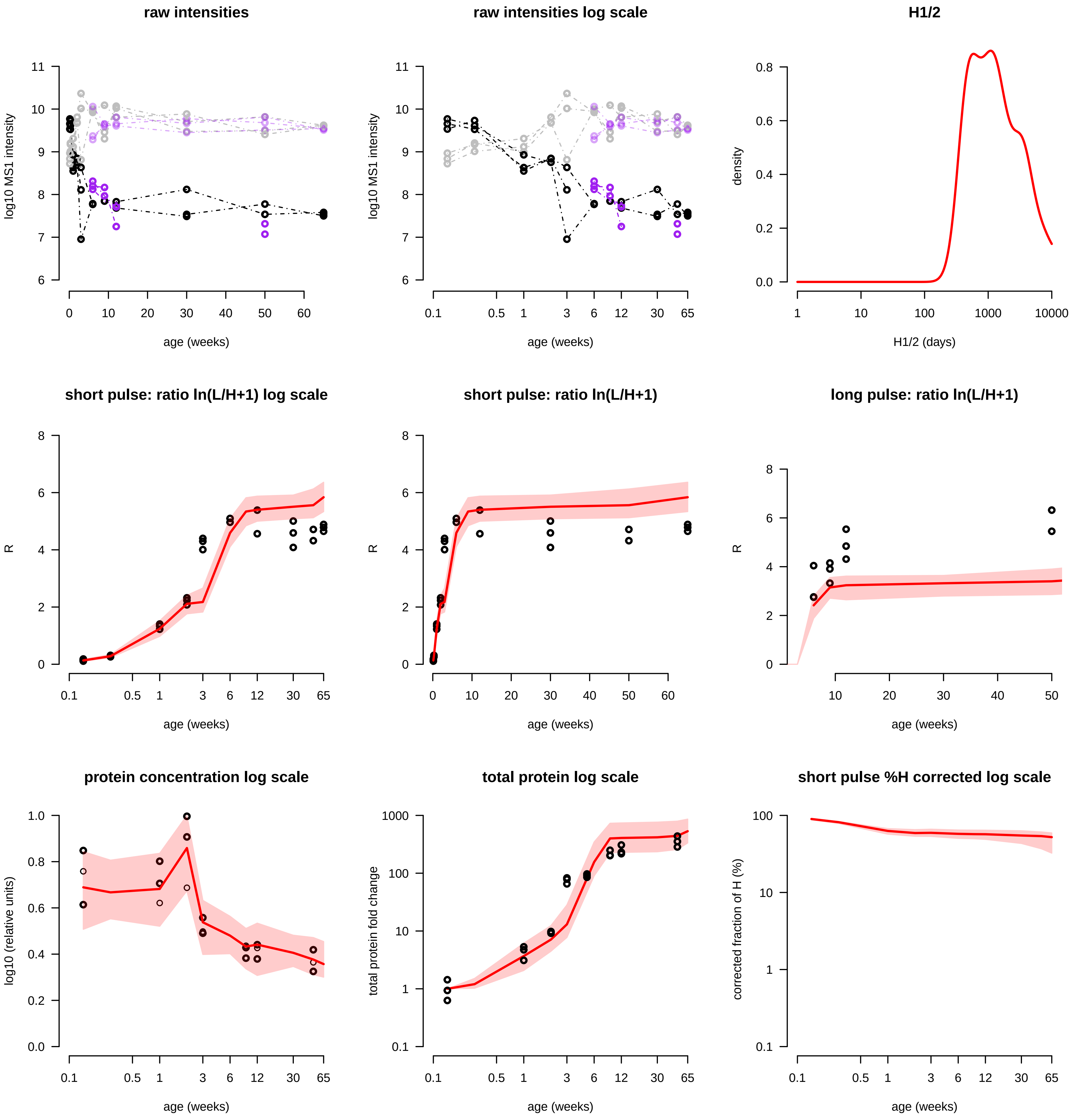

Q9D3D9

>sp|Q9D3D9|ATPD_MOUSE

ATP synthase subunit delta, mitochondrial

GN=Atp5f1d

Med H1/2: 59.3 days

Q5 H1/2: 27.2 days

Q95 H1/2: 189.4 days

reliable model fit: yes

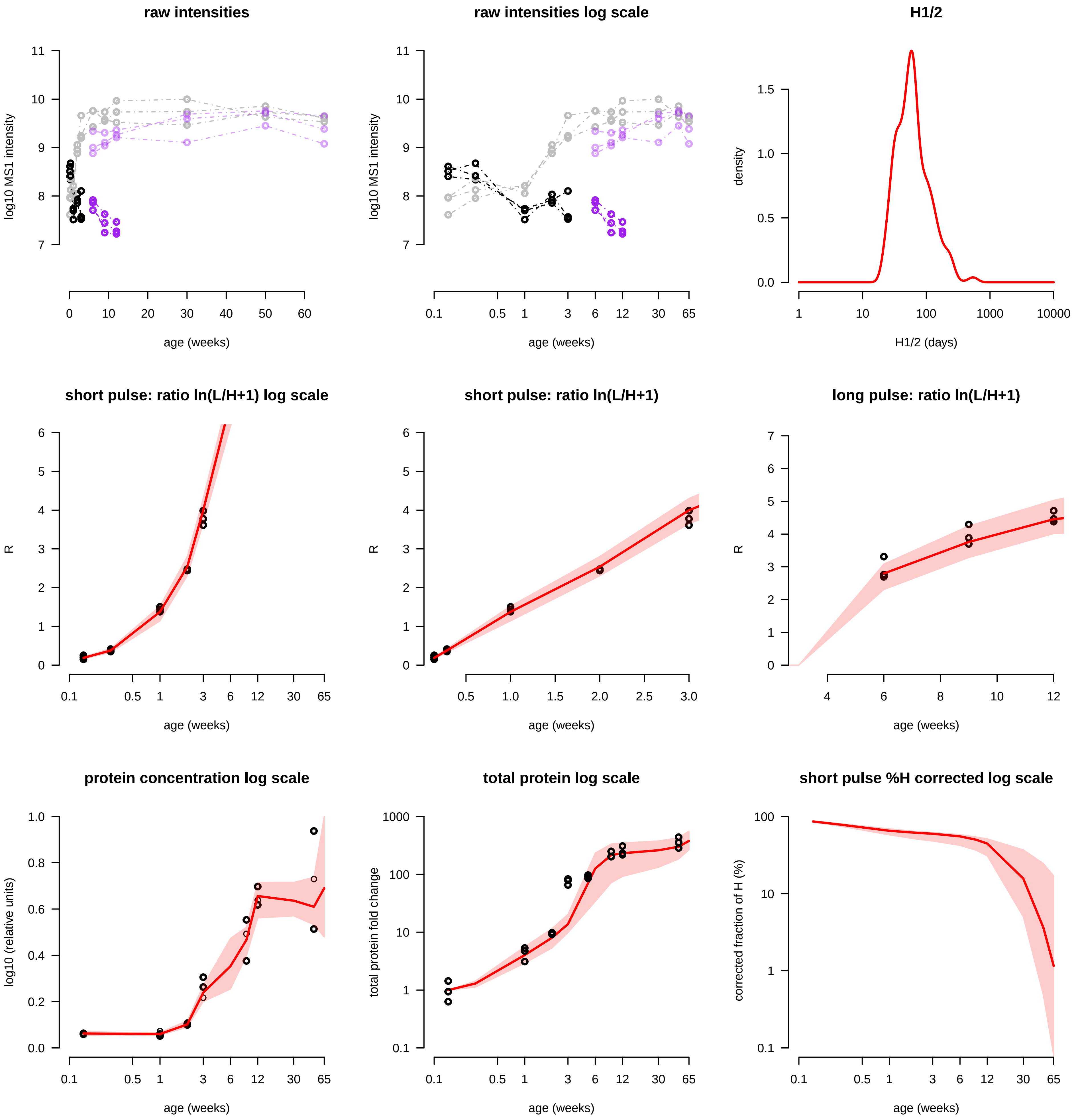

Q9Z2U1

>sp|Q9Z2U1|PSA5_MOUSE

Proteasome subunit alpha type−5

GN=Psma5

Med H1/2: 14.3 days

Q5 H1/2: 11.4 days

Q95 H1/2: 18.5 days

reliable model fit: yes

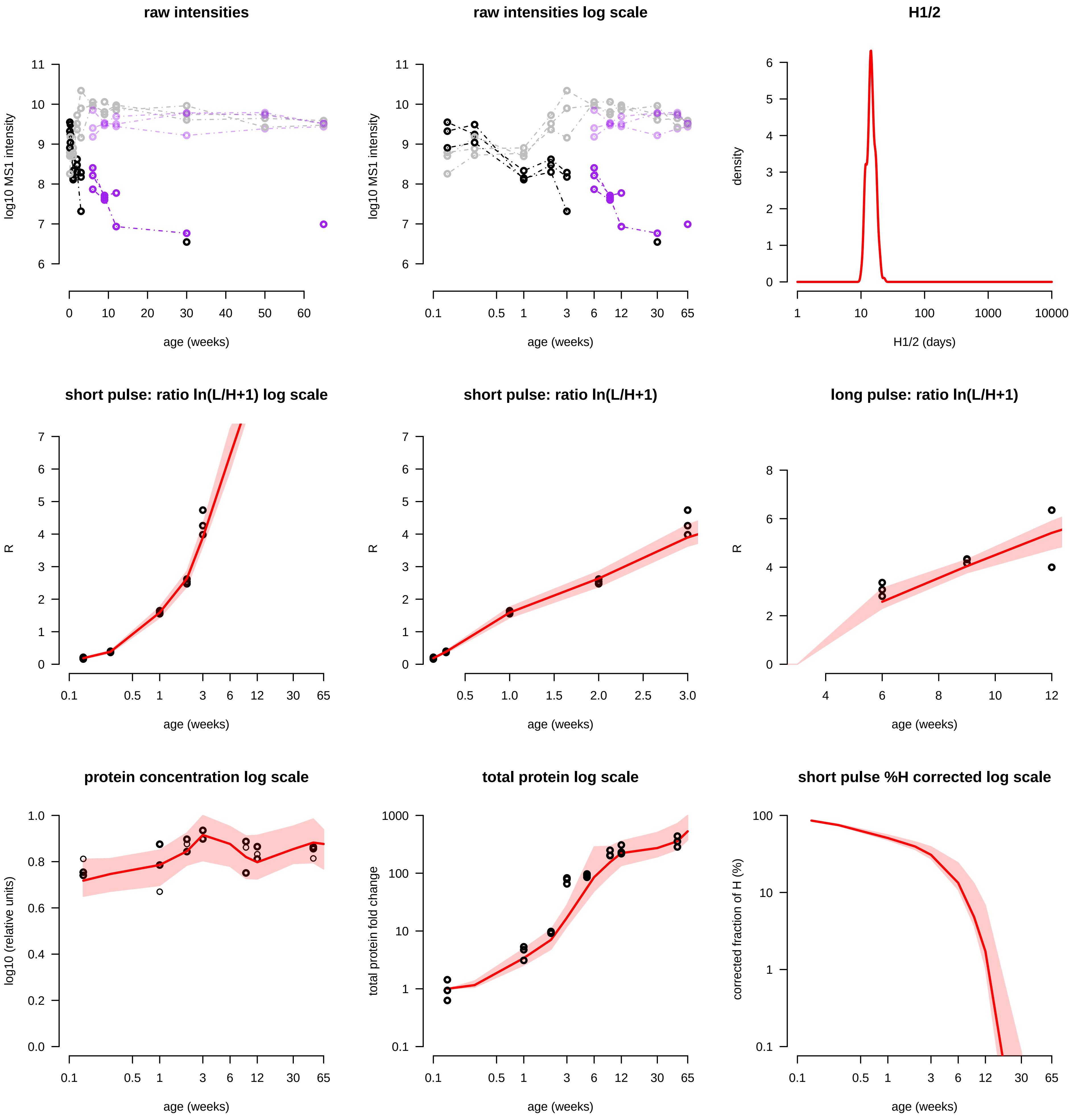

P27546

>sp|P27546|MAP4_MOUSE

Microtubule−associated protein 4

GN=Map4

Med H1/2: 93.5 days

Q5 H1/2: 54.5 days

Q95 H1/2: 260 days

reliable model fit: no

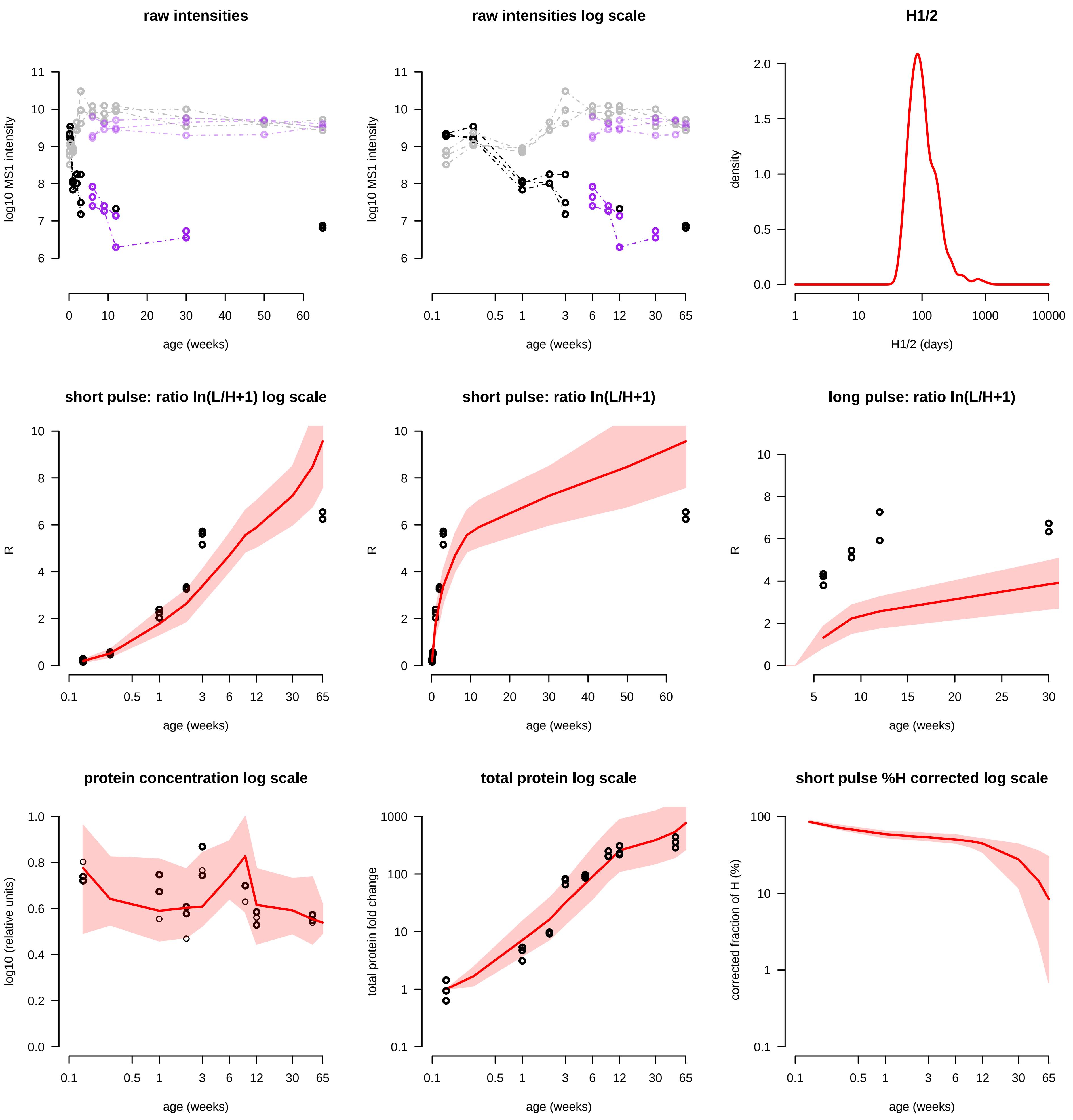

P58774−2

>sp|P58774−2|TPM2_MOUSE

Isoform 2 of Tropomyosin beta chain

GN=Tpm2

Med H1/2: 16.4 days

Q5 H1/2: 11.8 days

Q95 H1/2: 35.4 days

reliable model fit: yes

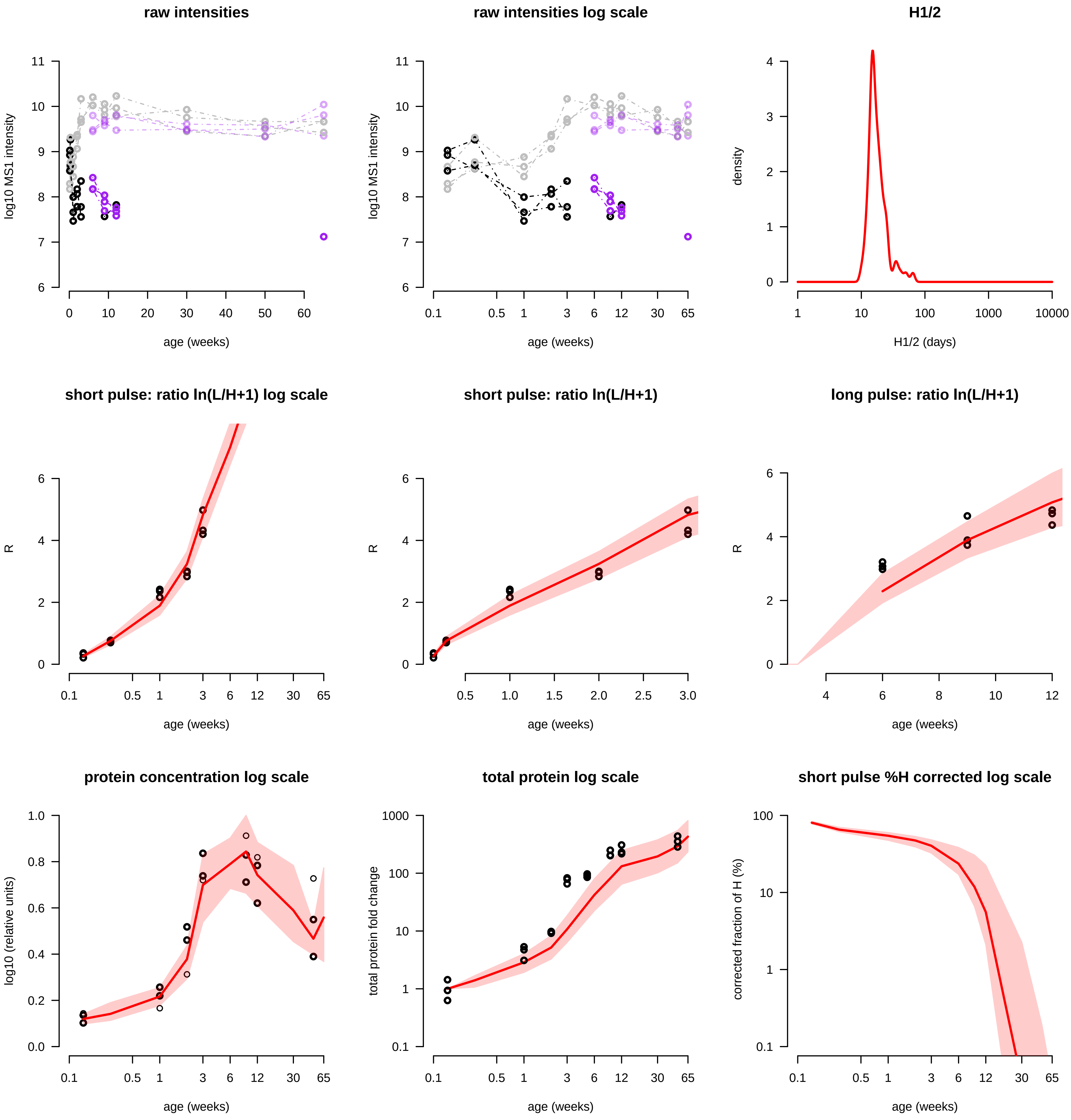

Q61147

>sp|Q61147|CERU_MOUSE

Ceruloplasmin

GN=Cp

Med H1/2: 33 days

Q5 H1/2: 21 days

Q95 H1/2: 67.5 days

reliable model fit: no

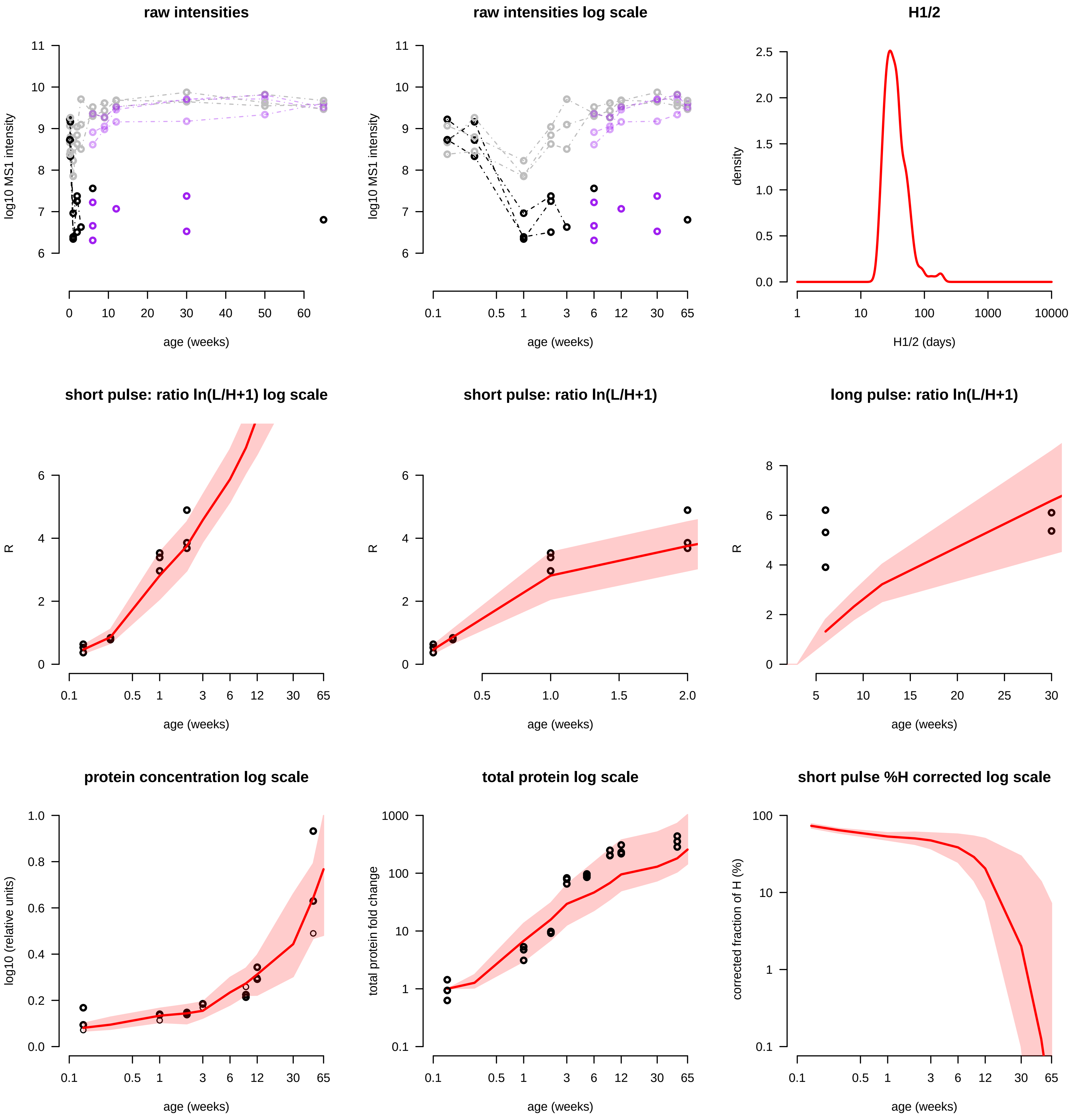

P97449

>sp|P97449|AMPN_MOUSE

Aminopeptidase N

GN=Anpep

Med H1/2: 101.4 days

Q5 H1/2: 68.5 days

Q95 H1/2: 191.4 days

reliable model fit: yes

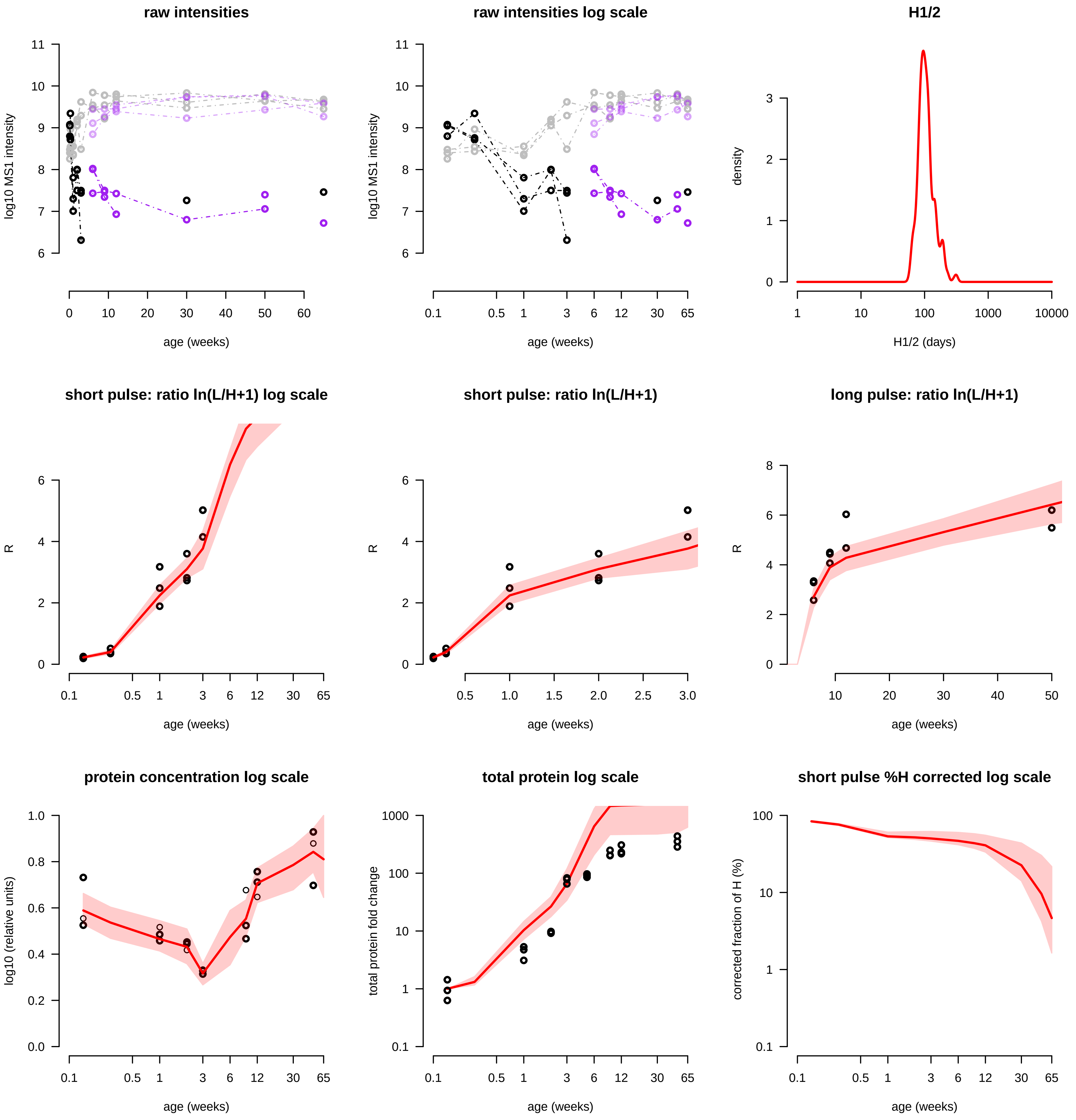

O35737

>sp|O35737|HNRH1_MOUSE

Heterogeneous nuclear ribonucleoprotein H

GN=Hnrnph1

Med H1/2: 193.2 days

Q5 H1/2: 110.6 days

Q95 H1/2: 606 days

reliable model fit: yes

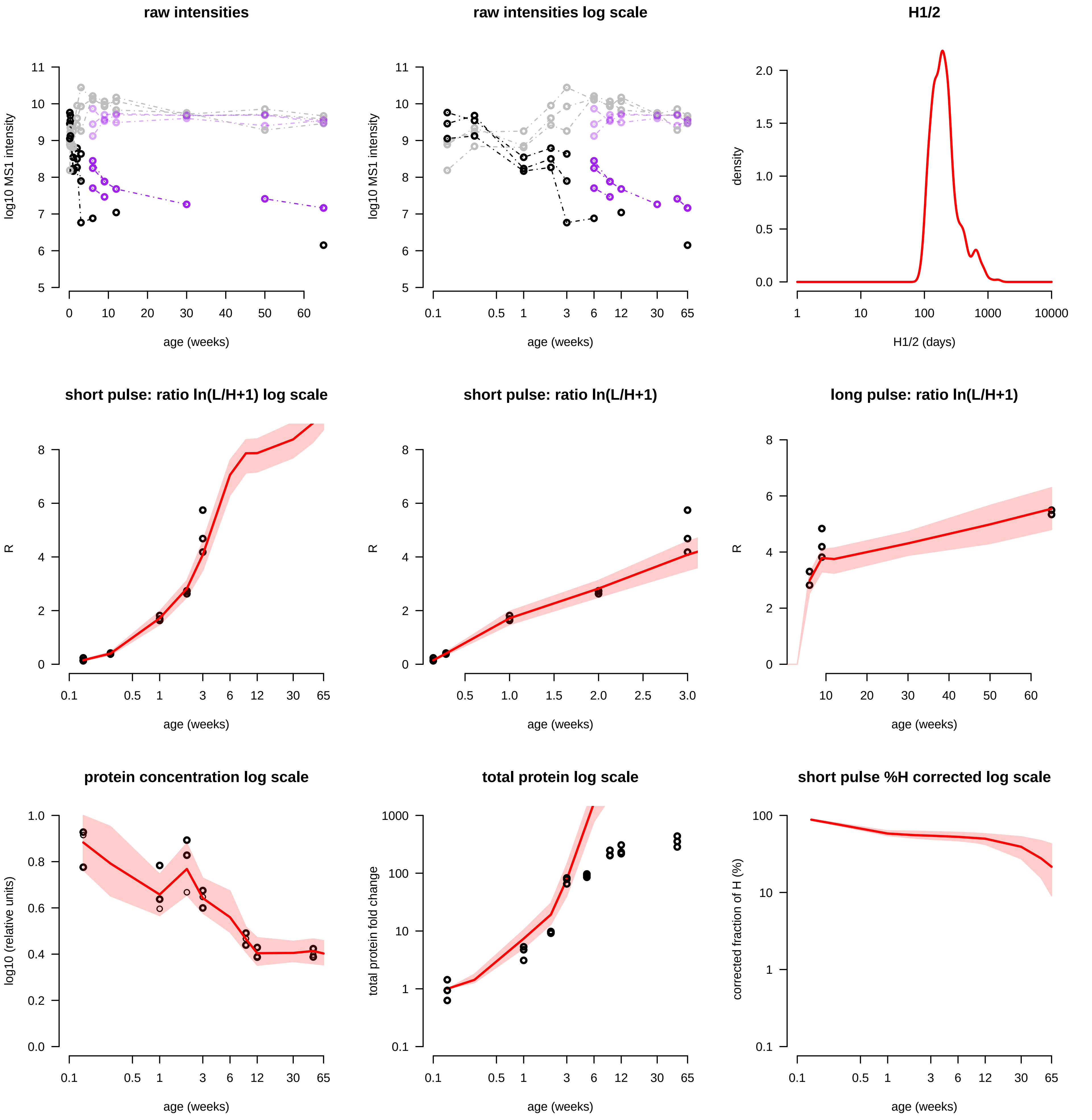

Q91VS7

>sp|Q91VS7|MGST1_MOUSE

Microsomal glutathione S−transferase 1

GN=Mgst1

Med H1/2: 18.6 days

Q5 H1/2: 13.6 days

Q95 H1/2: 43.7 days

reliable model fit: yes

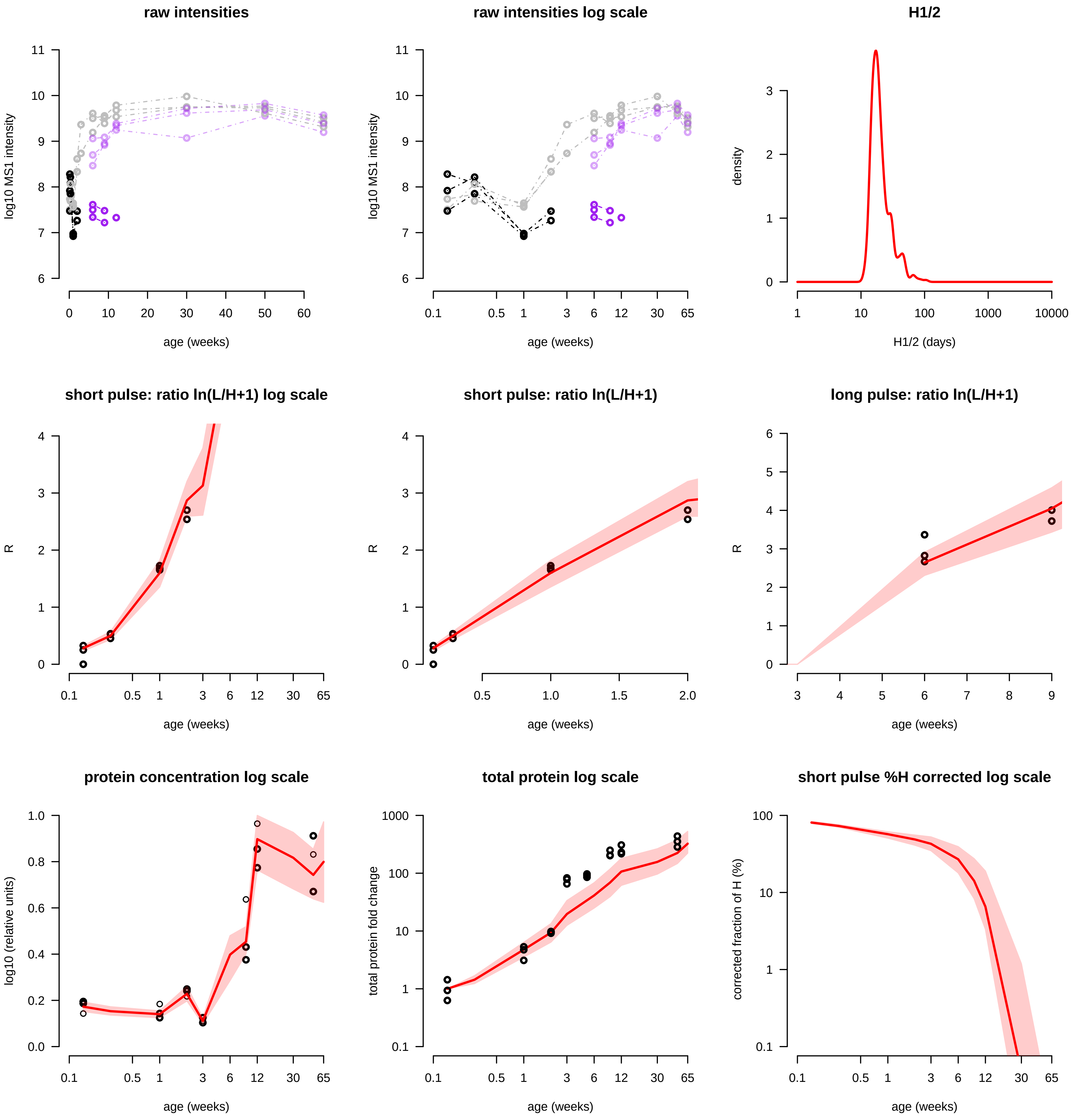

Q9D0F3

>sp|Q9D0F3|LMAN1_MOUSE

Protein ERGIC−53

GN=Lman1

Med H1/2: 4.3 days

Q5 H1/2: 3.5 days

Q95 H1/2: 5.2 days

reliable model fit: yes

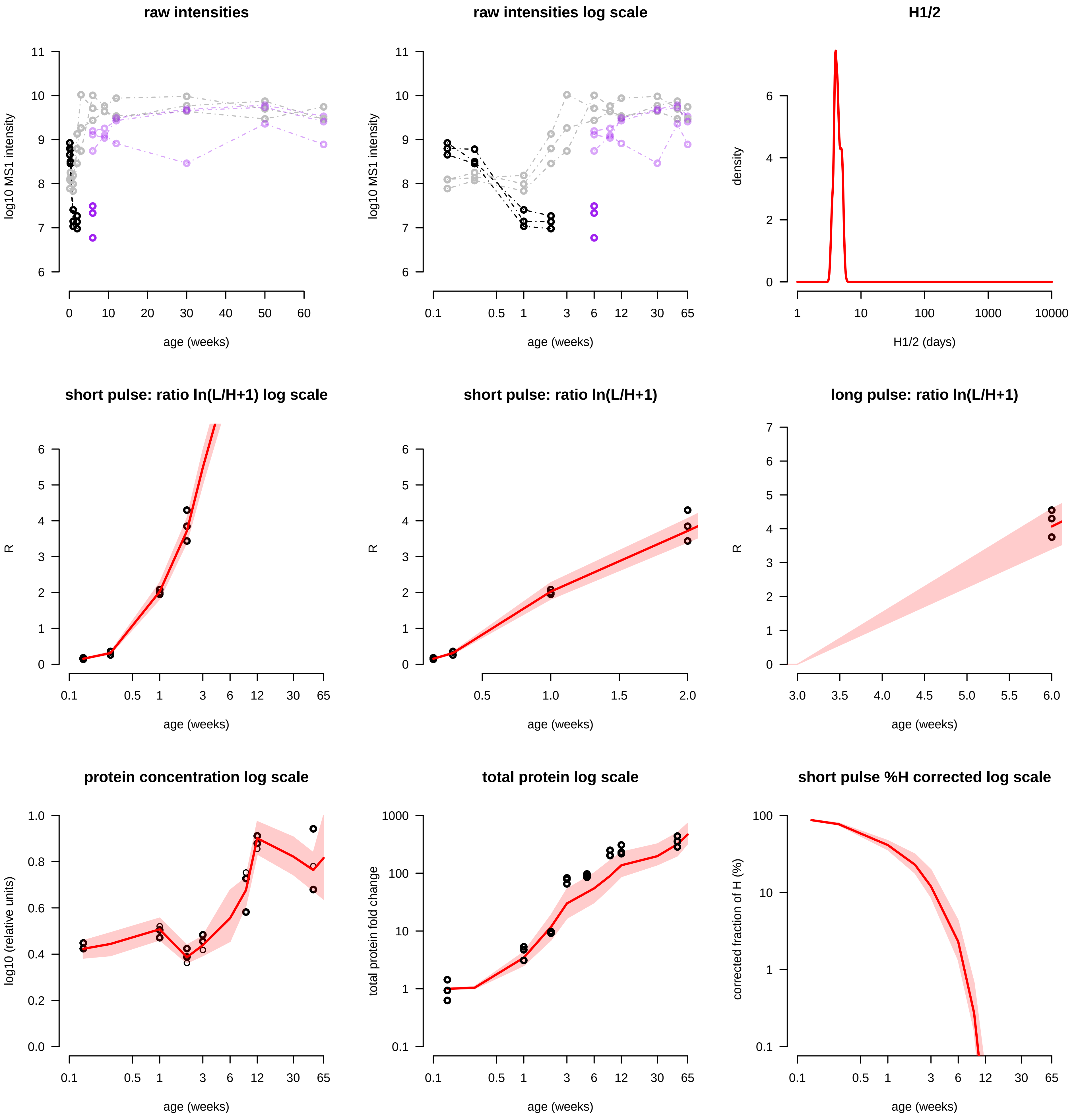

Q9D1D4;Q9D1D4−2

>sp|Q9D1D4|TMEDA_MOUSE

Transmembrane emp24 domain−containing protein 10

GN=Tmed10

Med H1/2: 699.8 days

Q5 H1/2: 218.7 days

Q95 H1/2: 7575.8 days

reliable model fit: yes

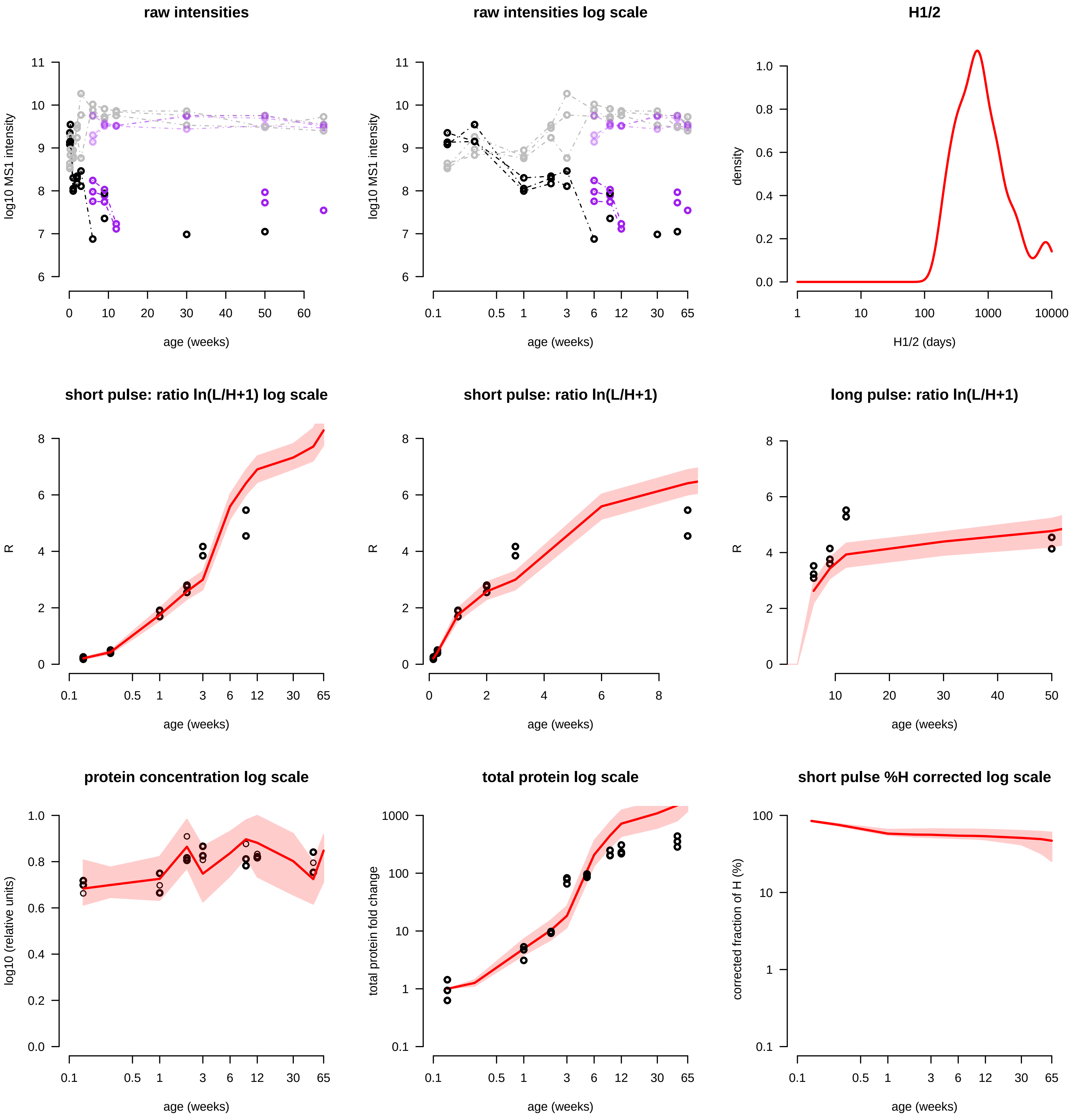

P10852

>sp|P10852|4F2_MOUSE

4F2 cell−surface antigen heavy chain

GN=Slc3a2

Med H1/2: 11.3 days

Q5   H1/2: 8.7 days

Q95 H1/2: 14.9 days

reliable model fit: yes

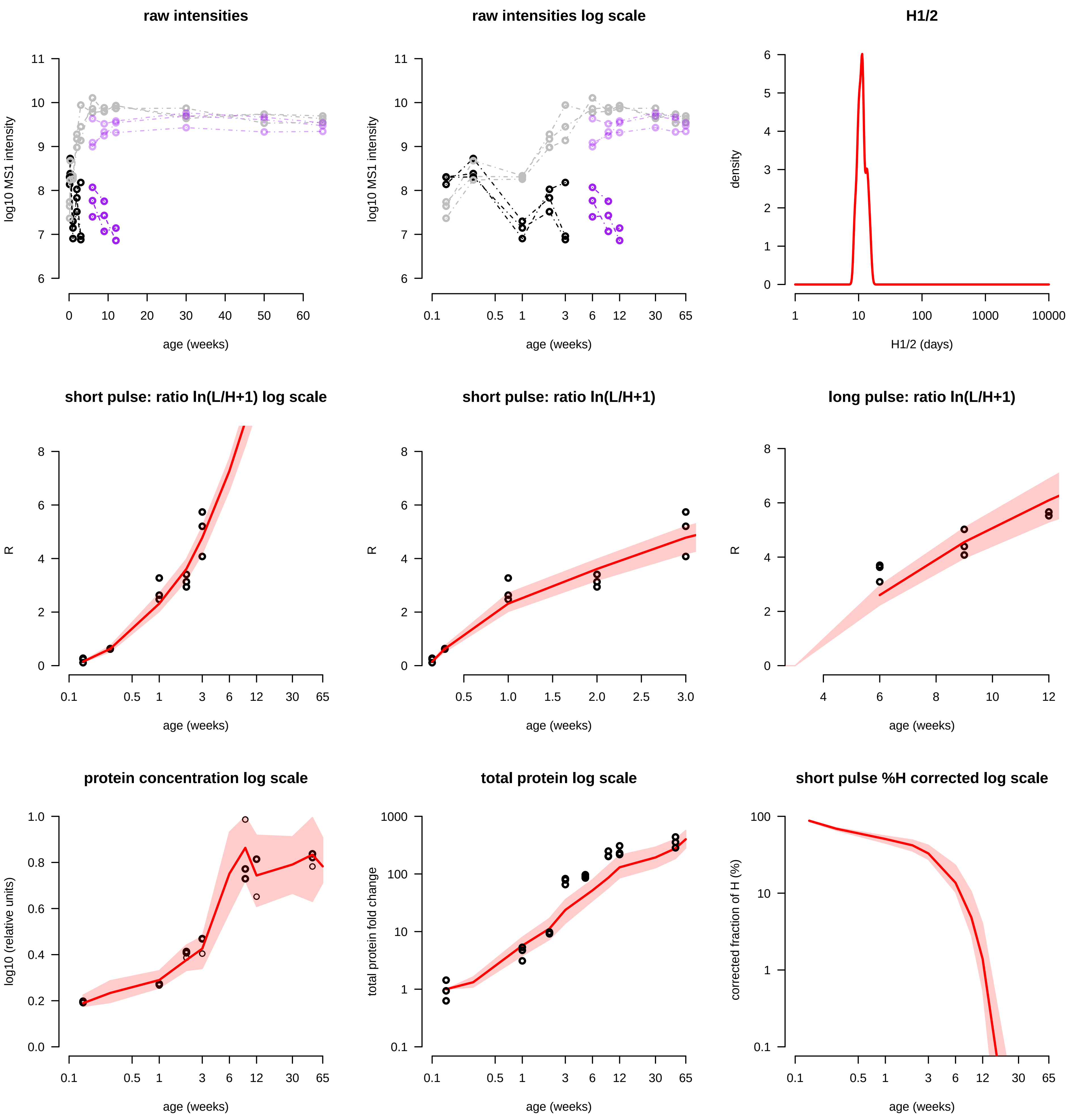

Q99020

>sp|Q99020|ROAA_MOUSE

Heterogeneous nuclear ribonucleoprotein A/B

GN=Hnrnpab

Med H1/2: 9.9 days

Q5 H1/2: 8.1 days

Q95 H1/2: 11.6 days

reliable model fit: yes

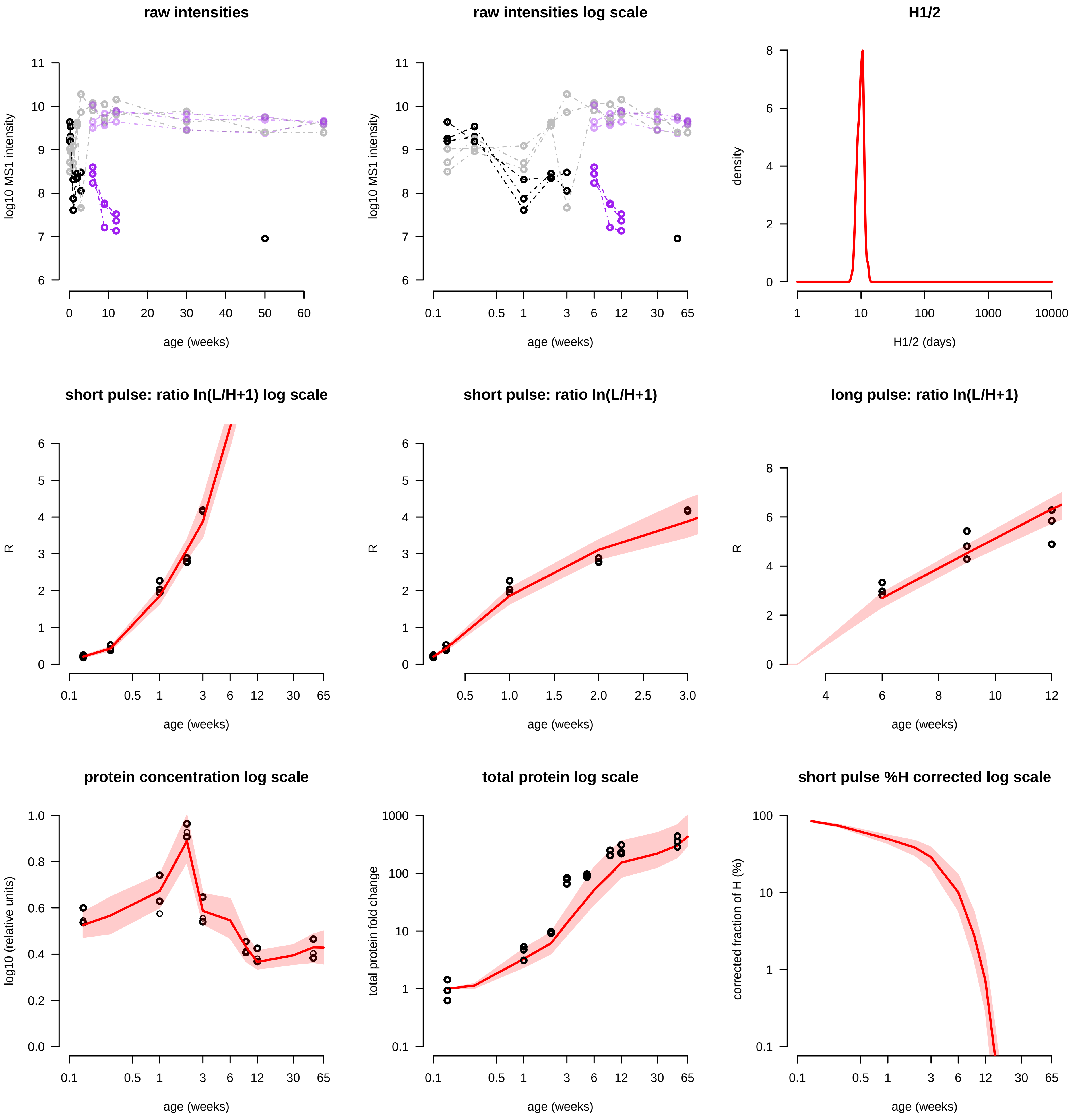

P11983;P11983−2

>sp|P11983|TCPA_MOUSE

T−complex protein 1 subunit alpha

GN=Tcp1

Med H1/2: 7 days

Q5 H1/2: 5.8 days

Q95 H1/2: 8.3 days

reliable model fit: yes

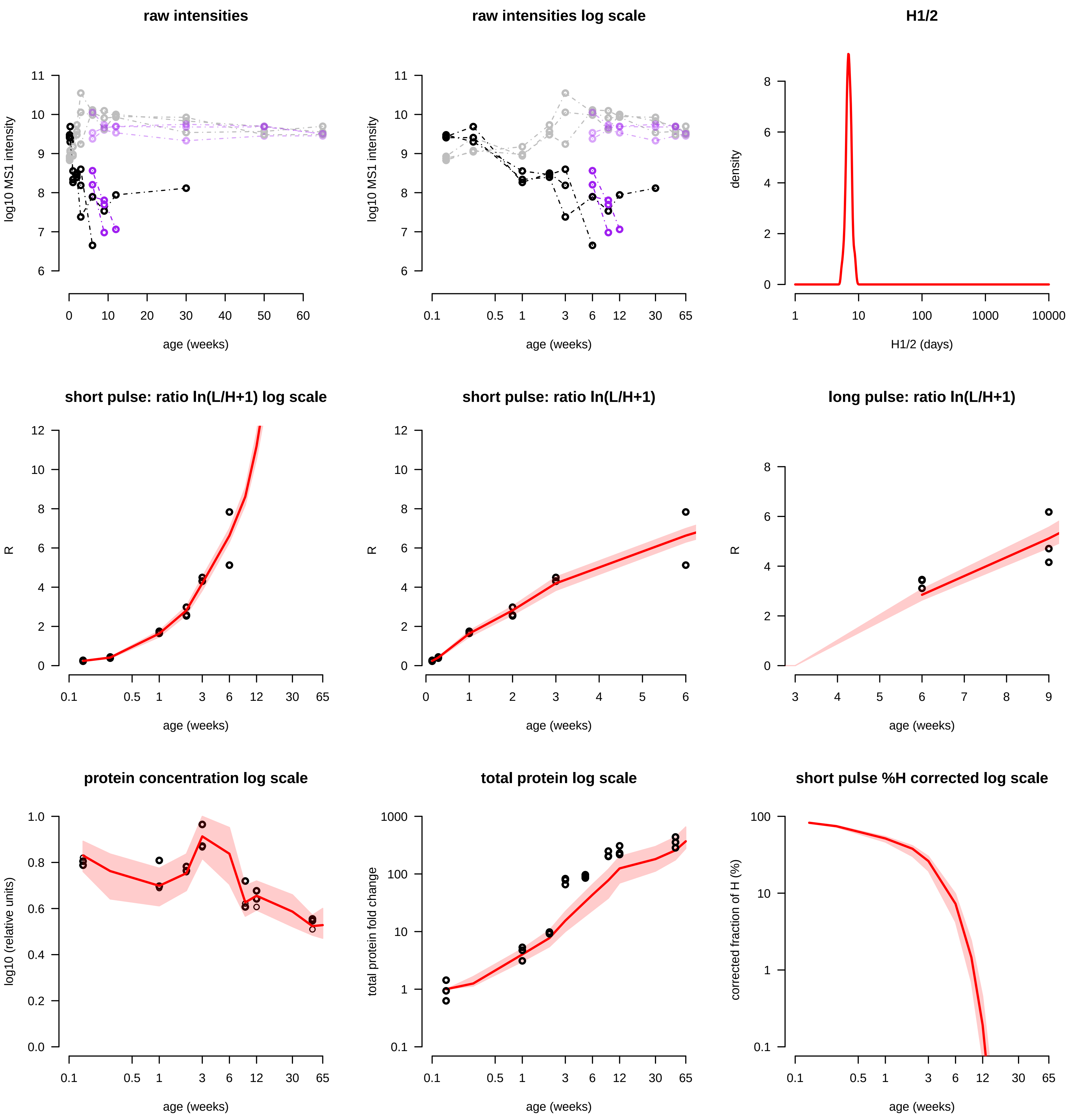

P47754

>sp|P47754|CAZA2_MOUSE

F−actin−capping protein subunit alpha−2

GN=Capza2

Med H1/2: 9.2 days

Q5 H1/2: 7.4 days

Q95 H1/2: 11.6 days

reliable model fit: yes

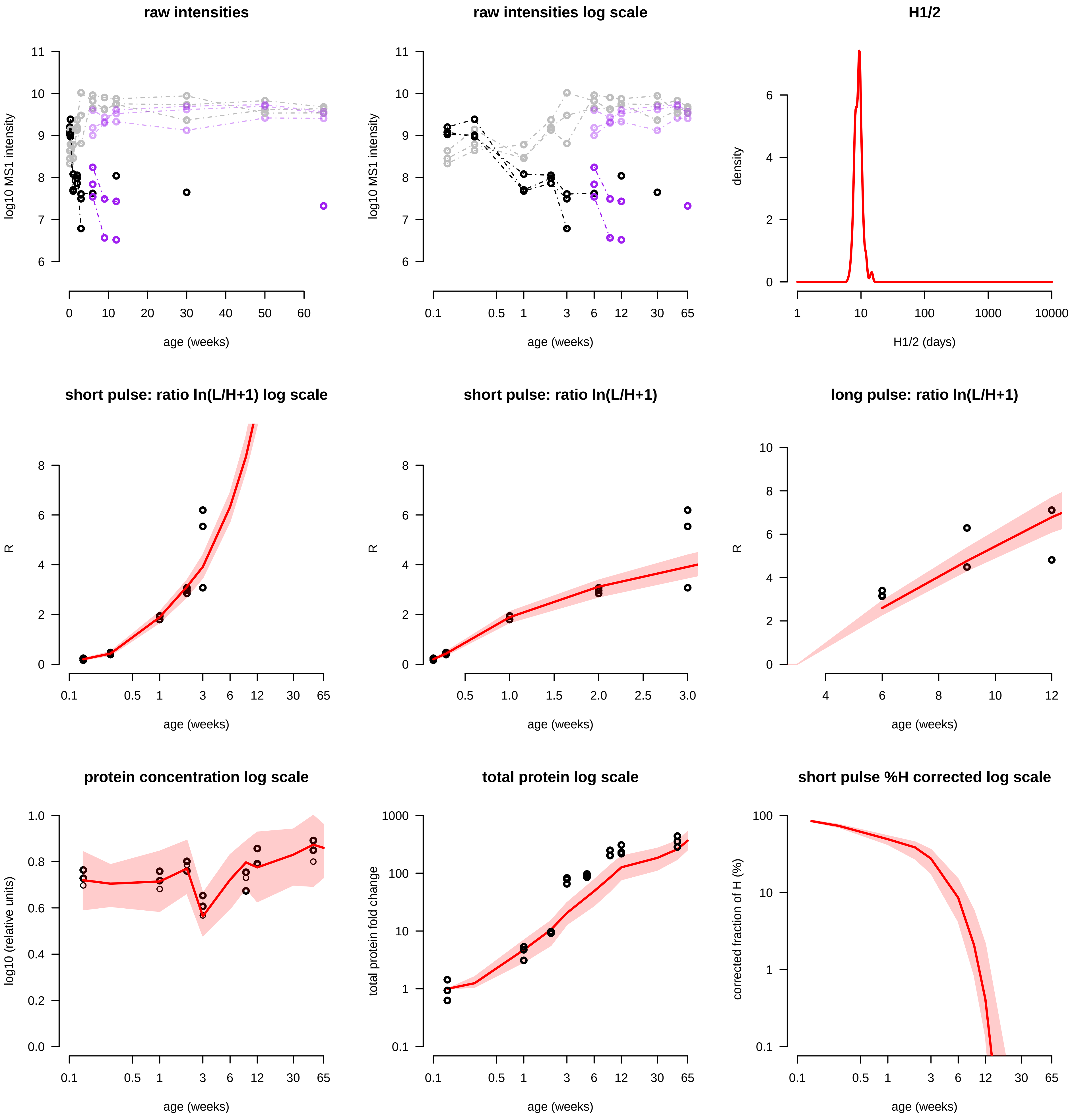

Q9Z2U0

>sp|Q9Z2U0|PSA7_MOUSE

Proteasome subunit alpha type−7

GN=Psma7

Med H1/2: 15.4 days

Q5 H1/2: 9.5 days

Q95 H1/2: 31.5 days

reliable model fit: yes

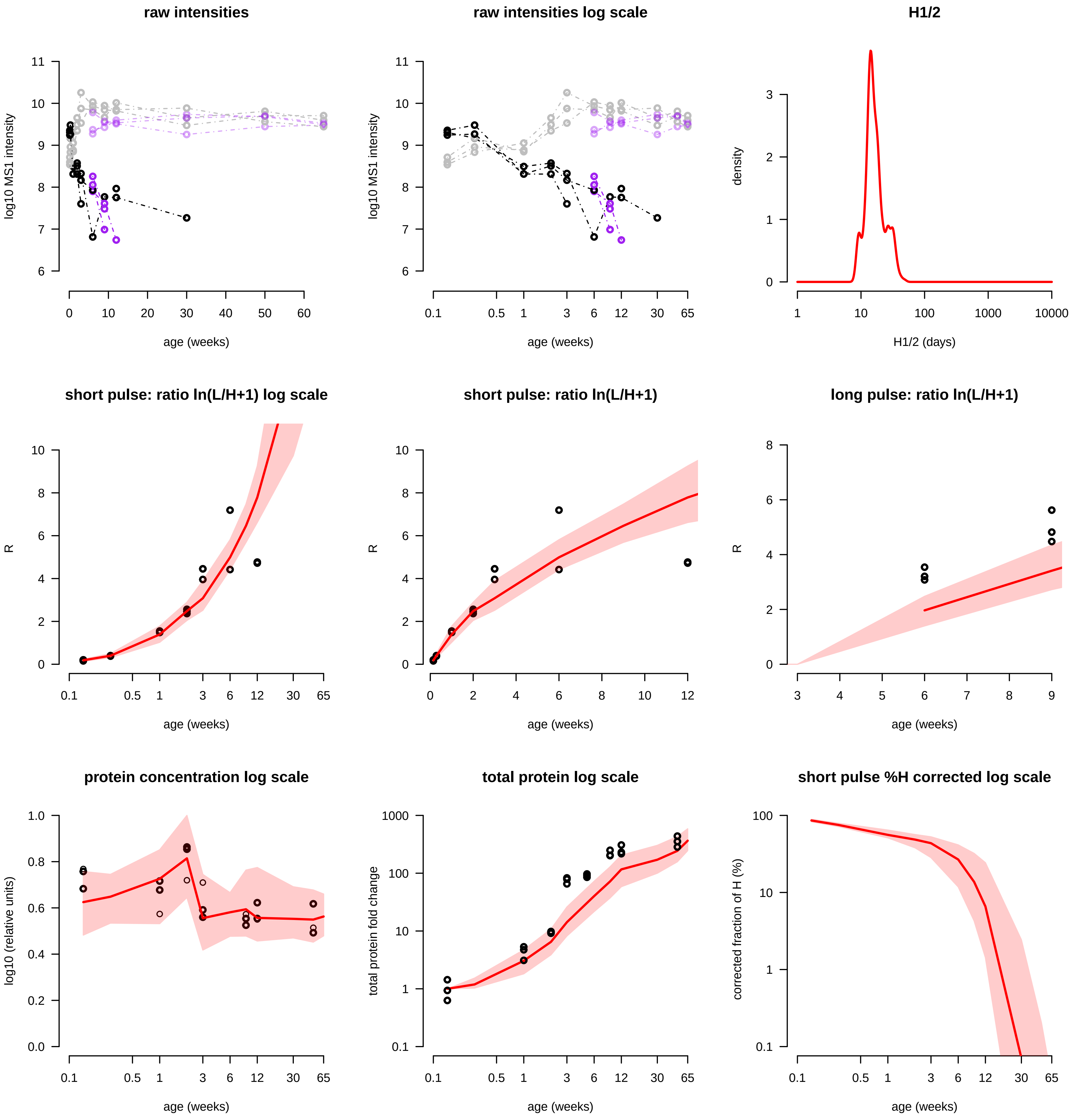

Q02788

>sp|Q02788|CO6A2_MOUSE

Collagen alpha−2(VI) chain

GN=Col6a2

Med H1/2: 9 days

Q5 H1/2: 7.2 days

Q95 H1/2: 12.1 days

reliable model fit: yes

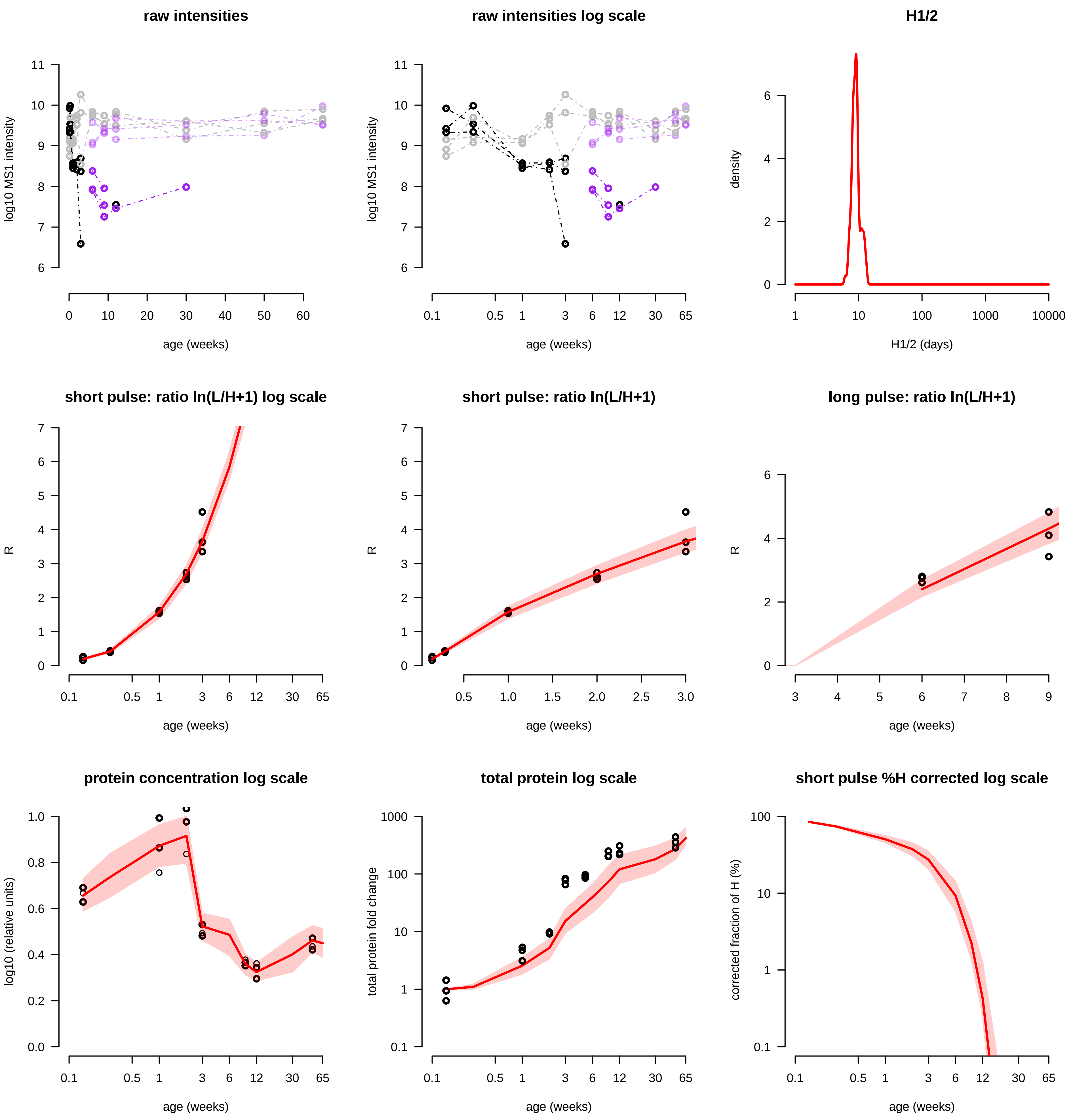

Q9CQ62

>sp|Q9CQ62|DECR_MOUSE

2,4−dienoyl−CoA reductase [(3E)−enoyl−CoA−producing], mitochondrial

GN=Decr1

Med H1/2: 241.7 days

Q5 H1/2: 74.3 days

Q95 H1/2: 1690.8 days

reliable model fit: no

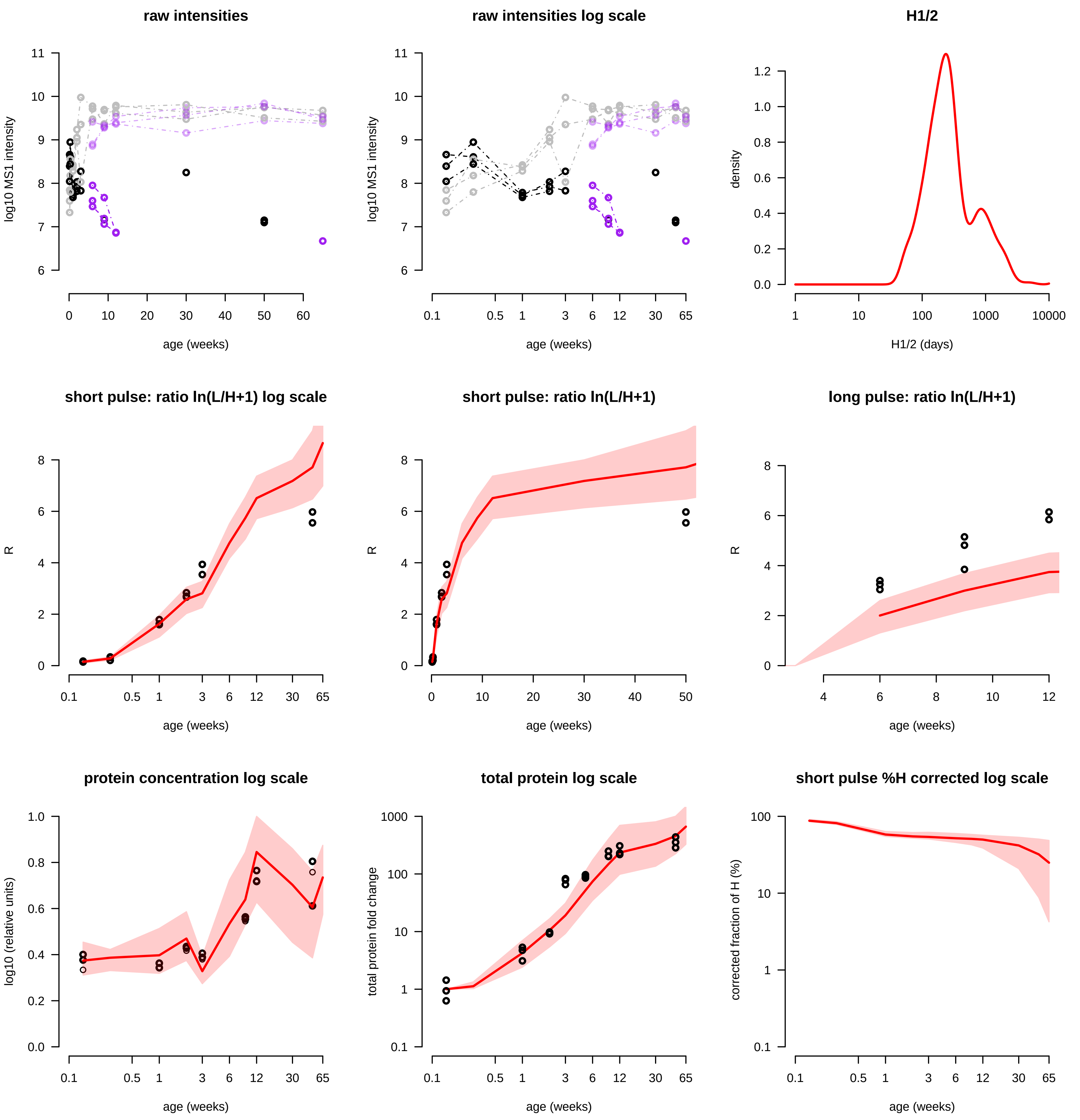

Q6PDN3;Q6PDN3−3

>sp|Q6PDN3|MYLK_MOUSE

Myosin light chain kinase, smooth muscle

GN=Mylk

Med H1/2: 8.3 days

Q5 H1/2: 6.8 days

Q95 H1/2: 10.4 days

reliable model fit: yes

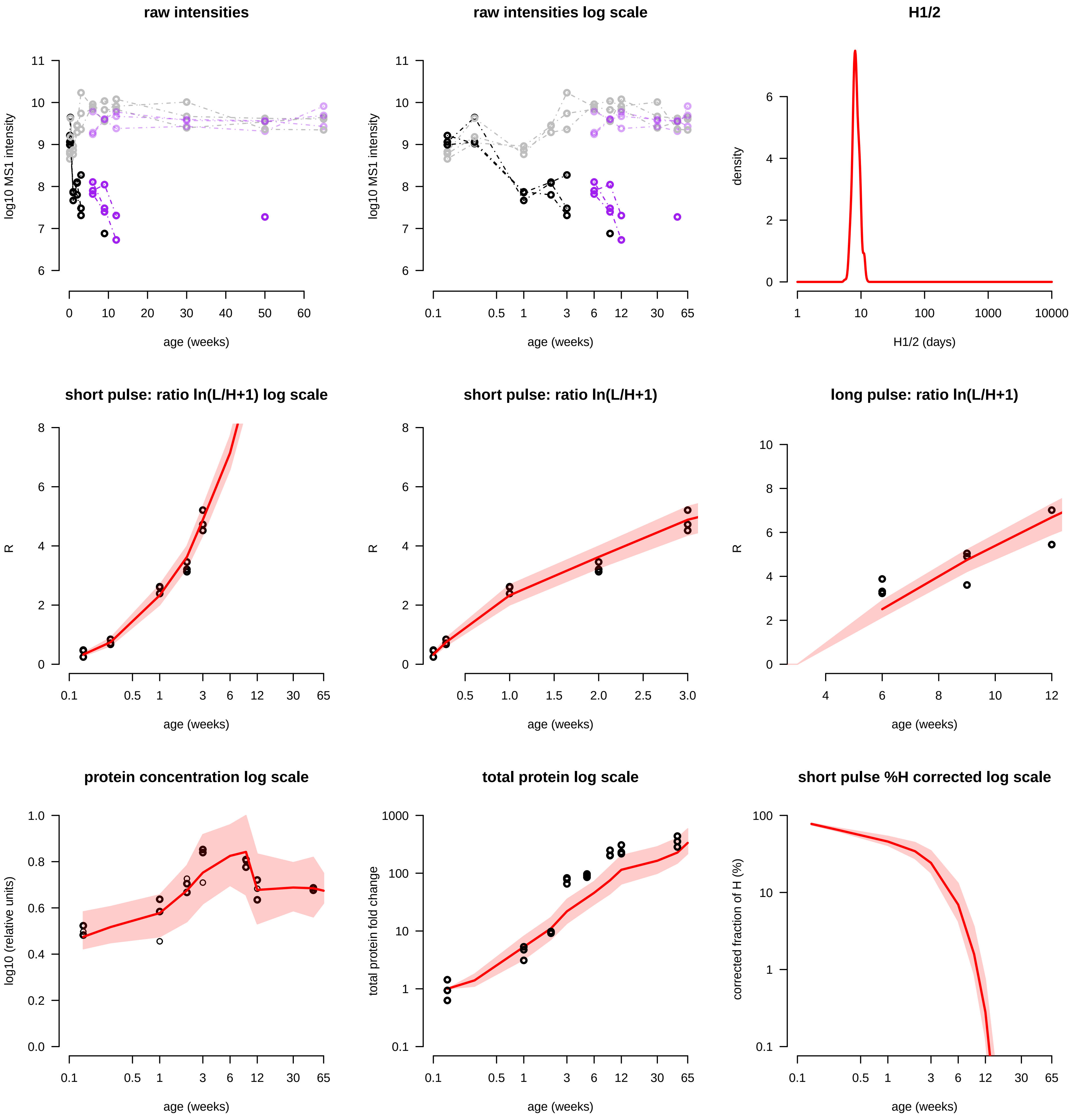

Q9CR62

>sp|Q9CR62|M2OM_MOUSE

Mitochondrial 2−oxoglutarate/malate carrier protein

GN=Slc25a11

Med H1/2: 8.7 days

Q5 H1/2: 6.7 days

Q95 H1/2: 11.3 days

reliable model fit: yes

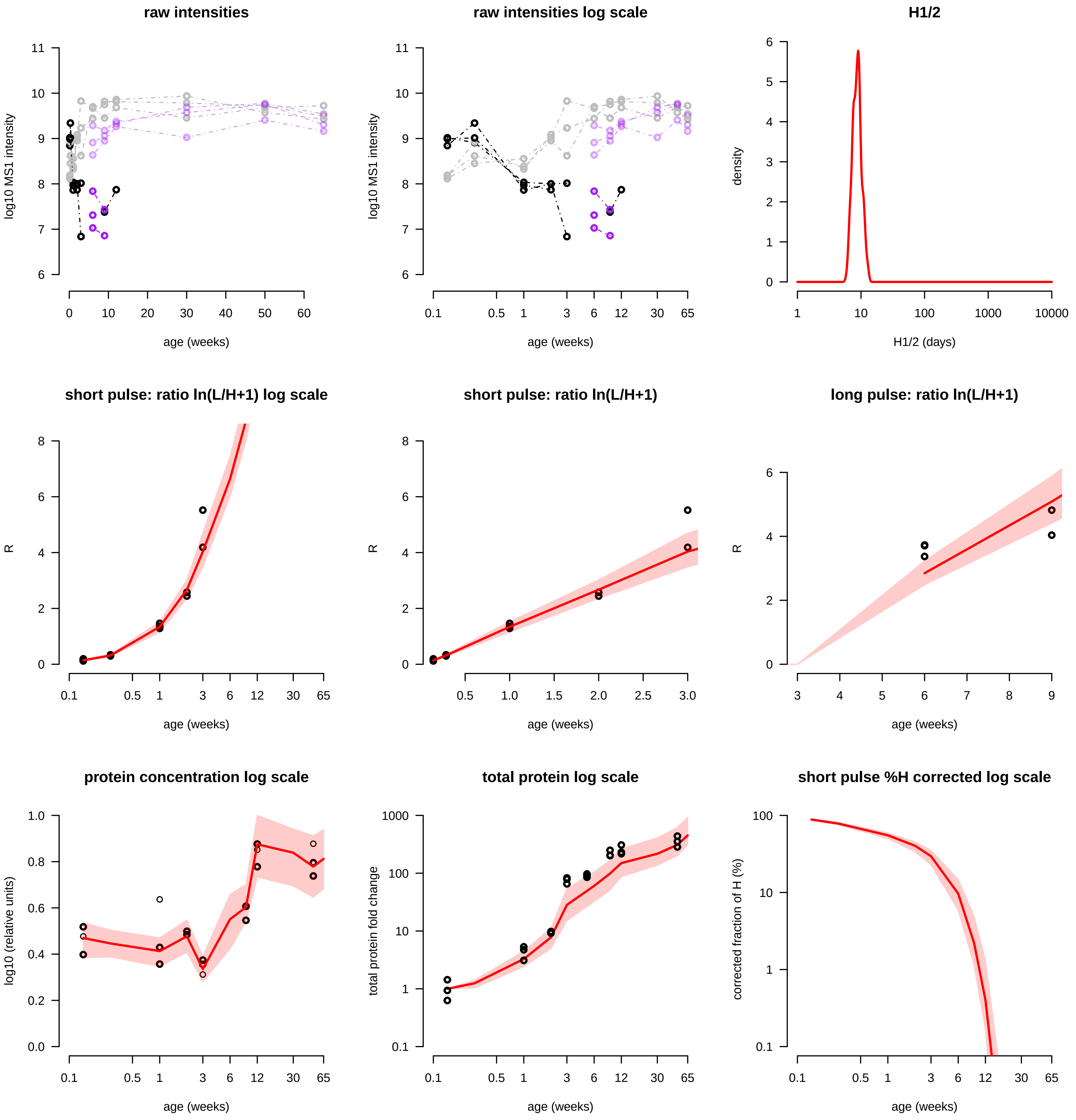

Q61656

>sp|Q61656|DDX5_MOUSE

Probable ATP−dependent RNA helicase DDX5

GN=Ddx5

Med H1/2: 169.3 days

Q5 H1/2: 85.7 days

Q95 H1/2: 466.6 days

reliable model fit: yes

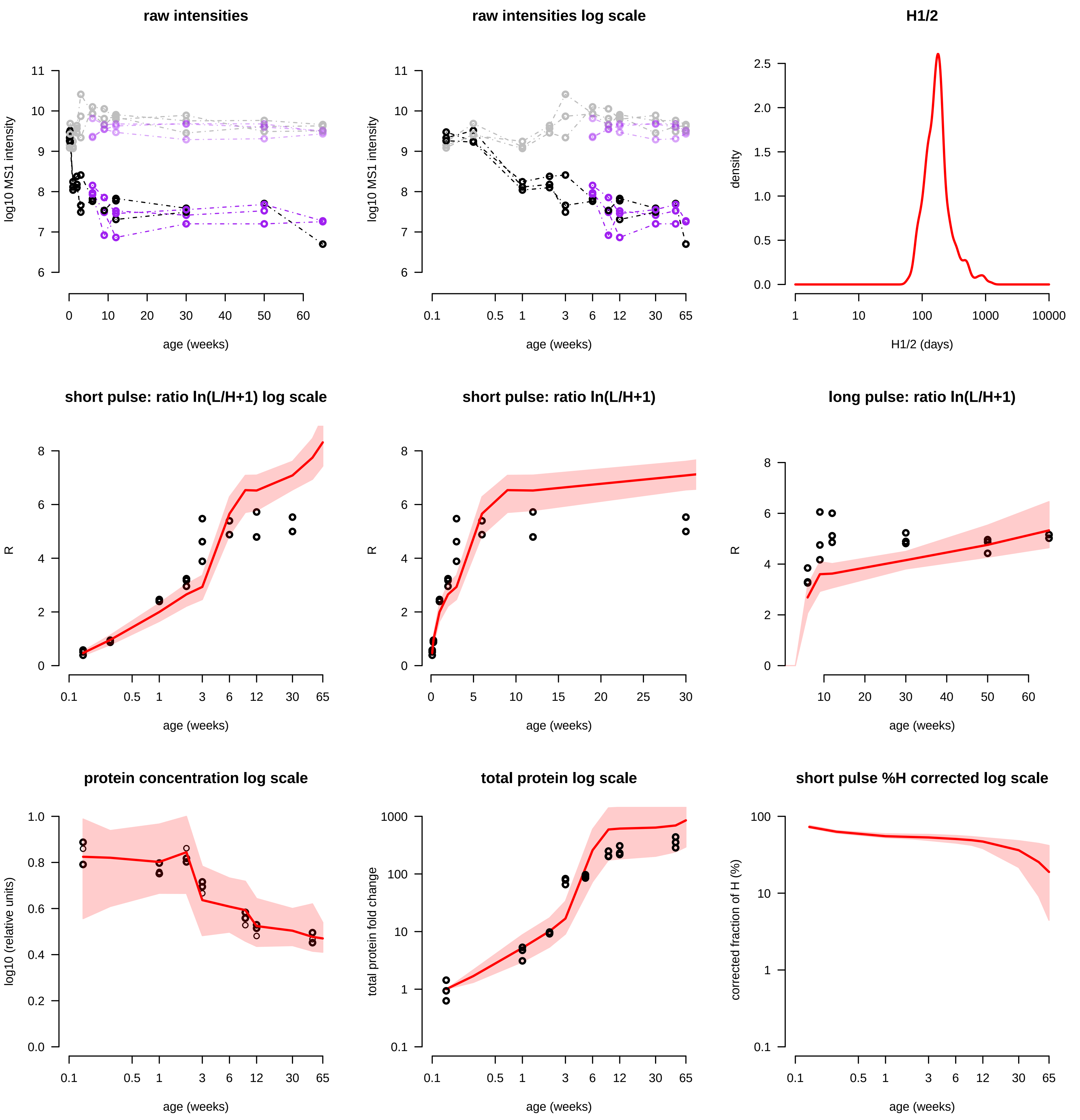

Q9CPQ8

>sp|Q9CPQ8|ATP5L_MOUSE

ATP synthase subunit g, mitochondrial

GN=Atp5mg

Med H1/2: 11 days

Q5 H1/2: 8.7 days

Q95 H1/2: 19.1 days

reliable model fit: yes

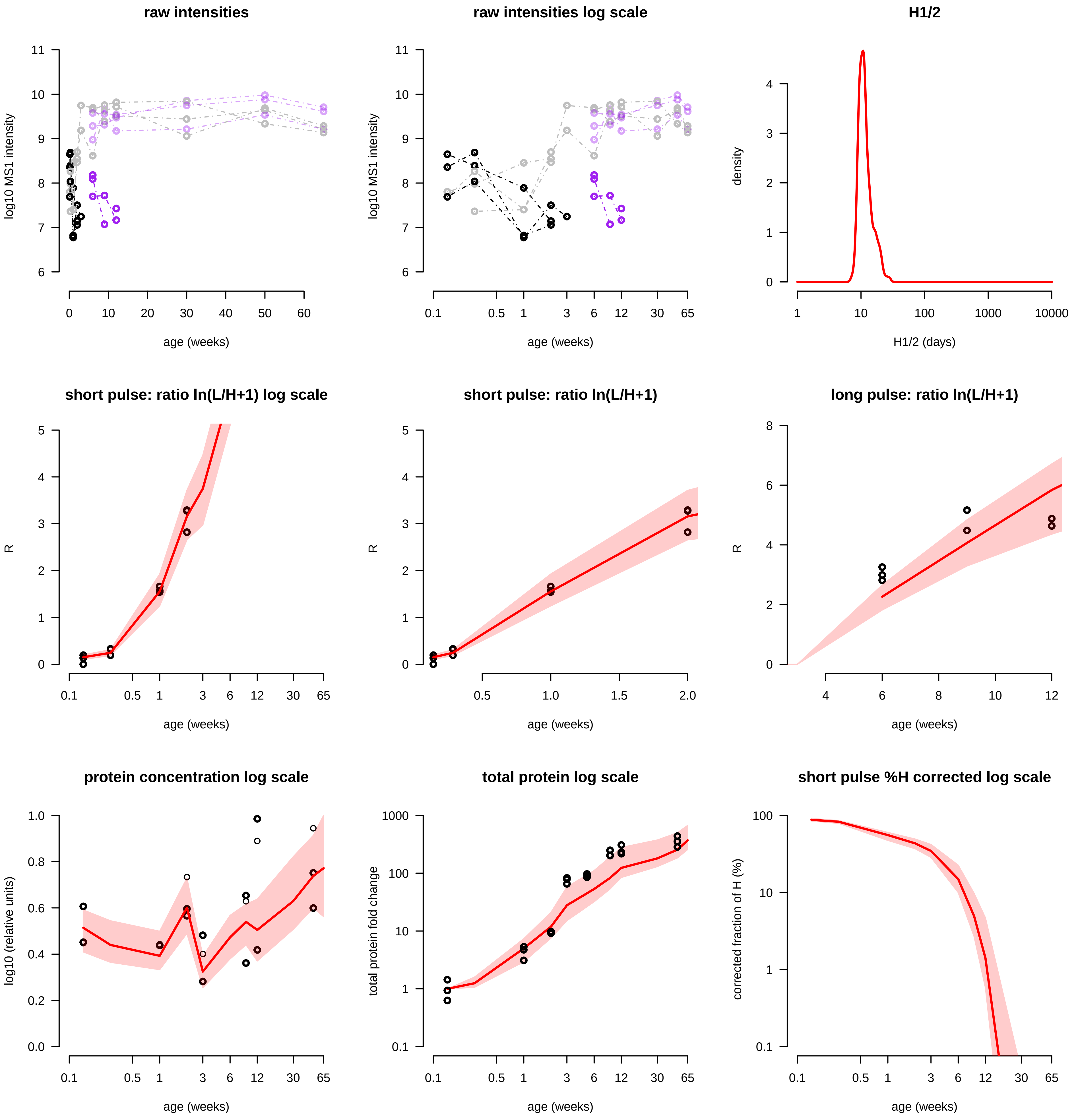

O08677;O08677−3

>sp|O08677|KNG1_MOUSE

Kininogen−1

GN=Kng1

Med H1/2: 5.8 days

Q5 H1/2: 4.6 days

Q95 H1/2: 7.1 days

reliable model fit: yes

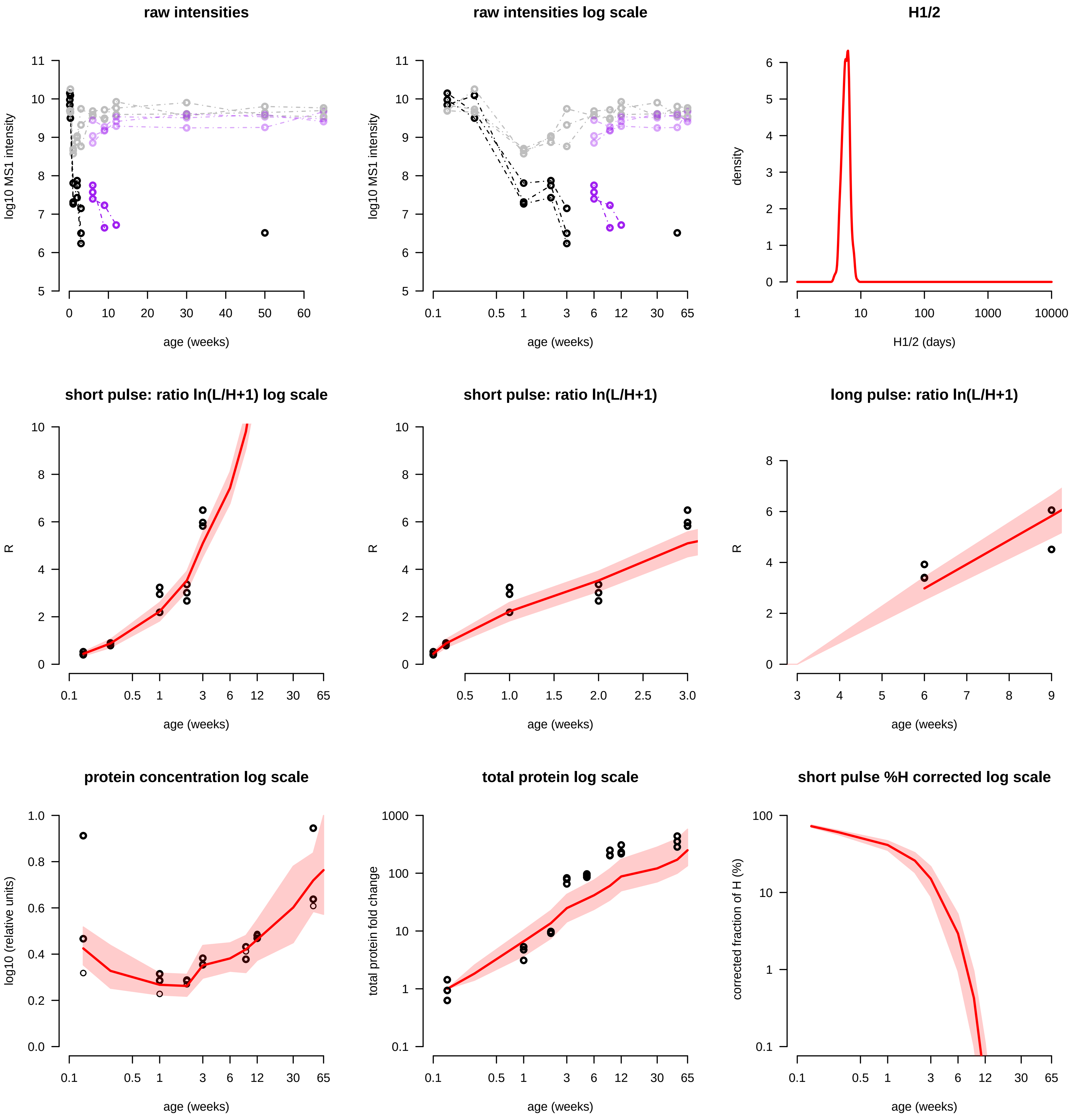

P00920

>sp|P00920|CAH2_MOUSE

Carbonic anhydrase 2

GN=Ca2

Med H1/2: 17.6 days

Q5 H1/2: 14.4 days

Q95 H1/2: 25 days

reliable model fit: yes

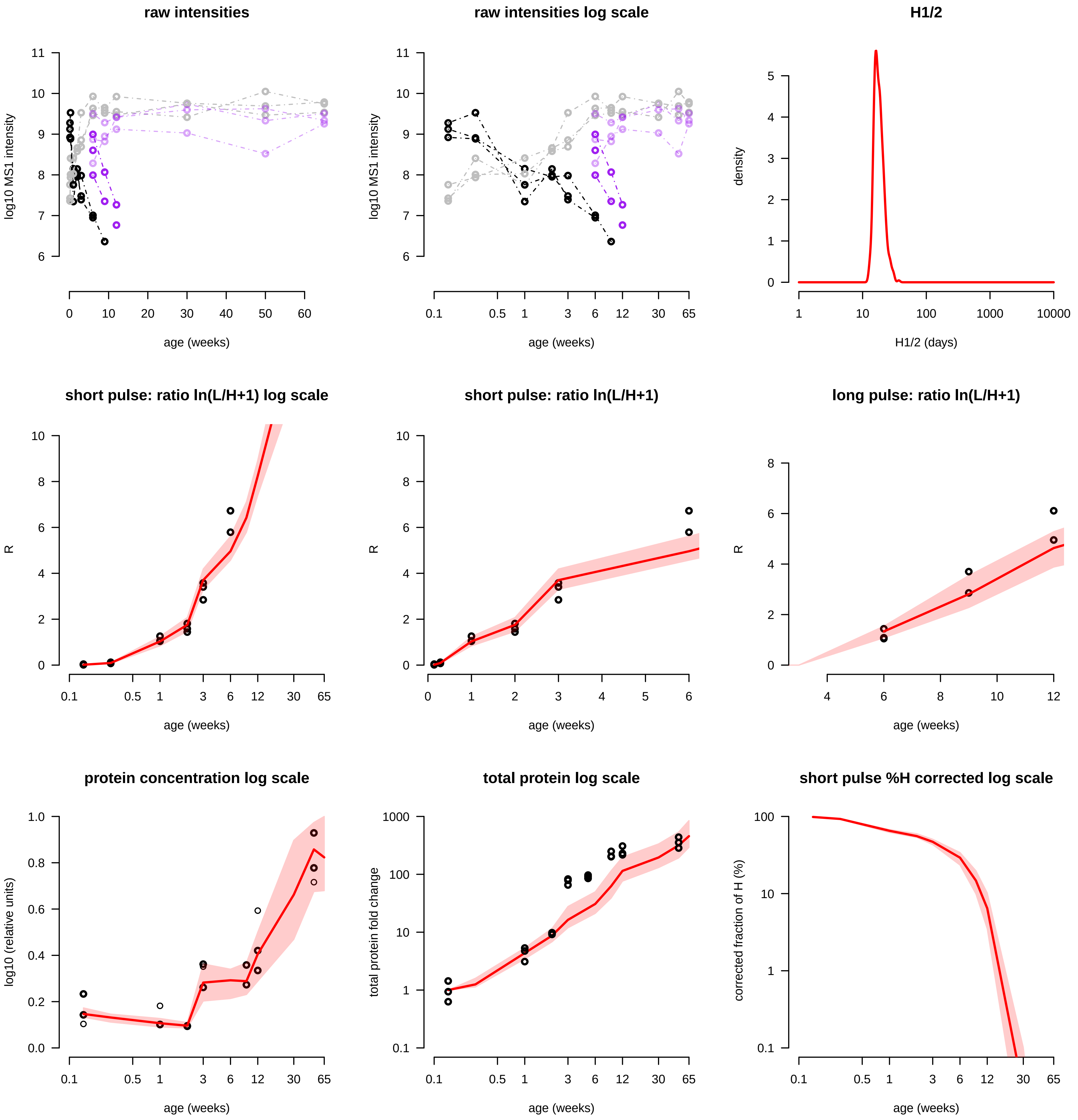

P56959

>sp|P56959|FUS_MOUSE

RNA−binding protein FUS

GN=Fus

Med H1/2: 8.2 days

Q5 H1/2: 6 days

Q95 H1/2: 11.8 days

reliable model fit: yes

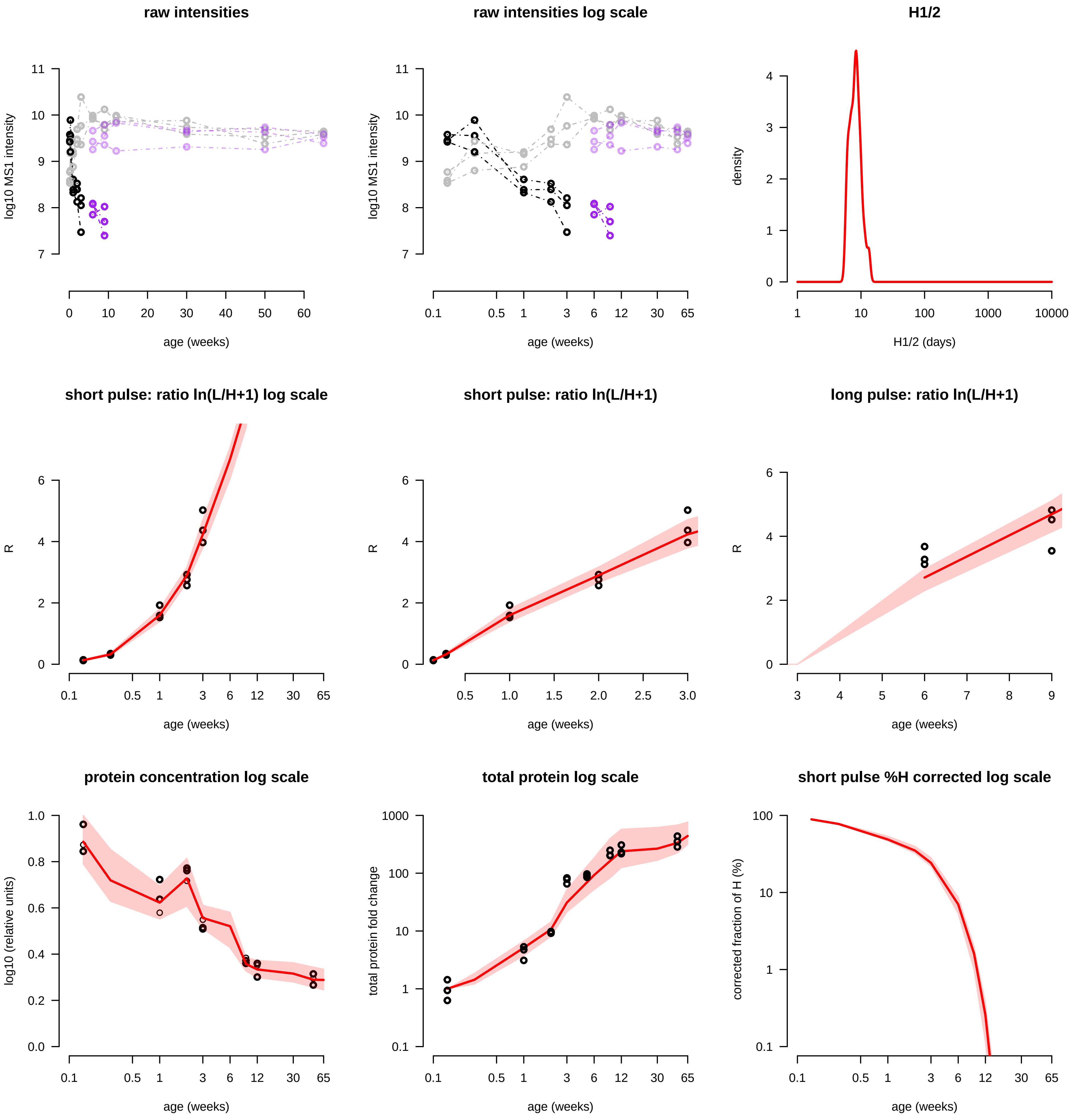

Q99K48

>sp|Q99K48|NONO_MOUSE

Non−POU domain−containing octamer−binding protein

GN=Nono

Med H1/2: 8.8 days

Q5 H1/2: 7.2 days

Q95 H1/2: 10.2 days

reliable model fit: yes

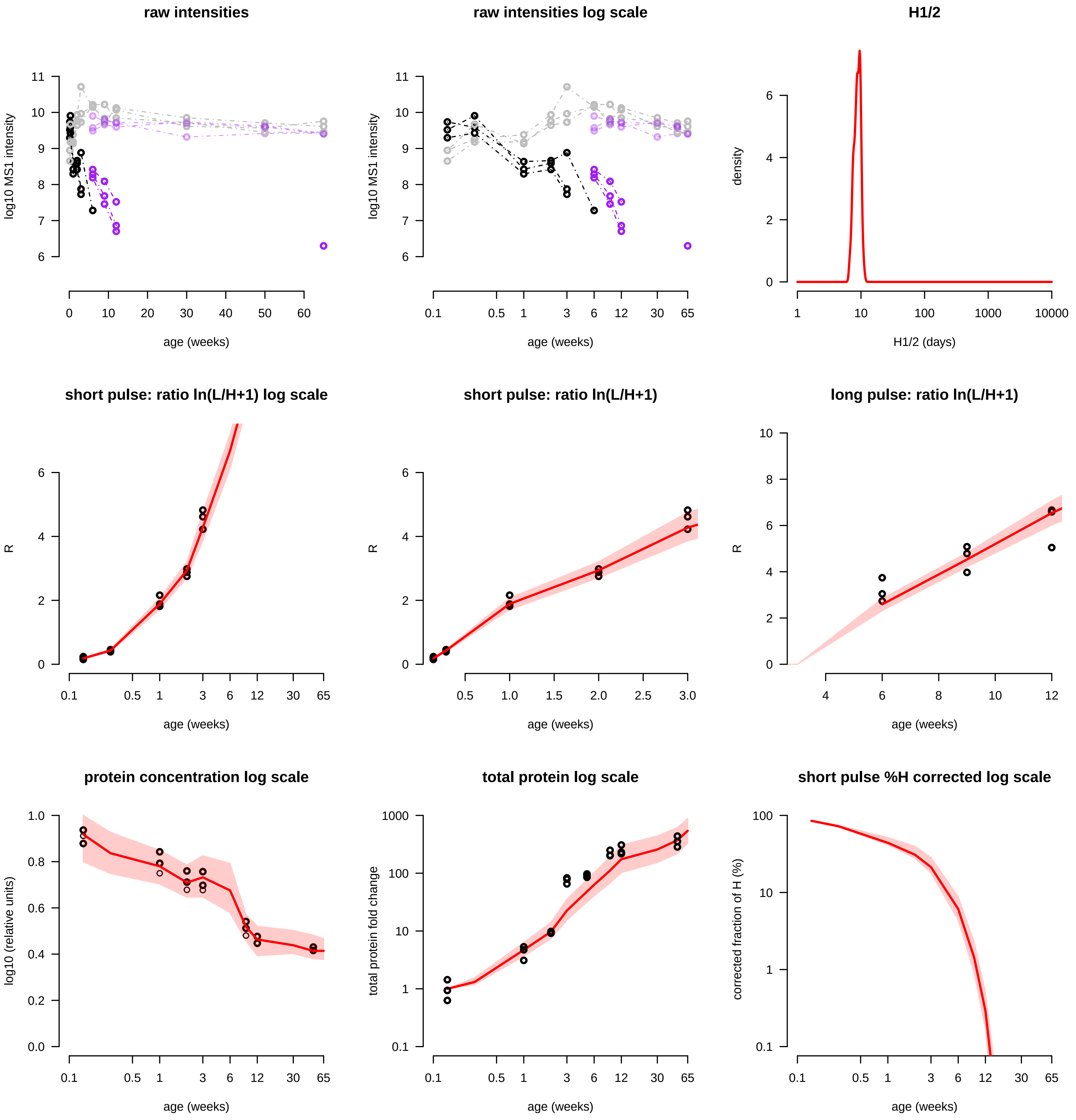

P10605

>sp|P10605|CATB_MOUSE

Cathepsin B

GN=Ctsb

Med H1/2: 7.8 days

Q5 H1/2: 6.5 days

Q95 H1/2: 10 days

reliable model fit: yes

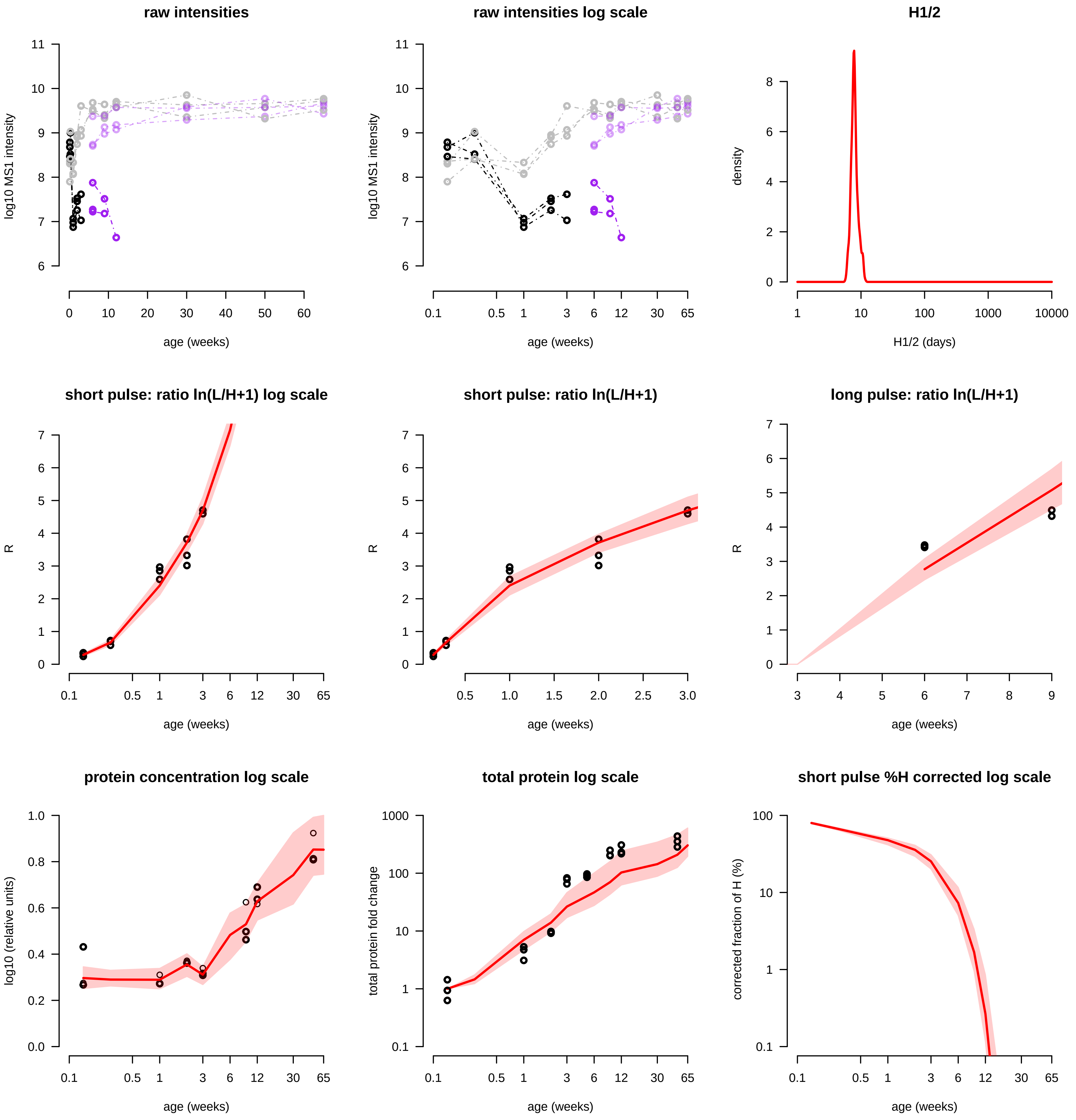

Q920A5

>sp|Q920A5|RISC_MOUSE

Retinoid−inducible serine carboxypeptidase

GN=Scpep1

Med H1/2: 1474.3 days

Q5 H1/2: 411.5 days

Q95 H1/2: 38487.3 days

reliable model fit: yes

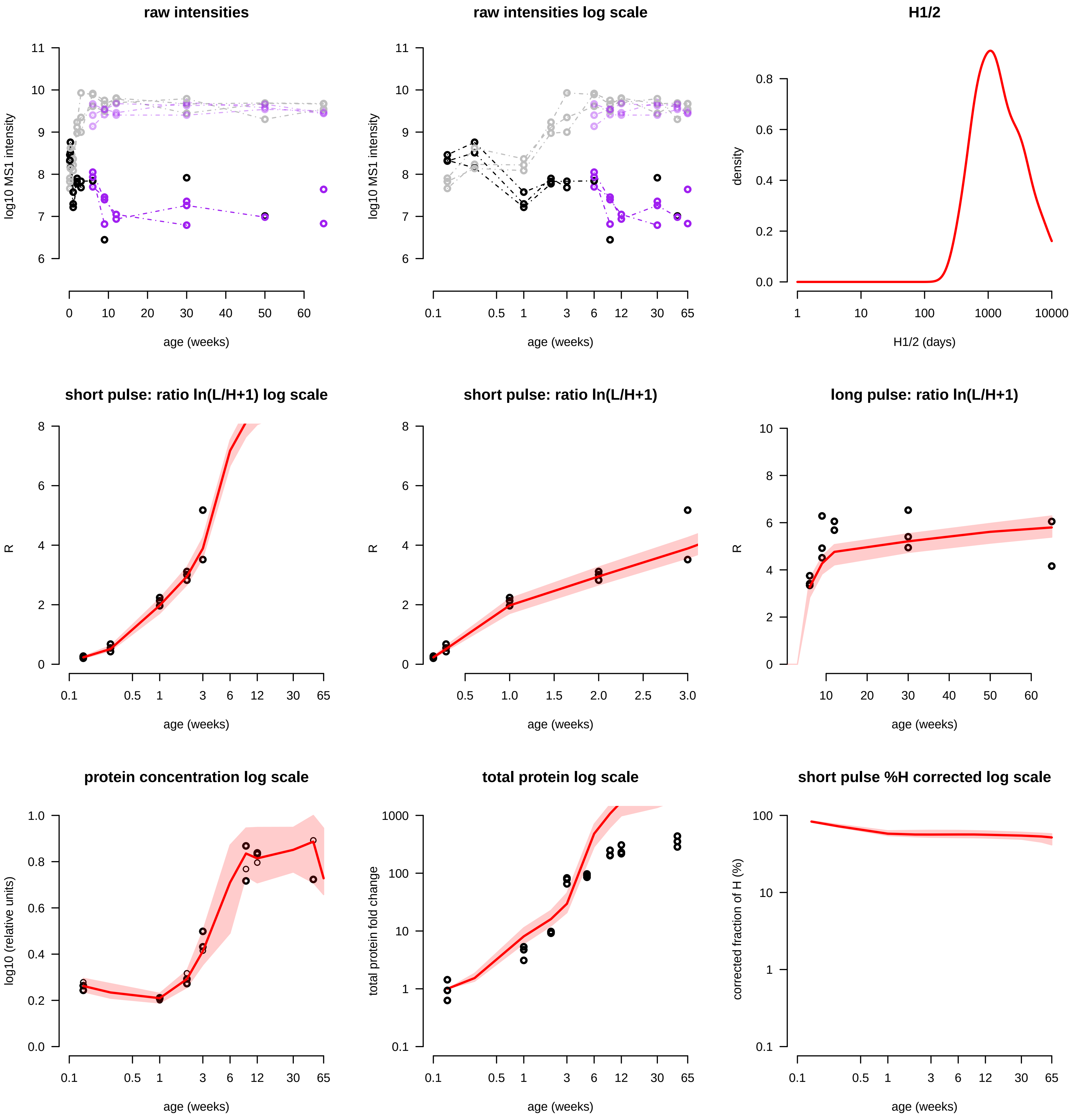

P32067

>sp|P32067|LA_MOUSE

Lupus La protein homolog

GN=Ssb

Med H1/2: 799.4 days

Q5 H1/2: 287.9 days

Q95 H1/2: 9035.7 days

reliable model fit: yes

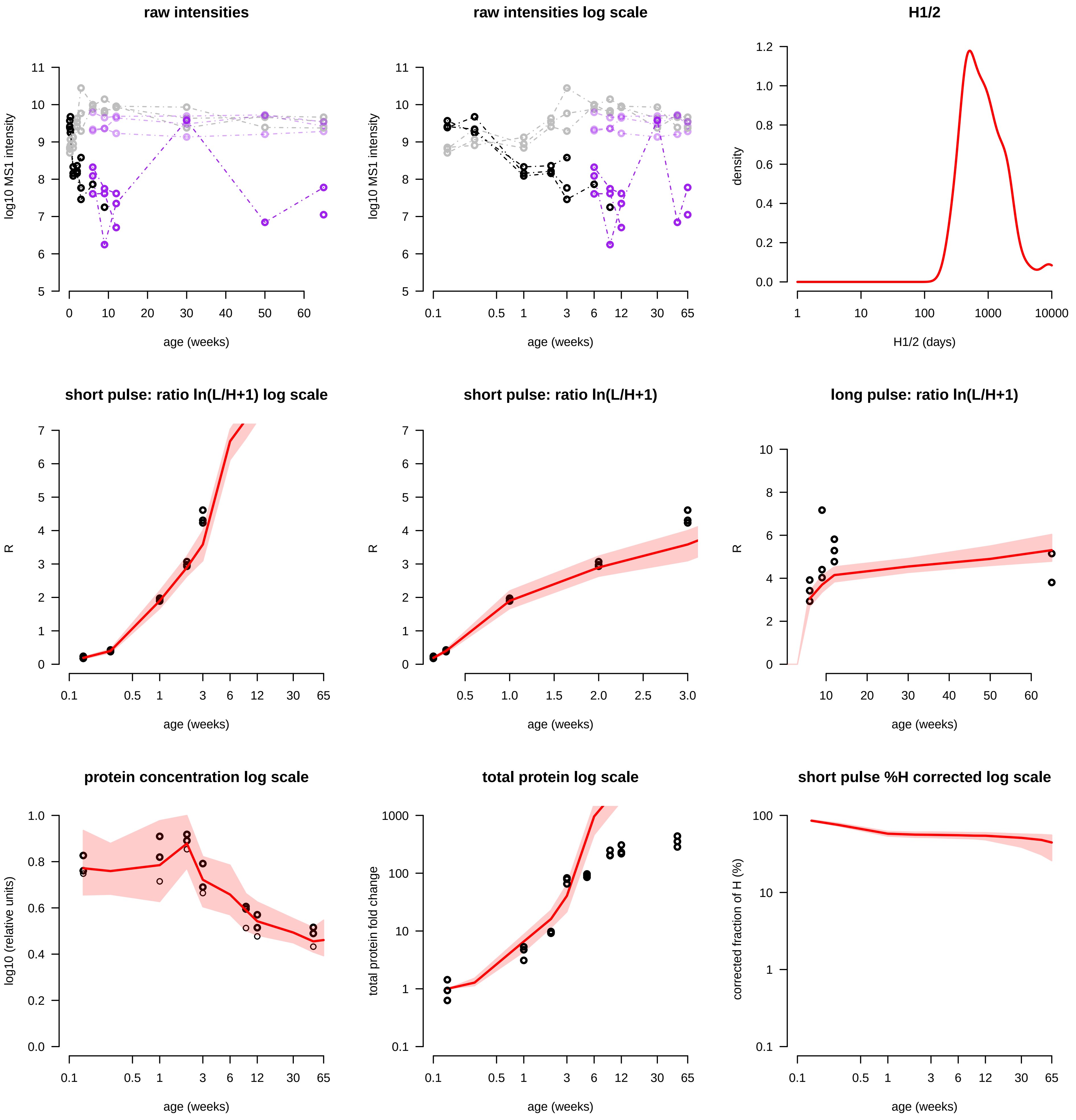

P82198

>sp|P82198|BGH3_MOUSE

Transforming growth factor−beta−induced protein ig−h3

GN=Tgfbi

Med H1/2: 6.9 days

Q5 H1/2: 5.5 days

Q95 H1/2: 9.3 days

reliable model fit: yes

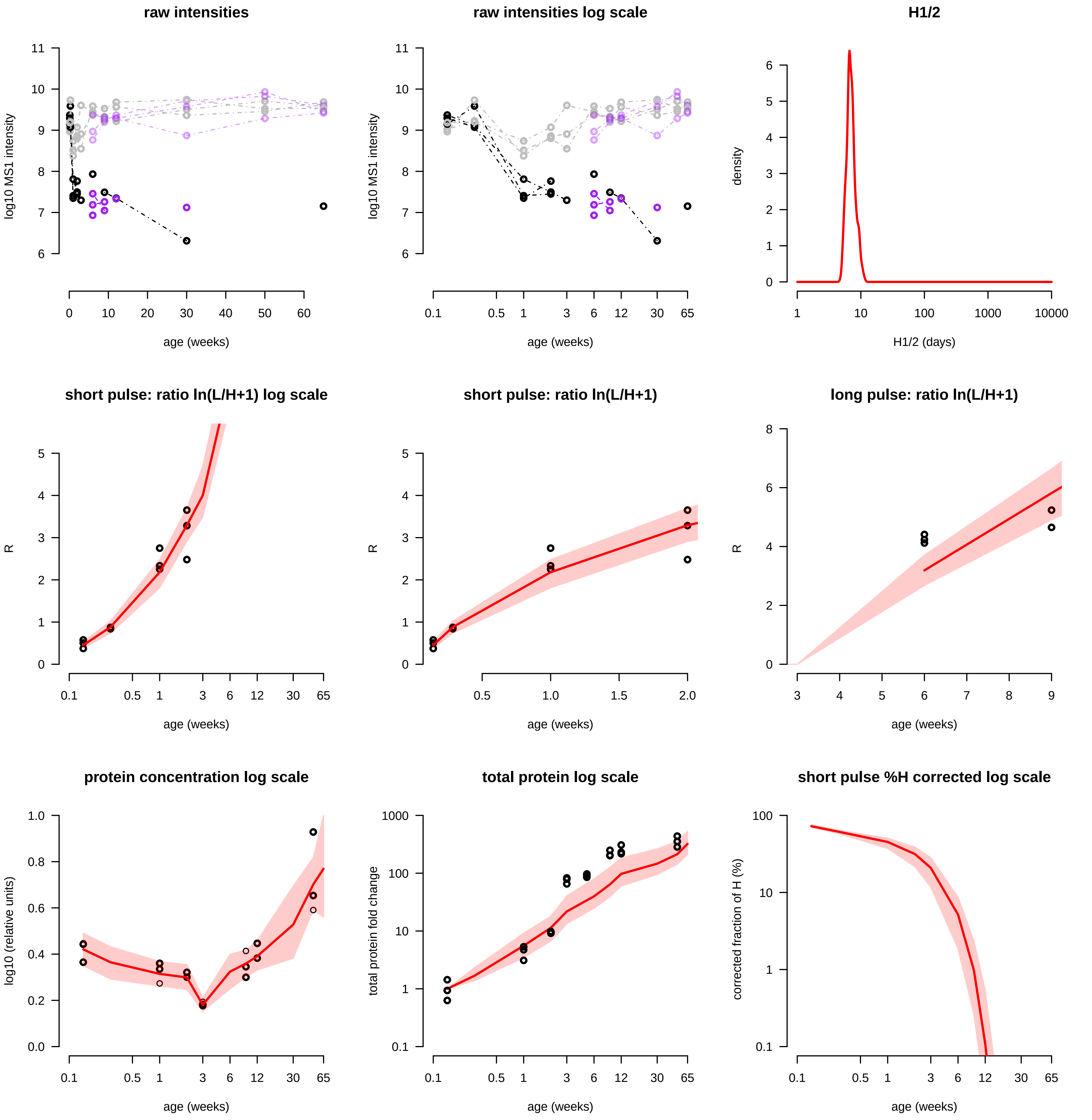

Q8BG32

>sp|Q8BG32|PSD11_MOUSE

26S proteasome non−ATPase regulatory subunit 11

GN=Psmd11

Med H1/2: 7.8 days

Q5 H1/2: 6.3 days

Q95 H1/2: 10.1 days

reliable model fit: yes

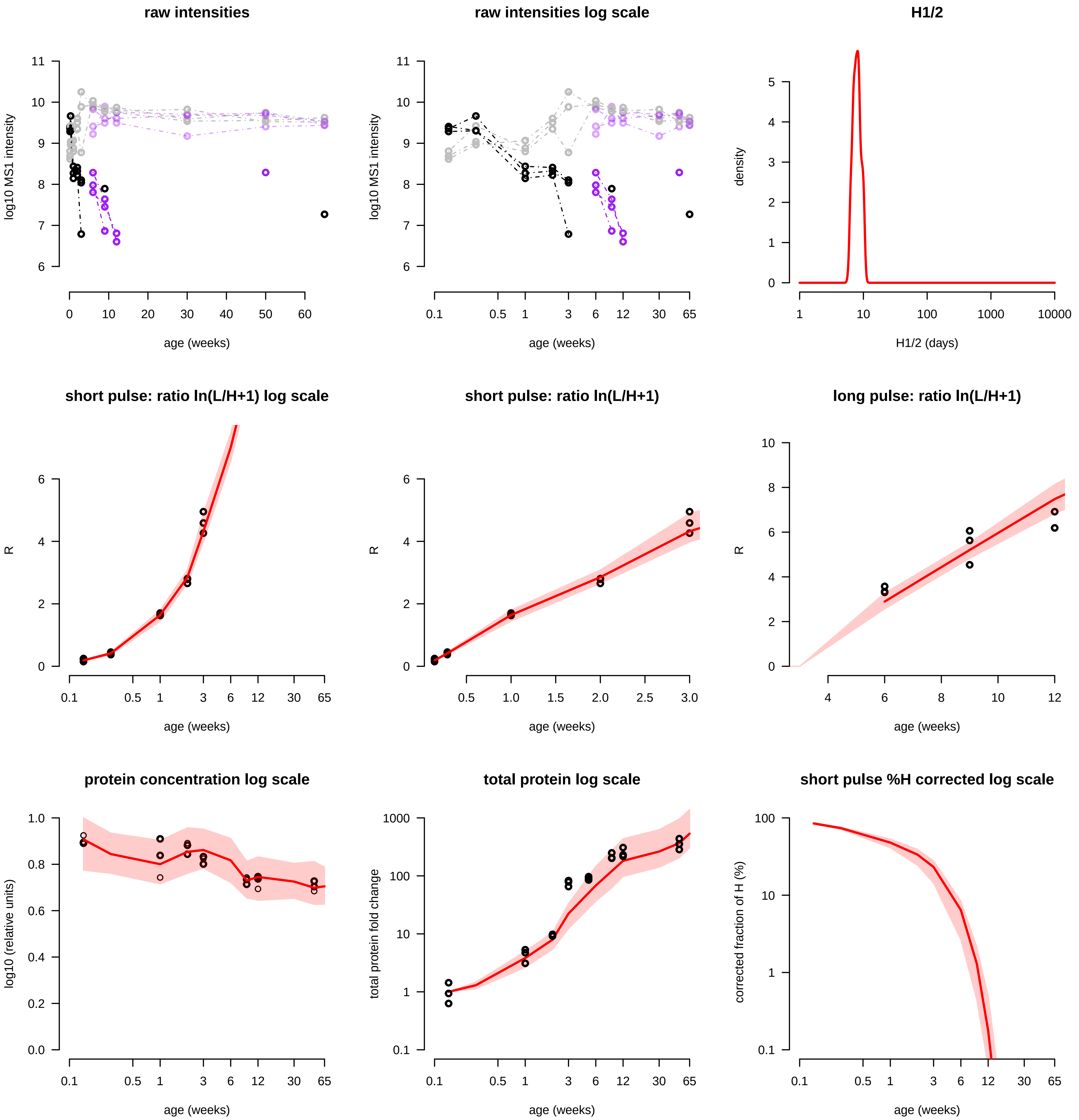

P51660

>sp|P51660|DHB4_MOUSE

Peroxisomal multifunctional enzyme type 2

GN=Hsd17b4

Med H1/2: 11 days

Q5 H1/2: 9 days

Q95 H1/2: 14.7 days

reliable model fit: yes

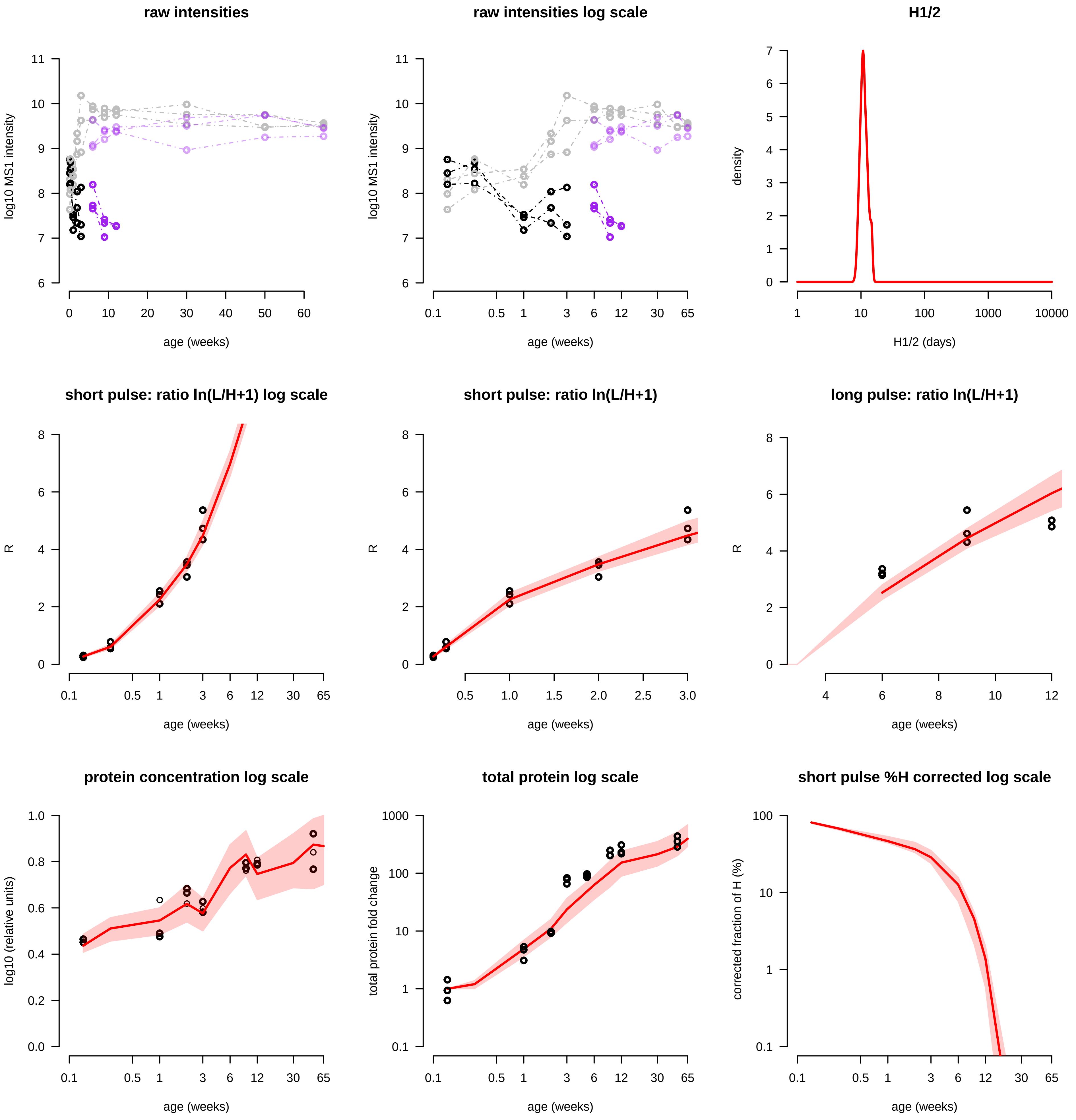

P62852

>sp|P62852|RS25_MOUSE

40S ribosomal protein S25

GN=Rps25

Med H1/2: 17.8 days

Q5 H1/2: 14 days

Q95 H1/2: 24.4 days

reliable model fit: yes

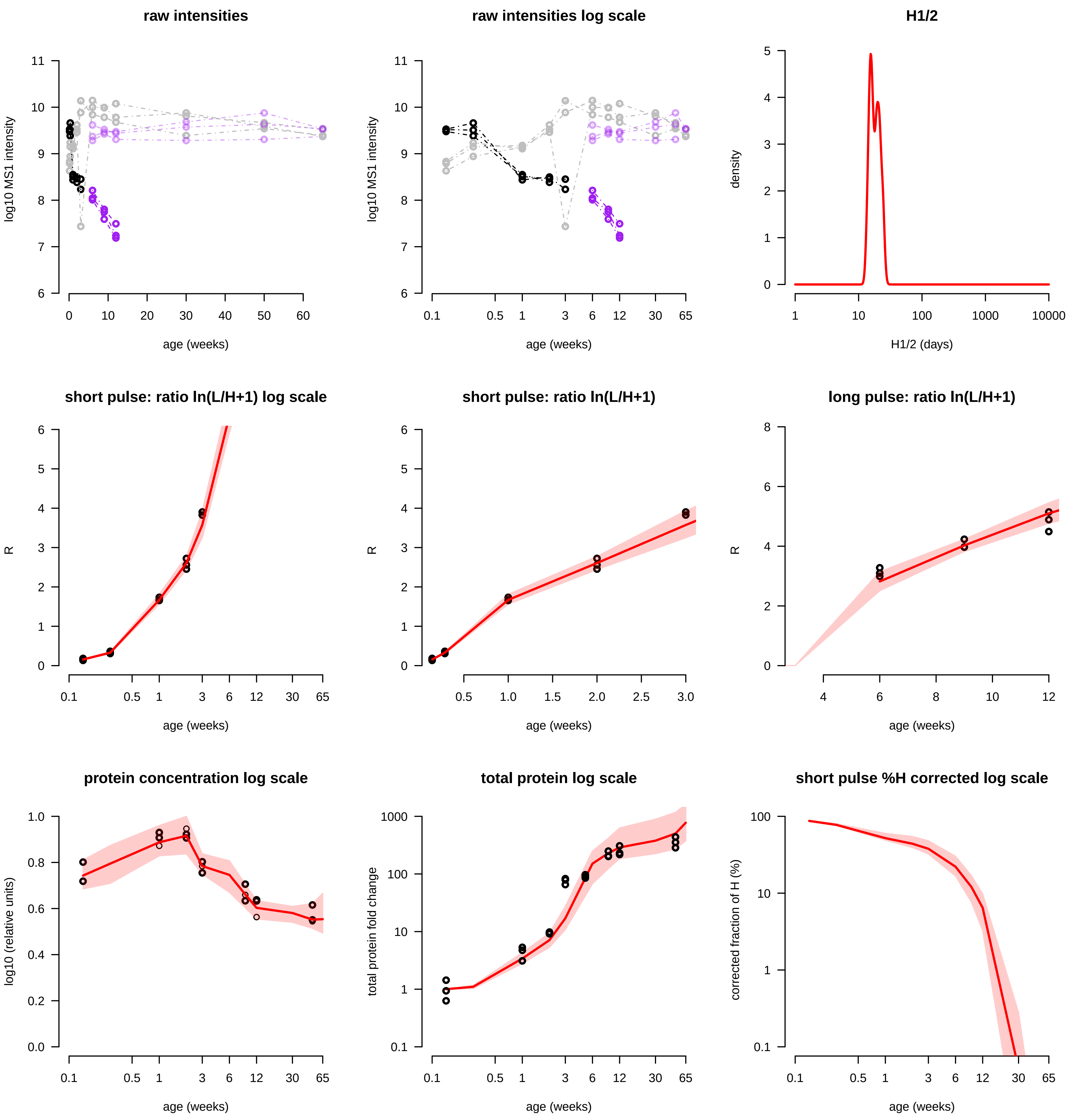

Q3U1J4

>sp|Q3U1J4|DDB1_MOUSE

DNA damage−binding protein 1

GN=Ddb1

Med H1/2: 6.3 days

Q5 H1/2: 5.1 days

Q95 H1/2: 7.6 days

reliable model fit: yes

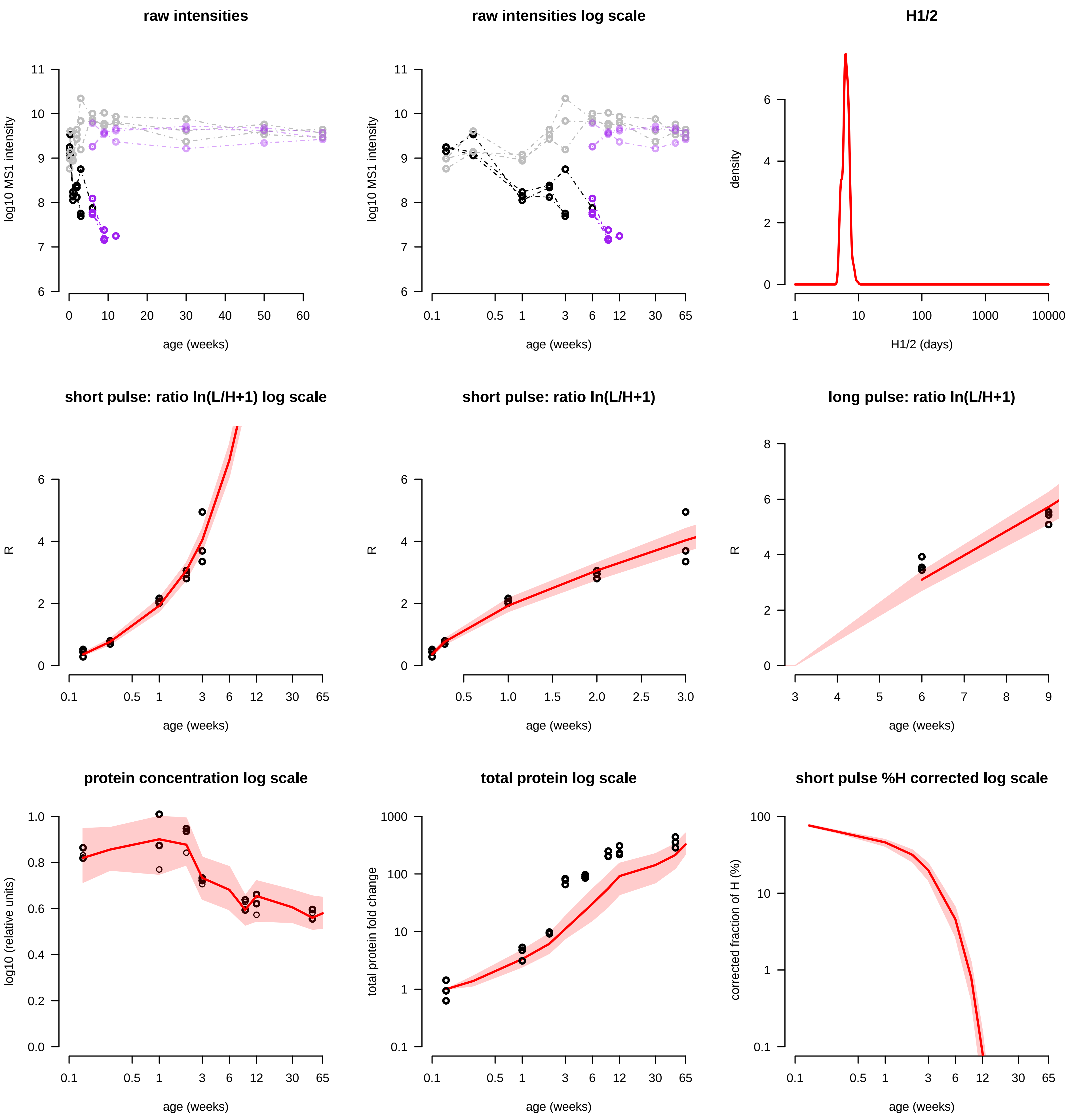

P23116

>sp|P23116|EIF3A_MOUSE

Eukaryotic translation initiation factor 3 subunit A

GN=Eif3a

Med H1/2: 1126 days

Q5 H1/2: 358.7 days

Q95 H1/2: 13962.3 days

reliable model fit: yes

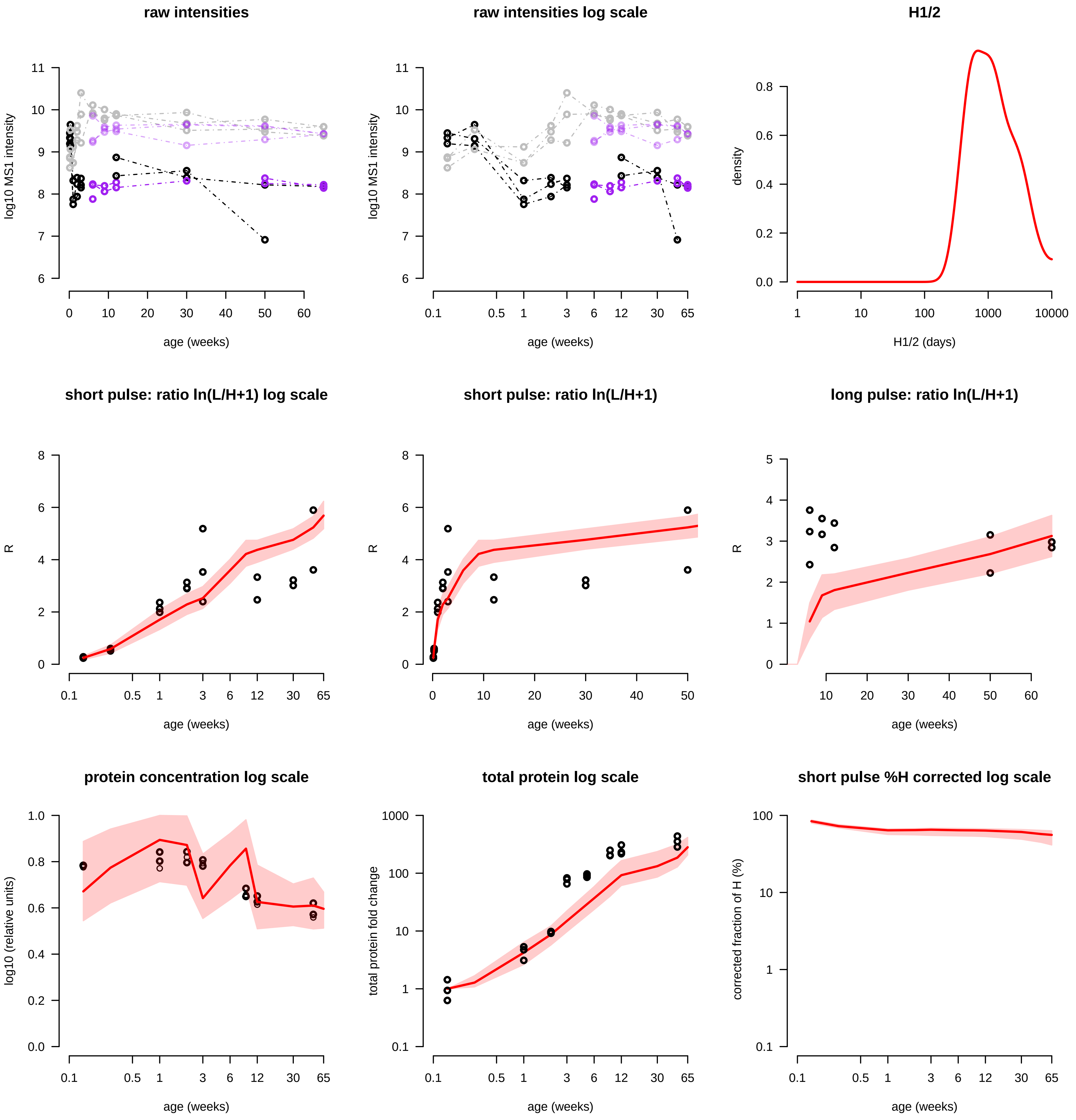

Q9Z2D6−2

>sp|Q9Z2D6−2|MECP2_MOUSE

Isoform B of Methyl−CpG−binding protein 2

GN=Mecp2

Med H1/2: 11.6 days

Q5 H1/2: 9.1 days

Q95 H1/2: 16.8 days

reliable model fit: yes

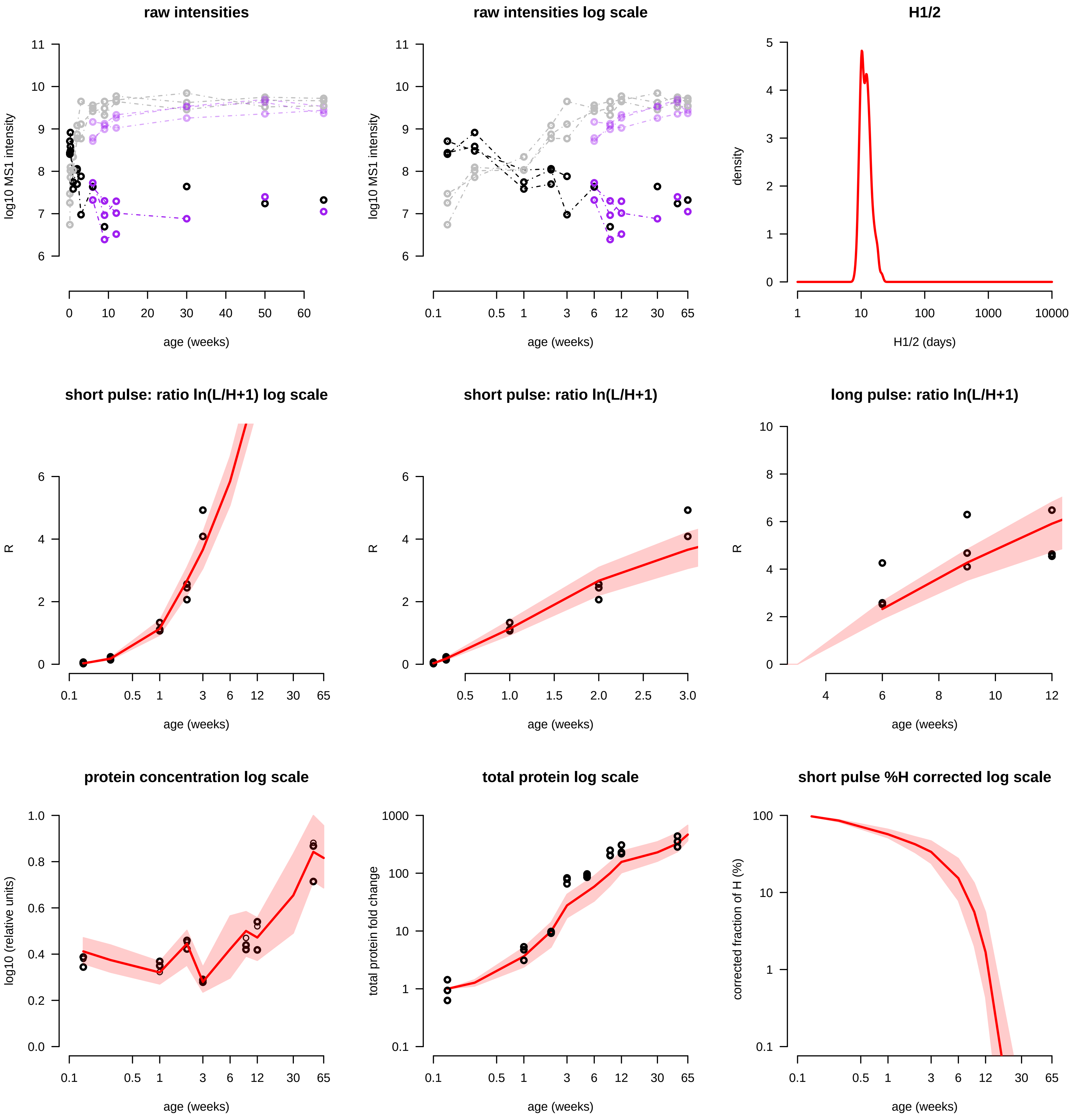

Q8BGQ7

>sp|Q8BGQ7|SYAC_MOUSE

Alanine−−tRNA ligase, cytoplasmic

GN=Aars1

Med H1/2: 4.4 days

Q5 H1/2: 3.7 days

Q95 H1/2: 5.5 days

reliable model fit: yes

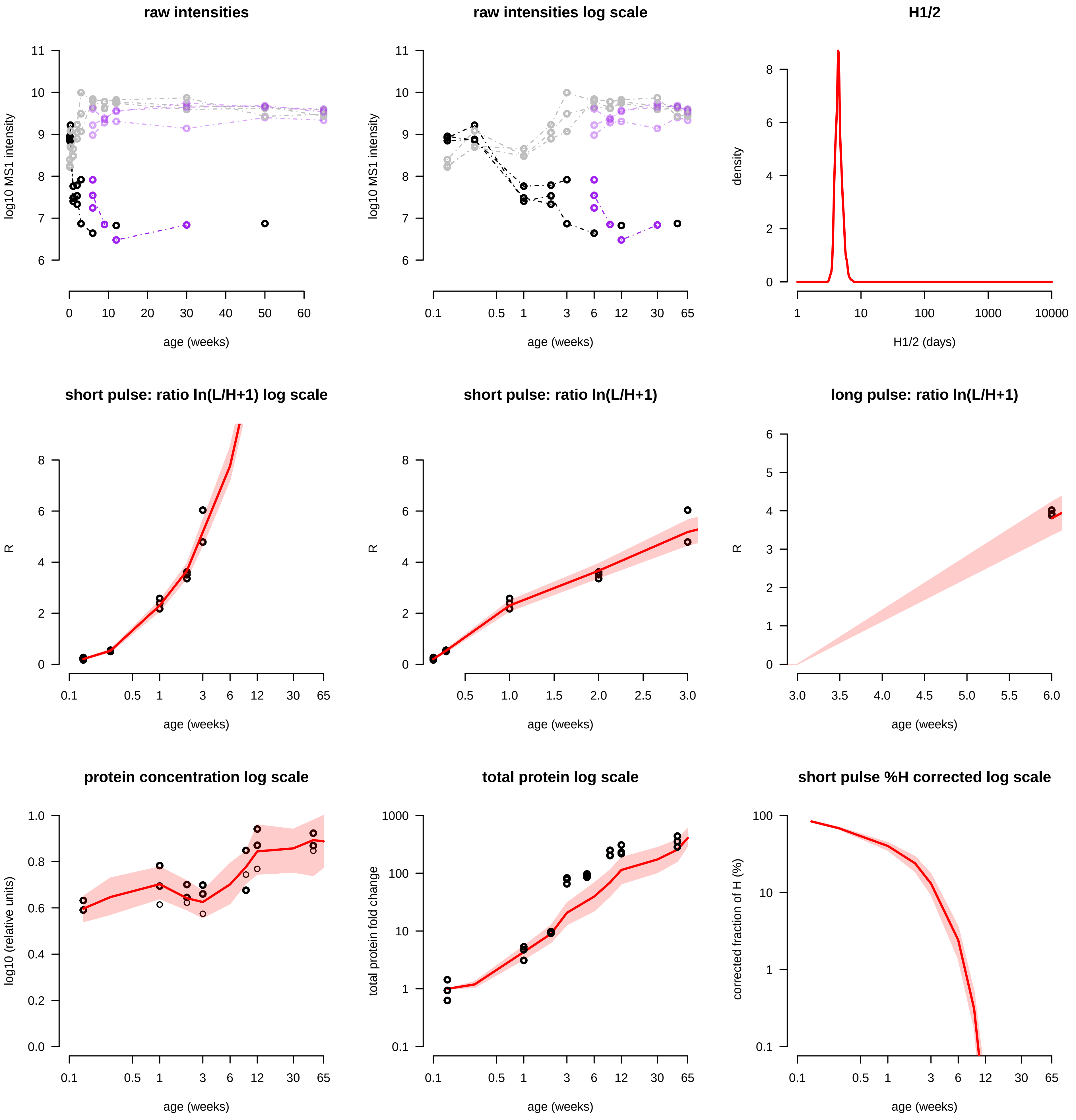

Q9D0J8

>sp|Q9D0J8|PTMS_MOUSE

Parathymosin

GN=Ptms

Med H1/2: 15.1 days

Q5 H1/2: 10.9 days

Q95 H1/2: 23.1 days

reliable model fit: yes

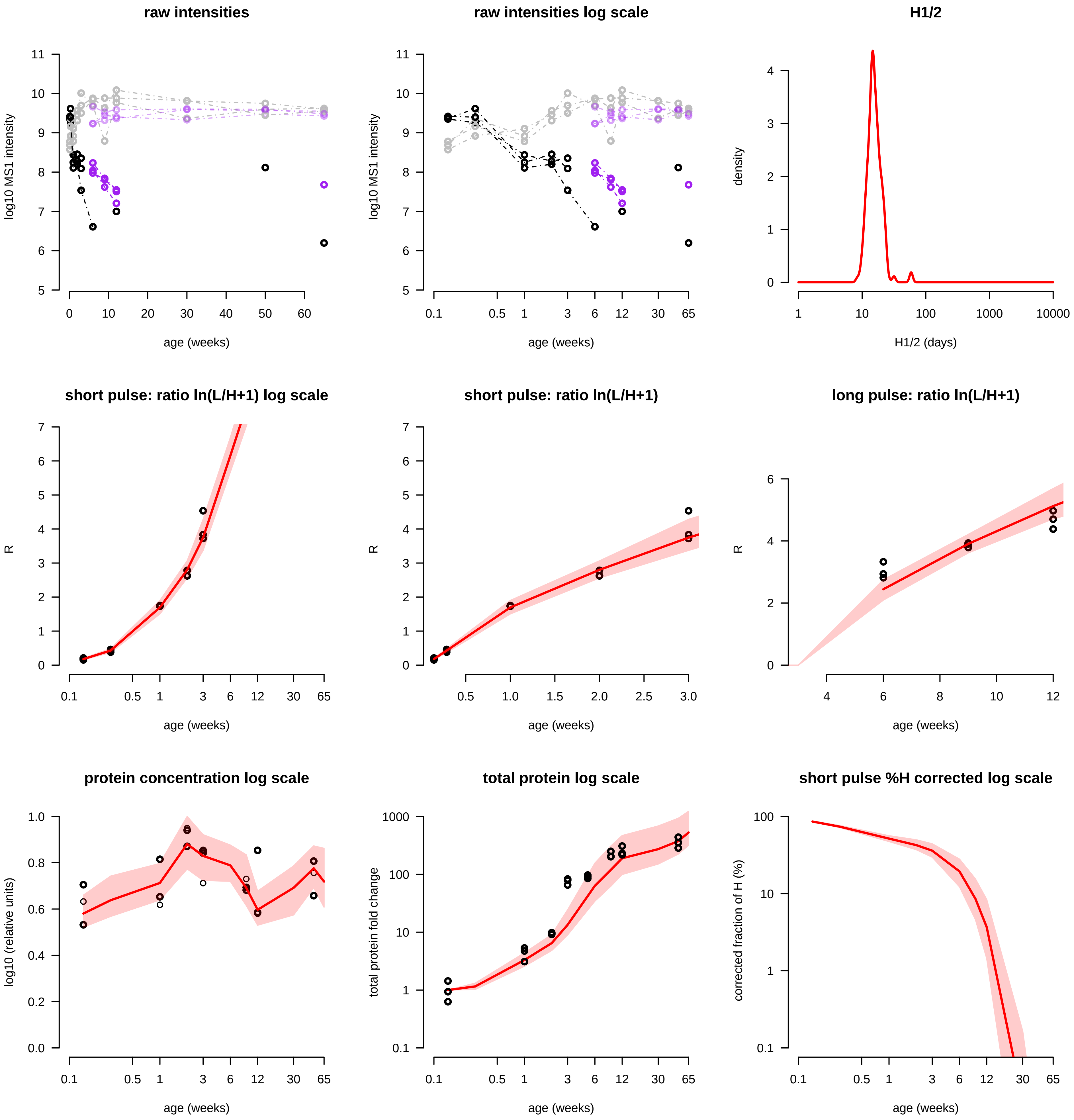

Q8BMF4

>sp|Q8BMF4|ODP2_MOUSE

sine−residue acetyltransferase component of pyruvate dehydrogenase complex

GN=Dlat

Med H1/2: 10.9 days

Q5 H1/2: 9 days

Q95 H1/2: 15 days

reliable model fit: yes

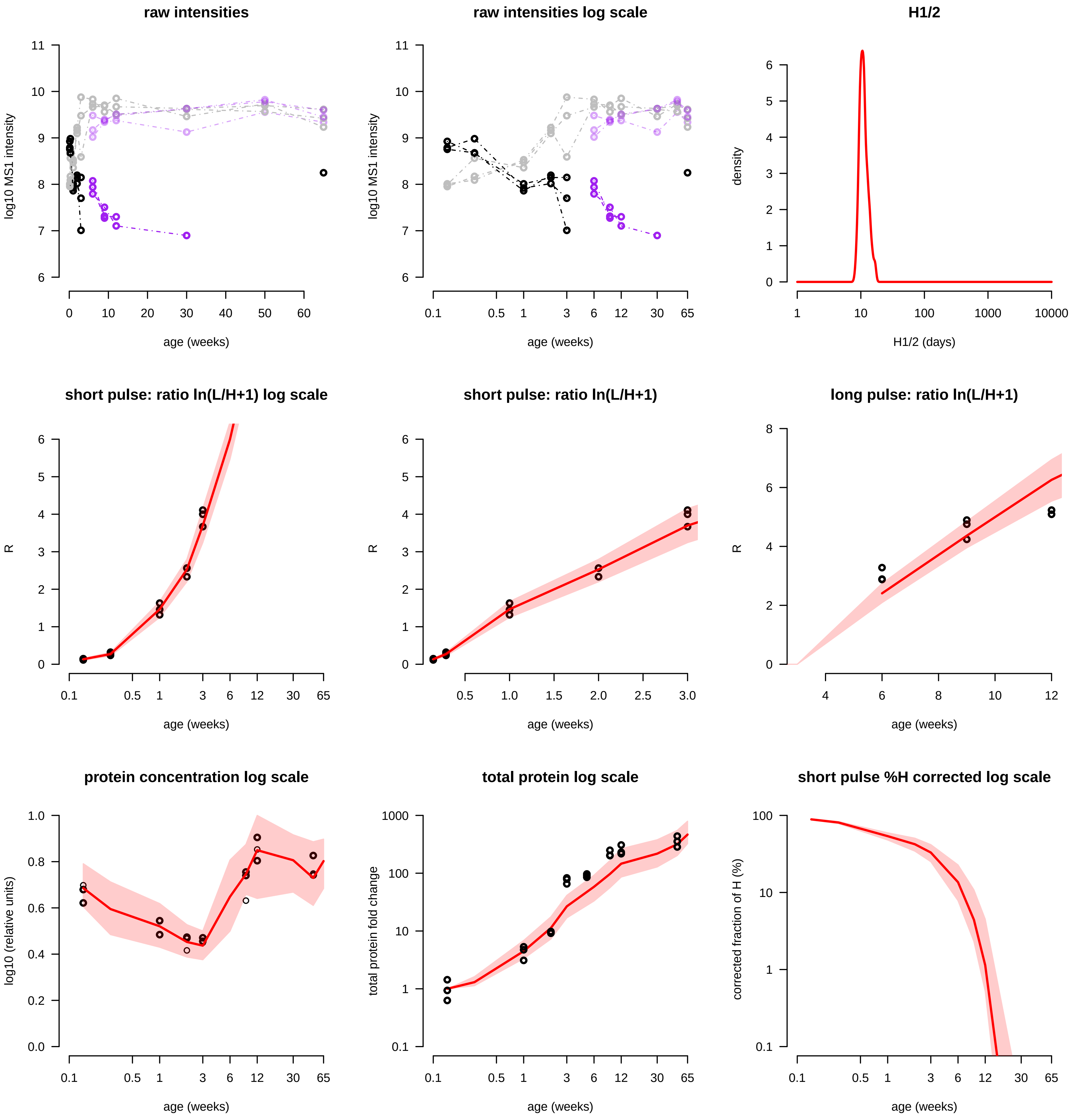

P29341

>sp|P29341|PABP1_MOUSE

Polyadenylate−binding protein 1

GN=Pabpc1

Med H1/2: 9.7 days

Q5 H1/2: 7.8 days

Q95 H1/2: 15.7 days

reliable model fit: yes

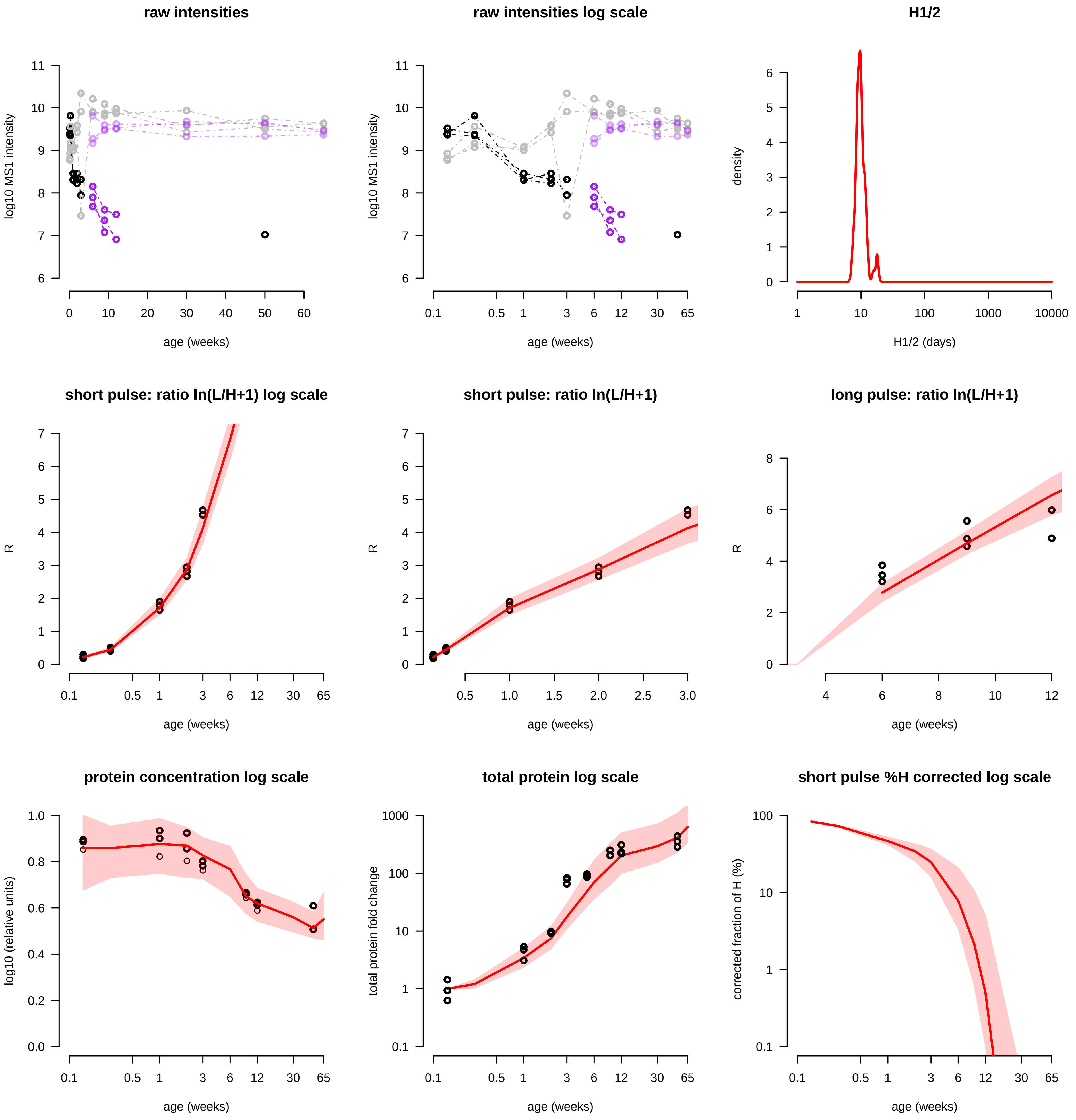

Q3THE2

>sp|Q3THE2|ML12B_MOUSE

Myosin regulatory light chain 12B

GN=Myl12b

Med H1/2: 7.1 days

Q5 H1/2: 5.7 days

Q95 H1/2: 10.6 days

reliable model fit: yes

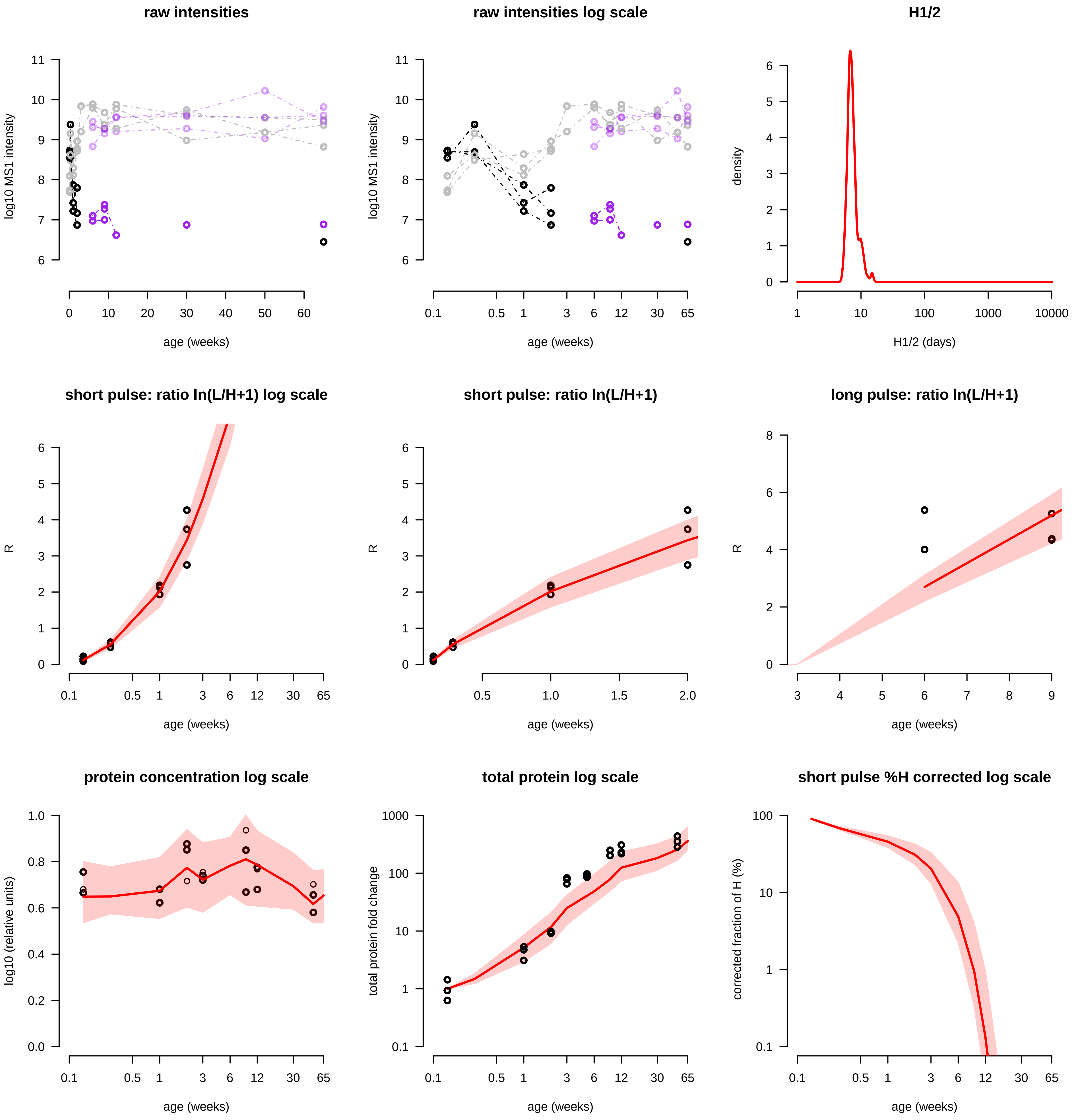

Q62465

>sp|Q62465|VAT1_MOUSE

Synaptic vesicle membrane protein VAT−1 homolog

GN=Vat1

Med H1/2: 724.3 days

Q5 H1/2: 156.5 days

Q95 H1/2: 7490.8 days

reliable model fit: yes

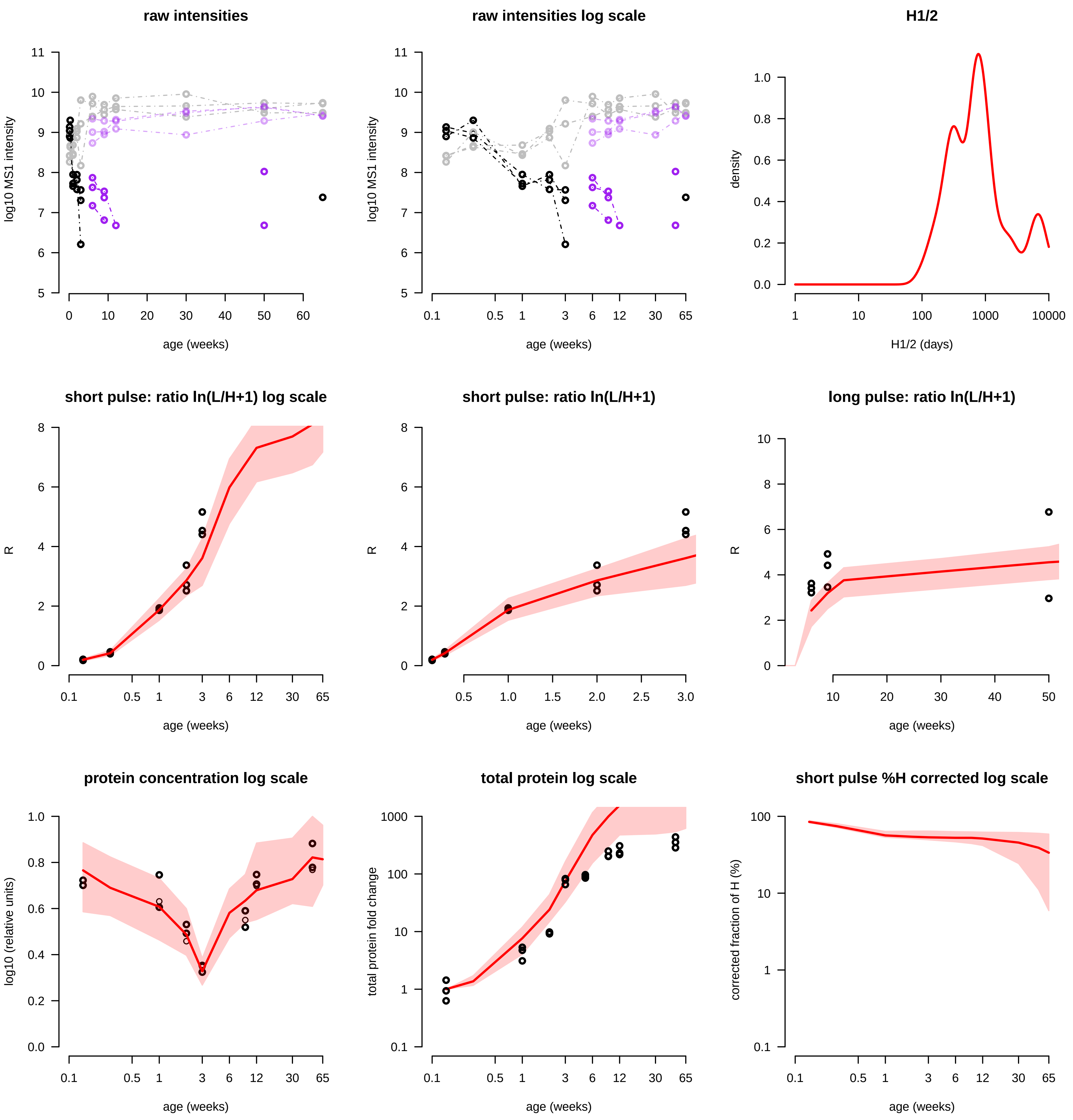

P62301

>sp|P62301|RS13_MOUSE

40S ribosomal protein S13

GN=Rps13

Med H1/2: 9.1 days

Q5 H1/2: 7.3 days

Q95 H1/2: 11.4 days

reliable model fit: yes

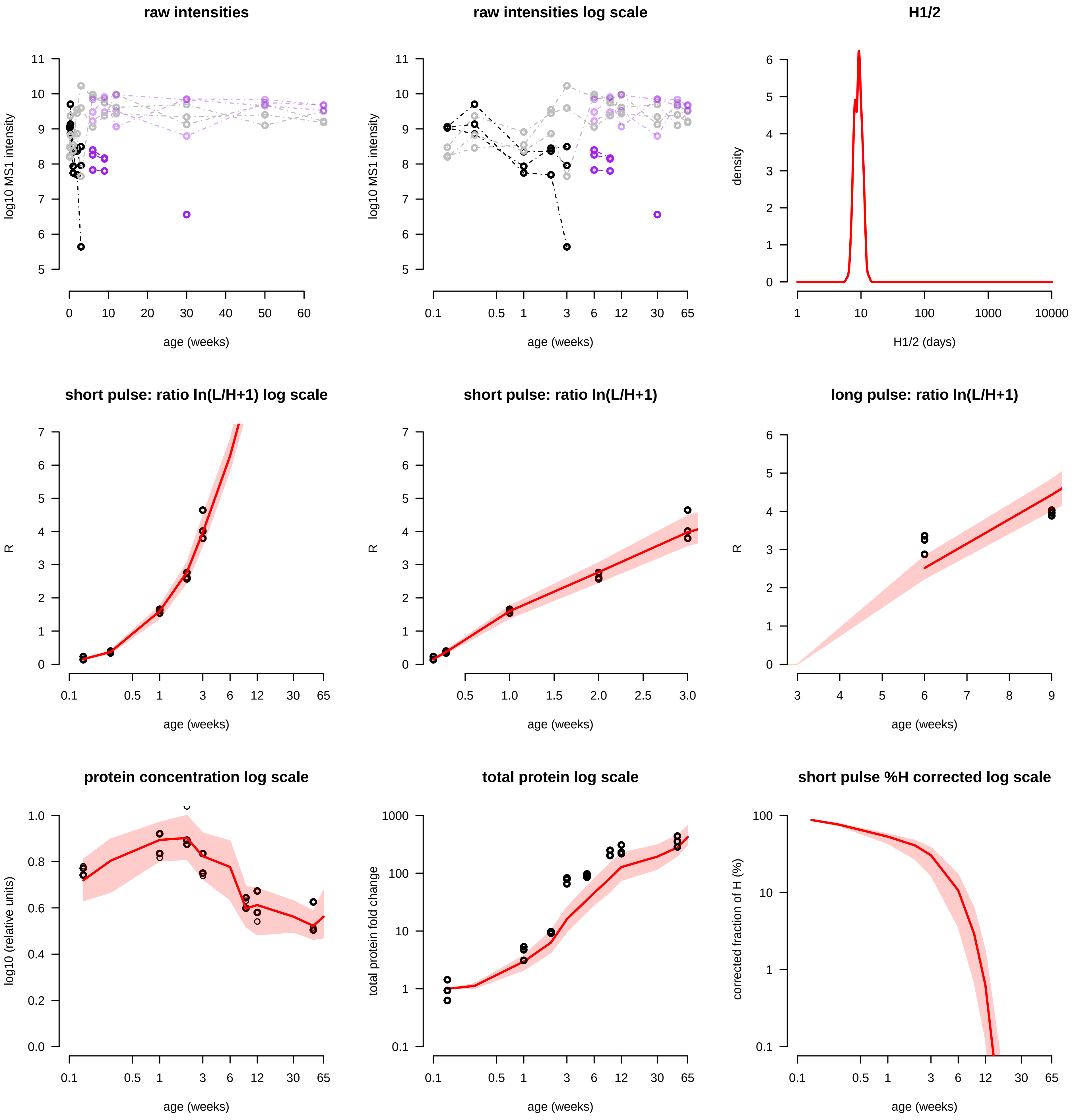

Q8VCW8

>sp|Q8VCW8|ACSF2_MOUSE

Medium−chain acyl−CoA ligase ACSF2, mitochondrial

GN=Acsf2

Med H1/2: 11.5 days

Q5 H1/2: 9.1 days

Q95 H1/2: 16.3 days

reliable model fit: yes

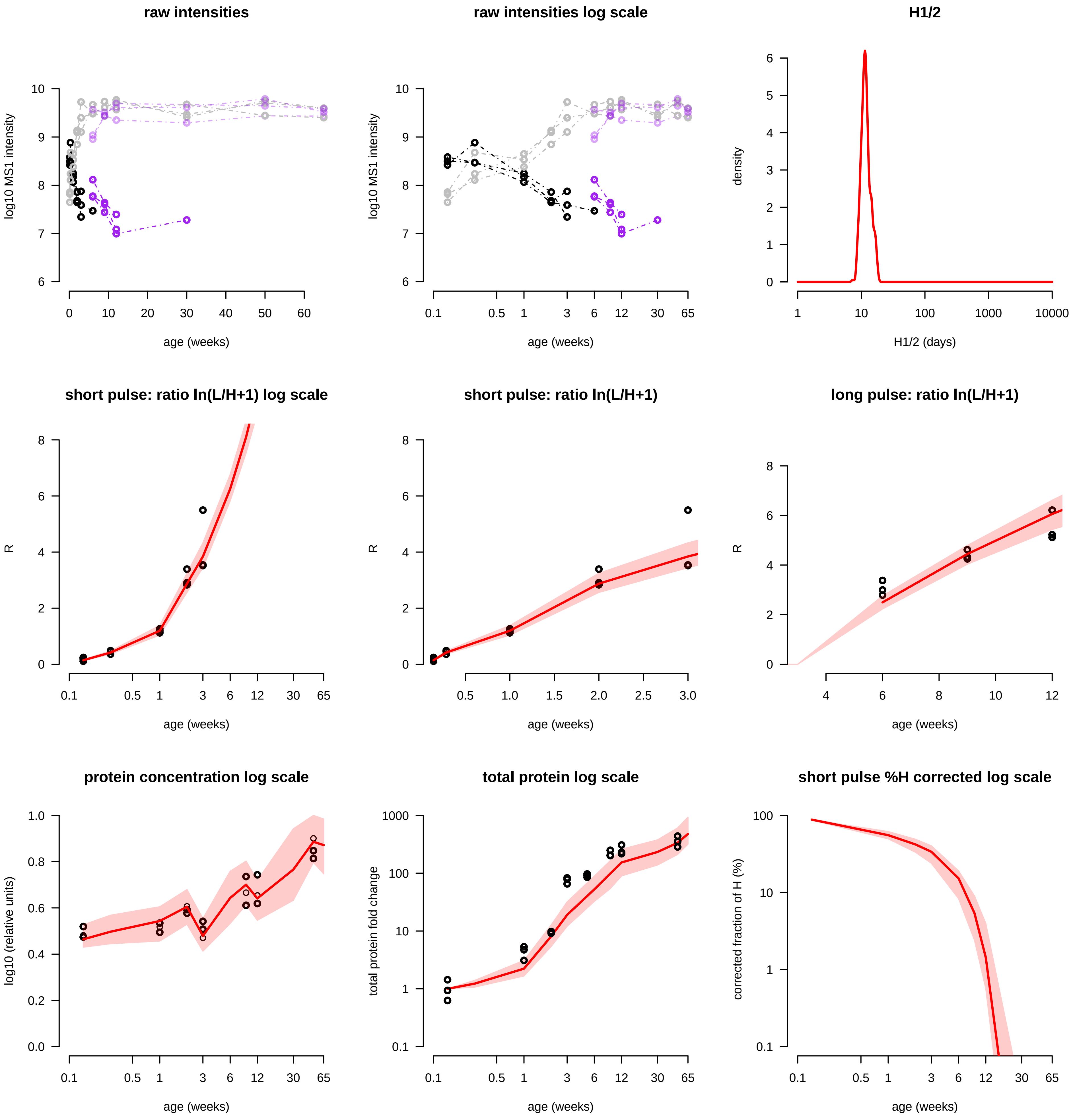

Q91V41

>sp|Q91V41|RAB14_MOUSE

Ras−related protein Rab−14

GN=Rab14

Med H1/2: 5.1 days

Q5 H1/2: 4.3 days

Q95 H1/2: 6.4 days

reliable model fit: yes

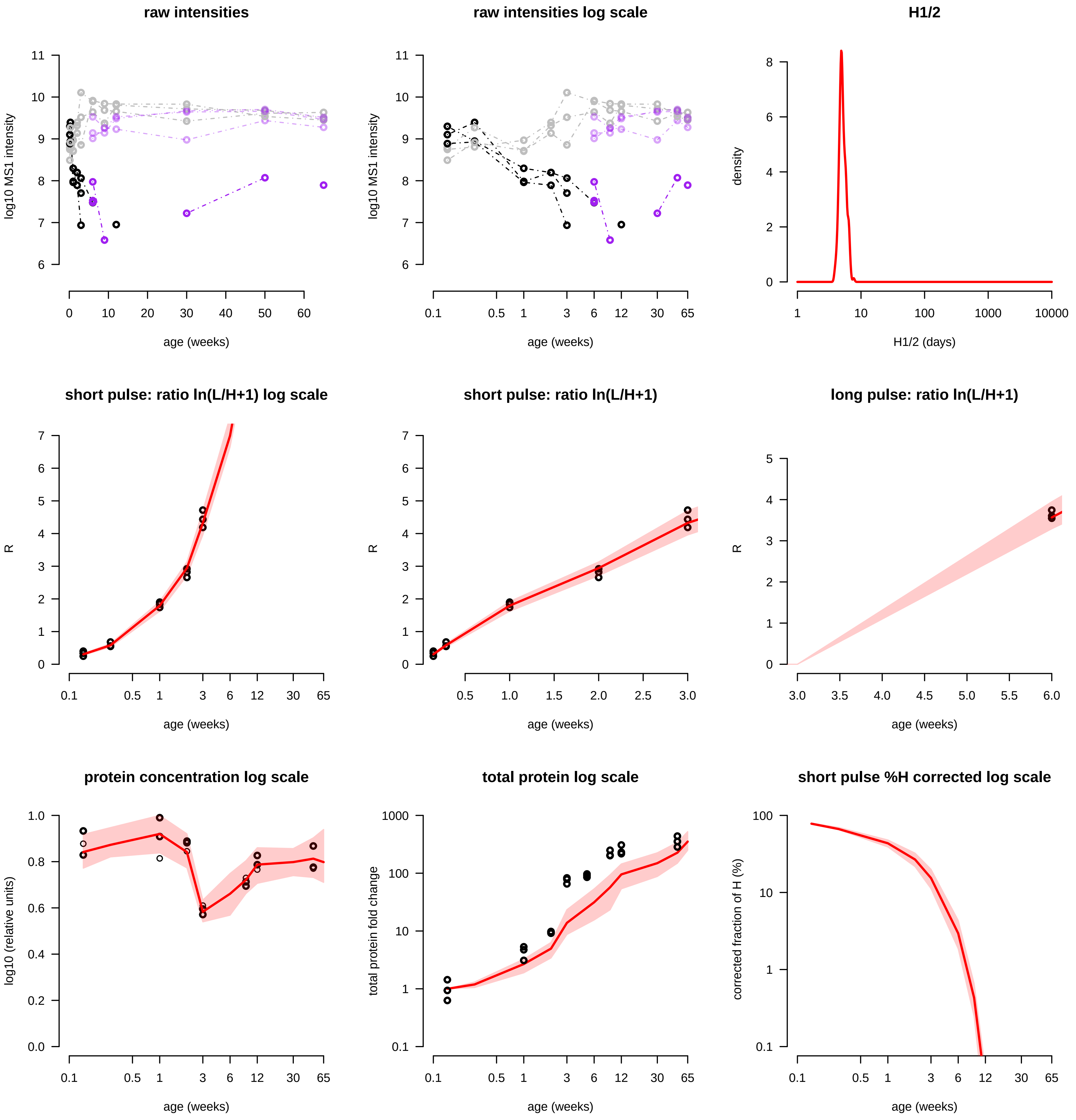

P80315

>sp|P80315|TCPD_MOUSE

T−complex protein 1 subunit delta

GN=Cct4

Med H1/2: 13.5 days

Q5 H1/2: 10.5 days

Q95 H1/2: 16.1 days

reliable model fit: yes

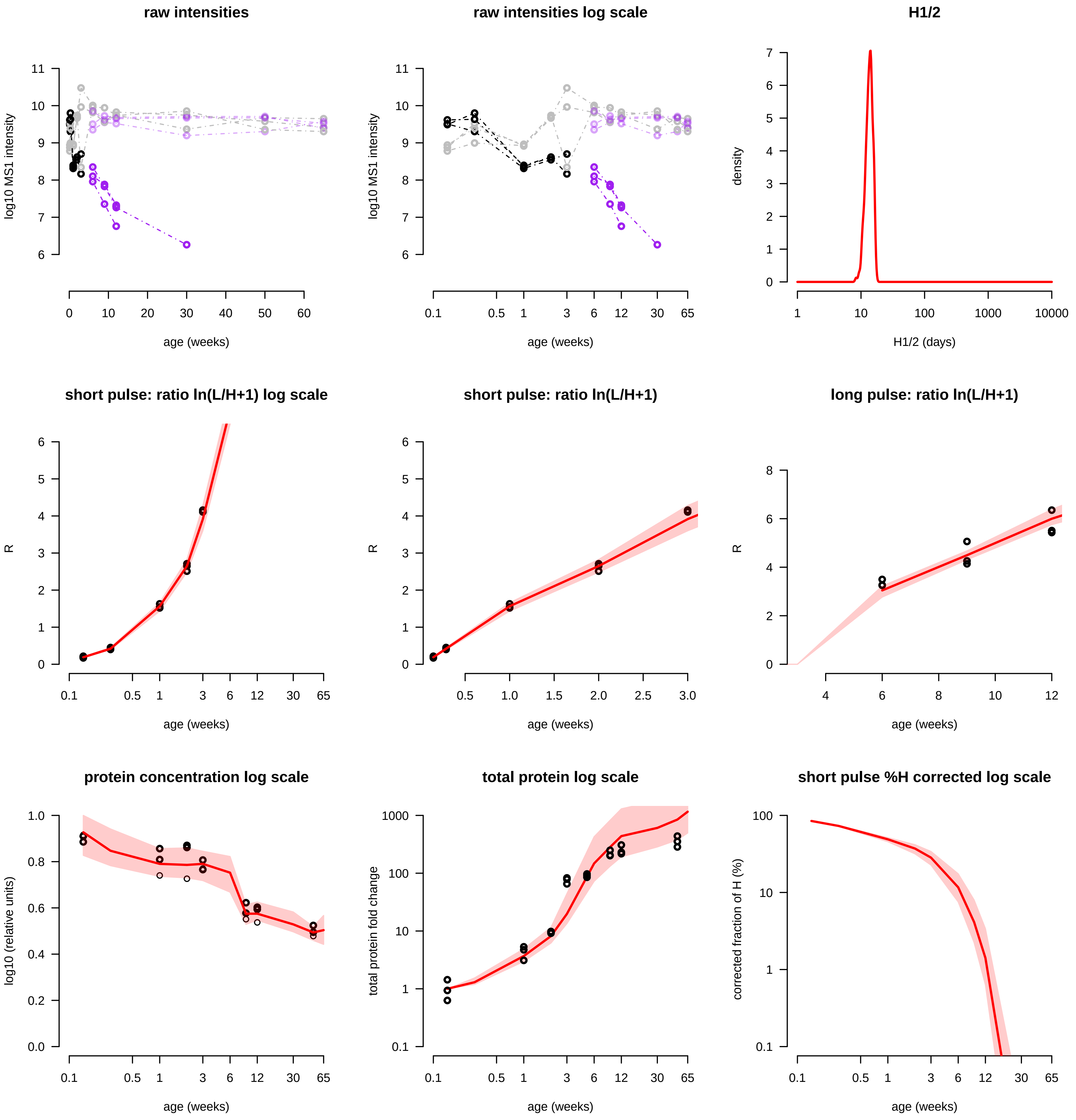

O70251

>sp|O70251|EF1B_MOUSE

Elongation factor 1−beta

GN=Eef1b

Med H1/2: 7.2 days

Q5 H1/2: 6.1 days

Q95 H1/2: 9.4 days

reliable model fit: yes

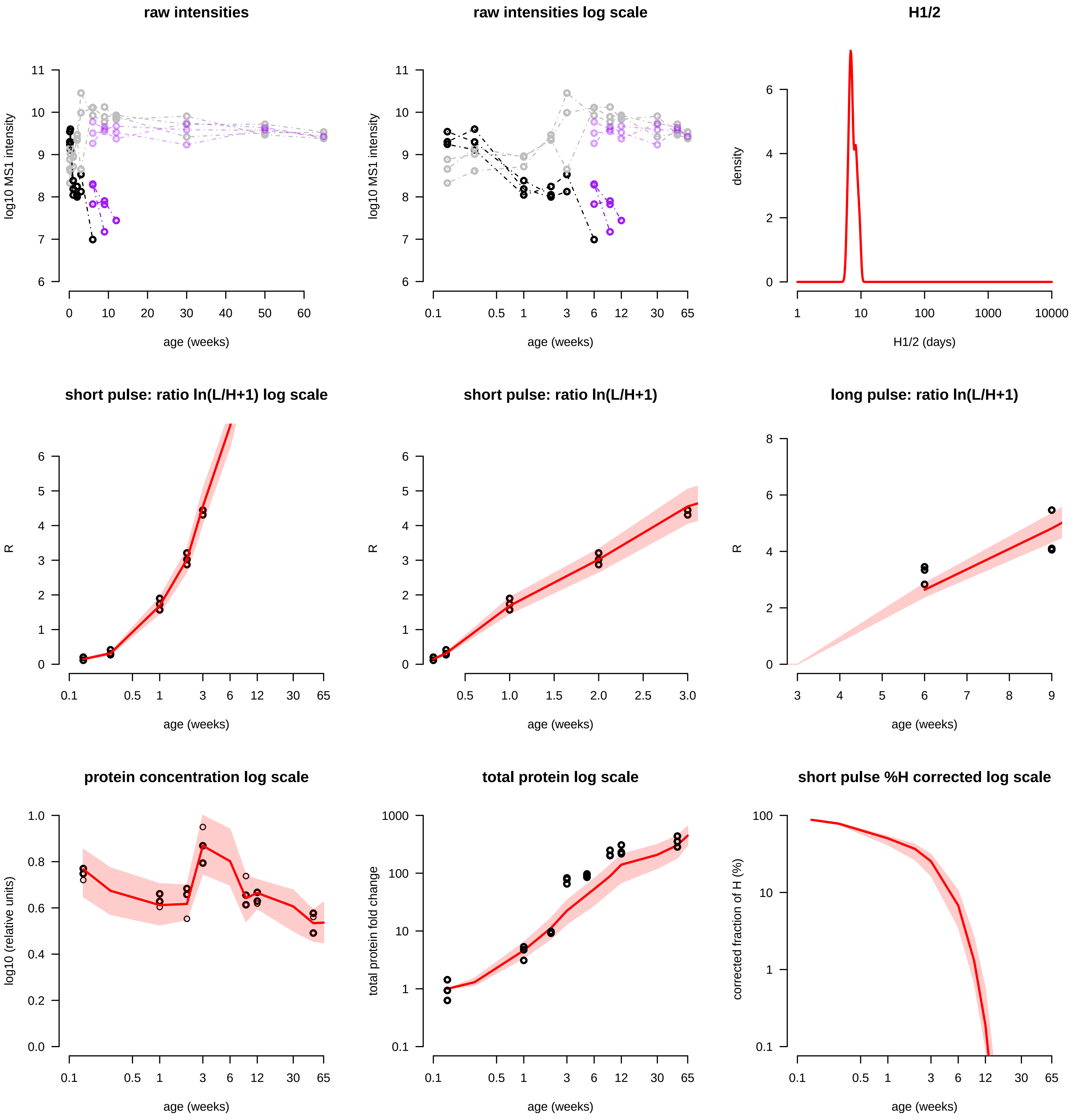

P01863;P01865

>sp|P01863|GCAA_MOUSE

Ig gamma−2A chain C region, A allele

GN=Ighg

Med H1/2: 81.6 days

Q5 H1/2: 34.7 days

Q95 H1/2: 685.9 days

reliable model fit: no

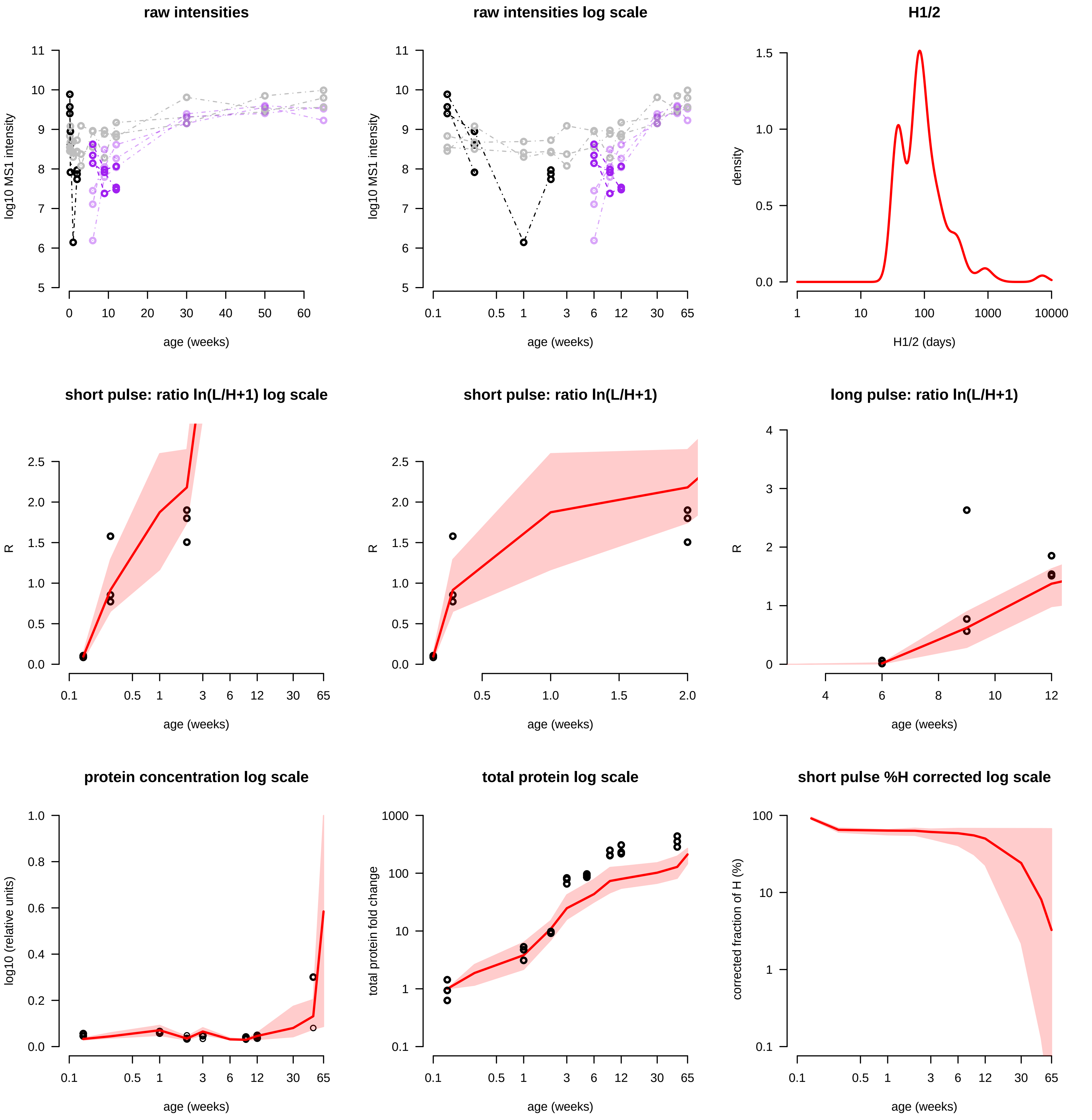

Q6P4T2

>sp|Q6P4T2|U520_MOUSE

U5 small nuclear ribonucleoprotein 200 kDa helicase

GN=Snrnp200

Med H1/2: 93.9 days

Q5 H1/2: 47.2 days

Q95 H1/2: 585 days

reliable model fit: yes

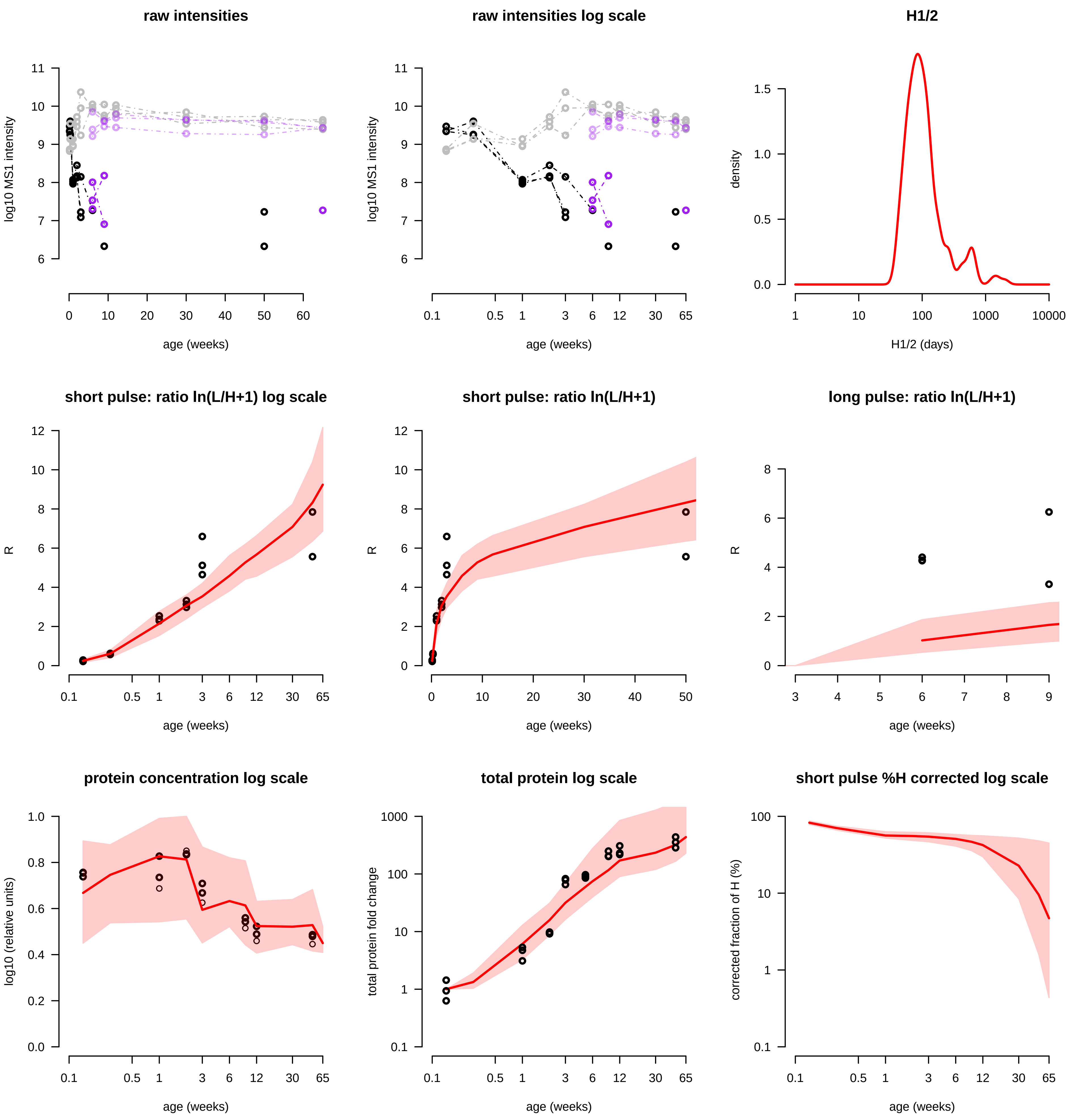

Q9CZW5

>sp|Q9CZW5|TOM70_MOUSE

Mitochondrial import receptor subunit TOM70

GN=Tomm70

Med H1/2: 100.9 days

Q5 H1/2: 66.5 days

Q95 H1/2: 197 days

reliable model fit: yes

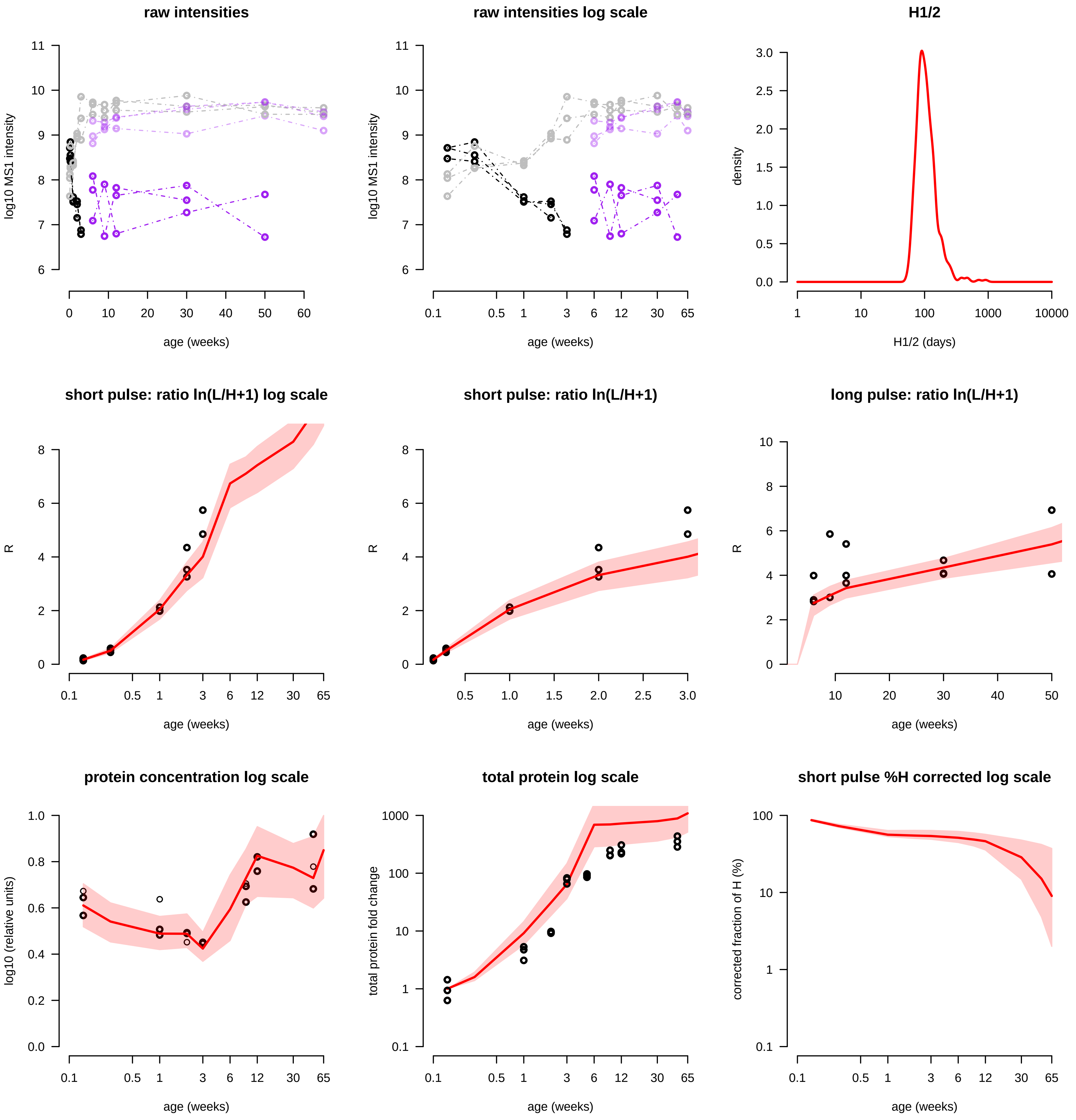

P22599

>sp|P22599|A1AT2_MOUSE

Alpha−1−antitrypsin 1−2

GN=Serpina1b

Med H1/2: 3.8 days

Q5 H1/2: 2.5 days

Q95 H1/2: 27 days

reliable model fit: yes

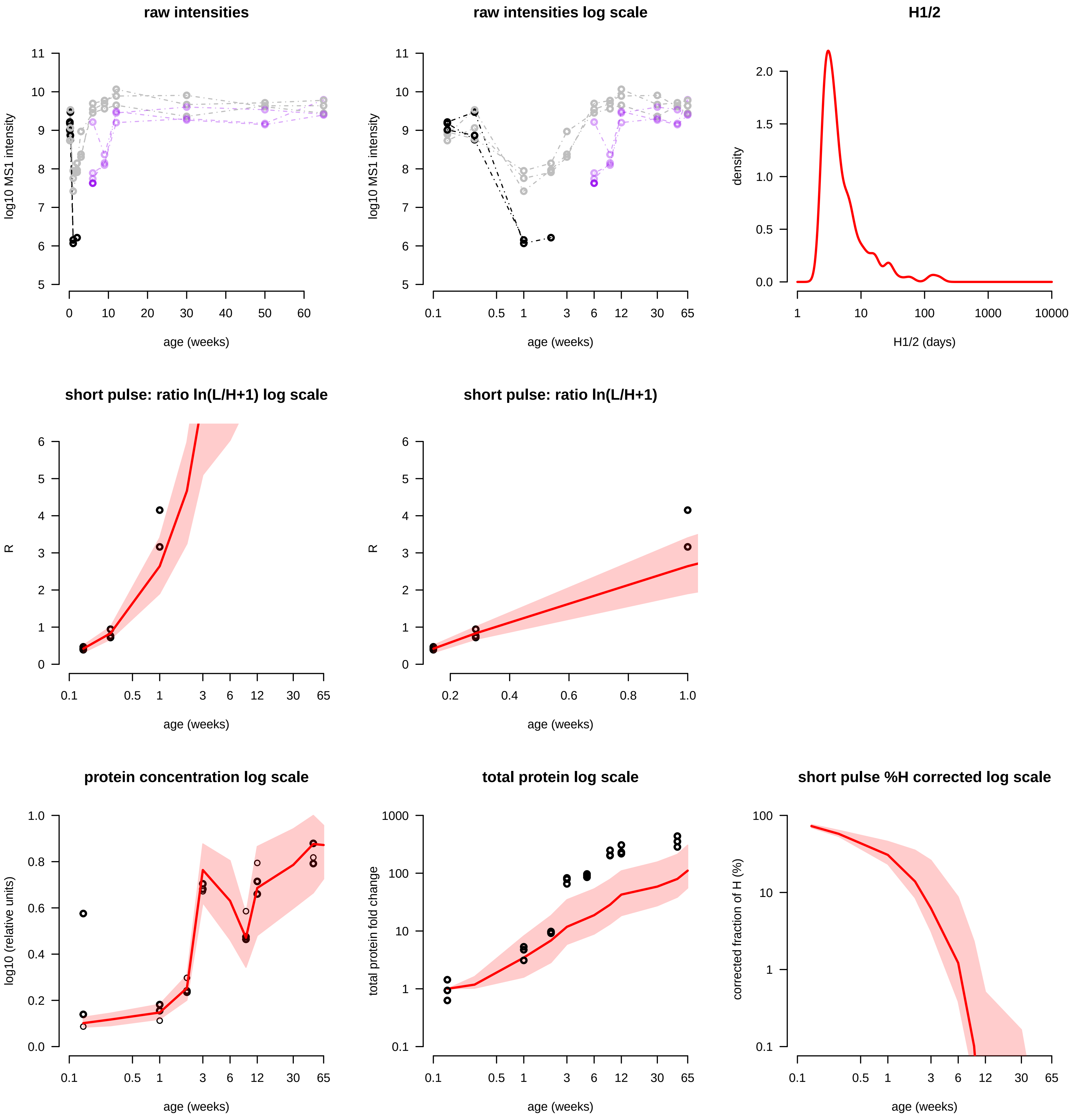

P53395

>sp|P53395|ODB2_MOUSE

ansferase component of branched−chain alpha−keto acid dehydrogenase compl

GN=Dbt

Med H1/2: 90.7 days

Q5 H1/2: 62.8 days

Q95 H1/2: 154.7 days

reliable model fit: yes

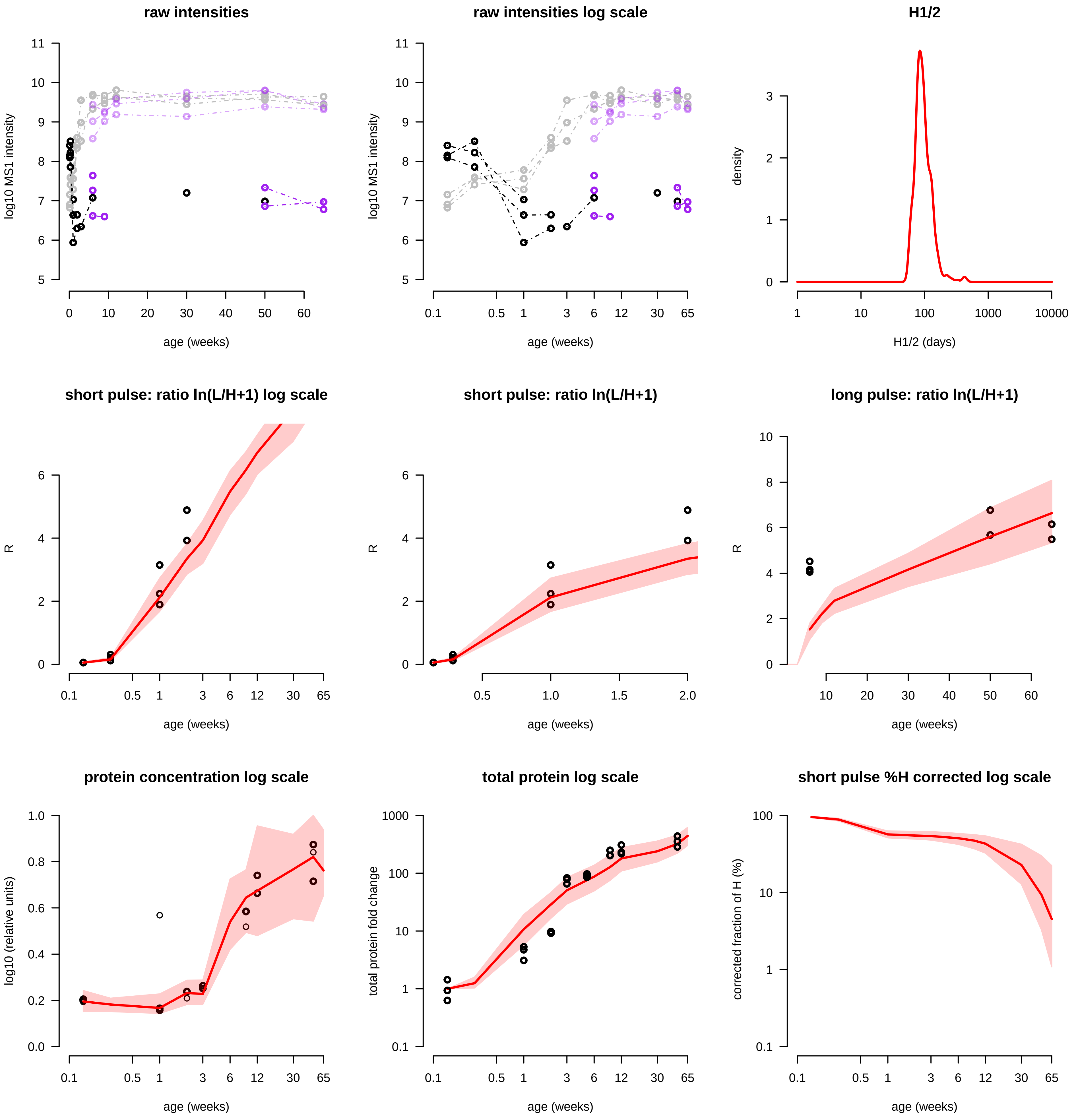

P35979

>sp|P35979|RL12_MOUSE

60S ribosomal protein L12

GN=Rpl12

Med H1/2: 11.4 days

Q5 H1/2: 9.2 days

Q95 H1/2: 15.8 days

reliable model fit: yes

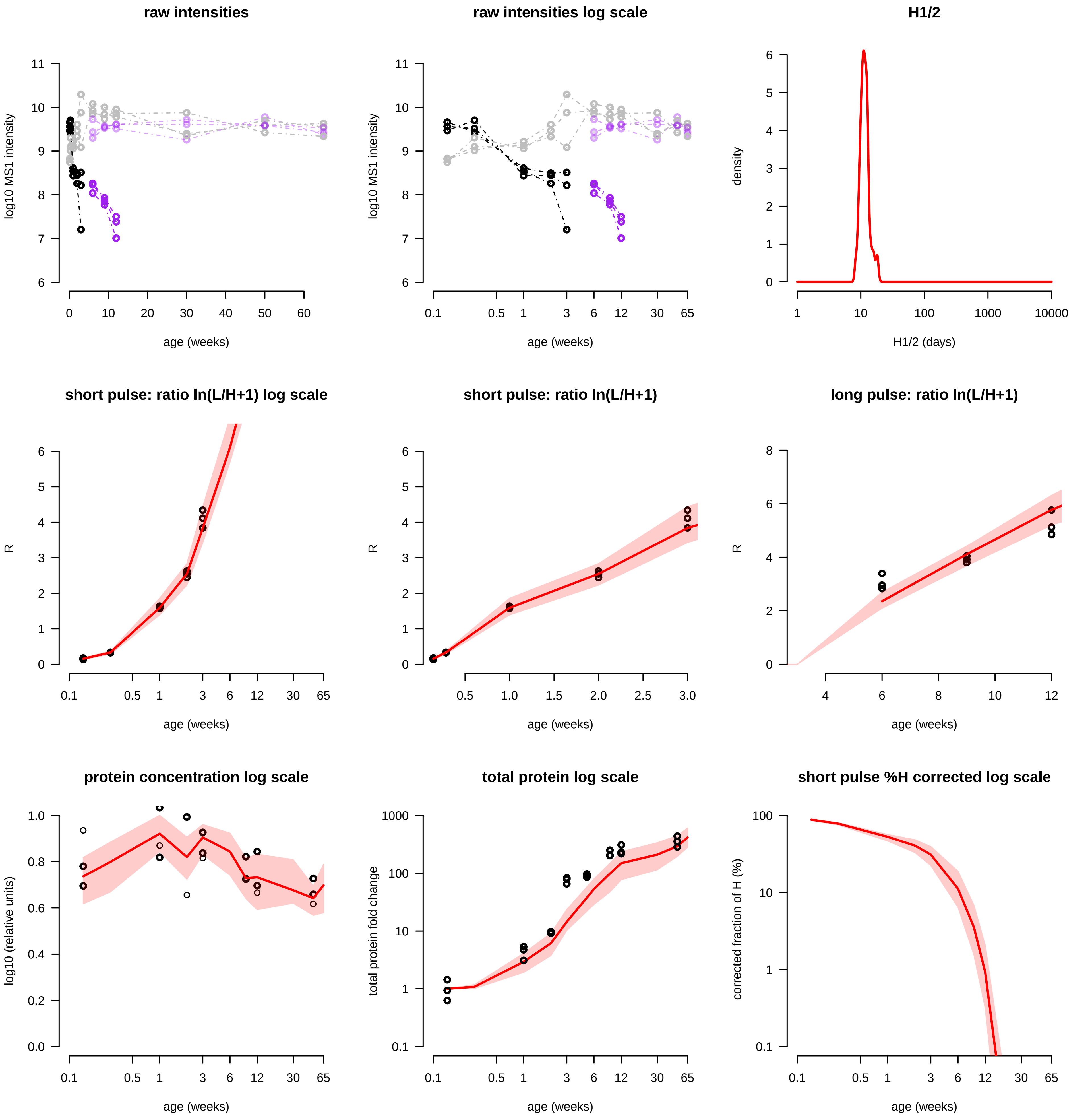

Q9D0F9

>sp|Q9D0F9|PGM1_MOUSE

Phosphoglucomutase−1

GN=Pgm1

Med H1/2: 9.2 days

Q5 H1/2: 7.6 days

Q95 H1/2: 11.8 days

reliable model fit: yes

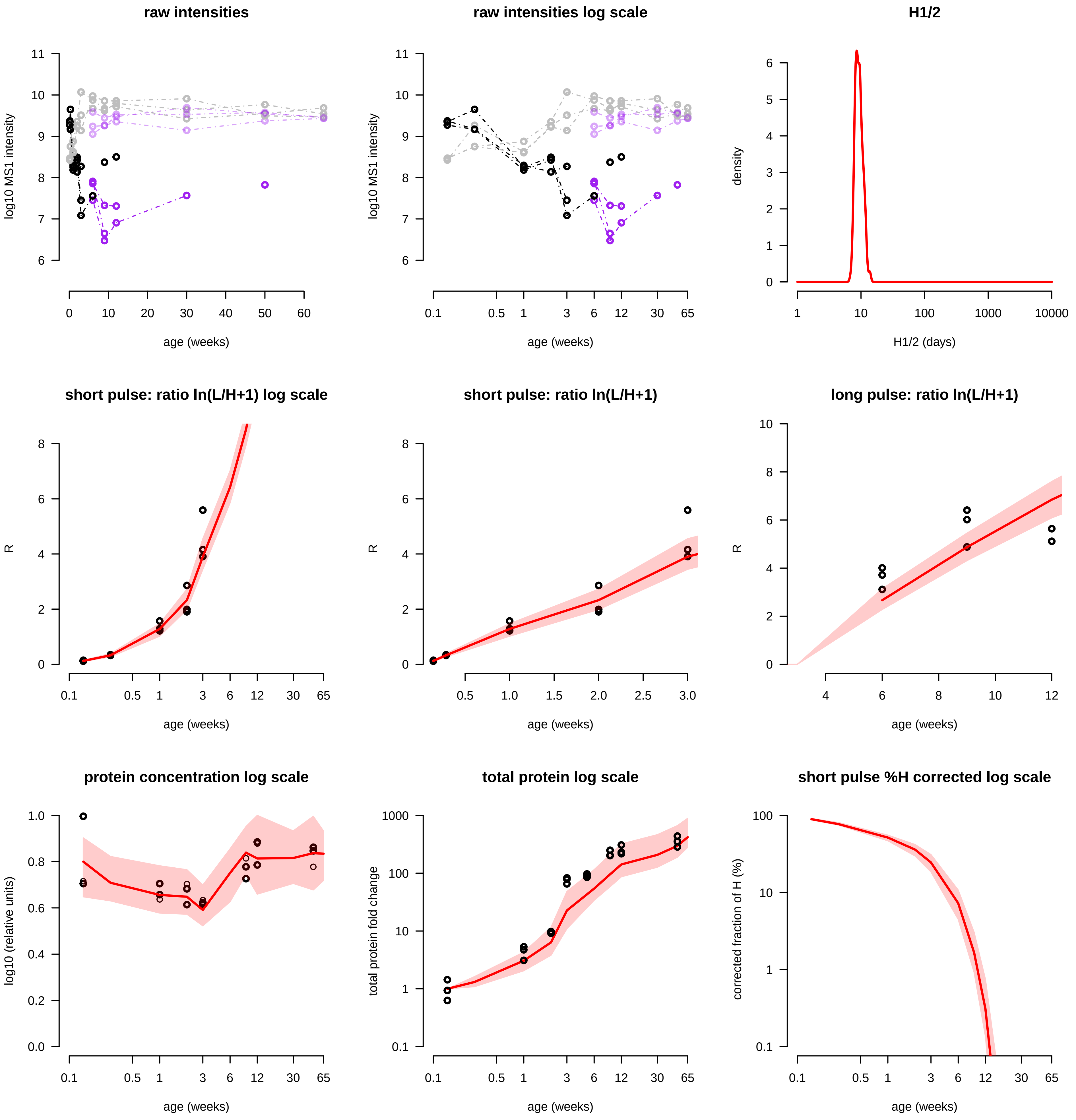

Q9WUM5

>sp|Q9WUM5|SUCA_MOUSE

Succinate−−CoA ligase [ADP/GDP−forming] subunit alpha, mitochondrial

GN=Suclg1

Med H1/2: 13.7 days

Q5 H1/2: 10.3 days

Q95 H1/2: 17.5 days

reliable model fit: yes

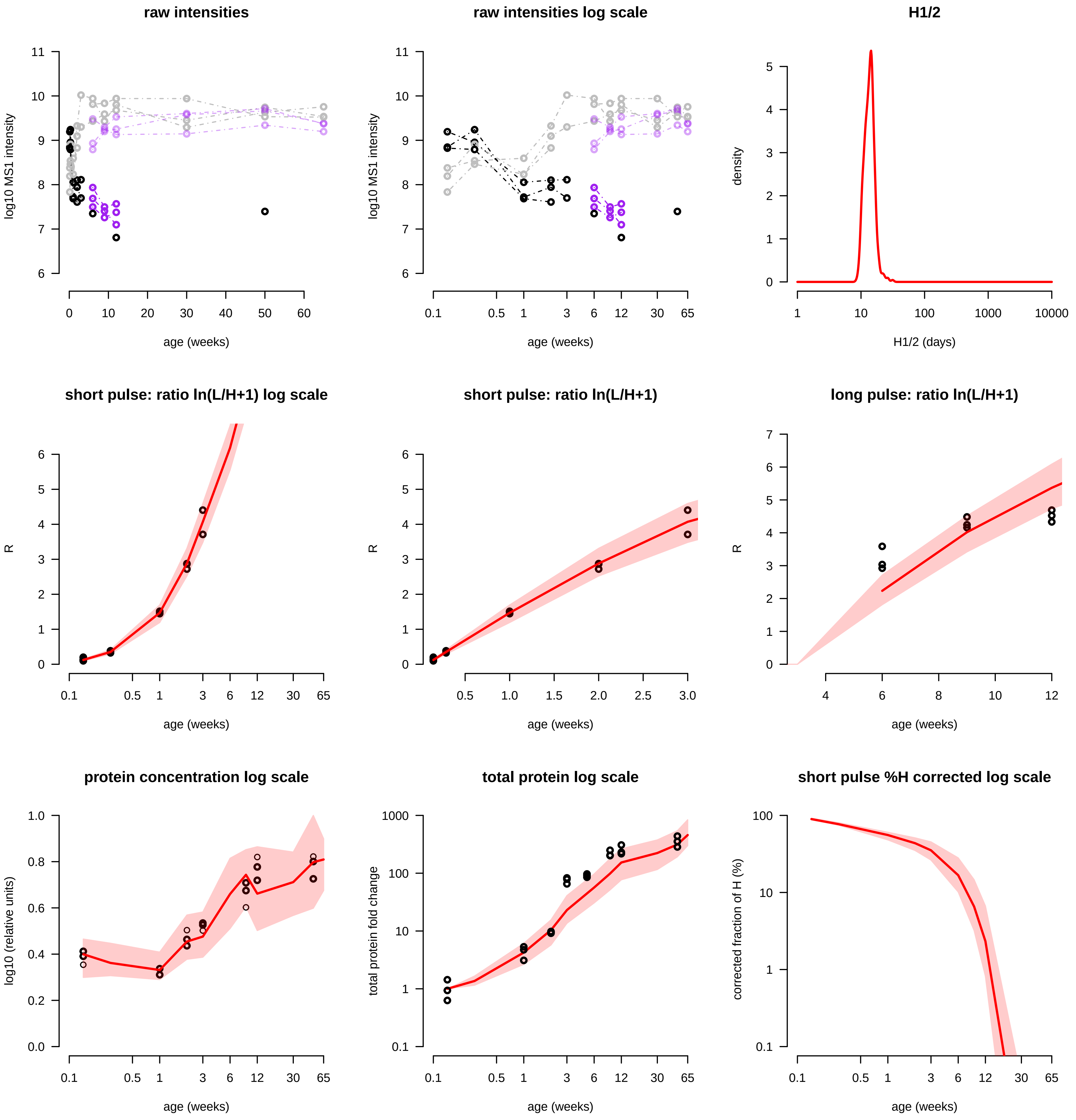

Q9D154

>sp|Q9D154|ILEUA_MOUSE

Leukocyte elastase inhibitor A

GN=Serpinb1a

Med H1/2: 7.5 days

Q5 H1/2: 6.2 days

Q95 H1/2: 9.6 days

reliable model fit: yes

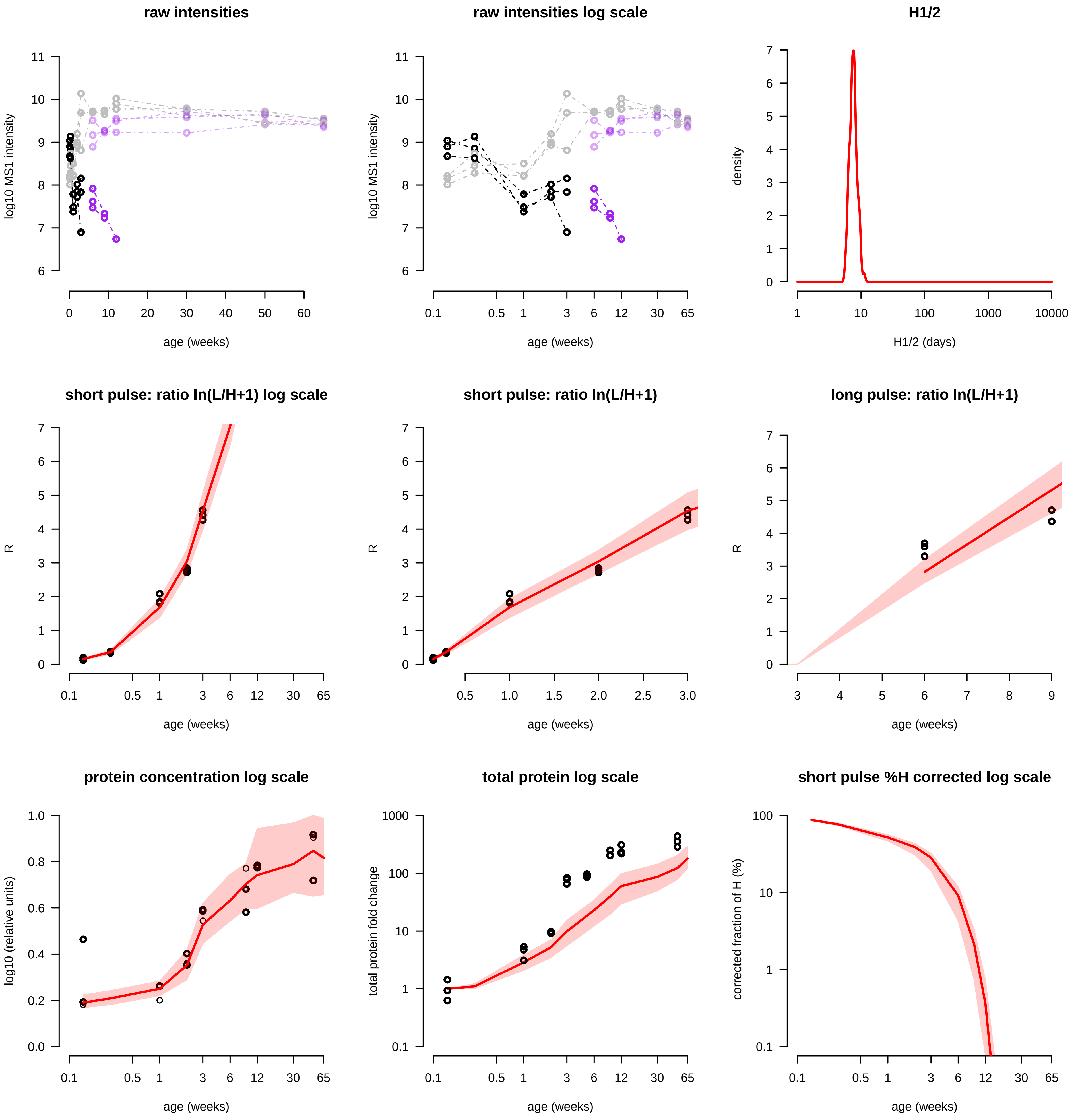

Q91VI7

>sp|Q91VI7|RINI_MOUSE

Ribonuclease inhibitor

GN=Rnh1

Med H1/2: 12 days

Q5 H1/2: 9.6 days

Q95 H1/2: 16.8 days

reliable model fit: yes

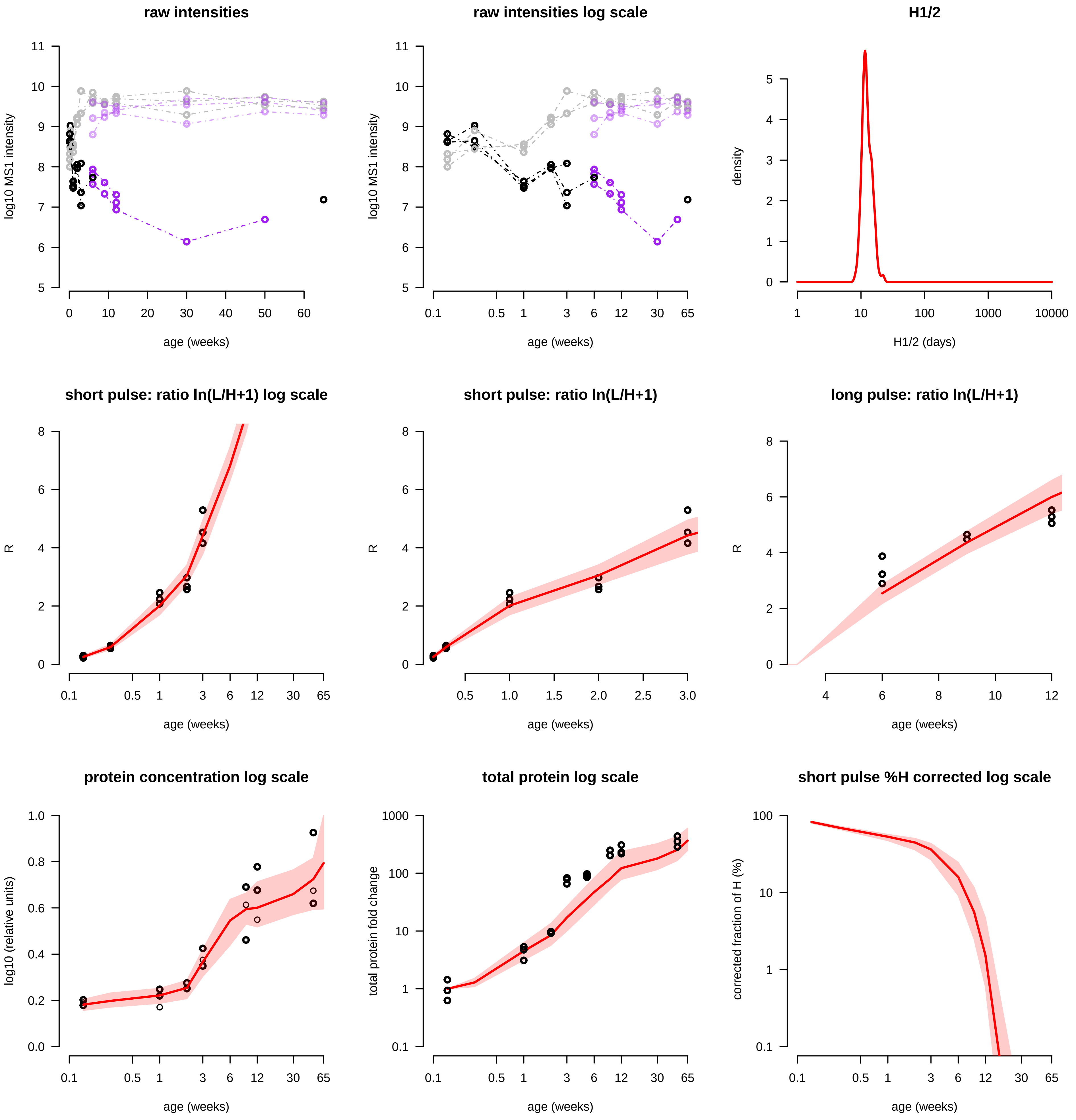

Q62167;P16381

>sp|Q62167|DDX3X_MOUSE

ATP−dependent RNA helicase DDX3X

GN=Ddx3x

Med H1/2: 6.4 days

Q5 H1/2: 5.2 days

Q95 H1/2: 8.4 days

reliable model fit: yes

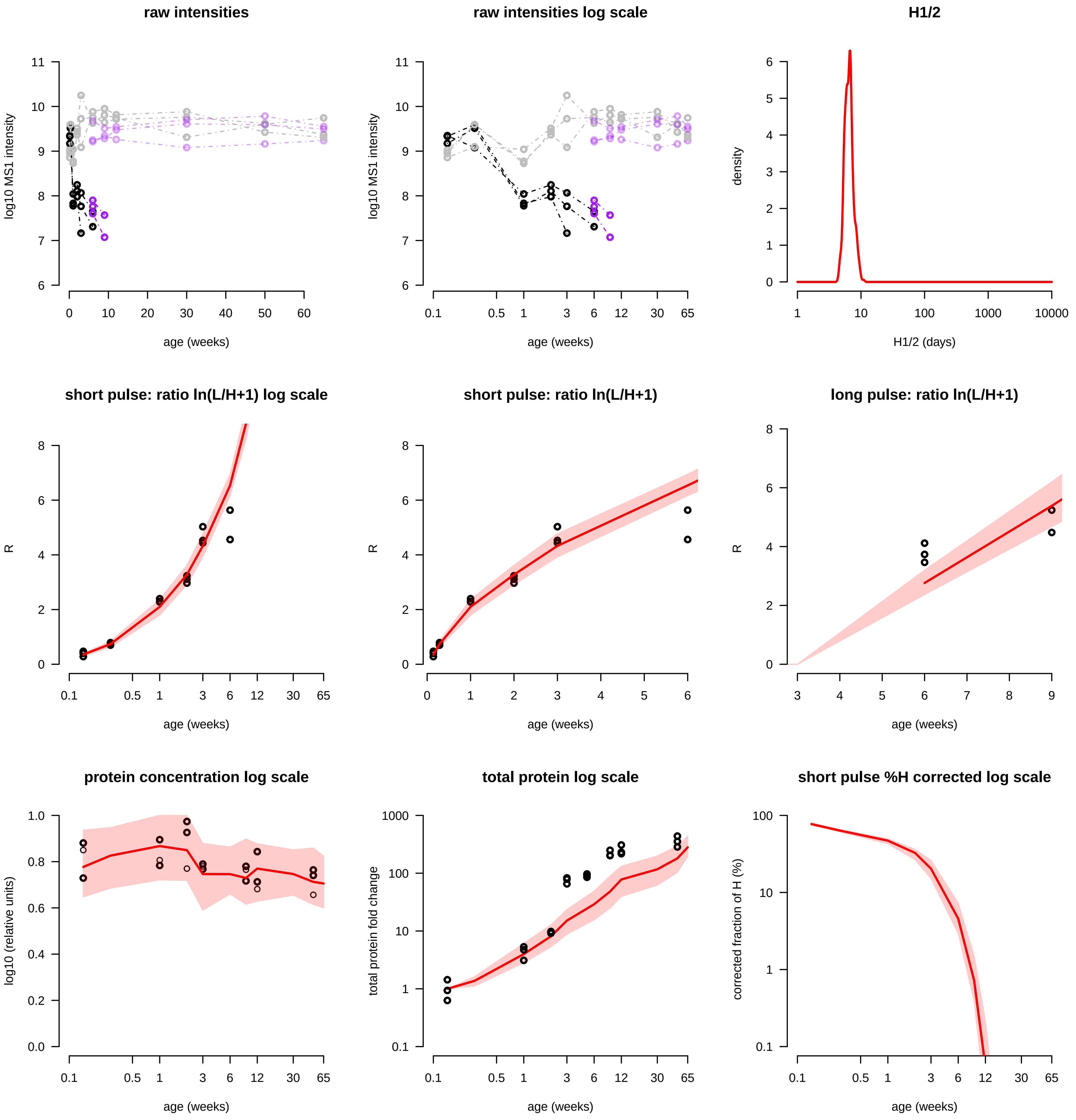

P28654

>sp|P28654|PGS2_MOUSE

Decorin

GN=Dcn

Med H1/2: 176.9 days

Q5 H1/2: 69.9 days

Q95 H1/2: 1237.9 days

reliable model fit: yes

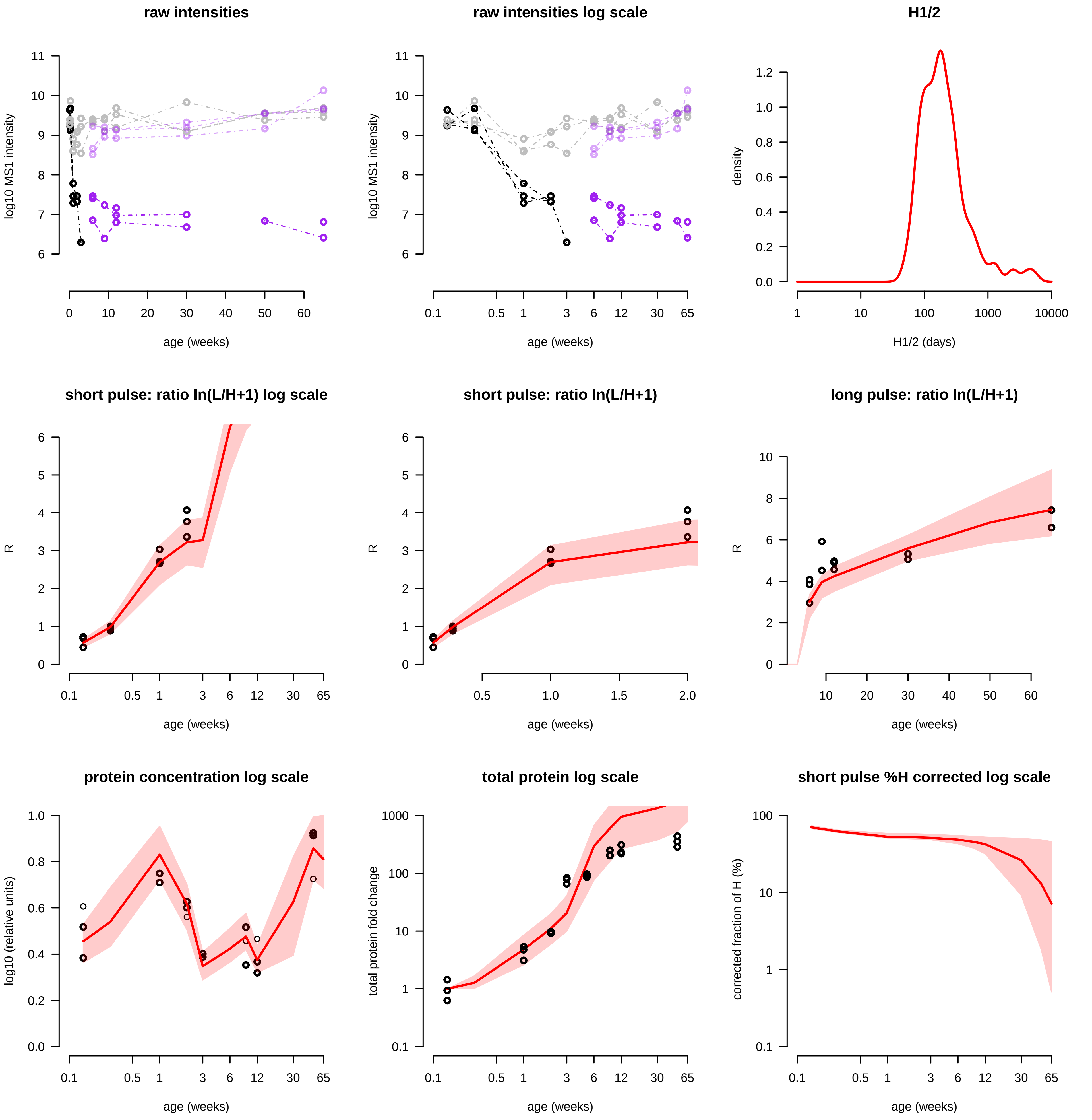

Q922R8

>sp|Q922R8|PDIA6_MOUSE

Protein disulfide−isomerase A6

GN=Pdia6

Med H1/2: 10.1 days

Q5 H1/2: 8.5 days

Q95 H1/2: 11.9 days

reliable model fit: yes

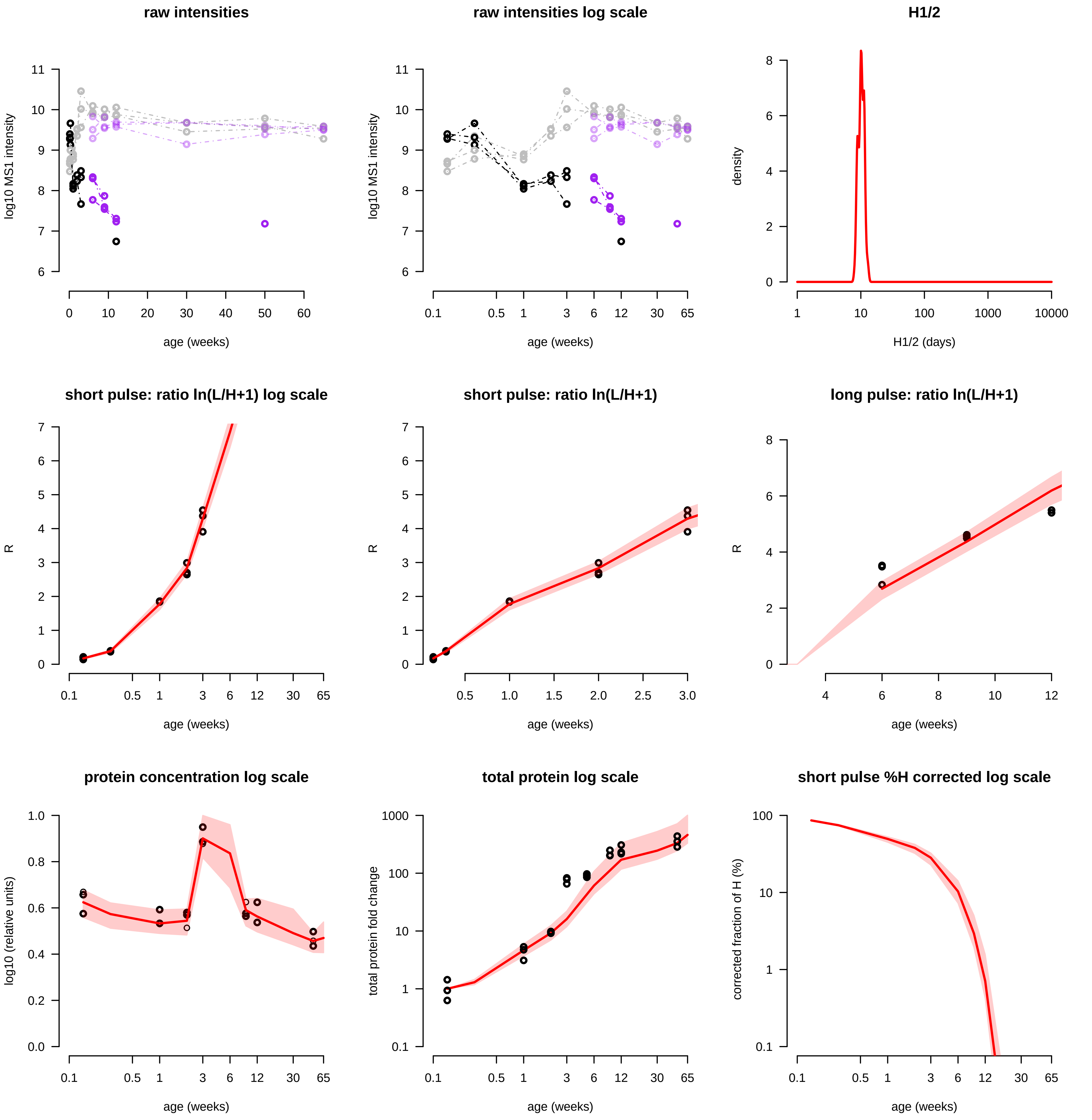

Q62426

>sp|Q62426|CYTB_MOUSE

Cystatin−B

GN=Cstb

Med H1/2: 109.1 days

Q5 H1/2: 57.1 days

Q95 H1/2: 570.4 days

reliable model fit: yes

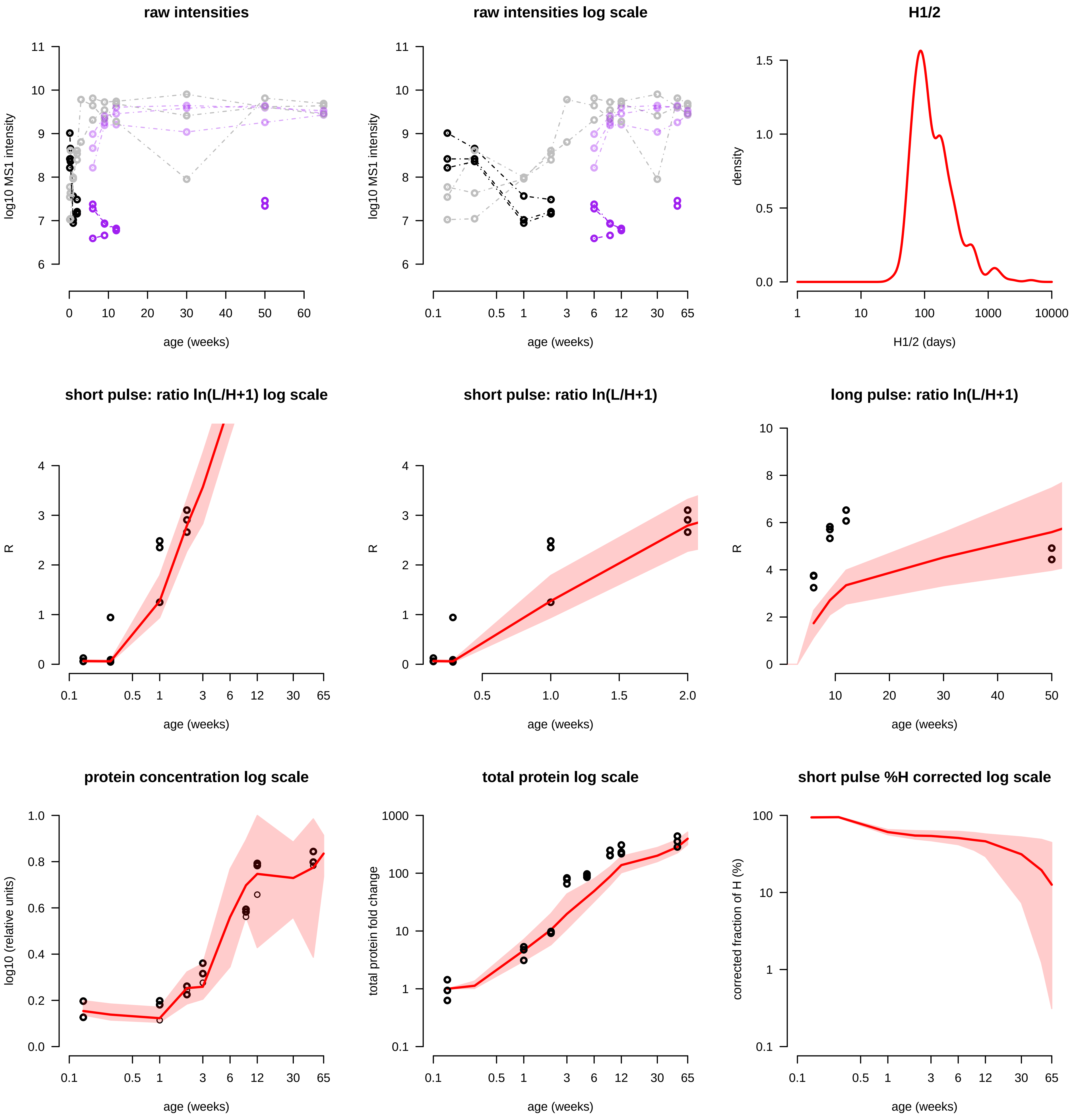

P51150

>sp|P51150|RAB7A_MOUSE

Ras−related protein Rab−7a

GN=Rab7a

Med H1/2: 10.3 days

Q5 H1/2: 7.9 days

Q95 H1/2: 13.3 days

reliable model fit: yes

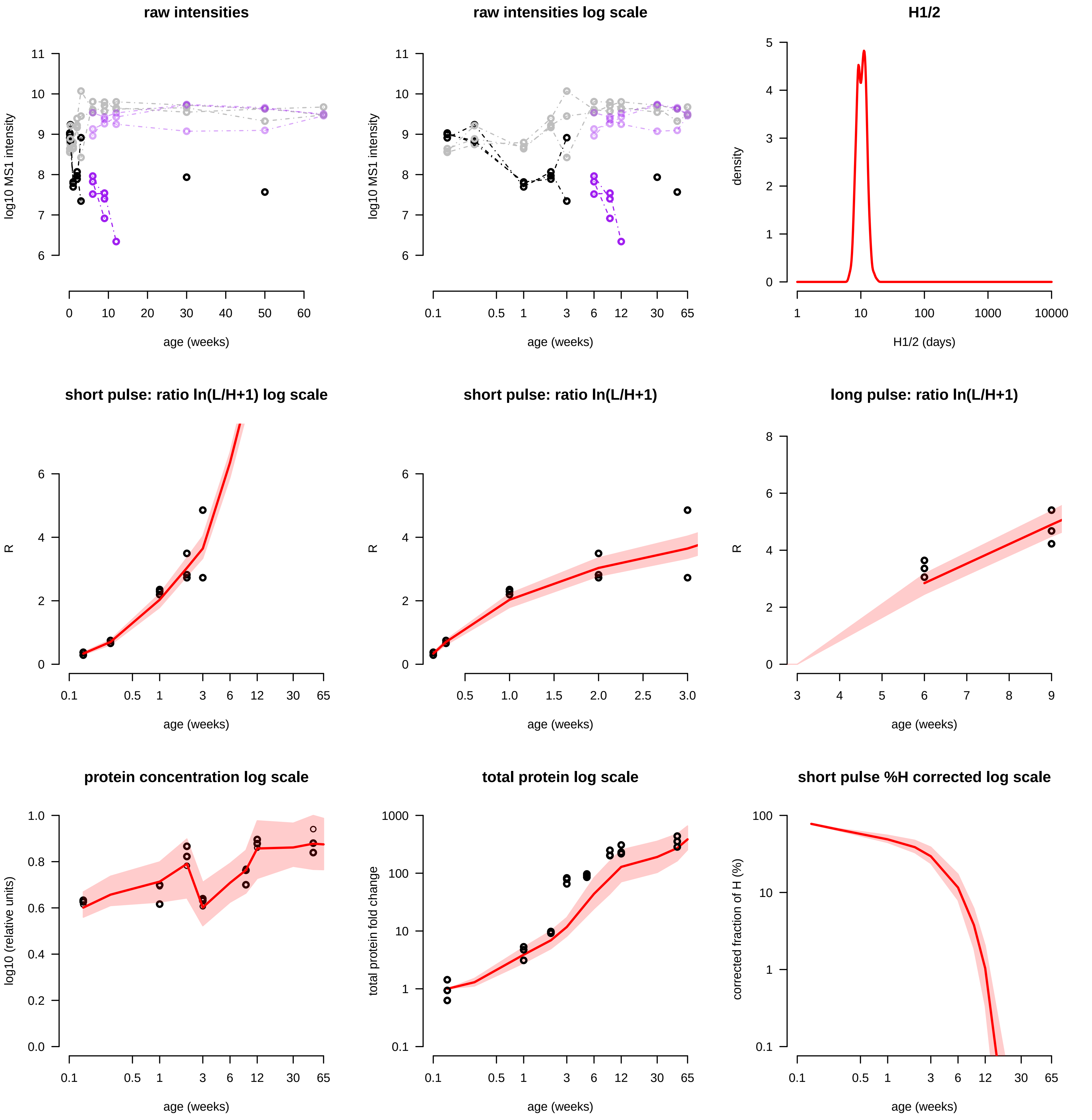

P28474

>sp|P28474|ADHX_MOUSE

Alcohol dehydrogenase class−3

GN=Adh5

Med H1/2: 920.8 days

Q5 H1/2: 242.6 days

Q95 H1/2: 20119.5 days

reliable model fit: yes

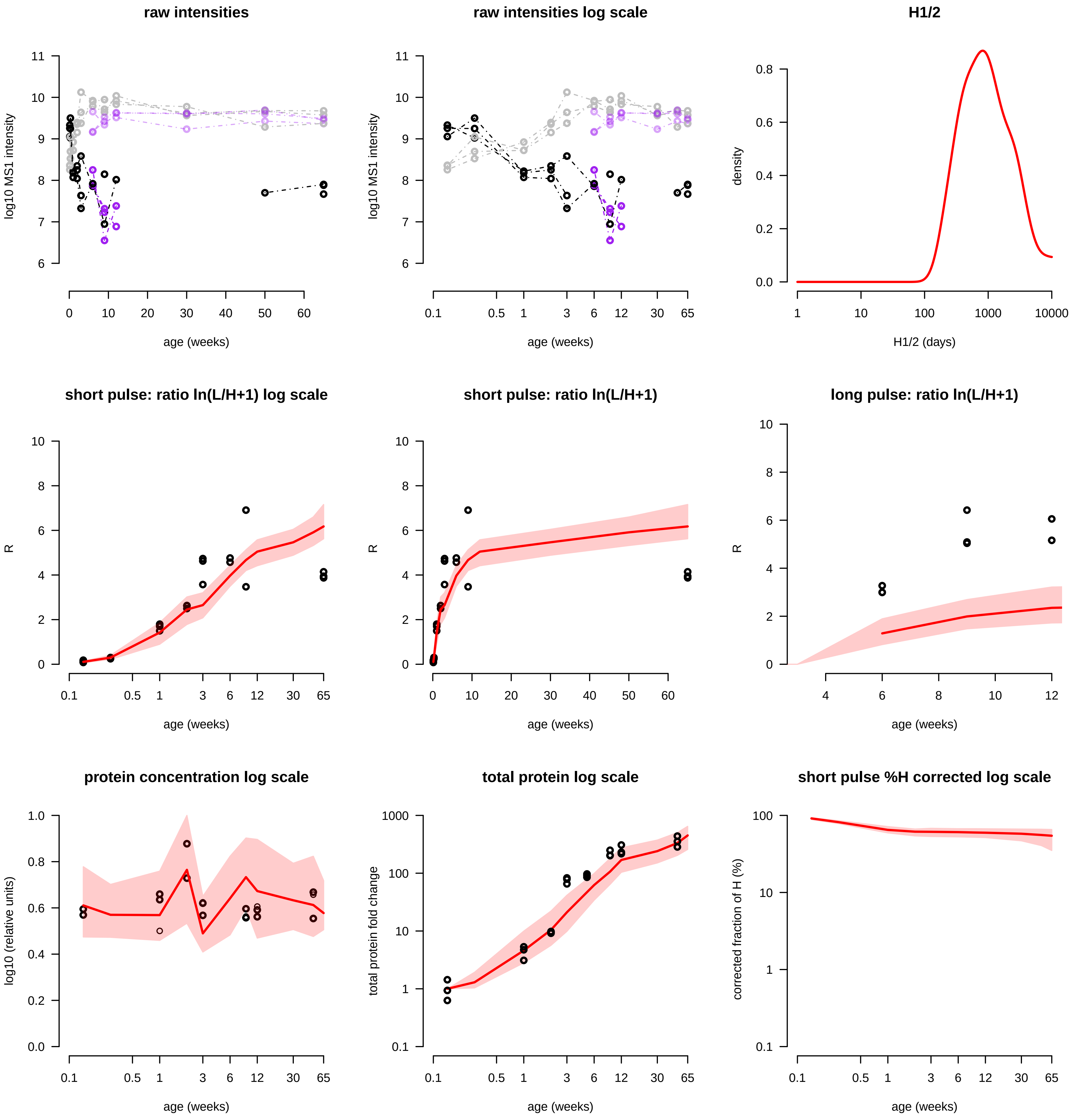

Q8VCT4

>sp|Q8VCT4|EST1D_MOUSE

Carboxylesterase 1D

GN=Ces1d

Med H1/2: 7.7 days

Q5 H1/2: 6.1 days

Q95 H1/2: 10.9 days

reliable model fit: yes

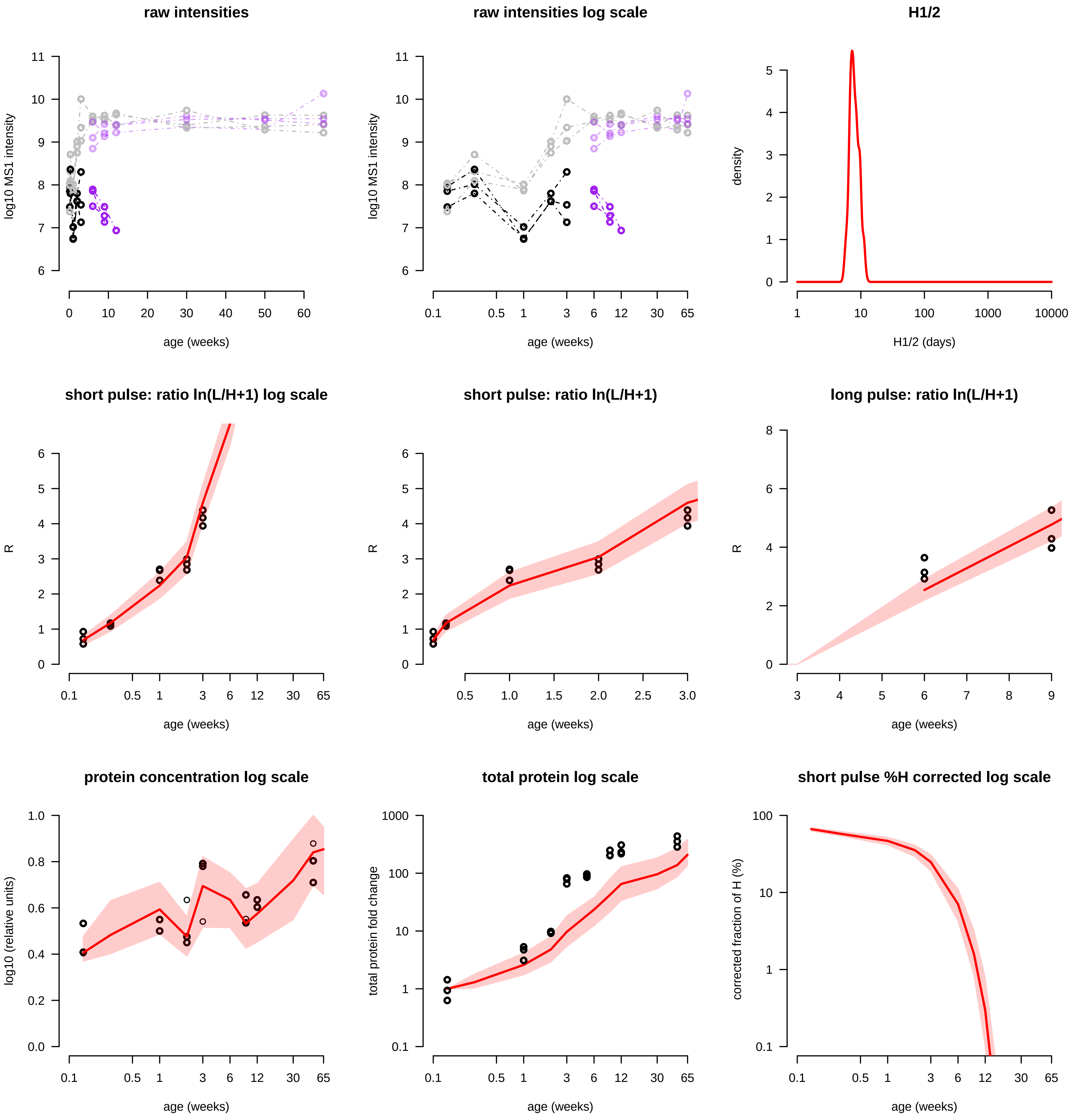

Q61768

>sp|Q61768|KINH_MOUSE

Kinesin−1 heavy chain

GN=Kif5b

Med H1/2: 3.7 days

Q5 H1/2: 3.2 days

Q95 H1/2: 4.4 days

reliable model fit: yes

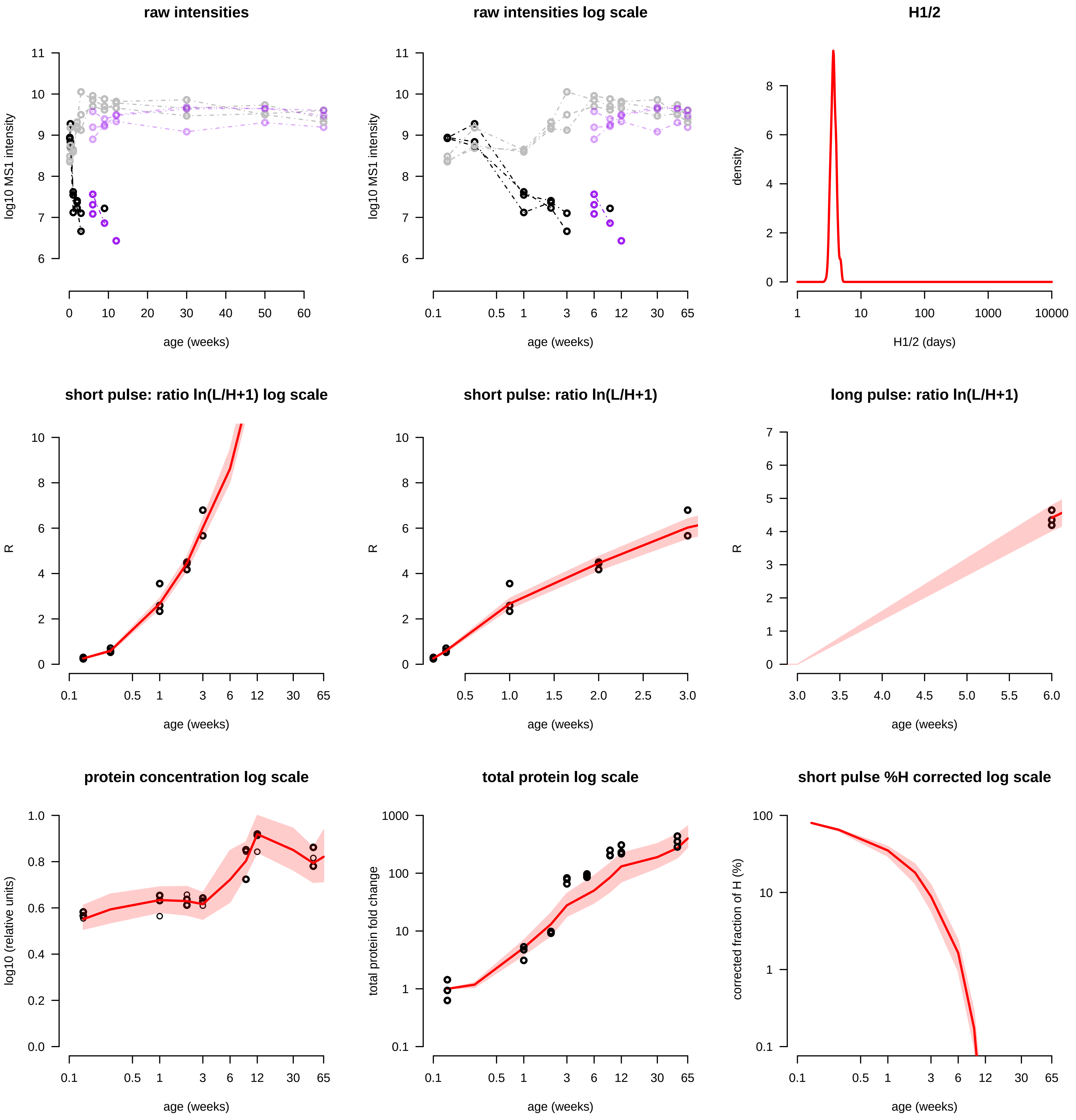

Q9EQK5

>sp|Q9EQK5|MVP_MOUSE

Major vault protein

GN=Mvp

Med H1/2: 245.6 days

Q5 H1/2: 122 days

Q95 H1/2: 1695.9 days

reliable model fit: yes

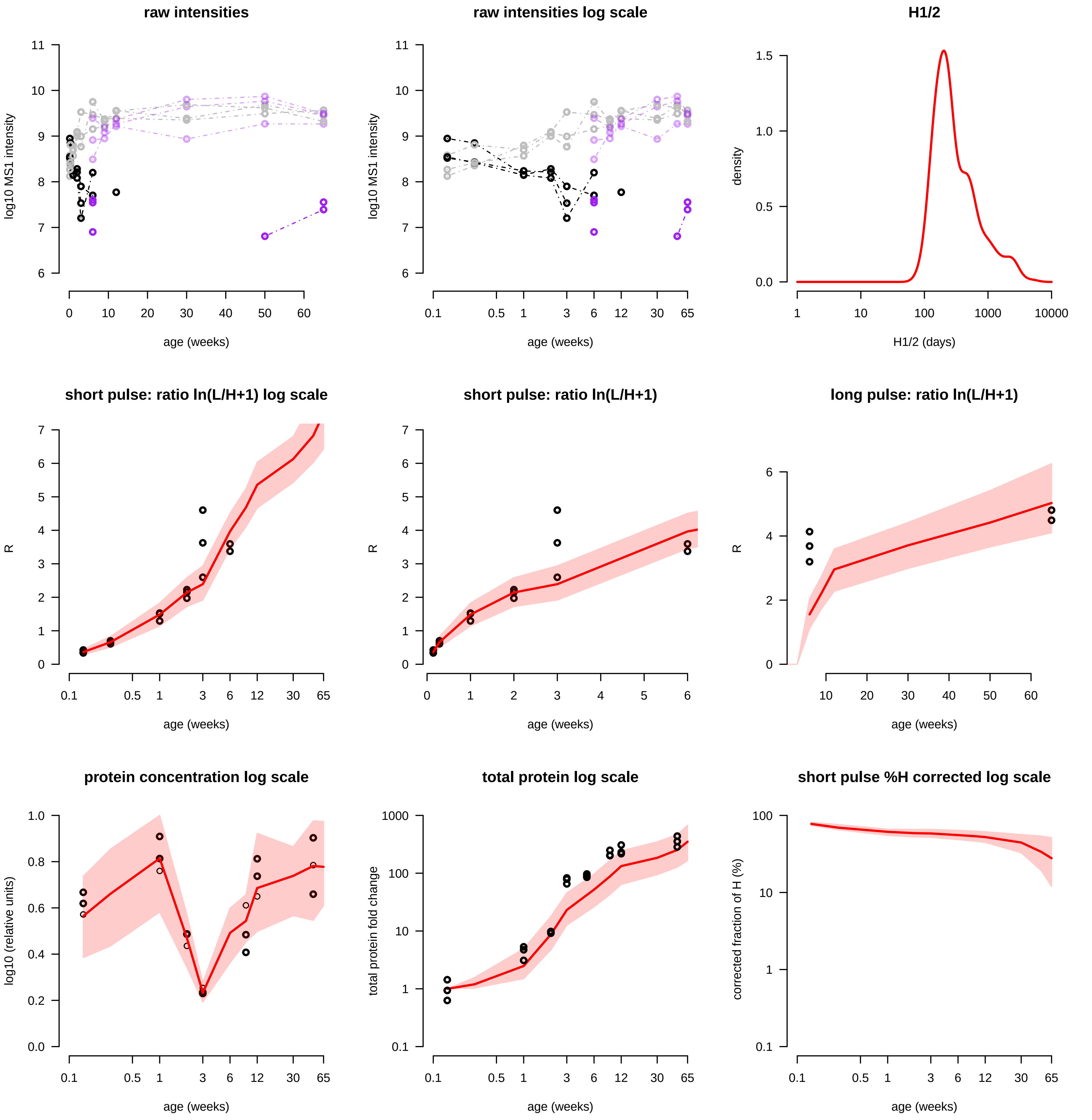

P63276

>sp|P63276|RS17_MOUSE

40S ribosomal protein S17

GN=Rps17

Med H1/2: 10.4 days

Q5 H1/2: 7.4 days

Q95 H1/2: 13.6 days

reliable model fit: yes

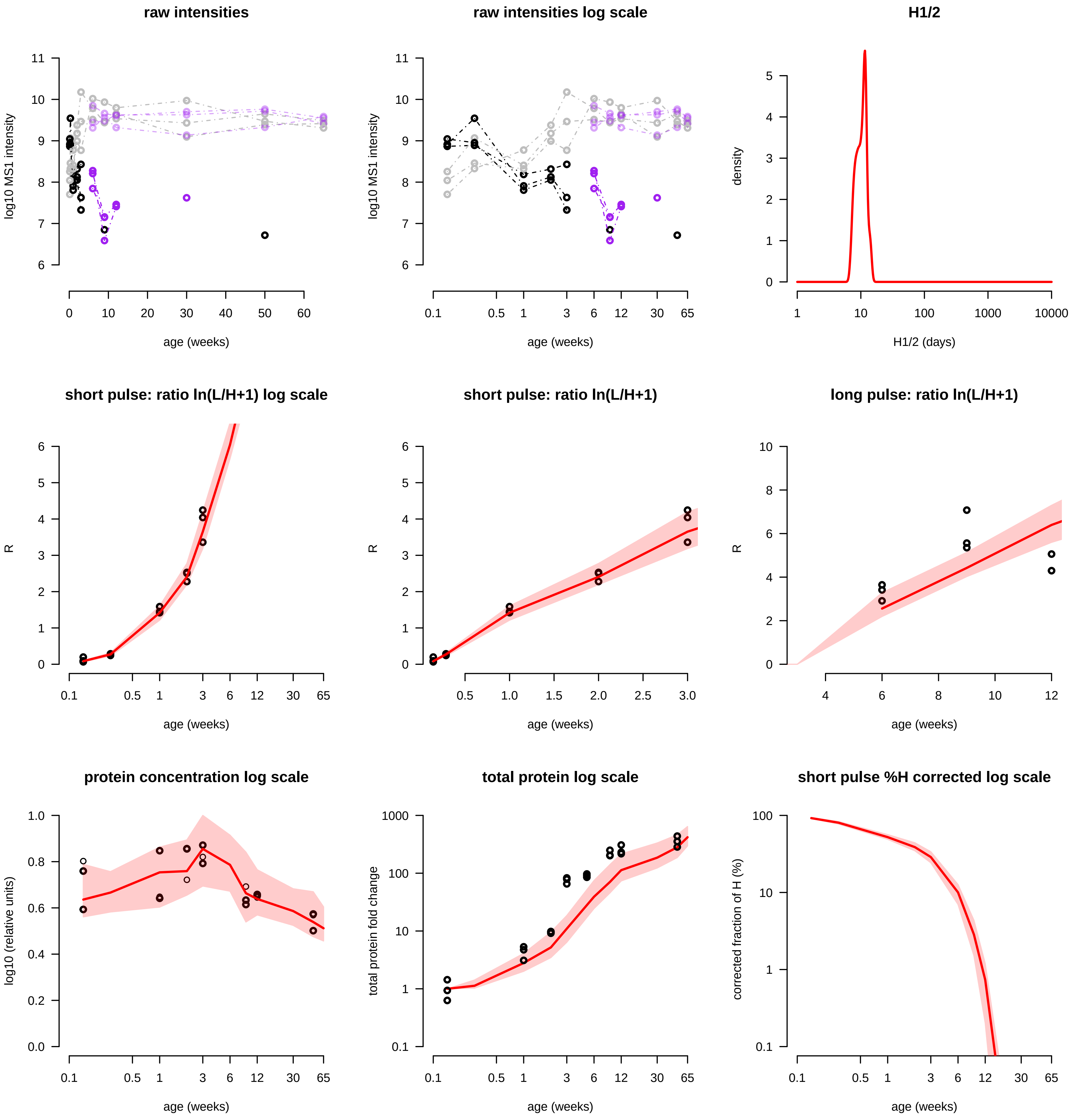

Q11011

>sp|Q11011|PSA_MOUSE

Puromycin−sensitive aminopeptidase

GN=Npepps

Med H1/2: 1088.3 days

Q5 H1/2: 298.8 days

Q95 H1/2: 25387.9 days

reliable model fit: yes

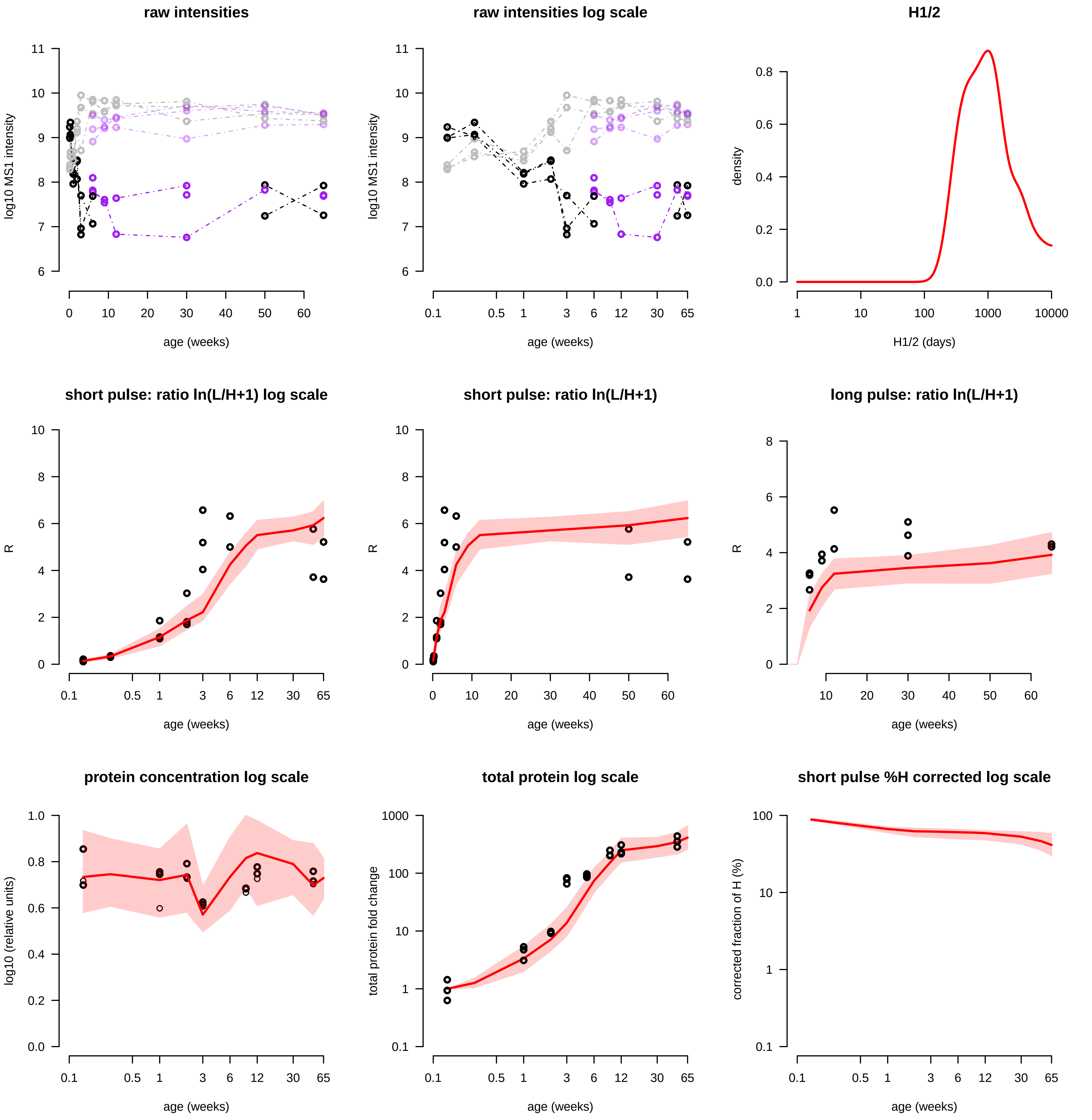

Q05793

>sp|Q05793|PGBM_MOUSE

Basement membrane−specific heparan sulfate proteoglycan core protein

GN=Hspg2

Med H1/2: 1304.2 days

Q5 H1/2: 300.1 days

Q95 H1/2: 12757.9 days

reliable model fit: no

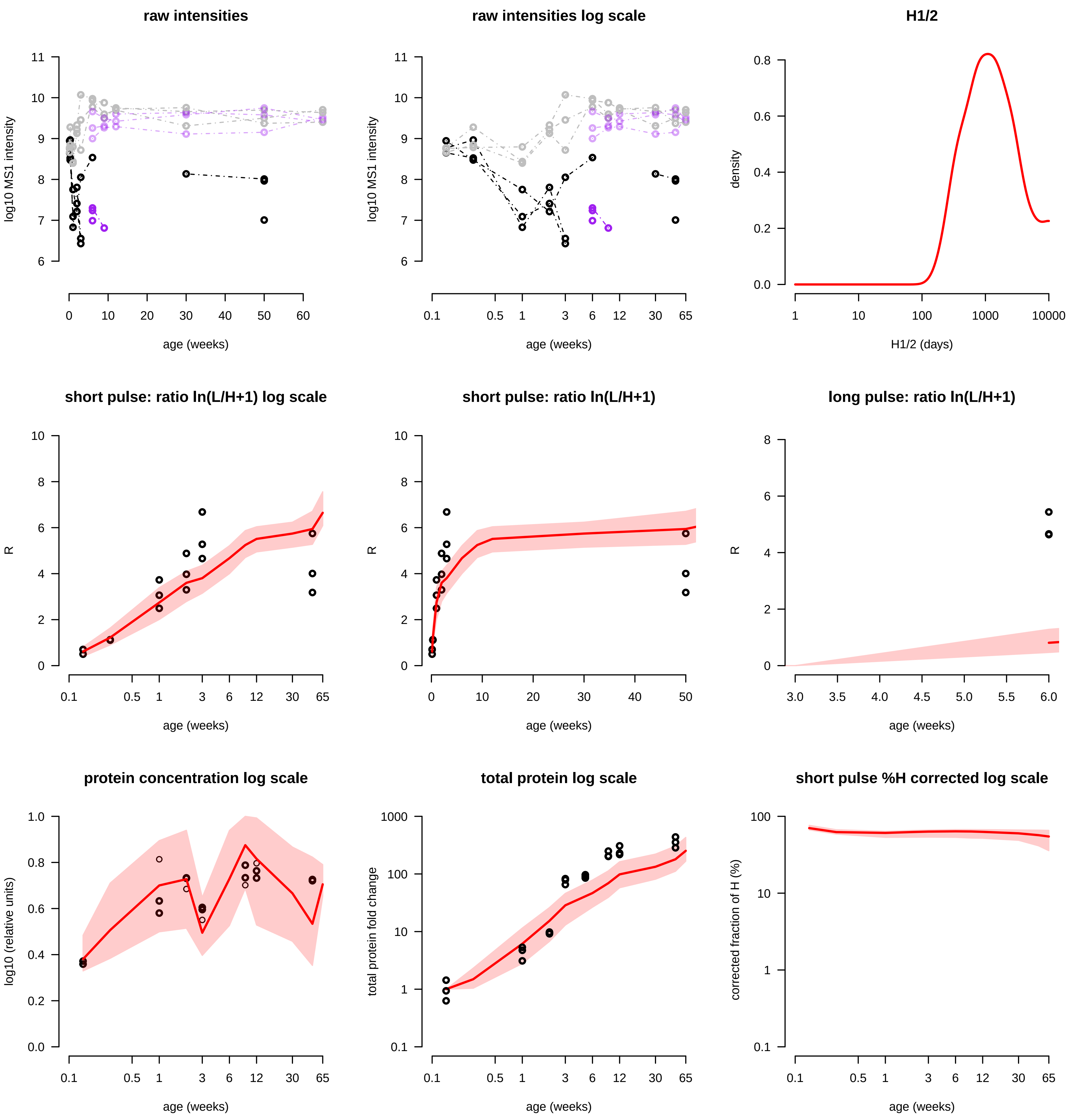

P50580;P50580−2

>sp|P50580|PA2G4_MOUSE

Proliferation−associated protein 2G4

GN=Pa2g4

Med H1/2: 9.4 days

Q5 H1/2: 8.1 days

Q95 H1/2: 11.2 days

reliable model fit: yes

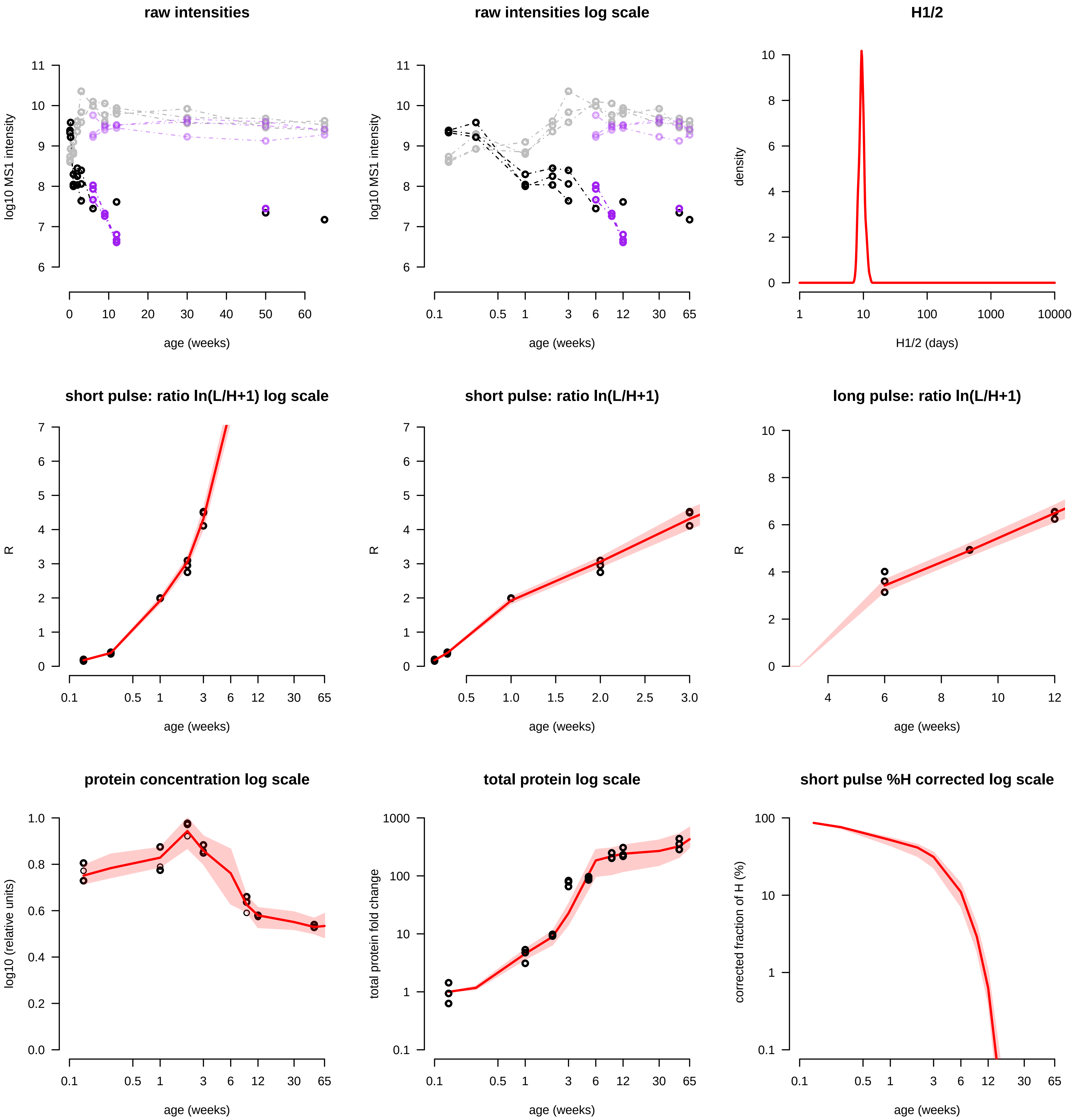

Q9CY58;Q9CY58−4

>sp|Q9CY58|PAIRB_MOUSE

Plasminogen activator inhibitor 1 RNA−binding protein

GN=Serbp1

Med H1/2: 6.6 days

Q5 H1/2: 5.8 days

Q95 H1/2: 9 days

reliable model fit: yes

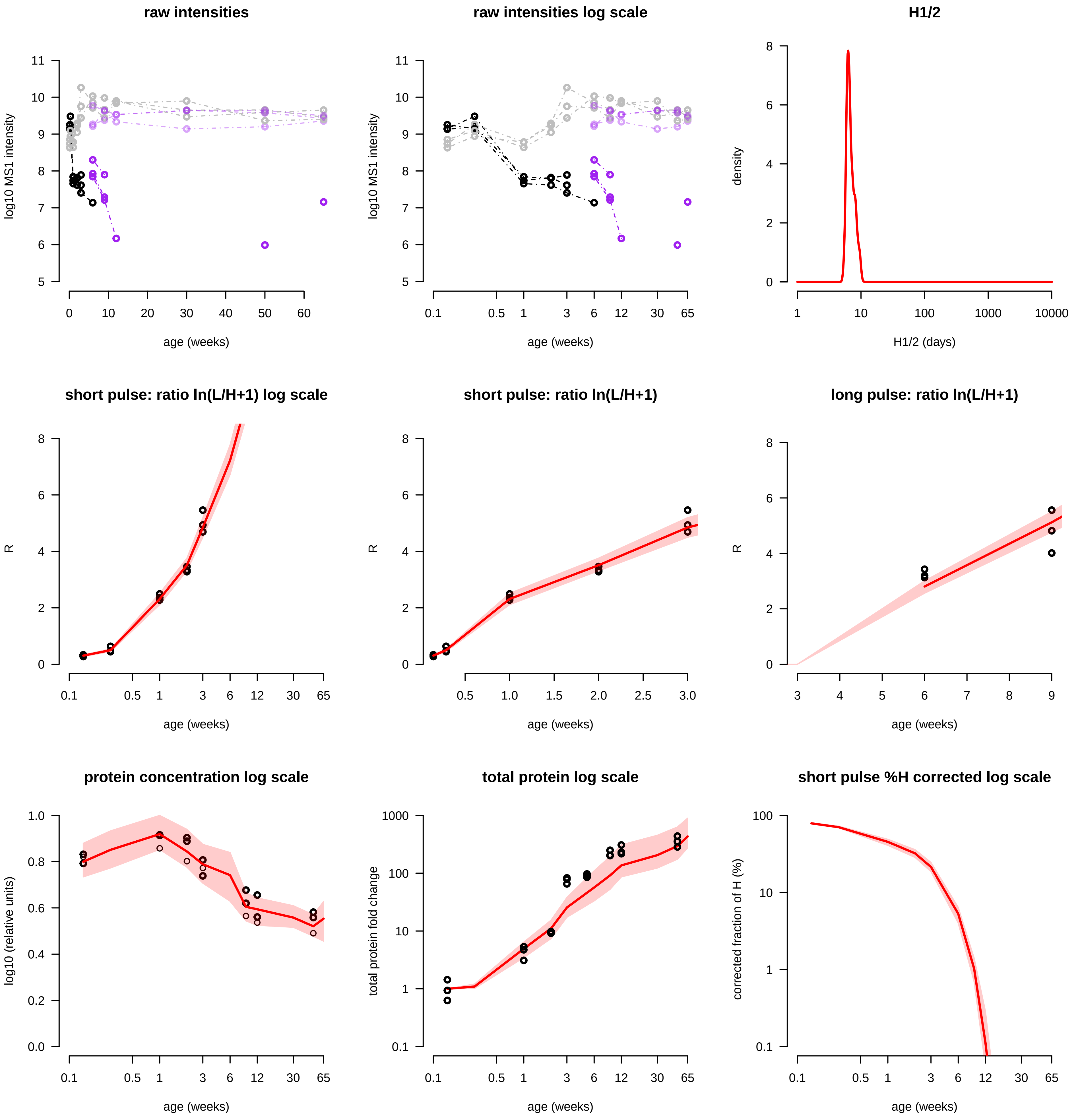

Q99MN9

>sp|Q99MN9|PCCB_MOUSE

Propionyl−CoA carboxylase beta chain, mitochondrial

GN=Pccb

Med H1/2: 9.1 days

Q5 H1/2: 6.4 days

Q95 H1/2: 17.8 days

reliable model fit: yes

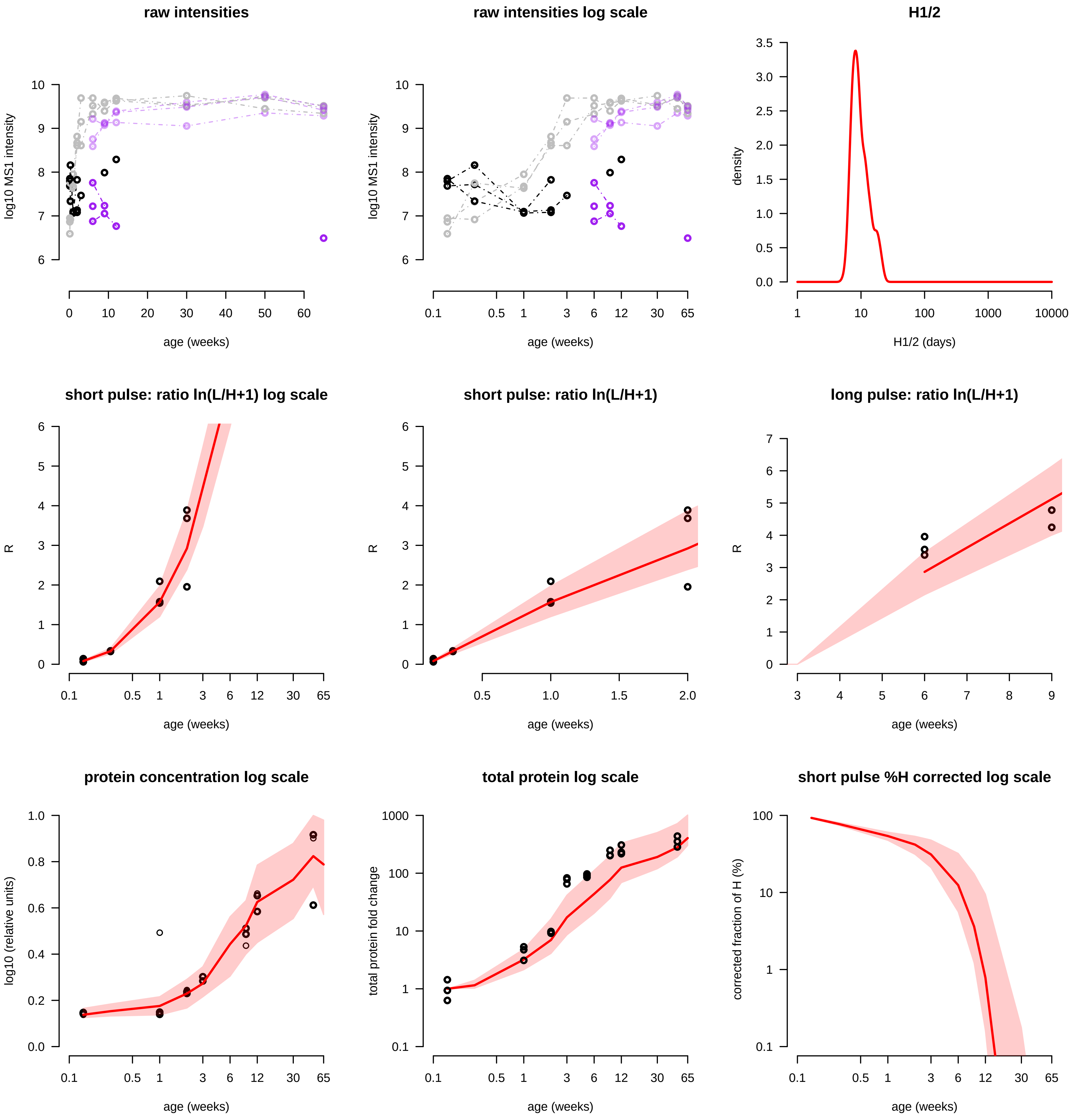

P61089

>sp|P61089|UBE2N_MOUSE

Ubiquitin−conjugating enzyme E2 N

GN=Ube2n

Med H1/2: 211.7 days

Q5 H1/2: 106.5 days

Q95 H1/2: 716.9 days

reliable model fit: yes

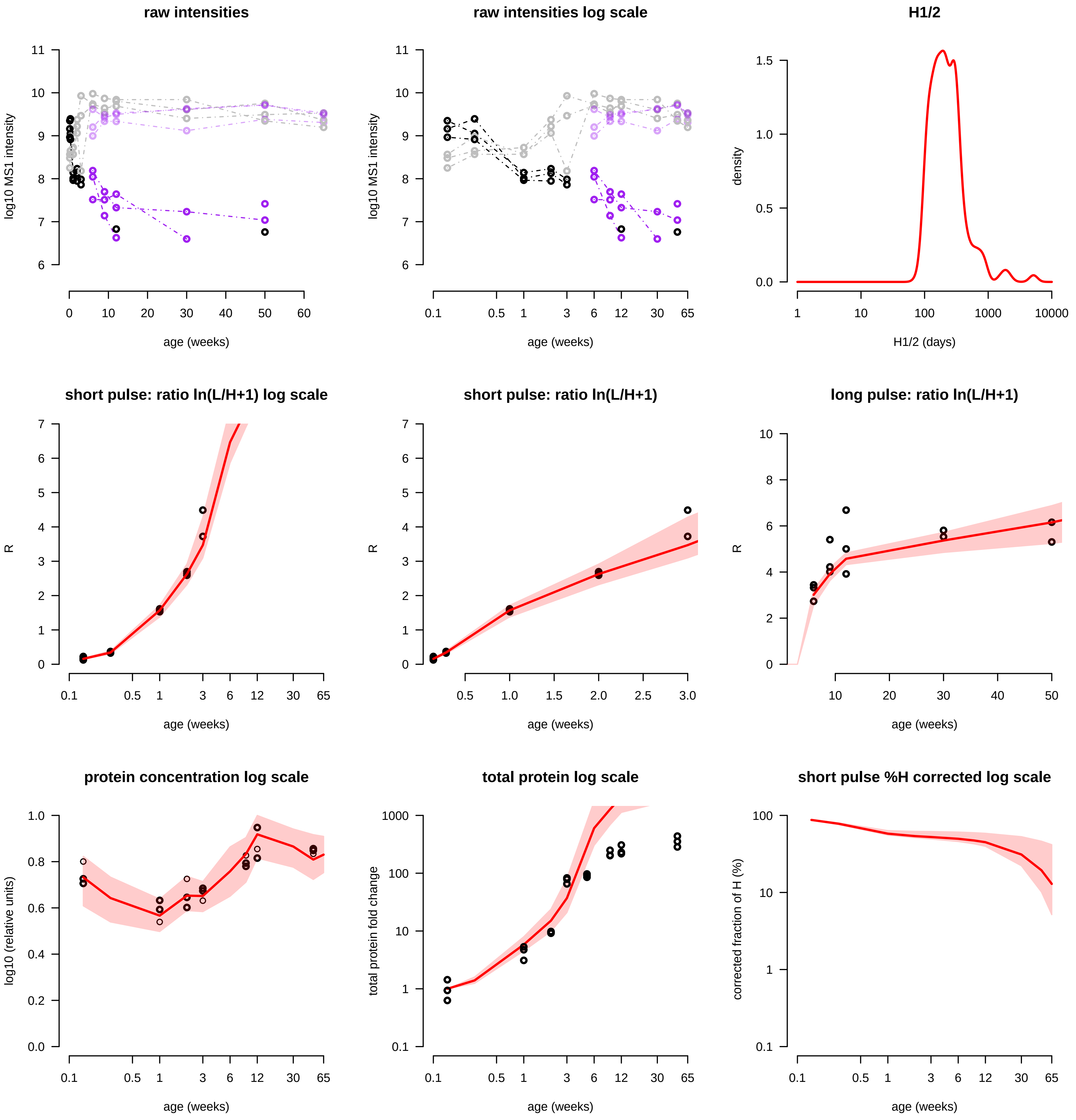

P21614

>sp|P21614|VTDB_MOUSE

Vitamin D−binding protein

GN=Gc

Med H1/2: 3.7 days

Q5 H1/2: 2.9 days

Q95 H1/2: 5 days

reliable model fit: yes

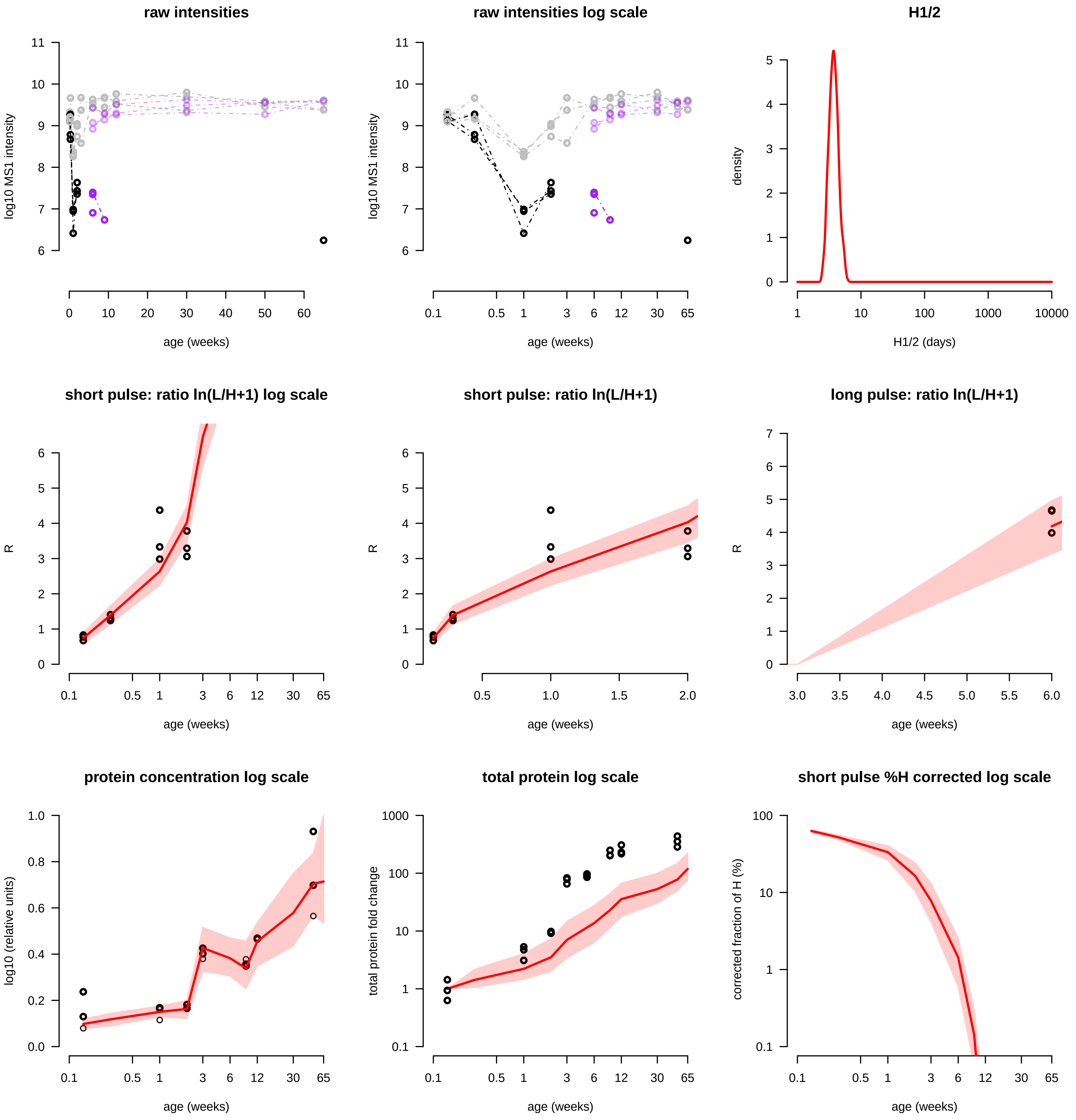

Q921F2

>sp|Q921F2|TADBP_MOUSE

TAR DNA−binding protein 43

GN=Tardbp

Med H1/2: 12 days

Q5 H1/2: 9.5 days

Q95 H1/2: 16.1 days

reliable model fit: yes

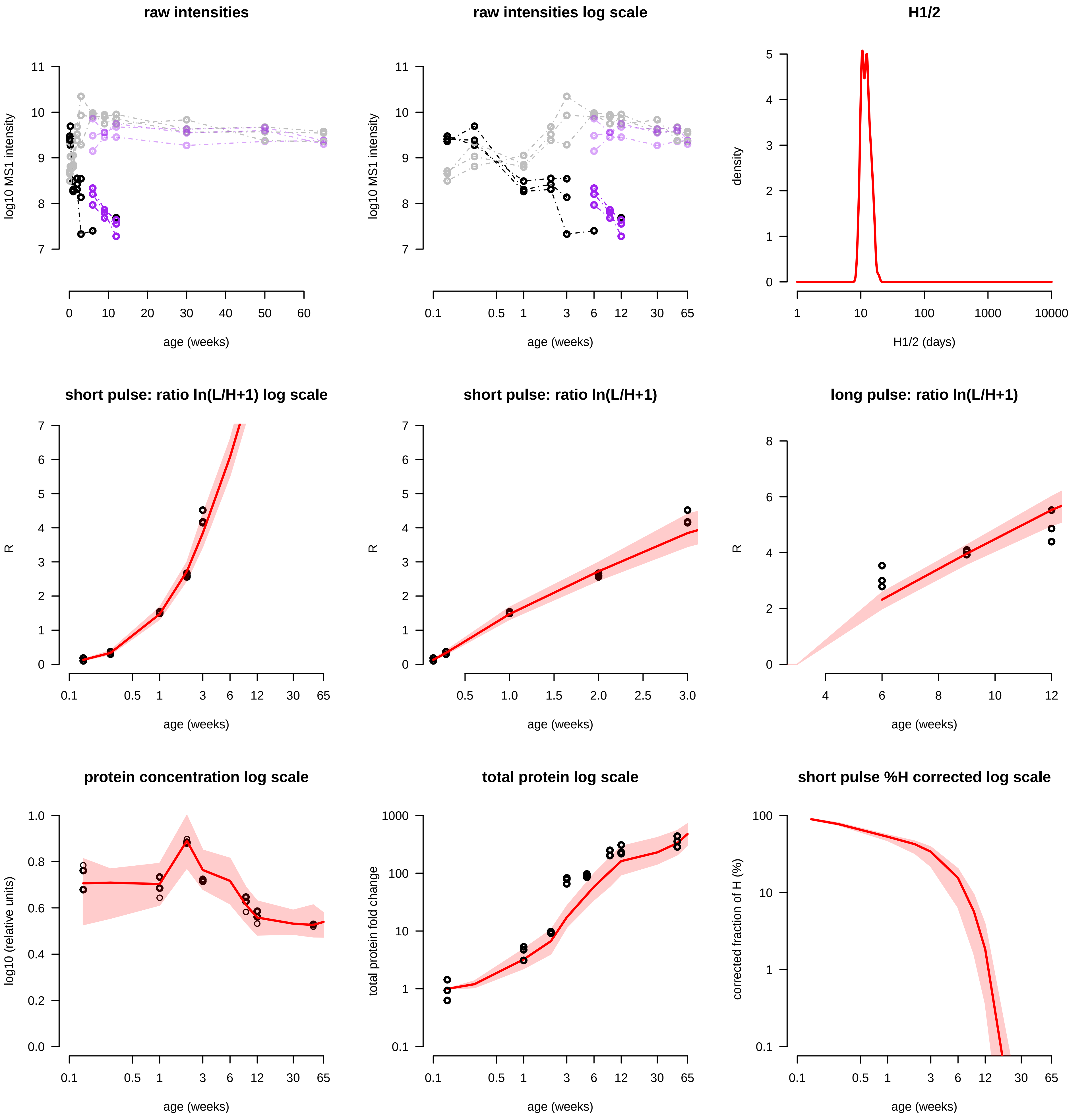

O08553

>sp|O08553|DPYL2_MOUSE

Dihydropyrimidinase−related protein 2

GN=Dpysl2

Med H1/2: 9.6 days

Q5 H1/2: 7.7 days

Q95 H1/2: 12.7 days

reliable model fit: yes

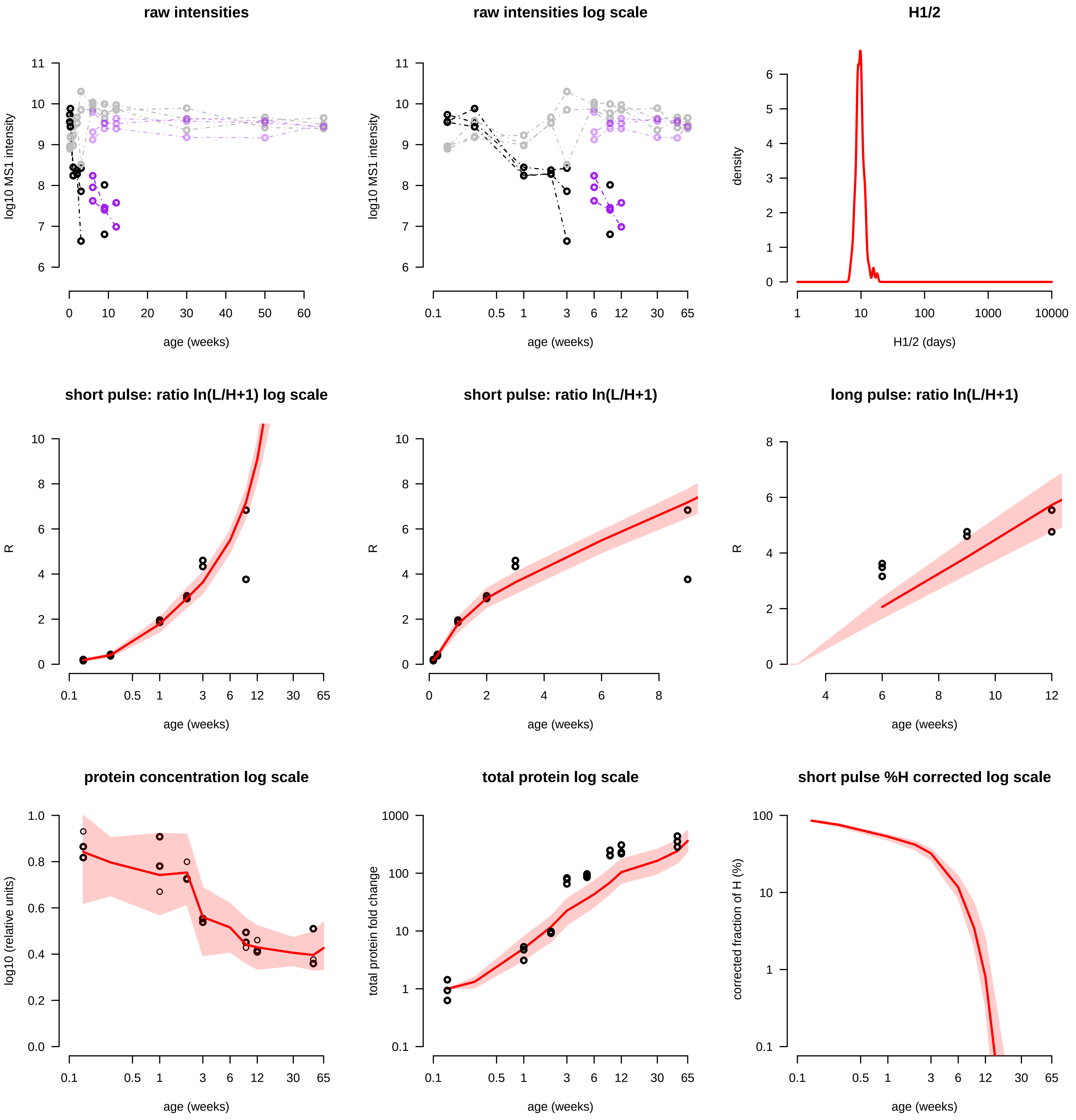

Q9CVB6

>sp|Q9CVB6|ARPC2_MOUSE

Actin−related protein 2/3 complex subunit 2

GN=Arpc2

Med H1/2: 183.6 days

Q5 H1/2: 79.3 days

Q95 H1/2: 1131.8 days

reliable model fit: yes

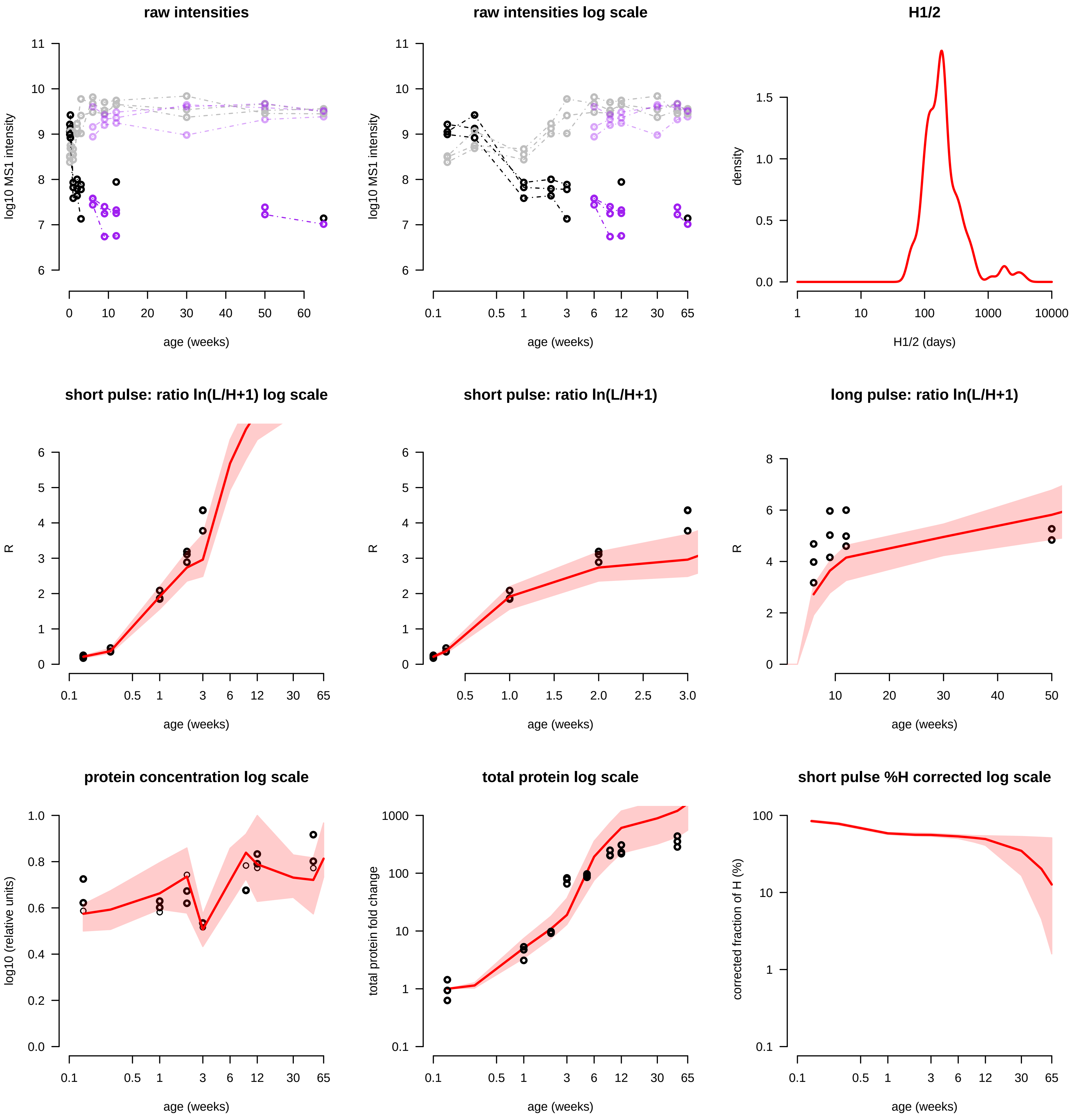

Q60598

>sp|Q60598|SRC8_MOUSE

Src substrate cortactin

GN=Cttn

Med H1/2: 934.5 days

Q5 H1/2: 246.1 days

Q95 H1/2: 14477.2 days

reliable model fit: yes

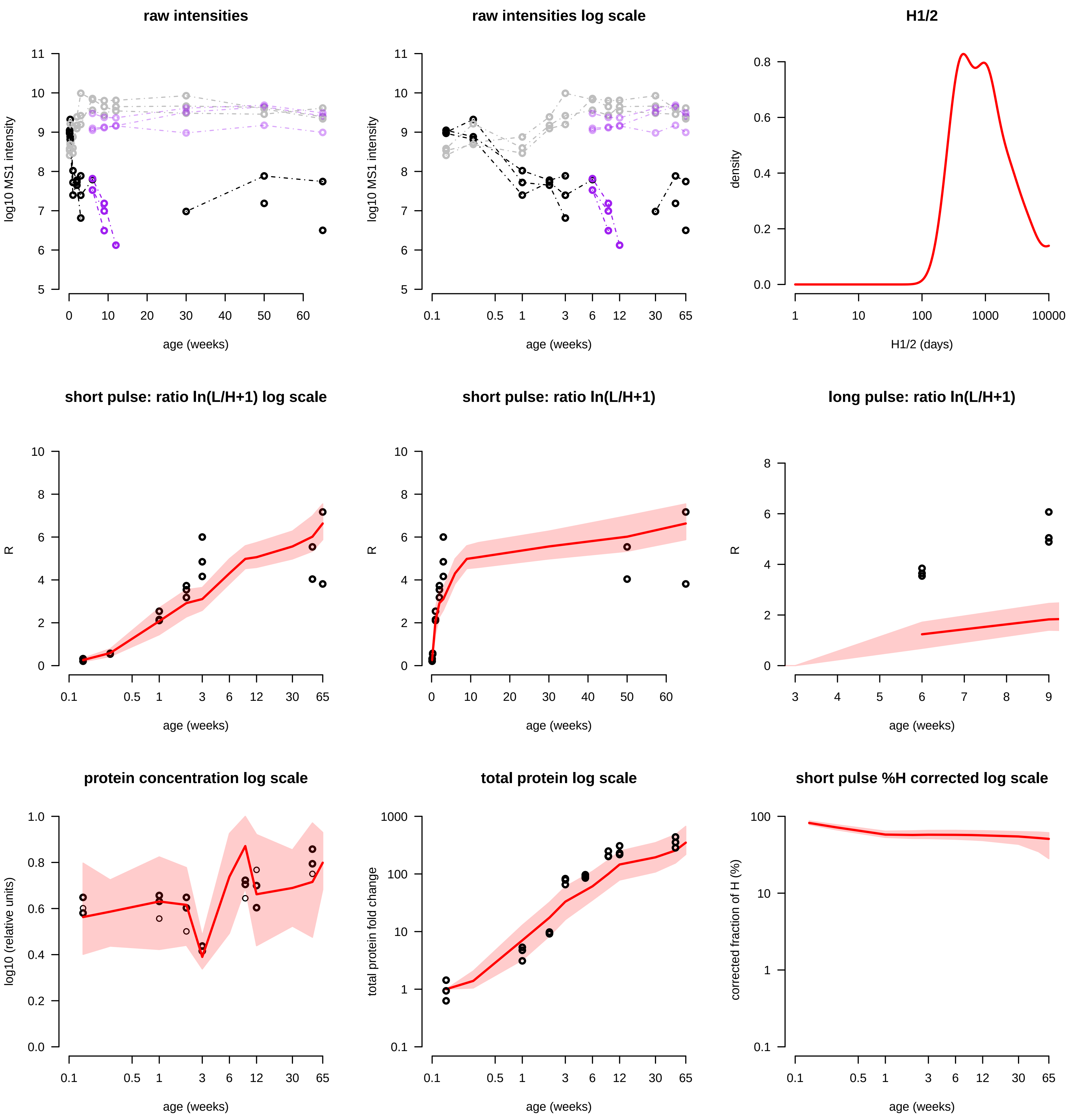

Q9Z1Z0;Q9Z1Z0−2;Q9Z1Z0−4

>sp|Q9Z1Z0|USO1_MOUSE

General vesicular transport factor p115

GN=Uso1

Med H1/2: 371.5 days

Q5 H1/2: 83.4 days

Q95 H1/2: 1693.6 days

reliable model fit: yes

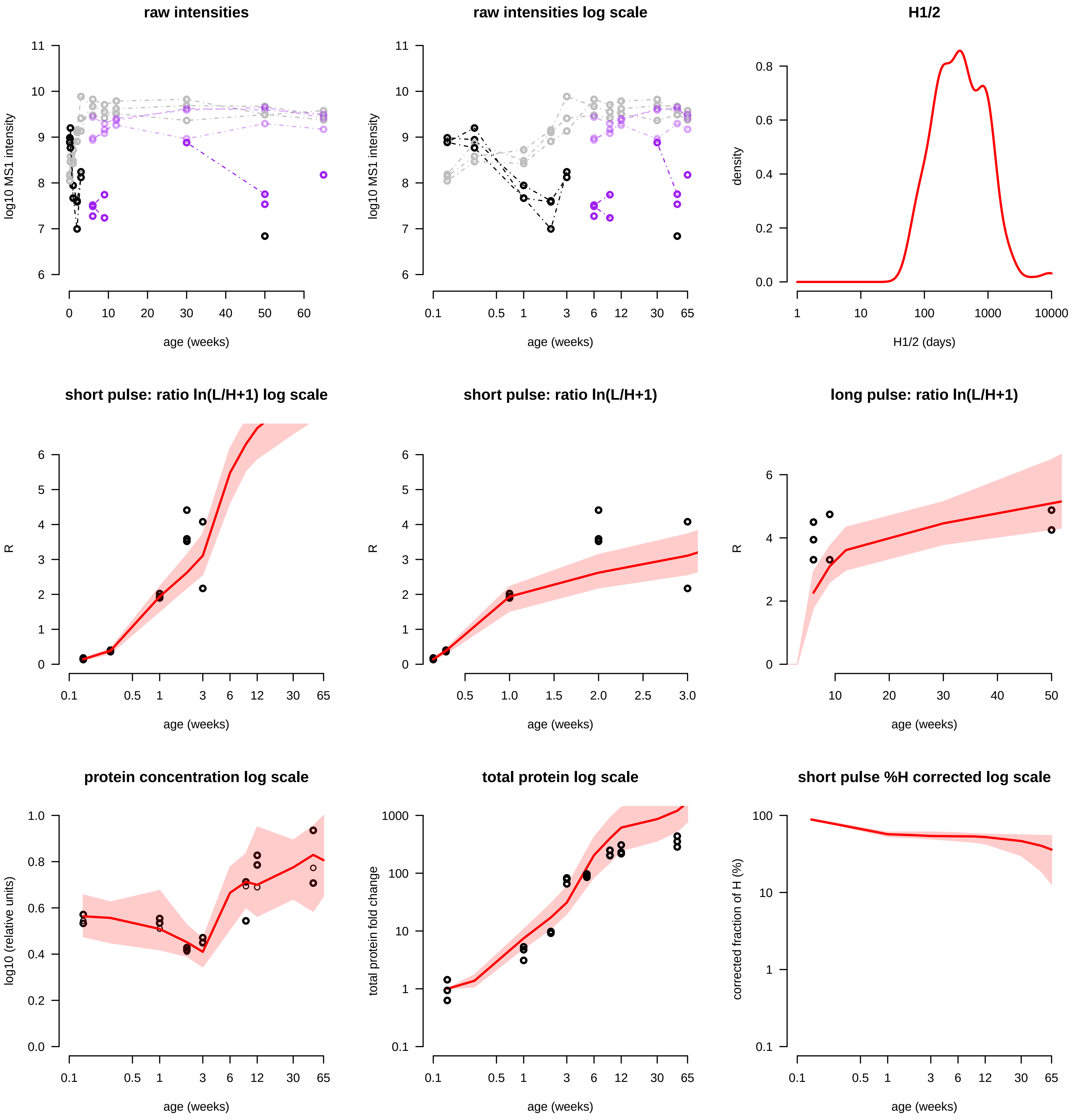

Q9CR57

>sp|Q9CR57|RL14_MOUSE

60S ribosomal protein L14

GN=Rpl14

Med H1/2: 1085.1 days

Q5 H1/2: 301.6 days

Q95 H1/2: 9477.5 days

reliable model fit: yes

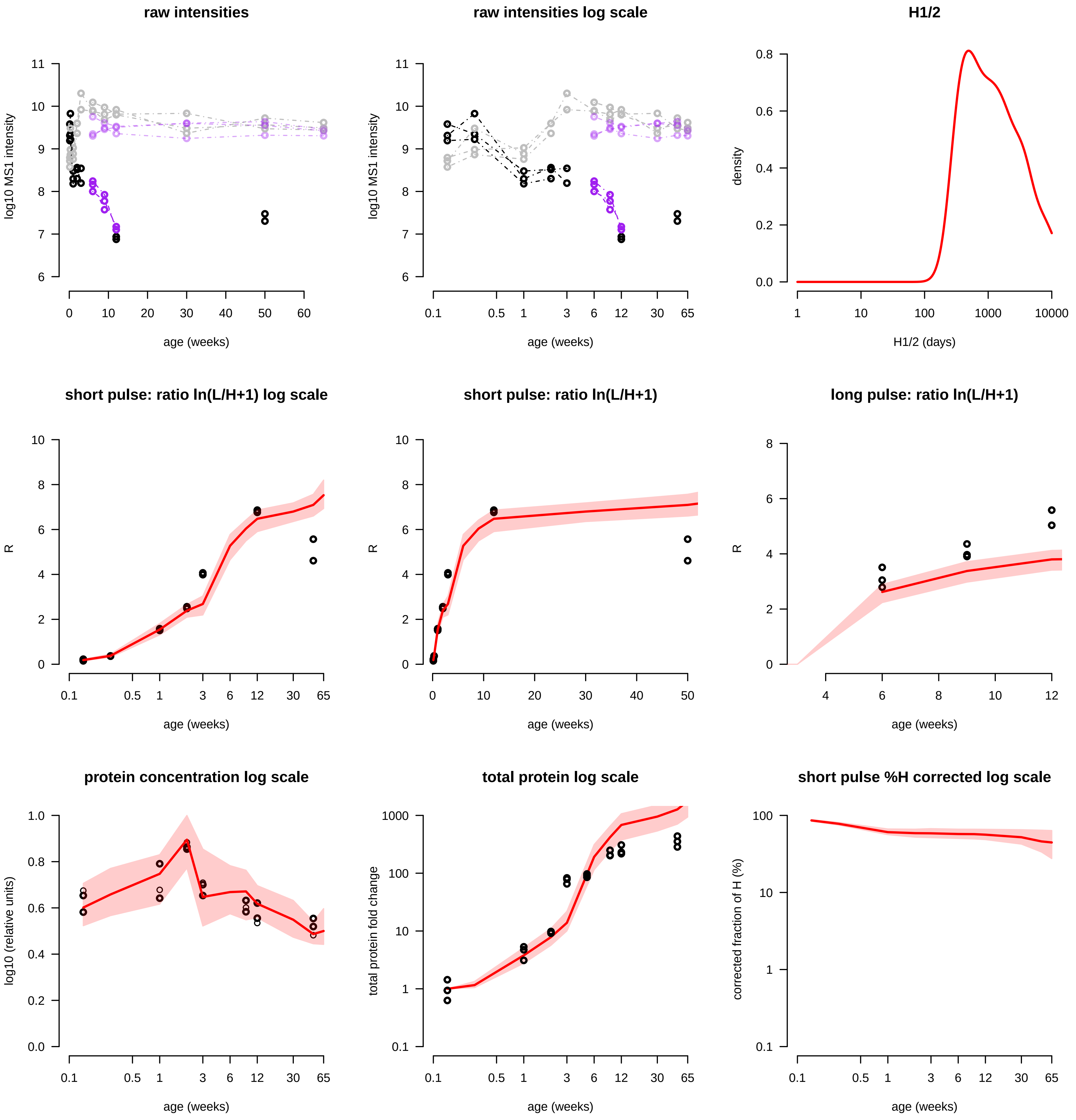

O09167

>sp|O09167|RL21_MOUSE

60S ribosomal protein L21

GN=Rpl21

Med H1/2: 9.9 days

Q5 H1/2: 8.1 days

Q95 H1/2: 13.5 days

reliable model fit: yes

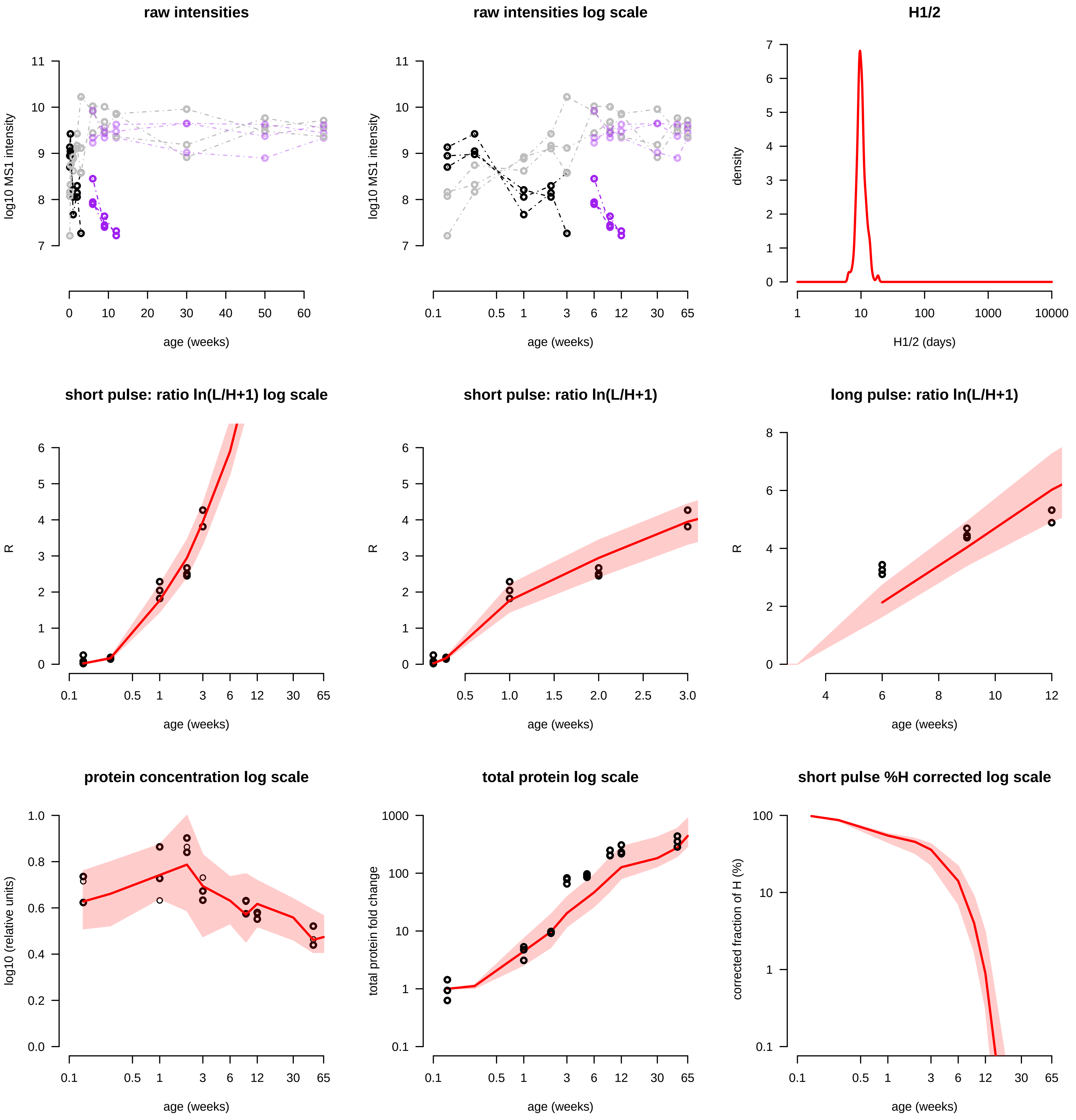

Q9DBL1

>sp|Q9DBL1|ACDSB_MOUSE

Short/branched chain specific acyl−CoA dehydrogenase, mitochondrial

GN=Acadsb

Med H1/2: 6.4 days

Q5 H1/2: 4.9 days

Q95 H1/2: 9.1 days

reliable model fit: yes

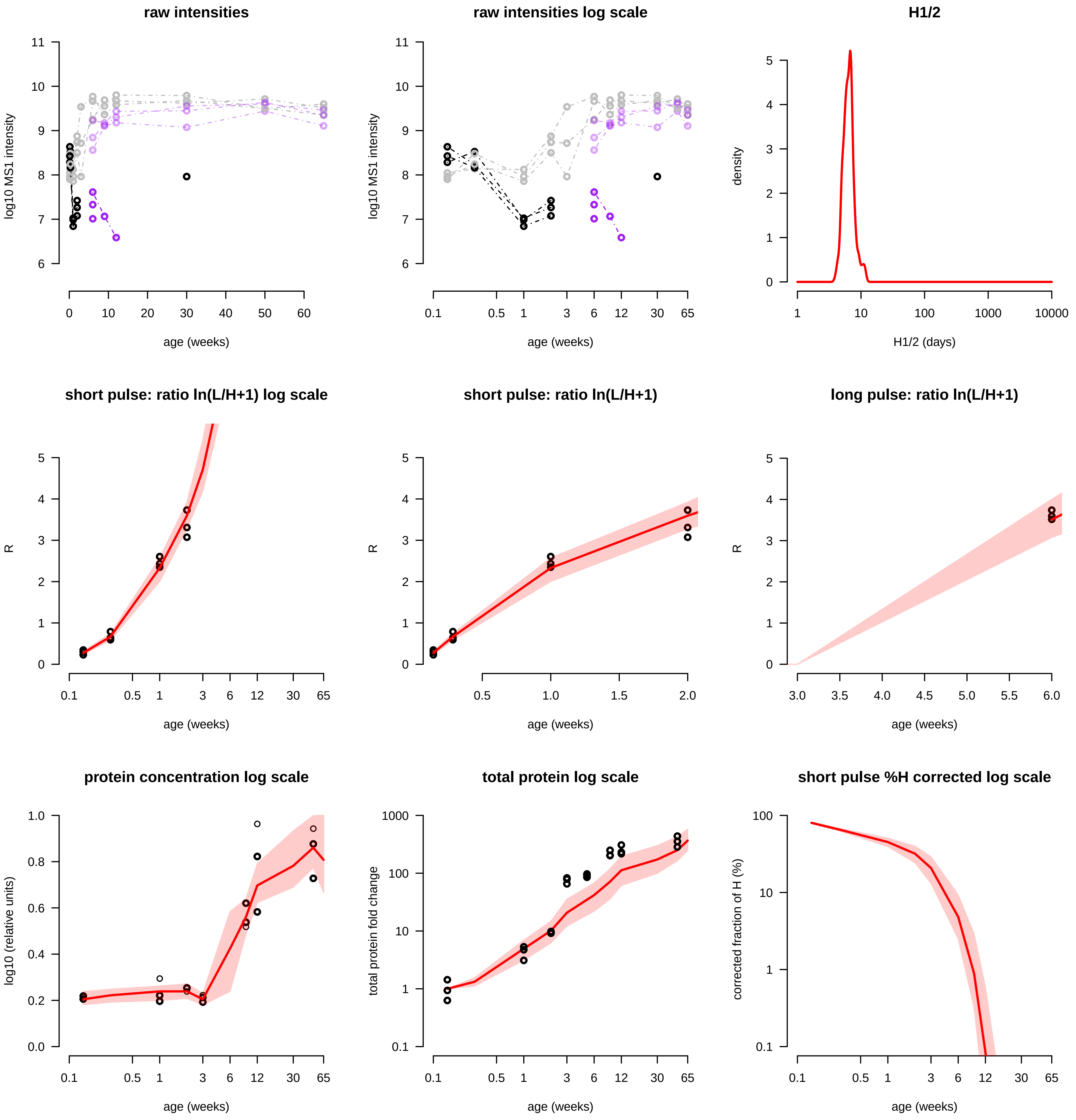

Q9D1A2

>sp|Q9D1A2|CNDP2_MOUSE

Cytosolic non−specific dipeptidase

GN=Cndp2

Med H1/2: 7.6 days

Q5 H1/2: 6.1 days

Q95 H1/2: 9.9 days

reliable model fit: yes

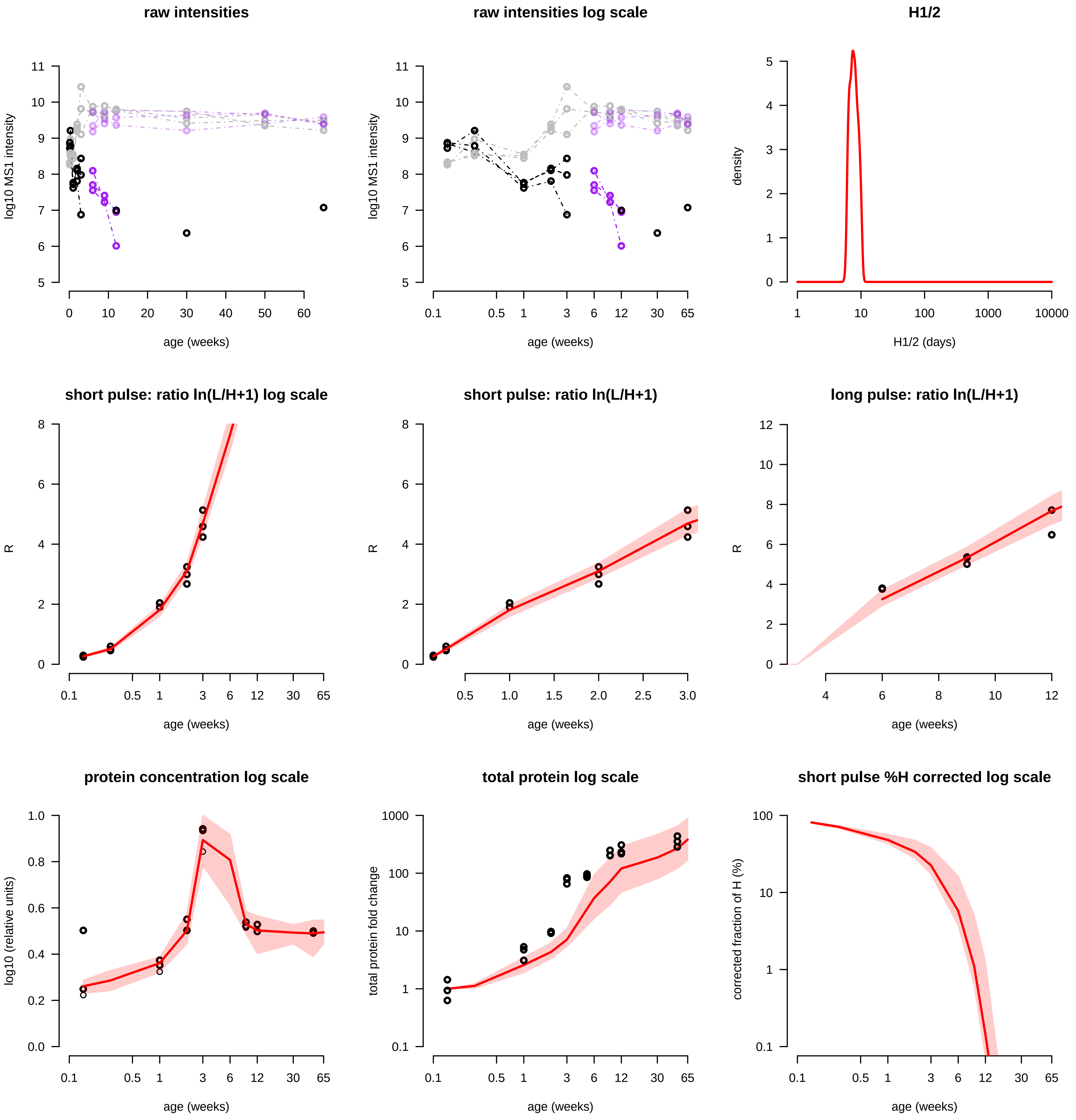

Q6P5E4

>sp|Q6P5E4|UGGG1_MOUSE

UDP−glucose:glycoprotein glucosyltransferase 1

GN=Uggt1

Med H1/2: 242.4 days

Q5 H1/2: 81.4 days

Q95 H1/2: 1373.3 days

reliable model fit: yes

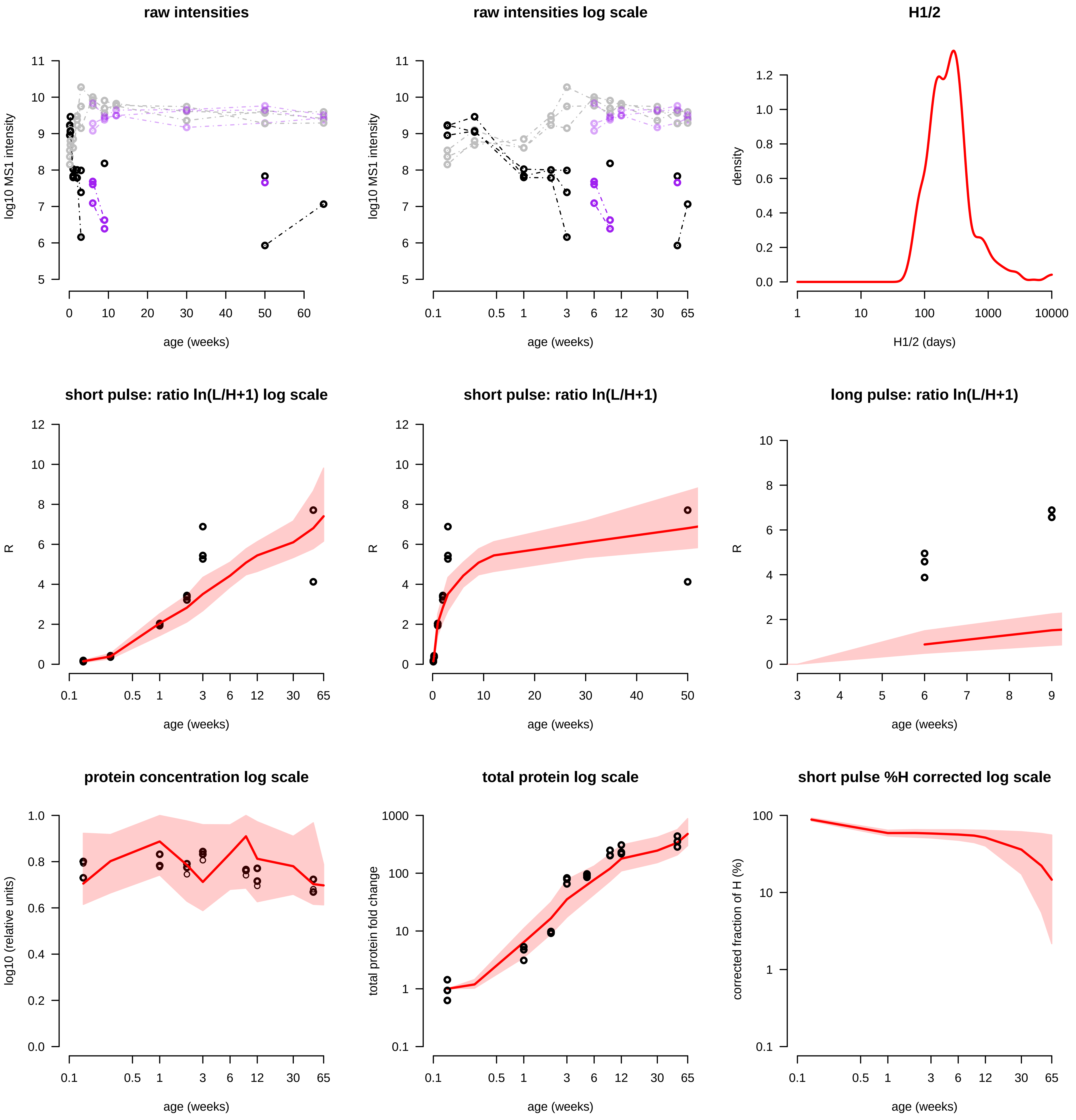

Q9D0M3−2;Q9D0M3

>sp|Q9D0M3−2|CY1_MOUSE

Isoform 2 of Cytochrome c1, heme protein, mitochondrial

GN=Cyc1

Med H1/2: 12.1 days

Q5 H1/2: 9.6 days

Q95 H1/2: 18.5 days

reliable model fit: yes

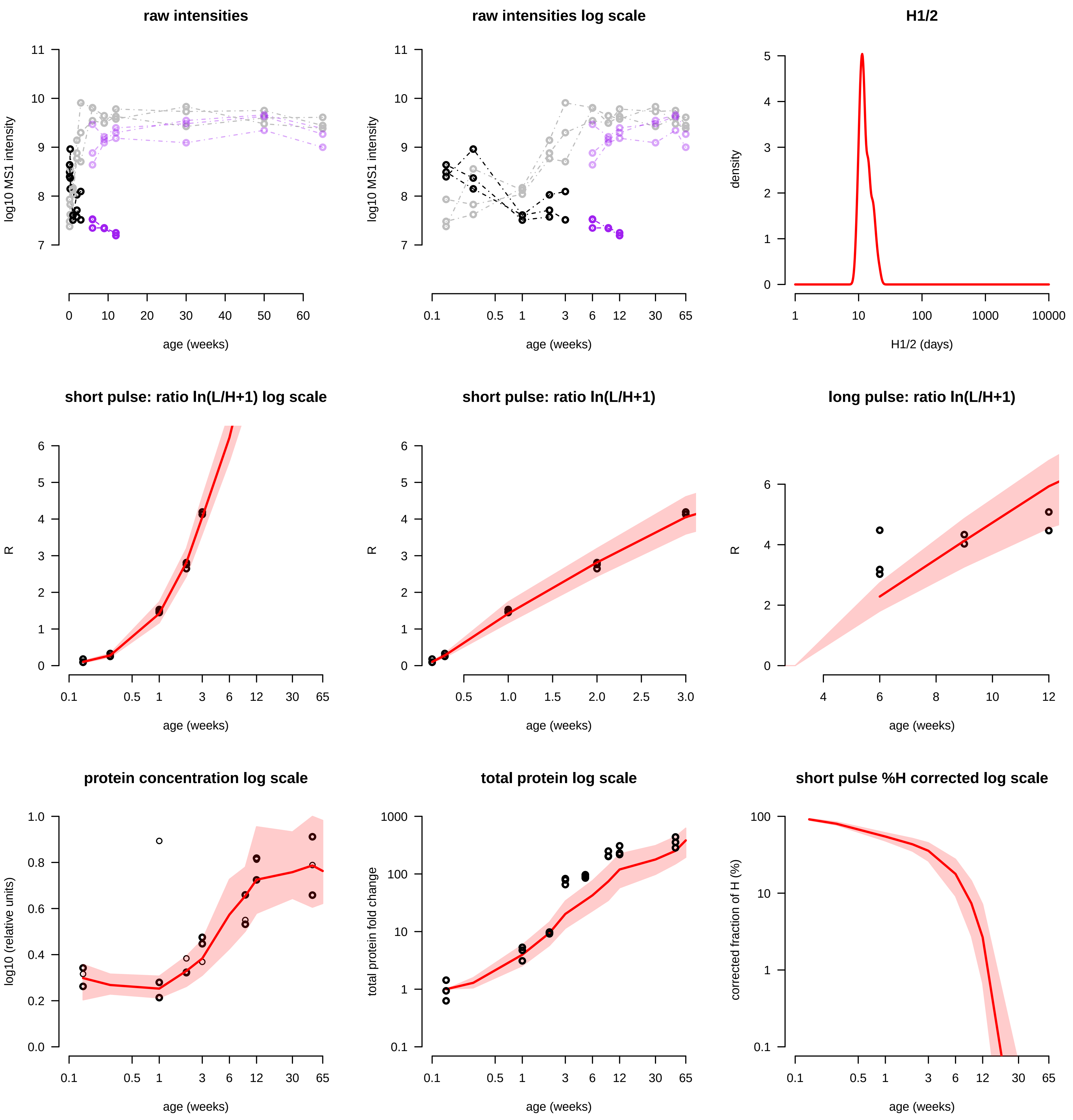

Q3TXS7

>sp|Q3TXS7|PSMD1_MOUSE

26S proteasome non−ATPase regulatory subunit 1

GN=Psmd1

Med H1/2: 106 days

Q5 H1/2: 67 days

Q95 H1/2: 229.9 days

reliable model fit: yes

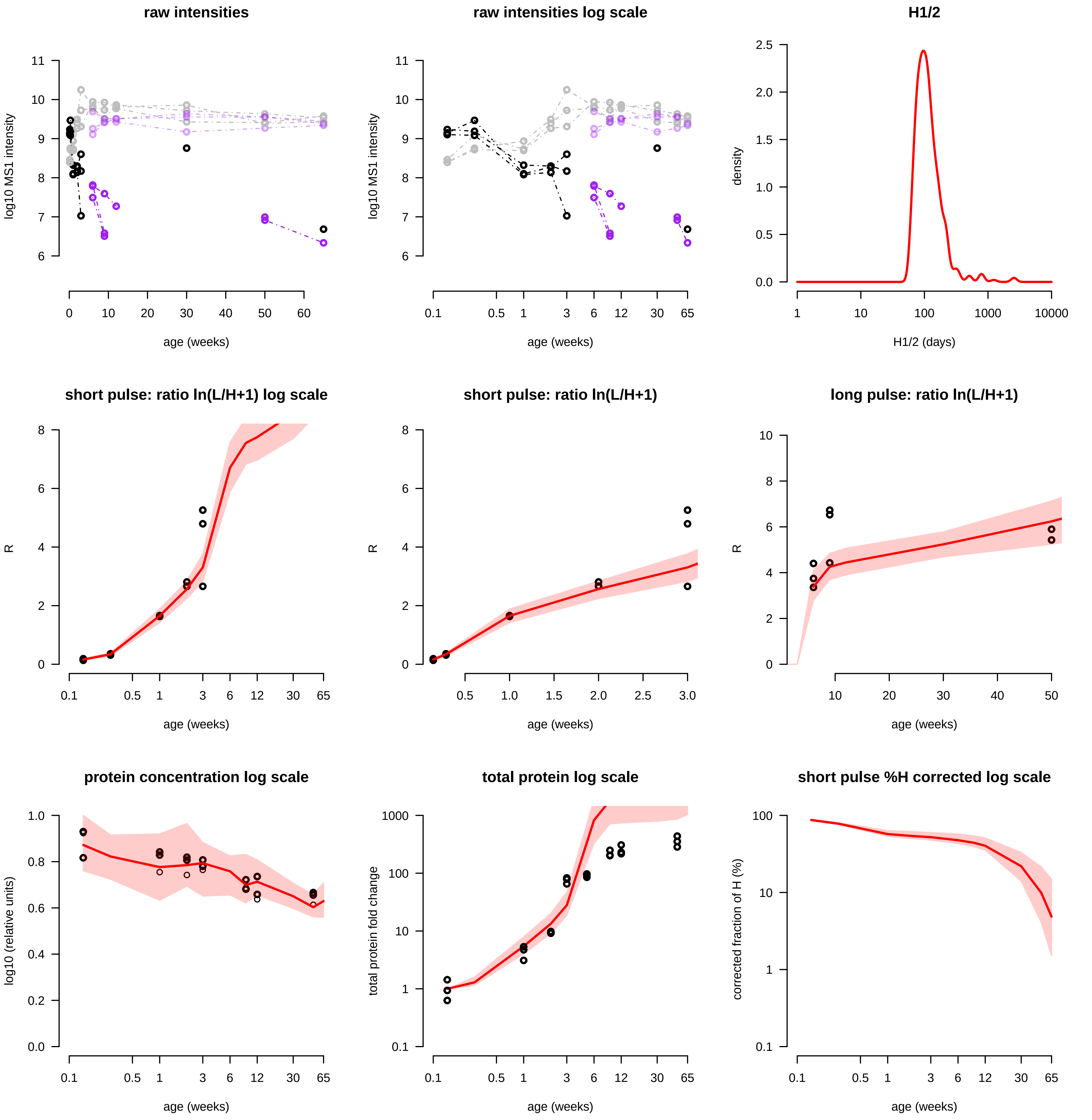

Q78IK4

>sp|Q78IK4|MIC27_MOUSE

MICOS complex subunit Mic27

GN=Apool

Med H1/2: 5.1 days

Q5 H1/2: 4 days

Q95 H1/2: 6.7 days

reliable model fit: yes

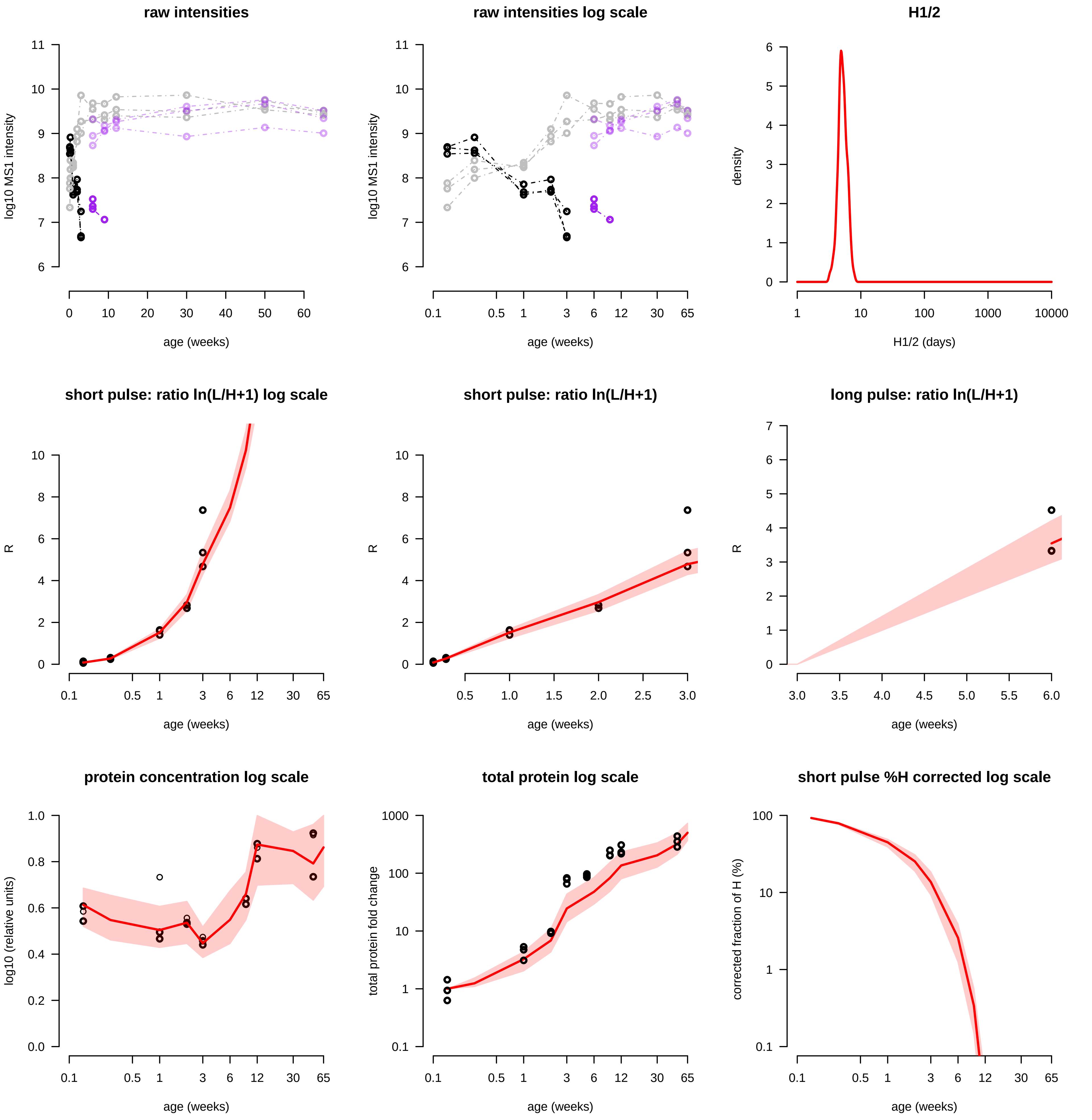

P84091

>sp|P84091|AP2M1_MOUSE

AP−2 complex subunit mu

GN=Ap2m1

Med H1/2: 6.8 days

Q5 H1/2: 6.3 days

Q95 H1/2: 7.8 days

reliable model fit: yes

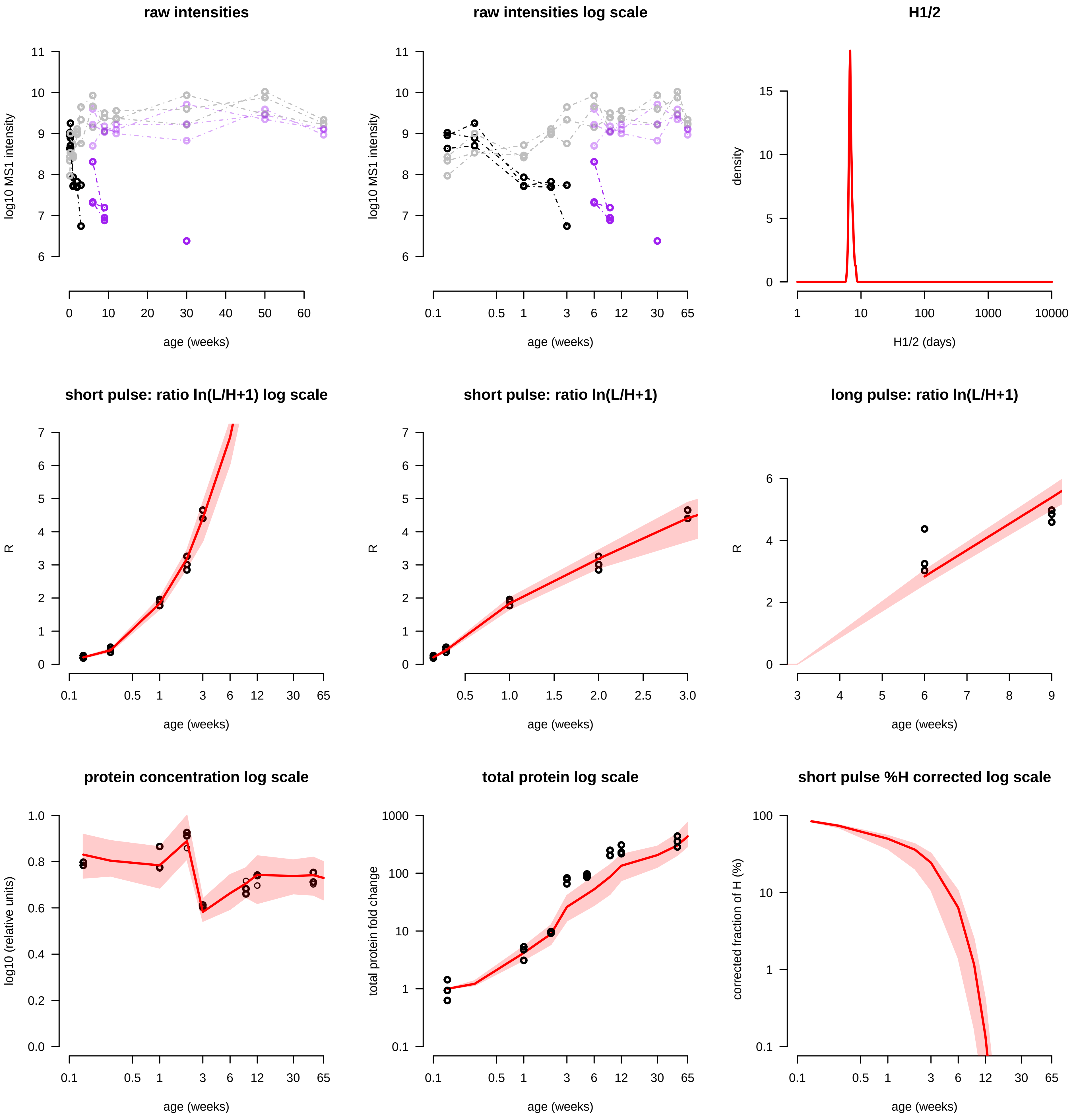

P51410

>sp|P51410|RL9_MOUSE

60S ribosomal protein L9

GN=Rpl9

Med H1/2: 12 days

Q5 H1/2: 9.9 days

Q95 H1/2: 16.5 days

reliable model fit: yes

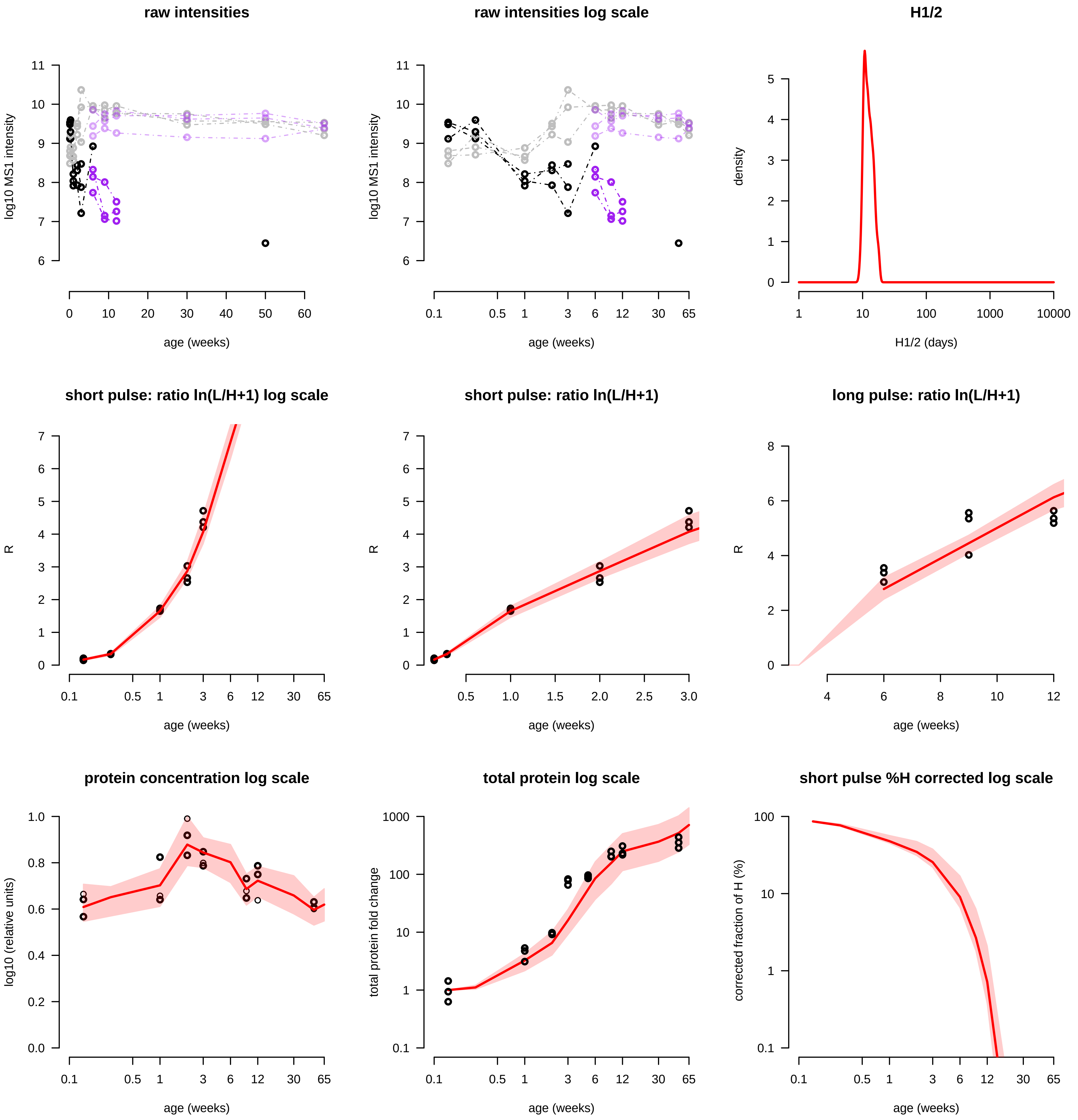

P58281−2;P58281

>sp|P58281−2|OPA1_MOUSE

Isoform 2 of Dynamin−like 120 kDa protein, mitochondrial

GN=Opa1

Med H1/2: 4.7 days

Q5 H1/2: 3.6 days

Q95 H1/2: 7.1 days

reliable model fit: yes

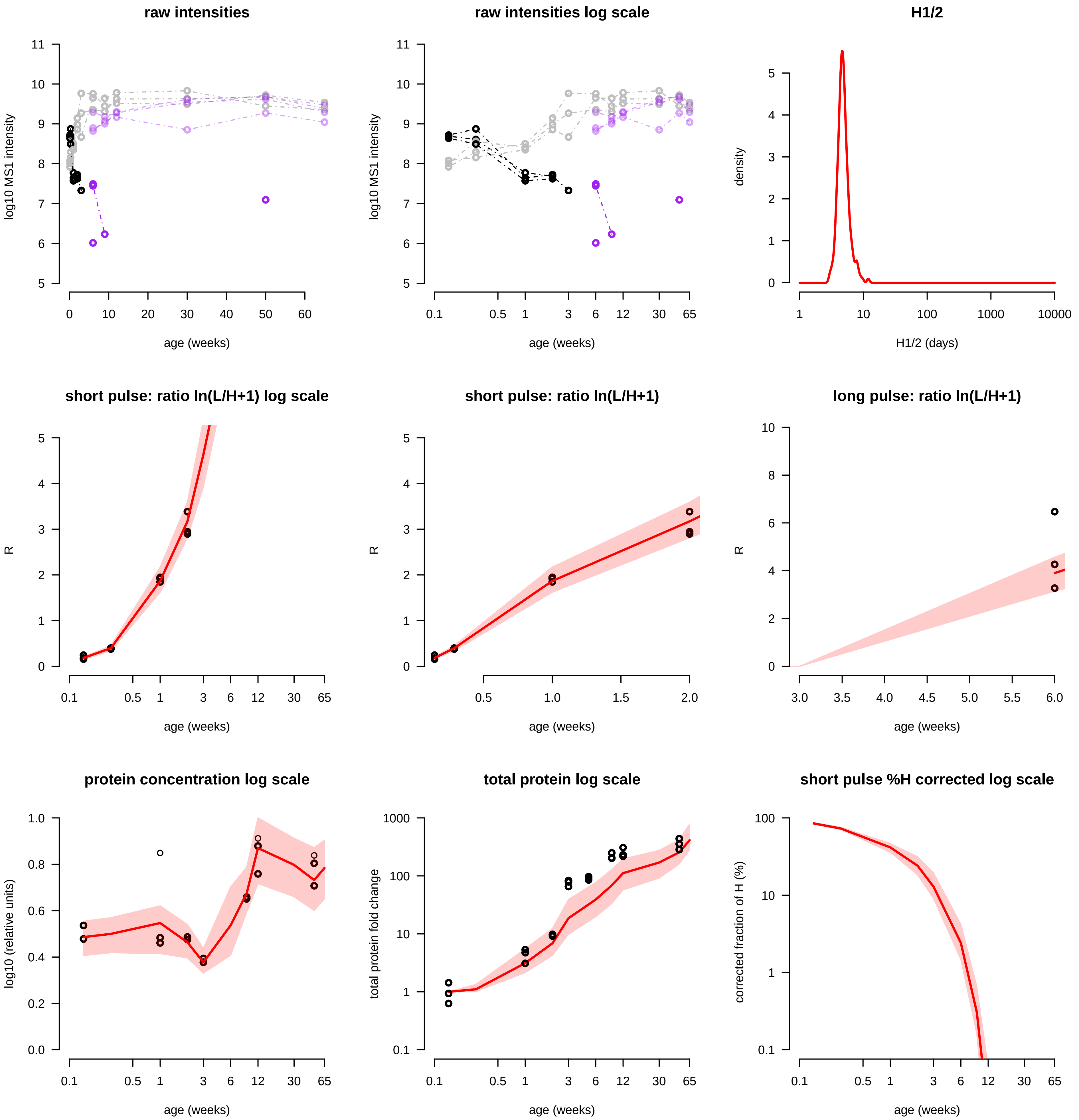

Q78PY7

>sp|Q78PY7|SND1_MOUSE

Staphylococcal nuclease domain−containing protein 1

GN=Snd1

Med H1/2: 124.9 days

Q5 H1/2: 78.6 days

Q95 H1/2: 248.9 days

reliable model fit: yes

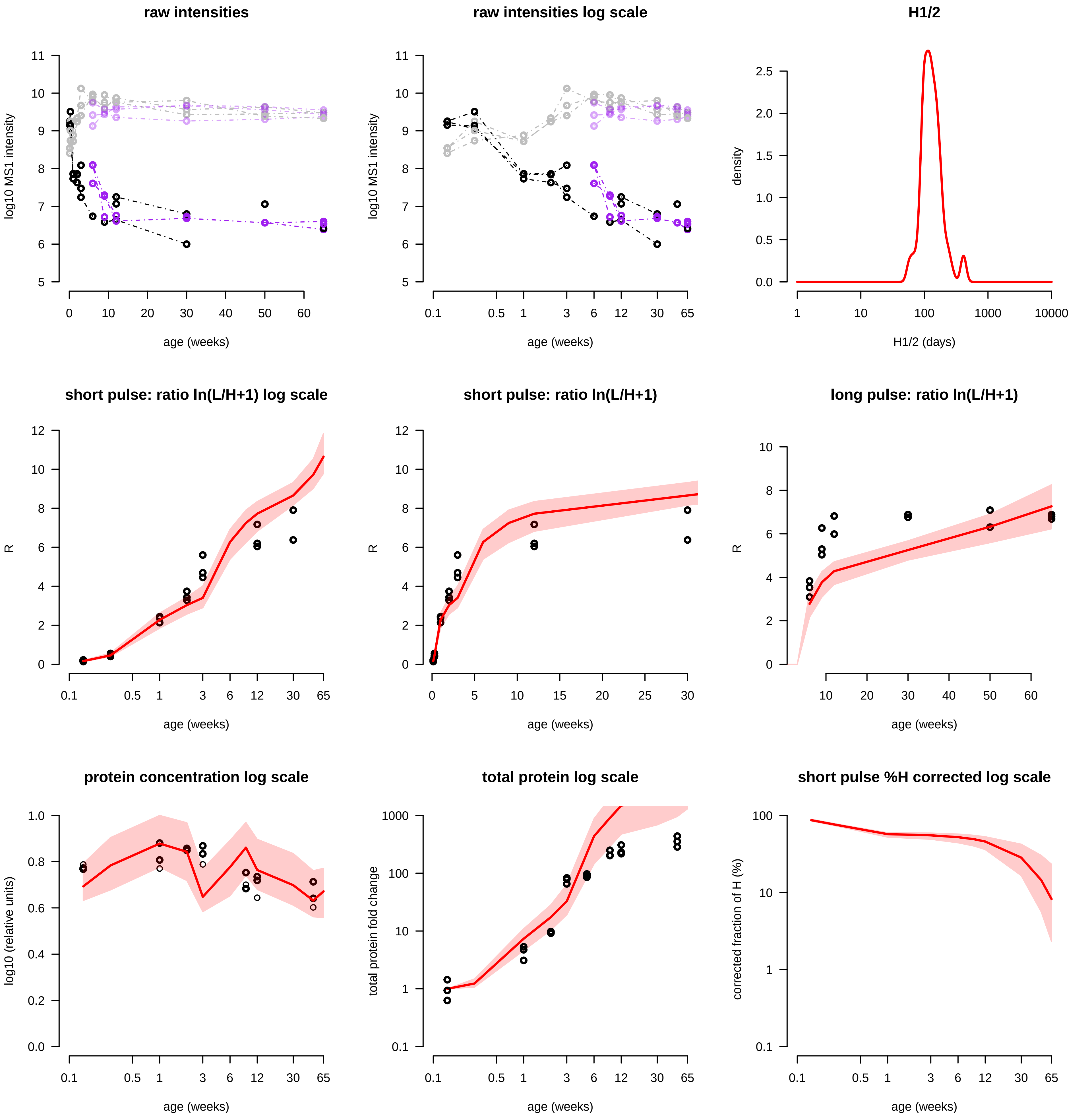

Q61335

>sp|Q61335|BAP31_MOUSE

B−cell receptor−associated protein 31

GN=Bcap31

Med H1/2: 73.4 days

Q5 H1/2: 46.6 days

Q95 H1/2: 213.9 days

reliable model fit: no

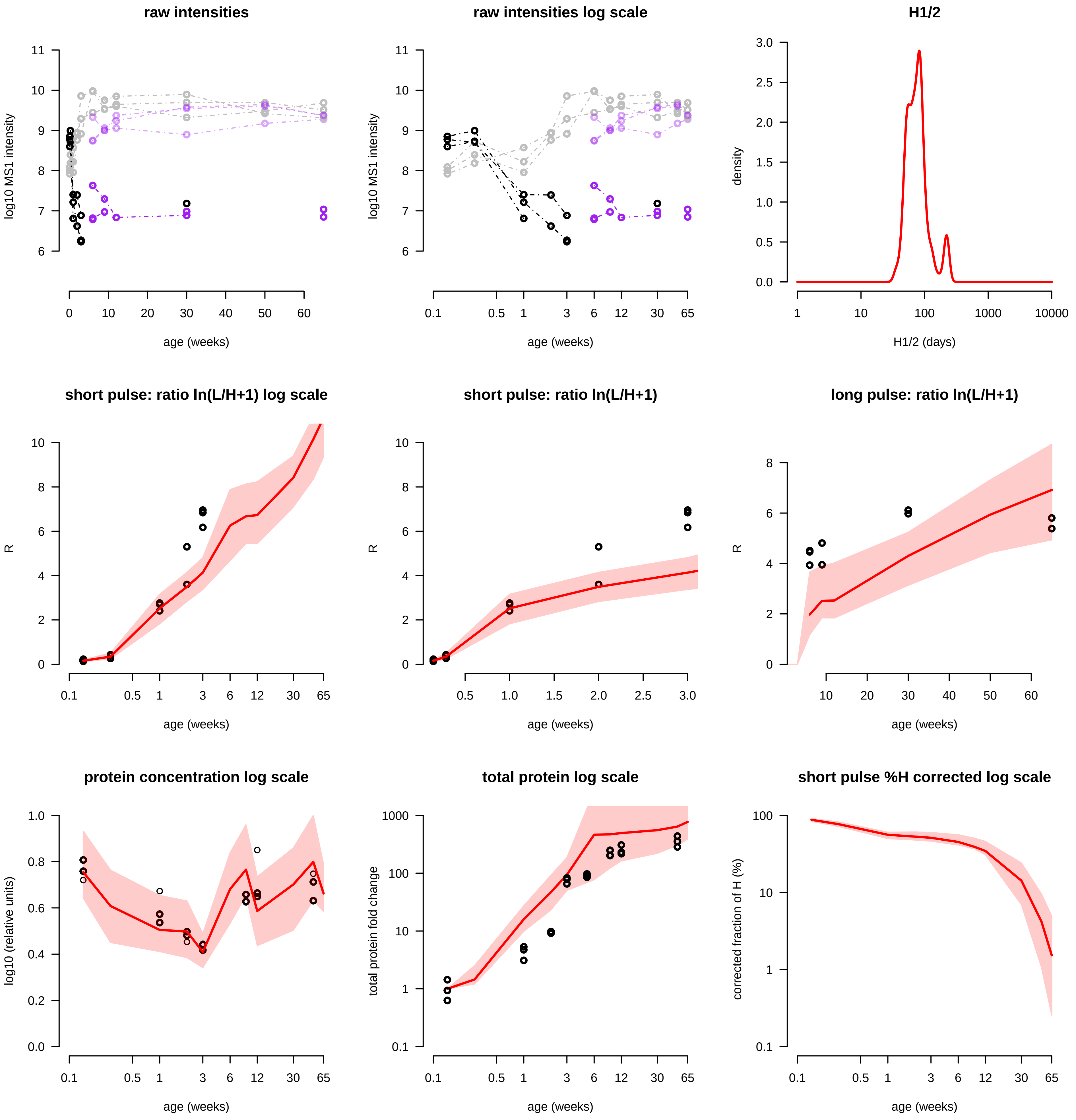

Q9JK53

>sp|Q9JK53|PRELP_MOUSE

Prolargin

GN=Prelp

Med H1/2: 8.7 days

Q5 H1/2: 7 days

Q95 H1/2: 11 days

reliable model fit: yes

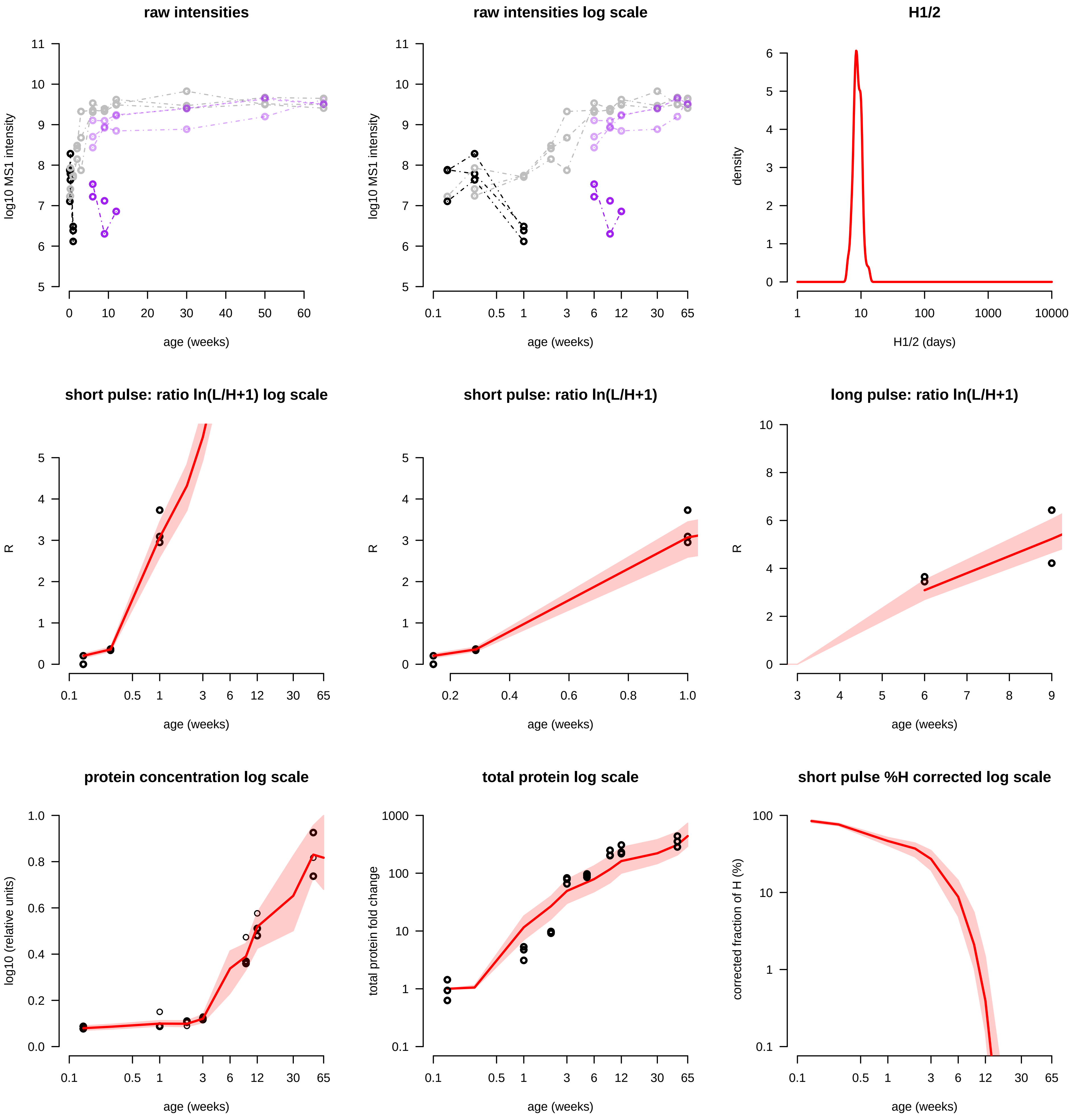

P28271

>sp|P28271|ACOC_MOUSE

Cytoplasmic aconitate hydratase

GN=Aco1

Med H1/2: 10.6 days

Q5 H1/2: 8.4 days

Q95 H1/2: 14.2 days

reliable model fit: yes

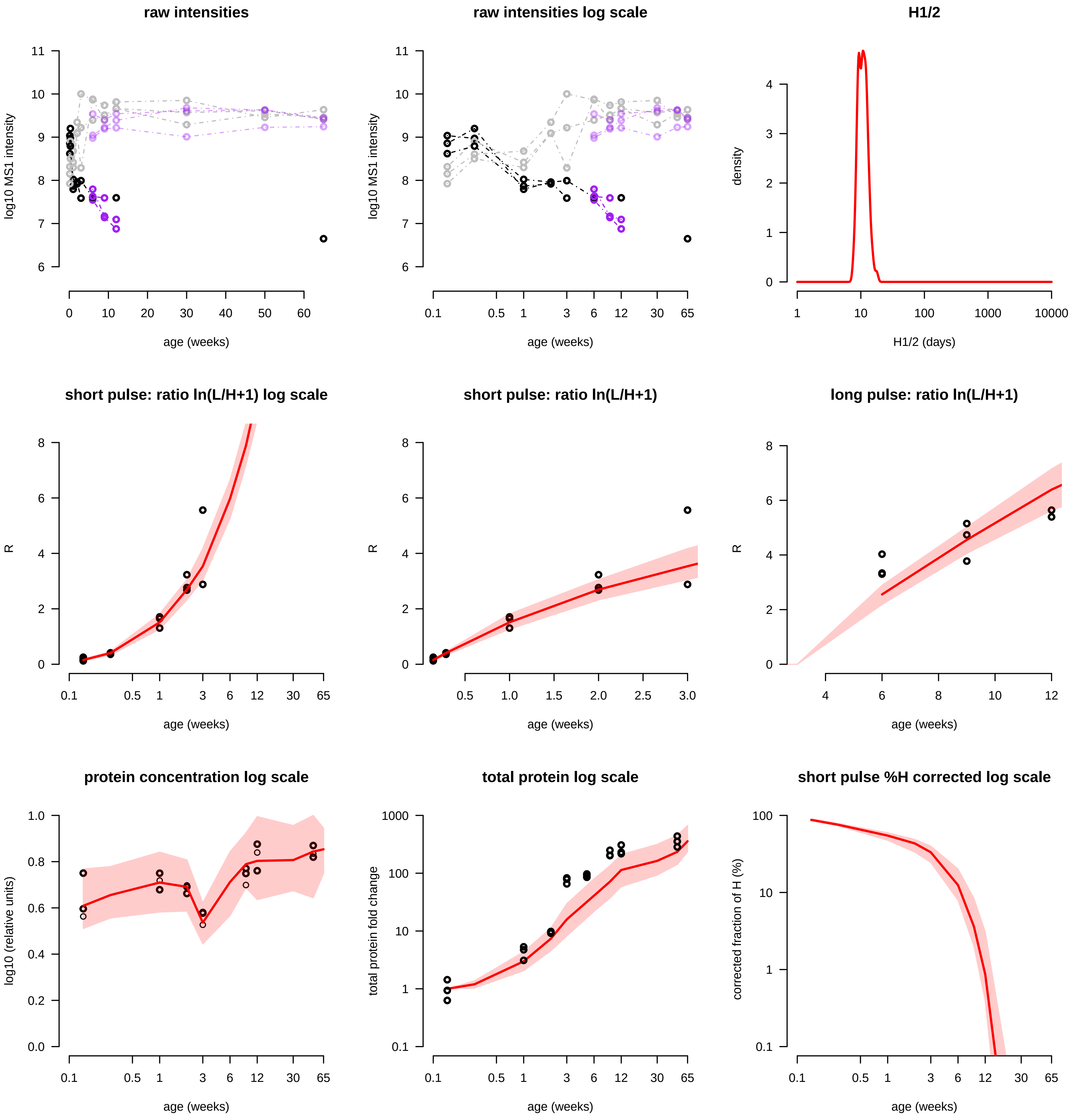

Q61233

>sp|Q61233|PLSL_MOUSE

Plastin−2

GN=Lcp1

Med H1/2: 6.9 days

Q5 H1/2: 5.5 days

Q95 H1/2: 9.6 days

reliable model fit: yes

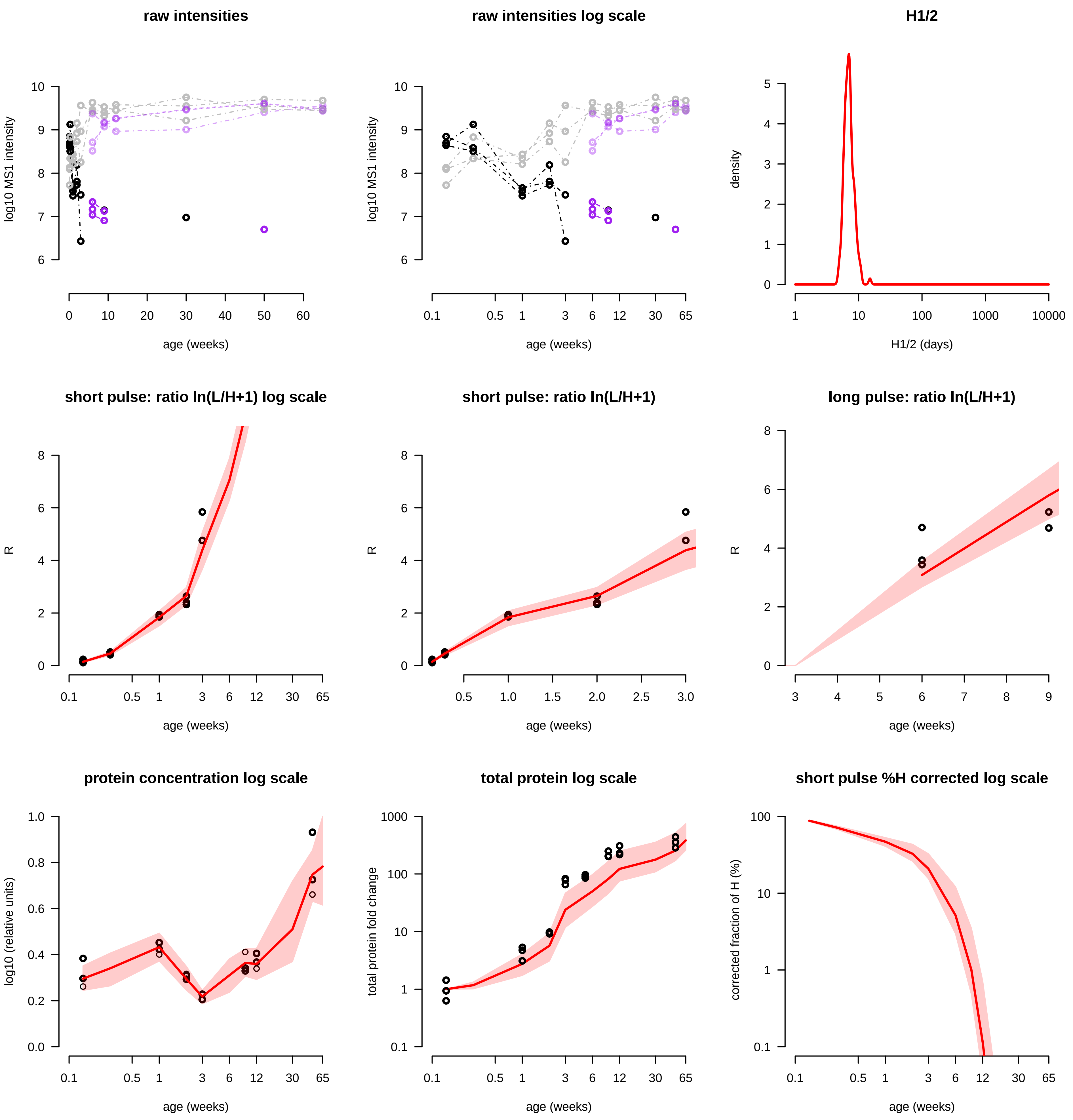

Q9Z2X1;Q9Z2X1−2

>sp|Q9Z2X1|HNRPF_MOUSE

Heterogeneous nuclear ribonucleoprotein F

GN=Hnrnpf

Med H1/2: 917.6 days

Q5 H1/2: 226.2 days

Q95 H1/2: 13419 days

reliable model fit: no

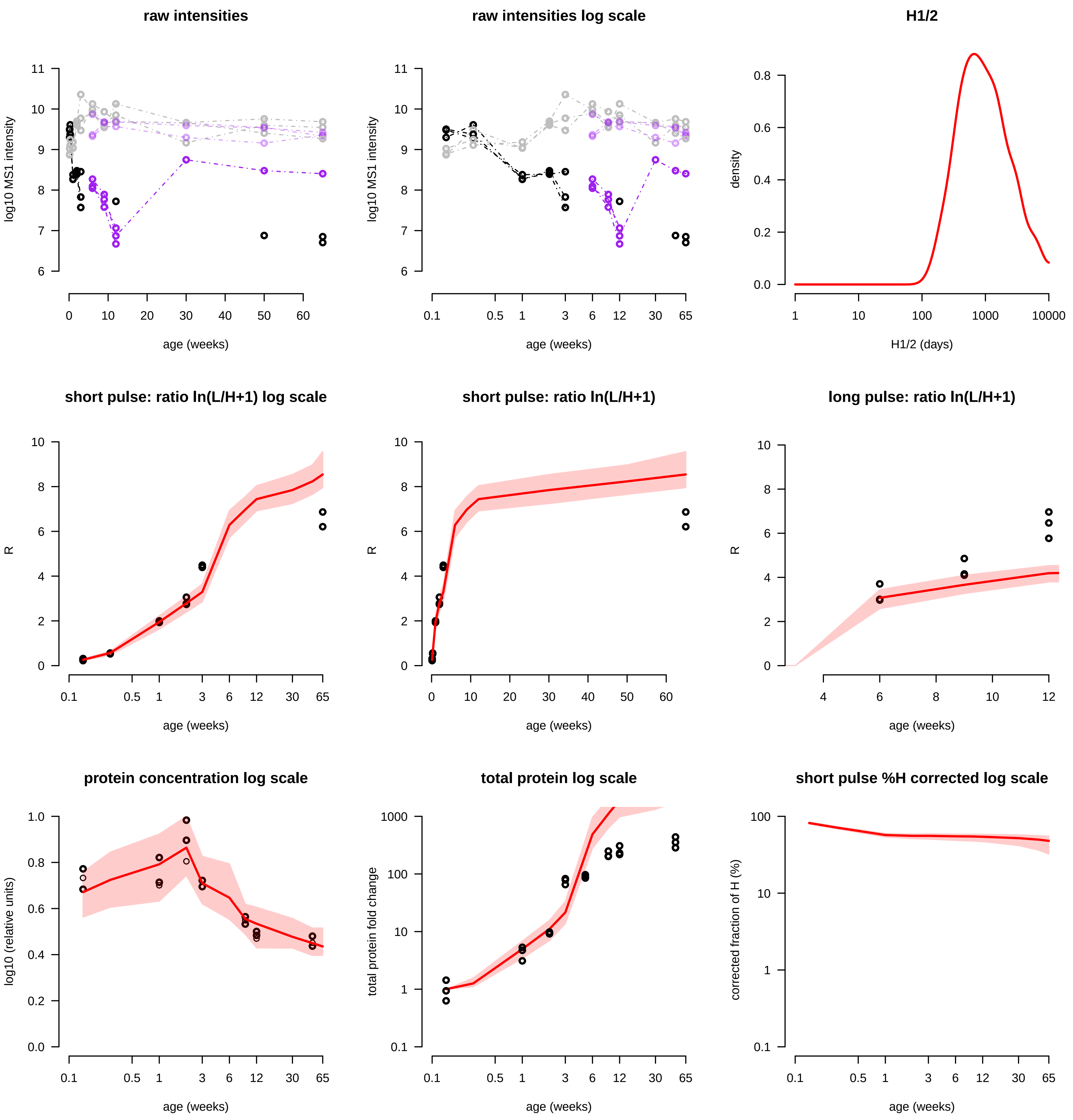

Q8JZU2

>sp|Q8JZU2|TXTP_MOUSE

Tricarboxylate transport protein, mitochondrial

GN=Slc25a1

Med H1/2: 11.7 days

Q5 H1/2: 9 days

Q95 H1/2: 16.4 days

reliable model fit: yes

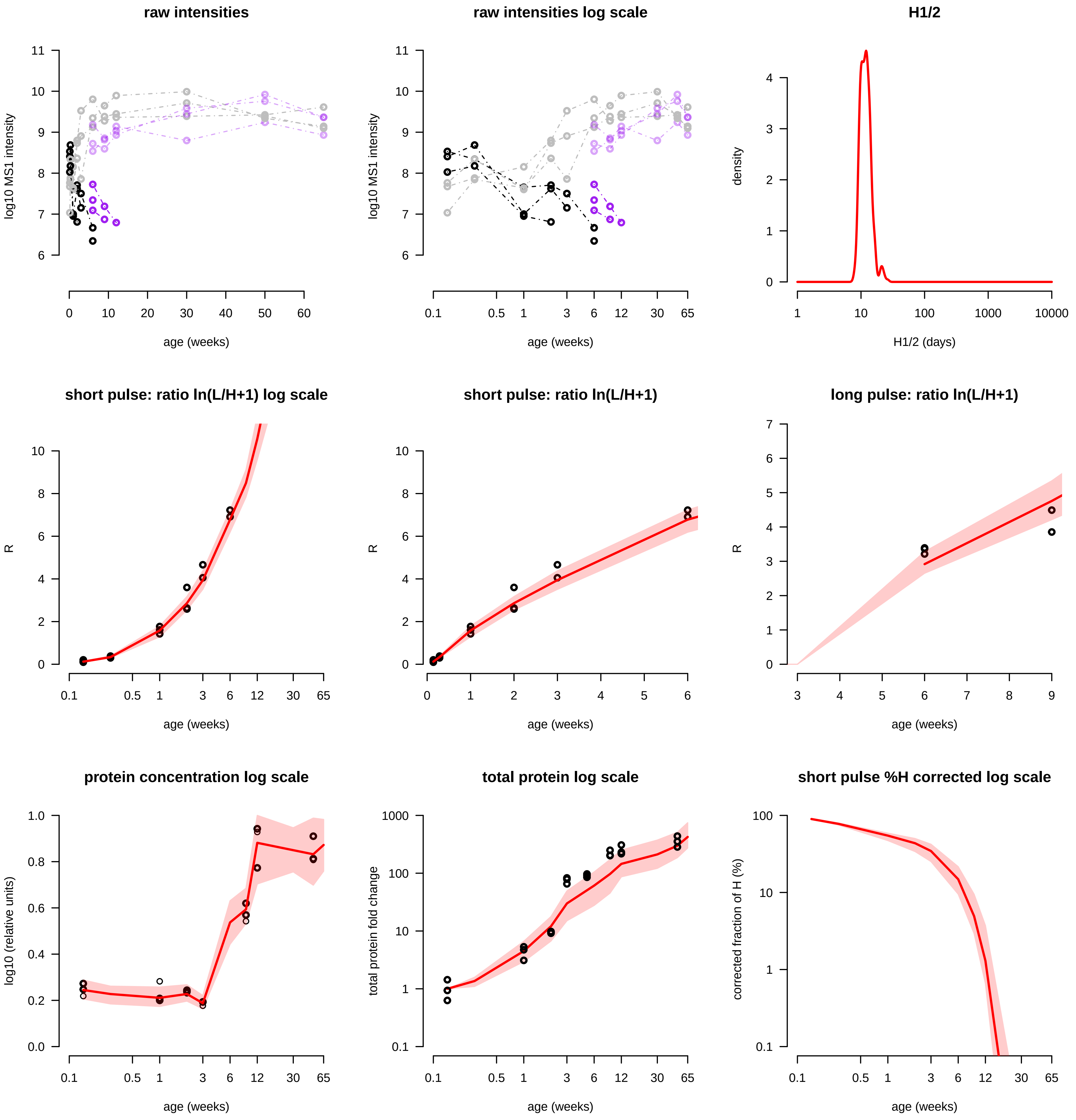

Q91VM5

>sp|Q91VM5|RMXL1_MOUSE

RNA binding motif protein, X−linked−like−1

GN=Rbmxl1

Med H1/2: 10.3 days

Q5 H1/2: 8.6 days

Q95 H1/2: 14 days

reliable model fit: yes

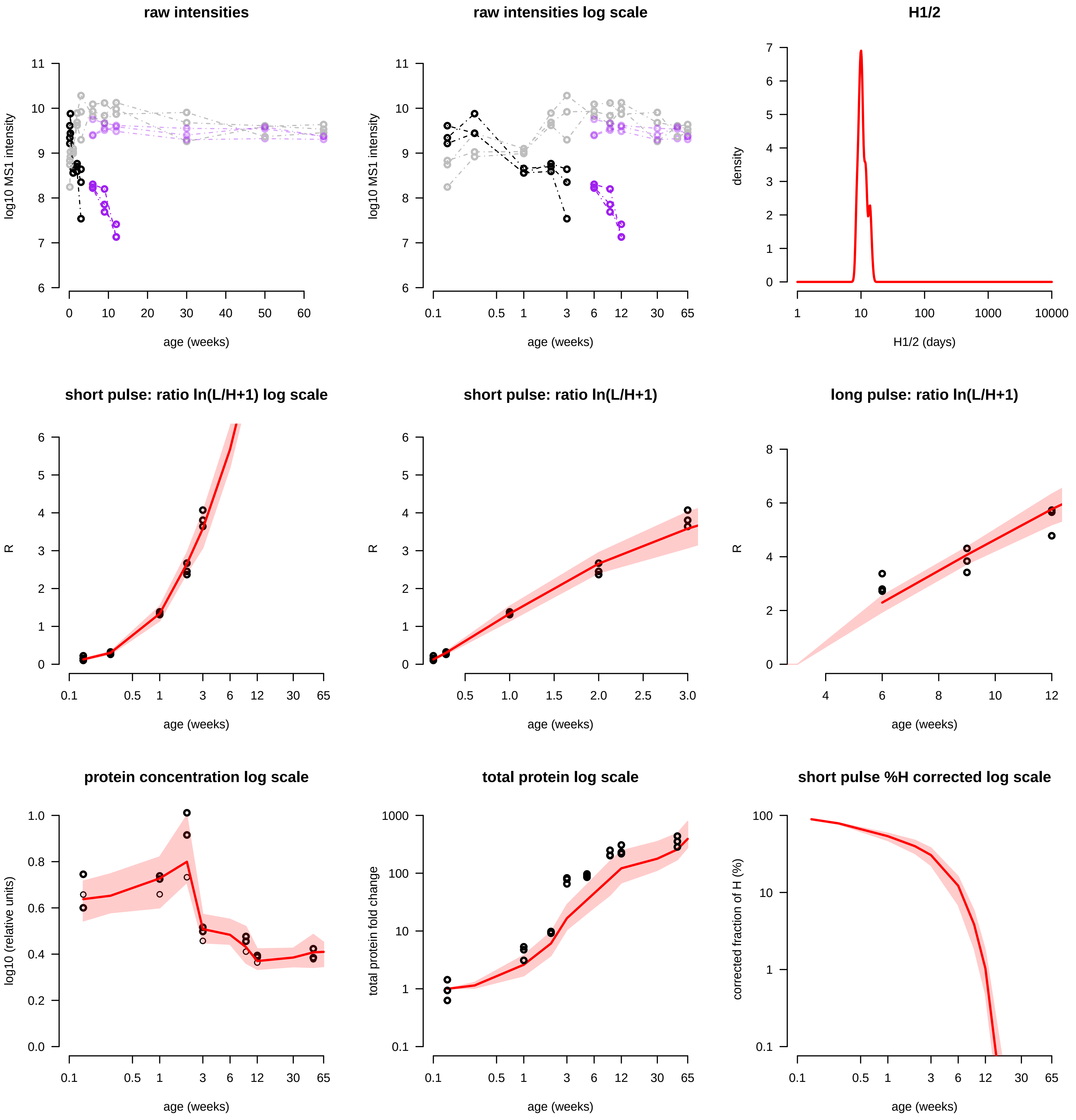

P16110

>sp|P16110|LEG3_MOUSE

Galectin−3

GN=Lgals3

Med H1/2: 66.7 days

Q5 H1/2: 25.5 days

Q95 H1/2: 491.8 days

reliable model fit: yes

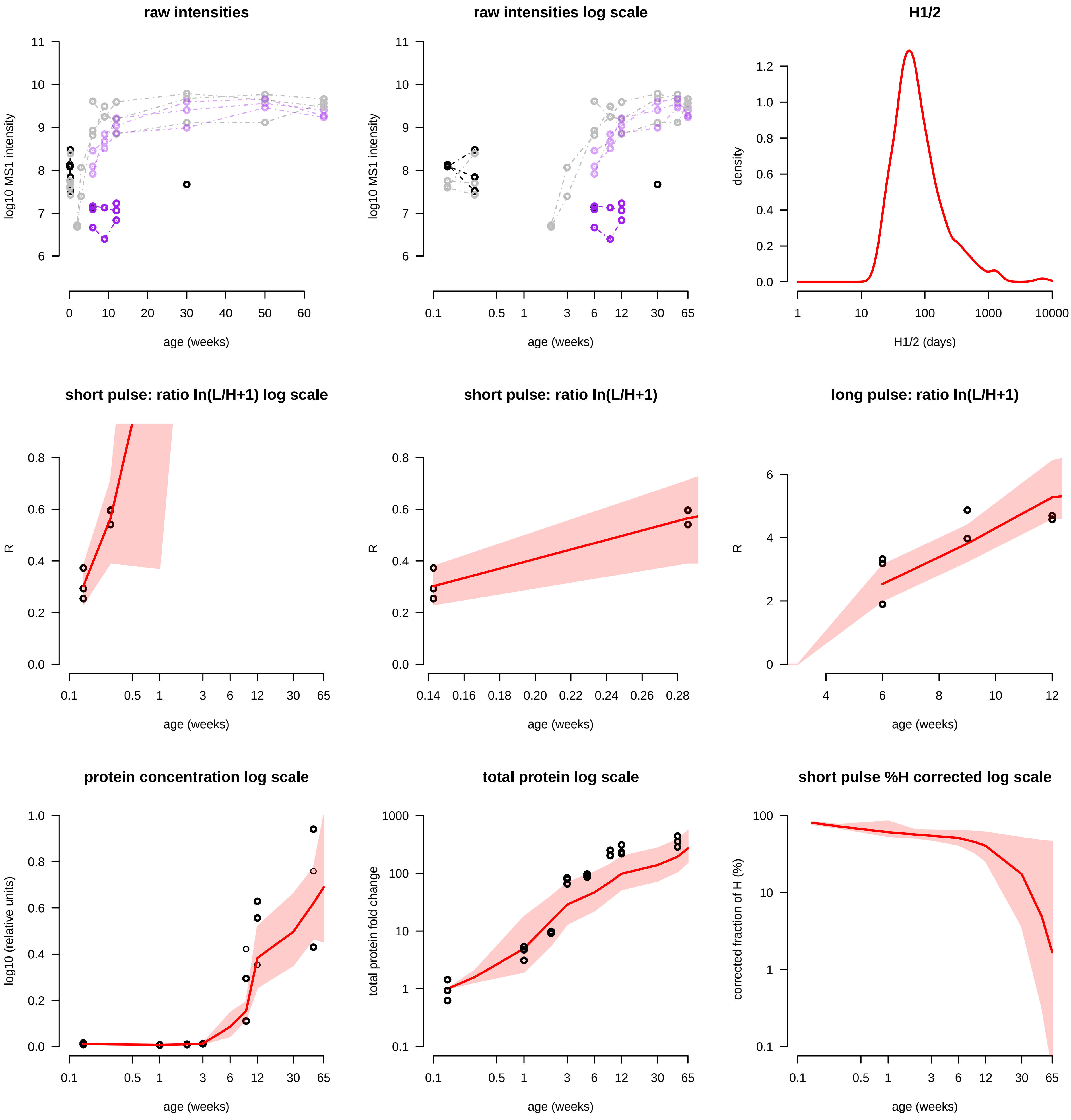

P35980

>sp|P35980|RL18_MOUSE

60S ribosomal protein L18

GN=Rpl18

Med H1/2: 16.6 days

Q5 H1/2: 12.9 days

Q95 H1/2: 24.5 days

reliable model fit: yes

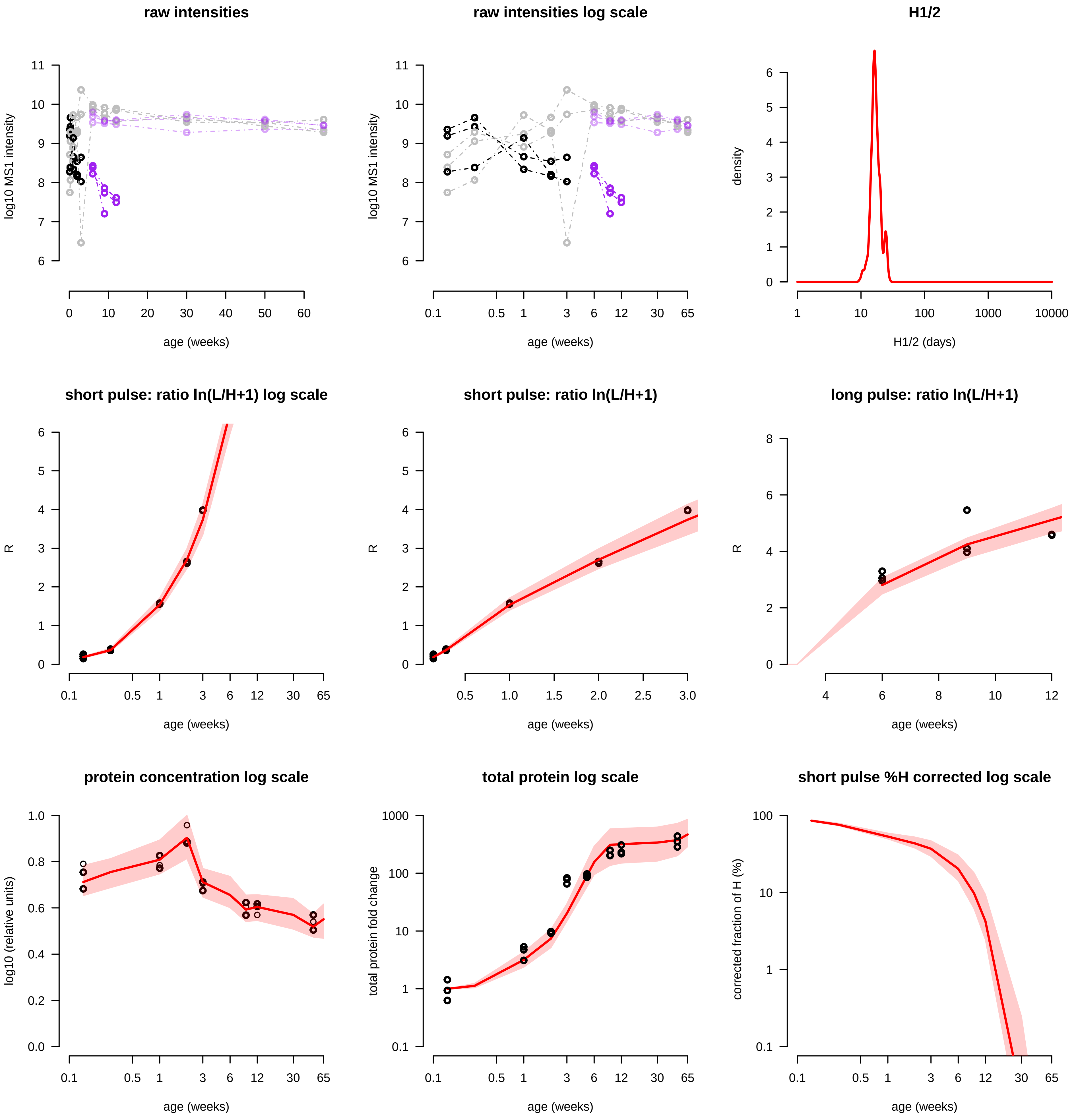

P60867

>sp|P60867|RS20_MOUSE

40S ribosomal protein S20

GN=Rps20

Med H1/2: 15.5 days

Q5 H1/2: 11.9 days

Q95 H1/2: 20.4 days

reliable model fit: yes

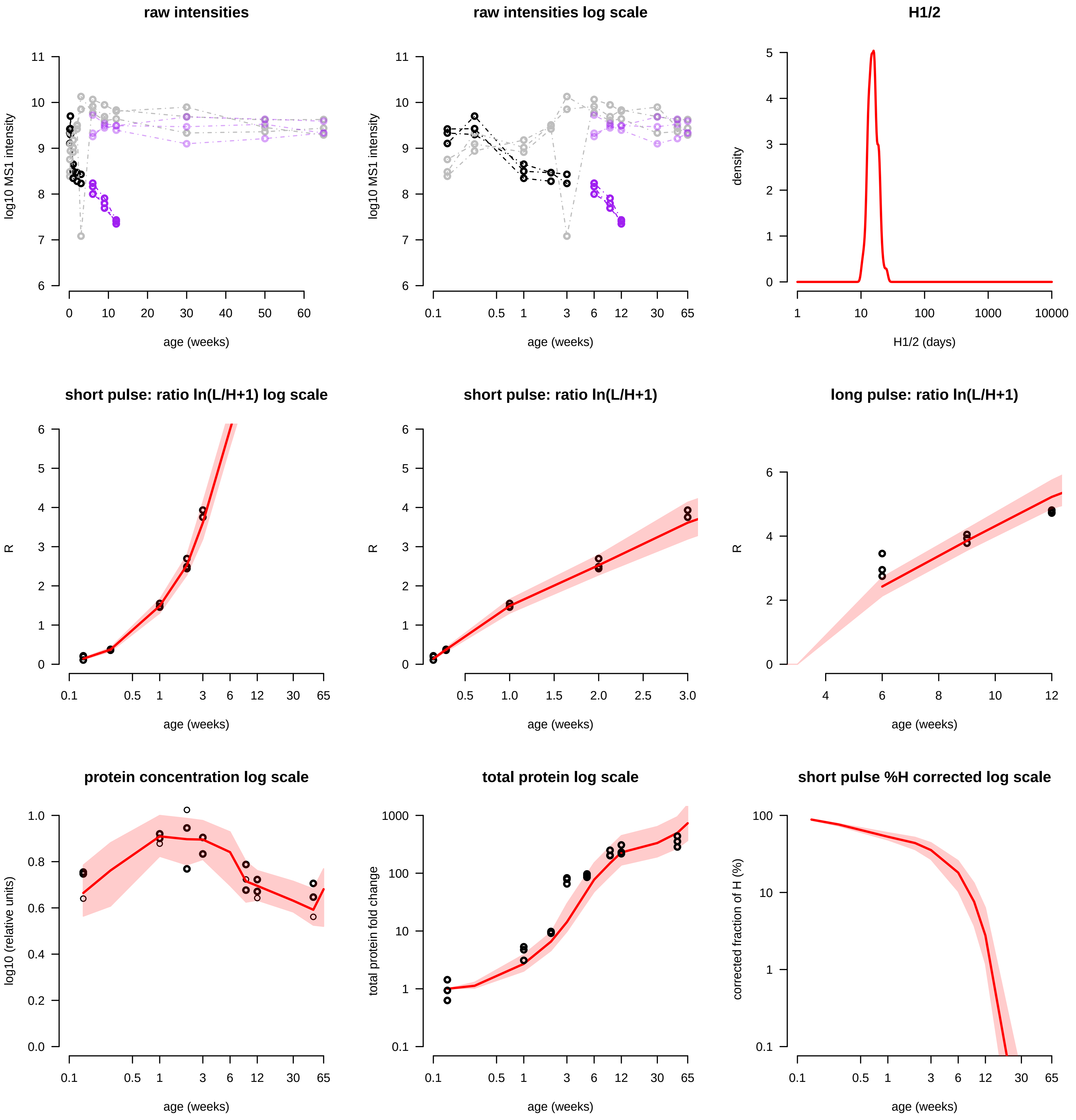

P47757−2;P47757−4

>sp|P47757−2|CAPZB_MOUSE

Isoform 2 of F−actin−capping protein subunit beta

GN=Capzb

Med H1/2: 11.7 days

Q5 H1/2: 9.6 days

Q95 H1/2: 15.1 days

reliable model fit: yes

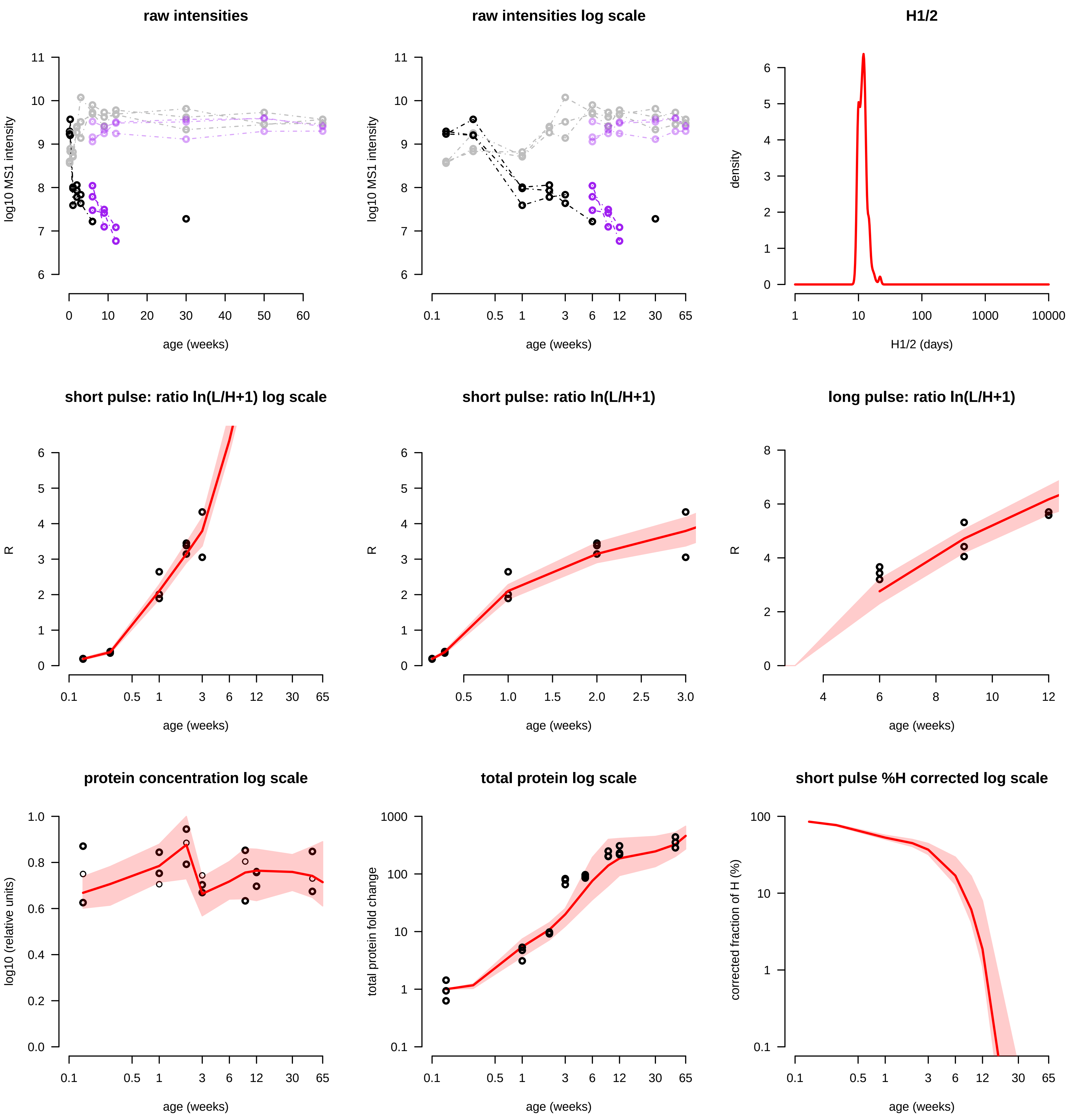

Q99L47

>sp|Q99L47|F10A1_MOUSE

Hsc70−interacting protein

GN=St13

Med H1/2: 10.1 days

Q5 H1/2: 7.3 days

Q95 H1/2: 13.3 days

reliable model fit: yes

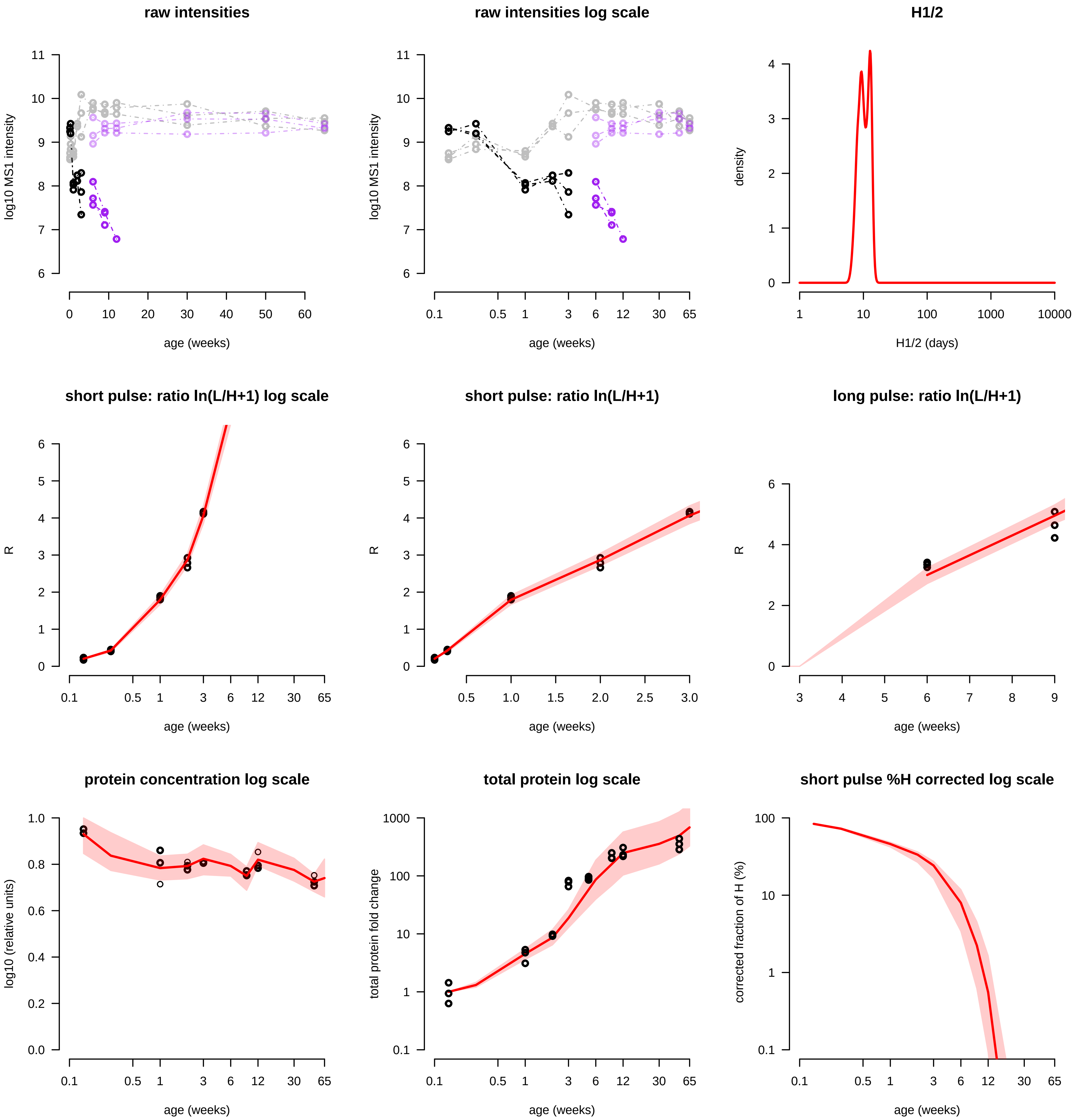

Q8QZS1

>sp|Q8QZS1|HIBCH_MOUSE

3−hydroxyisobutyryl−CoA hydrolase, mitochondrial

GN=Hibch

Med H1/2: 151.4 days

Q5 H1/2: 52.3 days

Q95 H1/2: 601.1 days

reliable model fit: no

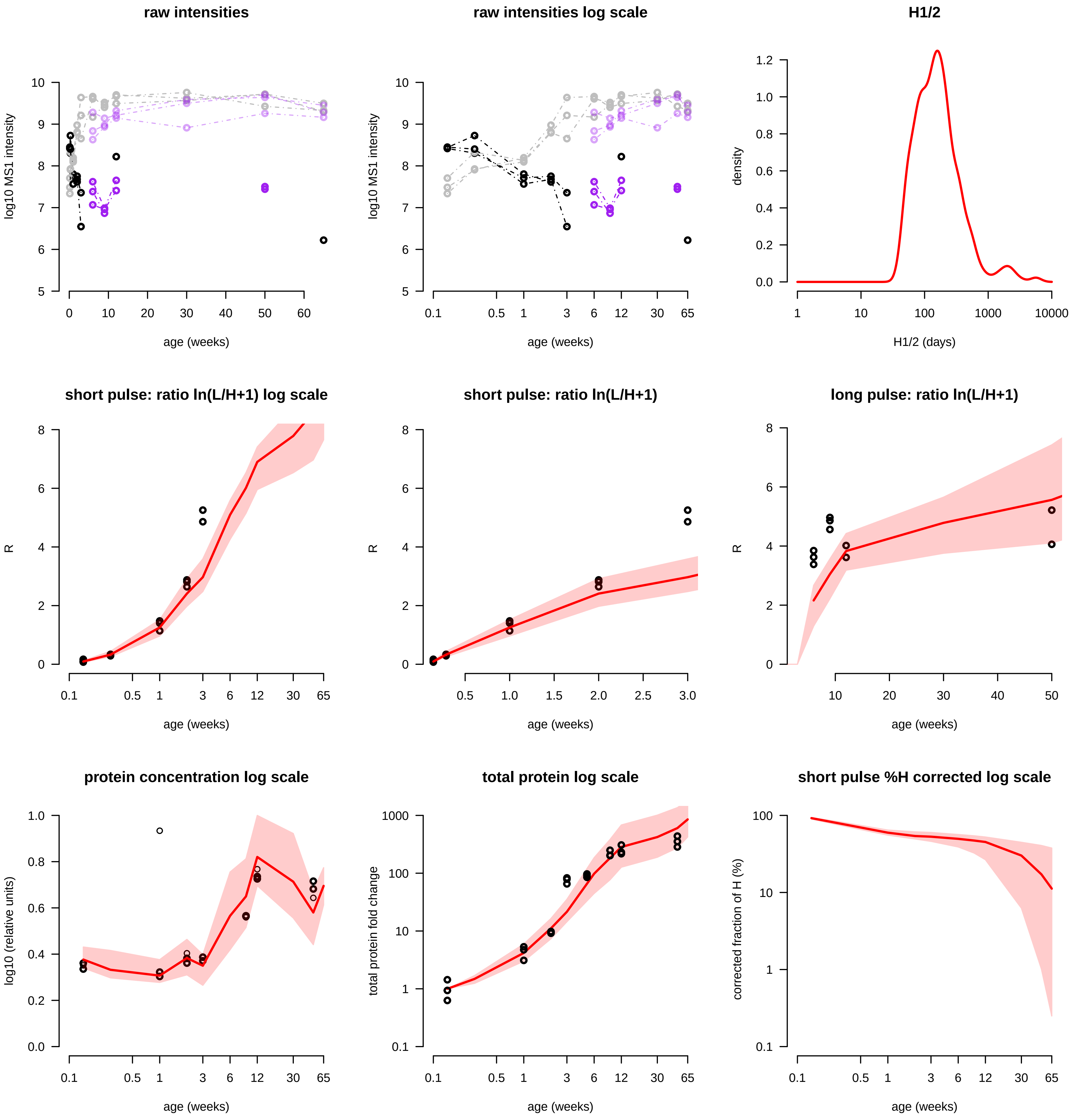

P21300

>sp|P21300|ALD1_MOUSE

Aldo−keto reductase family 1 member B7

GN=Akr1b7

Med H1/2: 5.4 days

Q5 H1/2: 3.7 days

Q95 H1/2: 10.8 days

reliable model fit: yes

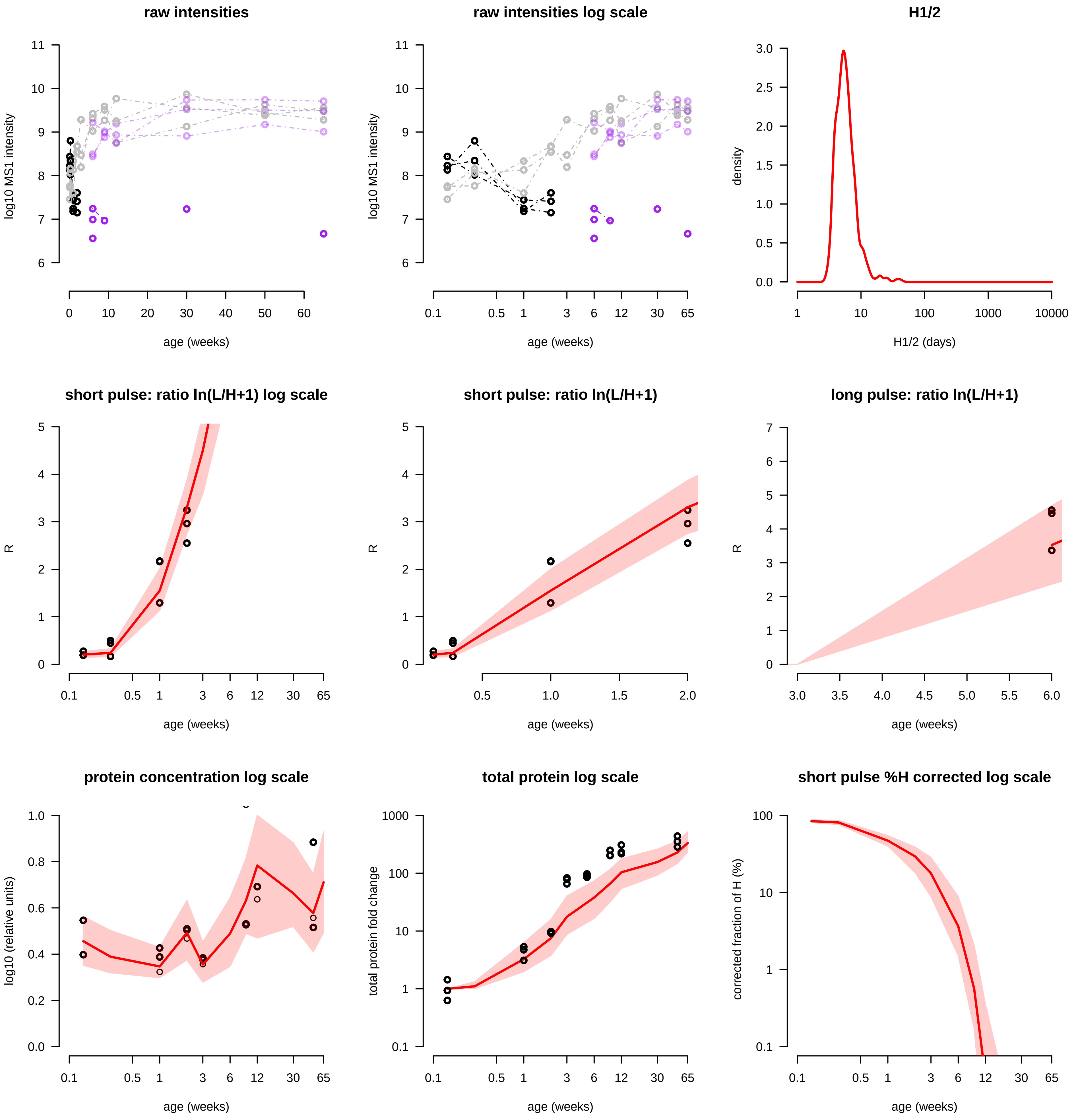

P09055;P09055−2

>sp|P09055|ITB1_MOUSE

Integrin beta−1

GN=Itgb1

Med H1/2: 7.2 days

Q5 H1/2: 5.9 days

Q95 H1/2: 9.1 days

reliable model fit: yes

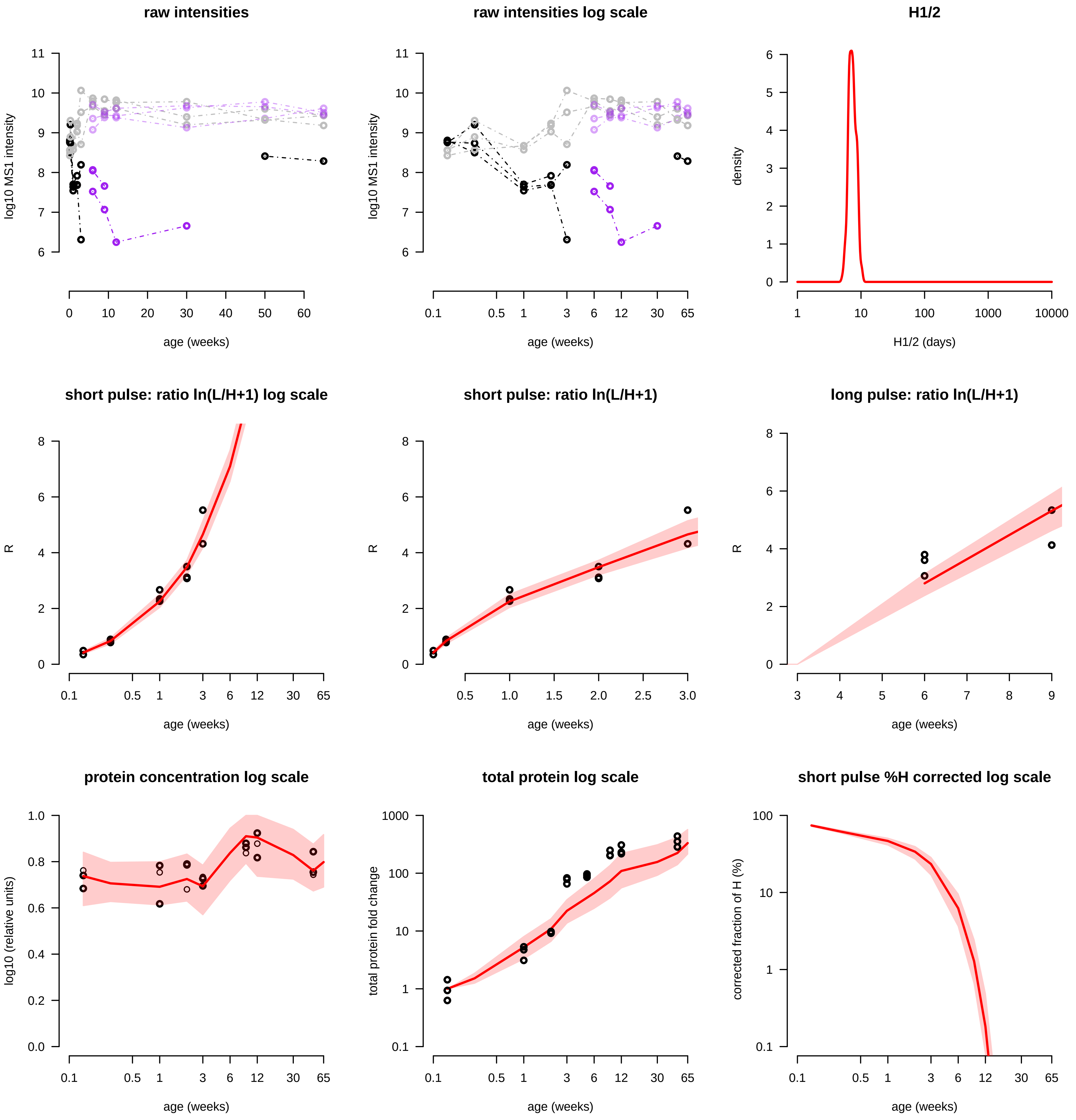

Q8K2C9

>sp|Q8K2C9|HACD3_MOUSE

Very−long−chain (3R)−3−hydroxyacyl−CoA dehydratase 3

GN=Hacd3

Med H1/2: 9.4 days

Q5 H1/2: 6.9 days

Q95 H1/2: 15.5 days

reliable model fit: yes

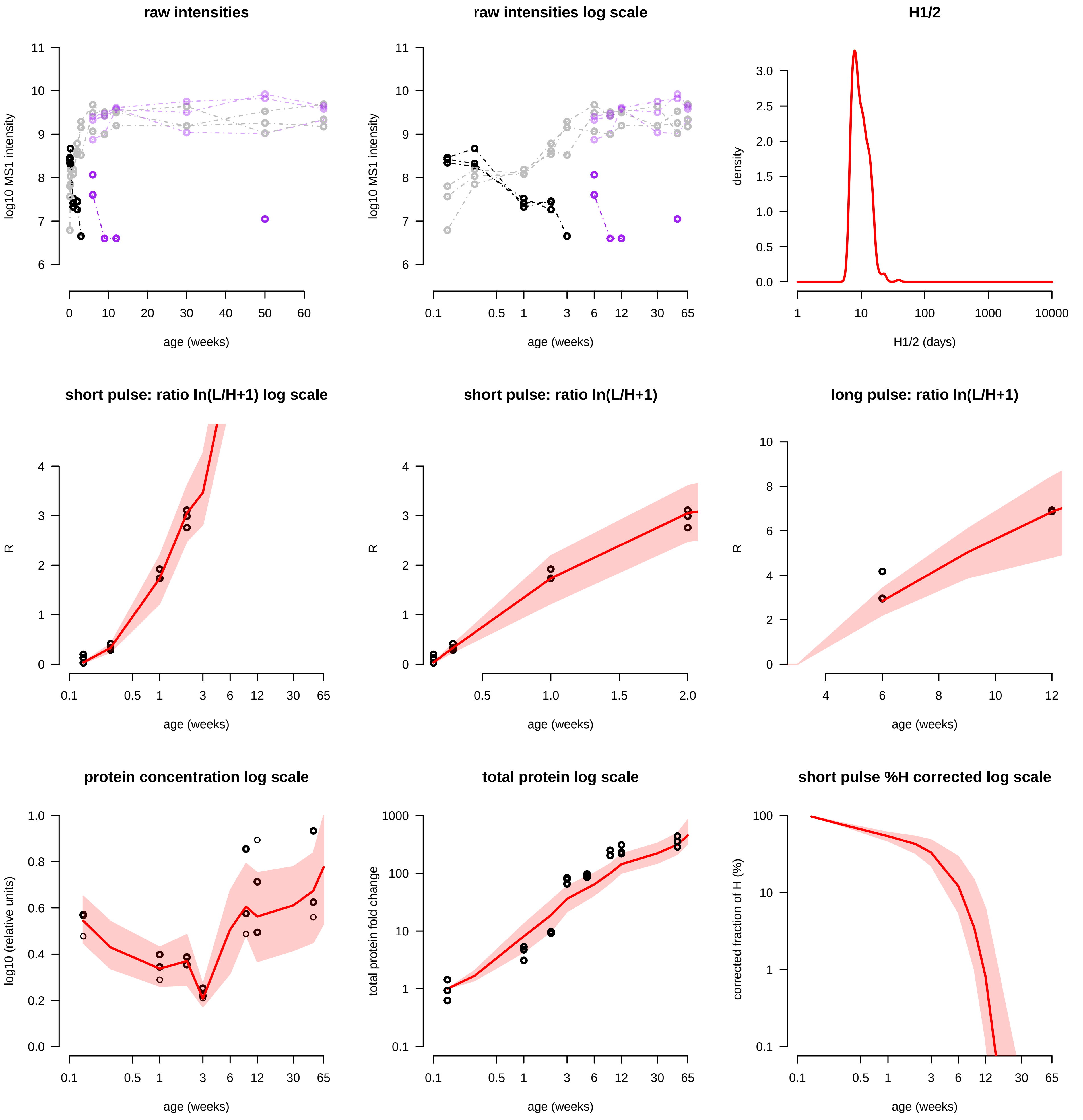

P97372

>sp|P97372|PSME2_MOUSE

Proteasome activator complex subunit 2

GN=Psme2

Med H1/2: 7.7 days

Q5 H1/2: 6.1 days

Q95 H1/2: 9.6 days

reliable model fit: yes

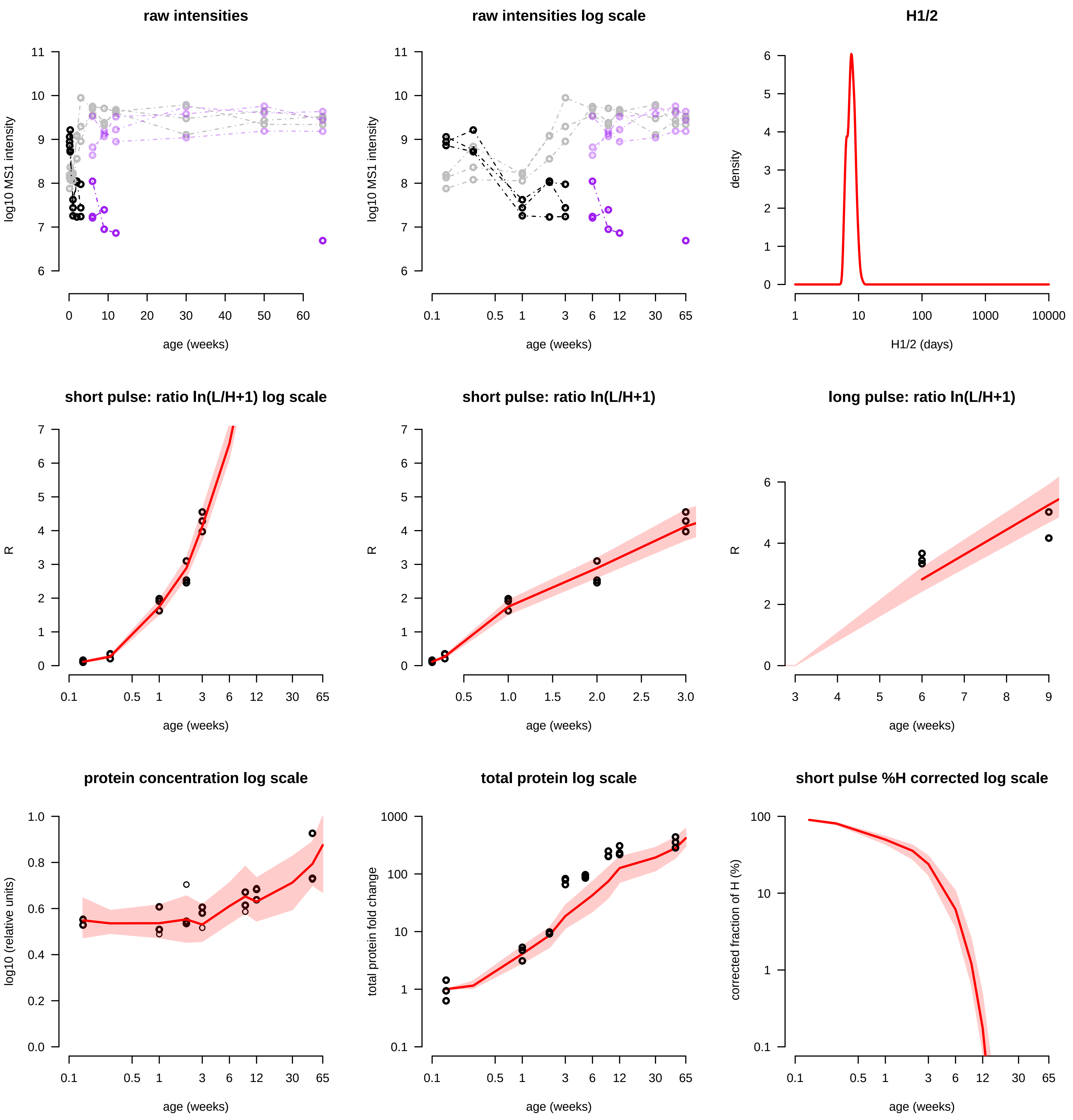

Q64378

>sp|Q64378|FKBP5_MOUSE

Peptidyl−prolyl cis−trans isomerase FKBP5

GN=Fkbp5

Med H1/2: 1764.1 days

Q5 H1/2: 377.1 days

Q95 H1/2: 17117.8 days

reliable model fit: yes

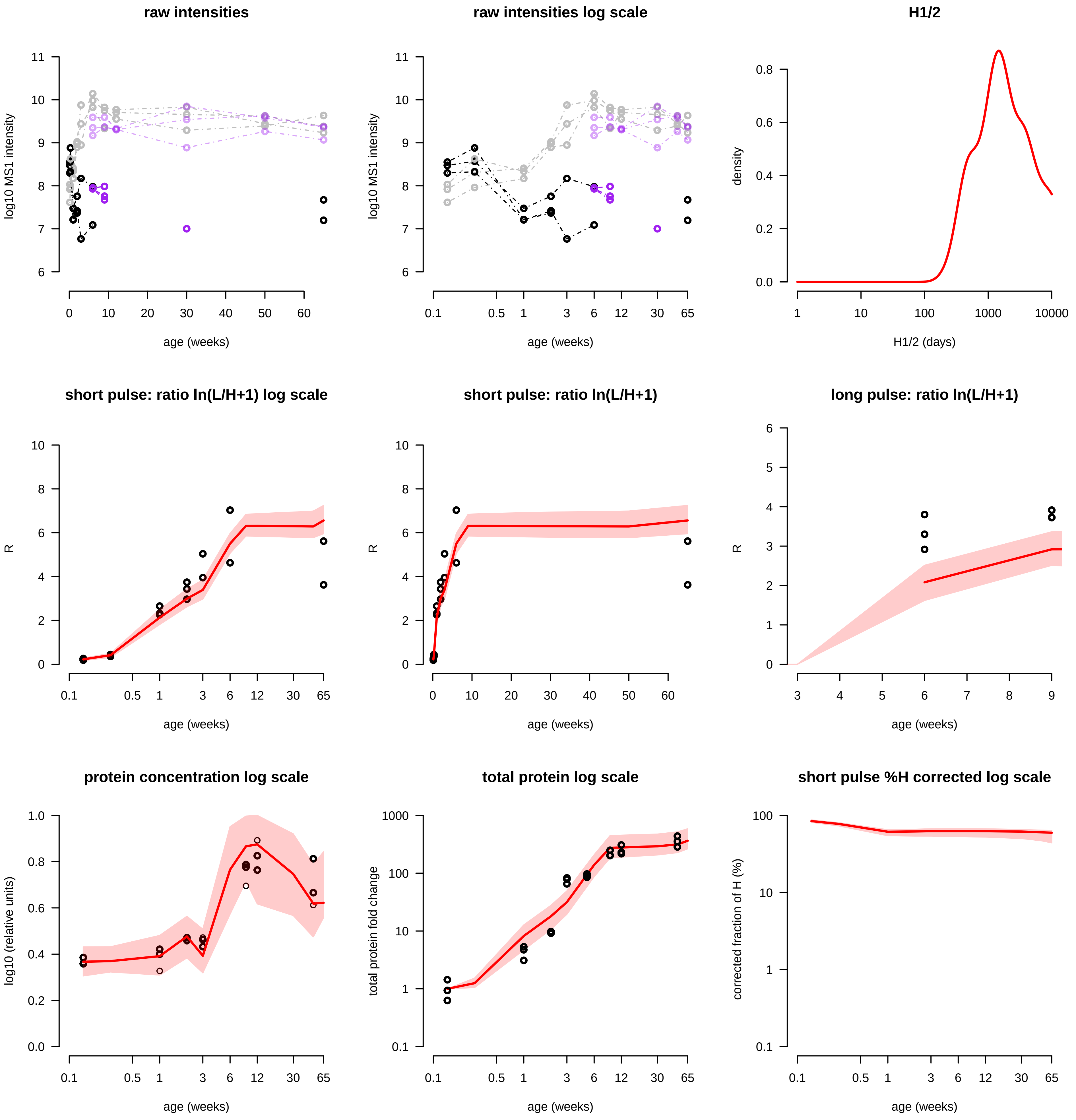

P31324

>sp|P31324|KAP3_MOUSE

cAMP−dependent protein kinase type II−beta regulatory subunit

GN=Prkar2b

Med H1/2: 7.6 days

Q5 H1/2: 6.2 days

Q95 H1/2: 10.5 days

reliable model fit: yes

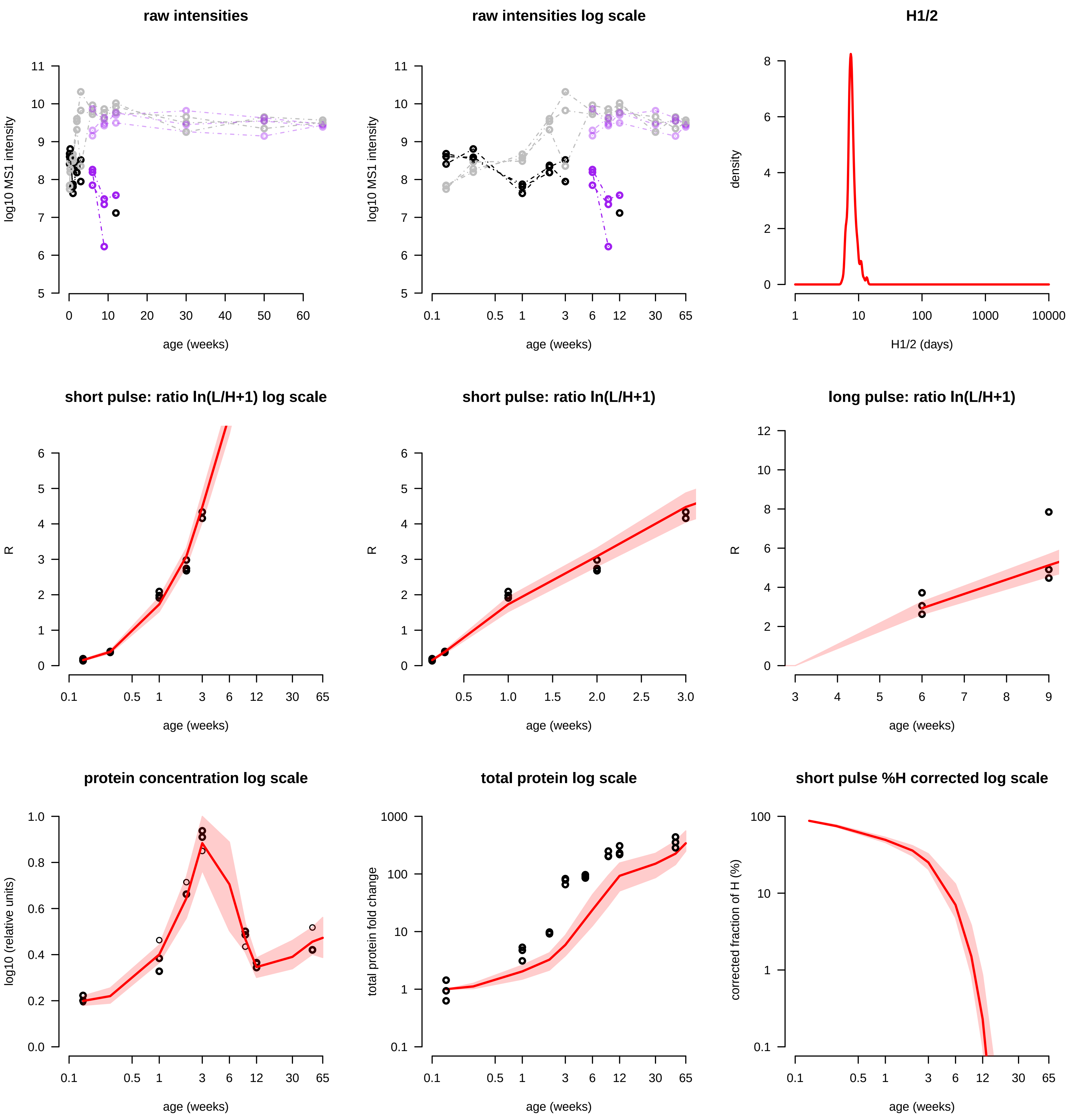

P28653

>sp|P28653|PGS1_MOUSE

Biglycan

GN=Bgn

Med H1/2: 7 days

Q5 H1/2: 5.7 days

Q95 H1/2: 9.5 days

reliable model fit: yes

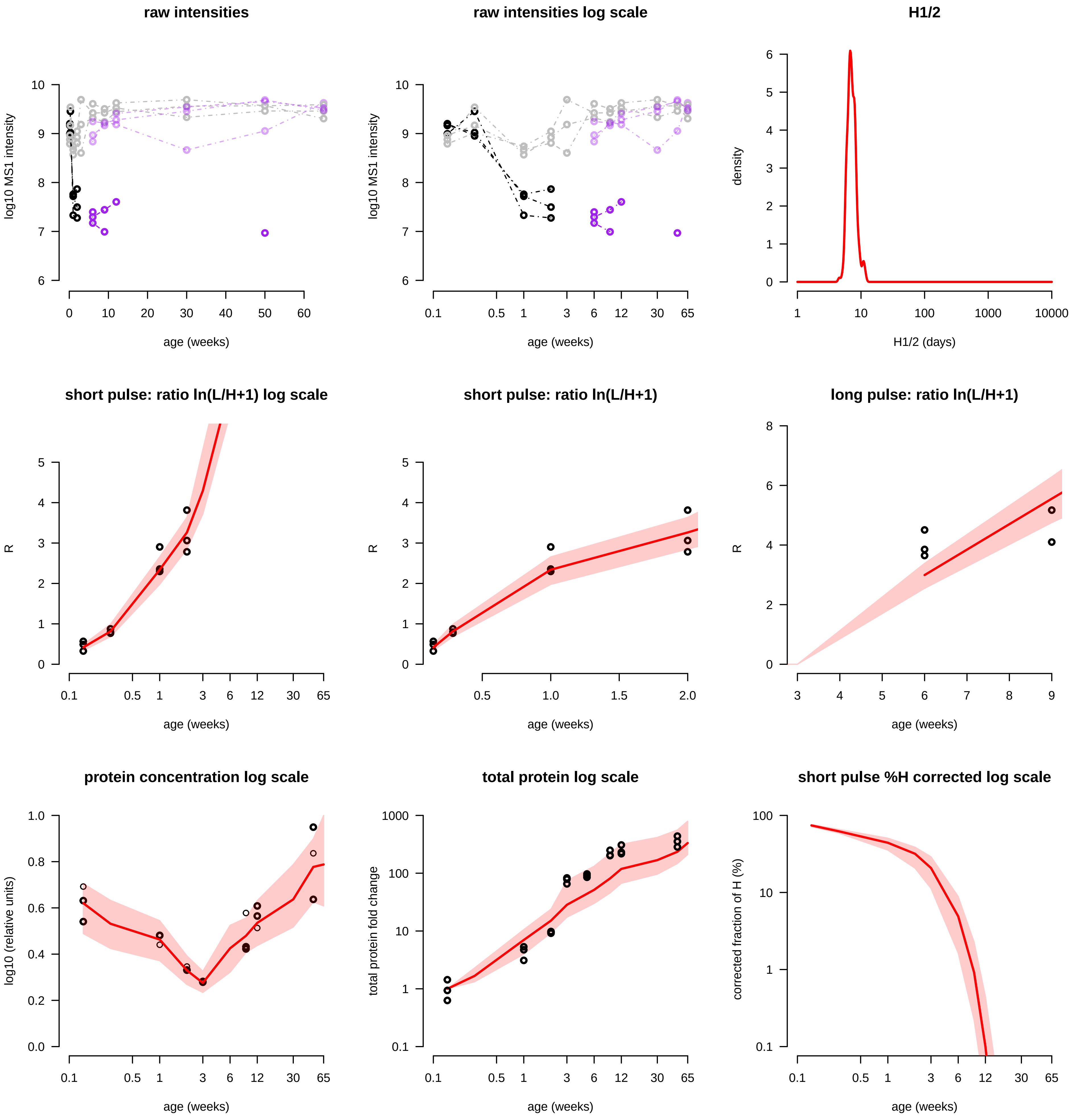

Q91YH5

>sp|Q91YH5|ATLA3_MOUSE

Atlastin−3

GN=Atl3

Med H1/2: 4529.1 days

Q5 H1/2: 1073 days

Q95 H1/2: 79834.4 days

reliable model fit: yes

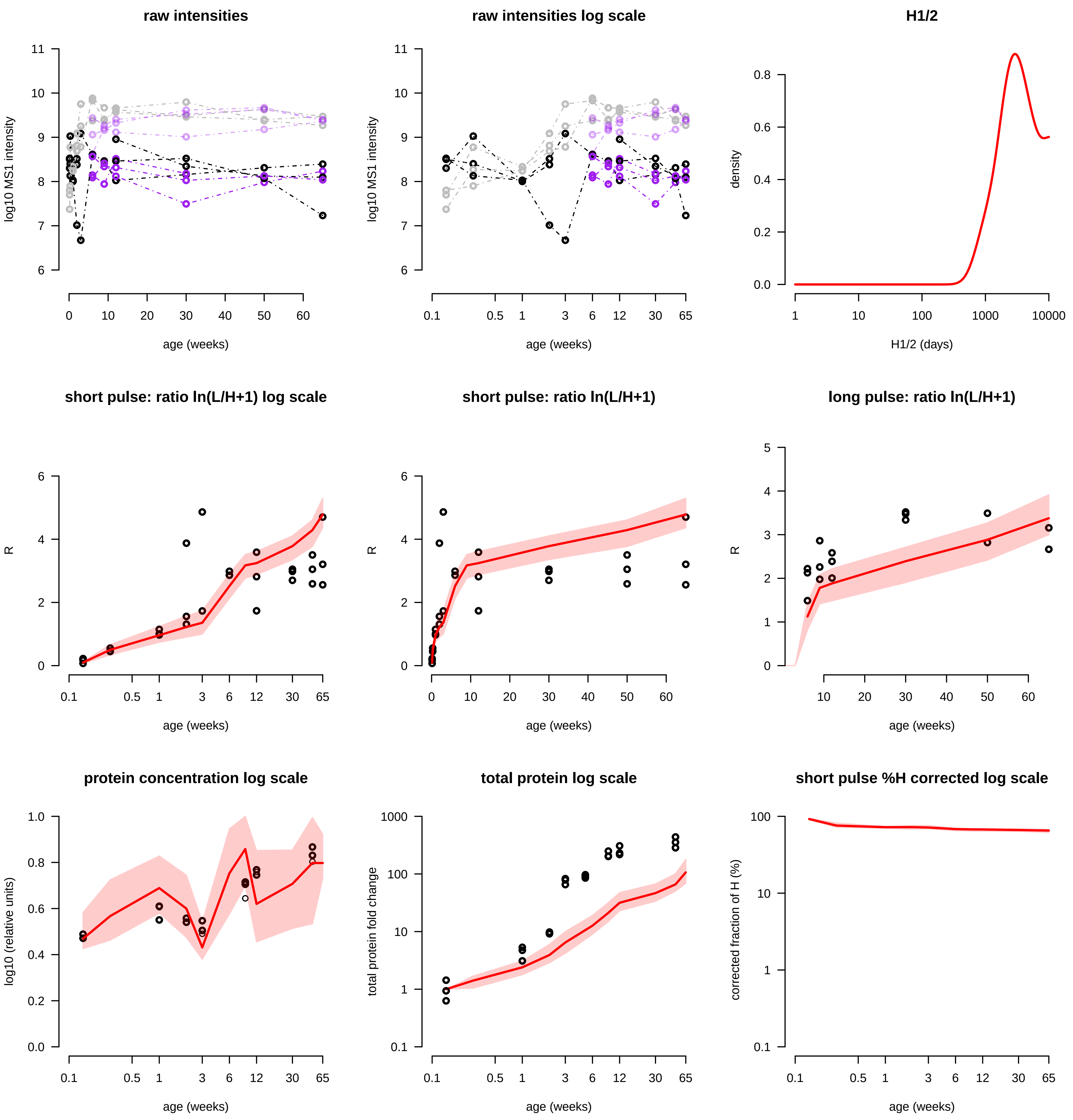

Q9EQH3

>sp|Q9EQH3|VPS35_MOUSE

Vacuolar protein sorting−associated protein 35

GN=Vps35

Med H1/2: 7.2 days

Q5 H1/2: 5.9 days

Q95 H1/2: 8.4 days

reliable model fit: yes

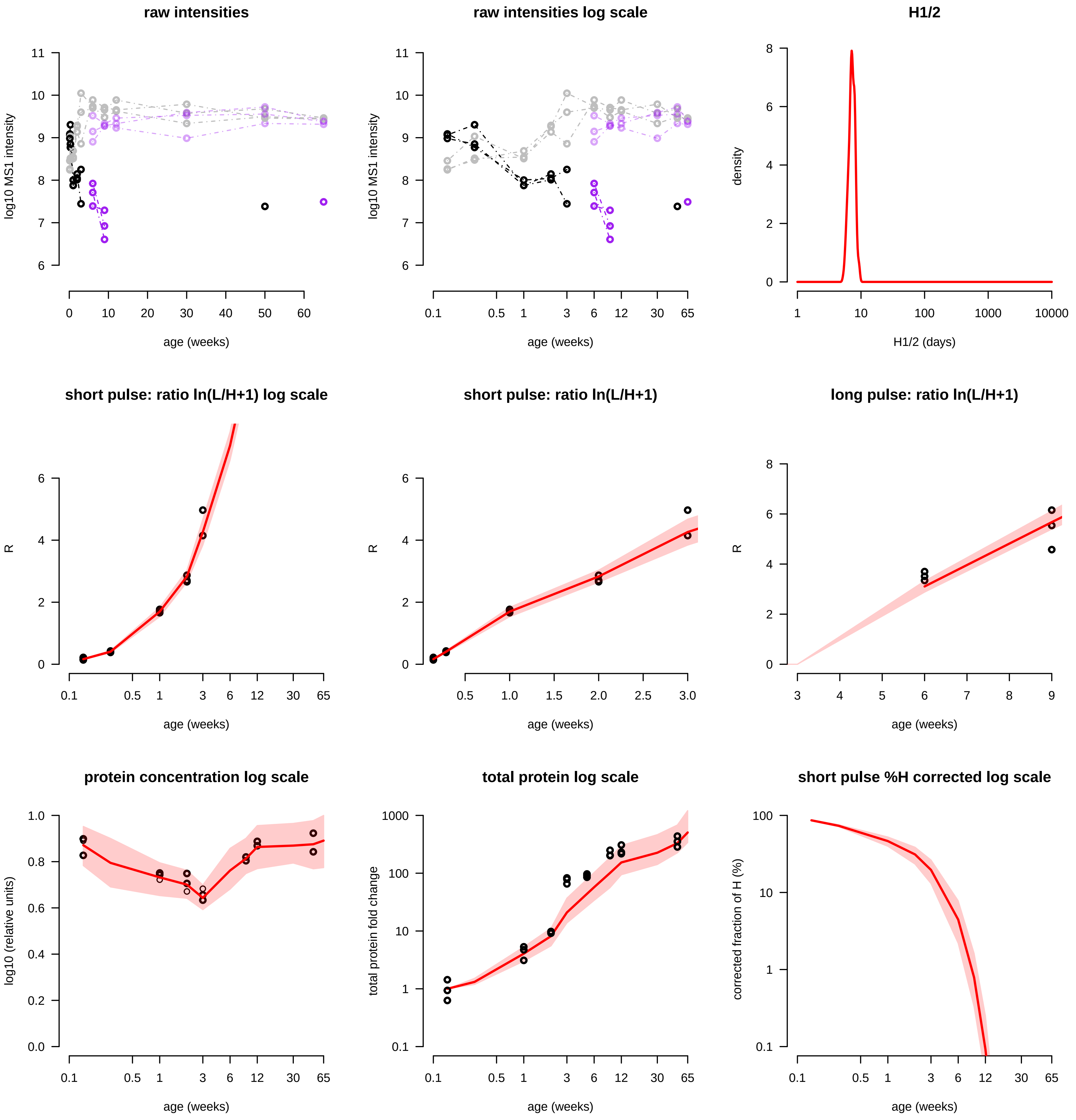

P30416

>sp|P30416|FKBP4_MOUSE

Peptidyl−prolyl cis−trans isomerase FKBP4

GN=Fkbp4

Med H1/2: 9.3 days

Q5 H1/2: 7.6 days

Q95 H1/2: 11.7 days

reliable model fit: yes

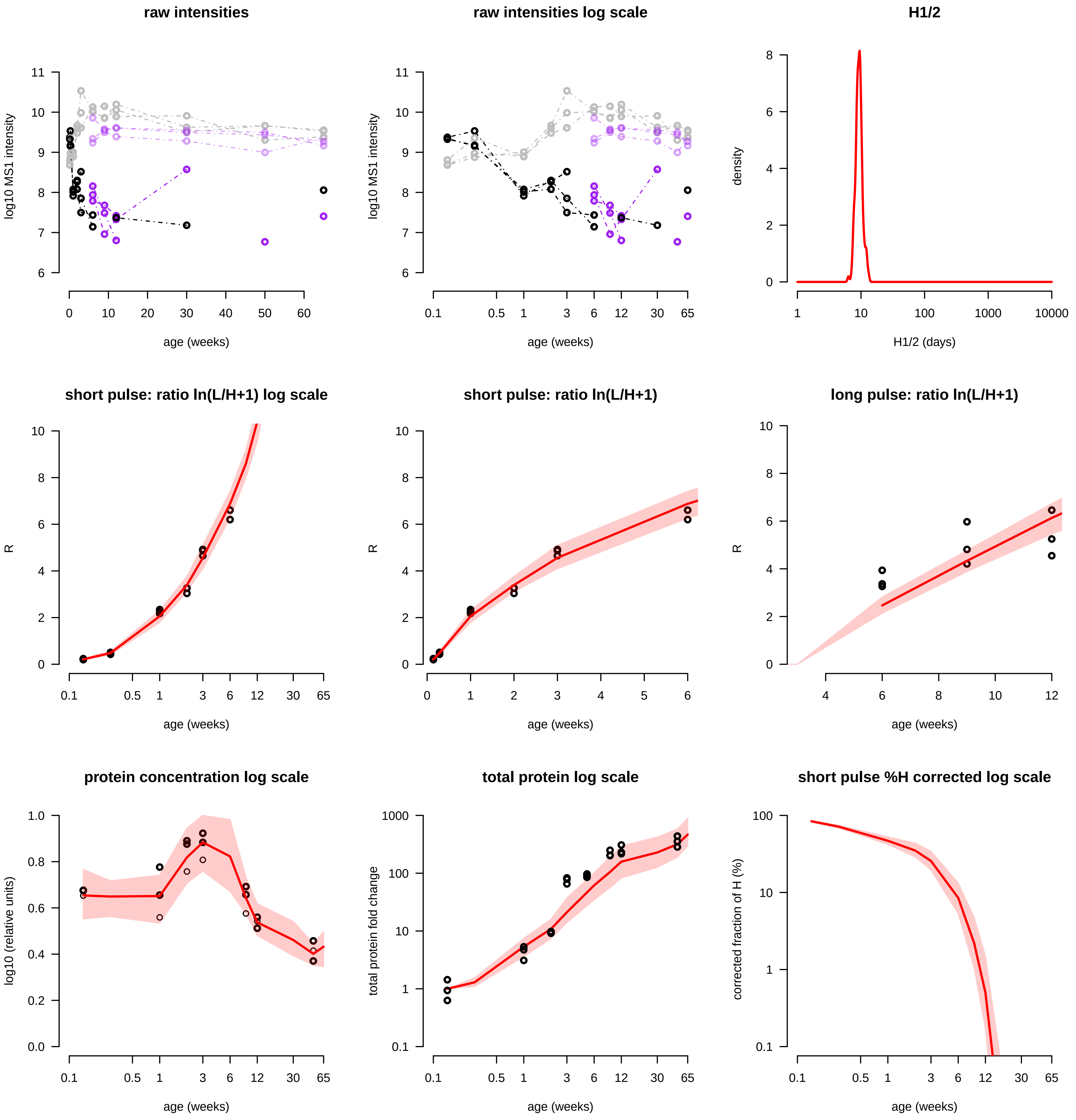

Q8K411−2;Q8K411;Q8K411−3

>sp|Q8K411−2|PREP_MOUSE

Isoform 2 of Presequence protease, mitochondrial

GN=Pitrm1

Med H1/2: 1153.2 days

Q5 H1/2: 279 days

Q95 H1/2: 17102 days

reliable model fit: yes

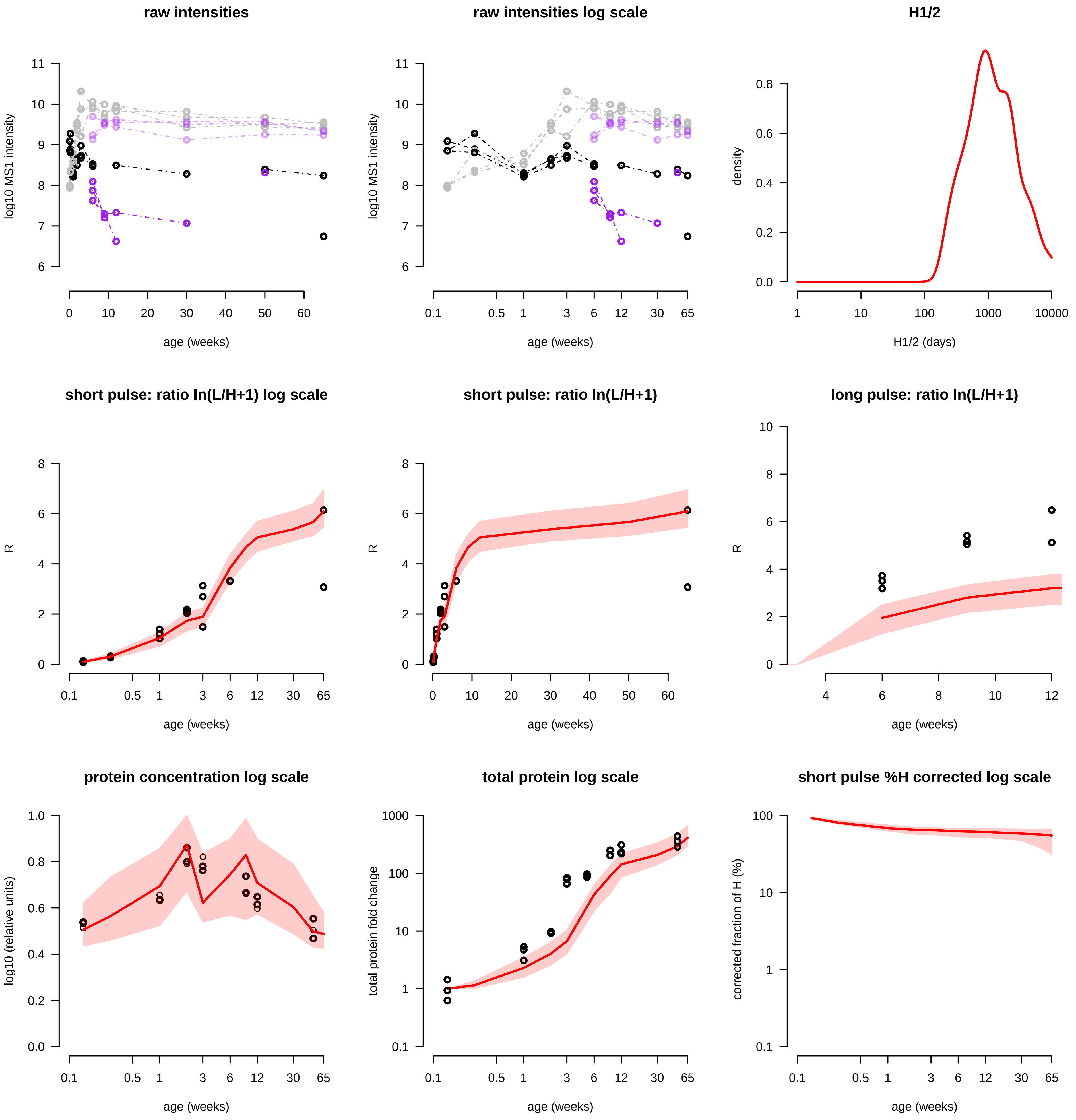

P53994

>sp|P53994|RAB2A_MOUSE

Ras−related protein Rab−2A

GN=Rab2a

Med H1/2: 54.4 days

Q5 H1/2: 41.4 days

Q95 H1/2: 85.2 days

reliable model fit: yes

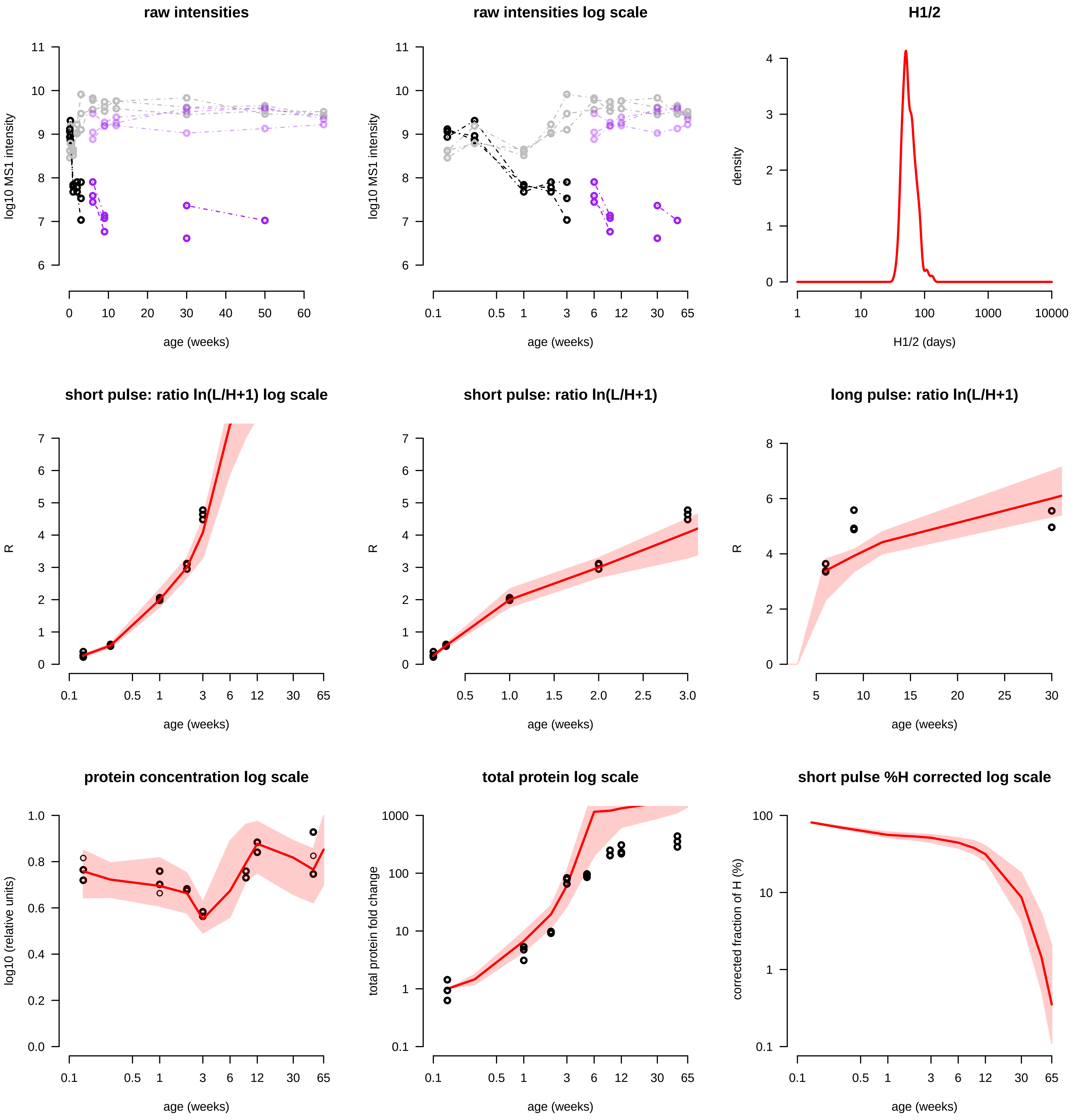

Q91WS0

>sp|Q91WS0|CISD1_MOUSE

CDGSH iron−sulfur domain−containing protein 1

GN=Cisd1

Med H1/2: 359.6 days

Q5 H1/2: 104.2 days

Q95 H1/2: 4306.7 days

reliable model fit: no

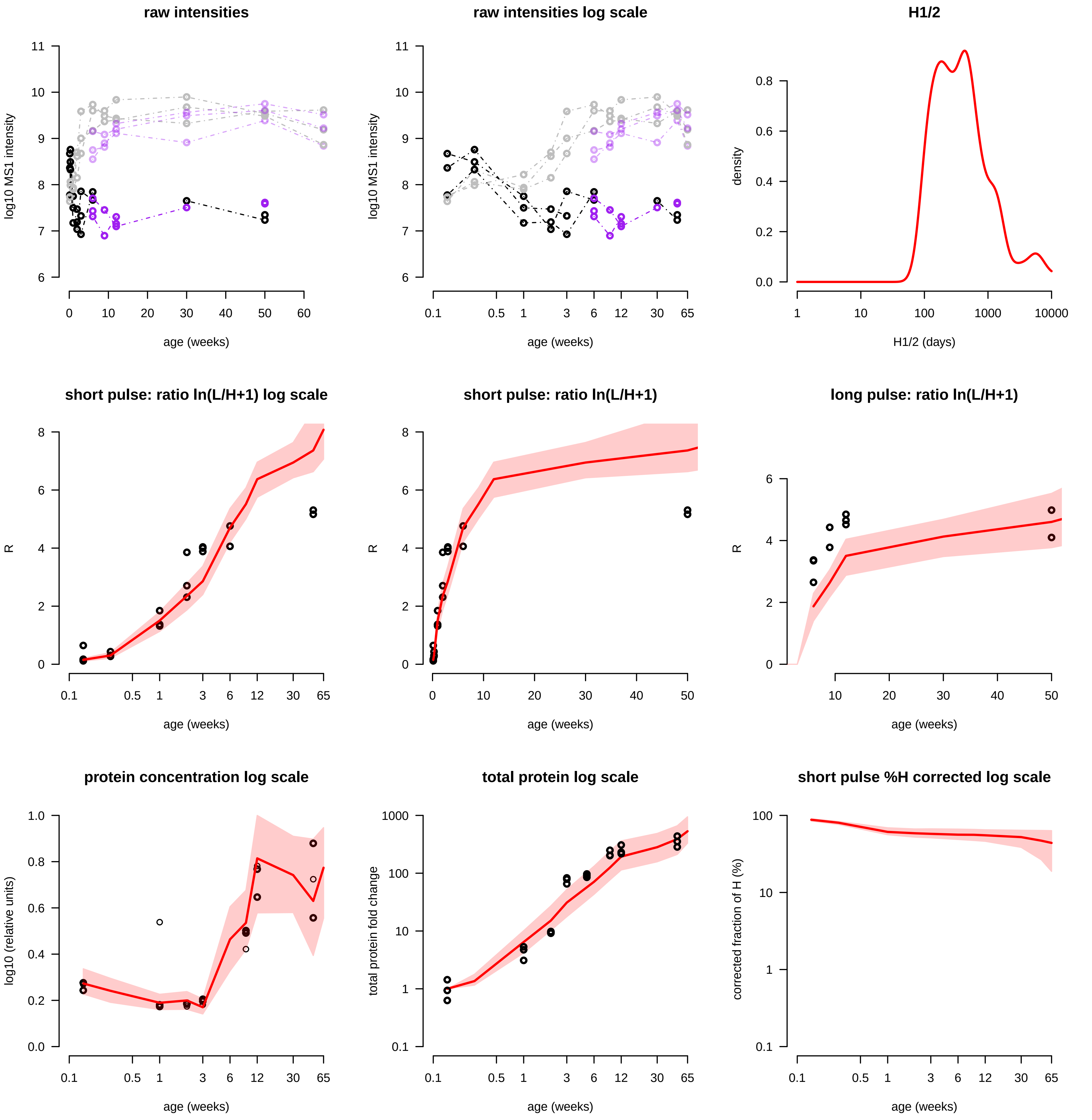

P11103;P11103−2

>sp|P11103|PARP1_MOUSE

Poly [ADP−ribose] polymerase 1

GN=Parp1

Med H1/2: 83.8 days

Q5 H1/2: 42.7 days

Q95 H1/2: 336.9 days

reliable model fit: no

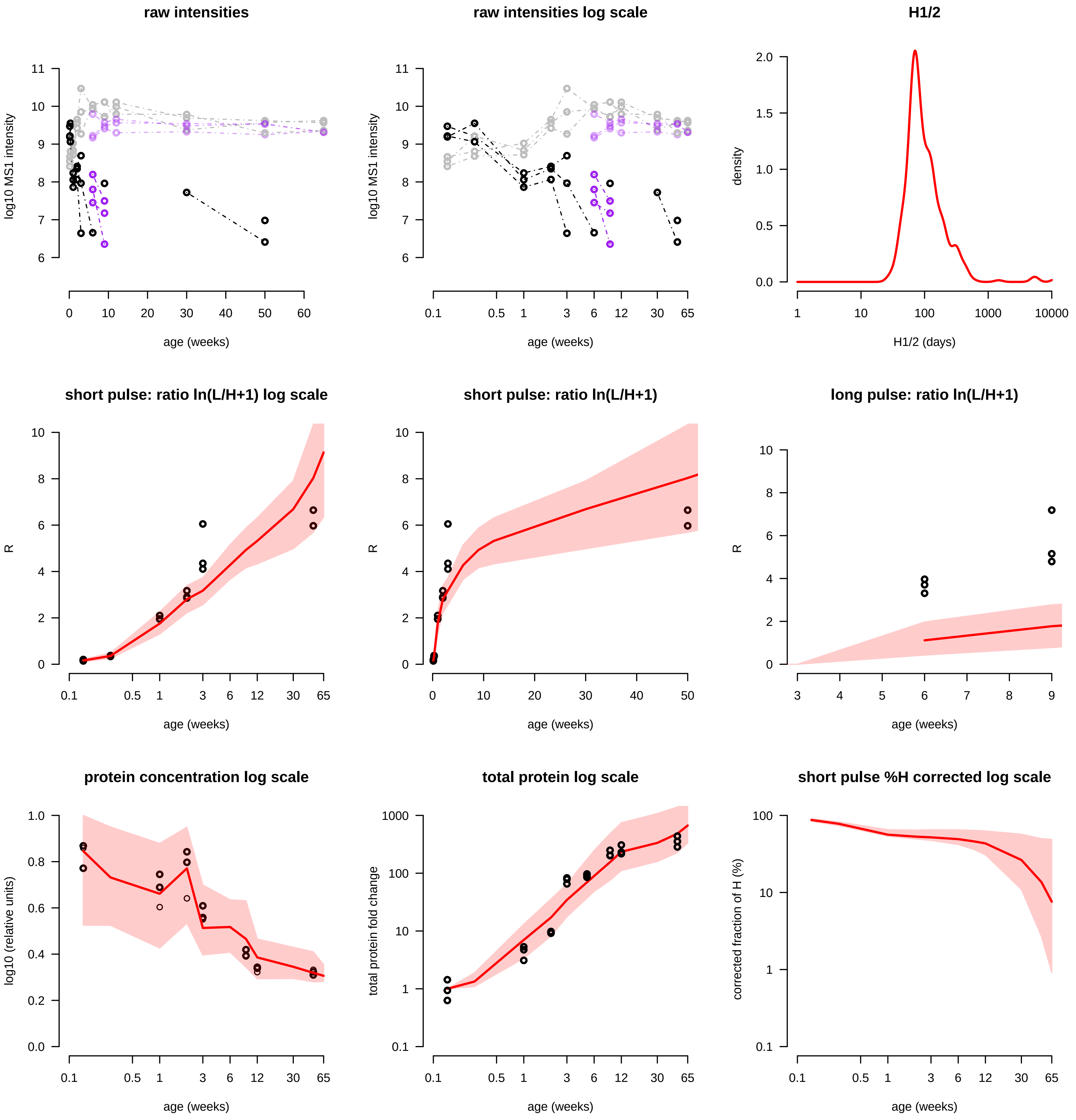

O70133−2

>sp|O70133−2|DHX9_MOUSE

Isoform 2 of ATP−dependent RNA helicase A

GN=Dhx9

Med H1/2: 6.7 days

Q5 H1/2: 5.7 days

Q95 H1/2: 9.1 days

reliable model fit: yes

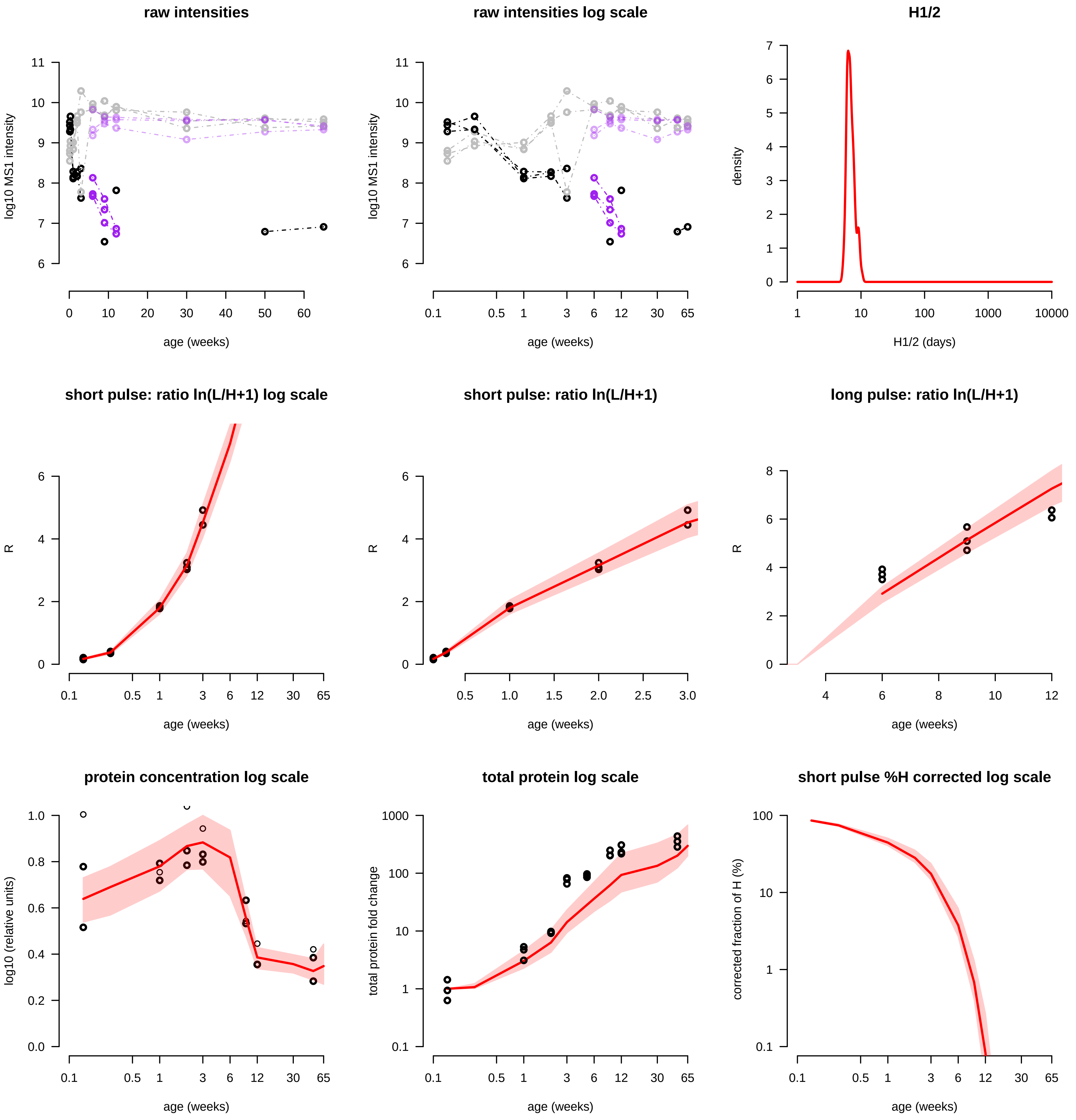

O54724

>sp|O54724|CAVN1_MOUSE

Caveolae−associated protein 1

GN=Cavin1

Med H1/2: 9.8 days

Q5 H1/2: 8 days

Q95 H1/2: 12.4 days

reliable model fit: yes

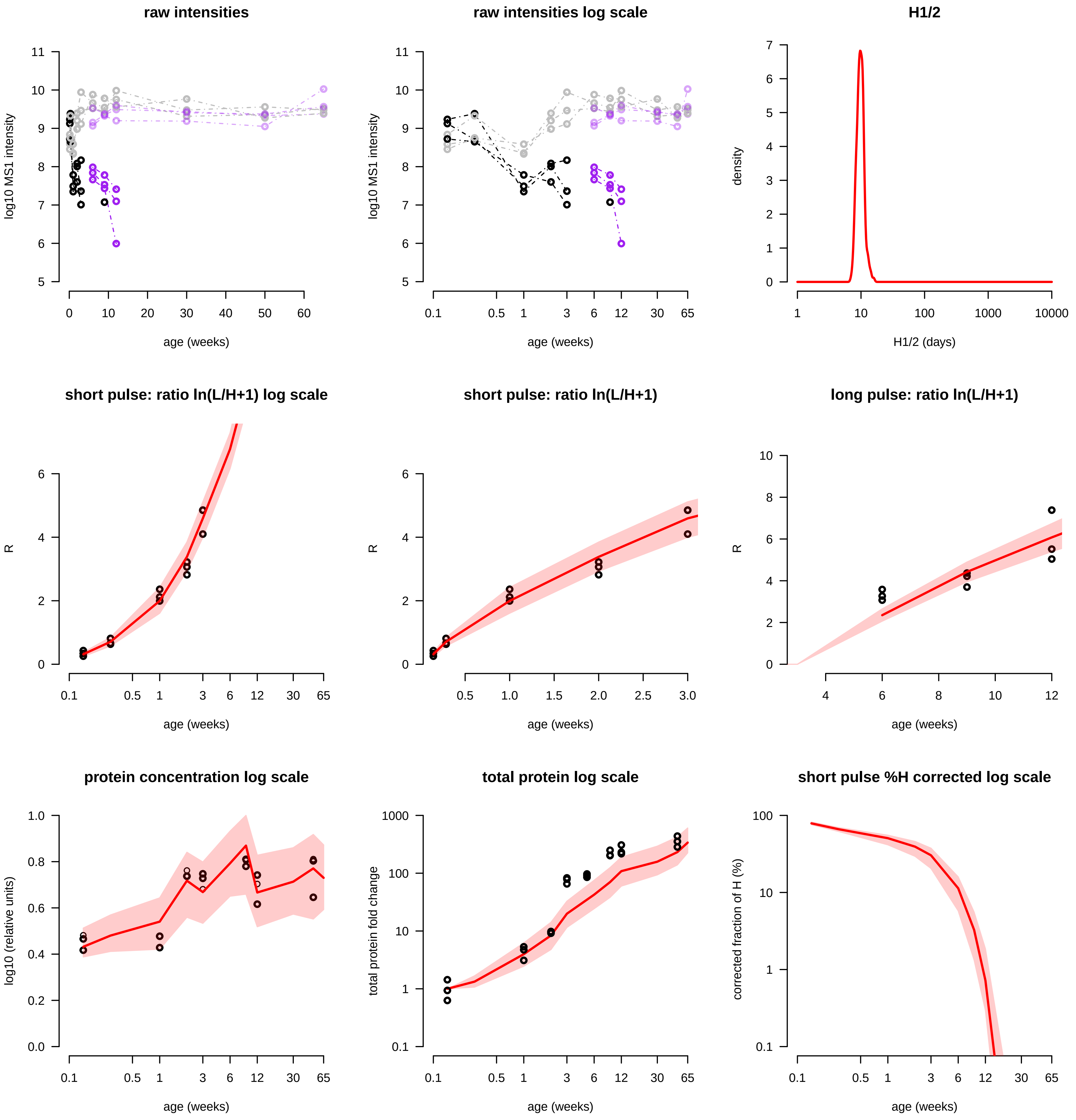

P42208

>sp|P42208|SEPT2_MOUSE

Septin−2

GN=Septin2

Med H1/2: 6.8 days

Q5 H1/2: 6.1 days

Q95 H1/2: 8.4 days

reliable model fit: yes

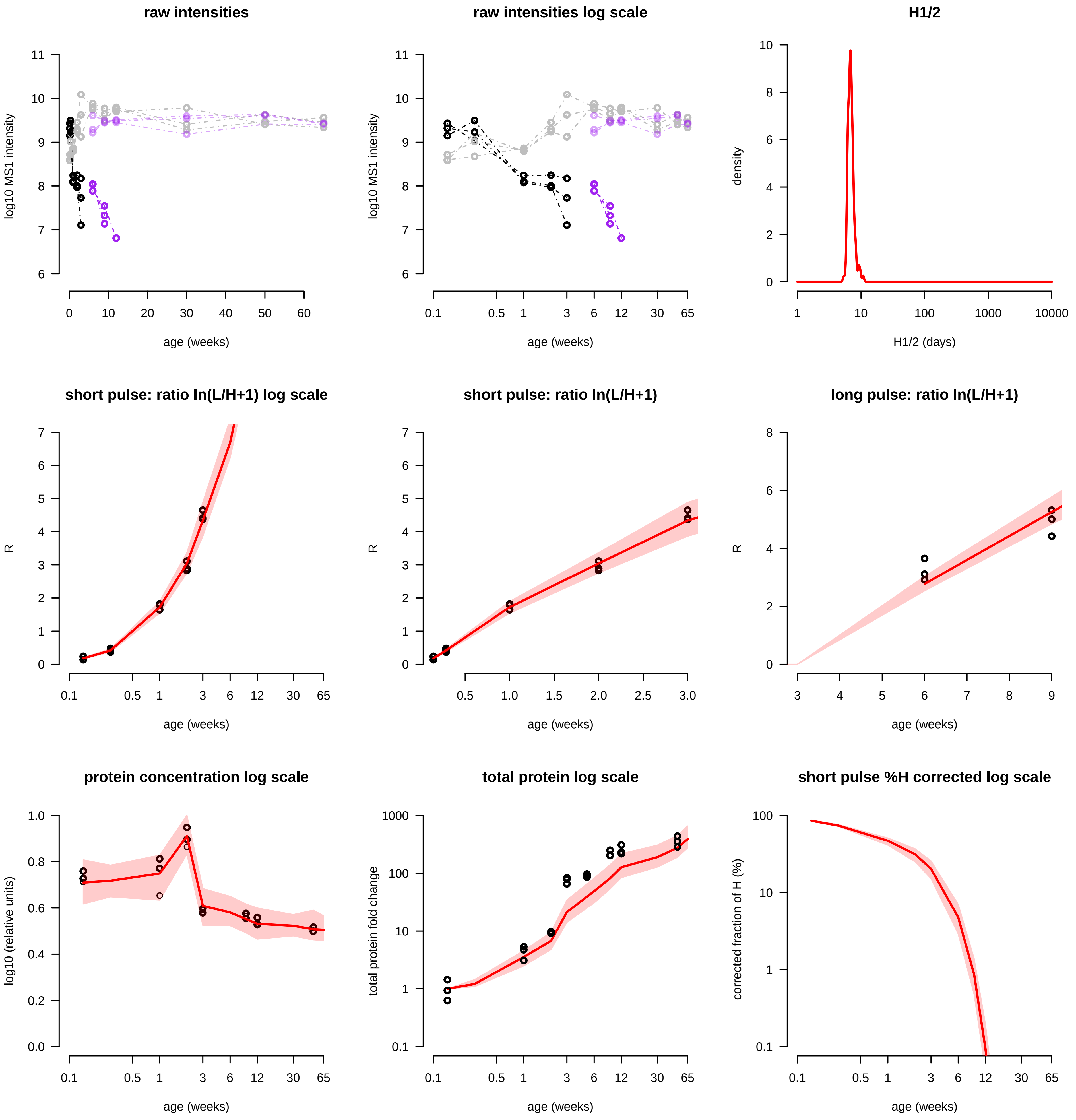

Q9QY76

>sp|Q9QY76|VAPB_MOUSE

Vesicle−associated membrane protein−associated protein B

GN=Vapb

Med H1/2: 4.4 days

Q5 H1/2: 3 days

Q95 H1/2: 6.5 days

reliable model fit: yes

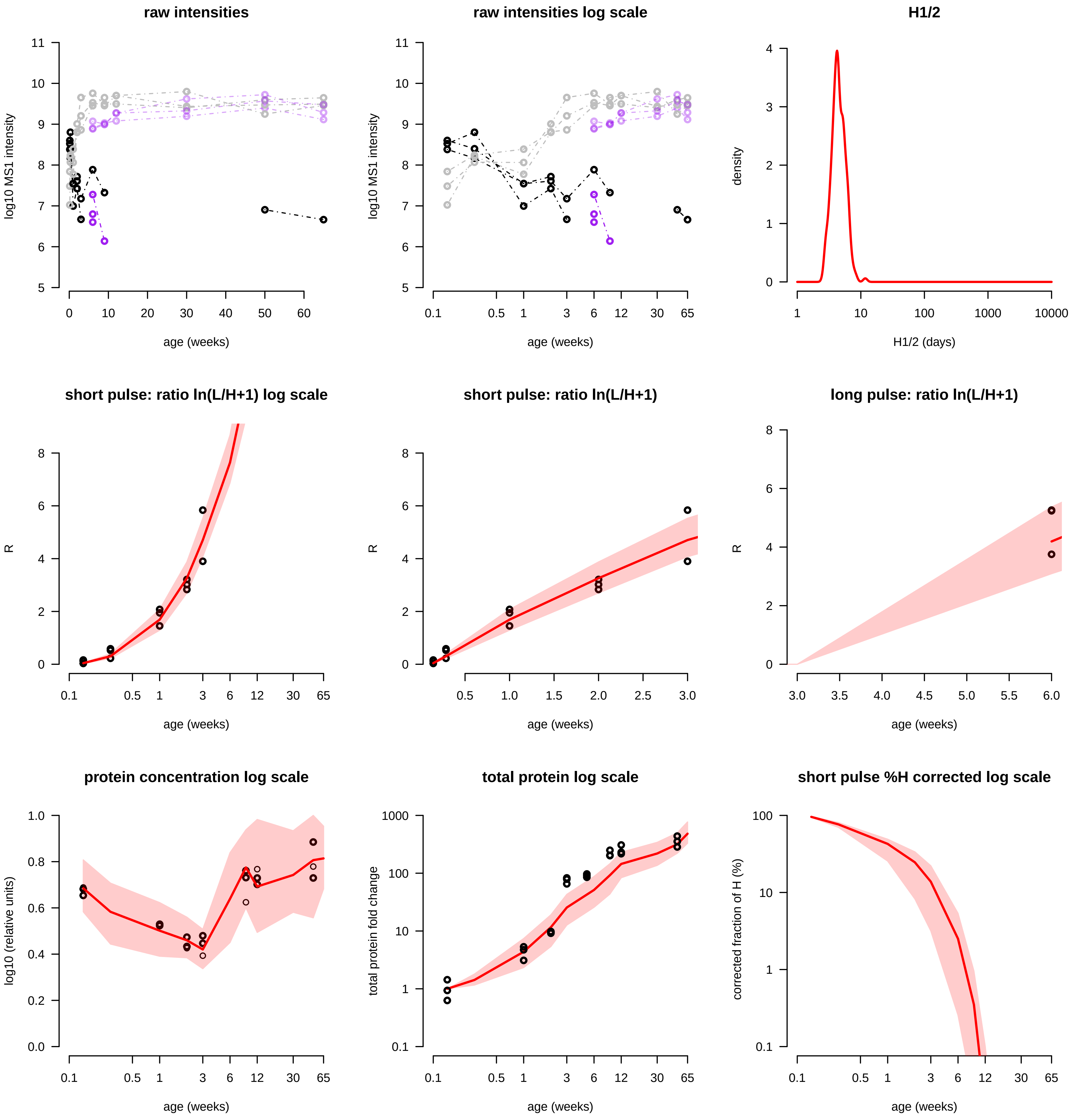

Q3UQ44

>sp|Q3UQ44|IQGA2_MOUSE

Ras GTPase−activating−like protein IQGAP2

GN=Iqgap2

Med H1/2: 9.6 days

Q5 H1/2: 6 days

Q95 H1/2: 14.3 days

reliable model fit: no

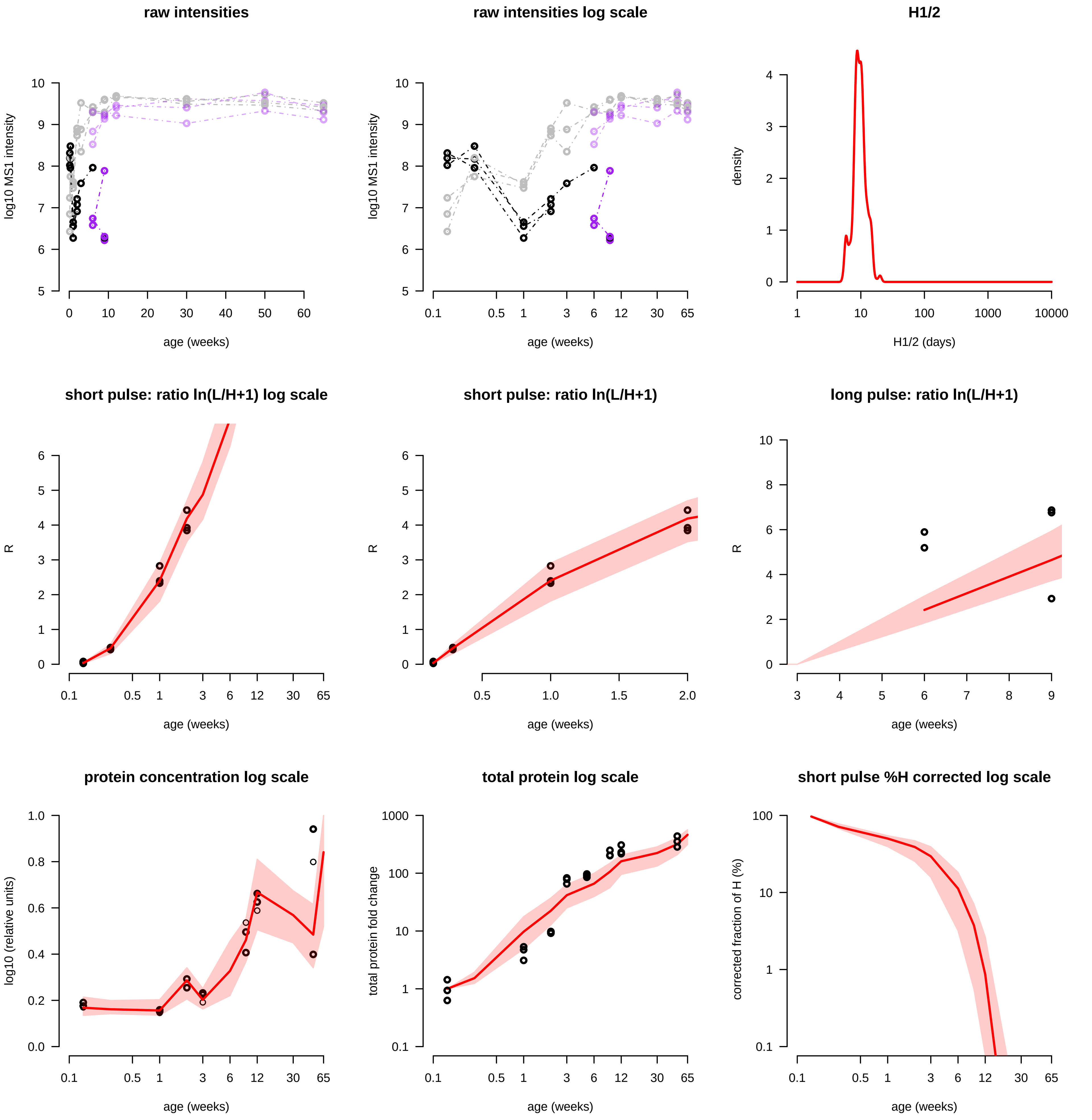

P62918

>sp|P62918|RL8_MOUSE

60S ribosomal protein L8

GN=Rpl8

Med H1/2: 14.9 days

Q5 H1/2: 9.7 days

Q95 H1/2: 21 days

reliable model fit: yes

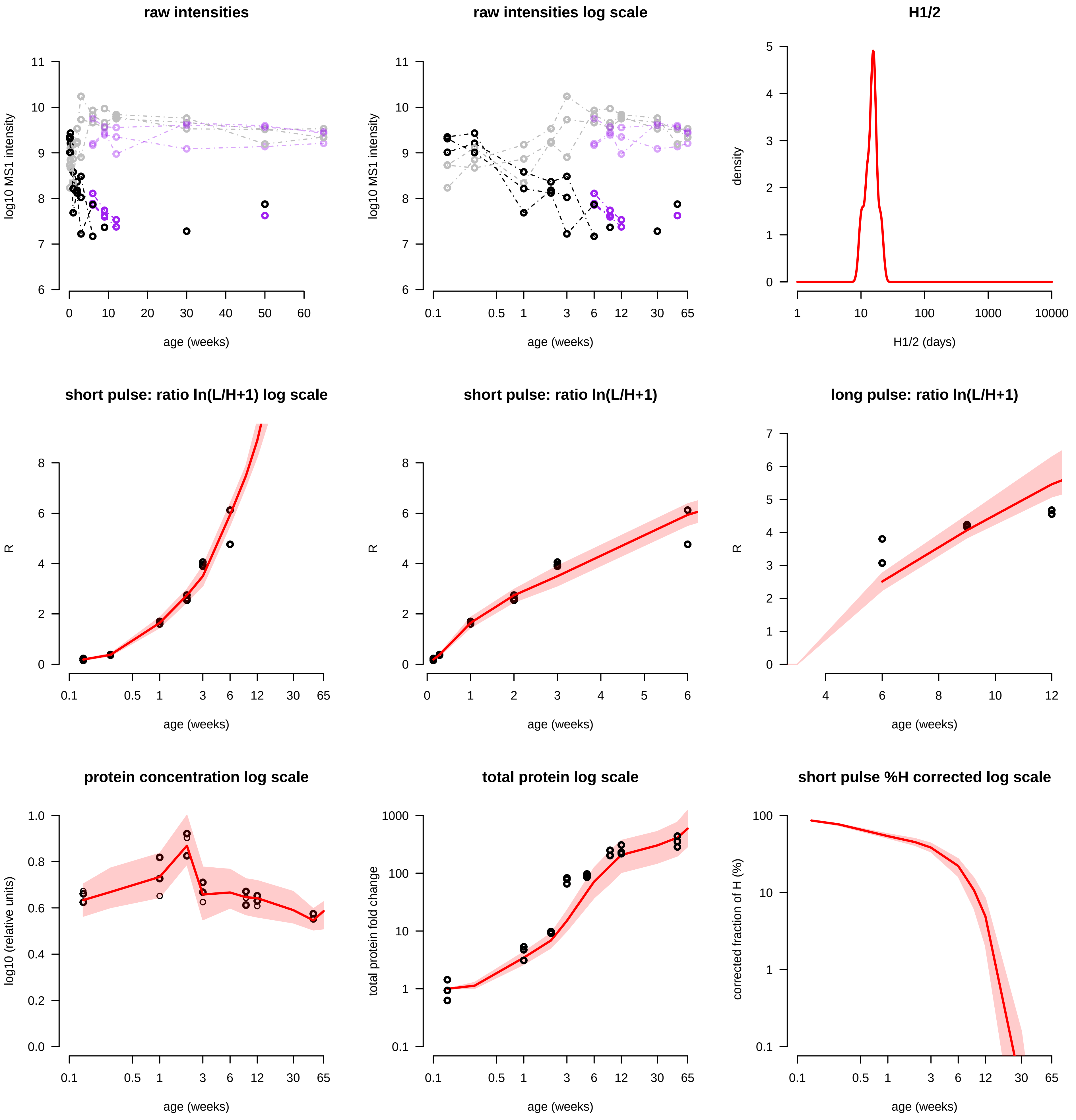

P55264

>sp|P55264|ADK_MOUSE

Adenosine kinase

GN=Adk

Med H1/2: 6.9 days

Q5 H1/2: 5.7 days

Q95 H1/2: 8.6 days

reliable model fit: yes

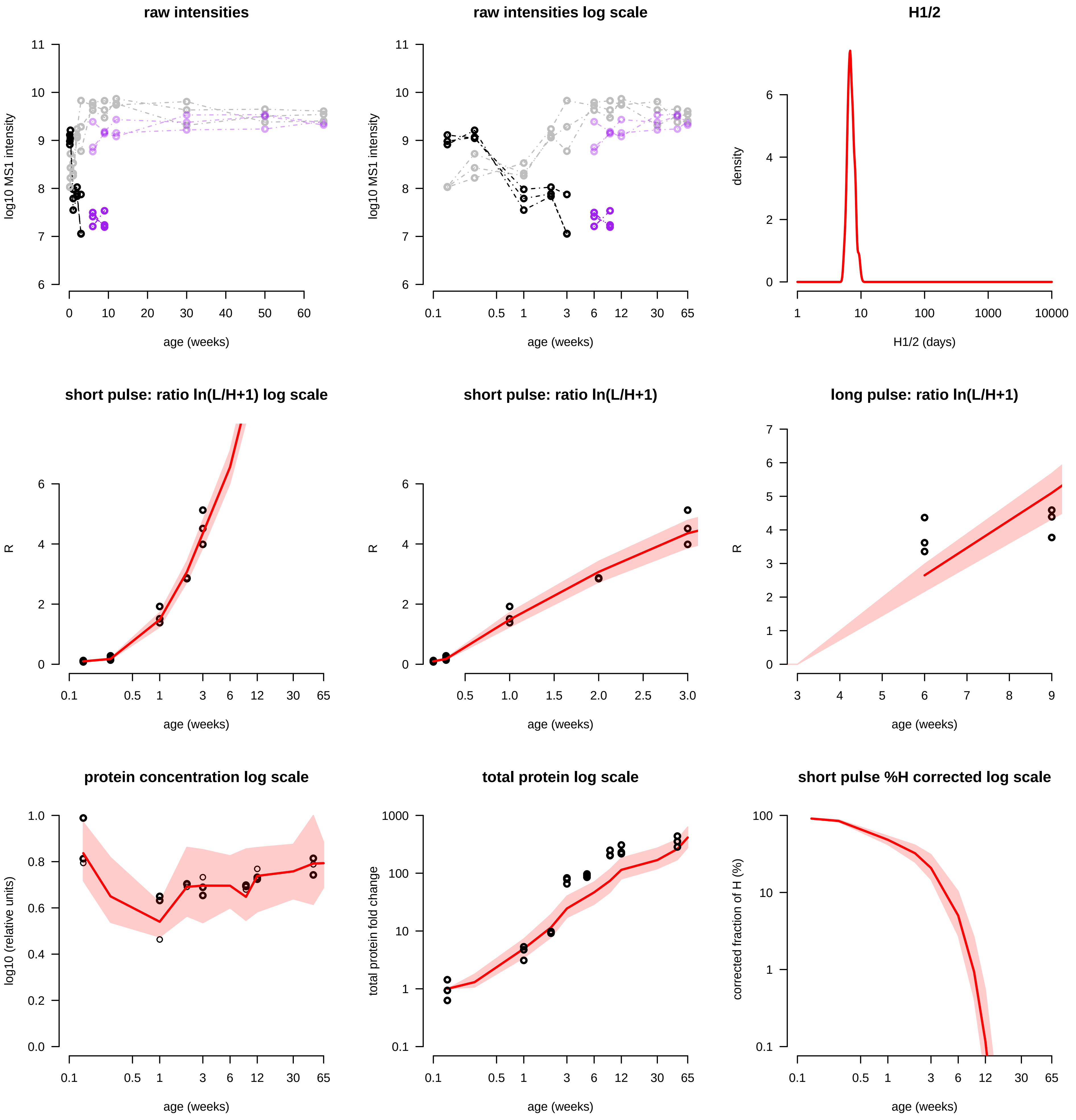

Q91VR5

>sp|Q91VR5|DDX1_MOUSE

ATP−dependent RNA helicase DDX1

GN=Ddx1

Med H1/2: 8.1 days

Q5 H1/2: 7 days

Q95 H1/2: 10.3 days

reliable model fit: yes

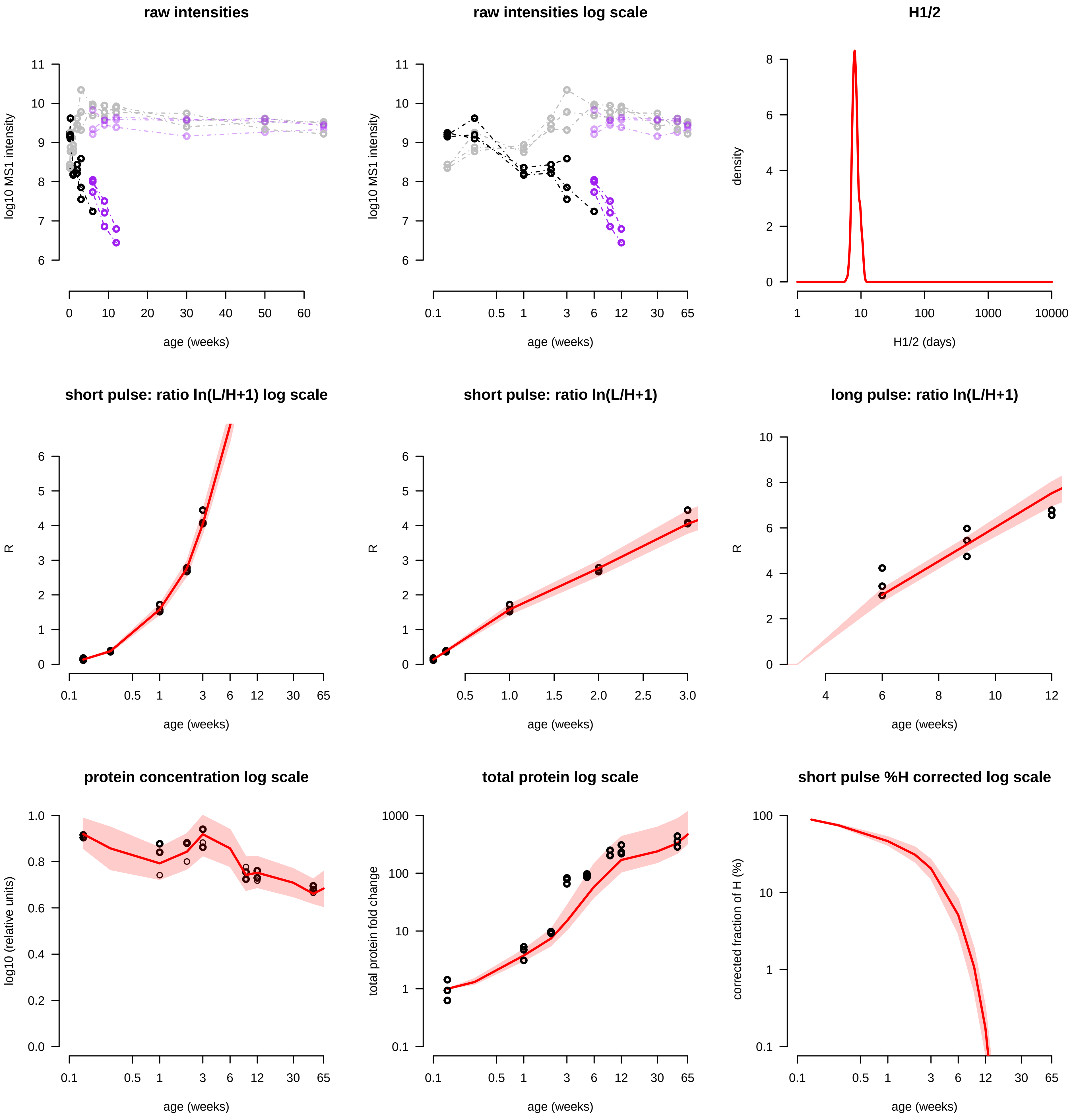

Q9DBG3;Q9DBG3−2

>sp|Q9DBG3|AP2B1_MOUSE

AP−2 complex subunit beta

GN=Ap2b1

Med H1/2: 5.4 days

Q5 H1/2: 4.7 days

Q95 H1/2: 6.4 days

reliable model fit: yes

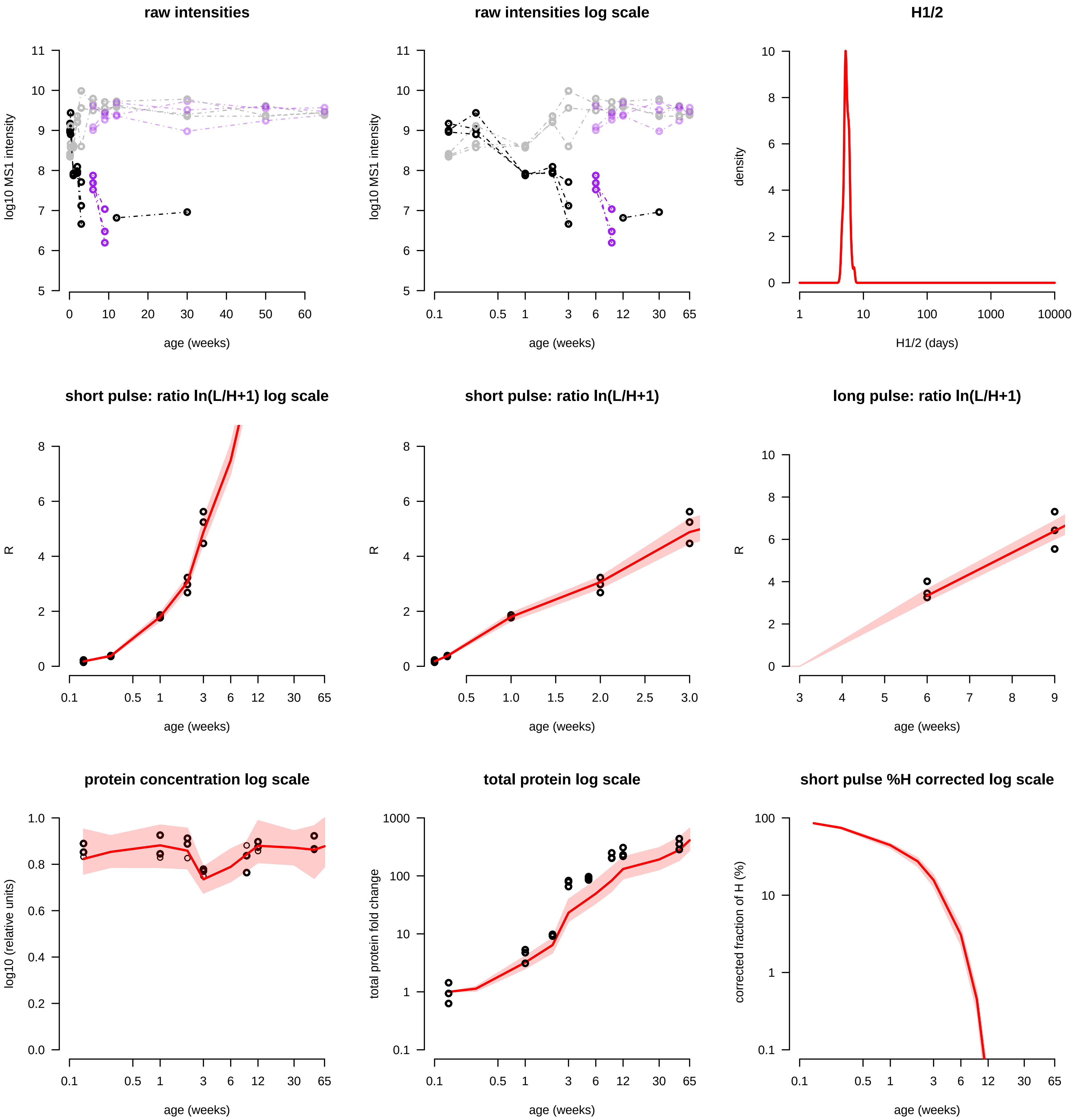

Q99PV0

>sp|Q99PV0|PRP8_MOUSE

Pre−mRNA−processing−splicing factor 8

GN=Prpf8

Med H1/2: 7.9 days

Q5 H1/2: 6.4 days

Q95 H1/2: 10 days

reliable model fit: no

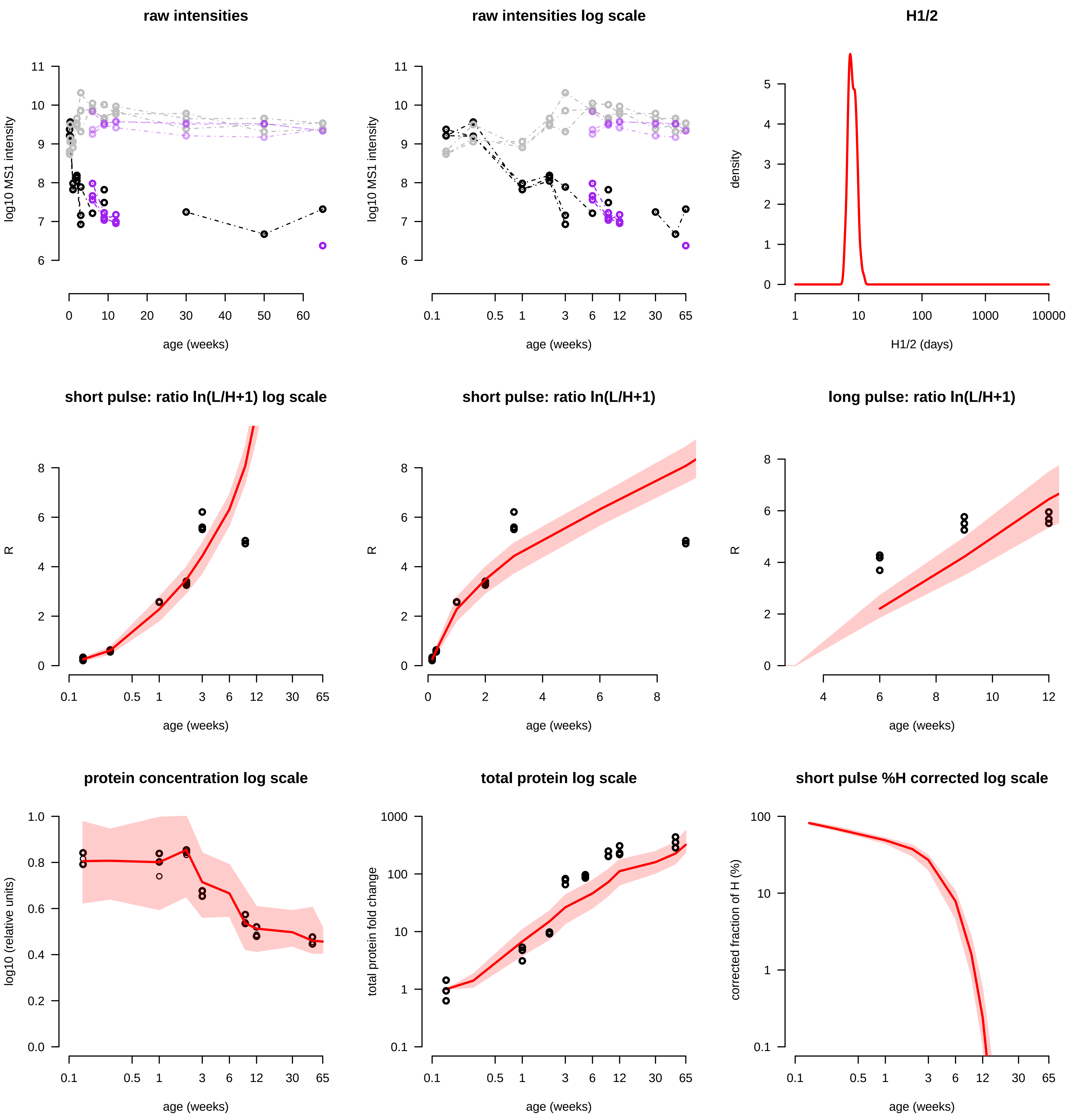

Q99NB9

>sp|Q99NB9|SF3B1_MOUSE

Splicing factor 3B subunit 1

GN=Sf3b1

Med H1/2: 115 days

Q5 H1/2: 54.1 days

Q95 H1/2: 265.5 days

reliable model fit: no

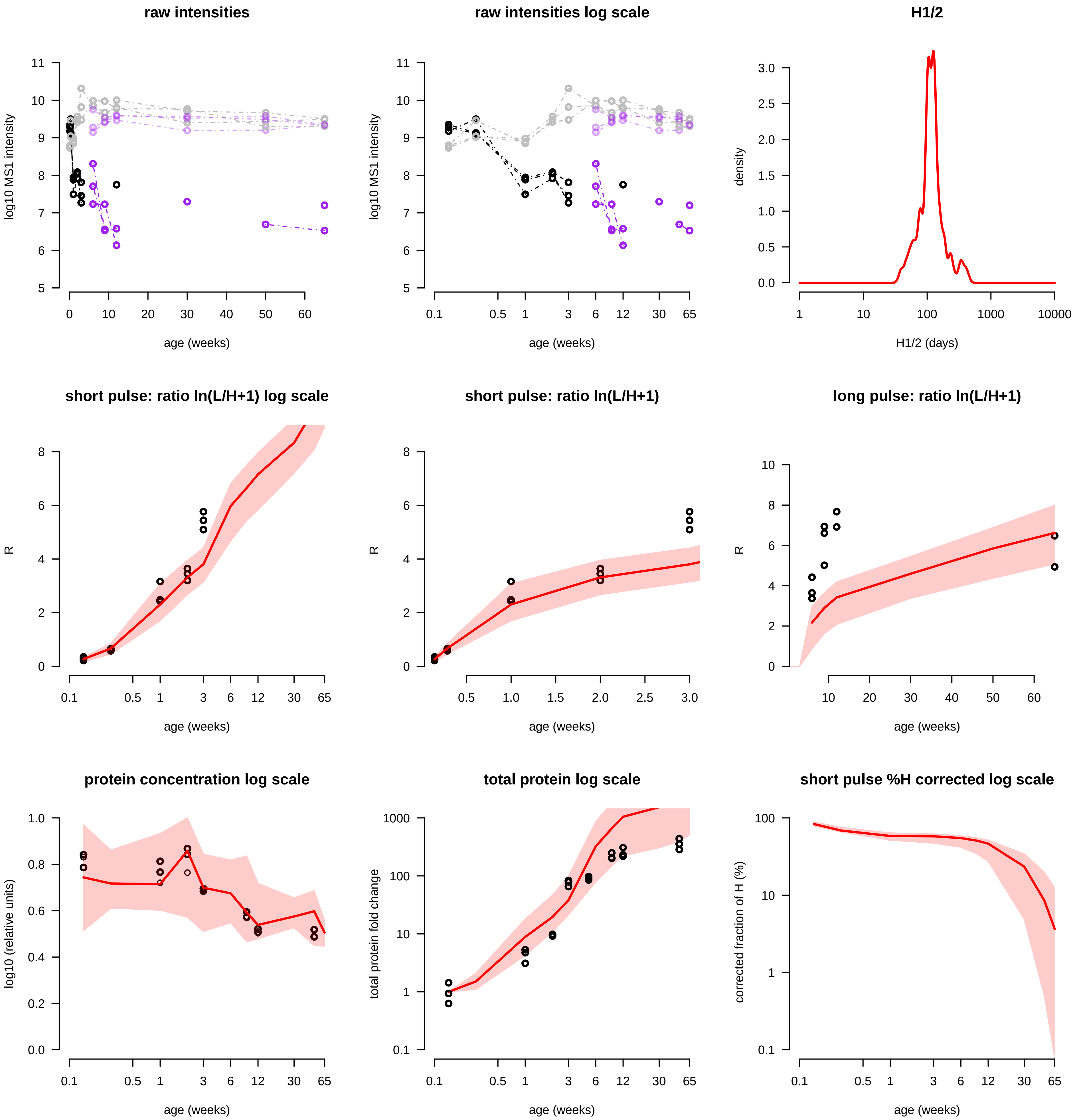

Q91W90

>sp|Q91W90|TXND5_MOUSE

Thioredoxin domain−containing protein 5

GN=Txndc5

Med H1/2: 5.8 days

Q5 H1/2: 4.9 days

Q95 H1/2: 7.2 days

reliable model fit: yes

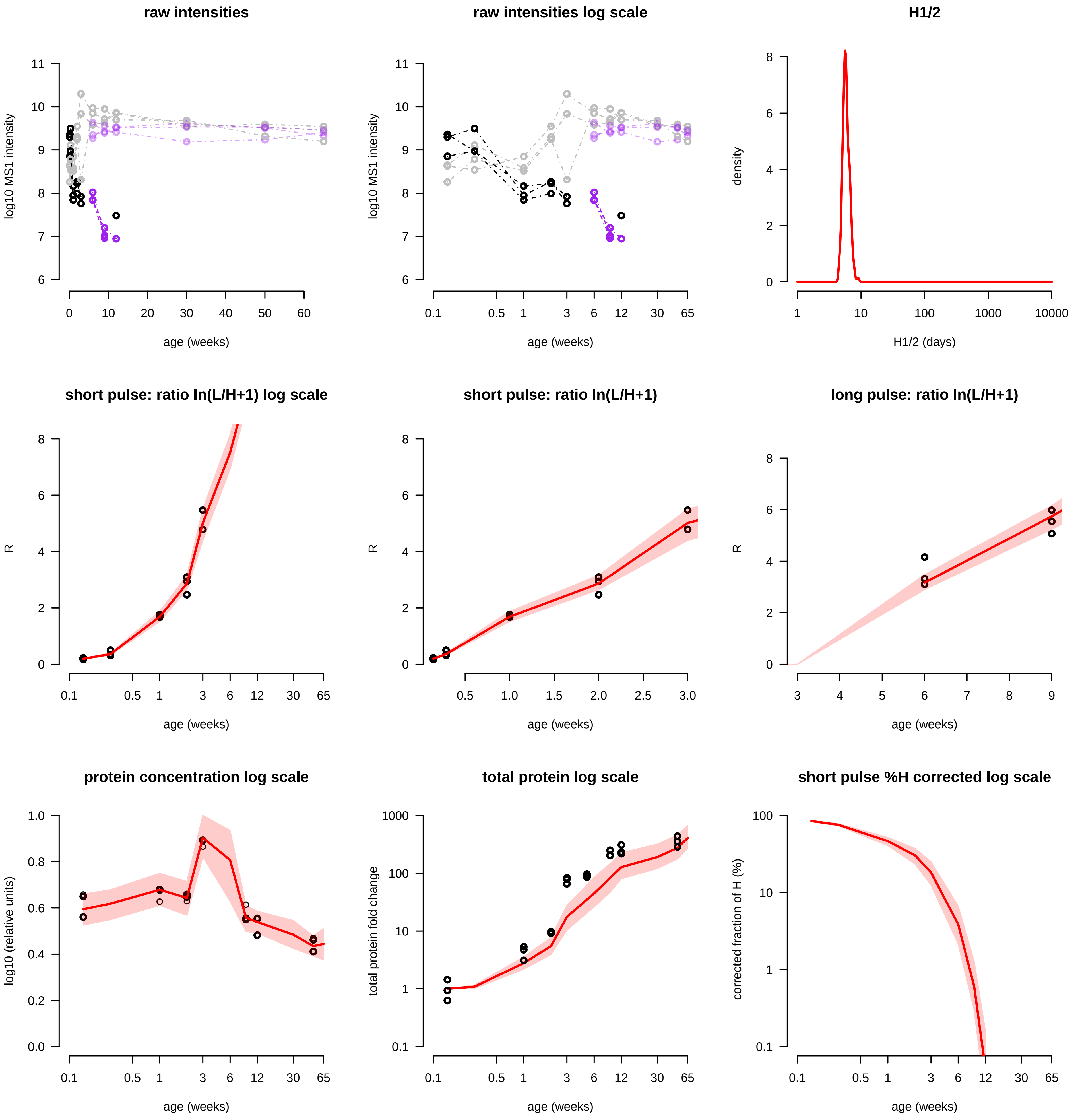

Q8VDM4

>sp|Q8VDM4|PSMD2_MOUSE

26S proteasome non−ATPase regulatory subunit 2

GN=Psmd2

Med H1/2: 5.1 days

Q5 H1/2: 4.4 days

Q95 H1/2: 6.2 days

reliable model fit: yes

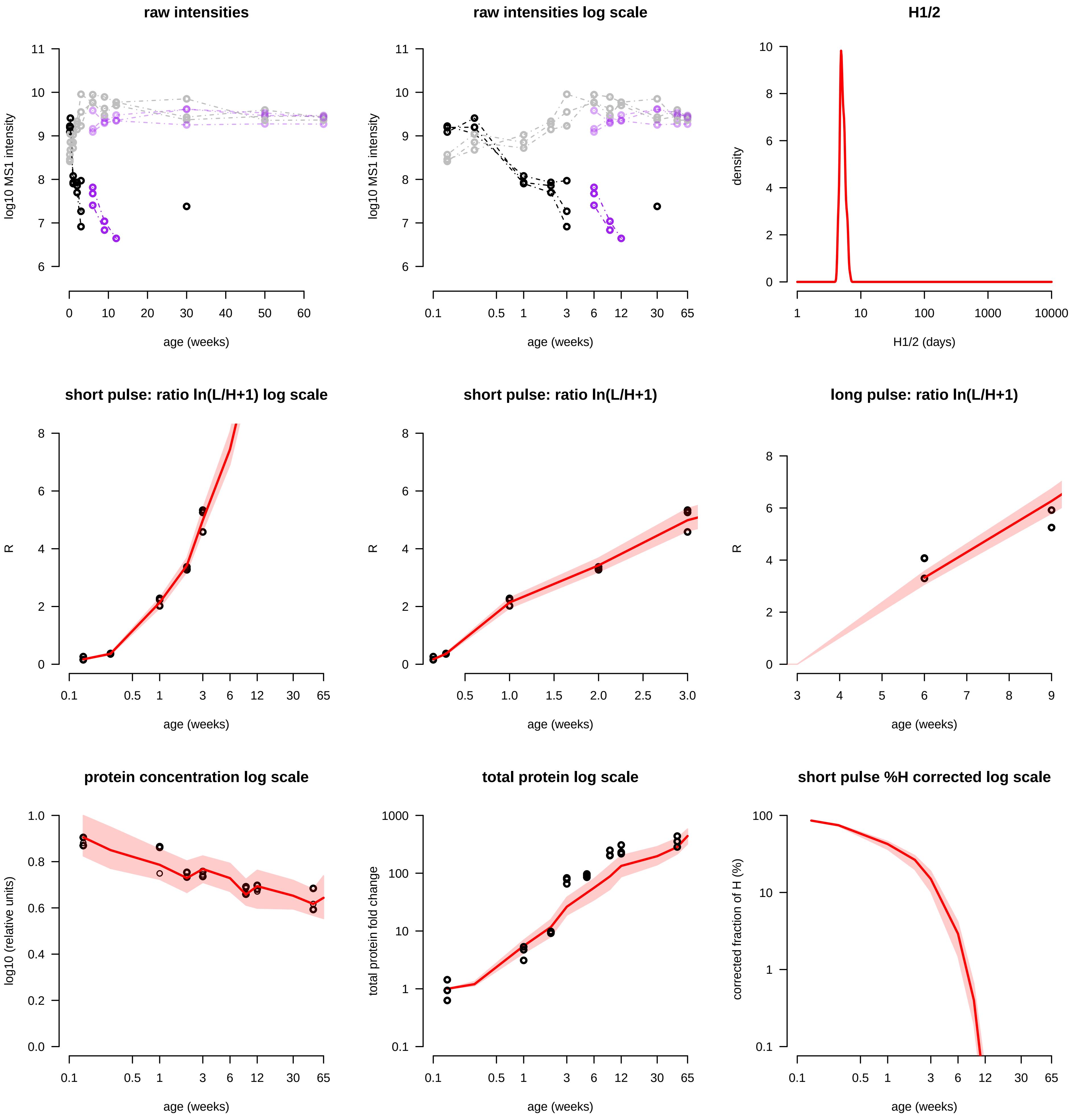

Q9CPQ1

>sp|Q9CPQ1|COX6C_MOUSE

Cytochrome c oxidase subunit 6C

GN=Cox6c

Med H1/2: 10.3 days

Q5 H1/2: 8.1 days

Q95 H1/2: 15.7 days

reliable model fit: yes

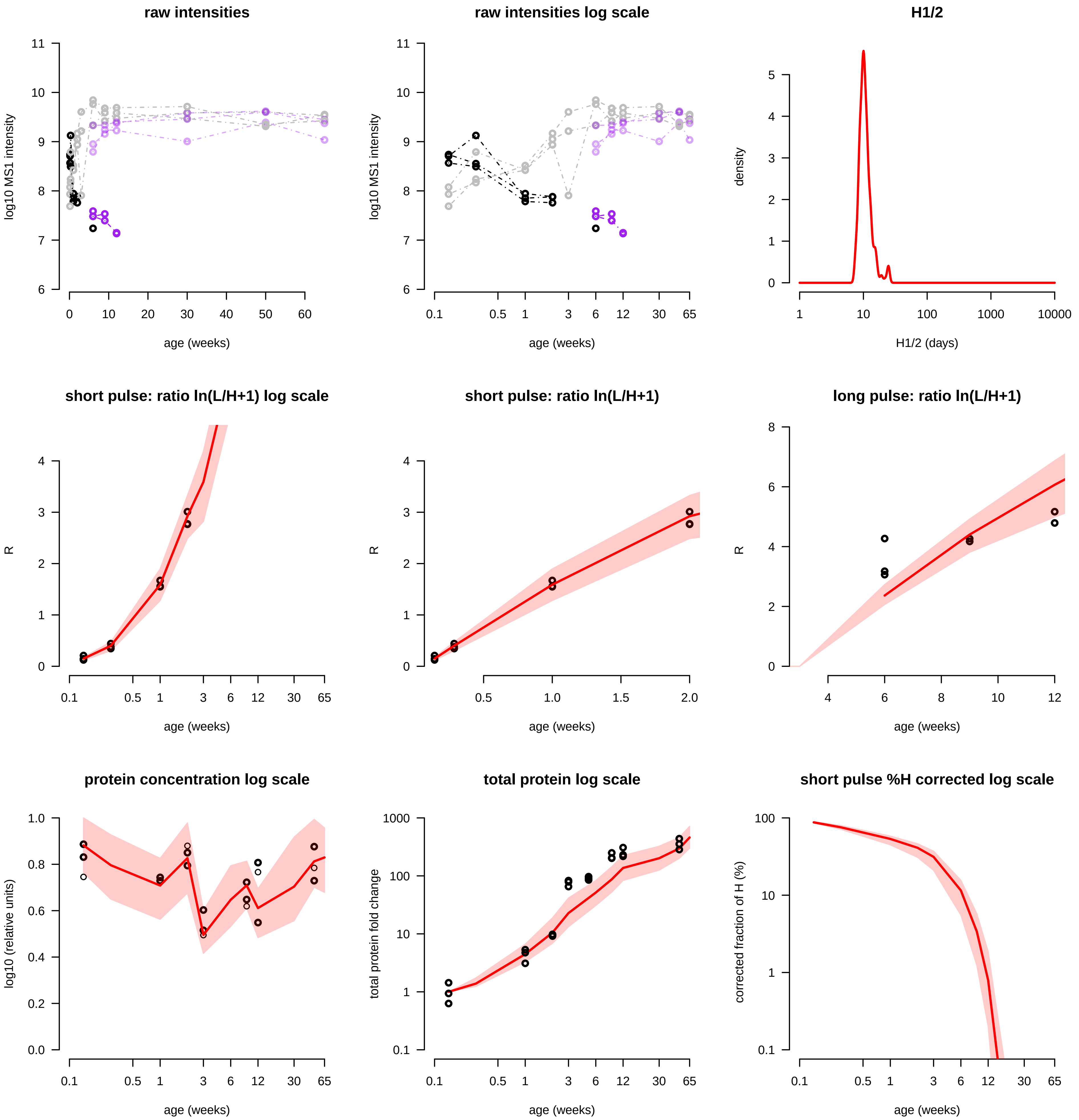

Q9EST5−2;Q9EST5

>sp|Q9EST5−2|AN32B_MOUSE

Isoform 2 of Acidic leucine−rich nuclear phosphoprotein 32 family member B

GN=Anp32b

Med H1/2: 648.2 days

Q5 H1/2: 220.8 days

Q95 H1/2: 4496.6 days

reliable model fit: no

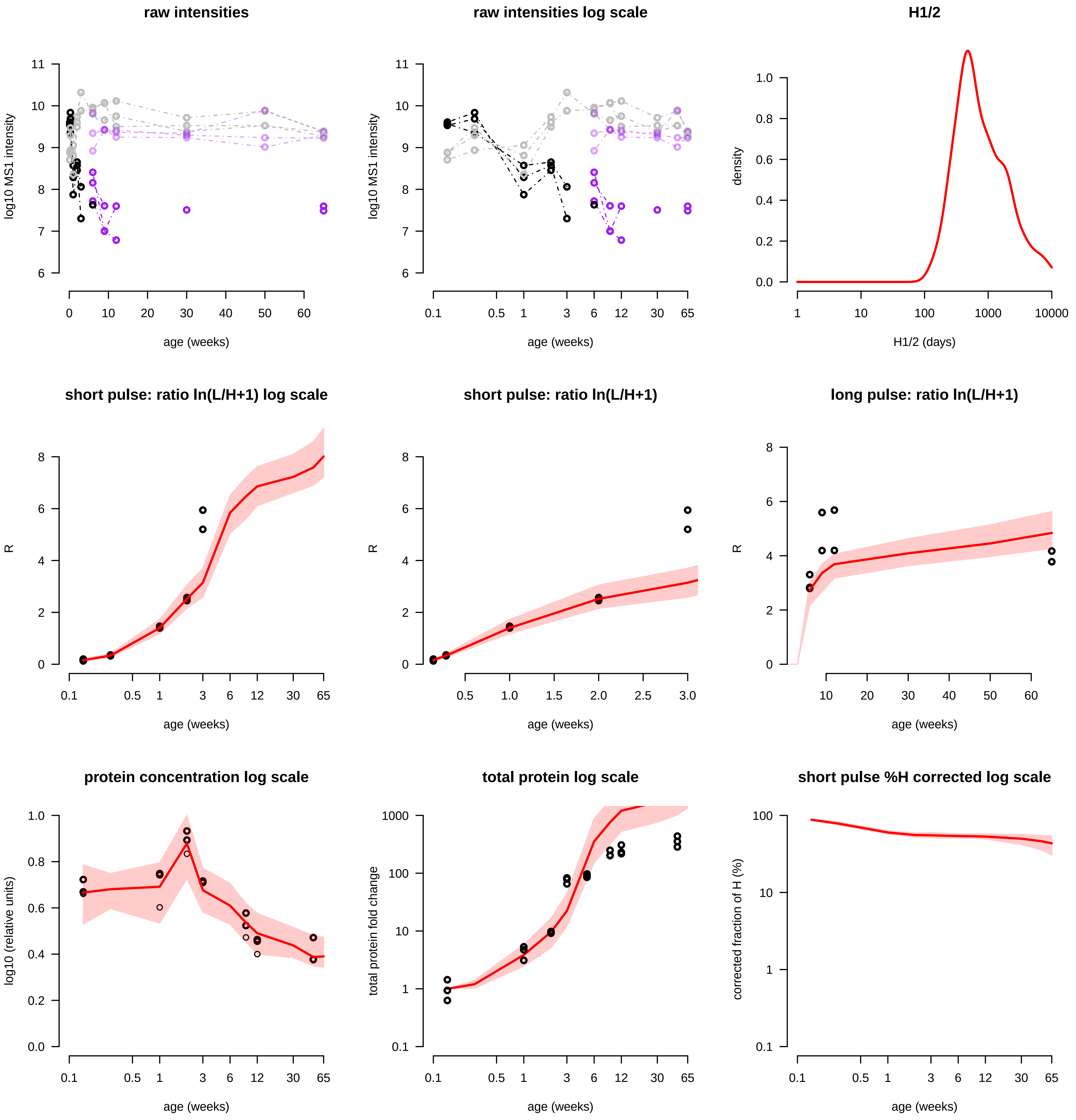

Q9R1P4

>sp|Q9R1P4|PSA1_MOUSE

Proteasome subunit alpha type−1

GN=Psma1

Med H1/2: 9.2 days

Q5 H1/2: 7.7 days

Q95 H1/2: 11.4 days

reliable model fit: yes

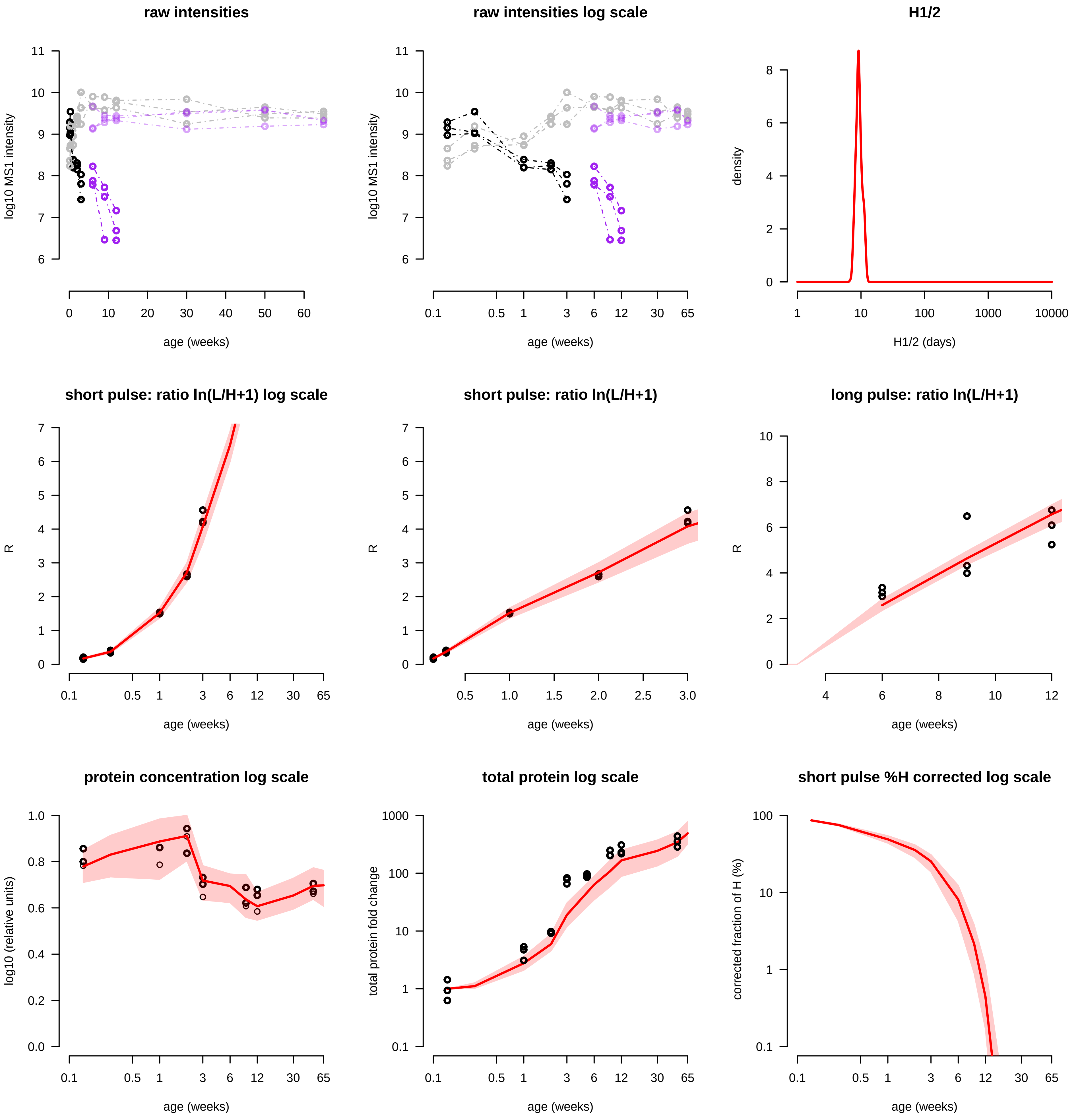

Q9JJI8

>sp|Q9JJI8|RL38_MOUSE

60S ribosomal protein L38

GN=Rpl38

Med H1/2: 6.5 days

Q5 H1/2: 5.1 days

Q95 H1/2: 8.7 days

reliable model fit: yes

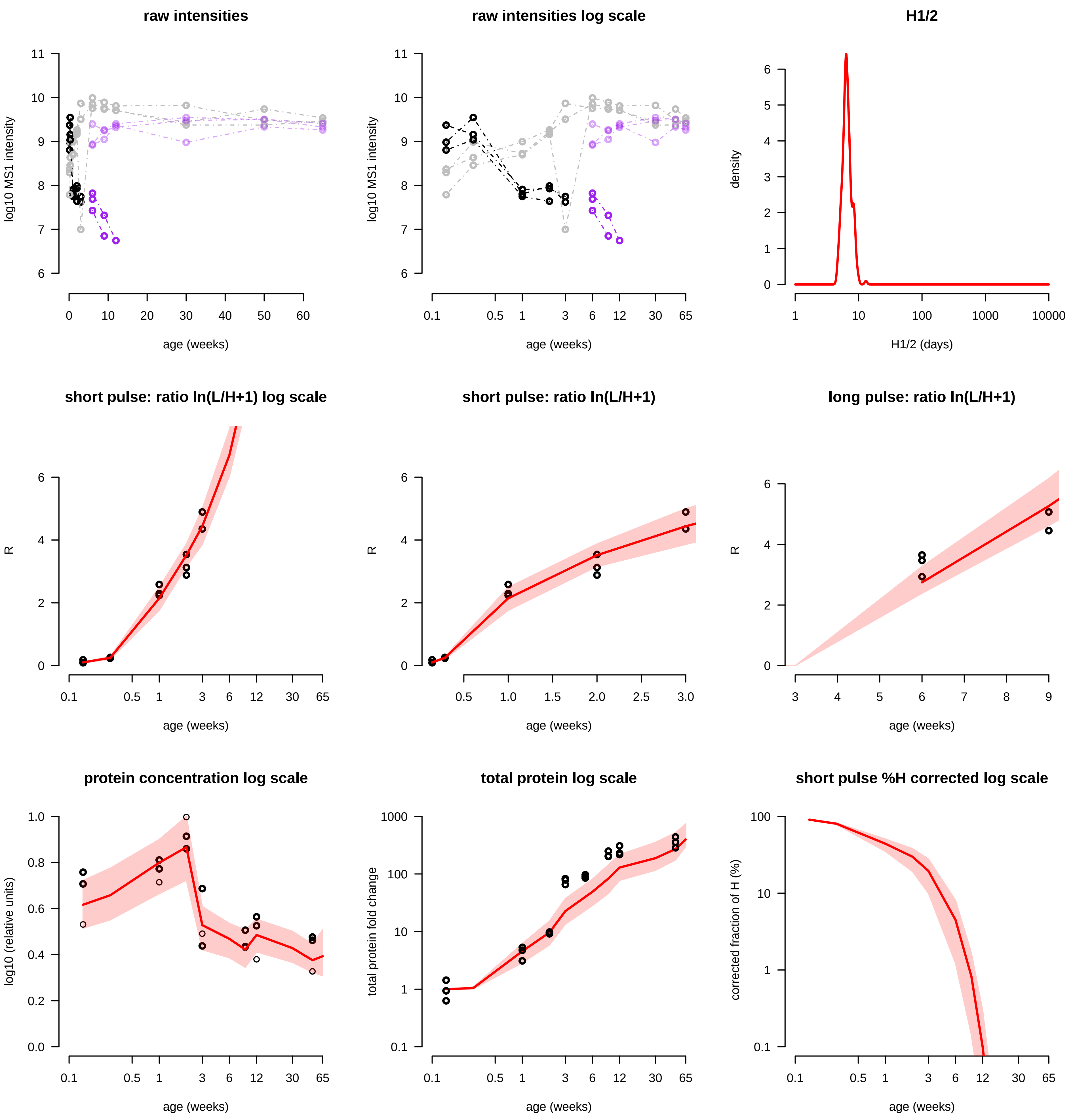

Q6ZWV3

>sp|Q6ZWV3|RL10_MOUSE

60S ribosomal protein L10

GN=Rpl10

Med H1/2: 7.6 days

Q5 H1/2: 6.2 days

Q95 H1/2: 9.4 days

reliable model fit: yes

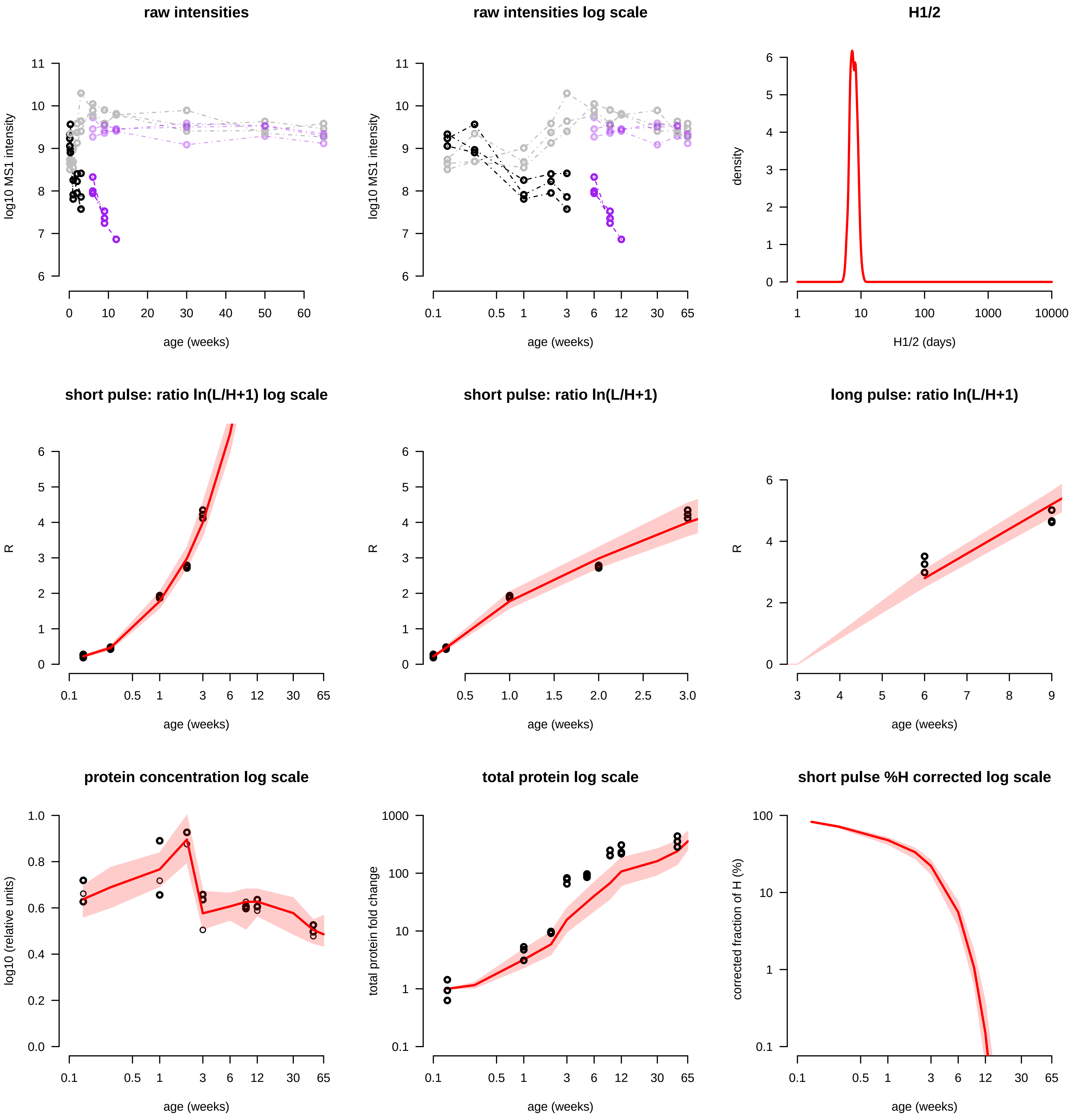

Q60710

>sp|Q60710|SAMH1_MOUSE

Deoxynucleoside triphosphate triphosphohydrolase SAMHD1

GN=Samhd1

Med H1/2: 718.9 days

Q5 H1/2: 173.1 days

Q95 H1/2: 8700.9 days

reliable model fit: no

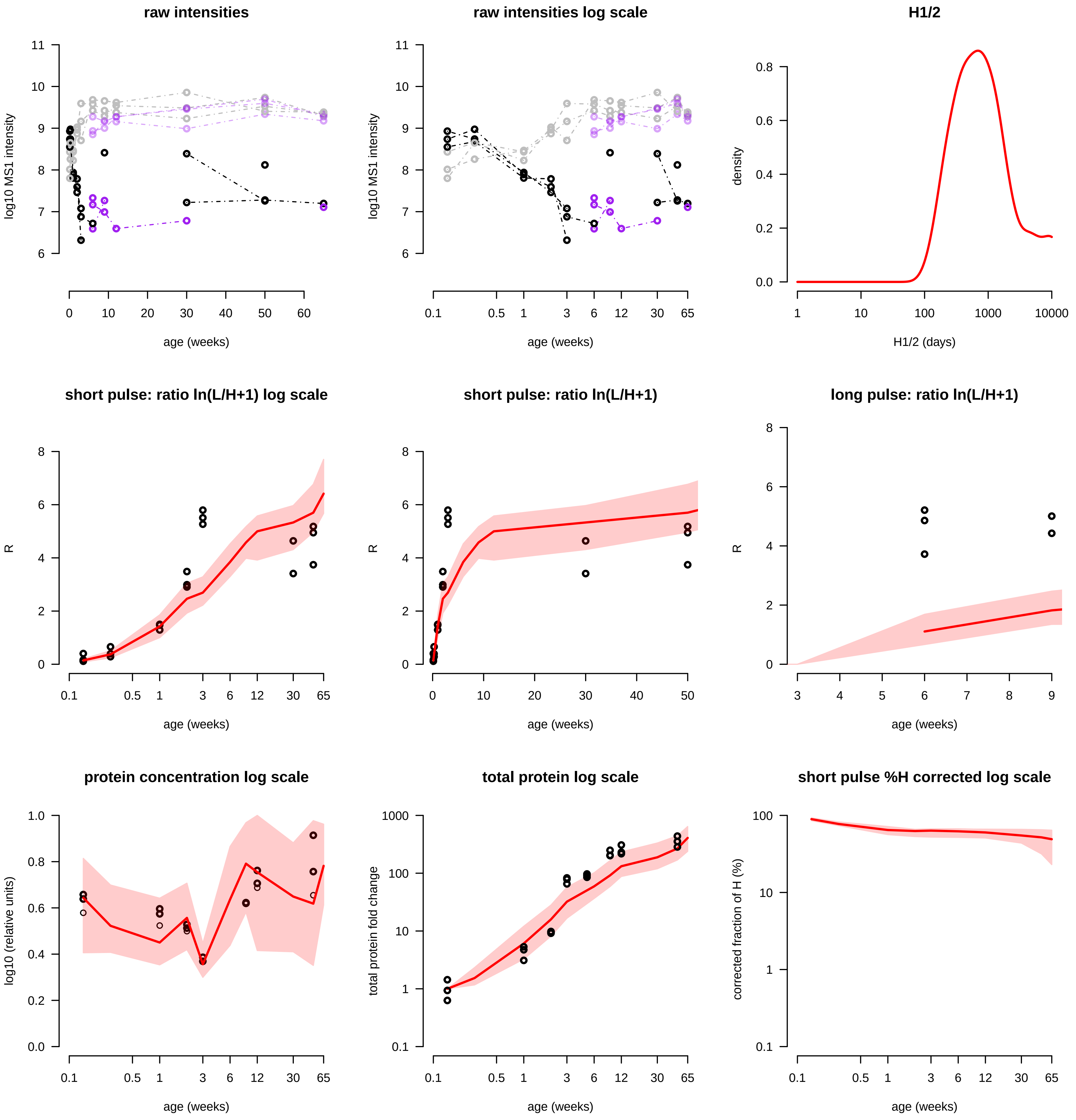

P30681

>sp|P30681|HMGB2_MOUSE

High mobility group protein B2

GN=Hmgb2

Med H1/2: 9.8 days

Q5 H1/2: 7.3 days

Q95 H1/2: 13.1 days

reliable model fit: yes

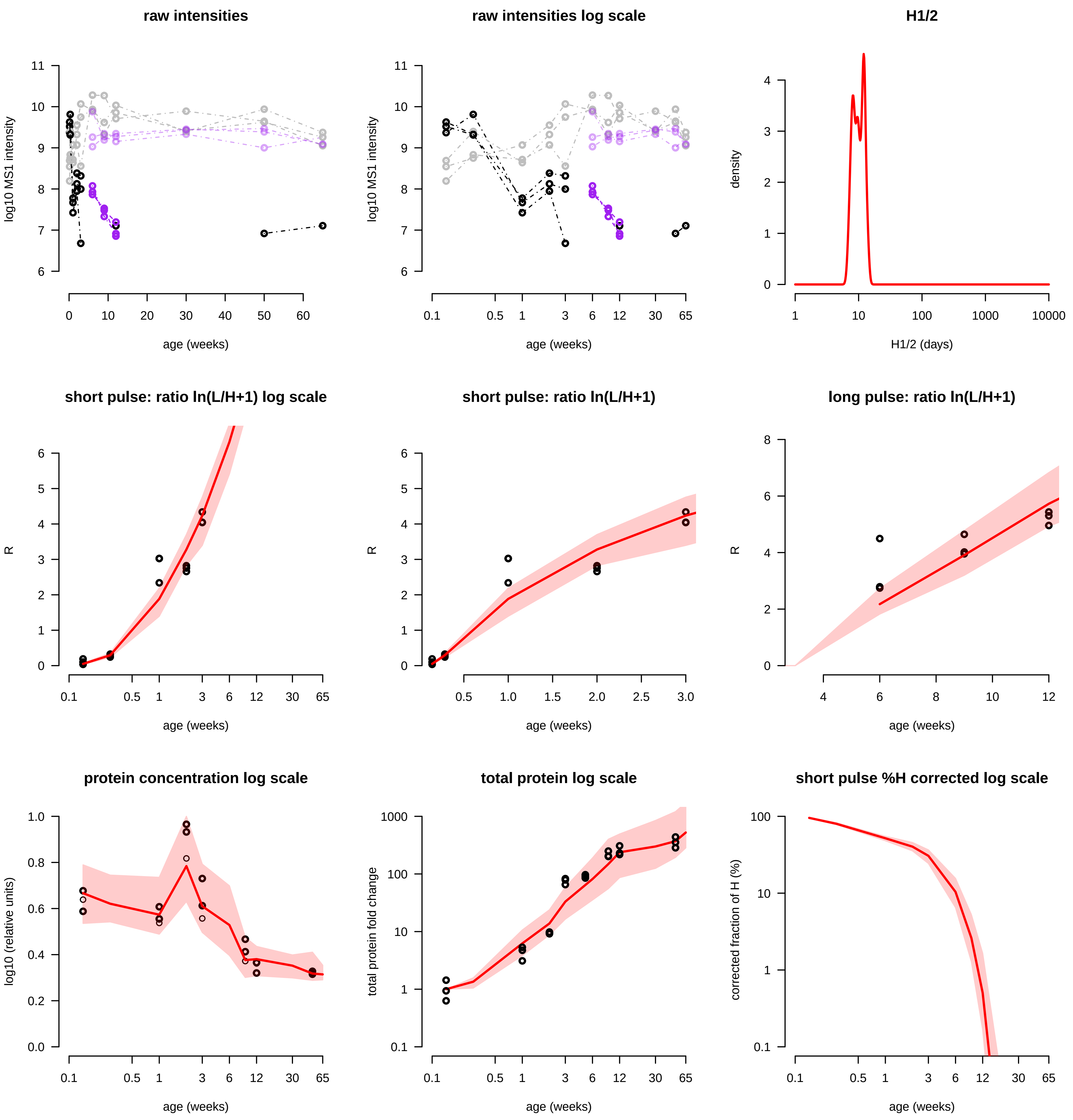

P26645

>sp|P26645|MARCS_MOUSE

Myristoylated alanine−rich C−kinase substrate

GN=Marcks

Med H1/2: 6.6 days

Q5 H1/2: 5.7 days

Q95 H1/2: 7.7 days

reliable model fit: yes

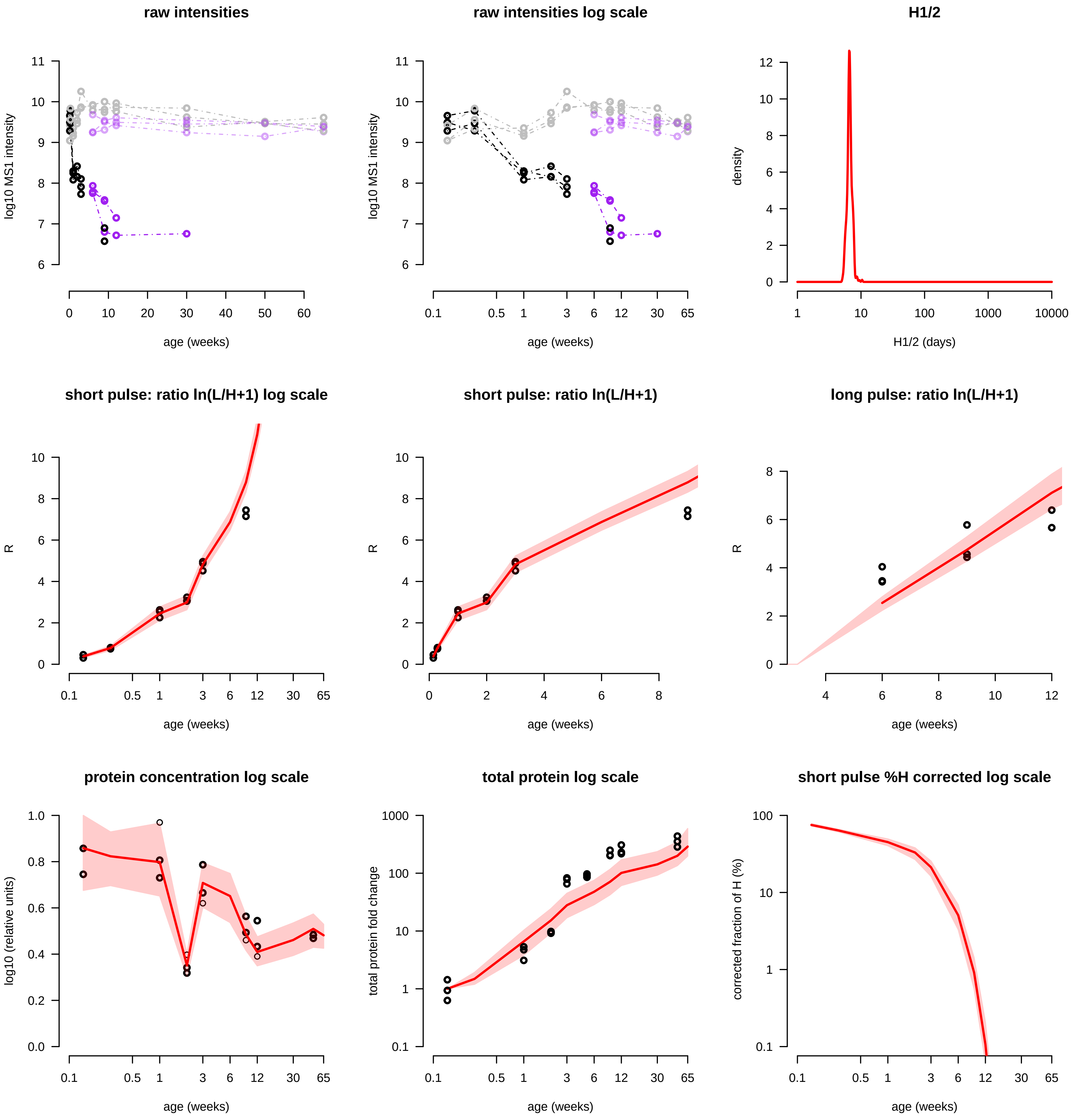

Q61207

>sp|Q61207|SAP_MOUSE

Prosaposin

GN=Psap

Med H1/2: 11.5 days

Q5 H1/2: 7.5 days

Q95 H1/2: 19.2 days

reliable model fit: yes

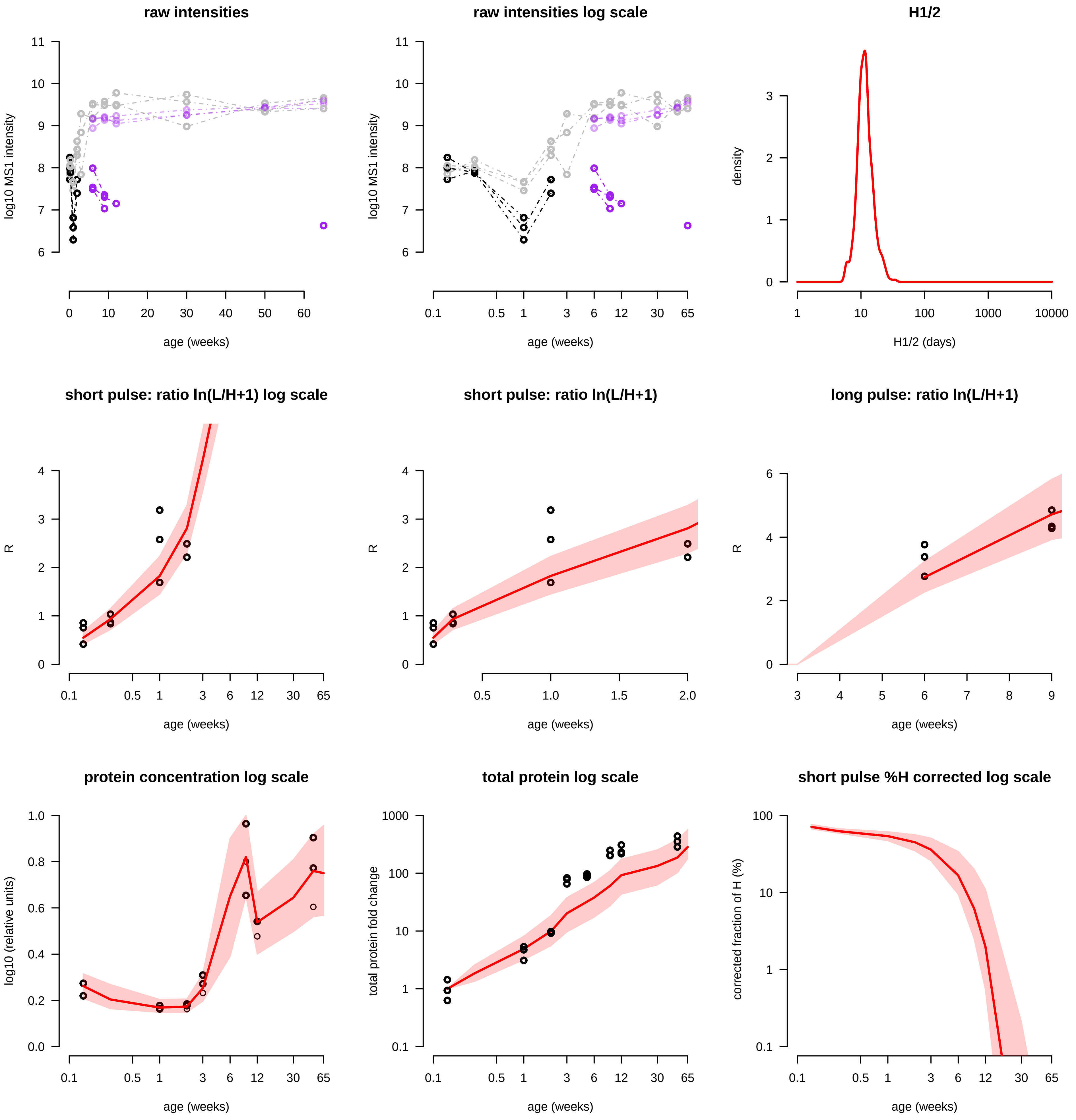

P35550

>sp|P35550|FBRL_MOUSE

rRNA 2'−O−methyltransferase fibrillarin

GN=Fbl

Med H1/2: 10.4 days

Q5 H1/2: 8.2 days

Q95 H1/2: 12.9 days

reliable model fit: yes

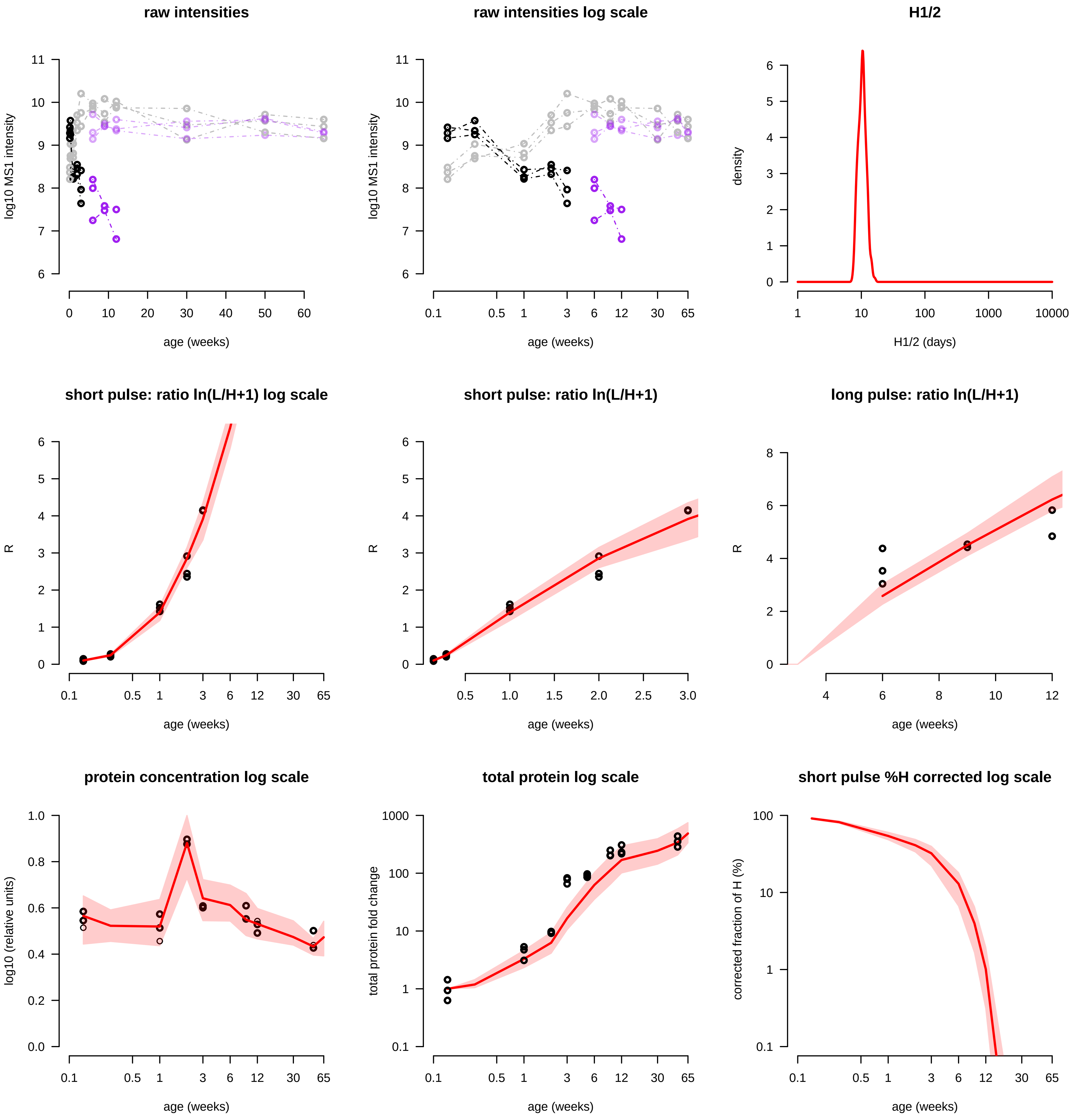

Q9D1R9

>sp|Q9D1R9|RL34_MOUSE

60S ribosomal protein L34

GN=Rpl34

Med H1/2: 9 days

Q5 H1/2: 7.4 days

Q95 H1/2: 11 days

reliable model fit: yes

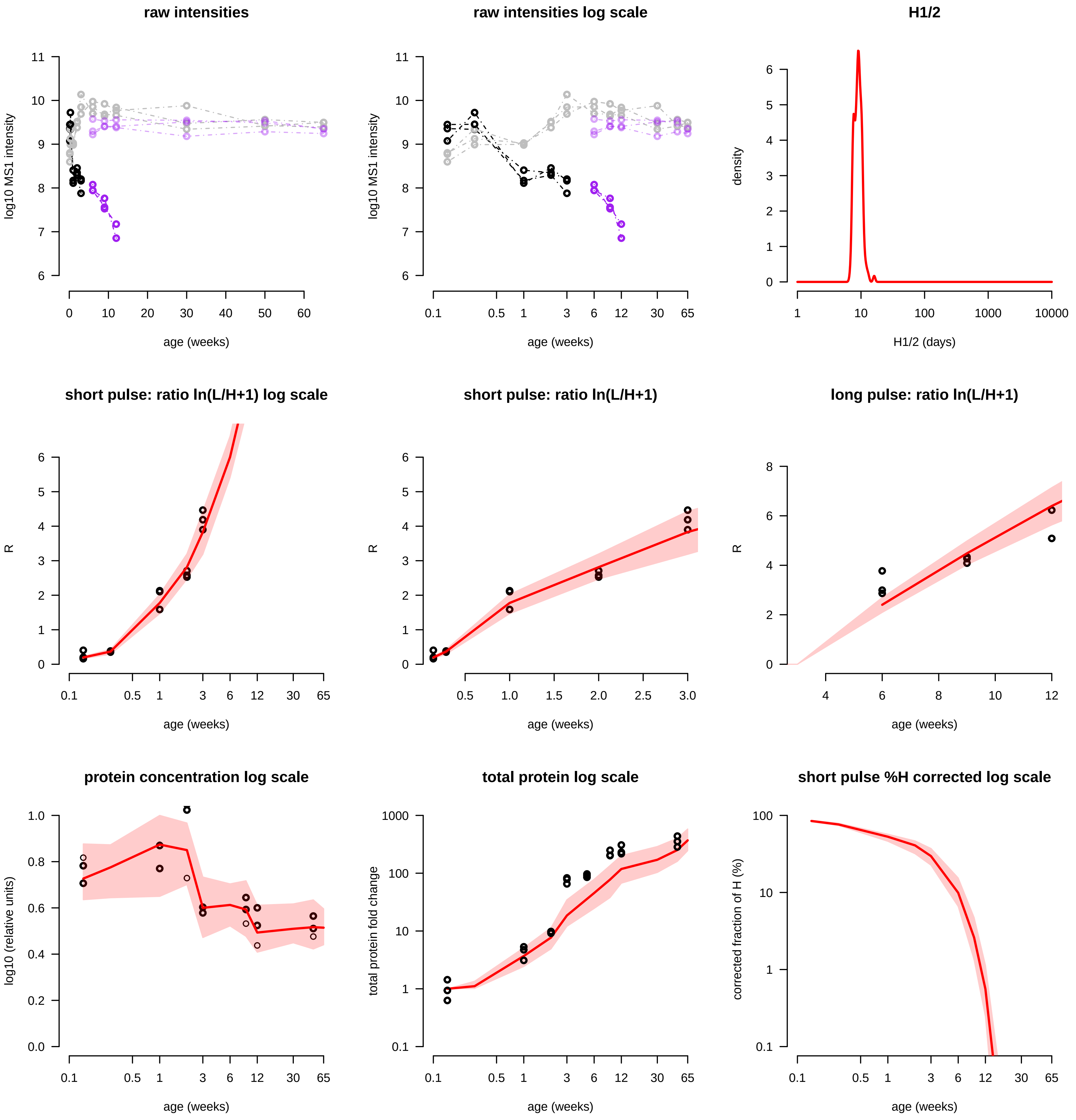

Q69ZN7;Q69ZN7−4

>sp|Q69ZN7|MYOF_MOUSE

Myoferlin

GN=Myof

Med H1/2: 10.9 days

Q5 H1/2: 8.3 days

Q95 H1/2: 14.1 days

reliable model fit: yes

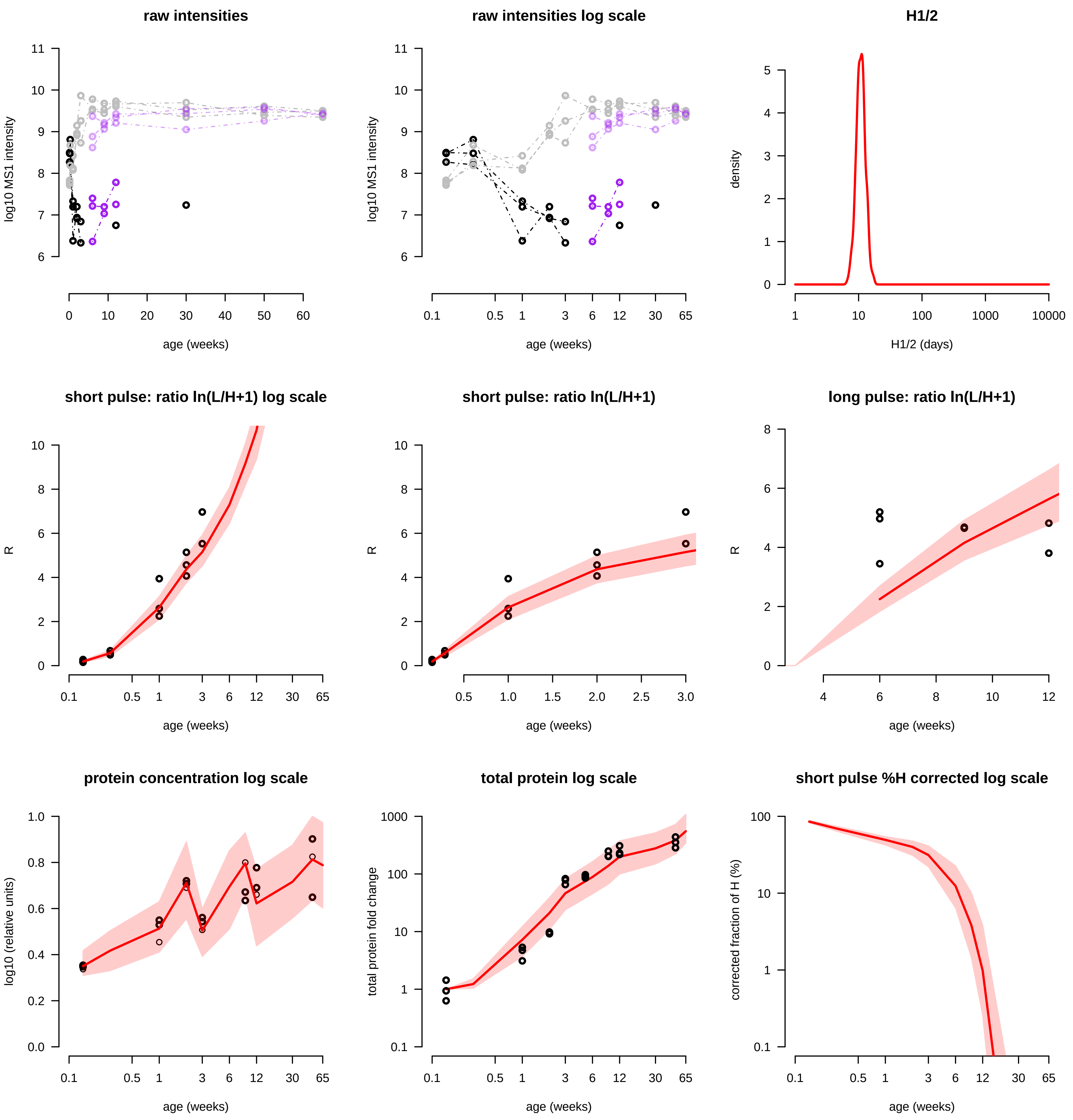

Q9DBF1−2

>sp|Q9DBF1−2|AL7A1_MOUSE

Isoform 2 of Alpha−aminoadipic semialdehyde dehydrogenase

GN=Aldh7a1

Med H1/2: 11.9 days

Q5 H1/2: 8.3 days

Q95 H1/2: 22.2 days

reliable model fit: no

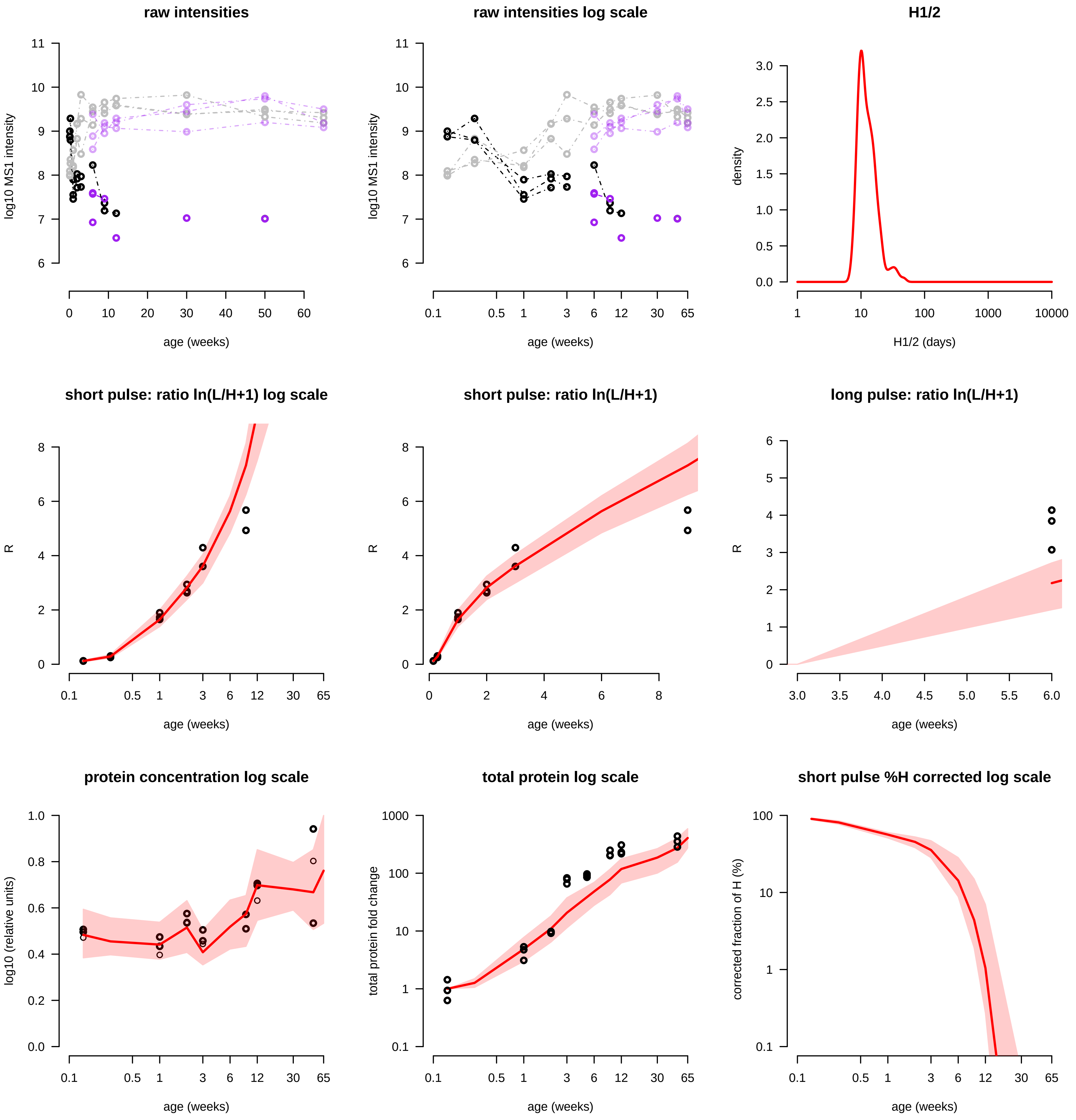

Q9D855

>sp|Q9D855|QCR7_MOUSE

Cytochrome b−c1 complex subunit 7

GN=Uqcrb

Med H1/2: 11.1 days

Q5 H1/2: 8.8 days

Q95 H1/2: 15.4 days

reliable model fit: yes

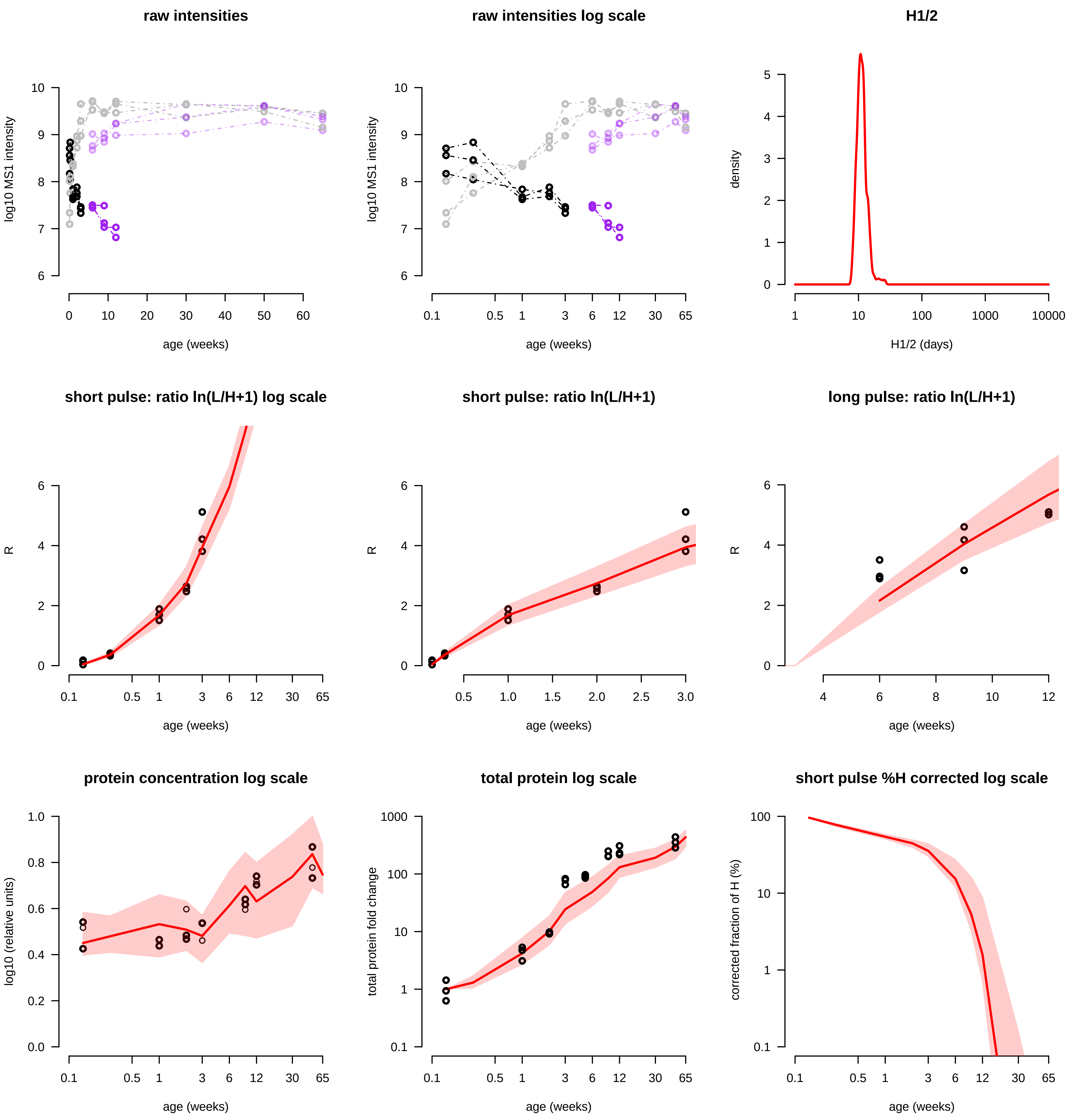

P68373

>sp|P68373|TBA1C_MOUSE

Tubulin alpha−1C chain

GN=Tuba1c

Med H1/2: 28 days

Q5 H1/2: 7.9 days

Q95 H1/2: 286.5 days

reliable model fit: yes

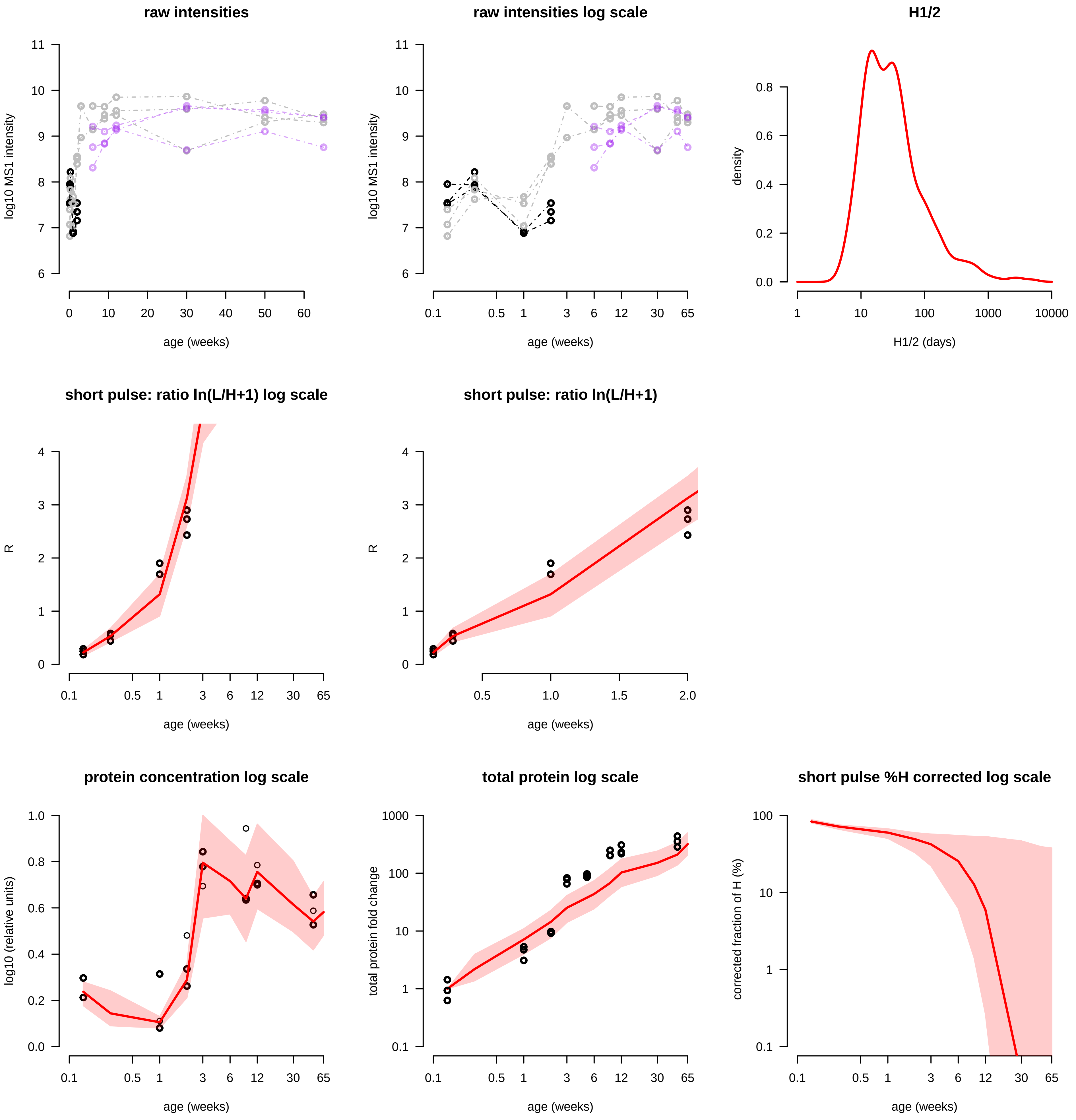

Q62318;Q62318−2

>sp|Q62318|TIF1B_MOUSE

Transcription intermediary factor 1−beta

GN=Trim28

Med H1/2: 572.7 days

Q5 H1/2: 106 days

Q95 H1/2: 5550.5 days

reliable model fit: yes

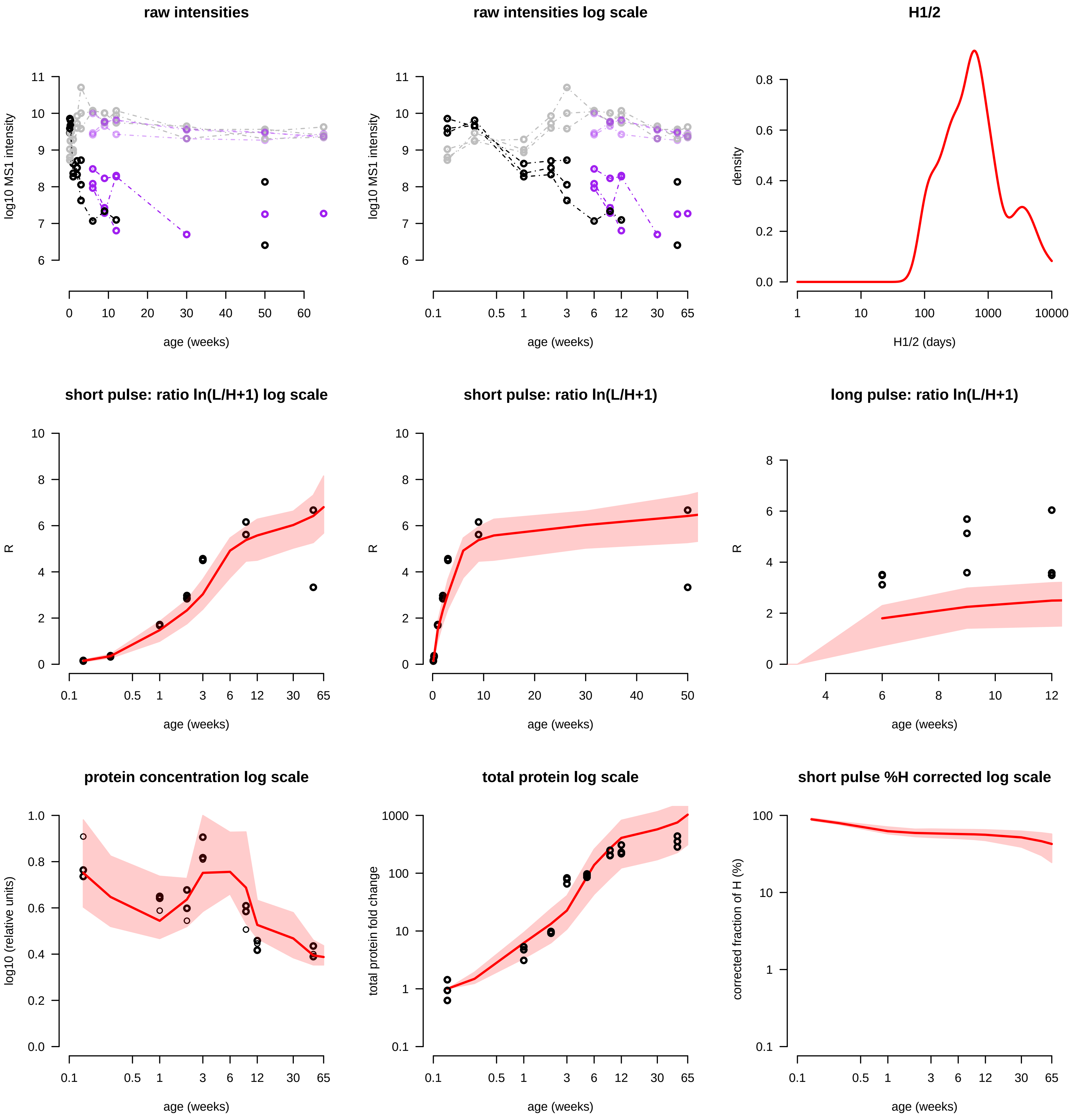

P52825

>sp|P52825|CPT2_MOUSE

Carnitine O−palmitoyltransferase 2, mitochondrial

GN=Cpt2

Med H1/2: 216.9 days

Q5 H1/2: 50.1 days

Q95 H1/2: 3616.9 days

reliable model fit: no

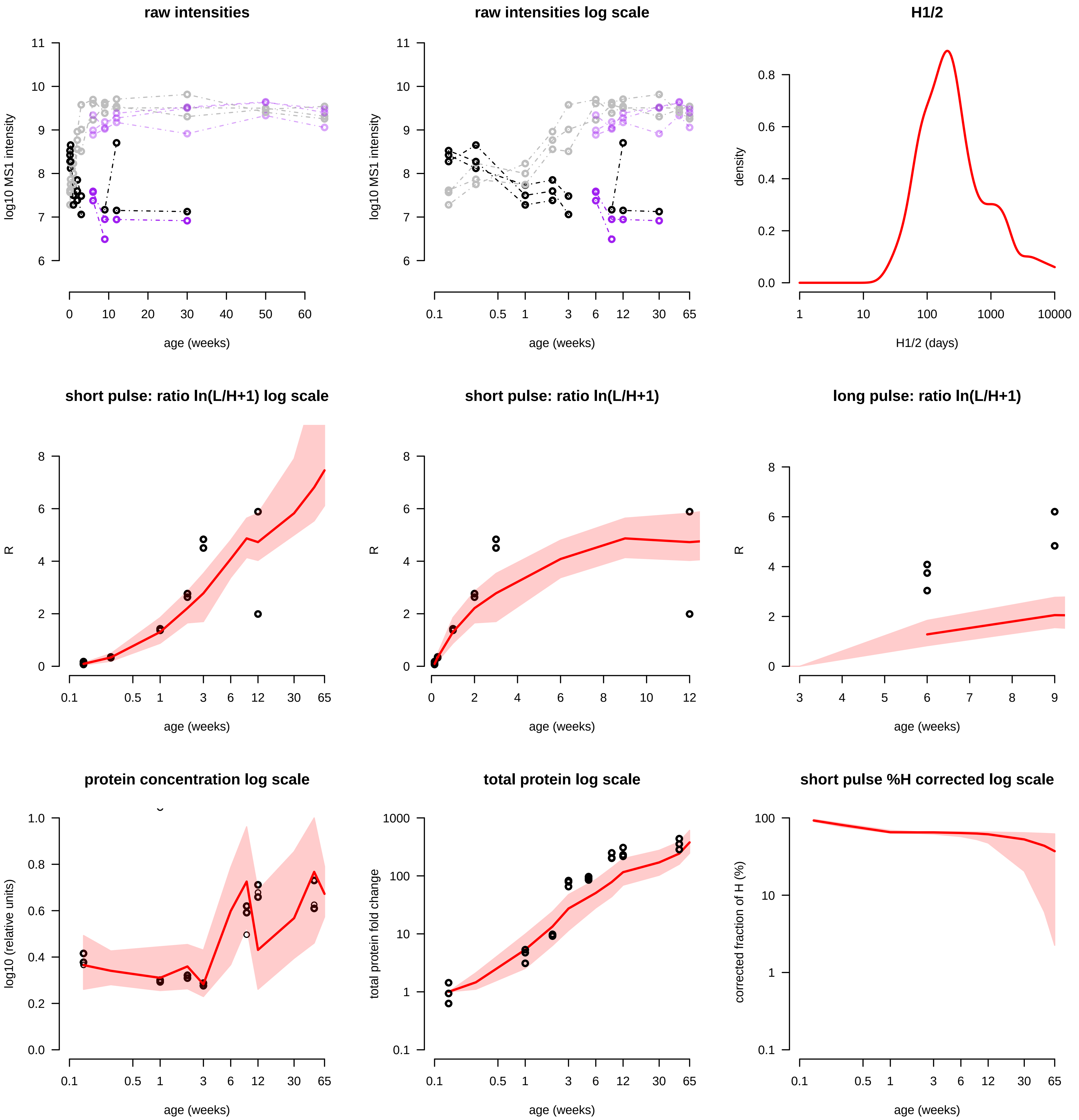

Q8R1I1

>sp|Q8R1I1|QCR9_MOUSE

Cytochrome b−c1 complex subunit 9

GN=Uqcr10

Med H1/2: 9.6 days

Q5 H1/2: 6.9 days

Q95 H1/2: 13.4 days

reliable model fit: yes

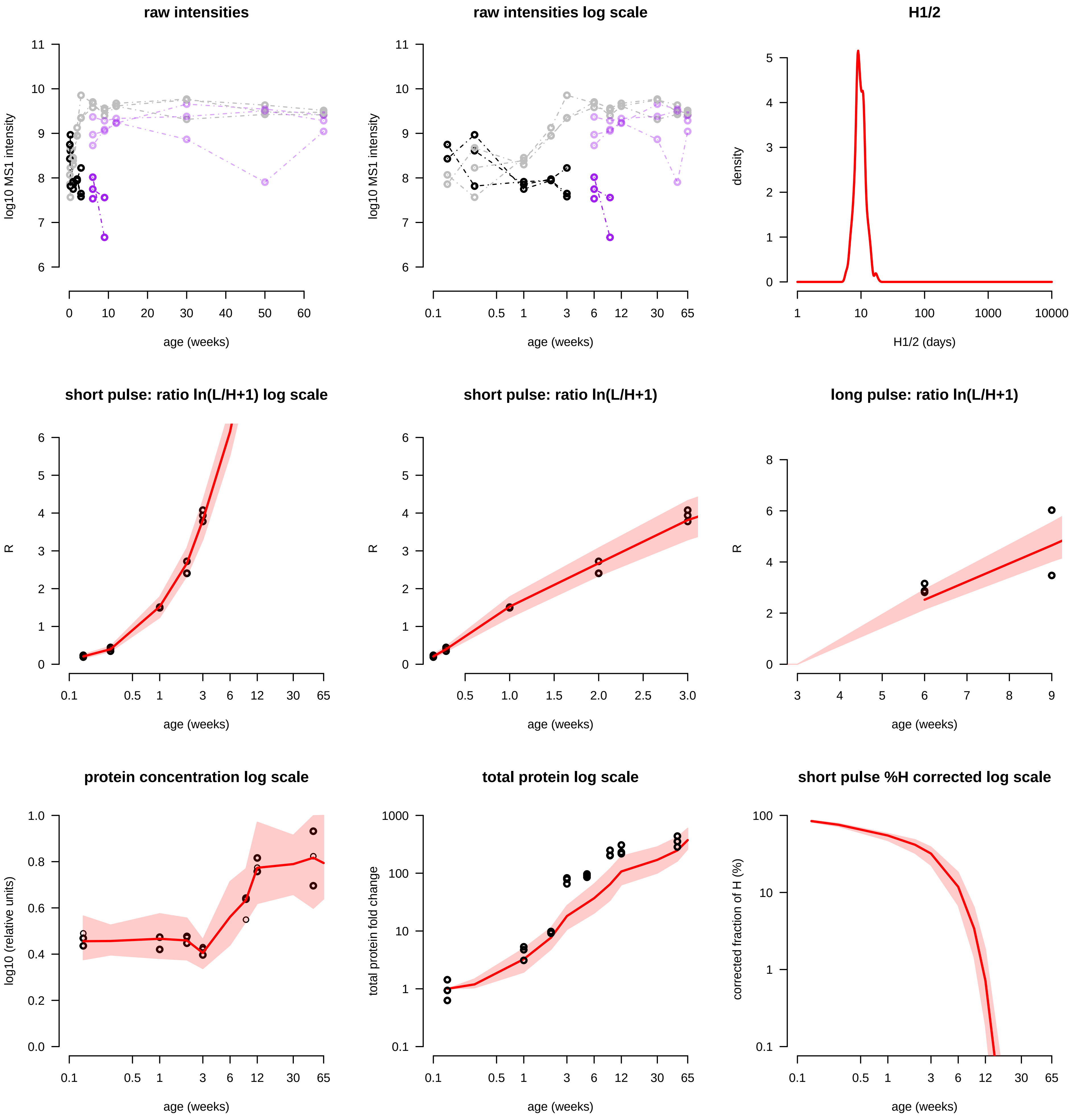

Q8C1B7−3;Q8C1B7−2;Q8C1B7

>sp|Q8C1B7−3|SEP11_MOUSE

Isoform 3 of Septin−11

GN=Septin11

Med H1/2: 14.3 days

Q5 H1/2: 9.9 days

Q95 H1/2: 27.4 days

reliable model fit: no

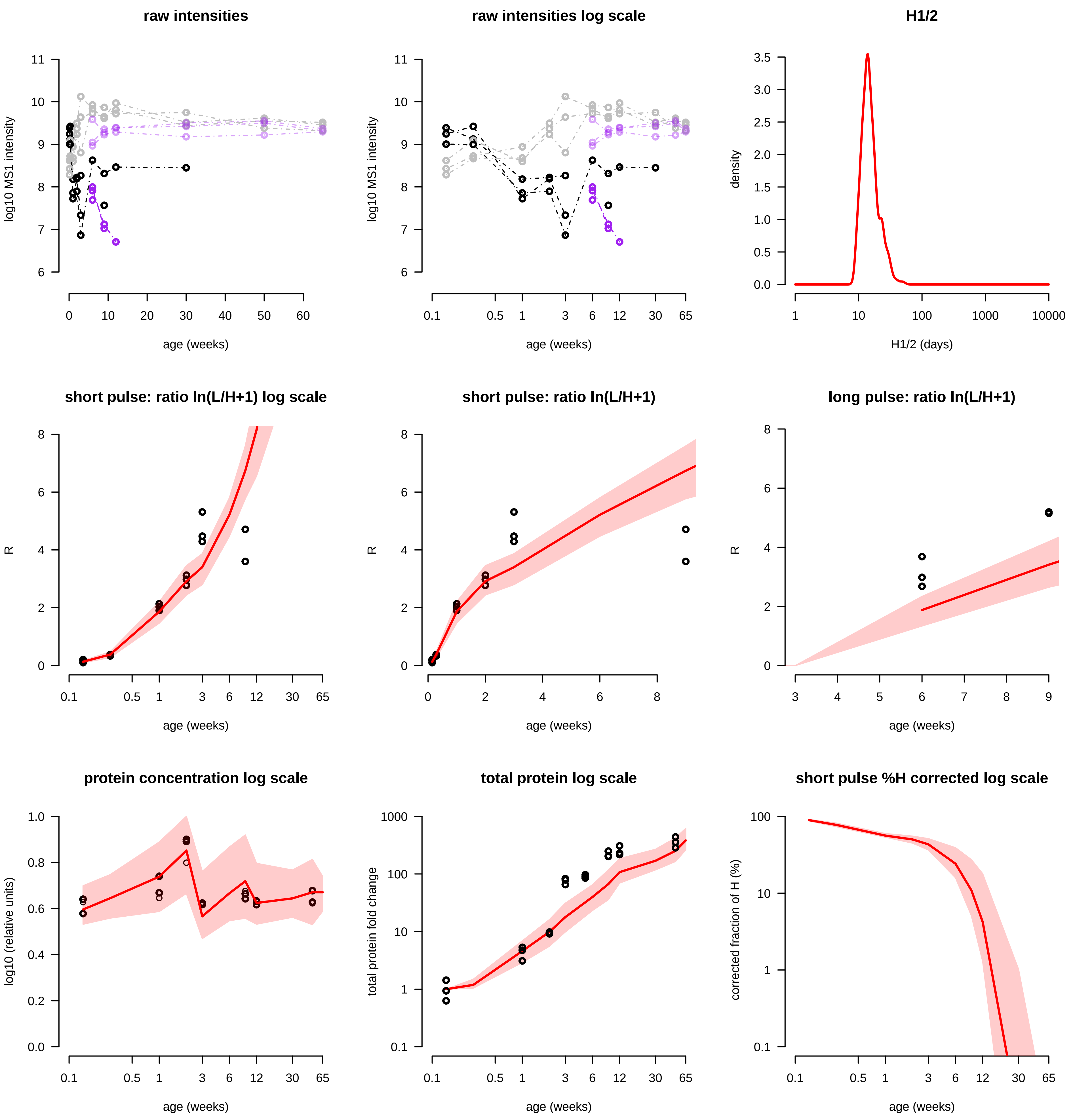

P68037

>sp|P68037|UB2L3_MOUSE

Ubiquitin−conjugating enzyme E2 L3

GN=Ube2l3

Med H1/2: 145.6 days

Q5 H1/2: 76.4 days

Q95 H1/2: 319.3 days

reliable model fit: yes

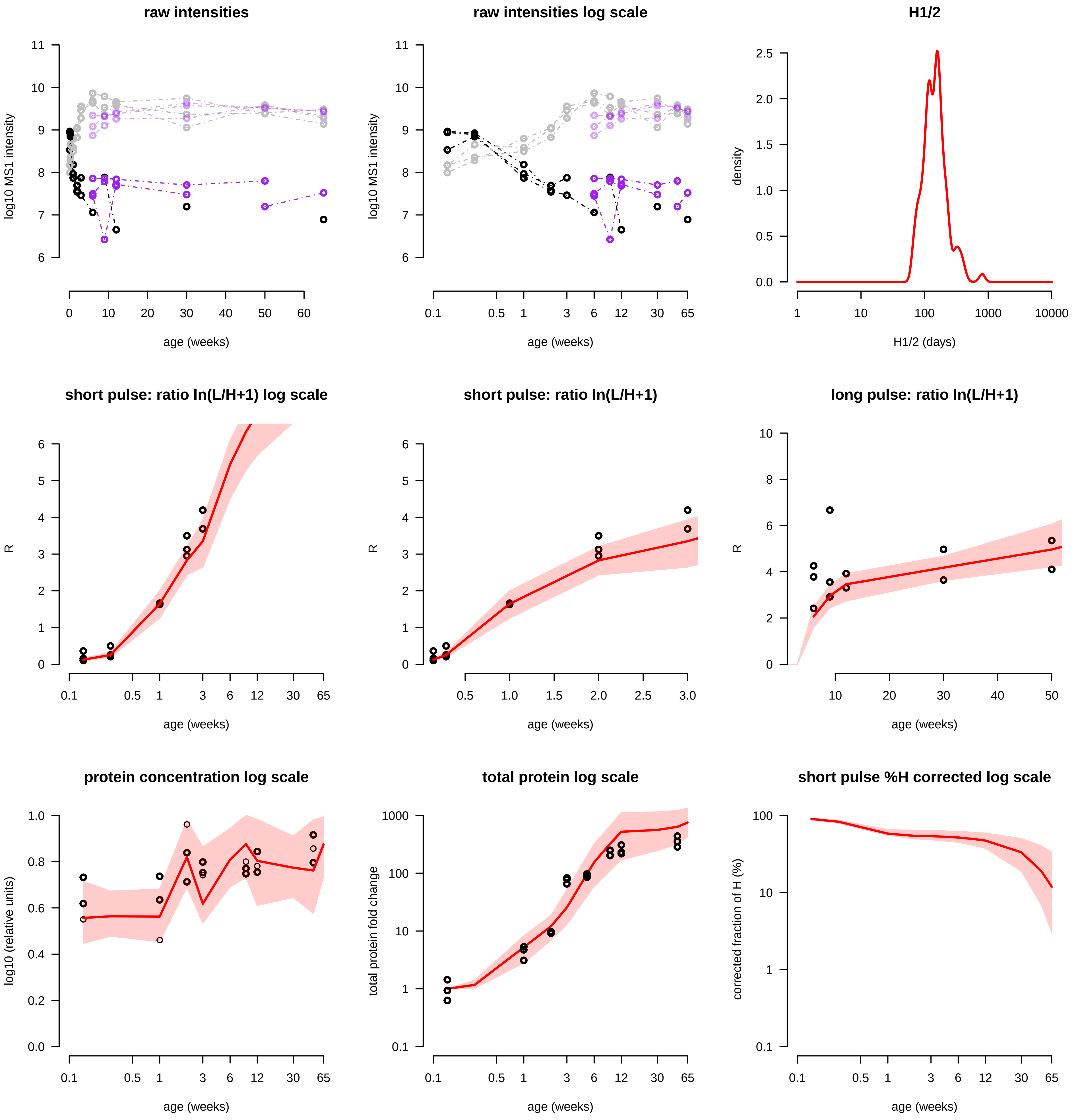

P08752

>sp|P08752|GNAI2_MOUSE

Guanine nucleotide−binding protein G(i) subunit alpha−2

GN=Gnai2

Med H1/2: 10.3 days

Q5 H1/2: 7.9 days

Q95 H1/2: 13.1 days

reliable model fit: yes

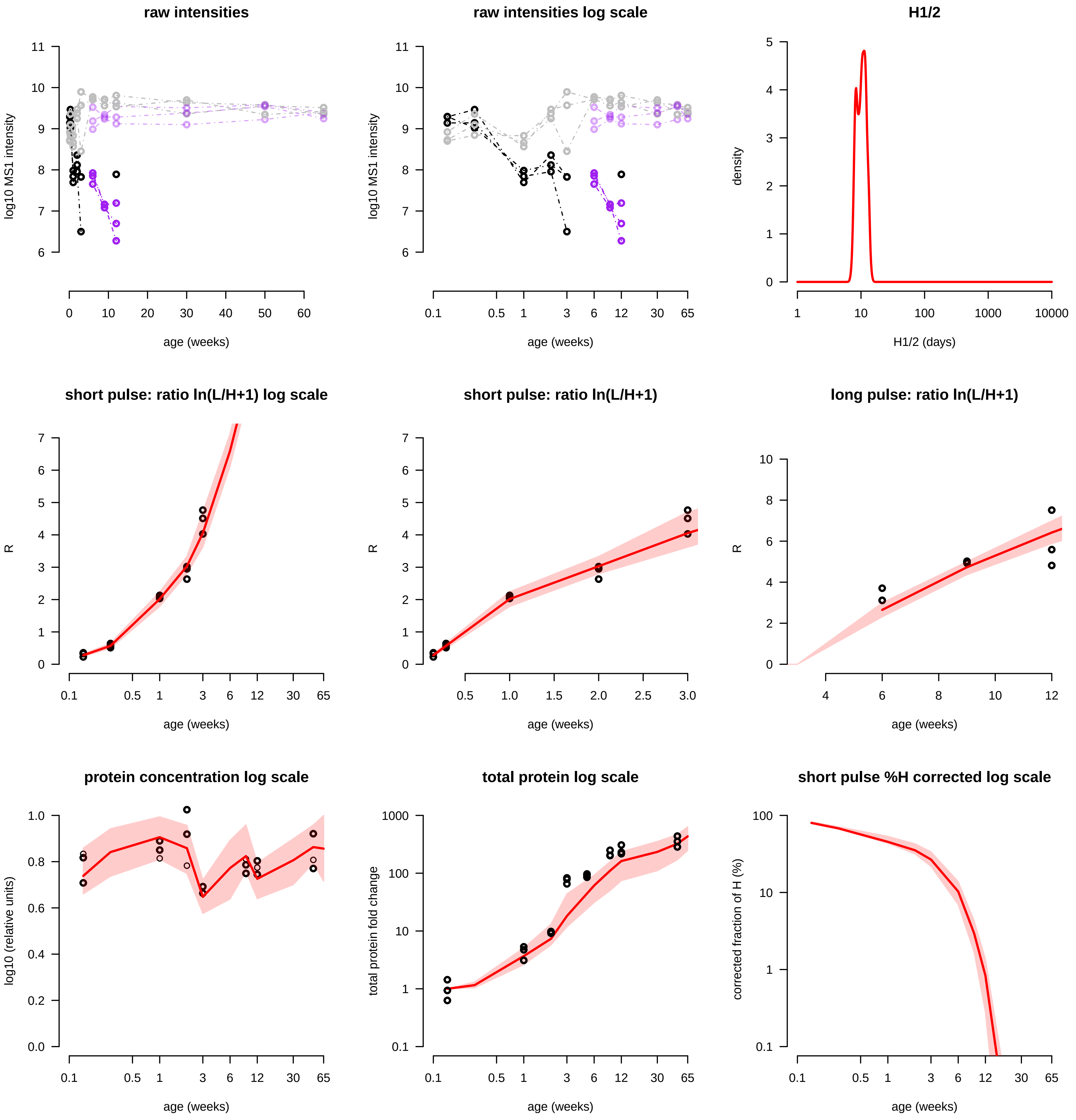

Q99L13

>sp|Q99L13|3HIDH_MOUSE

3−hydroxyisobutyrate dehydrogenase, mitochondrial

GN=Hibadh

Med H1/2: 15.8 days

Q5 H1/2: 11.8 days

Q95 H1/2: 26.4 days

reliable model fit: yes

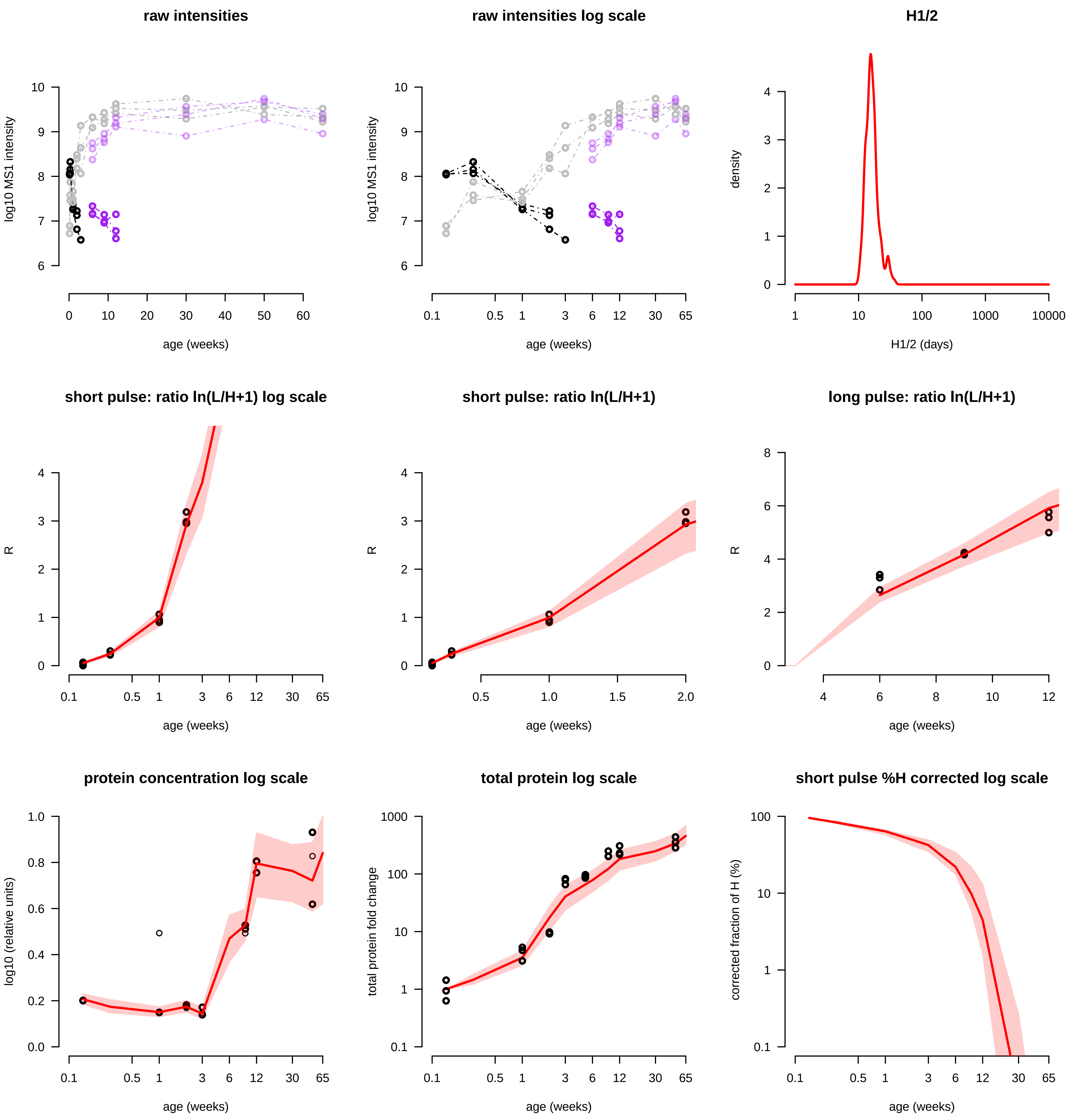

P62874

>sp|P62874|GBB1_MOUSE

Guanine nucleotide−binding protein G(I)/G(S)/G(T) subunit beta−1

GN=Gnb1

Med H1/2: 10.2 days

Q5 H1/2: 8.2 days

Q95 H1/2: 14.3 days

reliable model fit: yes

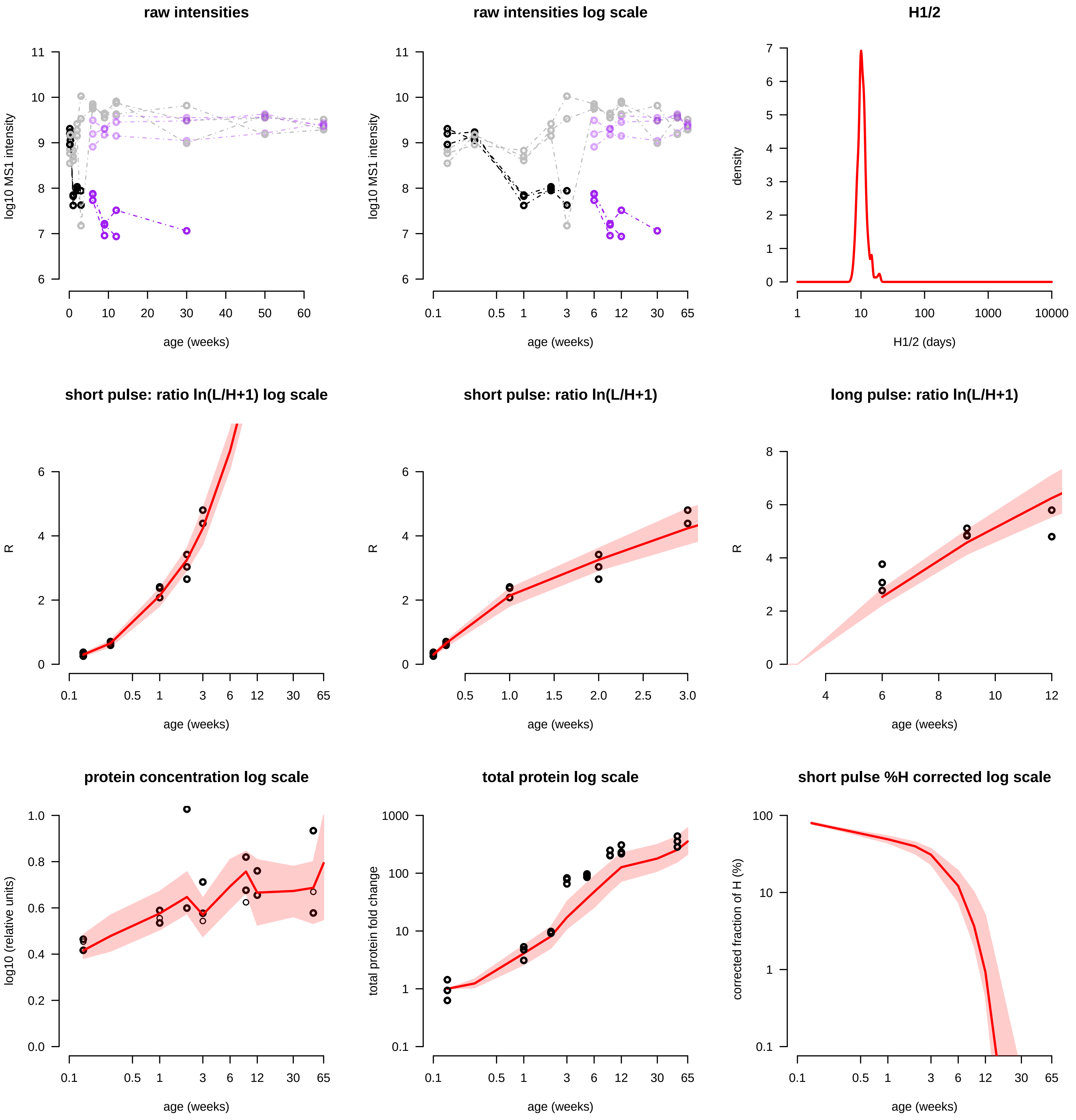

P43275

>sp|P43275|H11_MOUSE

Histone H1.1

GN=H1−1

Med H1/2: 11.5 days

Q5 H1/2: 9.8 days

Q95 H1/2: 16.5 days

reliable model fit: yes

raw intensities

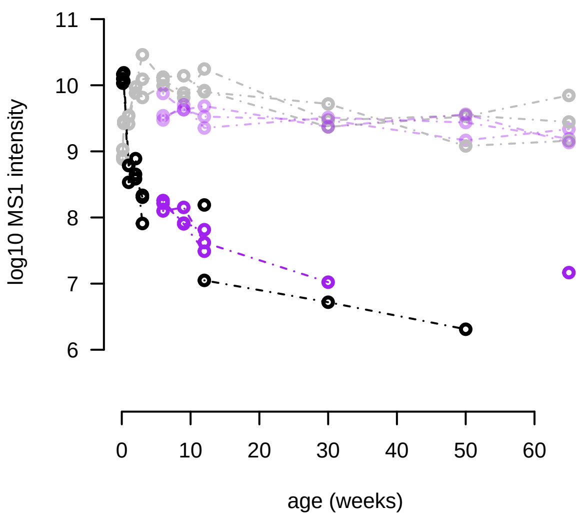

raw intensities log scale

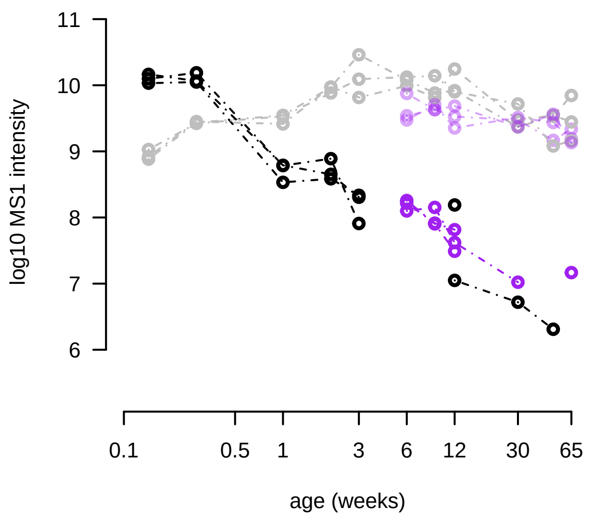

H1/2

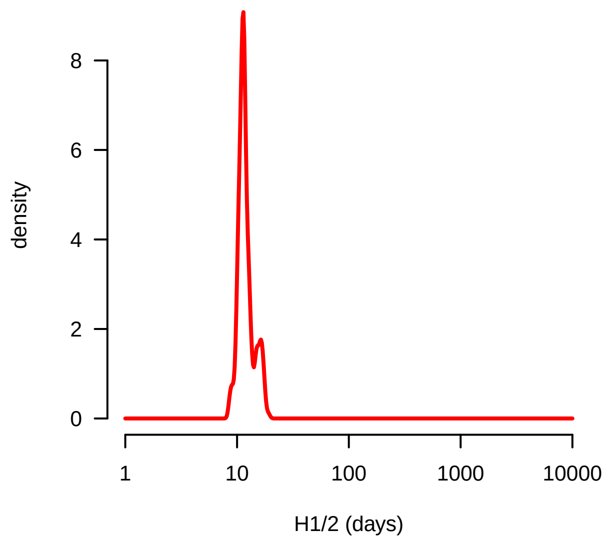

short pulse: ratio ln(L/H+1) log scale

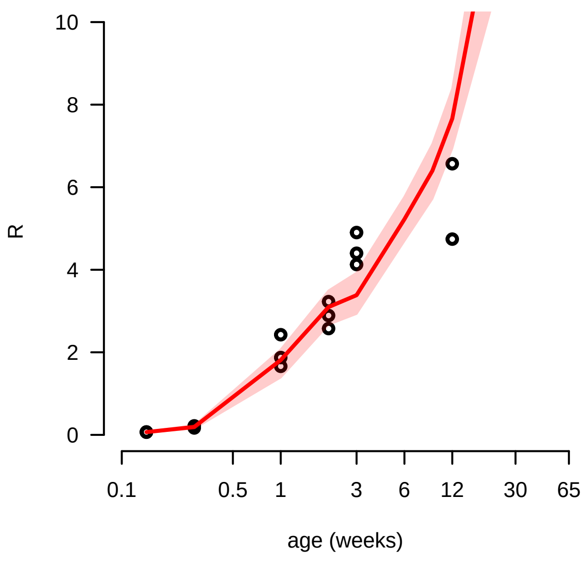

short pulse: ratio ln(L/H+1)

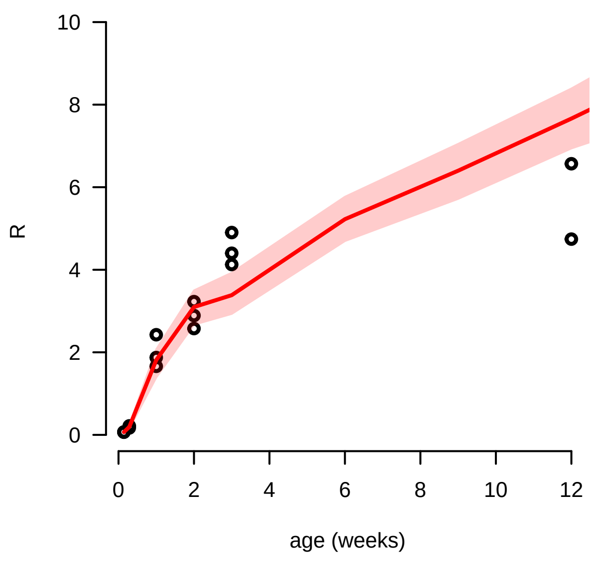

long pulse: ratio ln(L/H+1)

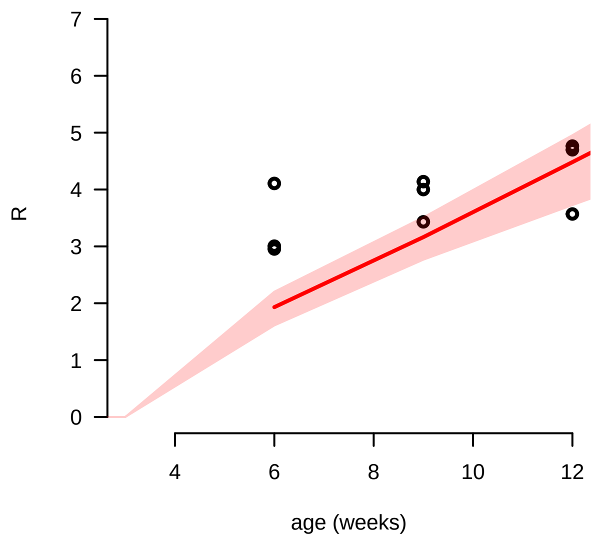

protein concentration log scale

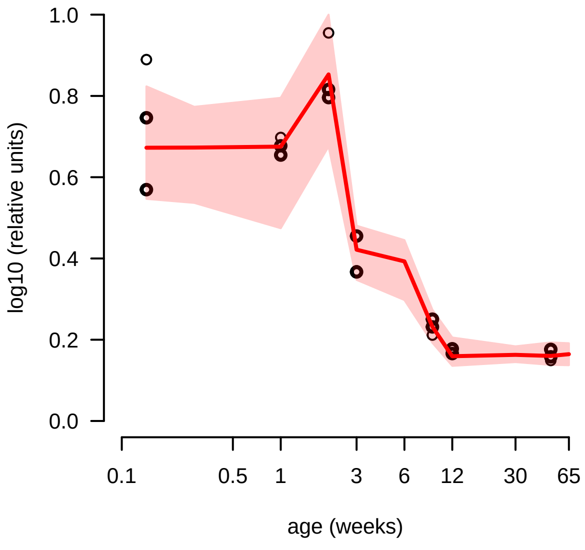

total protein log scale

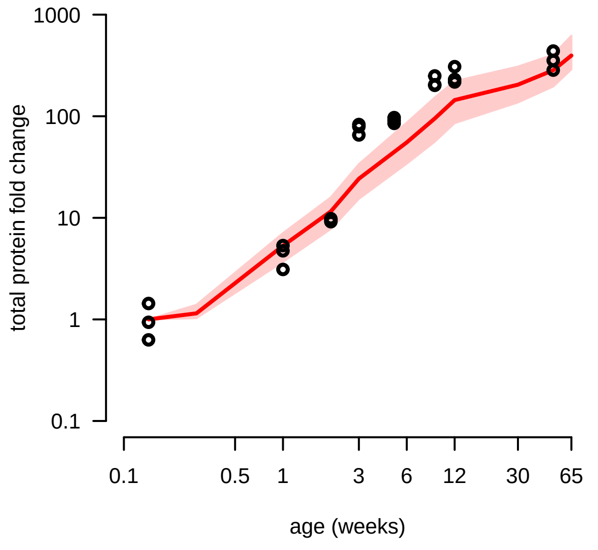

short pulse %H corrected log scale

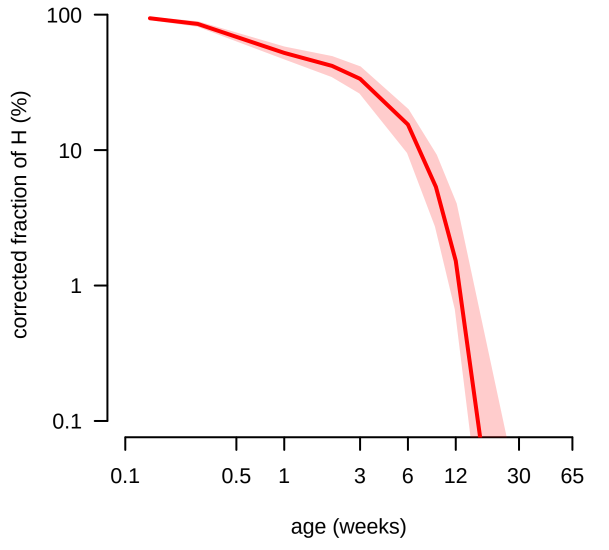

Q9D2G2

>sp|Q9D2G2|ODO2_MOUSE

−residue succinyltransferase component of 2−oxoglutarate dehydrogenase com

GN=Dlst

Med H1/2: 11.1 days

Q5 H1/2: 8.4 days

Q95 H1/2: 15.6 days

reliable model fit: yes

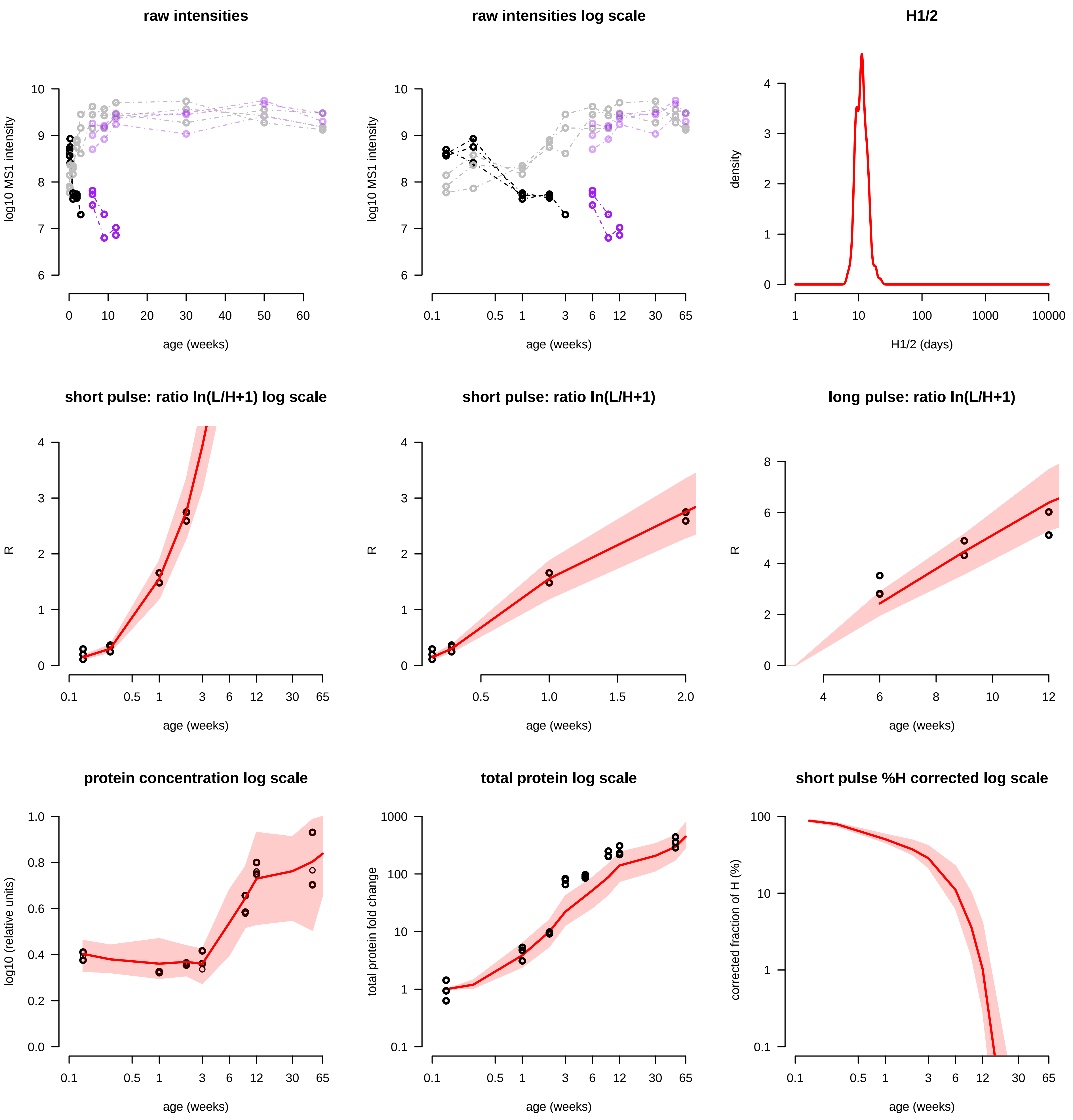

P60766

>sp|P60766|CDC42_MOUSE

Cell division control protein 42 homolog

GN=Cdc42

Med H1/2: 9 days

Q5 H1/2: 7.1 days

Q95 H1/2: 13.3 days

reliable model fit: yes

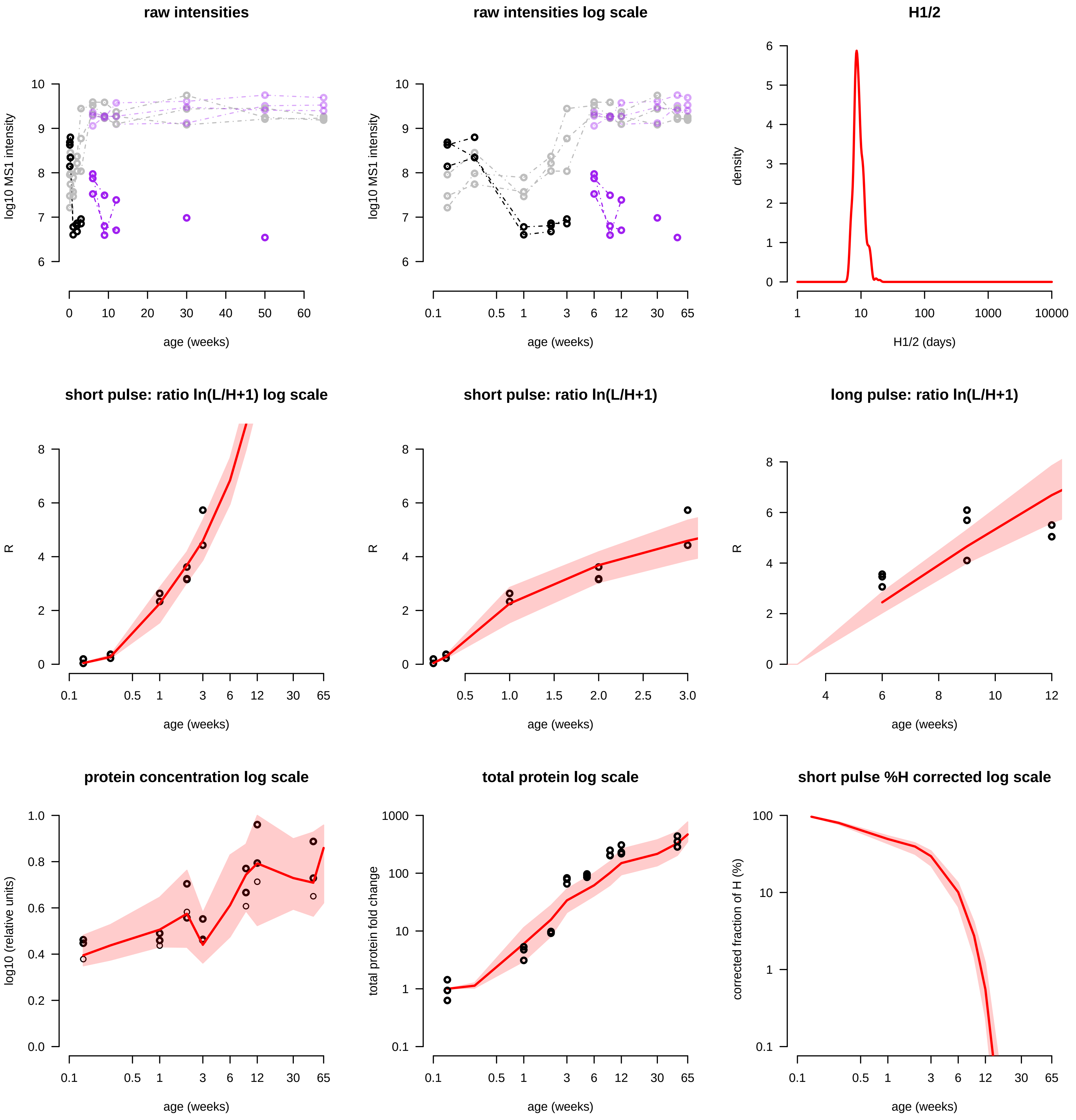

Q9WV32

>sp|Q9WV32|ARC1B_MOUSE

Actin−related protein 2/3 complex subunit 1B

GN=Arpc1b

Med H1/2: 7.4 days

Q5 H1/2: 6.4 days

Q95 H1/2: 8.8 days

reliable model fit: yes

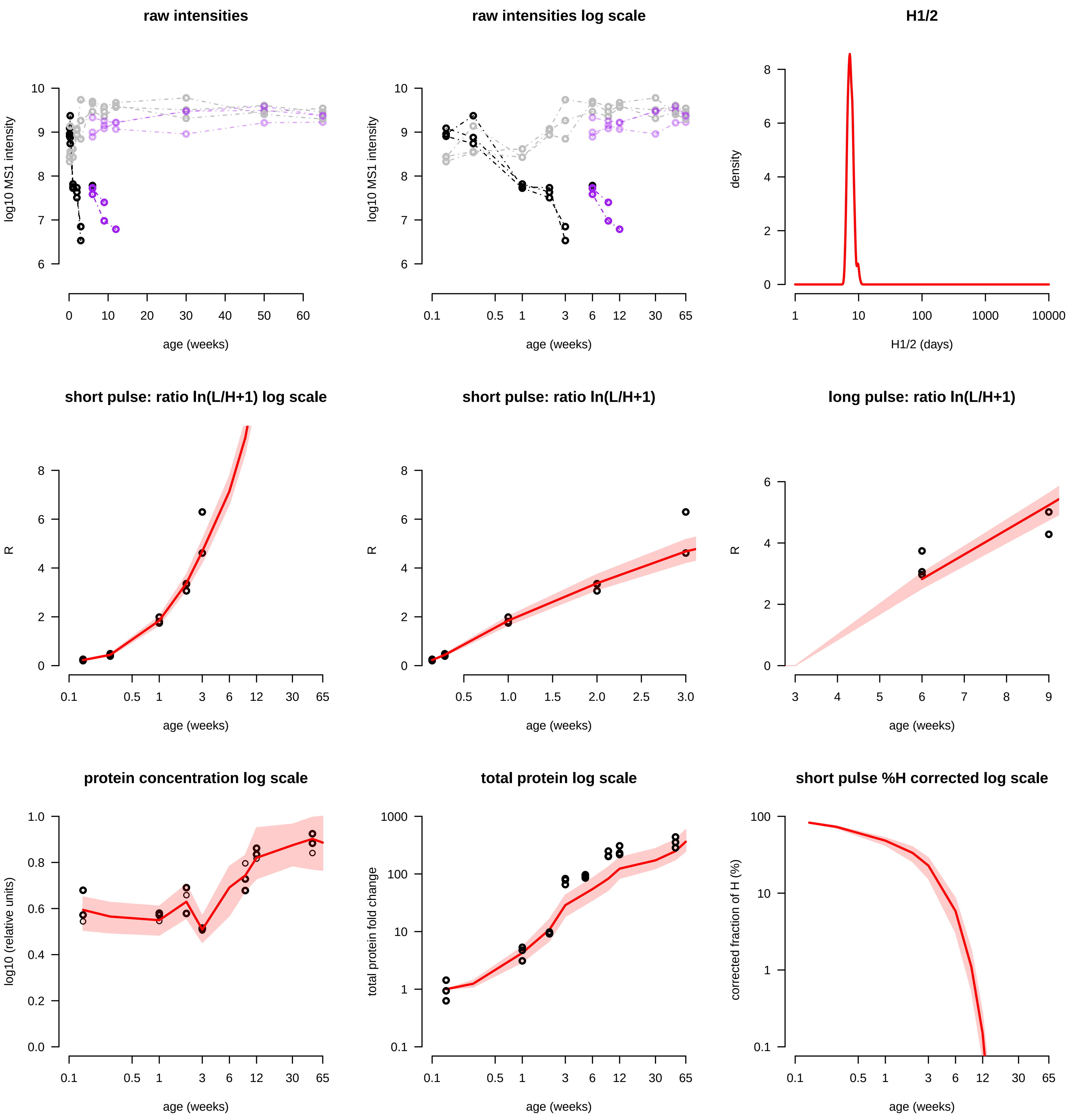

Q3UMF0−3;Q3UMF0

>sp|Q3UMF0−3|COBL1_MOUSE

Isoform 3 of Cordon−bleu protein−like 1

GN=Cobll1

Med H1/2: 3.4 days

Q5 H1/2: 2.6 days

Q95 H1/2: 6 days

reliable model fit: yes

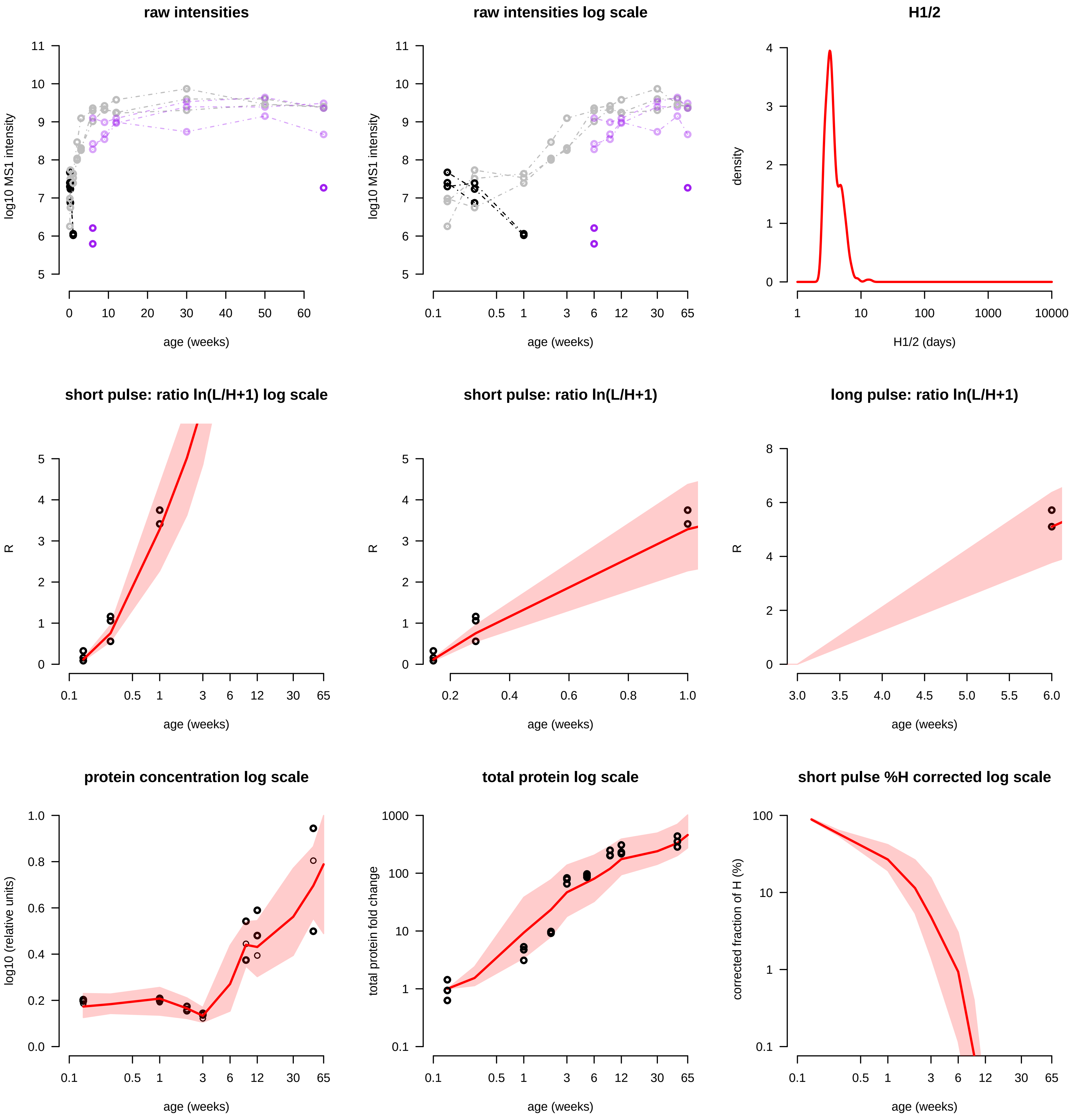

O55029

>sp|O55029|COPB2_MOUSE

Coatomer subunit beta'

GN=Copb2

Med H1/2: 8.3 days

Q5 H1/2: 6.8 days

Q95 H1/2: 10.1 days

reliable model fit: yes

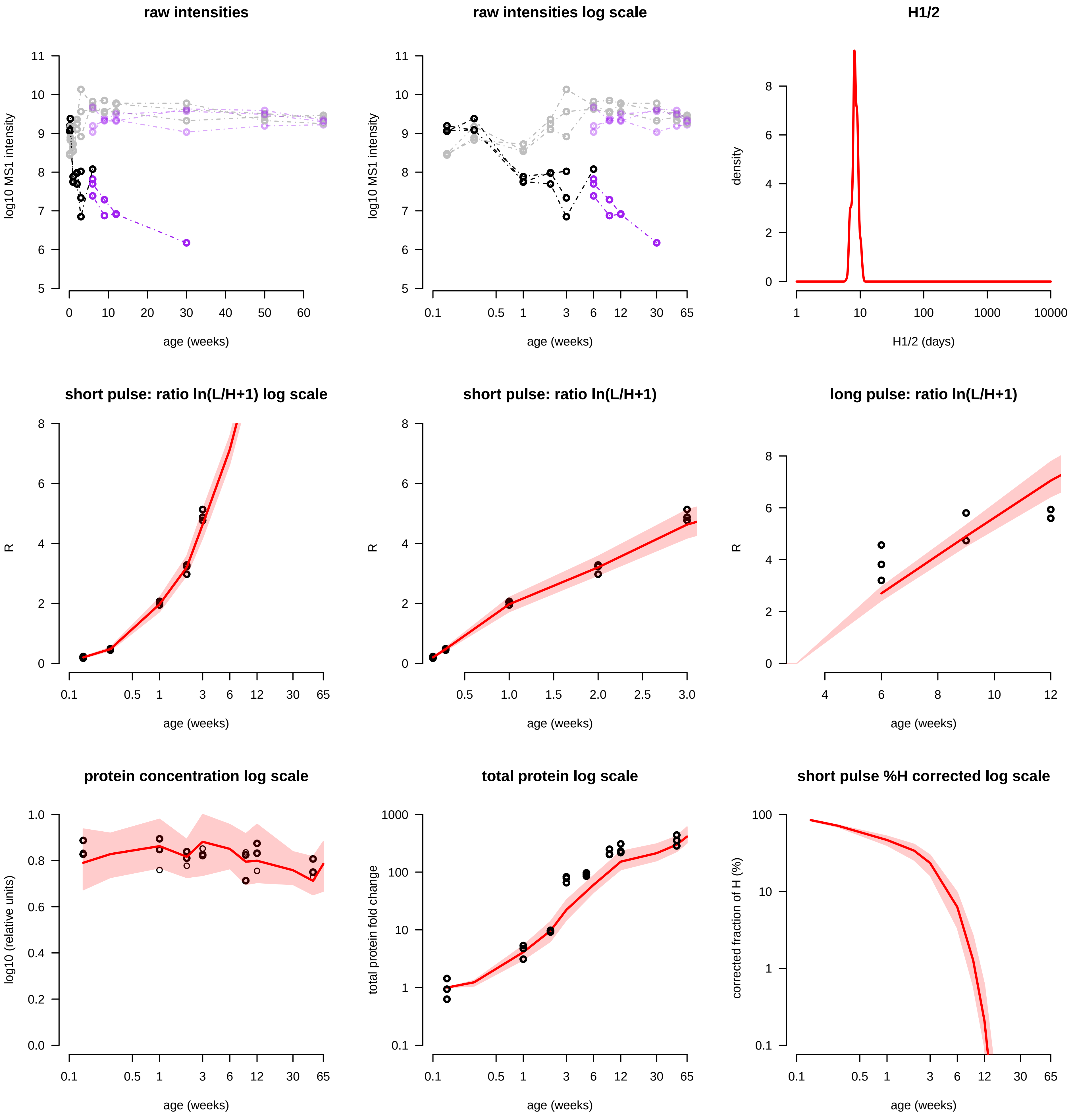

Q5XJY5

>sp|Q5XJY5|COPD_MOUSE

Coatomer subunit delta

GN=Arcn1

Med H1/2: 5.3 days

Q5 H1/2: 4.5 days

Q95 H1/2: 6.4 days

reliable model fit: yes

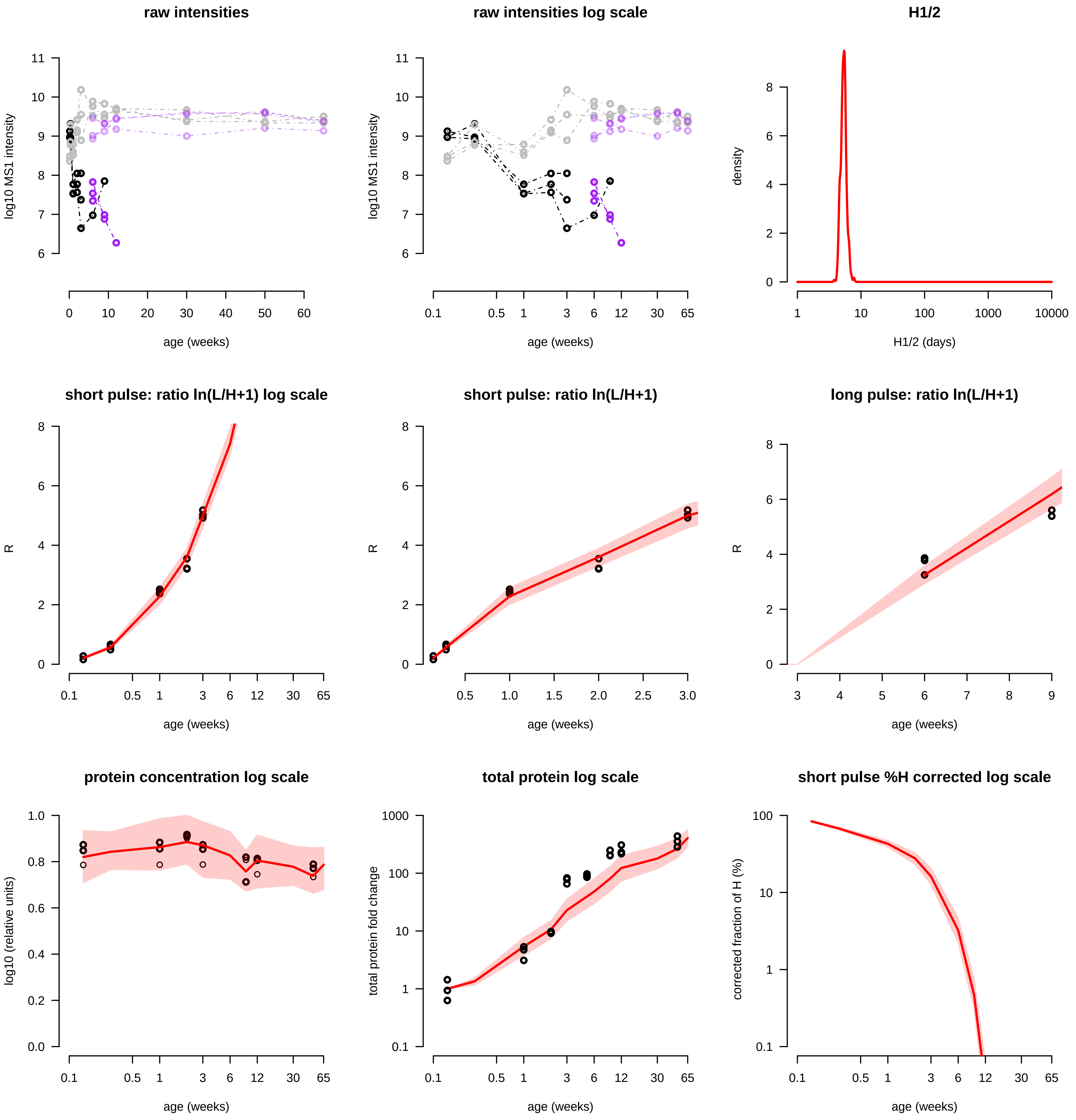

P05201

>sp|P05201|AATC_MOUSE

Aspartate aminotransferase, cytoplasmic

GN=Got1

Med H1/2: 8.9 days

Q5 H1/2: 7 days

Q95 H1/2: 10.7 days

reliable model fit: yes

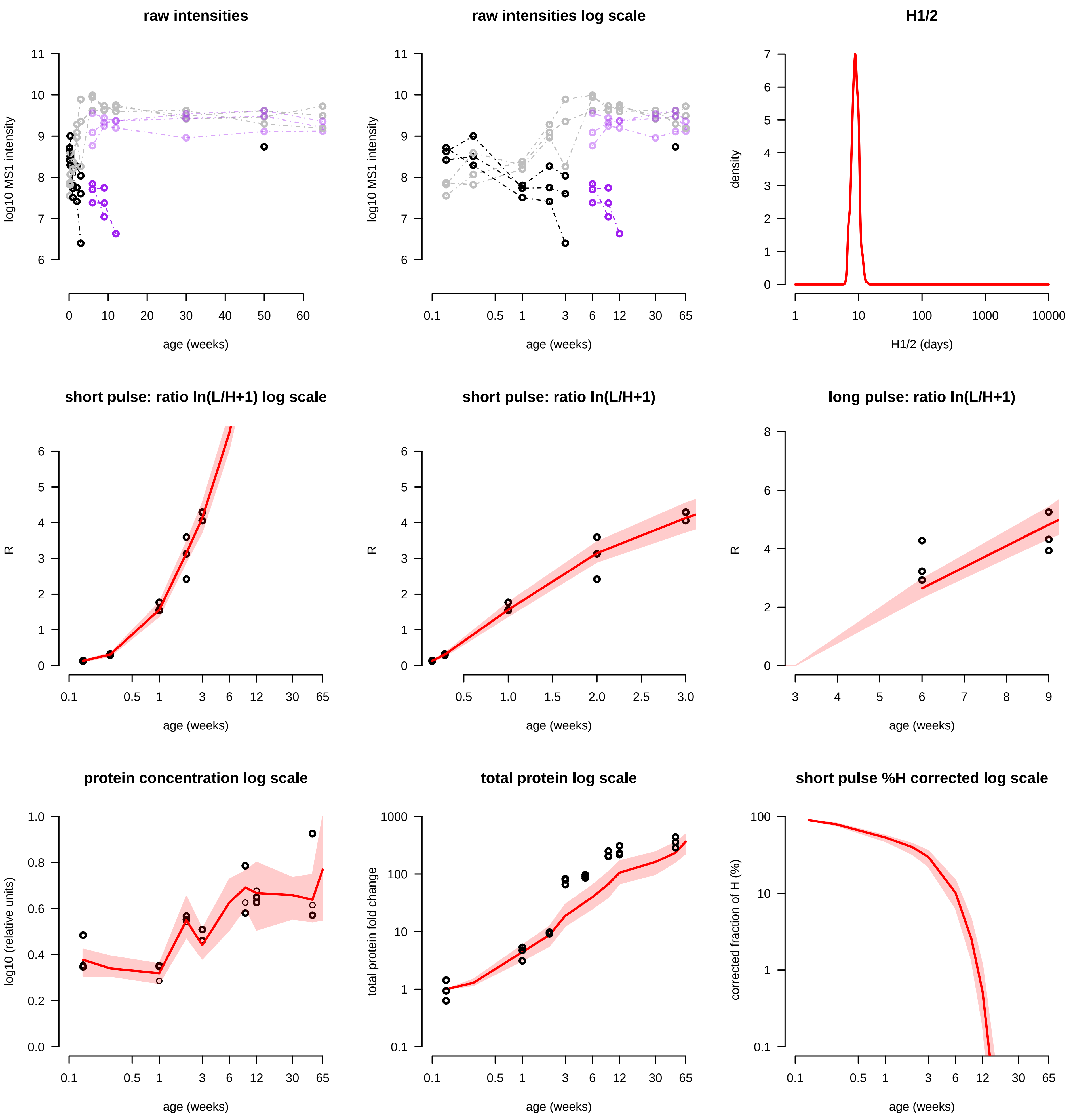

P62754

>sp|P62754|RS6_MOUSE

40S ribosomal protein S6

GN=Rps6

Med H1/2: 7.5 days

Q5 H1/2: 6.2 days

Q95 H1/2: 9.5 days

reliable model fit: yes

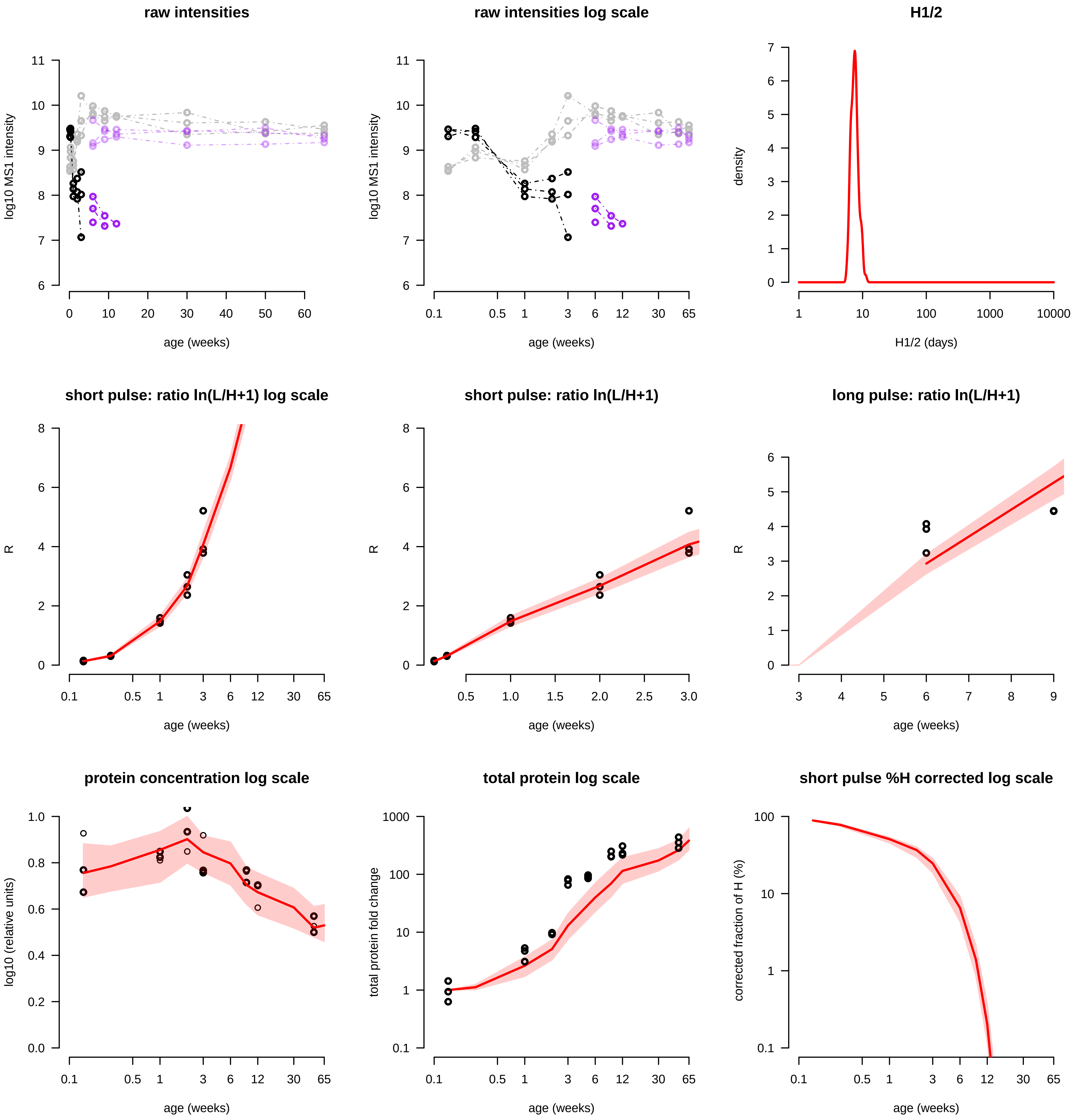

O54734

>sp|O54734|OST48_MOUSE

Dolichyl−diphosphooligosaccharide−−protein glycosyltransferase 48 kDa subuni

GN=Ddost

Med H1/2: 8.3 days

Q5 H1/2: 6.8 days

Q95 H1/2: 10.4 days

reliable model fit: yes

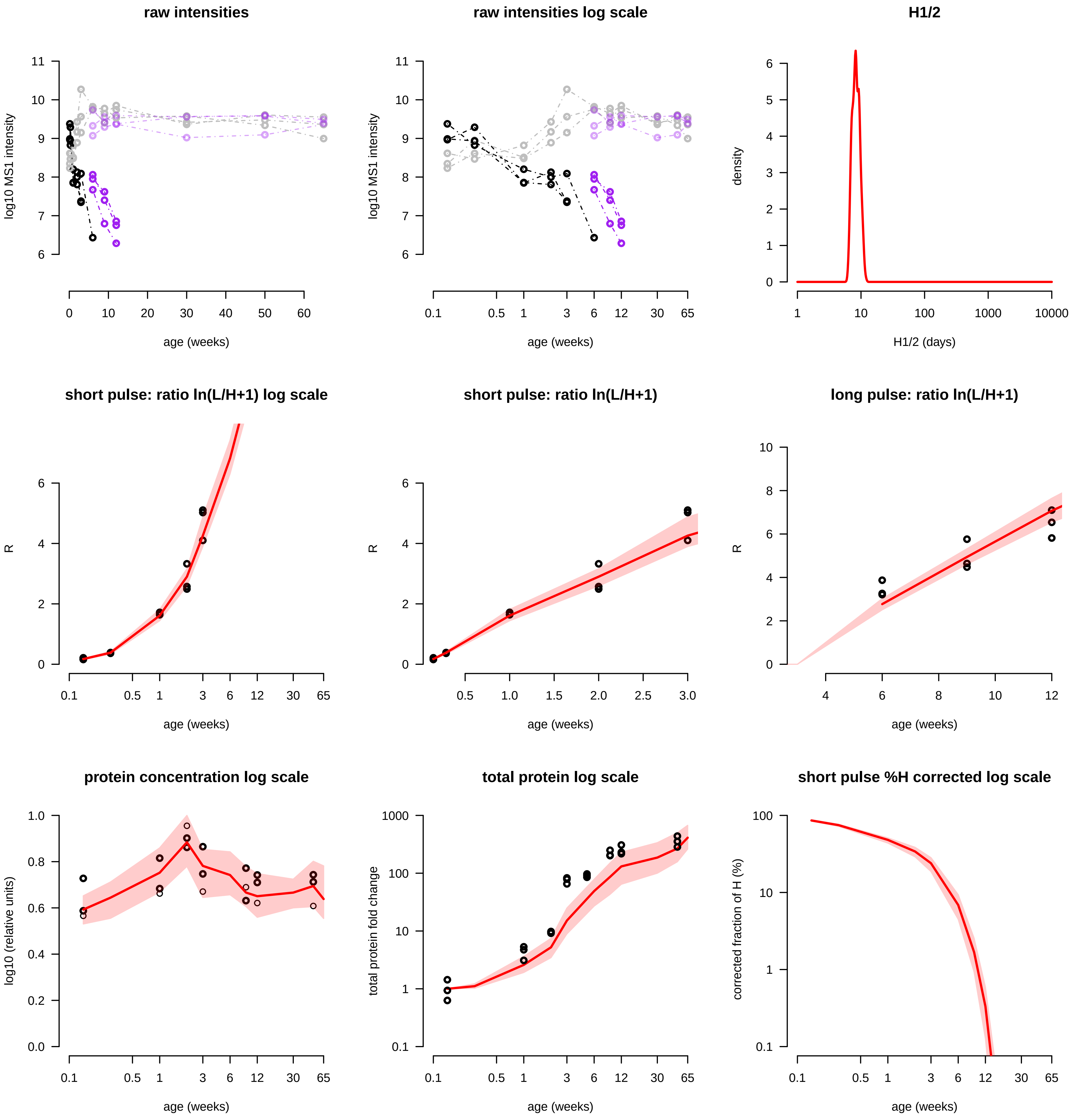

F6ZDS4

>sp|F6ZDS4|TPR_MOUSE

Nucleoprotein TPR

GN=Tpr

Med H1/2: 8.8 days

Q5 H1/2: 6.9 days

Q95 H1/2: 11.7 days

reliable model fit: no

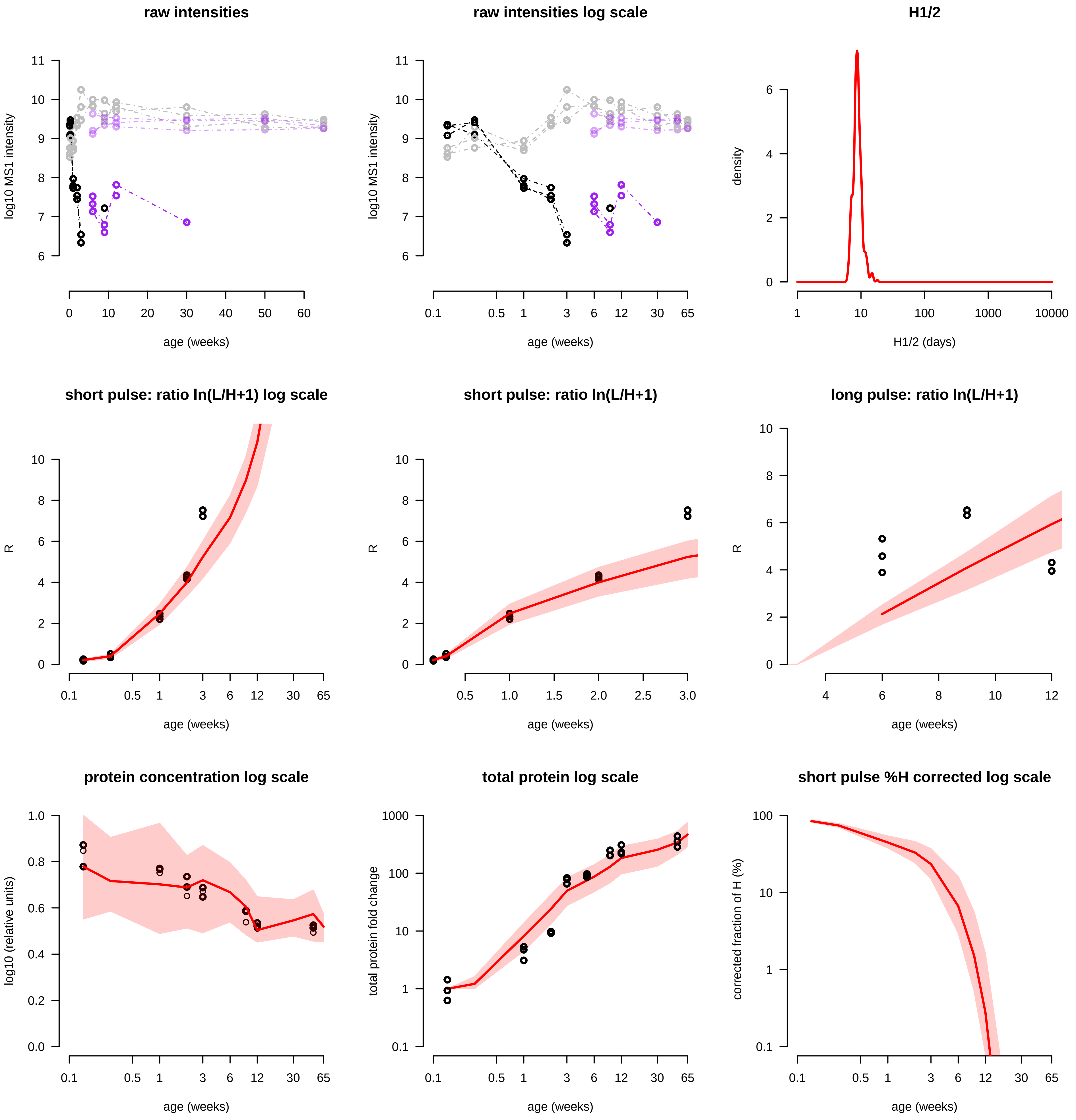

Q9QUJ7−2;Q9QUJ7

>sp|Q9QUJ7−2|ACSL4_MOUSE

Isoform Short of Long−chain−fatty−acid−−CoA ligase 4

GN=Acsl4

Med H1/2: 24 days

Q5 H1/2: 11.6 days

Q95 H1/2: 109.2 days

reliable model fit: yes

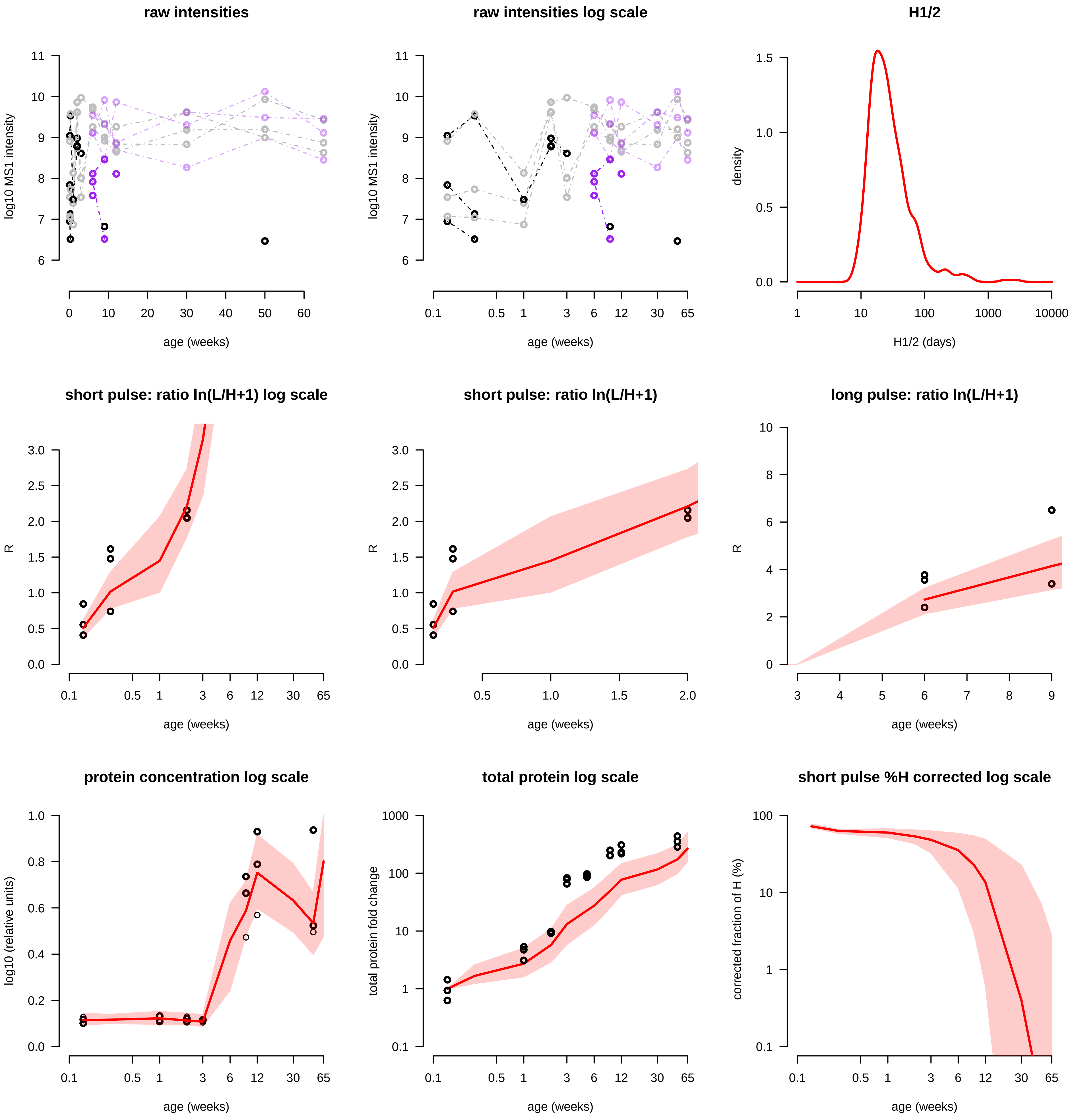

Q99JI6

>sp|Q99JI6|RAP1B_MOUSE

Ras−related protein Rap−1b

GN=Rap1b

Med H1/2: 10.9 days

Q5 H1/2: 8.9 days

Q95 H1/2: 14.3 days

reliable model fit: yes

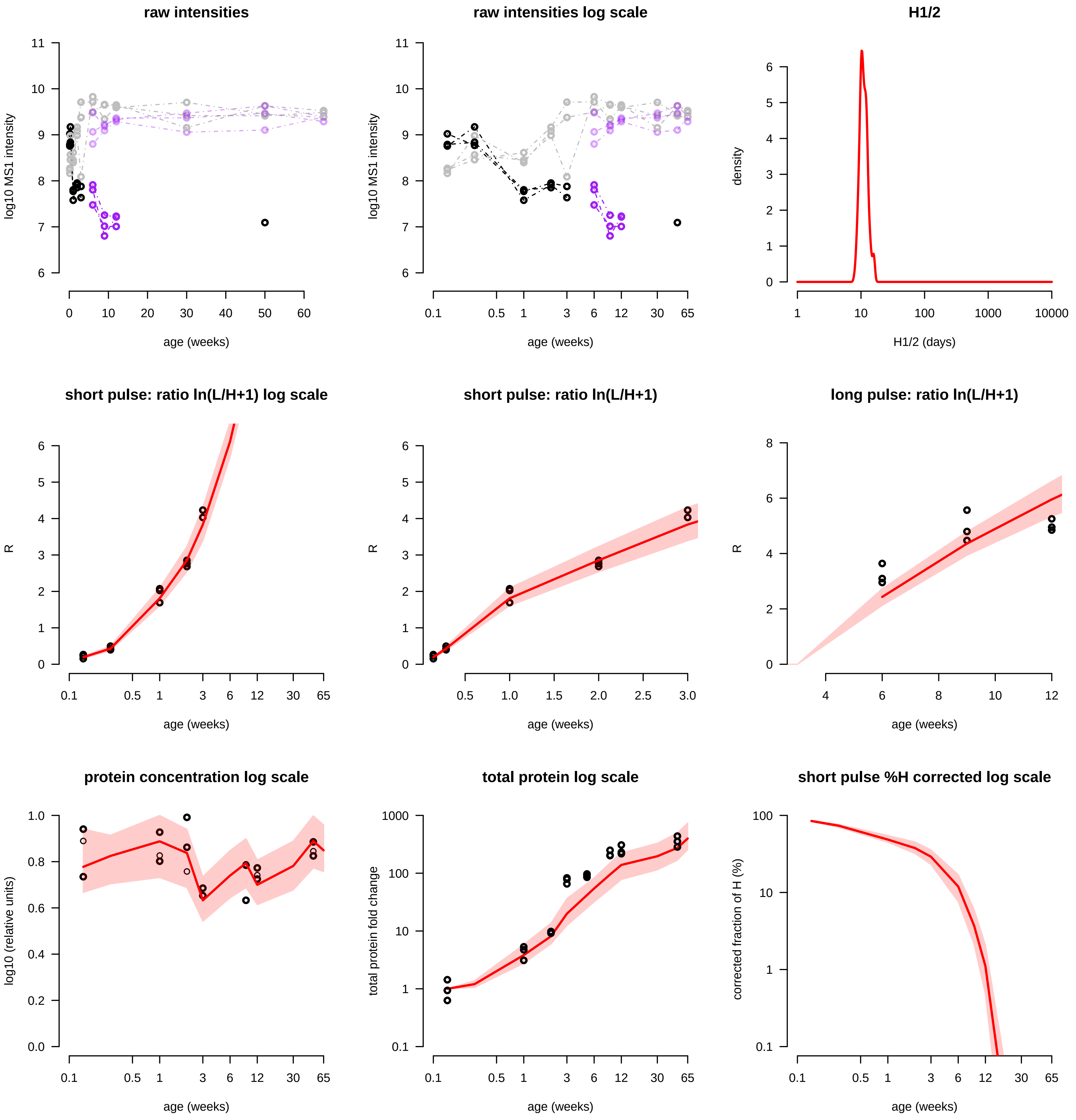

P57776;P57776−3

>sp|P57776|EF1D_MOUSE

Elongation factor 1−delta

GN=Eef1d

Med H1/2: 19.4 days

Q5 H1/2: 10.9 days

Q95 H1/2: 41.8 days

reliable model fit: yes

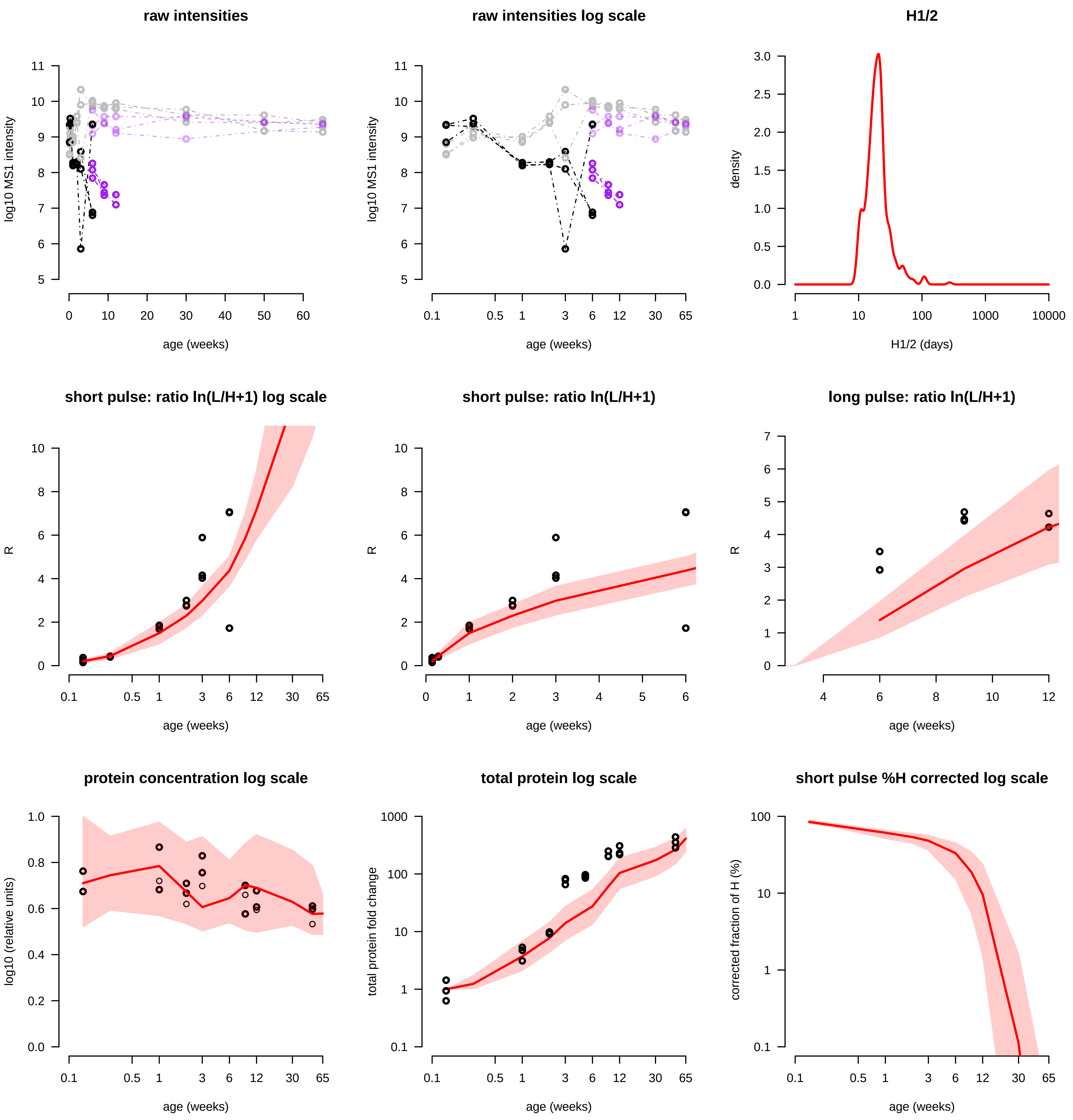

P62717

>sp|P62717|RL18A_MOUSE

60S ribosomal protein L18a

GN=Rpl18a

Med H1/2: 38.1 days

Q5 H1/2: 28.1 days

Q95 H1/2: 49.4 days

reliable model fit: yes

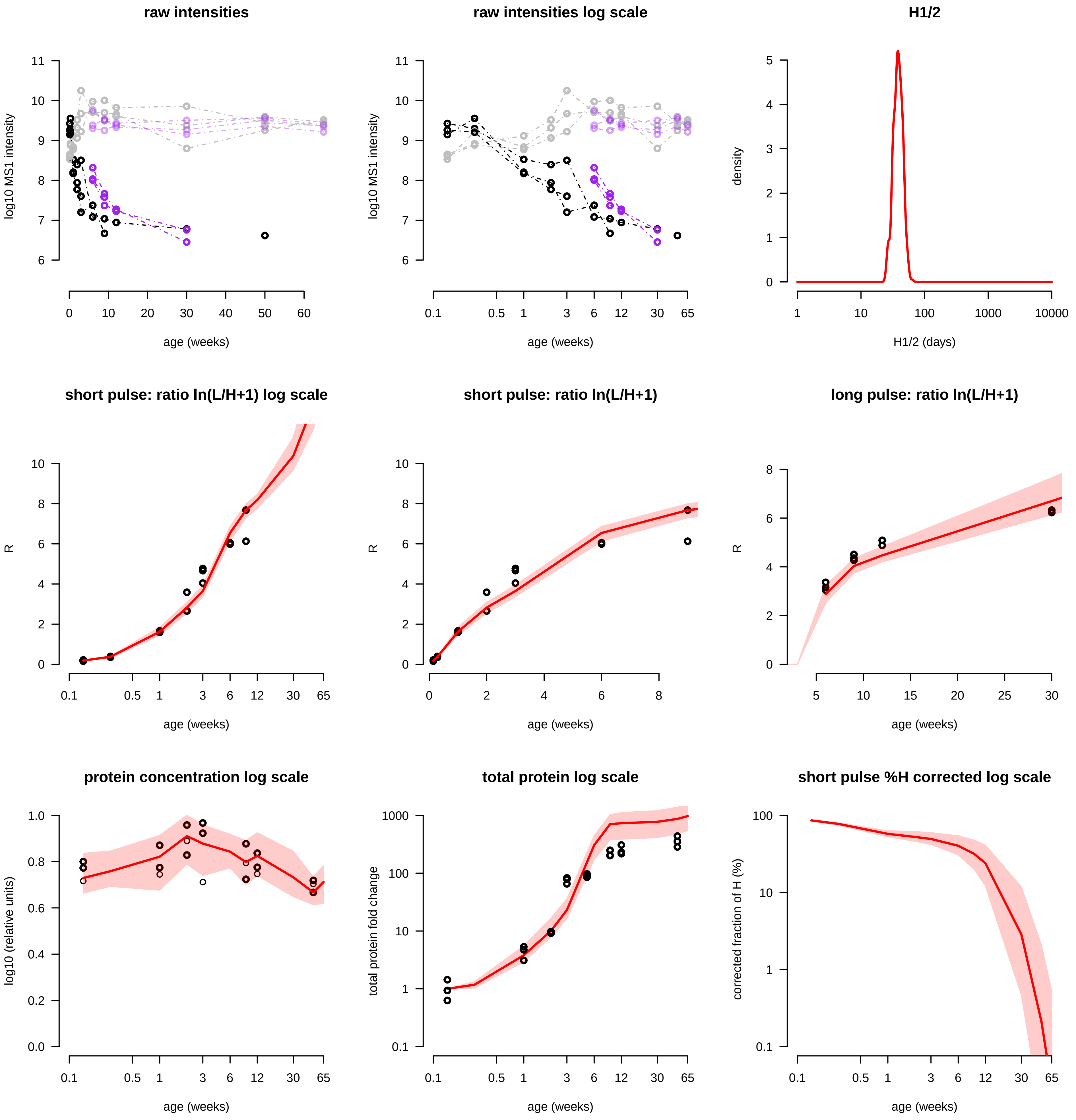

P14685

>sp|P14685|PSMD3_MOUSE

26S proteasome non−ATPase regulatory subunit 3

GN=Psmd3

Med H1/2: 25.8 days

Q5   H1/2: 13.8 days

Q95 H1/2: 73.2 days

reliable model fit: yes

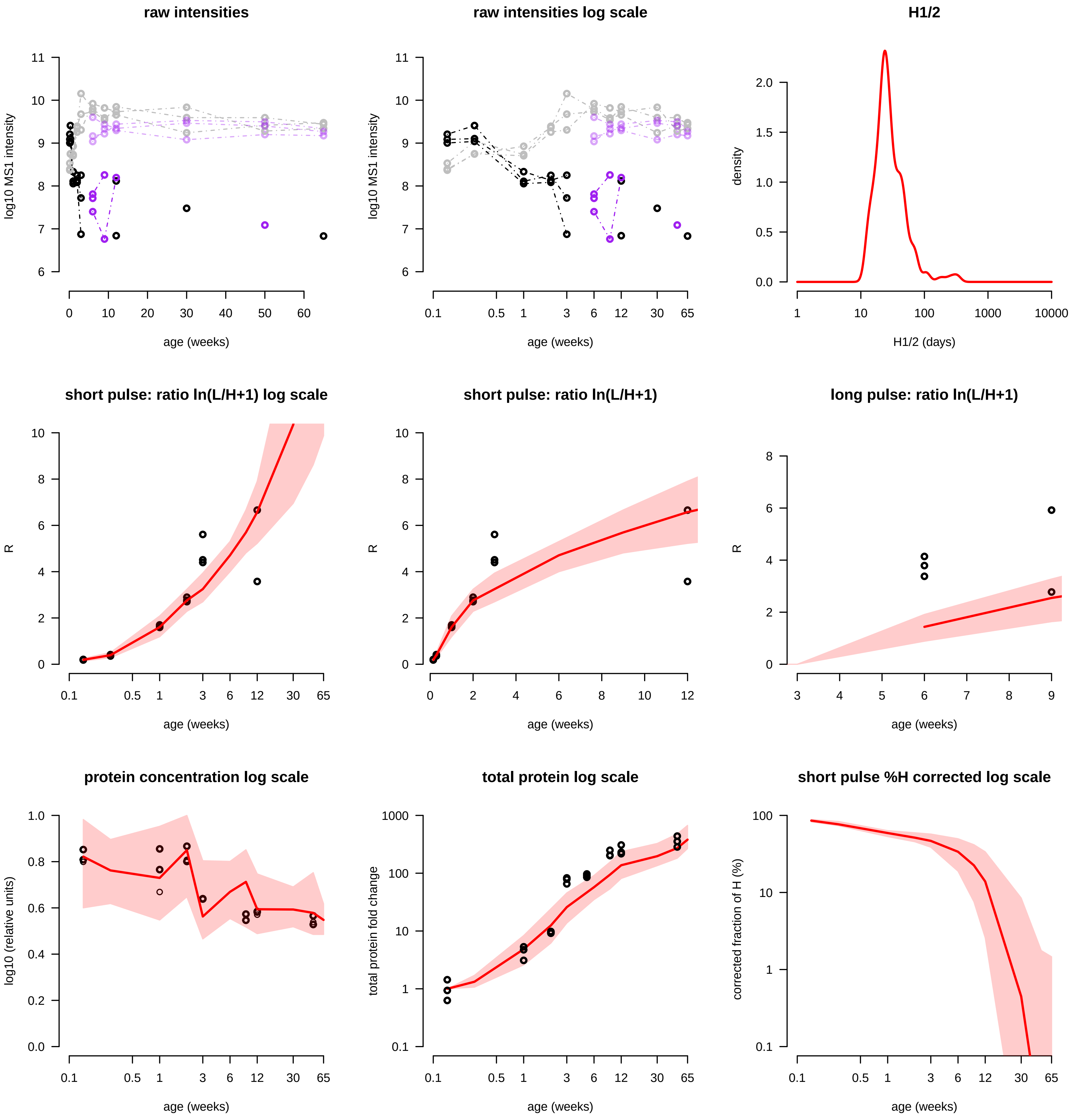

P12787

>sp|P12787|COX5A_MOUSE

Cytochrome c oxidase subunit 5A, mitochondrial

GN=Cox5a

Med H1/2: 8.9 days

Q5 H1/2: 6.6 days

Q95 H1/2: 13.4 days

reliable model fit: yes

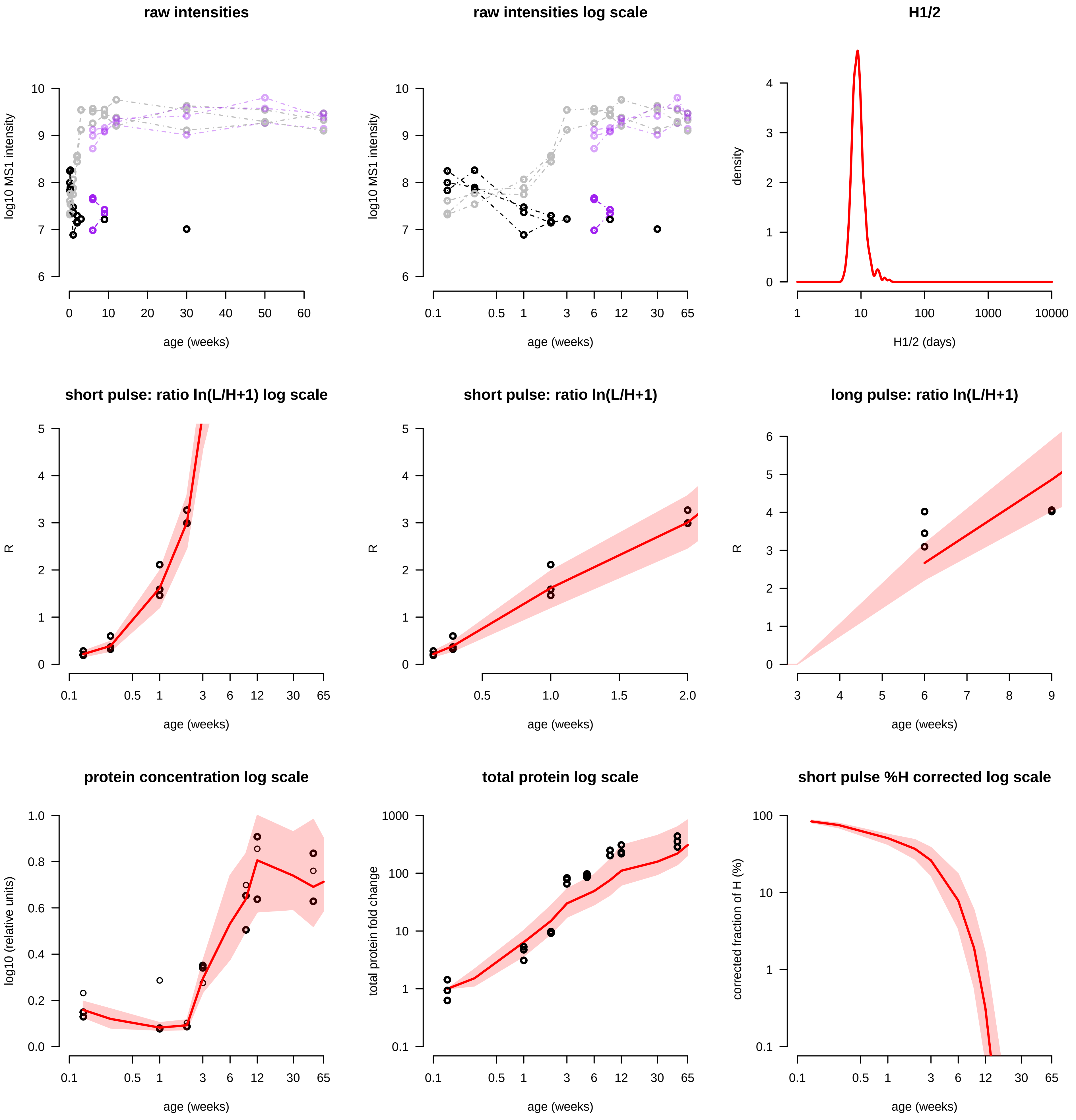

P14131

>sp|P14131|RS16_MOUSE

40S ribosomal protein S16

GN=Rps16

Med H1/2: 8.6 days

Q5 H1/2: 6.9 days

Q95 H1/2: 11 days

reliable model fit: yes

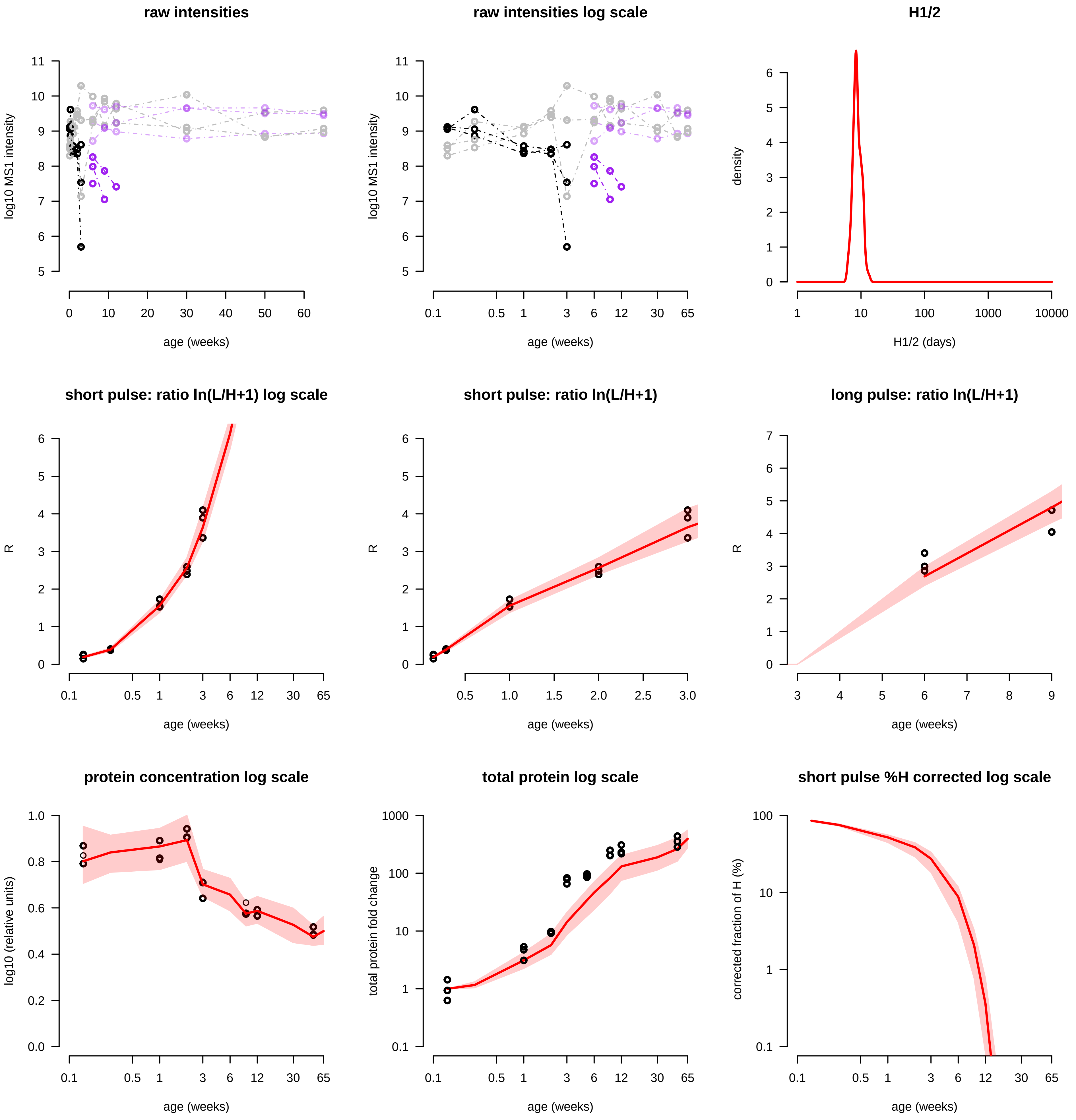

P31001

>sp|P31001|DESM_MOUSE

Desmin

GN=Des

Med H1/2: 6.3 days

Q5 H1/2: 4.8 days

Q95 H1/2: 8.7 days

reliable model fit: yes

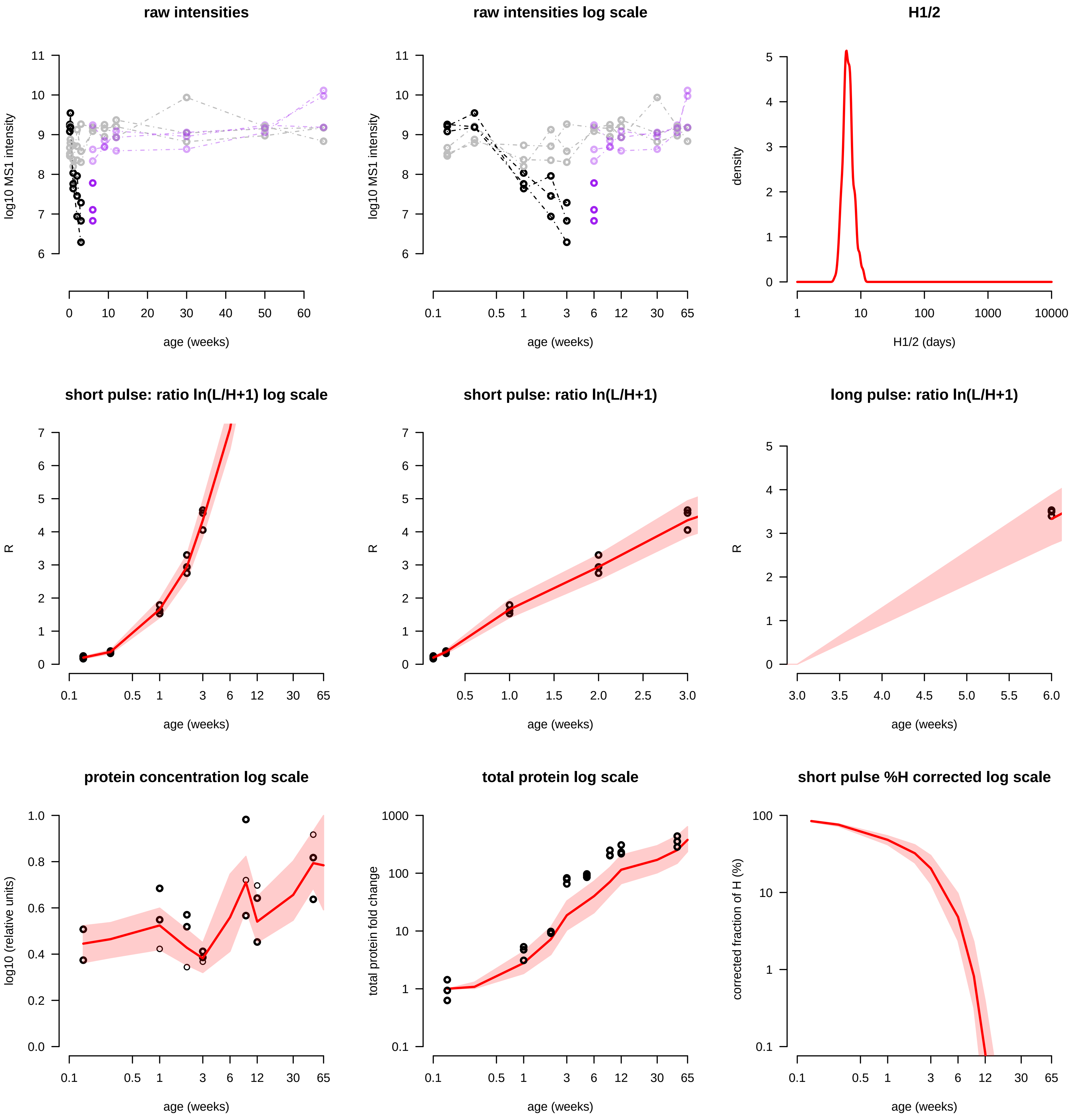

Q99LP6

>sp|Q99LP6|GRPE1_MOUSE

GrpE protein homolog 1, mitochondrial

GN=Grpel1

Med H1/2: 8.6 days

Q5 H1/2: 6.8 days

Q95 H1/2: 11.2 days

reliable model fit: yes

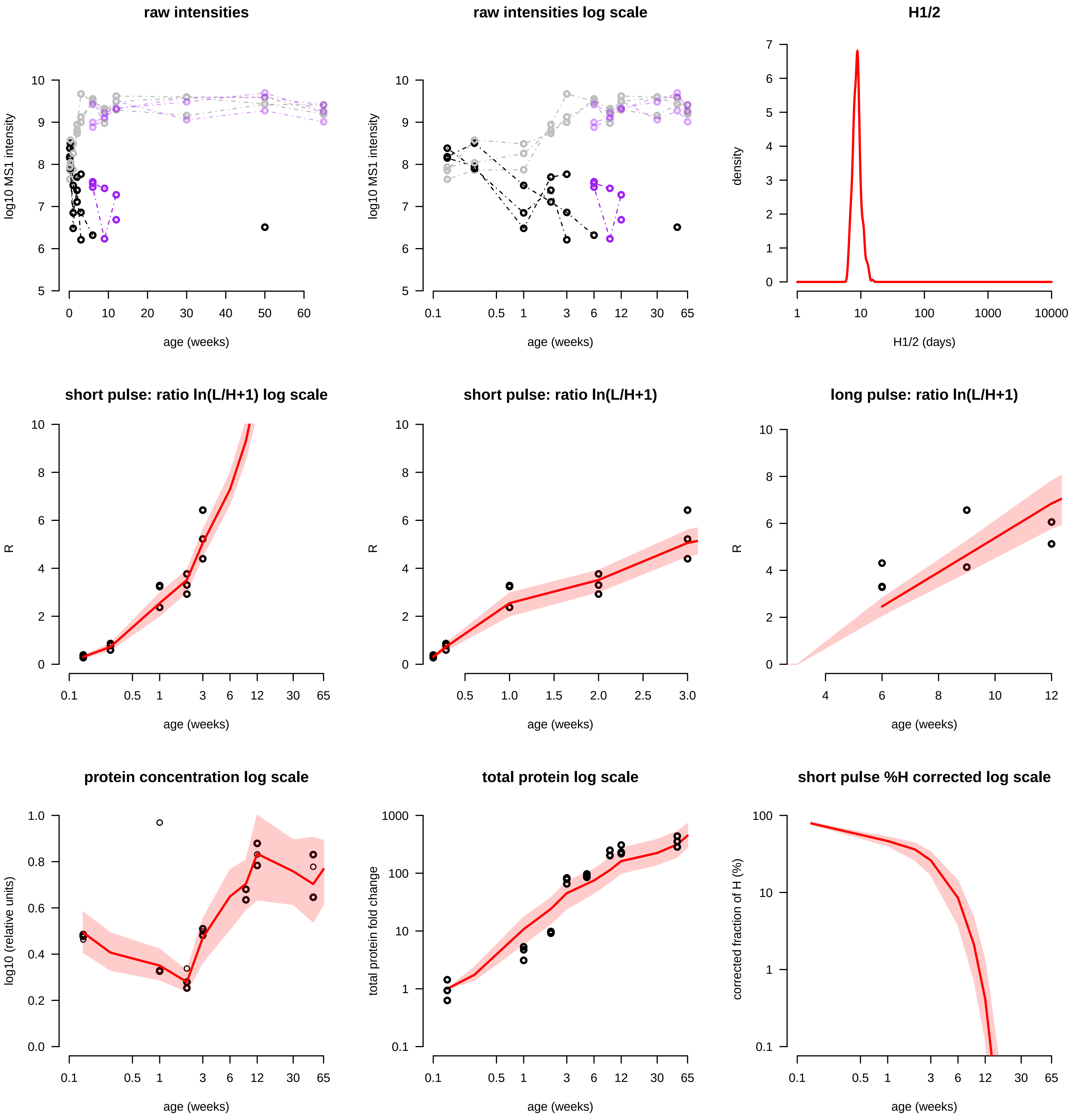

Q62188

>sp|Q62188|DPYL3_MOUSE

Dihydropyrimidinase−related protein 3

GN=Dpysl3

Med H1/2: 63.2 days

Q5 H1/2: 44.3 days

Q95 H1/2: 115.6 days

reliable model fit: yes

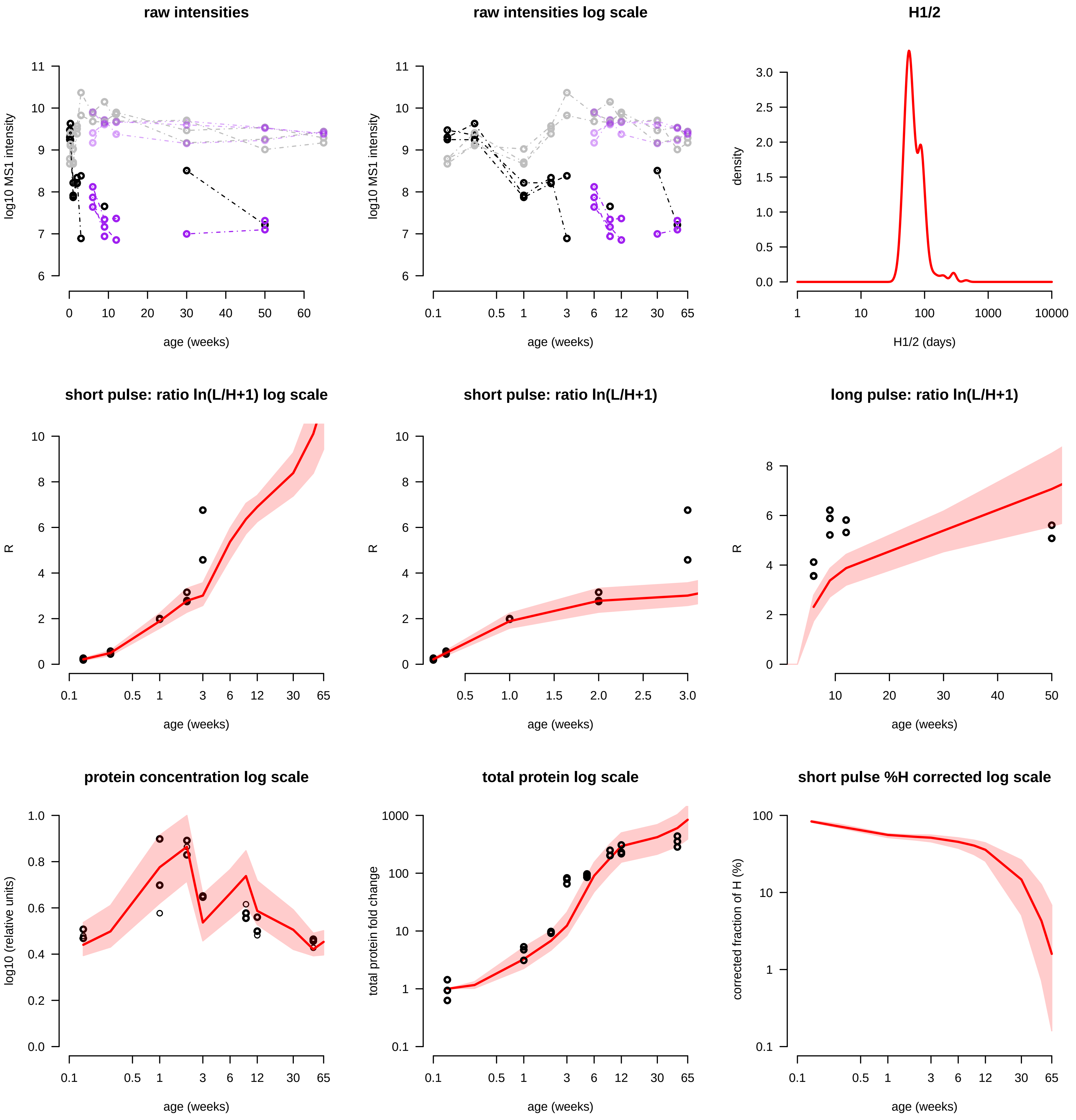

P62196

>sp|P62196|PRS8_MOUSE

26S proteasome regulatory subunit 8

GN=Psmc5

Med H1/2: 1898.4 days

Q5 H1/2: 527.2 days

Q95 H1/2: 27111.4 days

reliable model fit: yes

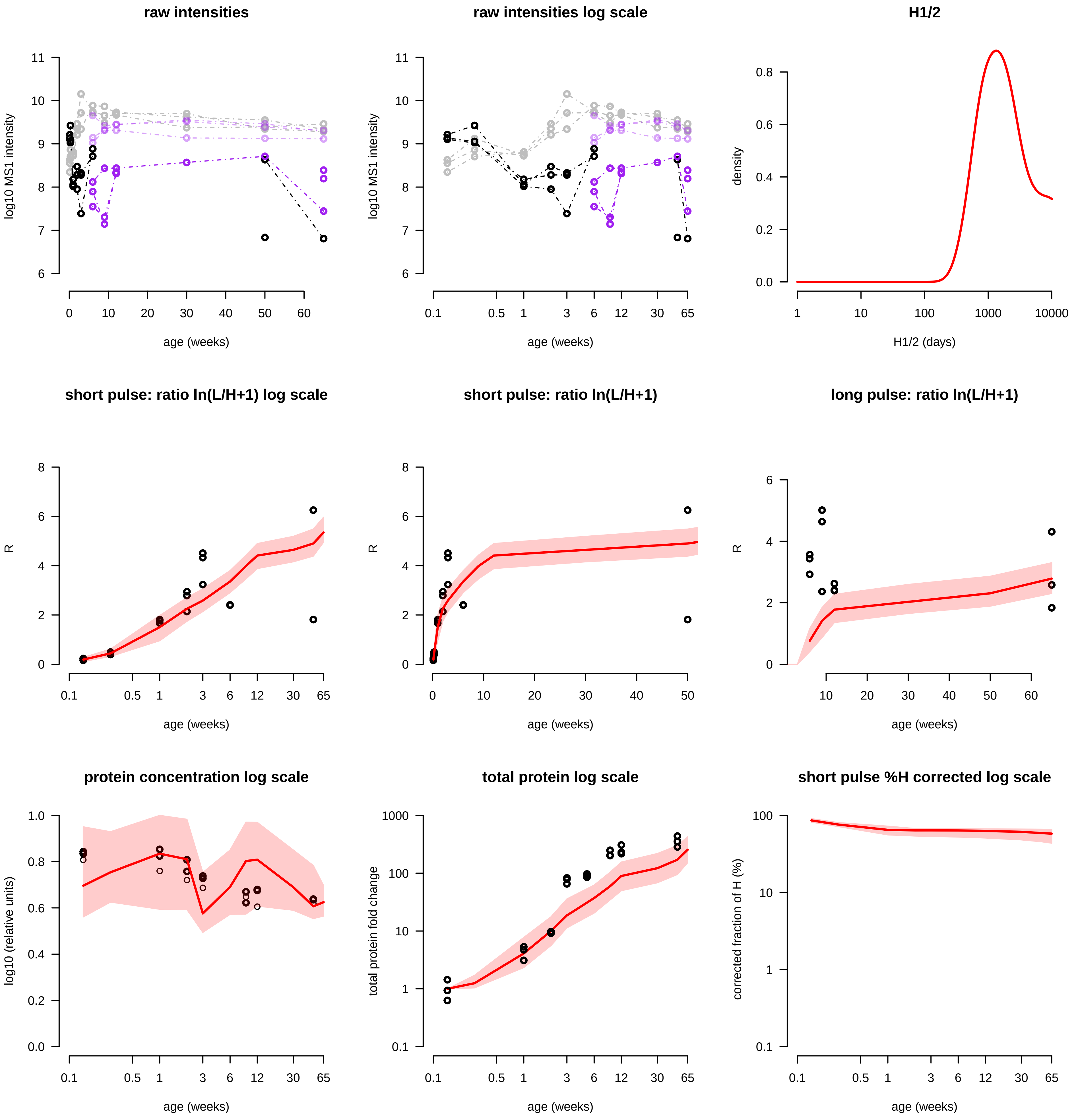

Q6ZWV7

>sp|Q6ZWV7|RL35_MOUSE

60S ribosomal protein L35

GN=Rpl35

Med H1/2: 17.3 days

Q5 H1/2: 13.4 days

Q95 H1/2: 25.5 days

reliable model fit: yes

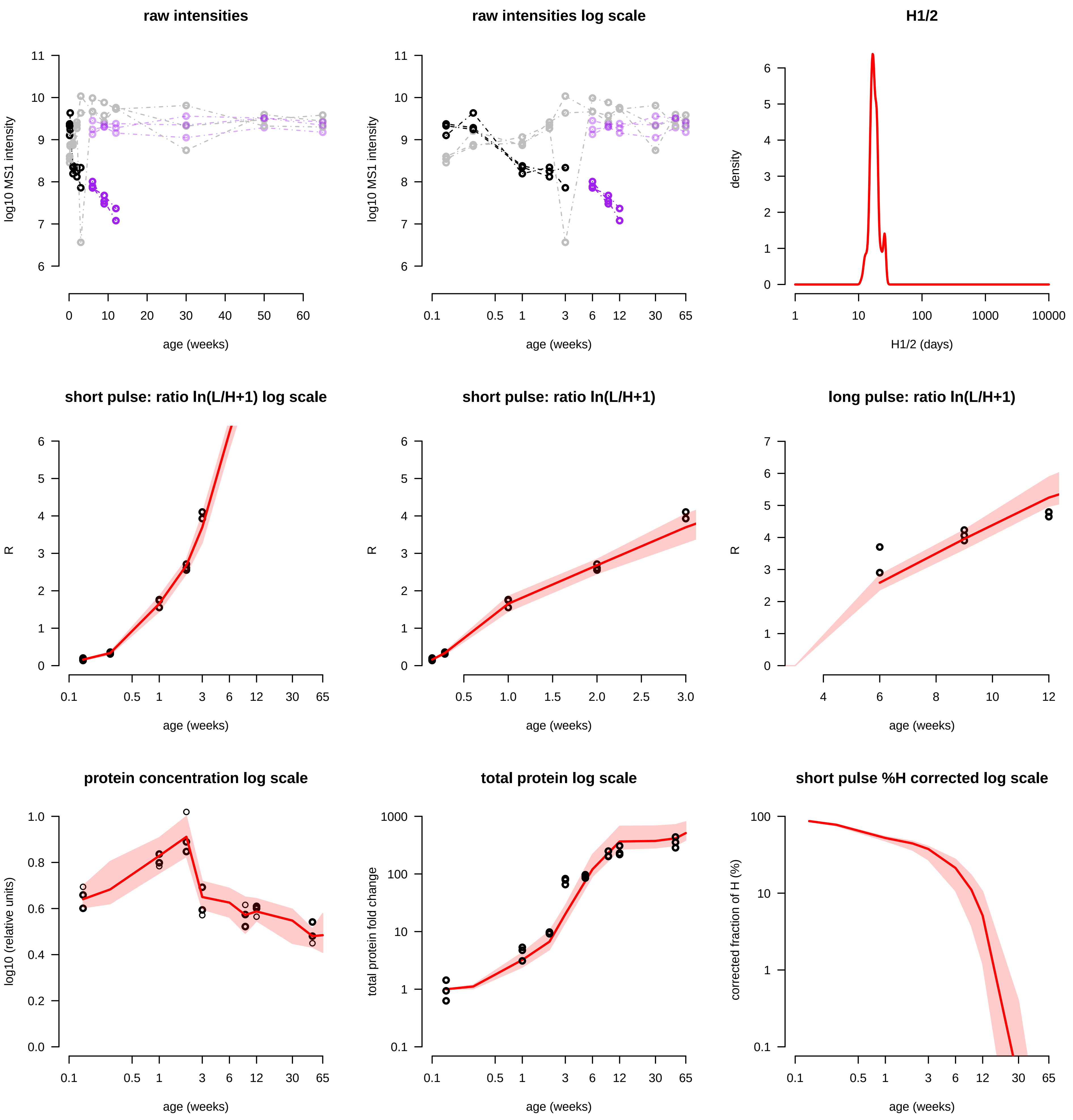

Q9CXI5

>sp|Q9CXI5|MANF_MOUSE

Mesencephalic astrocyte−derived neurotrophic factor

GN=Manf

Med H1/2: 10 days

Q5 H1/2: 7.9 days

Q95 H1/2: 17.9 days

reliable model fit: yes

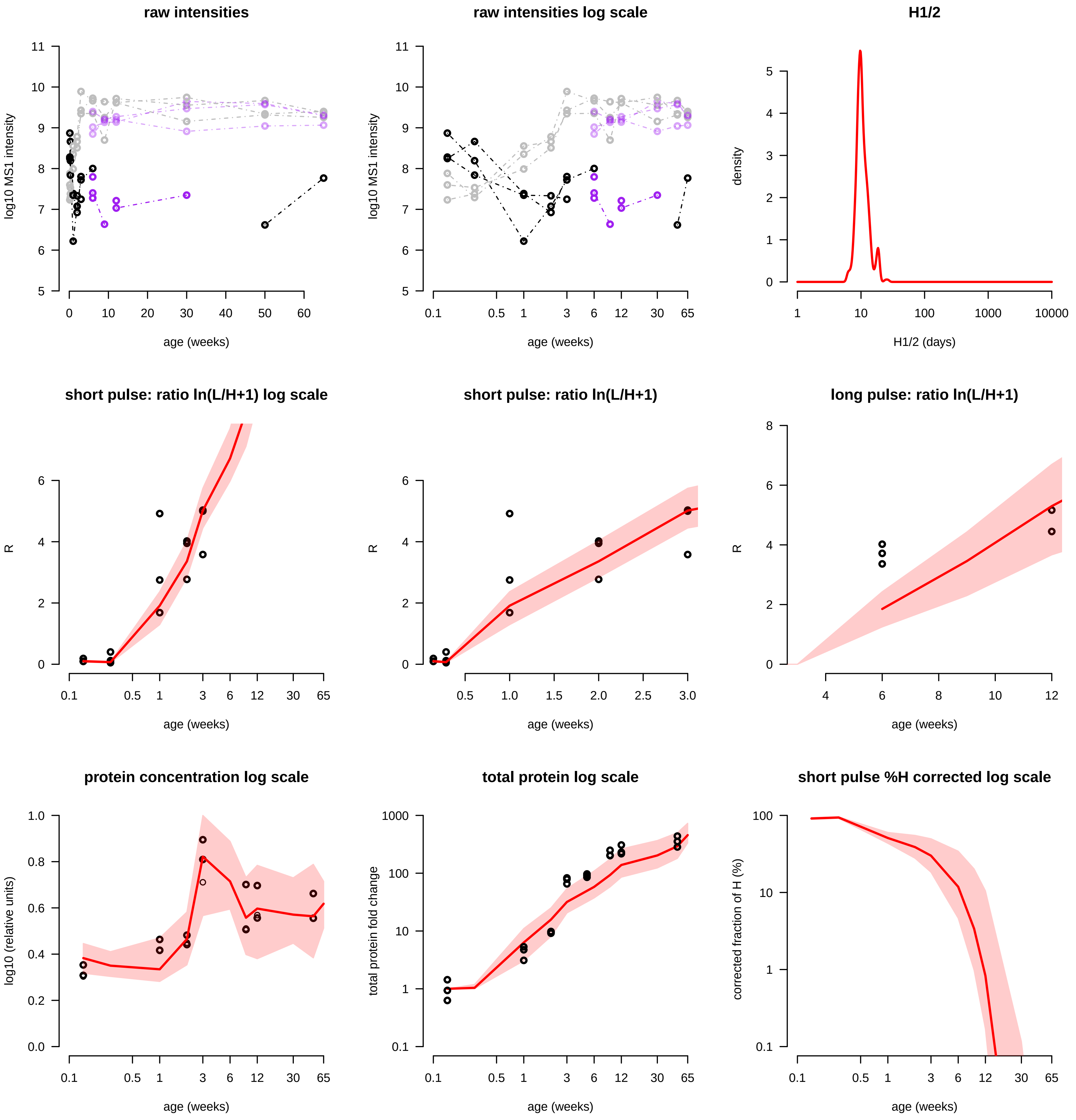

P70349

>sp|P70349|HINT1_MOUSE

Histidine triad nucleotide−binding protein 1

GN=Hint1

Med H1/2: 11.7 days

Q5 H1/2: 8.9 days

Q95 H1/2: 16.9 days

reliable model fit: yes

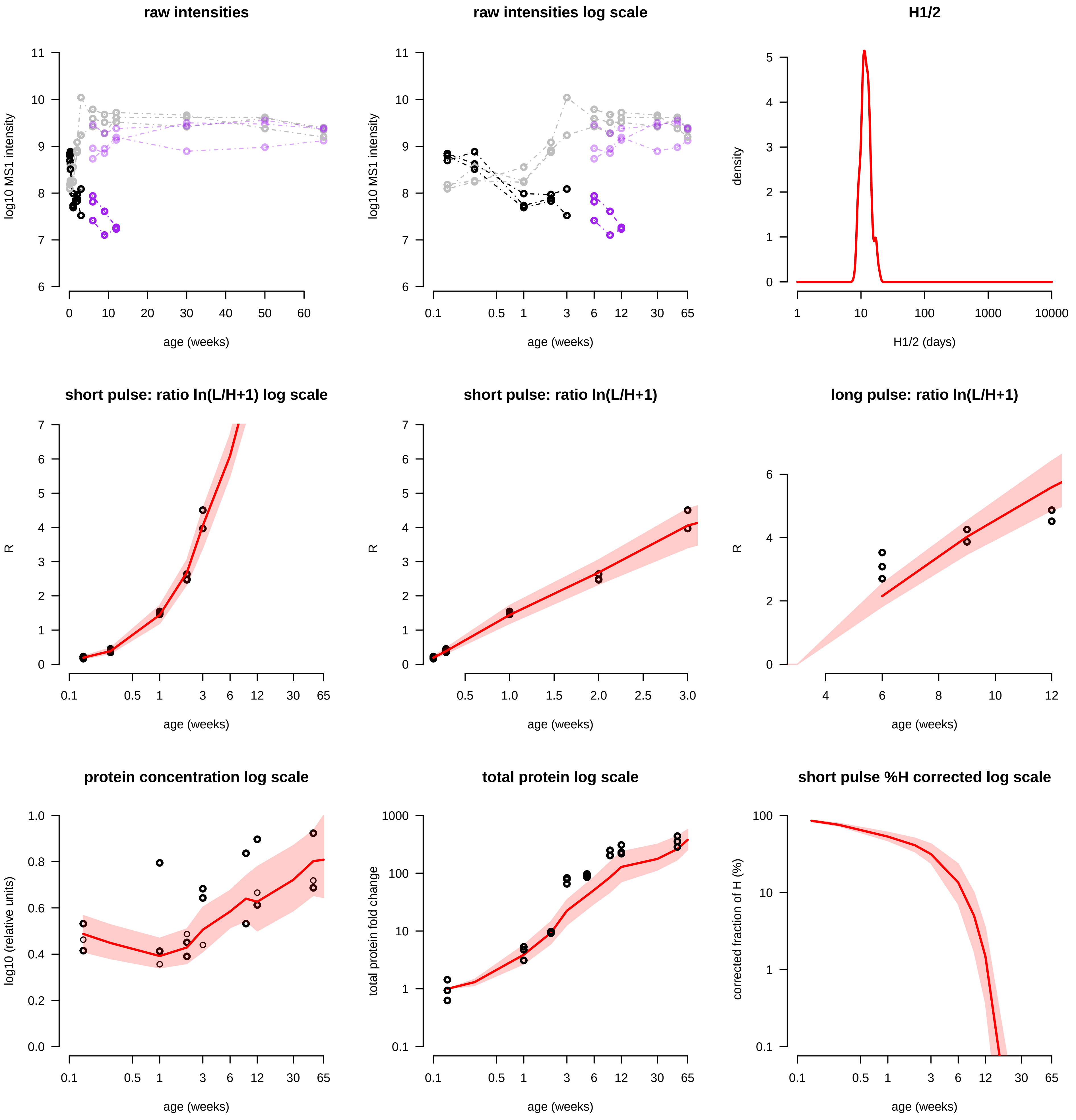

P61027

>sp|P61027|RAB10_MOUSE

Ras−related protein Rab−10

GN=Rab10

Med H1/2: 1644.3 days

Q5 H1/2: 479.5 days

Q95 H1/2: 27609.3 days

reliable model fit: yes

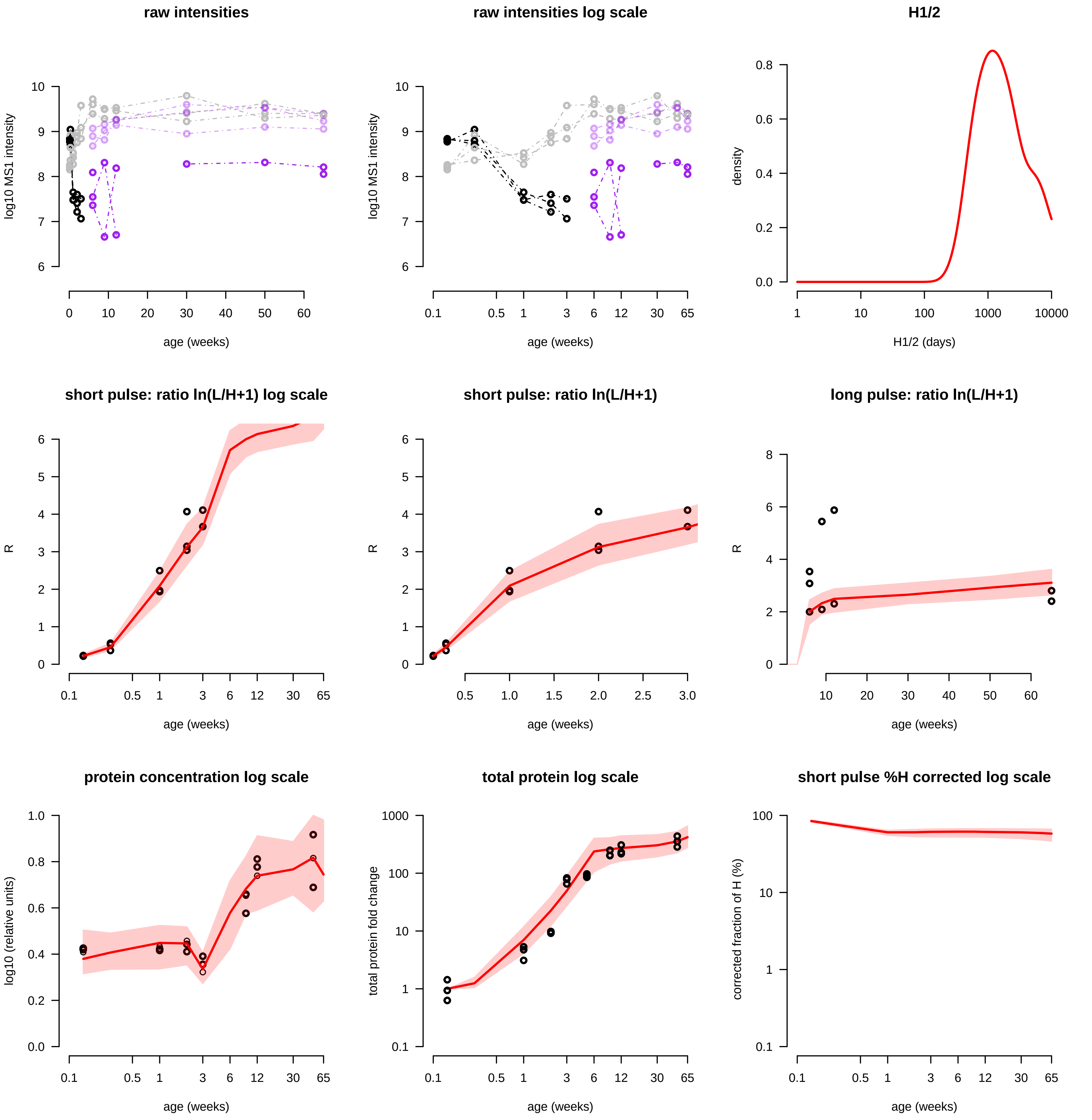

Q9CRB9

>sp|Q9CRB9|MIC19_MOUSE

MICOS complex subunit Mic19

GN=Chchd3

Med H1/2: 10.3 days

Q5 H1/2: 7.5 days

Q95 H1/2: 15.1 days

reliable model fit: yes

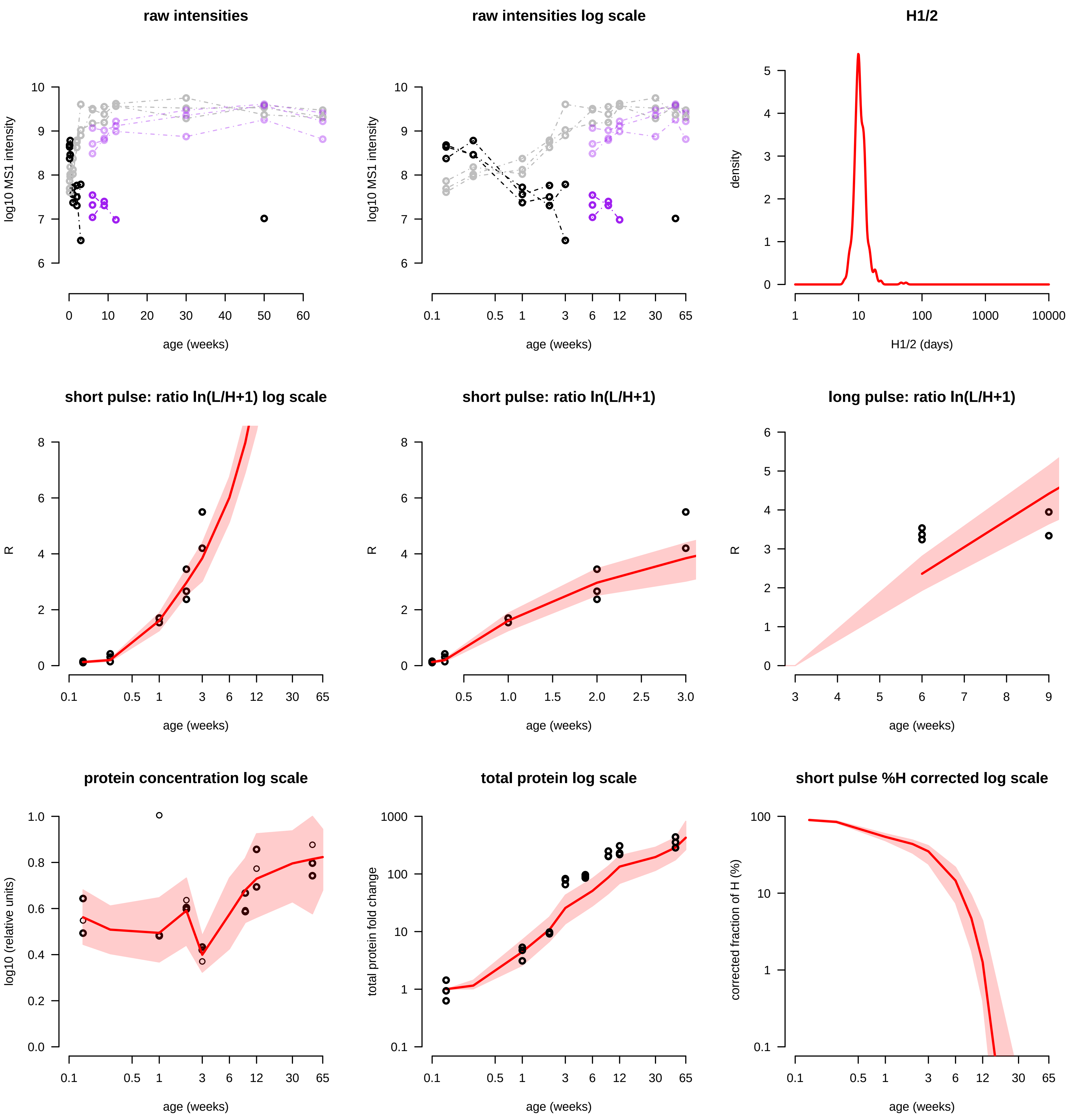

P48758

>sp|P48758|CBR1_MOUSE

Carbonyl reductase [NADPH] 1

GN=Cbr1

Med H1/2: 4.6 days

Q5 H1/2: 3.9 days

Q95 H1/2: 5.5 days

reliable model fit: yes

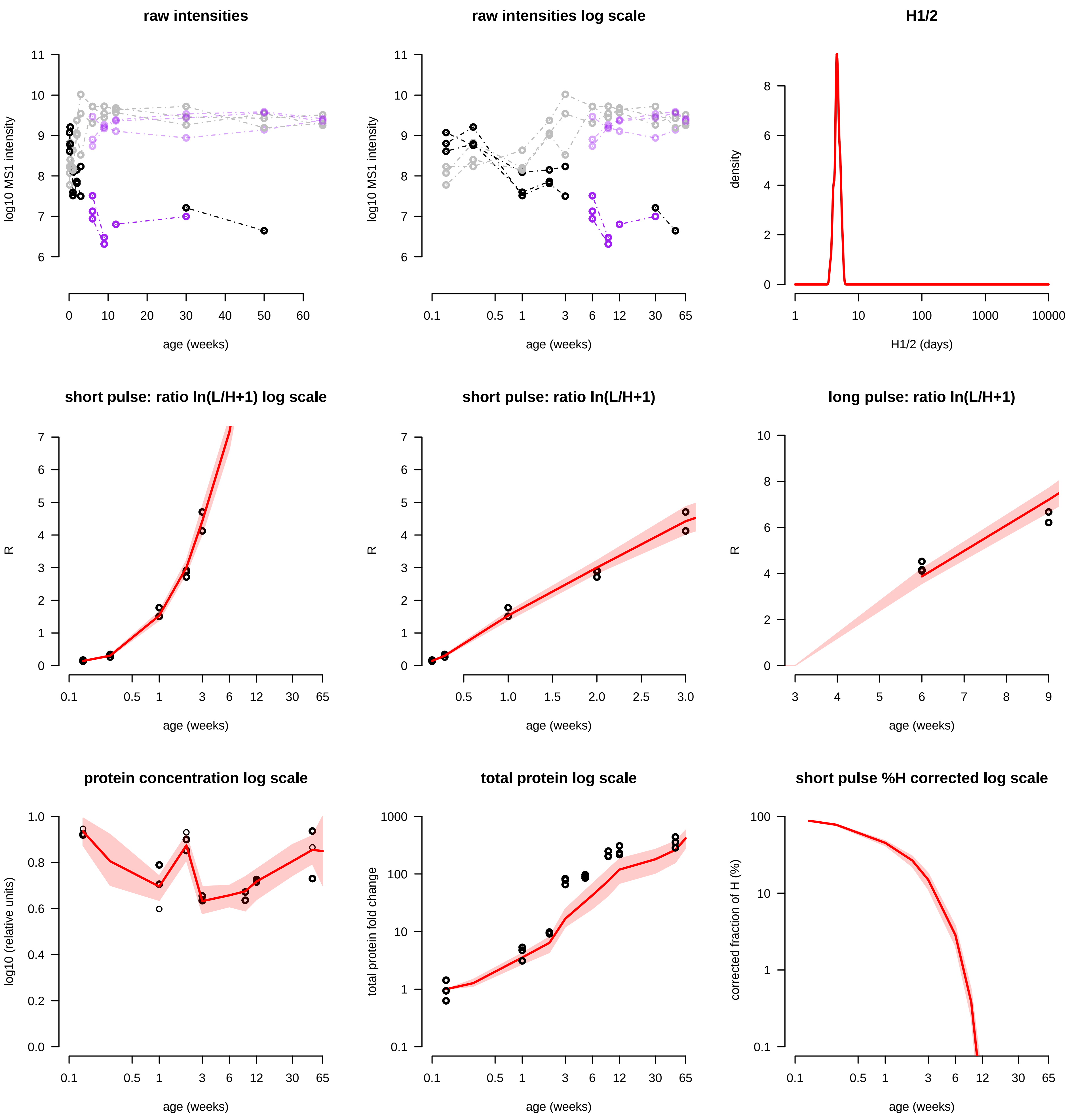

P03958

>sp|P03958|ADA_MOUSE

Adenosine deaminase

GN=Ada

Med H1/2: 6.7 days

Q5 H1/2: 3.1 days

Q95 H1/2: 56.5 days

reliable model fit: yes

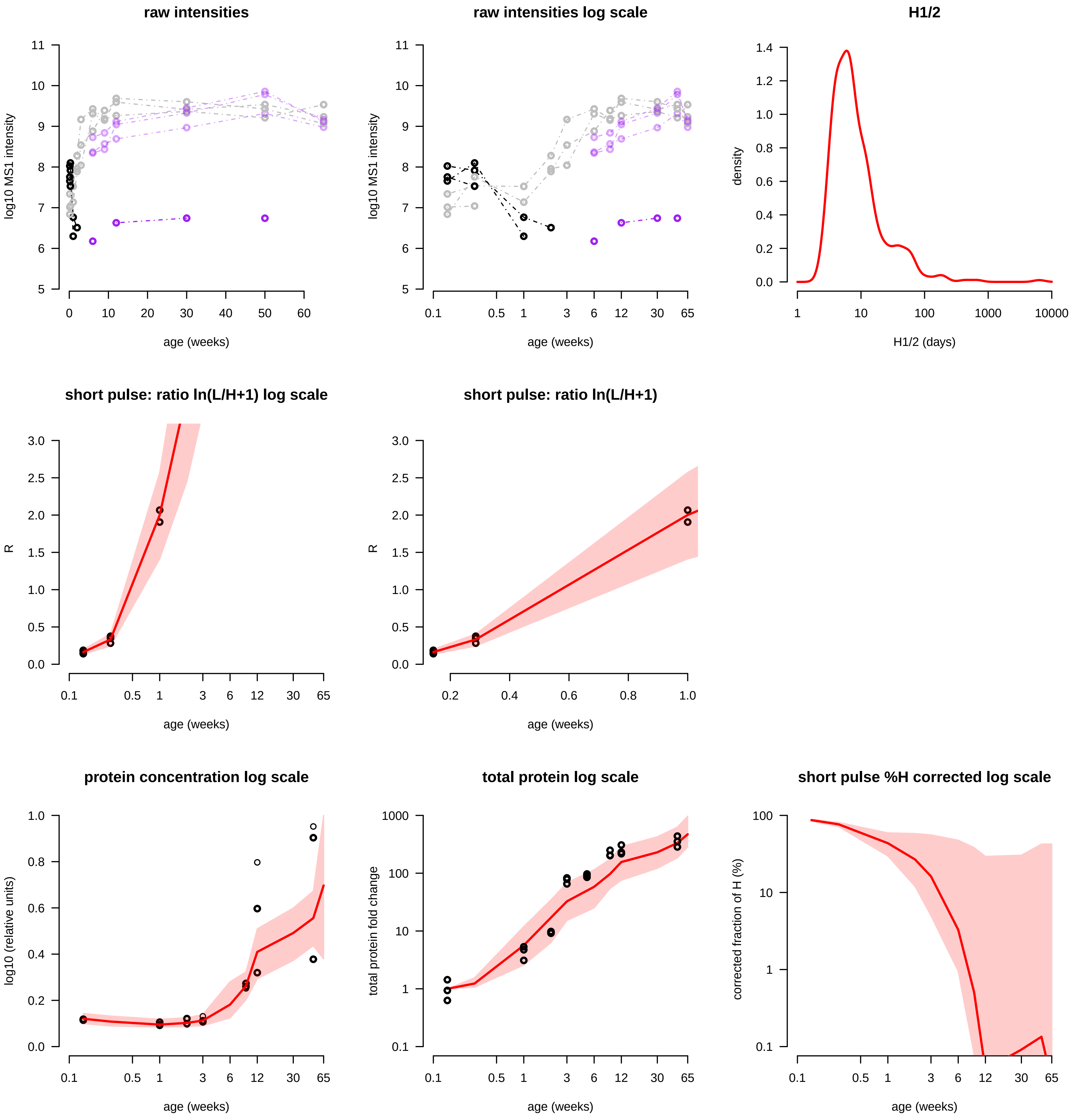

P50516;P50516−2

>sp|P50516|VATA_MOUSE

V−type proton ATPase catalytic subunit A

GN=Atp6v1a

Med H1/2: 5.5 days

Q5   H1/2: 4.6 days

Q95 H1/2: 7 days

reliable model fit: yes

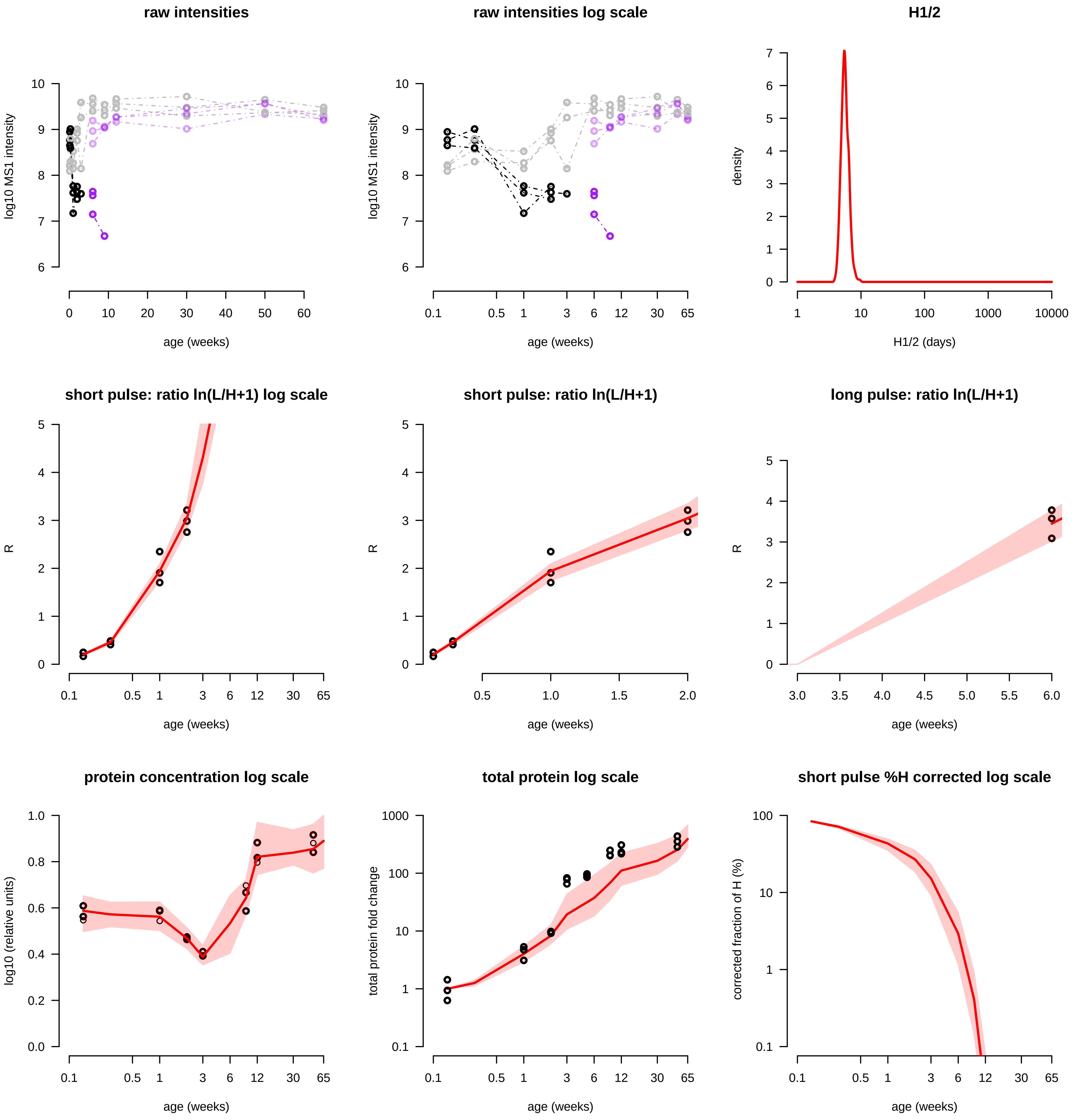

P62960

>sp|P62960|YBOX1_MOUSE

Y−box−binding protein 1

GN=Ybx1

Med H1/2: 7 days

Q5 H1/2: 5.8 days

Q95 H1/2: 8.7 days

reliable model fit: yes

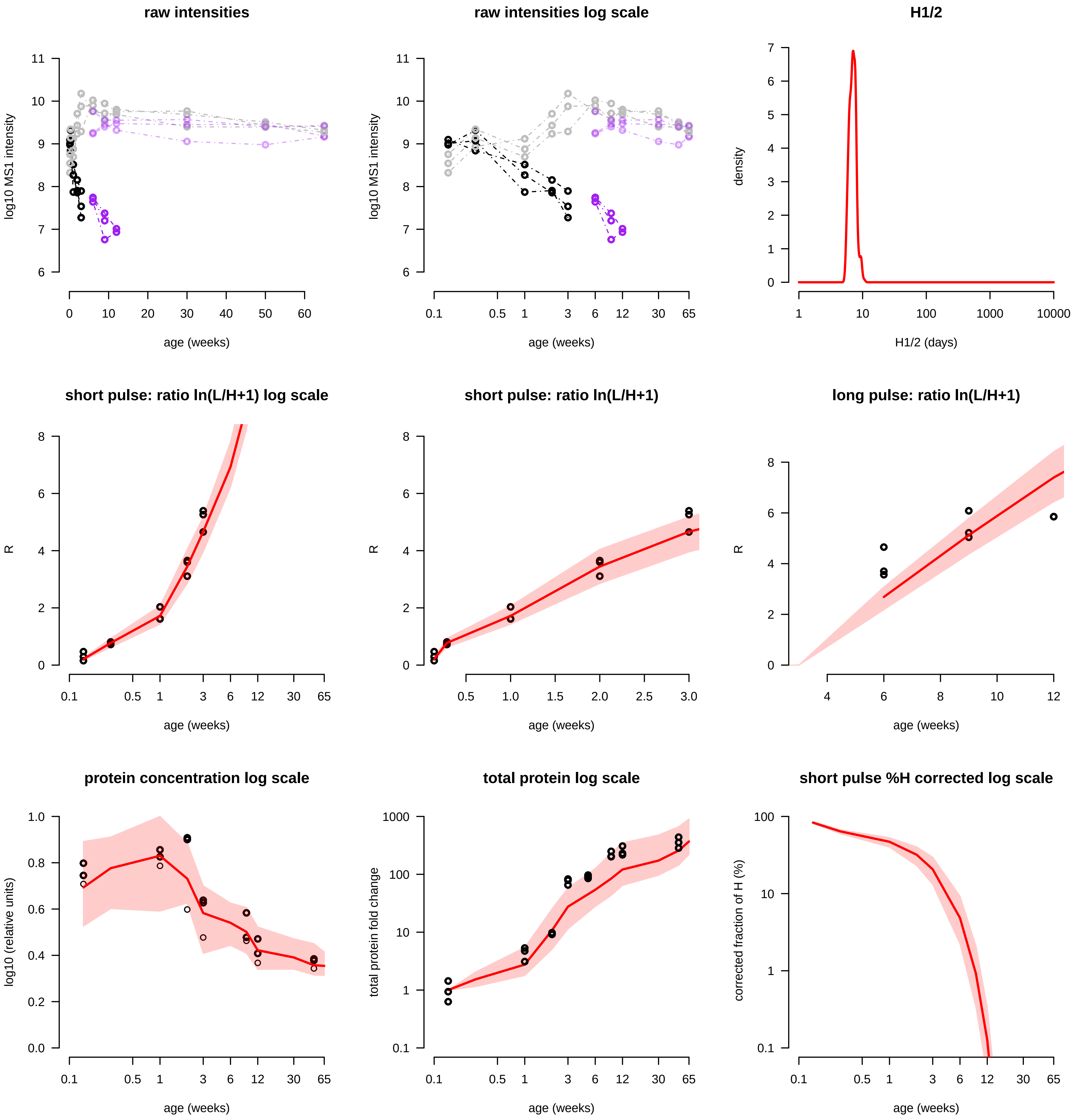

P16332

>sp|P16332|MUTA_MOUSE

Methylmalonyl−CoA mutase, mitochondrial

GN=Mmut

Med H1/2: 4.3 days

Q5 H1/2: 3.4 days

Q95 H1/2: 5.8 days

reliable model fit: yes

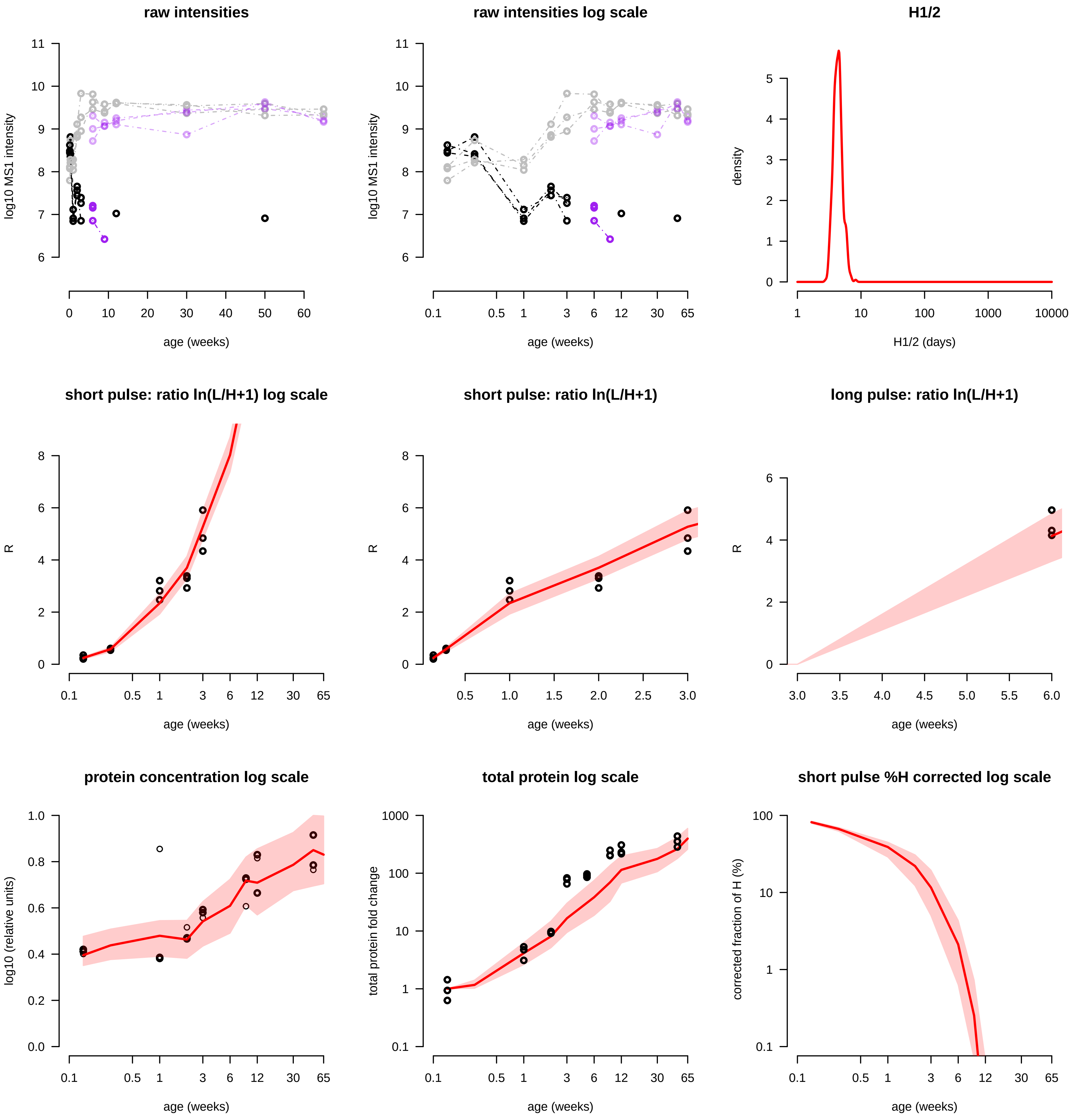

Q9CQ92

>sp|Q9CQ92|FIS1_MOUSE

Mitochondrial fission 1 protein

GN=Fis1

Med H1/2: 9.7 days

Q5 H1/2: 8.2 days

Q95 H1/2: 11.7 days

reliable model fit: yes

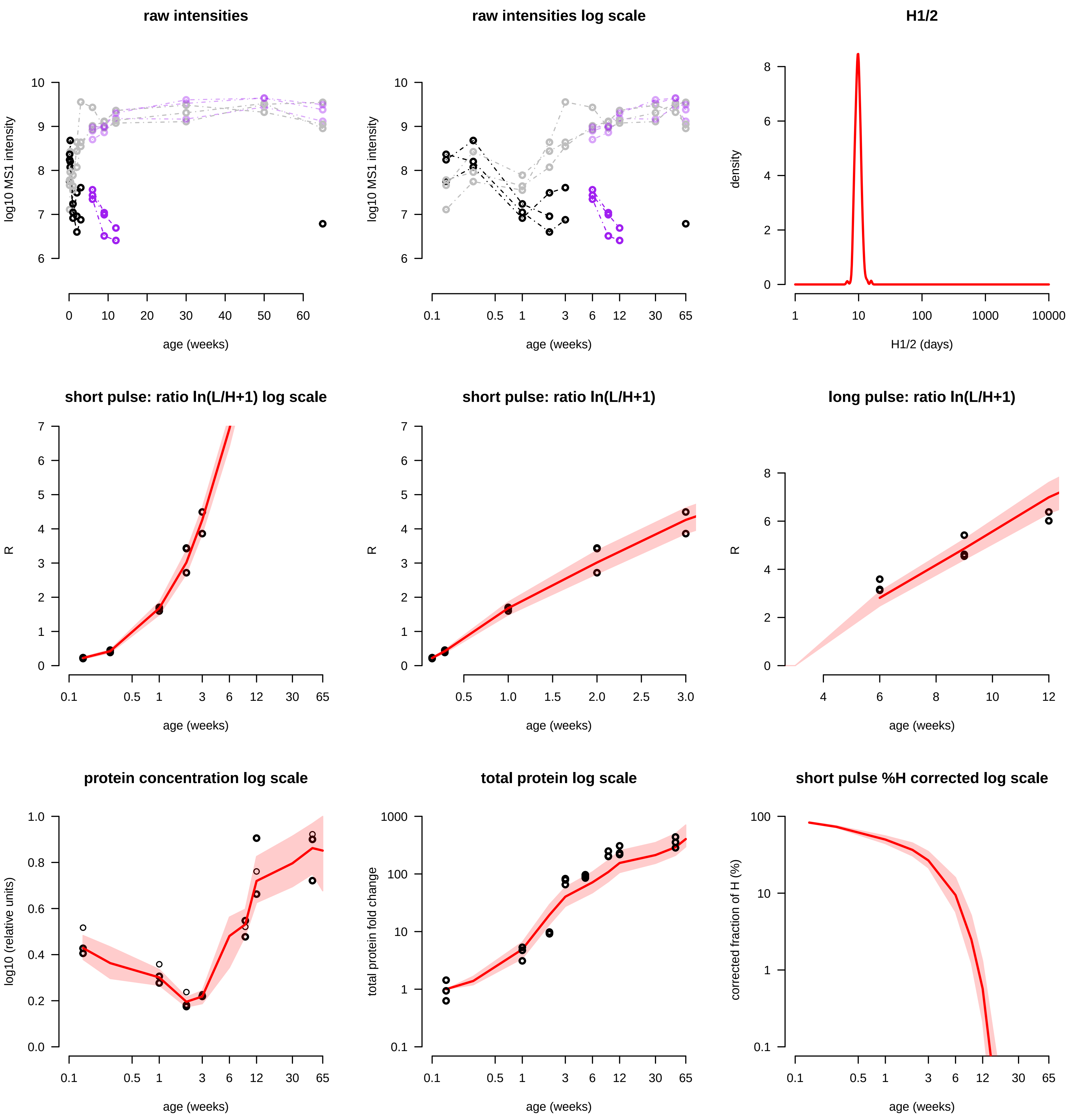

Q8CIB5

>sp|Q8CIB5|FERM2_MOUSE

Fermitin family homolog 2

GN=Fermt2

Med H1/2: 5.4 days

Q5 H1/2: 4.7 days

Q95 H1/2: 7.1 days

reliable model fit: yes

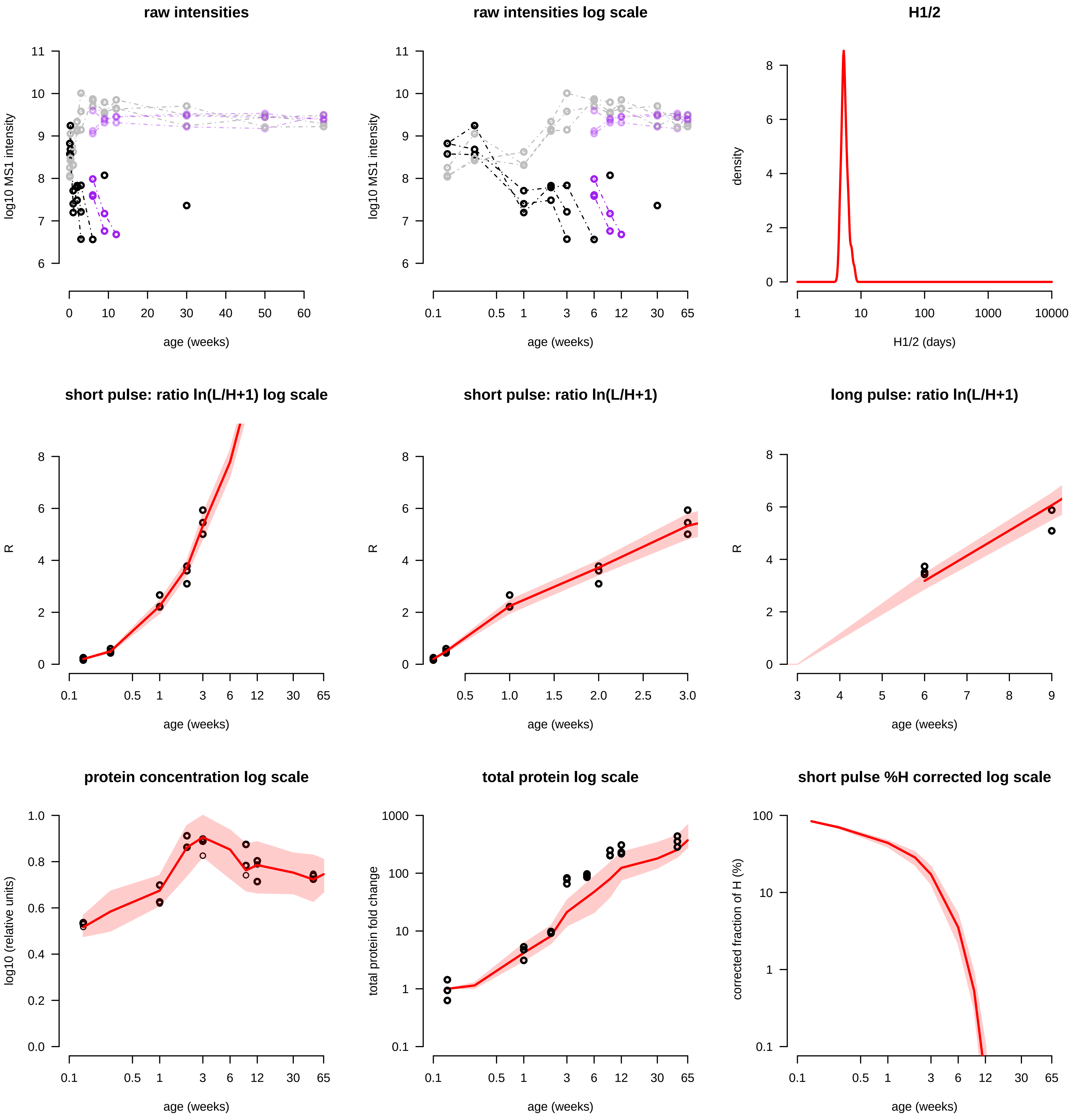

Q8CDN6

>sp|Q8CDN6|TXNL1_MOUSE

Thioredoxin−like protein 1

GN=Txnl1

Med H1/2: 5.9 days

Q5 H1/2: 4.7 days

Q95 H1/2: 7.9 days

reliable model fit: yes

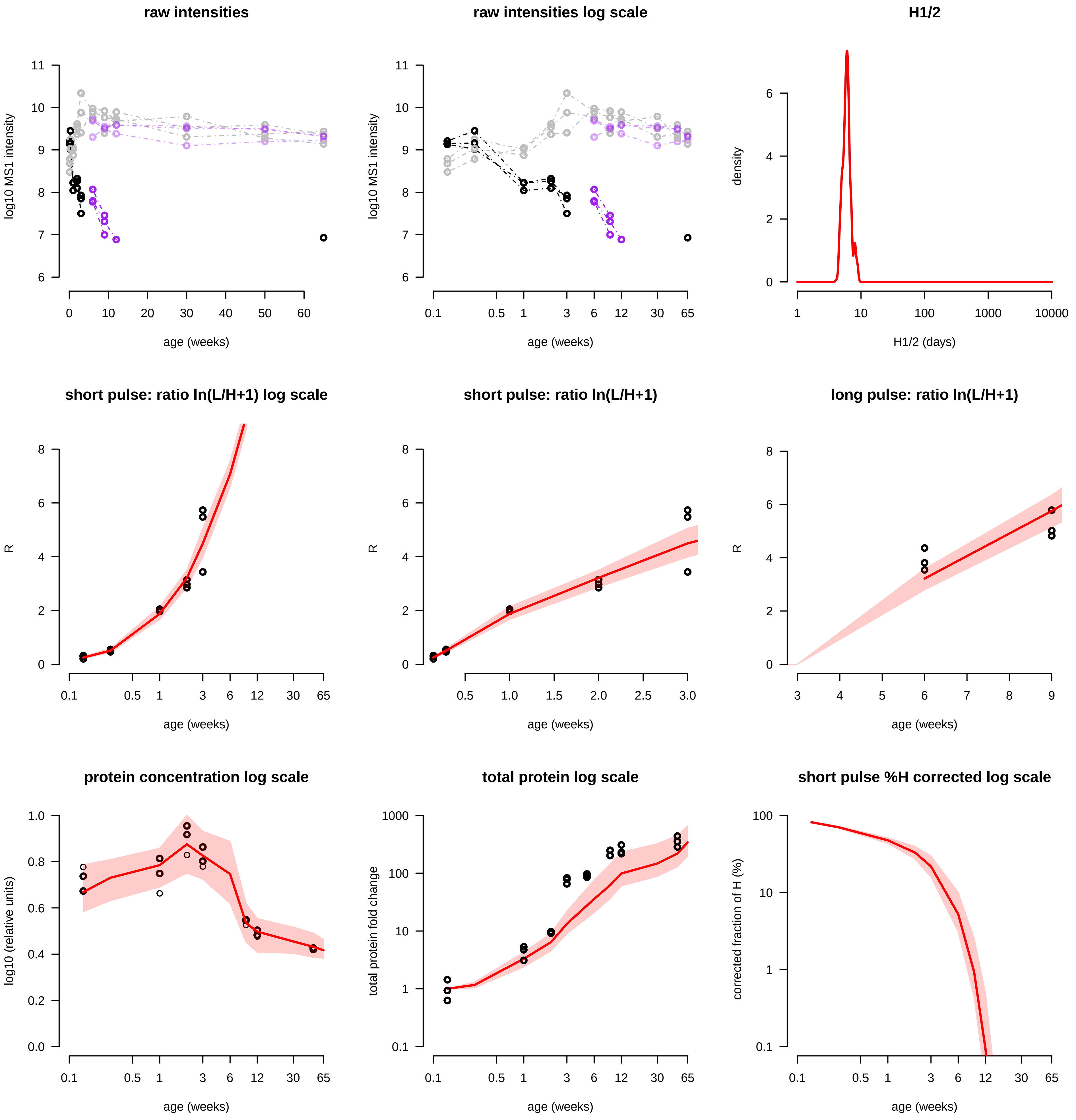

Q9WUA2

>sp|Q9WUA2|SYFB_MOUSE

Phenylalanine−−tRNA ligase beta subunit

GN=Farsb

Med H1/2: 9.2 days

Q5 H1/2: 7.4 days

Q95 H1/2: 11.1 days

reliable model fit: yes

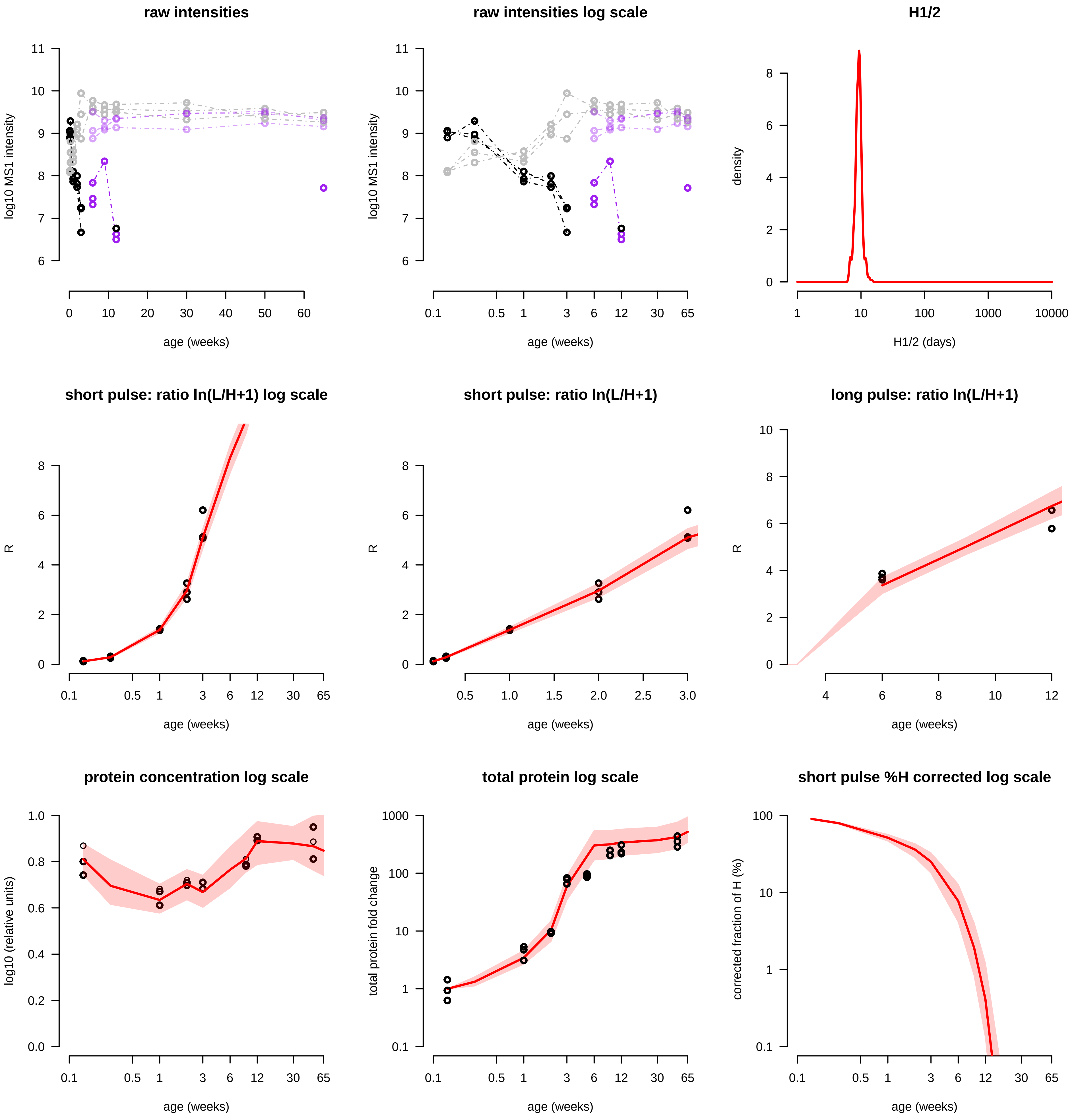

P17710−3;P17710−4;P17710;P17710−2

>sp|P17710−3|HXK1_MOUSE

Isoform HK1 of Hexokinase−1

GN=Hk1

Med H1/2: 5 days

Q5 H1/2: 4 days

Q95 H1/2: 6.8 days

reliable model fit: yes

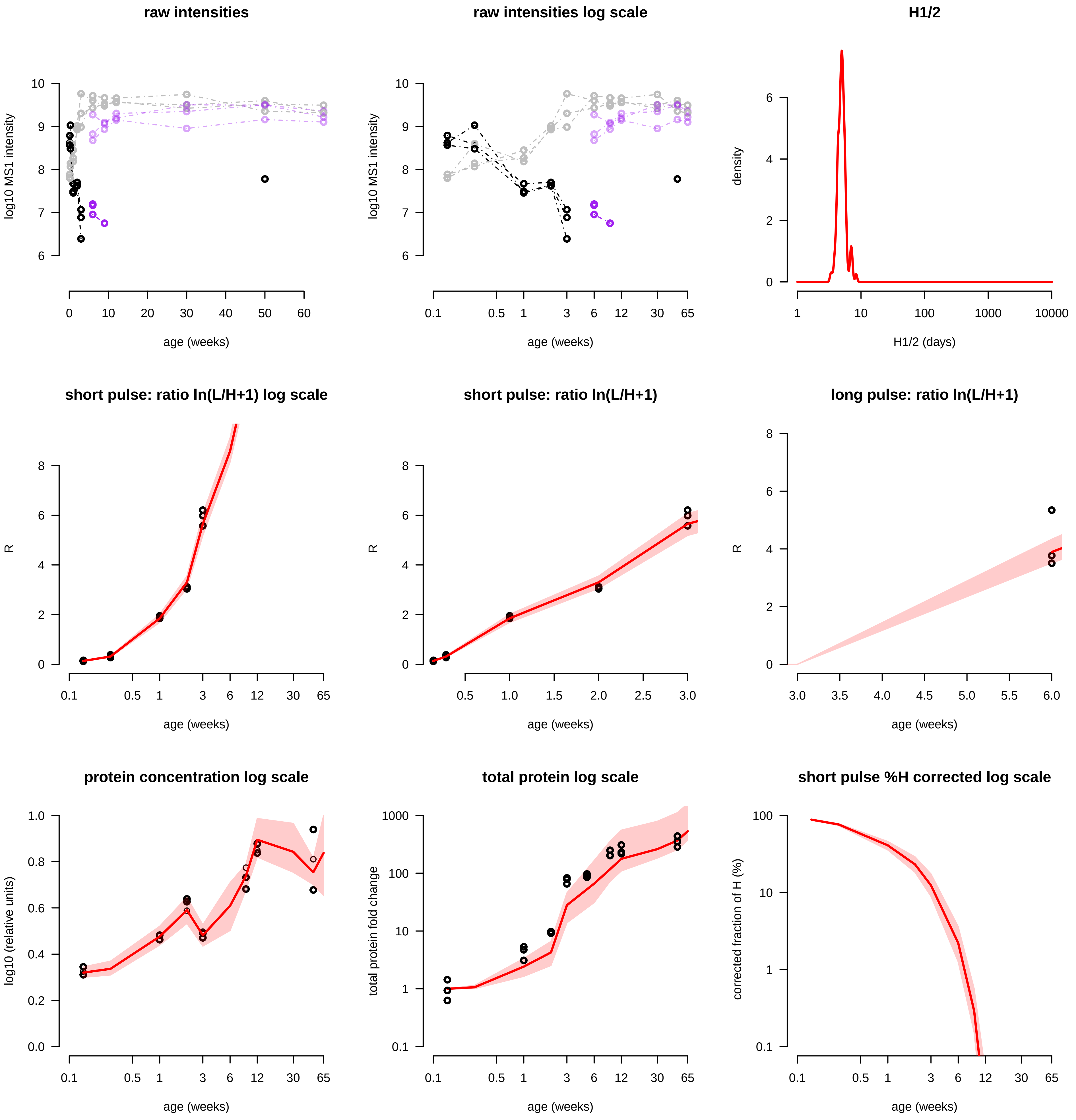

O55131

>sp|O55131|SEPT7_MOUSE

Septin−7

GN=Septin7

Med H1/2: 30.6 days

Q5 H1/2: 17.5 days

Q95 H1/2: 68.5 days

reliable model fit: no

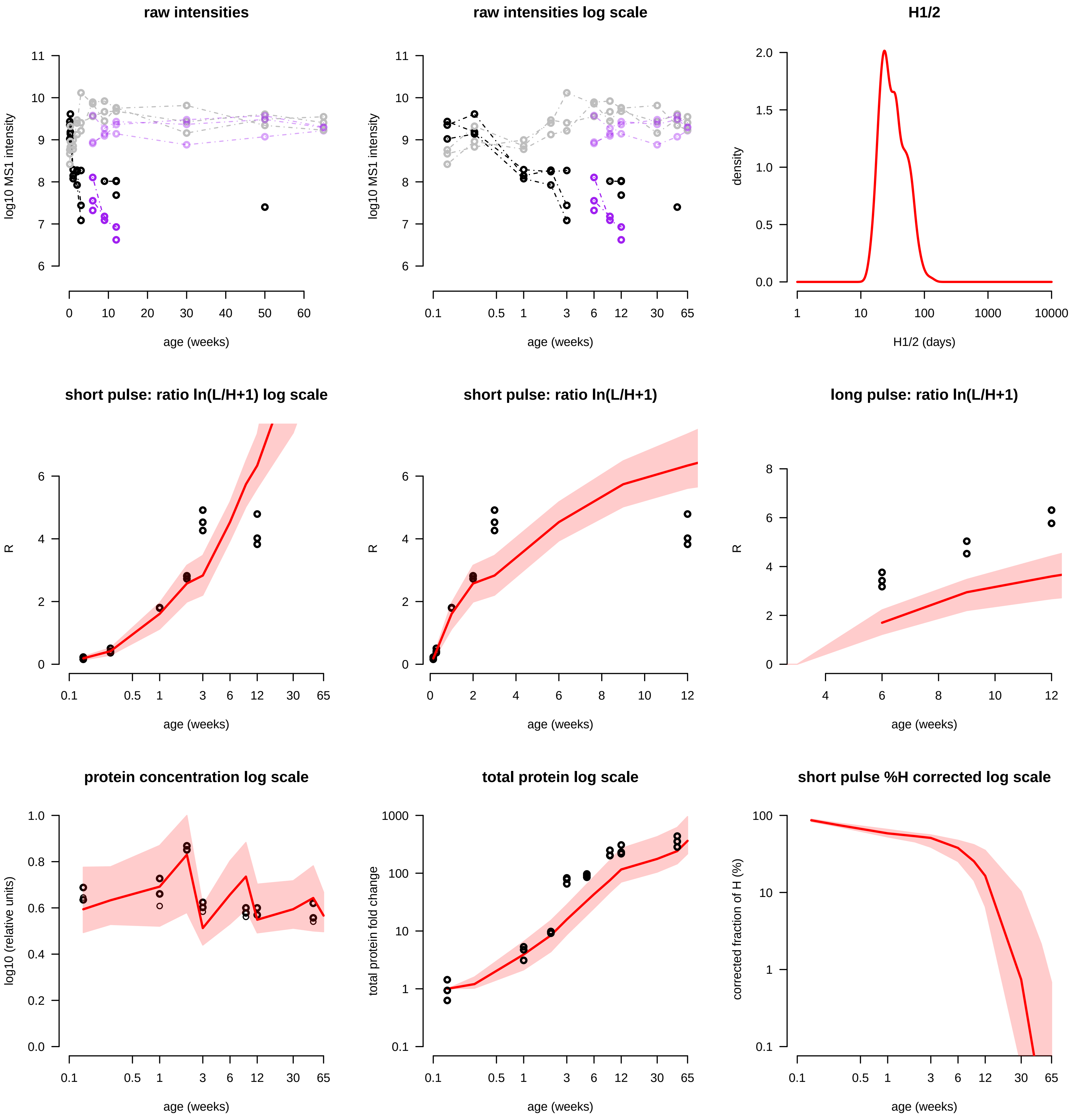

Q6P5F9

>sp|Q6P5F9|XPO1_MOUSE

Exportin−1

GN=Xpo1

Med H1/2: 8.6 days

Q5 H1/2: 7.5 days

Q95 H1/2: 10.3 days

reliable model fit: yes

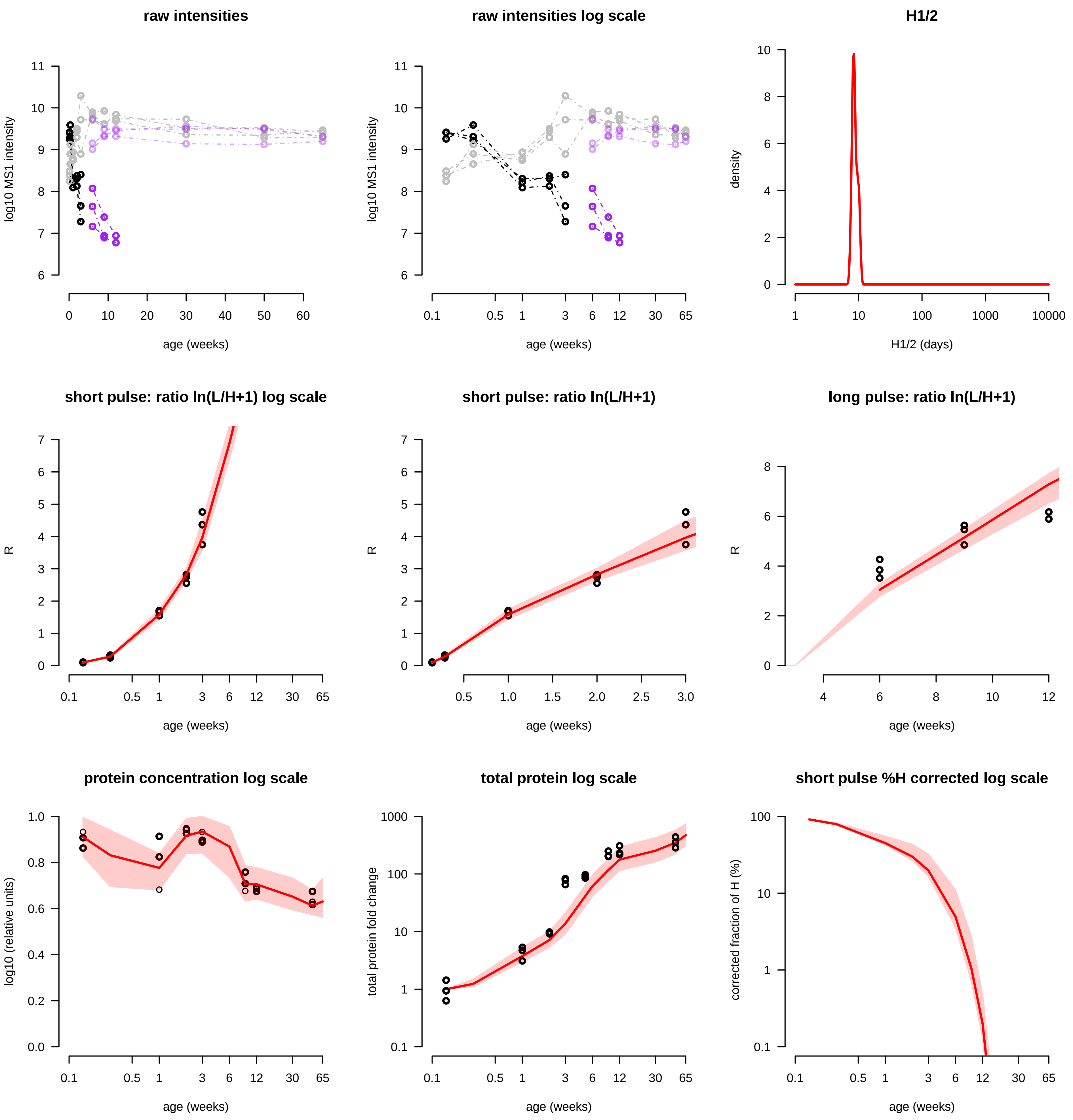

Q3UPL0;Q3UPL0−2

>sp|Q3UPL0|SC31A_MOUSE

Protein transport protein Sec31A

GN=Sec31a

Med H1/2: 4.7 days

Q5 H1/2: 4 days

Q95 H1/2: 5.6 days

reliable model fit: yes

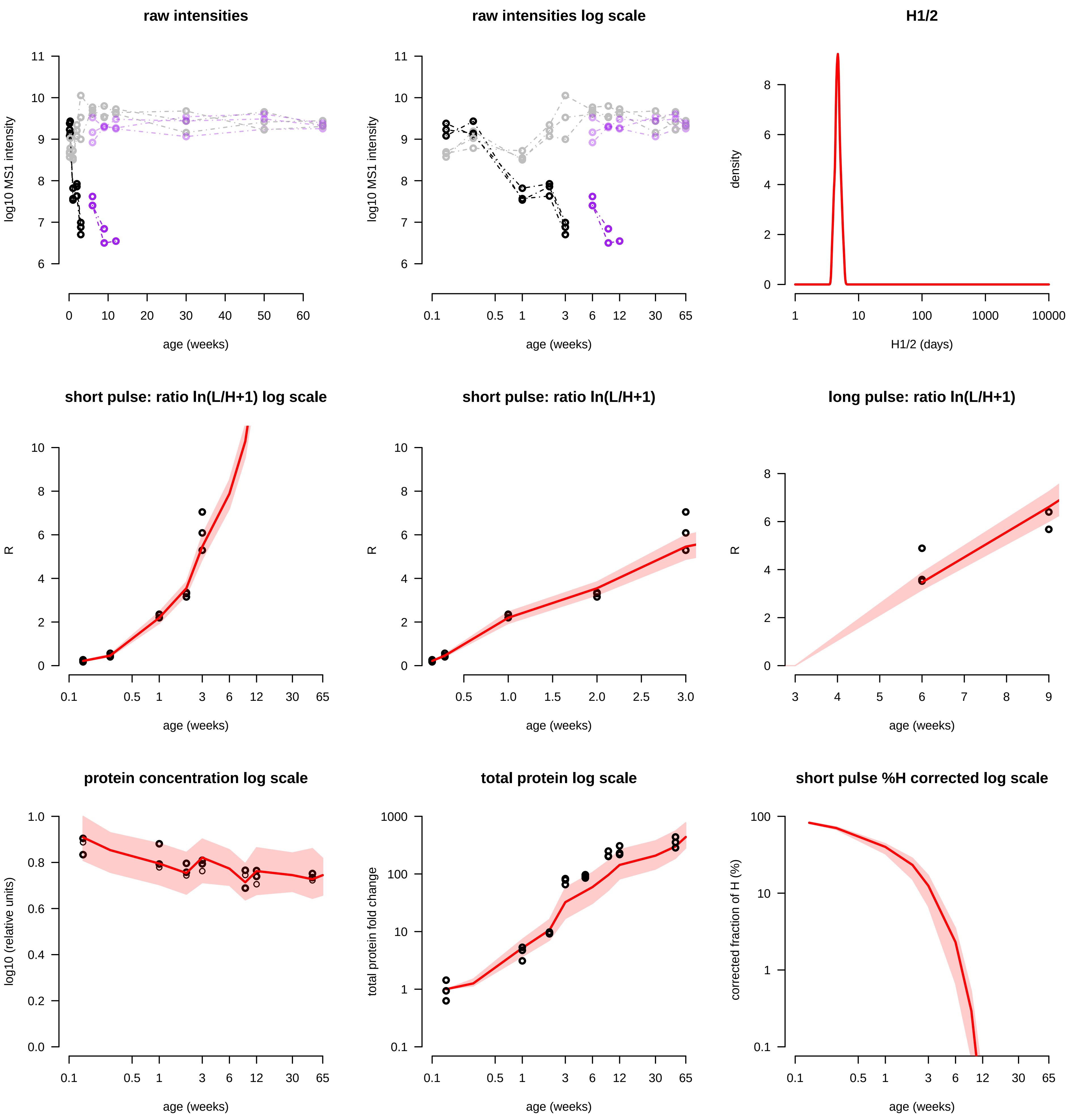

Q8BP67

>sp|Q8BP67|RL24_MOUSE

60S ribosomal protein L24

GN=Rpl24

Med H1/2: 17.1 days

Q5 H1/2: 12.1 days

Q95 H1/2: 22.3 days

reliable model fit: yes

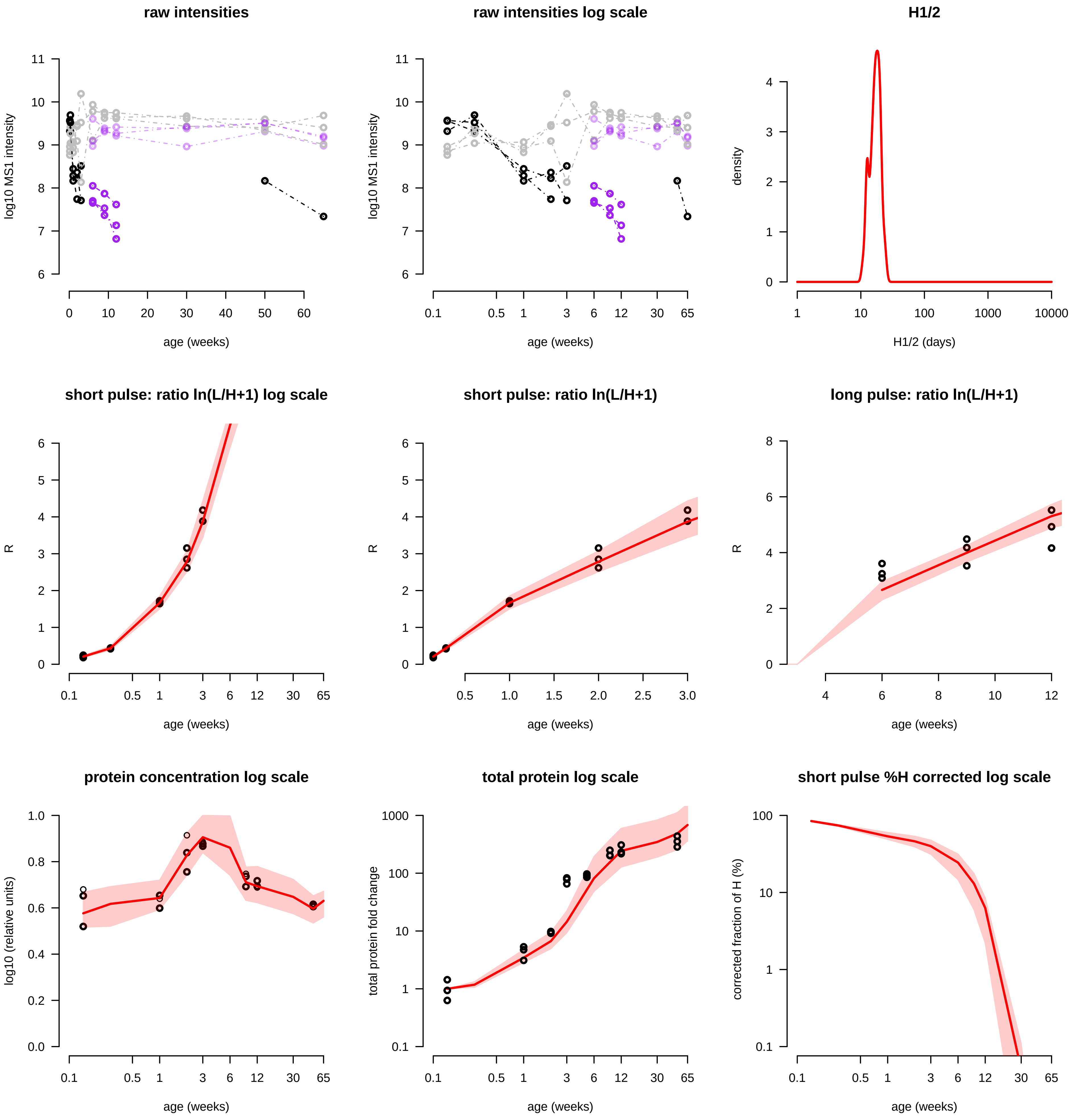

Q64511

>sp|Q64511|TOP2B_MOUSE

DNA topoisomerase 2−beta

GN=Top2b

Med H1/2: 5.1 days

Q5 H1/2: 4.1 days

Q95 H1/2: 6.5 days

reliable model fit: yes

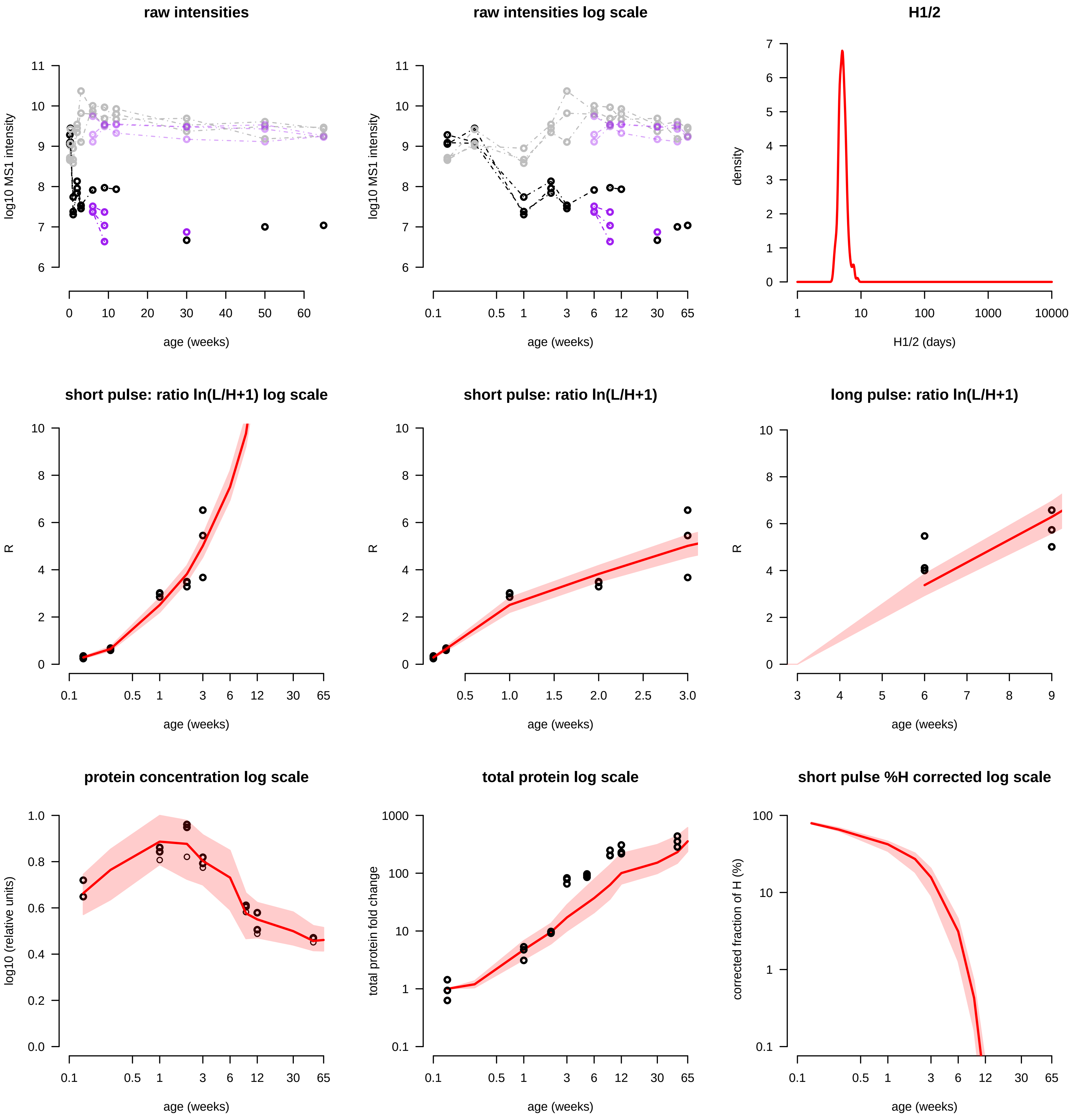

P13634

>sp|P13634|CAH1_MOUSE

Carbonic anhydrase 1

GN=Ca1

Med H1/2: 34.2 days

Q5 H1/2: 19.7 days

Q95 H1/2: 159.9 days

reliable model fit: yes

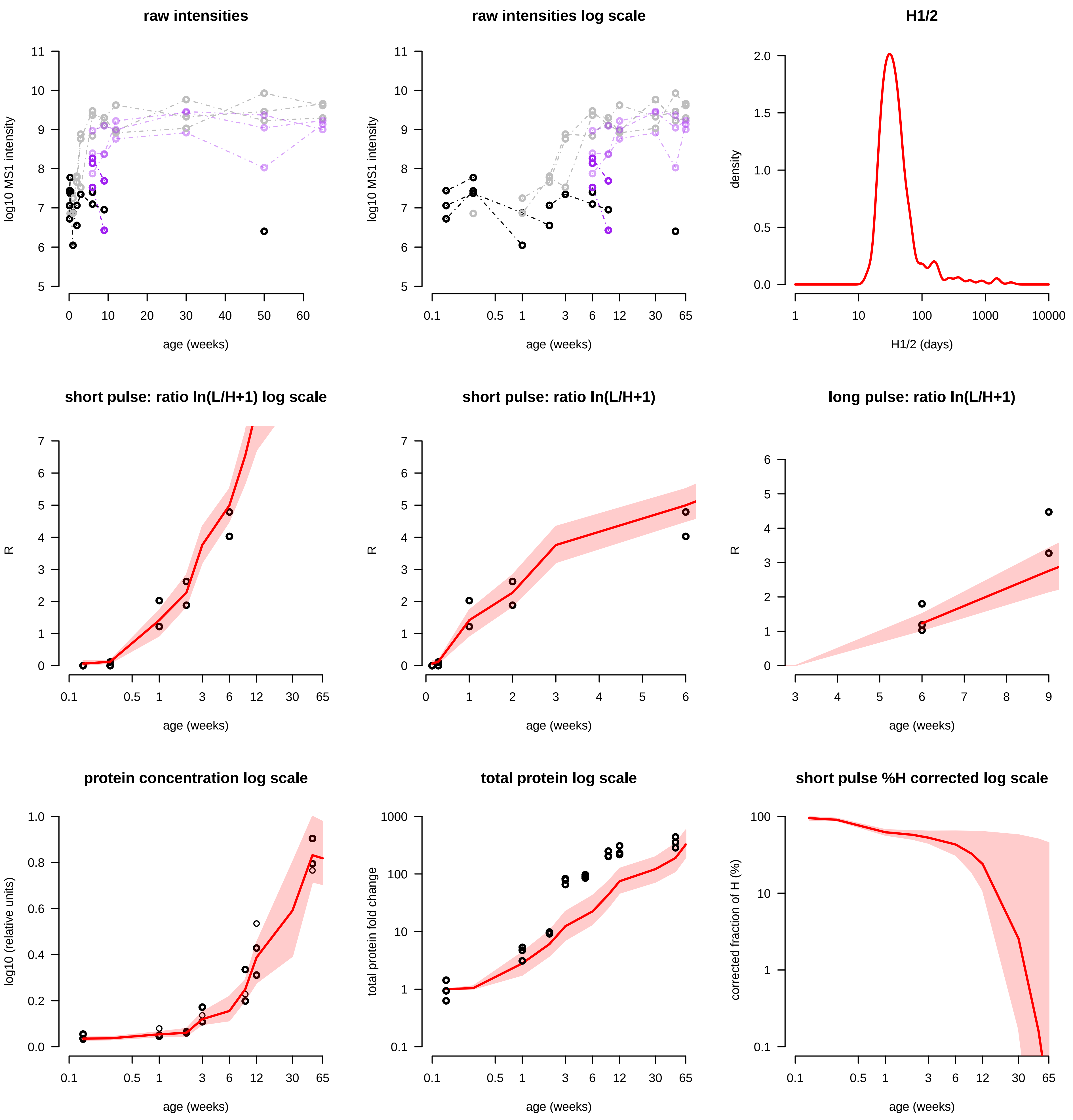

P00405

>sp|P00405|COX2_MOUSE

Cytochrome c oxidase subunit 2

GN=Mtco2

Med H1/2: 8.4 days

Q5 H1/2: 6.9 days

Q95 H1/2: 10.7 days

reliable model fit: yes

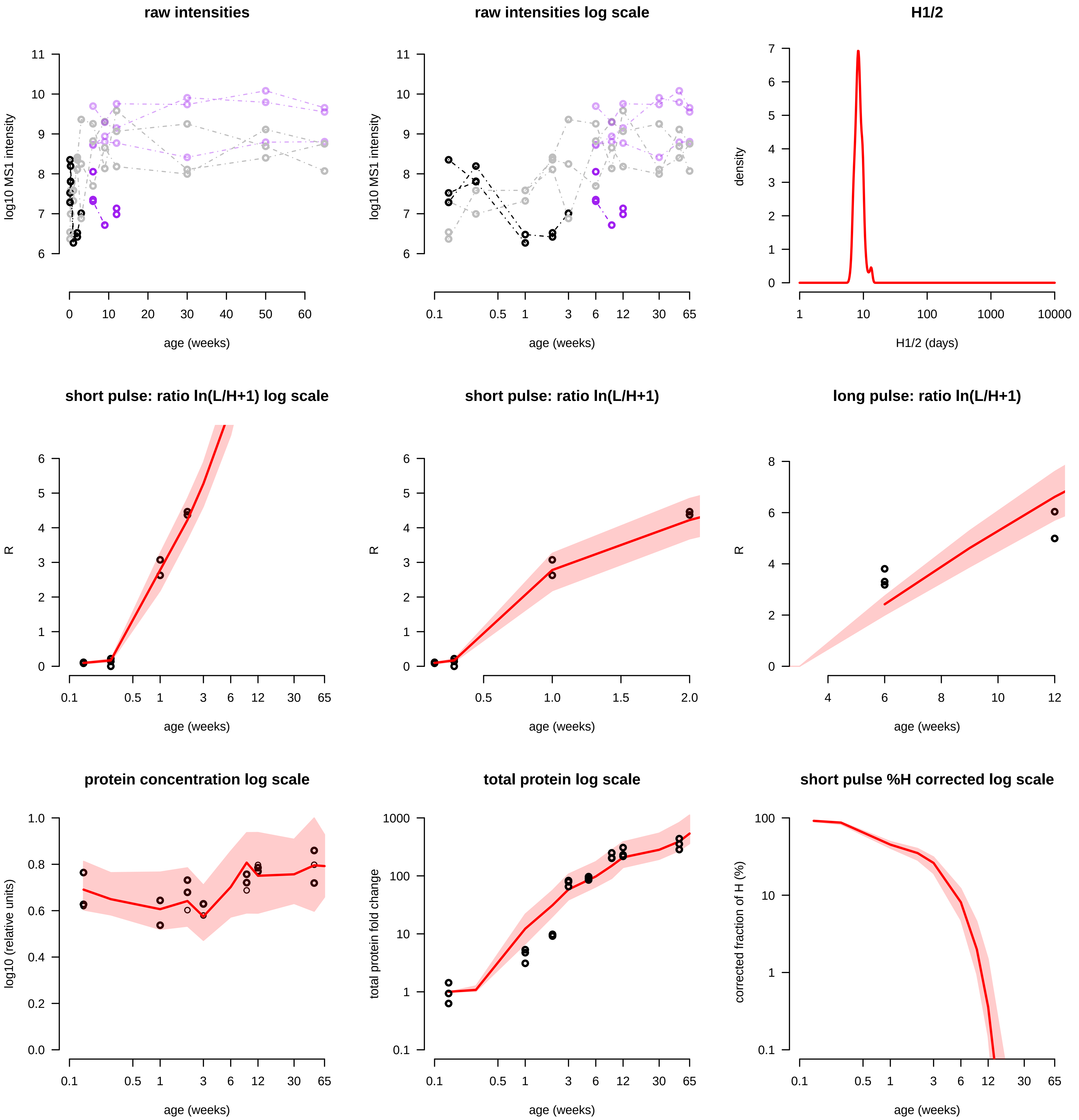

Q8BFZ9

>sp|Q8BFZ9|ERLN2_MOUSE

Erlin−2

GN=Erlin2

Med H1/2: 6.4 days

Q5 H1/2: 5.6 days

Q95 H1/2: 7.6 days

reliable model fit: yes

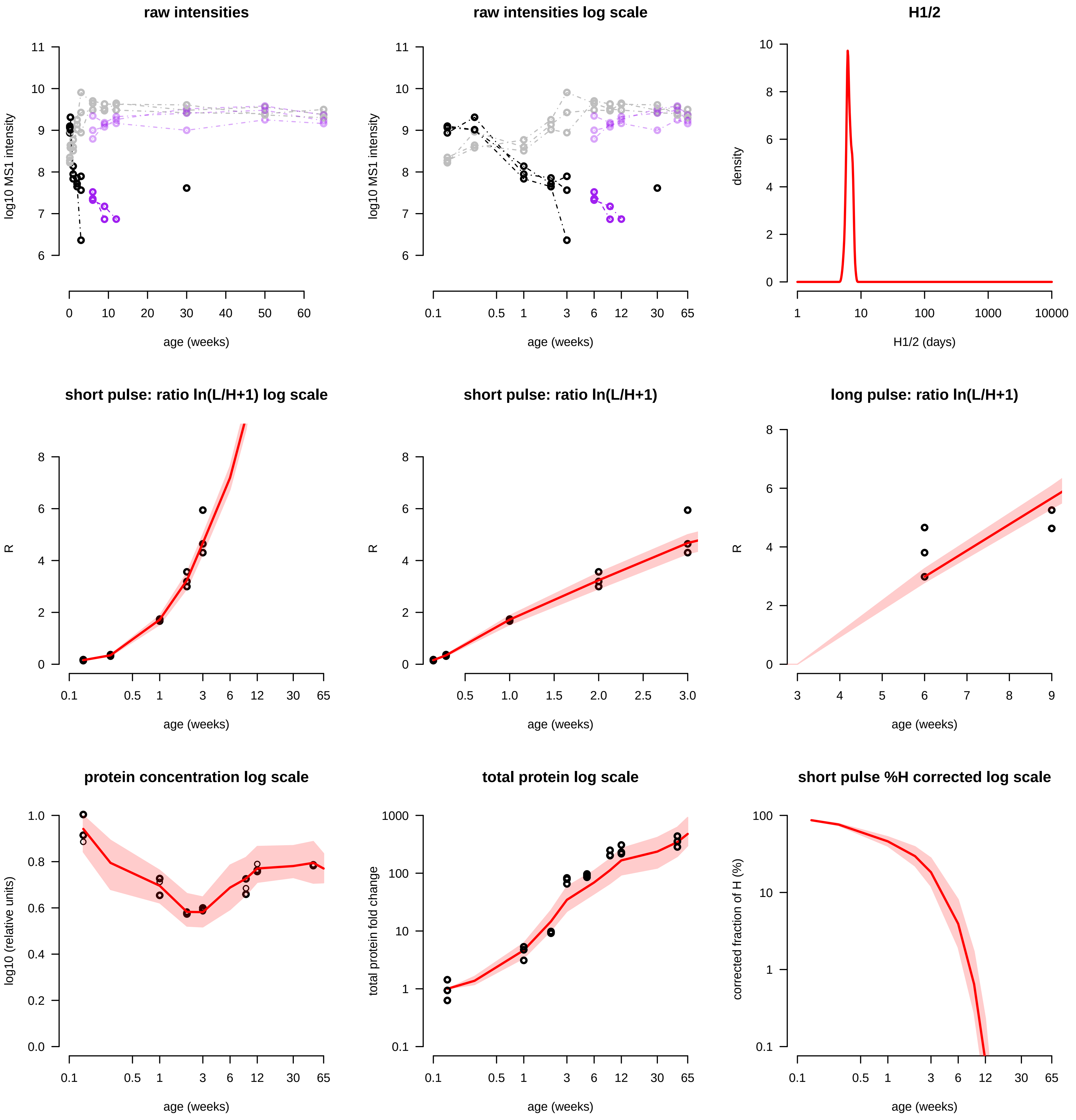

P62192

>sp|P62192|PRS4_MOUSE

26S proteasome regulatory subunit 4

GN=Psmc1

Med H1/2: 928.9 days

Q5 H1/2: 232.8 days

Q95 H1/2: 10620.6 days

reliable model fit: yes

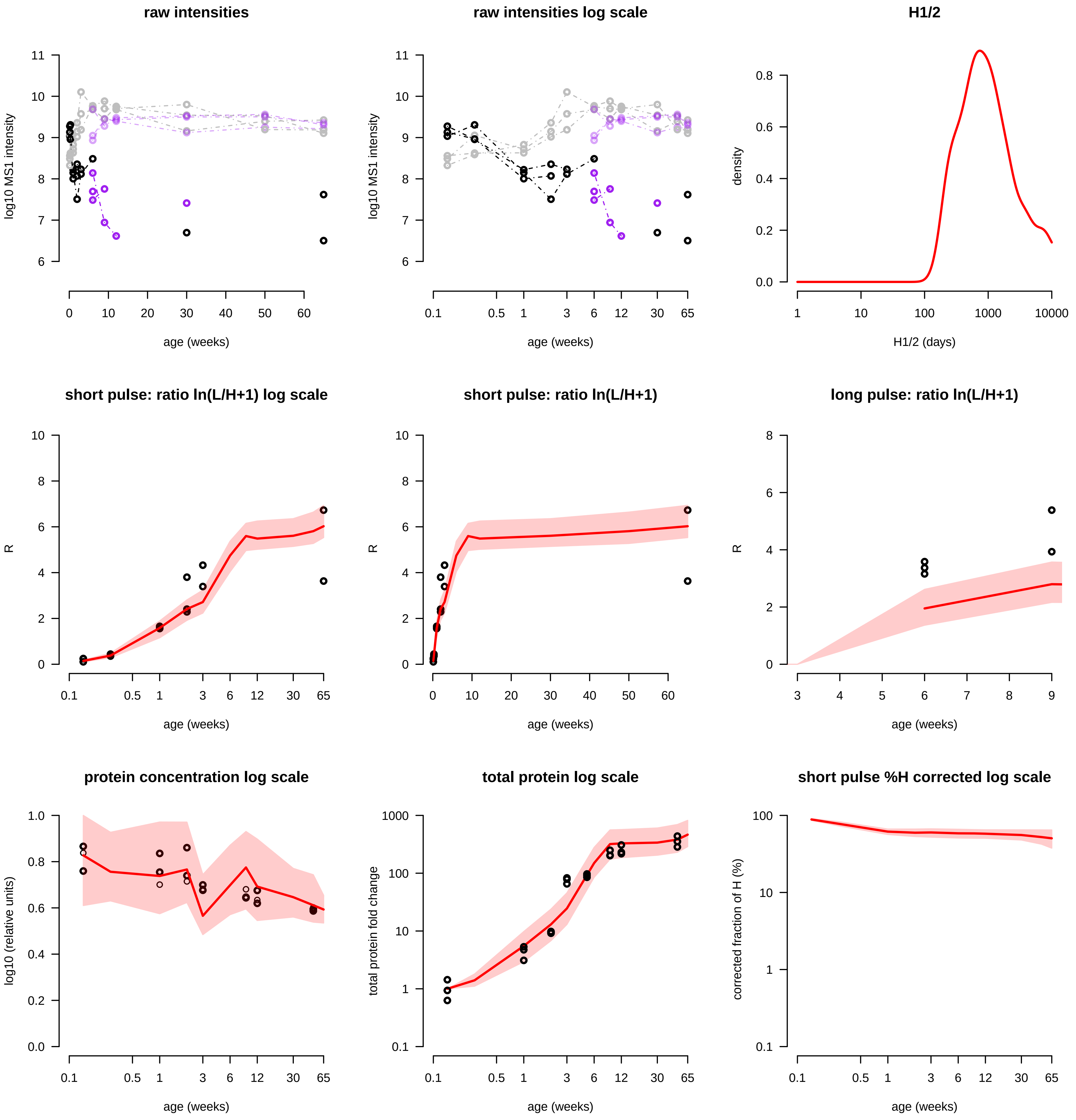

Q922D8

>sp|Q922D8|C1TC_MOUSE

C−1−tetrahydrofolate synthase, cytoplasmic

GN=Mthfd1

Med H1/2: 4.2 days

Q5 H1/2: 3.5 days

Q95 H1/2: 5.3 days

reliable model fit: yes

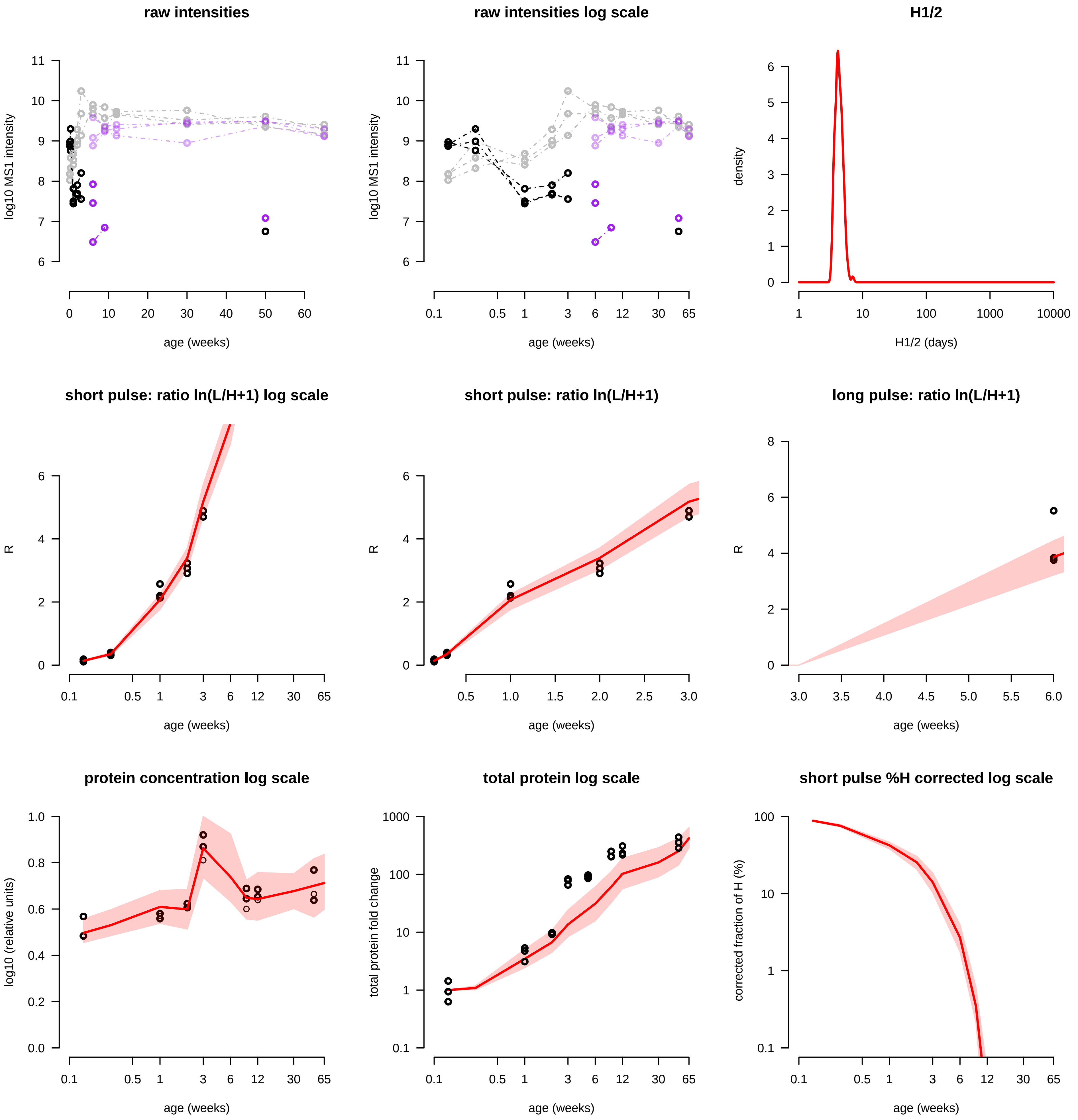

Q8BGH2

>sp|Q8BGH2|SAM50_MOUSE

Sorting and assembly machinery component 50 homolog

GN=Samm50

Med H1/2: 910.9 days

Q5 H1/2: 180.7 days

Q95 H1/2: 13944.2 days

reliable model fit: no

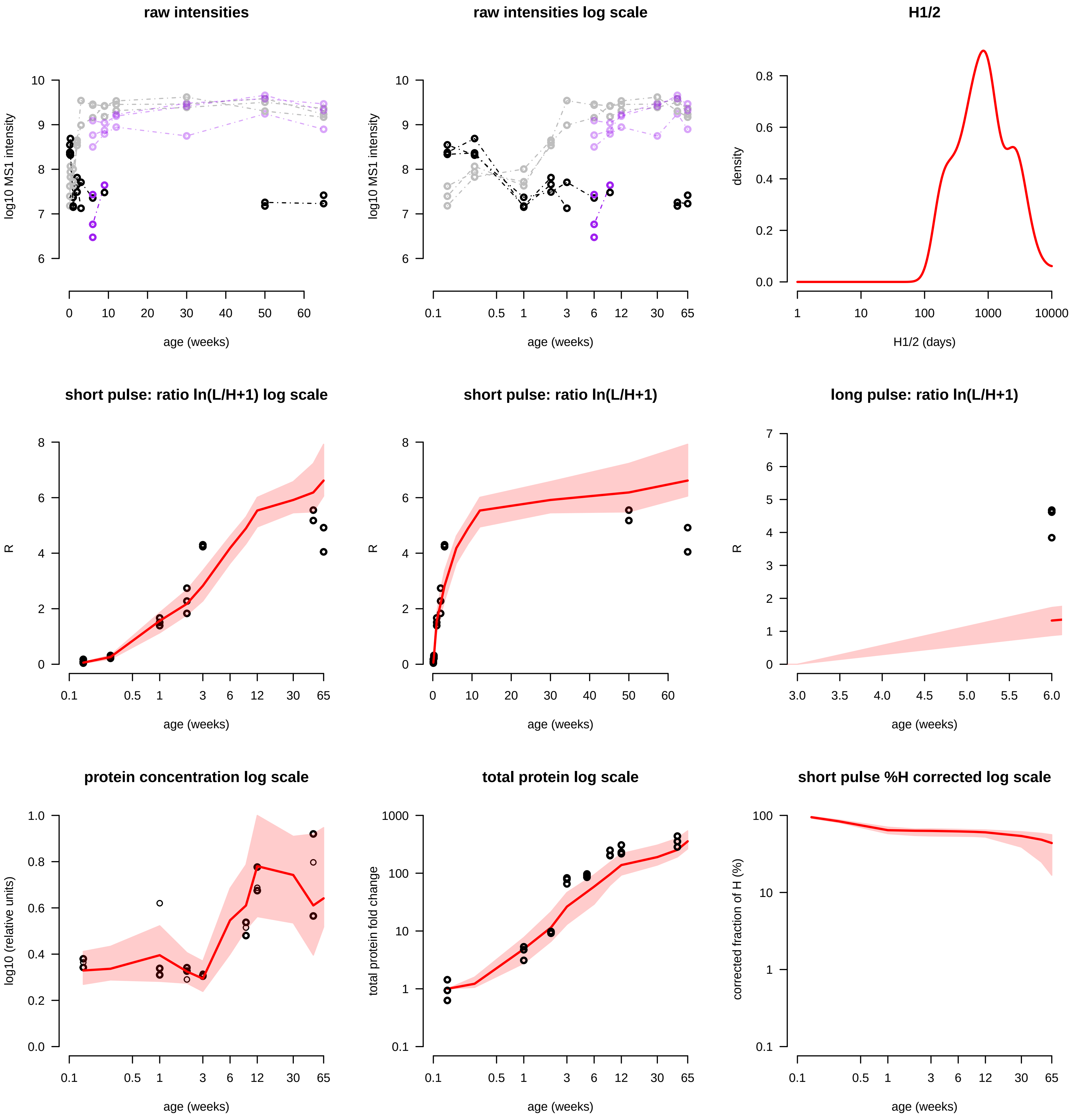

Q04750

>sp|Q04750|TOP1_MOUSE

DNA topoisomerase 1

GN=Top1

Med H1/2: 525.9 days

Q5 H1/2: 190.3 days

Q95 H1/2: 5457.8 days

reliable model fit: no

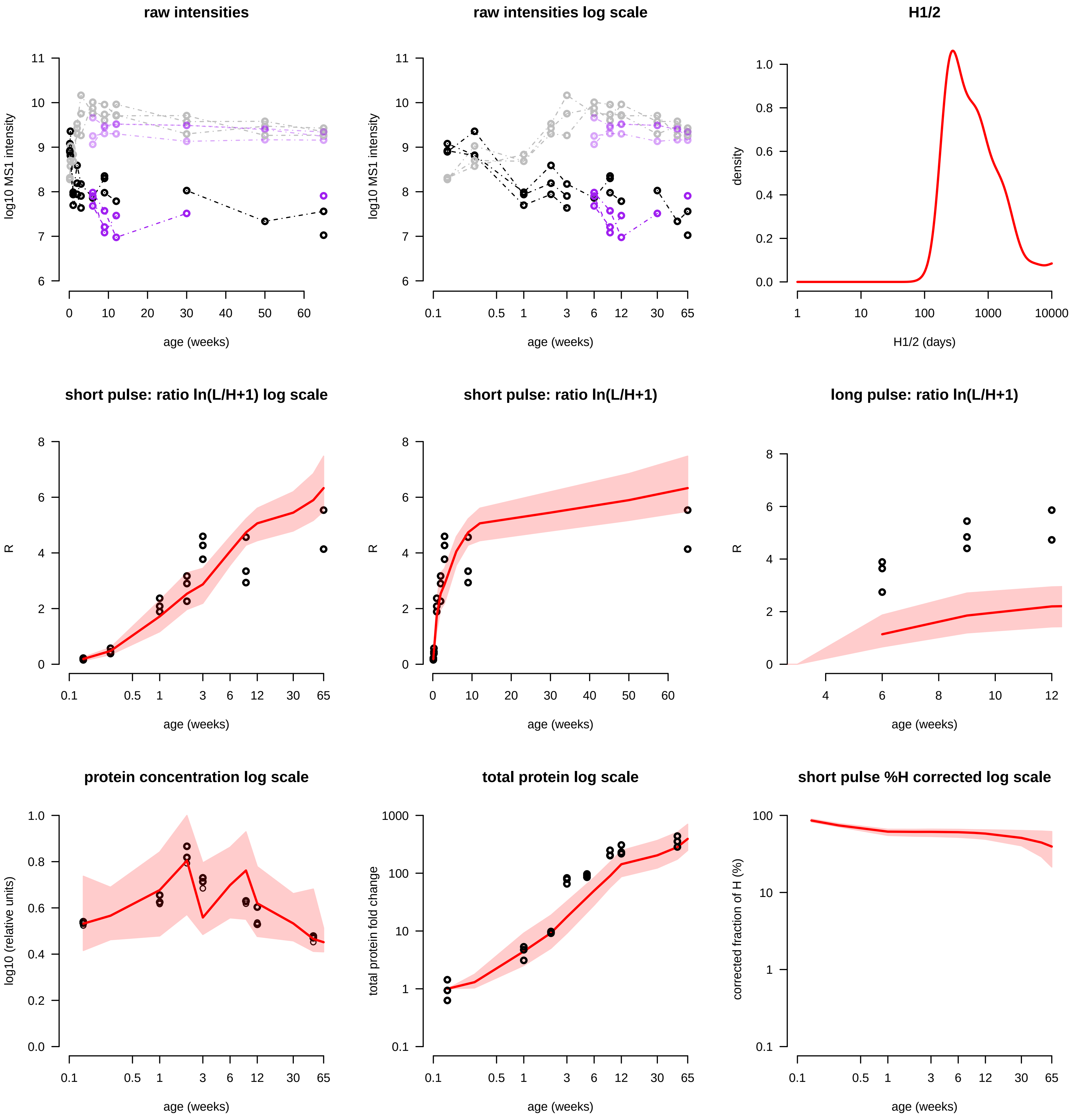

Q9D6R2

>sp|Q9D6R2|IDH3A_MOUSE

Isocitrate dehydrogenase [NAD] subunit alpha, mitochondrial

GN=Idh3a

Med H1/2: 732.8 days

Q5 H1/2: 167.5 days

Q95 H1/2: 5344.8 days

reliable model fit: no

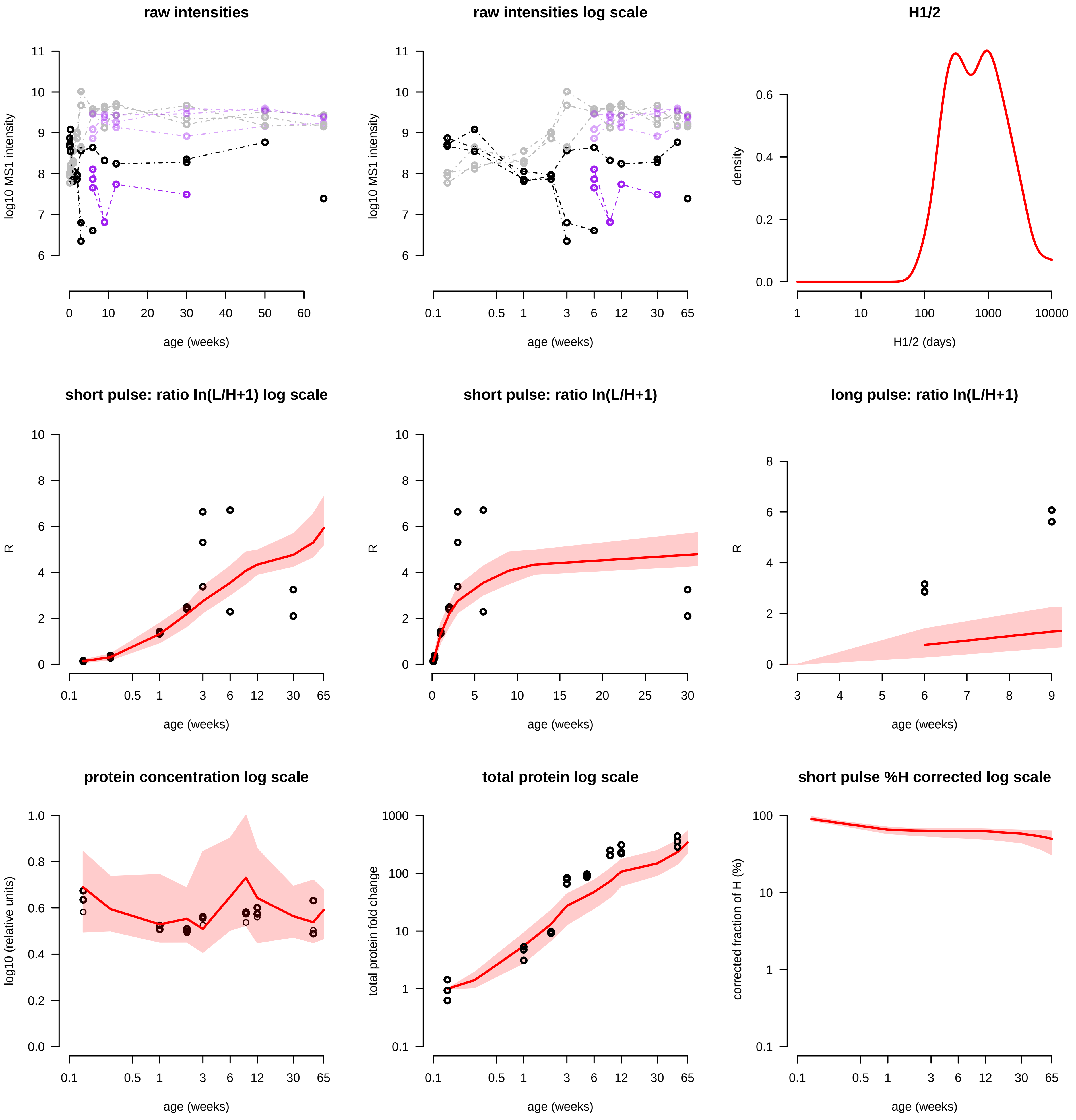

Q9Z0N1

>sp|Q9Z0N1|IF2G_MOUSE

Eukaryotic translation initiation factor 2 subunit 3, X−linked

GN=Eif2s3x

Med H1/2: 7.1 days

Q5 H1/2: 5.8 days

Q95 H1/2: 9.2 days

reliable model fit: yes

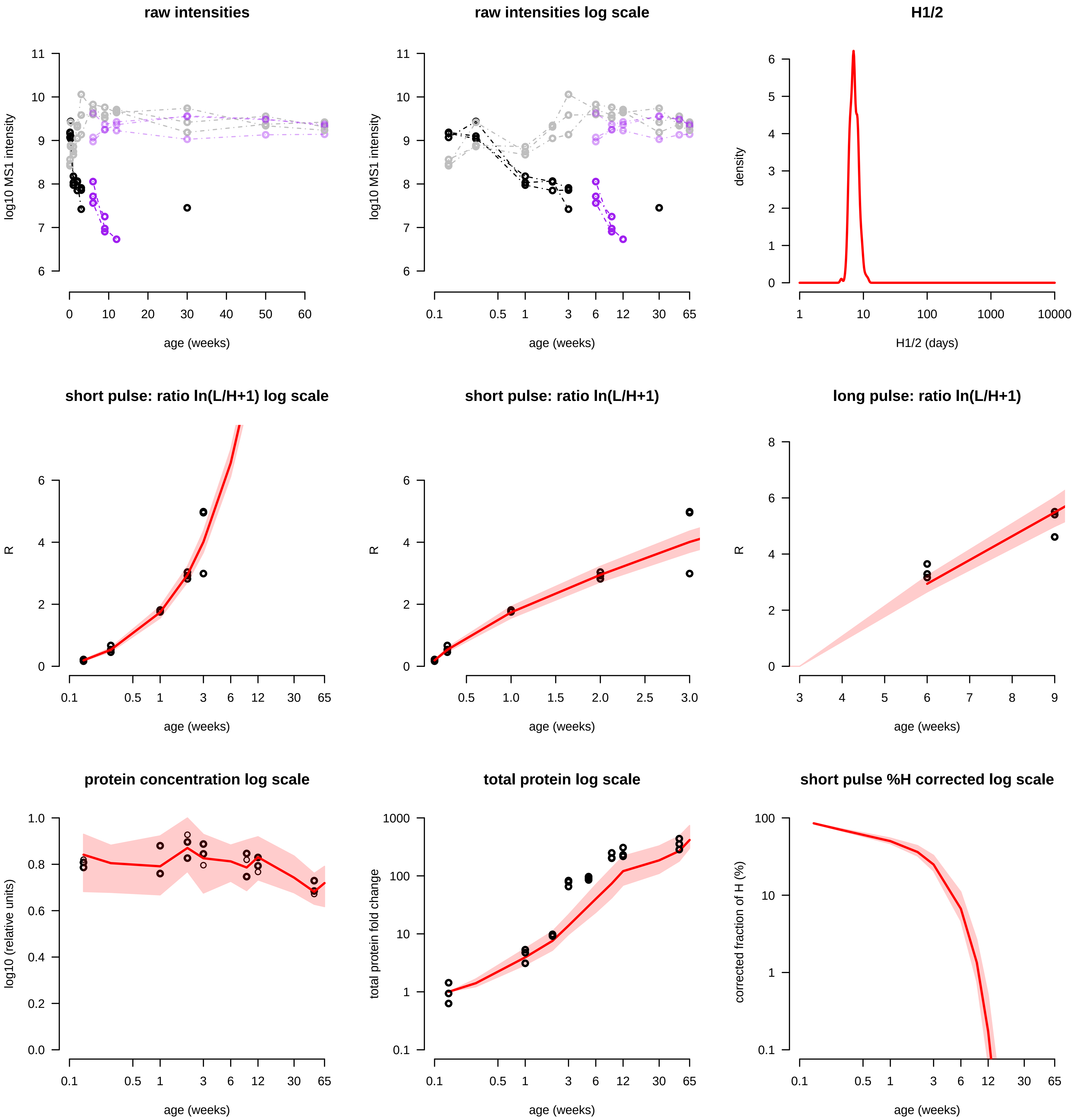

P56399

>sp|P56399|UBP5_MOUSE

Ubiquitin carboxyl−terminal hydrolase 5

GN=Usp5

Med H1/2: 5 days

Q5 H1/2: 4.3 days

Q95 H1/2: 5.9 days

reliable model fit: yes

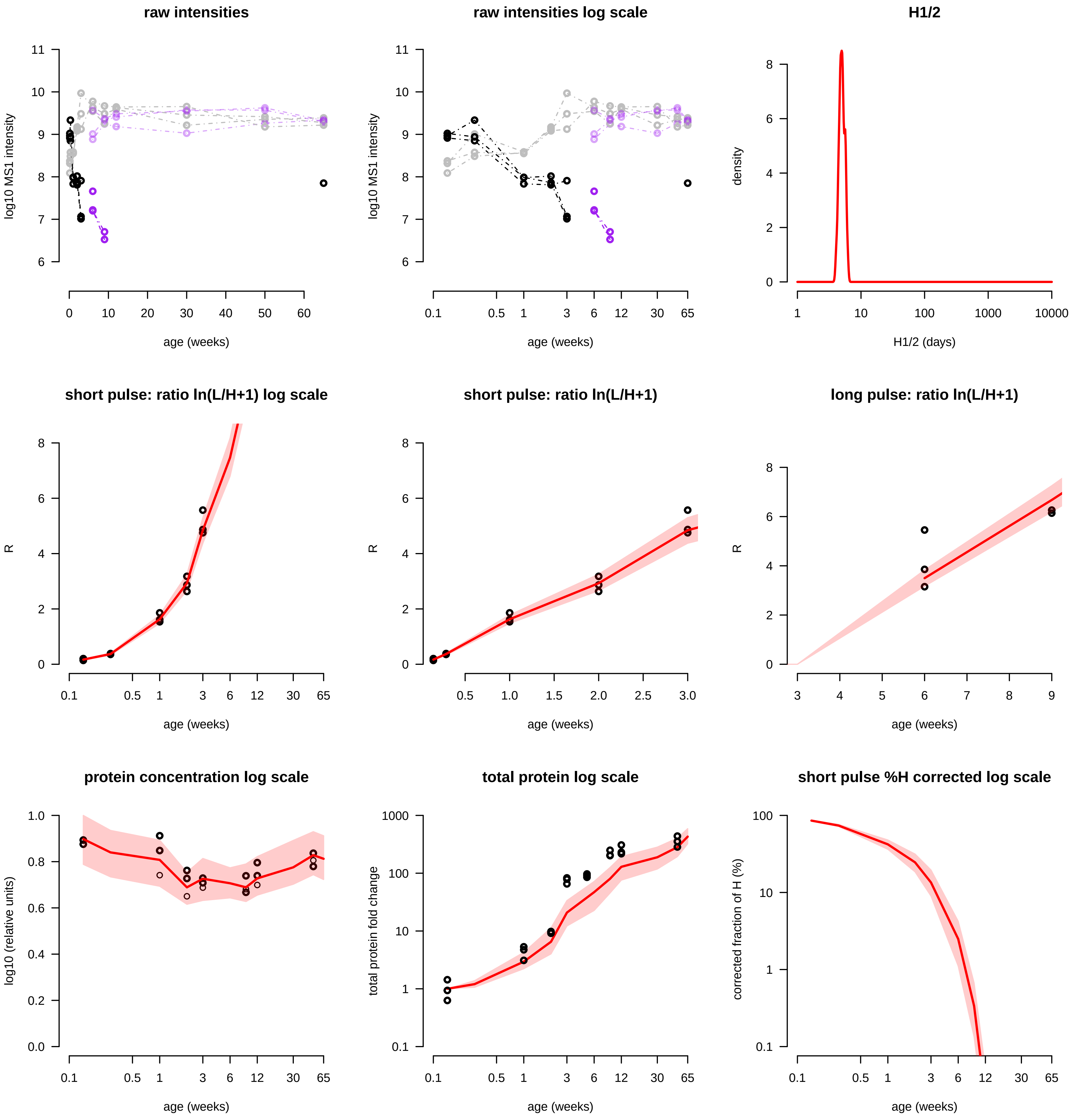

Q8BG05−2

>sp|Q8BG05−2|ROA3_MOUSE

Isoform 2 of Heterogeneous nuclear ribonucleoprotein A3

GN=Hnrnpa3

Med H1/2: 9.4 days

Q5 H1/2: 7.6 days

Q95 H1/2: 14 days

reliable model fit: yes

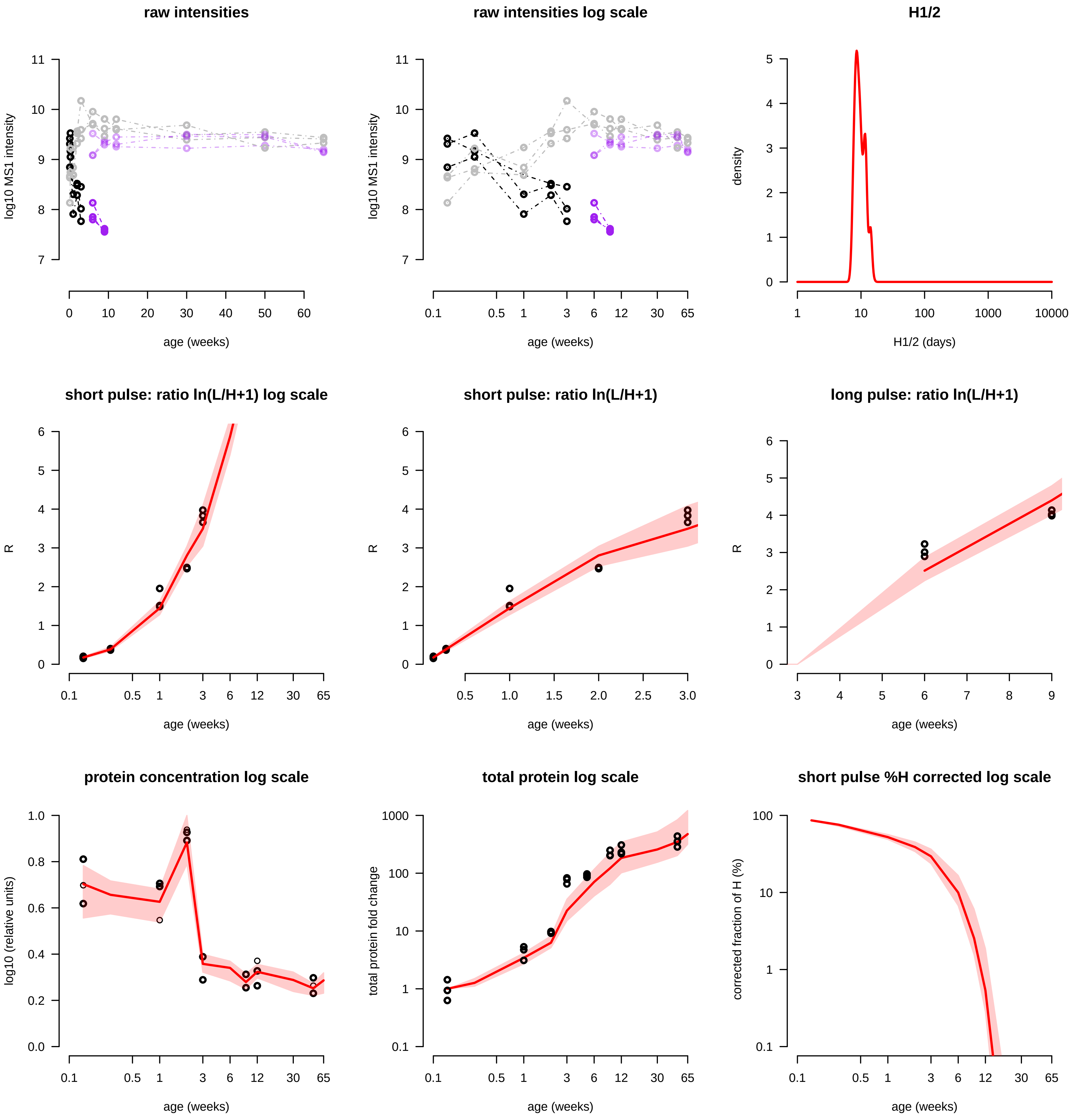

Q9QUI0

>sp|Q9QUI0|RHOA_MOUSE

Transforming protein RhoA

GN=Rhoa

Med H1/2: 9.2 days

Q5 H1/2: 7.4 days

Q95 H1/2: 11.5 days

reliable model fit: yes

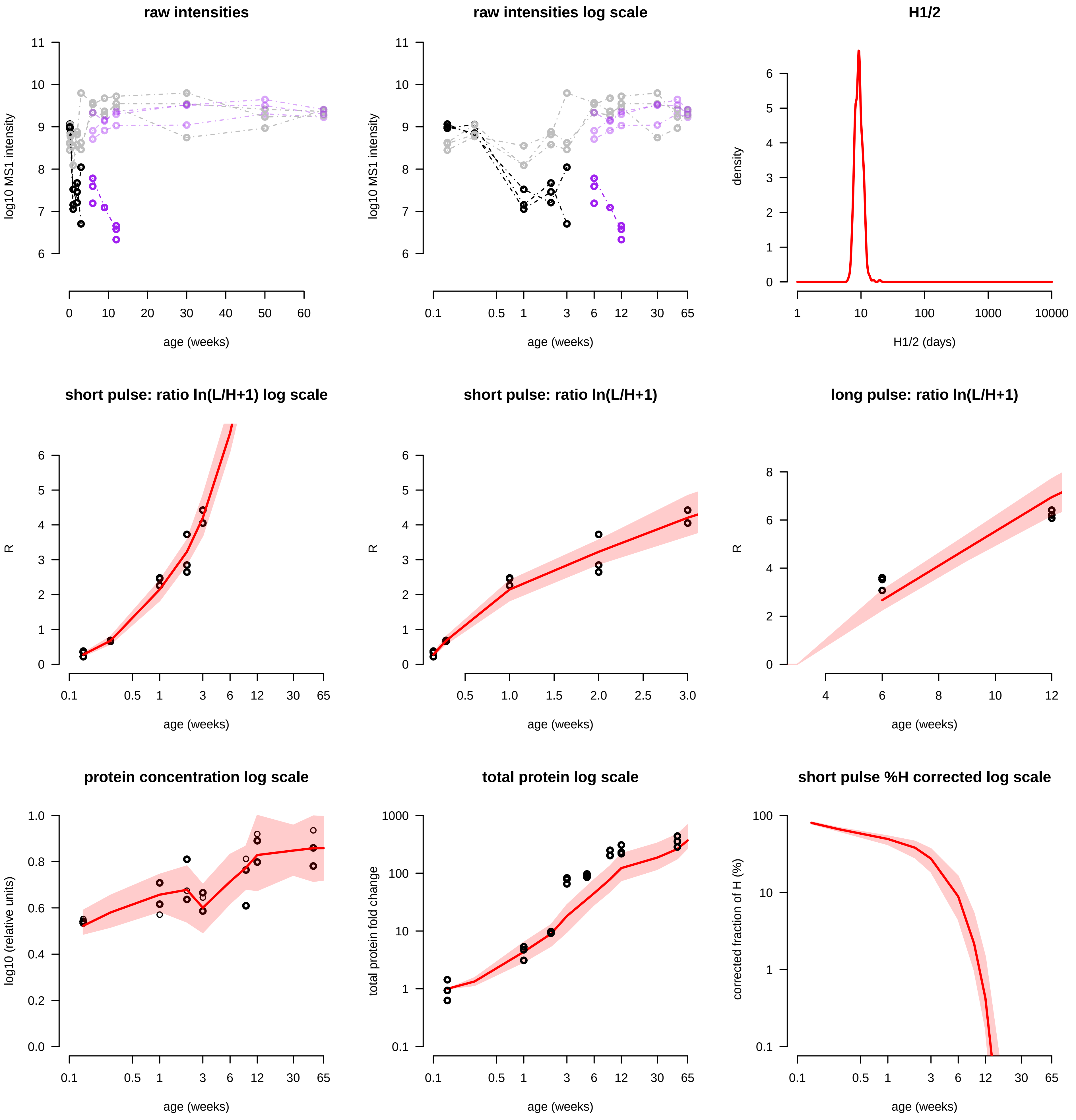

Q64331

>sp|Q64331|MYO6_MOUSE

Unconventional myosin−VI

GN=Myo6

Med H1/2: 5.2 days

Q5 H1/2: 4.3 days

Q95 H1/2: 6.5 days

reliable model fit: yes

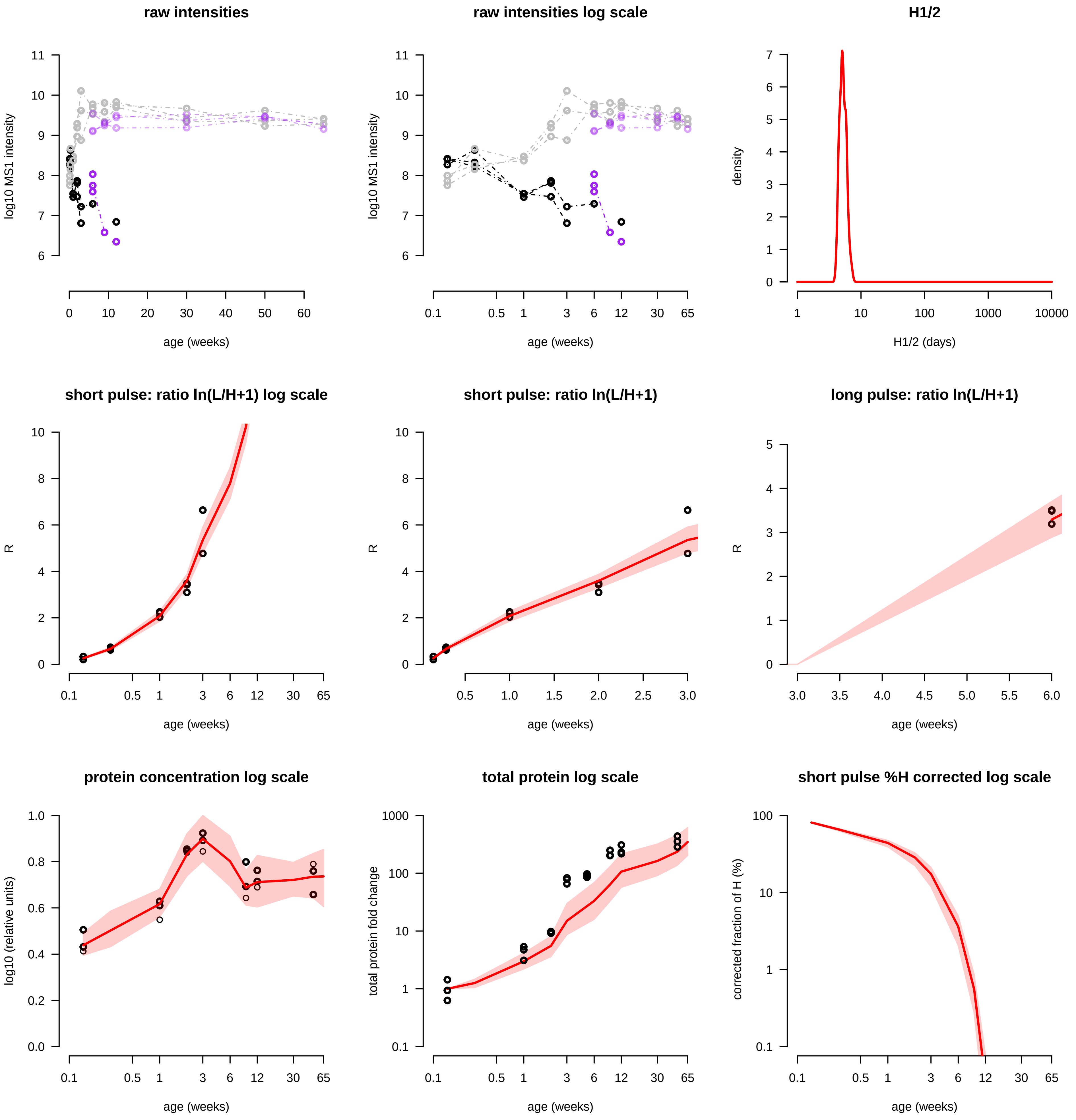

Q9WVA4

>sp|Q9WVA4|TAGL2_MOUSE

Transgelin−2

GN=Tagln2

Med H1/2: 809.3 days

Q5 H1/2: 177.5 days

Q95 H1/2: 11444.7 days

reliable model fit: no

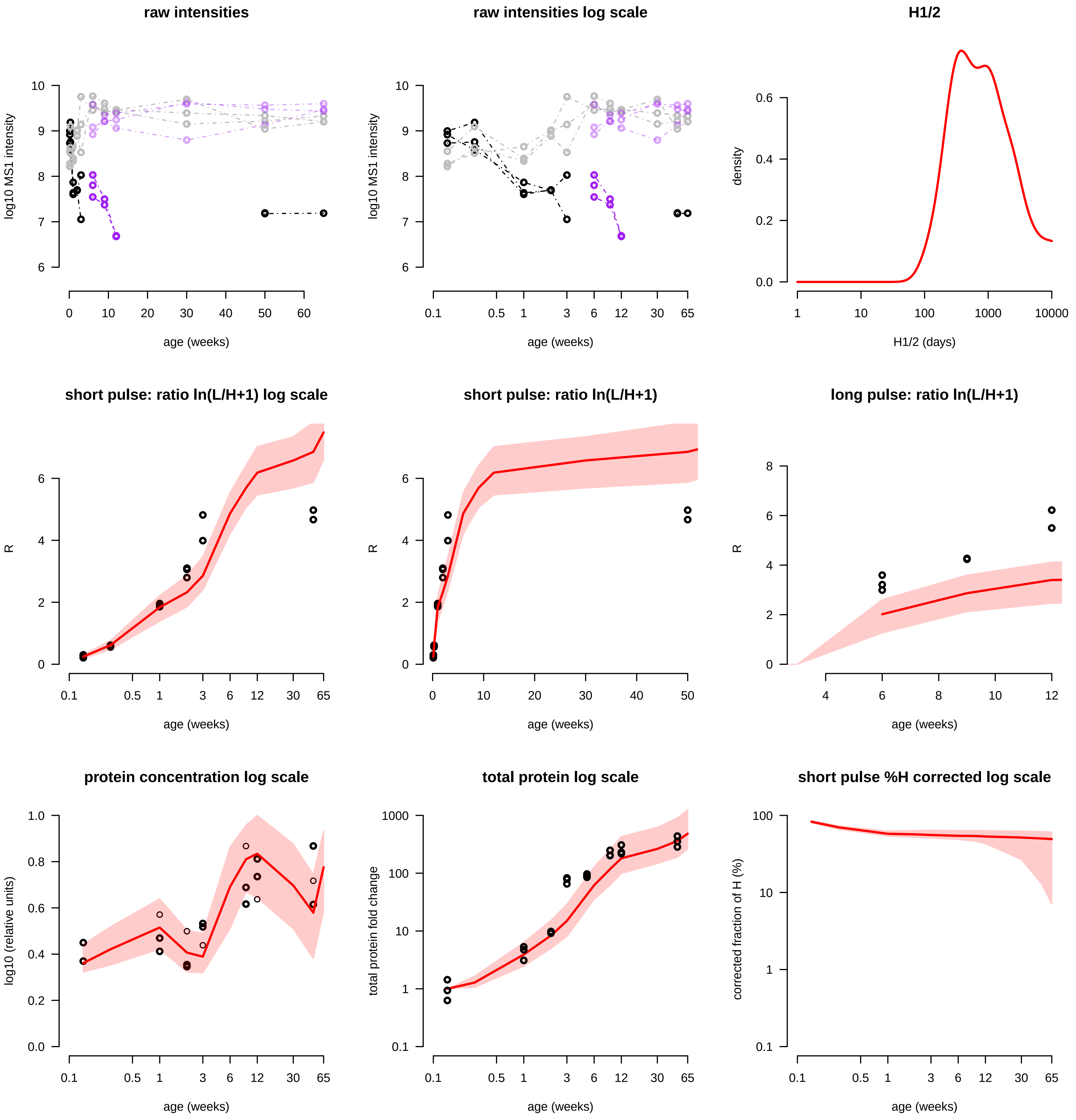

P26231

>sp|P26231|CTNA1_MOUSE

Catenin alpha−1

GN=Ctnna1

Med H1/2: 5.6 days

Q5 H1/2: 4.8 days

Q95 H1/2: 6.9 days

reliable model fit: yes

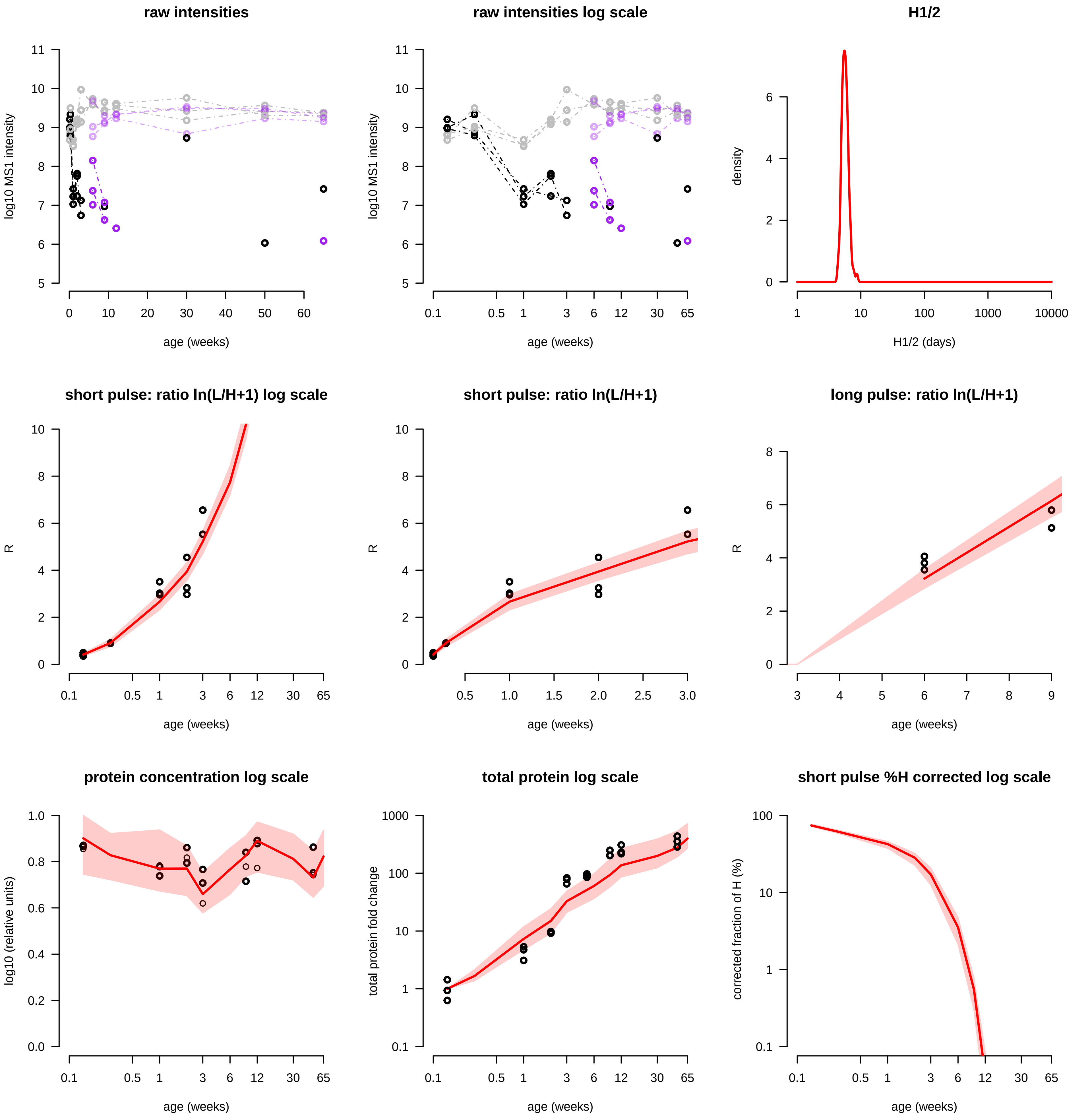

P62267

>sp|P62267|RS23_MOUSE

40S ribosomal protein S23

GN=Rps23

Med H1/2: 17 days

Q5 H1/2: 13 days

Q95 H1/2: 29.5 days

reliable model fit: yes

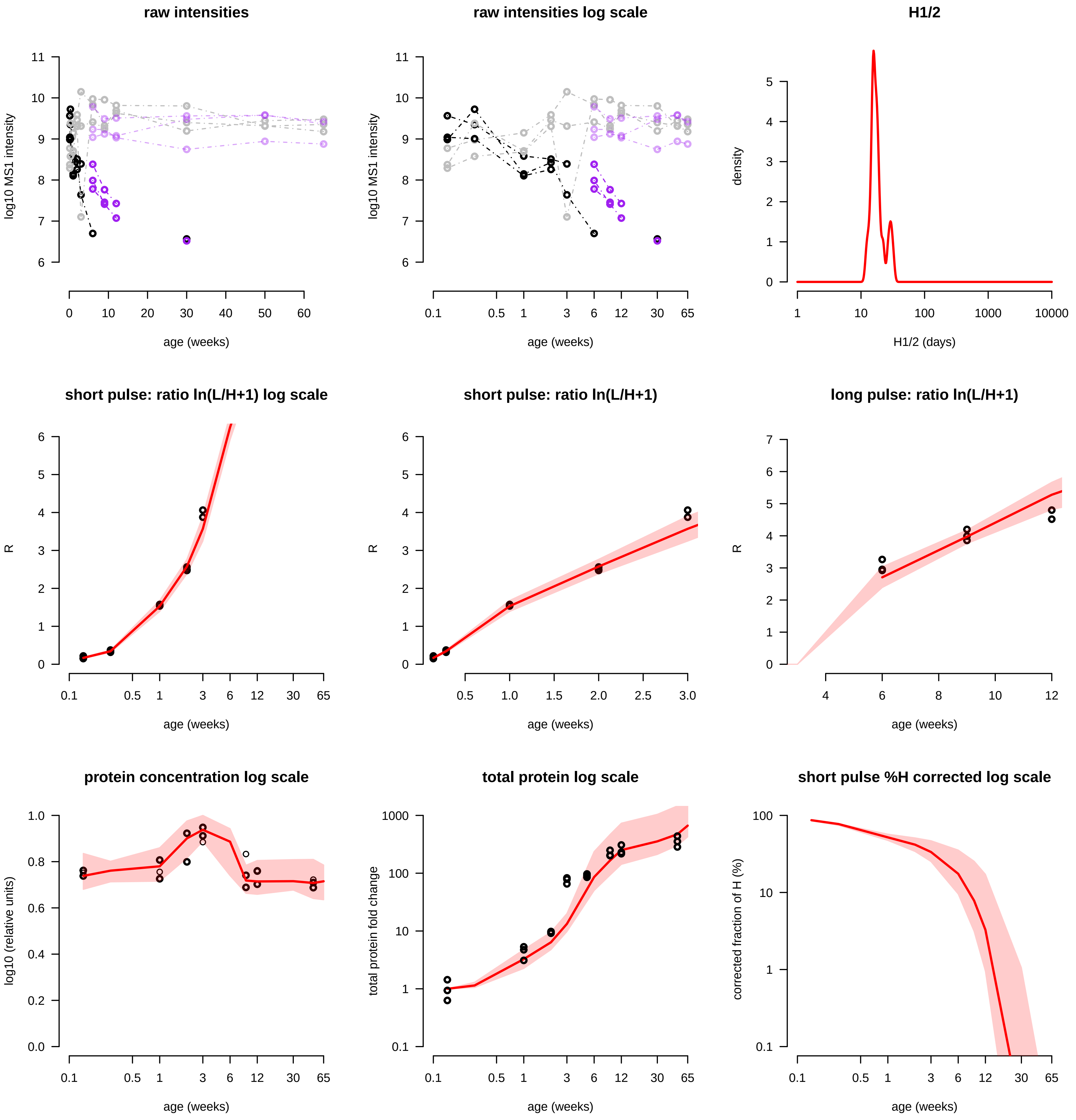

P39447

>sp|P39447|ZO1_MOUSE

Tight junction protein ZO−1

GN=Tjp1

Med H1/2: 4.2 days

Q5 H1/2: 3.5 days

Q95 H1/2: 4.9 days

reliable model fit: yes

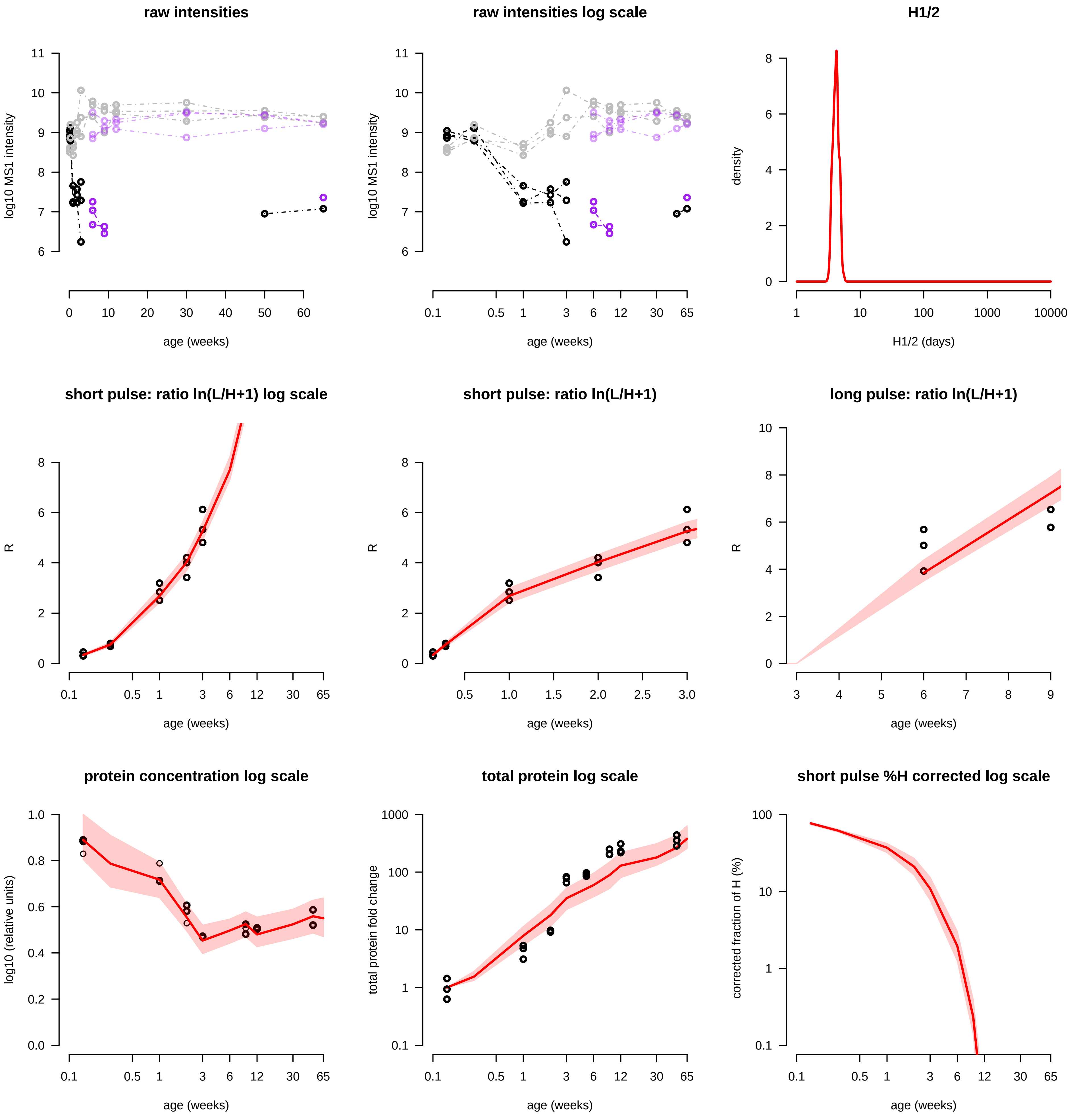

P62270

>sp|P62270|RS18_MOUSE

40S ribosomal protein S18

GN=Rps18

Med H1/2: 10.1 days

Q5 H1/2: 7.6 days

Q95 H1/2: 12.9 days

reliable model fit: yes

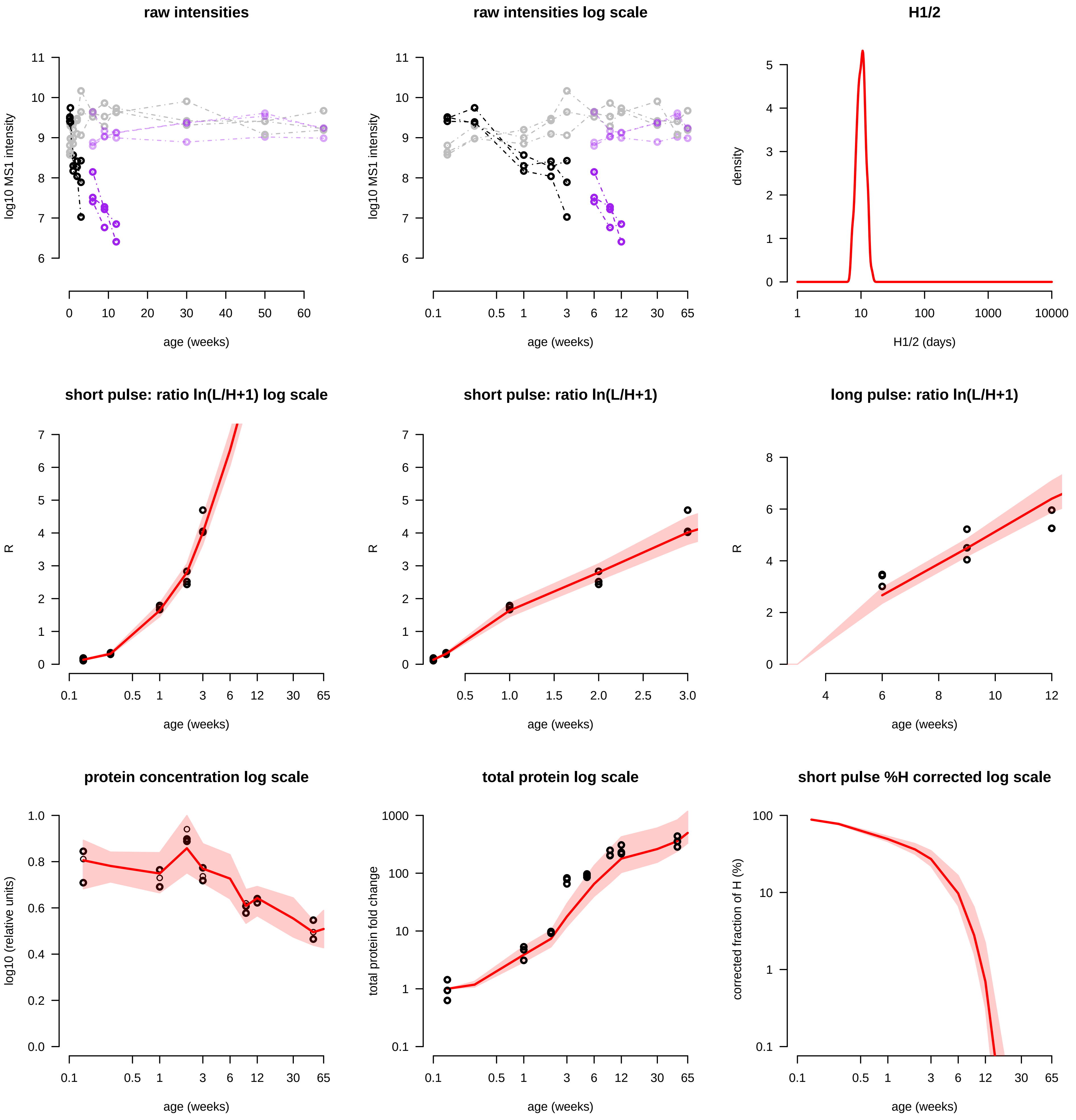

P35278

>sp|P35278|RAB5C_MOUSE

Ras−related protein Rab−5C

GN=Rab5c

Med H1/2: 11.5 days

Q5 H1/2: 9.1 days

Q95 H1/2: 15.7 days

reliable model fit: yes

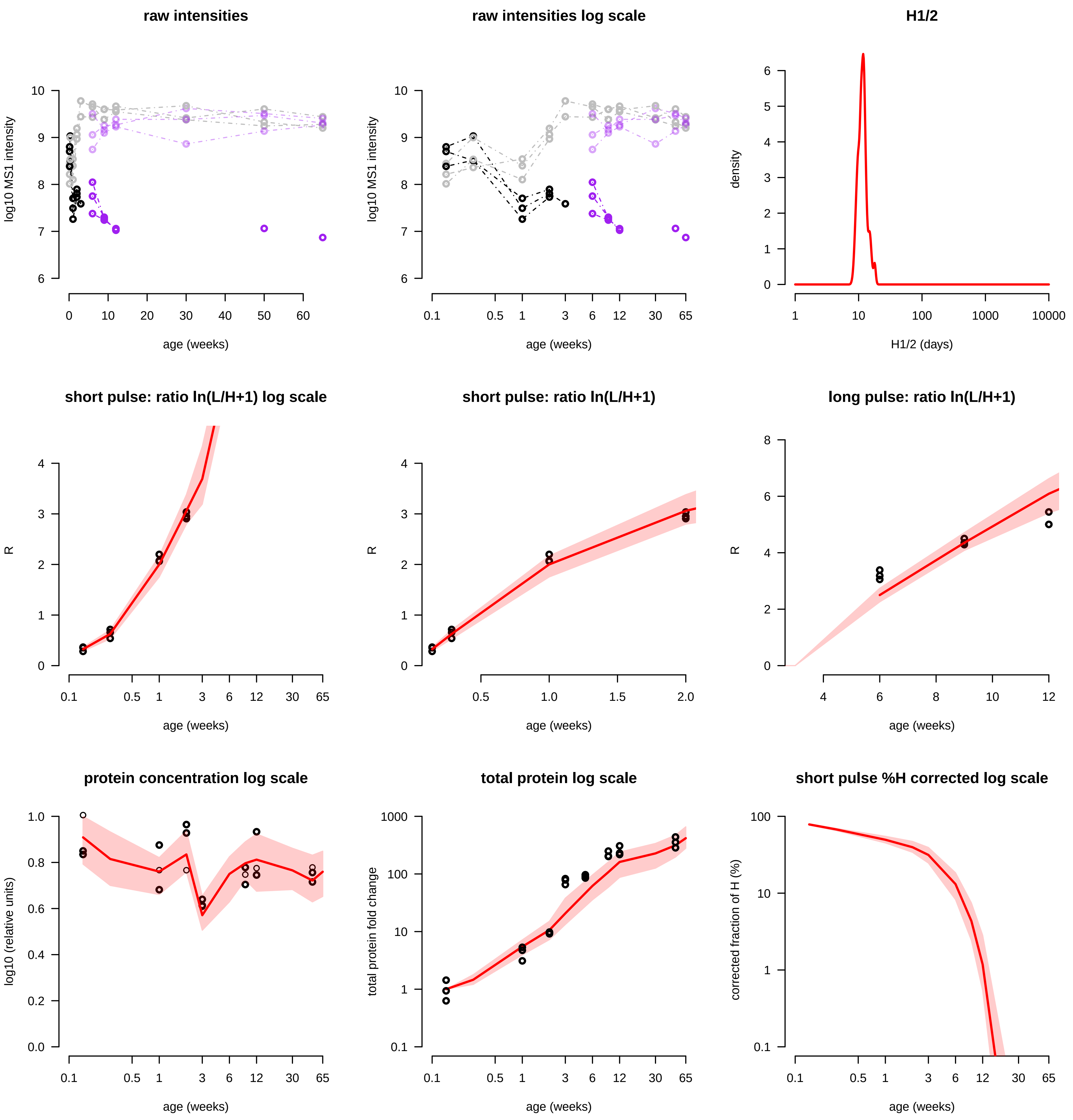

Q9QUM9

>sp|Q9QUM9|PSA6_MOUSE

Proteasome subunit alpha type−6

GN=Psma6

Med H1/2: 15 days

Q5 H1/2: 11.6 days

Q95 H1/2: 19.4 days

reliable model fit: yes

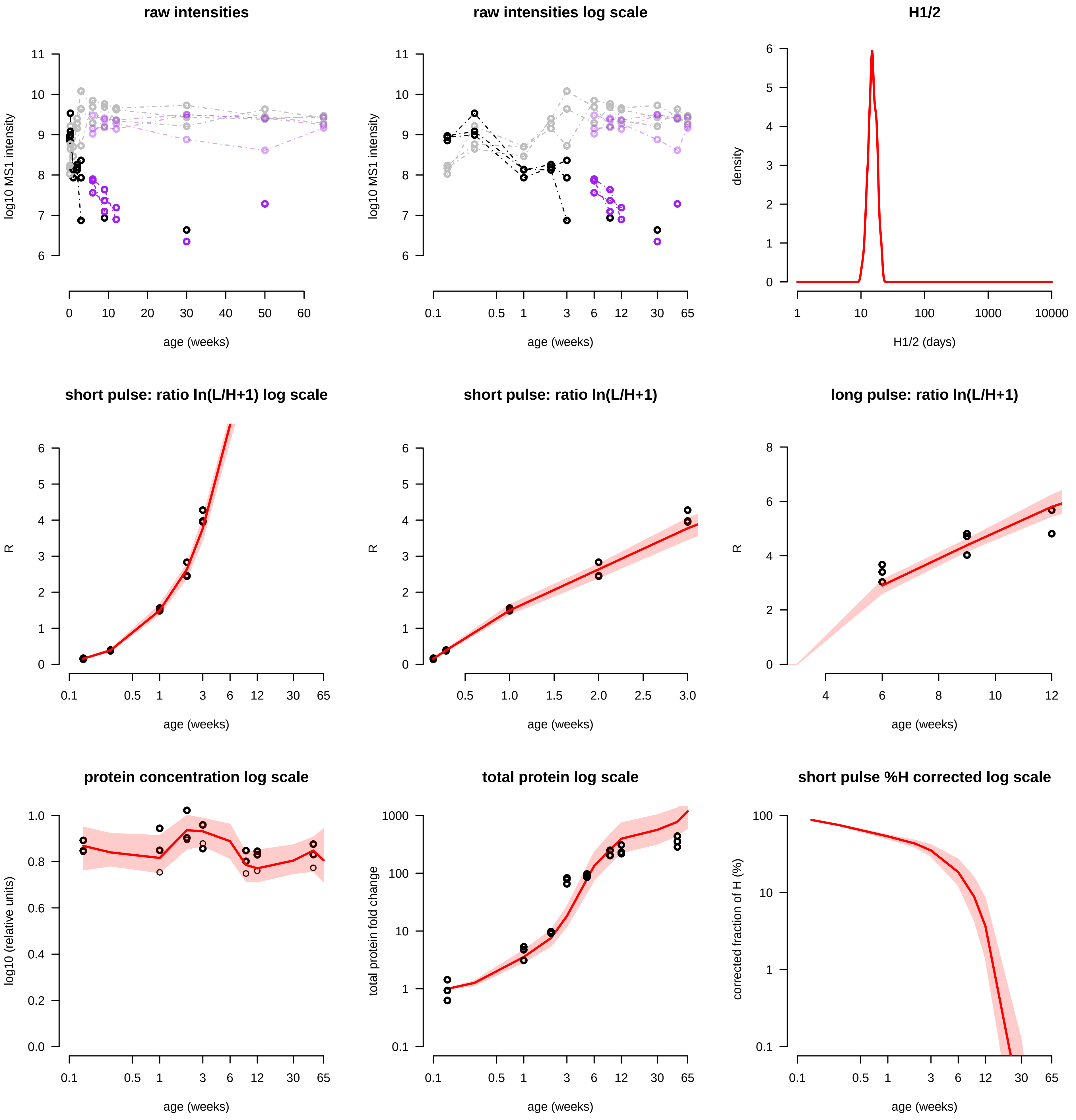

Q60865

>sp|Q60865|CAPR1_MOUSE

Caprin−1

GN=Caprin1

Med H1/2: 105.4 days

Q5 H1/2: 51.2 days

Q95 H1/2: 213.2 days

reliable model fit: yes

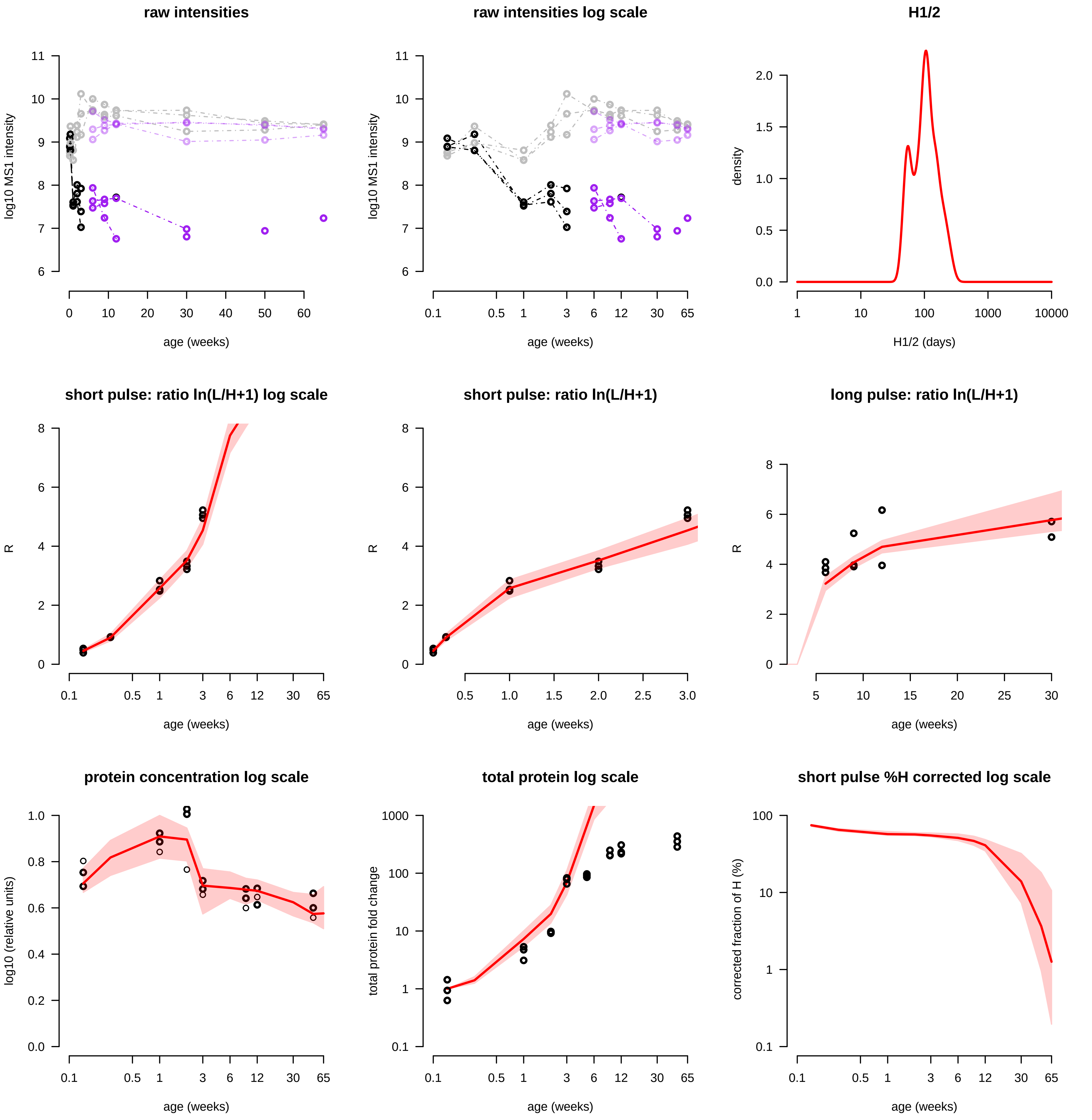

P21619;P21619−2

>sp|P21619|LMNB2_MOUSE

Lamin−B2

GN=Lmnb2

Med H1/2: 236.4 days

Q5 H1/2: 140.5 days

Q95 H1/2: 658.2 days

reliable model fit: yes

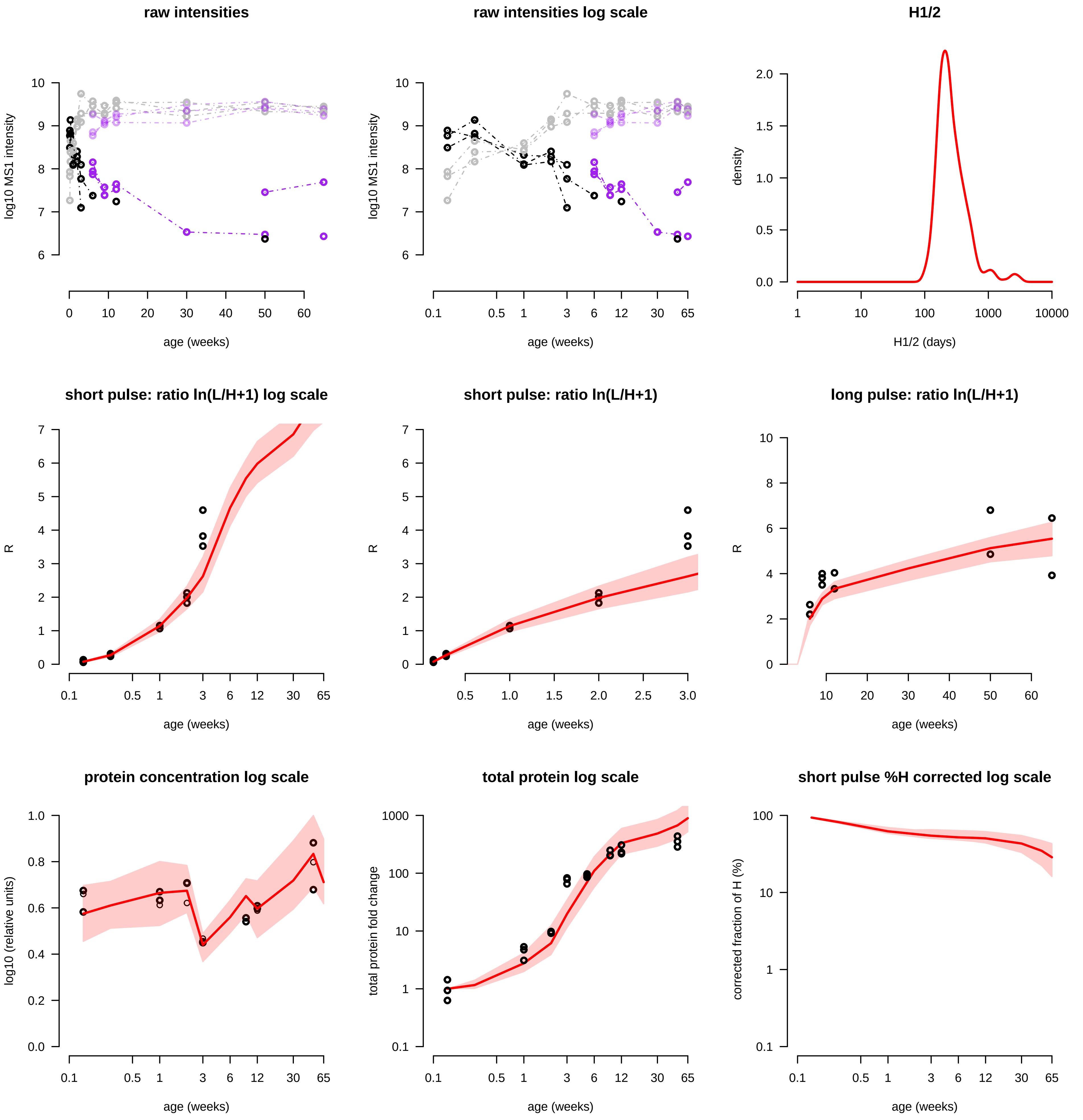

Q9QXX4

>sp|Q9QXX4|CMC2_MOUSE

Calcium−binding mitochondrial carrier protein Aralar2

GN=Slc25a13

Med H1/2: 11.8 days

Q5 H1/2: 8.4 days

Q95 H1/2: 14.8 days

reliable model fit: yes

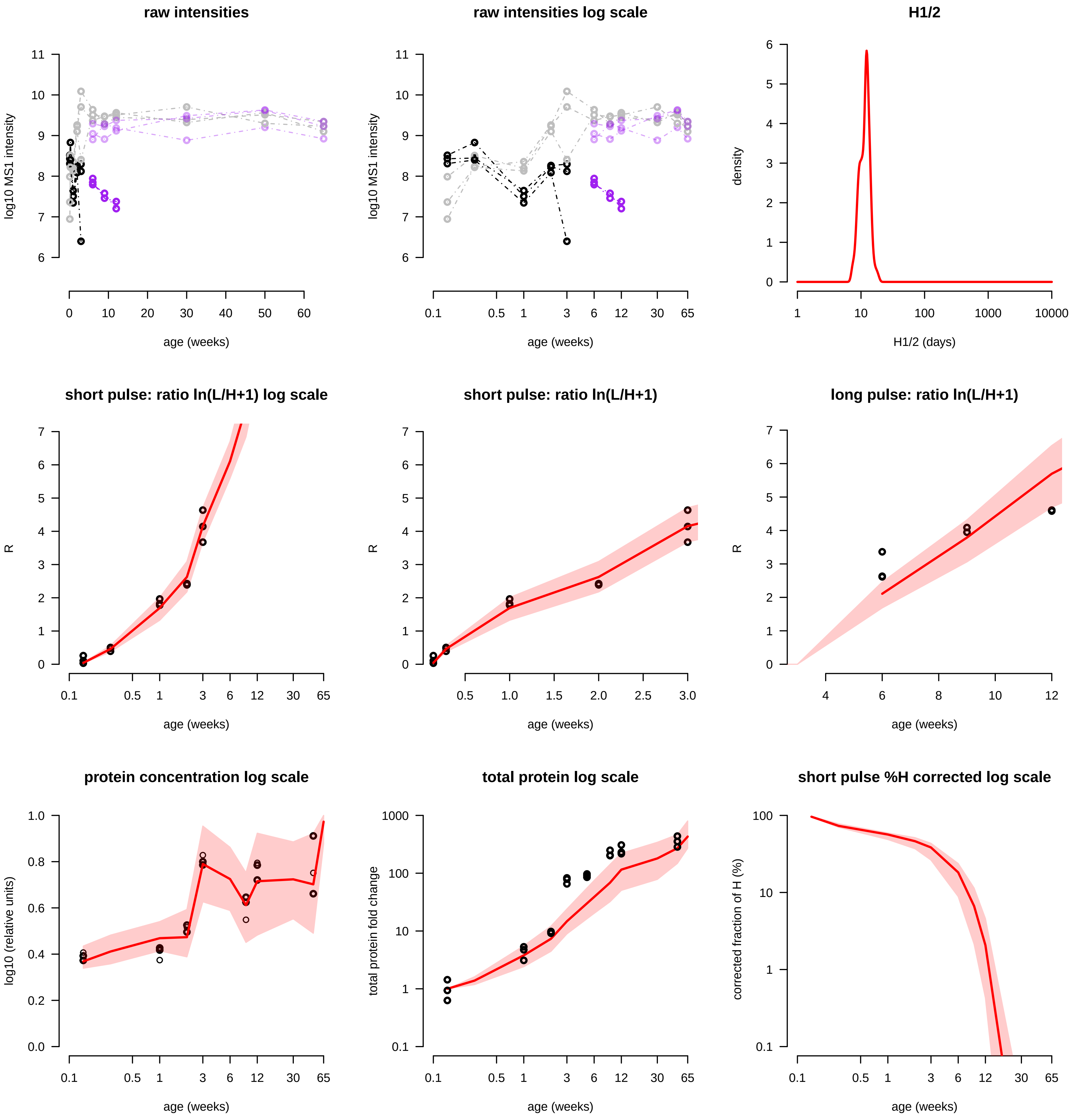

Q60973

>sp|Q60973|RBBP7_MOUSE

Histone−binding protein RBBP7

GN=Rbbp7

Med H1/2: 7.7 days

Q5 H1/2: 6.2 days

Q95 H1/2: 9.2 days

reliable model fit: yes

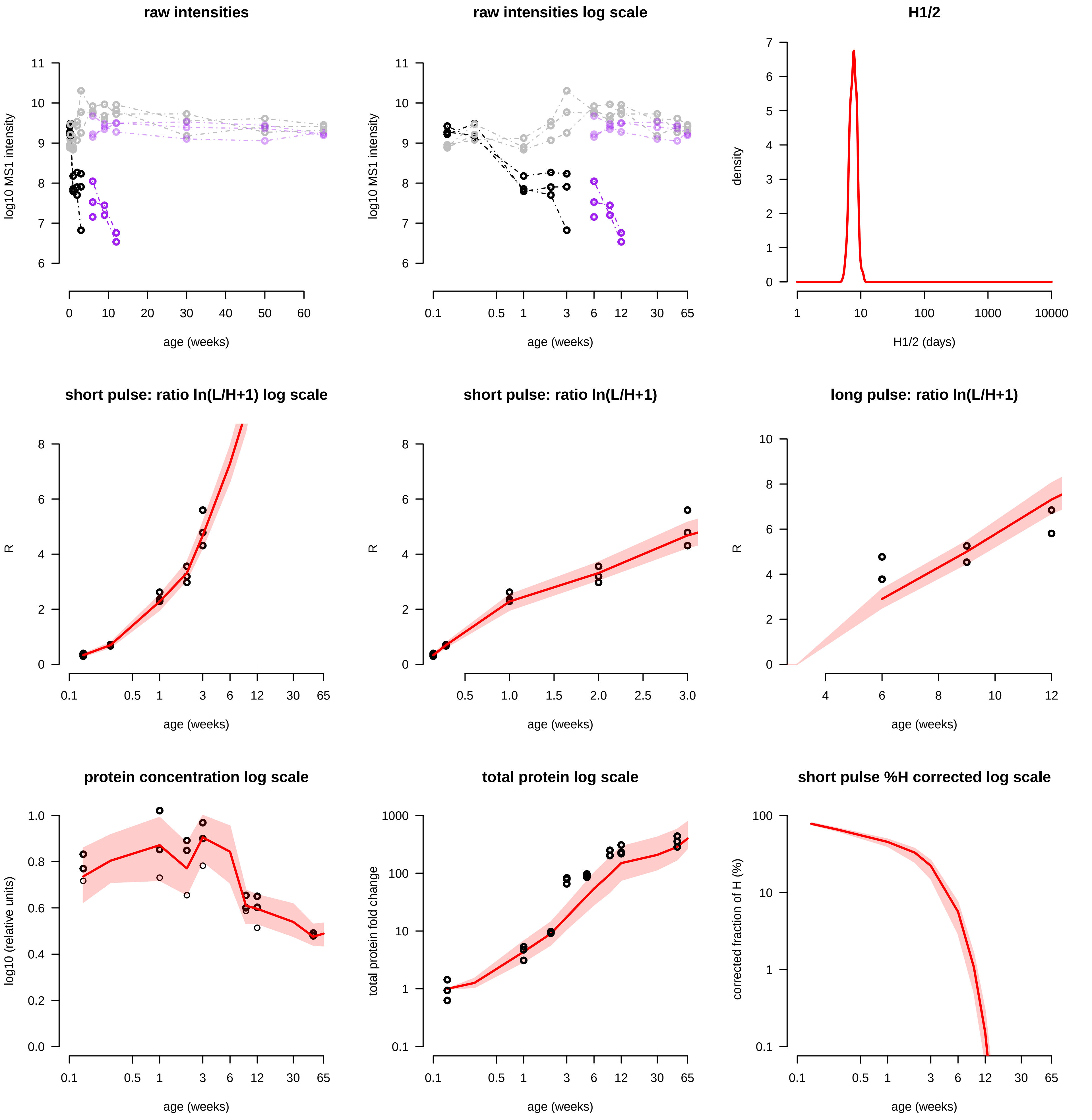

P20239

>sp|P20239|ZP2_MOUSE

Zona pellucida sperm−binding protein 2

GN=Zp2

Med H1/2: 186.2 days

Q5 H1/2: 123.3 days

Q95 H1/2: 443.8 days

reliable model fit: yes

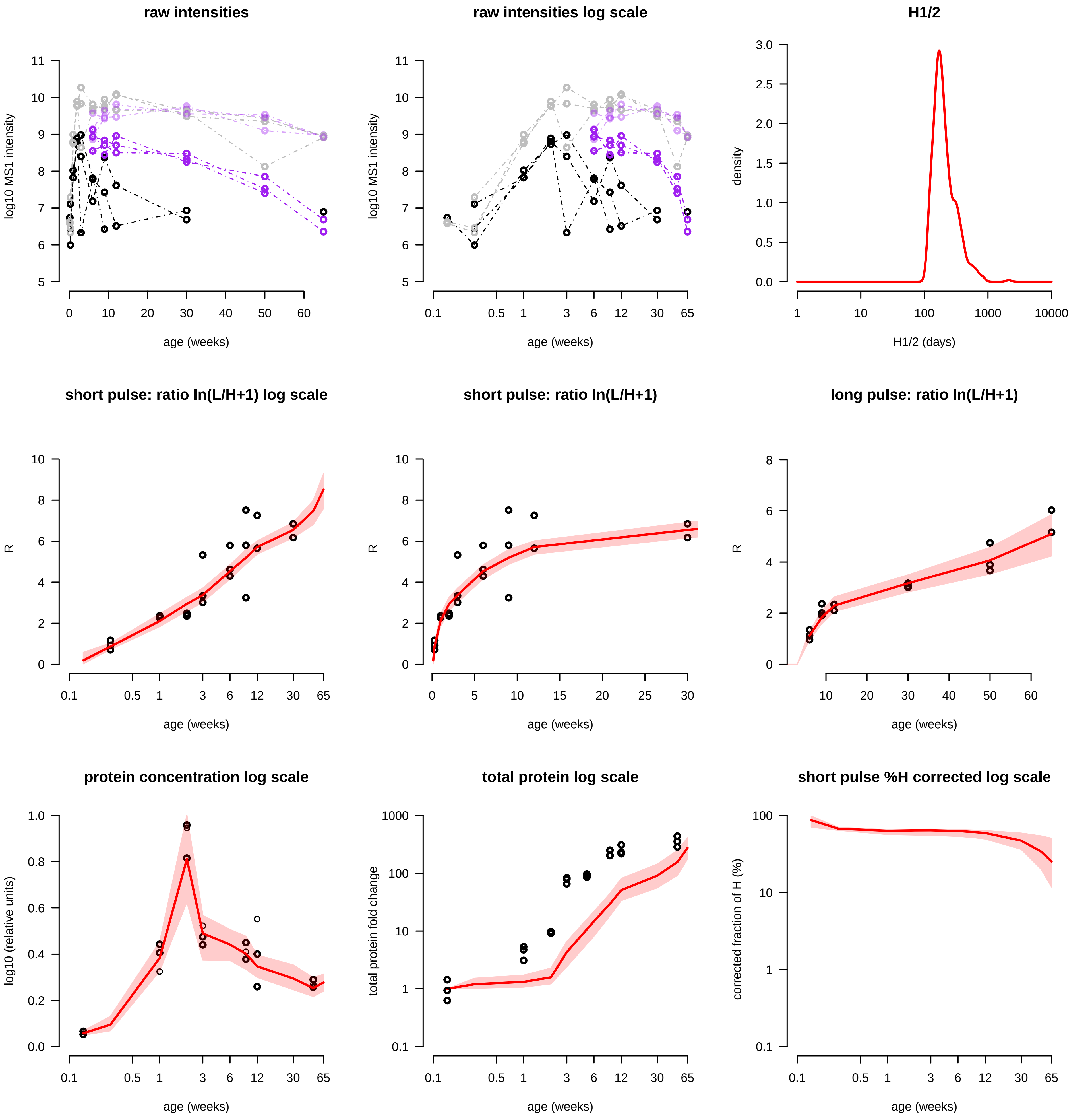

Q04447

>sp|Q04447|KCRB_MOUSE

Creatine kinase B−type

GN=Ckb

Med H1/2: 5.7 days

Q5 H1/2: 4.3 days

Q95 H1/2: 8.4 days

reliable model fit: yes

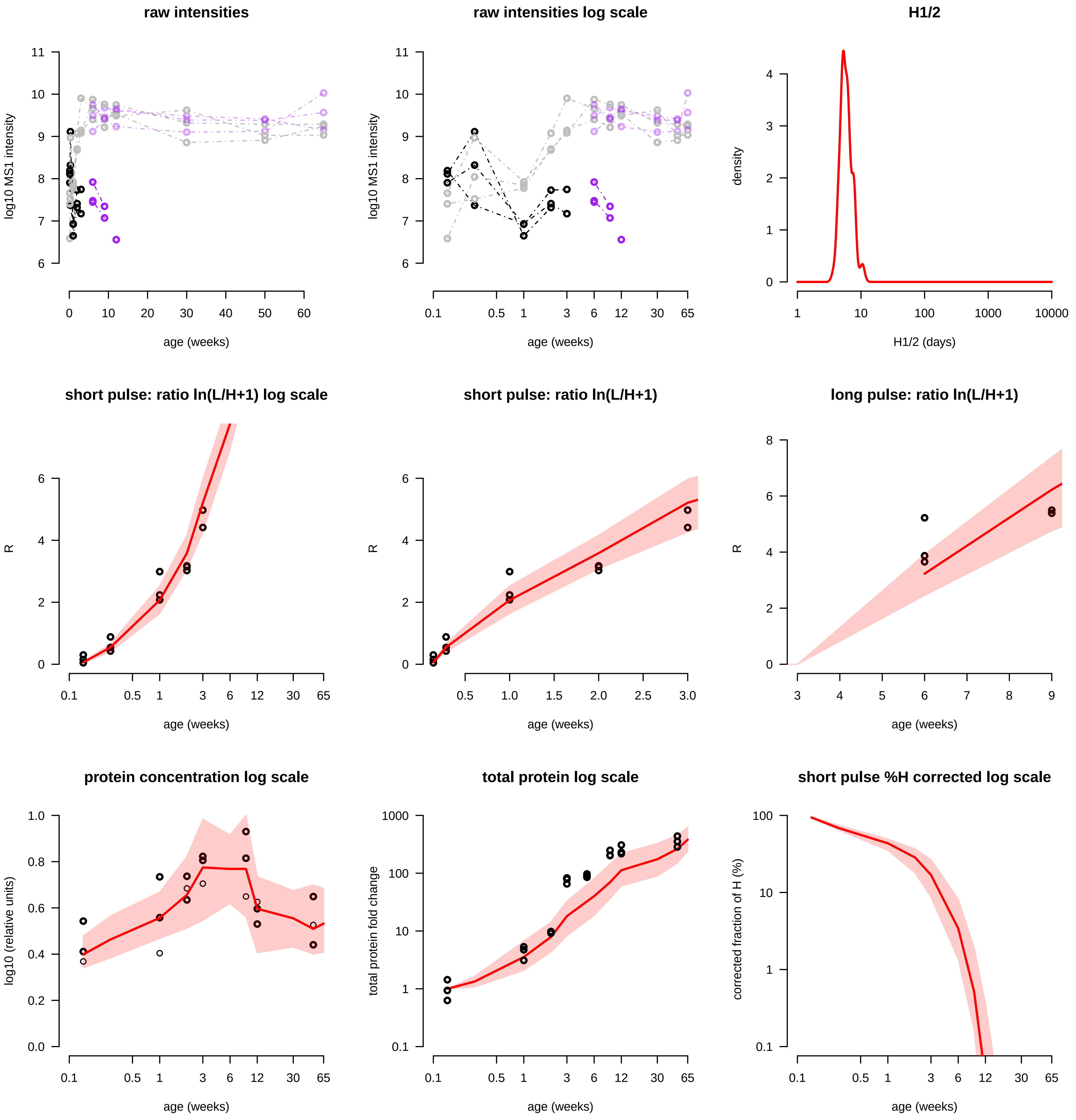

O88685

>sp|O88685|PRS6A_MOUSE

26S proteasome regulatory subunit 6A

GN=Psmc3

Med H1/2: 8.2 days

Q5 H1/2: 7.1 days

Q95 H1/2: 9.7 days

reliable model fit: no

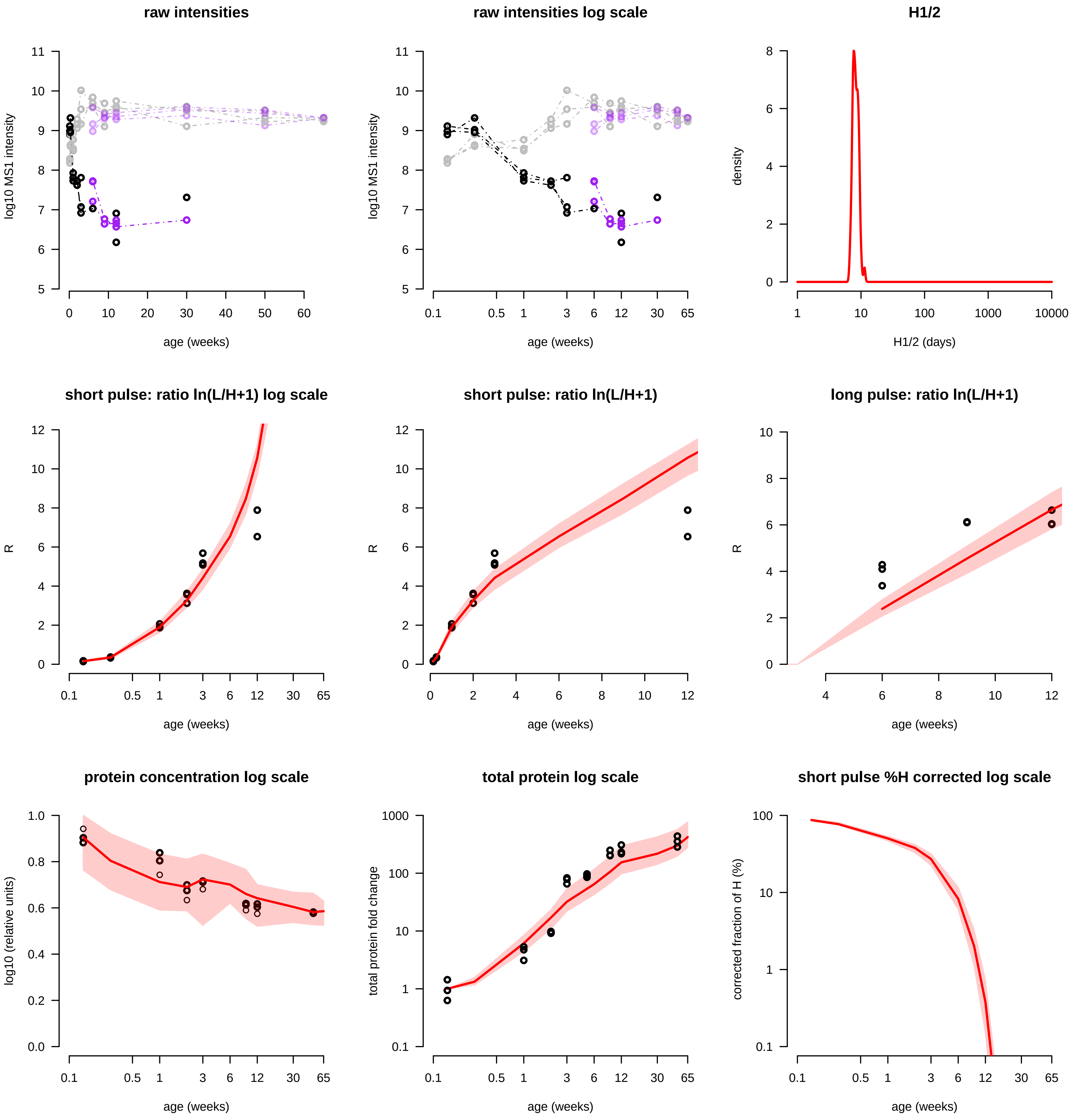

Q9CZM2

>sp|Q9CZM2|RL15_MOUSE

60S ribosomal protein L15

GN=Rpl15

Med H1/2: 9.7 days

Q5 H1/2: 7.1 days

Q95 H1/2: 12.2 days

reliable model fit: yes

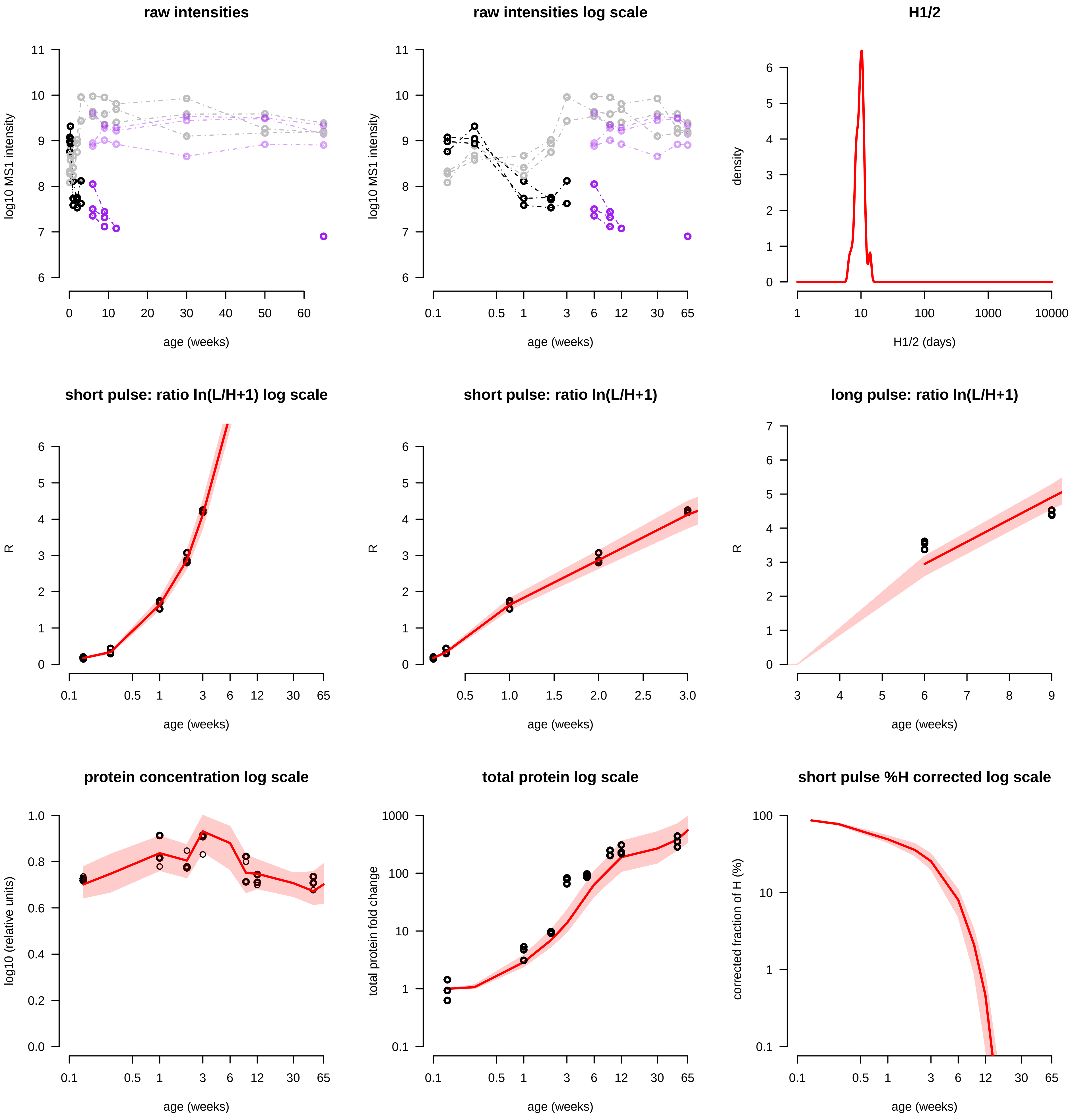

P46471

>sp|P46471|PRS7_MOUSE

26S proteasome regulatory subunit 7

GN=Psmc2

Med H1/2: 5.9 days

Q5 H1/2: 5.1 days

Q95 H1/2: 7.2 days

reliable model fit: yes

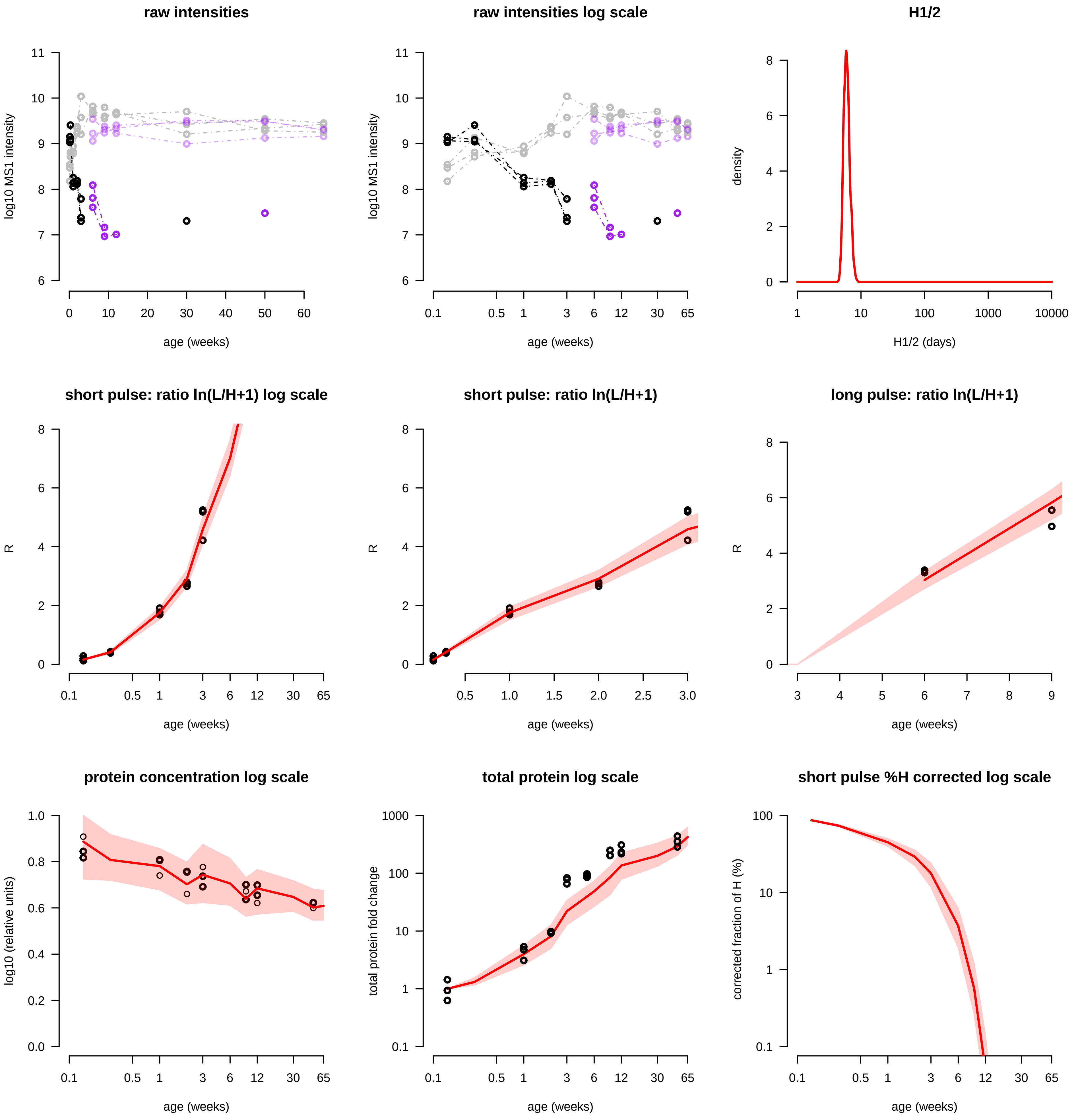

Q00612

>sp|Q00612|G6PD1_MOUSE

Glucose−6−phosphate 1−dehydrogenase X

GN=G6pdx

Med H1/2: 9.1 days

Q5 H1/2: 7.1 days

Q95 H1/2: 12.2 days

reliable model fit: yes

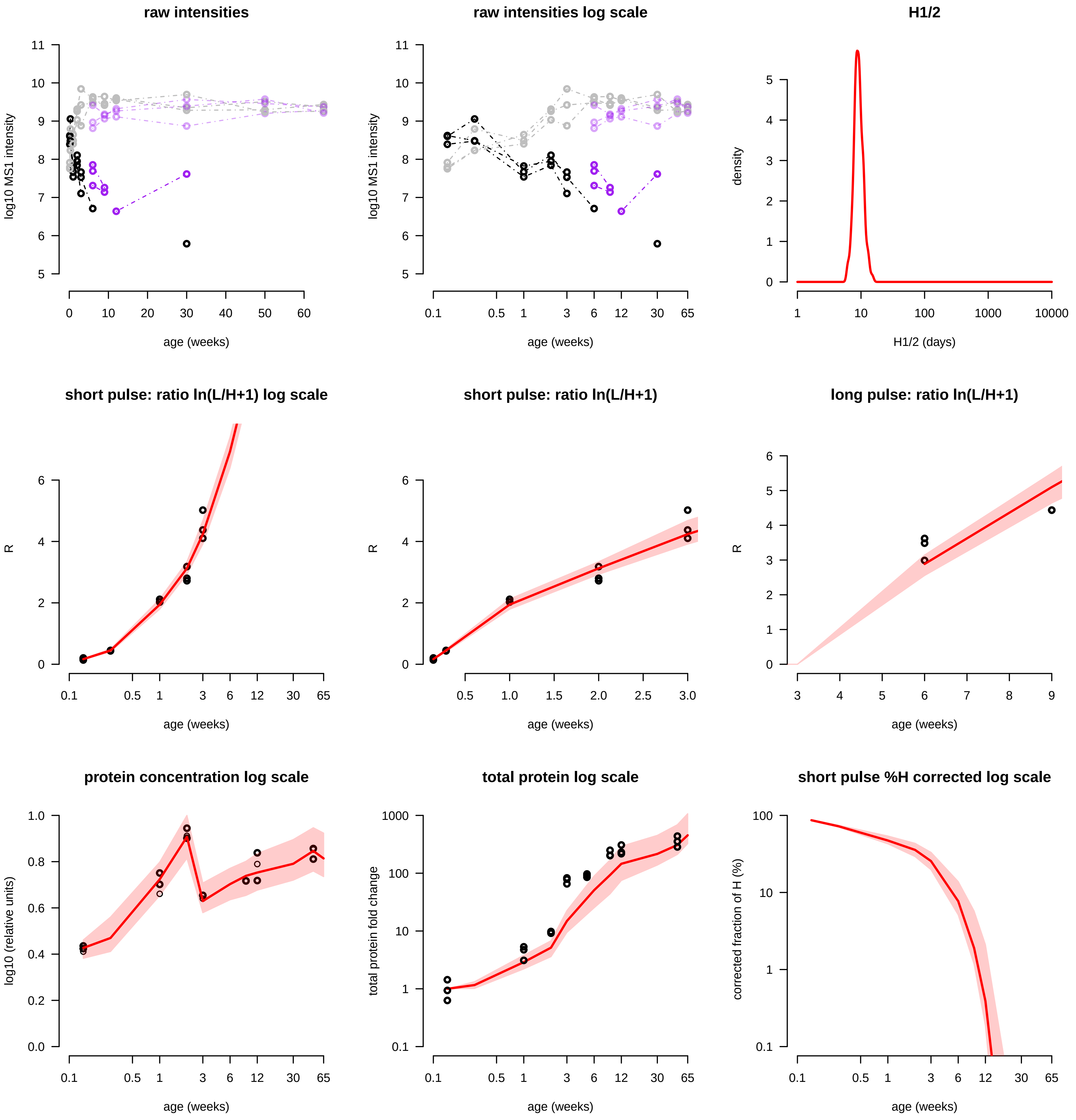

P62317

>sp|P62317|SMD2_MOUSE

Small nuclear ribonucleoprotein Sm D2

GN=Snrpd2

Med H1/2: 8.3 days

Q5 H1/2: 6.5 days

Q95 H1/2: 11 days

reliable model fit: yes

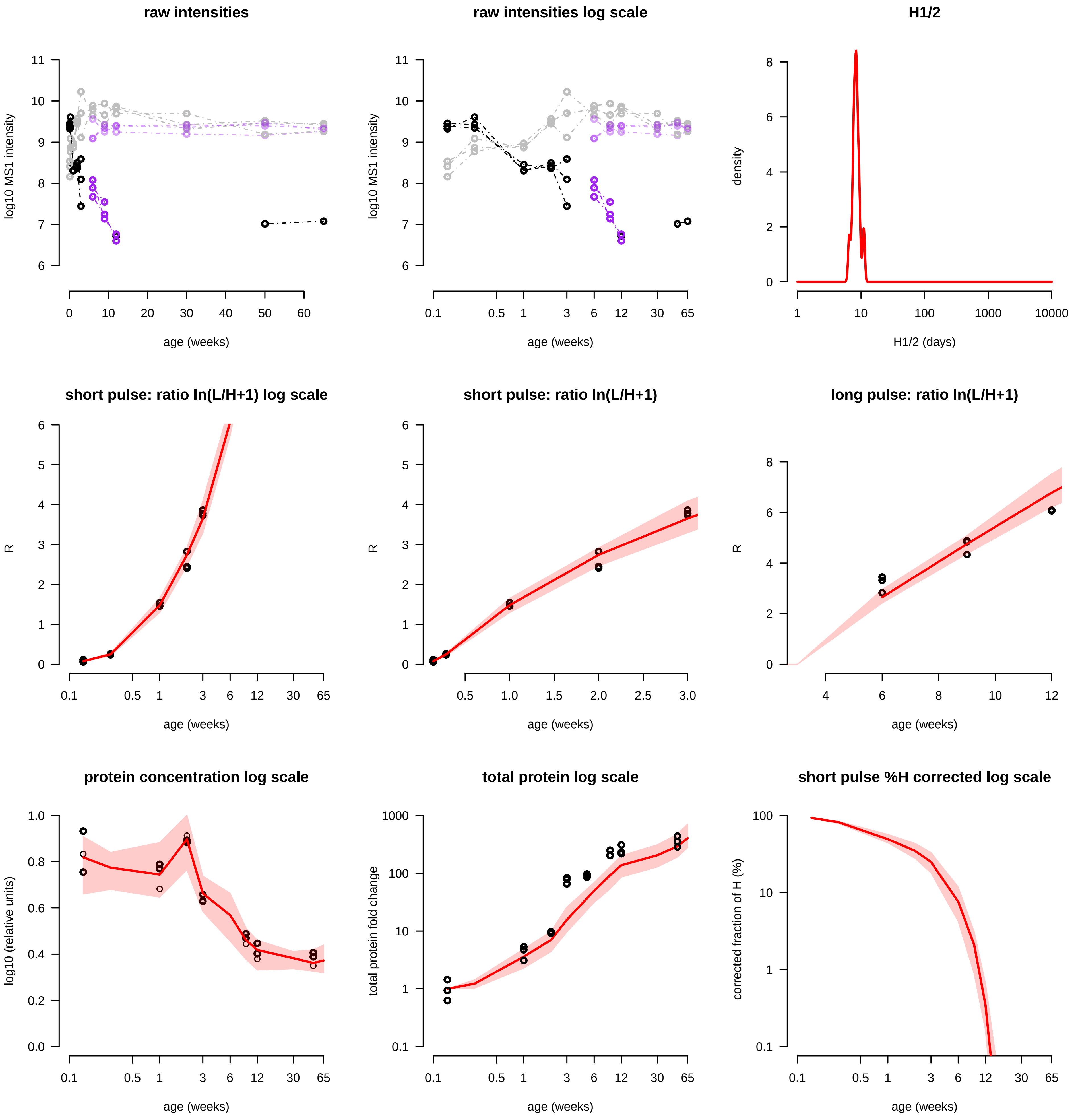

Q8K4Z5

>sp|Q8K4Z5|SF3A1_MOUSE

Splicing factor 3A subunit 1

GN=Sf3a1

Med H1/2: 5 days

Q5 H1/2: 4.4 days

Q95 H1/2: 5.8 days

reliable model fit: yes

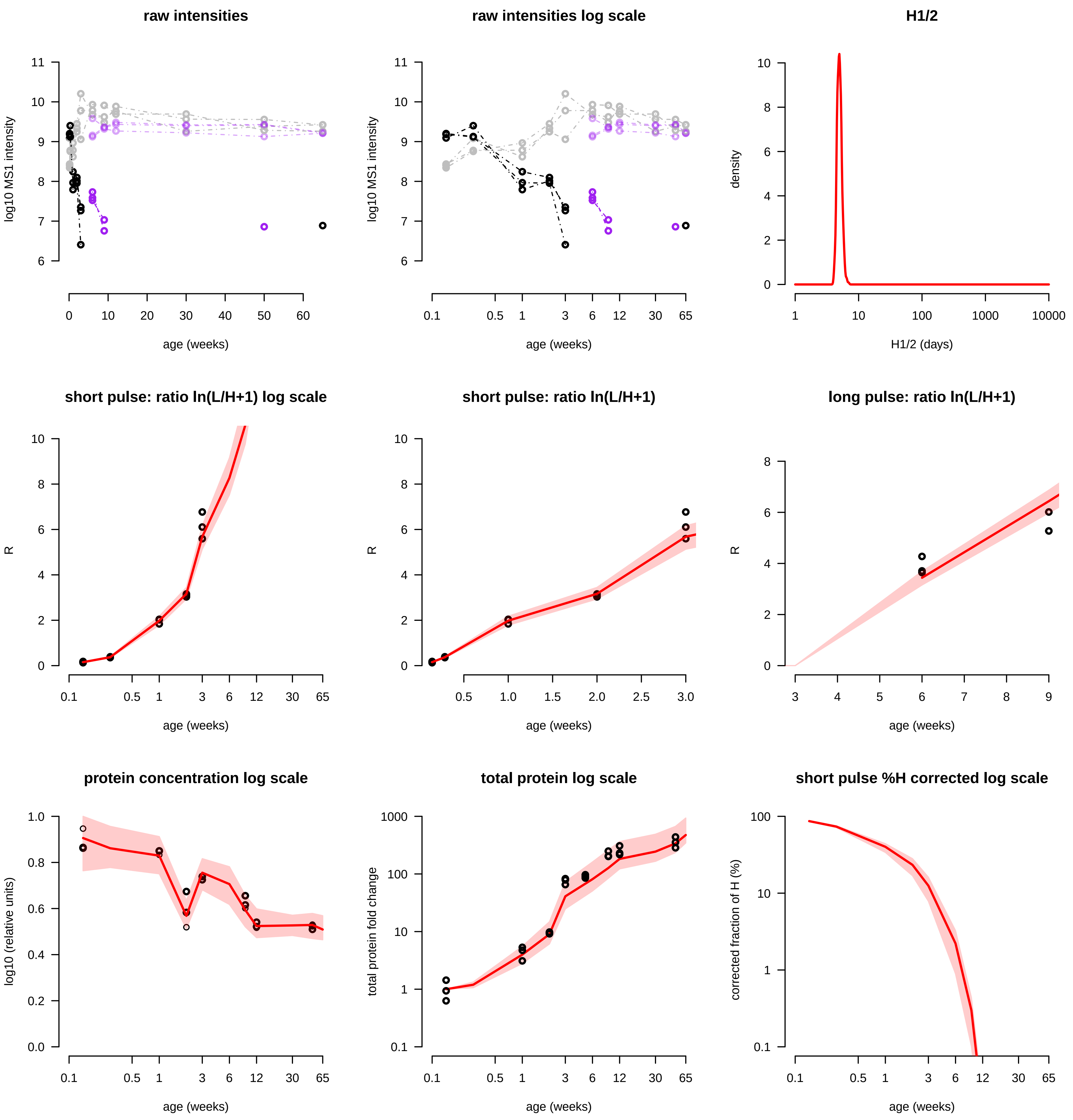

Q8JZQ2

>sp|Q8JZQ2|AFG32_MOUSE

AFG3−like protein 2

GN=Afg3l2

Med H1/2: 3.4 days

Q5 H1/2: 2.8 days

Q95 H1/2: 4.2 days

reliable model fit: yes

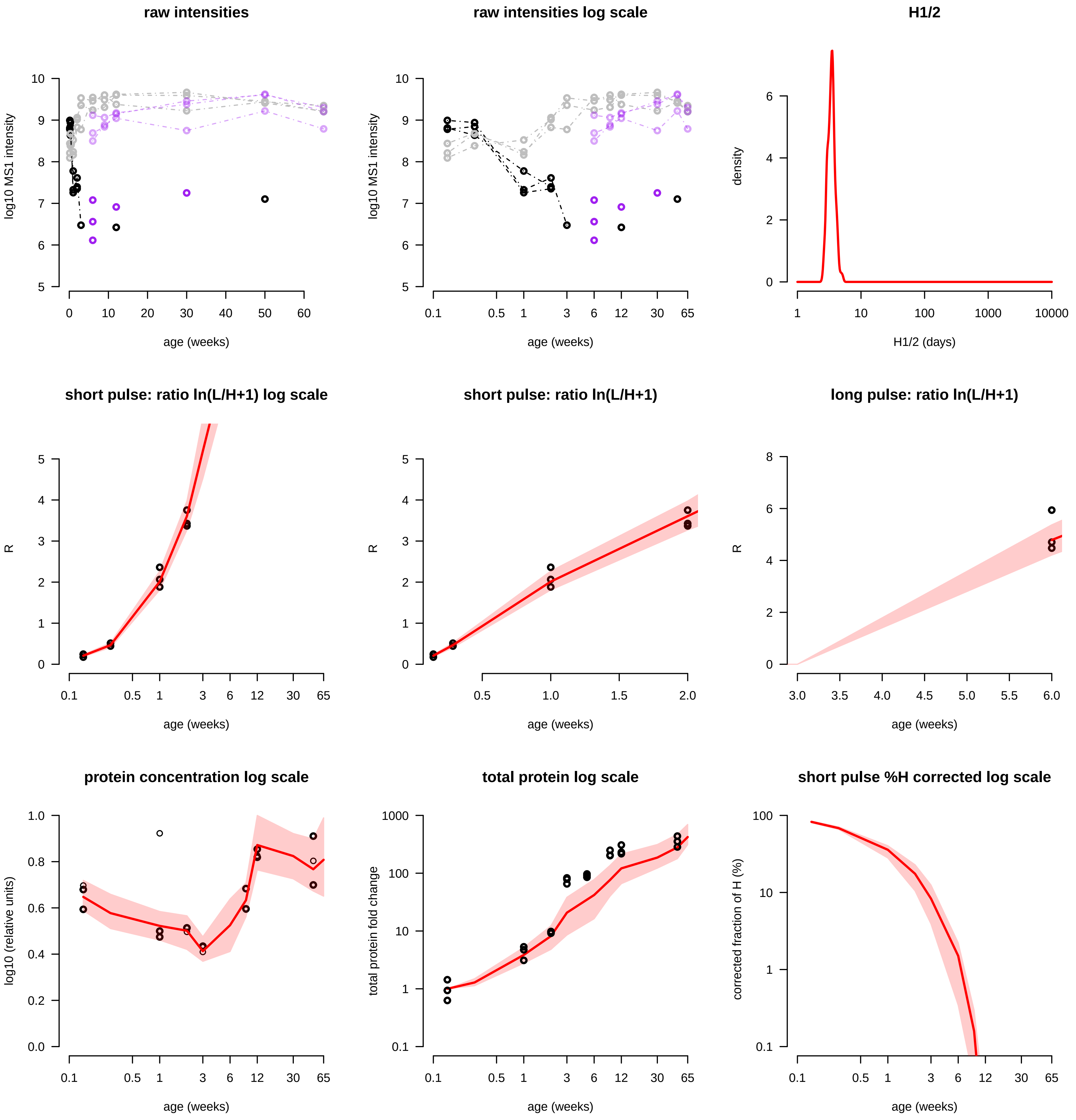

O08795−2;O08795

>sp|O08795−2|GLU2B_MOUSE

Isoform 2 of Glucosidase 2 subunit beta

GN=Prkcsh

Med H1/2: 7.3 days

Q5 H1/2: 6.3 days

Q95 H1/2: 9.4 days

reliable model fit: yes

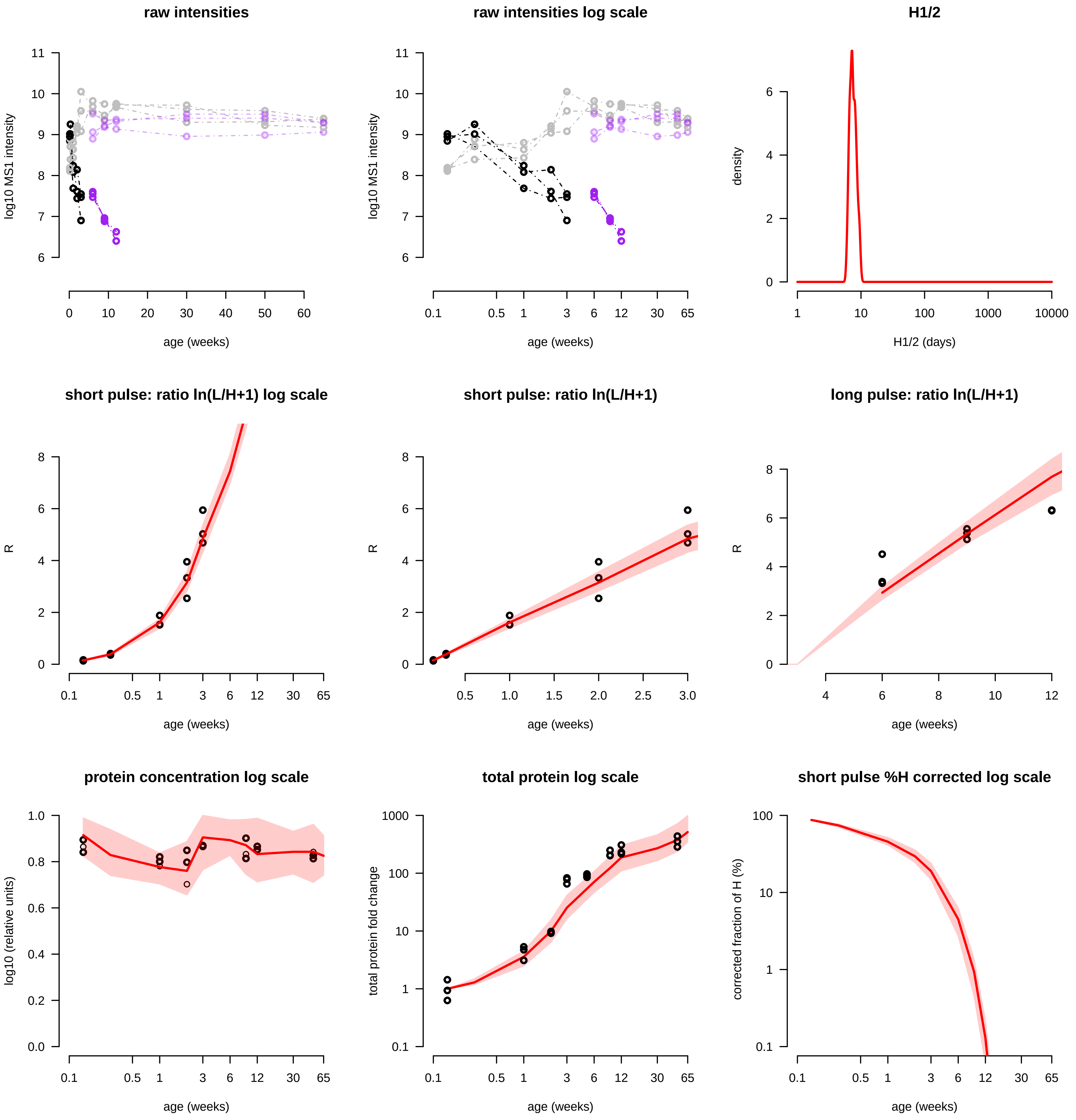

Q91ZJ5;Q91ZJ5−2

>sp|Q91ZJ5|UGPA_MOUSE

UTP−−glucose−1−phosphate uridylyltransferase

GN=Ugp2

Med H1/2: 615.4 days

Q5 H1/2: 186 days

Q95 H1/2: 5681.1 days

reliable model fit: no

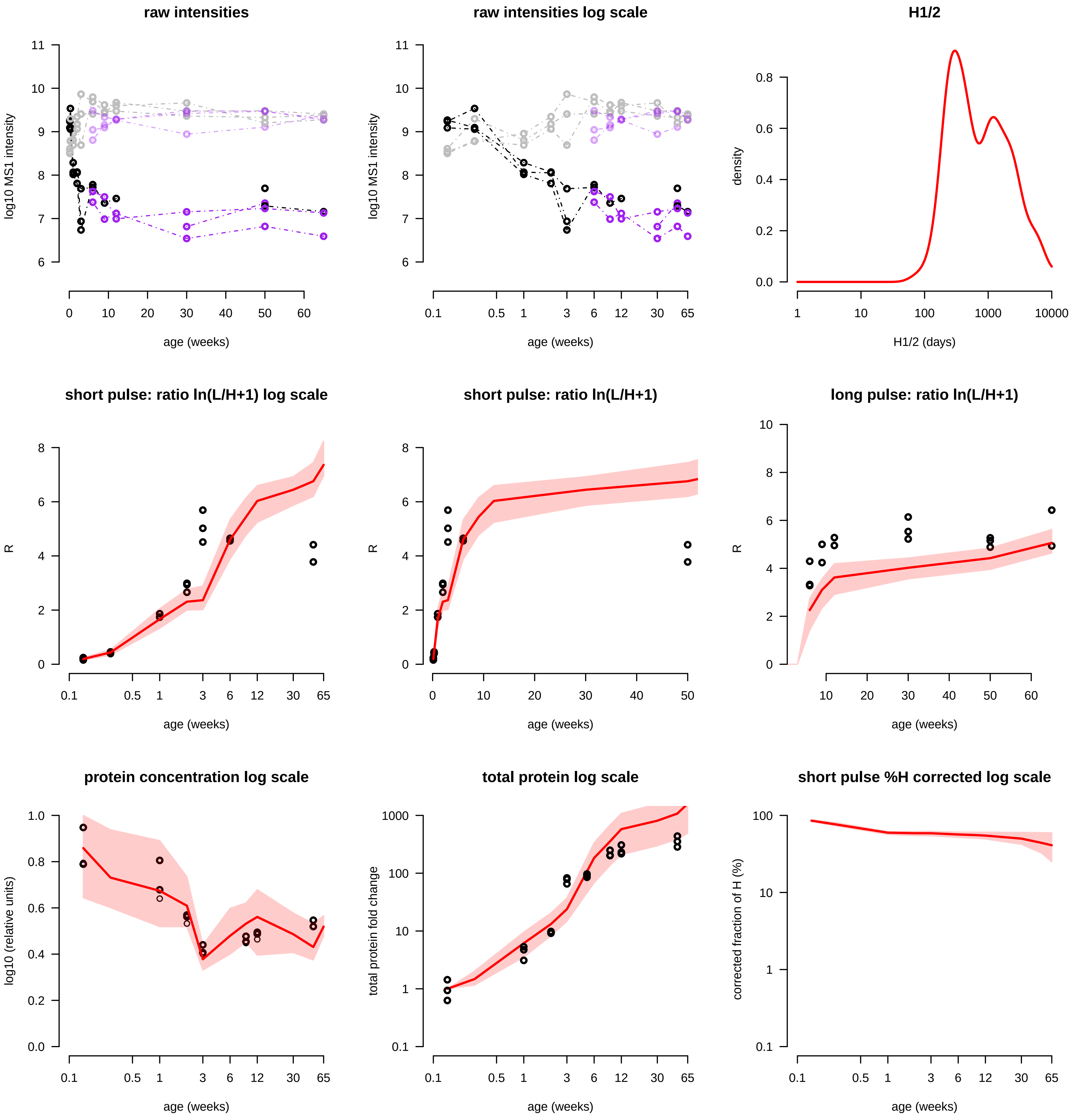

P54775

>sp|P54775|PRS6B_MOUSE

26S proteasome regulatory subunit 6B

GN=Psmc4

Med H1/2: 5 days

Q5 H1/2: 4 days

Q95 H1/2: 6.6 days

reliable model fit: yes

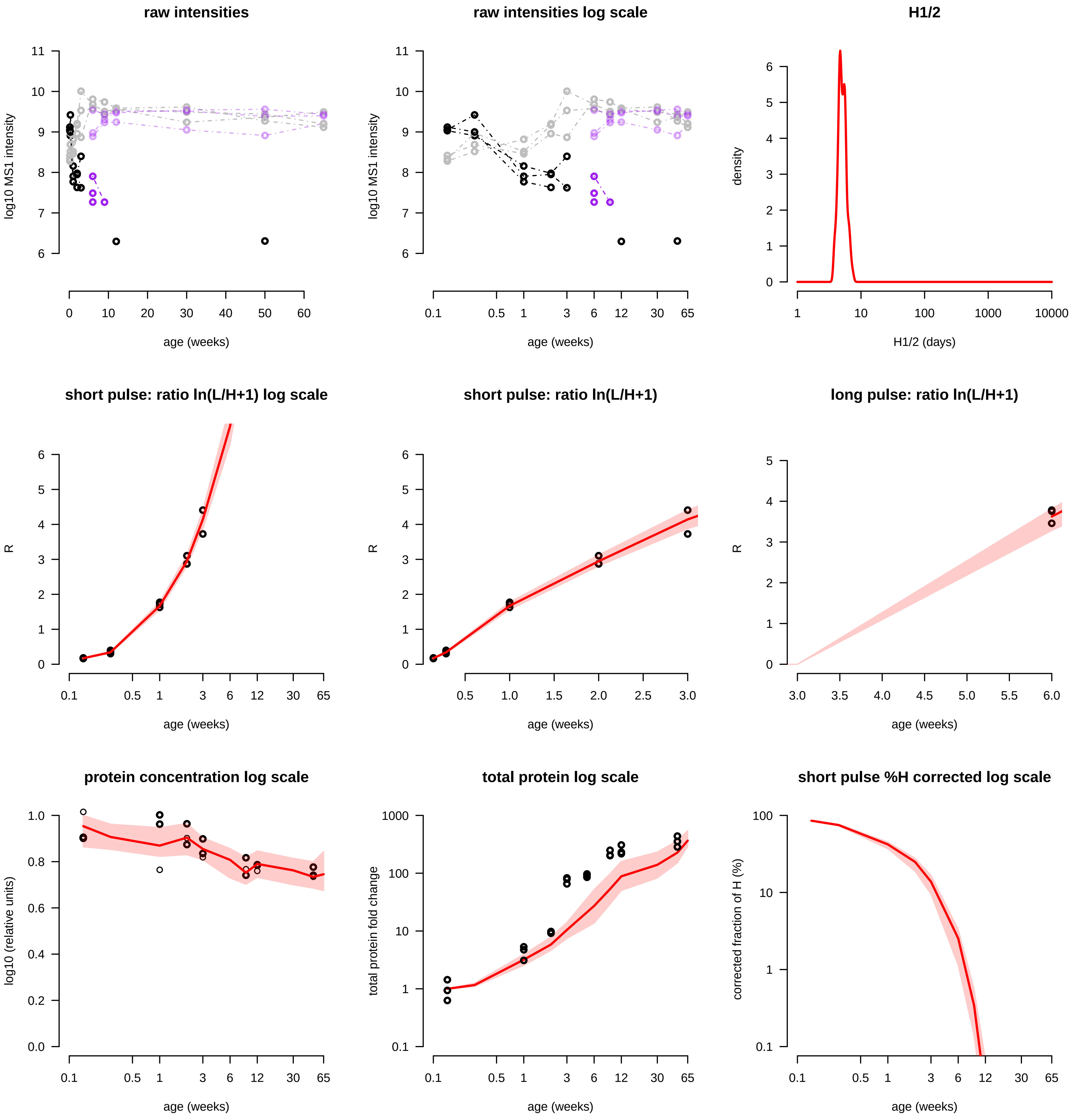

Q8BMK4

>sp|Q8BMK4|CKAP4_MOUSE

Cytoskeleton−associated protein 4

GN=Ckap4

Med H1/2: 5.7 days

Q5 H1/2: 5 days

Q95 H1/2: 6.5 days

reliable model fit: yes

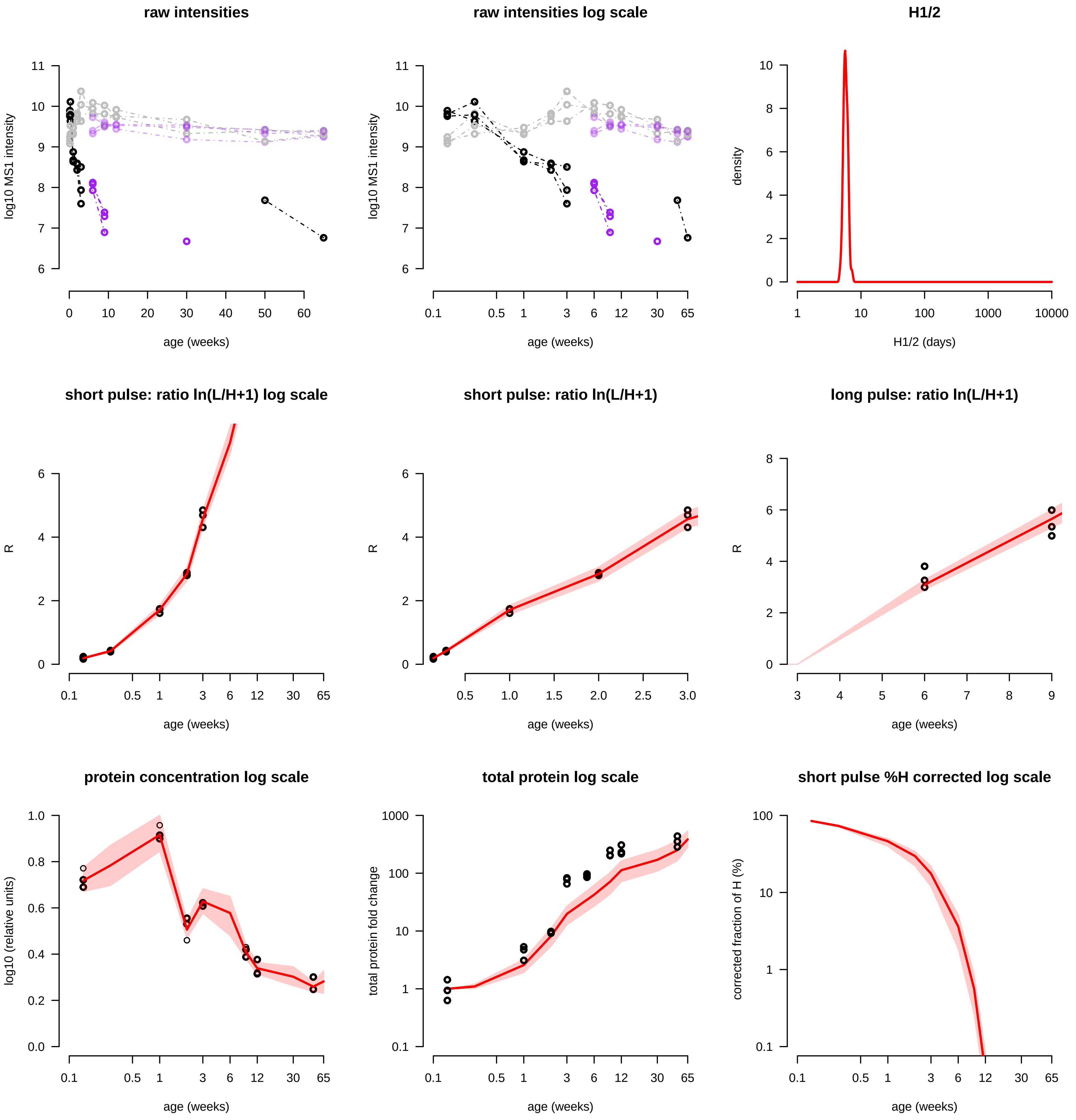

Q8CI94

>sp|Q8CI94|PYGB_MOUSE

Glycogen phosphorylase, brain form

GN=Pygb

Med H1/2: 5.9 days

Q5 H1/2: 5 days

Q95 H1/2: 7.8 days

reliable model fit: yes

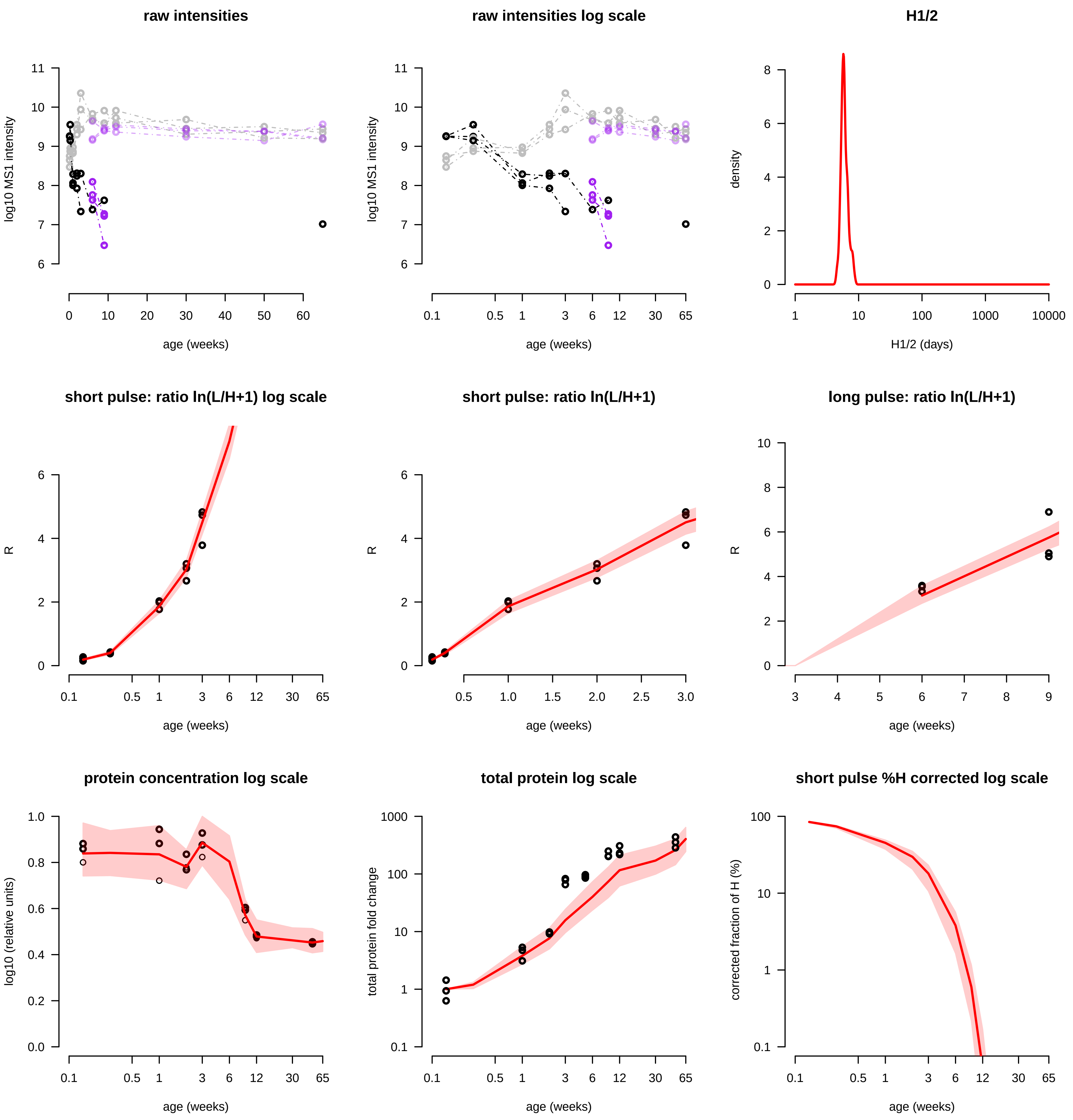

P26638

>sp|P26638|SYSC_MOUSE

Serine−−tRNA ligase, cytoplasmic

GN=Sars1

Med H1/2: 4.4 days

Q5 H1/2: 3.7 days

Q95 H1/2: 5.4 days

reliable model fit: yes

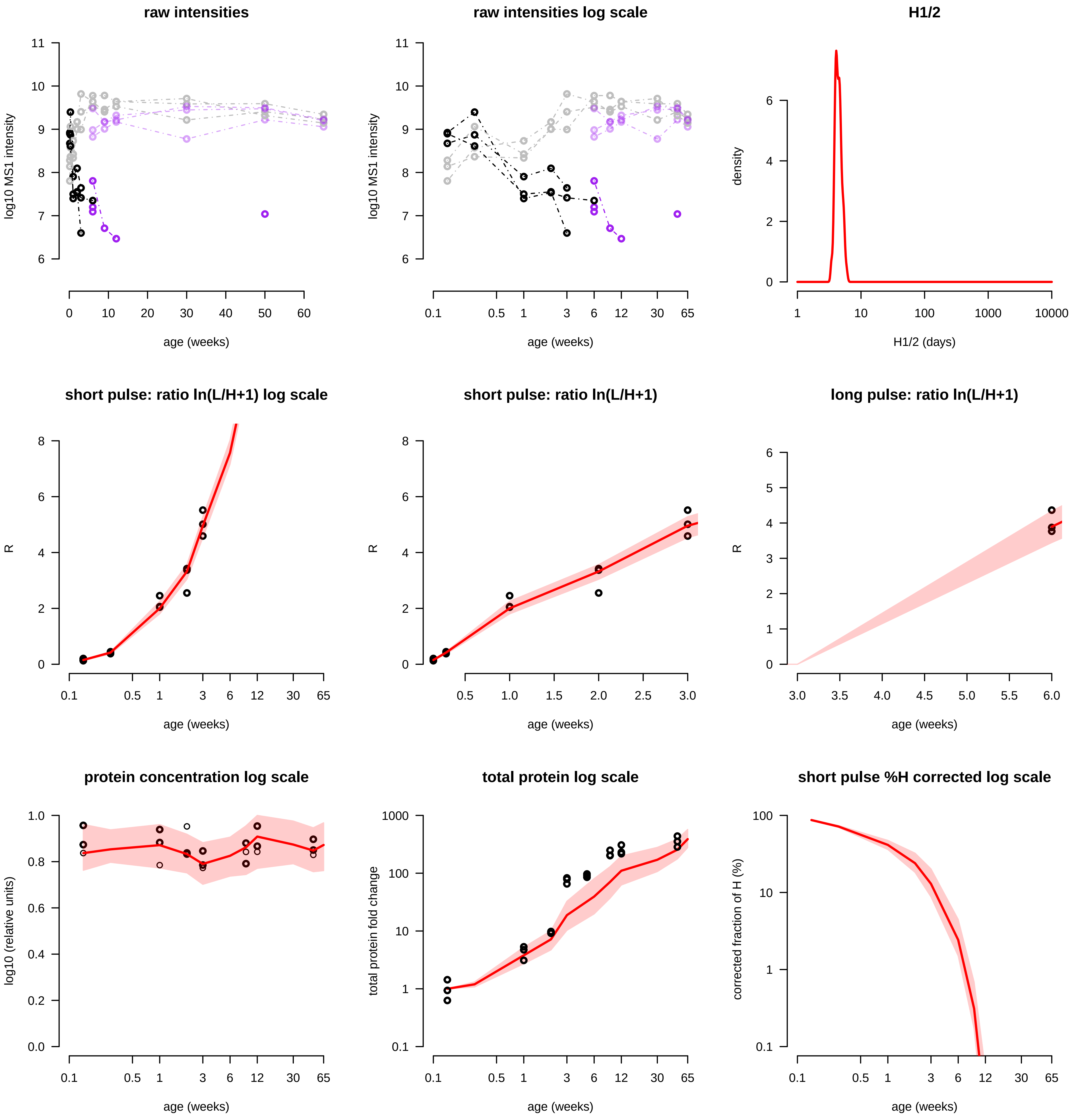

Q9QXZ0;Q9QXZ0−2

>sp|Q9QXZ0|MACF1_MOUSE

Microtubule−actin cross−linking factor 1

GN=Macf1

Med H1/2: 65.4 days

Q5 H1/2: 45.7 days

Q95 H1/2: 101.6 days

reliable model fit: yes

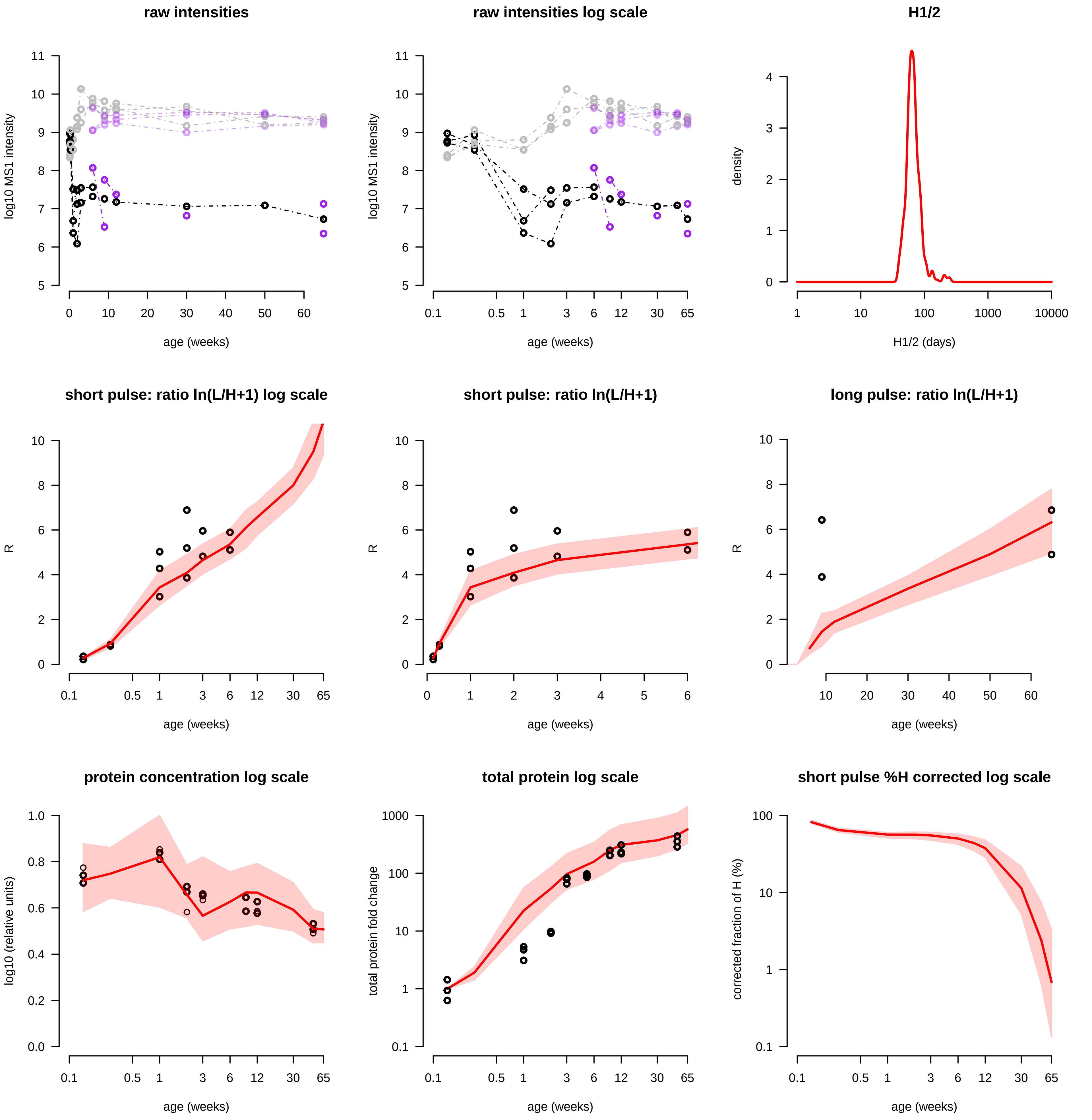

O35459

>sp|O35459|ECH1_MOUSE

Delta(3,5)−Delta(2,4)−dienoyl−CoA isomerase, mitochondrial

GN=Ech1

Med H1/2: 5.5 days

Q5 H1/2: 4 days

Q95 H1/2: 8.1 days

reliable model fit: yes

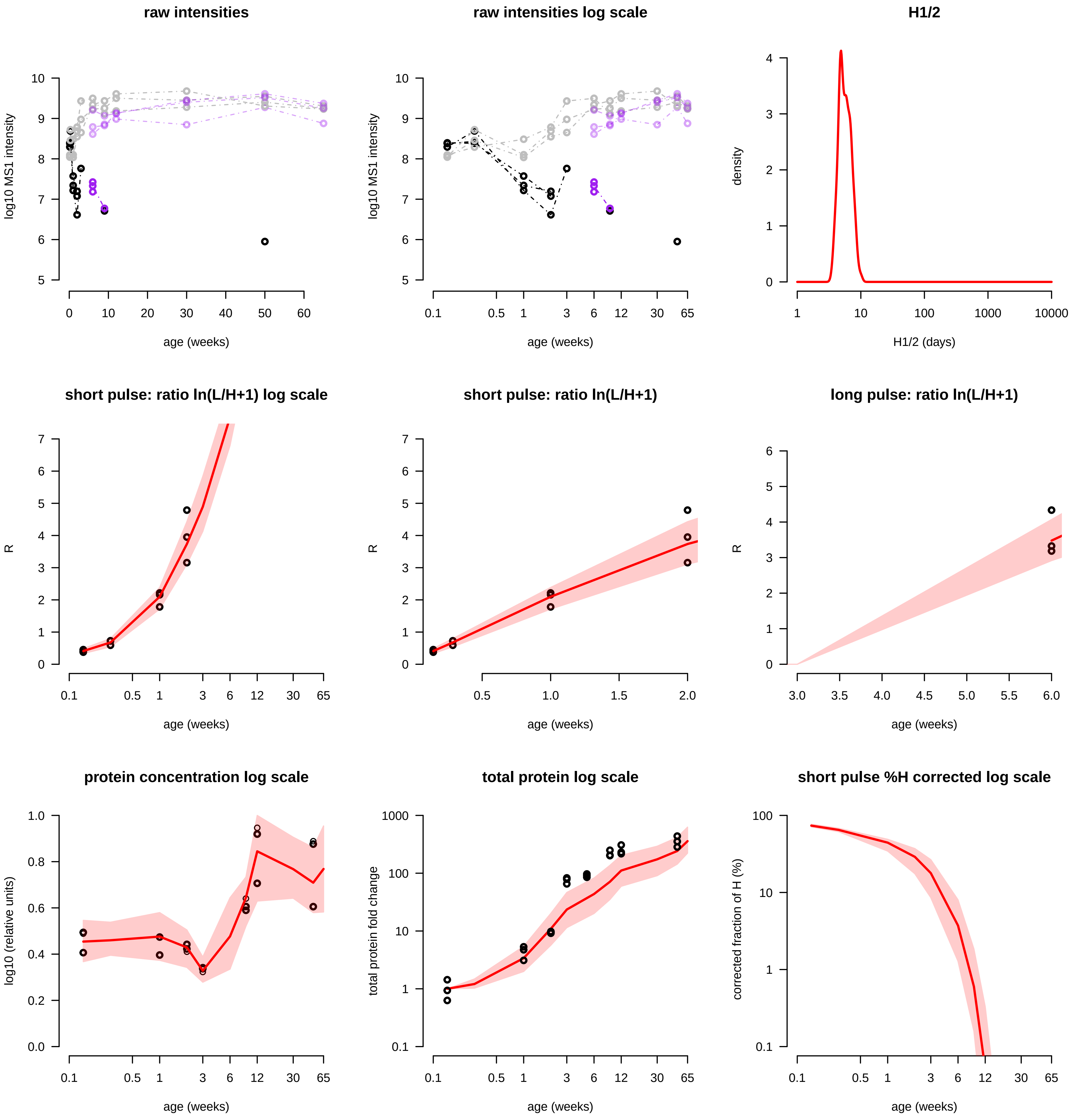

Q8BL66

>sp|Q8BL66|EEA1_MOUSE

Early endosome antigen 1

GN=Eea1

Med H1/2: 2.8 days

Q5 H1/2: 2.5 days

Q95 H1/2: 3.5 days

reliable model fit: yes

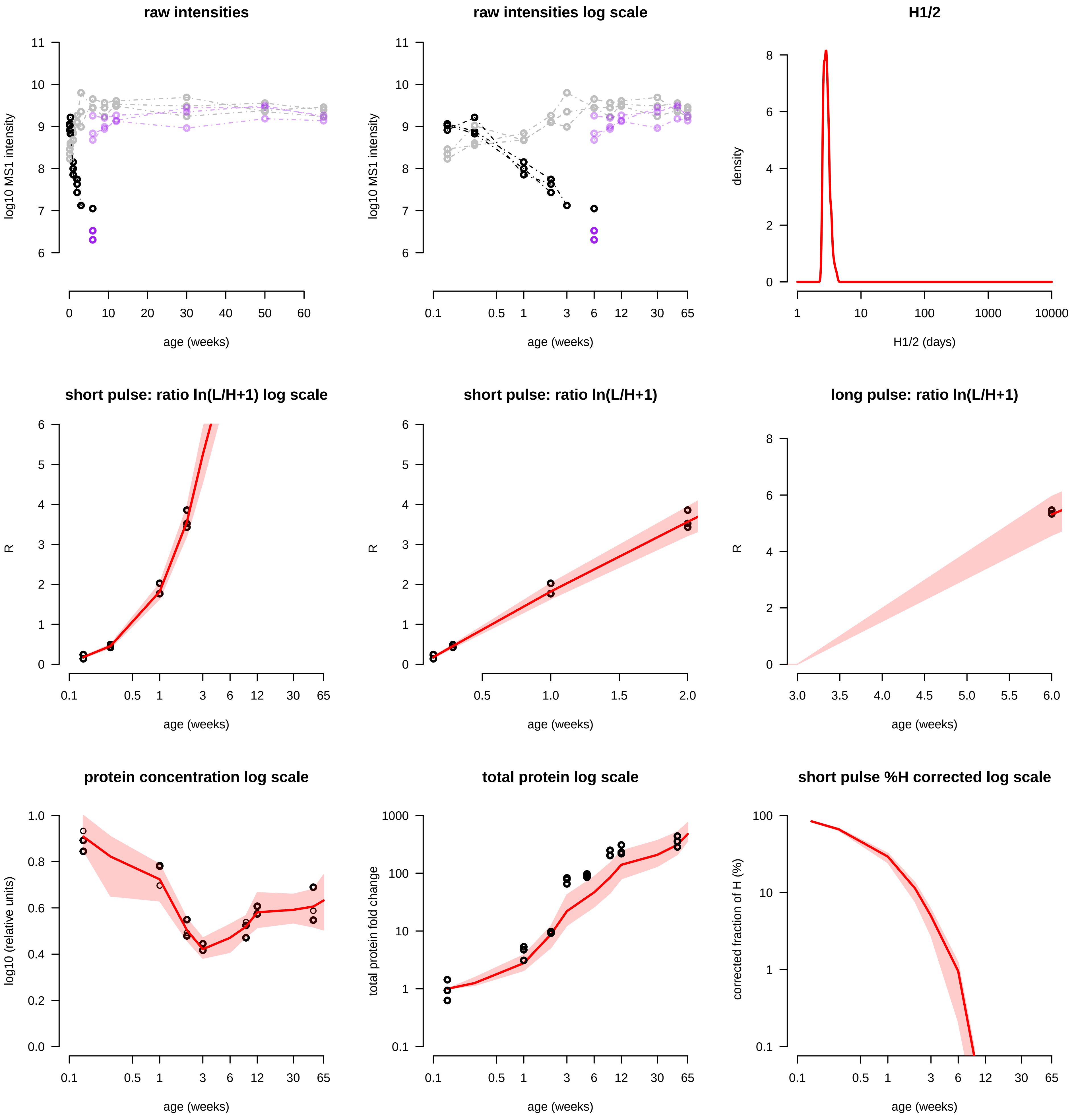

P23242

>sp|P23242|CXA1_MOUSE

Gap junction alpha−1 protein

GN=Gja1

Med H1/2: 7.1 days

Q5 H1/2: 5.4 days

Q95 H1/2: 10.6 days

reliable model fit: yes

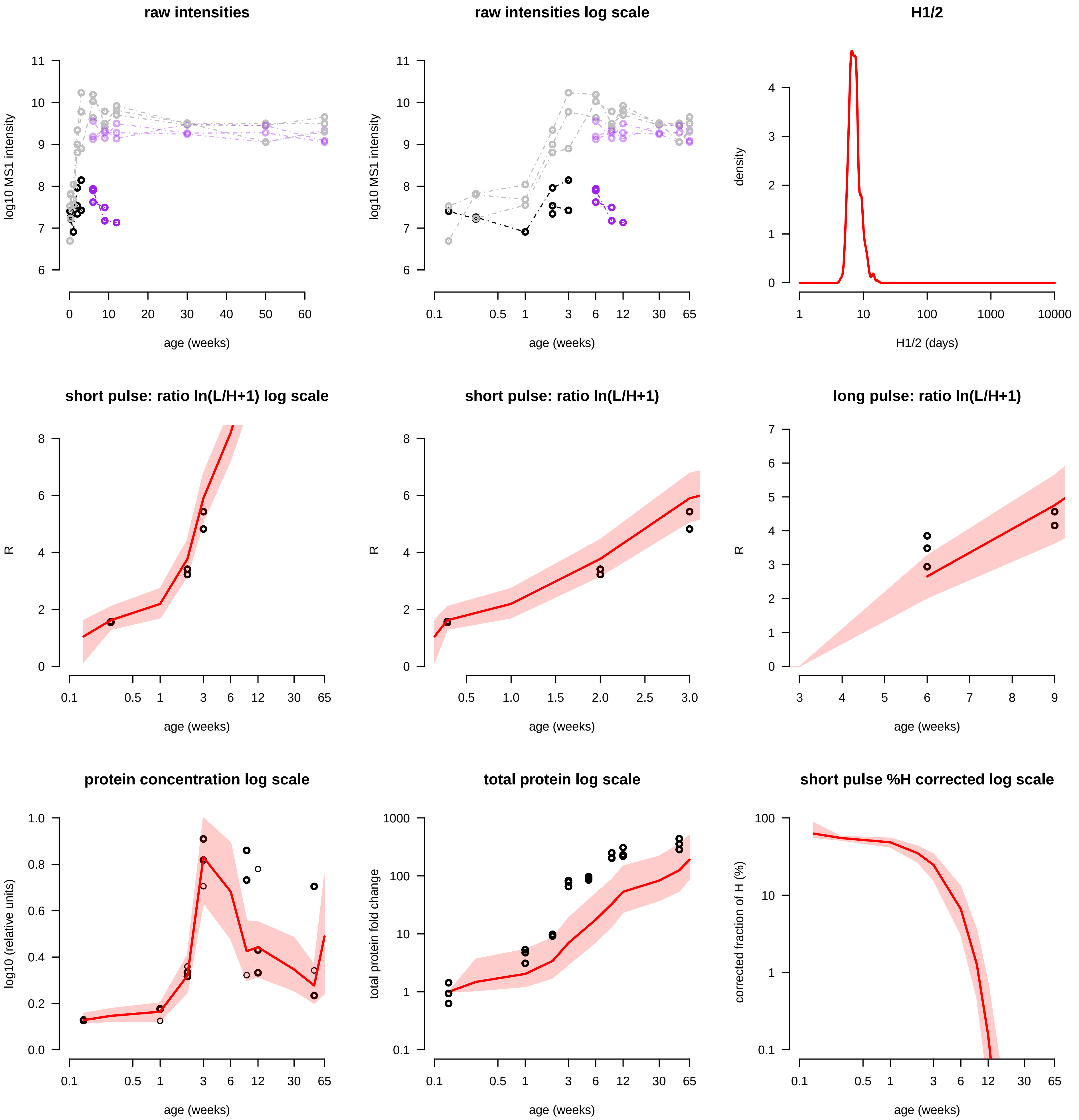

Q9CWJ9

>sp|Q9CWJ9|PUR9_MOUSE

Bifunctional purine biosynthesis protein ATIC

GN=Atic

Med H1/2: 816.4 days

Q5 H1/2: 193.4 days

Q95 H1/2: 12953.6 days

reliable model fit: no

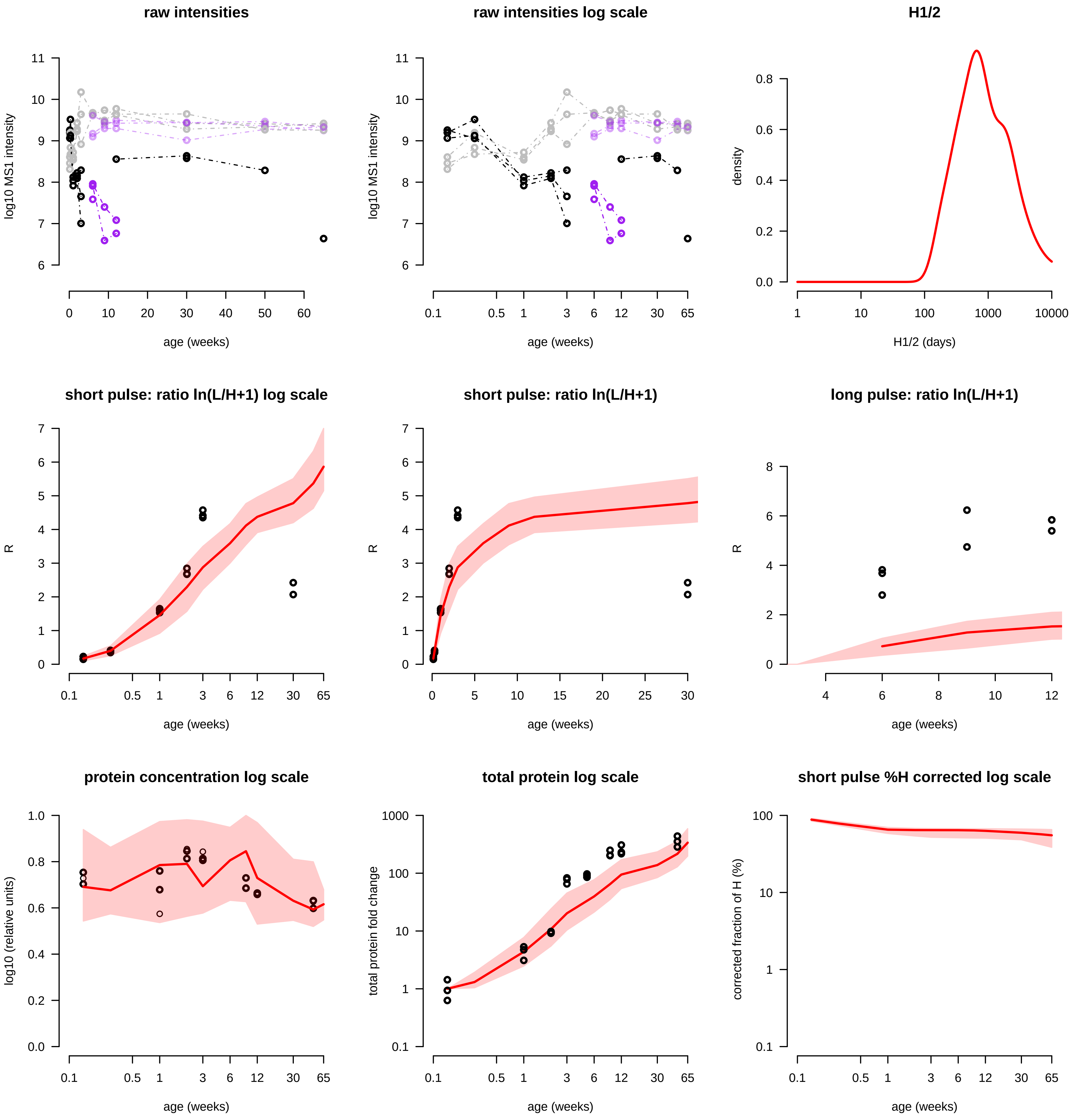

P46935

>sp|P46935|NEDD4_MOUSE

E3 ubiquitin−protein ligase NEDD4

GN=Nedd4

Med H1/2: 7.7 days

Q5 H1/2: 6.6 days

Q95 H1/2: 8.7 days

reliable model fit: yes

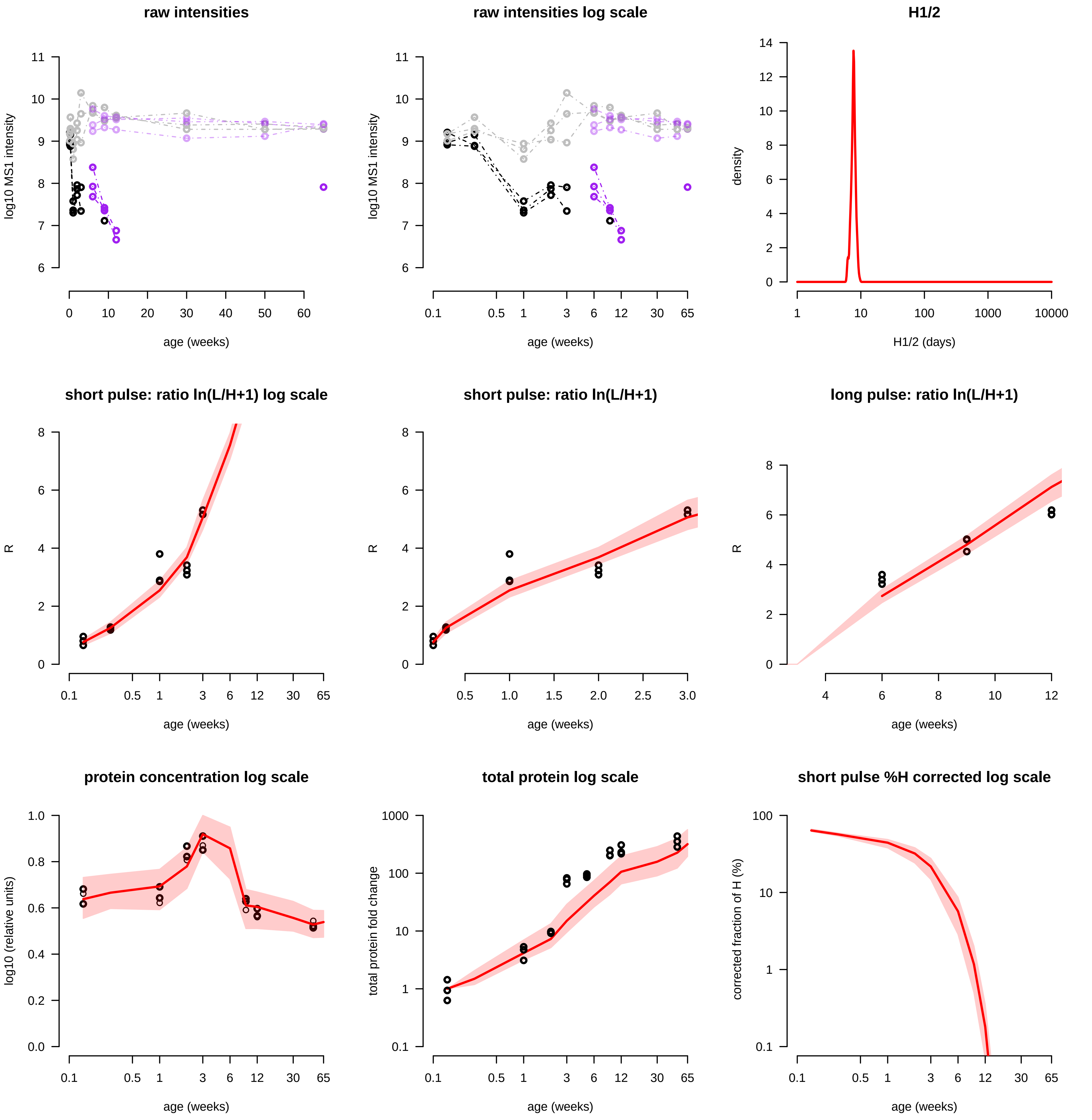

P97429

>sp|P97429|ANXA4_MOUSE

Annexin A4

GN=Anxa4

Med H1/2: 9.3 days

Q5 H1/2: 7.7 days

Q95 H1/2: 11.9 days

reliable model fit: yes

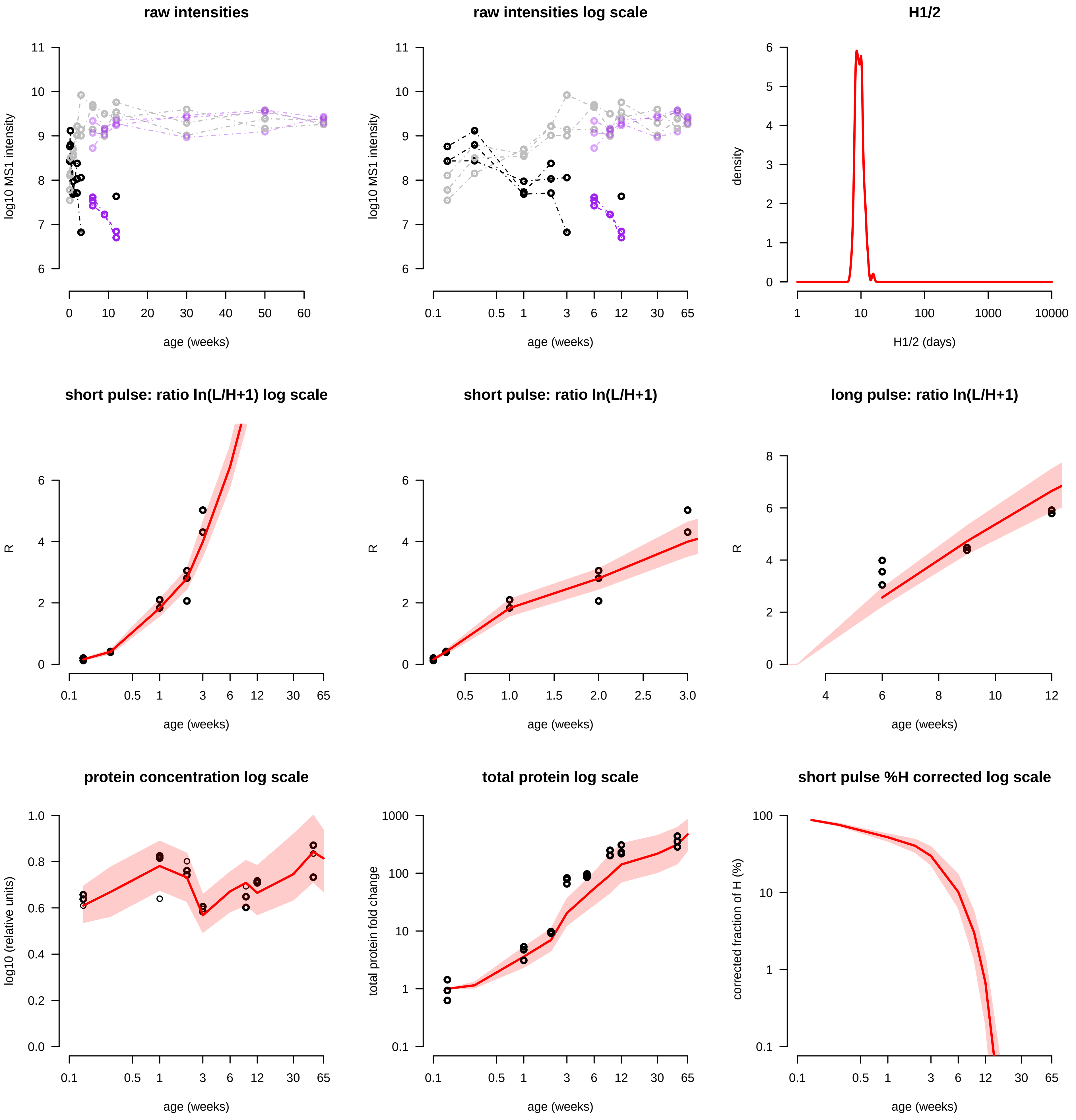

Q64521

>sp|Q64521|GPDM_MOUSE

Glycerol−3−phosphate dehydrogenase, mitochondrial

GN=Gpd2

Med H1/2: 7.1 days

Q5 H1/2: 6 days

Q95 H1/2: 9.2 days

reliable model fit: yes

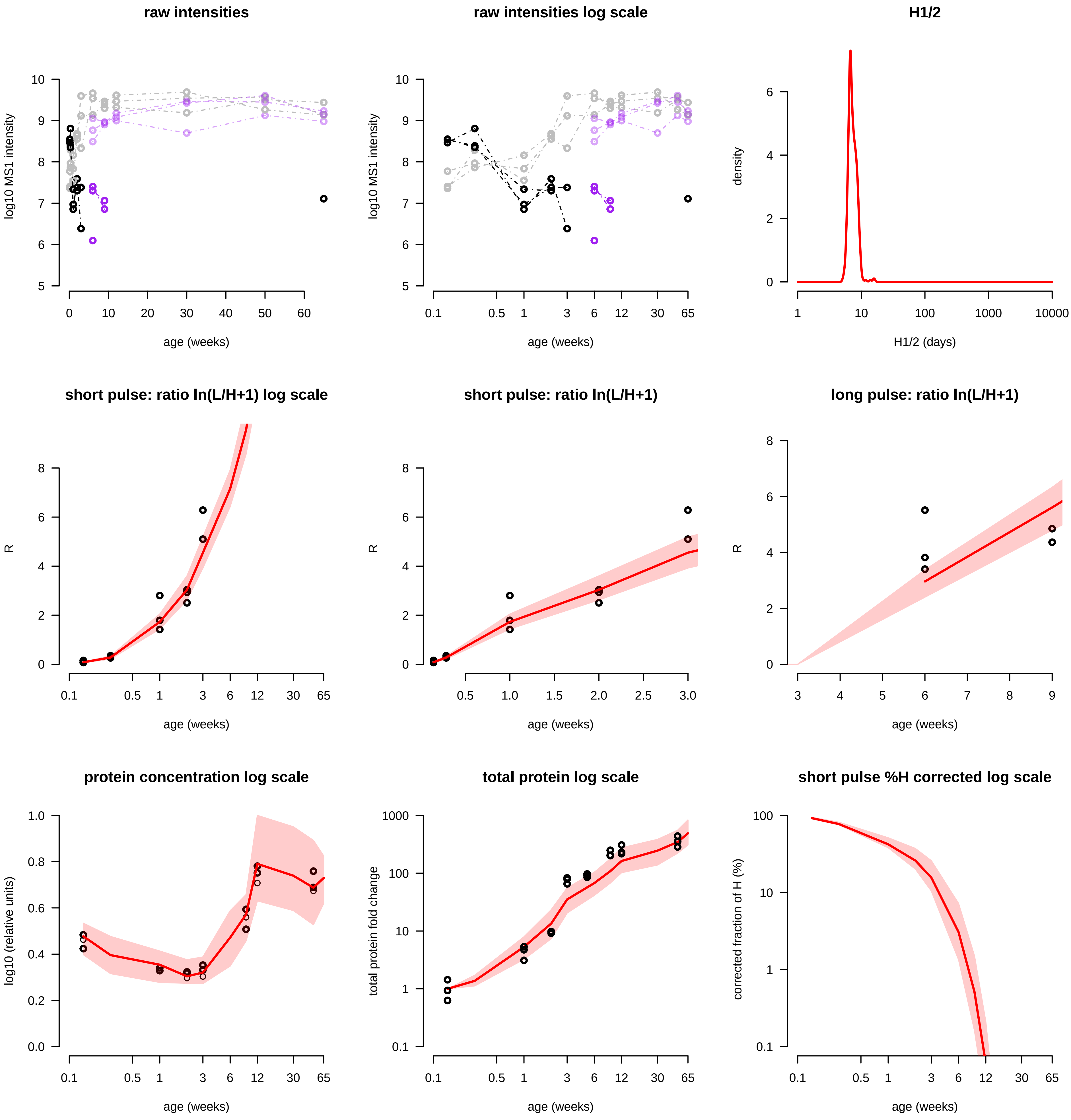

Q9CXY6

>sp|Q9CXY6|ILF2_MOUSE

Interleukin enhancer−binding factor 2

GN=Ilf2

Med H1/2: 6.8 days

Q5 H1/2: 5.5 days

Q95 H1/2: 9.2 days

reliable model fit: yes

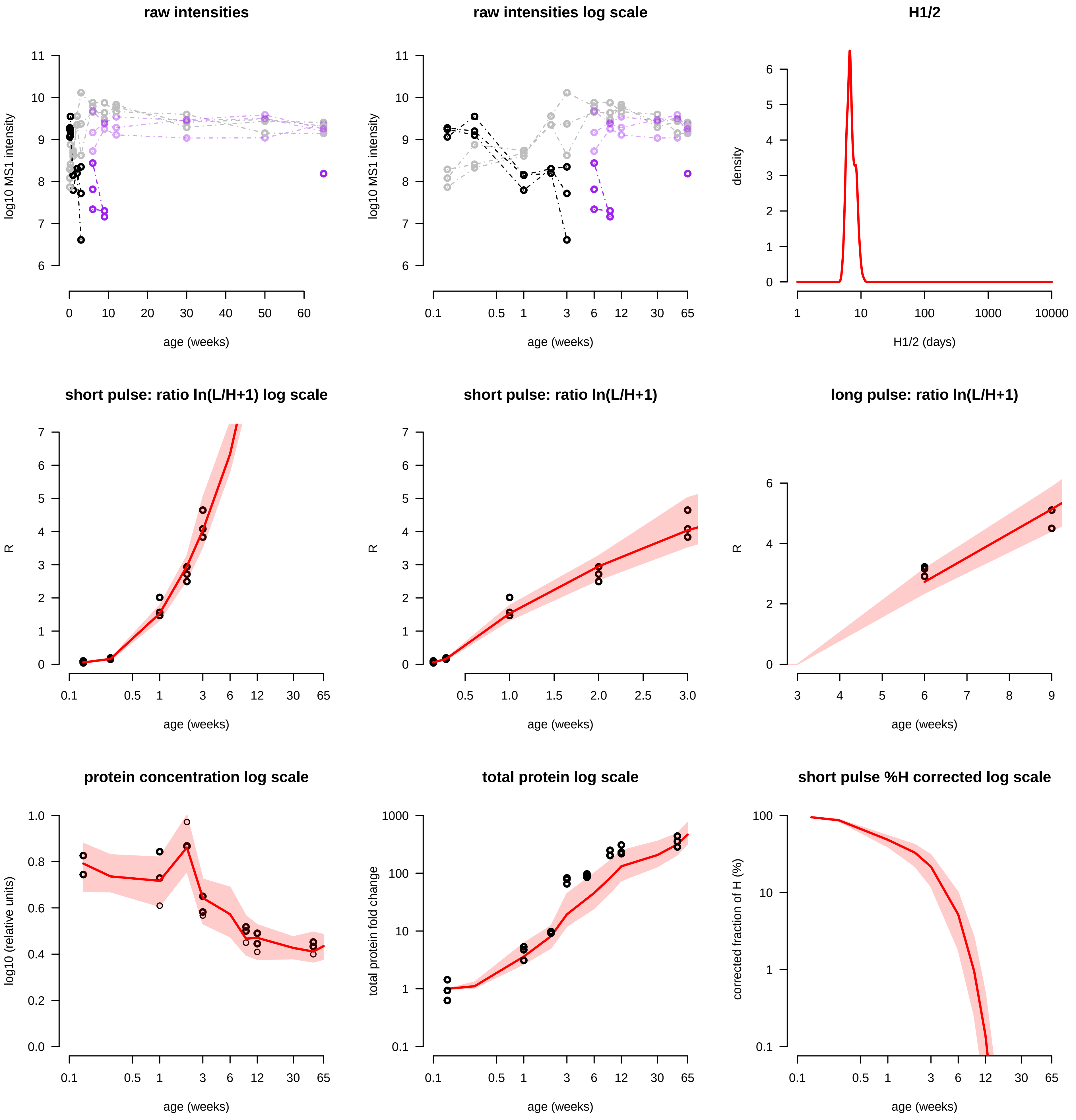

Q99LF4

>sp|Q99LF4|RTCB_MOUSE

RNA−splicing ligase RtcB homolog

GN=Rtcb

Med H1/2: 5.4 days

Q5 H1/2: 4.8 days

Q95 H1/2: 6.4 days

reliable model fit: yes

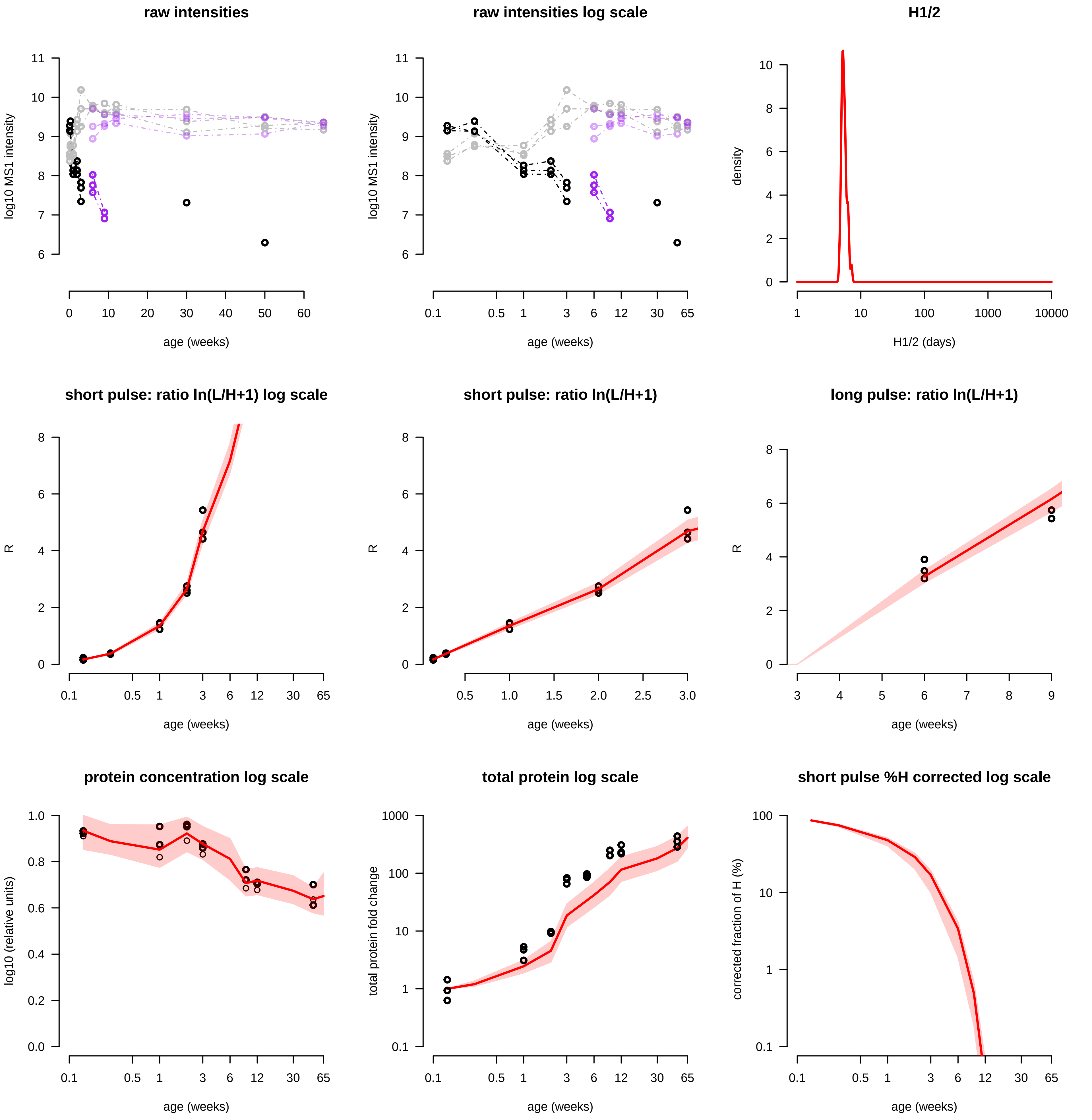

Q8BFW7;Q8BFW7−5;Q8BFW7−4

>sp|Q8BFW7|LPP_MOUSE

Lipoma−preferred partner homolog

GN=Lpp

Med H1/2: 296.4 days

Q5 H1/2: 99.9 days

Q95 H1/2: 1913.9 days

reliable model fit: yes

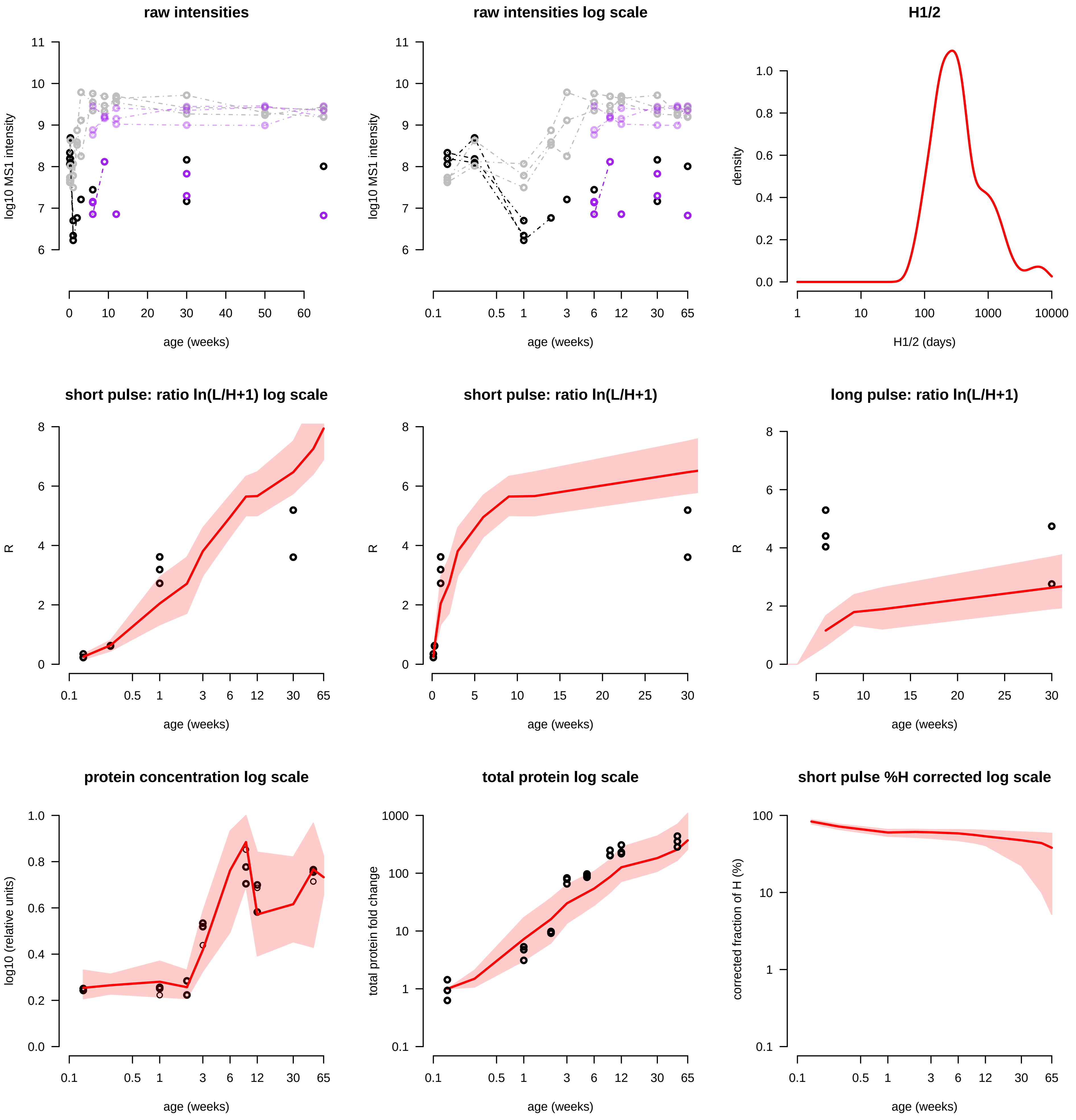

Q9DCJ9

>sp|Q9DCJ9|NPL_MOUSE

N−acetylneuraminate lyase

GN=Npl

Med H1/2: 444.8 days

Q5 H1/2: 162.6 days

Q95 H1/2: 7315.8 days

reliable model fit: yes

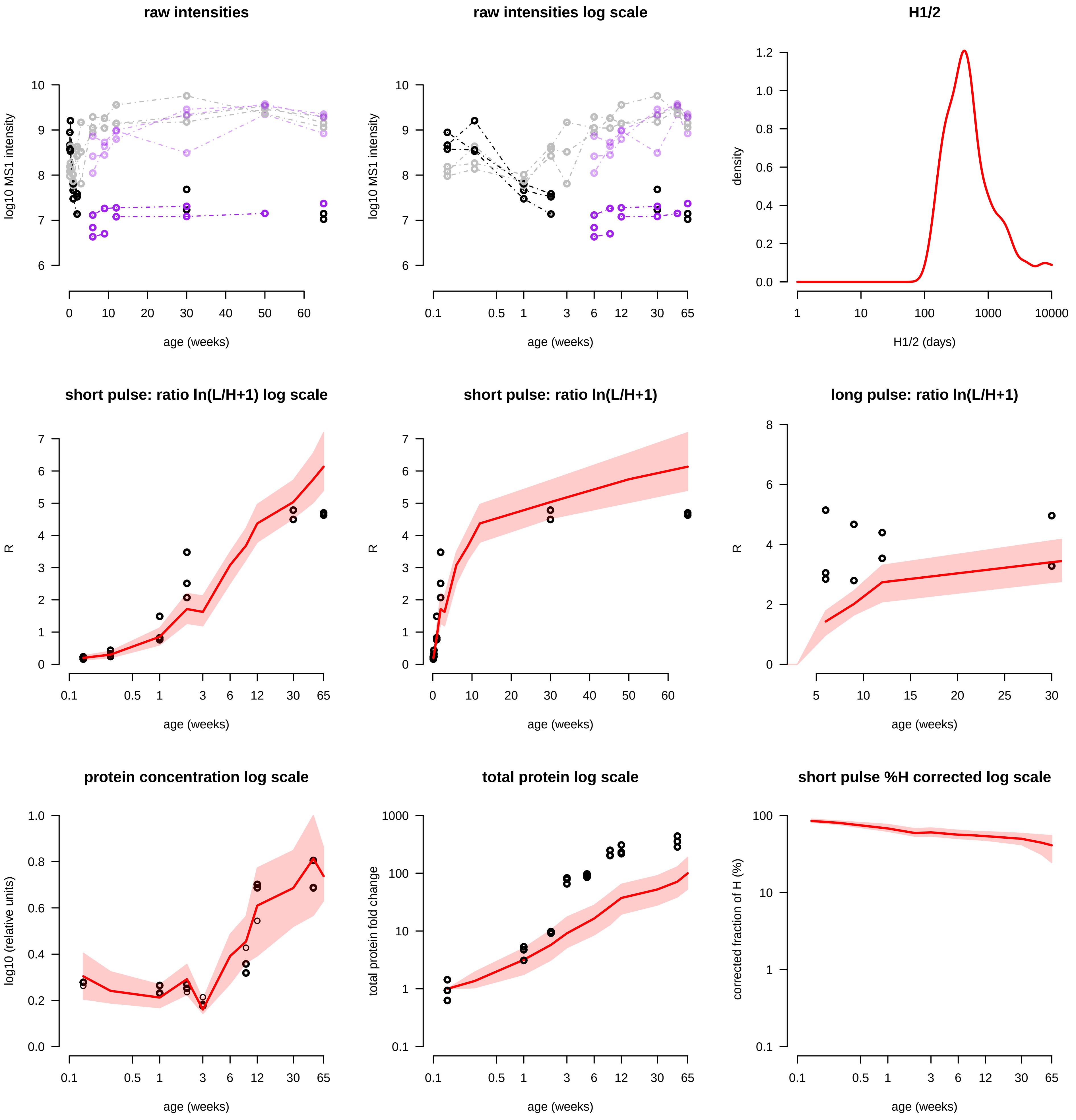

Q80UG5−3

>sp|Q80UG5−3|SEPT9_MOUSE

Isoform 3 of Septin−9

GN=Septin9

Med H1/2: 6.1 days

Q5 H1/2: 5.3 days

Q95 H1/2: 7 days

reliable model fit: yes

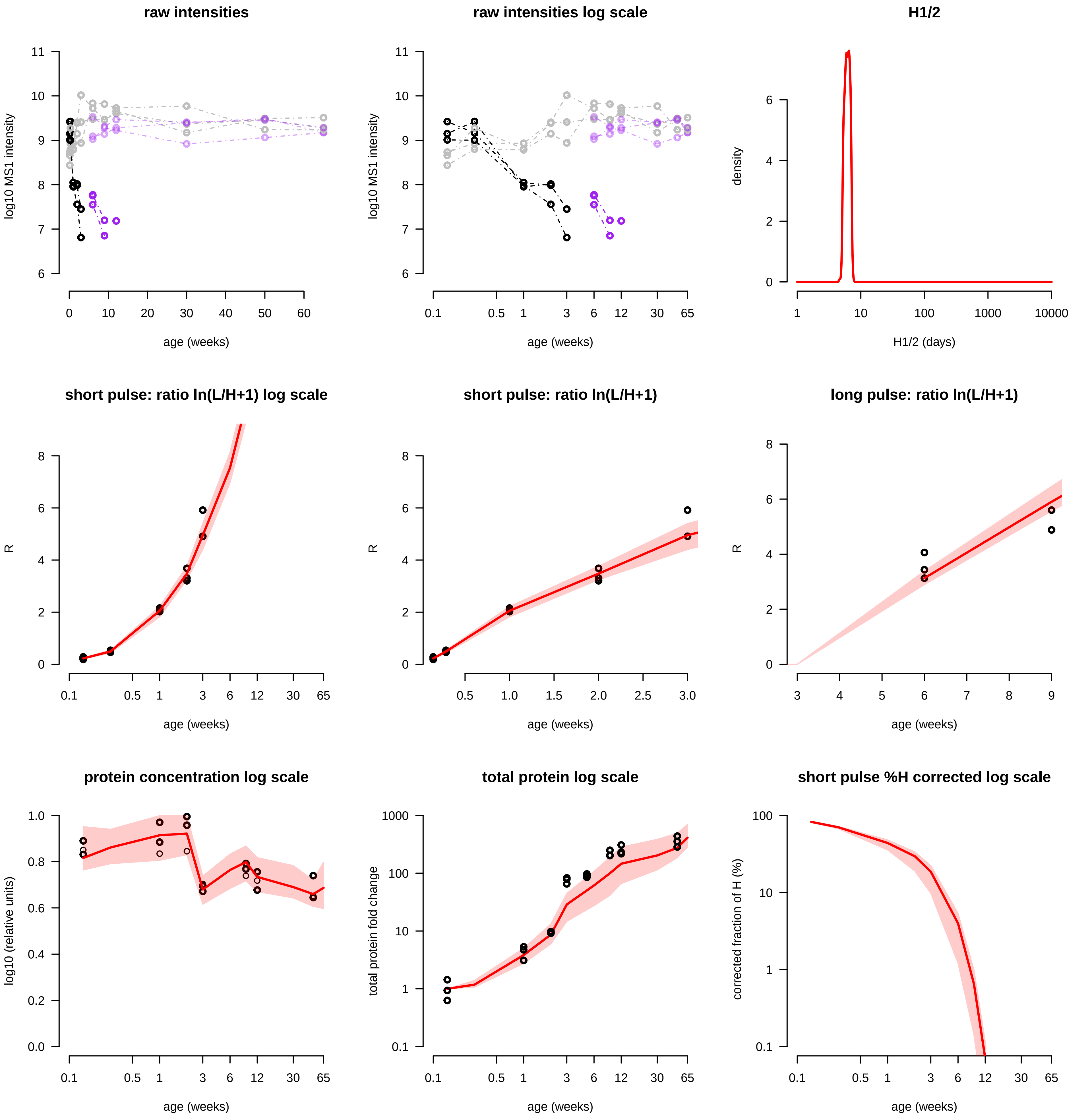

O70503

>sp|O70503|DHB12_MOUSE

Very−long−chain 3−oxoacyl−CoA reductase

GN=Hsd17b12

Med H1/2: 5.3 days

Q5 H1/2: 4.2 days

Q95 H1/2: 7.4 days

reliable model fit: yes

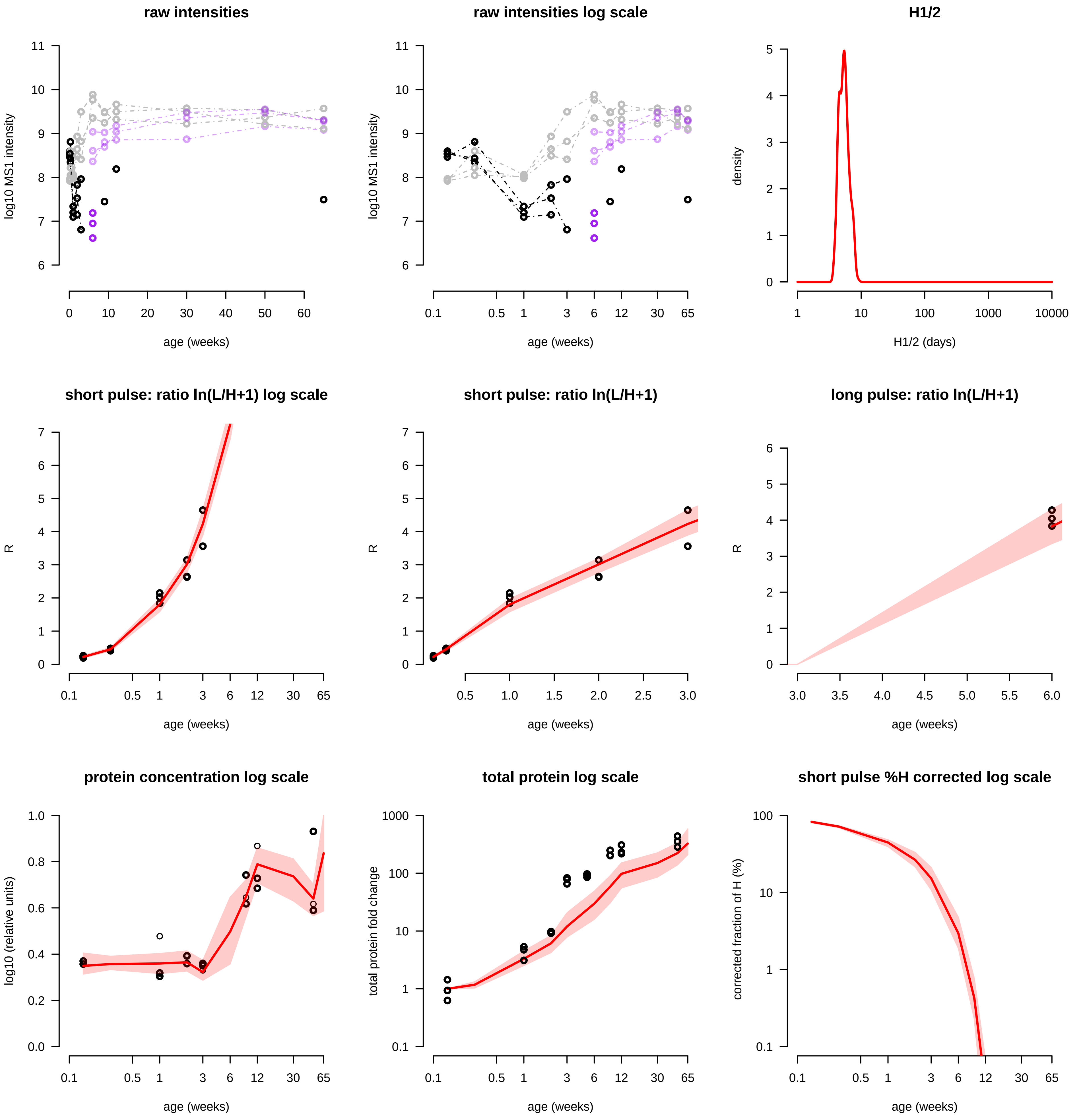

Q9Z1Z2

>sp|Q9Z1Z2|STRAP_MOUSE

Serine−threonine kinase receptor−associated protein

GN=Strap

Med H1/2: 4 days

Q5 H1/2: 3.2 days

Q95 H1/2: 4.8 days

reliable model fit: yes

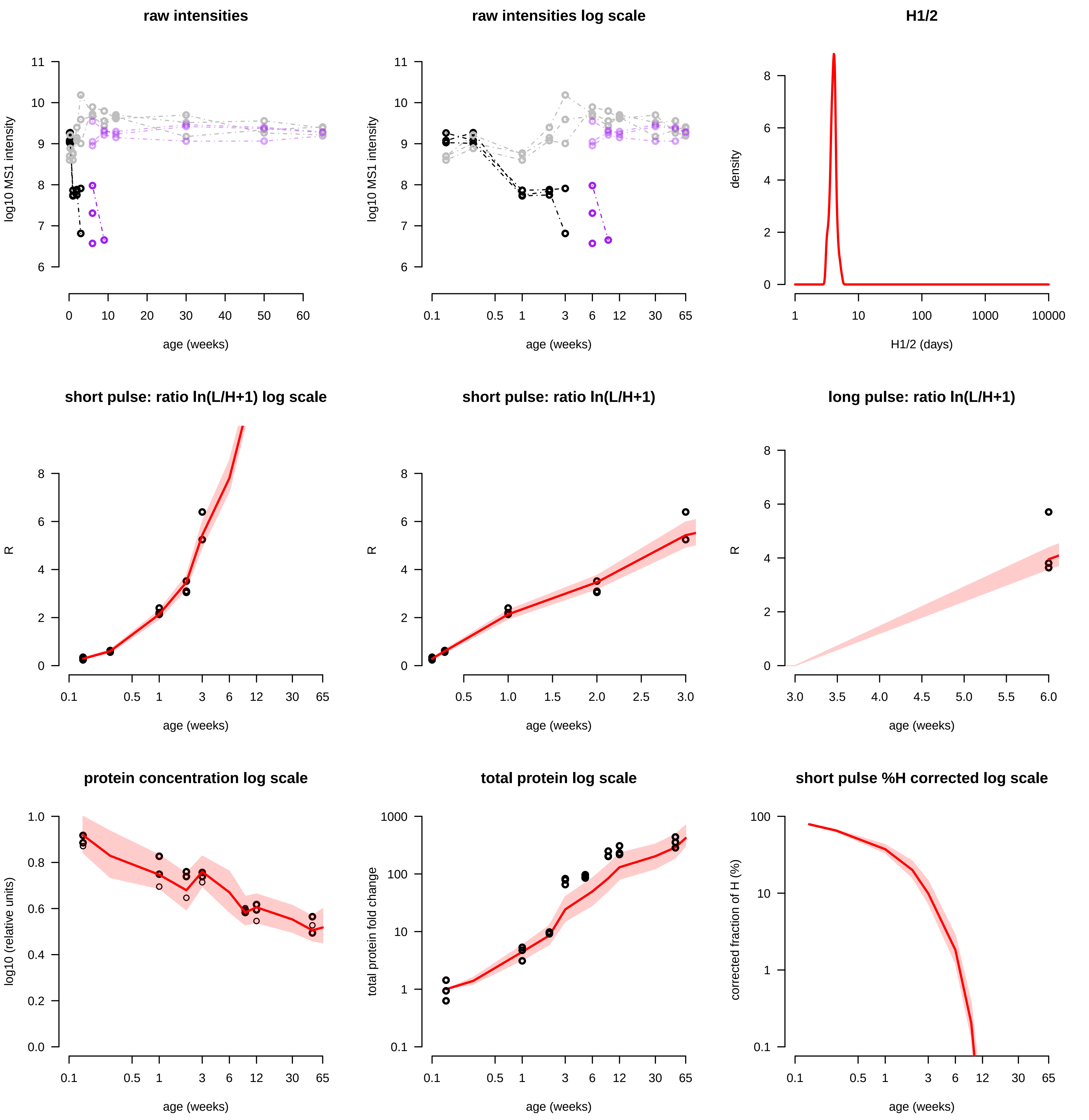

Q8JZQ9

>sp|Q8JZQ9|EIF3B_MOUSE

Eukaryotic translation initiation factor 3 subunit B

GN=Eif3b

Med H1/2: 153.6 days

Q5 H1/2: 44.8 days

Q95 H1/2: 2016.2 days

reliable model fit: yes

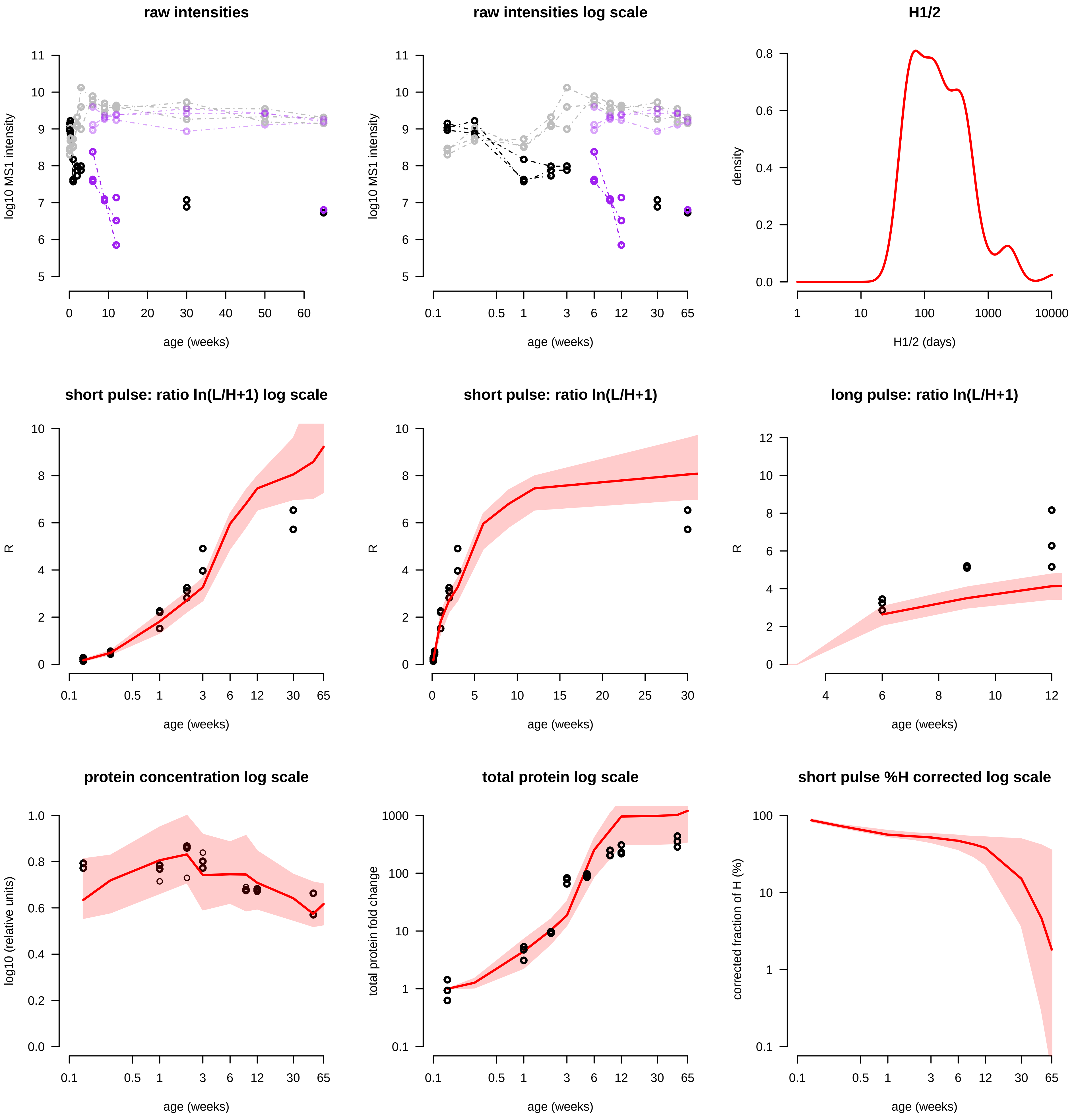

Q9D051

>sp|Q9D051|ODPB_MOUSE

Pyruvate dehydrogenase E1 component subunit beta, mitochondrial

GN=Pdhb

Med H1/2: 9.6 days

Q5 H1/2: 7.8 days

Q95 H1/2: 11.7 days

reliable model fit: yes

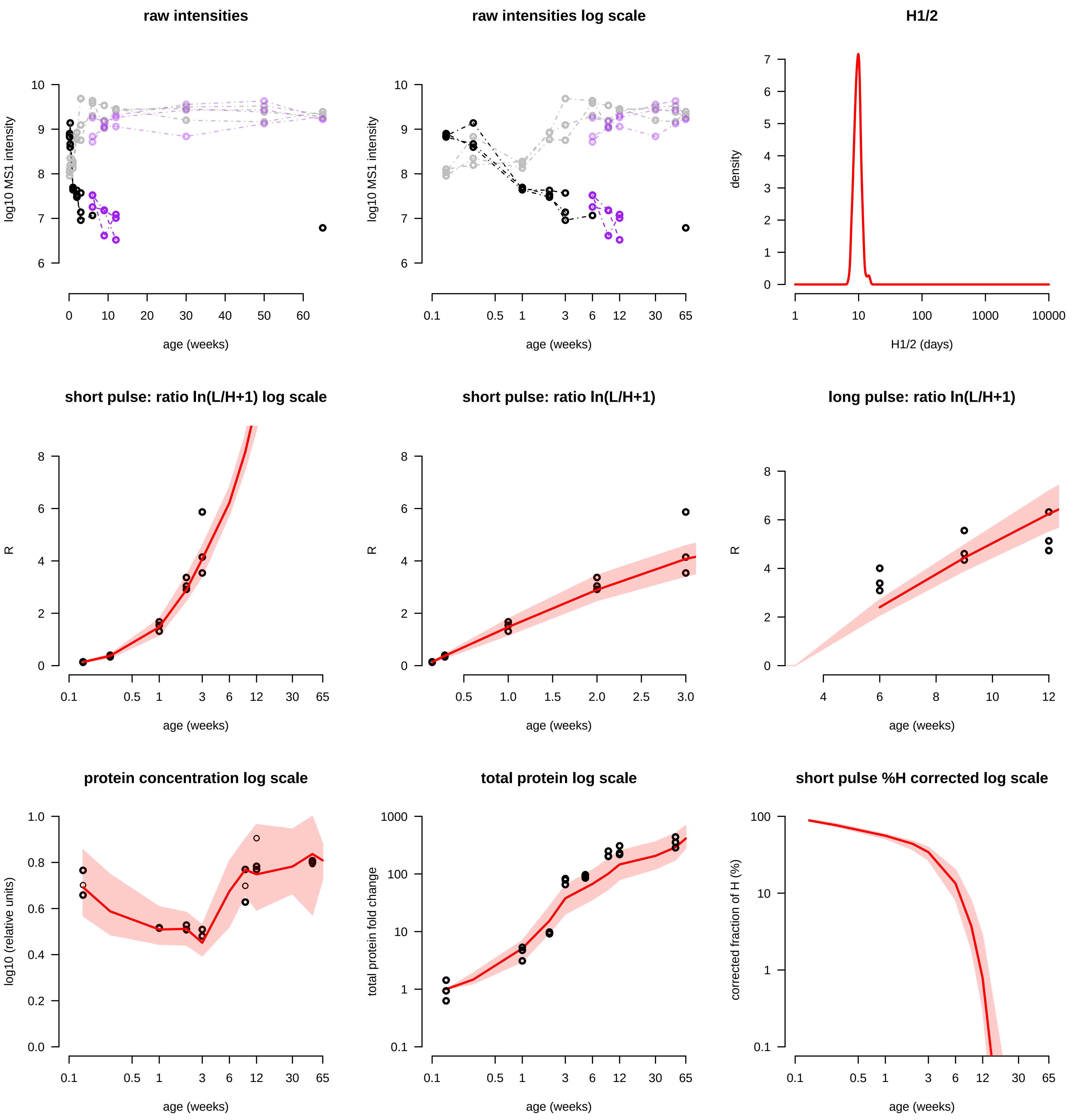

Q8R1G2

>sp|Q8R1G2|CMBL_MOUSE

Carboxymethylenebutenolidase homolog

GN=Cmbl

Med H1/2: 79.3 days

Q5 H1/2: 22.1 days

Q95 H1/2: 1801.8 days

reliable model fit: yes

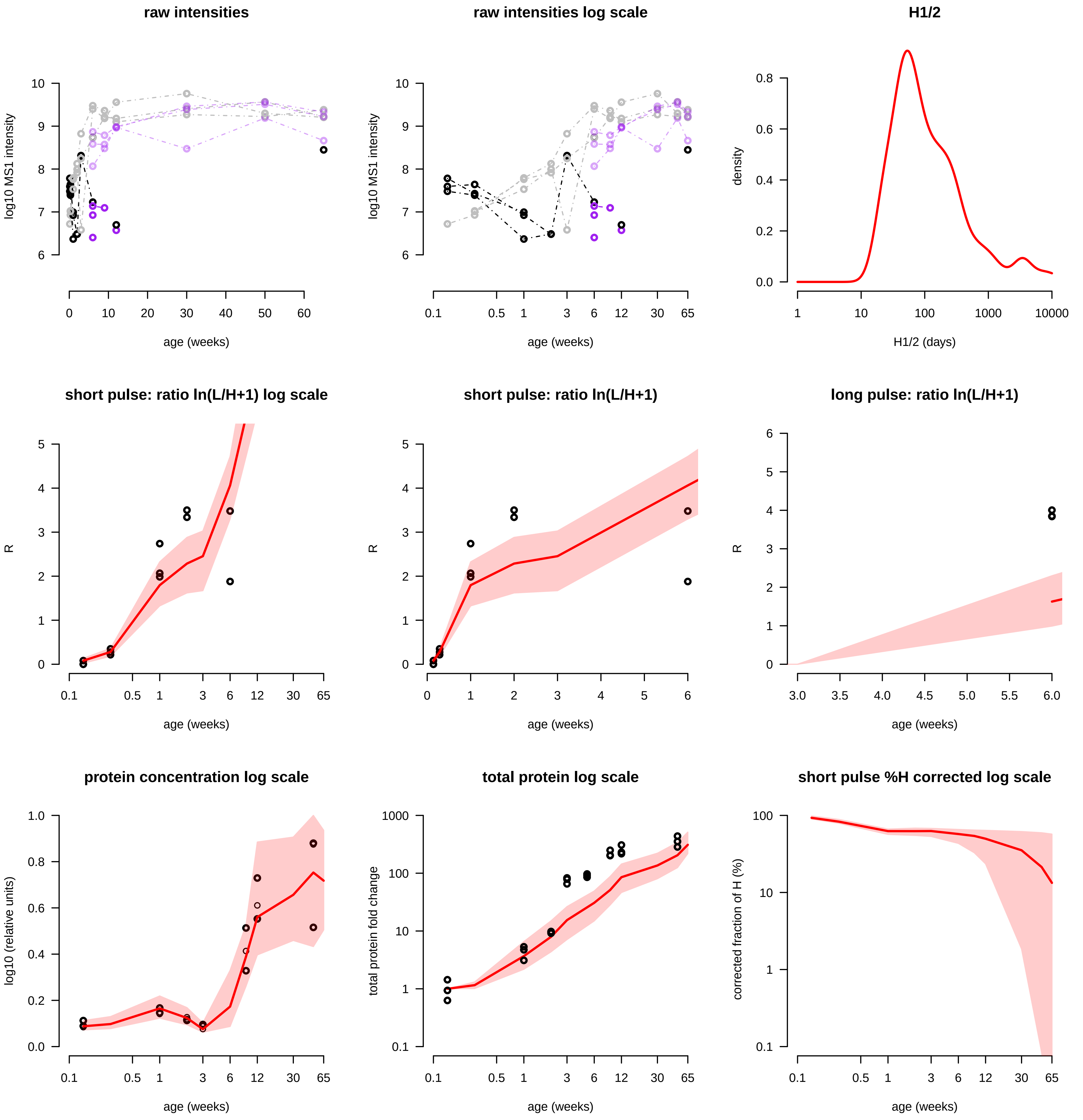

O35685

>sp|O35685|NUDC_MOUSE

Nuclear migration protein nudC

GN=Nudc

Med H1/2: 6.4 days

Q5 H1/2: 5.4 days

Q95 H1/2: 8.1 days

reliable model fit: yes

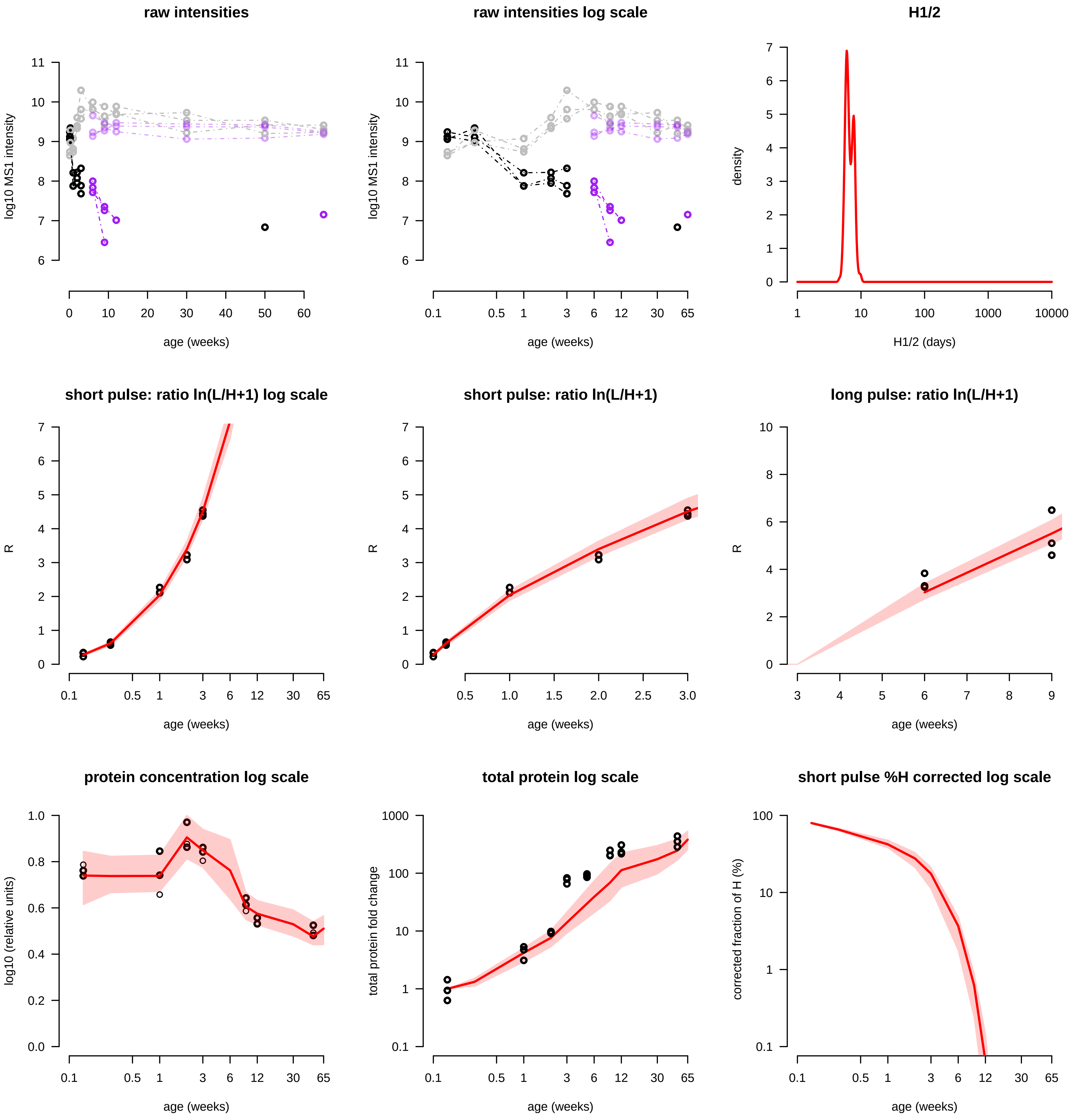

Q64514−2

>sp|Q64514−2|TPP2_MOUSE

Isoform Short of Tripeptidyl−peptidase 2

GN=Tpp2

Med H1/2: 996.3 days

Q5 H1/2: 331.6 days

Q95 H1/2: 12510.2 days

reliable model fit: yes

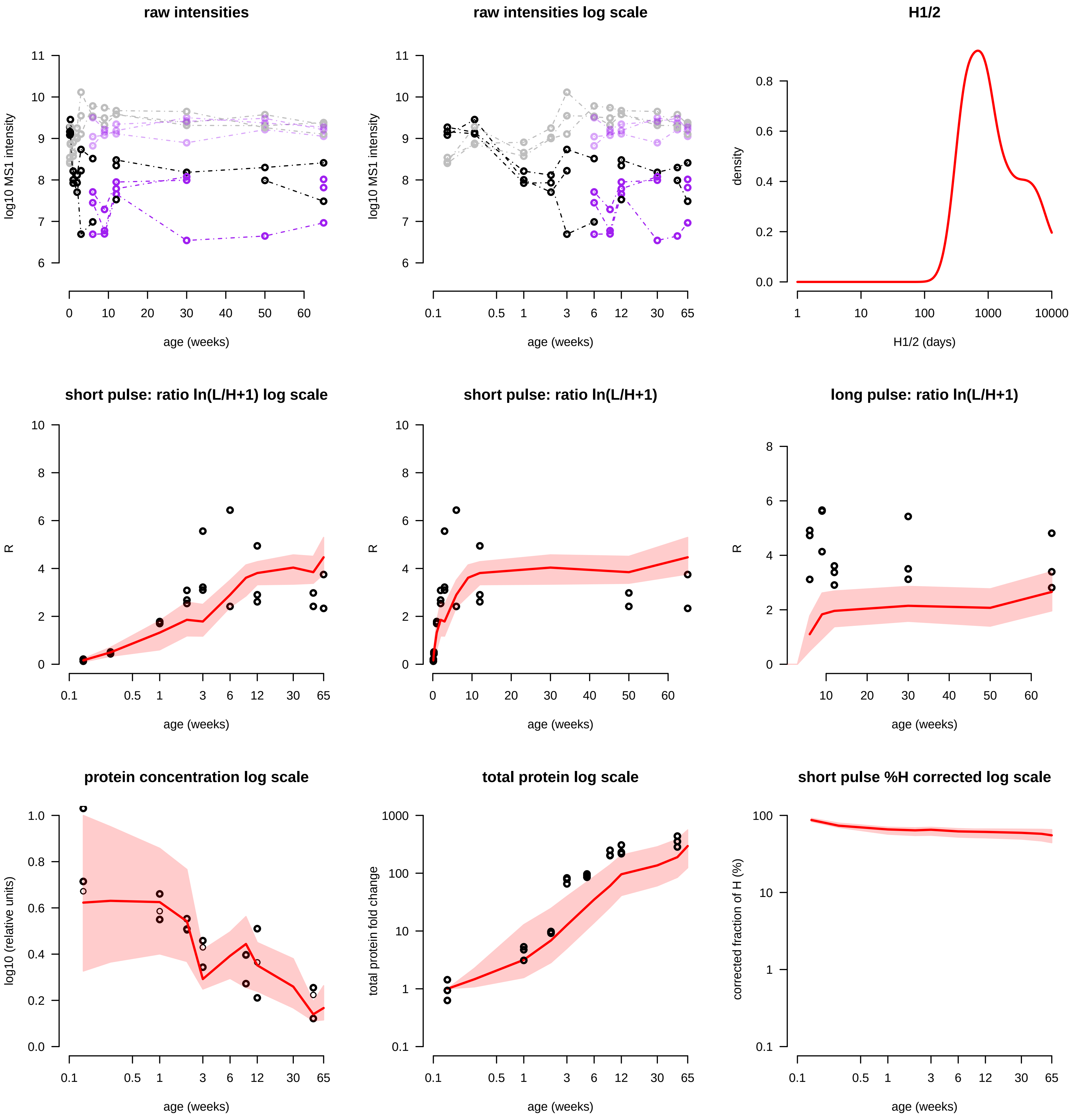

Q9JHR7

>sp|Q9JHR7|IDE_MOUSE

Insulin−degrading enzyme

GN=Ide

Med H1/2: 4 days

Q5 H1/2: 3.4 days

Q95 H1/2: 4.8 days

reliable model fit: yes

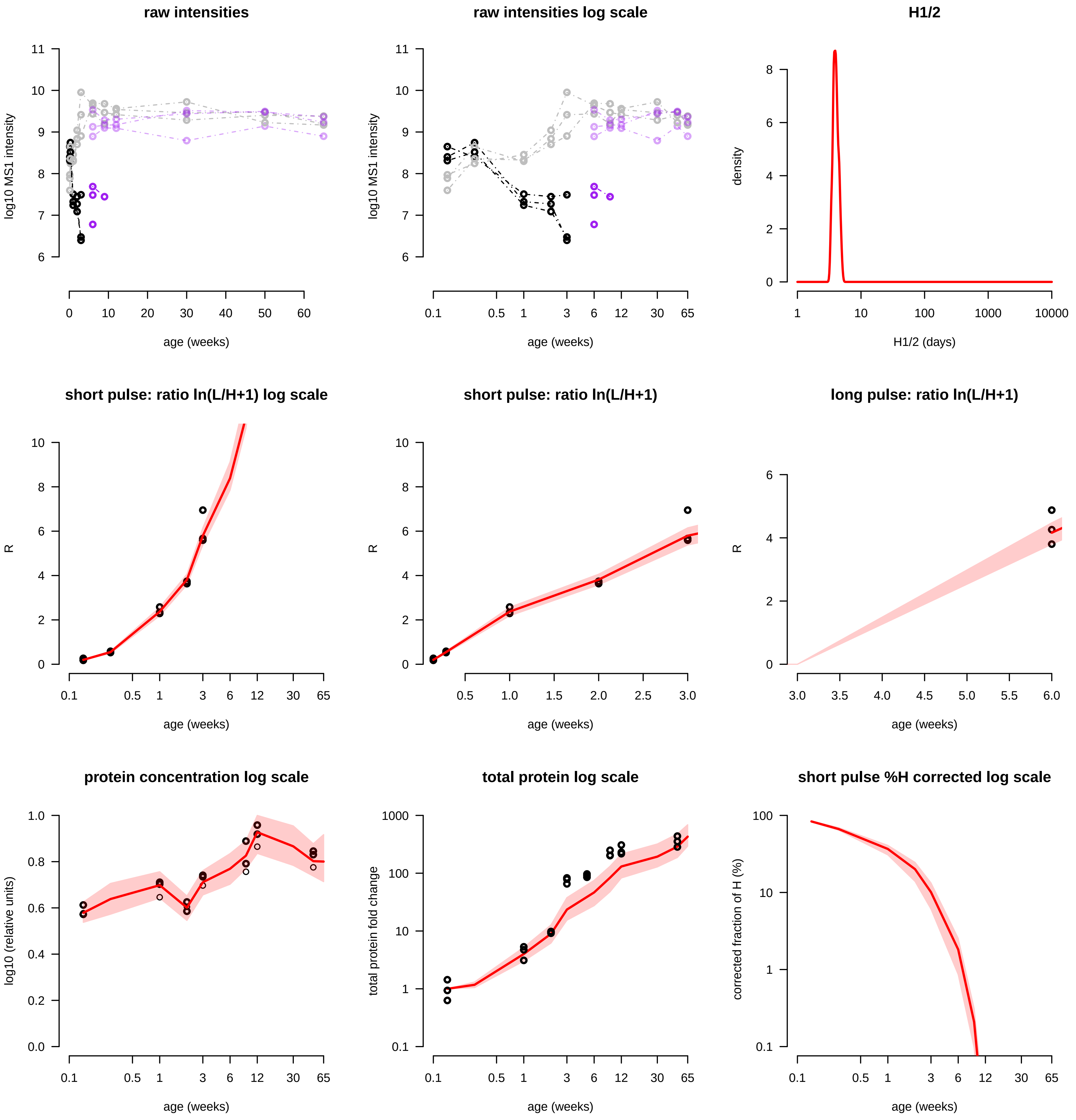

Q07417

>sp|Q07417|ACADS_MOUSE

Short−chain specific acyl−CoA dehydrogenase, mitochondrial

GN=Acads

Med H1/2: 9.3 days

Q5 H1/2: 6.7 days

Q95 H1/2: 15.3 days

reliable model fit: yes

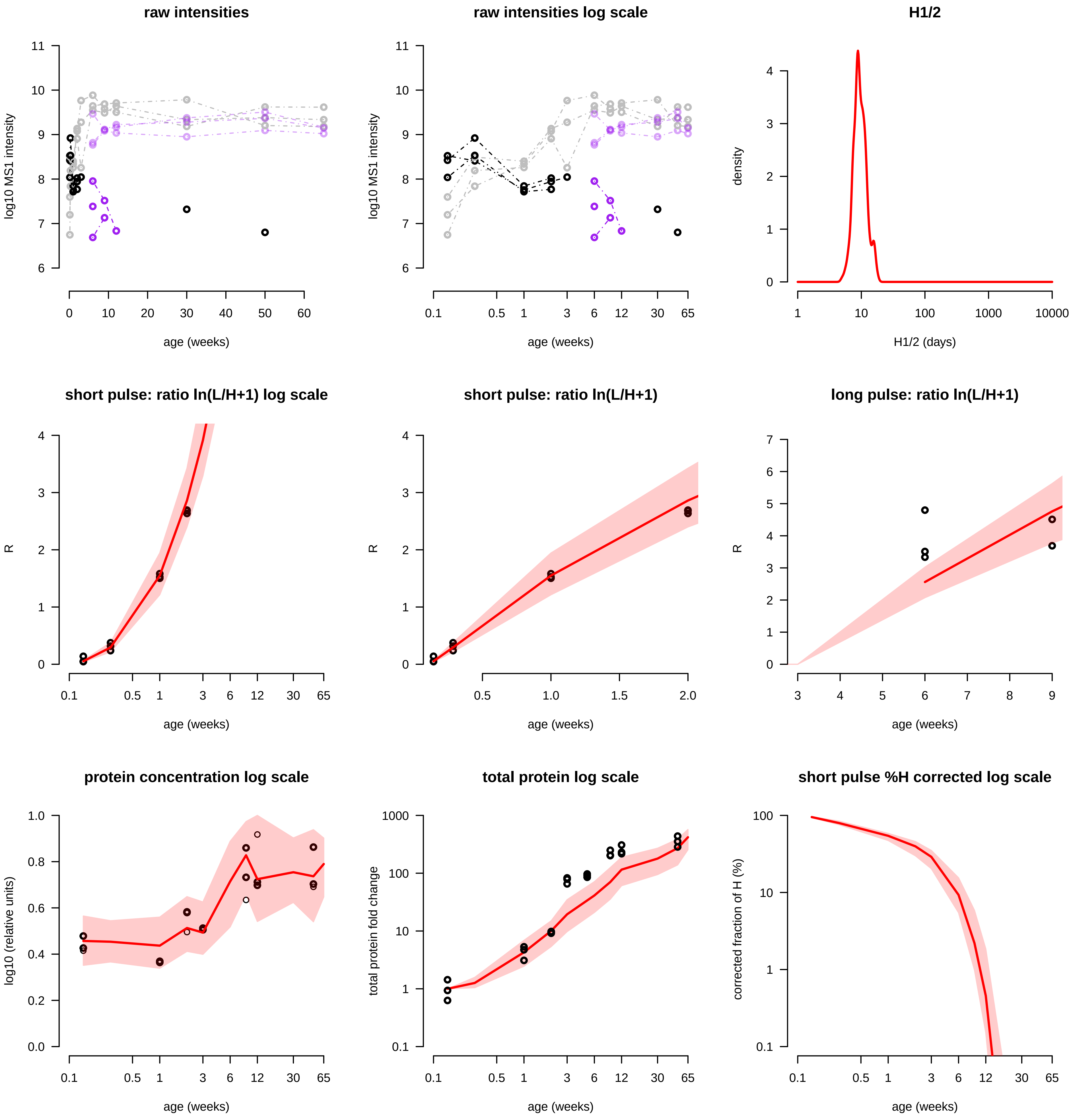

P05132;P05132−2

>sp|P05132|KAPCA_MOUSE

cAMP−dependent protein kinase catalytic subunit alpha

GN=Prkaca

Med H1/2: 7.1 days

Q5 H1/2: 6 days

Q95 H1/2: 9.3 days

reliable model fit: yes

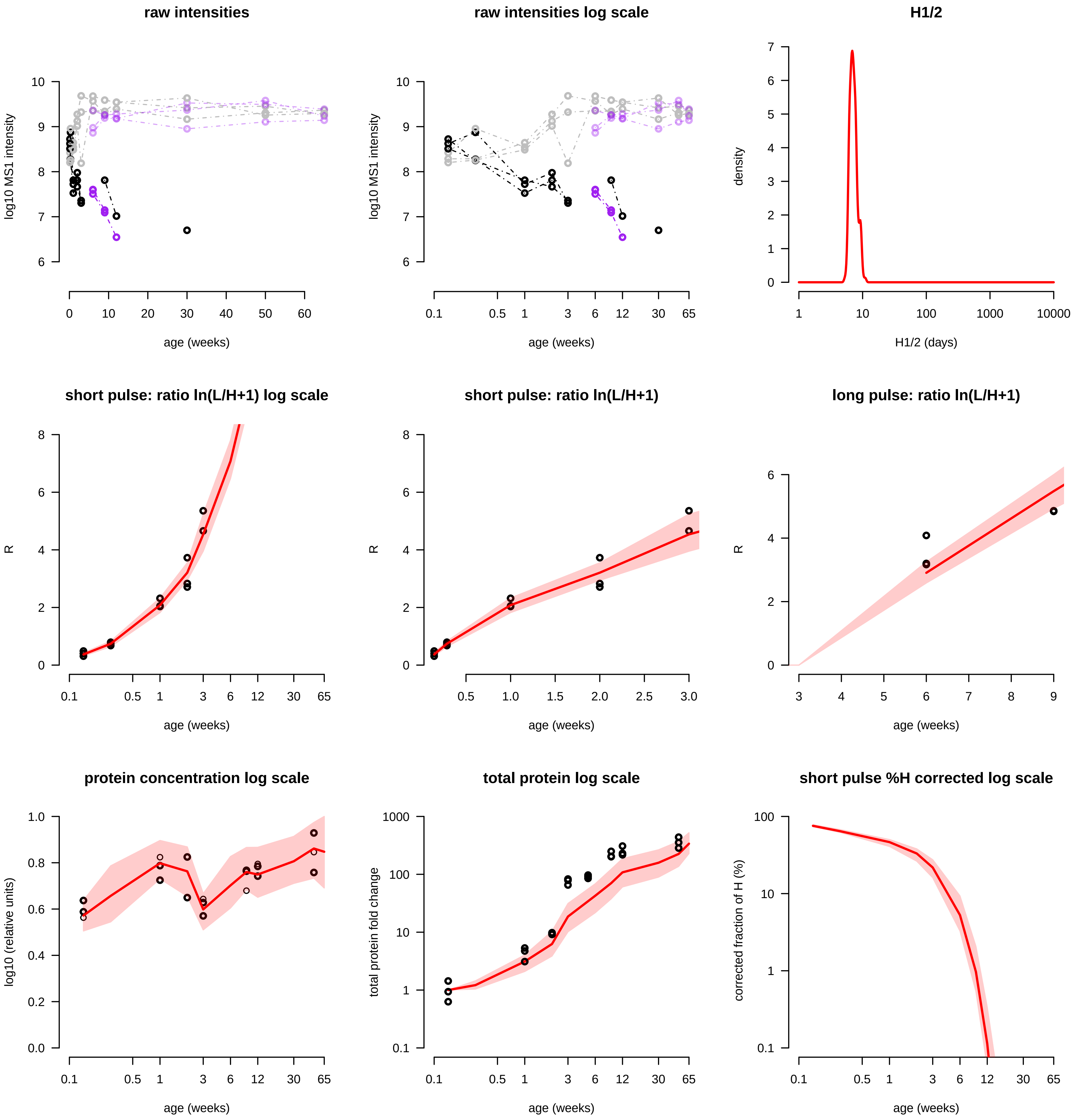

Q91V61−2;Q91V61

>sp|Q91V61−2|SFXN3_MOUSE

Isoform 2 of Sideroflexin−3

GN=Sfxn3

Med H1/2: 8 days

Q5 H1/2: 5.1 days

Q95 H1/2: 12.8 days

reliable model fit: yes

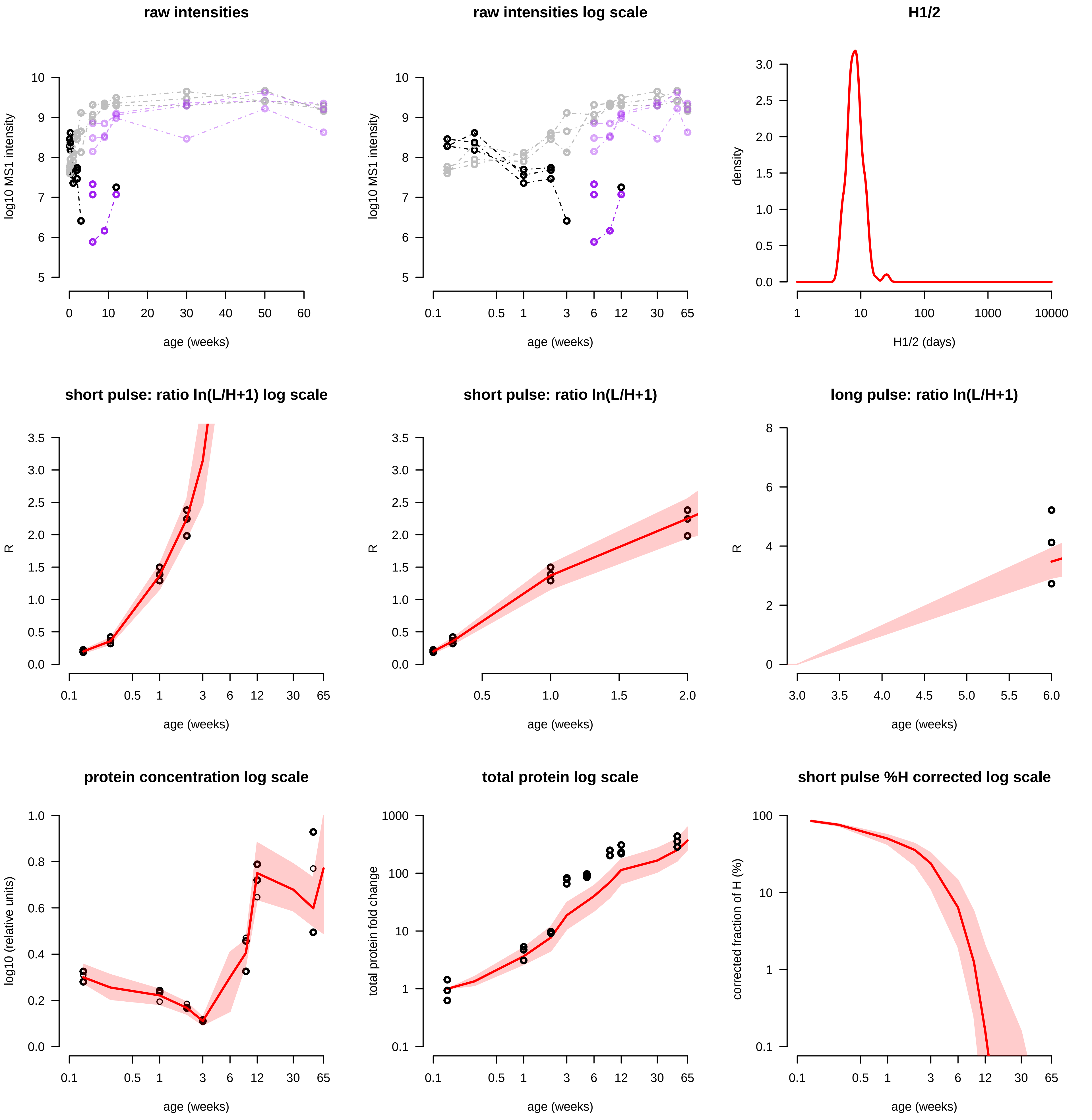

P42669

>sp|P42669|PURA_MOUSE

Transcriptional activator protein Pur−alpha

GN=Pura

Med H1/2: 8.2 days

Q5 H1/2: 7 days

Q95 H1/2: 11.3 days

reliable model fit: yes

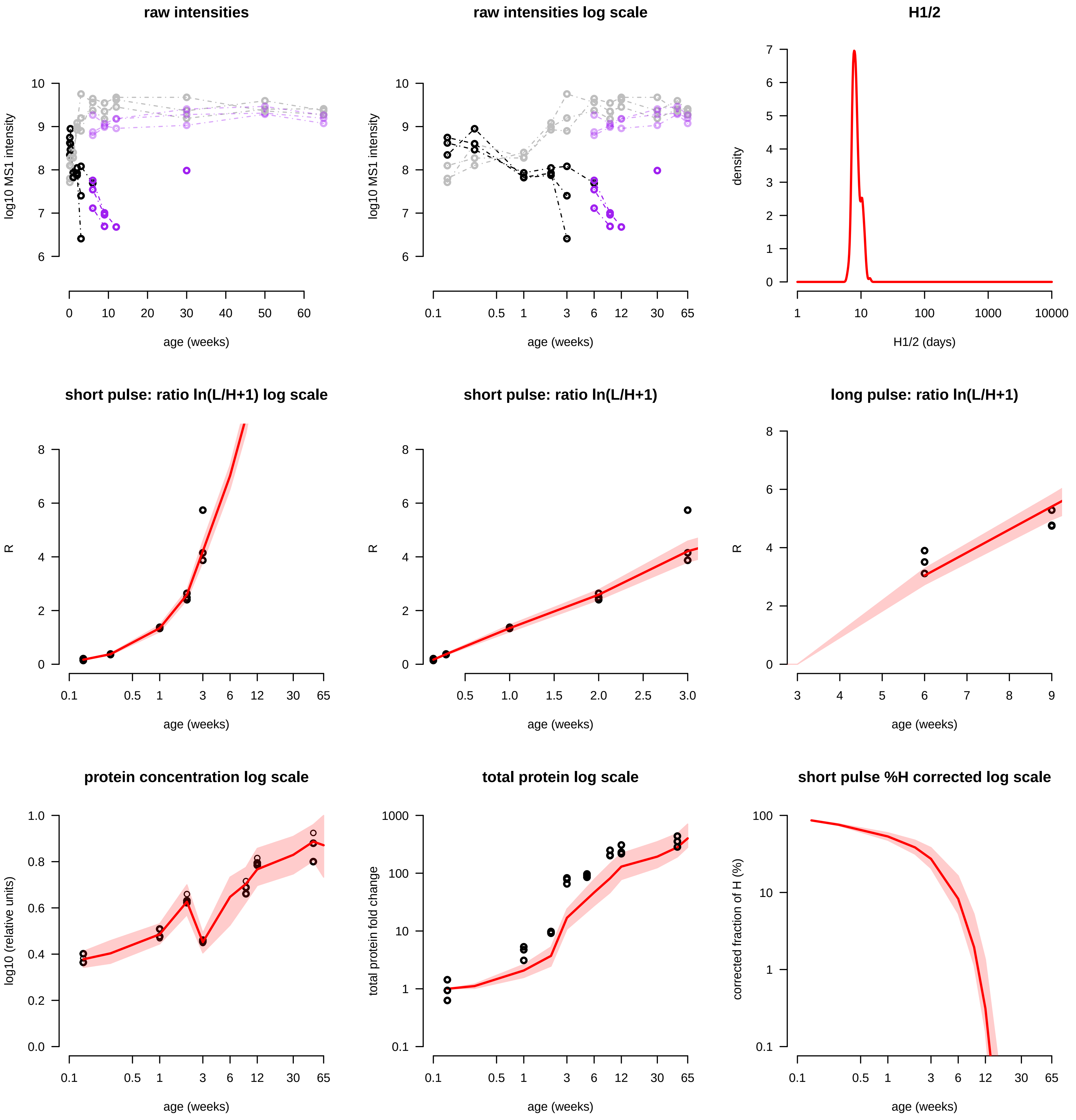

Q791V5

>sp|Q791V5|MTCH2_MOUSE

Mitochondrial carrier homolog 2

GN=Mtch2

Med H1/2: 7.4 days

Q5 H1/2: 5.7 days

Q95 H1/2: 14 days

reliable model fit: yes

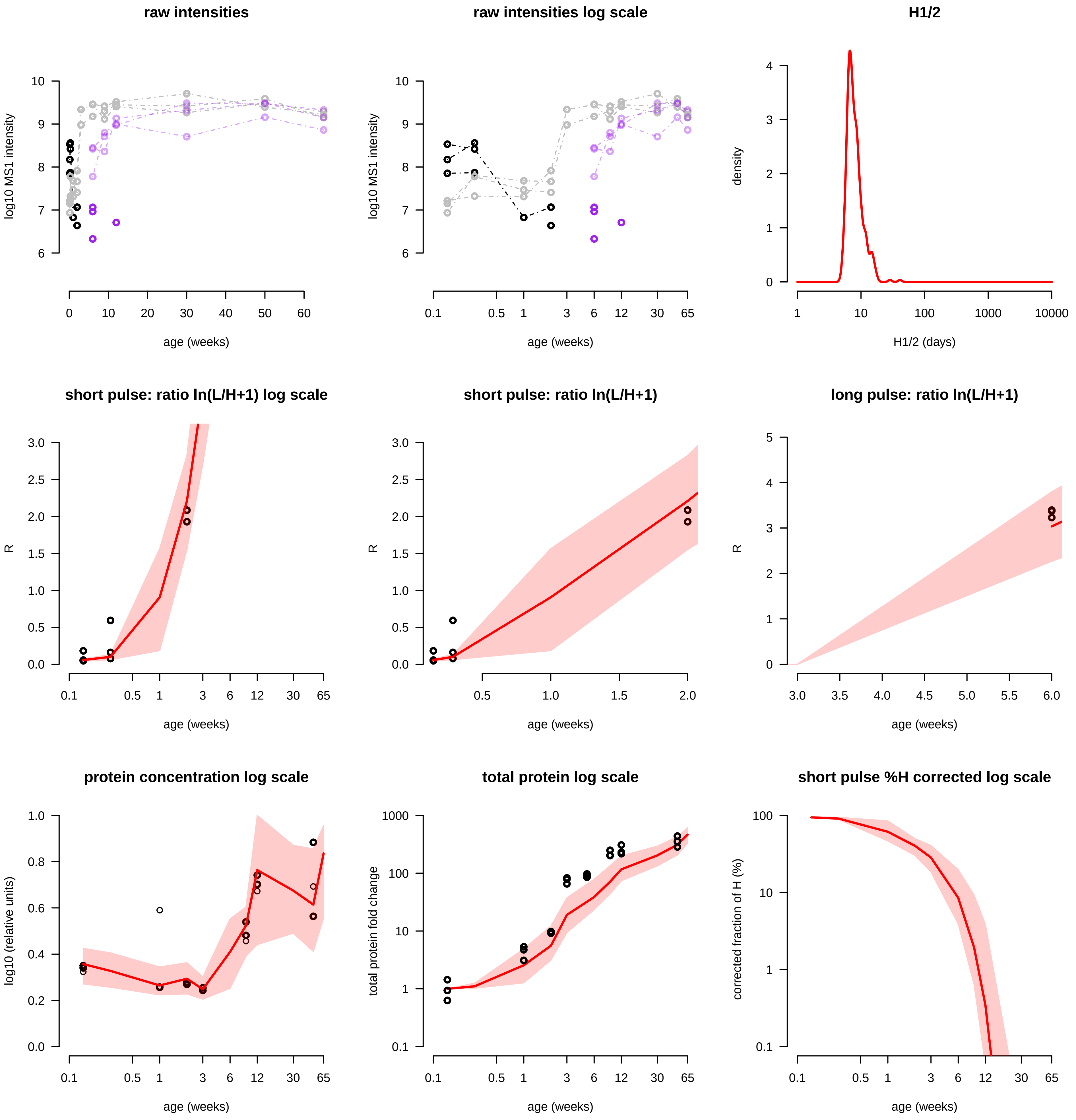

P61358

>sp|P61358|RL27_MOUSE

60S ribosomal protein L27

GN=Rpl27

Med H1/2: 223.8 days

Q5 H1/2: 115.3 days

Q95 H1/2: 1004.4 days

reliable model fit: yes

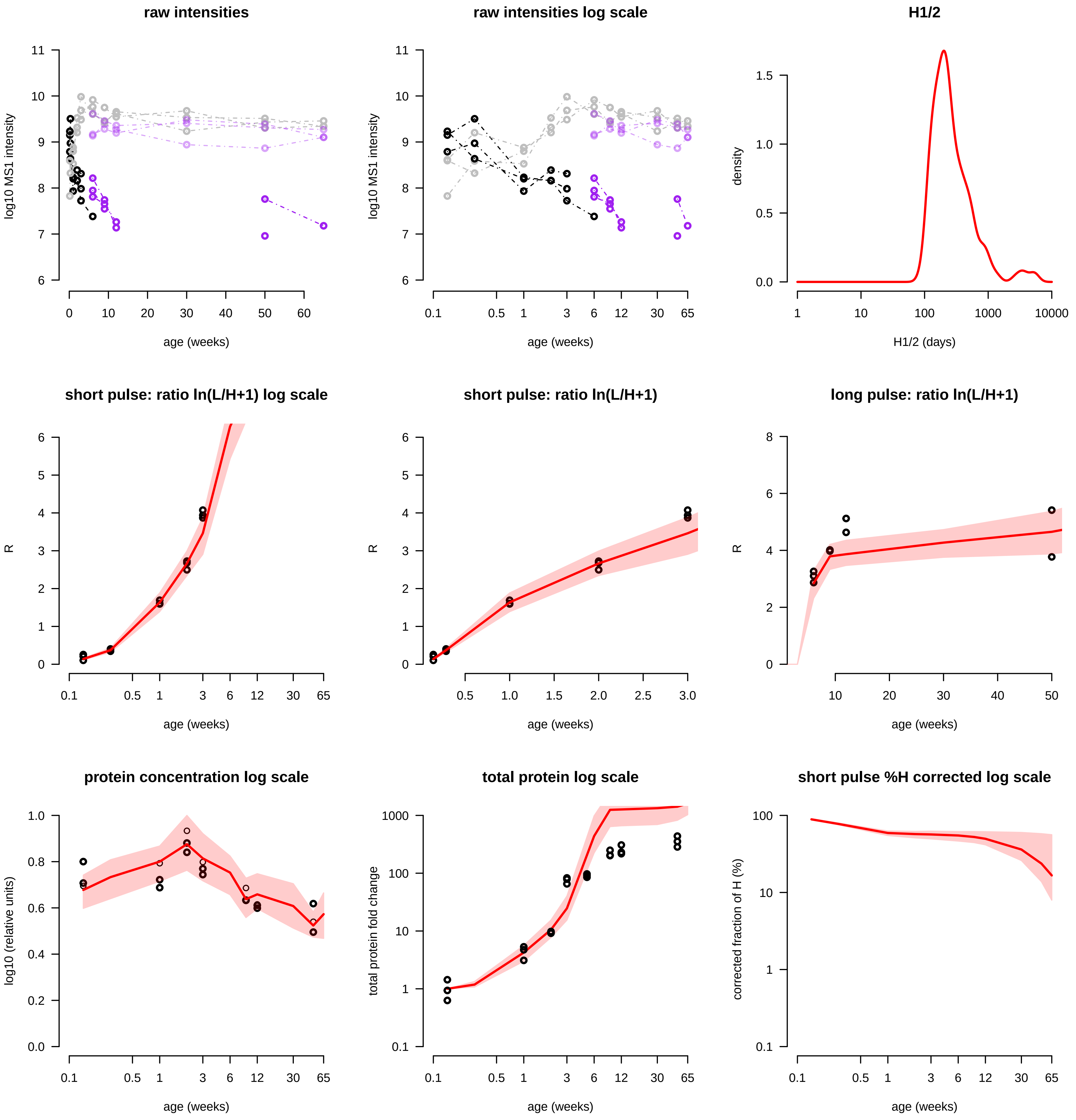

Q7TPV4

>sp|Q7TPV4|MBB1A_MOUSE

Myb−binding protein 1A

GN=Mybbp1a

Med H1/2: 6 days

Q5 H1/2: 5.3 days

Q95 H1/2: 7.1 days

reliable model fit: yes

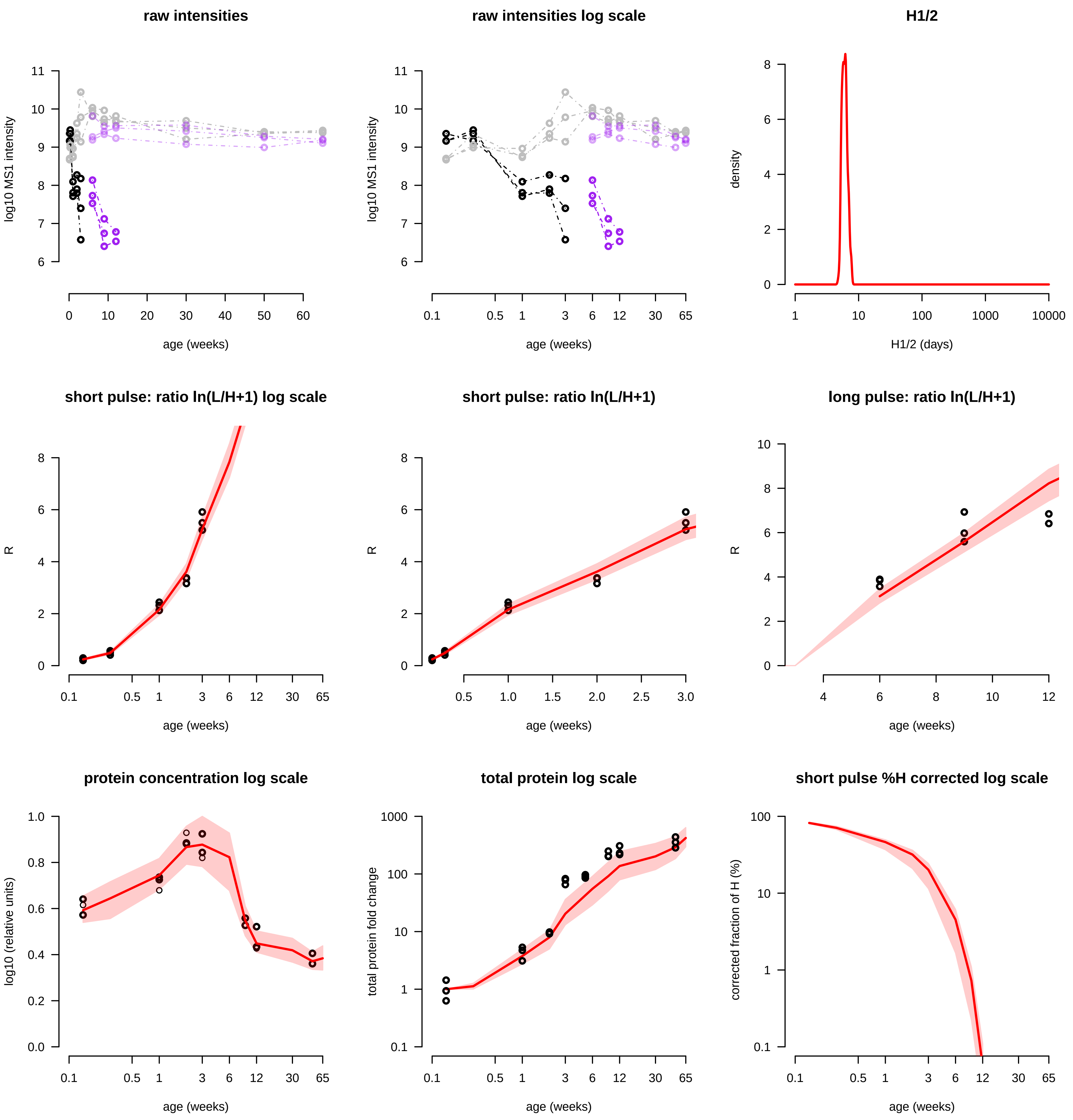

P61255

>sp|P61255|RL26_MOUSE

60S ribosomal protein L26

GN=Rpl26

Med H1/2: 7.6 days

Q5 H1/2: 6.3 days

Q95 H1/2: 9.8 days

reliable model fit: yes

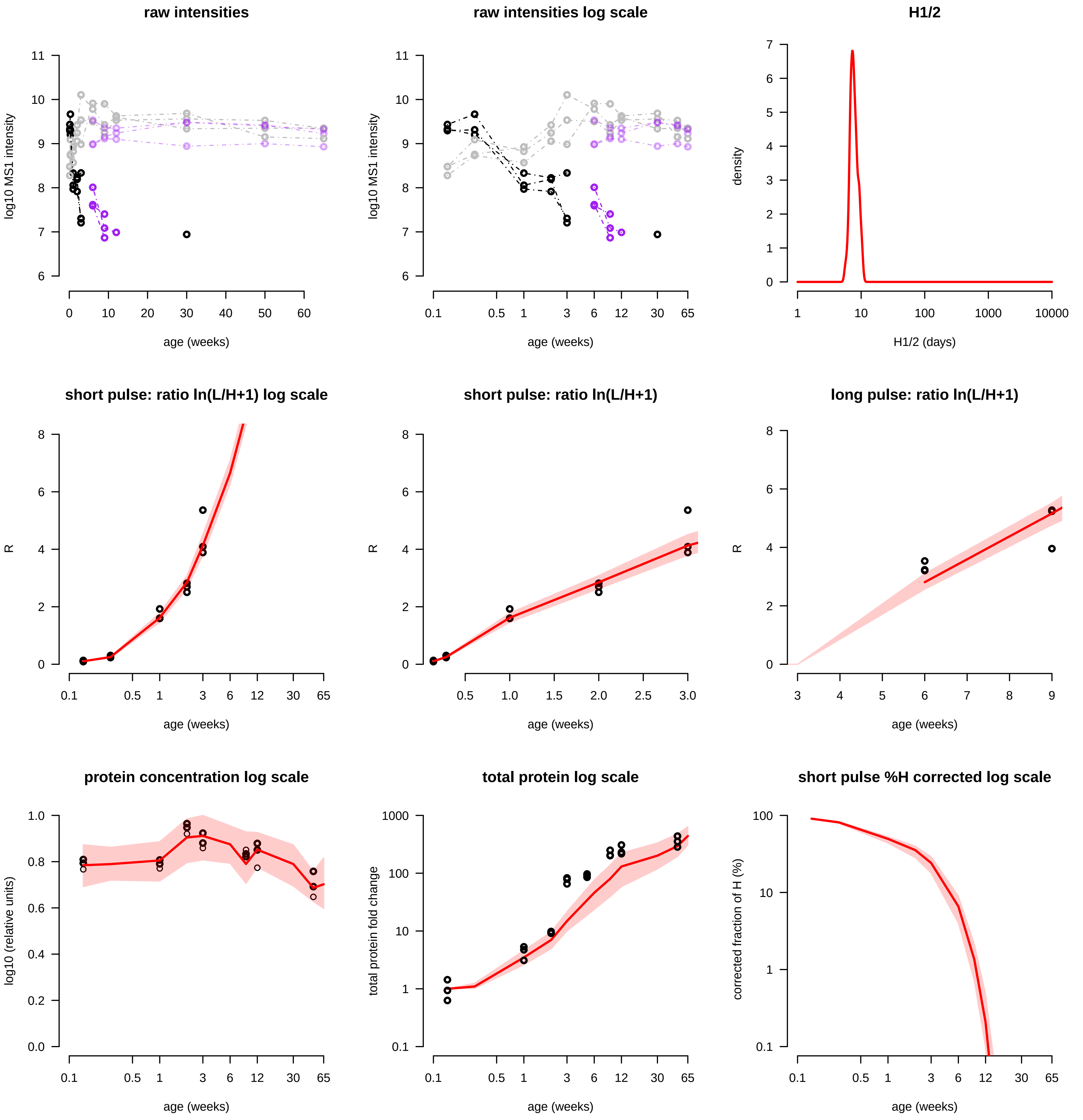

Q61990;Q61990−3

>sp|Q61990|PCBP2_MOUSE

Poly(rC)−binding protein 2

GN=Pcbp2

Med H1/2: 264.2 days

Q5 H1/2: 97.7 days

Q95 H1/2: 1888.6 days

reliable model fit: no

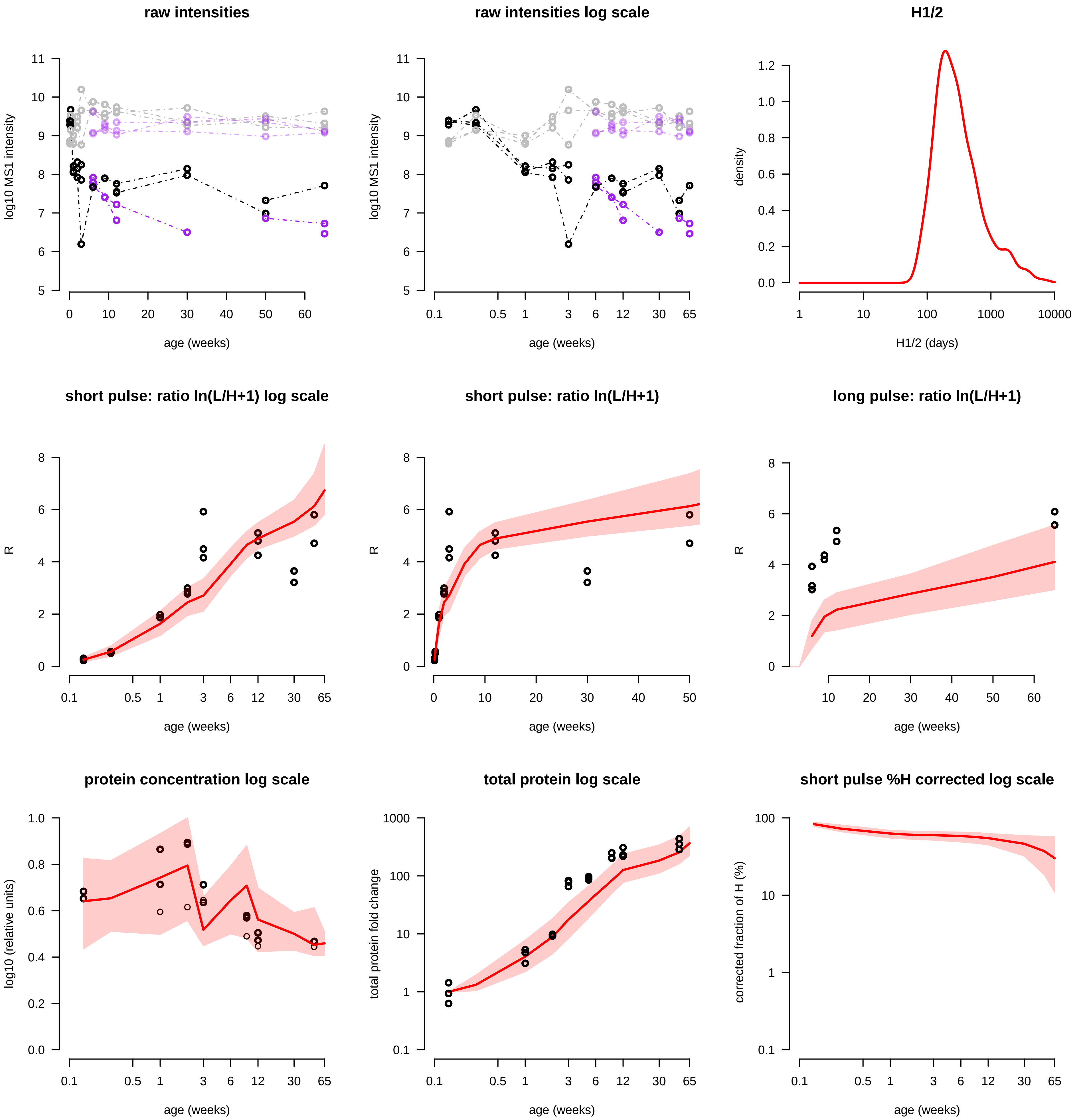

O70435

>sp|O70435|PSA3_MOUSE

Proteasome subunit alpha type−3

GN=Psma3

Med H1/2: 7.1 days

Q5 H1/2: 5.9 days

Q95 H1/2: 8.6 days

reliable model fit: yes

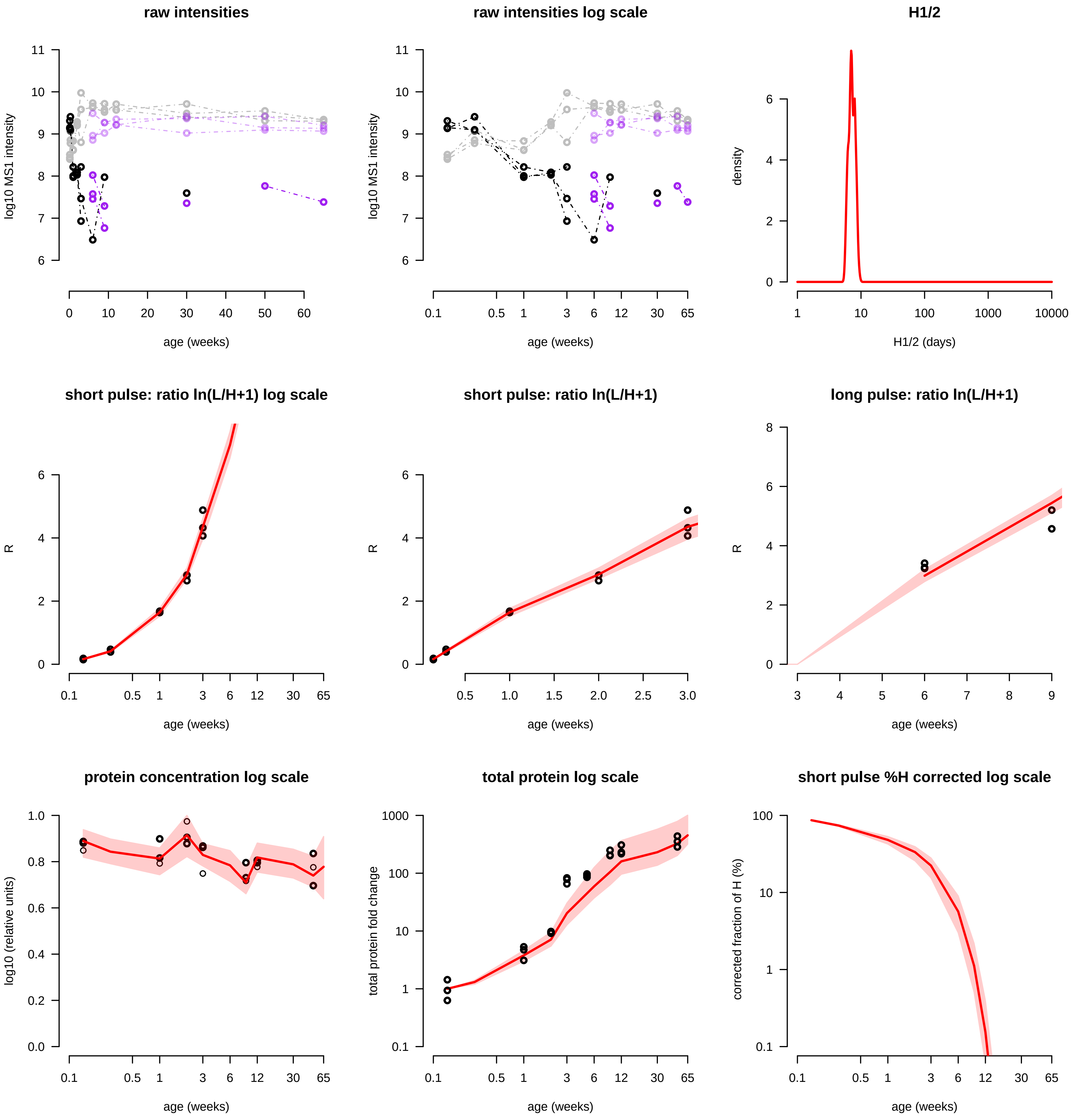

Q9JIF0−2;Q9JIF0

>sp|Q9JIF0−2|ANM1_MOUSE

Isoform 2 of Protein arginine N−methyltransferase 1

GN=Prmt1

Med H1/2: 5.5 days

Q5 H1/2: 4.6 days

Q95 H1/2: 6.9 days

reliable model fit: yes

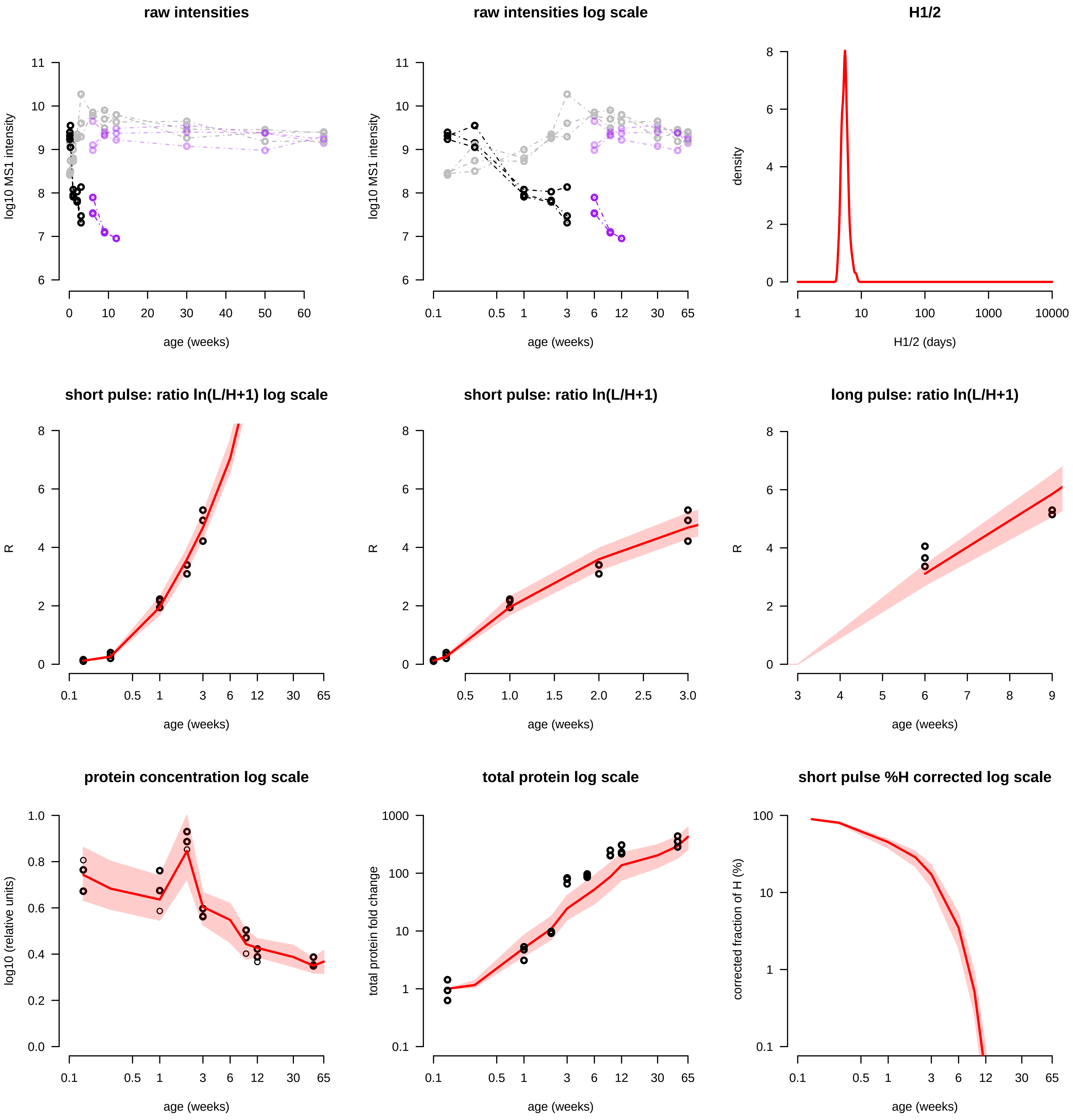

P62814

>sp|P62814|VATB2_MOUSE

V−type proton ATPase subunit B, brain isoform

GN=Atp6v1b2

Med H1/2: 18.8 days

Q5 H1/2: 13.6 days

Q95 H1/2: 34.7 days

reliable model fit: no

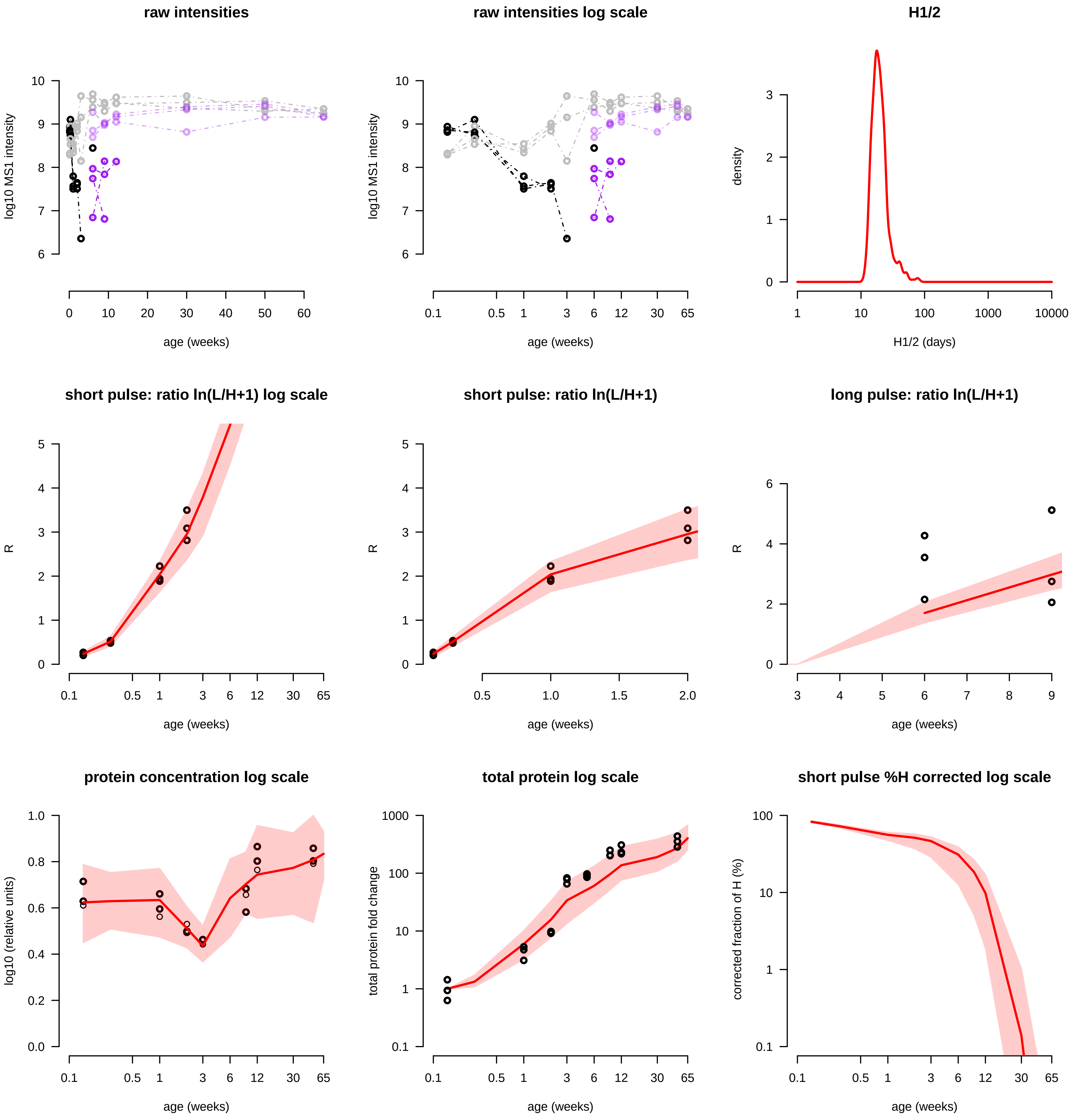

Q9CR16

>sp|Q9CR16|PPID_MOUSE

Peptidyl−prolyl cis−trans isomerase D

GN=Ppid

Med H1/2: 89.1 days

Q5 H1/2: 31.3 days

Q95 H1/2: 1051.2 days

reliable model fit: yes

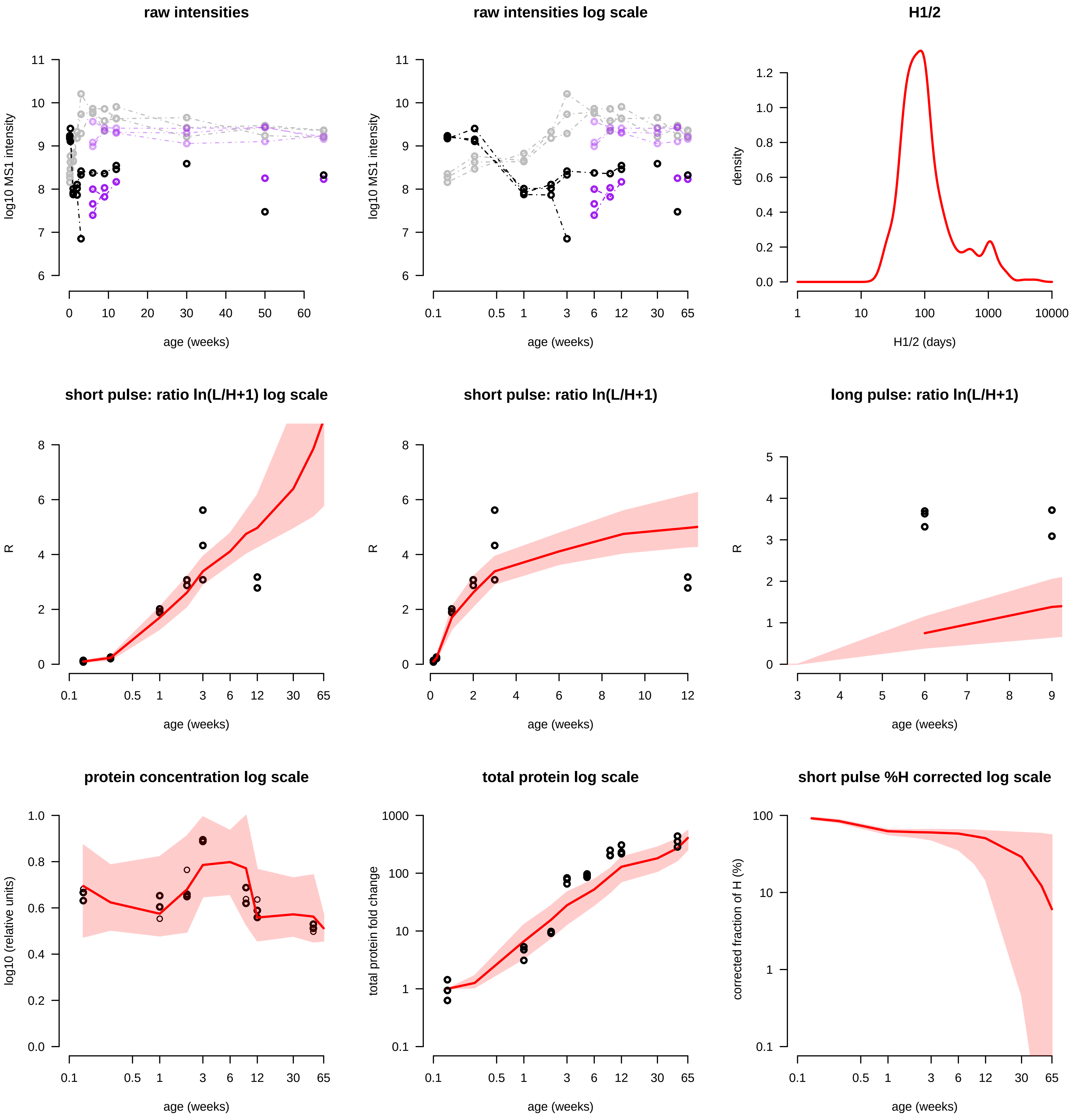

Q9QZE5

>sp|Q9QZE5|COPG1_MOUSE

Coatomer subunit gamma−1

GN=Copg1

Med H1/2: 123.8 days

Q5 H1/2: 69.6 days

Q95 H1/2: 405.9 days

reliable model fit: yes

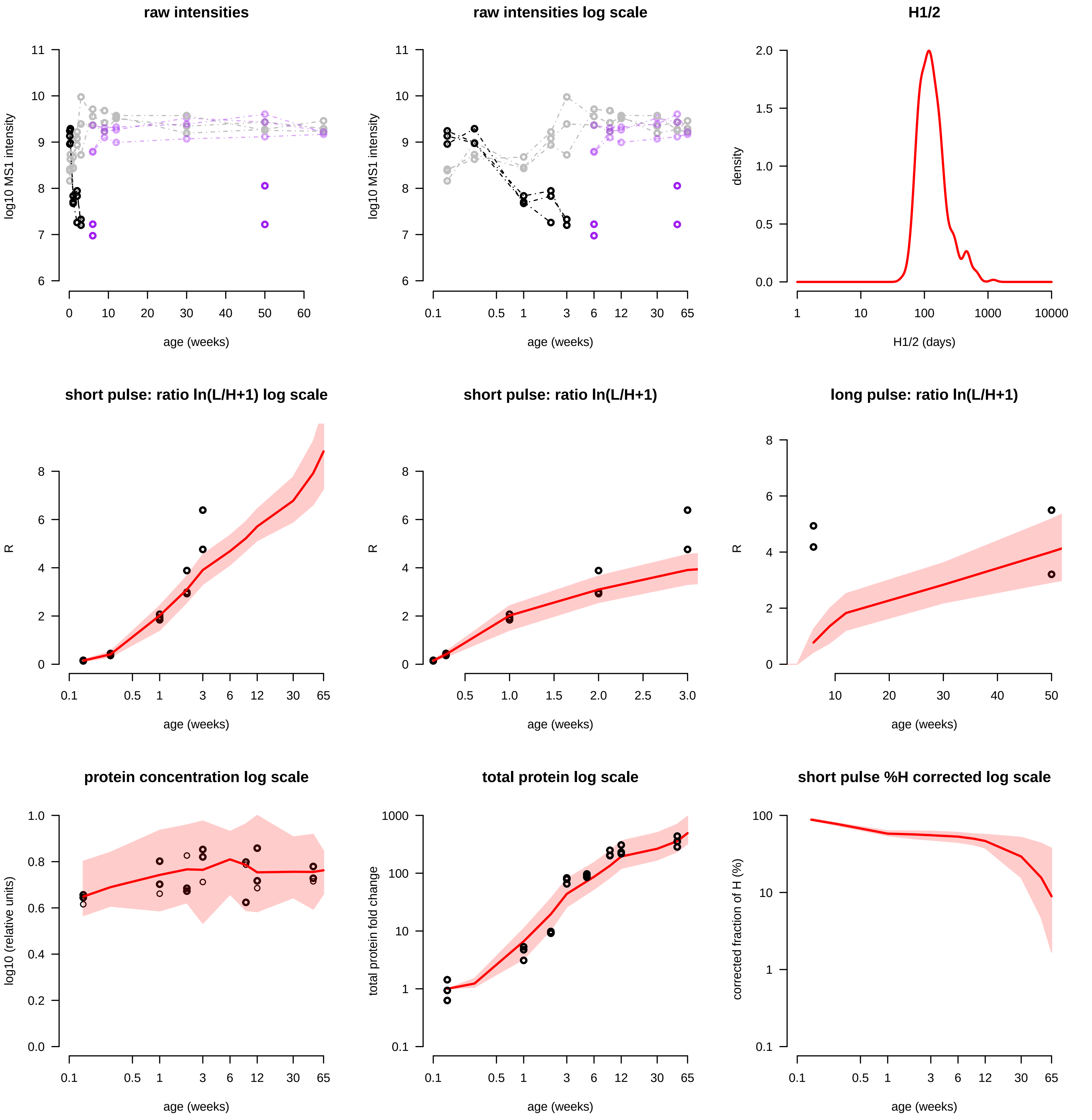

P19536

>sp|P19536|COX5B_MOUSE

Cytochrome c oxidase subunit 5B, mitochondrial

GN=Cox5b

Med H1/2: 13.1 days

Q5 H1/2: 9.3 days

Q95 H1/2: 18.9 days

reliable model fit: yes

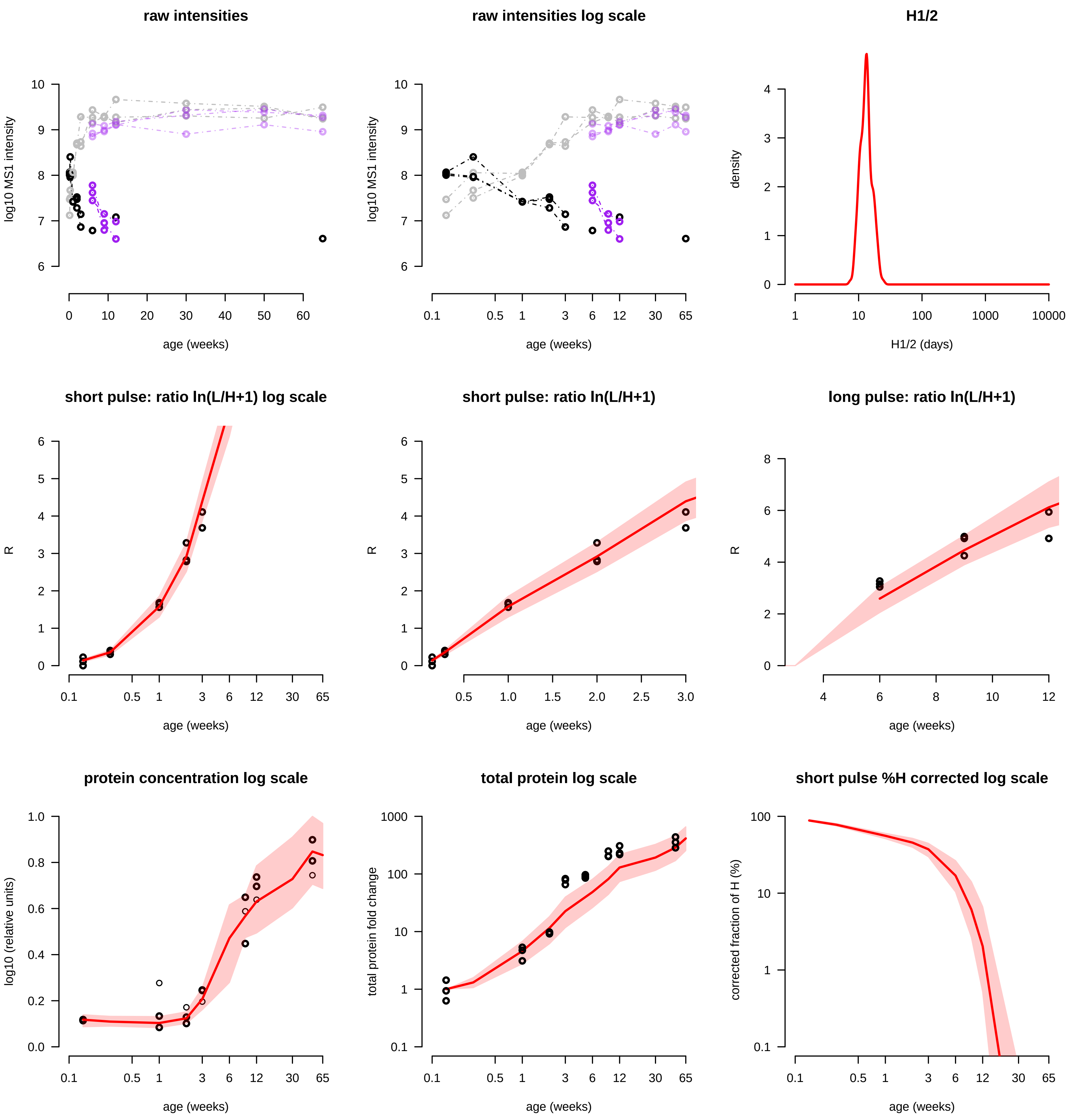

Q9CYH2

>sp|Q9CYH2|PXL2A_MOUSE

Peroxiredoxin−like 2A

GN=Prxl2a

Med H1/2: 7.8 days

Q5 H1/2: 6.3 days

Q95 H1/2: 10.7 days

reliable model fit: yes

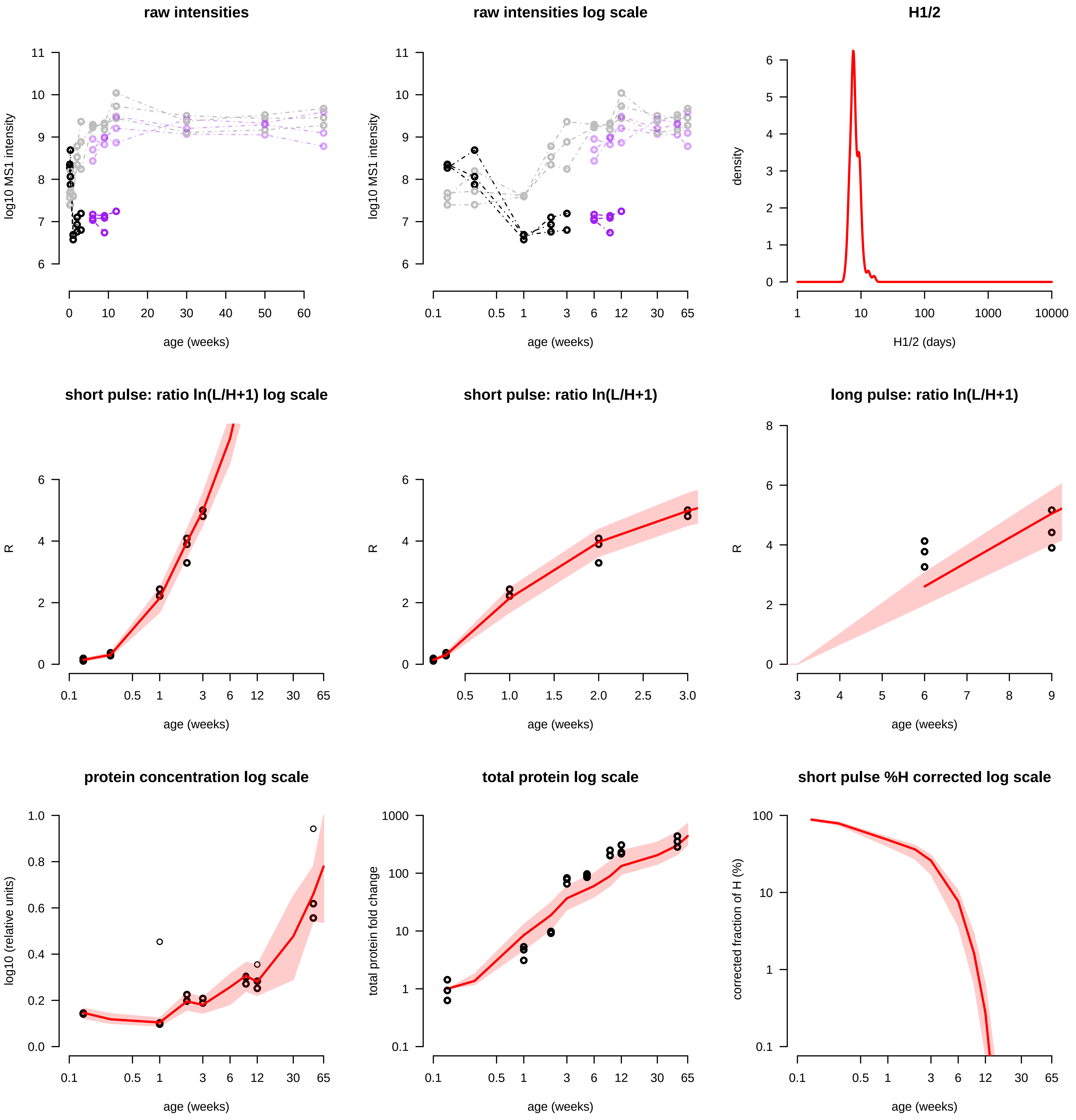

P41105

>sp|P41105|RL28_MOUSE

60S ribosomal protein L28

GN=Rpl28

Med H1/2: 863.2 days

Q5 H1/2: 321.5 days

Q95 H1/2: 12129.5 days

reliable model fit: yes

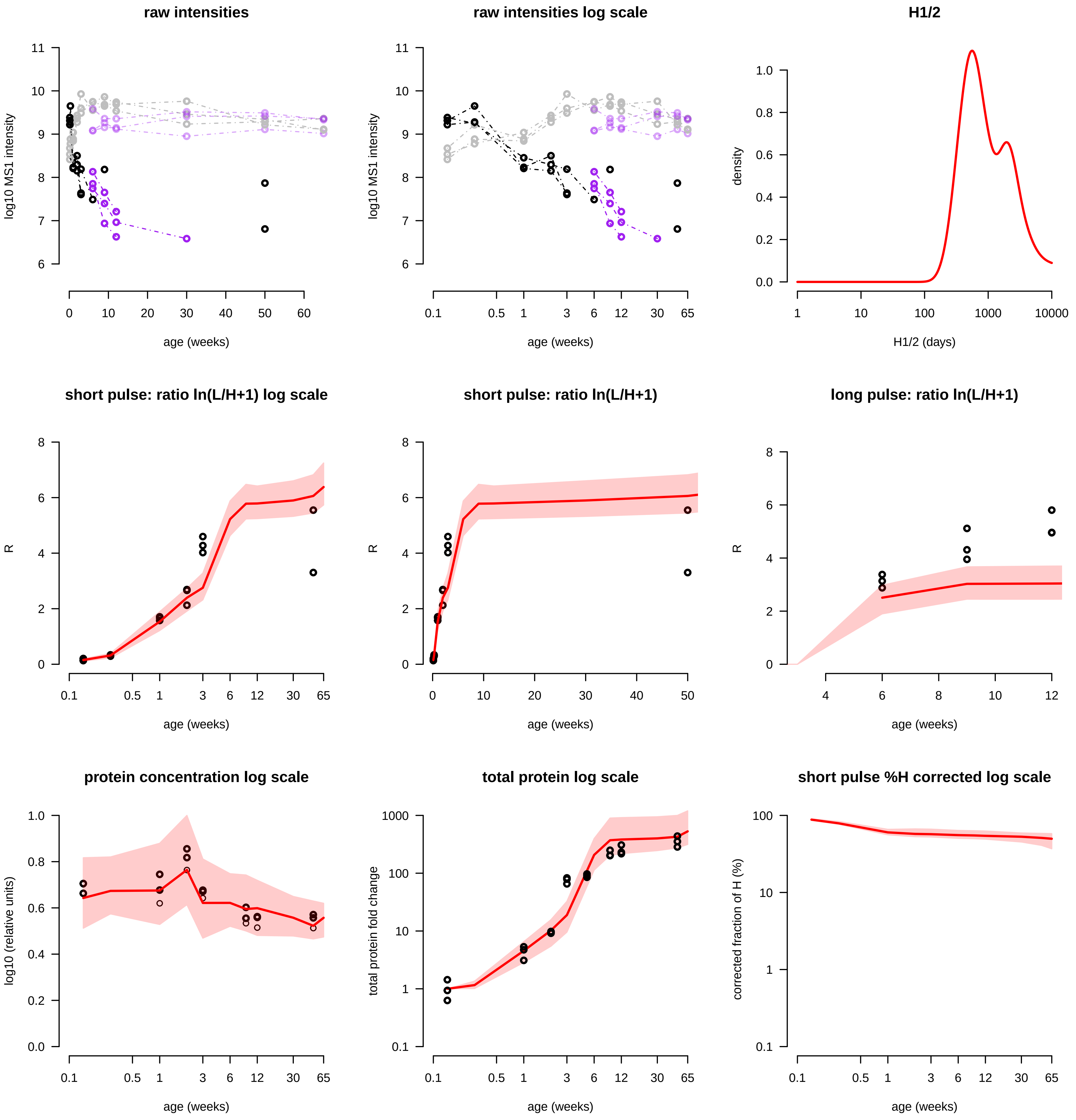

P60335

>sp|P60335|PCBP1_MOUSE

Poly(rC)−binding protein 1

GN=Pcbp1

Med H1/2: 8.5 days

Q5 H1/2: 6.5 days

Q95 H1/2: 11.2 days

reliable model fit: yes

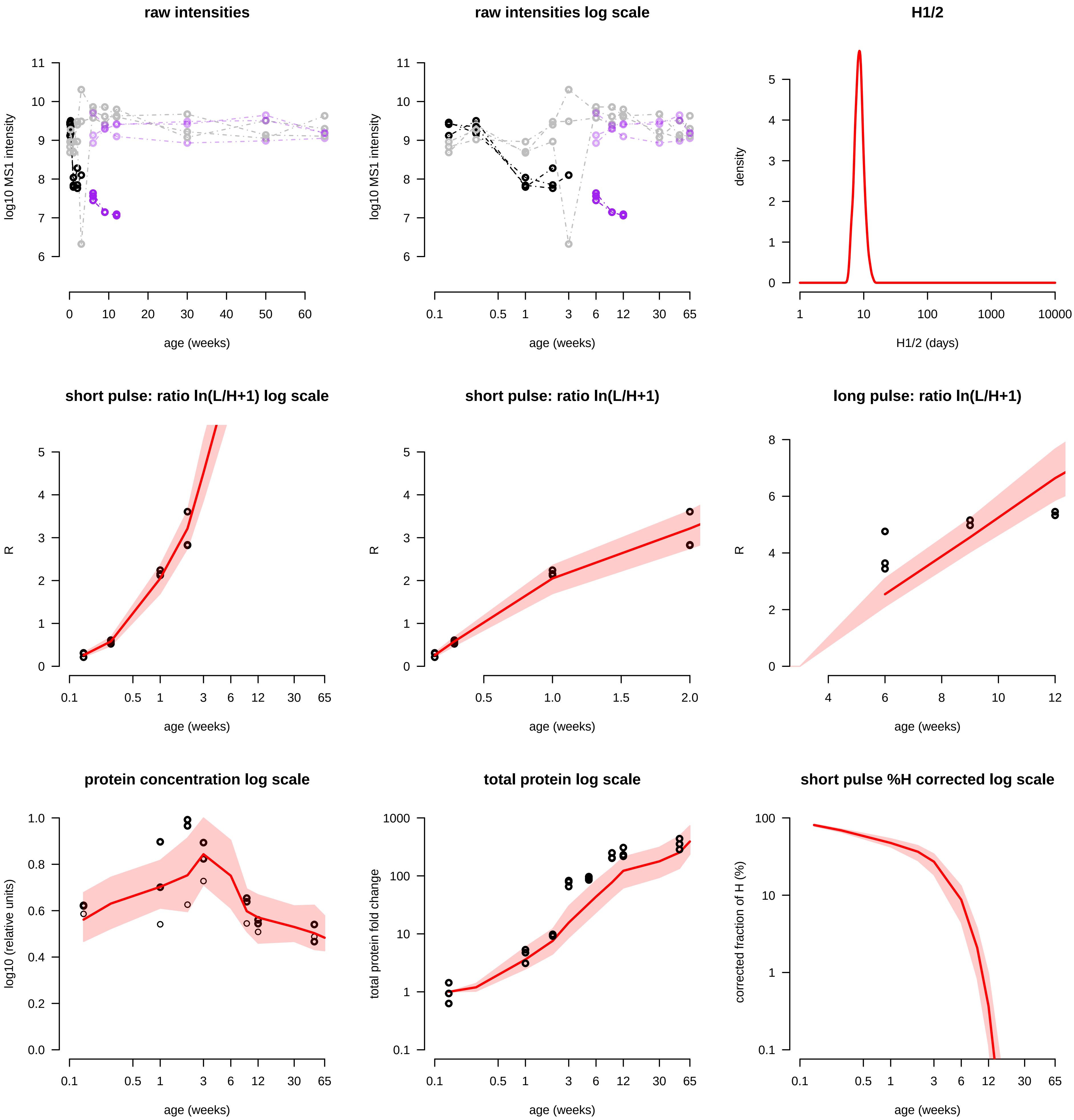

P17426−2;P17426

>sp|P17426−2|AP2A1_MOUSE

Isoform B of AP−2 complex subunit alpha−1

GN=Ap2a1

Med H1/2: 5.7 days

Q5 H1/2: 4.6 days

Q95 H1/2: 7.3 days

reliable model fit: yes

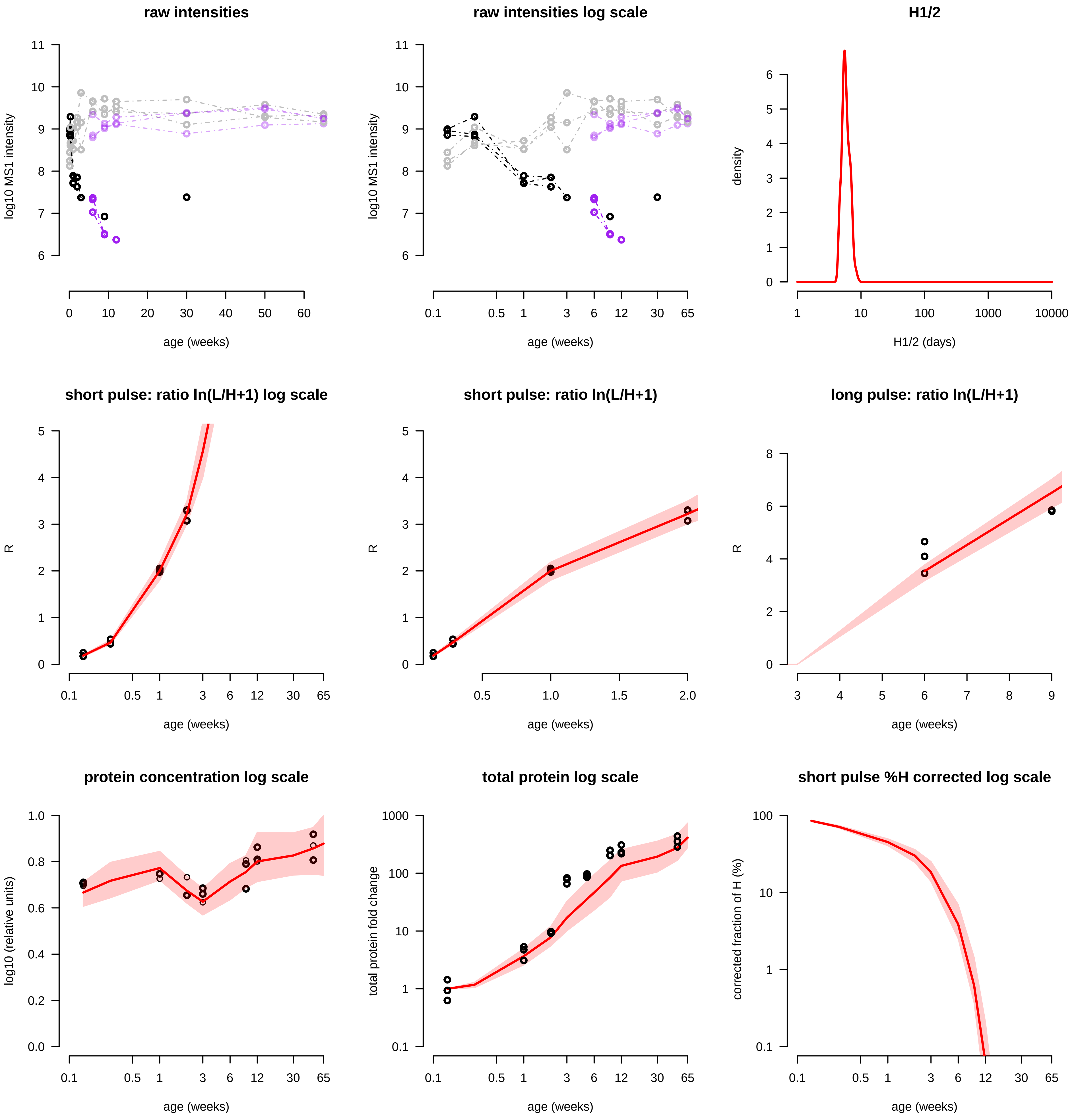

Q99JY9

>sp|Q99JY9|ARP3_MOUSE

Actin−related protein 3

GN=Actr3

Med H1/2: 3116.8 days

Q5 H1/2: 822.3 days

Q95 H1/2: 18807.7 days

reliable model fit: no

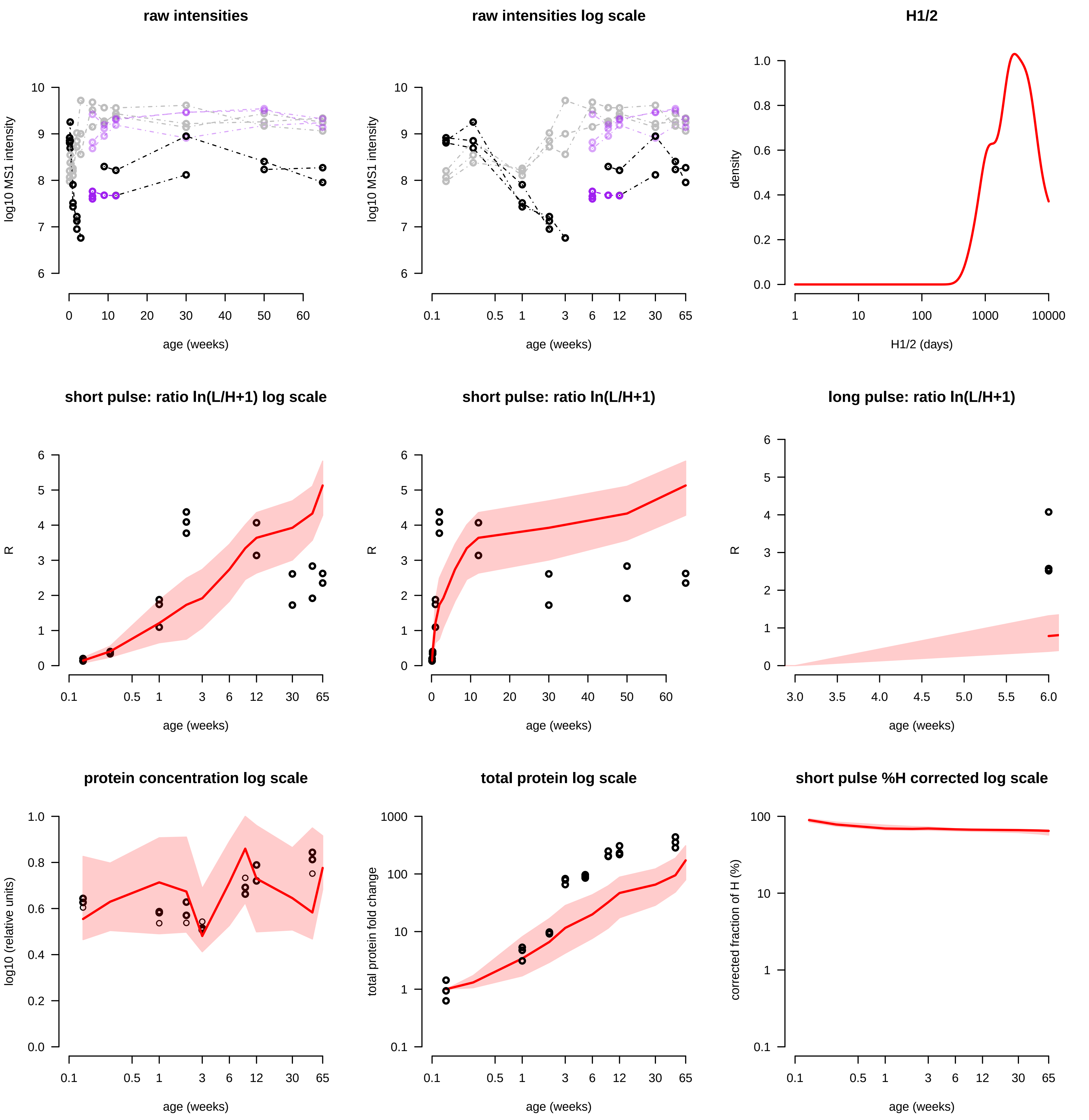

Q9ERU9

>sp|Q9ERU9|RBP2_MOUSE

E3 SUMO−protein ligase RanBP2

GN=Ranbp2

Med H1/2: 1032.7 days

Q5 H1/2: 319.5 days

Q95 H1/2: 11390.2 days

reliable model fit: yes

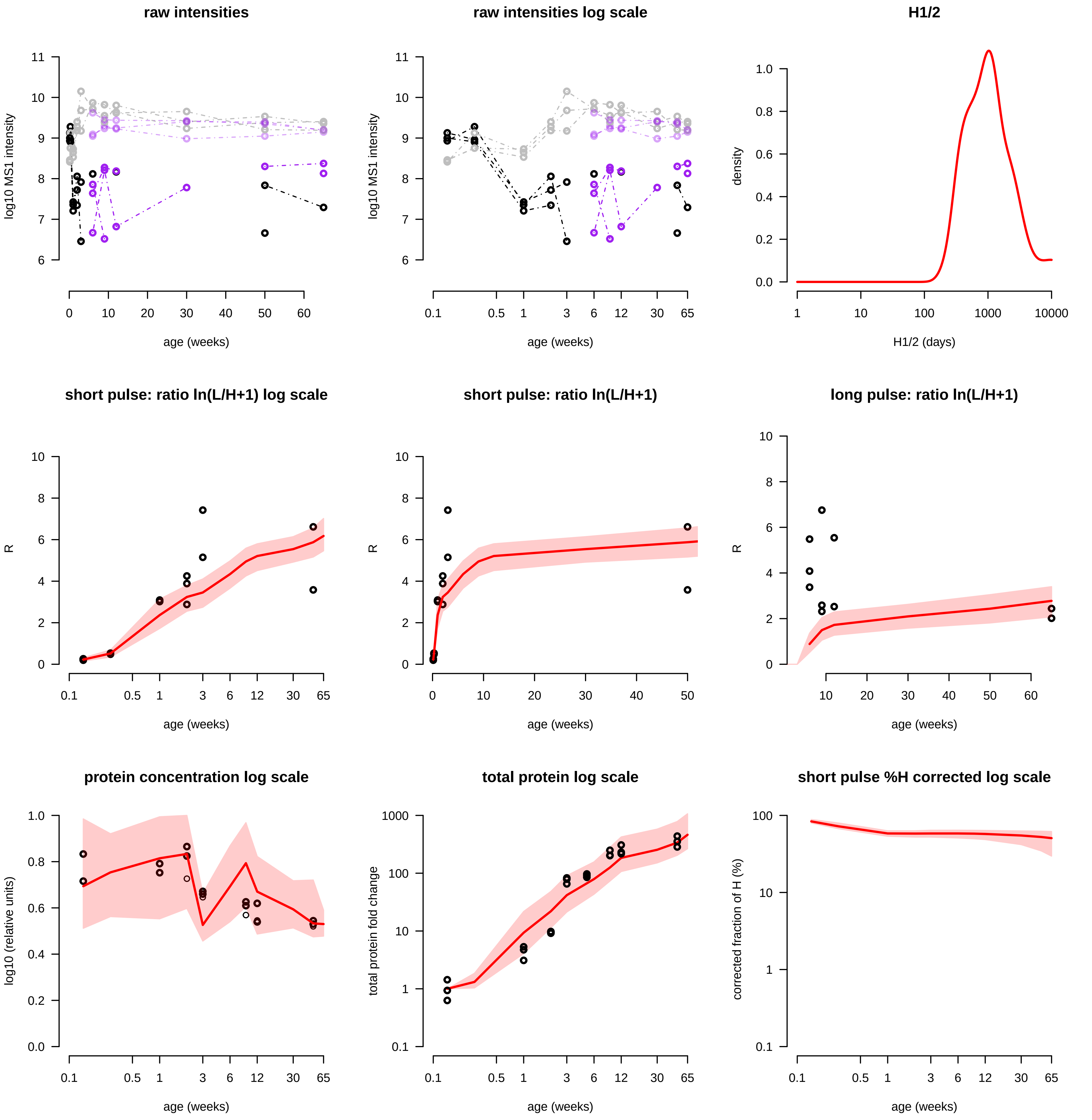

Q9WTI7;Q9WTI7−4

>sp|Q9WTI7|MYO1C_MOUSE

Unconventional myosin−Ic

GN=Myo1c

Med H1/2: 4.5 days

Q5 H1/2: 3.7 days

Q95 H1/2: 5.7 days

reliable model fit: yes

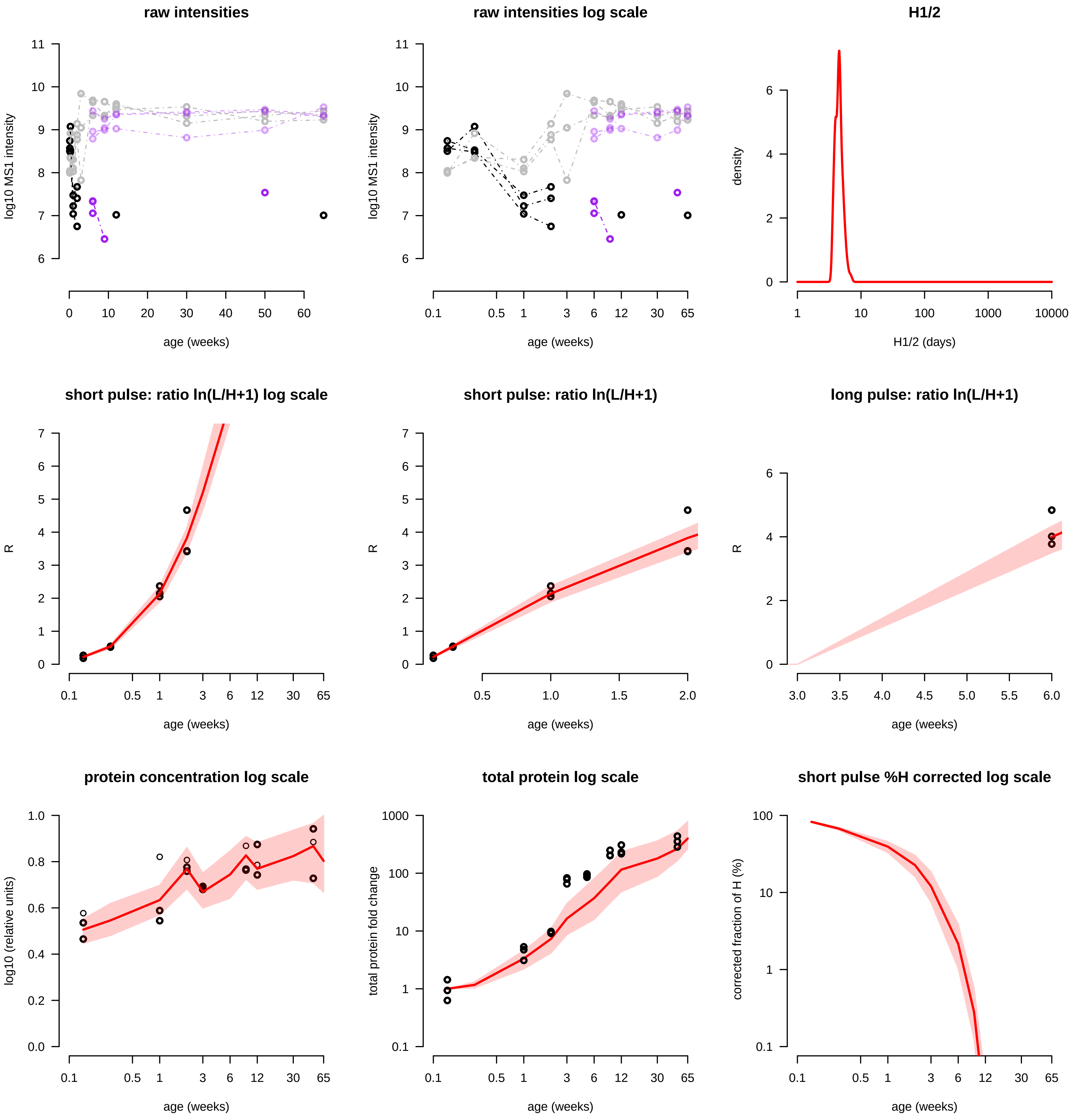

Q6A0A9

>sp|Q6A0A9|F120A_MOUSE

Constitutive coactivator of PPAR−gamma−like protein 1

GN=FAM120A

Med H1/2: 1534 days

Q5 H1/2: 401 days

Q95 H1/2: 14657.4 days

reliable model fit: no

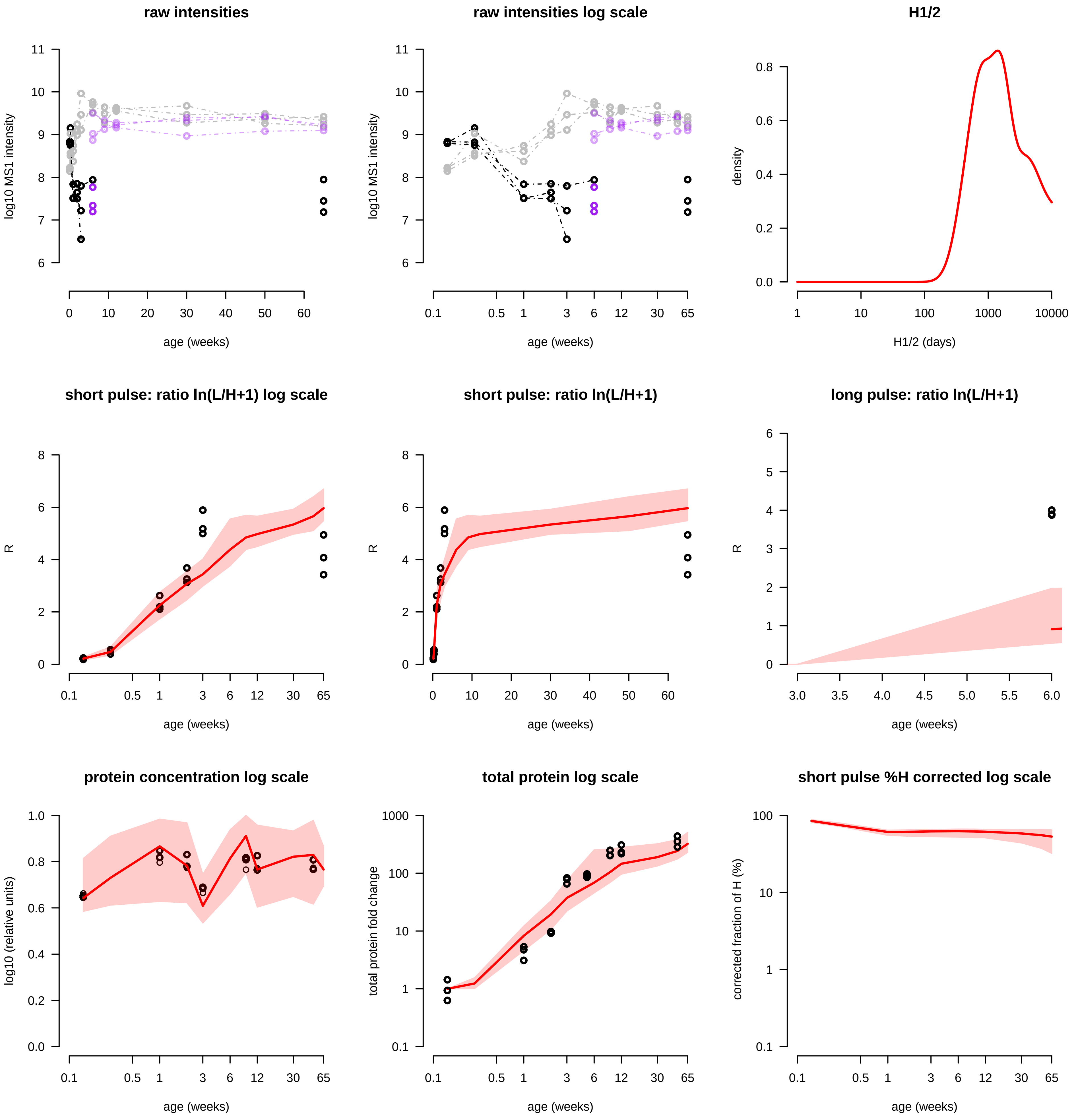

Q8K310

>sp|Q8K310|MATR3_MOUSE

Matrin−3

GN=Matr3

Med H1/2: 6.5 days

Q5 H1/2: 5.6 days

Q95 H1/2: 7.7 days

reliable model fit: yes

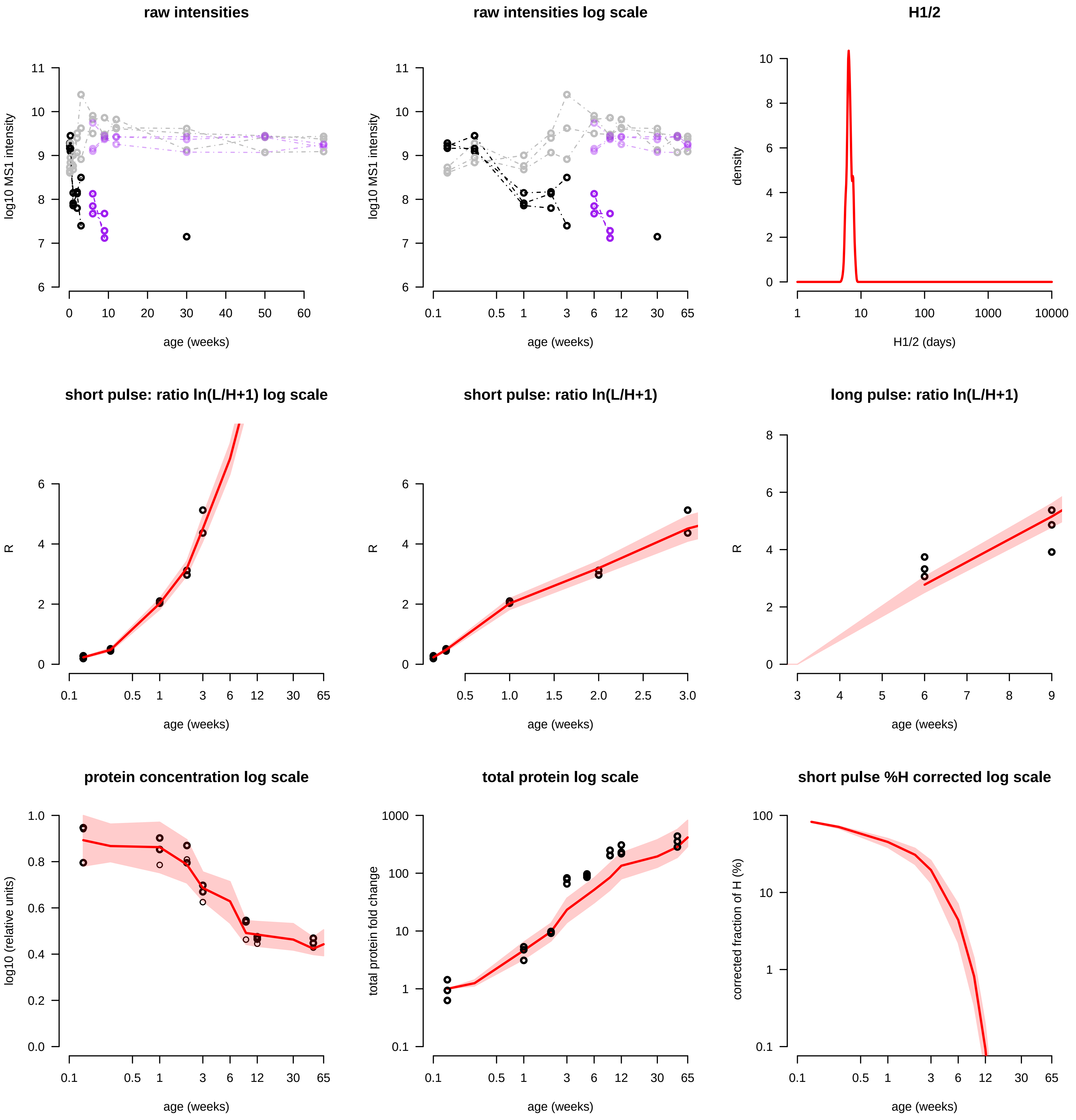

Q8BKC5

>sp|Q8BKC5|IPO5_MOUSE

Importin−5

GN=Ipo5

Med H1/2: 44.1 days

Q5 H1/2: 25.5 days

Q95 H1/2: 192.3 days

reliable model fit: no

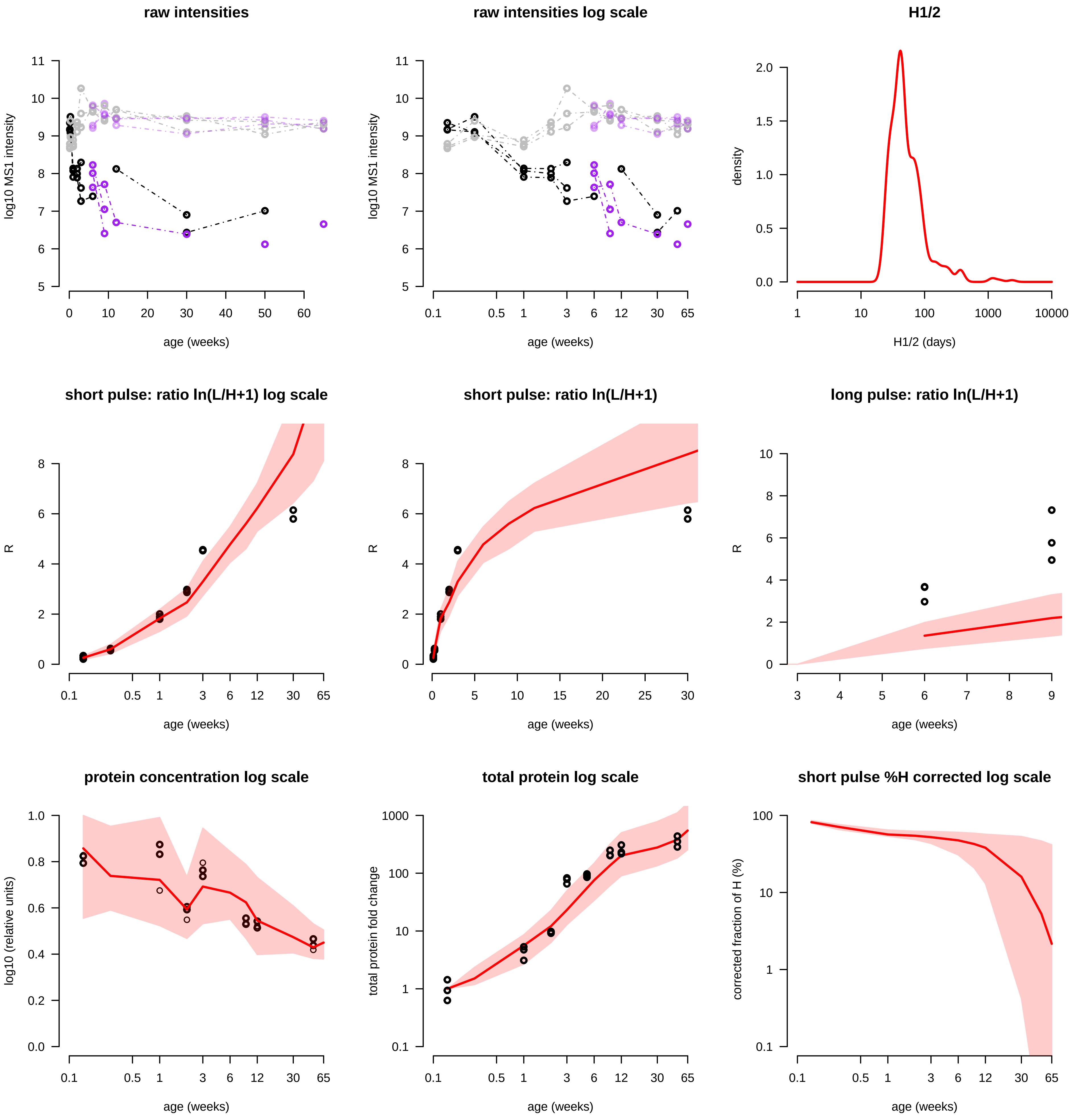

Q99KP6;Q99KP6−2;Q99KP6−3

>sp|Q99KP6|PRP19_MOUSE

Pre−mRNA−processing factor 19

GN=Prpf19

Med H1/2: 5.6 days

Q5 H1/2: 4.7 days

Q95 H1/2: 6.8 days

reliable model fit: yes

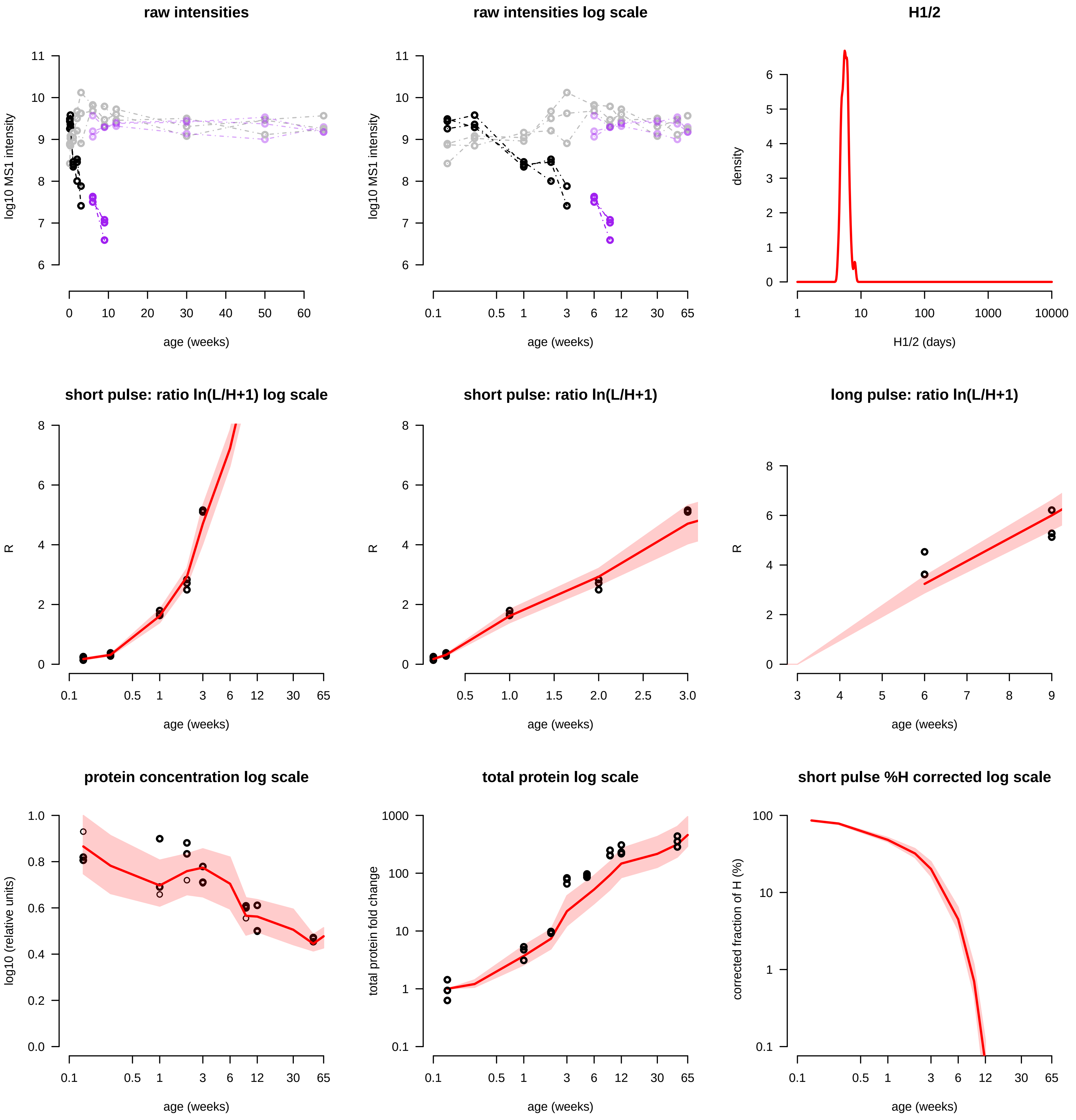

Q921M3;Q921M3−2

>sp|Q921M3|SF3B3_MOUSE

Splicing factor 3B subunit 3

GN=Sf3b3

Med H1/2: 6.4 days

Q5 H1/2: 5.6 days

Q95 H1/2: 8.9 days

reliable model fit: yes

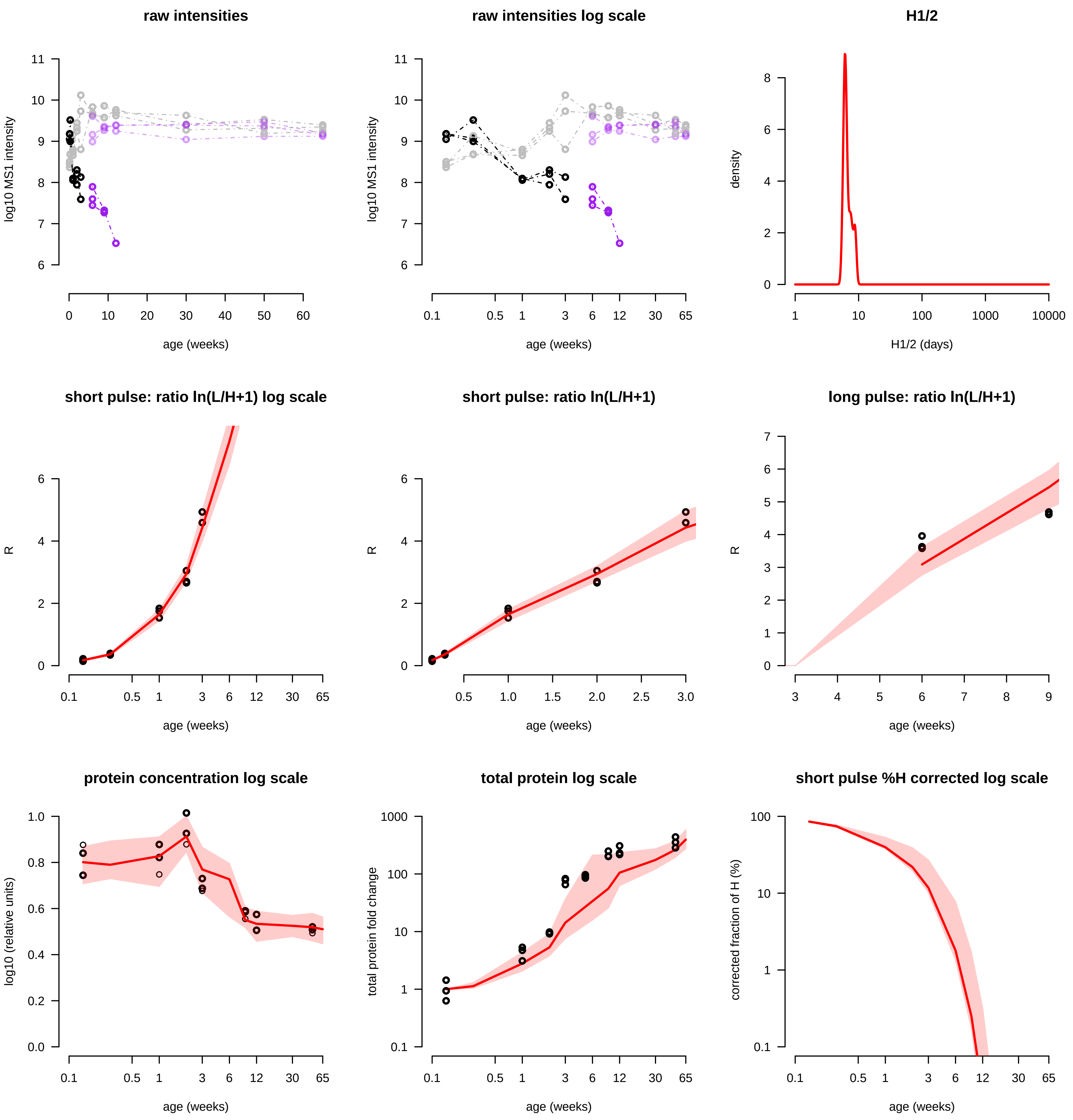

P40630;P40630−2

>sp|P40630|TFAM_MOUSE

Transcription factor A, mitochondrial

GN=Tfam

Med H1/2: 12.3 days

Q5 H1/2: 3.9 days

Q95 H1/2: 135 days

reliable model fit: yes

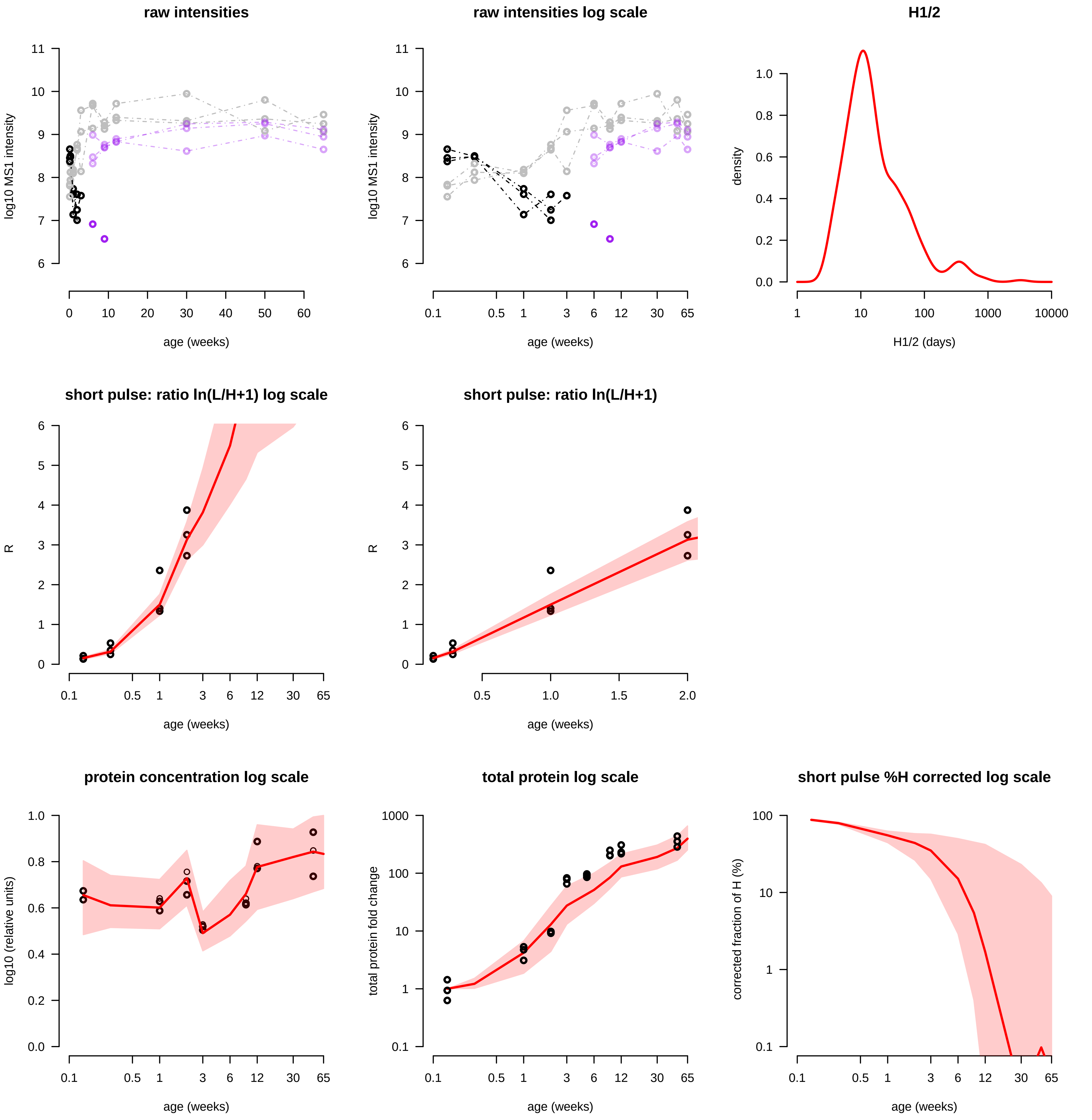

Q9CU62

>sp|Q9CU62|SMC1A_MOUSE

Structural maintenance of chromosomes protein 1A

GN=Smc1a

Med H1/2: 205.8 days

Q5 H1/2: 79.6 days

Q95 H1/2: 1186.1 days

reliable model fit: no

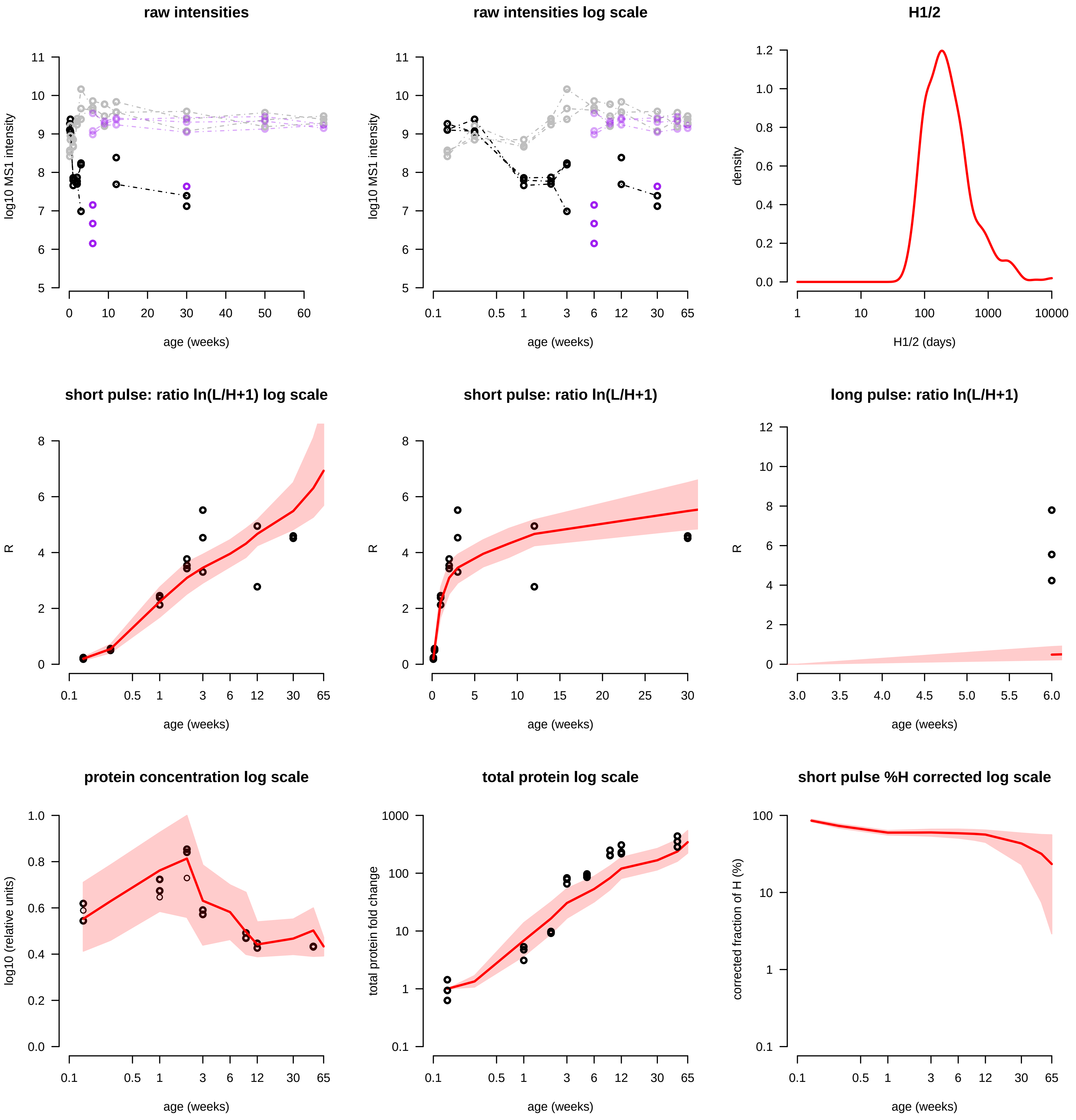

Q8C0C7

>sp|Q8C0C7|SYFA_MOUSE

Phenylalanine−−tRNA ligase alpha subunit

GN=Farsa

Med H1/2: 3.7 days

Q5 H1/2: 3 days

Q95 H1/2: 4.5 days

reliable model fit: yes

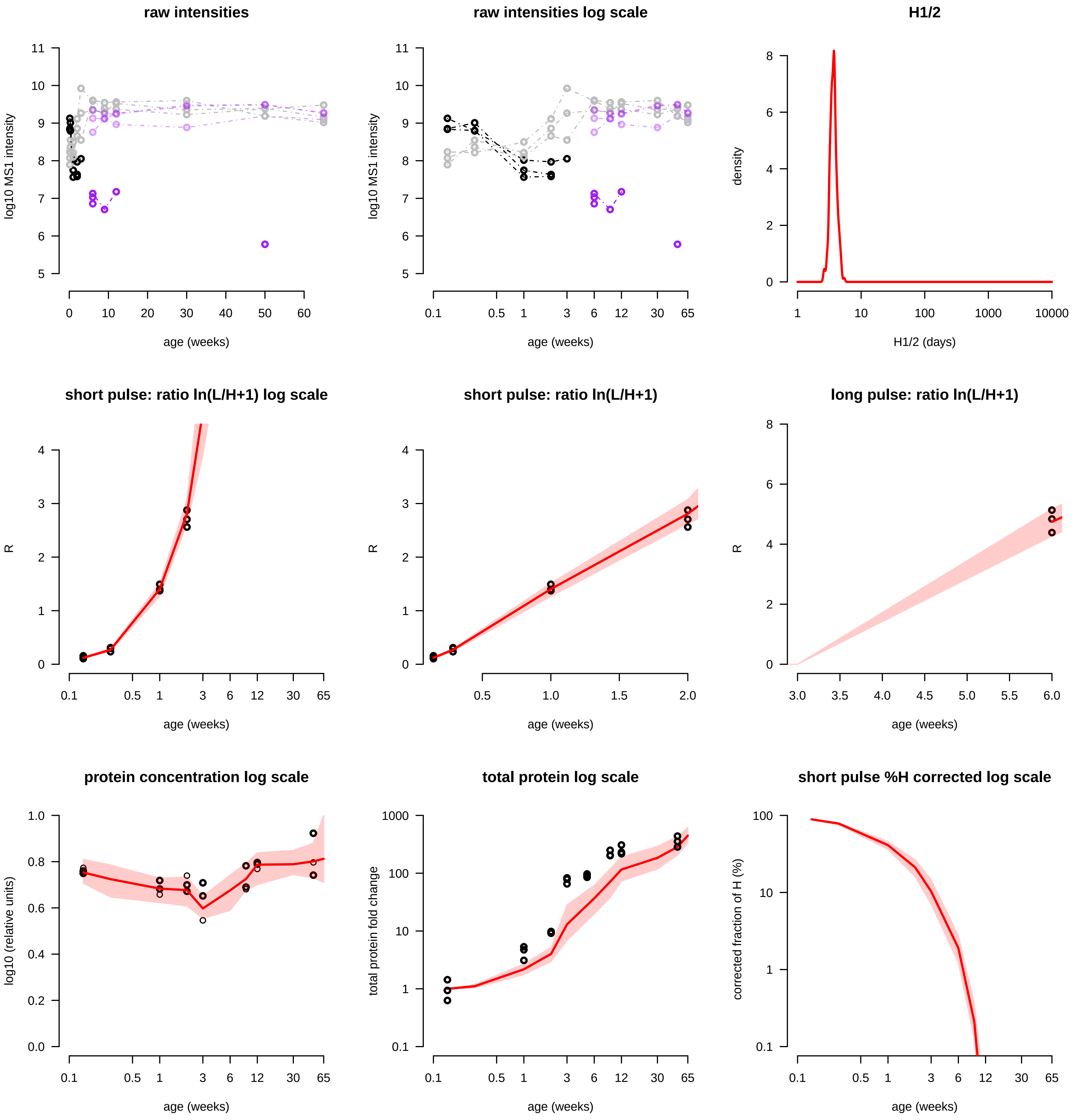

Q9D7N9

>sp|Q9D7N9|APMAP_MOUSE

Adipocyte plasma membrane−associated protein

GN=Apmap

Med H1/2: 7.3 days

Q5 H1/2: 3.2 days

Q95 H1/2: 47.4 days

reliable model fit: yes

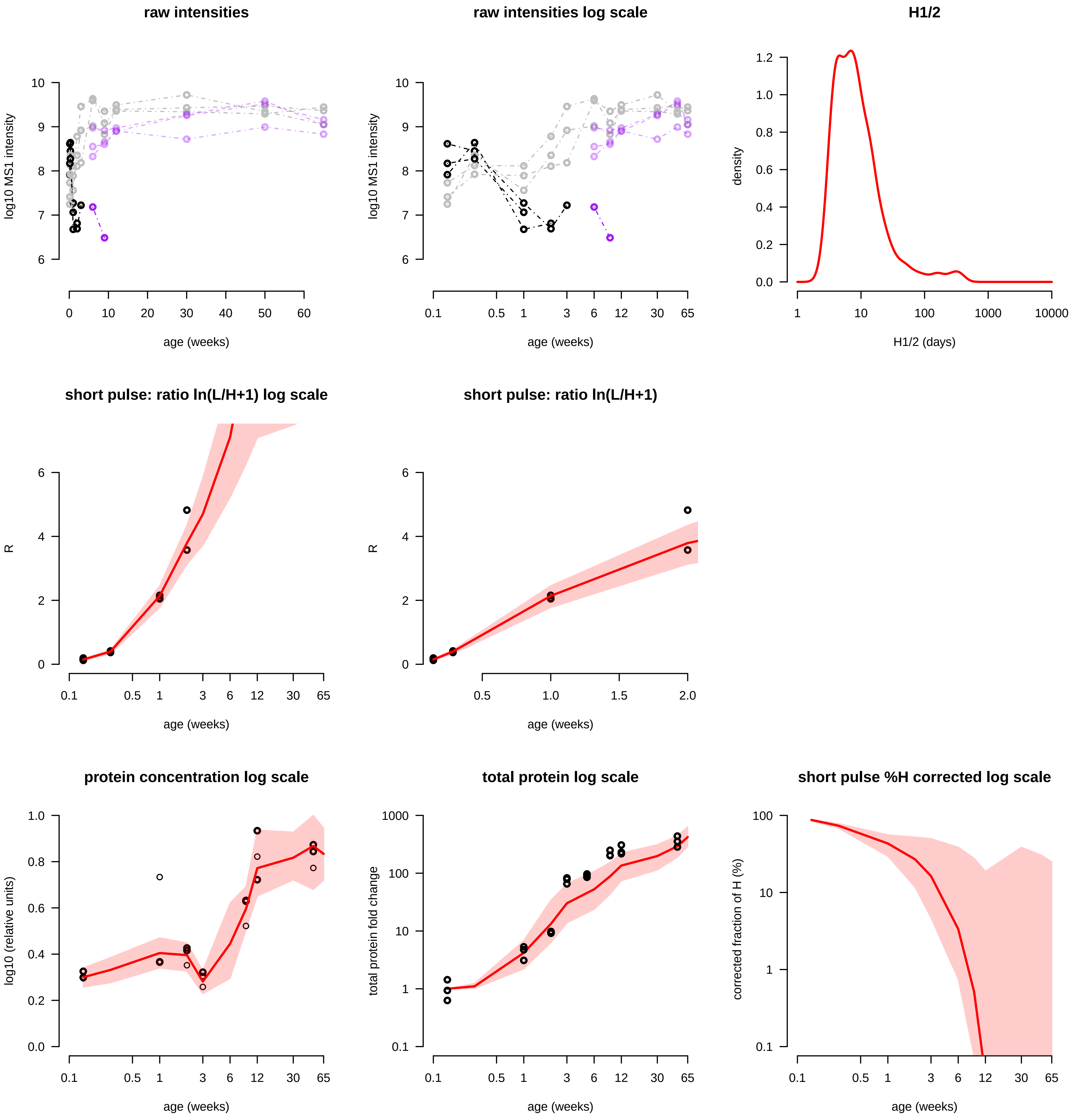

Q8R480

>sp|Q8R480|NUP85_MOUSE

Nuclear pore complex protein Nup85

GN=Nup85

Med H1/2: 14 days

Q5 H1/2: 10.8 days

Q95 H1/2: 20.2 days

reliable model fit: yes

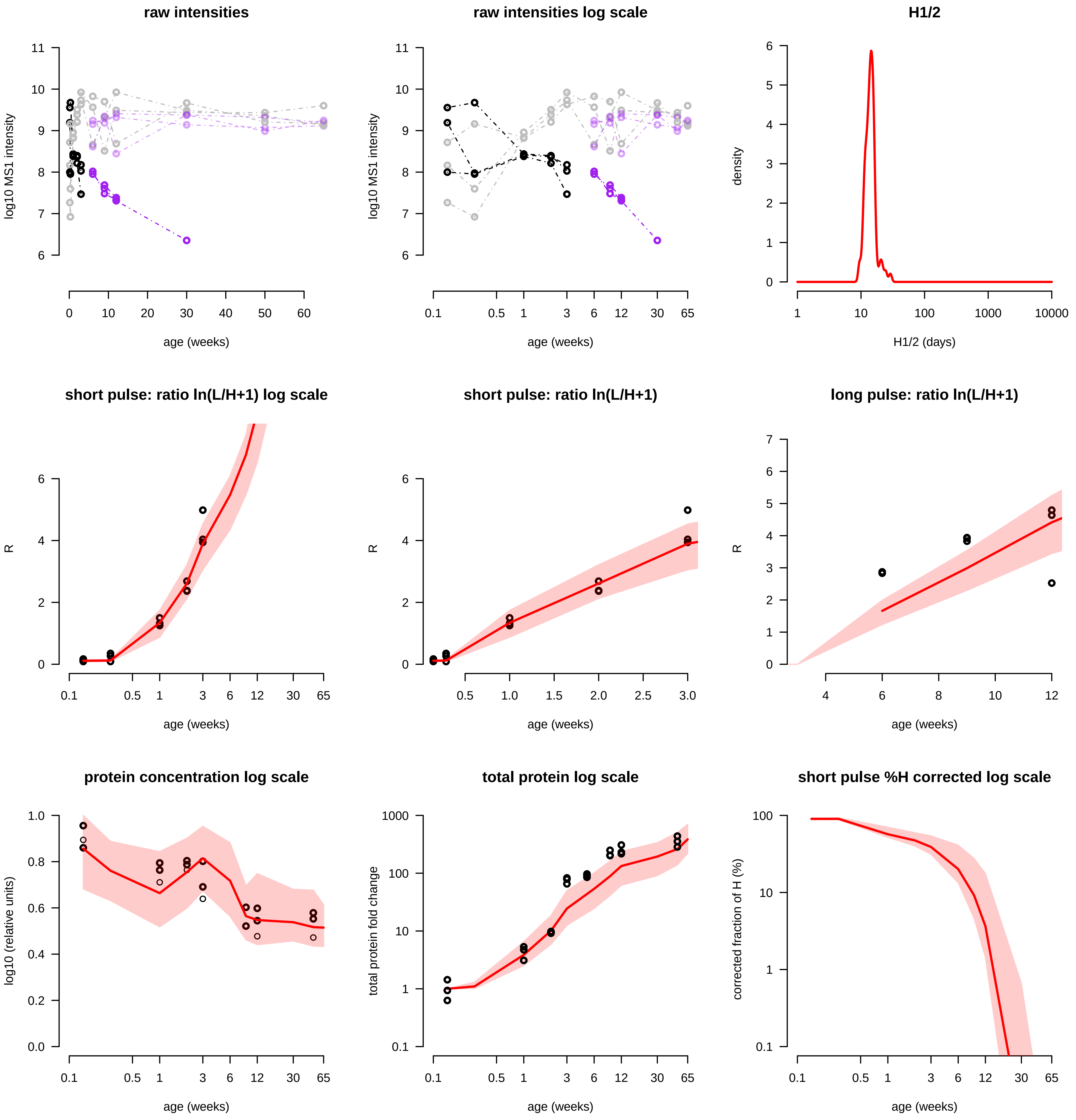

Q9CY27

>sp|Q9CY27|TECR_MOUSE

Very−long−chain enoyl−CoA reductase

GN=Tecr

Med H1/2: 8.1 days

Q5 H1/2: 6.2 days

Q95 H1/2: 11.4 days

reliable model fit: yes

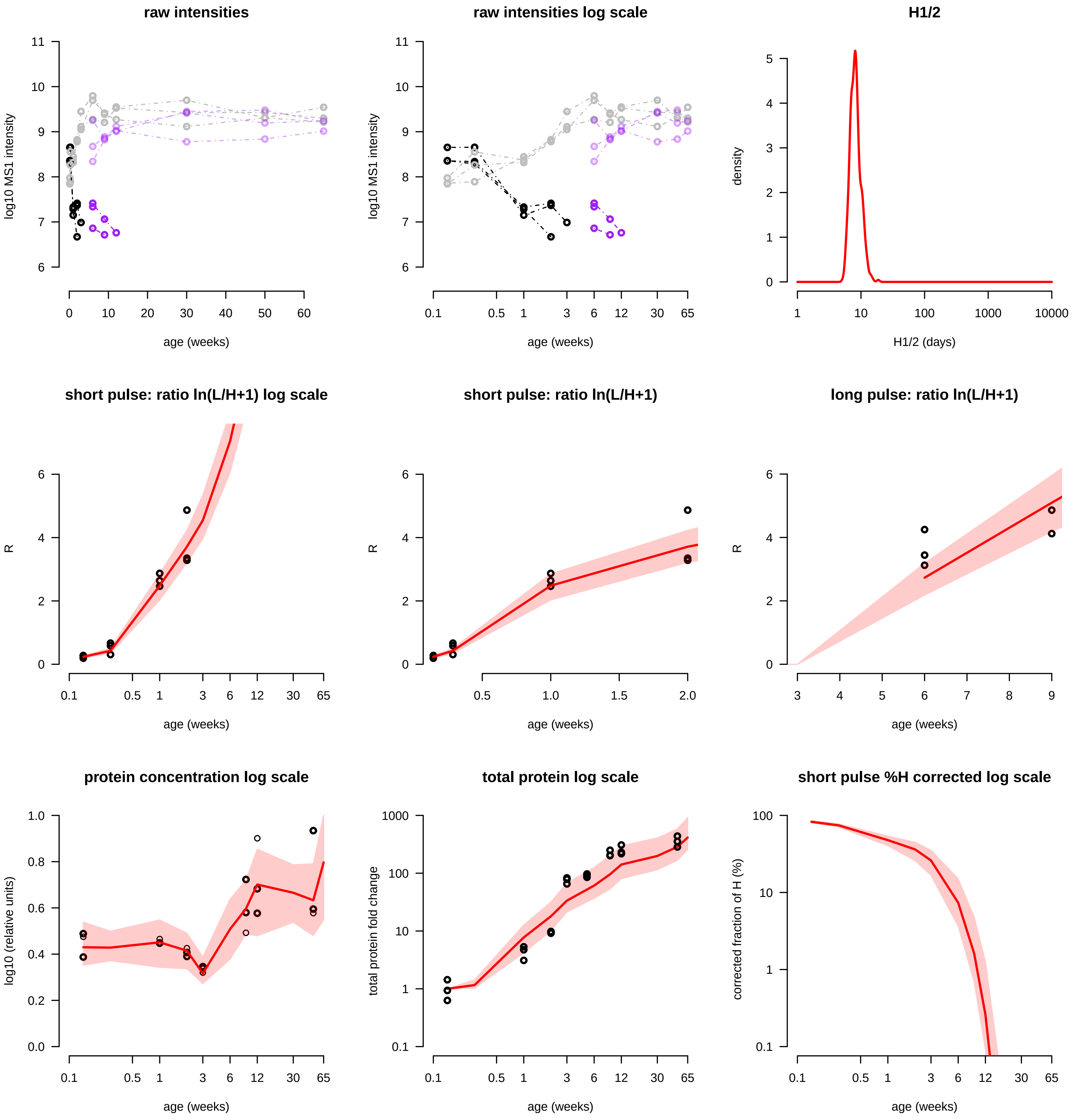

Q9R1P1

>sp|Q9R1P1|PSB3_MOUSE

Proteasome subunit beta type−3

GN=Psmb3

Med H1/2: 8.1 days

Q5 H1/2: 7 days

Q95 H1/2: 10.4 days

reliable model fit: yes

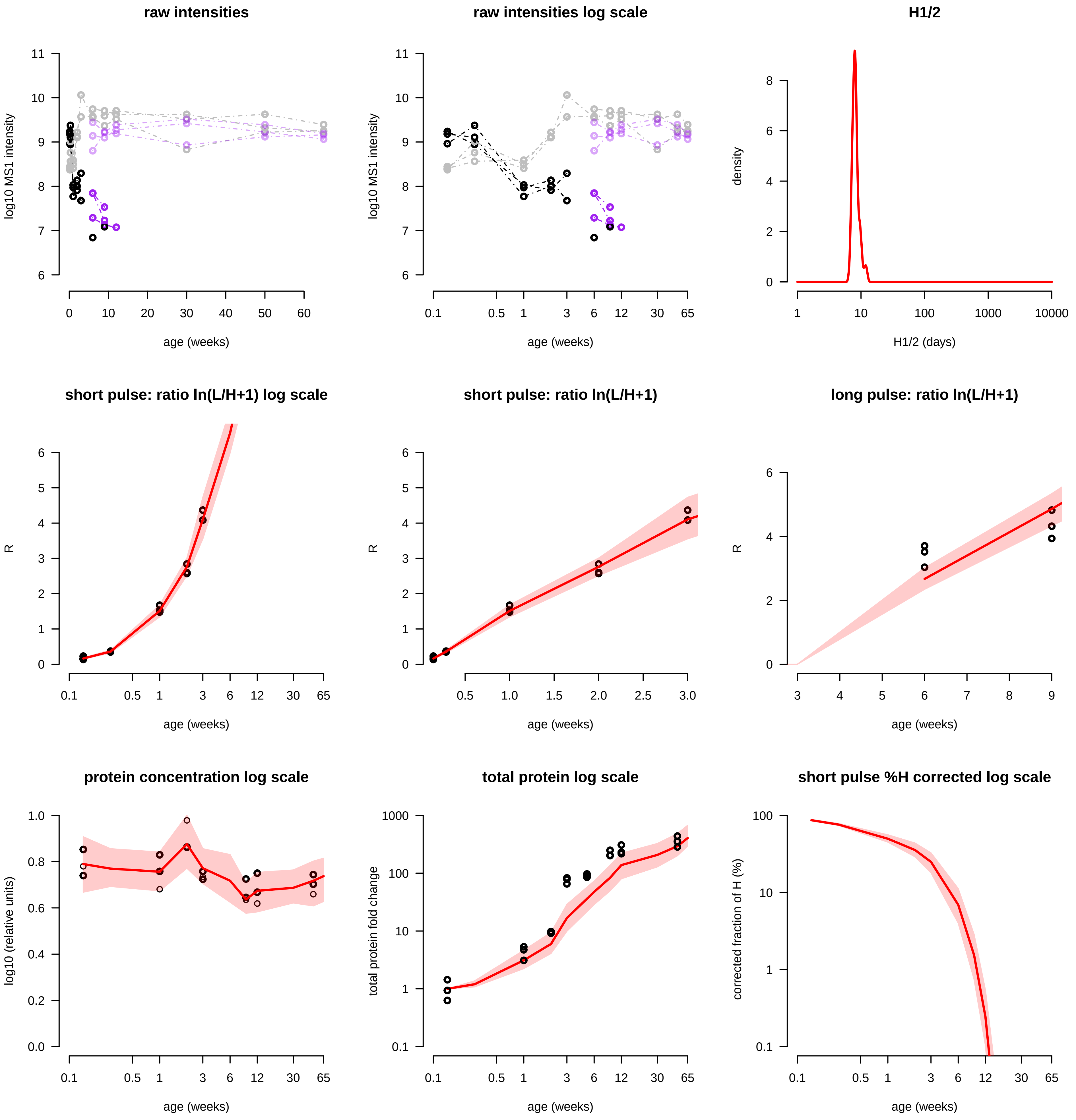

Q8R0X7

>sp|Q8R0X7|SGPL1_MOUSE

Sphingosine−1−phosphate lyase 1

GN=Sgpl1

Med H1/2: 6 days

Q5 H1/2: 4.9 days

Q95 H1/2: 7.5 days

reliable model fit: yes

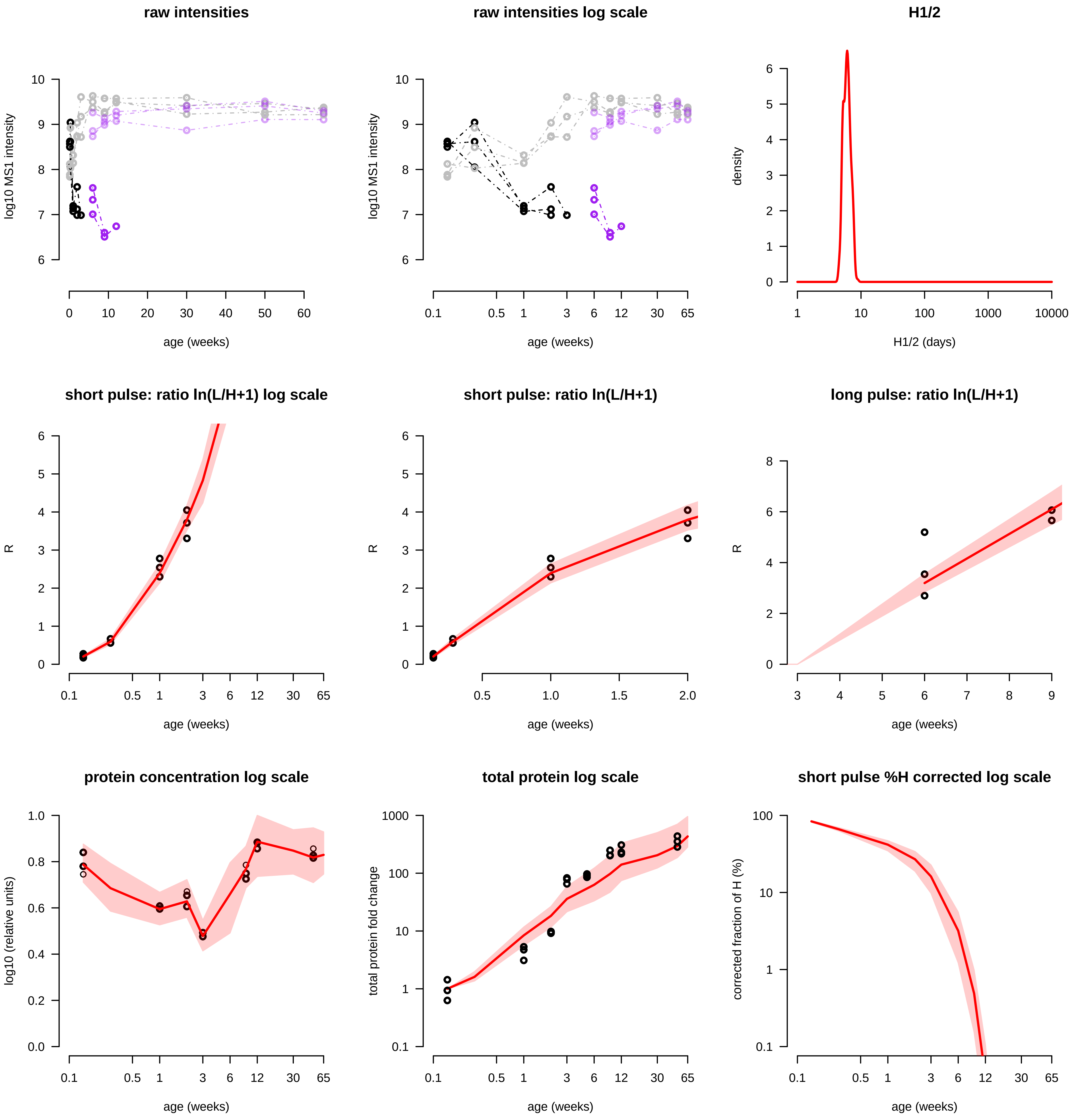

Q922Q1

>sp|Q922Q1|MARC2_MOUSE

Mitochondrial amidoxime reducing component 2

GN=Mtarc2

Med H1/2: 1617.8 days

Q5 H1/2: 344.4 days

Q95 H1/2: 11249.5 days

reliable model fit: yes

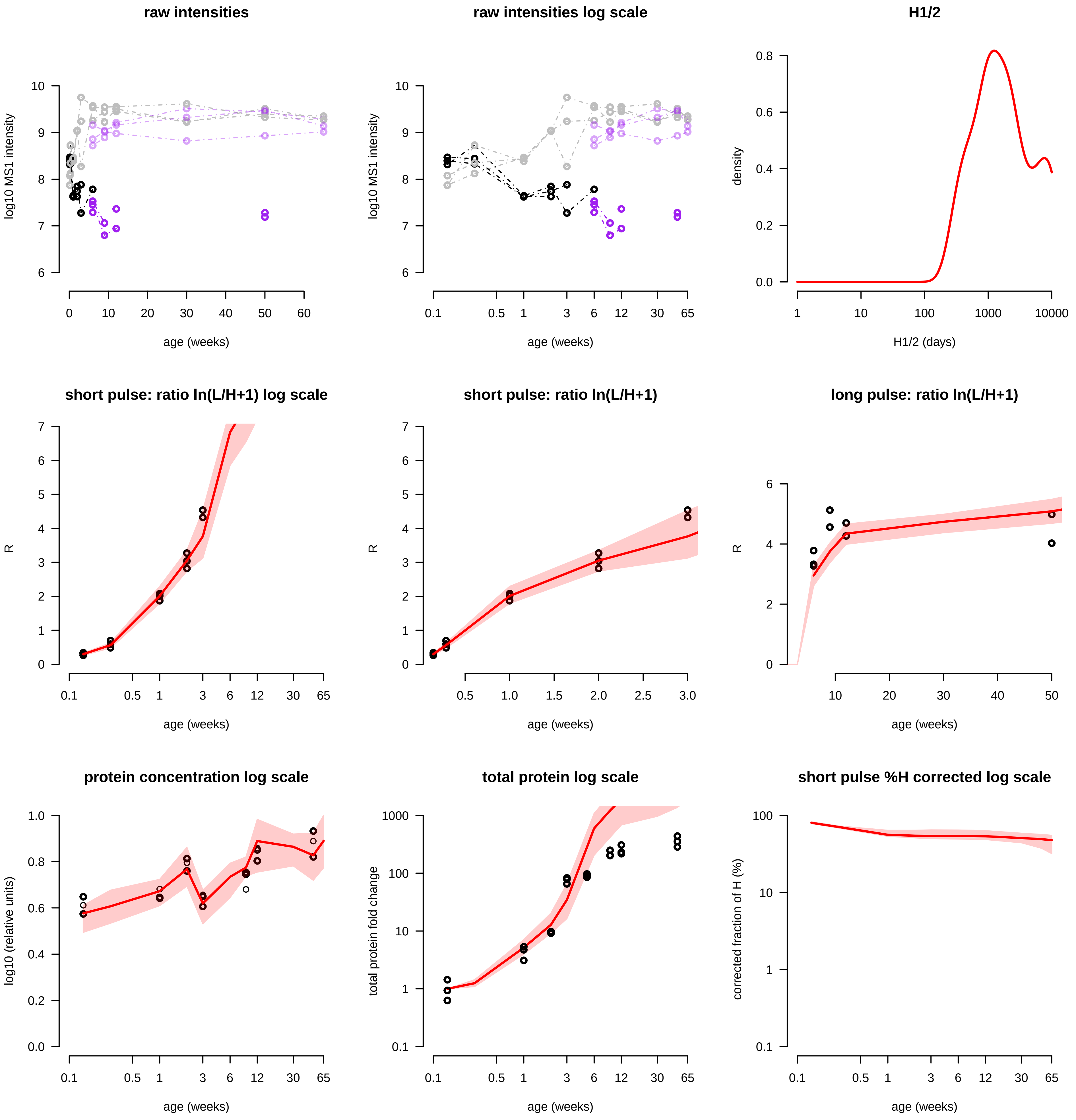

Q8BH59

>sp|Q8BH59|CMC1_MOUSE

Calcium−binding mitochondrial carrier protein Aralar1

GN=Slc25a12

Med H1/2: 230.3 days

Q5 H1/2: 93.1 days

Q95 H1/2: 1701.9 days

reliable model fit: no

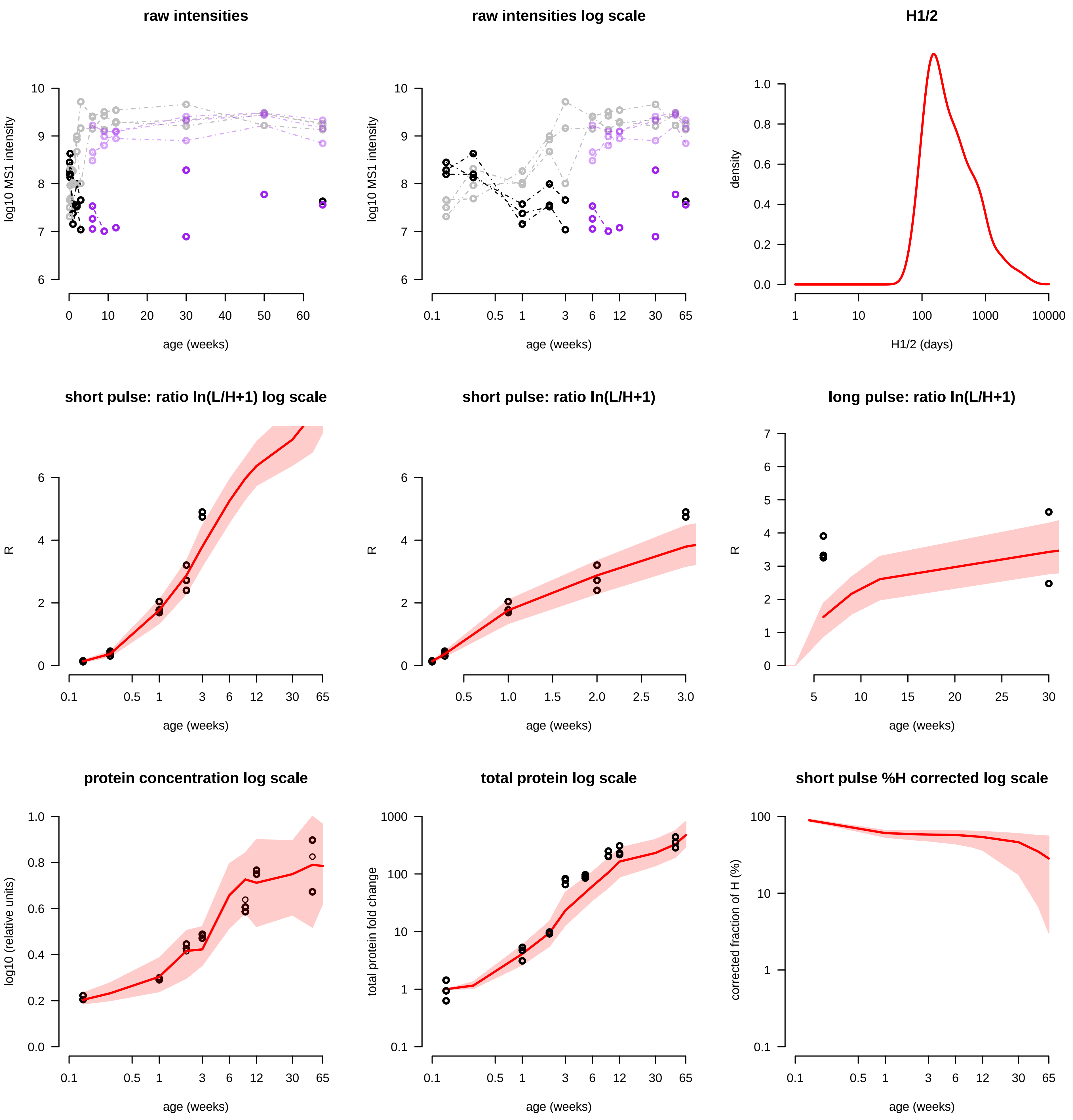

O08810

>sp|O08810|U5S1_MOUSE

116 kDa U5 small nuclear ribonucleoprotein component

GN=Eftud2

Med H1/2: 12.3 days

Q5 H1/2: 9.9 days

Q95 H1/2: 19.4 days

reliable model fit: yes

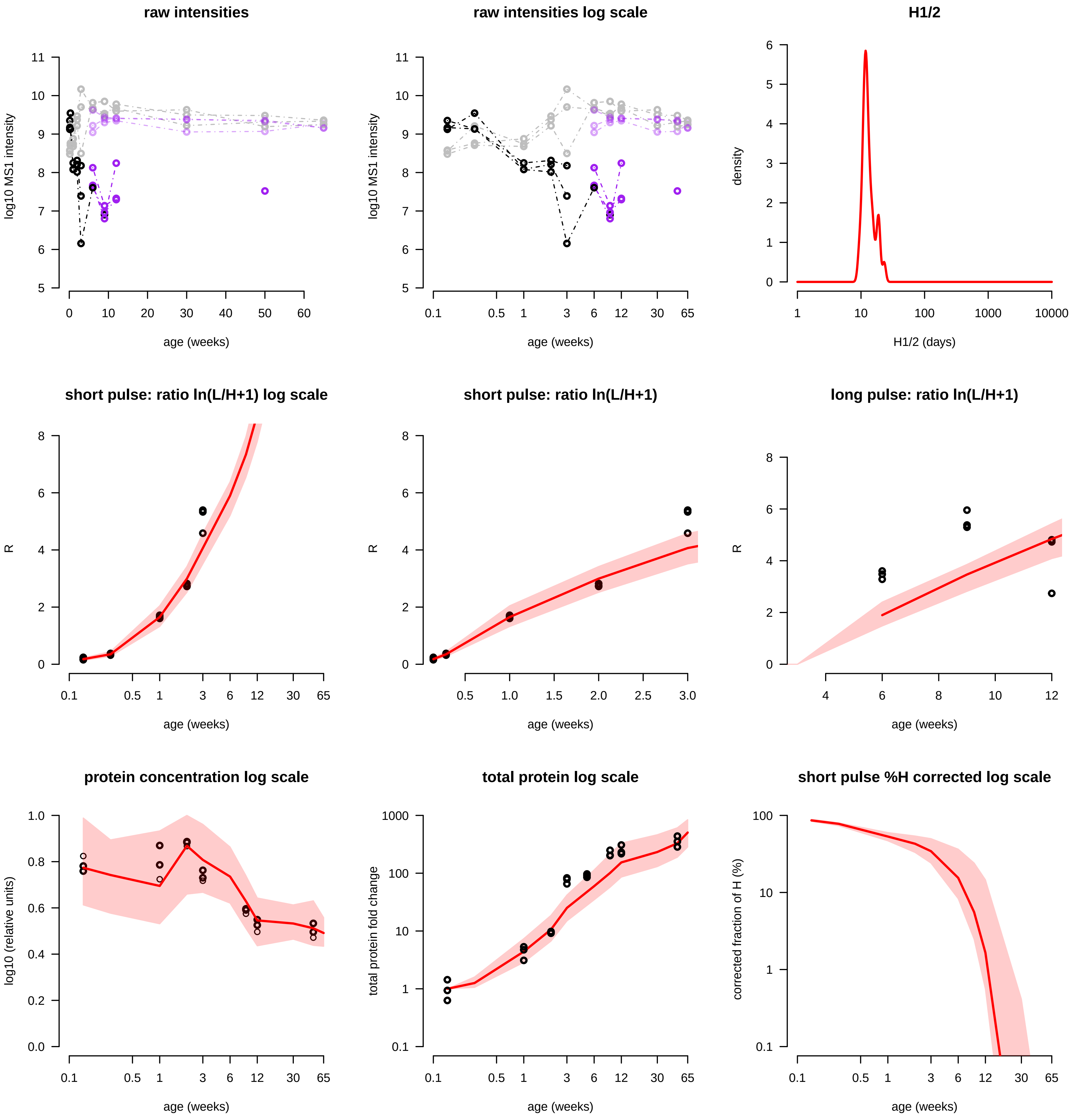

P26369

>sp|P26369|U2AF2_MOUSE

Splicing factor U2AF 65 kDa subunit

GN=U2af2

Med H1/2: 8 days

Q5 H1/2: 6.6 days

Q95 H1/2: 9.9 days

reliable model fit: yes

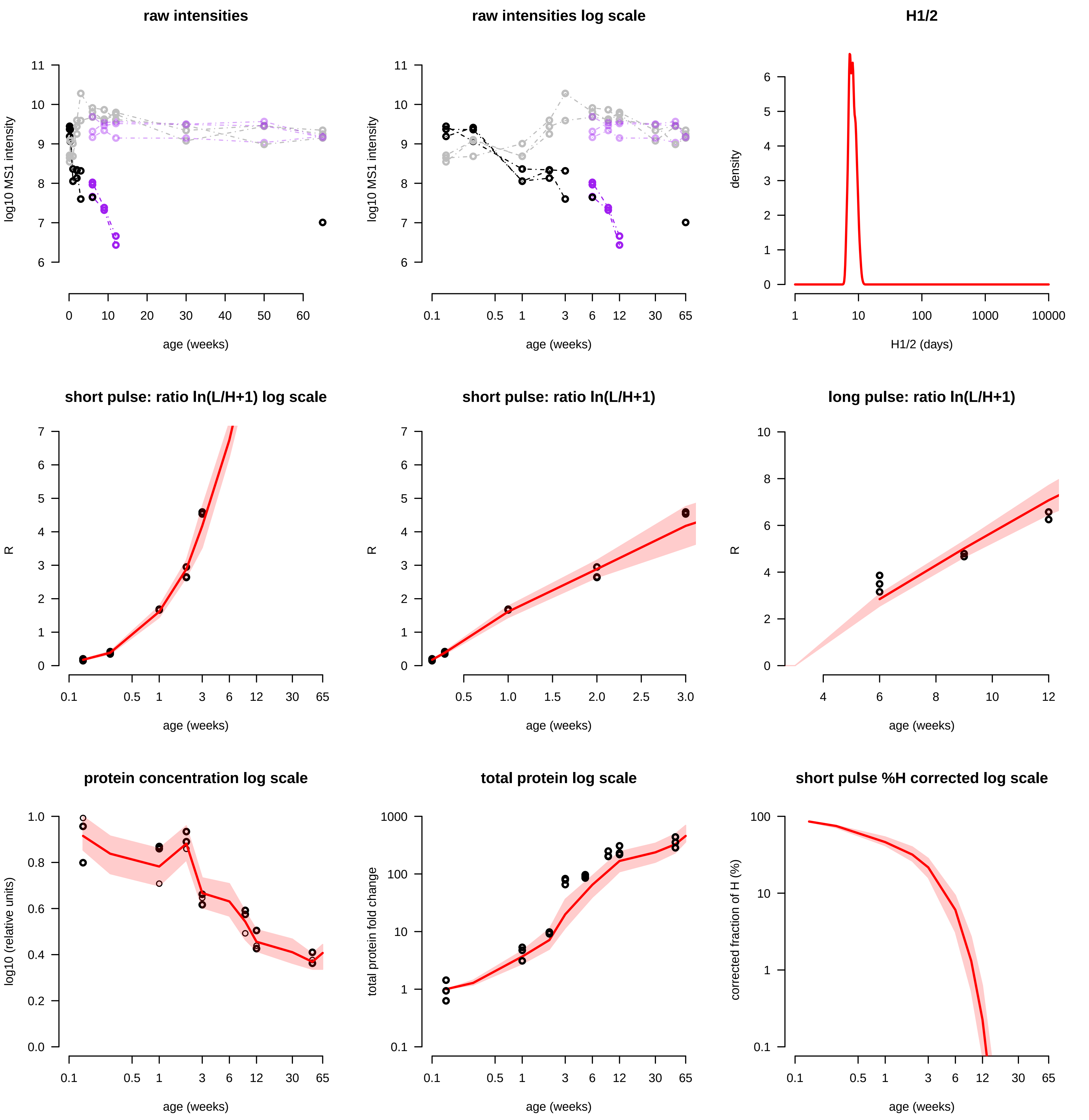

P70441

>sp|P70441|NHRF1_MOUSE

Na(+)/H(+) exchange regulatory cofactor NHE−RF1

GN=Slc9a3r1

Med H1/2: 4 days

Q5 H1/2: 2.5 days

Q95 H1/2: 26.6 days

reliable model fit: no

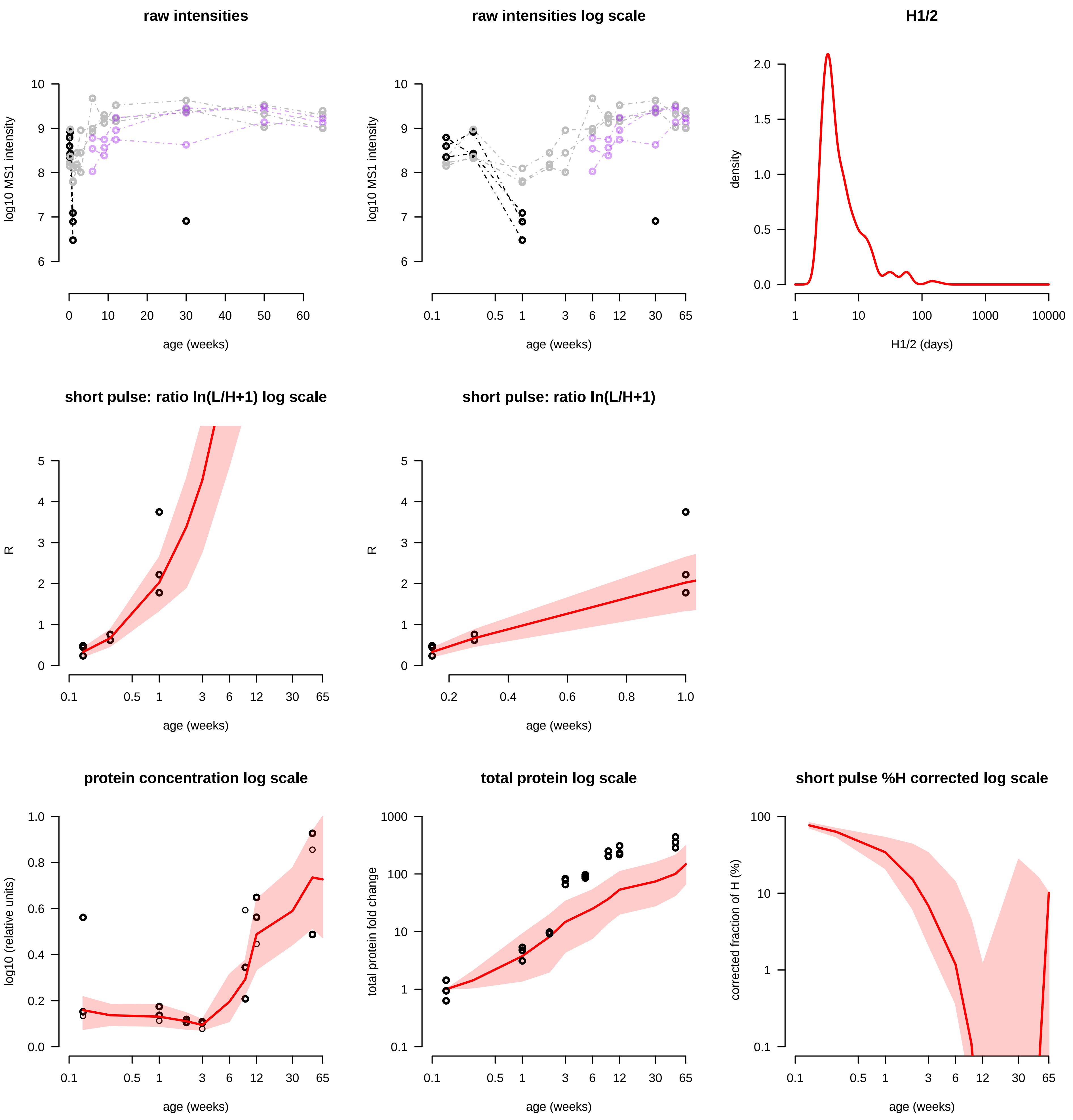

P49817

>sp|P49817|CAV1_MOUSE

Caveolin−1

GN=Cav1

Med H1/2: 5.8 days

Q5 H1/2: 4.5 days

Q95 H1/2: 7 days

reliable model fit: yes

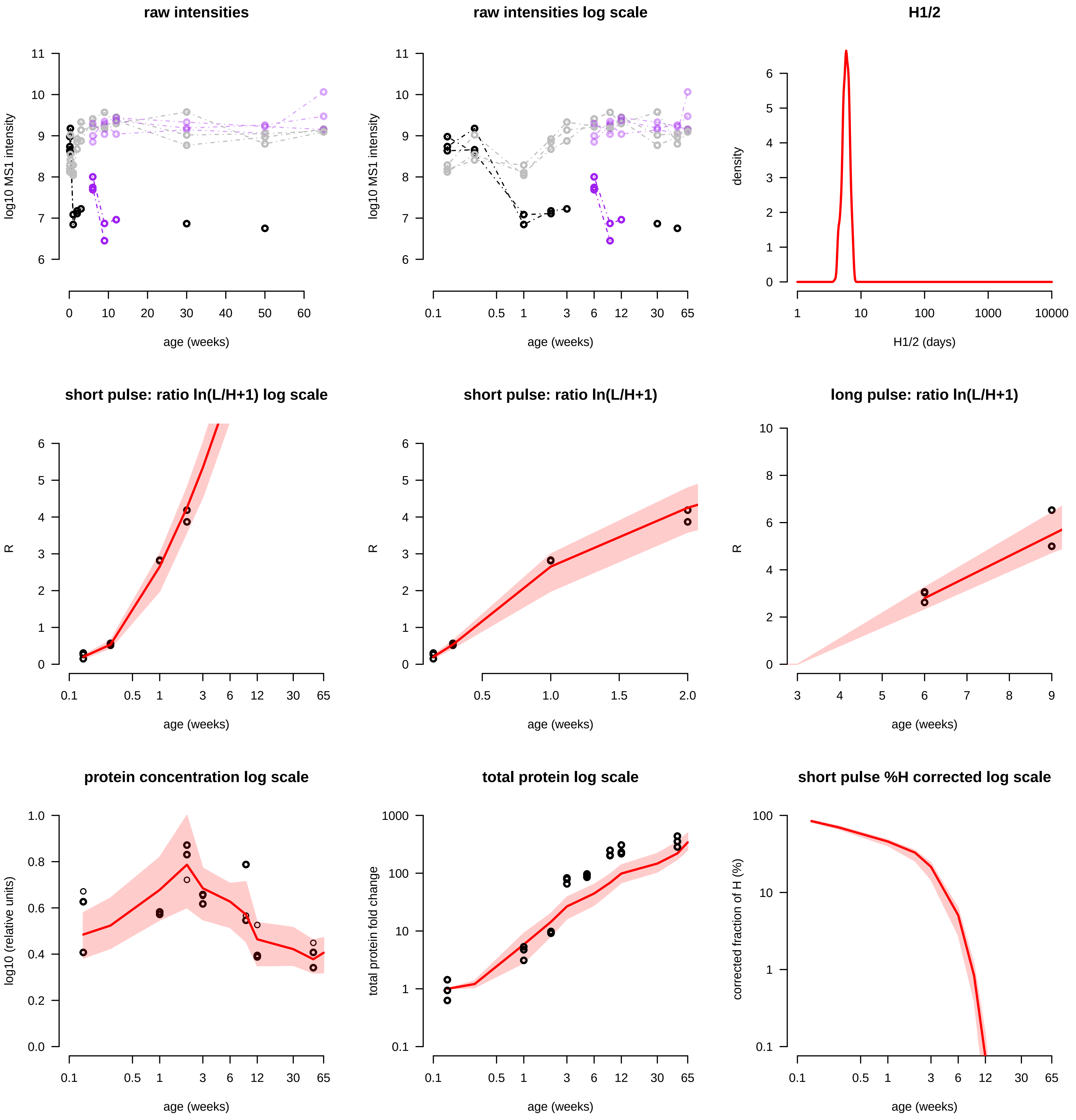

Q9D0K2

>sp|Q9D0K2|SCOT1_MOUSE

Succinyl−CoA:3−ketoacid coenzyme A transferase 1, mitochondrial

GN=Oxct1

Med H1/2: 496.6 days

Q5 H1/2: 189 days

Q95 H1/2: 5766.2 days

reliable model fit: no

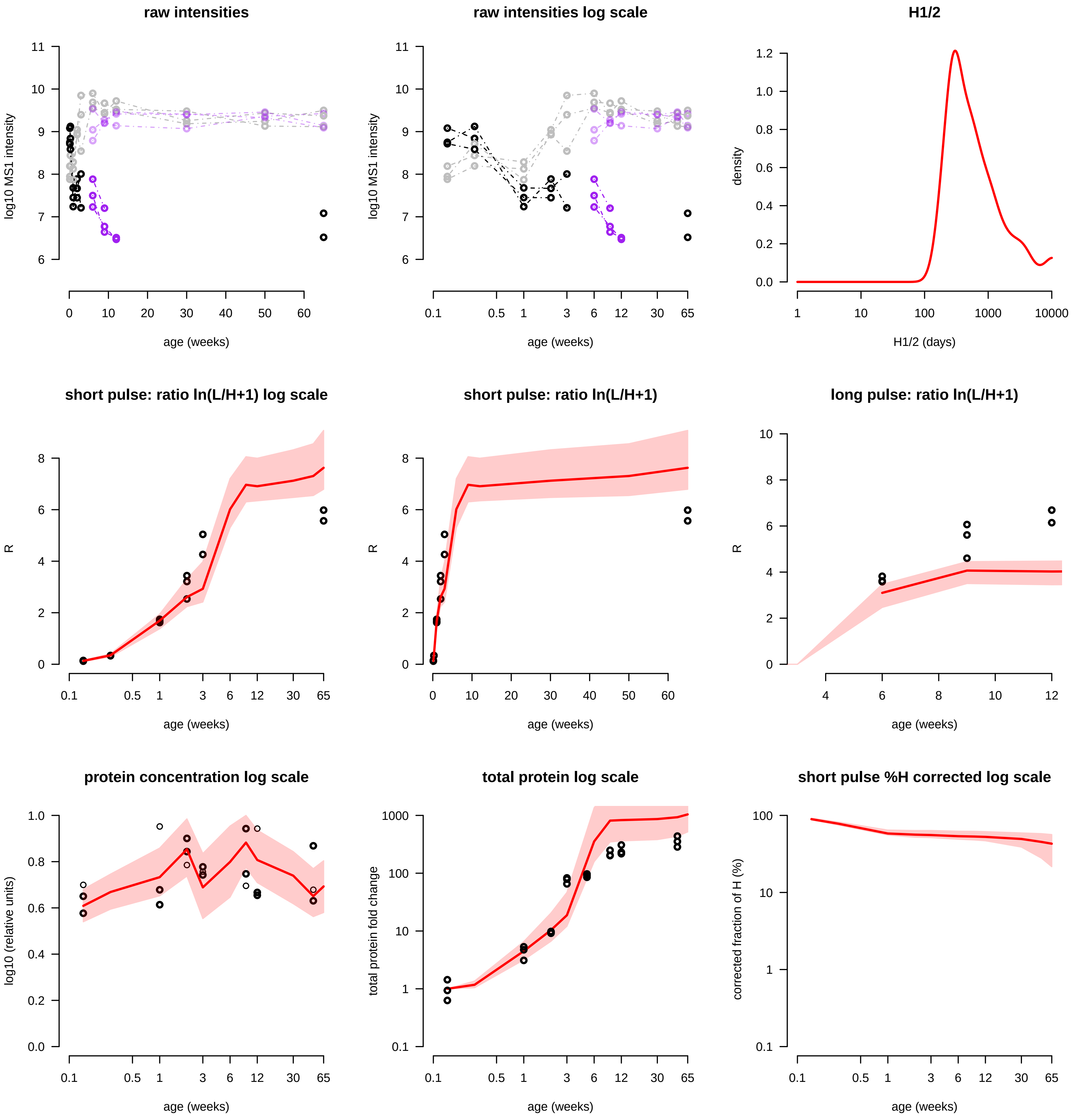

Q00PI9

>sp|Q00PI9|HNRL2_MOUSE

Heterogeneous nuclear ribonucleoprotein U−like protein 2

GN=Hnrnpul2

Med H1/2: 5.1 days

Q5 H1/2: 4.4 days

Q95 H1/2: 6.5 days

reliable model fit: yes

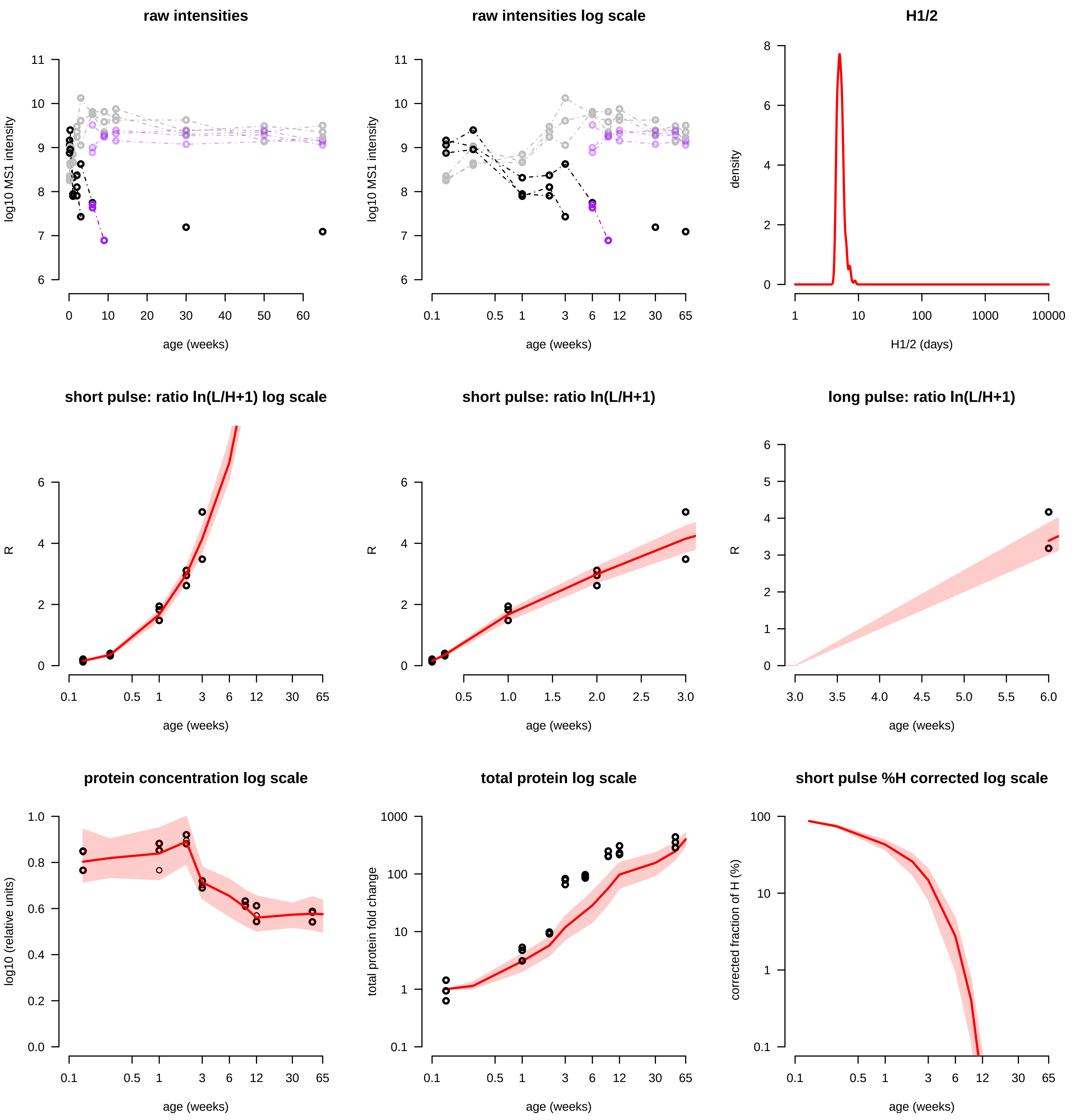

Q61696;P17879

>sp|Q61696|HS71A_MOUSE

Heat shock 70 kDa protein 1A

GN=Hspa1a

Med H1/2: 6.7 days

Q5 H1/2: 5.3 days

Q95 H1/2: 8.7 days

reliable model fit: yes

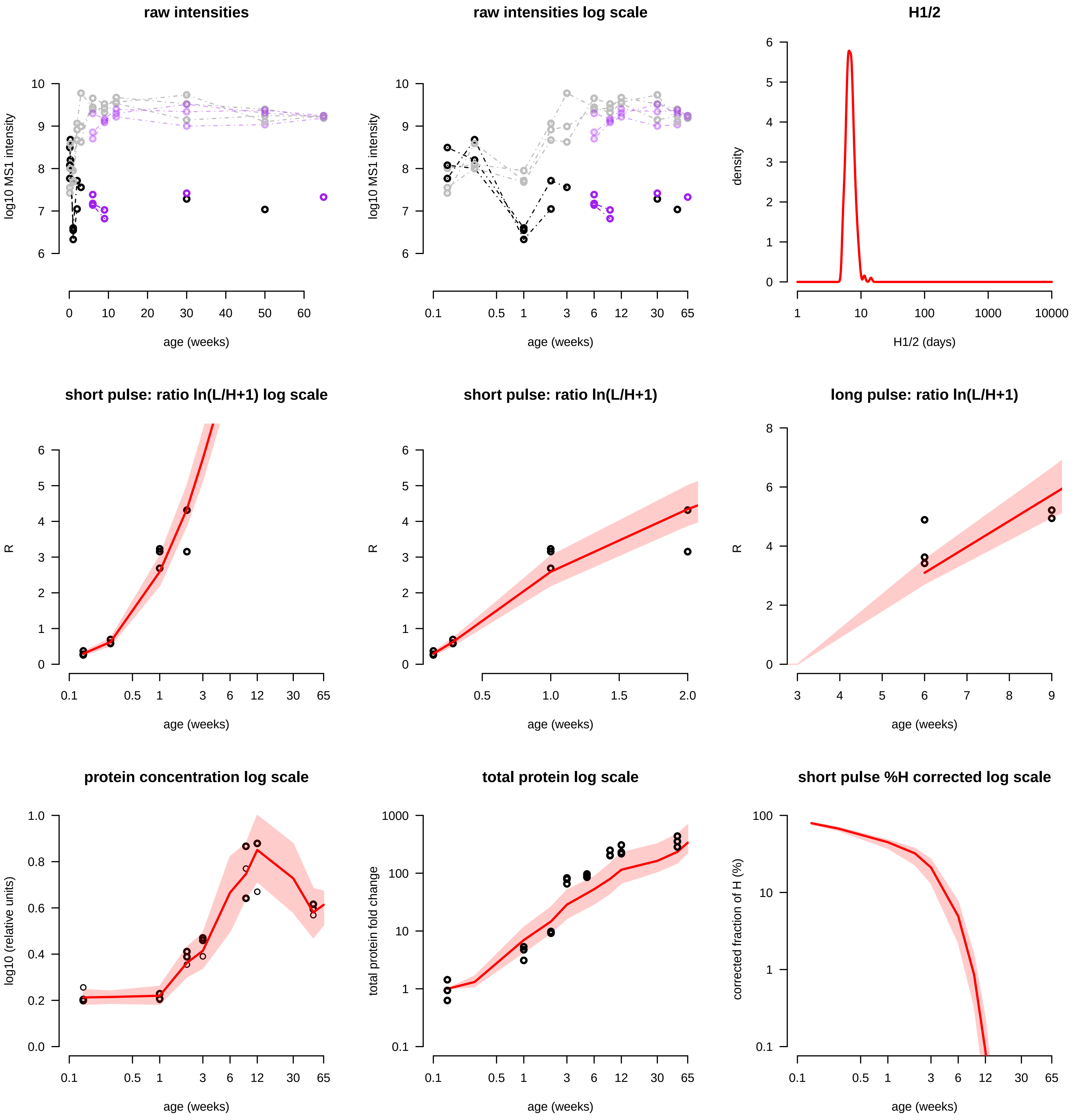

Q9CQX8

>sp|Q9CQX8|RT36_MOUSE

28S ribosomal protein S36, mitochondrial

GN=Mrps36

Med H1/2: 16.7 days

Q5 H1/2: 6.3 days

Q95 H1/2: 100.7 days

reliable model fit: yes

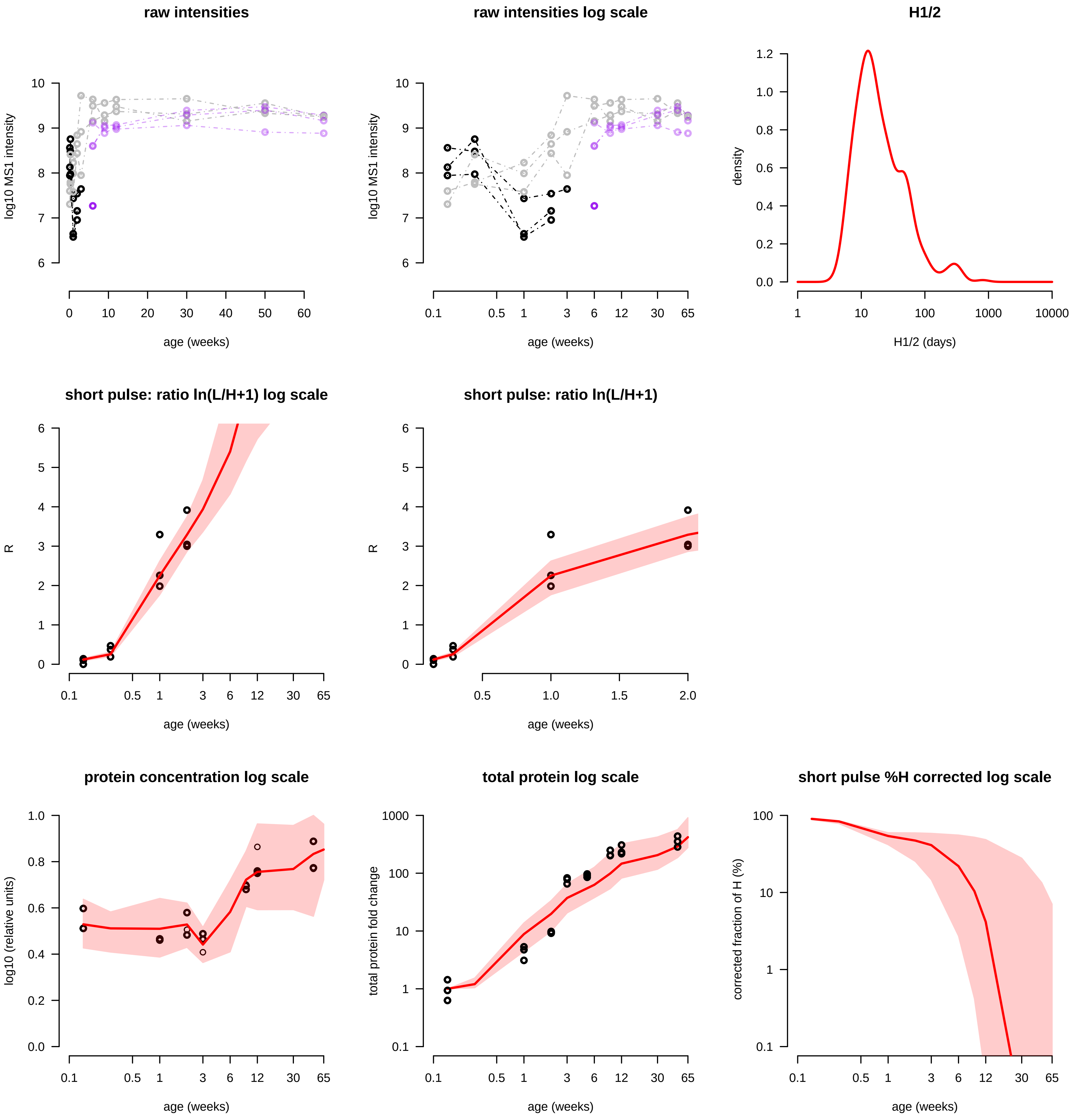

Q9CQC6

>sp|Q9CQC6|BZW1_MOUSE

Basic leucine zipper and W2 domain−containing protein 1

GN=Bzw1

Med H1/2: 7.9 days

Q5 H1/2: 6.4 days

Q95 H1/2: 10.9 days

reliable model fit: yes

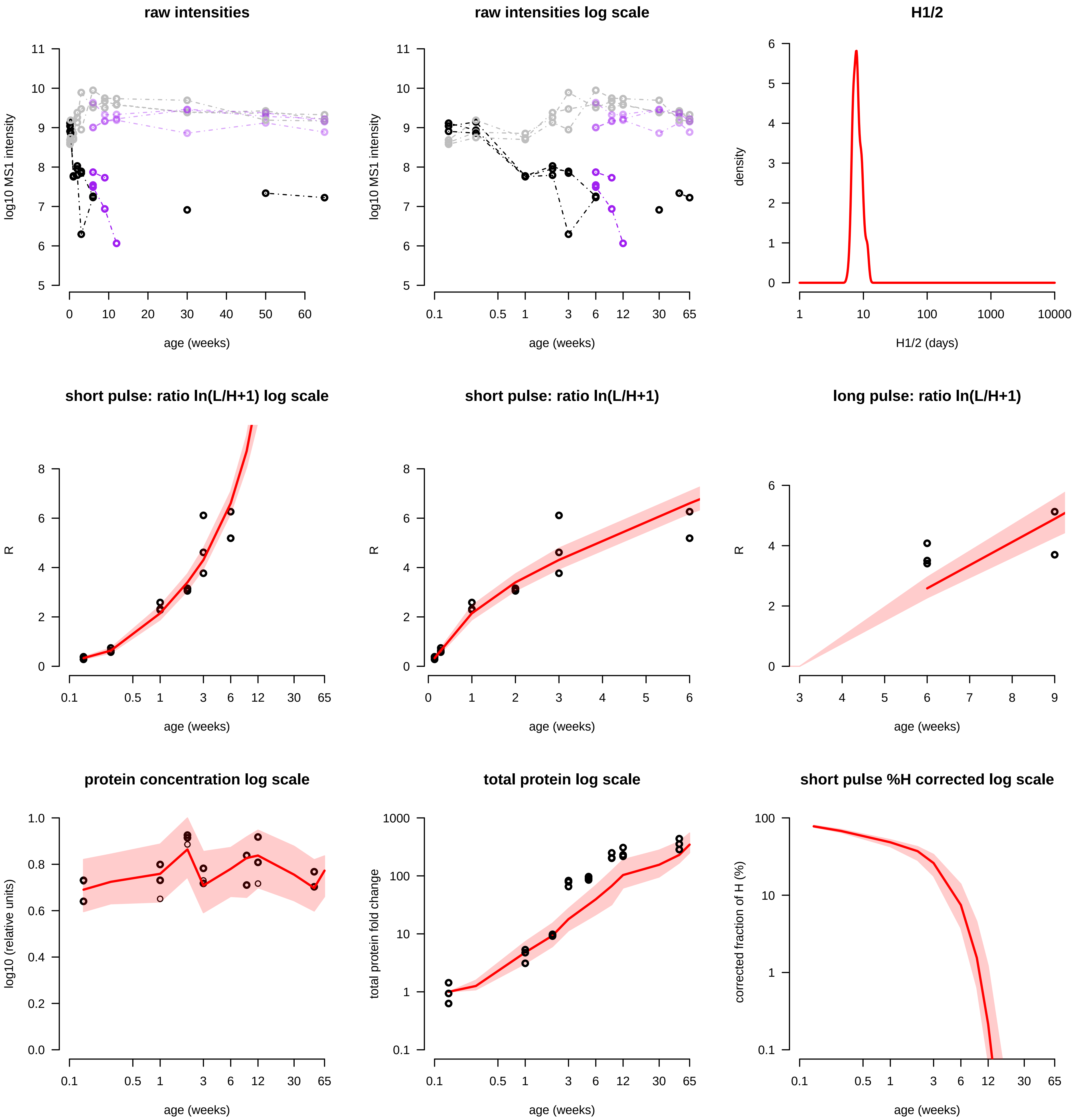

Q9D6Z1

>sp|Q9D6Z1|NOP56_MOUSE

Nucleolar protein 56

GN=Nop56

Med H1/2: 21.3 days

Q5 H1/2: 14 days

Q95 H1/2: 44.2 days

reliable model fit: yes

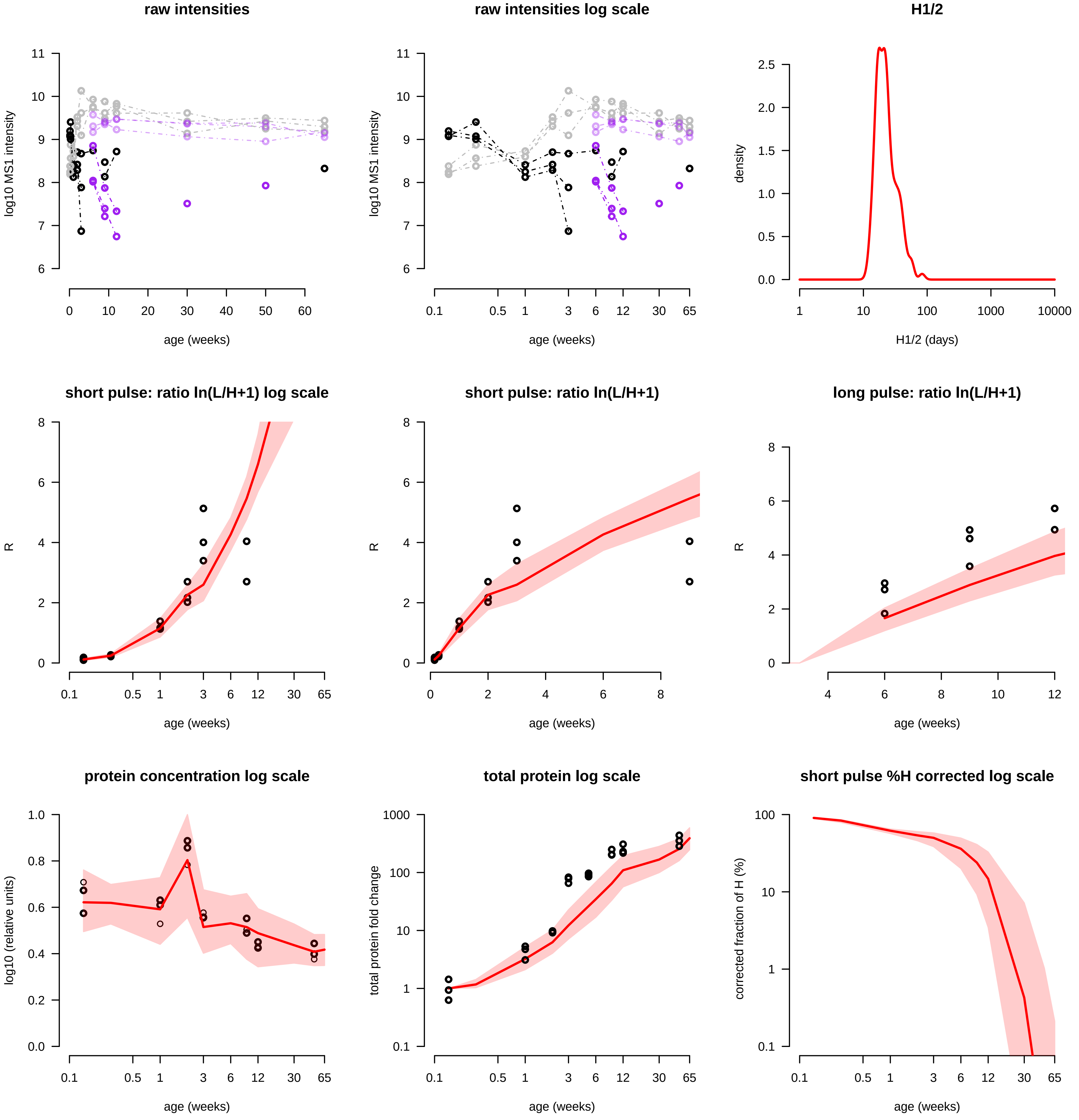

P97479;P97479−2

>sp|P97479|MYO7A_MOUSE

Unconventional myosin−VIIa

GN=Myo7a

Med H1/2: 394.3 days

Q5 H1/2: 114.6 days

Q95 H1/2: 3411.2 days

reliable model fit: no

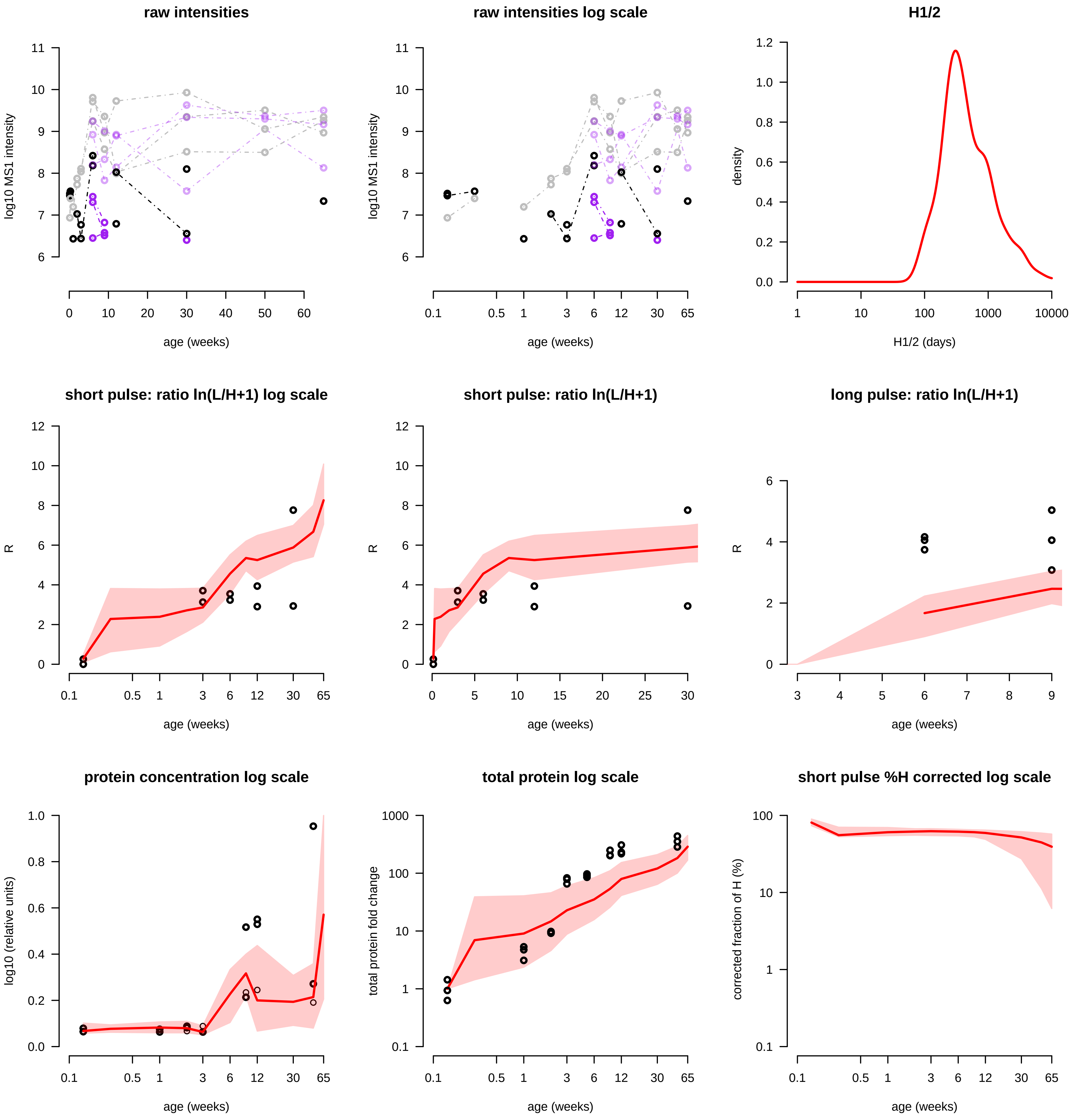

Q9Z1Q5

>sp|Q9Z1Q5|CLIC1_MOUSE

Chloride intracellular channel protein 1

GN=Clic1

Med H1/2: 7.4 days

Q5 H1/2: 6.4 days

Q95 H1/2: 9.2 days

reliable model fit: yes

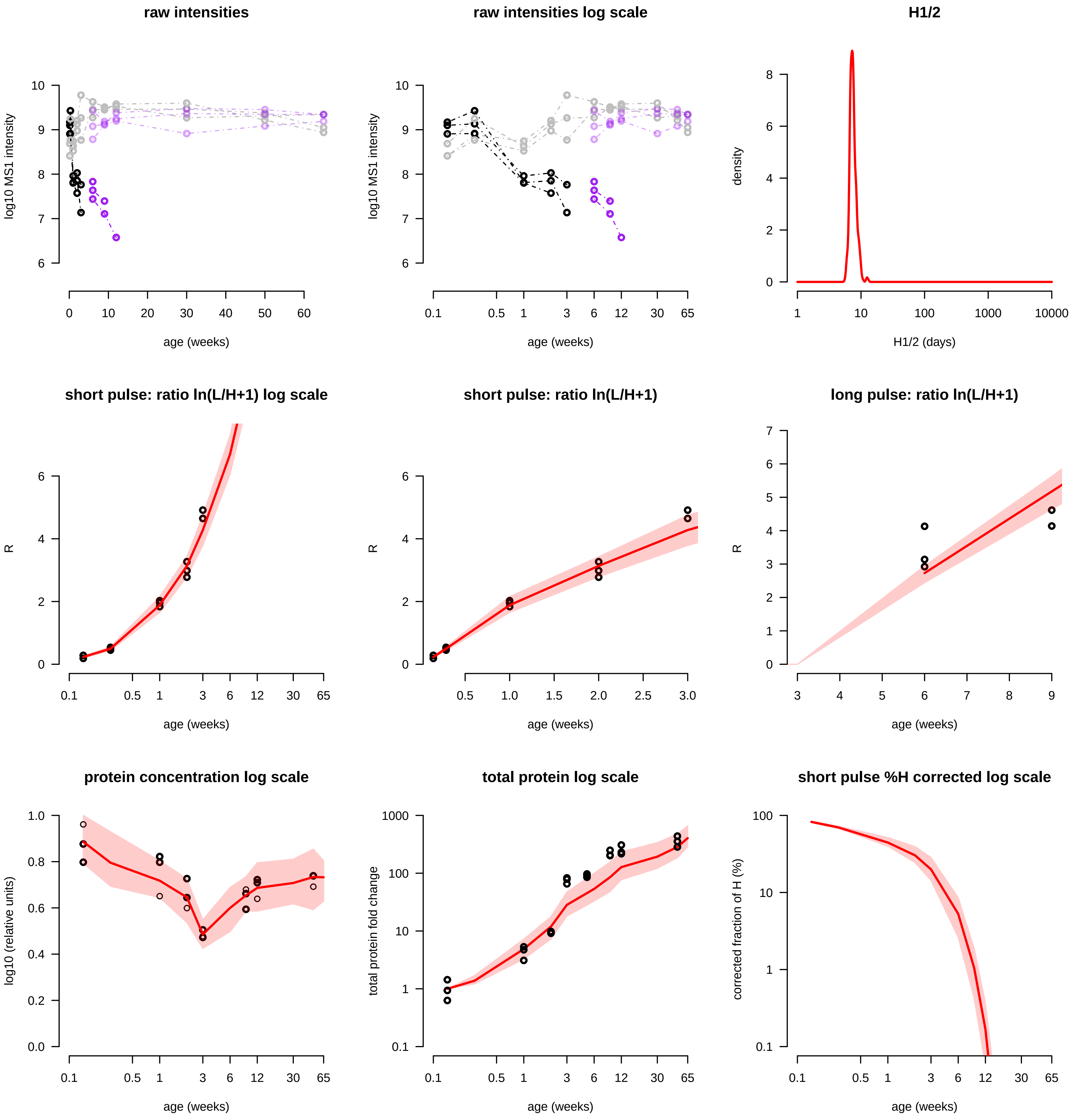

Q9R1P0

>sp|Q9R1P0|PSA4_MOUSE

Proteasome subunit alpha type−4

GN=Psma4

Med H1/2: 7.5 days

Q5 H1/2: 6 days

Q95 H1/2: 9.9 days

reliable model fit: yes

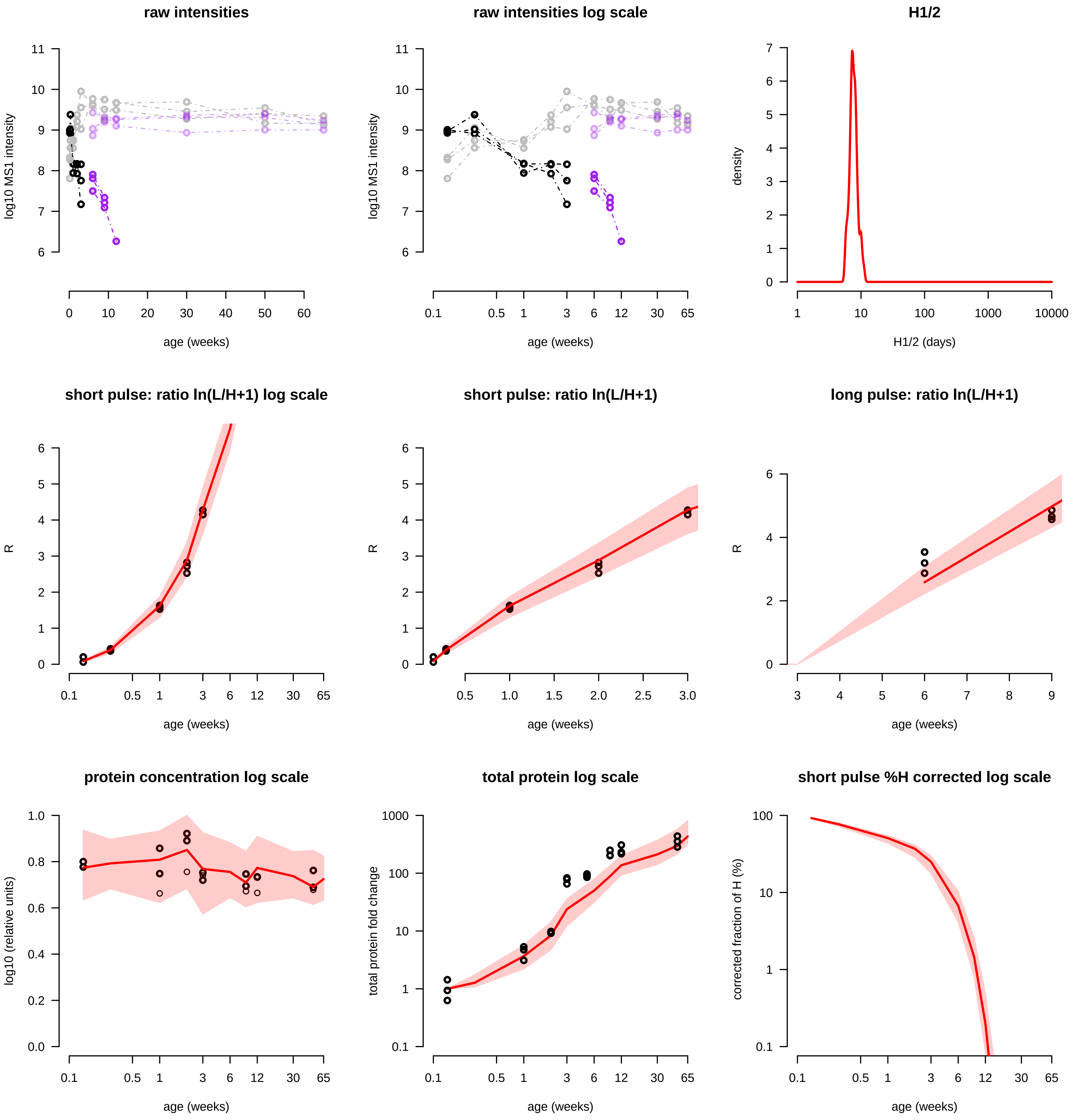

Q61595;Q61595−15

>sp|Q61595|KTN1_MOUSE

Kinectin

GN=Ktn1

Med H1/2: 4.5 days

Q5 H1/2: 3.8 days

Q95 H1/2: 5.4 days

reliable model fit: yes

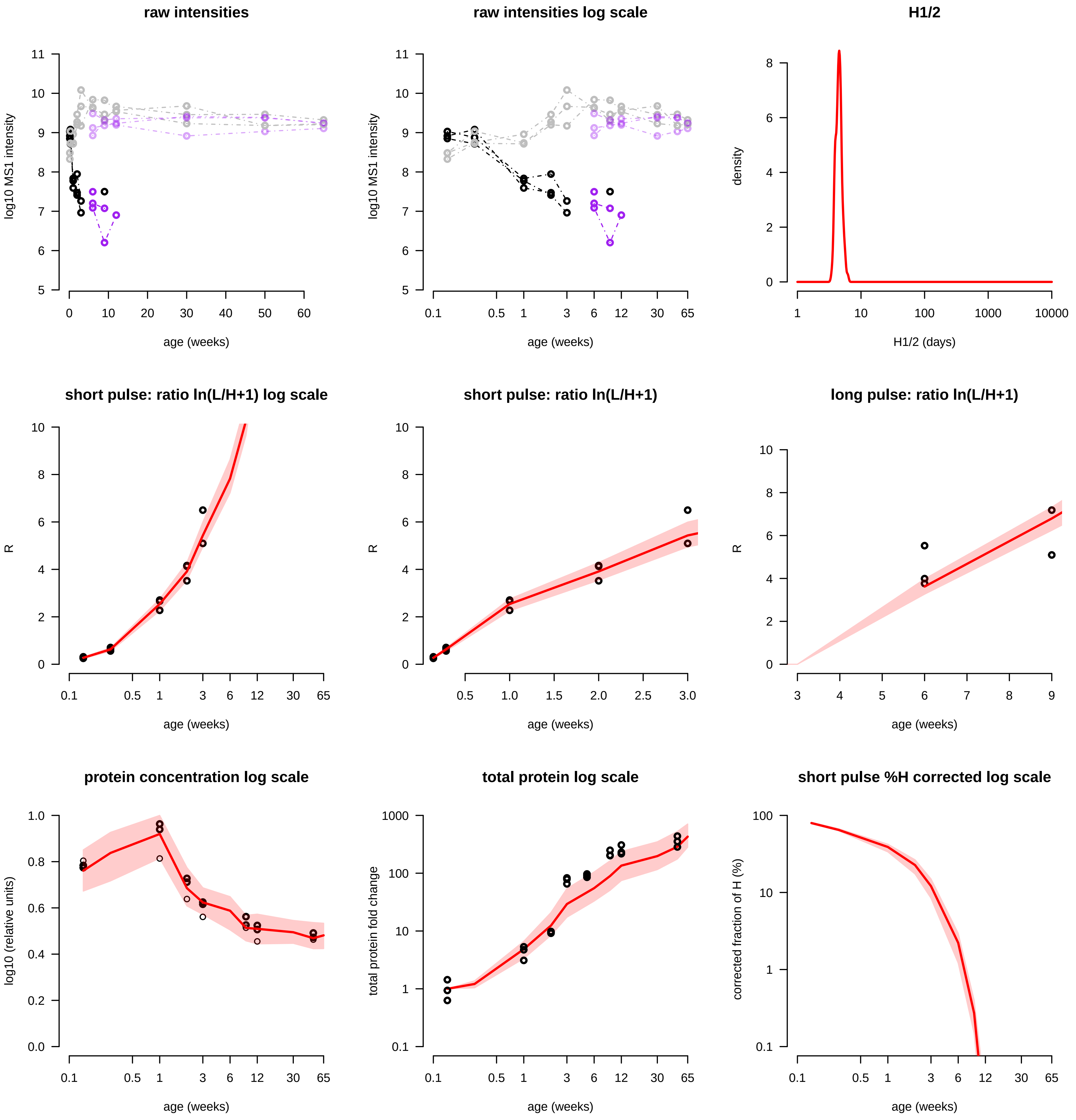

Q61166

>sp|Q61166|MARE1_MOUSE

Microtubule−associated protein RP/EB family member 1

GN=Mapre1

Med H1/2: 10.1 days

Q5 H1/2: 8.1 days

Q95 H1/2: 13.8 days

reliable model fit: yes

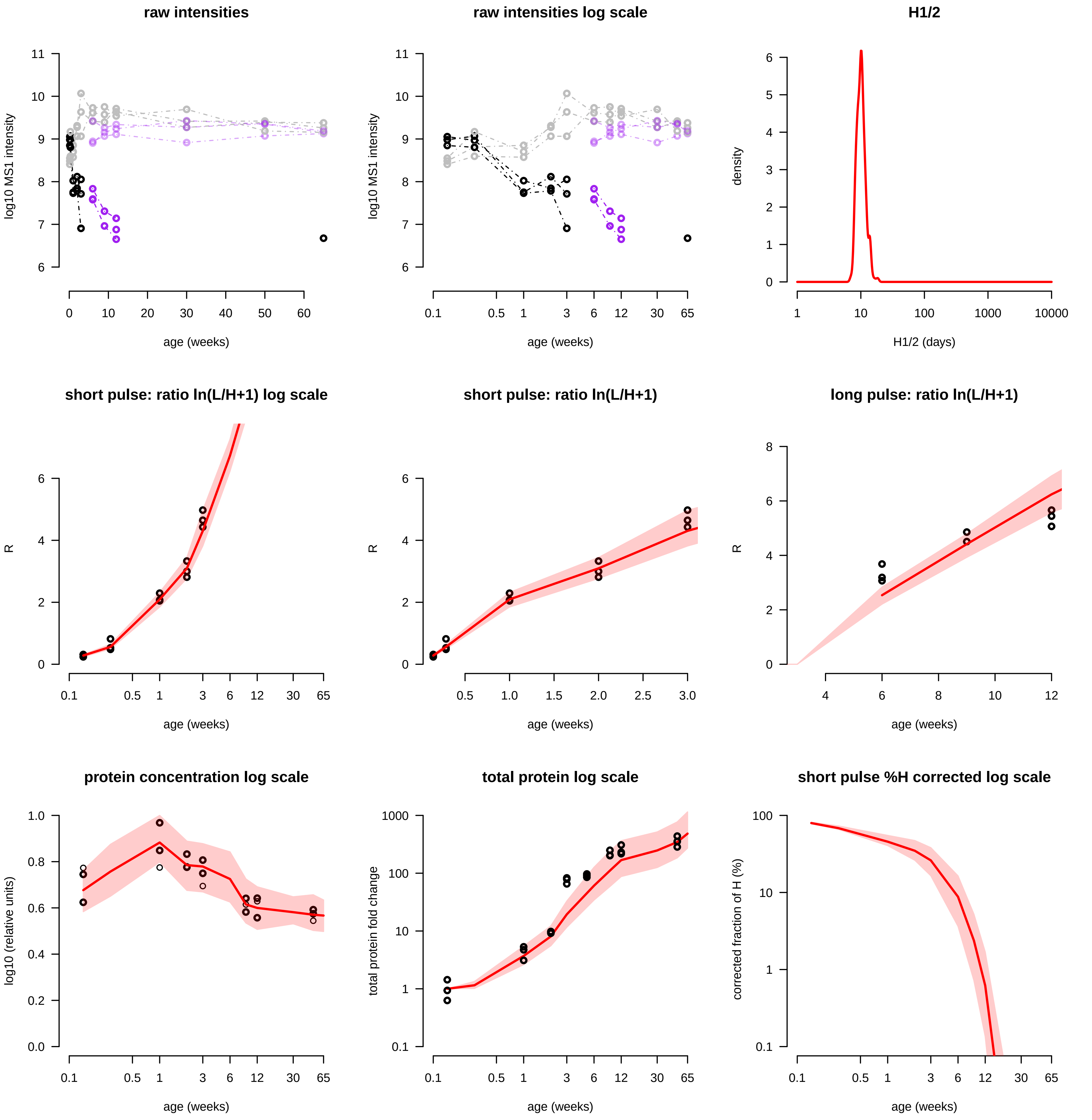

Q922B2

>sp|Q922B2|SYDC_MOUSE

Aspartate−−tRNA ligase, cytoplasmic

GN=Dars1

Med H1/2: 4.5 days

Q5 H1/2: 4 days

Q95 H1/2: 5.3 days

reliable model fit: yes

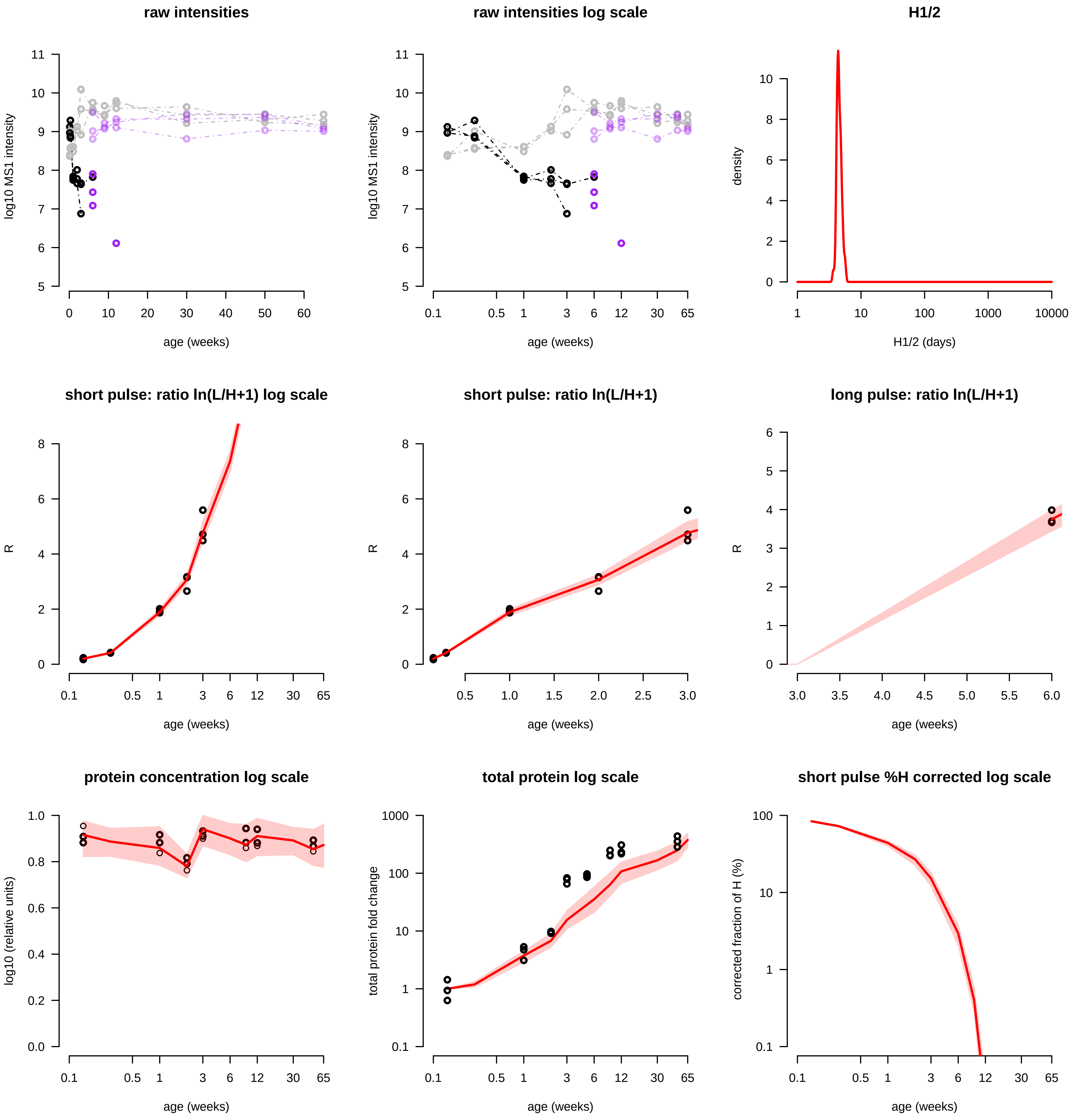

Q9CXS4

>sp|Q9CXS4|CENPV_MOUSE

Centromere protein V

GN=Cenpv

Med H1/2: 23.5 days

Q5 H1/2: 15 days

Q95 H1/2: 44.6 days

reliable model fit: yes

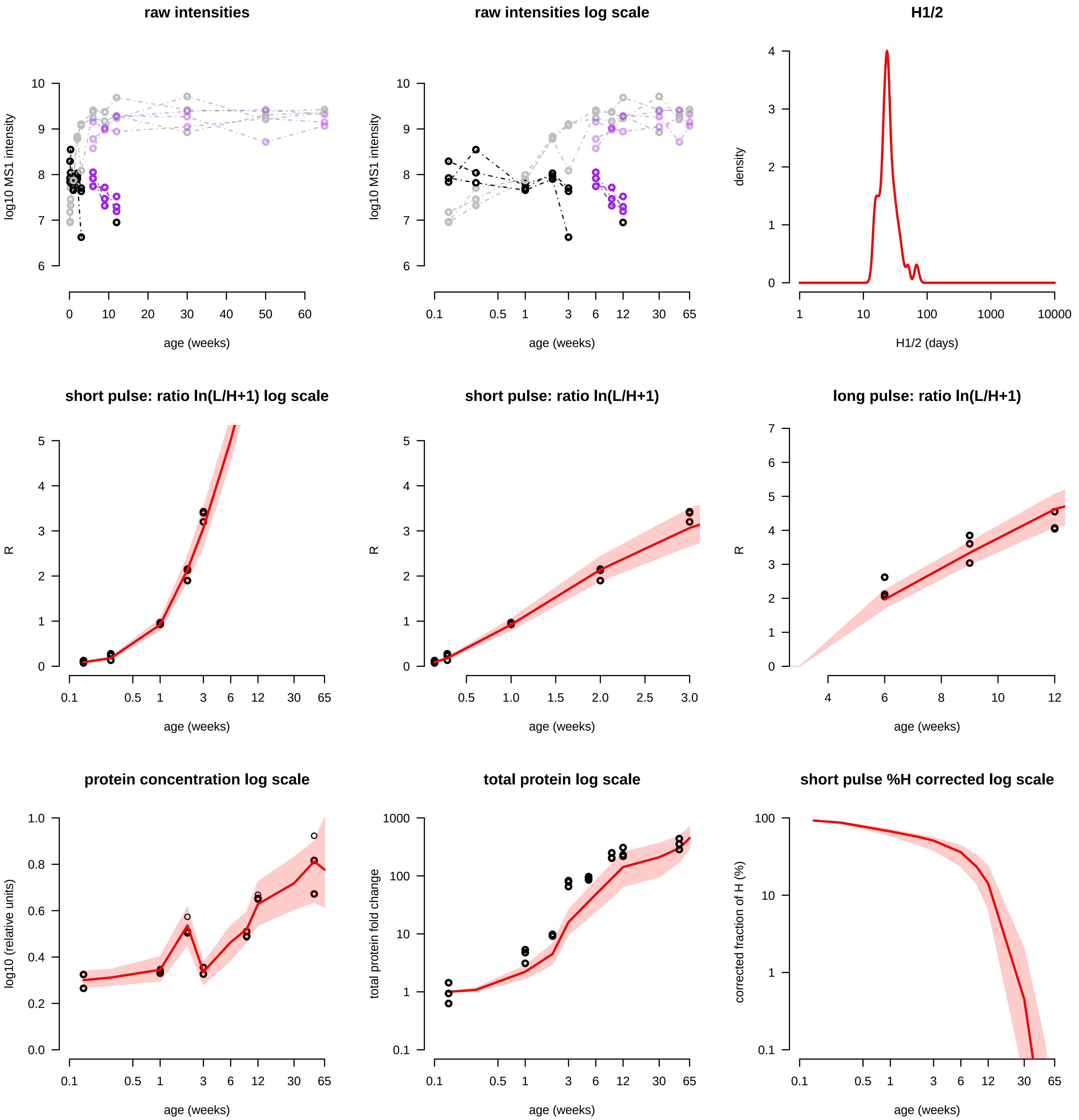

Q9R1P3

>sp|Q9R1P3|PSB2_MOUSE

Proteasome subunit beta type−2

GN=Psmb2

Med H1/2: 5.2 days

Q5 H1/2: 3.8 days

Q95 H1/2: 7.1 days

reliable model fit: yes

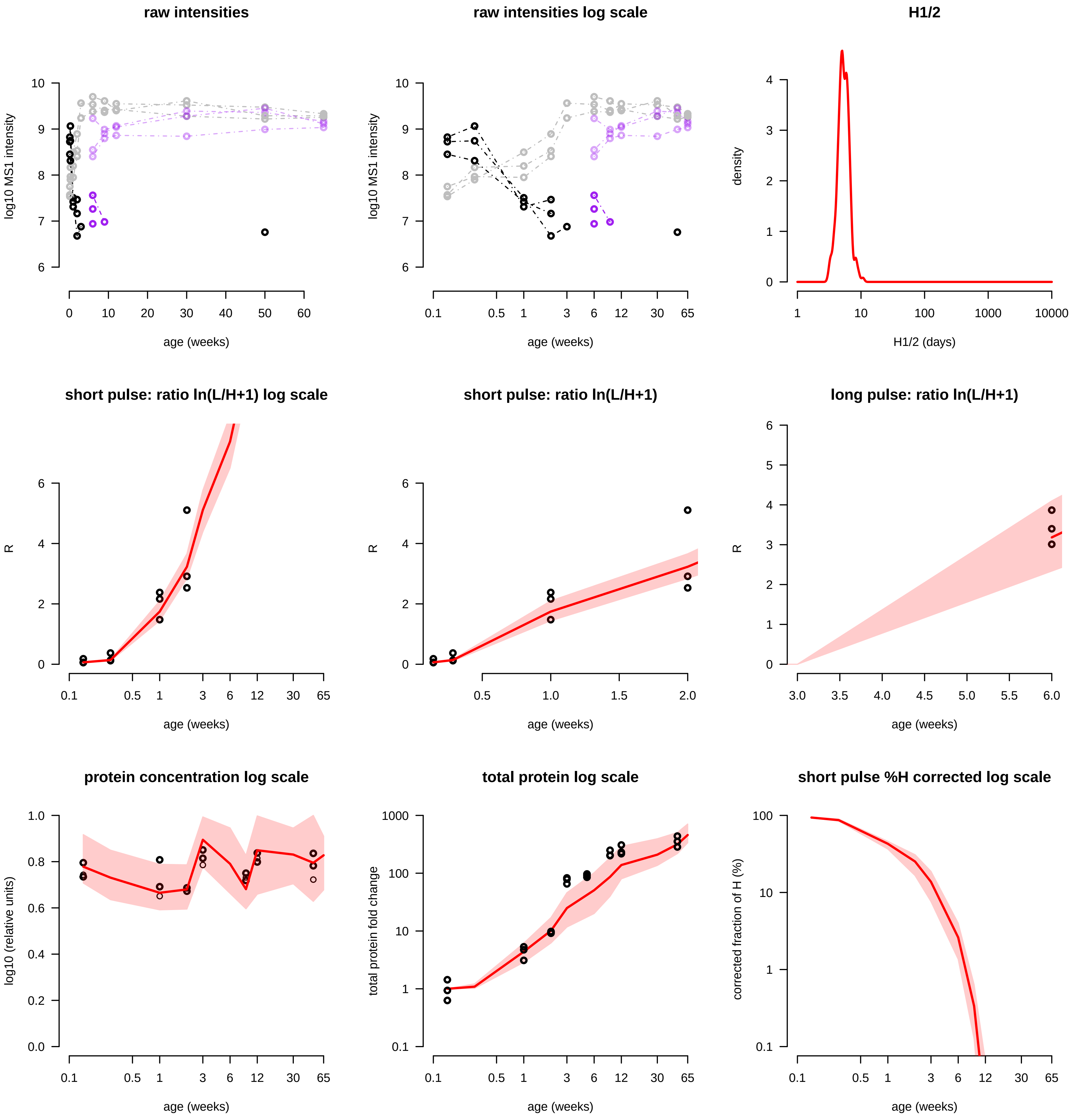

P68510

>sp|P68510|1433F_MOUSE

14−3−3 protein eta

GN=Ywhah

Med H1/2: 6.7 days

Q5 H1/2: 5.8 days

Q95 H1/2: 8.2 days

reliable model fit: yes

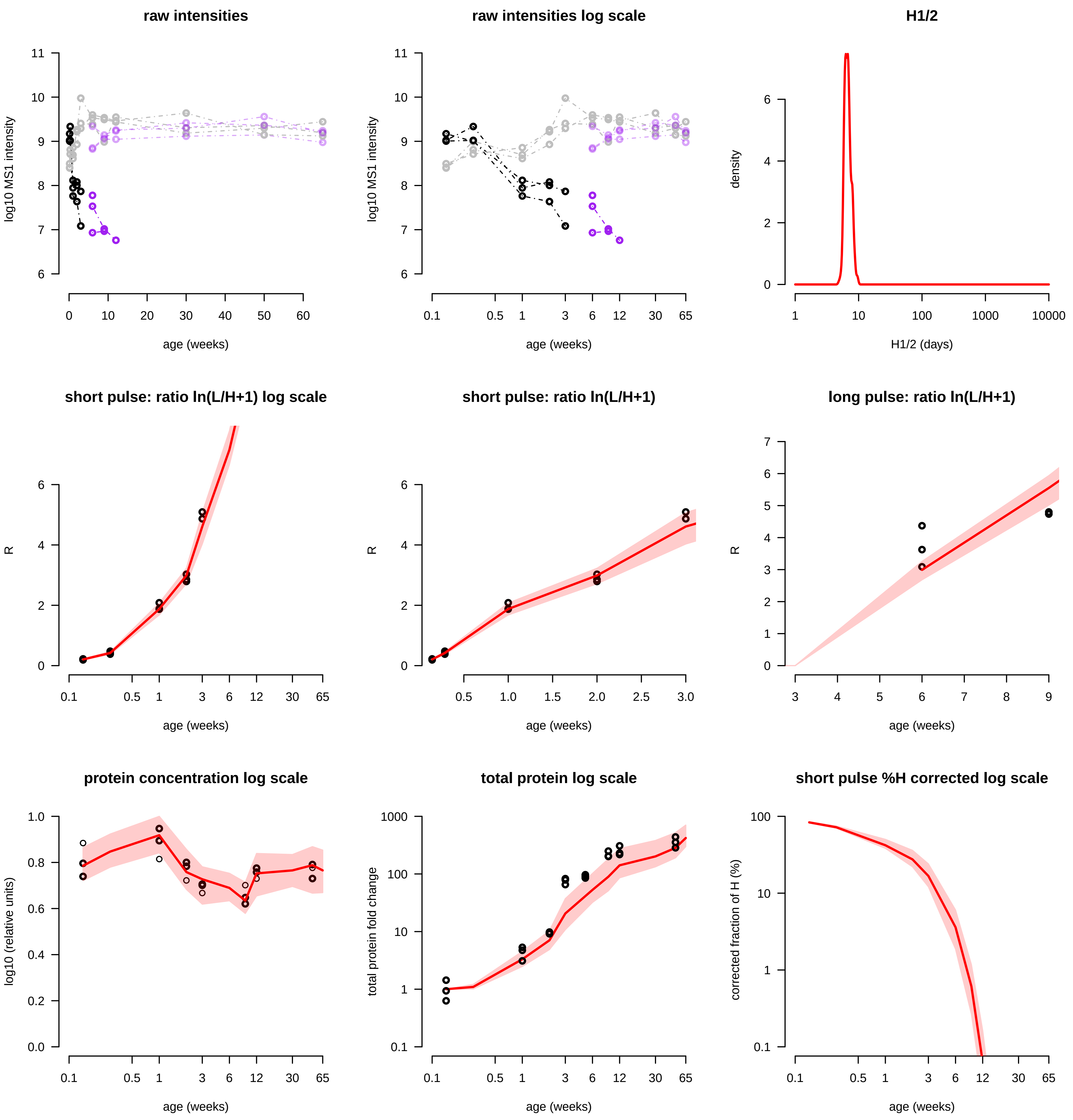

P62849−2;P62849−3;P62849

>sp|P62849−2|RS24_MOUSE

Isoform 2 of 40S ribosomal protein S24

GN=Rps24

Med H1/2: 6.2 days

Q5 H1/2: 5.1 days

Q95 H1/2: 7.4 days

reliable model fit: yes

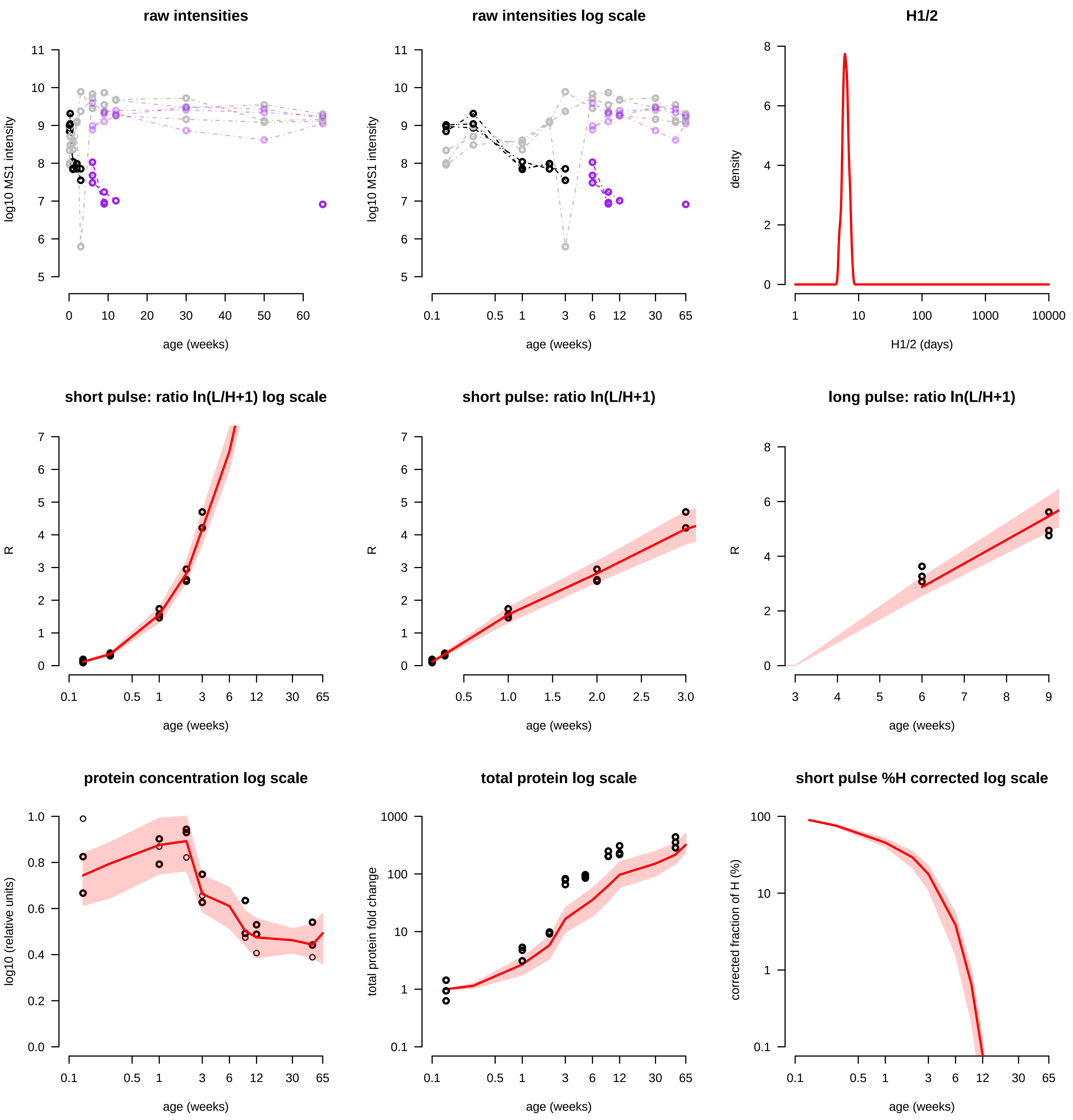

P41216

>sp|P41216|ACSL1_MOUSE

Long−chain−fatty−acid−−CoA ligase 1

GN=Acsl1

Med H1/2: 64 days

Q5 H1/2: 39.8 days

Q95 H1/2: 141.6 days

reliable model fit: yes

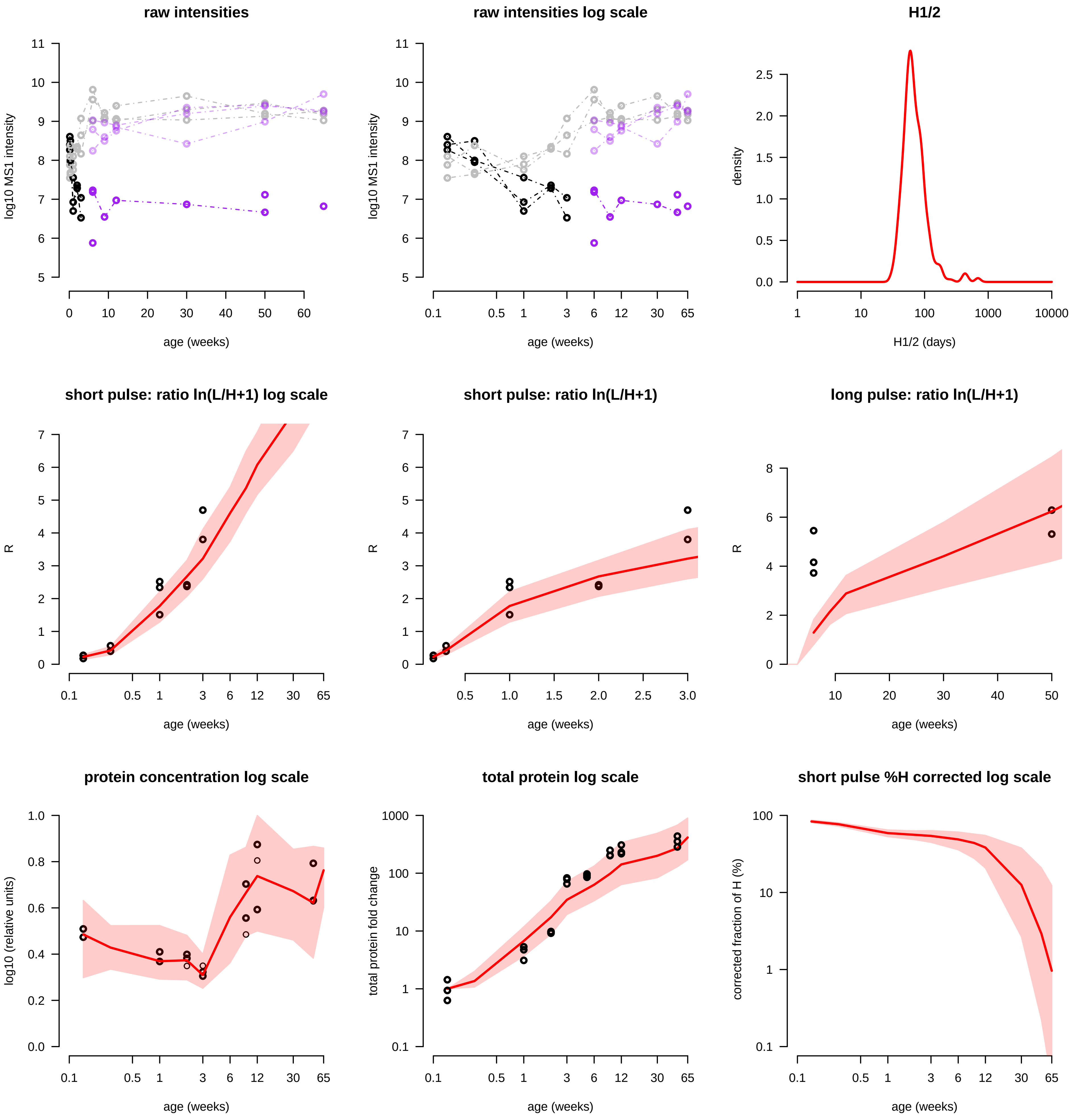

P70670

>sp|P70670|NACAM_MOUSE

Nascent polypeptide−associated complex subunit alpha, muscle−specific form

GN=Naca

Med H1/2: 10.3 days

Q5 H1/2: 8 days

Q95 H1/2: 13.2 days

reliable model fit: yes

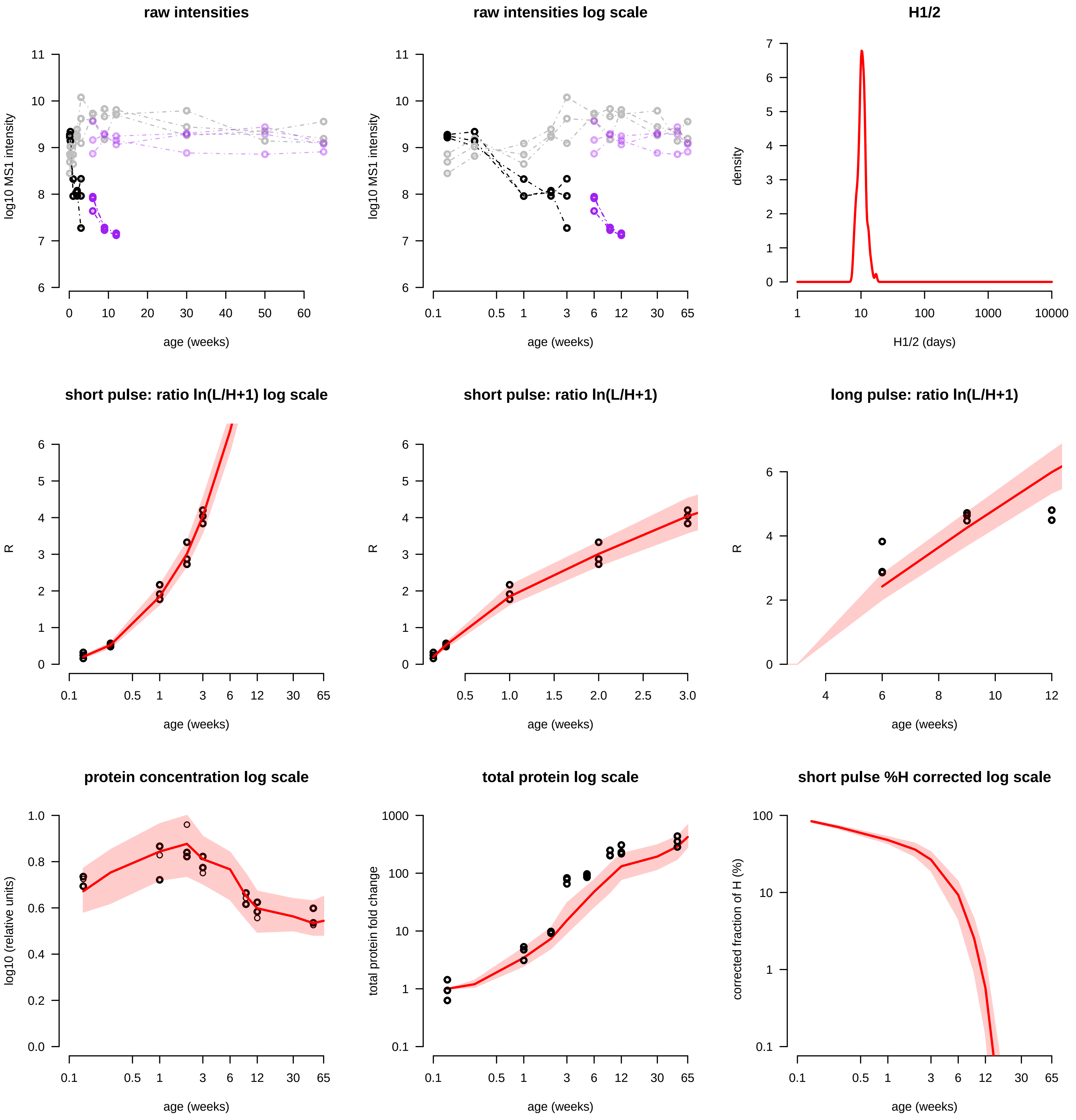

Q91ZX7

>sp|Q91ZX7|LRP1_MOUSE

Prolow−density lipoprotein receptor−related protein 1

GN=Lrp1

Med H1/2: 2086.5 days

Q5 H1/2: 467.7 days

Q95 H1/2: 22085 days

reliable model fit: yes

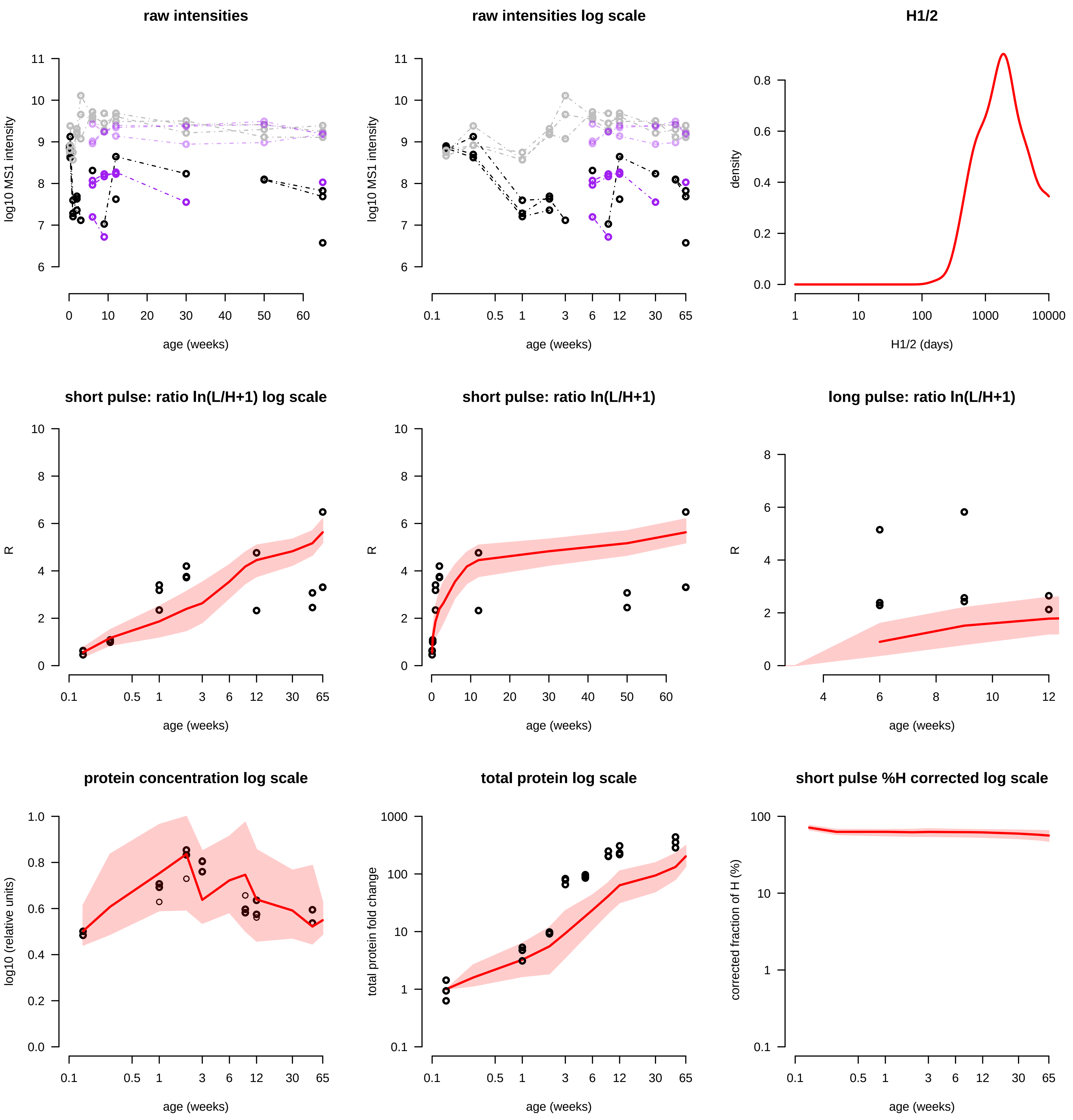

O89023

>sp|O89023|TPP1_MOUSE

Tripeptidyl−peptidase 1

GN=Tpp1

Med H1/2: 7.8 days

Q5 H1/2: 6.6 days

Q95 H1/2: 9.6 days

reliable model fit: yes

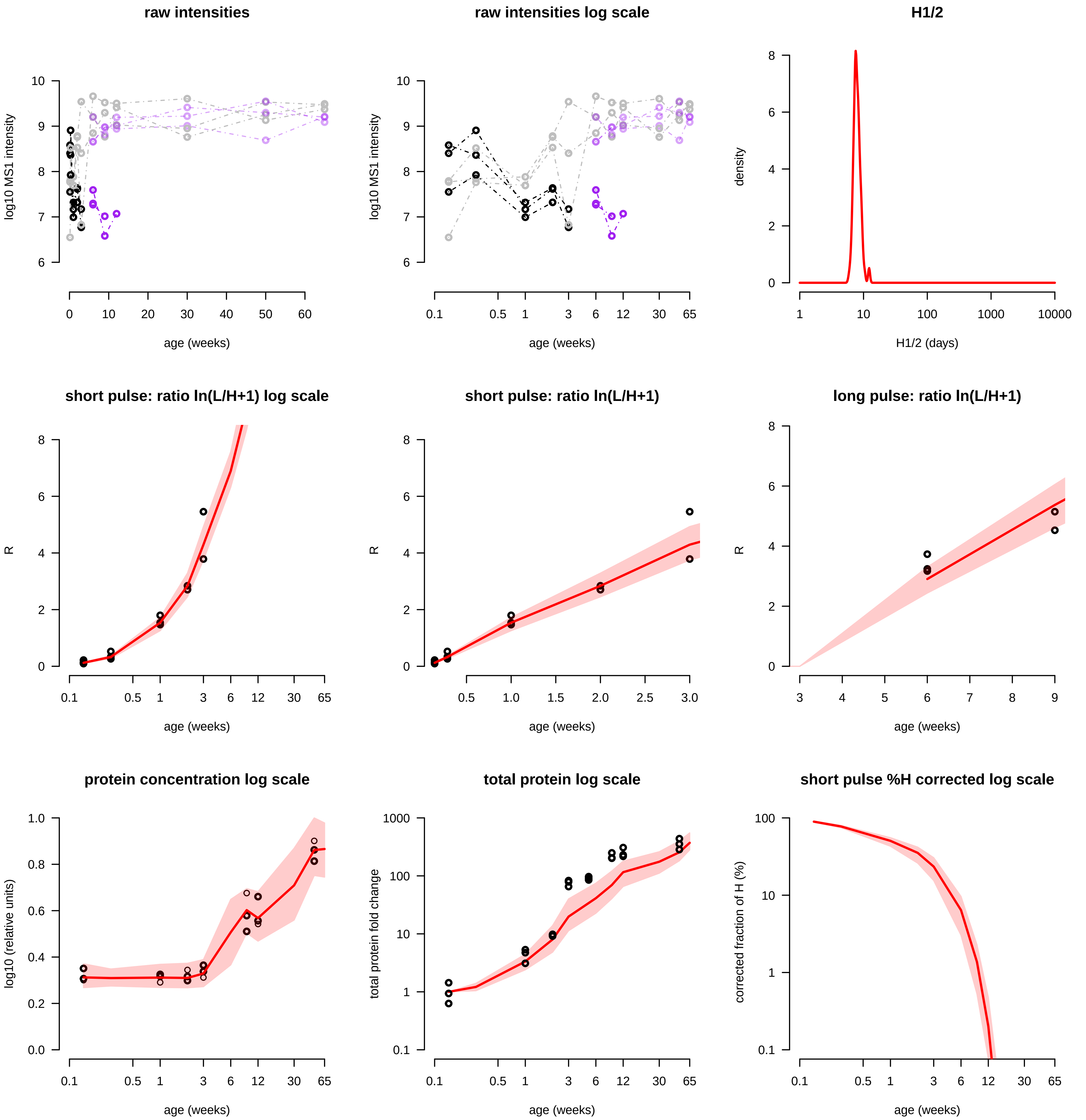

Q60972

>sp|Q60972|RBBP4_MOUSE

Histone−binding protein RBBP4

GN=Rbbp4

Med H1/2: 7 days

Q5 H1/2: 6 days

Q95 H1/2: 8.9 days

reliable model fit: yes

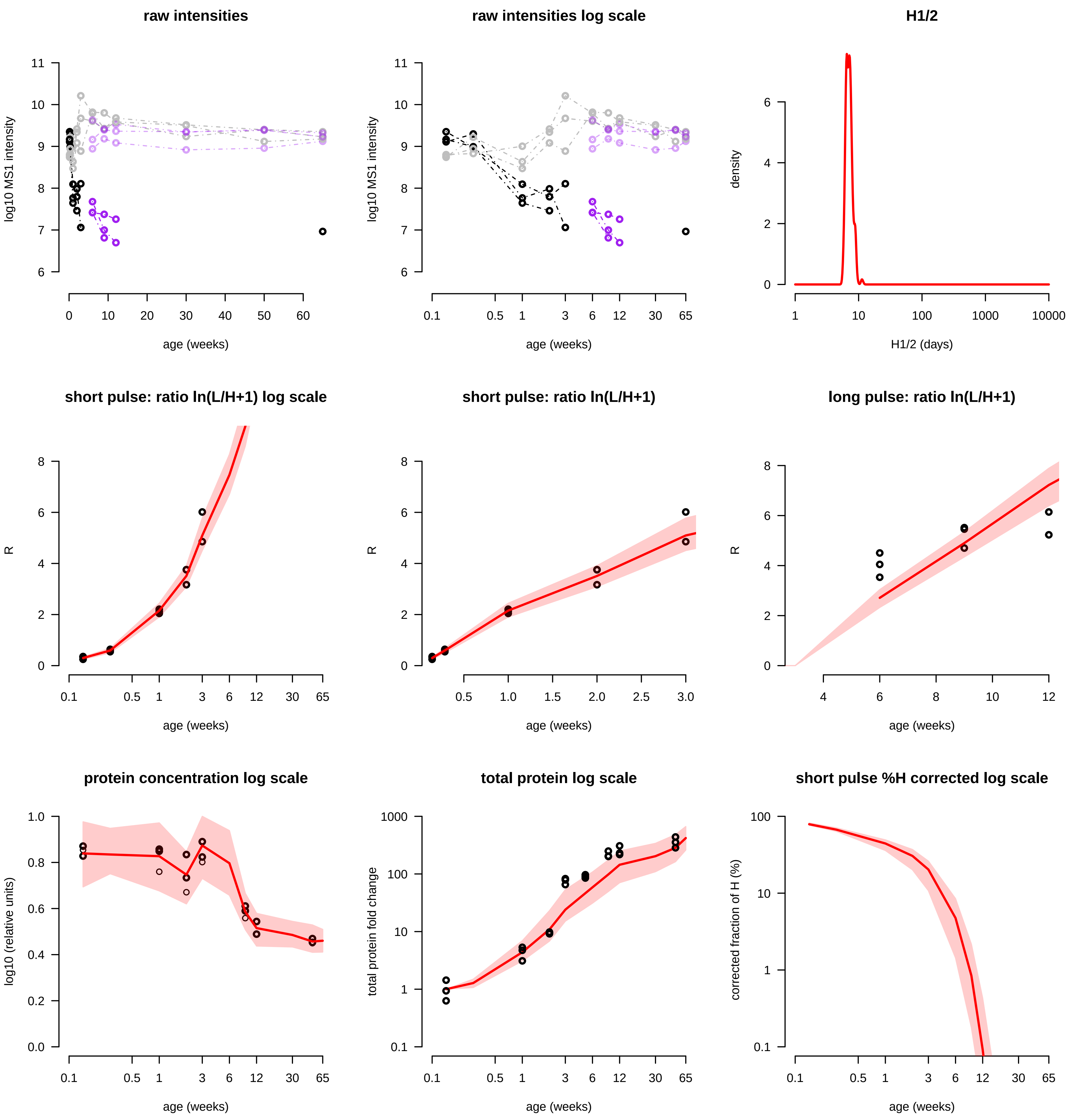

A2AX52

>sp|A2AX52|CO6A4_MOUSE

Collagen alpha−4(VI) chain

GN=Col6a4

Med H1/2: 5.6 days

Q5 H1/2: 3.8 days

Q95 H1/2: 8.7 days

reliable model fit: yes

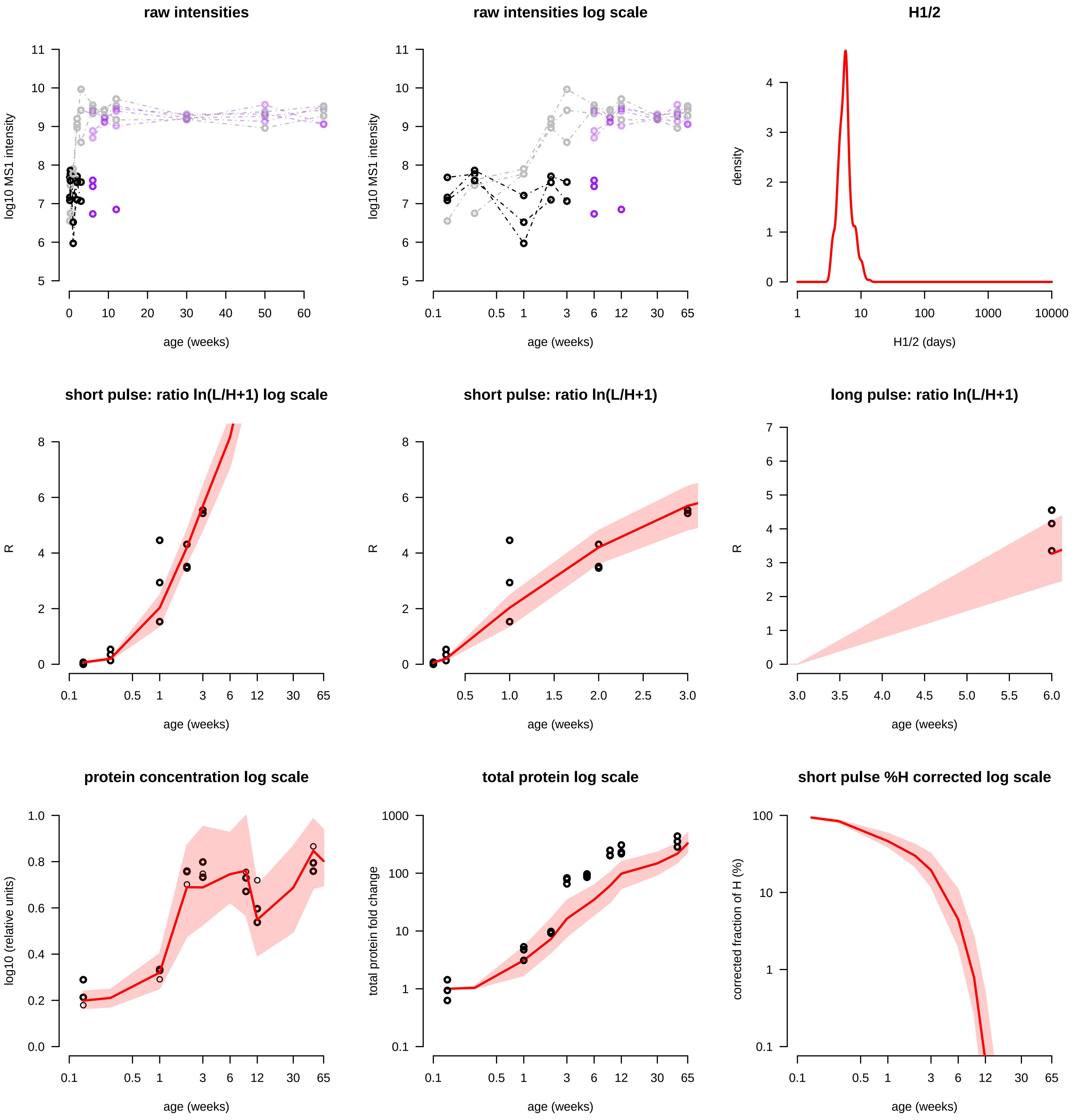

Q9Z0J0

>sp|Q9Z0J0|NPC2_MOUSE

NPC intracellular cholesterol transporter 2

GN=Npc2

Med H1/2: 7.6 days

Q5 H1/2: 5.6 days

Q95 H1/2: 10.3 days

reliable model fit: yes

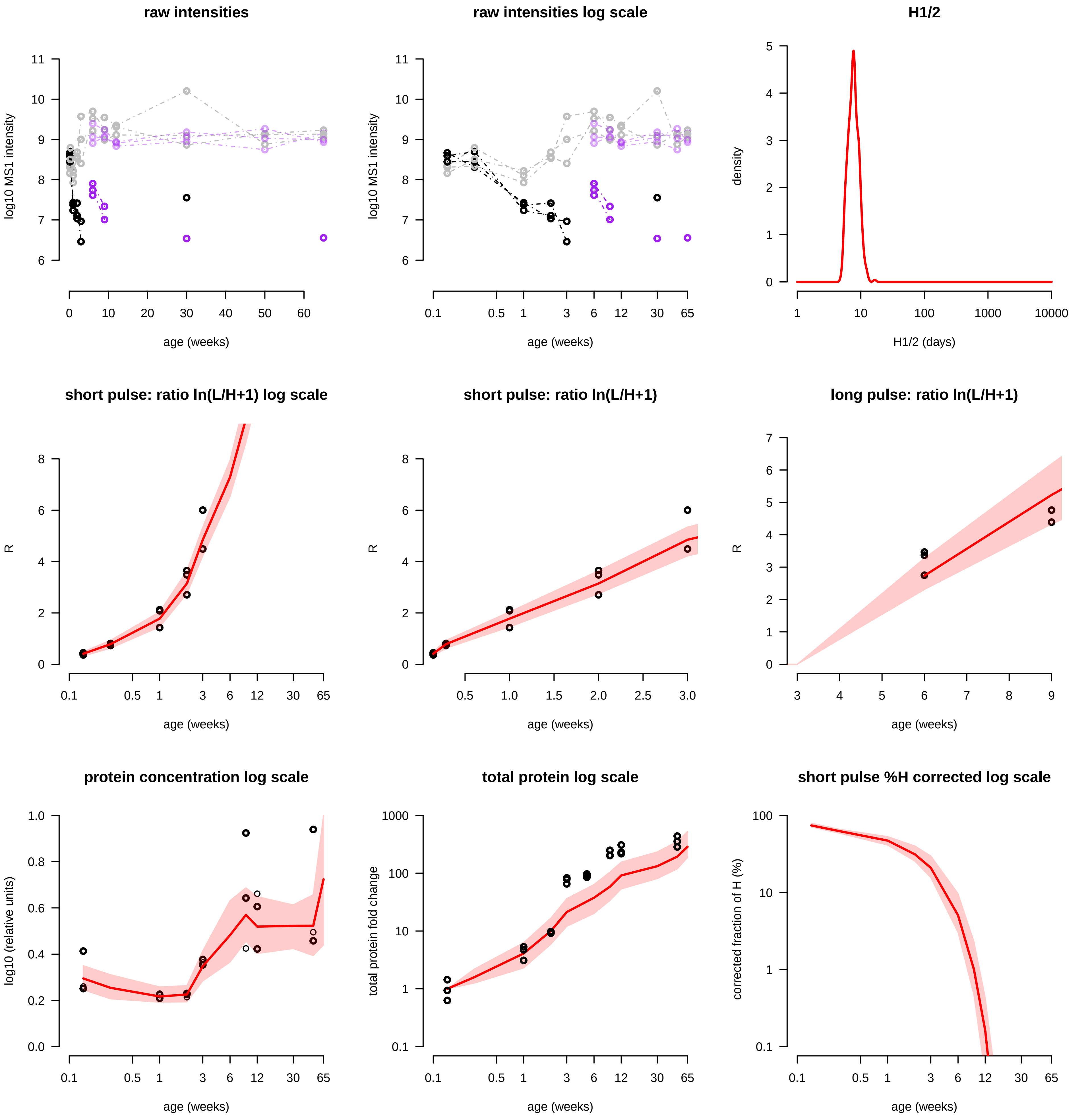

P53810

>sp|P53810|PIPNA_MOUSE

Phosphatidylinositol transfer protein alpha isoform

GN=Pitpna

Med H1/2: 181.1 days

Q5 H1/2: 101 days

Q95 H1/2: 705.9 days

reliable model fit: yes

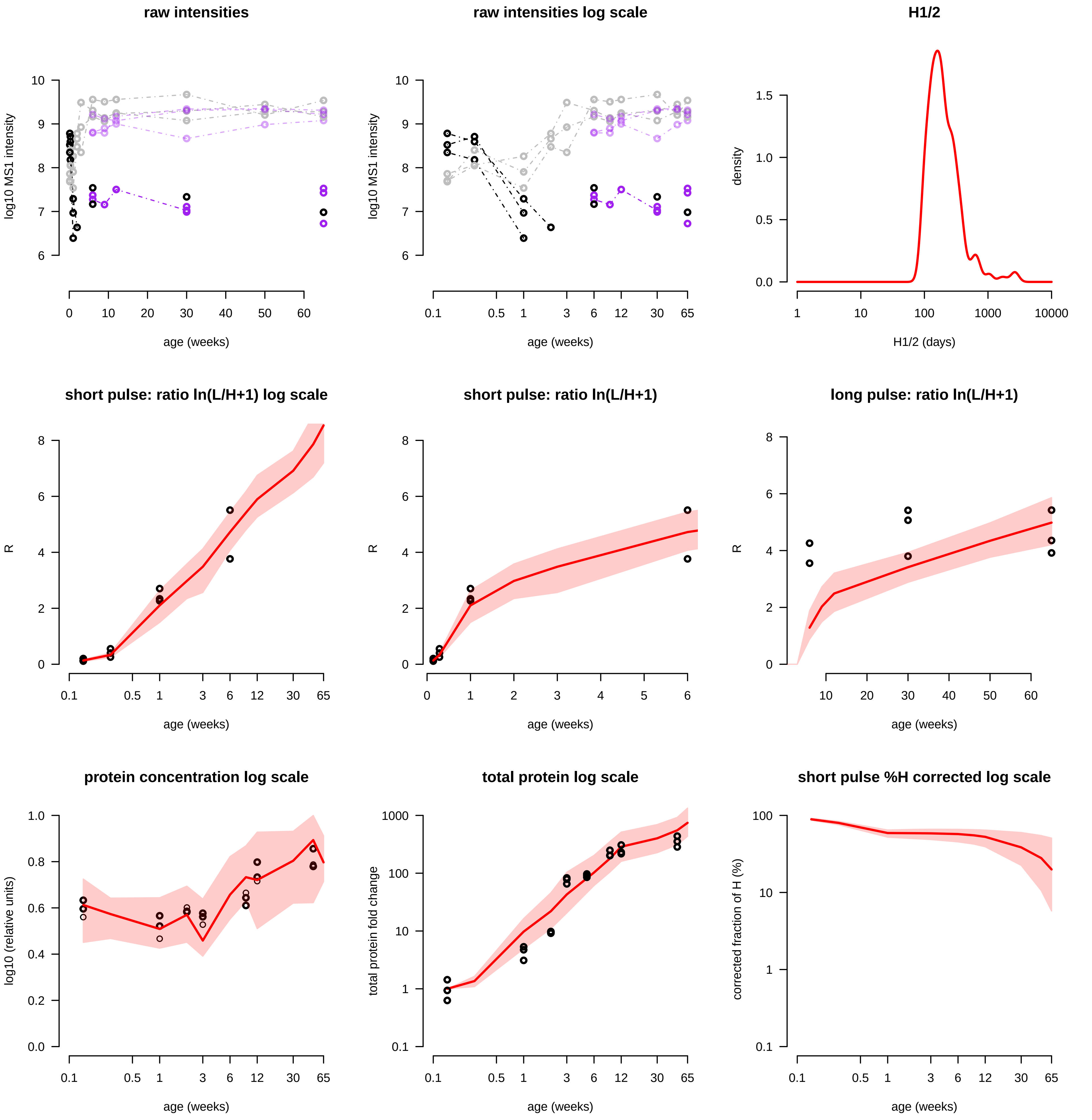

O09131

>sp|O09131|GSTO1_MOUSE

Glutathione S−transferase omega−1

GN=Gsto1

Med H1/2: 1758.6 days

Q5 H1/2: 437.9 days

Q95 H1/2: 39287.2 days

reliable model fit: no

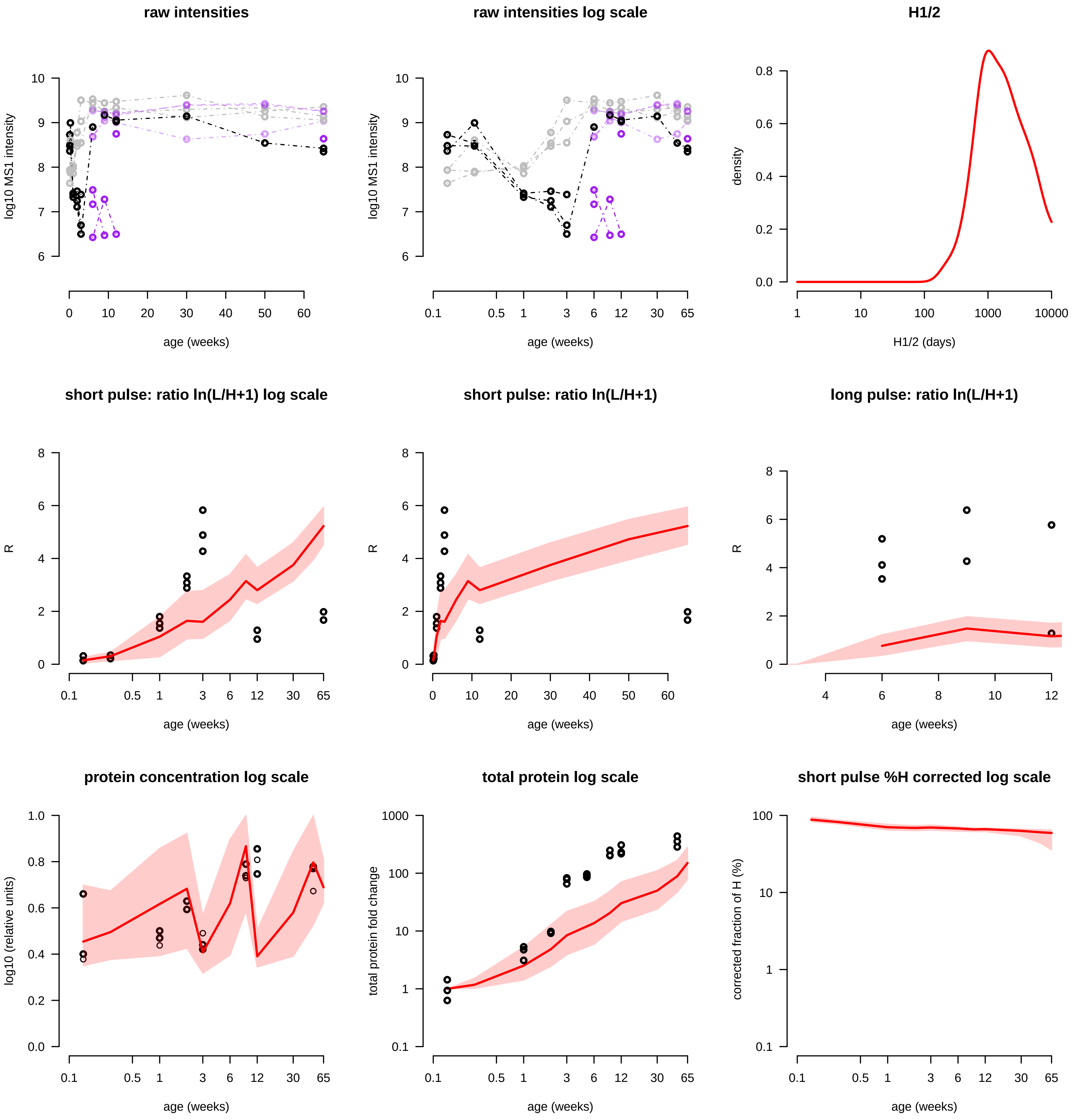

P70372

>sp|P70372|ELAV1_MOUSE

ELAV−like protein 1

GN=Elavl1

Med H1/2: 6.1 days

Q5 H1/2: 5.5 days

Q95 H1/2: 7 days

reliable model fit: yes

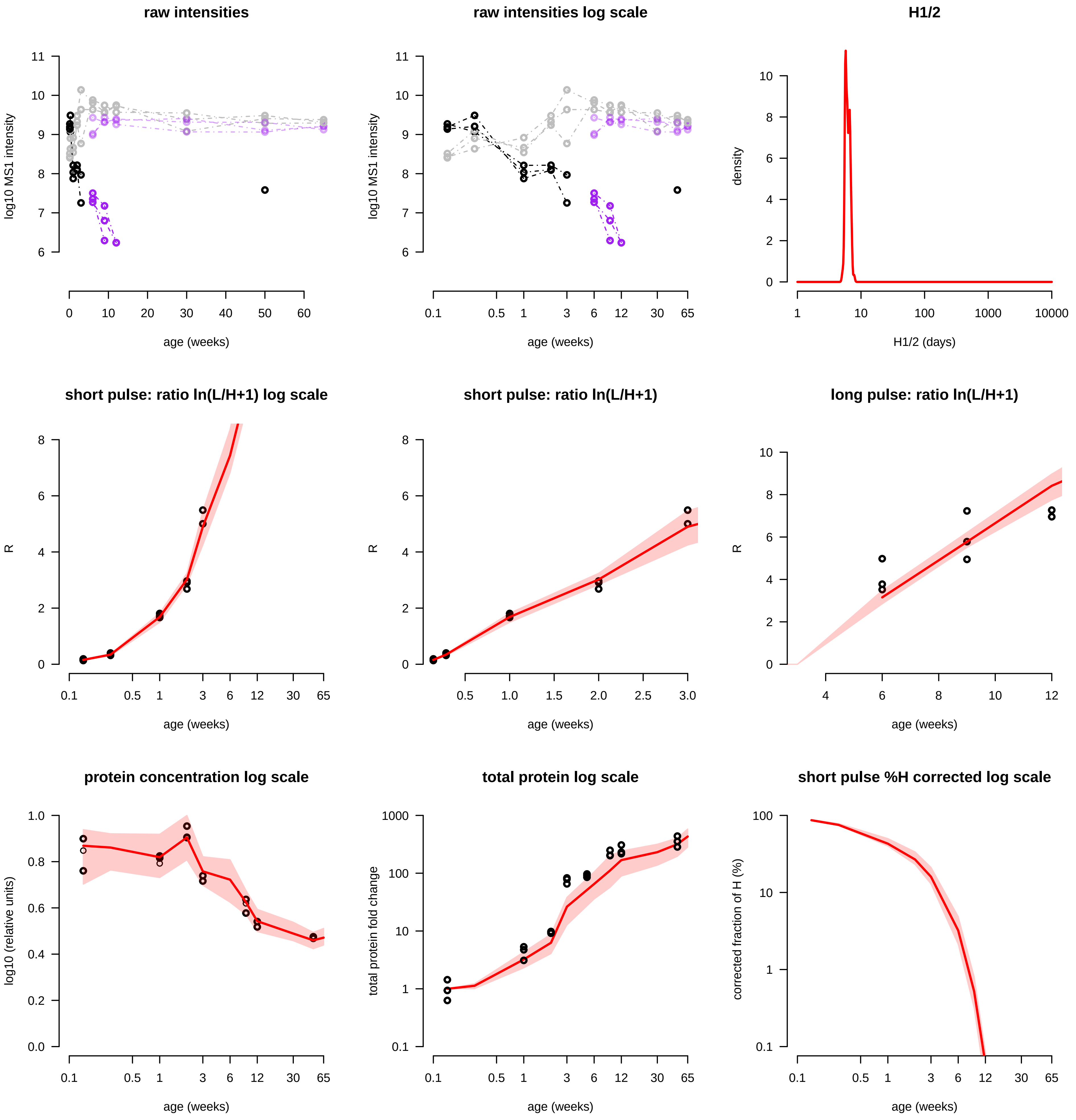

Q8BVI4

>sp|Q8BVI4|DHPR_MOUSE

Dihydropteridine reductase

GN=Qdpr

Med H1/2: 7.3 days

Q5 H1/2: 6.1 days

Q95 H1/2: 9.3 days

reliable model fit: yes

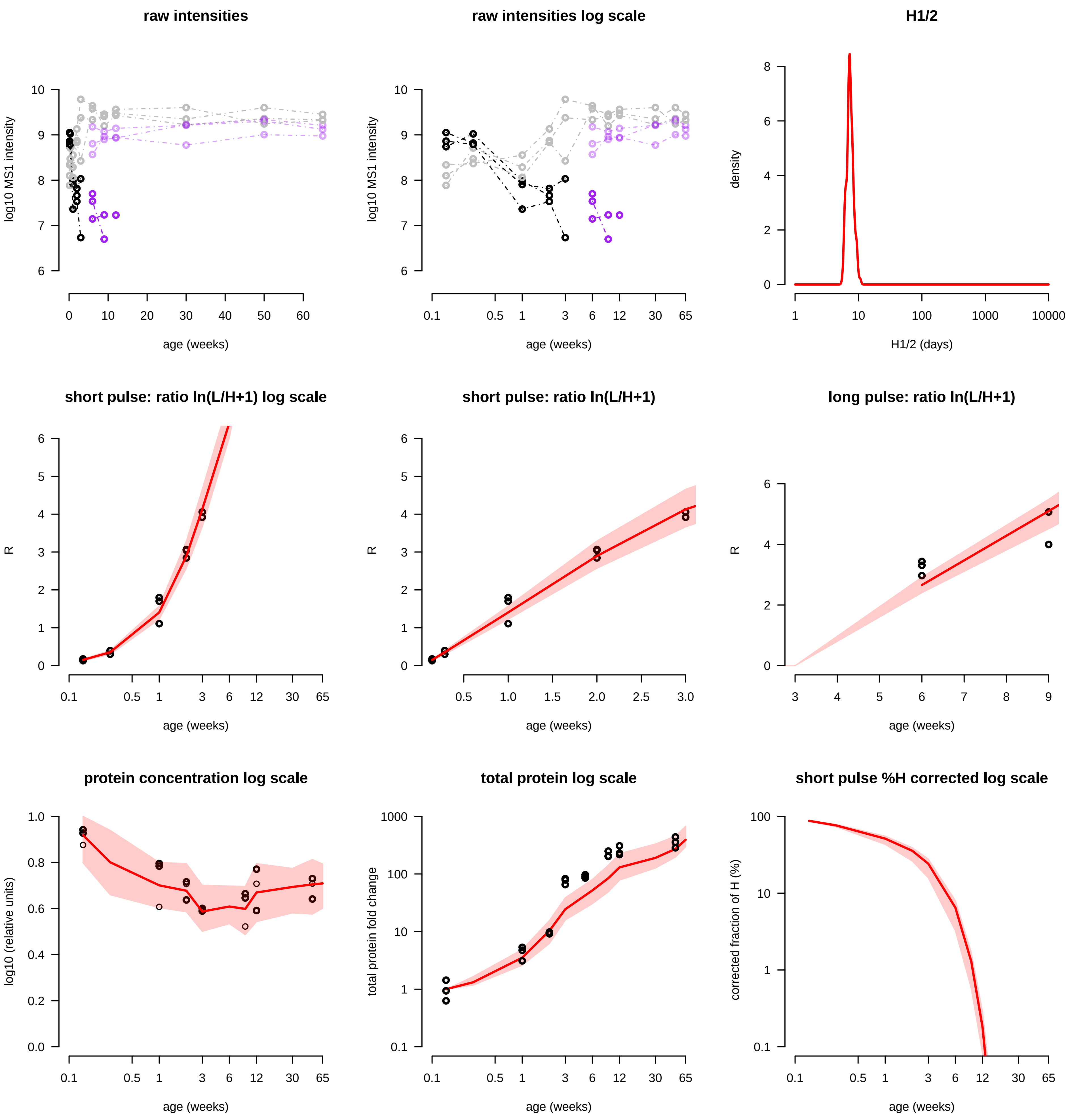

Q8K1M6−3

>sp|Q8K1M6−3|DNM1L_MOUSE

Isoform 3 of Dynamin−1−like protein

GN=Dnm1l

Med H1/2: 4.3 days

Q5 H1/2: 3.6 days

Q95 H1/2: 5.3 days

reliable model fit: yes

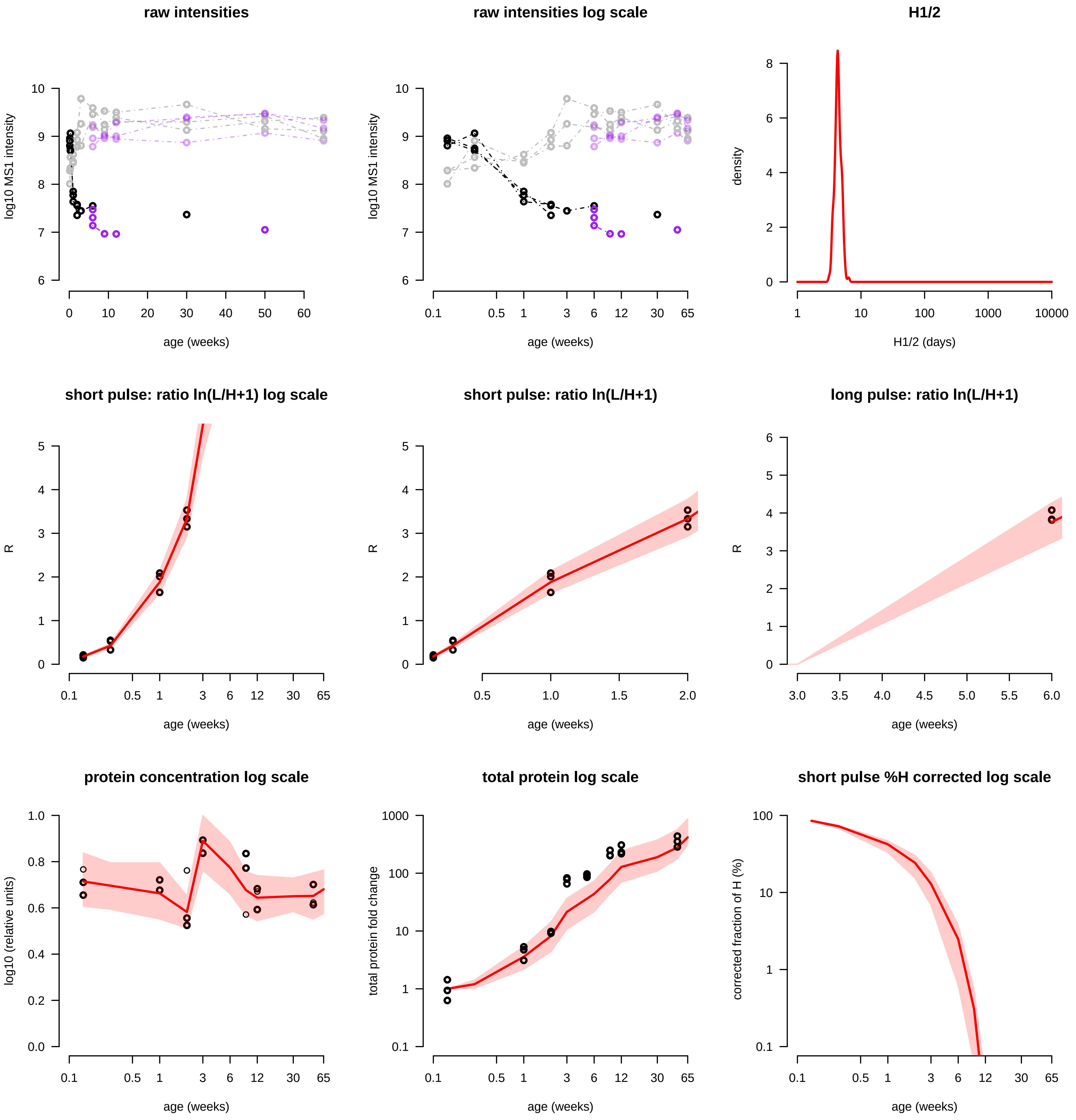

P06909

>sp|P06909|CFAH_MOUSE

Complement factor H

GN=Cfh

Med H1/2: 3.7 days

Q5 H1/2: 2.7 days

Q95 H1/2: 5 days

reliable model fit: yes

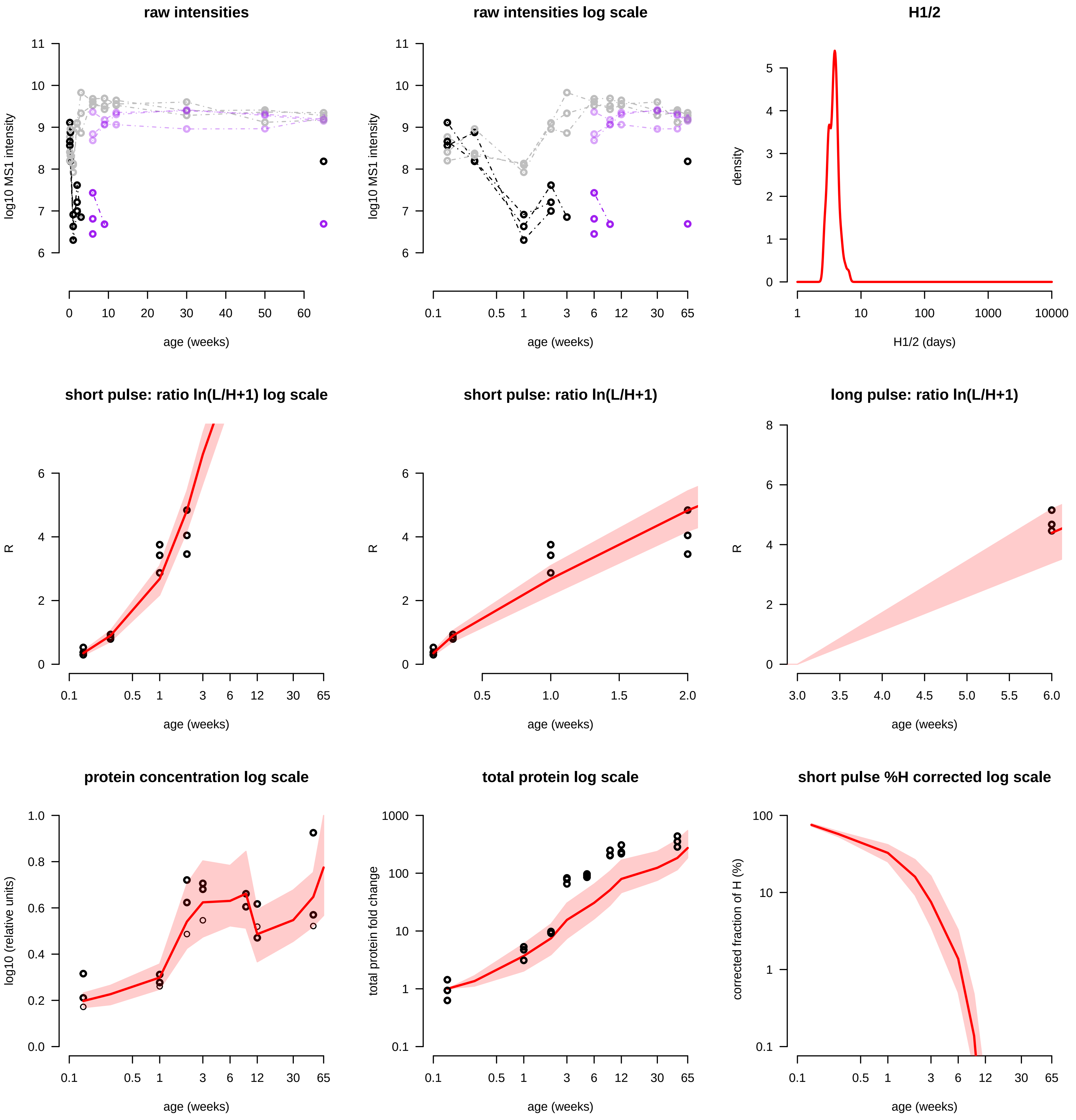

Q9QYG0−2

>sp|Q9QYG0−2|NDRG2_MOUSE

Isoform 2 of Protein NDRG2

GN=Ndrg2

Med H1/2: 5.6 days

Q5 H1/2: 4 days

Q95 H1/2: 9.1 days

reliable model fit: yes

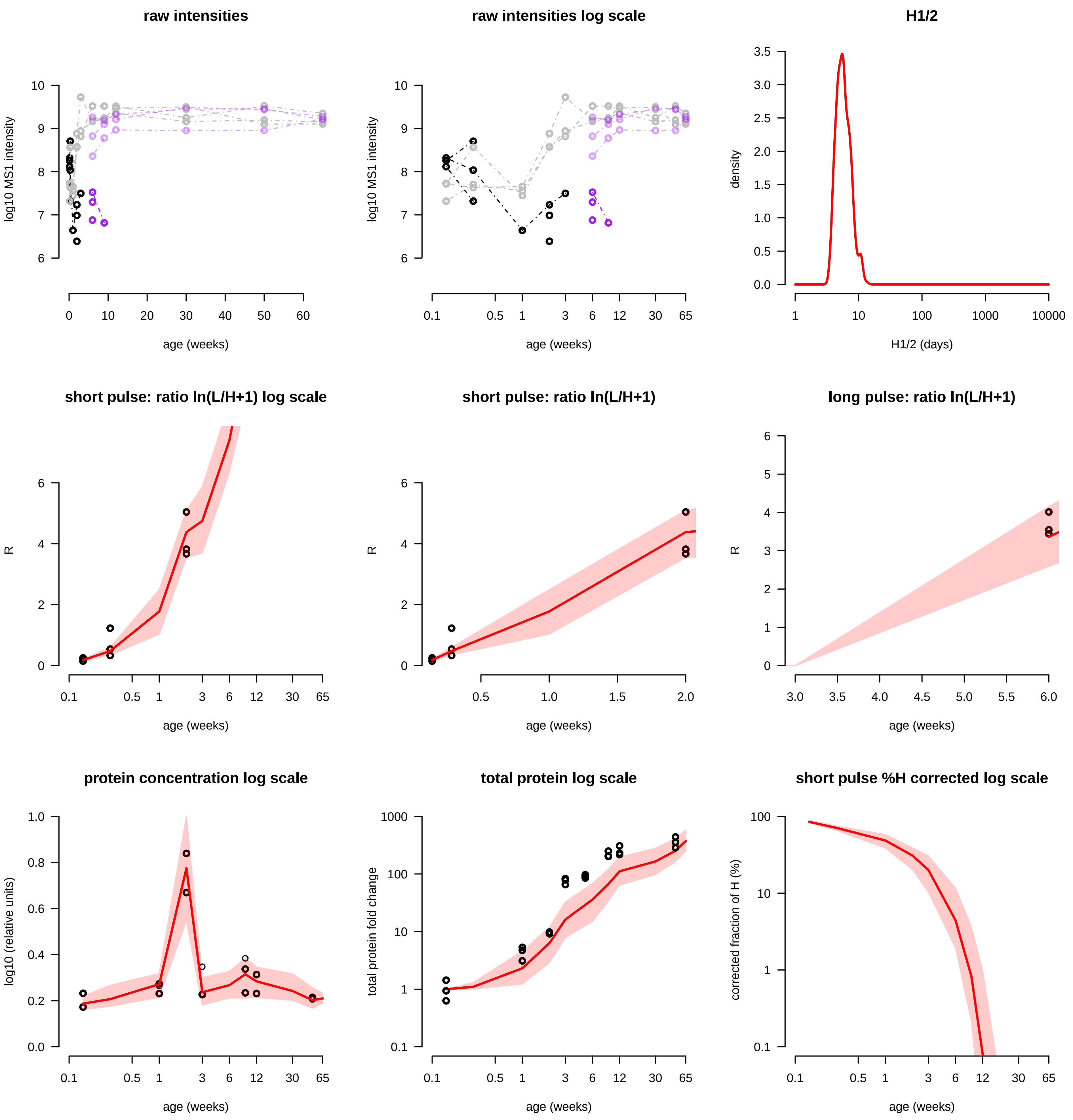

Q8BP47

>sp|Q8BP47|SYNC_MOUSE

Asparagine−−tRNA ligase, cytoplasmic

GN=NARS1

Med H1/2: 4.9 days

Q5 H1/2: 4 days

Q95 H1/2: 6.4 days

reliable model fit: yes

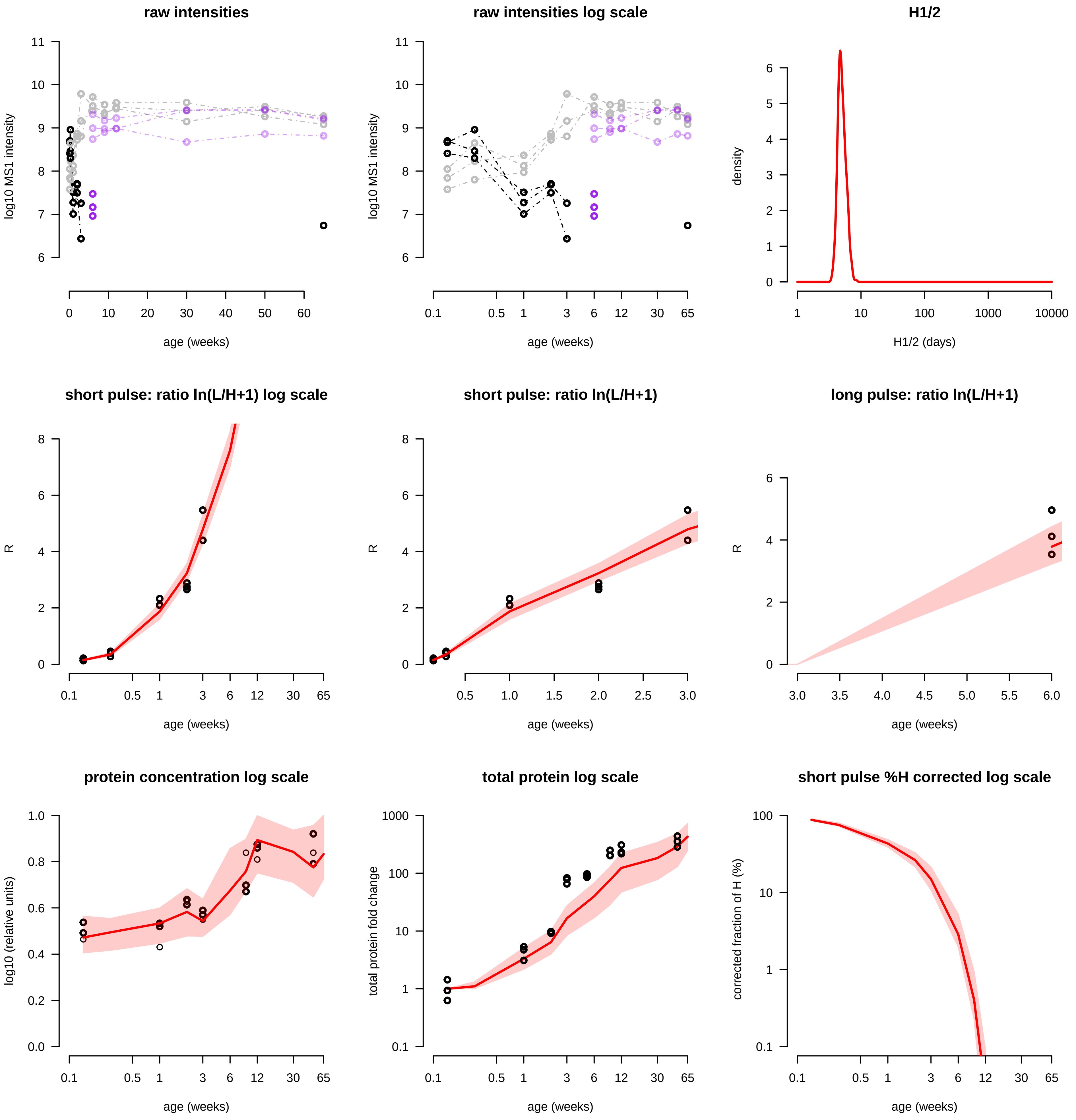

Q9Z0E6

>sp|Q9Z0E6|GBP2_MOUSE

Guanylate−binding protein 2

GN=Gbp2

Med H1/2: 6.2 days

Q5 H1/2: 4.7 days

Q95 H1/2: 8.9 days

reliable model fit: yes

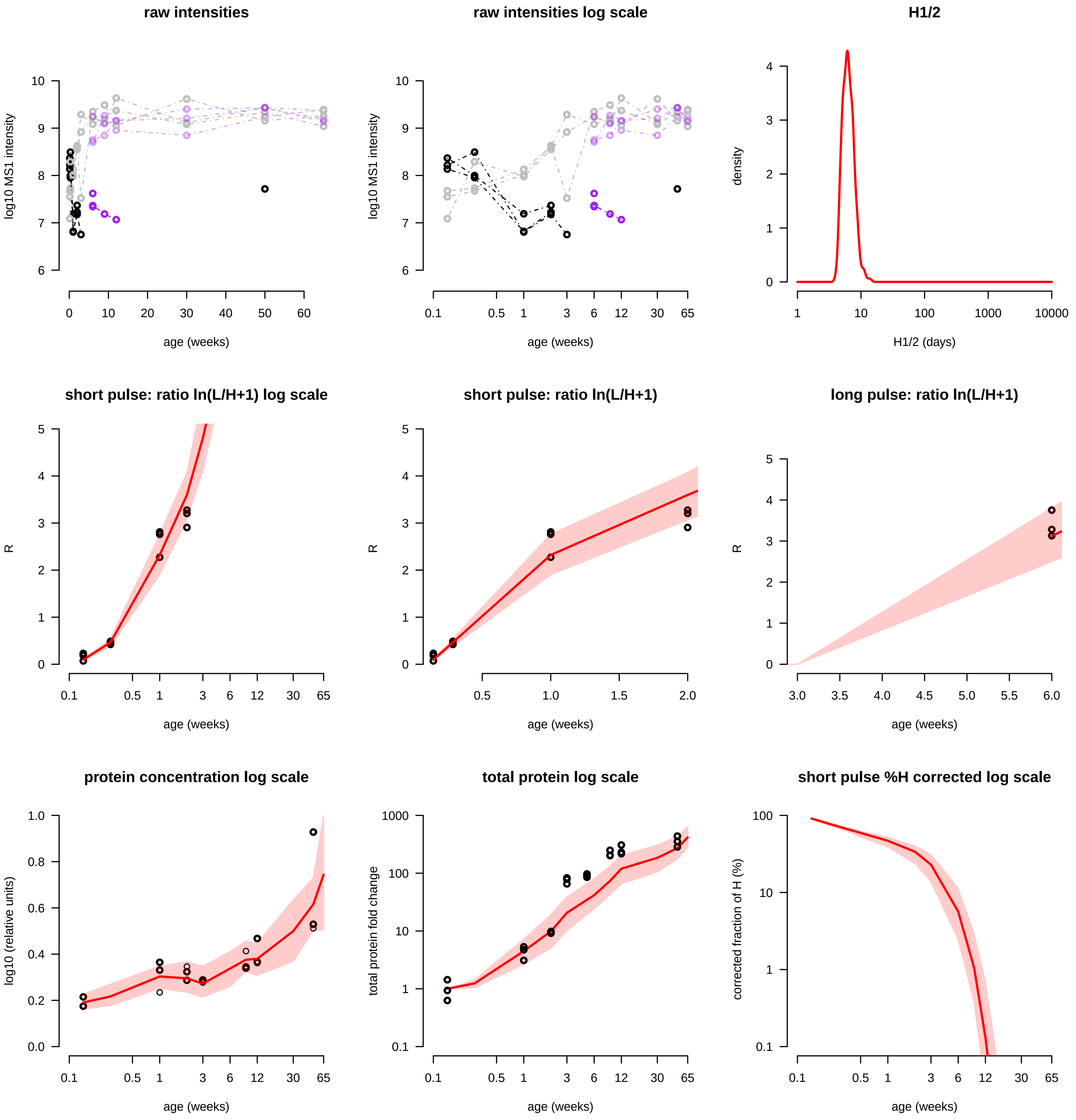

Q8QZY1

>sp|Q8QZY1|EIF3L_MOUSE

Eukaryotic translation initiation factor 3 subunit L

GN=Eif3l

Med H1/2: 127.6 days

Q5 H1/2: 50.4 days

Q95 H1/2: 935.3 days

reliable model fit: no

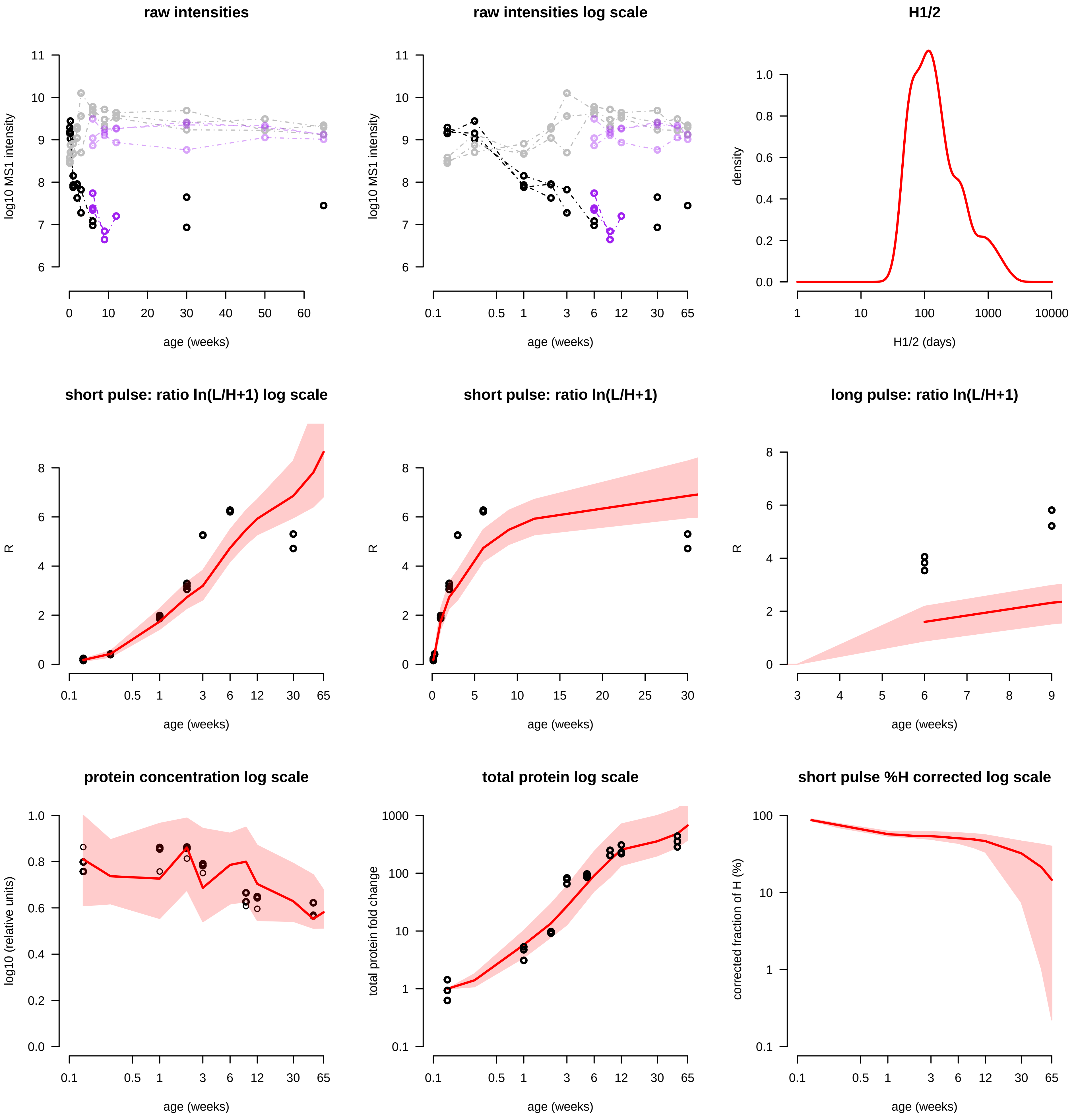

P11438

>sp|P11438|LAMP1_MOUSE

Lysosome−associated membrane glycoprotein 1

GN=Lamp1

Med H1/2: 8.2 days

Q5 H1/2: 6.8 days

Q95 H1/2: 11 days

reliable model fit: yes

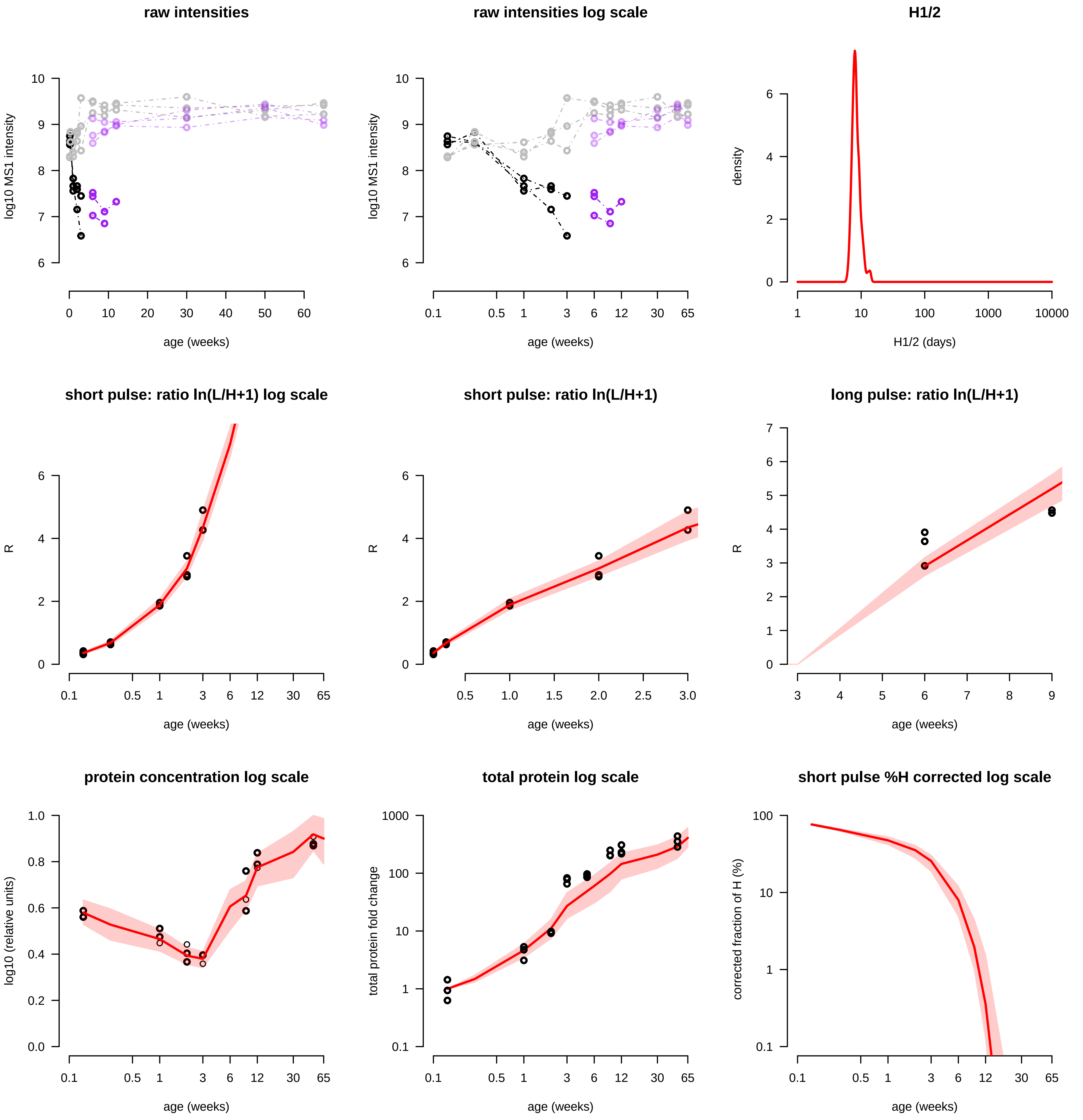

Q8JZN5

>sp|Q8JZN5|ACAD9_MOUSE

Complex I assembly factor ACAD9, mitochondrial

GN=Acad9

Med H1/2: 4.7 days

Q5 H1/2: 3.7 days

Q95 H1/2: 5.7 days

reliable model fit: yes

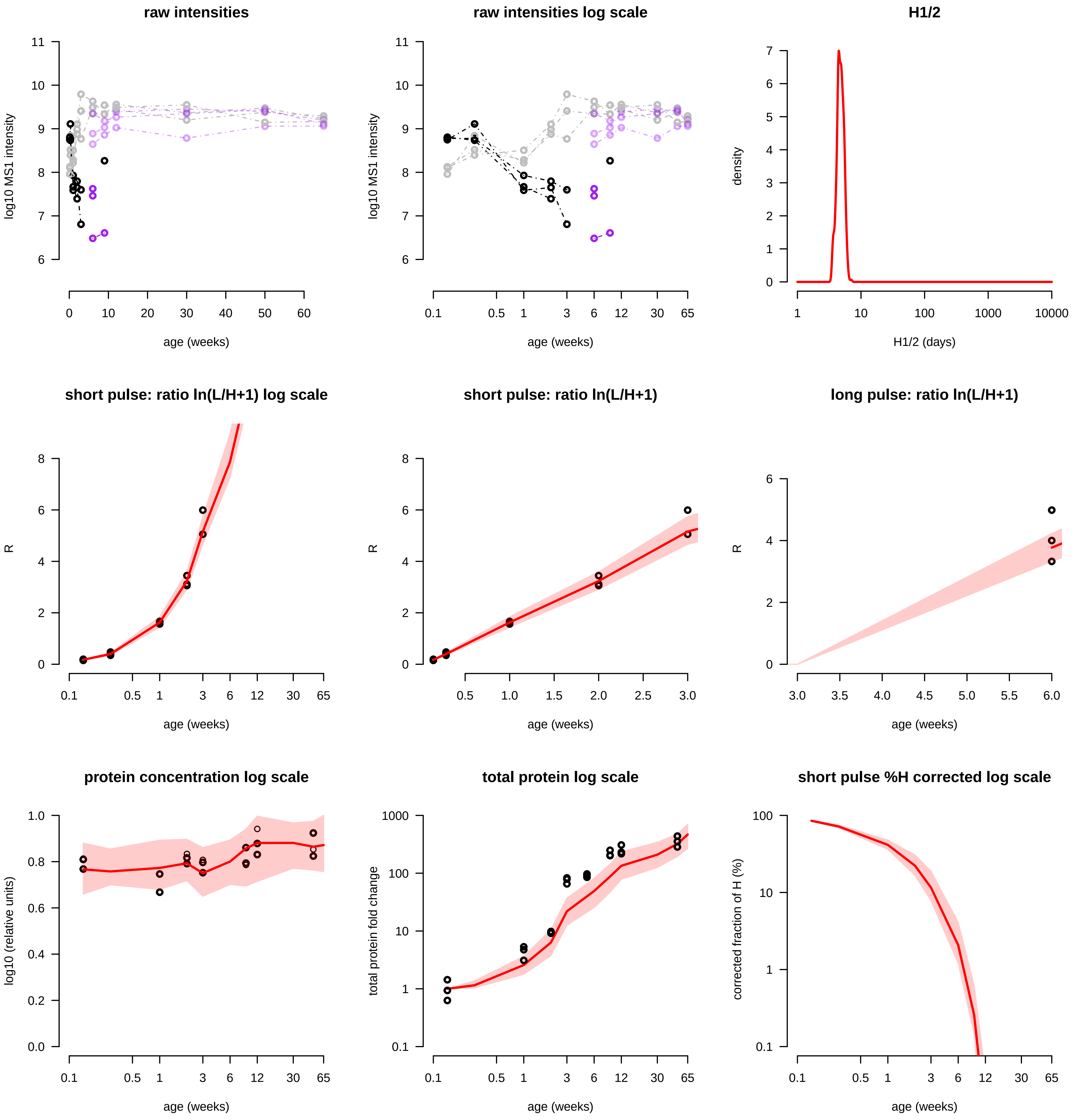

Q9CPY7;Q9CPY7−2

>sp|Q9CPY7|AMPL_MOUSE

Cytosol aminopeptidase

GN=Lap3

Med H1/2: 8.3 days

Q5 H1/2: 6.9 days

Q95 H1/2: 10.7 days

reliable model fit: yes

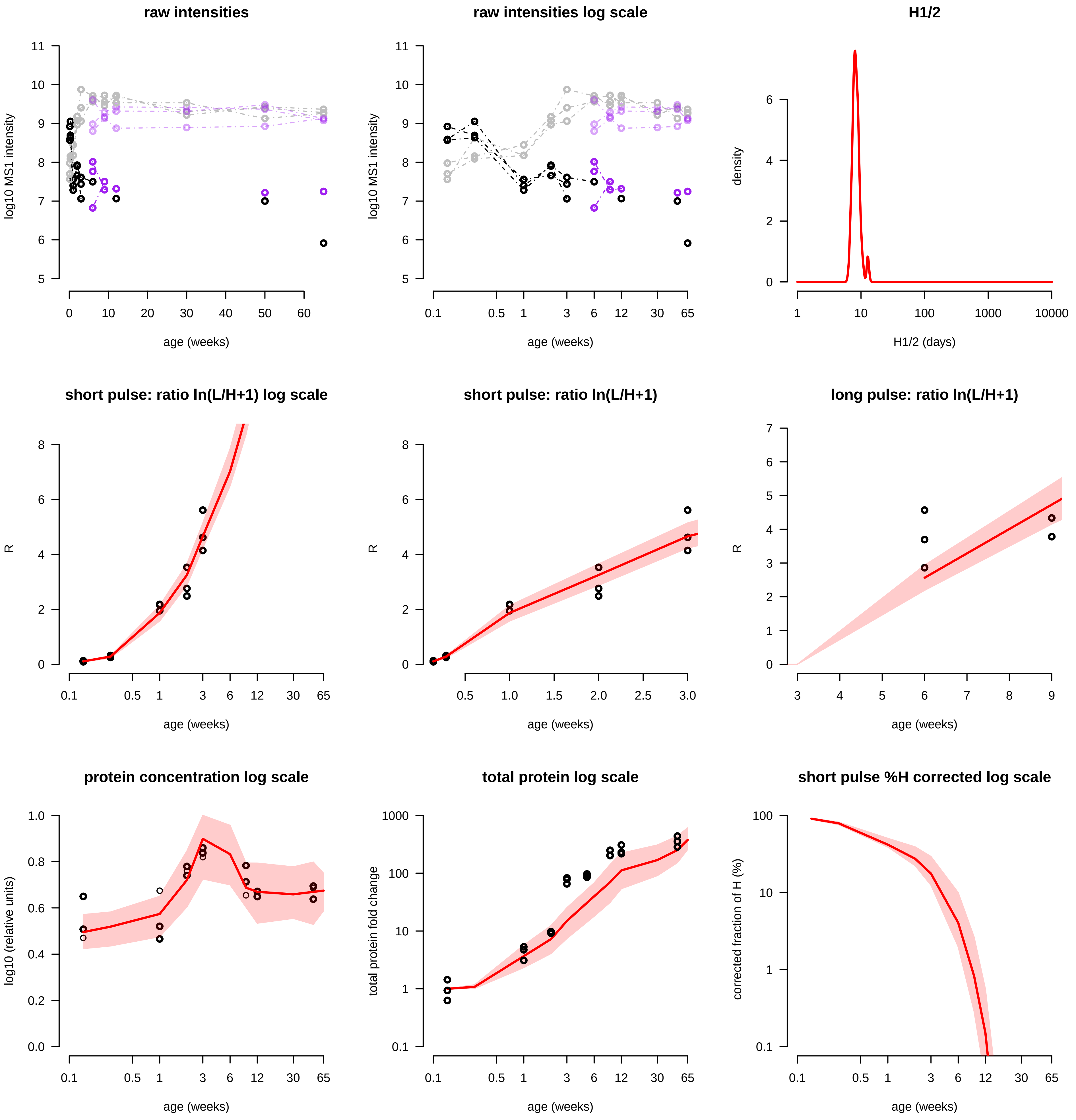

P56382

>sp|P56382|ATP5E_MOUSE

ATP synthase subunit epsilon, mitochondrial

GN=Atp5f1e

Med H1/2: 19.2 days

Q5 H1/2: 13.9 days

Q95 H1/2: 42.5 days

reliable model fit: yes

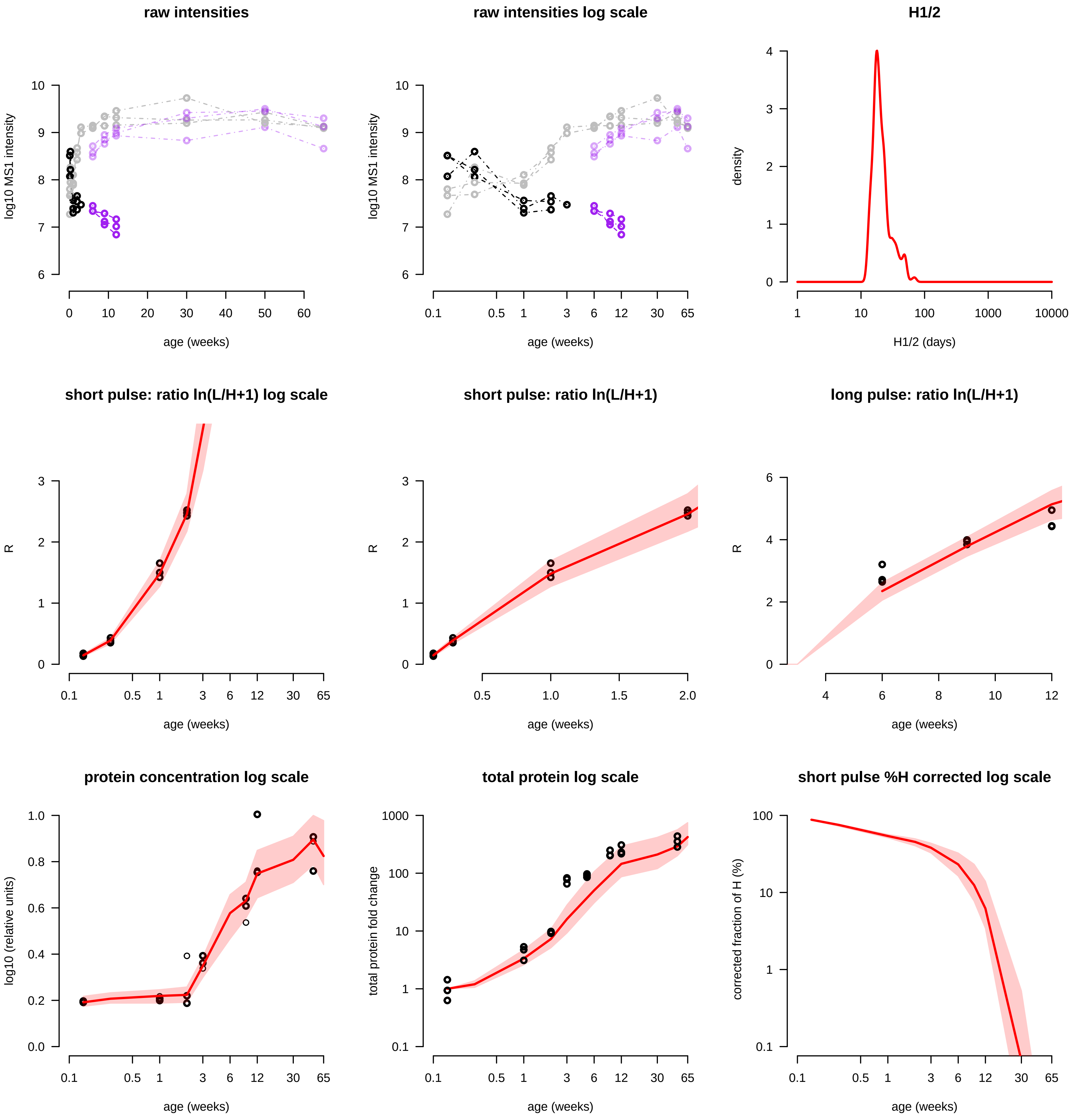

P60122

>sp|P60122|RUVB1_MOUSE

RuvB−like 1

GN=Ruvbl1

Med H1/2: 7.5 days

Q5 H1/2: 6.1 days

Q95 H1/2: 8.5 days

reliable model fit: yes

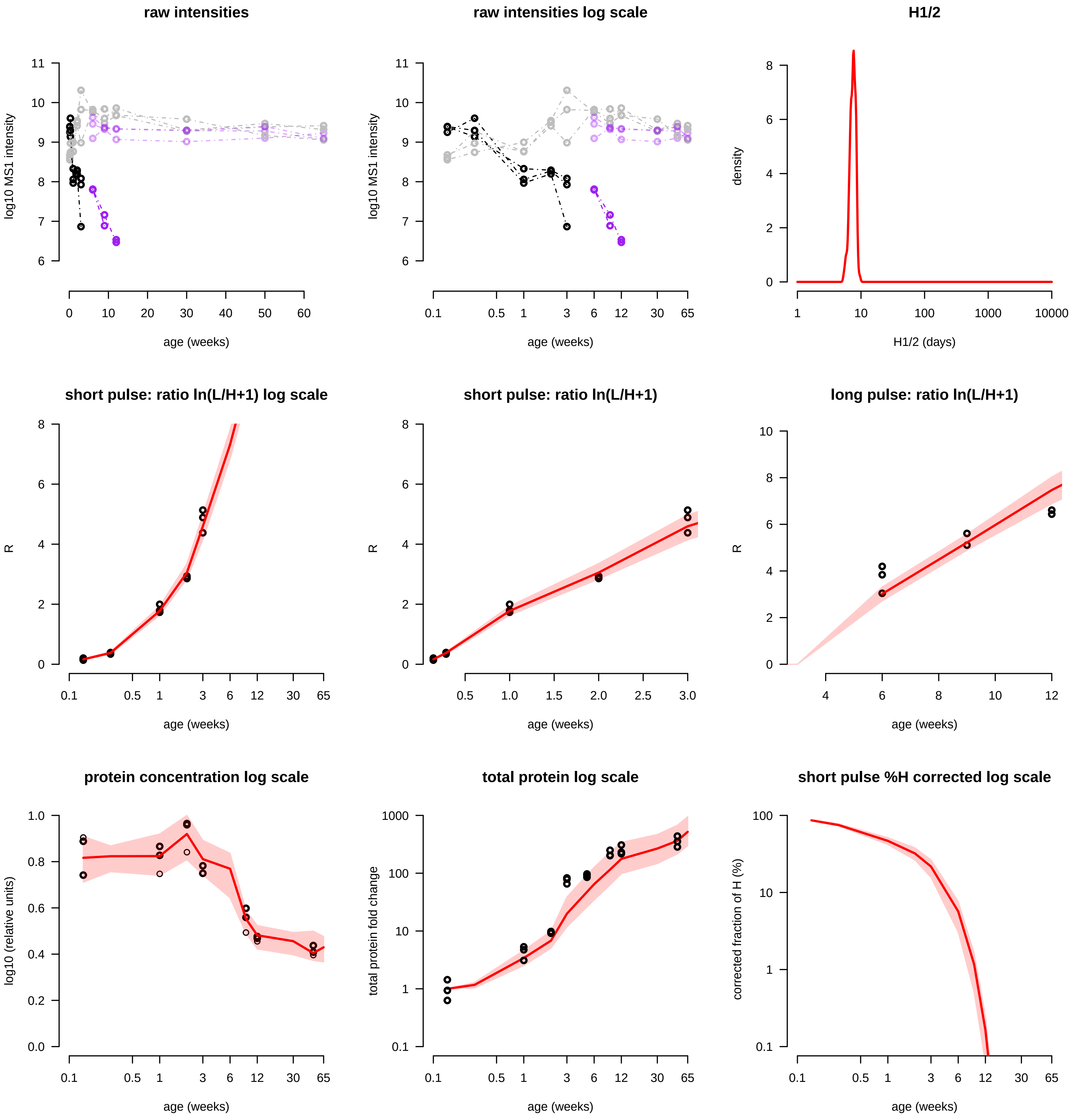

Q7TQI3

>sp|Q7TQI3|OTUB1_MOUSE

Ubiquitin thioesterase OTUB1

GN=Otub1

Med H1/2: 7.6 days

Q5 H1/2: 6.3 days

Q95 H1/2: 9.6 days

reliable model fit: yes

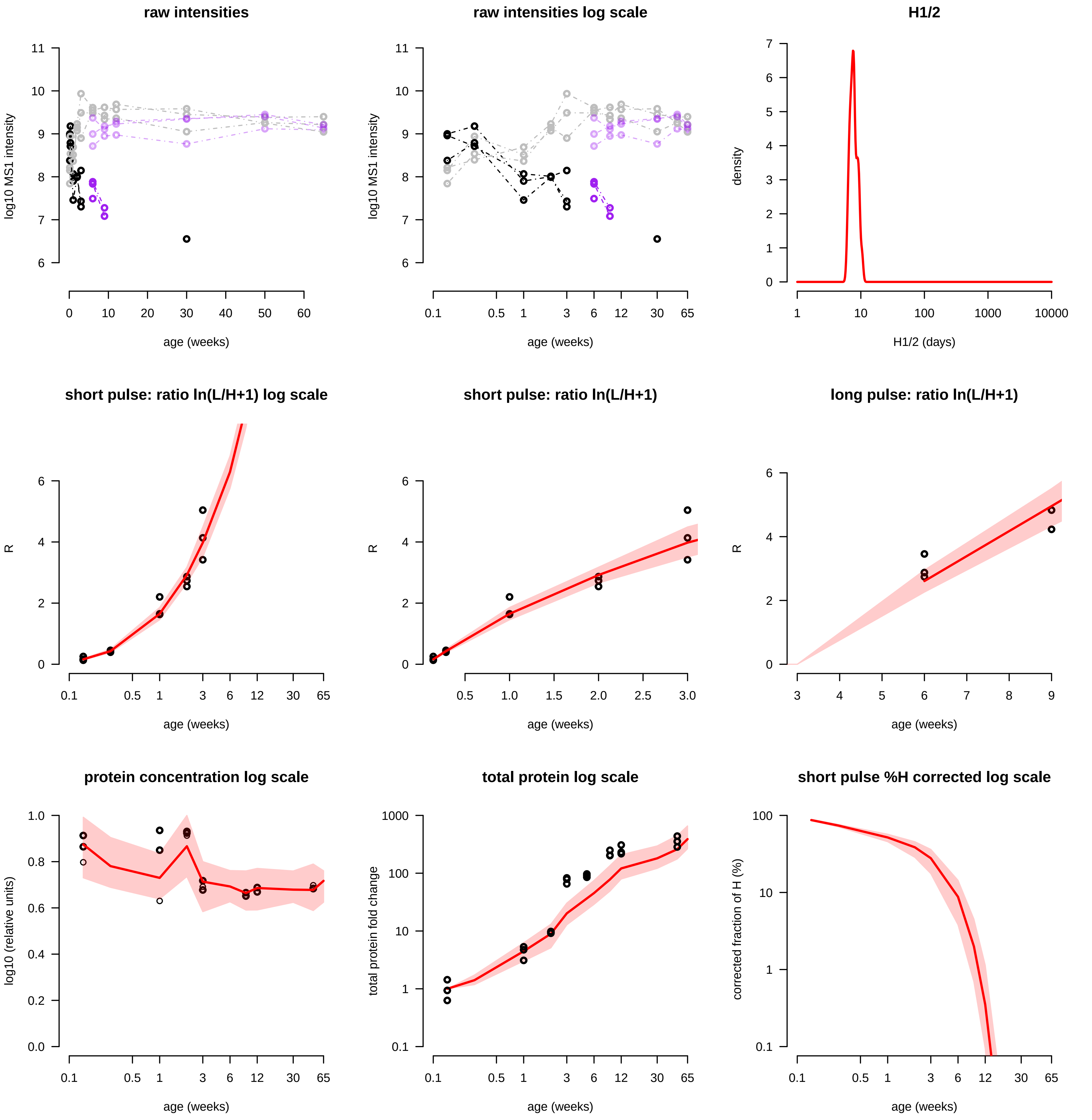

Q62446

>sp|Q62446|FKBP3_MOUSE

Peptidyl−prolyl cis−trans isomerase FKBP3

GN=Fkbp3

Med H1/2: 139.6 days

Q5 H1/2: 64.6 days

Q95 H1/2: 910.5 days

reliable model fit: no

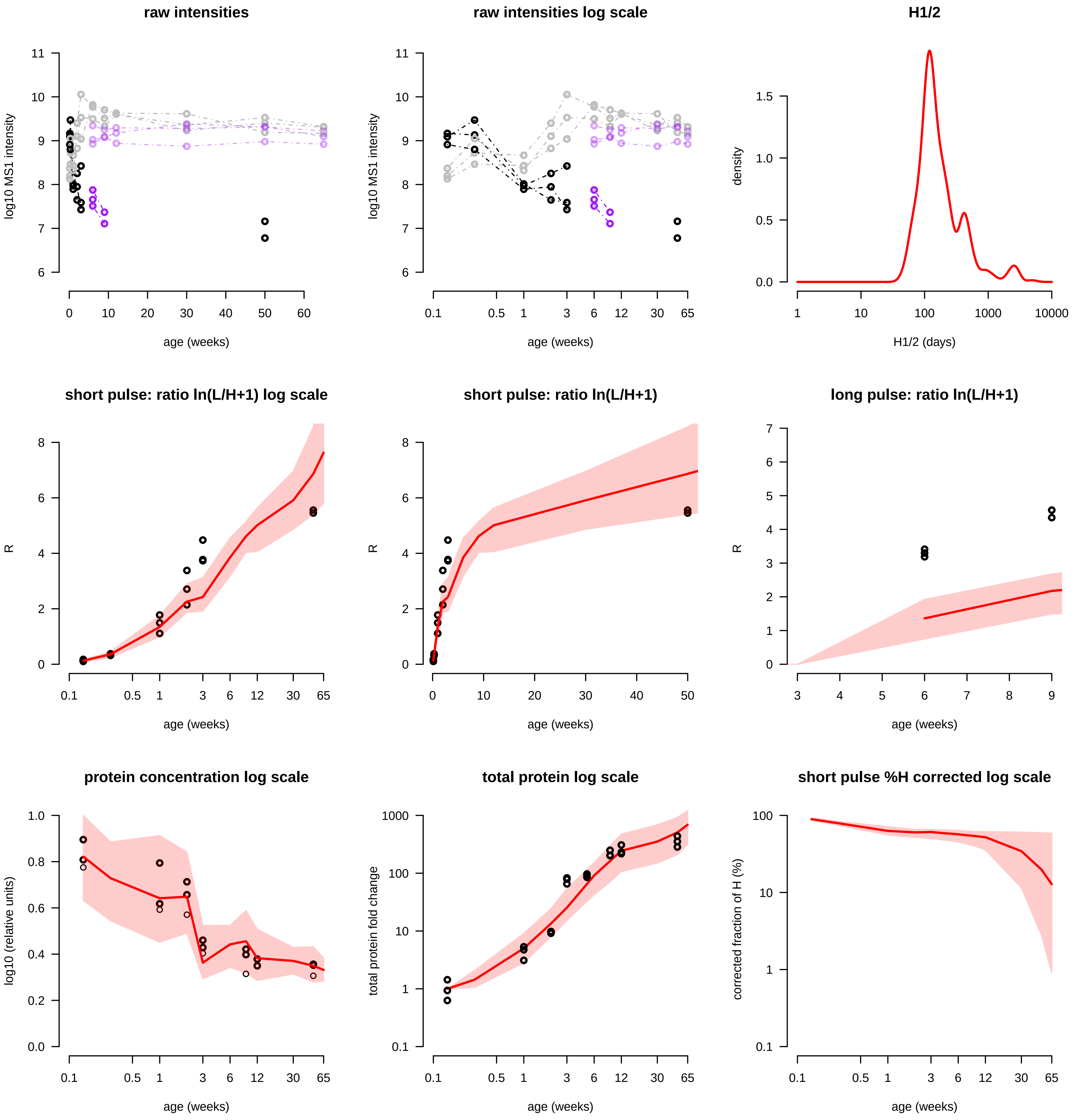

Q91WQ3

>sp|Q91WQ3|SYYC_MOUSE

Tyrosine−−tRNA ligase, cytoplasmic

GN=Yars1

Med H1/2: 15.8 days

Q5 H1/2: 10.5 days

Q95 H1/2: 28.2 days

reliable model fit: yes

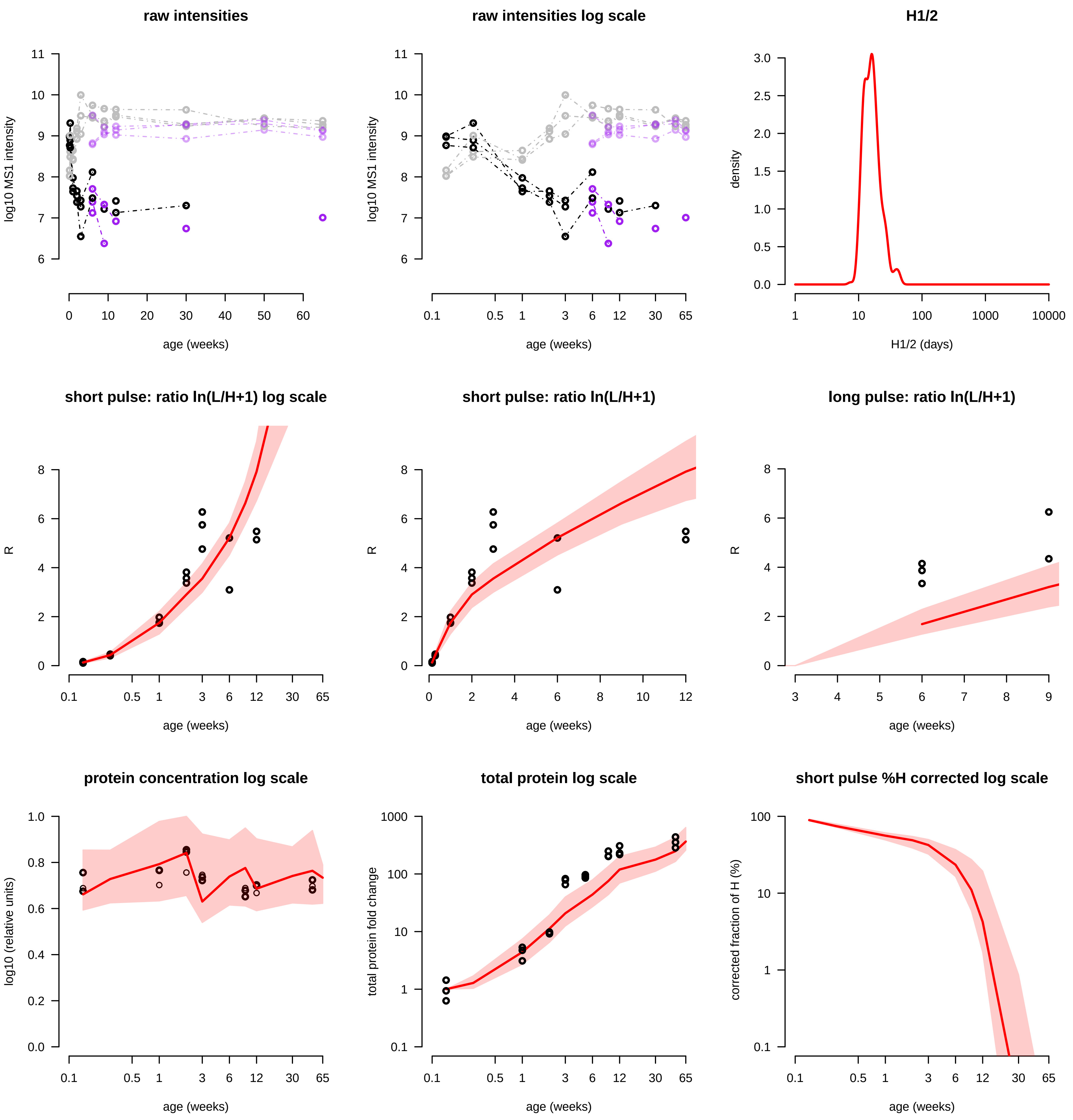

Q8R164

>sp|Q8R164|BPHL_MOUSE

Valacyclovir hydrolase

GN=Bphl

Med H1/2: 6.9 days

Q5 H1/2: 5.4 days

Q95 H1/2: 10.1 days

reliable model fit: yes

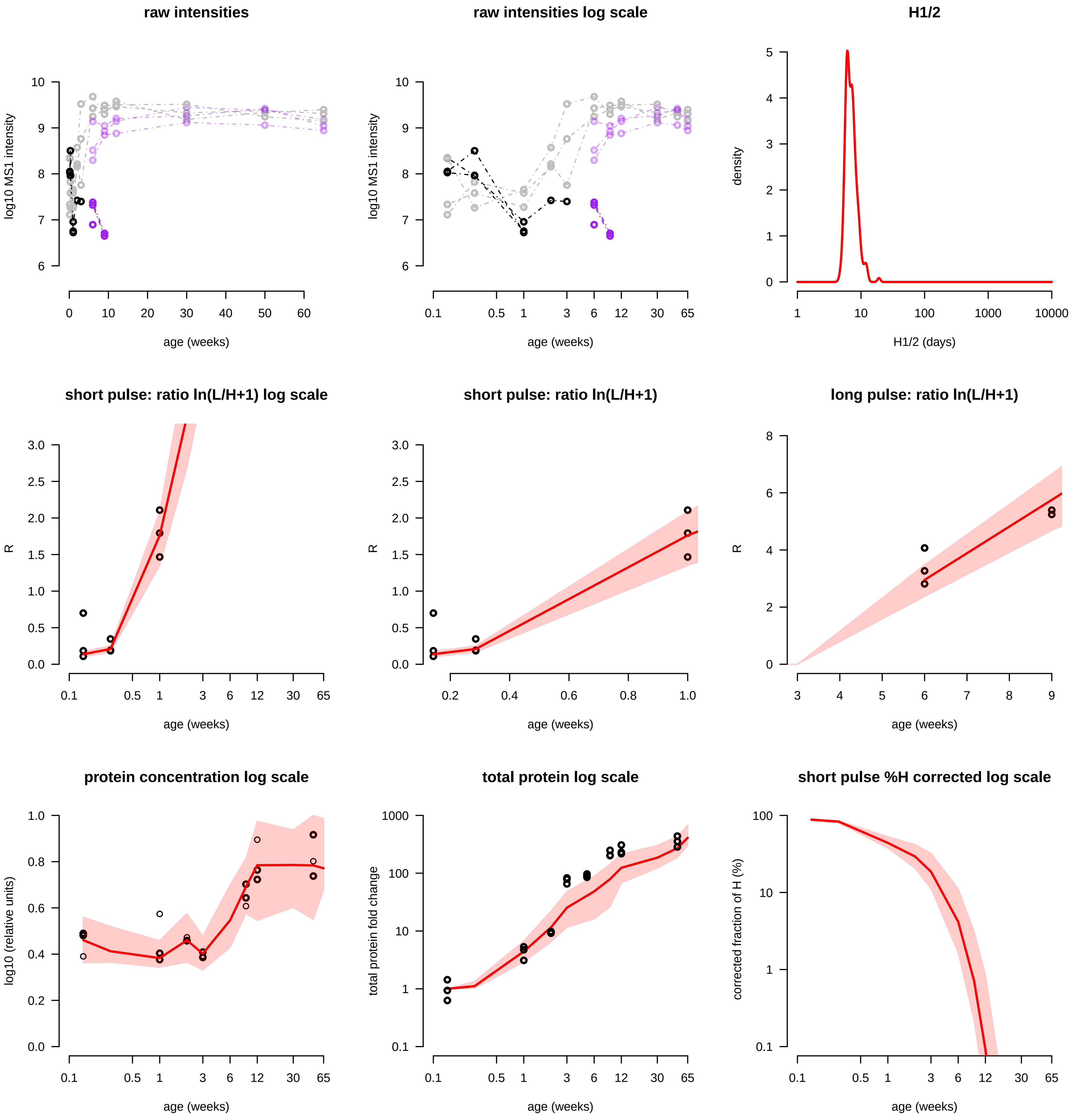

O35286

>sp|O35286|DHX15_MOUSE

Pre−mRNA−splicing factor ATP−dependent RNA helicase DHX15

GN=Dhx15

Med H1/2: 380.8 days

Q5 H1/2: 119.2 days

Q95 H1/2: 3152.6 days

reliable model fit: yes

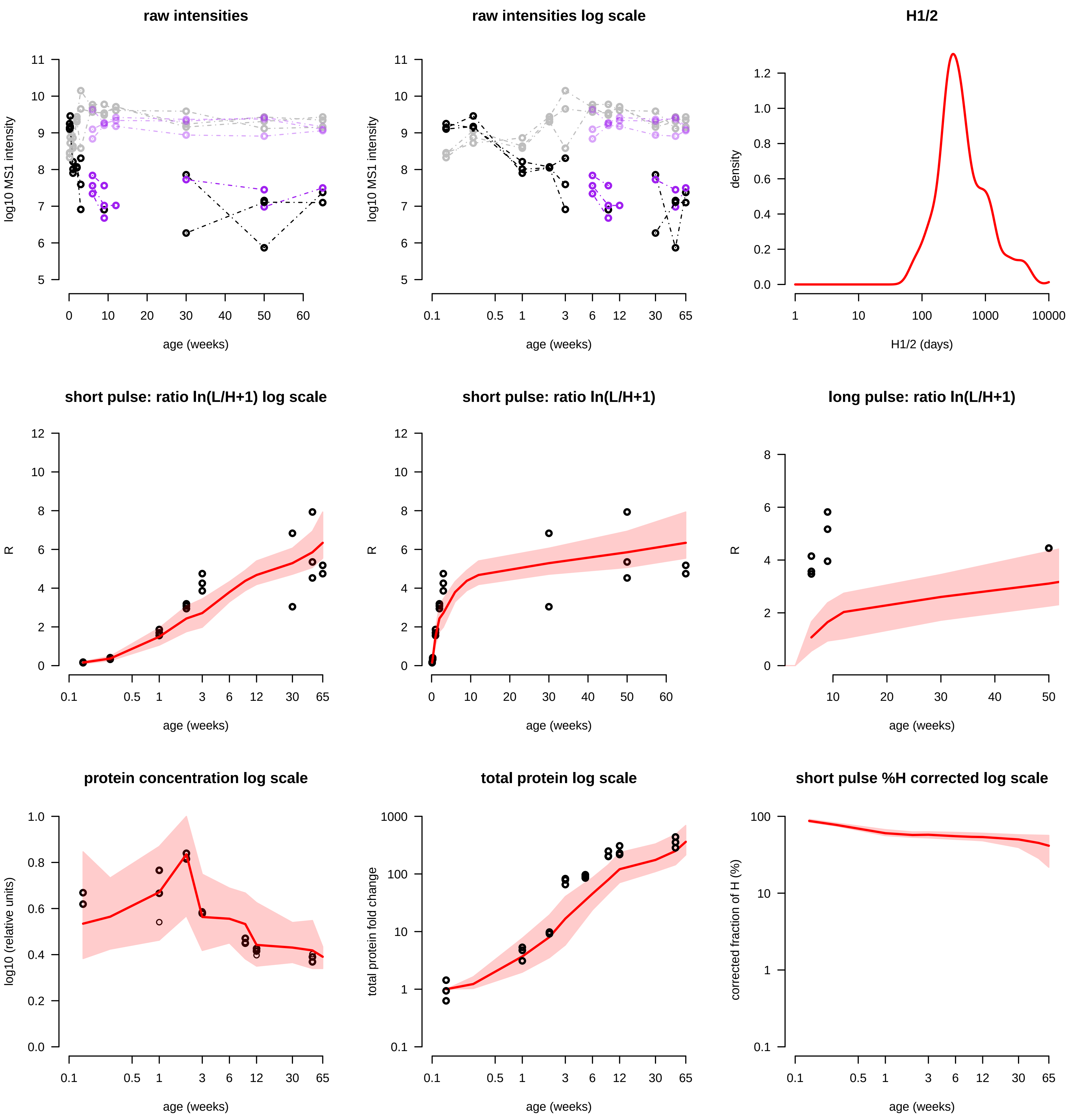

P97450

>sp|P97450|ATP5J_MOUSE

ATP synthase−coupling factor 6, mitochondrial

GN=Atp5pf

Med H1/2: 8.4 days

Q5   H1/2: 4.1 days

Q95 H1/2: 19.2 days

reliable model fit: no

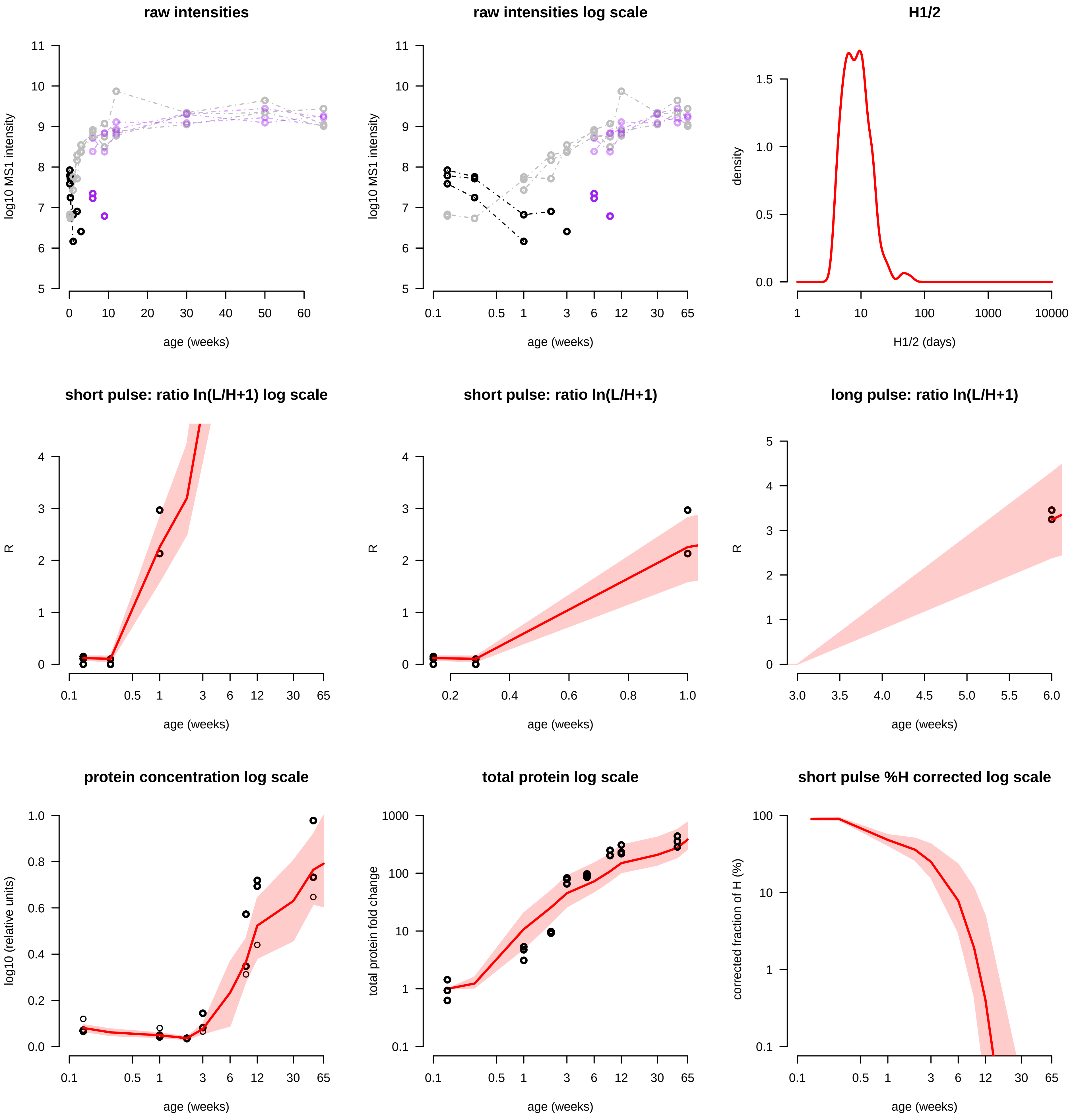

P70168

>sp|P70168|IMB1_MOUSE

Importin subunit beta−1

GN=Kpnb1

Med H1/2: 6.5 days

Q5 H1/2: 5.6 days

Q95 H1/2: 8.4 days

reliable model fit: yes

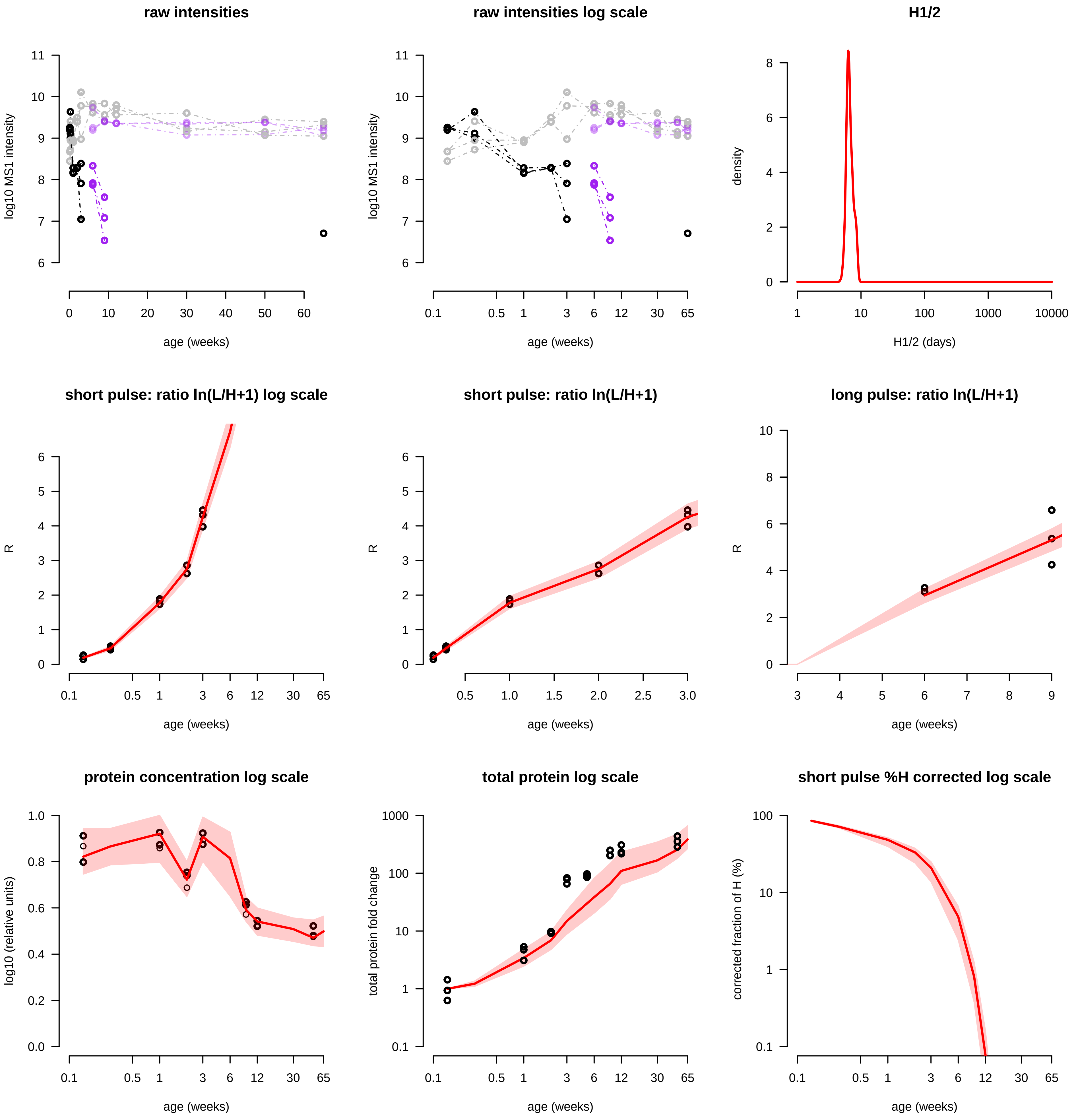

Q6PDQ2

>sp|Q6PDQ2|CHD4_MOUSE

Chromodomain−helicase−DNA−binding protein 4

GN=Chd4

Med H1/2: 25.9 days

Q5 H1/2: 19.2 days

Q95 H1/2: 39.3 days

reliable model fit: no

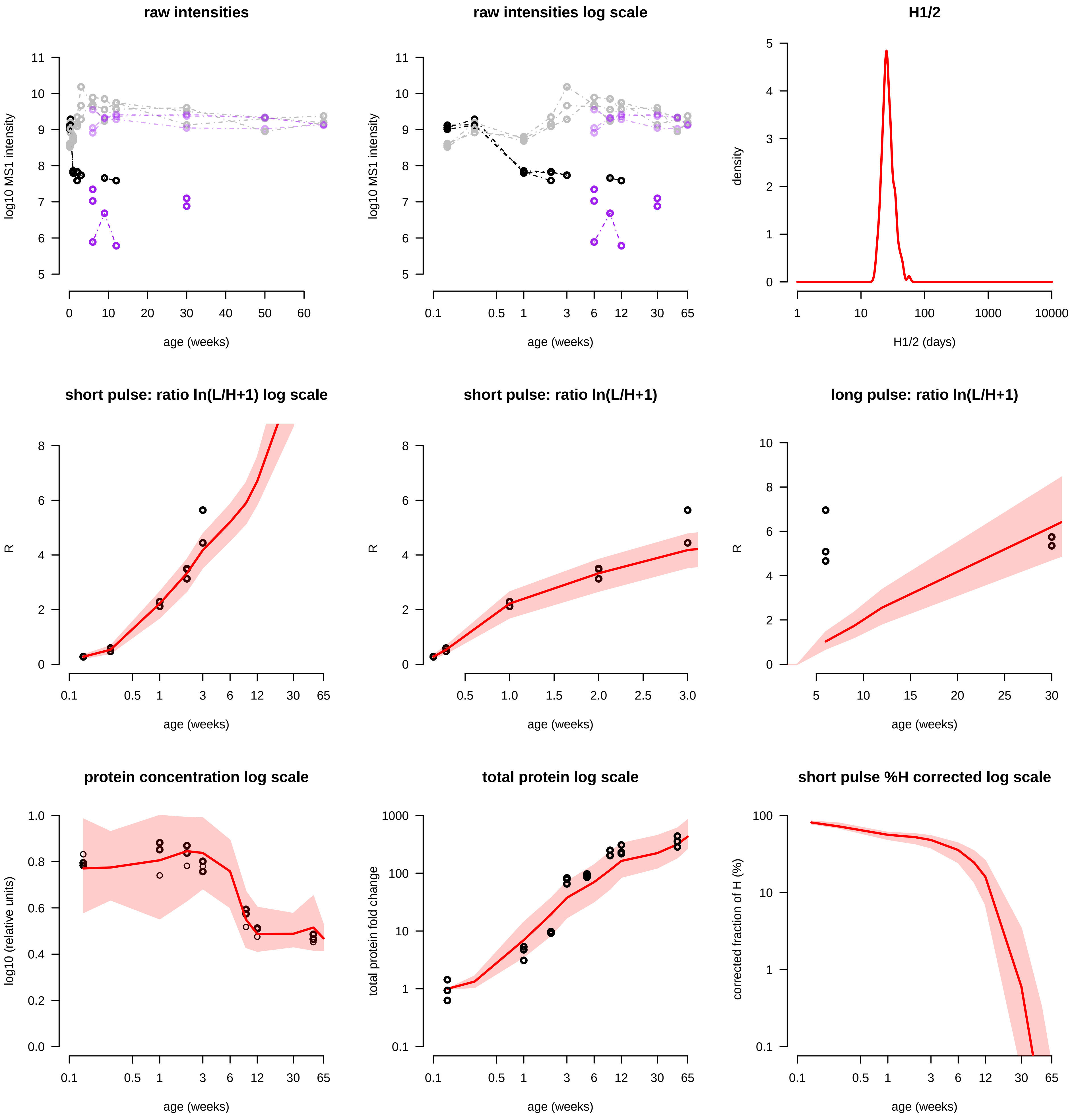

O35857

>sp|O35857|TIM44_MOUSE

Mitochondrial import inner membrane translocase subunit TIM44

GN=Timm44

Med H1/2: 14.2 days

Q5 H1/2: 11 days

Q95 H1/2: 18.7 days

reliable model fit: yes

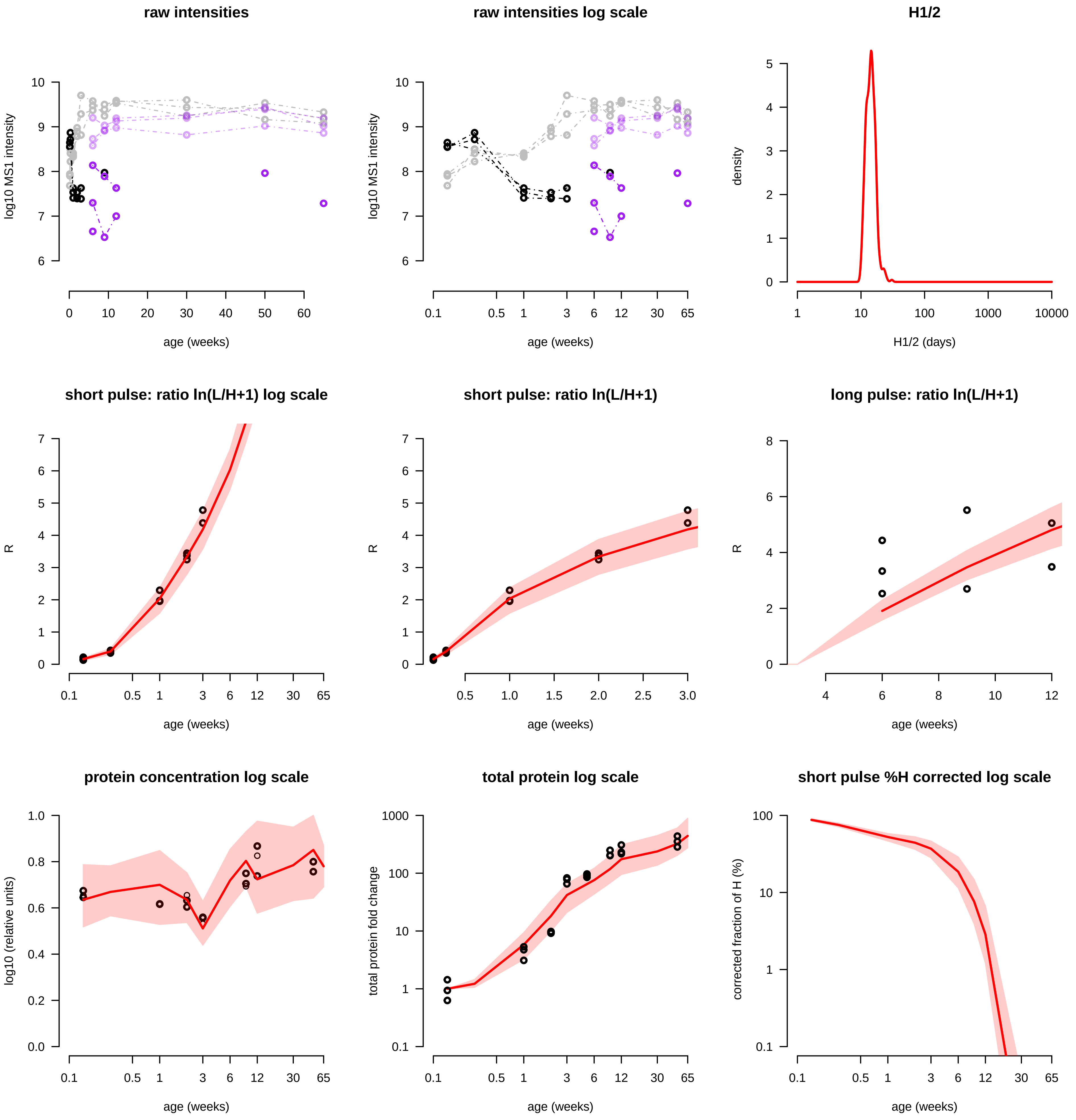

P23198

>sp|P23198|CBX3_MOUSE

Chromobox protein homolog 3

GN=Cbx3

Med H1/2: 9.6 days

Q5 H1/2: 7.2 days

Q95 H1/2: 15.9 days

reliable model fit: yes

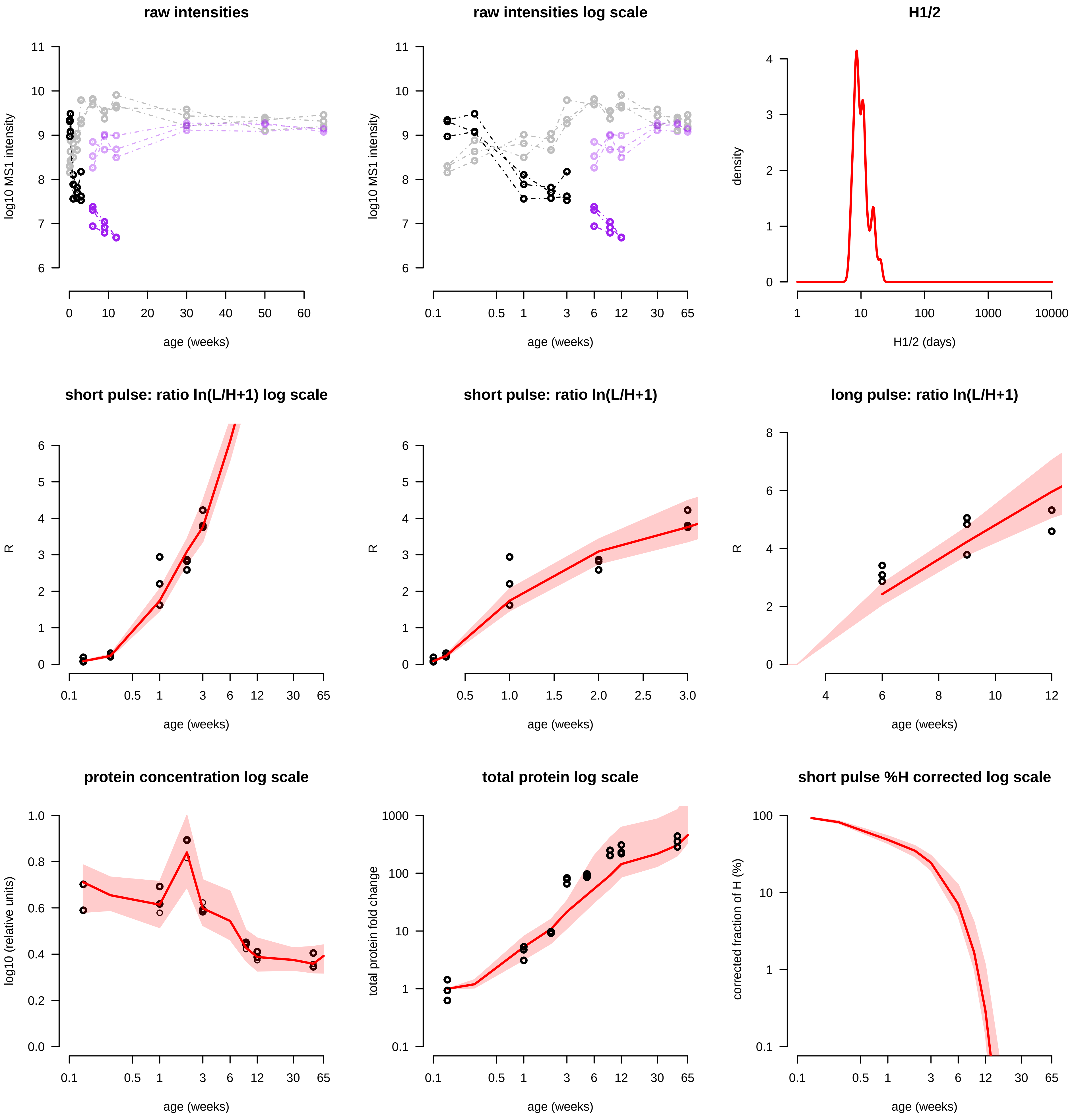

Q8CIN4

>sp|Q8CIN4|PAK2_MOUSE

Serine/threonine−protein kinase PAK 2

GN=Pak2

Med H1/2: 5.6 days

Q5 H1/2: 4.6 days

Q95 H1/2: 10.7 days

reliable model fit: yes

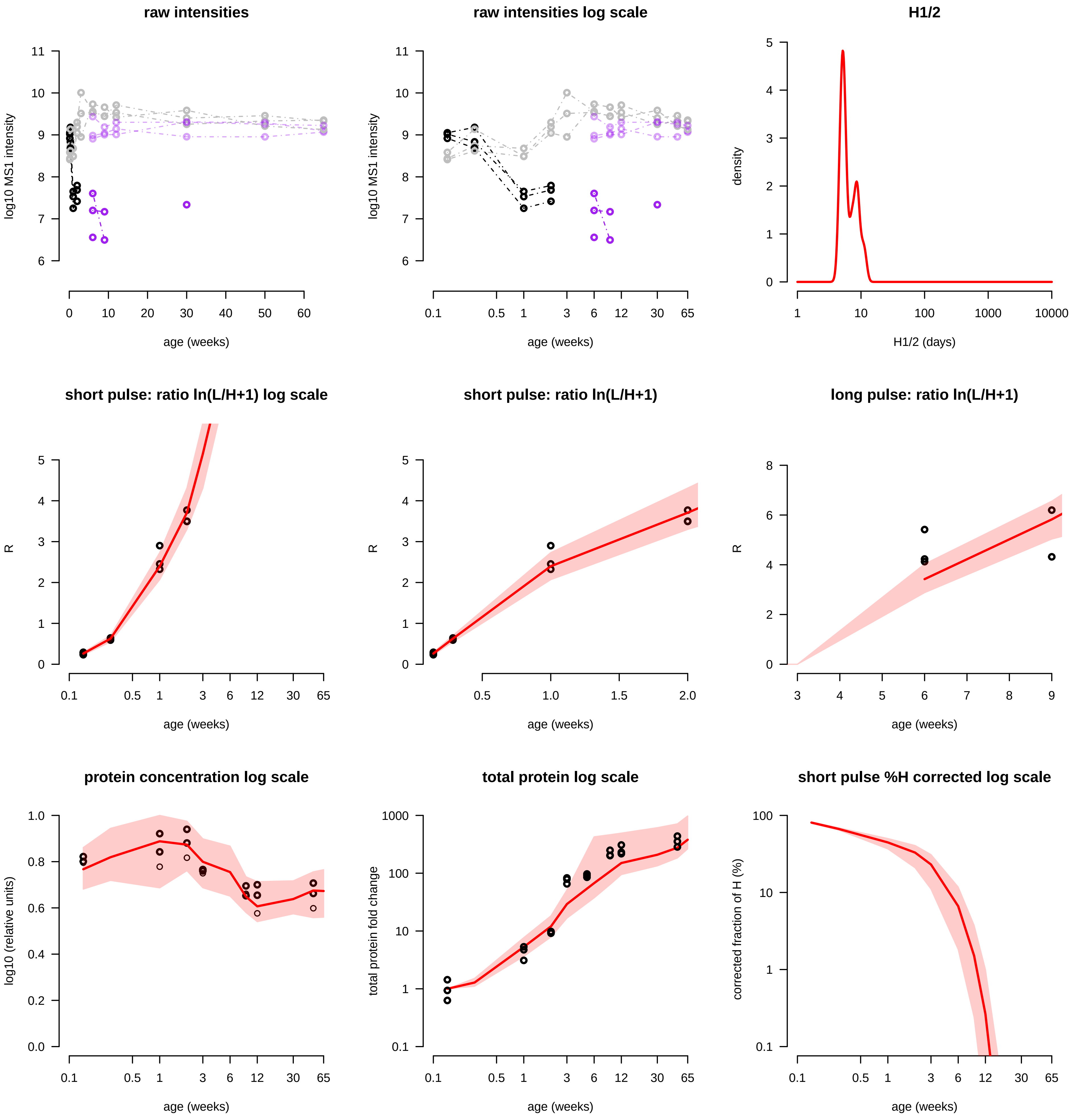

P61957

>sp|P61957|SUMO2_MOUSE

Small ubiquitin−related modifier 2

GN=Sumo2

Med H1/2: 11.1 days

Q5 H1/2: 8.3 days

Q95 H1/2: 15.3 days

reliable model fit: yes

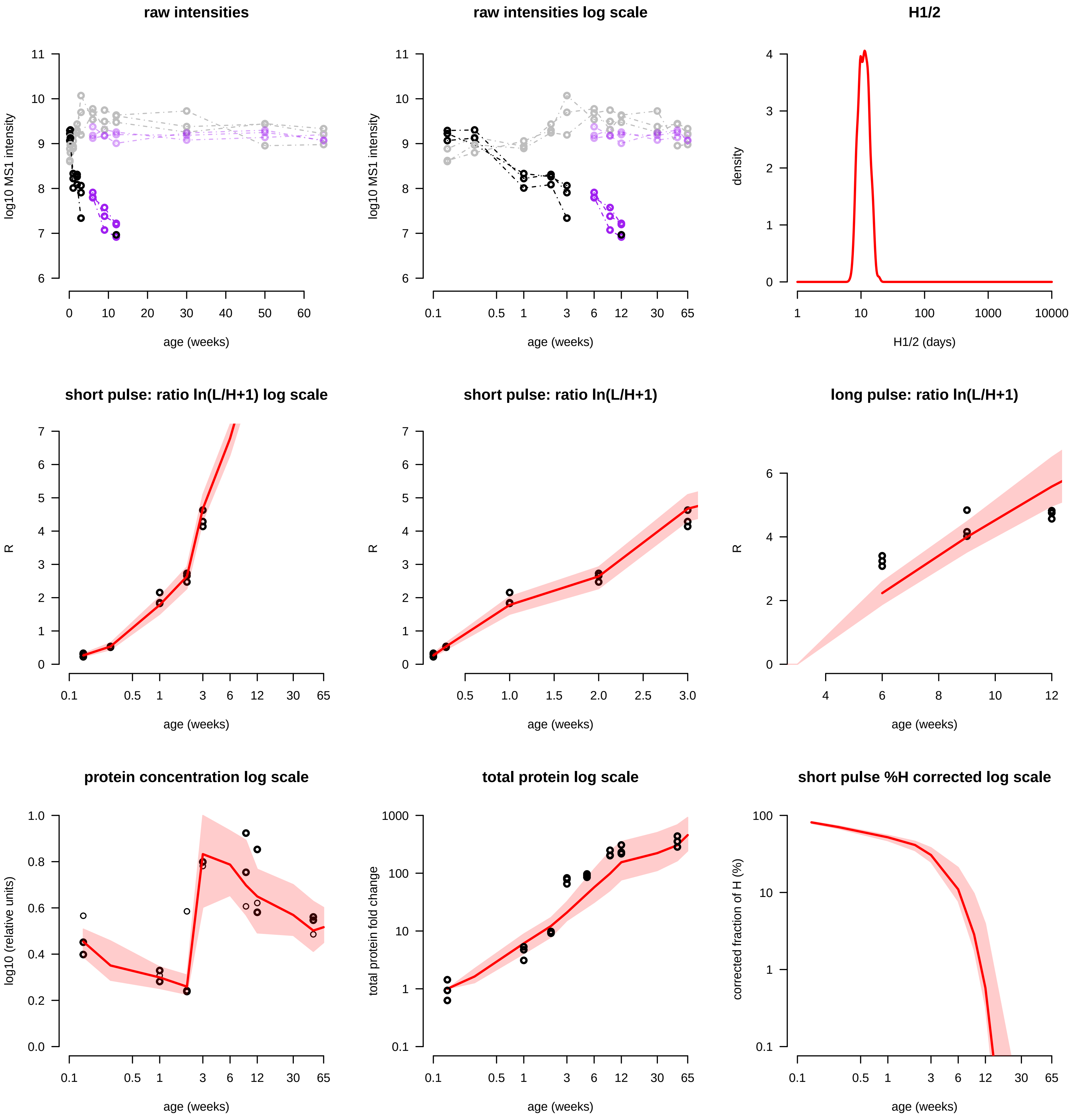

Q9DBH5

>sp|Q9DBH5|LMAN2_MOUSE

Vesicular integral−membrane protein VIP36

GN=Lman2

Med H1/2: 7.4 days

Q5 H1/2: 5.9 days

Q95 H1/2: 9.5 days

reliable model fit: yes

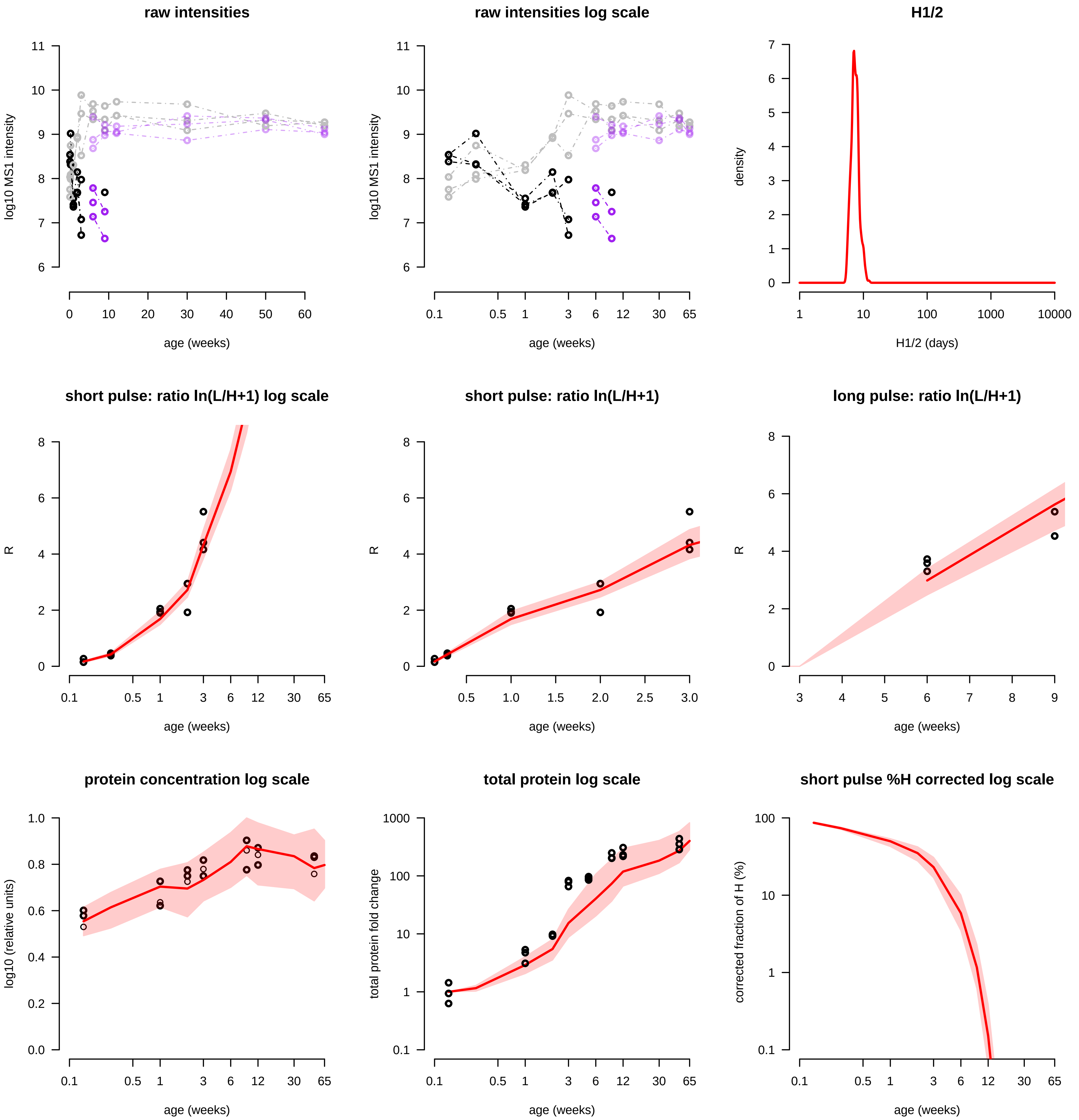

Q9WVJ2

>sp|Q9WVJ2|PSD13_MOUSE

26S proteasome non−ATPase regulatory subunit 13

GN=Psmd13

Med H1/2: 5.4 days

Q5 H1/2: 4.5 days

Q95 H1/2: 6.4 days

reliable model fit: yes

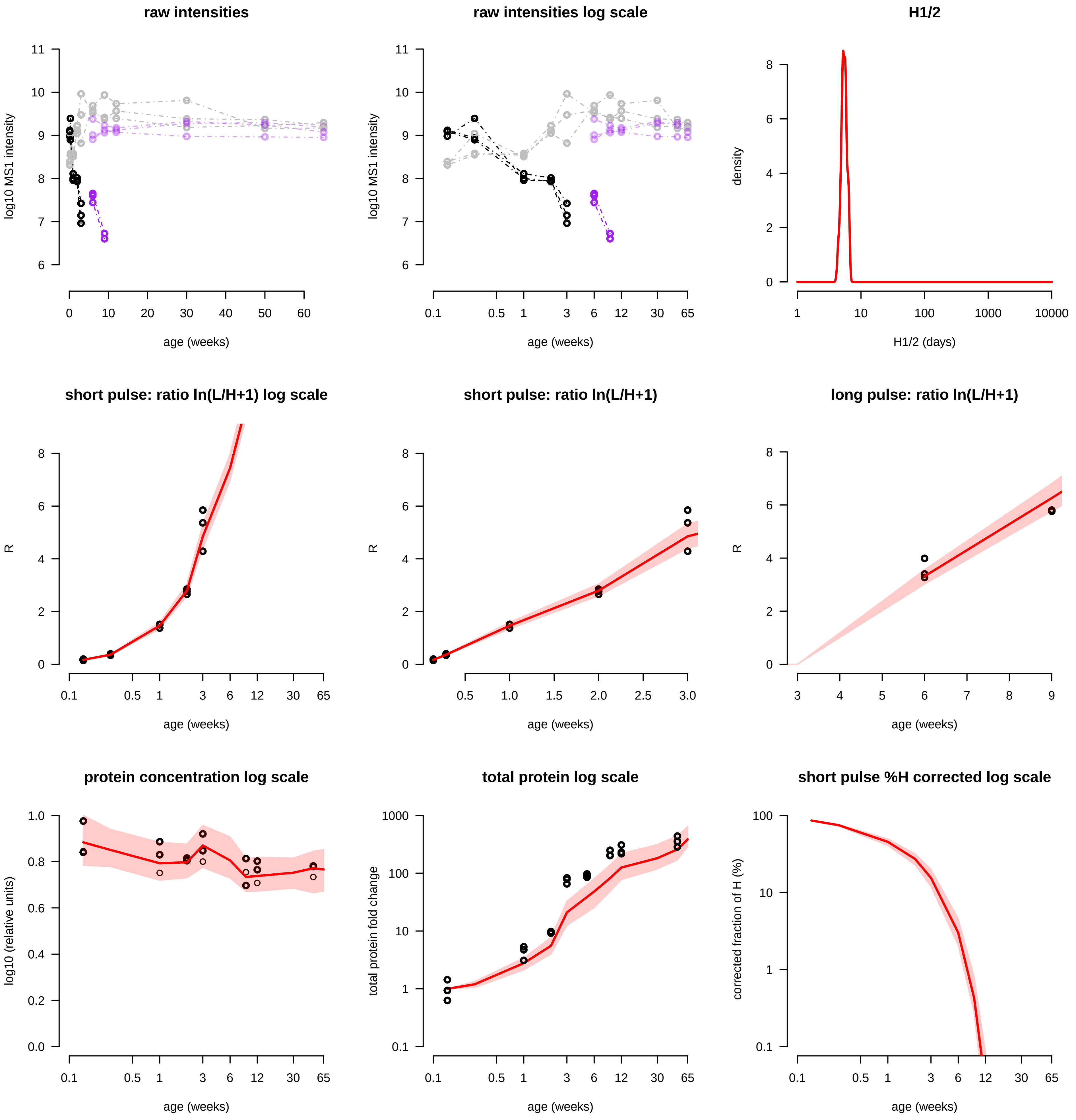

Q60749

>sp|Q60749|KHDR1_MOUSE

KH domain−containing, RNA−binding, signal transduction−associated protein 1

GN=Khdrbs1

Med H1/2: 6.5 days

Q5 H1/2: 5.3 days

Q95 H1/2: 7.7 days

reliable model fit: yes

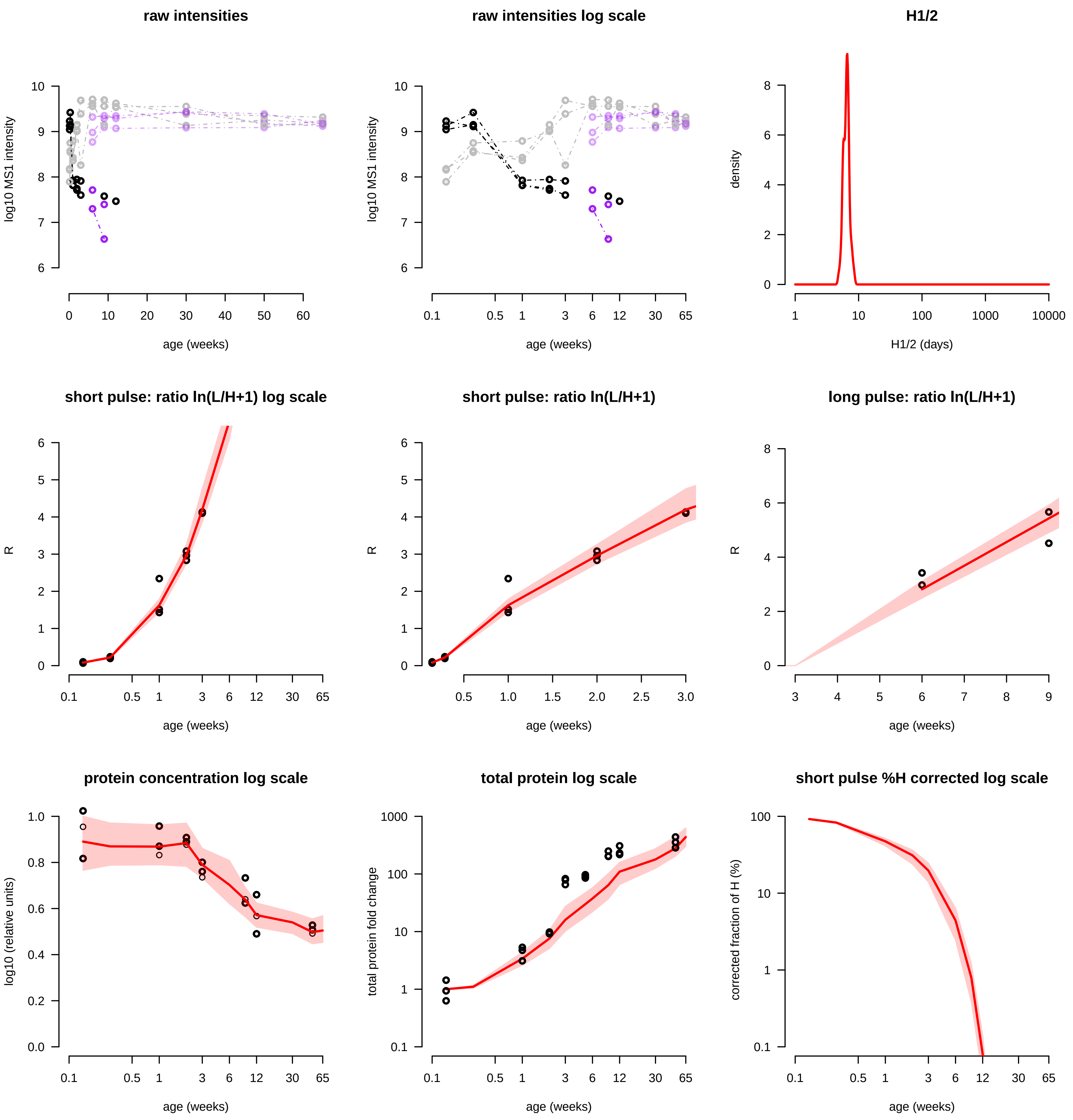

Q9WV80

>sp|Q9WV80|SNX1_MOUSE

Sorting nexin−1

GN=Snx1

Med H1/2: 8.2 days

Q5 H1/2: 6.9 days

Q95 H1/2: 11.1 days

reliable model fit: yes

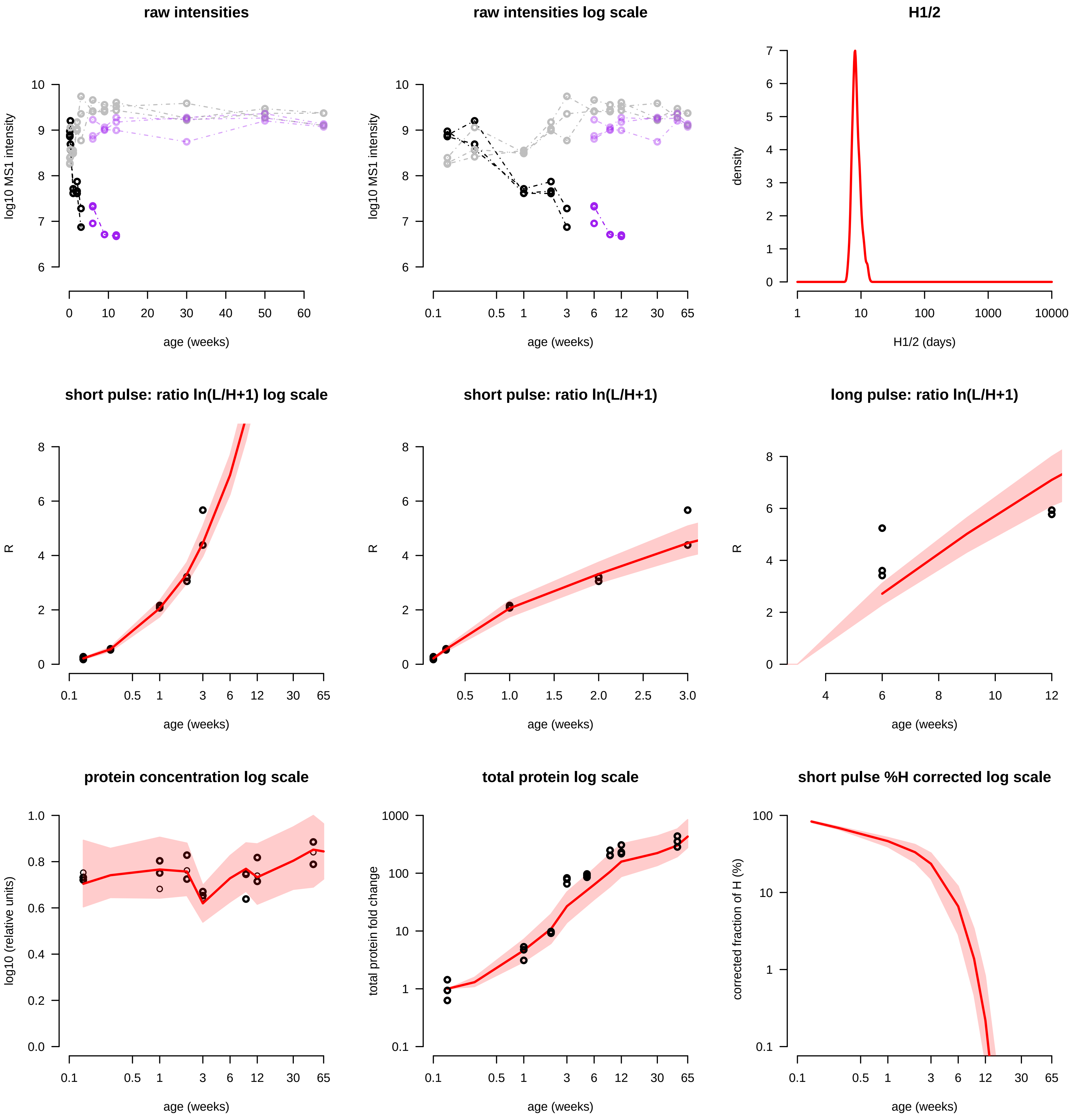

Q99K51

>sp|Q99K51|PLST_MOUSE

Plastin−3

GN=Pls3

Med H1/2: 162.8 days

Q5 H1/2: 82.3 days

Q95 H1/2: 346.1 days

reliable model fit: yes

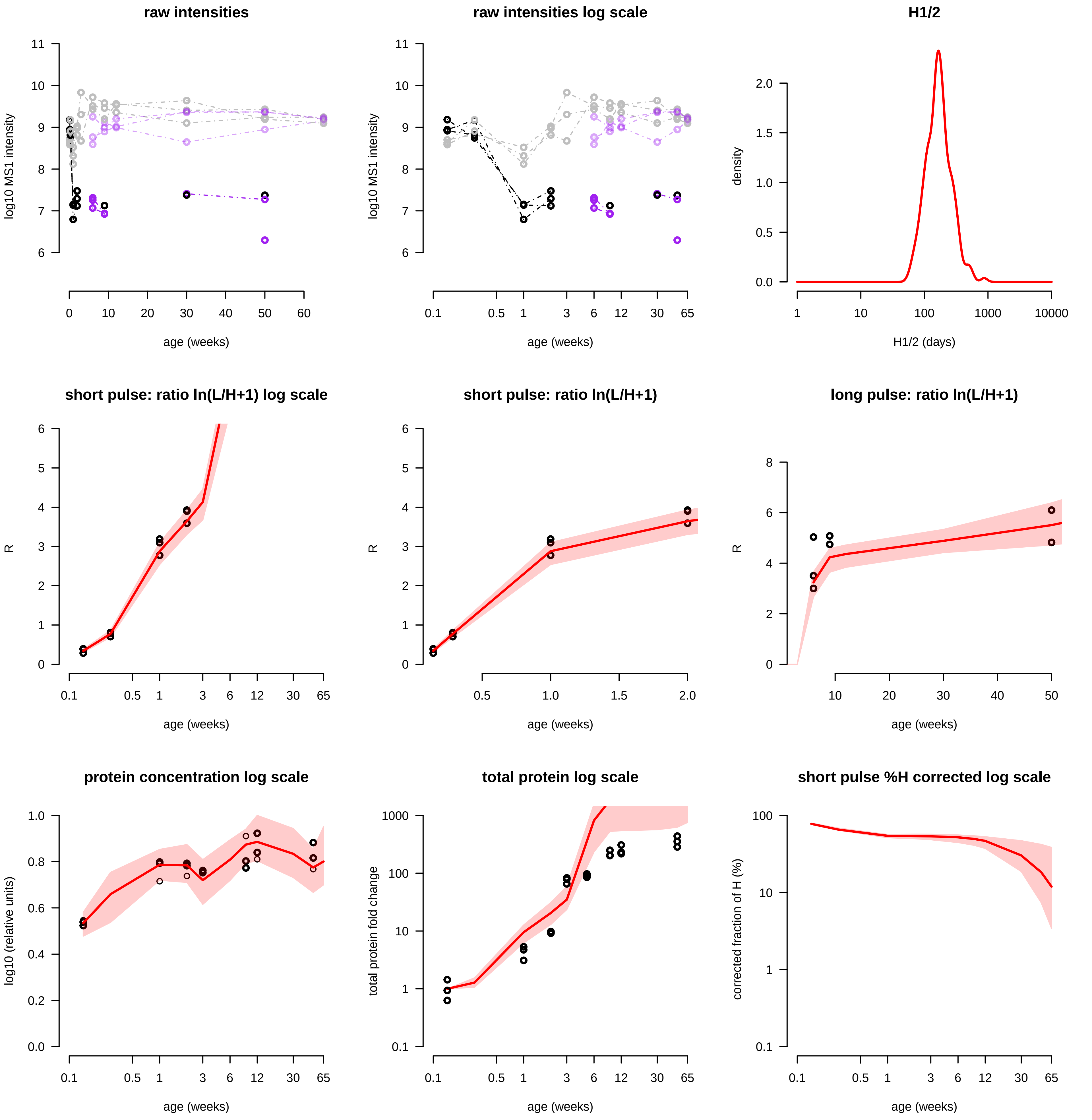

P17563

>sp|P17563|SBP1_MOUSE

Methanethiol oxidase

GN=Selenbp1

Med H1/2: 5.3 days

Q5 H1/2: 4.4 days

Q95 H1/2: 6.4 days

reliable model fit: yes

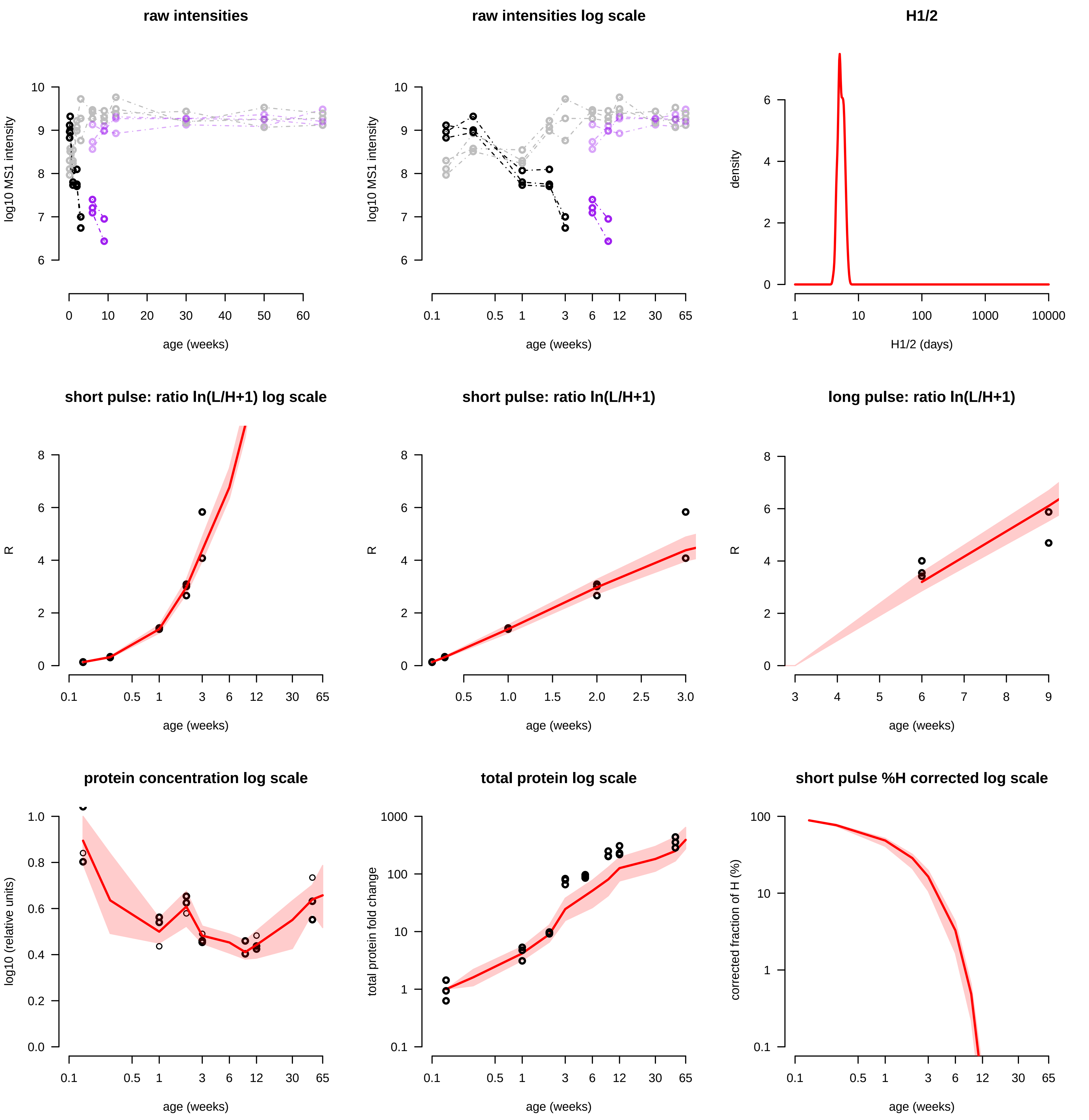

Q6NZJ6

>sp|Q6NZJ6|IF4G1_MOUSE

Eukaryotic translation initiation factor 4 gamma 1

GN=Eif4g1

Med H1/2: 5.1 days

Q5 H1/2: 4.2 days

Q95 H1/2: 6.2 days

reliable model fit: yes

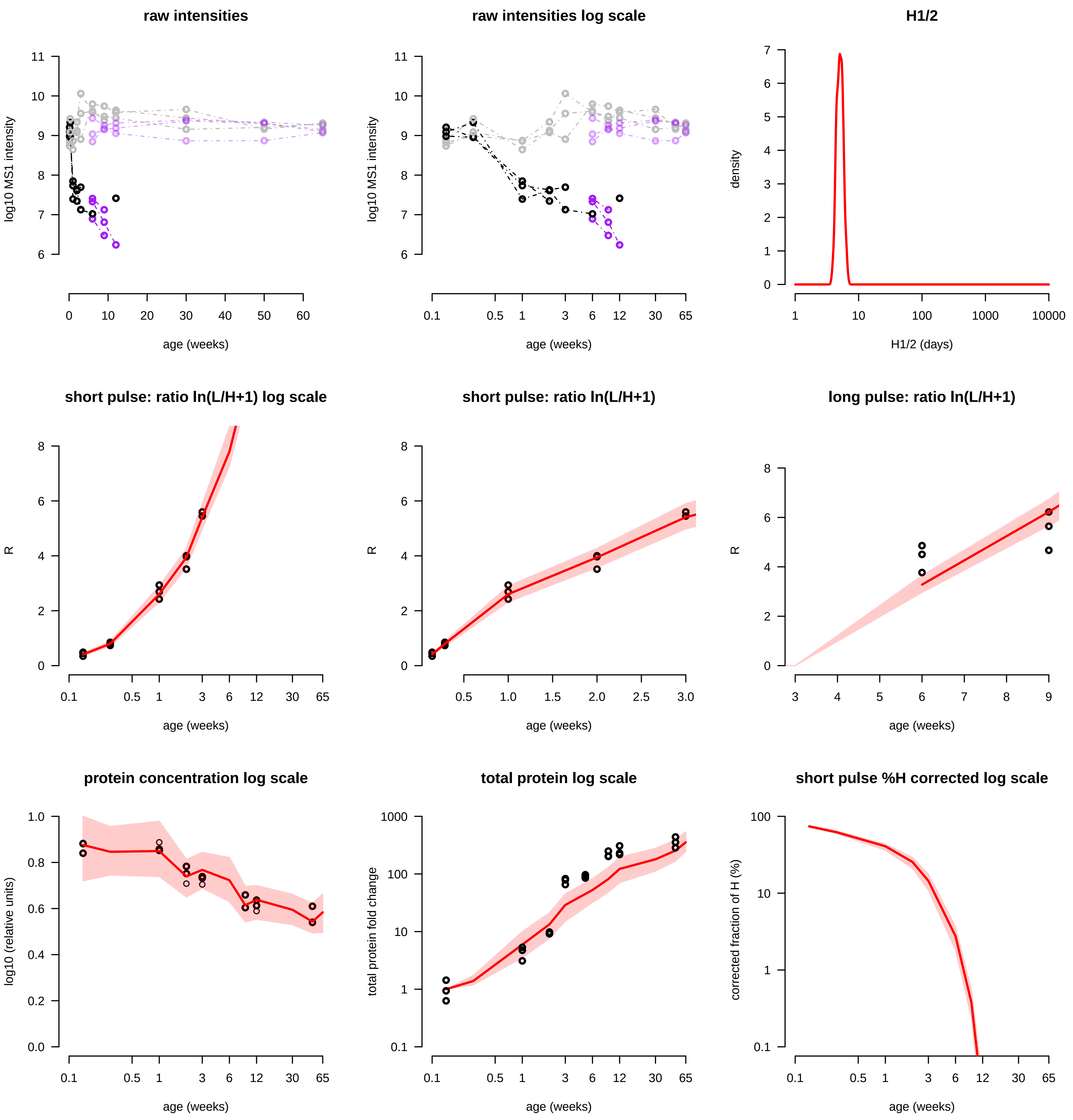

Q9D0R2

>sp|Q9D0R2|SYTC_MOUSE

Threonine−−tRNA ligase 1, cytoplasmic

GN=Tars1

Med H1/2: 4.3 days

Q5 H1/2: 3.4 days

Q95 H1/2: 5.7 days

reliable model fit: yes

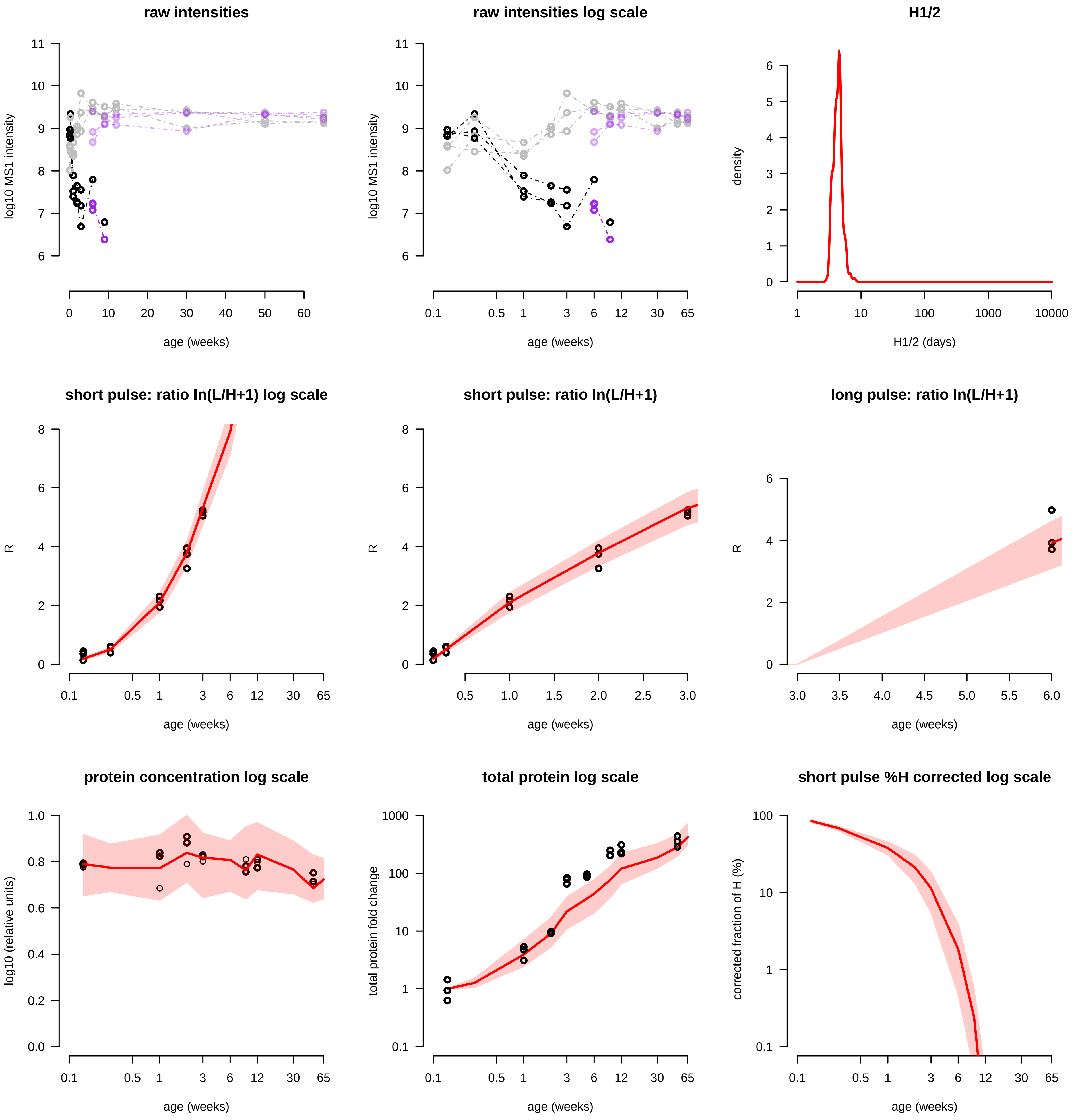

Q61699

>sp|Q61699|HS105_MOUSE

Heat shock protein 105 kDa

GN=Hsph1

Med H1/2: 221.7 days

Q5 H1/2: 57.4 days

Q95 H1/2: 1528.1 days

reliable model fit: yes

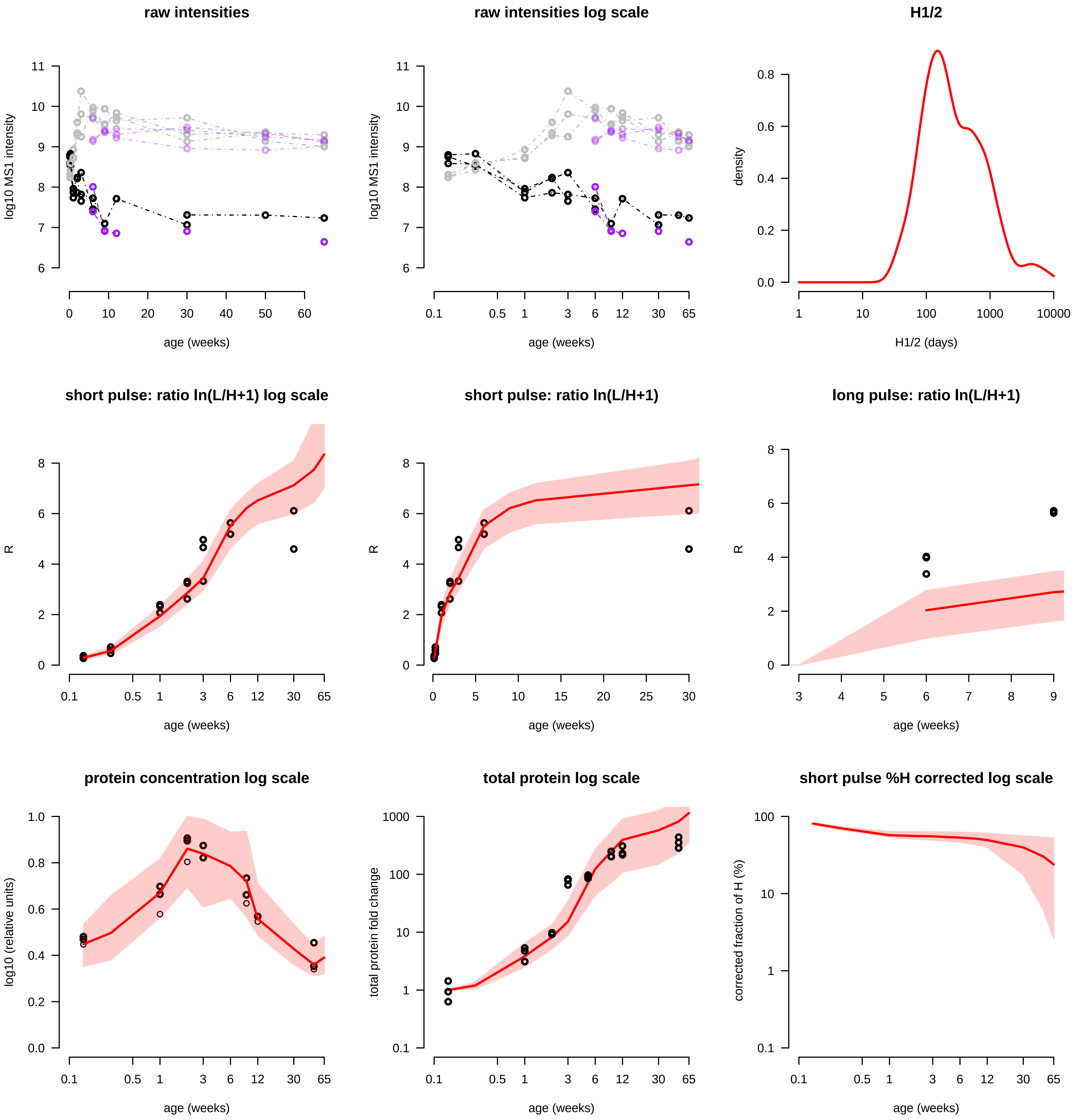

Q9R0Y5;Q9R0Y5−2

>sp|Q9R0Y5|KAD1_MOUSE

Adenylate kinase isoenzyme 1

GN=Ak1

Med H1/2: 70.3 days

Q5 H1/2: 22.1 days

Q95 H1/2: 476.3 days

reliable model fit: yes

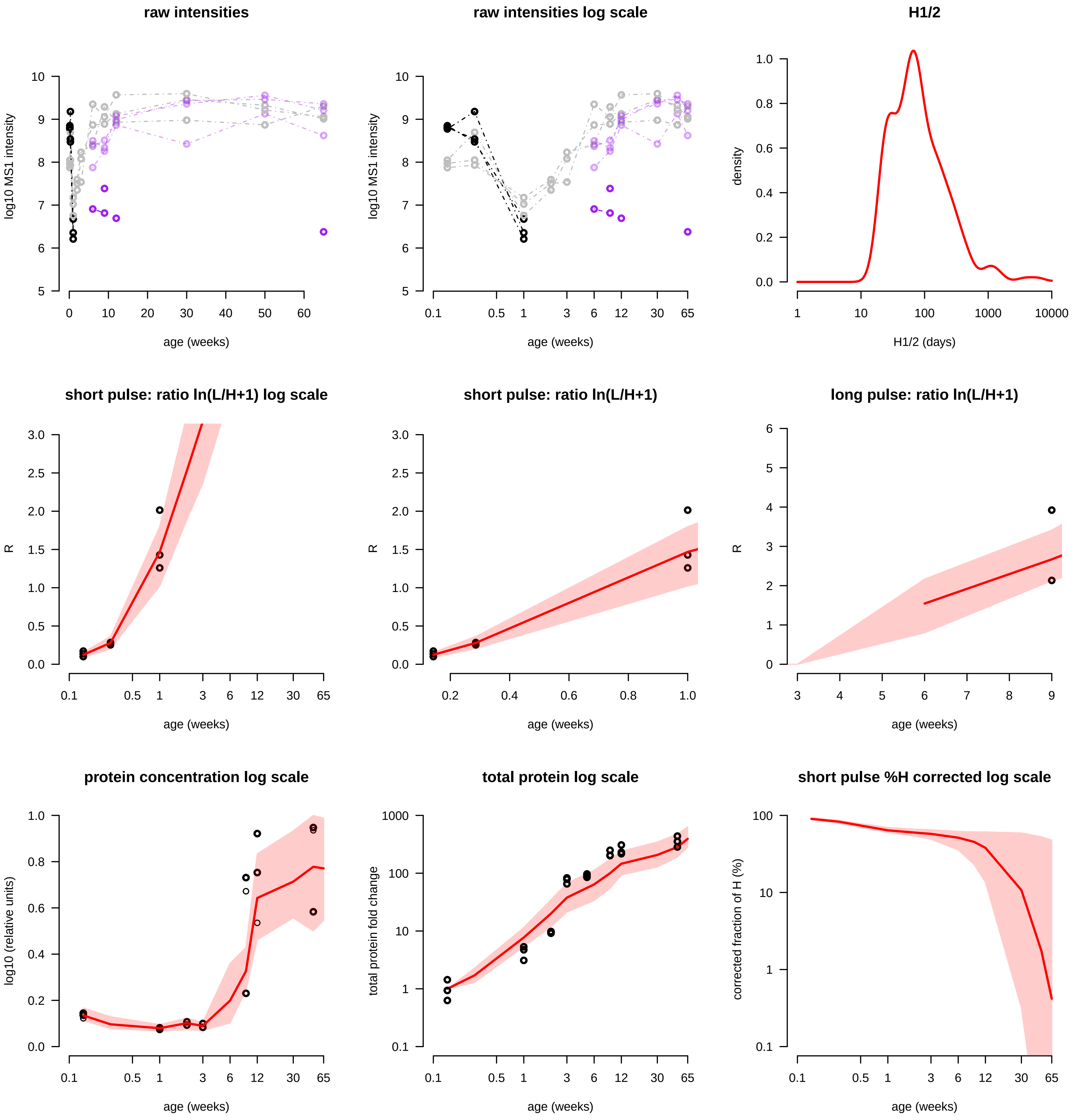

Q9EP69

>sp|Q9EP69|SAC1_MOUSE

Phosphatidylinositol−3−phosphatase SAC1

GN=Sacm1l

Med H1/2: 6.4 days

Q5 H1/2: 5.3 days

Q95 H1/2: 8.8 days

reliable model fit: yes

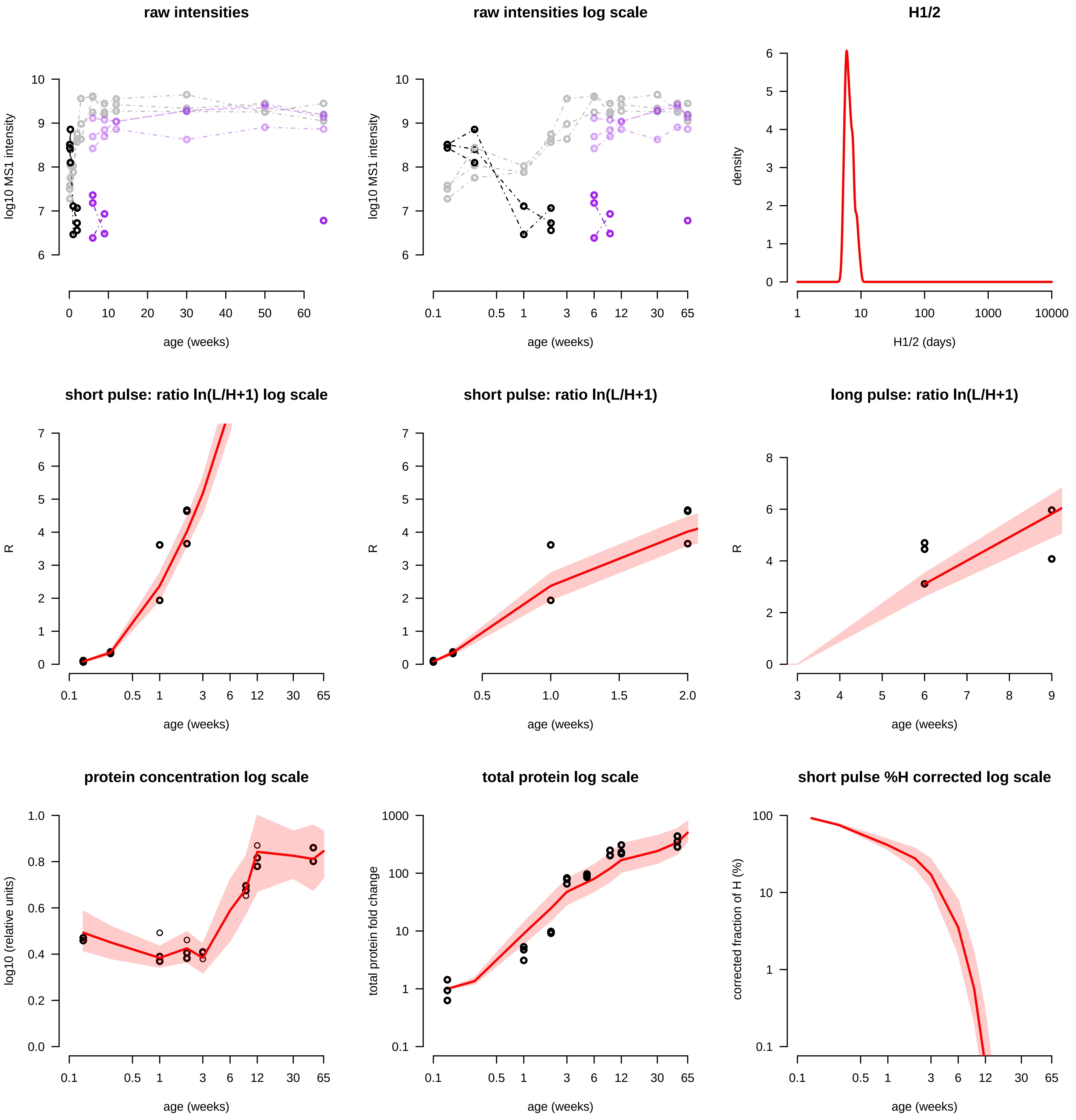

Q9JKB3

>sp|Q9JKB3|YBOX3_MOUSE

Y−box−binding protein 3

GN=Ybx3

Med H1/2: 7.2 days

Q5   H1/2: 6.1 days

Q95 H1/2: 9.1 days

reliable model fit: yes

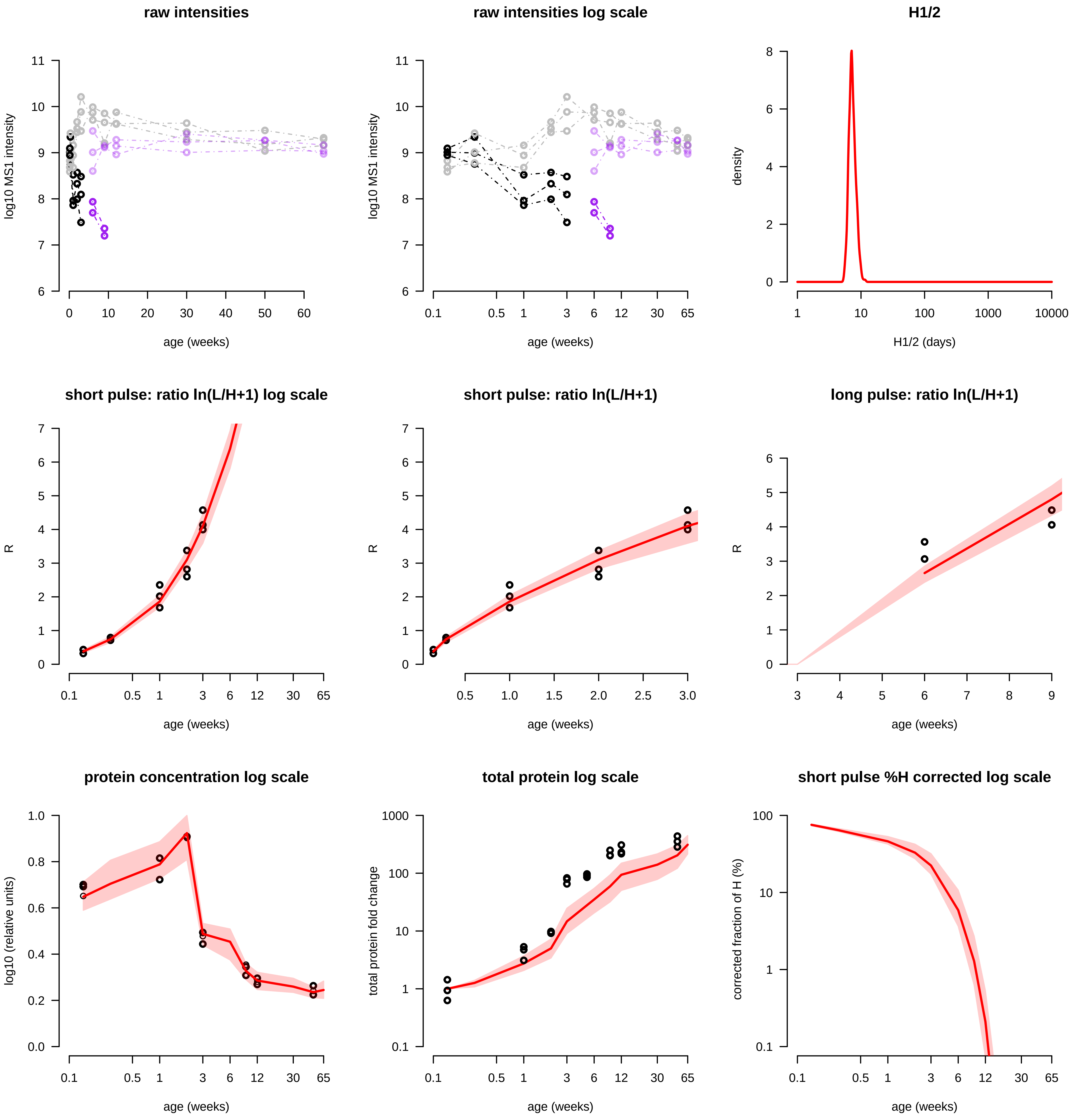

Q8C1A5

>sp|Q8C1A5|THOP1_MOUSE

Thimet oligopeptidase

GN=Thop1

Med H1/2: 7.3 days

Q5 H1/2: 6.2 days

Q95 H1/2: 9 days

reliable model fit: yes

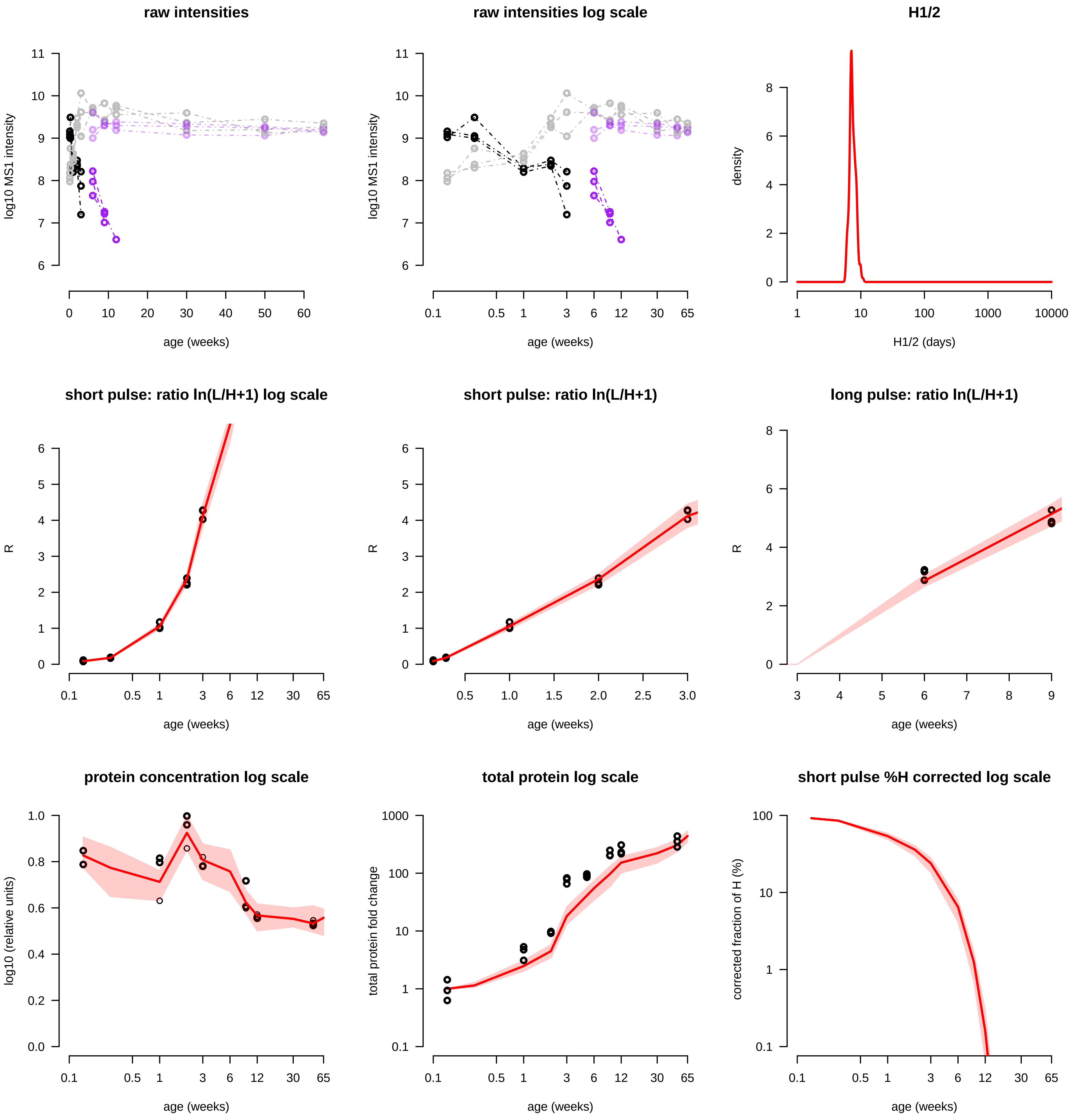

E9PVA8

>sp|E9PVA8|GCN1_MOUSE

eIF−2−alpha kinase activator GCN1

GN=Gcn1

Med H1/2: 82.7 days

Q5 H1/2: 56.3 days

Q95 H1/2: 126.2 days

reliable model fit: no

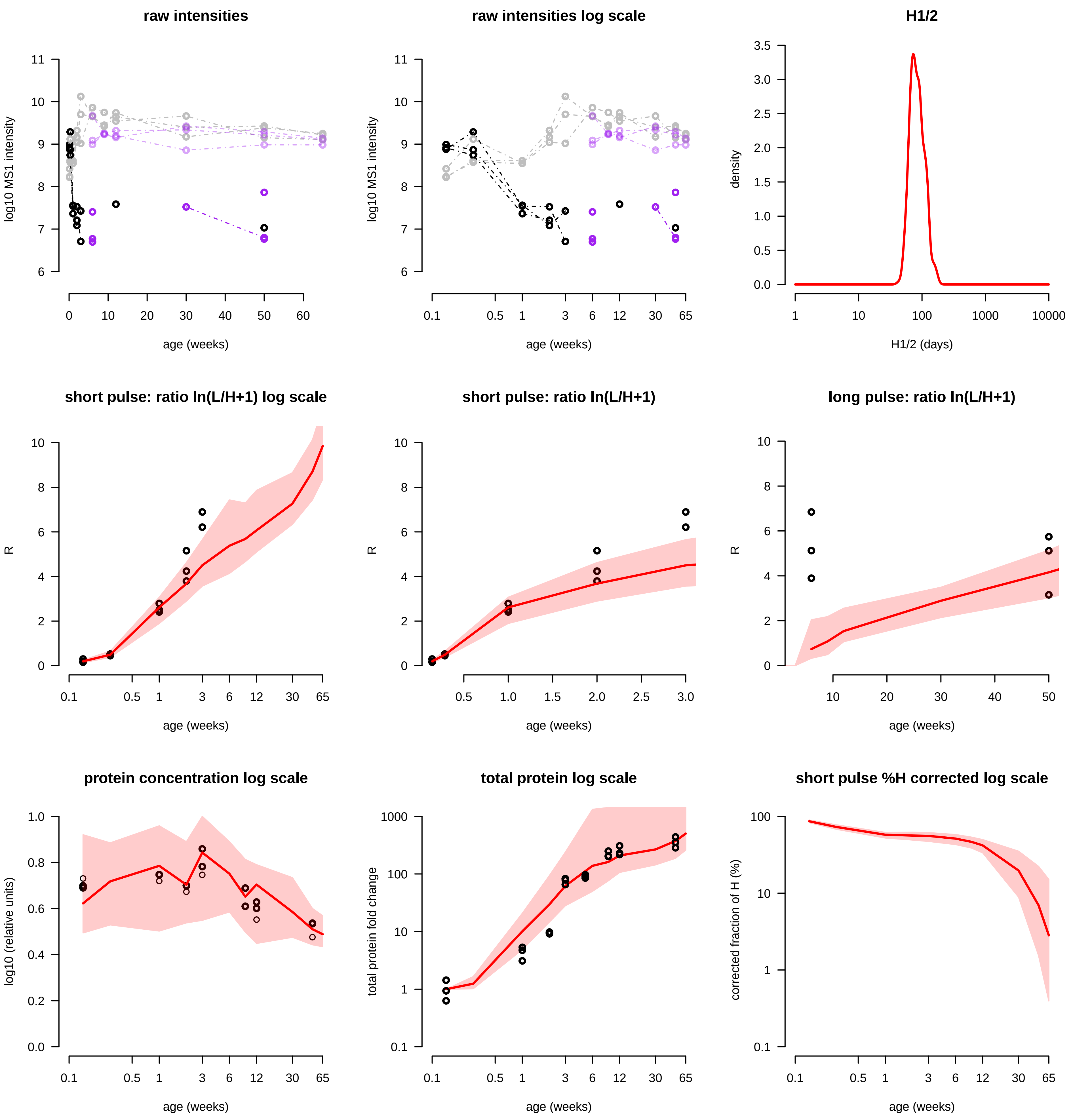

Q9D1G1

>sp|Q9D1G1|RAB1B_MOUSE

Ras−related protein Rab−1B

GN=Rab1b

Med H1/2: 12.2 days

Q5 H1/2: 9.3 days

Q95 H1/2: 16.3 days

reliable model fit: yes

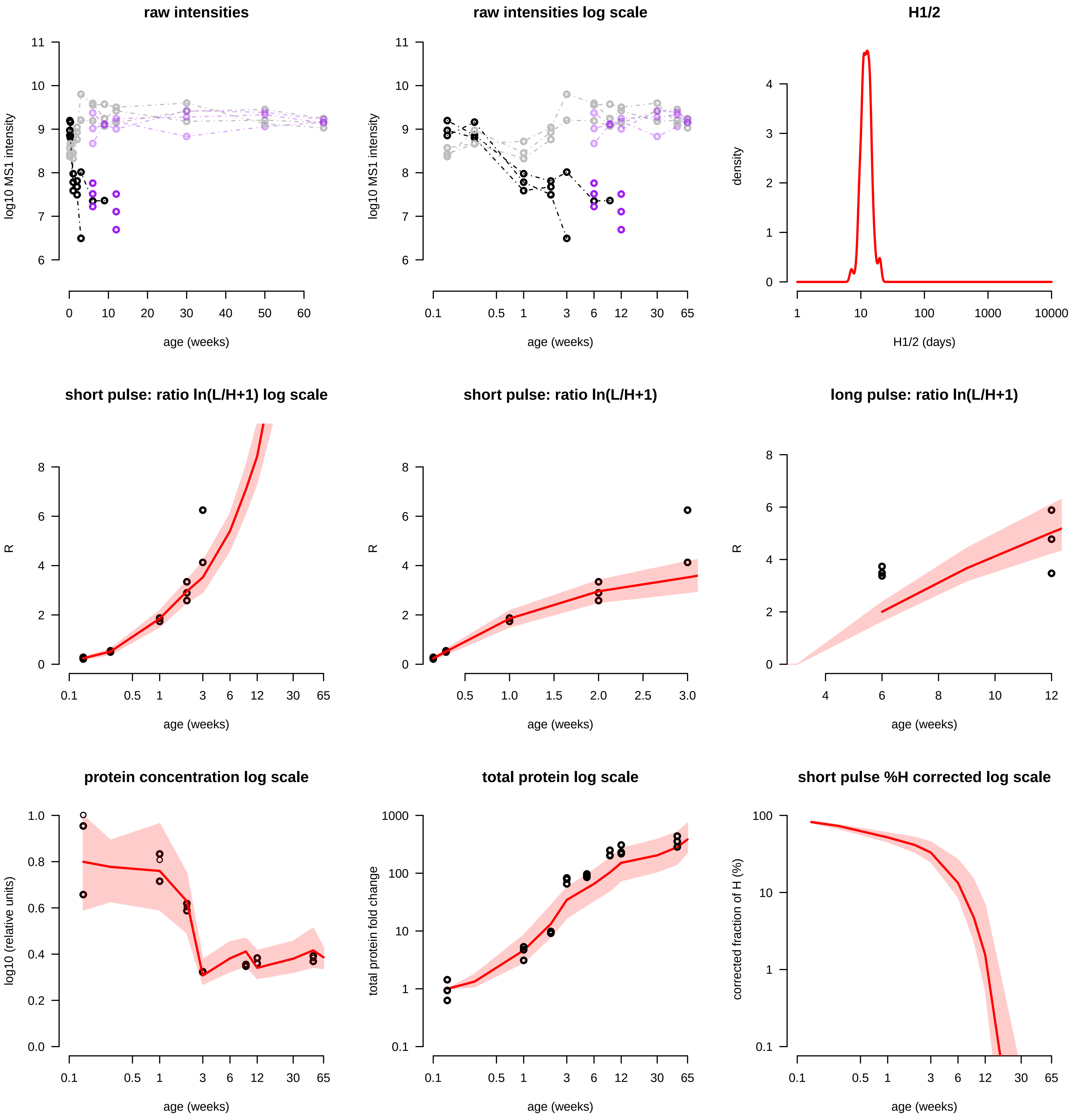

Q9CW03

>sp|Q9CW03|SMC3_MOUSE

Structural maintenance of chromosomes protein 3

GN=Smc3

Med H1/2: 16 days

Q5 H1/2: 8.8 days

Q95 H1/2: 45.2 days

reliable model fit: no

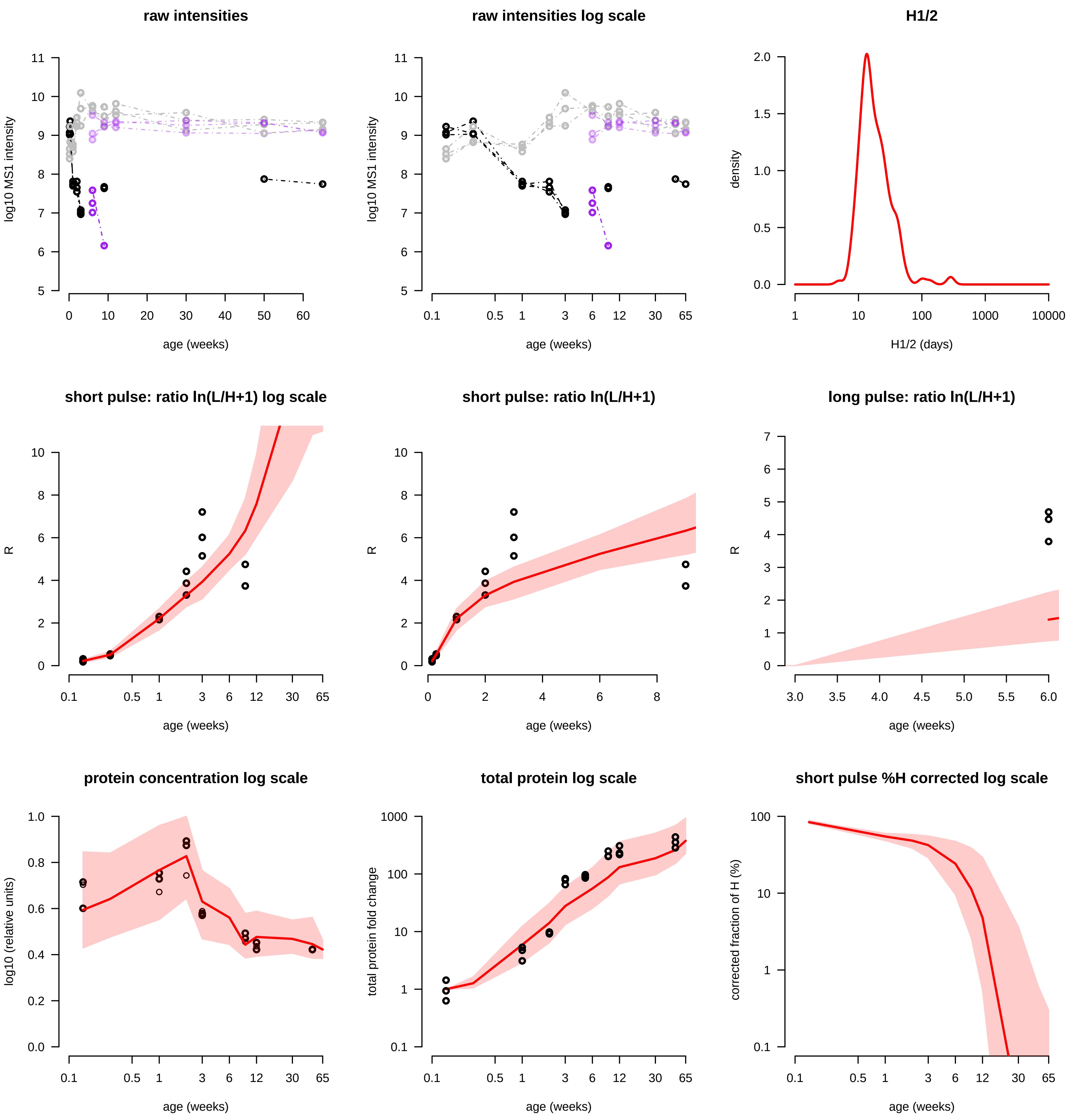

P02468

>sp|P02468|LAMC1_MOUSE

Laminin subunit gamma−1

GN=Lamc1

Med H1/2: 5.2 days

Q5 H1/2: 4.4 days

Q95 H1/2: 6.5 days

reliable model fit: yes

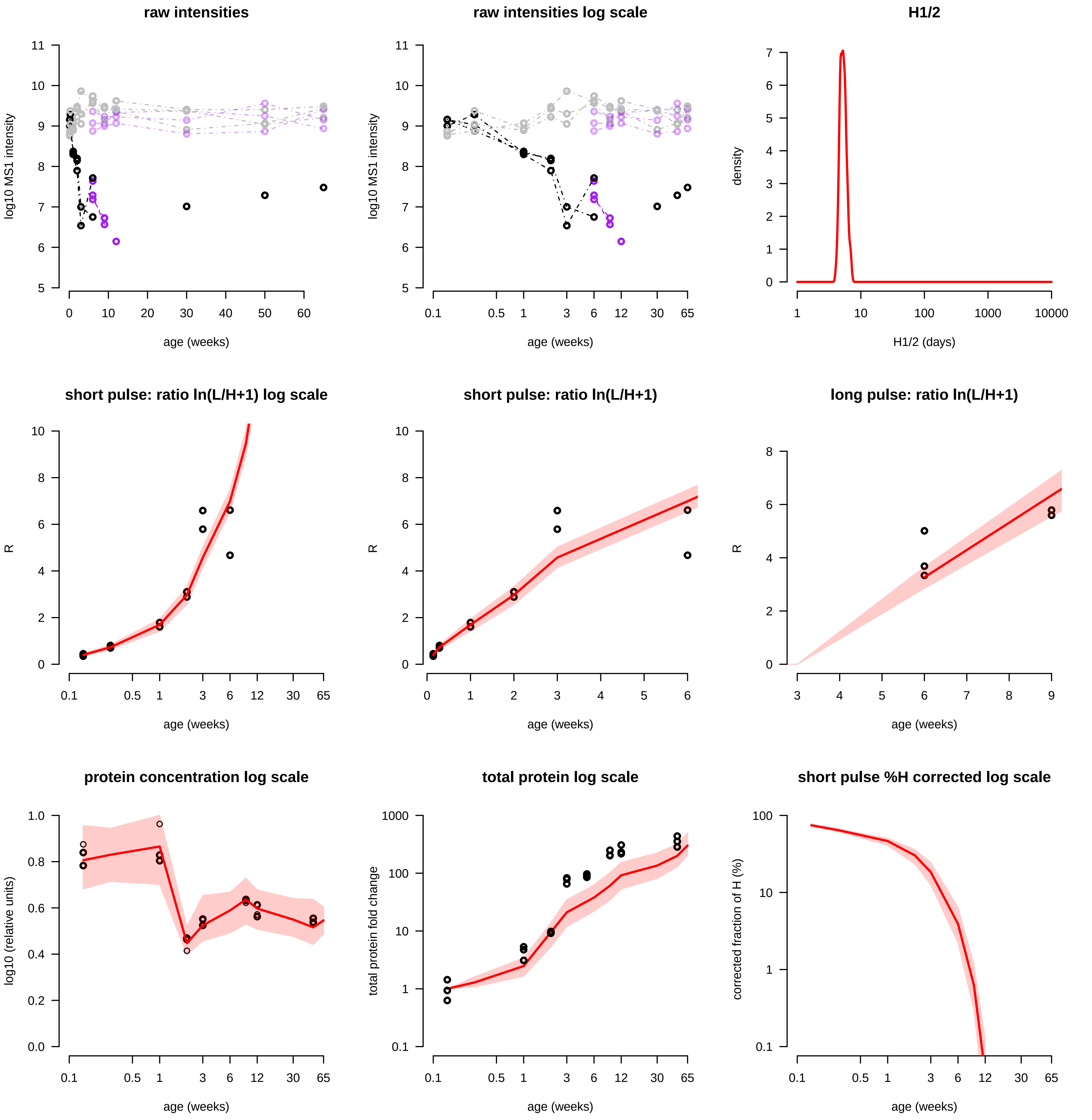

Q9D819

>sp|Q9D819|IPYR_MOUSE

Inorganic pyrophosphatase

GN=Ppa1

Med H1/2: 6.3 days

Q5 H1/2: 5.2 days

Q95 H1/2: 7.4 days

reliable model fit: yes

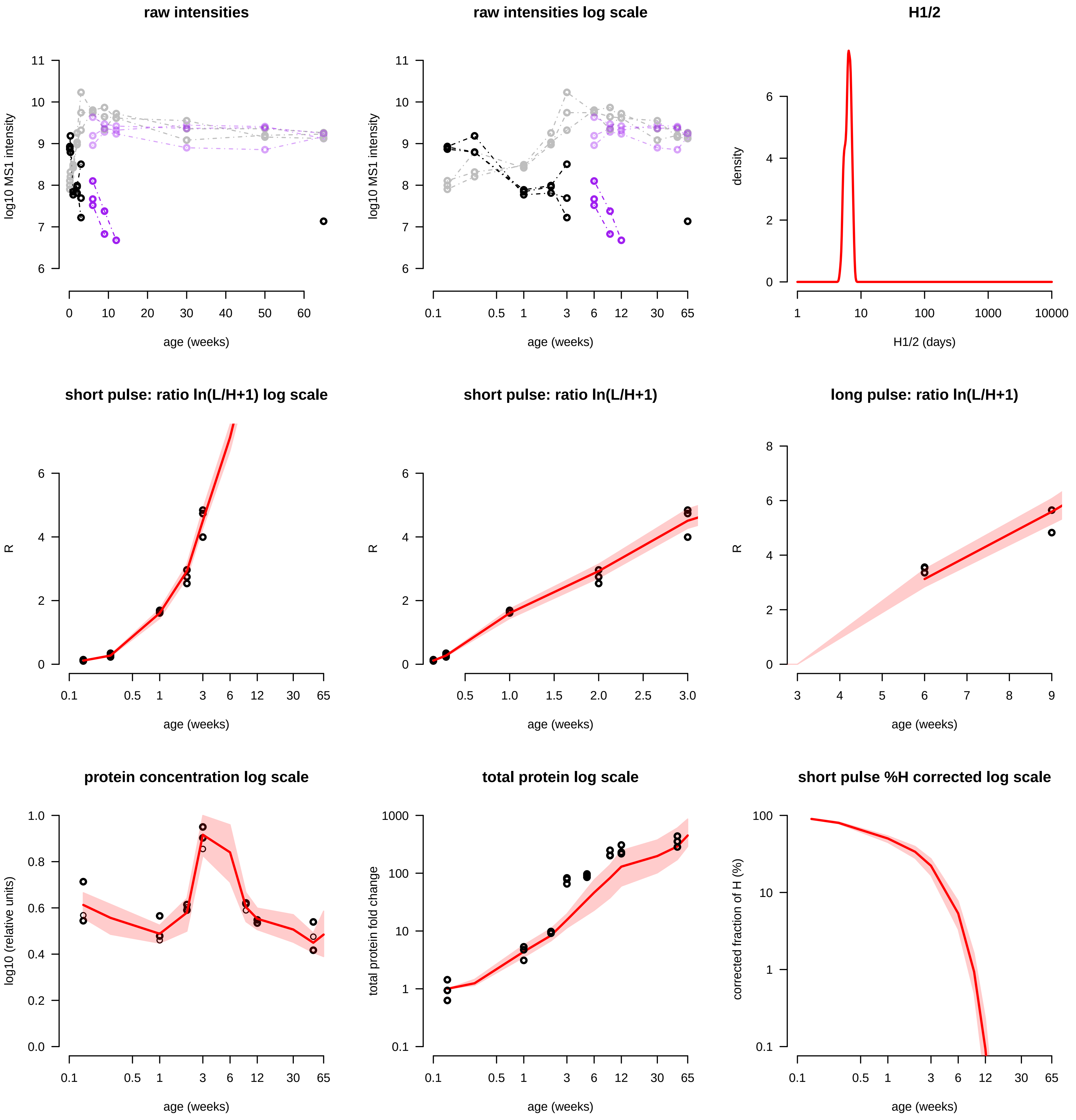

P63037

>sp|P63037|DNJA1_MOUSE

DnaJ homolog subfamily A member 1

GN=Dnaja1

Med H1/2: 214.1 days

Q5 H1/2: 62.7 days

Q95 H1/2: 2221.4 days

reliable model fit: yes

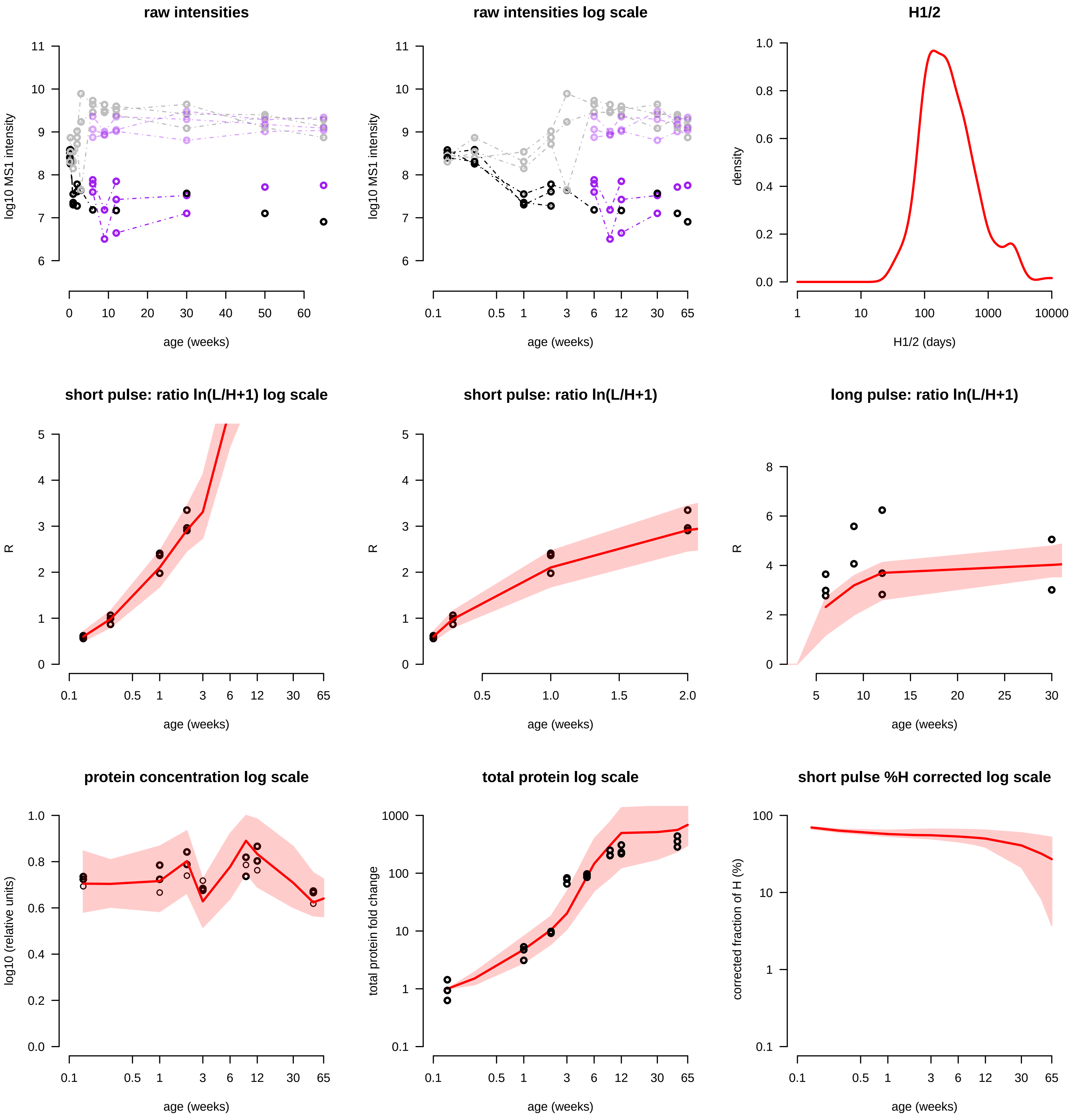

Q8BWY3

>sp|Q8BWY3|ERF1_MOUSE

Eukaryotic peptide chain release factor subunit 1

GN=Etf1

Med H1/2: 8.7 days

Q5 H1/2: 5.1 days

Q95 H1/2: 16.1 days

reliable model fit: no

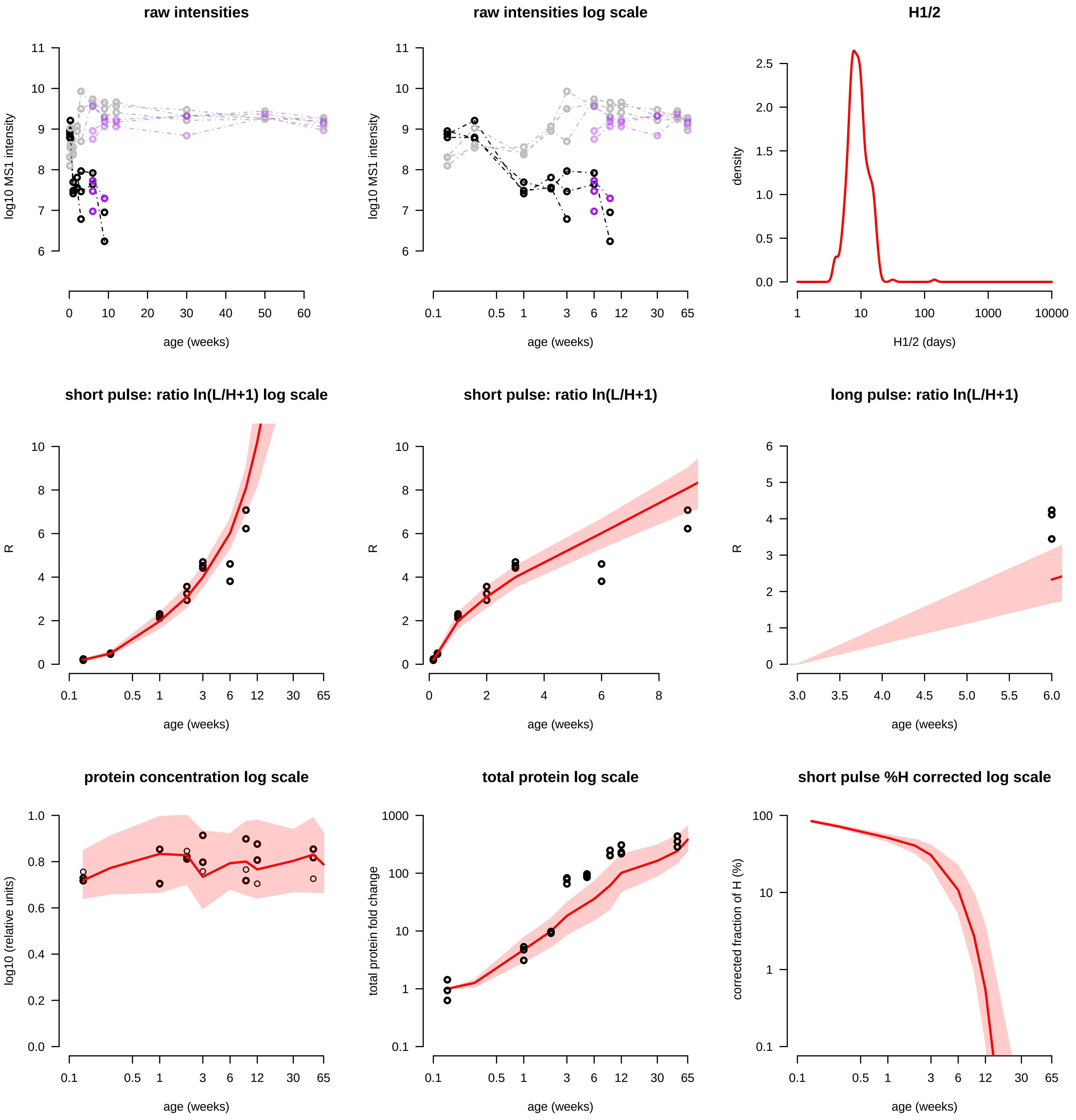

P39054

>sp|P39054|DYN2_MOUSE

Dynamin−2

GN=Dnm2

Med H1/2: 4.2 days

Q5 H1/2: 3.8 days

Q95 H1/2: 4.8 days

reliable model fit: yes

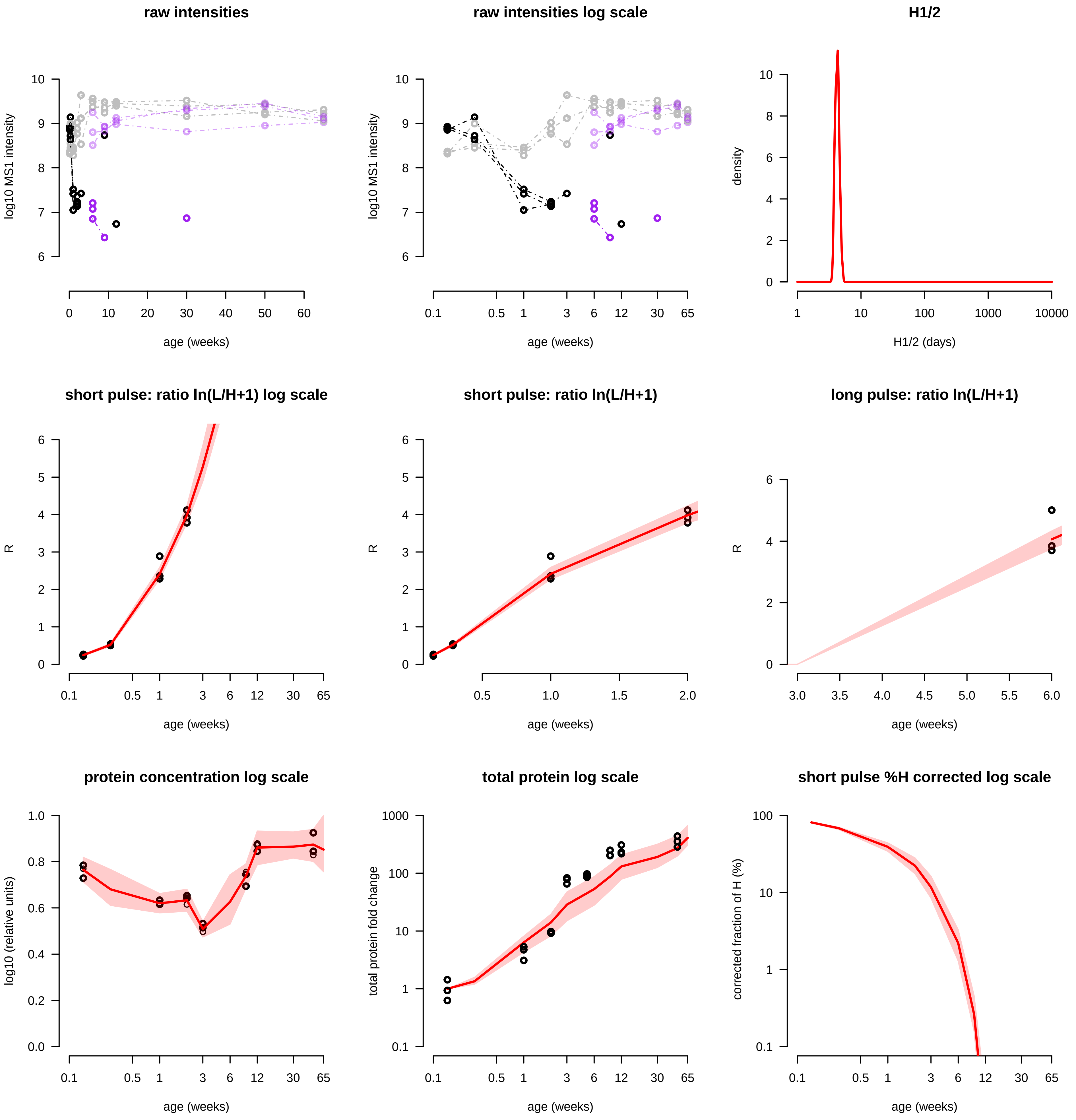

P83882

>sp|P83882|RL36A_MOUSE

60S ribosomal protein L36a

GN=Rpl36a

Med H1/2: 6.9 days

Q5 H1/2: 5.6 days

Q95 H1/2: 8.7 days

reliable model fit: yes

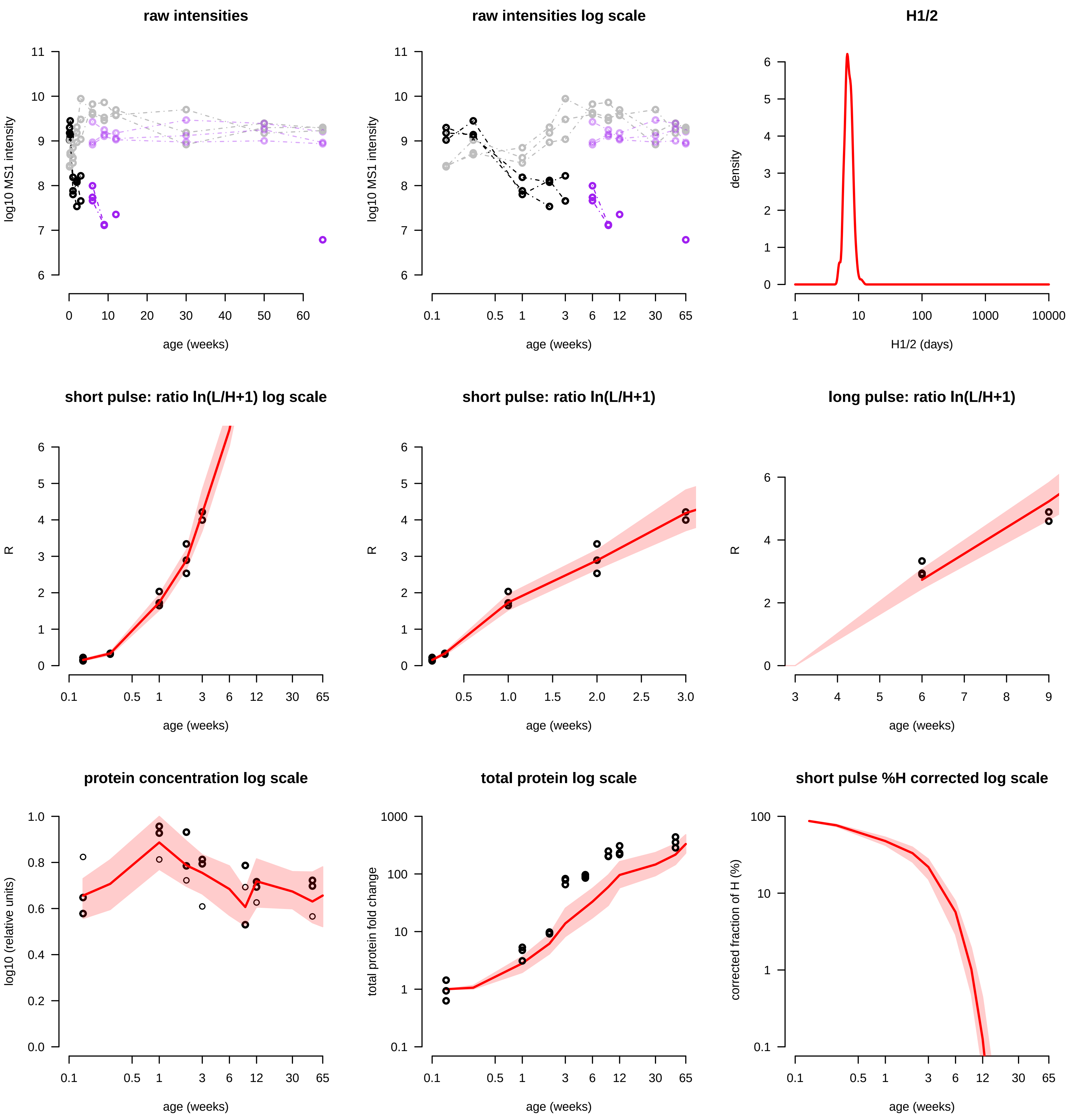

Q9Z1X4−3;Q9Z1X4;Q9Z1X4−2

>sp|Q9Z1X4−3|ILF3_MOUSE

Isoform 3 of Interleukin enhancer−binding factor 3

GN=Ilf3

Med H1/2: 457.4 days

Q5 H1/2: 164.6 days

Q95 H1/2: 4392.5 days

reliable model fit: yes

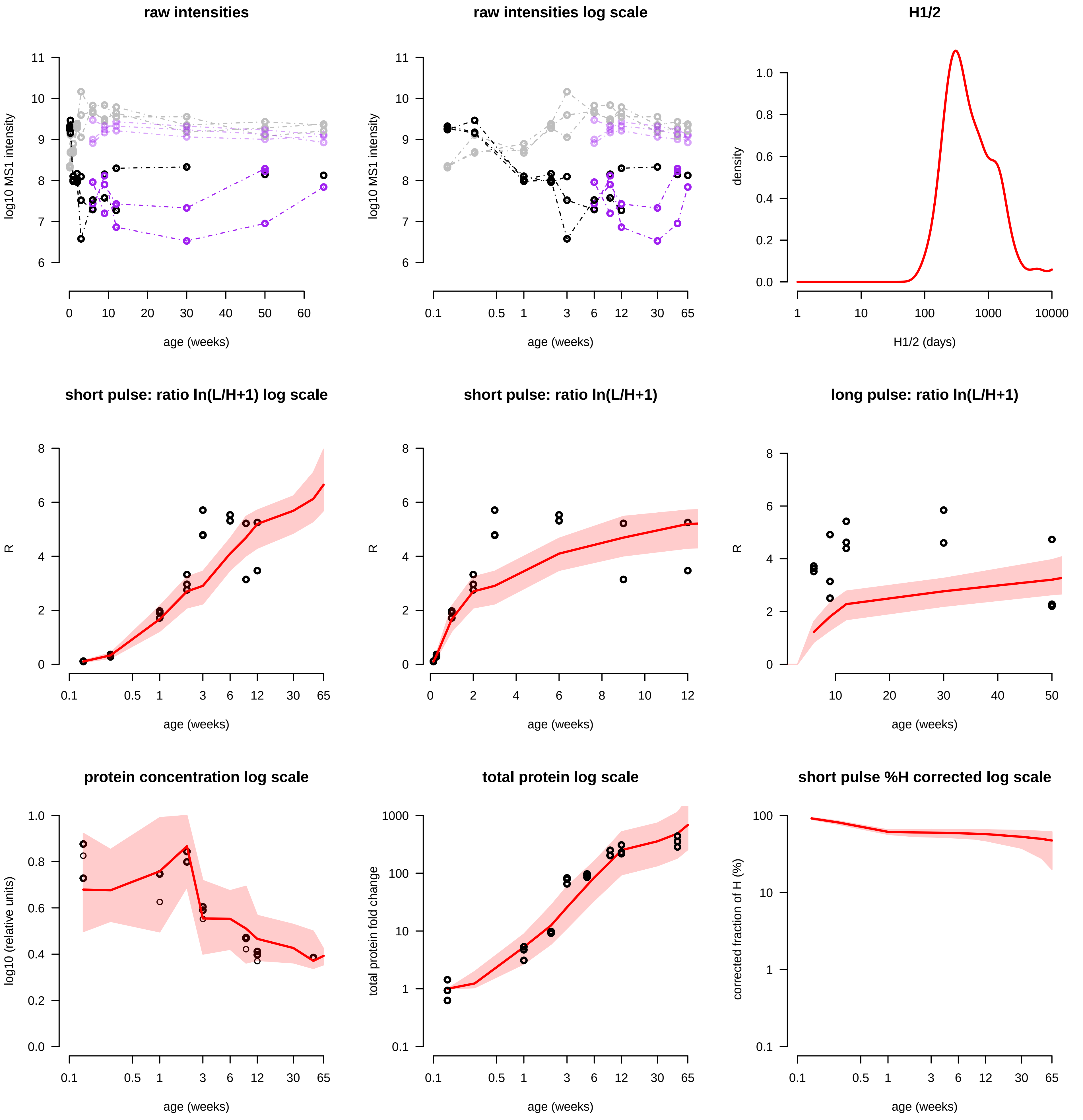

P63001

>sp|P63001|RAC1_MOUSE

Ras−related C3 botulinum toxin substrate 1

GN=Rac1

Med H1/2: 4.5 days

Q5 H1/2: 3.8 days

Q95 H1/2: 5.5 days

reliable model fit: yes

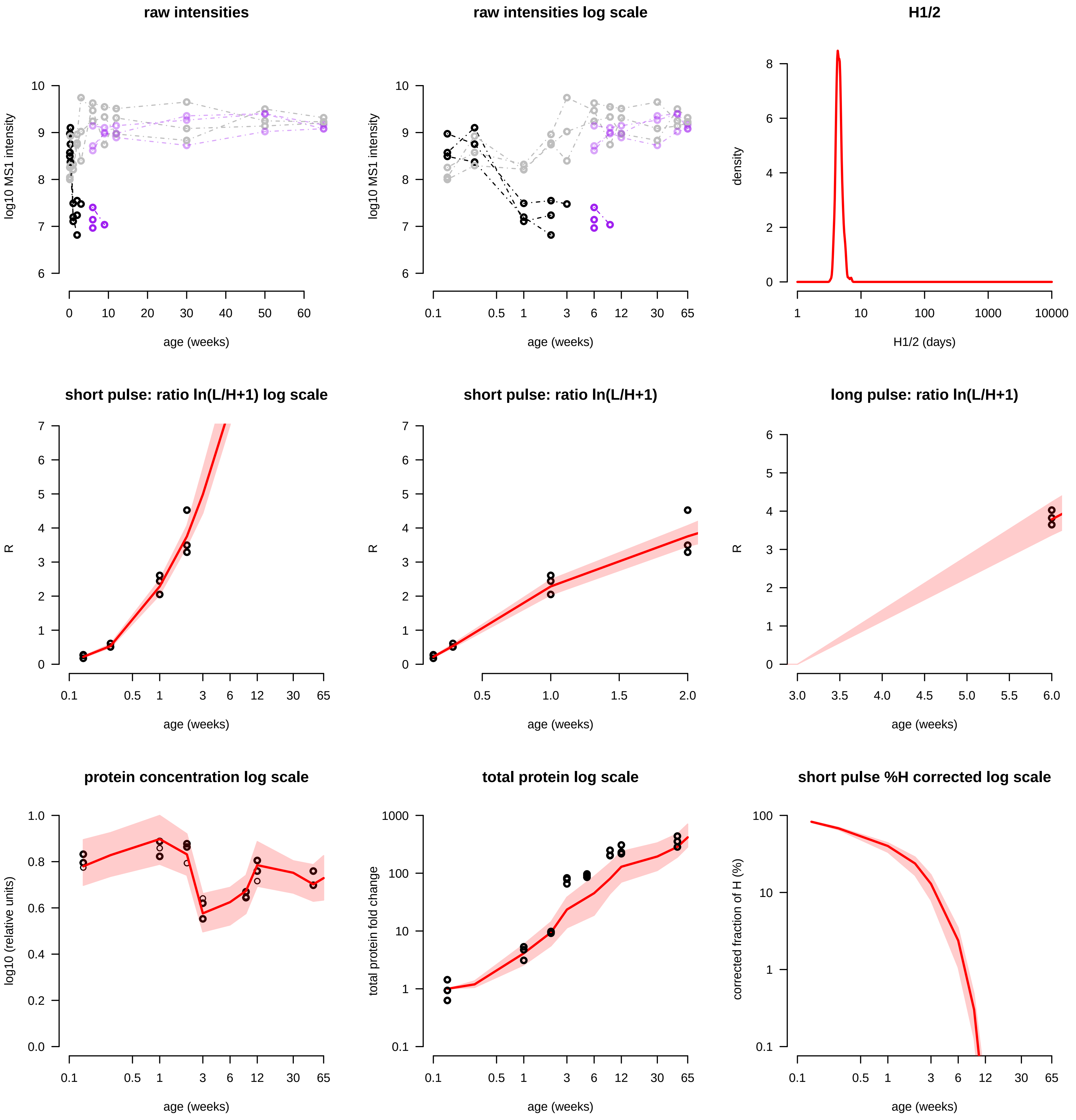

Q05816

>sp|Q05816|FABP5_MOUSE

Fatty acid−binding protein 5

GN=Fabp5

Med H1/2: 440.8 days

Q5 H1/2: 105.9 days

Q95 H1/2: 5405.1 days

reliable model fit: no

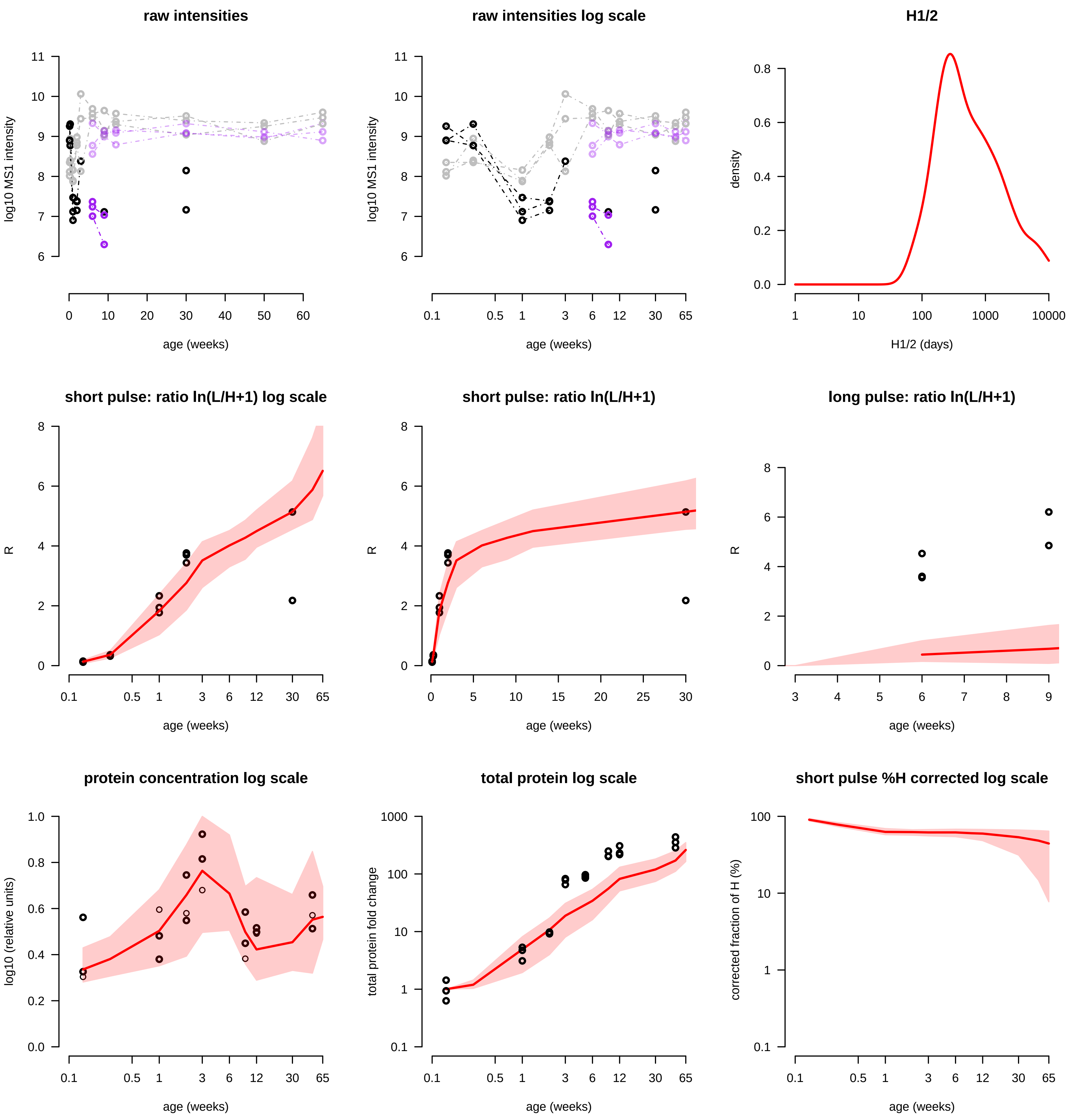

Q922Q8

>sp|Q922Q8|LRC59_MOUSE

Leucine−rich repeat−containing protein 59

GN=Lrrc59

Med H1/2: 6.2 days

Q5 H1/2: 5 days

Q95 H1/2: 7.6 days

reliable model fit: yes

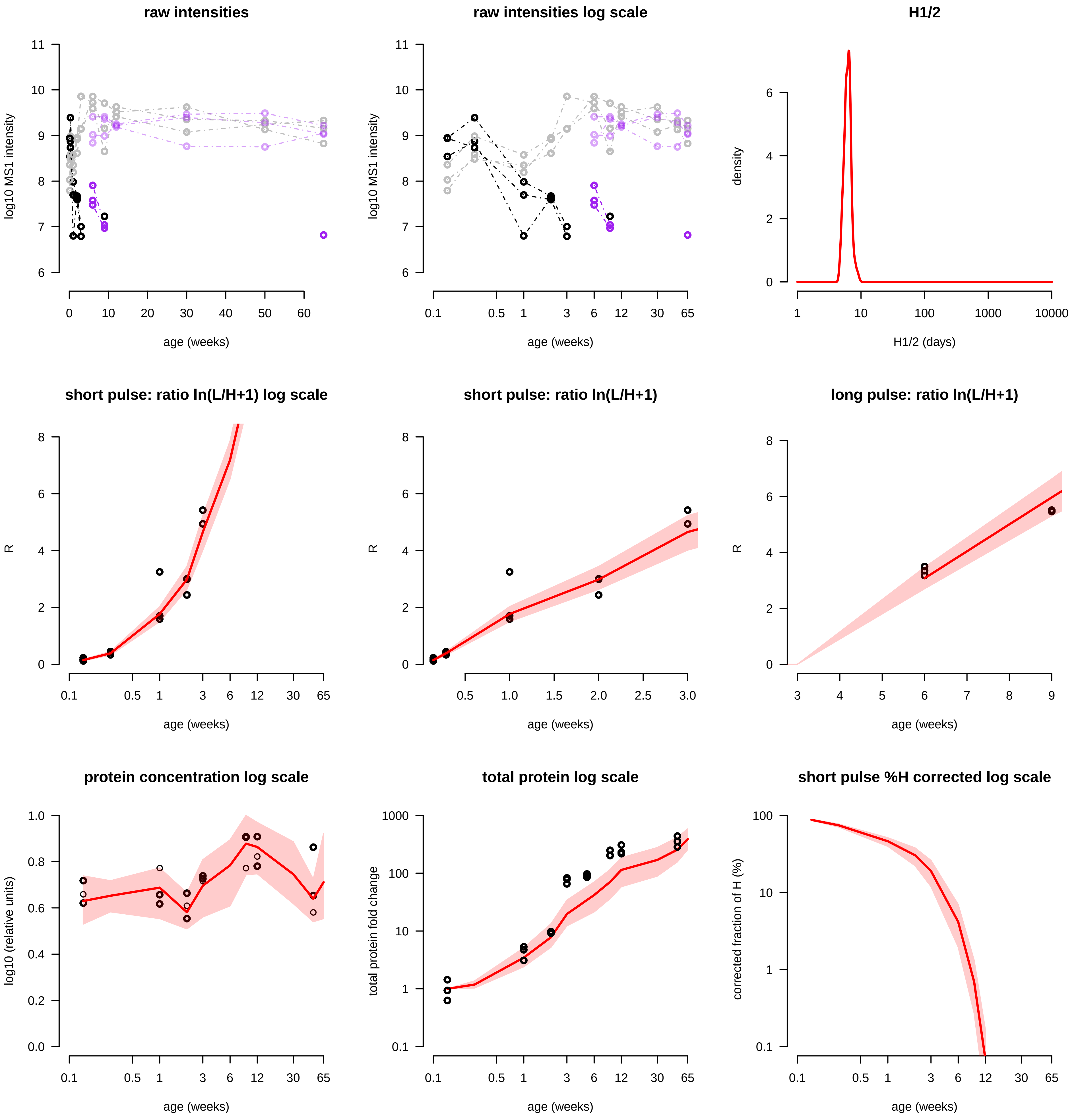

Q8BHD7

>sp|Q8BHD7|PTBP3_MOUSE

Polypyrimidine tract−binding protein 3

GN=Ptbp3

Med H1/2: 13.8 days

Q5 H1/2: 12.1 days

Q95 H1/2: 18.2 days

reliable model fit: yes

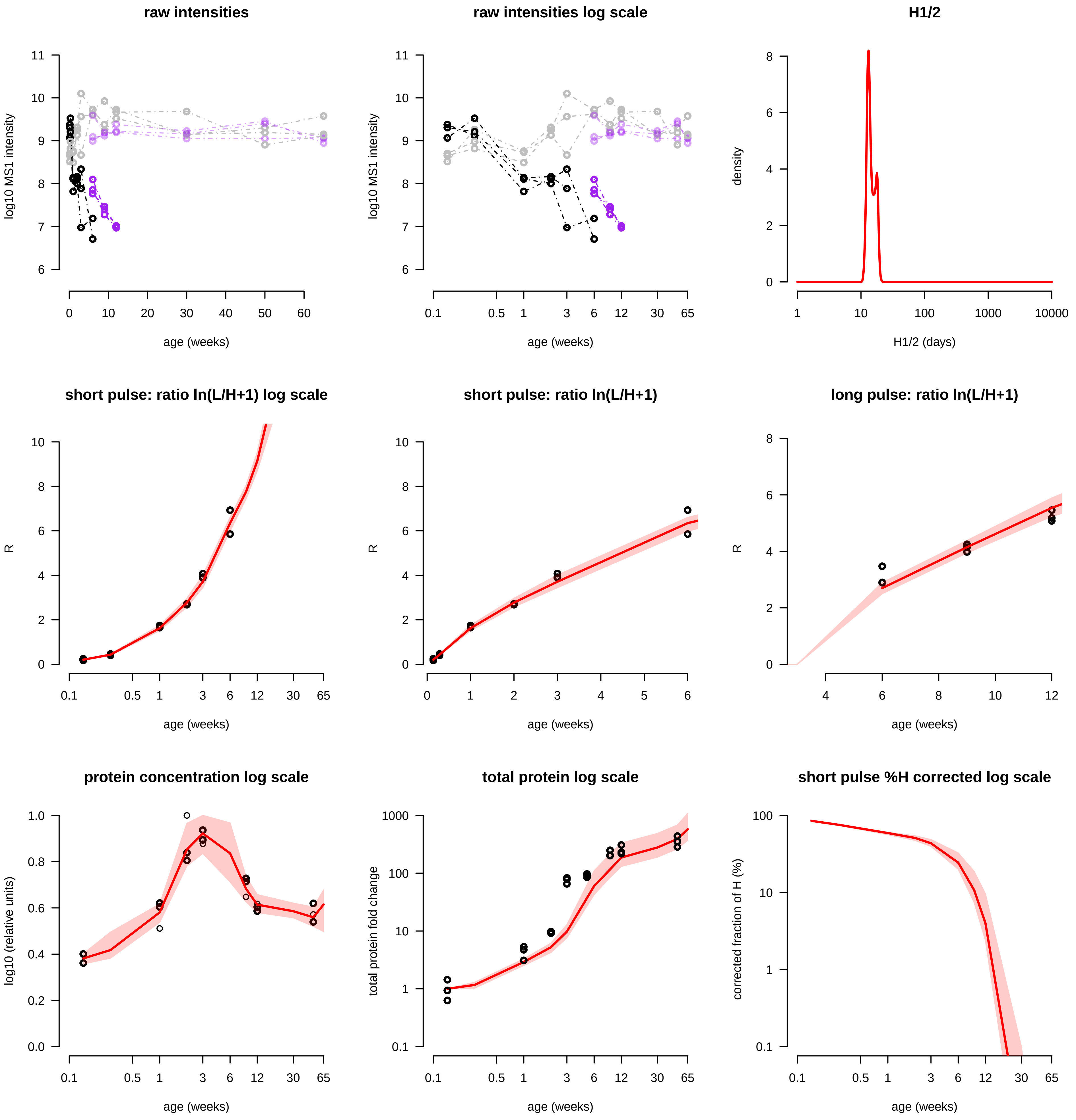

Q68FL6

>sp|Q68FL6|SYMC_MOUSE

Methionine−−tRNA ligase, cytoplasmic

GN=Mars1

Med H1/2: 5.7 days

Q5 H1/2: 5 days

Q95 H1/2: 7.3 days

reliable model fit: yes

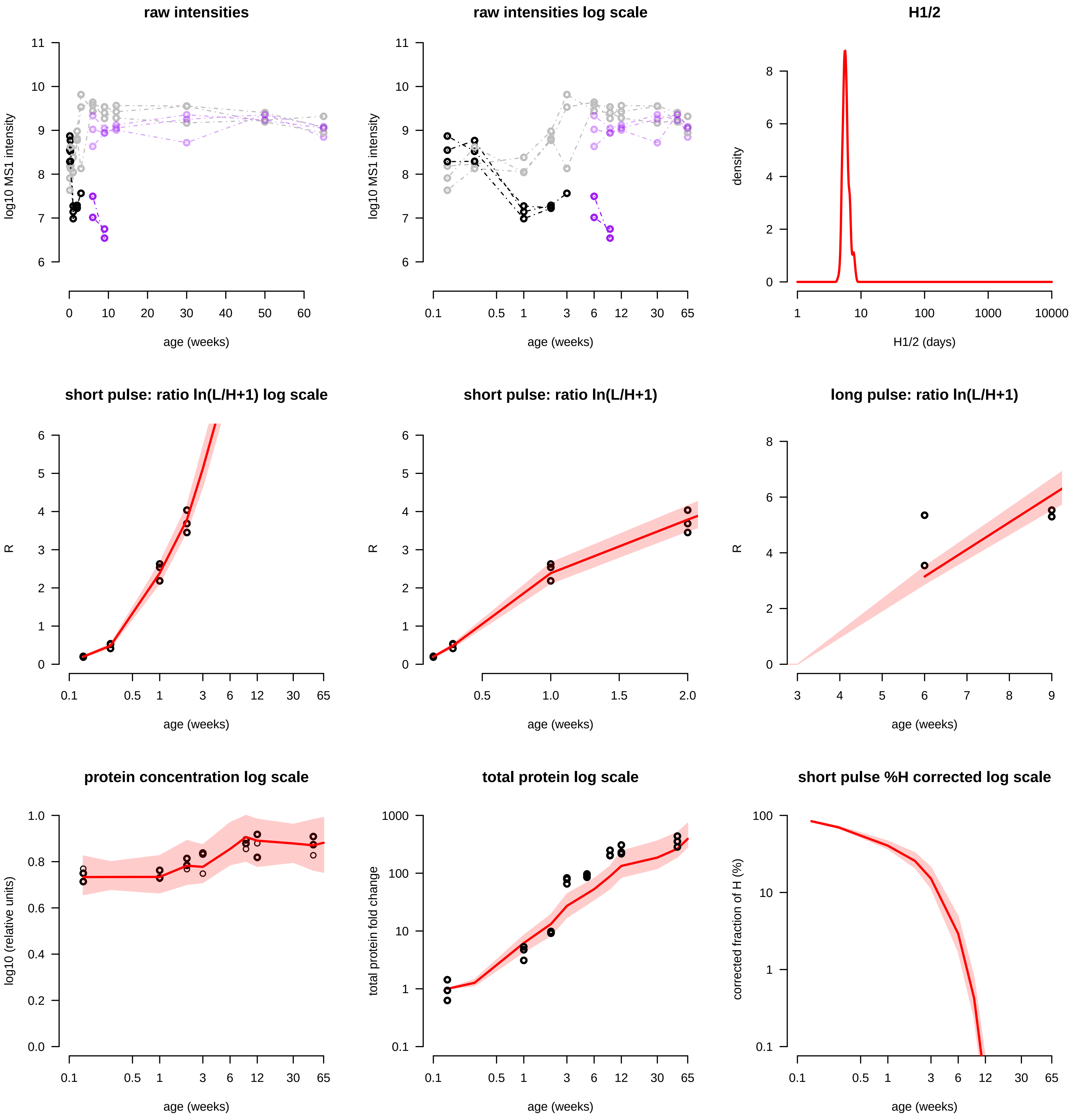

P63323

>sp|P63323|RS12_MOUSE

40S ribosomal protein S12

GN=Rps12

Med H1/2: 9.6 days

Q5 H1/2: 7.5 days

Q95 H1/2: 13.6 days

reliable model fit: yes

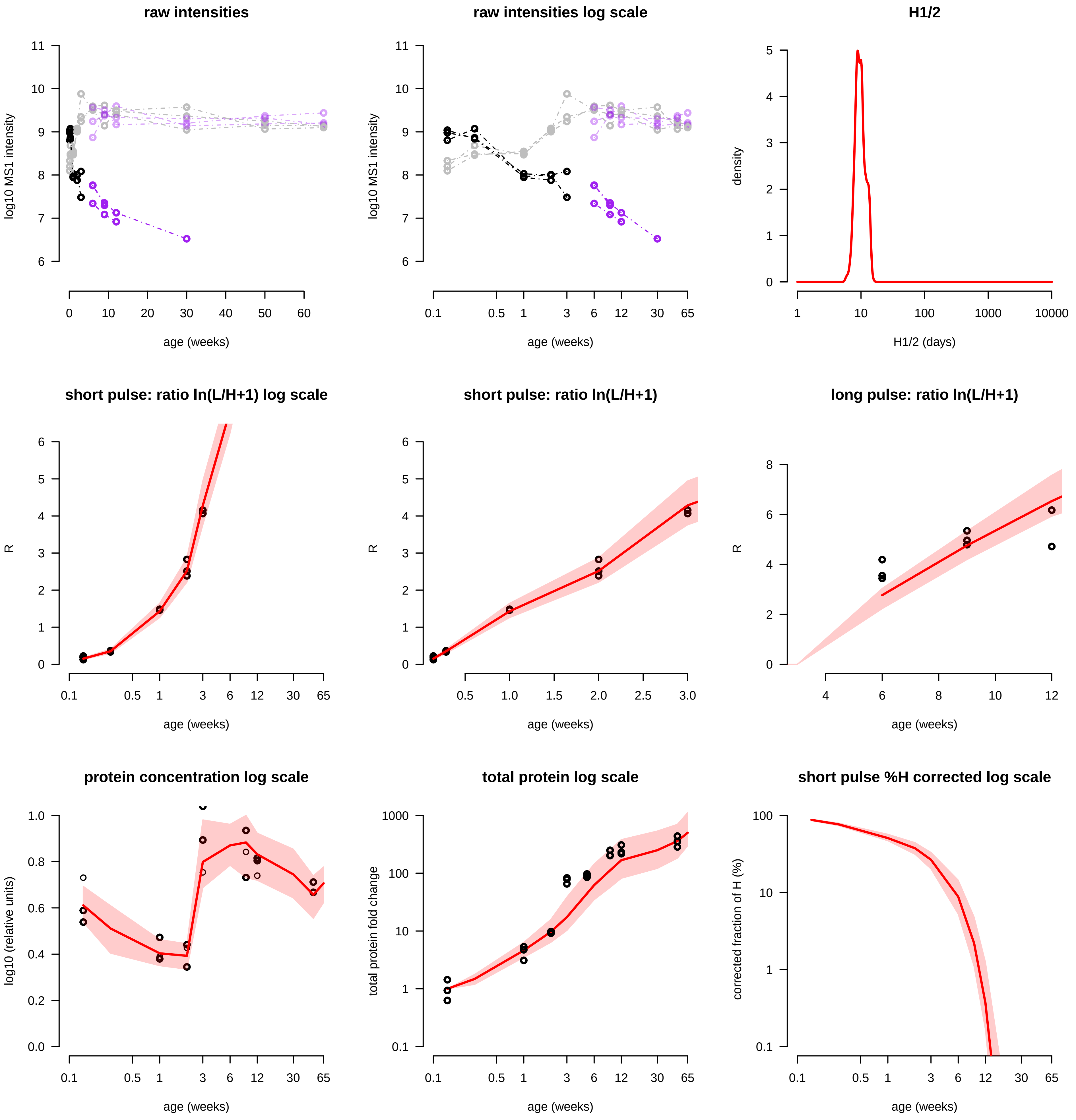

P63005;P63005−2

>sp|P63005|LIS1_MOUSE

Platelet−activating factor acetylhydrolase IB subunit beta

GN=Pafah1b1

Med H1/2: 4.6 days

Q5 H1/2: 3.7 days

Q95 H1/2: 6.1 days

reliable model fit: yes

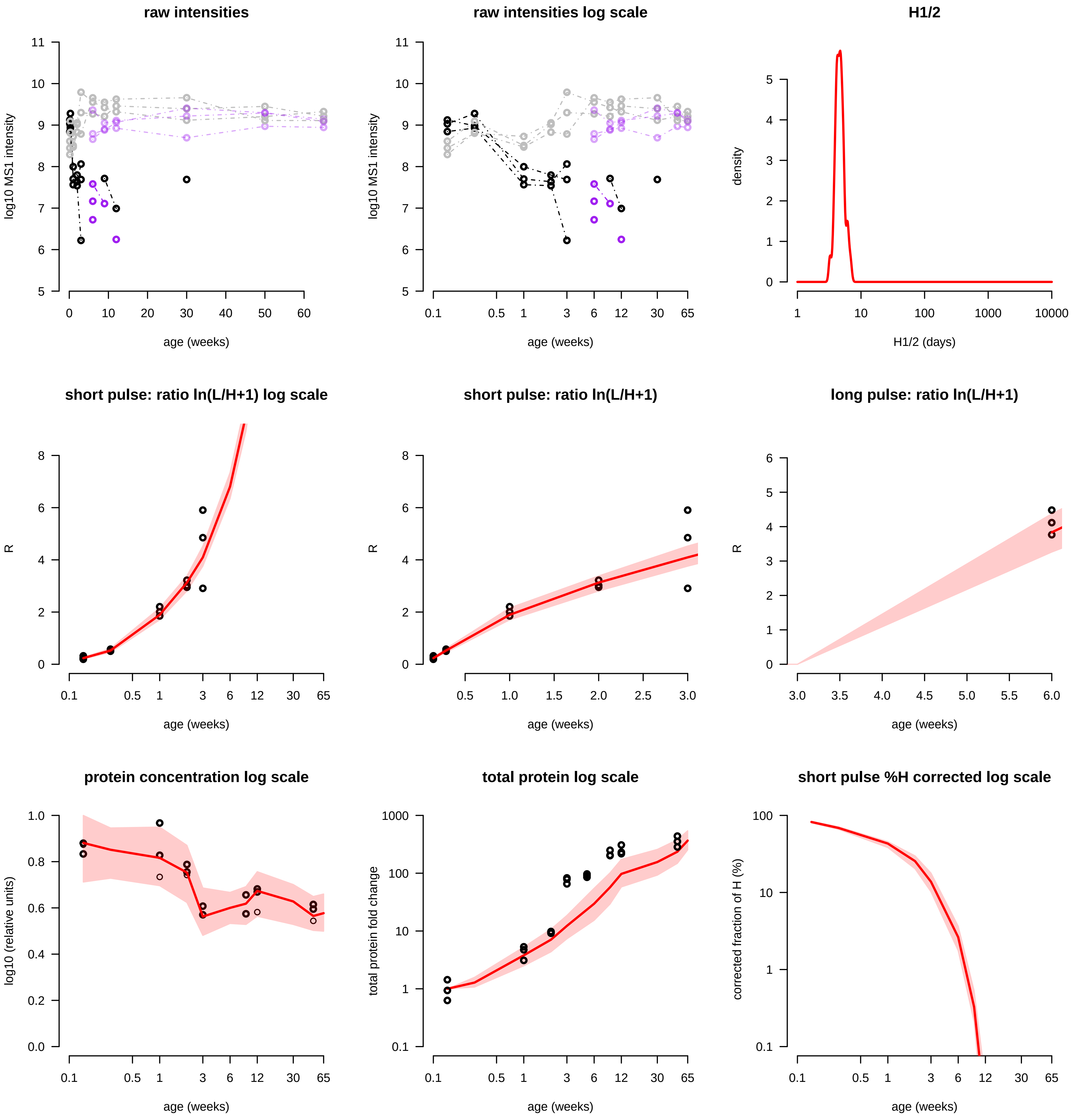

Q60737

>sp|Q60737|CSK21_MOUSE

Casein kinase II subunit alpha

GN=Csnk2a1

Med H1/2: 5.3 days

Q5 H1/2: 4.3 days

Q95 H1/2: 6.5 days

reliable model fit: yes

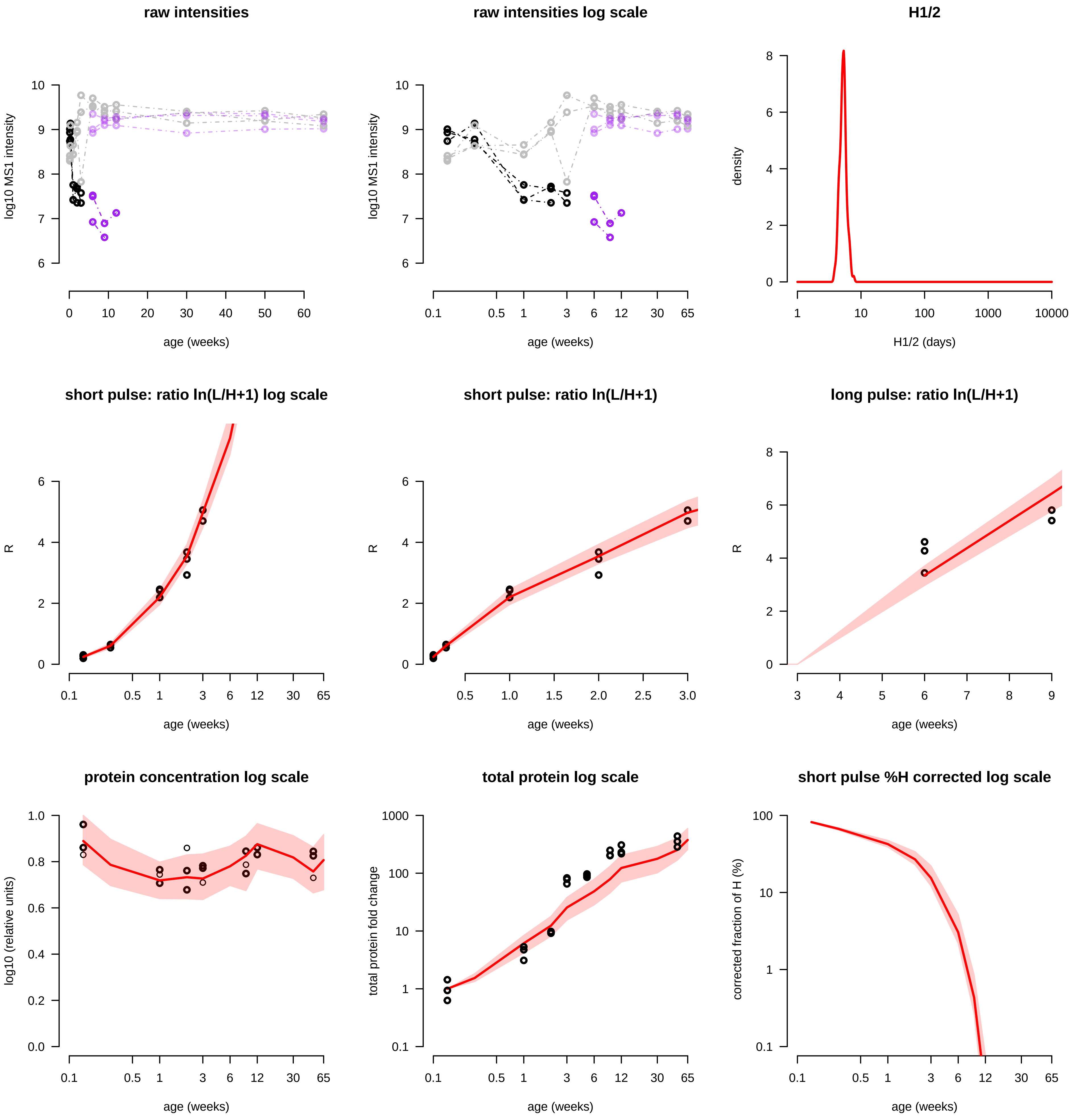

O35855

>sp|O35855|BCAT2_MOUSE

Branched−chain−amino−acid aminotransferase, mitochondrial

GN=Bcat2

Med H1/2: 3.9 days

Q5 H1/2: 3.3 days

Q95 H1/2: 4.8 days

reliable model fit: yes

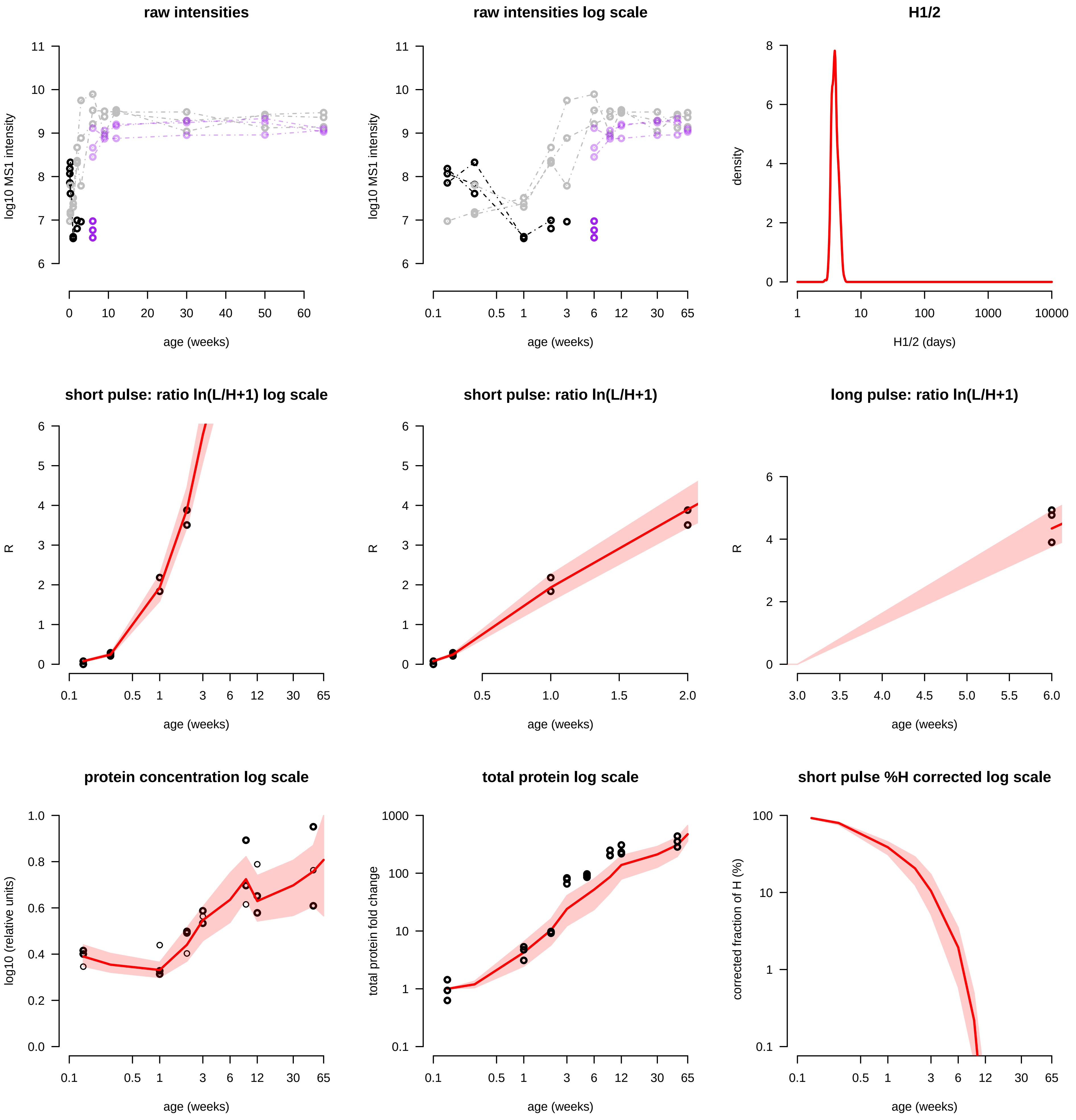

Q9R0A0

>sp|Q9R0A0|PEX14_MOUSE

Peroxisomal membrane protein PEX14

GN=Pex14

Med H1/2: 3.8 days

Q5 H1/2: 3.1 days

Q95 H1/2: 4.7 days

reliable model fit: yes

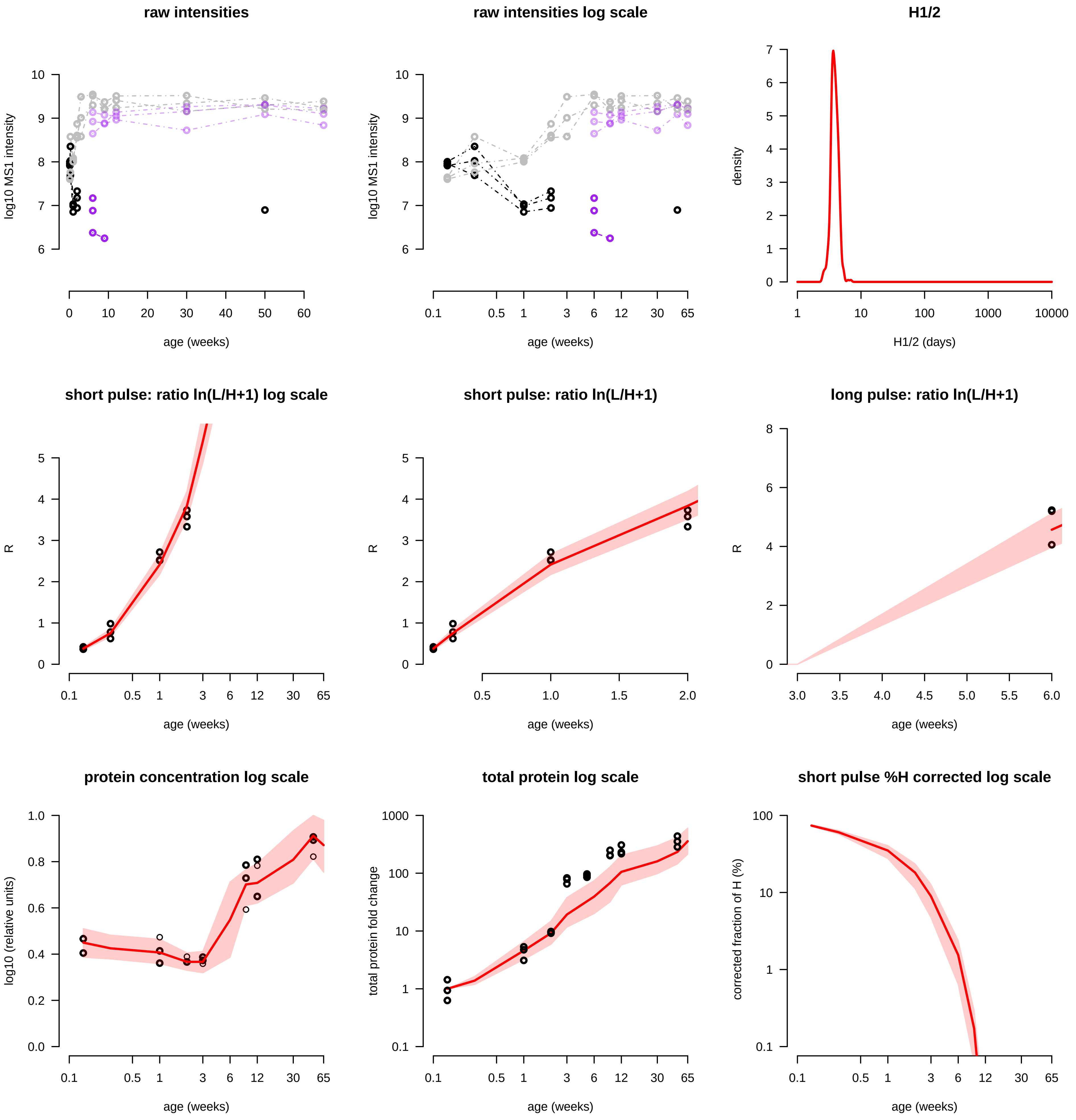

P34022

>sp|P34022|RANG_MOUSE

Ran−specific GTPase−activating protein

GN=Ranbp1

Med H1/2: 5.8 days

Q5 H1/2: 5.1 days

Q95 H1/2: 7 days

reliable model fit: yes

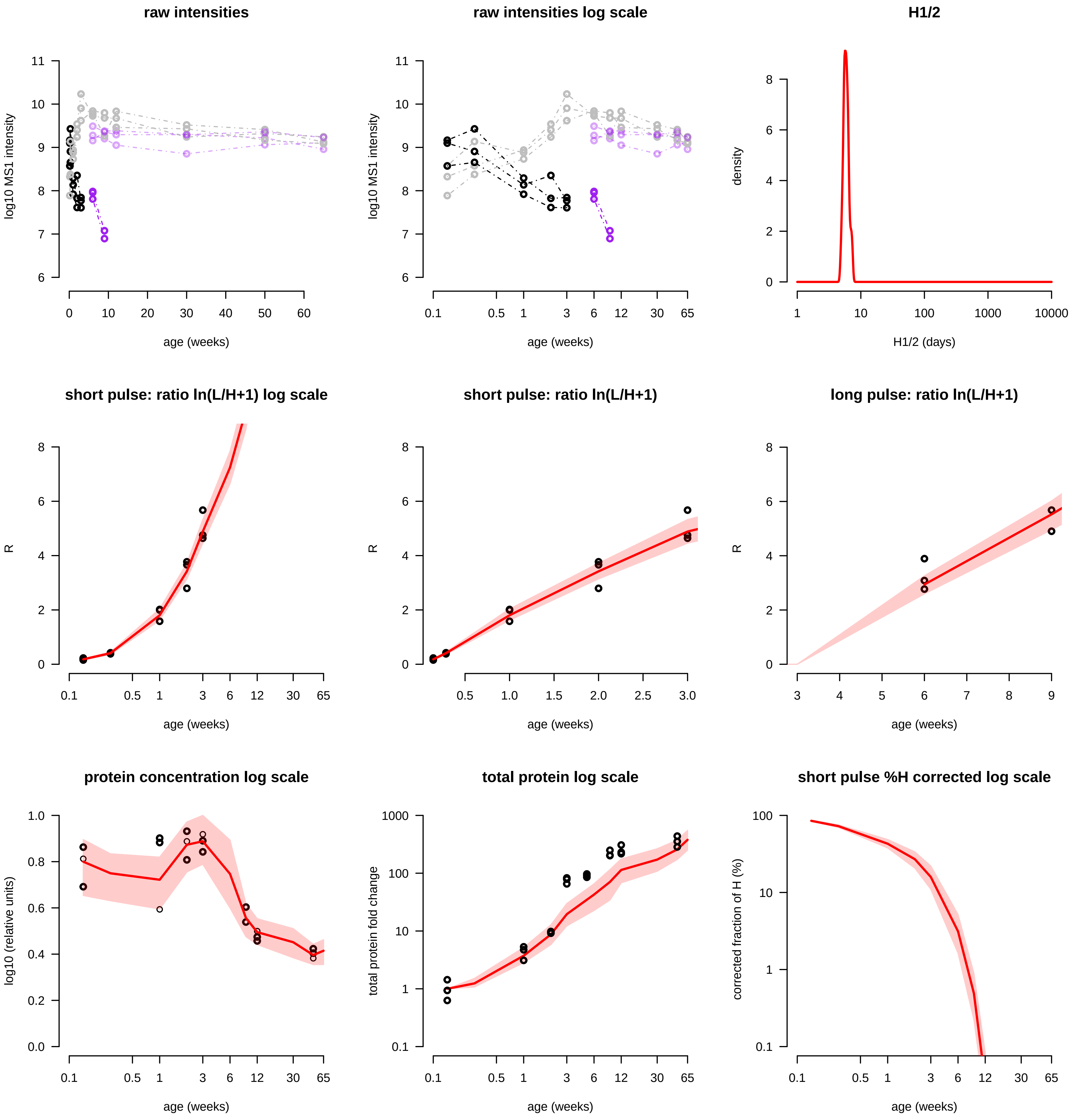

P61161

>sp|P61161|ARP2_MOUSE

Actin−related protein 2

GN=Actr2

Med H1/2: 9.7 days

Q5 H1/2: 7.5 days

Q95 H1/2: 13.1 days

reliable model fit: yes

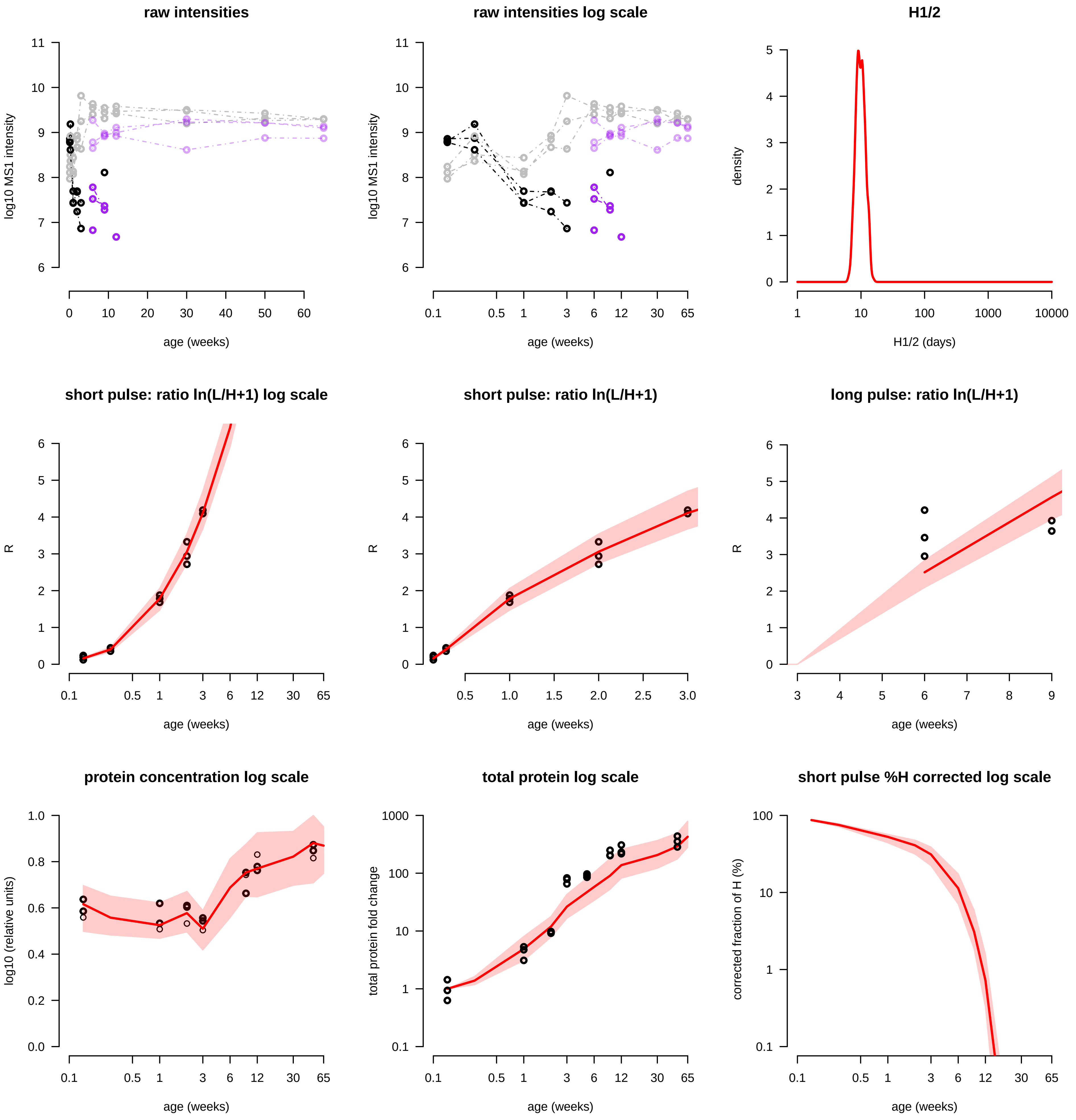

Q80W54

>sp|Q80W54|FACE1_MOUSE

CAAX prenyl protease 1 homolog

GN=Zmpste24

Med H1/2: 5.7 days

Q5 H1/2: 4.5 days

Q95 H1/2: 7.8 days

reliable model fit: yes

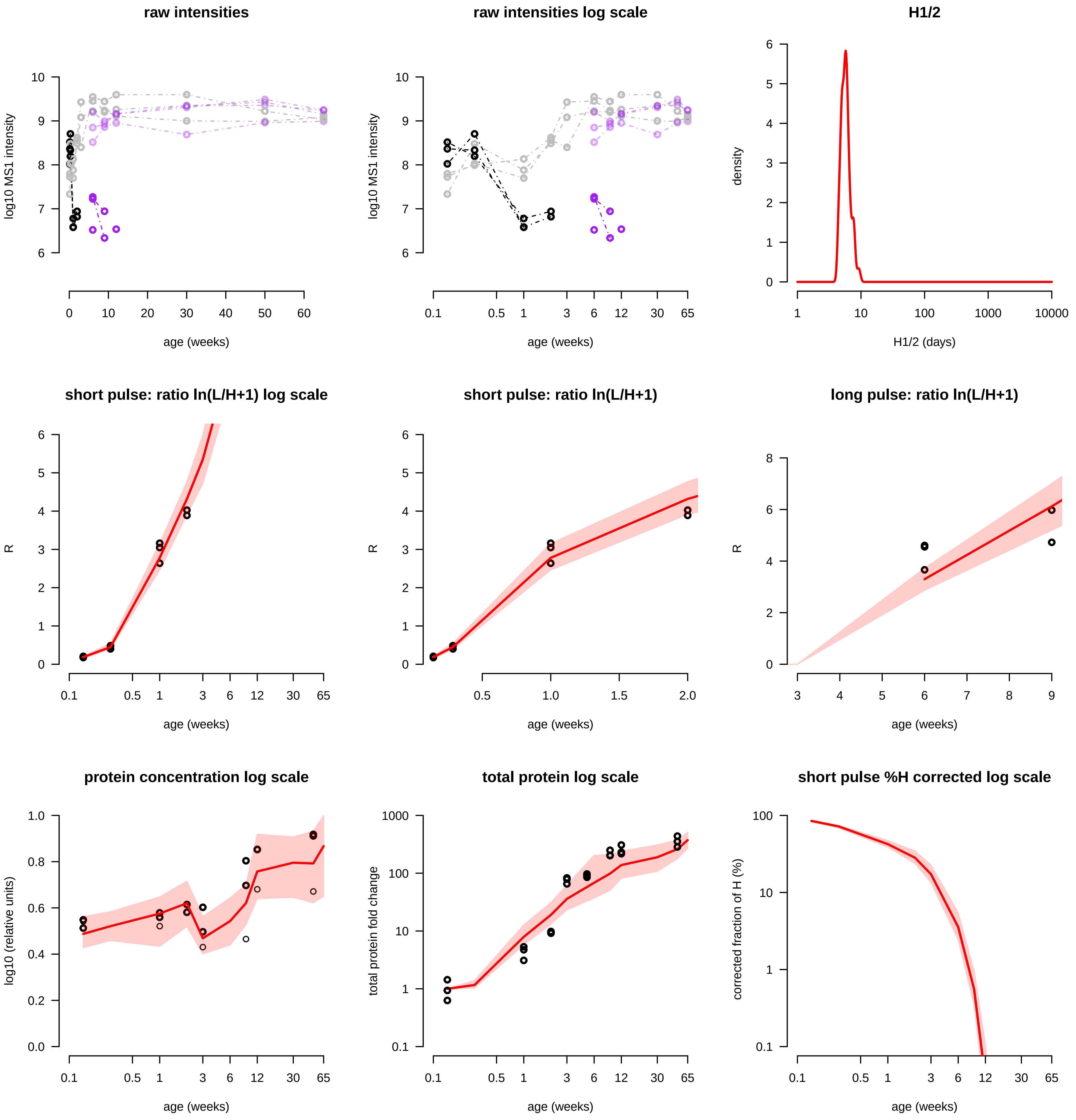

Q99L45

>sp|Q99L45|IF2B_MOUSE

Eukaryotic translation initiation factor 2 subunit 2

GN=Eif2s2

Med H1/2: 774.9 days

Q5 H1/2: 171.1 days

Q95 H1/2: 13004.6 days

reliable model fit: no

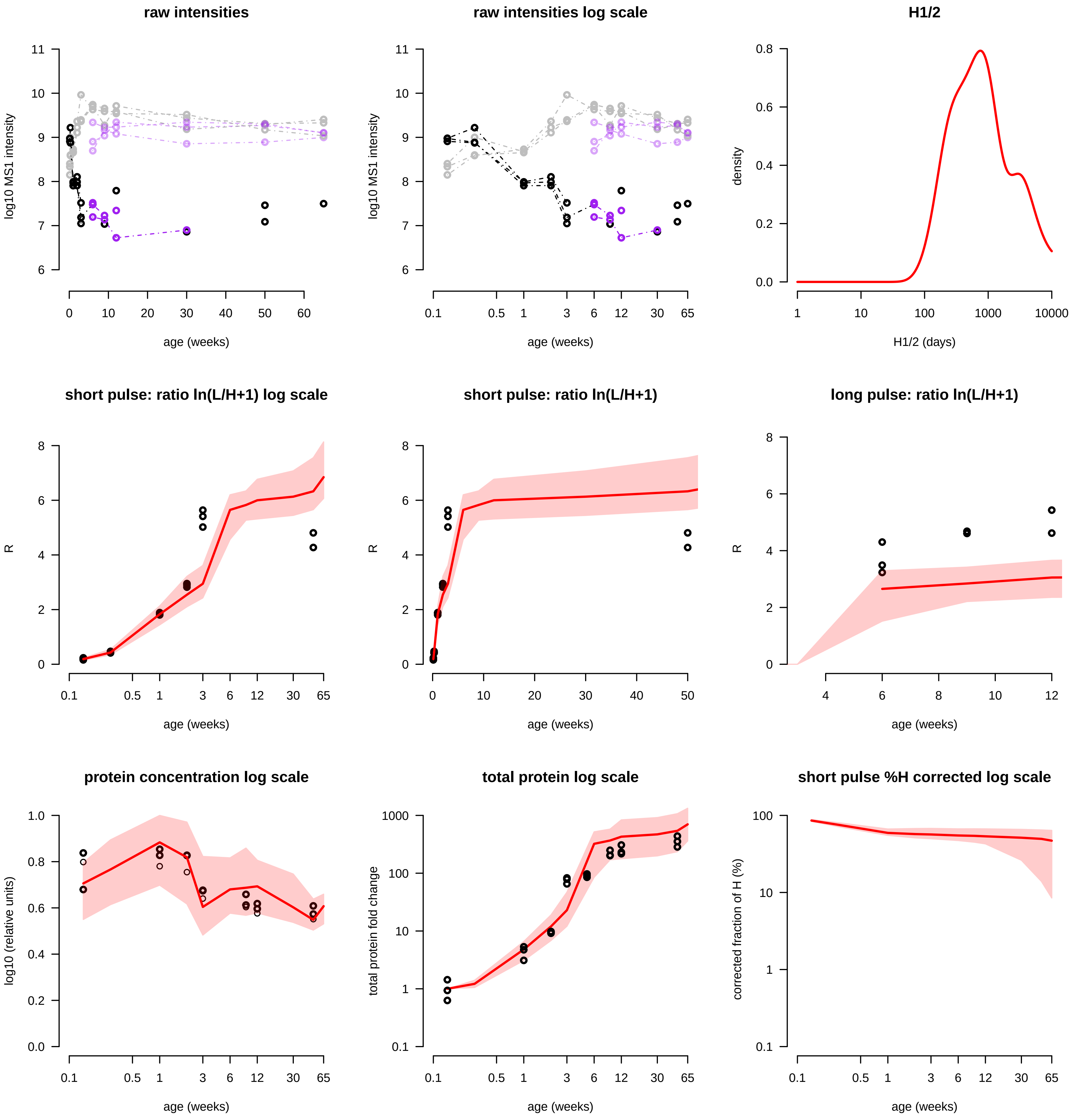

O88958

>sp|O88958|GNPI1_MOUSE

Glucosamine−6−phosphate isomerase 1

GN=Gnpda1

Med H1/2: 5.6 days

Q5 H1/2: 4.5 days

Q95 H1/2: 7.8 days

reliable model fit: yes

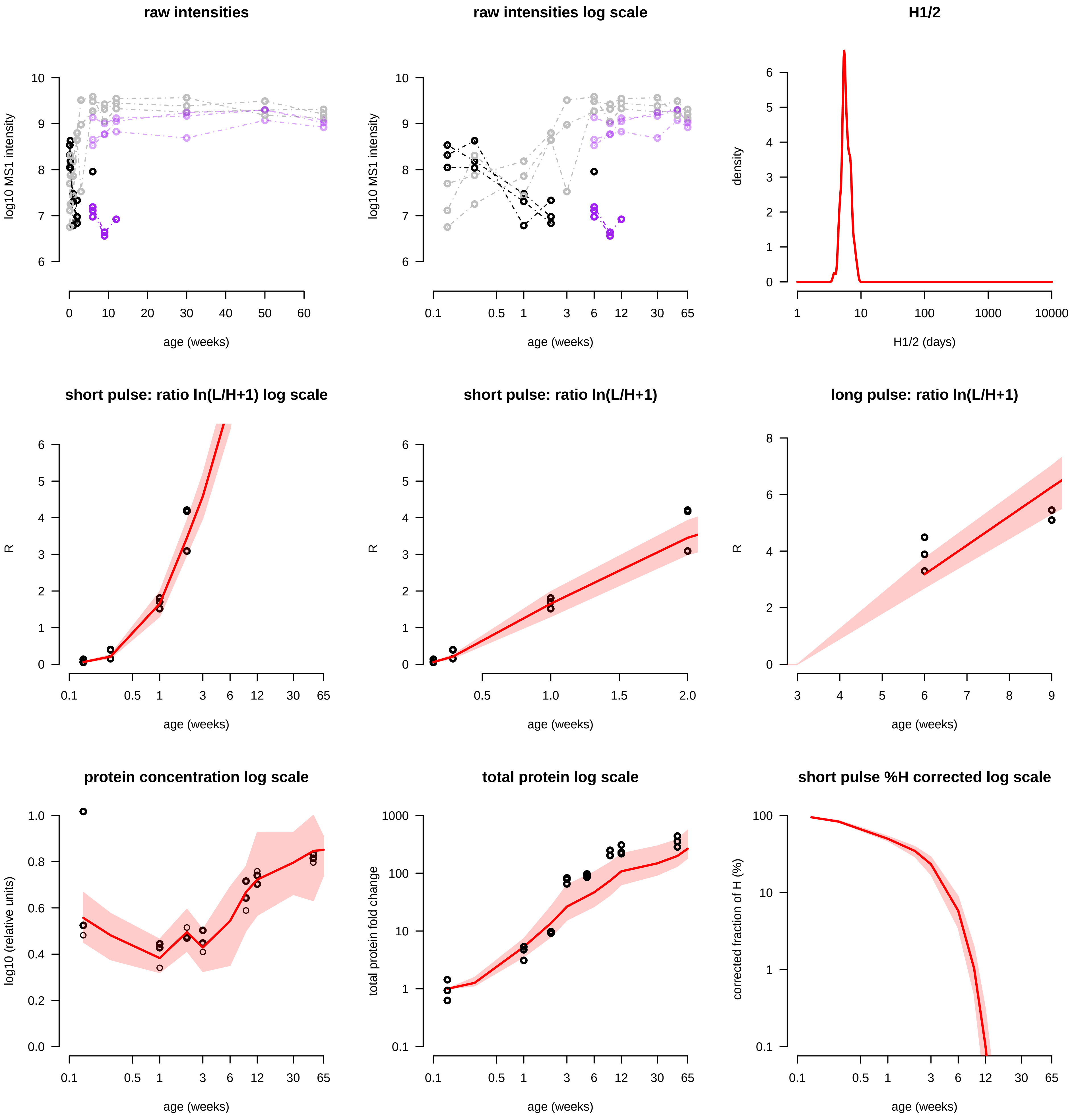

P61222

>sp|P61222|ABCE1_MOUSE

ATP−binding cassette sub−family E member 1

GN=Abce1

Med H1/2: 3.5 days

Q5 H1/2: 3 days

Q95 H1/2: 4.2 days

reliable model fit: yes

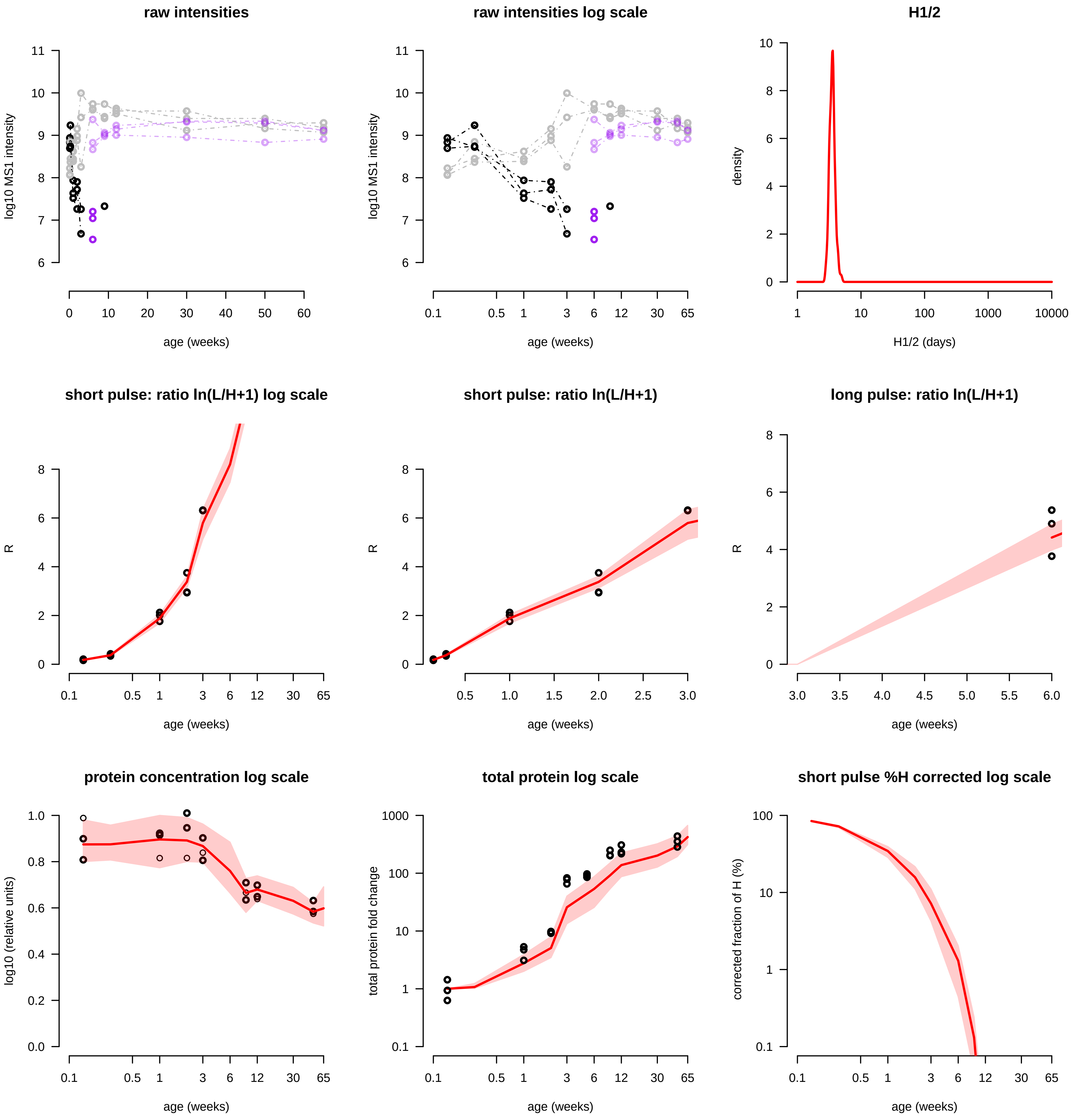

P97855

>sp|P97855|G3BP1_MOUSE

Ras GTPase−activating protein−binding protein 1

GN=G3bp1

Med H1/2: 11.8 days

Q5 H1/2: 8.7 days

Q95 H1/2: 16.2 days

reliable model fit: yes

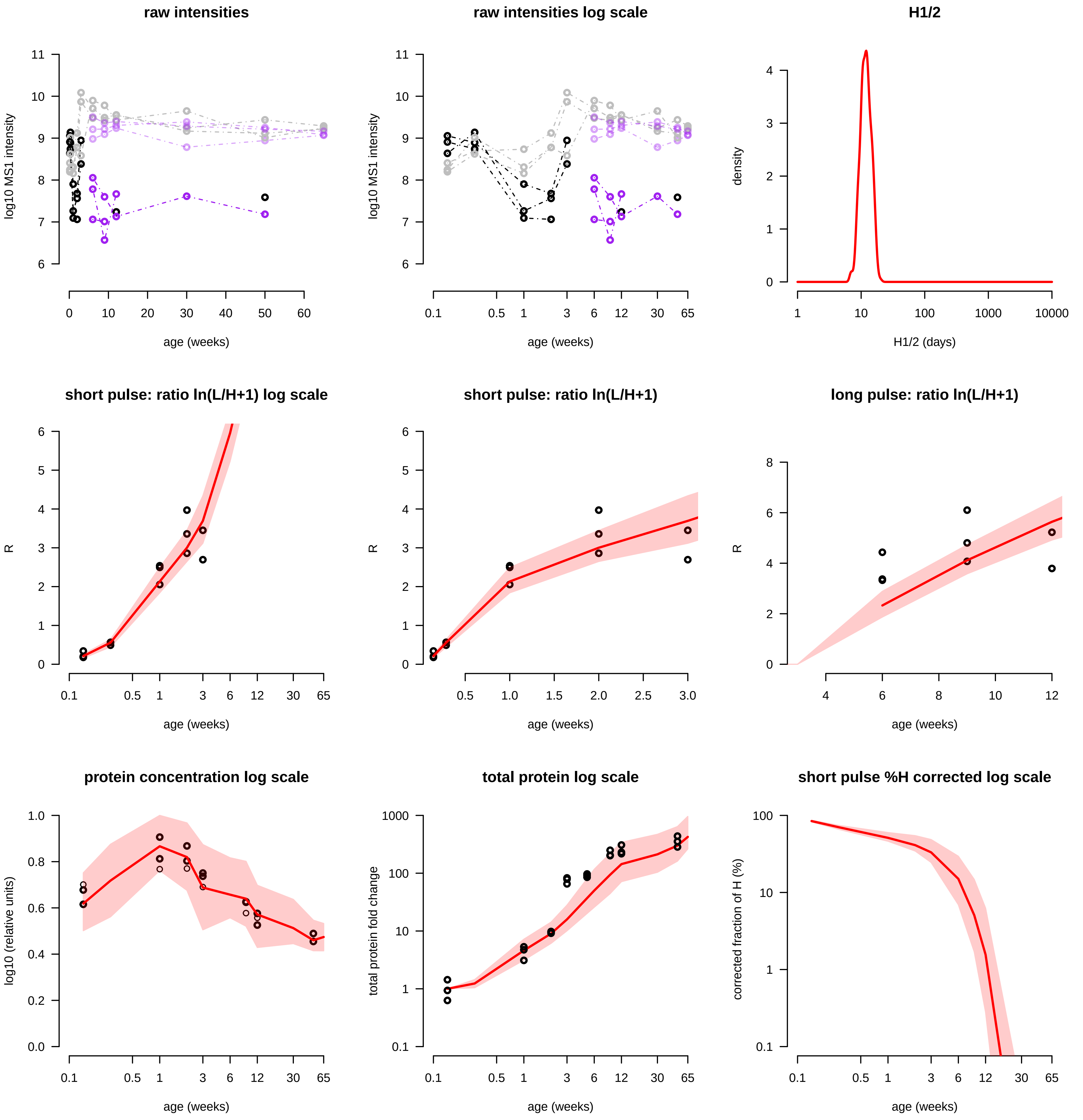

Q6ZWU9

>sp|Q6ZWU9|RS27_MOUSE

40S ribosomal protein S27

GN=Rps27

Med H1/2: 8 days

Q5 H1/2: 6.7 days

Q95 H1/2: 10.2 days

reliable model fit: yes

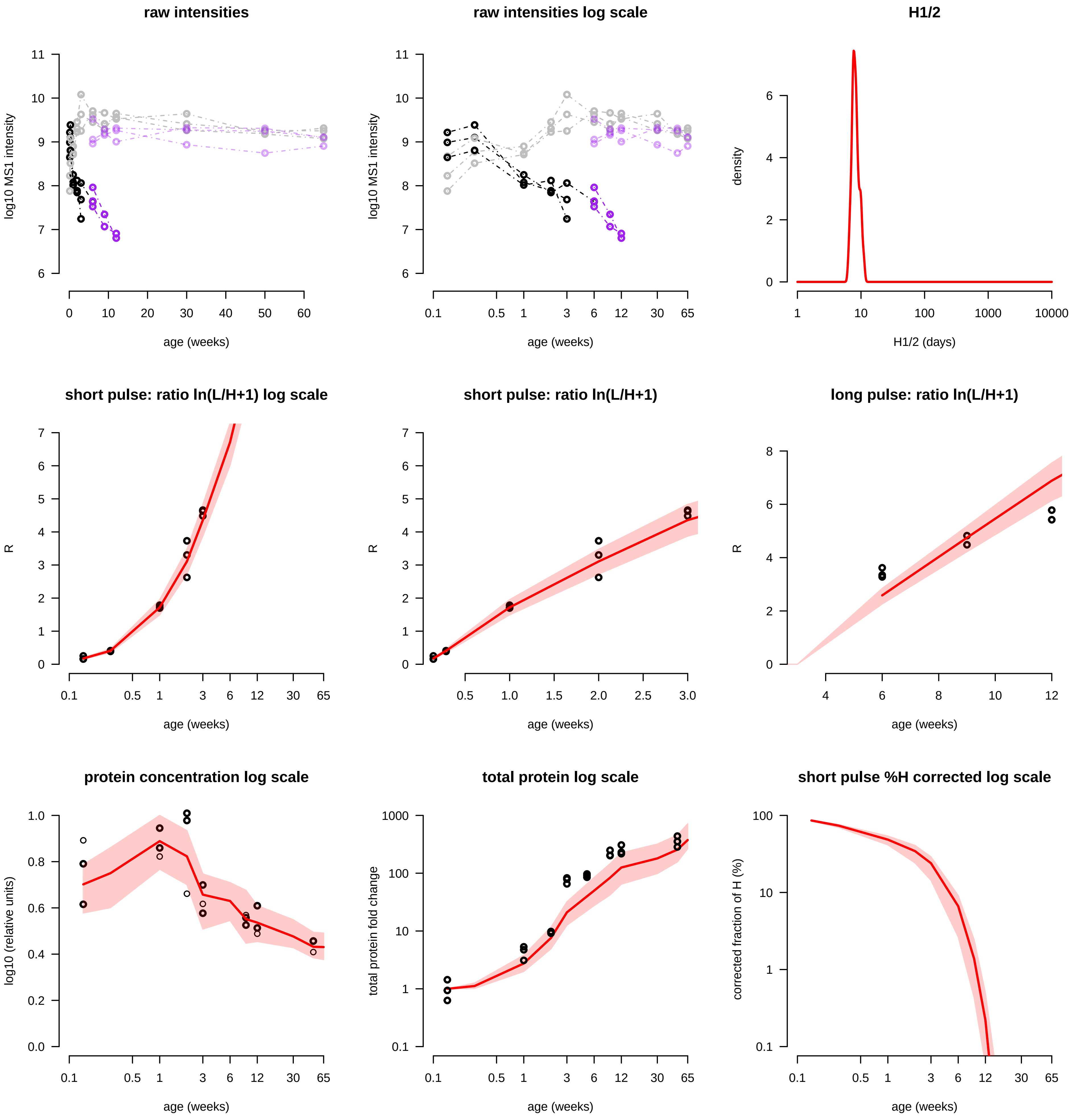

A6X935;A6X935−2

>sp|A6X935|ITIH4_MOUSE

Inter alpha−trypsin inhibitor, heavy chain 4

GN=Itih4

Med H1/2: 18.2 days

Q5 H1/2: 13 days

Q95 H1/2: 29.3 days

reliable model fit: yes

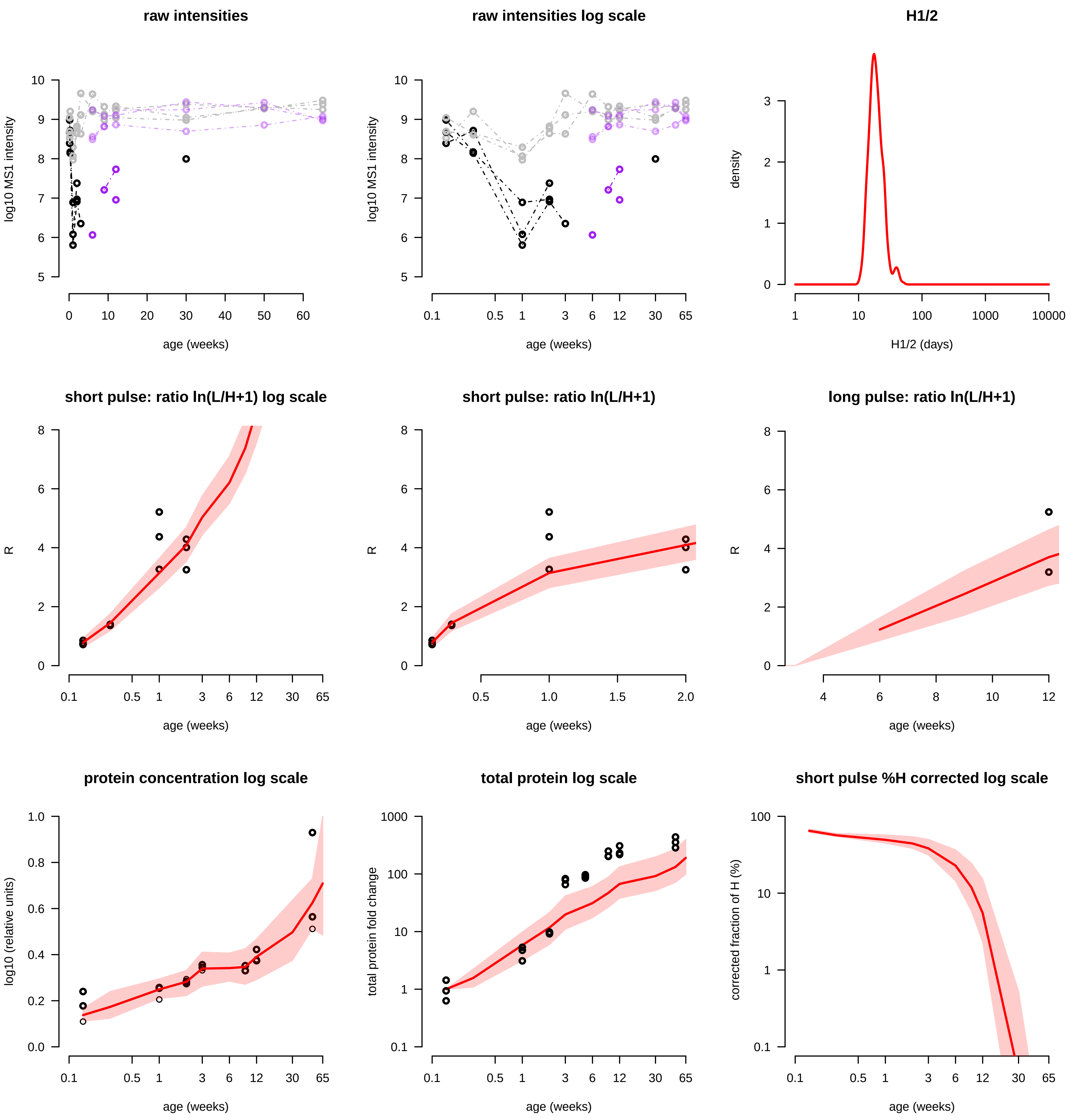

Q9JIK5

>sp|Q9JIK5|DDX21_MOUSE

Nucleolar RNA helicase 2

GN=Ddx21

Med H1/2: 5.3 days

Q5 H1/2: 4.6 days

Q95 H1/2: 6.7 days

reliable model fit: yes

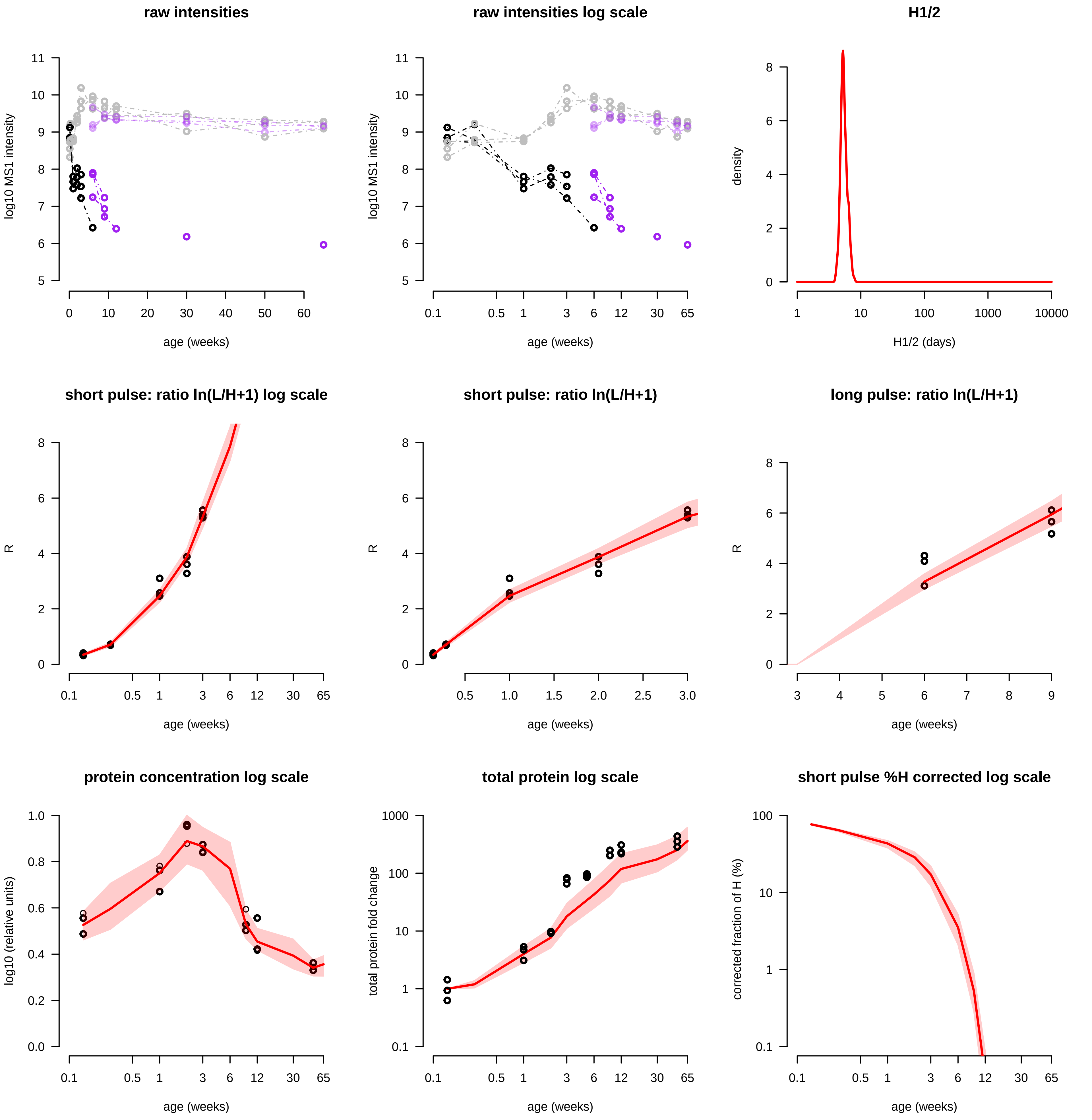

P50396

>sp|P50396|GDIA_MOUSE

Rab GDP dissociation inhibitor alpha

GN=Gdi1

Med H1/2: 7 days

Q5 H1/2: 5.2 days

Q95 H1/2: 9.3 days

reliable model fit: yes

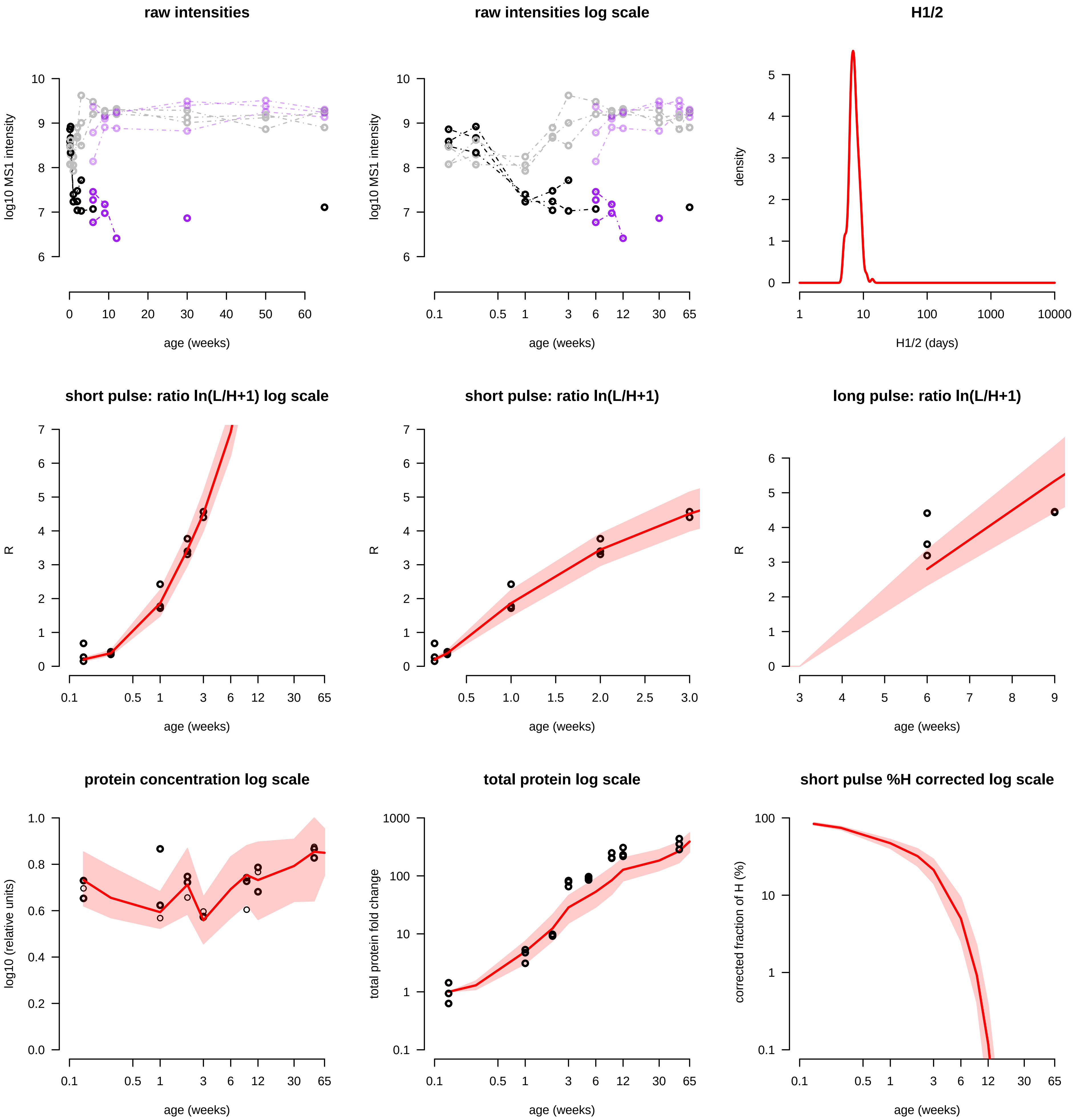

O55222

>sp|O55222|ILK_MOUSE

Integrin−linked protein kinase

GN=Ilk

Med H1/2: 4.8 days

Q5 H1/2: 4 days

Q95 H1/2: 5.8 days

reliable model fit: yes

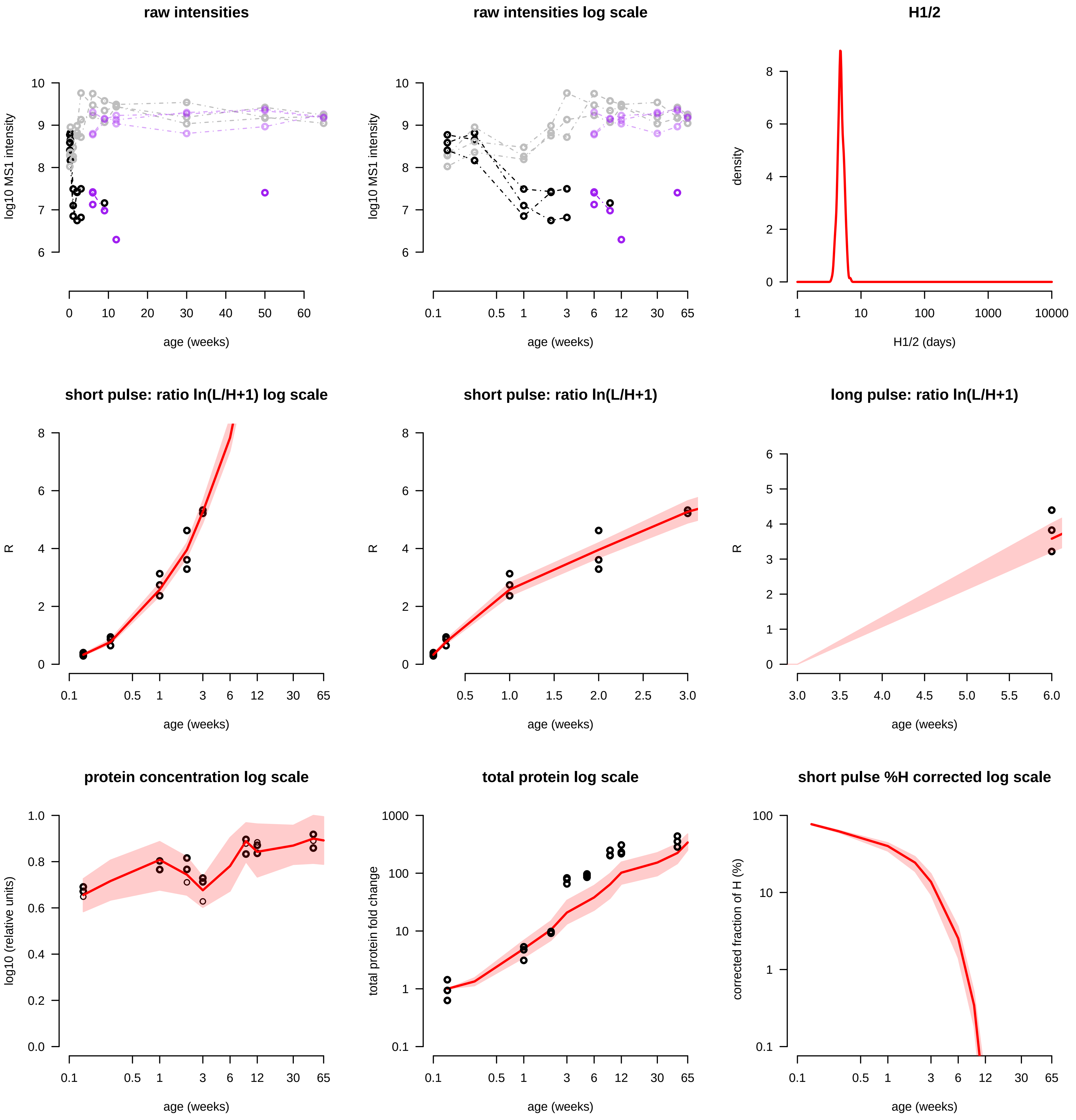

Q9CXR1

>sp|Q9CXR1|DHRS7_MOUSE

Dehydrogenase/reductase SDR family member 7

GN=Dhrs7

Med H1/2: 5 days

Q5 H1/2: 3.1 days

Q95 H1/2: 15.3 days

reliable model fit: yes

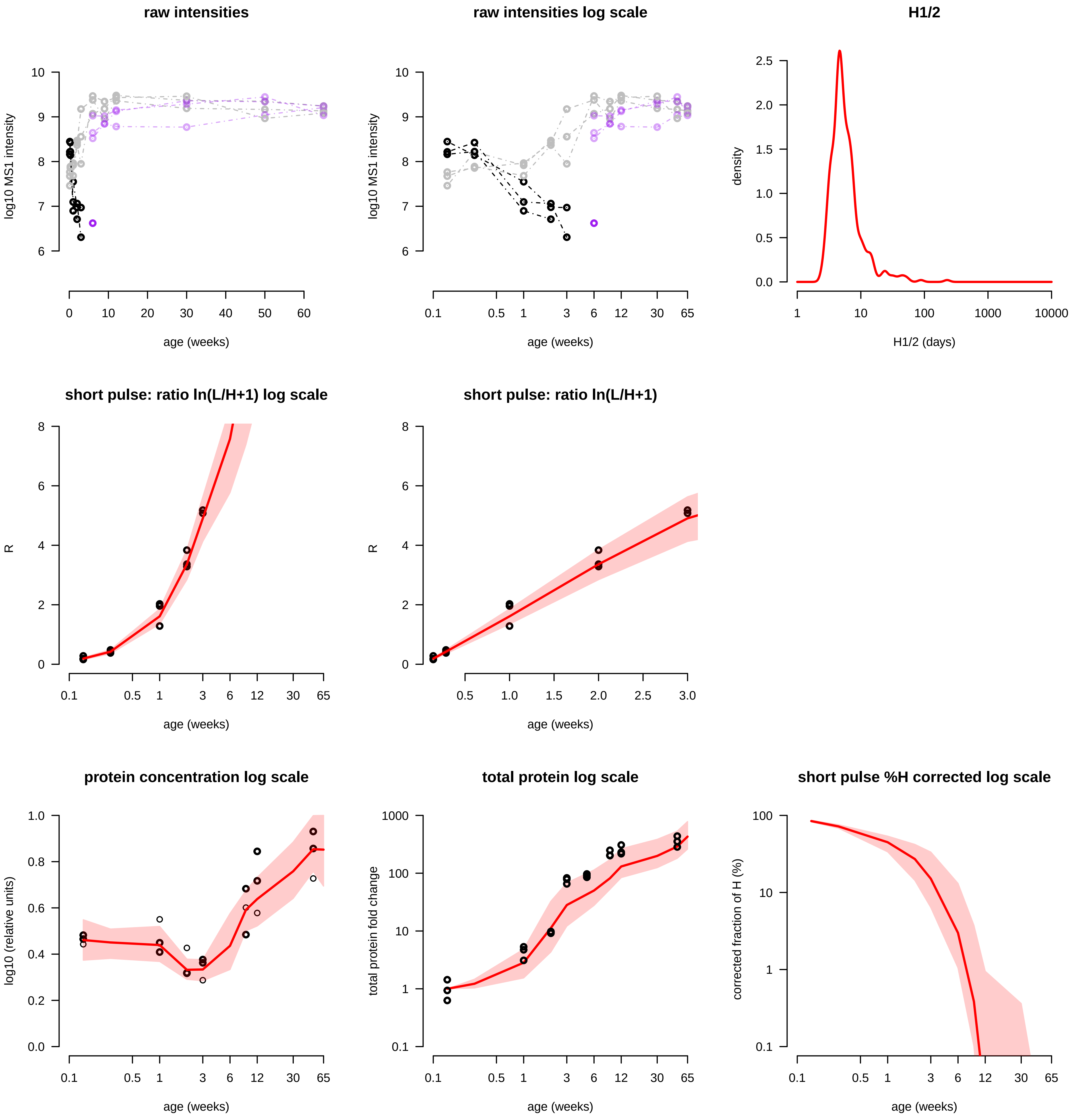

Q9D0I9

>sp|Q9D0I9|SYRC_MOUSE

Arginine−−tRNA ligase, cytoplasmic

GN=Rars1

Med H1/2: 5 days

Q5 H1/2: 4.5 days

Q95 H1/2: 6.2 days

reliable model fit: yes

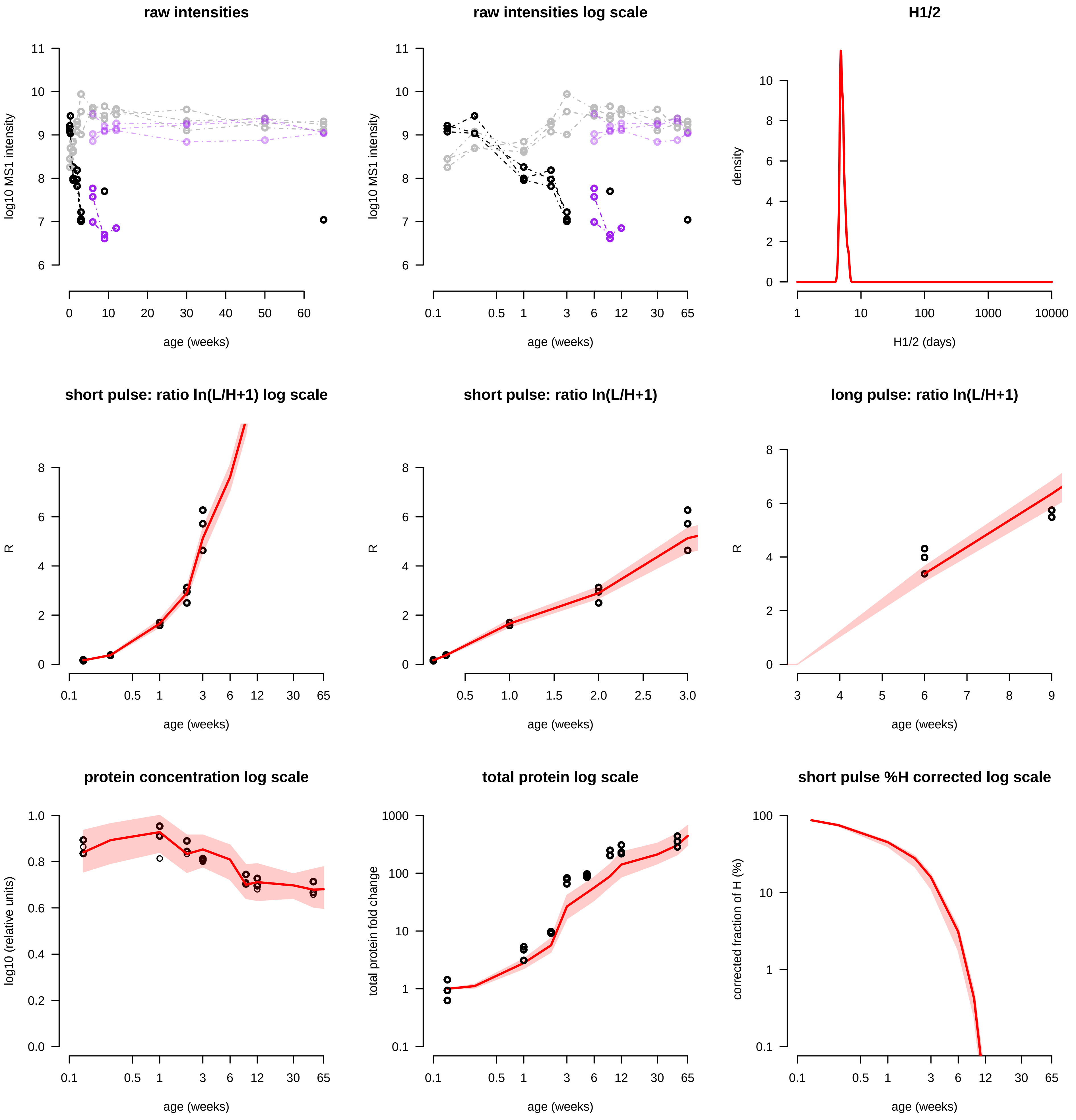

O88531

>sp|O88531|PPT1_MOUSE

Palmitoyl−protein thioesterase 1

GN=Ppt1

Med H1/2: 9.9 days

Q5 H1/2: 6.8 days

Q95 H1/2: 16.7 days

reliable model fit: yes

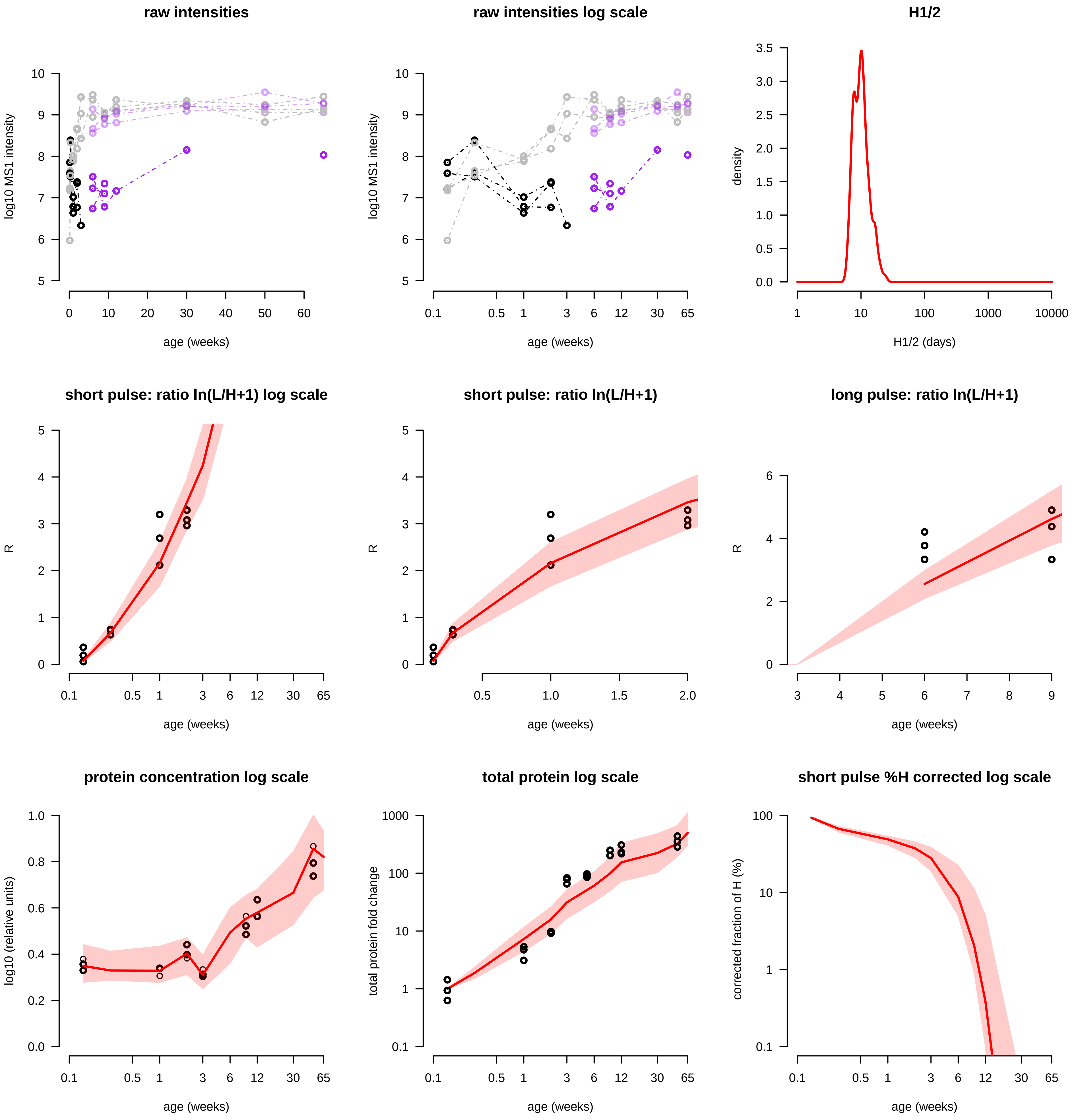

Q05D44

>sp|Q05D44|IF2P_MOUSE

Eukaryotic translation initiation factor 5B

GN=Eif5b

Med H1/2: 408.9 days

Q5 H1/2: 168 days

Q95 H1/2: 7265.1 days

reliable model fit: no

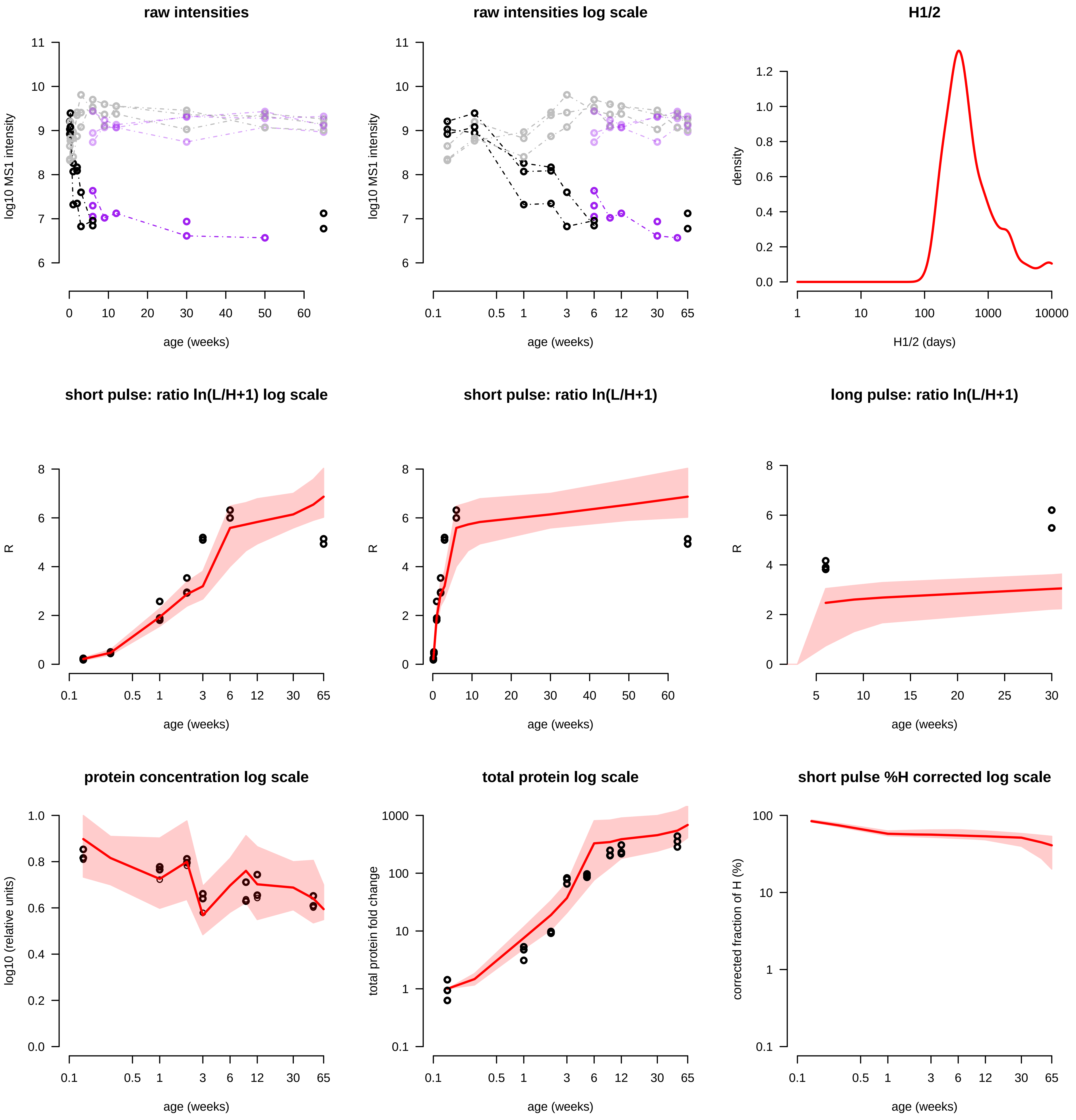

P28352

>sp|P28352|APEX1_MOUSE

DNA−(apurinic or apyrimidinic site) endonuclease

GN=Apex1

Med H1/2: 12.1 days

Q5 H1/2: 9.3 days

Q95 H1/2: 19.5 days

reliable model fit: yes

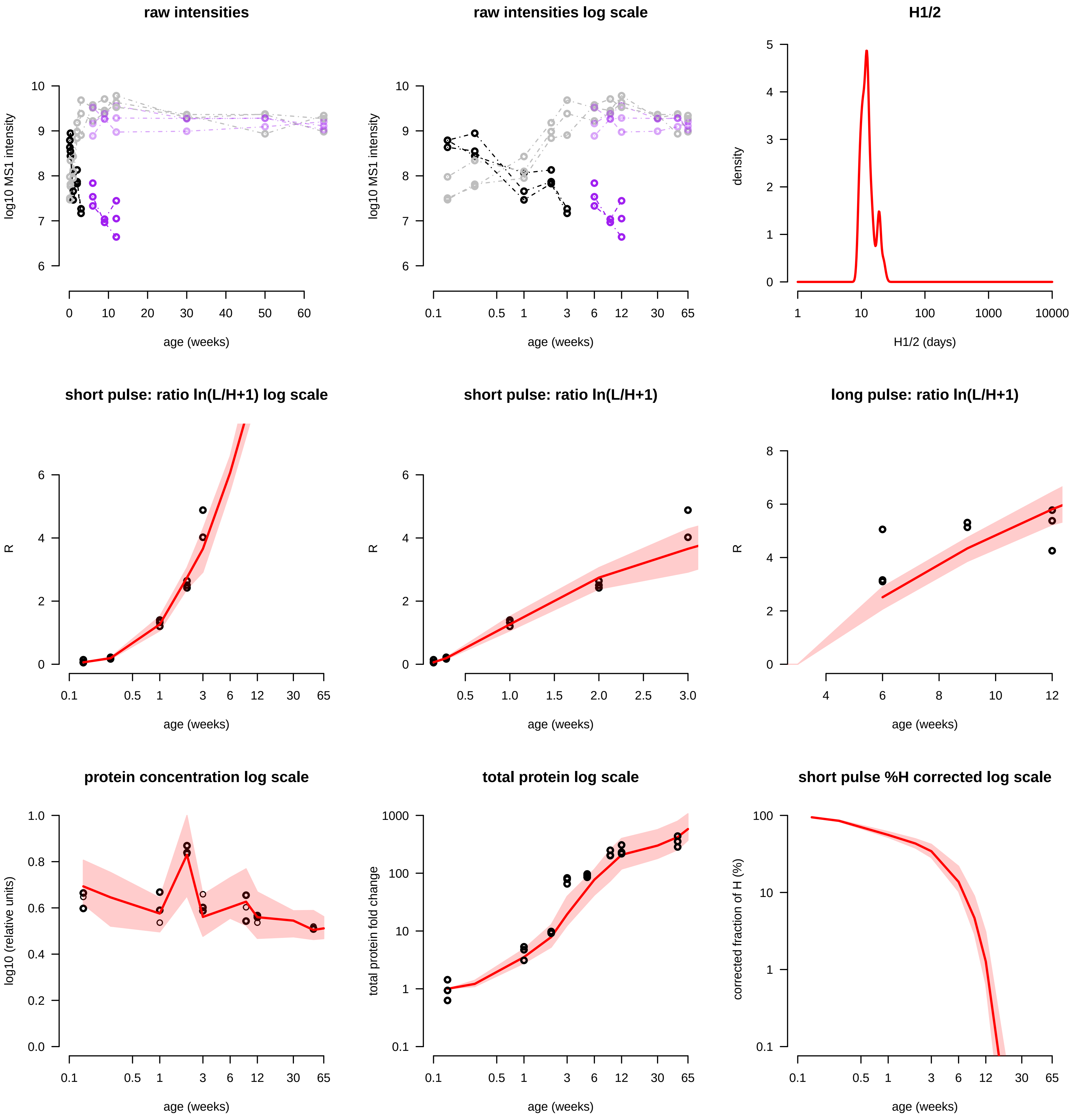

Q91VD9

>sp|Q91VD9|NDUS1_MOUSE

NADH−ubiquinone oxidoreductase 75 kDa subunit, mitochondrial

GN=Ndufs1

Med H1/2: 626.8 days

Q5 H1/2: 227.6 days

Q95 H1/2: 7508.5 days

reliable model fit: no

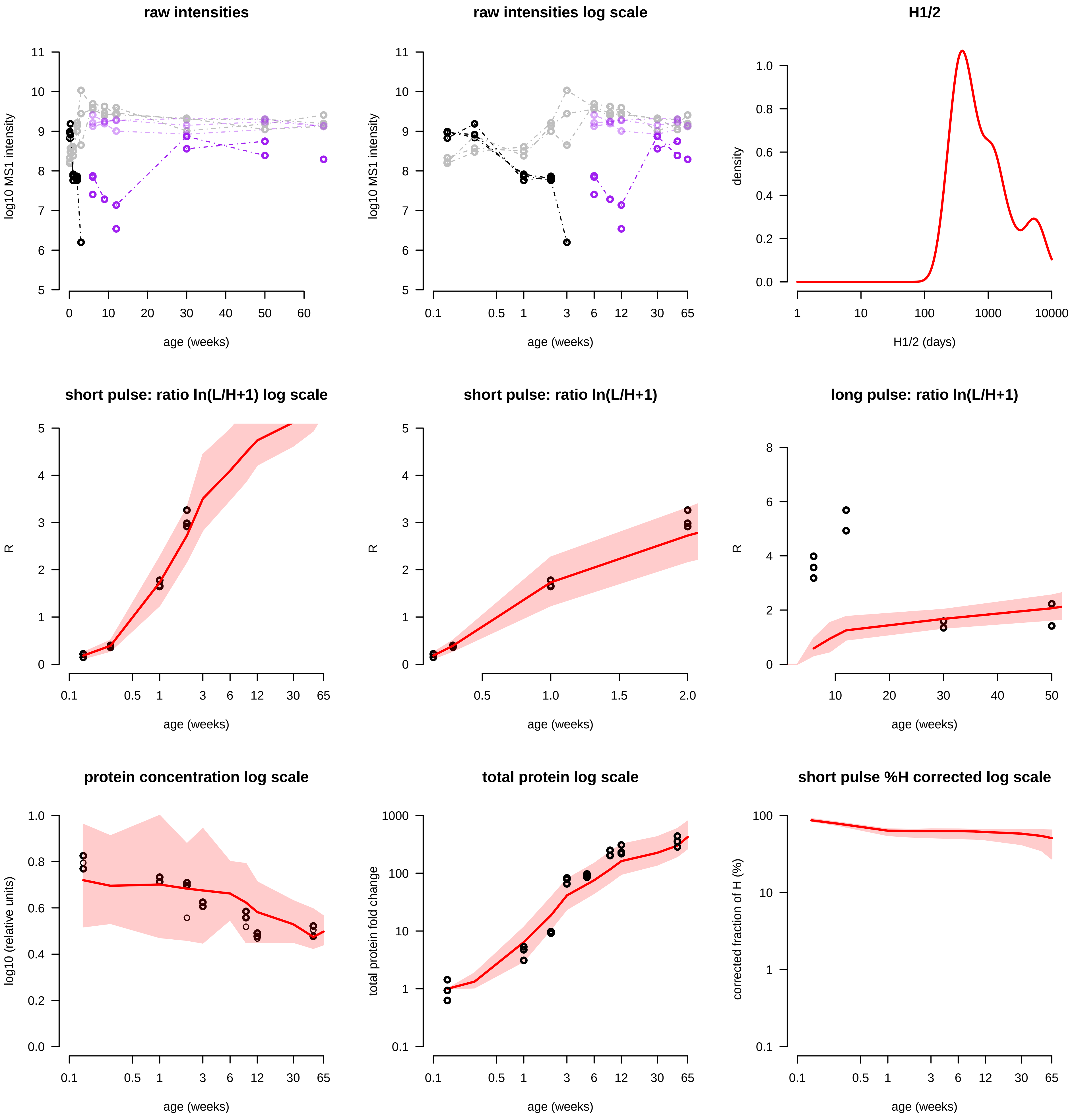

Q9WUR2−2

>sp|Q9WUR2−2|ECI2_MOUSE

Isoform 2 of Enoyl−CoA delta isomerase 2

GN=Eci2

Med H1/2: 4.6 days

Q5 H1/2: 3.8 days

Q95 H1/2: 6.2 days

reliable model fit: yes

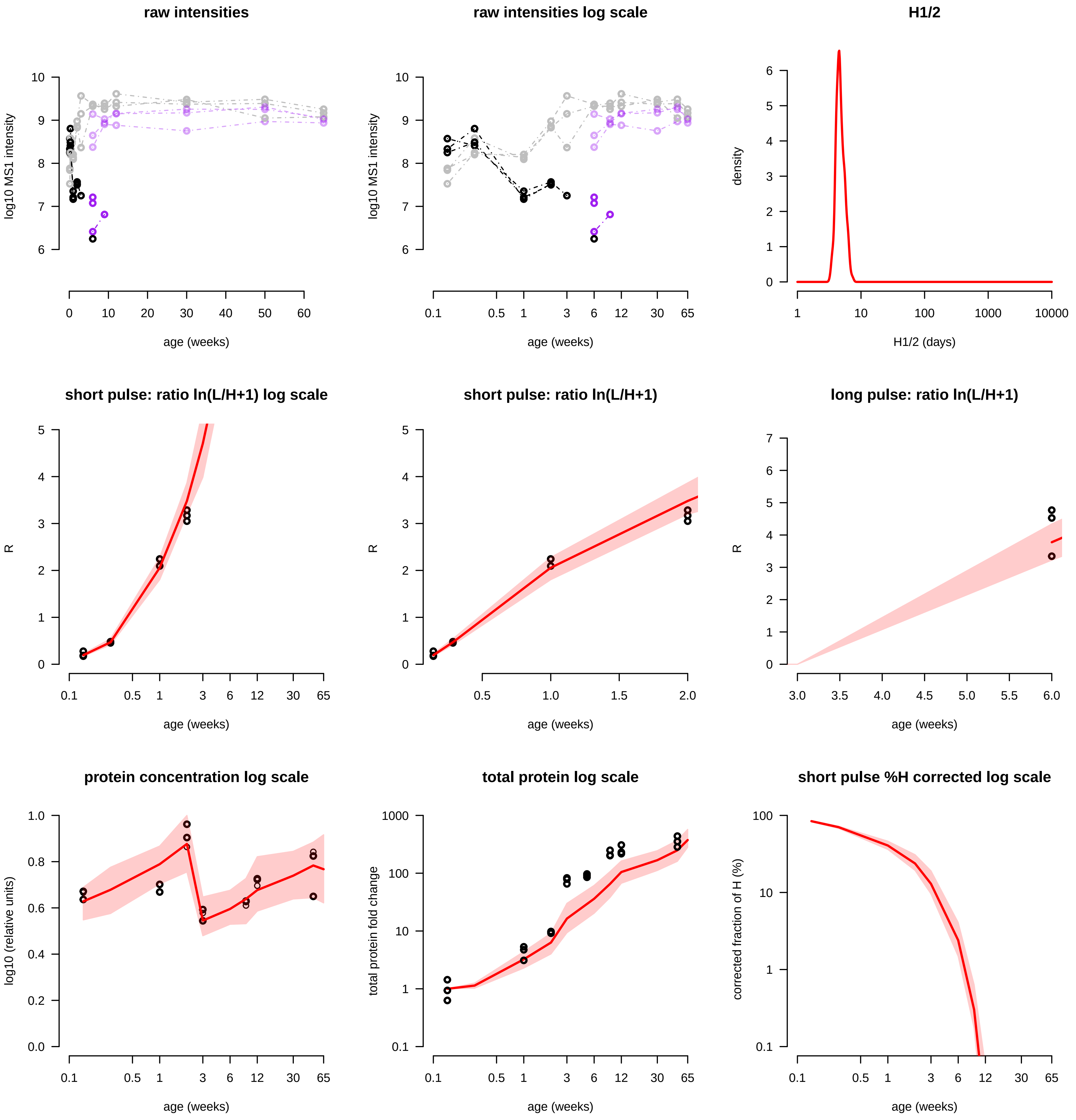

Q9D8W5

>sp|Q9D8W5|PSD12_MOUSE

26S proteasome non−ATPase regulatory subunit 12

GN=Psmd12

Med H1/2: 4.5 days

Q5 H1/2: 3.8 days

Q95 H1/2: 5.7 days

reliable model fit: yes

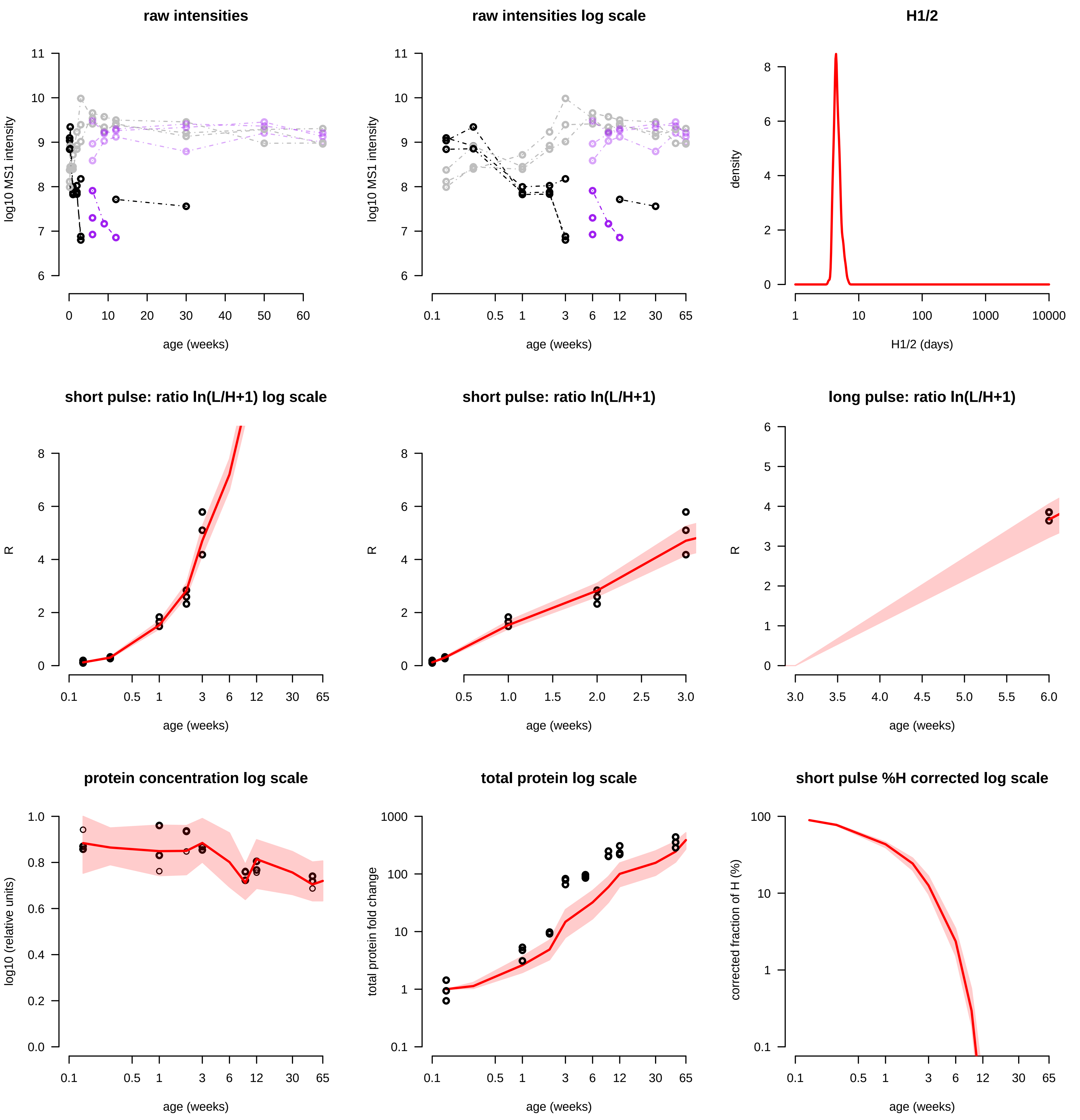

P56391

>sp|P56391|CX6B1_MOUSE

Cytochrome c oxidase subunit 6B1

GN=Cox6b1

Med H1/2: 12.6 days

Q5 H1/2: 9.6 days

Q95 H1/2: 29.3 days

reliable model fit: yes

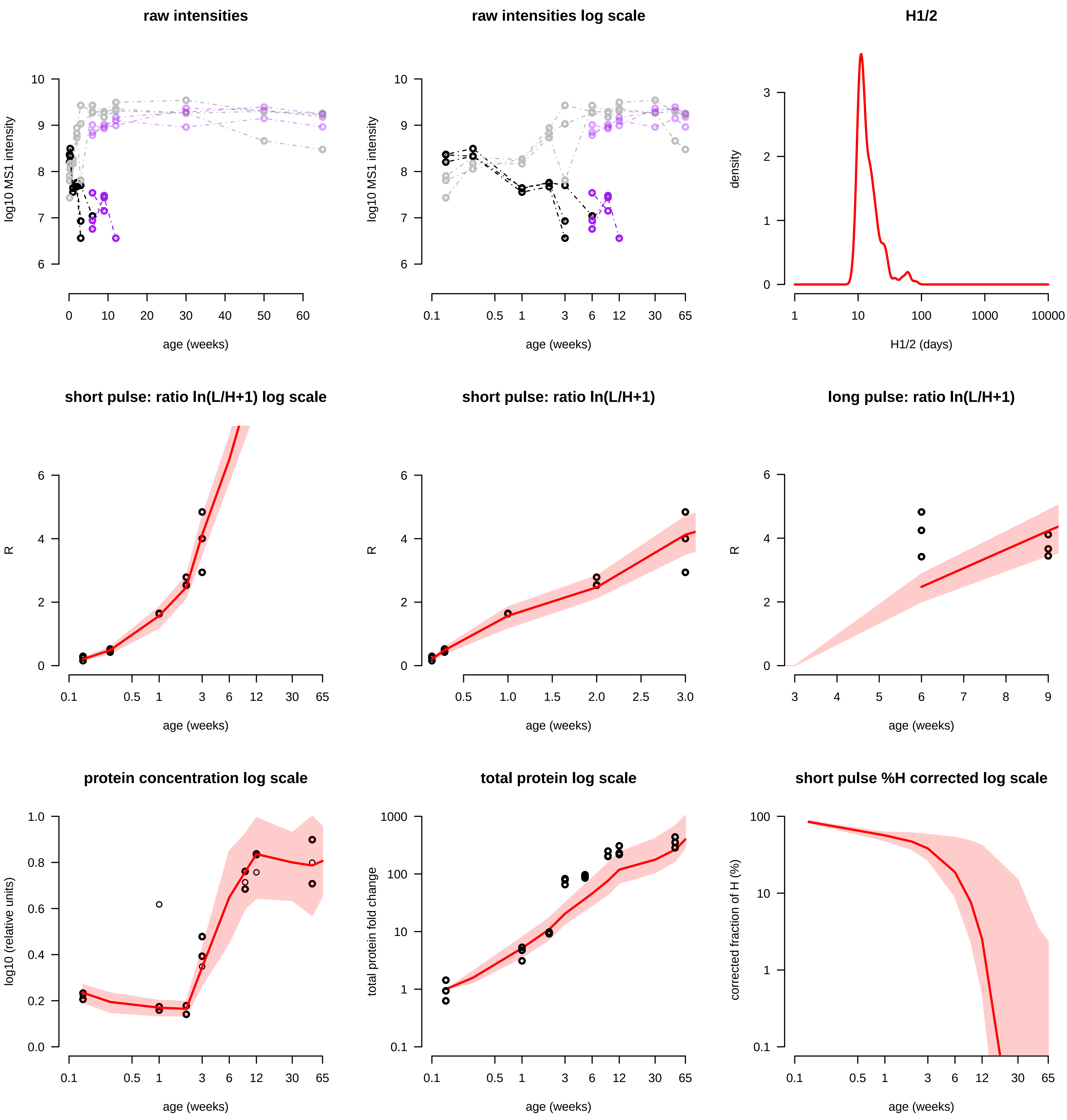

O55142

>sp|O55142|RL35A_MOUSE

60S ribosomal protein L35a

GN=Rpl35a

Med H1/2: 8.9 days

Q5 H1/2: 7.4 days

Q95 H1/2: 11.3 days

reliable model fit: yes

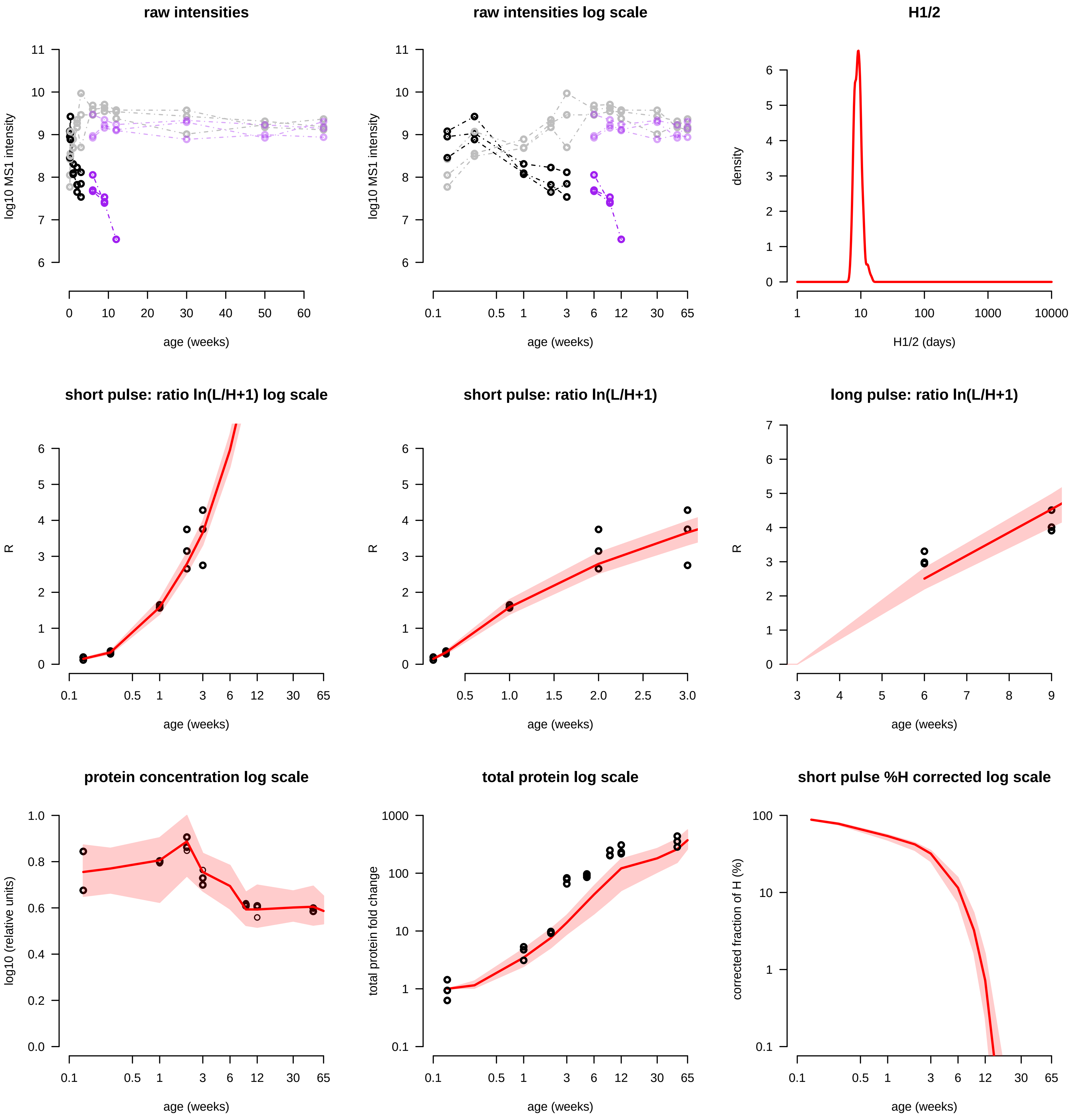

Q9R1J0

>sp|Q9R1J0|NSDHL_MOUSE

Sterol−4−alpha−carboxylate 3−dehydrogenase, decarboxylating

GN=Nsdhl

Med H1/2: 239.1 days

Q5 H1/2: 71.9 days

Q95 H1/2: 1635.4 days

reliable model fit: yes

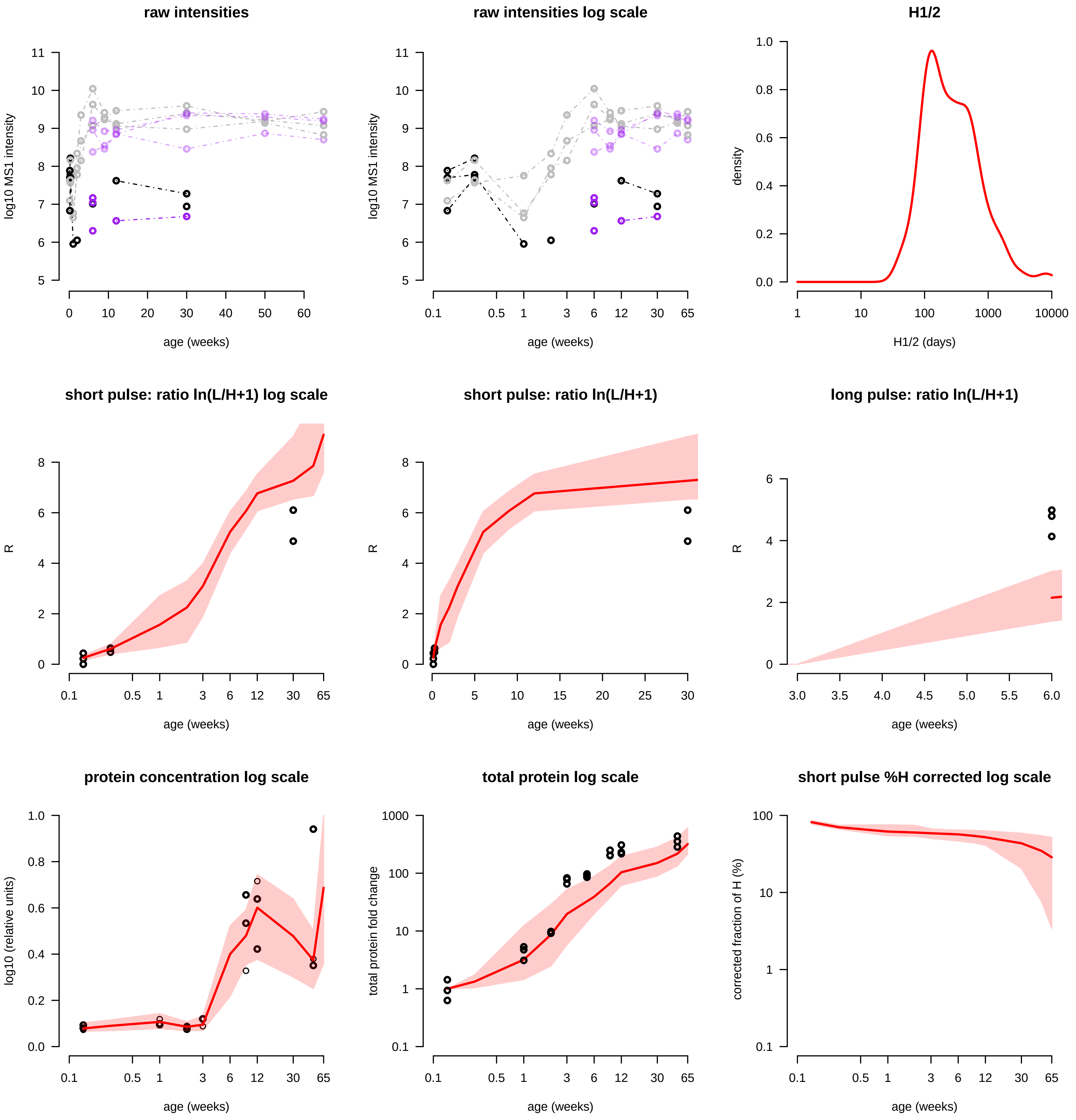

Q9CXW3

>sp|Q9CXW3|CYBP_MOUSE

Calcyclin−binding protein

GN=Cacybp

Med H1/2: 6.8 days

Q5 H1/2: 5.2 days

Q95 H1/2: 9.8 days

reliable model fit: yes

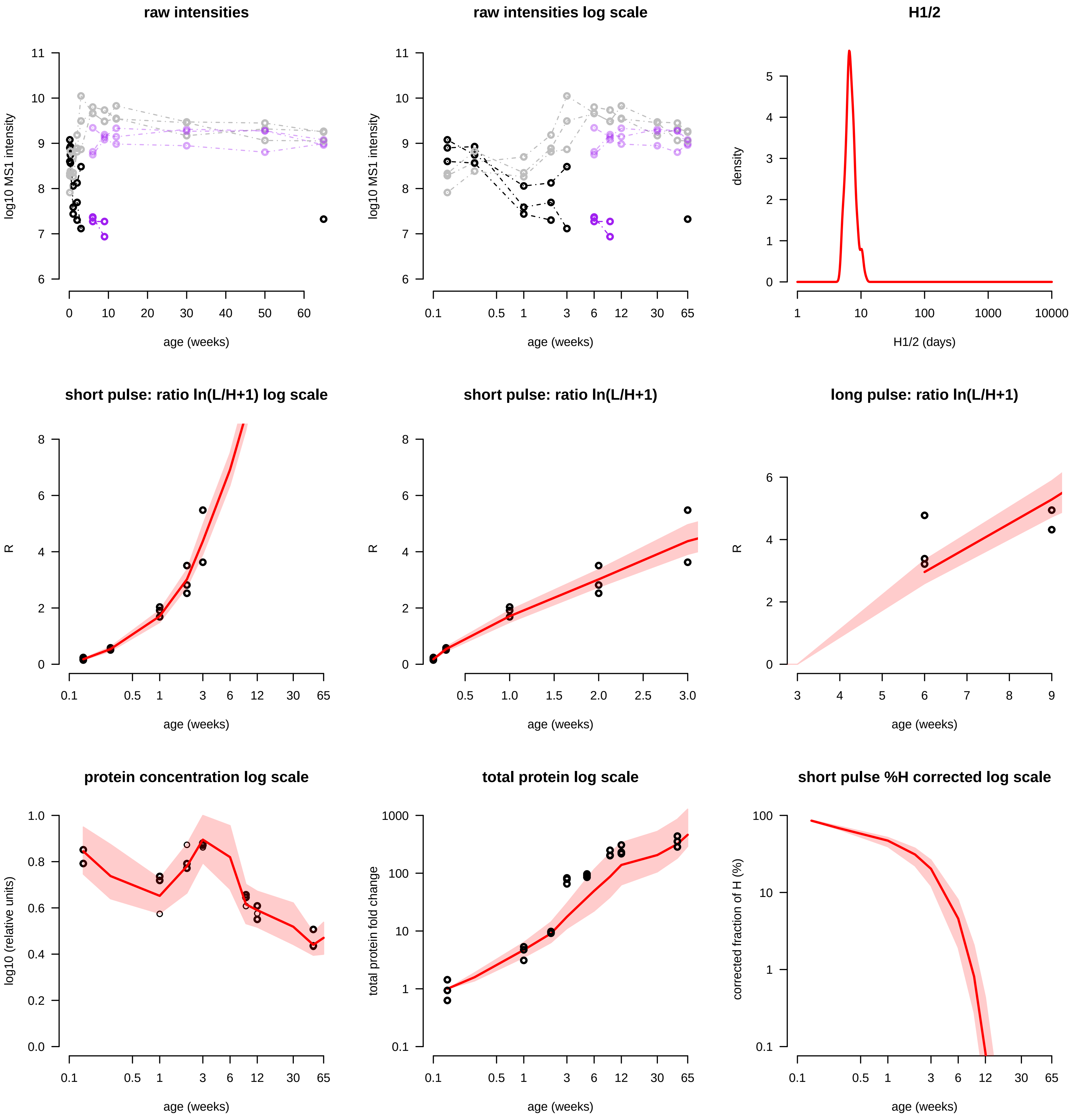

Q9EPU0;Q9EPU0−2

>sp|Q9EPU0|RENT1_MOUSE

Regulator of nonsense transcripts 1

GN=Upf1

Med H1/2: 4.4 days

Q5 H1/2: 3.6 days

Q95 H1/2: 5.3 days

reliable model fit: yes

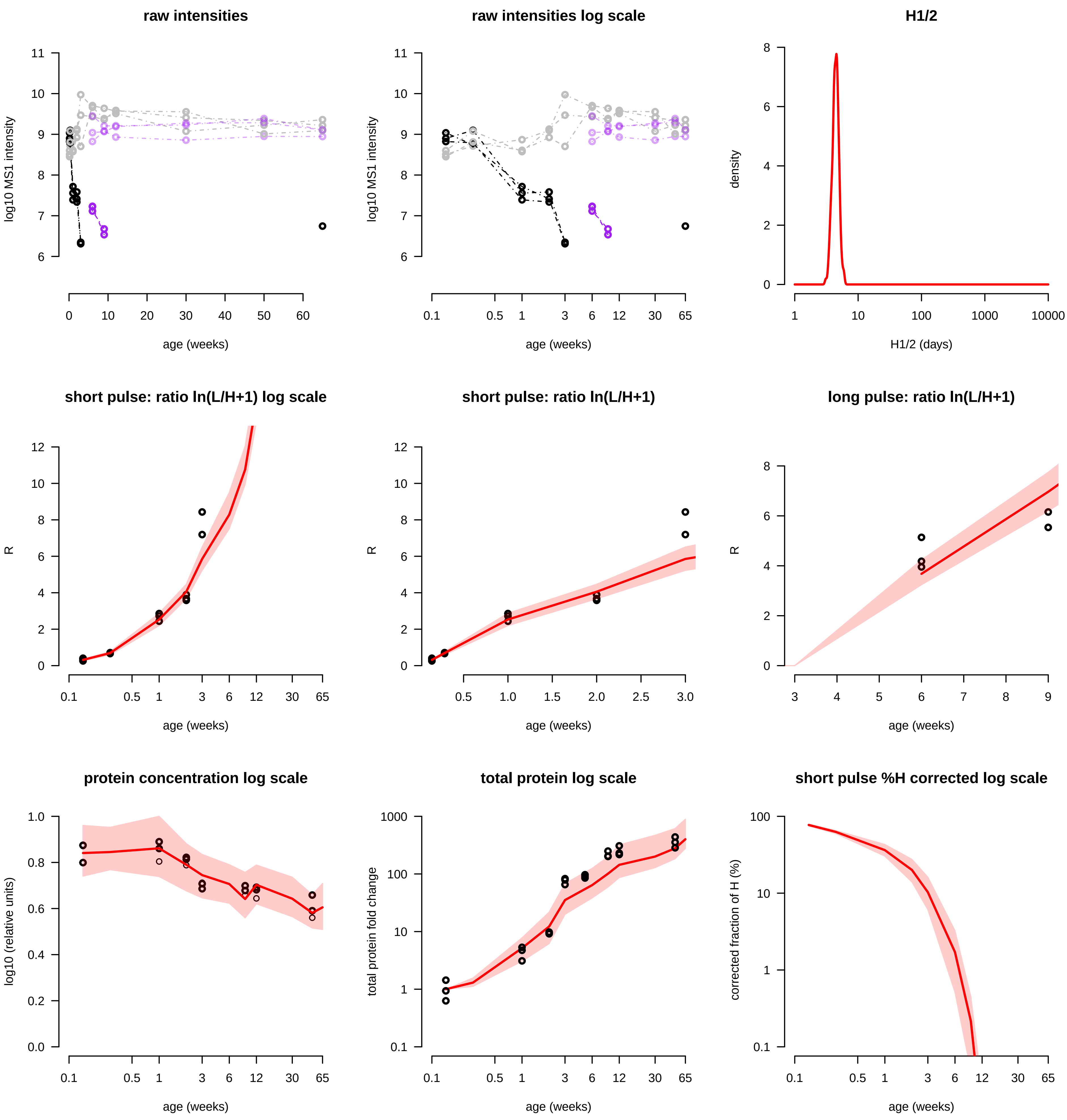

Q9EQ06;Q9EQ06−2

>sp|Q9EQ06|DHB11_MOUSE

Estradiol 17−beta−dehydrogenase 11

GN=Hsd17b11

Med H1/2: 13.1 days

Q5 H1/2: 10.1 days

Q95 H1/2: 21.5 days

reliable model fit: yes

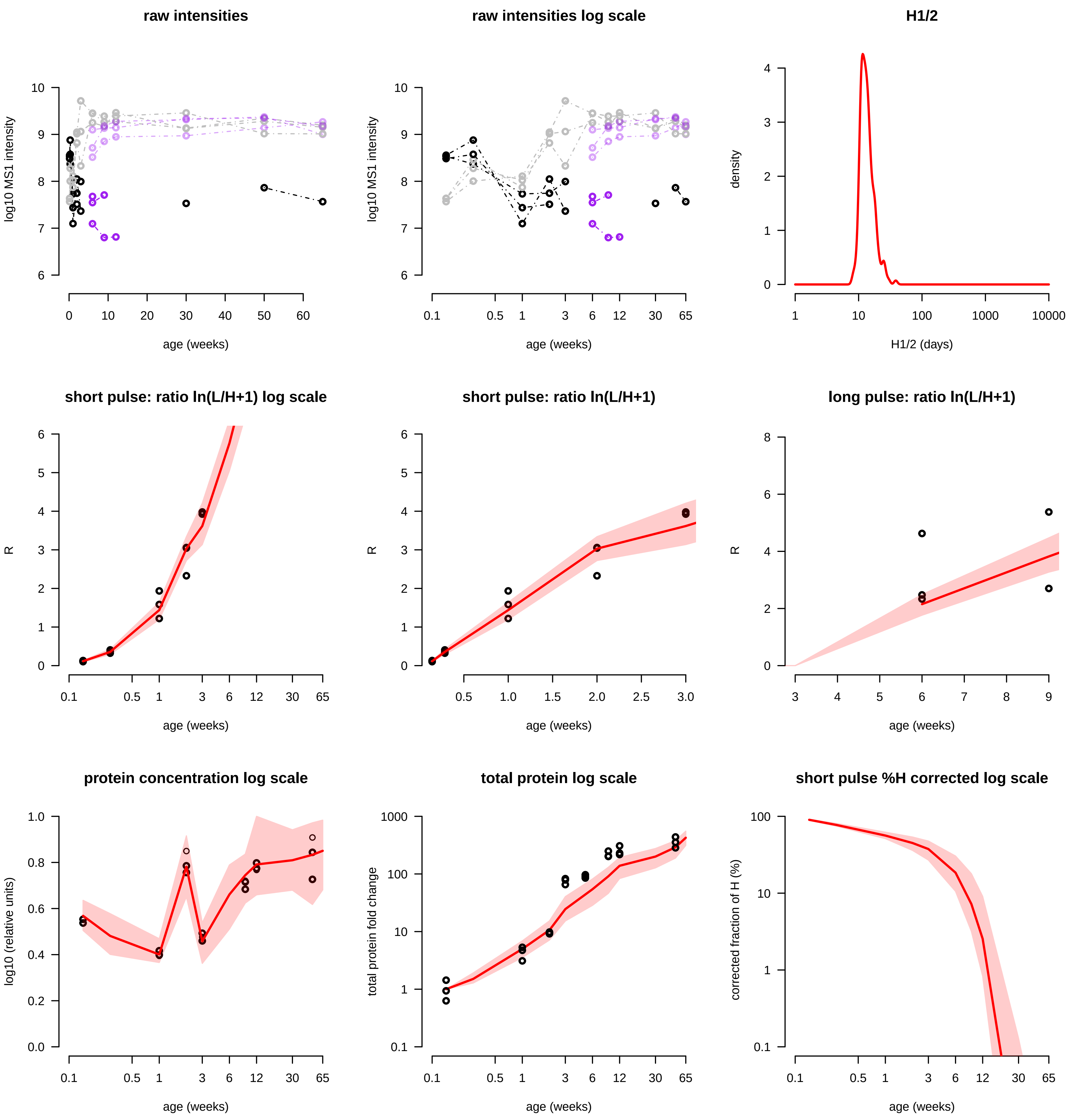

P10761

>sp|P10761|ZP3_MOUSE

Zona pellucida sperm−binding protein 3

GN=Zp3

Med H1/2: 557.1 days

Q5 H1/2: 179.7 days

Q95 H1/2: 5798.5 days

reliable model fit: yes

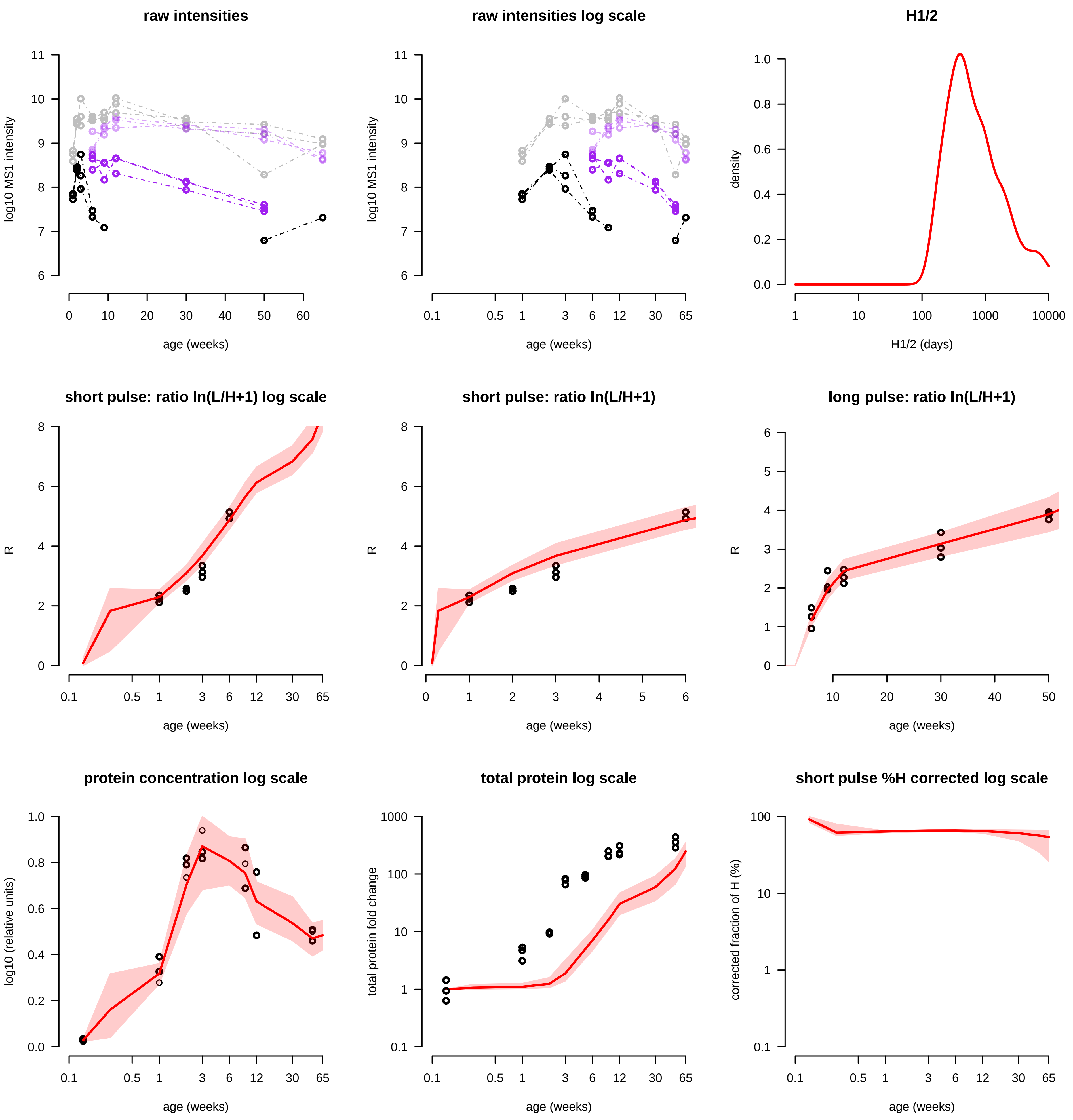

Q9Z0S1

>sp|Q9Z0S1|BPNT1_MOUSE

3'(2'),5'−bisphosphate nucleotidase 1

GN=Bpnt1

Med H1/2: 8.6 days

Q5 H1/2: 6.9 days

Q95 H1/2: 11.4 days

reliable model fit: yes

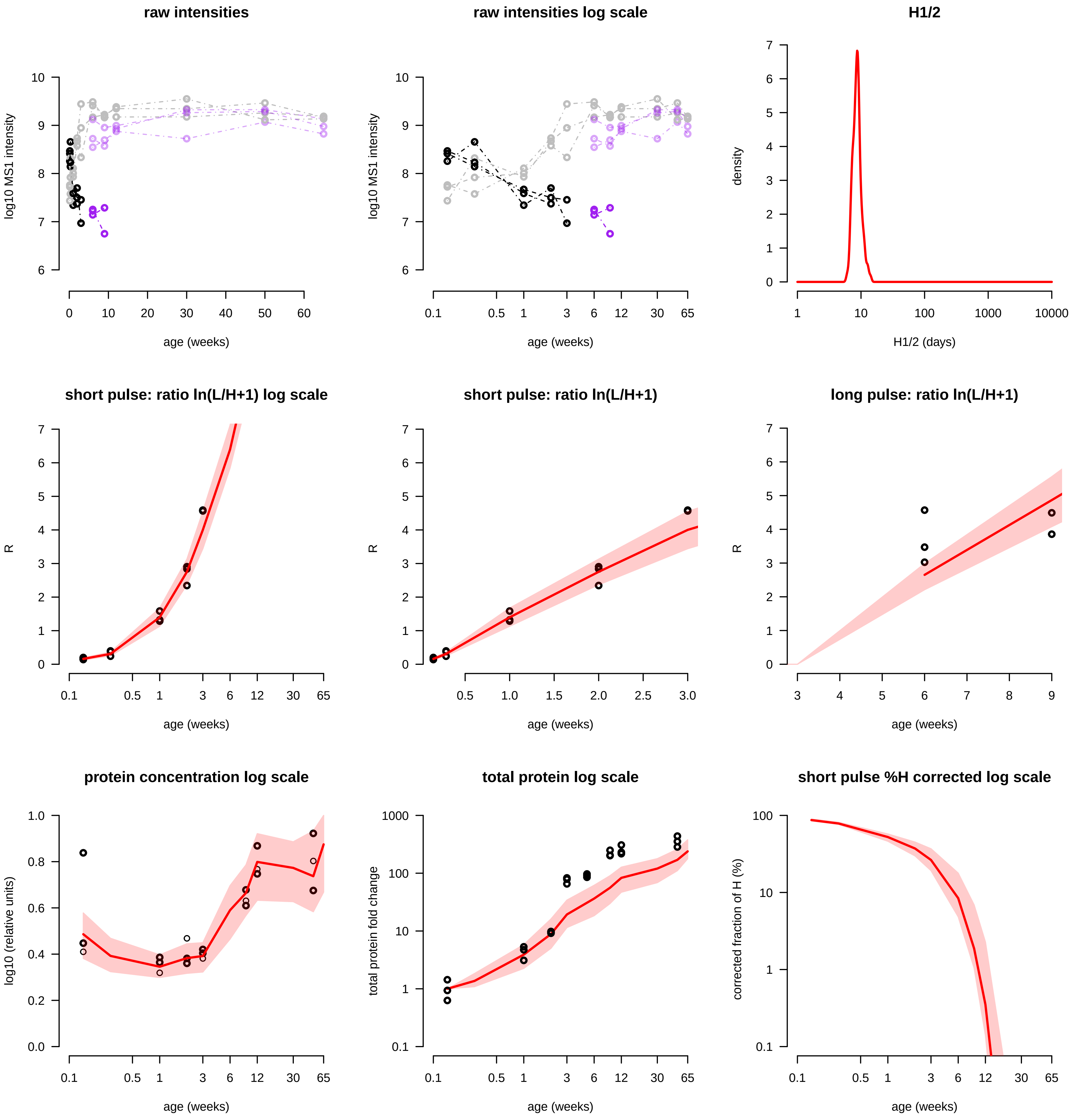

P04919;P04919−2

>sp|P04919|B3AT_MOUSE

Band 3 anion transport protein

GN=Slc4a1

Med H1/2: 34.4 days

Q5 H1/2: 20.4 days

Q95 H1/2: 207 days

reliable model fit: no

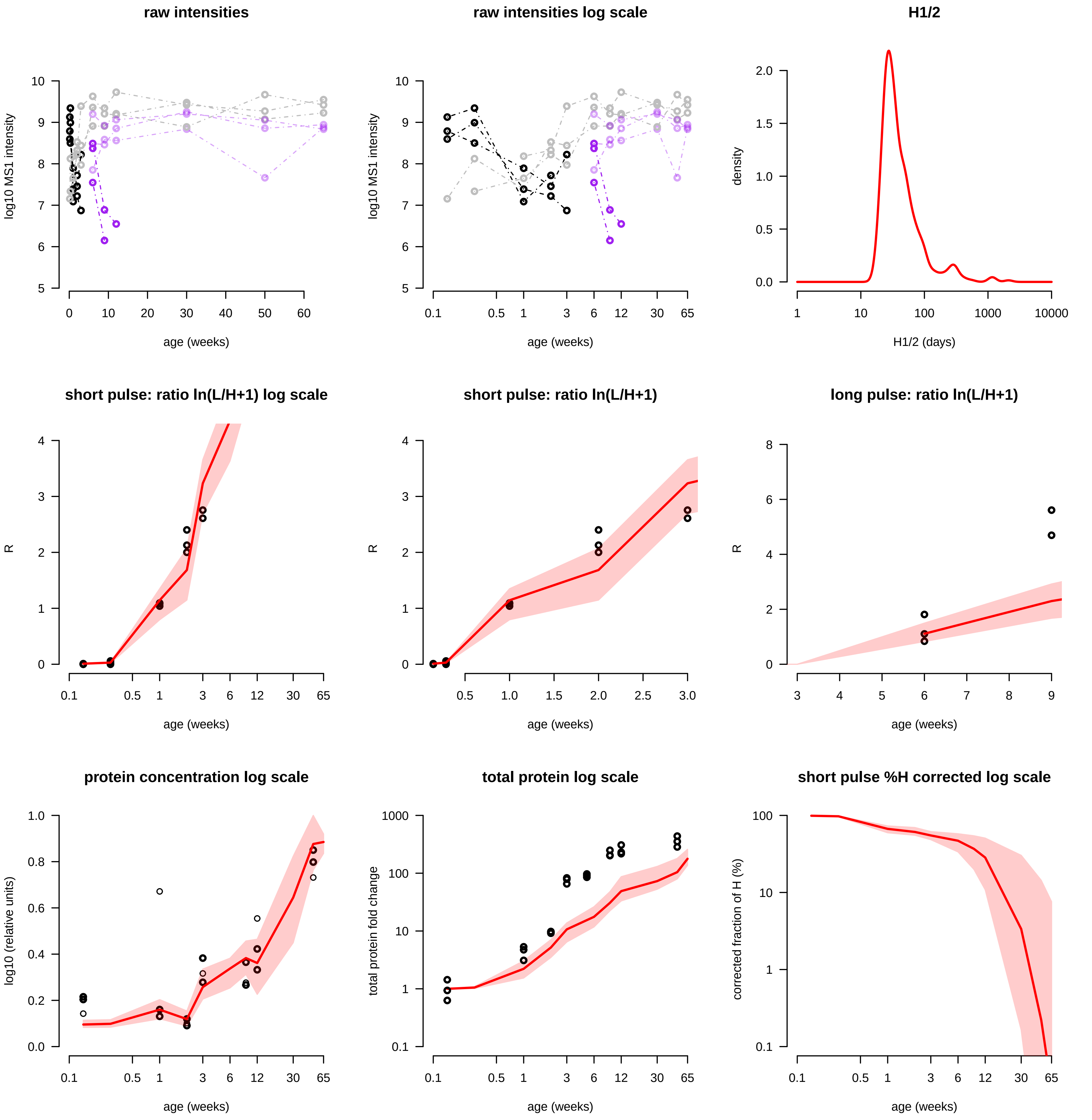

Q9Z2H5−2

>sp|Q9Z2H5−2|E41L1_MOUSE

Isoform 2 of Band 4.1−like protein 1

GN=Epb41l1

Med H1/2: 3.4 days

Q5 H1/2: 2.7 days

Q95 H1/2: 5.2 days

reliable model fit: no

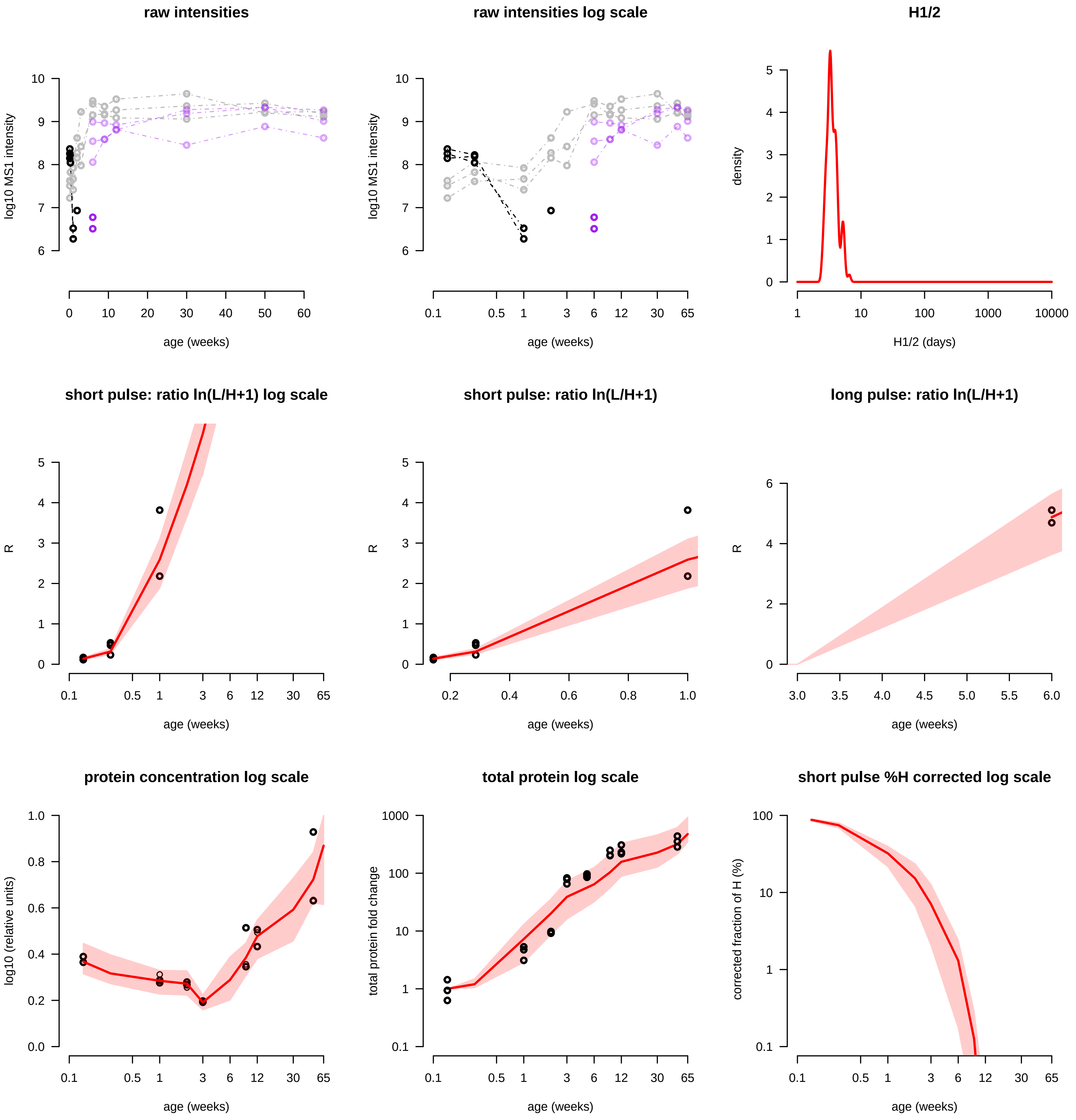

Q9CZB0

>sp|Q9CZB0|C560_MOUSE

Succinate dehydrogenase cytochrome b560 subunit, mitochondrial

GN=Sdhc

Med H1/2: 8.8 days

Q5 H1/2: 6.6 days

Q95 H1/2: 12.4 days

reliable model fit: yes

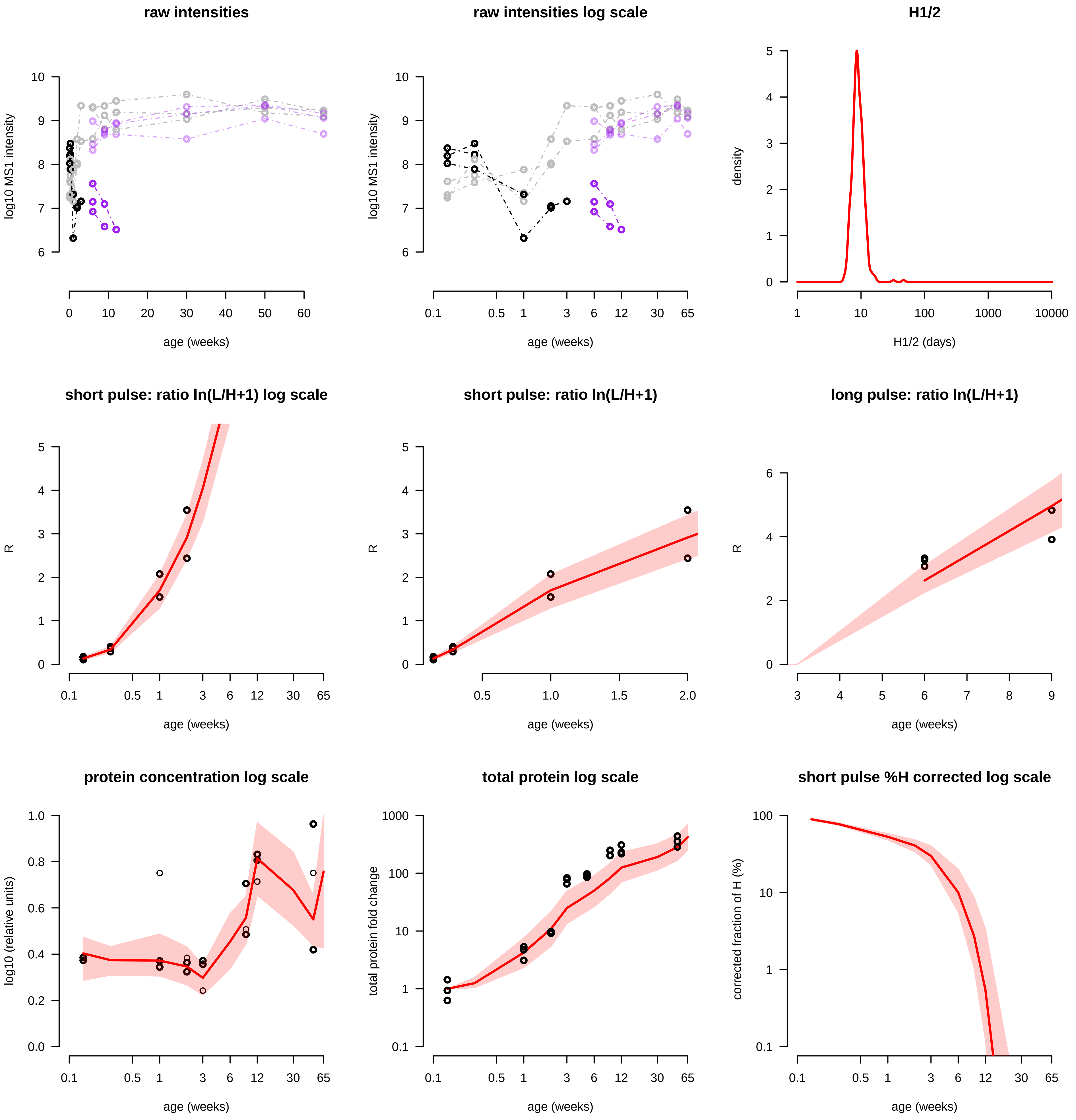

Q3UMC0

>sp|Q3UMC0|AFG2H_MOUSE

ATPase family protein 2 homolog

GN=Spata5

Med H1/2: 13.2 days

Q5 H1/2: 10.8 days

Q95 H1/2: 16.9 days

reliable model fit: yes

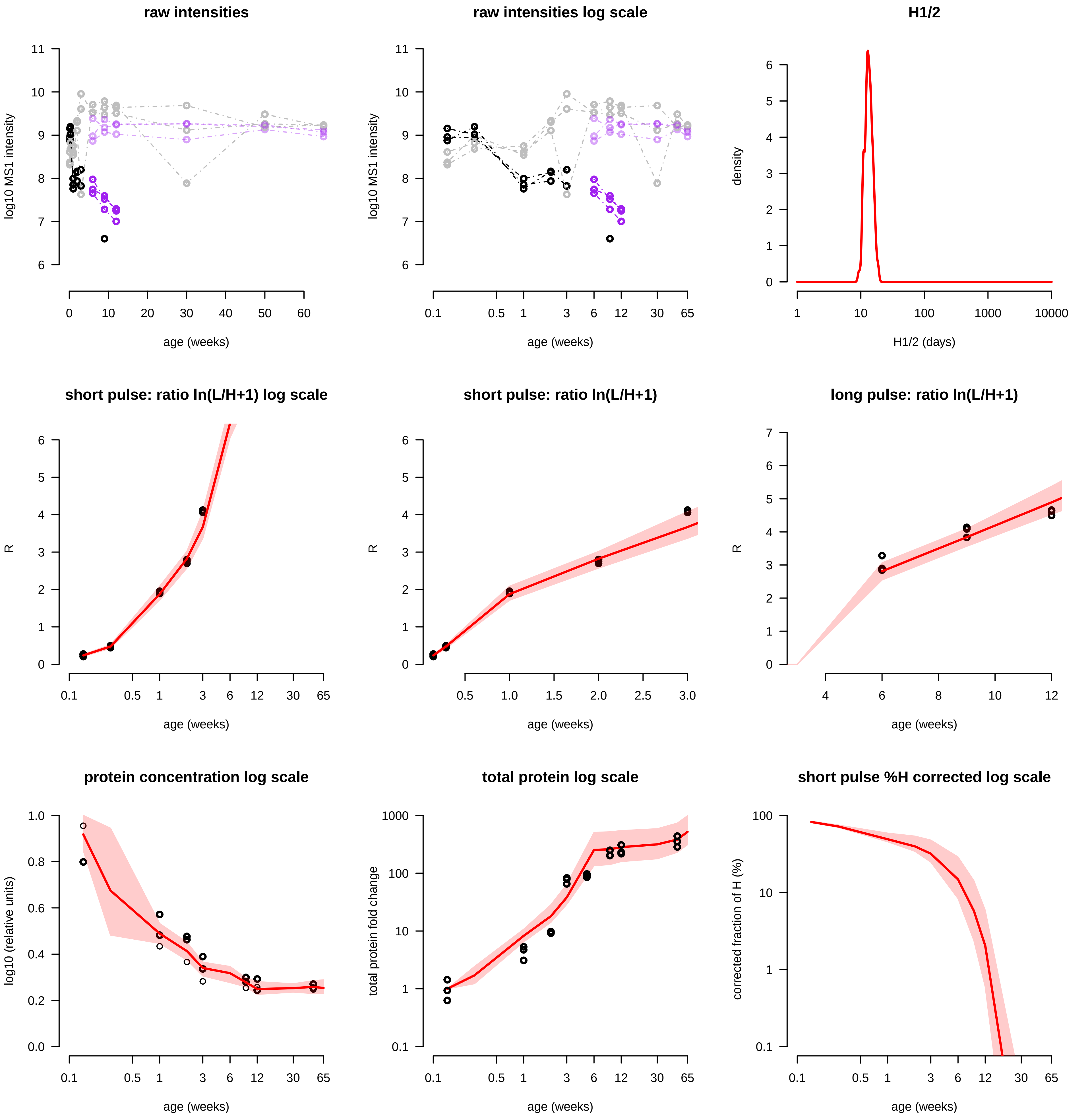

Q99KE1

>sp|Q99KE1|MAOM_MOUSE

NAD−dependent malic enzyme, mitochondrial

GN=Me2

Med H1/2: 15.3 days

Q5 H1/2: 11.2 days

Q95 H1/2: 23.5 days

reliable model fit: yes

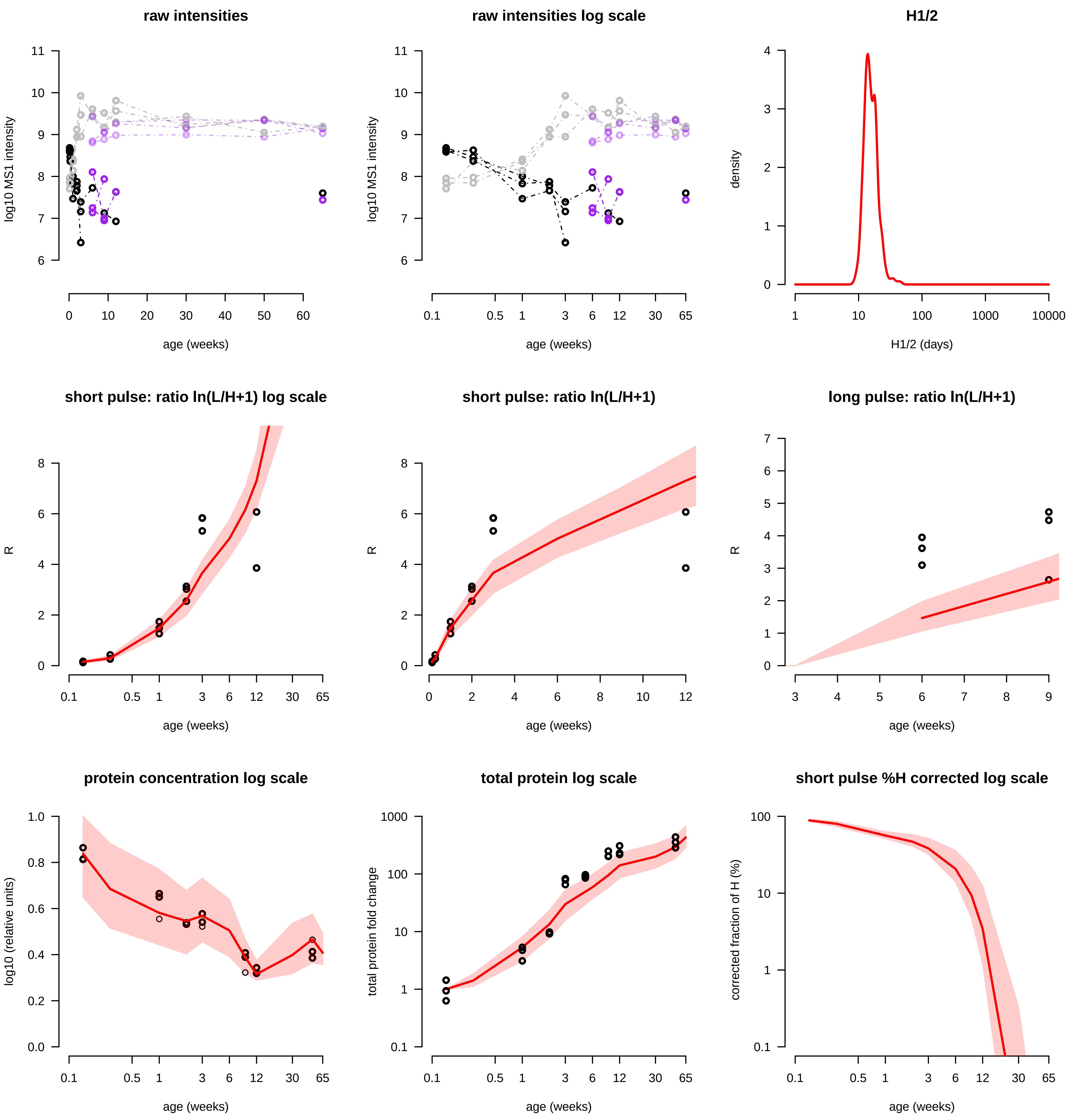

Q99JF8

>sp|Q99JF8|PSIP1_MOUSE

PC4 and SFRS1−interacting protein

GN=Psip1

Med H1/2: 651.9 days

Q5 H1/2: 279.9 days

Q95 H1/2: 4701.7 days

reliable model fit: no

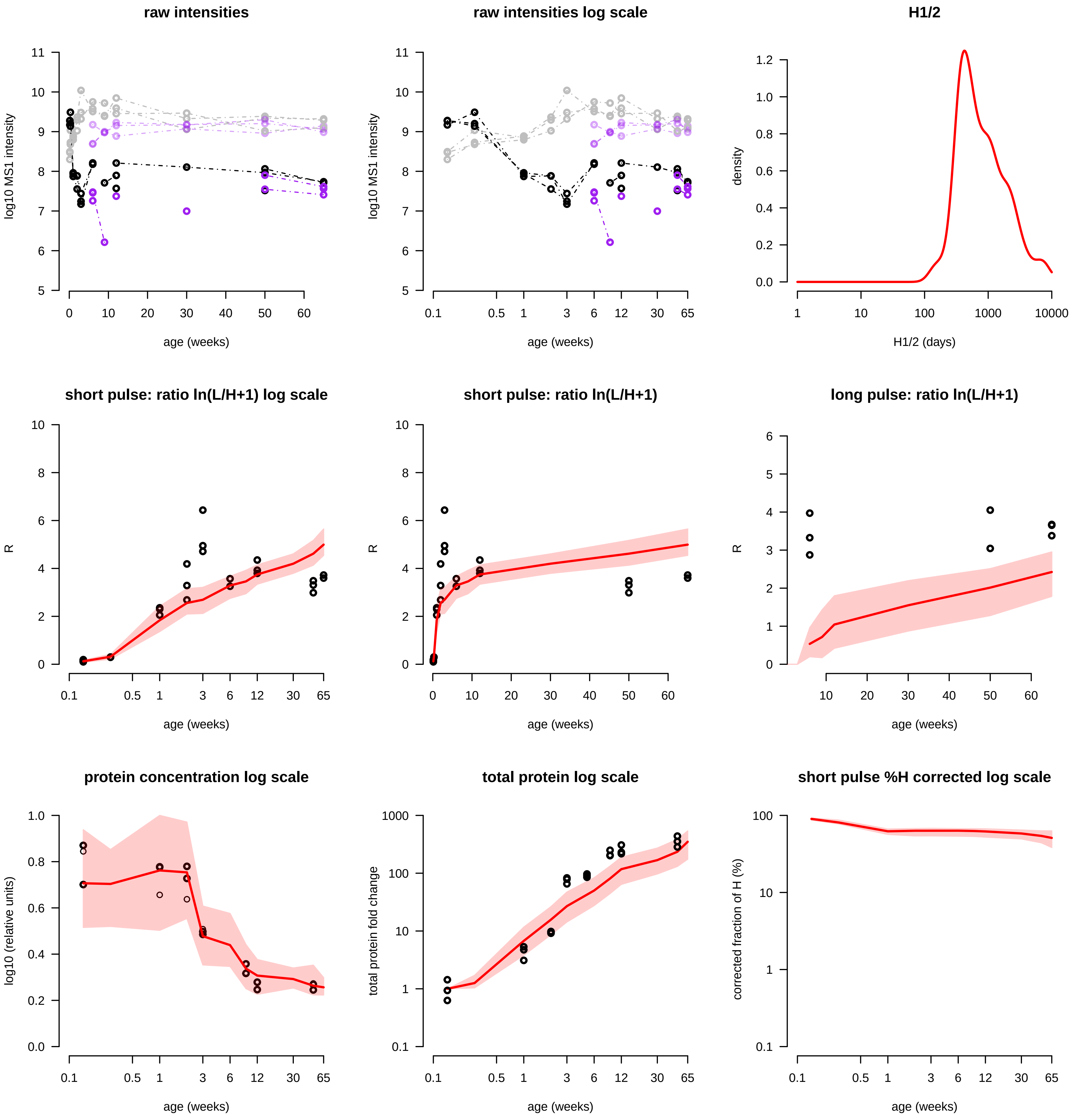

Q91YW3

>sp|Q91YW3|DNJC3_MOUSE

DnaJ homolog subfamily C member 3

GN=Dnajc3

Med H1/2: 4.6 days

Q5 H1/2: 3.7 days

Q95 H1/2: 6.3 days

reliable model fit: yes

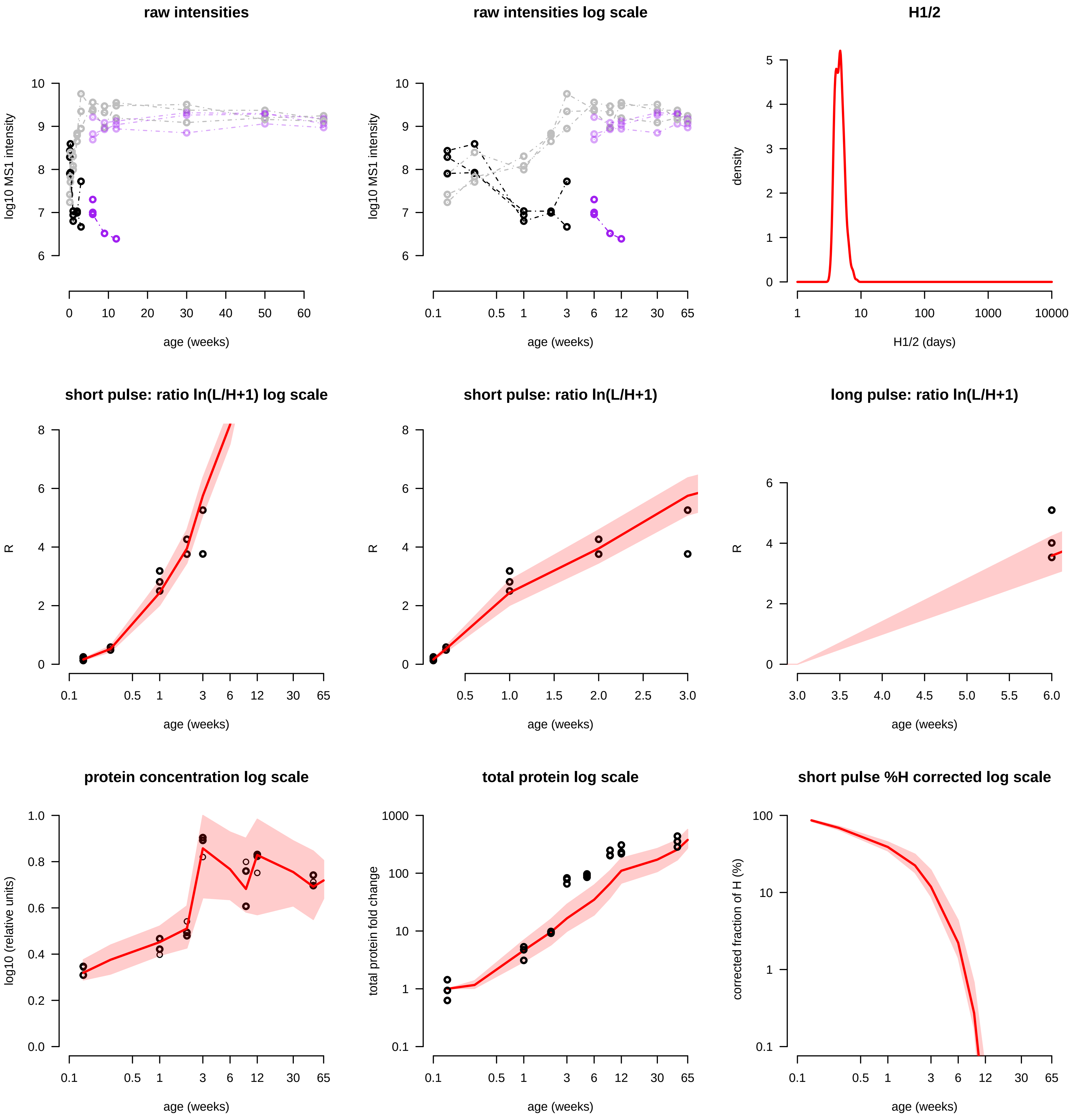

Q3ULJ0;Q3ULJ0−2

>sp|Q3ULJ0|GPD1L_MOUSE

Glycerol−3−phosphate dehydrogenase 1−like protein

GN=Gpd1l

Med H1/2: 4 days

Q5 H1/2: 3.2 days

Q95 H1/2: 5.6 days

reliable model fit: yes

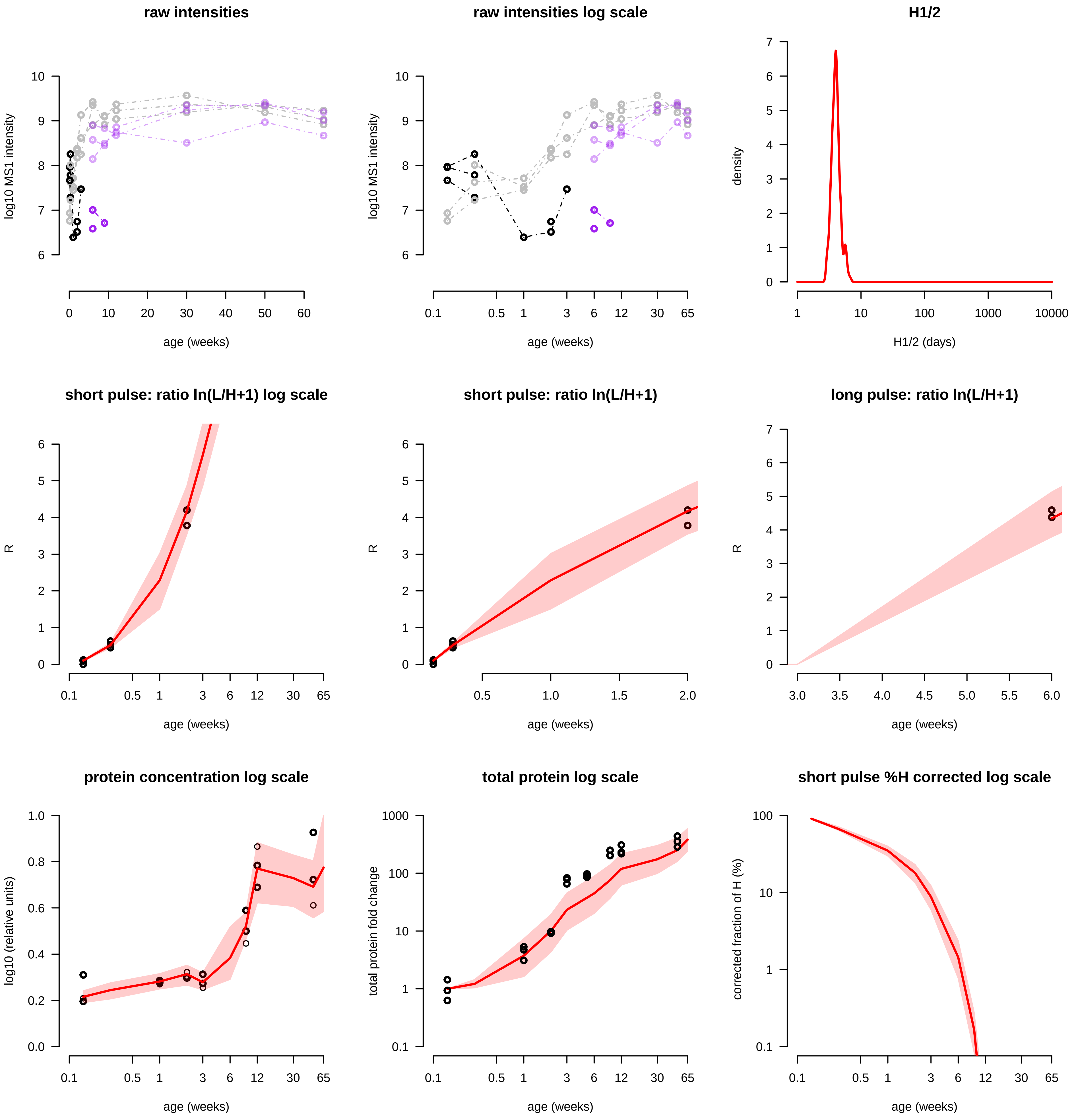

O08532;O08532−2;O08532−5

>sp|O08532|CA2D1_MOUSE

Voltage−dependent calcium channel subunit alpha−2/delta−1

GN=Cacna2d1

Med H1/2: 10.3 days

Q5 H1/2: 5.9 days

Q95 H1/2: 21.5 days

reliable model fit: yes

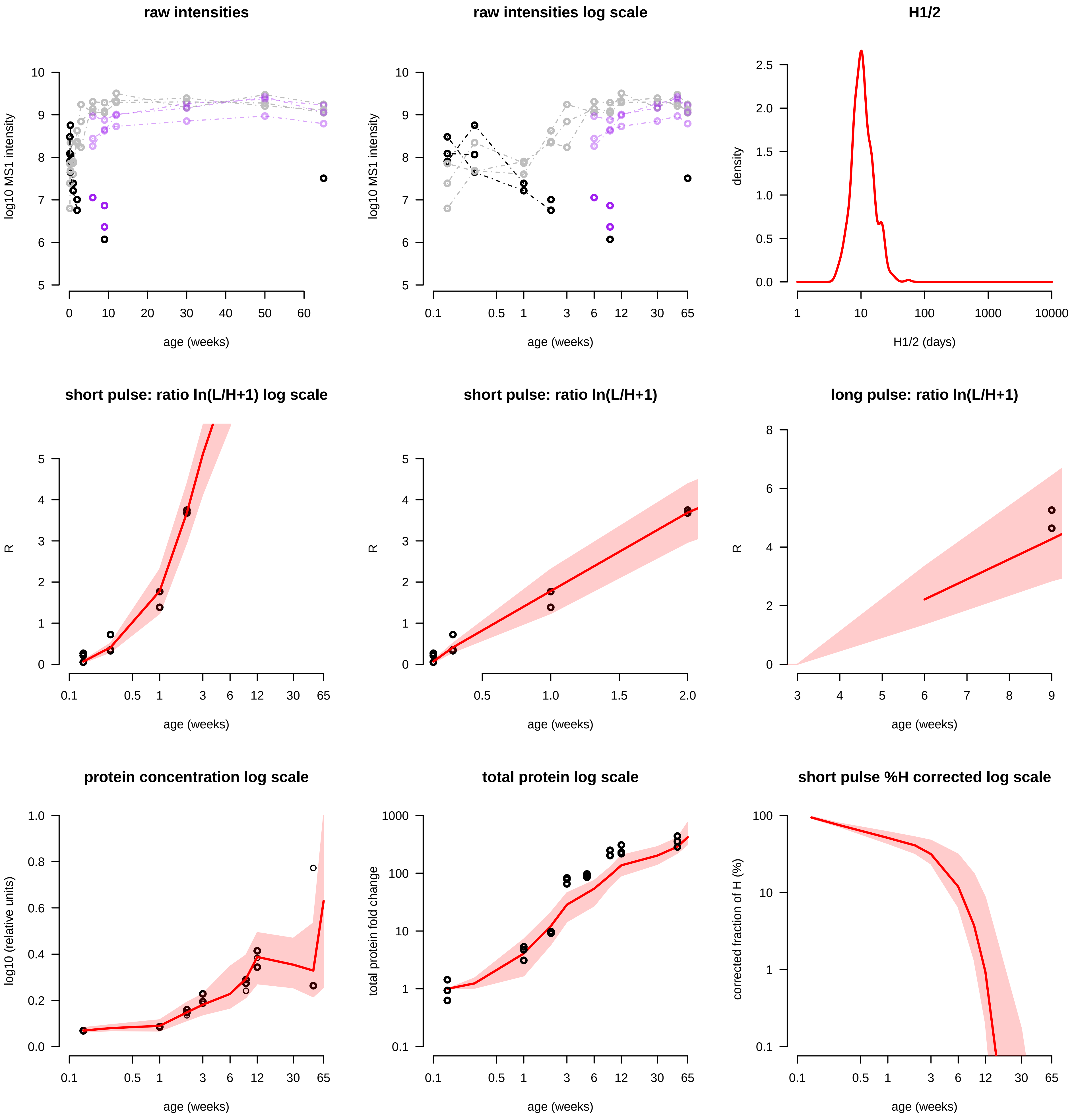

Q501J6;Q501J6−2

>sp|Q501J6|DDX17_MOUSE

Probable ATP−dependent RNA helicase DDX17

GN=Ddx17

Med H1/2: 98.6 days

Q5 H1/2: 41.6 days

Q95 H1/2: 1247.4 days

reliable model fit: yes

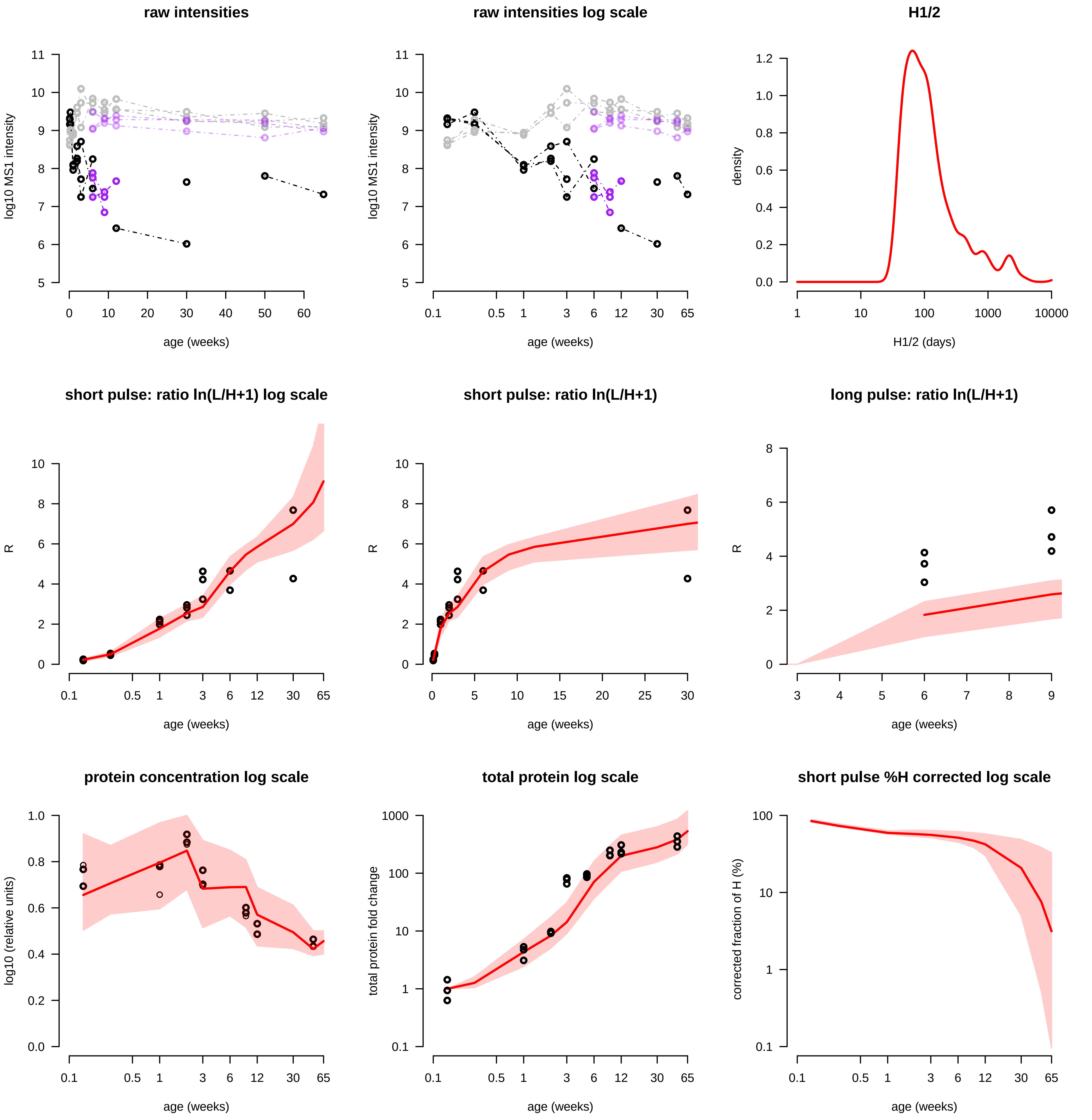

A2AN08−3;A2AN08

>sp|A2AN08−3|UBR4_MOUSE

Isoform 3 of E3 ubiquitin−protein ligase UBR4

GN=Ubr4

Med H1/2: 3.8 days

Q5 H1/2: 3.1 days

Q95 H1/2: 4.7 days

reliable model fit: yes

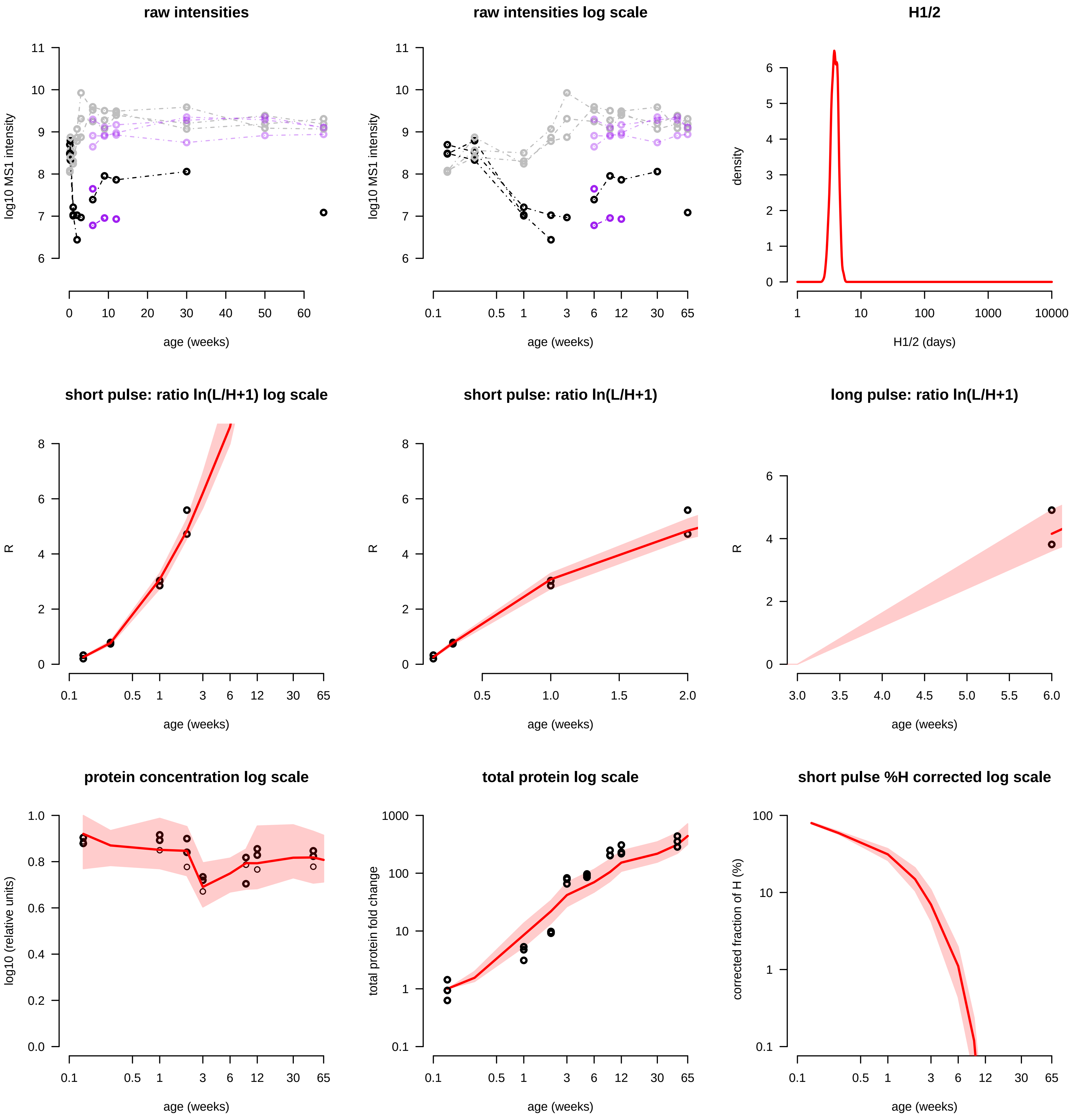

Q9Z1Q9

>sp|Q9Z1Q9|SYVC_MOUSE

Valine−−tRNA ligase

GN=Vars1

Med H1/2: 5.1 days

Q5 H1/2: 4.2 days

Q95 H1/2: 6.2 days

reliable model fit: yes

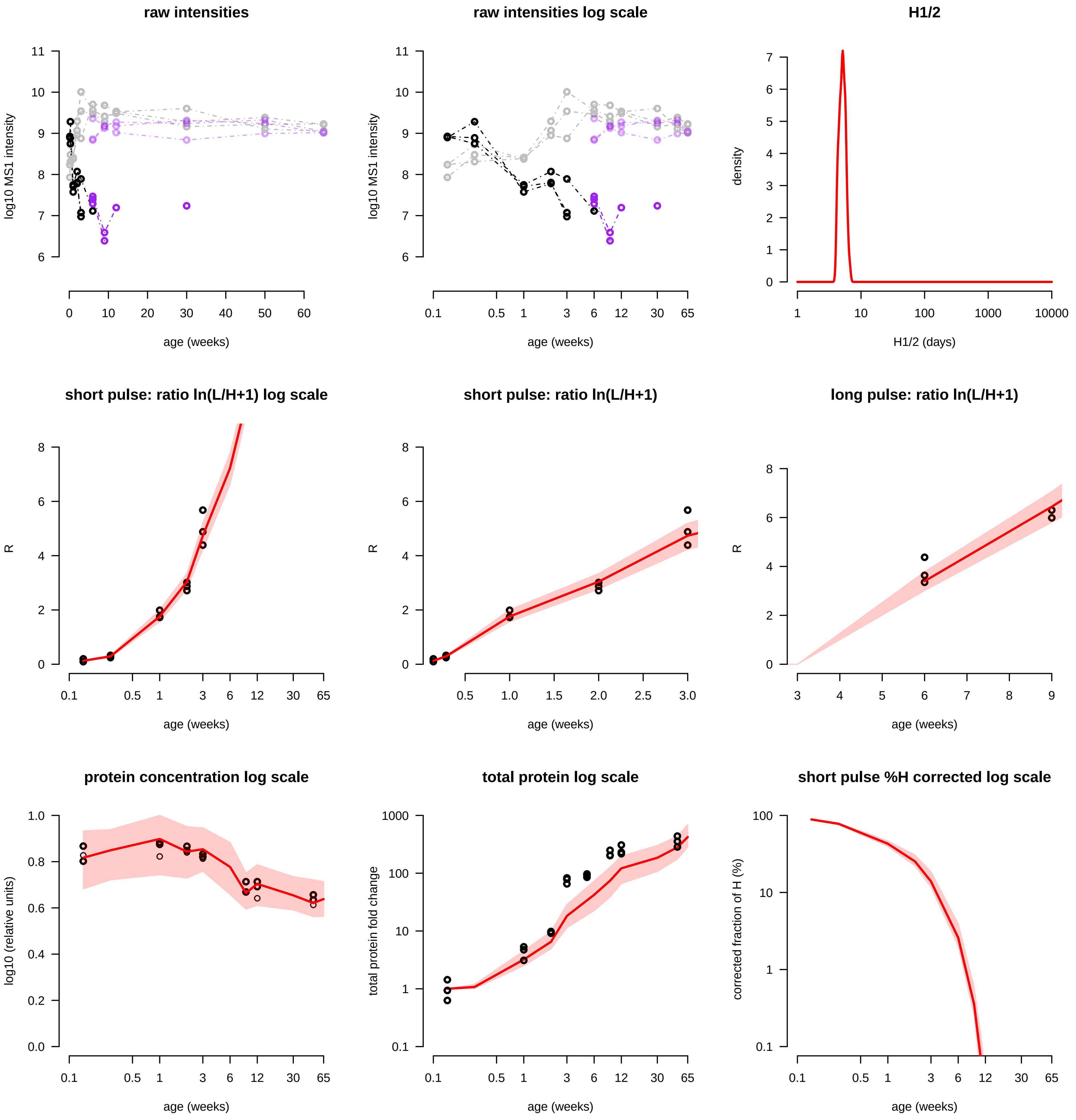

Q9QYA2

>sp|Q9QYA2|TOM40_MOUSE

Mitochondrial import receptor subunit TOM40 homolog

GN=Tomm40

Med H1/2: 7.4 days

Q5 H1/2: 6.1 days

Q95 H1/2: 9.5 days

reliable model fit: yes

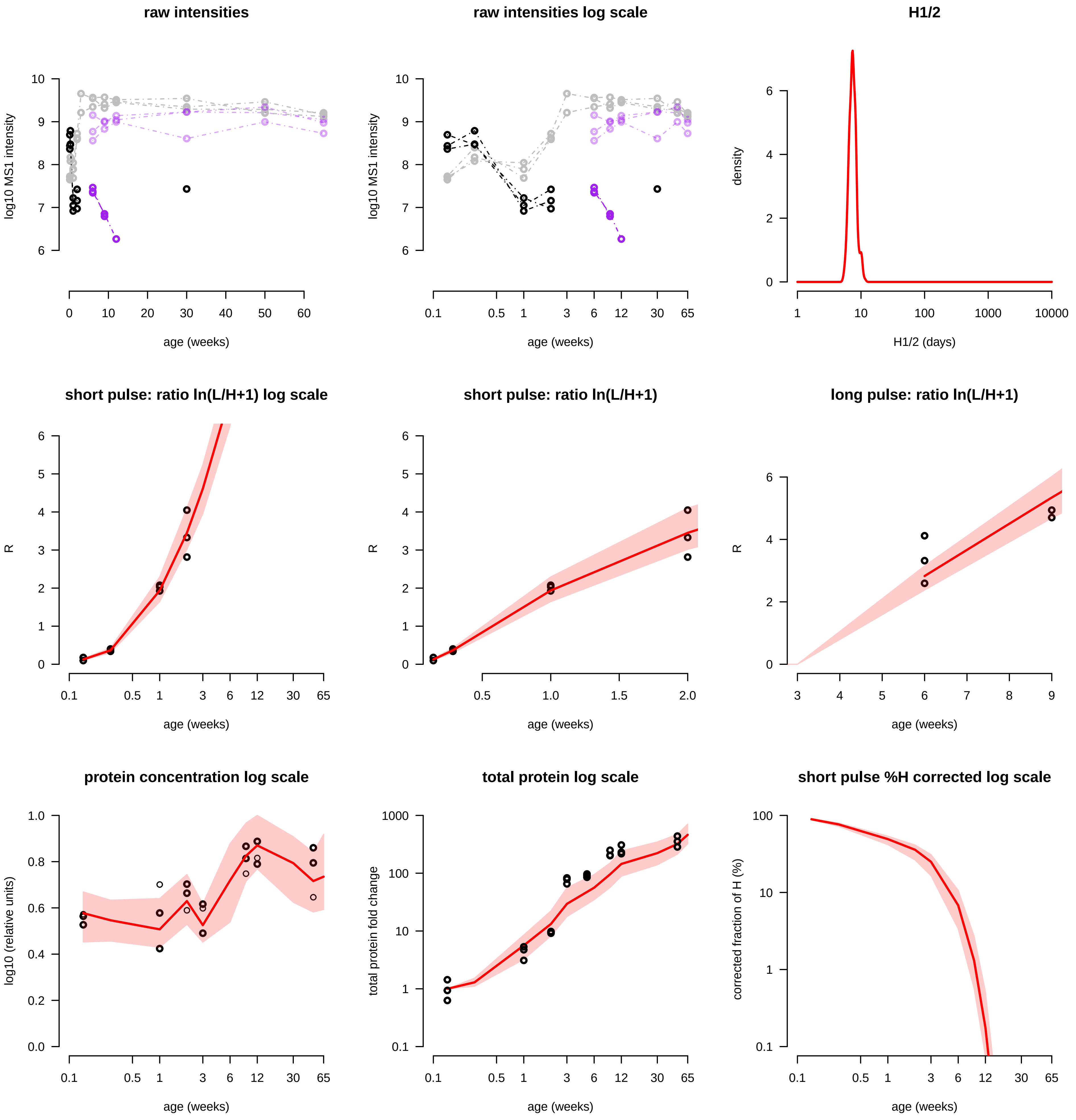

Q6ZWX6

>sp|Q6ZWX6|IF2A_MOUSE

Eukaryotic translation initiation factor 2 subunit 1

GN=Eif2s1

Med H1/2: 128.1 days

Q5 H1/2: 48.2 days

Q95 H1/2: 786.8 days

reliable model fit: no

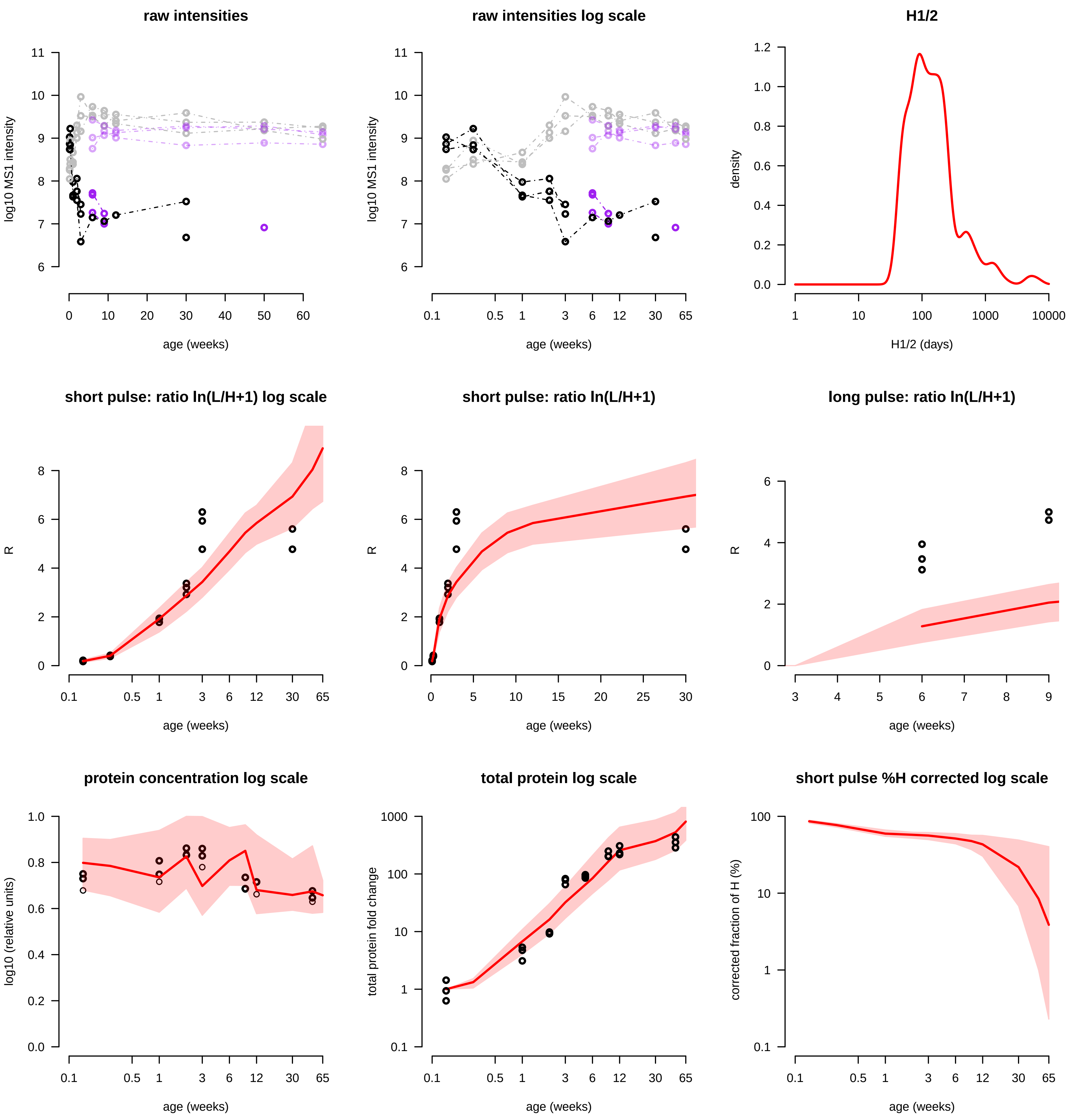

P63085

>sp|P63085|MK01_MOUSE

Mitogen−activated protein kinase 1

GN=Mapk1

Med H1/2: 4.1 days

Q5 H1/2: 3.5 days

Q95 H1/2: 5.2 days

reliable model fit: yes

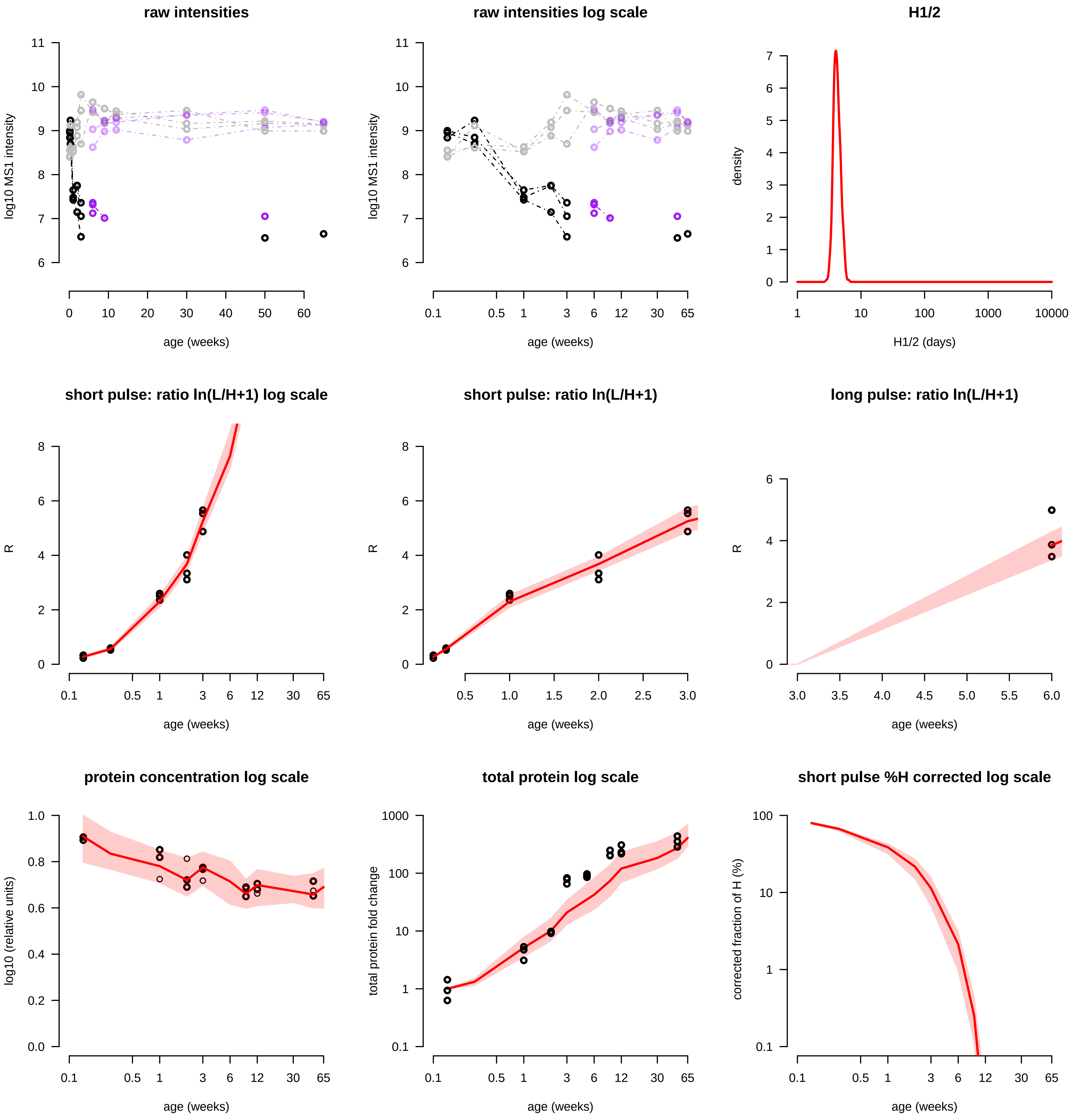

O08756

>sp|O08756|HCD2_MOUSE

3−hydroxyacyl−CoA dehydrogenase type−2

GN=Hsd17b10

Med H1/2: 11.4 days

Q5 H1/2: 9.1 days

Q95 H1/2: 14.6 days

reliable model fit: yes

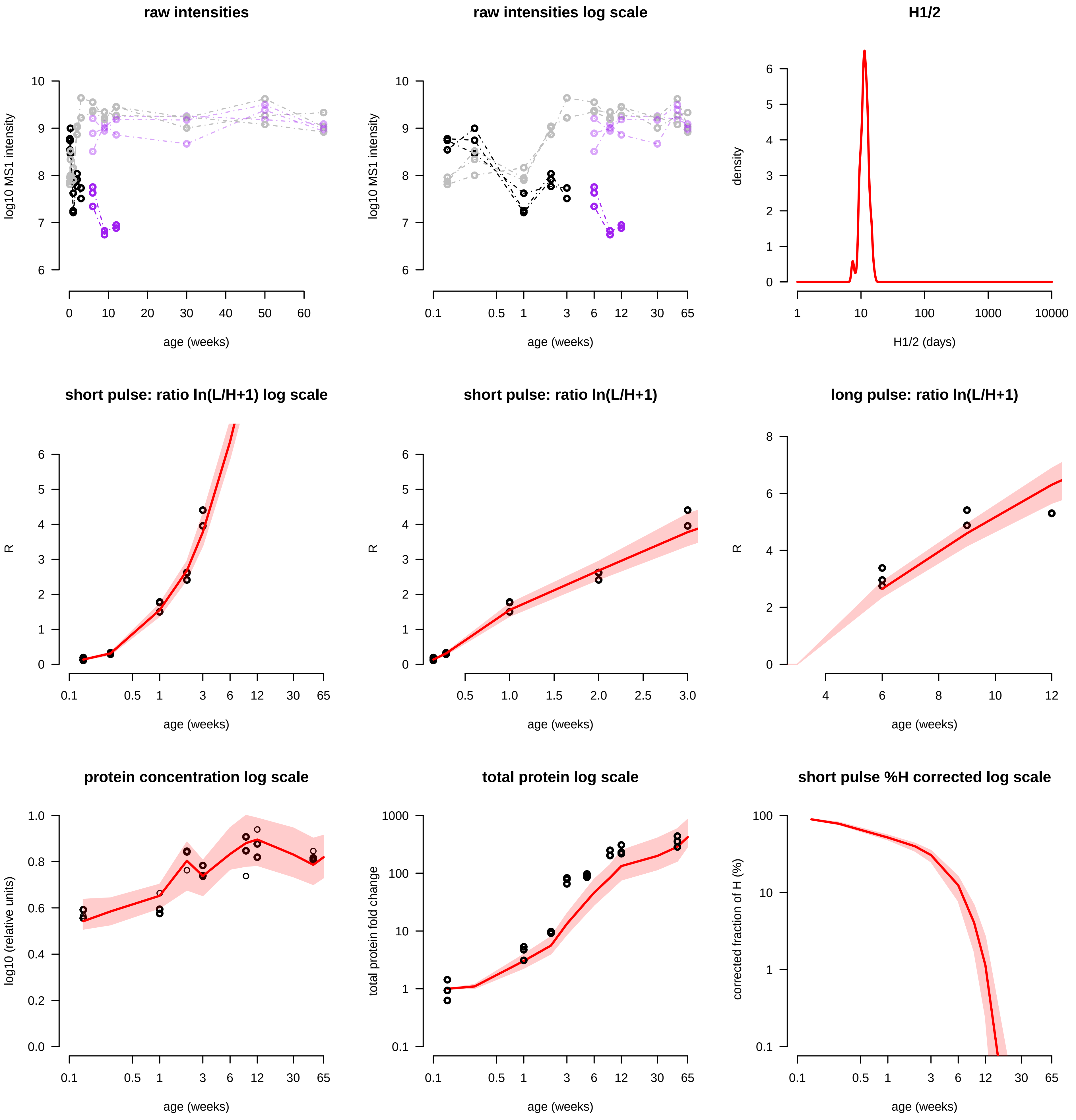

Q3U0V1

>sp|Q3U0V1|FUBP2_MOUSE

Far upstream element−binding protein 2

GN=Khsrp

Med H1/2: 5.2 days

Q5 H1/2: 4.7 days

Q95 H1/2: 6.2 days

reliable model fit: yes

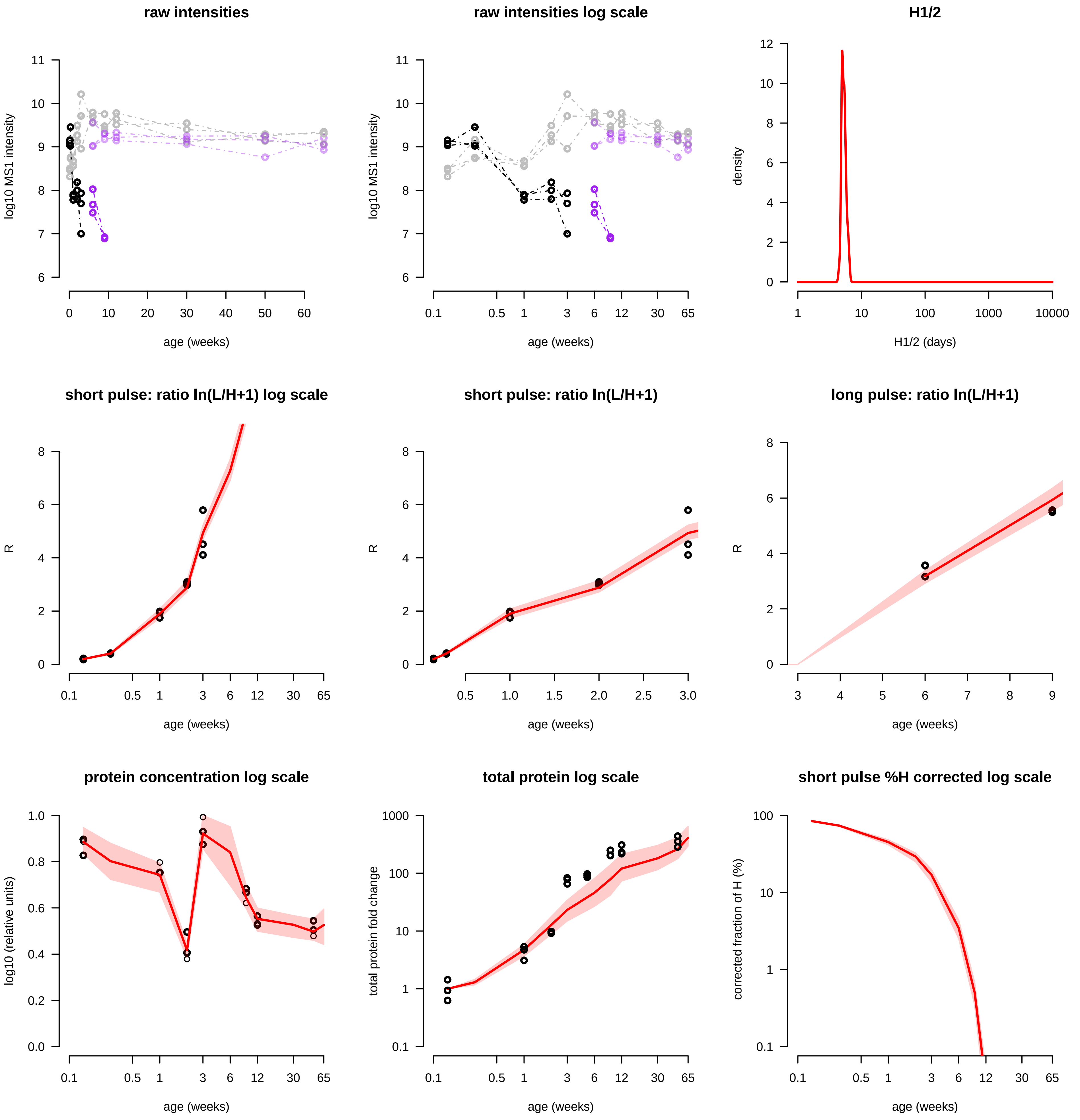

Q8R050−2;Q8R050

>sp|Q8R050−2|ERF3A_MOUSE

soform 2 of Eukaryotic peptide chain release factor GTP−binding subunit ERF3/

GN=Gspt1

Med H1/2: 6.1 days

Q5 H1/2: 5 days

Q95 H1/2: 7.6 days

reliable model fit: yes

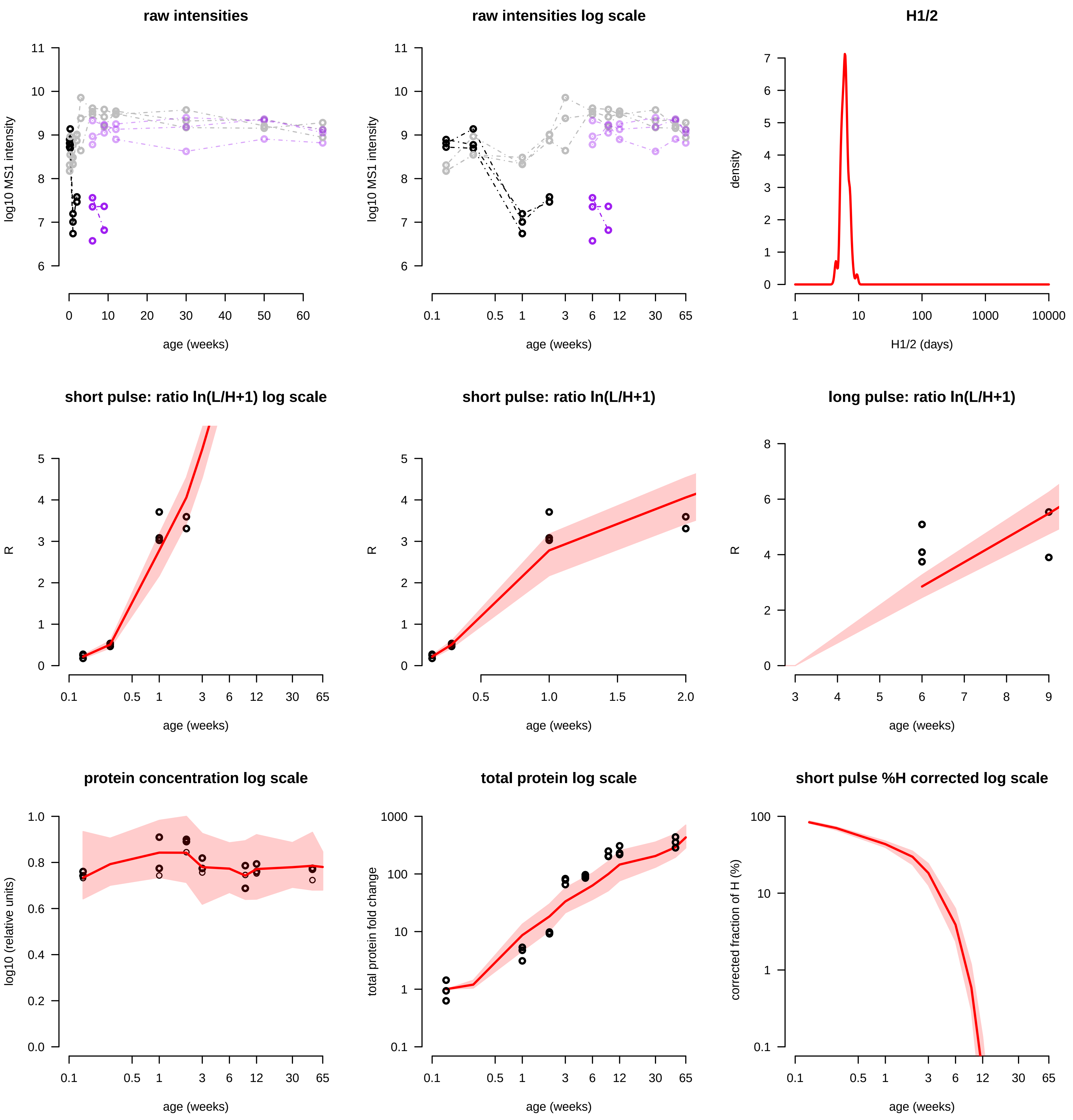

P17427

>sp|P17427|AP2A2_MOUSE

AP−2 complex subunit alpha−2

GN=Ap2a2

Med H1/2: 4.1 days

Q5 H1/2: 3.6 days

Q95 H1/2: 4.9 days

reliable model fit: yes

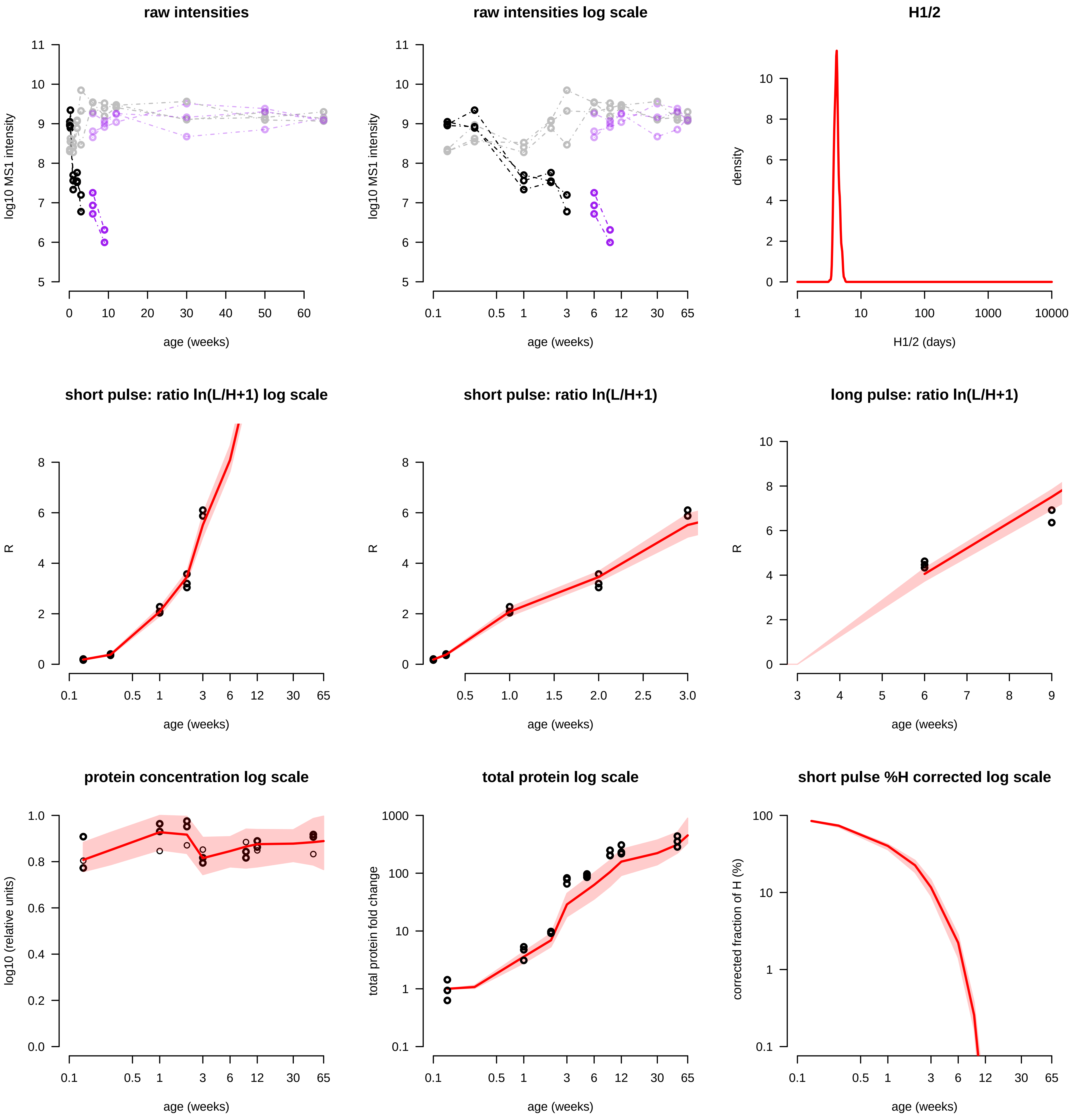

O08547

>sp|O08547|SC22B_MOUSE

Vesicle−trafficking protein SEC22b

GN=Sec22b

Med H1/2: 4.5 days

Q5 H1/2: 3.6 days

Q95 H1/2: 5.2 days

reliable model fit: yes

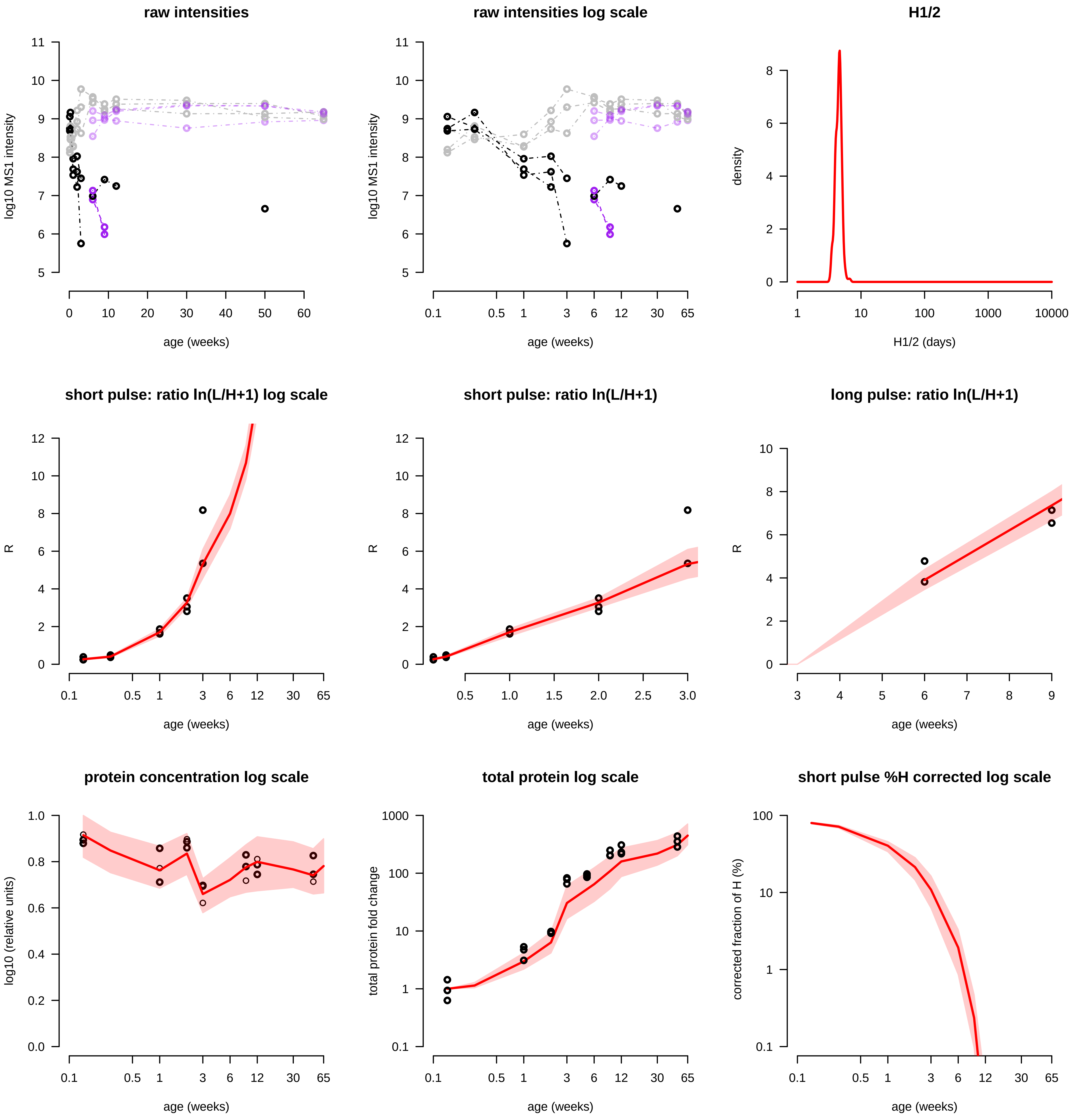

Q78IK2

>sp|Q78IK2|ATPMK_MOUSE

ATP synthase membrane subunit K, mitochondrial

GN=Atp5mk

Med H1/2: 12.7 days

Q5 H1/2: 10.3 days

Q95 H1/2: 25.3 days

reliable model fit: yes

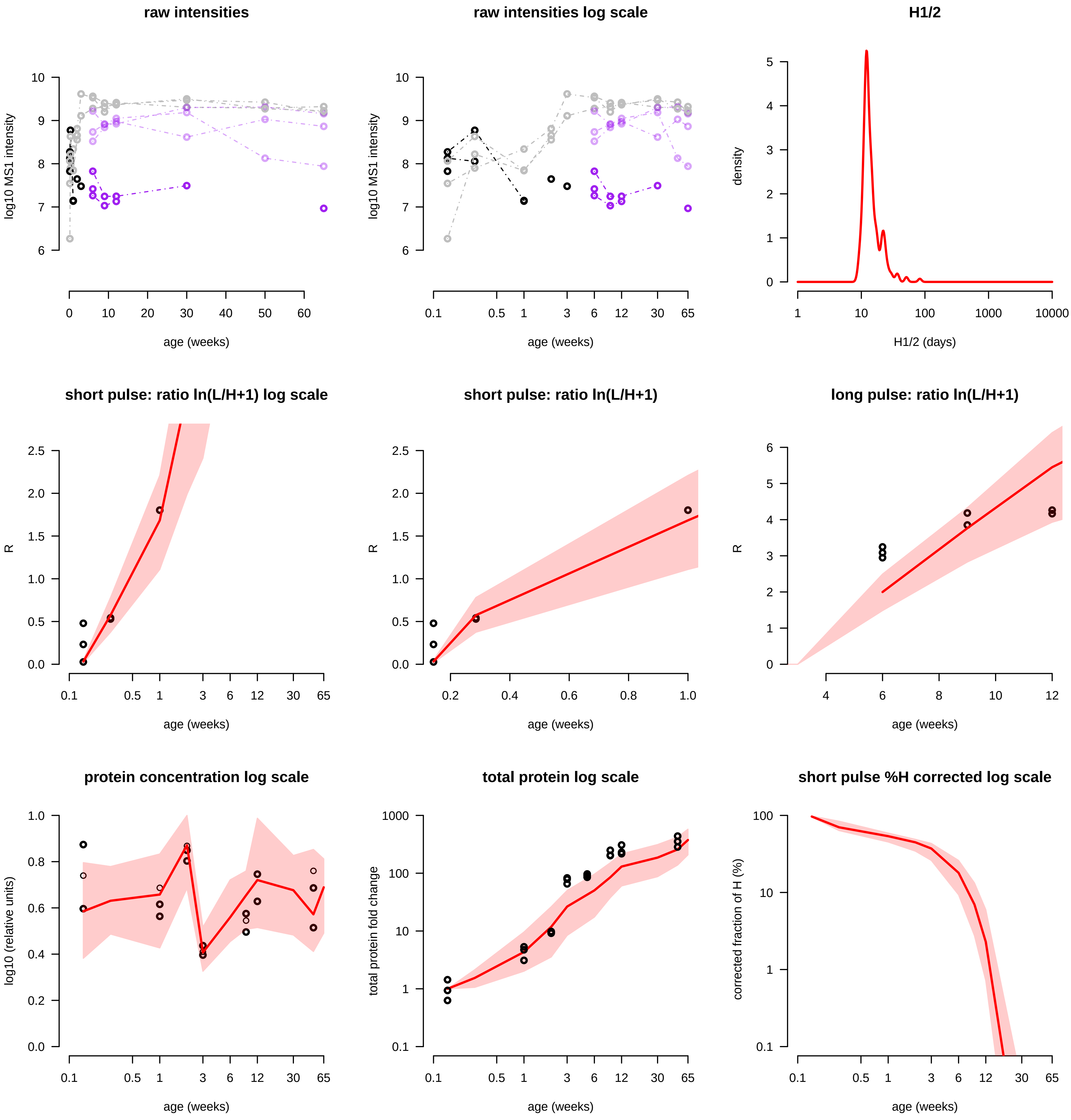

P13745;P10648

>sp|P13745|GSTA1_MOUSE

Glutathione S−transferase A1

GN=Gsta1

Med H1/2: 802.9 days

Q5 H1/2: 172.7 days

Q95 H1/2: 9791.2 days

reliable model fit: no

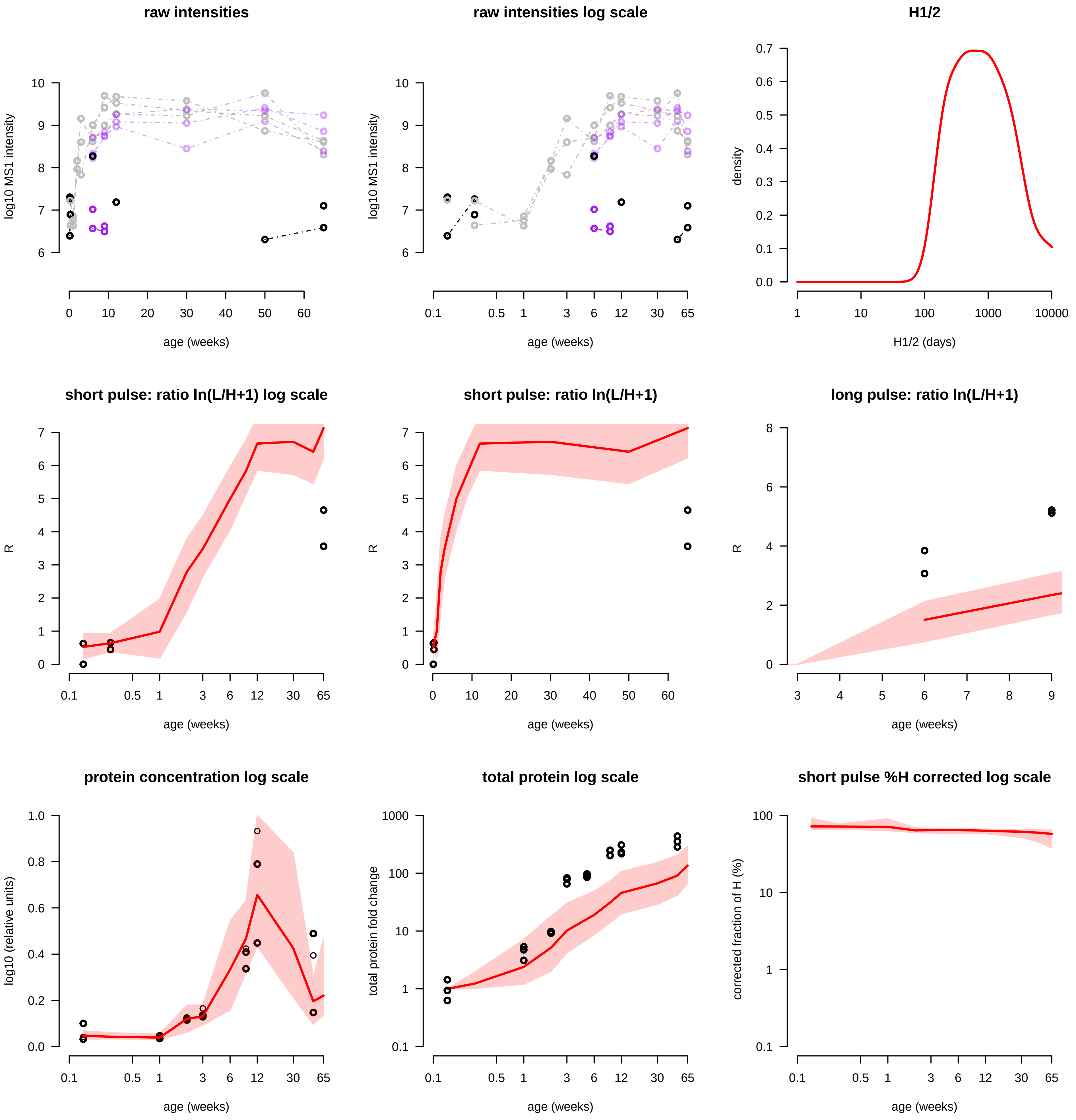

Q6URW6;Q6URW6−2;Q6URW6−3

>sp|Q6URW6|MYH14_MOUSE

Myosin−14

GN=Myh14

Med H1/2: 380 days

Q5   H1/2: 121.4 days

Q95 H1/2: 3437.6 days

reliable model fit: yes

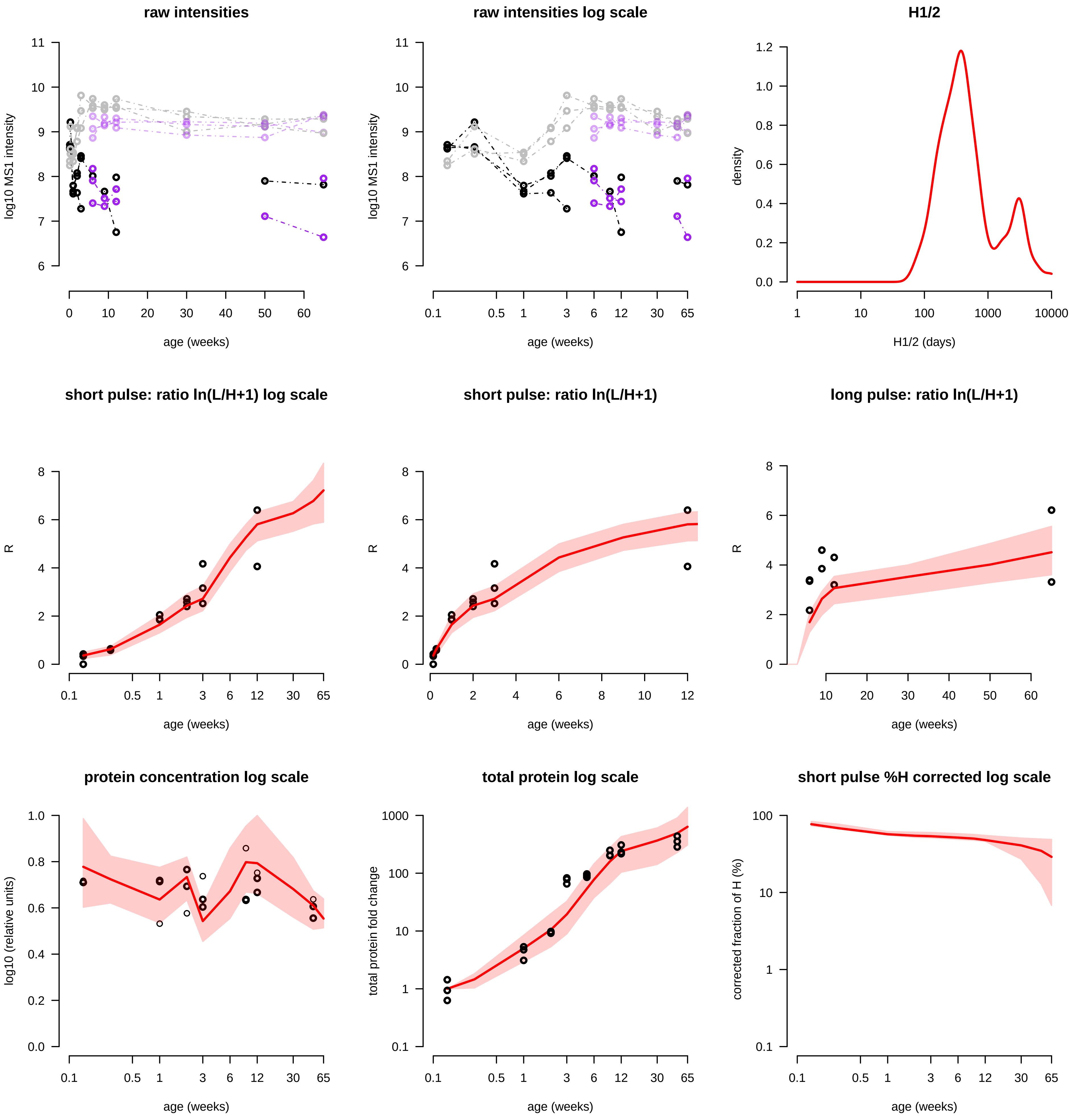

P56395

>sp|P56395|CYB5_MOUSE

Cytochrome b5

GN=Cyb5a

Med H1/2: 10.6 days

Q5 H1/2: 7.9 days

Q95 H1/2: 15 days

reliable model fit: yes

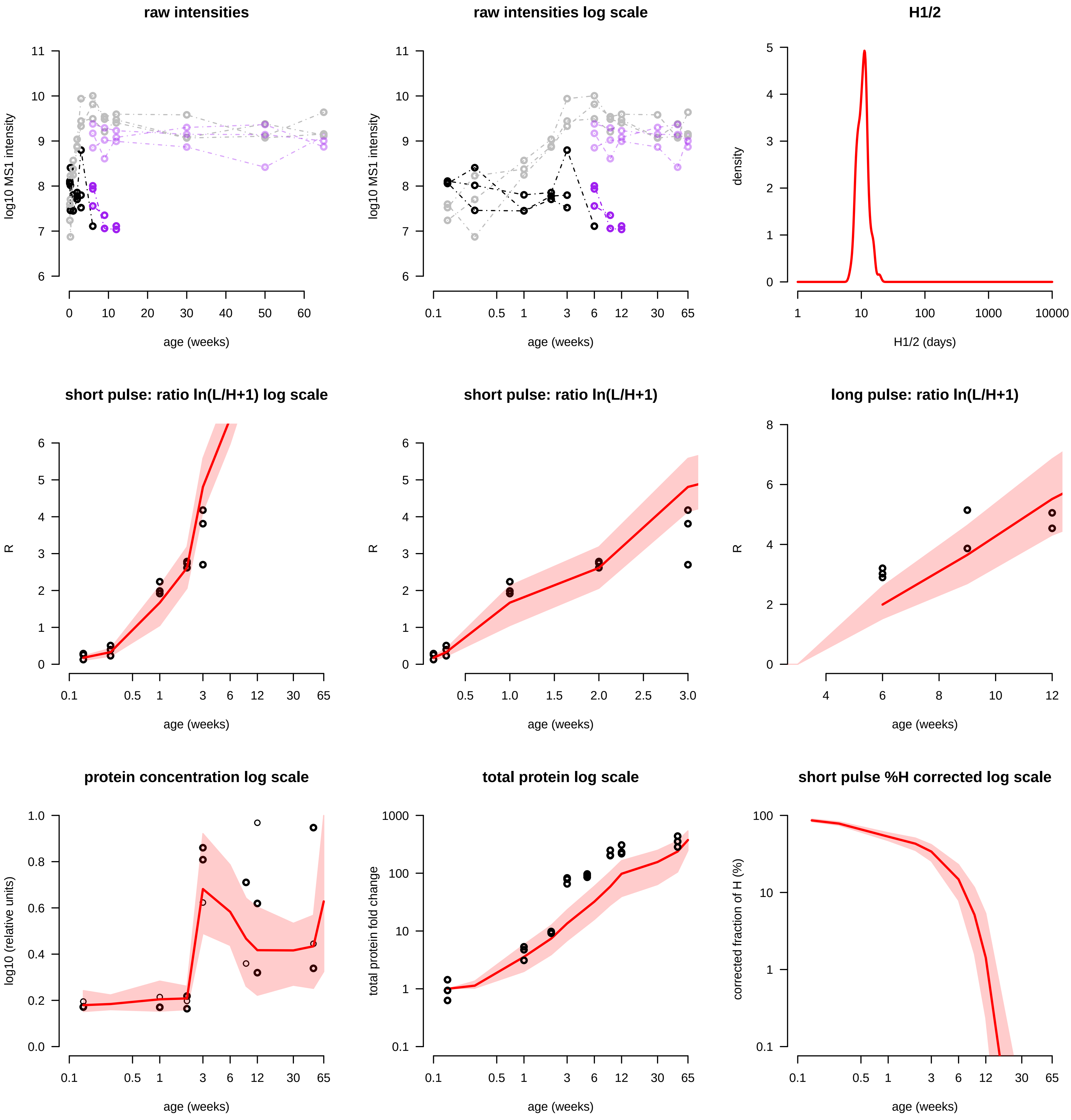

Q9CPU0

>sp|Q9CPU0|LGUL_MOUSE

Lactoylglutathione lyase

GN=Glo1

Med H1/2: 11.5 days

Q5 H1/2: 8.2 days

Q95 H1/2: 17.7 days

reliable model fit: yes

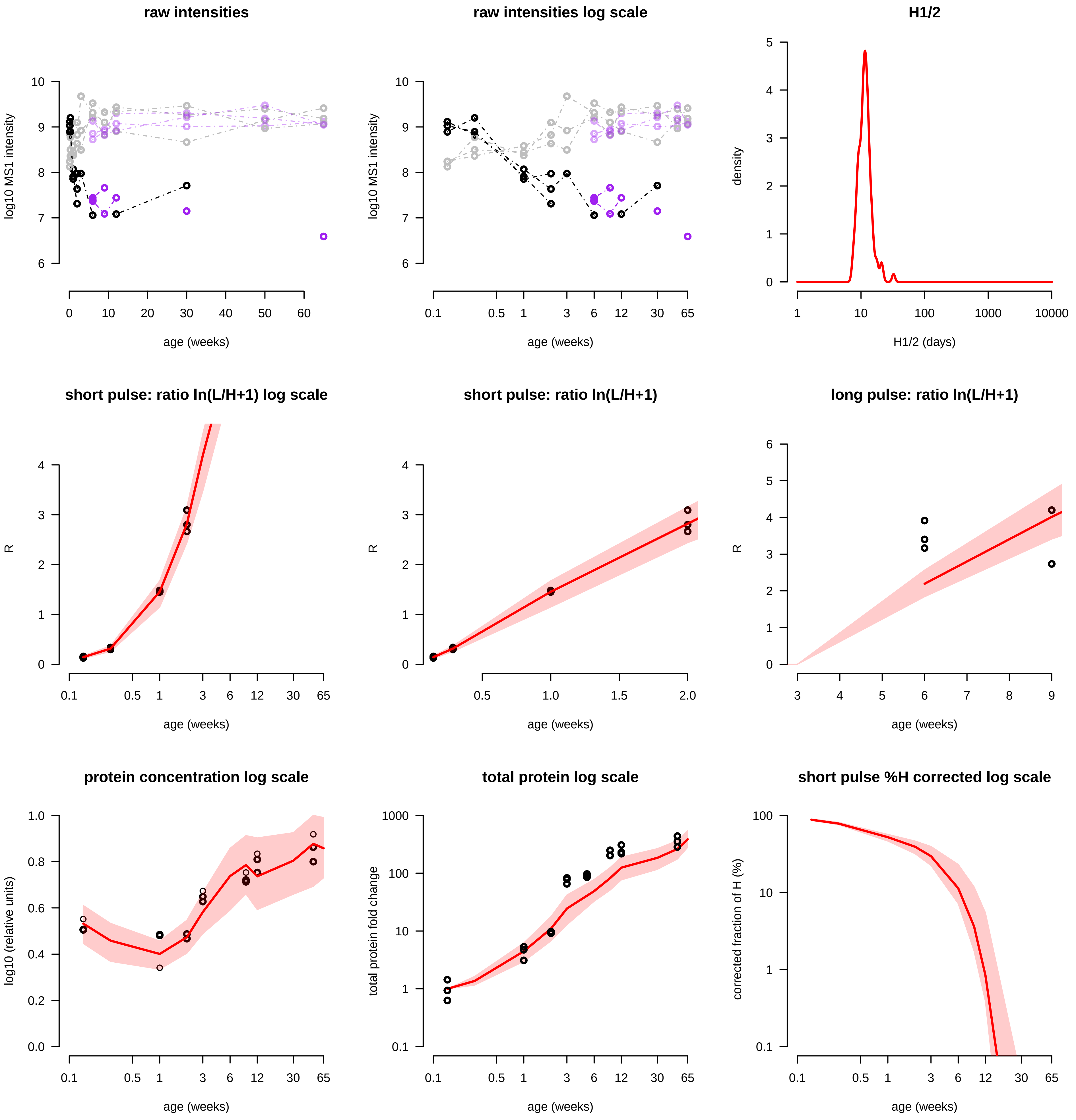

O35841

>sp|O35841|API5_MOUSE

Apoptosis inhibitor 5

GN=Api5

Med H1/2: 503 days

Q5 H1/2: 115.3 days

Q95 H1/2: 6736.9 days

reliable model fit: no

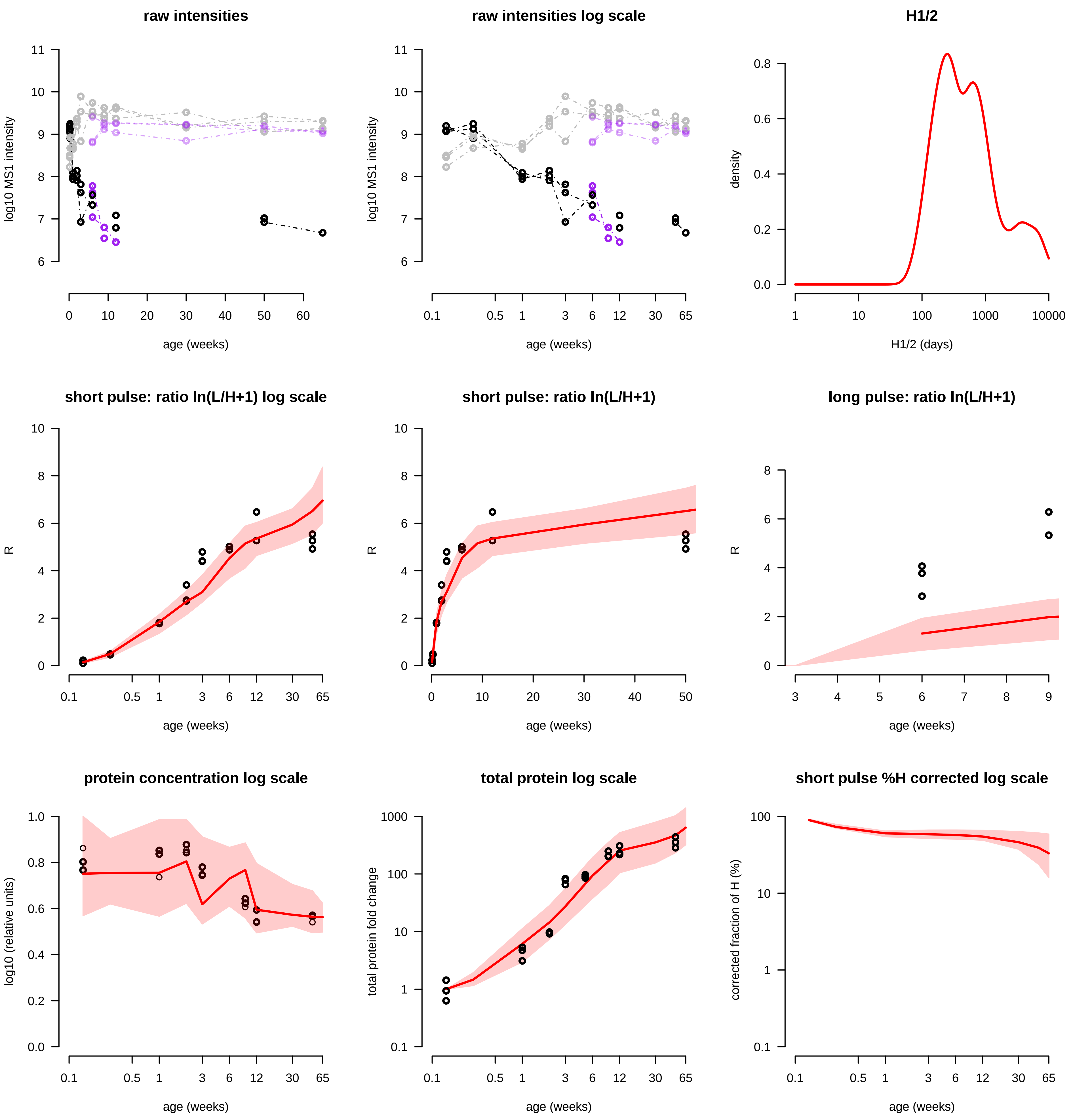

Q8BRT1

>sp|Q8BRT1|CLAP2_MOUSE

CLIP−associating protein 2

GN=Clasp2

Med H1/2: 3.6 days

Q5 H1/2: 3 days

Q95 H1/2: 4.8 days

reliable model fit: yes

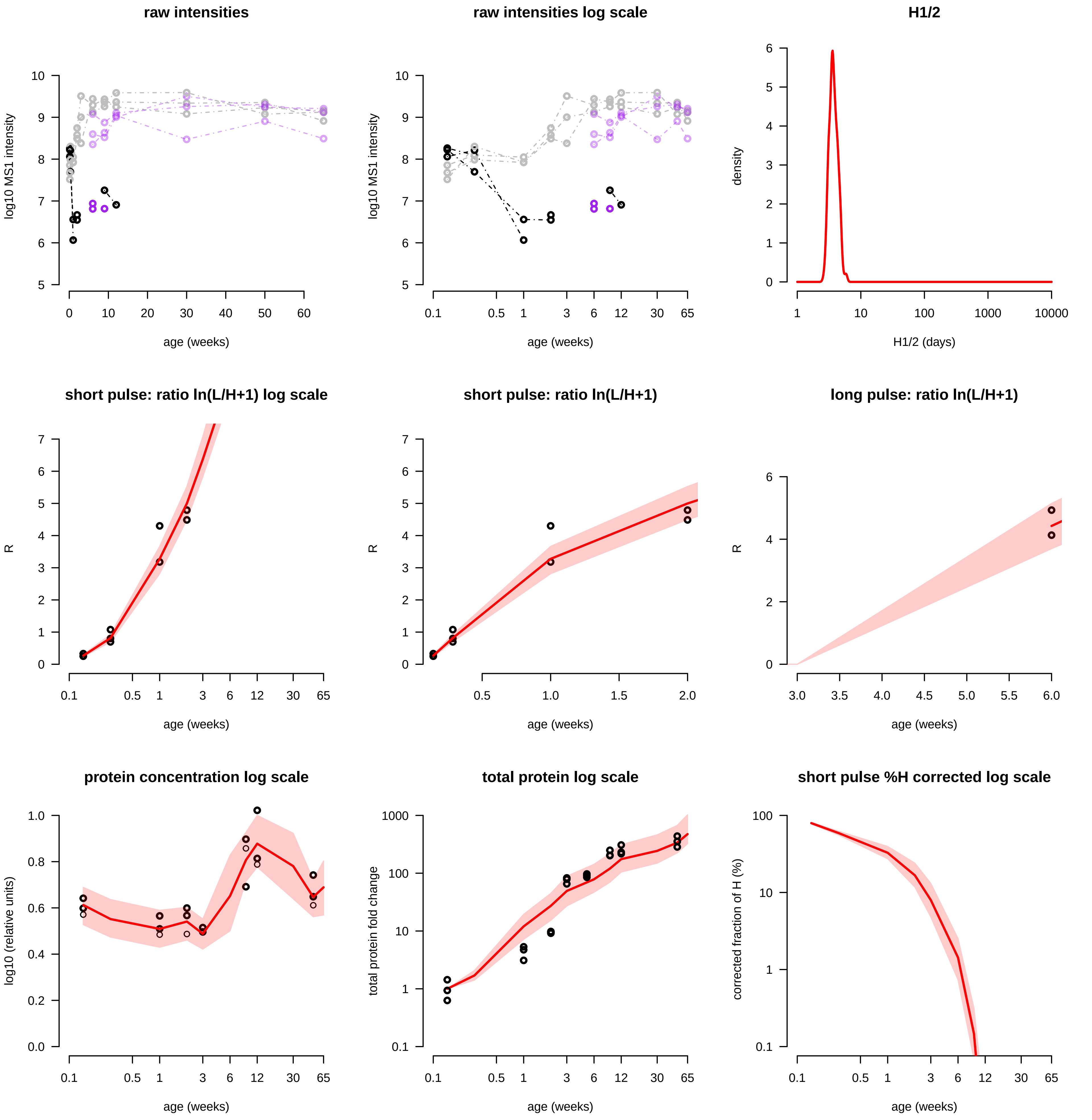

Q8R016

>sp|Q8R016|BLMH_MOUSE

Bleomycin hydrolase

GN=Blmh

Med H1/2: 816.2 days

Q5 H1/2: 246.2 days

Q95 H1/2: 9925.6 days

reliable model fit: yes

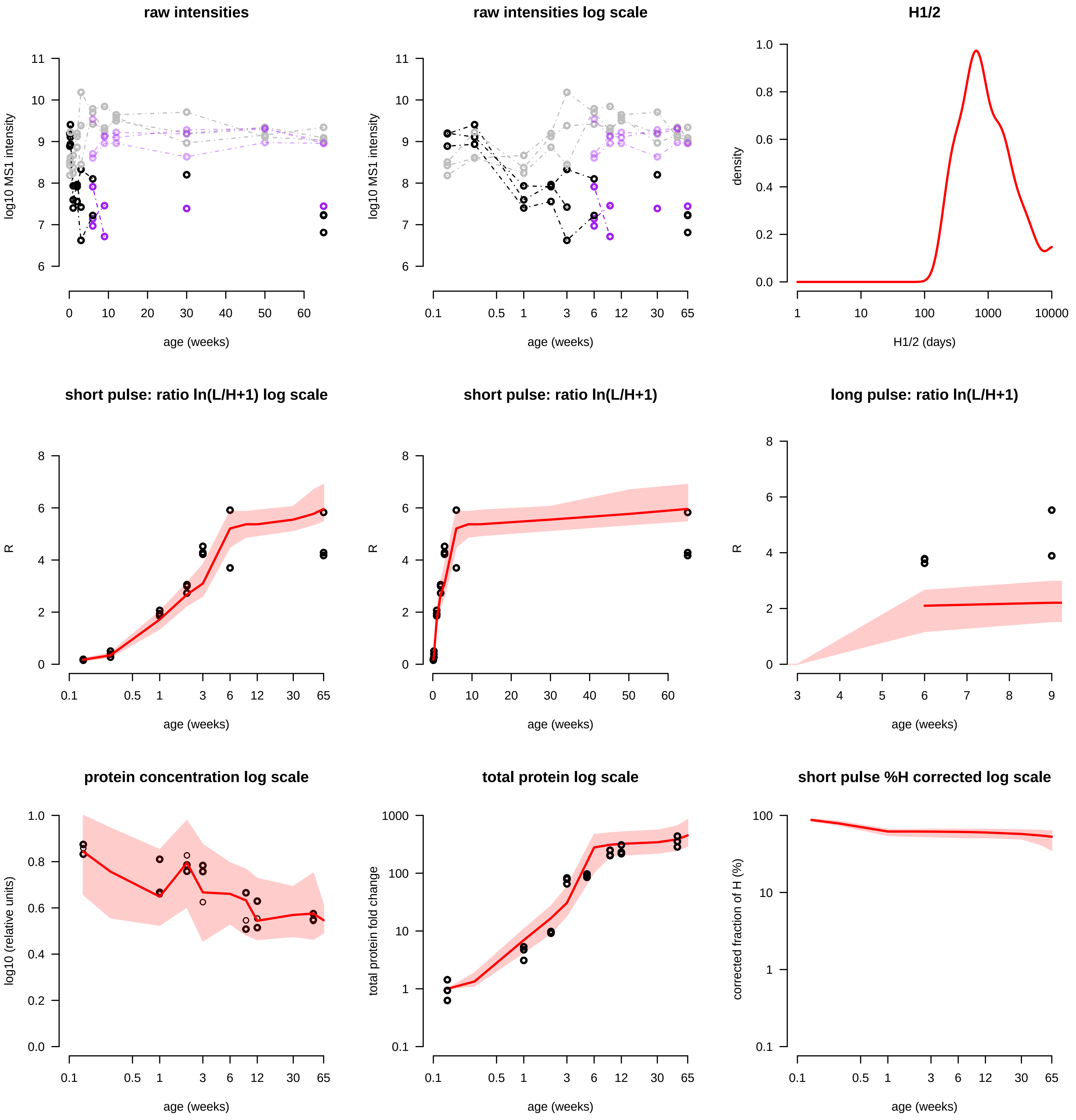

Q9CR68

>sp|Q9CR68|UCRI_MOUSE

Cytochrome b−c1 complex subunit Rieske, mitochondrial

GN=Uqcrfs1

Med H1/2: 9.1 days

Q5 H1/2: 7.3 days

Q95 H1/2: 13.3 days

reliable model fit: yes

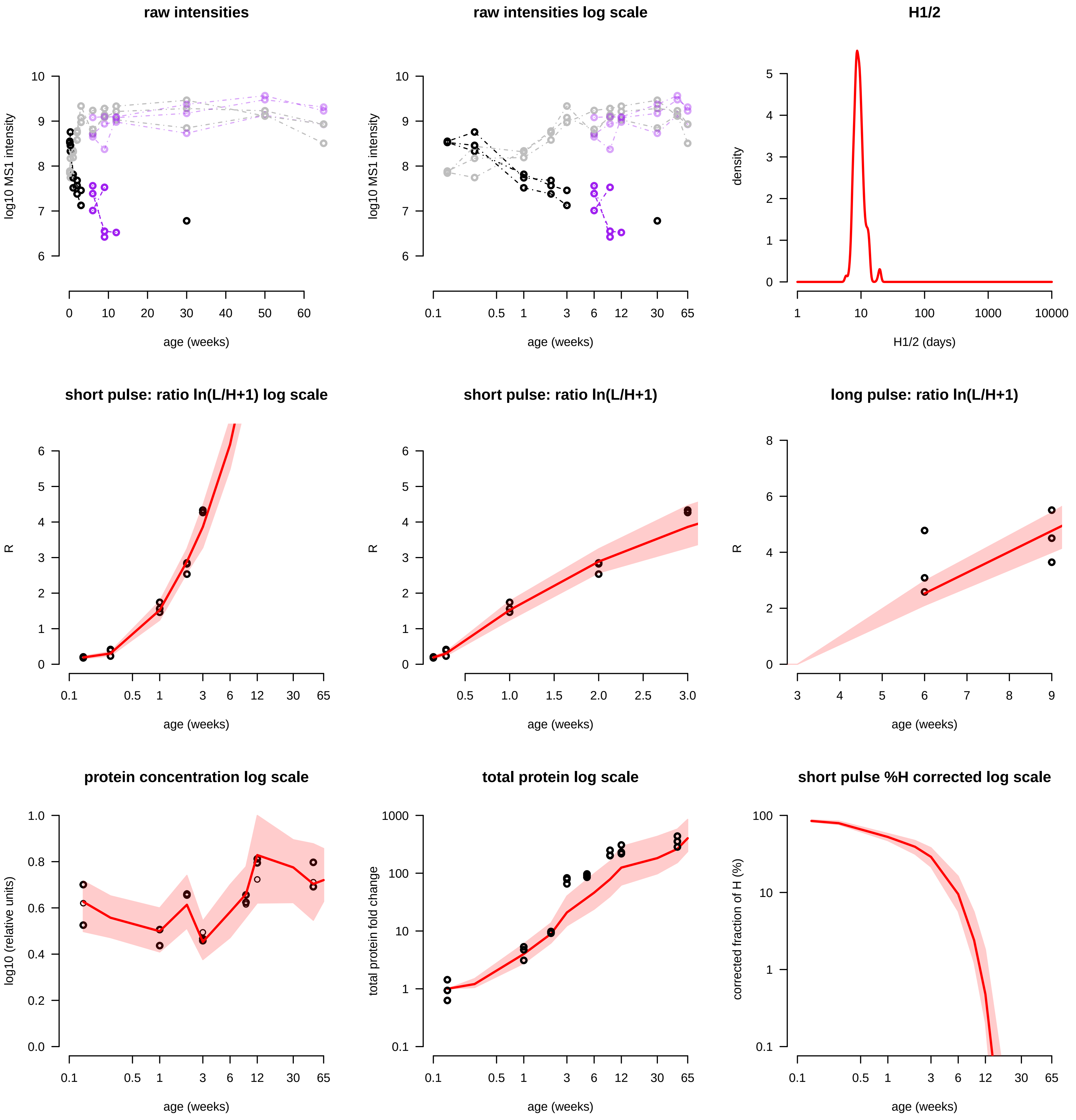

P62331

>sp|P62331|ARF6_MOUSE

ADP−ribosylation factor 6

GN=Arf6

Med H1/2: 5.1 days

Q5 H1/2: 4.3 days

Q95 H1/2: 6.6 days

reliable model fit: yes

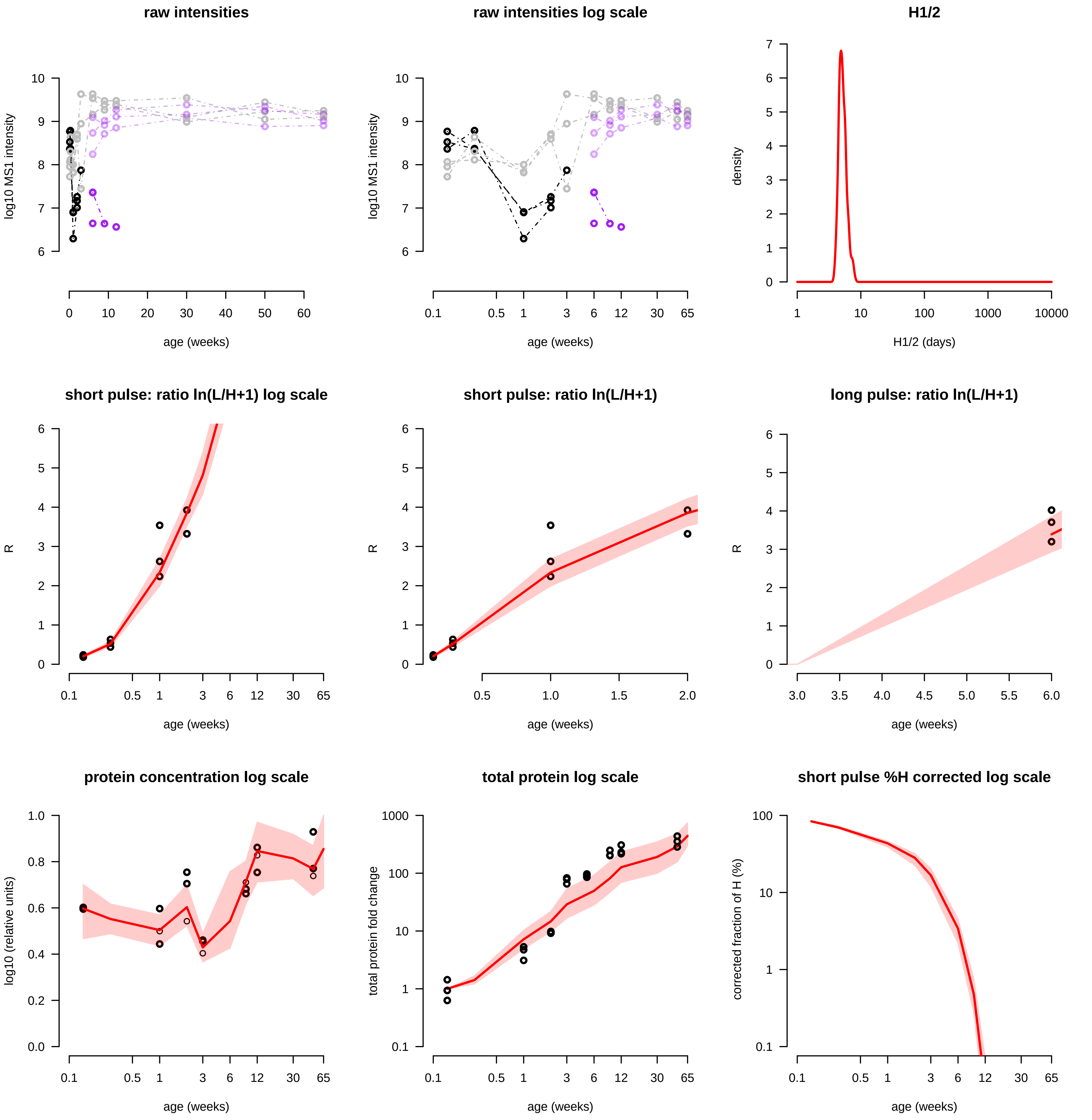

P62843

>sp|P62843|RS15_MOUSE

40S ribosomal protein S15

GN=Rps15

Med H1/2: 53.6 days

Q5 H1/2: 11.8 days

Q95 H1/2: 372.2 days

reliable model fit: yes

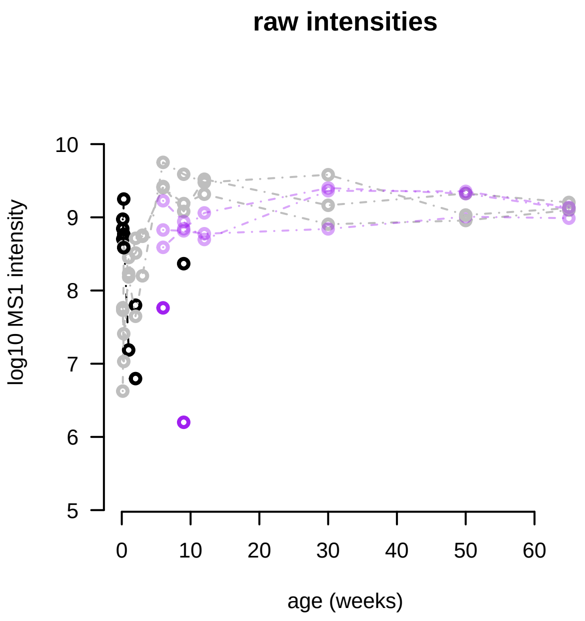

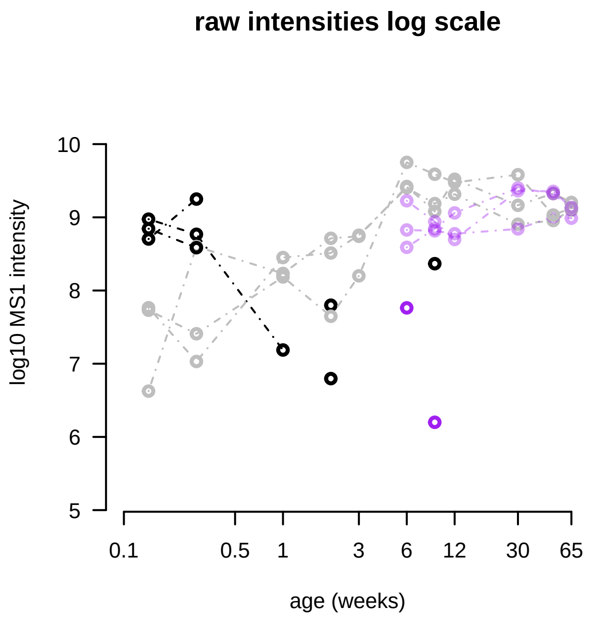

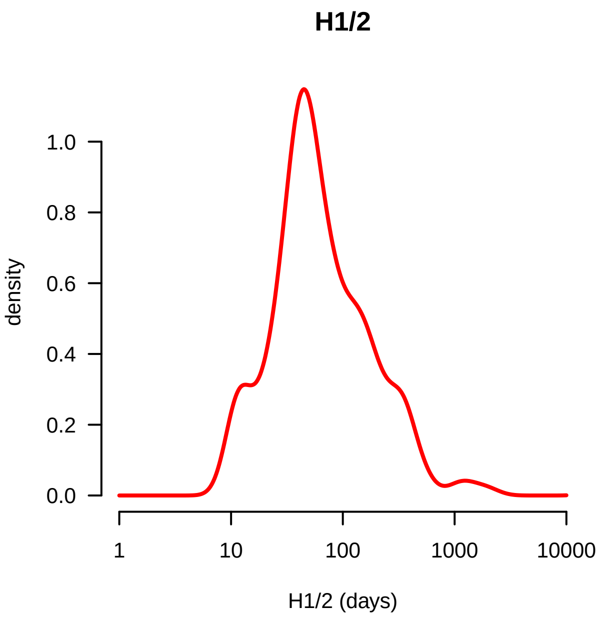

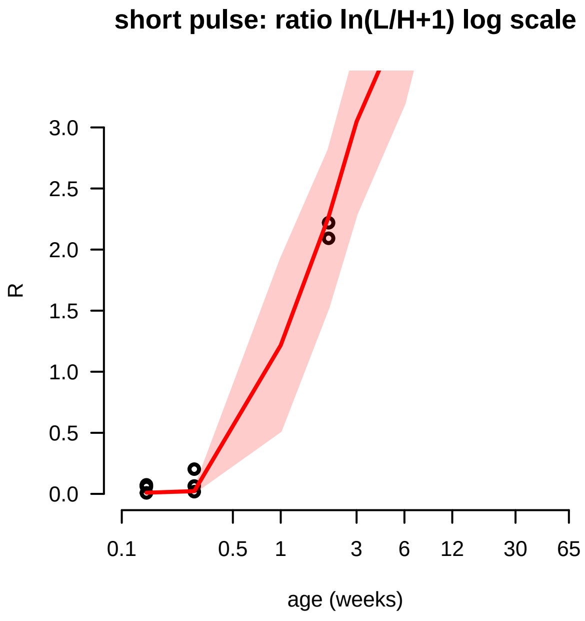

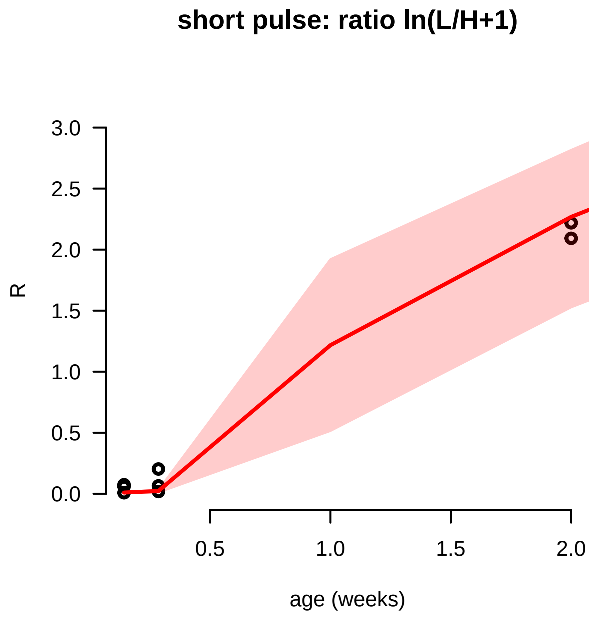

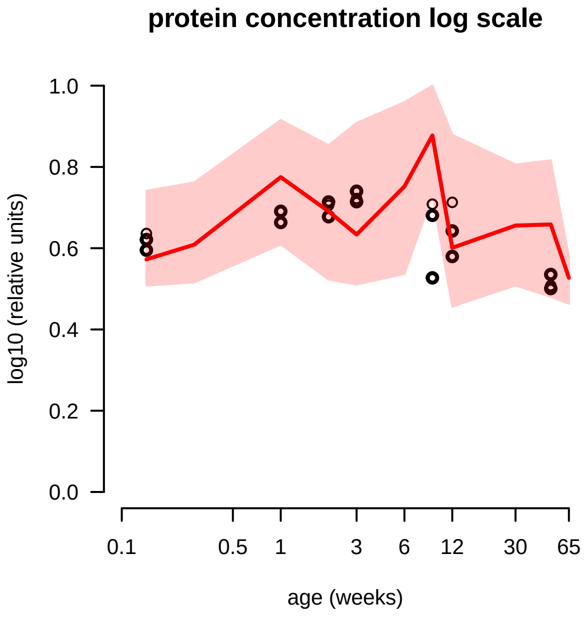

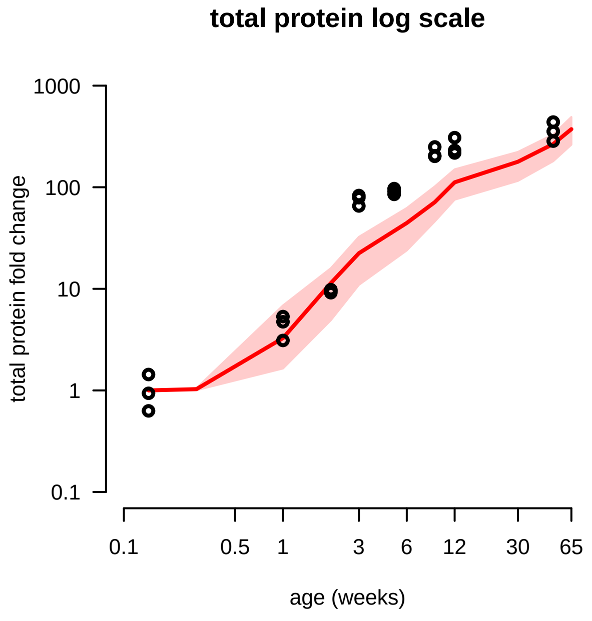

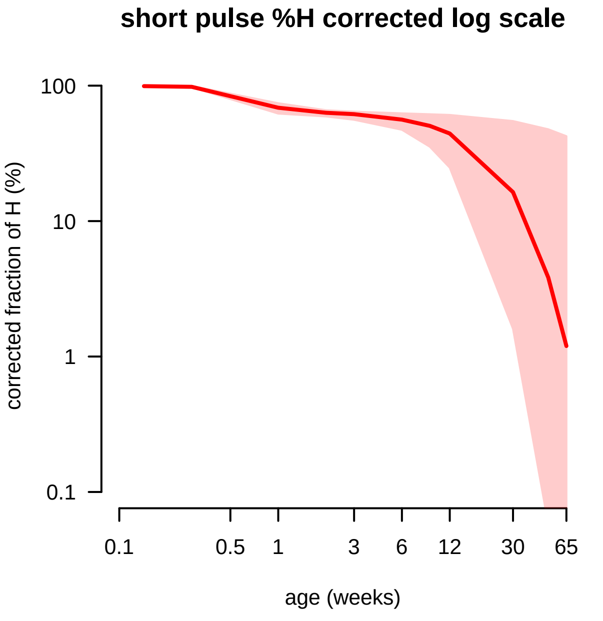

Q9D023

>sp|Q9D023|MPC2_MOUSE

Mitochondrial pyruvate carrier 2

GN=Mpc2

Med H1/2: 8.1 days

Q5 H1/2: 5.5 days

Q95 H1/2: 13.8 days

reliable model fit: yes

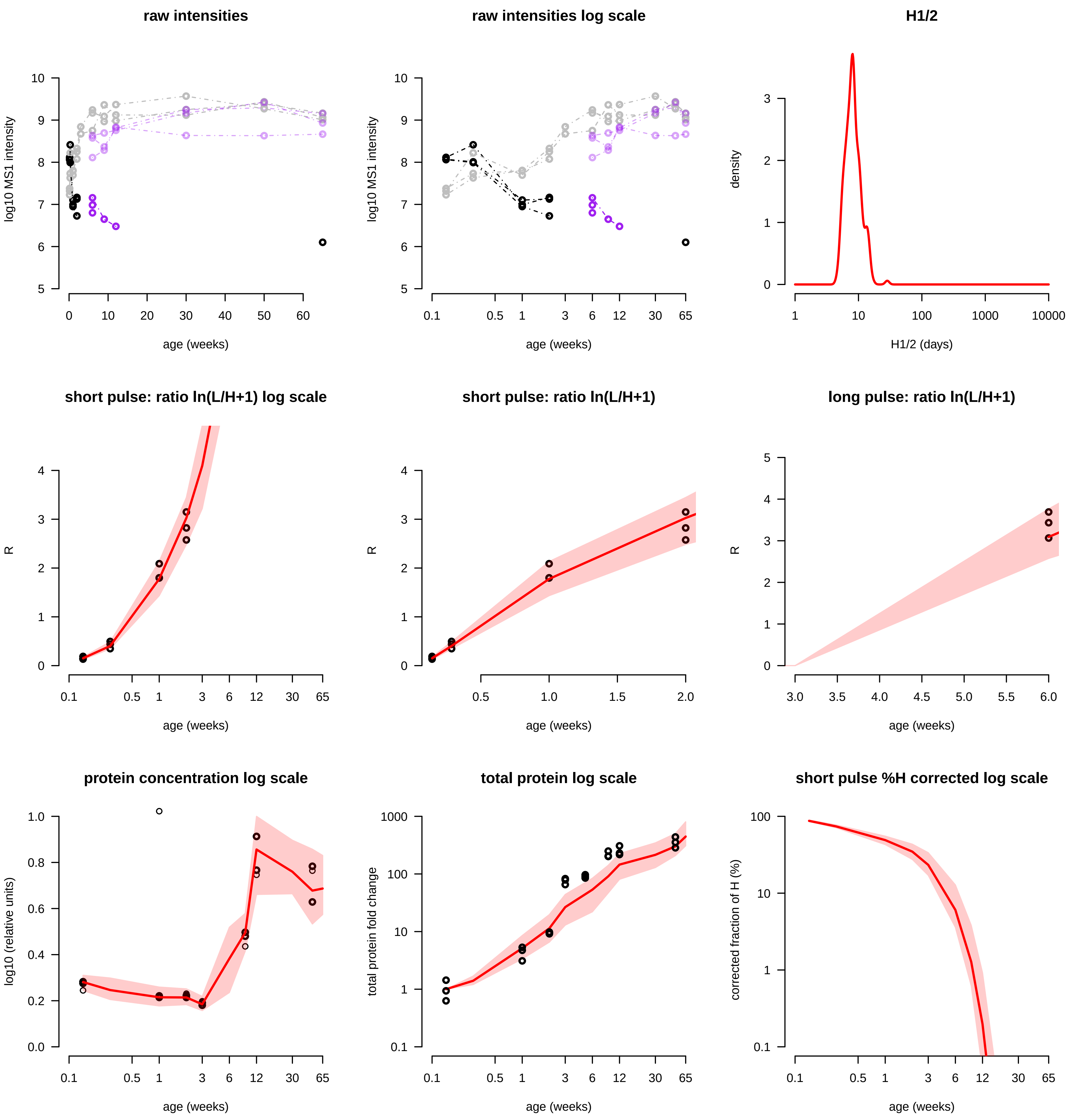

Q62348

>sp|Q62348|TSN_MOUSE

Translin

GN=Tsn

Med H1/2: 8.5 days

Q5 H1/2: 6.8 days

Q95 H1/2: 11.6 days

reliable model fit: yes

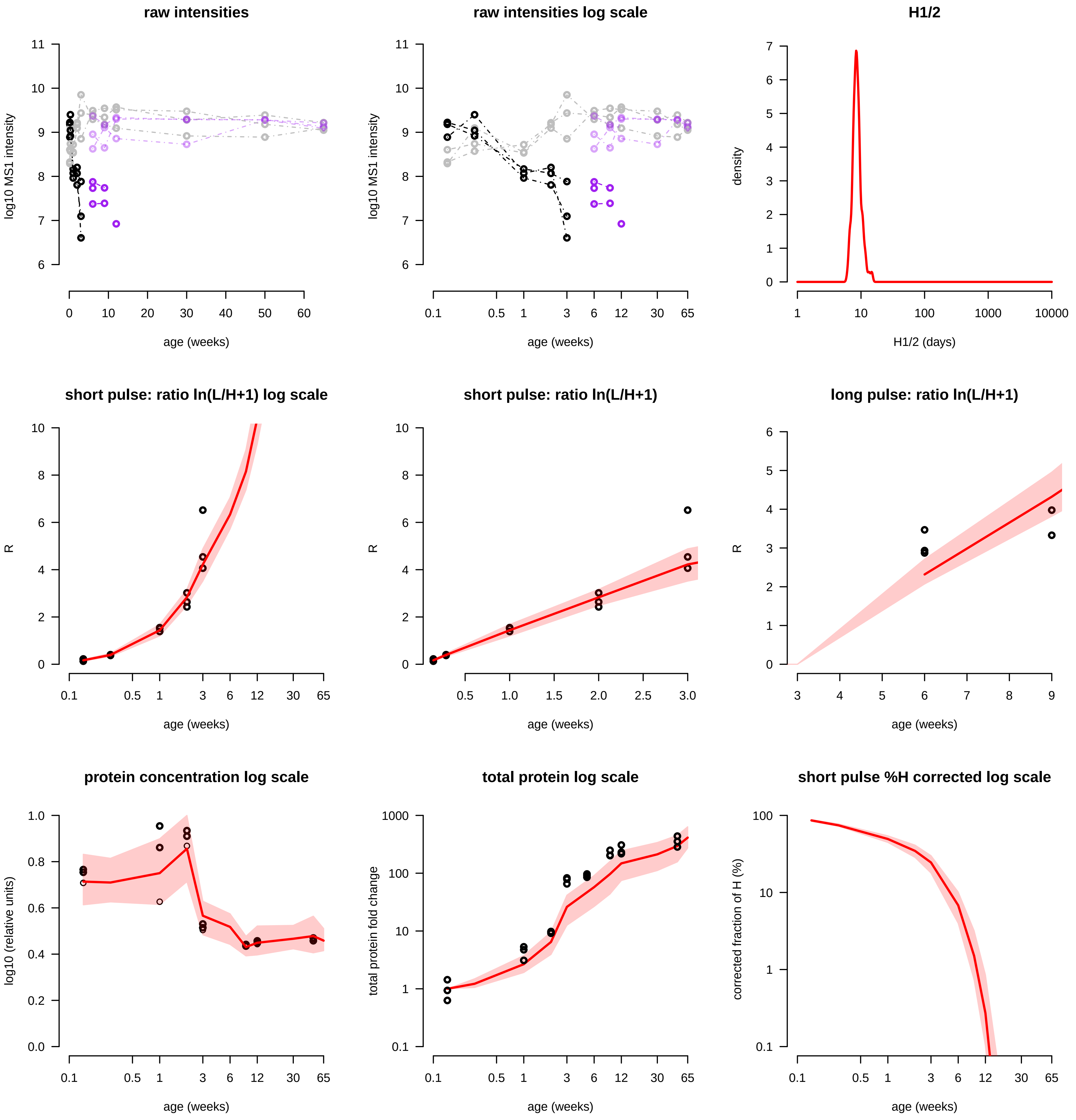

Q91WK2

>sp|Q91WK2|EIF3H_MOUSE

Eukaryotic translation initiation factor 3 subunit H

GN=Eif3h

Med H1/2: 1137.6 days

Q5 H1/2: 355.4 days

Q95 H1/2: 25087.3 days

reliable model fit: no

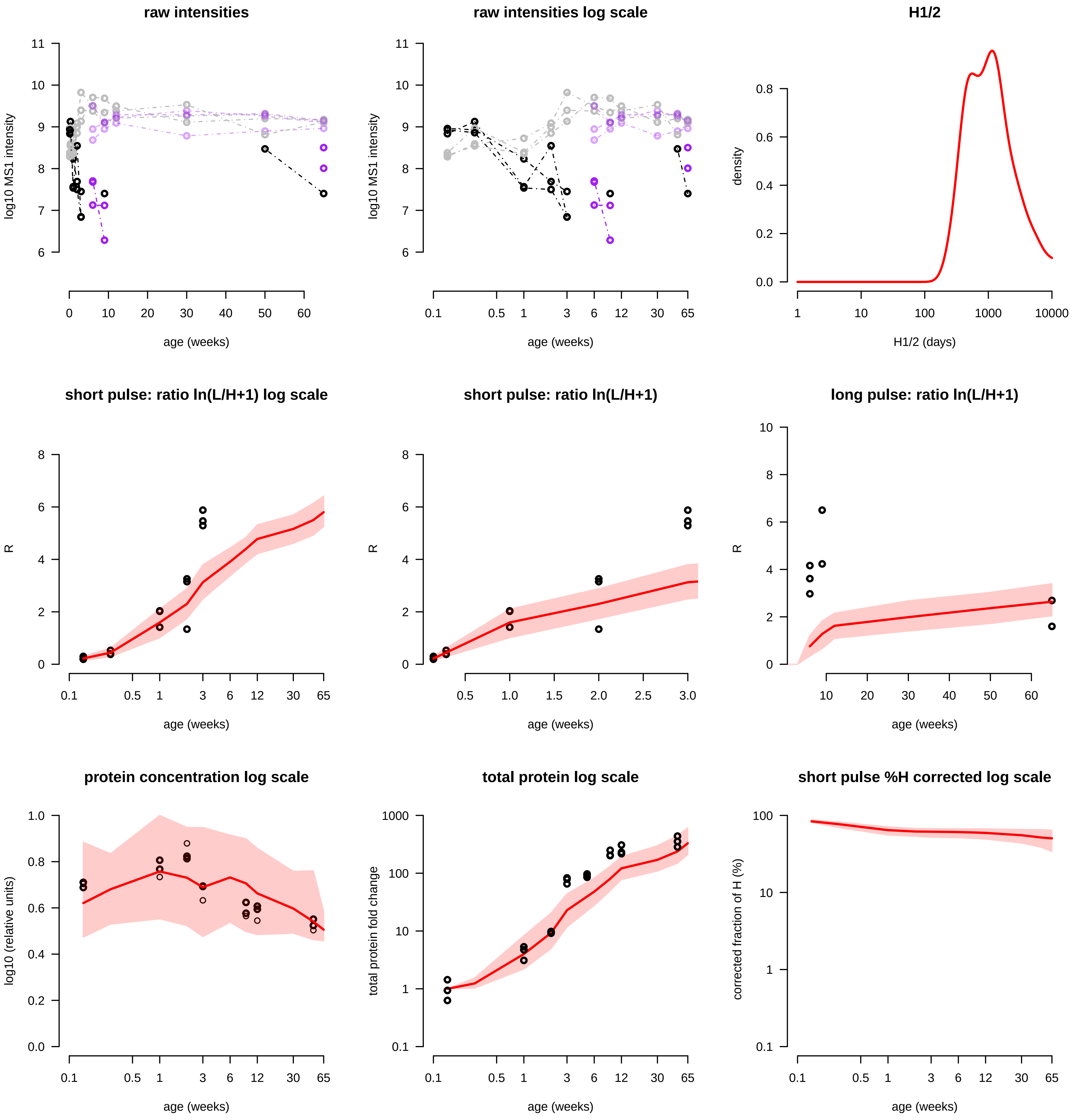

Q8BX70;Q8BX70−3;Q8BX70−2

>sp|Q8BX70|VP13C_MOUSE

Vacuolar protein sorting−associated protein 13C

GN=Vps13c

Med H1/2: 1105.1 days

Q5 H1/2: 317 days

Q95 H1/2: 10991.9 days

reliable model fit: no

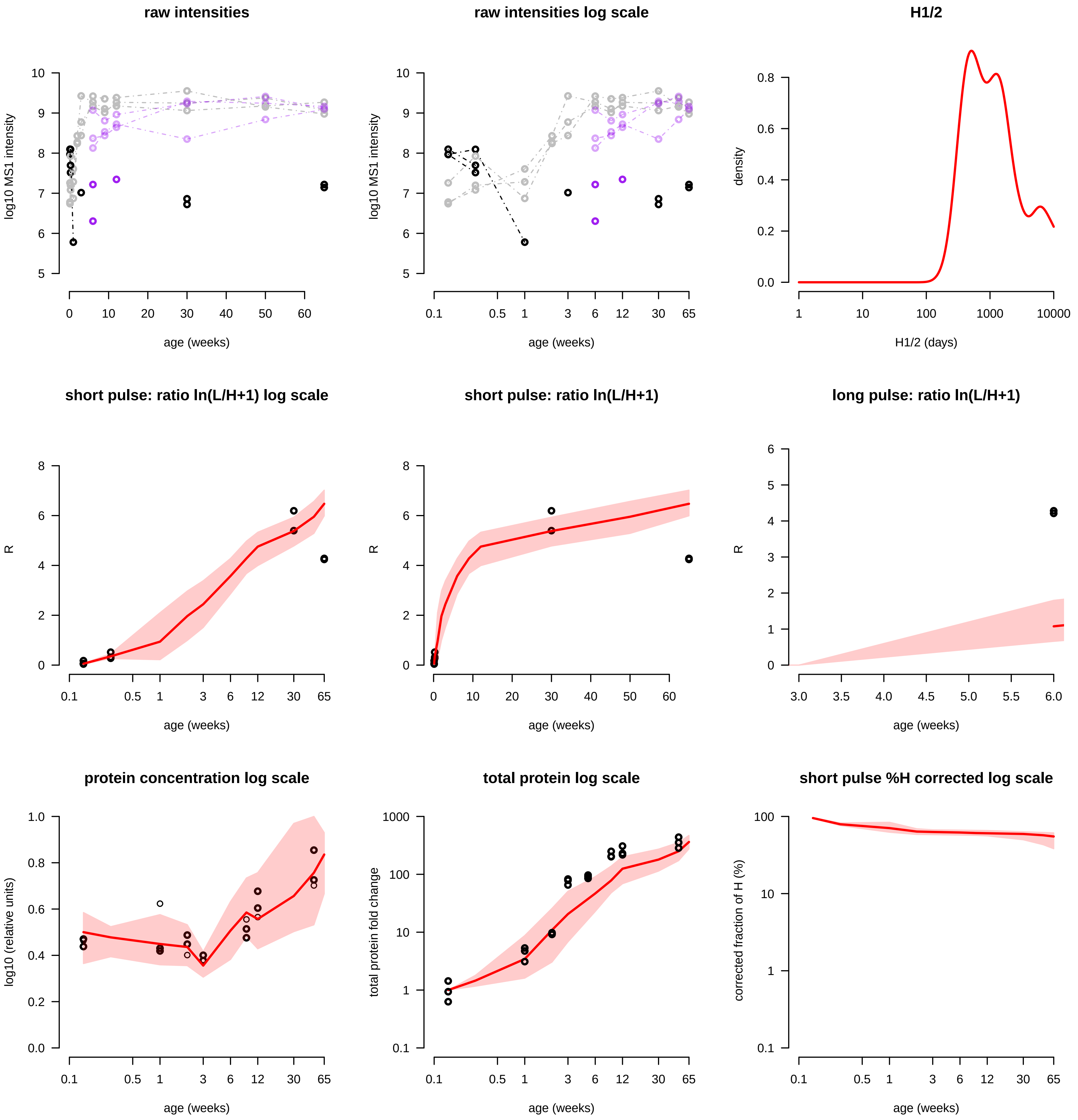

Q61543

>sp|Q61543|GSLG1_MOUSE

Golgi apparatus protein 1

GN=Glg1

Med H1/2: 4.7 days

Q5 H1/2: 4 days

Q95 H1/2: 5.8 days

reliable model fit: yes

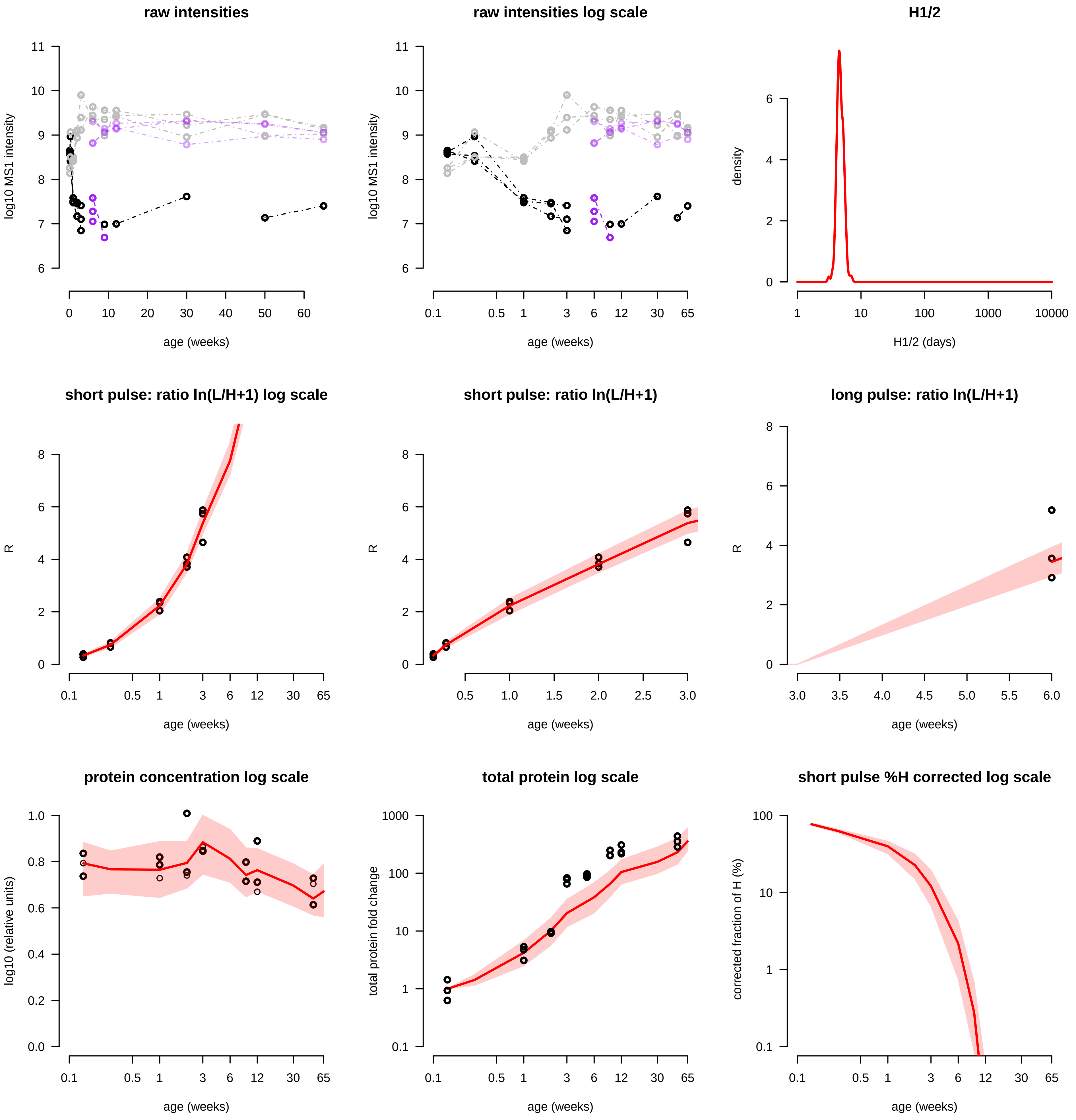

Q01730

>sp|Q01730|RSU1_MOUSE

Ras suppressor protein 1

GN=Rsu1

Med H1/2: 6.7 days

Q5 H1/2: 5.7 days

Q95 H1/2: 8.3 days

reliable model fit: yes

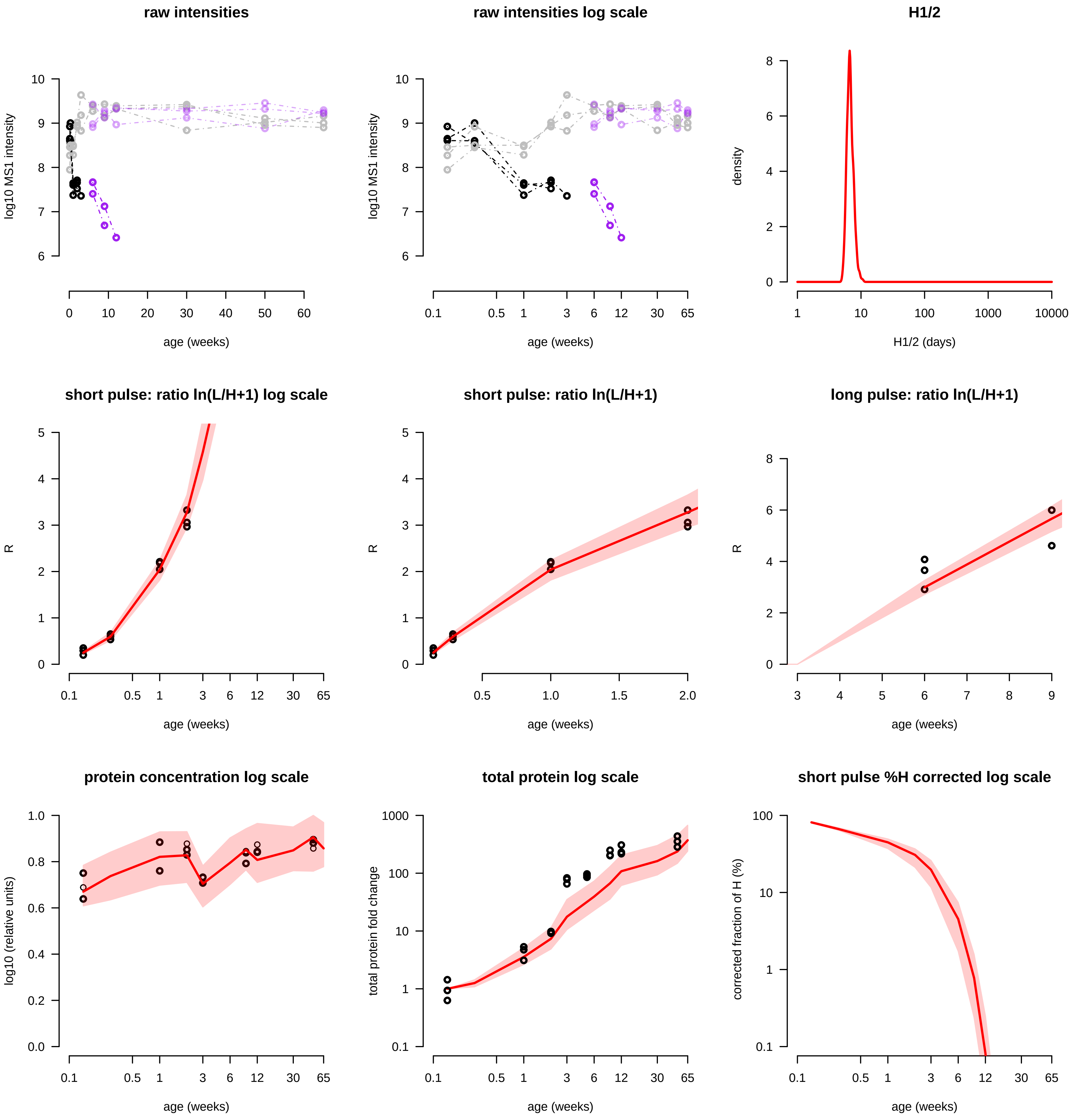

P59325

>sp|P59325|IF5_MOUSE

Eukaryotic translation initiation factor 5

GN=Eif5

Med H1/2: 4.3 days

Q5 H1/2: 3.6 days

Q95 H1/2: 5.1 days

reliable model fit: yes

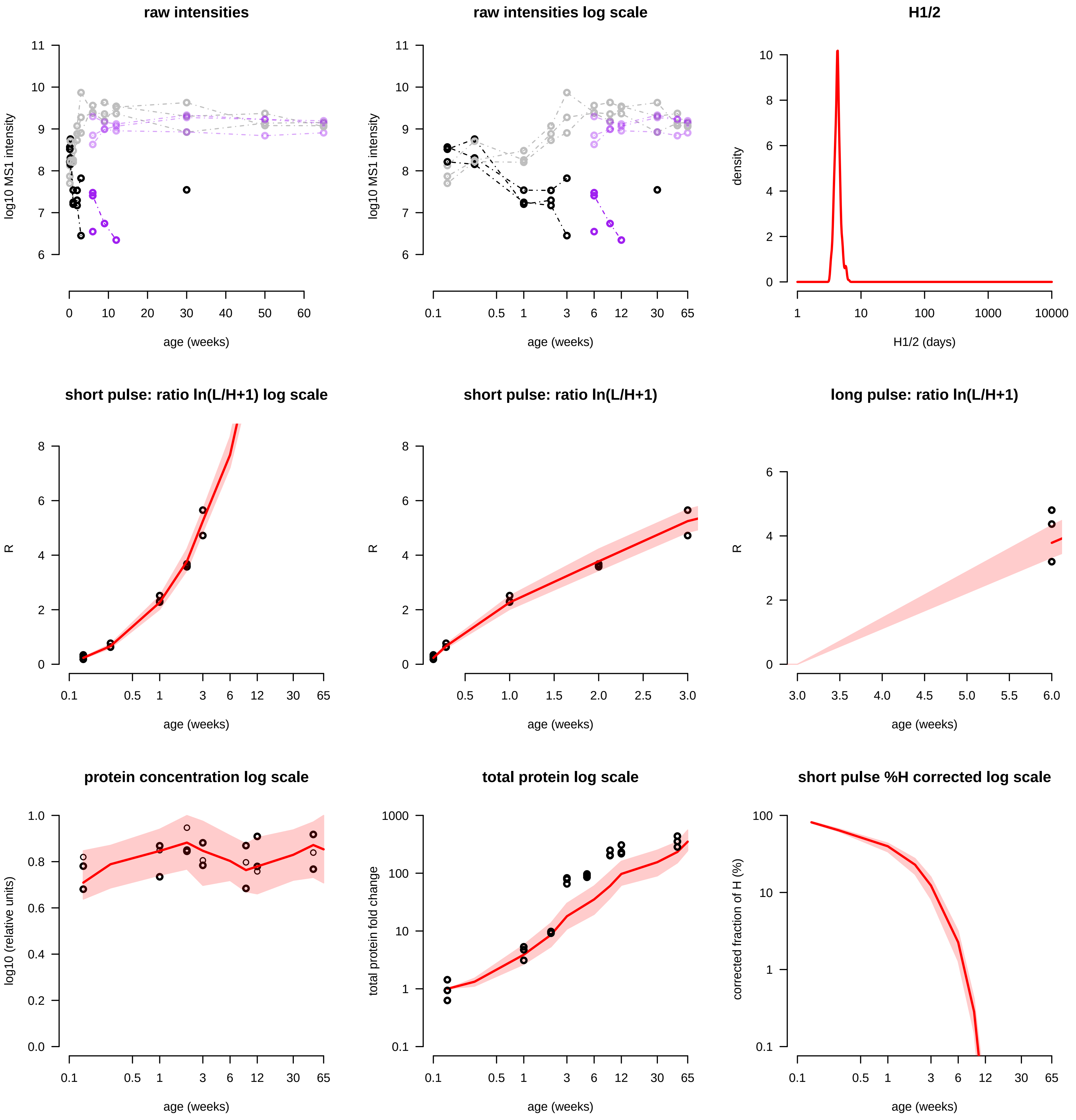

O70423;O70423−2

>sp|O70423|AOC3_MOUSE

Membrane primary amine oxidase

GN=Aoc3

Med H1/2: 12.7 days

Q5 H1/2: 9.5 days

Q95 H1/2: 18.6 days

reliable model fit: yes

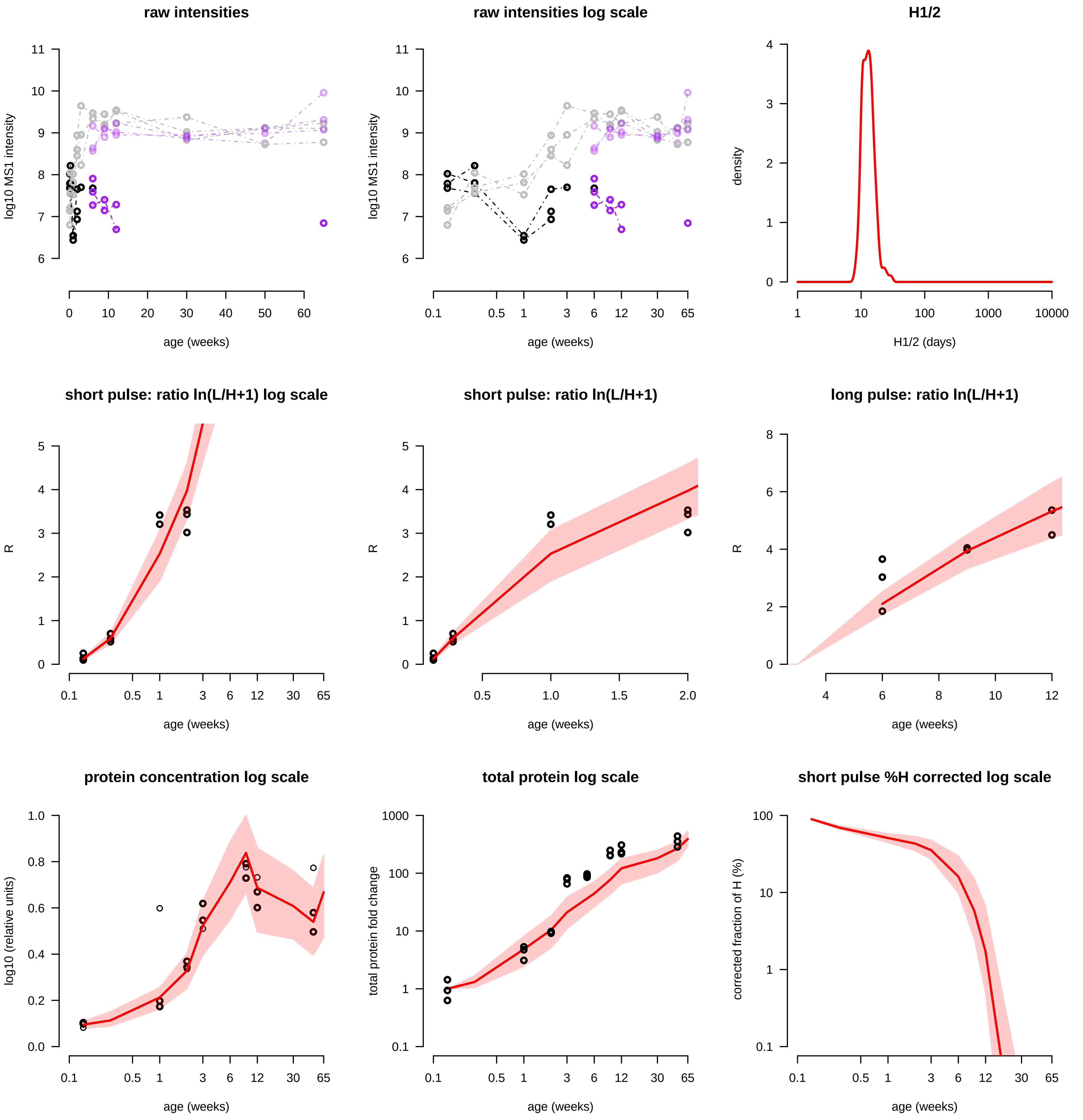

Q922J3;Q922J3−2

>sp|Q922J3|CLIP1_MOUSE

CAP−Gly domain−containing linker protein 1

GN=Clip1

Med H1/2: 14.3 days

Q5 H1/2: 11.9 days

Q95 H1/2: 20.1 days

reliable model fit: yes

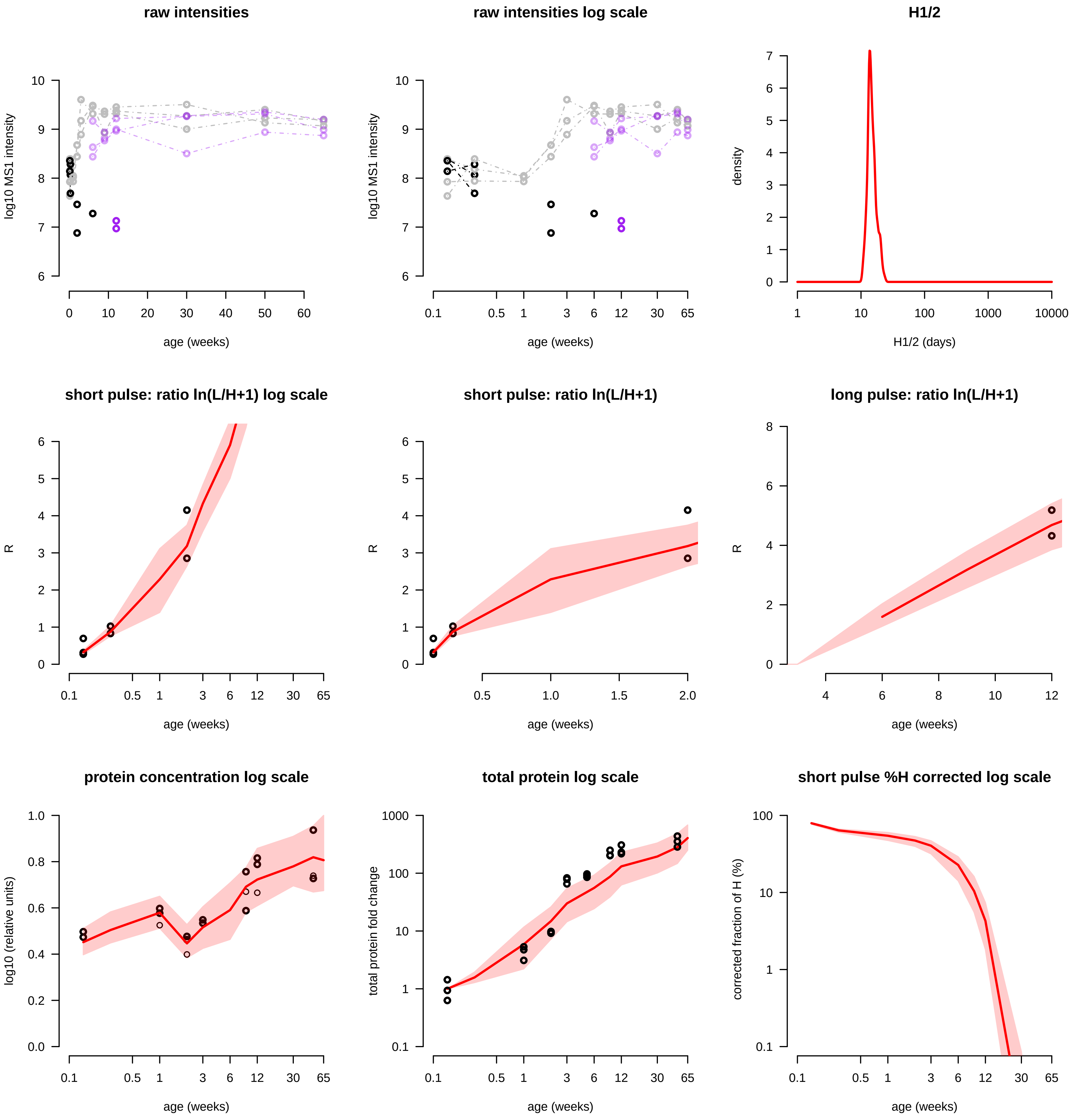

Q3UTJ2−2

>sp|Q3UTJ2−2|SRBS2_MOUSE

Isoform 2 of Sorbin and SH3 domain−containing protein 2

GN=Sorbs2

Med H1/2: 7.5 days

Q5 H1/2: 6.3 days

Q95 H1/2: 9.6 days

reliable model fit: yes

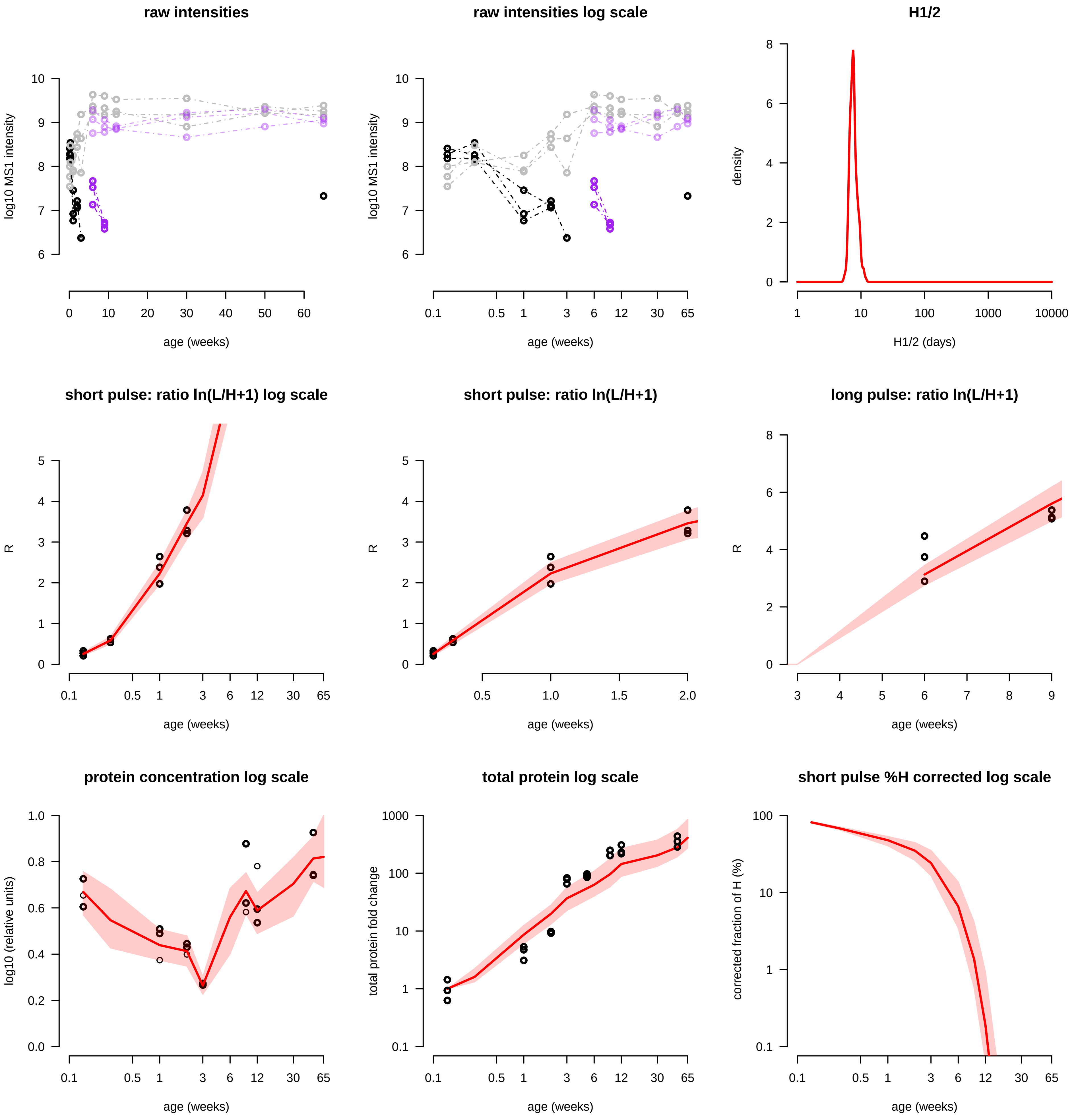

Q6PDG5−2;Q6PDG5

>sp|Q6PDG5−2|SMRC2_MOUSE

Isoform 2 of SWI/SNF complex subunit SMARCC2

GN=Smarcc2

Med H1/2: 6.4 days

Q5 H1/2: 5.2 days

Q95 H1/2: 8.2 days

reliable model fit: yes

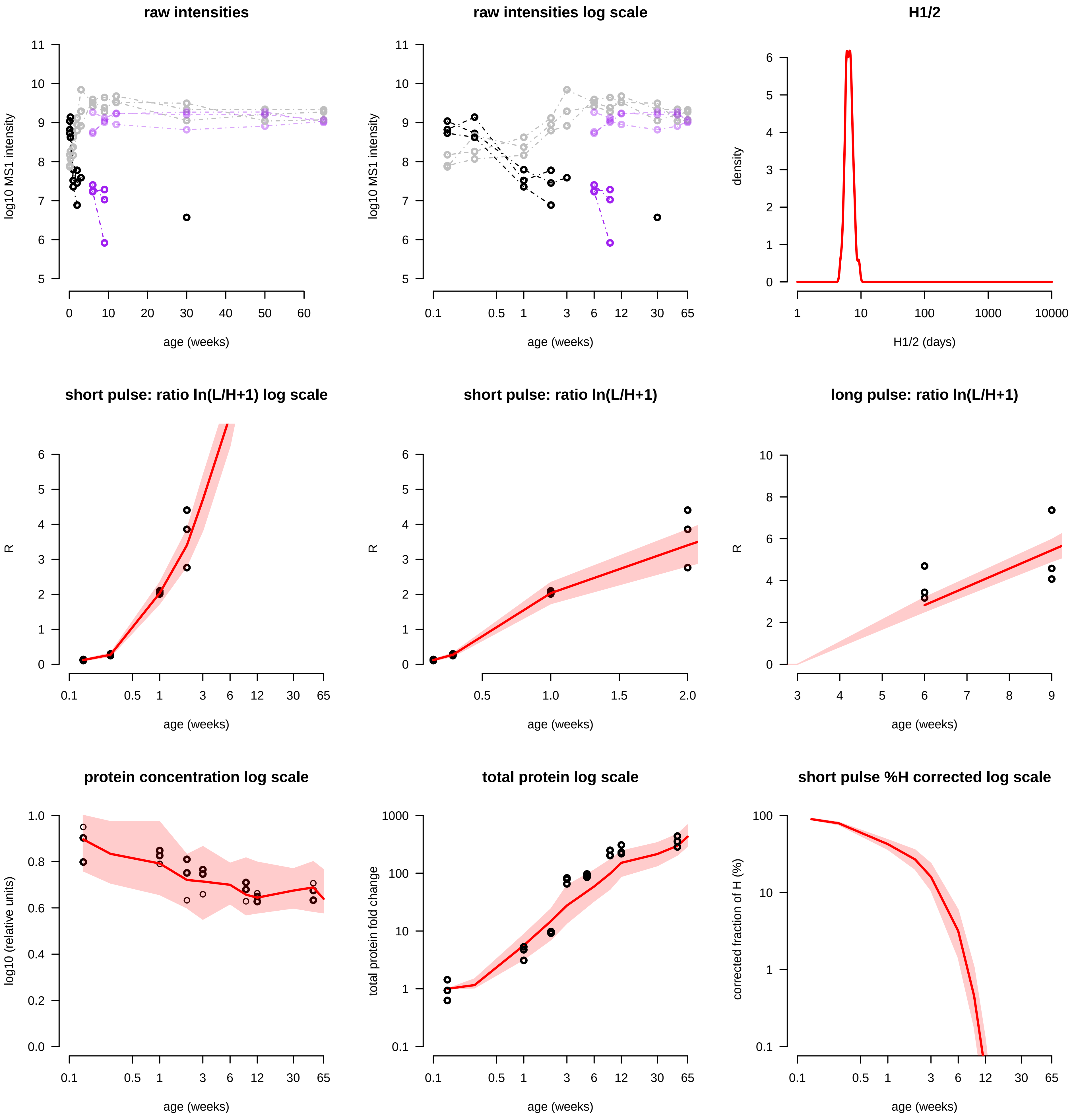

Q9WUB3

>sp|Q9WUB3|PYGM_MOUSE

Glycogen phosphorylase, muscle form

GN=Pygm

Med H1/2: 8.4 days

Q5 H1/2: 6.8 days

Q95 H1/2: 10.5 days

reliable model fit: yes

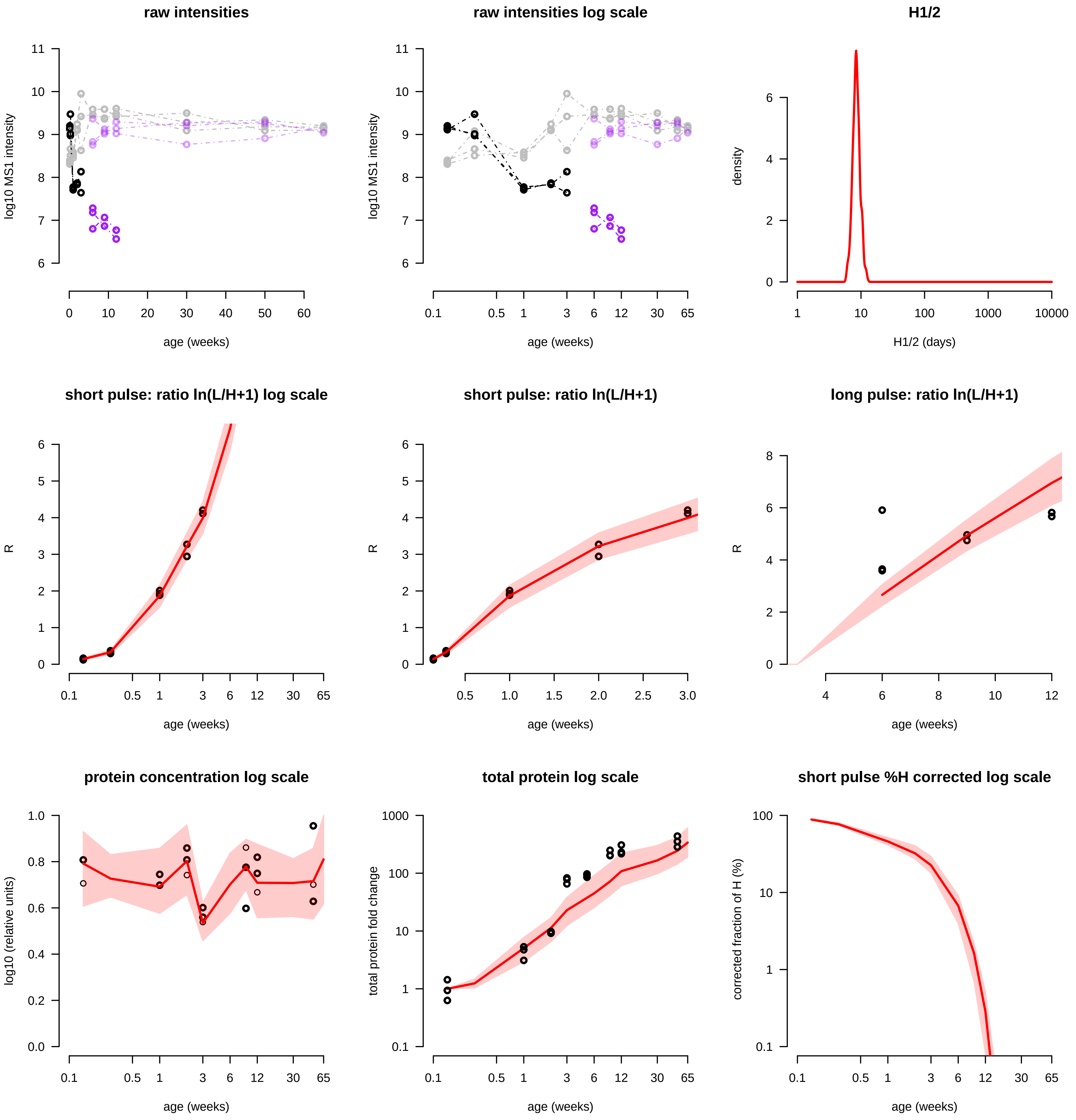

Q9DBP5

>sp|Q9DBP5|KCY_MOUSE

UMP−CMP kinase

GN=Cmpk1

Med H1/2: 6.9 days

Q5 H1/2: 5.7 days

Q95 H1/2: 10.2 days

reliable model fit: yes

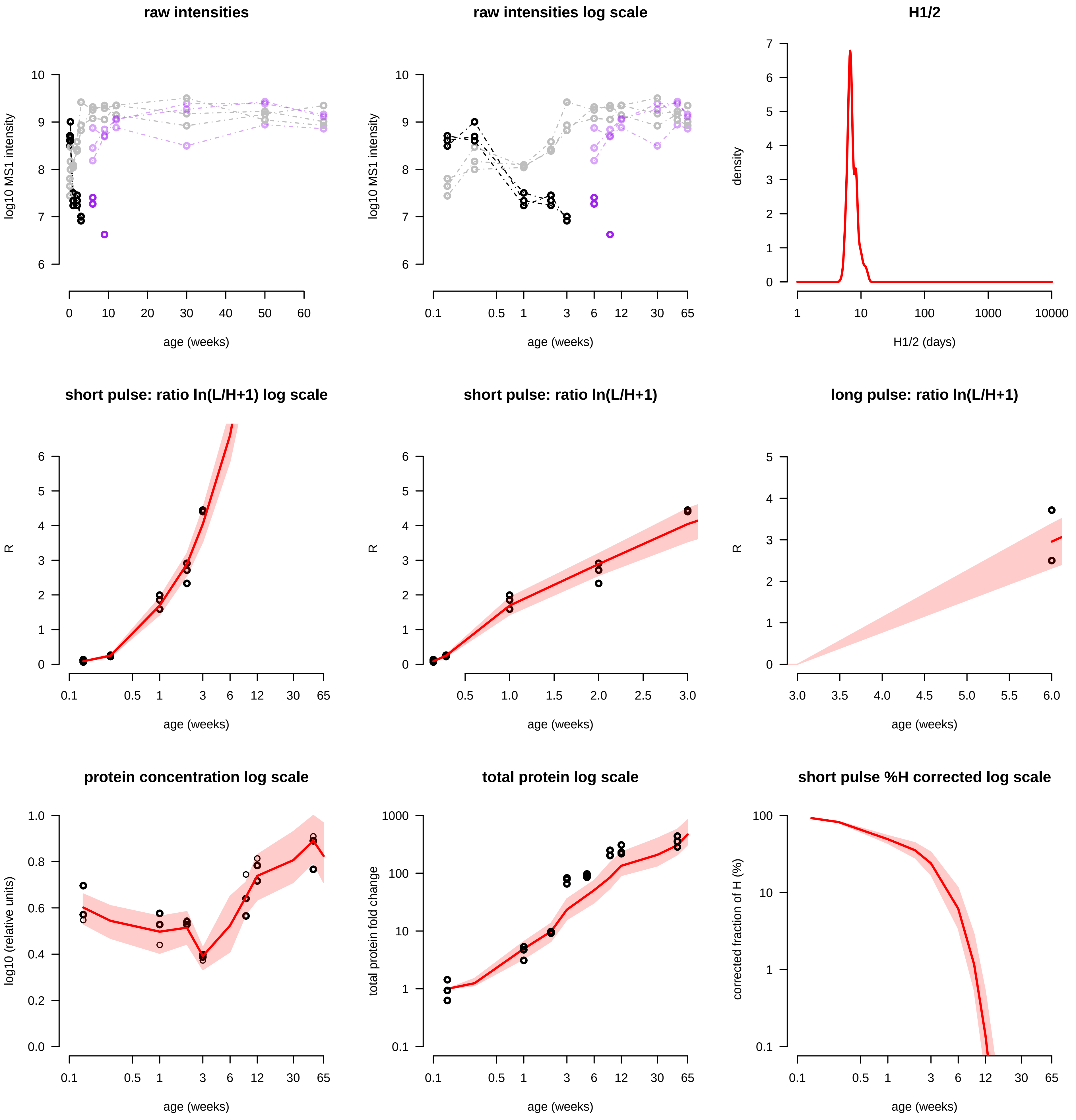

P55096

>sp|P55096|ABCD3_MOUSE

ATP−binding cassette sub−family D member 3

GN=Abcd3

Med H1/2: 7.7 days

Q5 H1/2: 6.3 days

Q95 H1/2: 9.7 days

reliable model fit: yes

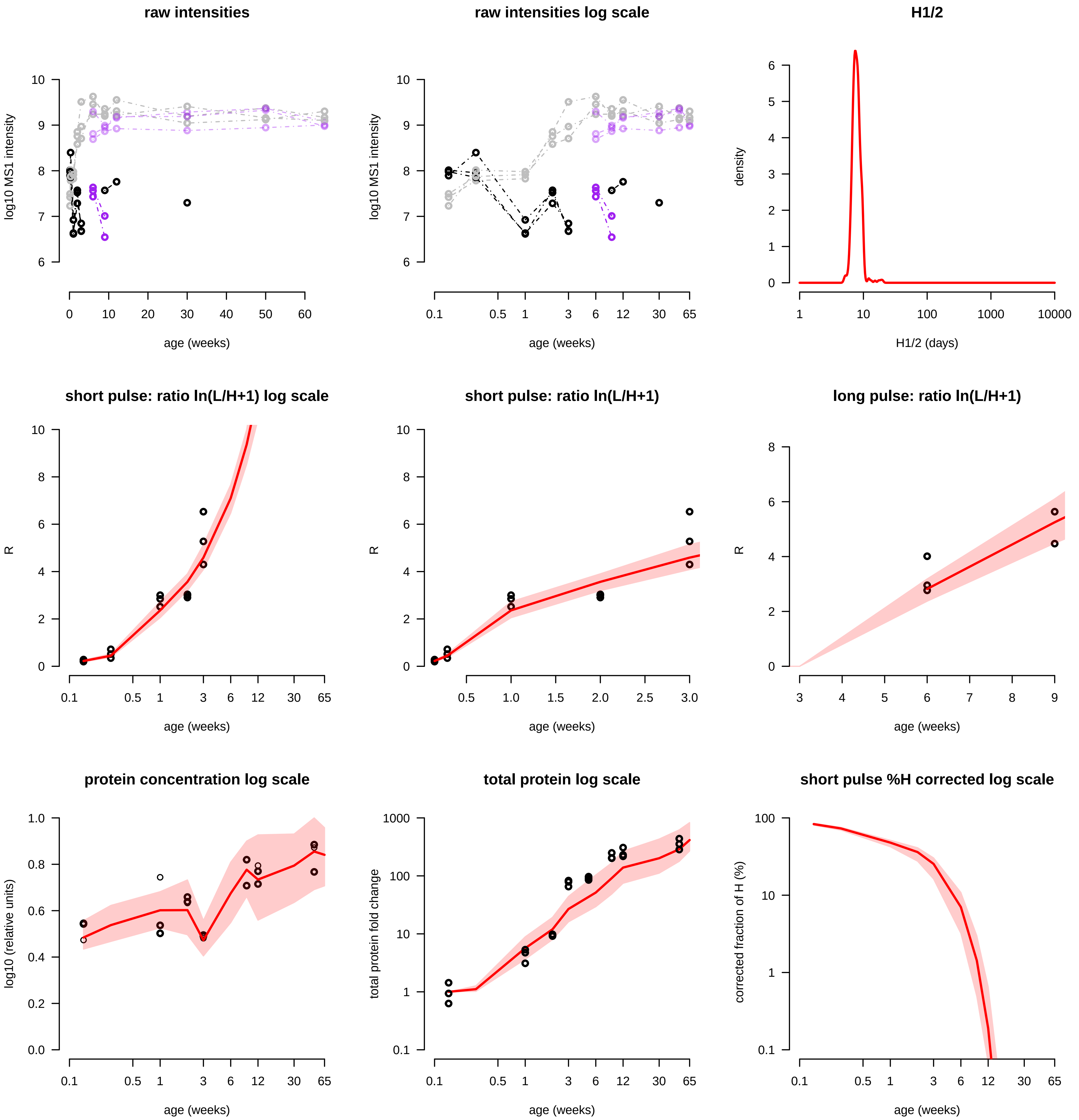

O88428

>sp|O88428|PAPS2_MOUSE

Bifunctional 3'−phosphoadenosine 5'−phosphosulfate synthase 2

GN=Papss2

Med H1/2: 633.7 days

Q5 H1/2: 249.4 days

Q95 H1/2: 5330.9 days

reliable model fit: yes

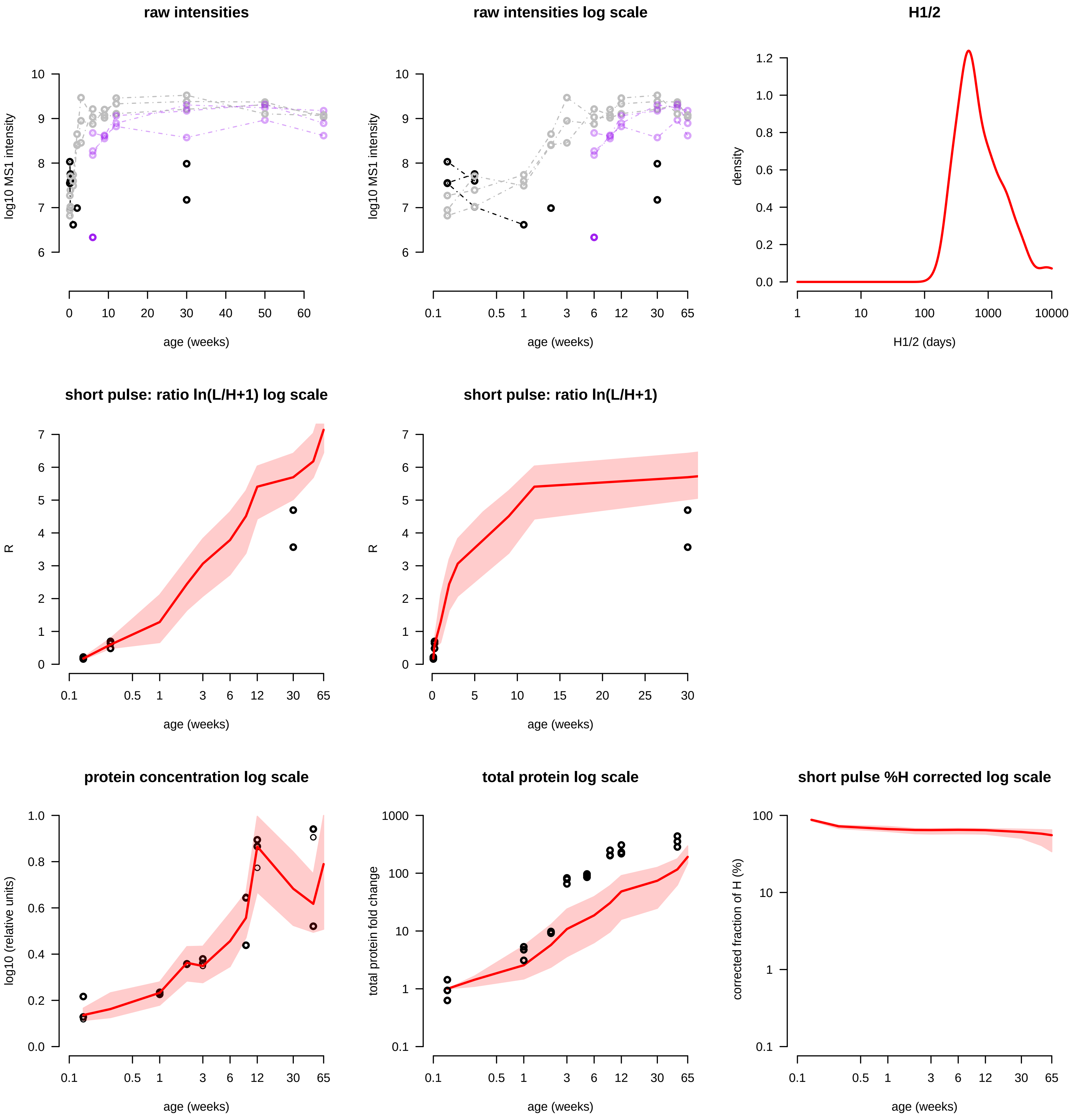

P47791−2

>sp|P47791−2|GSHR_MOUSE

Isoform Cytoplasmic of Glutathione reductase, mitochondrial

GN=Gsr

Med H1/2: 5.2 days

Q5 H1/2: 3.9 days

Q95 H1/2: 6.7 days

reliable model fit: yes

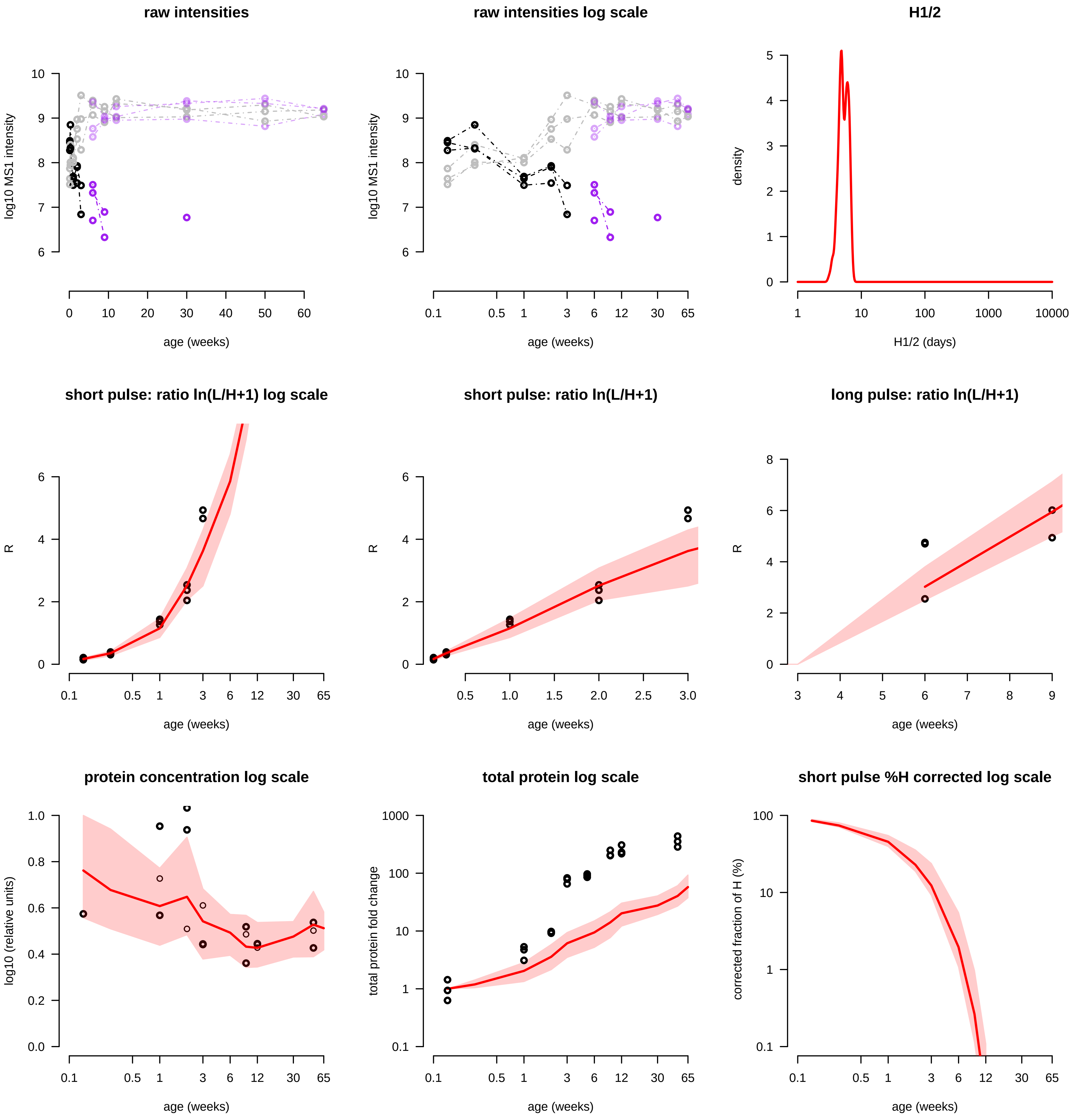

Q60648

>sp|Q60648|SAP3_MOUSE

Ganglioside GM2 activator

GN=Gm2a

Med H1/2: 8.8 days

Q5 H1/2: 6.6 days

Q95 H1/2: 13.1 days

reliable model fit: yes

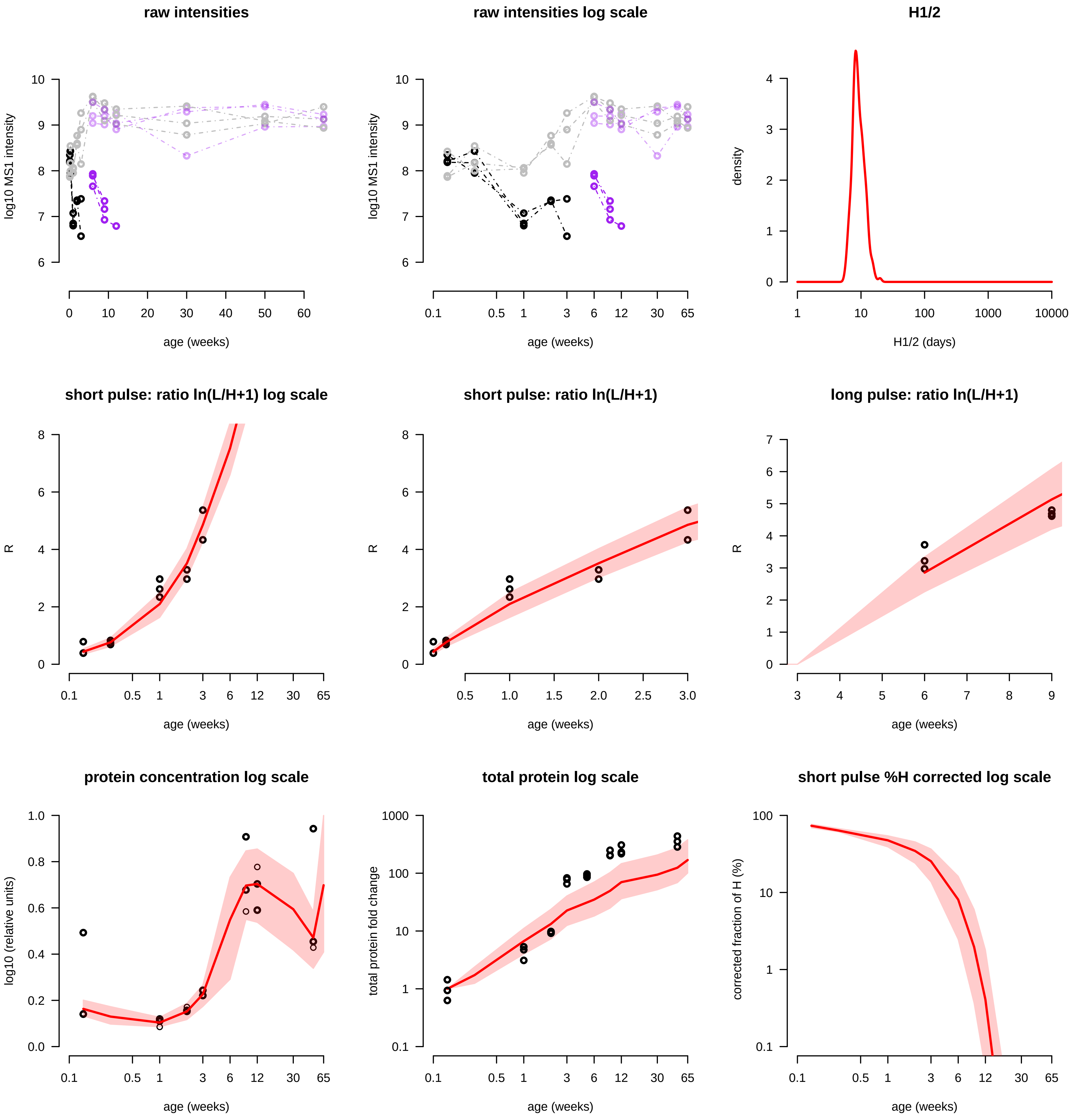

Q70IV5

>sp|Q70IV5|SYNEM_MOUSE

Synemin

GN=Synm

Med H1/2: 937.7 days

Q5 H1/2: 262.9 days

Q95 H1/2: 12653.9 days

reliable model fit: no

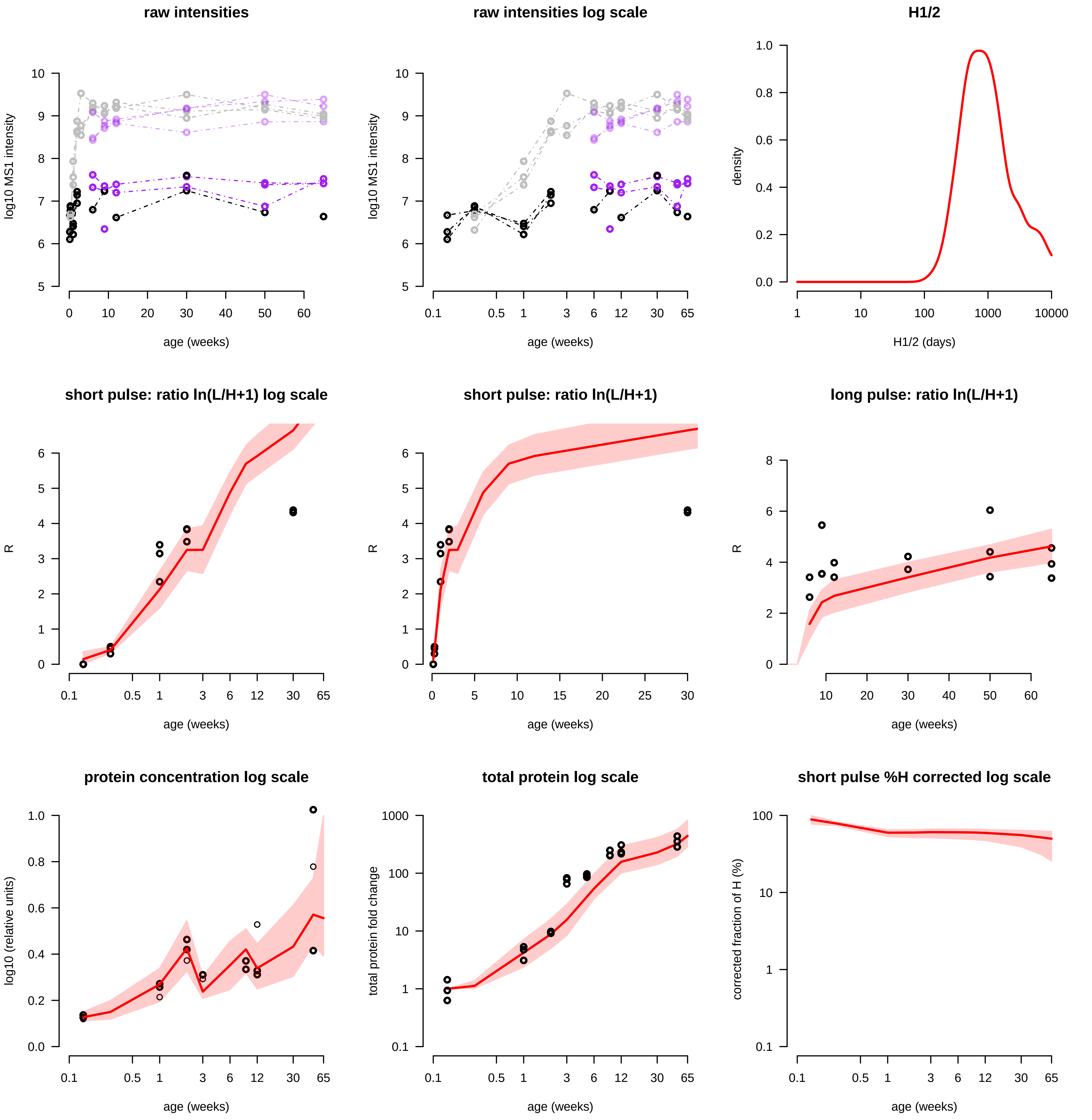

Q9Z2Z6

>sp|Q9Z2Z6|MCAT_MOUSE

Mitochondrial carnitine/acylcarnitine carrier protein

GN=Slc25a20

Med H1/2: 112.8 days

Q5 H1/2: 81.3 days

Q95 H1/2: 223.1 days

reliable model fit: yes

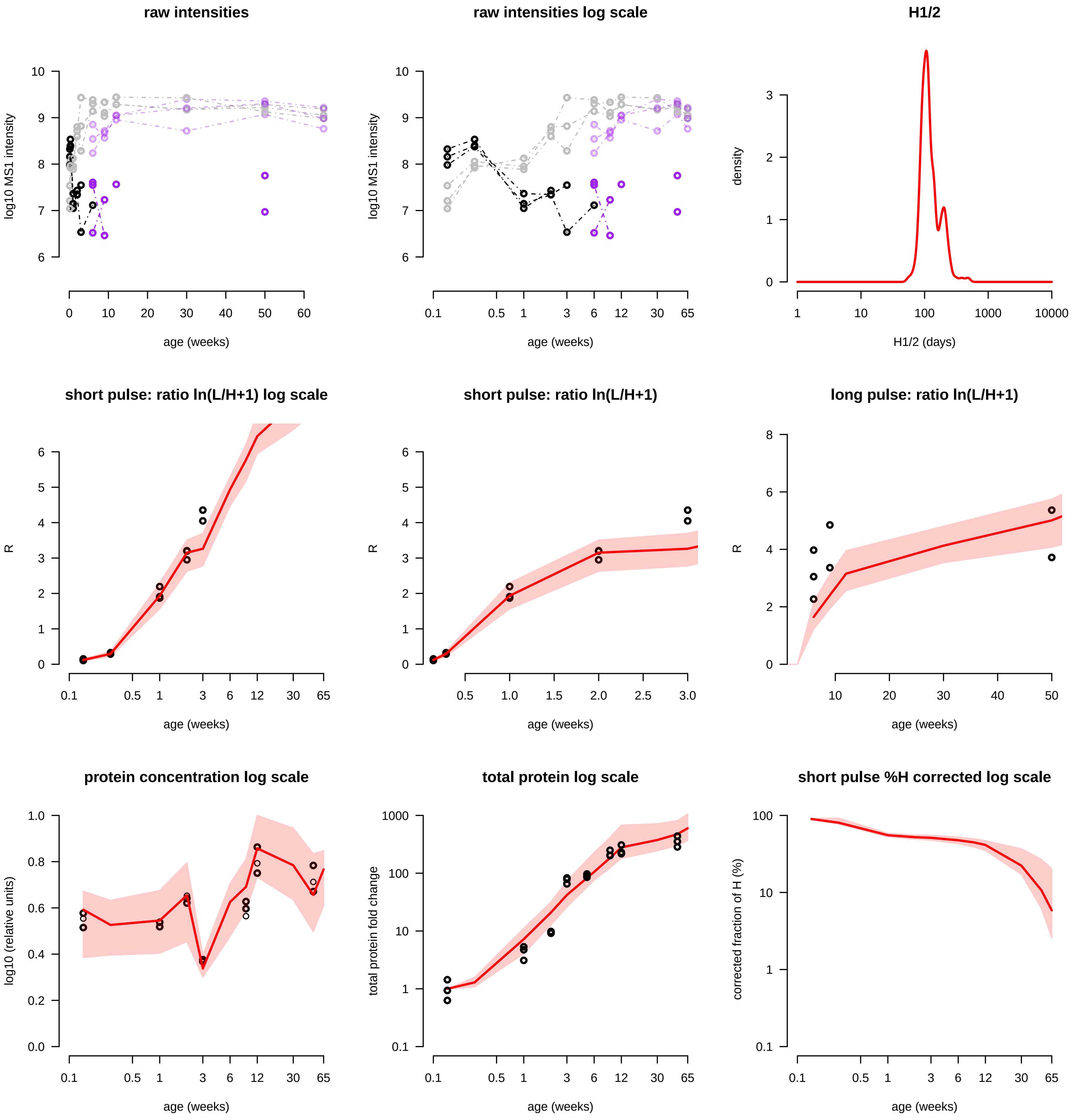

P47857−3;P47857−2;P47857

>sp|P47857−3|PFKAM_MOUSE

Isoform 3 of ATP−dependent 6−phosphofructokinase, muscle type

GN=Pfkm

Med H1/2: 729.7 days

Q5 H1/2: 228.9 days

Q95 H1/2: 8398.7 days

reliable model fit: yes

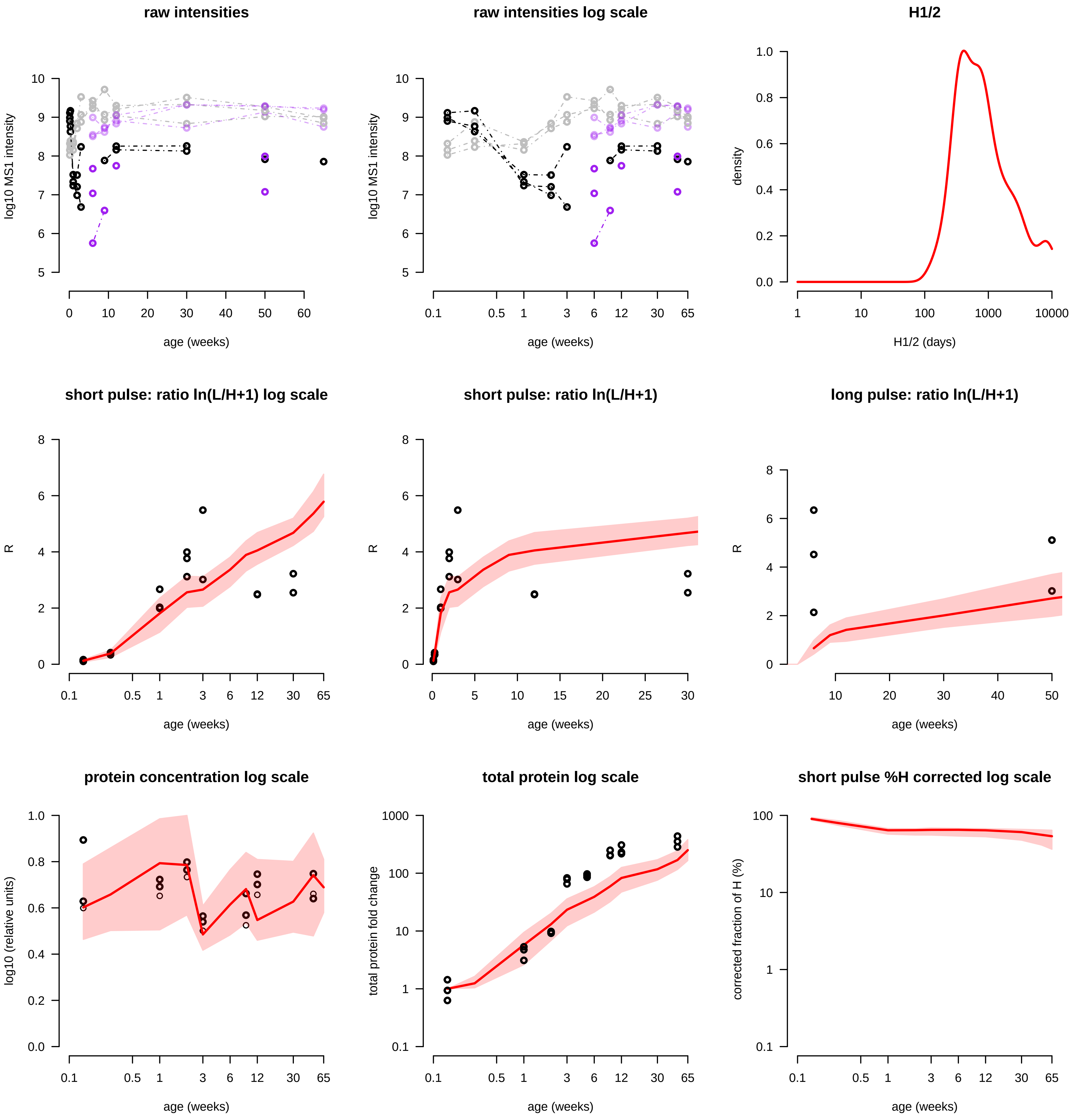

Q9QUR6

>sp|Q9QUR6|PPCE_MOUSE

Prolyl endopeptidase

GN=Prep

Med H1/2: 5 days

Q5 H1/2: 4.5 days

Q95 H1/2: 6.1 days

reliable model fit: yes

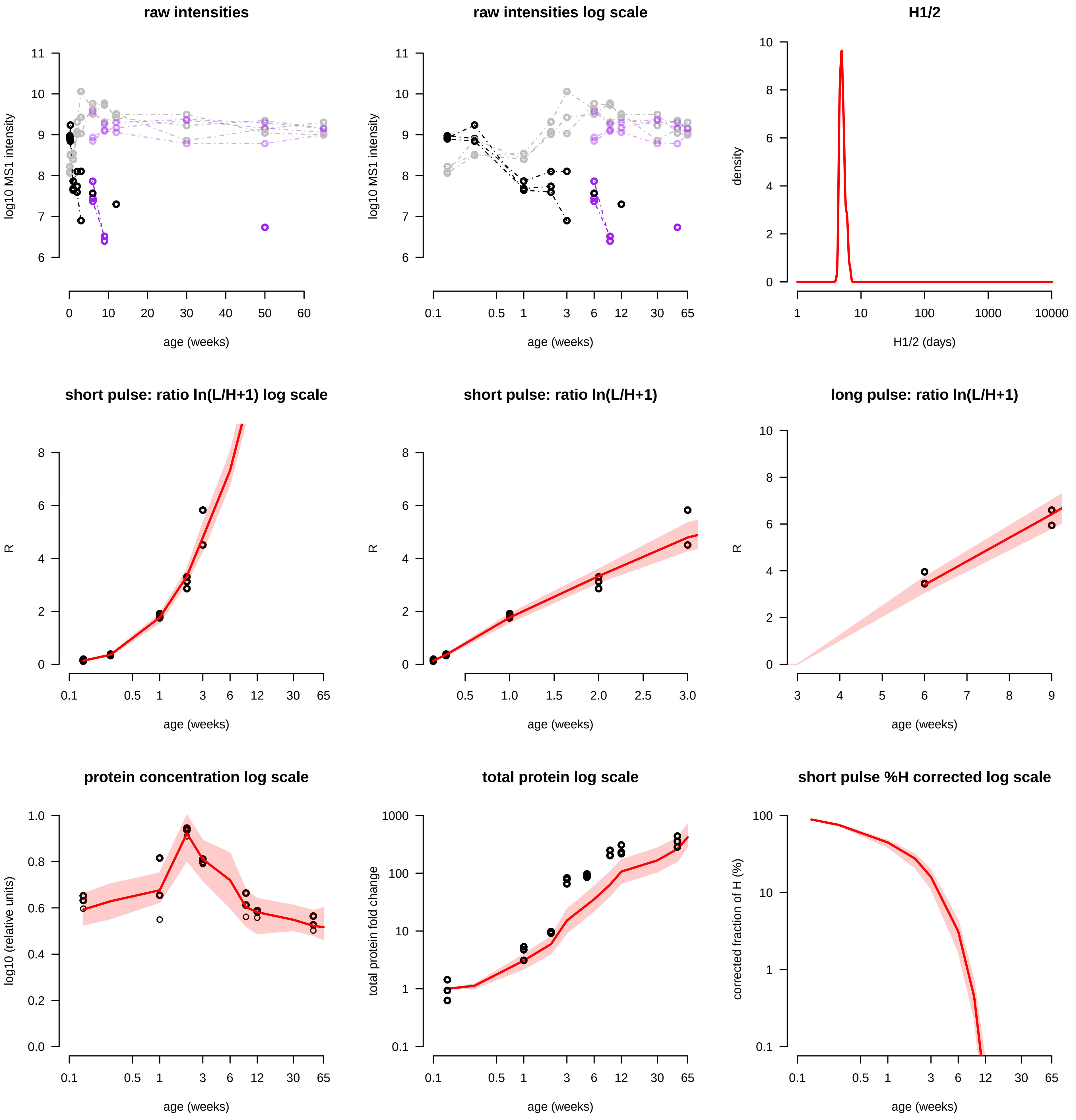

O89079

>sp|O89079|COPE_MOUSE

Coatomer subunit epsilon

GN=Cope

Med H1/2: 5 days

Q5 H1/2: 4.4 days

Q95 H1/2: 5.9 days

reliable model fit: yes

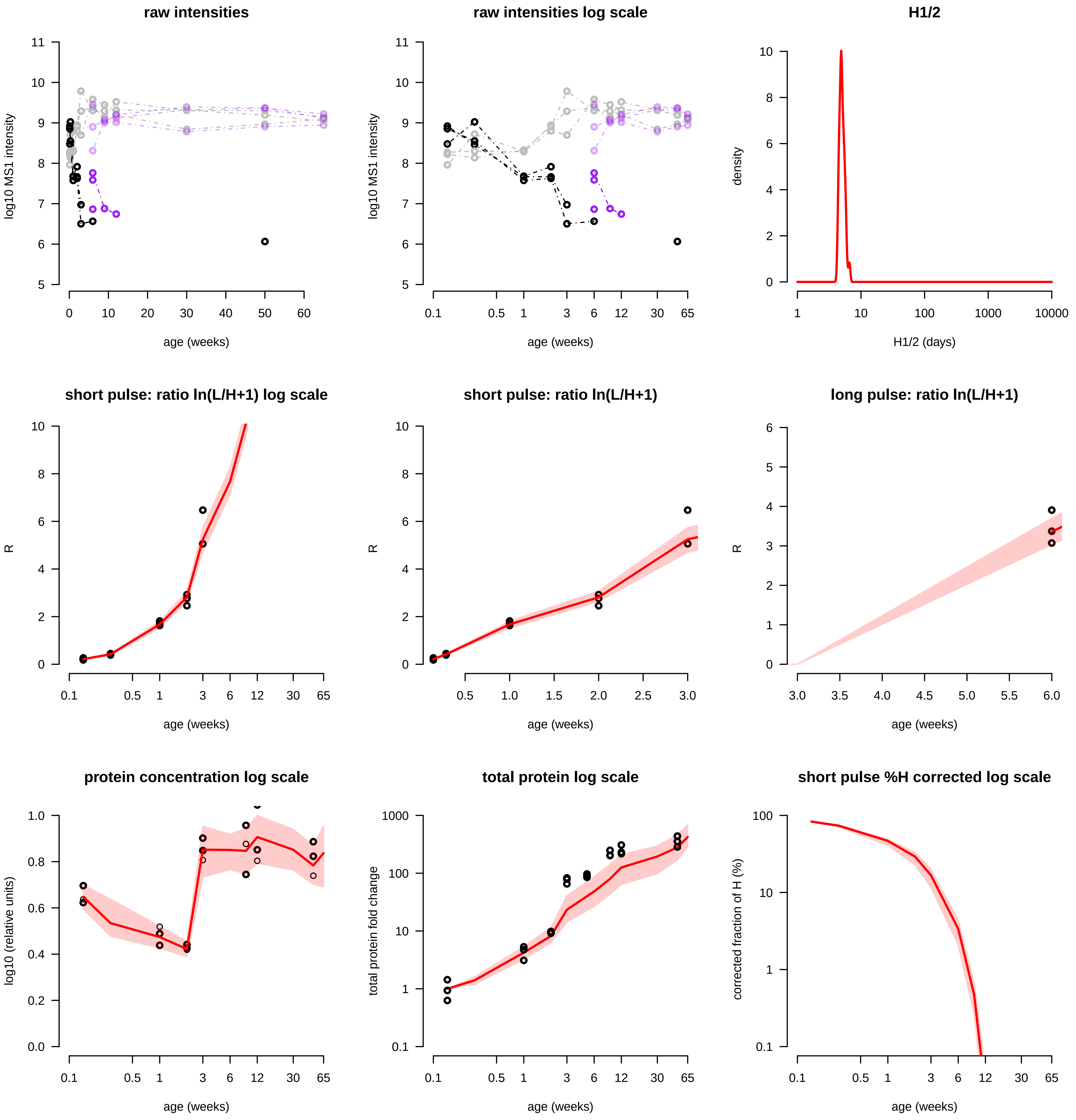

Q99KF1

>sp|Q99KF1|TMED9_MOUSE

Transmembrane emp24 domain−containing protein 9

GN=Tmed9

Med H1/2: 5.9 days

Q5 H1/2: 4.7 days

Q95 H1/2: 8.2 days

reliable model fit: yes

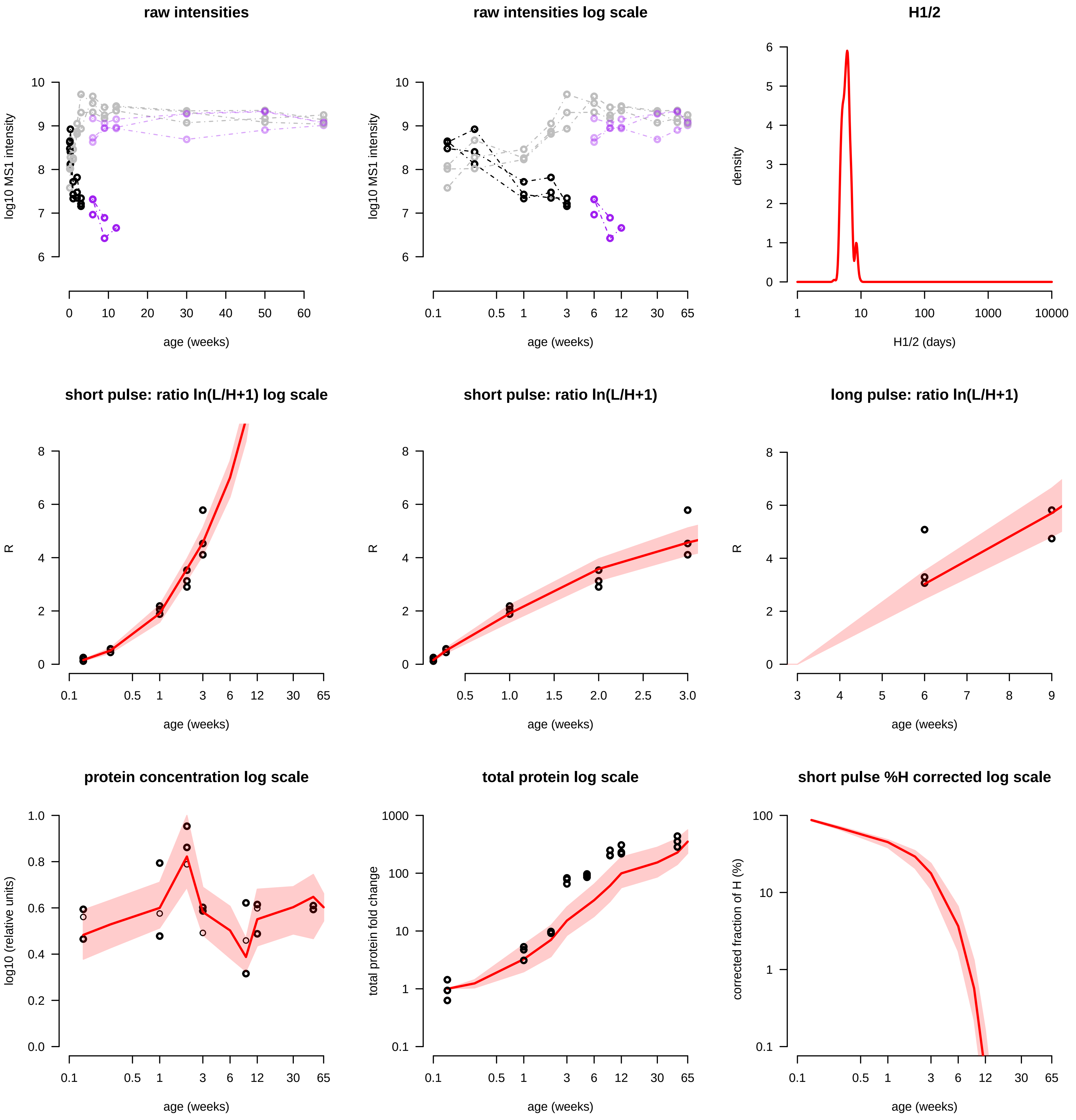

P62137

>sp|P62137|PP1A_MOUSE

Serine/threonine−protein phosphatase PP1−alpha catalytic subunit

GN=Ppp1ca

Med H1/2: 11.3 days

Q5 H1/2: 8.1 days

Q95 H1/2: 16.4 days

reliable model fit: yes

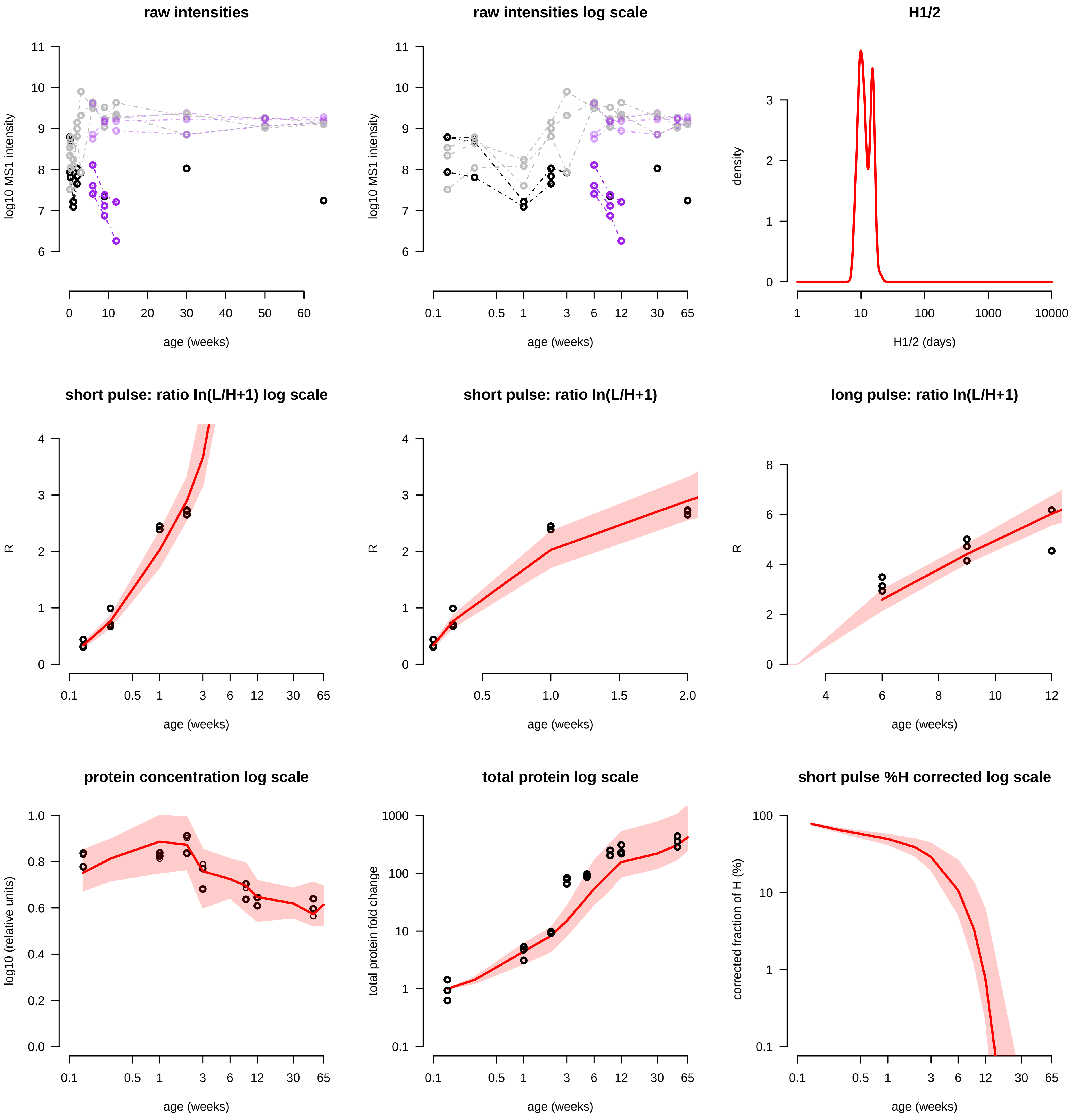

Q9JHW2

>sp|Q9JHW2|NIT2_MOUSE

Omega−amidase NIT2

GN=Nit2

Med H1/2: 474.7 days

Q5 H1/2: 168.8 days

Q95 H1/2: 5478 days

reliable model fit: no

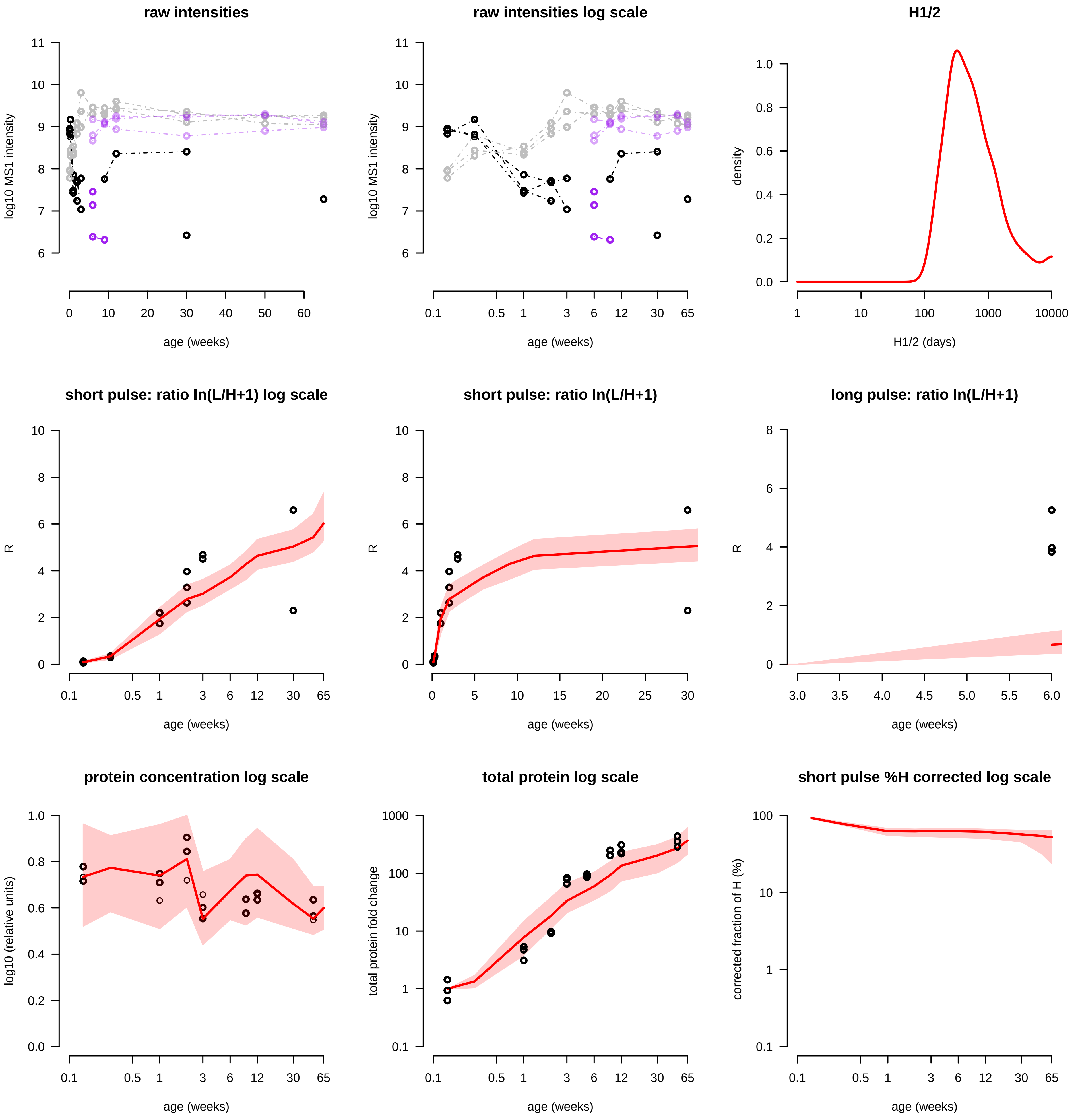

P62889

>sp|P62889|RL30_MOUSE

60S ribosomal protein L30

GN=Rpl30

Med H1/2: 3.6 days

Q5 H1/2: 2.9 days

Q95 H1/2: 5 days

reliable model fit: yes

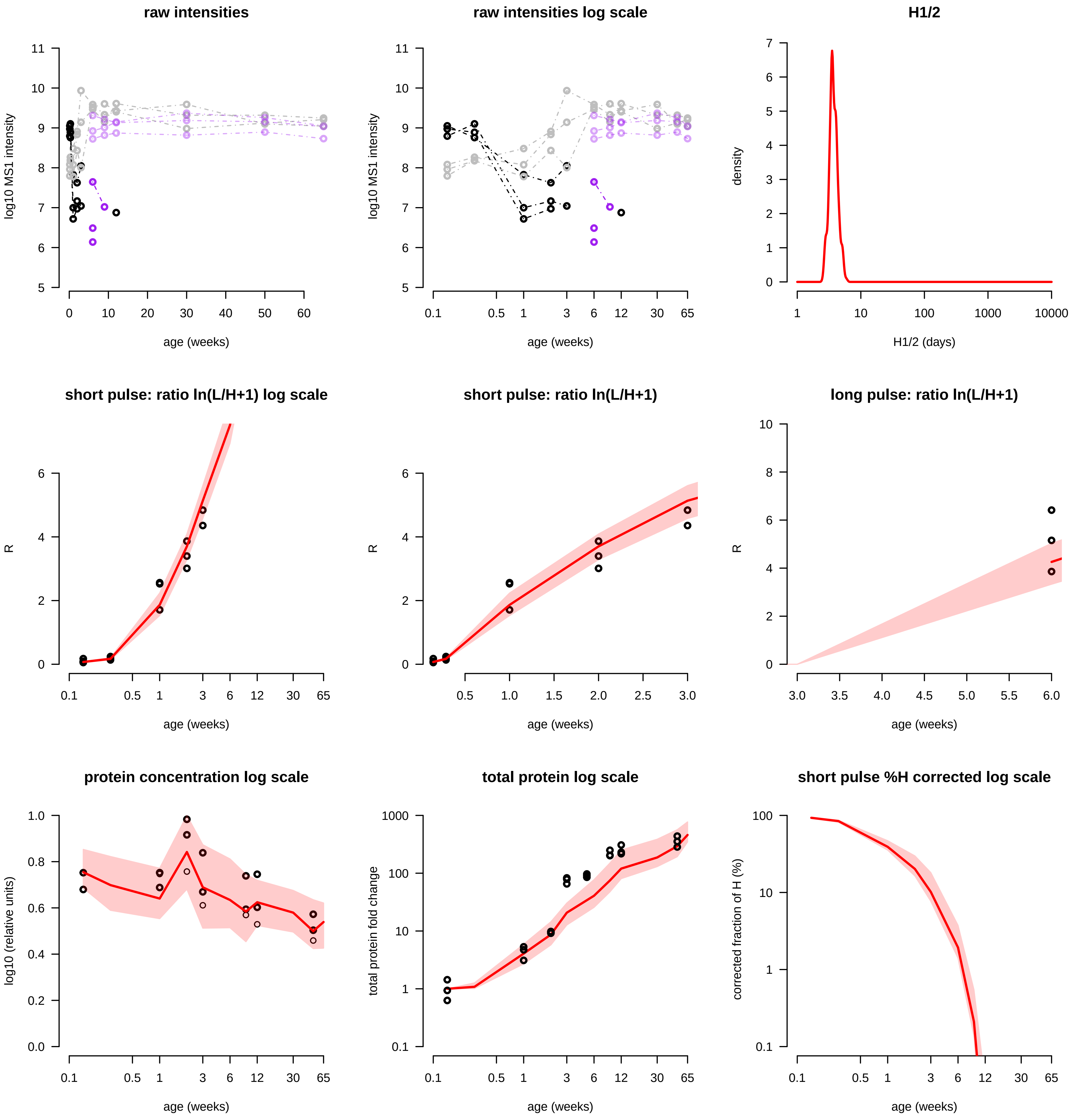

Q02819

>sp|Q02819|NUCB1_MOUSE

Nucleobindin−1

GN=Nucb1

Med H1/2: 3.7 days

Q5 H1/2: 3.1 days

Q95 H1/2: 4.6 days

reliable model fit: yes

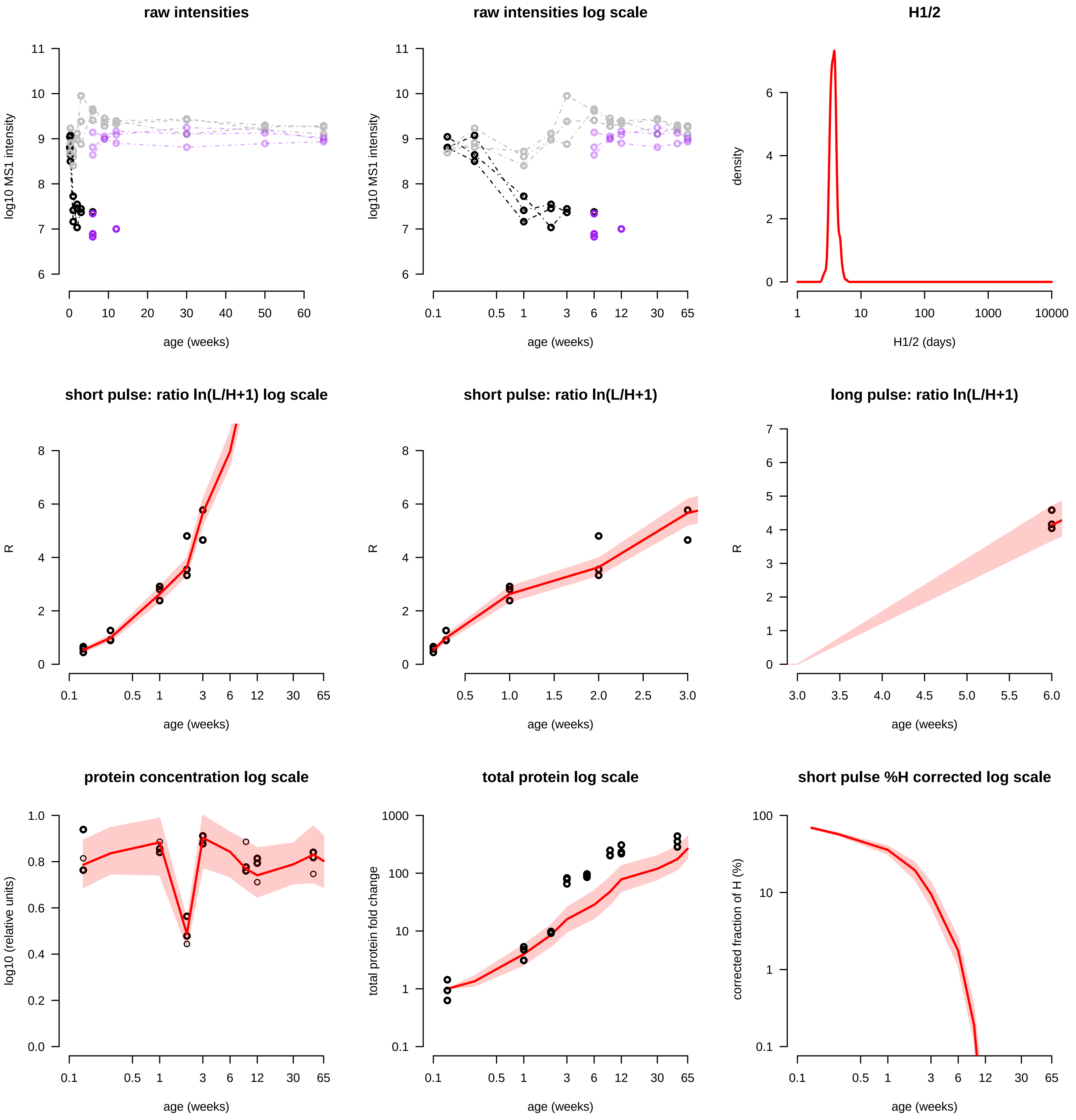

P62334

>sp|P62334|PRS10_MOUSE

26S proteasome regulatory subunit 10B

GN=Psmc6

Med H1/2: 7.4 days

Q5 H1/2: 6.1 days

Q95 H1/2: 9.1 days

reliable model fit: yes

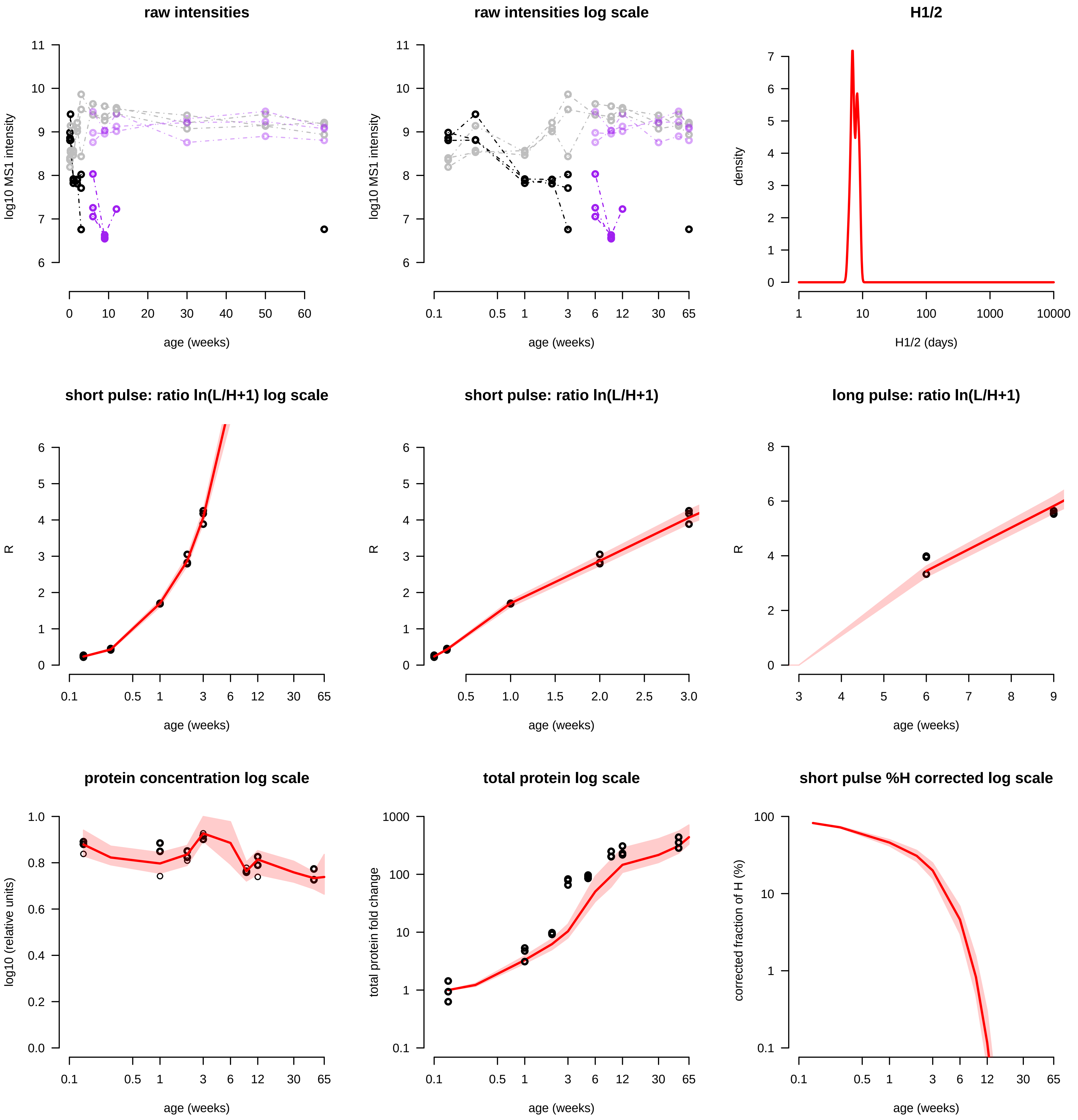

Q8JZK9

>sp|Q8JZK9|HMCS1_MOUSE

Hydroxymethylglutaryl−CoA synthase, cytoplasmic

GN=Hmgcs1

Med H1/2: 166.2 days

Q5 H1/2: 87 days

Q95 H1/2: 835.4 days

reliable model fit: yes

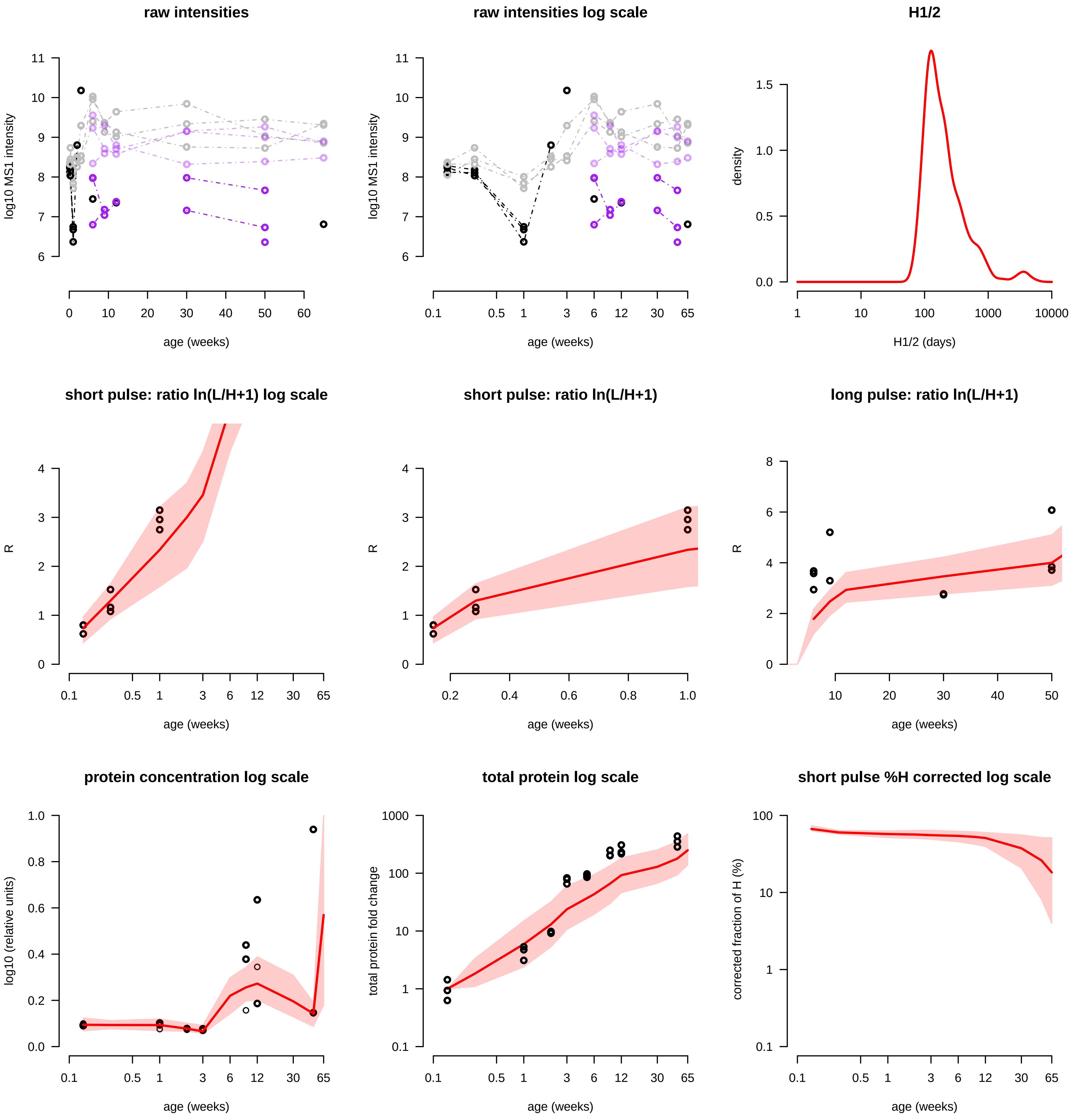

Q99MN1

>sp|Q99MN1|SYK_MOUSE

Lysine−−tRNA ligase

GN=Kars1

Med H1/2: 514.2 days

Q5 H1/2: 135.2 days

Q95 H1/2: 3306.1 days

reliable model fit: yes

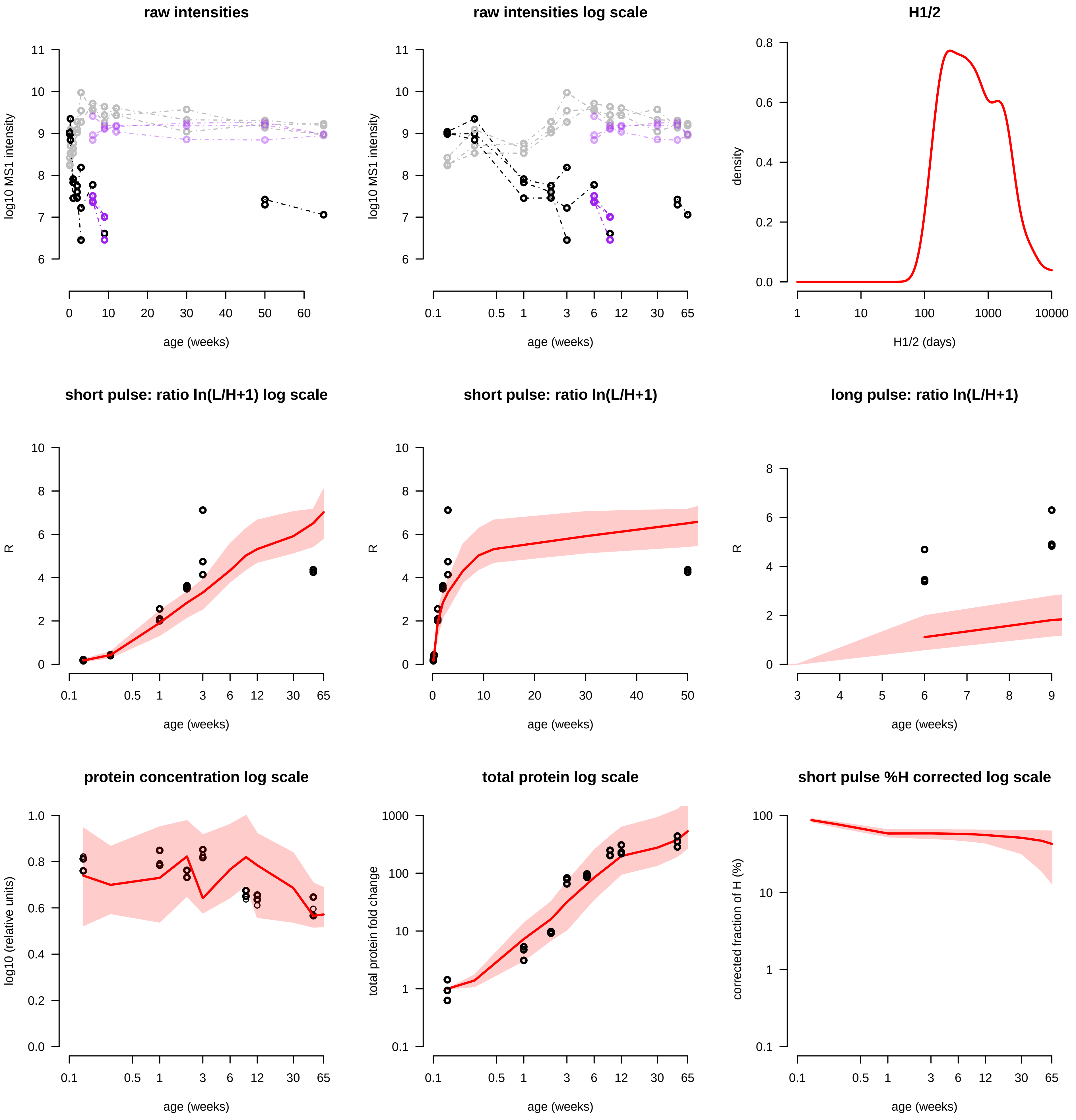

P62315

>sp|P62315|SMD1_MOUSE

Small nuclear ribonucleoprotein Sm D1

GN=Snrpd1

Med H1/2: 10.5 days

Q5 H1/2: 8.4 days

Q95 H1/2: 13.1 days

reliable model fit: yes

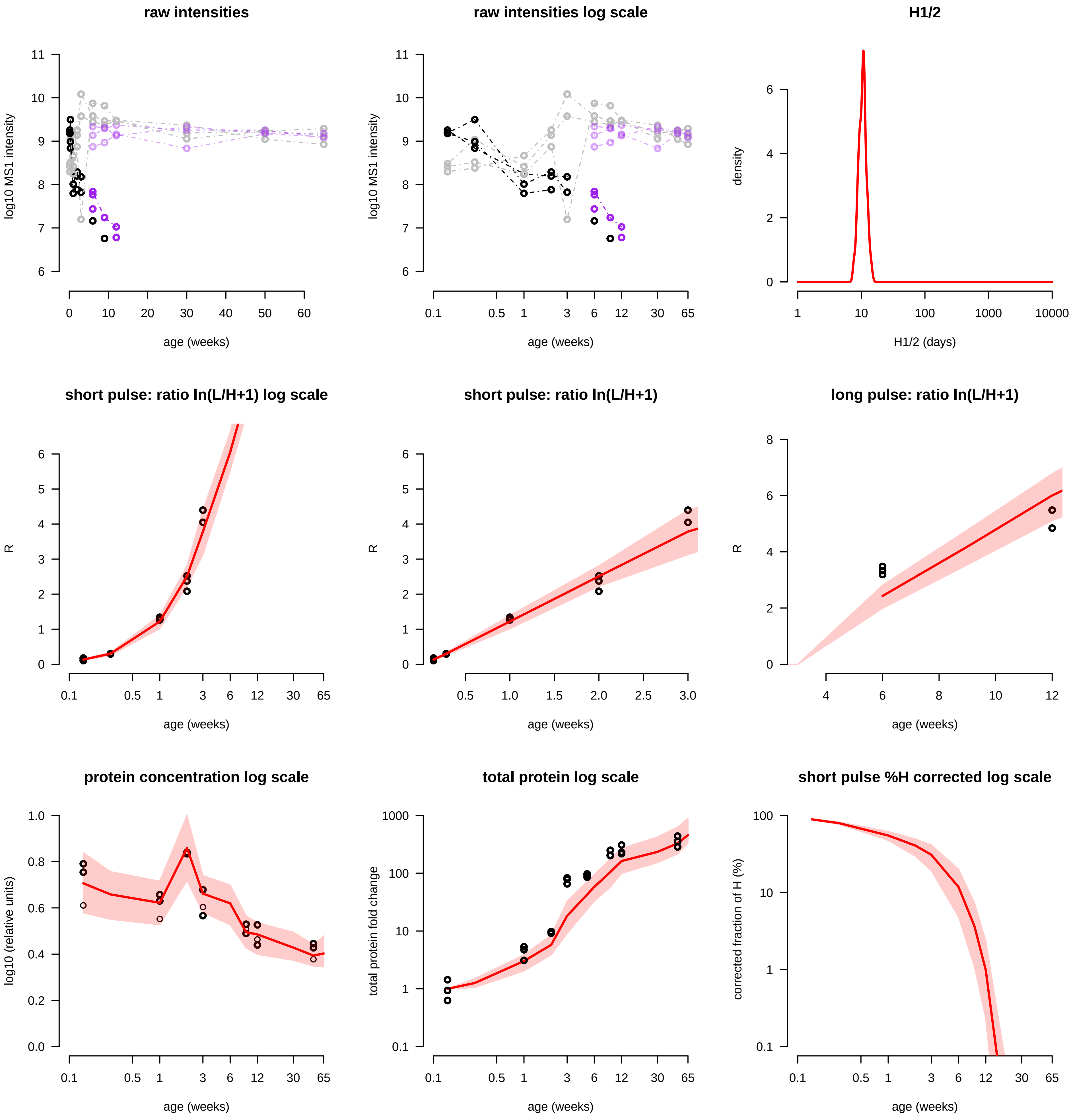

Q99KK7

>sp|Q99KK7|DPP3_MOUSE

Dipeptidyl peptidase 3

GN=Dpp3

Med H1/2: 141.7 days

Q5 H1/2: 64.7 days

Q95 H1/2: 465.6 days

reliable model fit: yes

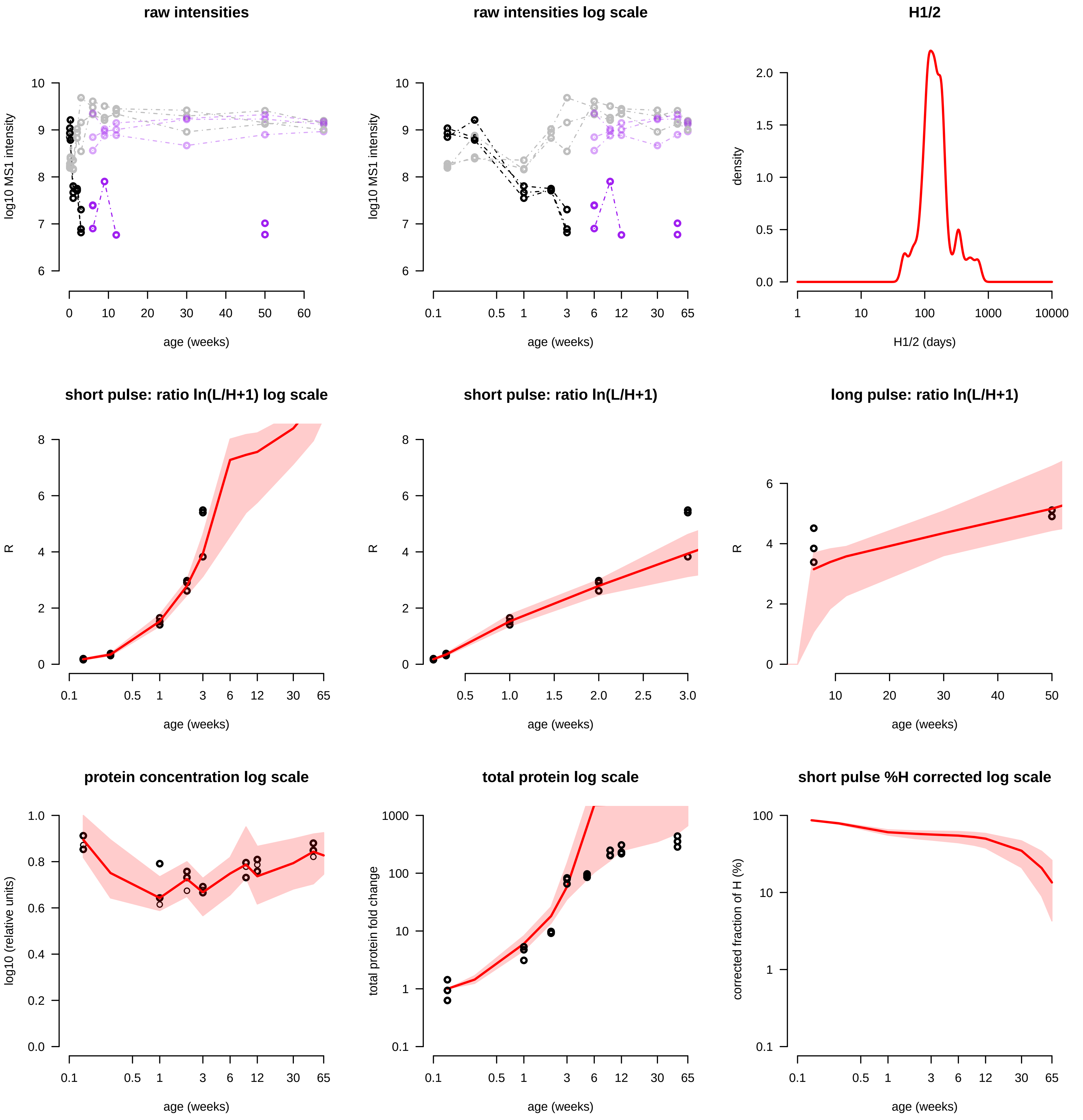

Q9Z1T1

>sp|Q9Z1T1|AP3B1_MOUSE

AP−3 complex subunit beta−1

GN=Ap3b1

Med H1/2: 7.5 days

Q5 H1/2: 5.9 days

Q95 H1/2: 9.8 days

reliable model fit: yes

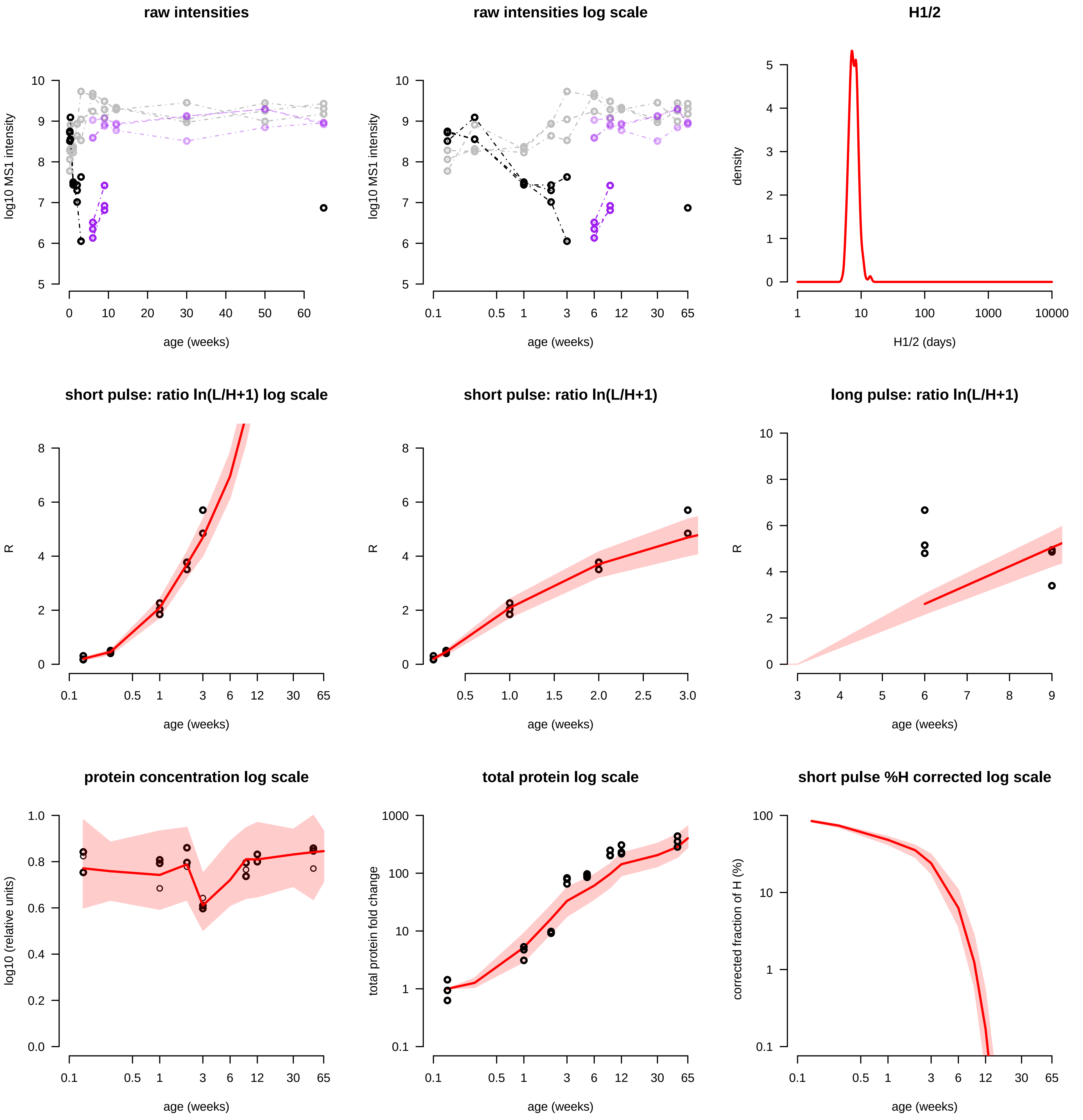

Q9R0Q7

>sp|Q9R0Q7|TEBP_MOUSE

Prostaglandin E synthase 3

GN=Ptges3

Med H1/2: 4.4 days

Q5 H1/2: 3.7 days

Q95 H1/2: 5.7 days

reliable model fit: yes

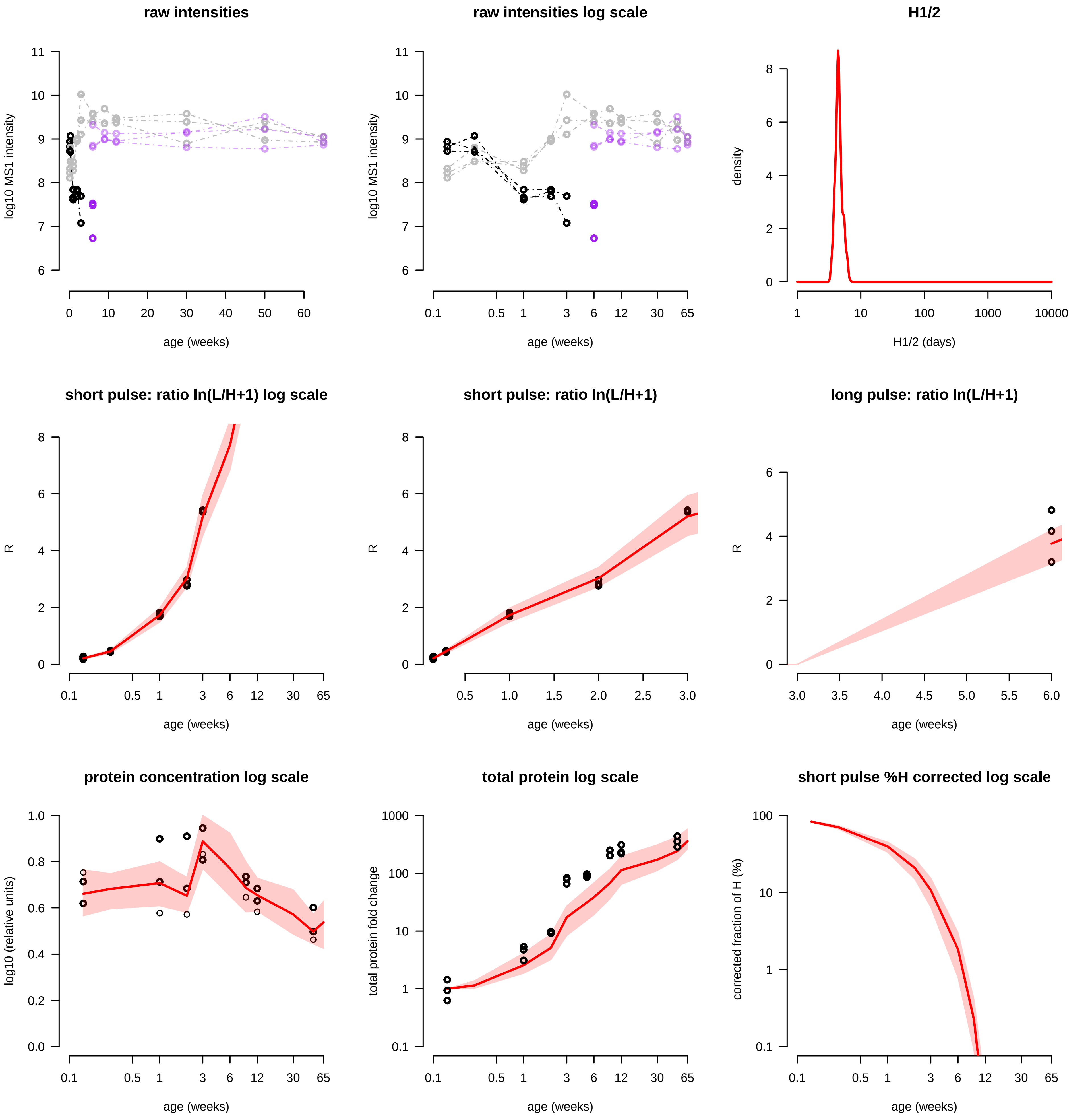

O54774

>sp|O54774|AP3D1_MOUSE

AP−3 complex subunit delta−1

GN=Ap3d1

Med H1/2: 3.9 days

Q5 H1/2: 3 days

Q95 H1/2: 5.2 days

reliable model fit: yes

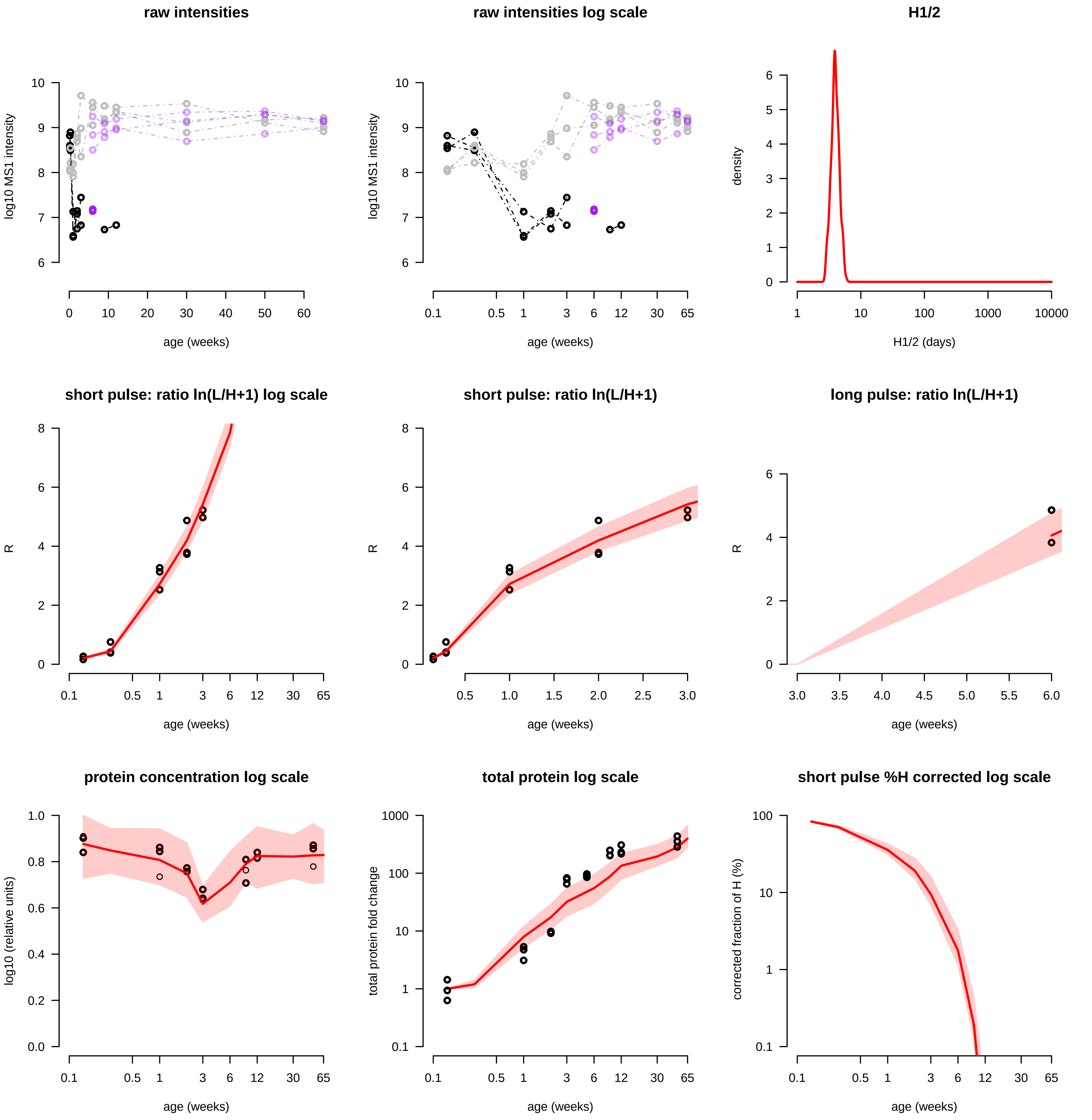

Q3UEB3−2;Q3UEB3;Q3UEB3−3

>sp|Q3UEB3−2|PUF60_MOUSE

Isoform 2 of Poly(U)−binding−splicing factor PUF60

GN=Puf60

Med H1/2: 4.6 days

Q5 H1/2: 3.8 days

Q95 H1/2: 5.6 days

reliable model fit: yes

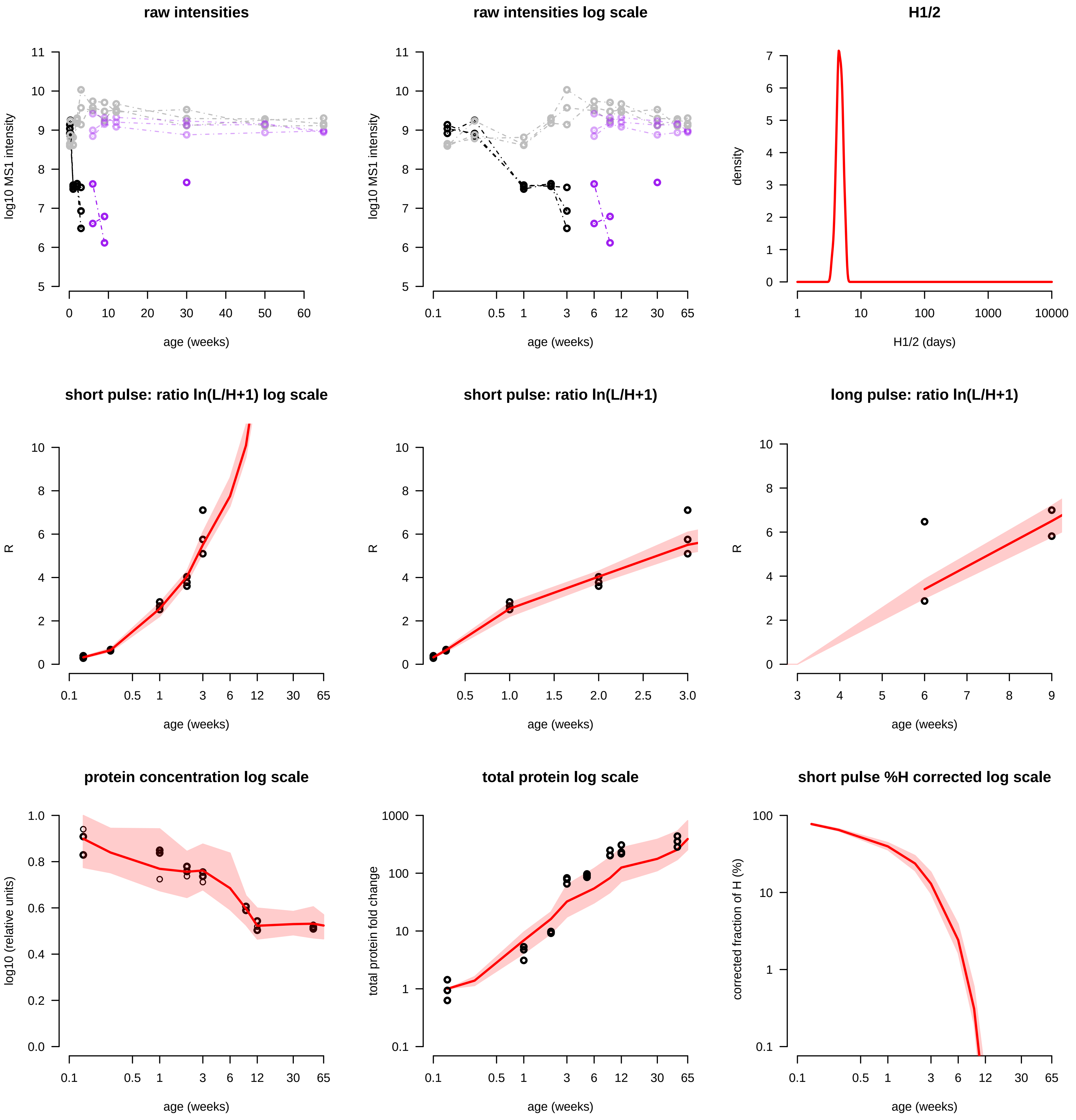

Q8CAY6

>sp|Q8CAY6|THIC_MOUSE

Acetyl−CoA acetyltransferase, cytosolic

GN=Acat2

Med H1/2: 4.6 days

Q5 H1/2: 3.6 days

Q95 H1/2: 6.1 days

reliable model fit: yes

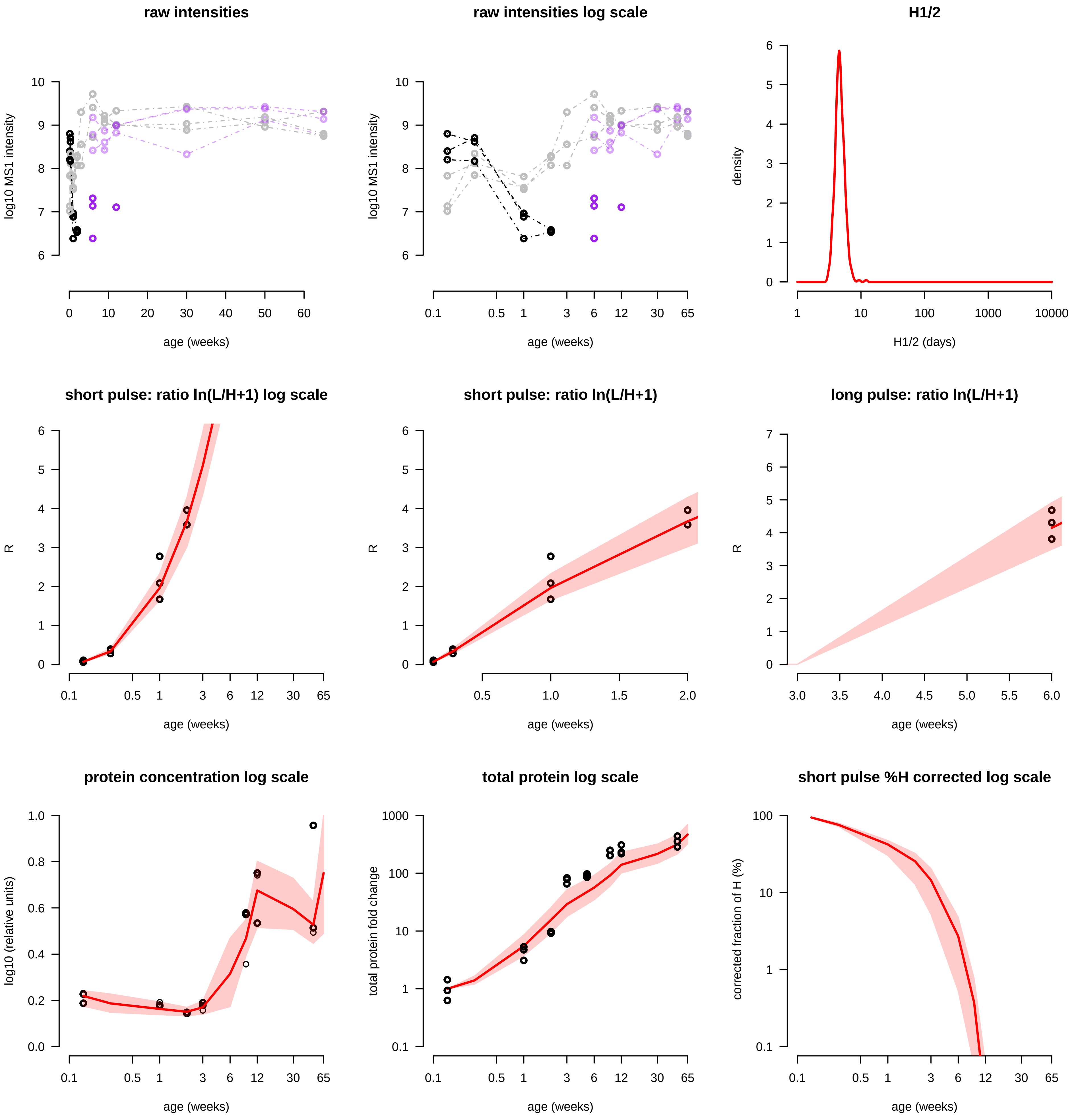

P31938

>sp|P31938|MP2K1_MOUSE

Dual specificity mitogen−activated protein kinase kinase 1

GN=Map2k1

Med H1/2: 11.4 days

Q5 H1/2: 8.9 days

Q95 H1/2: 13.7 days

reliable model fit: yes

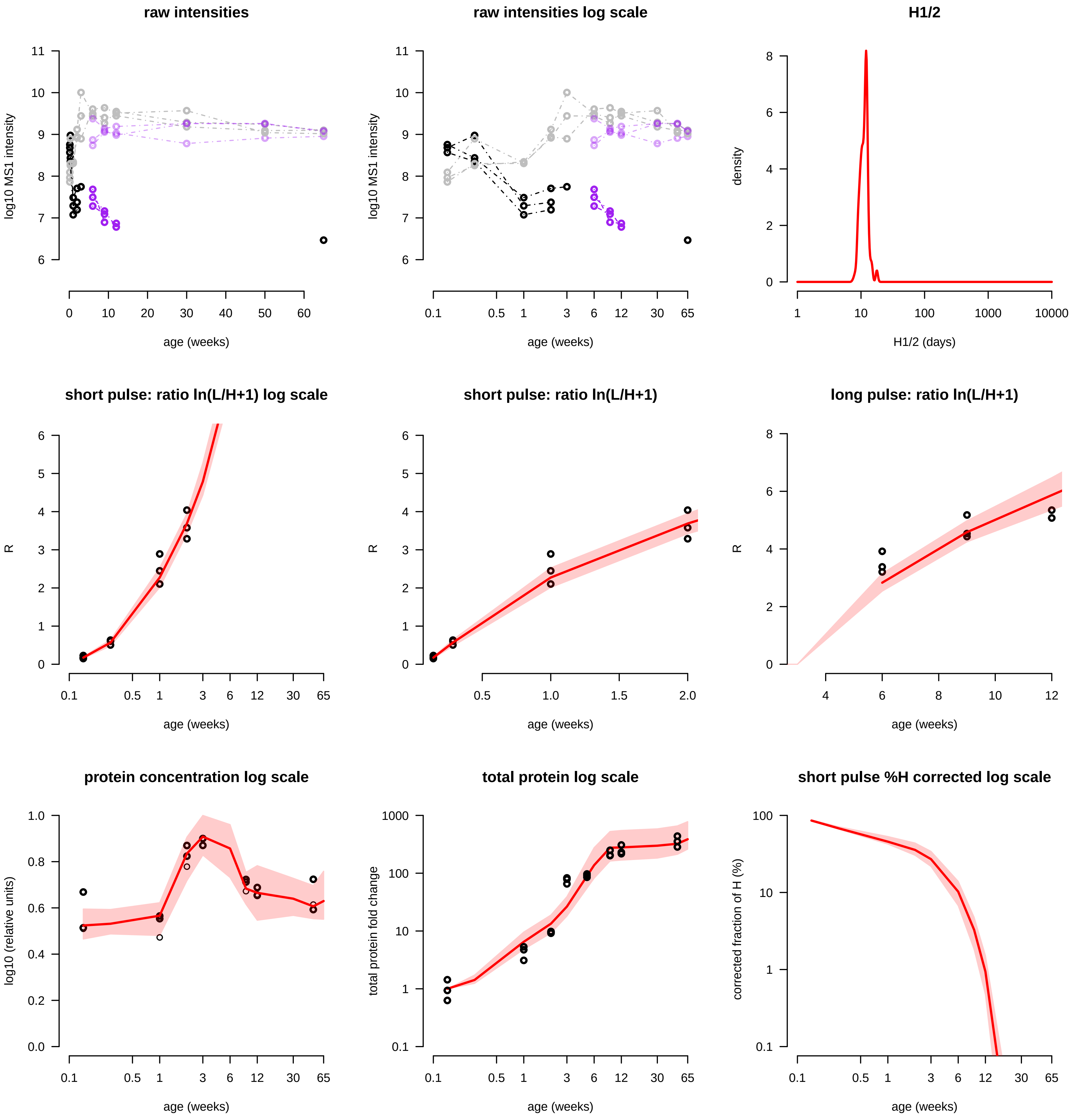

Q5FWK3

>sp|Q5FWK3|RHG01_MOUSE

Rho GTPase−activating protein 1

GN=Arhgap1

Med H1/2: 5.3 days

Q5 H1/2: 4.6 days

Q95 H1/2: 6.3 days

reliable model fit: yes

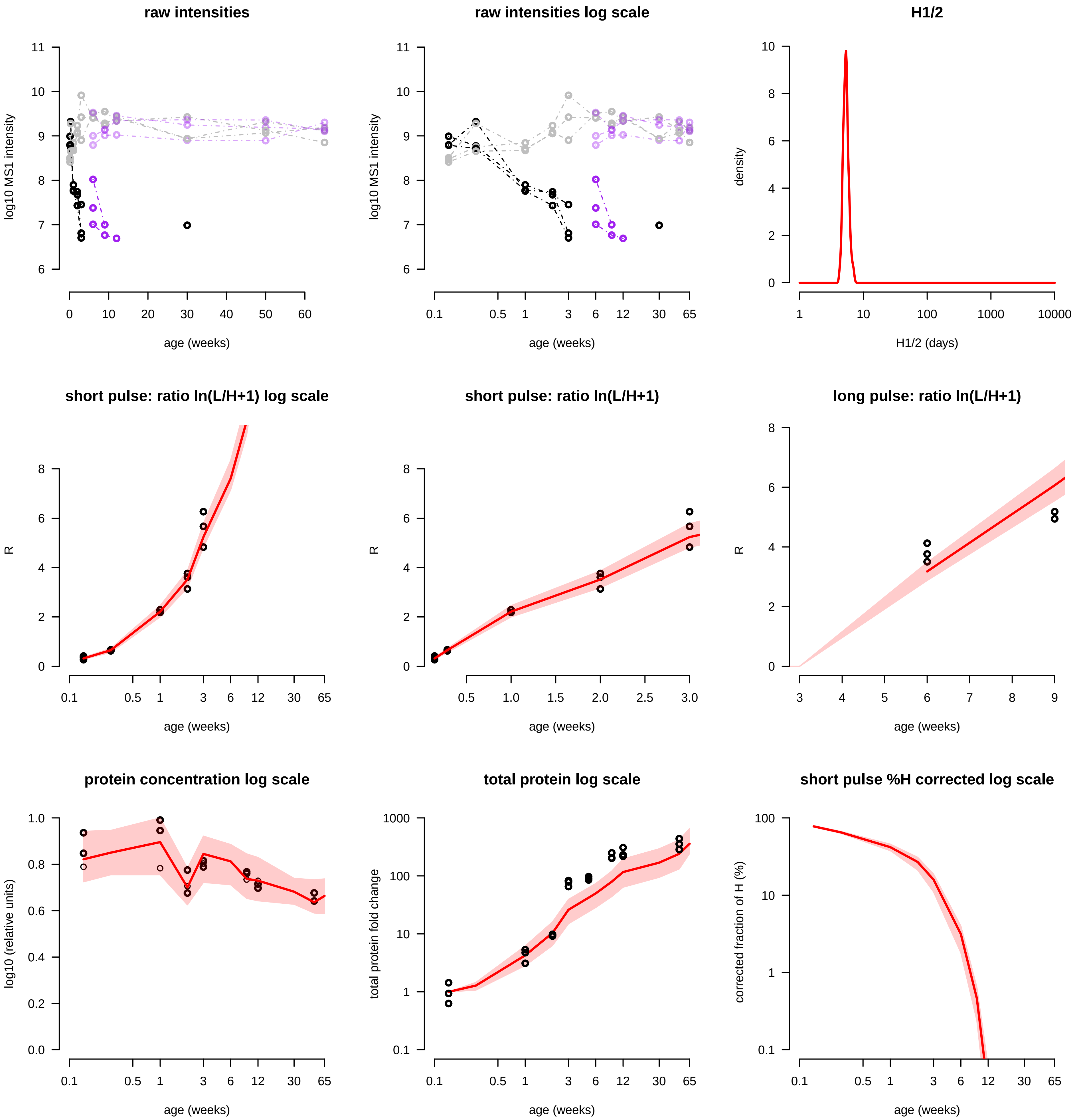

P67984

>sp|P67984|RL22_MOUSE

60S ribosomal protein L22

GN=Rpl22

Med H1/2: 8.6 days

Q5 H1/2: 7.3 days

Q95 H1/2: 12 days

reliable model fit: no

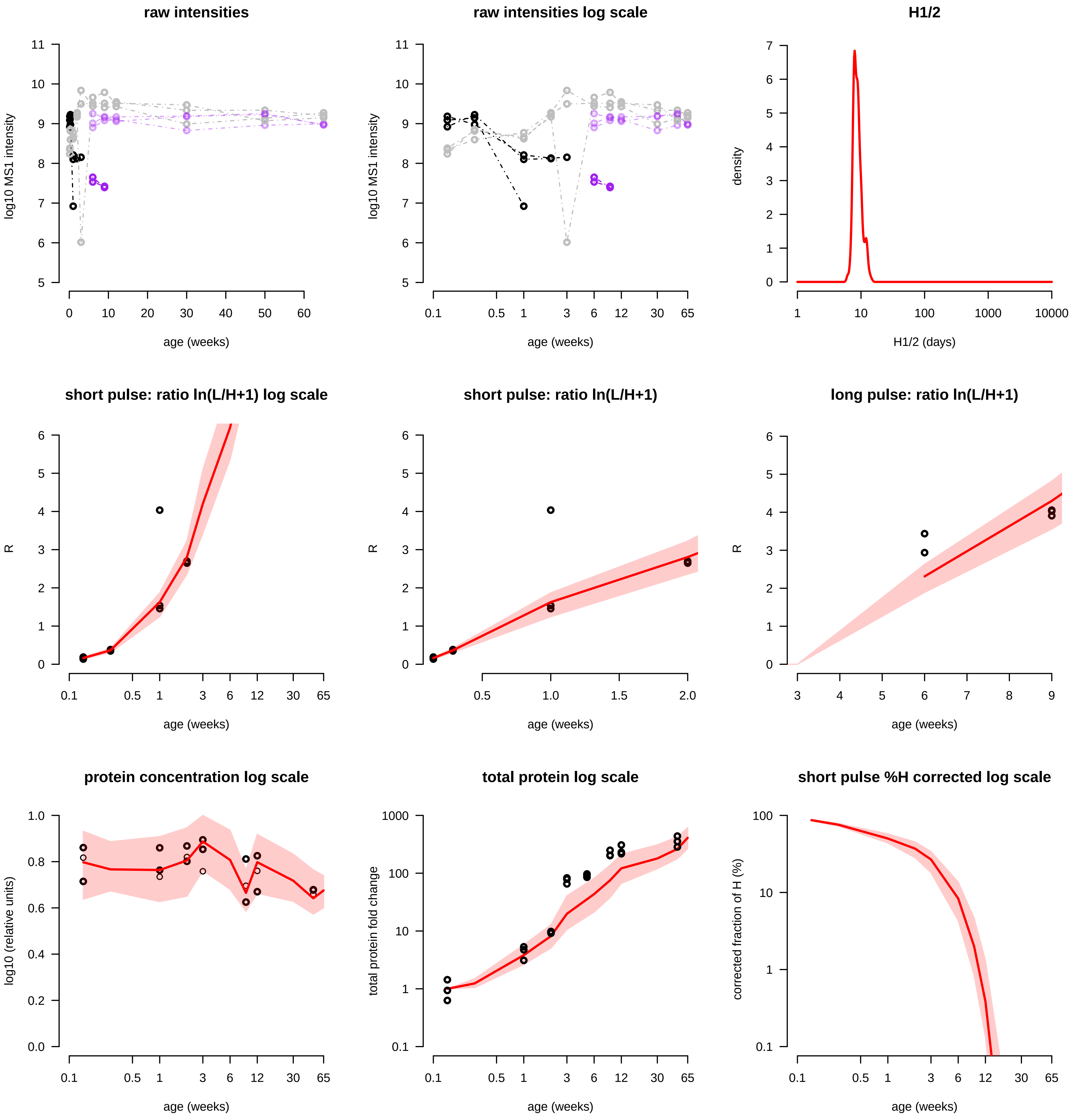

Q99J77

>sp|Q99J77|SIAS_MOUSE

Sialic acid synthase

GN=Nans

Med H1/2: 9.7 days

Q5 H1/2: 7.9 days

Q95 H1/2: 12 days

reliable model fit: yes

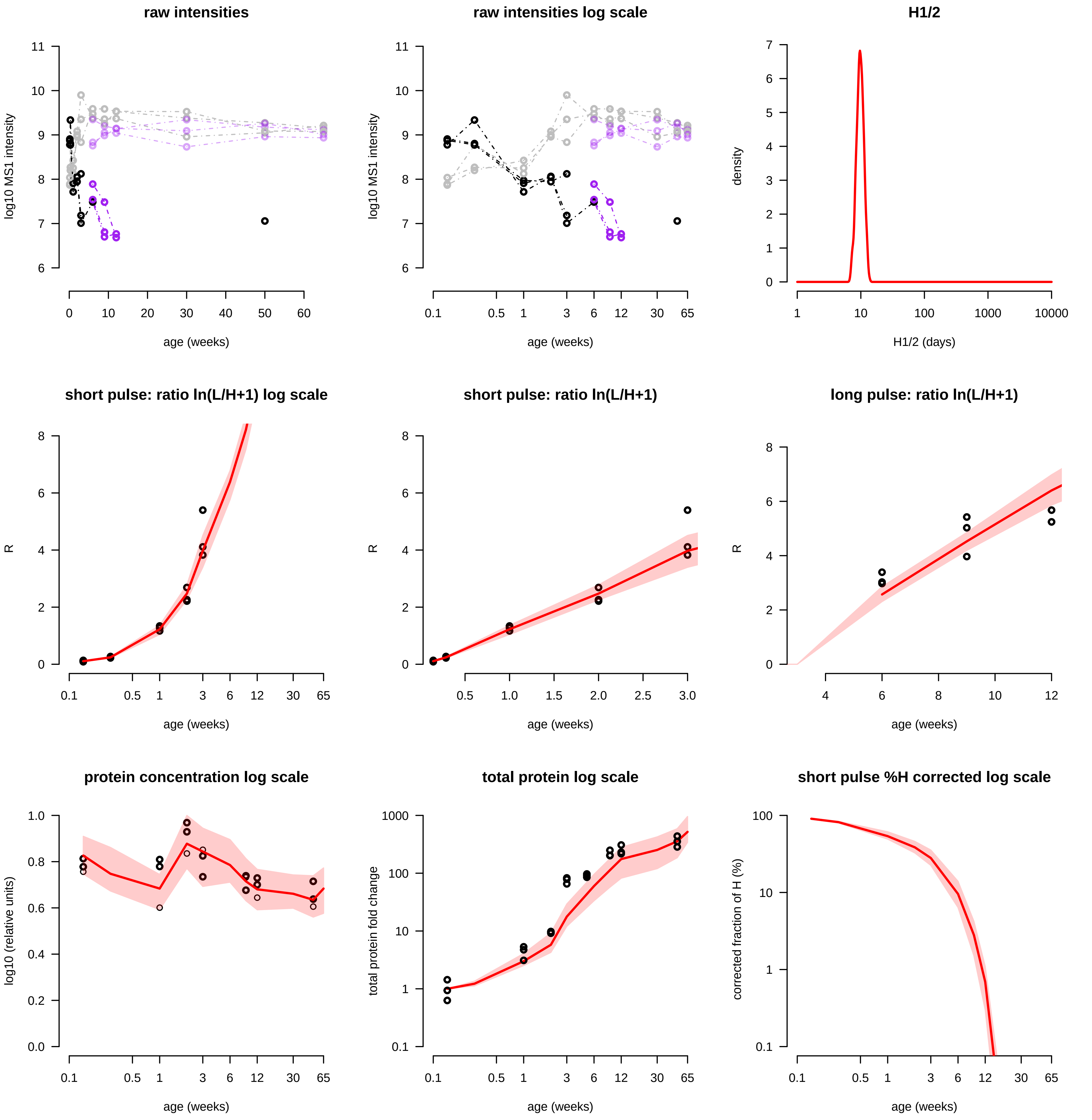

Q2TPA8

>sp|Q2TPA8|HSDL2_MOUSE

Hydroxysteroid dehydrogenase−like protein 2

GN=Hsdl2

Med H1/2: 7.9 days

Q5 H1/2: 4.1 days

Q95 H1/2: 53.8 days

reliable model fit: yes

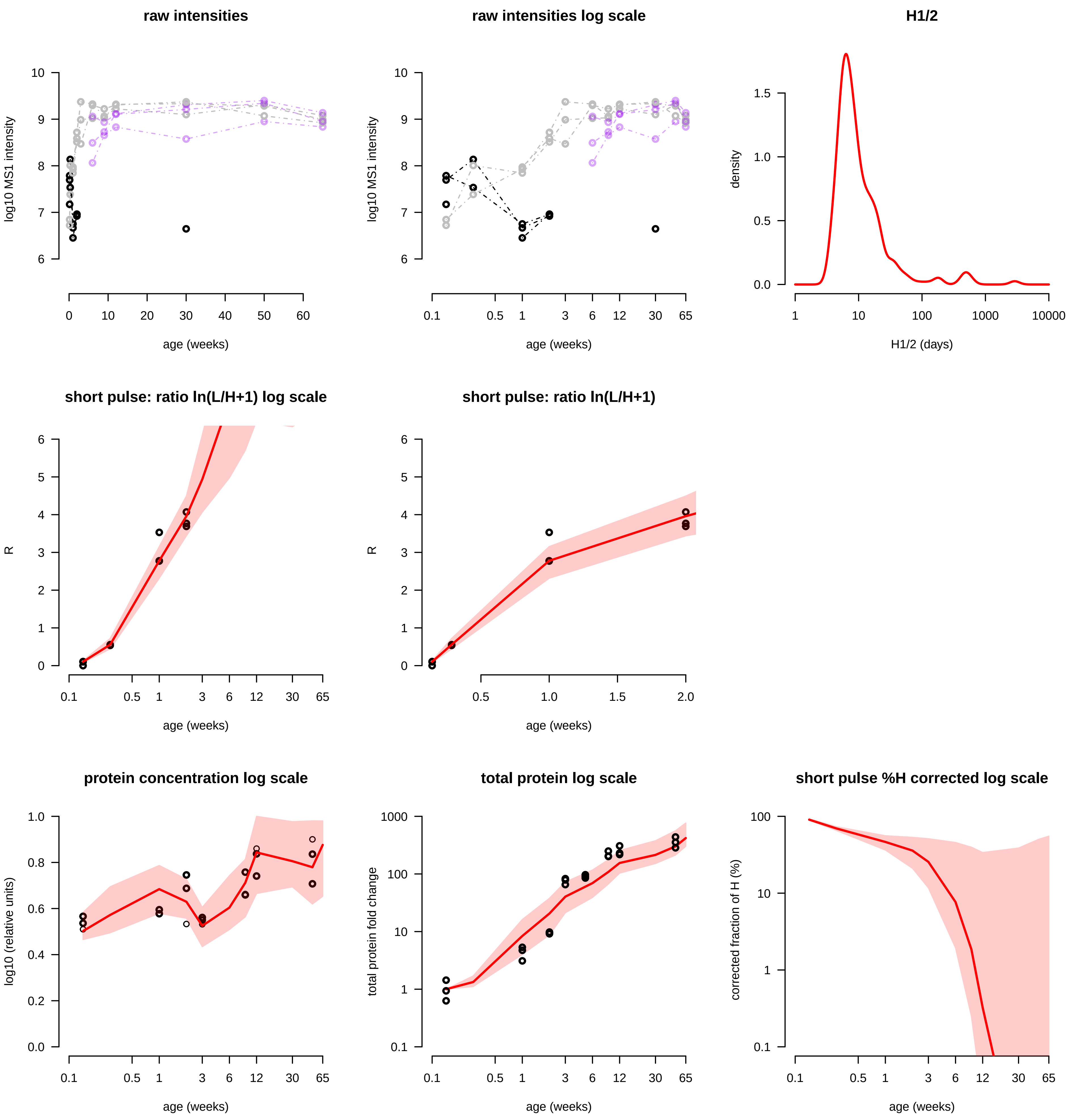

Q61191

>sp|Q61191|HCFC1_MOUSE

Host cell factor 1

GN=Hcfc1

Med H1/2: 360.8 days

Q5 H1/2: 154.3 days

Q95 H1/2: 2011.3 days

reliable model fit: no

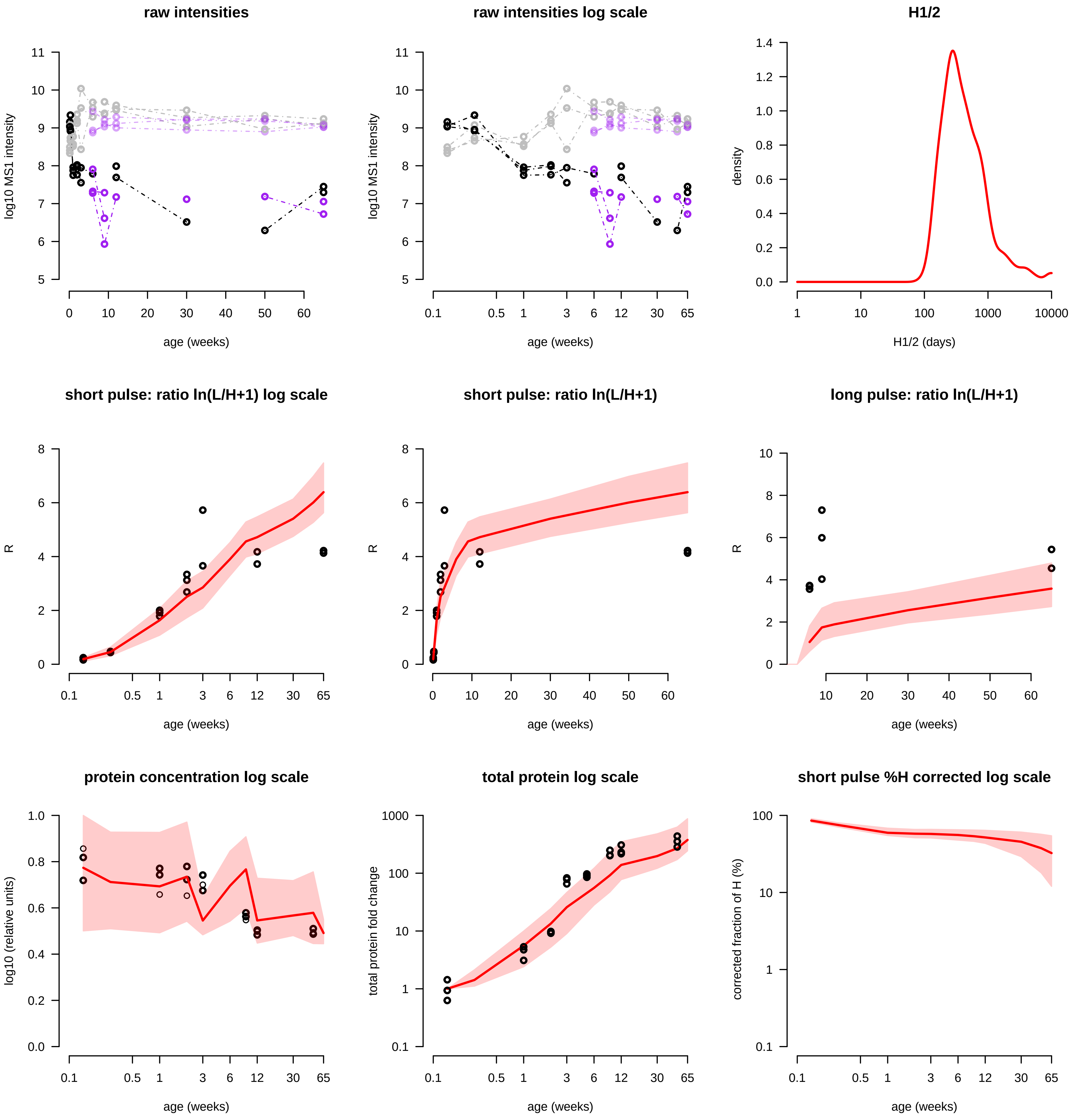

O55023

>sp|O55023|IMPA1_MOUSE

Inositol monophosphatase 1

GN=Impa1

Med H1/2: 5.8 days

Q5 H1/2: 4.6 days

Q95 H1/2: 8 days

reliable model fit: yes

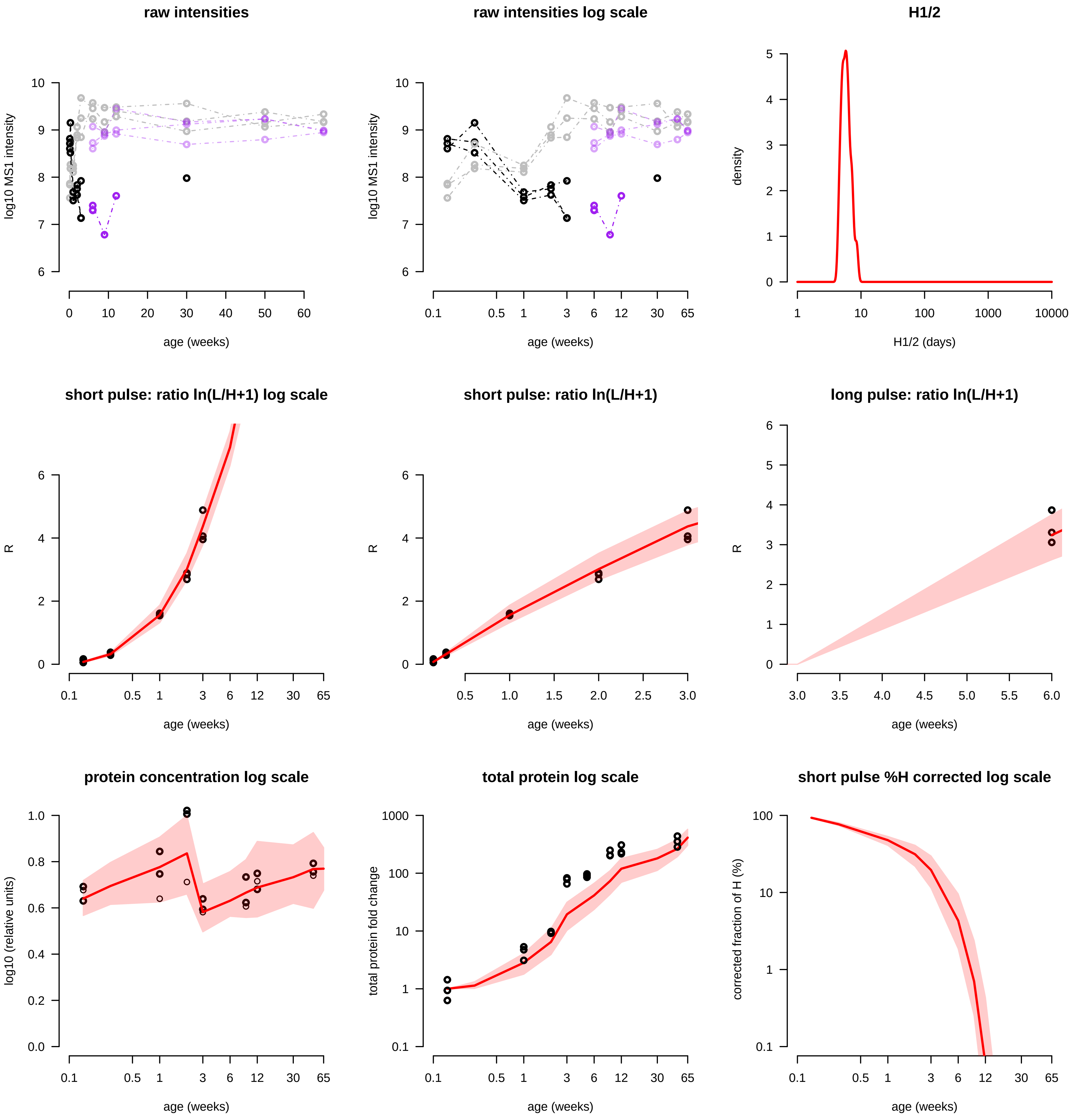

P61514

>sp|P61514|RL37A_MOUSE

60S ribosomal protein L37a

GN=Rpl37a

Med H1/2: 29.7 days

Q5 H1/2: 12.7 days

Q95 H1/2: 176.8 days

reliable model fit: yes

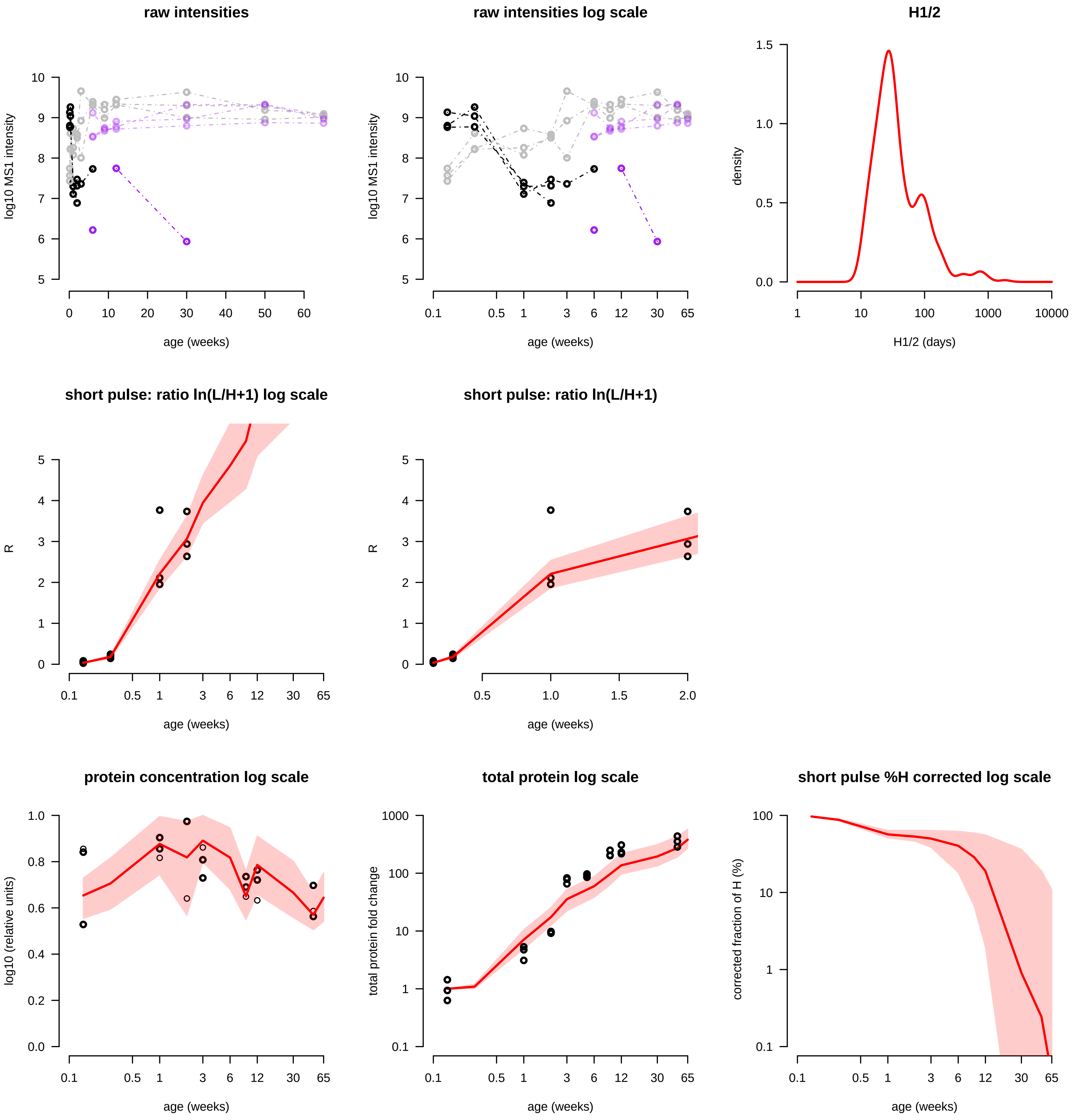

Q9DBR7

>sp|Q9DBR7|MYPT1_MOUSE

Protein phosphatase 1 regulatory subunit 12A

GN=Ppp1r12a

Med H1/2: 208.9 days

Q5 H1/2: 84.1 days

Q95 H1/2: 1512.8 days

reliable model fit: no

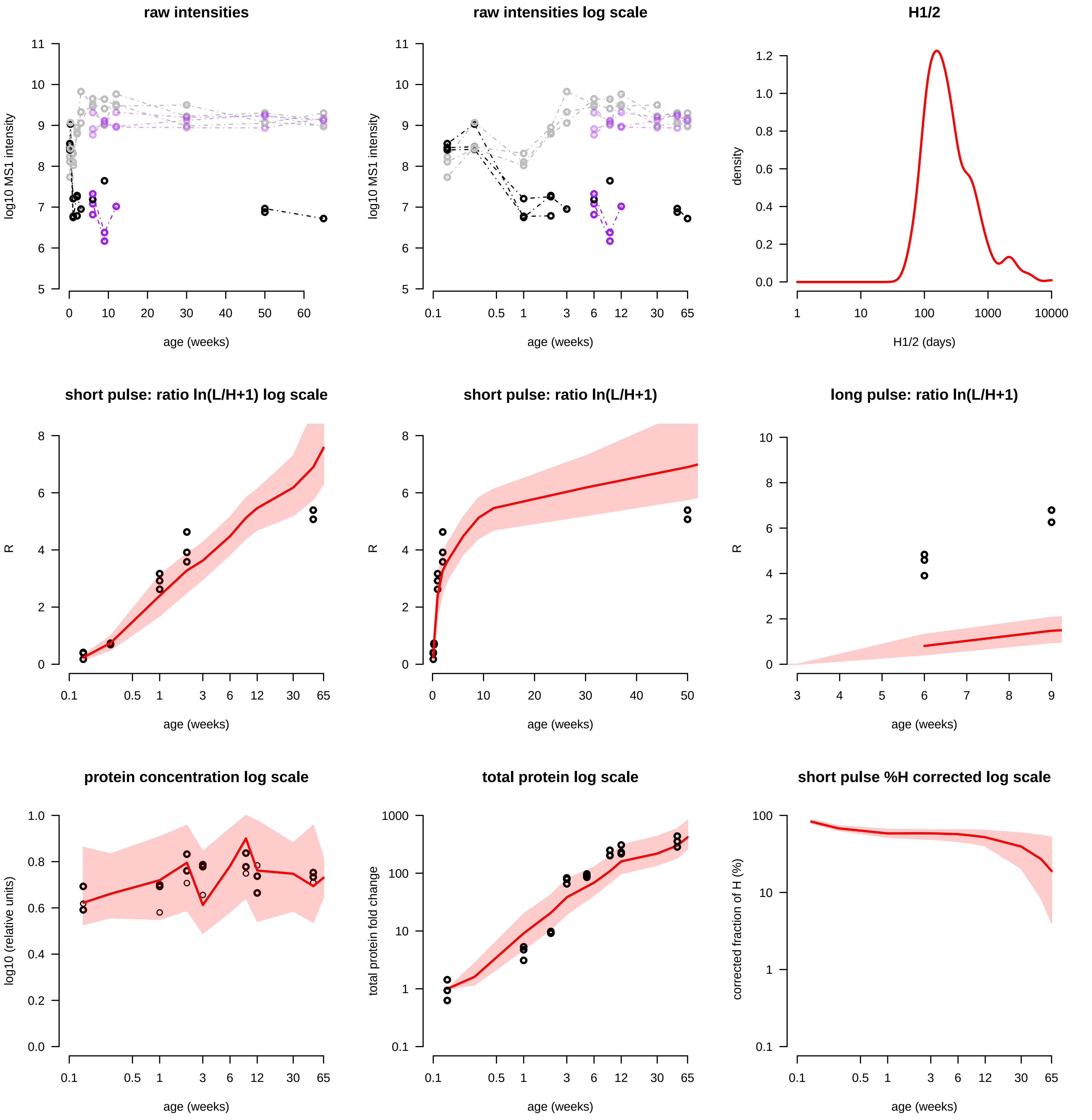

Q61703

>sp|Q61703|ITIH2_MOUSE

Inter−alpha−trypsin inhibitor heavy chain H2

GN=Itih2

Med H1/2: 204.6 days

Q5 H1/2: 102.7 days

Q95 H1/2: 3253.6 days

reliable model fit: no

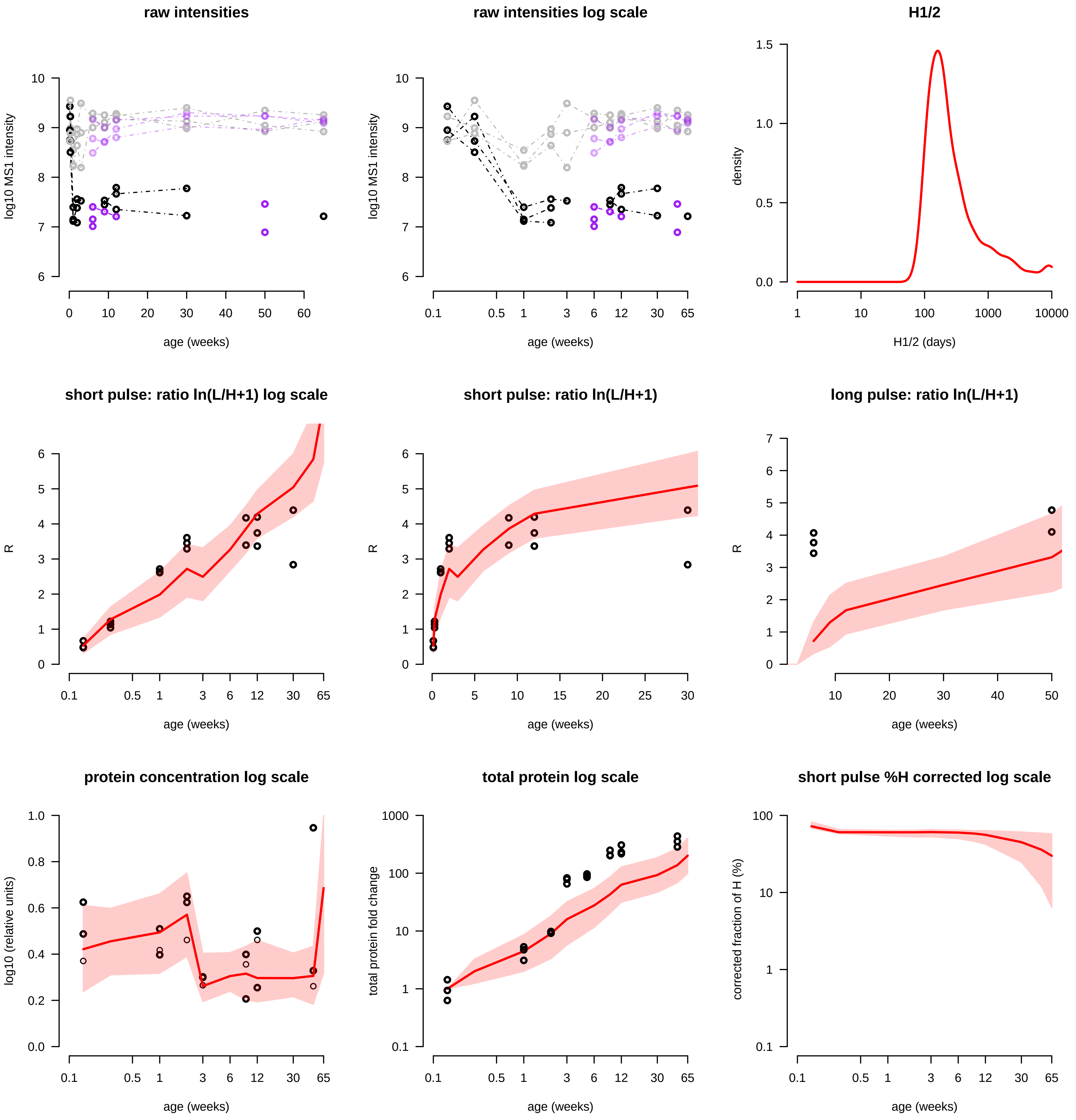

Q9CQ65

>sp|Q9CQ65|MTAP_MOUSE

S−methyl−5'−thioadenosine phosphorylase

GN=Mtap

Med H1/2: 4 days

Q5 H1/2: 2.9 days

Q95 H1/2: 6.6 days

reliable model fit: yes

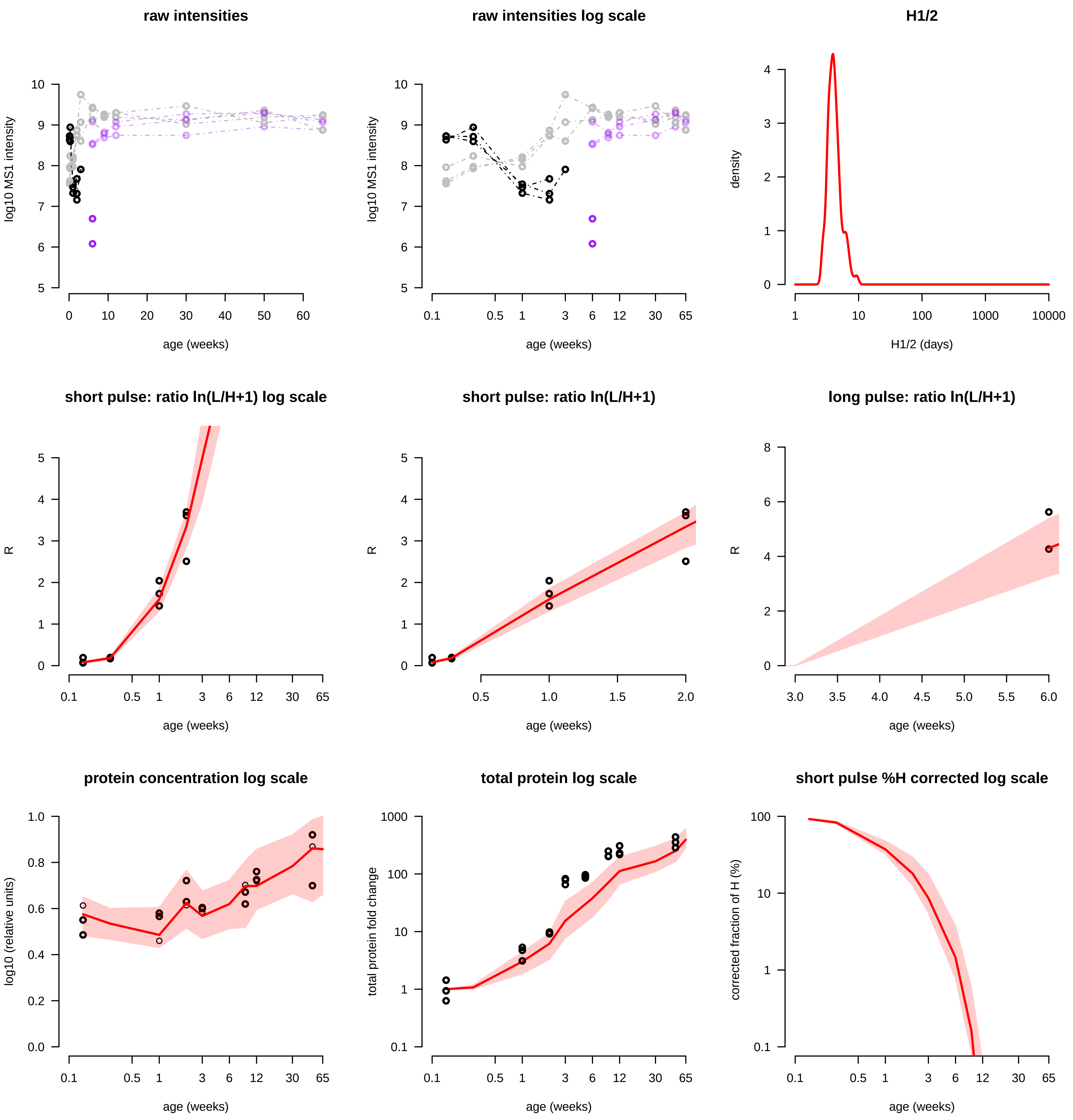

Q9QZD9

>sp|Q9QZD9|EIF3I_MOUSE

Eukaryotic translation initiation factor 3 subunit I

GN=Eif3i

Med H1/2: 6.5 days

Q5 H1/2: 5.6 days

Q95 H1/2: 8.1 days

reliable model fit: yes

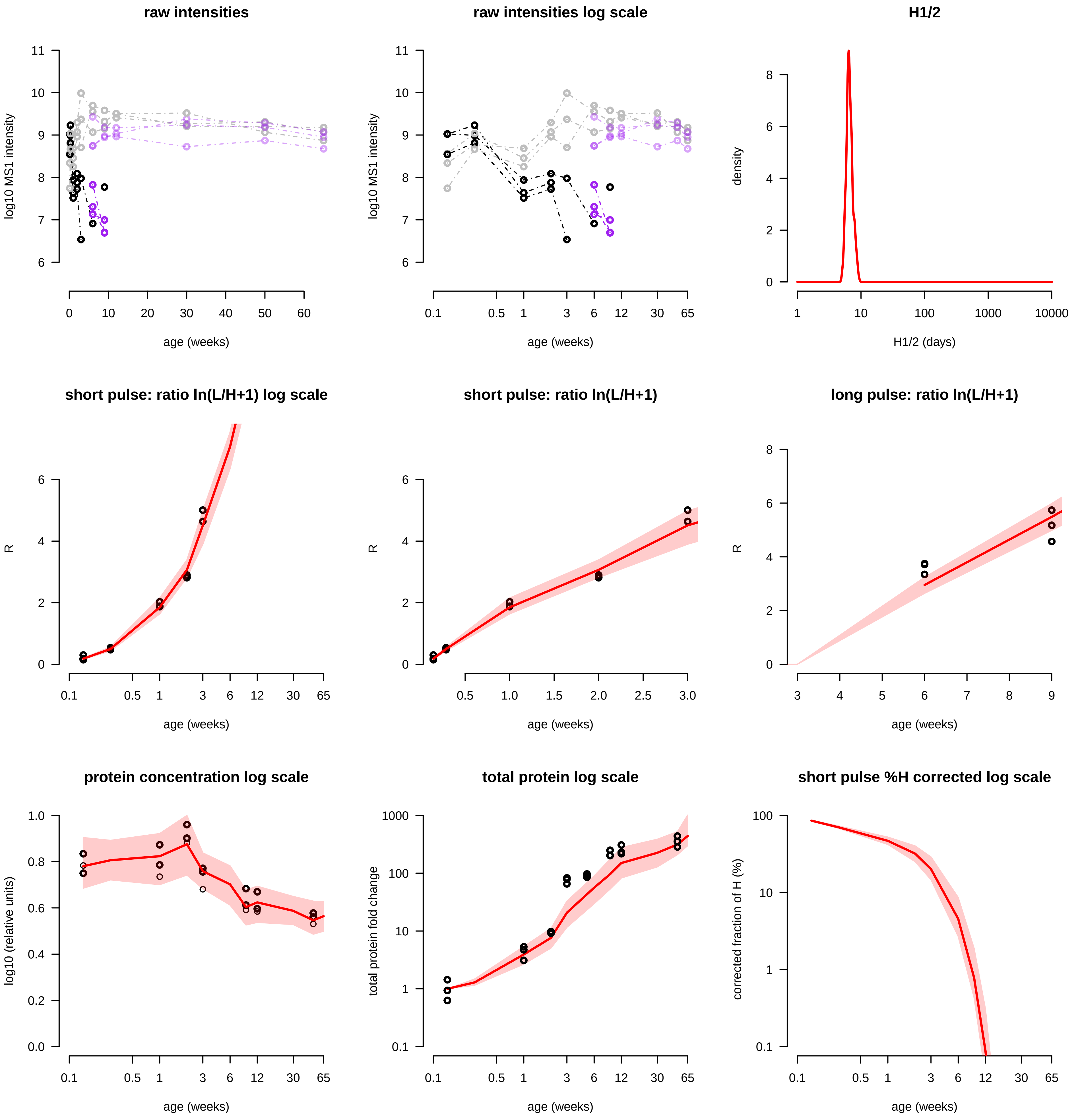

Q8BWF0

>sp|Q8BWF0|SSDH_MOUSE

Succinate−semialdehyde dehydrogenase, mitochondrial

GN=Aldh5a1

Med H1/2: 314.6 days

Q5 H1/2: 153.9 days

Q95 H1/2: 988.3 days

reliable model fit: yes

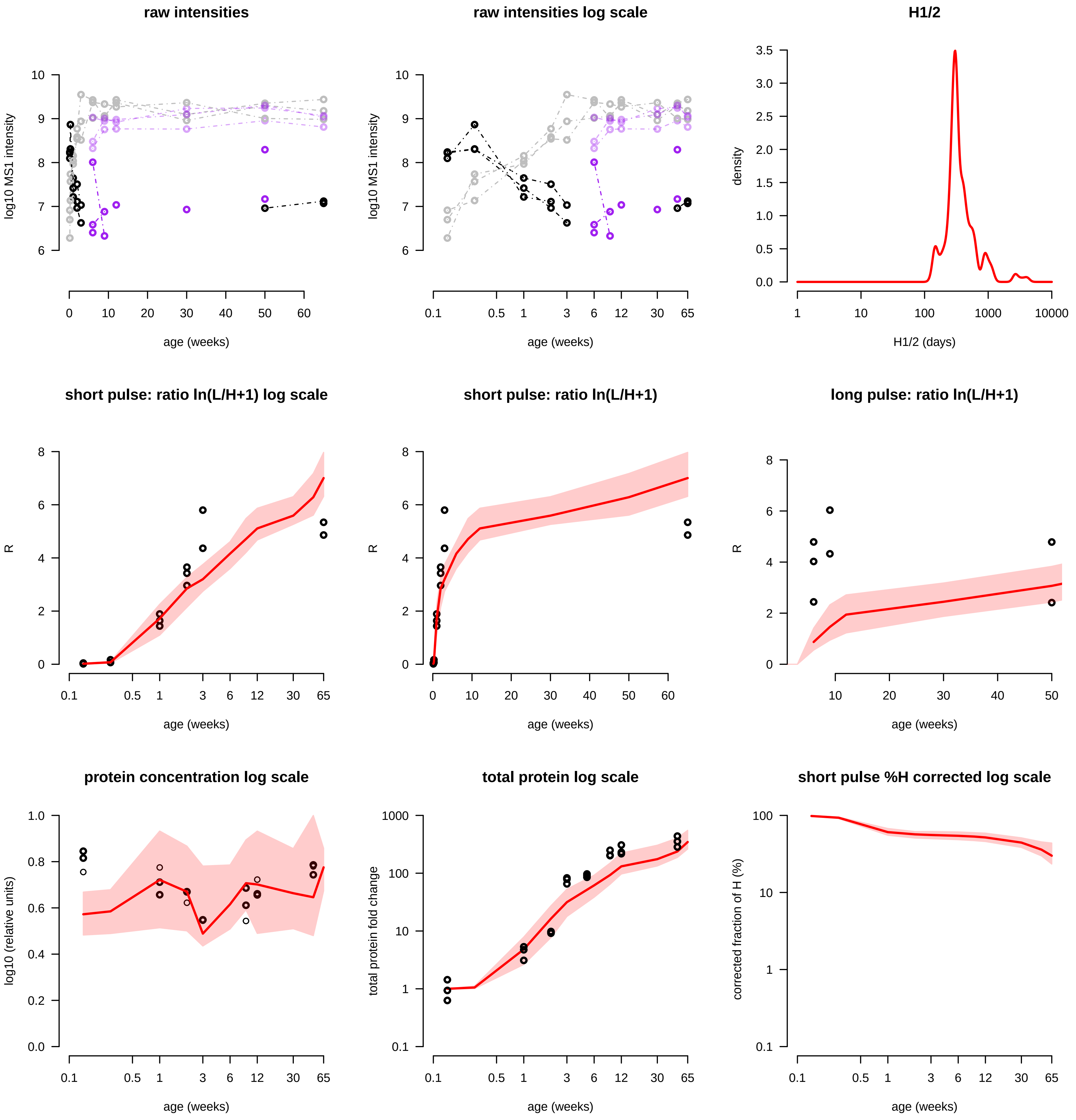

P61202;P61202−2

>sp|P61202|CSN2_MOUSE

COP9 signalosome complex subunit 2

GN=Cops2

Med H1/2: 4.2 days

Q5 H1/2: 3.7 days

Q95 H1/2: 5.4 days

reliable model fit: yes

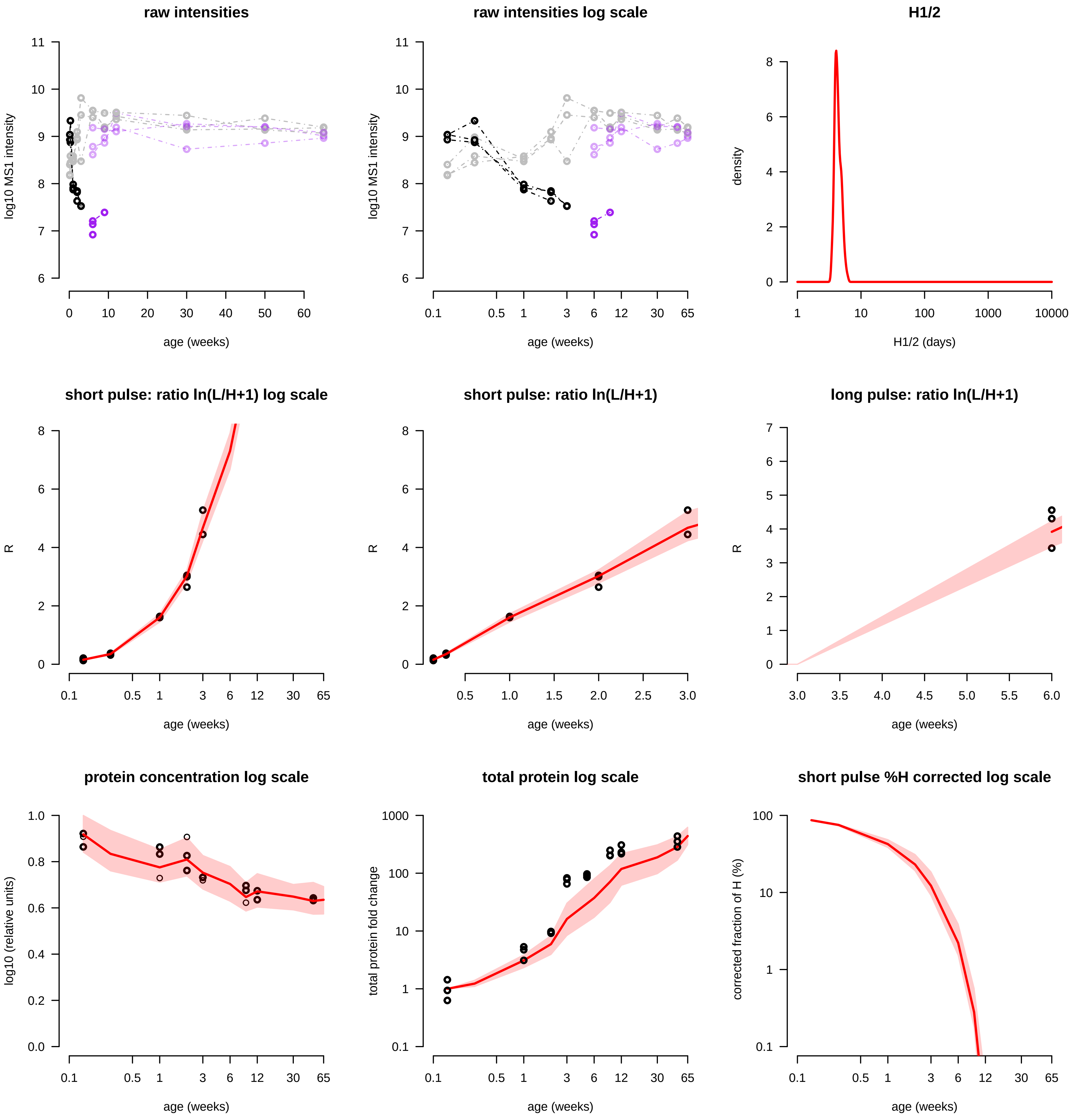

Q52KB6;Q52KB6−3;Q52KB6−2

>sp|Q52KB6|C2CD3_MOUSE

C2 domain−containing protein 3

GN=C2cd3

Med H1/2: 10.7 days

Q5 H1/2: 8.4 days

Q95 H1/2: 15.6 days

reliable model fit: yes

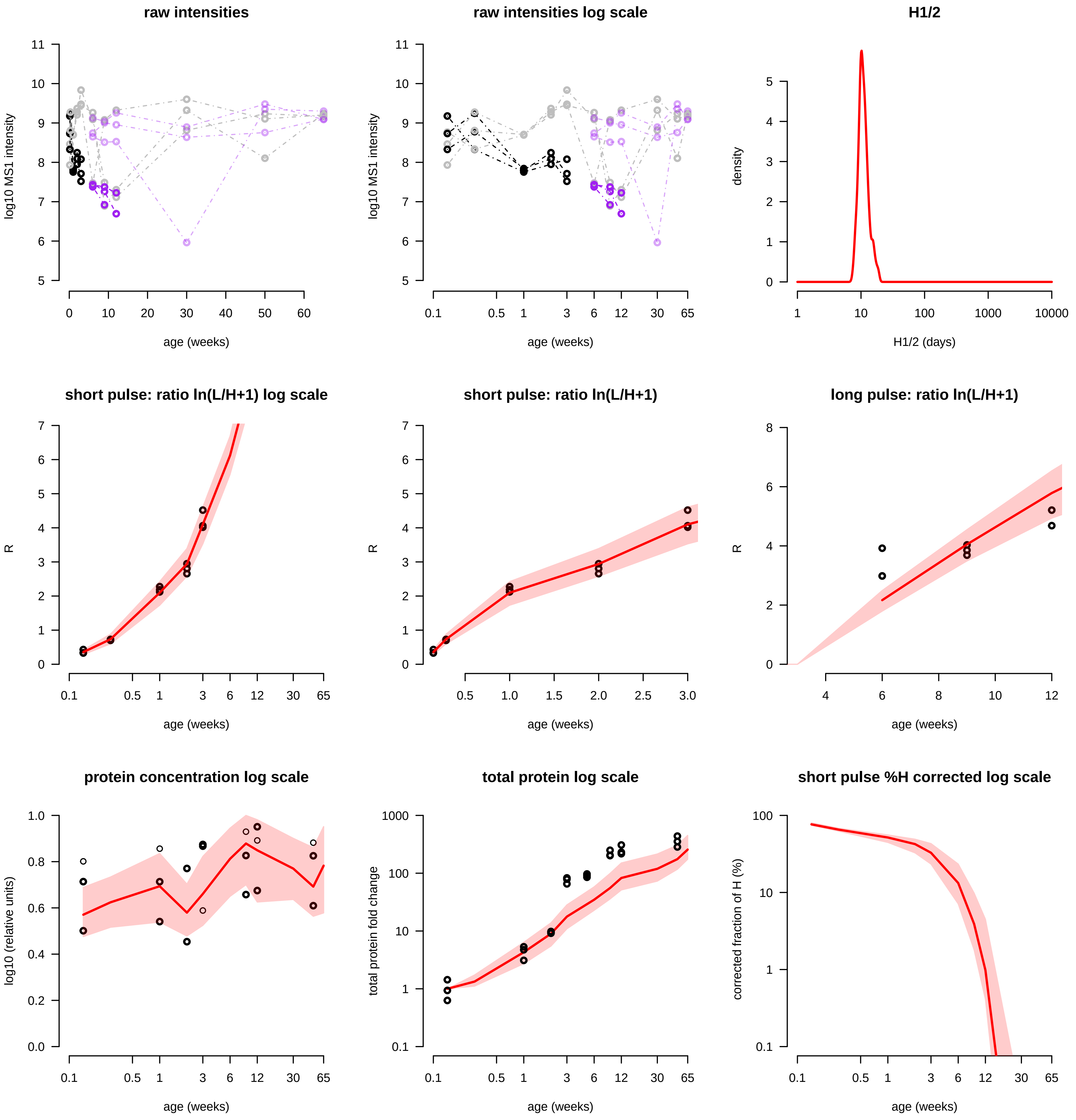

Q9CZ30;Q9CZ30−2

>sp|Q9CZ30|OLA1_MOUSE

Obg−like ATPase 1

GN=Ola1

Med H1/2: 5.9 days

Q5 H1/2: 4.8 days

Q95 H1/2: 7 days

reliable model fit: yes

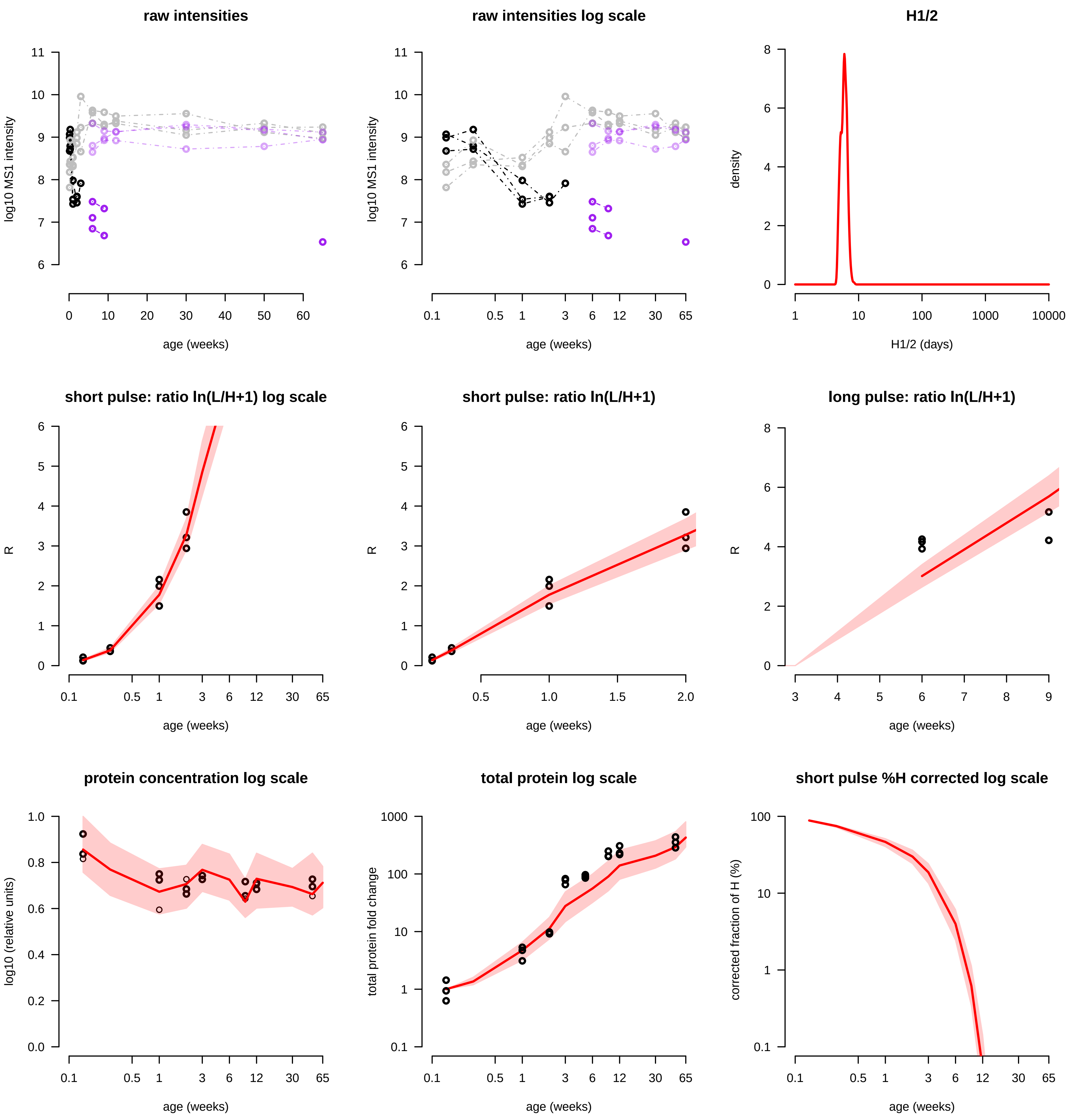

P99026

>sp|P99026|PSB4_MOUSE

Proteasome subunit beta type−4

GN=Psmb4

Med H1/2: 9 days

Q5 H1/2: 6.8 days

Q95 H1/2: 13 days

reliable model fit: yes

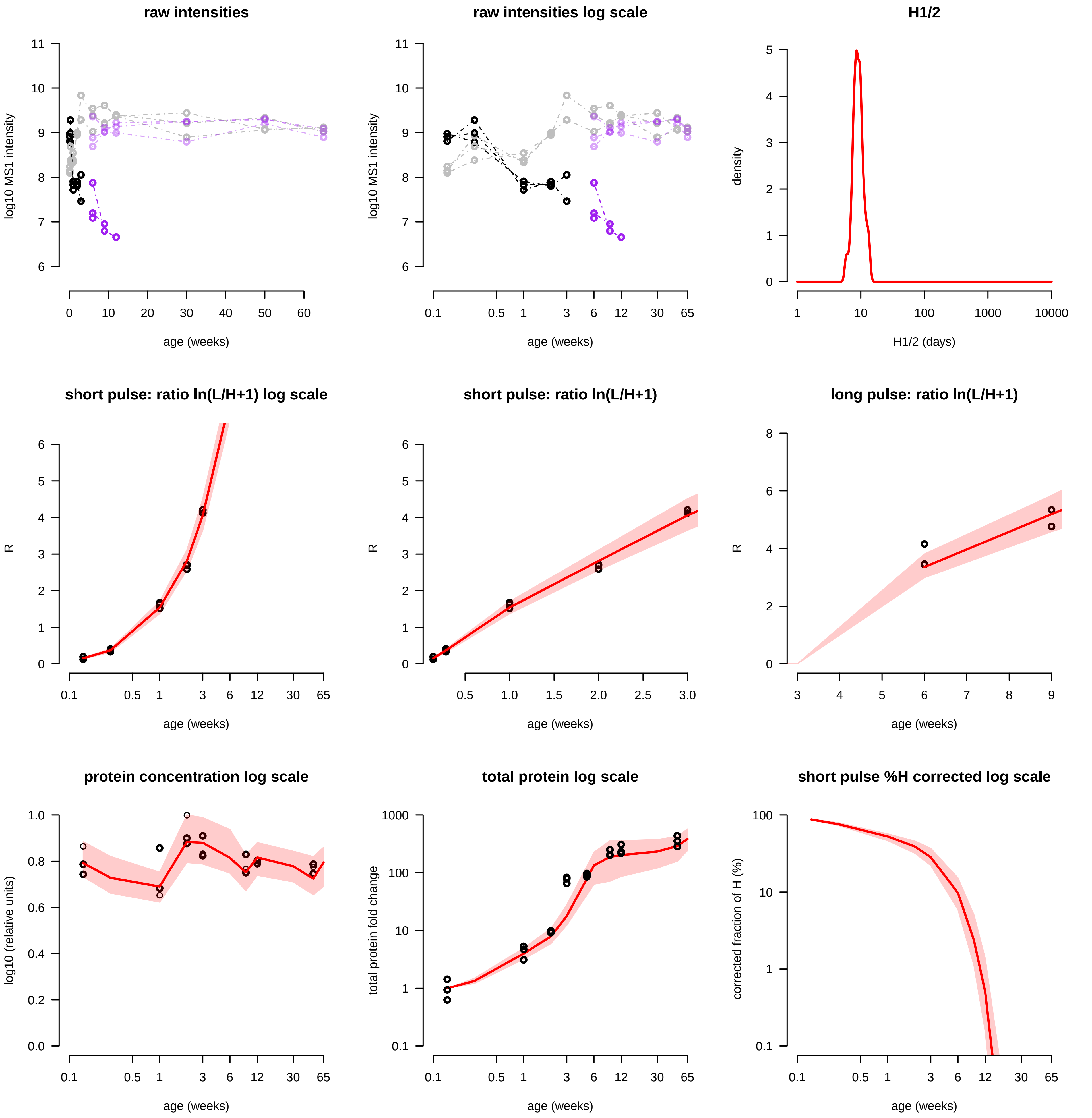

Q9EPC1

>sp|Q9EPC1|PARVA_MOUSE

Alpha−parvin

GN=Parva

Med H1/2: 5.5 days

Q5 H1/2: 4.5 days

Q95 H1/2: 7.4 days

reliable model fit: yes

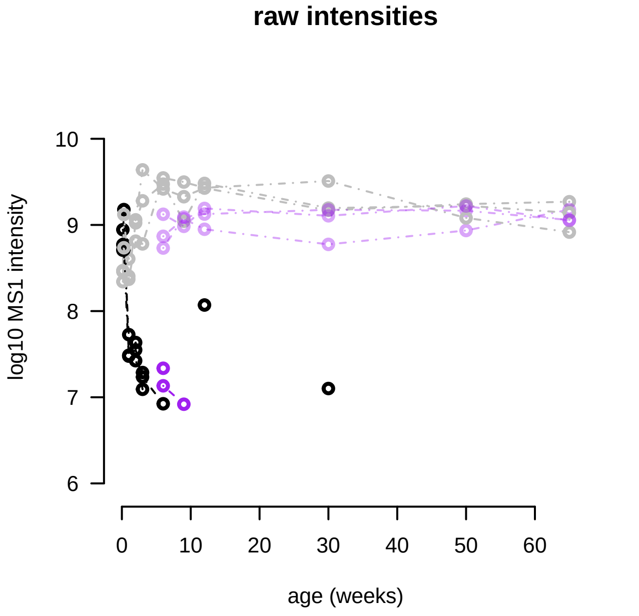

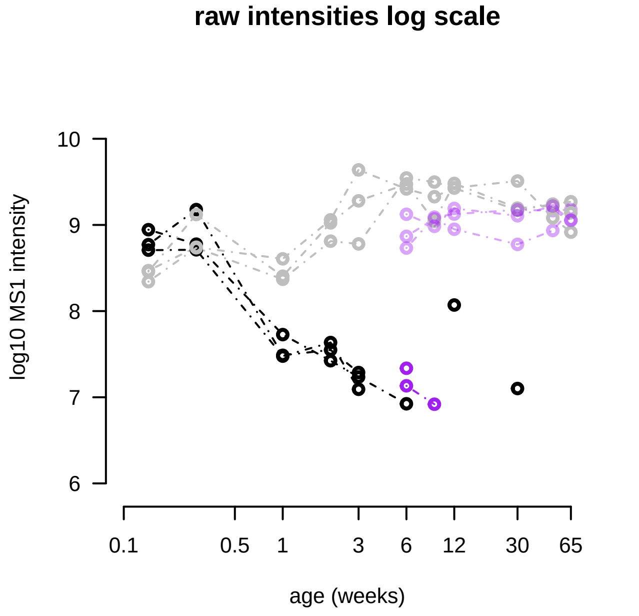

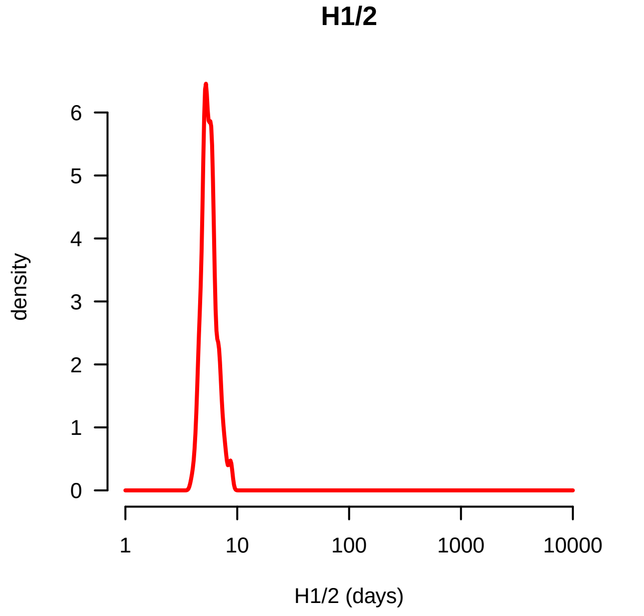

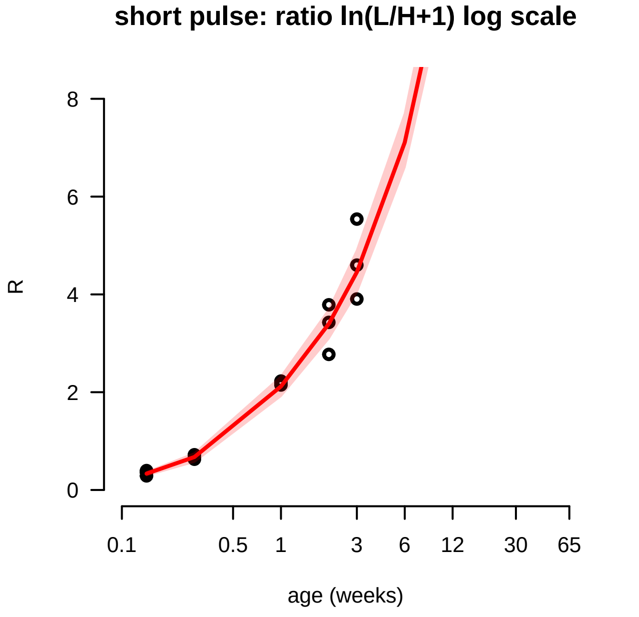

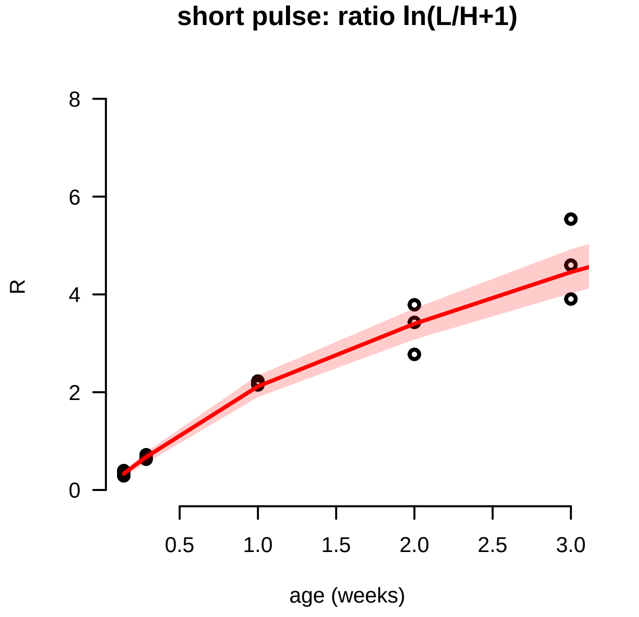

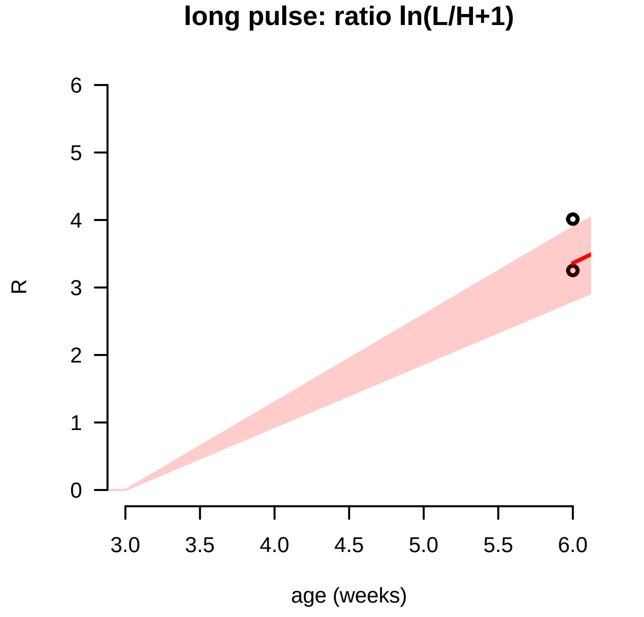

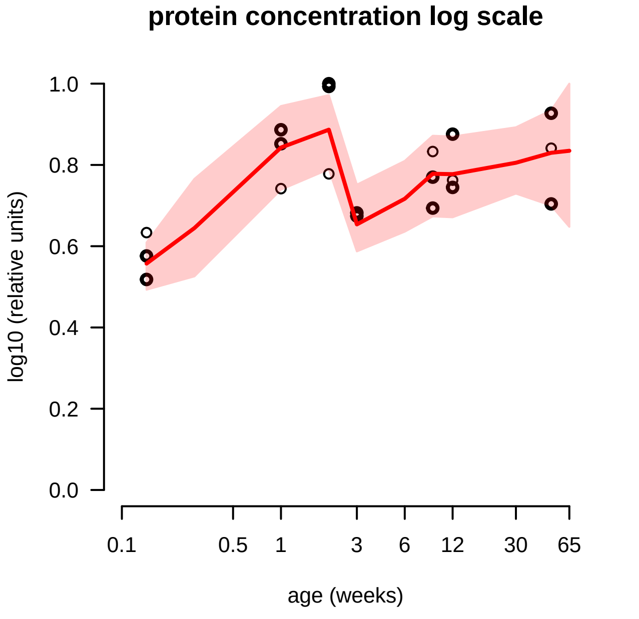

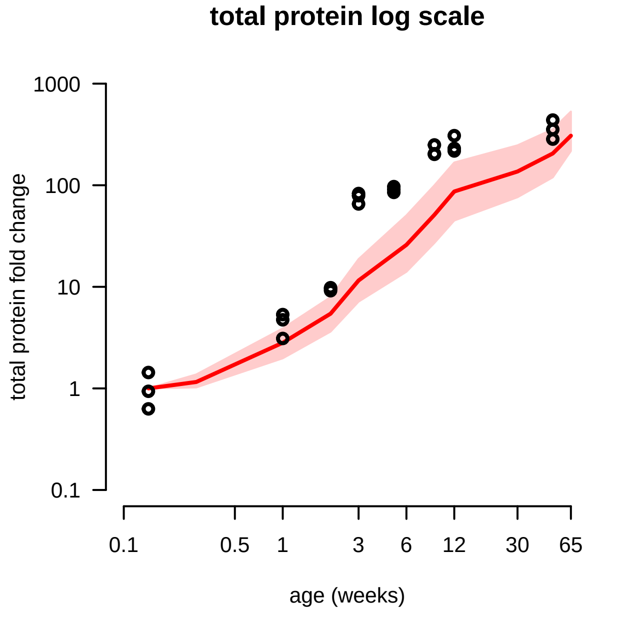

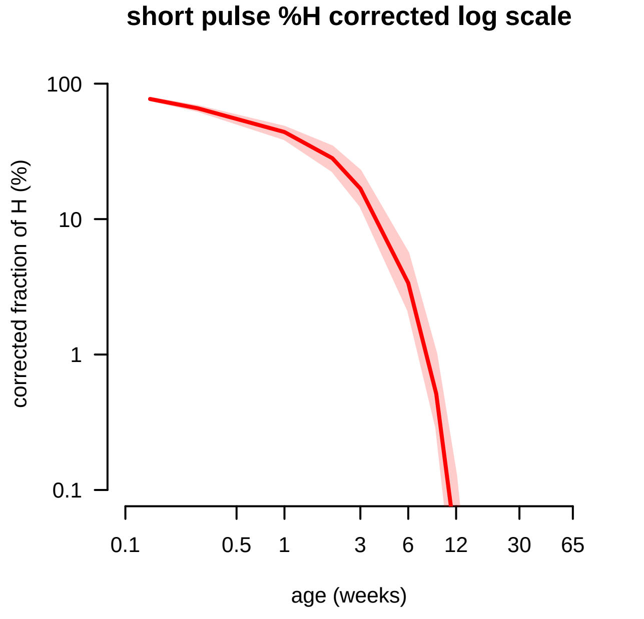

Q6DFW4

>sp|Q6DFW4|NOP58_MOUSE

Nucleolar protein 58

GN=Nop58

Med H1/2: 6.8 days

Q5 H1/2: 5.4 days

Q95 H1/2: 8.6 days

reliable model fit: yes

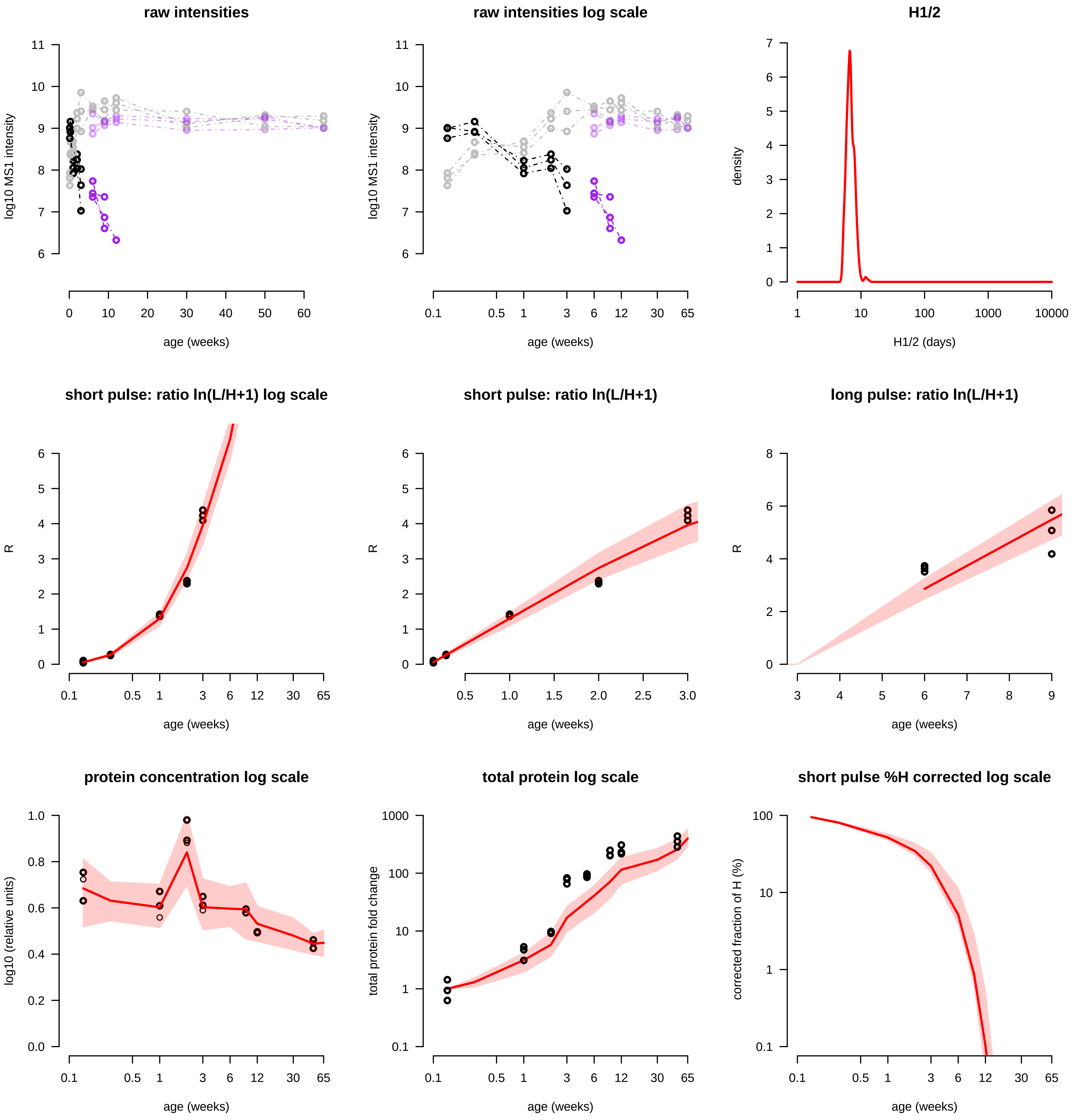

Q8R1B4

>sp|Q8R1B4|EIF3C_MOUSE

Eukaryotic translation initiation factor 3 subunit C

GN=Eif3c

Med H1/2: 29.4 days

Q5 H1/2: 19.3 days

Q95 H1/2: 43.6 days

reliable model fit: no

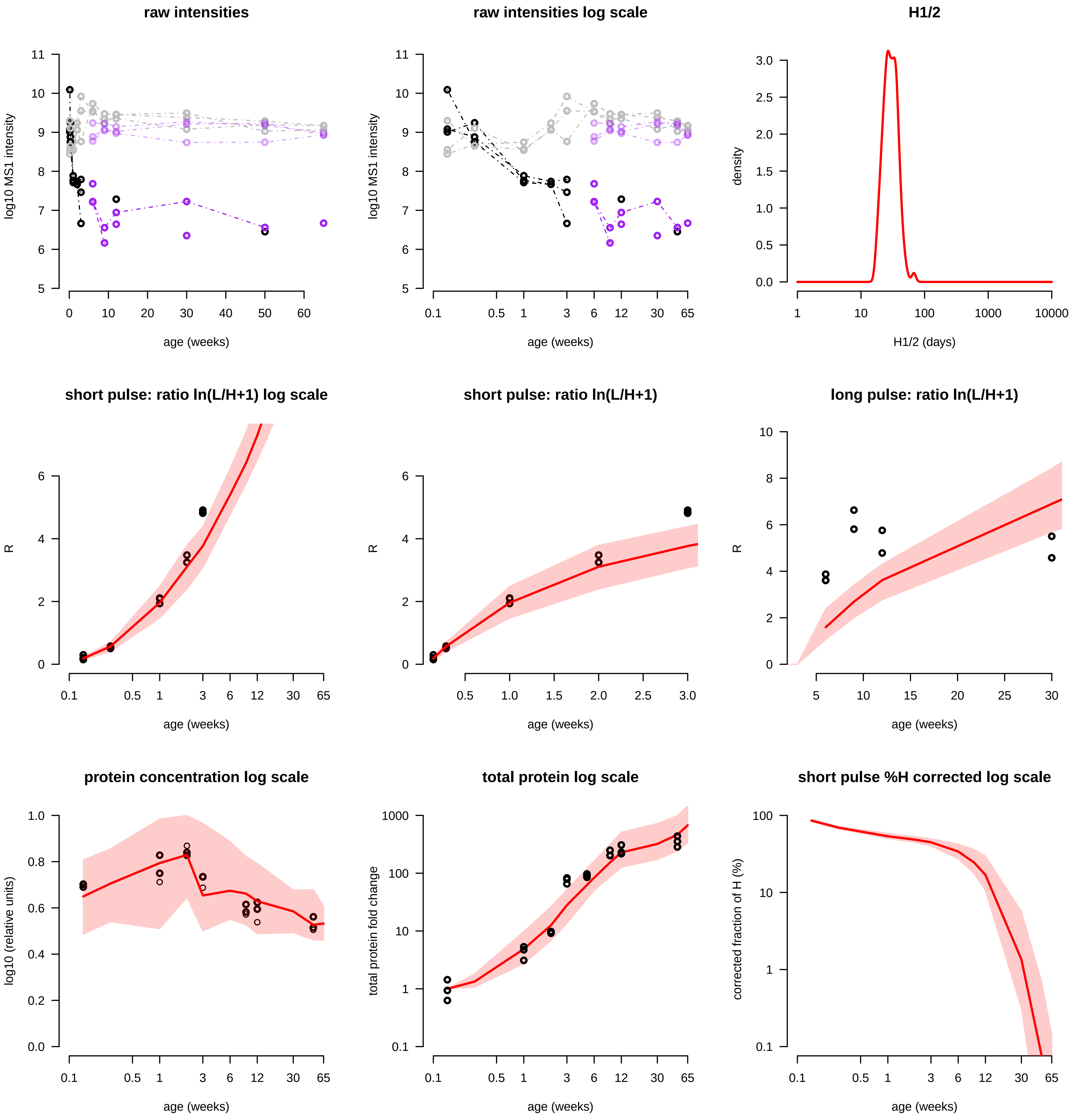

O35226−2;O35226−5

>sp|O35226−2|PSMD4_MOUSE

Isoform Rpn10B of 26S proteasome non−ATPase regulatory subunit 4

GN=Psmd4

Med H1/2: 4.7 days

Q5 H1/2: 3.9 days

Q95 H1/2: 5.6 days

reliable model fit: yes

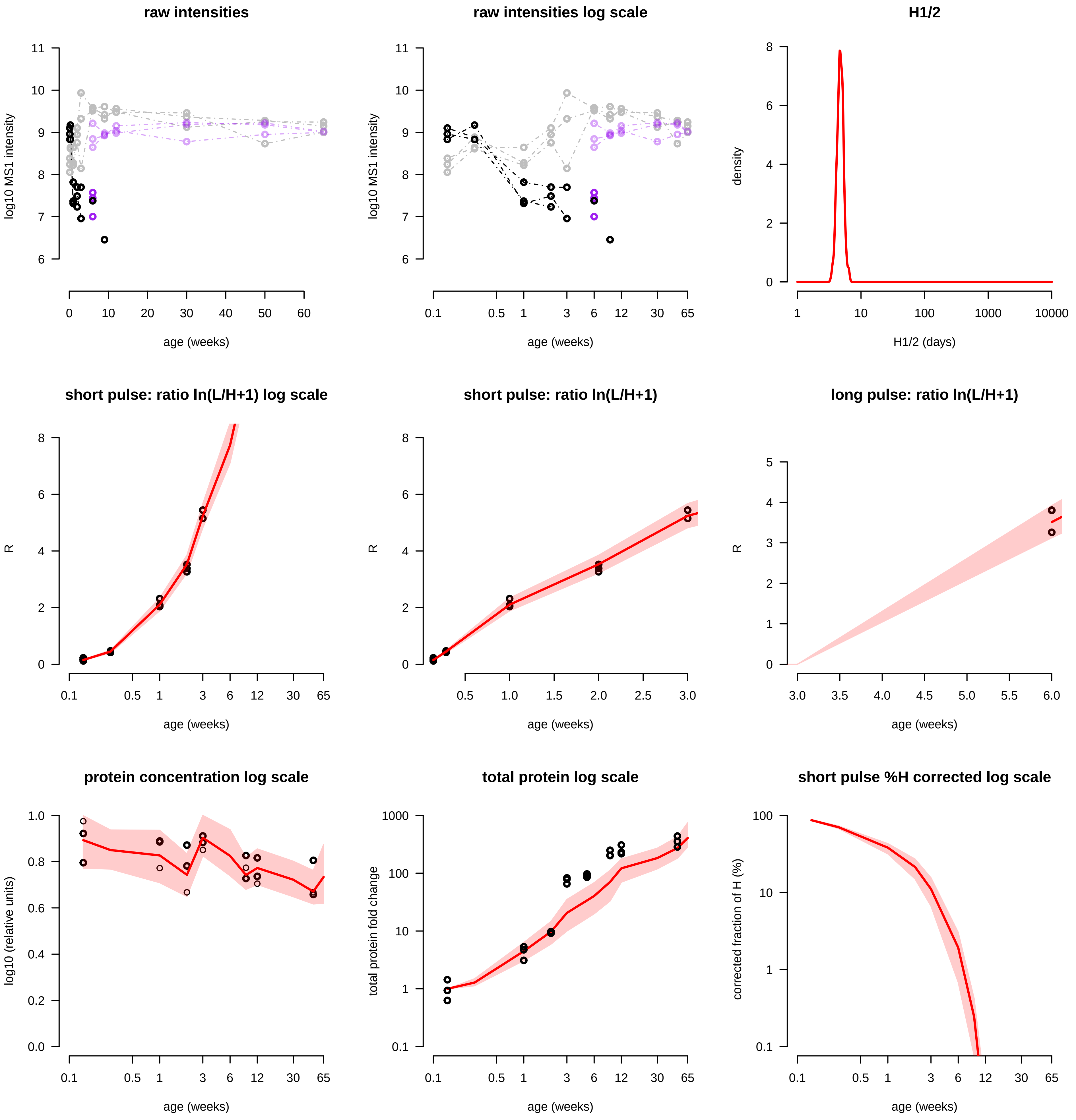

Q8K4Z3

>sp|Q8K4Z3|NNRE_MOUSE

NAD(P)H−hydrate epimerase

GN=Naxe

Med H1/2: 6.1 days

Q5 H1/2: 4.9 days

Q95 H1/2: 8.2 days

reliable model fit: yes

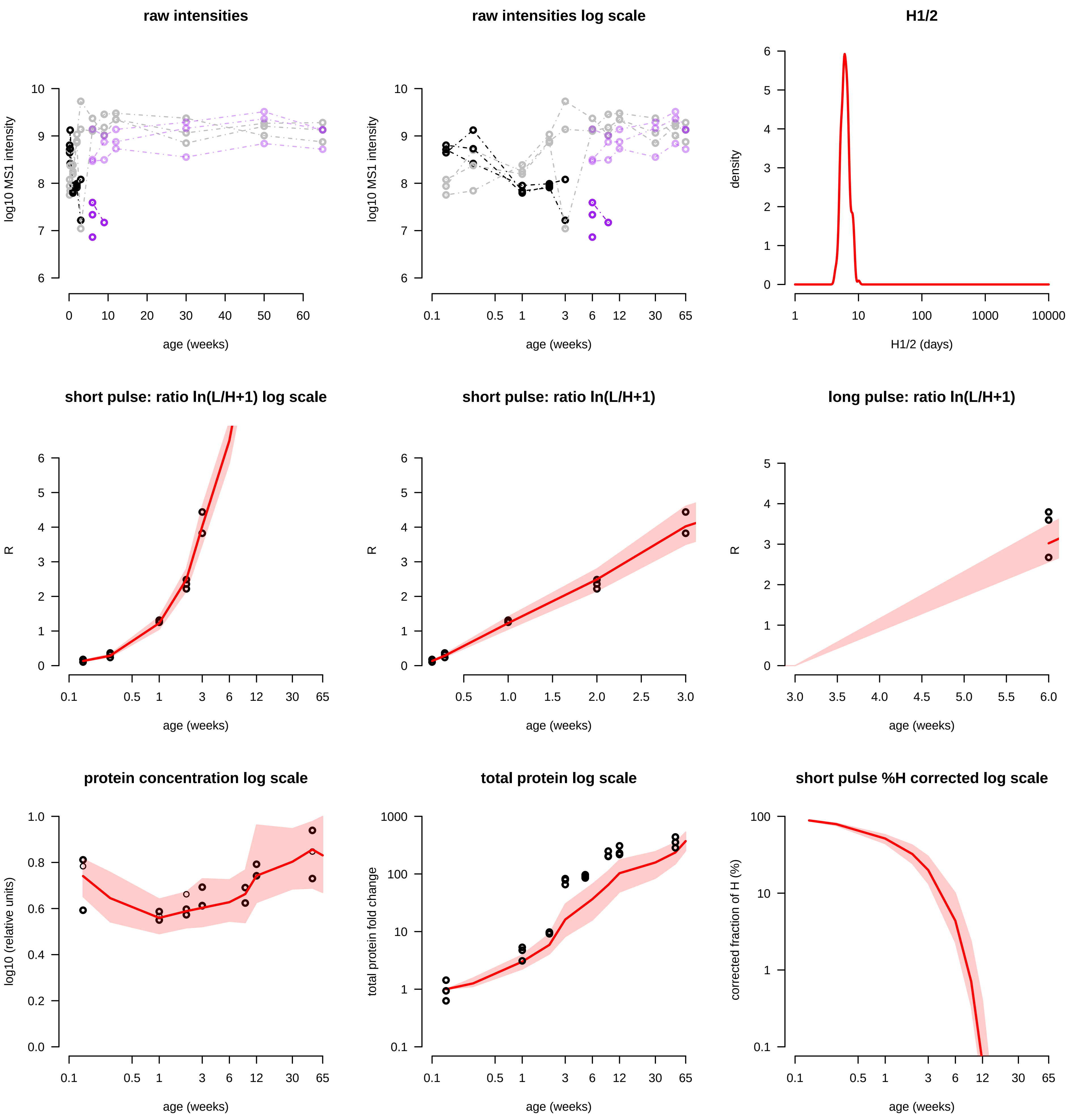

Q9D8Y0

>sp|Q9D8Y0|EFHD2_MOUSE

EF−hand domain−containing protein D2

GN=Efhd2

Med H1/2: 1613.4 days

Q5 H1/2: 375.7 days

Q95 H1/2: 33738.4 days

reliable model fit: yes

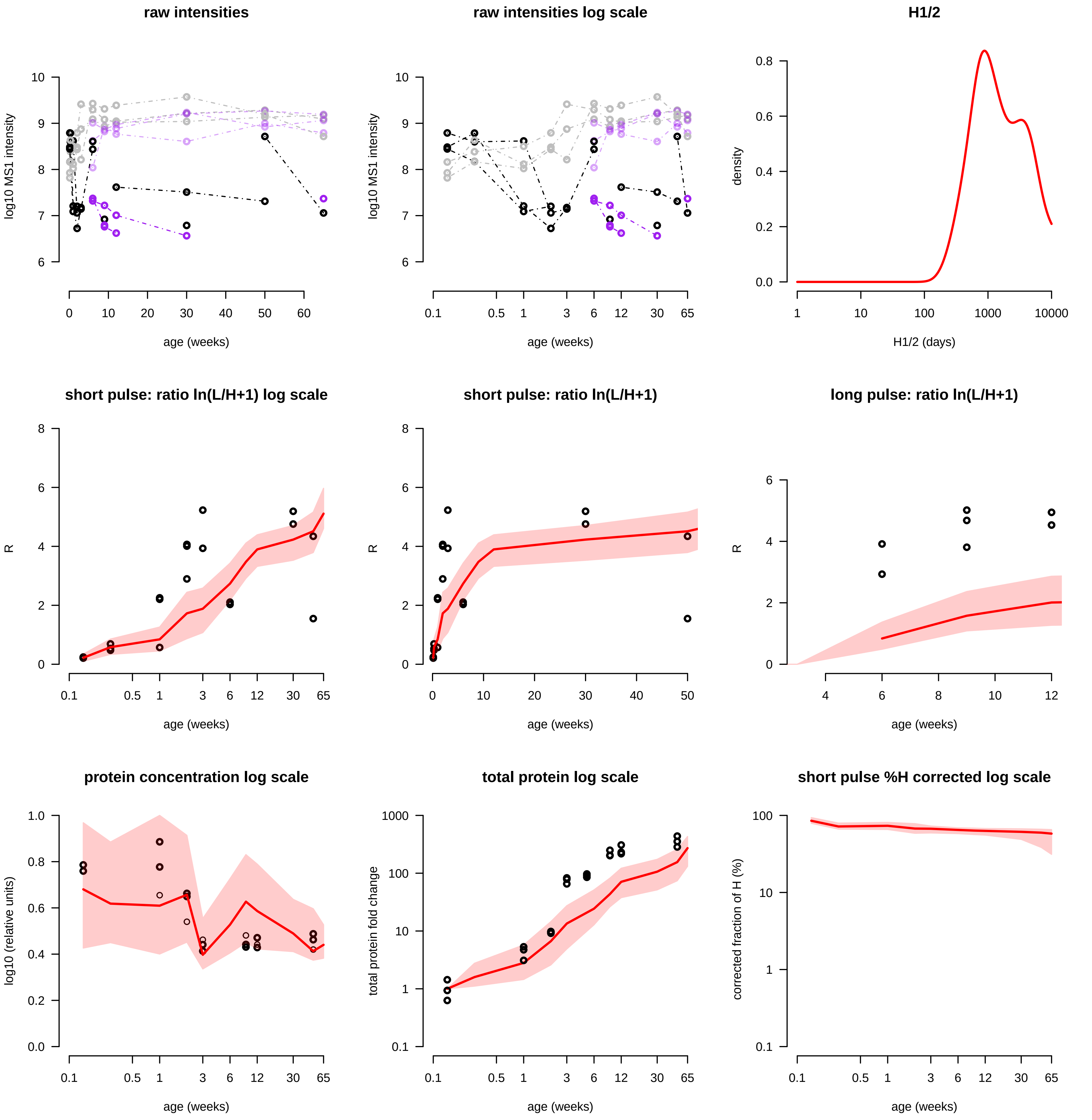

P70404

>sp|P70404|IDHG1_MOUSE

Isocitrate dehydrogenase [NAD] subunit gamma 1, mitochondrial

GN=Idh3g

Med H1/2: 3.9 days

Q5 H1/2: 3.2 days

Q95 H1/2: 5 days

reliable model fit: yes

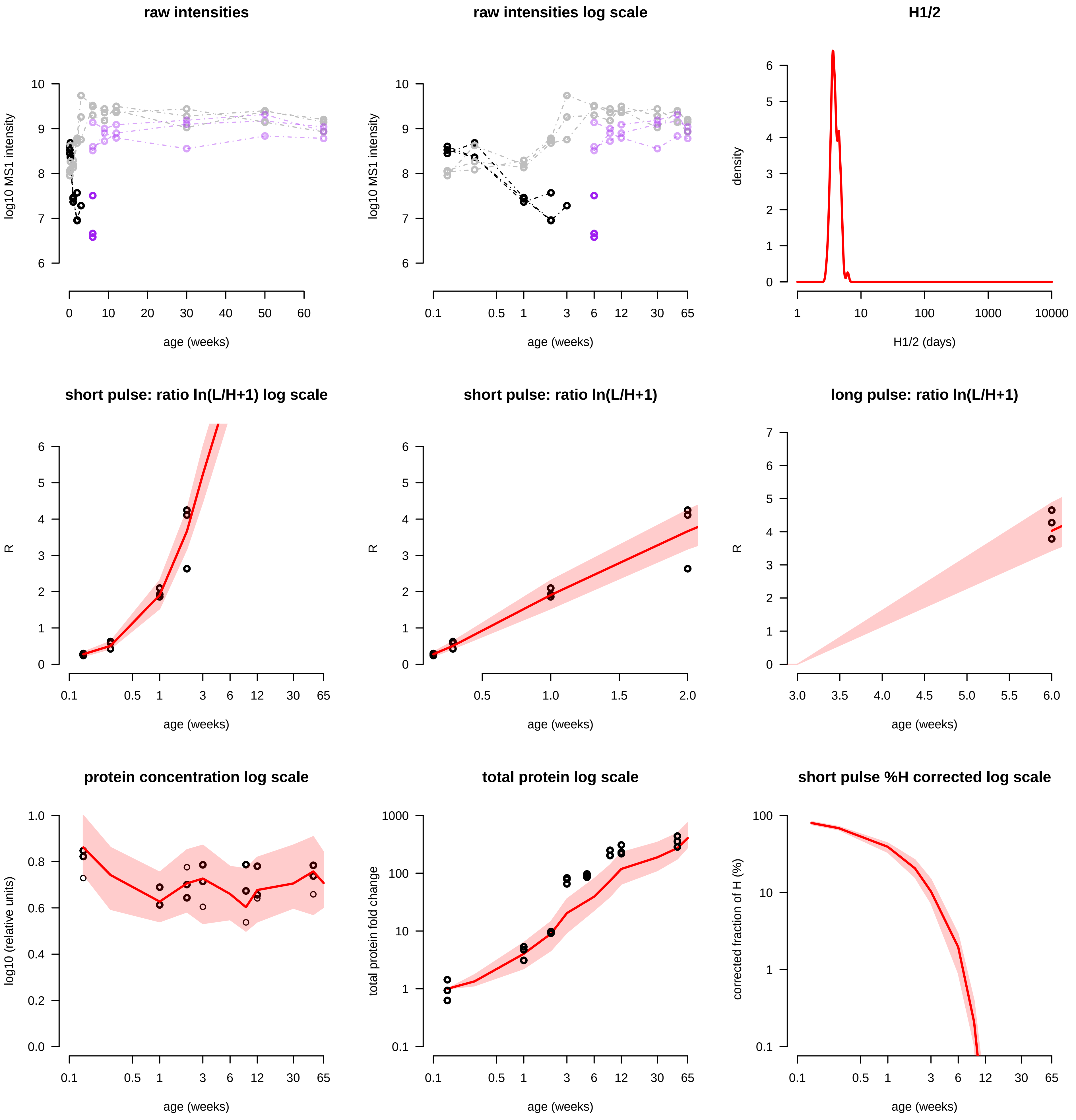

Q9JM76

>sp|Q9JM76|ARPC3_MOUSE

Actin−related protein 2/3 complex subunit 3

GN=Arpc3

Med H1/2: 6.6 days

Q5 H1/2: 5.6 days

Q95 H1/2: 8.2 days

reliable model fit: yes

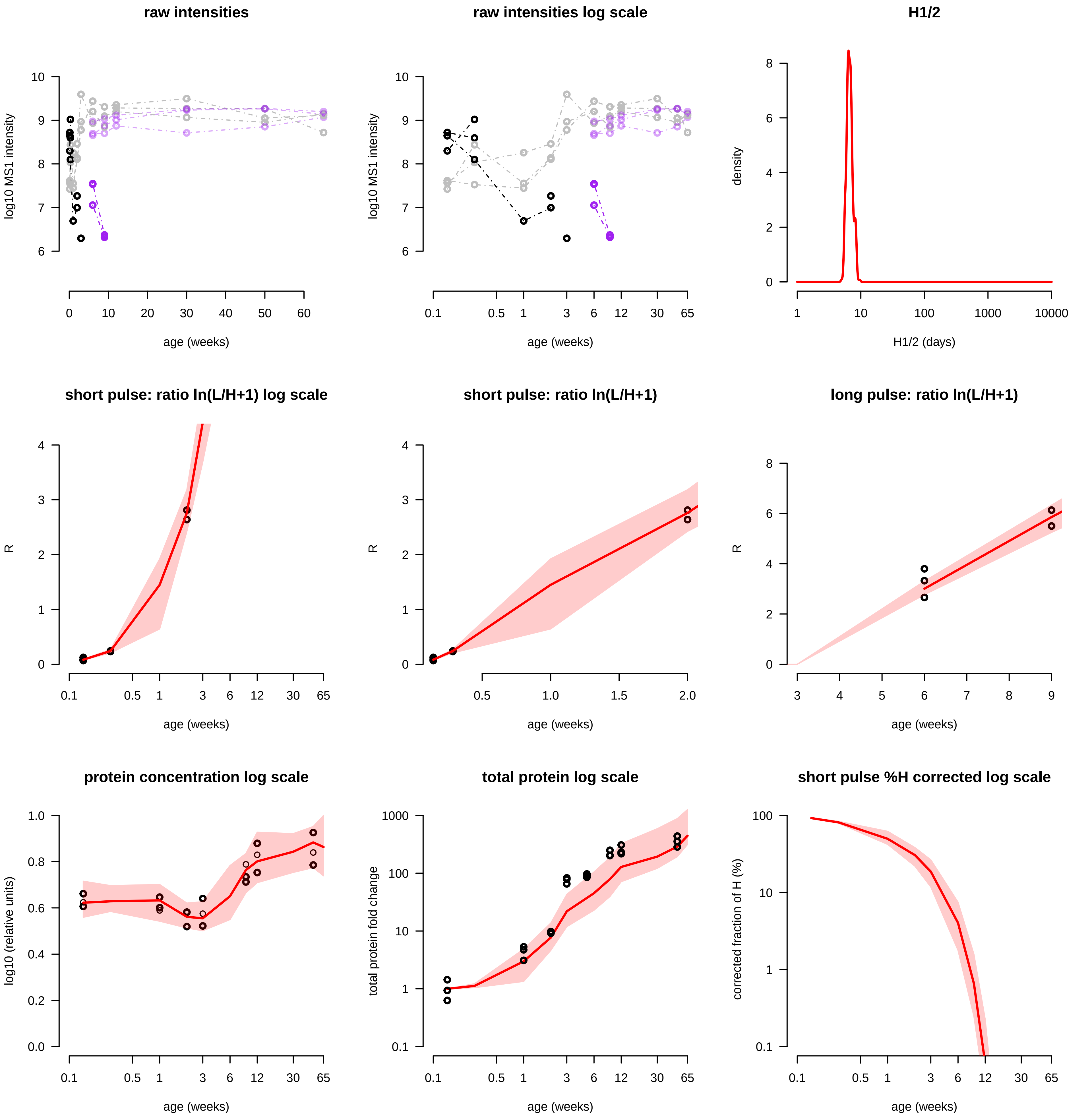

Q99J99

>sp|Q99J99|THTM_MOUSE

3−mercaptopyruvate sulfurtransferase

GN=Mpst

Med H1/2: 5.1 days

Q5 H1/2: 4.1 days

Q95 H1/2: 6.5 days

reliable model fit: yes

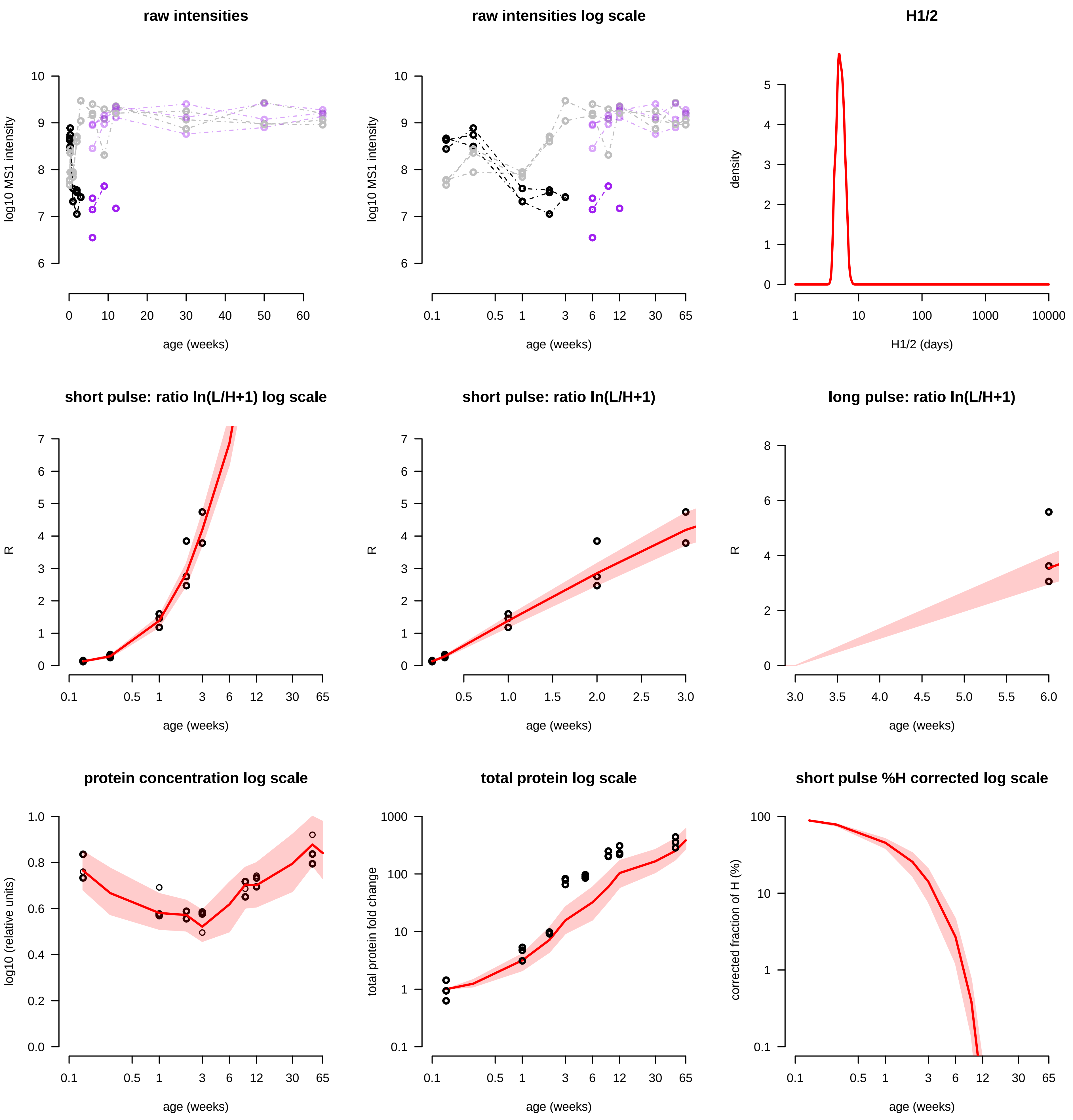

O08529

>sp|O08529|CAN2_MOUSE

Calpain−2 catalytic subunit

GN=Capn2

Med H1/2: 174.7 days

Q5 H1/2: 54.8 days

Q95 H1/2: 1595.4 days

reliable model fit: no

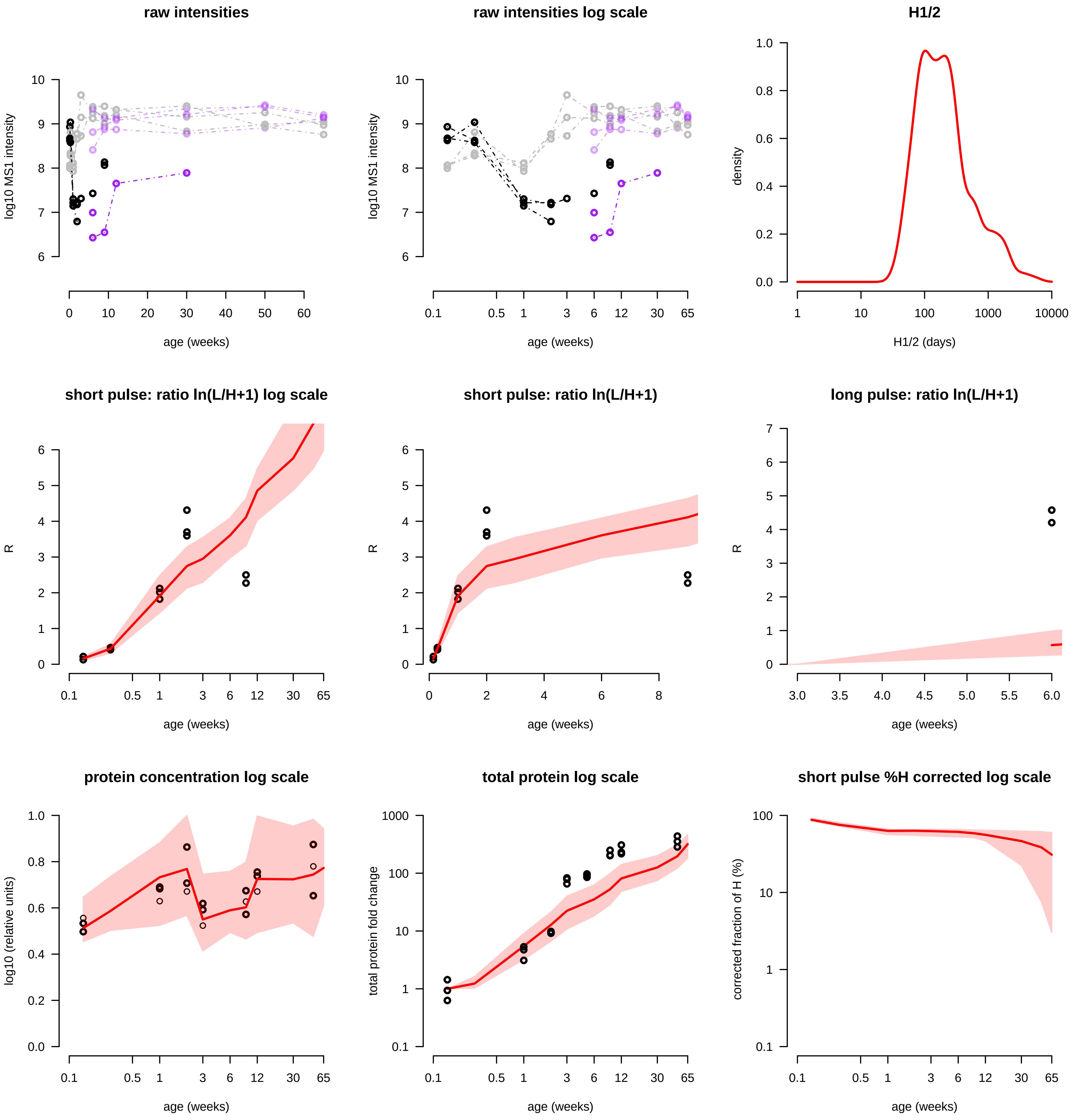

Q9EP71

>sp|Q9EP71|RAI14_MOUSE

Ankycorbin

GN=Rai14

Med H1/2: 143.1 days

Q5 H1/2: 93.2 days

Q95 H1/2: 242.8 days

reliable model fit: yes

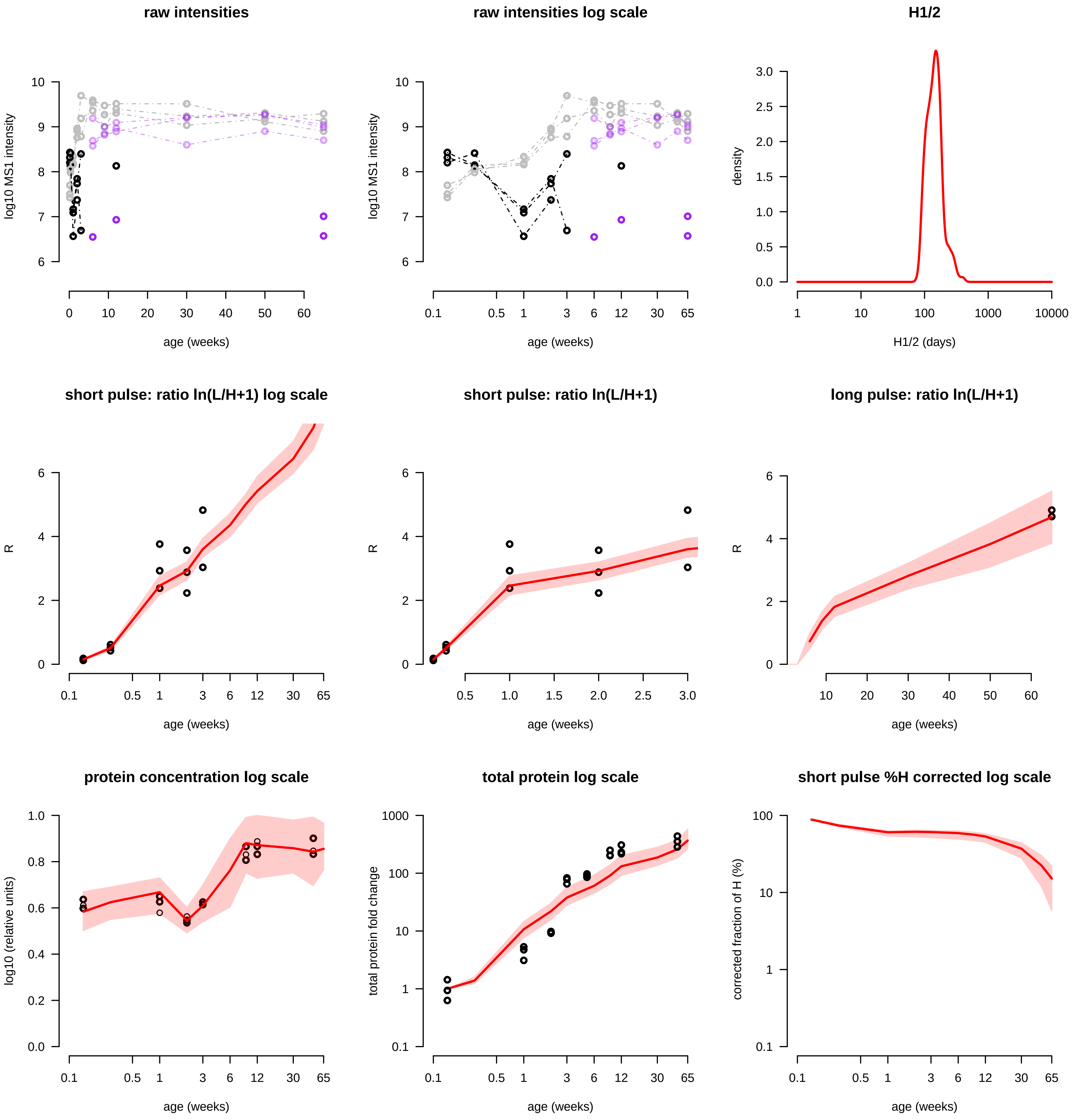

Q8VHX6;Q8VHX6−2

>sp|Q8VHX6|FLNC_MOUSE

Filamin−C

GN=Flnc

Med H1/2: 6.1 days

Q5 H1/2: 5 days

Q95 H1/2: 7.4 days

reliable model fit: yes

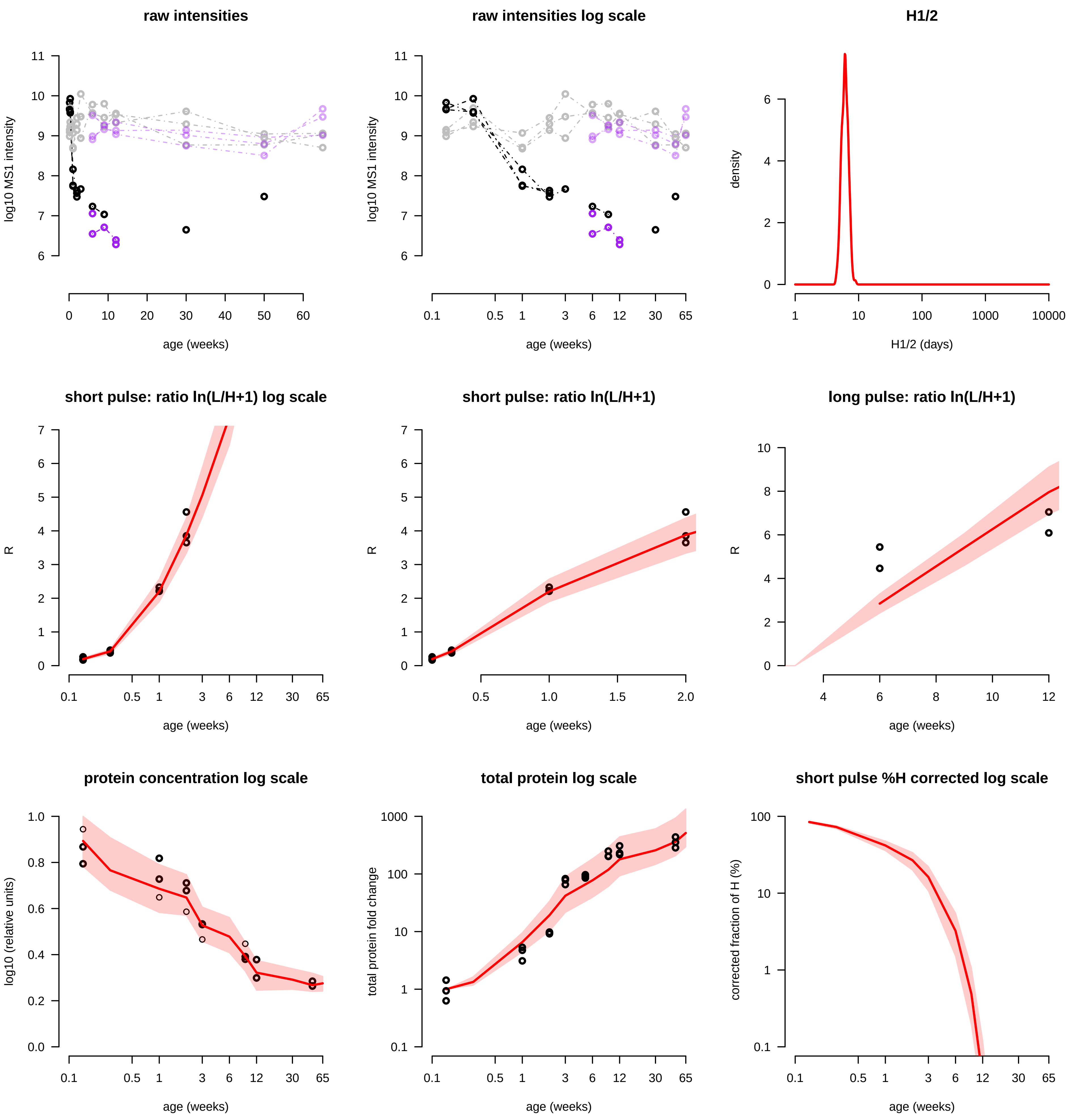

Q9D1Q6

>sp|Q9D1Q6|ERP44_MOUSE

Endoplasmic reticulum resident protein 44

GN=Erp44

Med H1/2: 4.9 days

Q5 H1/2: 4.2 days

Q95 H1/2: 5.8 days

reliable model fit: yes

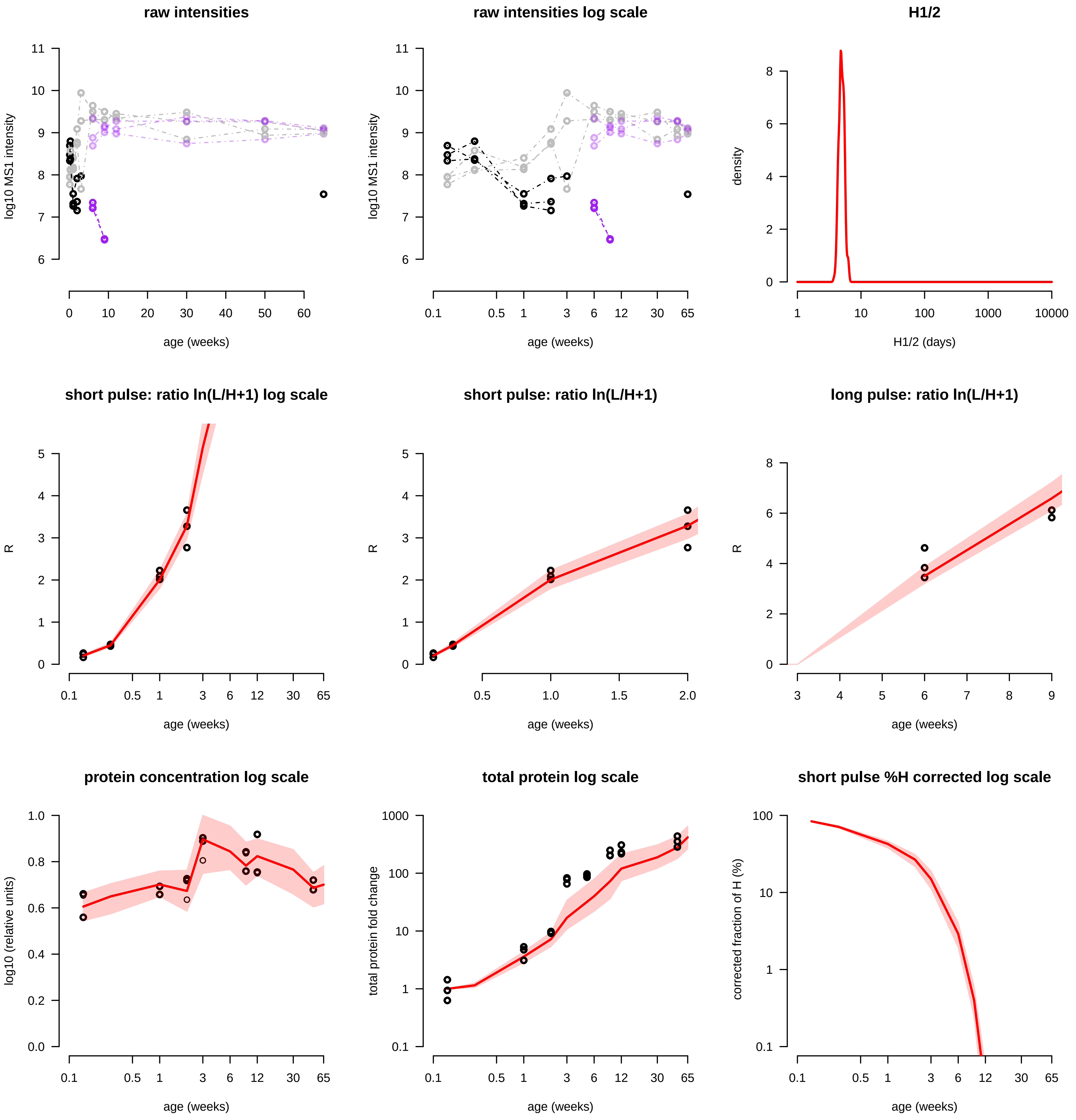

Q8BML9

>sp|Q8BML9|SYQ_MOUSE

Glutamine−−tRNA ligase

GN=Qars1

Med H1/2: 23 days

Q5 H1/2: 11.4 days

Q95 H1/2: 93.3 days

reliable model fit: yes

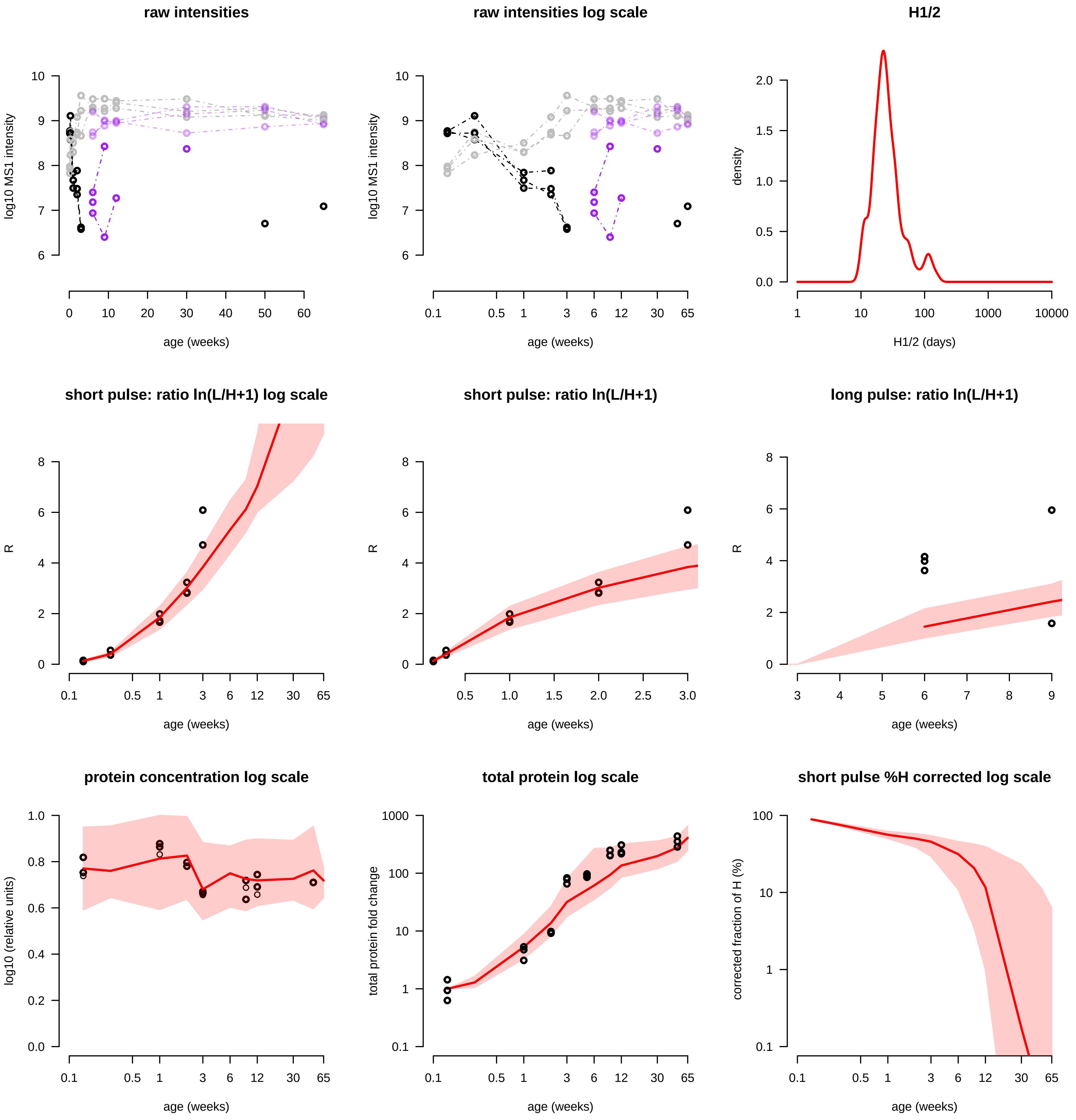

Q9DAR7

>sp|Q9DAR7|DCPS_MOUSE

m7GpppX diphosphatase

GN=Dcps

Med H1/2: 6.7 days

Q5 H1/2: 5.4 days

Q95 H1/2: 8.1 days

reliable model fit: yes

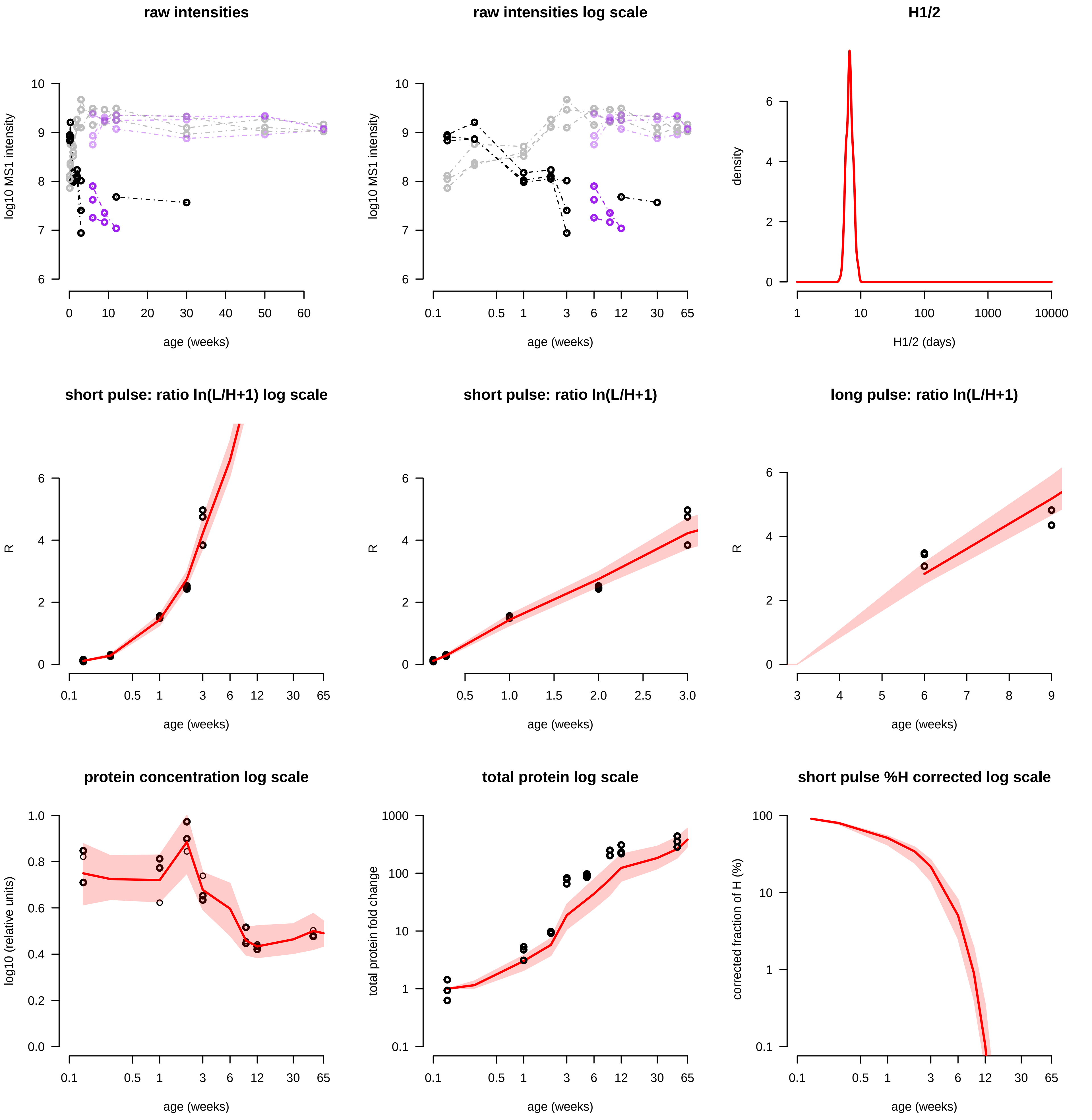

Q64105

>sp|Q64105|SPRE_MOUSE

Sepiapterin reductase

GN=Spr

Med H1/2: 10.1 days

Q5 H1/2: 7.5 days

Q95 H1/2: 13.8 days

reliable model fit: yes

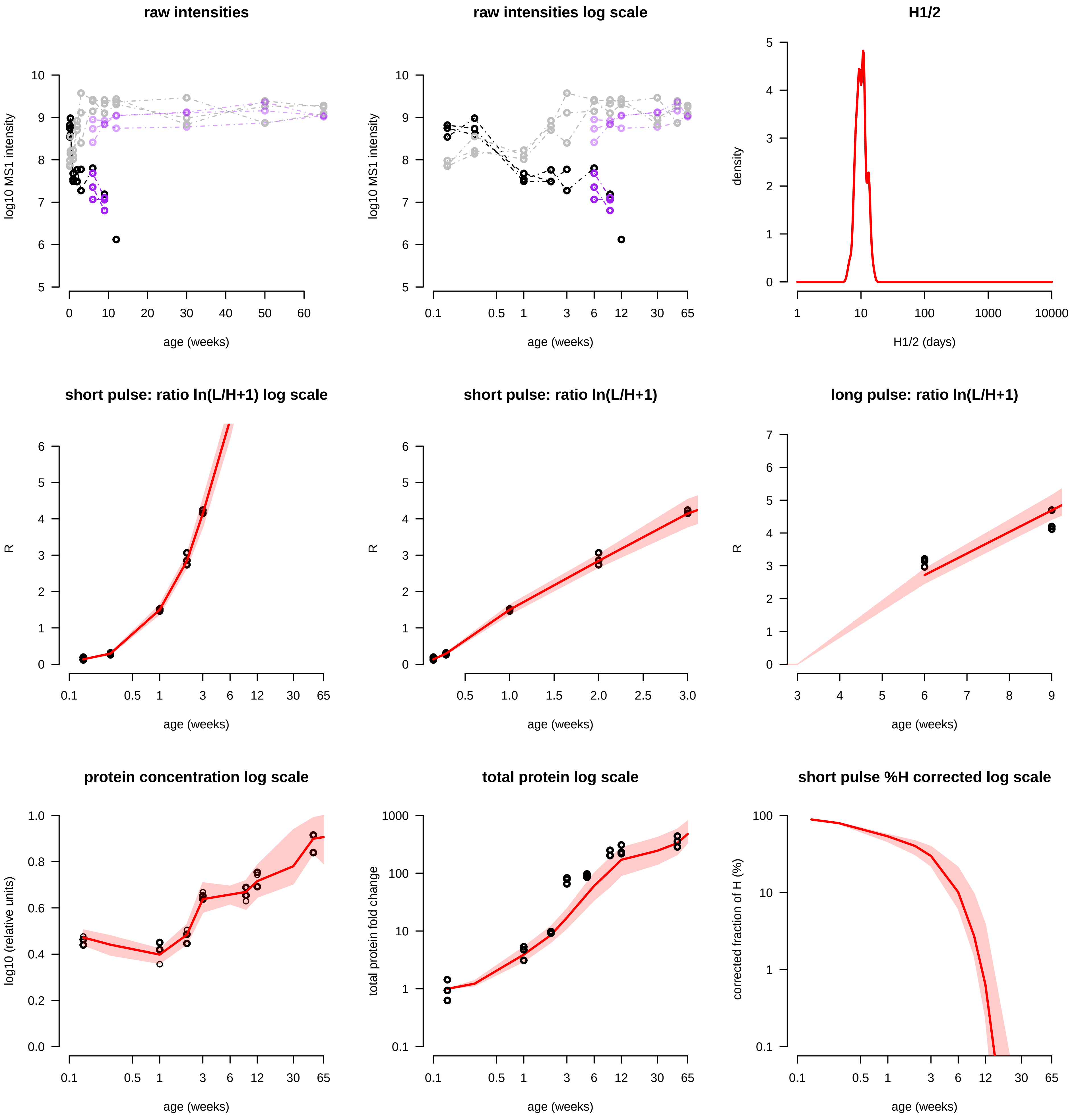

Q7TSV4

>sp|Q7TSV4|PGM2_MOUSE

Phosphoglucomutase−2

GN=Pgm2

Med H1/2: 9.8 days

Q5 H1/2: 6.8 days

Q95 H1/2: 15.7 days

reliable model fit: yes

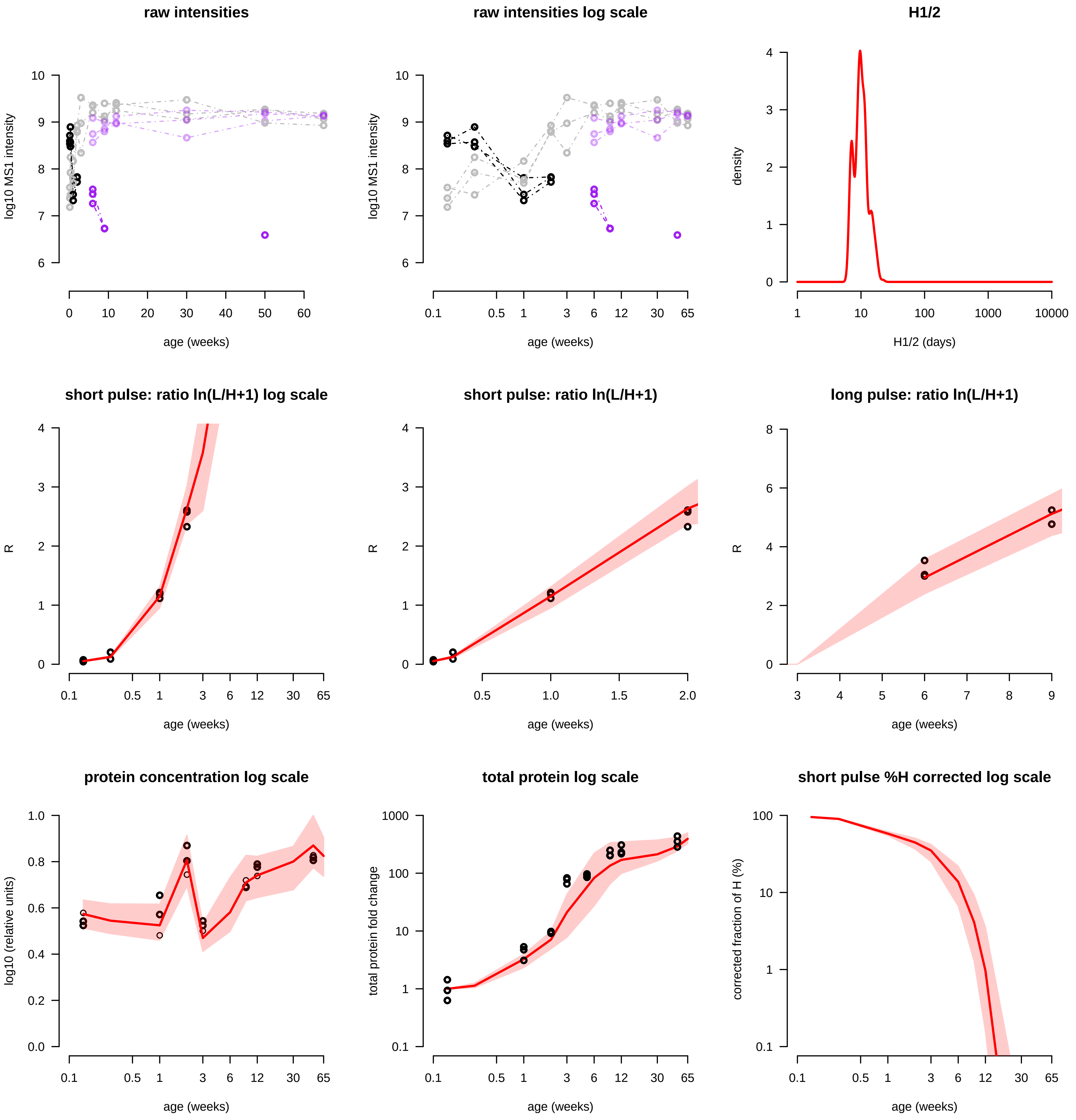

Q8R1Q8

>sp|Q8R1Q8|DC1L1_MOUSE

Cytoplasmic dynein 1 light intermediate chain 1

GN=Dync1li1

Med H1/2: 4.4 days

Q5 H1/2: 3.7 days

Q95 H1/2: 5.3 days

reliable model fit: yes

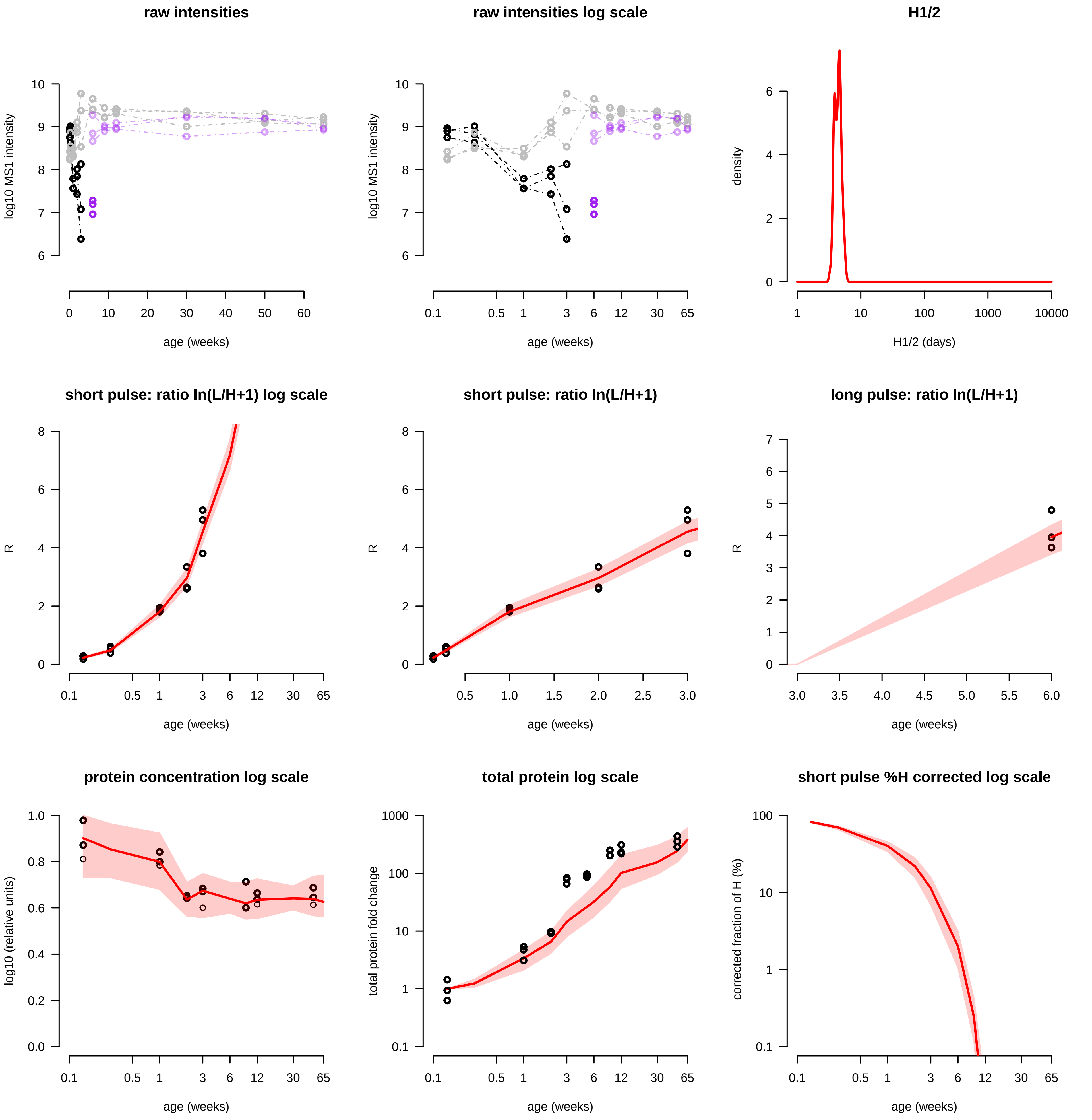

O70310

>sp|O70310|NMT1_MOUSE

Glycylpeptide N−tetradecanoyltransferase 1

GN=Nmt1

Med H1/2: 89 days

Q5 H1/2: 30.4 days

Q95 H1/2: 813 days

reliable model fit: yes

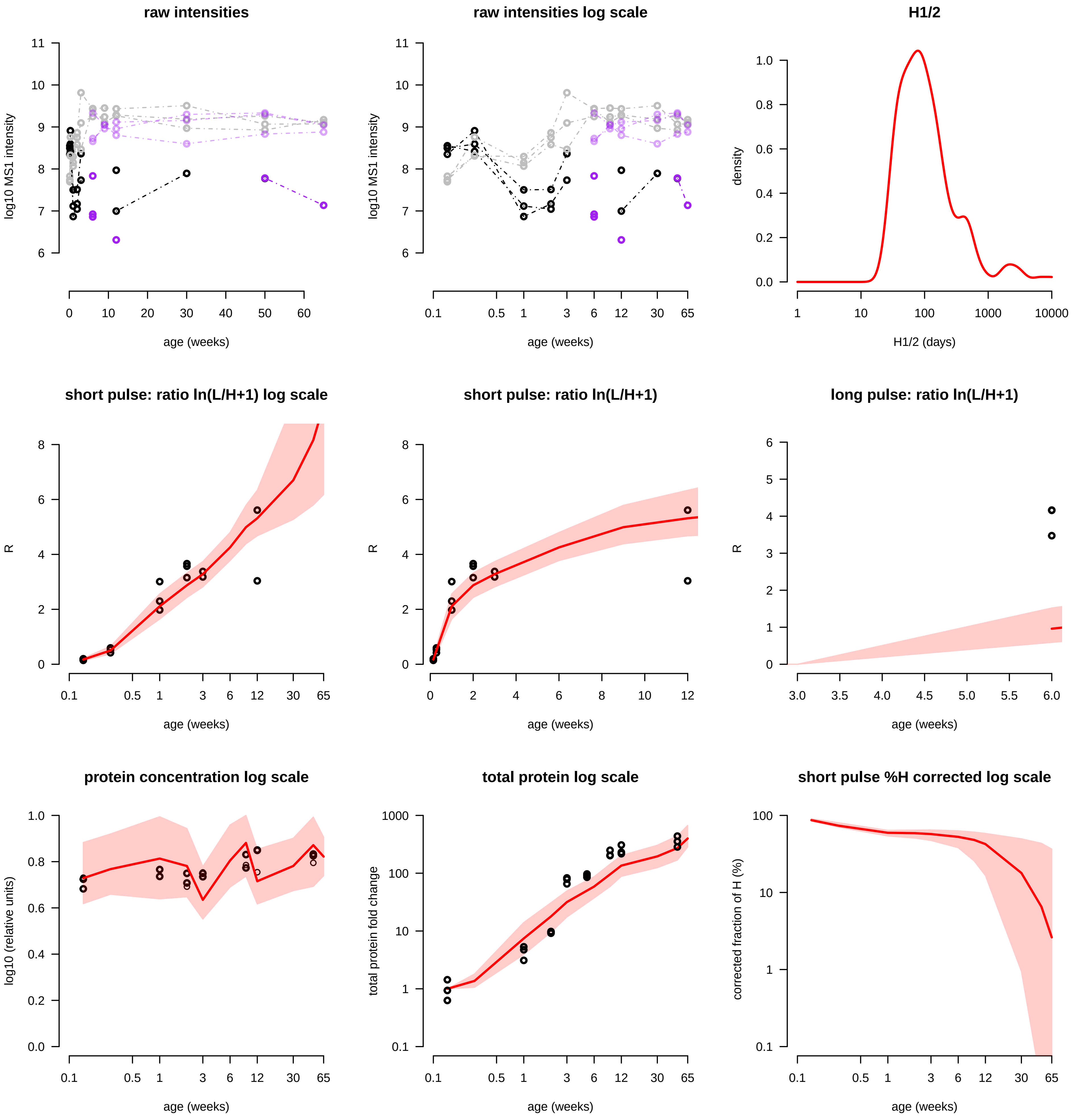

Q8CHT0

>sp|Q8CHT0|AL4A1_MOUSE

Delta−1−pyrroline−5−carboxylate dehydrogenase, mitochondrial

GN=Aldh4a1

Med H1/2: 5 days

Q5 H1/2: 3.6 days

Q95 H1/2: 8.1 days

reliable model fit: yes

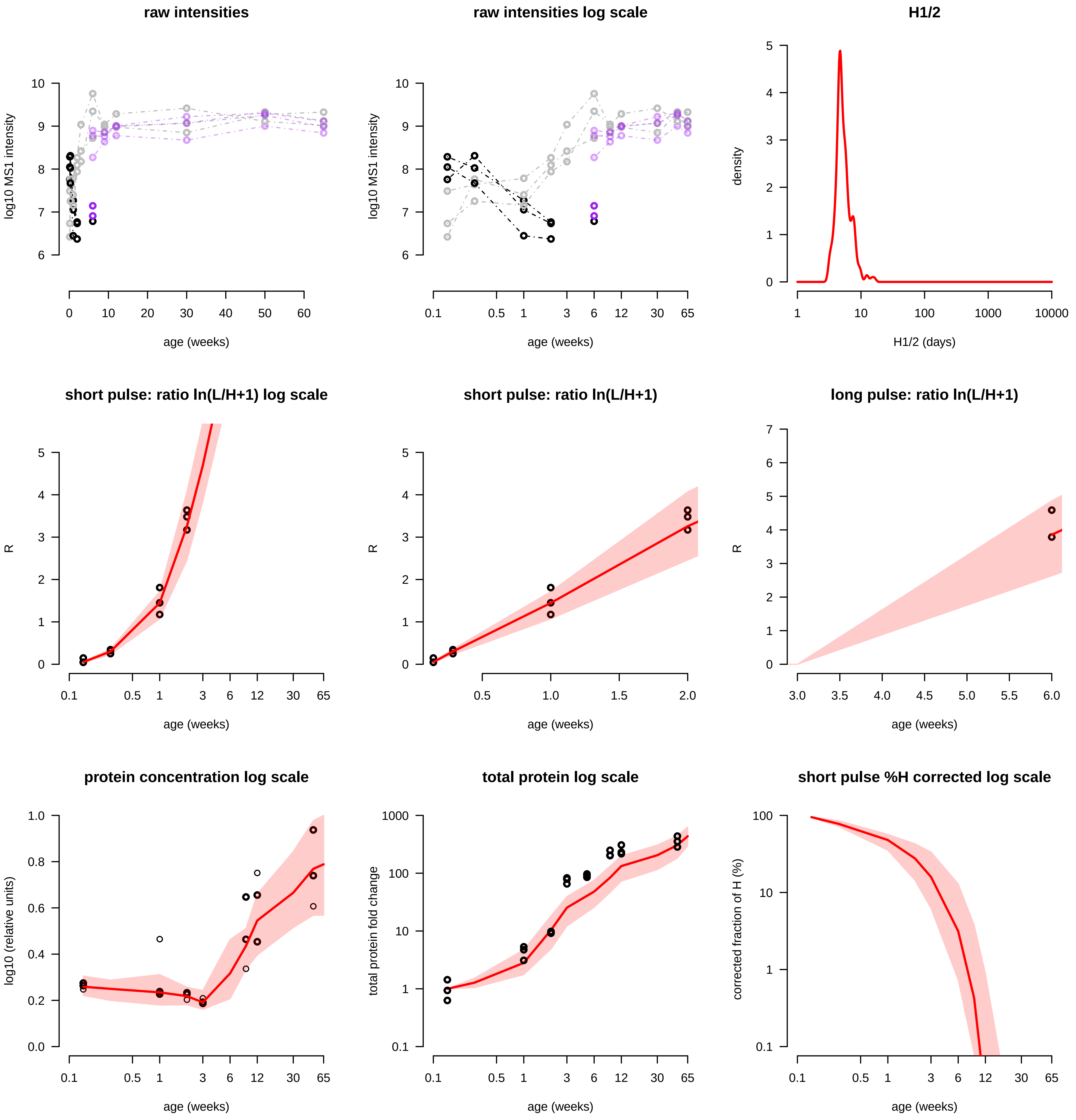

Q9CQM9

>sp|Q9CQM9|GLRX3_MOUSE

Glutaredoxin−3

GN=Glrx3

Med H1/2: 11.7 days

Q5 H1/2: 9.2 days

Q95 H1/2: 17.8 days

reliable model fit: yes

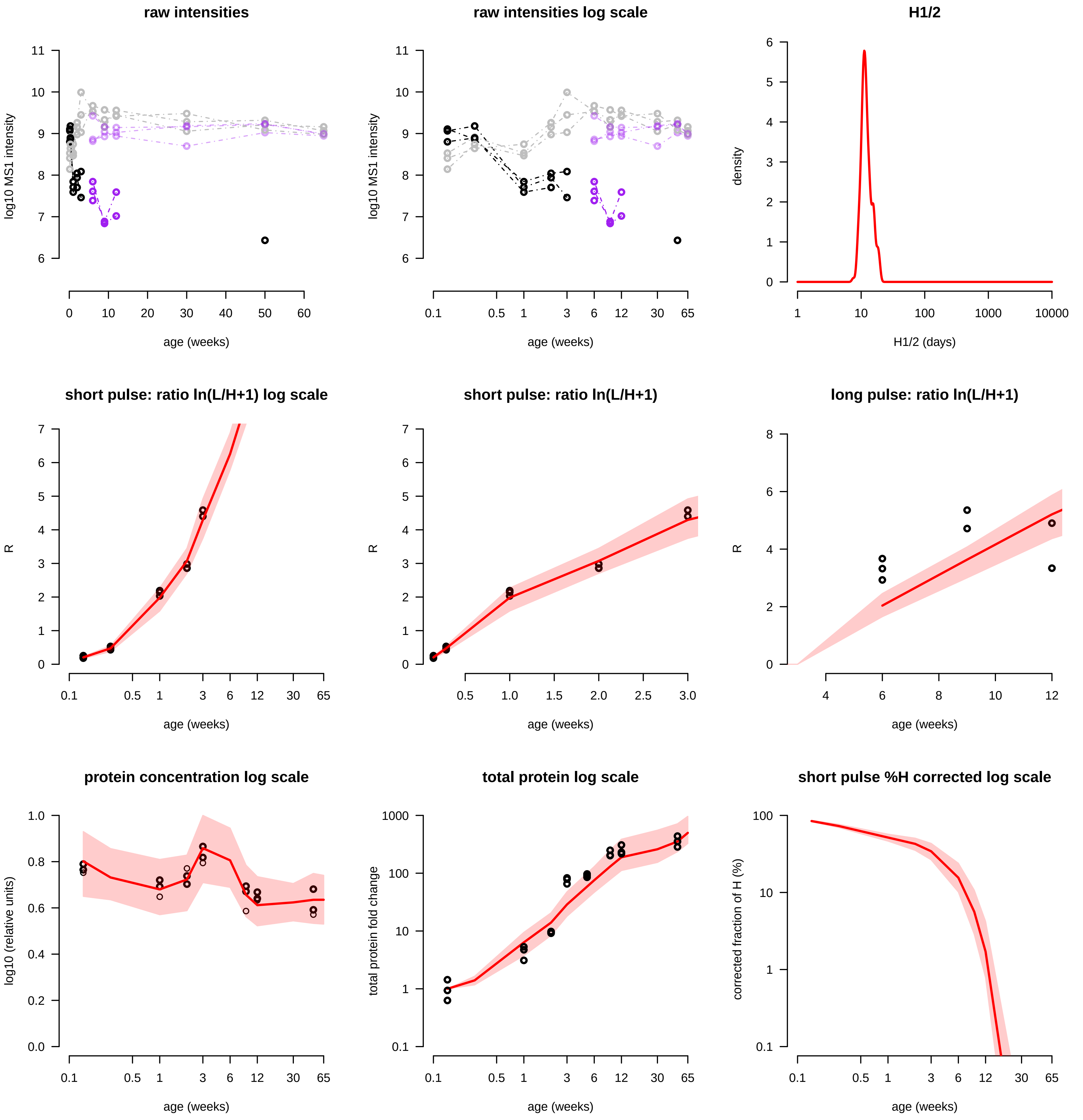

Q8CI59−2

>sp|Q8CI59−2|STEA3_MOUSE

Isoform 2 of Metalloreductase STEAP3

GN=Steap3

Med H1/2: 3.6 days

Q5 H1/2: 2.6 days

Q95 H1/2: 7.9 days

reliable model fit: yes

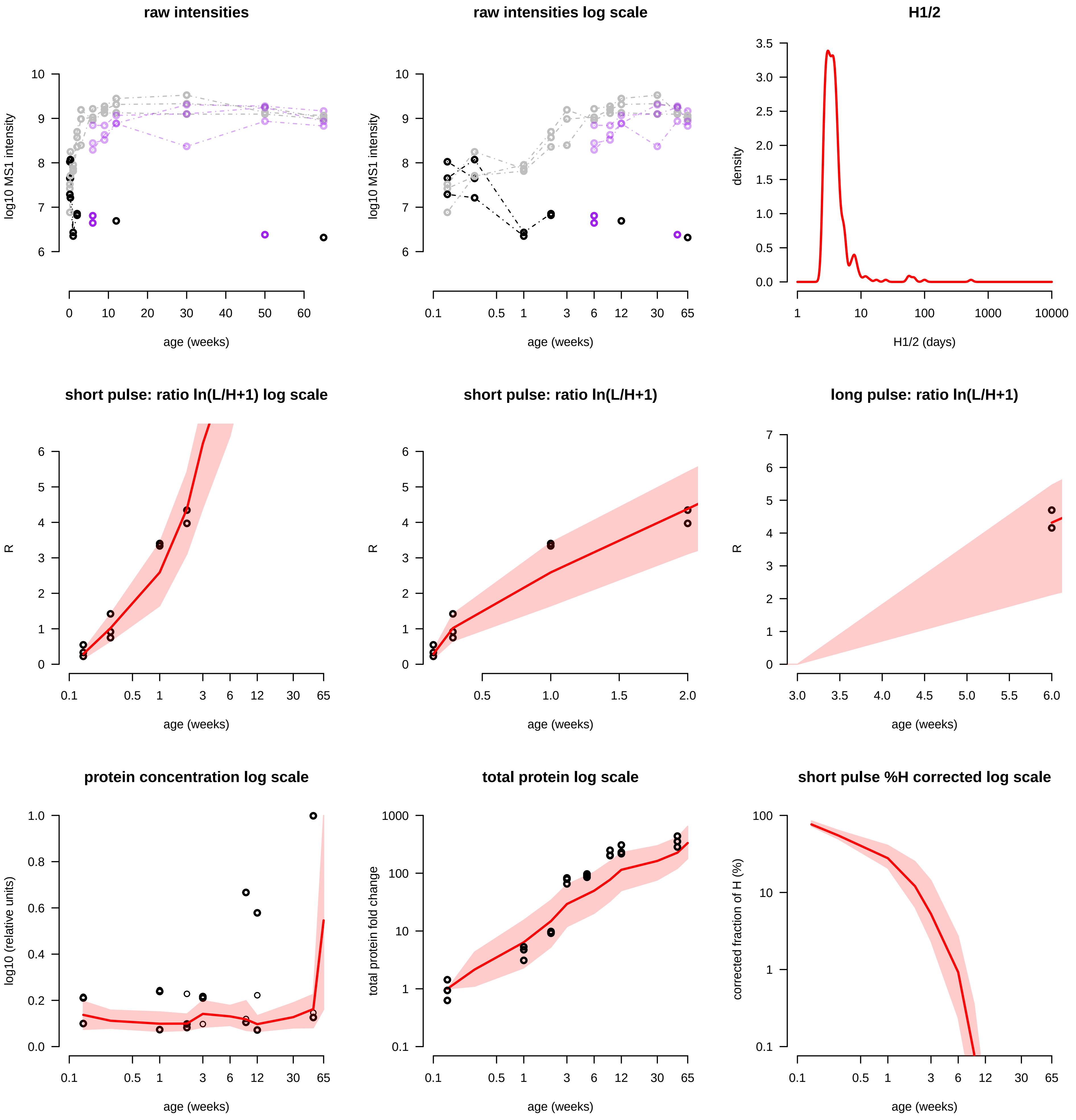

Q9WTM5

>sp|Q9WTM5|RUVB2_MOUSE

RuvB−like 2

GN=Ruvbl2

Med H1/2: 6 days

Q5 H1/2: 5 days

Q95 H1/2: 7.8 days

reliable model fit: yes

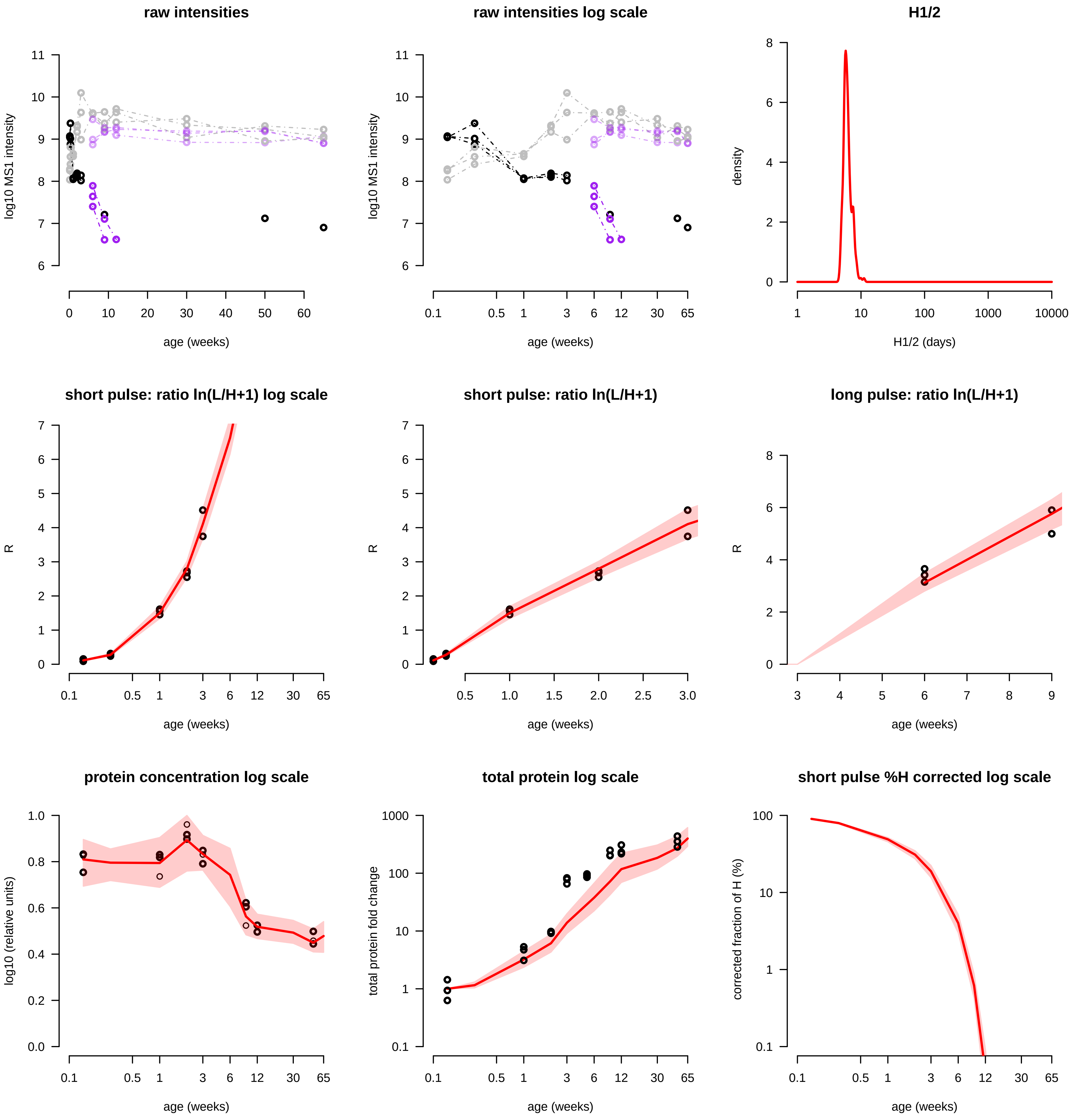

P62264

>sp|P62264|RS14_MOUSE

40S ribosomal protein S14

GN=Rps14

Med H1/2: 11.7 days

Q5 H1/2: 9.1 days

Q95 H1/2: 16.3 days

reliable model fit: yes

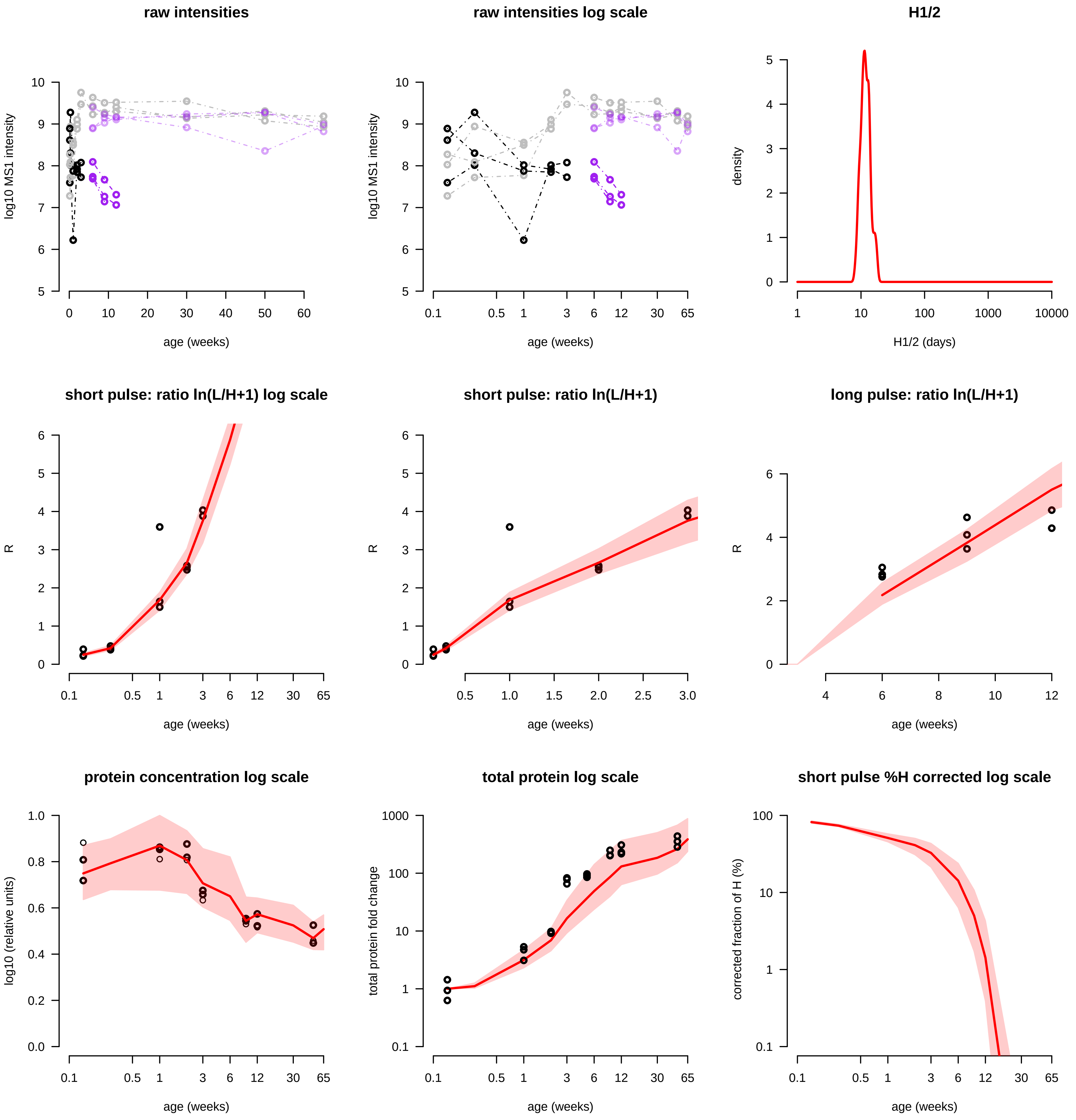

P45591

>sp|P45591|COF2_MOUSE

Cofilin−2

GN=Cfl2

Med H1/2: 19.3 days

Q5 H1/2: 11.8 days

Q95 H1/2: 41.5 days

reliable model fit: yes

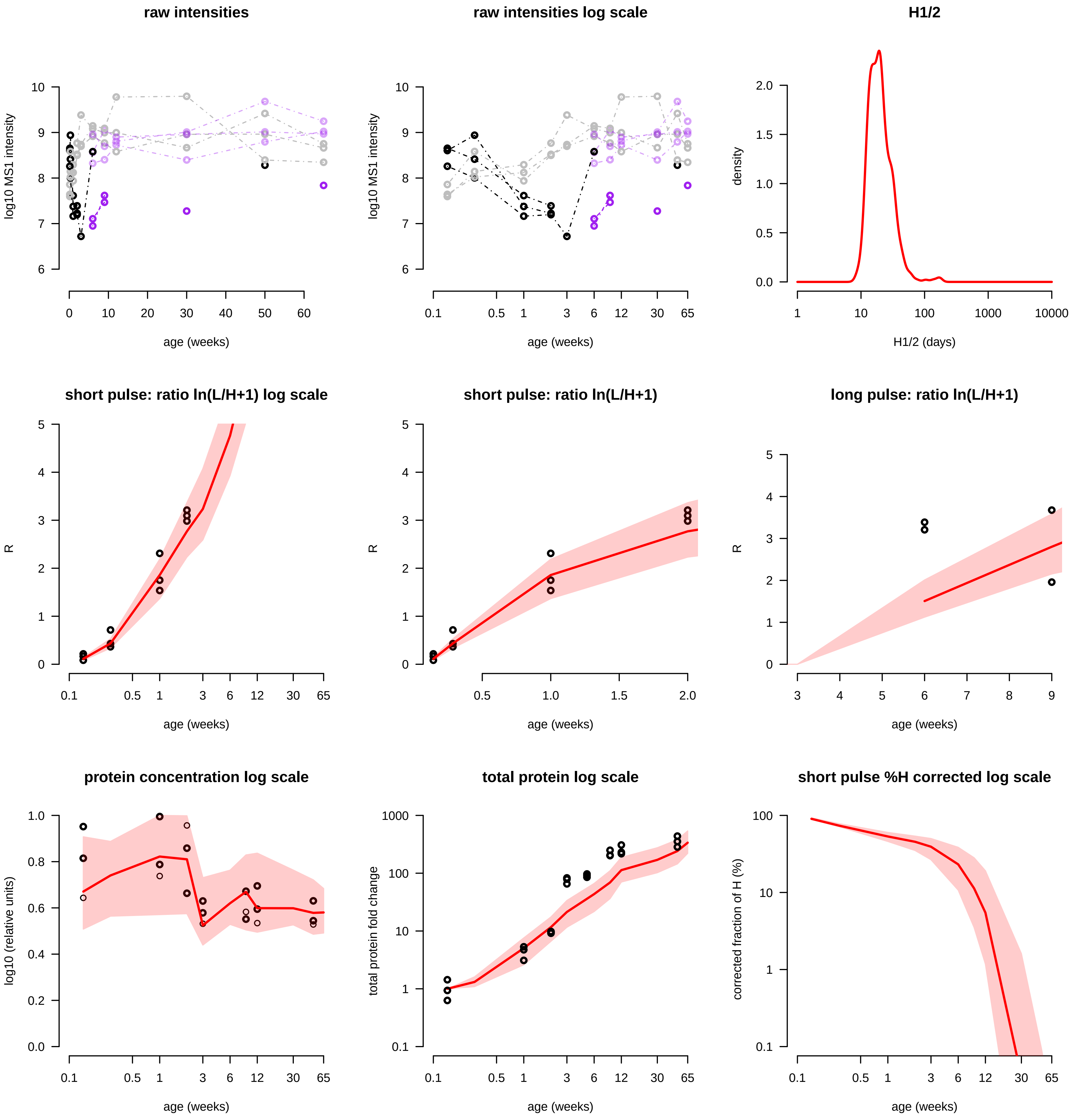

P97461

>sp|P97461|RS5_MOUSE

40S ribosomal protein S5

GN=Rps5

Med H1/2: 10.2 days

Q5 H1/2: 7.9 days

Q95 H1/2: 14.8 days

reliable model fit: yes

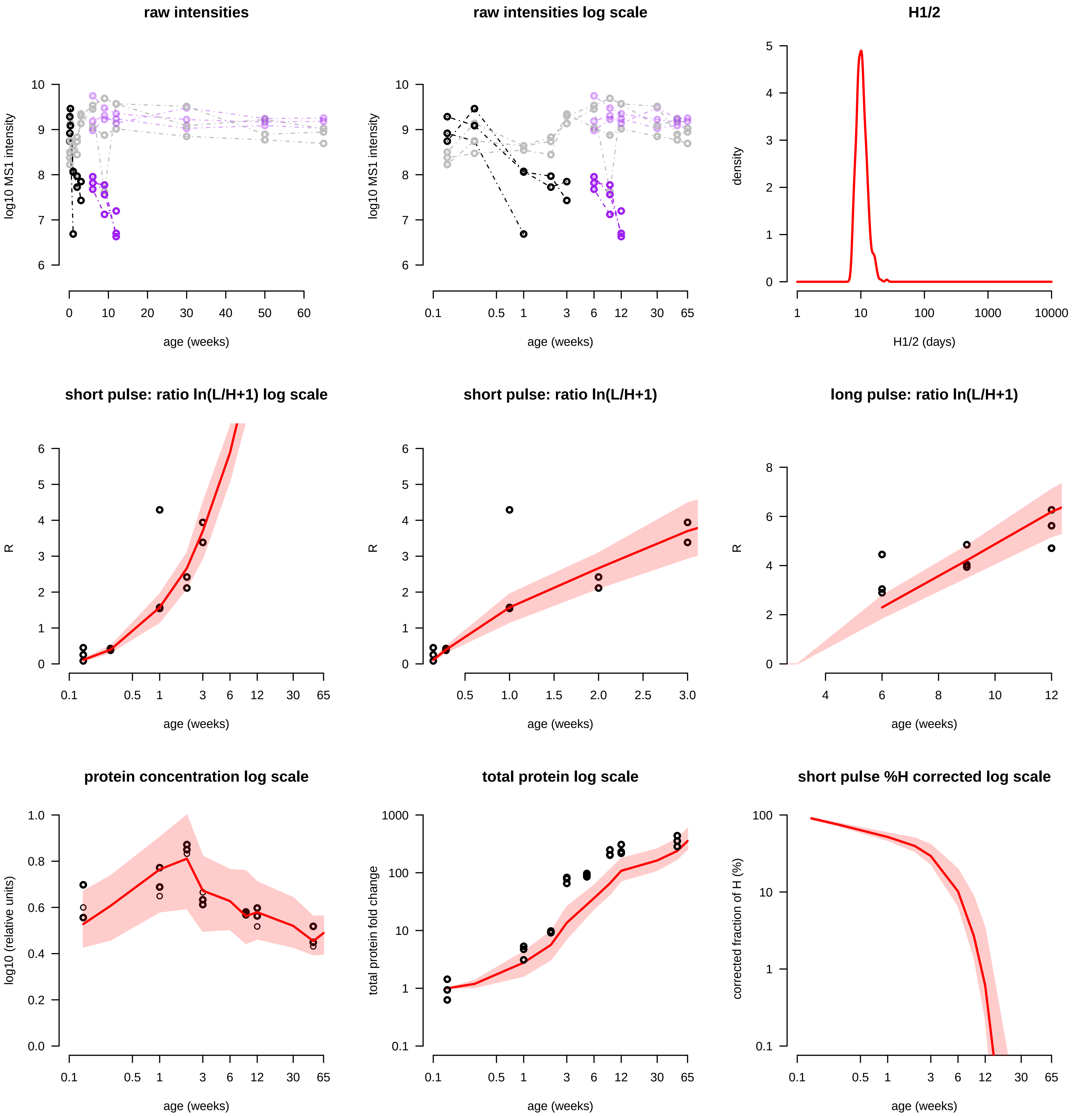

Q9DAW9

>sp|Q9DAW9|CNN3_MOUSE

Calponin−3

GN=Cnn3

Med H1/2: 5.5 days

Q5 H1/2: 4.4 days

Q95 H1/2: 6.9 days

reliable model fit: yes

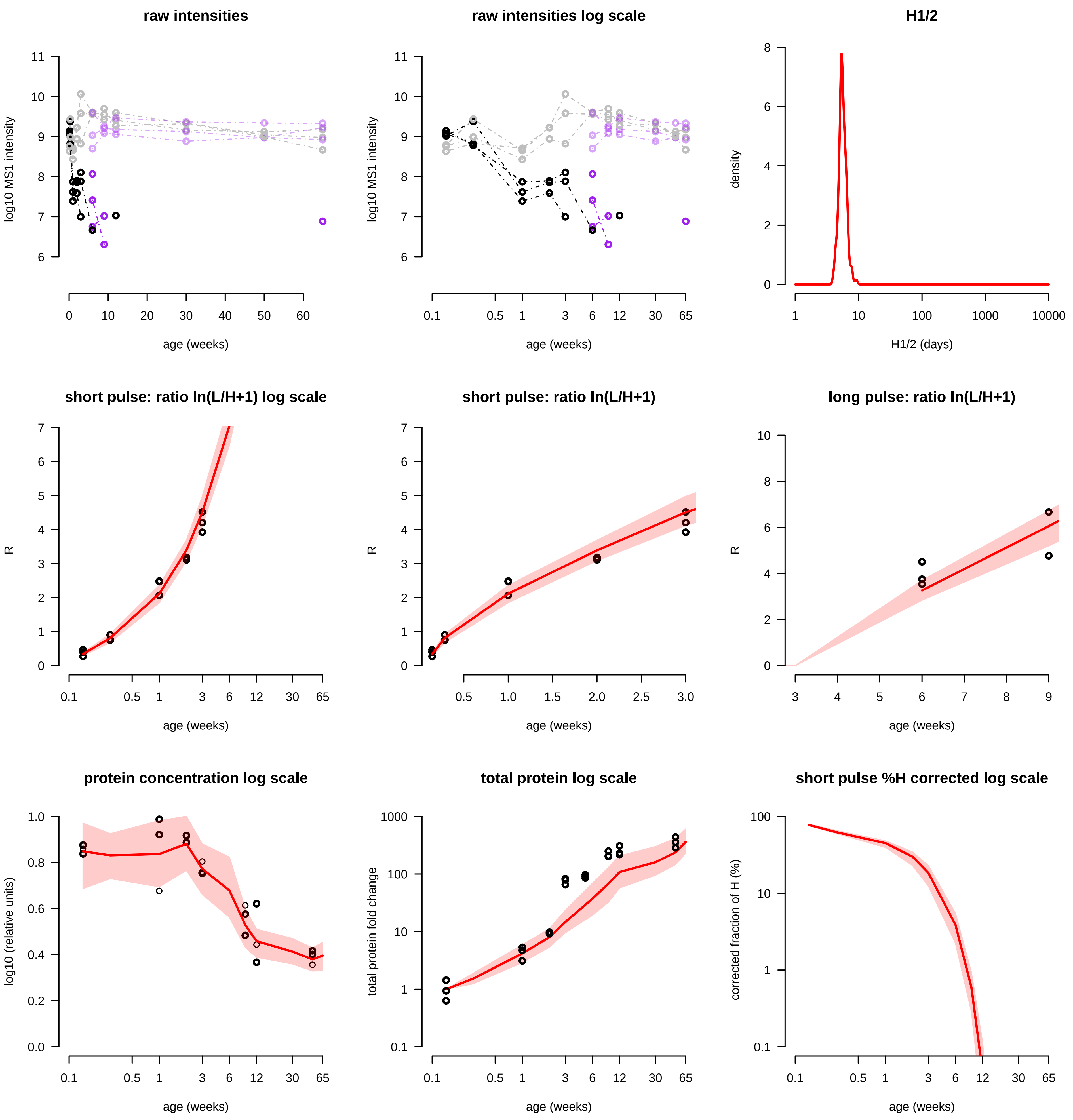

Q8R146−2;Q8R146

>sp|Q8R146−2|APEH_MOUSE

Isoform 2 of Acylamino−acid−releasing enzyme

GN=Apeh

Med H1/2: 4794.6 days

Q5 H1/2: 1135.1 days

Q95 H1/2: 42811.4 days

reliable model fit: no

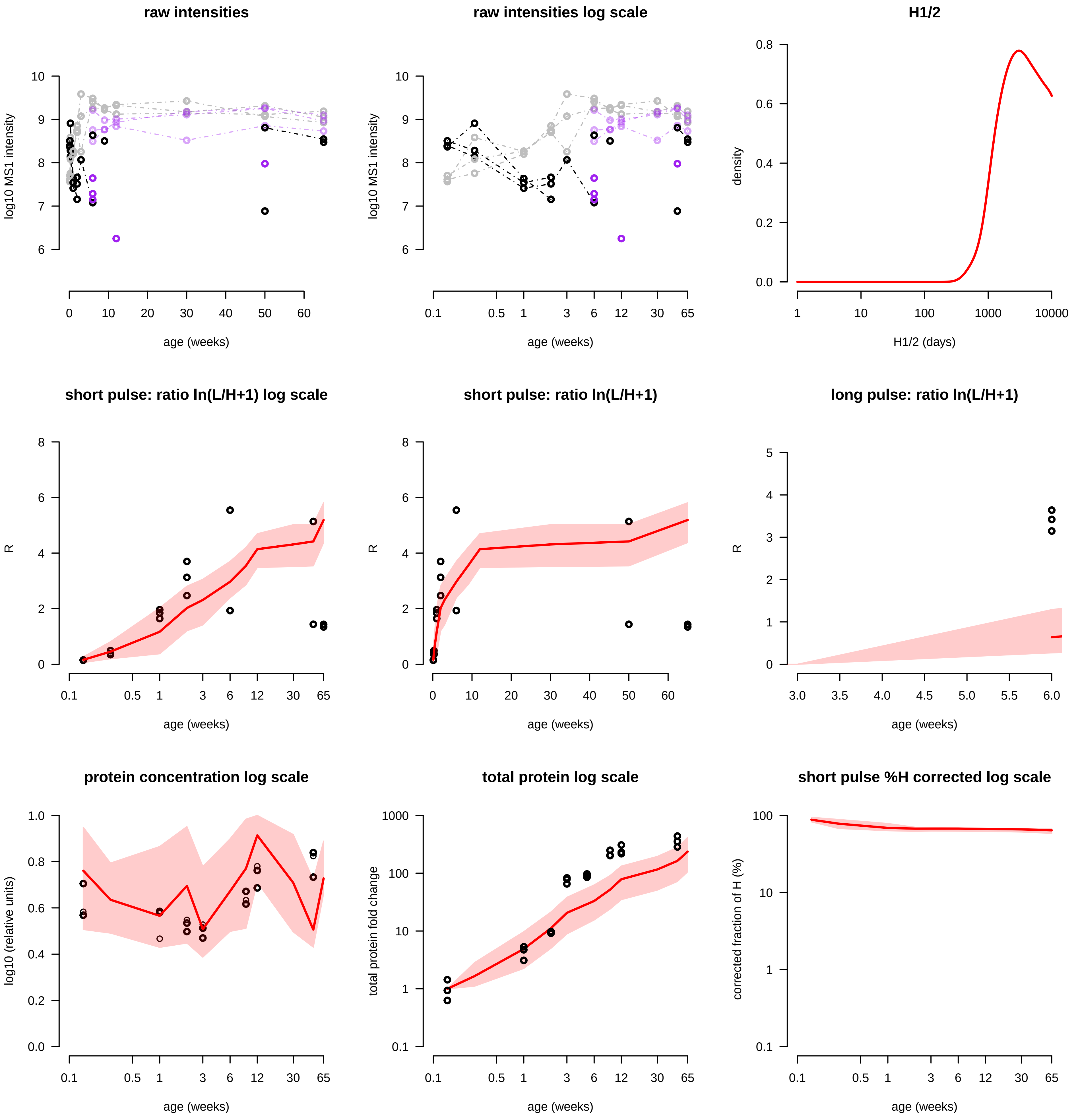

Q9WVE8

>sp|Q9WVE8|PACN2_MOUSE

Protein kinase C and casein kinase substrate in neurons protein 2

GN=Pacsin2

Med H1/2: 3.9 days

Q5 H1/2: 3.1 days

Q95 H1/2: 4.8 days

reliable model fit: yes

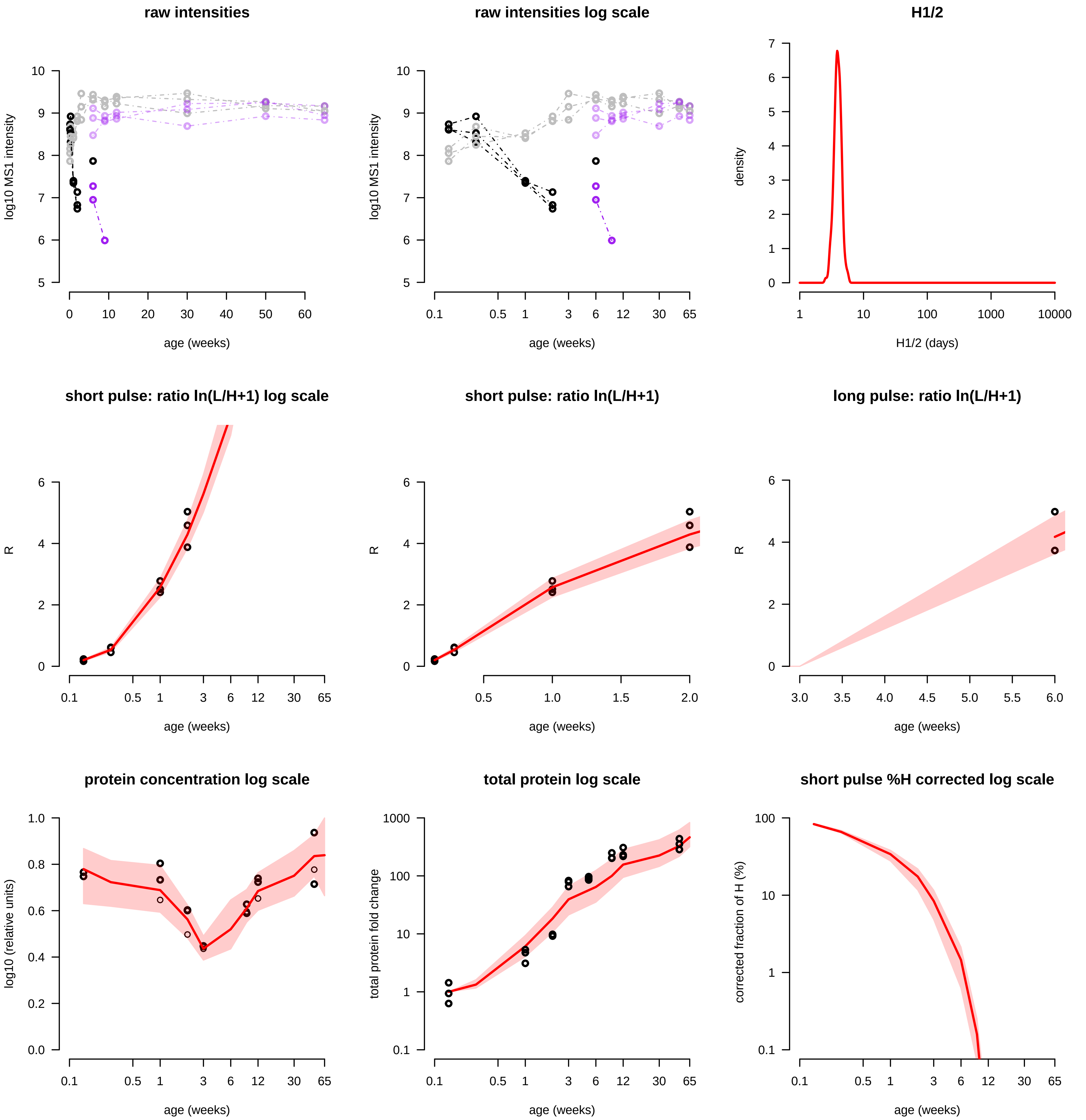

Q9QYJ0

>sp|Q9QYJ0|DNJA2_MOUSE

DnaJ homolog subfamily A member 2

GN=Dnaja2

Med H1/2: 149.1 days

Q5 H1/2: 68.2 days

Q95 H1/2: 930.2 days

reliable model fit: yes

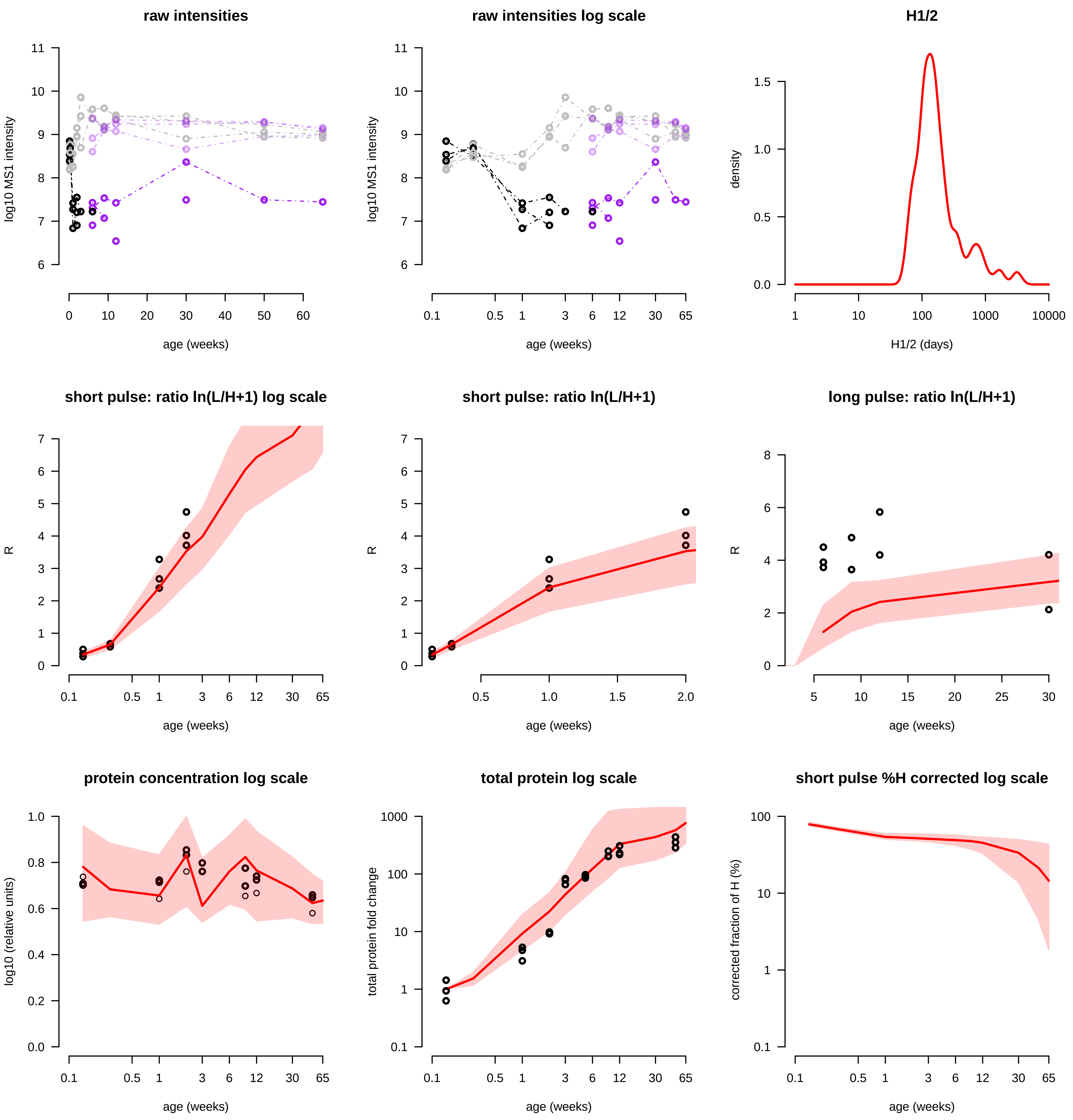

Q920Q6;Q920Q6−3;Q920Q6−2

>sp|Q920Q6|MSI2H_MOUSE

RNA−binding protein Musashi homolog 2

GN=Msi2

Med H1/2: 7.2 days

Q5 H1/2: 5.8 days

Q95 H1/2: 9.3 days

reliable model fit: yes

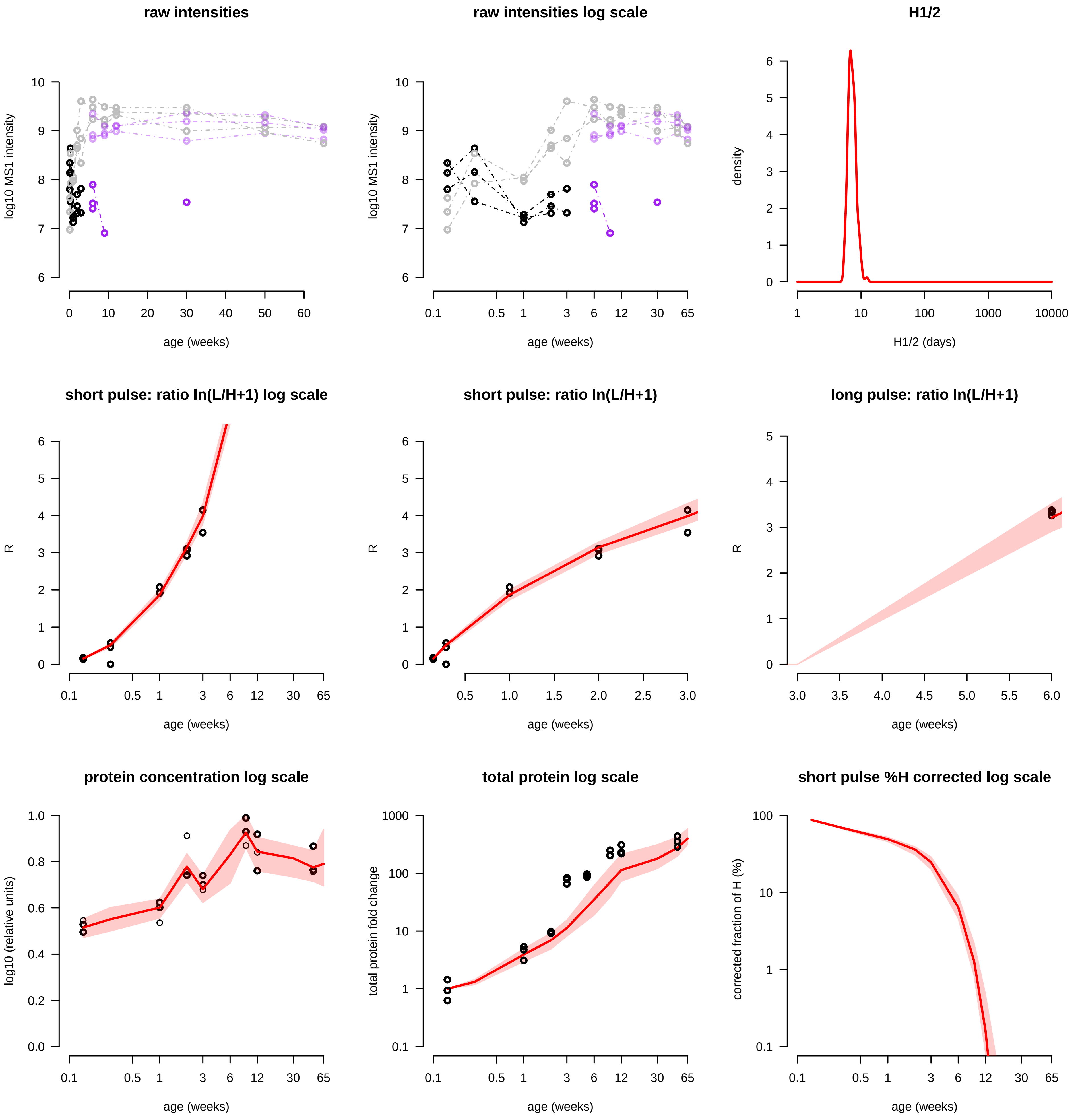

Q3THK7

>sp|Q3THK7|GUAA_MOUSE

GMP synthase [glutamine−hydrolyzing]

GN=Gmps

Med H1/2: 198.1 days

Q5 H1/2: 92.6 days

Q95 H1/2: 846.3 days

reliable model fit: yes

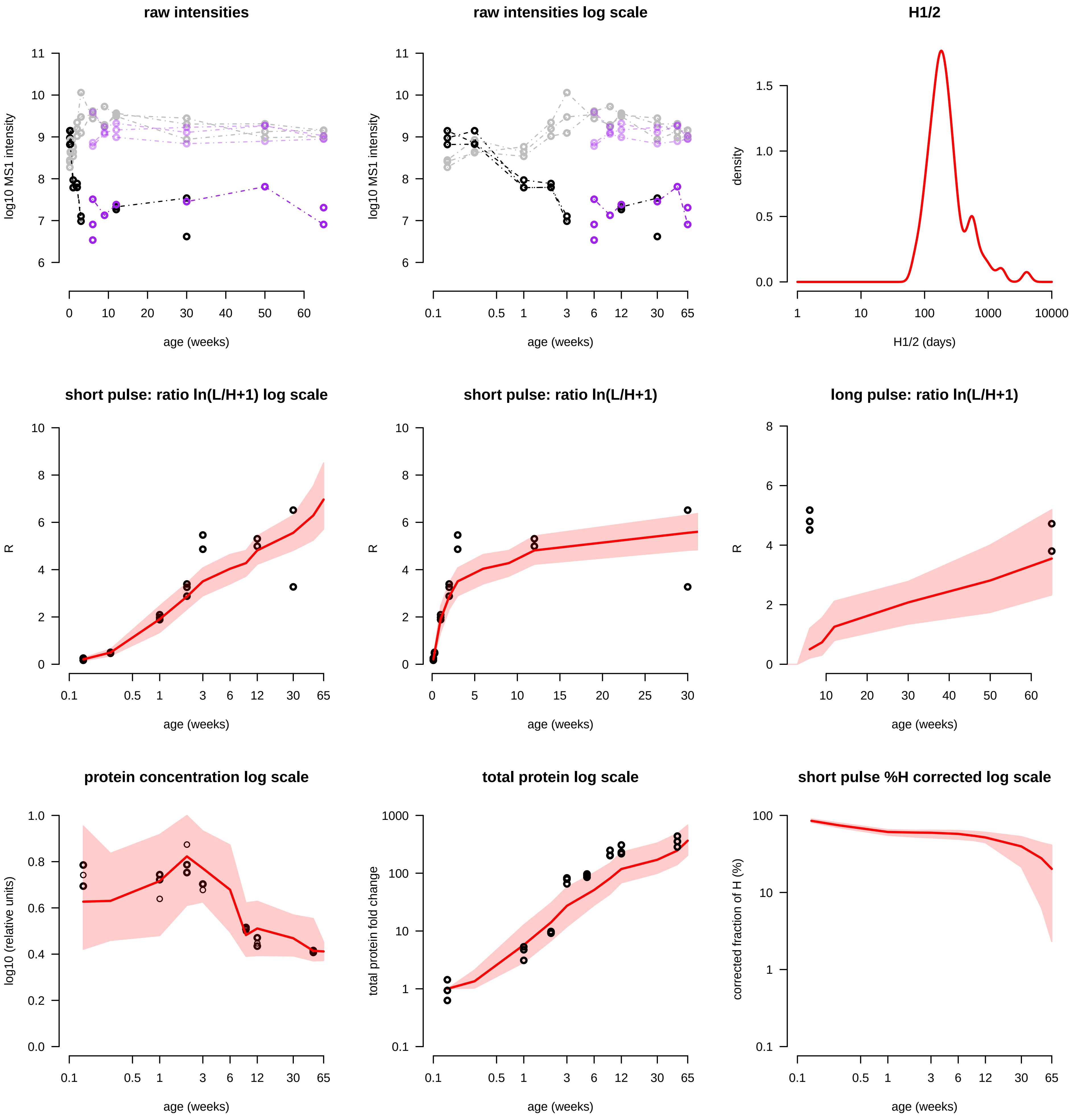

Q9D5T0

>sp|Q9D5T0|ATAD1_MOUSE

Outer mitochondrial transmembrane helix translocase

GN=Atad1

Med H1/2: 5.3 days

Q5 H1/2: 4.3 days

Q95 H1/2: 7 days

reliable model fit: yes

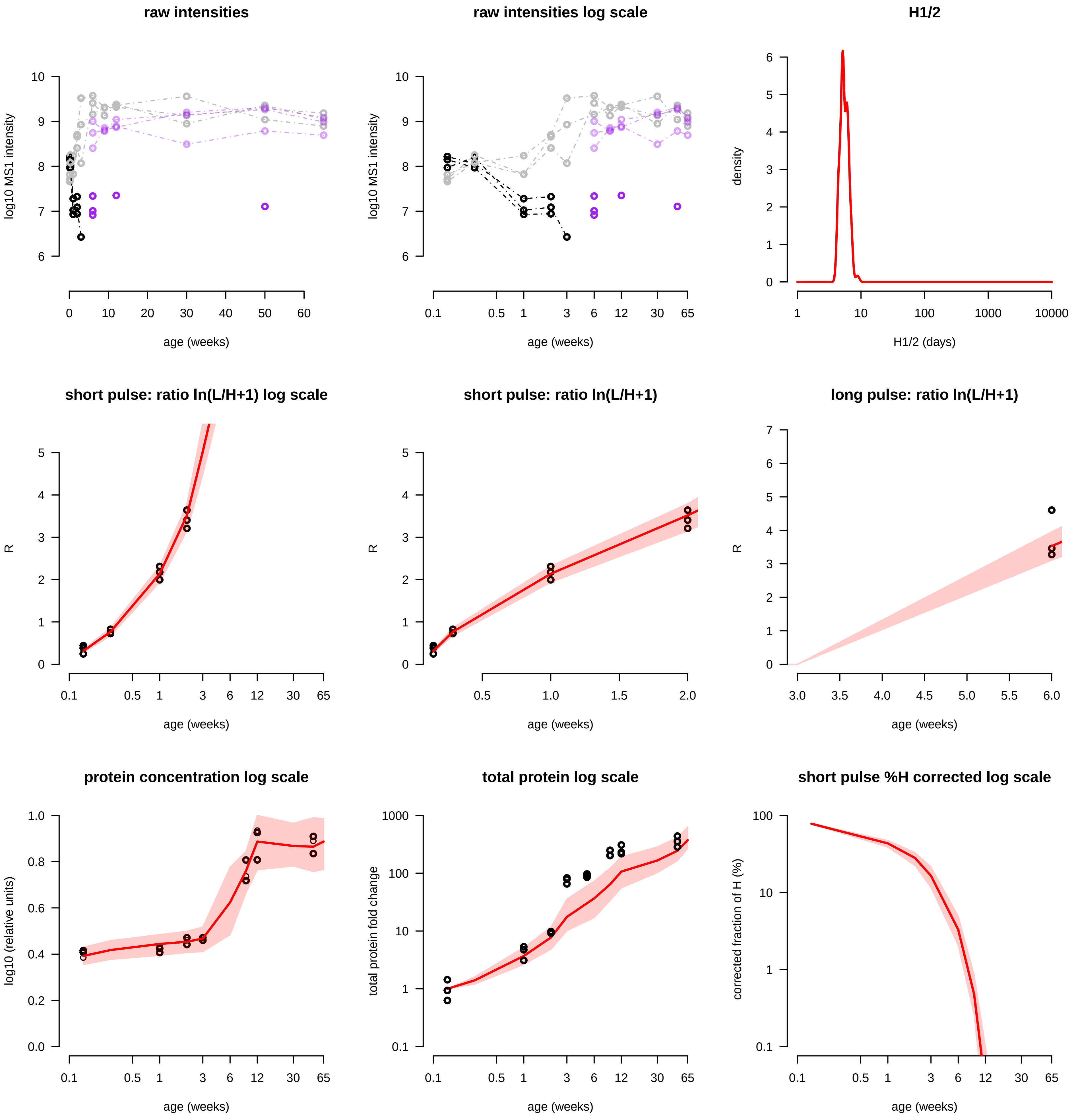

Q99KJ8

>sp|Q99KJ8|DCTN2_MOUSE

Dynactin subunit 2

GN=Dctn2

Med H1/2: 4.4 days

Q5 H1/2: 3.7 days

Q95 H1/2: 5.2 days

reliable model fit: yes

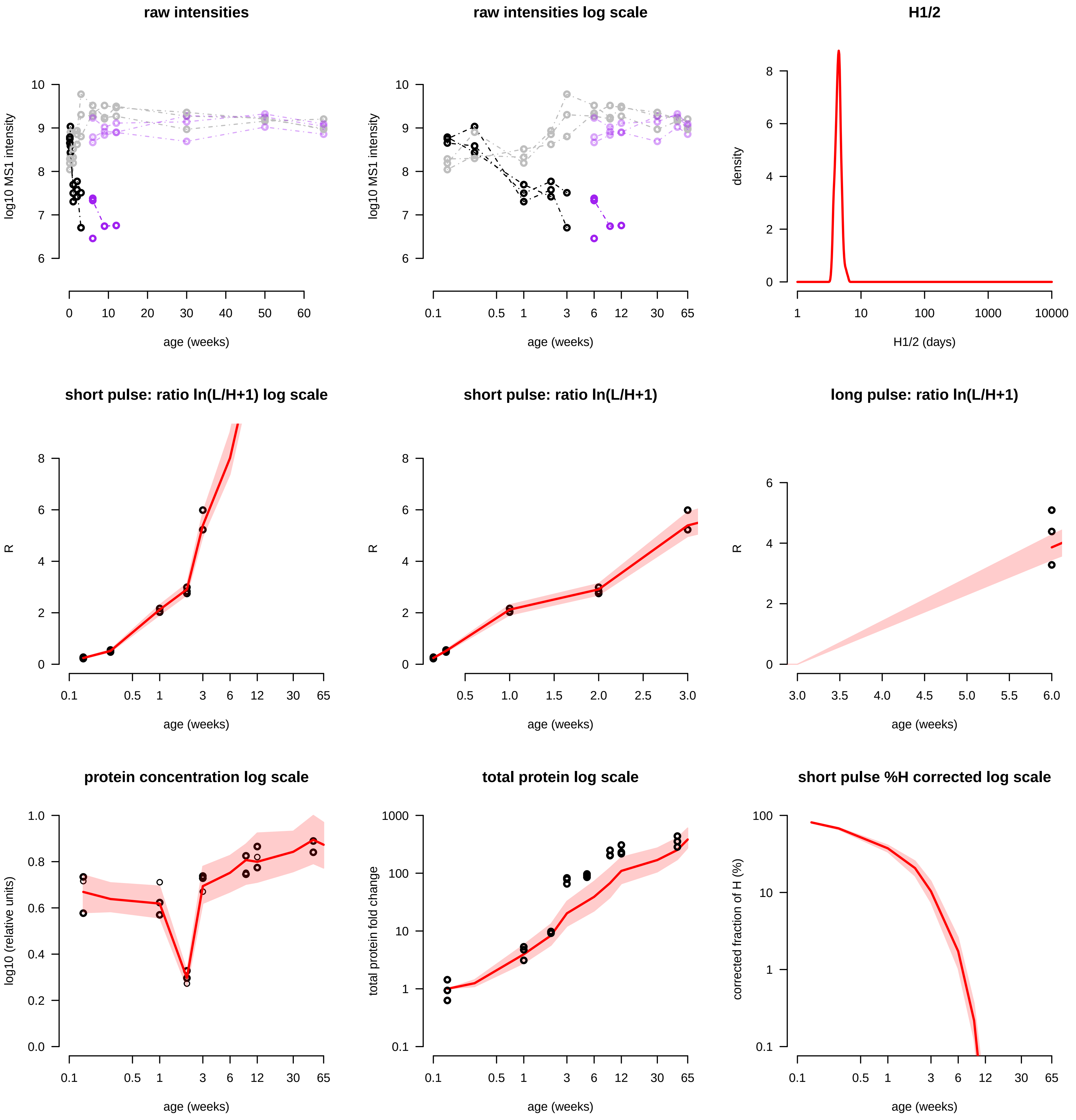

Q6ZQI3

>sp|Q6ZQI3|MLEC_MOUSE

Malectin

GN=Mlec

Med H1/2: 6 days

Q5 H1/2: 4.7 days

Q95 H1/2: 8 days

reliable model fit: yes

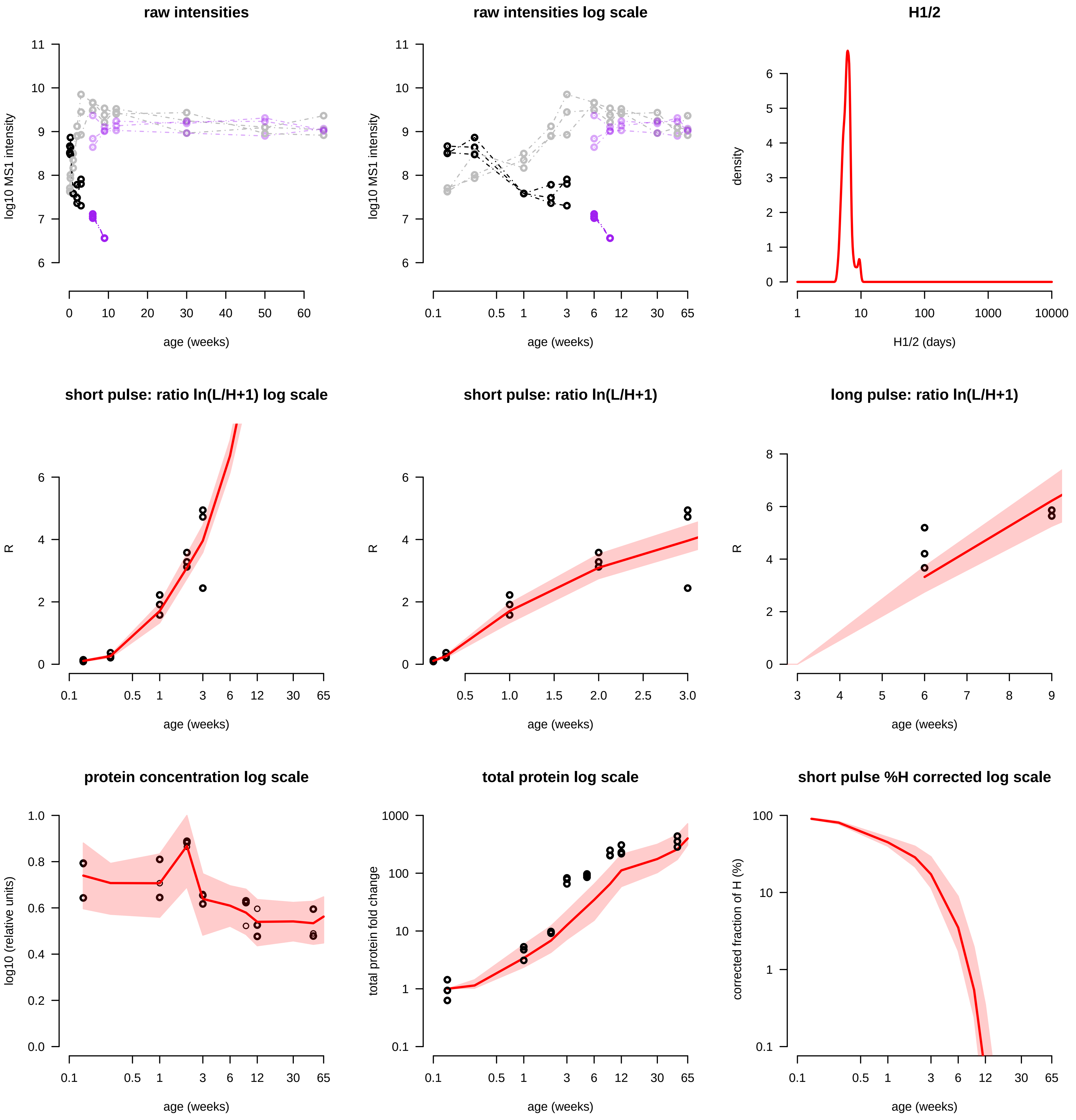

Q9R1T2;Q9R1T2−2

>sp|Q9R1T2|SAE1_MOUSE

SUMO−activating enzyme subunit 1

GN=Sae1

Med H1/2: 4.2 days

Q5 H1/2: 3.5 days

Q95 H1/2: 5.3 days

reliable model fit: yes

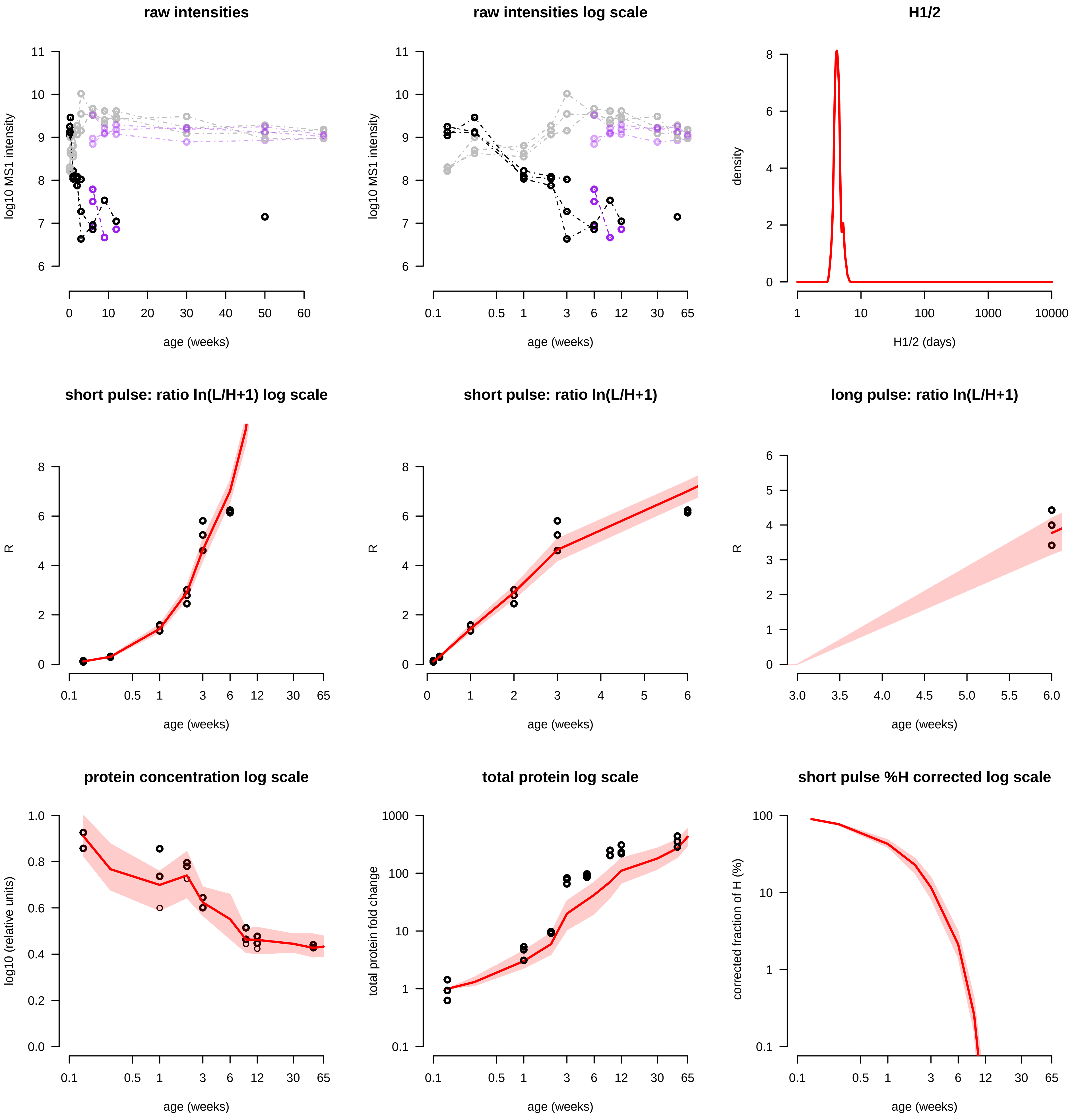

Q9DC61

>sp|Q9DC61|MPPA_MOUSE

Mitochondrial−processing peptidase subunit alpha

GN=Pmpca

Med H1/2: 5 days

Q5 H1/2: 3.6 days

Q95 H1/2: 8.7 days

reliable model fit: yes

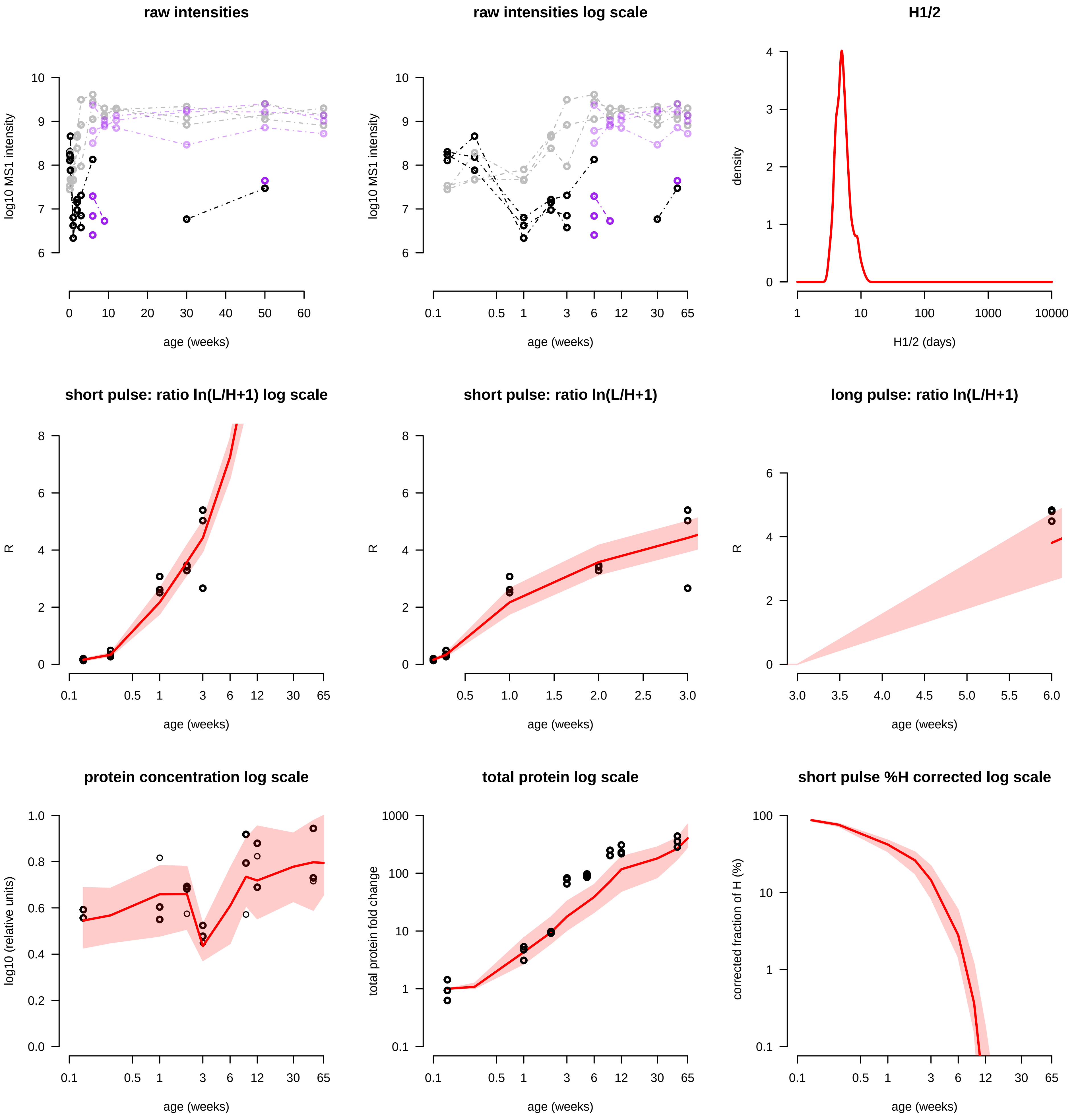

Q00897

>sp|Q00897|A1AT4_MOUSE

Alpha−1−antitrypsin 1−4

GN=Serpina1d

Med H1/2: 9.4 days

Q5 H1/2: 6.9 days

Q95 H1/2: 13.5 days

reliable model fit: yes

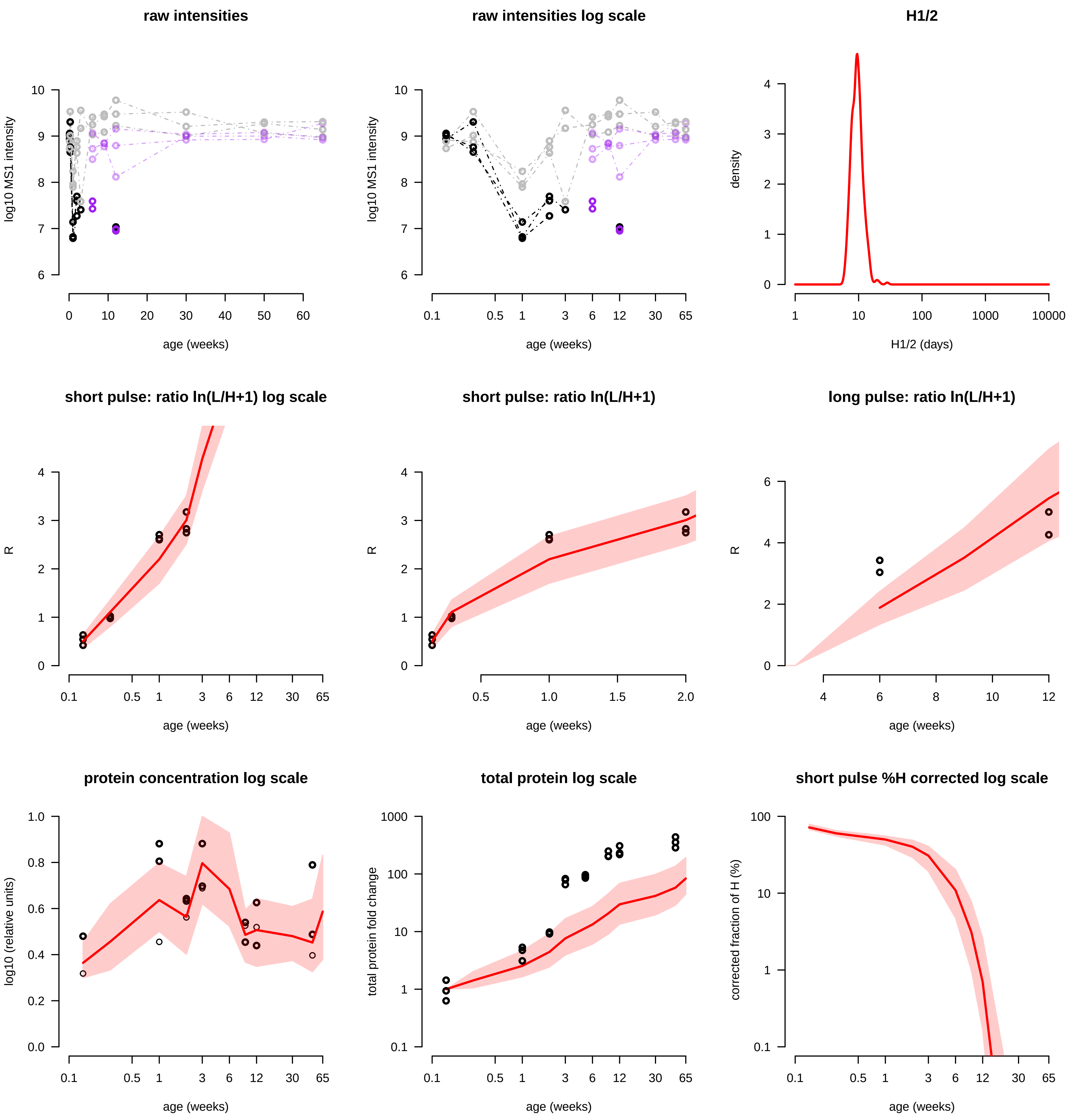

P46460

>sp|P46460|NSF_MOUSE

Vesicle−fusing ATPase

GN=Nsf

Med H1/2: 5.1 days

Q5 H1/2: 4.1 days

Q95 H1/2: 6.9 days

reliable model fit: yes

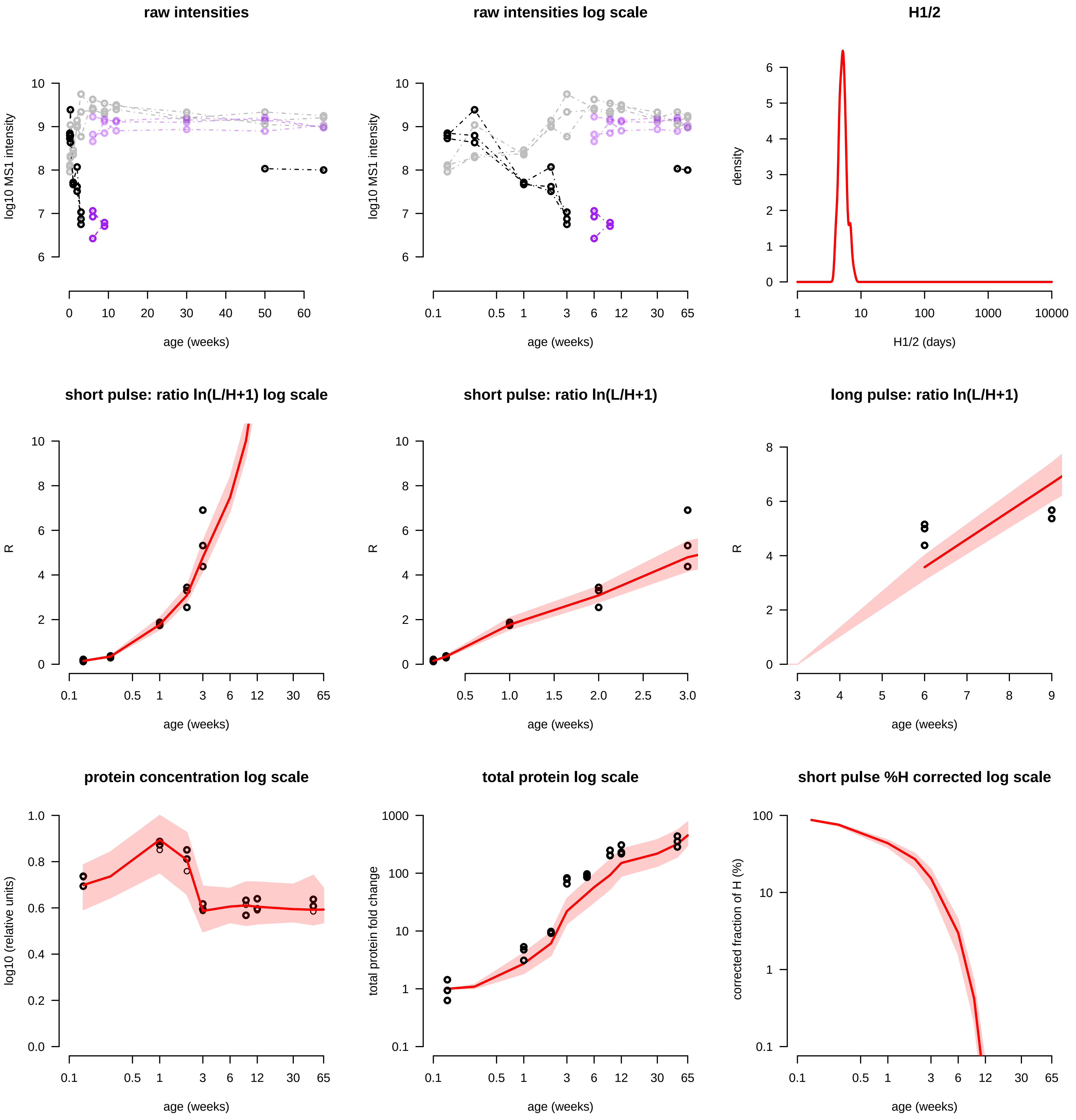

P62245

>sp|P62245|RS15A_MOUSE

40S ribosomal protein S15a

GN=Rps15a

Med H1/2: 8.5 days

Q5 H1/2: 7 days

Q95 H1/2: 10.9 days

reliable model fit: yes

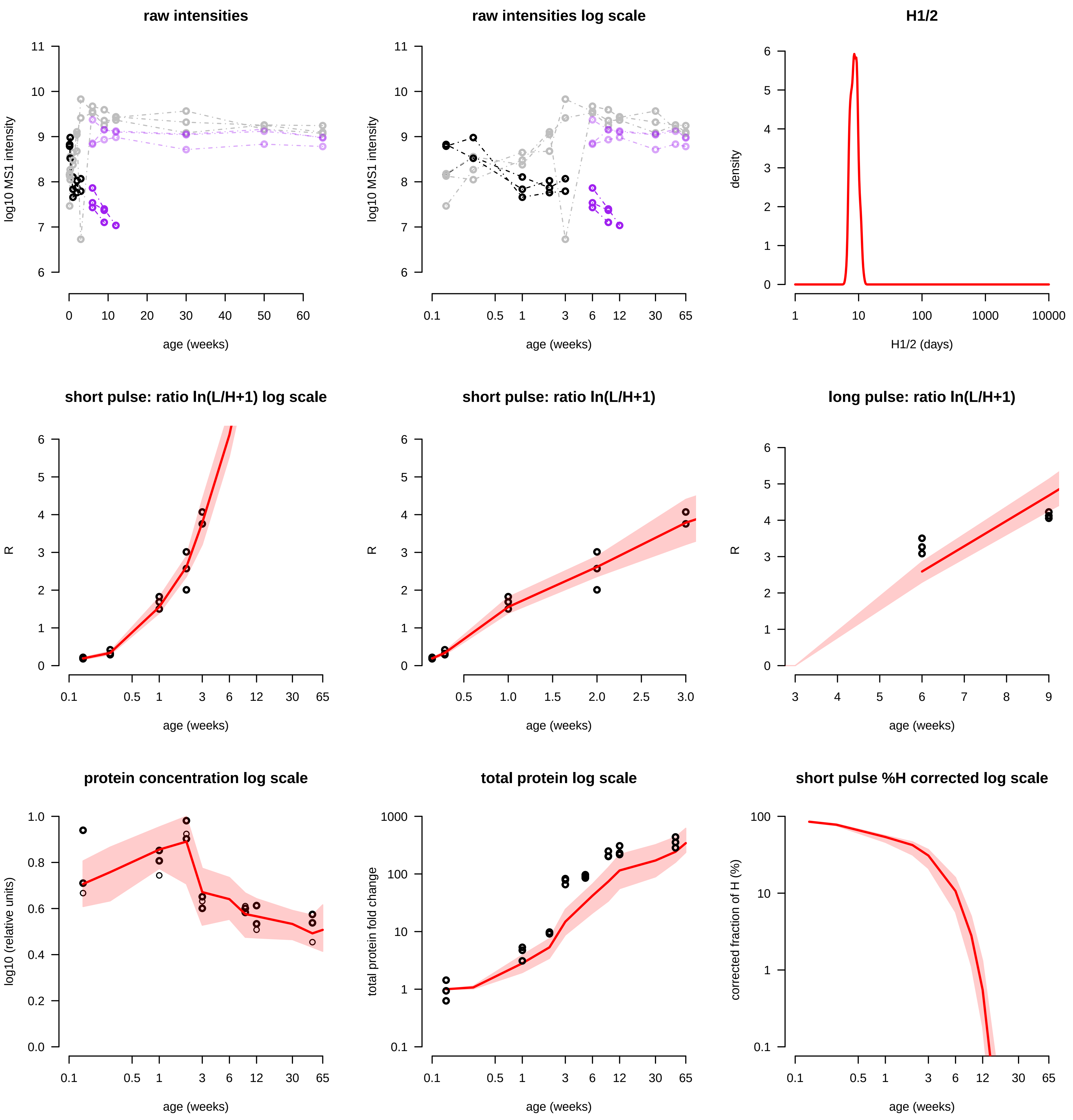

Q9Z1F9

>sp|Q9Z1F9|SAE2_MOUSE

SUMO−activating enzyme subunit 2

GN=Uba2

Med H1/2: 5.2 days

Q5 H1/2: 4.7 days

Q95 H1/2: 6.1 days

reliable model fit: yes

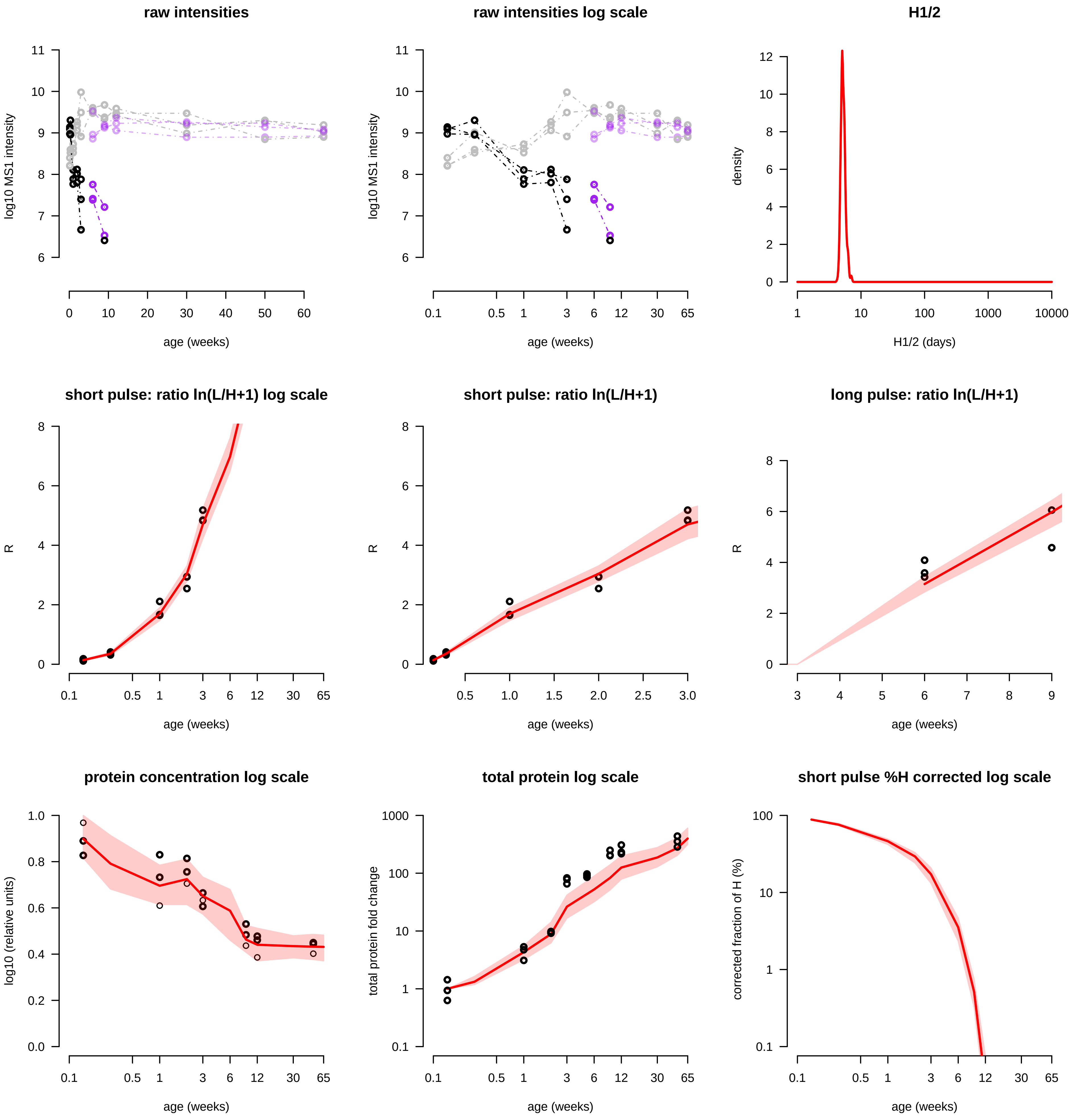

P11087;P11087−2

>sp|P11087|CO1A1_MOUSE

Collagen alpha−1(I) chain

GN=Col1a1

Med H1/2: 7.2 days

Q5 H1/2: 5.4 days

Q95 H1/2: 11.7 days

reliable model fit: yes

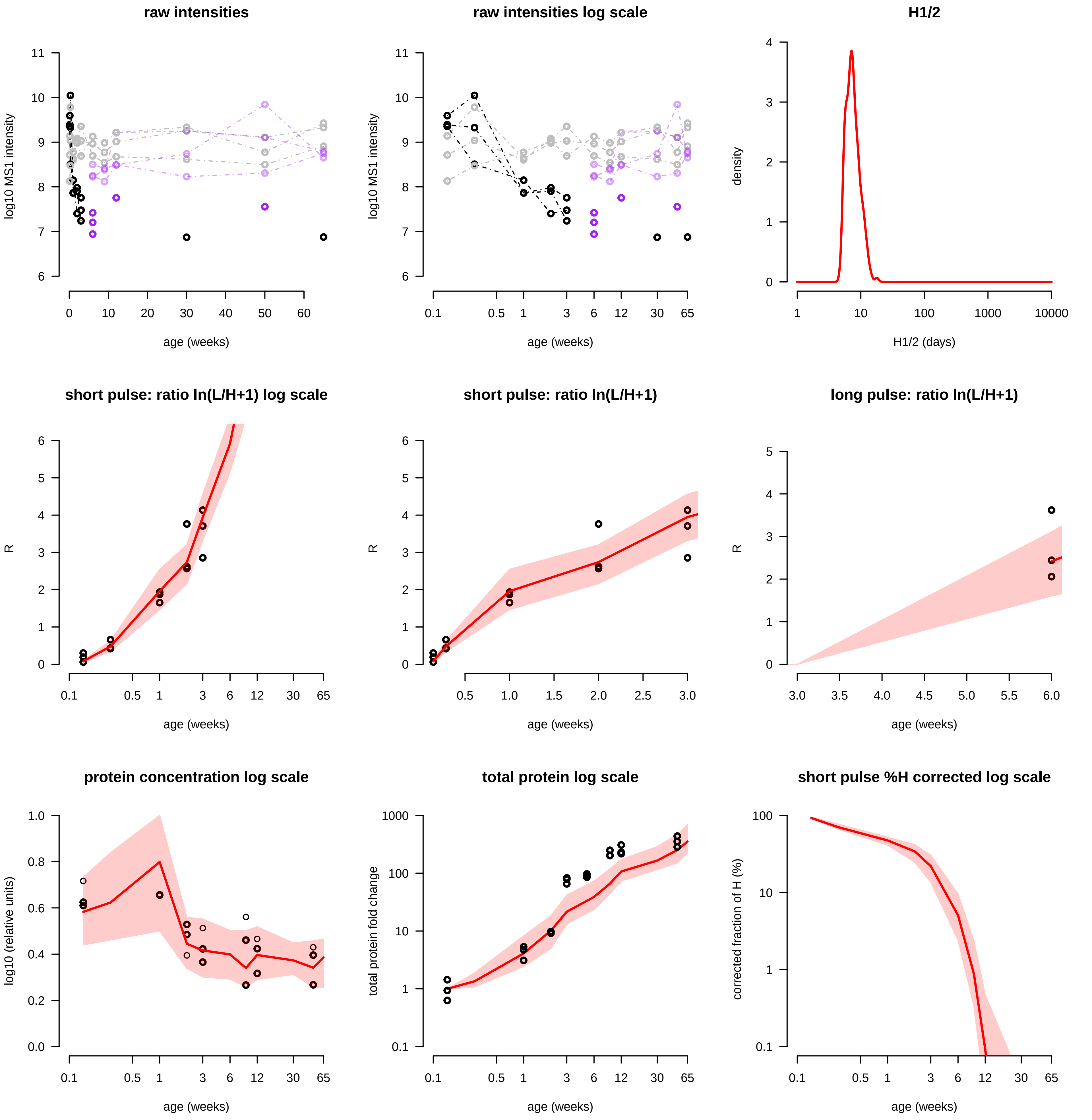

Q4VA53

>sp|Q4VA53|PDS5B_MOUSE

Sister chromatid cohesion protein PDS5 homolog B

GN=Pds5b

Med H1/2: 5.4 days

Q5 H1/2: 4.4 days

Q95 H1/2: 6.6 days

reliable model fit: yes

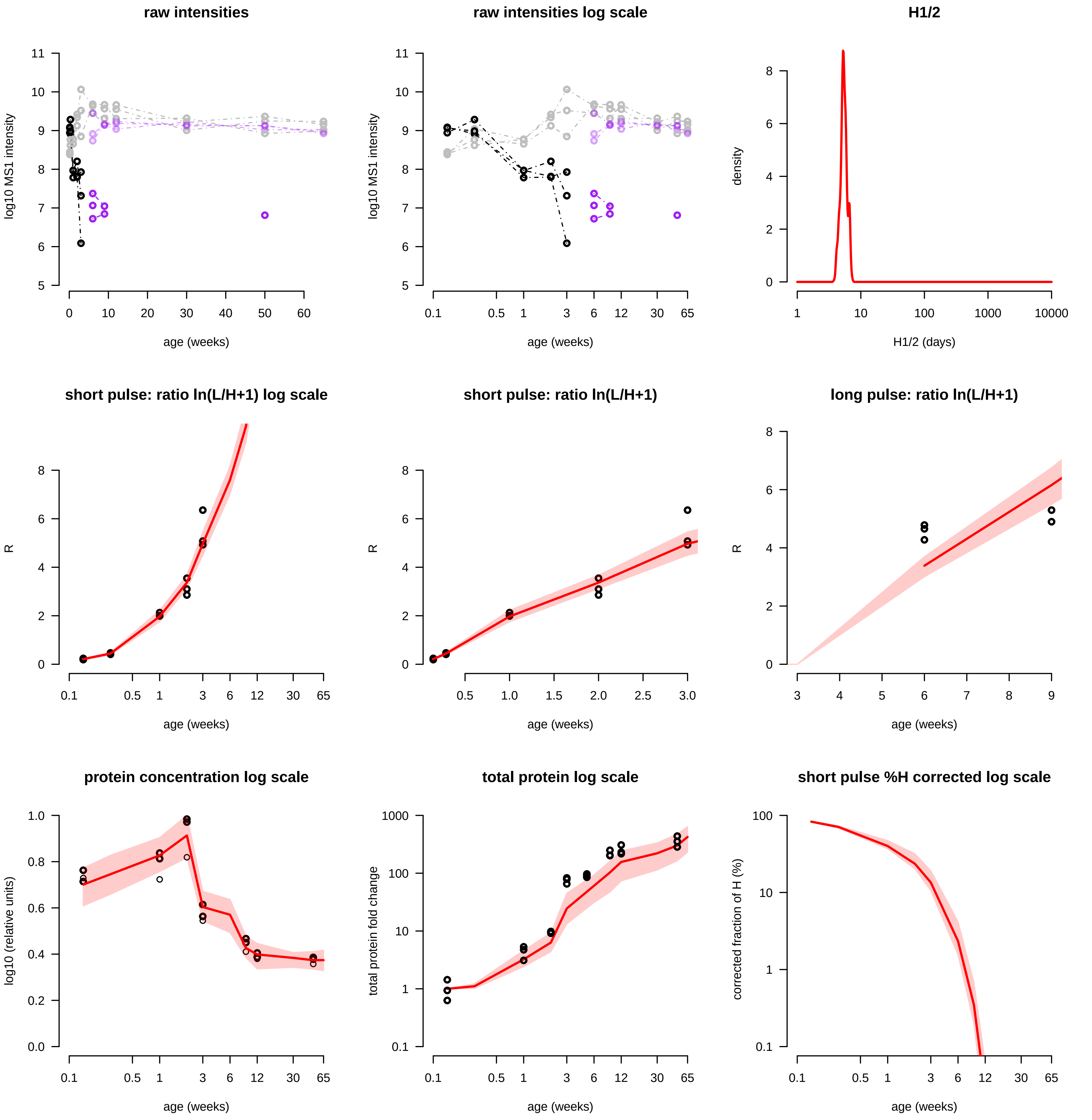

Q8BMJ2

>sp|Q8BMJ2|SYLC_MOUSE

Leucine−−tRNA ligase, cytoplasmic

GN=Lars1

Med H1/2: 13.5 days

Q5 H1/2: 9 days

Q95 H1/2: 25.8 days

reliable model fit: yes

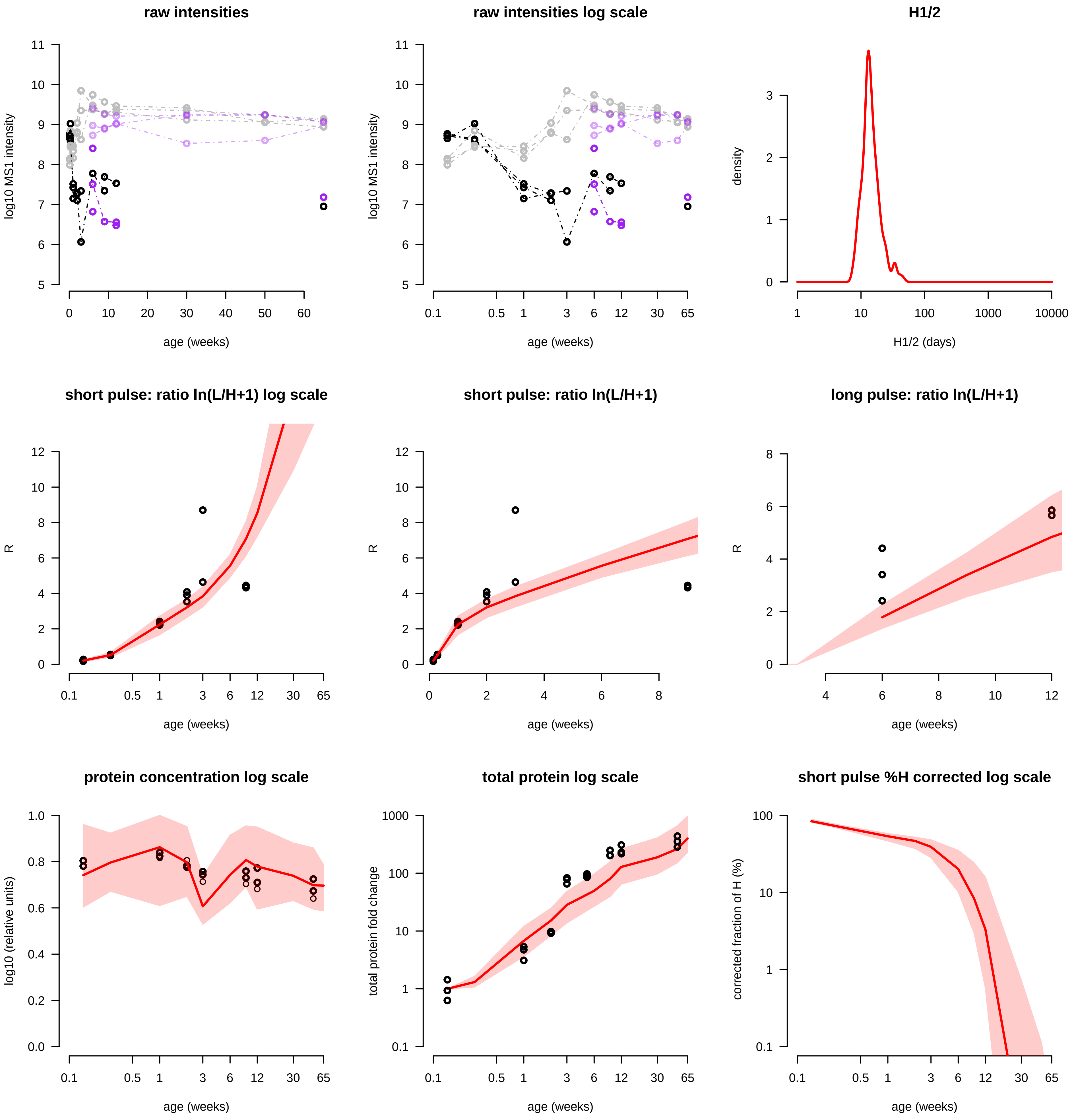

P31230

>sp|P31230|AIMP1_MOUSE

Aminoacyl tRNA synthase complex−interacting multifunctional protein 1

GN=Aimp1

Med H1/2: 5.1 days

Q5 H1/2: 4.3 days

Q95 H1/2: 6.2 days

reliable model fit: yes

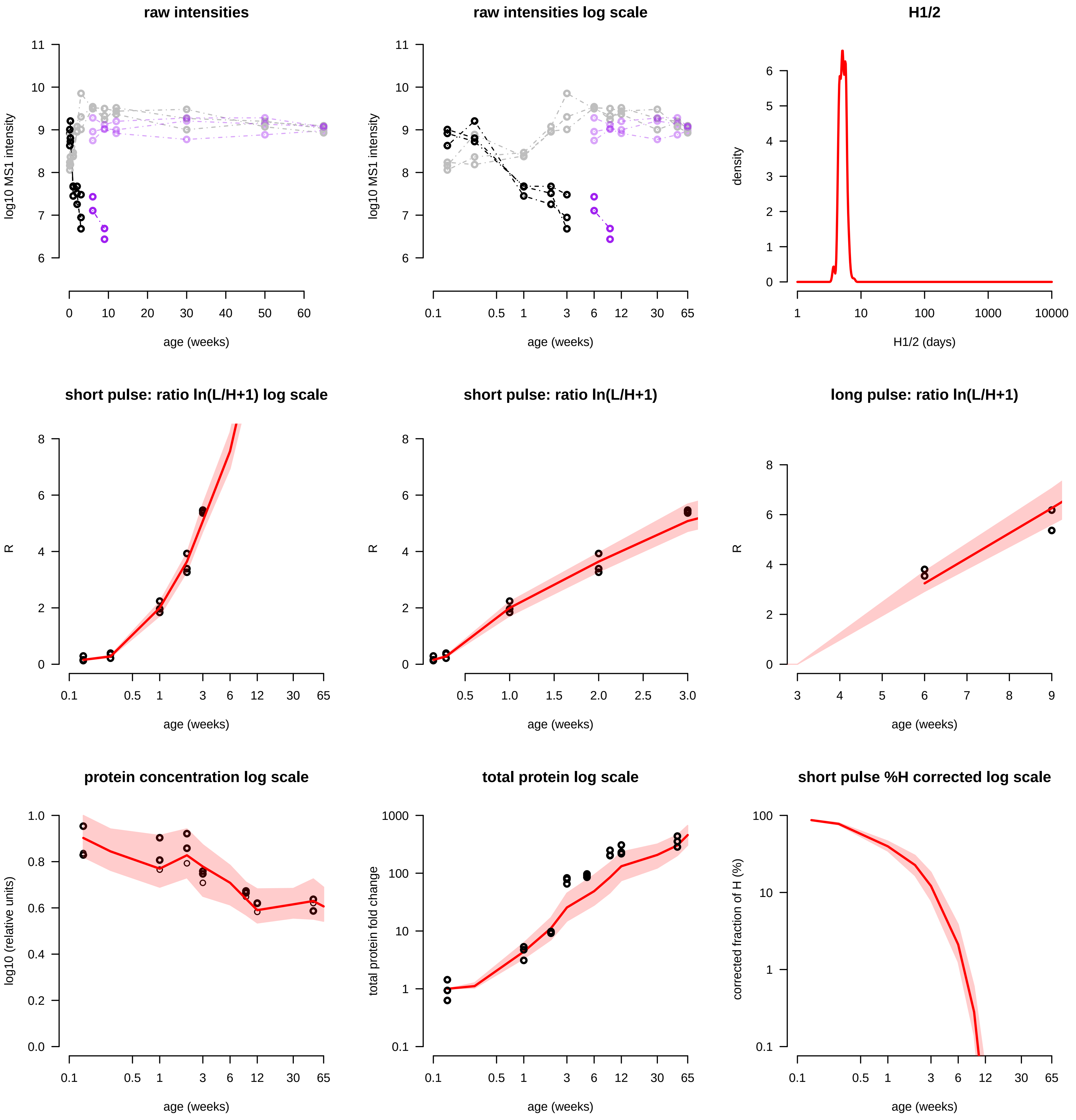

P01897

>sp|P01897|HA1L_MOUSE

H−2 class I histocompatibility antigen, L−D alpha chain

GN=H2−L

Med H1/2: 6 days

Q5 H1/2: 4.1 days

Q95 H1/2: 9.5 days

reliable model fit: yes

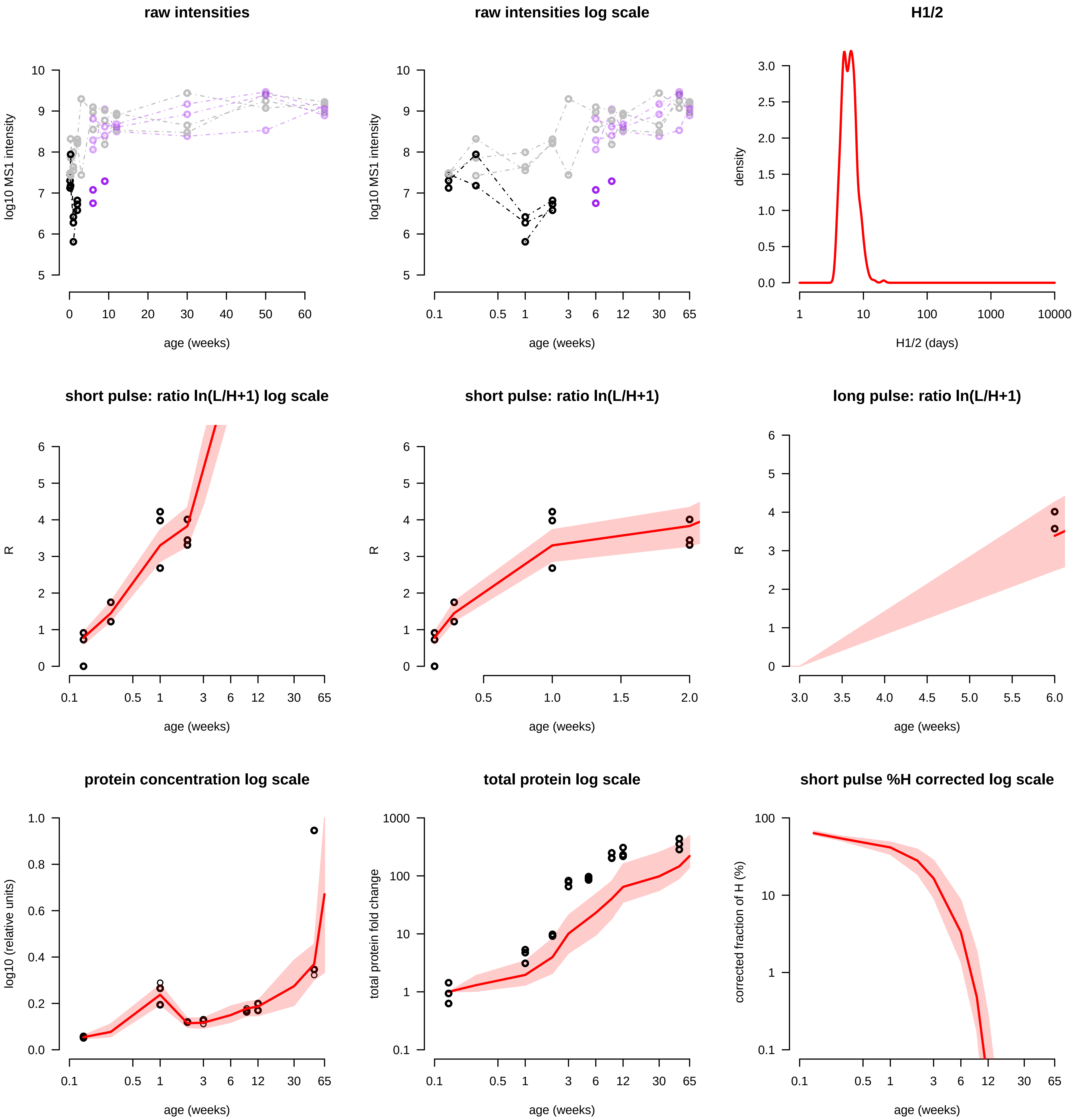

Q9QZD8

>sp|Q9QZD8|DIC_MOUSE

Mitochondrial dicarboxylate carrier

GN=Slc25a10

Med H1/2: 6.3 days

Q5 H1/2: 3 days

Q95 H1/2: 33.1 days

reliable model fit: yes

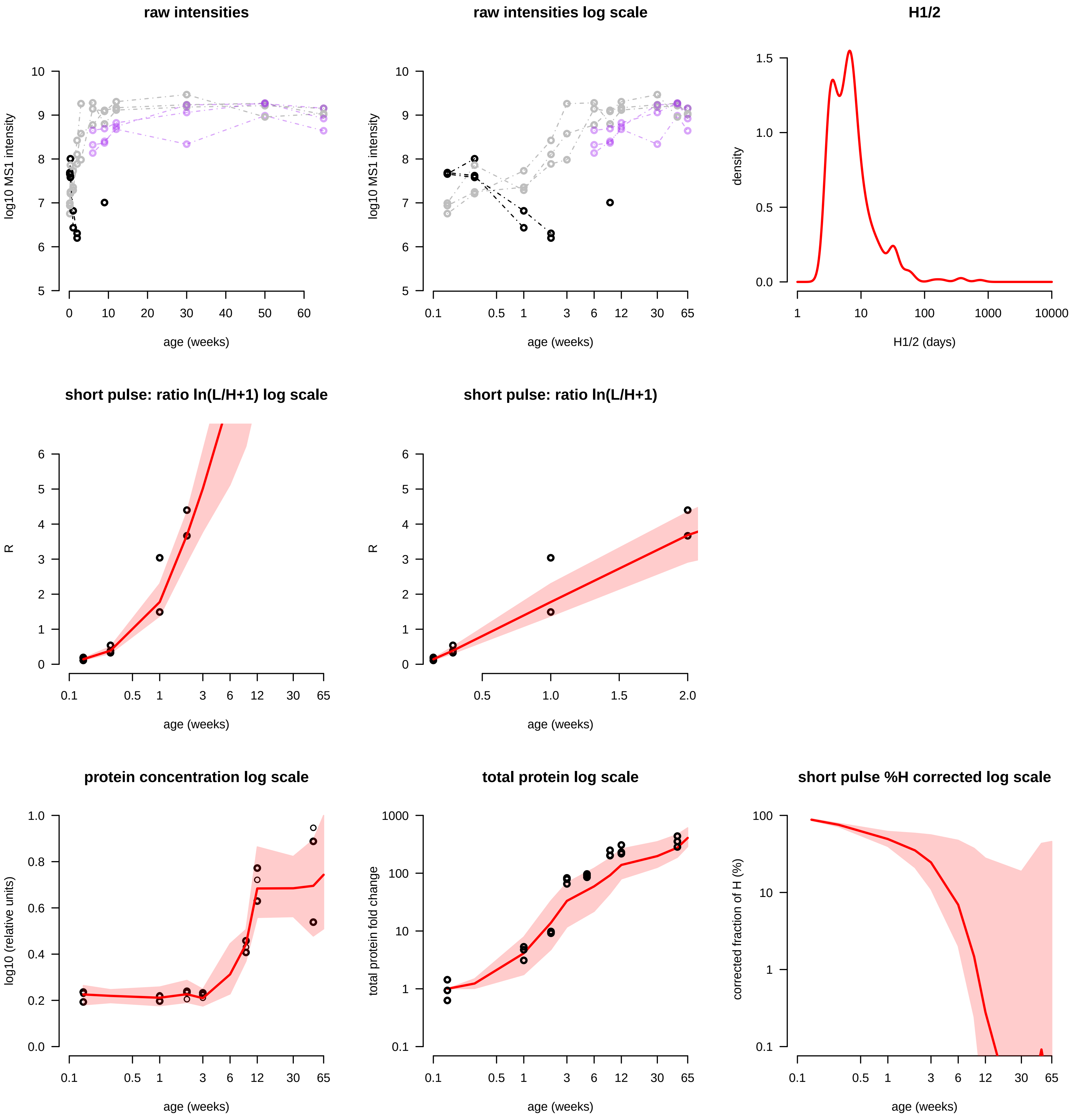

Q7TNV0

>sp|Q7TNV0|DEK_MOUSE

Protein DEK

GN=Dek

Med H1/2: 6.2 days

Q5 H1/2: 5.1 days

Q95 H1/2: 7.8 days

reliable model fit: yes

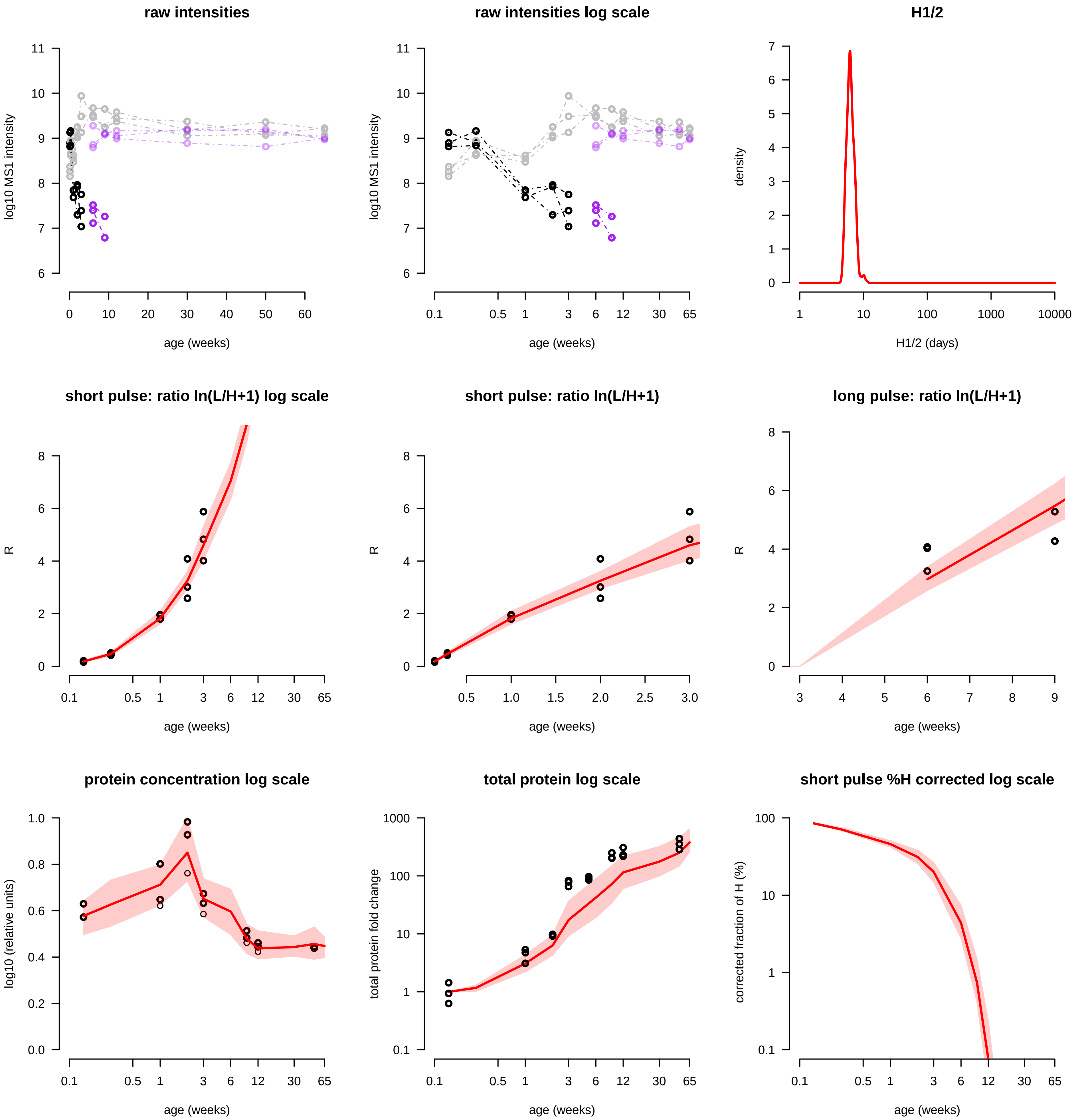

P10630;P10630−2

>sp|P10630|IF4A2_MOUSE

Eukaryotic initiation factor 4A−II

GN=Eif4a2

Med H1/2: 4.4 days

Q5 H1/2: 3.5 days

Q95 H1/2: 6.2 days

reliable model fit: yes

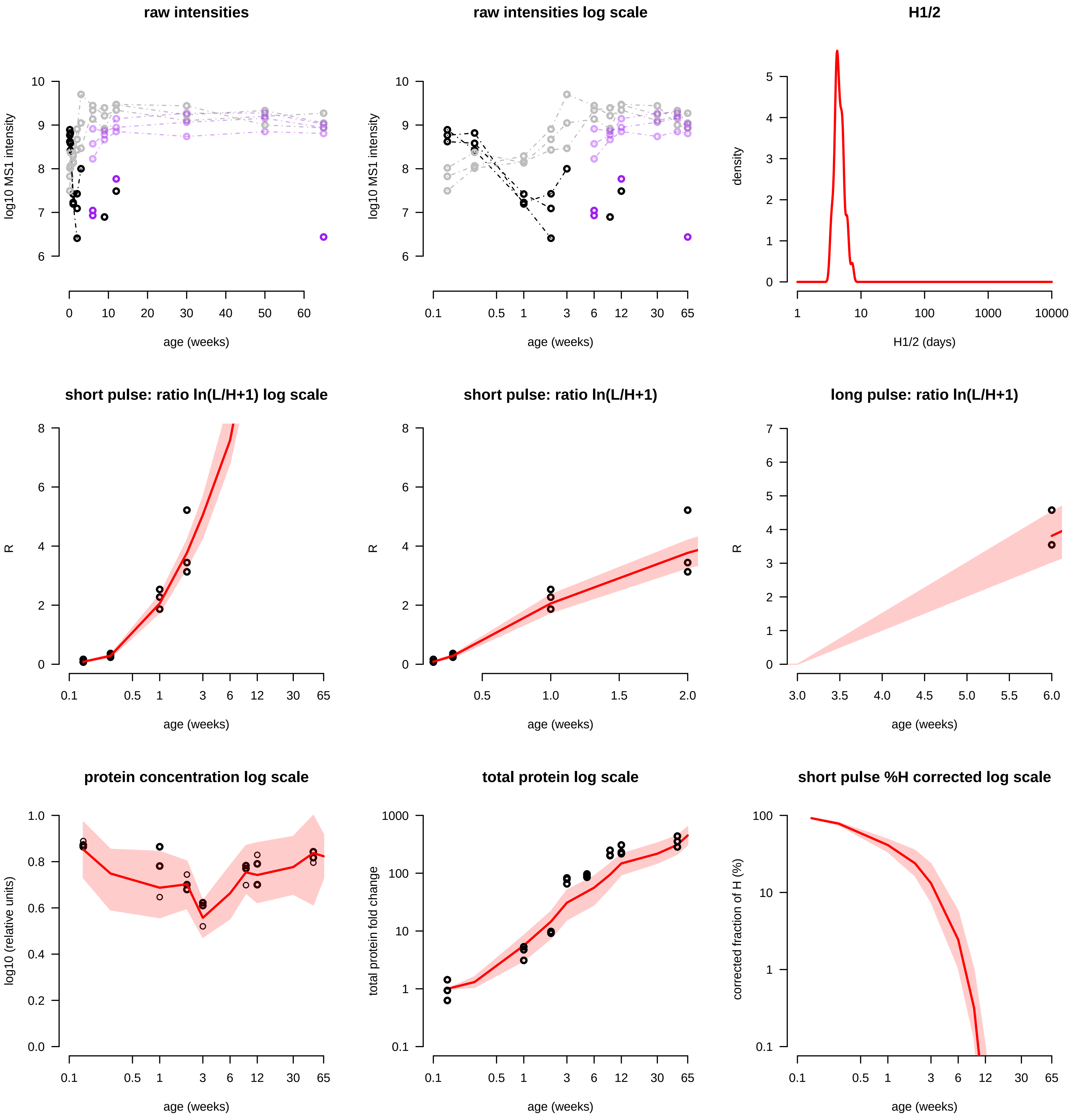

P49586

>sp|P49586|PCY1A_MOUSE

Choline−phosphate cytidylyltransferase A

GN=Pcyt1a

Med H1/2: 1902.4 days

Q5 H1/2: 455.4 days

Q95 H1/2: 30779.8 days

reliable model fit: no

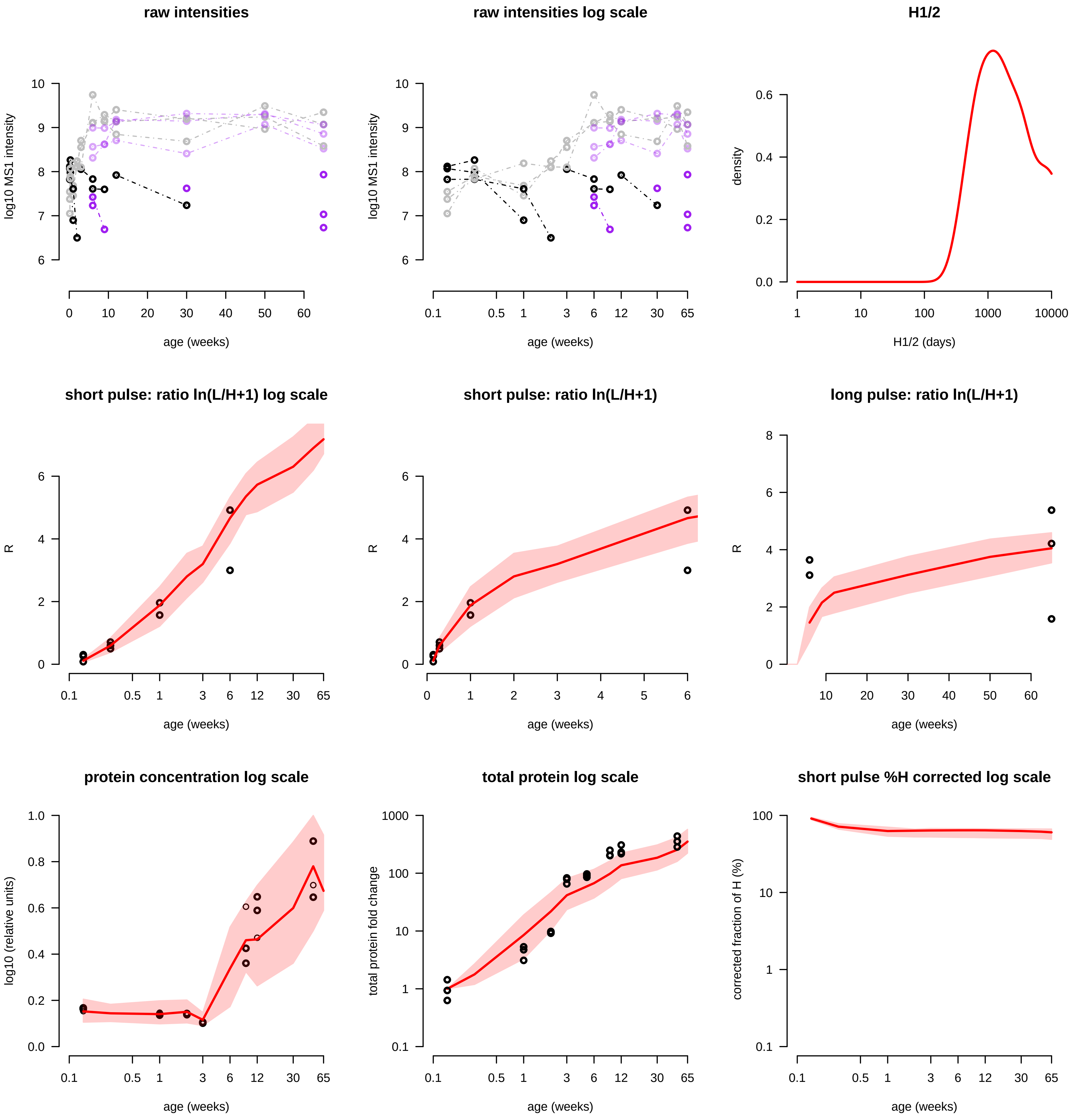

P83917

>sp|P83917|CBX1_MOUSE

Chromobox protein homolog 1

GN=Cbx1

Med H1/2: 9.1 days

Q5 H1/2: 7 days

Q95 H1/2: 12.7 days

reliable model fit: yes

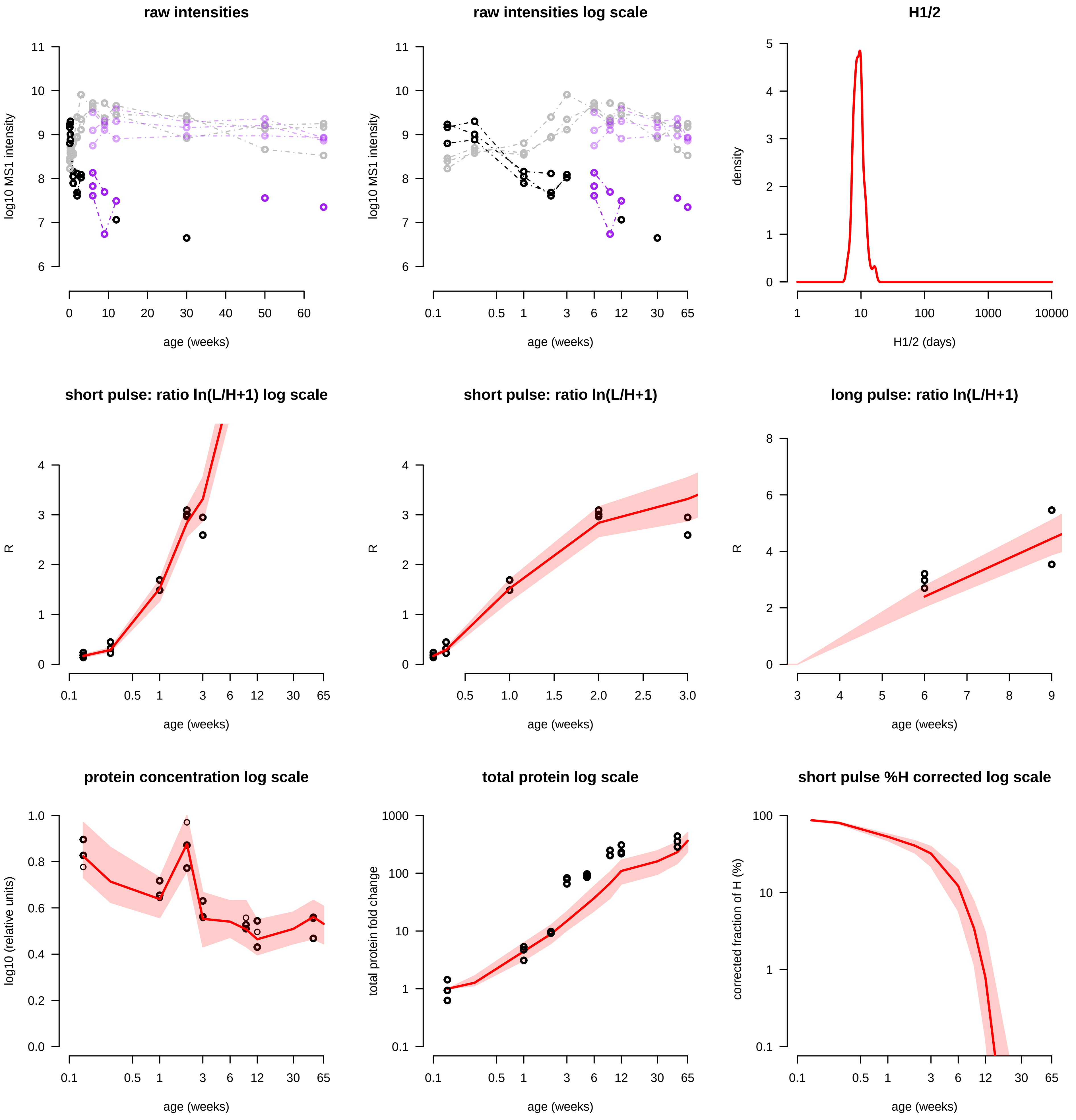

Q9D0M5

>sp|Q9D0M5|DYL2_MOUSE

Dynein light chain 2, cytoplasmic

GN=Dynll2

Med H1/2: 14.7 days

Q5 H1/2: 11.3 days

Q95 H1/2: 19.2 days

reliable model fit: yes

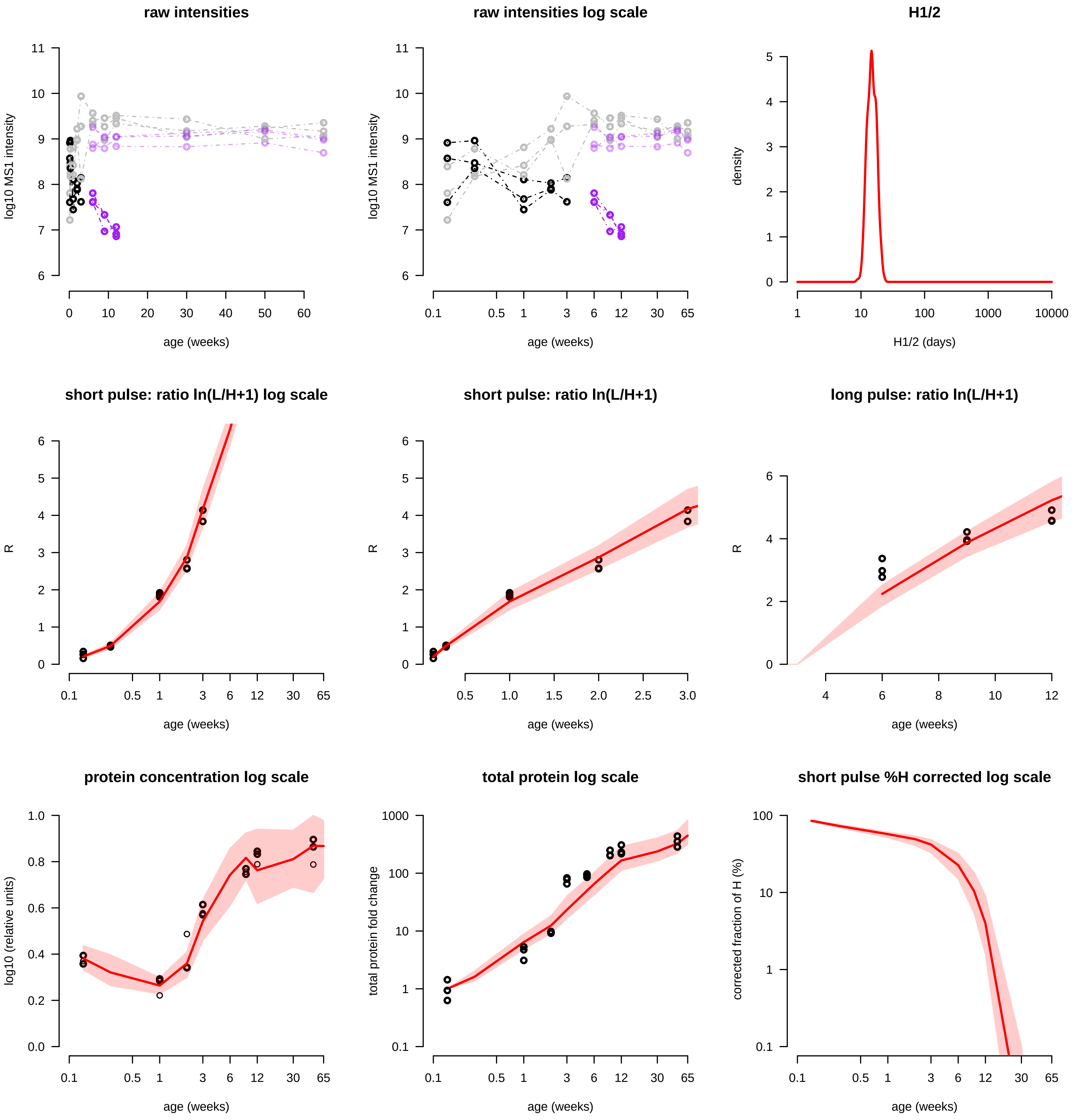

Q62189

>sp|Q62189|SNRPA_MOUSE

U1 small nuclear ribonucleoprotein A

GN=Snrpa

Med H1/2: 4 days

Q5 H1/2: 3.3 days

Q95 H1/2: 4.7 days

reliable model fit: yes

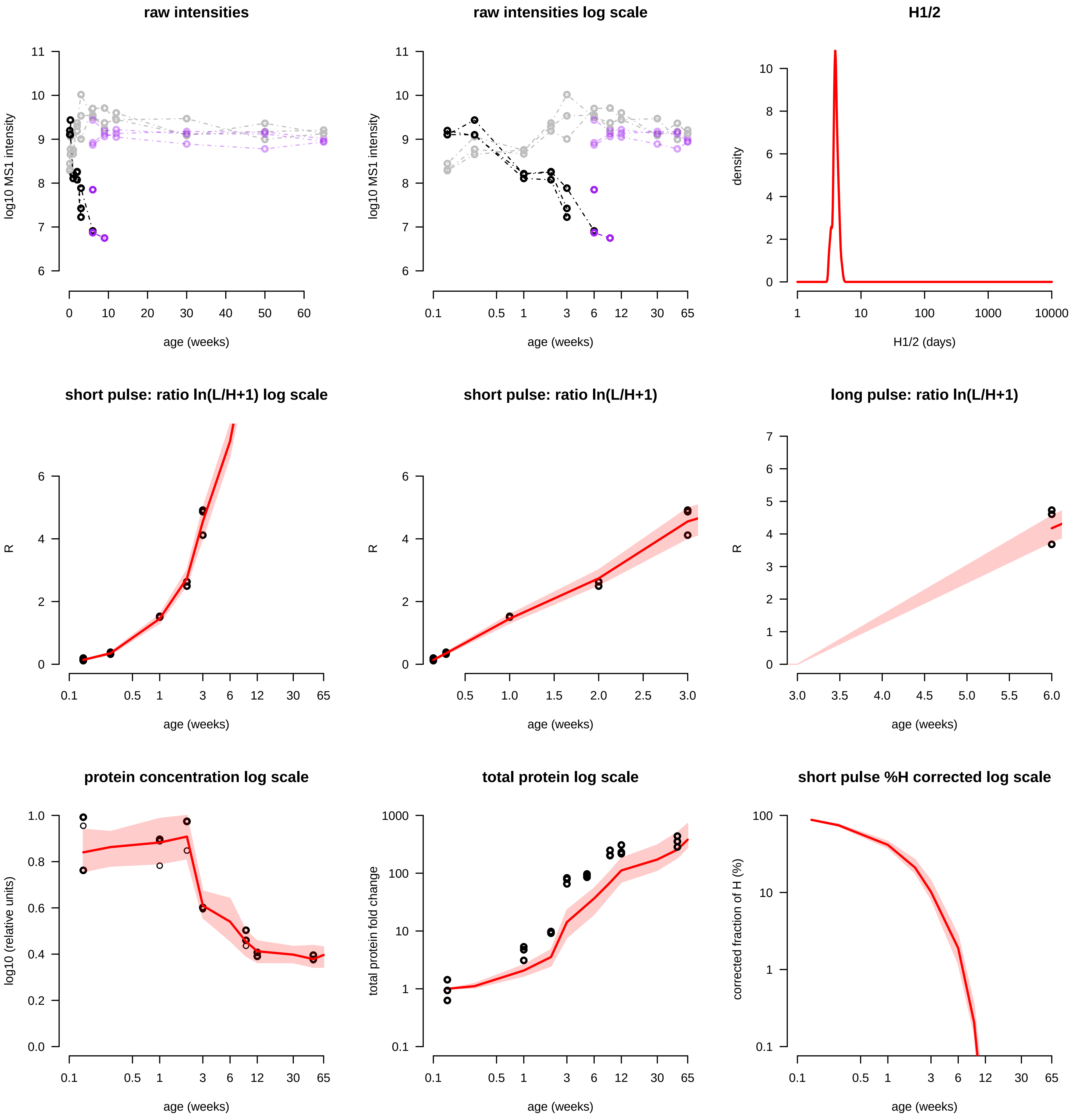

Q99JI4

>sp|Q99JI4|PSMD6_MOUSE

26S proteasome non−ATPase regulatory subunit 6

GN=Psmd6

Med H1/2: 344.6 days

Q5 H1/2: 140.1 days

Q95 H1/2: 1347.6 days

reliable model fit: yes

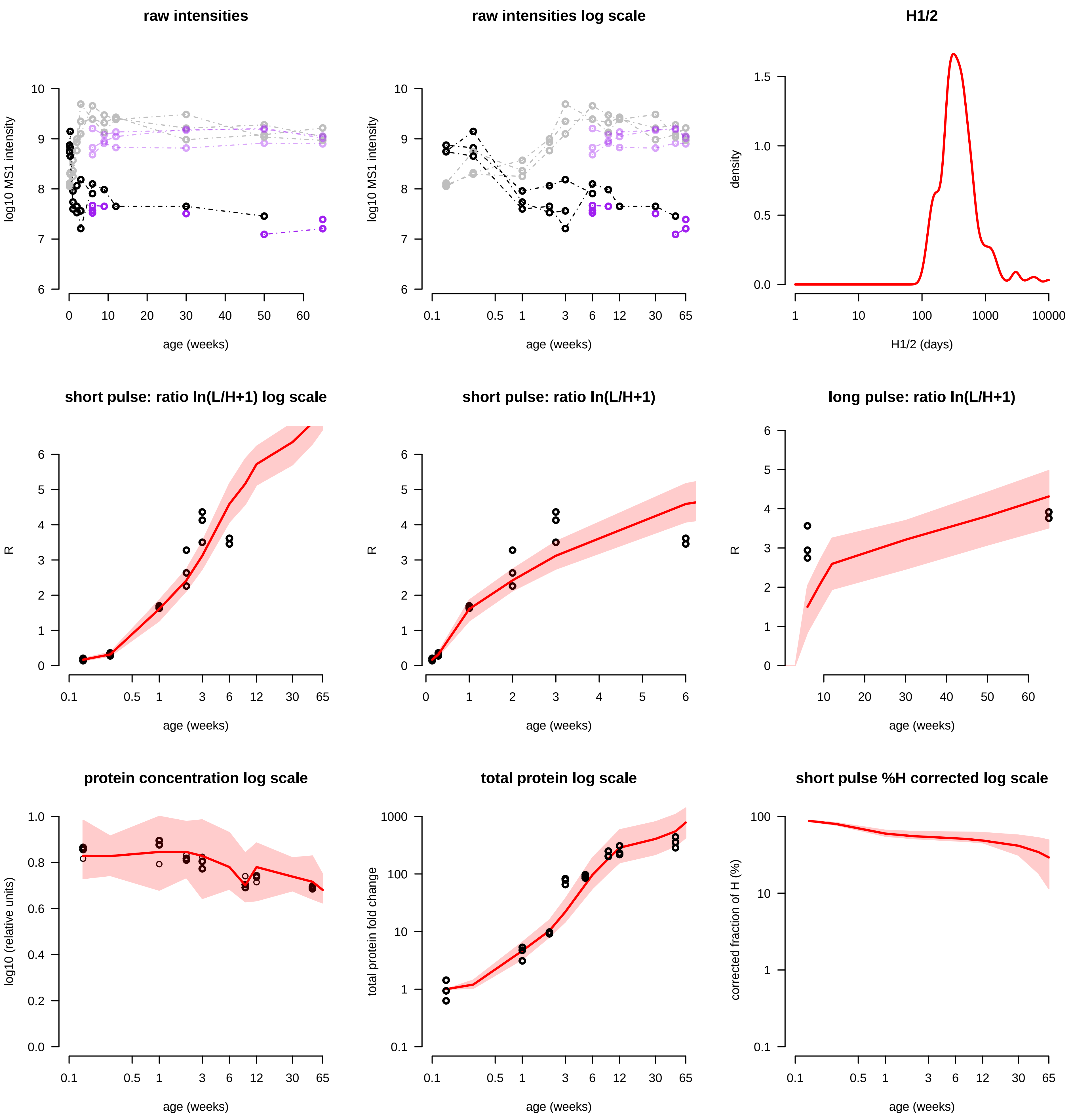

P70398

>sp|P70398|USP9X_MOUSE

Probable ubiquitin carboxyl−terminal hydrolase FAF−X

GN=Usp9x

Med H1/2: 3.2 days

Q5 H1/2: 2.8 days

Q95 H1/2: 3.9 days

reliable model fit: yes

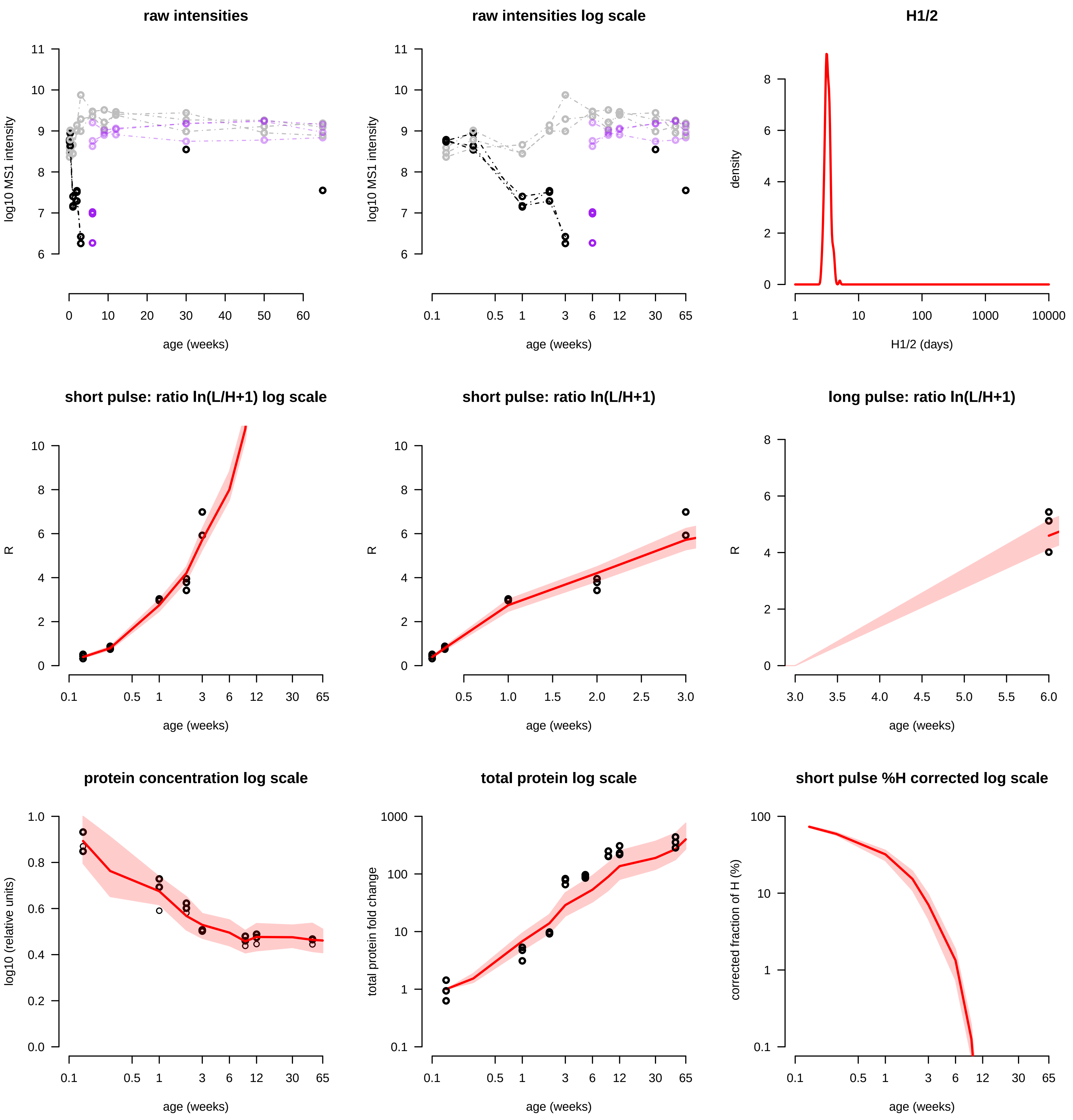

P58710

>sp|P58710|GGLO_MOUSE

L−gulonolactone oxidase

GN=Gulo

Med H1/2: 11.2 days

Q5 H1/2: 8.8 days

Q95 H1/2: 15 days

reliable model fit: yes

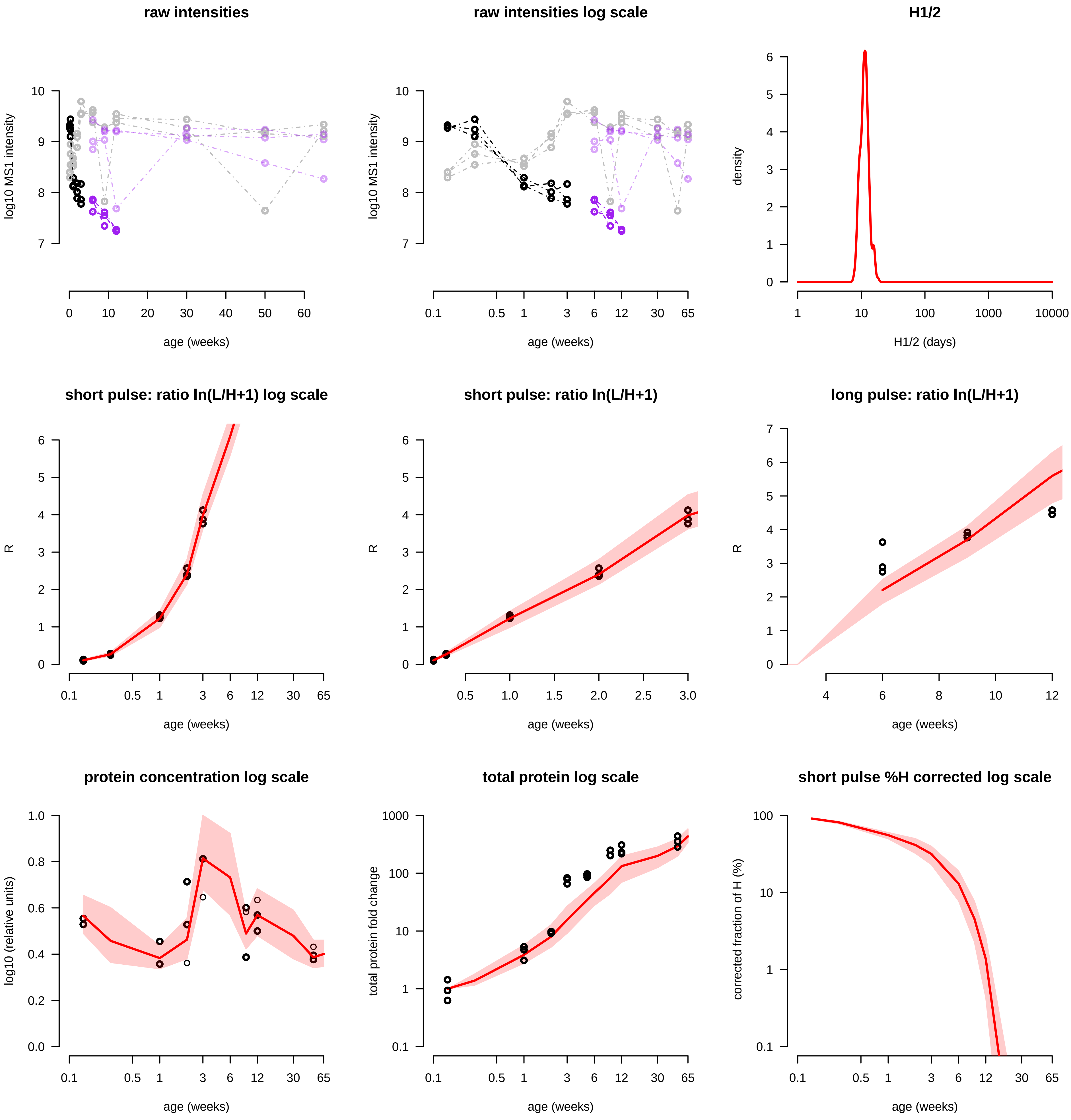

Q8CIM3

>sp|Q8CIM3|D2HDH_MOUSE

D−2−hydroxyglutarate dehydrogenase, mitochondrial

GN=D2hgdh

Med H1/2: 786.1 days

Q5 H1/2: 209.4 days

Q95 H1/2: 11066 days

reliable model fit: no

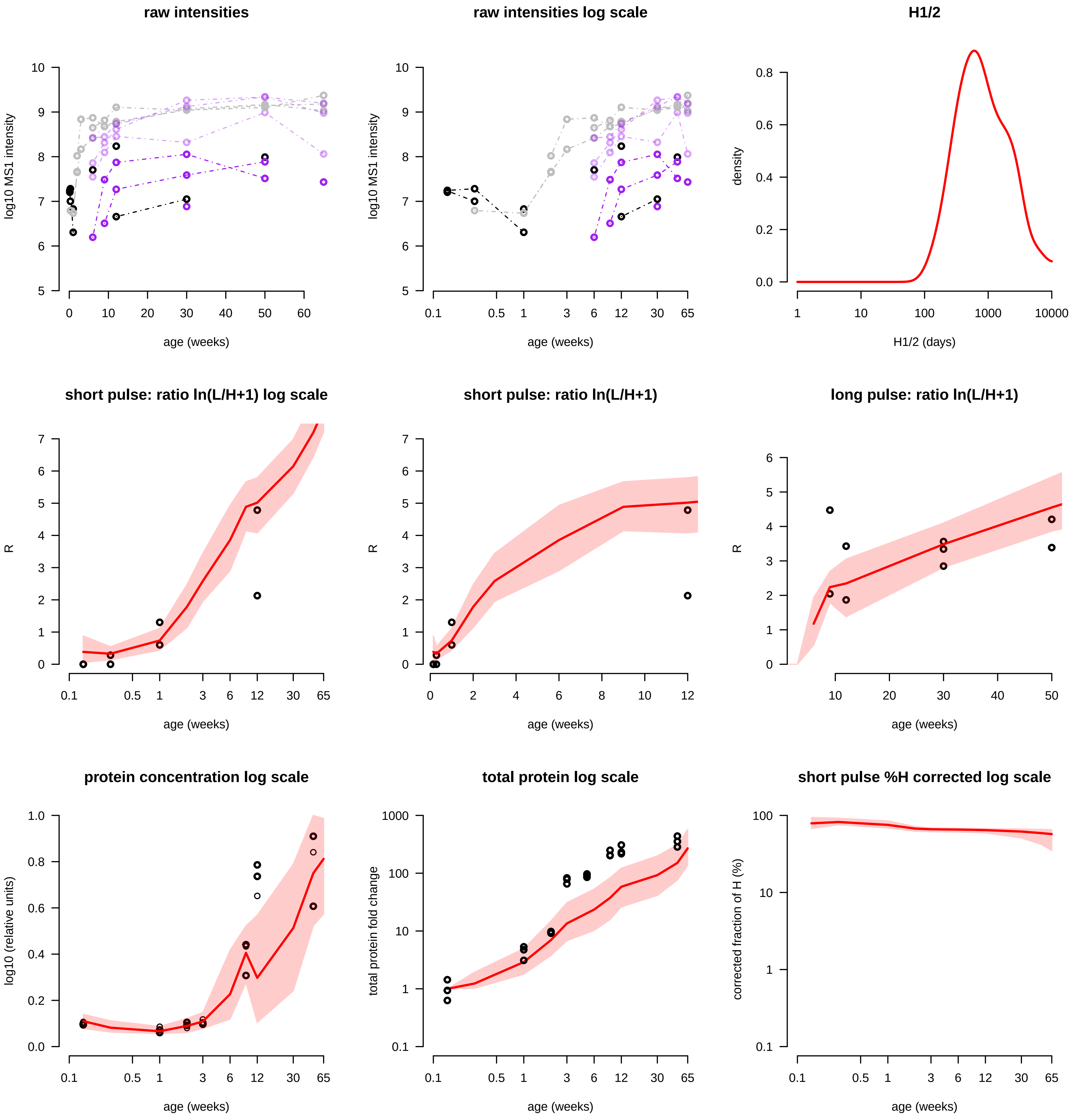

Q8BGC4

>sp|Q8BGC4|PTGR3_MOUSE

Prostaglandin reductase−3

GN=Zadh2

Med H1/2: 22.5 days

Q5 H1/2: 14.4 days

Q95 H1/2: 47.2 days

reliable model fit: yes

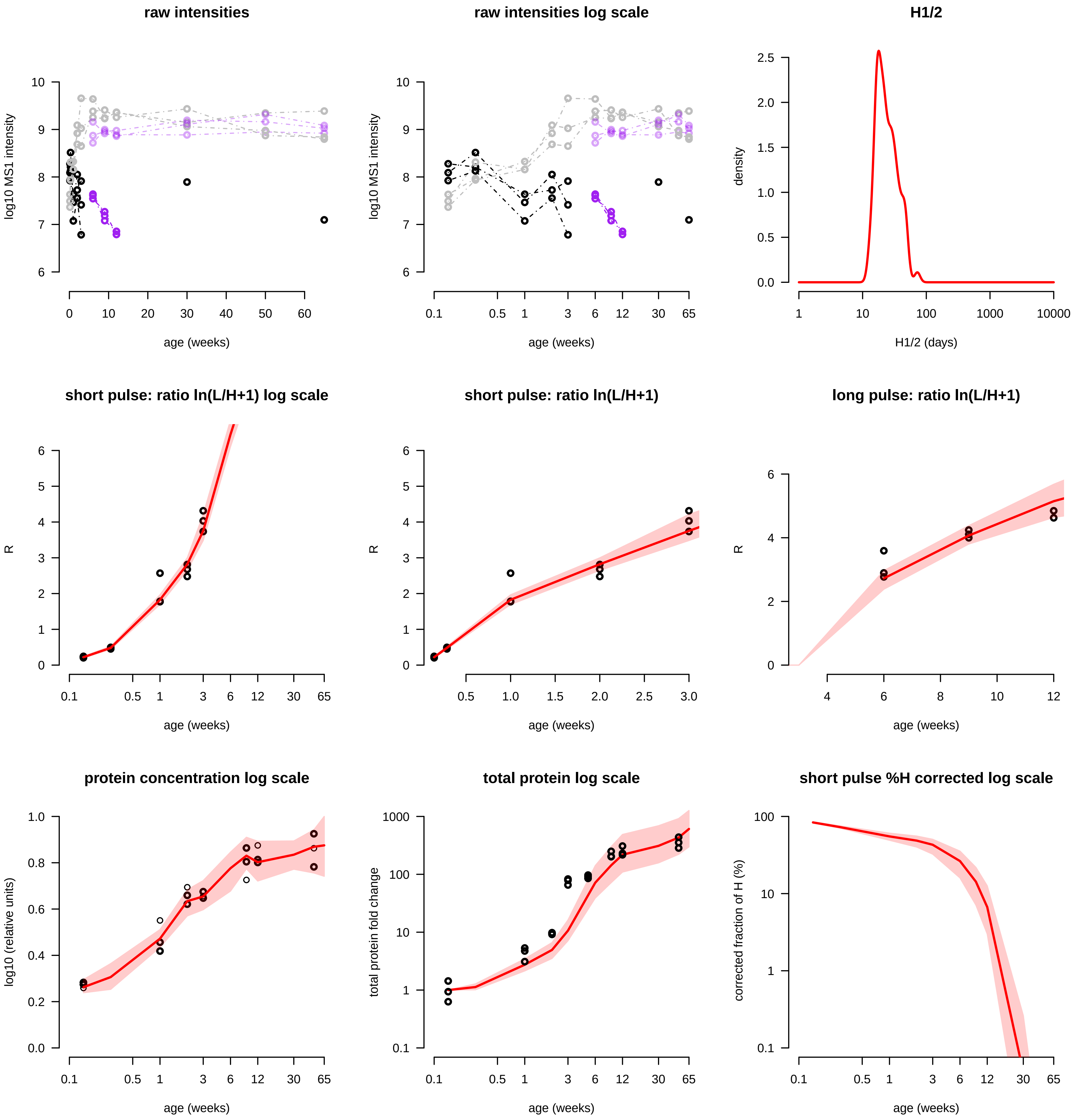

P04186

>sp|P04186|CFAB_MOUSE

Complement factor B

GN=Cfb

Med H1/2: 4.7 days

Q5 H1/2: 3.6 days

Q95 H1/2: 6.1 days

reliable model fit: yes

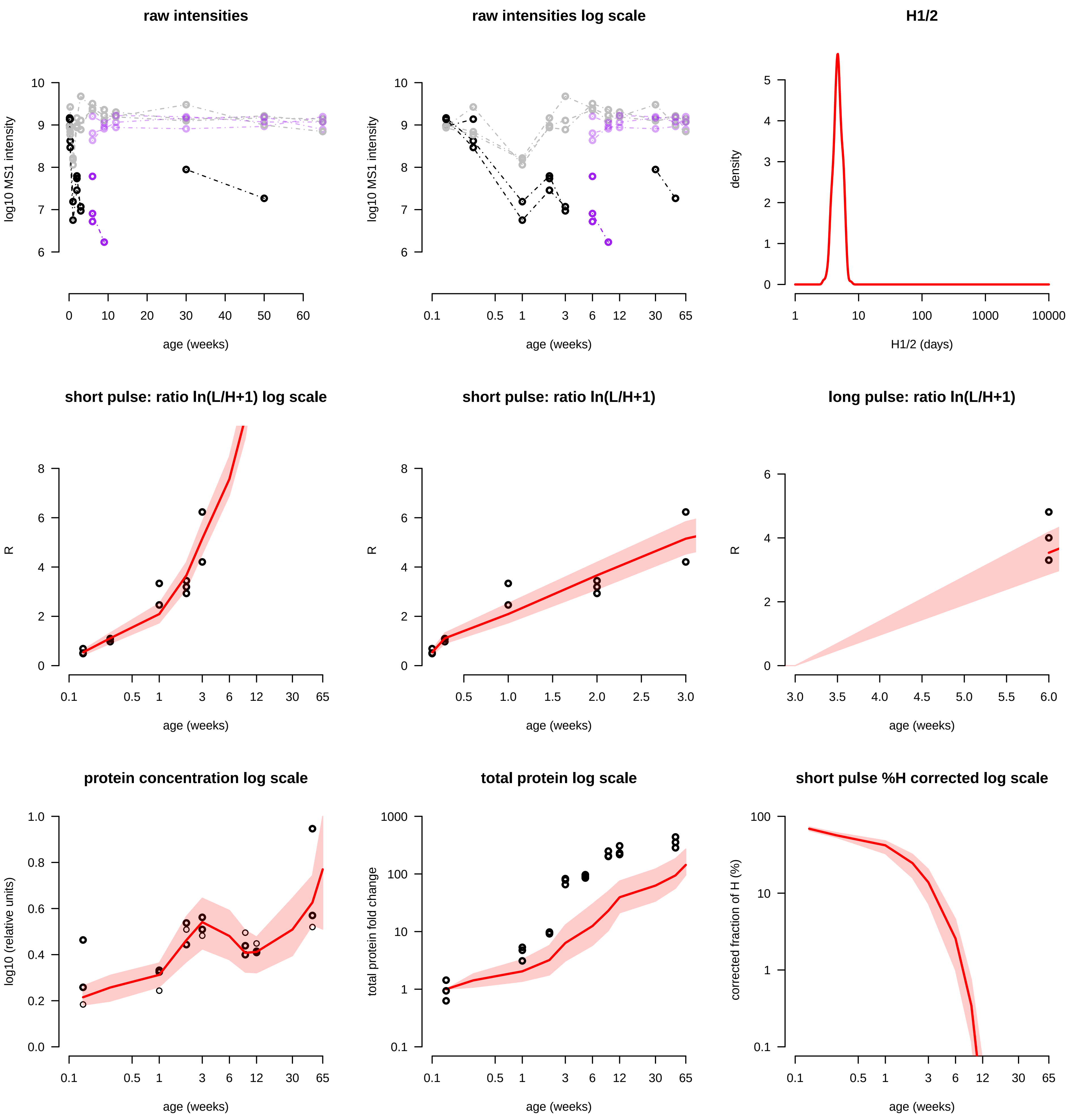

Q3TC72

>sp|Q3TC72|FAHD2_MOUSE

Fumarylacetoacetate hydrolase domain−containing protein 2A

GN=Fahd2

Med H1/2: 12 days

Q5 H1/2: 3 days

Q95 H1/2: 107.3 days

reliable model fit: yes

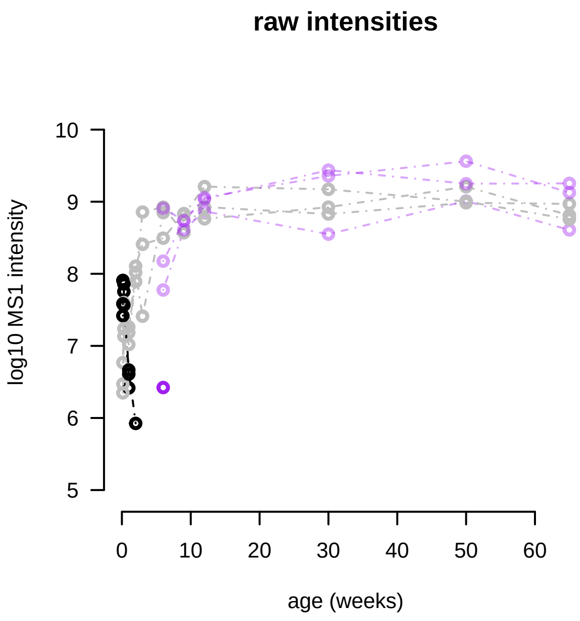

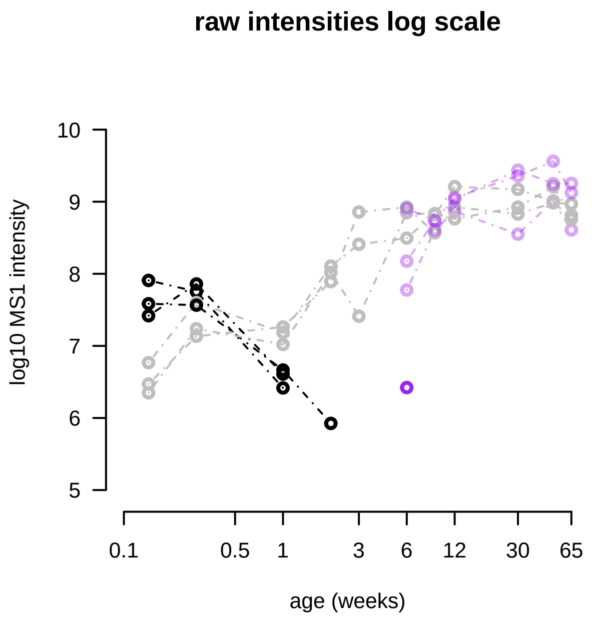

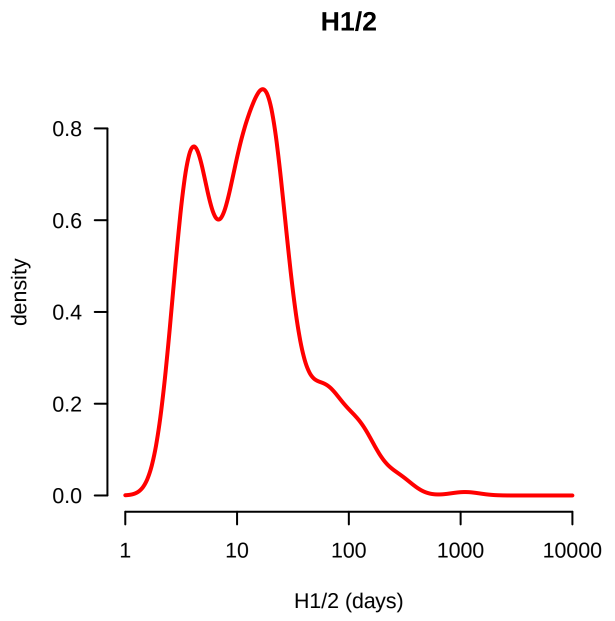

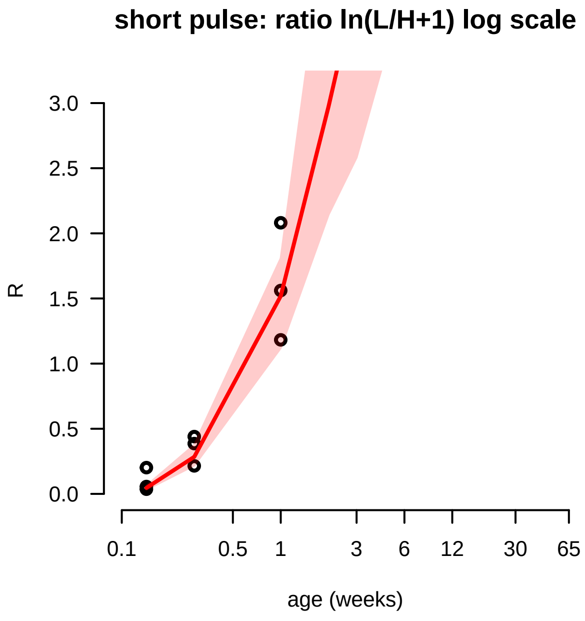

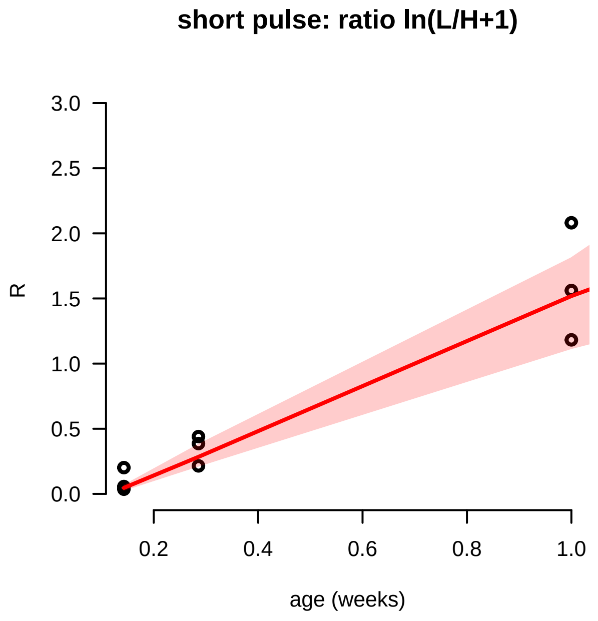

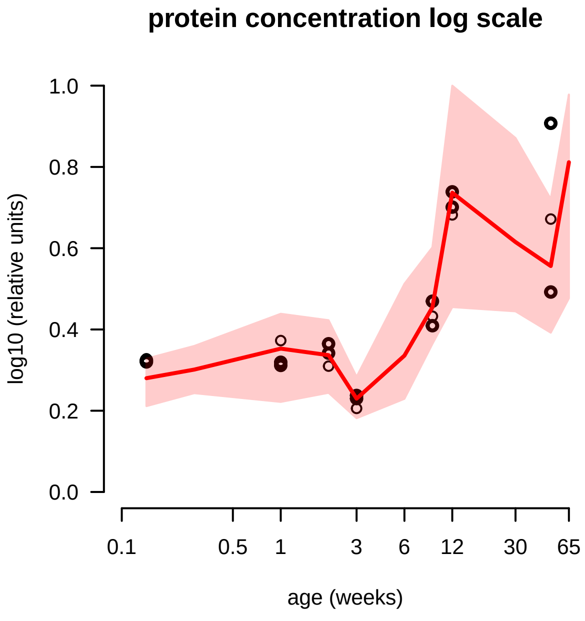

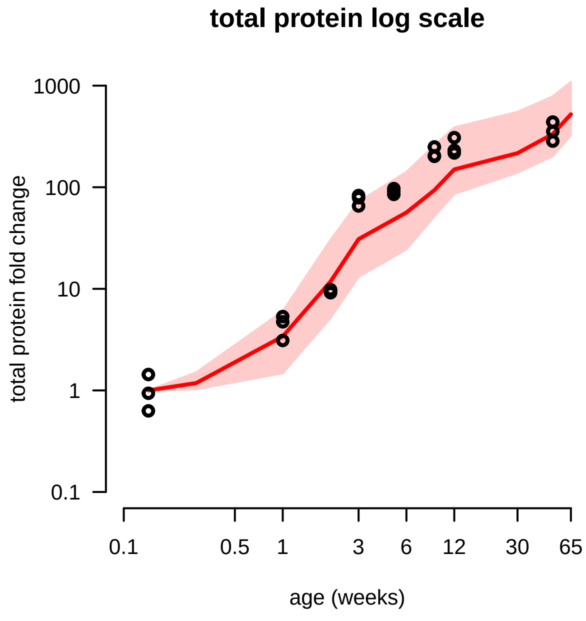

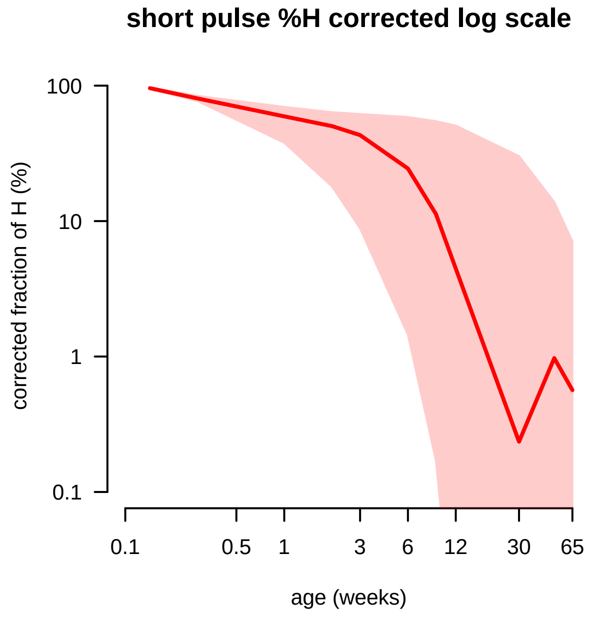

Q91VC3

>sp|Q91VC3|IF4A3_MOUSE

Eukaryotic initiation factor 4A−III

GN=Eif4a3

Med H1/2: 5.9 days

Q5 H1/2: 5.1 days

Q95 H1/2: 7.4 days

reliable model fit: yes

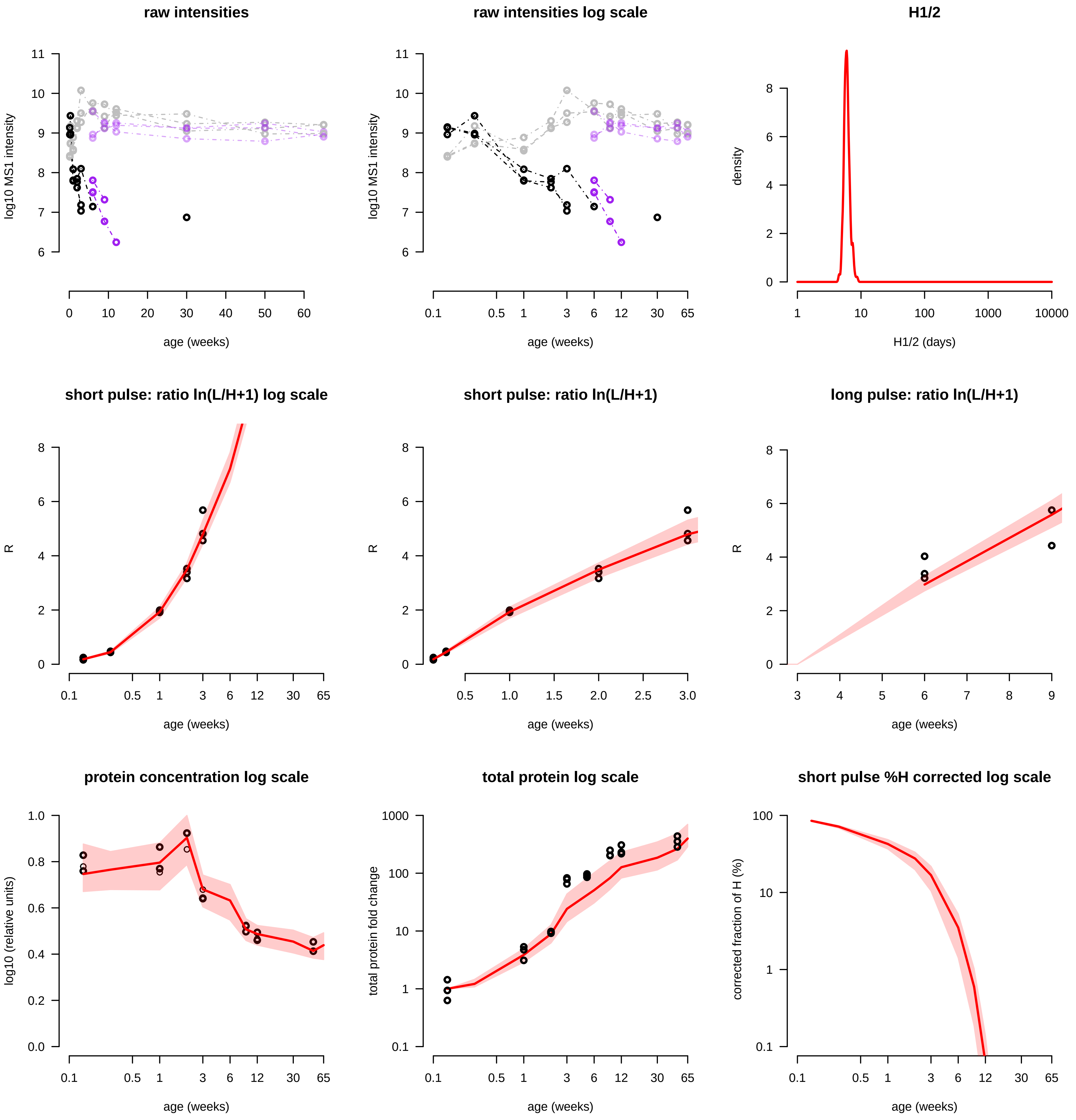

P47915

>sp|P47915|RL29_MOUSE

60S ribosomal protein L29

GN=Rpl29

Med H1/2: 15.7 days

Q5 H1/2: 11.7 days

Q95 H1/2: 25.3 days

reliable model fit: yes

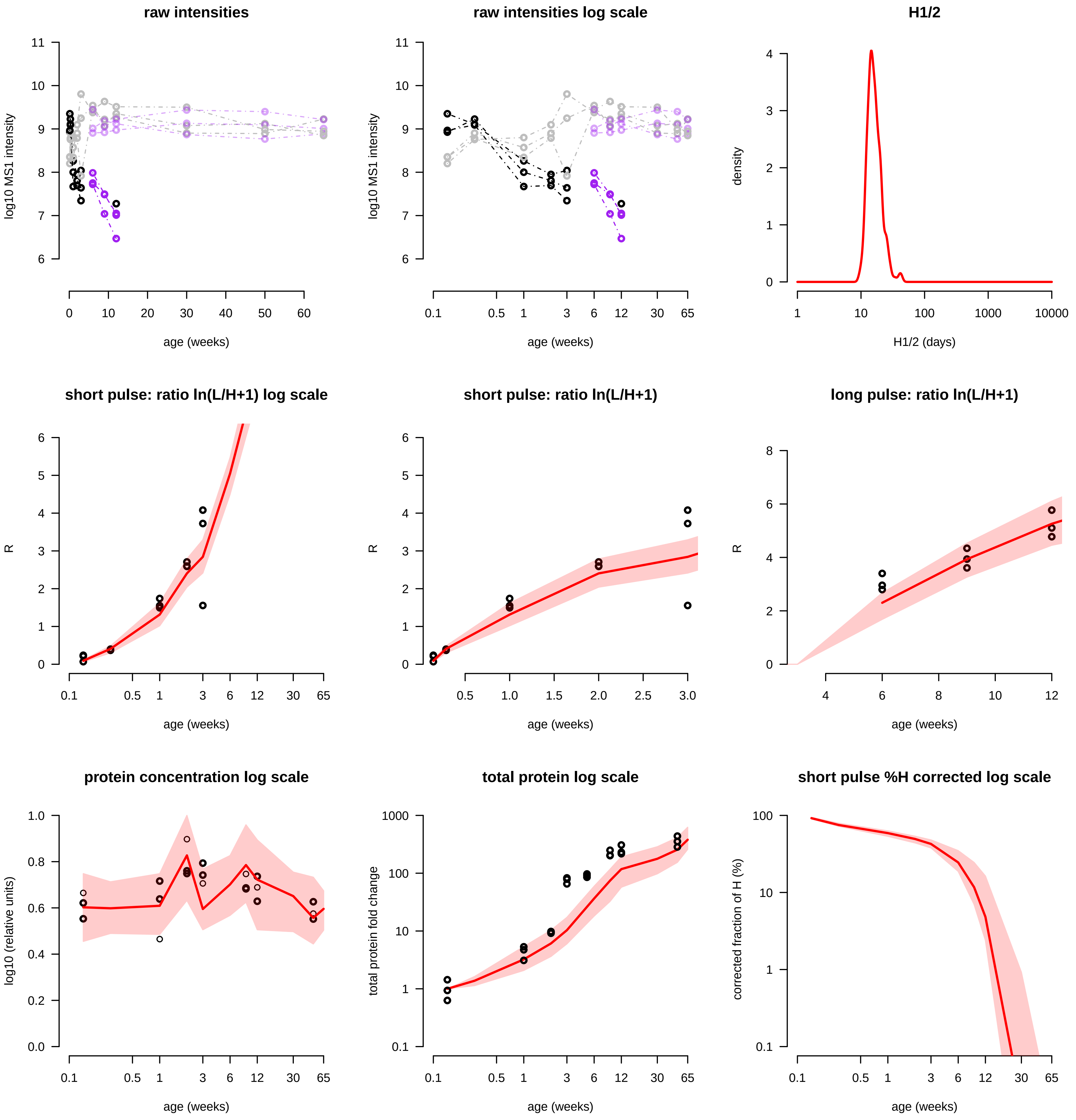

Q8R2Y8

>sp|Q8R2Y8|PTH2_MOUSE

Peptidyl−tRNA hydrolase 2, mitochondrial

GN=Ptrh2

Med H1/2: 17.1 days

Q5 H1/2: 11.2 days

Q95 H1/2: 35.6 days

reliable model fit: yes

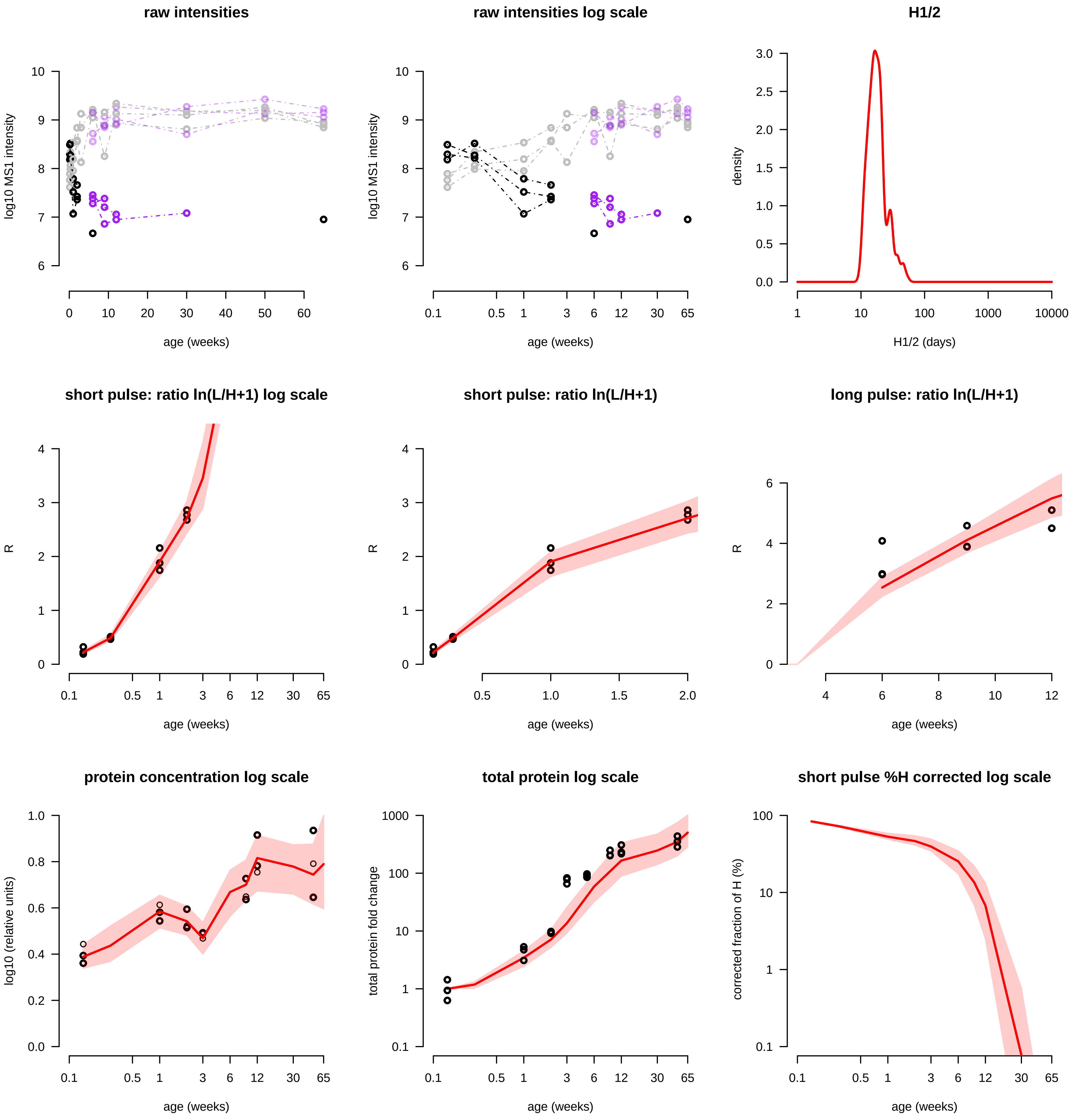

P70336;P70336−2

>sp|P70336|ROCK2_MOUSE

Rho−associated protein kinase 2

GN=Rock2

Med H1/2: 231 days

Q5 H1/2: 137.9 days

Q95 H1/2: 793.6 days

reliable model fit: yes

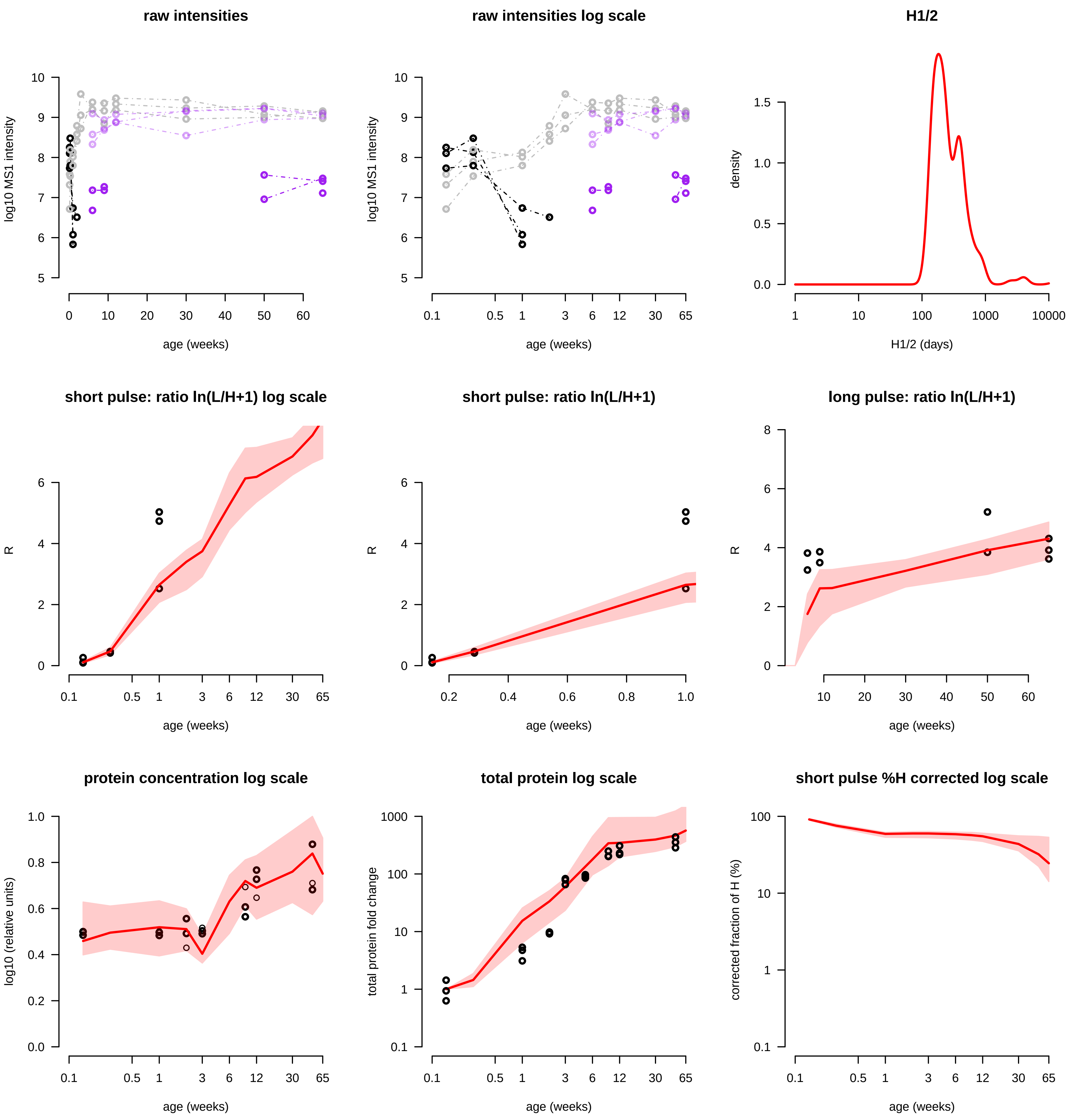

P26040

>sp|P26040|EZRI_MOUSE

Ezrin

GN=Ezr

Med H1/2: 4.8 days

Q5   H1/2: 3.8 days

Q95 H1/2: 6 days

reliable model fit: yes

raw intensities

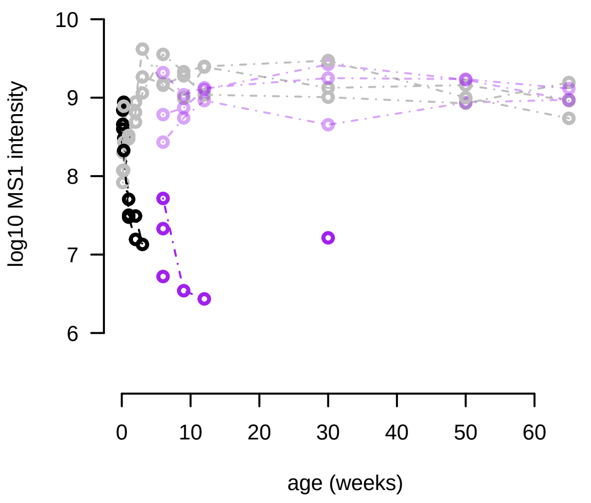

raw intensities log scale

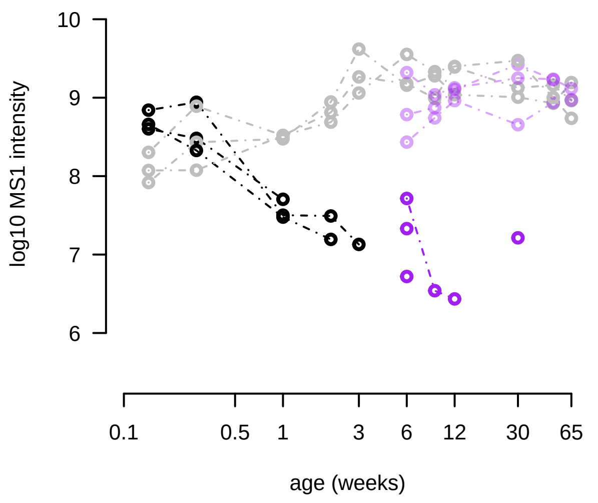

H1/2

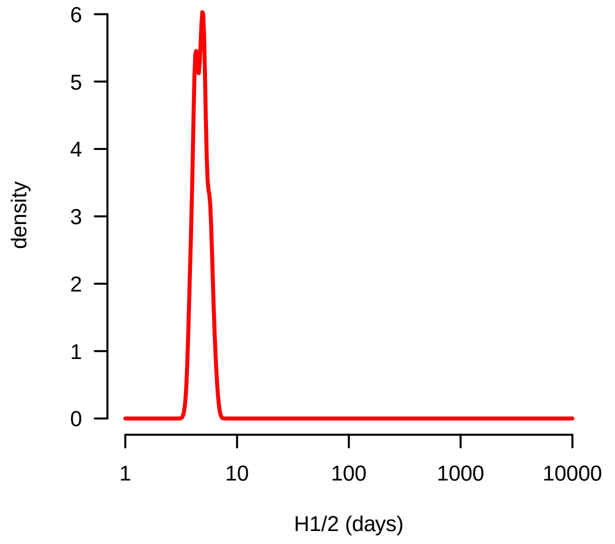

short pulse: ratio ln(L/H+1) log scale

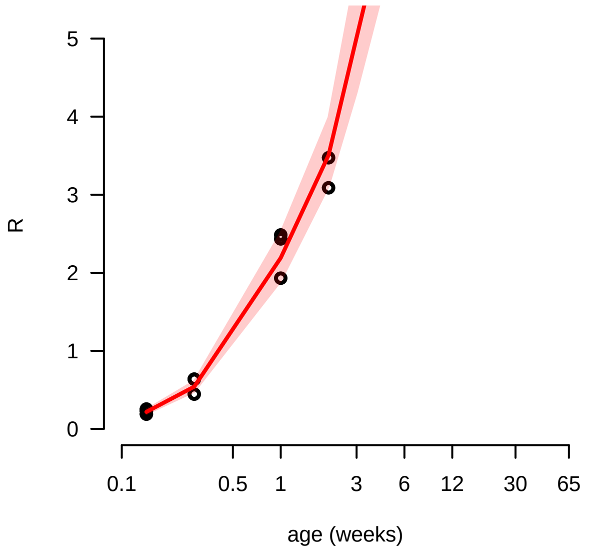

short pulse: ratio ln(L/H+1)

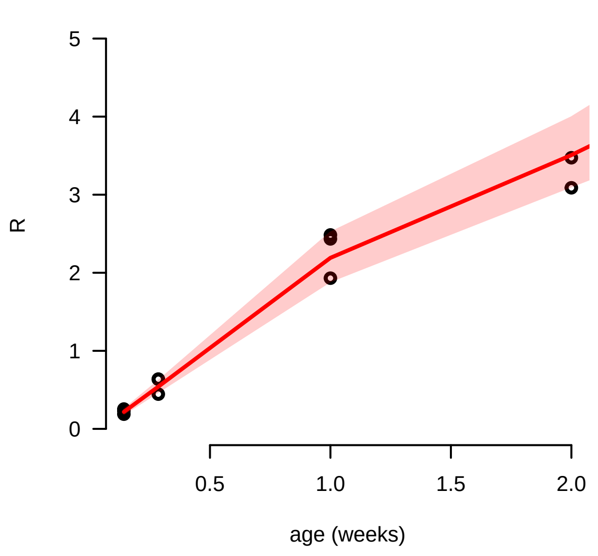

long pulse: ratio ln(L/H+1)

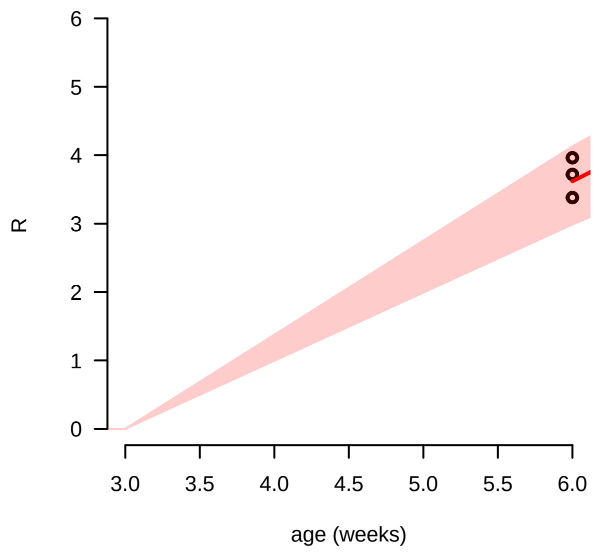

protein concentration log scale

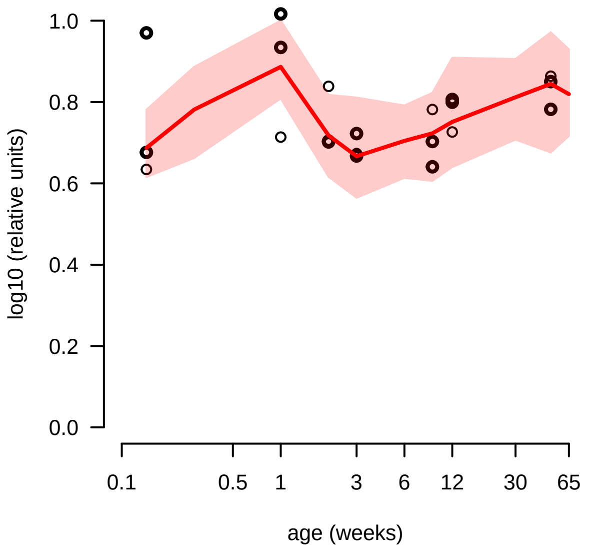

total protein log scale

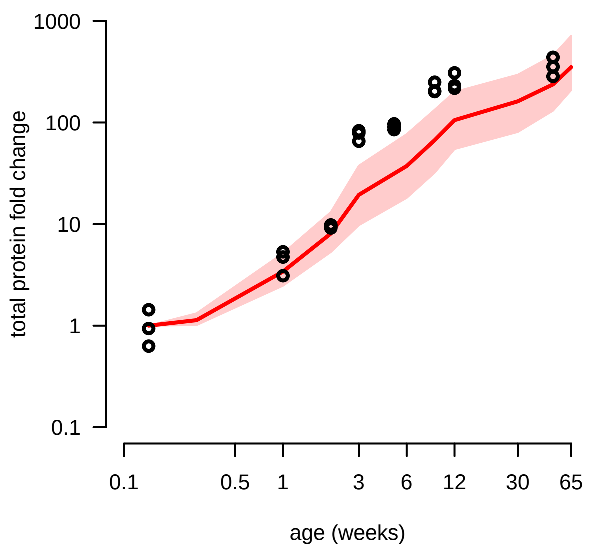

short pulse %H corrected log scale

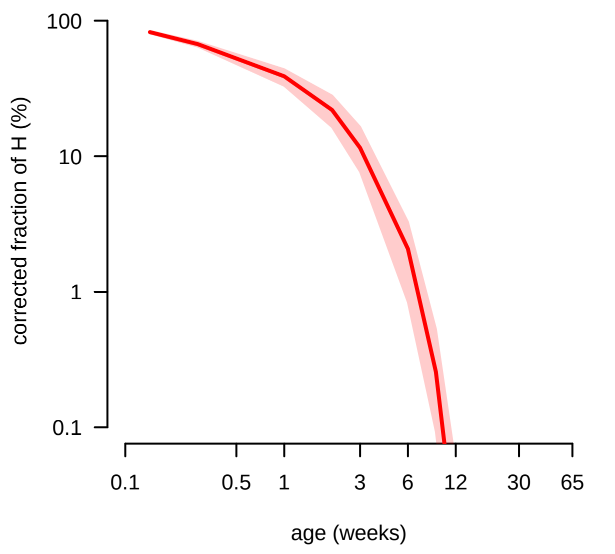

Q6PDI5;Q6PDI5−2

>sp|Q6PDI5|ECM29_MOUSE

Proteasome adapter and scaffold protein ECM29

GN=Ecpas

Med H1/2: 3.7 days

Q5 H1/2: 3.1 days

Q95 H1/2: 4.5 days

reliable model fit: yes

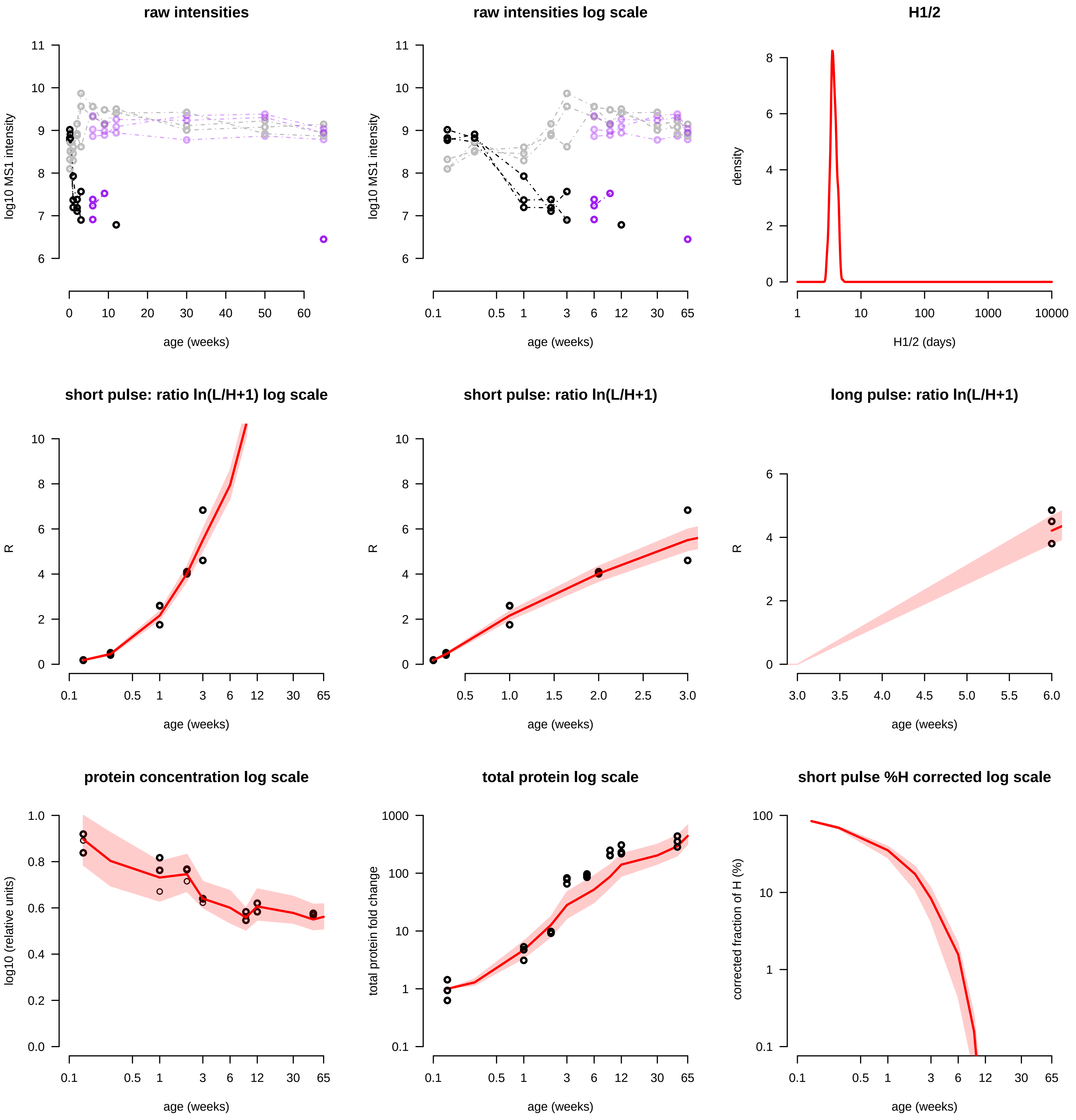

P11881−8;P11881−6;P11881−4;P11881−2

>sp|P11881−8|ITPR1_MOUSE

Isoform 8 of Inositol 1,4,5−trisphosphate receptor type 1

GN=Itpr1

Med H1/2: 6.8 days

Q5 H1/2: 3.4 days

Q95 H1/2: 31.2 days

reliable model fit: yes

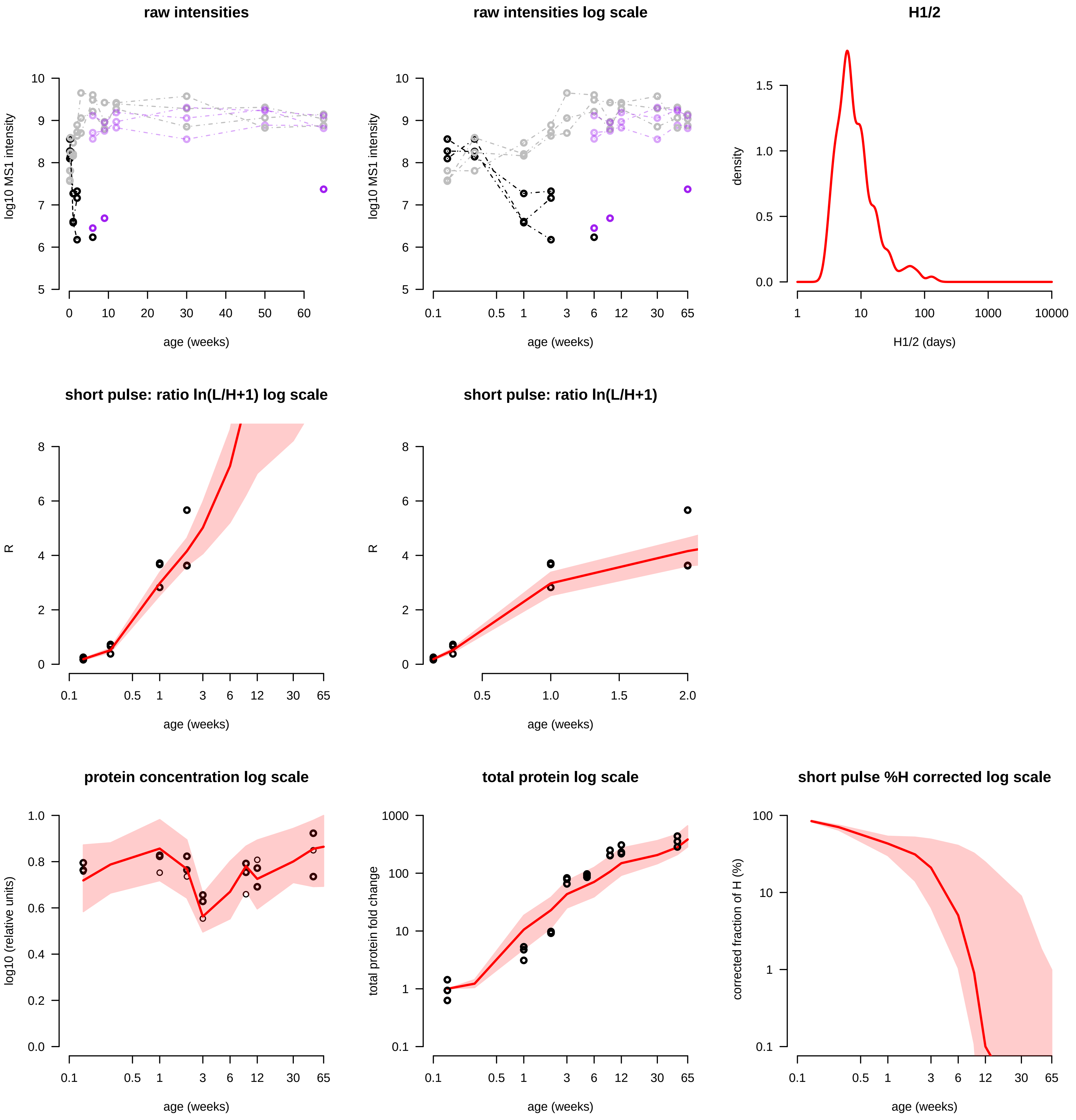

Q9CQH7

>sp|Q9CQH7|BT3L4_MOUSE

Transcription factor BTF3 homolog 4

GN=Btf3l4

Med H1/2: 4 days

Q5 H1/2: 2.8 days

Q95 H1/2: 5.9 days

reliable model fit: yes

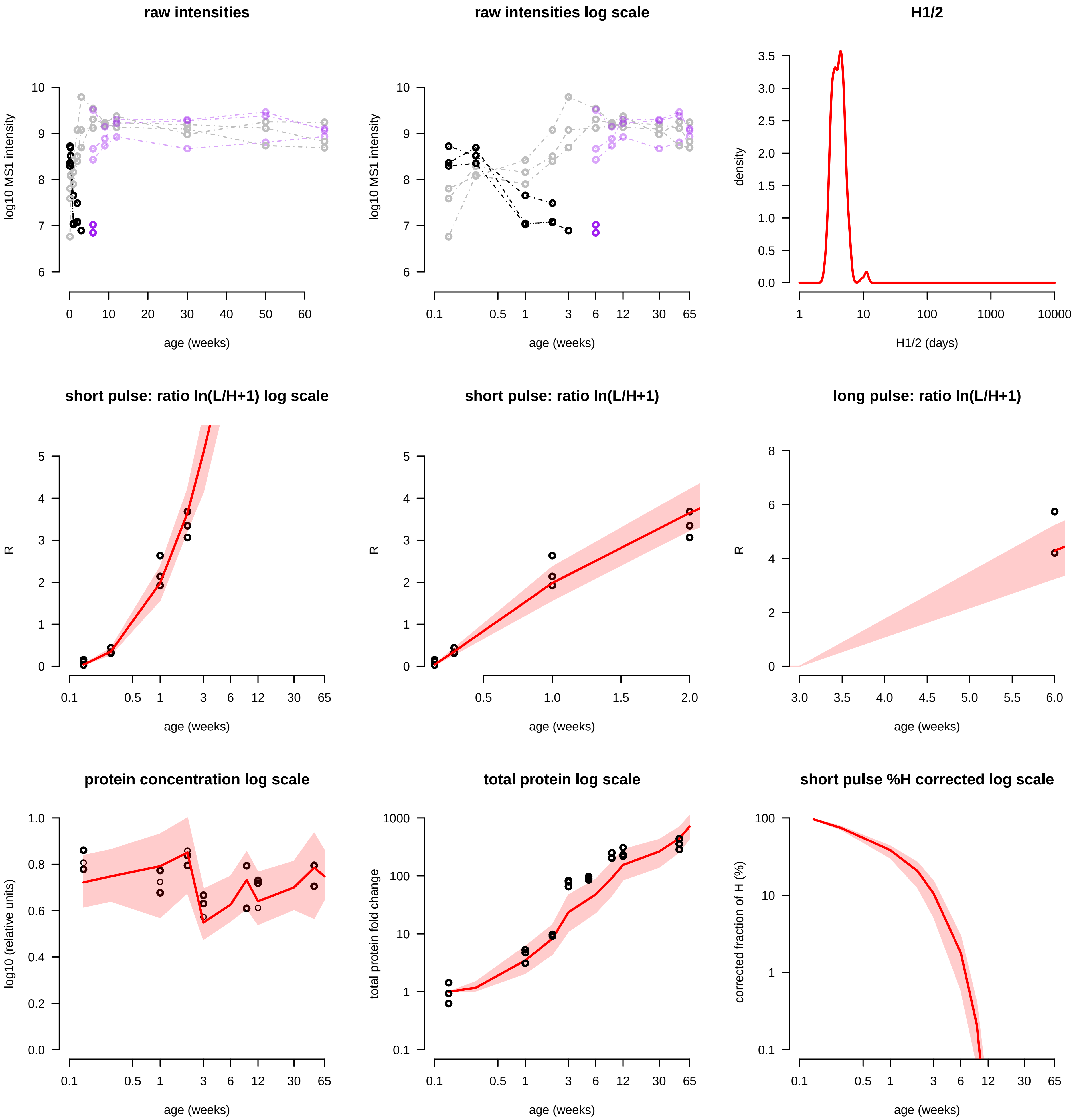

O88544

>sp|O88544|CSN4_MOUSE

COP9 signalosome complex subunit 4

GN=Cops4

Med H1/2: 6.3 days

Q5 H1/2: 5.1 days

Q95 H1/2: 8.3 days

reliable model fit: yes

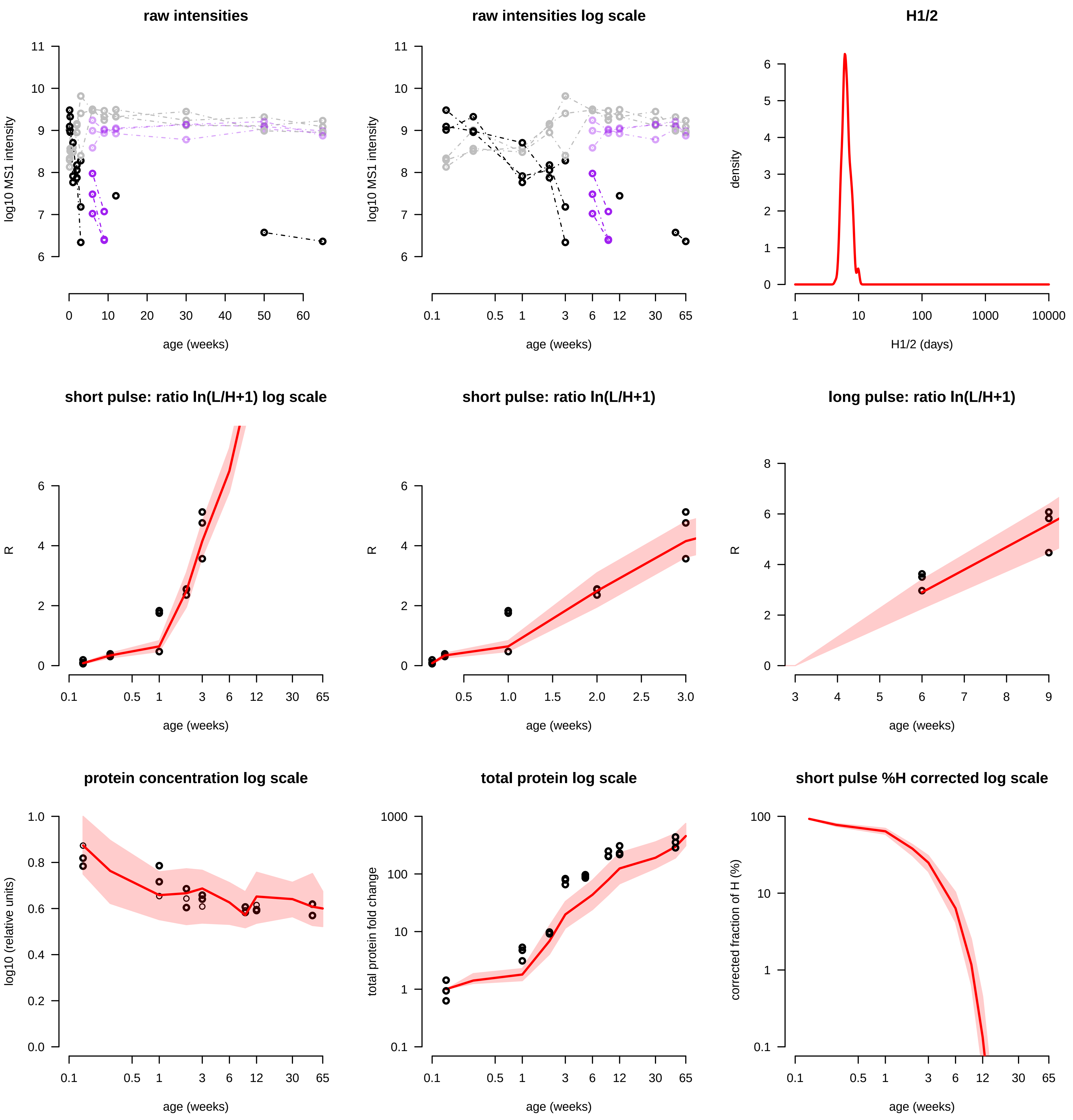

P63325

>sp|P63325|RS10_MOUSE

40S ribosomal protein S10

GN=Rps10

Med H1/2: 7.4 days

Q5 H1/2: 6.1 days

Q95 H1/2: 9.4 days

reliable model fit: yes

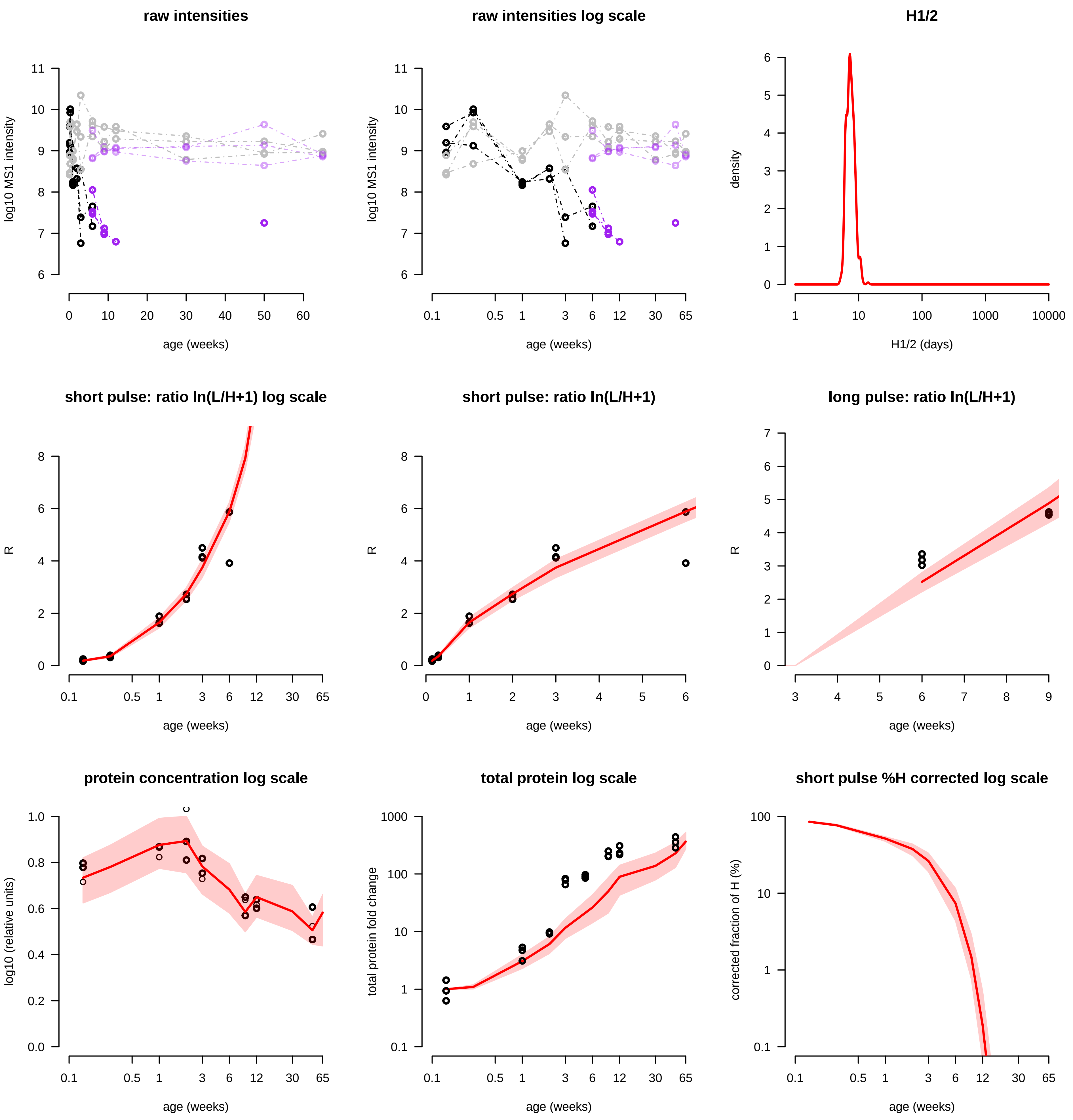

Q9DCS3

>sp|Q9DCS3|MECR_MOUSE

Enoyl−[acyl−carrier−protein] reductase, mitochondrial

GN=Mecr

Med H1/2: 14.6 days

Q5 H1/2: 10.6 days

Q95 H1/2: 22.4 days

reliable model fit: yes

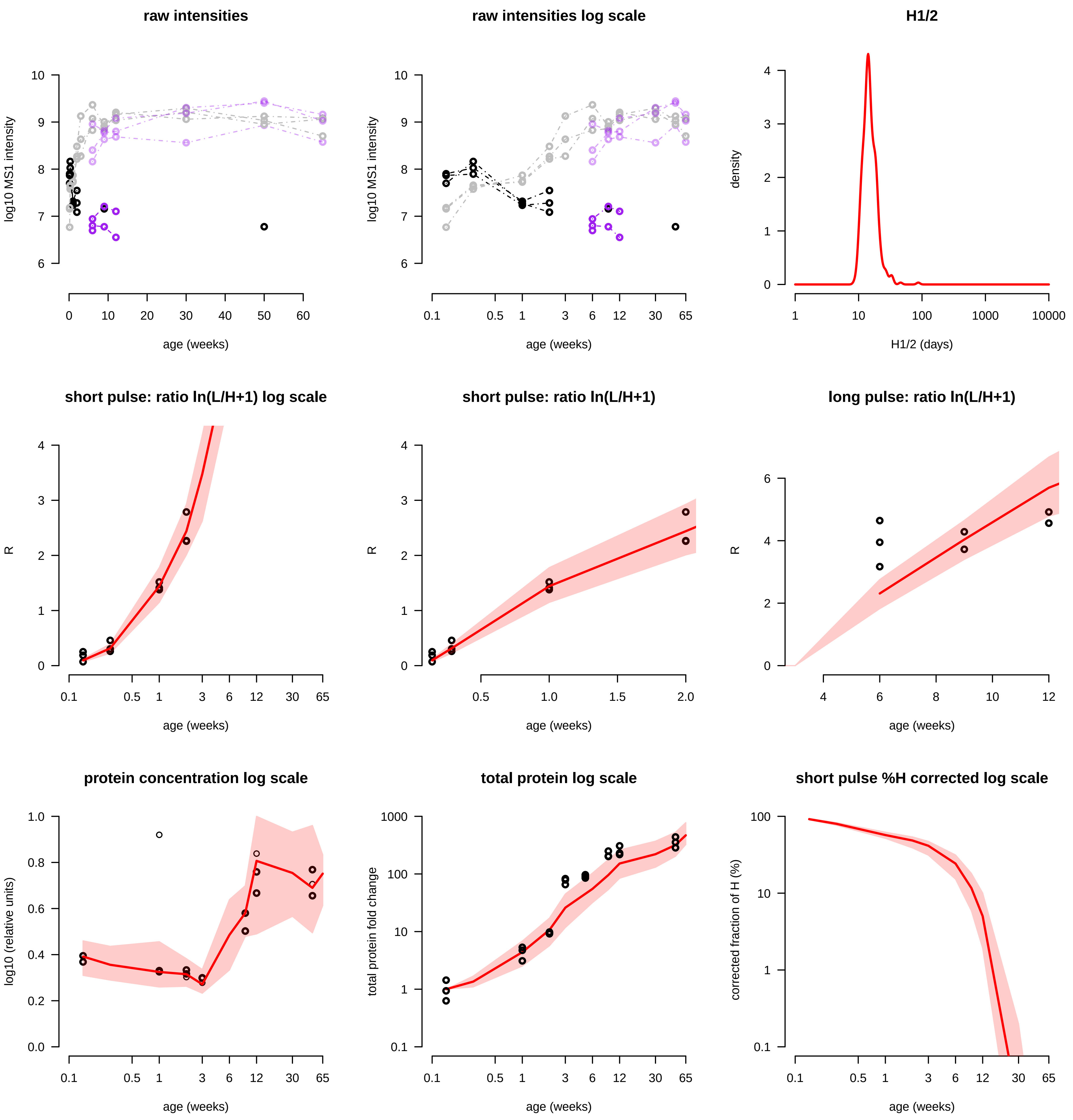

Q62087

>sp|Q62087|PON3_MOUSE

Serum paraoxonase/lactonase 3

GN=Pon3

Med H1/2: 10.6 days

Q5 H1/2: 7.6 days

Q95 H1/2: 16 days

reliable model fit: yes

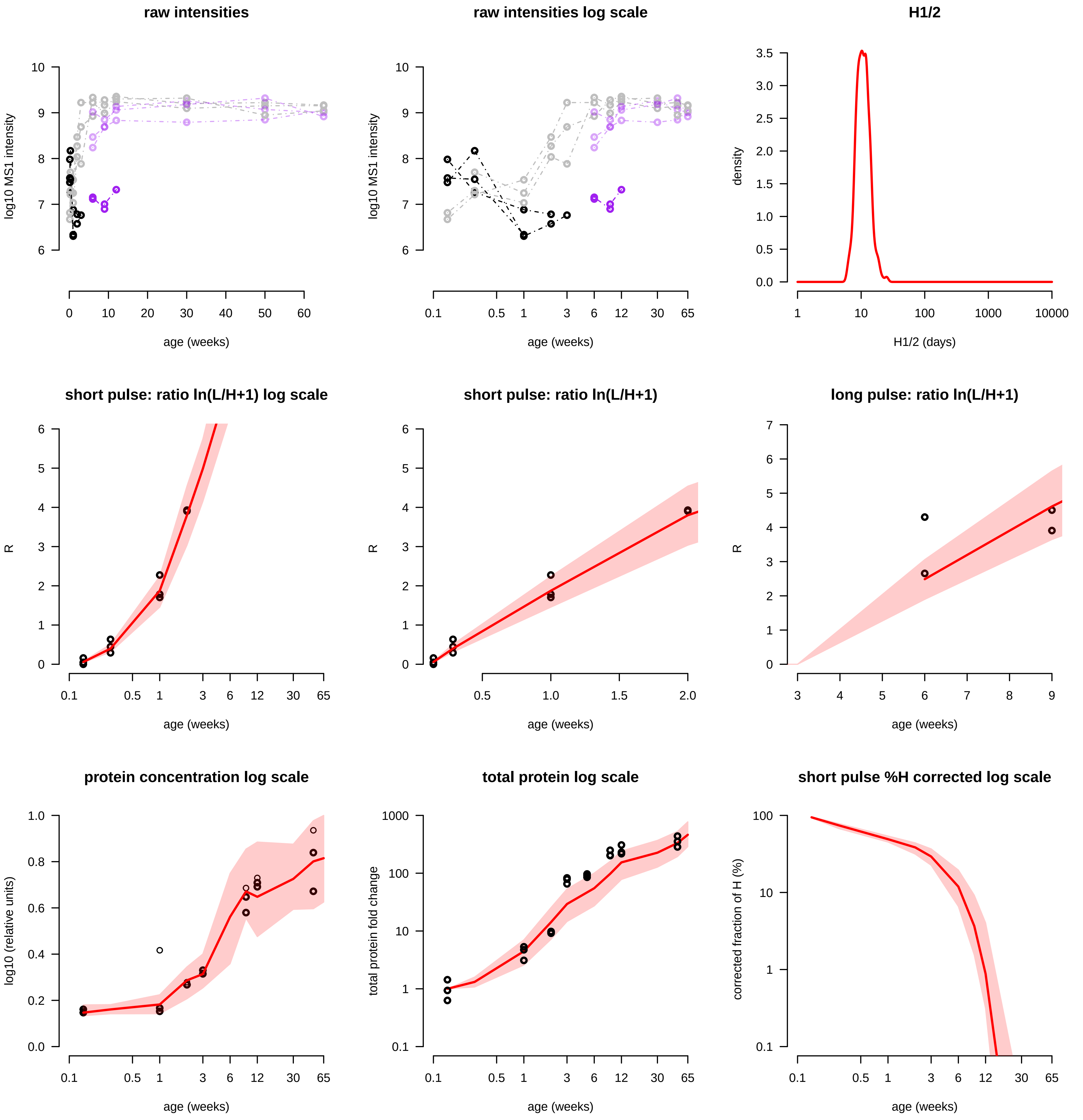

O70325−2;O70325;O70325−3

>sp|O70325−2|GPX4_MOUSE

Isoform Cytoplasmic of Phospholipid hydroperoxide glutathione peroxidase

GN=Gpx4

Med H1/2: 5.1 days

Q5 H1/2: 3.5 days

Q95 H1/2: 8.9 days

reliable model fit: yes

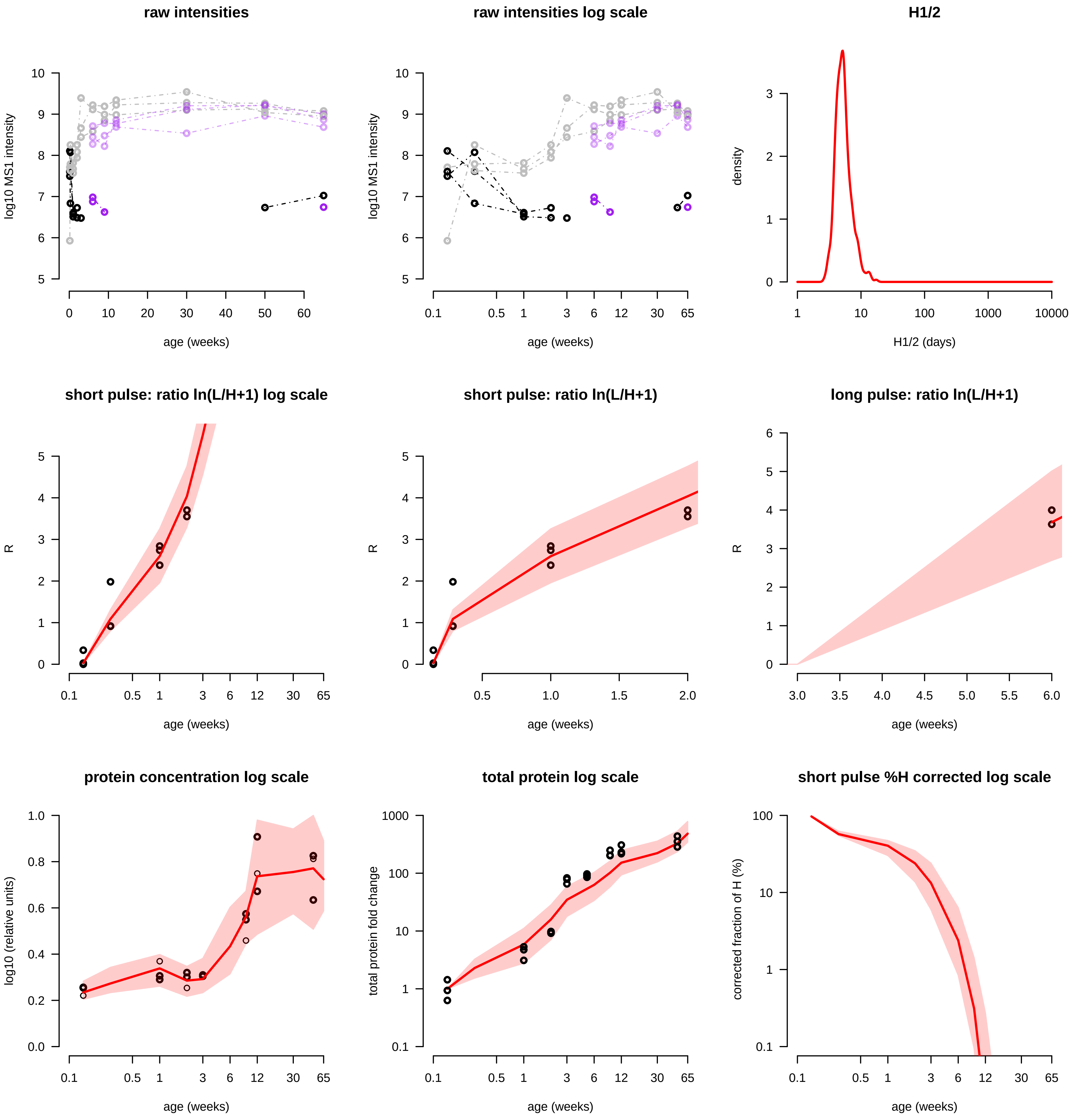

Q9CZ42−3;Q9CZ42

>sp|Q9CZ42−3|NNRD_MOUSE

Isoform 3 of ATP−dependent (S)−NAD(P)H−hydrate dehydratase

GN=Naxd

Med H1/2: 7.4 days

Q5 H1/2: 6.3 days

Q95 H1/2: 9.4 days

reliable model fit: yes

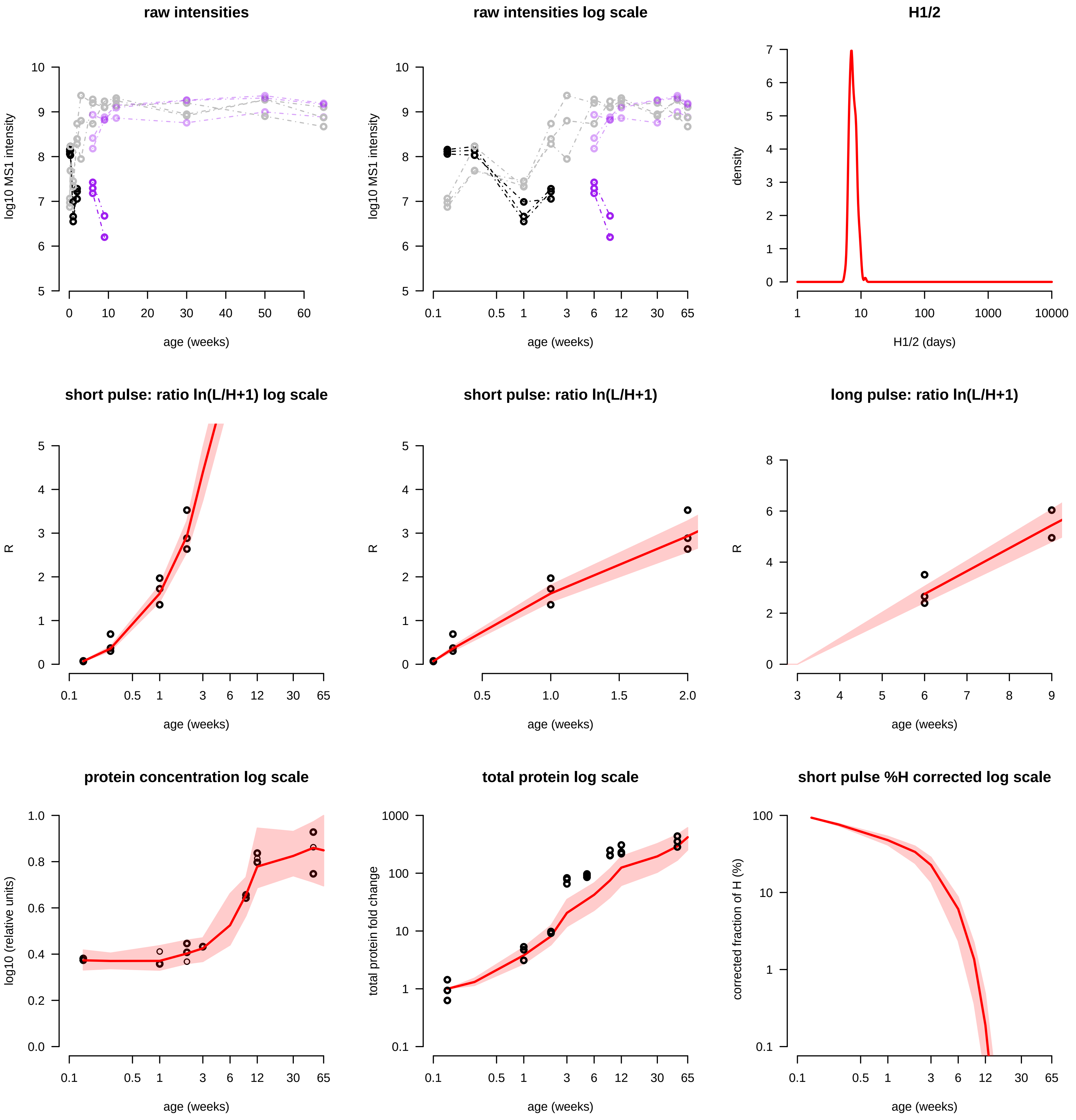

P60229

>sp|P60229|EIF3E_MOUSE

Eukaryotic translation initiation factor 3 subunit E

GN=Eif3e

Med H1/2: 5 days

Q5 H1/2: 4.5 days

Q95 H1/2: 5.6 days

reliable model fit: yes

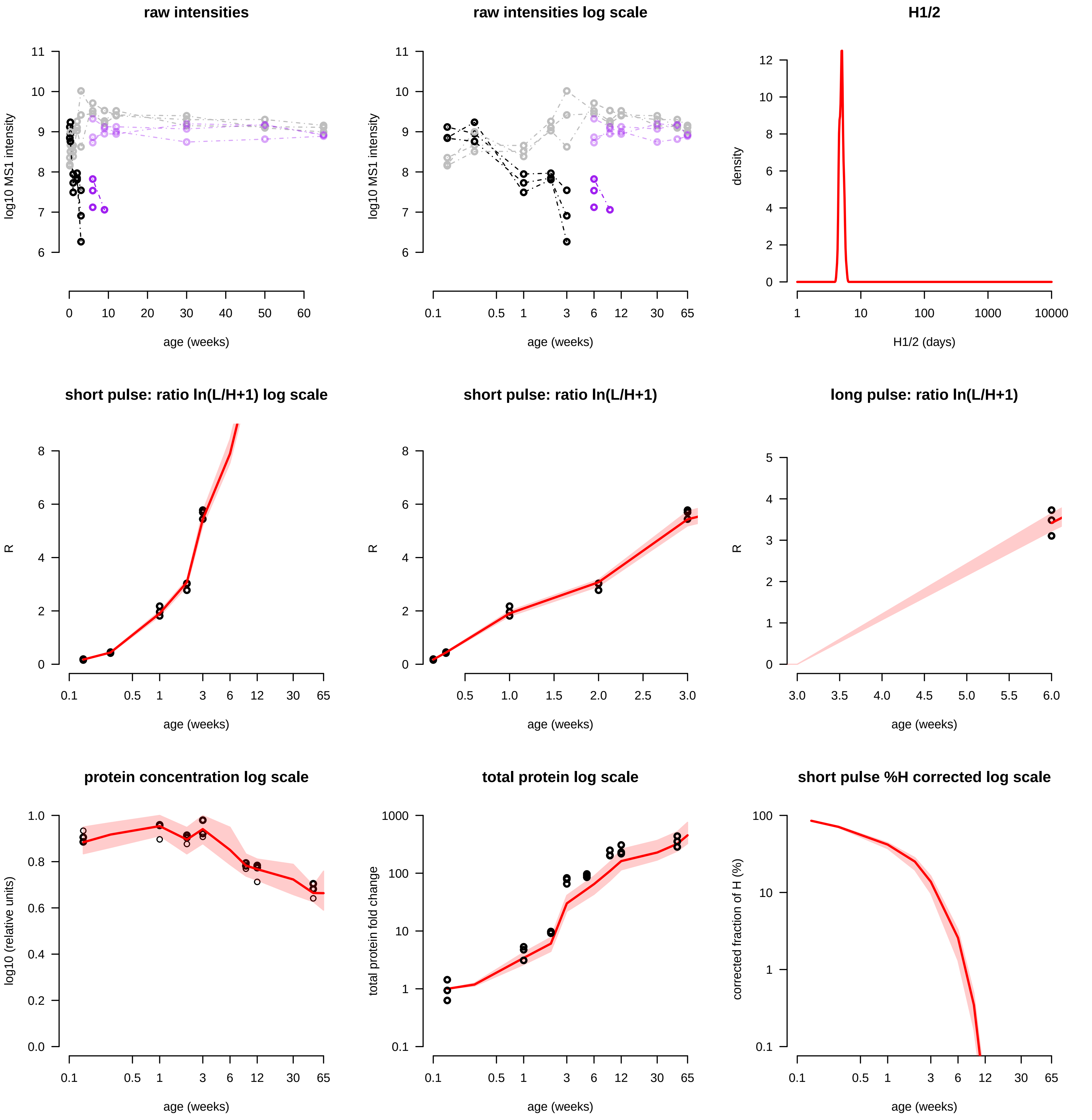

O08908

>sp|O08908|P85B_MOUSE

Phosphatidylinositol 3−kinase regulatory subunit beta

GN=Pik3r2

Med H1/2: 84.2 days

Q5 H1/2: 22.7 days

Q95 H1/2: 1221.6 days

reliable model fit: no

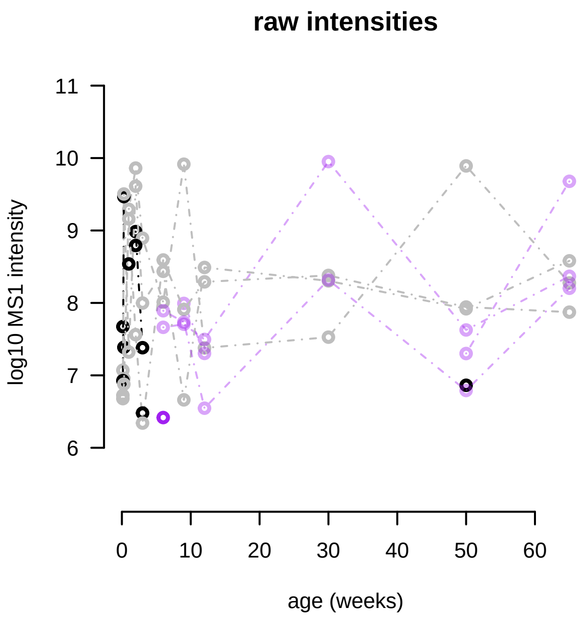

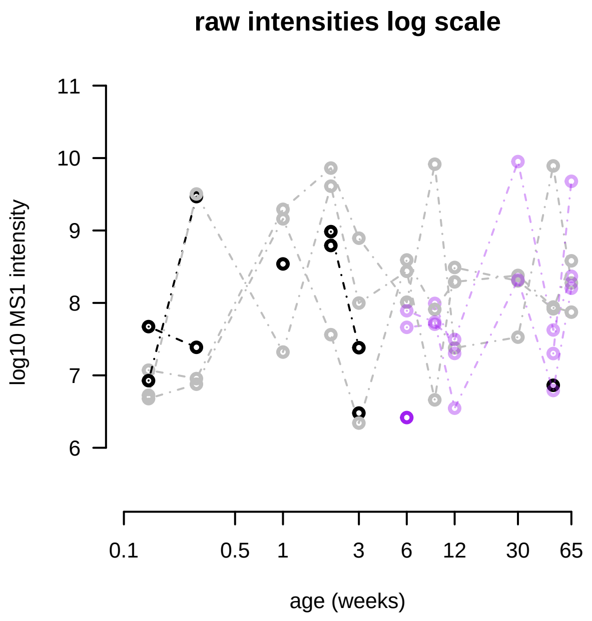

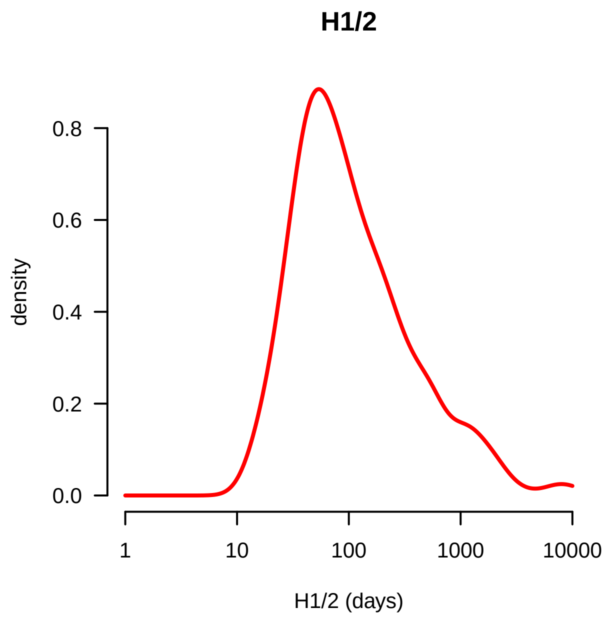

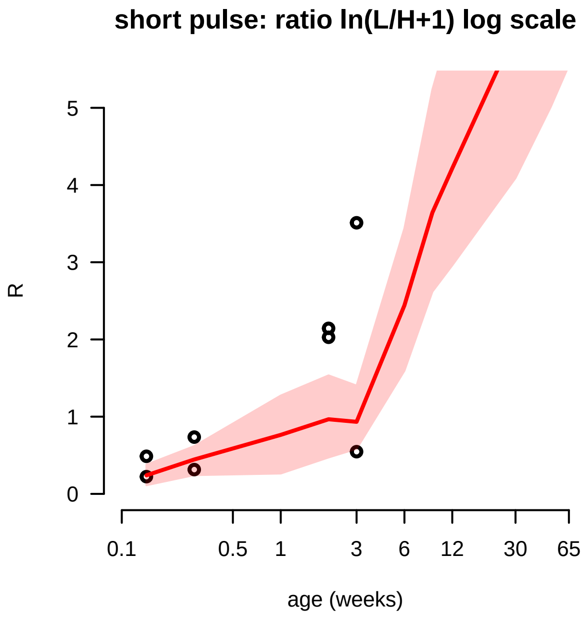

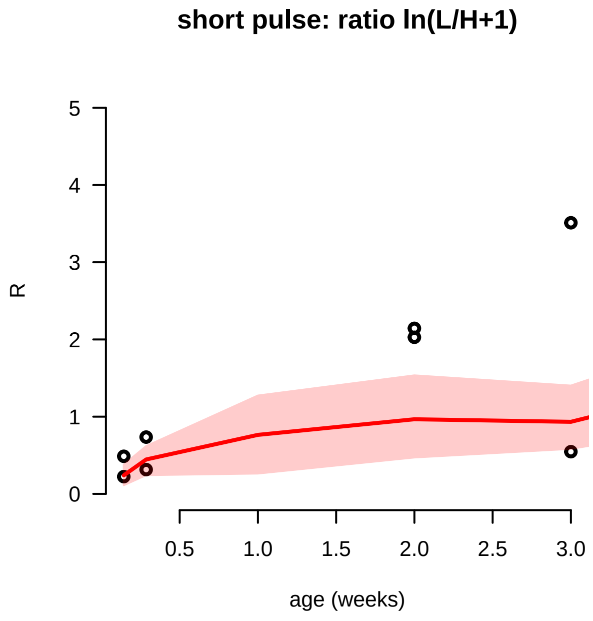

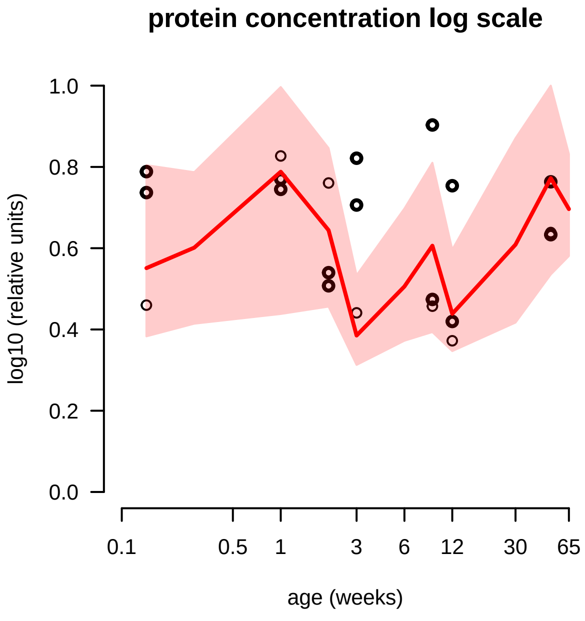

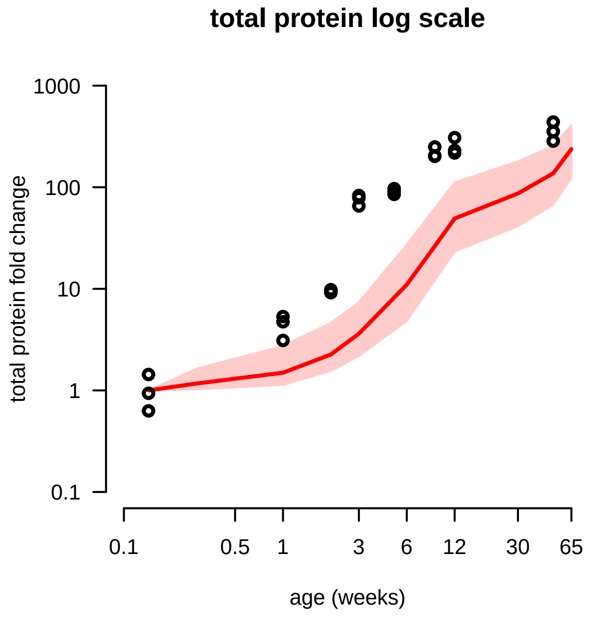

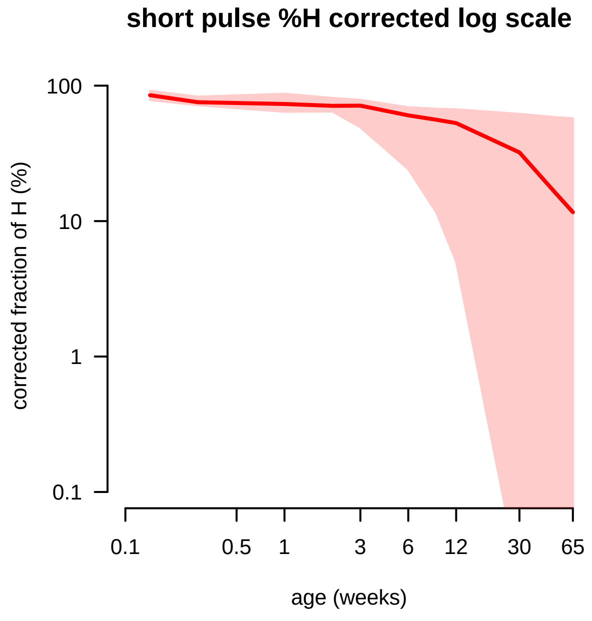

Q9DCJ5

>sp|Q9DCJ5|NDUA8_MOUSE

NADH dehydrogenase [ubiquinone] 1 alpha subcomplex subunit 8

GN=Ndufa8

Med H1/2: 5.4 days

Q5 H1/2: 4.1 days

Q95 H1/2: 7.3 days

reliable model fit: yes

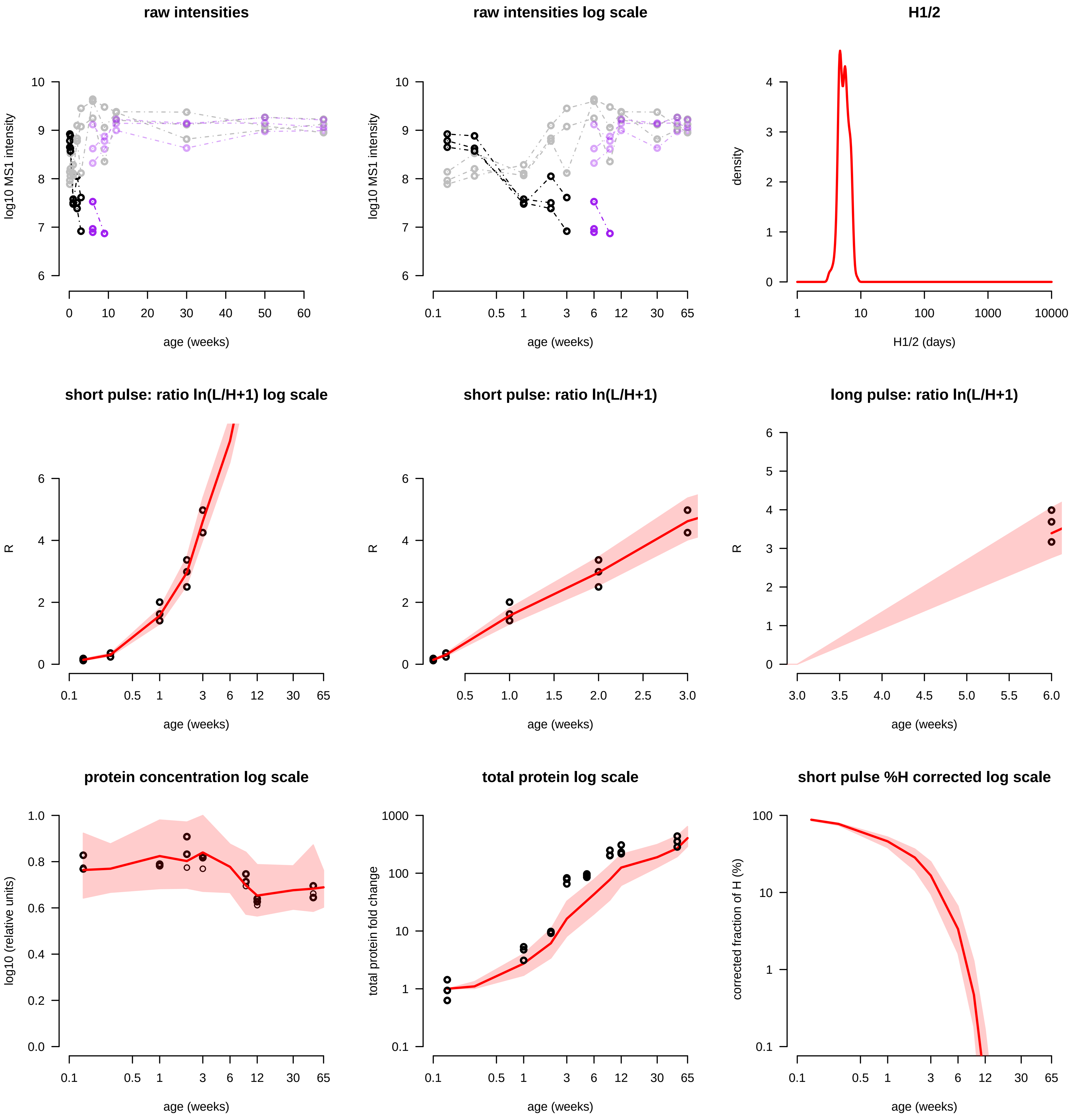

P12382

>sp|P12382|PFKAL_MOUSE

ATP−dependent 6−phosphofructokinase, liver type

GN=Pfkl

Med H1/2: 6.7 days

Q5 H1/2: 5.6 days

Q95 H1/2: 8.7 days

reliable model fit: yes

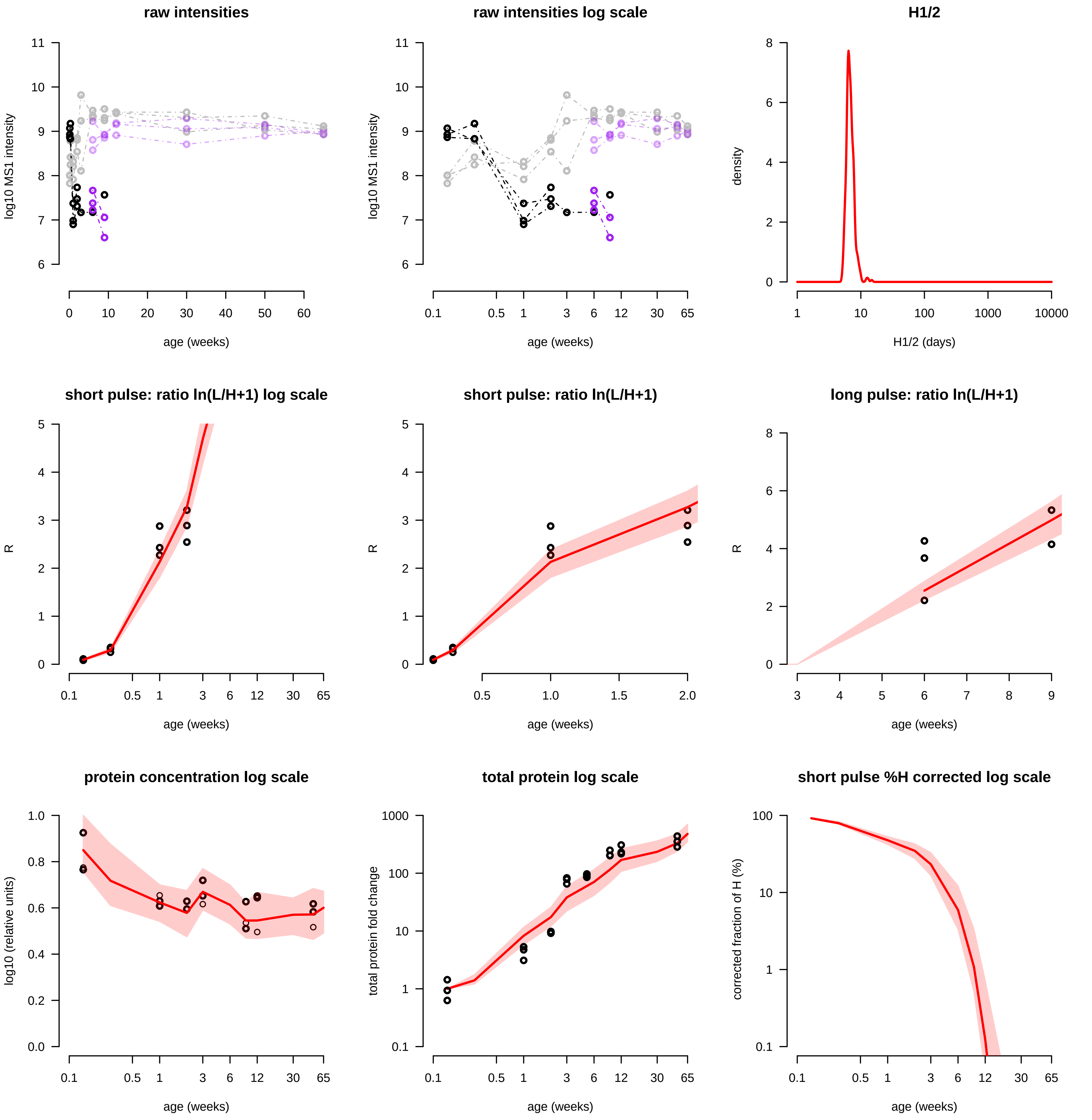

P97370

>sp|P97370|AT1B3_MOUSE

Sodium/potassium−transporting ATPase subunit beta−3

GN=Atp1b3

Med H1/2: 5 days

Q5 H1/2: 4 days

Q95 H1/2: 7.3 days

reliable model fit: yes

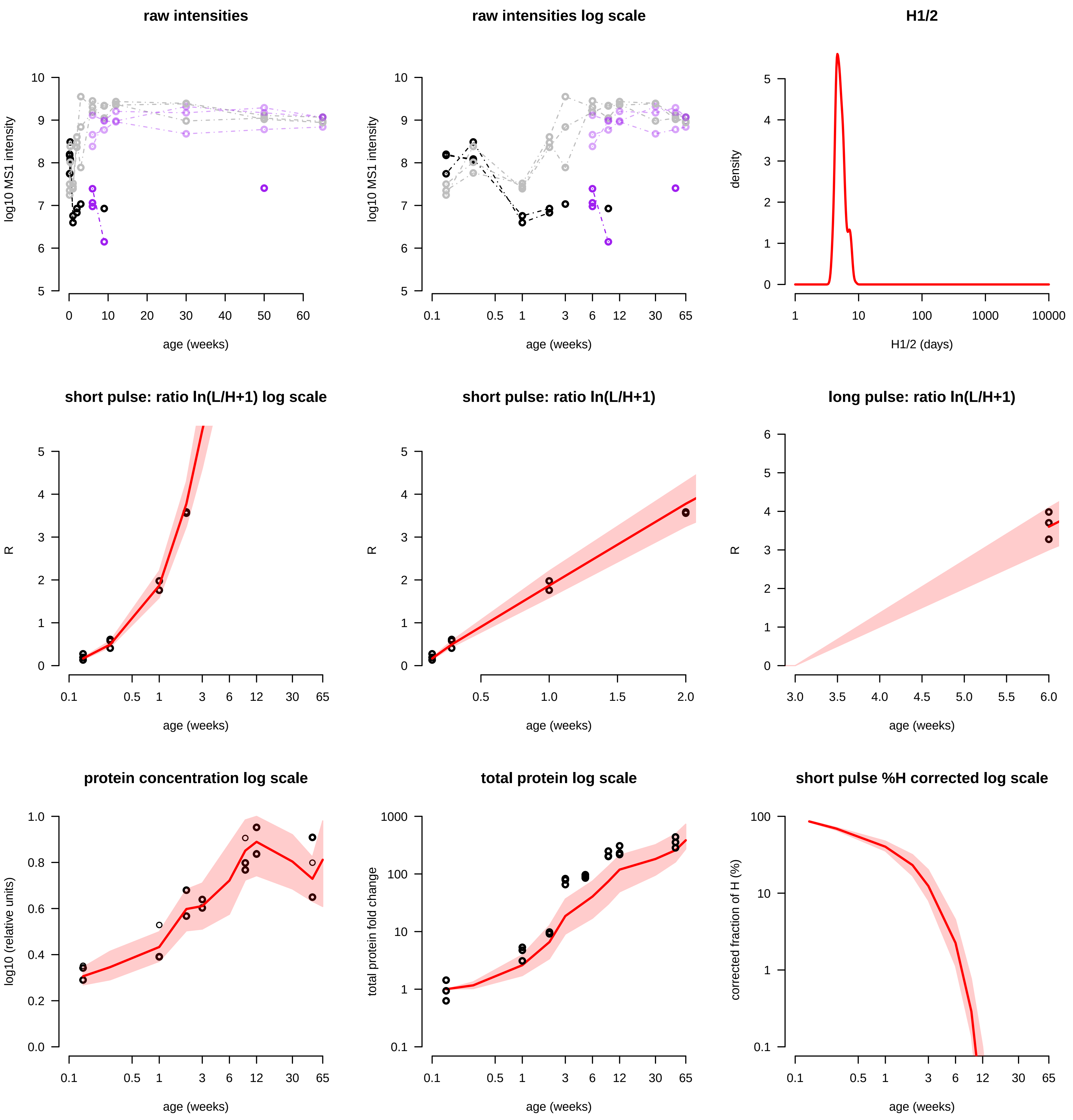

Q7TMY8;Q7TMY8−4;Q7TMY8−2

>sp|Q7TMY8|HUWE1_MOUSE

E3 ubiquitin−protein ligase HUWE1

GN=Huwe1

Med H1/2: 3.9 days

Q5 H1/2: 2.5 days

Q95 H1/2: 10.4 days

reliable model fit: yes

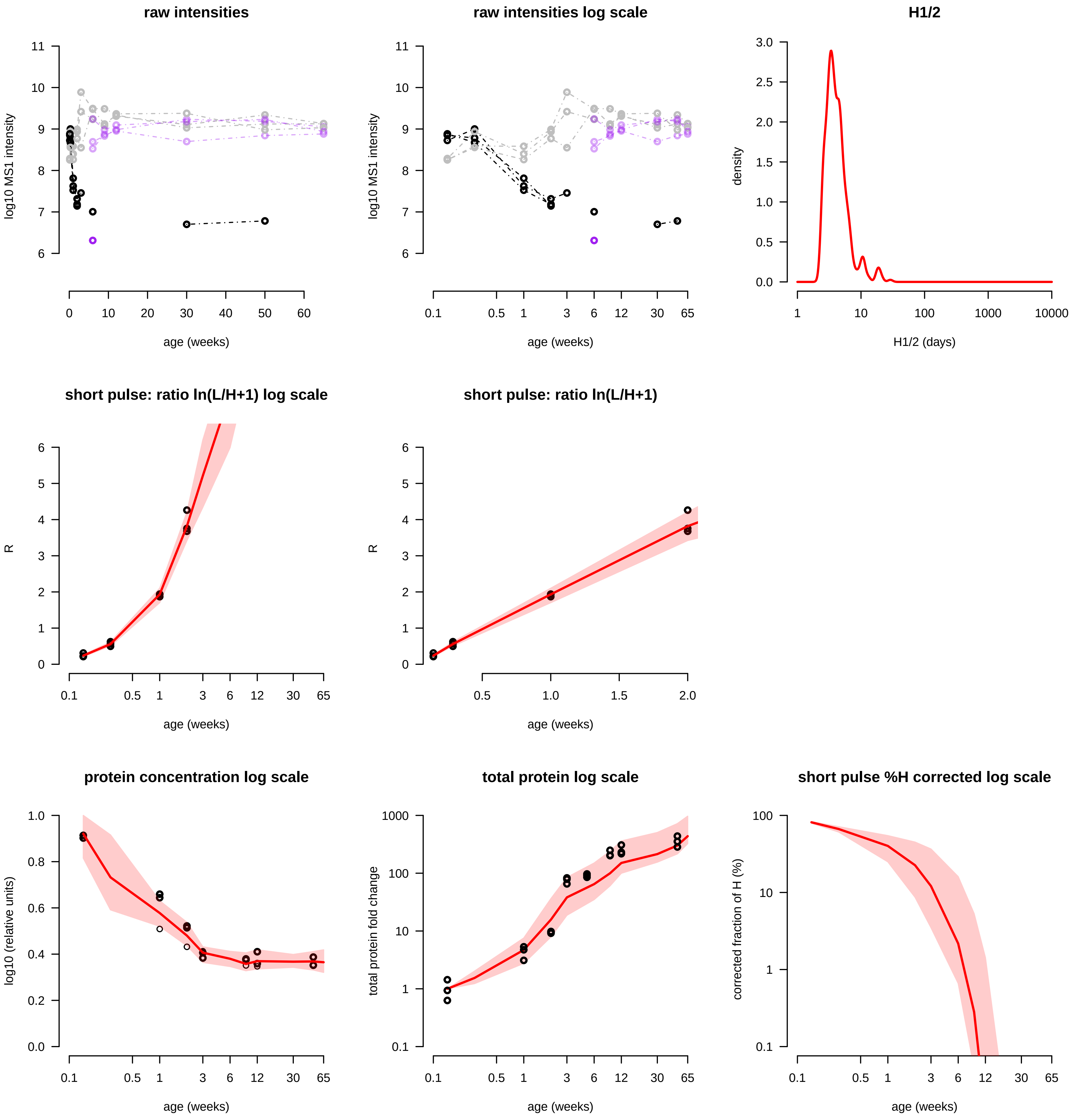

P00493

>sp|P00493|HPRT_MOUSE

Hypoxanthine−guanine phosphoribosyltransferase

GN=Hprt1

Med H1/2: 5.2 days

Q5 H1/2: 4.1 days

Q95 H1/2: 8.2 days

reliable model fit: yes

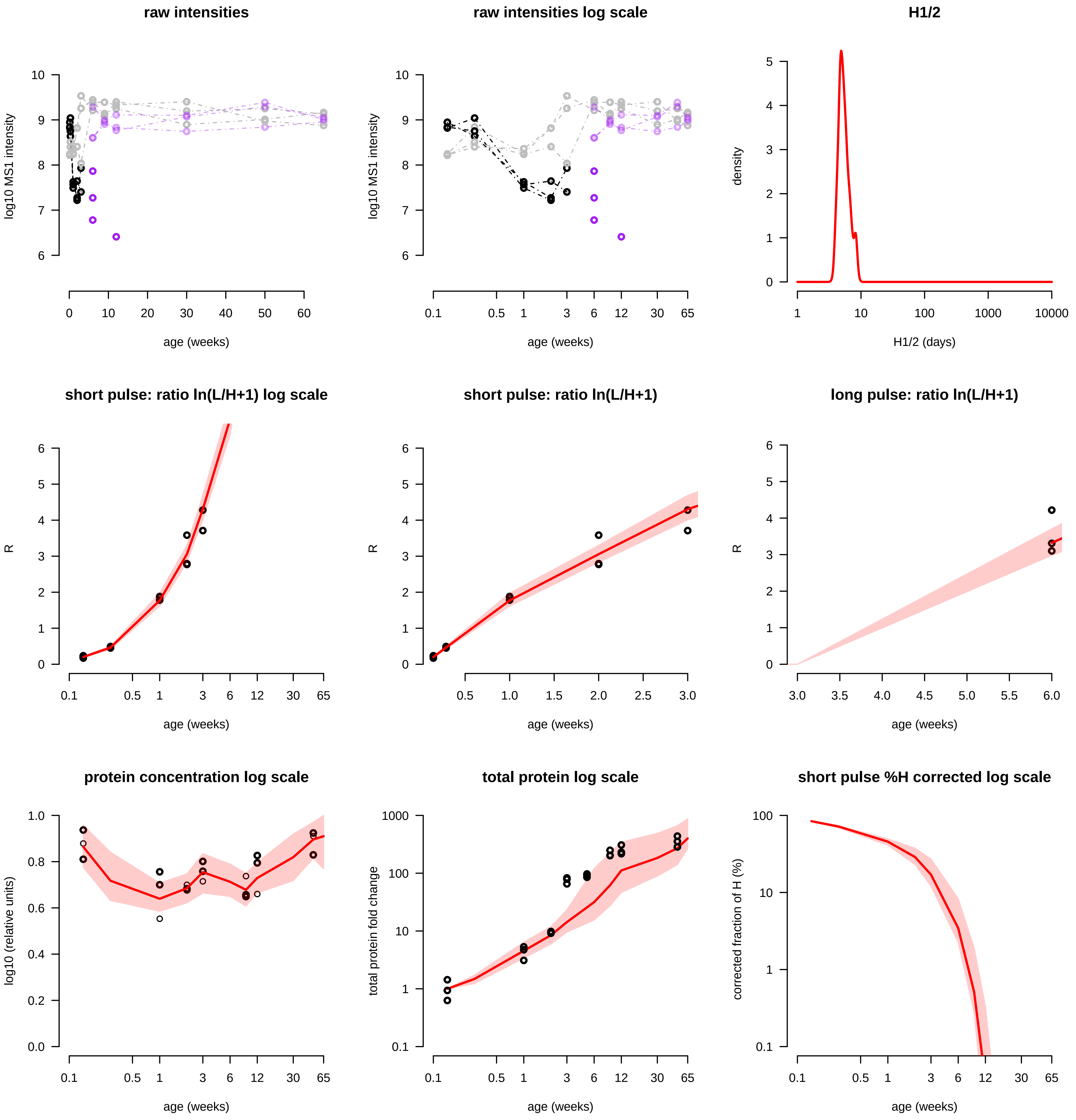

P54822

>sp|P54822|PUR8_MOUSE

Adenylosuccinate lyase

GN=Adsl

Med H1/2: 25 days

Q5 H1/2: 14 days

Q95 H1/2: 72.9 days

reliable model fit: yes

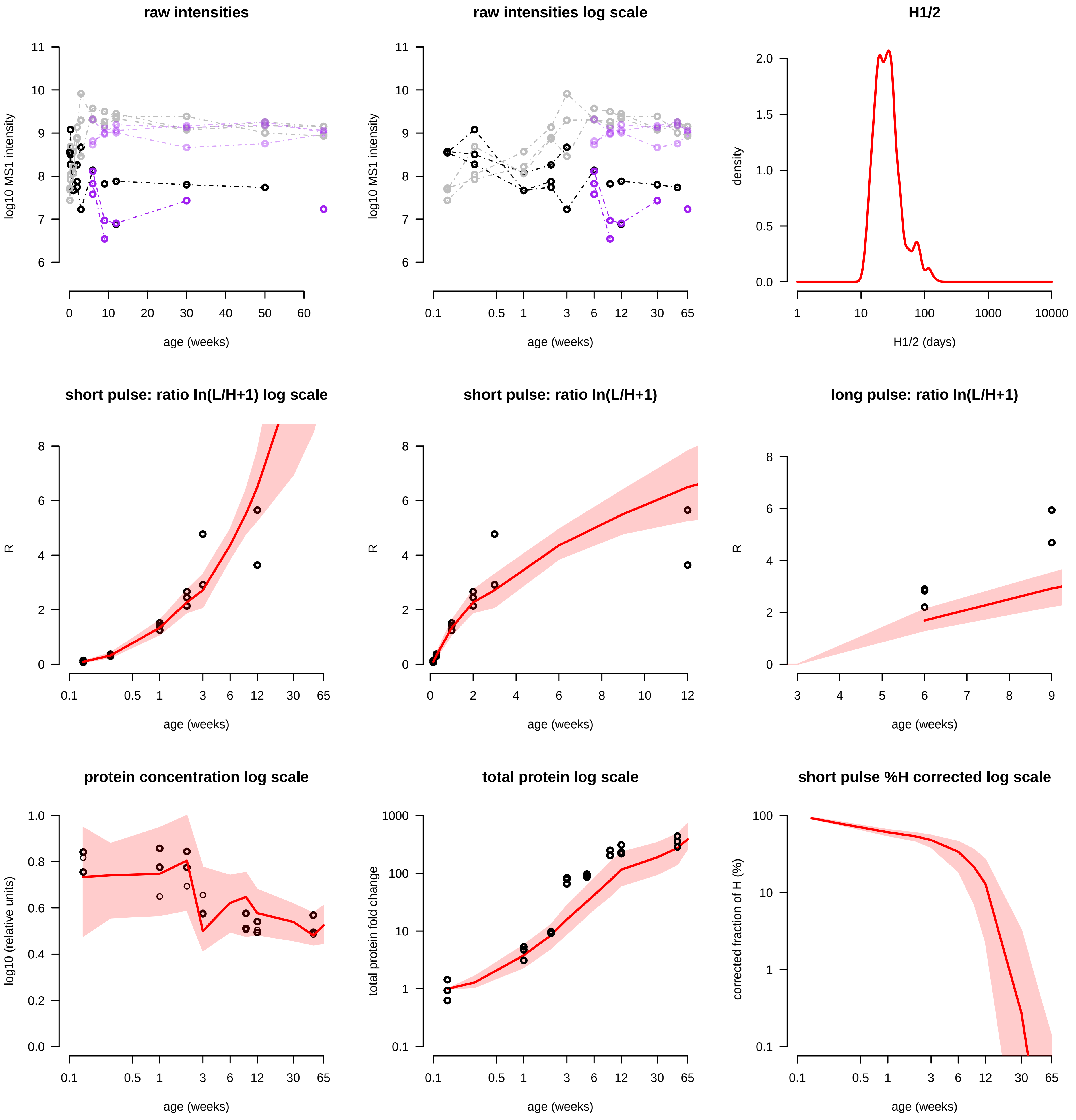

:SZ8−5;Q9ESZ8−4;Q9ESZ8−3;Q9ESZ8−6;Q9ESZ8−2;Q9E

>sp|Q9ESZ8−5|GTF2I_MOUSE

Isoform 5 of General transcription factor II−I

GN=Gtf2i

Med H1/2: 5.5 days

Q5 H1/2: 4.5 days

Q95 H1/2: 6.7 days

reliable model fit: yes

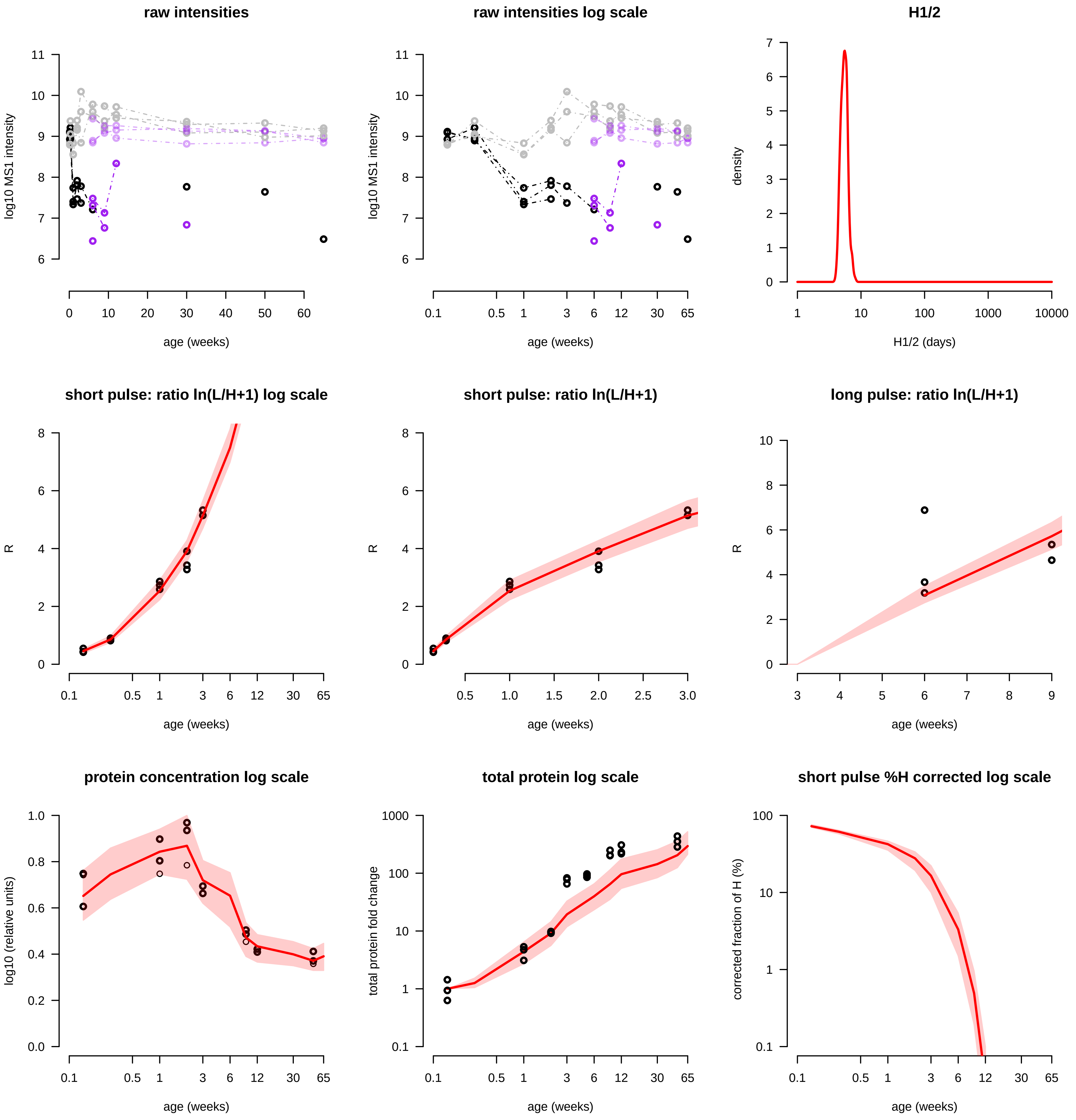

Q9ESX5

>sp|Q9ESX5|DKC1_MOUSE

H/ACA ribonucleoprotein complex subunit DKC1

GN=Dkc1

Med H1/2: 6.5 days

Q5 H1/2: 5.6 days

Q95 H1/2: 7.6 days

reliable model fit: yes

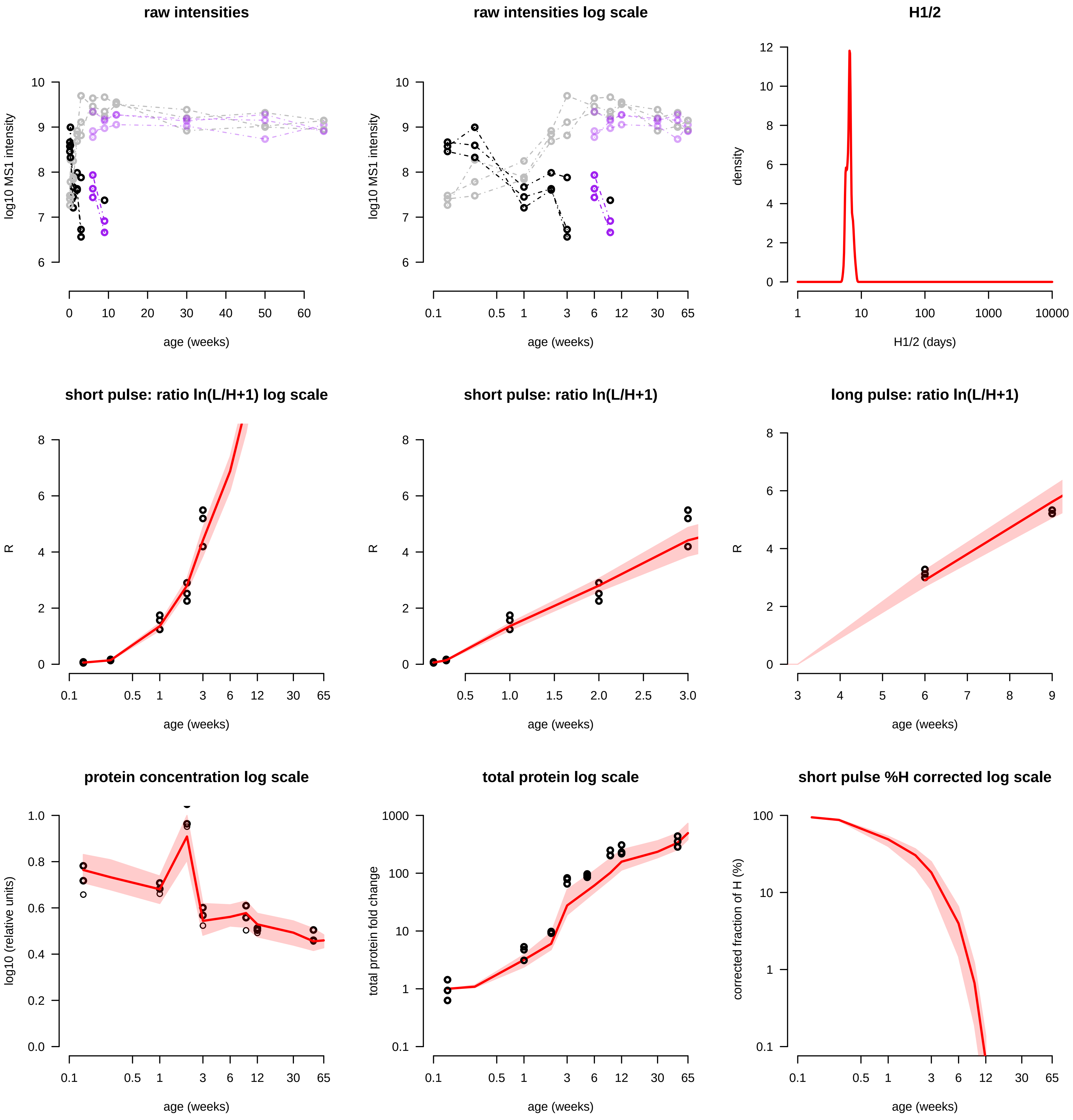

Q64012−2;Q64012

>sp|Q64012−2|RALY_MOUSE

Isoform 1 of RNA−binding protein Raly

GN=Raly

Med H1/2: 7 days

Q5 H1/2: 5.4 days

Q95 H1/2: 8.7 days

reliable model fit: yes

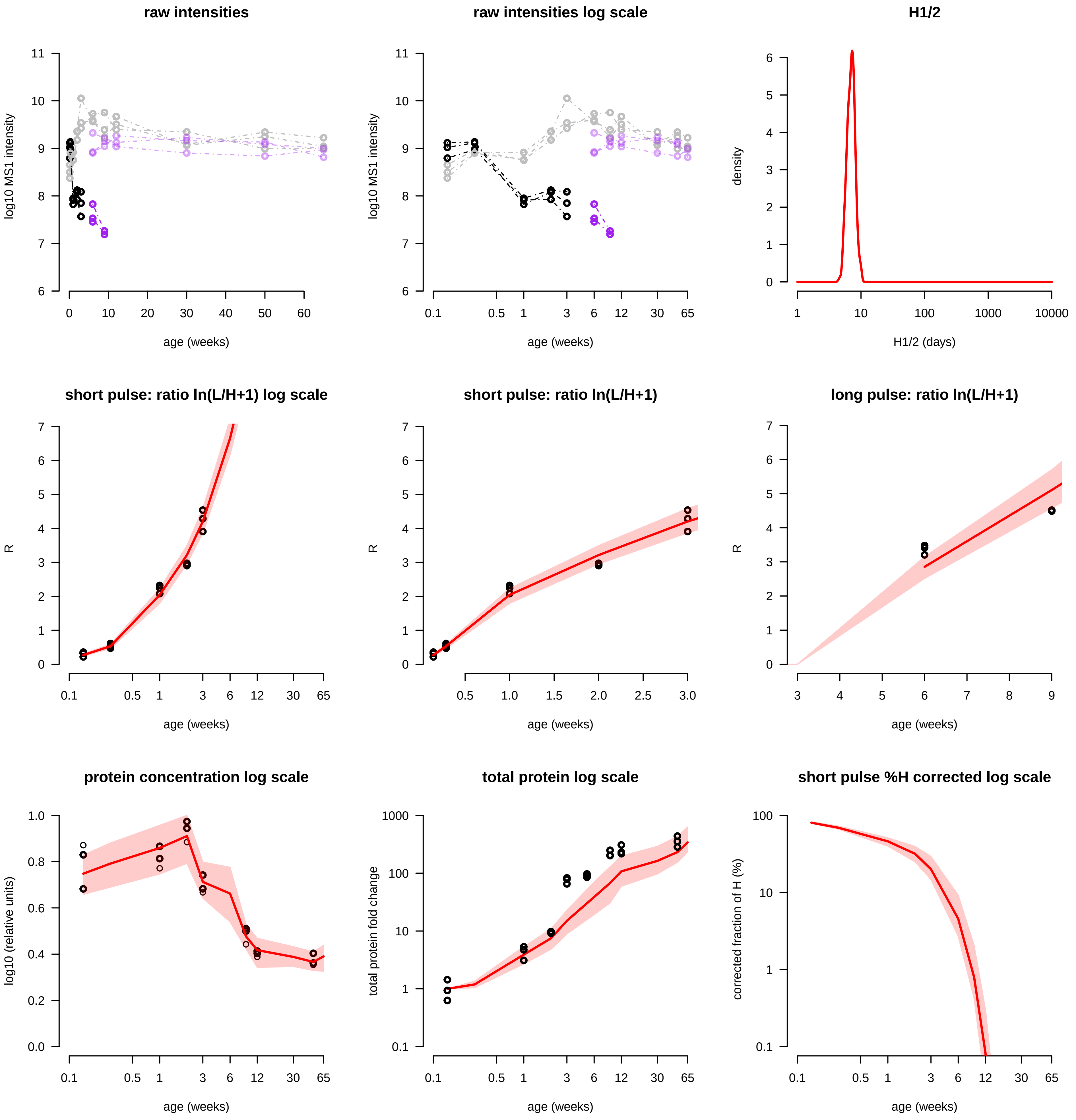

Q9CZD3

>sp|Q9CZD3|GARS_MOUSE

Glycine−−tRNA ligase

GN=Gars1

Med H1/2: 6.8 days

Q5 H1/2: 4.8 days

Q95 H1/2: 12.5 days

reliable model fit: yes

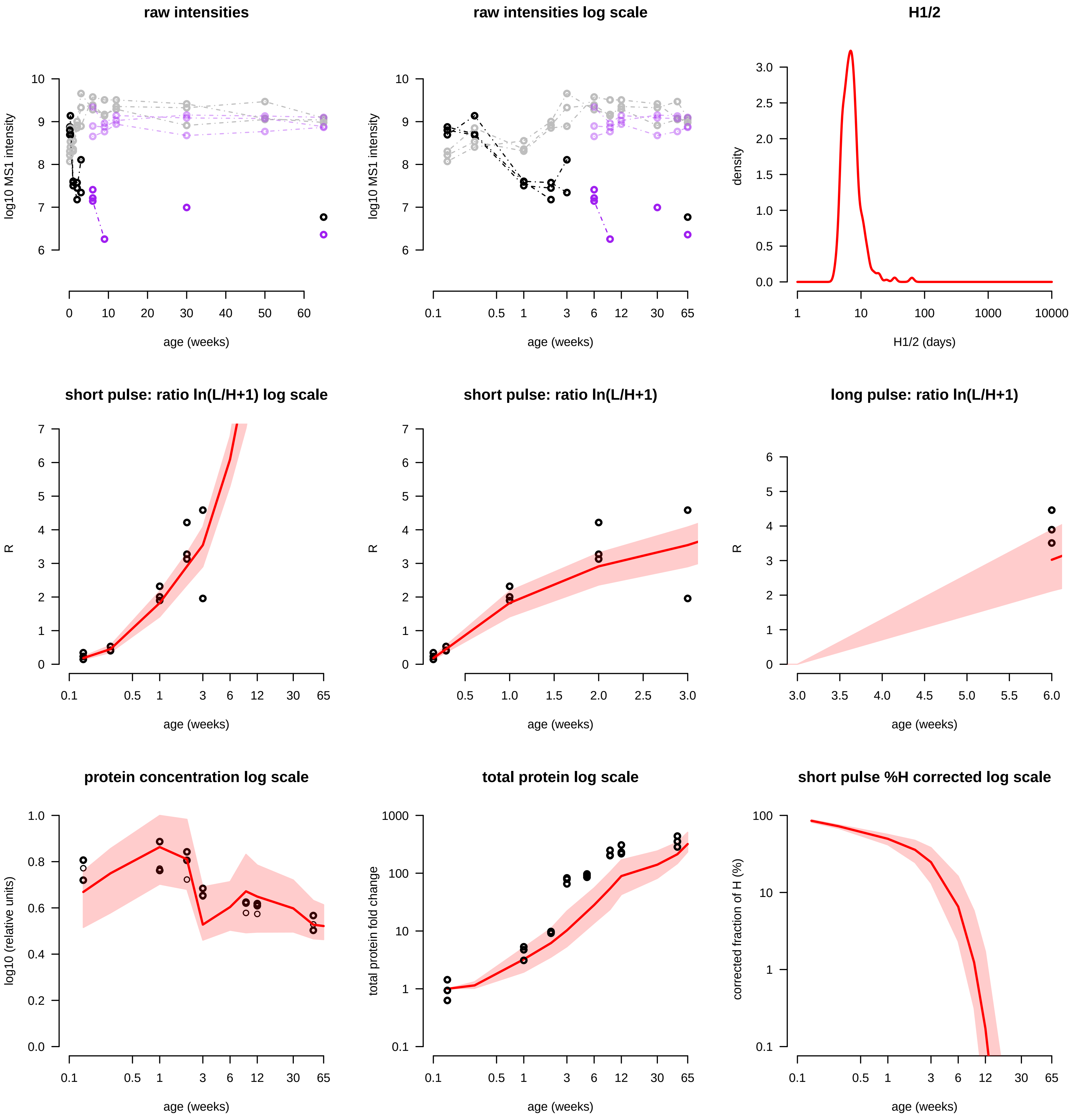

Q8C129

>sp|Q8C129|LCAP_MOUSE

Leucyl−cystinyl aminopeptidase

GN=Lnpep

Med H1/2: 10.8 days

Q5 H1/2: 4 days

Q95 H1/2: 82.6 days

reliable model fit: yes

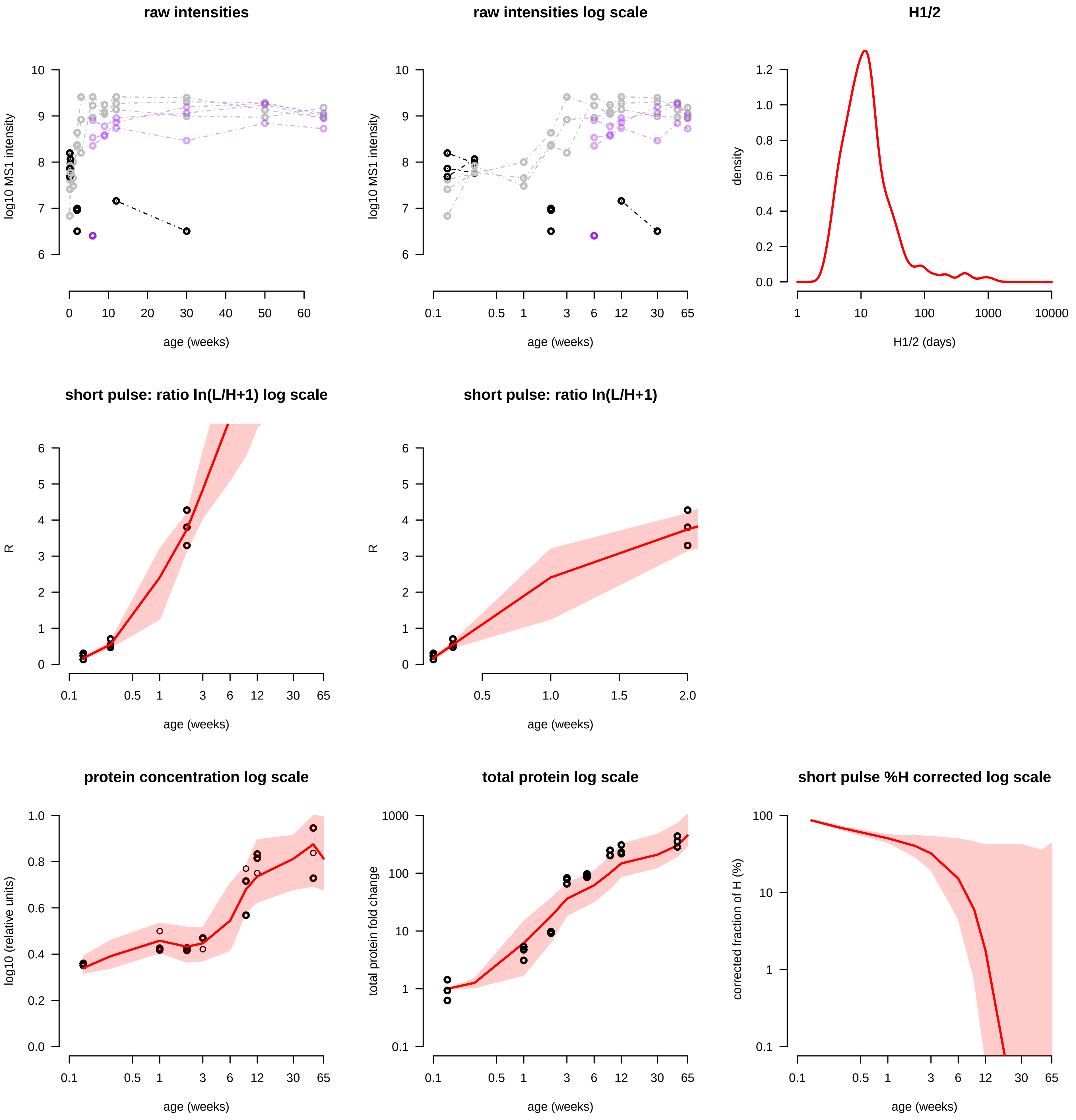

P63280

>sp|P63280|UBC9_MOUSE

SUMO−conjugating enzyme UBC9

GN=Ube2i

Med H1/2: 8.3 days

Q5 H1/2: 7.3 days

Q95 H1/2: 9.7 days

reliable model fit: yes

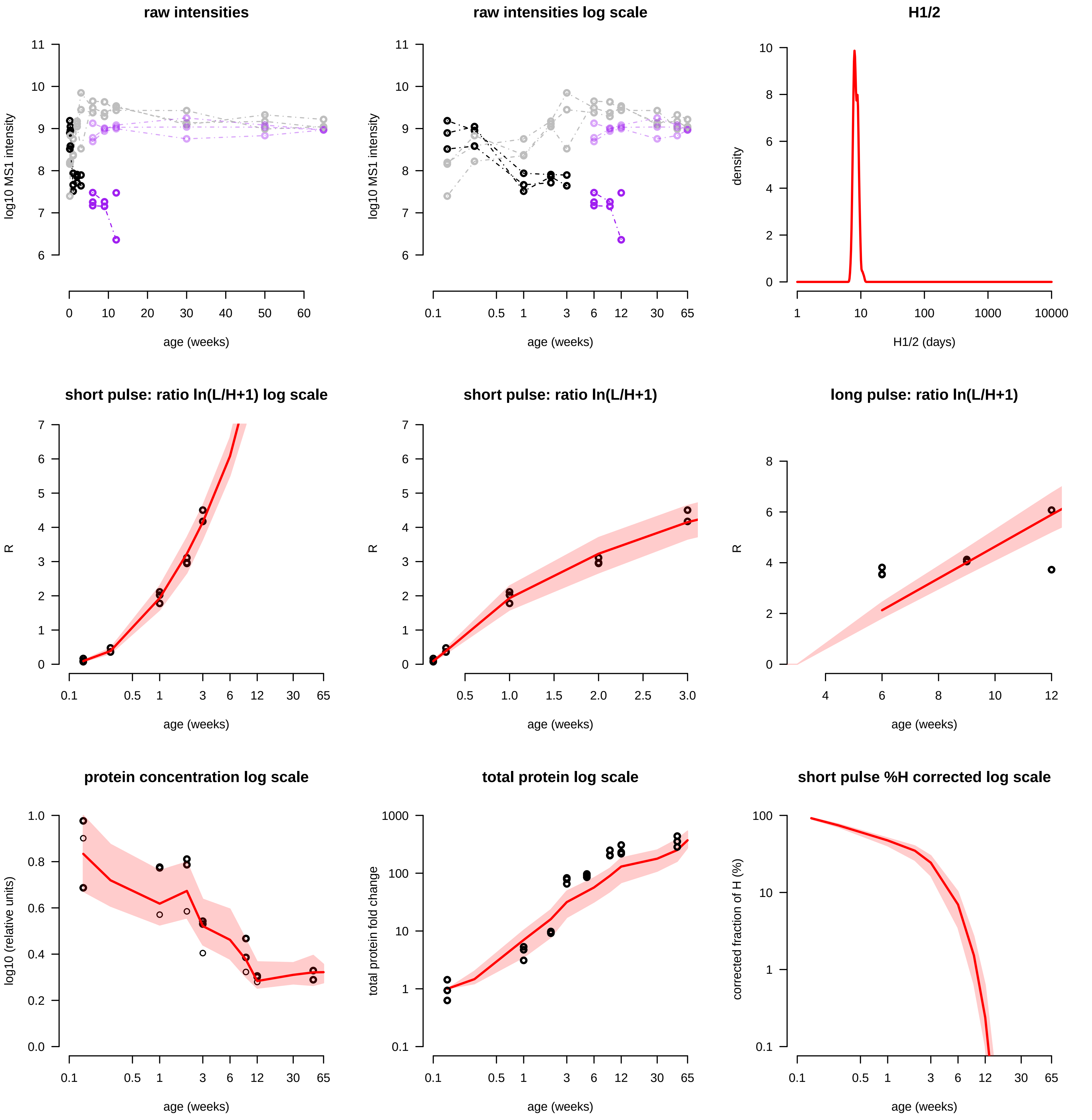

P36536

>sp|P36536|SAR1A_MOUSE

GTP−binding protein SAR1a

GN=Sar1a

Med H1/2: 11.1 days

Q5 H1/2: 8.7 days

Q95 H1/2: 14.7 days

reliable model fit: yes

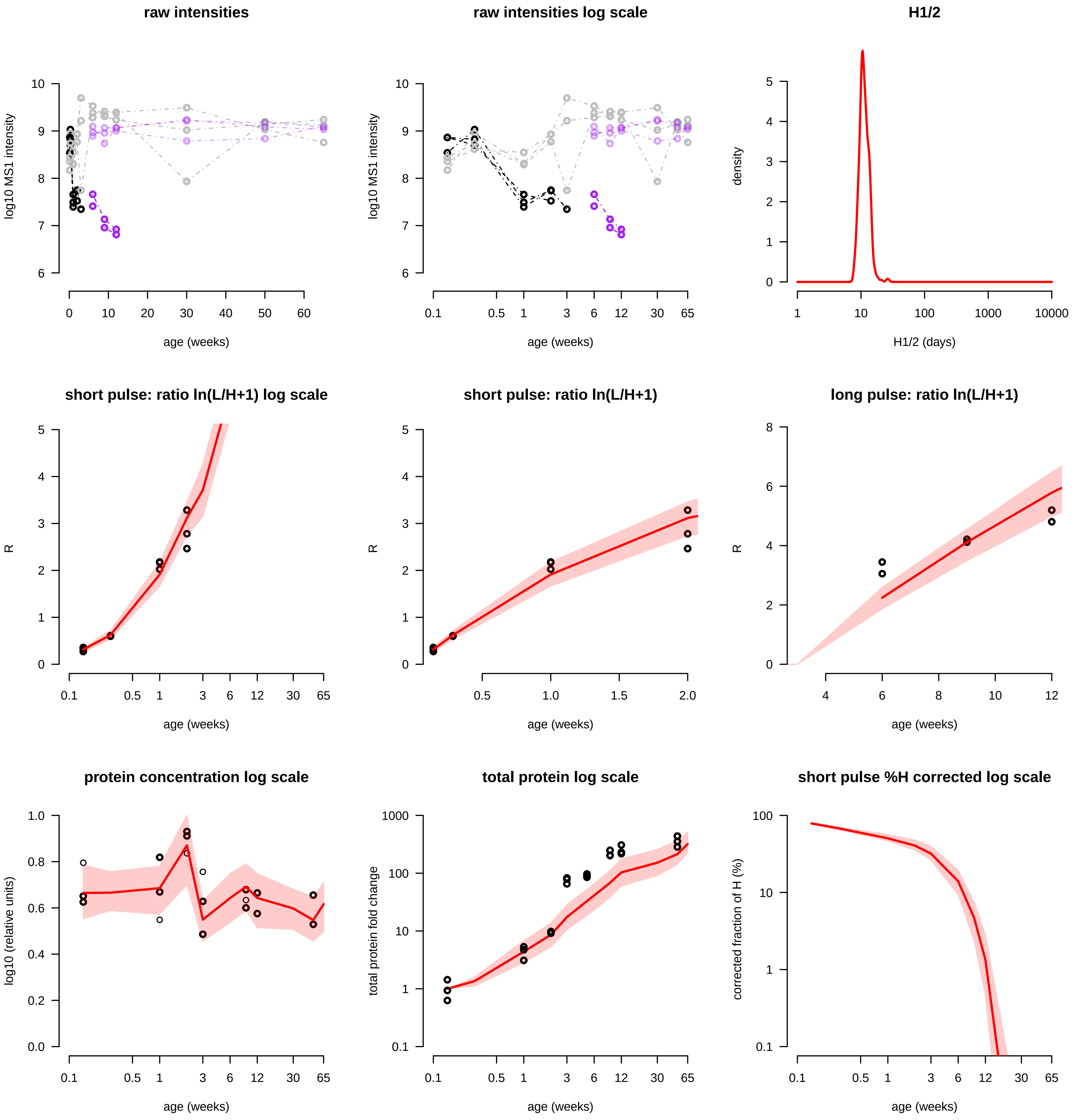

Q9DB05

>sp|Q9DB05|SNAA_MOUSE

Alpha−soluble NSF attachment protein

GN=Napa

Med H1/2: 5.9 days

Q5 H1/2: 5 days

Q95 H1/2: 7.4 days

reliable model fit: yes

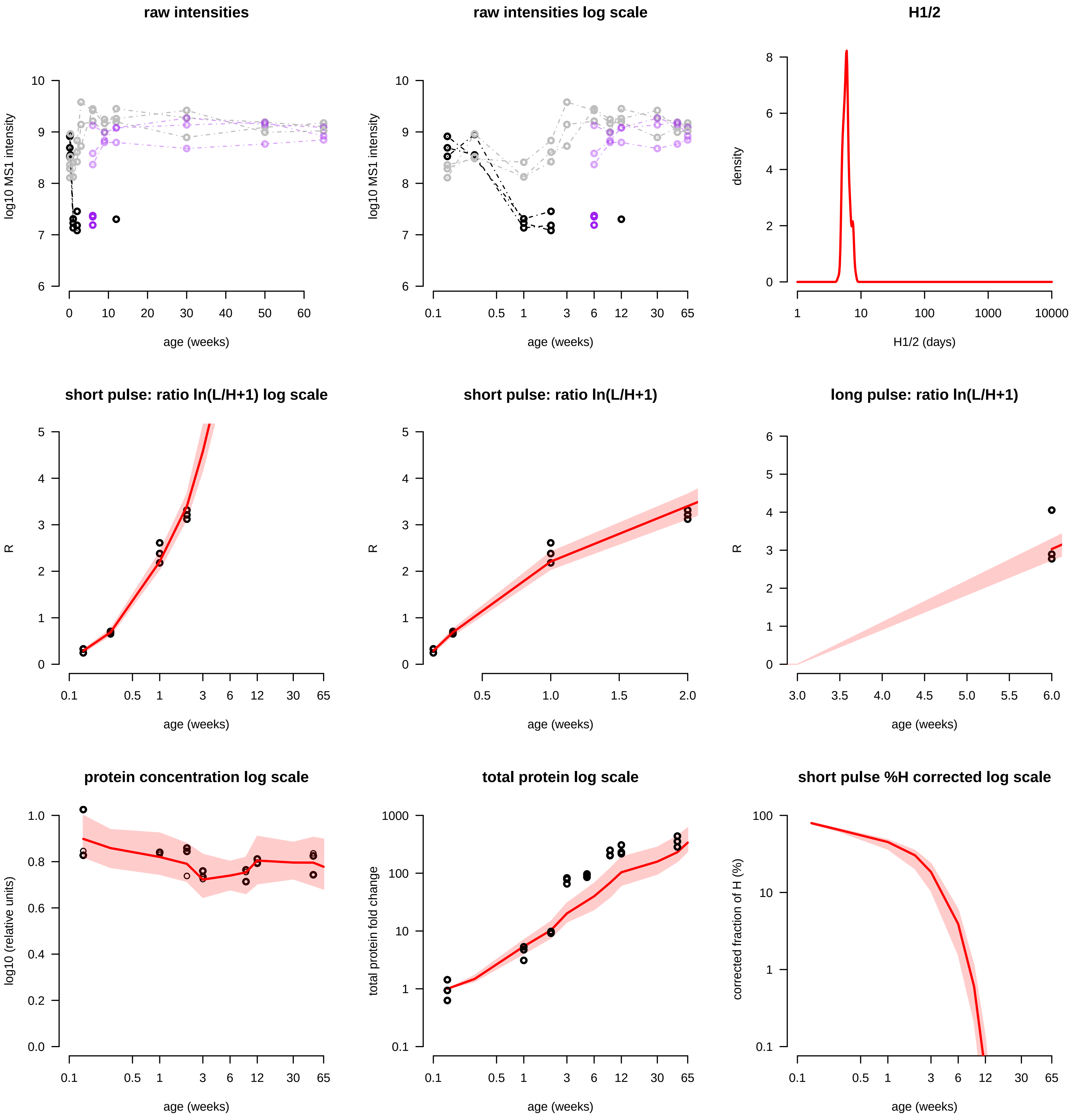

Q8R1V4

>sp|Q8R1V4|TMED4_MOUSE

Transmembrane emp24 domain−containing protein 4

GN=Tmed4

Med H1/2: 6 days

Q5 H1/2: 4.8 days

Q95 H1/2: 7.8 days

reliable model fit: yes

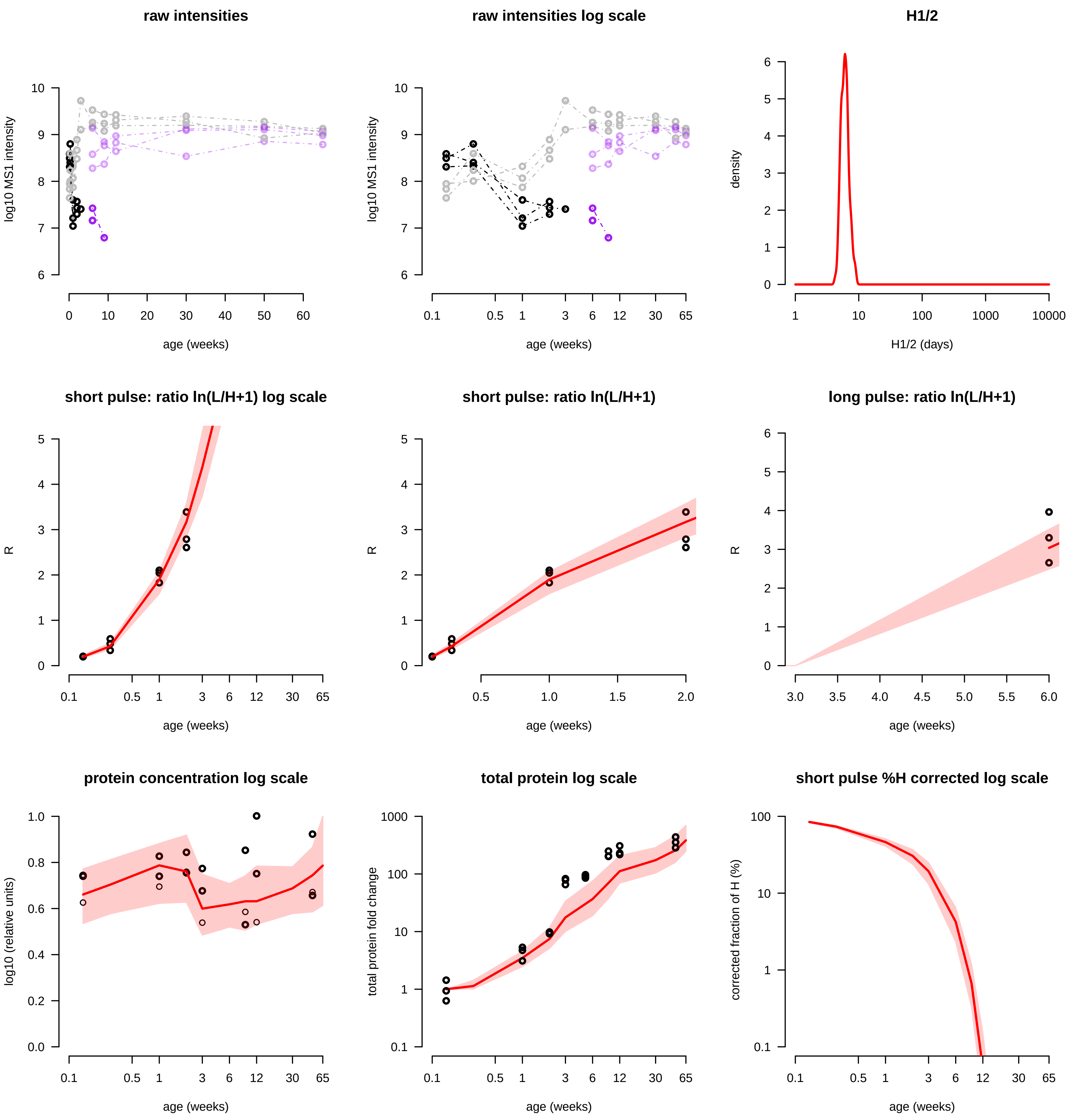

Q06890

>sp|Q06890|CLUS_MOUSE

Clusterin

GN=Clu

Med H1/2: 4.6 days

Q5 H1/2: 3.4 days

Q95 H1/2: 6.2 days

reliable model fit: yes

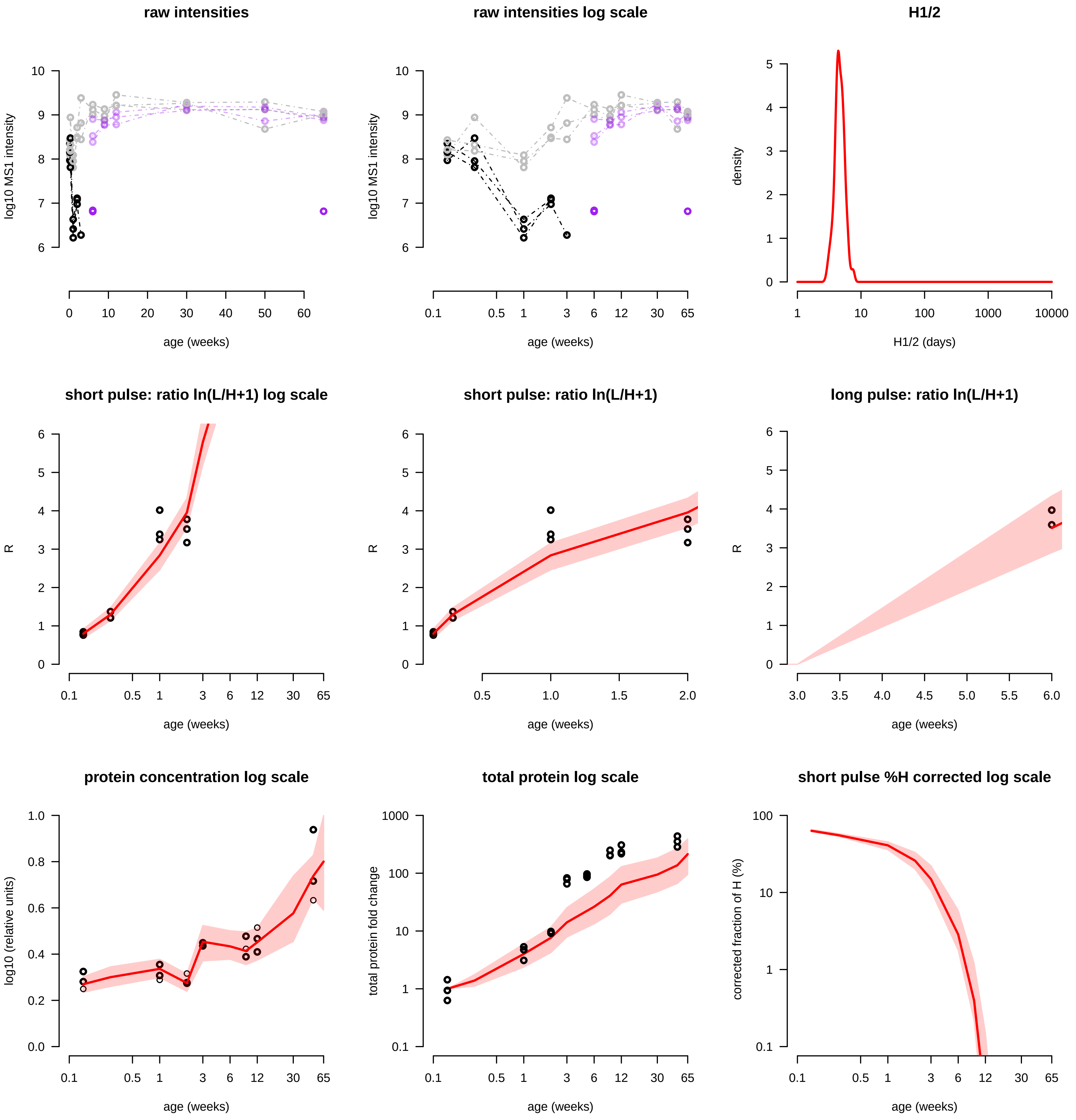

Q9DBG5

>sp|Q9DBG5|PLIN3_MOUSE

Perilipin−3

GN=Plin3

Med H1/2: 4.2 days

Q5 H1/2: 3.7 days

Q95 H1/2: 5.2 days

reliable model fit: yes

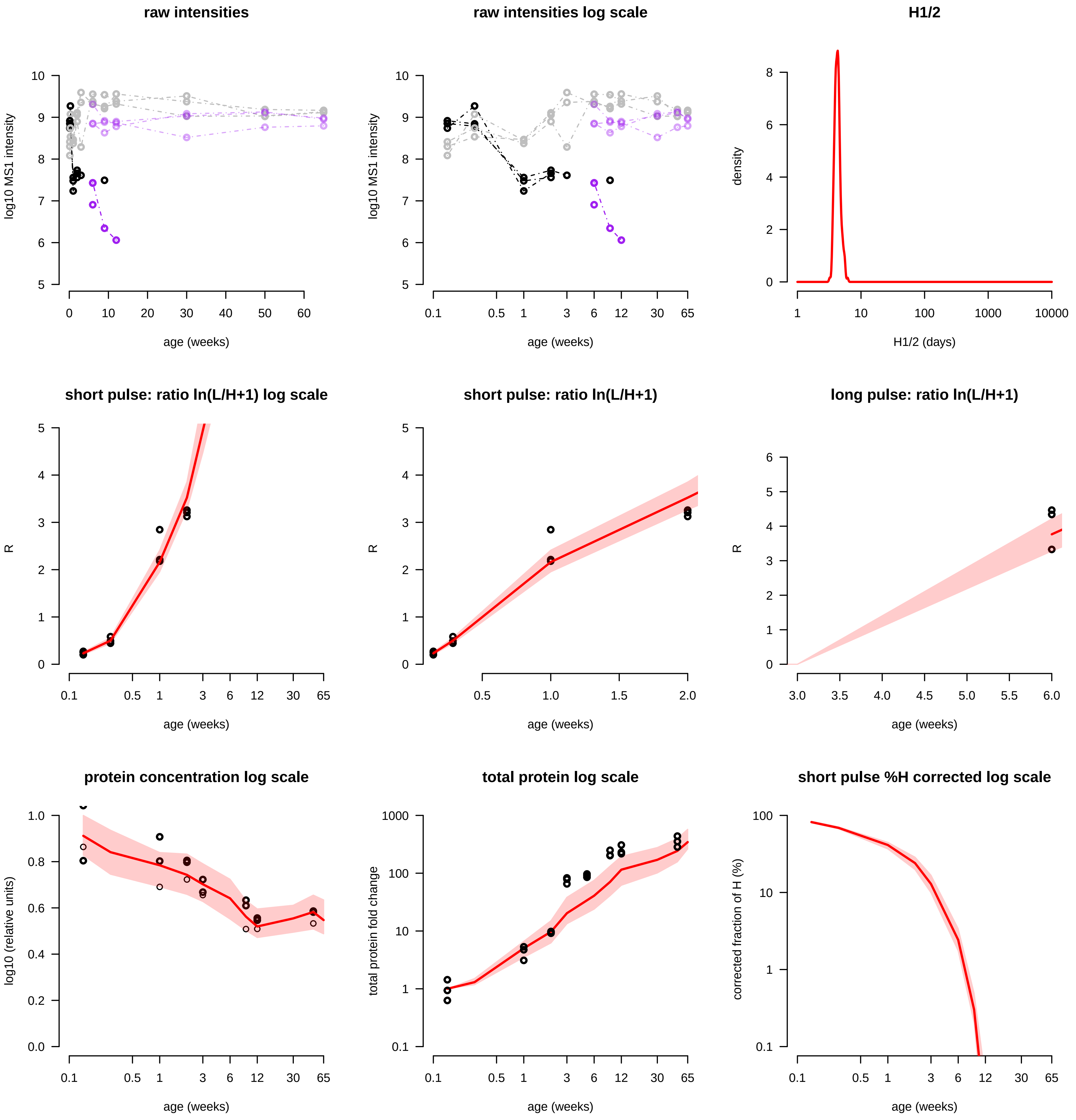

O08788−2;O08788

>sp|O08788−2|DCTN1_MOUSE

Isoform 2 of Dynactin subunit 1

GN=Dctn1

Med H1/2: 6.1 days

Q5 H1/2: 3.5 days

Q95 H1/2: 16.5 days

reliable model fit: yes

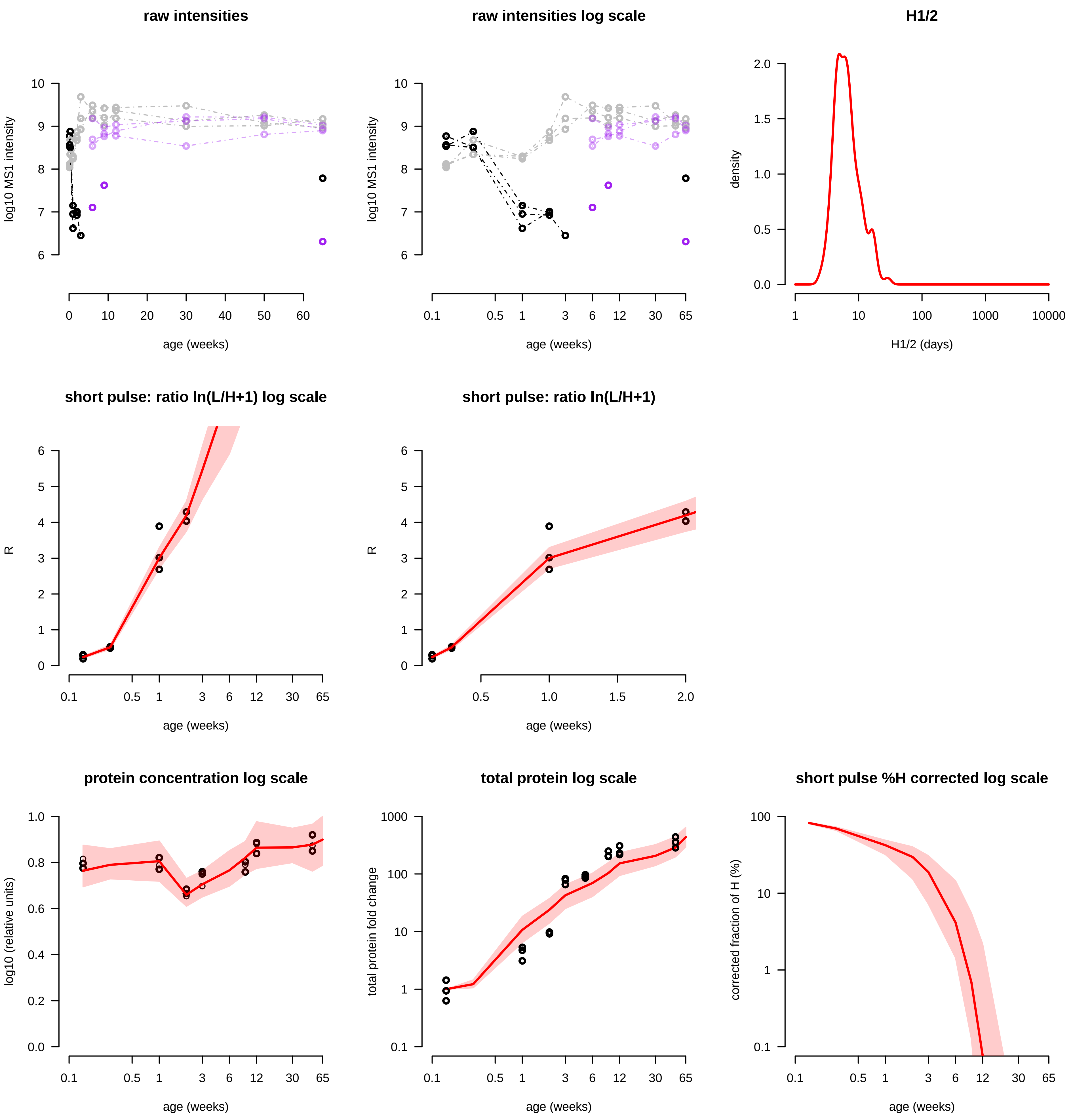

P59999

>sp|P59999|ARPC4_MOUSE

Actin−related protein 2/3 complex subunit 4

GN=Arpc4

Med H1/2: 10.6 days

Q5 H1/2: 8.5 days

Q95 H1/2: 14.9 days

reliable model fit: yes

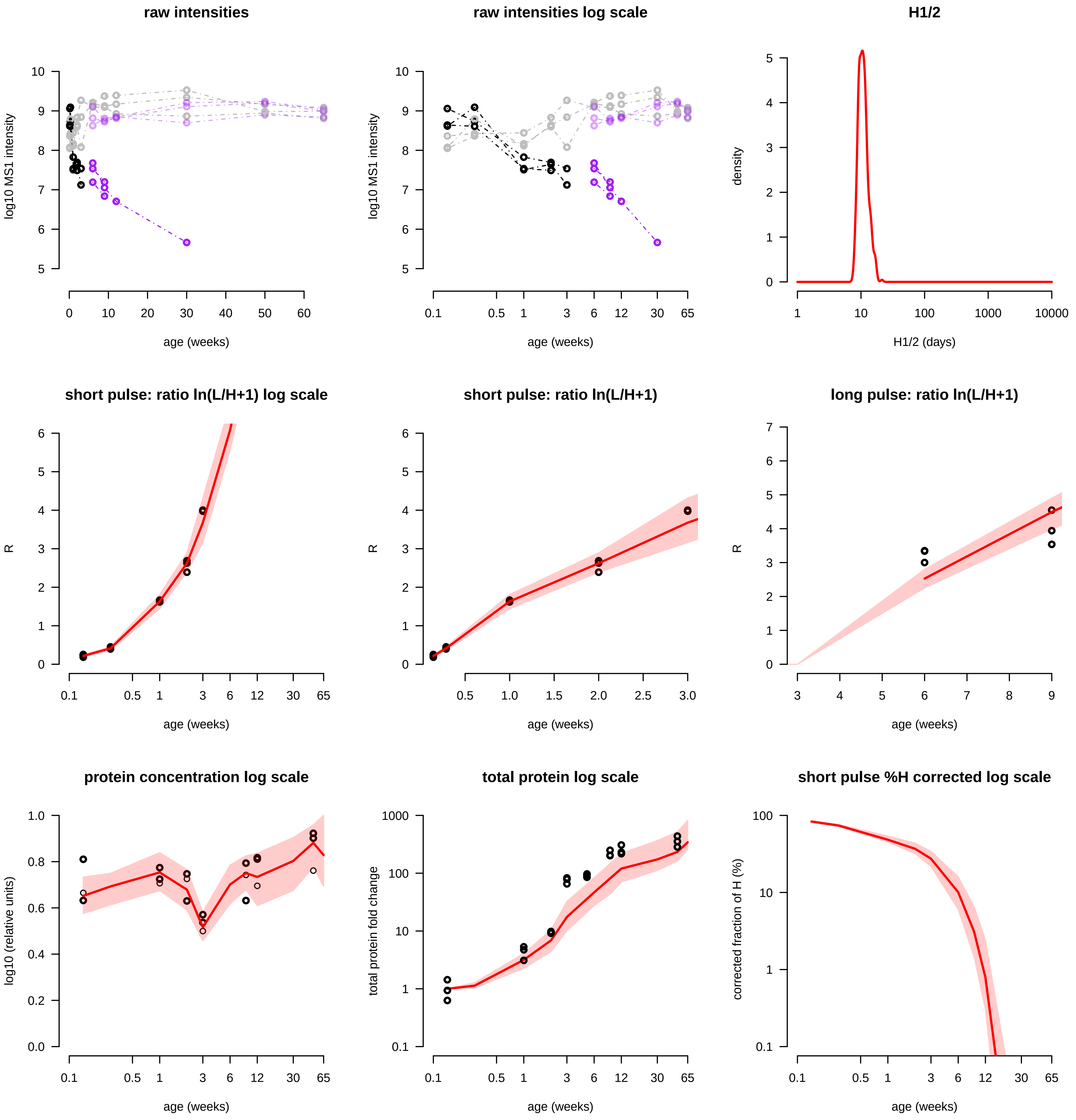

Q9WTX5

>sp|Q9WTX5|SKP1_MOUSE

S−phase kinase−associated protein 1

GN=Skp1

Med H1/2: 7.2 days

Q5 H1/2: 5.7 days

Q95 H1/2: 9.1 days

reliable model fit: yes

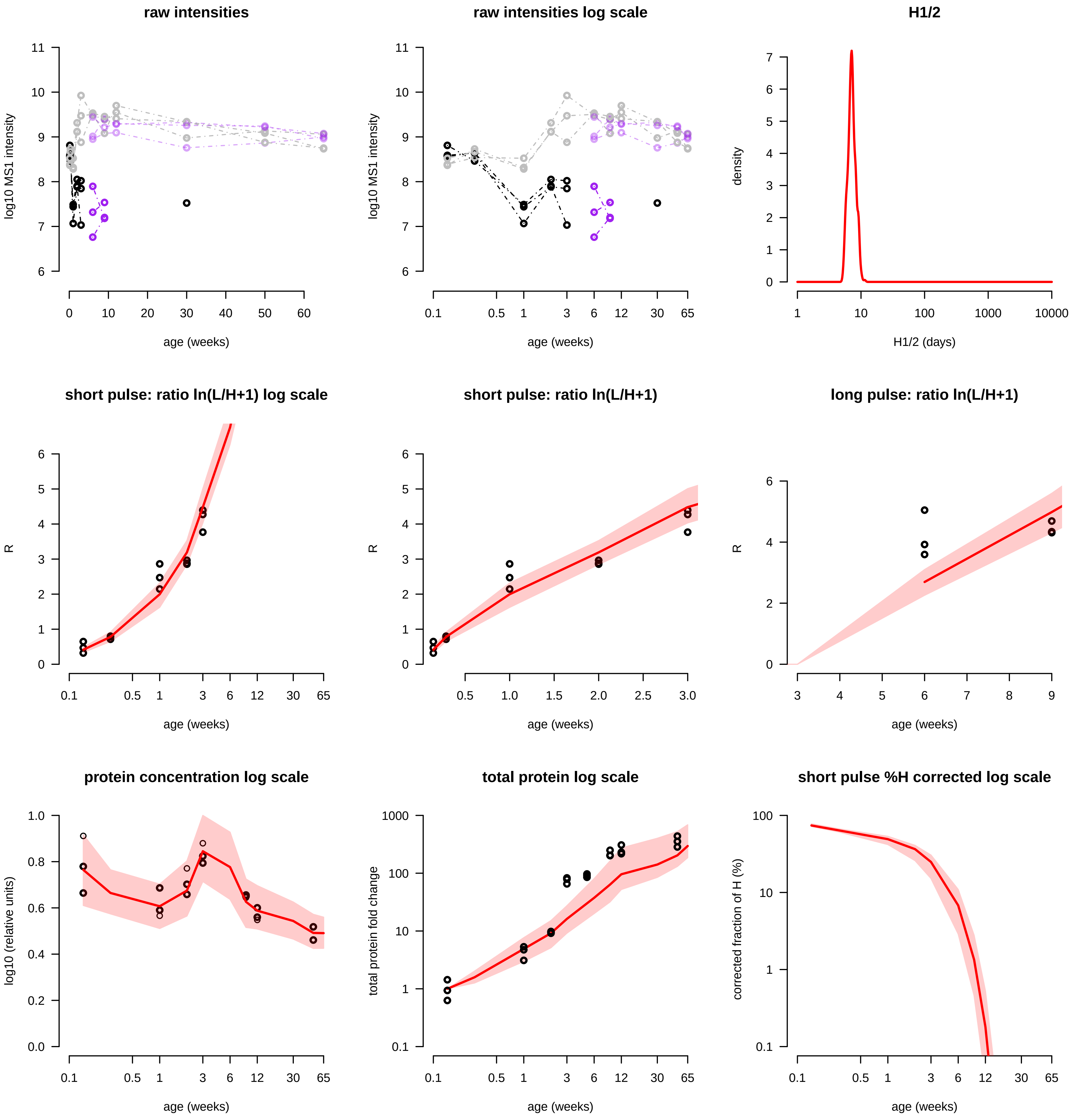

P62869

>sp|P62869|ELOB_MOUSE

Elongin−B

GN=Elob

Med H1/2: 5 days

Q5 H1/2: 4 days

Q95 H1/2: 7.1 days

reliable model fit: yes

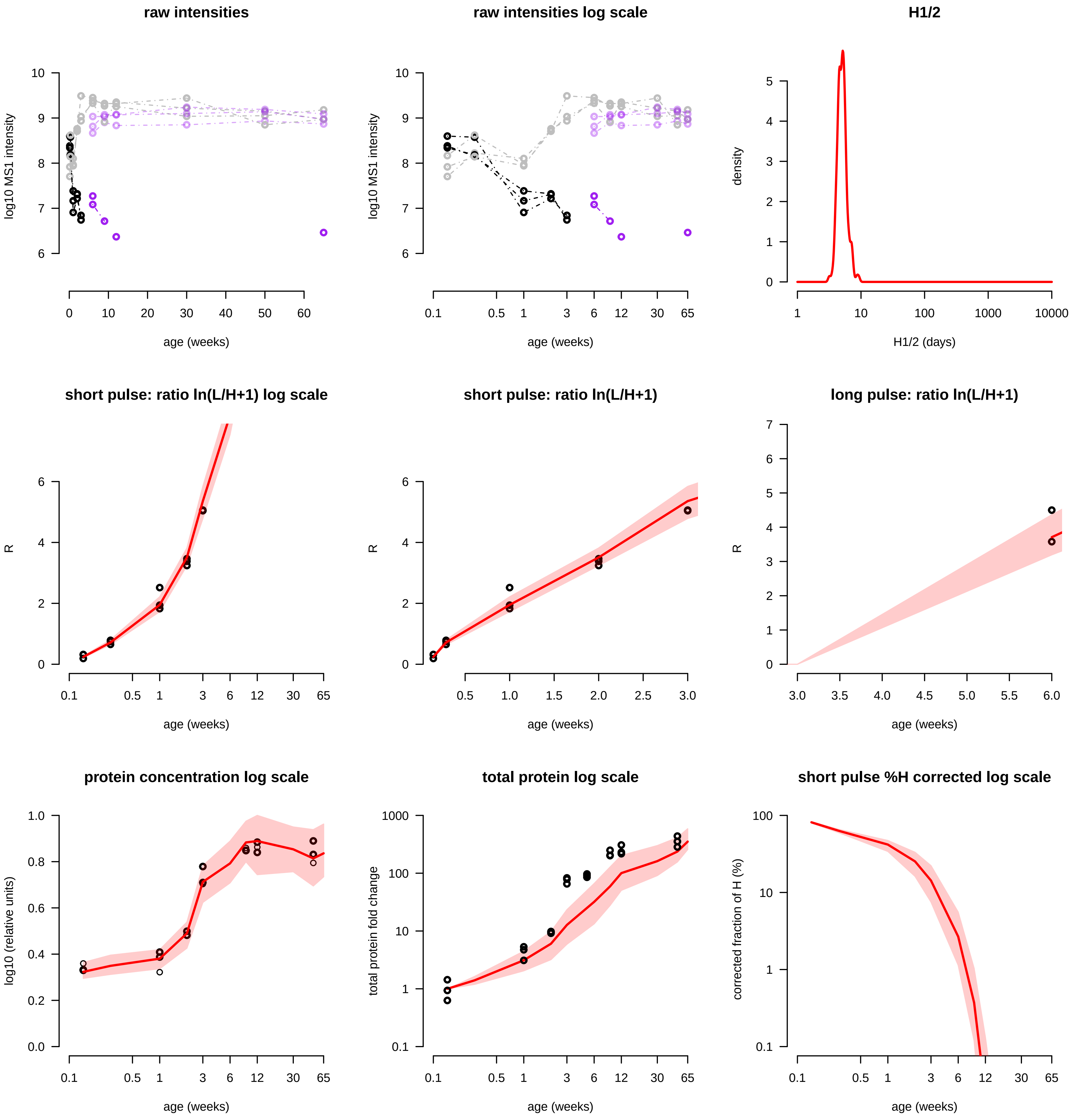

Q61081

>sp|Q61081|CDC37_MOUSE

Hsp90 co−chaperone Cdc37

GN=Cdc37

Med H1/2: 7.8 days

Q5 H1/2: 6.5 days

Q95 H1/2: 13.3 days

reliable model fit: yes

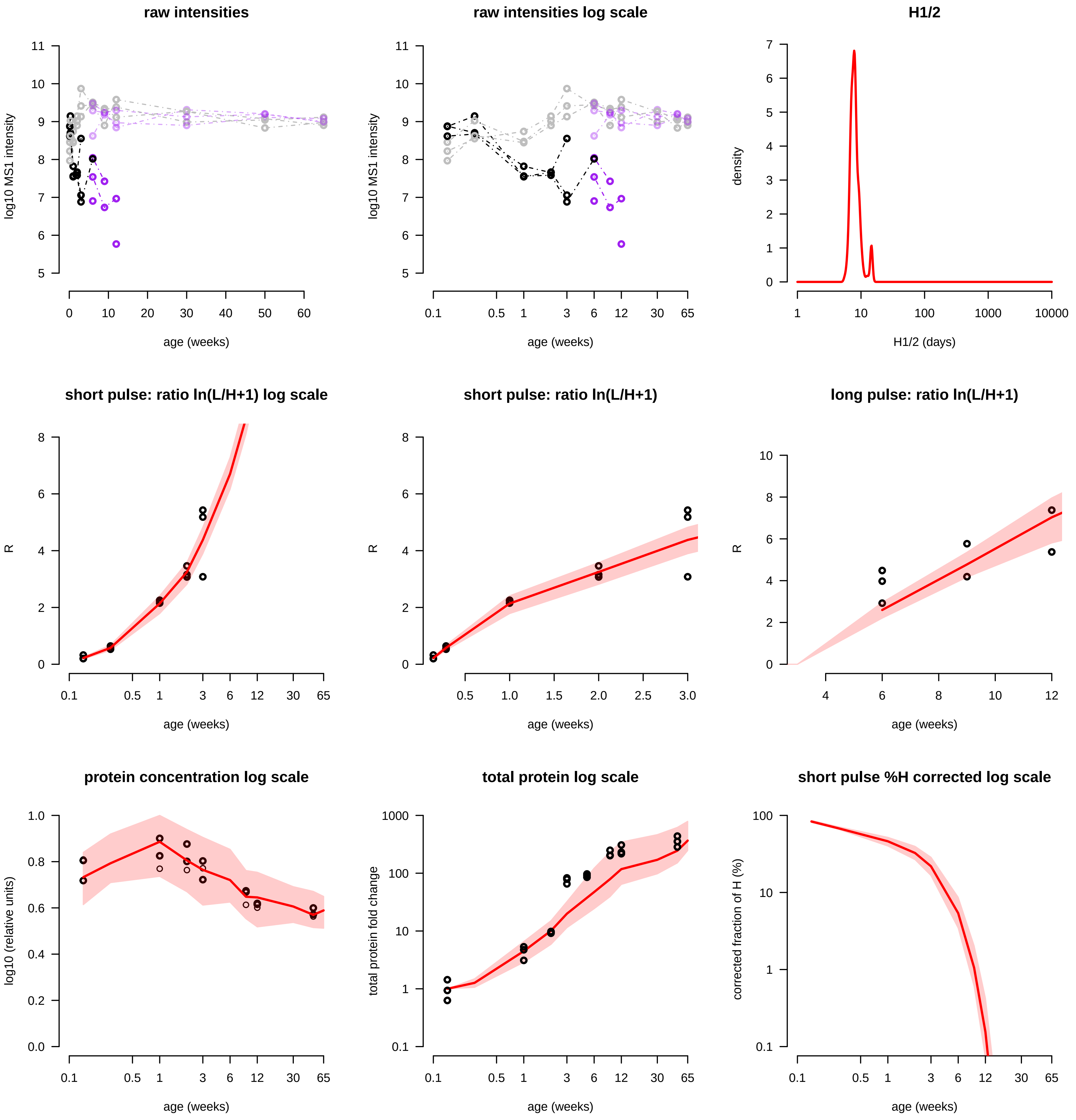

Q9JHJ0

>sp|Q9JHJ0|TMOD3_MOUSE

Tropomodulin−3

GN=Tmod3

Med H1/2: 12.2 days

Q5 H1/2: 9.5 days

Q95 H1/2: 20.5 days

reliable model fit: yes

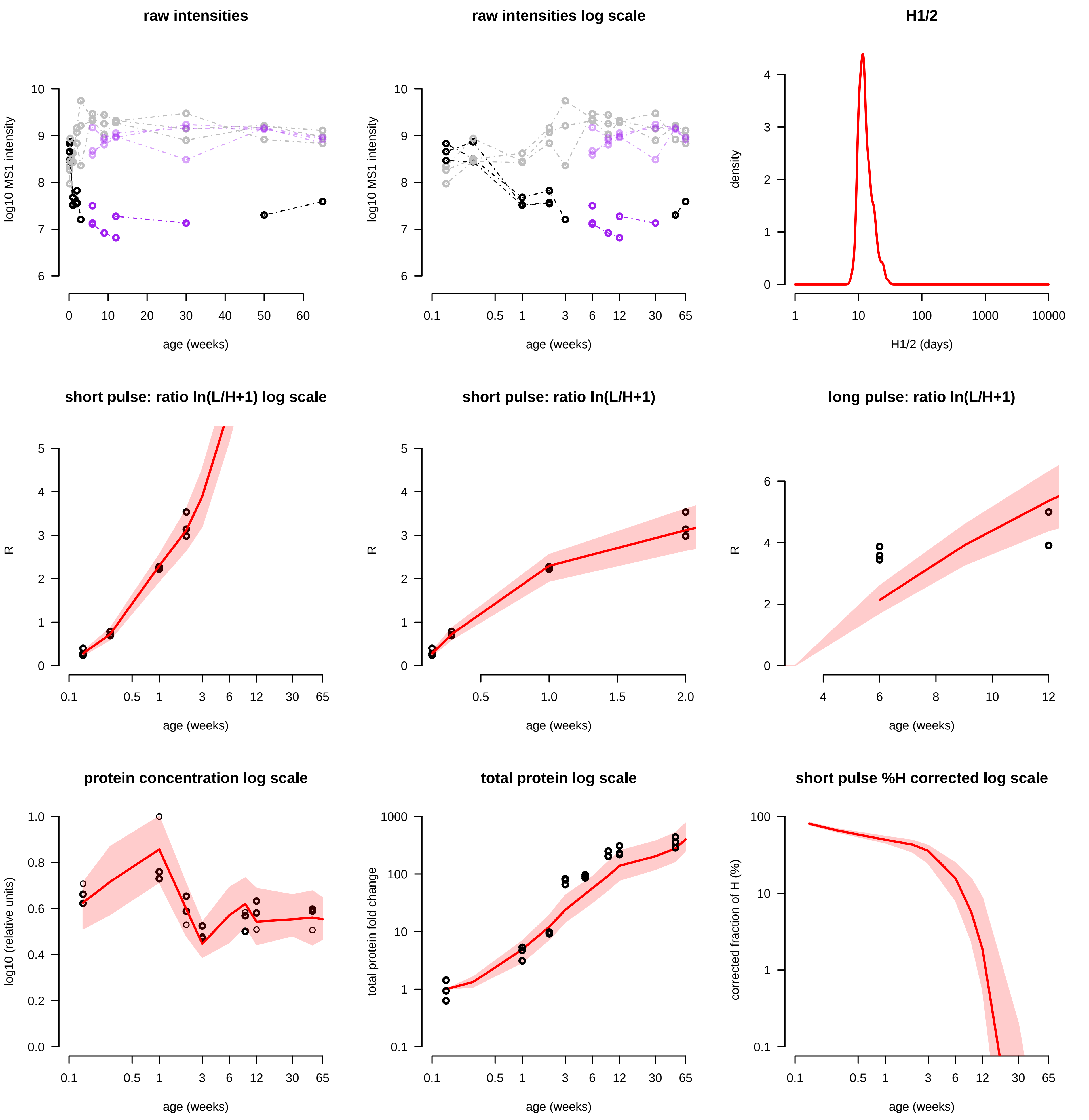

Q9EQH2

>sp|Q9EQH2|ERAP1_MOUSE

Endoplasmic reticulum aminopeptidase 1

GN=Erap1

Med H1/2: 4.3 days

Q5 H1/2: 3.3 days

Q95 H1/2: 6.2 days

reliable model fit: yes

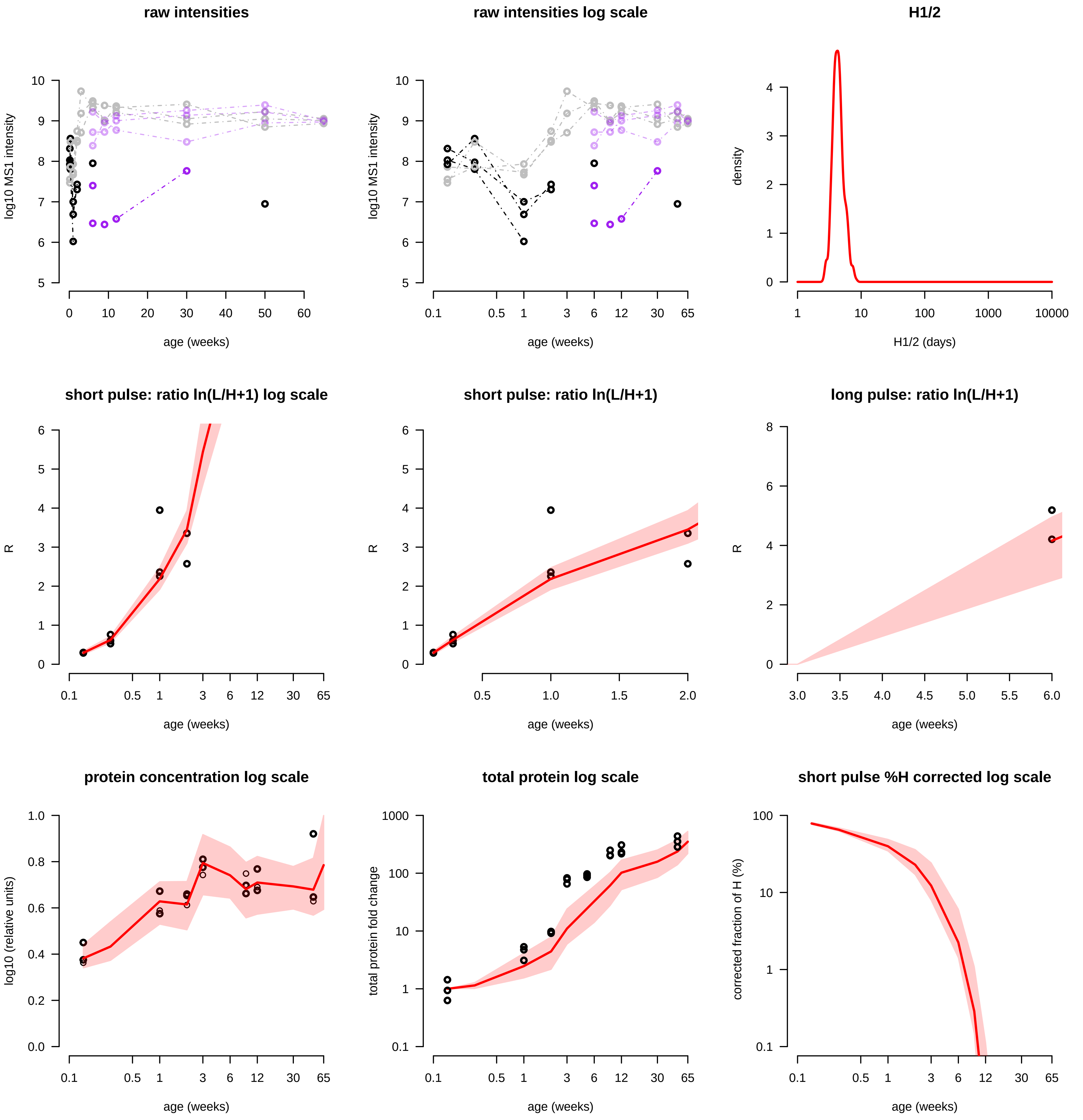

Q01149

>sp|Q01149|CO1A2_MOUSE

Collagen alpha−2(I) chain

GN=Col1a2

Med H1/2: 202.9 days

Q5 H1/2: 140.5 days

Q95 H1/2: 405.9 days

reliable model fit: yes

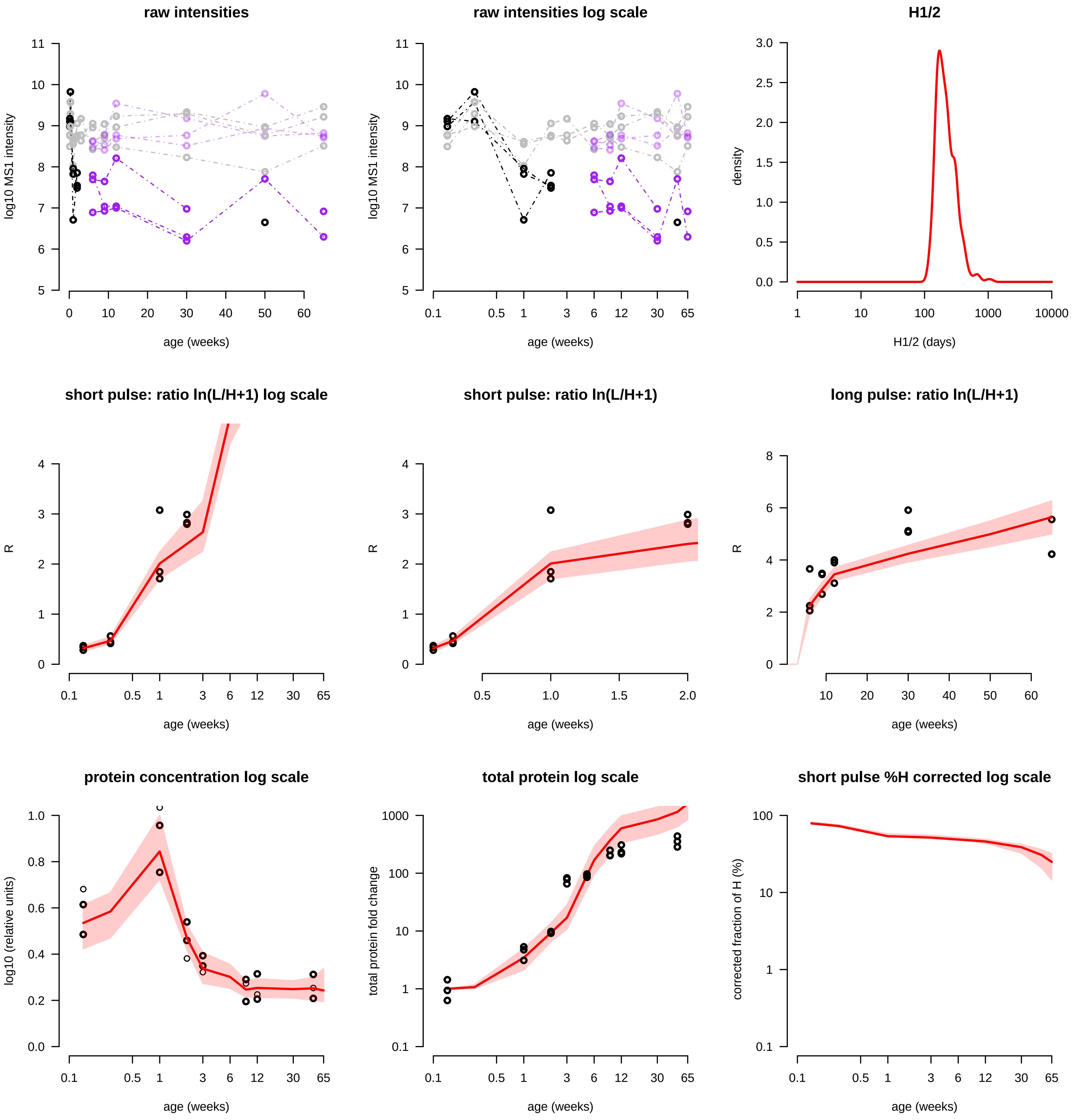

Q9QZ88;Q9QZ88−2

>sp|Q9QZ88|VPS29_MOUSE

Vacuolar protein sorting−associated protein 29

GN=Vps29

Med H1/2: 7.4 days

Q5 H1/2: 6.2 days

Q95 H1/2: 9.7 days

reliable model fit: yes

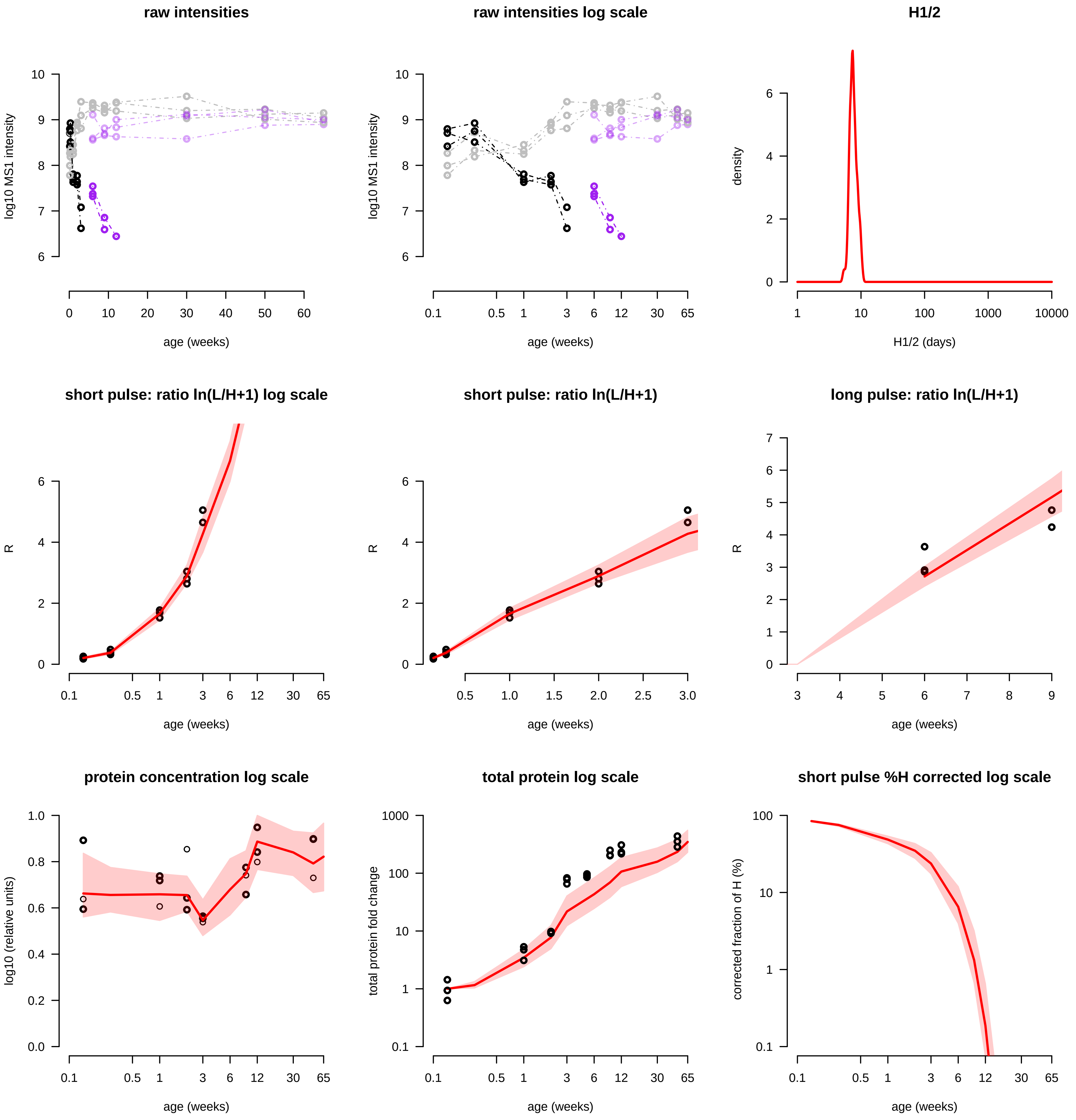

P47856−2;P47856

>sp|P47856−2|GFPT1_MOUSE

soforn 2 of Glutamine−−fructose−6−phosphate aminotransferase [isomerizing] 1

GN=Gfpt1

Med H1/2: 4 days

Q5 H1/2: 3.3 days

Q95 H1/2: 5 days

reliable model fit: yes

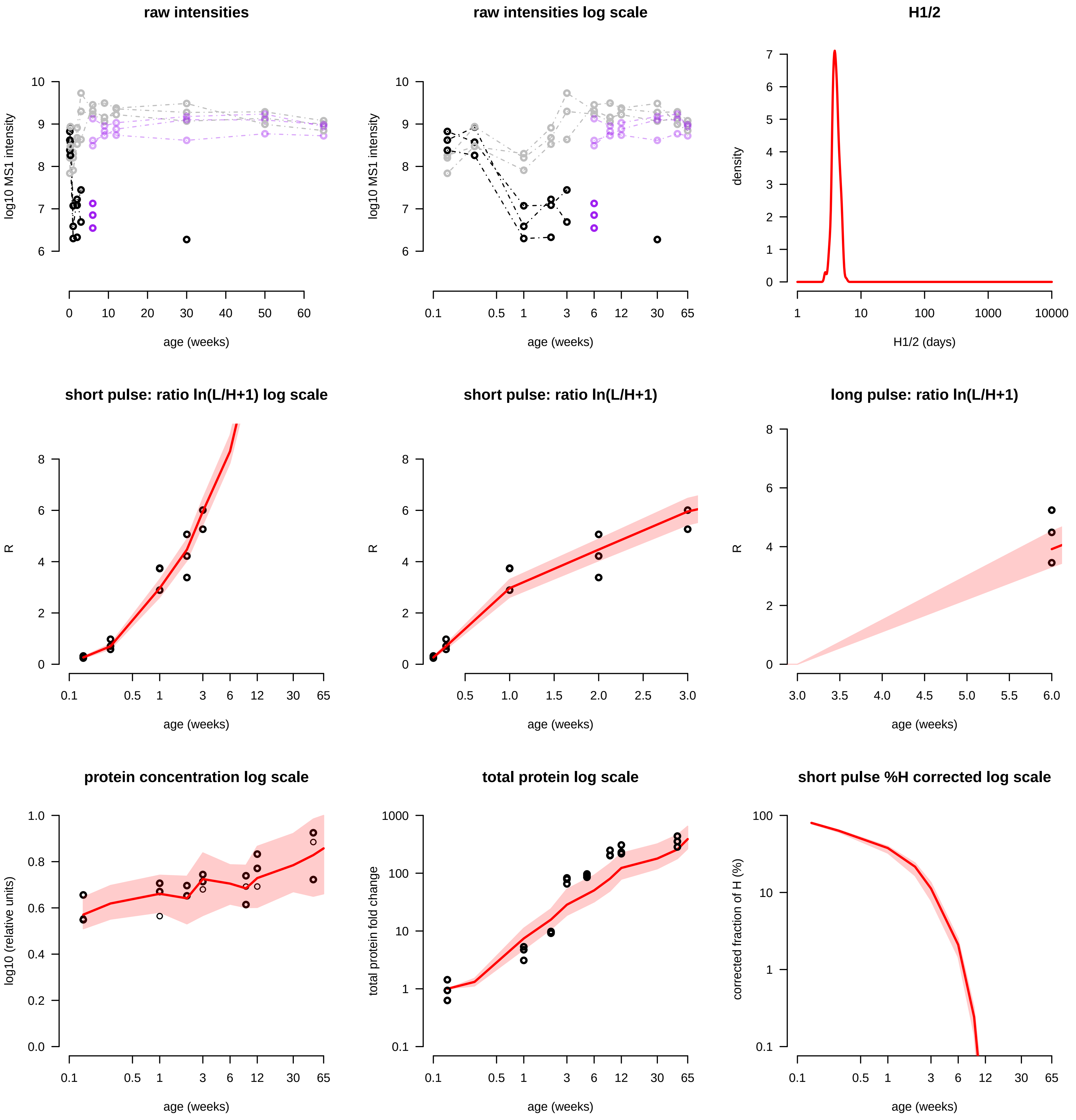

P47753

>sp|P47753|CAZA1_MOUSE

F−actin−capping protein subunit alpha−1

GN=Capza1

Med H1/2: 7.1 days

Q5 H1/2: 5.5 days

Q95 H1/2: 9.9 days

reliable model fit: yes

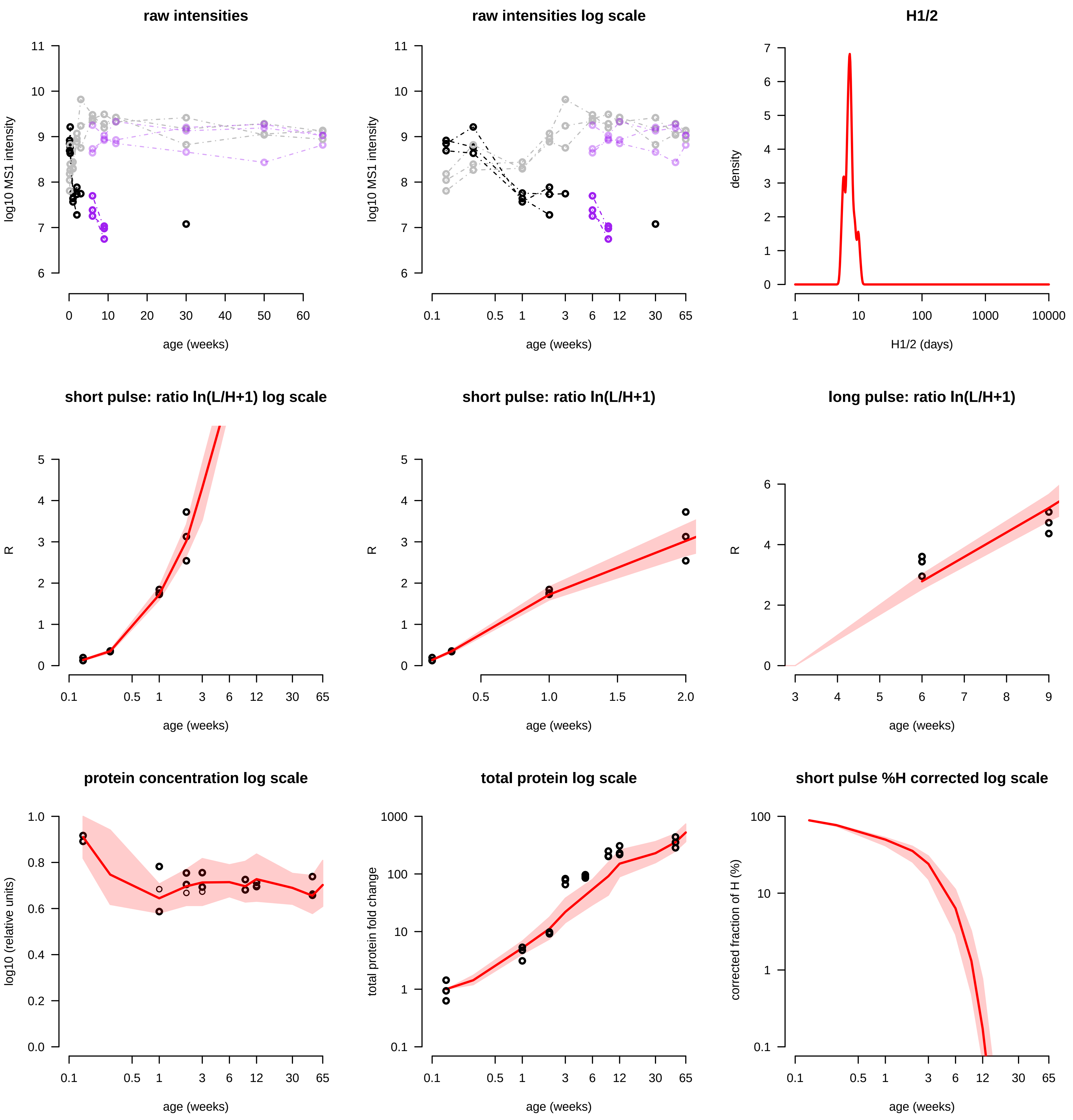

Q9ERE7

>sp|Q9ERE7|MESD_MOUSE

LRP chaperone MESD

GN=Mesd

Med H1/2: 15.1 days

Q5 H1/2: 5.3 days

Q95 H1/2: 70.5 days

reliable model fit: yes

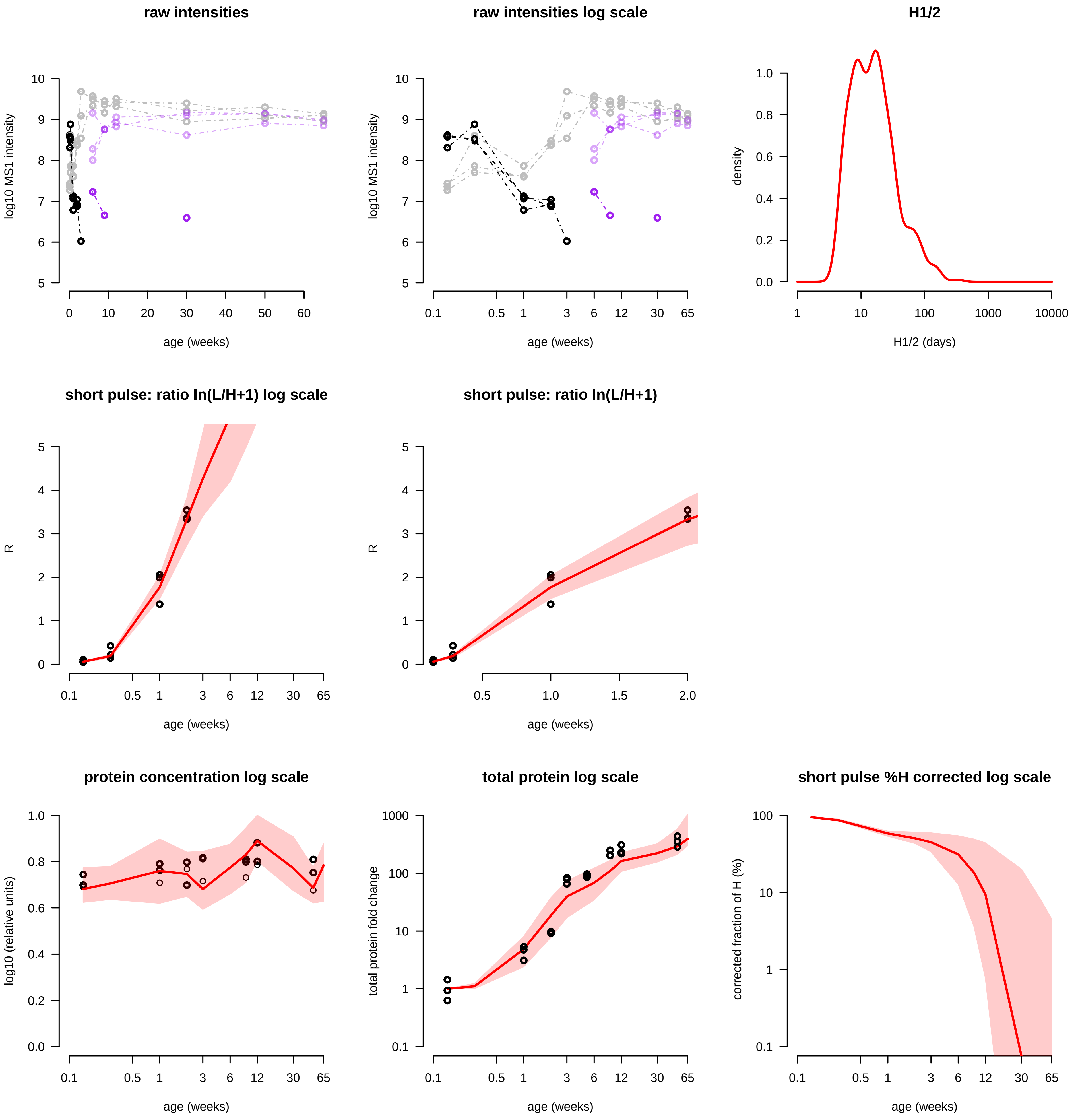

Q9JMA1

>sp|Q9JMA1|UBP14_MOUSE

Ubiquitin carboxyl−terminal hydrolase 14

GN=Usp14

Med H1/2: 81.7 days

Q5 H1/2: 48.5 days

Q95 H1/2: 214.2 days

reliable model fit: yes

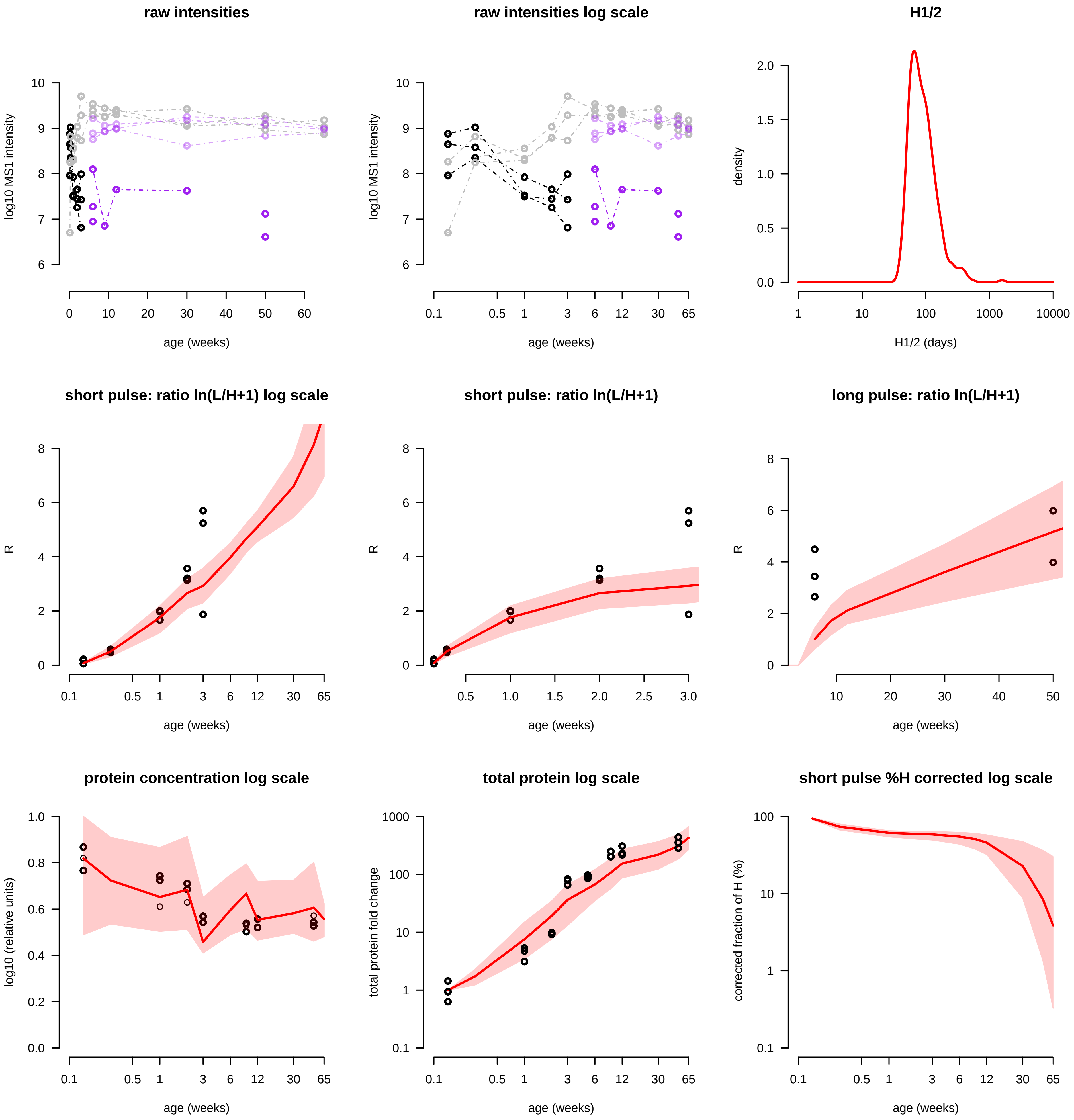

Q80UM7

>sp|Q80UM7|MOGS_MOUSE

Mannosyl−oligosaccharide glucosidase

GN=Mogs

Med H1/2: 6.9 days

Q5 H1/2: 5.1 days

Q95 H1/2: 8.8 days

reliable model fit: yes

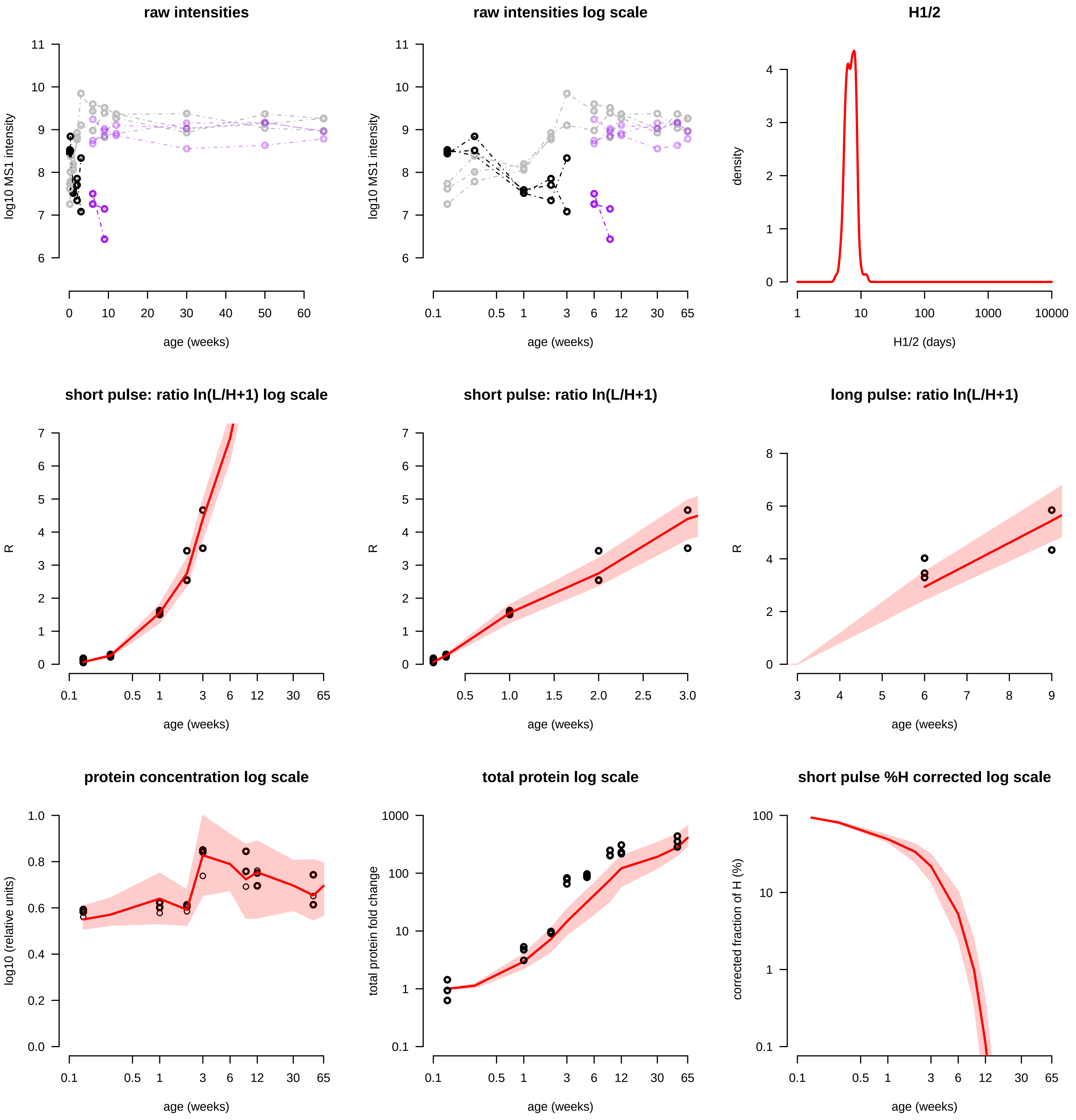

P47199

>sp|P47199|QOR_MOUSE

Quinone oxidoreductase

GN=Cryz

Med H1/2: 5.6 days

Q5 H1/2: 4.6 days

Q95 H1/2: 6.8 days

reliable model fit: yes

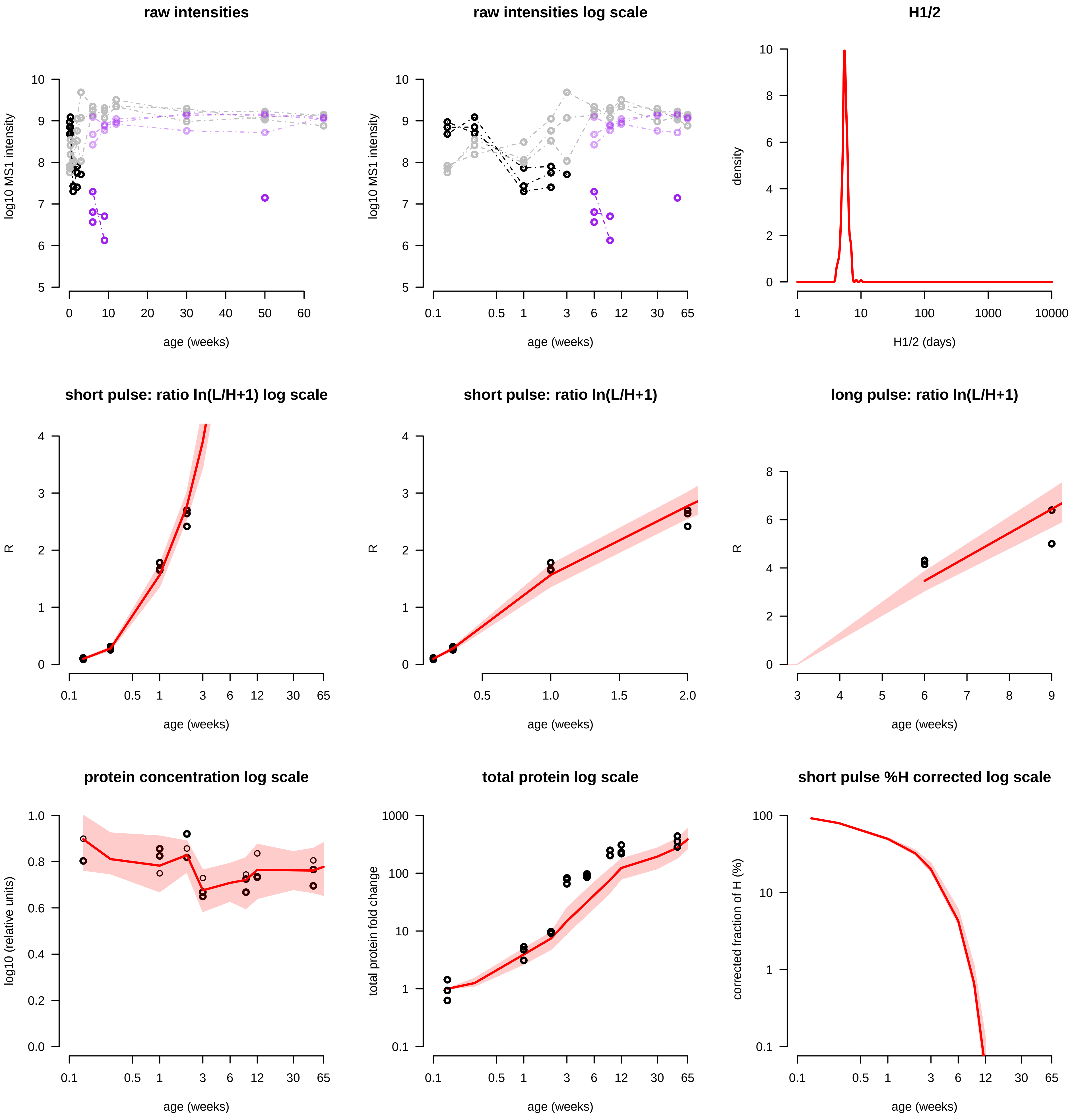

Q99KV1

>sp|Q99KV1|DJB11_MOUSE

DnaJ homolog subfamily B member 11

GN=Dnajb11

Med H1/2: 545.6 days

Q5 H1/2: 66.3 days

Q95 H1/2: 5059.2 days

reliable model fit: no

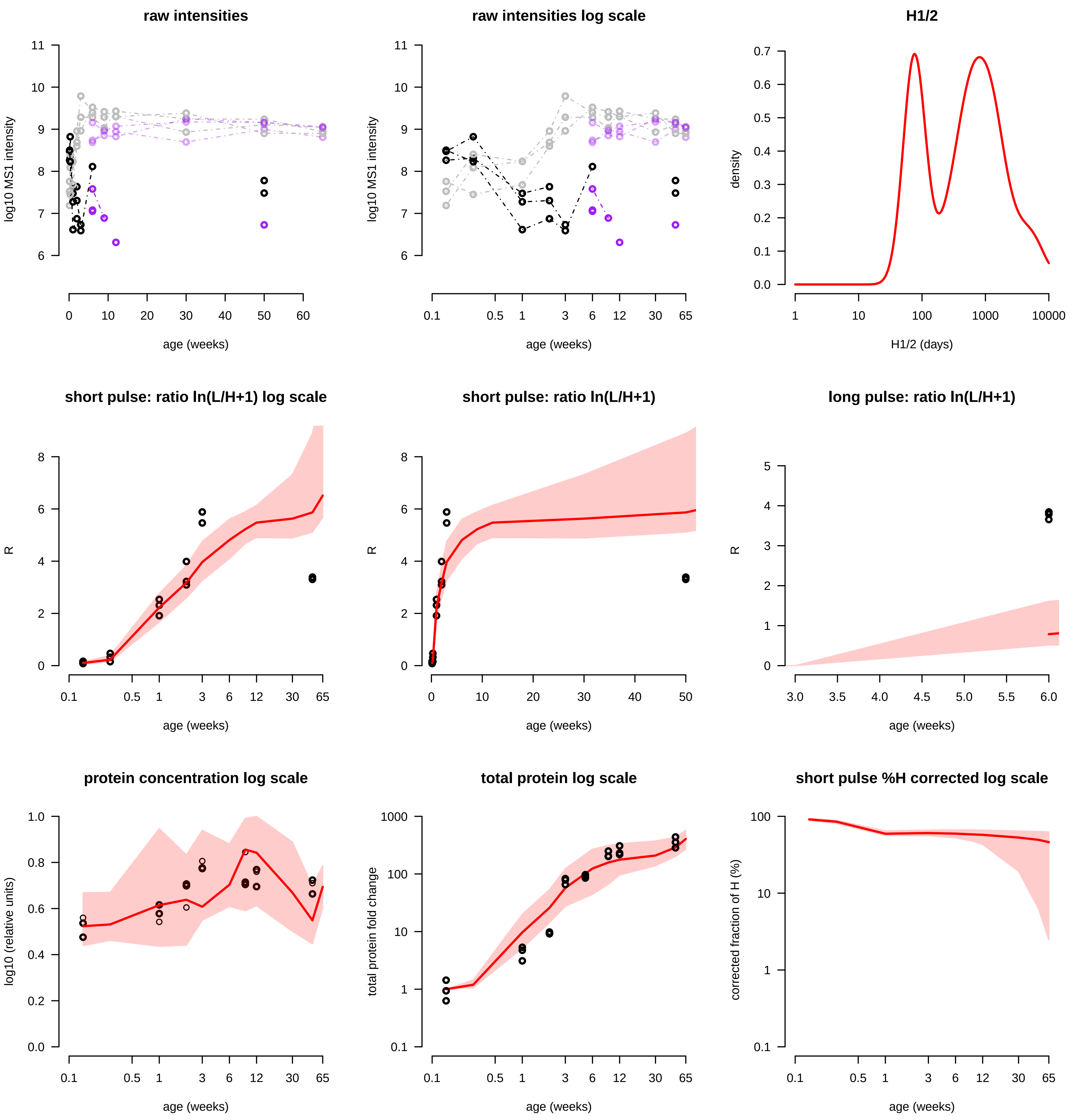

Q8BHG1

>sp|Q8BHG1|NRDC_MOUSE

Nardilysin

GN=Nrdc

Med H1/2: 2784 days

Q5 H1/2: 556.6 days

Q95 H1/2: 24345.8 days

reliable model fit: no

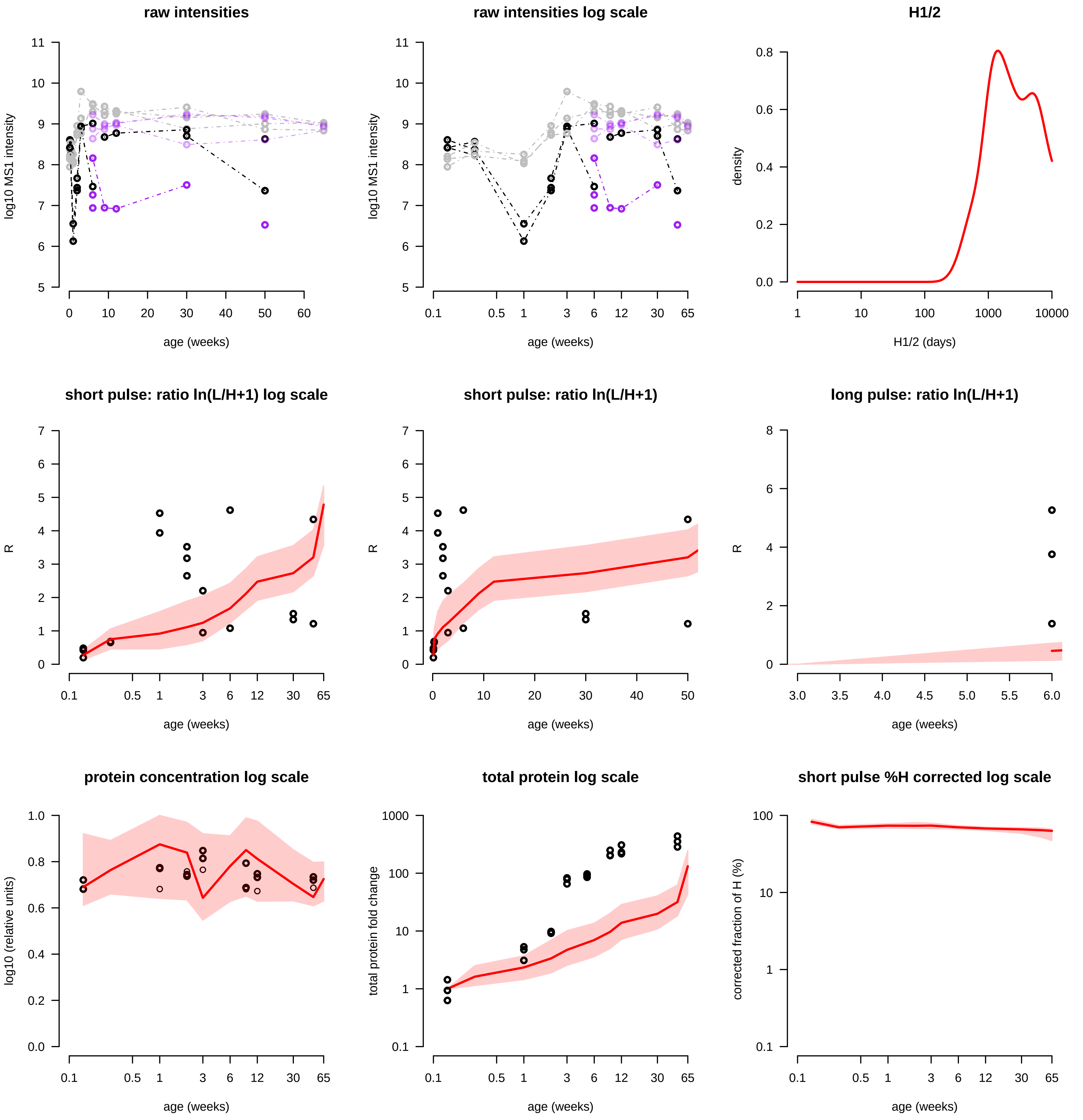

P97822;P97822−2

>sp|P97822|AN32E_MOUSE

Acidic leucine−rich nuclear phosphoprotein 32 family member E

GN=Anp32e

Med H1/2: 5.3 days

Q5 H1/2: 4.3 days

Q95 H1/2: 6.7 days

reliable model fit: yes

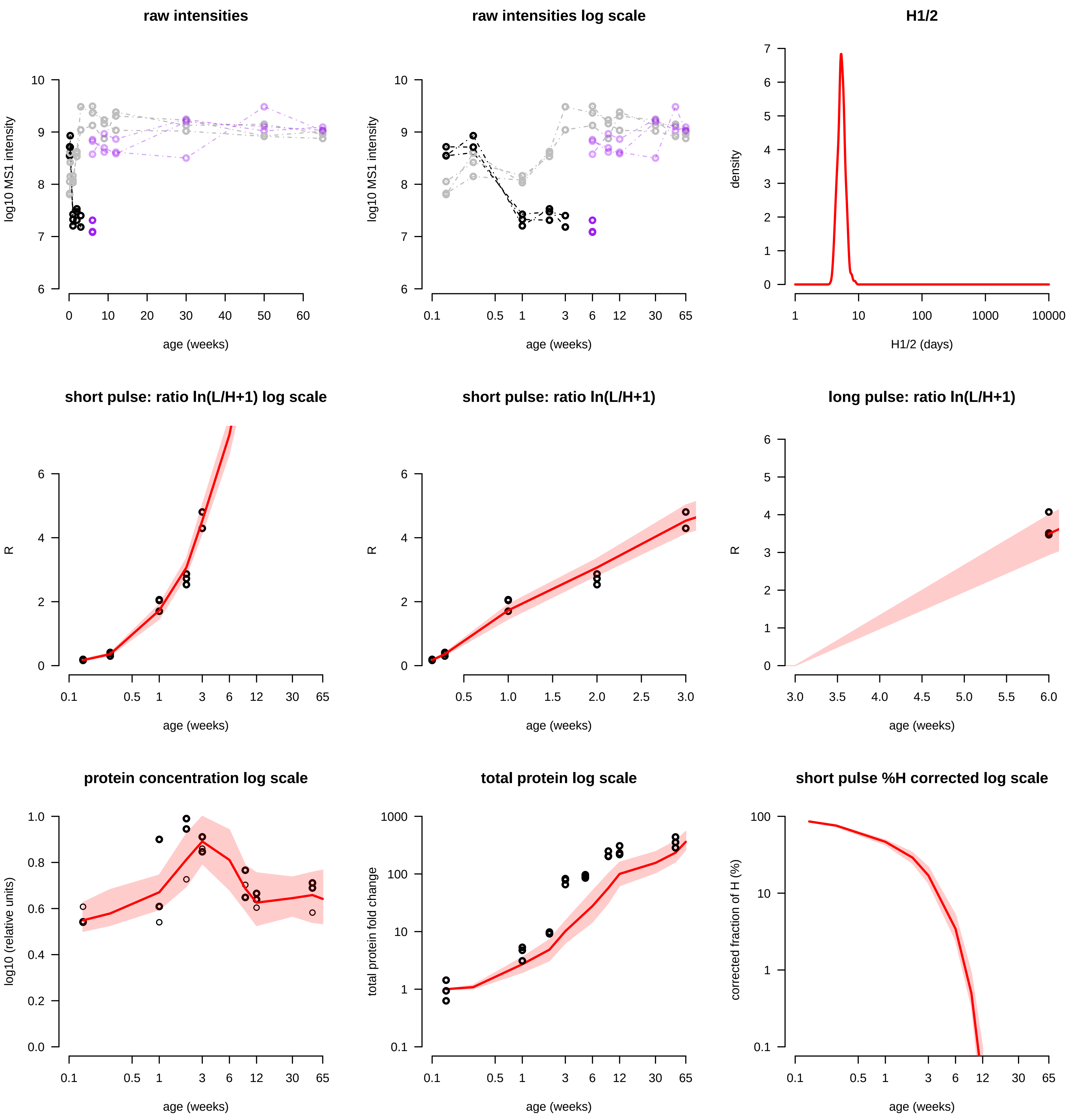

Q9D8U8

>sp|Q9D8U8|SNX5_MOUSE

Sorting nexin−5

GN=Snx5

Med H1/2: 2927.6 days

Q5 H1/2: 697.3 days

Q95 H1/2: 25088.4 days

reliable model fit: no

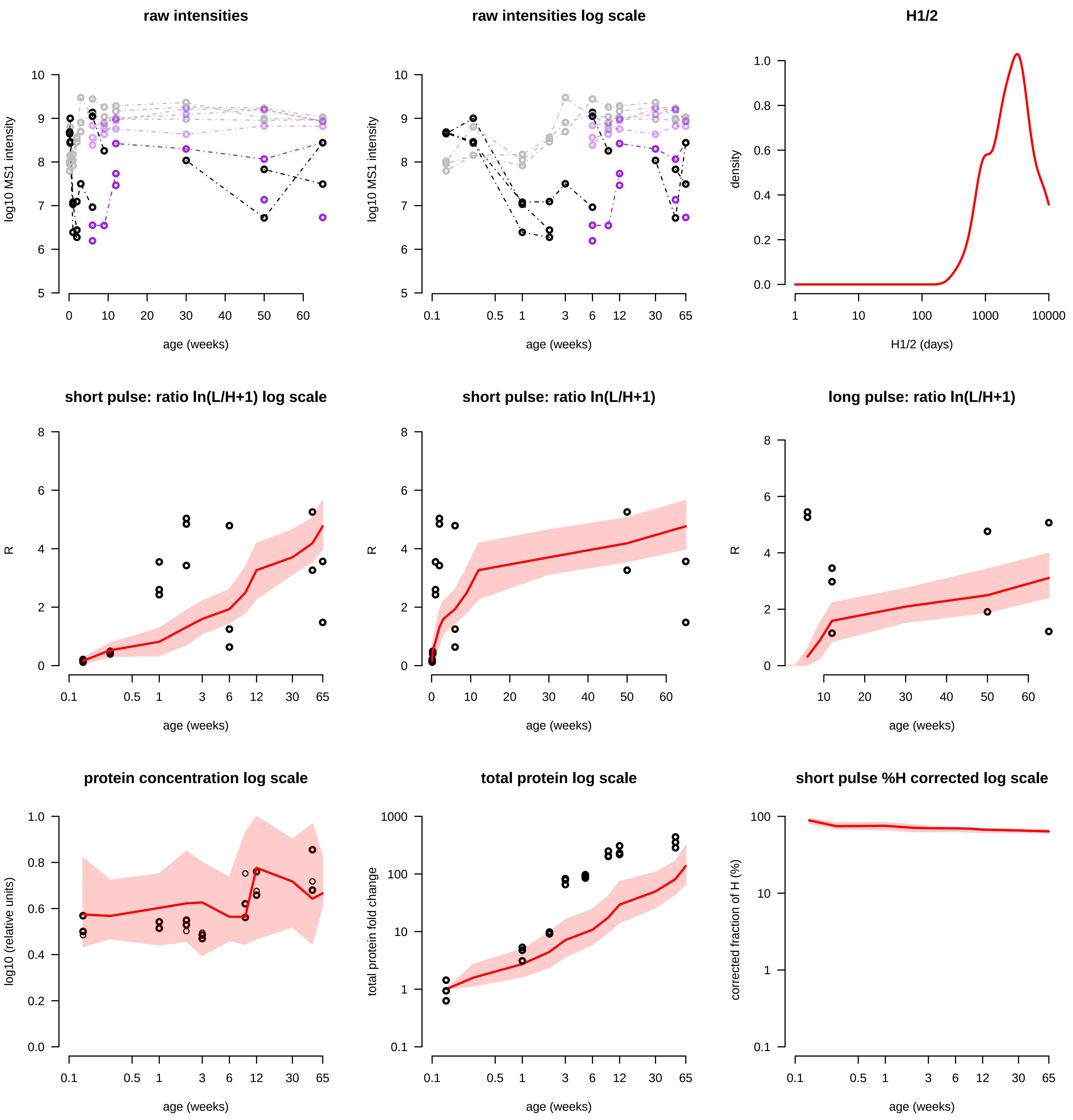

O55234

>sp|O55234|PSB5_MOUSE

Proteasome subunit beta type−5

GN=Psmb5

Med H1/2: 262.6 days

Q5 H1/2: 56.8 days

Q95 H1/2: 3255 days

reliable model fit: yes

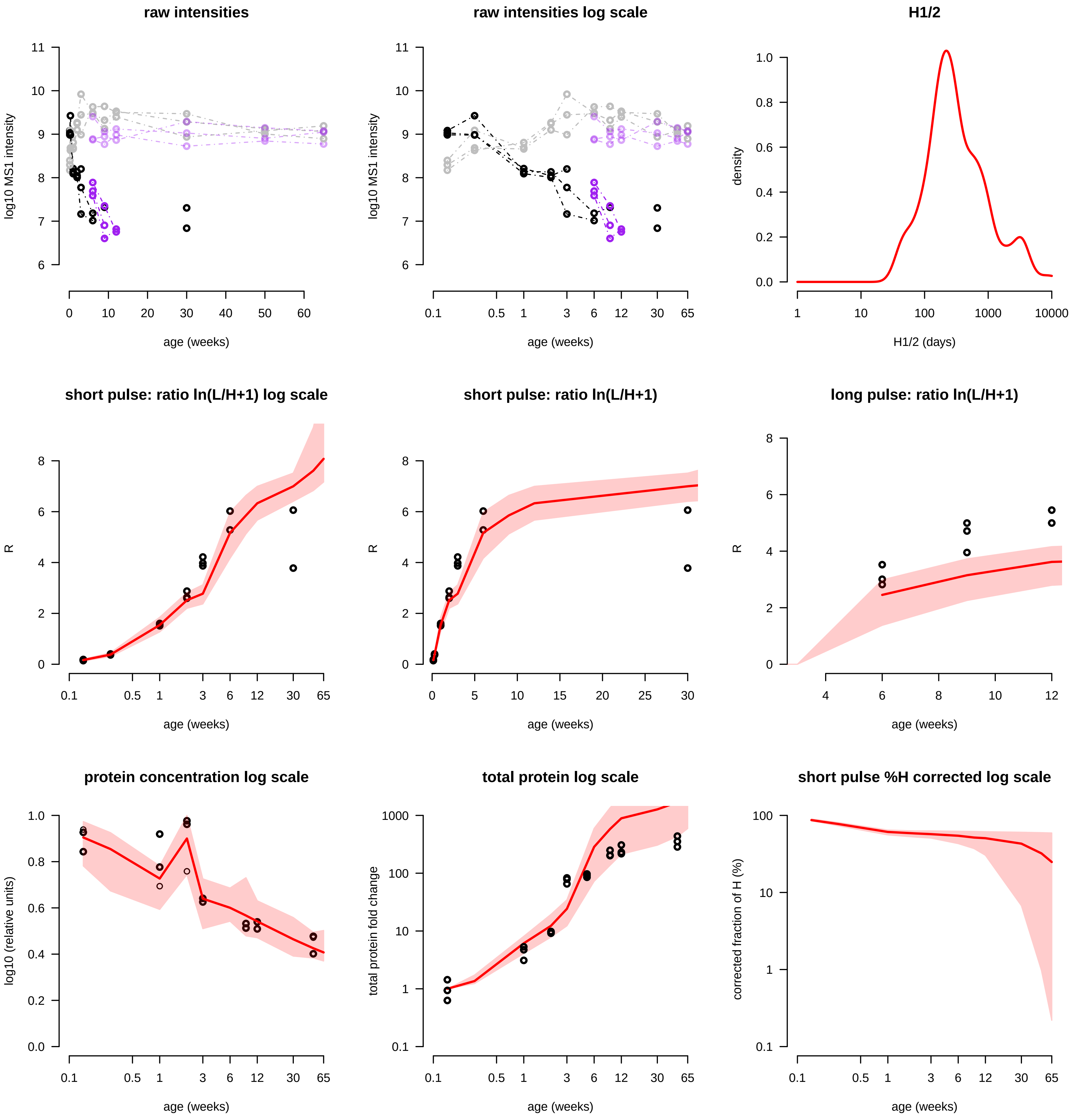

Q8BI84

>sp|Q8BI84|TGO1_MOUSE

Transport and Golgi organization protein 1 homolog

GN=Mia3

Med H1/2: 2.8 days

Q5 H1/2: 2.5 days

Q95 H1/2: 3.3 days

reliable model fit: yes

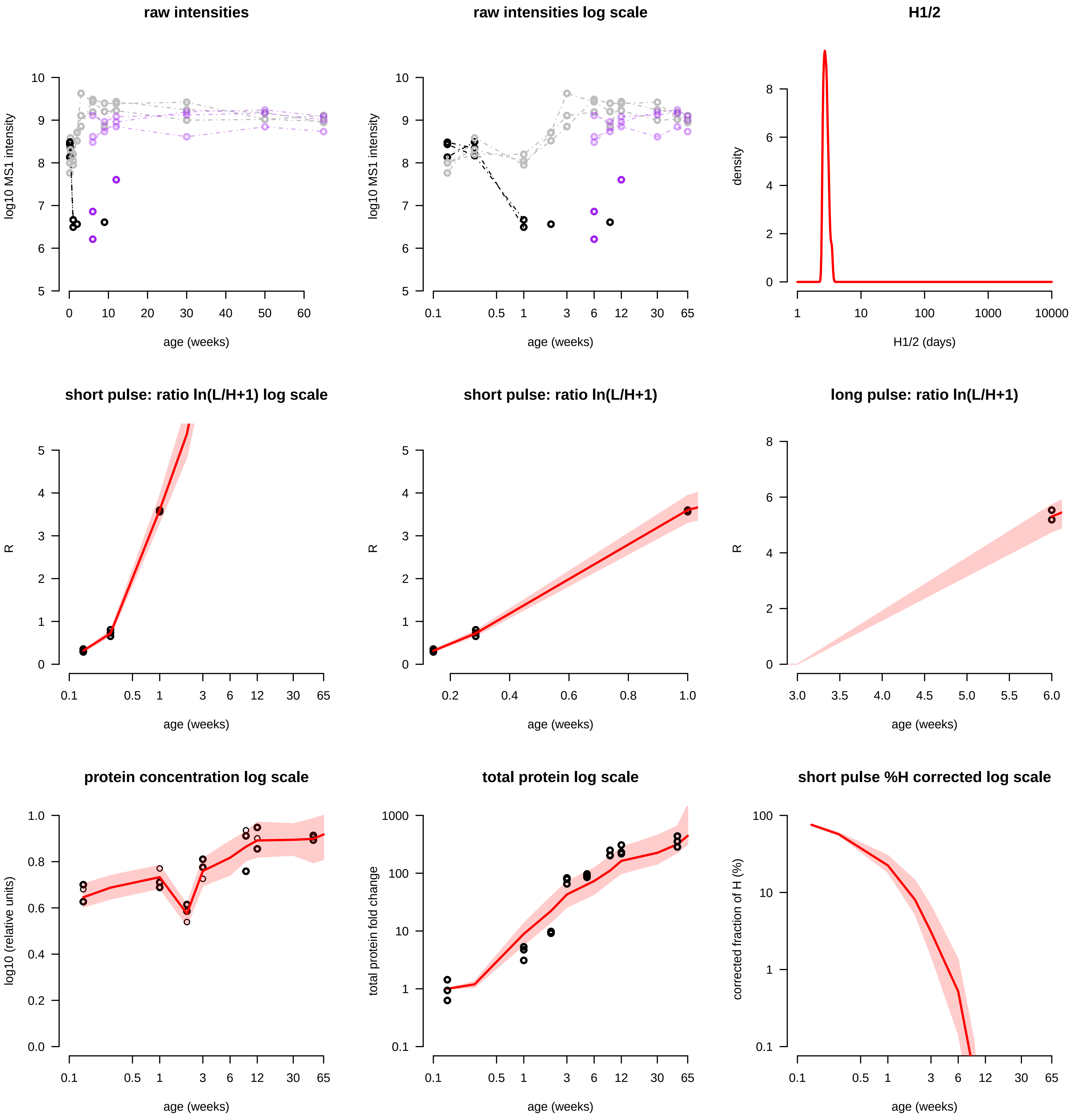

P50518

>sp|P50518|VATE1_MOUSE

V−type proton ATPase subunit E 1

GN=Atp6v1e1

Med H1/2: 661.2 days

Q5 H1/2: 235.8 days

Q95 H1/2: 12670.5 days

reliable model fit: yes

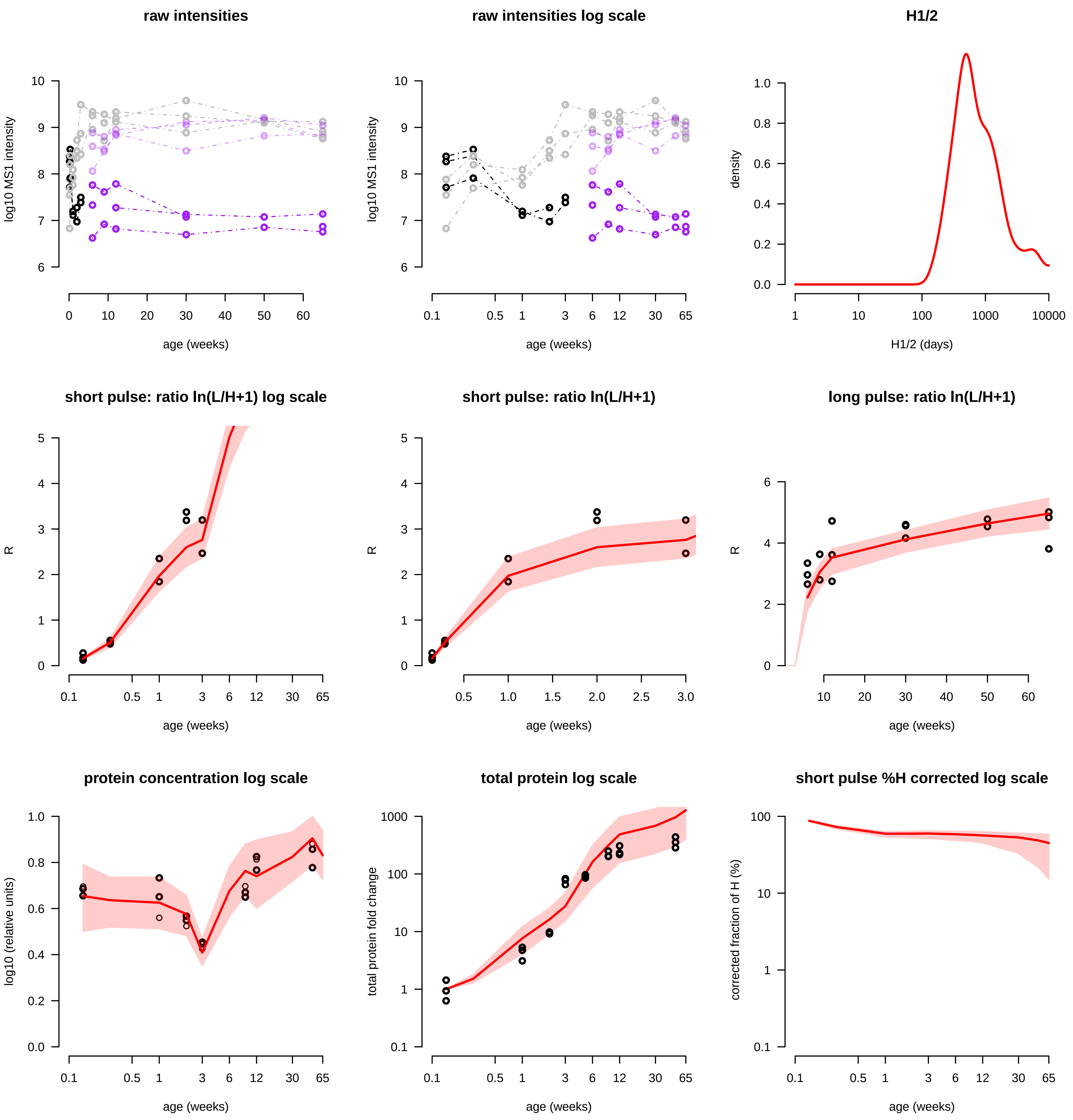

Q9CQF3

>sp|Q9CQF3|CPSF5_MOUSE

Cleavage and polyadenylation specificity factor subunit 5

GN=Nudt21

Med H1/2: 4.7 days

Q5 H1/2: 3.8 days

Q95 H1/2: 5.9 days

reliable model fit: yes

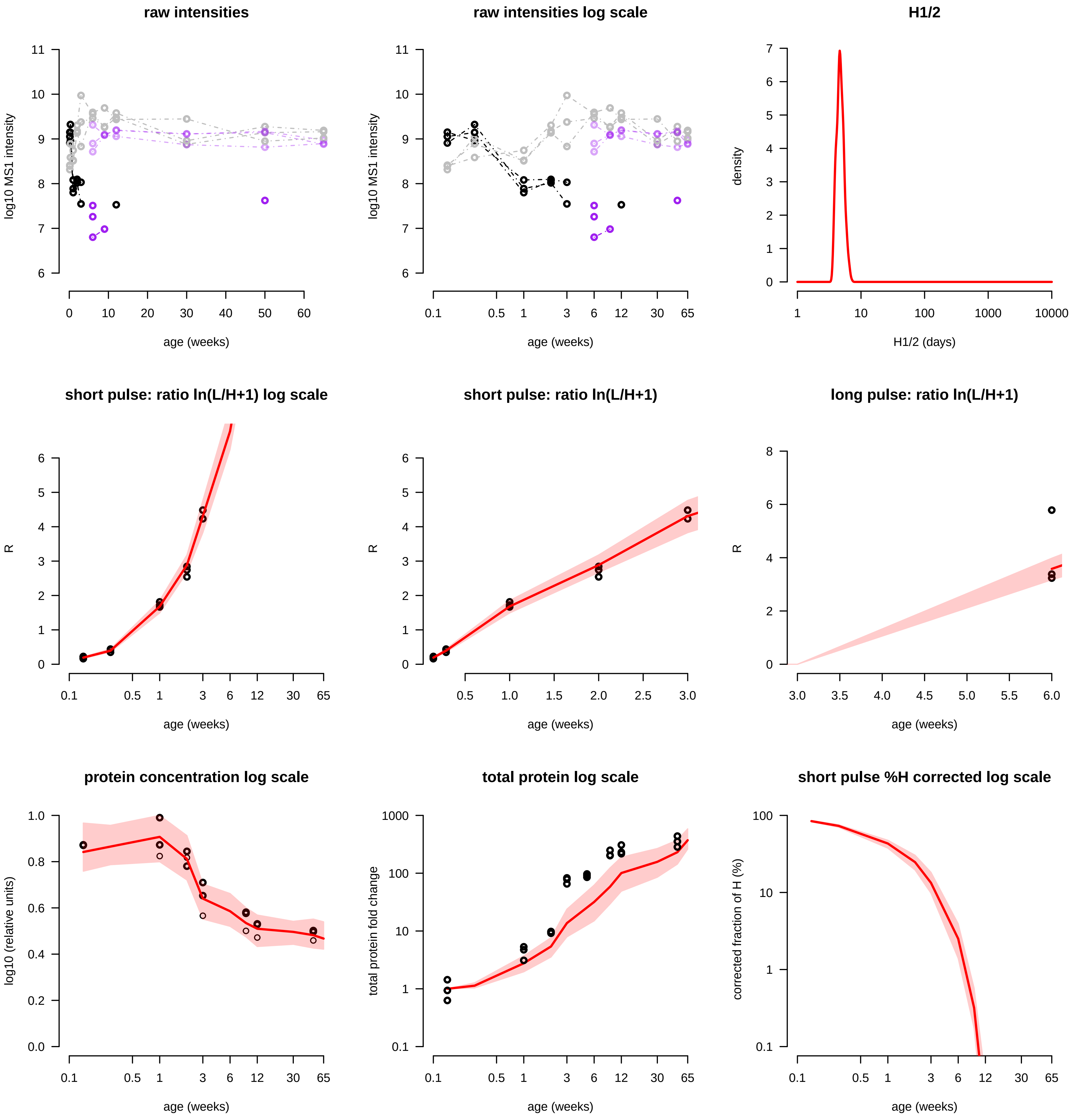

Q61545

>sp|Q61545|EWS_MOUSE

RNA−binding protein EWS

GN=Ewsr1

Med H1/2: 9.4 days

Q5 H1/2: 7.8 days

Q95 H1/2: 12.8 days

reliable model fit: yes

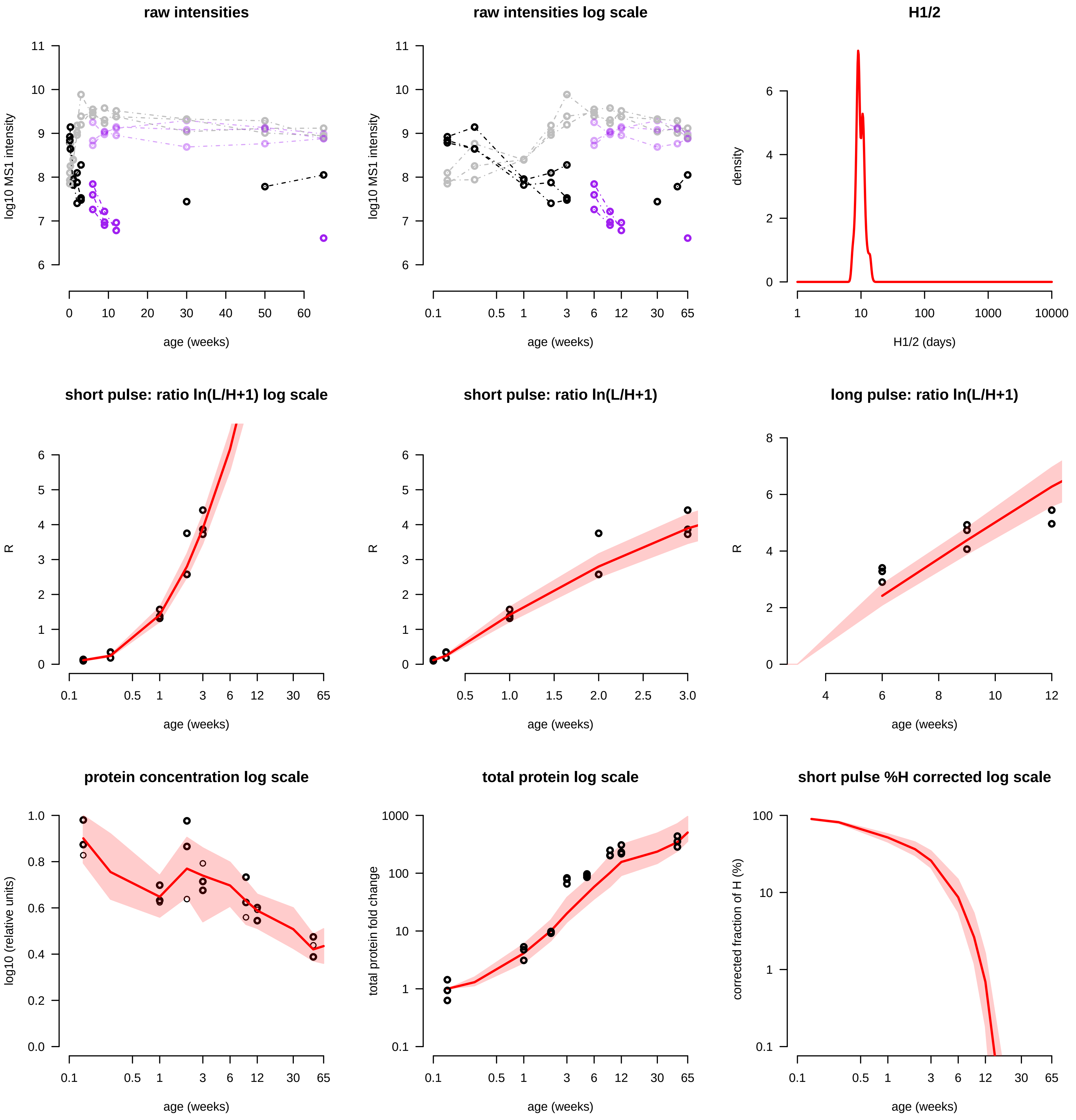

Q8VI84

>sp|Q8VI84|NOC3L_MOUSE

Nucleolar complex protein 3 homolog

GN=Noc3l

Med H1/2: 10.5 days

Q5 H1/2: 8.7 days

Q95 H1/2: 13.6 days

reliable model fit: yes

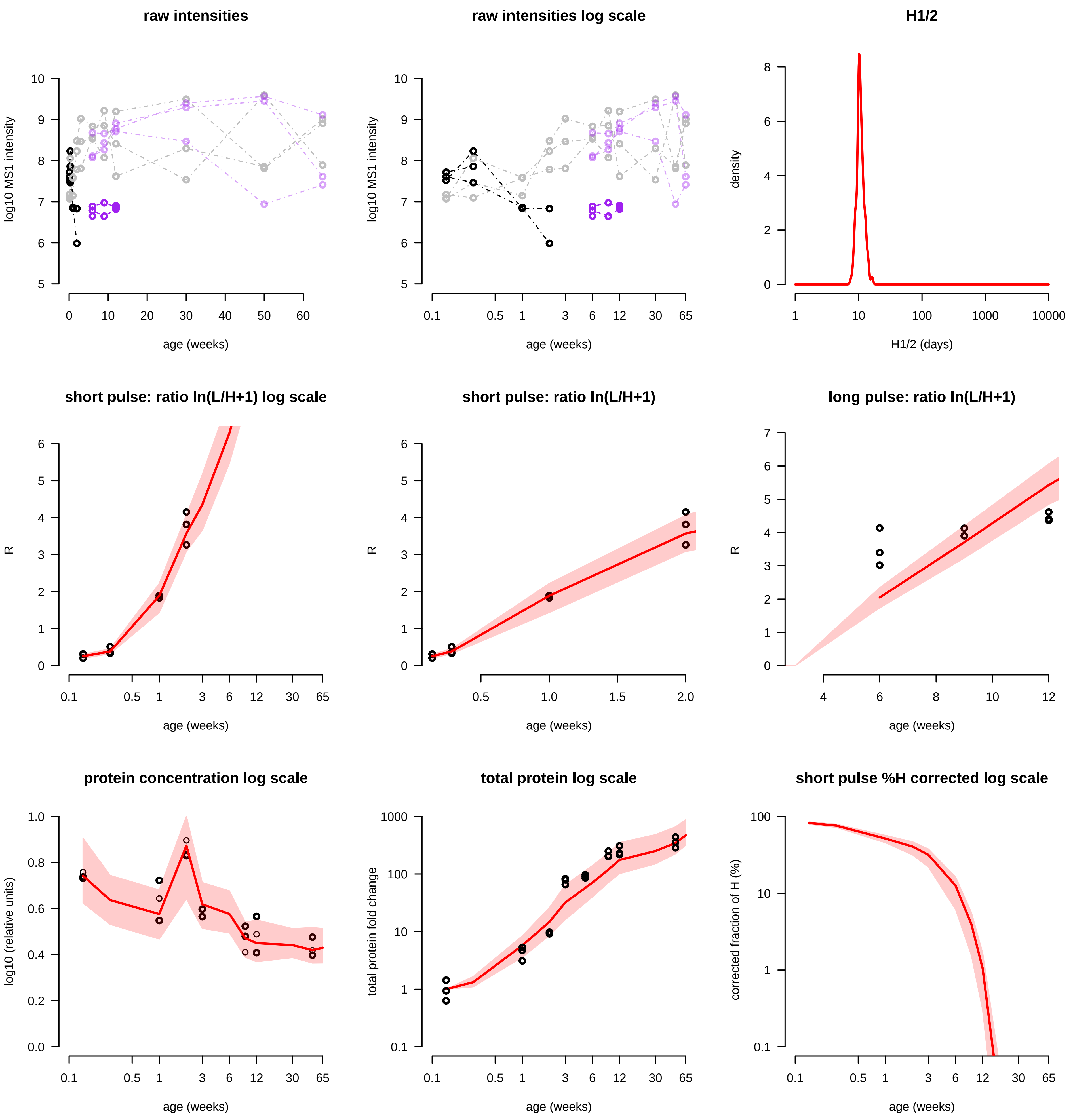

Q6P6M7

>sp|Q6P6M7|SPCS_MOUSE

O−phosphoseryl−tRNA(Sec) selenium transferase

GN=Sepsecs

Med H1/2: 7.9 days

Q5 H1/2: 6.6 days

Q95 H1/2: 9.8 days

reliable model fit: yes

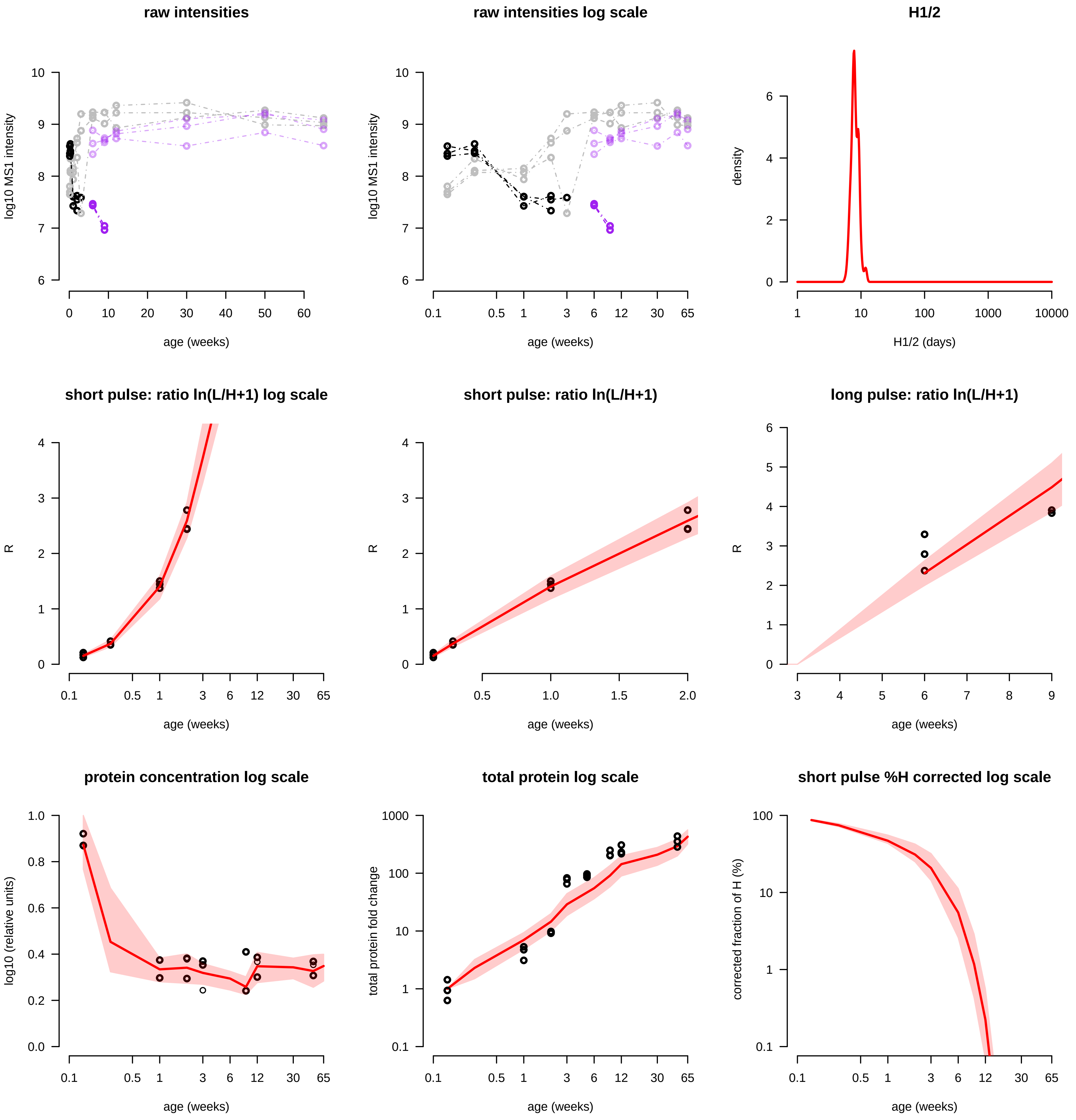

6Y3−6;Q7M6Y3−5;Q7M6Y3;Q7M6Y3−2;Q7M6Y3−3;Q7M6Y

>sp|Q7M6Y3−6|PICAL_MOUSE

Isoform 6 of Phosphatidylinositol−binding clathrin assembly protein

GN=Picalm

Med H1/2: 8.7 days

Q5 H1/2: 7.3 days

Q95 H1/2: 10.6 days

reliable model fit: yes

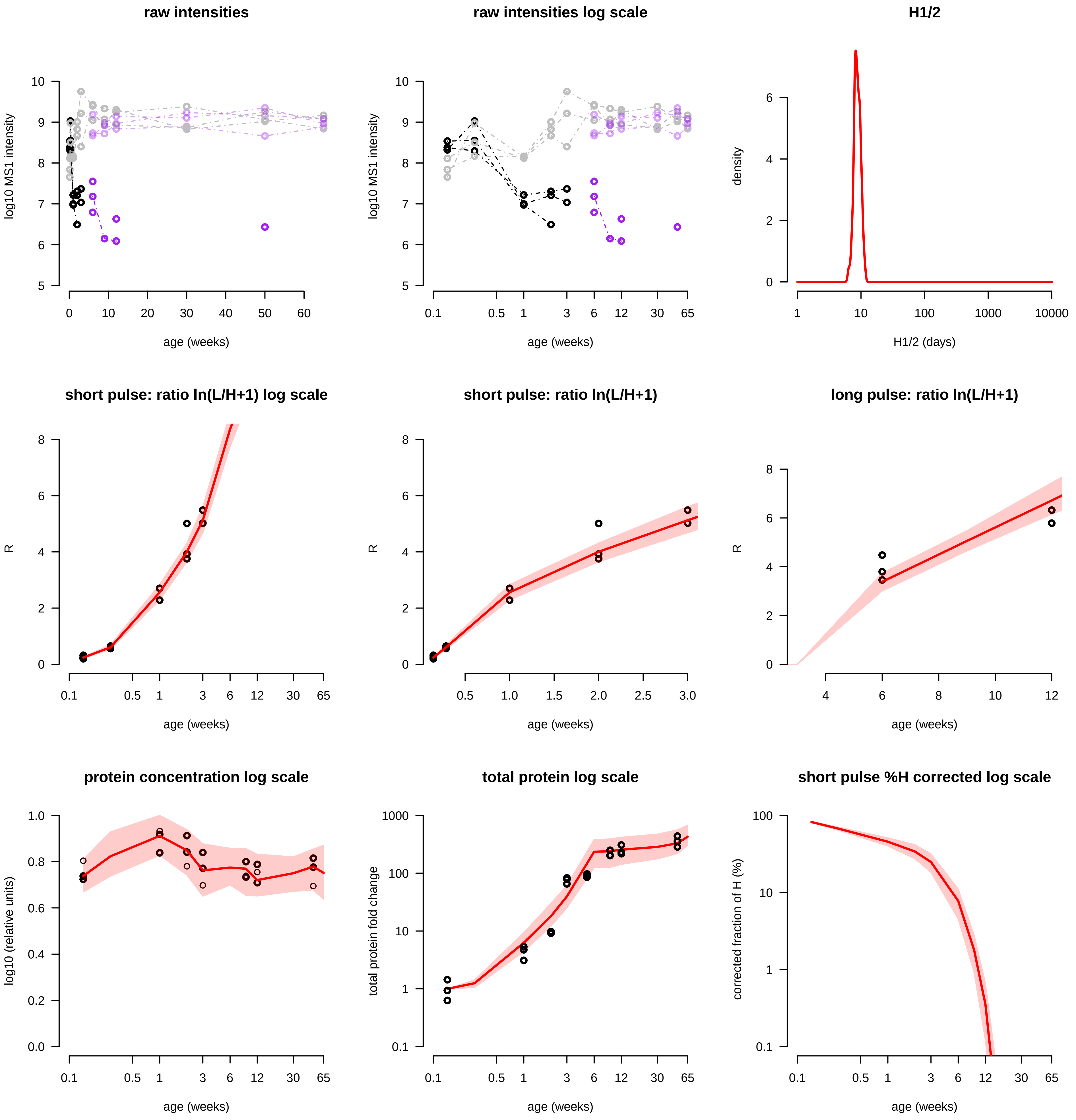

Q8BRF7;Q8BRF7−3

>sp|Q8BRF7|SCFD1_MOUSE

Sec1 family domain−containing protein 1

GN=Scfd1

Med H1/2: 7.2 days

Q5 H1/2: 5.8 days

Q95 H1/2: 10.2 days

reliable model fit: yes

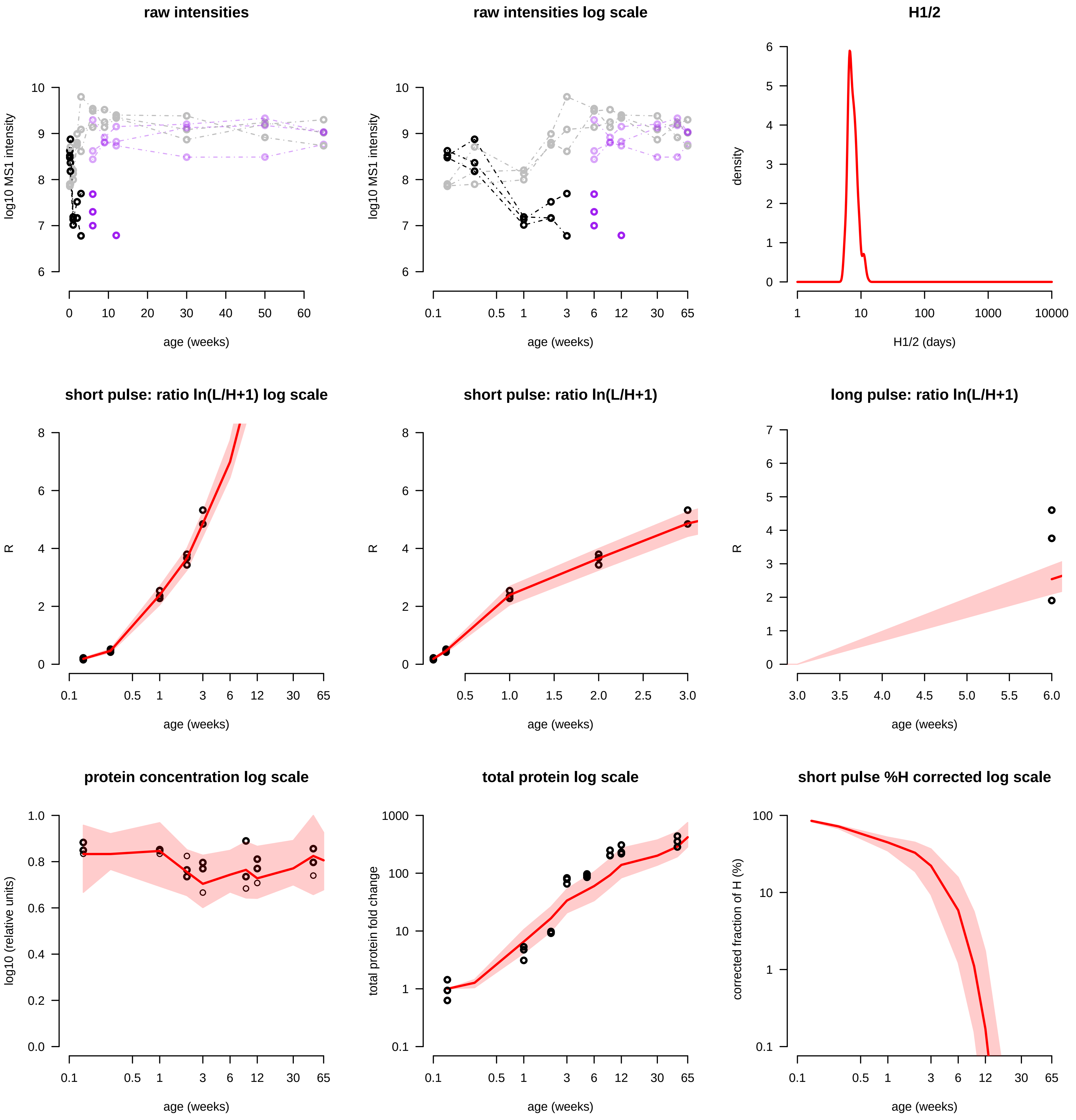

Q99LB2

>sp|Q99LB2|DHRS4_MOUSE

Dehydrogenase/reductase SDR family member 4

GN=Dhrs4

Med H1/2: 5.3 days

Q5 H1/2: 4 days

Q95 H1/2: 7.4 days

reliable model fit: yes

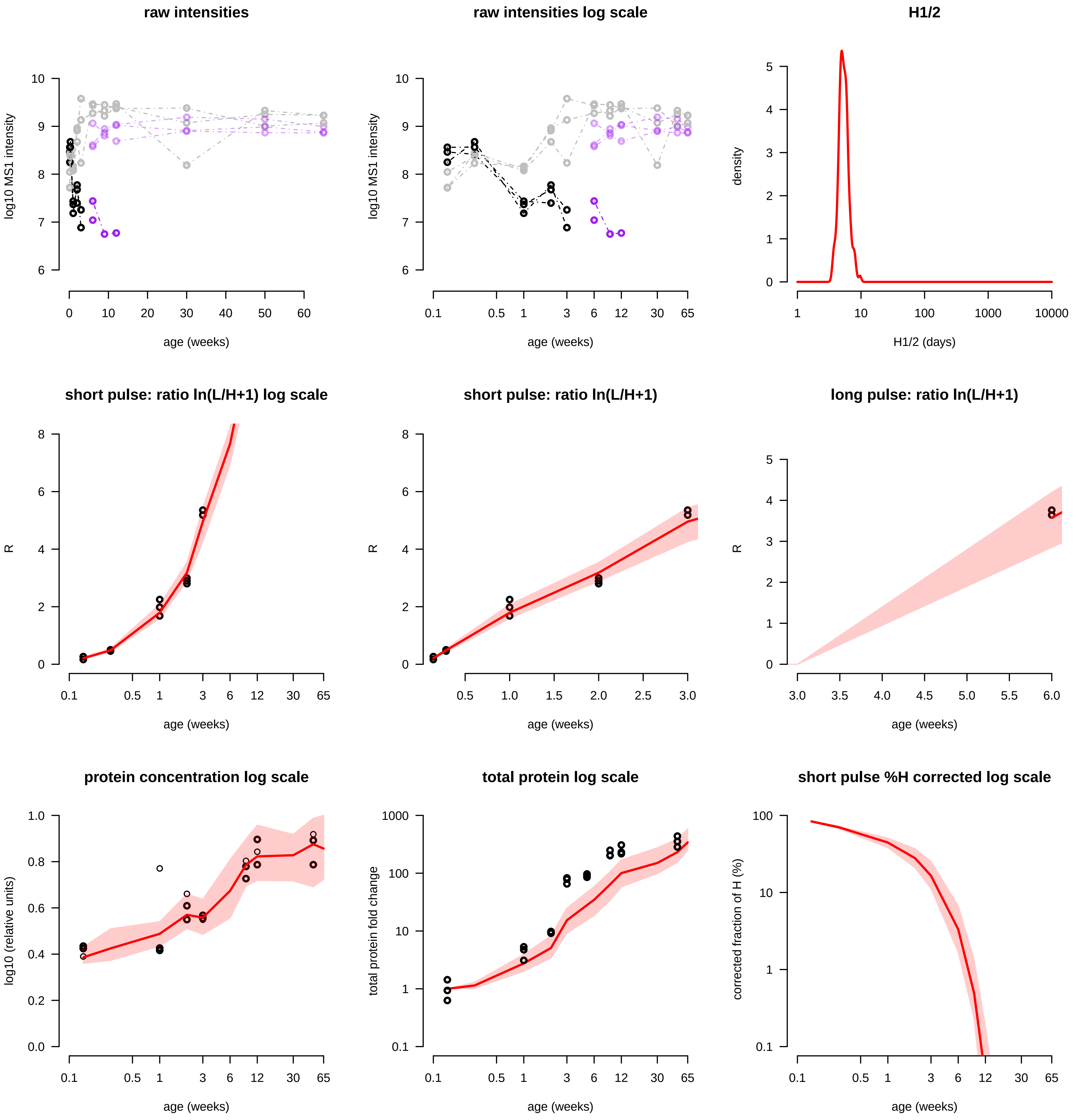

Q61554

>sp|Q61554|FBN1_MOUSE

Fibrillin−1

GN=Fbn1

Med H1/2: 2141.7 days

Q5 H1/2: 570 days

Q95 H1/2: 31507.5 days

reliable model fit: yes

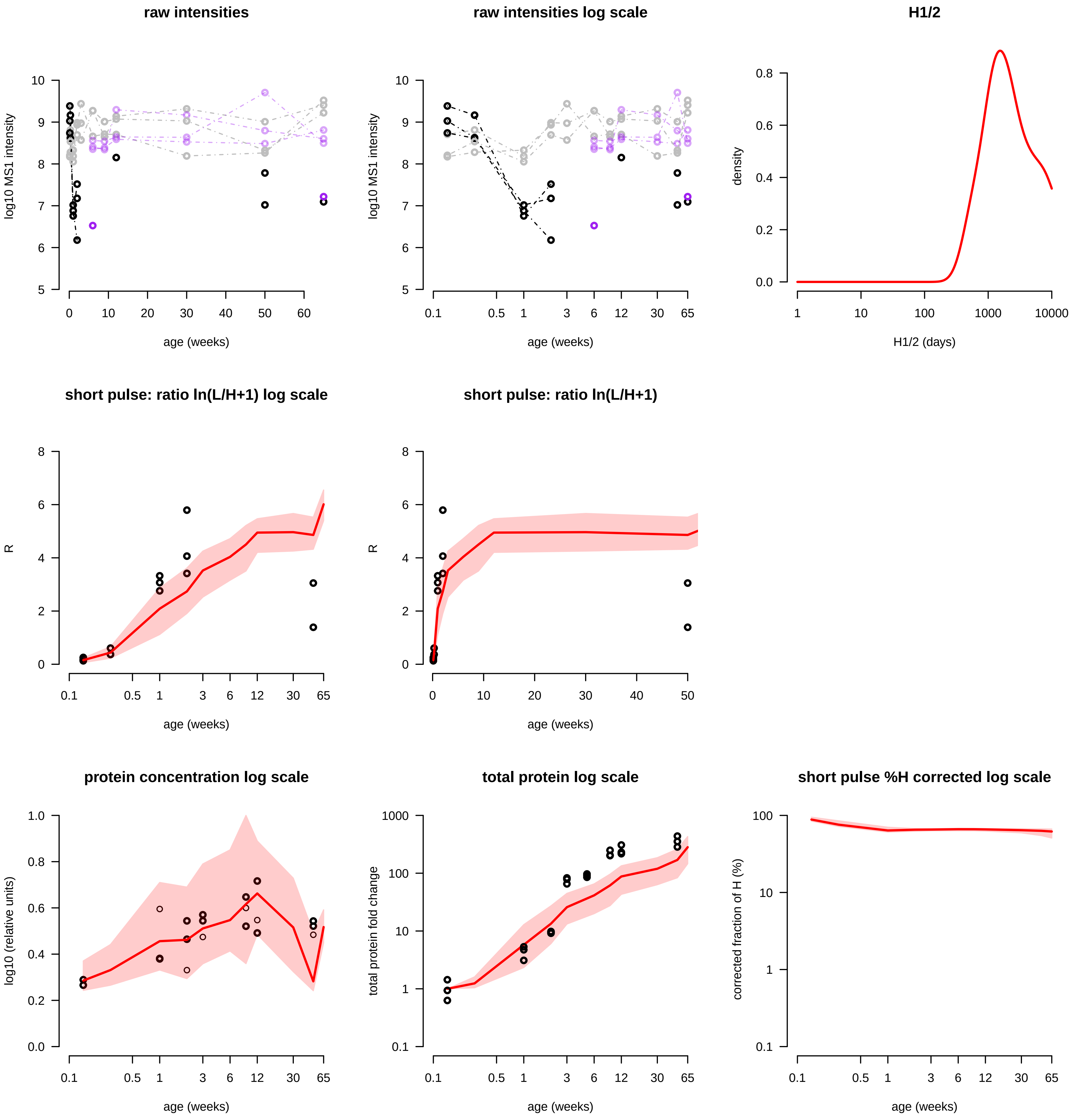

P35293

>sp|P35293|RAB18_MOUSE

Ras−related protein Rab−18

GN=Rab18

Med H1/2: 7.1 days

Q5 H1/2: 5.8 days

Q95 H1/2: 9.4 days

reliable model fit: yes

raw intensities

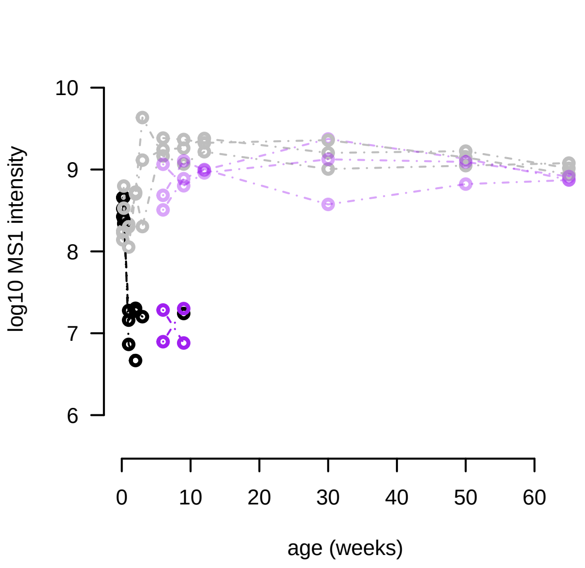

raw intensities log scale

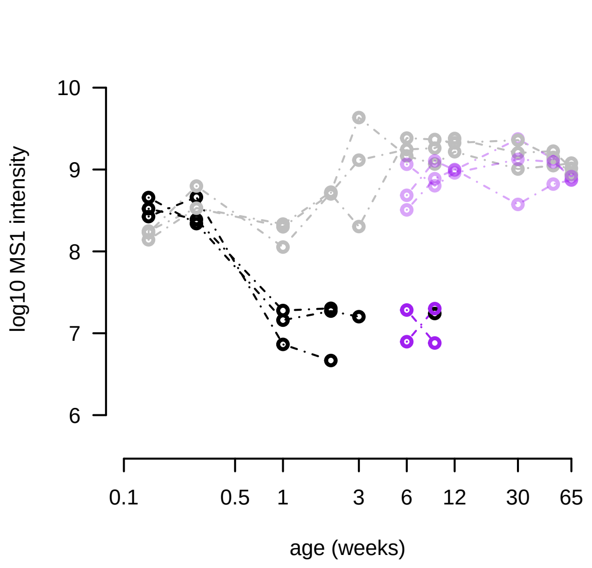

H1/2

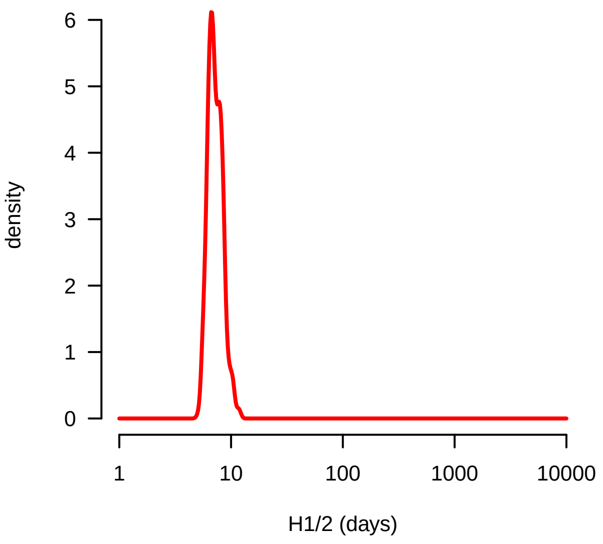

short pulse: ratio ln(L/H+1) log scale

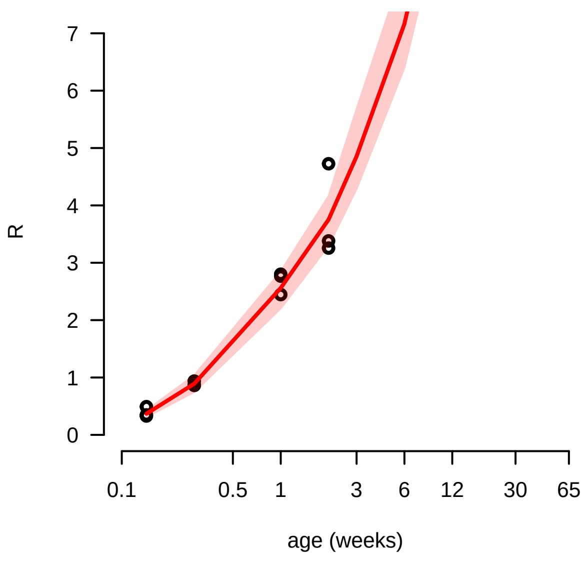

short pulse: ratio ln(L/H+1)

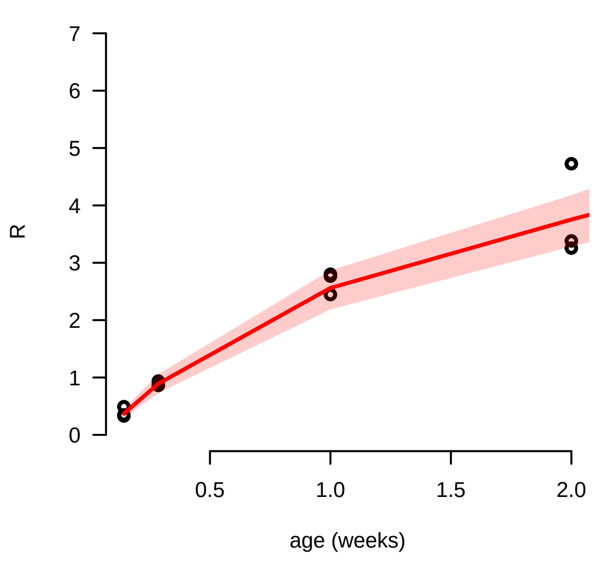

long pulse: ratio ln(L/H+1)

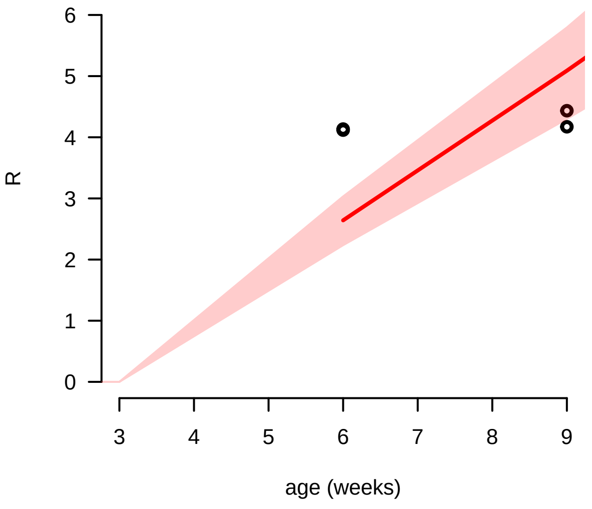

protein concentration log scale

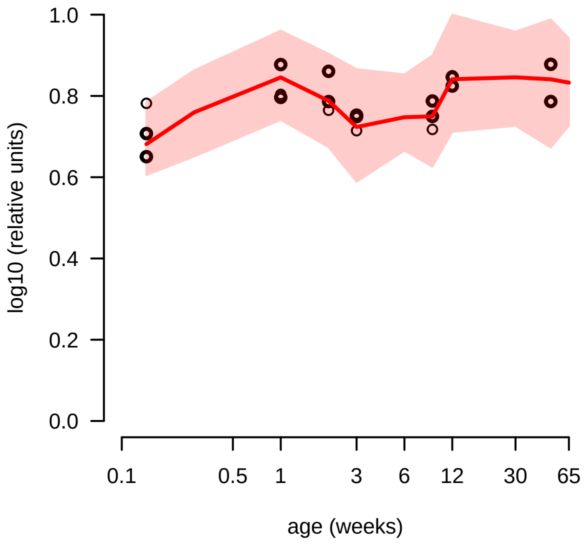

total protein log scale

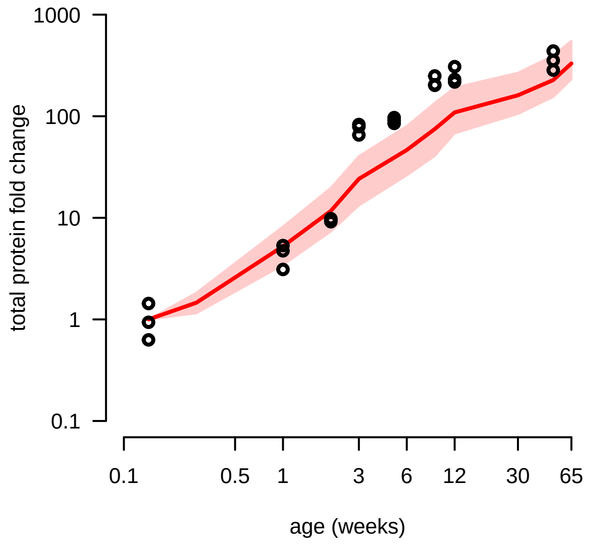

short pulse %H corrected log scale

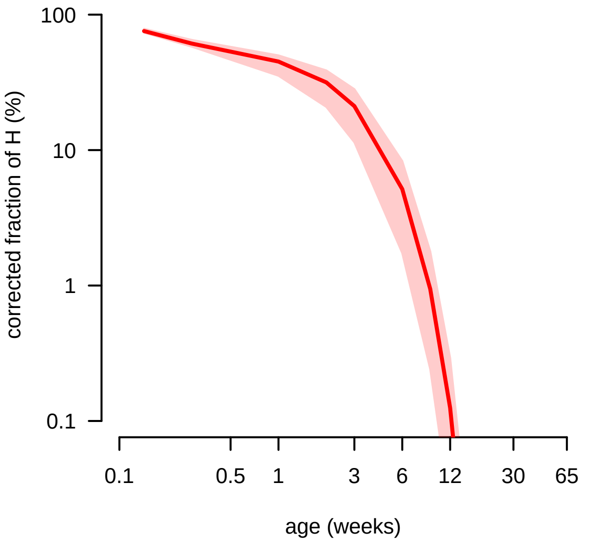

Q6GQT9

>sp|Q6GQT9|NOMO1_MOUSE

Nodal modulator 1

GN=Nomo1

Med H1/2: 290.2 days

Q5 H1/2: 124.3 days

Q95 H1/2: 2045.6 days

reliable model fit: yes

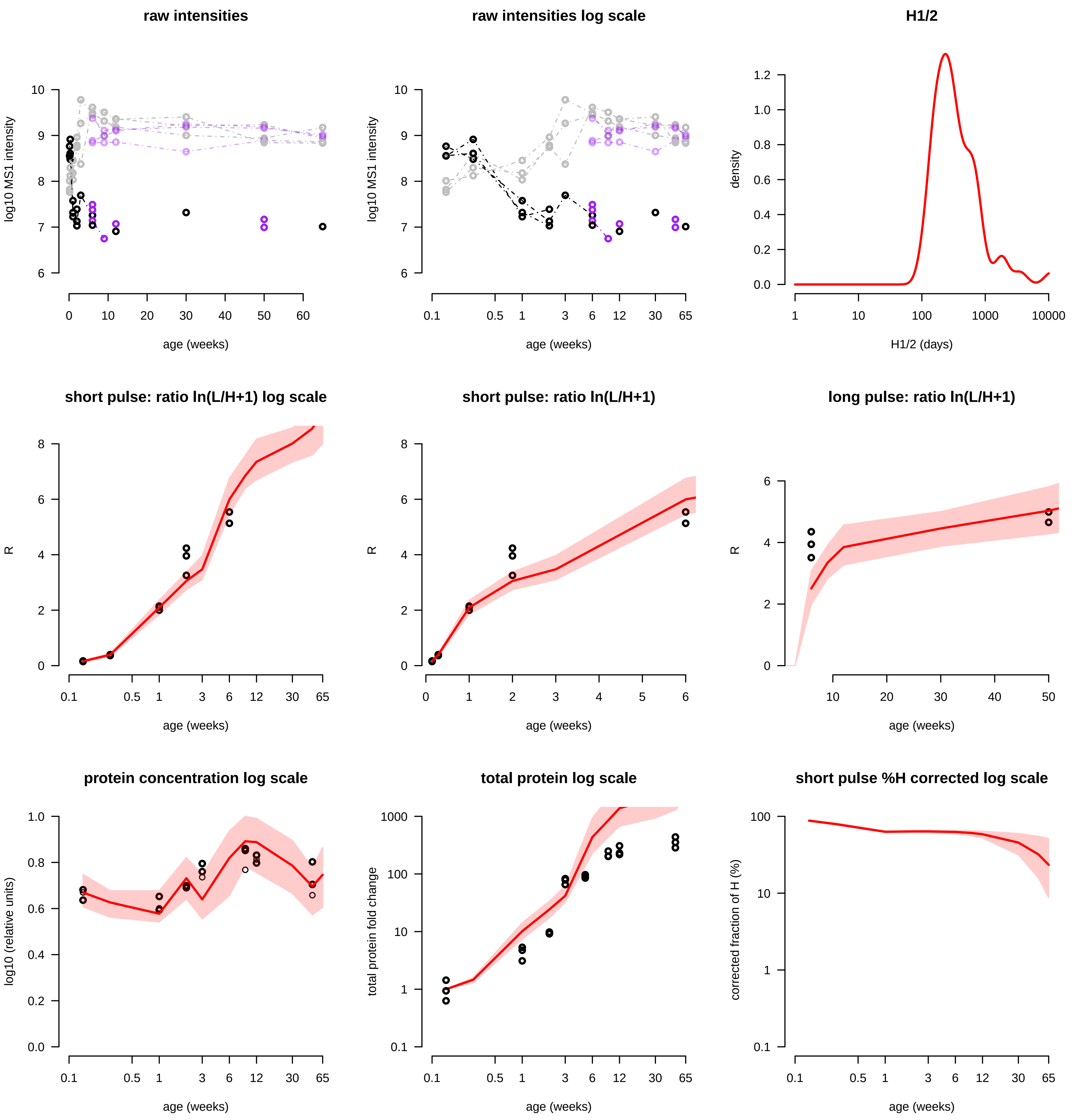

P24547

>sp|P24547|IMDH2_MOUSE

Inosine−5'−monophosphate dehydrogenase 2

GN=Impdh2

Med H1/2: 4.3 days

Q5 H1/2: 3.7 days

Q95 H1/2: 5.4 days

reliable model fit: yes

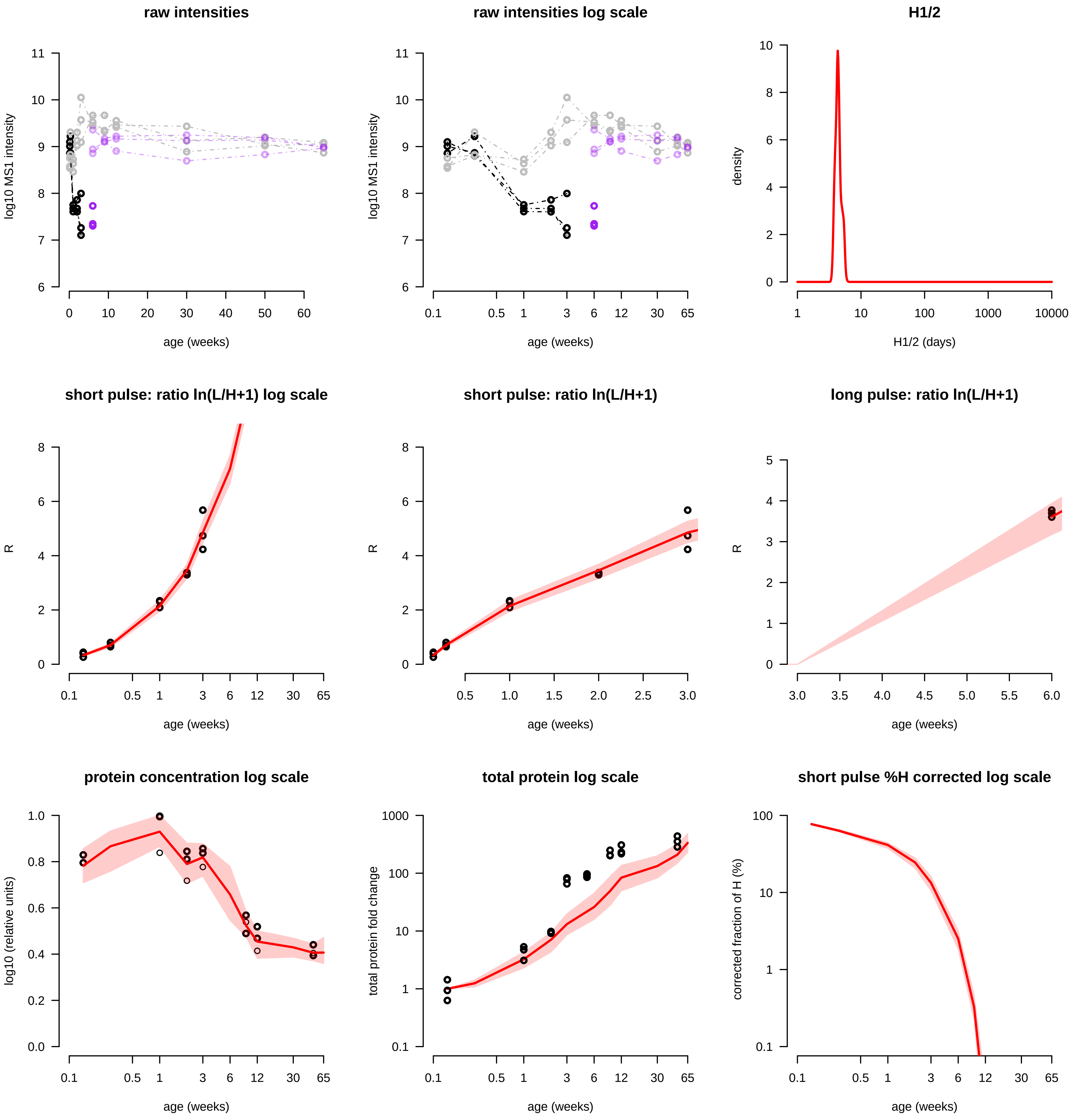

O88738;O88738−3;O88738−2

>sp|O88738|BIRC6_MOUSE

Baculoviral IAP repeat−containing protein 6

GN=Birc6

Med H1/2: 826.6 days

Q5 H1/2: 214 days

Q95 H1/2: 22026.2 days

reliable model fit: yes

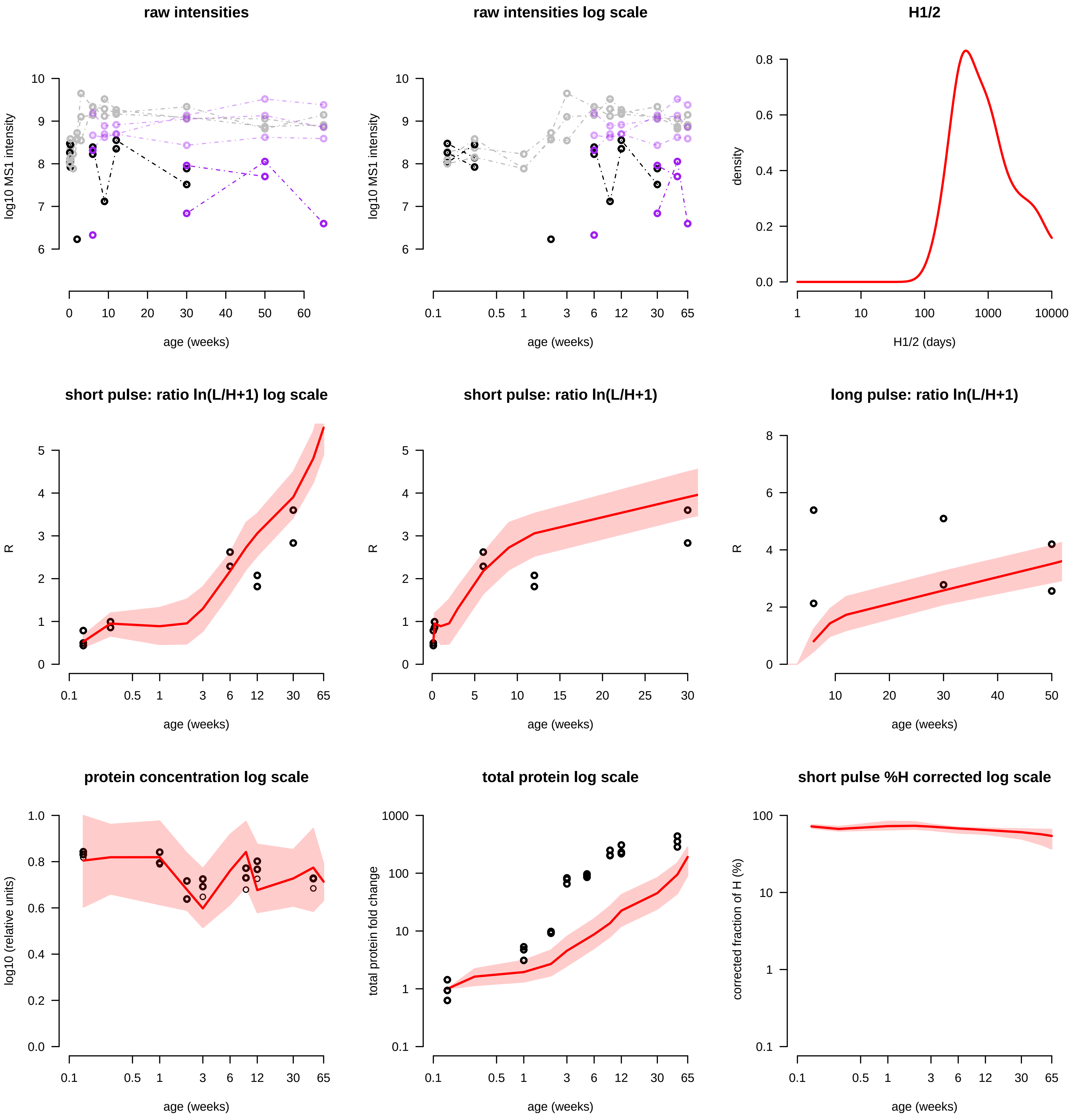

P61327;Q9CQL1

>sp|P61327|MGN_MOUSE

Protein mago nashi homolog

GN=Magoh

Med H1/2: 1056.4 days

Q5 H1/2: 334.1 days

Q95 H1/2: 10396.1 days

reliable model fit: no

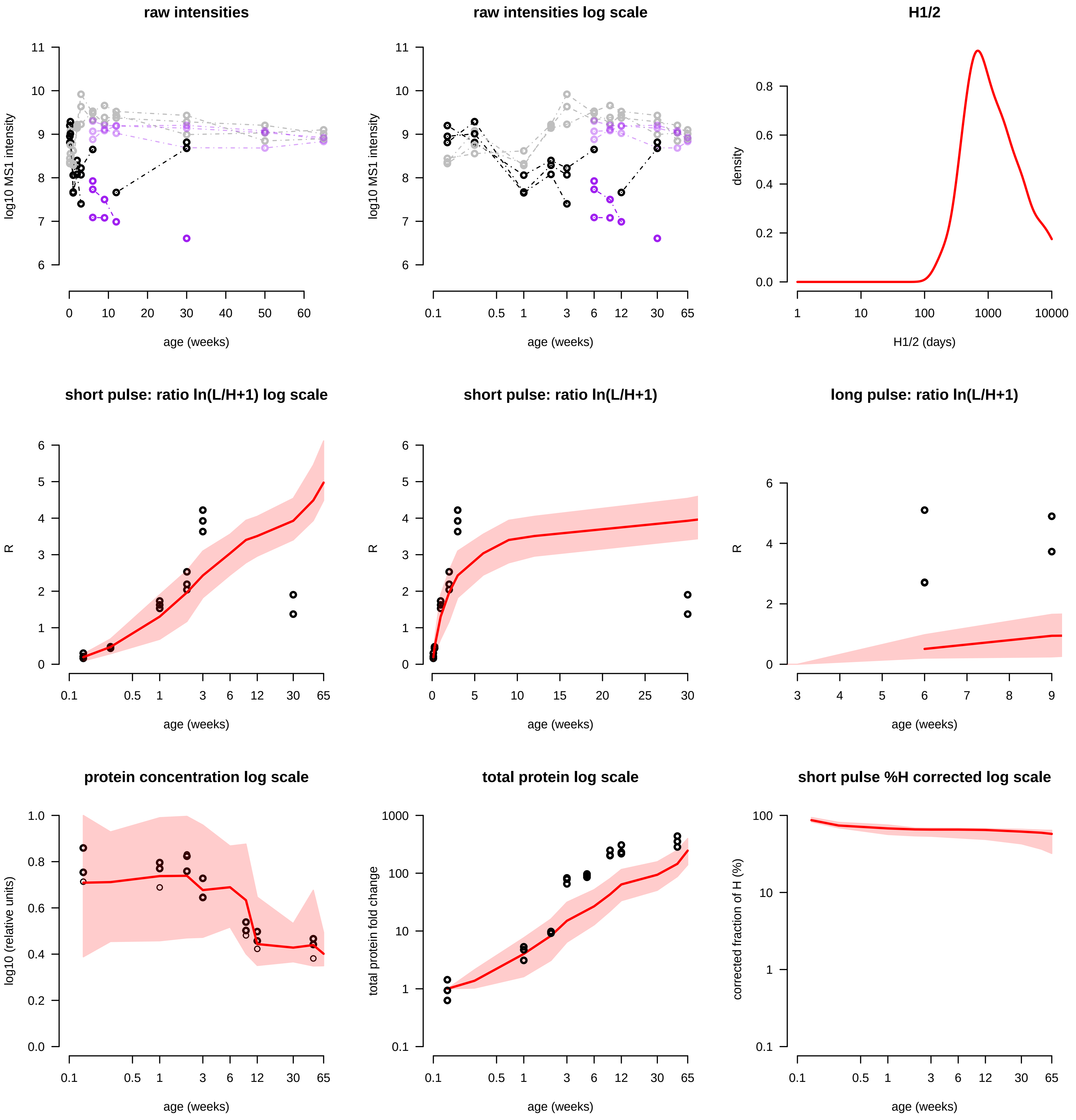

Q569Z5;Q569Z5−2

>sp|Q569Z5|DDX46_MOUSE

Probable ATP−dependent RNA helicase DDX46

GN=Ddx46

Med H1/2: 4.2 days

Q5 H1/2: 3.5 days

Q95 H1/2: 5 days

reliable model fit: yes

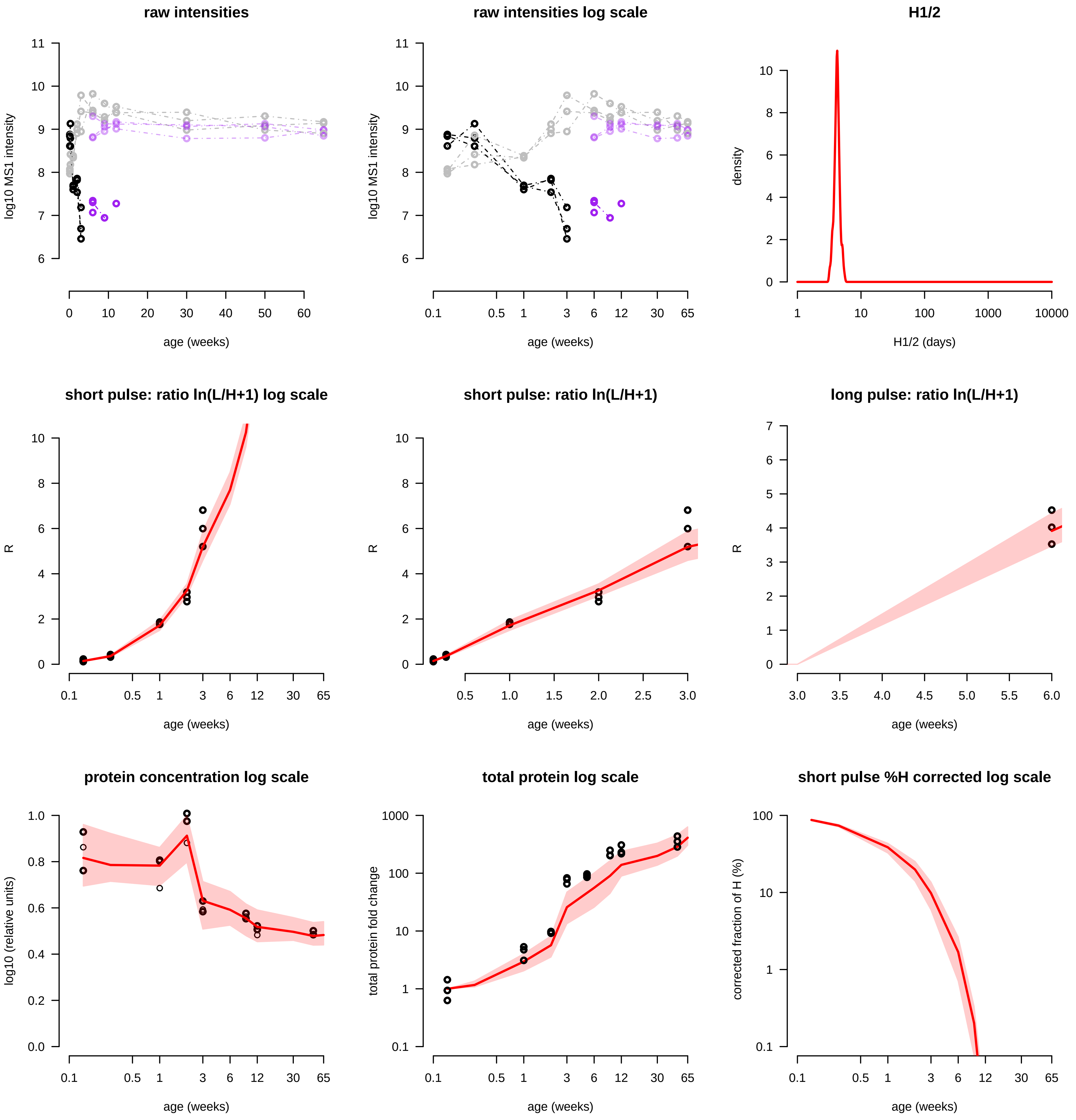

Q9JHU9

>sp|Q9JHU9|INO1_MOUSE

Inositol−3−phosphate synthase 1

GN=Isyna1

Med H1/2: 361.6 days

Q5 H1/2: 133.3 days

Q95 H1/2: 2857.9 days

reliable model fit: yes

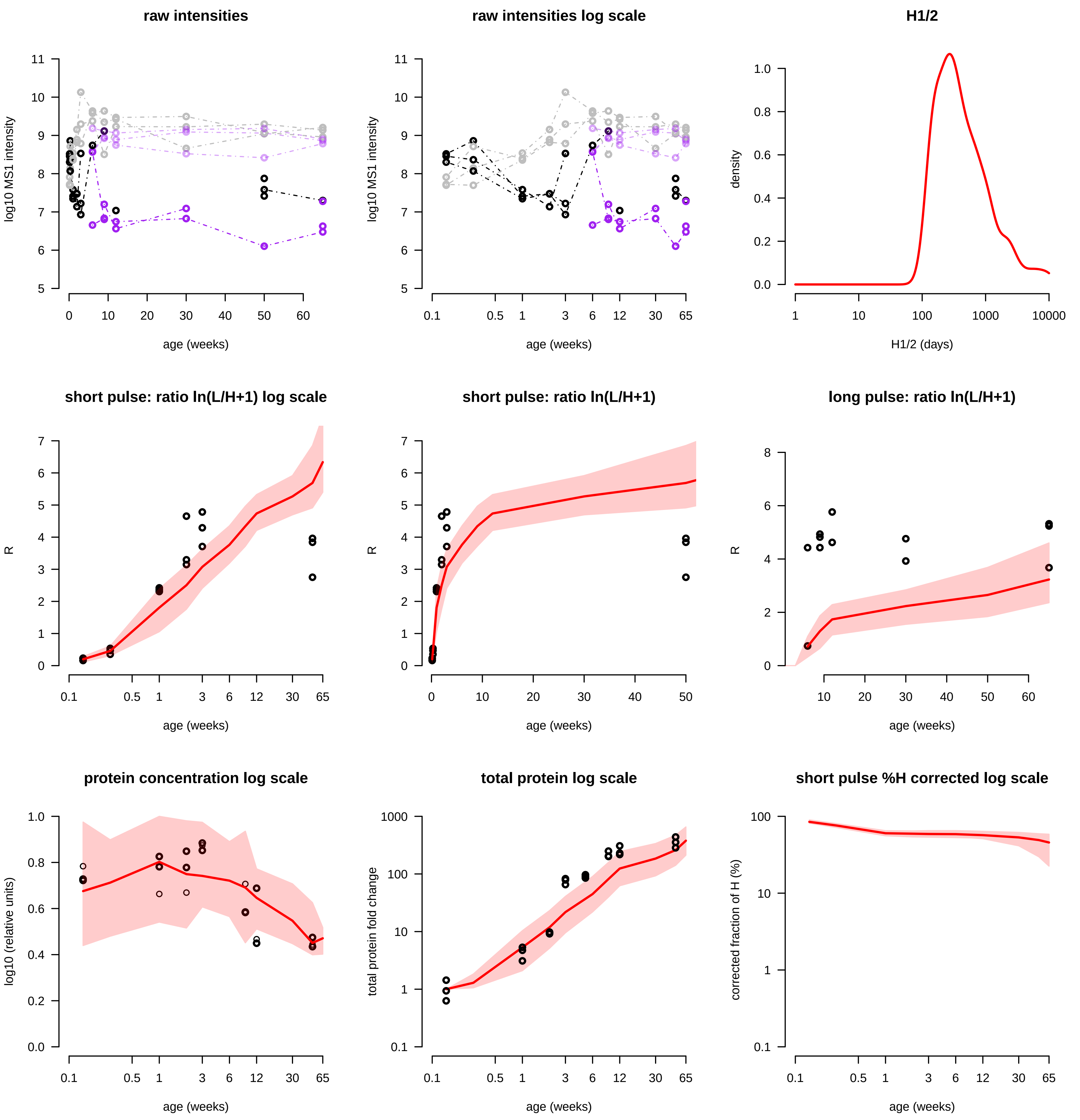

Q9Z1J3

>sp|Q9Z1J3|NFS1_MOUSE

Cysteine desulfurase, mitochondrial

GN=Nfs1

Med H1/2: 9 days

Q5 H1/2: 7.2 days

Q95 H1/2: 11.3 days

reliable model fit: yes

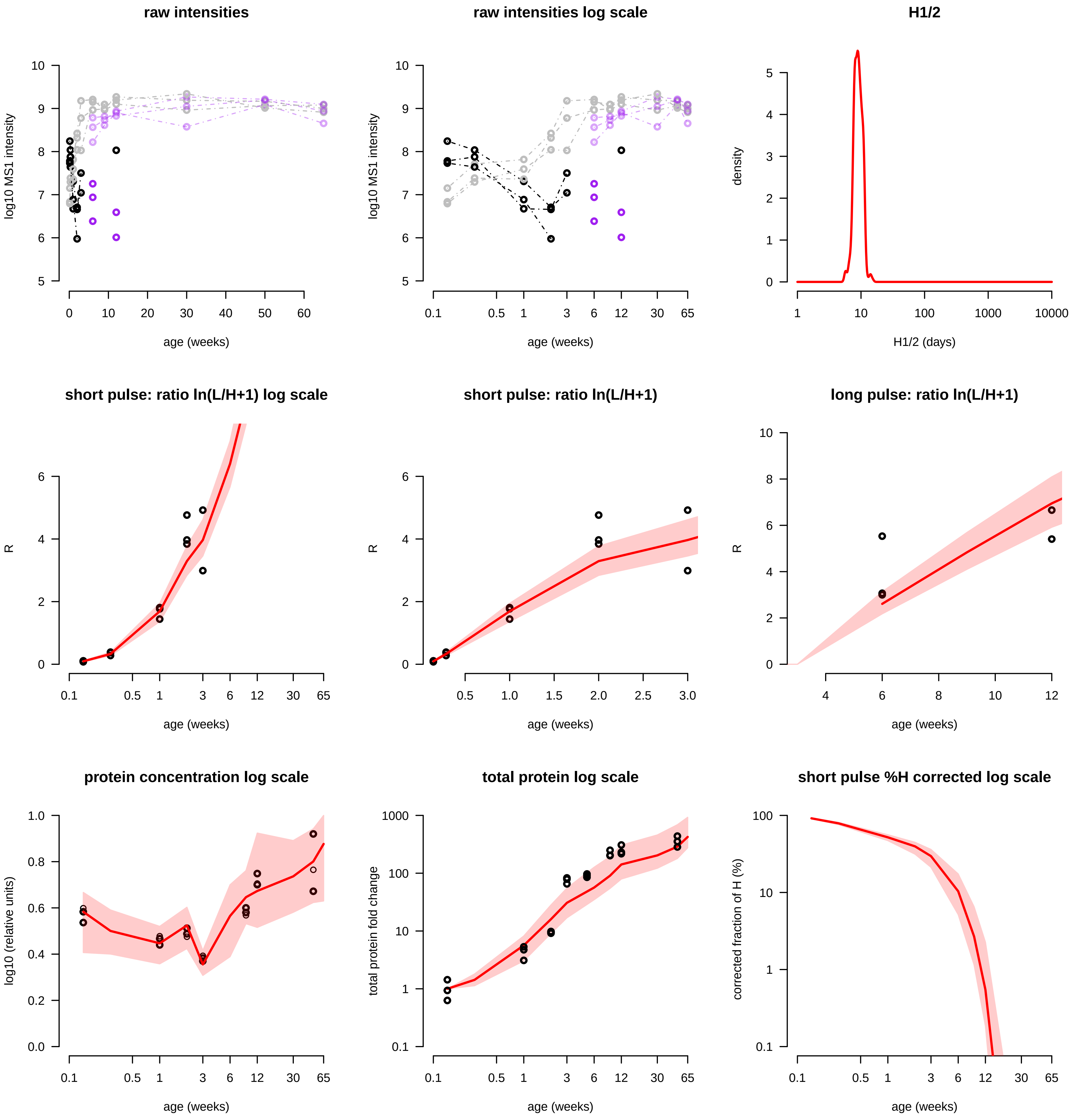

Q8BX02;Q8BX02−2

>sp|Q8BX02|KANK2_MOUSE

KN motif and ankyrin repeat domain−containing protein 2

GN=Kank2

Med H1/2: 4.6 days

Q5   H1/2: 3.8 days

Q95 H1/2: 6 days

reliable model fit: yes

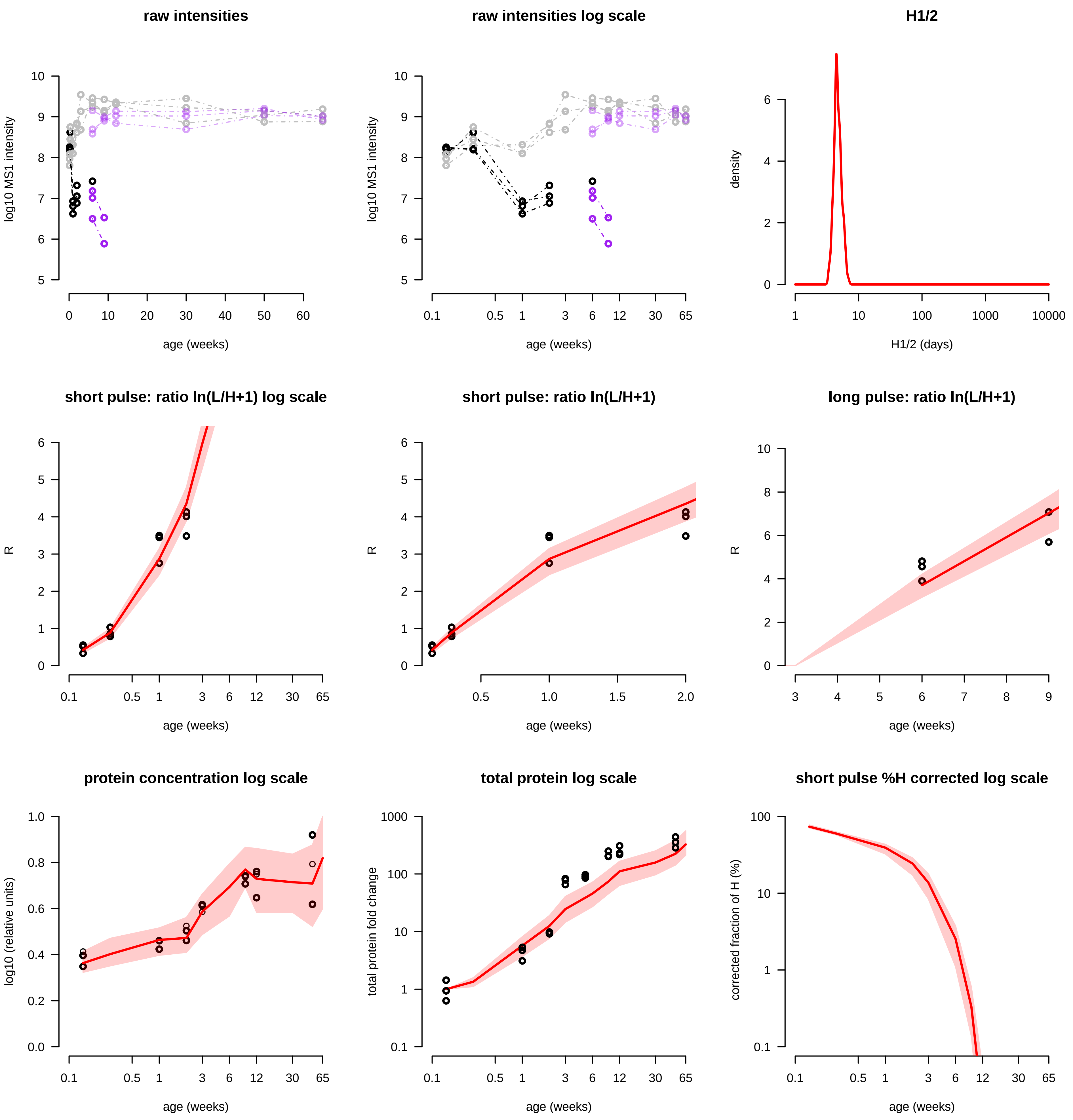

P47740

>sp|P47740|AL3A2_MOUSE

Aldehyde dehydrogenase family 3 member A2

GN=Aldh3a2

Med H1/2: 5 days

Q5 H1/2: 4.1 days

Q95 H1/2: 6.2 days

reliable model fit: yes

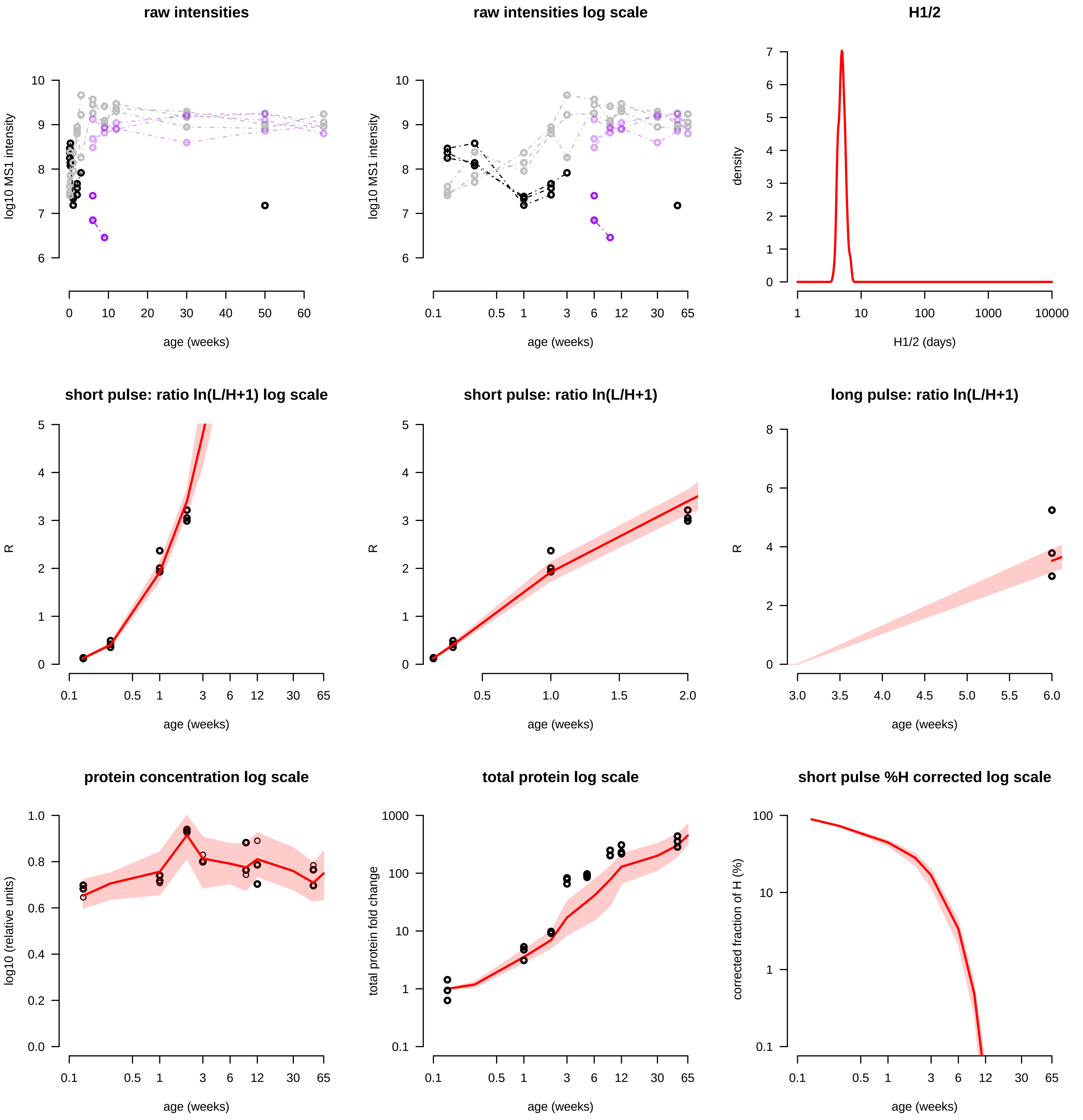

Q91YI0

>sp|Q91YI0|ARLY_MOUSE

Argininosuccinate lyase

GN=Asl

Med H1/2: 1478.2 days

Q5 H1/2: 185.1 days

Q95 H1/2: 10496.5 days

reliable model fit: no

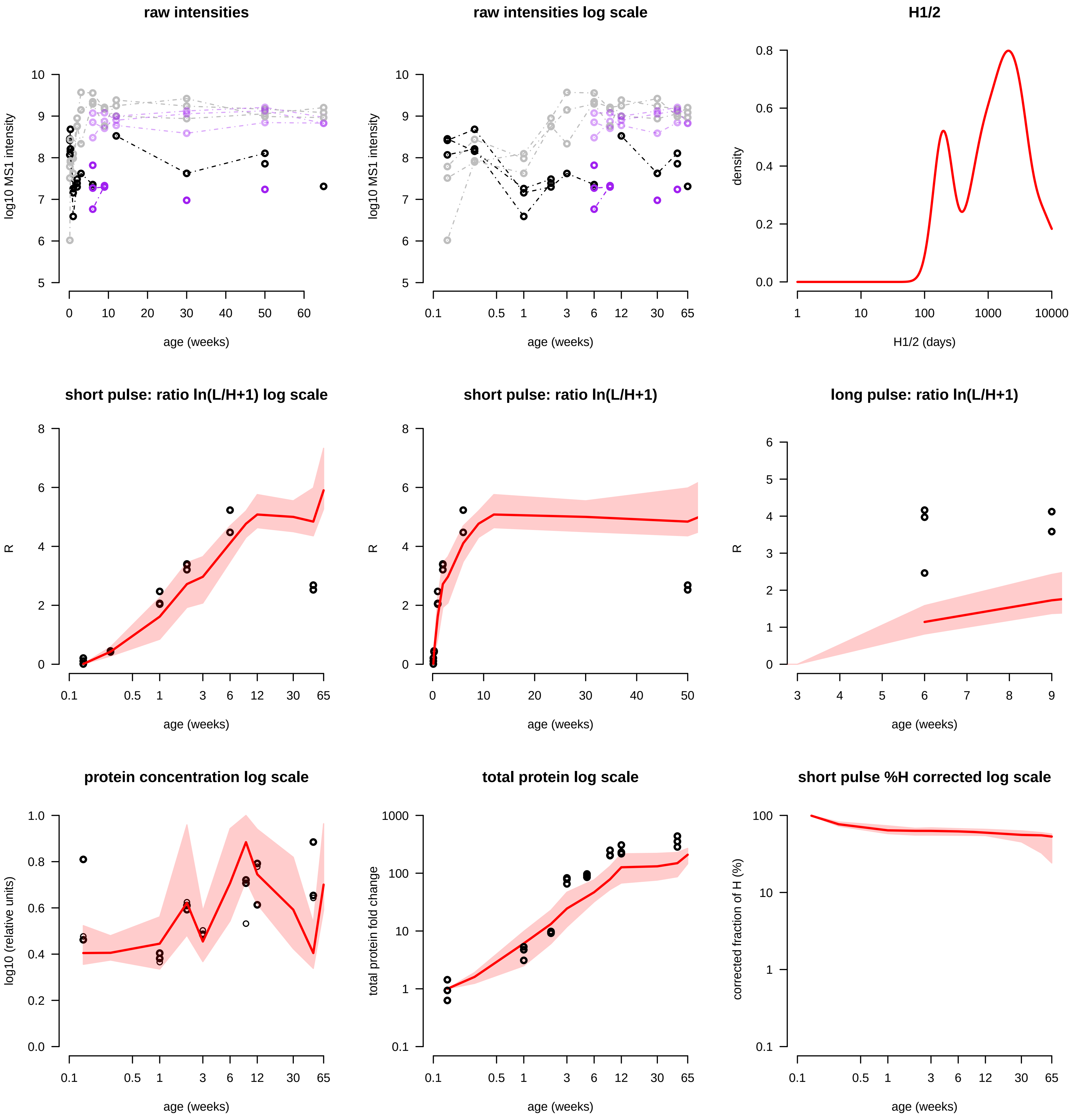

P23780

>sp|P23780|BGAL_MOUSE

Beta−galactosidase

GN=Glb1

Med H1/2: 4.1 days

Q5 H1/2: 3 days

Q95 H1/2: 6 days

reliable model fit: yes

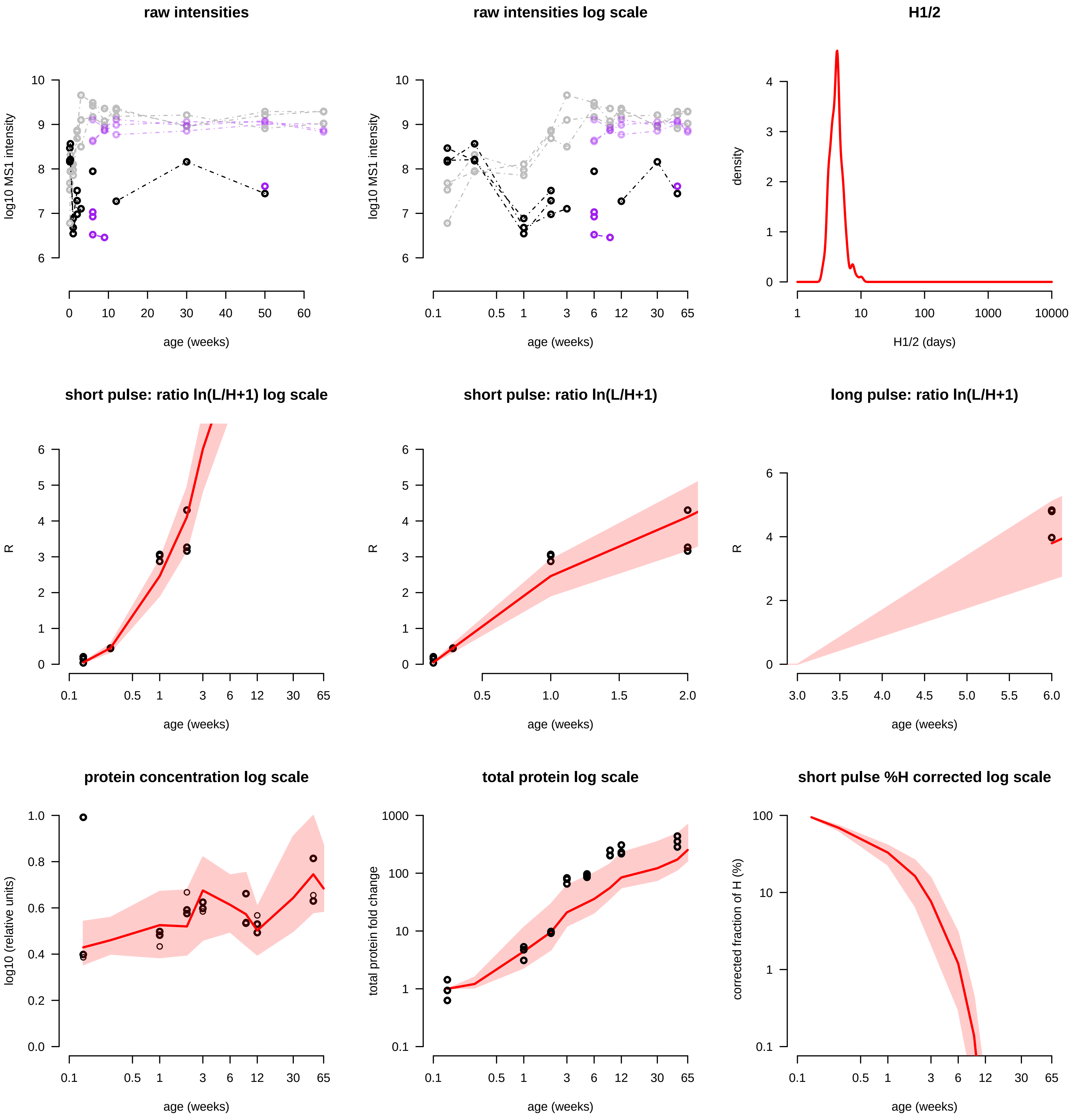

Q91ZW3

>sp|Q91ZW3|SMCA5_MOUSE

-related matrix−associated actin−dependent regulator of chromatin subfamily A

GN=Smarca5

Med H1/2: 5.2 days

Q5 H1/2: 4.3 days

Q95 H1/2: 6.6 days

reliable model fit: yes

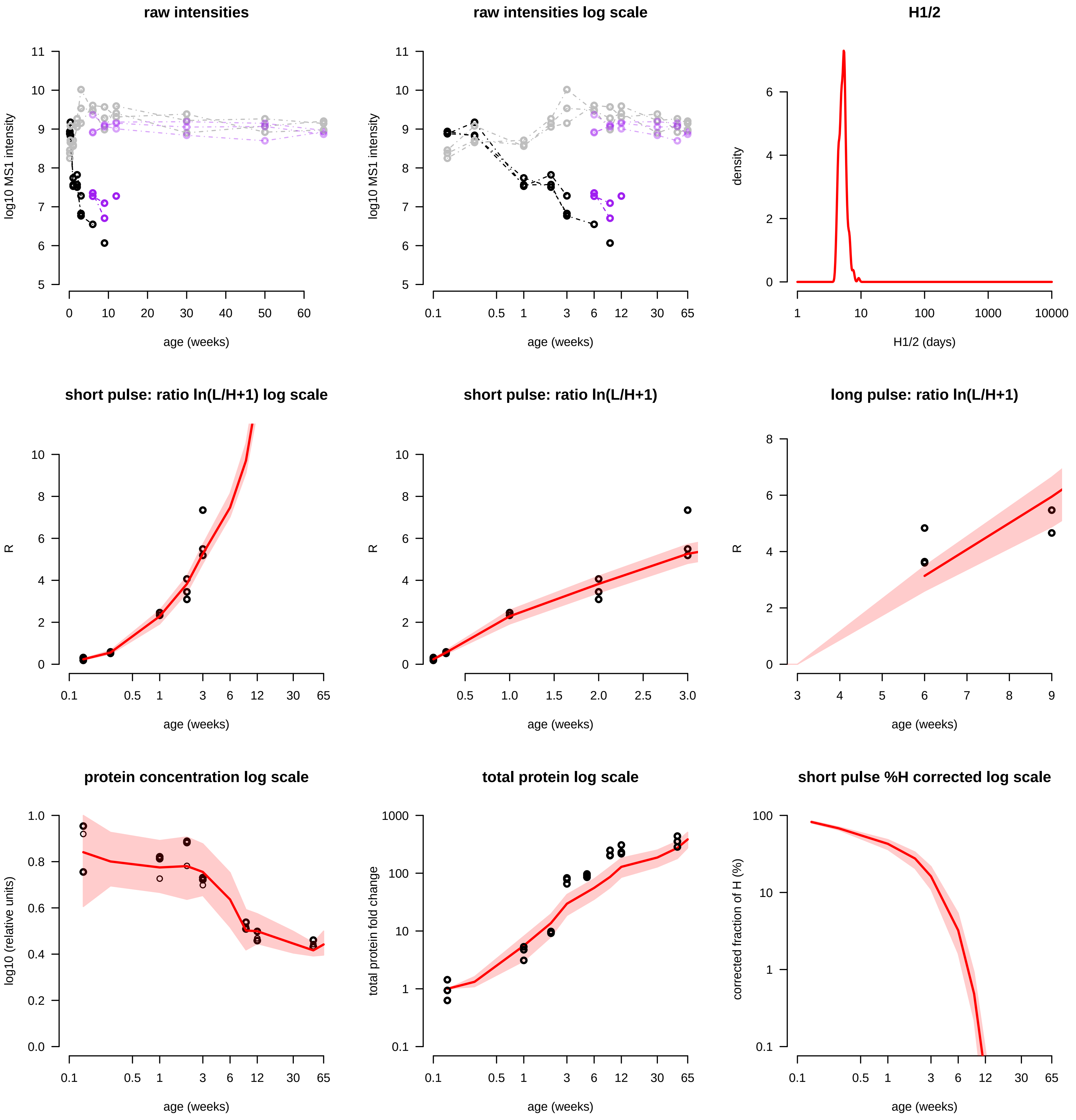

Q9ESW4

>sp|Q9ESW4|AGK_MOUSE

Acylglycerol kinase, mitochondrial

GN=Agk

Med H1/2: 6.1 days

Q5 H1/2: 4.6 days

Q95 H1/2: 8.2 days

reliable model fit: yes

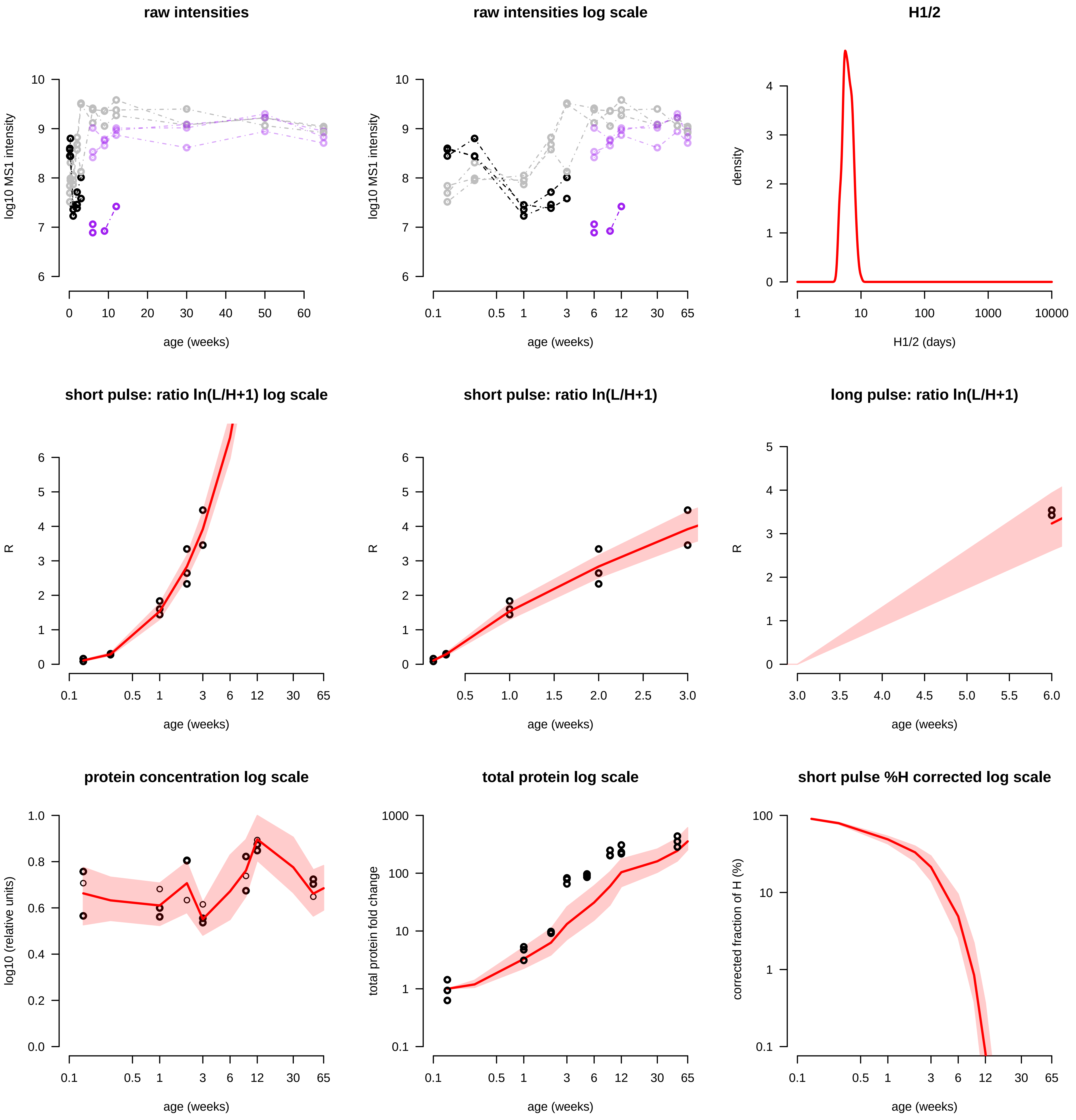

A2AGT5;A2AGT5−3;A2AGT5−2

>sp|A2AGT5|CKAP5_MOUSE

Cytoskeleton−associated protein 5

GN=Ckap5

Med H1/2: 265 days

Q5 H1/2: 113 days

Q95 H1/2: 2240.1 days

reliable model fit: no

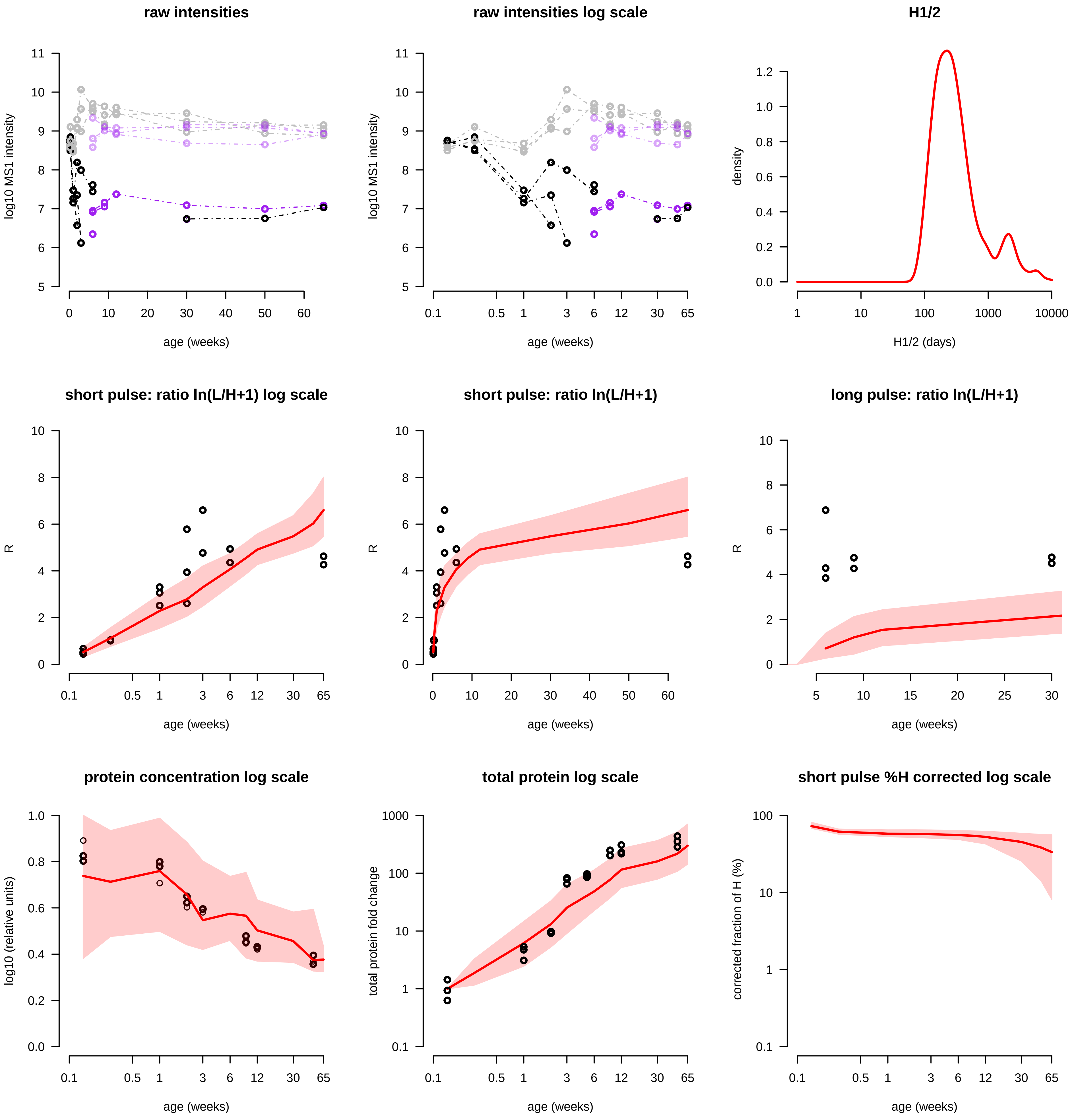

Q8VDM6

>sp|Q8VDM6|HNRL1_MOUSE

Heterogeneous nuclear ribonucleoprotein U−like protein 1

GN=Hnrnpul1

Med H1/2: 7.1 days

Q5 H1/2: 5 days

Q95 H1/2: 11.3 days

reliable model fit: yes

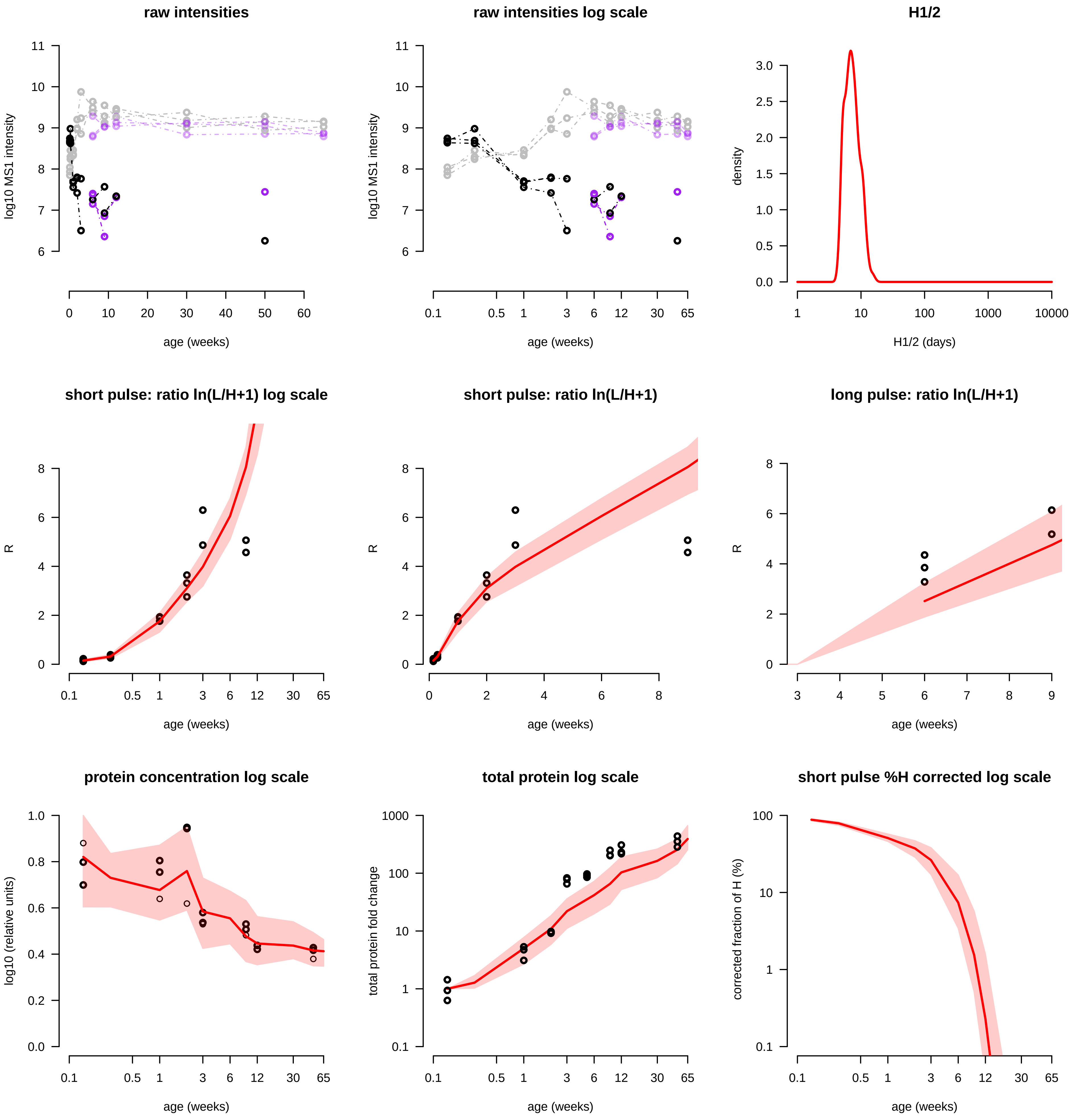

Q9JMG7;Q9JMG7−2

>sp|Q9JMG7|HDGR3_MOUSE

Hepatoma−derived growth factor−related protein 3

GN=Hdgfl3

Med H1/2: 9.8 days

Q5 H1/2: 6.6 days

Q95 H1/2: 14.8 days

reliable model fit: yes

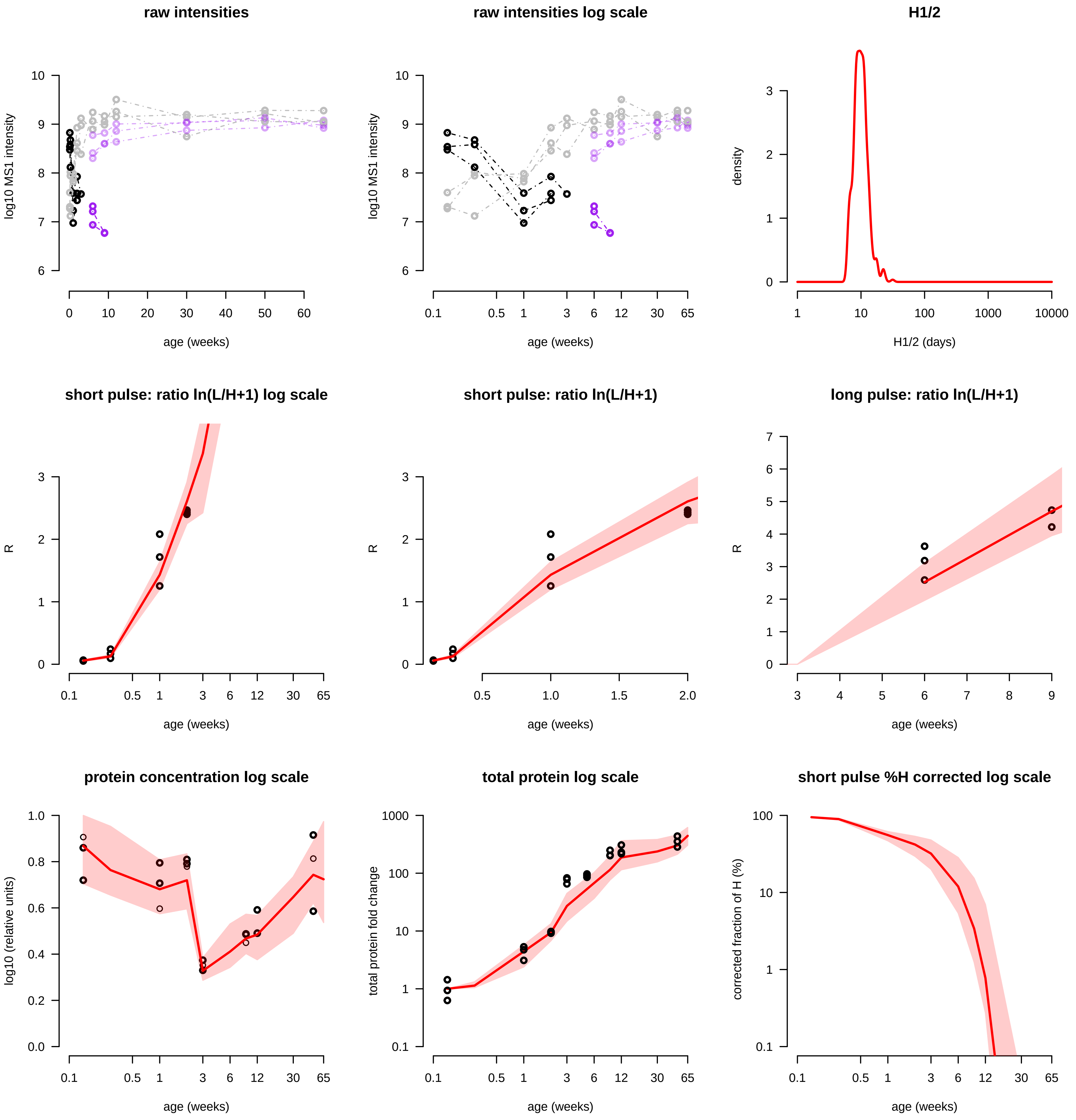

P70195

>sp|P70195|PSB7_MOUSE

Proteasome subunit beta type−7

GN=Psmb7

Med H1/2: 5.1 days

Q5 H1/2: 4.4 days

Q95 H1/2: 6.1 days

reliable model fit: yes

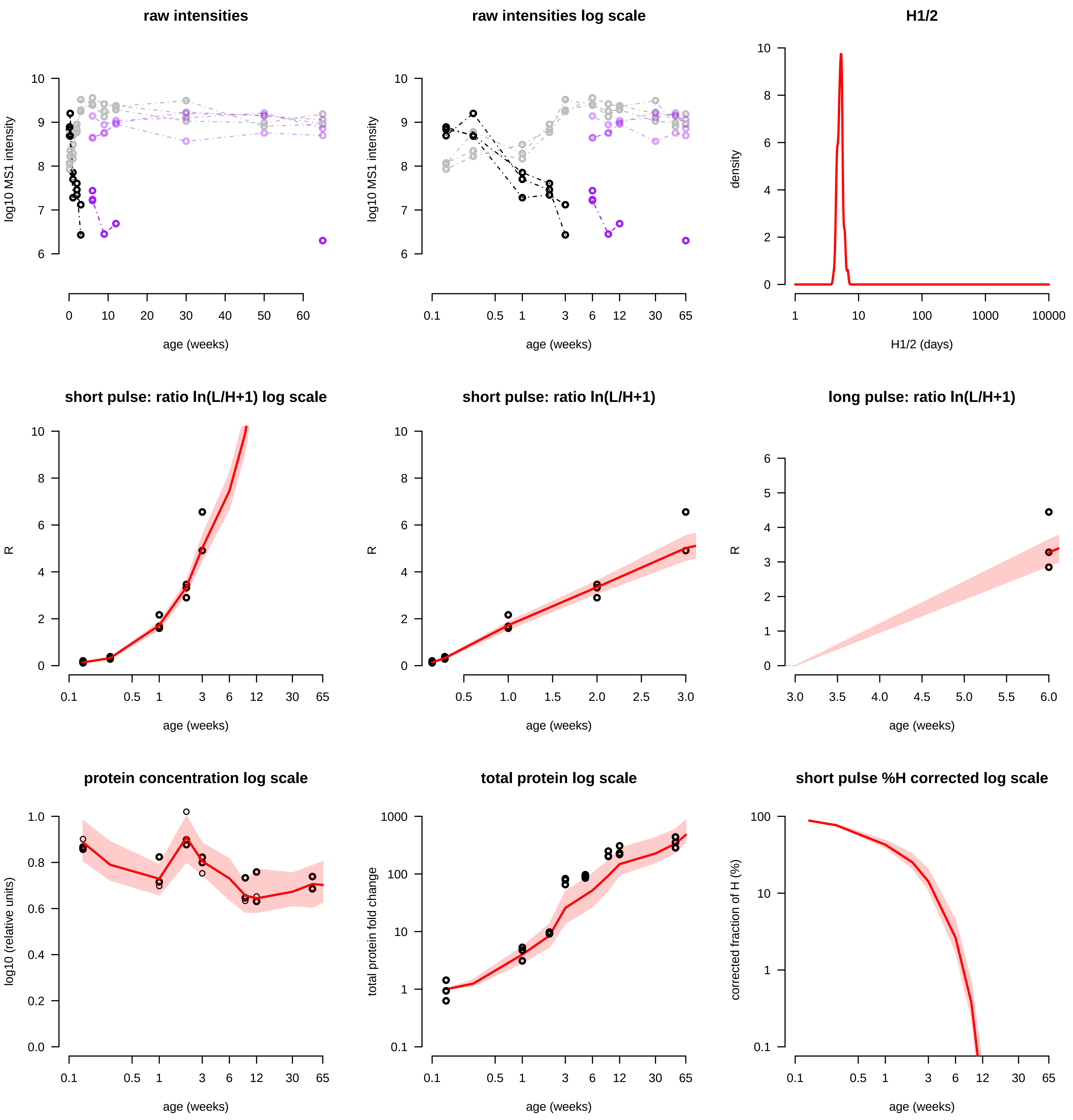

Q9QYR9

>sp|Q9QYR9|ACOT2_MOUSE

Acyl−coenzyme A thioesterase 2, mitochondrial

GN=Acot2

Med H1/2: 7.4 days

Q5 H1/2: 5.8 days

Q95 H1/2: 9.8 days

reliable model fit: yes

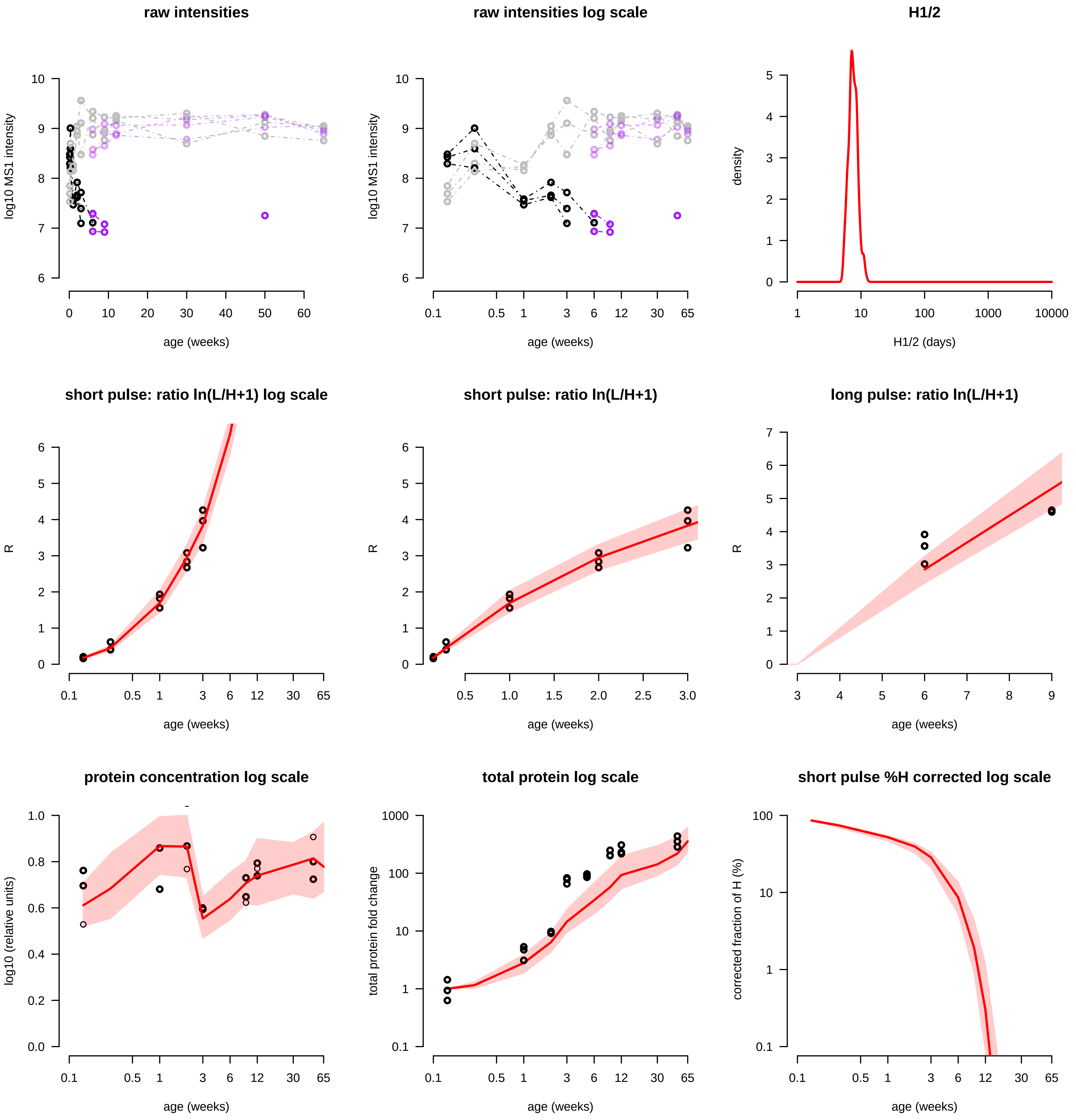

Q61033

>sp|Q61033|LAP2A_MOUSE

Lamina−associated polypeptide 2, isoforms alpha/zeta

GN=Tmpo

Med H1/2: 11.1 days

Q5 H1/2: 8.8 days

Q95 H1/2: 16.7 days

reliable model fit: yes

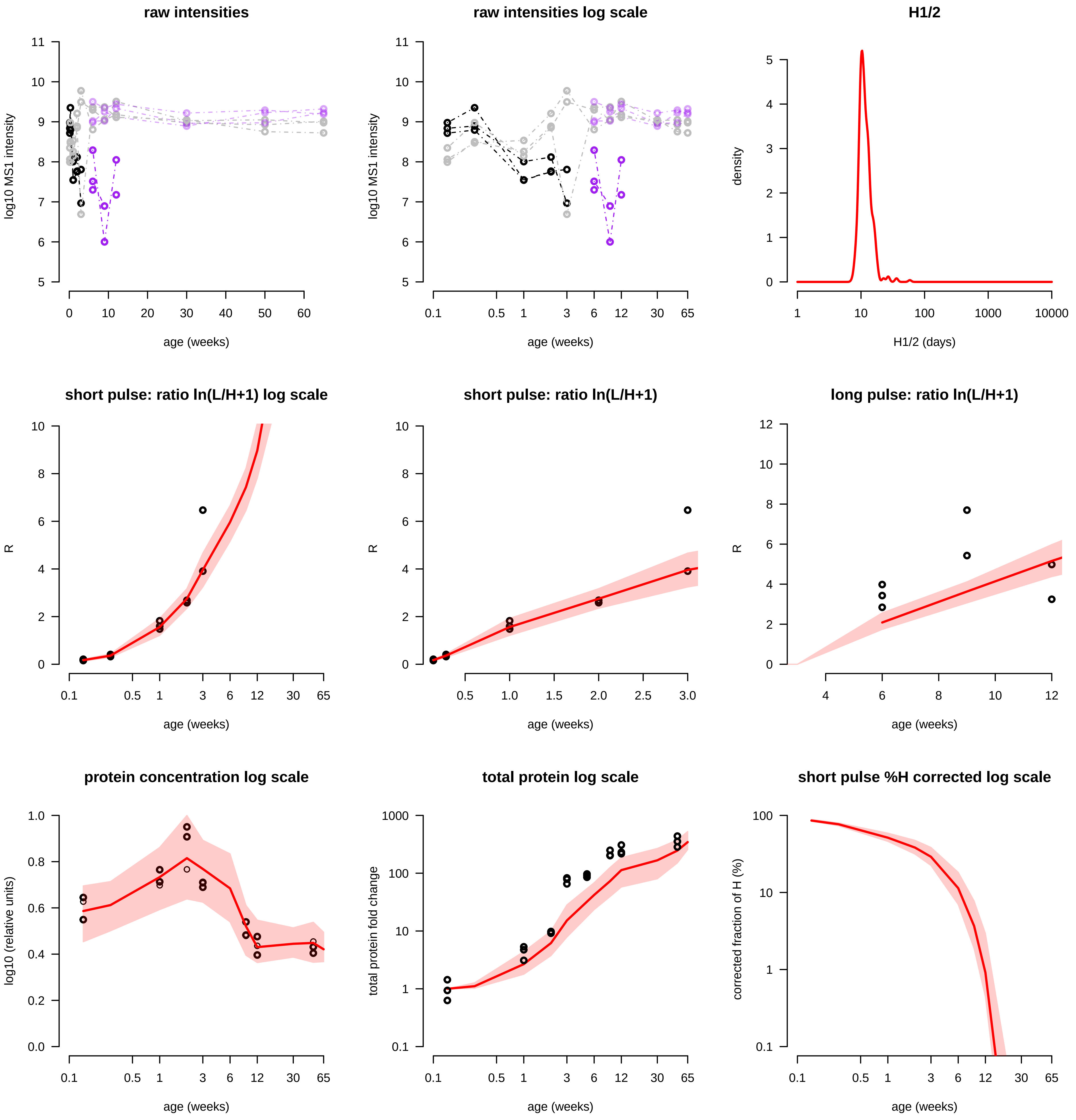

Q91V64

>sp|Q91V64|ISOC1_MOUSE

Isochorismatase domain−containing protein 1

GN=Isoc1

Med H1/2: 5.3 days

Q5 H1/2: 4.6 days

Q95 H1/2: 6.4 days

reliable model fit: yes

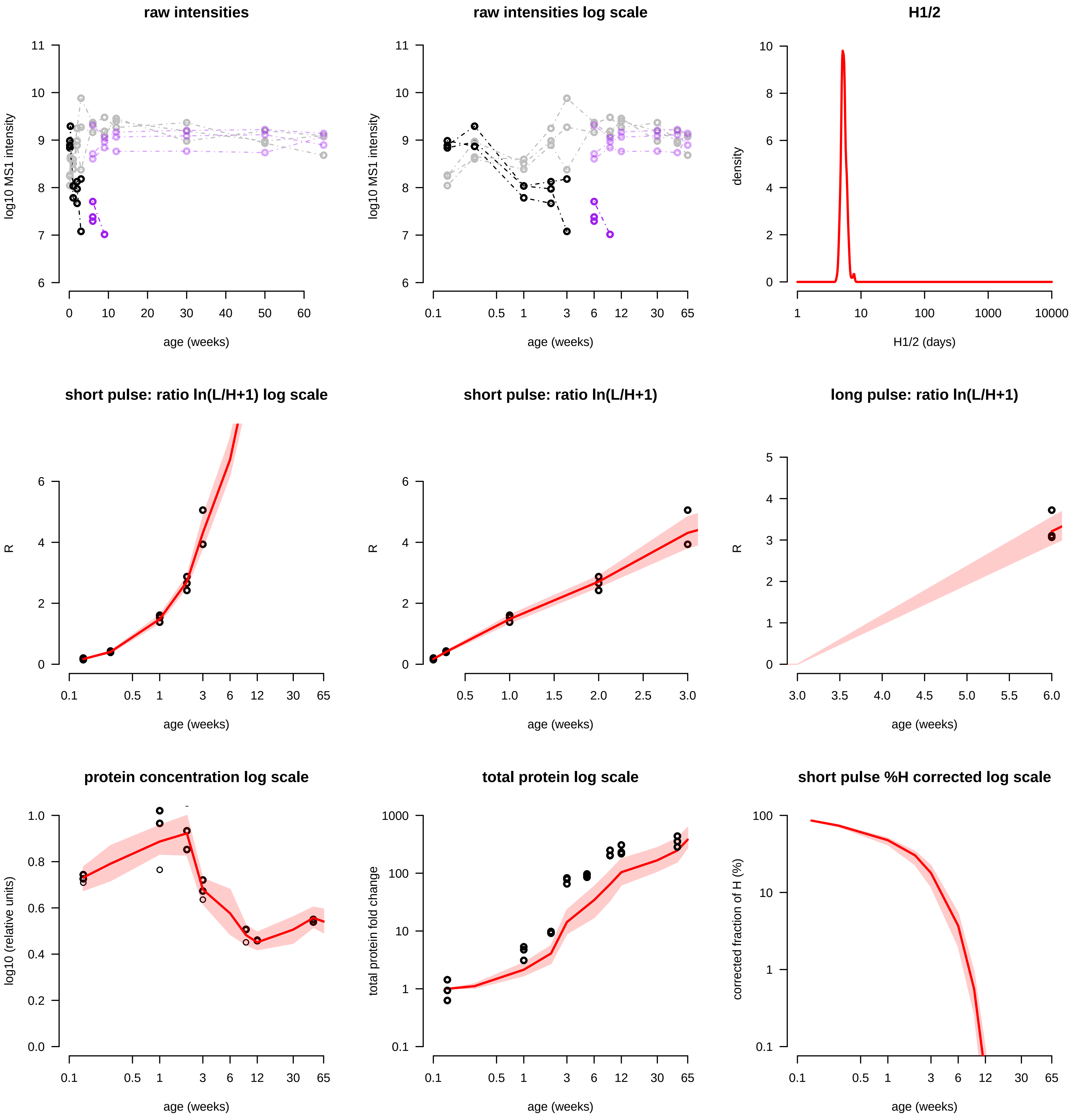

P27612

>sp|P27612|PLAP_MOUSE

Phospholipase A−2−activating protein

GN=Plaa

Med H1/2: 3.5 days

Q5 H1/2: 2.9 days

Q95 H1/2: 4.5 days

reliable model fit: yes

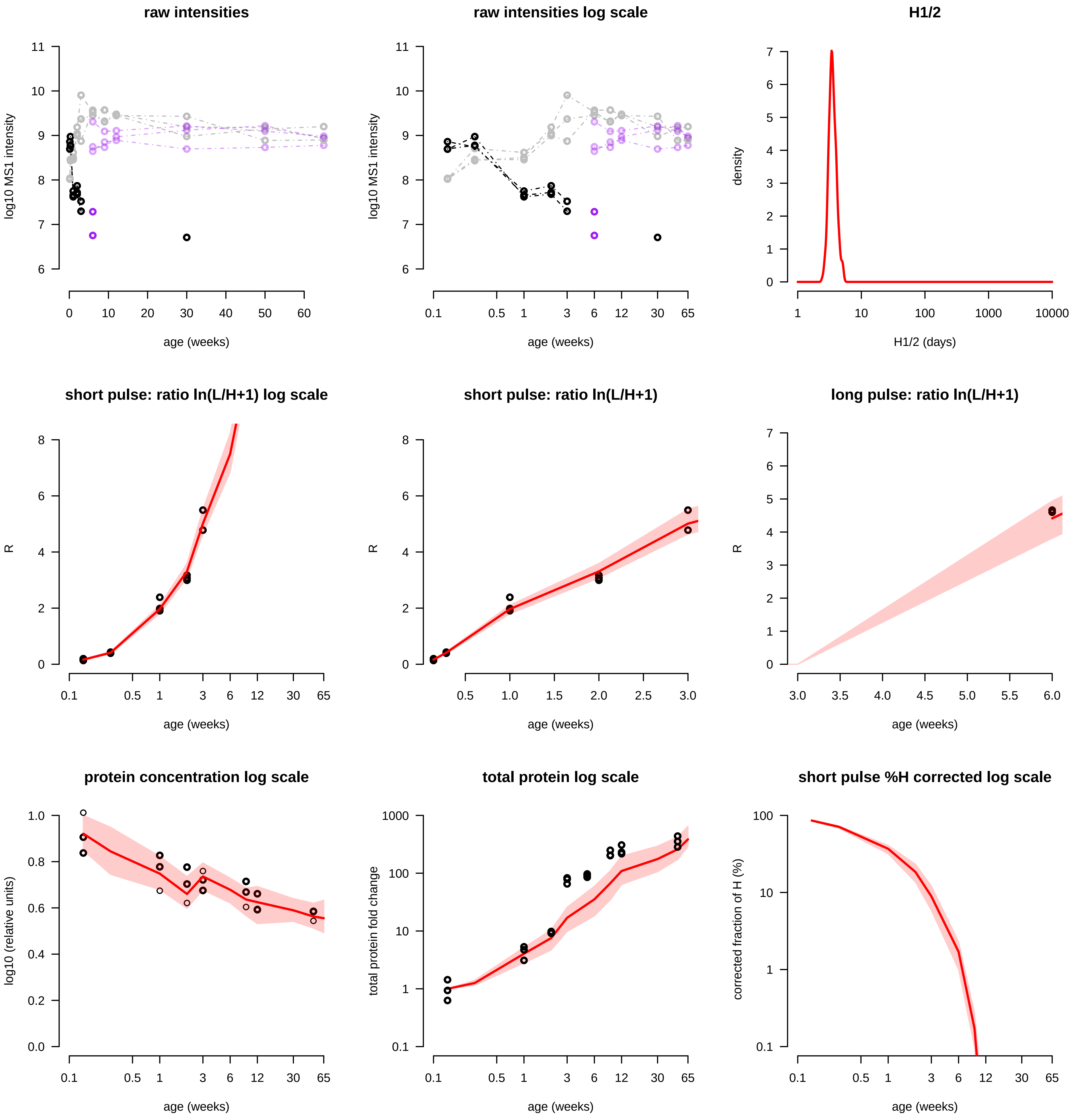

Q9CYZ2

>sp|Q9CYZ2|TPD54_MOUSE

Tumor protein D54

GN=Tpd52l2

Med H1/2: 4.1 days

Q5 H1/2: 3.3 days

Q95 H1/2: 5.4 days

reliable model fit: no

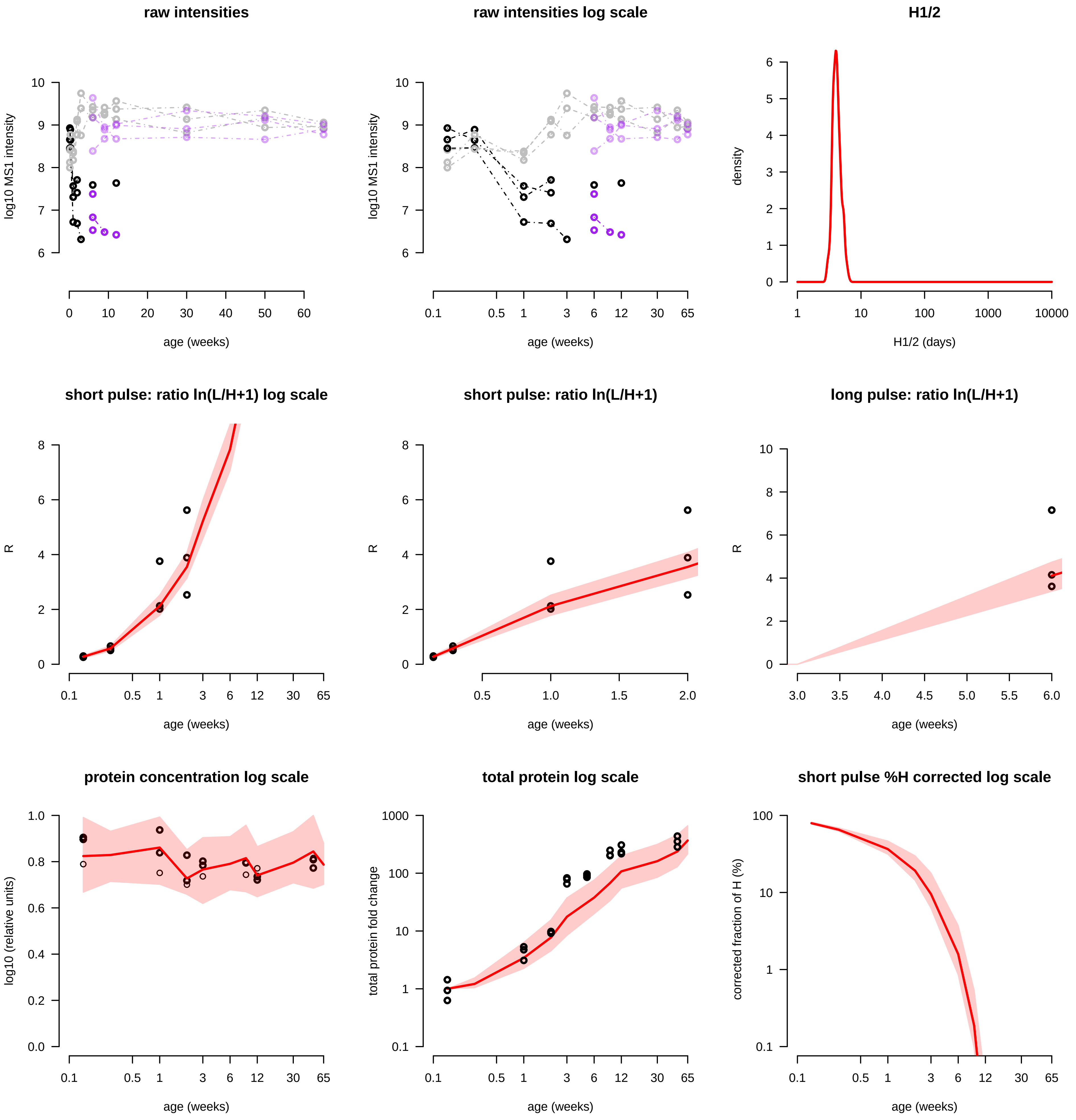

Q71LX4

>sp|Q71LX4|TLN2_MOUSE

Talin−2

GN=Tln2

Med H1/2: 935.9 days

Q5 H1/2: 302 days

Q95 H1/2: 14926.5 days

reliable model fit: yes

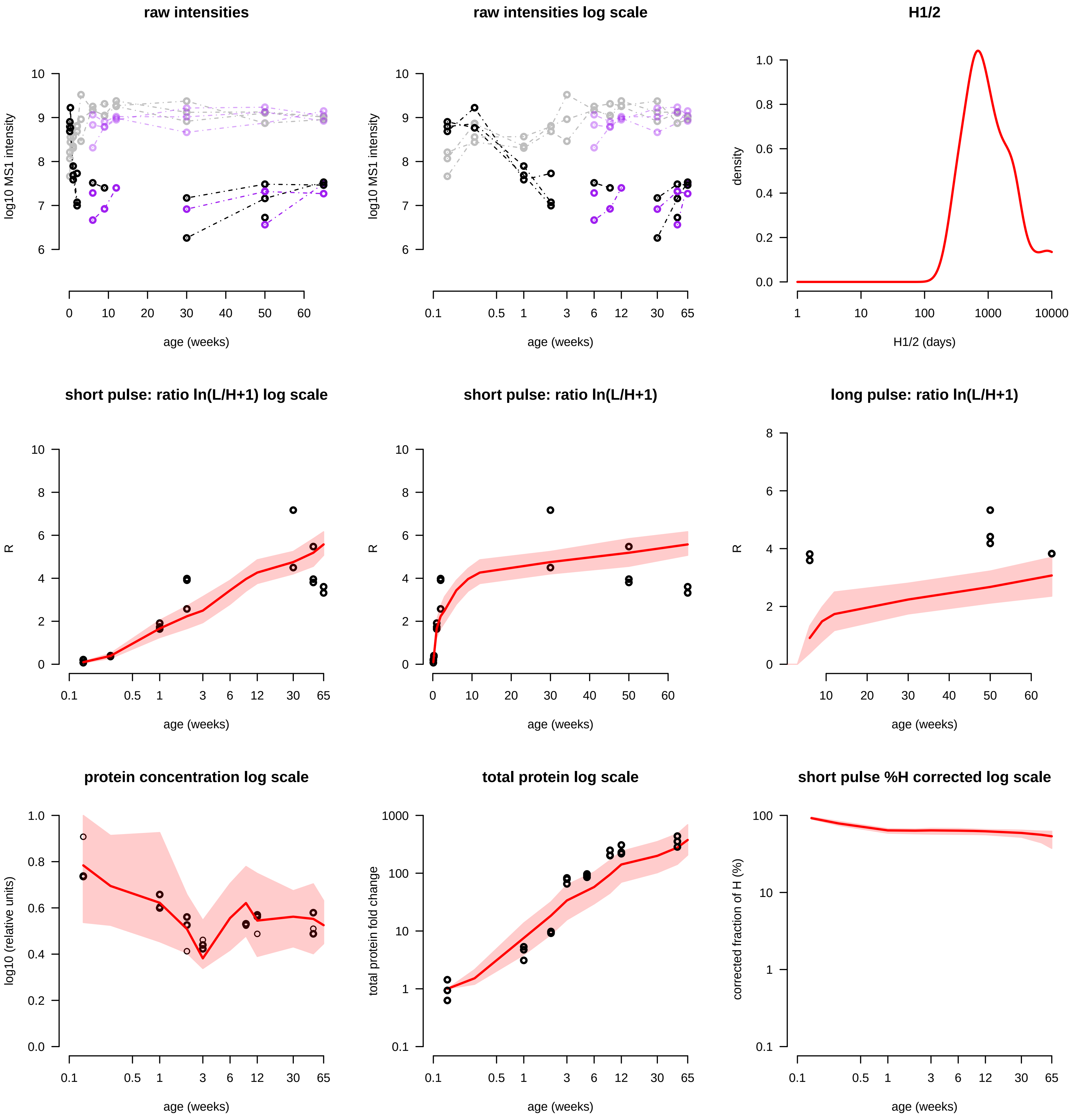

Q8BSY0

>sp|Q8BSY0|ASPH_MOUSE

Aspartyl/asparaginyl beta−hydroxylase

GN=Asph

Med H1/2: 4.2 days

Q5 H1/2: 3.6 days

Q95 H1/2: 5.3 days

reliable model fit: yes

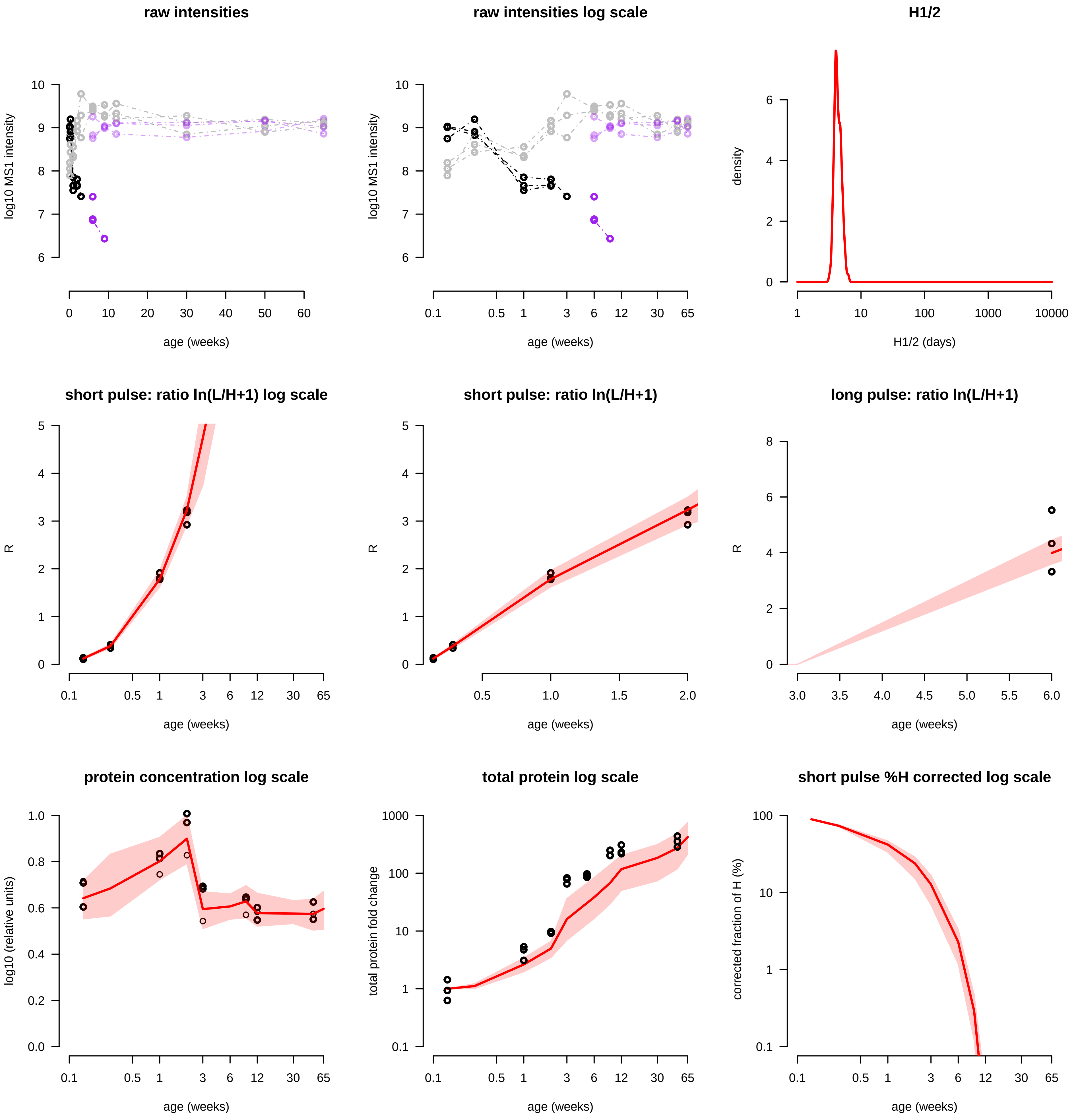

Q80UM3

>sp|Q80UM3|NAA15_MOUSE

N−alpha−acetyltransferase 15, NatA auxiliary subunit

GN=Naa15

Med H1/2: 53.8 days

Q5 H1/2: 40.2 days

Q95 H1/2: 77.6 days

reliable model fit: no

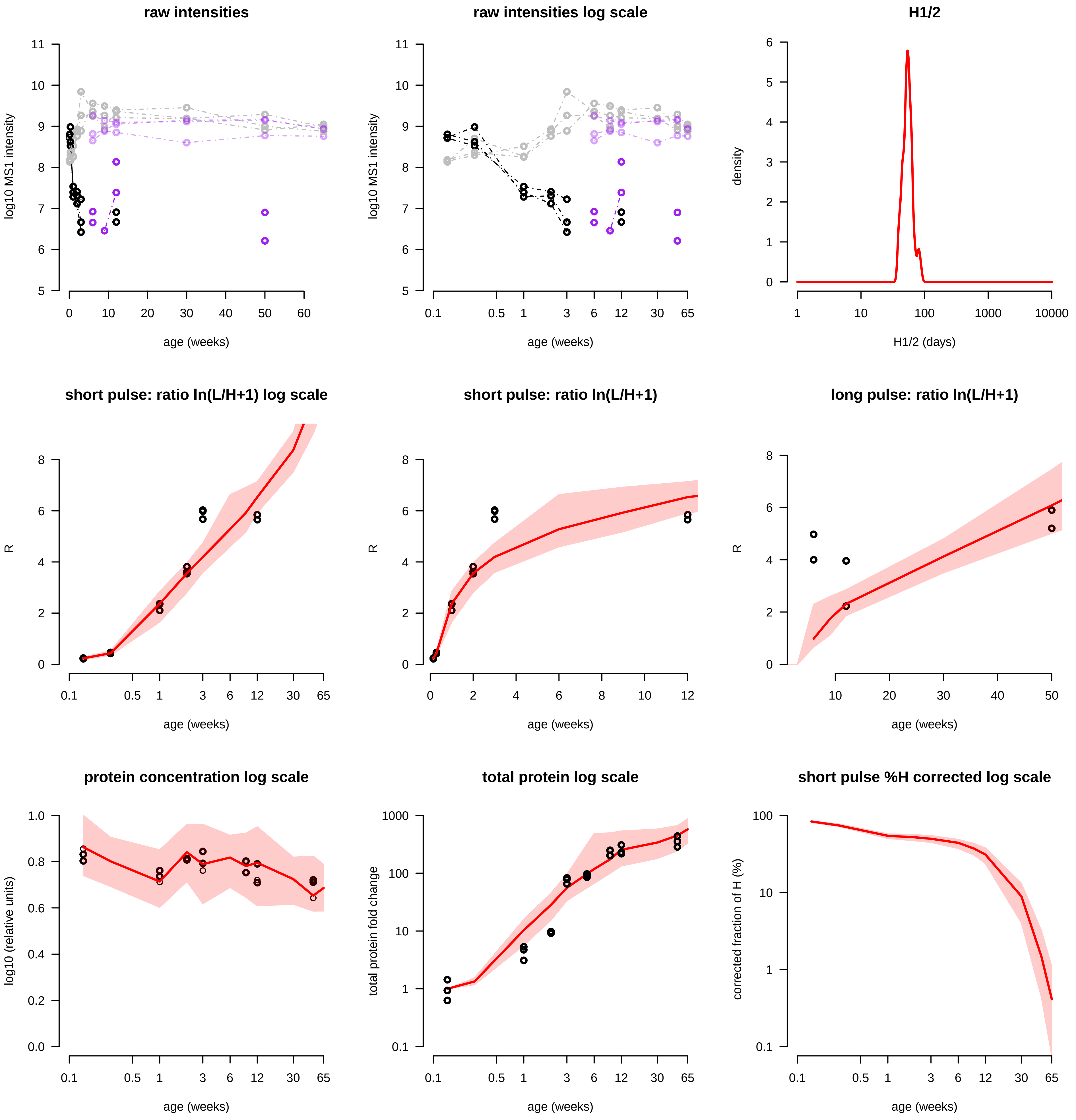

Q569Z6

>sp|Q569Z6|TR150_MOUSE

Thyroid hormone receptor−associated protein 3

GN=Thrap3

Med H1/2: 9.4 days

Q5 H1/2: 7.9 days

Q95 H1/2: 12 days

reliable model fit: yes

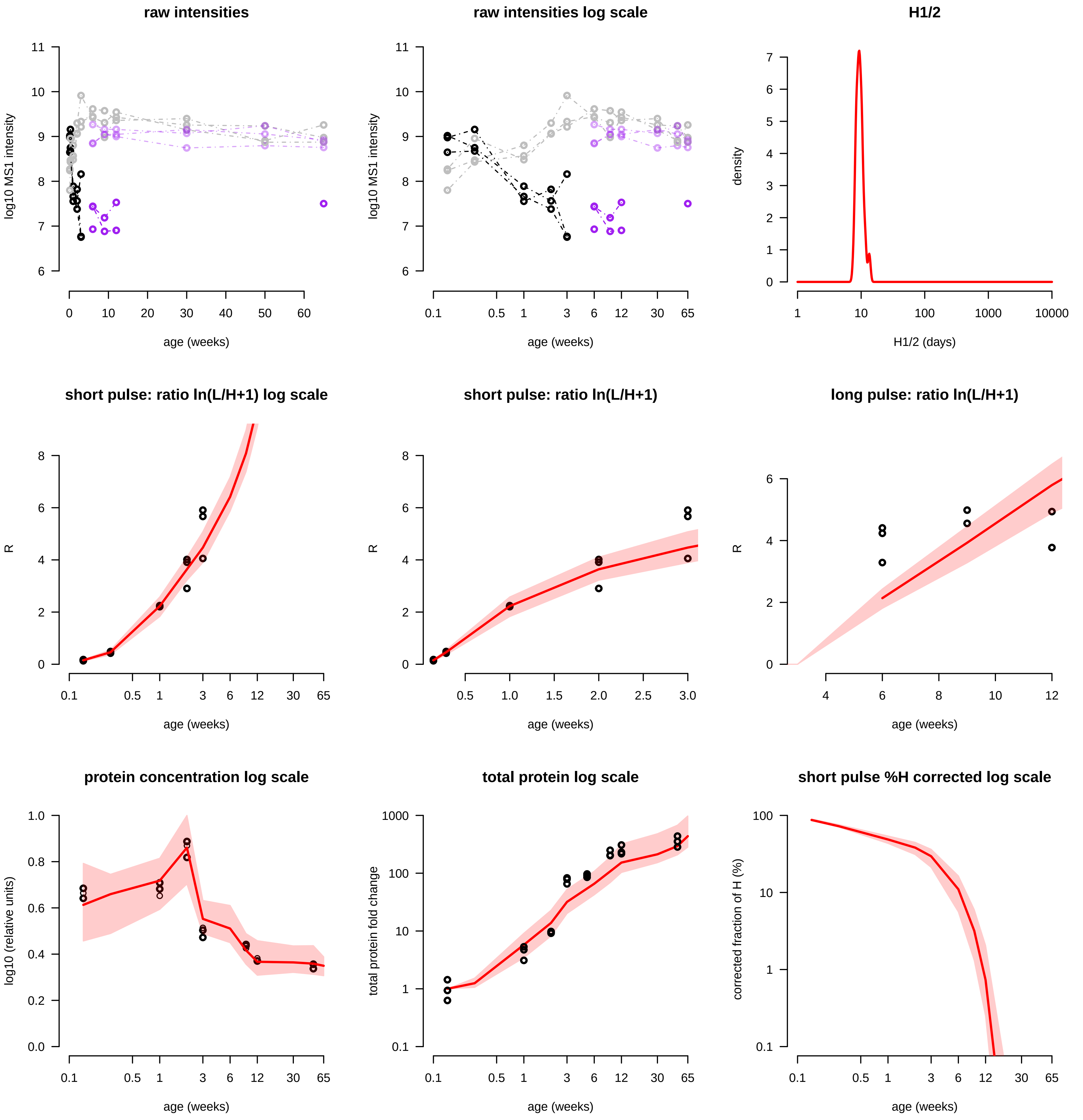

P27046

>sp|P27046|MA2A1_MOUSE

Alpha−mannosidase 2

GN=Man2a1

Med H1/2: 2705.3 days

Q5 H1/2: 850.5 days

Q95 H1/2: 29215.7 days

reliable model fit: no

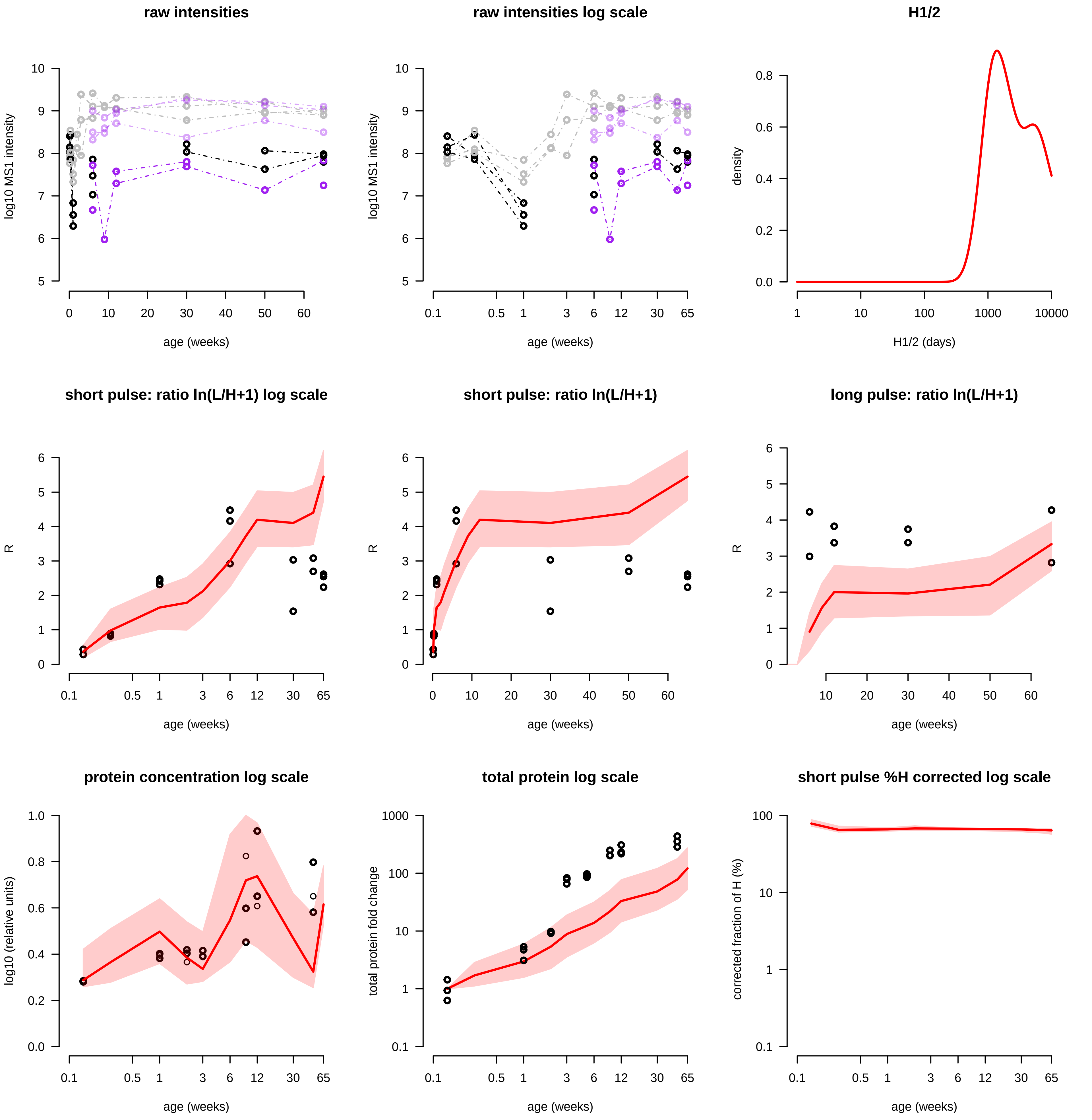

Q9WVL0

>sp|Q9WVL0|MAAI_MOUSE

Maleylacetoacetate isomerase

GN=Gstz1

Med H1/2: 6.4 days

Q5 H1/2: 5 days

Q95 H1/2: 8.3 days

reliable model fit: yes

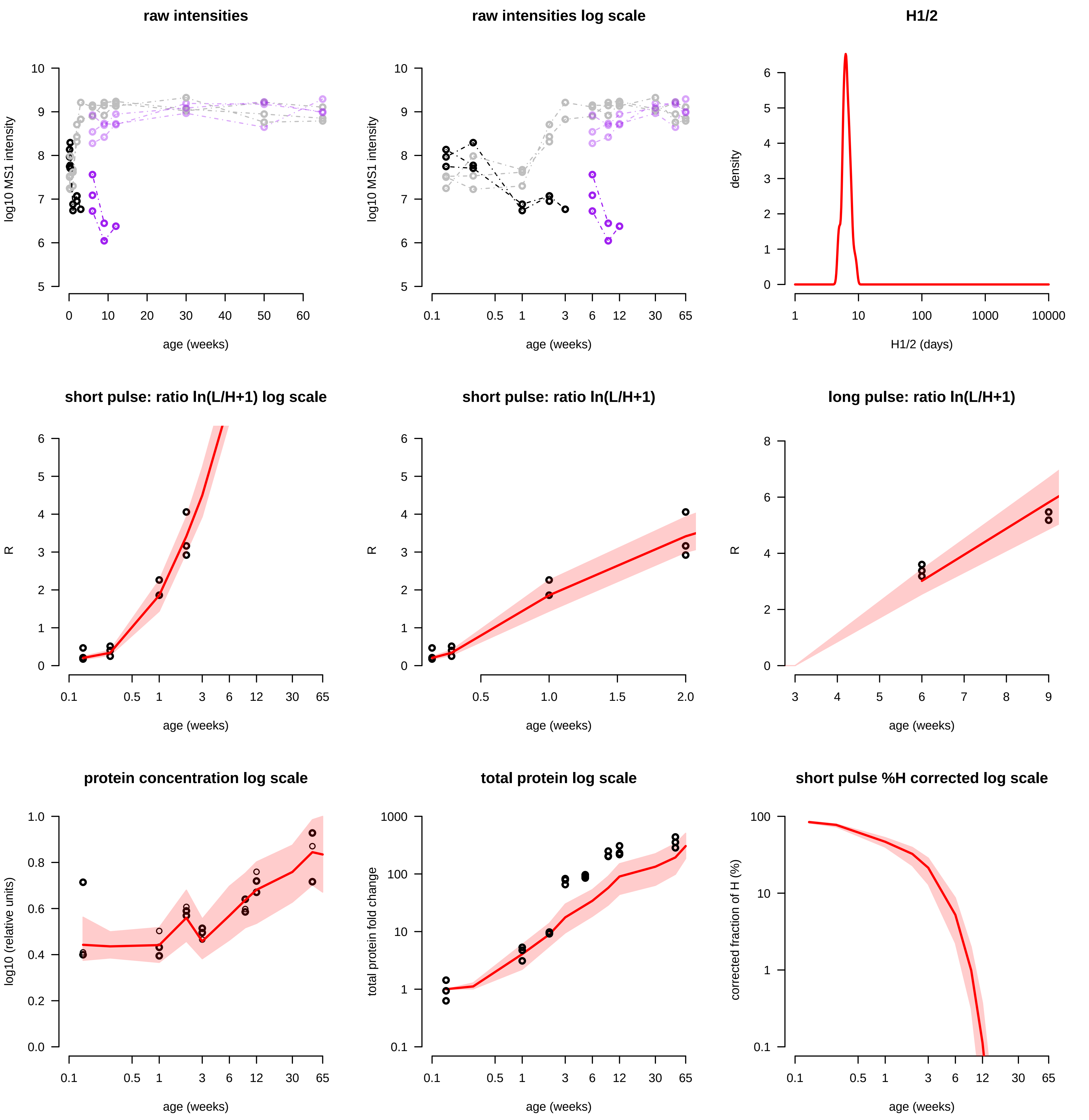

P28656

>sp|P28656|NP1L1_MOUSE

Nucleosome assembly protein 1−like 1

GN=Nap1l1

Med H1/2: 5.7 days

Q5 H1/2: 4.8 days

Q95 H1/2: 6.9 days

reliable model fit: yes

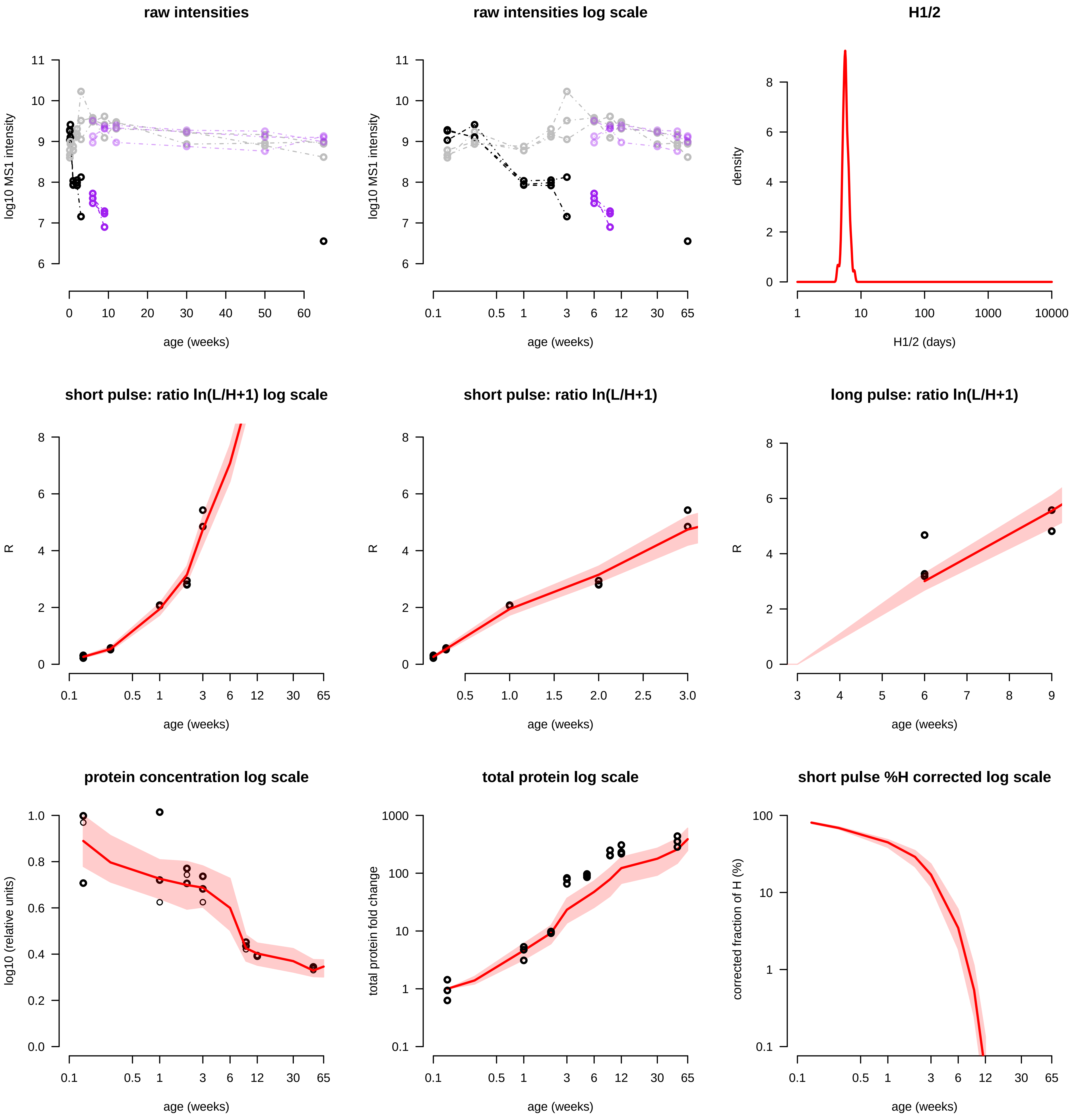

Q9WVK4

>sp|Q9WVK4|EHD1_MOUSE

EH domain−containing protein 1

GN=Ehd1

Med H1/2: 5.4 days

Q5 H1/2: 3 days

Q95 H1/2: 20.9 days

reliable model fit: yes

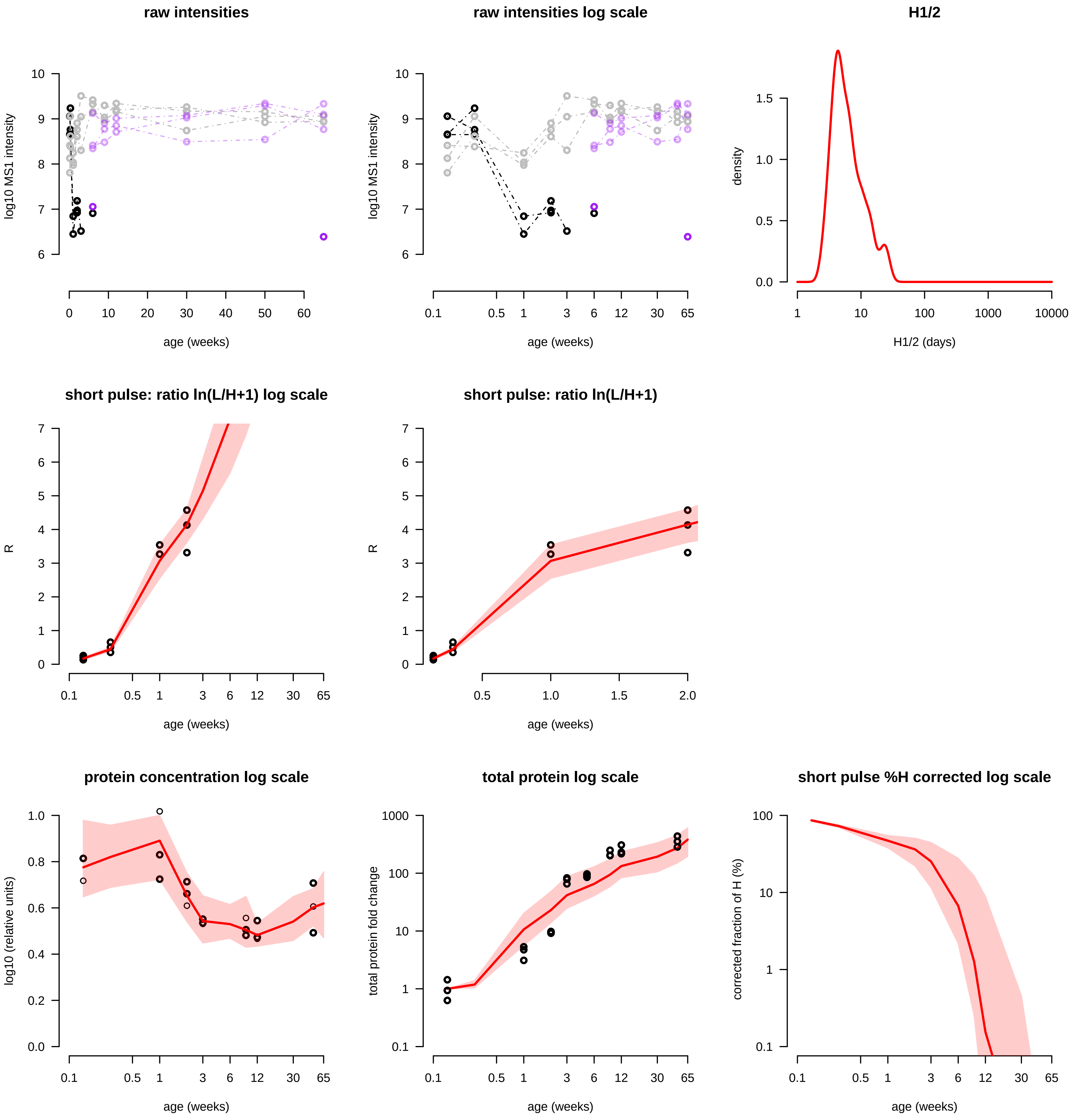

Q9EQP2

>sp|Q9EQP2|EHD4_MOUSE

EH domain−containing protein 4

GN=Ehd4

Med H1/2: 14.2 days

Q5 H1/2: 10.9 days

Q95 H1/2: 20.4 days

reliable model fit: yes

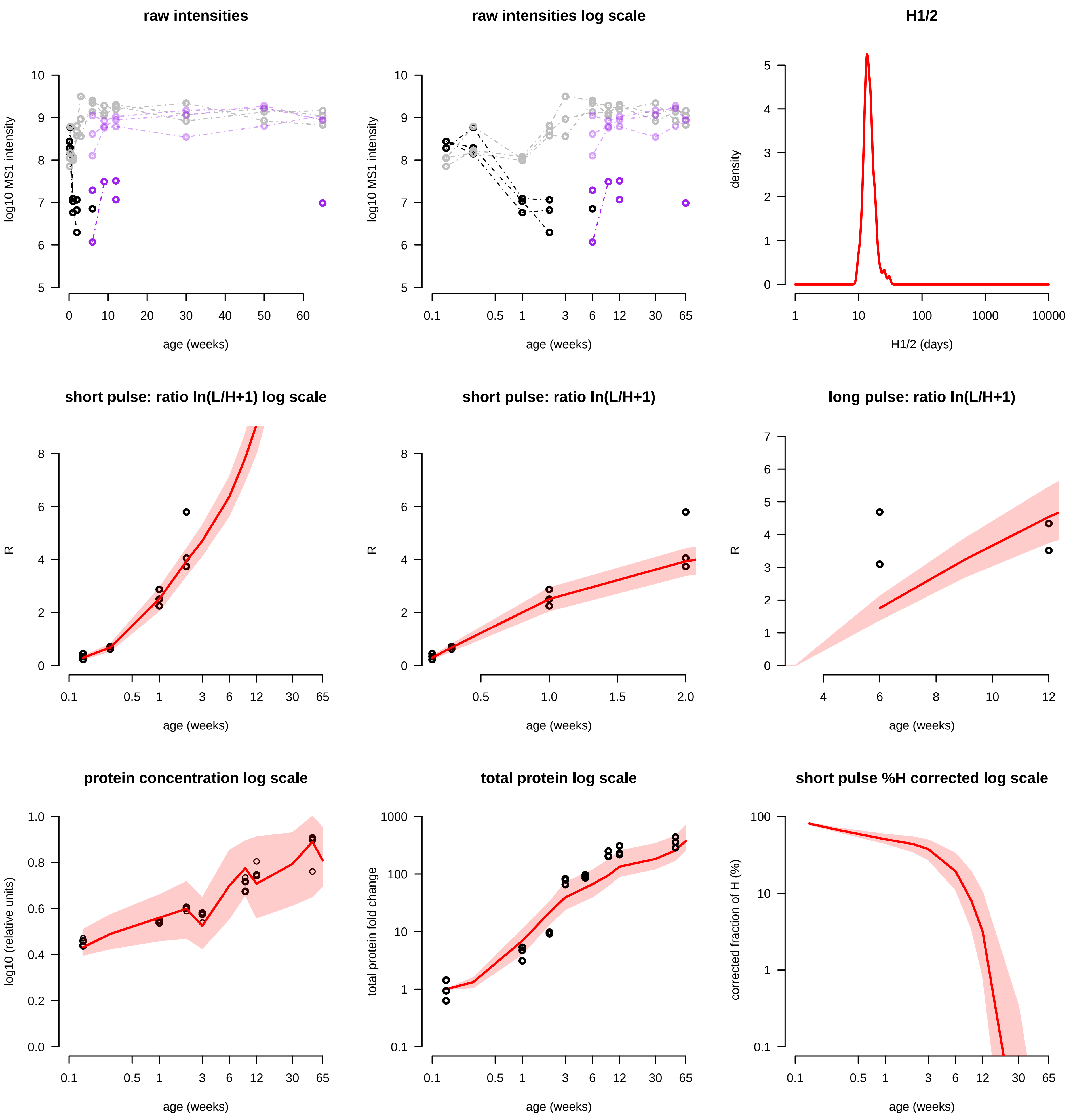

P57759

>sp|P57759|ERP29_MOUSE

Endoplasmic reticulum resident protein 29

GN=Erp29

Med H1/2: 6.8 days

Q5 H1/2: 5.1 days

Q95 H1/2: 9.5 days

reliable model fit: yes

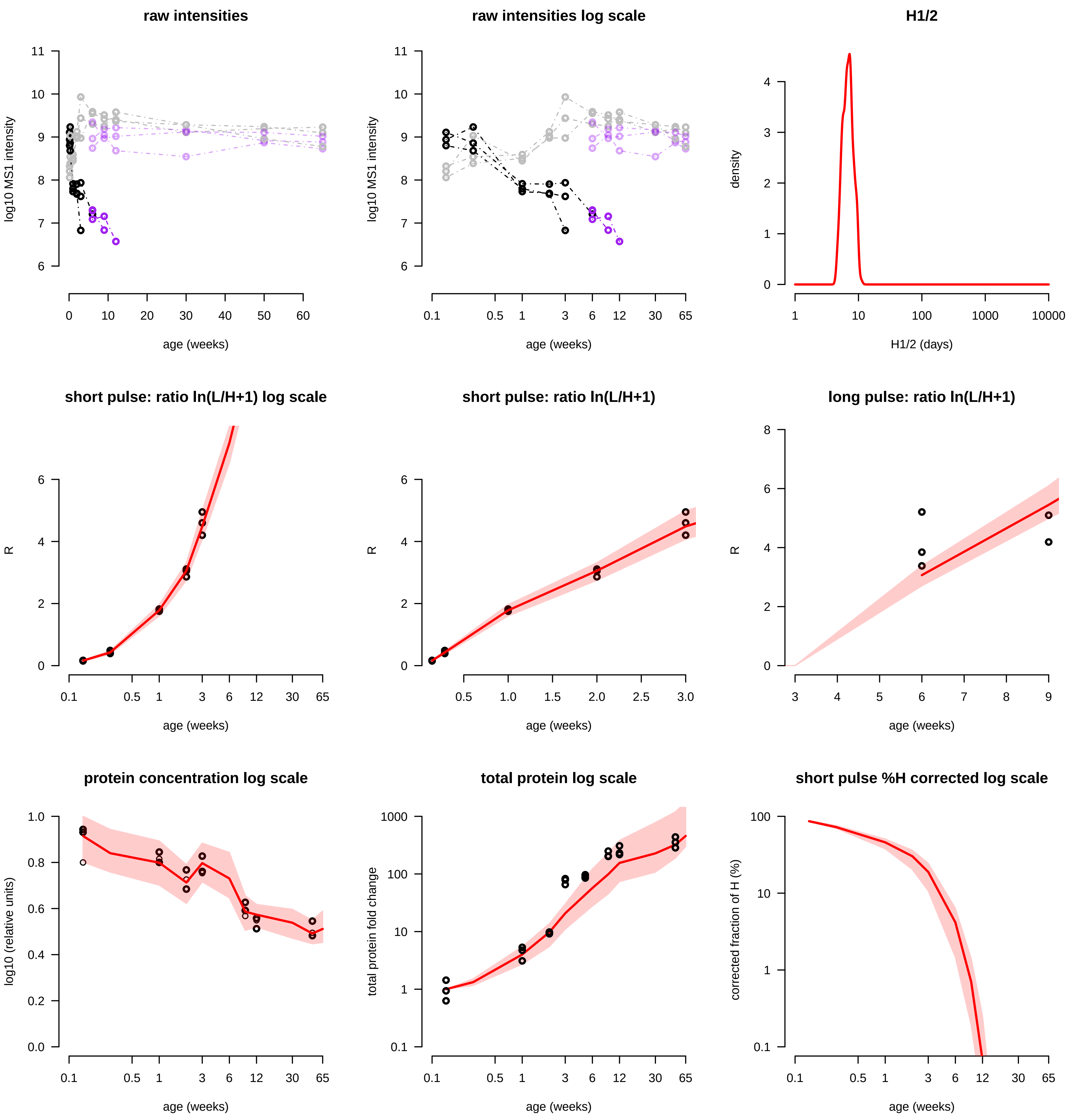

Q9D1J3

>sp|Q9D1J3|SARNP_MOUSE

SAP domain−containing ribonucleoprotein

GN=Sarnp

Med H1/2: 5.6 days

Q5 H1/2: 4.6 days

Q95 H1/2: 8.1 days

reliable model fit: yes

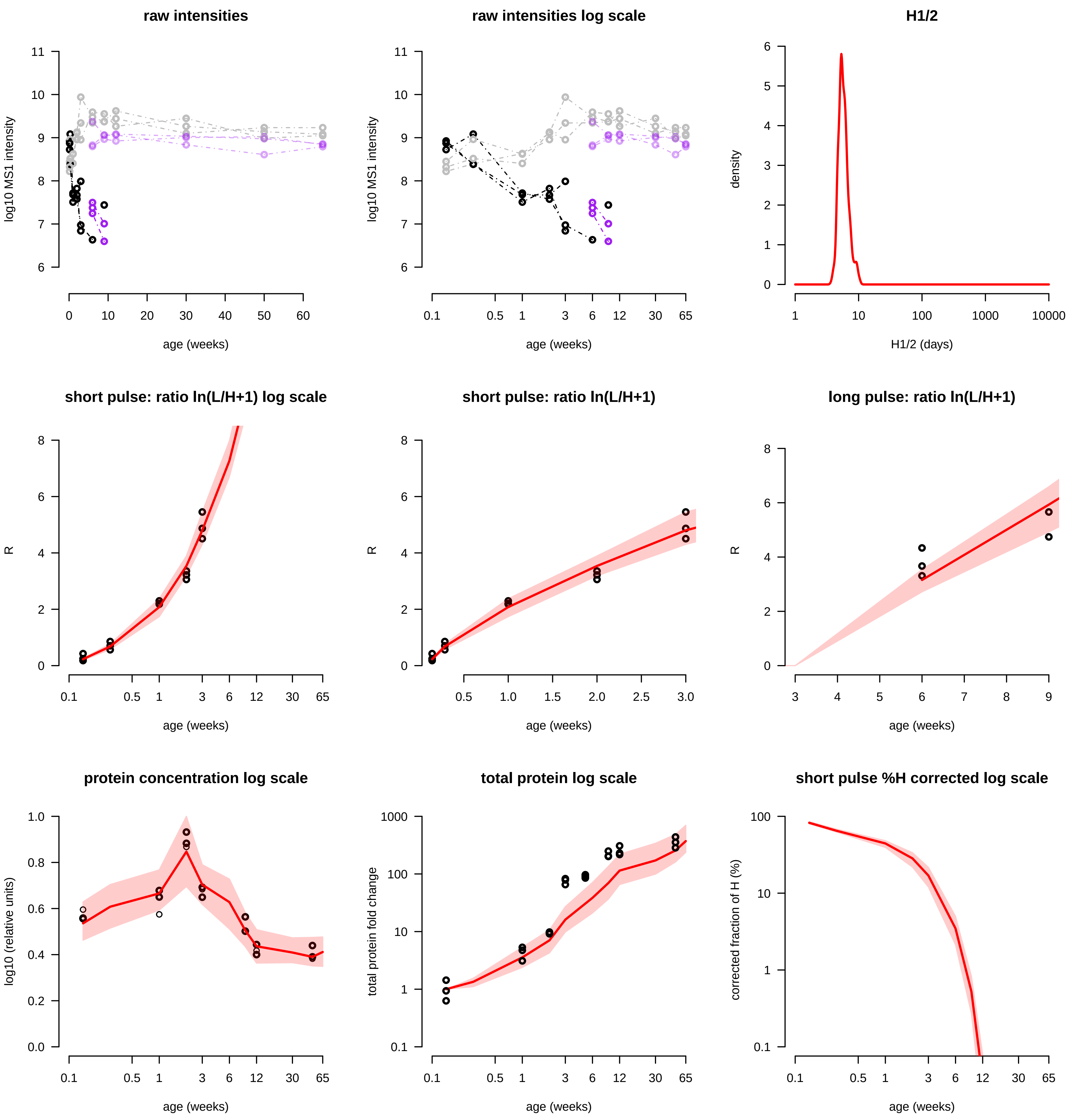

Q80YR5

>sp|Q80YR5|SAFB2_MOUSE

Scaffold attachment factor B2

GN=Safb2

Med H1/2: 4.1 days

Q5 H1/2: 3.4 days

Q95 H1/2: 4.9 days

reliable model fit: yes

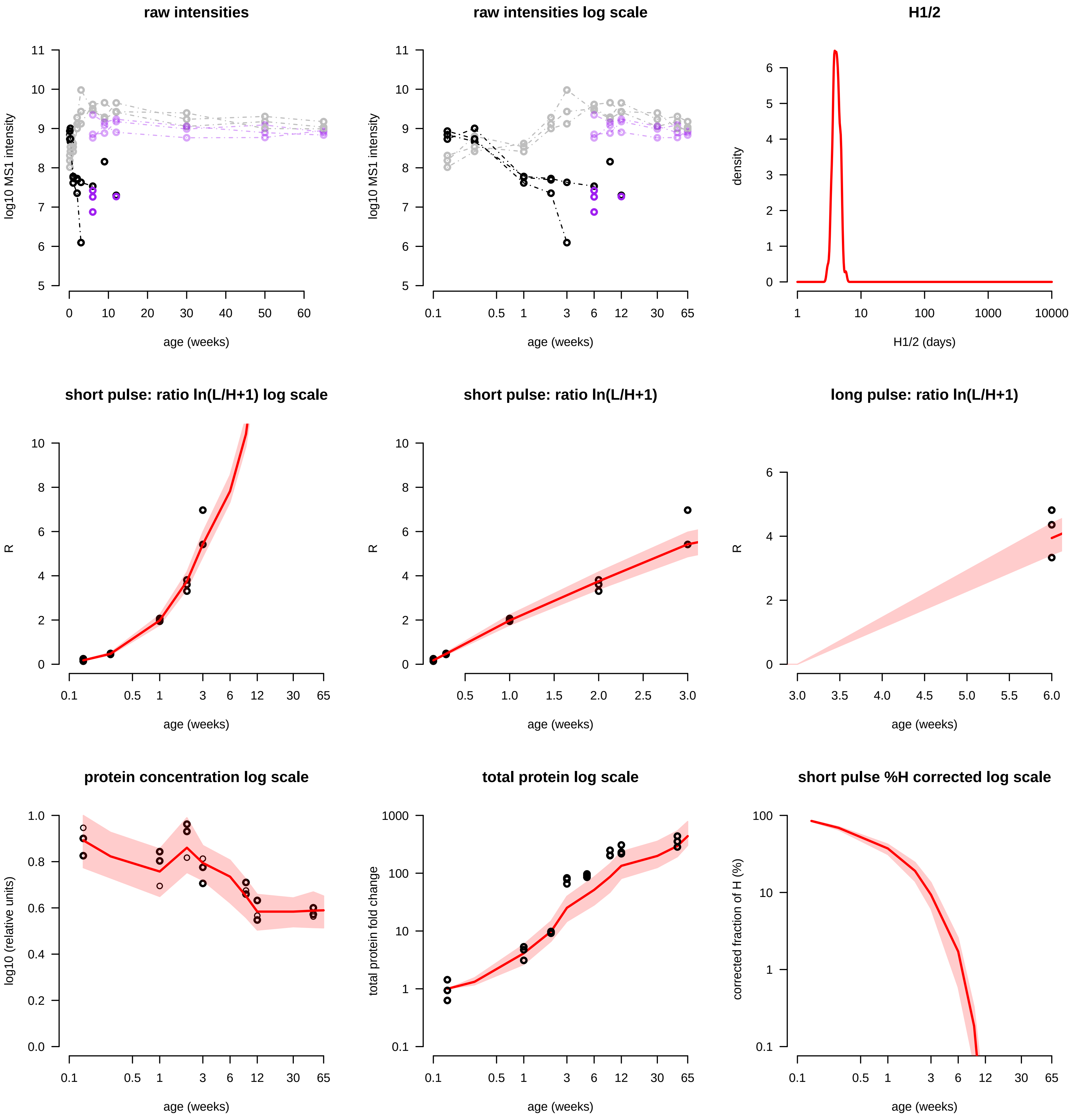

Q9DC16

>sp|Q9DC16|ERGI1_MOUSE

Endoplasmic reticulum−Golgi intermediate compartment protein 1

GN=Ergic1

Med H1/2: 7.7 days

Q5 H1/2: 5.9 days

Q95 H1/2: 9.5 days

reliable model fit: yes

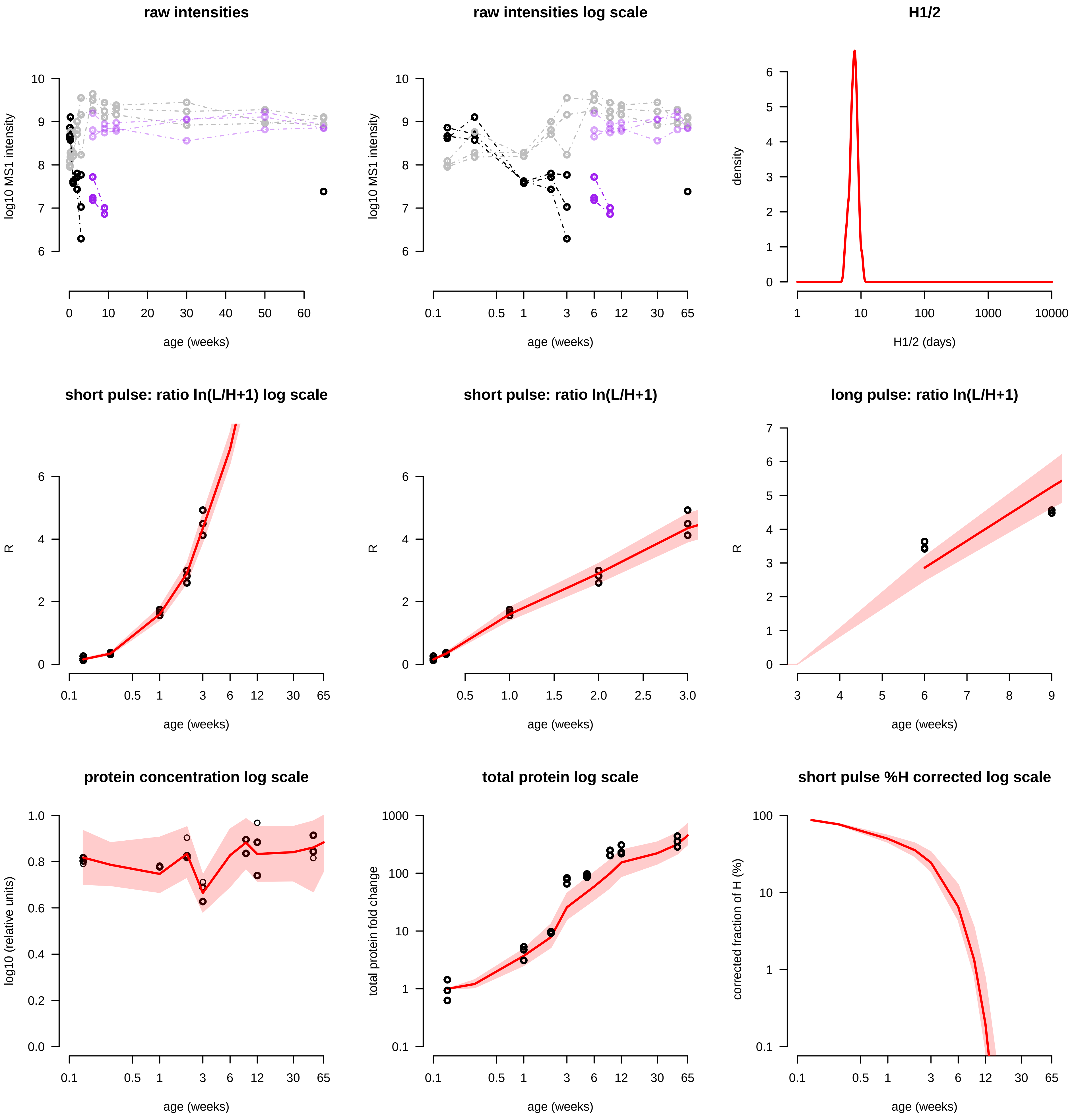

Q3B7Z2;Q3B7Z2−2

>sp|Q3B7Z2|OSBP1_MOUSE

Oxysterol−binding protein 1

GN=Osbp

Med H1/2: 3.9 days

Q5 H1/2: 2.5 days

Q95 H1/2: 9.7 days

reliable model fit: yes

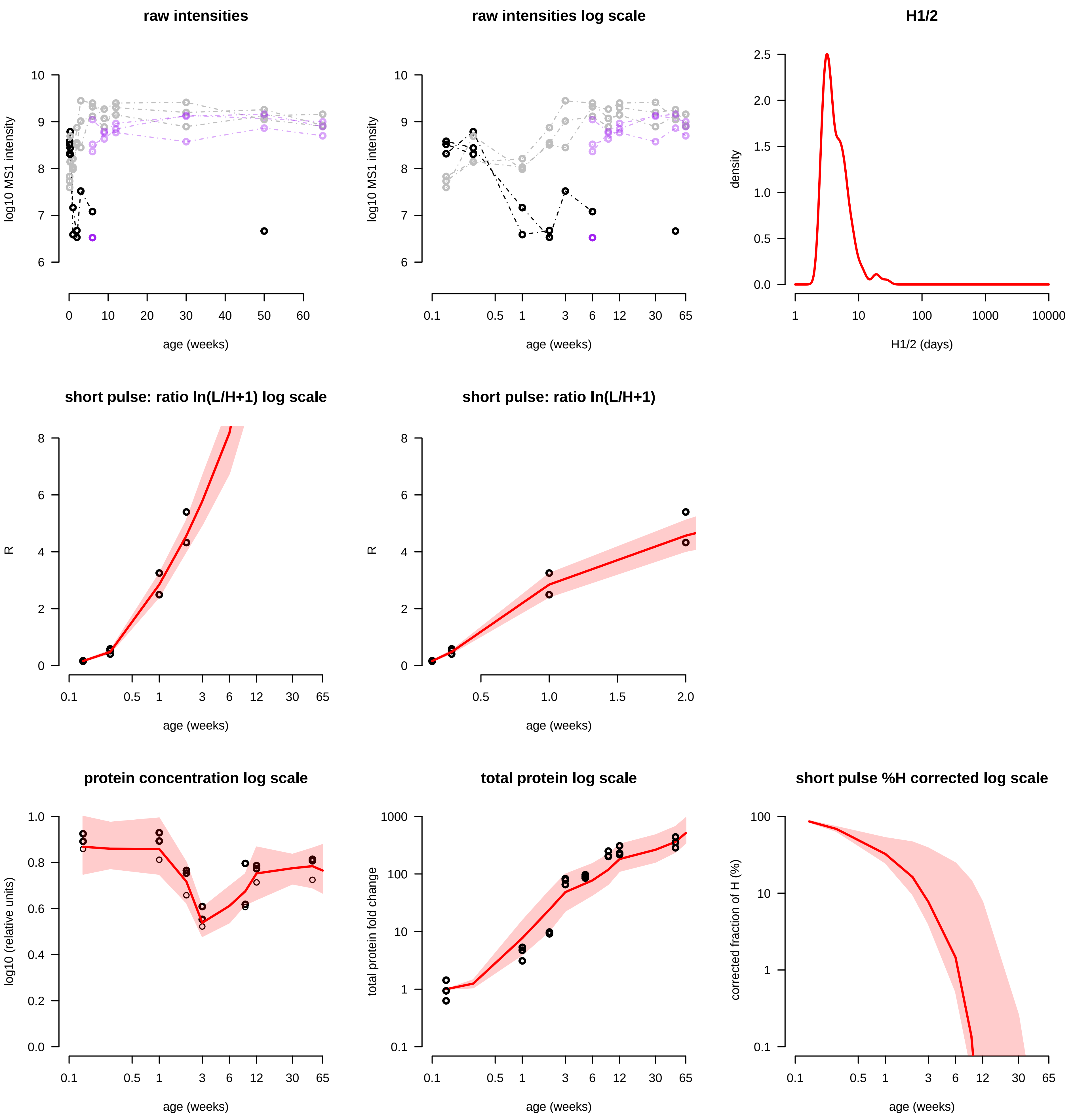

P20918

>sp|P20918|PLMN_MOUSE

Plasminogen

GN=Plg

Med H1/2: 3.7 days

Q5 H1/2: 2.7 days

Q95 H1/2: 5.2 days

reliable model fit: yes

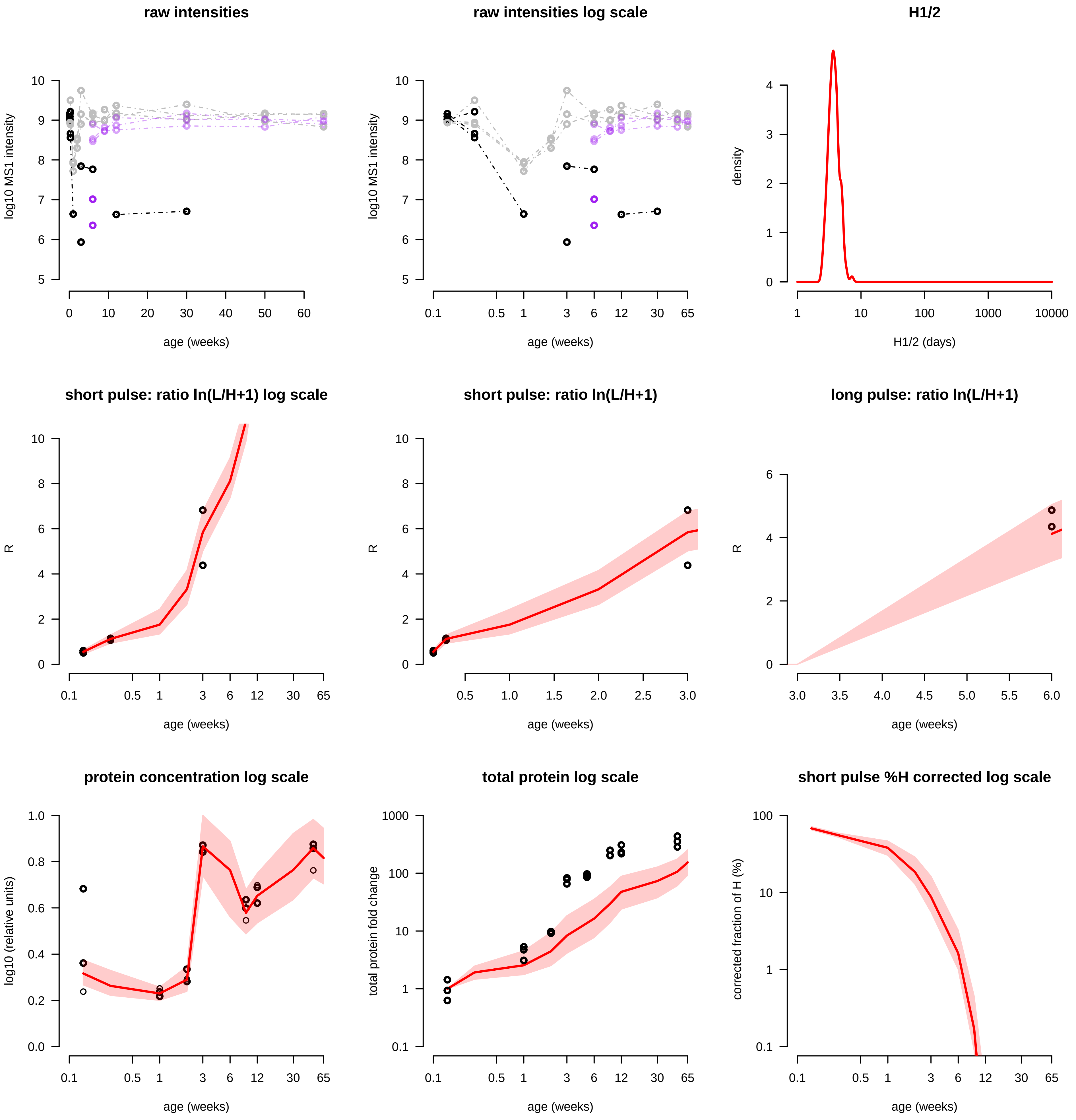

Q8K010

>sp|Q8K010|OPLA_MOUSE

5−oxoprolinase

GN=Oplah

Med H1/2: 6.7 days

Q5 H1/2: 3.3 days

Q95 H1/2: 29.2 days

reliable model fit: yes

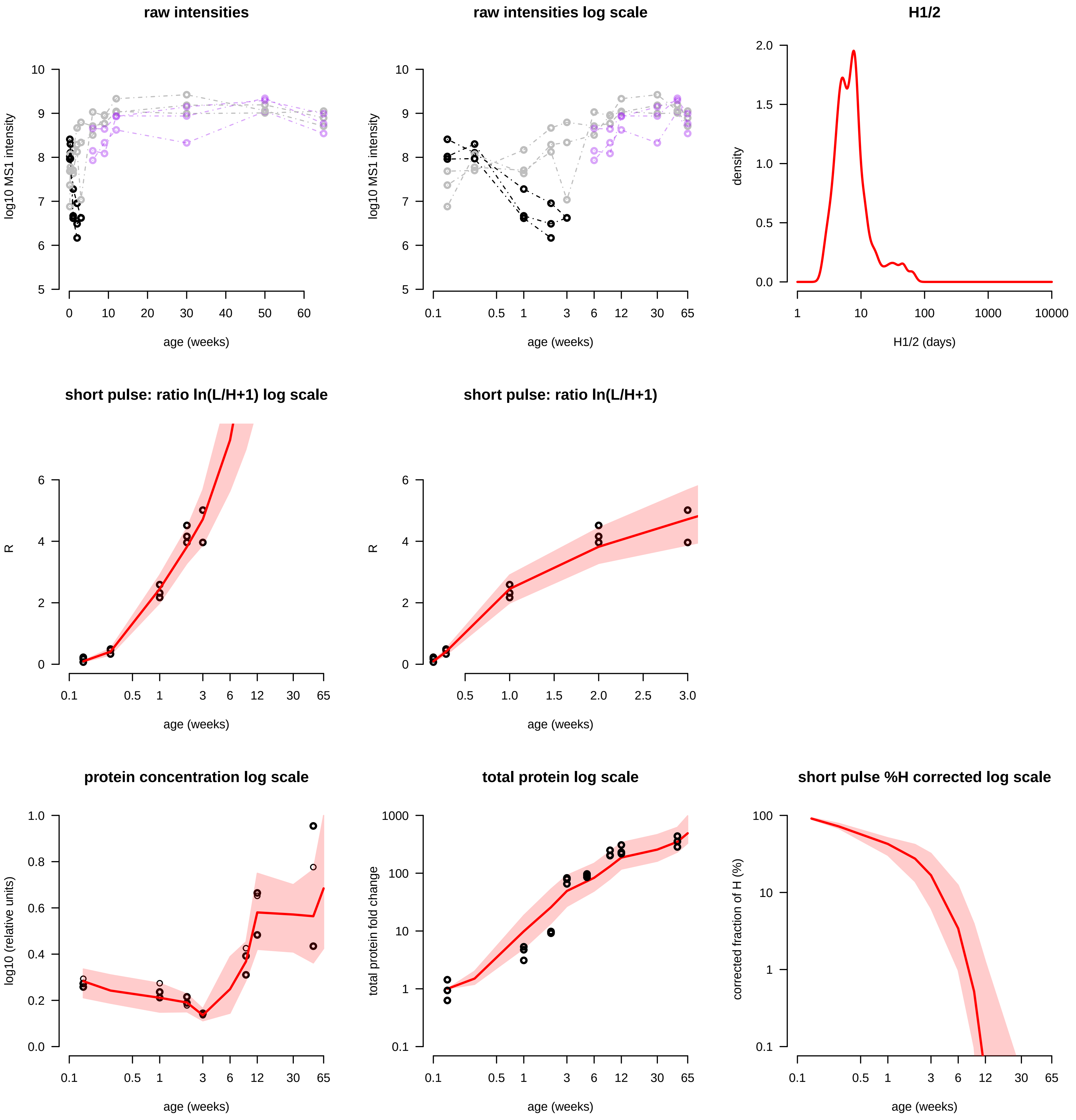

P43406

>sp|P43406|ITAV_MOUSE

Integrin alpha−V

GN=Itgav

Med H1/2: 66.5 days

Q5   H1/2: 46.9 days

Q95 H1/2: 96.7 days

reliable model fit: no

raw intensities

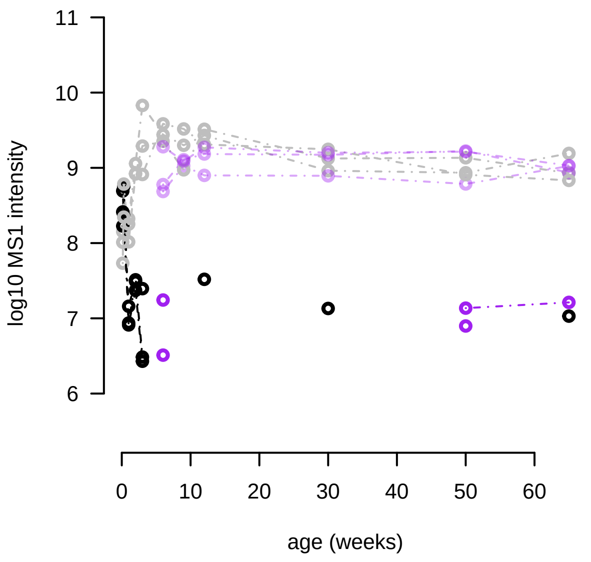

raw intensities log scale

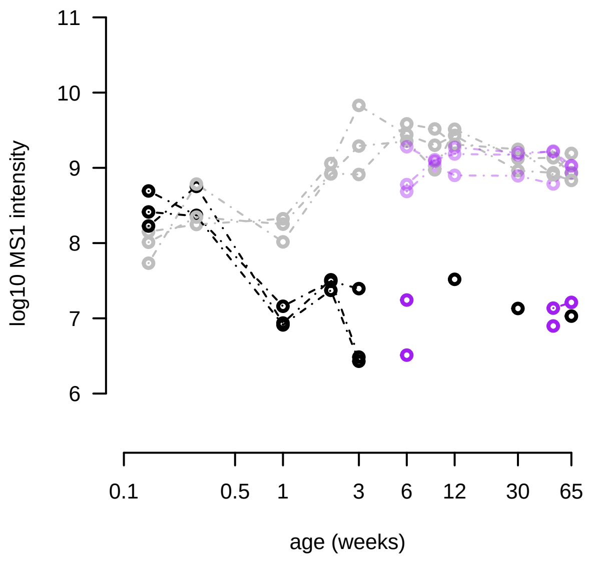

H1/2

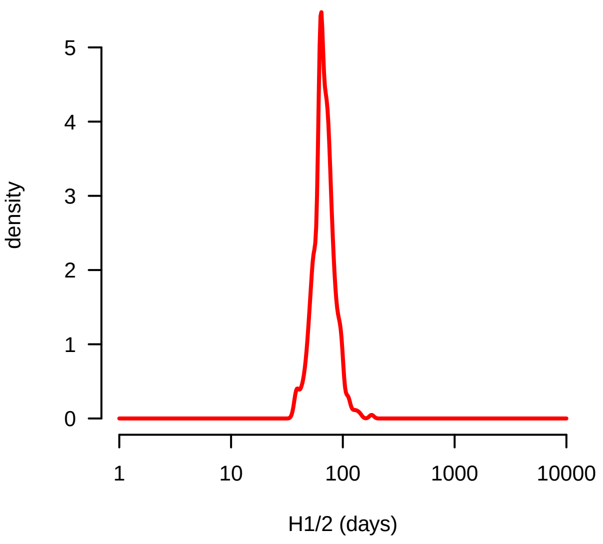

short pulse: ratio ln(L/H+1) log scale

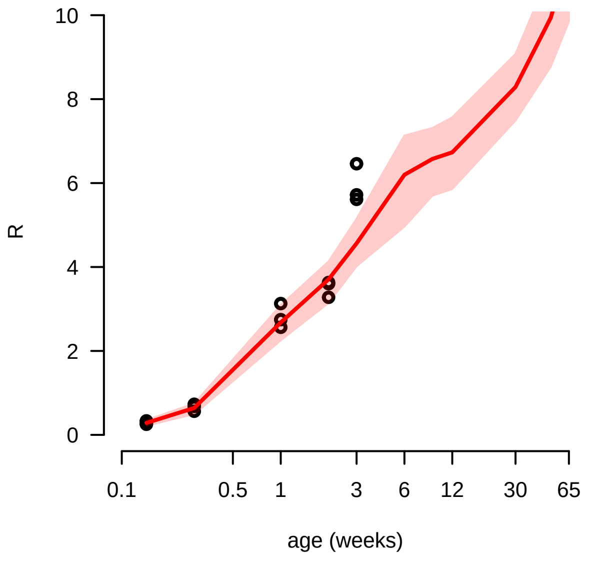

short pulse: ratio ln(L/H+1)

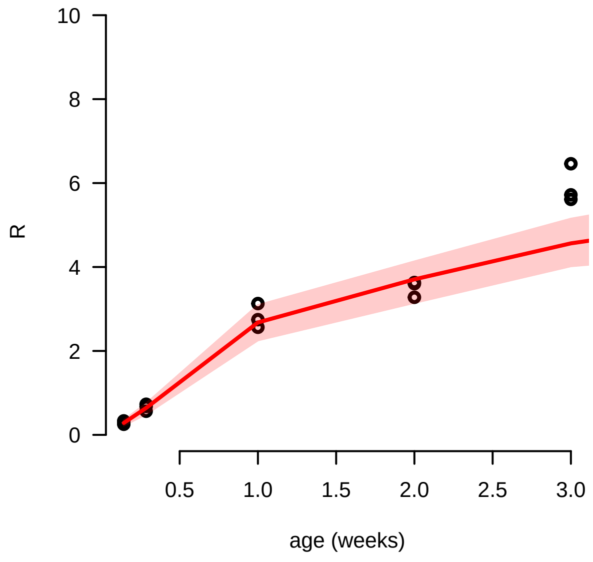

long pulse: ratio ln(L/H+1)

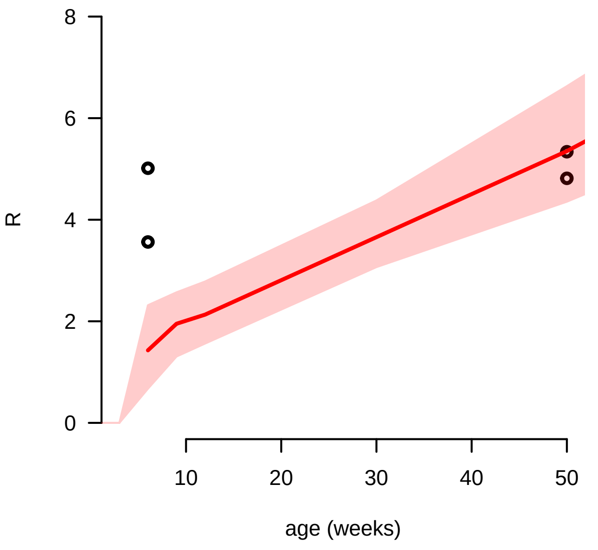

protein concentration log scale

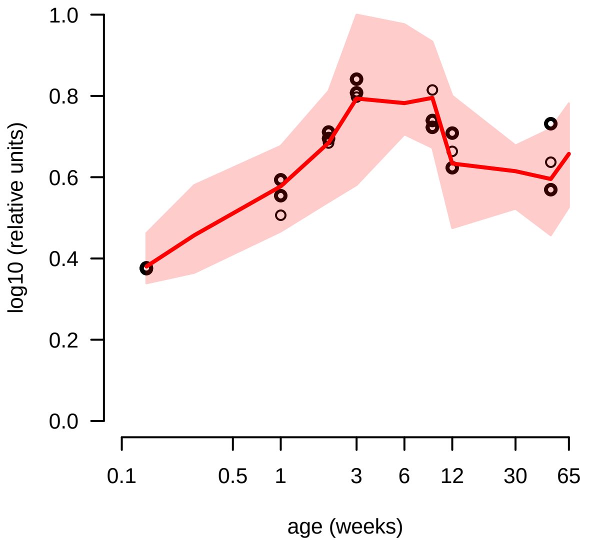

total protein log scale

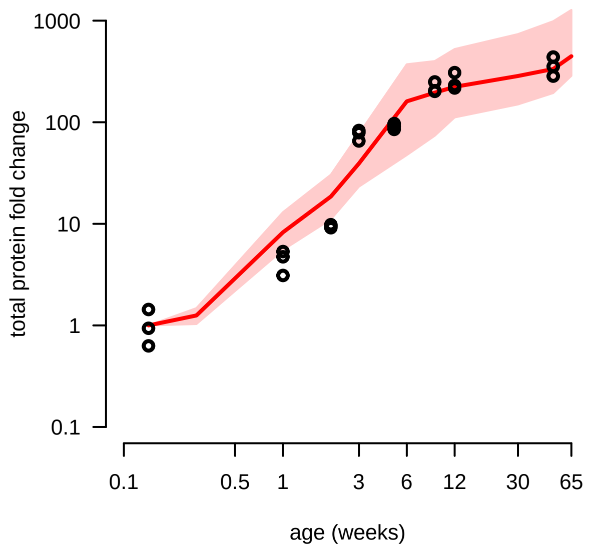

short pulse %H corrected log scale

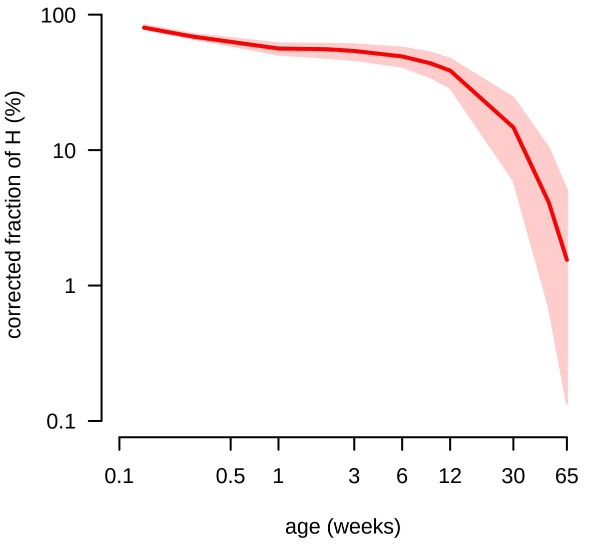

Q91YR1

>sp|Q91YR1|TWF1_MOUSE

Twinfilin−1

GN=Twf1

Med H1/2: 4.8 days

Q5 H1/2: 3.9 days

Q95 H1/2: 6 days

reliable model fit: yes

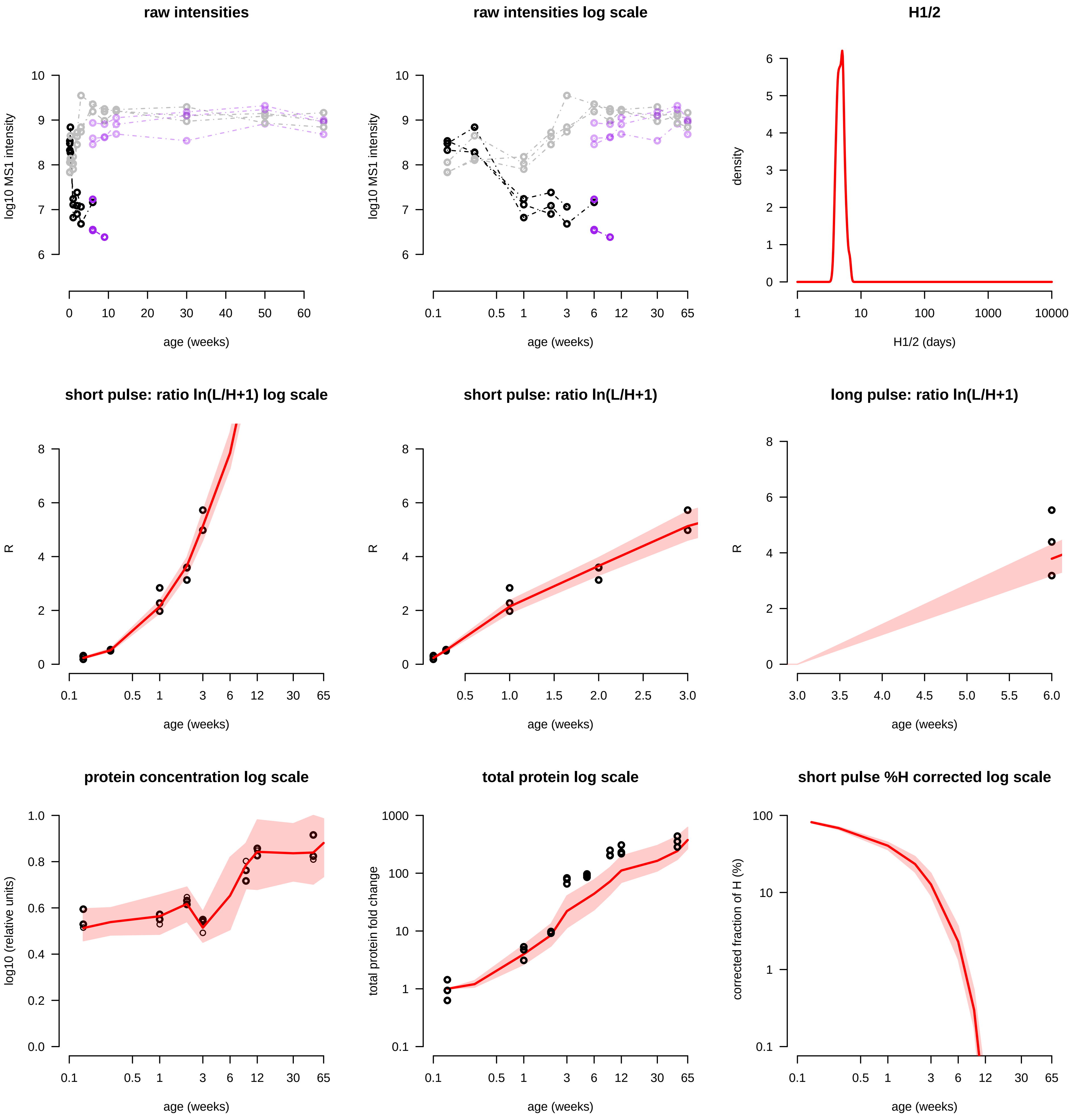

Q6P1F6

>sp|Q6P1F6|2ABA_MOUSE

rine/threonine−protein phosphatase 2A 55 kDa regulatory subunit B alpha isofo

GN=Ppp2r2a

Med H1/2: 6 days

Q5 H1/2: 4.7 days

Q95 H1/2: 7.5 days

reliable model fit: yes

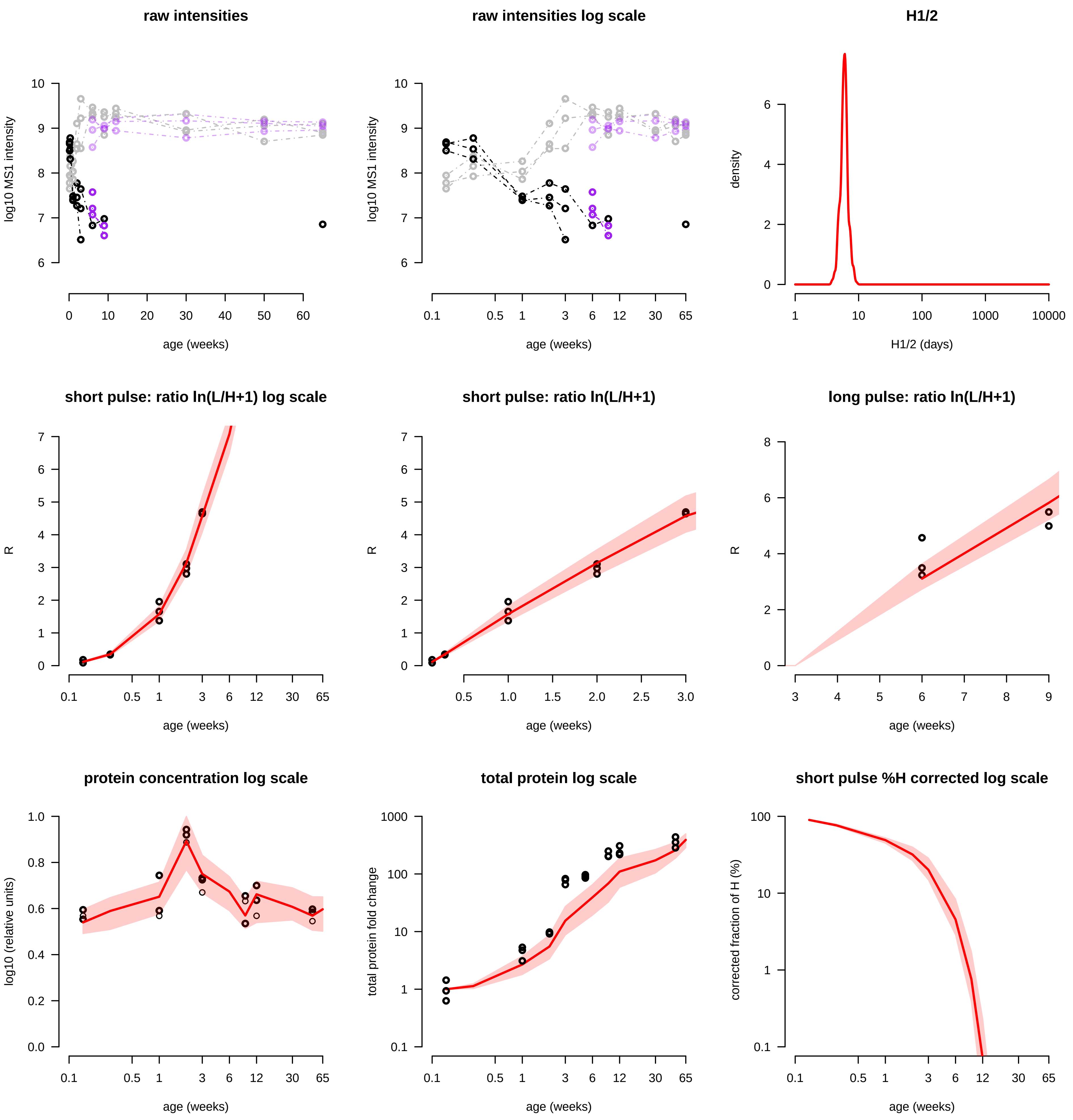

Q9CZN7−2;Q9CZN7

>sp|Q9CZN7−2|GLYM_MOUSE

Isoform 2 of Serine hydroxymethyltransferase, mitochondrial

GN=Shmt2

Med H1/2: 6.8 days

Q5 H1/2: 5.7 days

Q95 H1/2: 9.1 days

reliable model fit: yes

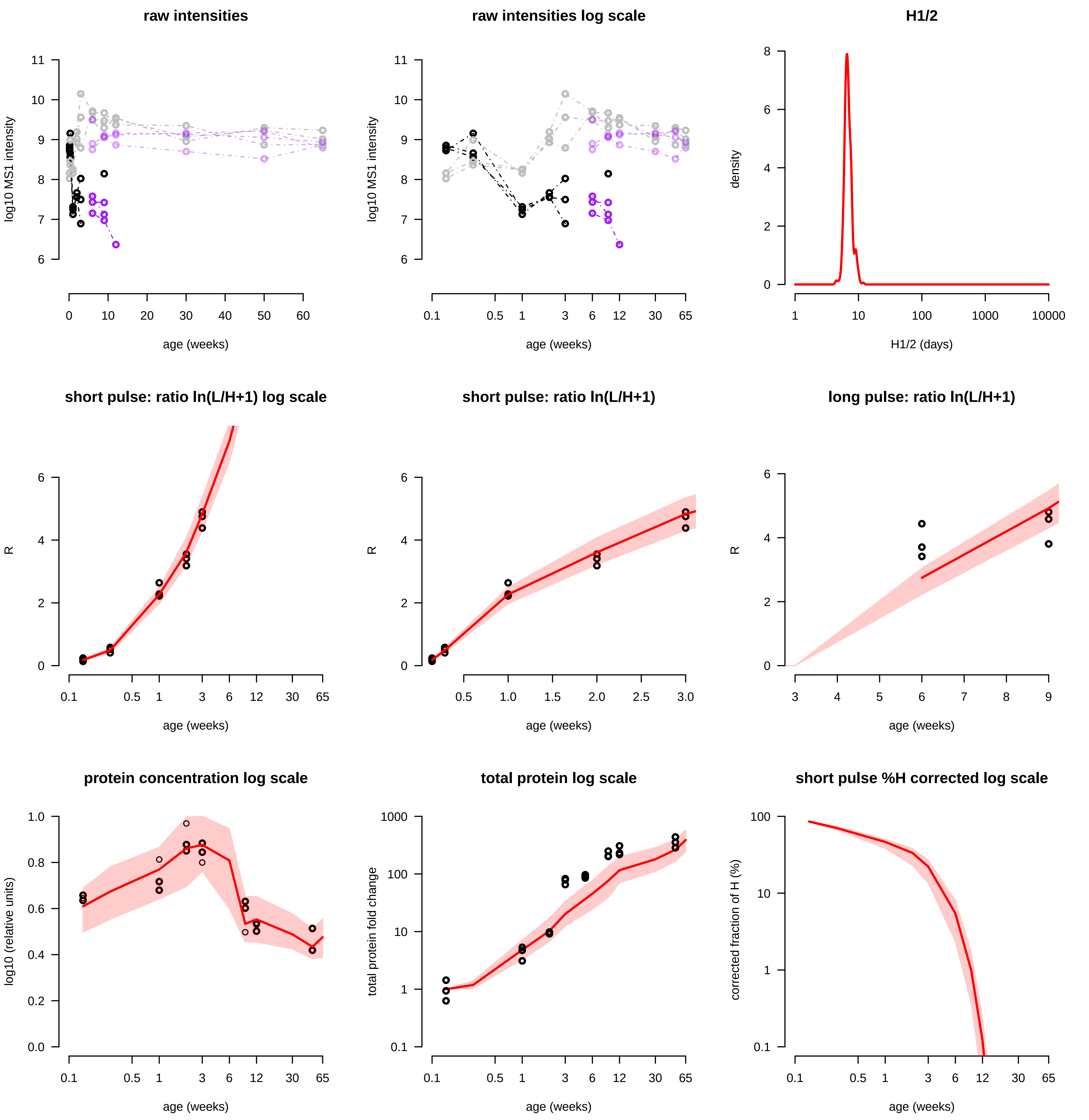

Q91V12−2

>sp|Q91V12−2|BACH_MOUSE

Isoform A of Cytosolic acyl coenzyme A thioester hydrolase

GN=Acot7

Med H1/2: 12.1 days

Q5 H1/2: 8.9 days

Q95 H1/2: 23.7 days

reliable model fit: yes

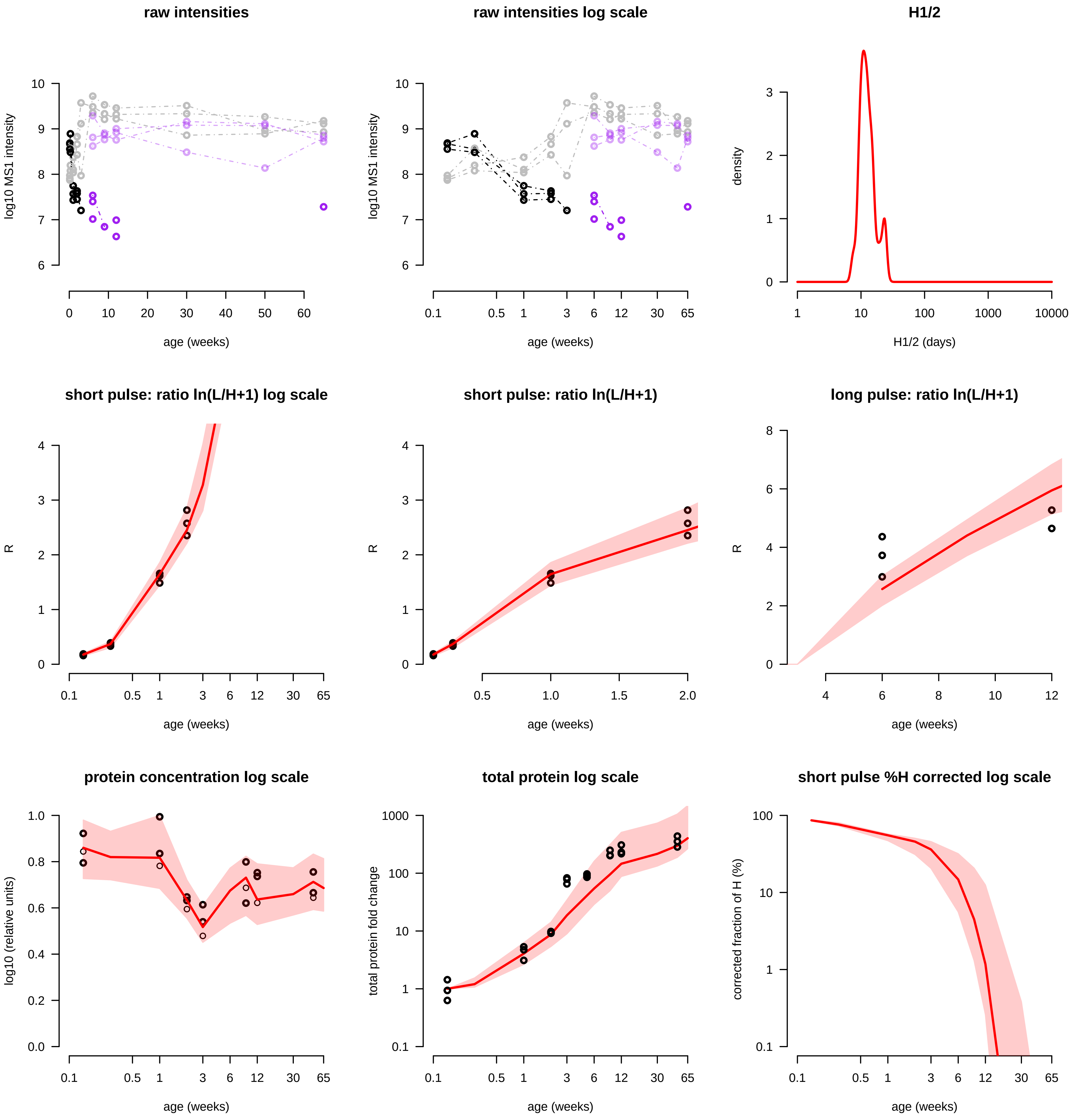

Q7TQK5

>sp|Q7TQK5|CCD93_MOUSE

Coiled−coil domain−containing protein 93

GN=Ccdc93

Med H1/2: 17.6 days

Q5 H1/2: 13.4 days

Q95 H1/2: 26.3 days

reliable model fit: yes

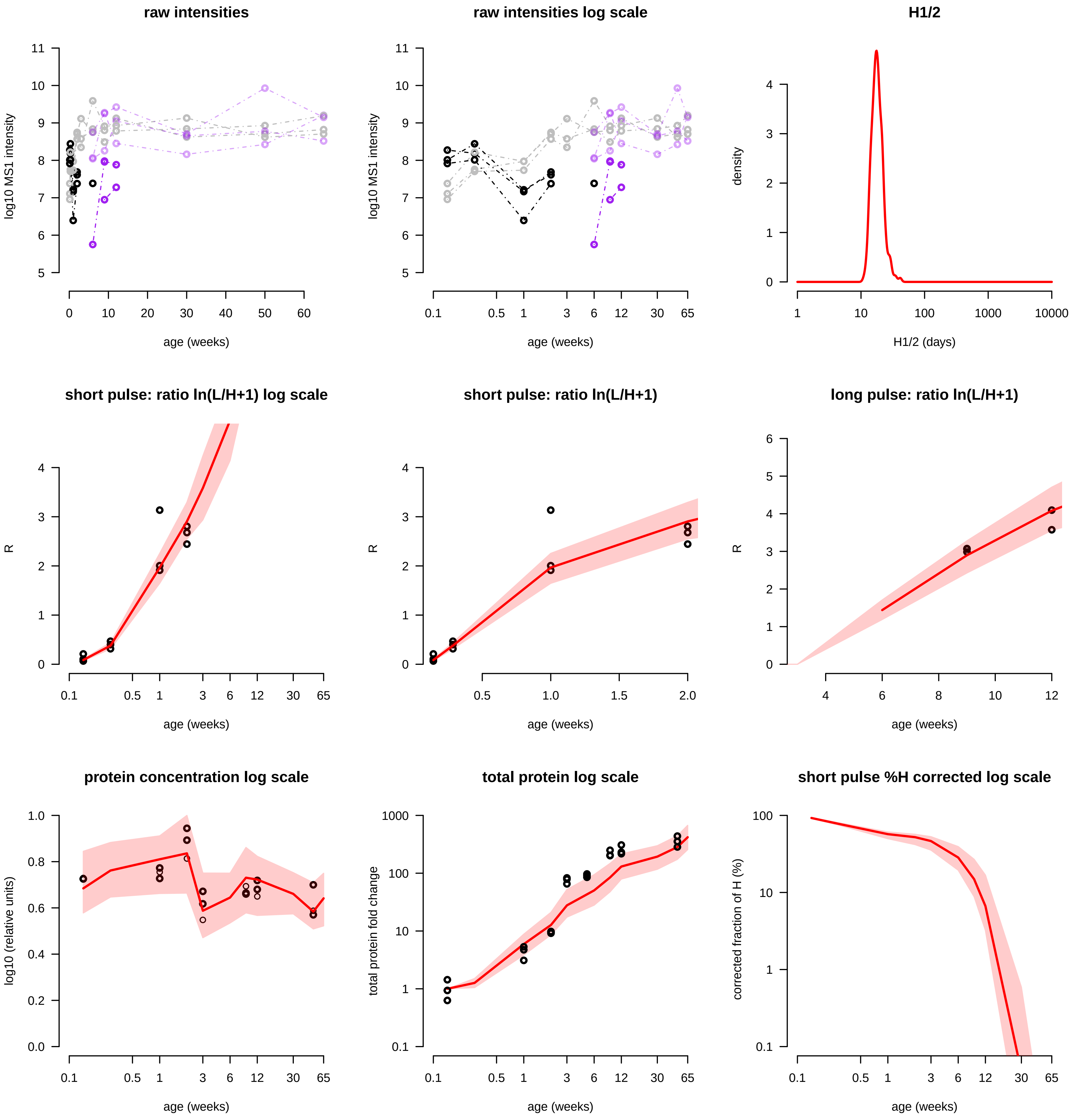

P02469

>sp|P02469|LAMB1_MOUSE

Laminin subunit beta−1

GN=Lamb1

Med H1/2: 6.4 days

Q5 H1/2: 5.3 days

Q95 H1/2: 8.2 days

reliable model fit: yes

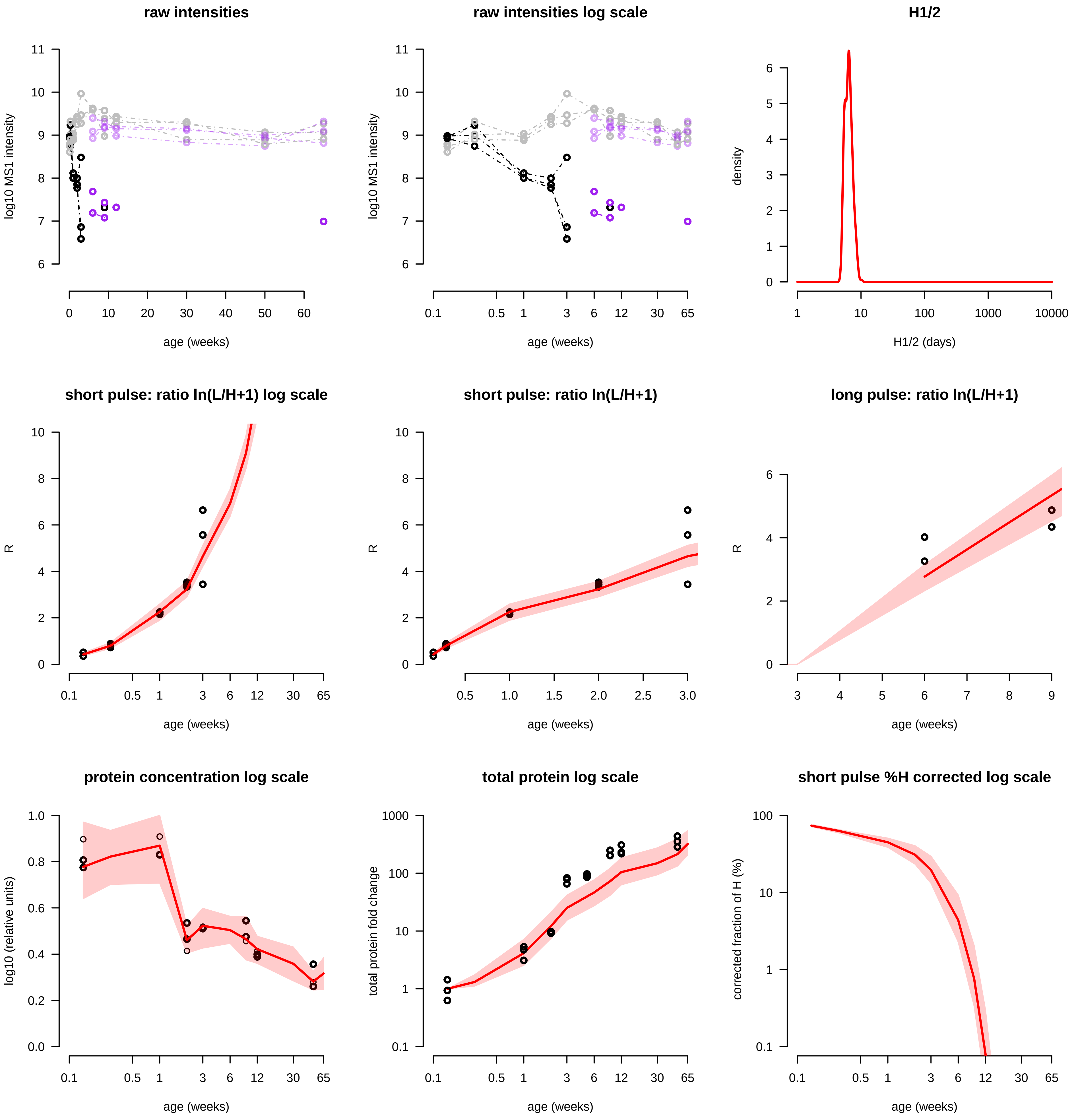

Q6PDM2;Q6PDM2−3;Q6PDM2−2

>sp|Q6PDM2|SRSF1_MOUSE

Serine/arginine−rich splicing factor 1

GN=Srsf1

Med H1/2: 5.3 days

Q5 H1/2: 4.4 days

Q95 H1/2: 7.4 days

reliable model fit: yes

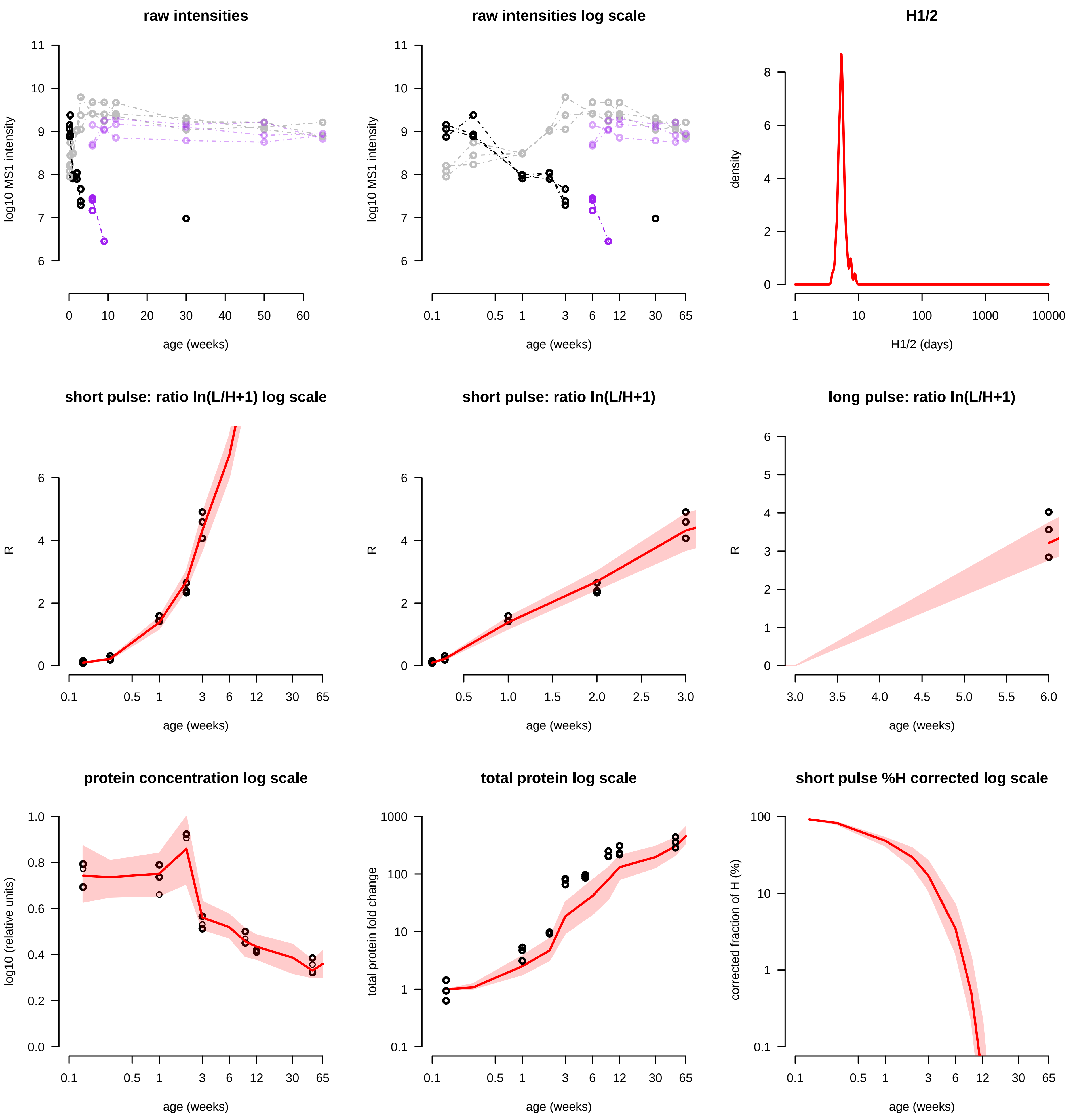

Q99JB2

>sp|Q99JB2|STML2_MOUSE

Stomatin−like protein 2, mitochondrial

GN=Stoml2

Med H1/2: 6.2 days

Q5 H1/2: 5.1 days

Q95 H1/2: 9.3 days

reliable model fit: yes

raw intensities

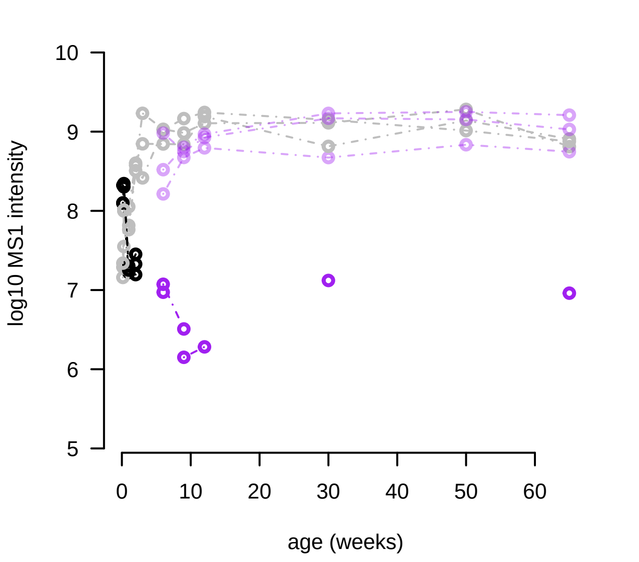

raw intensities log scale

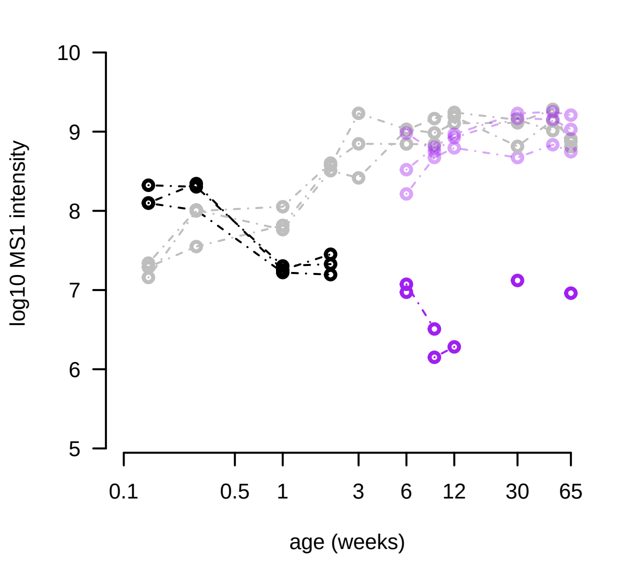

H1/2

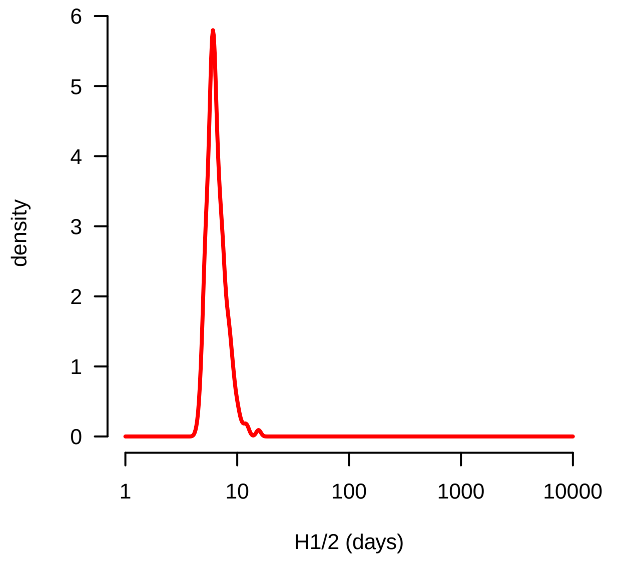

short pulse: ratio ln(L/H+1) log scale

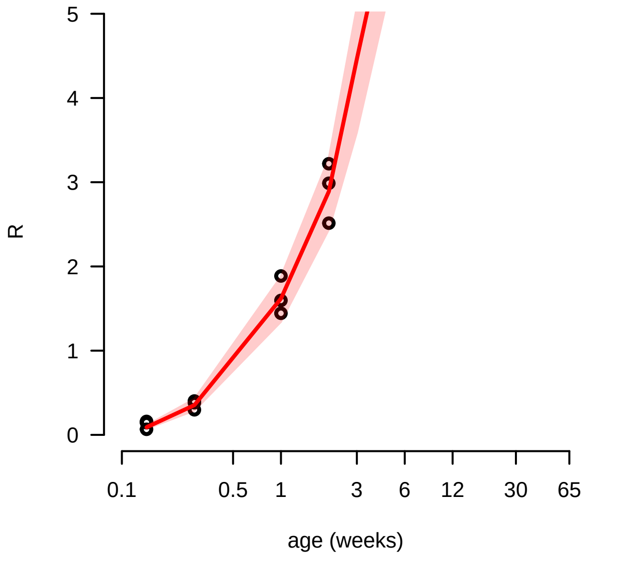

short pulse: ratio ln(L/H+1)

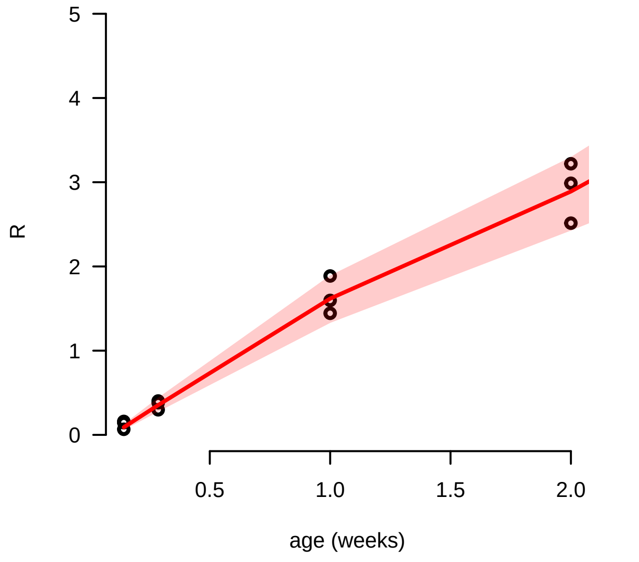

long pulse: ratio ln(L/H+1)

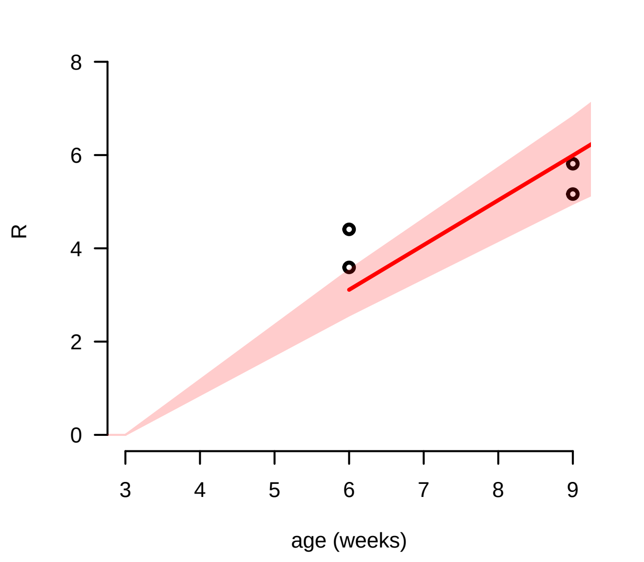

protein concentration log scale

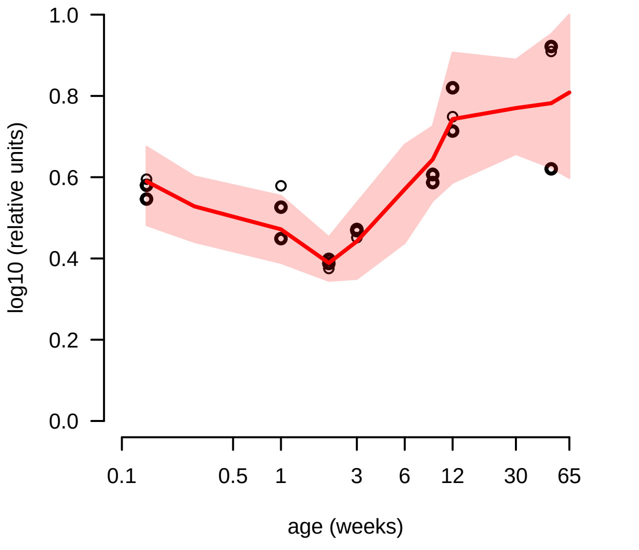

total protein log scale

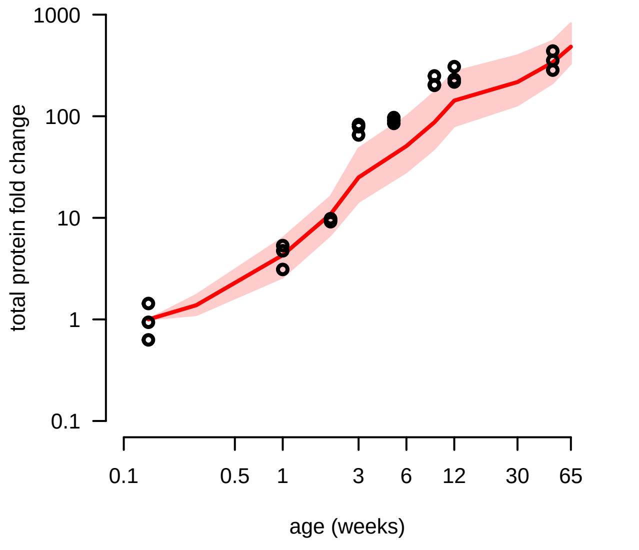

short pulse %H corrected log scale

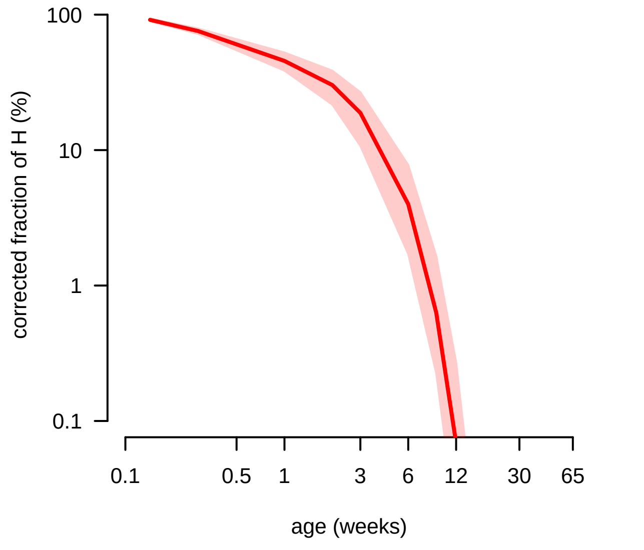

Q9WTX6

>sp|Q9WTX6|CUL1_MOUSE

Cullin−1

GN=Cul1

Med H1/2: 4.2 days

Q5 H1/2: 3.4 days

Q95 H1/2: 5.3 days

reliable model fit: yes

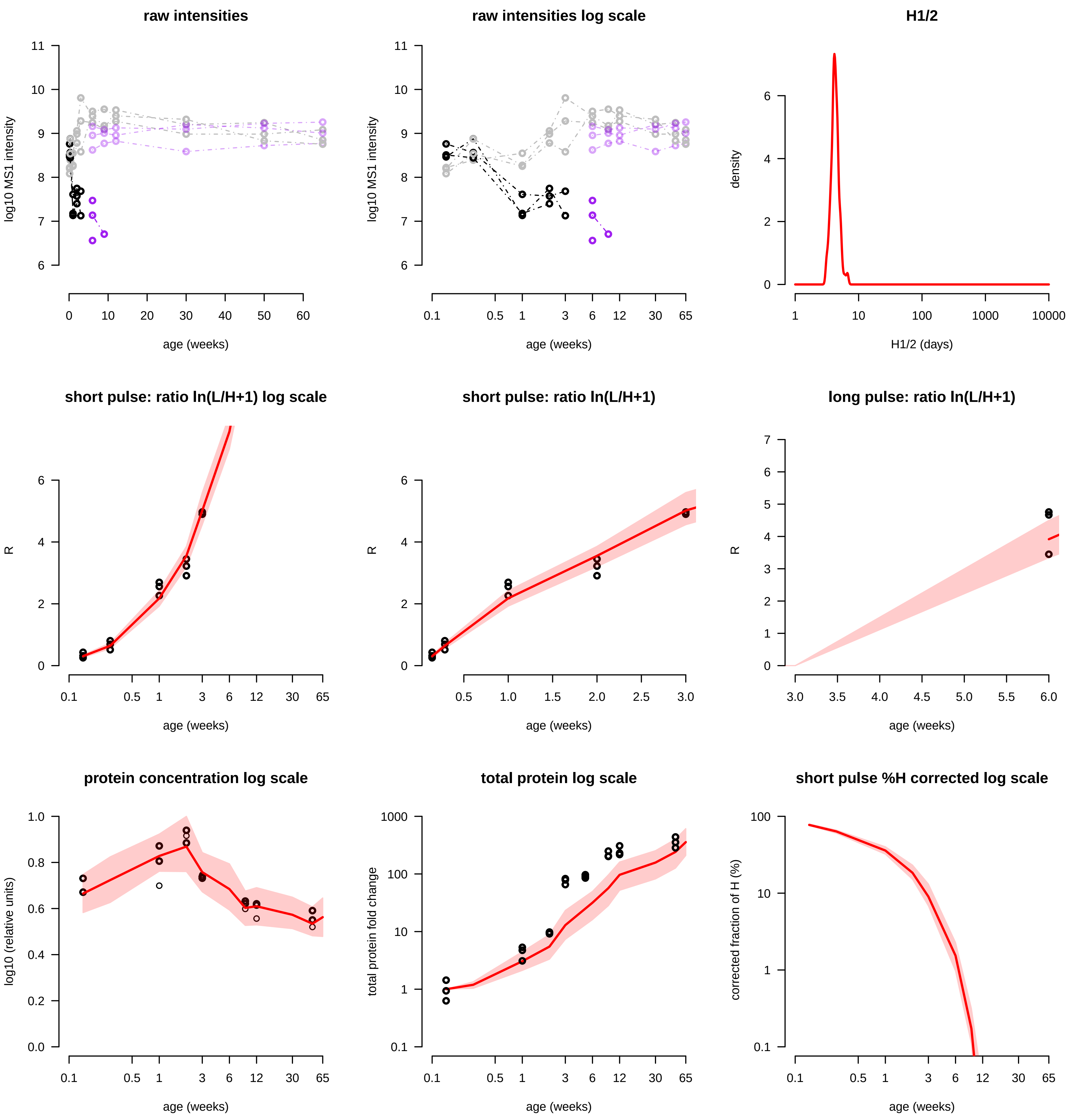

Q61510

>sp|Q61510|TRI25_MOUSE

E3 ubiquitin/ISG15 ligase TRIM25

GN=Trim25

Med H1/2: 9.5 days

Q5 H1/2: 7.2 days

Q95 H1/2: 13.1 days

reliable model fit: yes

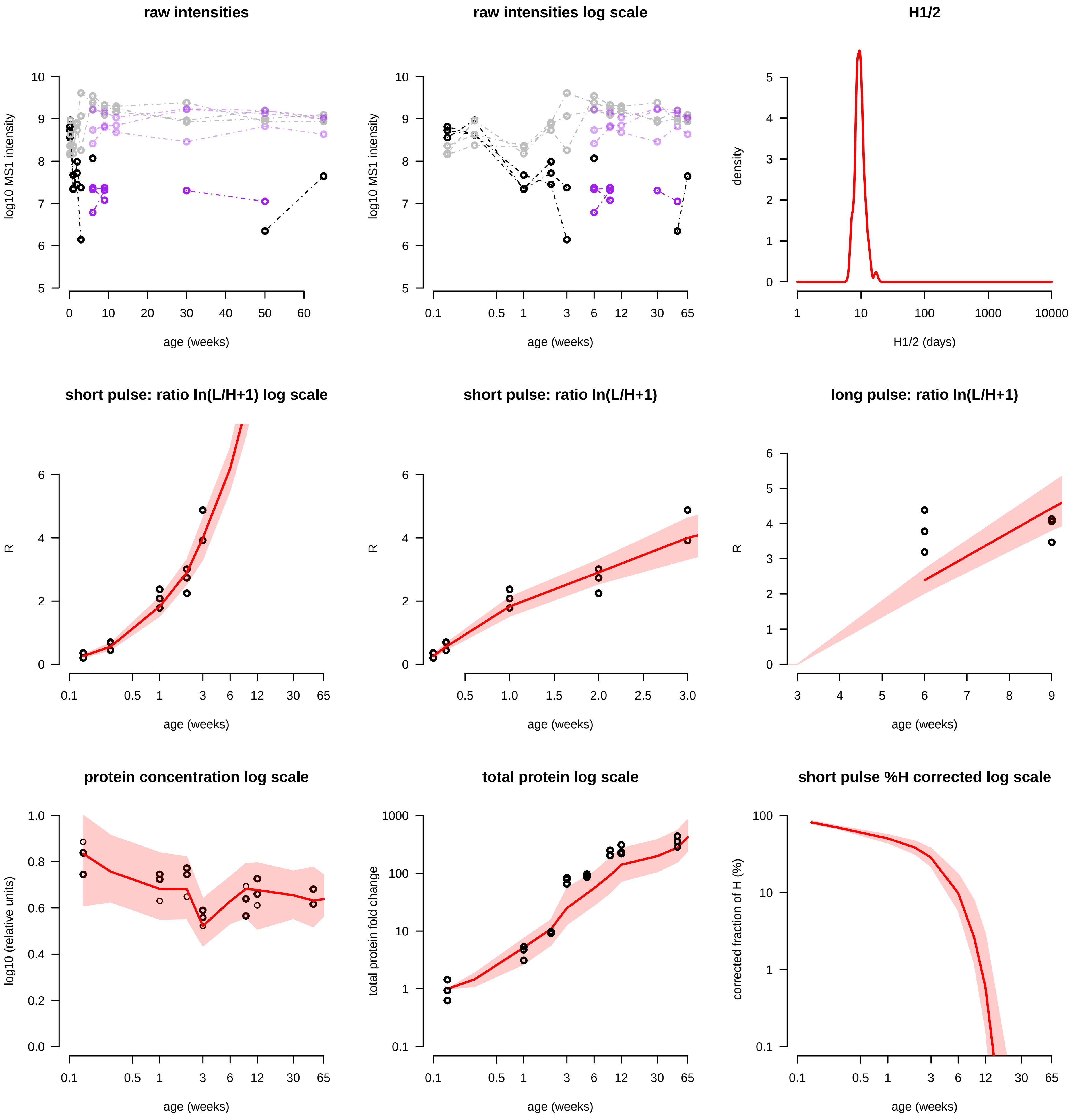

Q61702

>sp|Q61702|ITIH1_MOUSE

Inter−alpha−trypsin inhibitor heavy chain H1

GN=Itih1

Med H1/2: 3.3 days

Q5 H1/2: 2.6 days

Q95 H1/2: 6.9 days

reliable model fit: yes

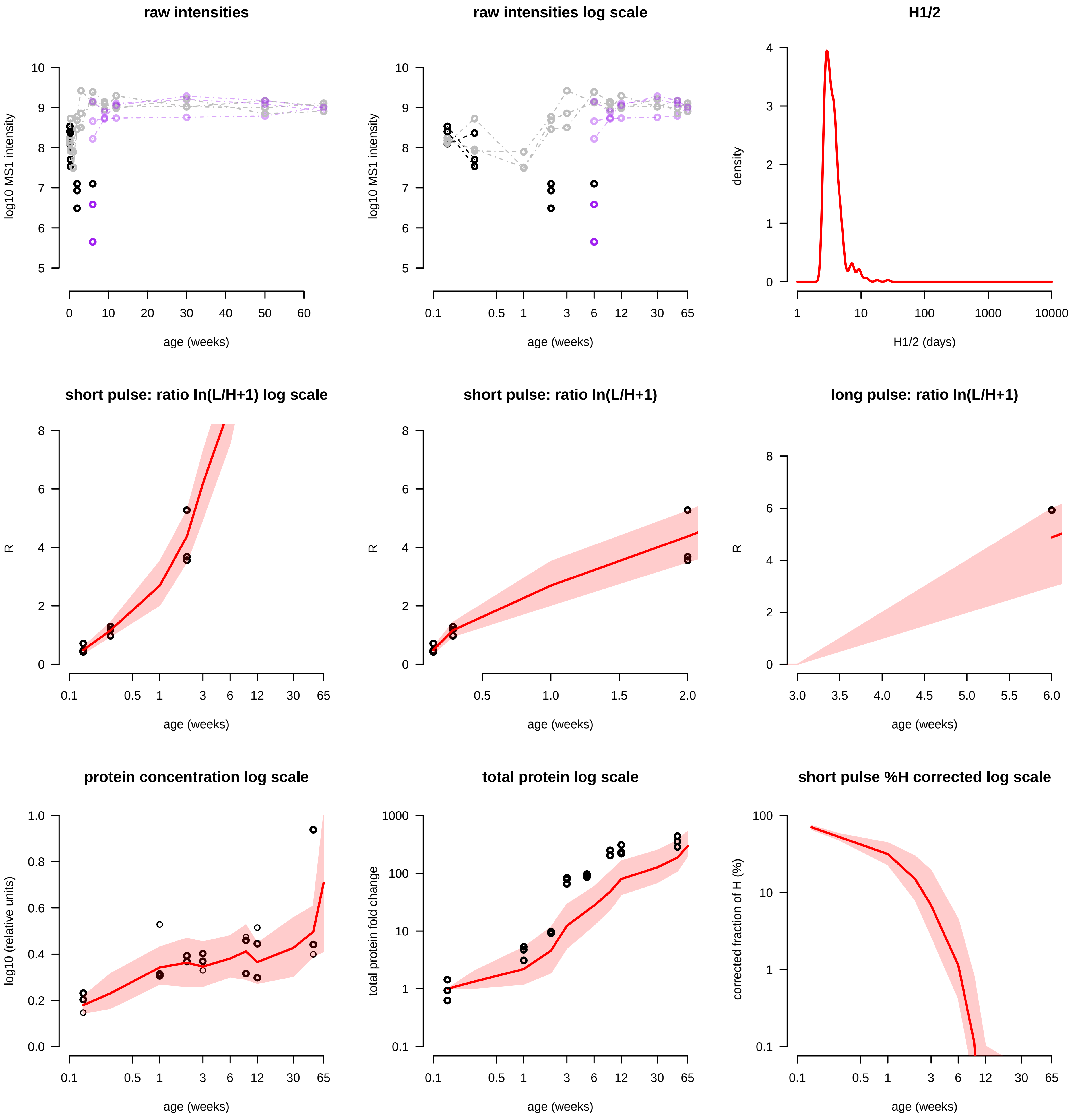

O08663

>sp|O08663|MAP2_MOUSE

Methionine aminopeptidase 2

GN=Metap2

Med H1/2: 3.2 days

Q5 H1/2: 2.5 days

Q95 H1/2: 4.2 days

reliable model fit: yes

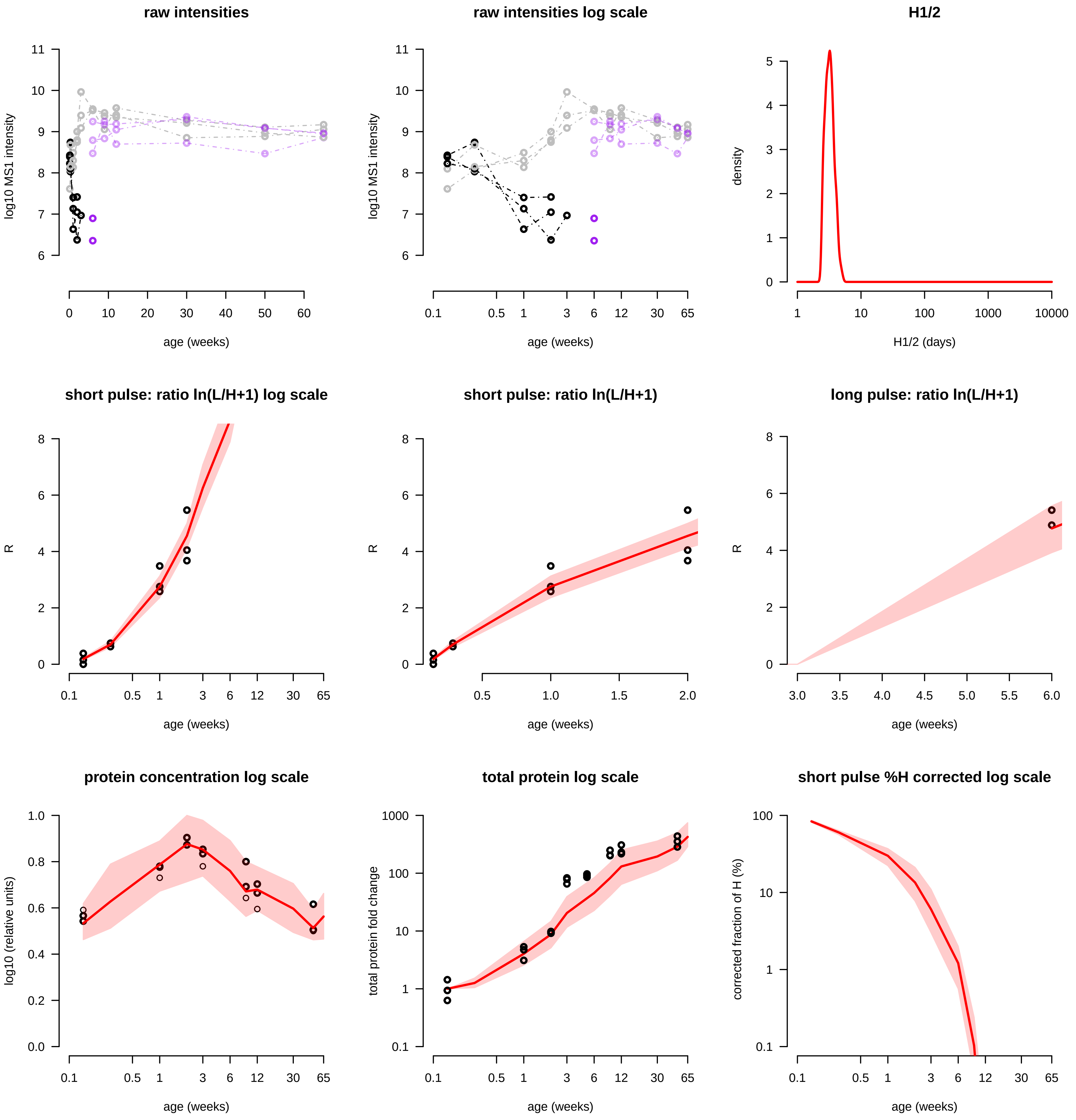

O70492

>sp|O70492|SNX3_MOUSE

Sorting nexin−3

GN=Snx3

Med H1/2: 10.9 days

Q5 H1/2: 8.4 days

Q95 H1/2: 13.6 days

reliable model fit: yes

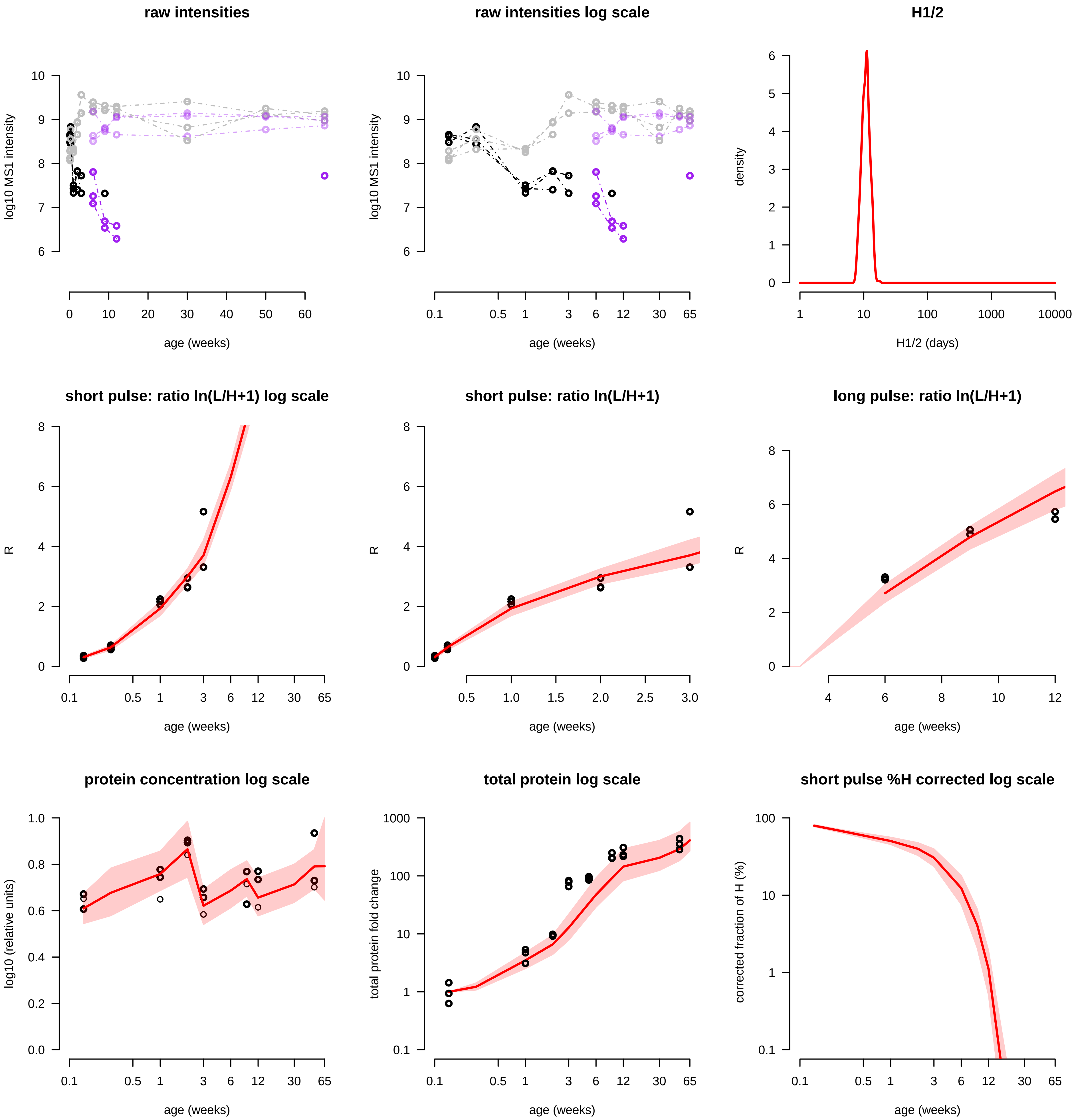

O08807

>sp|O08807|PRDX4_MOUSE

Peroxiredoxin−4

GN=Prdx4

Med H1/2: 5.5 days

Q5 H1/2: 4.8 days

Q95 H1/2: 6.5 days

reliable model fit: yes

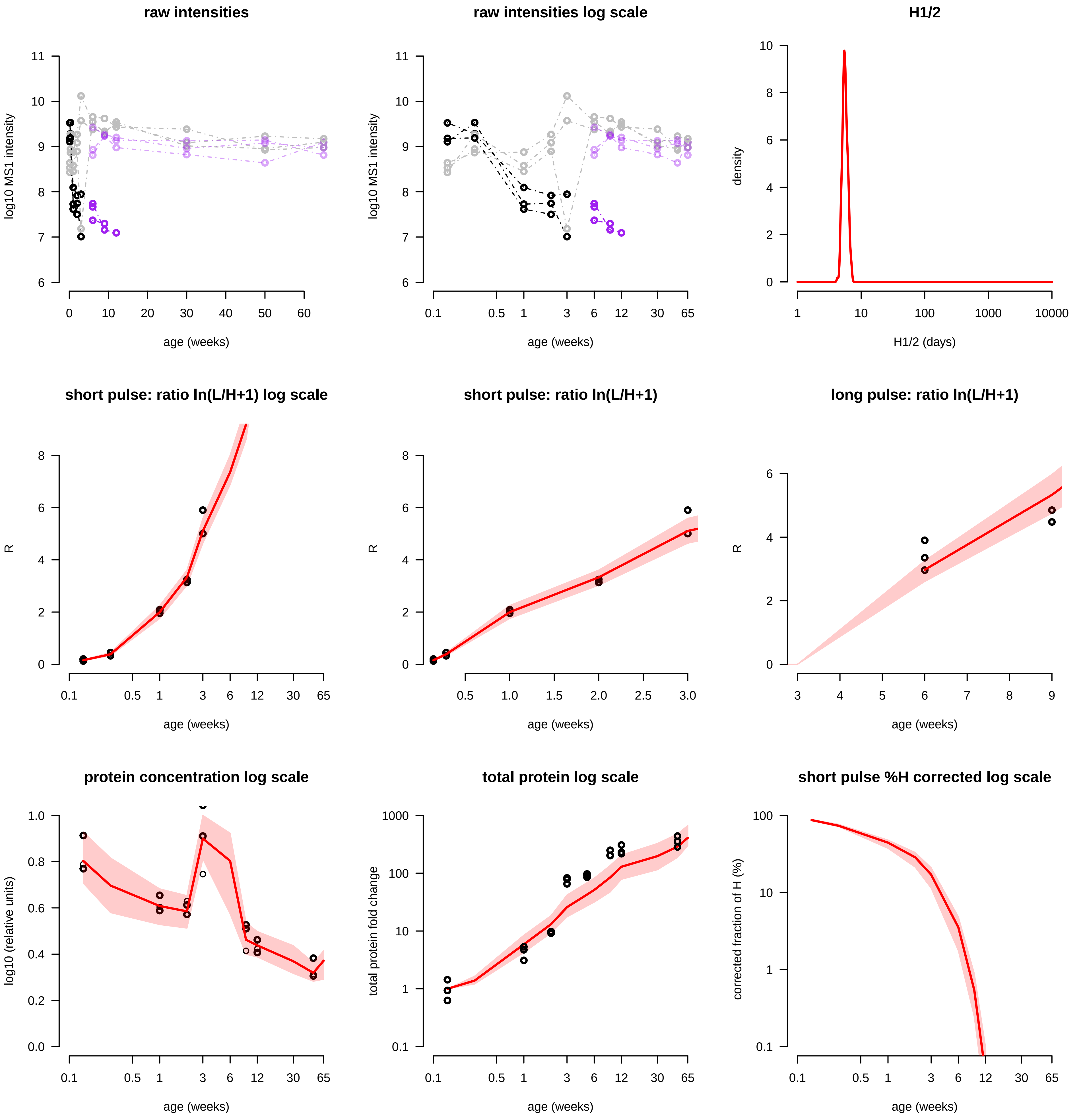

P54728

>sp|P54728|RD23B_MOUSE

UV excision repair protein RAD23 homolog B

GN=Rad23b

Med H1/2: 5.9 days

Q5 H1/2: 5 days

Q95 H1/2: 7.2 days

reliable model fit: yes

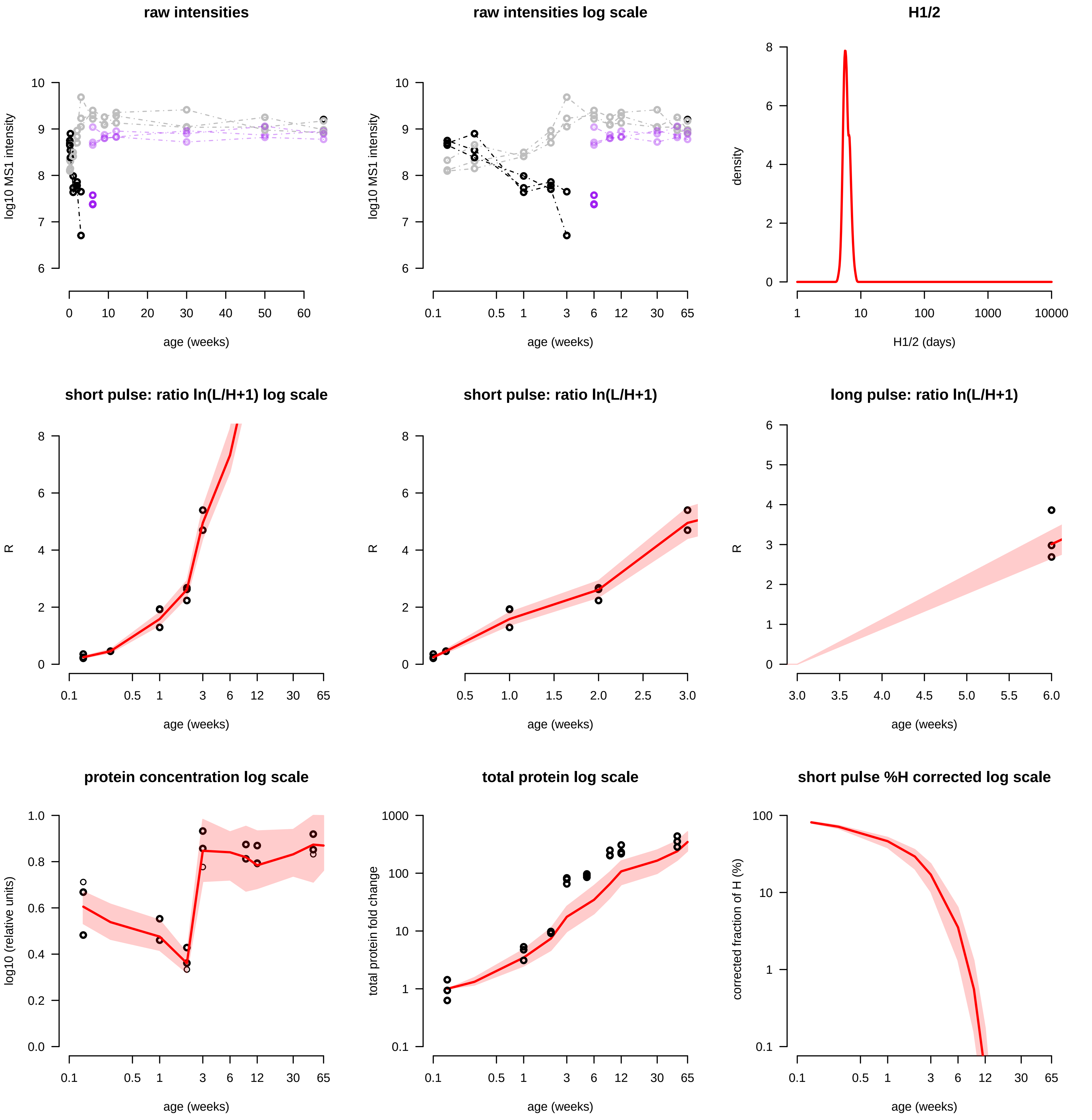

O08997

>sp|O08997|ATOX1_MOUSE

Copper transport protein ATOX1

GN=Atox1

Med H1/2: 8.7 days

Q5 H1/2: 5.1 days

Q95 H1/2: 19.2 days

reliable model fit: no

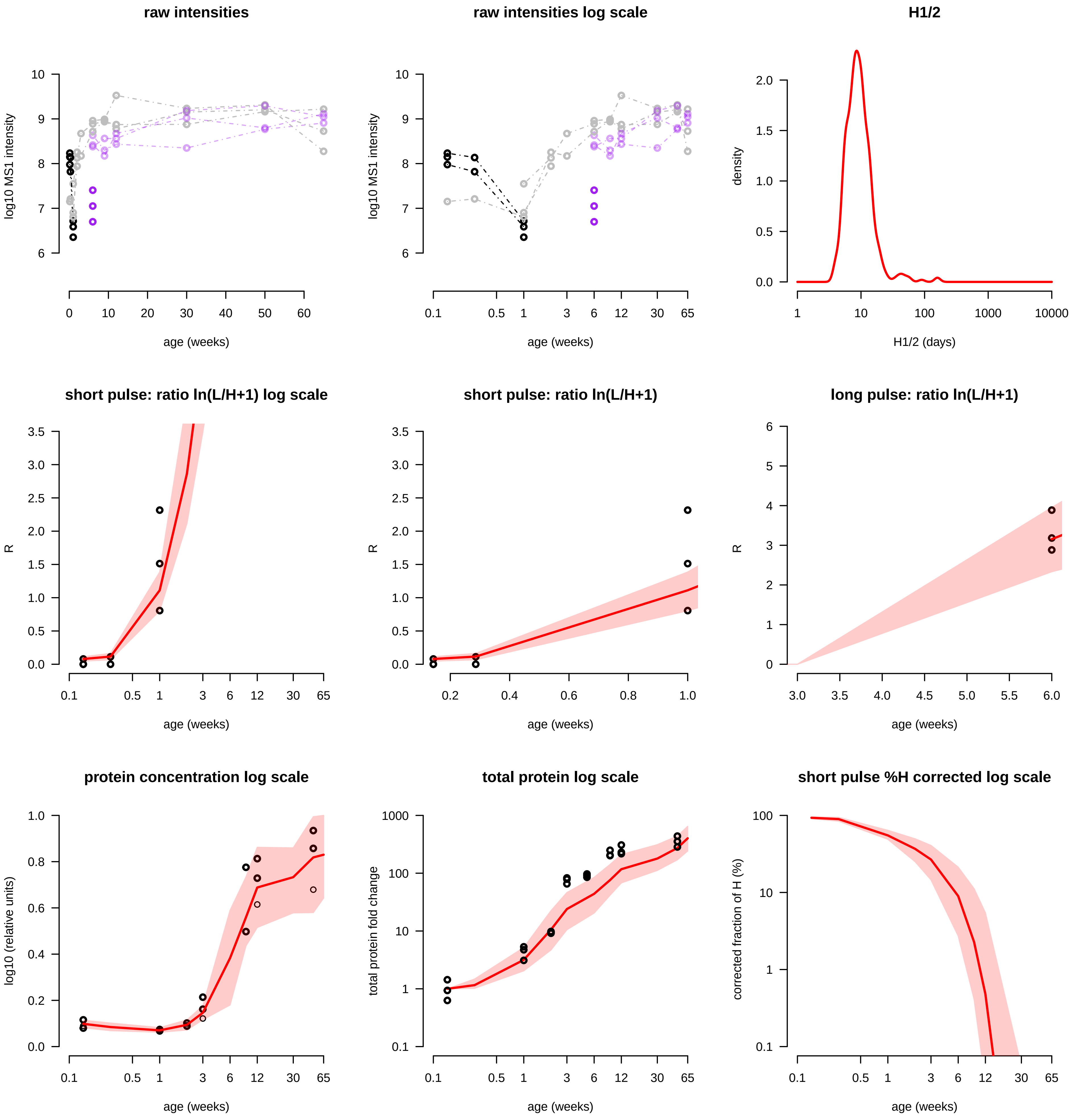

Q6NZB0

>sp|Q6NZB0|DNJC8_MOUSE

DnaJ homolog subfamily C member 8

GN=Dnajc8

Med H1/2: 10.5 days

Q5 H1/2: 8.5 days

Q95 H1/2: 14.3 days

reliable model fit: yes

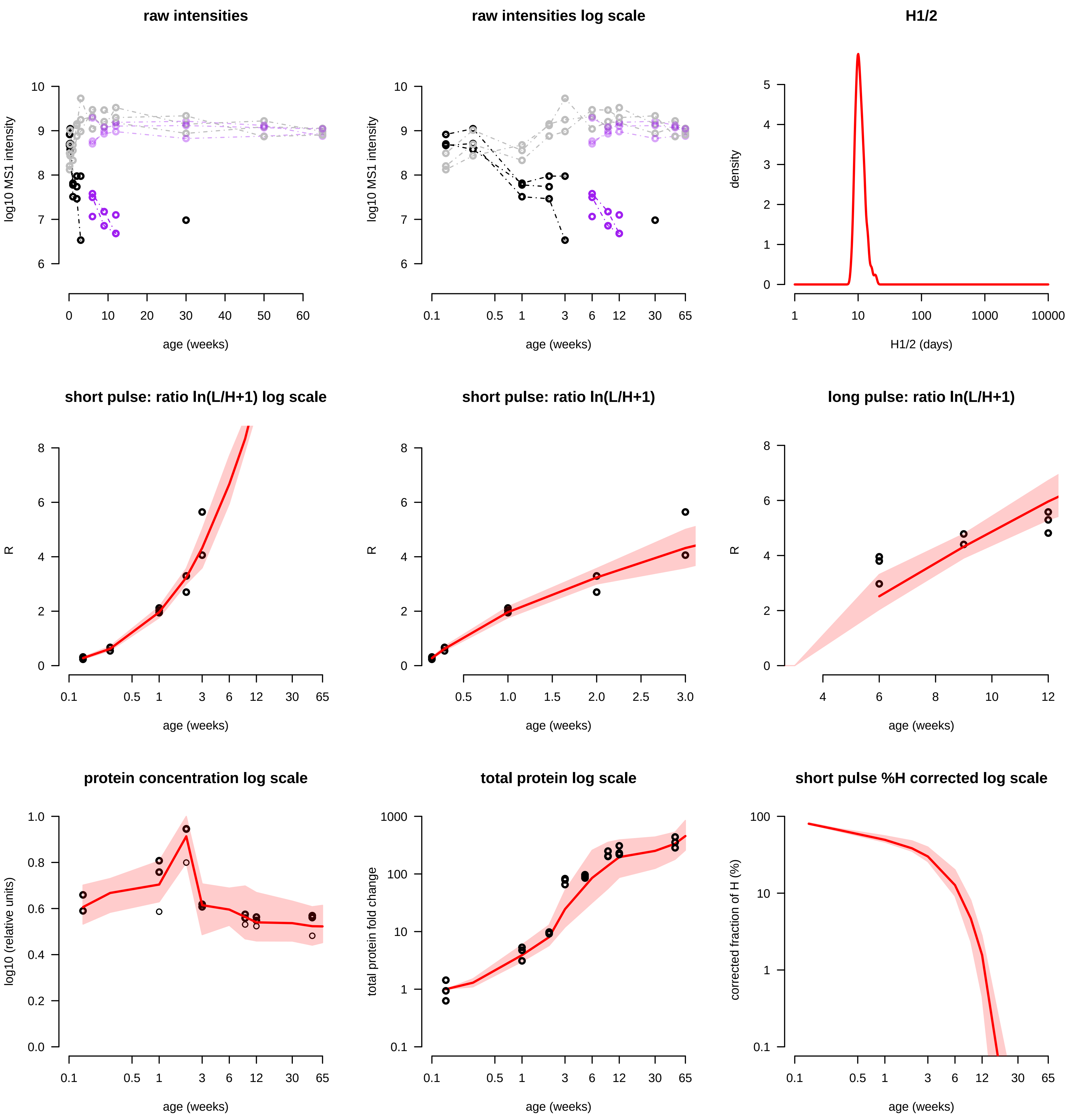

Q7TT50

>sp|Q7TT50|MRCKB_MOUSE

Serine/threonine−protein kinase MRCK beta

GN=Cdc42bpb

Med H1/2: 4.8 days

Q5 H1/2: 2.8 days

Q95 H1/2: 12.6 days

reliable model fit: yes

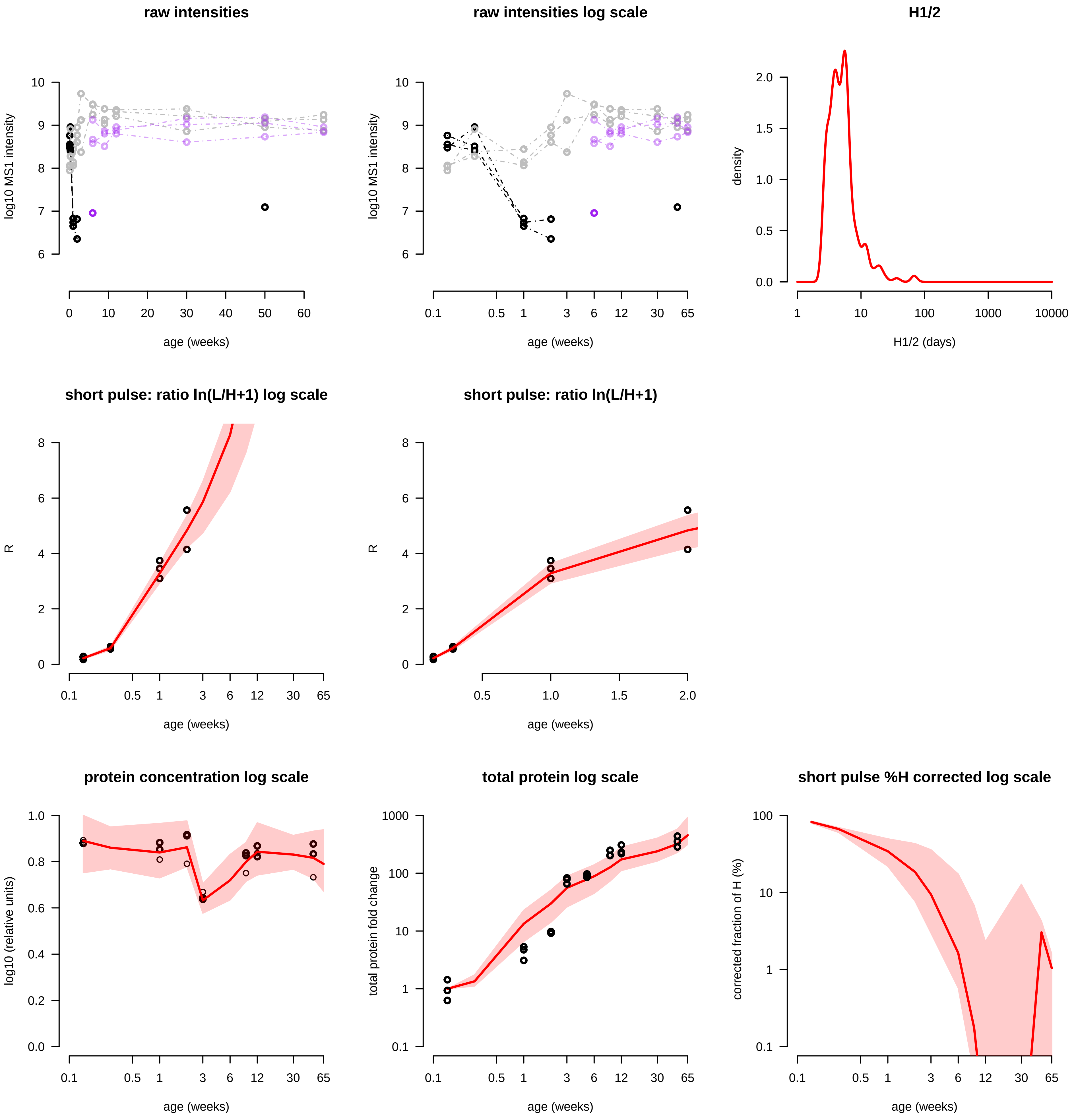

O54962

>sp|O54962|BAF_MOUSE

Barrier−to−autointegration factor

GN=Banf1

Med H1/2: 6 days

Q5 H1/2: 4.4 days

Q95 H1/2: 12.2 days

reliable model fit: yes

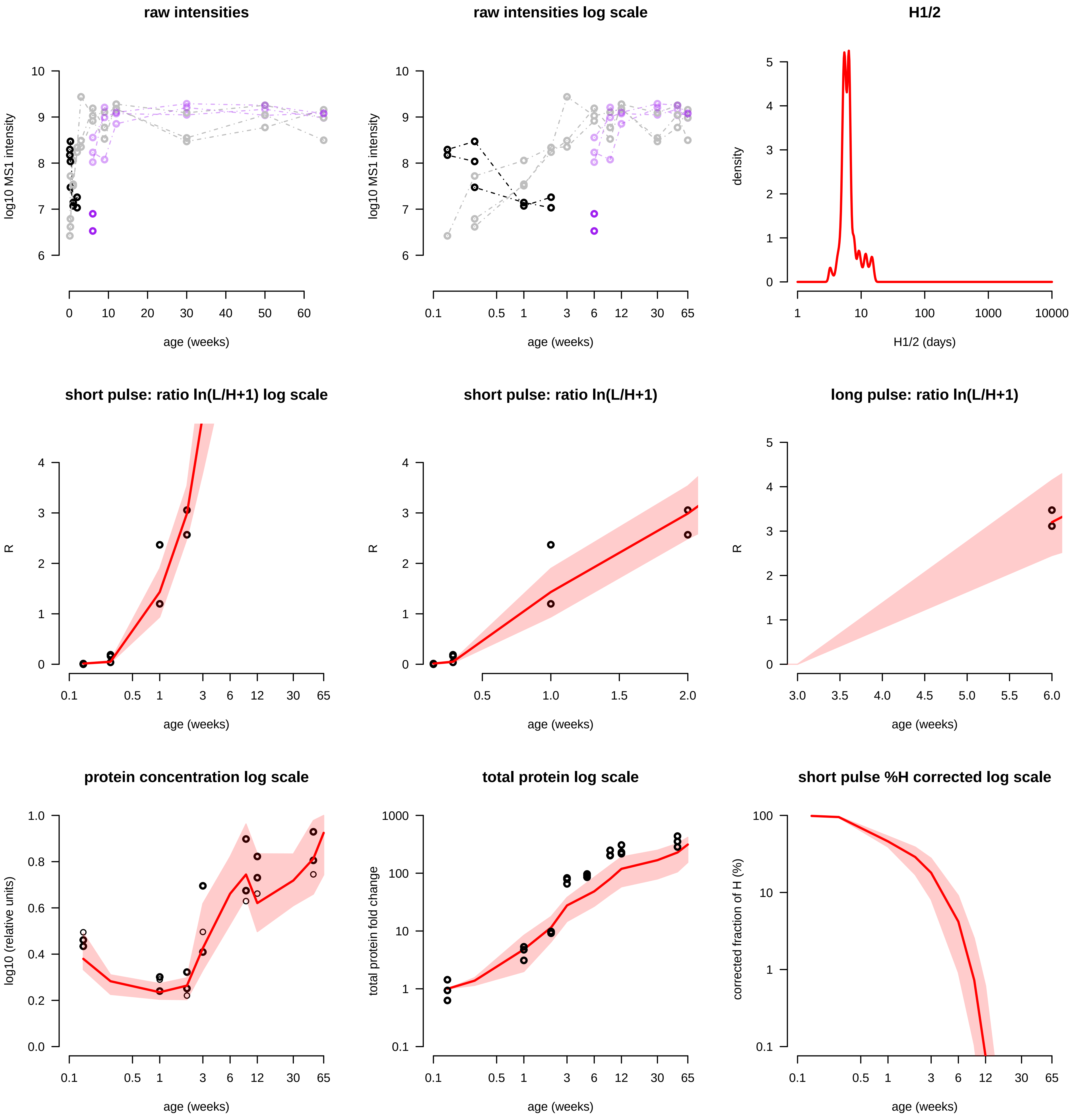

Q78ZA7

>sp|Q78ZA7|NP1L4_MOUSE

Nucleosome assembly protein 1−like 4

GN=Nap1l4

Med H1/2: 6.4 days

Q5 H1/2: 5.3 days

Q95 H1/2: 7.7 days

reliable model fit: yes

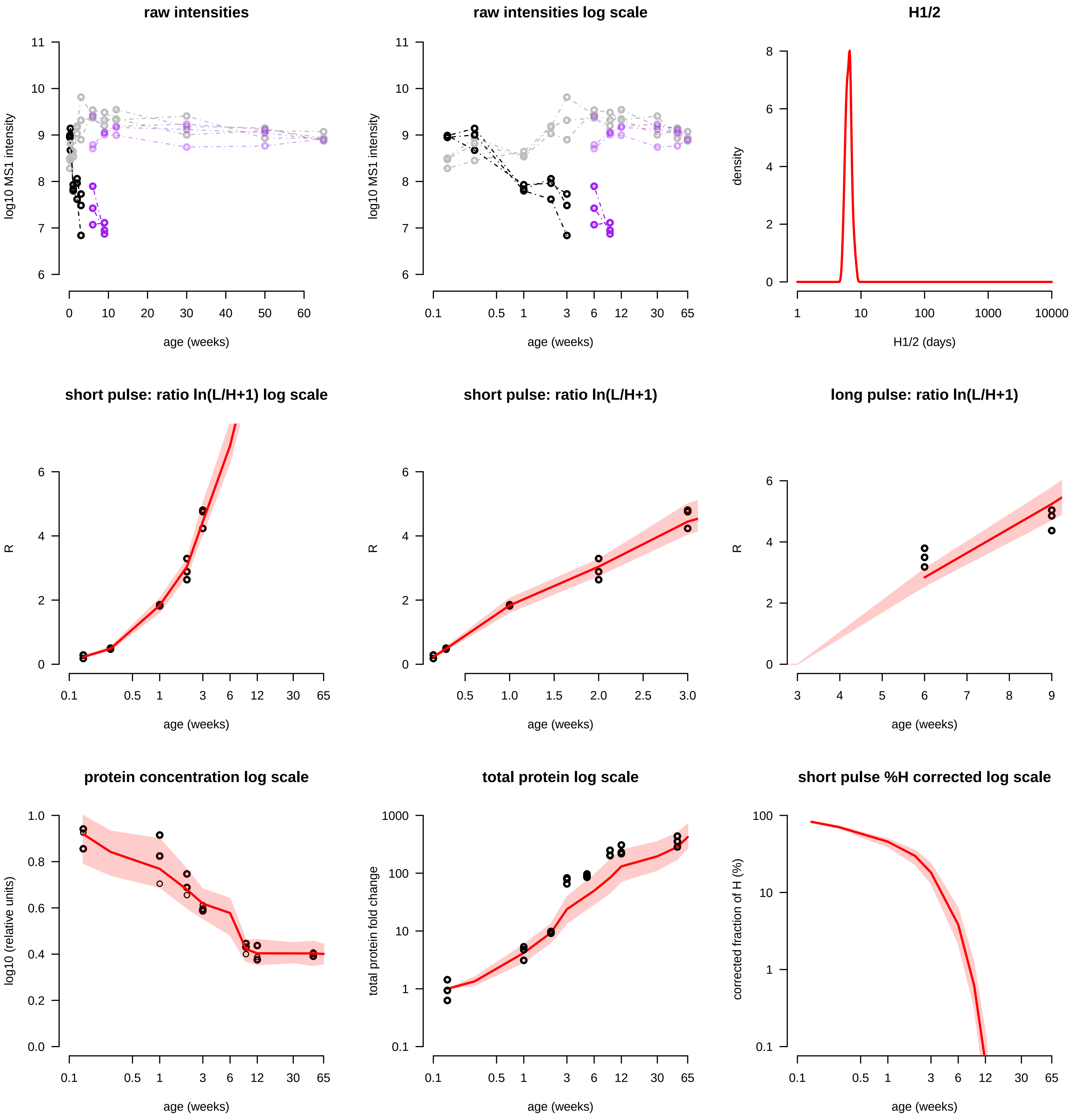

Q91YT0

>sp|Q91YT0|NDUV1_MOUSE

NADH dehydrogenase [ubiquinone] flavoprotein 1, mitochondrial

GN=Ndufv1

Med H1/2: 5.3 days

Q5 H1/2: 3.8 days

Q95 H1/2: 7.6 days

reliable model fit: yes

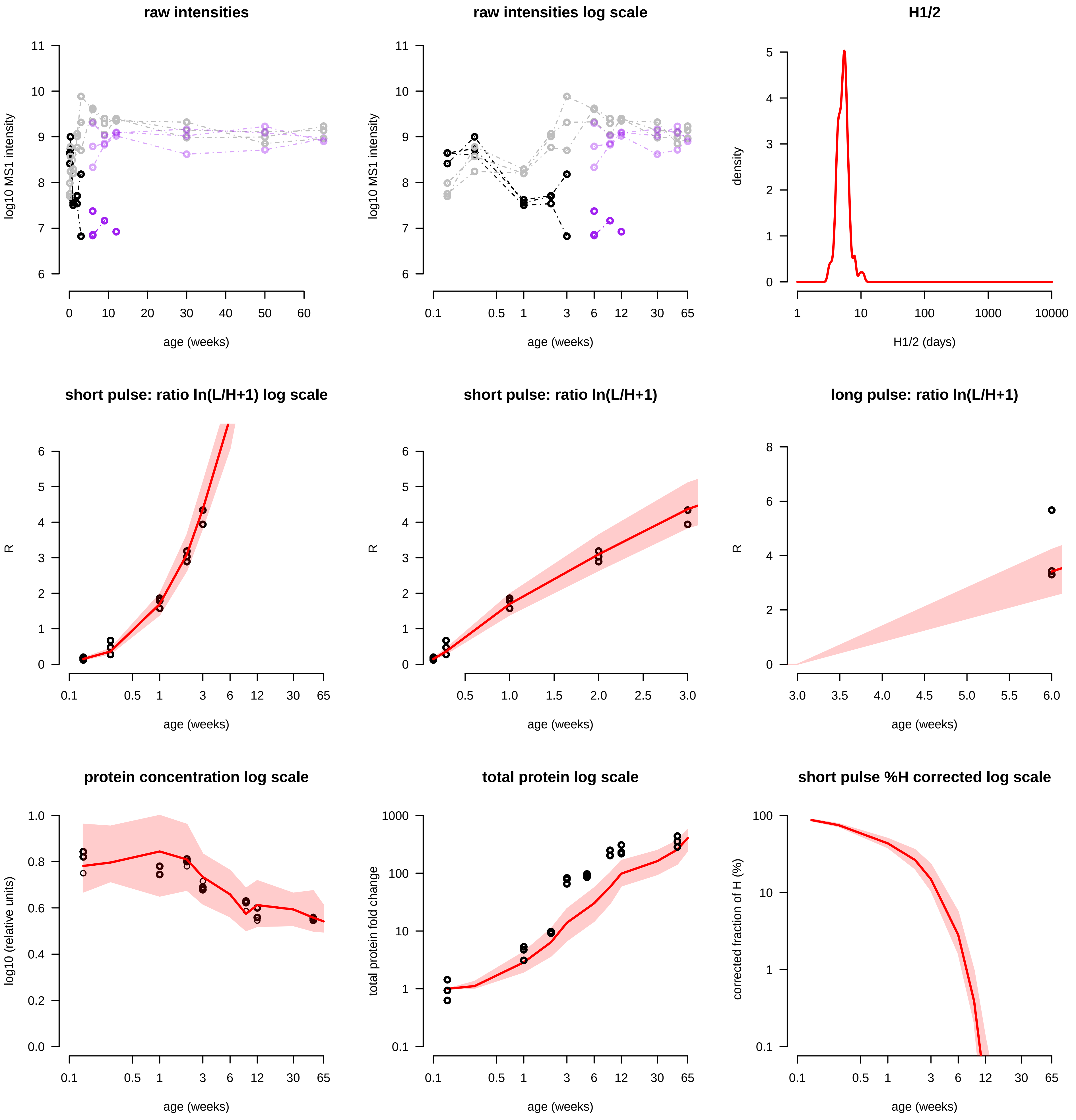

Q9CX34

>sp|Q9CX34|SGT1_MOUSE

Protein SGT1 homolog

GN=Sugt1

Med H1/2: 98.2 days

Q5 H1/2: 43.3 days

Q95 H1/2: 3837.8 days

reliable model fit: no

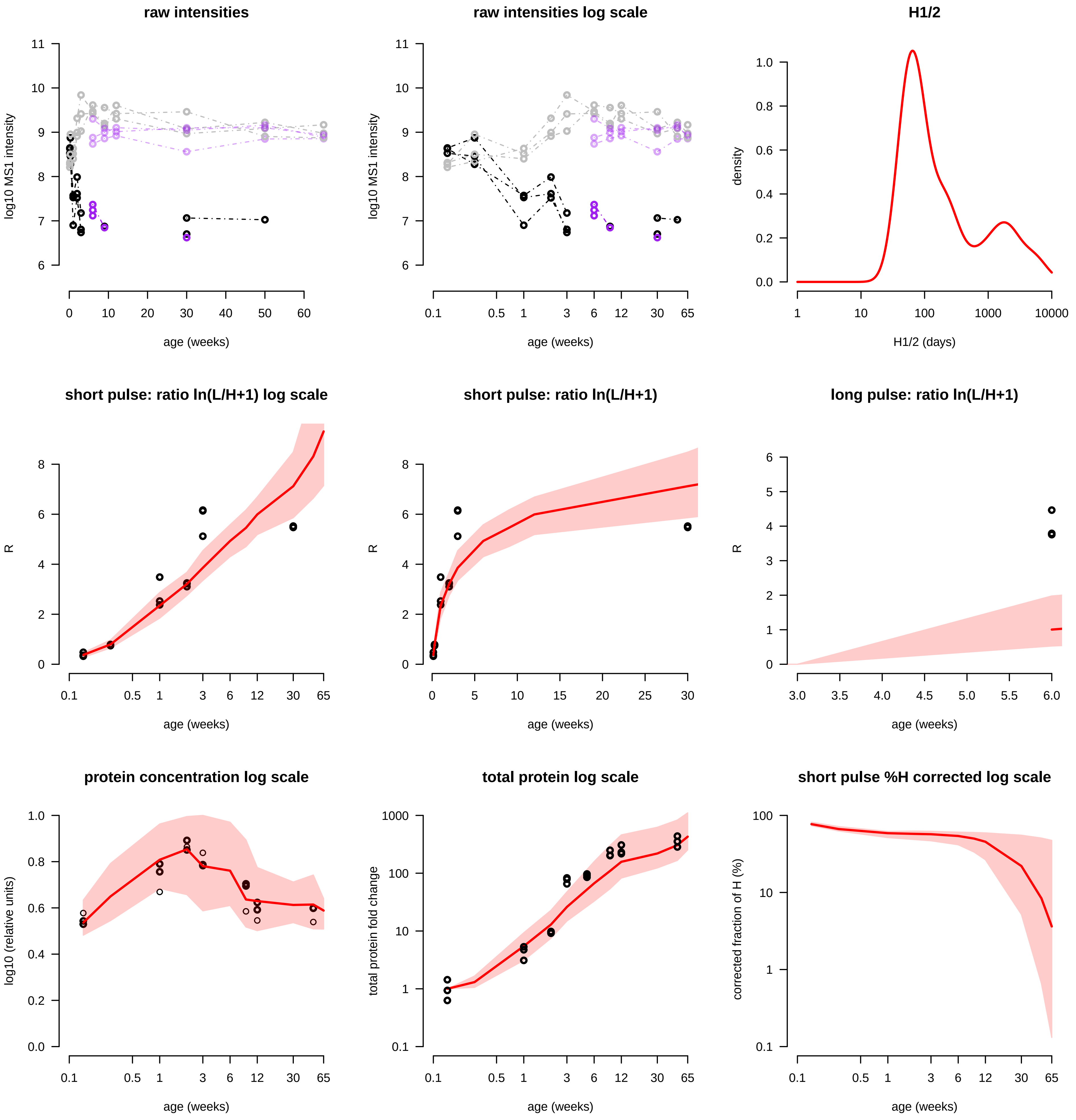

P46735;P46735−2

>sp|P46735|MYO1B_MOUSE

Unconventional myosin−Ib

GN=Myo1b

Med H1/2: 1302.9 days

Q5 H1/2: 283.6 days

Q95 H1/2: 15789.4 days

reliable model fit: yes

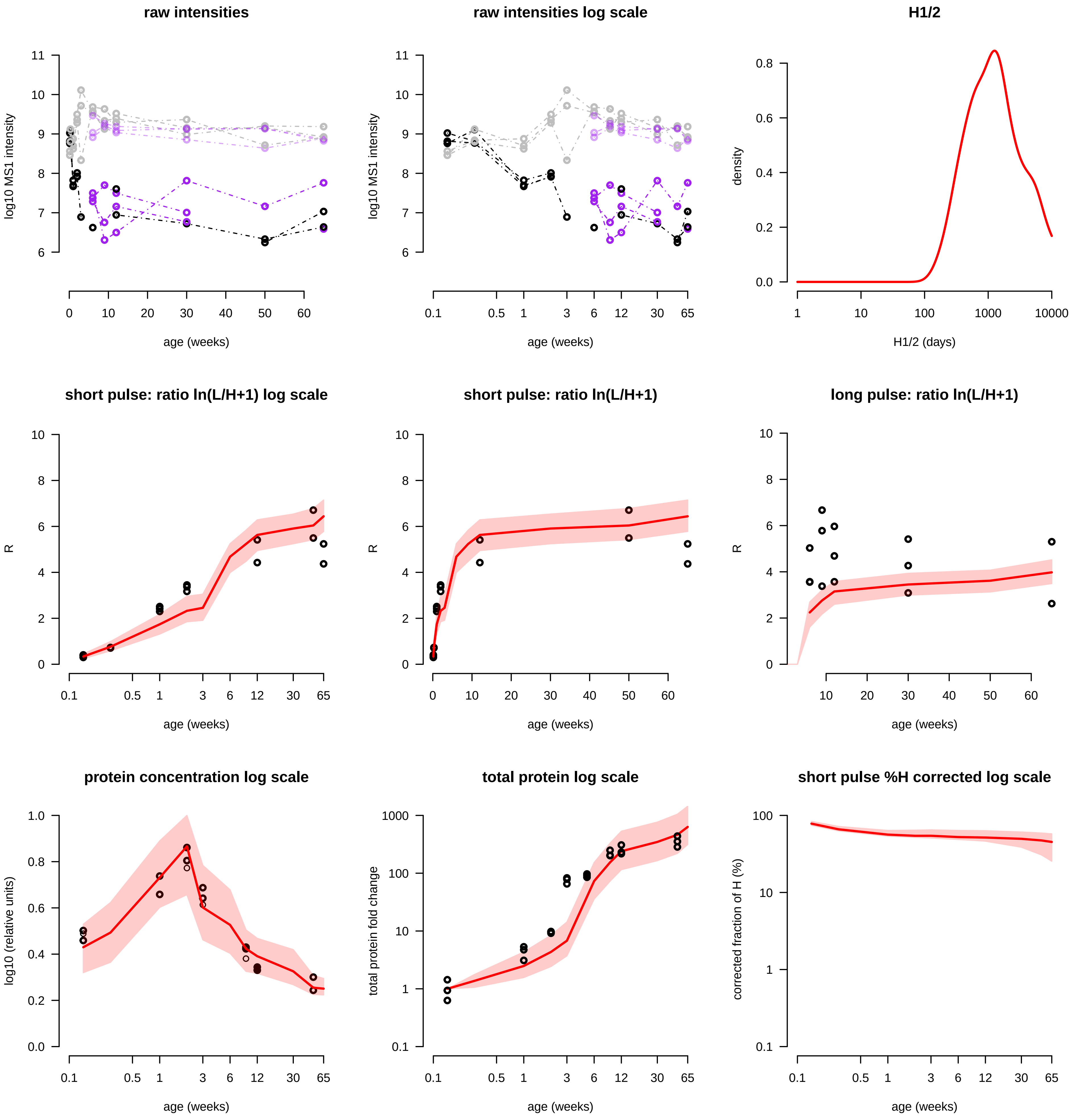

P46978

>sp|P46978|STT3A_MOUSE

Dolichyl−diphosphooligosaccharide−−protein glycosyltransferase subunit STT3A

GN=Stt3a

Med H1/2: 5.2 days

Q5 H1/2: 3.9 days

Q95 H1/2: 7 days

reliable model fit: yes

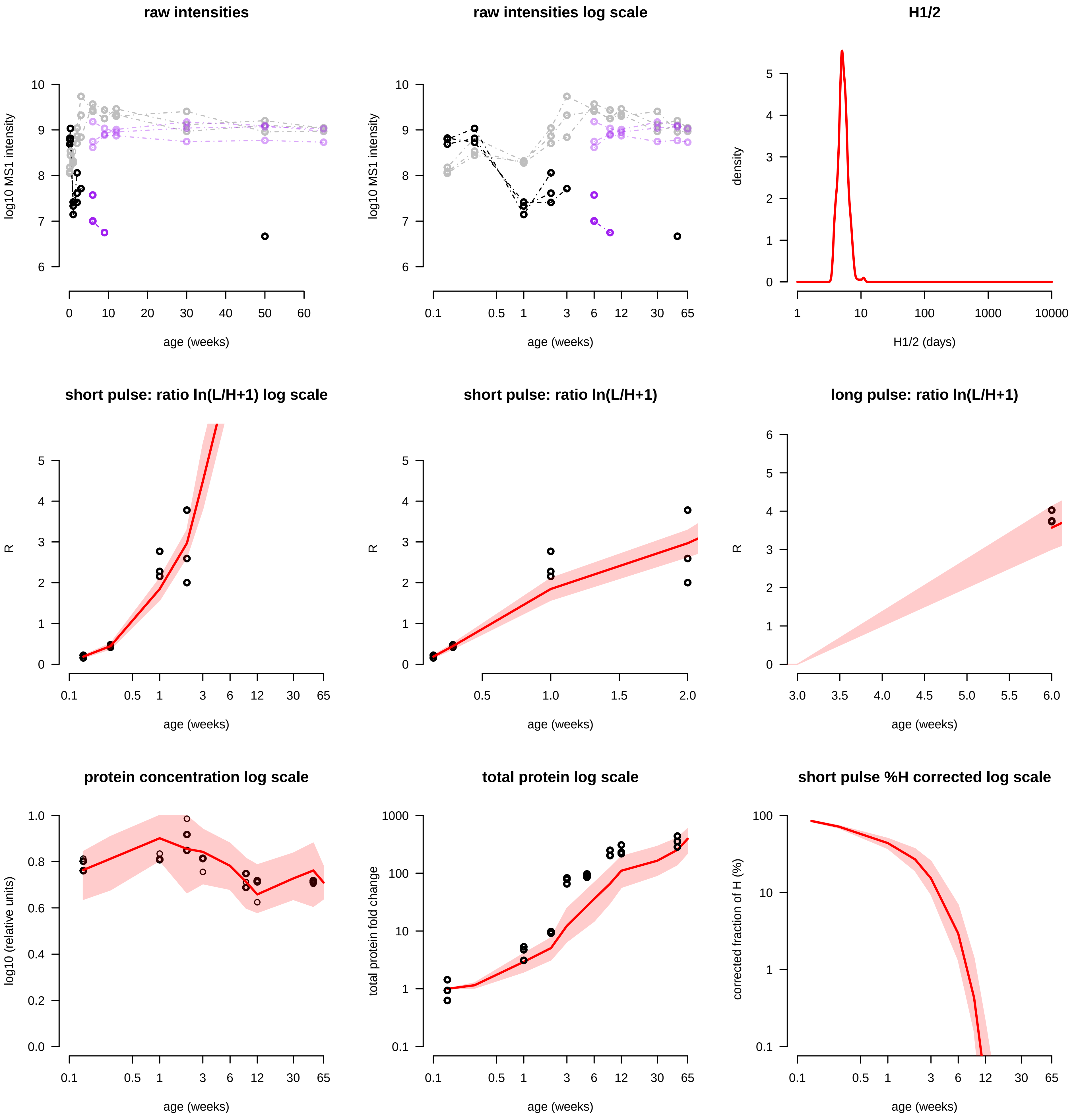

P42227

>sp|P42227|STAT3_MOUSE

Signal transducer and activator of transcription 3

GN=Stat3

Med H1/2: 3.9 days

Q5 H1/2: 3.1 days

Q95 H1/2: 4.9 days

reliable model fit: yes

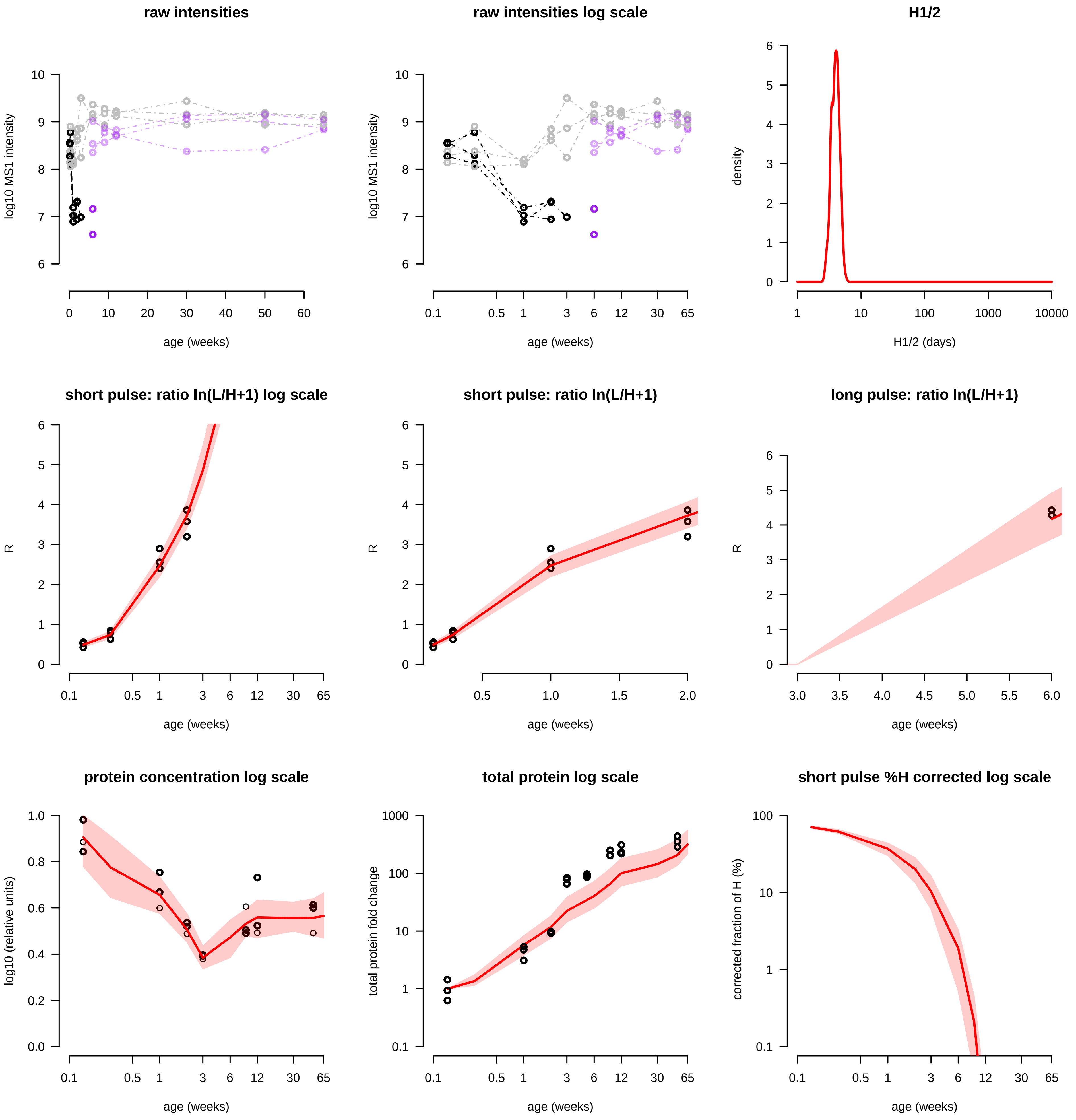

P18653

>sp|P18653|KS6A1_MOUSE

Ribosomal protein S6 kinase alpha−1

GN=Rps6ka1

Med H1/2: 4.1 days

Q5 H1/2: 3.2 days

Q95 H1/2: 5.5 days

reliable model fit: yes

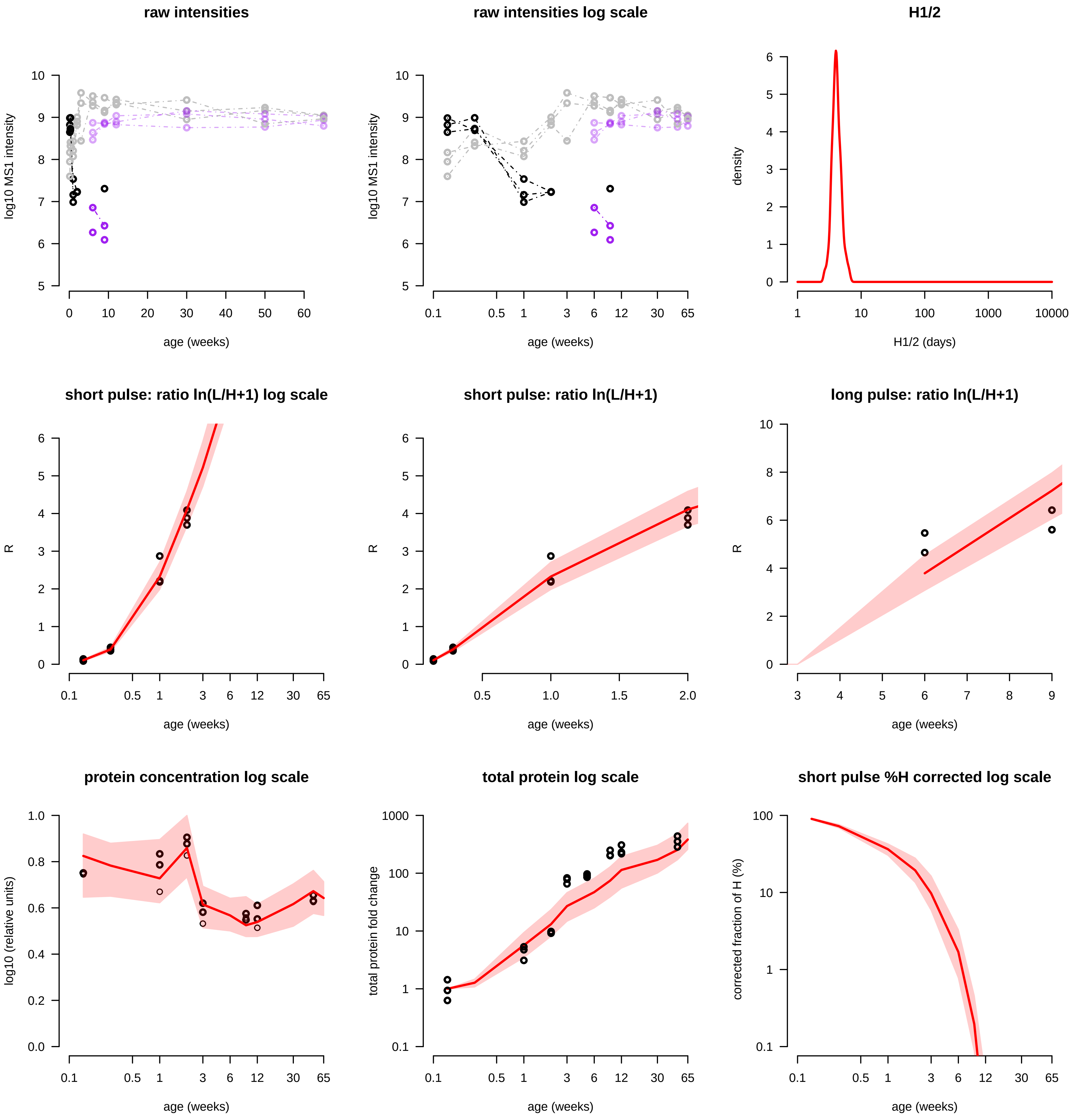

Q91WJ8

>sp|Q91WJ8|FUBP1_MOUSE

Far upstream element−binding protein 1

GN=Fubp1

Med H1/2: 4.8 days

Q5 H1/2: 3.9 days

Q95 H1/2: 5.9 days

reliable model fit: yes

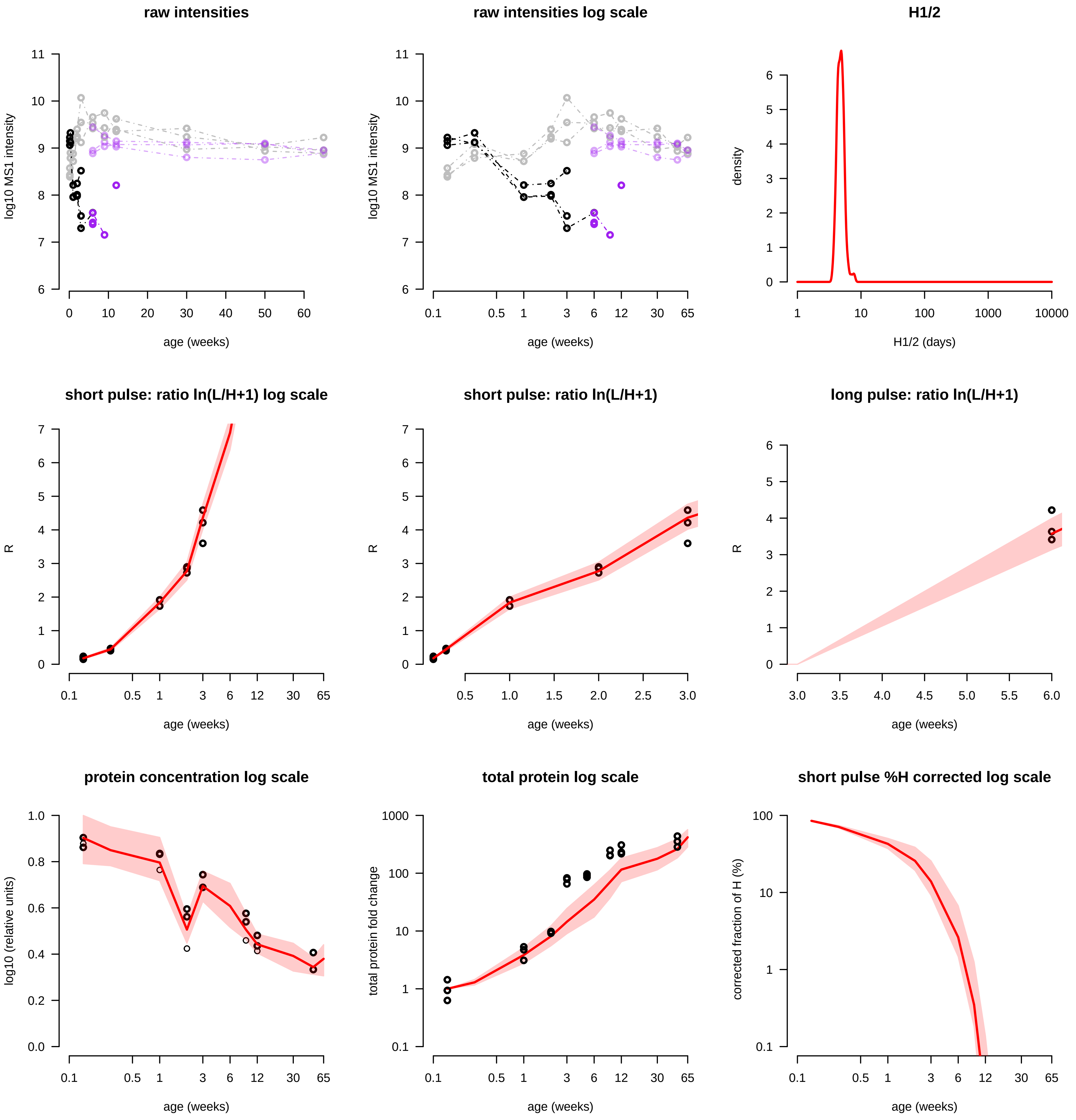

P26516

>sp|P26516|PSMD7_MOUSE

26S proteasome non−ATPase regulatory subunit 7

GN=Psmd7

Med H1/2: 7.2 days

Q5 H1/2: 5.9 days

Q95 H1/2: 9.3 days

reliable model fit: yes

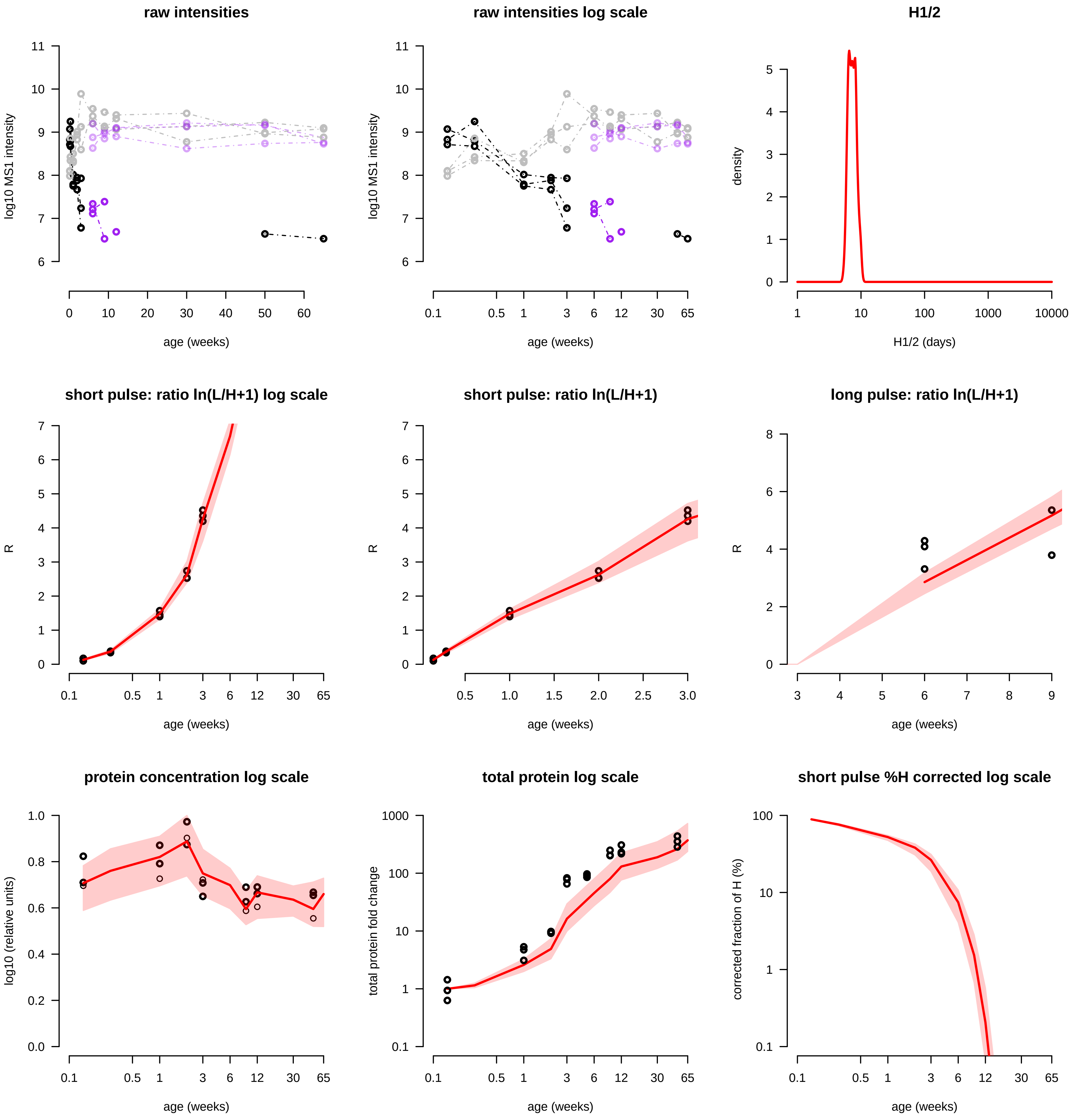

Q6P1B1

>sp|Q6P1B1|XPP1_MOUSE

Xaa−Pro aminopeptidase 1

GN=Xpnpep1

Med H1/2: 6.4 days

Q5 H1/2: 5.1 days

Q95 H1/2: 9.1 days

reliable model fit: yes

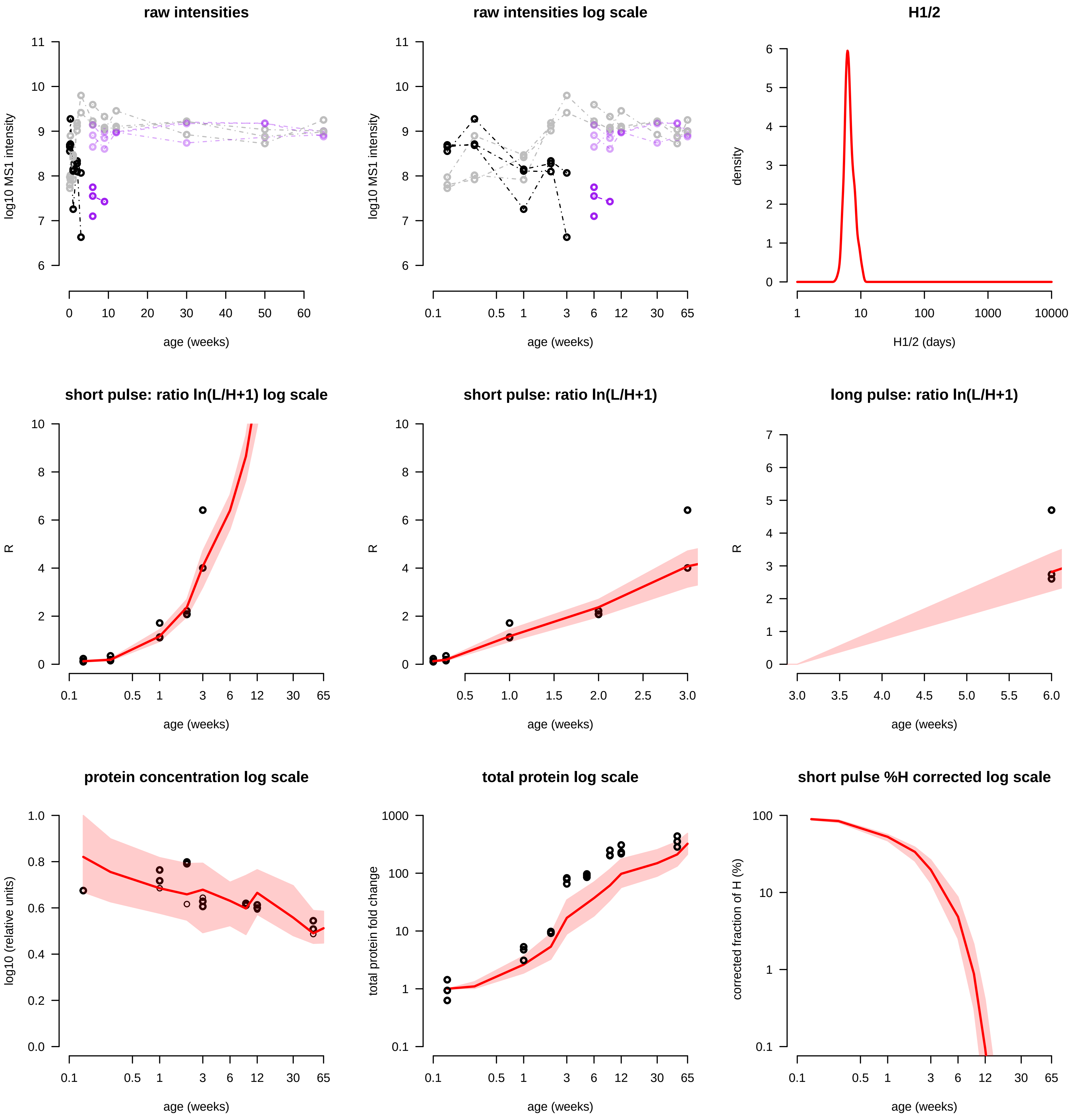

Q6A4J8−2

>sp|Q6A4J8−2|UBP7_MOUSE

Isoform 2 of Ubiquitin carboxyl−terminal hydrolase 7

GN=Usp7

Med H1/2: 59.1 days

Q5 H1/2: 42.1 days

Q95 H1/2: 129.8 days

reliable model fit: no

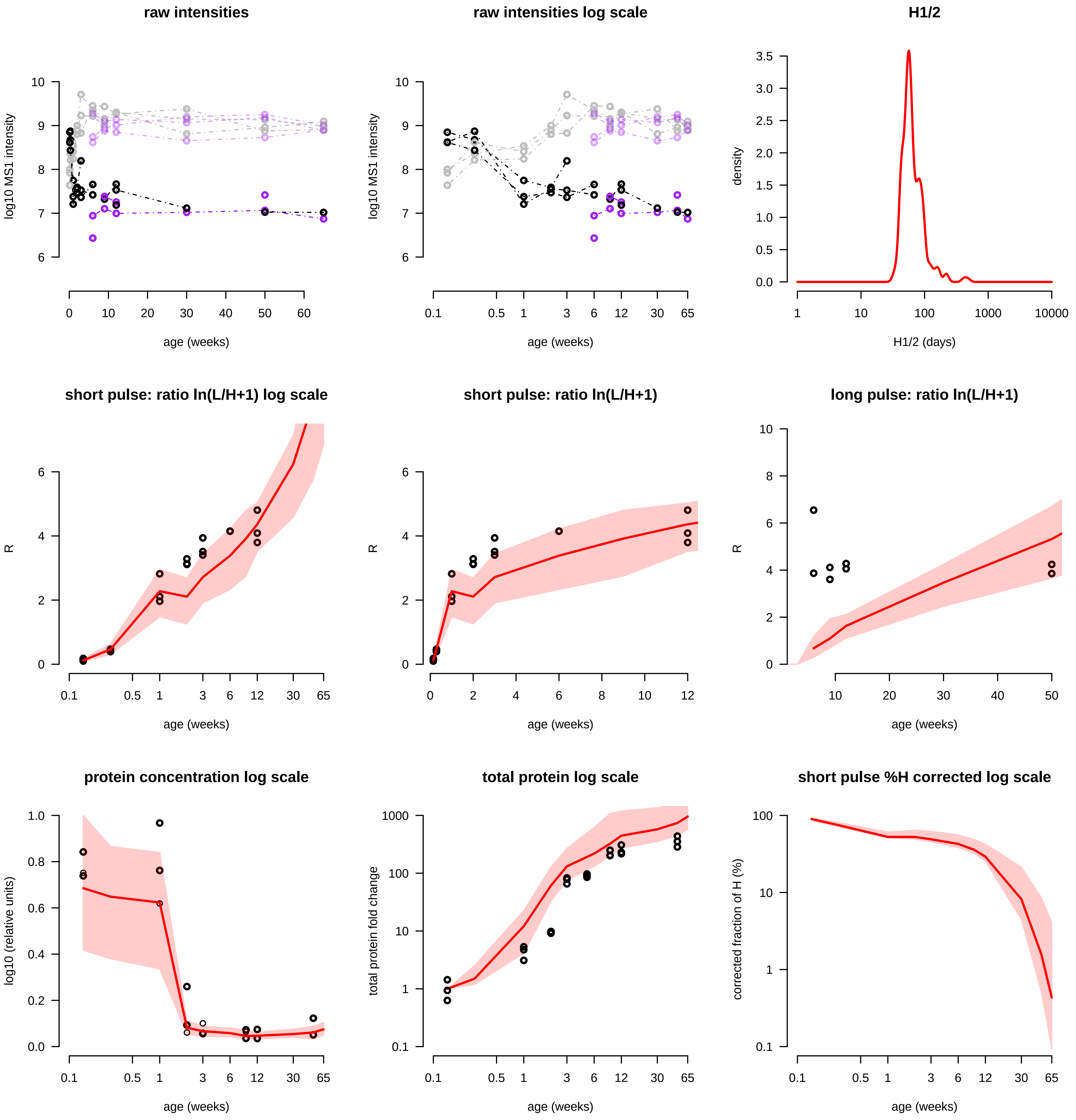

E9Q3L2

>sp|E9Q3L2|PI4KA_MOUSE

Phosphatidylinositol 4−kinase alpha

GN=Pi4ka

Med H1/2: 395.3 days

Q5 H1/2: 134.7 days

Q95 H1/2: 3797.6 days

reliable model fit: no

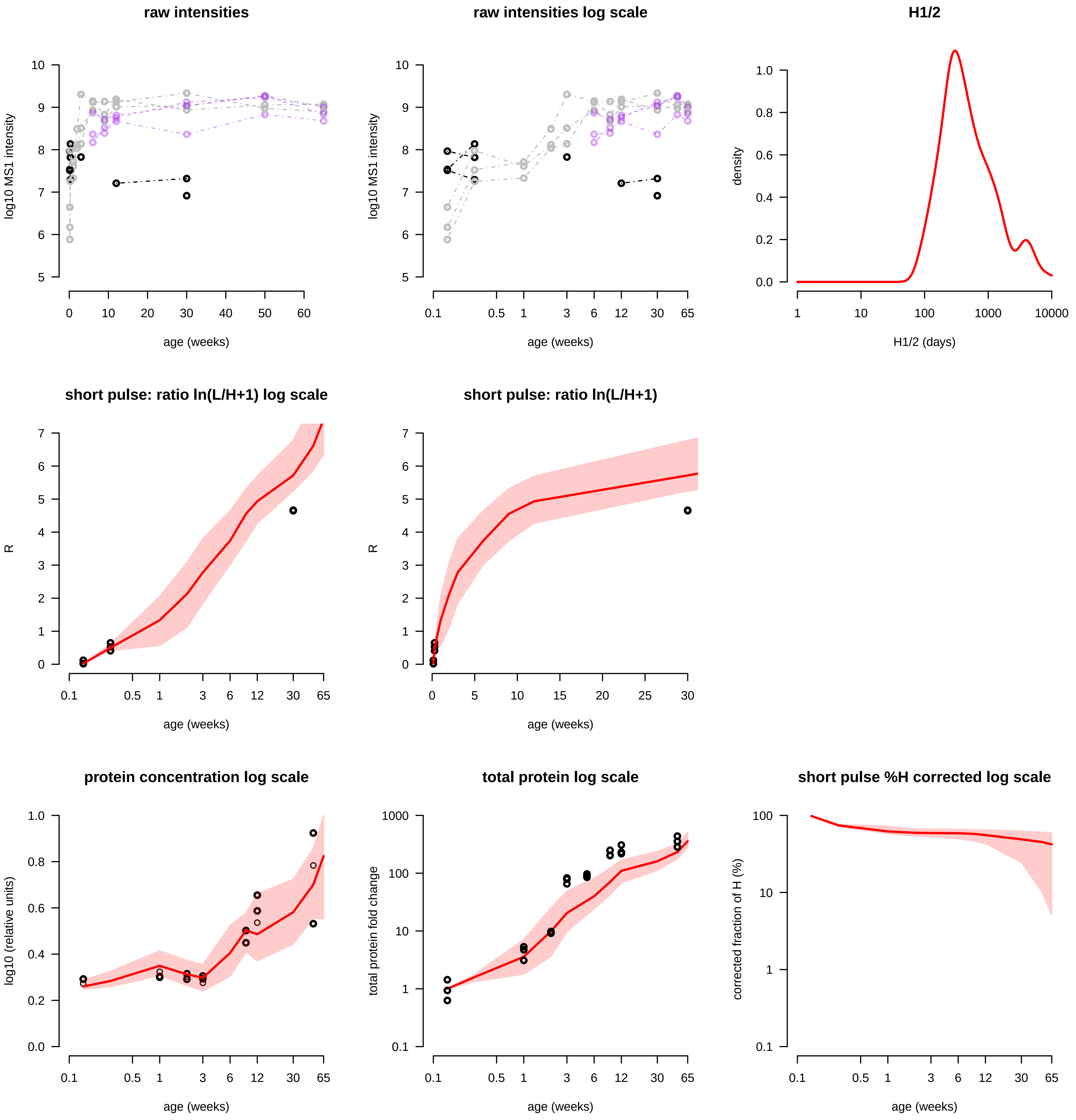

Q02248

>sp|Q02248|CTNB1_MOUSE

Catenin beta−1

GN=Ctnnb1

Med H1/2: 3.4 days

Q5 H1/2: 2.9 days

Q95 H1/2: 4.1 days

reliable model fit: yes

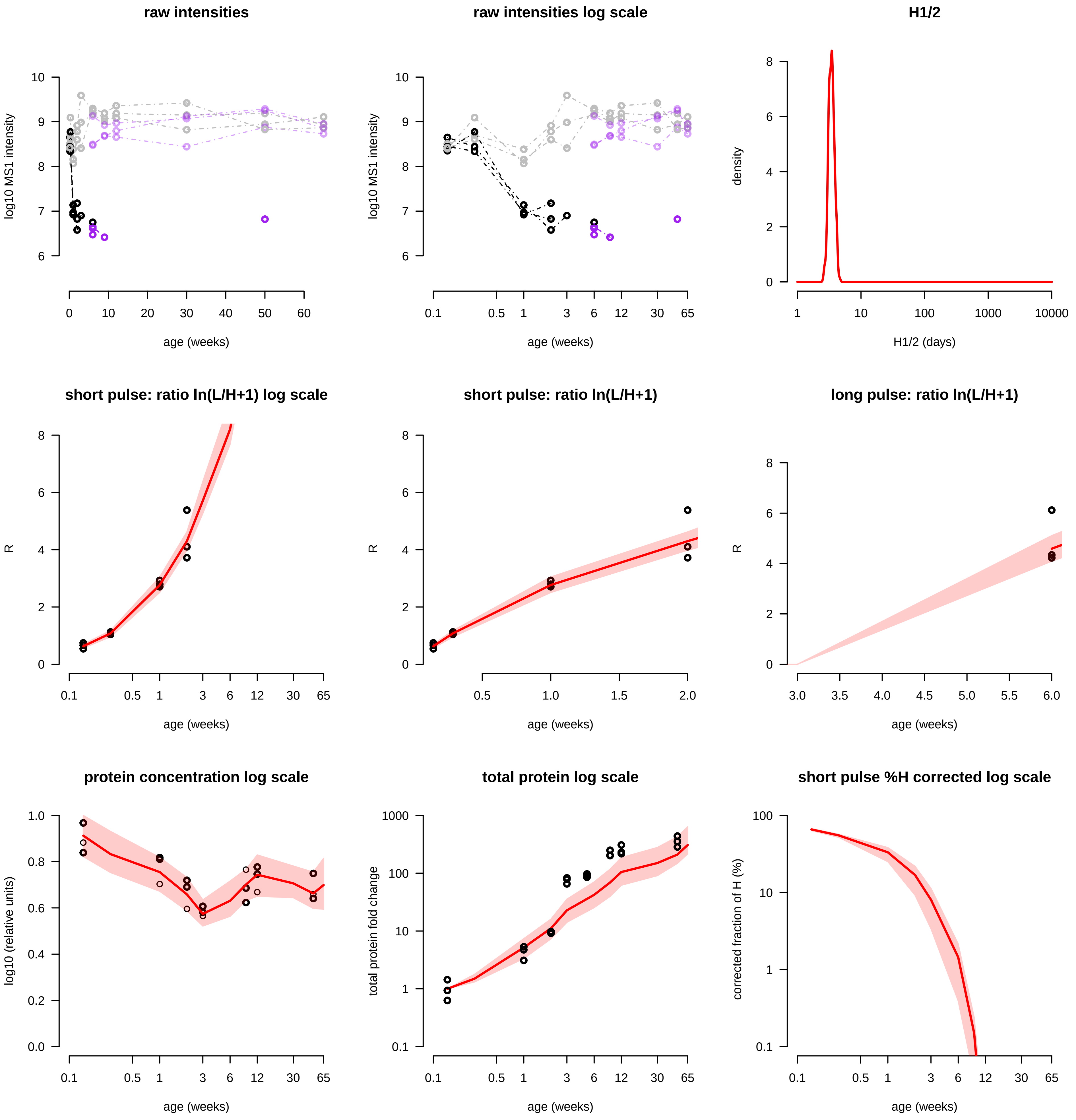

Q9Z1D1

>sp|Q9Z1D1|EIF3G_MOUSE

Eukaryotic translation initiation factor 3 subunit G

GN=Eif3g

Med H1/2: 3.4 days

Q5 H1/2: 2.8 days

Q95 H1/2: 4.5 days

reliable model fit: yes

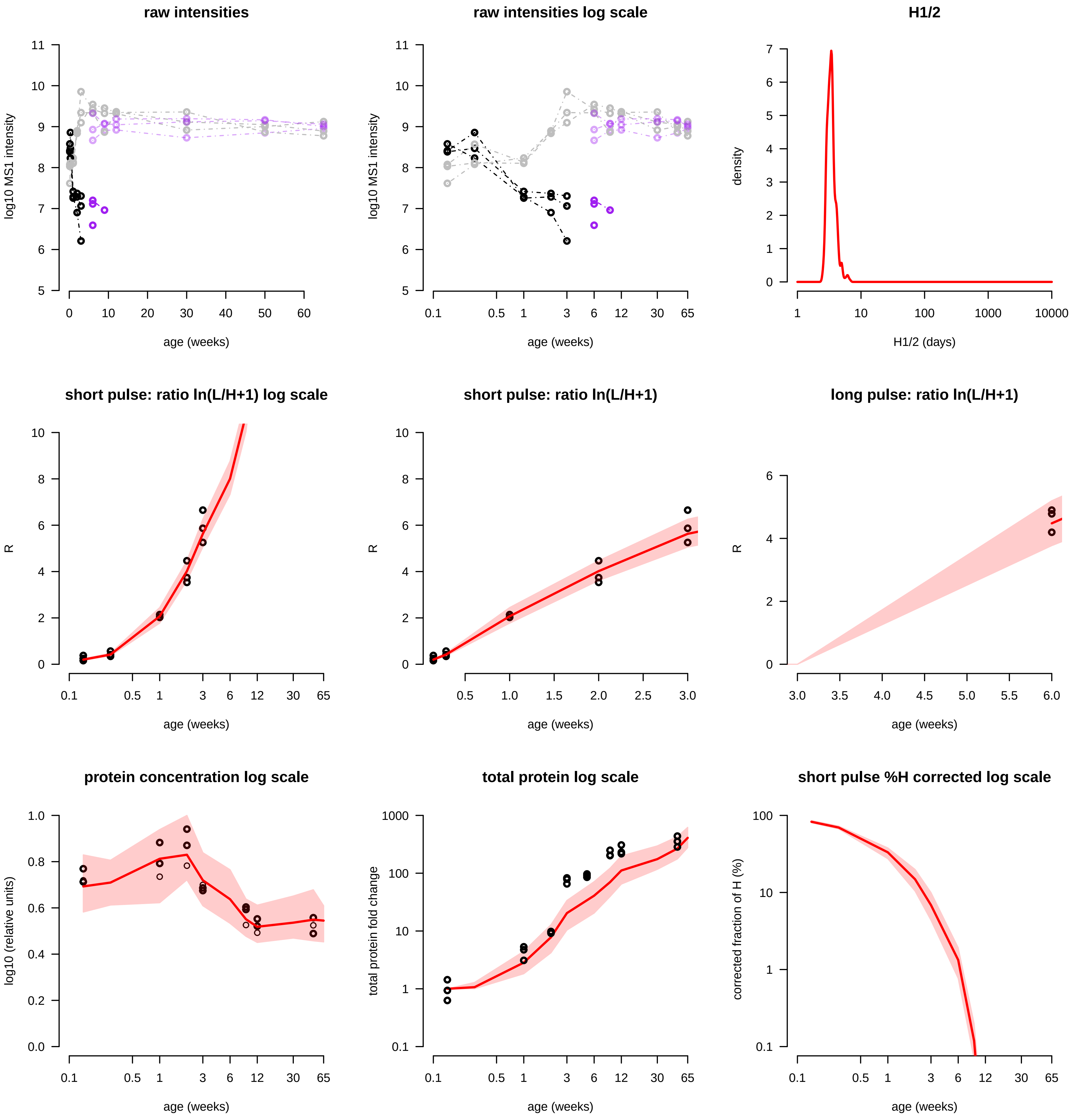

Q8BJW6;Q8BJW6−2

>sp|Q8BJW6|EIF2A_MOUSE

Eukaryotic translation initiation factor 2A

GN=Eif2a

Med H1/2: 3.4 days

Q5 H1/2: 2.9 days

Q95 H1/2: 4 days

reliable model fit: yes

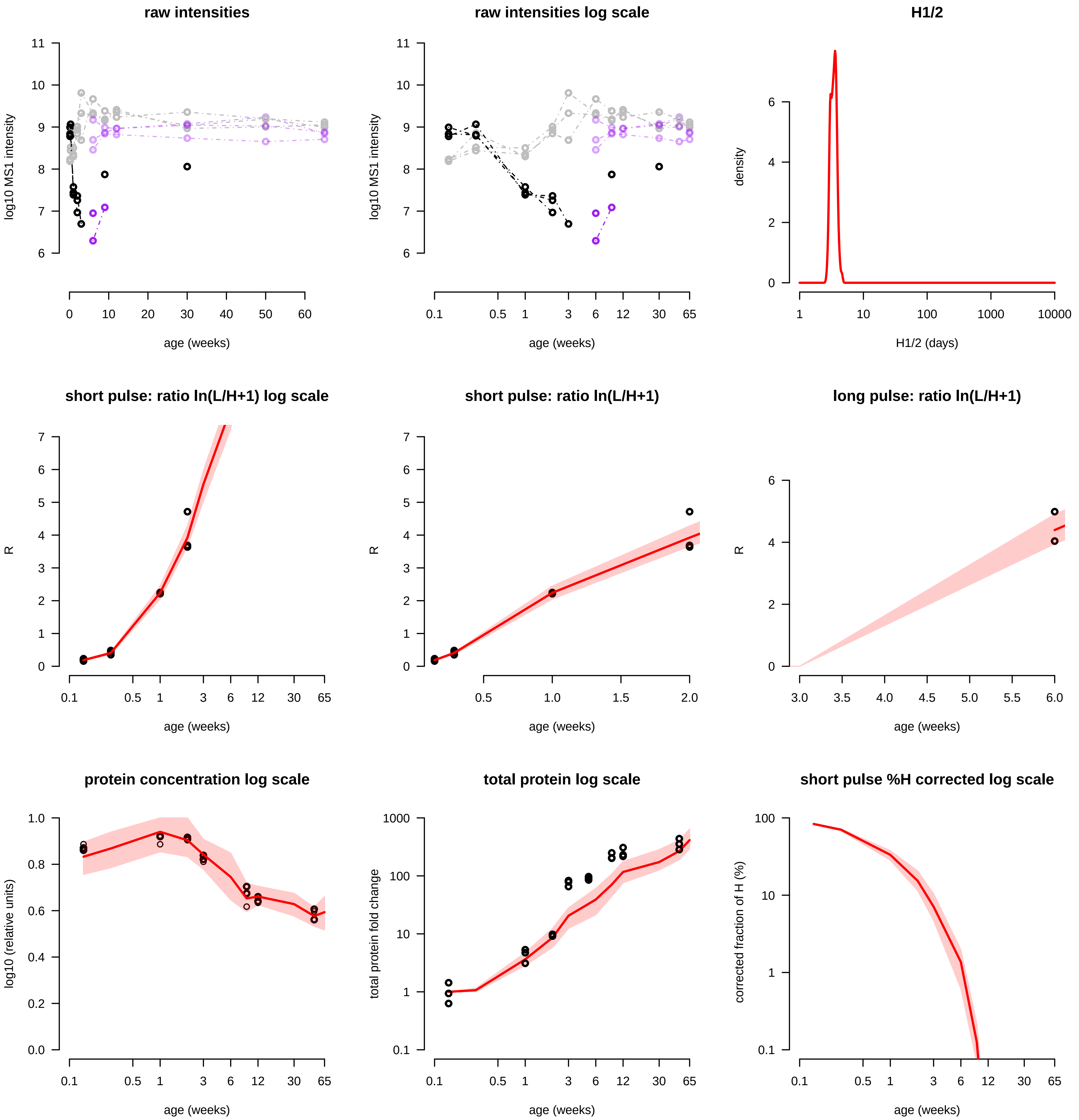

Q9CX80

>sp|Q9CX80|CYGB_MOUSE

Cytoglobin

GN=Cygb

Med H1/2: 5.3 days

Q5 H1/2: 3.7 days

Q95 H1/2: 8.2 days

reliable model fit: yes

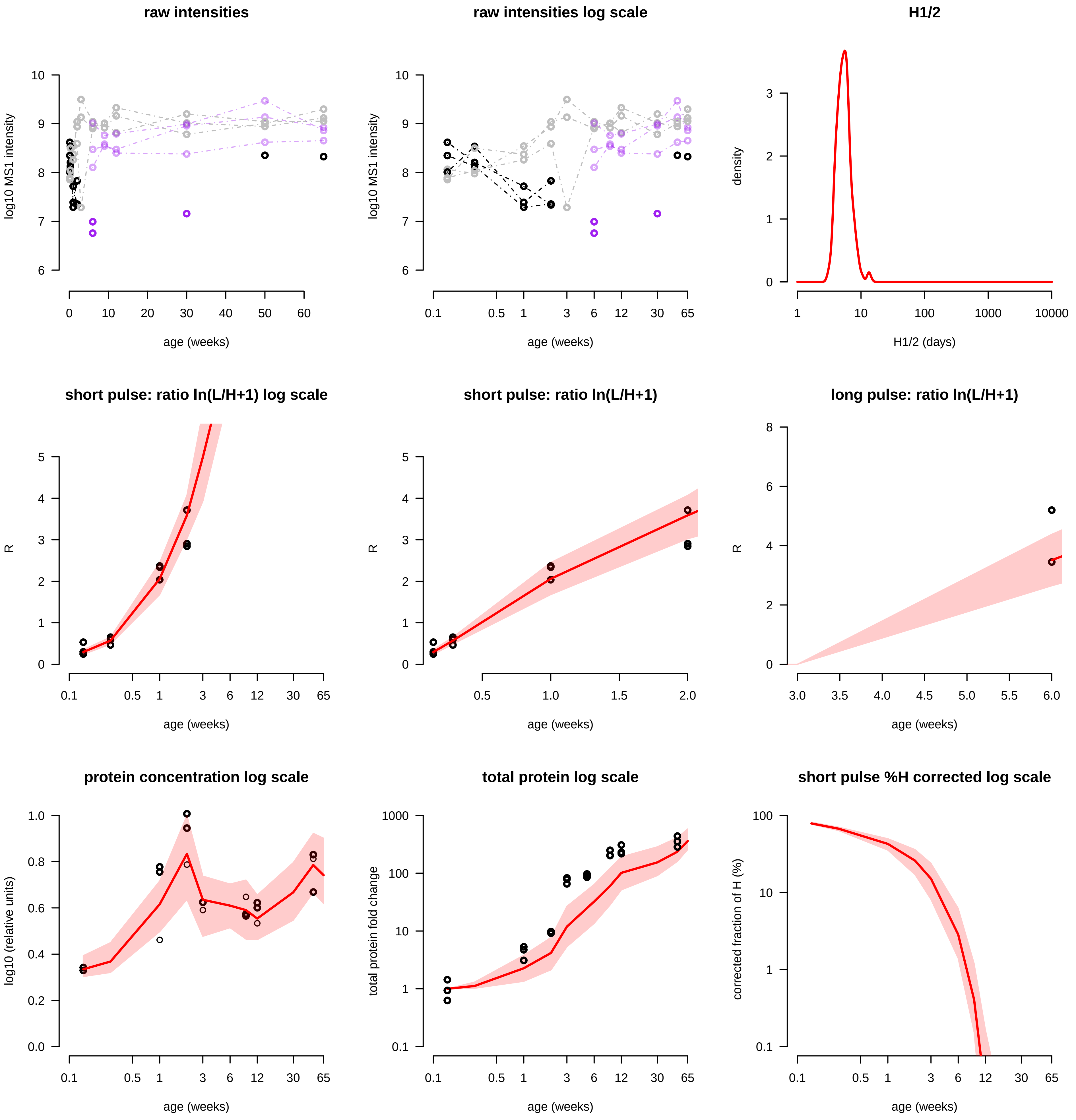

Q62148

>sp|Q62148|AL1A2_MOUSE

Retinal dehydrogenase 2

GN=Aldh1a2

Med H1/2: 3.9 days

Q5 H1/2: 3.1 days

Q95 H1/2: 4.5 days

reliable model fit: yes

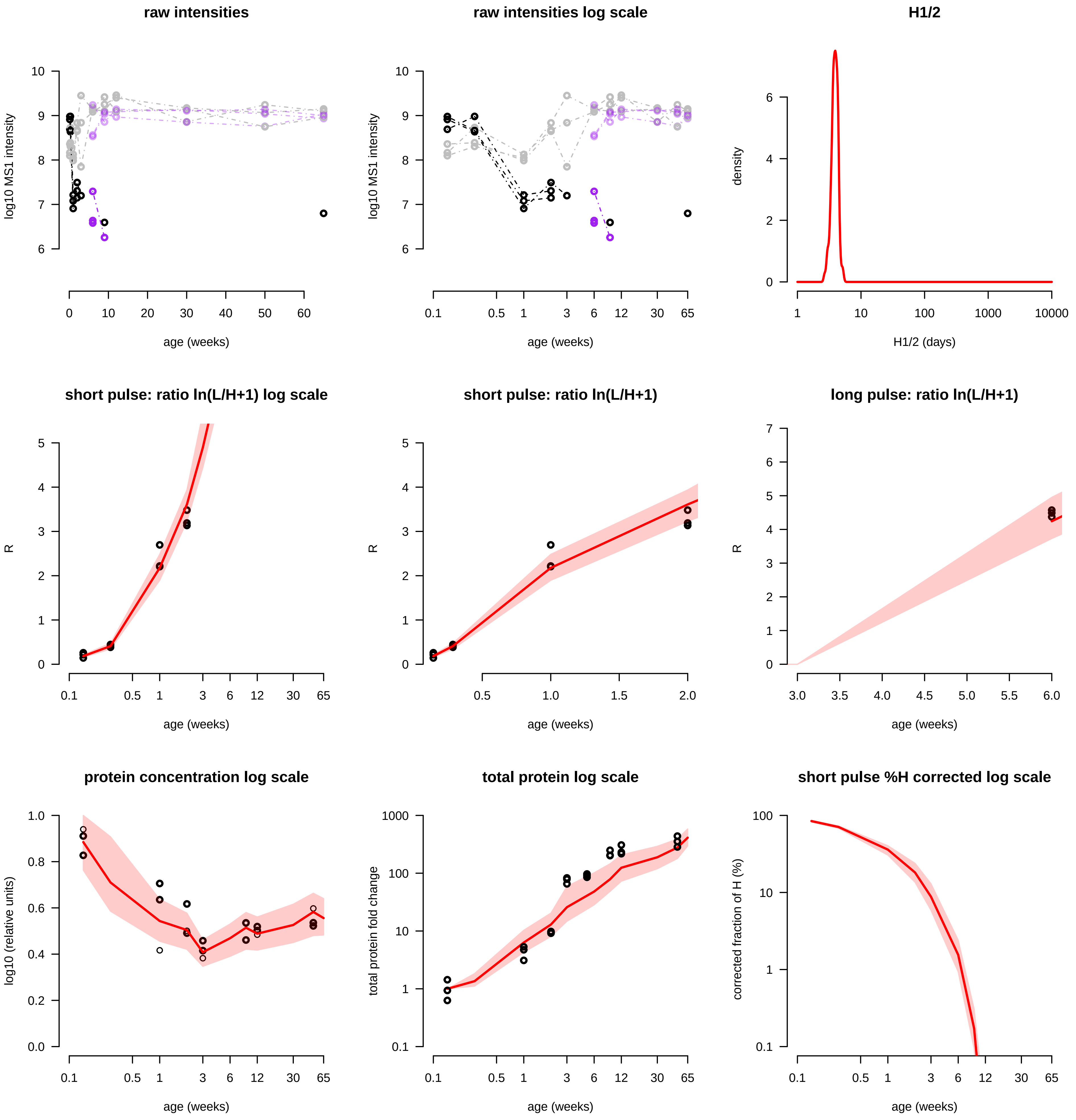

Q9D892

>sp|Q9D892|ITPA_MOUSE

Inosine triphosphate pyrophosphatase

GN=Itpa

Med H1/2: 4.4 days

Q5 H1/2: 3.5 days

Q95 H1/2: 5.8 days

reliable model fit: yes

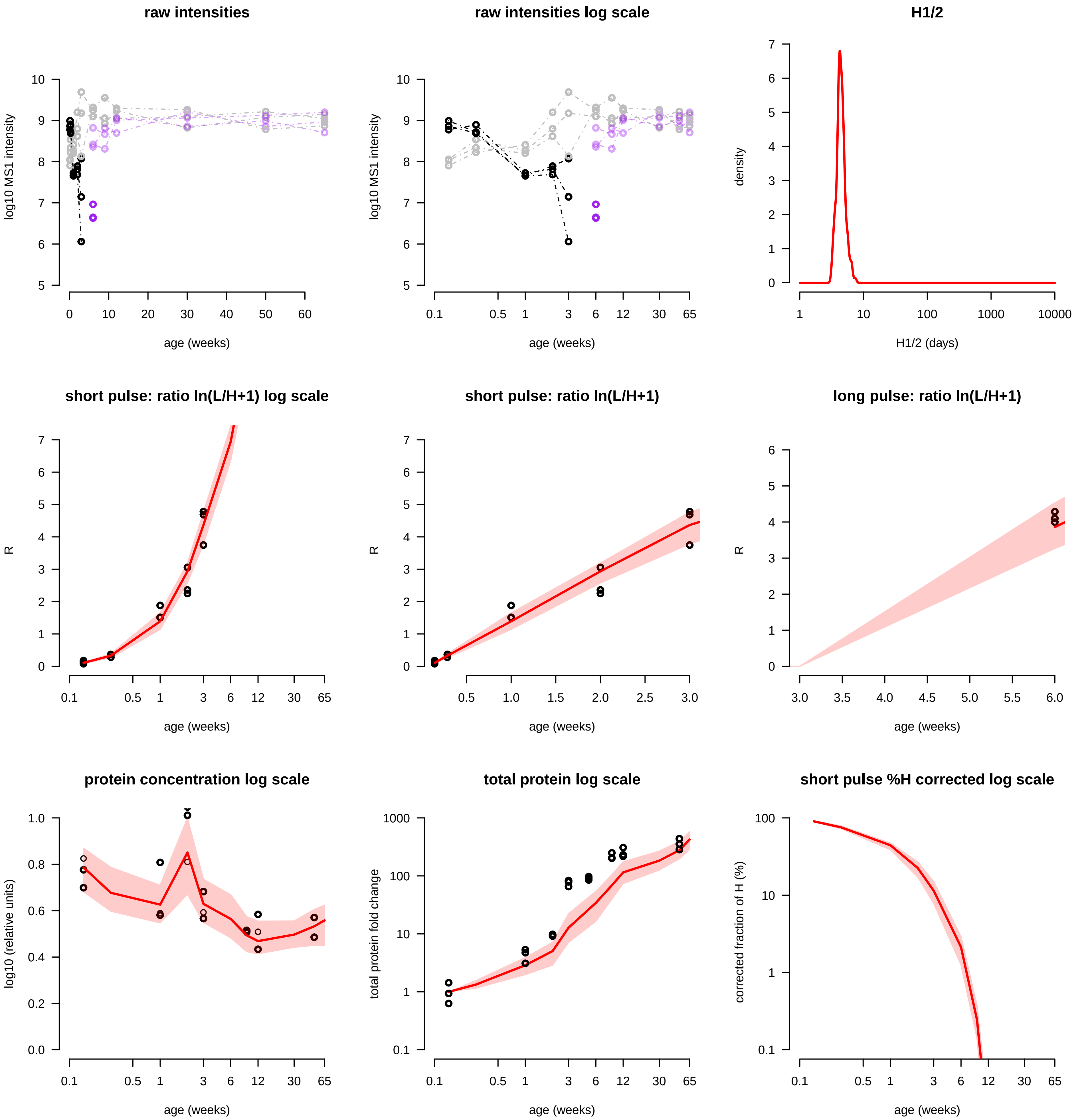

Q61247

>sp|Q61247|A2AP_MOUSE

Alpha−2−antiplasmin

GN=Serpinf2

Med H1/2: 7.8 days

Q5 H1/2: 3.4 days

Q95 H1/2: 34.1 days

reliable model fit: yes

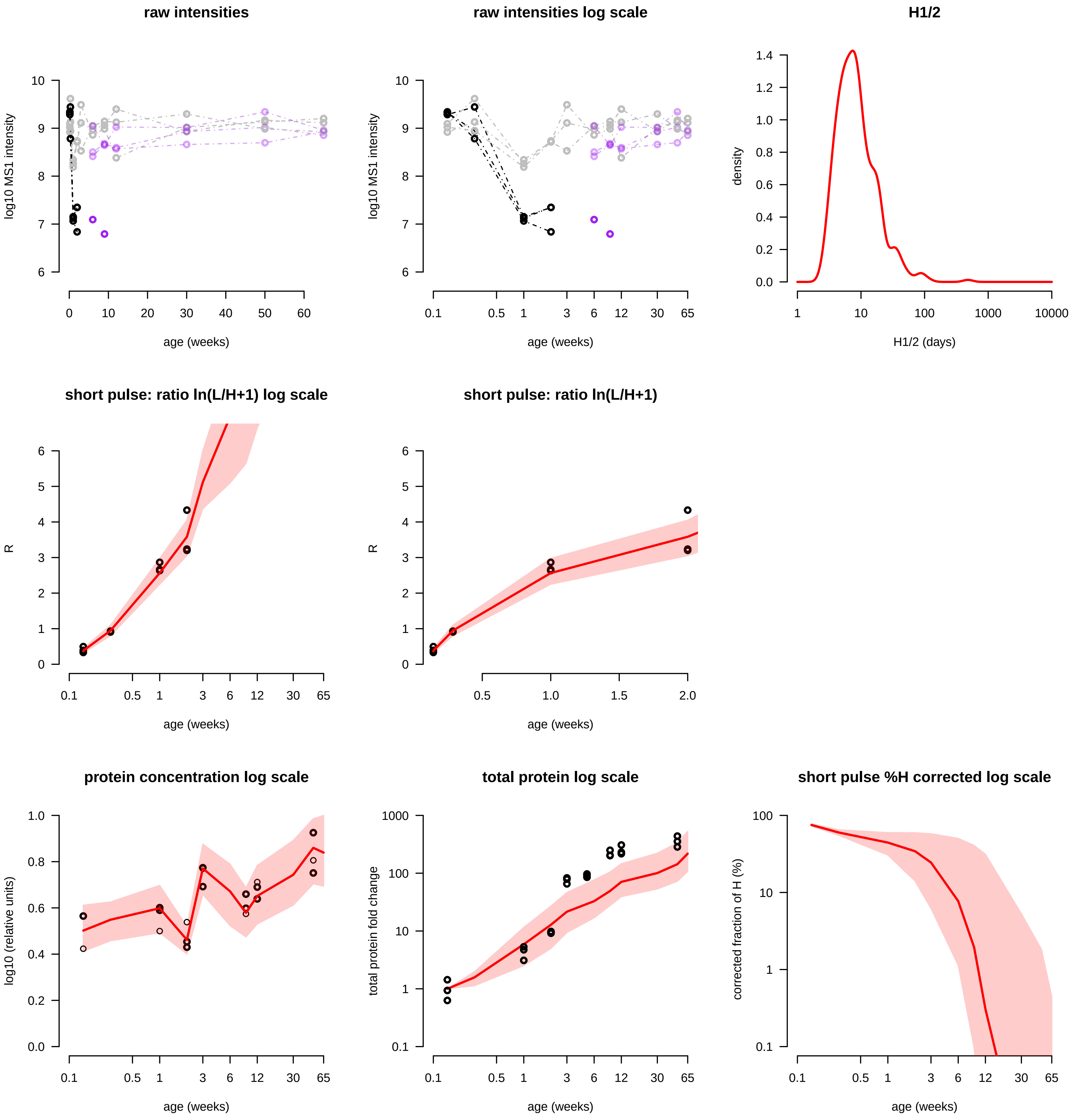

Q920B9

>sp|Q920B9|SP16H_MOUSE

FACT complex subunit SPT16

GN=Supt16h

Med H1/2: 3.9 days

Q5 H1/2: 3.3 days

Q95 H1/2: 4.5 days

reliable model fit: yes

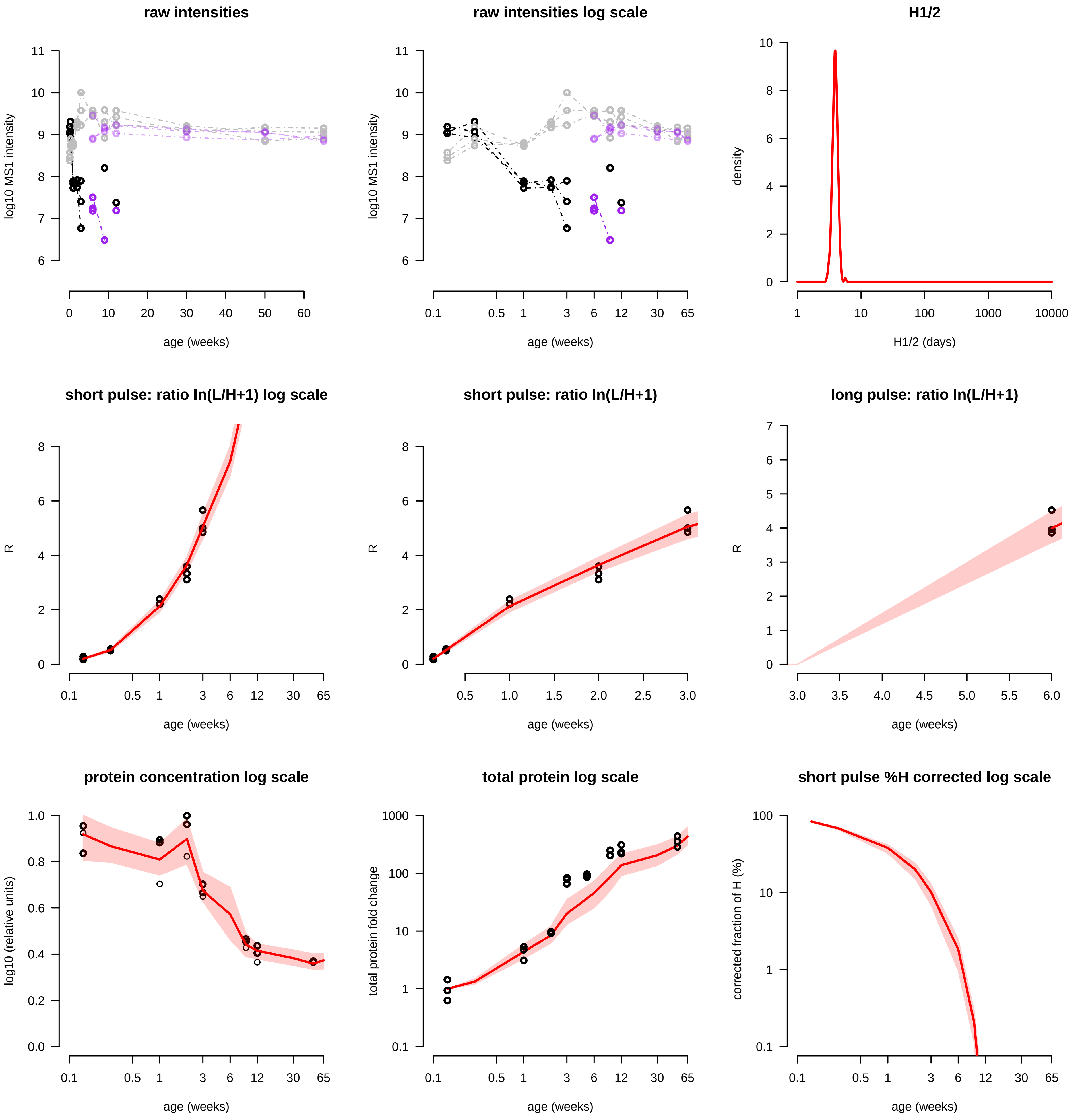

Q8CH18;Q8CH18−3

>sp|Q8CH18|CCAR1_MOUSE

Cell division cycle and apoptosis regulator protein 1

GN=Ccar1

Med H1/2: 3.9 days

Q5 H1/2: 3.2 days

Q95 H1/2: 4.6 days

reliable model fit: yes

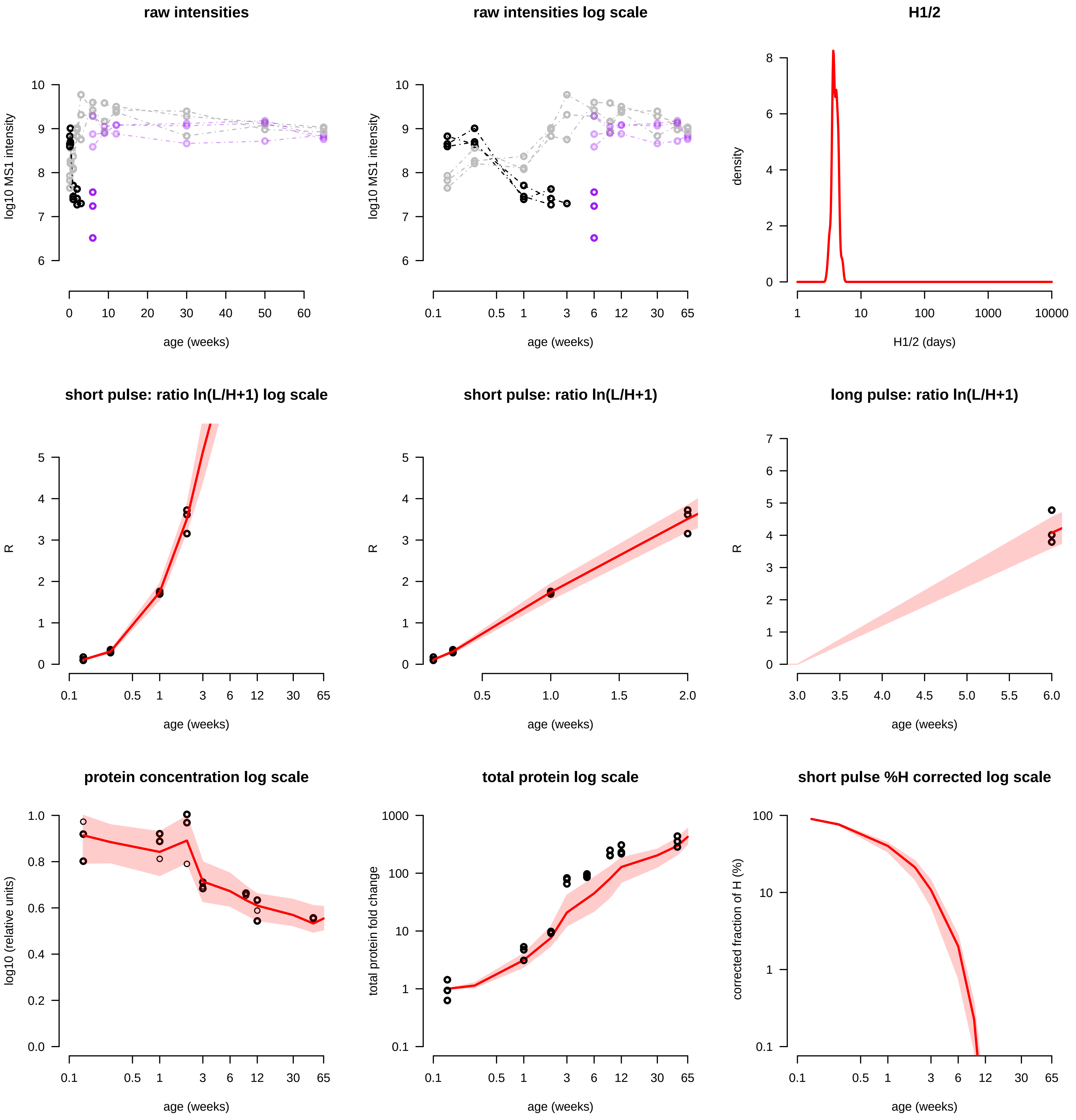

O54984

>sp|O54984|GET3_MOUSE

ATPase GET3

GN=Get3

Med H1/2: 4.9 days

Q5 H1/2: 3.6 days

Q95 H1/2: 8.4 days

reliable model fit: yes

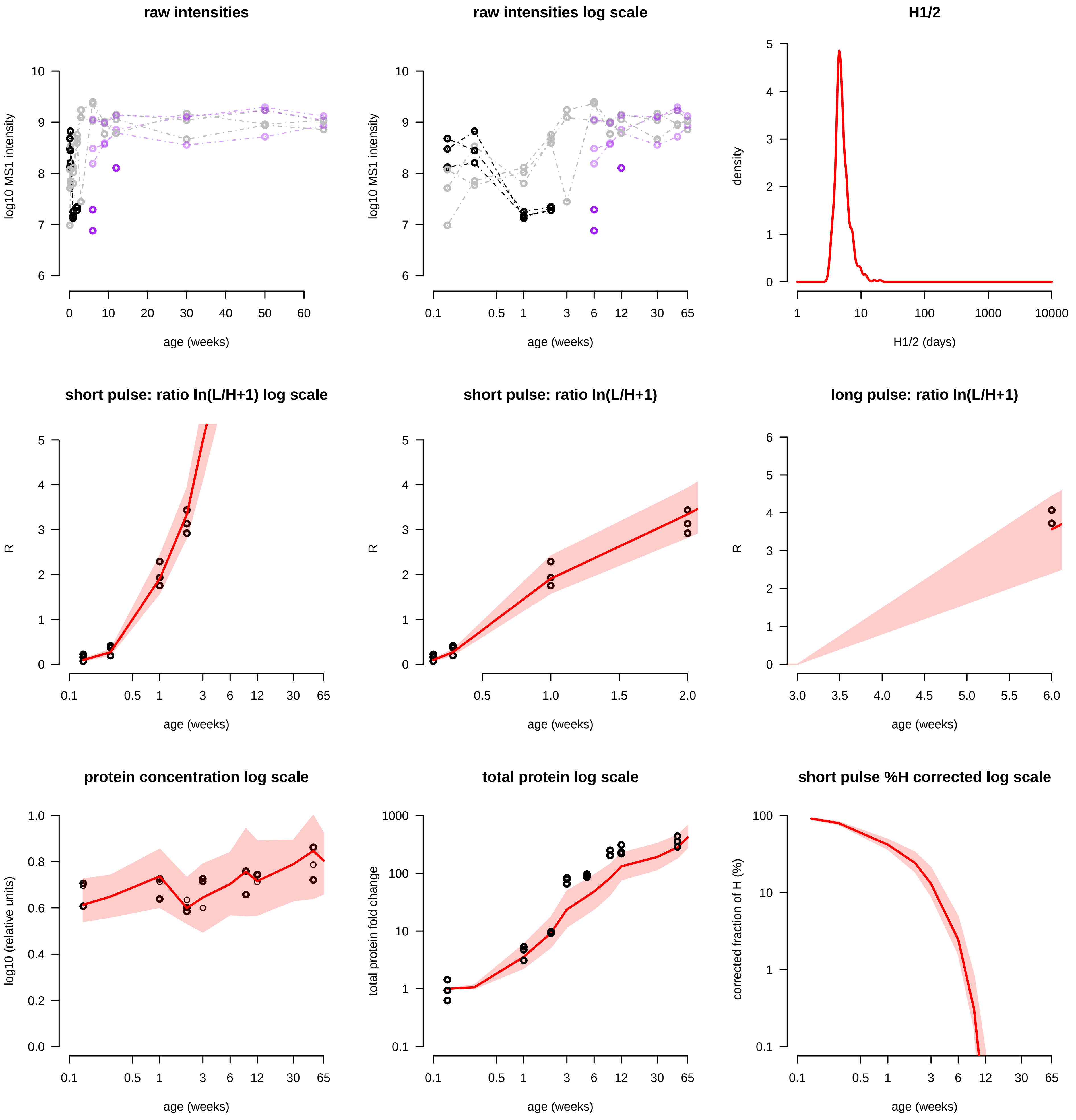

Q9ERG0;Q9ERG0−2

>sp|Q9ERG0|LIMA1_MOUSE

LIM domain and actin−binding protein 1

GN=Lima1

Med H1/2: 1508.3 days

Q5 H1/2: 409.8 days

Q95 H1/2: 13274.2 days

reliable model fit: yes

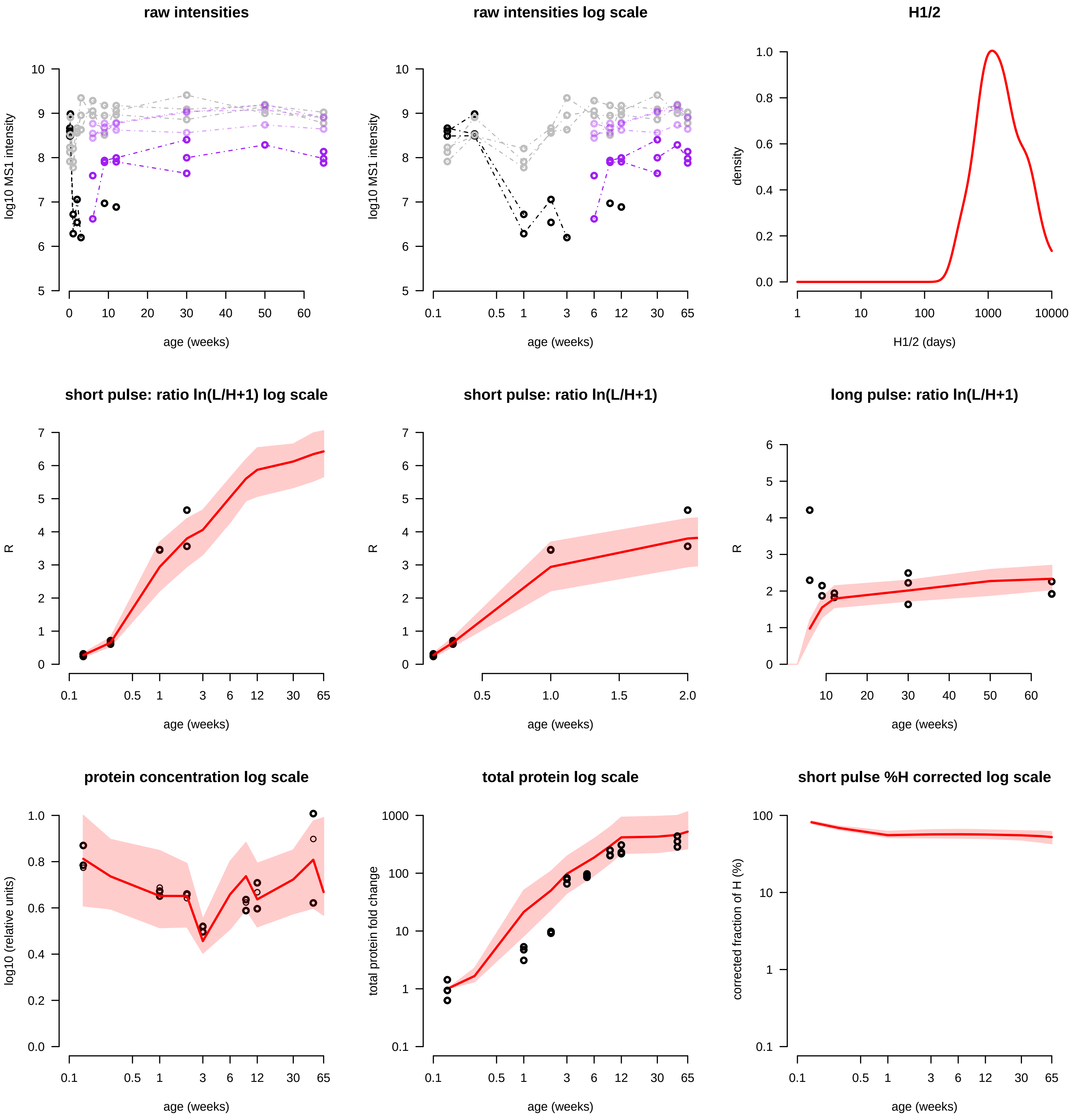

Q9D1P4

>sp|Q9D1P4|CHRD1_MOUSE

Cysteine and histidine−rich domain−containing protein 1

GN=Chordc1

Med H1/2: 7.3 days

Q5 H1/2: 5.8 days

Q95 H1/2: 10.3 days

reliable model fit: yes

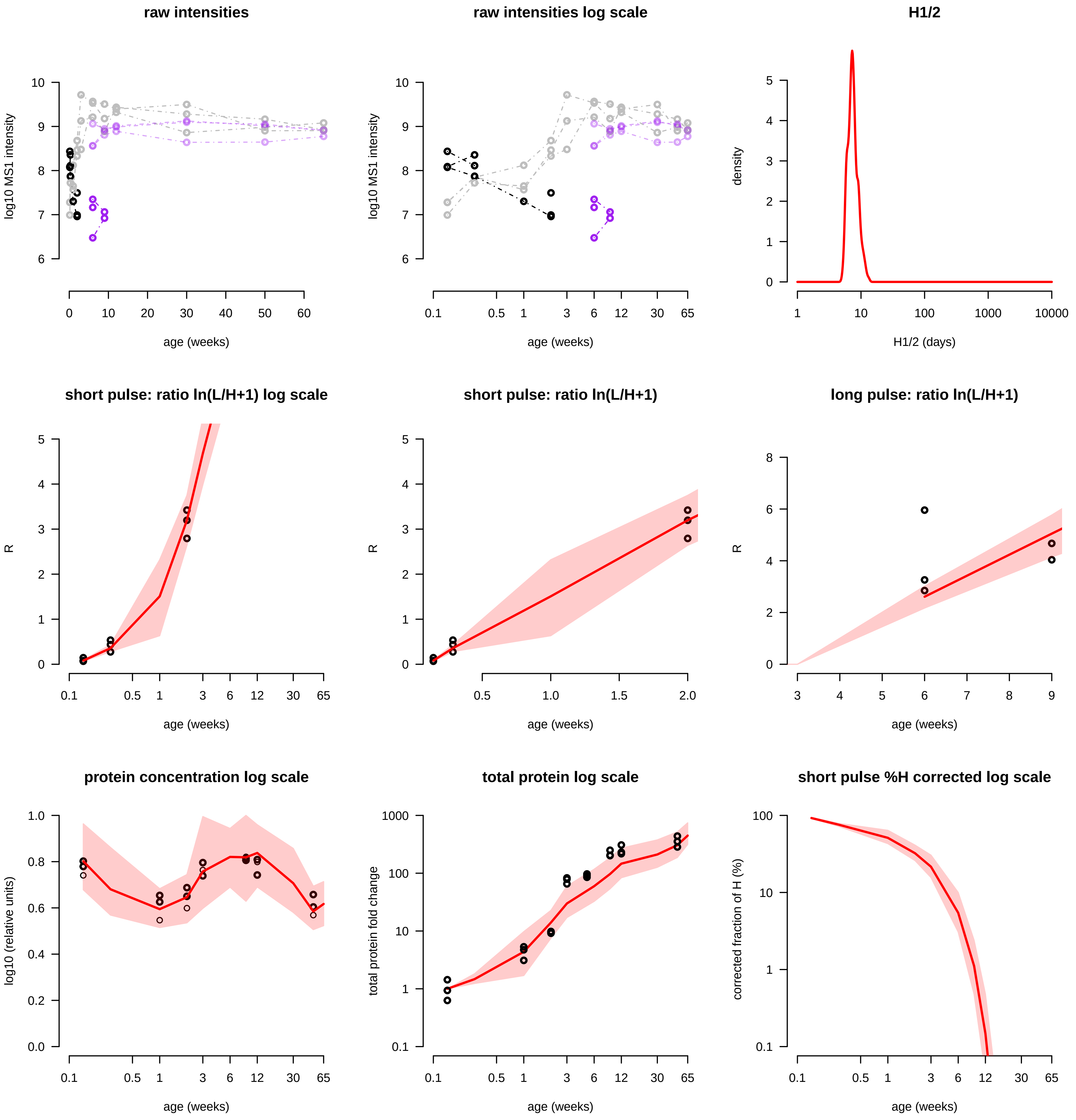

Q9CWK8

>sp|Q9CWK8|SNX2_MOUSE

Sorting nexin−2

GN=Snx2

Med H1/2: 281 days

Q5 H1/2: 123 days

Q95 H1/2: 7678.3 days

reliable model fit: yes

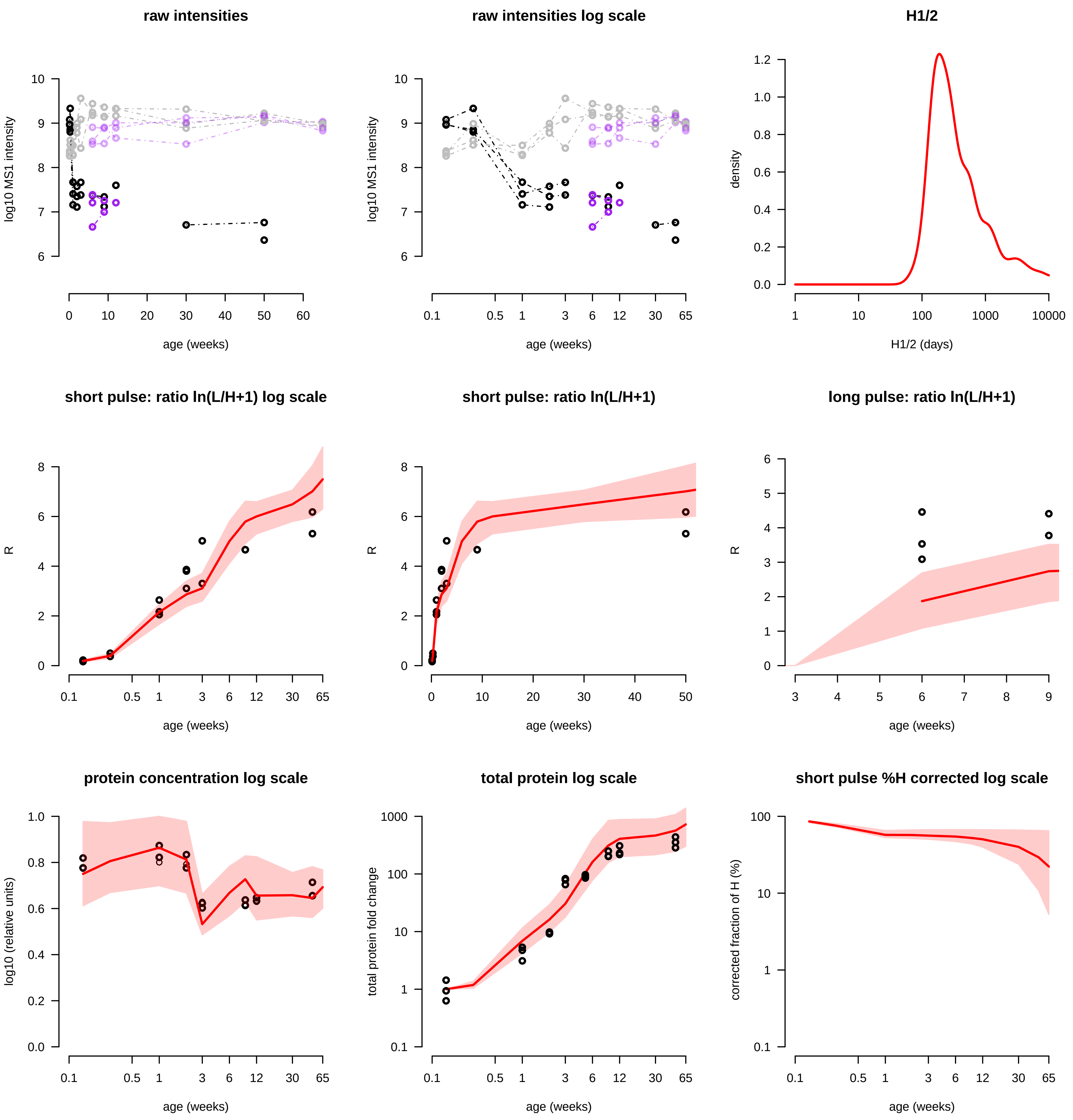

Q9R0Q3

>sp|Q9R0Q3|TMED2_MOUSE

Transmembrane emp24 domain−containing protein 2

GN=Tmed2

Med H1/2: 2112.3 days

Q5 H1/2: 579.1 days

Q95 H1/2: 34844.3 days

reliable model fit: yes

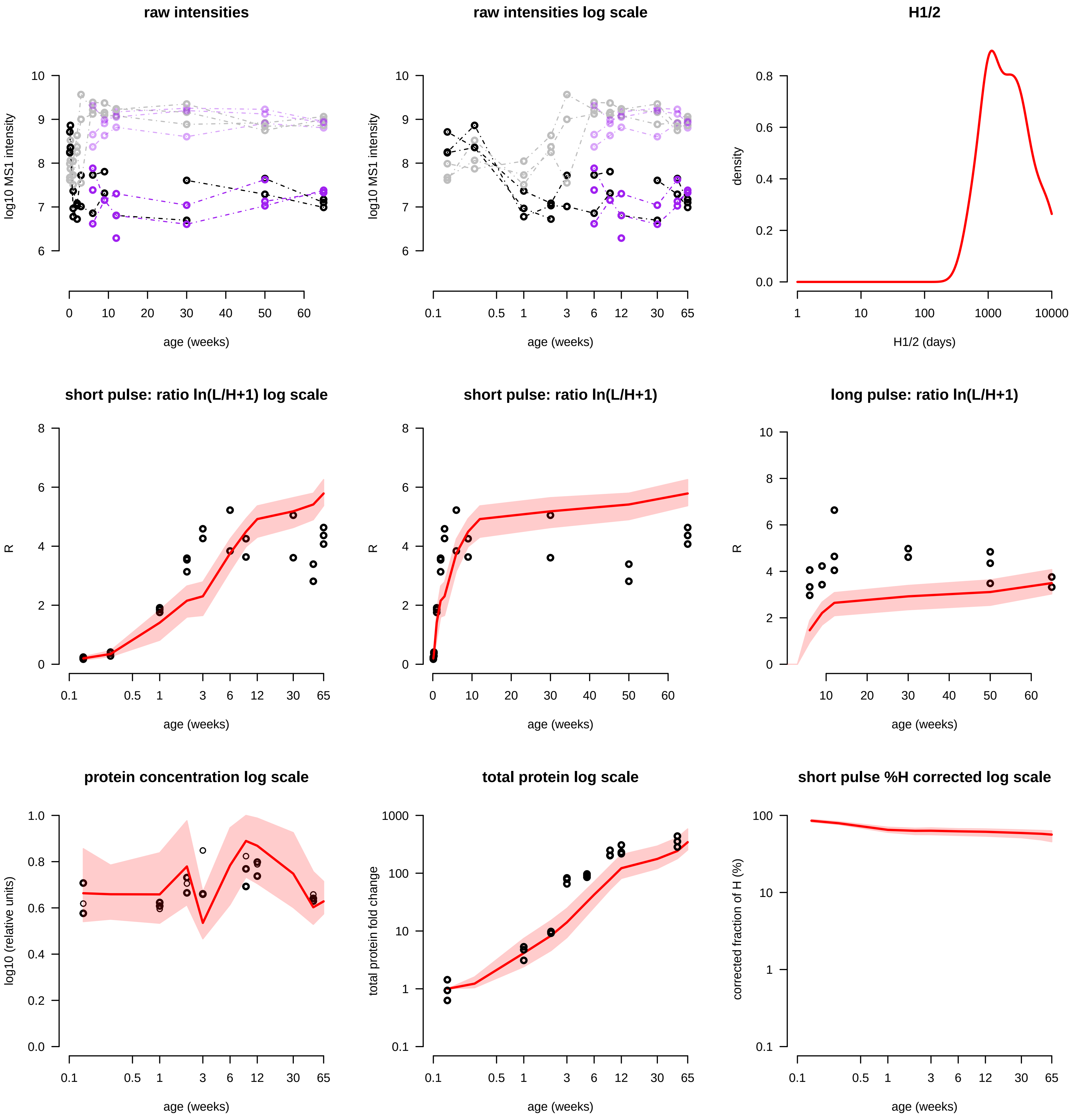

Q9CPT4

>sp|Q9CPT4|MYDGF_MOUSE

Myeloid−derived growth factor

GN=Mydgf

Med H1/2: 6.6 days

Q5 H1/2: 4.8 days

Q95 H1/2: 9.1 days

reliable model fit: yes

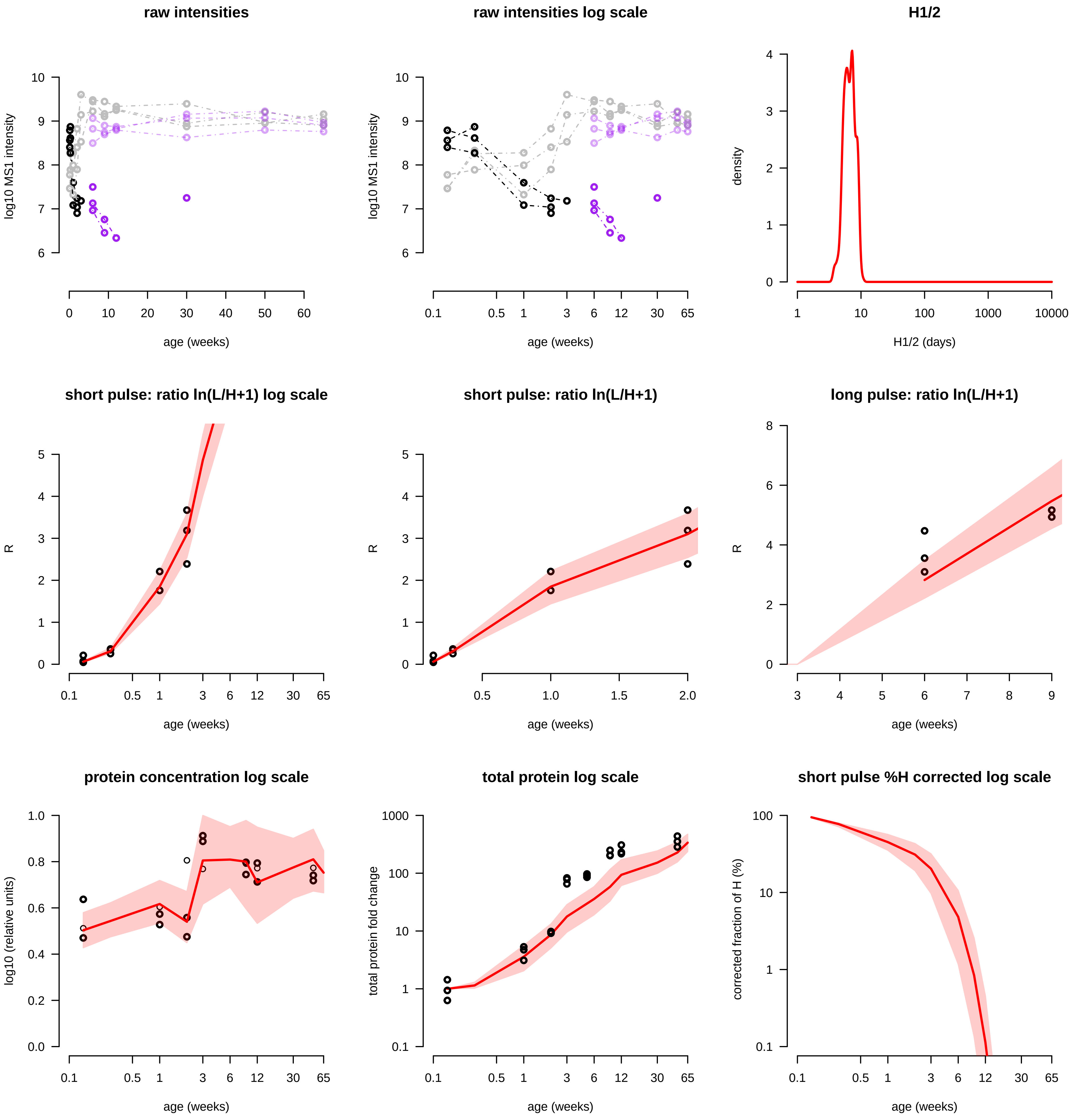

Q8VDP4

>sp|Q8VDP4|CCAR2_MOUSE

Cell cycle and apoptosis regulator protein 2

GN=Ccar2

Med H1/2: 5.7 days

Q5 H1/2: 4.1 days

Q95 H1/2: 9.7 days

reliable model fit: yes

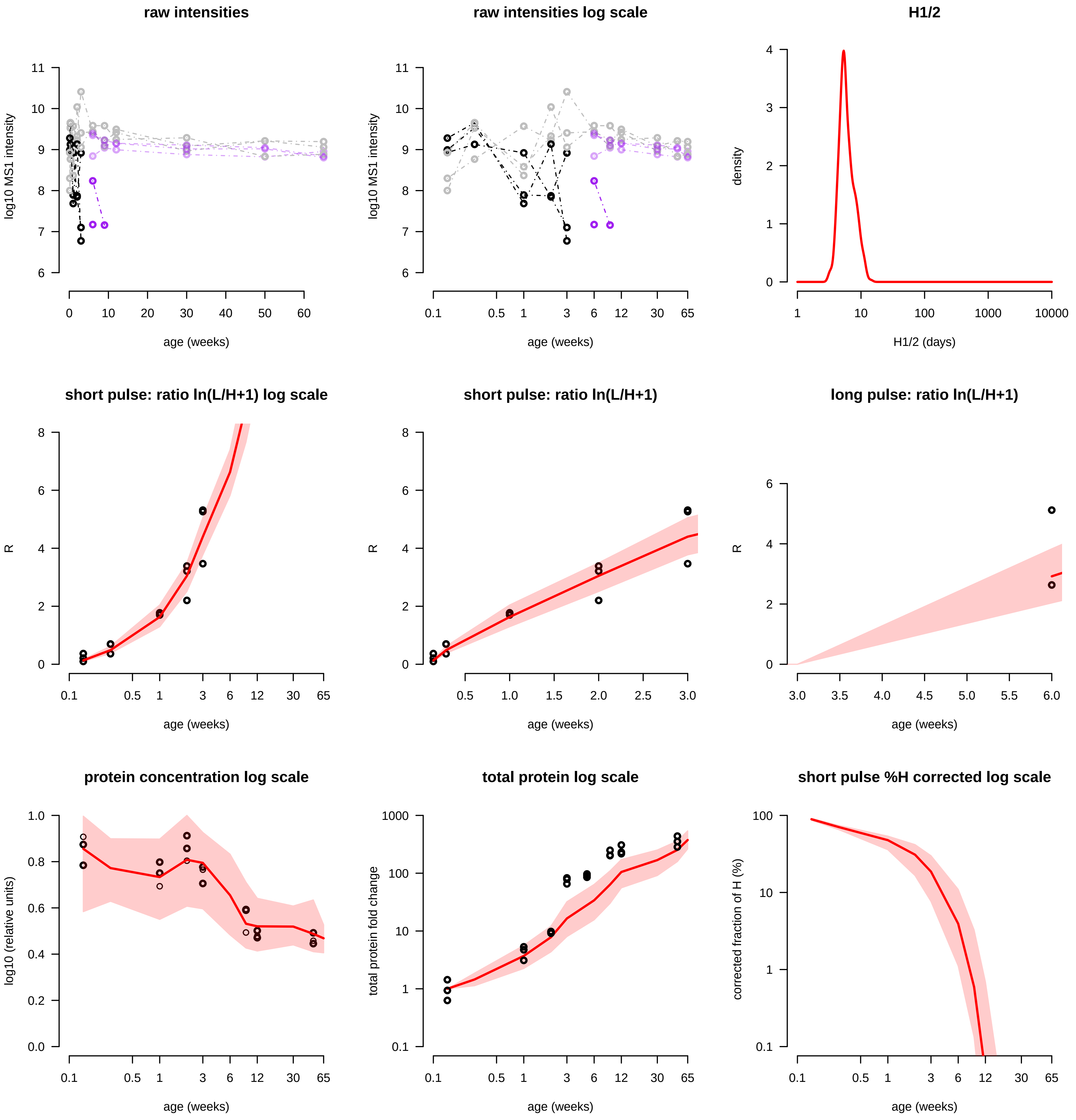

Q8BU30

>sp|Q8BU30|SYIC_MOUSE

Isoleucine−−tRNA ligase, cytoplasmic

GN=Iars1

Med H1/2: 773.9 days

Q5 H1/2: 239.1 days

Q95 H1/2: 7211.3 days

reliable model fit: no

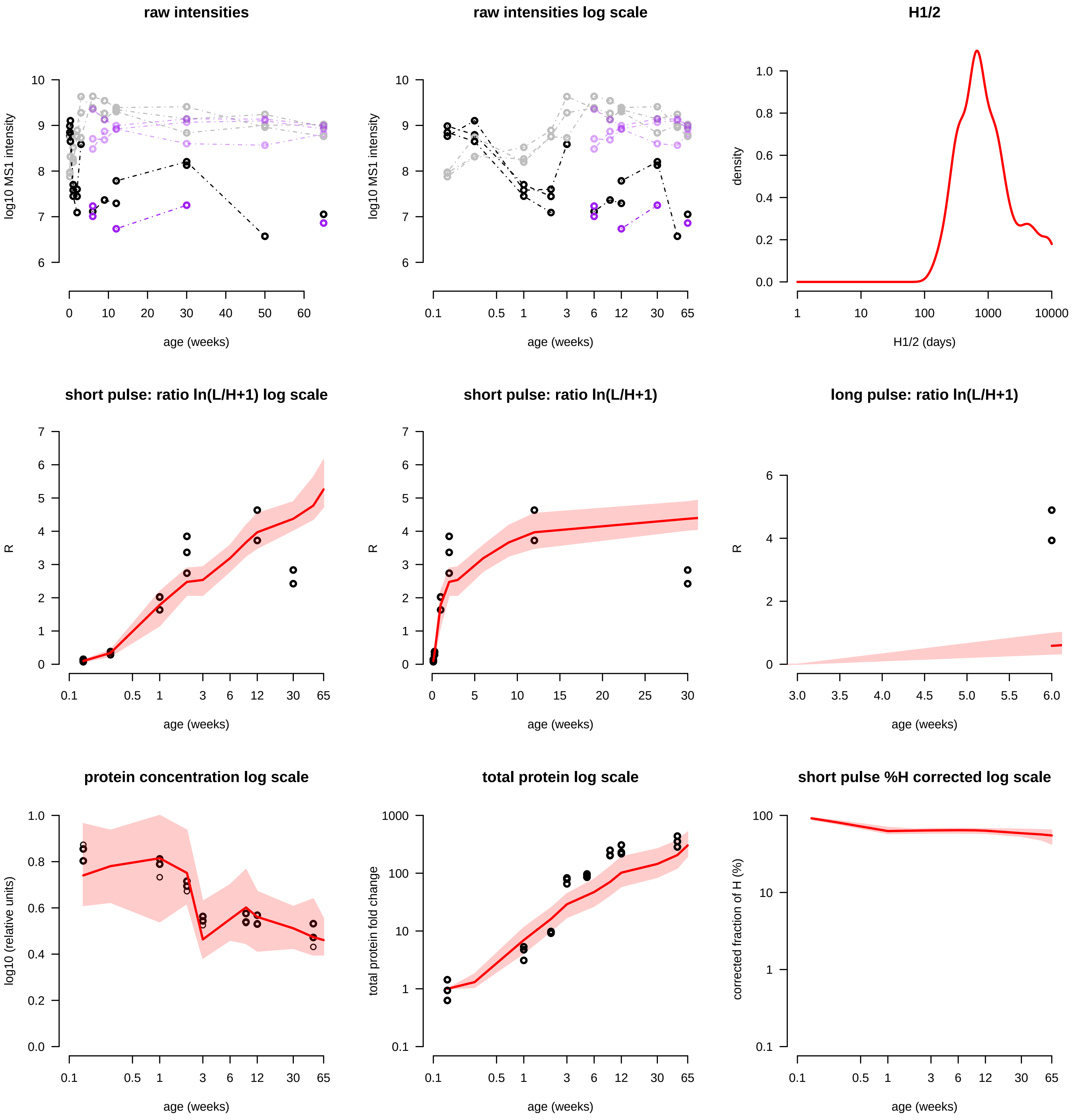

Q9CPW4

>sp|Q9CPW4|ARPC5_MOUSE

Actin−related protein 2/3 complex subunit 5

GN=Arpc5

Med H1/2: 7.9 days

Q5 H1/2: 6.2 days

Q95 H1/2: 11.2 days

reliable model fit: yes

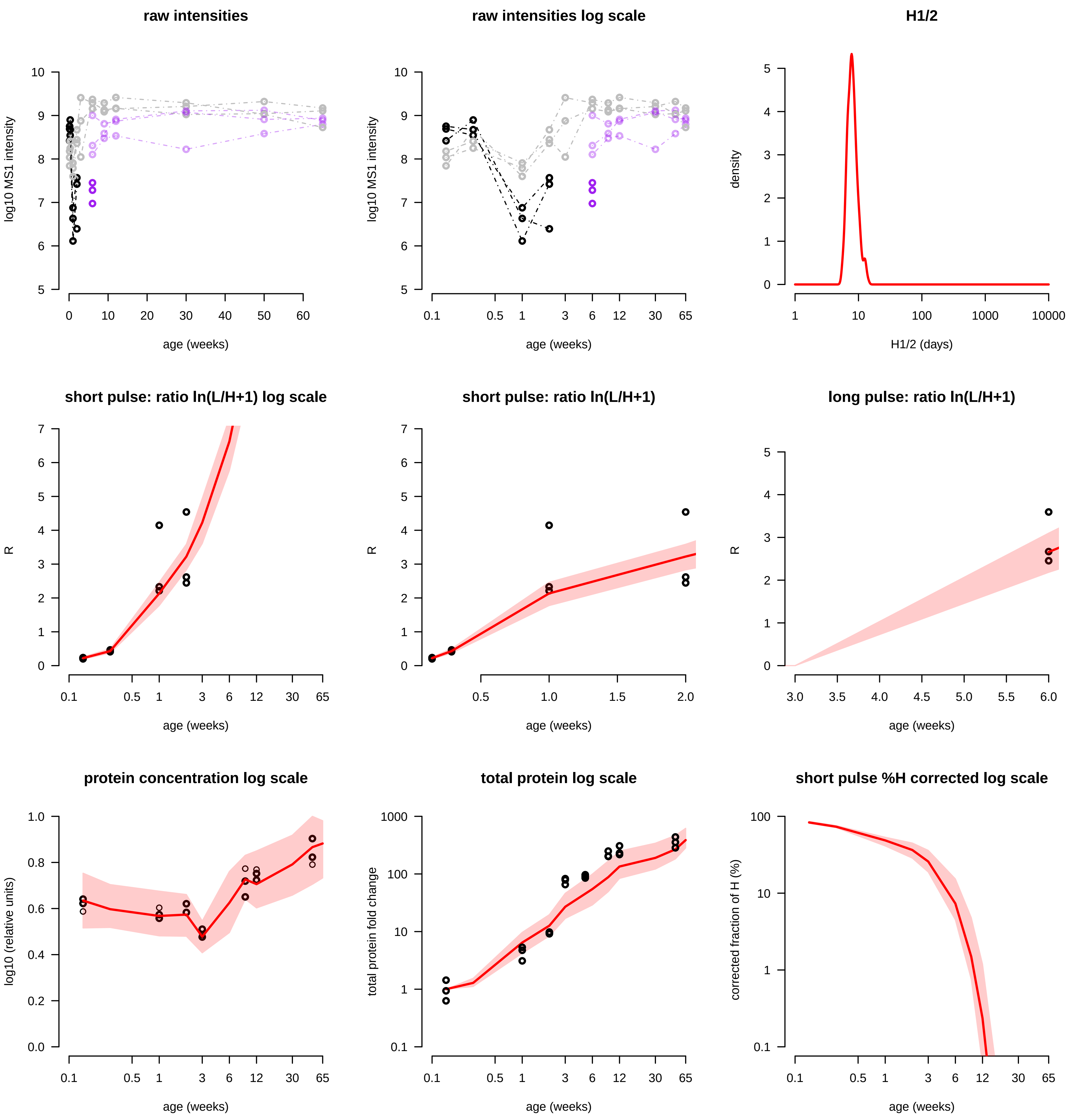

Q9JJU8

>sp|Q9JJU8|SH3L1_MOUSE

SH3 domain−binding glutamic acid−rich−like protein

GN=Sh3bgrl

Med H1/2: 4.5 days

Q5 H1/2: 3.4 days

Q95 H1/2: 5.6 days

reliable model fit: yes

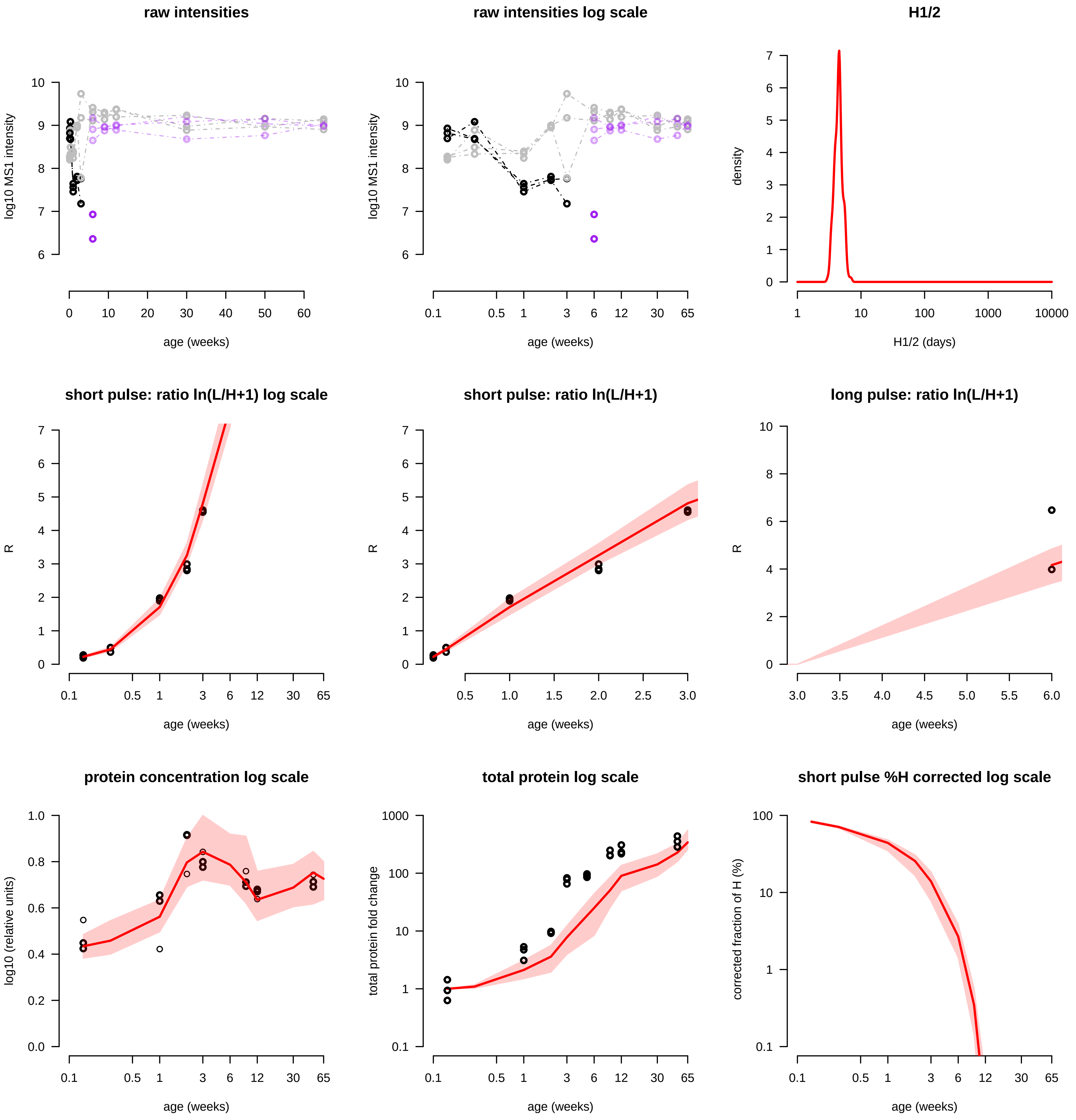

Q9D7B6

>sp|Q9D7B6|ACAD8_MOUSE

Isobutyryl−CoA dehydrogenase, mitochondrial

GN=Acad8

Med H1/2: 5.3 days

Q5 H1/2: 3.9 days

Q95 H1/2: 7.4 days

reliable model fit: yes

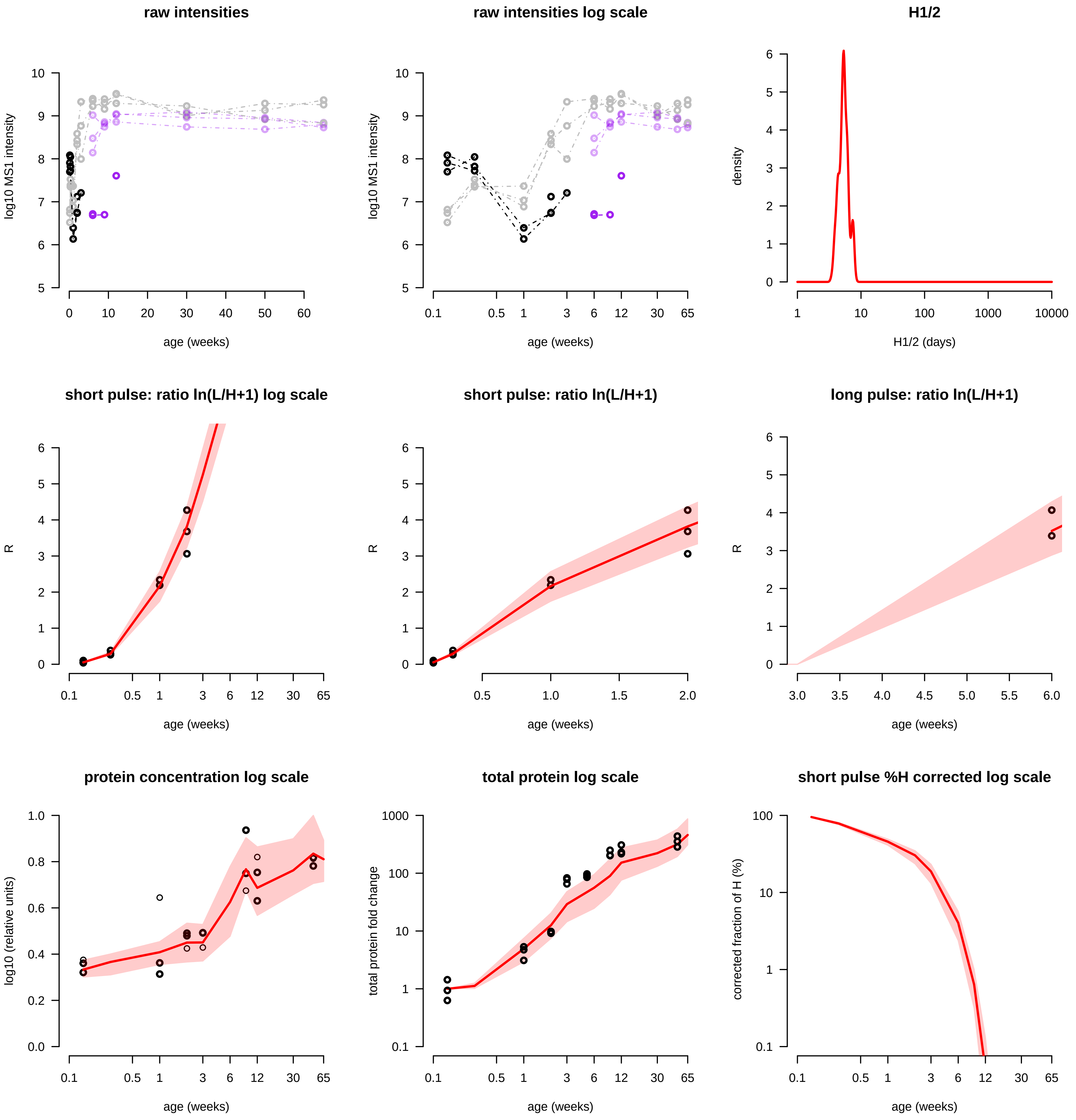

Q91W50

>sp|Q91W50|CSDE1_MOUSE

Cold shock domain−containing protein E1

GN=Csde1

Med H1/2: 506.4 days

Q5 H1/2: 160.8 days

Q95 H1/2: 7288.9 days

reliable model fit: no

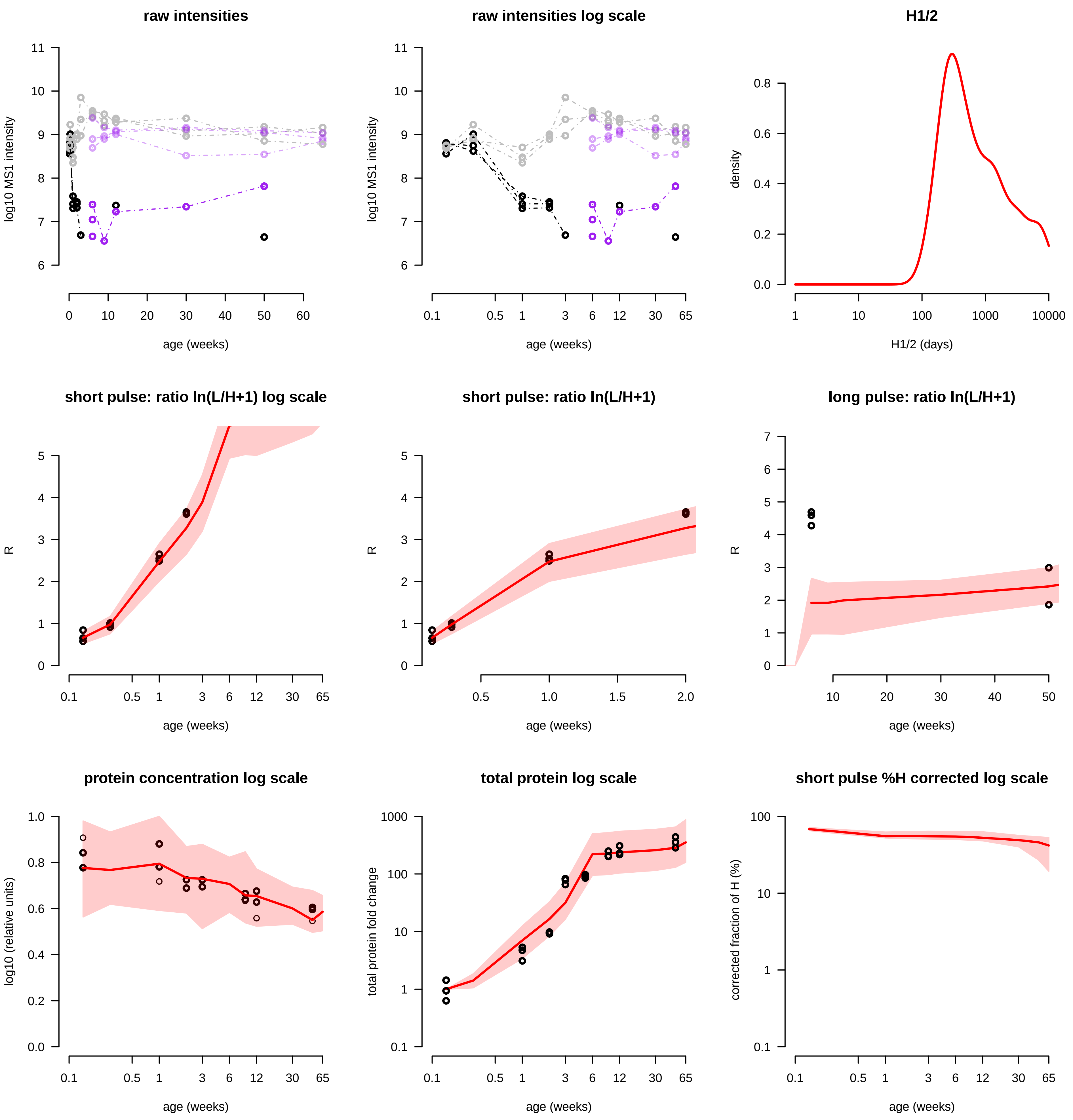

P58044

>sp|P58044|IDI1_MOUSE

Isopentenyl−diphosphate Delta−isomerase 1

GN=Idi1

Med H1/2: 6.3 days

Q5 H1/2: 4.2 days

Q95 H1/2: 15.8 days

reliable model fit: yes

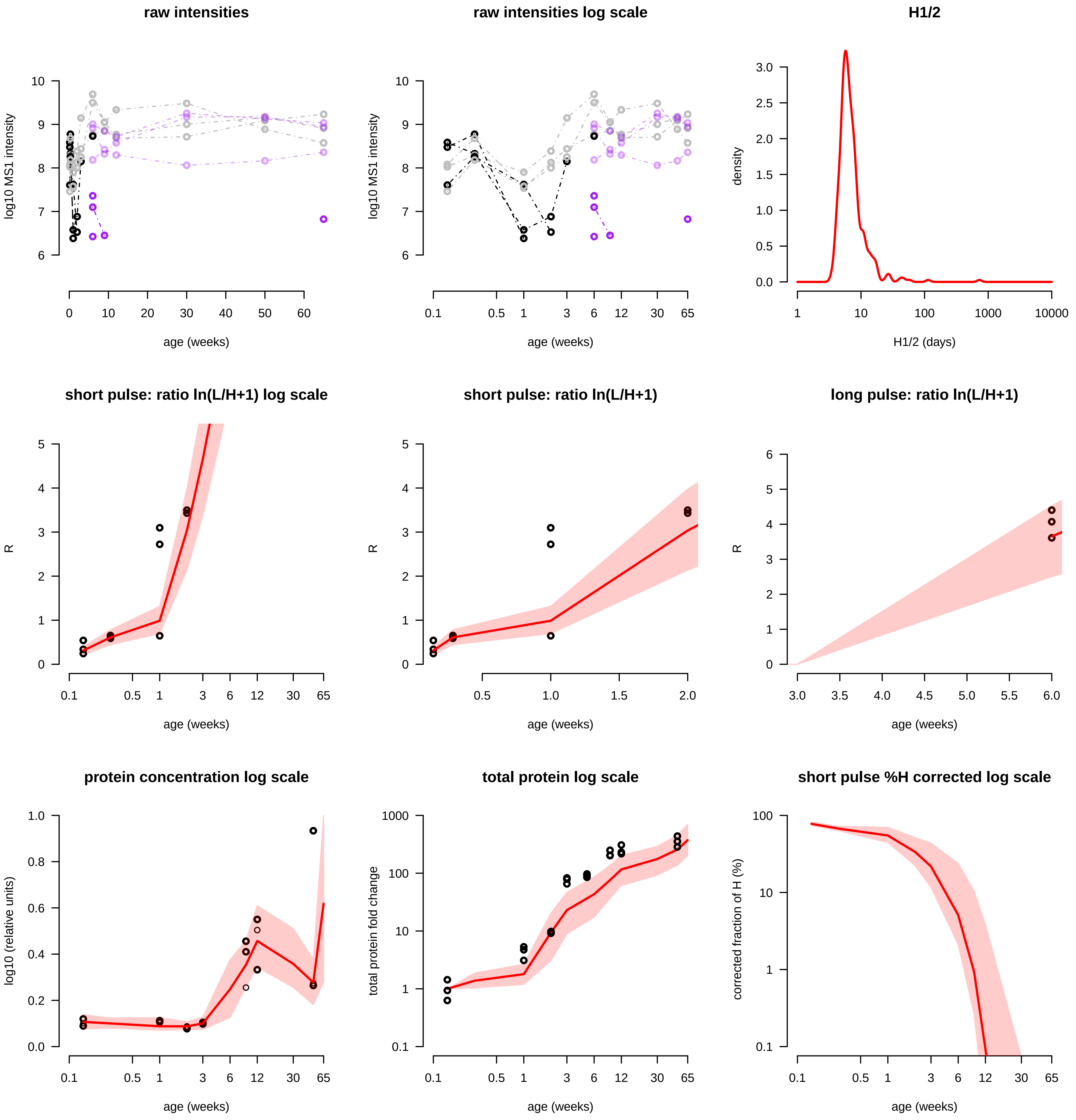

Q9CQI3

>sp|Q9CQI3|GMFB_MOUSE

Glia maturation factor beta

GN=Gmfb

Med H1/2: 4.6 days

Q5 H1/2: 3.7 days

Q95 H1/2: 5.8 days

reliable model fit: yes

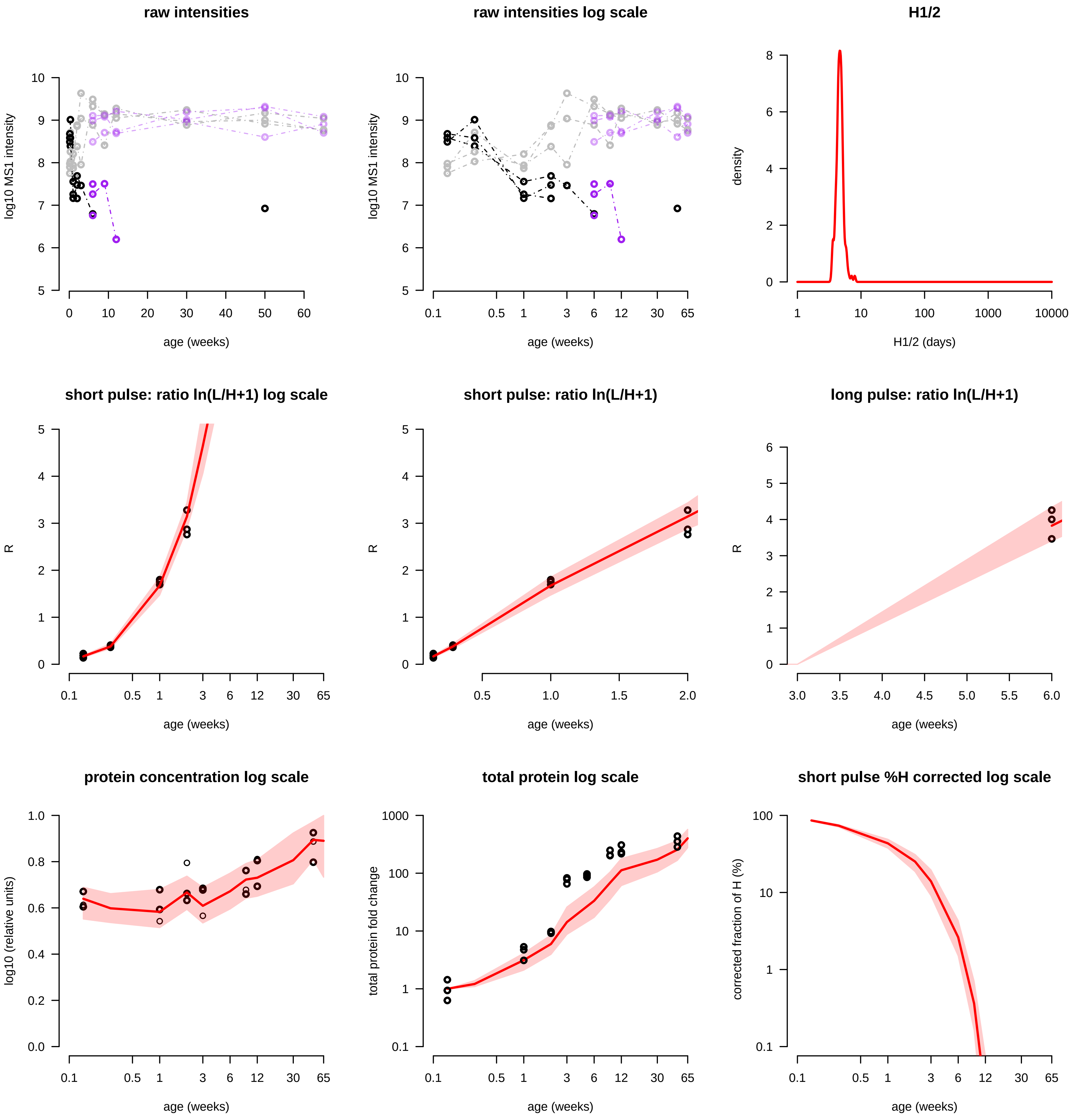

P48428

>sp|P48428|TBCA_MOUSE

Tubulin−specific chaperone A

GN=Tbca

Med H1/2: 7.8 days

Q5 H1/2: 6.3 days

Q95 H1/2: 9.7 days

reliable model fit: yes

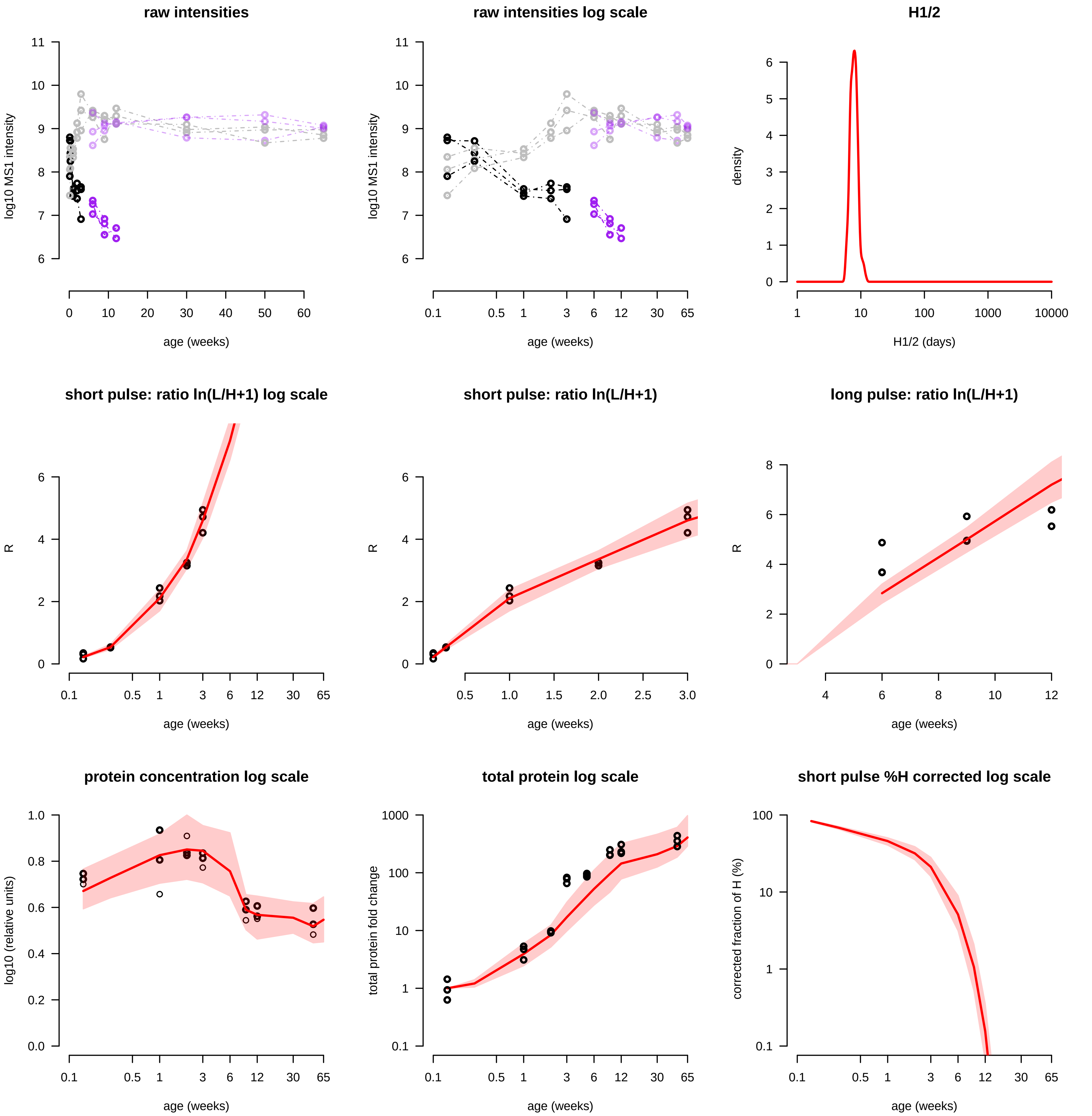

Q8VI36;Q8VI36−2

>sp|Q8VI36|PAXI_MOUSE

Paxillin

GN=Pxn

Med H1/2: 21.7 days

Q5 H1/2: 14.2 days

Q95 H1/2: 38.4 days

reliable model fit: no

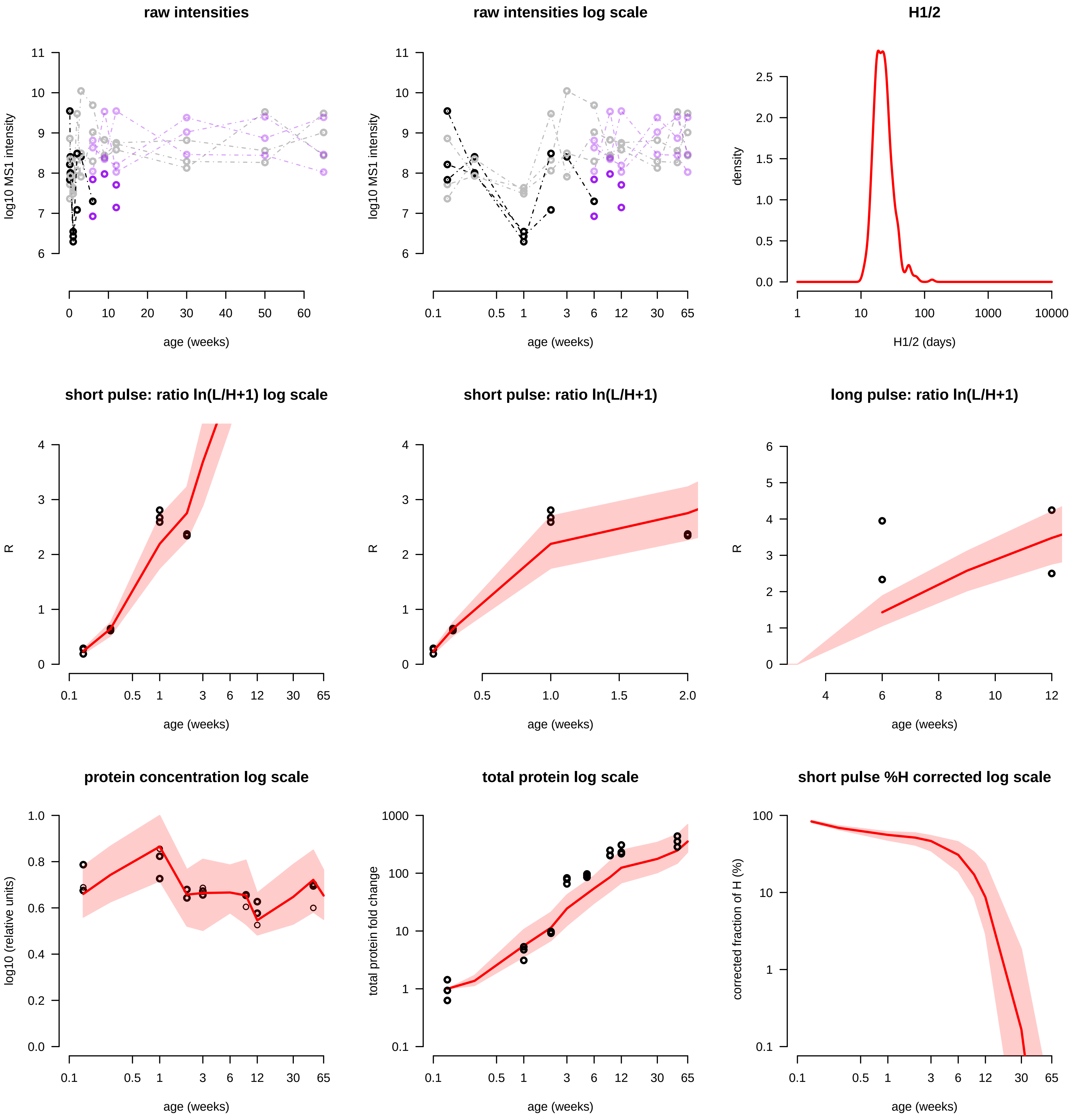

Q810B6

>sp|Q810B6|ANFY1_MOUSE

Rabankyrin−5

GN=Ankfy1

Med H1/2: 4423.2 days

Q5 H1/2: 1204.6 days

Q95 H1/2: 54336.2 days

reliable model fit: yes

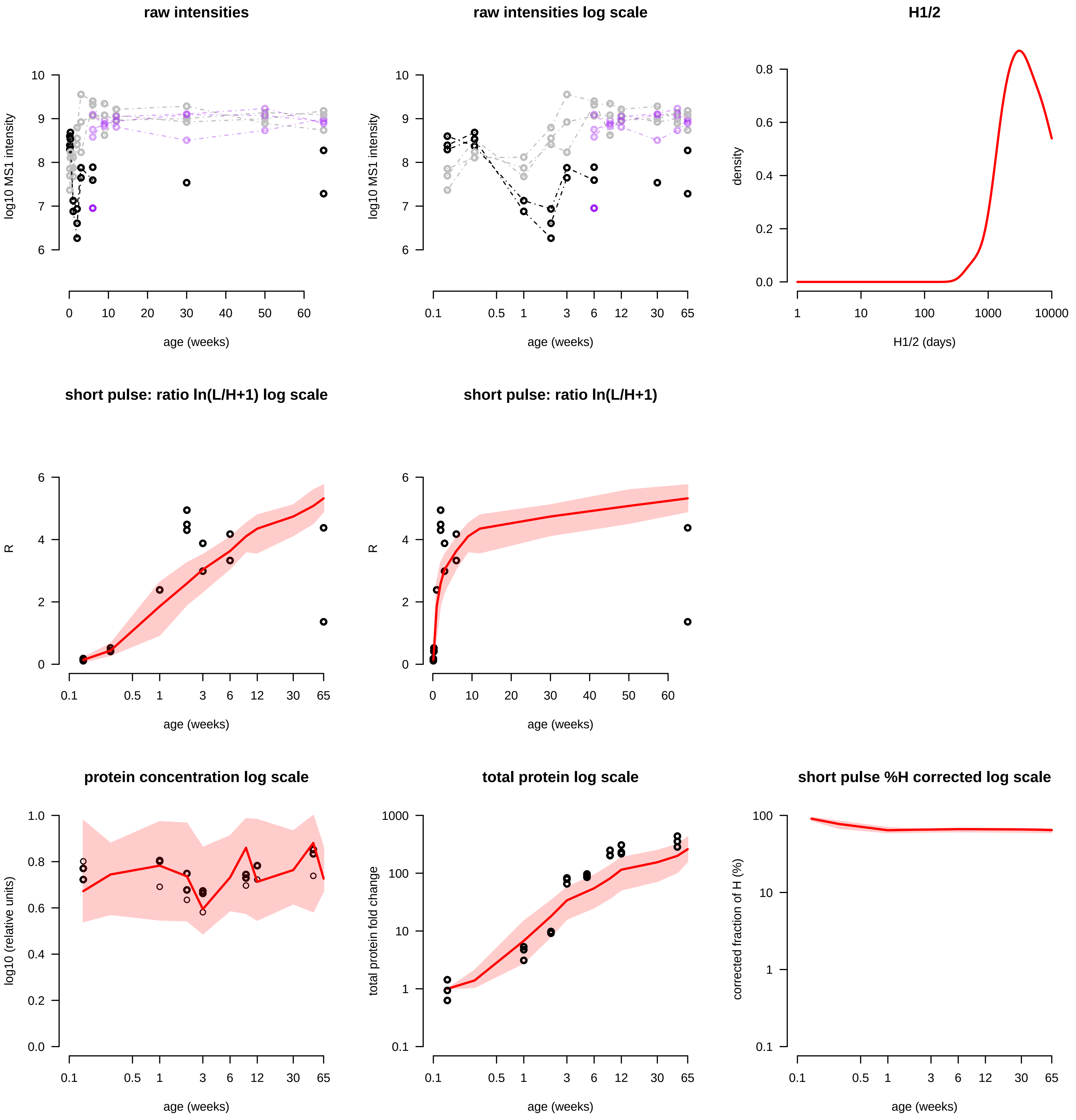

Q01339

>sp|Q01339|APOH_MOUSE

Beta−2−glycoprotein 1

GN=Apoh

Med H1/2: 110.6 days

Q5 H1/2: 37.3 days

Q95 H1/2: 1038.5 days

reliable model fit: yes

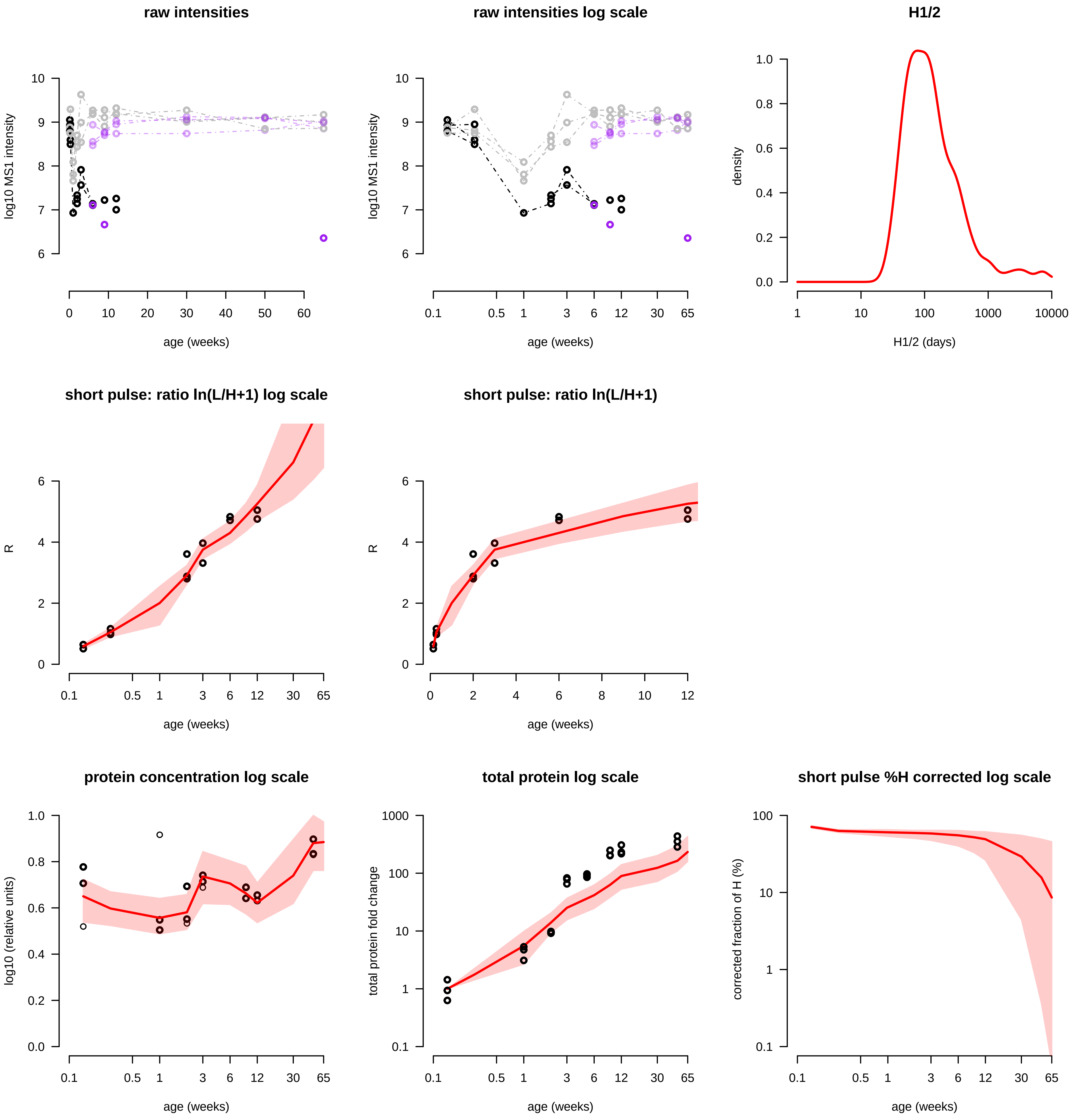

Q8CHP8

>sp|Q8CHP8|PGP_MOUSE

Glycerol−3−phosphate phosphatase

GN=Pgp

Med H1/2: 5.7 days

Q5 H1/2: 4.5 days

Q95 H1/2: 9 days

reliable model fit: yes

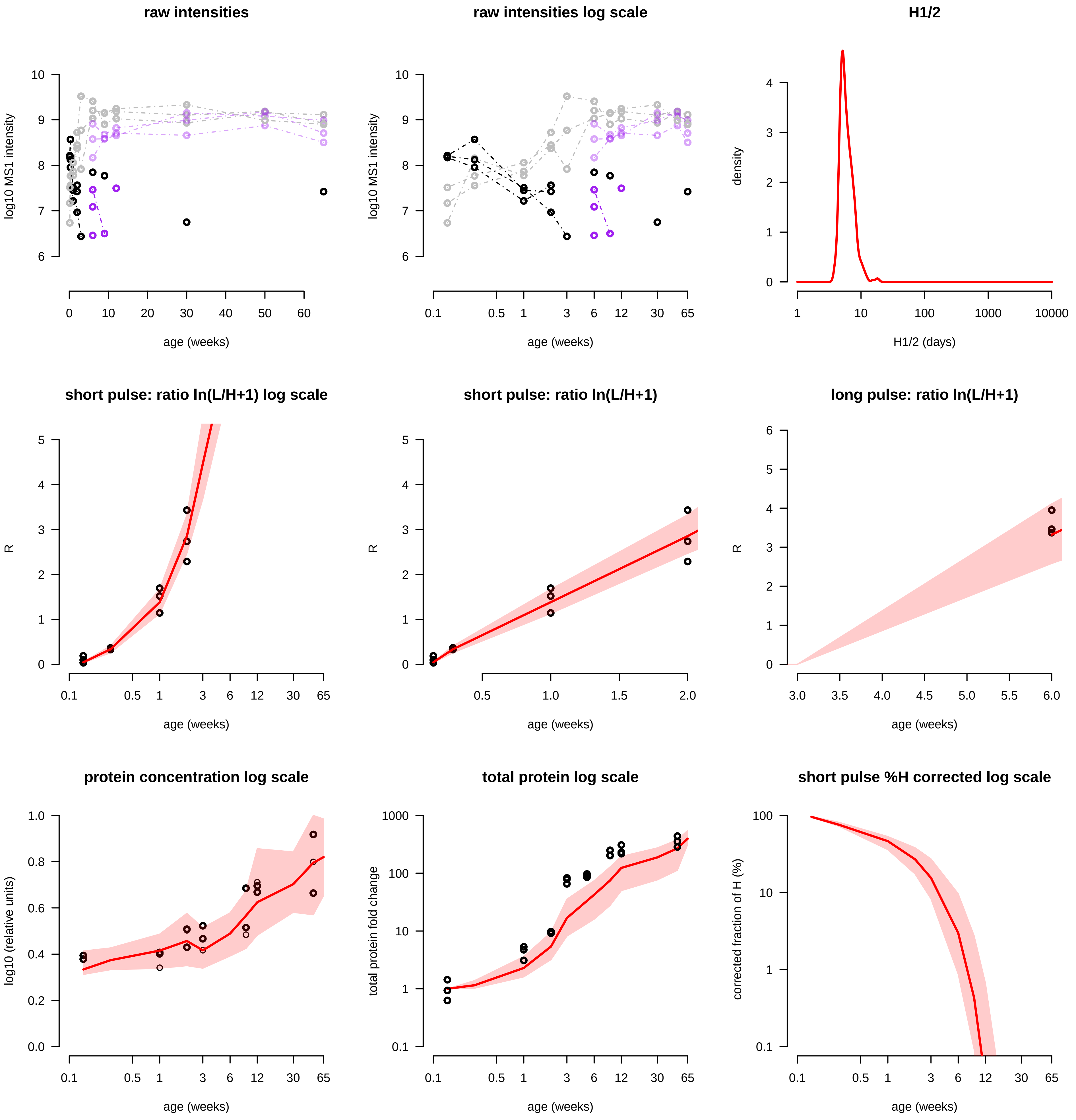

P97447

>sp|P97447|FHL1_MOUSE

Four and a half LIM domains protein 1

GN=Fhl1

Med H1/2: 8.1 days

Q5 H1/2: 6.6 days

Q95 H1/2: 10 days

reliable model fit: yes

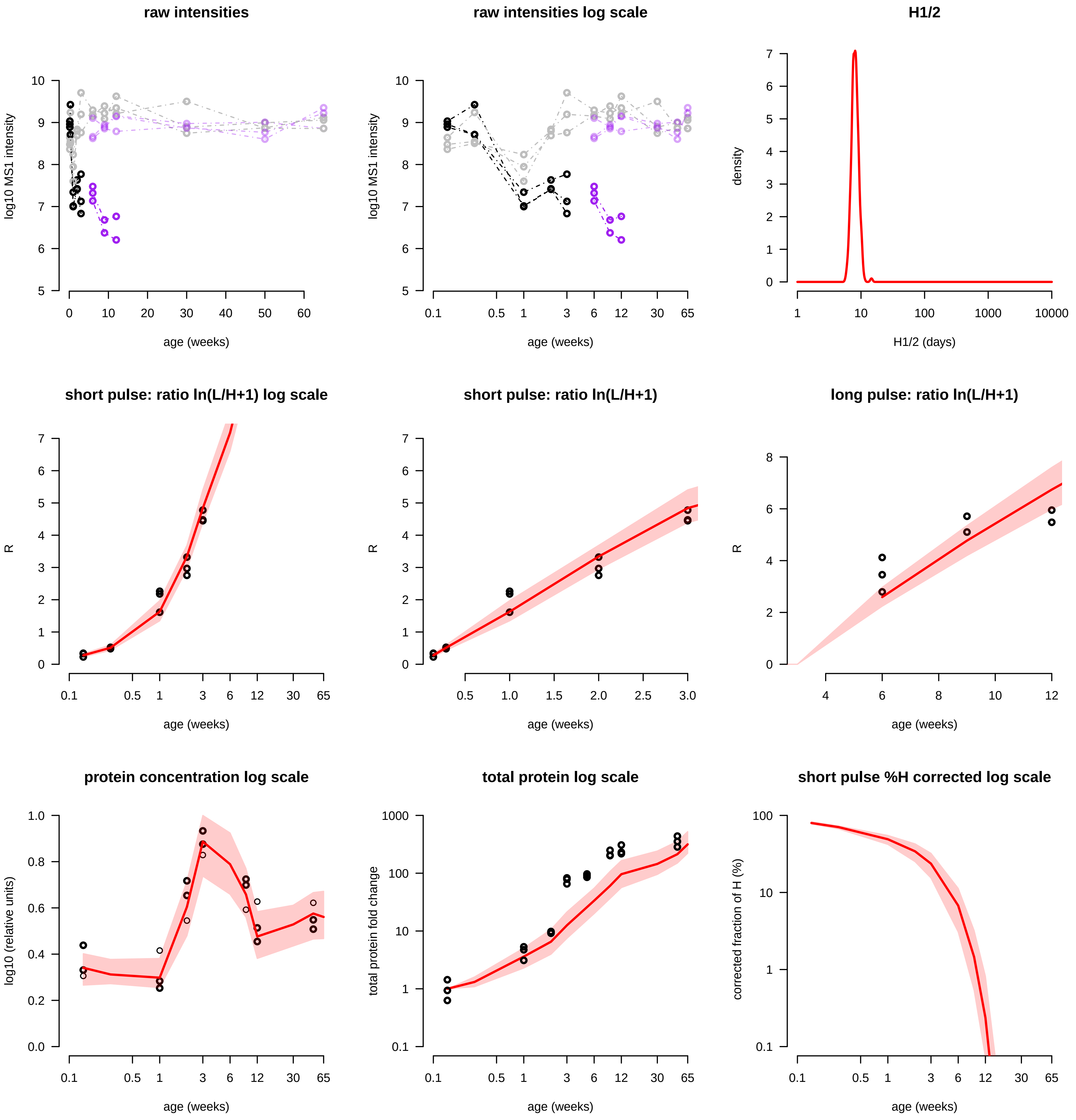

P14576

>sp|P14576|SRP54_MOUSE

Signal recognition particle 54 kDa protein

GN=Srp54

Med H1/2: 4 days

Q5 H1/2: 3.4 days

Q95 H1/2: 4.8 days

reliable model fit: yes

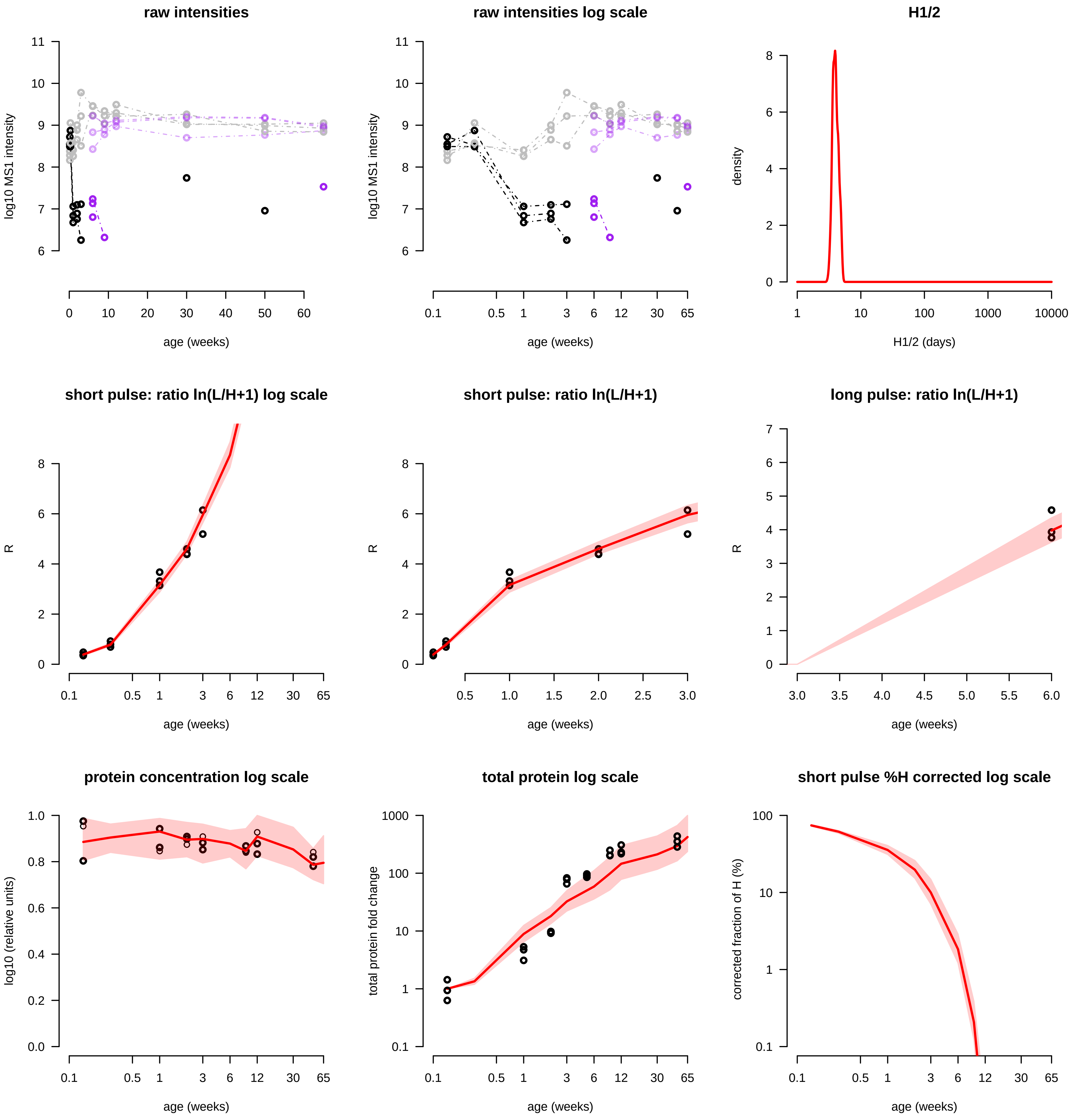

P49722

>sp|P49722|PSA2_MOUSE

Proteasome subunit alpha type−2

GN=Psma2

Med H1/2: 7.6 days

Q5 H1/2: 6 days

Q95 H1/2: 10.4 days

reliable model fit: yes

raw intensities

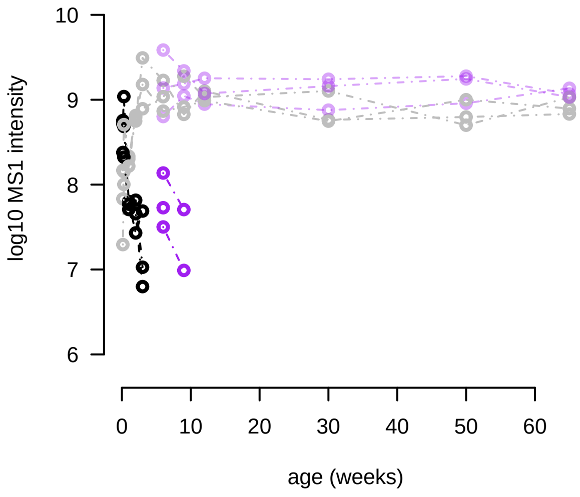

raw intensities log scale

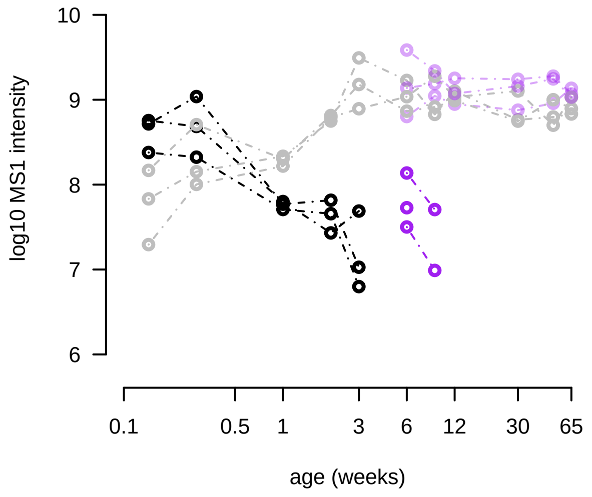

H1/2

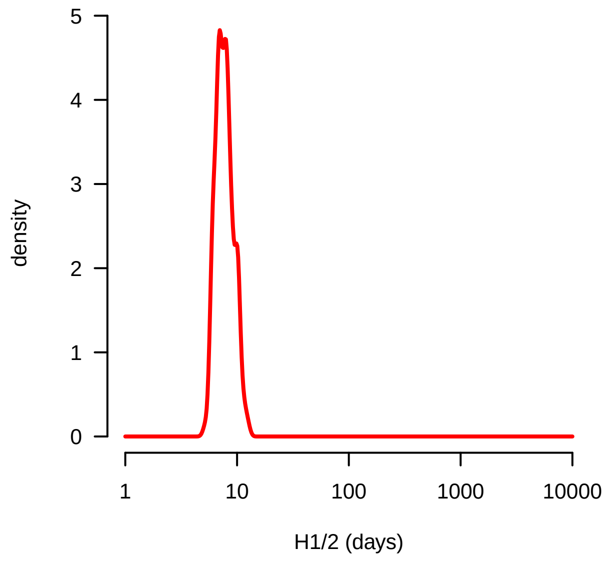

short pulse: ratio ln(L/H+1) log scale

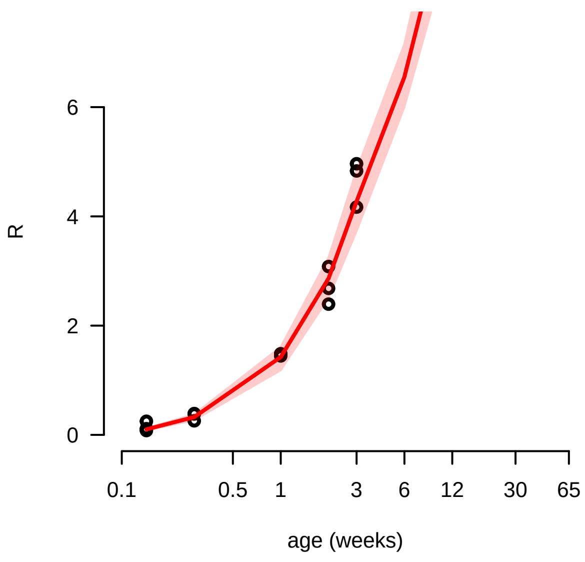

short pulse: ratio ln(L/H+1)

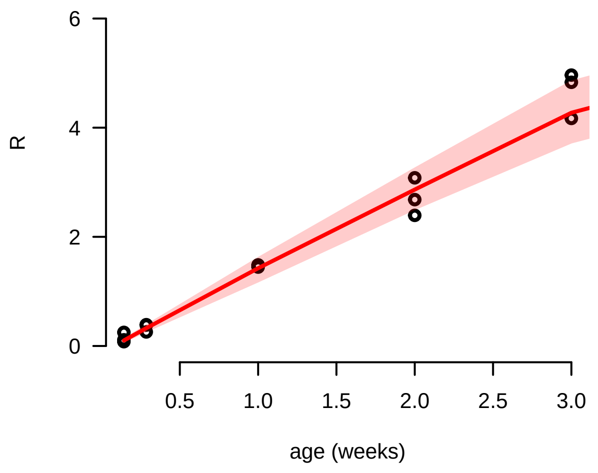

long pulse: ratio ln(L/H+1)

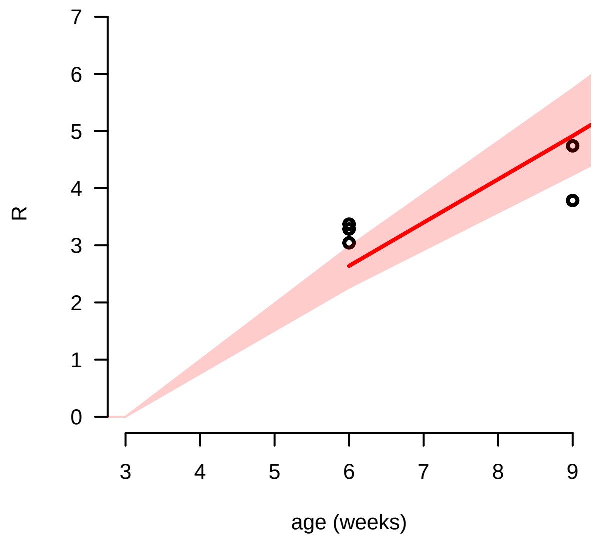

protein concentration log scale

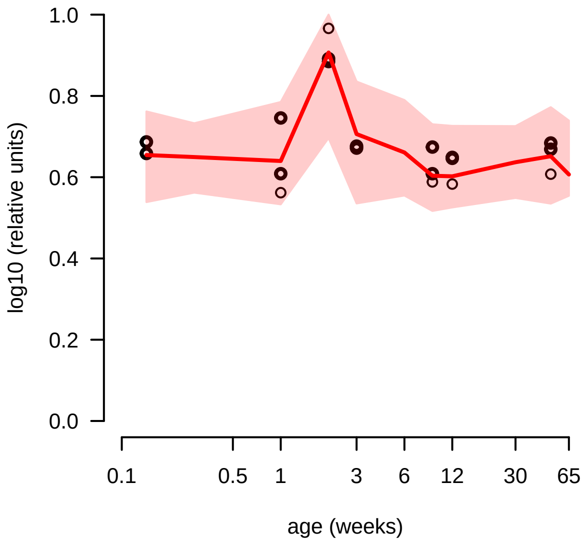

total protein log scale

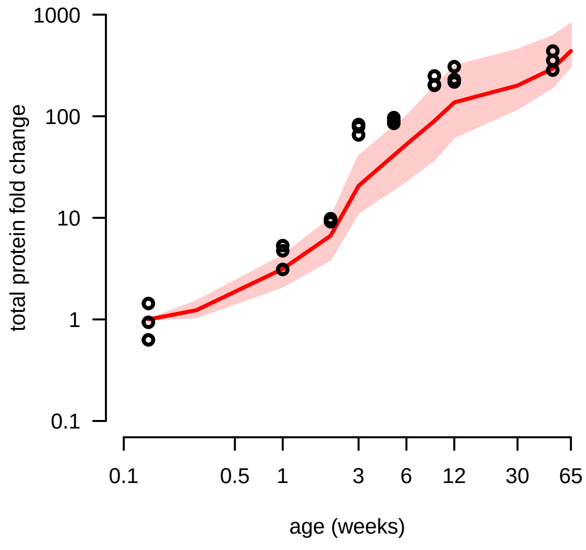

short pulse %H corrected log scale

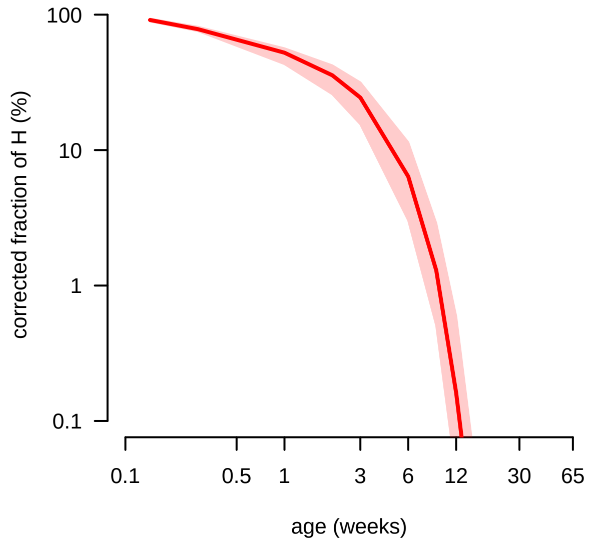

Q8C7R4

>sp|Q8C7R4|UBA6_MOUSE

Ubiquitin−like modifier−activating enzyme 6

GN=Uba6

Med H1/2: 63.6 days

Q5 H1/2: 53.9 days

Q95 H1/2: 79.2 days

reliable model fit: yes

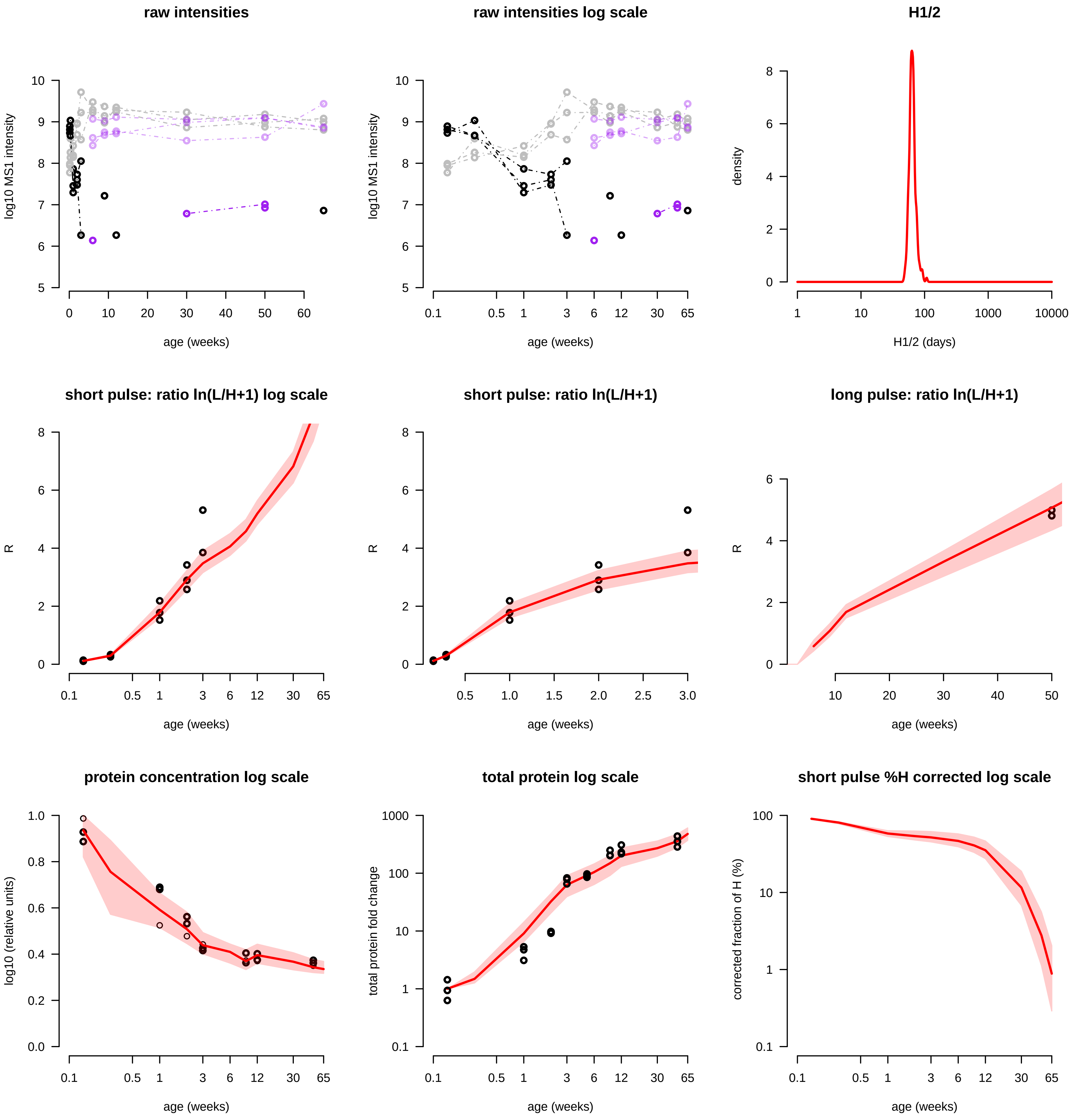

Q99KN9;Q99KN9−2

>sp|Q99KN9|EPN4_MOUSE

Clathrin interactor 1

GN=Clint1

Med H1/2: 4.7 days

Q5 H1/2: 3.7 days

Q95 H1/2: 5.8 days

reliable model fit: yes

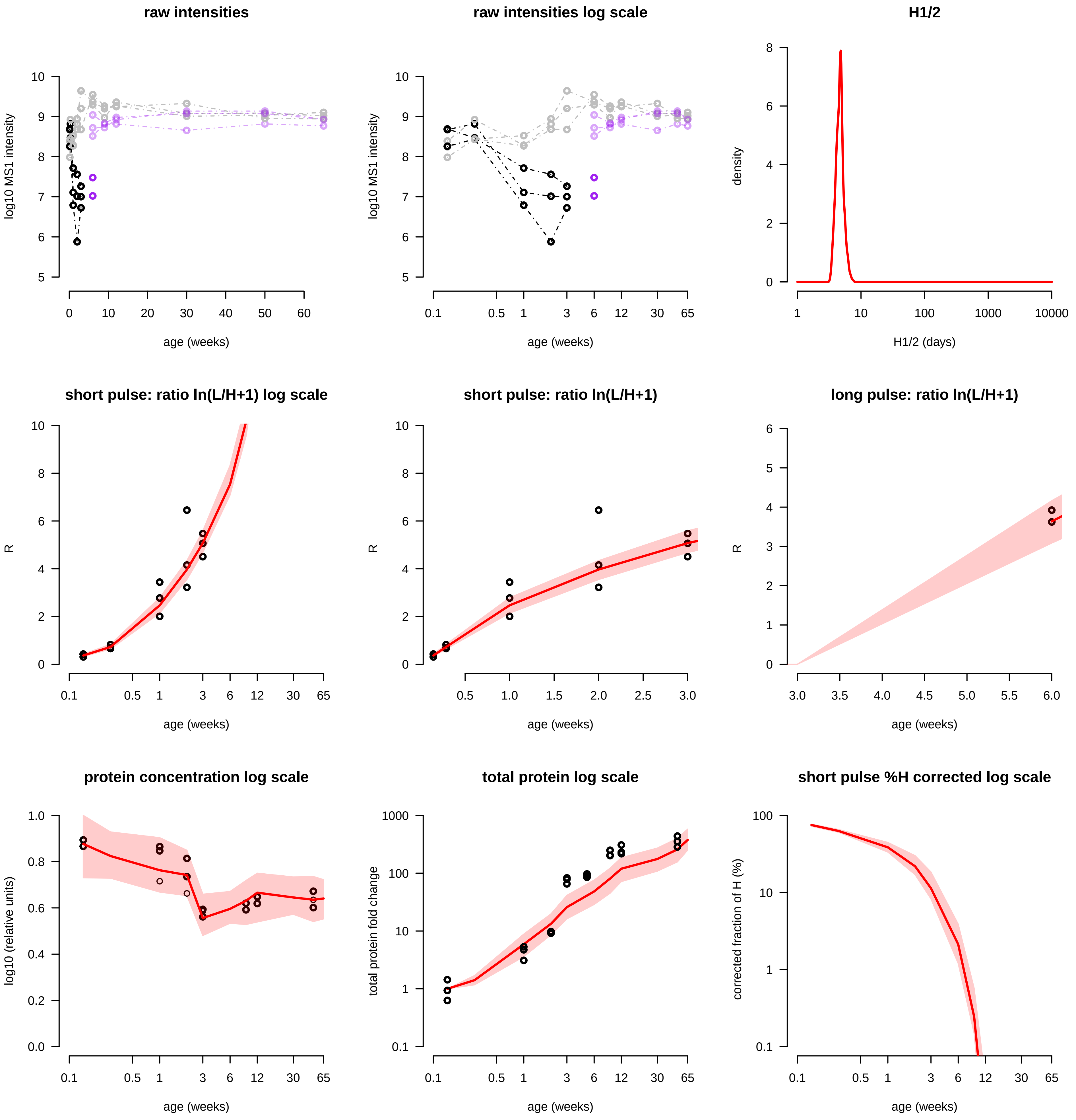

Q64152−2

>sp|Q64152−2|BTF3_MOUSE

Isoform 2 of Transcription factor BTF3

GN=Btf3

Med H1/2: 7.5 days

Q5 H1/2: 6.3 days

Q95 H1/2: 9.4 days

reliable model fit: yes

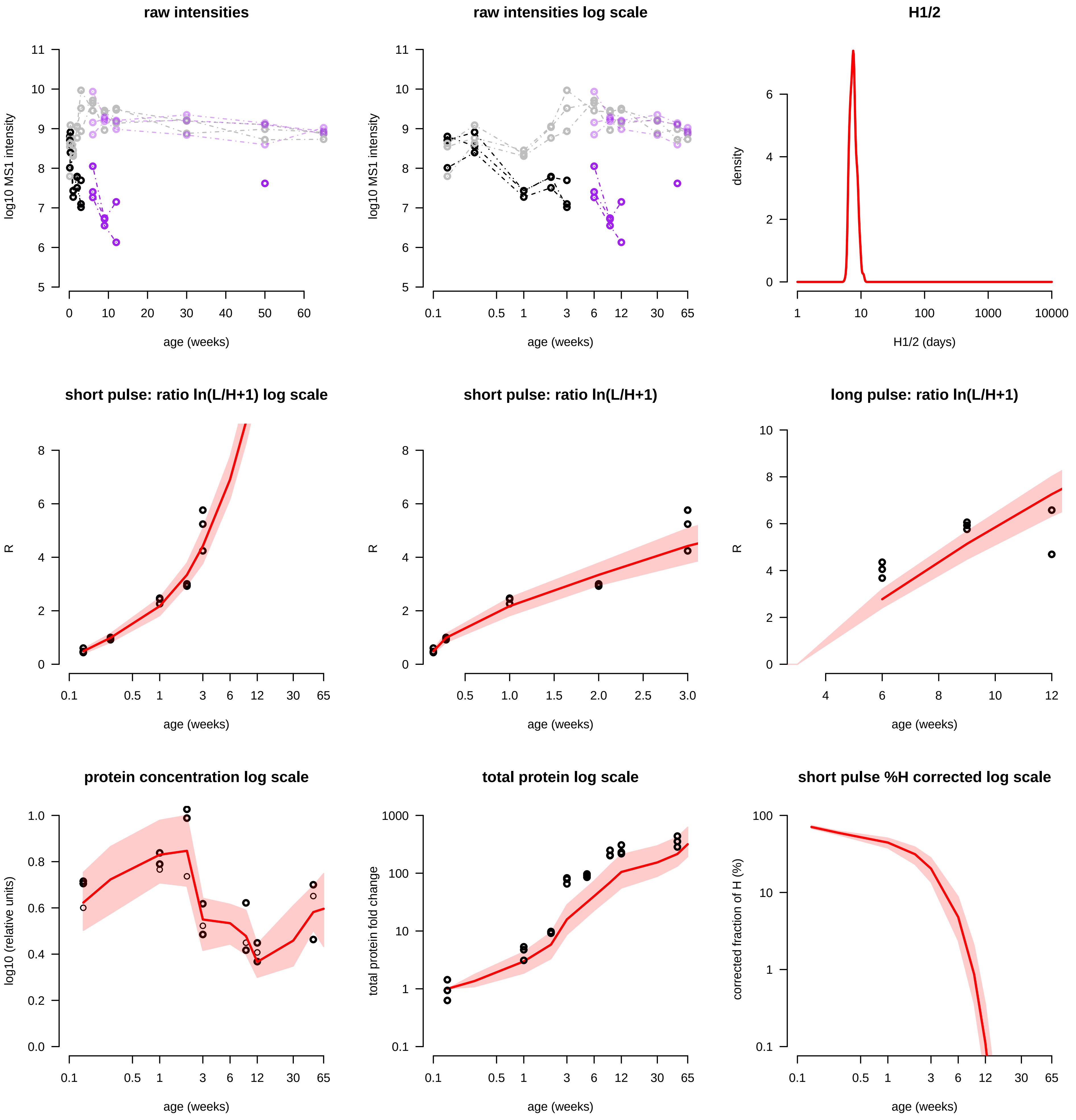

Q9WUU7

>sp|Q9WUU7|CATZ_MOUSE

Cathepsin Z

GN=Ctsz

Med H1/2: 6.7 days

Q5 H1/2: 5.9 days

Q95 H1/2: 8.2 days

reliable model fit: yes

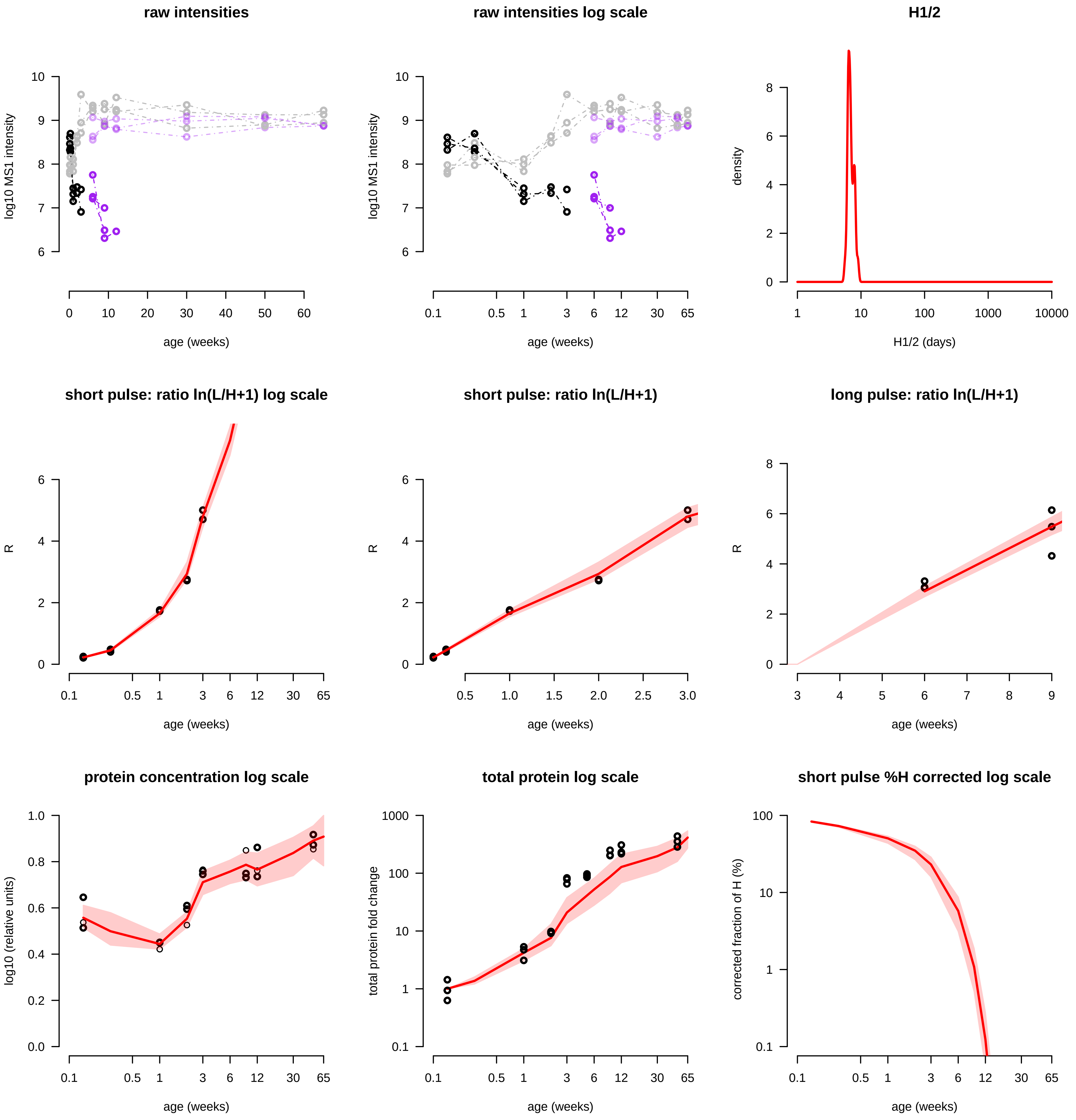

P56379

>sp|P56379|ATP68_MOUSE

ATP synthase subunit ATP5MJ, mitochondrial

GN=Atp5mj

Med H1/2: 12.8 days

Q5 H1/2: 5.3 days

Q95 H1/2: 54.6 days

reliable model fit: yes

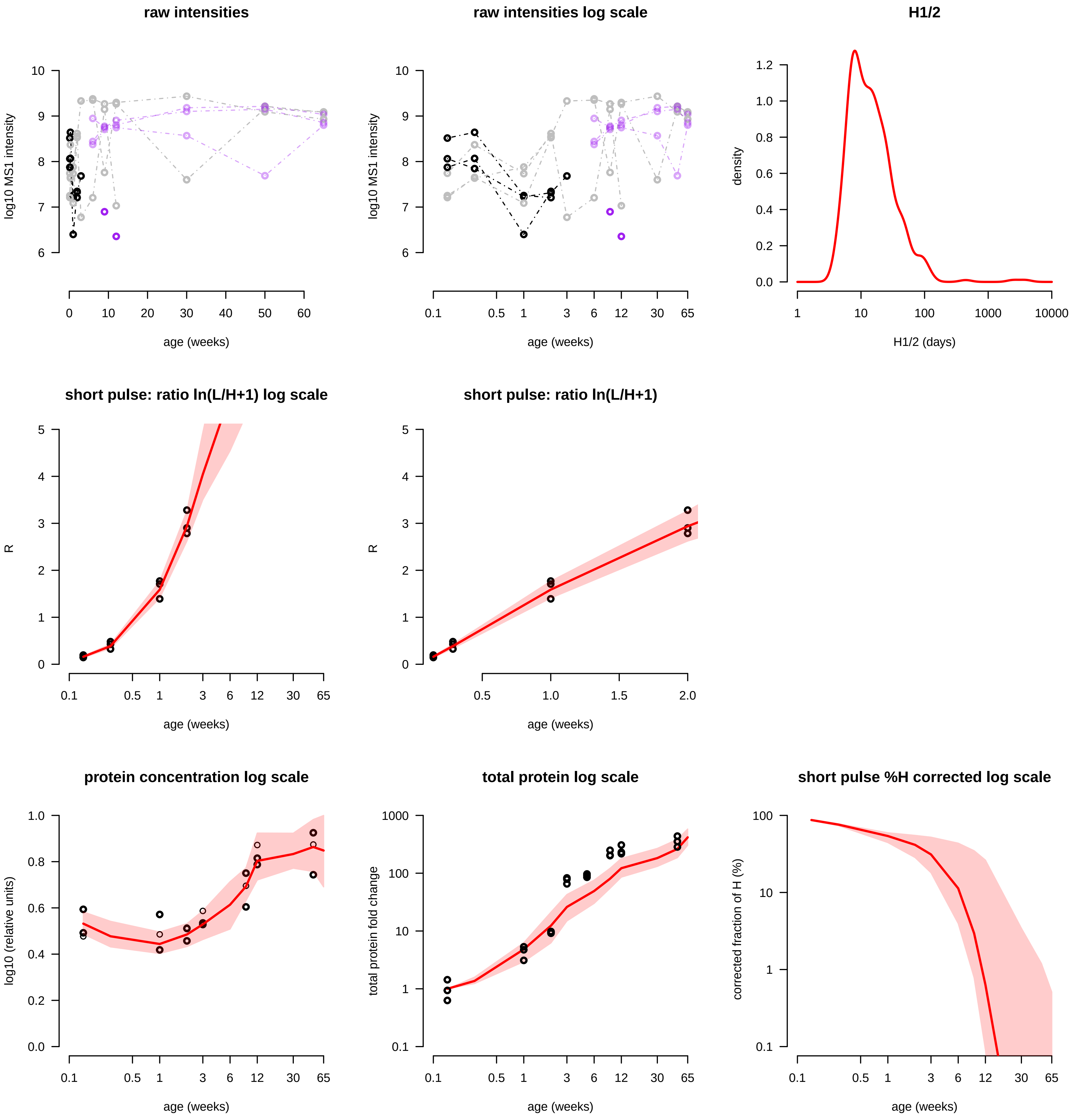

P28660;P28660−2

>sp|P28660|NCKP1_MOUSE

Nck−associated protein 1

GN=Nckap1

Med H1/2: 1126.8 days

Q5 H1/2: 192.5 days

Q95 H1/2: 9791.6 days

reliable model fit: no

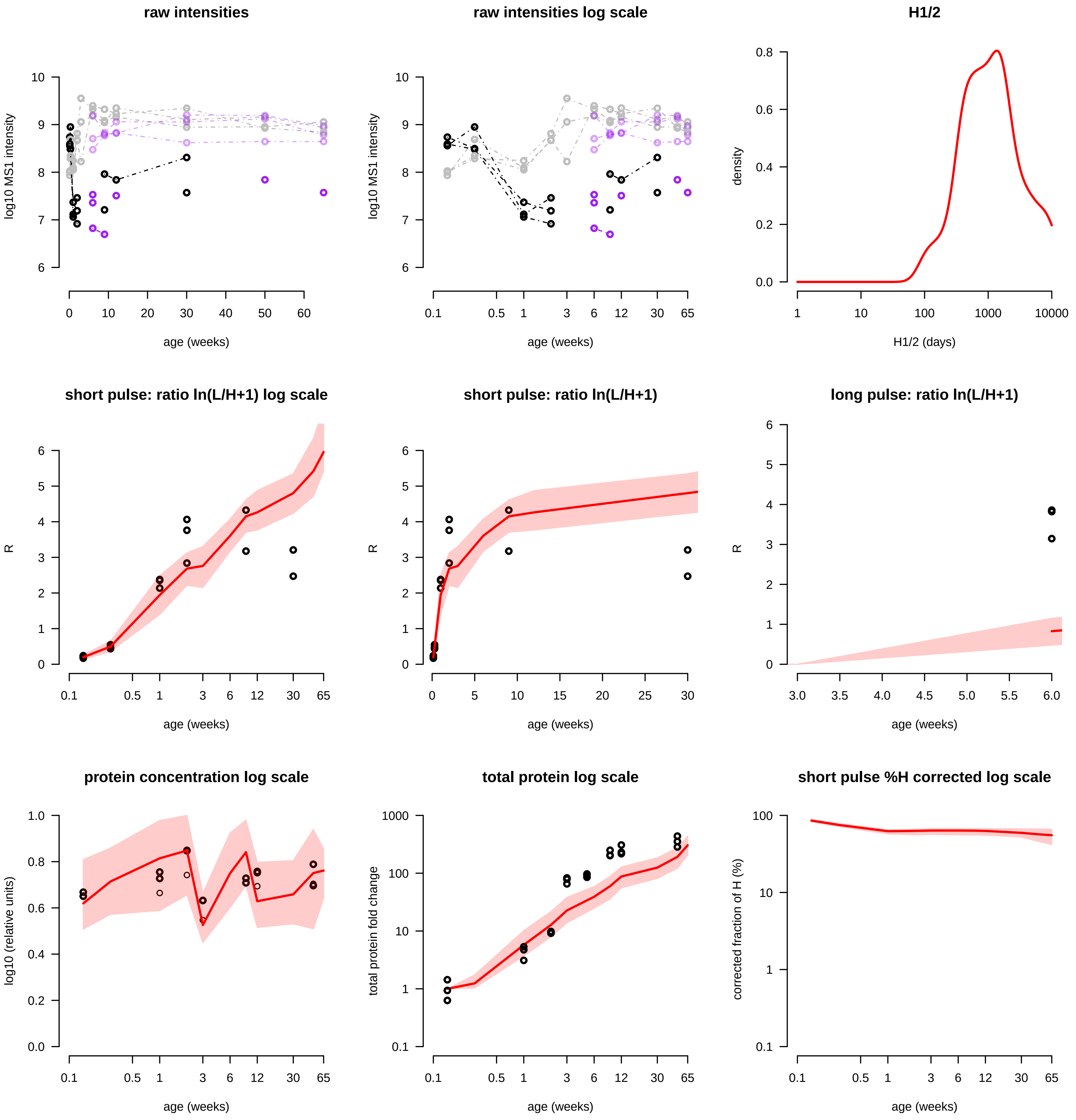

Q9D517

>sp|Q9D517|PLCC_MOUSE

1−acyl−sn−glycerol−3−phosphate acyltransferase gamma

GN=Agpat3

Med H1/2: 158 days

Q5 H1/2: 43.5 days

Q95 H1/2: 1062.9 days

reliable model fit: no

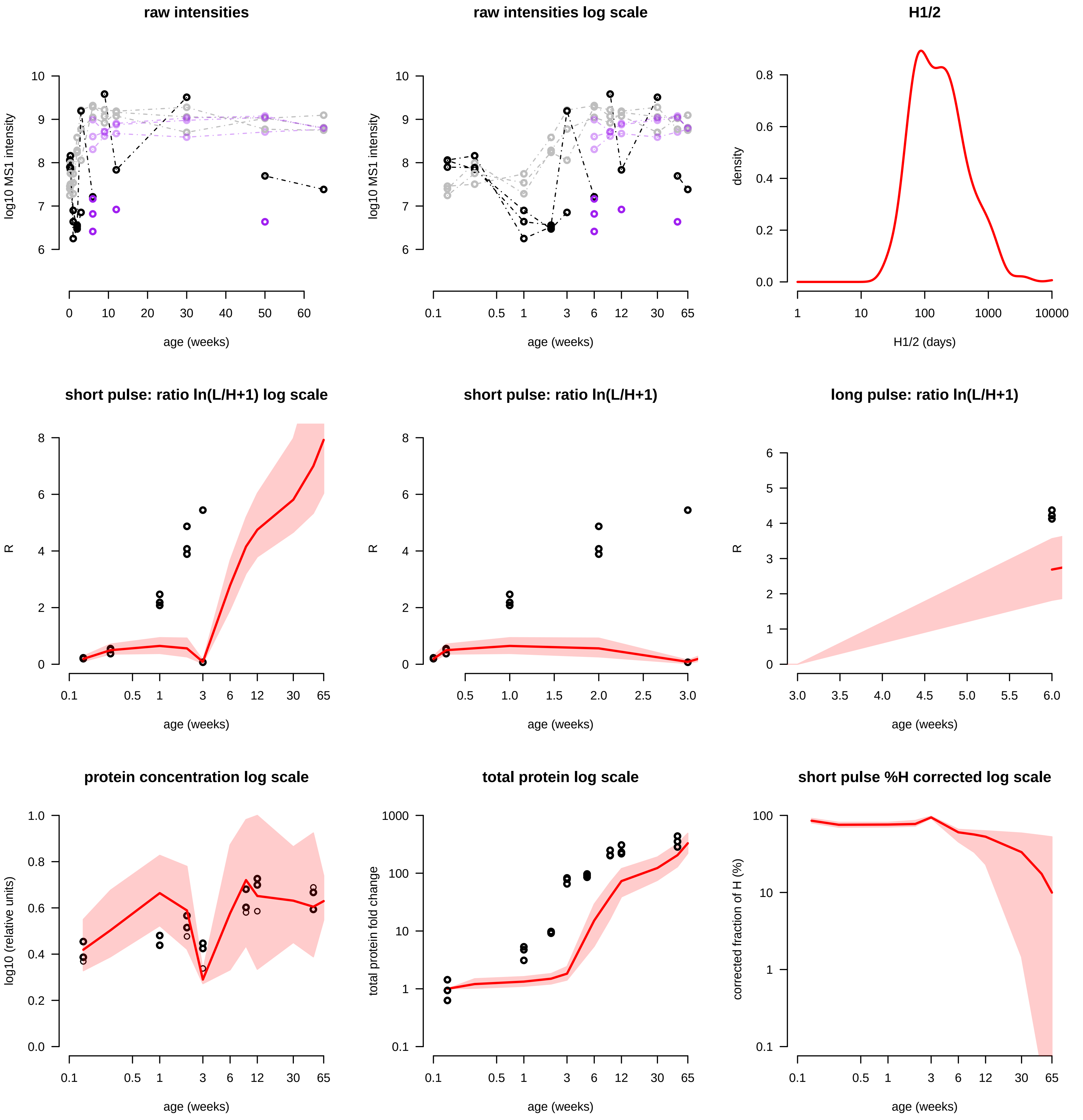

Q8BK64

>sp|Q8BK64|AHSA1_MOUSE

Activator of 90 kDa heat shock protein ATPase homolog 1

GN=Ahsa1

Med H1/2: 3.9 days

Q5 H1/2: 3.3 days

Q95 H1/2: 5 days

reliable model fit: yes

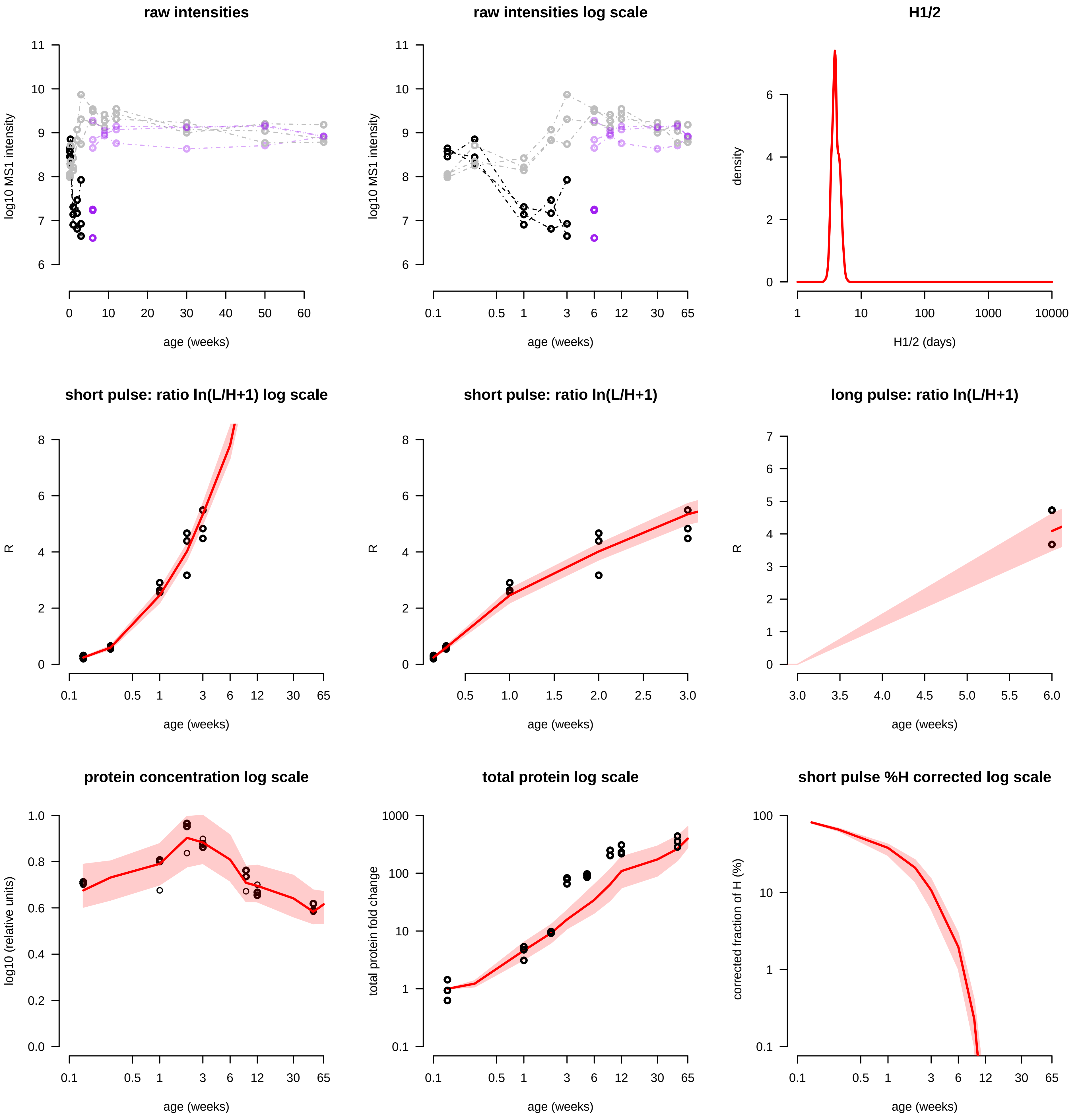

Q5SYD0;Q5SYD0−2

>sp|Q5SYD0|MYO1D_MOUSE

Unconventional myosin−Id

GN=Myo1d

Med H1/2: 5.9 days

Q5 H1/2: 2.6 days

Q95 H1/2: 42.2 days

reliable model fit: yes

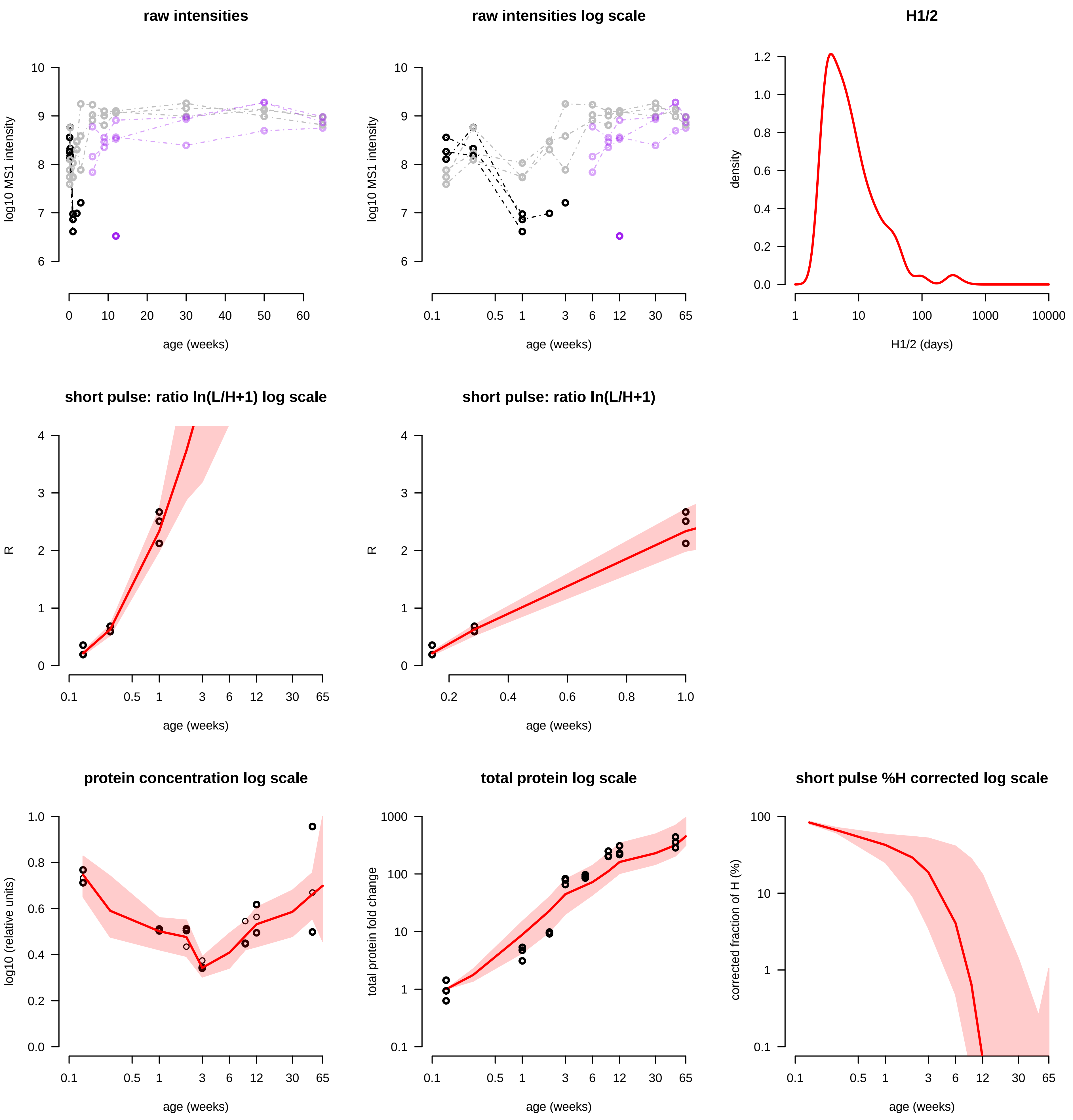

O70589−5;O70589;O70589−3;O70589−2

>sp|O70589−5|CSKP_MOUSE

Isoform 5 of Peripheral plasma membrane protein CASK

GN=Cask

Med H1/2: 31.7 days

Q5 H1/2: 7.6 days

Q95 H1/2: 404.6 days

reliable model fit: yes

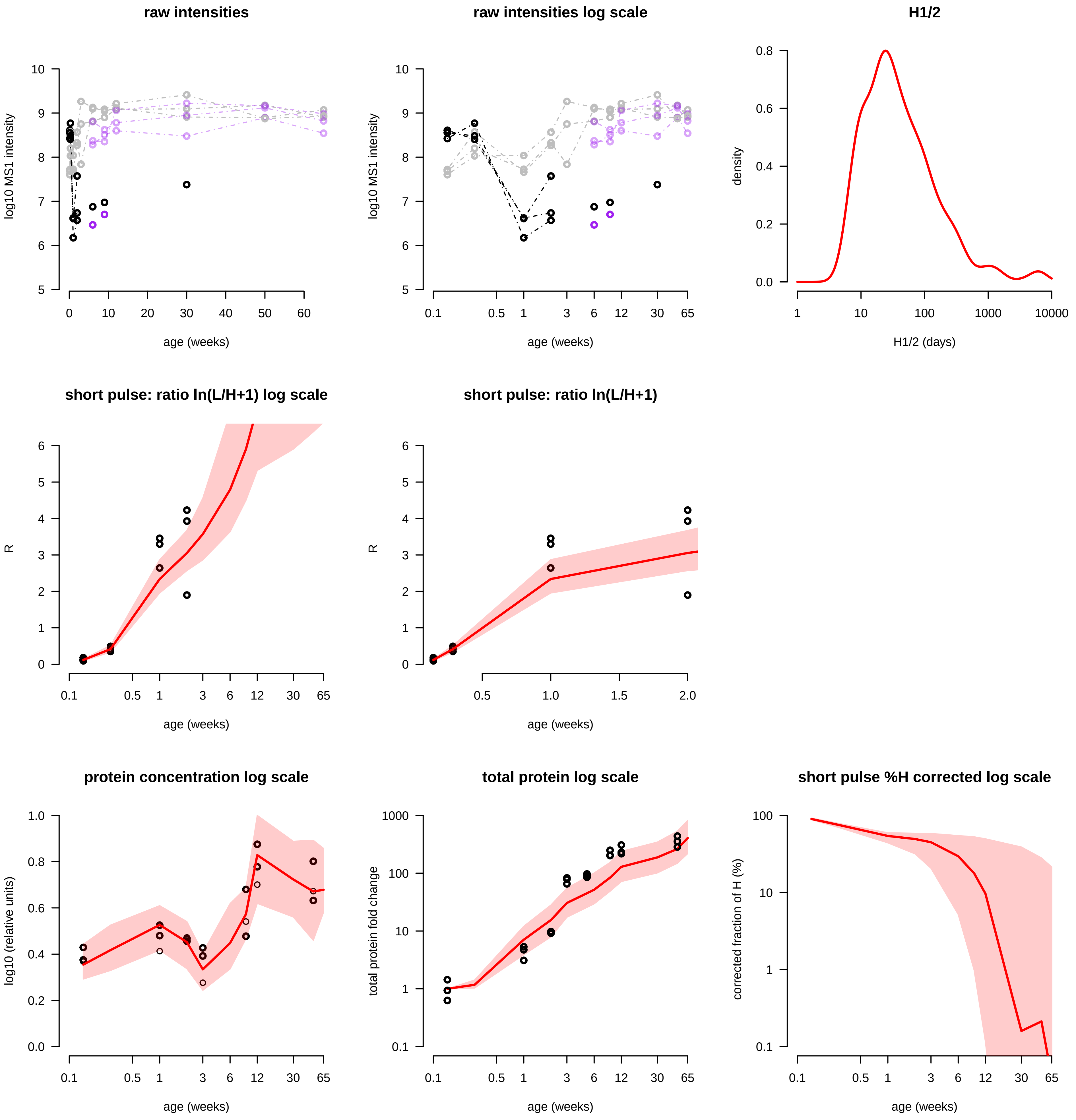

P84089

>sp|P84089|ERH_MOUSE

Enhancer of rudimentary homolog

GN=Erh

Med H1/2: 14.4 days

Q5 H1/2: 11.6 days

Q95 H1/2: 20.1 days

reliable model fit: yes

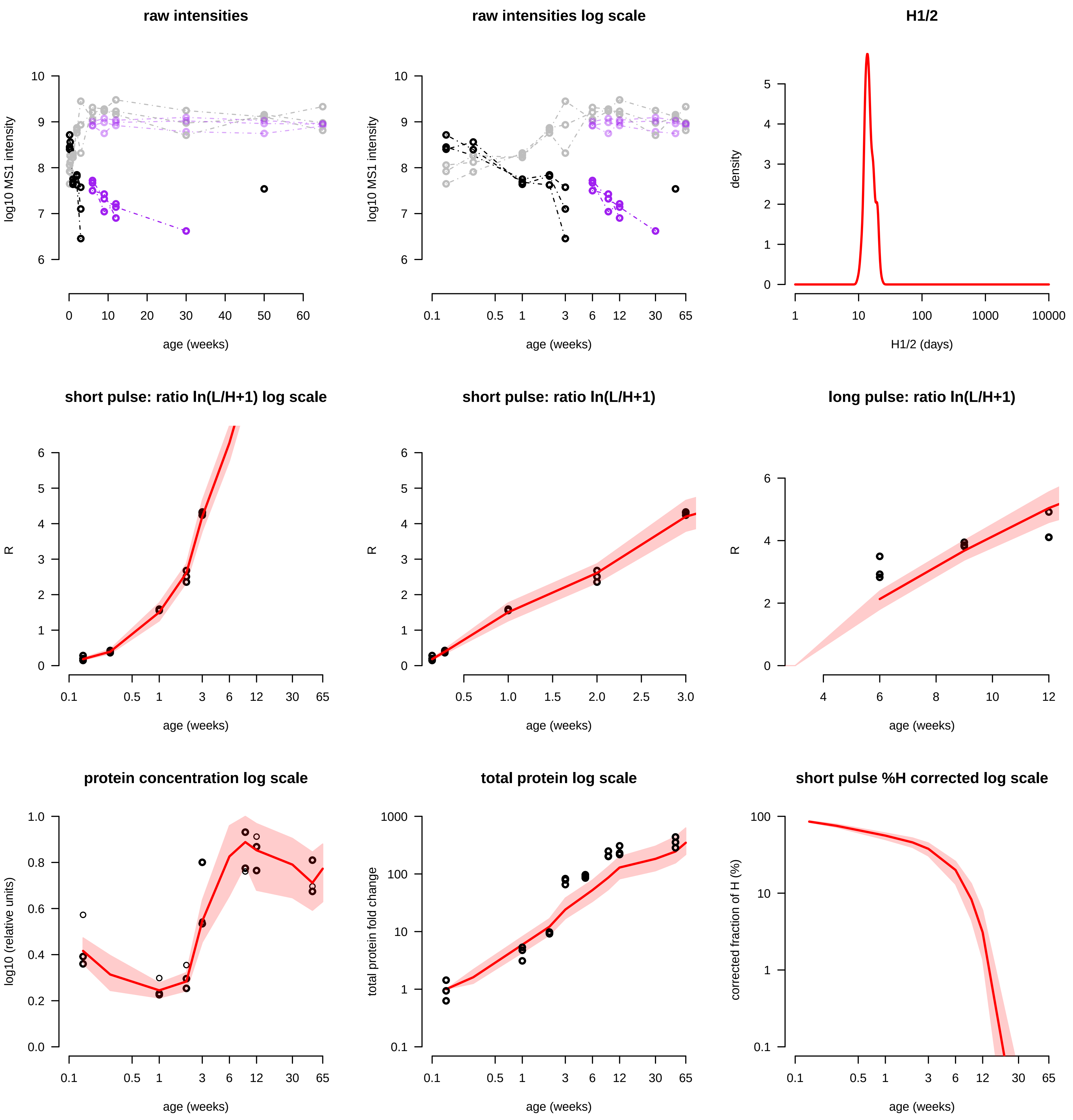

P97290

>sp|P97290|IC1_MOUSE

Plasma protease C1 inhibitor

GN=Serping1

Med H1/2: 15.7 days

Q5 H1/2: 8.8 days

Q95 H1/2: 58.6 days

reliable model fit: yes

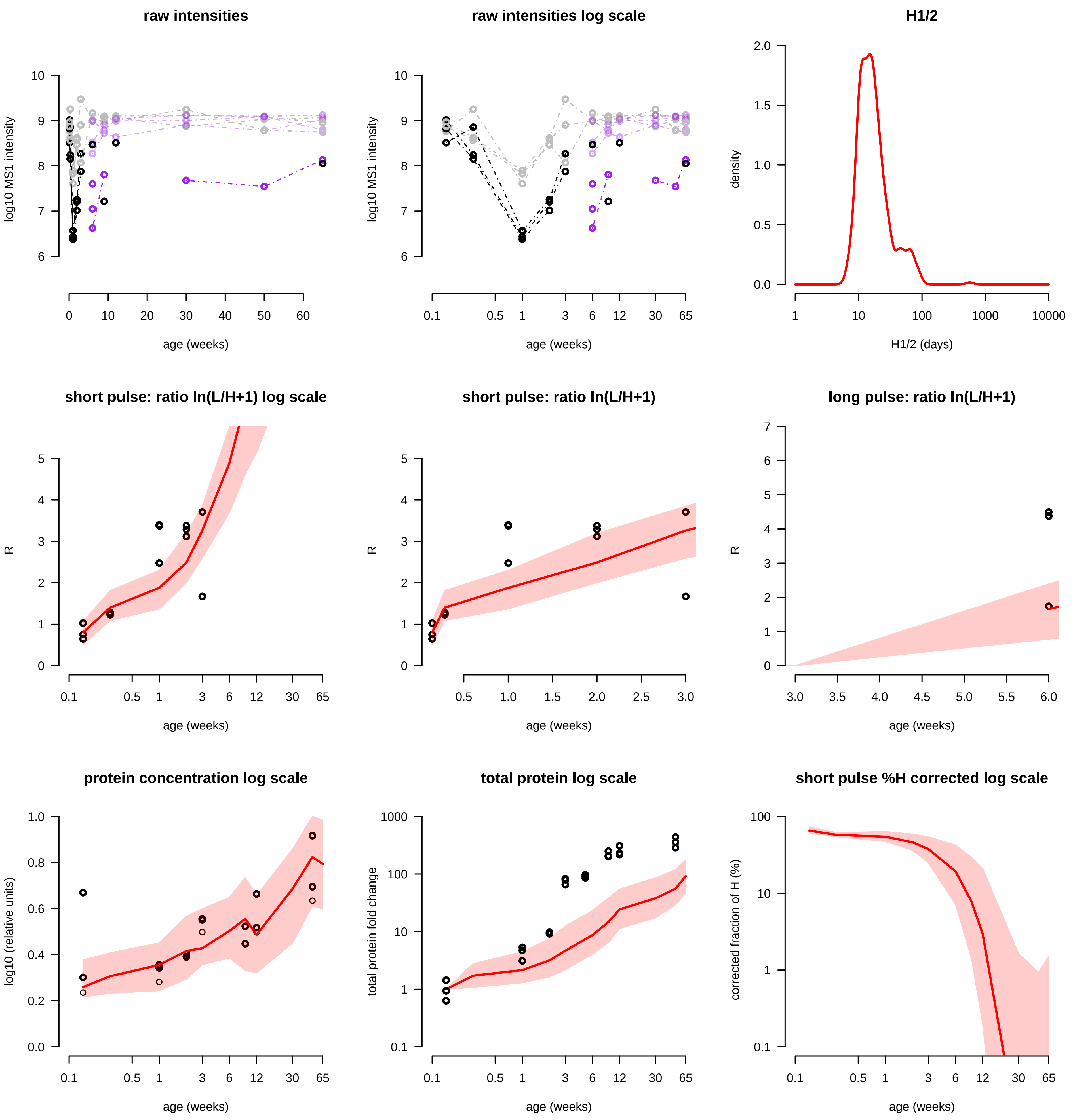

Q62448

>sp|Q62448|IF4G2_MOUSE

Eukaryotic translation initiation factor 4 gamma 2

GN=Eif4g2

Med H1/2: 3.5 days

Q5 H1/2: 3 days

Q95 H1/2: 4.3 days

reliable model fit: yes

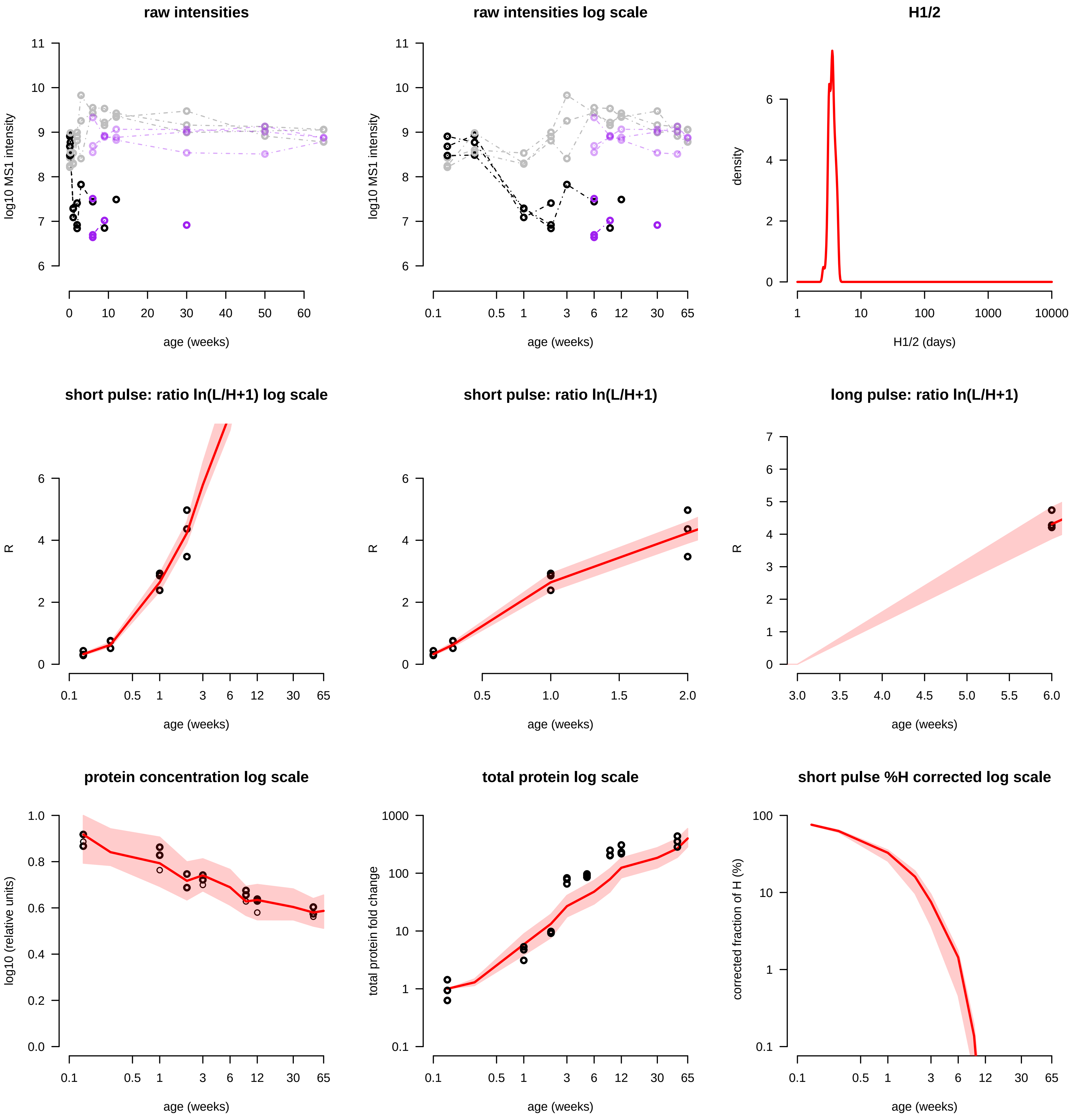

O35350

>sp|O35350|CAN1_MOUSE

Calpain−1 catalytic subunit

GN=Capn1

Med H1/2: 4.2 days

Q5 H1/2: 3.4 days

Q95 H1/2: 5.5 days

reliable model fit: yes

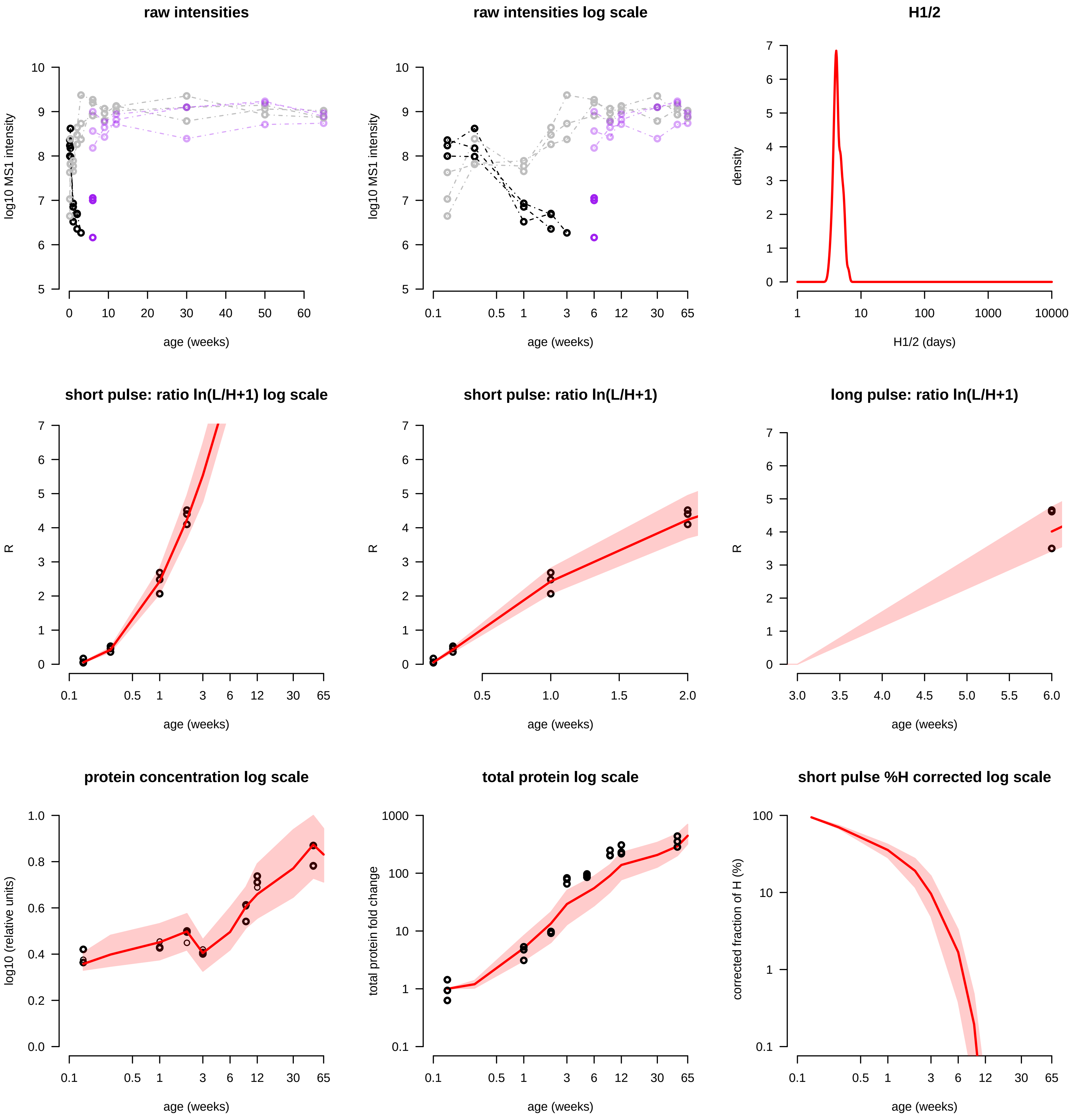

Q99MR6−2;Q99MR6;Q99MR6−3;Q99MR6−4

>sp|Q99MR6−2|SRRT_MOUSE

Isoform B of Serrate RNA effector molecule homolog

GN=Srrt

Med H1/2: 601.7 days

Q5 H1/2: 244 days

Q95 H1/2: 7914.7 days

reliable model fit: yes

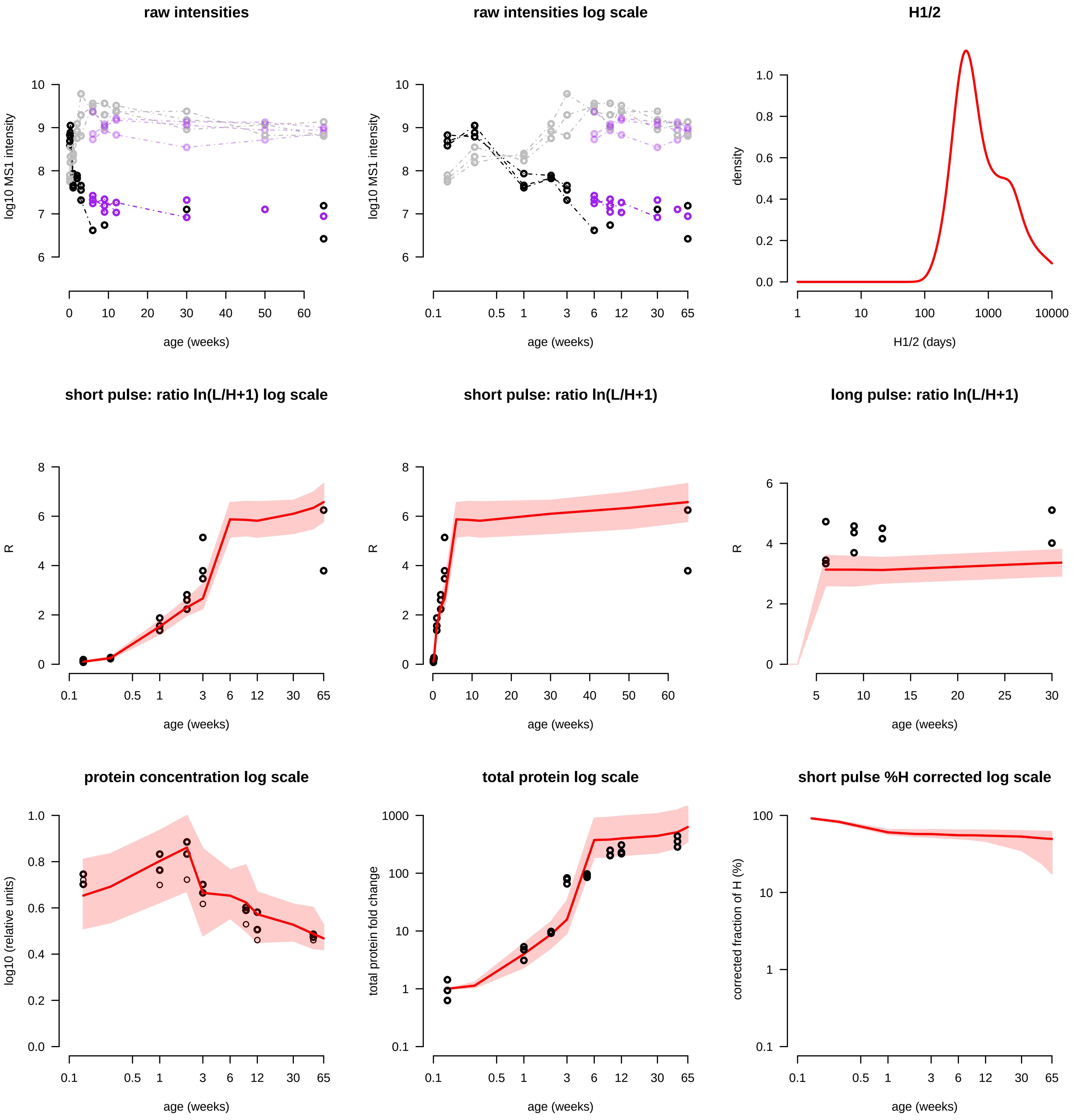

P61971

>sp|P61971|NTF2_MOUSE

Nuclear transport factor 2

GN=Nutf2

Med H1/2: 6.5 days

Q5 H1/2: 4.3 days

Q95 H1/2: 10.1 days

reliable model fit: yes

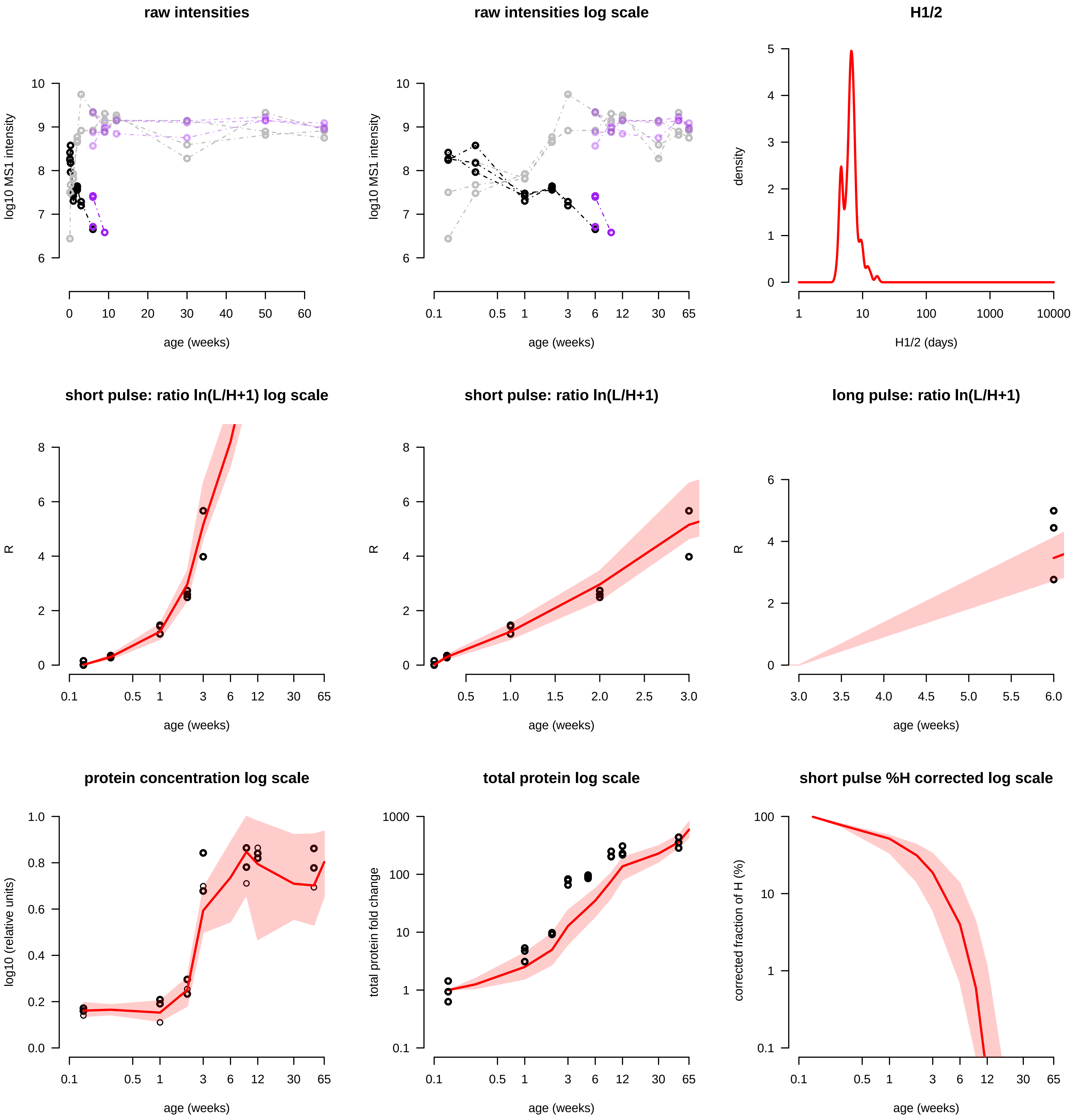

Q9D554

>sp|Q9D554|SF3A3_MOUSE

Splicing factor 3A subunit 3

GN=Sf3a3

Med H1/2: 4.9 days

Q5 H1/2: 4 days

Q95 H1/2: 6.3 days

reliable model fit: yes

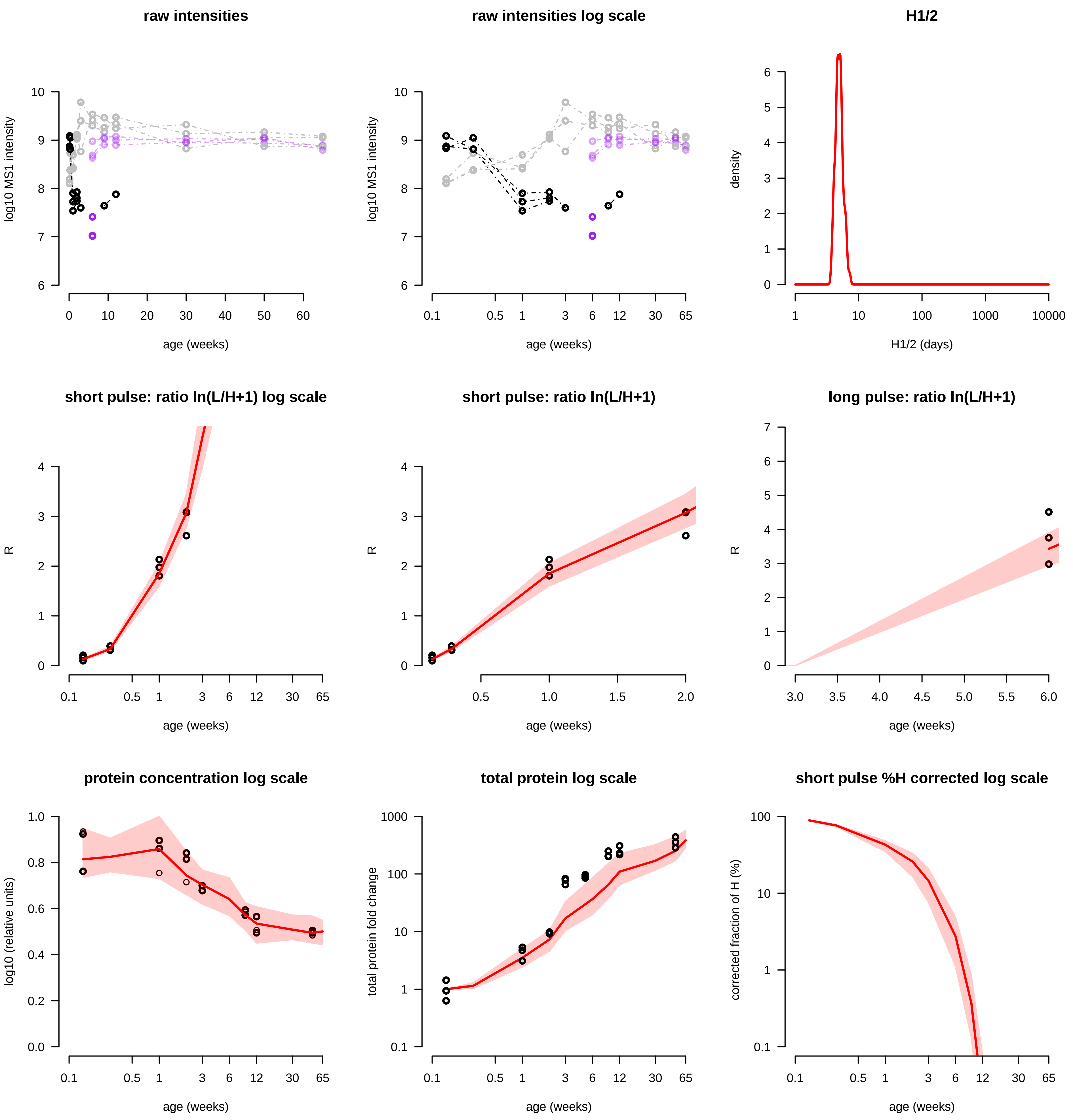

Q62165

>sp|Q62165|DAG1_MOUSE

Dystroglycan

GN=Dag1

Med H1/2: 10.6 days

Q5 H1/2: 3.2 days

Q95 H1/2: 81.1 days

reliable model fit: yes

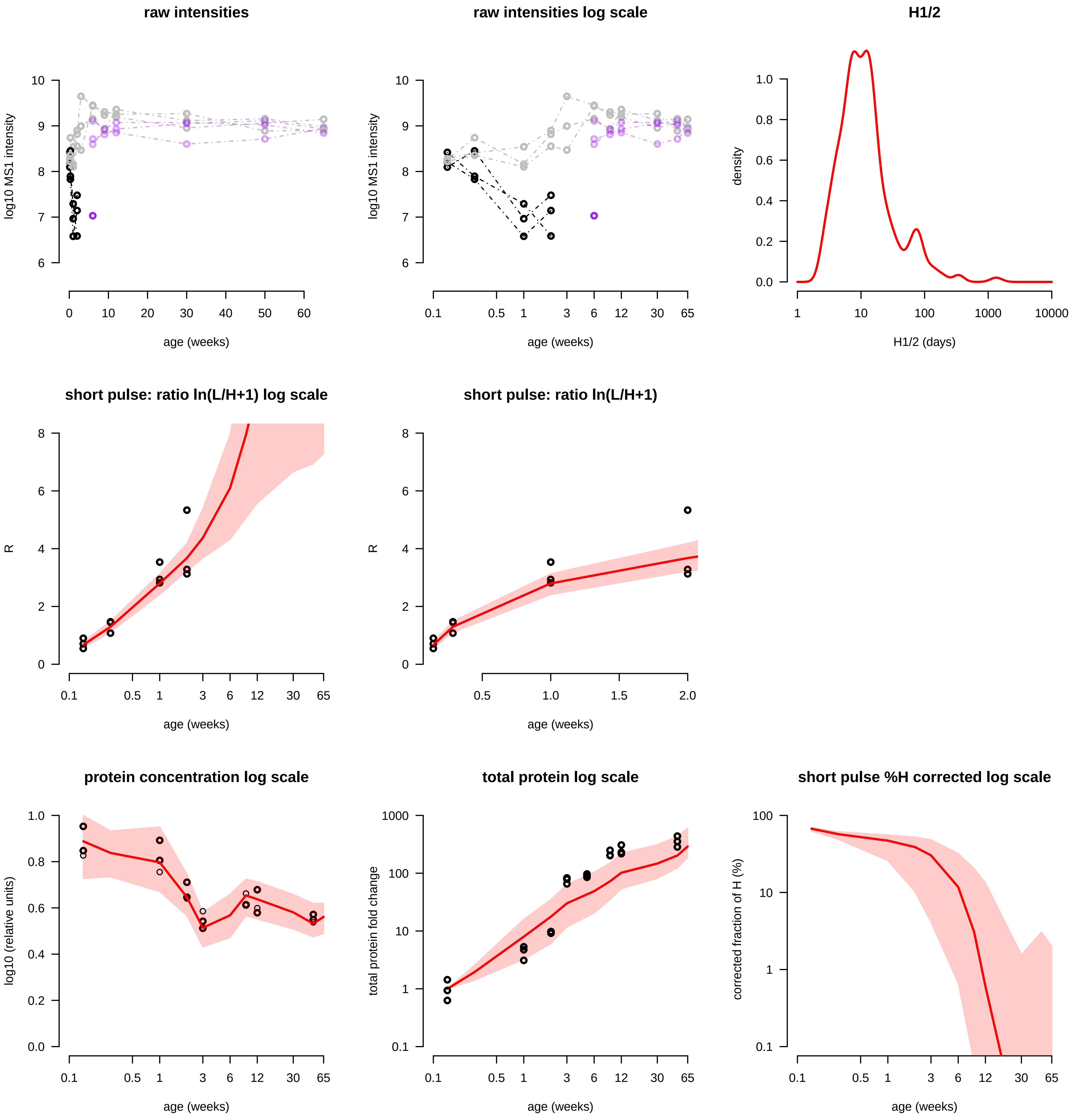

Q00915

>sp|Q00915|RET1_MOUSE

Retinol−binding protein 1

GN=Rbp1

Med H1/2: 255.8 days

Q5 H1/2: 80.3 days

Q95 H1/2: 1427.8 days

reliable model fit: yes

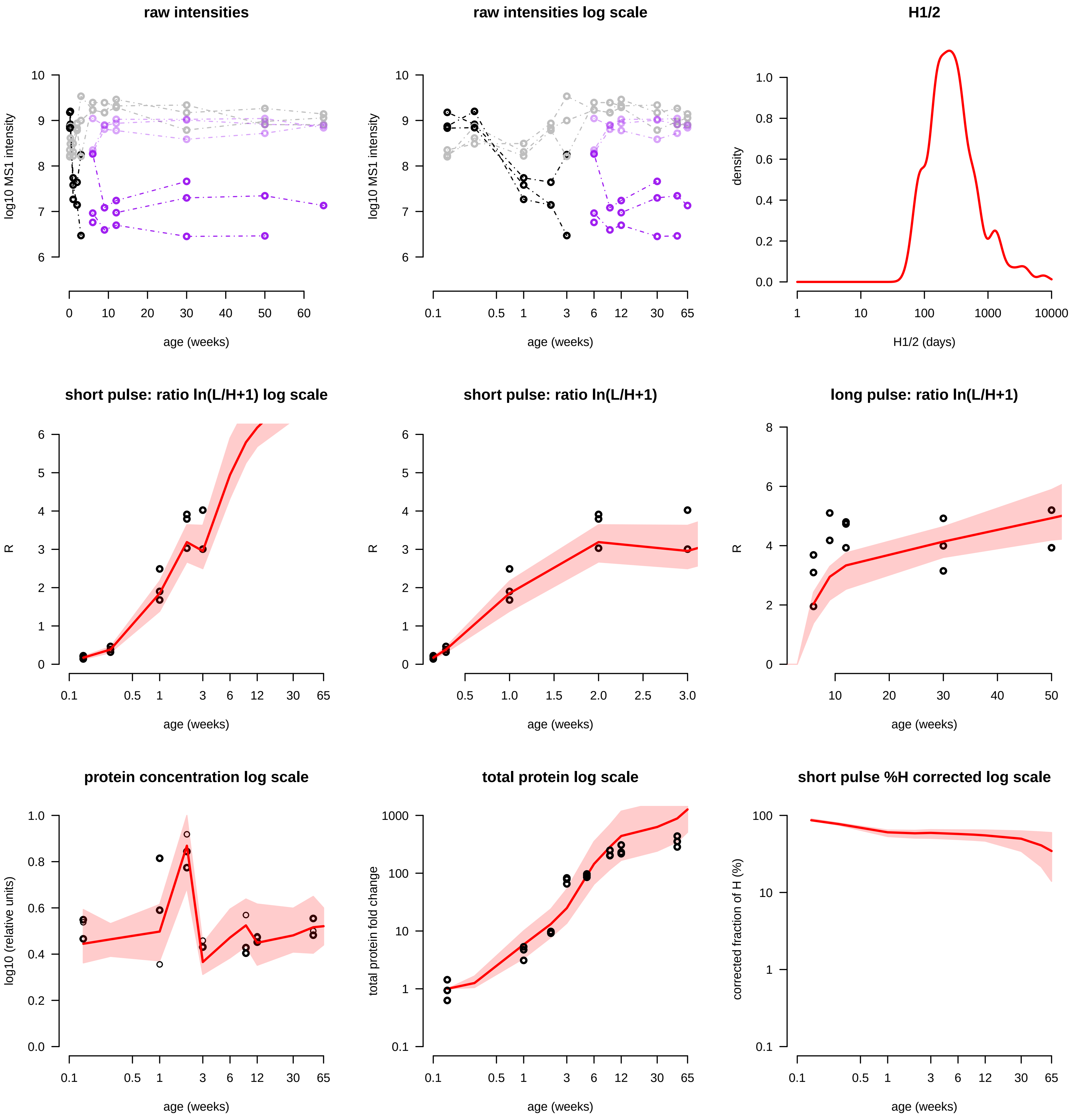

P54823

>sp|P54823|DDX6_MOUSE

Probable ATP−dependent RNA helicase DDX6

GN=Ddx6

Med H1/2: 7.5 days

Q5 H1/2: 5.9 days

Q95 H1/2: 10.4 days

reliable model fit: no

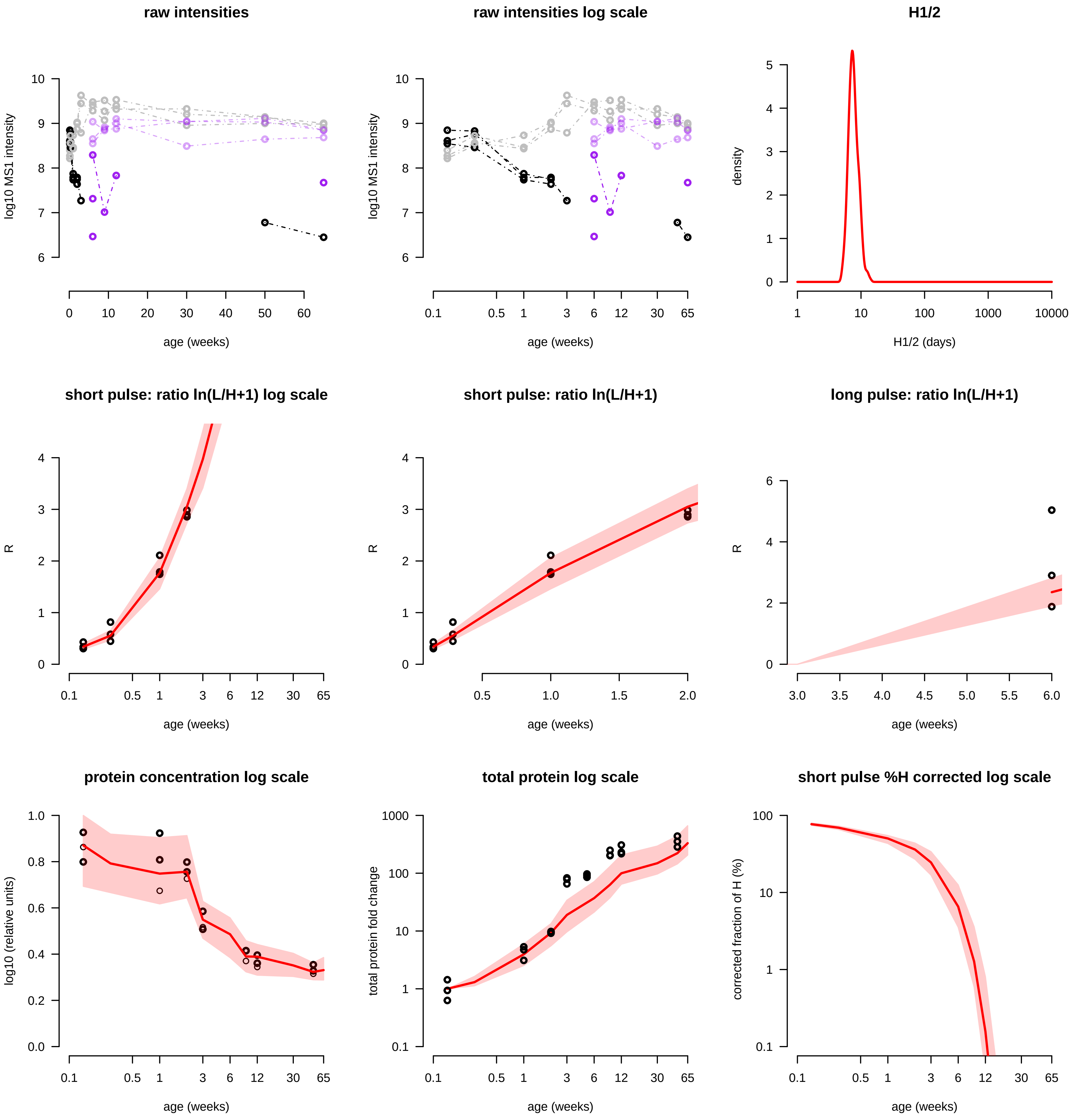

Q9DBC7

>sp|Q9DBC7|KAP0_MOUSE

cAMP−dependent protein kinase type I−alpha regulatory subunit

GN=Prkar1a

Med H1/2: 8.6 days

Q5 H1/2: 6.3 days

Q95 H1/2: 12.8 days

reliable model fit: yes

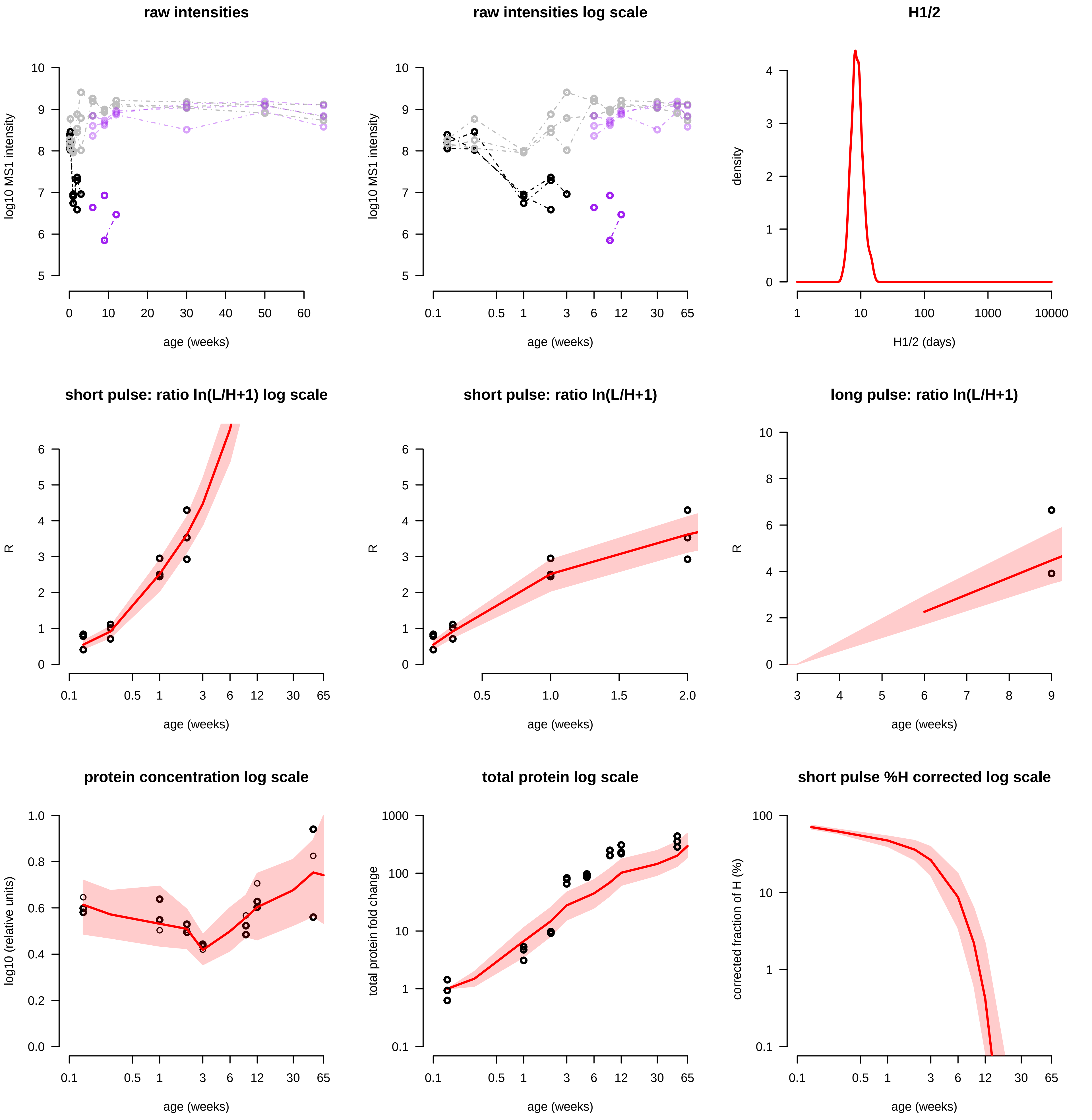

Q8BK67

>sp|Q8BK67|RCC2_MOUSE

Protein RCC2

GN=Rcc2

Med H1/2: 6.3 days

Q5 H1/2: 5.1 days

Q95 H1/2: 8.3 days

reliable model fit: yes

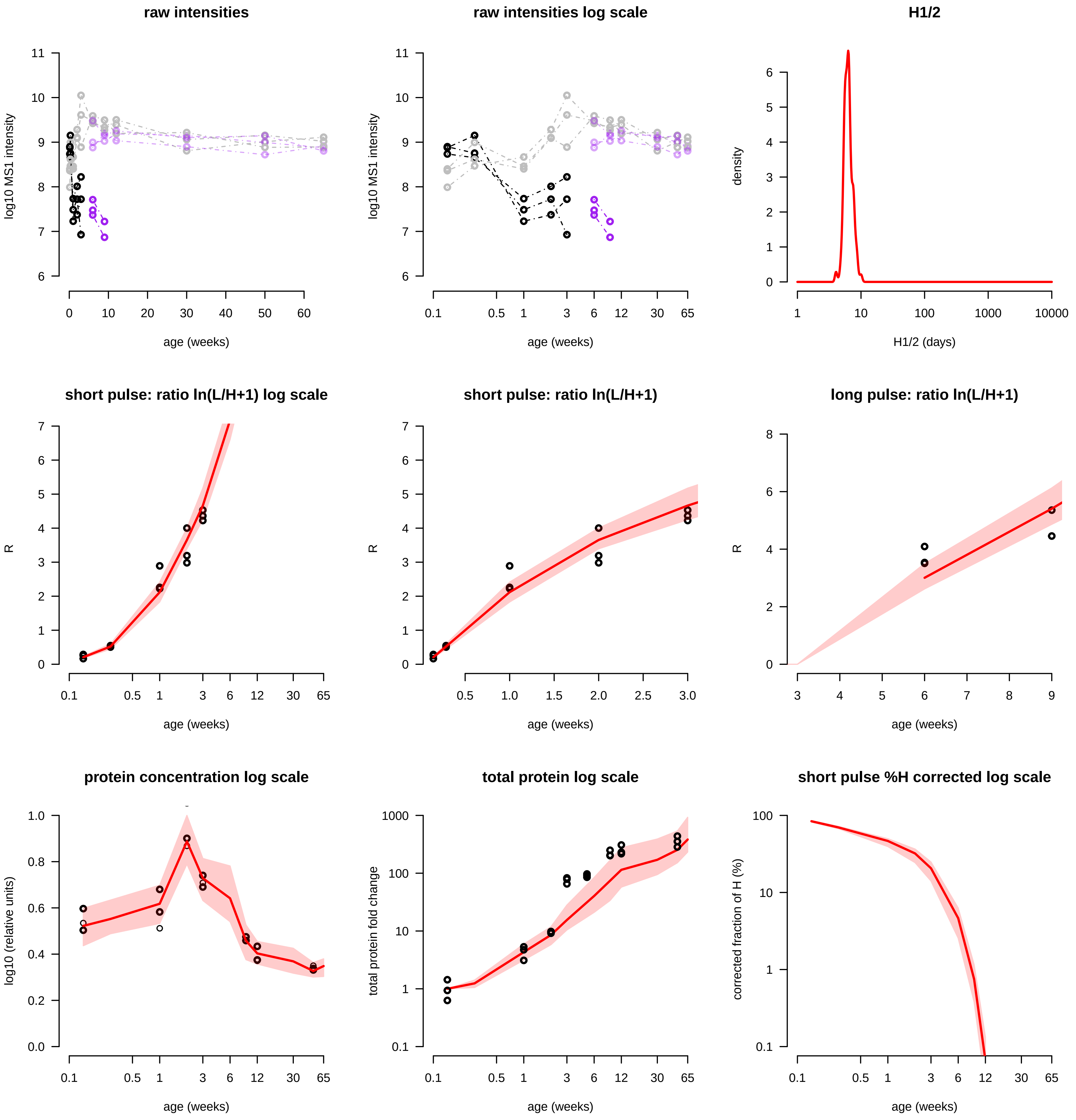

Q8K1J6

>sp|Q8K1J6|TRNT1_MOUSE

CCA tRNA nucleotidyltransferase 1, mitochondrial

GN=Trnt1

Med H1/2: 7.7 days

Q5 H1/2: 5.5 days

Q95 H1/2: 12.3 days

reliable model fit: yes

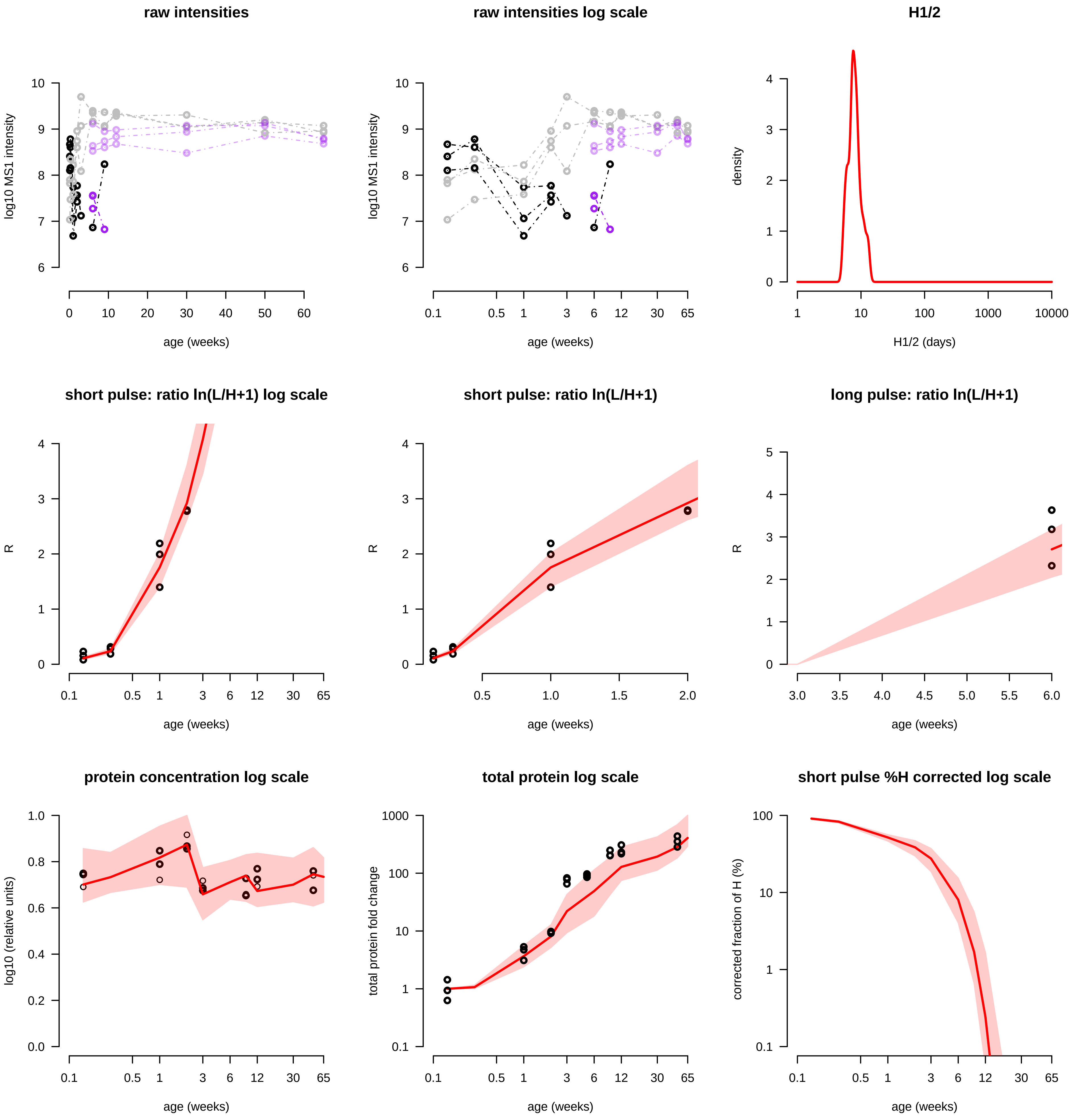

Q9WUM4

>sp|Q9WUM4|COR1C_MOUSE

Coronin−1C

GN=Coro1c

Med H1/2: 5.5 days

Q5 H1/2: 4.7 days

Q95 H1/2: 7 days

reliable model fit: yes

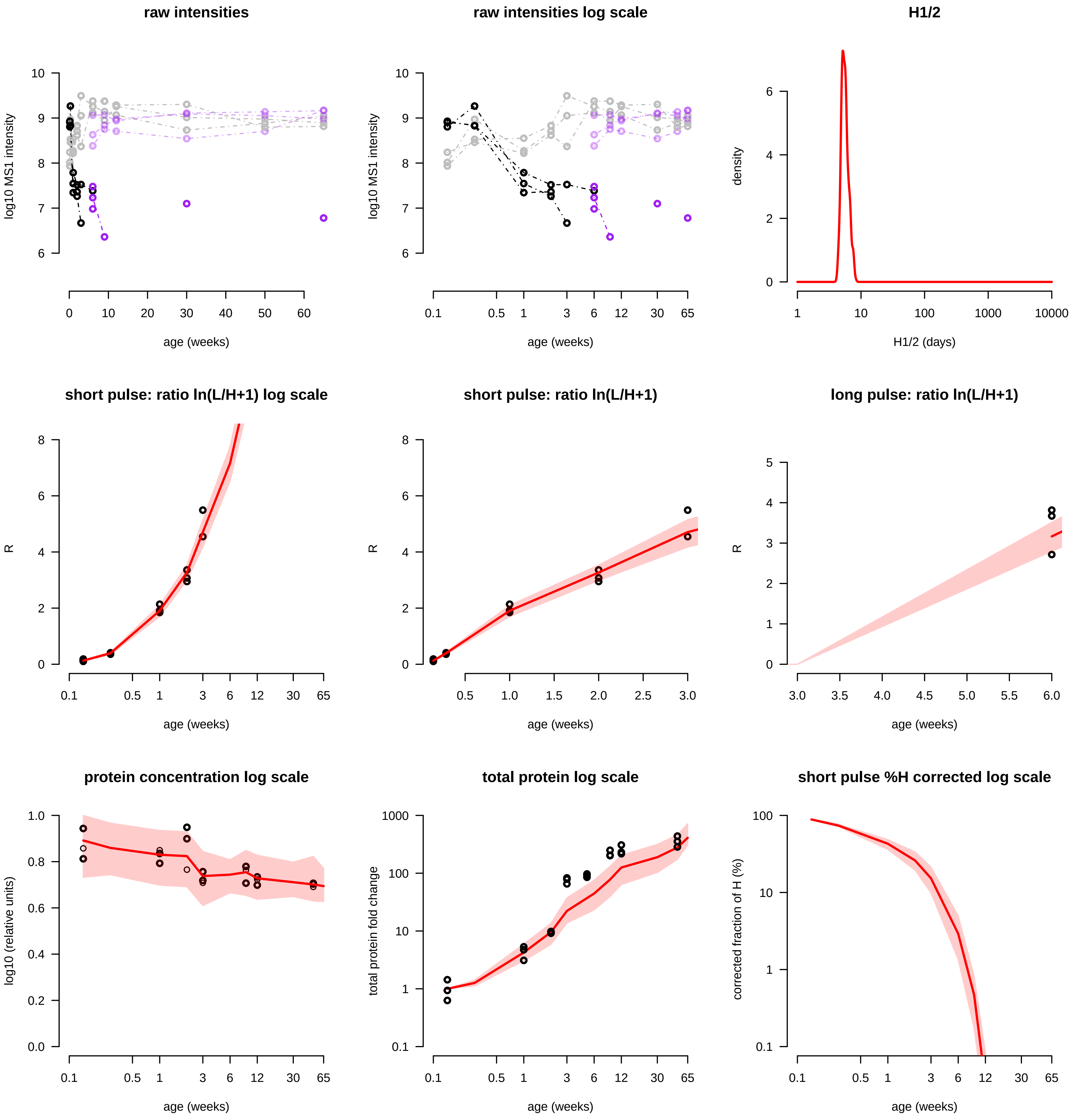

Q9CZ44;Q9CZ44−3;Q9CZ44−2

>sp|Q9CZ44|NSF1C_MOUSE

NSFL1 cofactor p47

GN=Nsfl1c

Med H1/2: 9 days

Q5 H1/2: 6.3 days

Q95 H1/2: 14.5 days

reliable model fit: yes

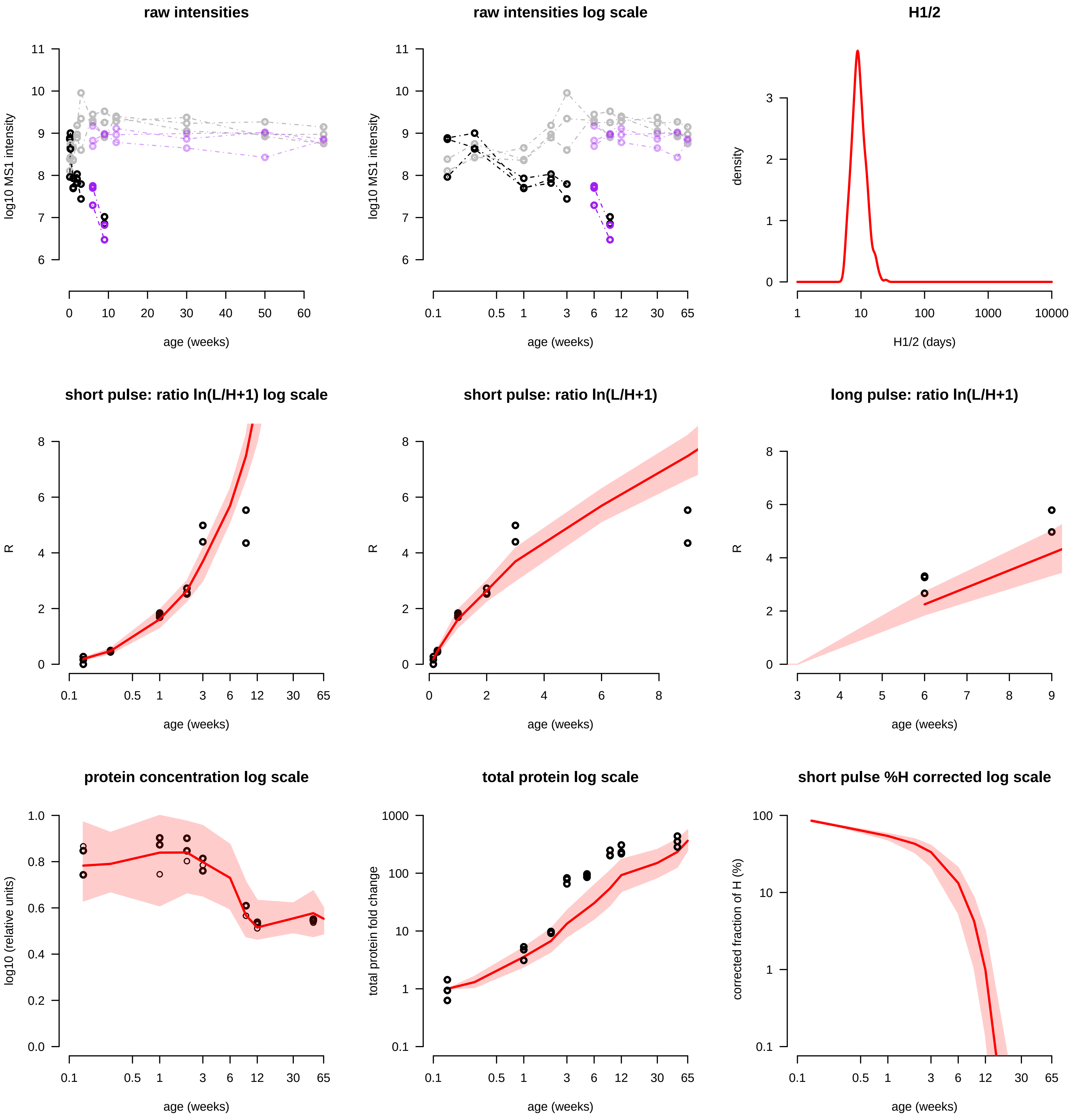

Q3U7R1;Q3U7R1−2

>sp|Q3U7R1|ESYT1_MOUSE

Extended synaptotagmin−1

GN=Esyt1

Med H1/2: 5.4 days

Q5 H1/2: 4.5 days

Q95 H1/2: 6.5 days

reliable model fit: yes

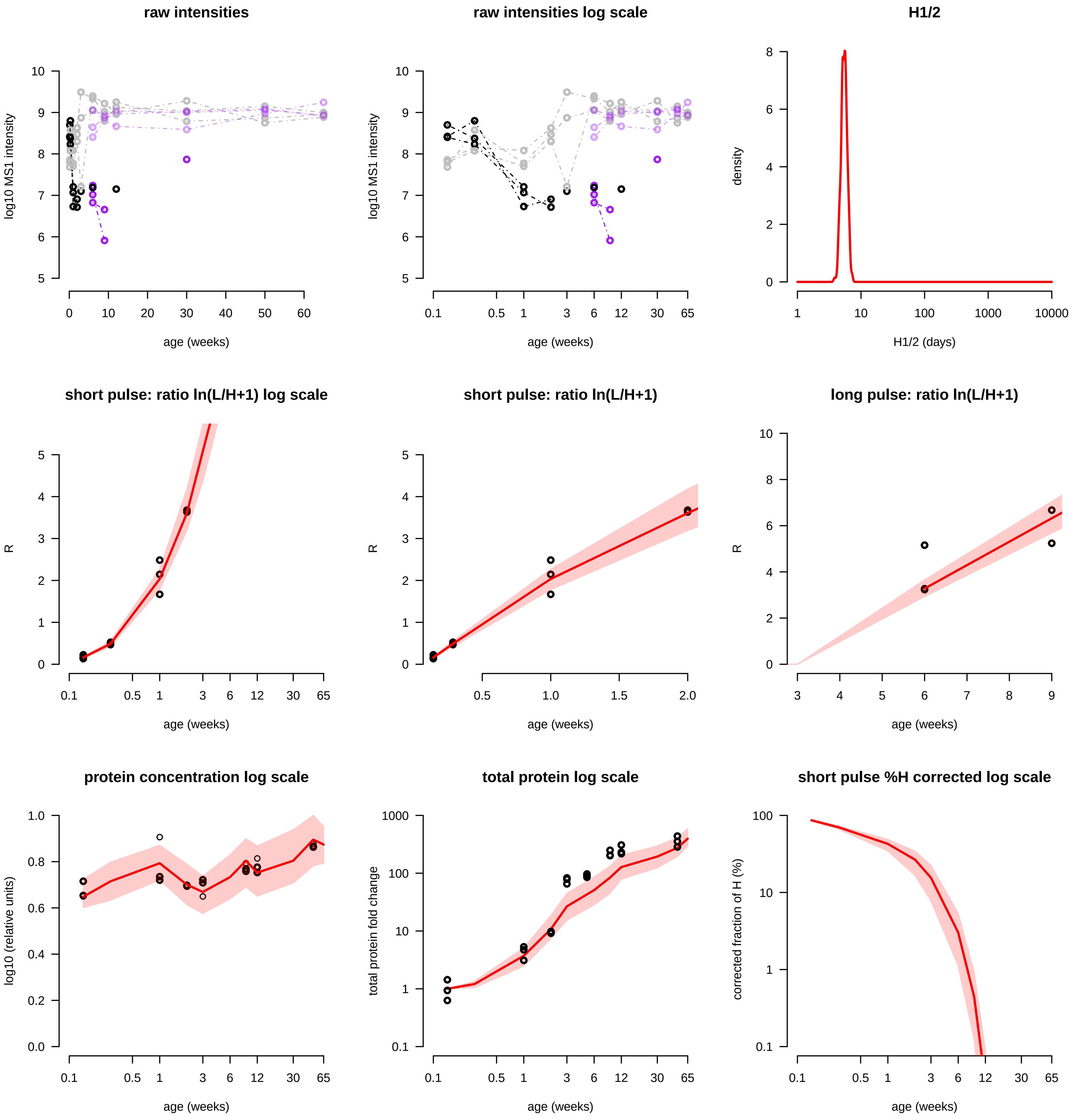

Q06138

>sp|Q06138|CAB39_MOUSE

Calcium−binding protein 39

GN=Cab39

Med H1/2: 117.1 days

Q5 H1/2: 69 days

Q95 H1/2: 269.4 days

reliable model fit: no

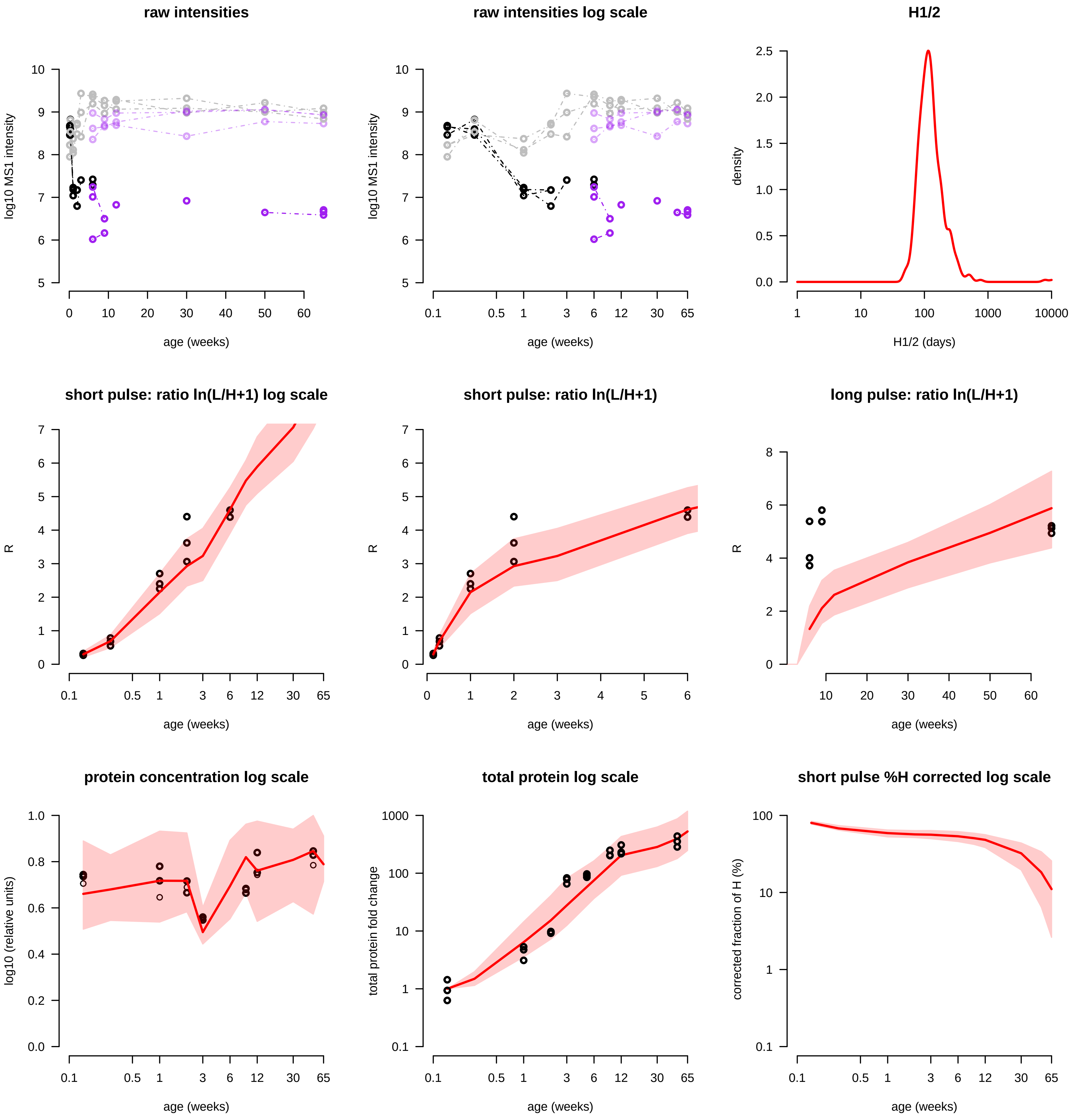

P83940

>sp|P83940|ELOC_MOUSE

Elongin−C

GN=Eloc

Med H1/2: 12 days

Q5 H1/2: 8.7 days

Q95 H1/2: 17.3 days

reliable model fit: yes

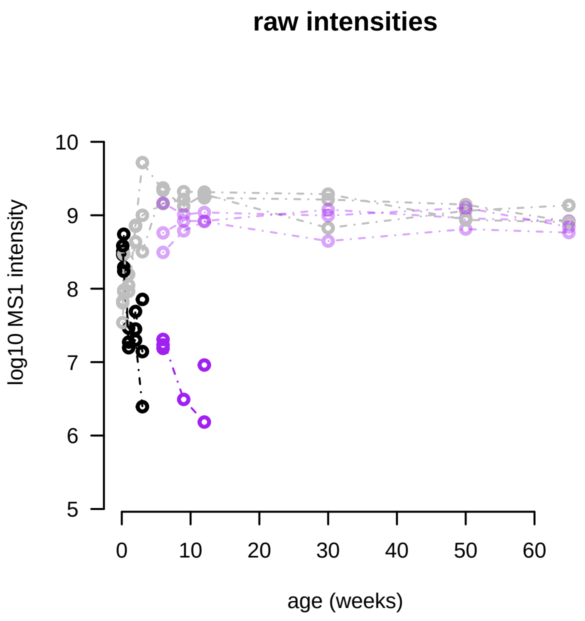

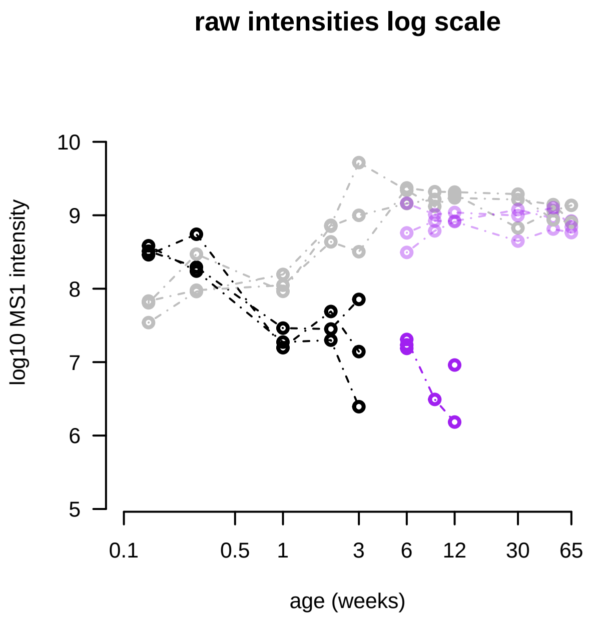

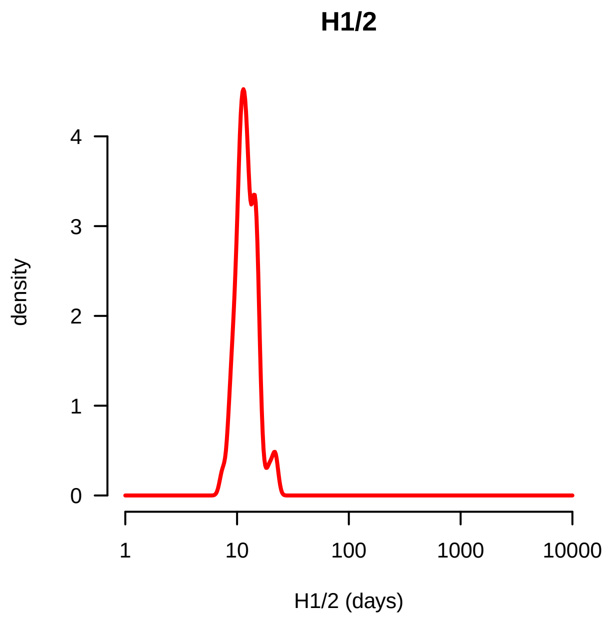

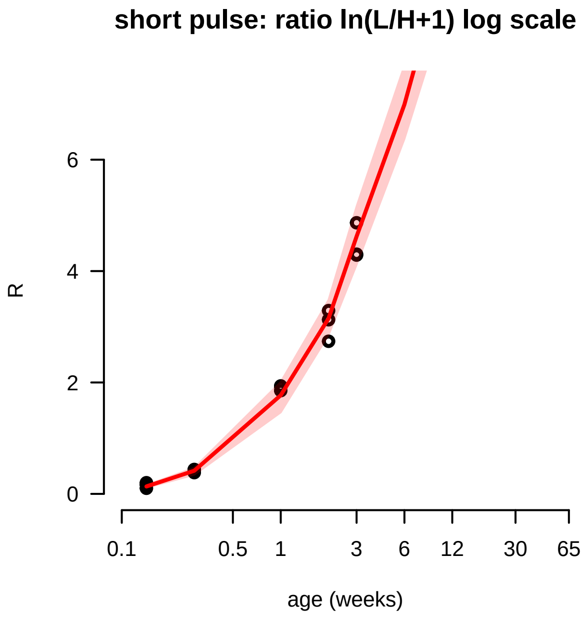

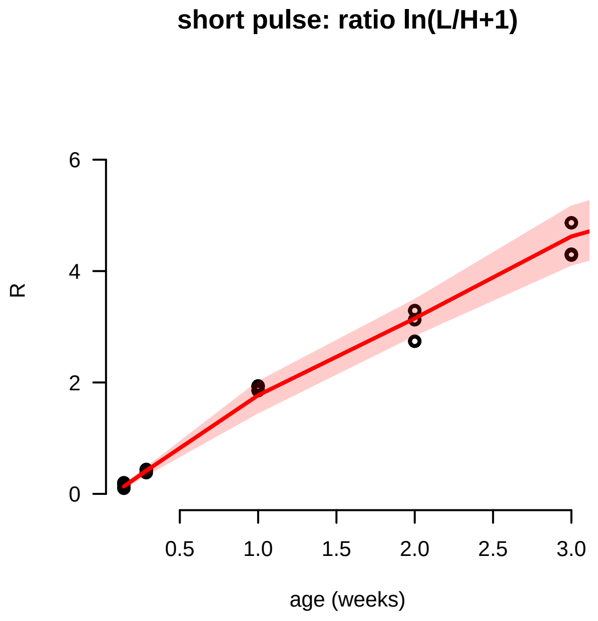

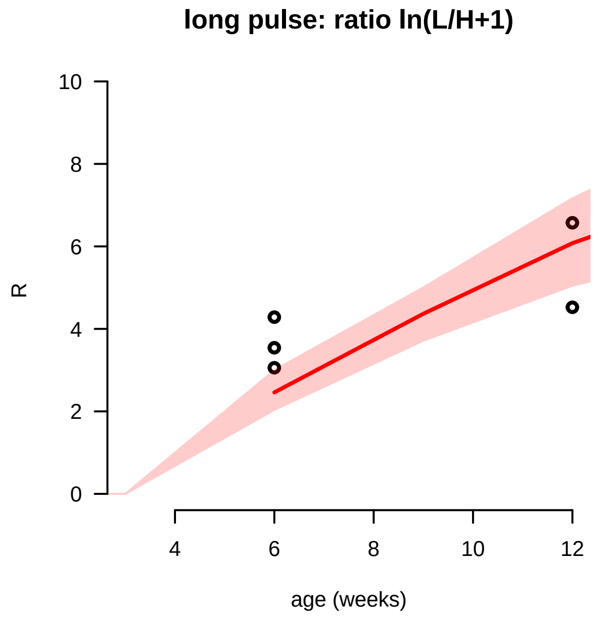

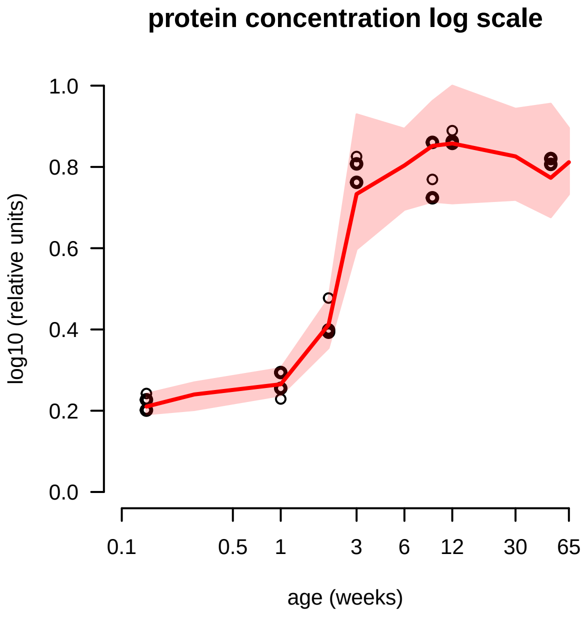

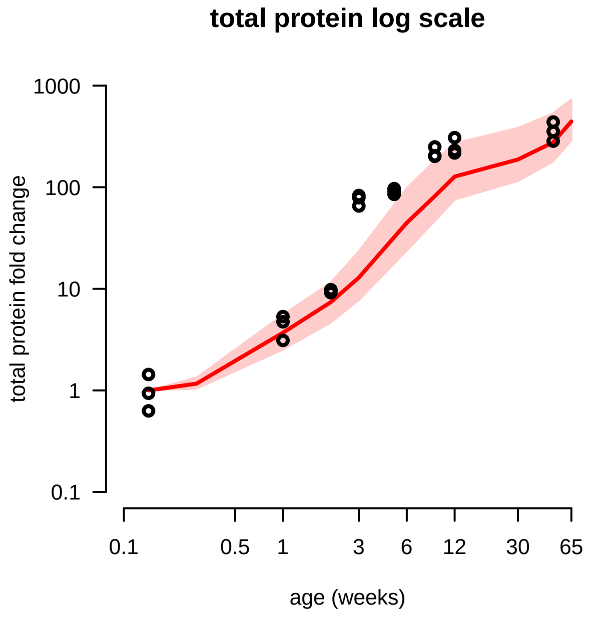

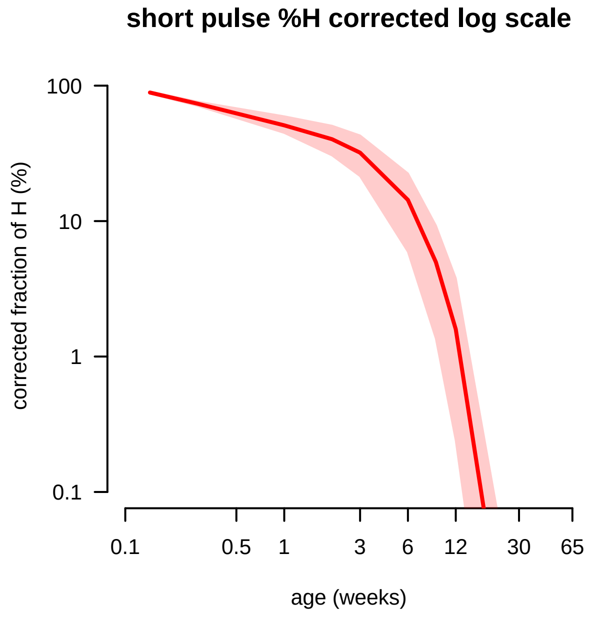

Q9DCL9

>sp|Q9DCL9|PUR6_MOUSE

Multifunctional protein ADE2

GN=Paics

Med H1/2: 3.7 days

Q5 H1/2: 3 days

Q95 H1/2: 4.6 days

reliable model fit: yes

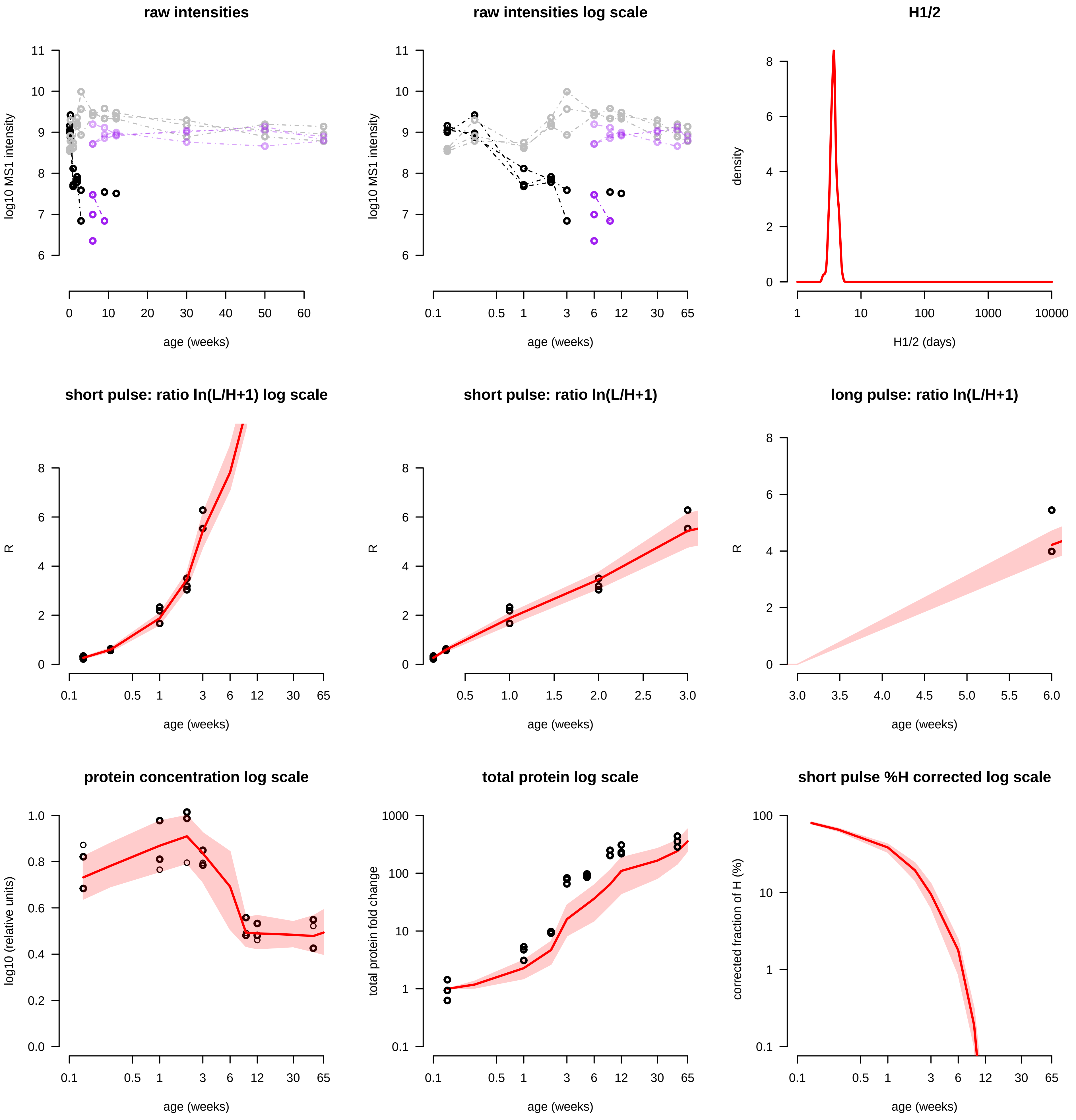

Q6P8X1

>sp|Q6P8X1|SNX6_MOUSE

Sorting nexin−6

GN=Snx6

Med H1/2: 245.5 days

Q5 H1/2: 119.4 days

Q95 H1/2: 1489.1 days

reliable model fit: no

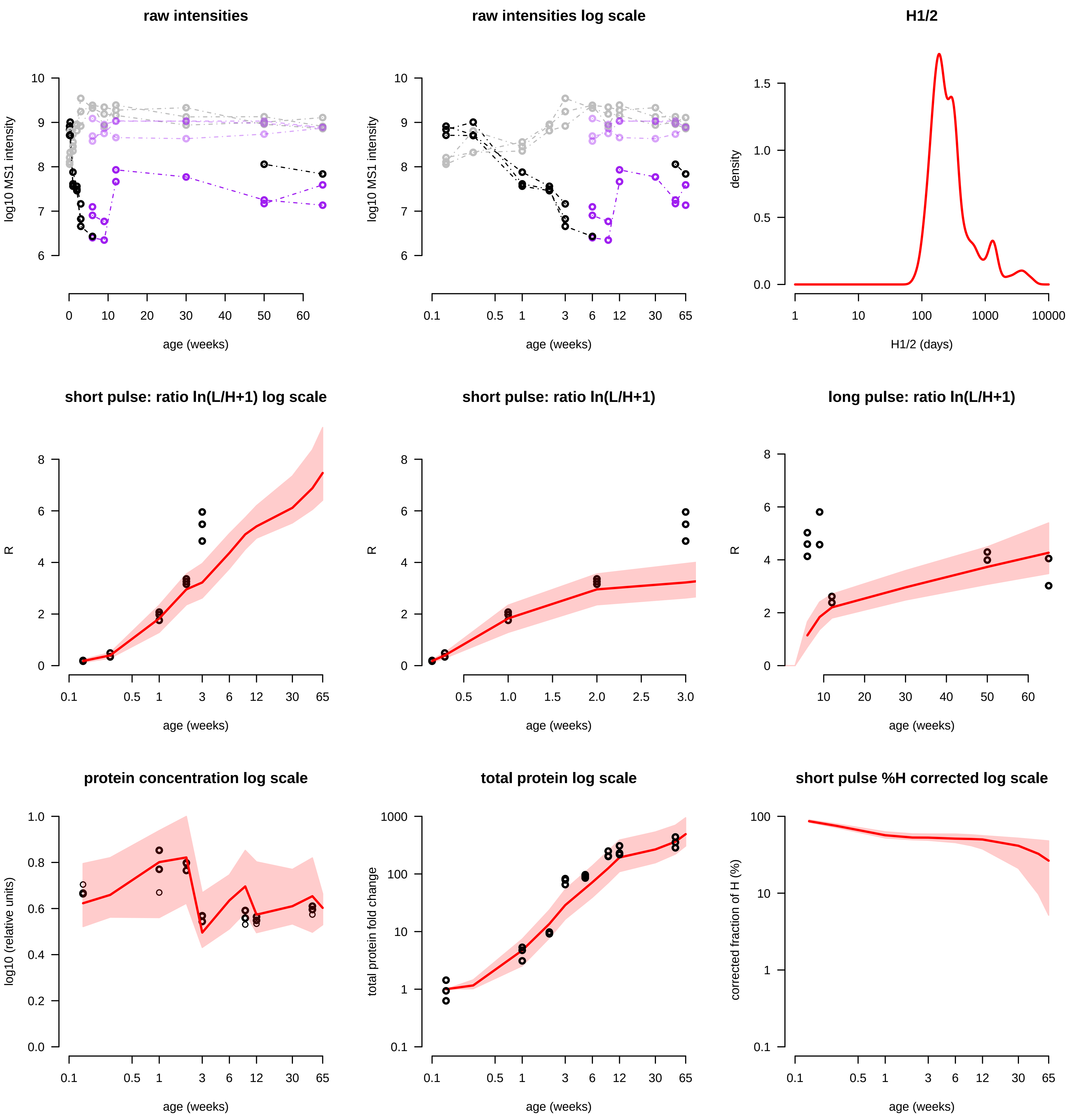

A2A432;A2A432−2

>sp|A2A432|CUL4B_MOUSE

Cullin−4B

GN=Cul4b

Med H1/2: 4.8 days

Q5 H1/2: 3.8 days

Q95 H1/2: 5.8 days

reliable model fit: yes

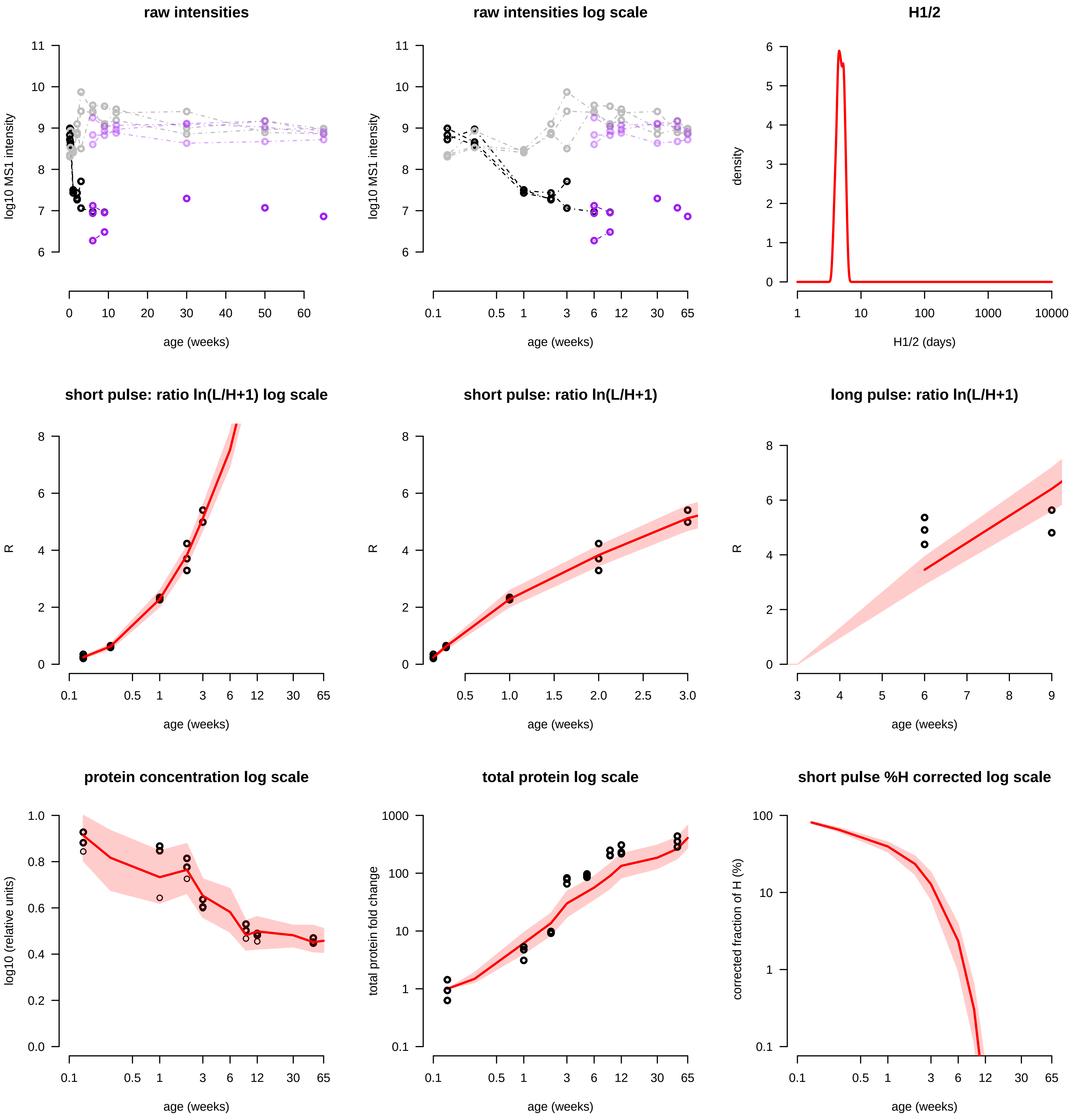

Q9Z329;Q9Z329−3;Q9Z329−2

>sp|Q9Z329|ITPR2_MOUSE

Inositol 1,4,5−trisphosphate receptor type 2

GN=Itpr2

Med H1/2: 11.2 days

Q5 H1/2: 7.7 days

Q95 H1/2: 18.1 days

reliable model fit: yes

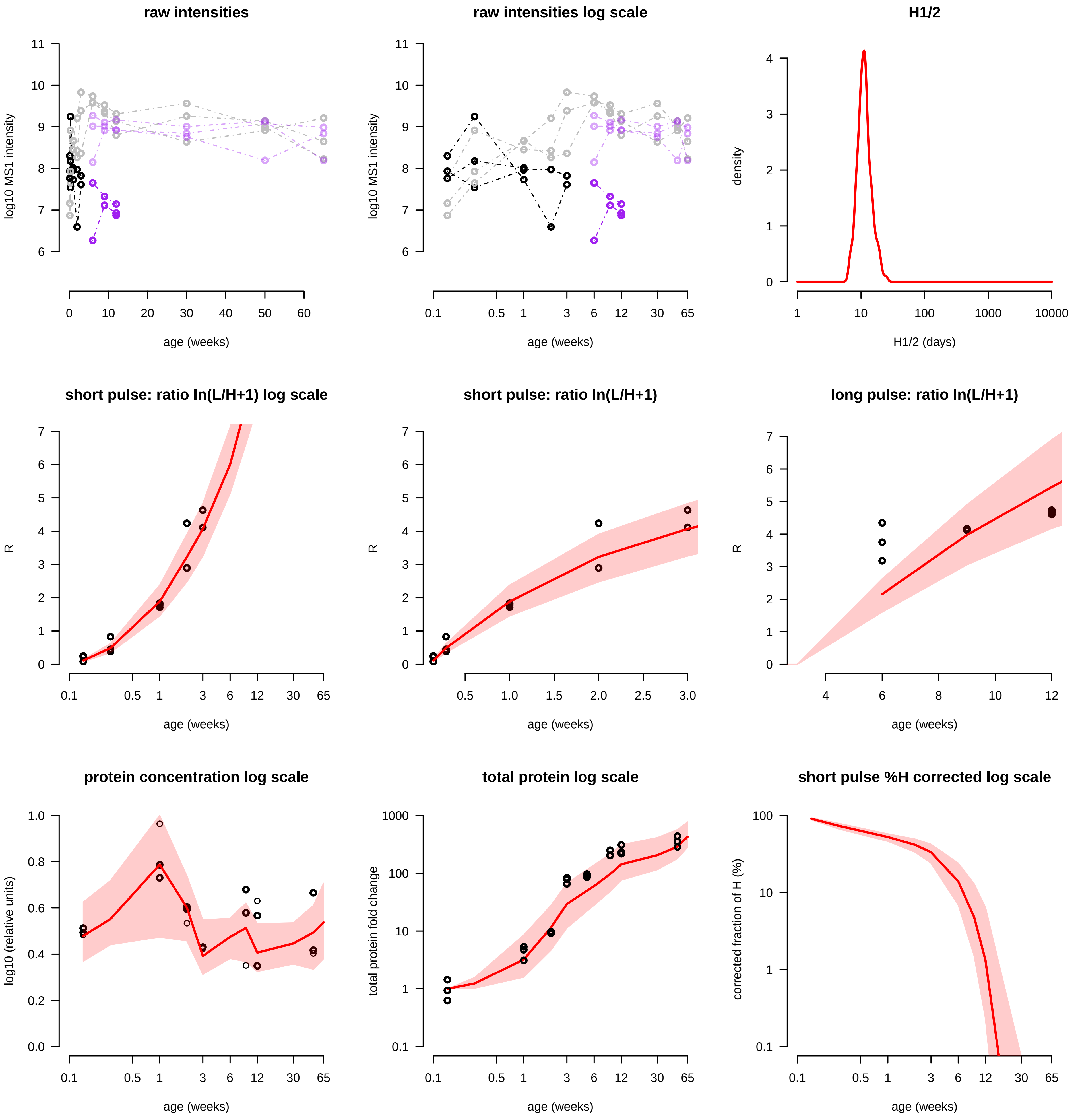

Q8BLF1

>sp|Q8BLF1|NCEH1_MOUSE

Neutral cholesterol ester hydrolase 1

GN=Nceh1

Med H1/2: 9.1 days

Q5 H1/2: 5.9 days

Q95 H1/2: 29.9 days

reliable model fit: no

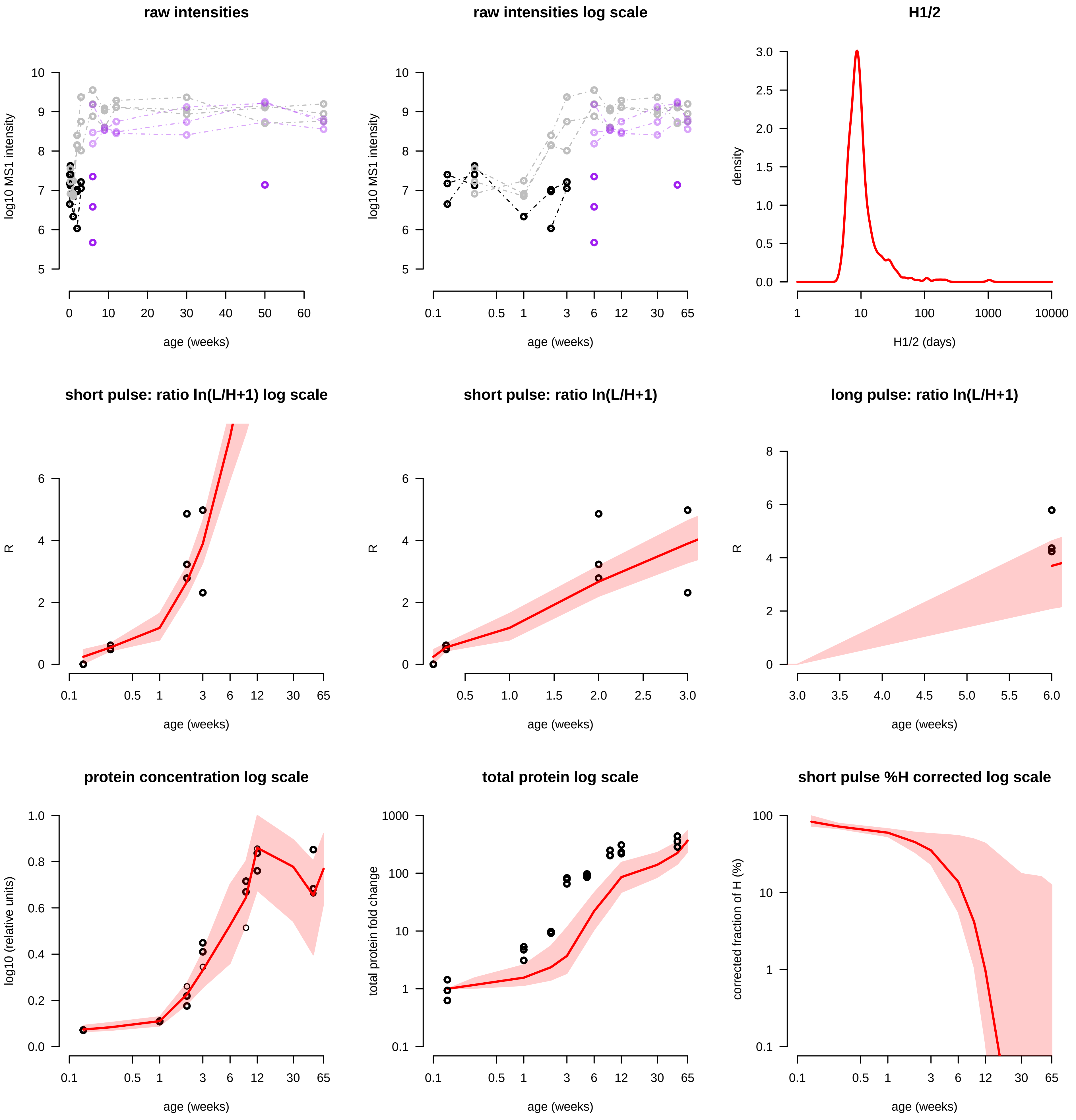

Q9CRD0;Q9CRD0−3;Q9CRD0−2

>sp|Q9CRD0|OCAD1_MOUSE

OCIA domain−containing protein 1

GN=Ociad1

Med H1/2: 5.4 days

Q5 H1/2: 4.2 days

Q95 H1/2: 7.6 days

reliable model fit: yes

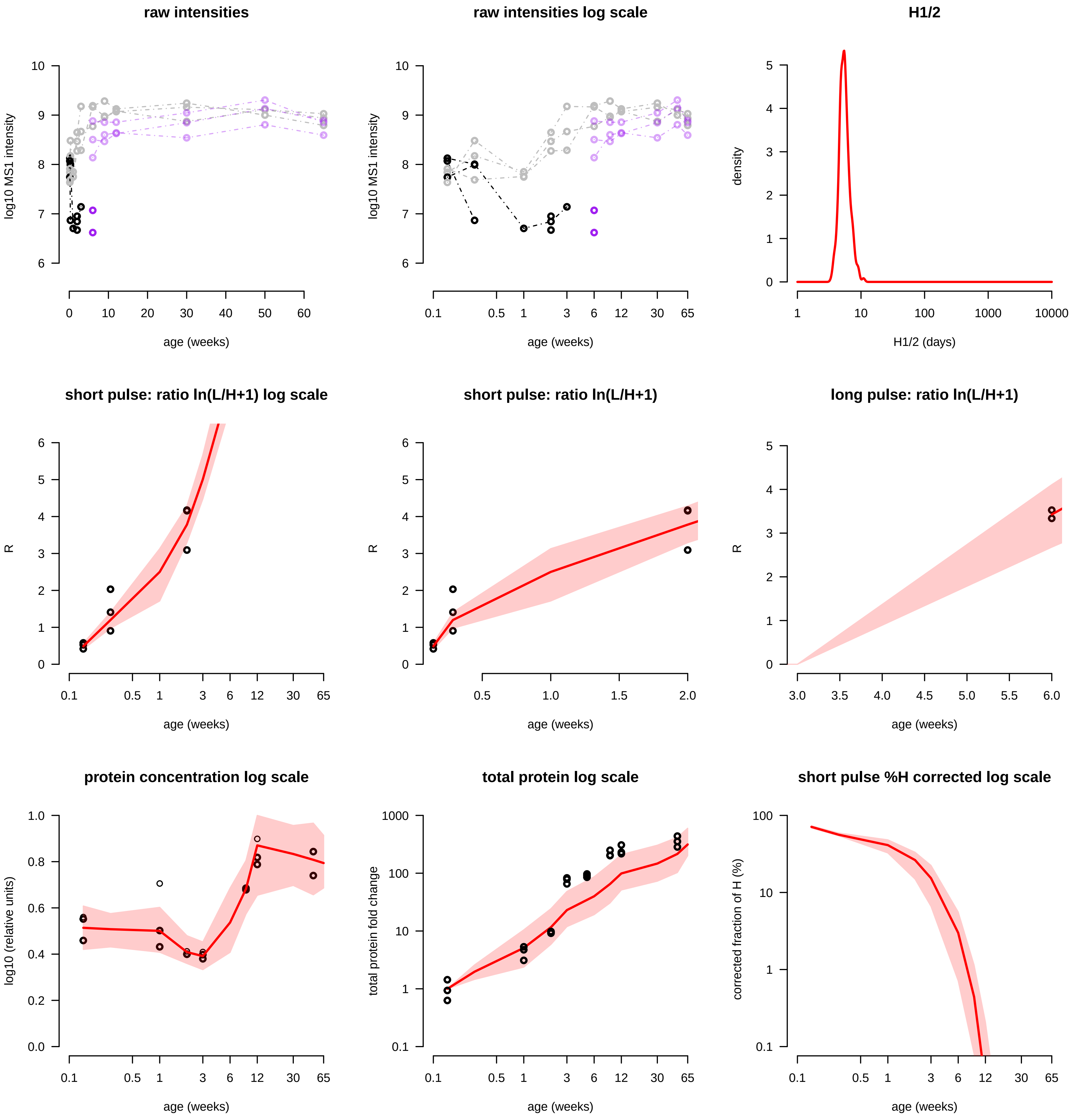

P97434−2

>sp|P97434−2|MPRIP_MOUSE

Isoform 2 of Myosin phosphatase Rho−interacting protein

GN=Mprip

Med H1/2: 3.9 days

Q5 H1/2: 2.5 days

Q95 H1/2: 13.7 days

reliable model fit: yes

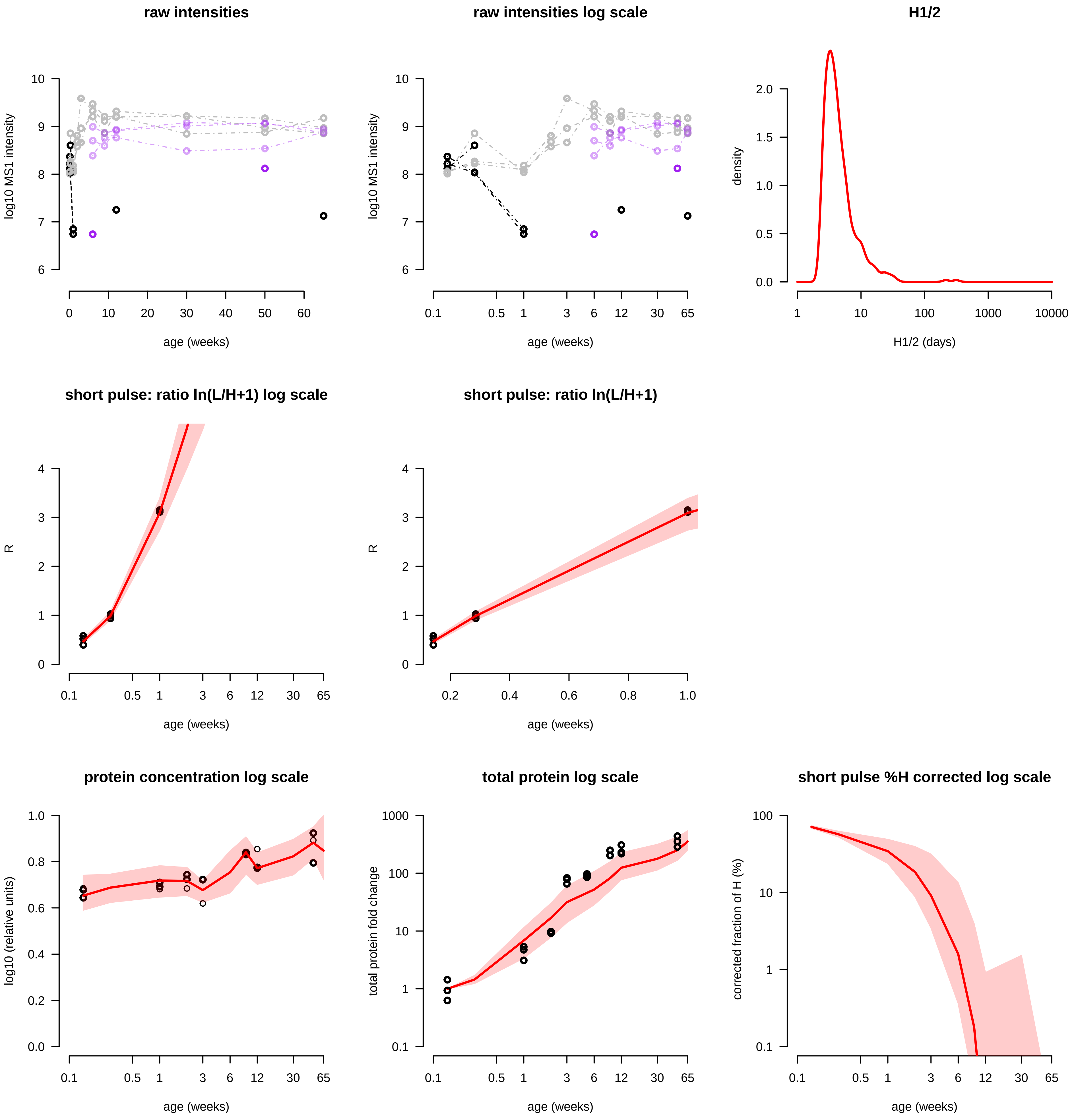

P42230

>sp|P42230|STA5A_MOUSE

Signal transducer and activator of transcription 5A

GN=Stat5a

Med H1/2: 3.4 days

Q5 H1/2: 2.7 days

Q95 H1/2: 4.6 days

reliable model fit: yes

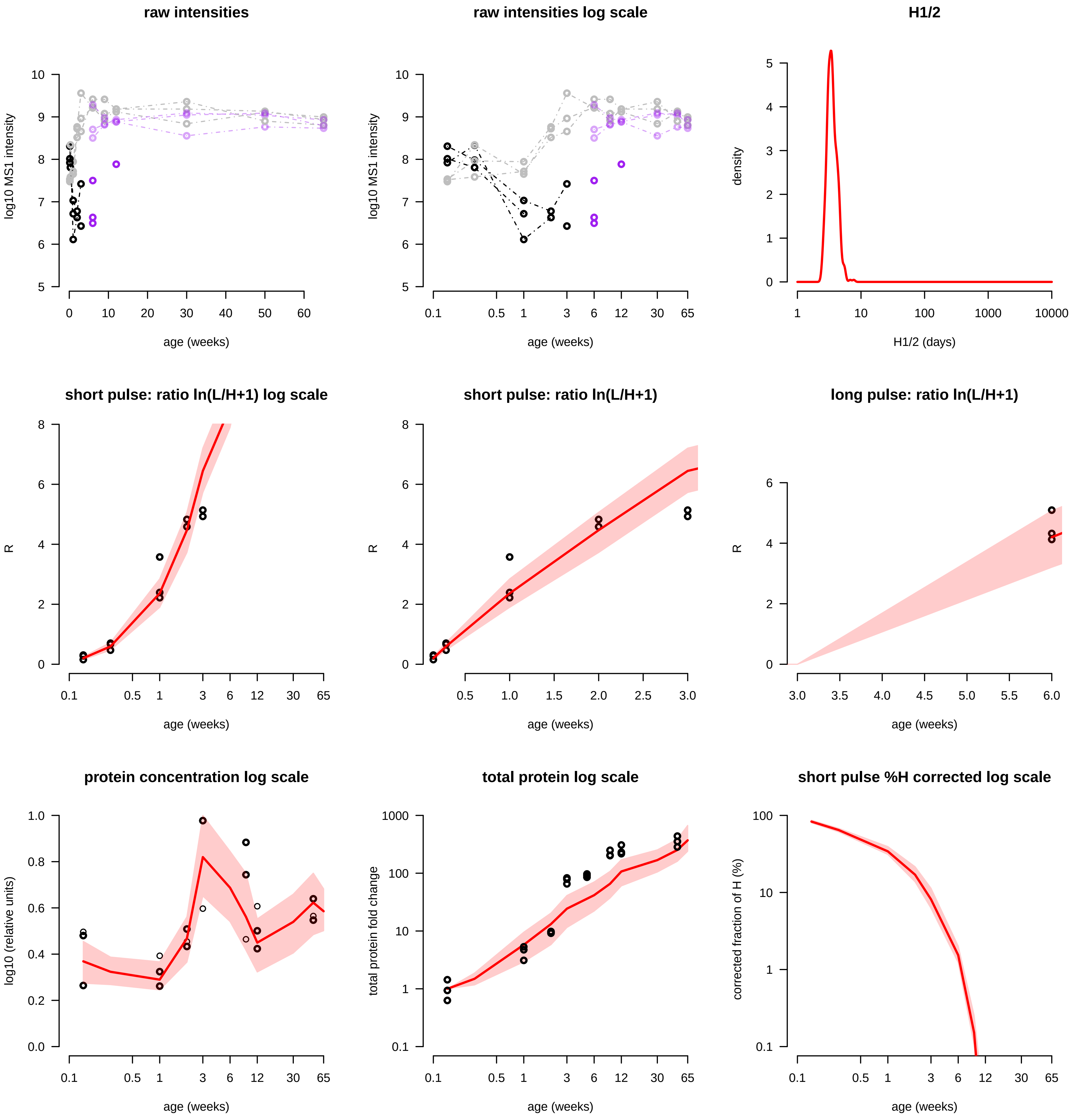

Q61029;Q61029−2;Q61029−4;Q61033−2

>sp|Q61029|LAP2B_MOUSE

Lamina−associated polypeptide 2, isoforms beta/delta/epsilon/gamma

GN=Tmpo

Med H1/2: 7.2 days

Q5 H1/2: 5.9 days

Q95 H1/2: 9.7 days

reliable model fit: yes

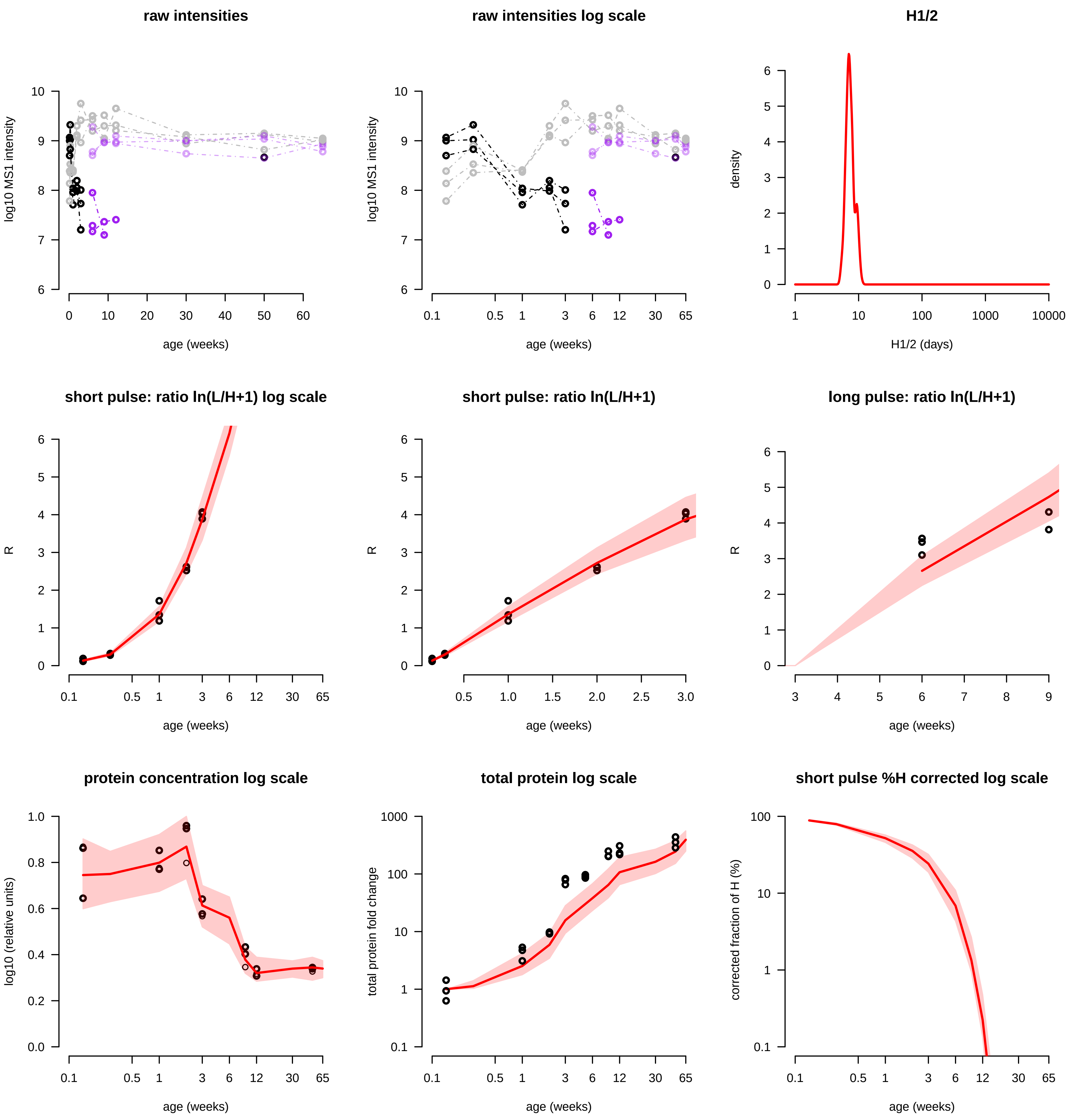

Q62376;Q62376−2

>sp|Q62376|RU17_MOUSE

U1 small nuclear ribonucleoprotein 70 kDa

GN=Snrnp70

Med H1/2: 40.8 days

Q5 H1/2: 30.4 days

Q95 H1/2: 65.7 days

reliable model fit: yes

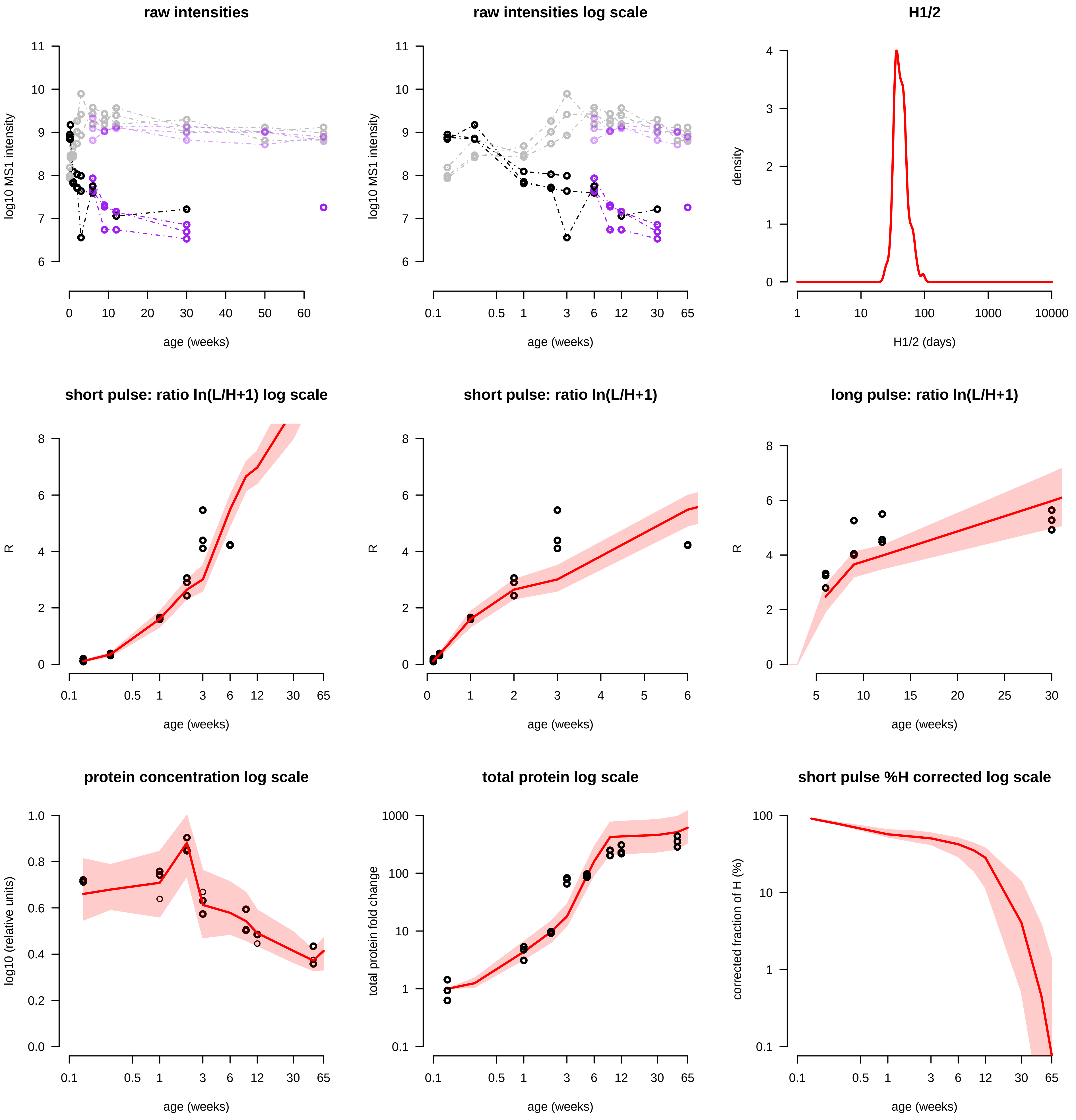

Q61035

>sp|Q61035|HARS1_MOUSE

Histidine−−tRNA ligase, cytoplasmic

GN=Hars1

Med H1/2: 3.1 days

Q5 H1/2: 2.6 days

Q95 H1/2: 3.6 days

reliable model fit: yes

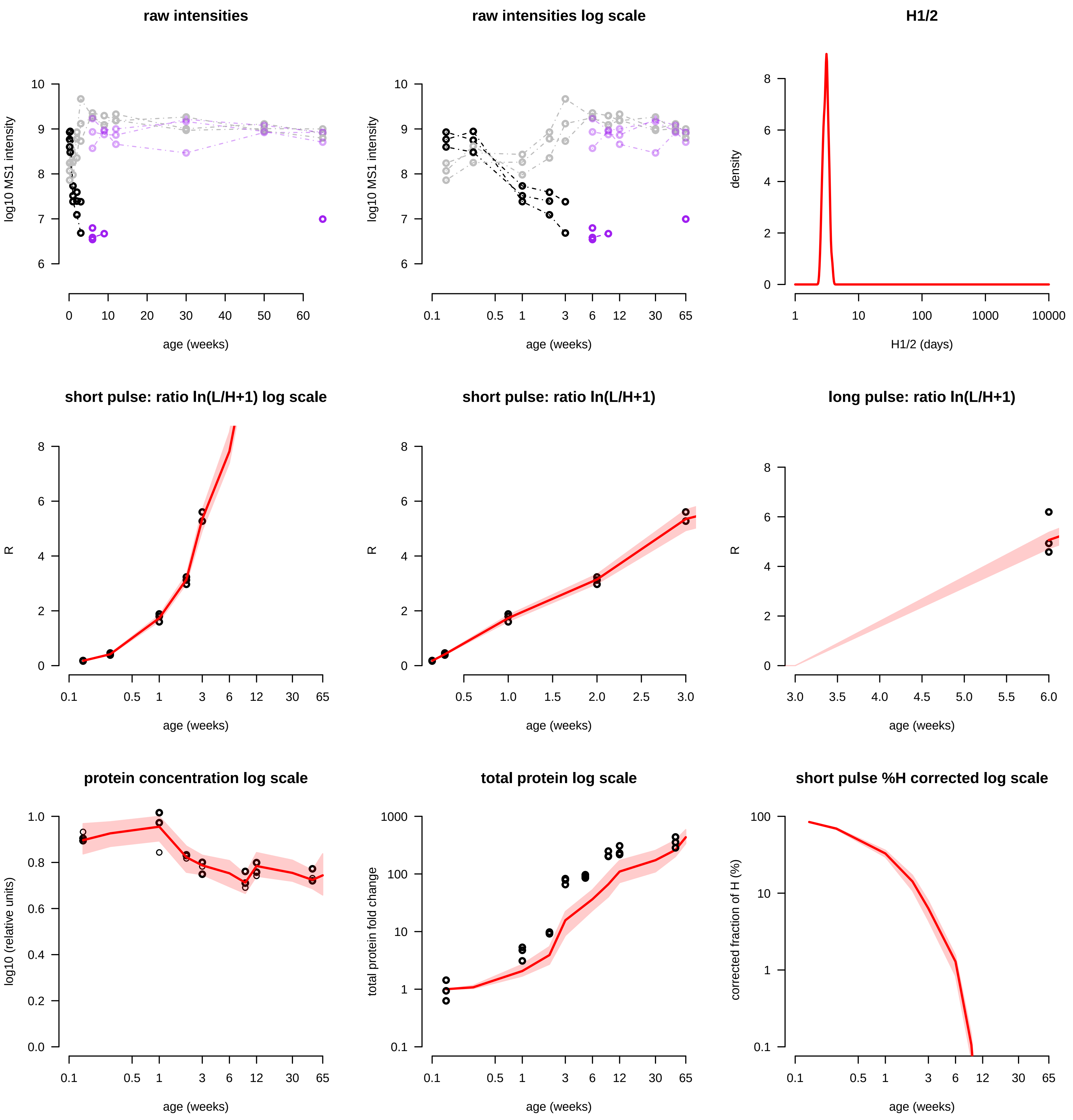

P35585

>sp|P35585|AP1M1_MOUSE

AP−1 complex subunit mu−1

GN=Ap1m1

Med H1/2: 4.4 days

Q5 H1/2: 3.5 days

Q95 H1/2: 5.6 days

reliable model fit: yes

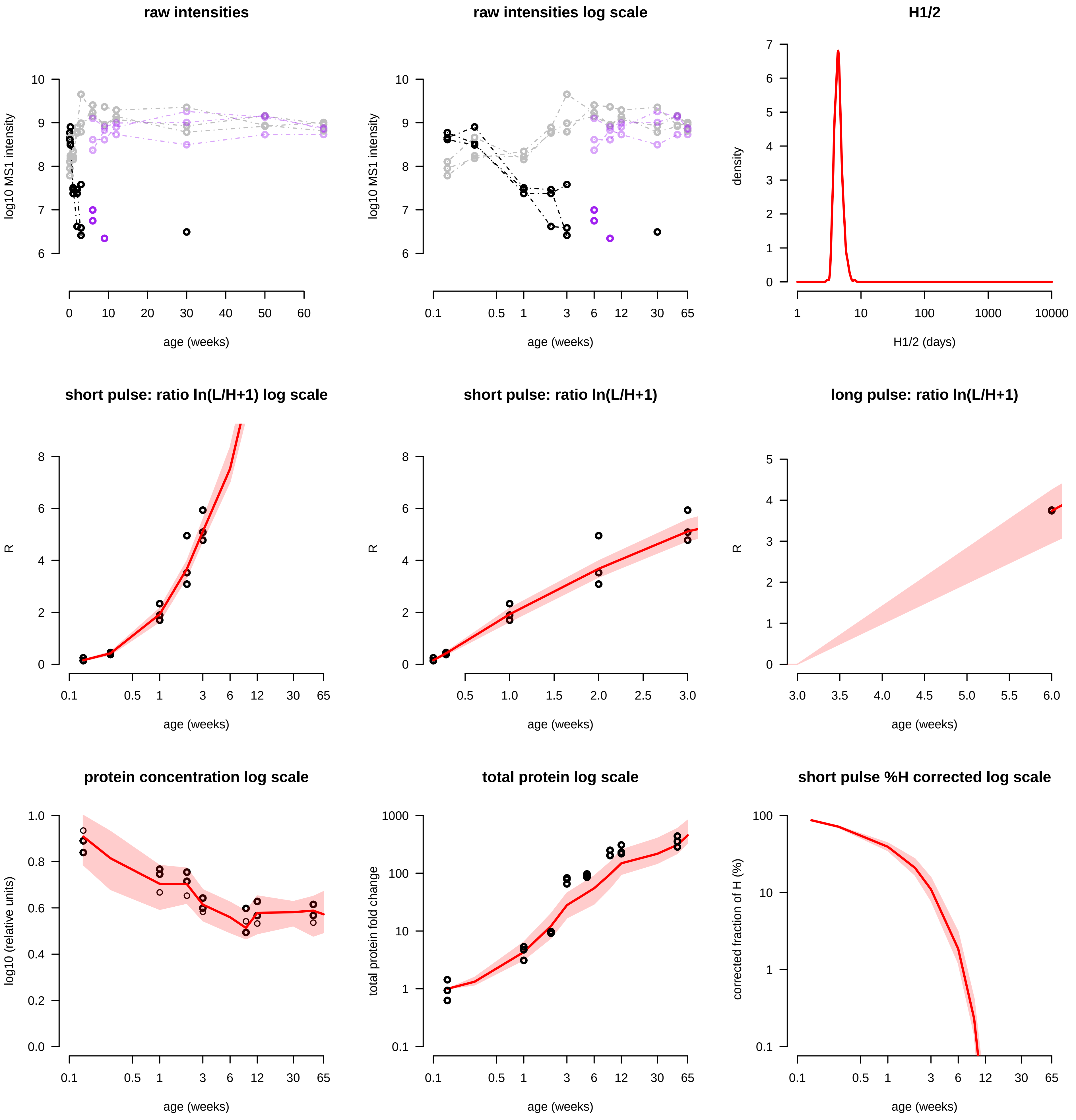

Q9CQW2

>sp|Q9CQW2|ARL8B_MOUSE

ADP−ribosylation factor−like protein 8B

GN=Arl8b

Med H1/2: 13.1 days

Q5 H1/2: 5.1 days

Q95 H1/2: 79 days

reliable model fit: yes

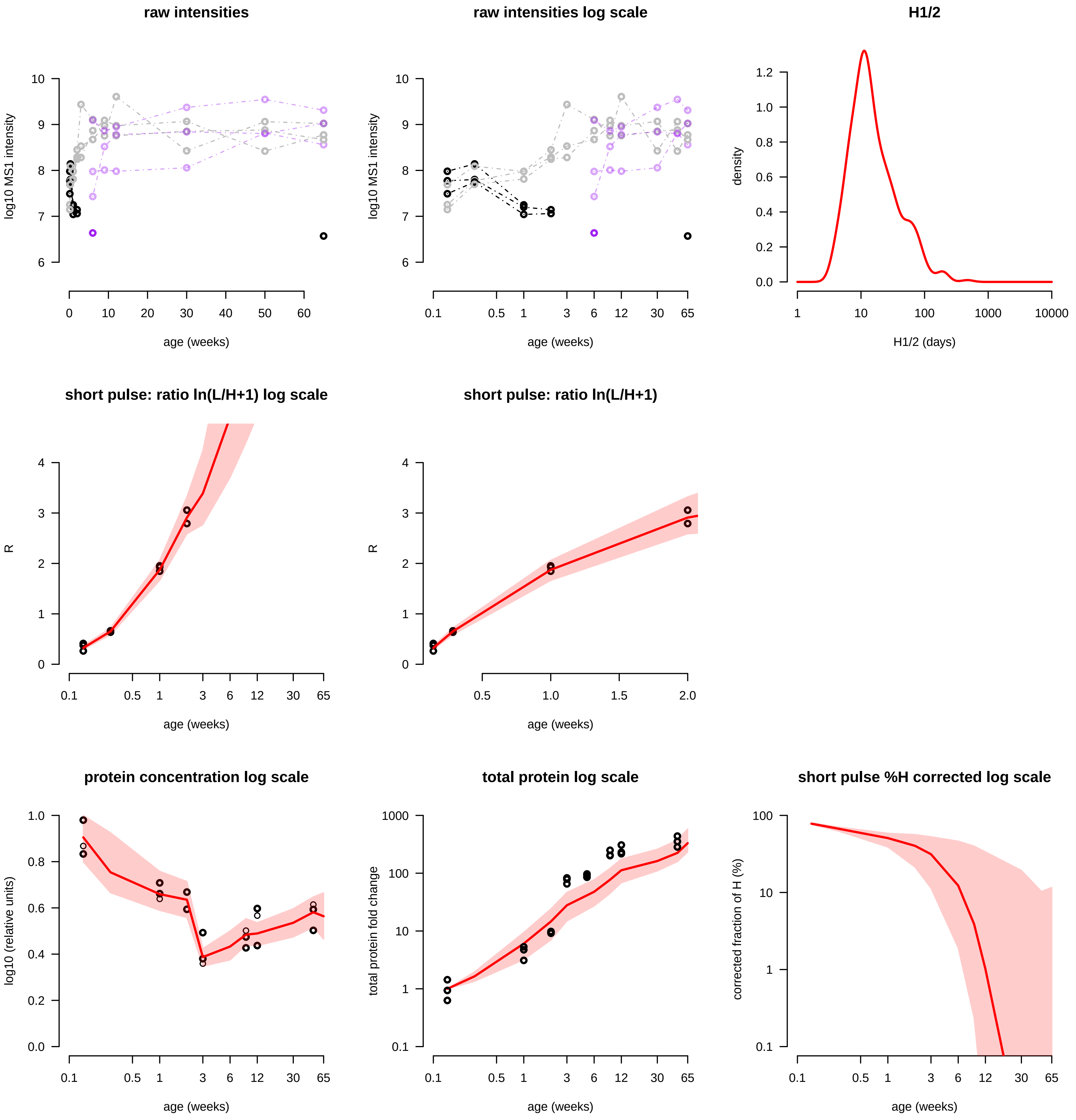

Q62422

>sp|Q62422|OSTF1_MOUSE

Osteoclast−stimulating factor 1

GN=Ostf1

Med H1/2: 7.1 days

Q5 H1/2: 5.3 days

Q95 H1/2: 10.4 days

reliable model fit: yes

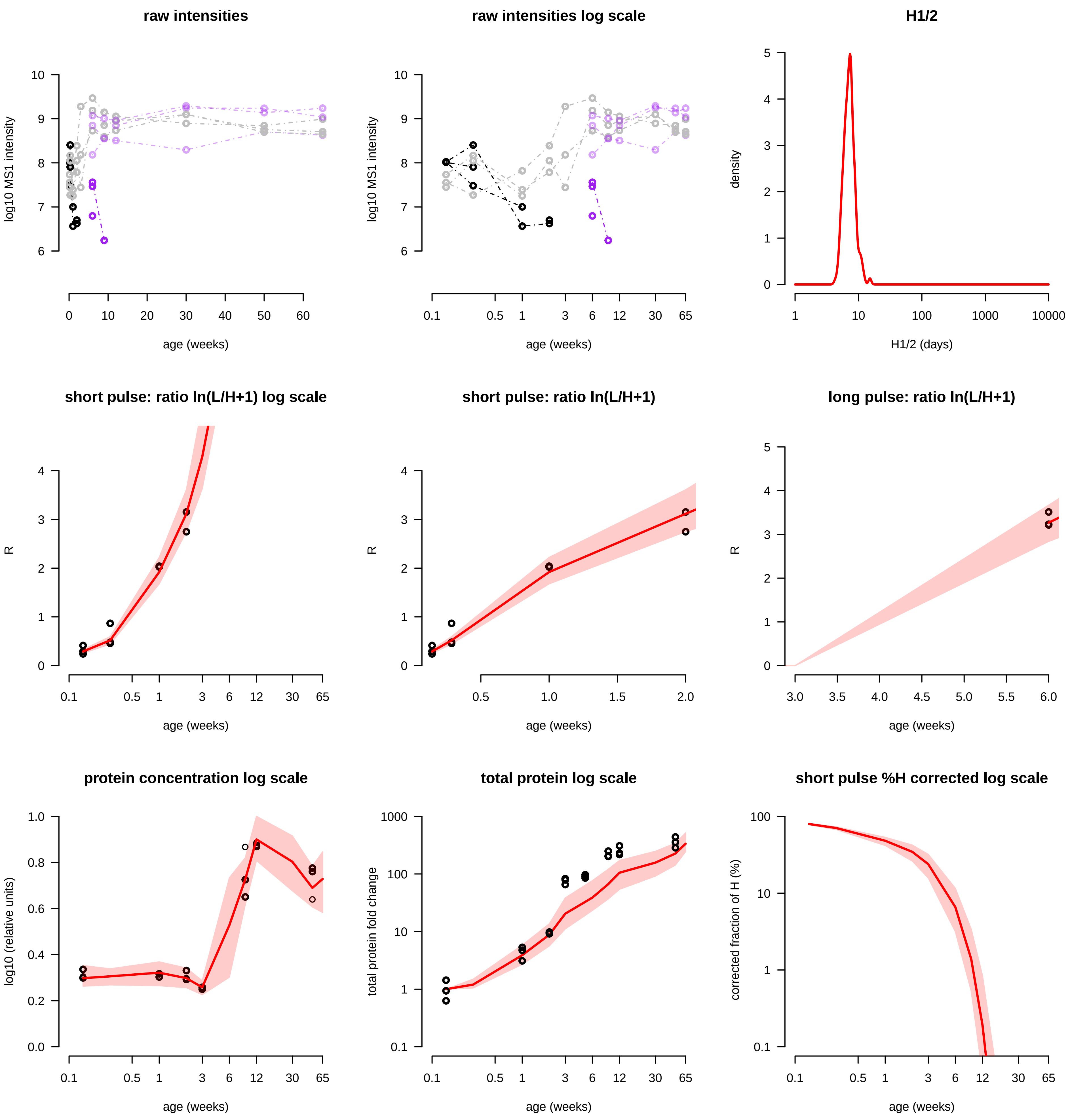

Q99LM2

>sp|Q99LM2|CK5P3_MOUSE

CDK5 regulatory subunit−associated protein 3

GN=Cdk5rap3

Med H1/2: 6.3 days

Q5 H1/2: 2.9 days

Q95 H1/2: 25.2 days

reliable model fit: yes

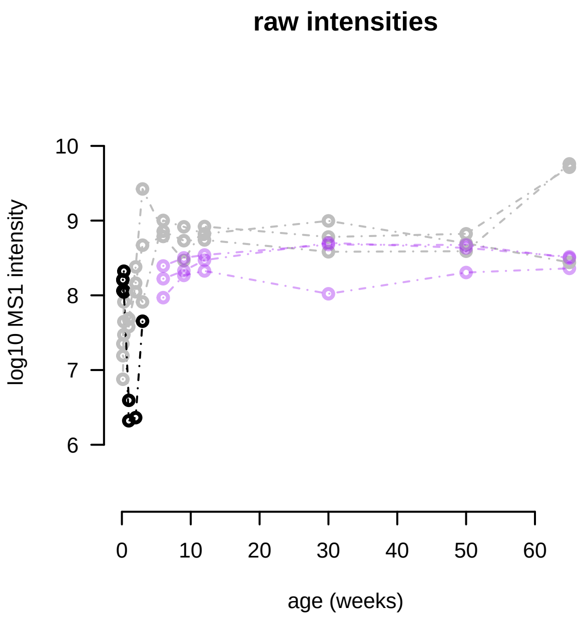

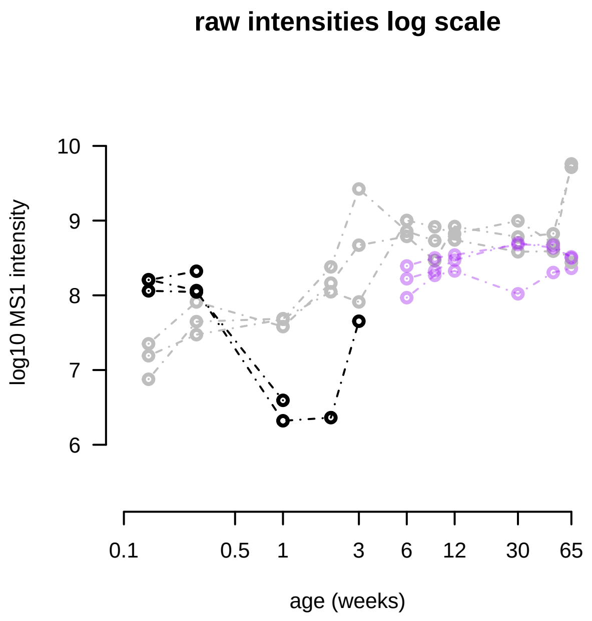

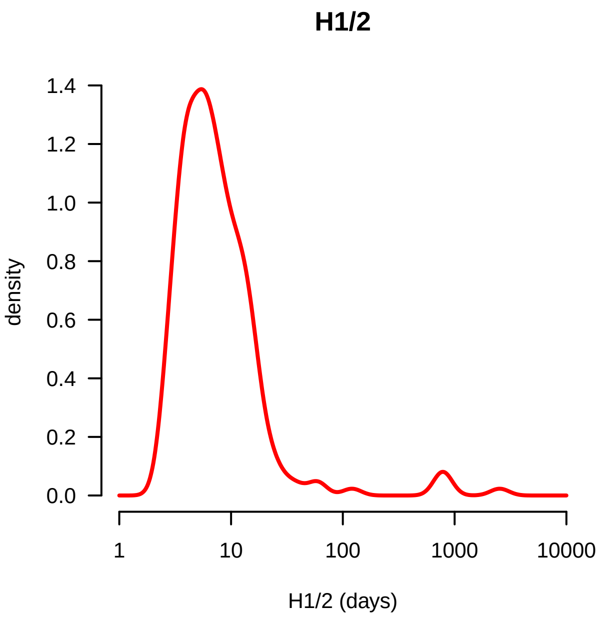

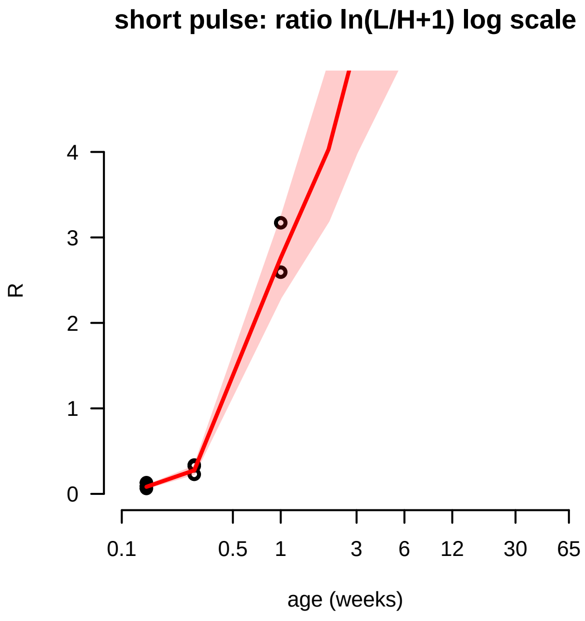

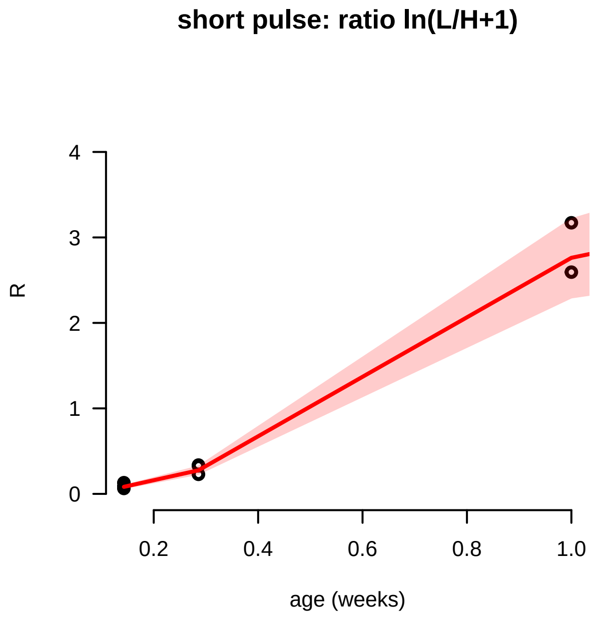

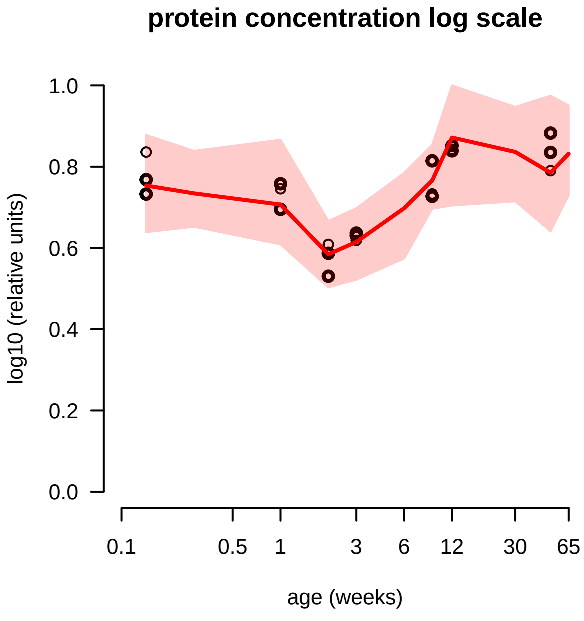

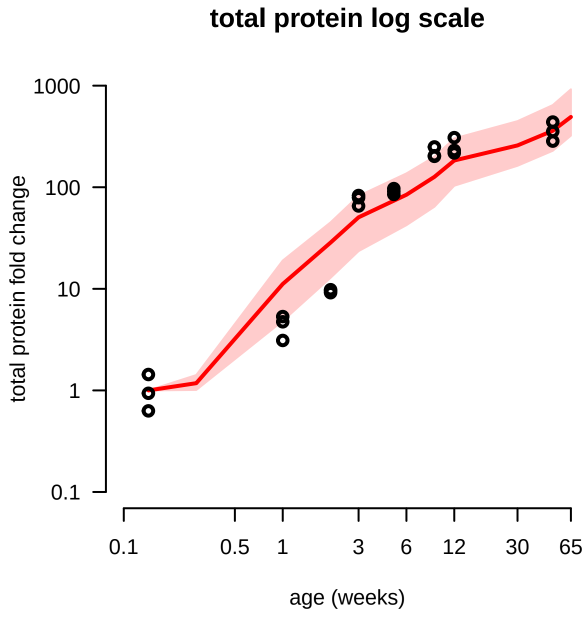

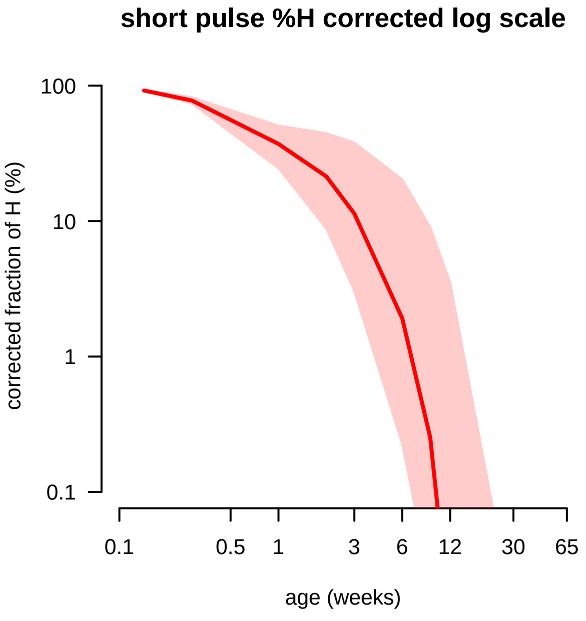

Q62009;Q62009−3;Q62009−4

>sp|Q62009|POSTN_MOUSE

Periostin

GN=Postn

Med H1/2: 4.2 days

Q5 H1/2: 2.6 days

Q95 H1/2: 20.1 days

reliable model fit: yes

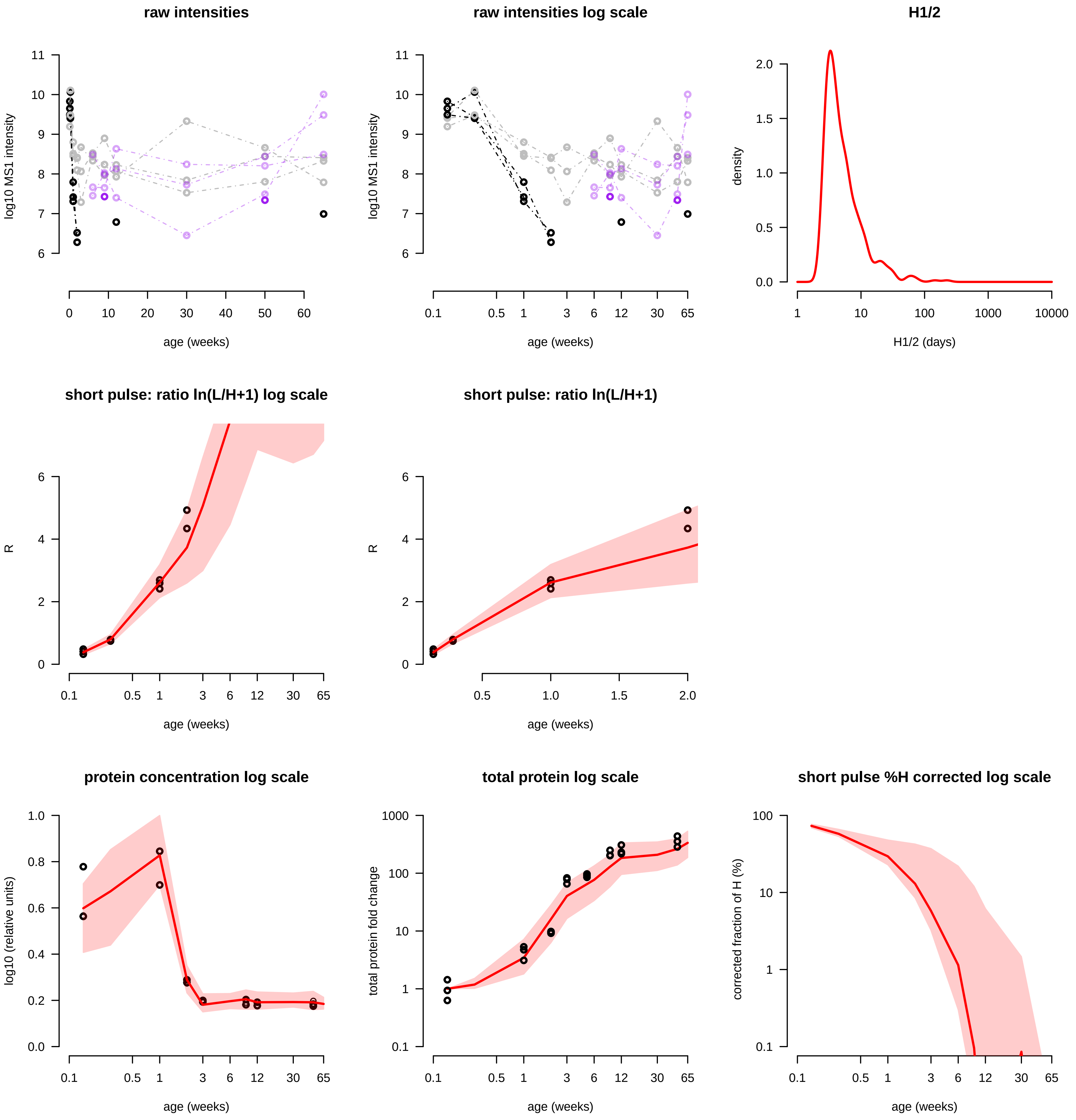

Q8VCR7

>sp|Q8VCR7|ABHEB_MOUSE

Protein ABHD14B

GN=Abhd14b

Med H1/2: 20.4 days

Q5 H1/2: 8.3 days

Q95 H1/2: 131.3 days

reliable model fit: yes

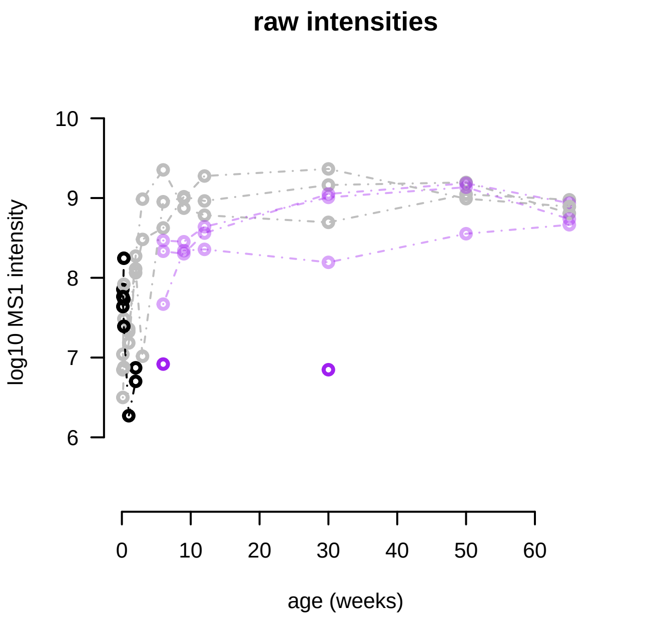

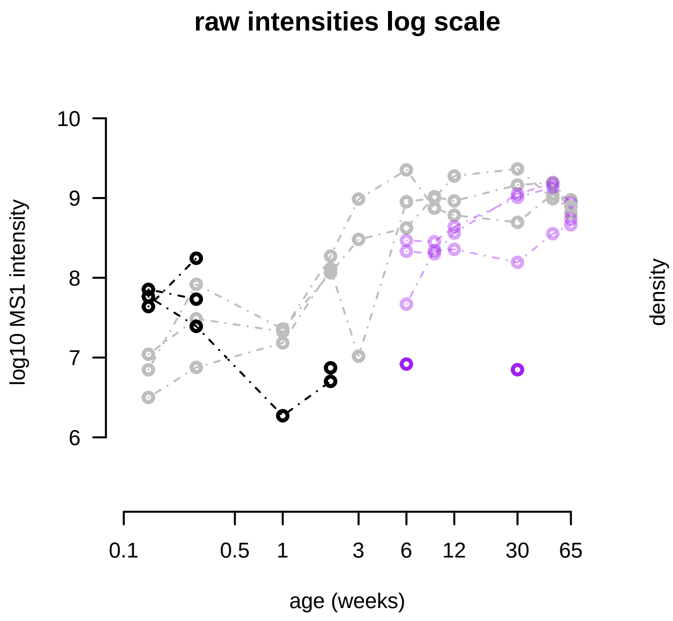

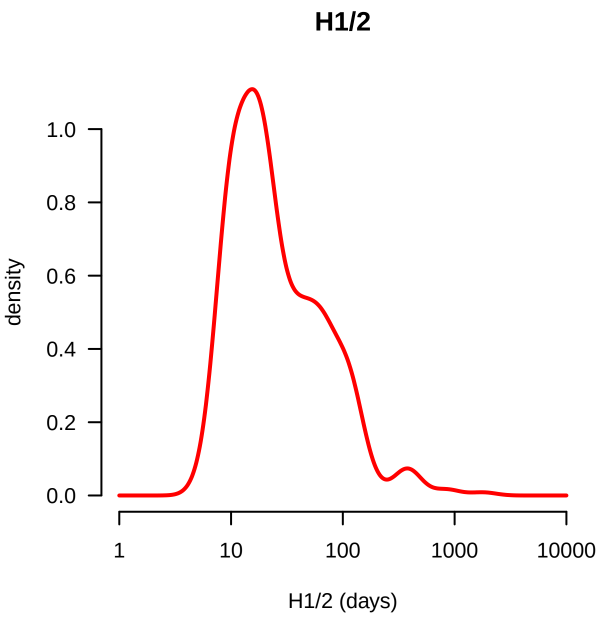

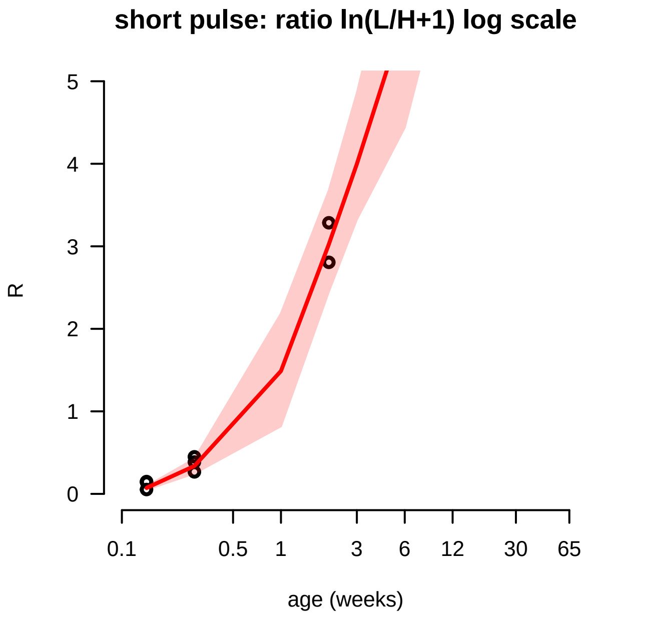

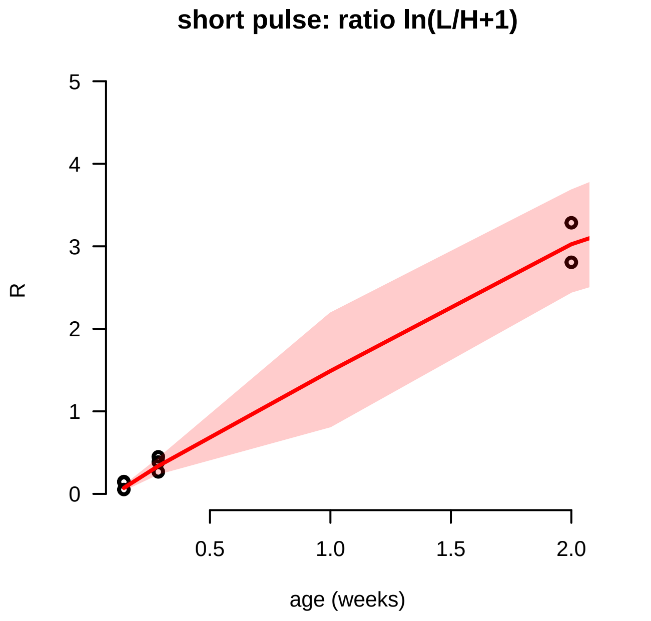

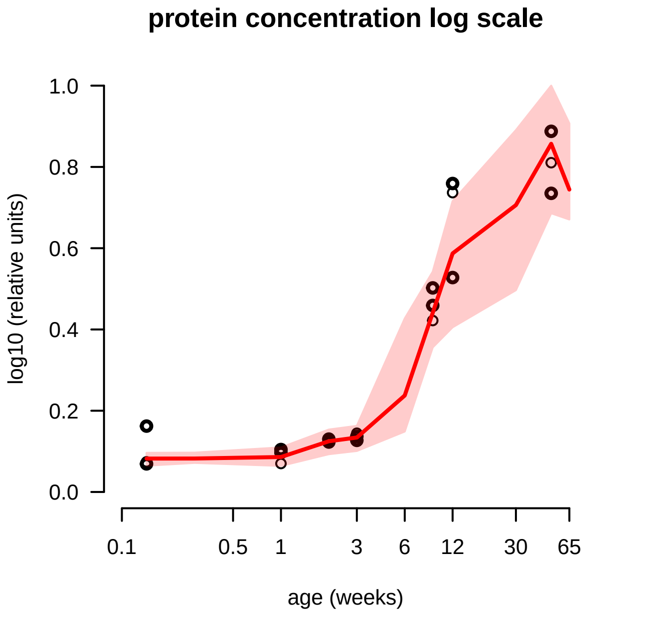

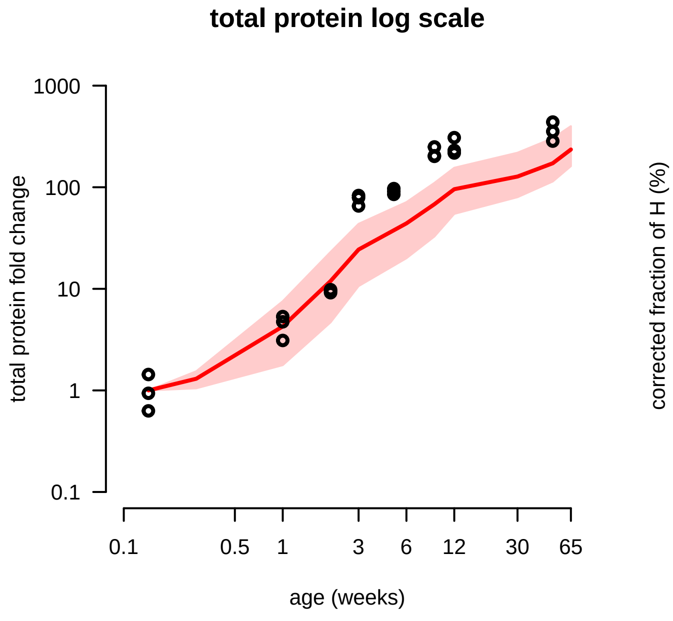

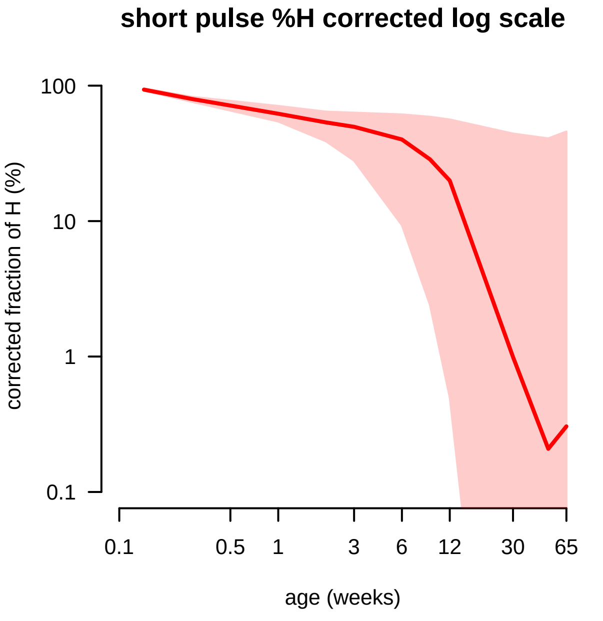

Q62000

>sp|Q62000|MIME_MOUSE

Mimecan

GN=Ogn

Med H1/2: 2211.5 days

Q5 H1/2: 449.6 days

Q95 H1/2: 35353.7 days

reliable model fit: no

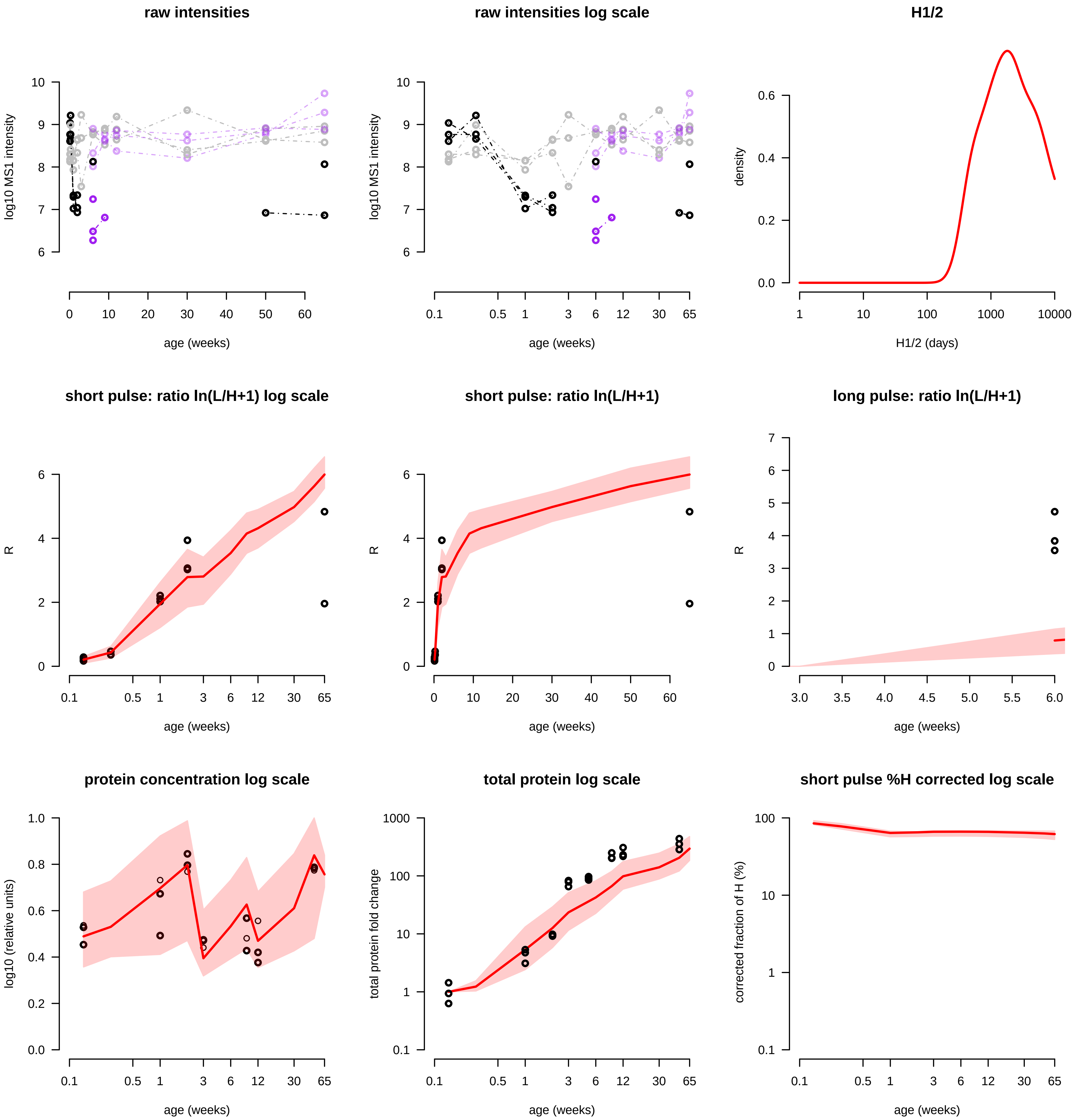

O70311

>sp|O70311|NMT2_MOUSE

Glycylpeptide N−tetradecanoyltransferase 2

GN=Nmt2

Med H1/2: 6.9 days

Q5 H1/2: 5.5 days

Q95 H1/2: 9.4 days

reliable model fit: yes

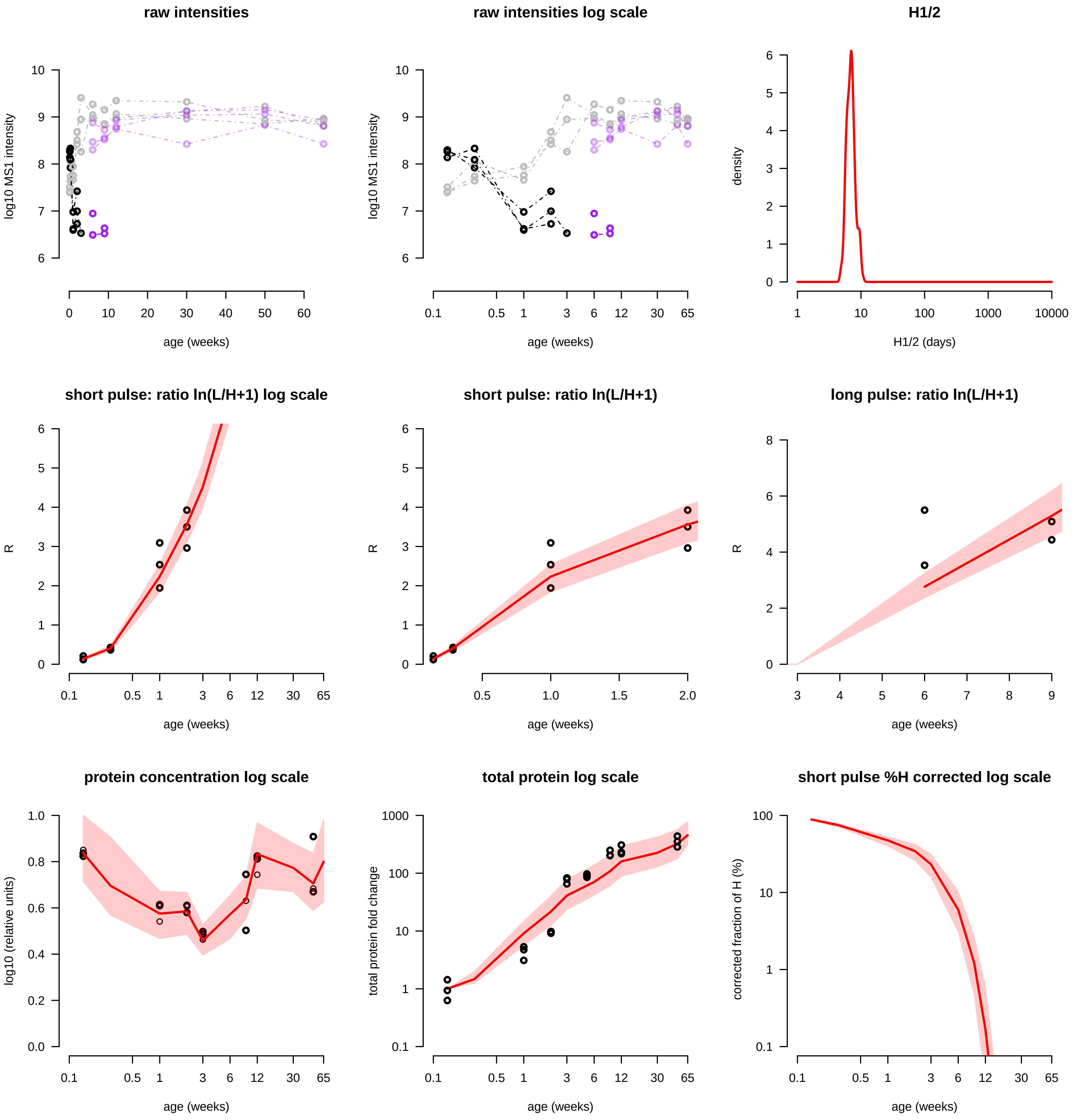

Q921F4

>sp|Q921F4|HNRLL_MOUSE

Heterogeneous nuclear ribonucleoprotein L−like

GN=Hnrnpll

Med H1/2: 3.8 days

Q5 H1/2: 3.1 days

Q95 H1/2: 4.6 days

reliable model fit: yes

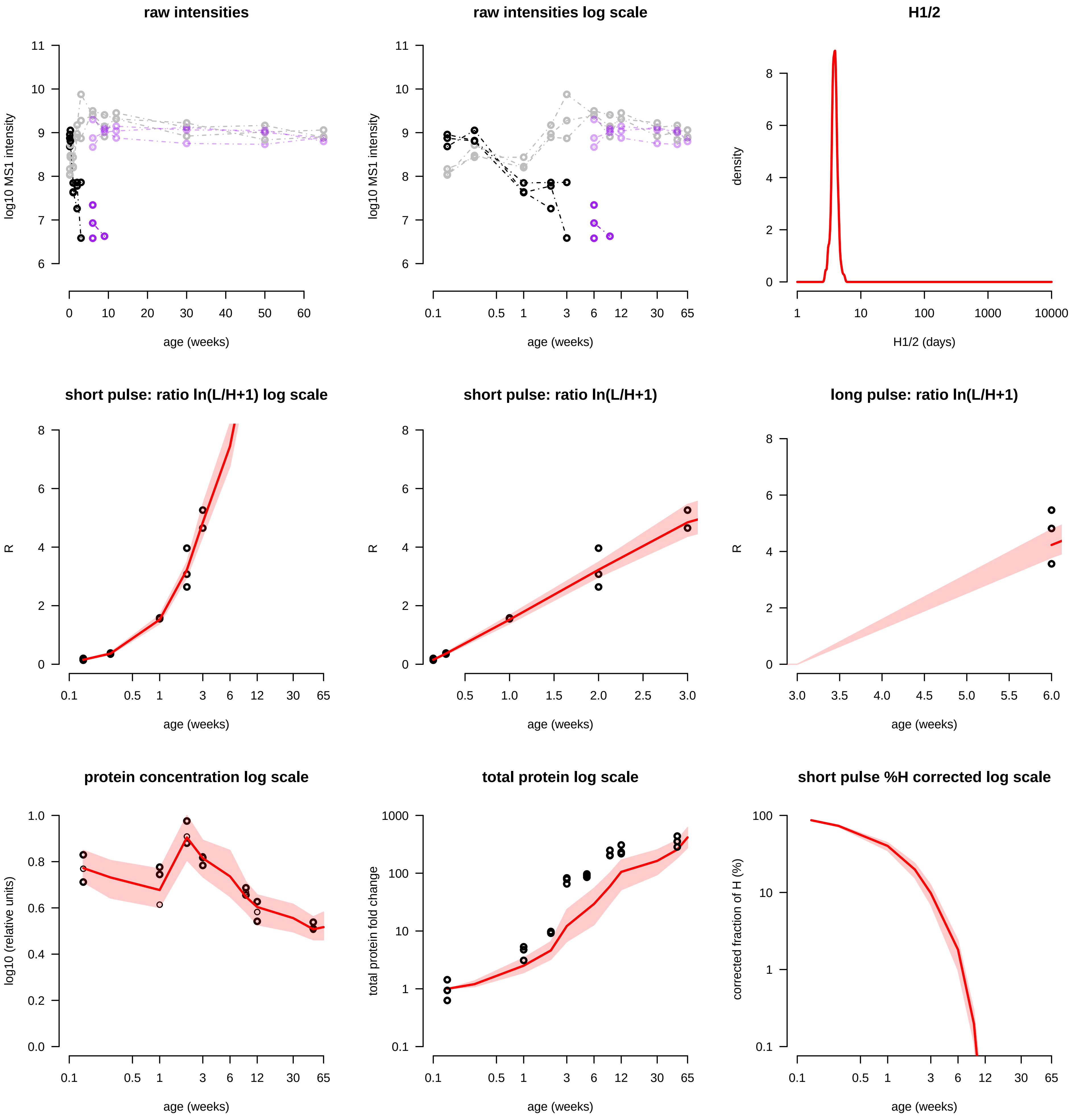

P97494

>sp|P97494|GSH1_MOUSE

Glutamate−−cysteine ligase catalytic subunit

GN=Gclc

Med H1/2: 3.8 days

Q5 H1/2: 3 days

Q95 H1/2: 6.1 days

reliable model fit: yes

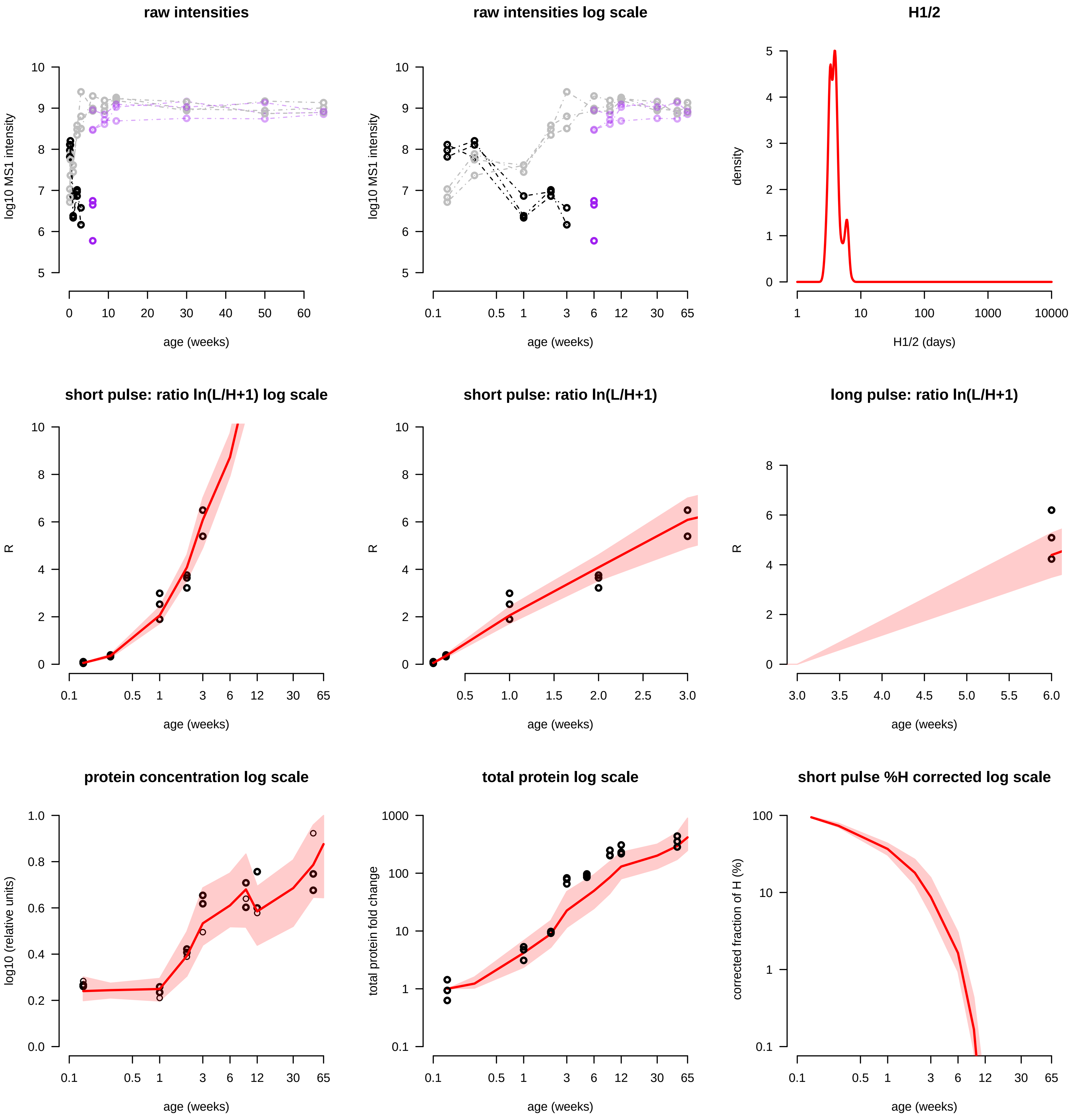

Q9JLI8

>sp|Q9JLI8|SART3_MOUSE

Squamous cell carcinoma antigen recognized by T−cells 3

GN=Sart3

Med H1/2: 2.9 days

Q5 H1/2: 2.5 days

Q95 H1/2: 4 days

reliable model fit: yes

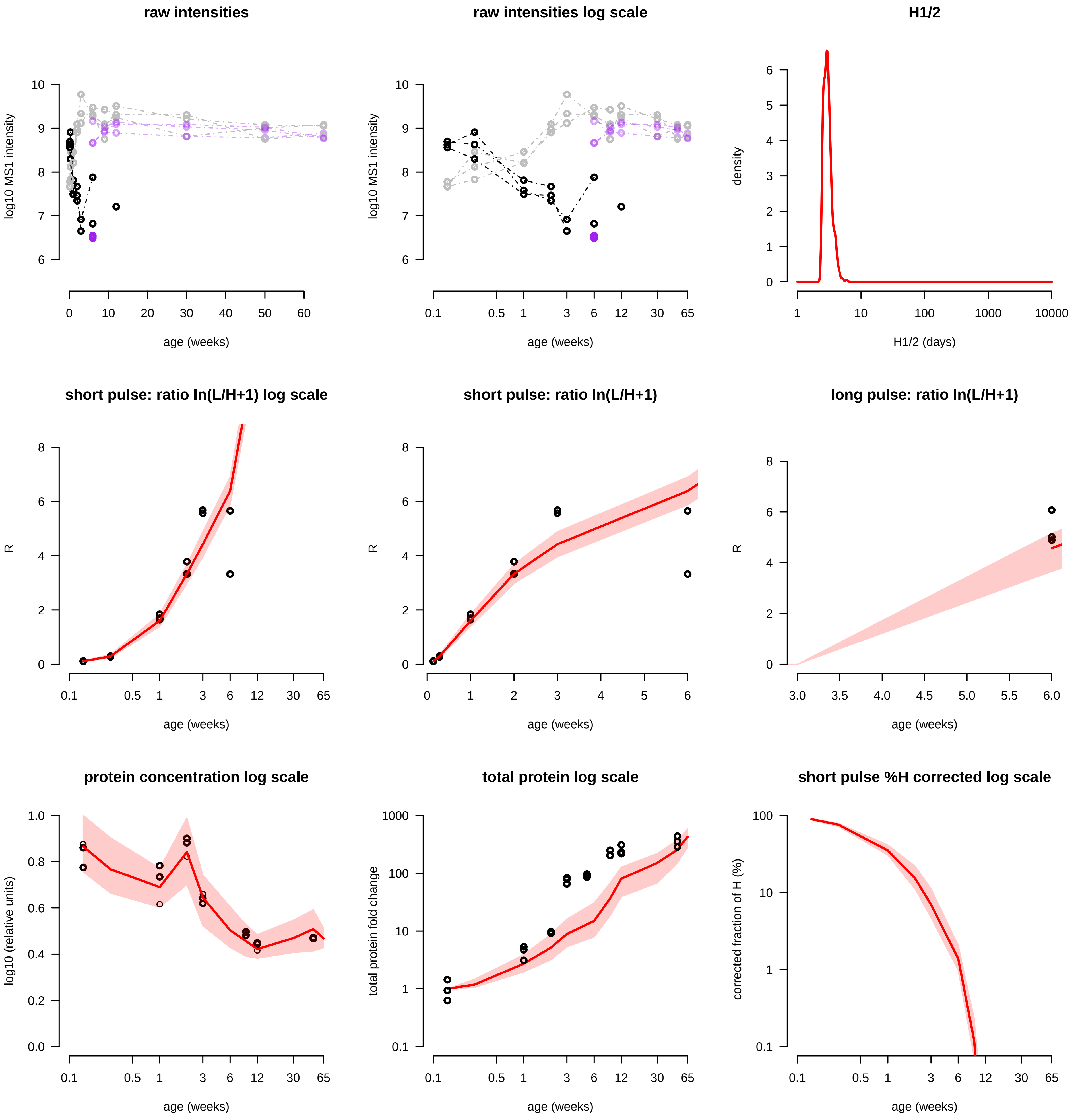

Q7TNC4−4;Q7TNC4;Q7TNC4−2;Q7TNC4−3

>sp|Q7TNC4−4|LC7L2_MOUSE

Isoform 4 of Putative RNA−binding protein Luc7−like 2

GN=Luc7l2

Med H1/2: 7.5 days

Q5 H1/2: 6.2 days

Q95 H1/2: 10.1 days

reliable model fit: yes

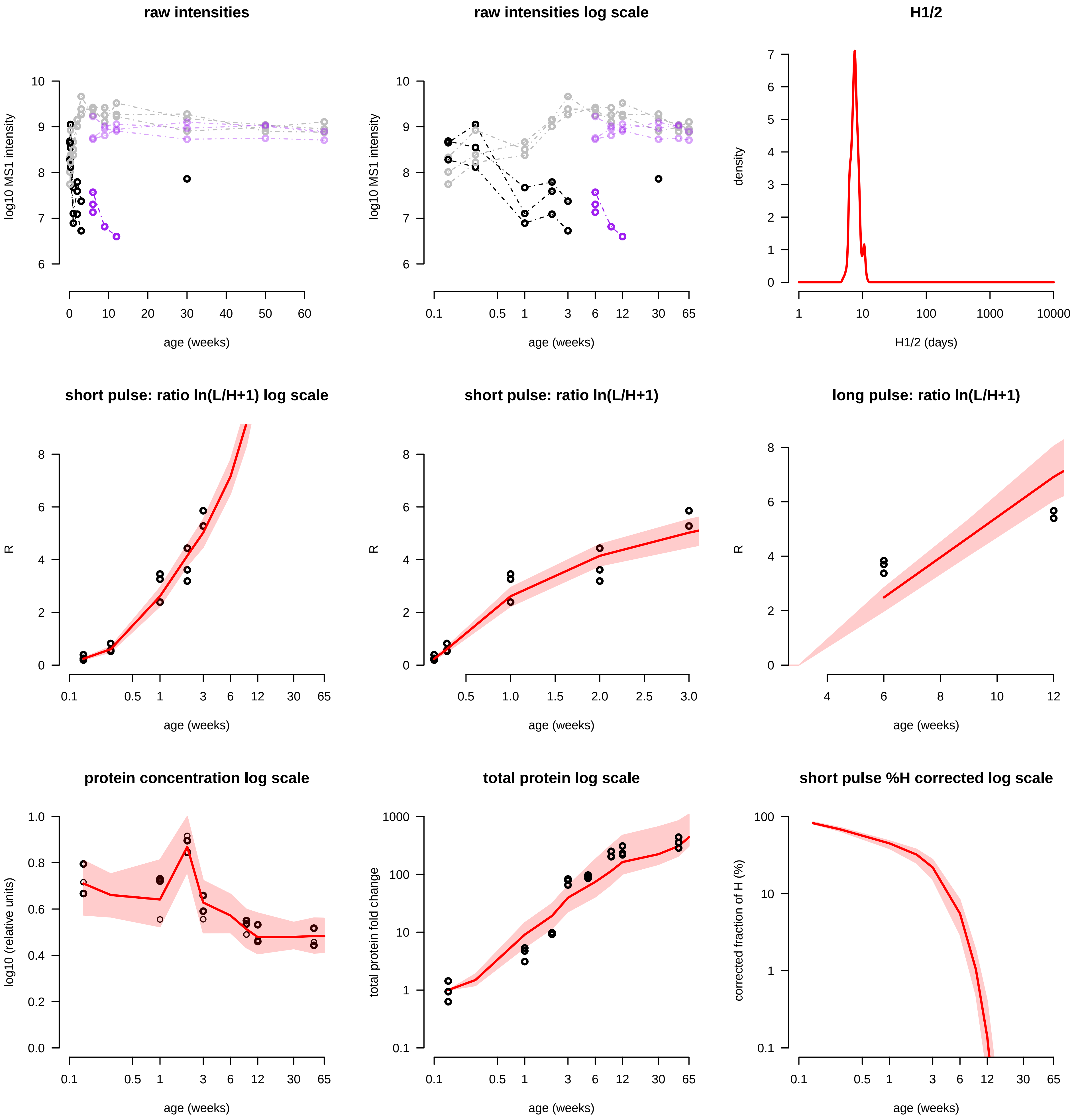

Q9D5V6

>sp|Q9D5V6|SYAP1_MOUSE

Synapse−associated protein 1

GN=Syap1

Med H1/2: 3.1 days

Q5 H1/2: 2.5 days

Q95 H1/2: 6.4 days

reliable model fit: yes

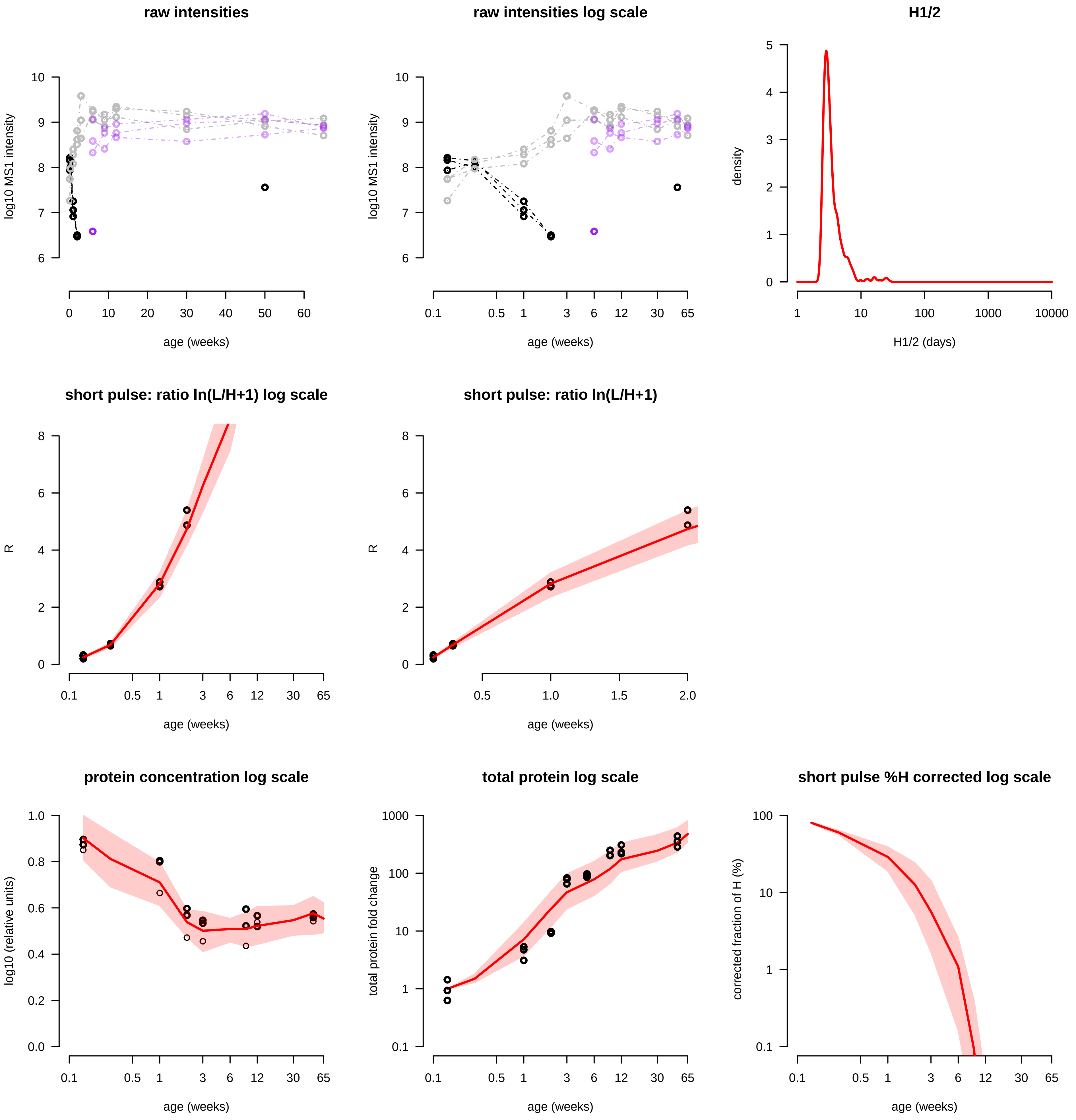

Q922F4

>sp|Q922F4|TBB6_MOUSE

Tubulin beta−6 chain

GN=Tubb6

Med H1/2: 6.1 days

Q5 H1/2: 5 days

Q95 H1/2: 8.8 days

reliable model fit: yes

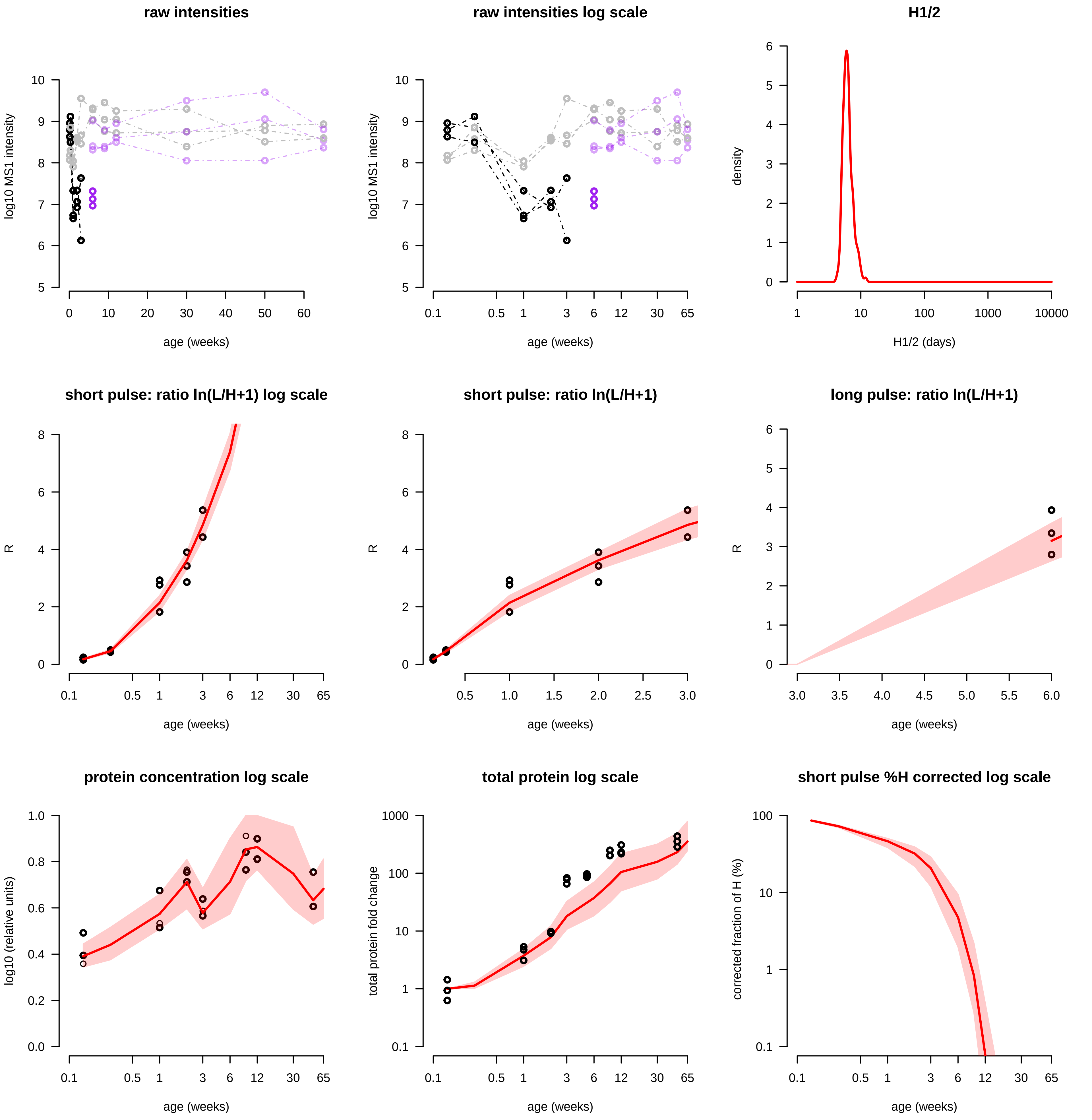

Q3TZZ7;Q3TZZ7−2

>sp|Q3TZZ7|ESYT2_MOUSE

Extended synaptotagmin−2

GN=Esyt2

Med H1/2: 4.2 days

Q5   H1/2: 3.3 days

Q95 H1/2: 5.9 days

reliable model fit: yes

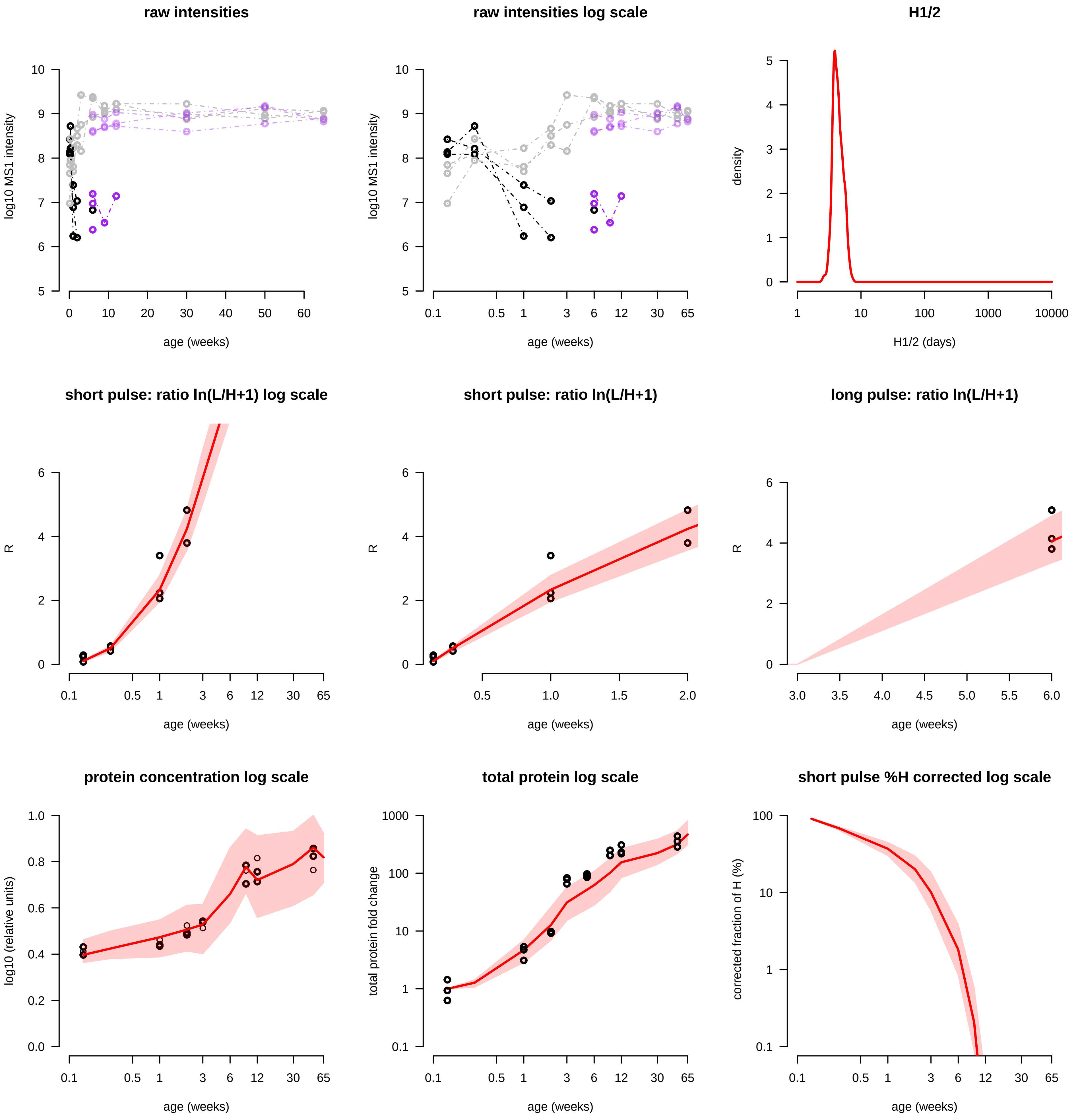

Q8BT60

>sp|Q8BT60|CPNE3_MOUSE

Copine−3

GN=Cpne3

Med H1/2: 7.9 days

Q5 H1/2: 6.2 days

Q95 H1/2: 10.8 days

reliable model fit: yes

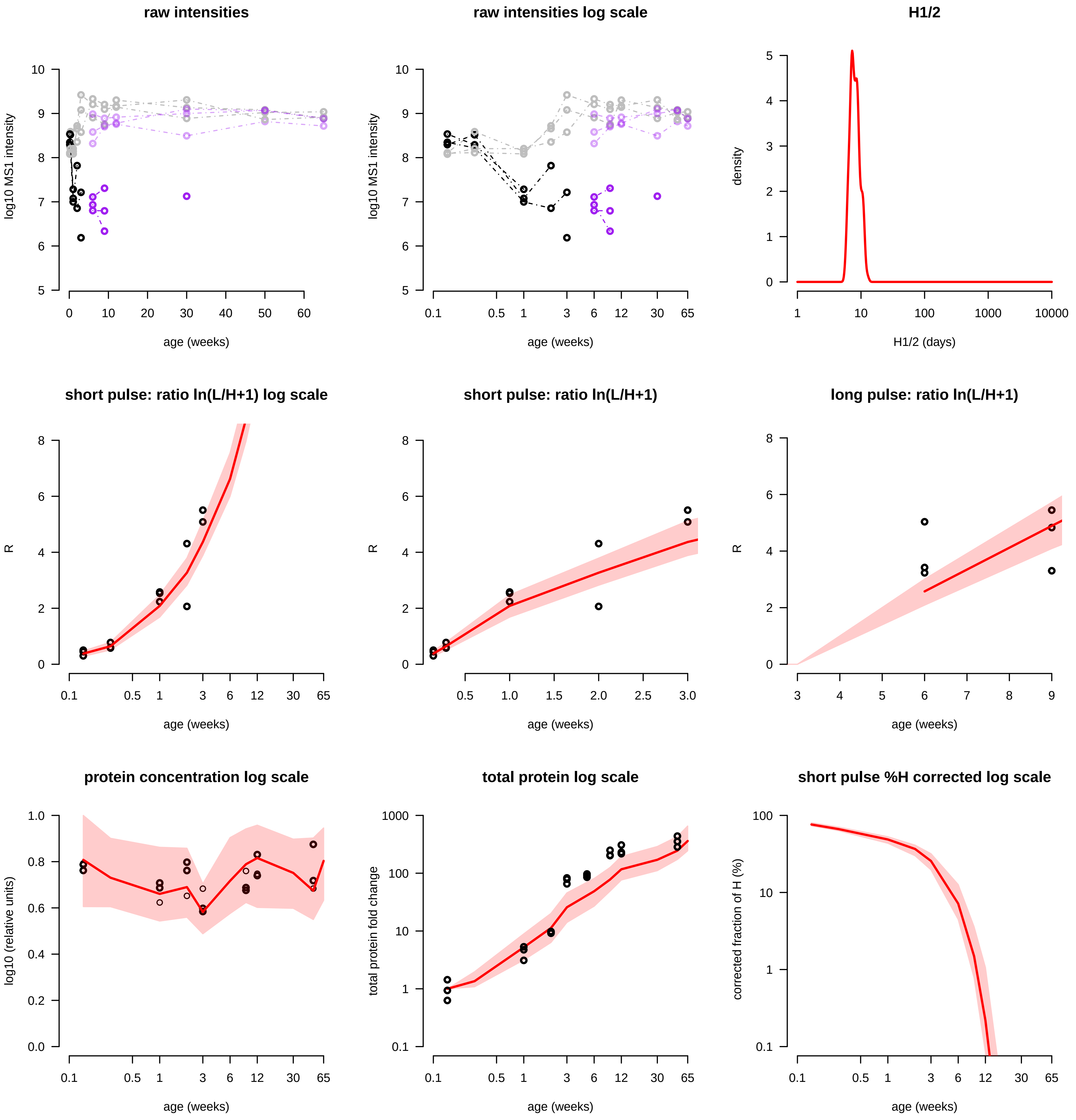

Q8BVE3

>sp|Q8BVE3|VATH_MOUSE

V−type proton ATPase subunit H

GN=Atp6v1h

Med H1/2: 22.7 days

Q5   H1/2: 7 days

Q95 H1/2: 243 days

reliable model fit: no

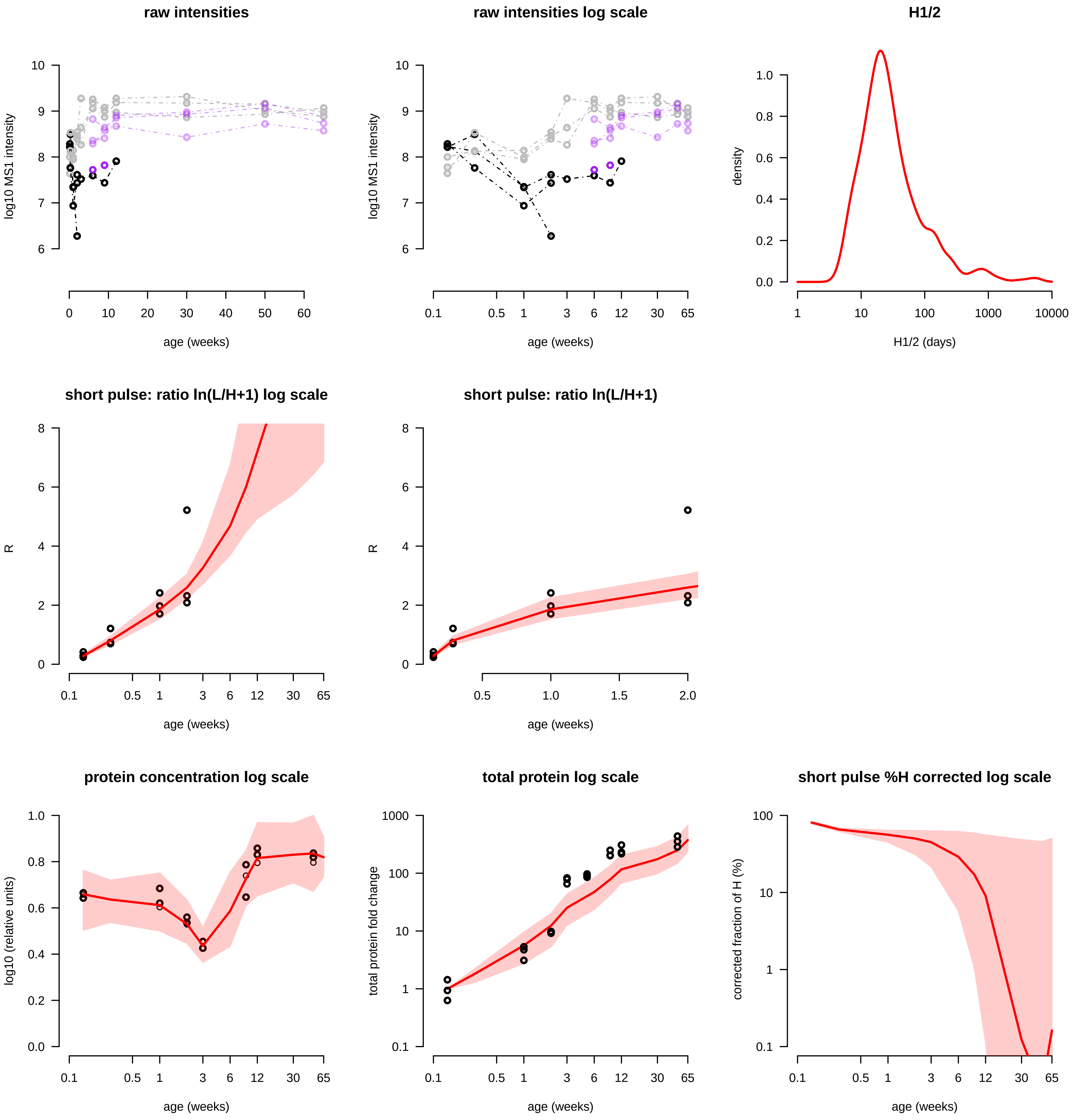

O88487

>sp|O88487|DC1I2_MOUSE

Cytoplasmic dynein 1 intermediate chain 2

GN=Dync1i2

Med H1/2: 7 days

Q5 H1/2: 5.9 days

Q95 H1/2: 8.9 days

reliable model fit: yes

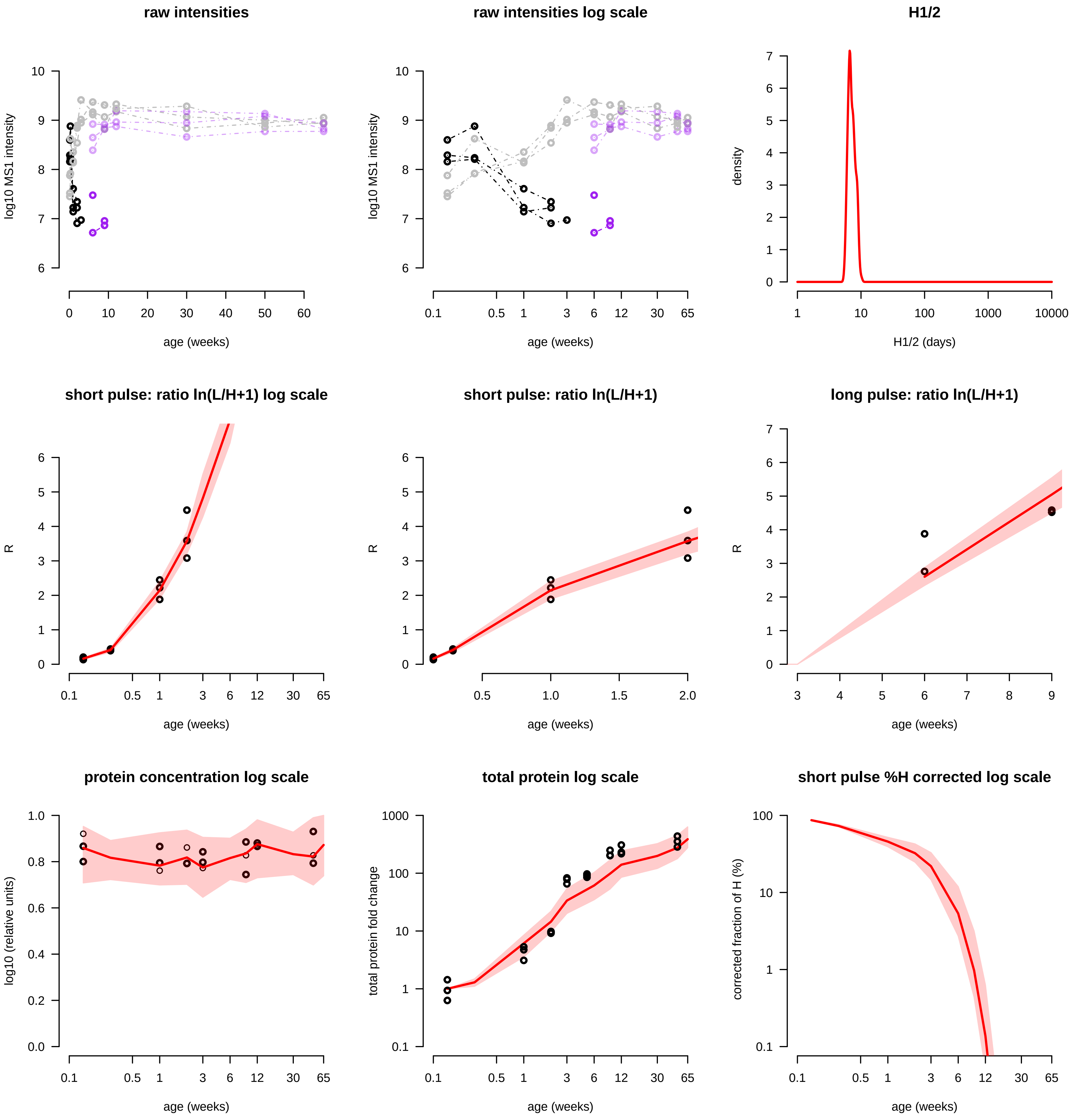

O88851

>sp|O88851|RBBP9_MOUSE

Putative hydrolase RBBP9

GN=Rbbp9

Med H1/2: 6.5 days

Q5 H1/2: 3.5 days

Q95 H1/2: 81.7 days

reliable model fit: yes

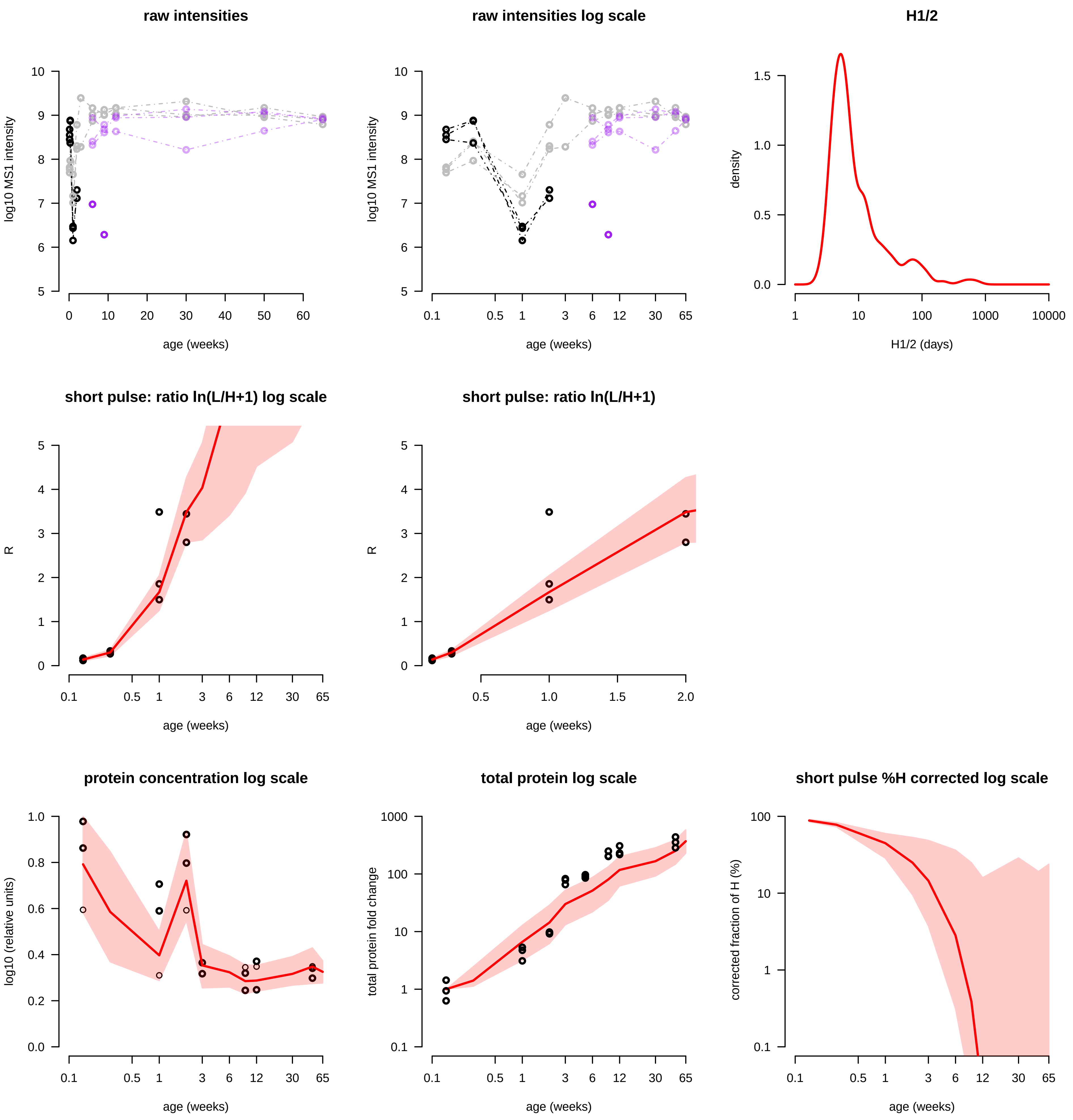

O35969;O35969−2

>sp|O35969|GAMT_MOUSE

Guanidinoacetate N−methyltransferase

GN=Gamt

Med H1/2: 6.8 days

Q5 H1/2: 4.9 days

Q95 H1/2: 10 days

reliable model fit: yes

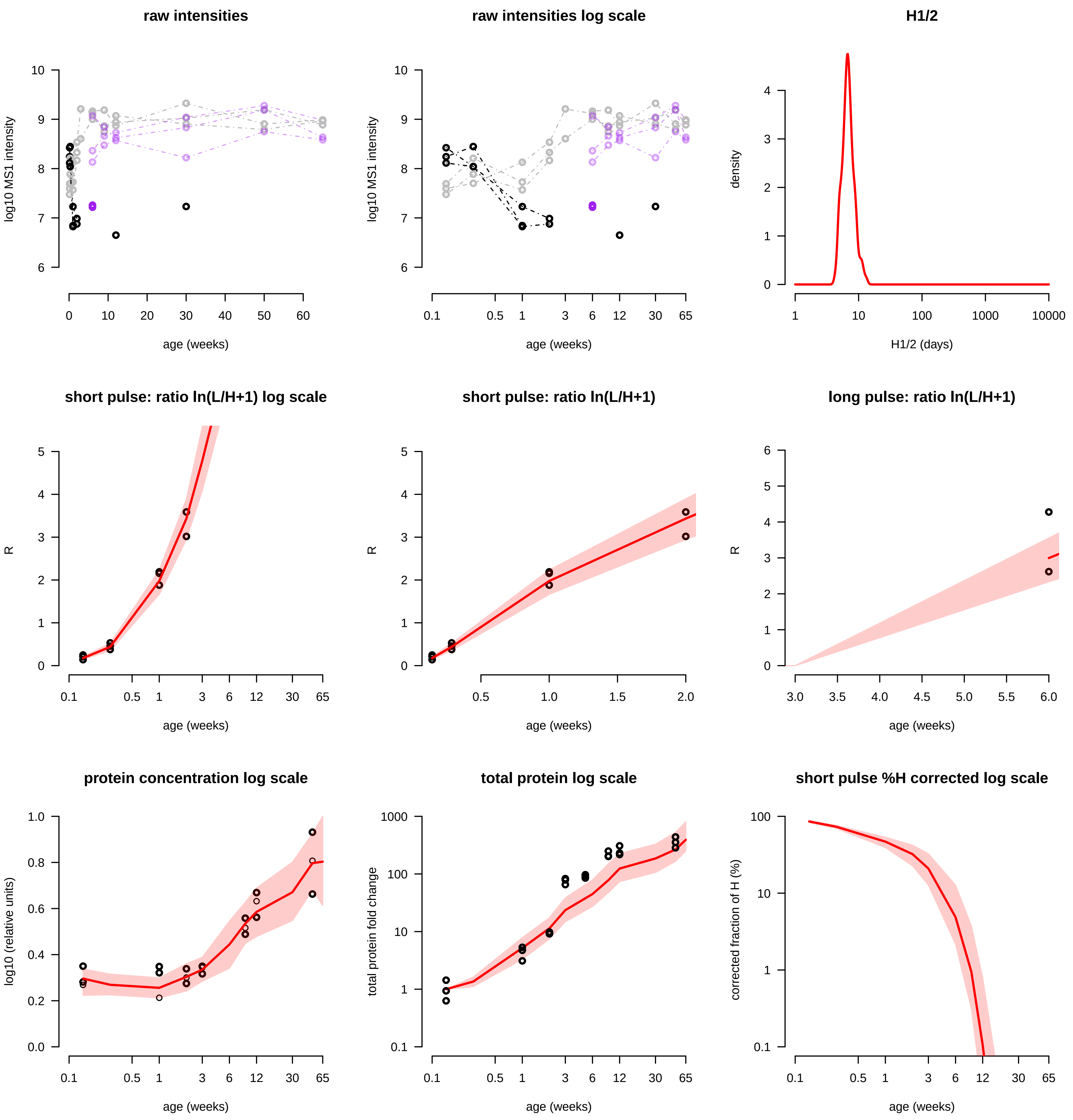

O55201;O55201−2

>sp|O55201|SPT5H_MOUSE

Transcription elongation factor SPT5

GN=Supt5h

Med H1/2: 3.6 days

Q5 H1/2: 3.1 days

Q95 H1/2: 4.5 days

reliable model fit: yes

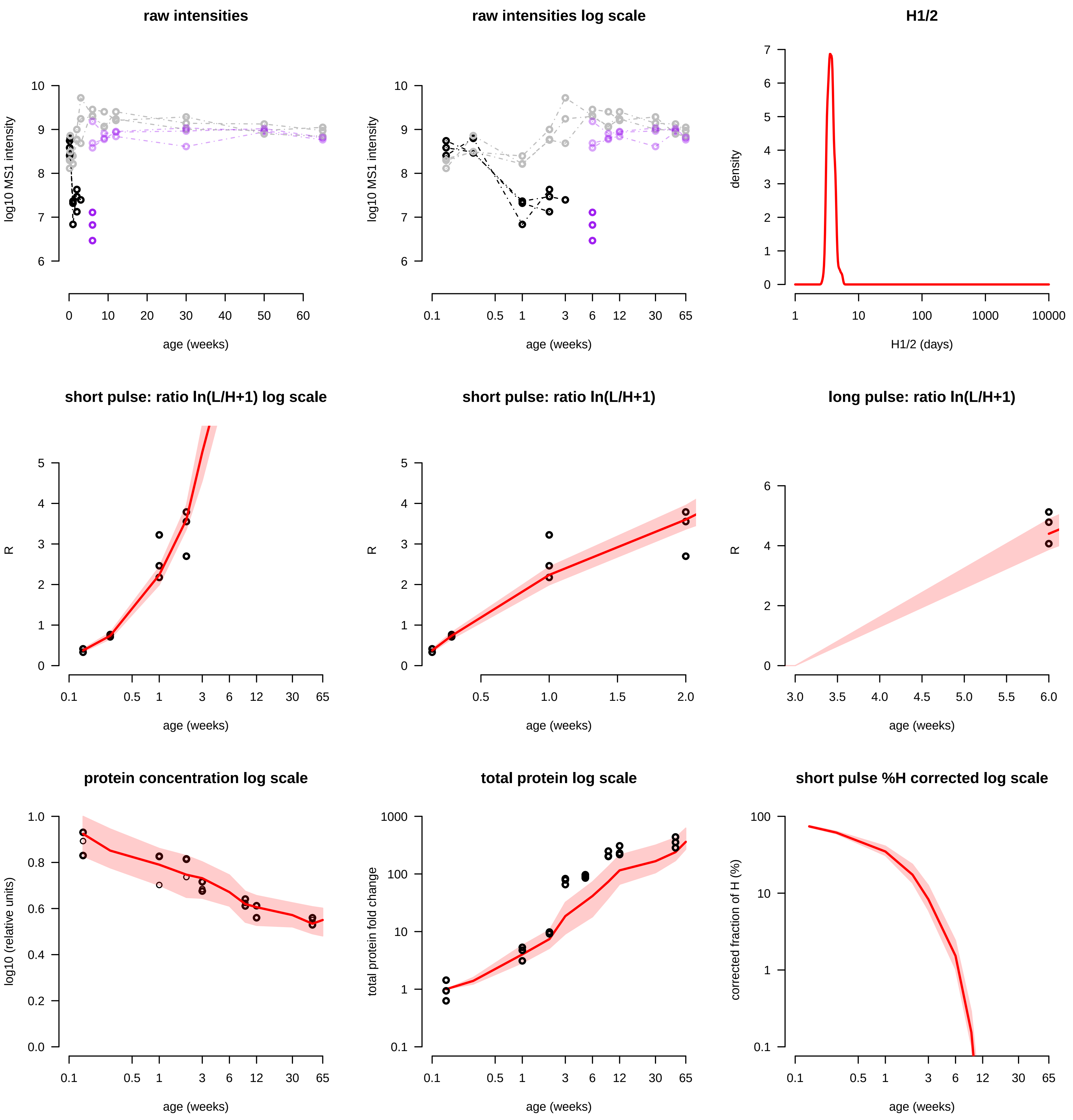

P17918

>sp|P17918|PCNA_MOUSE

Proliferating cell nuclear antigen

GN=Pcna

Med H1/2: 4.6 days

Q5 H1/2: 3.7 days

Q95 H1/2: 5.9 days

reliable model fit: yes

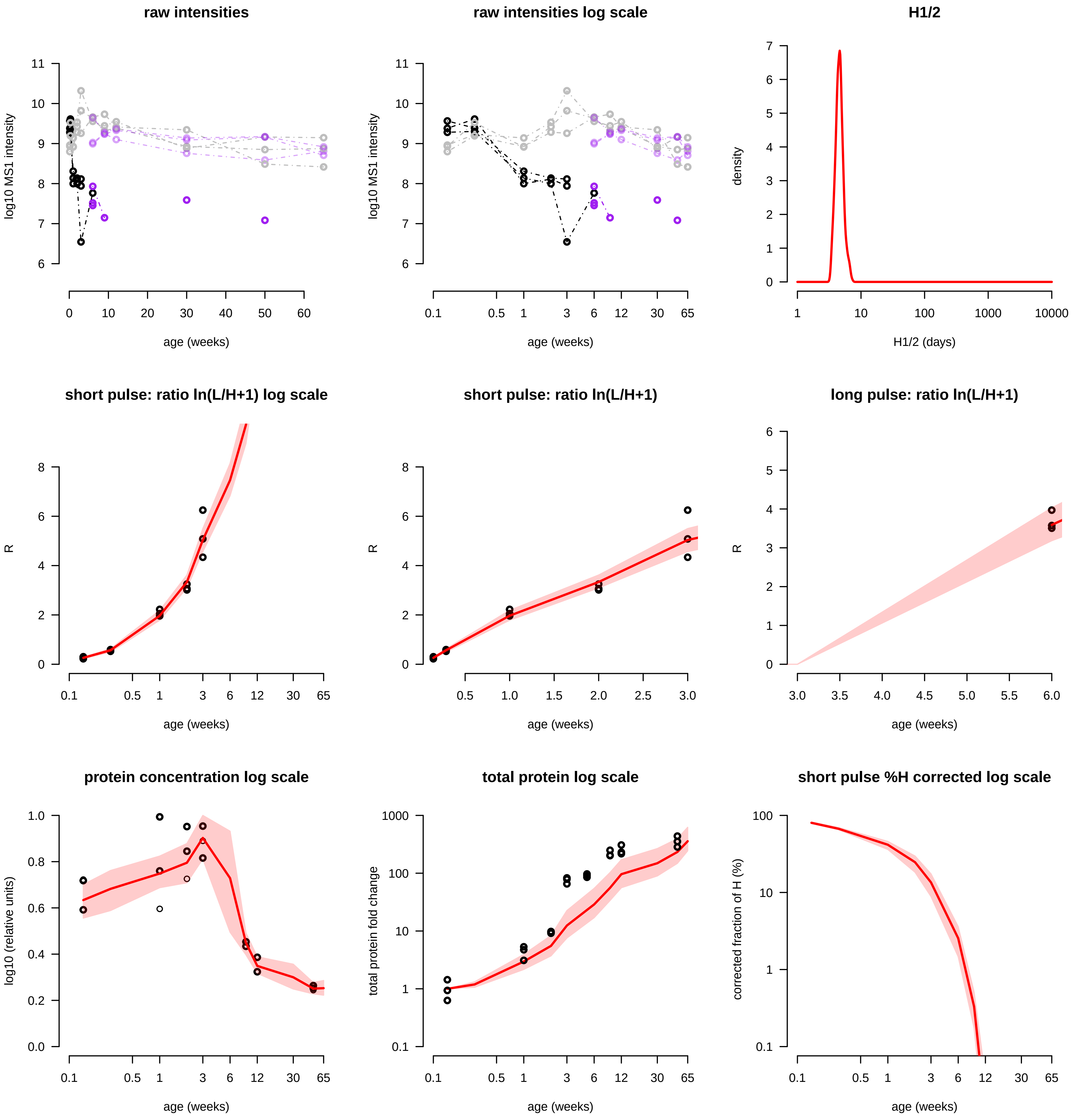

Q80SW1

>sp|Q80SW1|SAHH2_MOUSE

S−adenosylhomocysteine hydrolase−like protein 1

GN=Ahcyl1

Med H1/2: 9.2 days

Q5 H1/2: 4 days

Q95 H1/2: 74.4 days

reliable model fit: yes

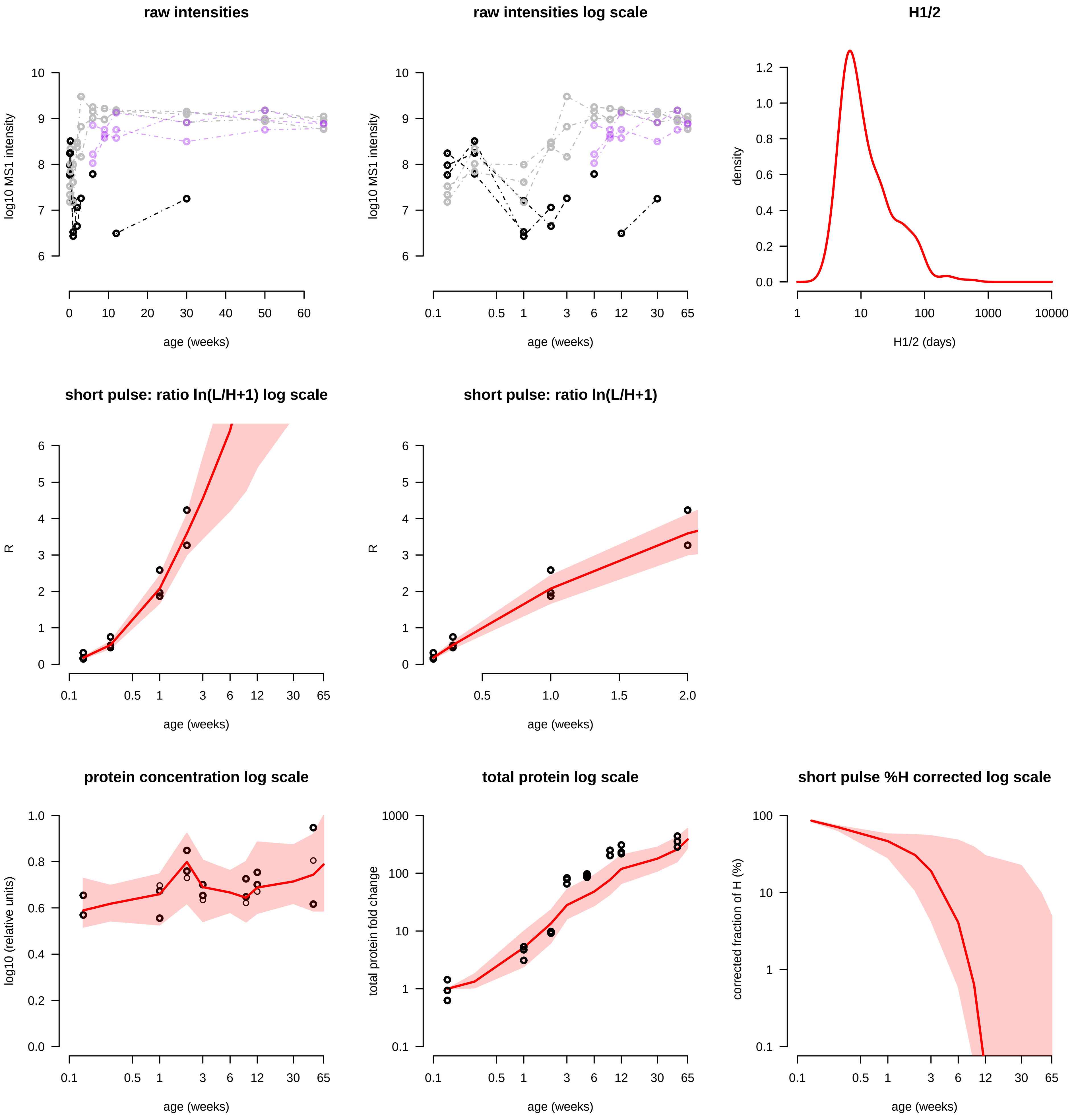

O88545

>sp|O88545|CSN6_MOUSE

COP9 signalosome complex subunit 6

GN=Cops6

Med H1/2: 6.3 days

Q5 H1/2: 5.1 days

Q95 H1/2: 7.3 days

reliable model fit: yes

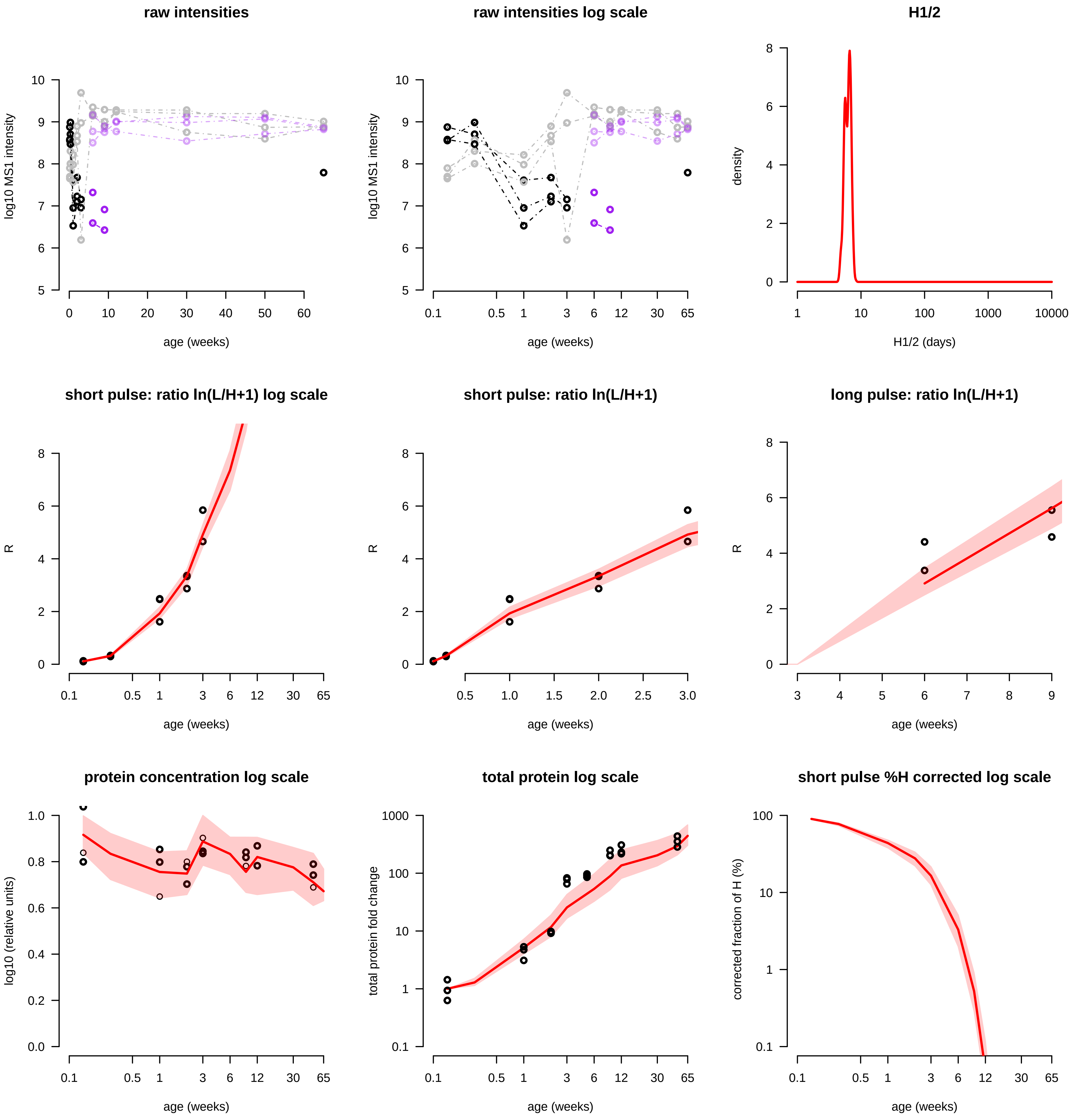

O08583;O08583−2

>sp|O08583|THOC4_MOUSE

THO complex subunit 4

GN=Alyref

Med H1/2: 428 days

Q5 H1/2: 125 days

Q95 H1/2: 4743.8 days

reliable model fit: no

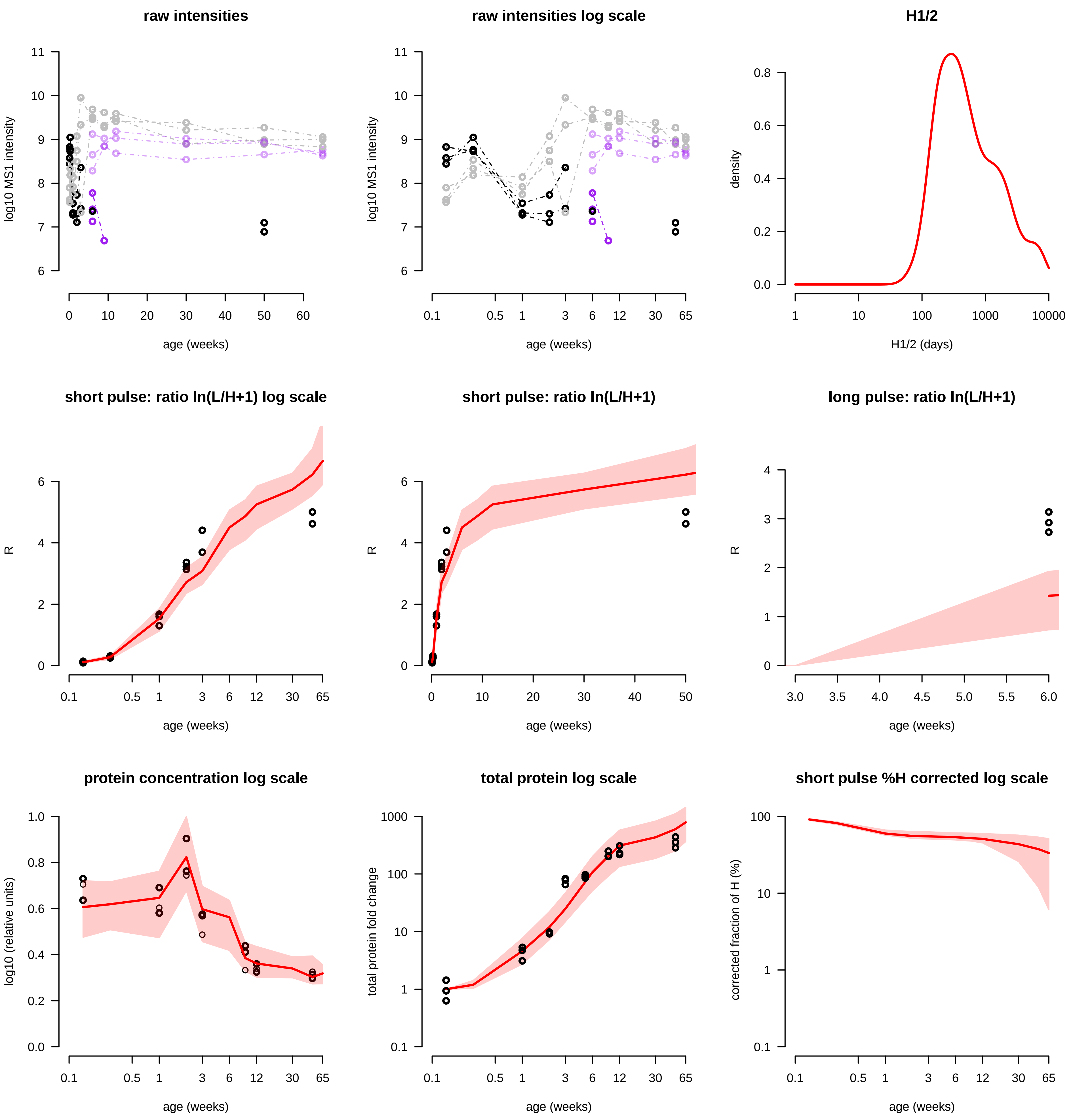

D3Z7P3

>sp|D3Z7P3|GLSK_MOUSE

Glutaminase kidney isoform, mitochondrial

GN=Gls

Med H1/2: 4.4 days

Q5 H1/2: 3.1 days

Q95 H1/2: 6.8 days

reliable model fit: yes

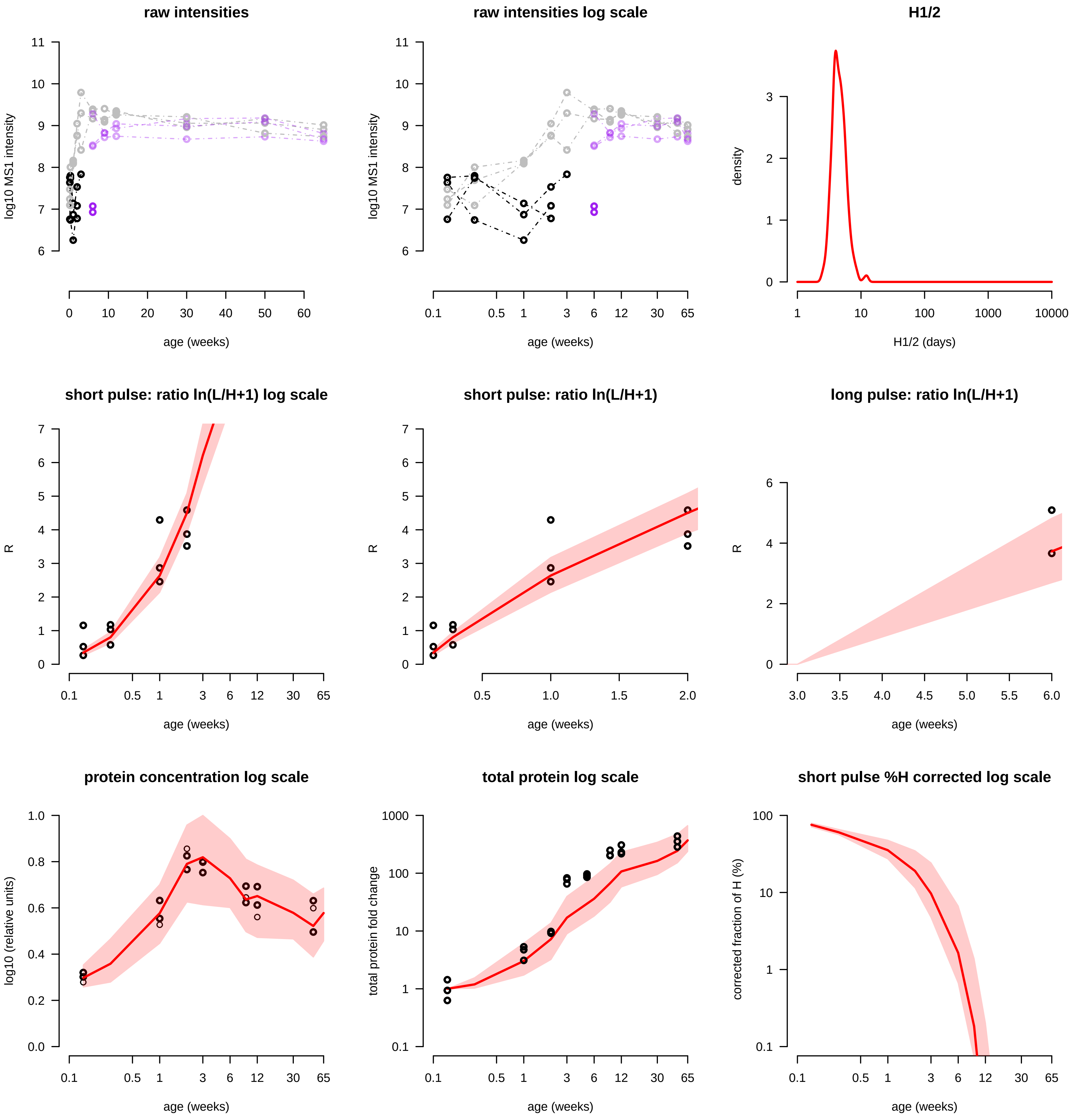

Q8CGF7−2;Q8CGF7;Q8CGF7−3

>sp|Q8CGF7−2|TCRG1_MOUSE

Isoform 2 of Transcription elongation regulator 1

GN=Tcerg1

Med H1/2: 13.7 days

Q5 H1/2: 5.9 days

Q95 H1/2: 72.9 days

reliable model fit: yes

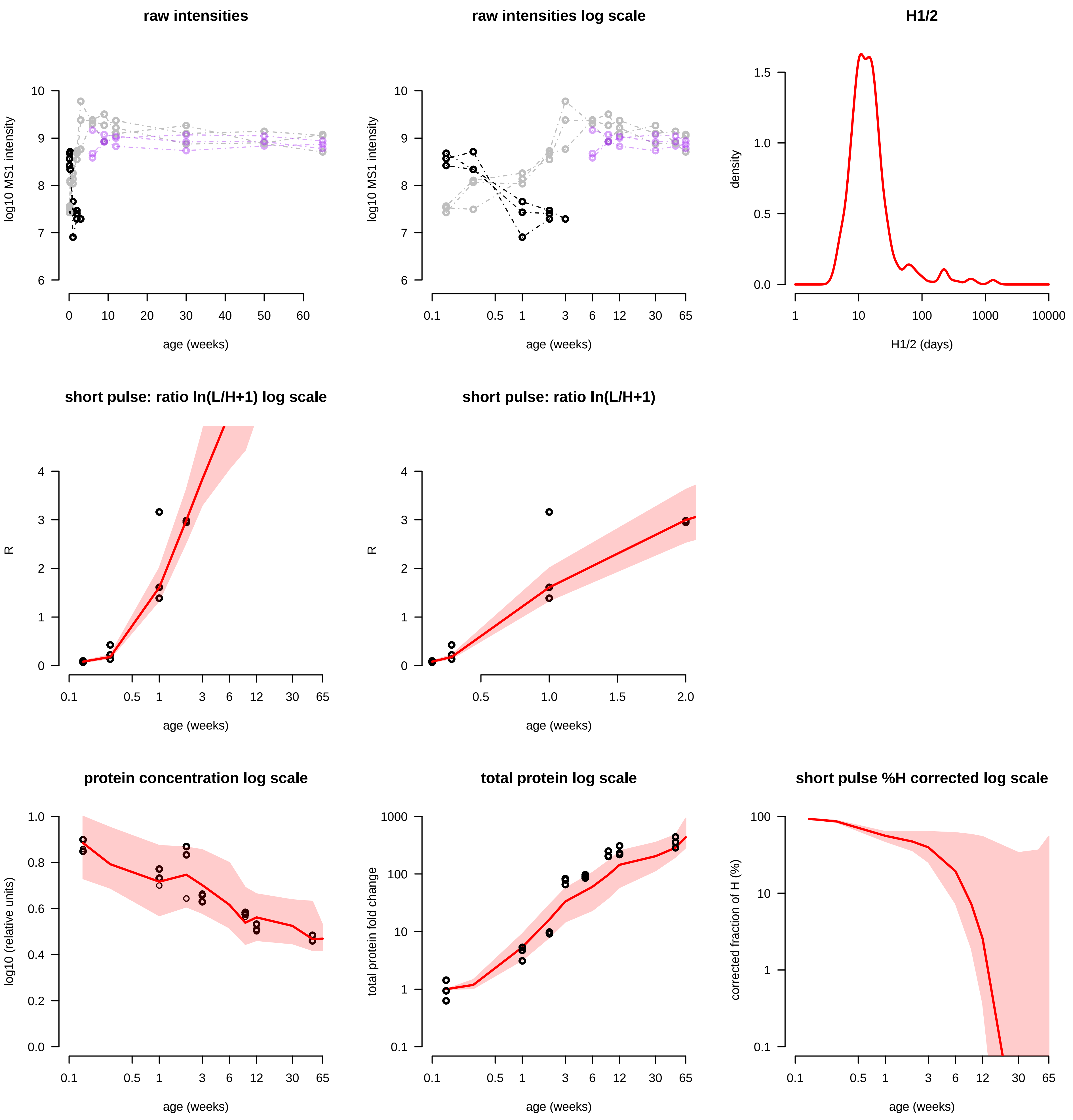

P14602;P14602−2;P14602−3

>sp|P14602|HSPB1_MOUSE

Heat shock protein beta−1

GN=Hspb1

Med H1/2: 4.5 days

Q5 H1/2: 3.4 days

Q95 H1/2: 6.9 days

reliable model fit: yes

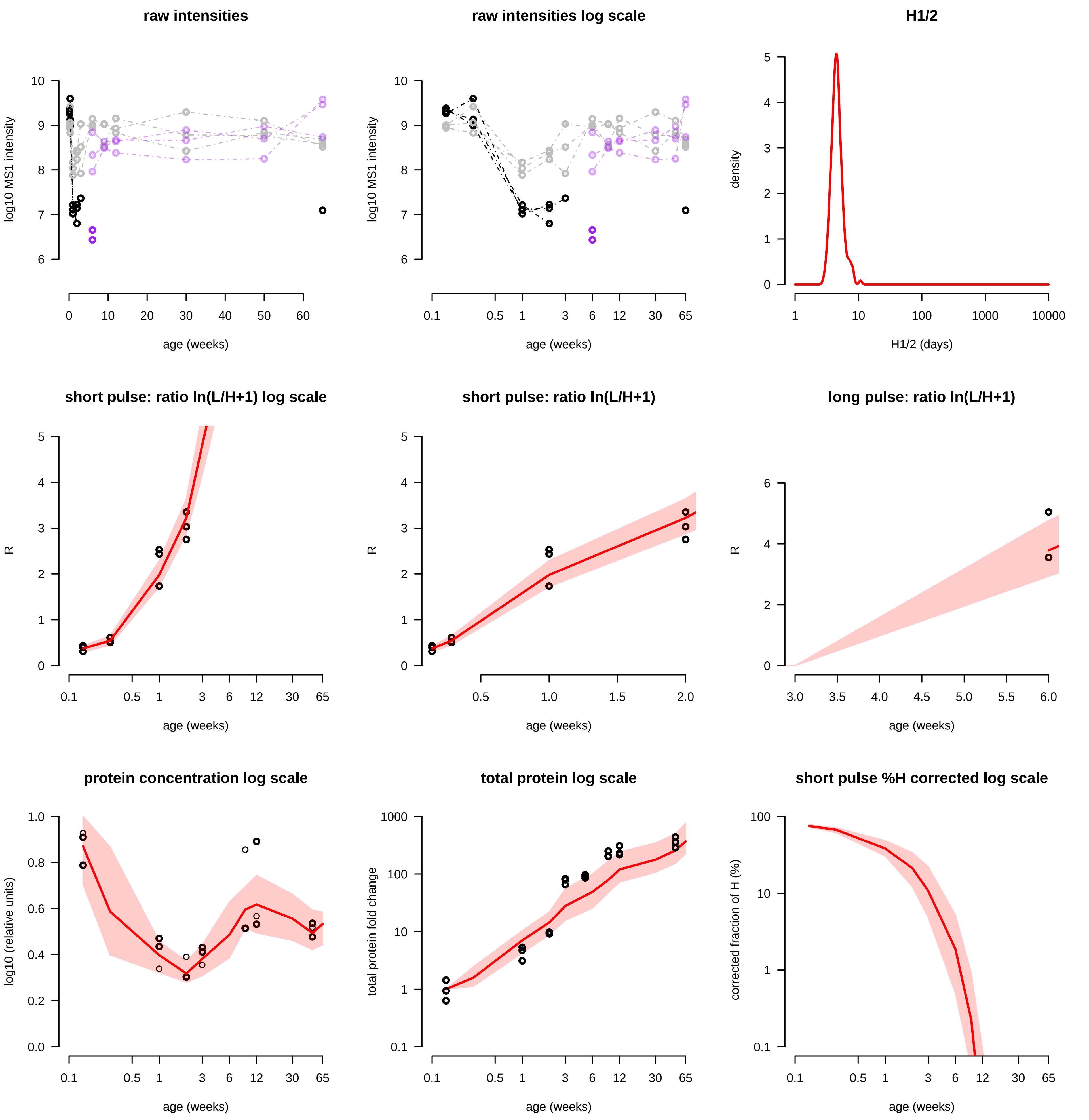

Q9D8B3

>sp|Q9D8B3|CHM4B_MOUSE

Charged multivesicular body protein 4b

GN=Chmp4b

Med H1/2: 10.1 days

Q5 H1/2: 8.3 days

Q95 H1/2: 12.8 days

reliable model fit: yes

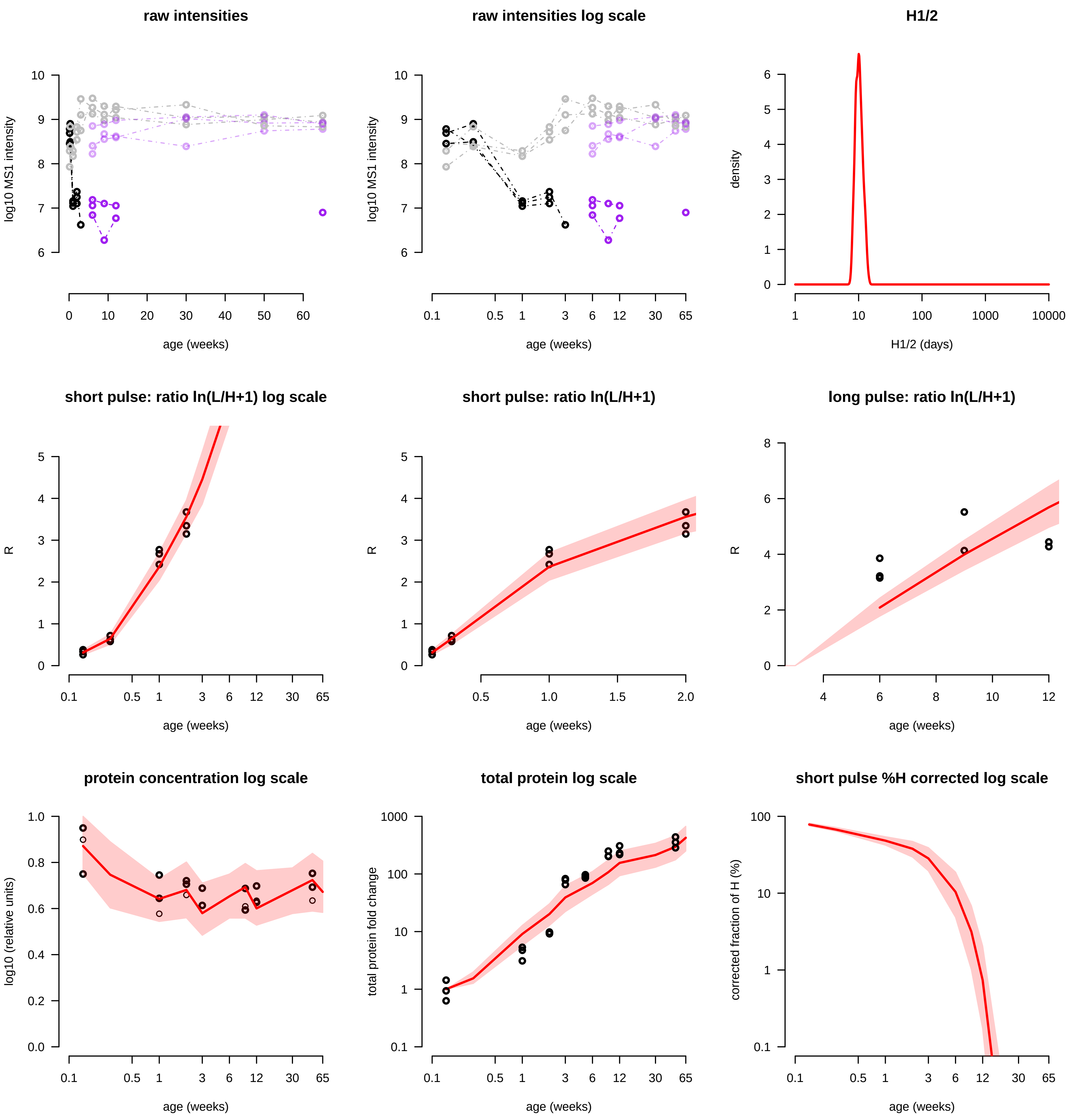

O35593

>sp|O35593|PSDE_MOUSE

26S proteasome non−ATPase regulatory subunit 14

GN=Psmd14

Med H1/2: 5.1 days

Q5 H1/2: 4.2 days

Q95 H1/2: 7 days

reliable model fit: yes

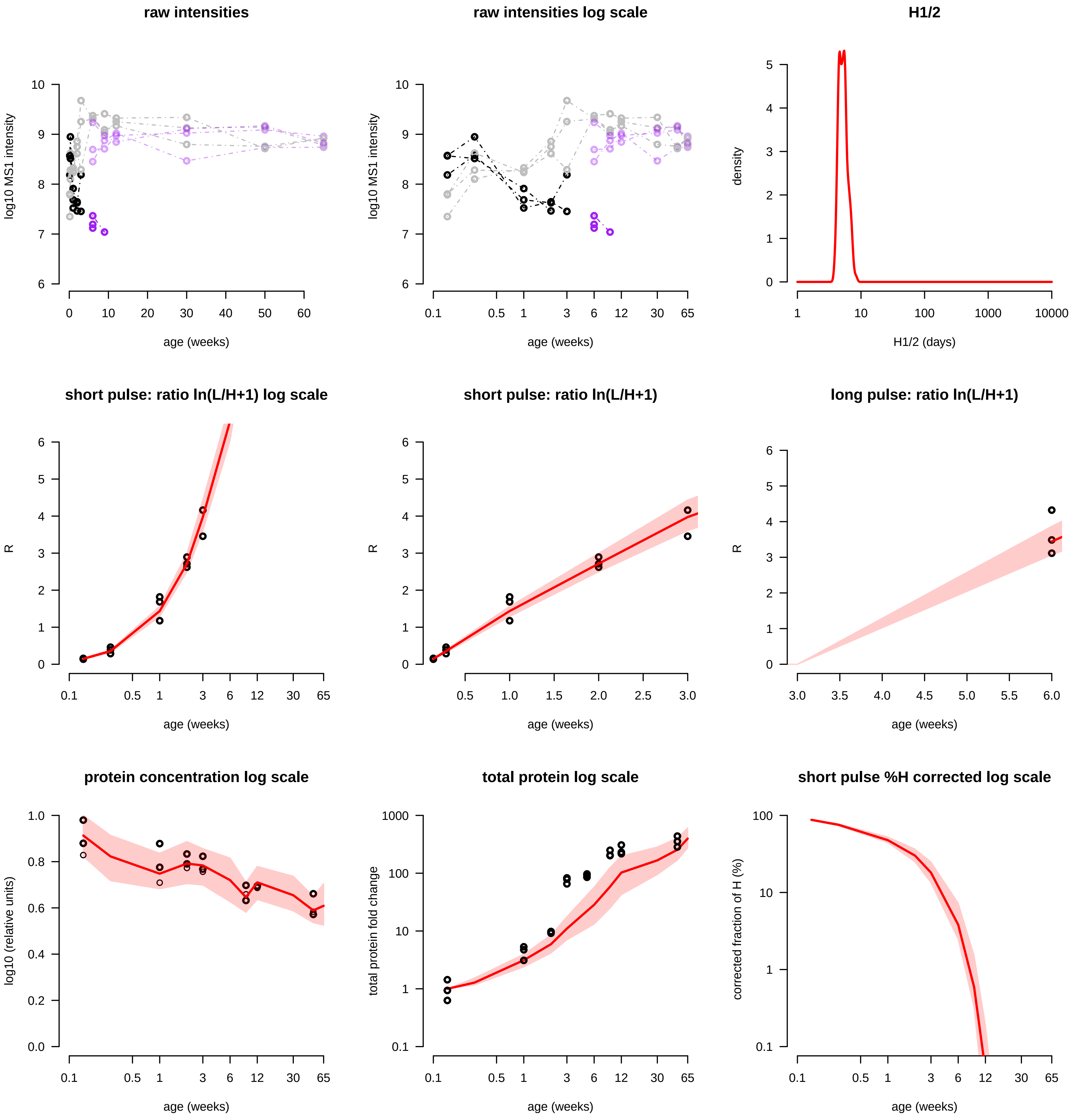

P35486

>sp|P35486|ODPA_MOUSE

yruvate dehydrogenase E1 component subunit alpha, somatic form, mitochondri

GN=Pdha1

Med H1/2: 31.5 days

Q5 H1/2: 7.1 days

Q95 H1/2: 469 days

reliable model fit: yes

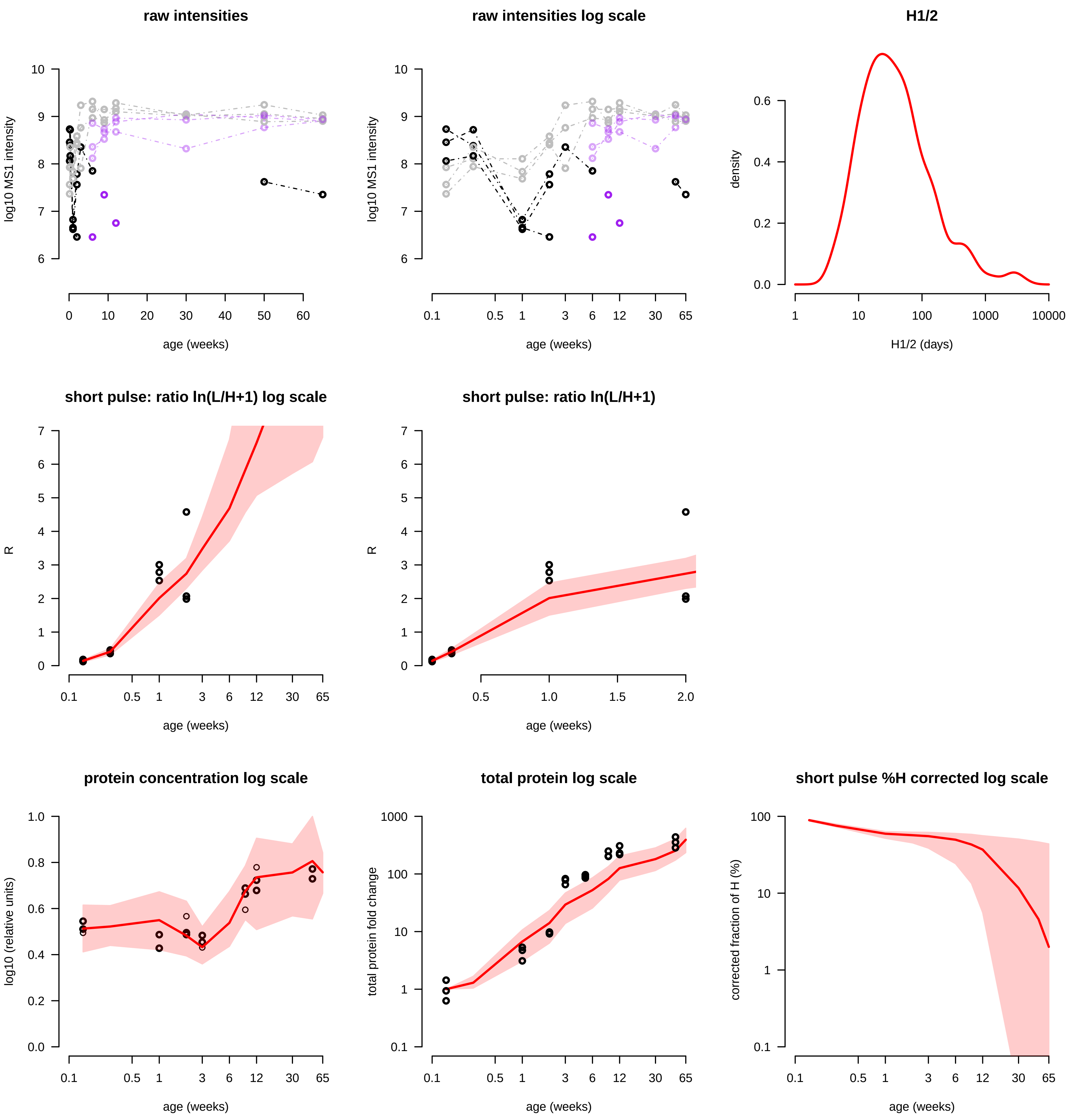

Q921T2;Q921T2−2;Q921T2−3

>sp|Q921T2|TOIP1_MOUSE

Torsin−1A−interacting protein 1

GN=Tor1aip1

Med H1/2: 3.8 days

Q5 H1/2: 3.2 days

Q95 H1/2: 4.7 days

reliable model fit: yes

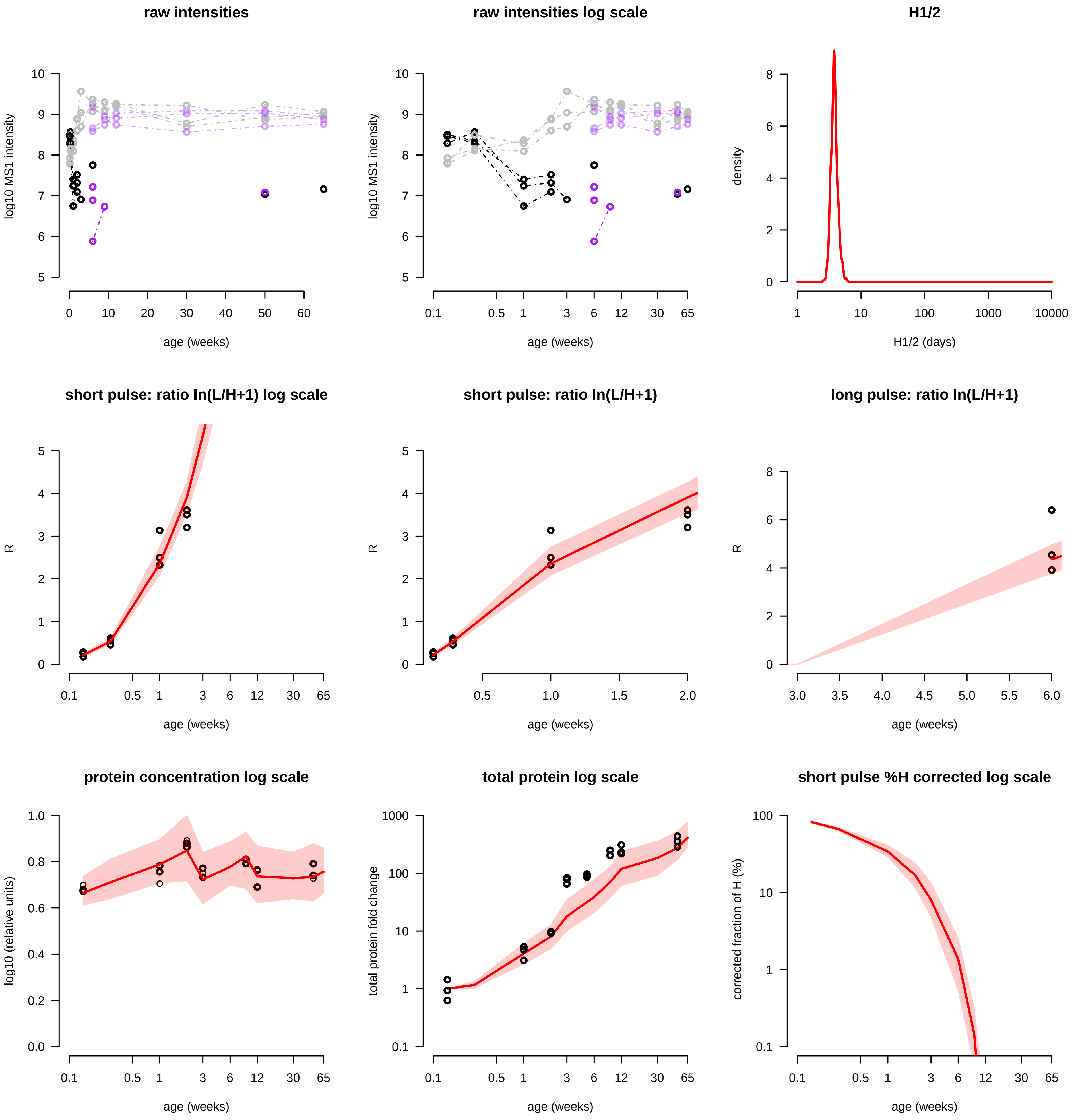

P61750

>sp|P61750|ARF4_MOUSE

ADP−ribosylation factor 4

GN=Arf4

Med H1/2: 10.4 days

Q5 H1/2: 7.9 days

Q95 H1/2: 12.8 days

reliable model fit: yes

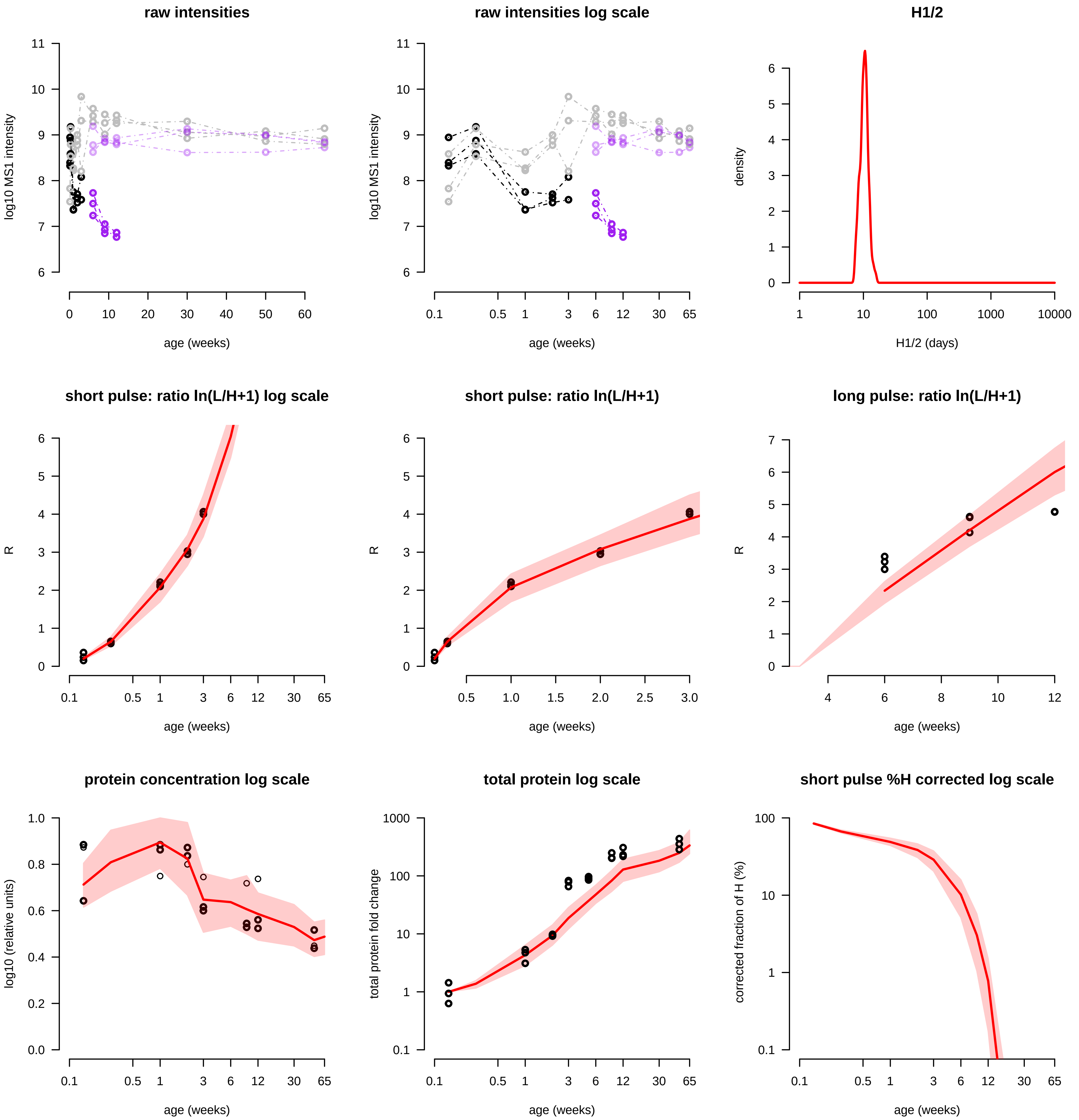

P62911

>sp|P62911|RL32_MOUSE

60S ribosomal protein L32

GN=Rpl32

Med H1/2: 36.1 days

Q5 H1/2: 19.8 days

Q95 H1/2: 160.6 days

reliable model fit: no

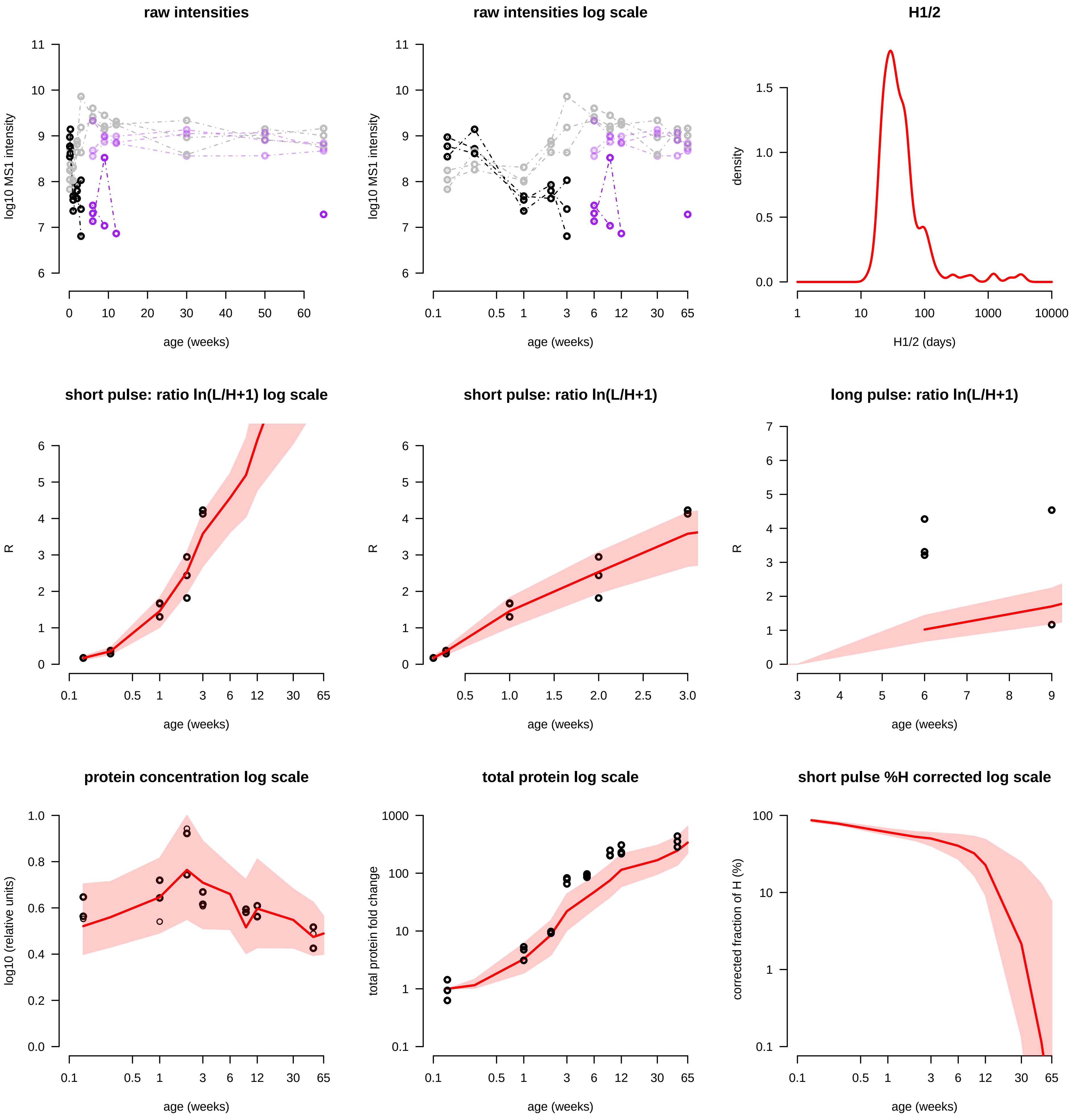

Q7TMB8;Q7TMB8−2

>sp|Q7TMB8|CYFP1_MOUSE

Cytoplasmic FMR1−interacting protein 1

GN=Cyfip1

Med H1/2: 25.5 days

Q5 H1/2: 15.7 days

Q95 H1/2: 66.4 days

reliable model fit: yes

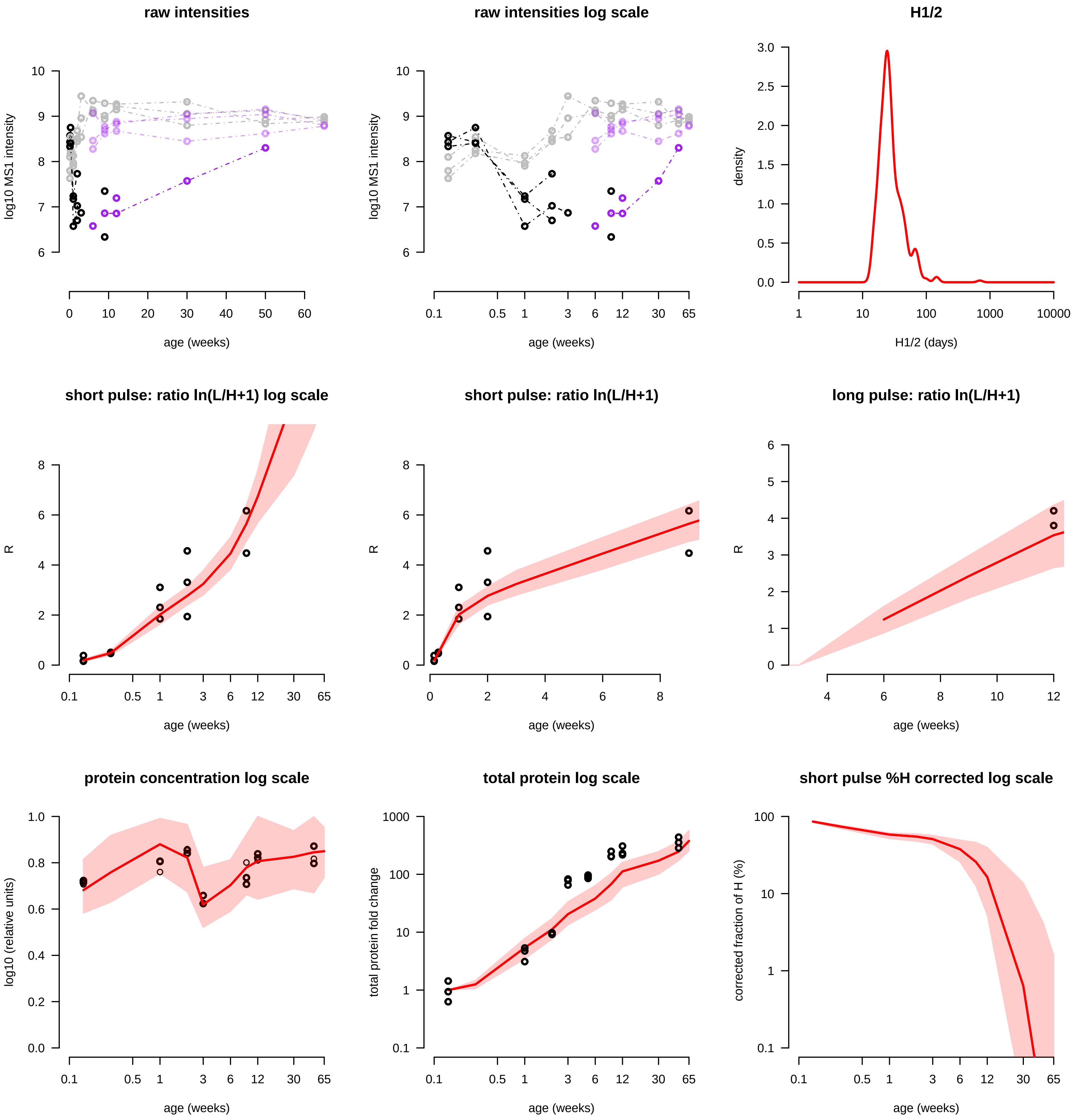

Q91V76

>sp|Q91V76|CK054_MOUSE

Ester hydrolase C11orf54 homolog

Med H1/2: 6.2 days

Q5 H1/2: 4.6 days

Q95 H1/2: 8.3 days

reliable model fit: yes

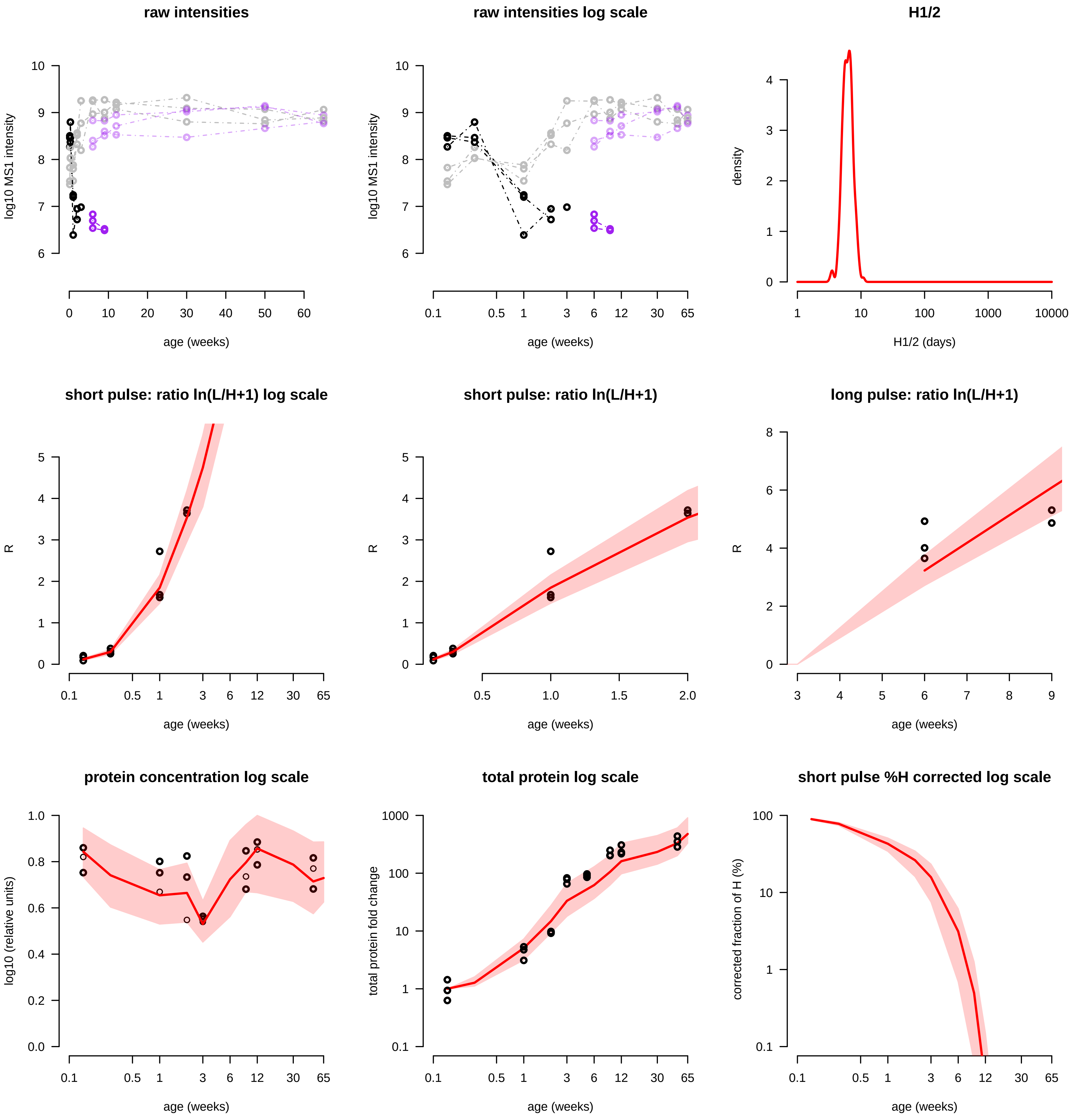

Q8K370

>sp|Q8K370|ACD10_MOUSE

Acyl−CoA dehydrogenase family member 10

GN=Acad10

Med H1/2: 3.8 days

Q5 H1/2: 2.8 days

Q95 H1/2: 5.2 days

reliable model fit: yes

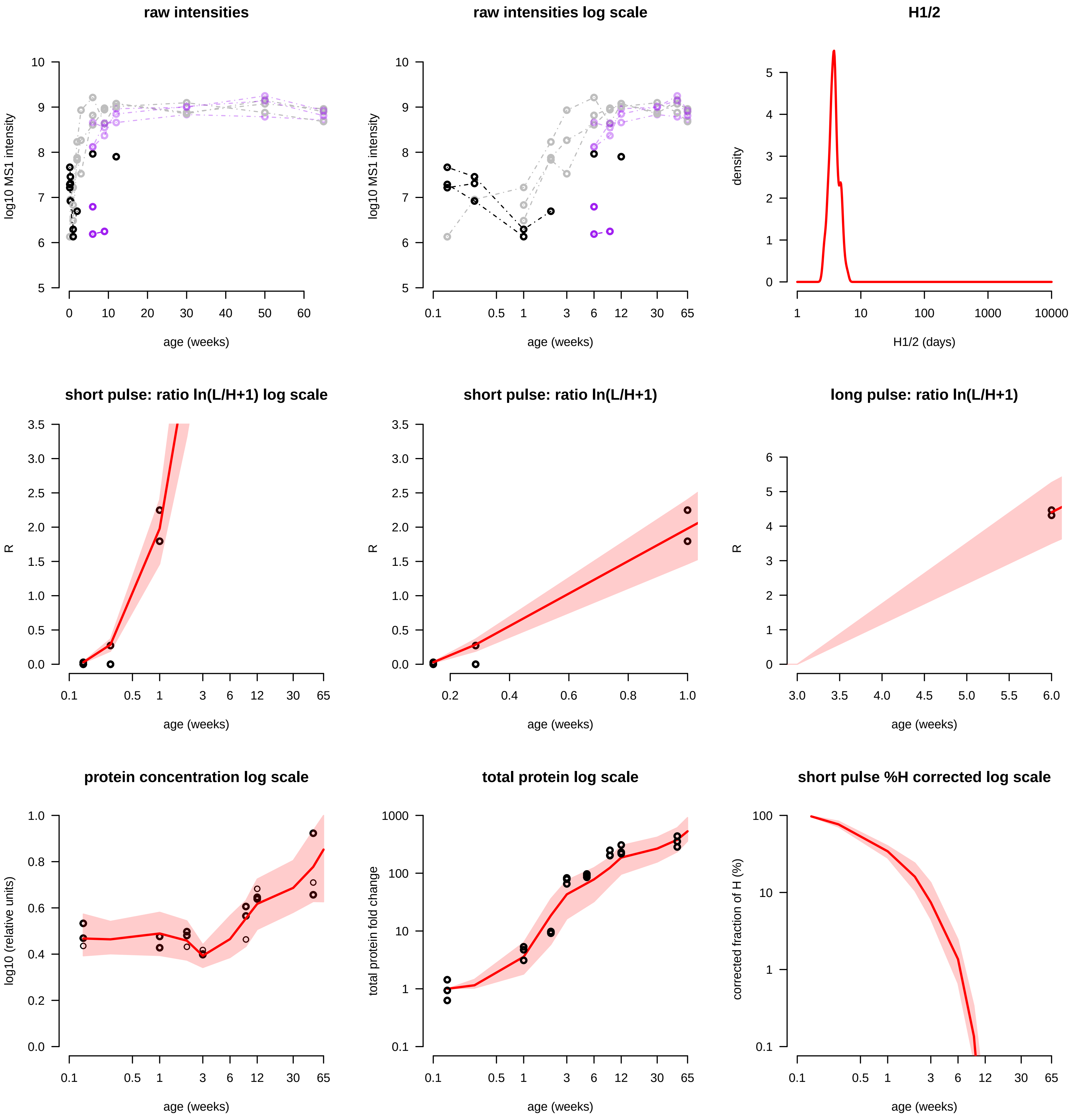

O35945

>sp|O35945|AL1A7_MOUSE

Aldehyde dehydrogenase, cytosolic 1

GN=Aldh1a7

Med H1/2: 14.6 days

Q5 H1/2: 10.8 days

Q95 H1/2: 19.6 days

reliable model fit: yes

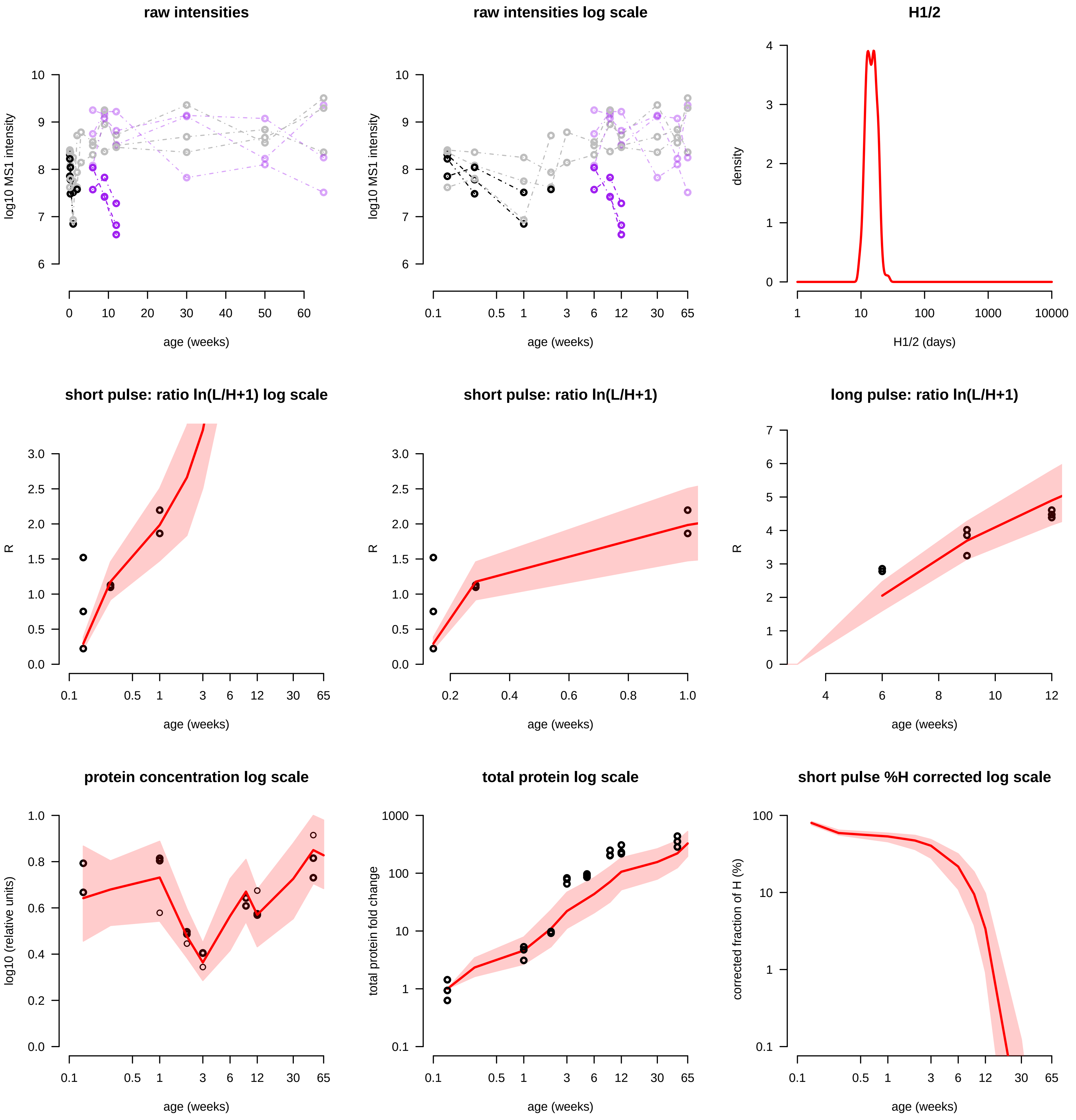

Q8JZN7

>sp|Q8JZN7|MIRO2_MOUSE

Mitochondrial Rho GTPase 2

GN=Rhot2

Med H1/2: 4.9 days

Q5 H1/2: 3.8 days

Q95 H1/2: 9.9 days

reliable model fit: yes

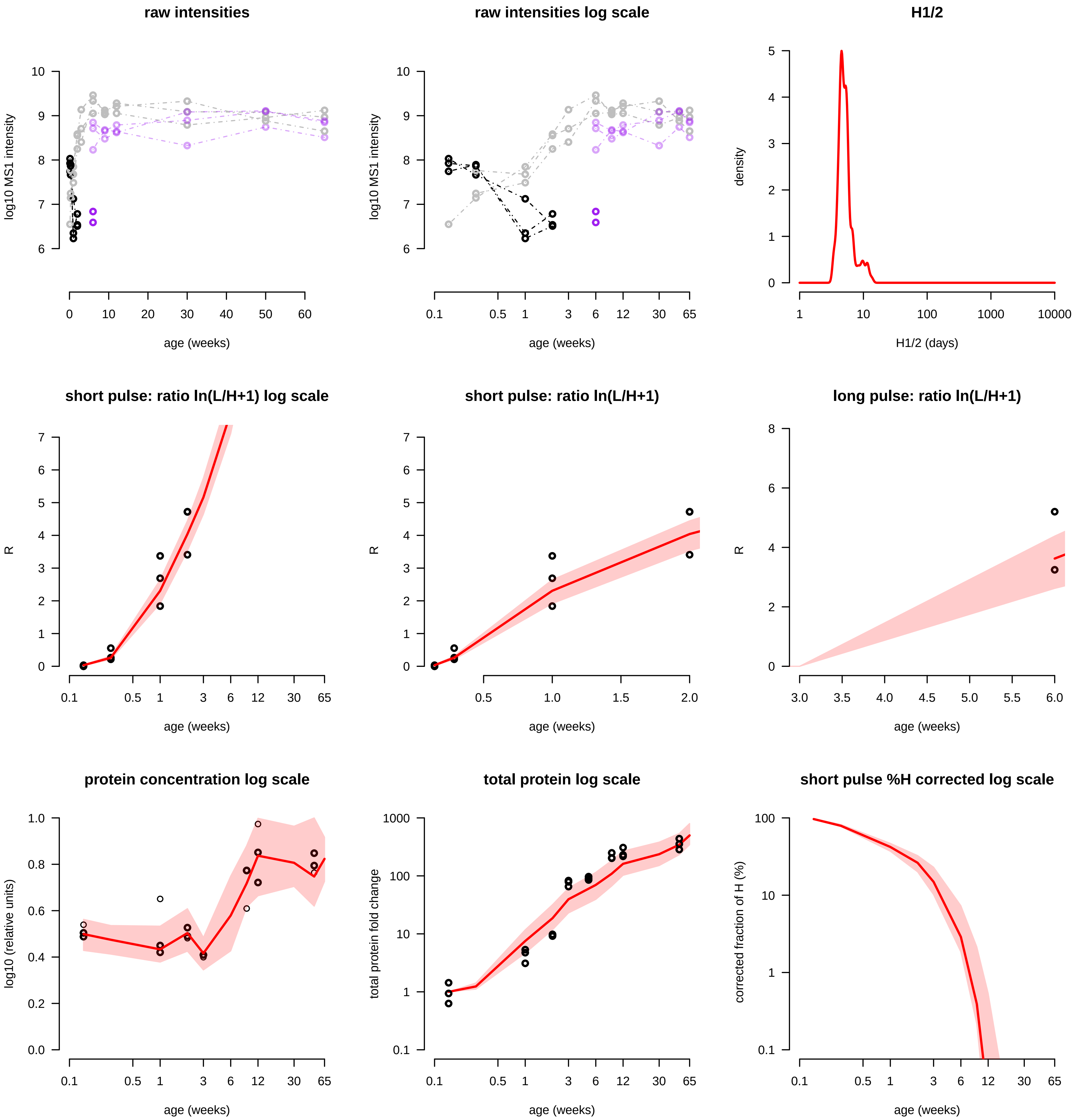

Q8K1R3;Q8K1R3−2

>sp|Q8K1R3|PNPT1_MOUSE

Polyribonucleotide nucleotidyltransferase 1, mitochondrial

GN=Pnpt1

Med H1/2: 4.7 days

Q5 H1/2: 3.4 days

Q95 H1/2: 7.1 days

reliable model fit: yes

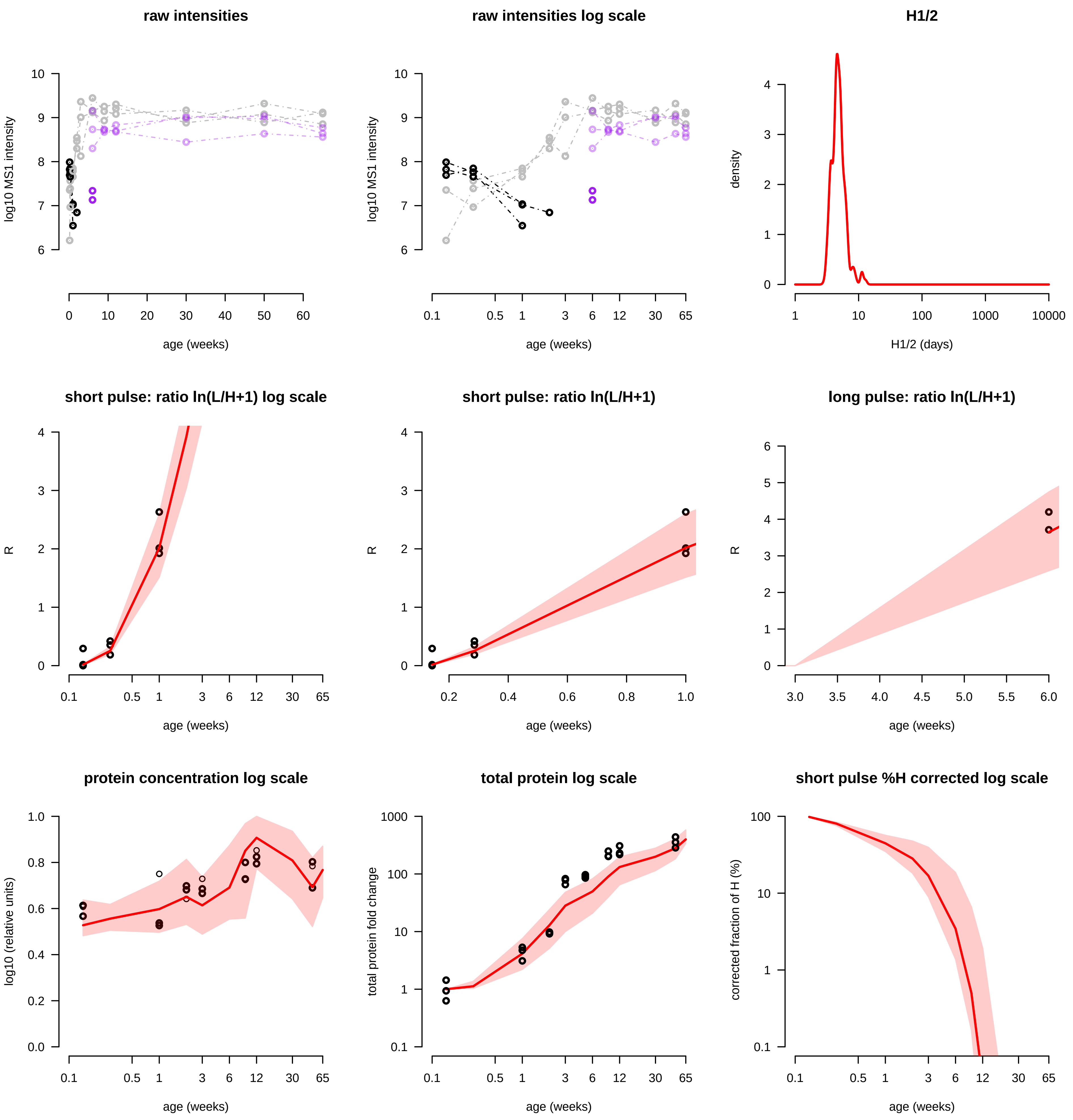

Q9JIX8−4;Q9JIX8

>sp|Q9JIX8−4|ACINU_MOUSE

Isoform 4 of Apoptotic chromatin condensation inducer in the nucleus

GN=Acin1

Med H1/2: 4.3 days

Q5 H1/2: 3.6 days

Q95 H1/2: 5.5 days

reliable model fit: yes

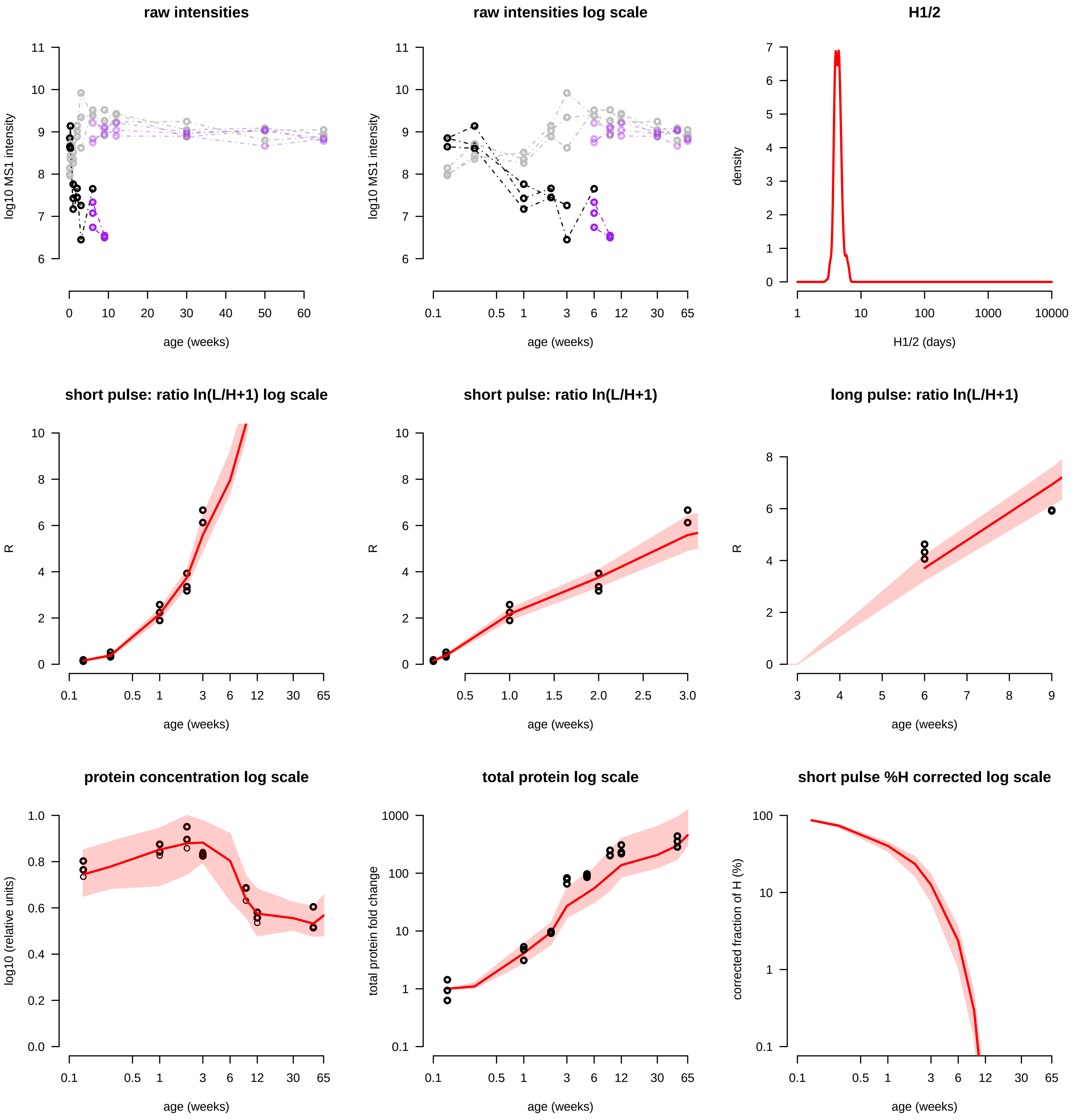

O88696

>sp|O88696|CLPP_MOUSE

ATP−dependent Clp protease proteolytic subunit, mitochondrial

GN=Clpp

Med H1/2: 5.8 days

Q5 H1/2: 4.2 days

Q95 H1/2: 9.3 days

reliable model fit: yes

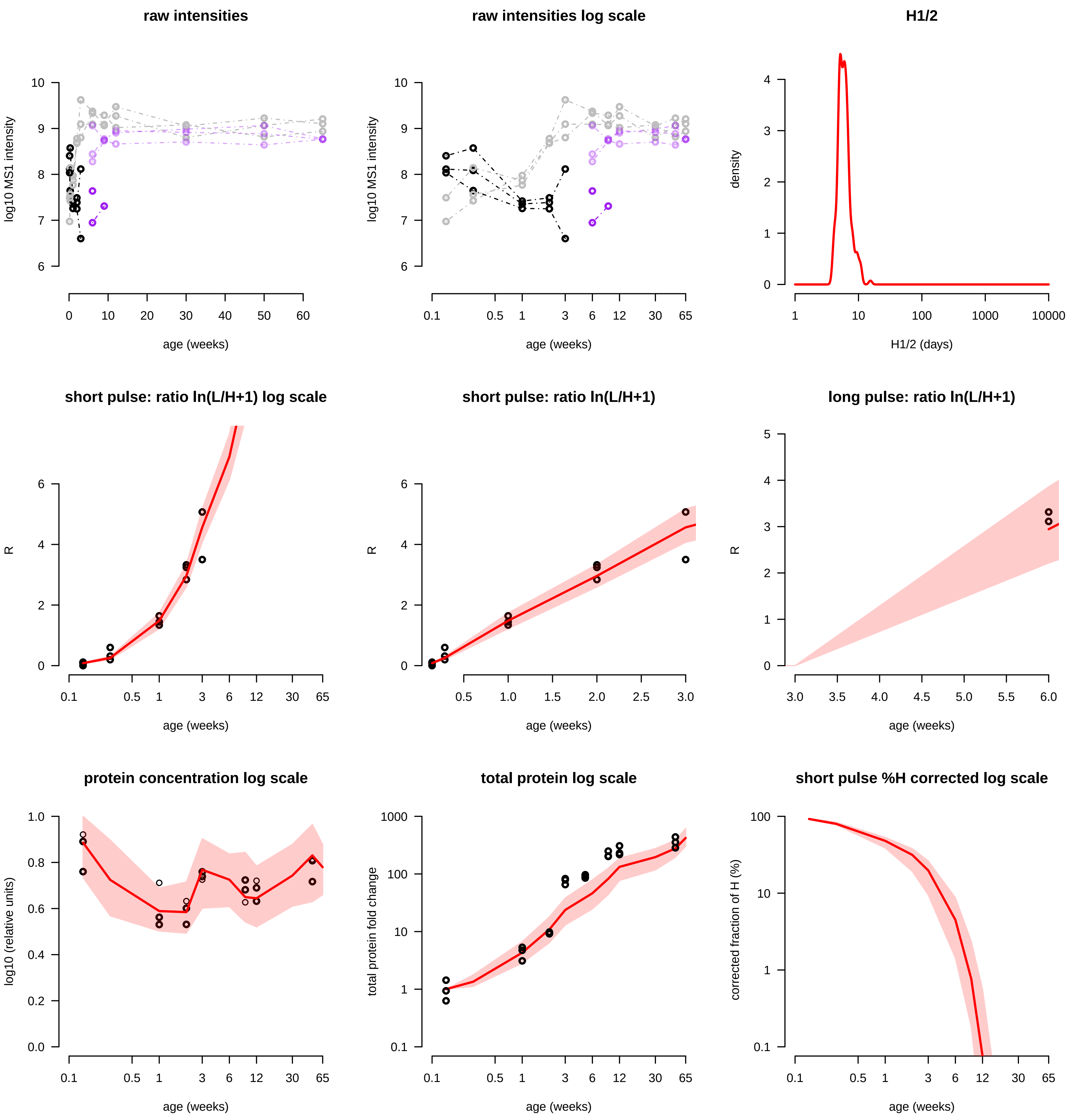

Q9Z2Y8

>sp|Q9Z2Y8|PLPHP_MOUSE

Pyridoxal phosphate homeostasis protein

GN=Plpbp

Med H1/2: 746.1 days

Q5 H1/2: 179.9 days

Q95 H1/2: 5470.7 days

reliable model fit: no

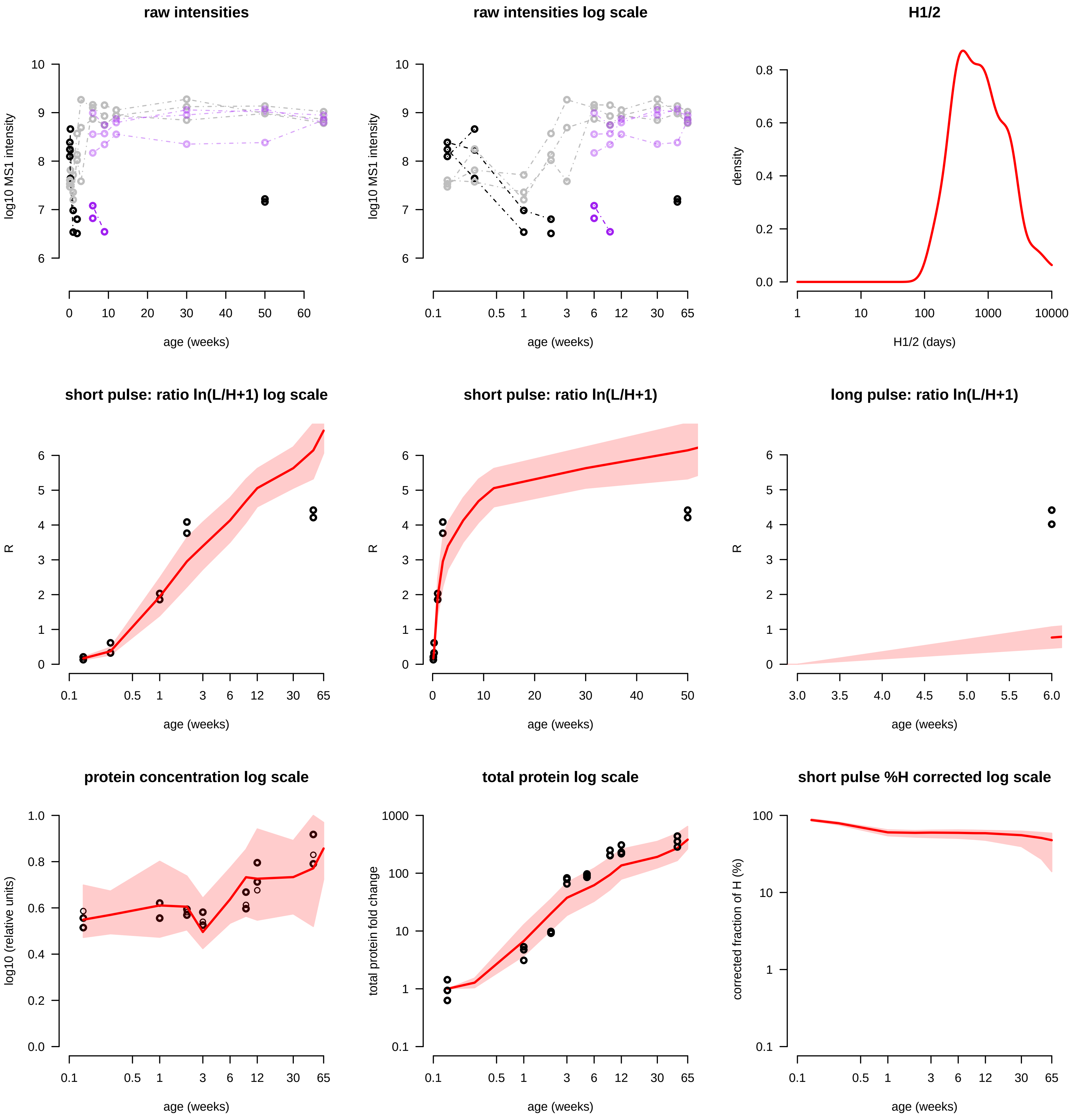

P53811

>sp|P53811|PIPNB_MOUSE

Phosphatidylinositol transfer protein beta isoform

GN=Pitpnb

Med H1/2: 4.1 days

Q5 H1/2: 3.3 days

Q95 H1/2: 4.8 days

reliable model fit: yes

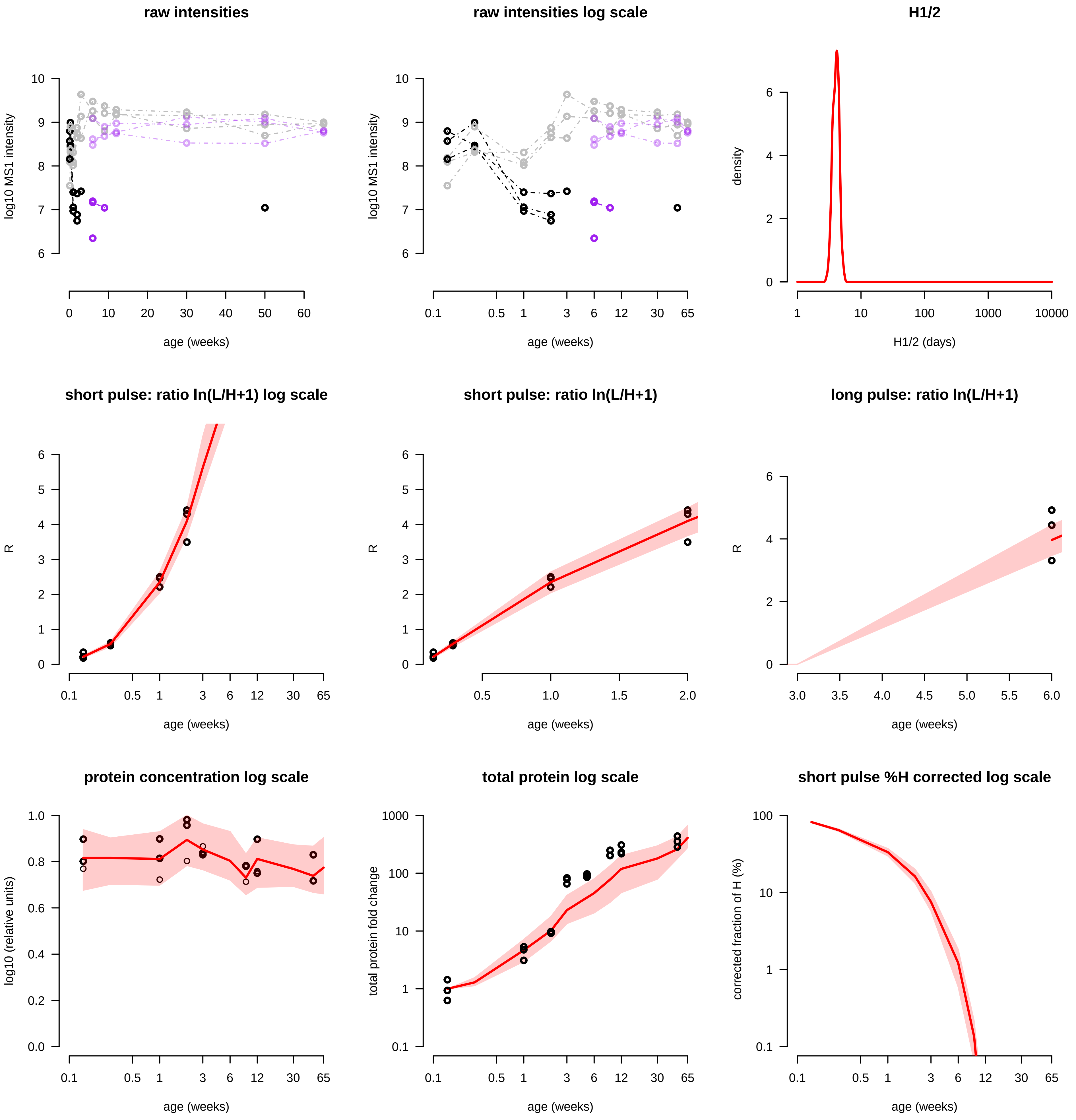

Q9ET30

>sp|Q9ET30|TM9S3_MOUSE

Transmembrane 9 superfamily member 3

GN=Tm9sf3

Med H1/2: 8.1 days

Q5 H1/2: 3.7 days

Q95 H1/2: 66.3 days

reliable model fit: yes

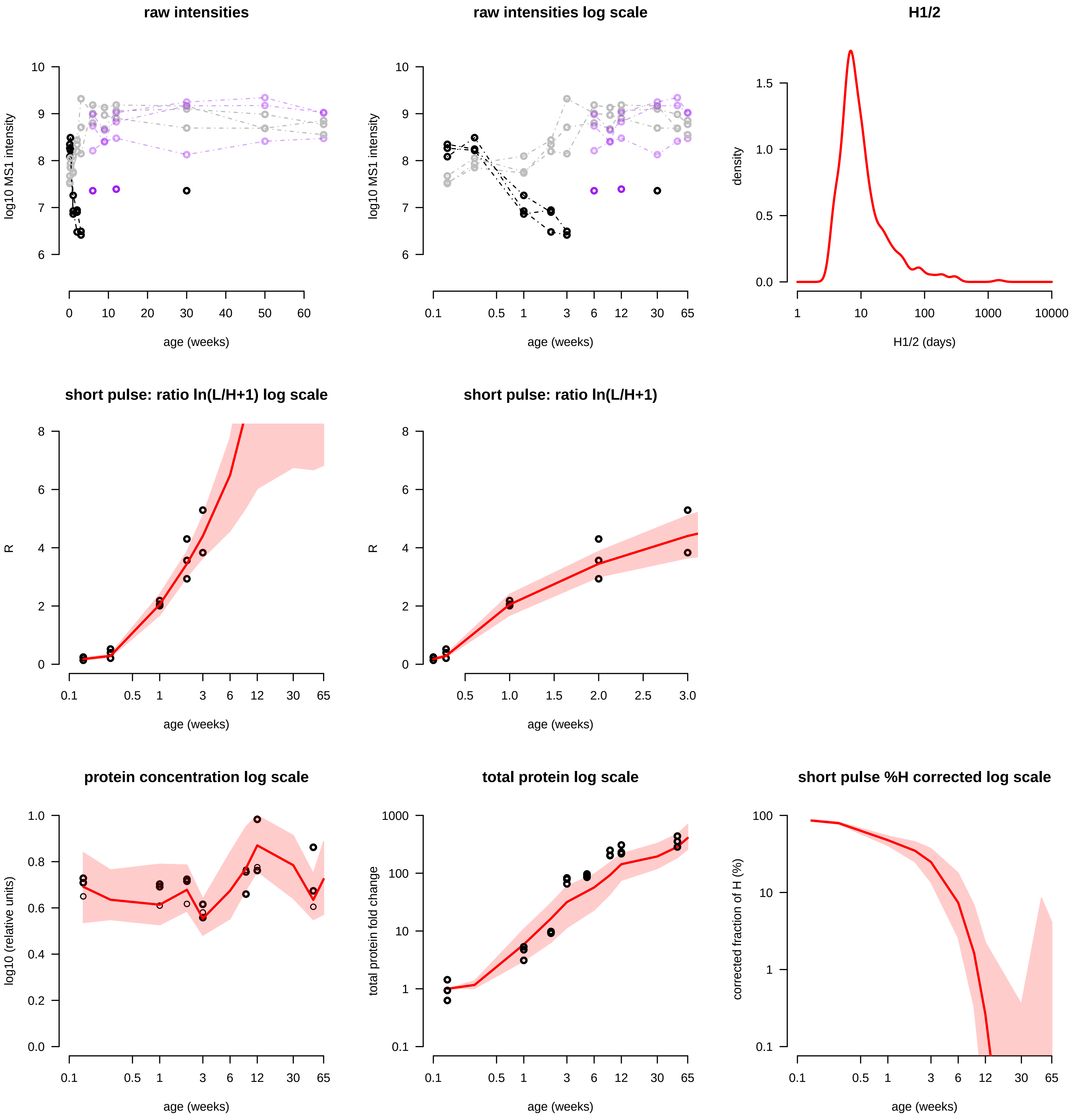

O89053

>sp|O89053|COR1A_MOUSE

Coronin−1A

GN=Coro1a

Med H1/2: 6.3 days

Q5 H1/2: 4.2 days

Q95 H1/2: 10 days

reliable model fit: yes

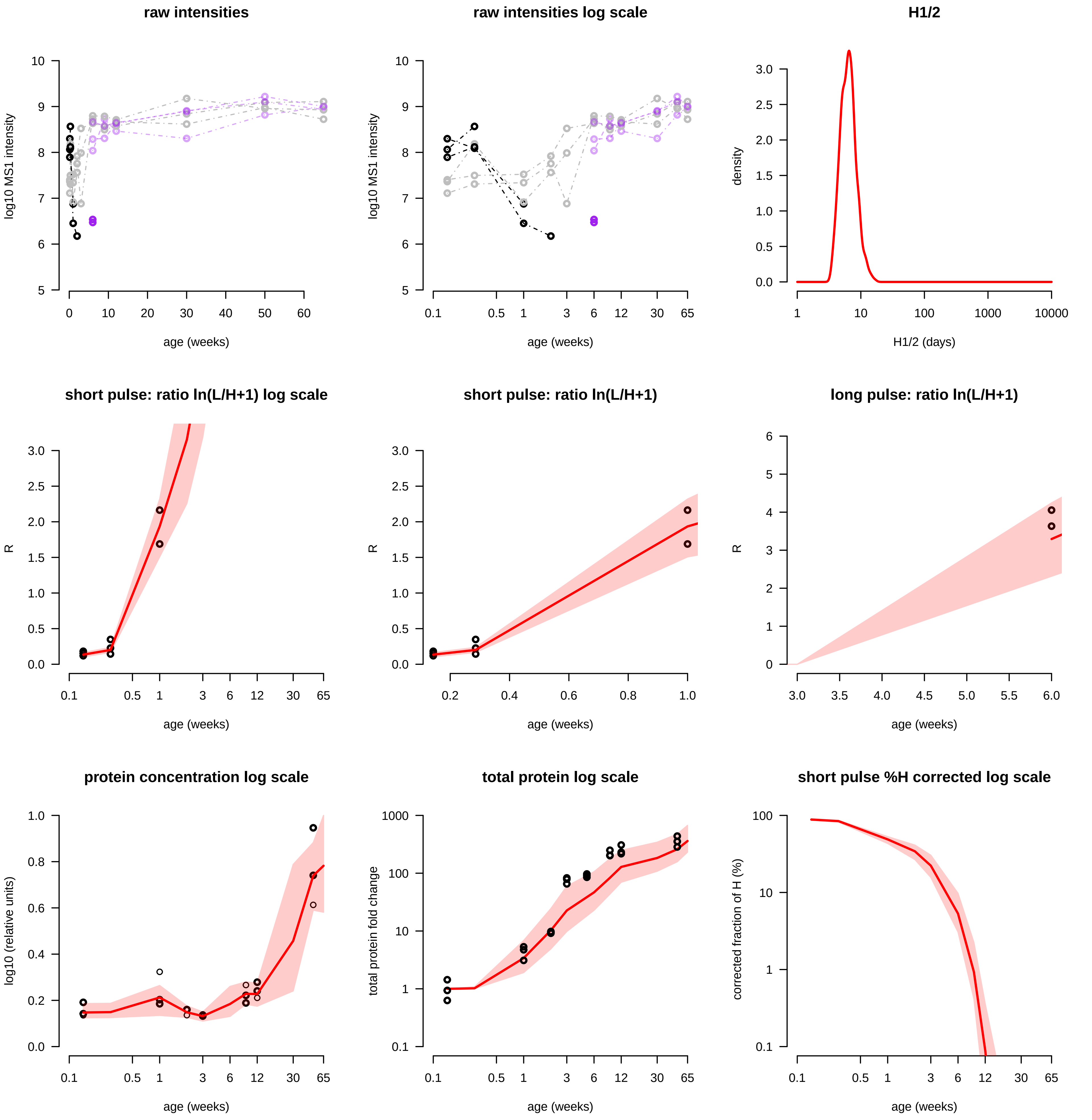

Q61792

>sp|Q61792|LASP1_MOUSE

LIM and SH3 domain protein 1

GN=Lasp1

Med H1/2: 1801.7 days

Q5 H1/2: 420.3 days

Q95 H1/2: 44414.9 days

reliable model fit: yes

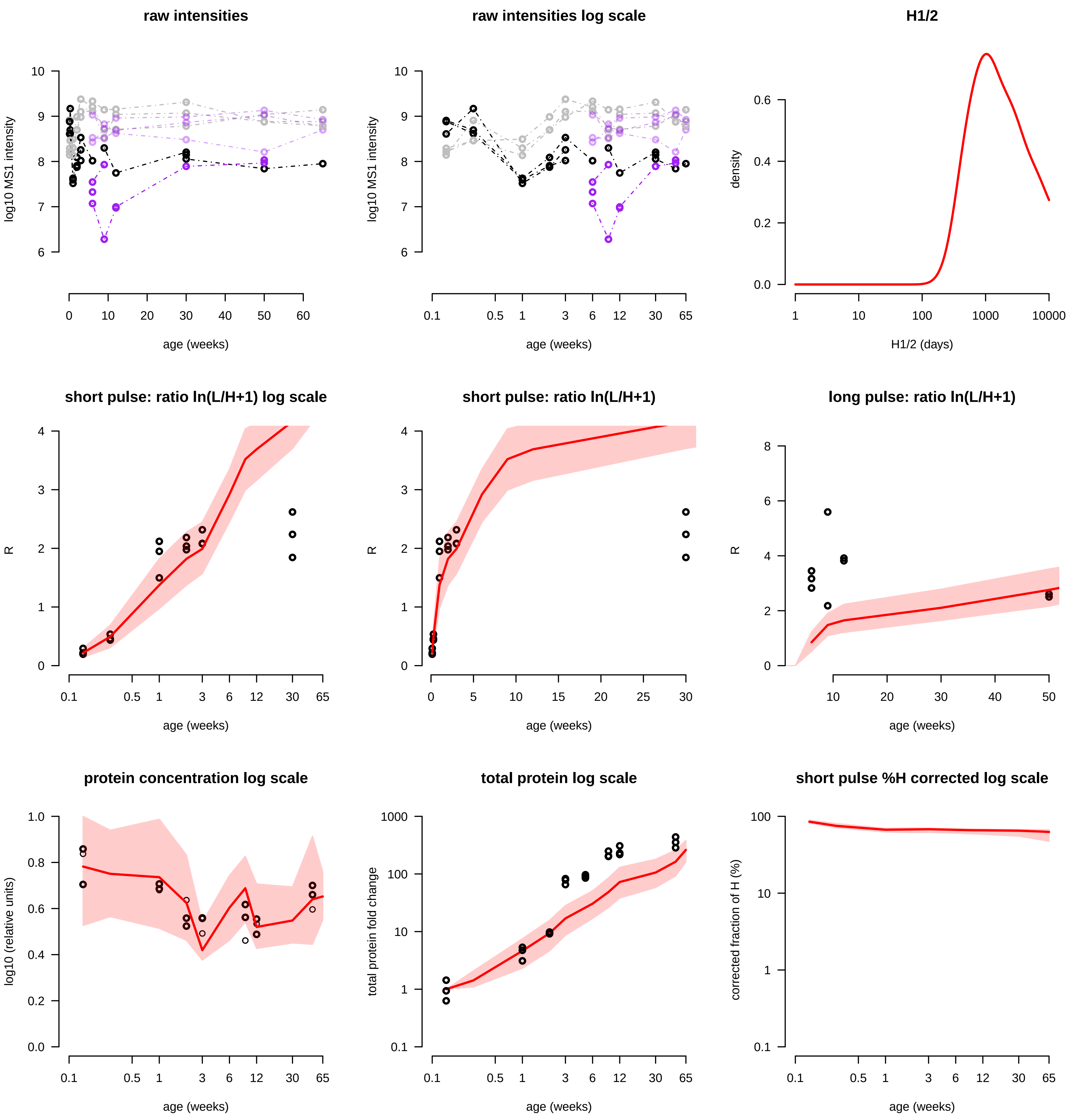

G5E829

>sp|G5E829|AT2B1_MOUSE

Plasma membrane calcium−transporting ATPase 1

GN=Atp2b1

Med H1/2: 4.1 days

Q5 H1/2: 3.4 days

Q95 H1/2: 4.7 days

reliable model fit: yes

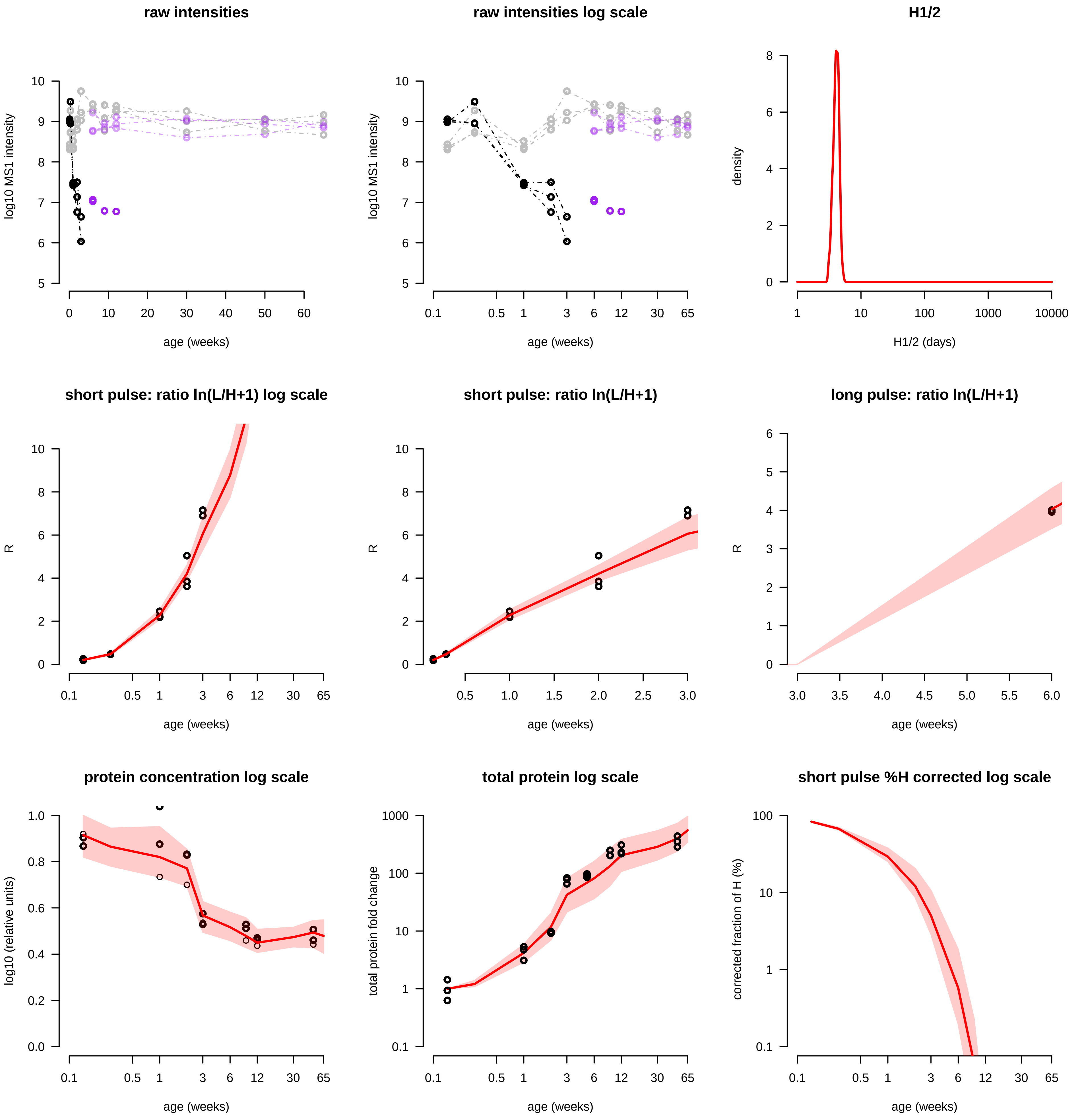

Q61553

>sp|Q61553|FSCN1_MOUSE

Fascin

GN=Fscn1

Med H1/2: 869.6 days

Q5 H1/2: 265.1 days

Q95 H1/2: 14012.1 days

reliable model fit: yes

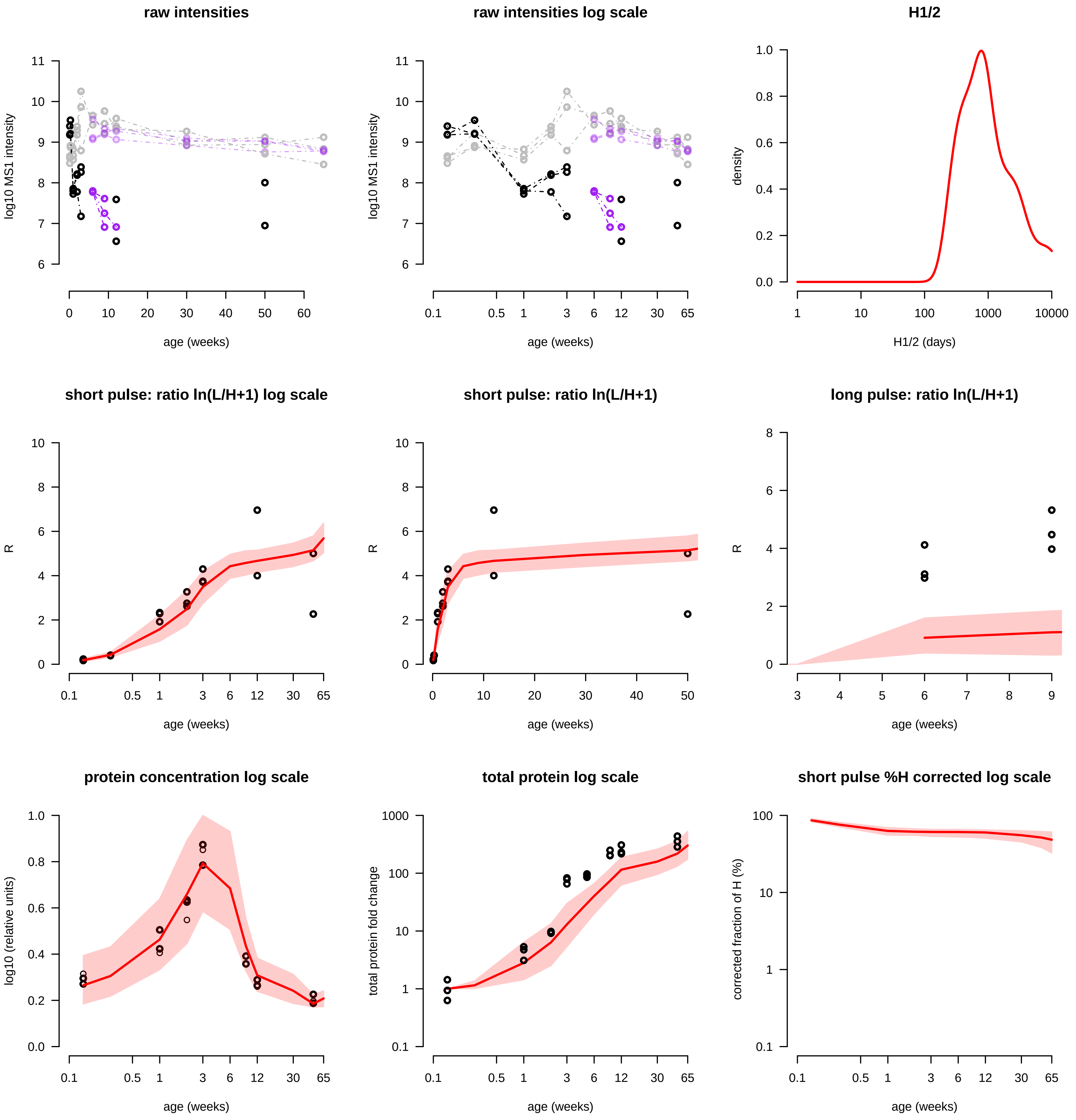

Q05186

>sp|Q05186|RCN1_MOUSE

Reticulocalbin−1

GN=Rcn1

Med H1/2: 114.6 days

Q5 H1/2: 53.5 days

Q95 H1/2: 596.7 days

reliable model fit: yes

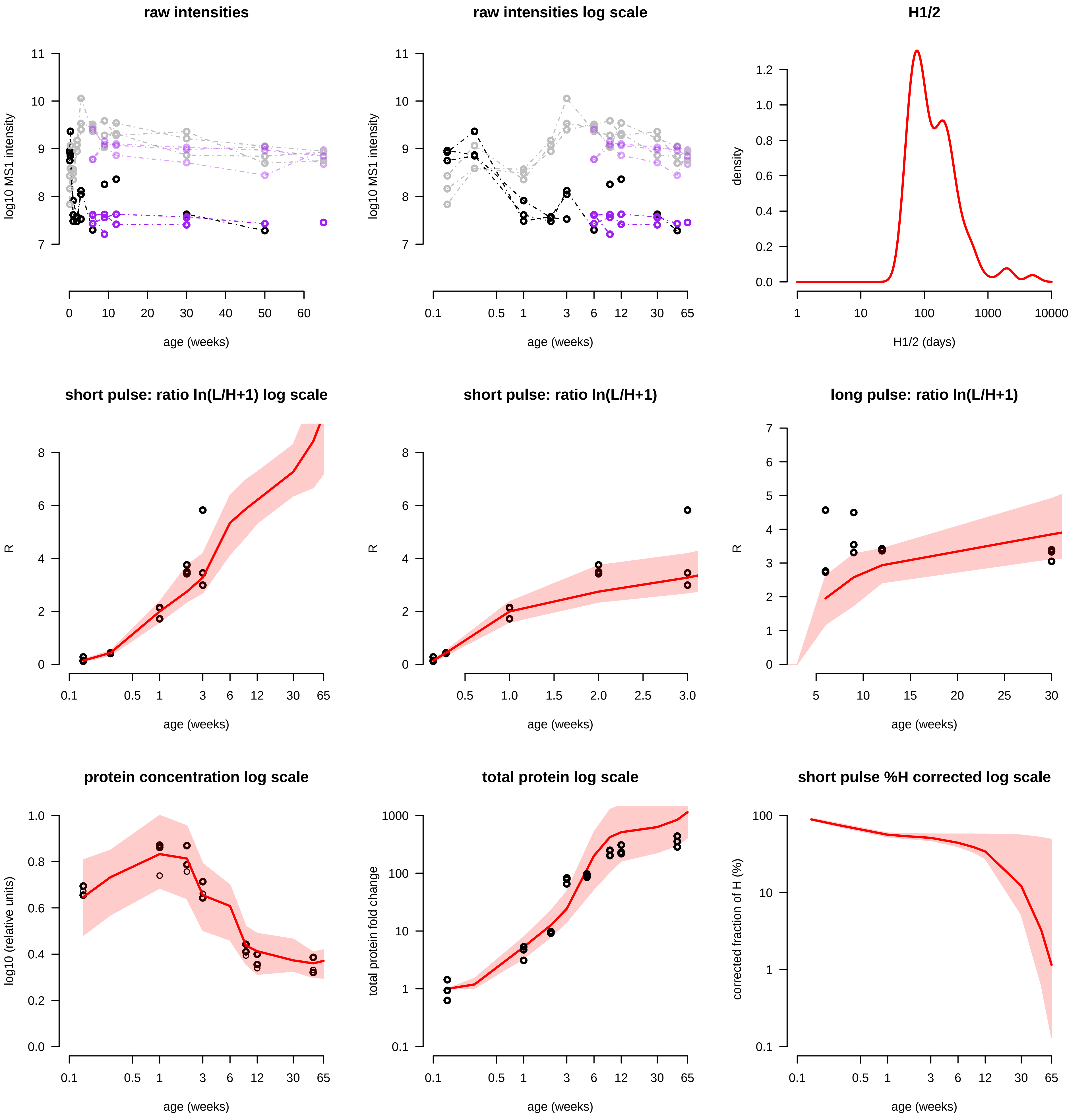

Q8K3V4

>sp|Q8K3V4|PADI6_MOUSE

Protein−arginine deiminase type−6

GN=Padi6

Med H1/2: 21.6 days

Q5 H1/2: 14.3 days

Q95 H1/2: 36.3 days

reliable model fit: yes

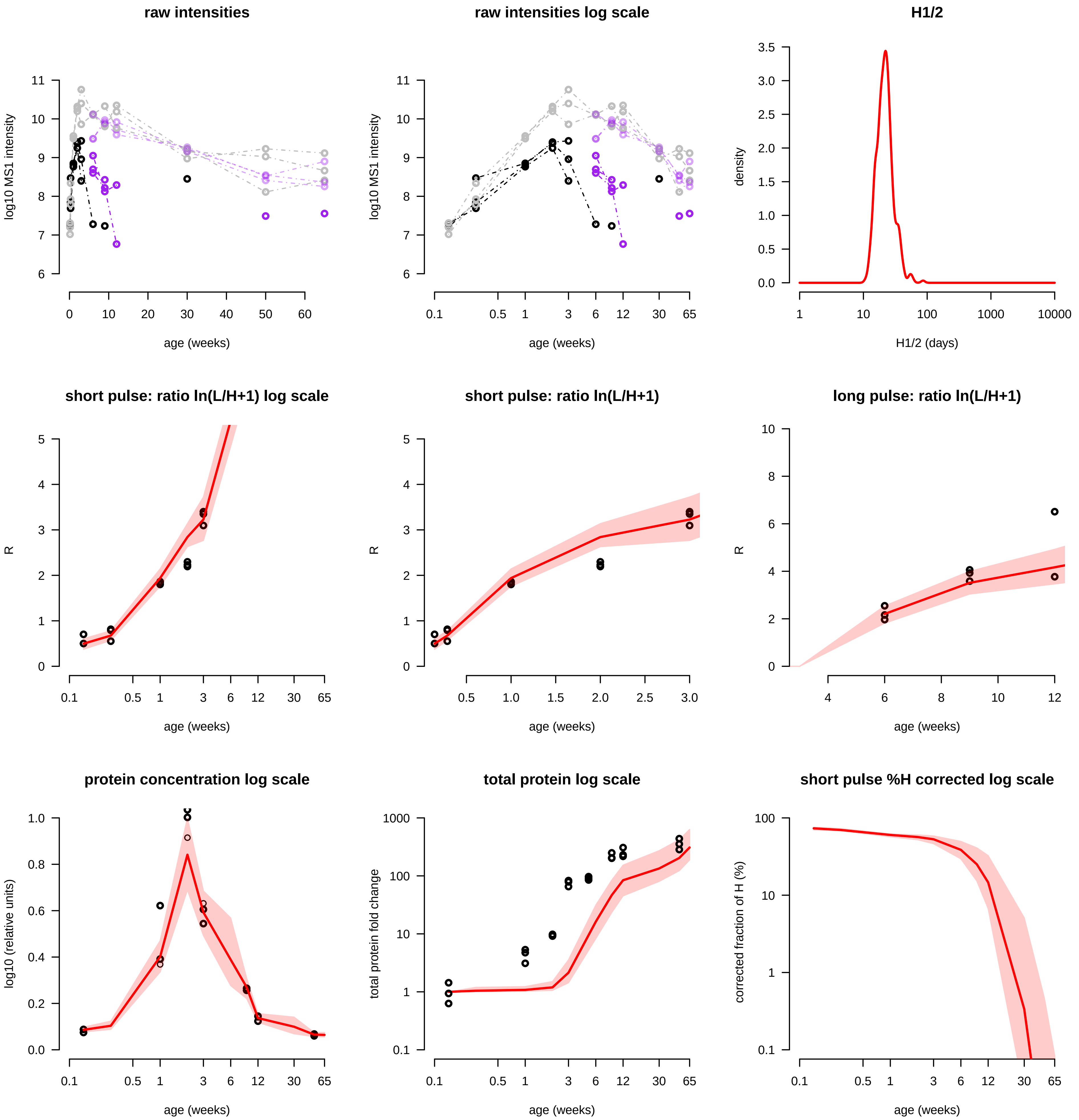

Q64471

>sp|Q64471|GSTT1_MOUSE

Glutathione S−transferase theta−1

GN=Gstt1

Med H1/2: 5.7 days

Q5 H1/2: 4.3 days

Q95 H1/2: 8 days

reliable model fit: yes

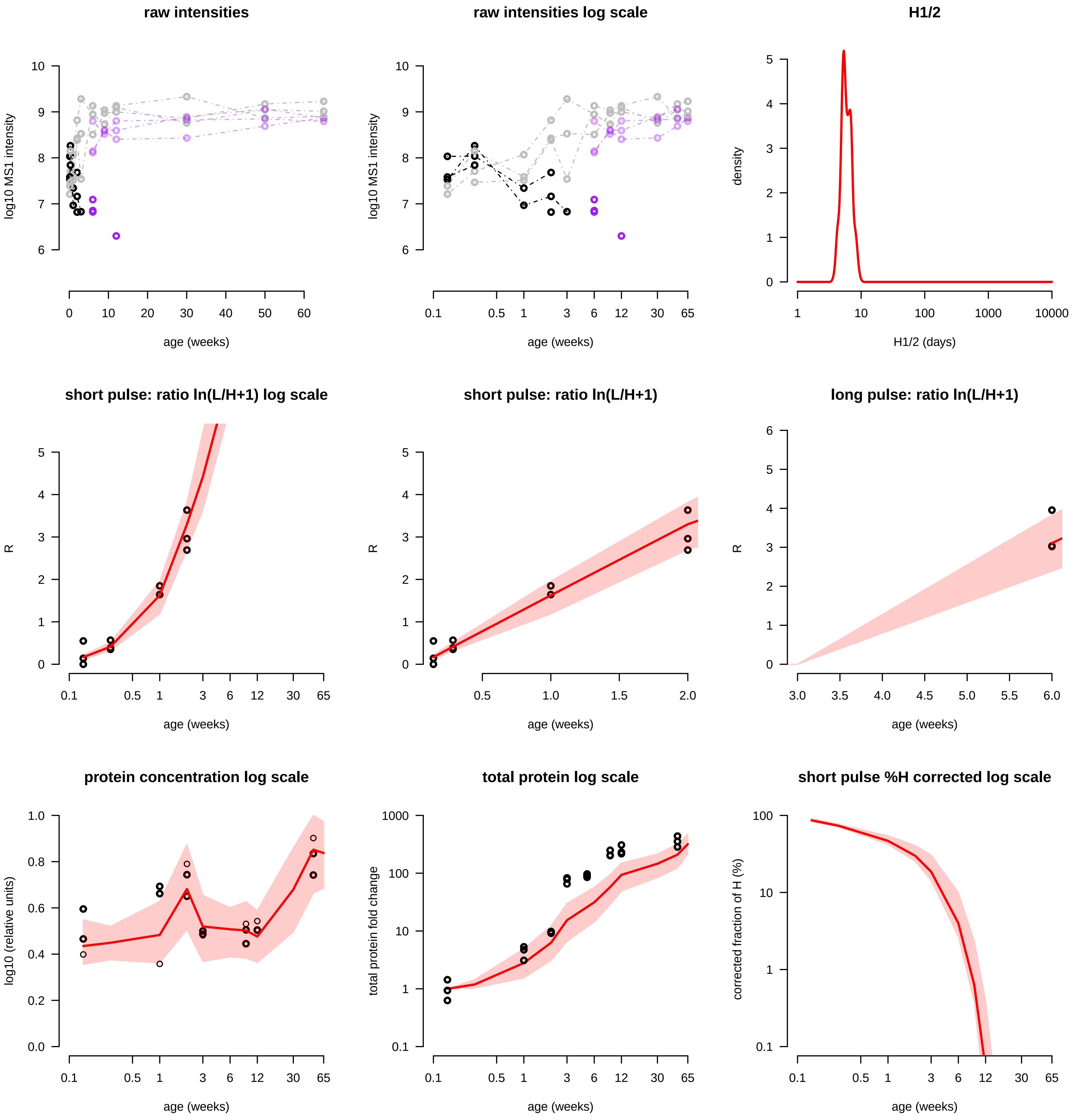

P47934

>sp|P47934|CACP_MOUSE

Carnitine O−acetyltransferase

GN=Crat

Med H1/2: 9.7 days

Q5 H1/2: 4 days

Q95 H1/2: 62.7 days

reliable model fit: yes

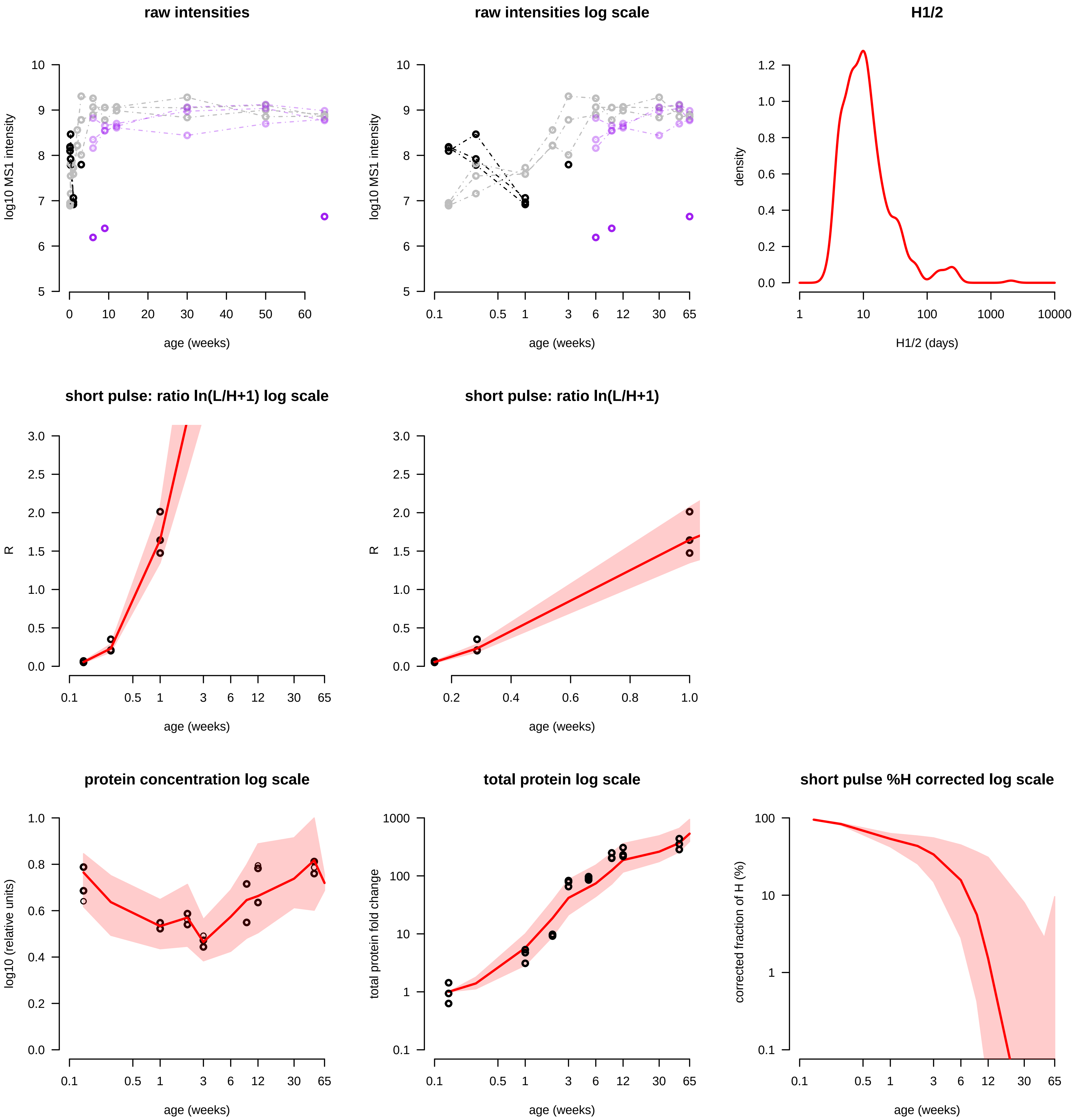

P62855

>sp|P62855|RS26_MOUSE

40S ribosomal protein S26

GN=Rps26

Med H1/2: 6.5 days

Q5 H1/2: 5.2 days

Q95 H1/2: 8.9 days

reliable model fit: yes

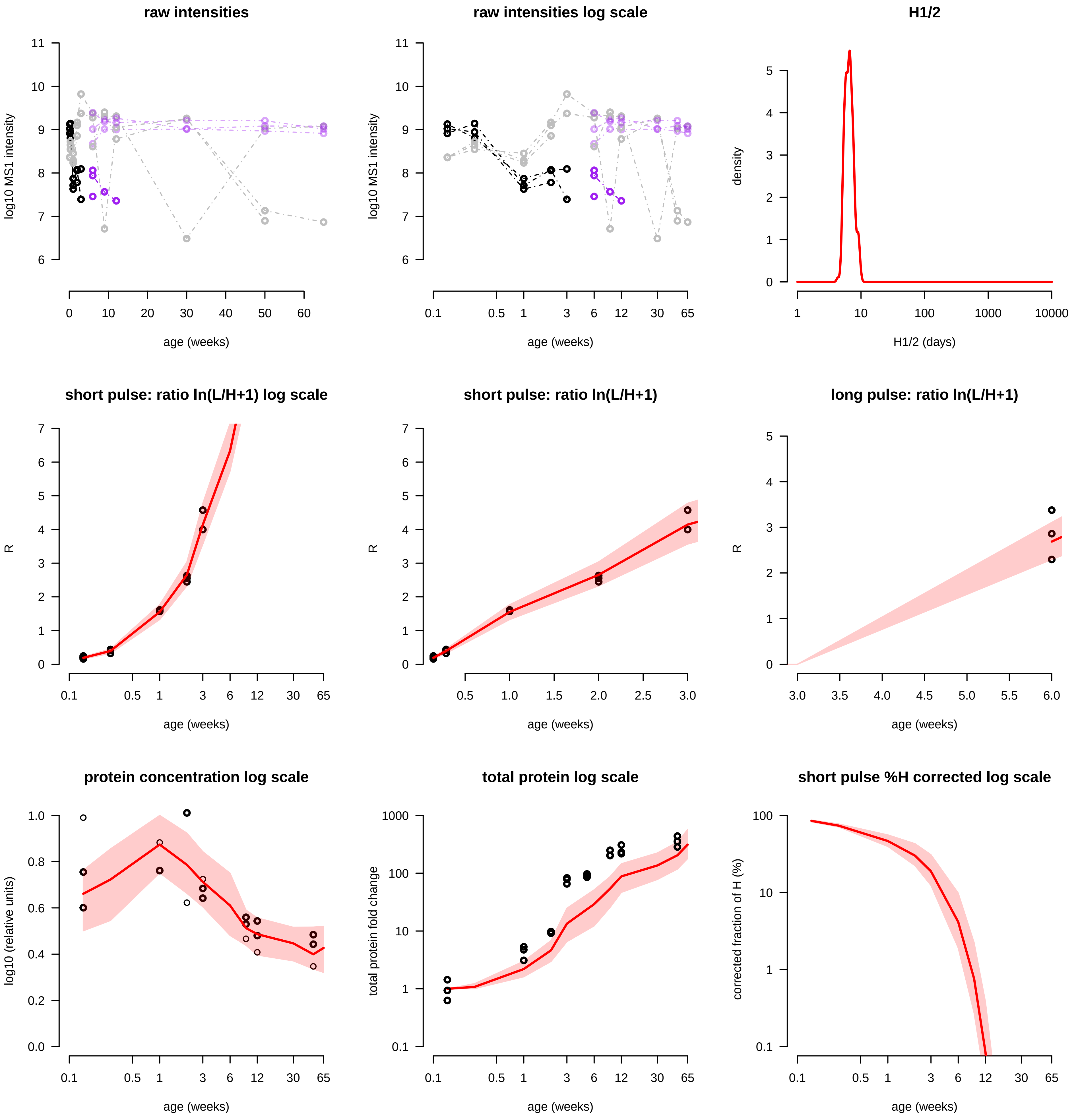

O54879

>sp|O54879|HMGB3_MOUSE

High mobility group protein B3

GN=Hmgb3

Med H1/2: 6.7 days

Q5 H1/2: 5.4 days

Q95 H1/2: 9.1 days

reliable model fit: yes

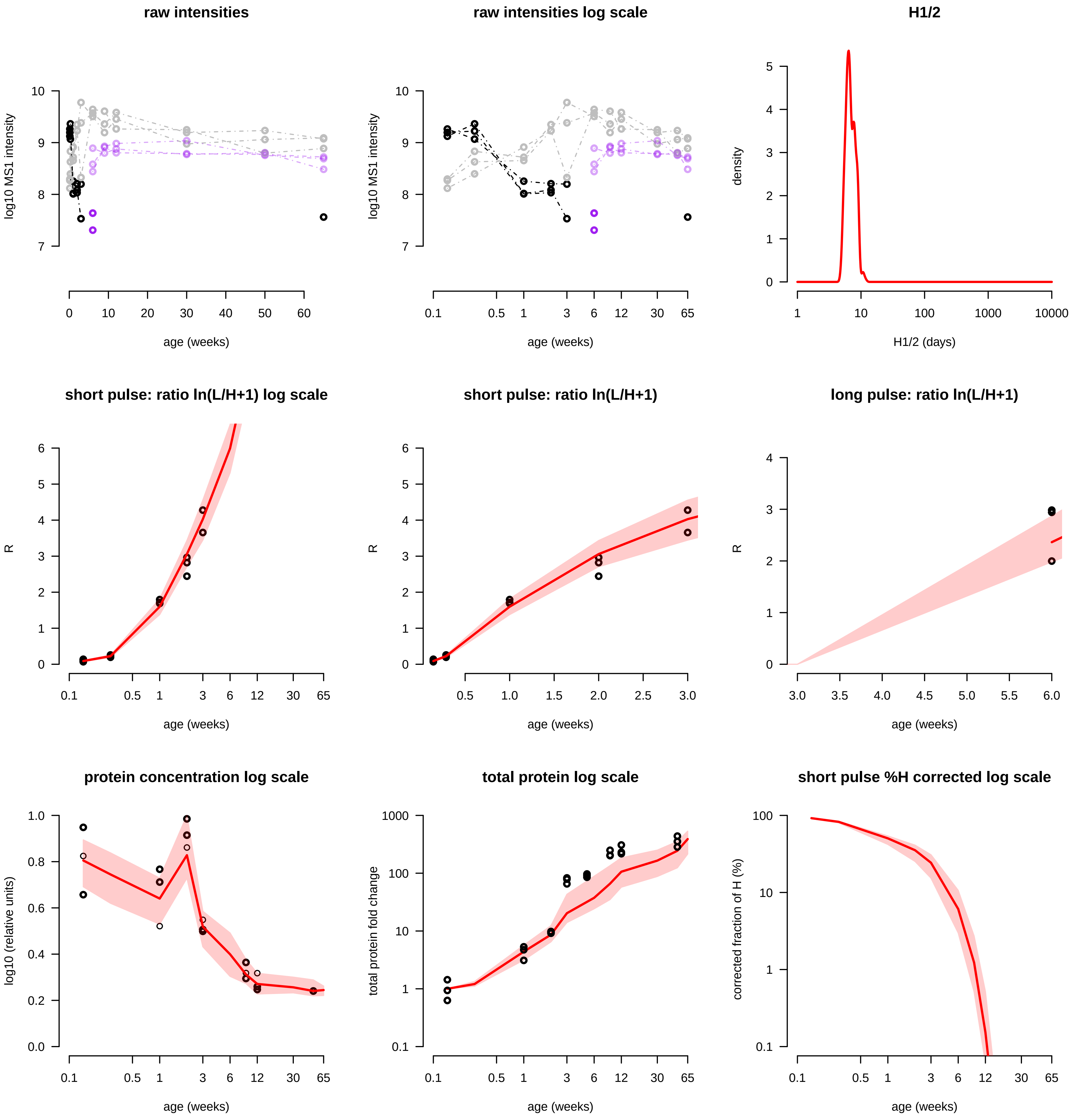

Q9CQU0

>sp|Q9CQU0|TXD12_MOUSE

Thioredoxin domain−containing protein 12

GN=Txndc12

Med H1/2: 7.2 days

Q5 H1/2: 5.9 days

Q95 H1/2: 8.8 days

reliable model fit: yes

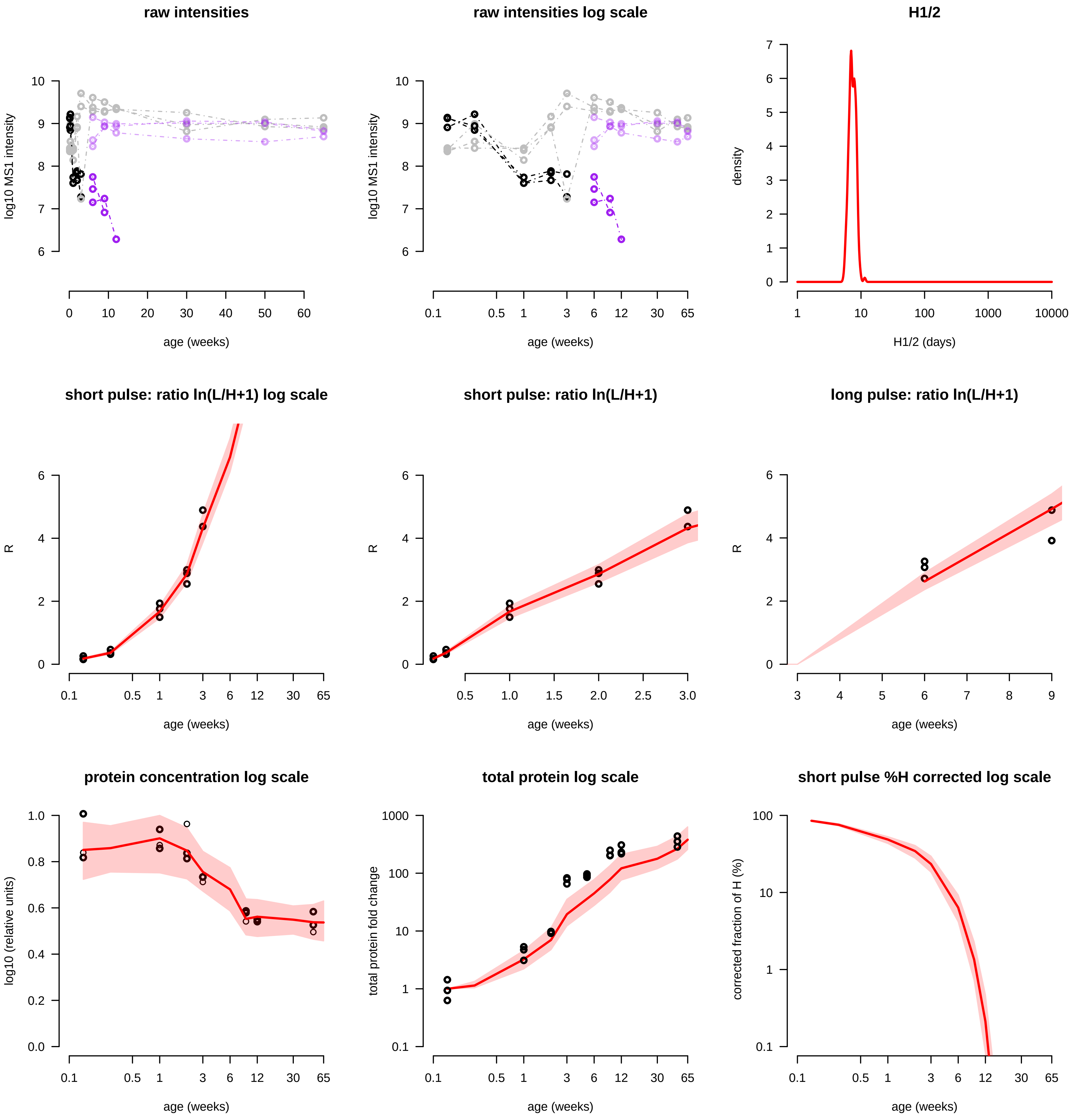

Q64213;Q64213−2;Q64213−3

>sp|Q64213|SF01_MOUSE

Splicing factor 1

GN=Sf1

Med H1/2: 4.5 days

Q5 H1/2: 3.8 days

Q95 H1/2: 5.9 days

reliable model fit: yes

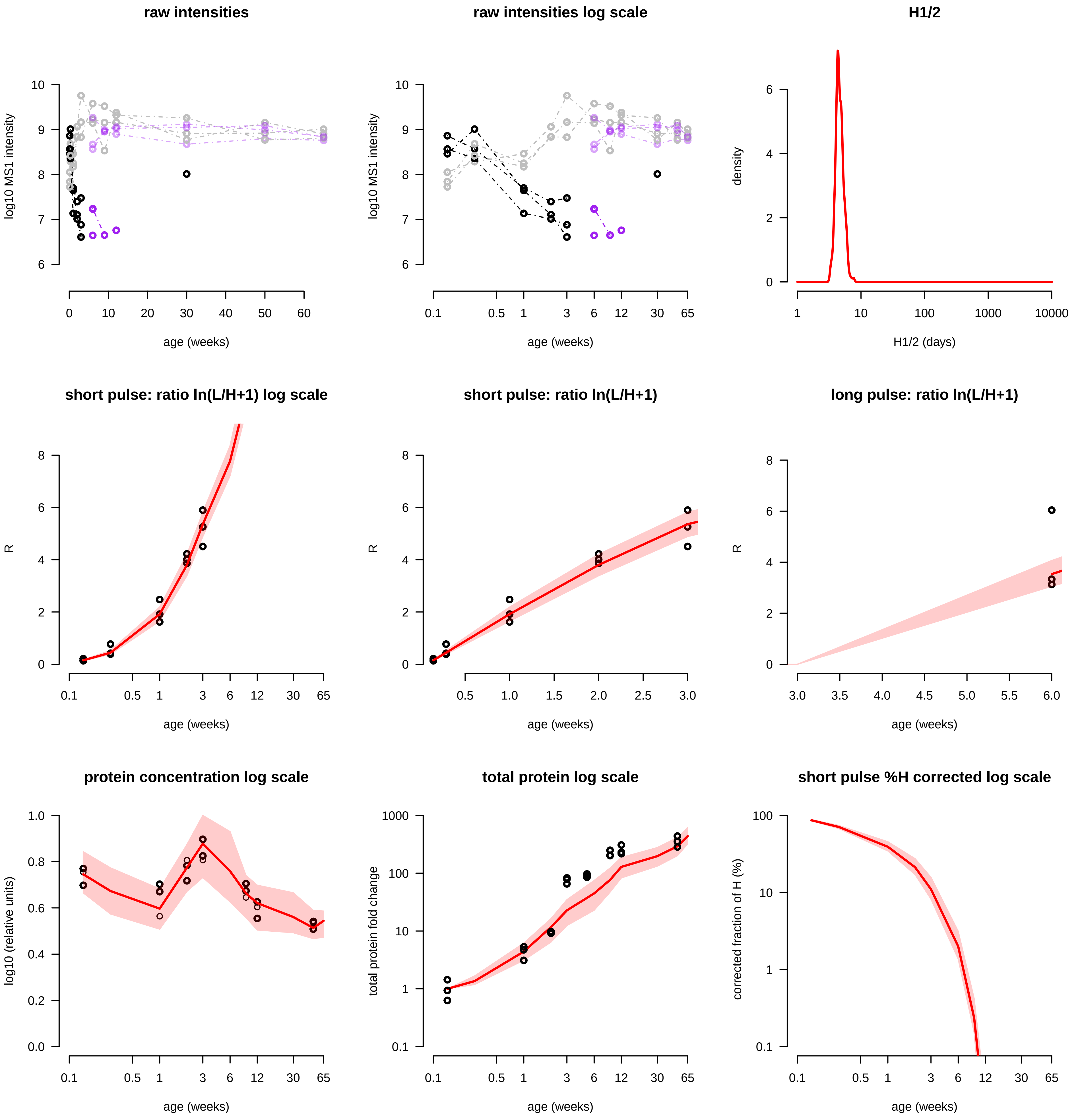

Q9CZR2

>sp|Q9CZR2|NALD2_MOUSE

N−acetylated−alpha−linked acidic dipeptidase 2

GN=Naalad2

Med H1/2: 5.4 days

Q5 H1/2: 3.9 days

Q95 H1/2: 7.7 days

reliable model fit: yes

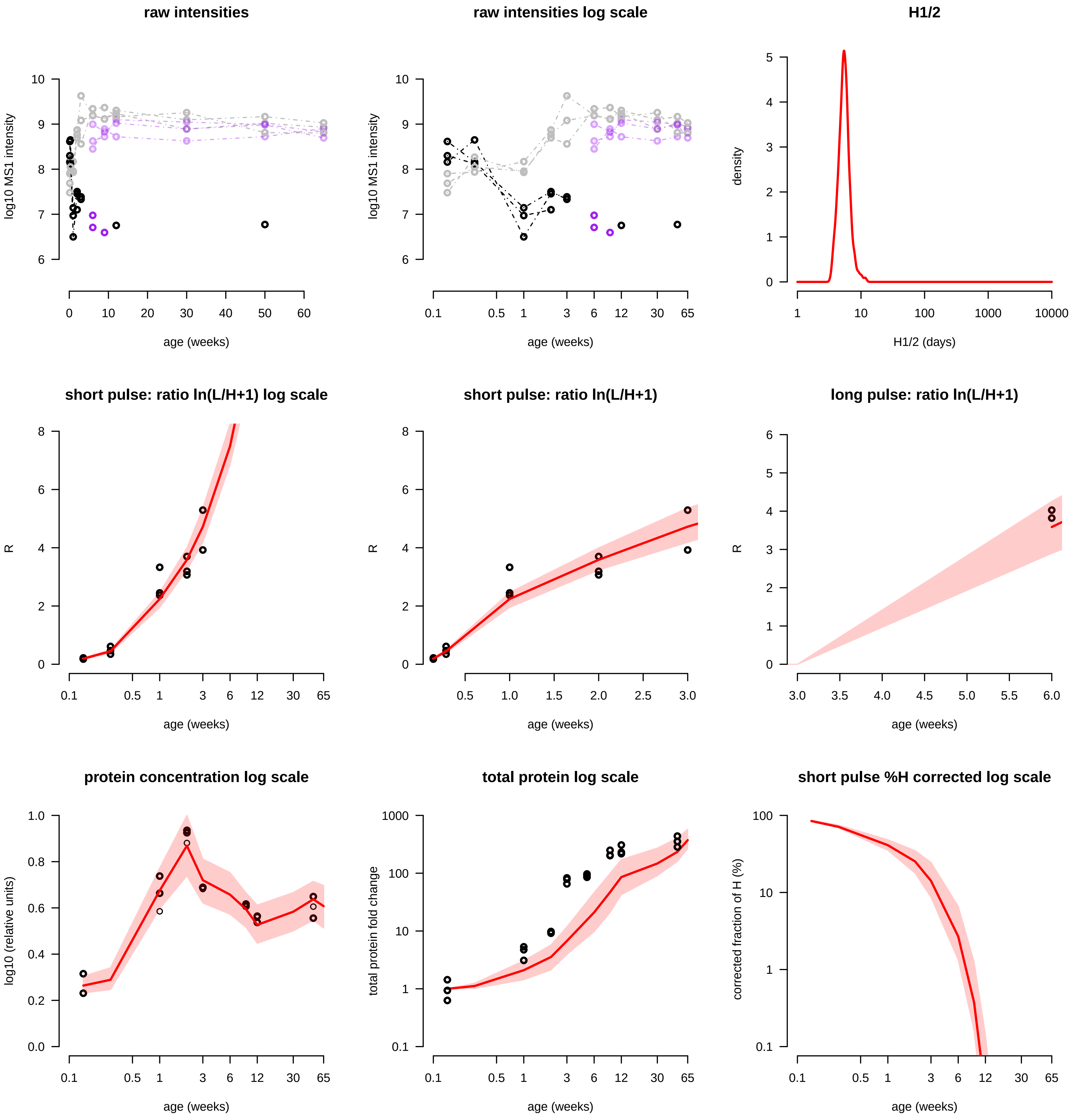

Q9D832

>sp|Q9D832|DNJB4_MOUSE

DnaJ homolog subfamily B member 4

GN=Dnajb4

Med H1/2: 54.8 days

Q5 H1/2: 20.9 days

Q95 H1/2: 385.6 days

reliable model fit: no

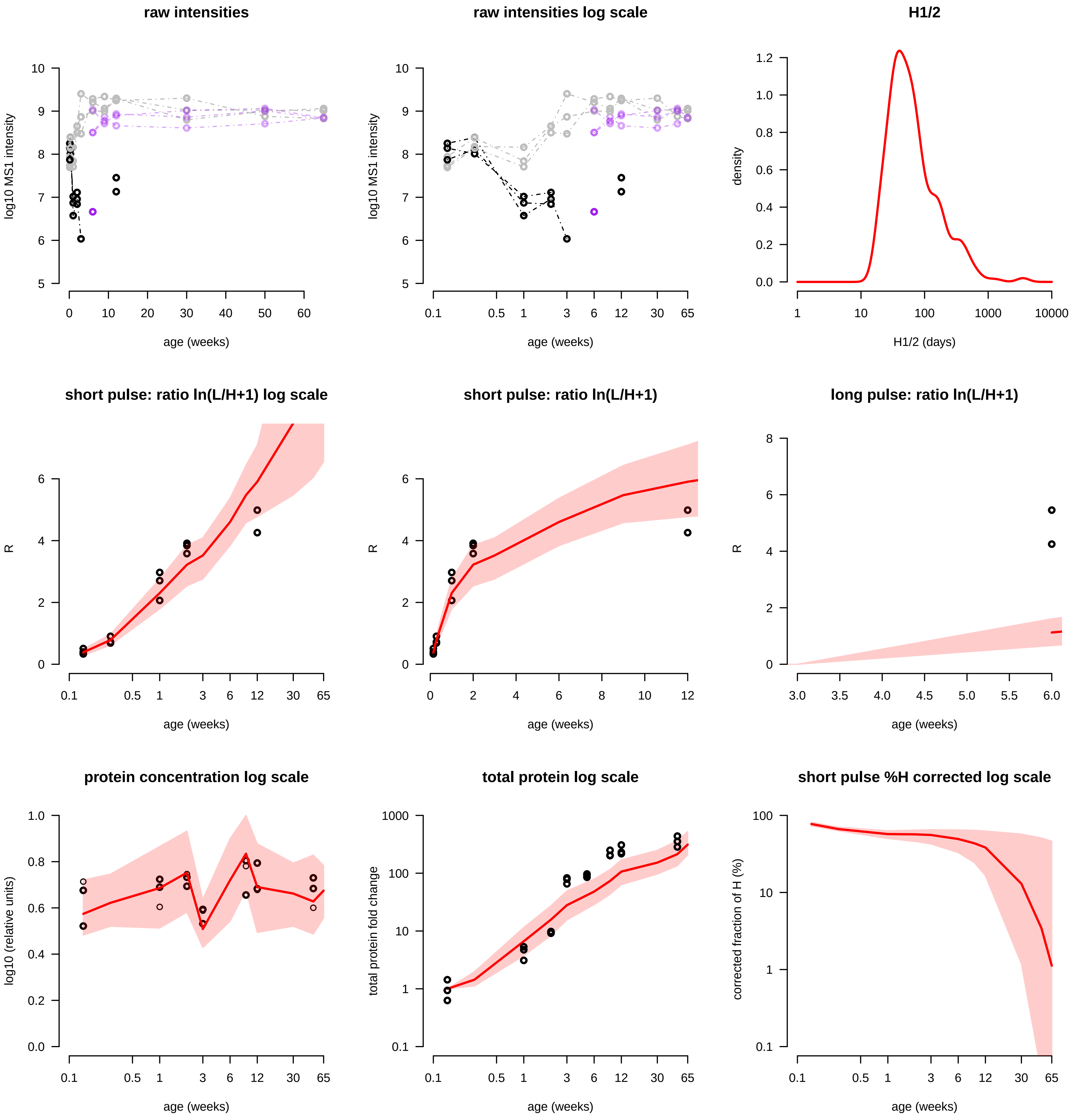

Q60967

>sp|Q60967|PAPS1_MOUSE

Bifunctional 3'−phosphoadenosine 5'−phosphosulfate synthase 1

GN=Papss1

Med H1/2: 3.1 days

Q5 H1/2: 2.5 days

Q95 H1/2: 4.2 days

reliable model fit: yes

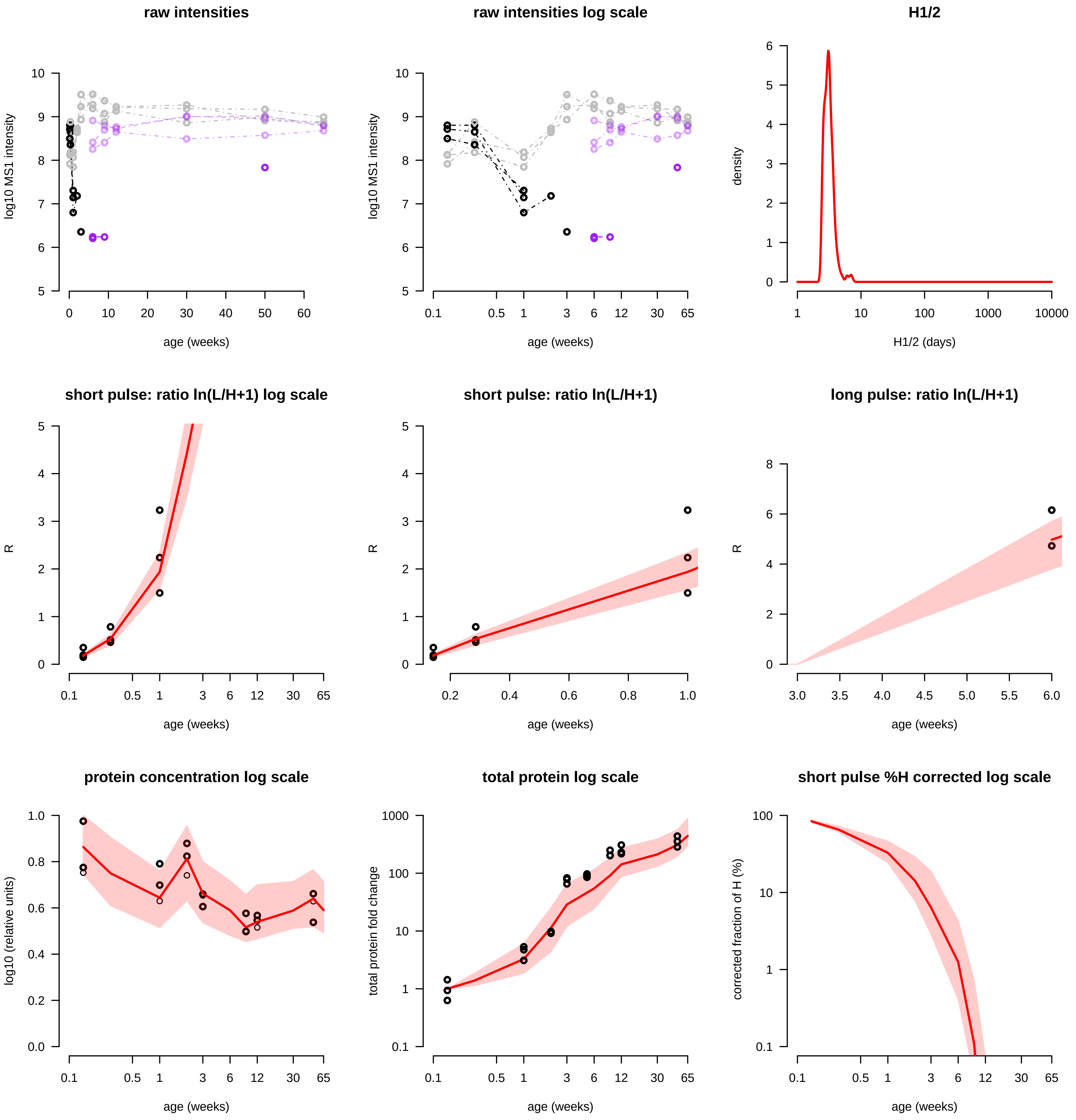

O35639

>sp|O35639|ANXA3_MOUSE

Annexin A3

GN=Anxa3

Med H1/2: 5.3 days

Q5 H1/2: 3.9 days

Q95 H1/2: 7.4 days

reliable model fit: yes

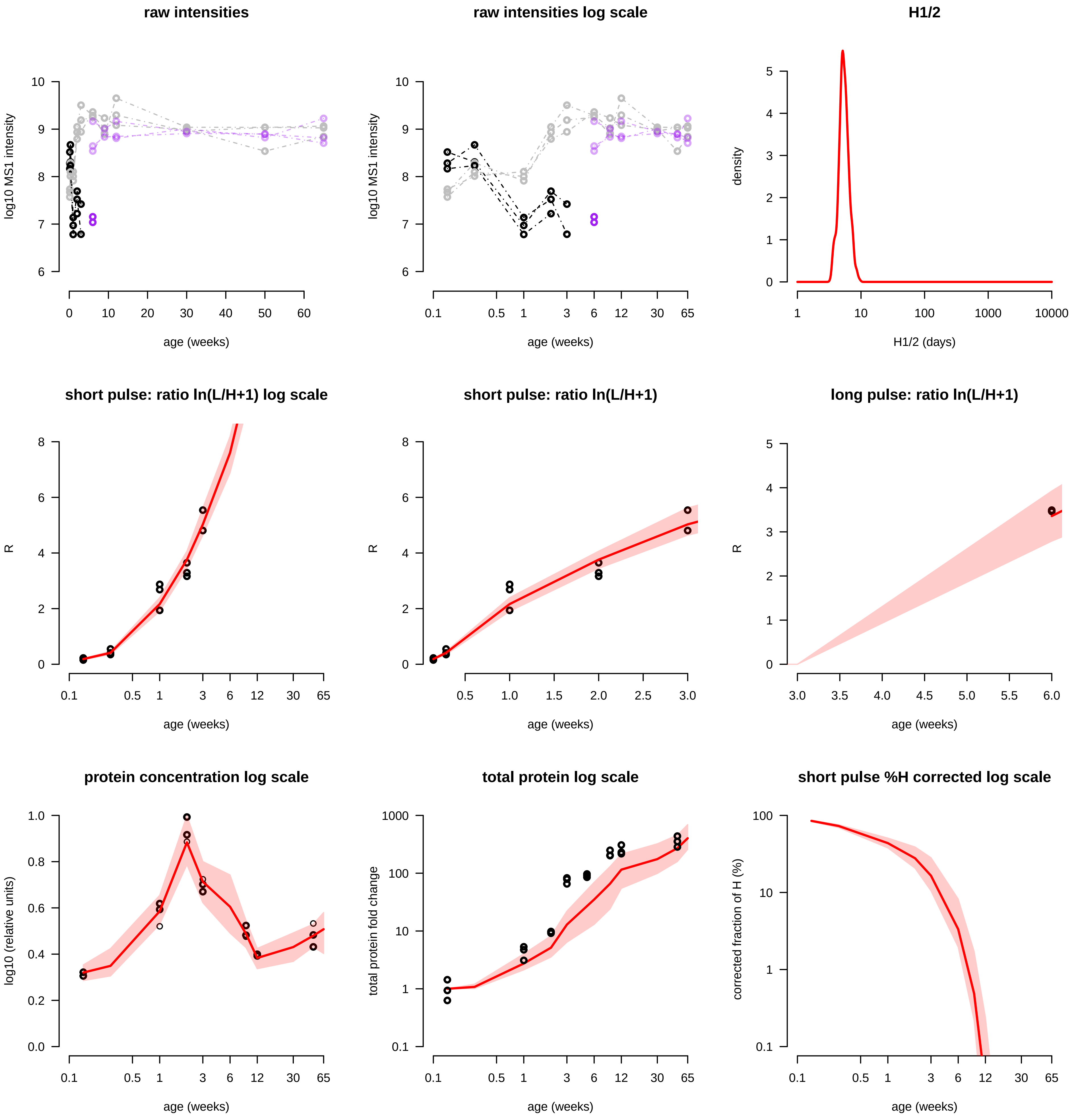

Q9DAK9

>sp|Q9DAK9|PHP14_MOUSE

14 kDa phosphohistidine phosphatase

GN=Phpt1

Med H1/2: 5.6 days

Q5 H1/2: 4.7 days

Q95 H1/2: 7.2 days

reliable model fit: yes

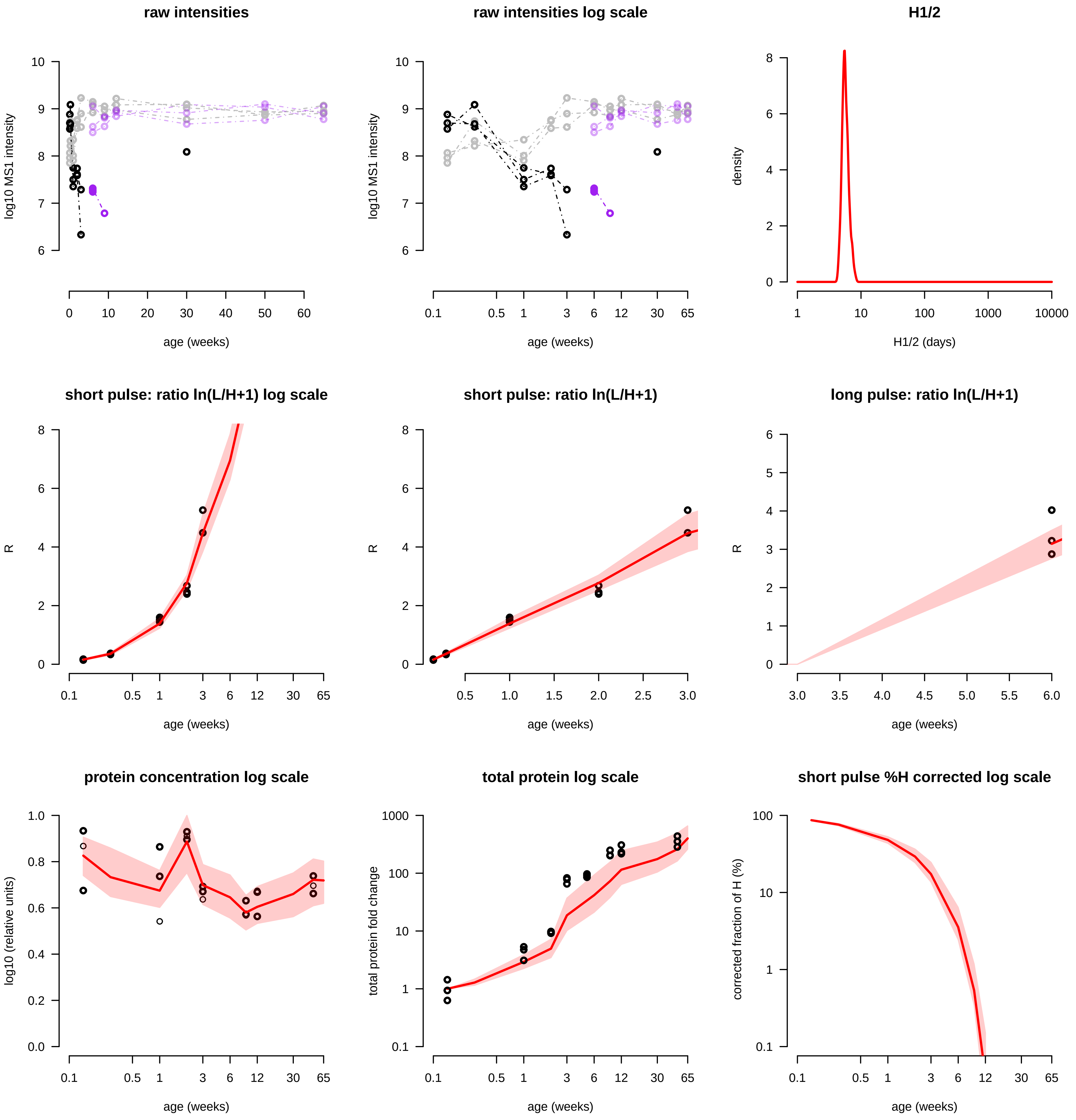

Q9WUM3

>sp|Q9WUM3|COR1B_MOUSE

Coronin−1B

GN=Coro1b

Med H1/2: 5 days

Q5 H1/2: 3.8 days

Q95 H1/2: 6.8 days

reliable model fit: no

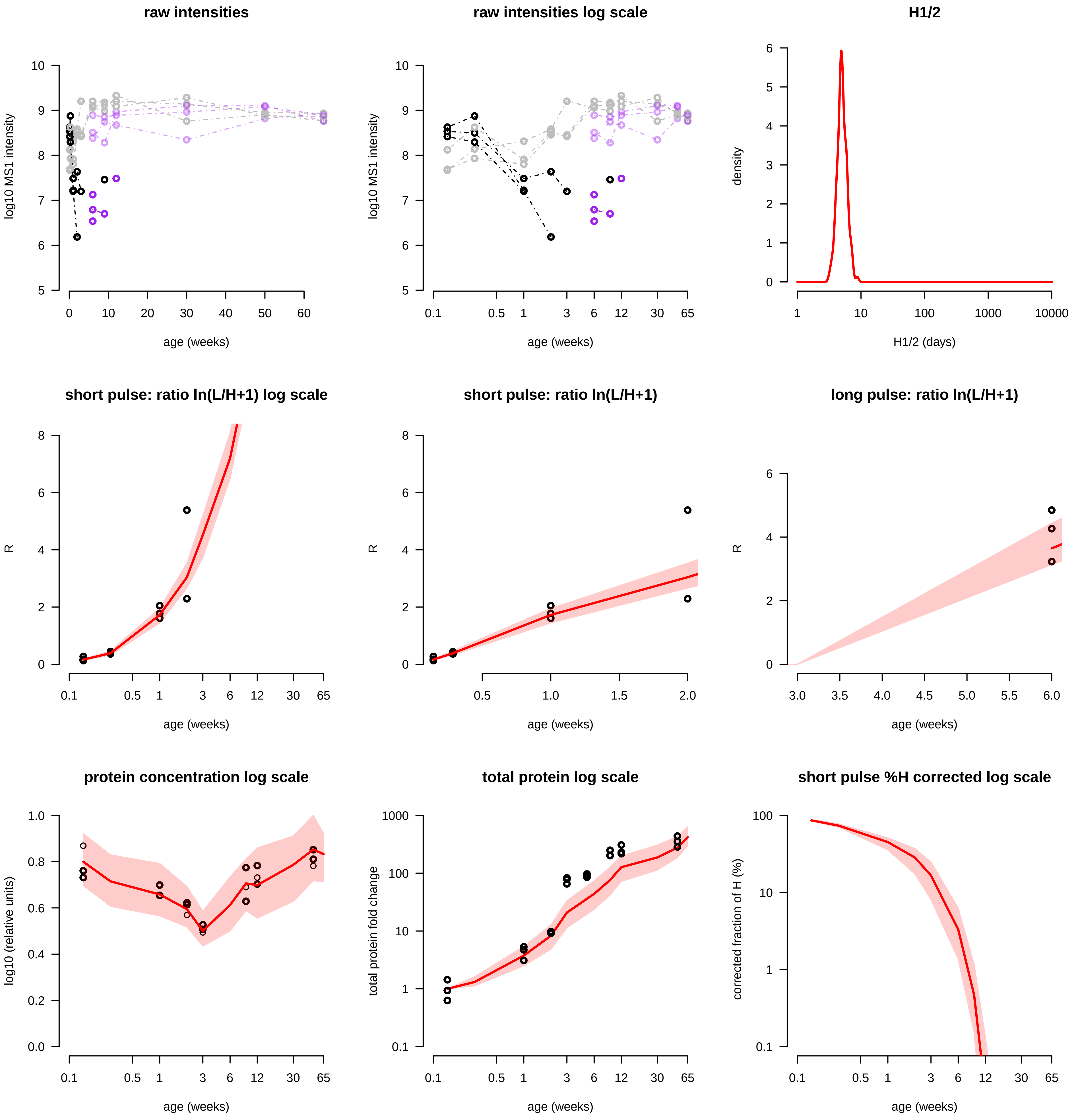

P55258

>sp|P55258|RAB8A_MOUSE

Ras−related protein Rab−8A

GN=Rab8a

Med H1/2: 6.1 days

Q5   H1/2: 5 days

Q95 H1/2: 7.9 days

reliable model fit: yes

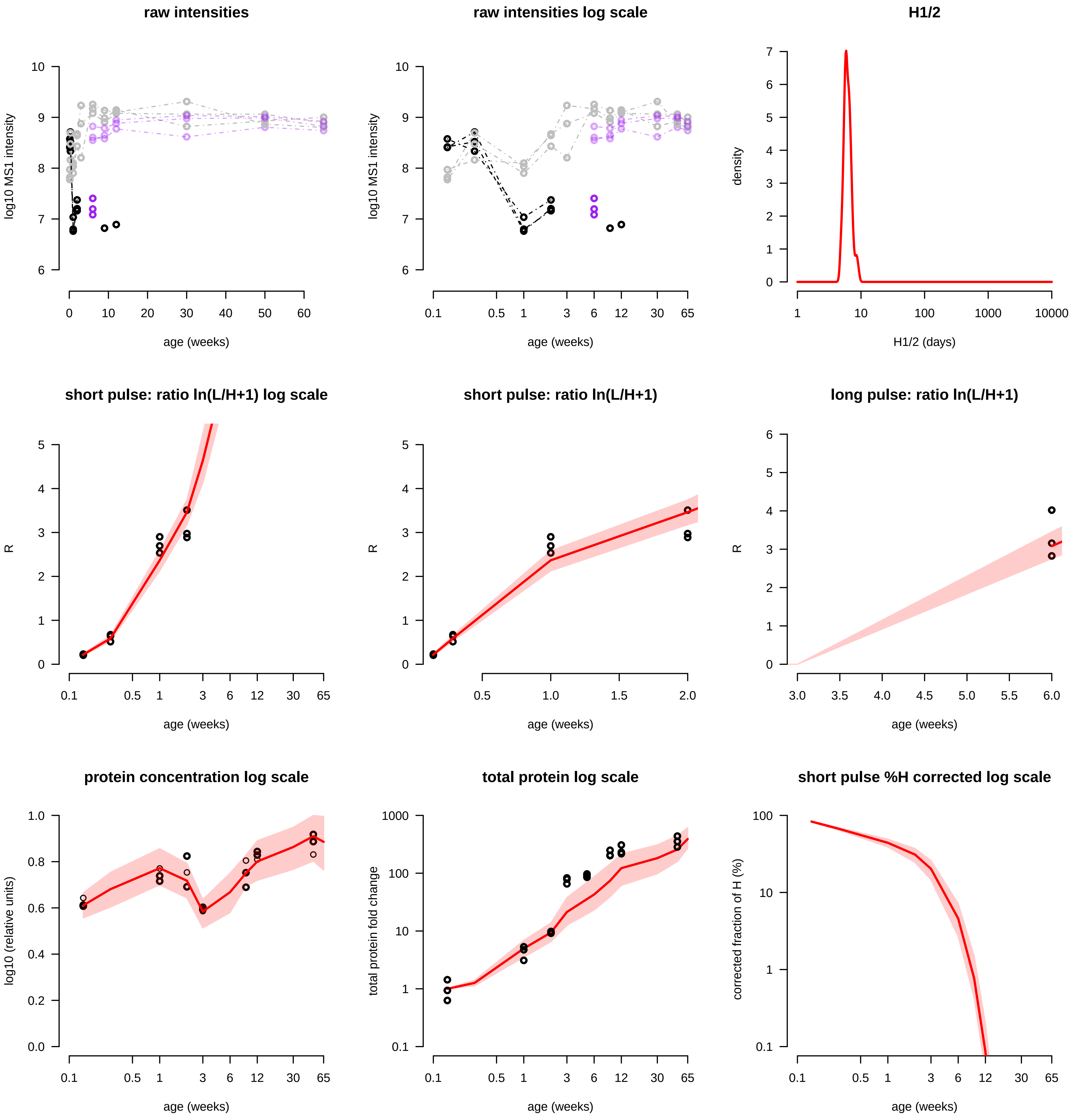

Q9Z2W0

>sp|Q9Z2W0|DNPEP_MOUSE

Aspartyl aminopeptidase

GN=Dnpep

Med H1/2: 4.8 days

Q5 H1/2: 3.9 days

Q95 H1/2: 7.1 days

reliable model fit: yes

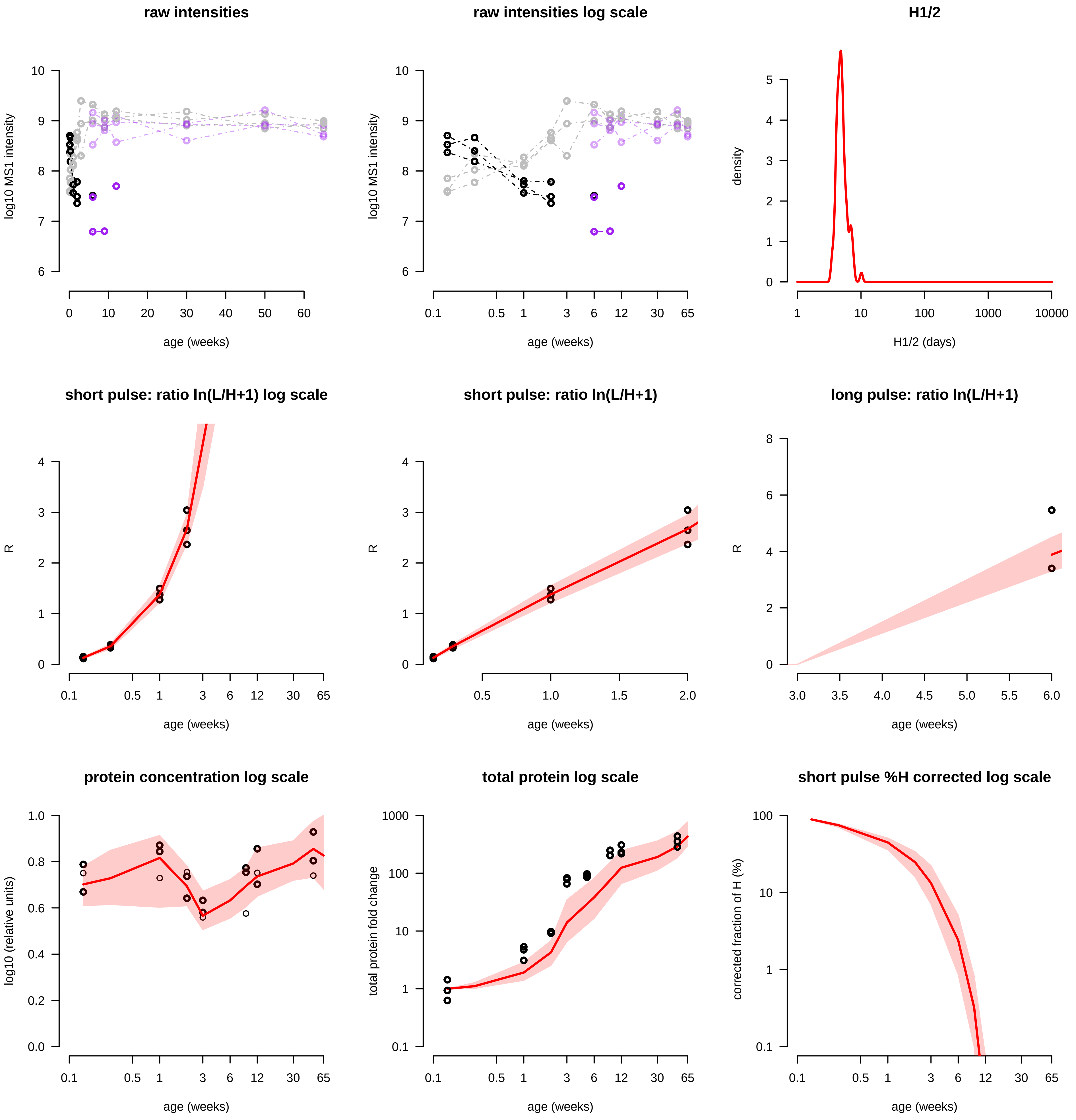

Q924Z4

>sp|Q924Z4|CERS2_MOUSE

Ceramide synthase 2

GN=Cers2

Med H1/2: 6.2 days

Q5 H1/2: 4.8 days

Q95 H1/2: 8.8 days

reliable model fit: yes

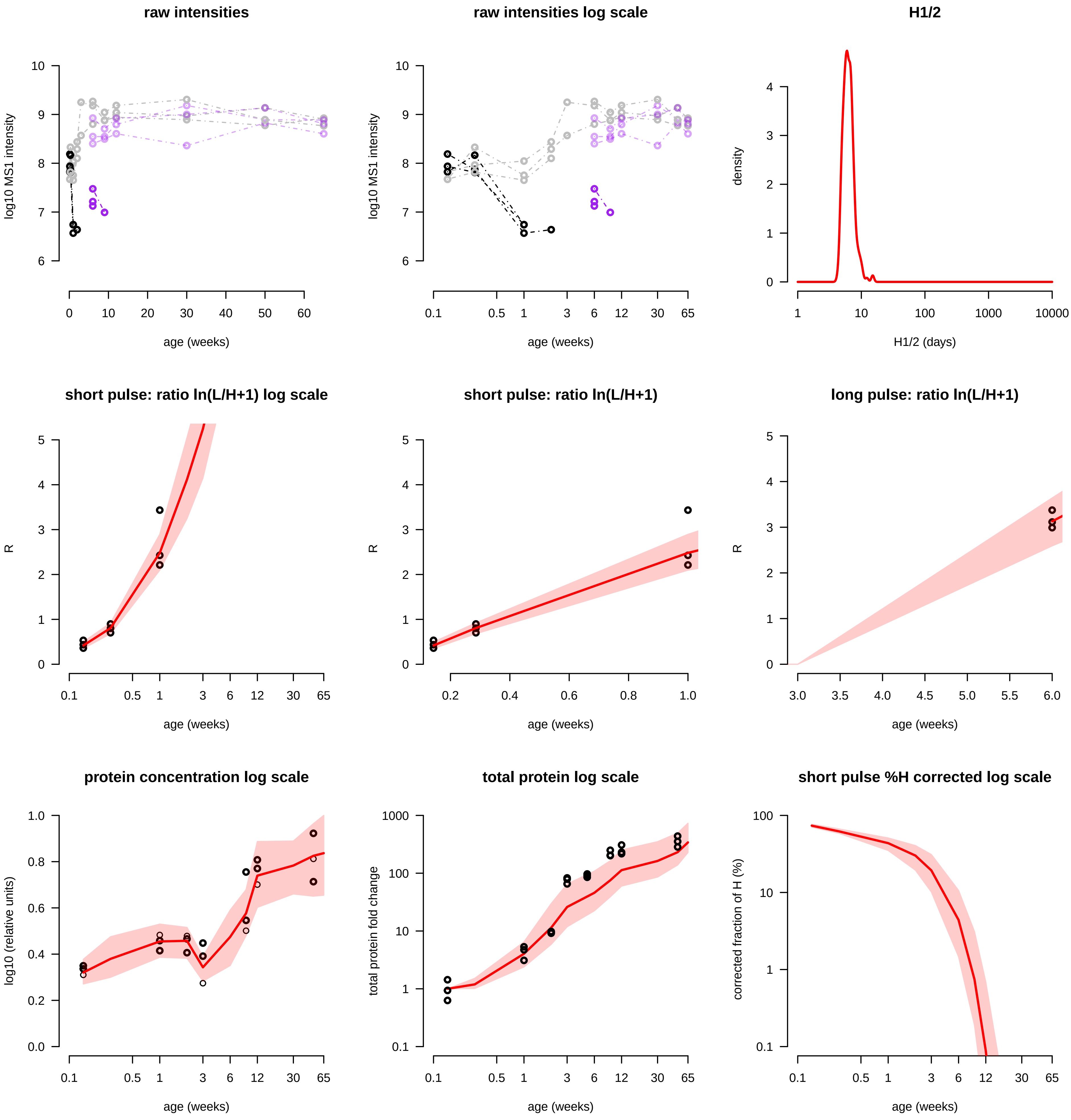

P25976;P25976−2

>sp|P25976|UBF1_MOUSE

Nucleolar transcription factor 1

GN=Ubtf

Med H1/2: 4.1 days

Q5 H1/2: 3.5 days

Q95 H1/2: 5.1 days

reliable model fit: yes

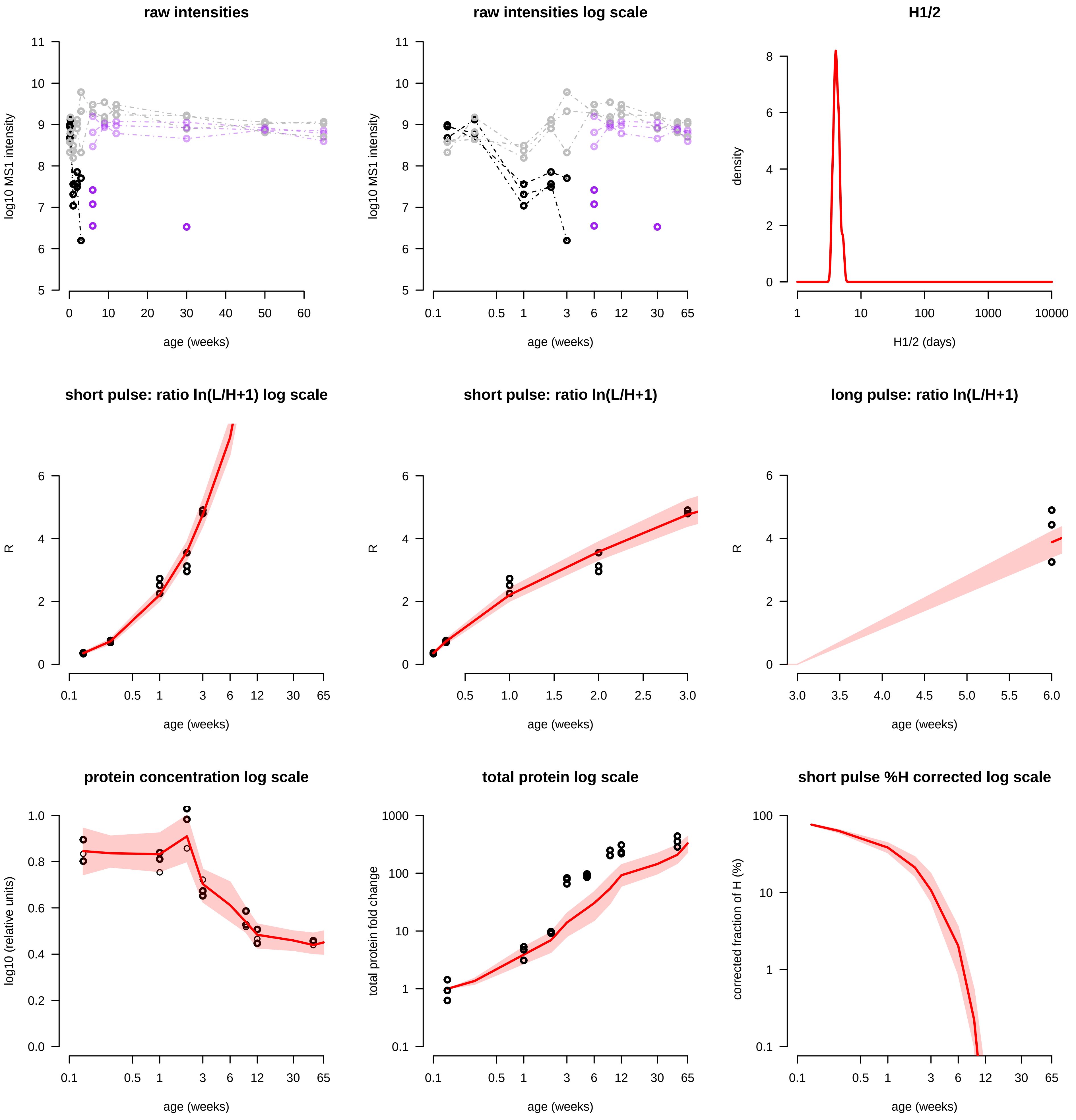

Q8R404

>sp|Q8R404|MIC13_MOUSE

MICOS complex subunit MIC13

GN=Micos13

Med H1/2: 4.8 days

Q5 H1/2: 3.7 days

Q95 H1/2: 6.9 days

reliable model fit: yes

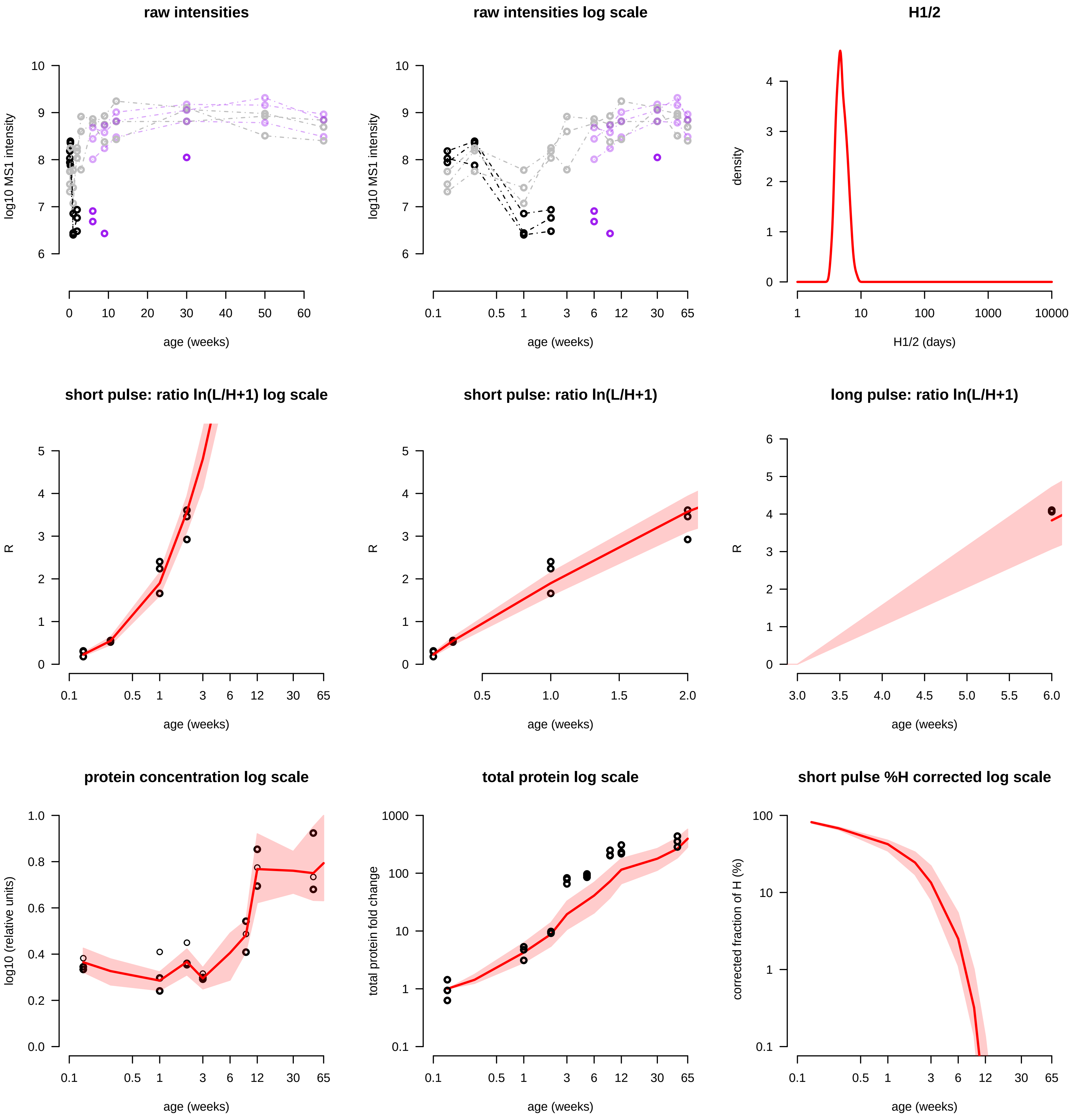

Q8BFY9;Q8BFY9−2

>sp|Q8BFY9|TNPO1_MOUSE

Transportin−1

GN=Tnpo1

Med H1/2: 10.5 days

Q5 H1/2: 7.2 days

Q95 H1/2: 16.3 days

reliable model fit: yes

raw intensities

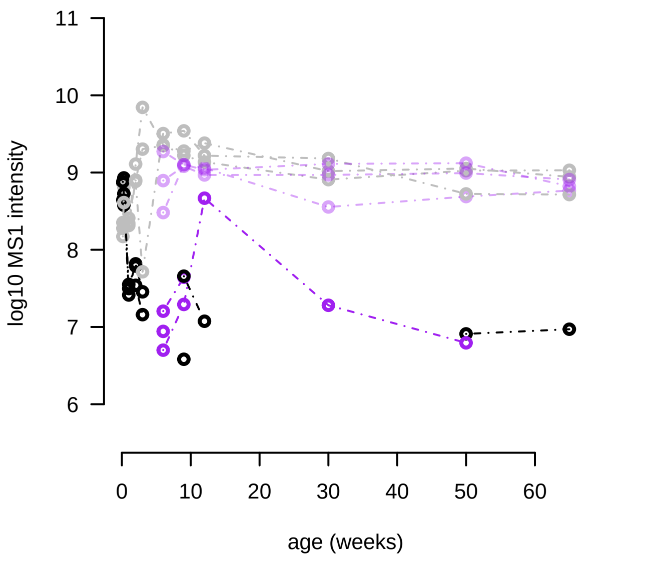

raw intensities log scale

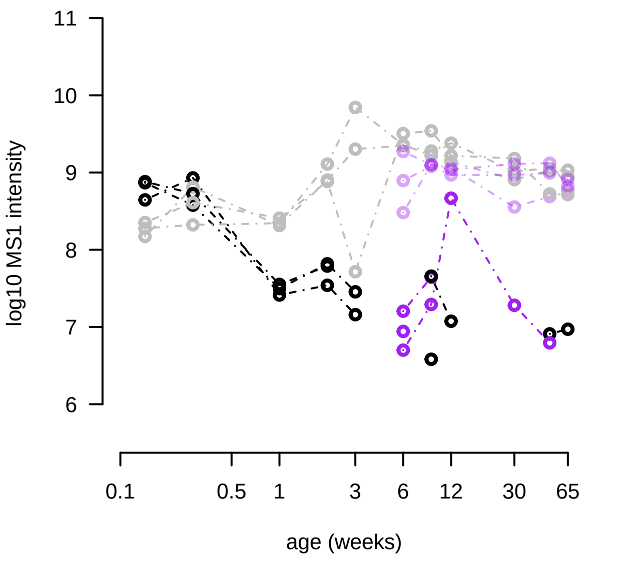

H1/2

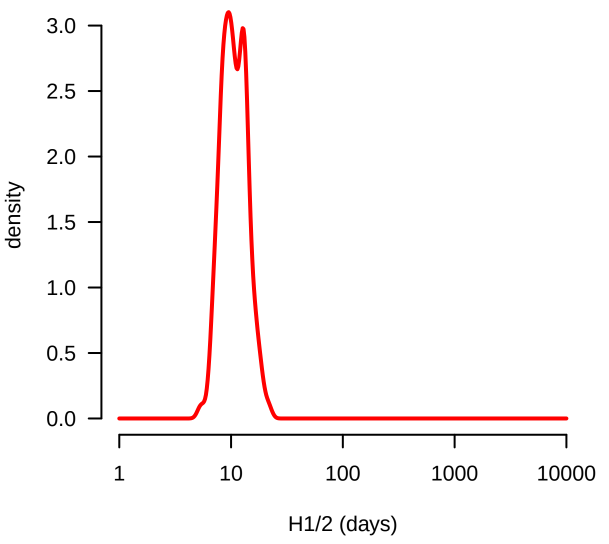

short pulse: ratio ln(L/H+1) log scale

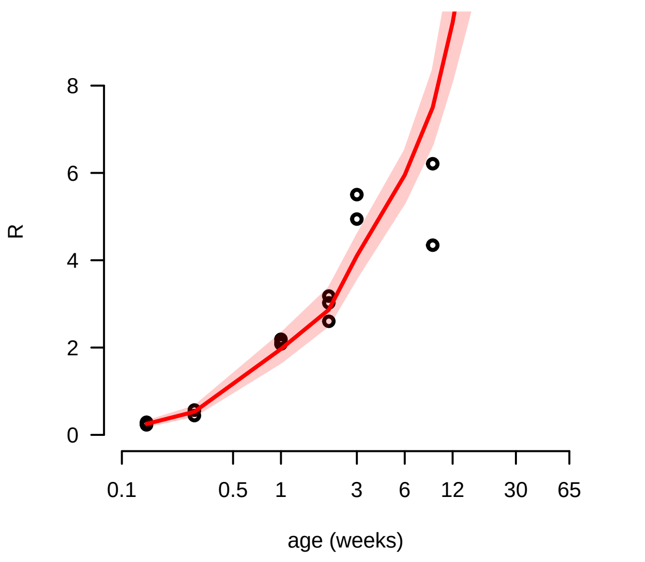

short pulse: ratio ln(L/H+1)

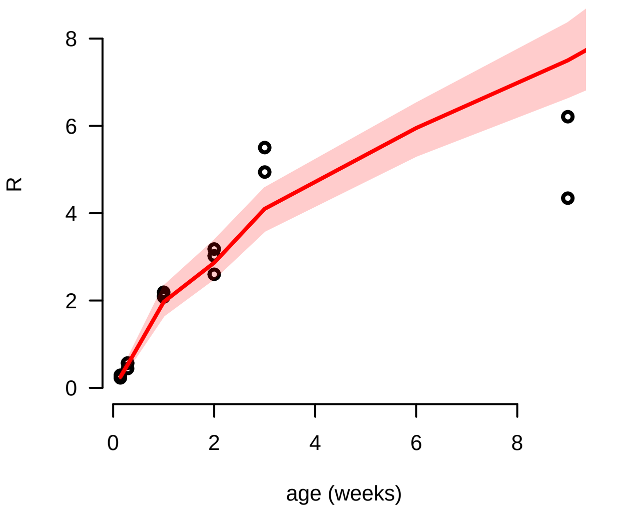

long pulse: ratio ln(L/H+1)

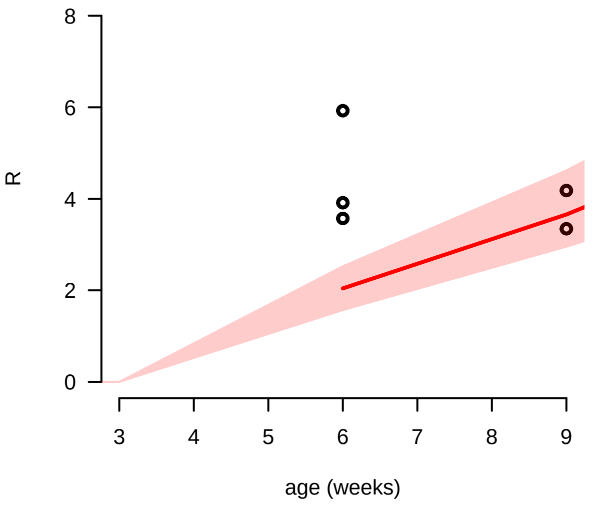

protein concentration log scale

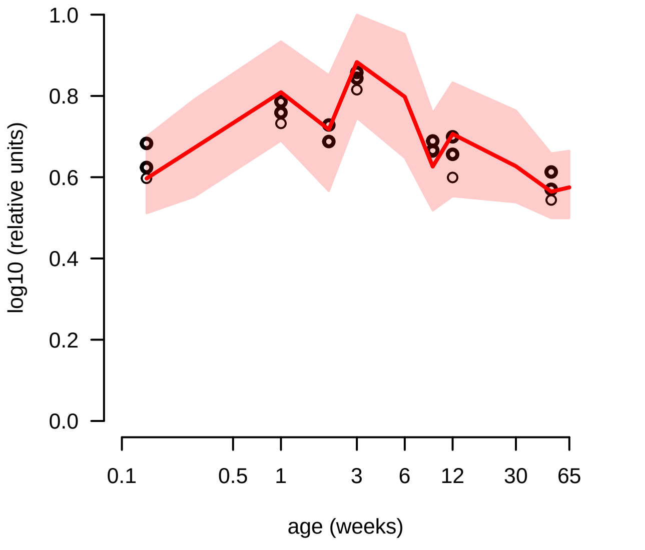

total protein log scale

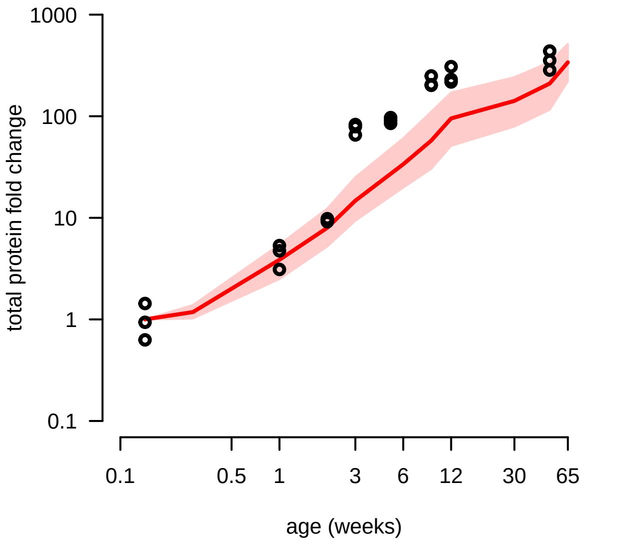

short pulse %H corrected log scale

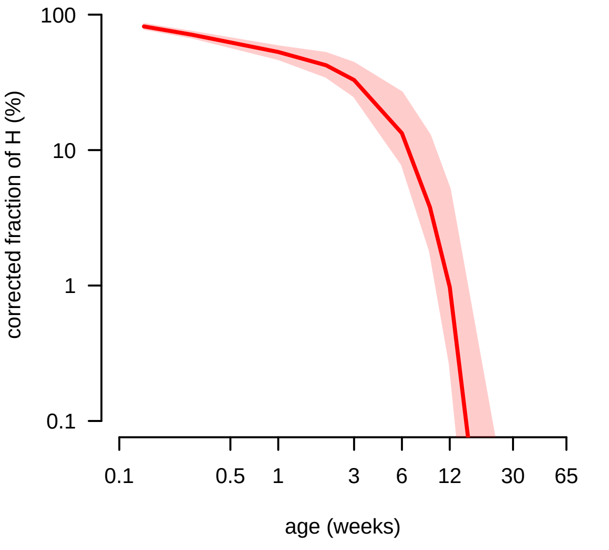

P17665

>sp|P17665|COX7C_MOUSE

Cytochrome c oxidase subunit 7C, mitochondrial

GN=Cox7c

Med H1/2: 18.7 days

Q5 H1/2: 6.9 days

Q95 H1/2: 262.7 days

reliable model fit: no

raw intensities

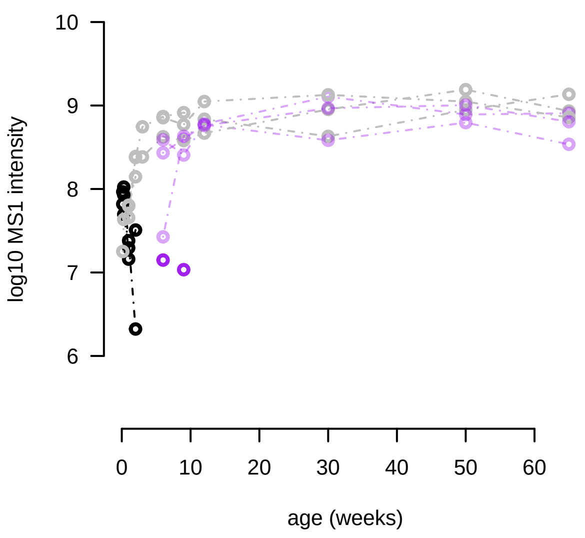

raw intensities log scale

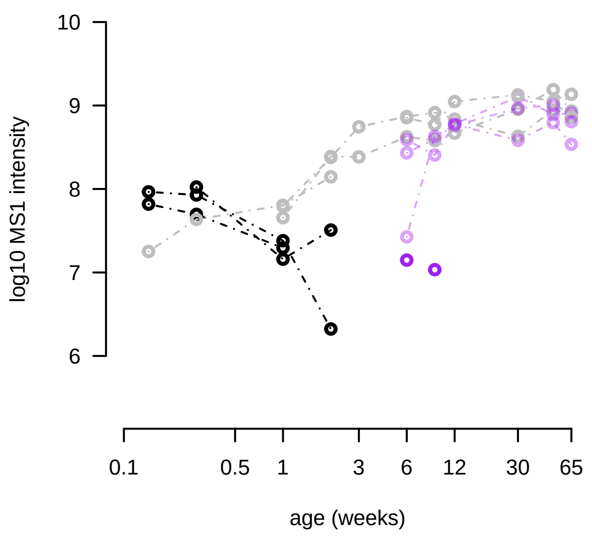

H1/2

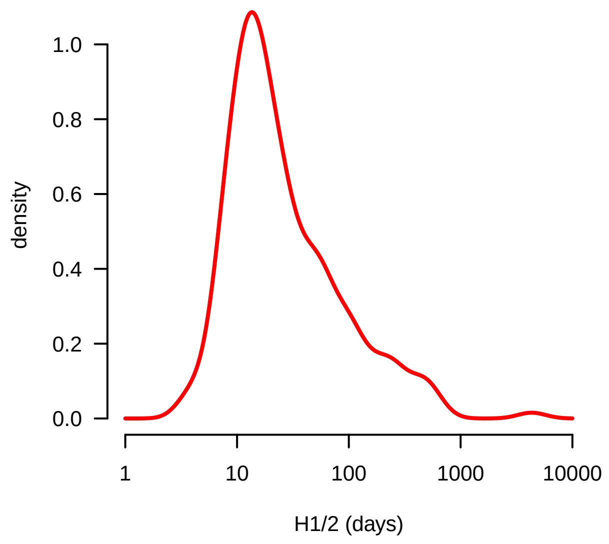

short pulse: ratio ln(L/H+1) log scale

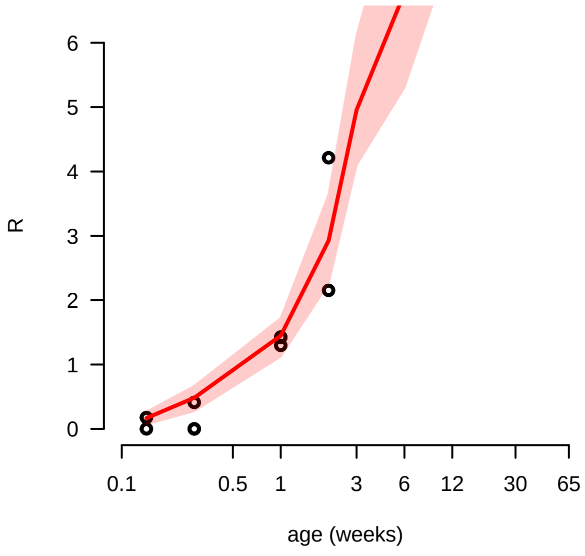

short pulse: ratio ln(L/H+1)

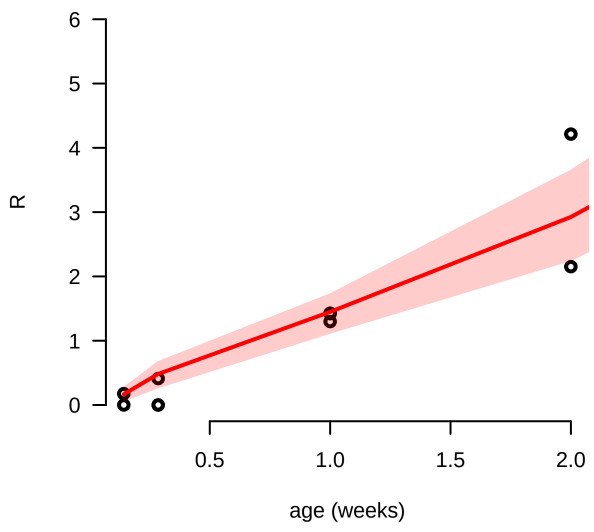

protein concentration log scale

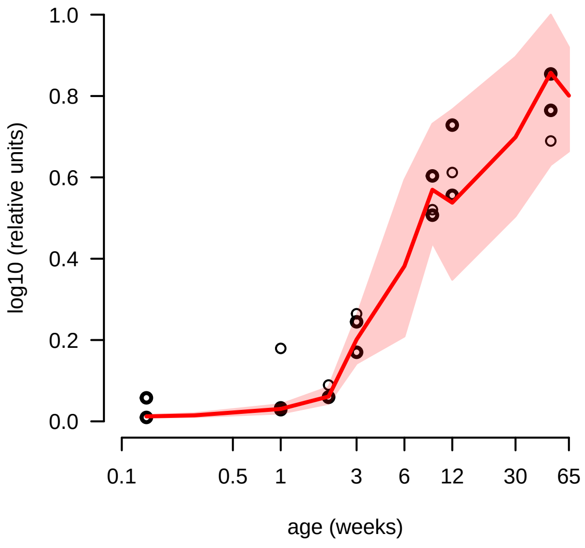

total protein log scale

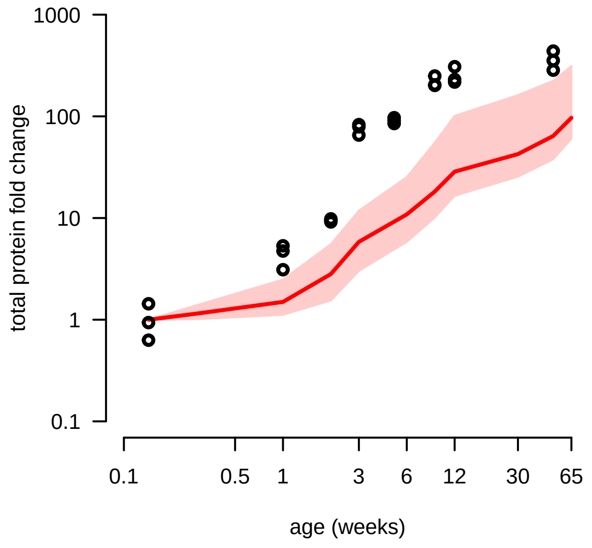

short pulse %H corrected log scale

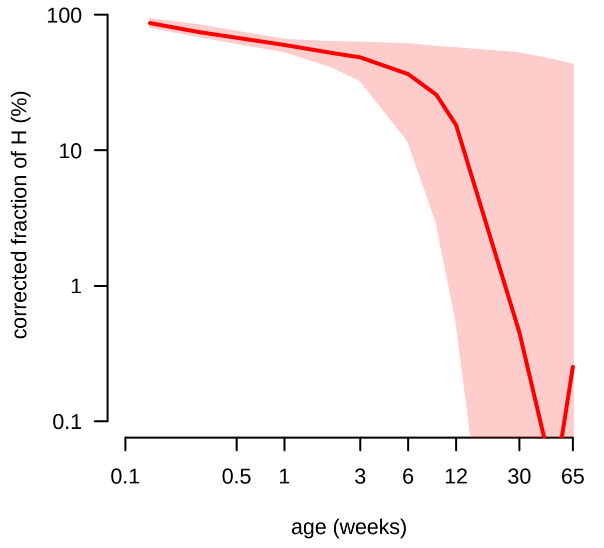

P61164

>sp|P61164|ACTZ_MOUSE

Alpha−centractin

GN=Actr1a

Med H1/2: 4.6 days

Q5 H1/2: 3.5 days

Q95 H1/2: 6 days

reliable model fit: yes

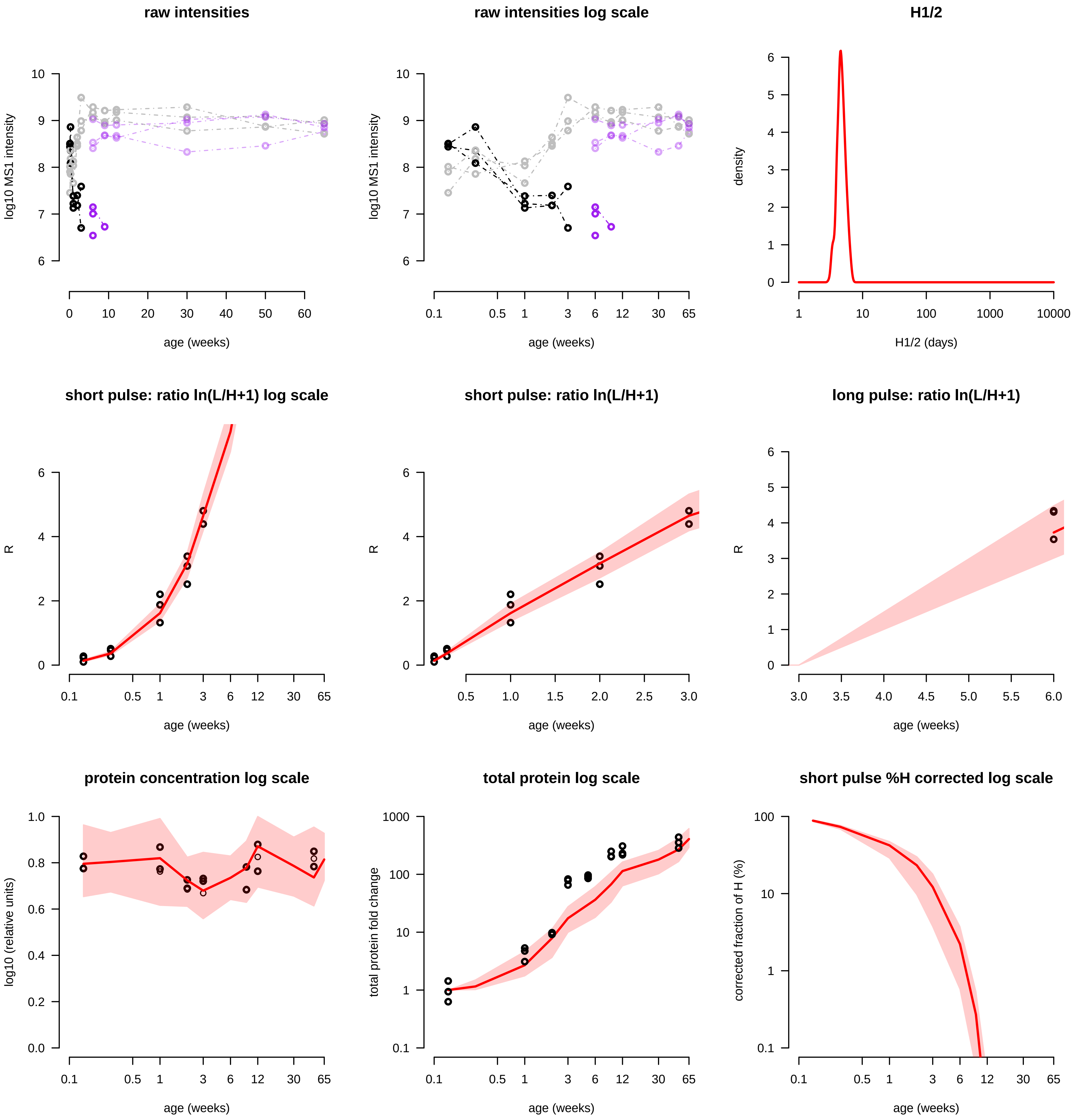

Q9CY64

>sp|Q9CY64|BIEA_MOUSE

Biliverdin reductase A

GN=Blvra

Med H1/2: 5.8 days

Q5 H1/2: 4.7 days

Q95 H1/2: 7.4 days

reliable model fit: yes

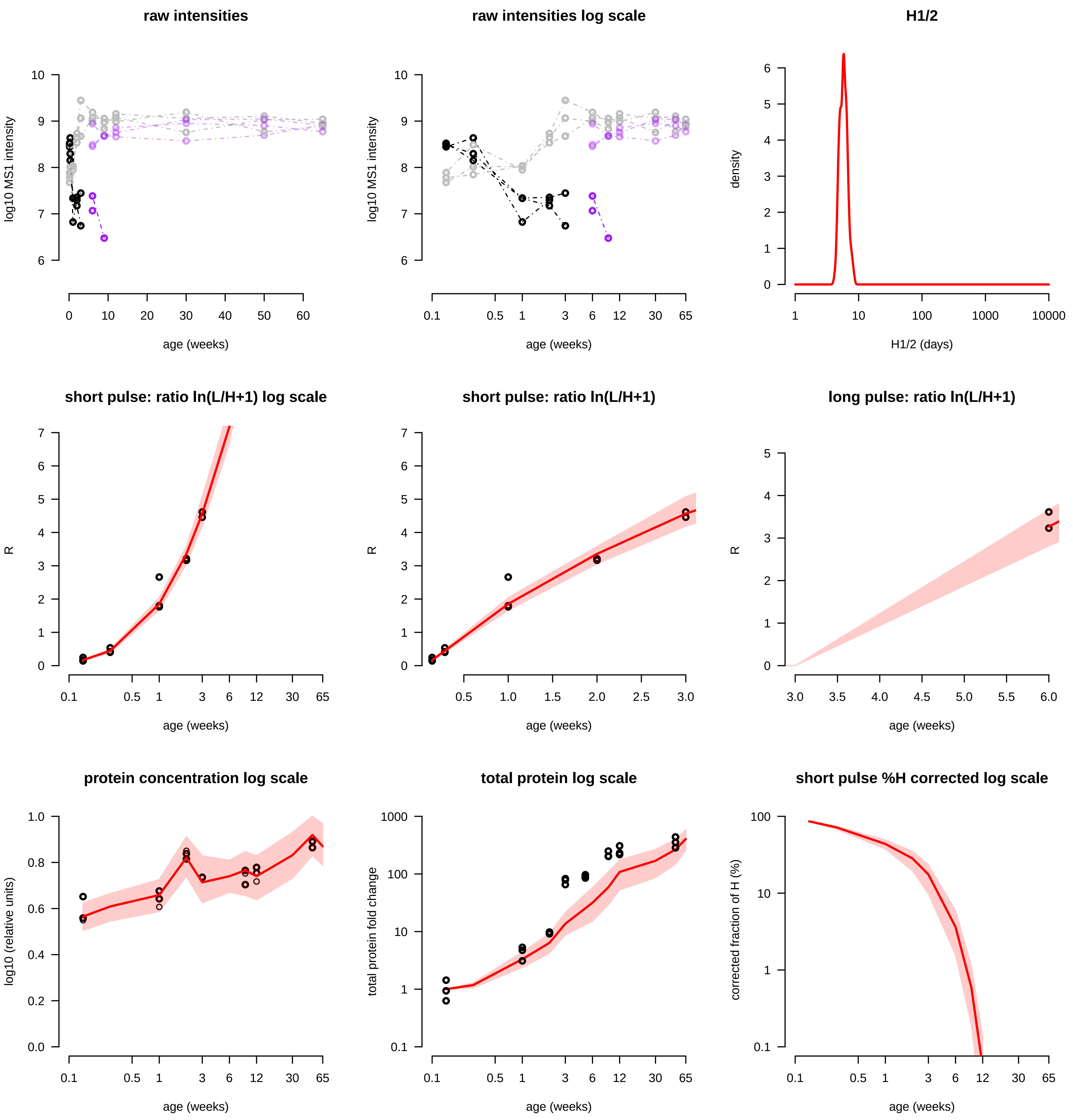

P30115

>sp|P30115|GSTA3_MOUSE

Glutathione S−transferase A3

GN=Gsta3

Med H1/2: 9 days

Q5 H1/2: 3.2 days

Q95 H1/2: 68.8 days

reliable model fit: yes

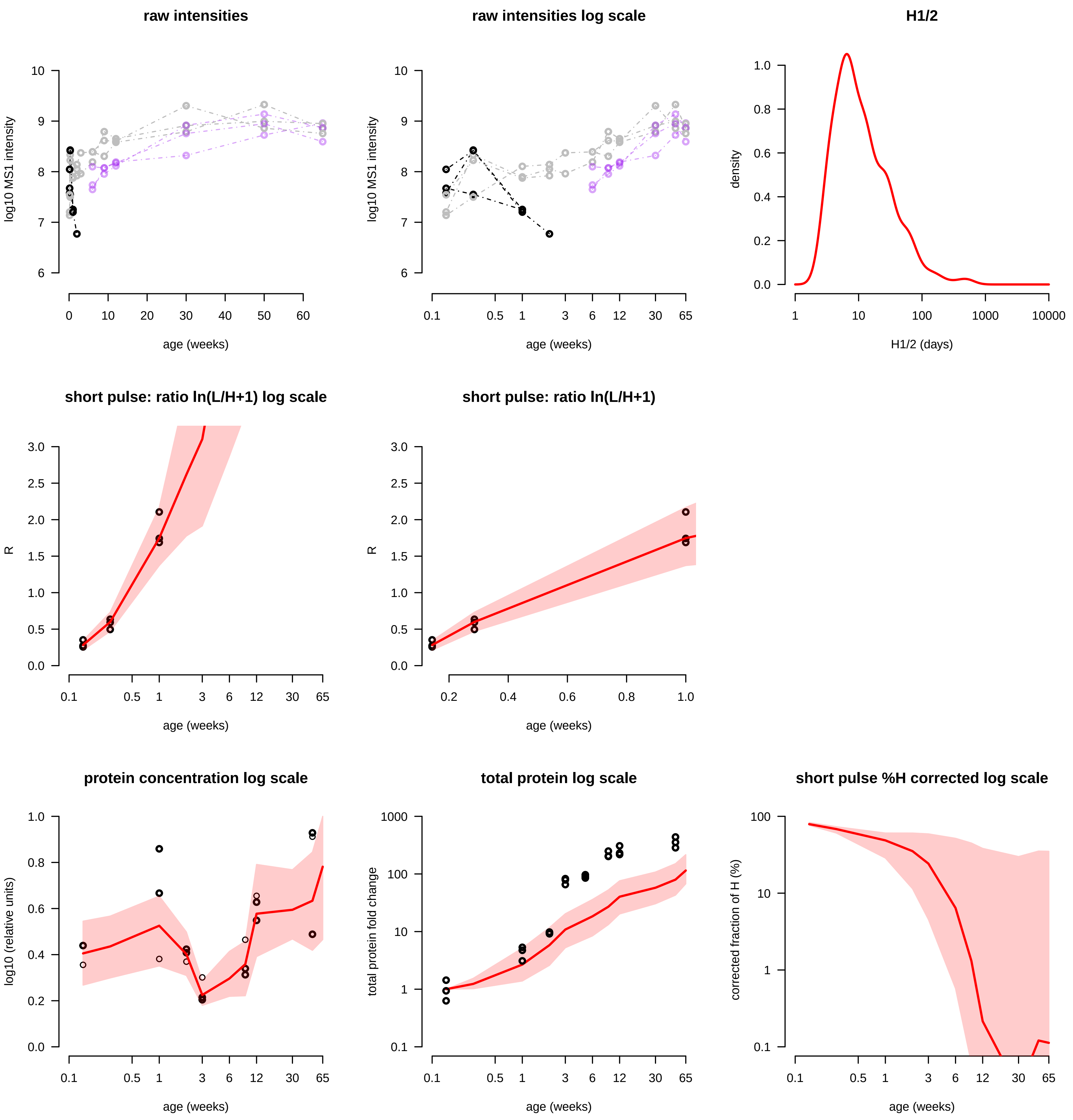

P16675

>sp|P16675|PPGB_MOUSE

Lysosomal protective protein

GN=Ctsa

Med H1/2: 5.7 days

Q5 H1/2: 4.6 days

Q95 H1/2: 8.1 days

reliable model fit: no

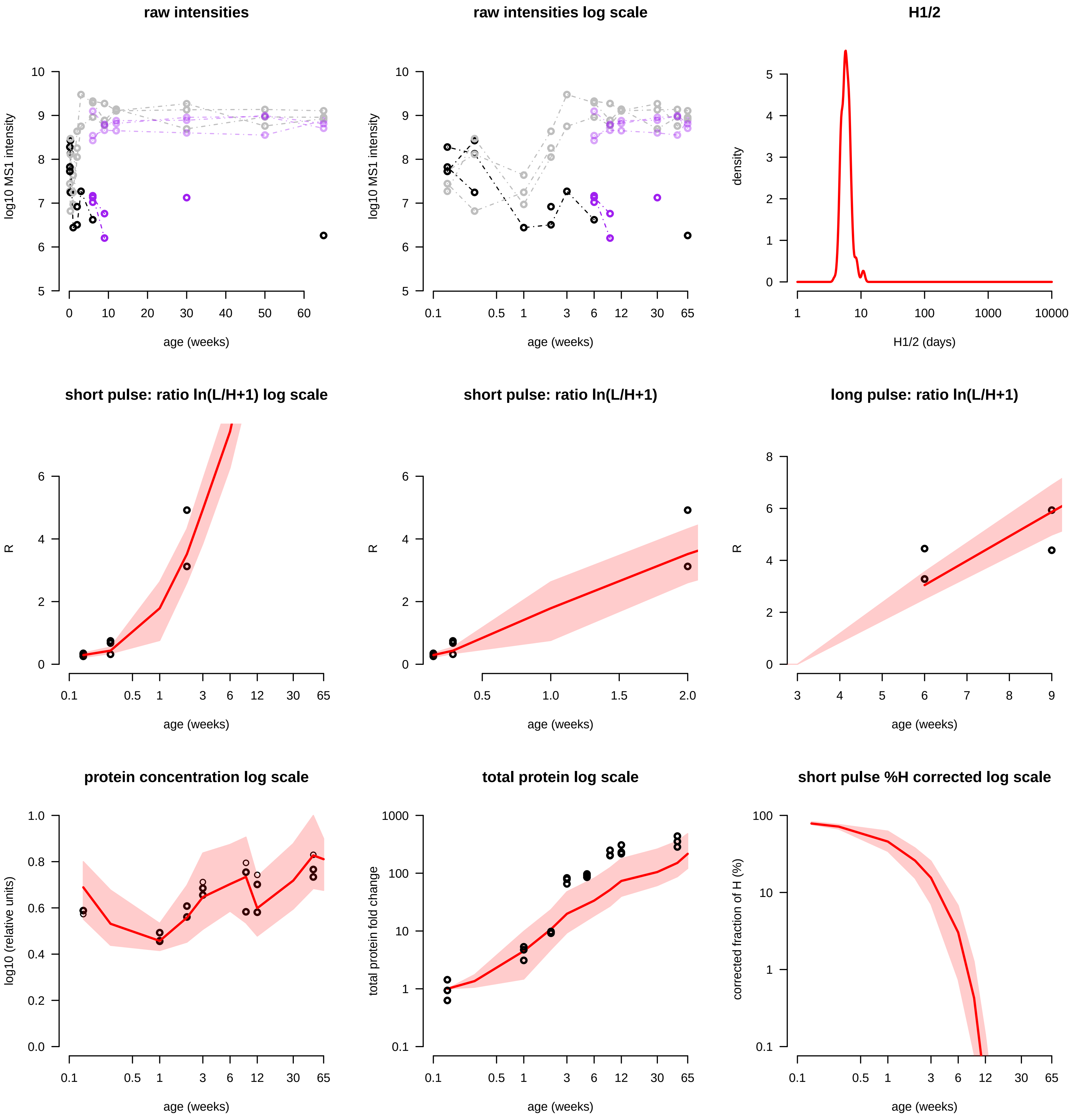

P58389

>sp|P58389|PTPA_MOUSE

Serine/threonine−protein phosphatase 2A activator

GN=Ptpa

Med H1/2: 19.9 days

Q5 H1/2: 8.5 days

Q95 H1/2: 235.4 days

reliable model fit: yes

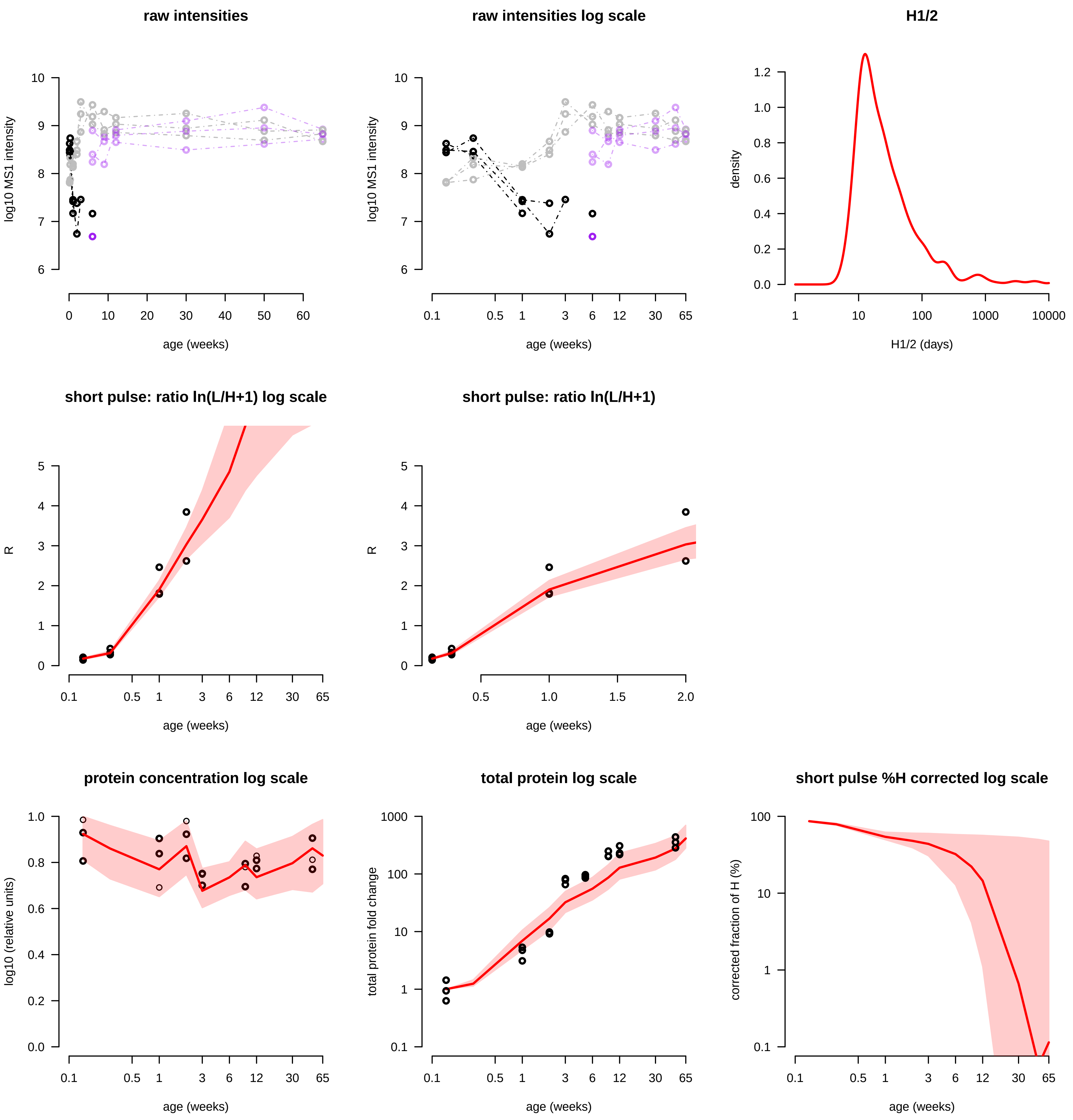

Q791T5−2;Q791T5

>sp|Q791T5−2|MTCH1_MOUSE

Isoform 2 of Mitochondrial carrier homolog 1

GN=Mtch1

Med H1/2: 11.1 days

Q5 H1/2: 3.9 days

Q95 H1/2: 155.1 days

reliable model fit: yes

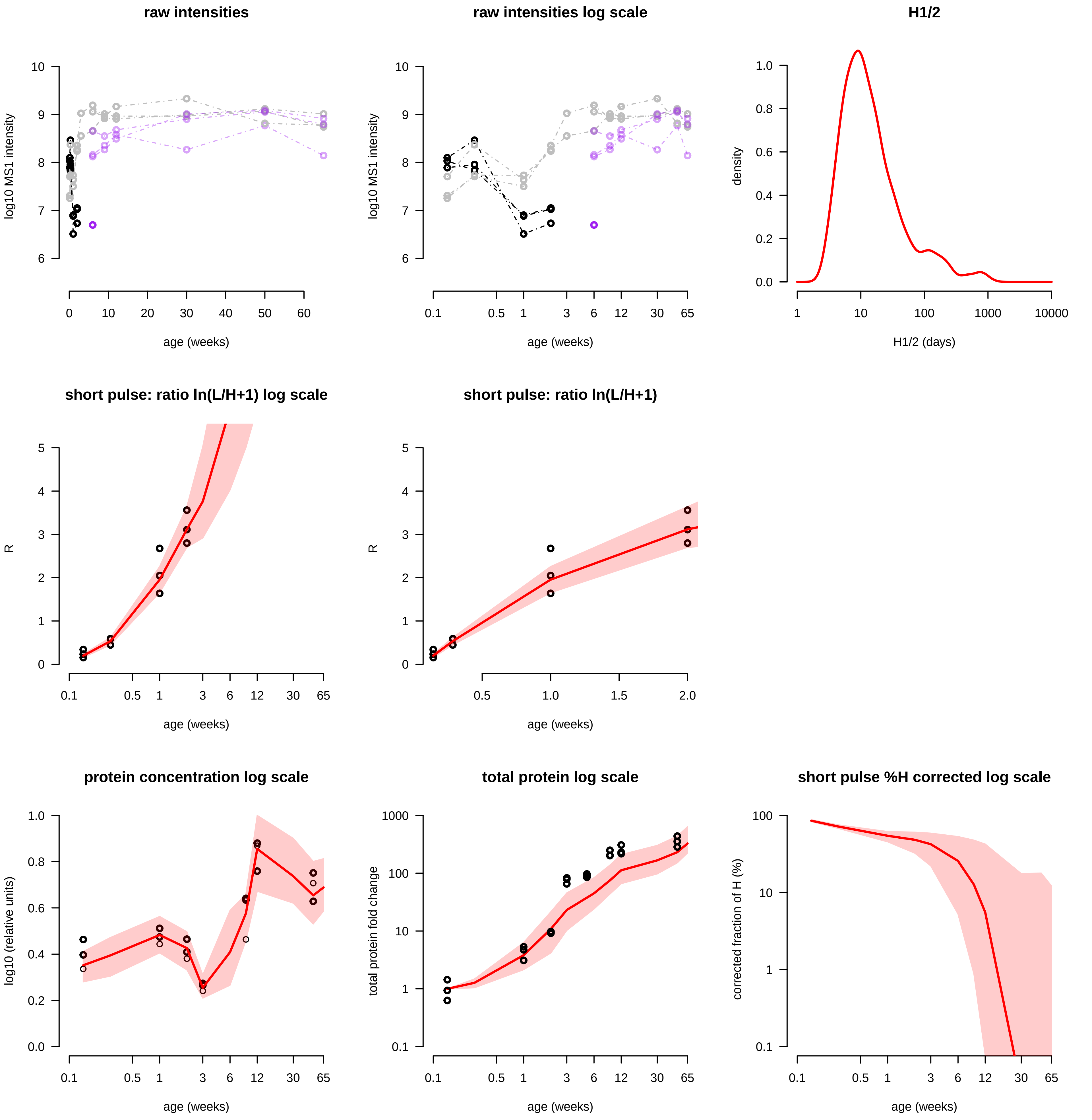

P50429;P50429−2

>sp|P50429|ARSB_MOUSE

Arylsulfatase B

GN=Arsb

Med H1/2: 6.9 days

Q5 H1/2: 4.9 days

Q95 H1/2: 11.4 days

reliable model fit: yes

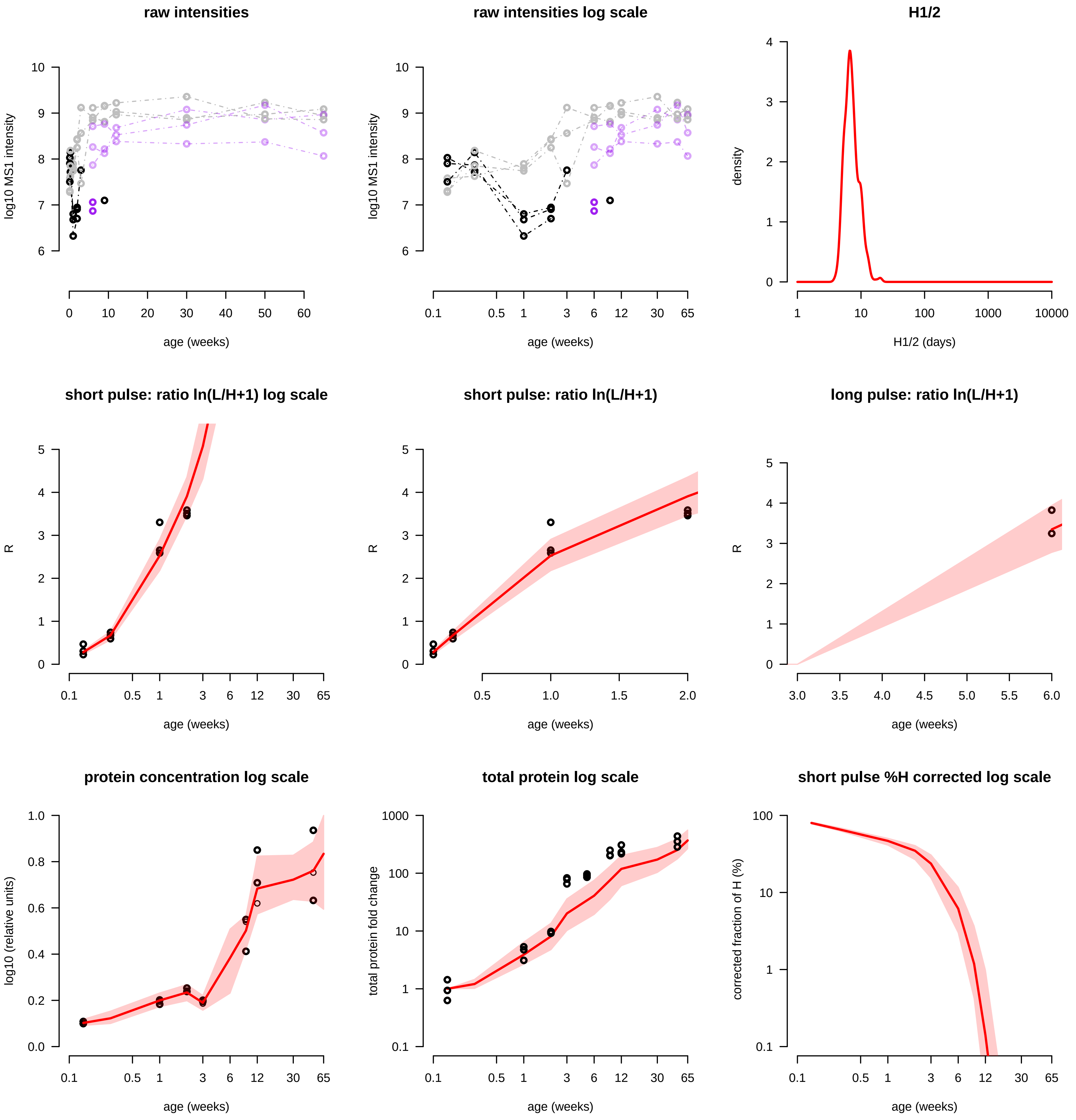

P70122

>sp|P70122|SBDS_MOUSE

Ribosome maturation protein SBDS

GN=Sbds

Med H1/2: 30.6 days

Q5 H1/2: 9.1 days

Q95 H1/2: 329.6 days

reliable model fit: no

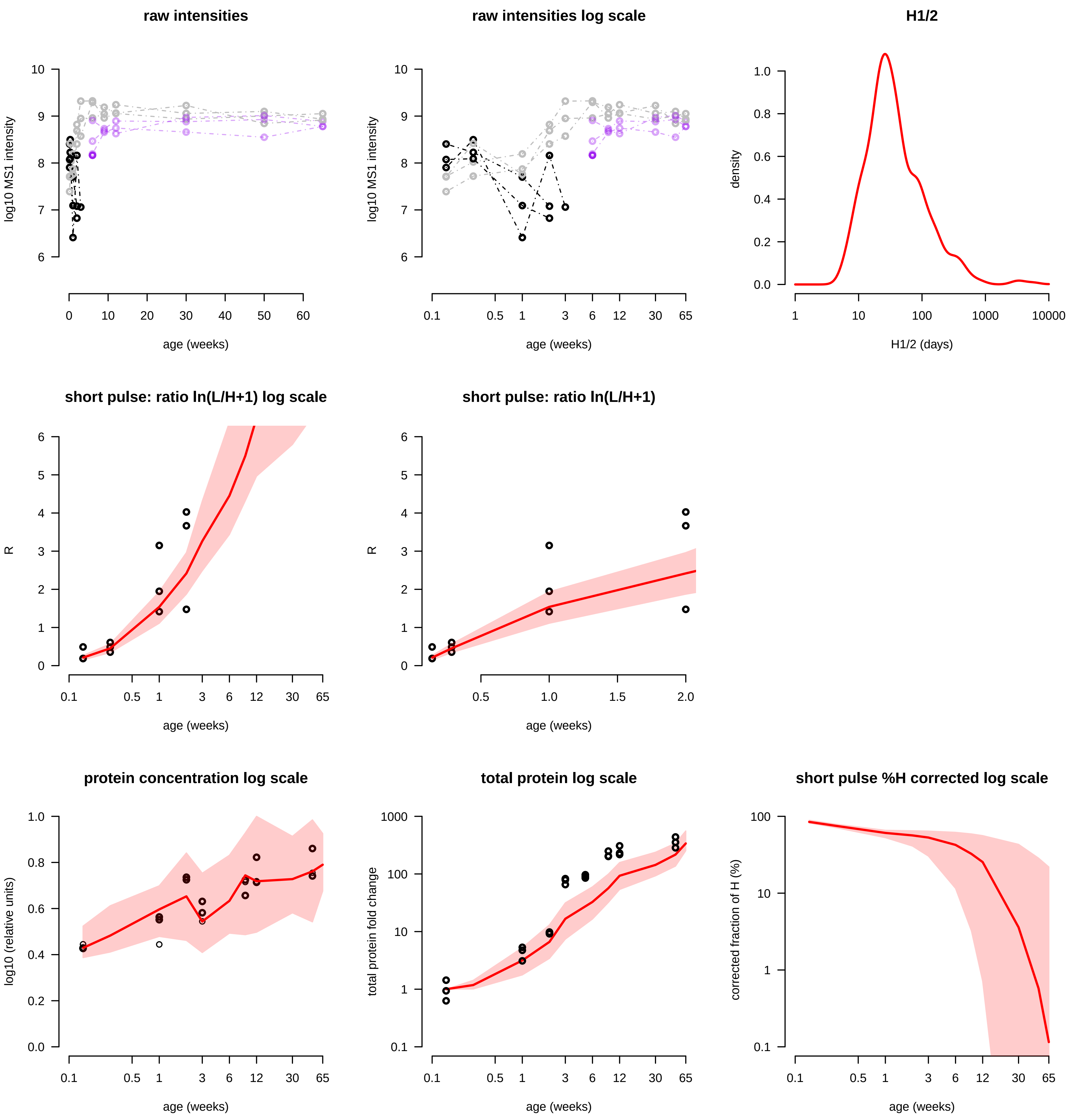

P18654

>sp|P18654|KS6A3_MOUSE

Ribosomal protein S6 kinase alpha−3

GN=Rps6ka3

Med H1/2: 8.4 days

Q5 H1/2: 5.8 days

Q95 H1/2: 13.1 days

reliable model fit: no

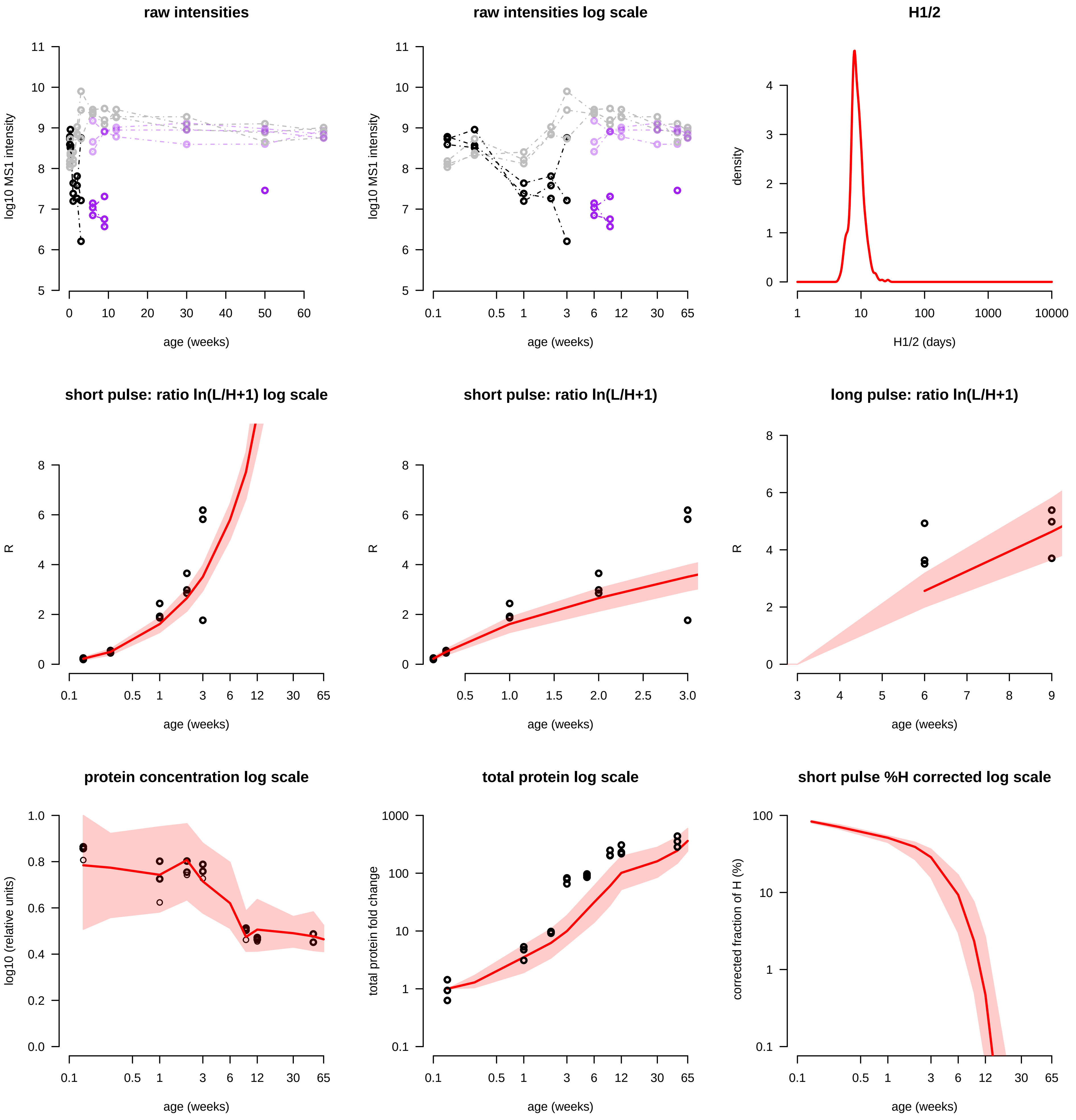

P32921−2

>sp|P32921−2|SYWC_MOUSE

Isoform 2 of Tryptophan−−tRNA ligase, cytoplasmic

GN=Wars1

Med H1/2: 3.5 days

Q5 H1/2: 3 days

Q95 H1/2: 4.2 days

reliable model fit: yes

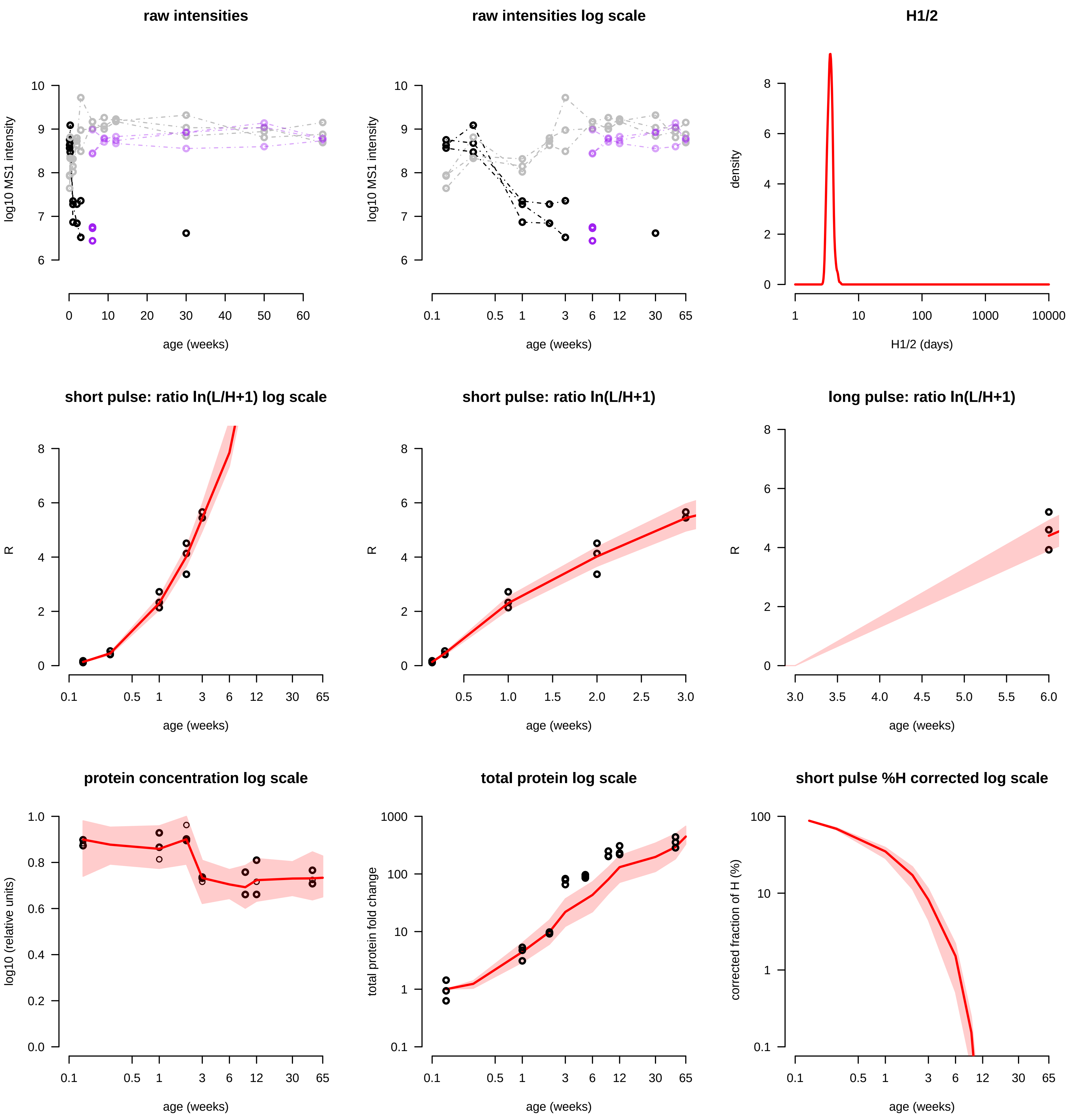

P40336;P40336−2

>sp|P40336|VP26A_MOUSE

Vacuolar protein sorting−associated protein 26A

GN=Vps26a

Med H1/2: 3.9 days

Q5 H1/2: 3.5 days

Q95 H1/2: 4.7 days

reliable model fit: yes

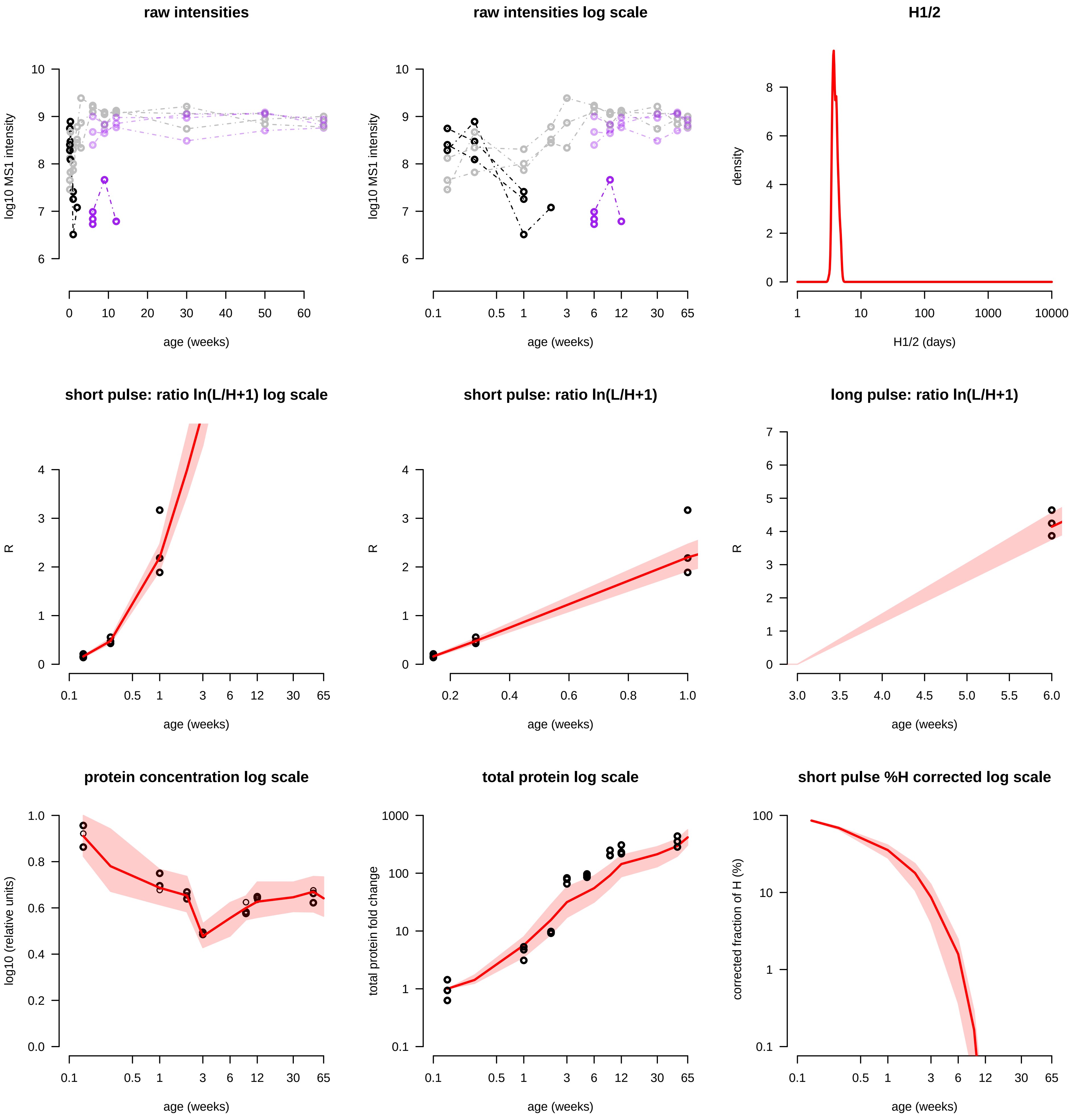

Q08091;Q08091−2

>sp|Q08091|CNN1_MOUSE

Calponin−1

GN=Cnn1

Med H1/2: 7.5 days

Q5 H1/2: 4.8 days

Q95 H1/2: 13.3 days

reliable model fit: yes

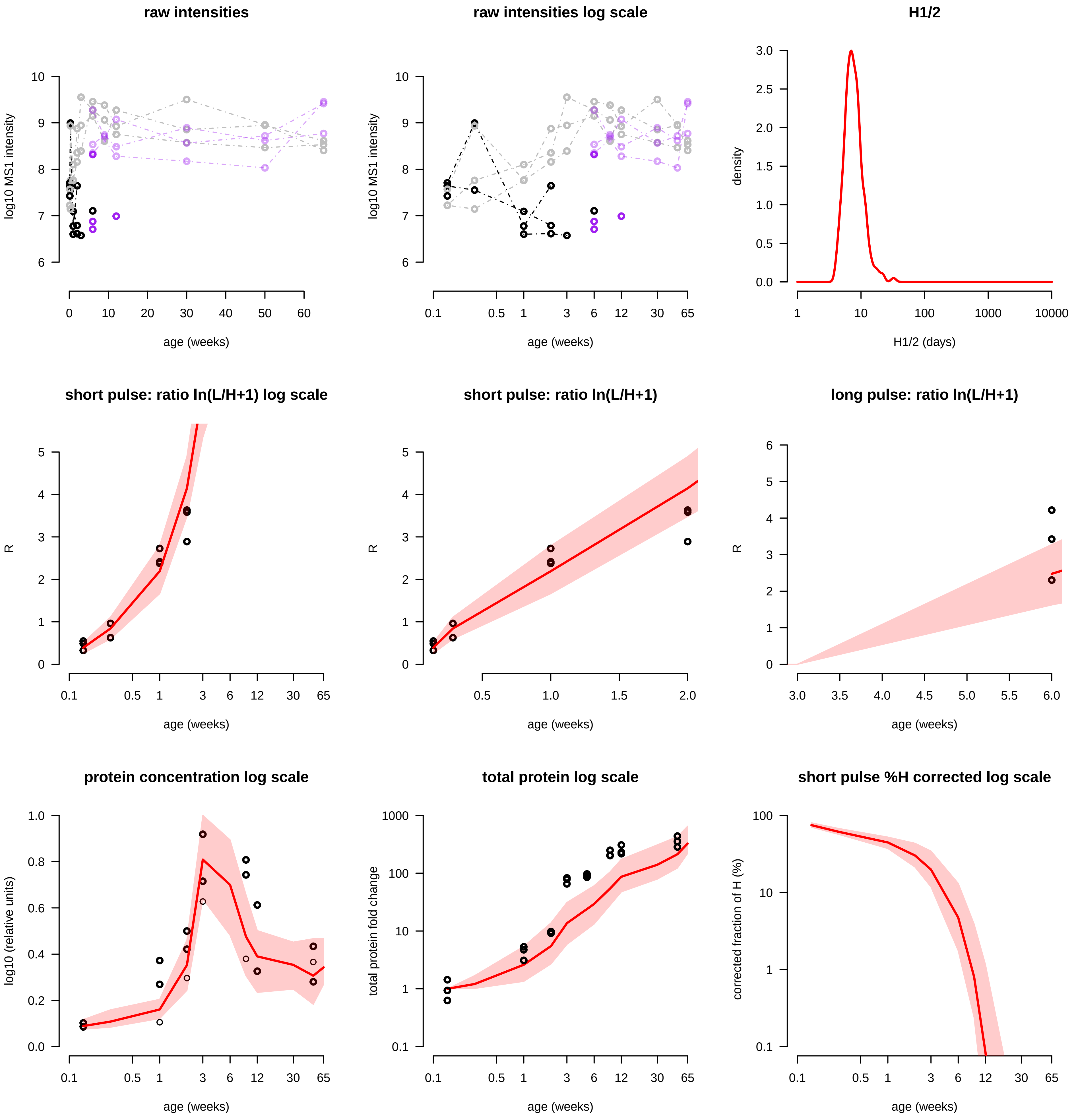

P97379

>sp|P97379|G3BP2_MOUSE

Ras GTPase−activating protein−binding protein 2

GN=G3bp2

Med H1/2: 4.3 days

Q5 H1/2: 3.7 days

Q95 H1/2: 5.2 days

reliable model fit: yes

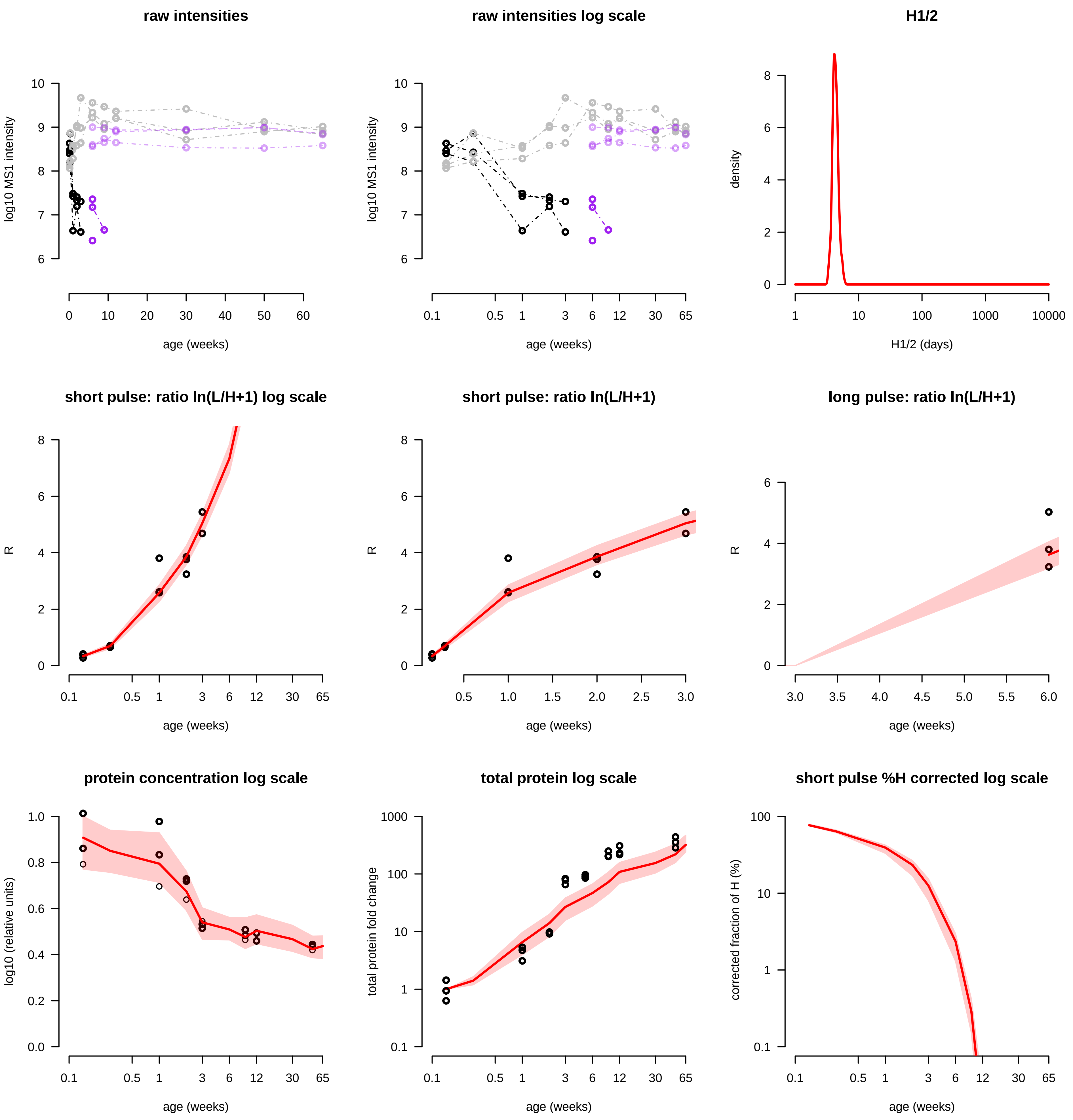

G3X9K3

>sp|G3X9K3|BIG1_MOUSE

Brefeldin A−inhibited guanine nucleotide−exchange protein 1

GN=Arfgef1

Med H1/2: 3100.8 days

Q5 H1/2: 977.8 days

Q95 H1/2: 63217.7 days

reliable model fit: yes

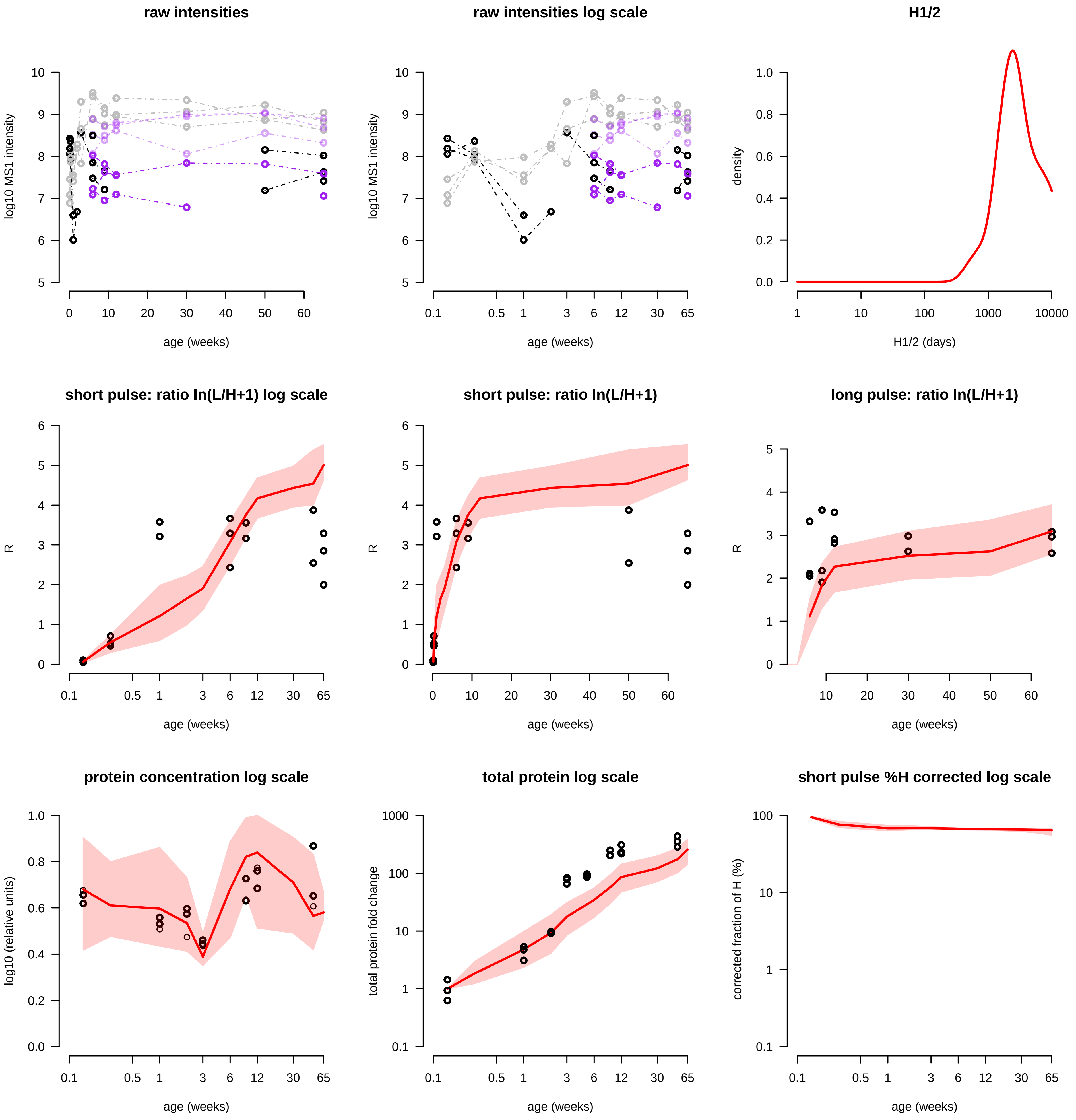

P61211

>sp|P61211|ARL1_MOUSE

ADP−ribosylation factor−like protein 1

GN=Arl1

Med H1/2: 7.7 days

Q5 H1/2: 6.2 days

Q95 H1/2: 9.9 days

reliable model fit: yes

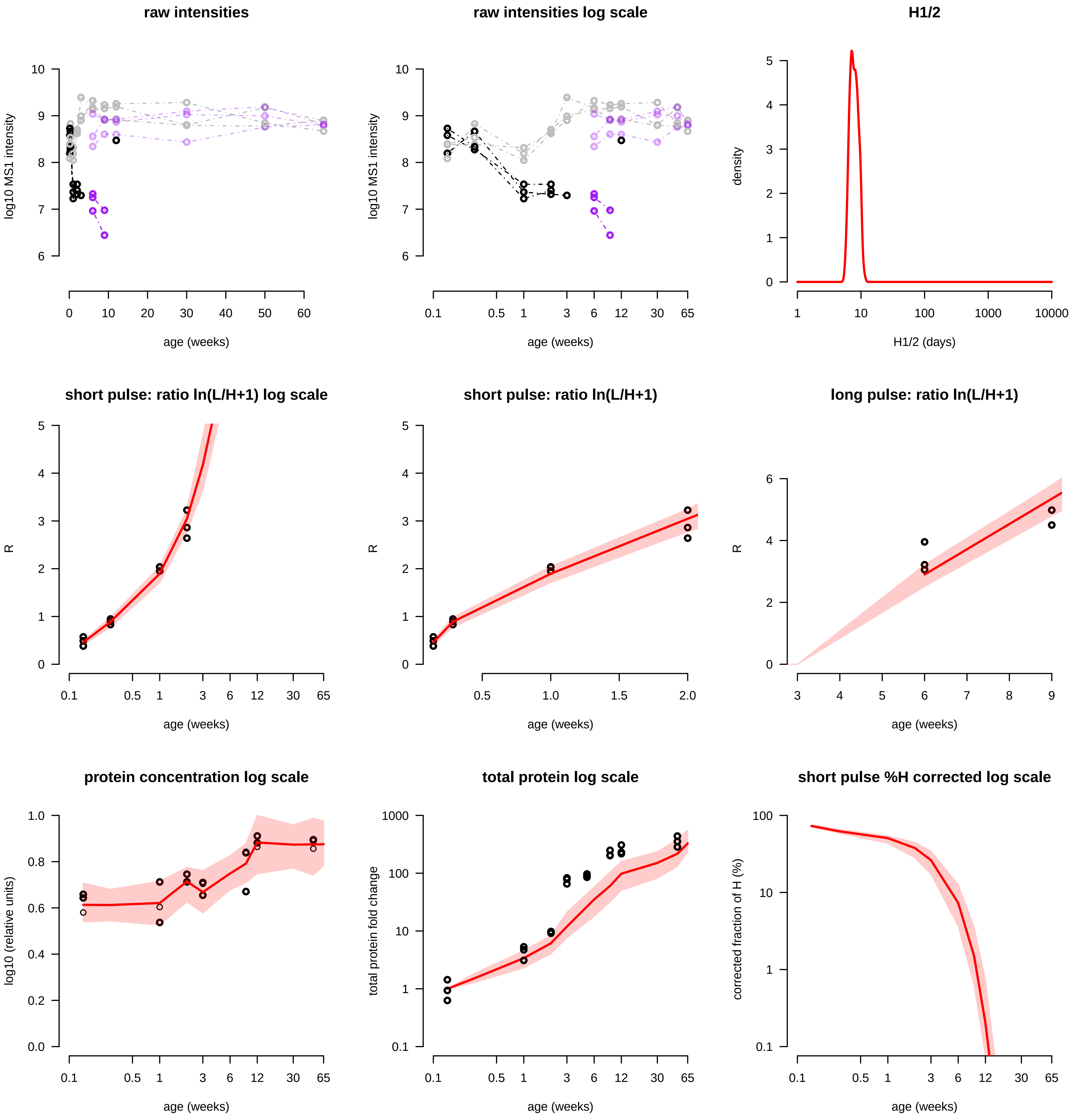

Q9JLV5

>sp|Q9JLV5|CUL3_MOUSE

Cullin−3

GN=Cul3

Med H1/2: 146.1 days

Q5 H1/2: 84.8 days

Q95 H1/2: 406.6 days

reliable model fit: yes

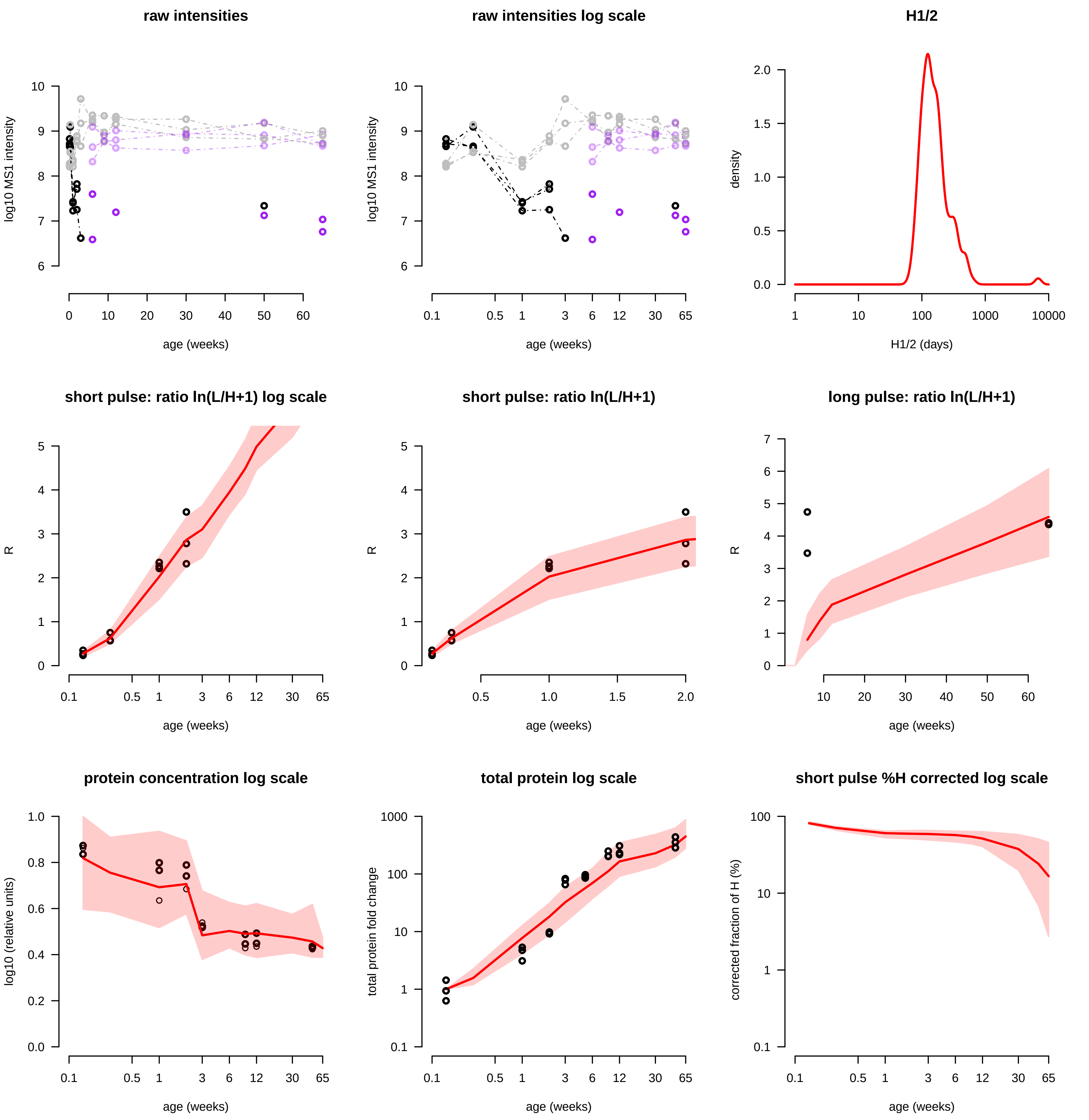

P22315

>sp|P22315|HEMH_MOUSE

Ferrochelatase, mitochondrial

GN=Fech

Med H1/2: 5.4 days

Q5 H1/2: 4.2 days

Q95 H1/2: 7.3 days

reliable model fit: yes

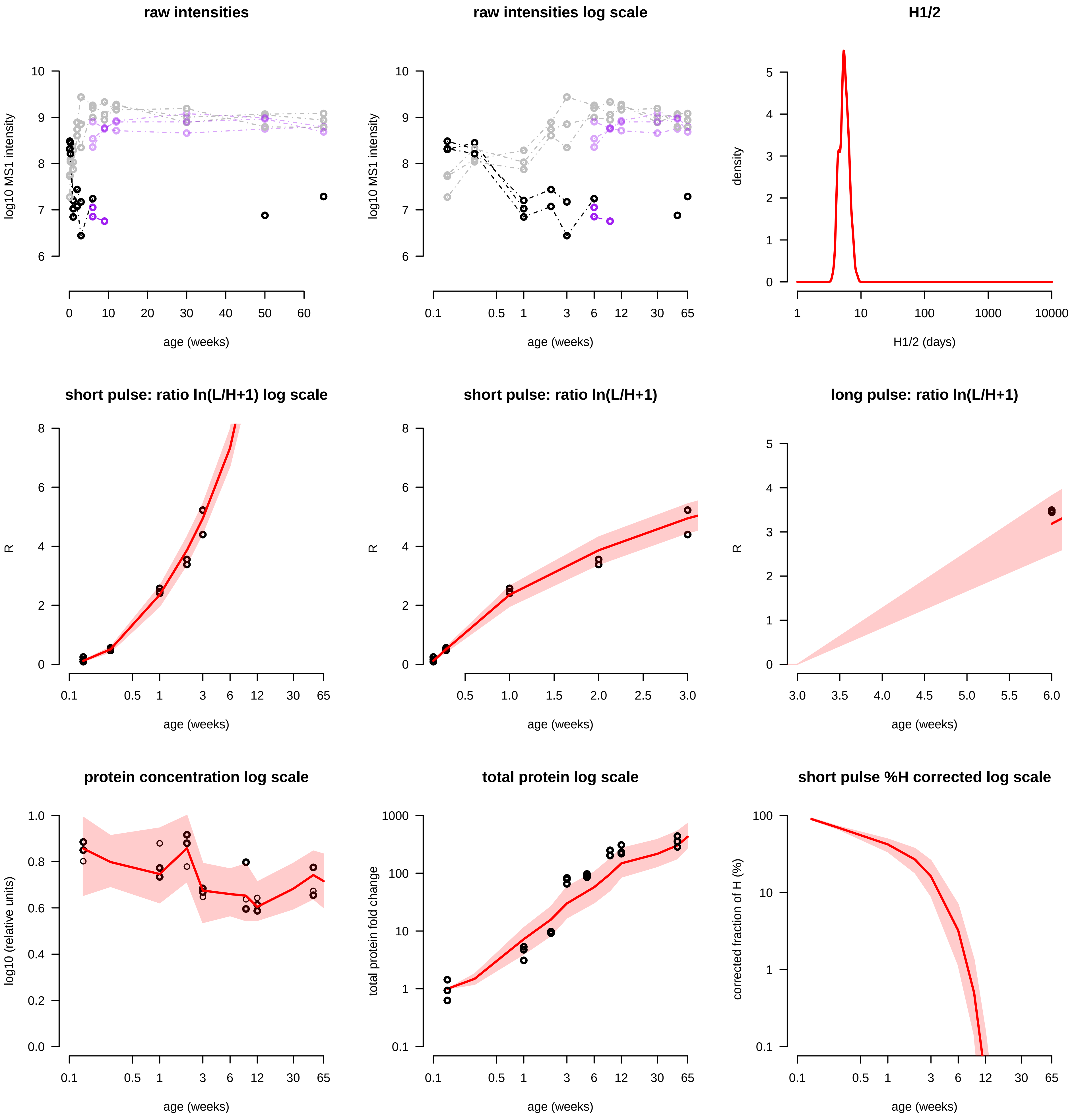

Q8BJU0

>sp|Q8BJU0|SGTA_MOUSE

Small glutamine−rich tetratricopeptide repeat−containing protein alpha

GN=Sgta

Med H1/2: 6 days

Q5   H1/2: 5.1 days

Q95 H1/2: 7.1 days

reliable model fit: yes

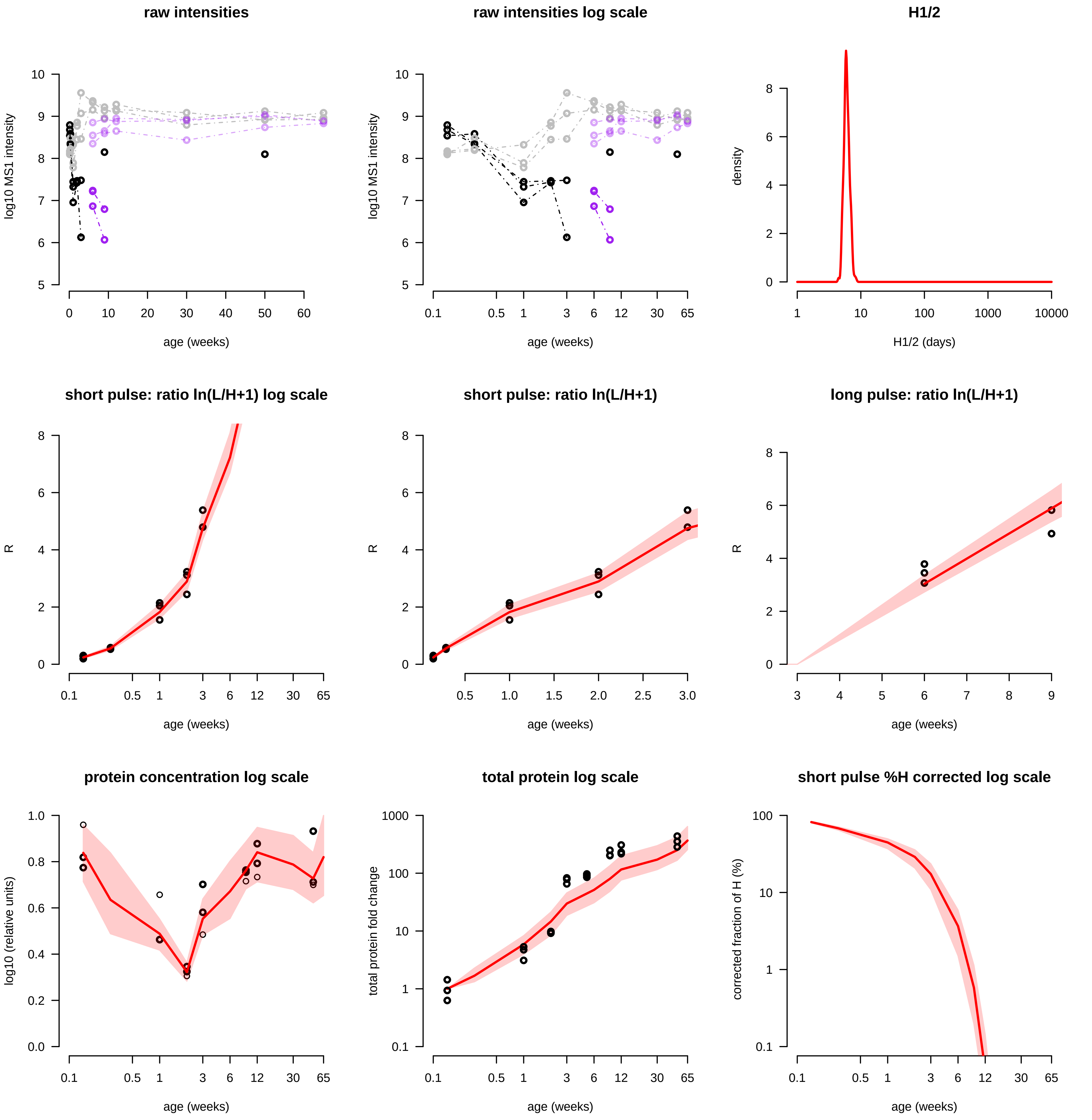

Q9QYB1

>sp|Q9QYB1|CLIC4_MOUSE

Chloride intracellular channel protein 4

GN=Clic4

Med H1/2: 37.8 days

Q5 H1/2: 8.9 days

Q95 H1/2: 373.7 days

reliable model fit: yes

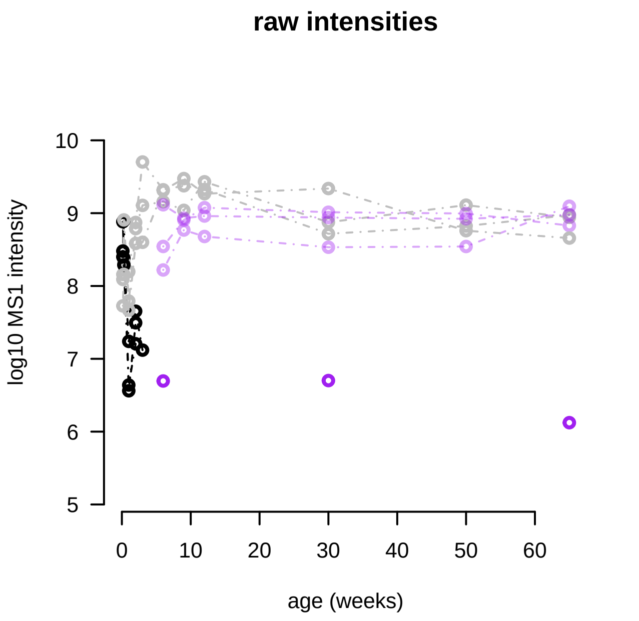

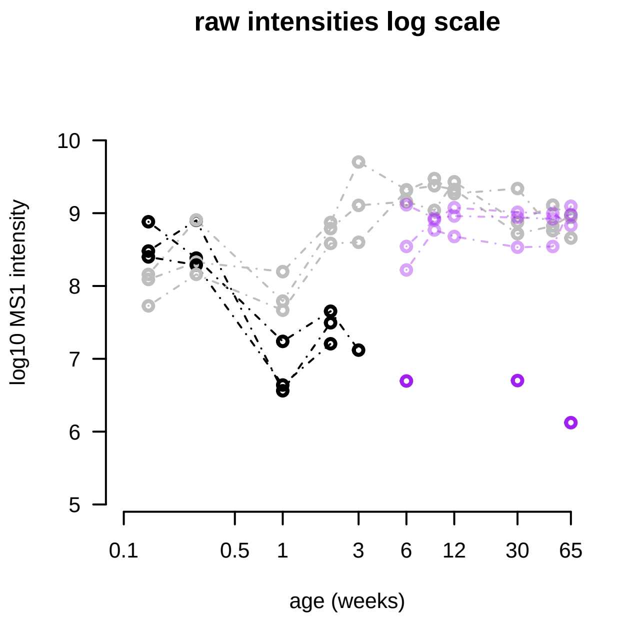

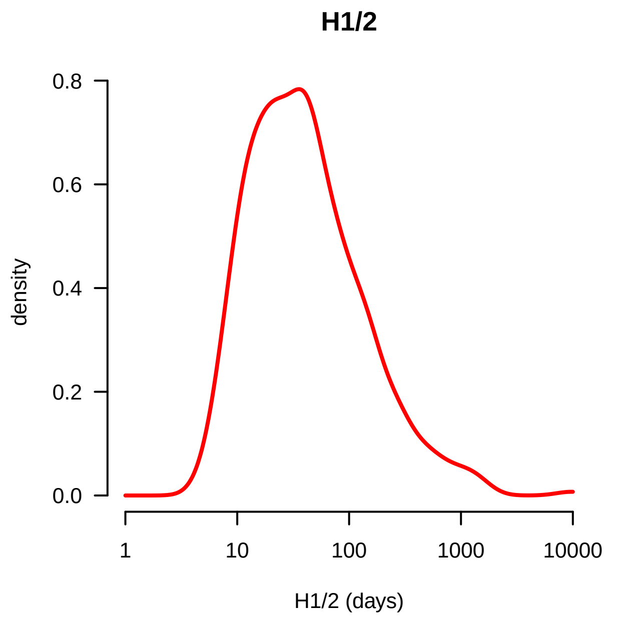

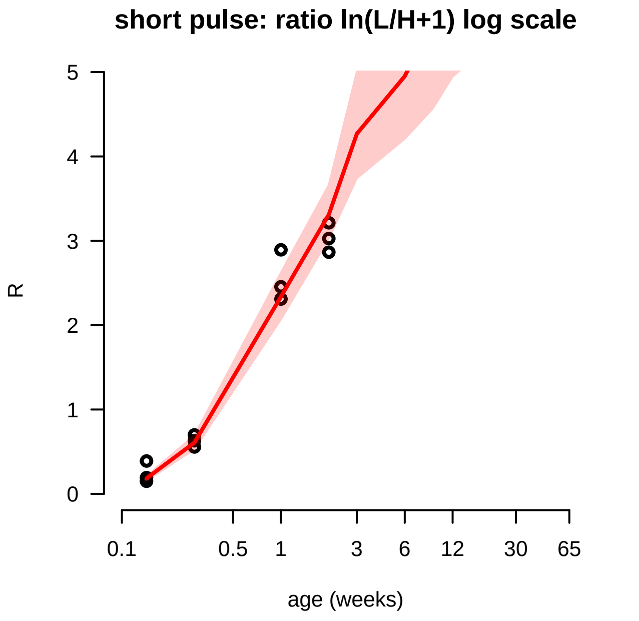

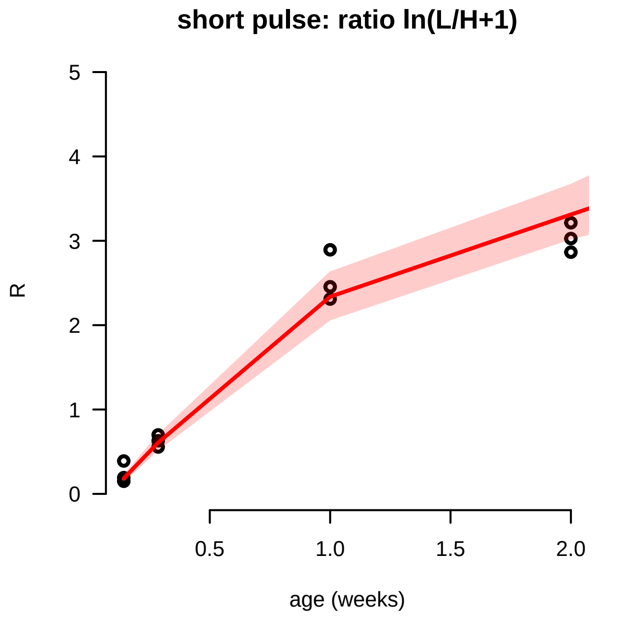

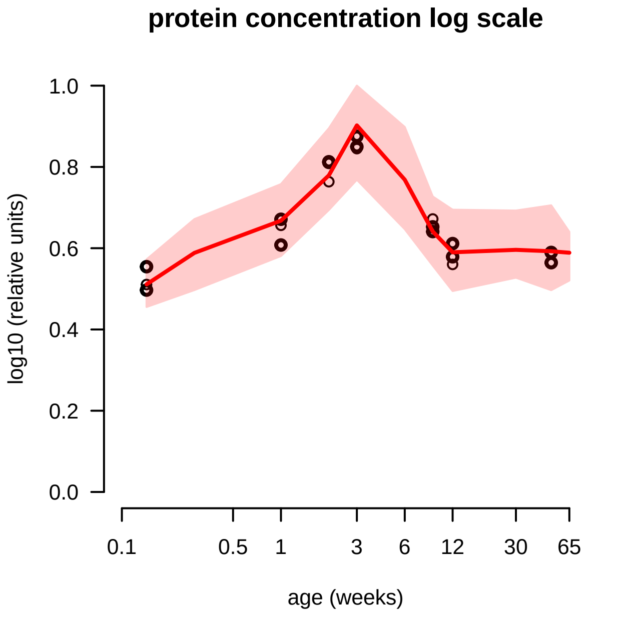

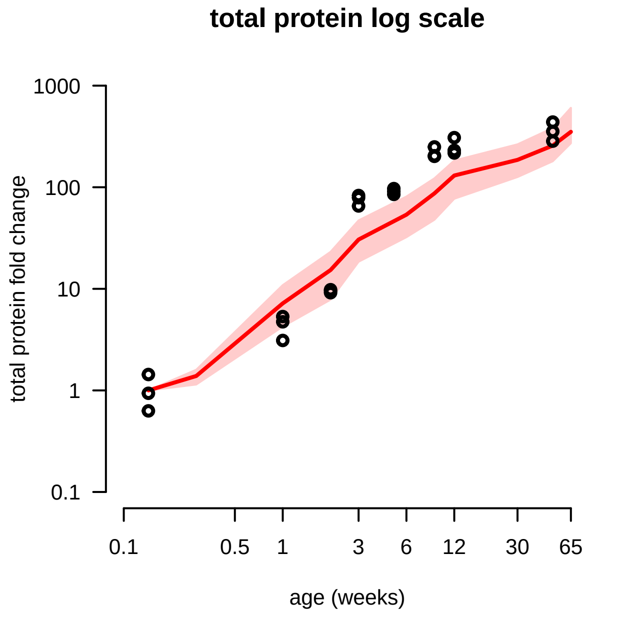

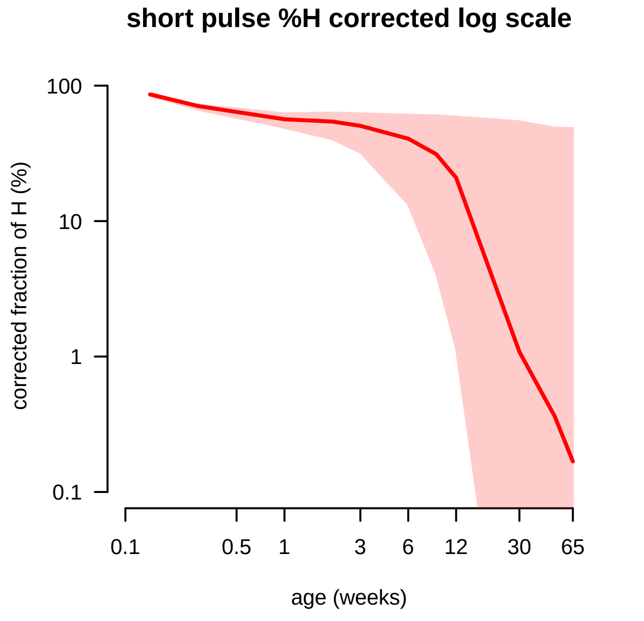

P10518

>sp|P10518|HEM2_MOUSE

Delta−aminolevulinic acid dehydratase

GN=Alad

Med H1/2: 6.2 days

Q5 H1/2: 5 days

Q95 H1/2: 8 days

reliable model fit: yes

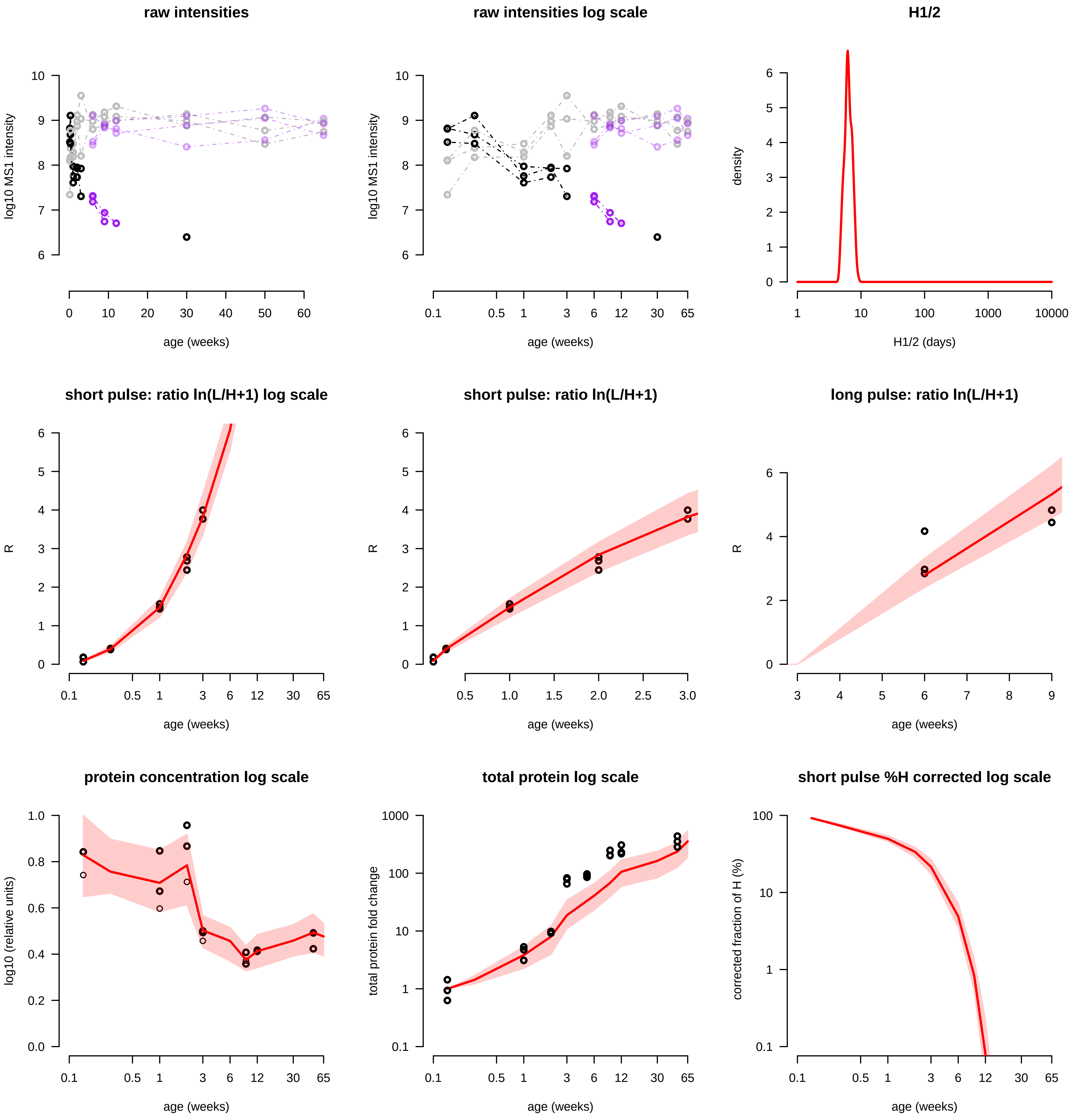

Q5SSZ5

>sp|Q5SSZ5|TENS3_MOUSE

Tensin−3

GN=Tns3

Med H1/2: 3949.2 days

Q5 H1/2: 928.7 days

Q95 H1/2: 28920.5 days

reliable model fit: yes

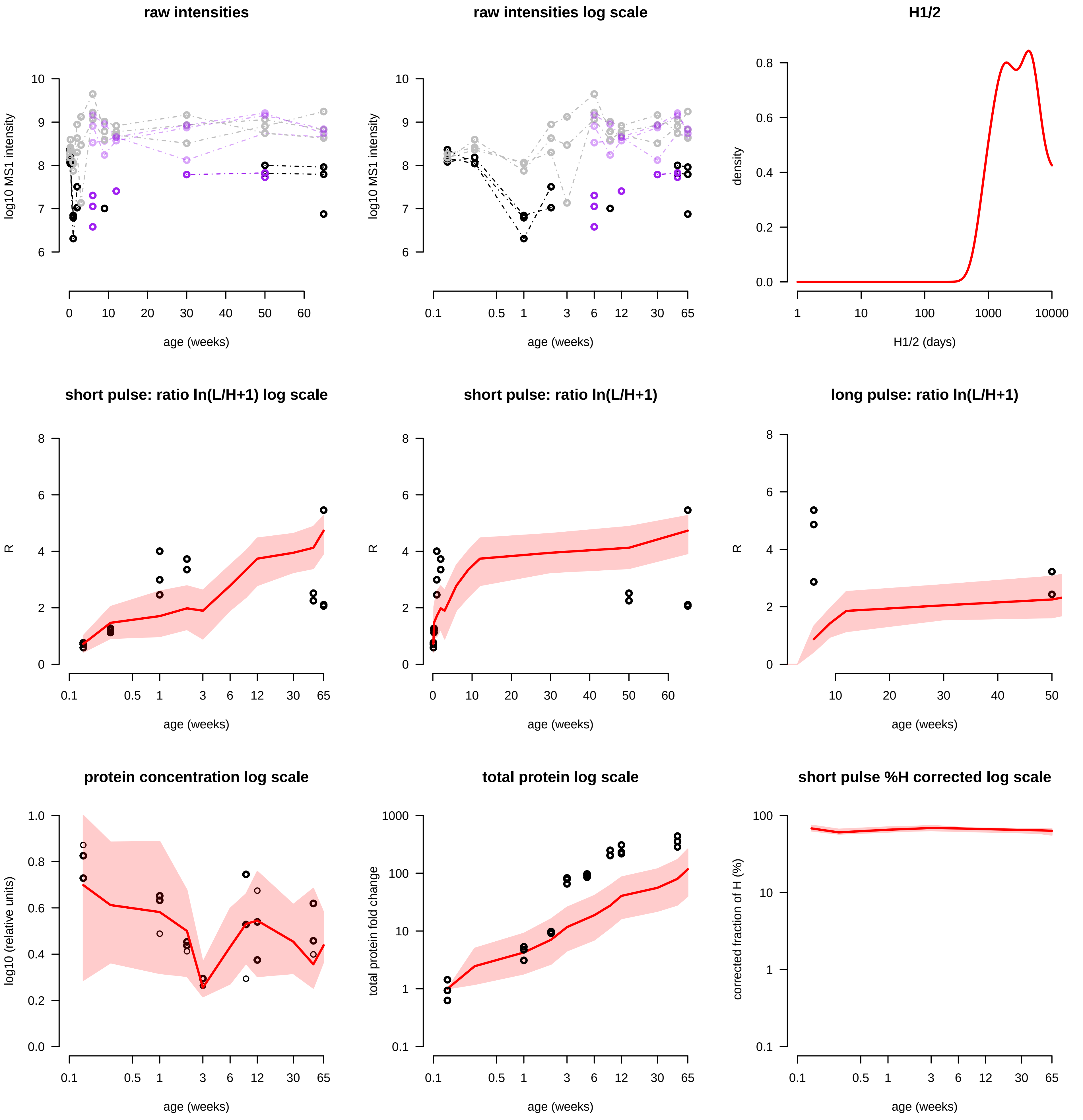

P63073

>sp|P63073|IF4E_MOUSE

Eukaryotic translation initiation factor 4E

GN=Eif4e

Med H1/2: 6.1 days

Q5 H1/2: 5 days

Q95 H1/2: 7.4 days

reliable model fit: yes

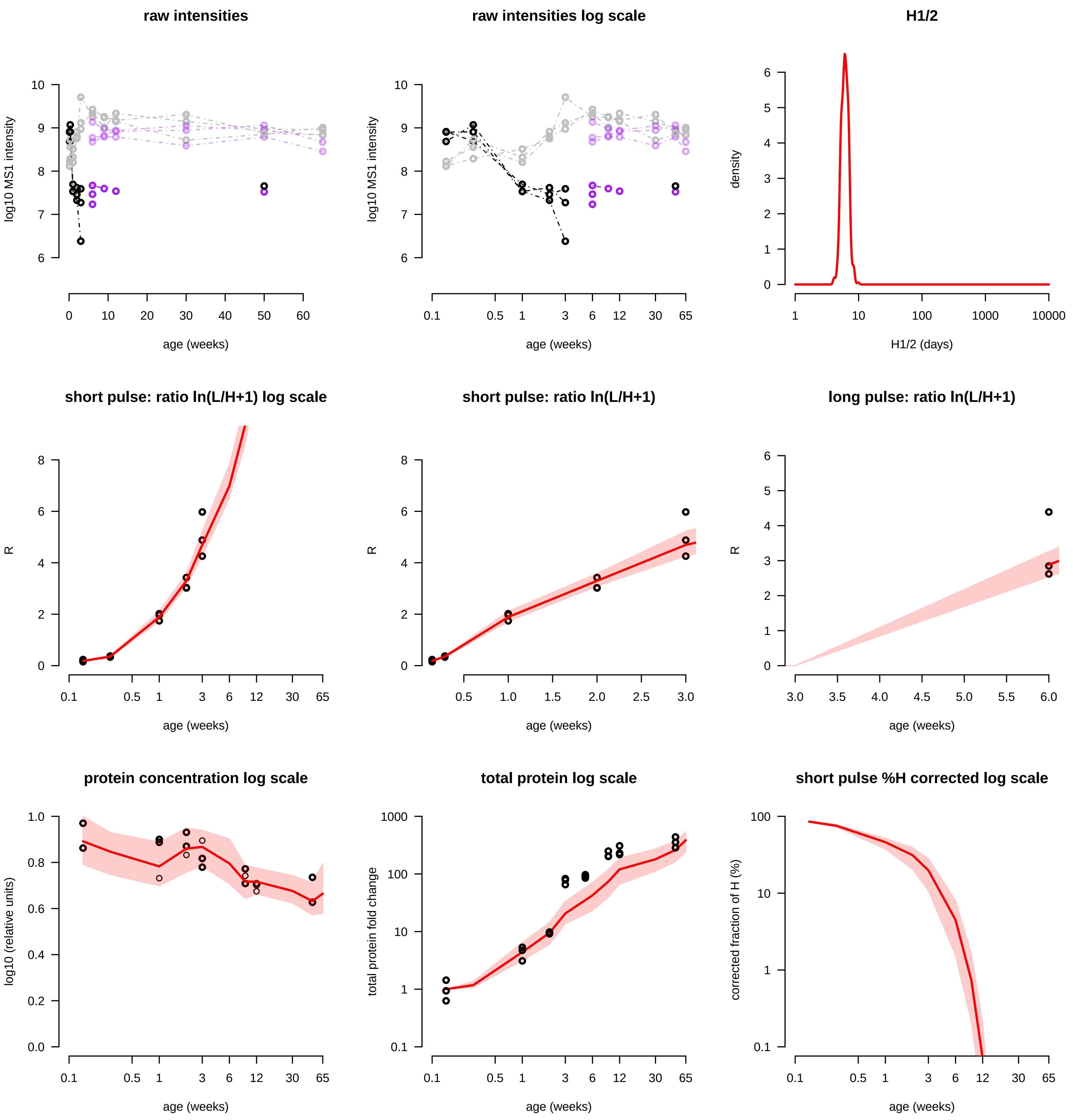

P08032

>sp|P08032|SPTA1_MOUSE

Spectrin alpha chain, erythrocytic 1

GN=Spta1

Med H1/2: 14.5 days

Q5 H1/2: 10.4 days

Q95 H1/2: 26 days

reliable model fit: yes

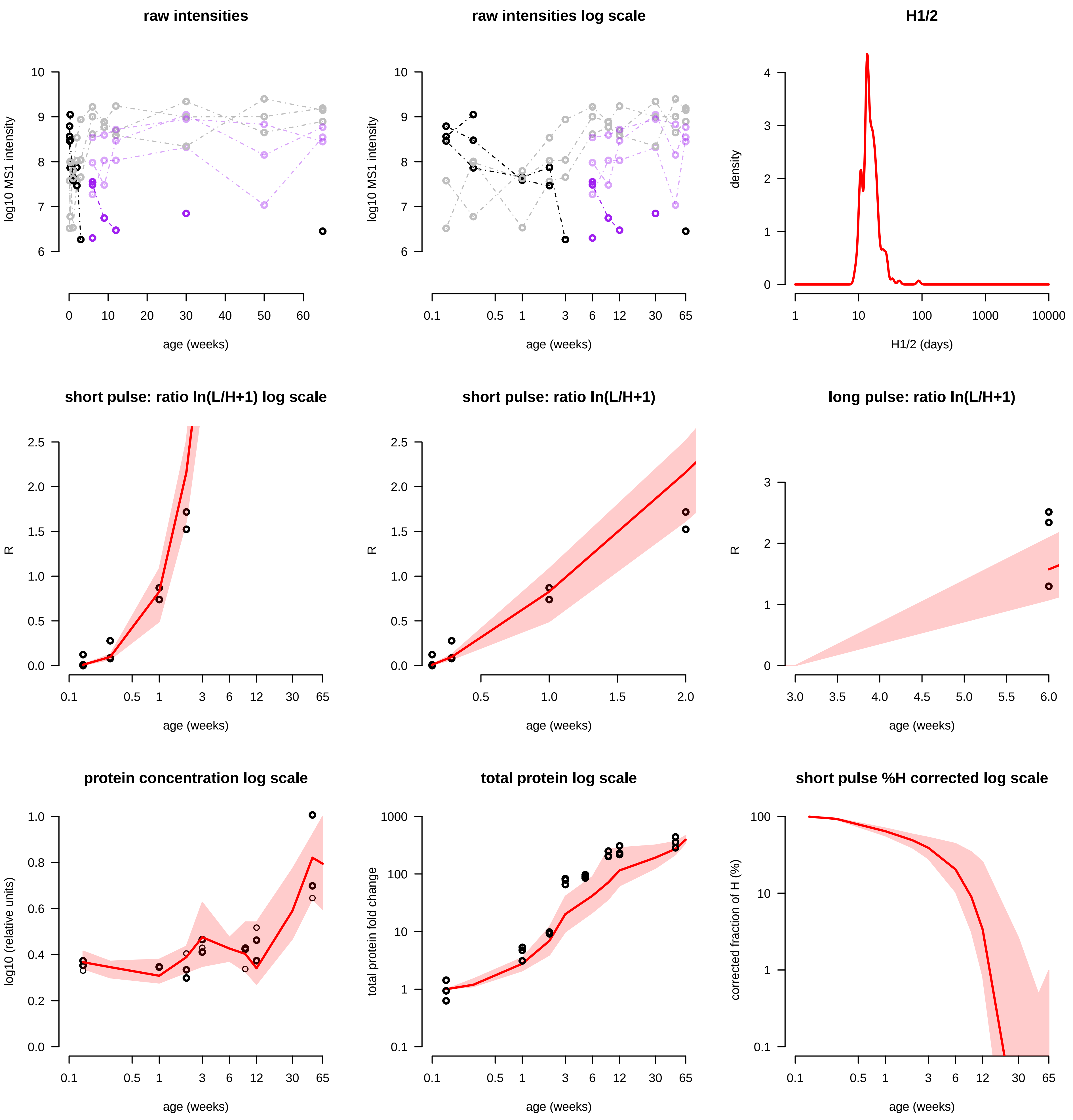

P21279

>sp|P21279|GNAQ_MOUSE

Guanine nucleotide−binding protein G(q) subunit alpha

GN=Gnaq

Med H1/2: 5.5 days

Q5 H1/2: 4.4 days

Q95 H1/2: 7.2 days

reliable model fit: yes

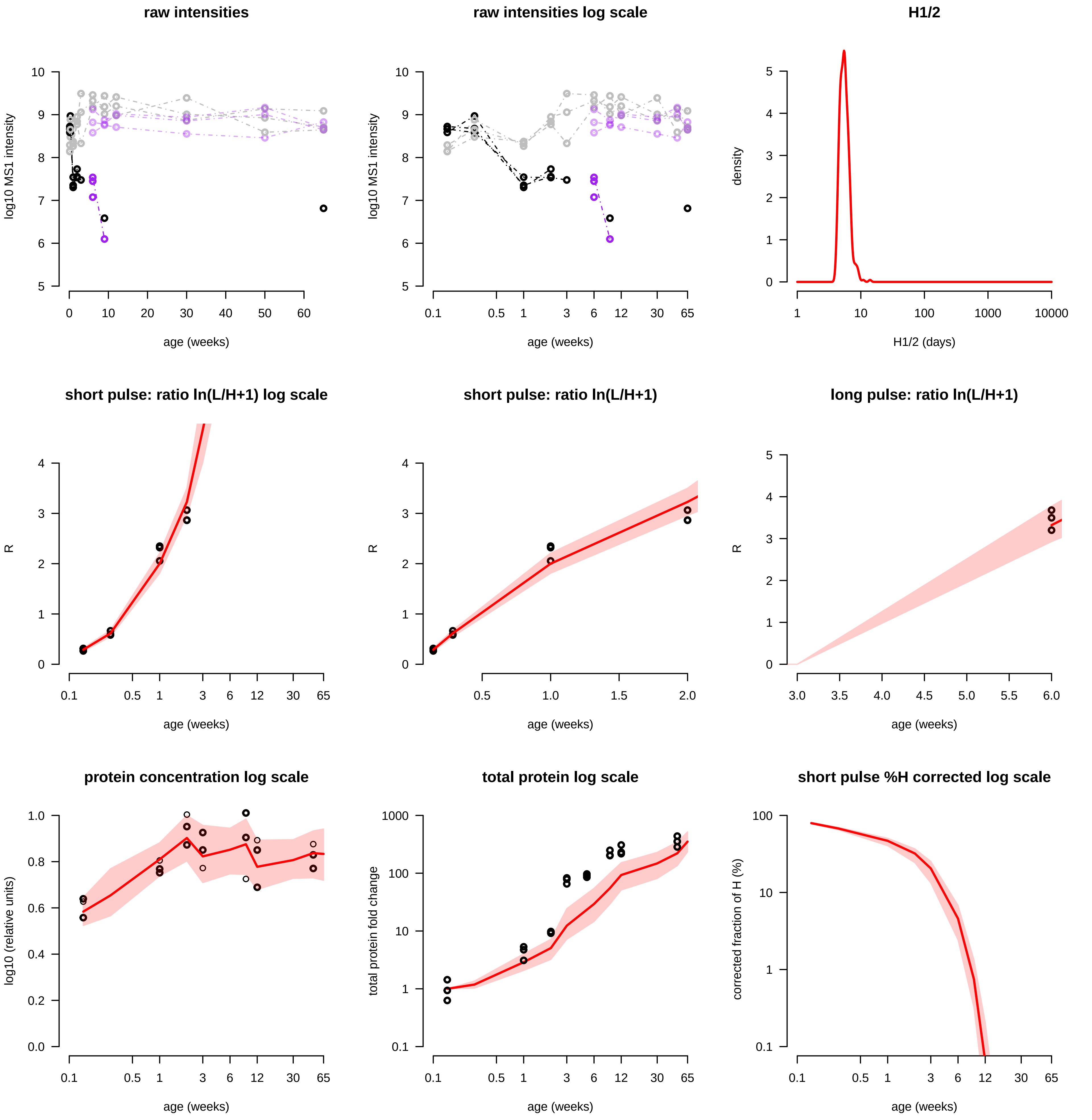

P70290

>sp|P70290|EM55_MOUSE

55 kDa erythrocyte membrane protein

GN=Mpp1

Med H1/2: 5.9 days

Q5 H1/2: 4.7 days

Q95 H1/2: 8.2 days

reliable model fit: yes

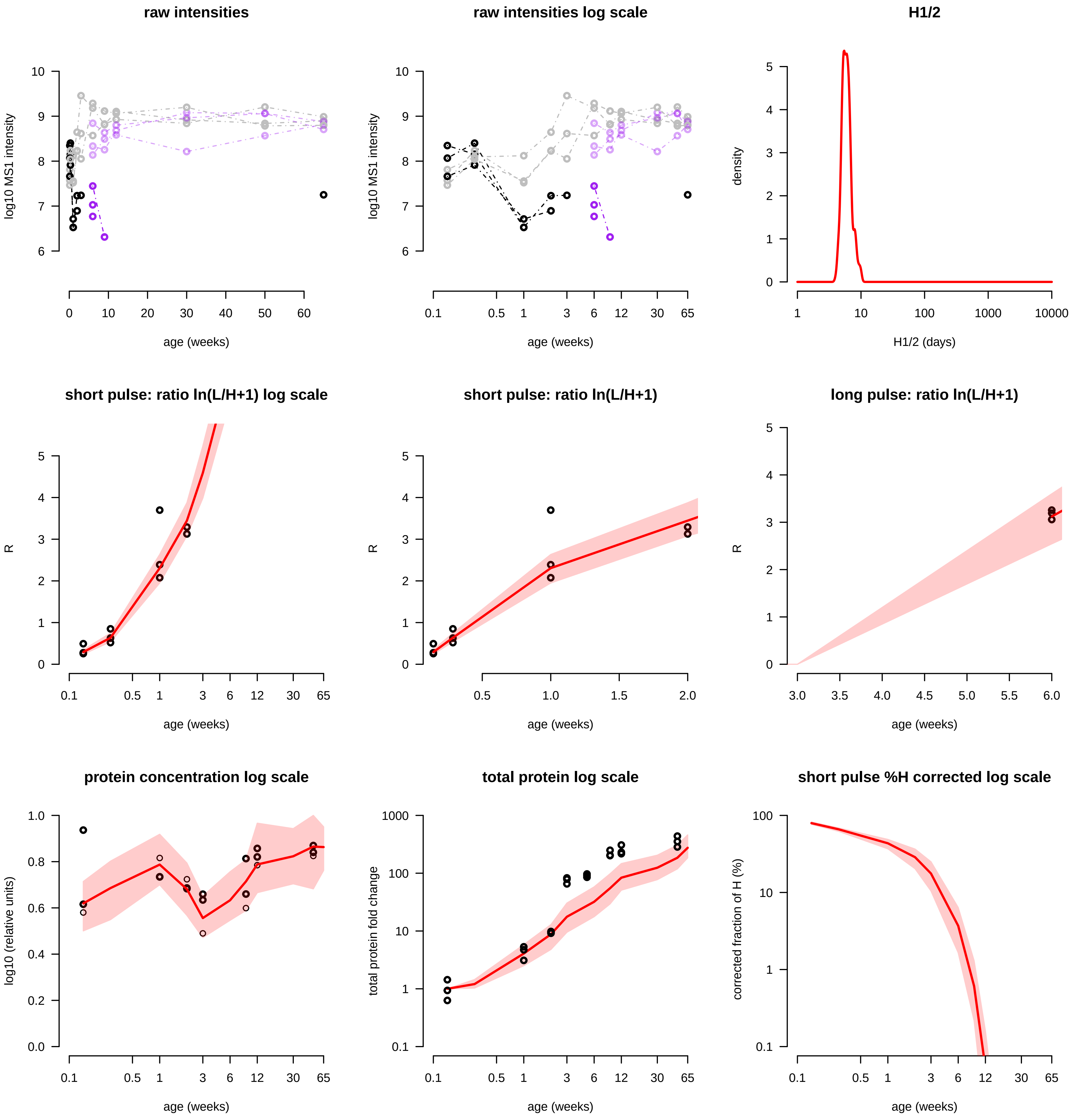

Q923T9−3

>sp|Q923T9−3|KCC2G_MOUSE

soform 3 of Calcium/calmodulin−dependent protein kinase type II subunit gamm

GN=Camk2g

Med H1/2: 4.5 days

Q5 H1/2: 3.2 days

Q95 H1/2: 7.2 days

reliable model fit: yes

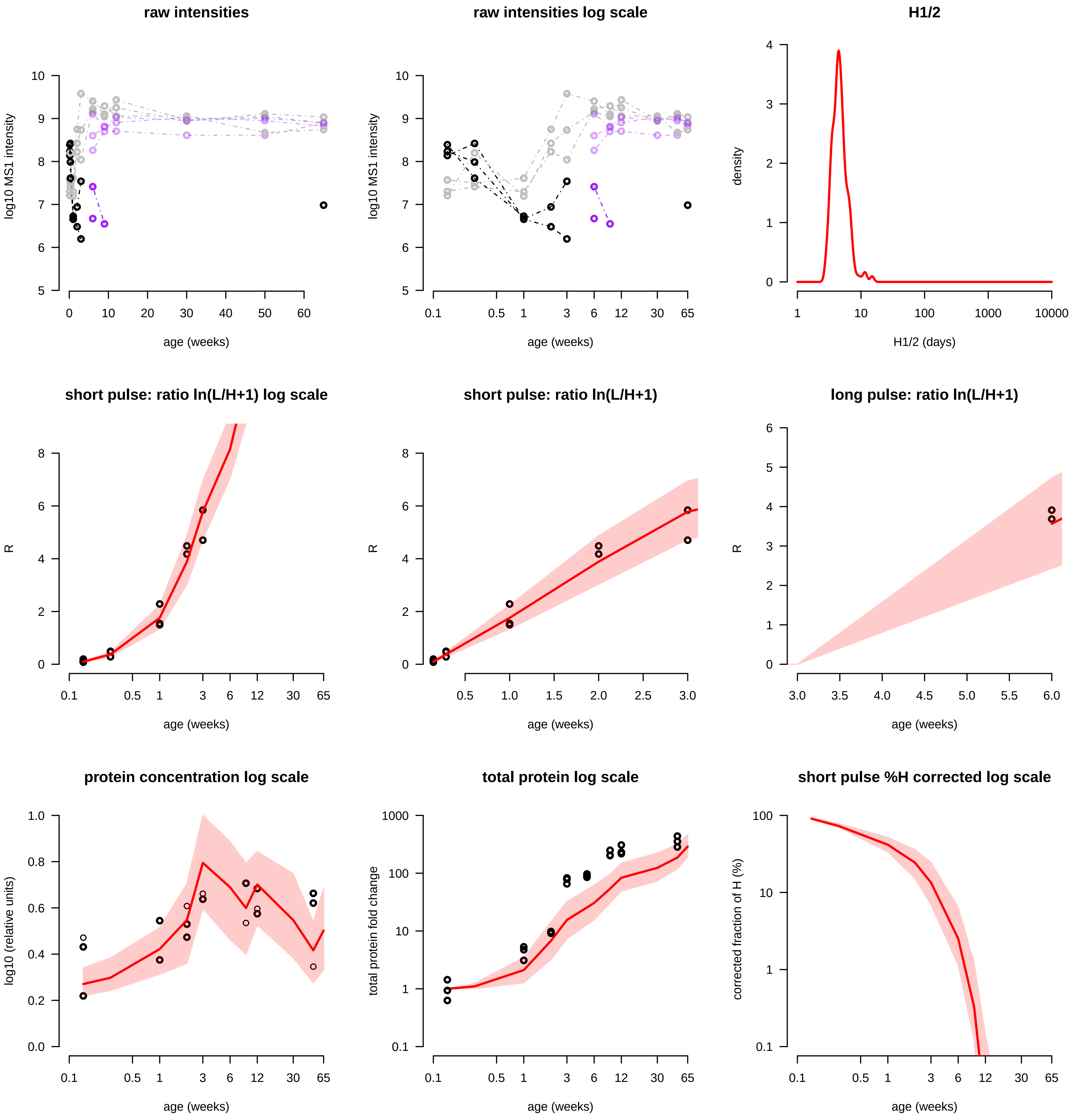

Q8VHE0

>sp|Q8VHE0|SEC63_MOUSE

Translocation protein SEC63 homolog

GN=Sec63

Med H1/2: 12 days

Q5 H1/2: 6.5 days

Q95 H1/2: 73.1 days

reliable model fit: yes

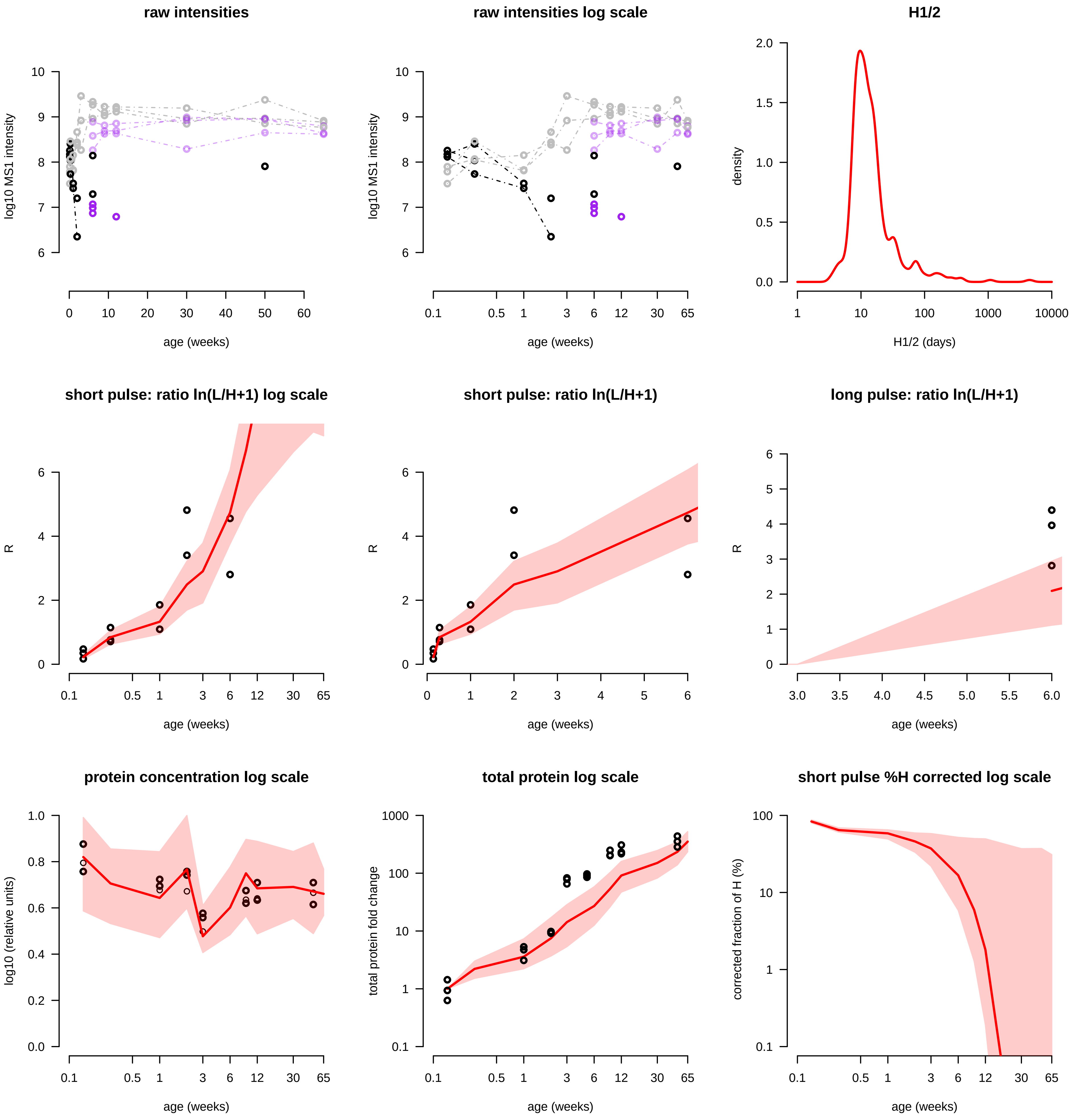

Q9R0X4;Q32MW3

>sp|Q9R0X4|ACOT9_MOUSE

Acyl−coenzyme A thioesterase 9, mitochondrial

GN=Acot9

Med H1/2: 4.7 days

Q5 H1/2: 3.4 days

Q95 H1/2: 6.2 days

reliable model fit: yes

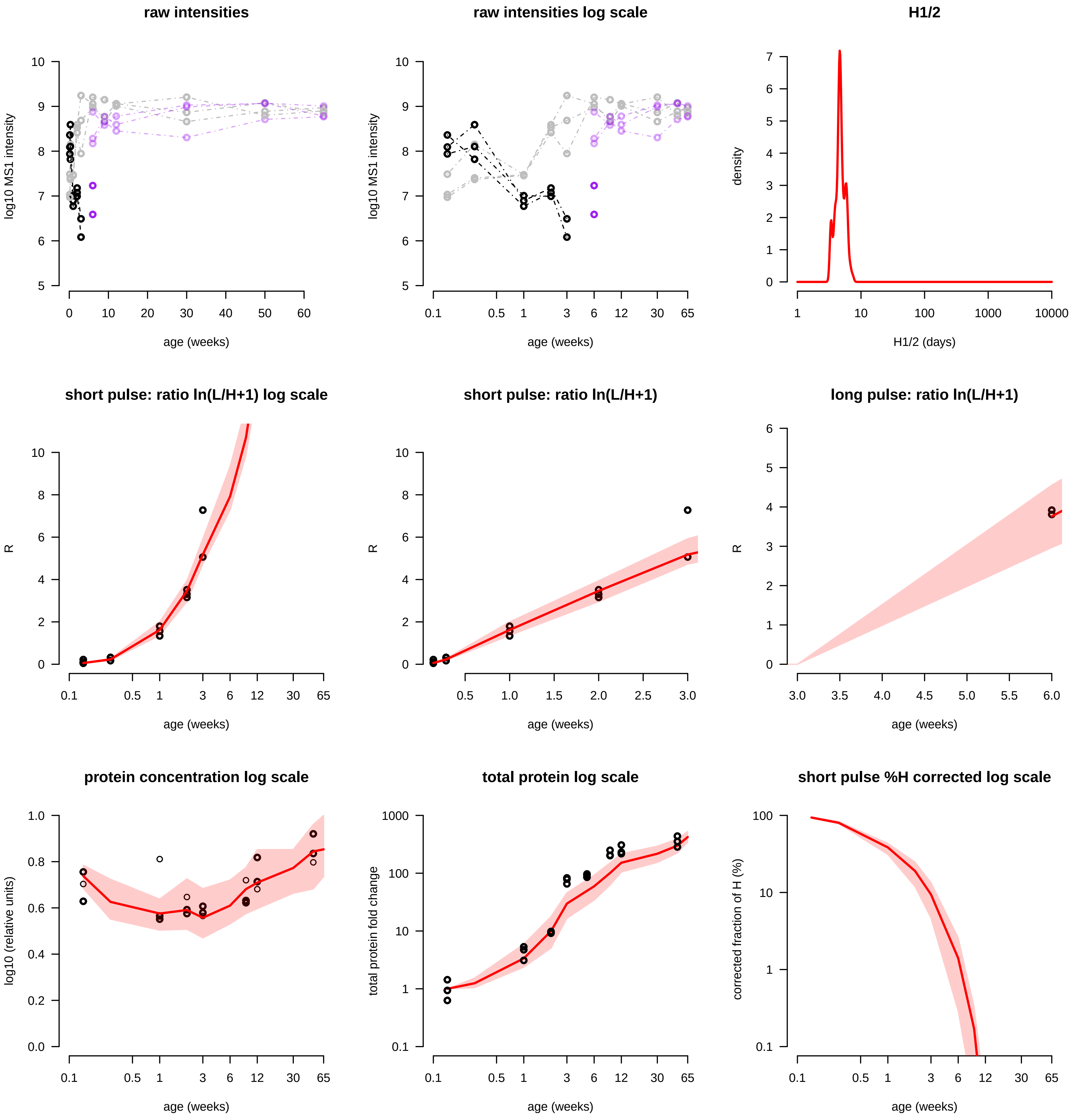

Q9CQE8

>sp|Q9CQE8|RTRAF_MOUSE

RNA transcription, translation and transport factor protein

GN=RTRAF

Med H1/2: 5.1 days

Q5 H1/2: 4.1 days

Q95 H1/2: 6.6 days

reliable model fit: yes

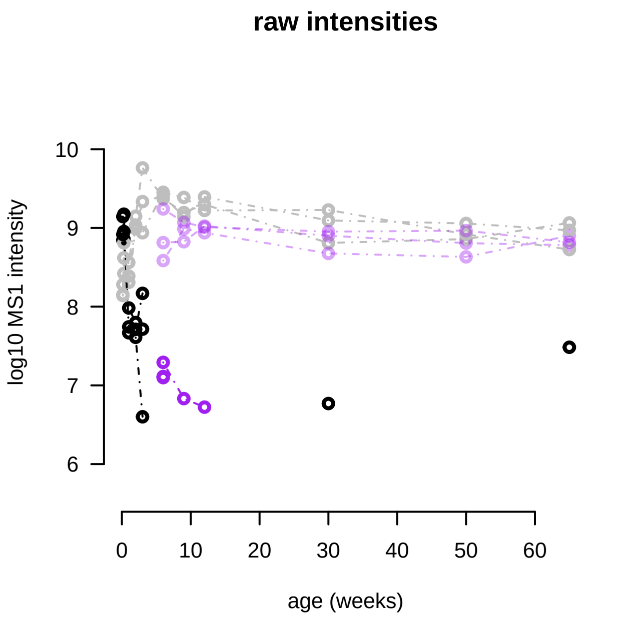

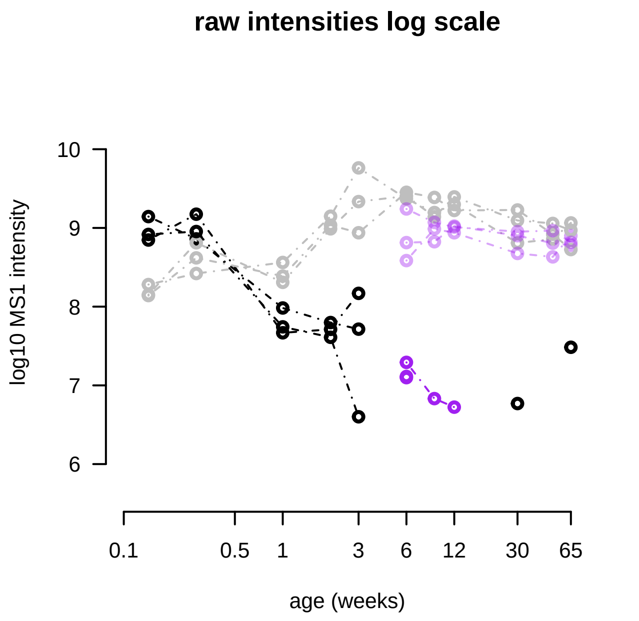

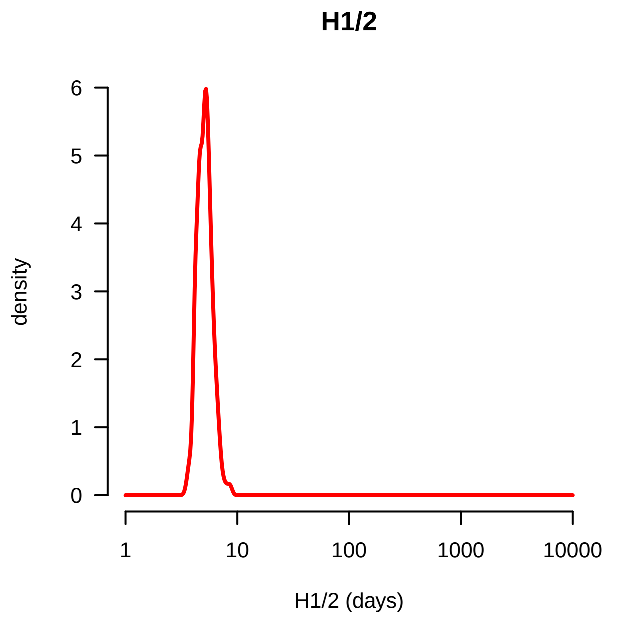

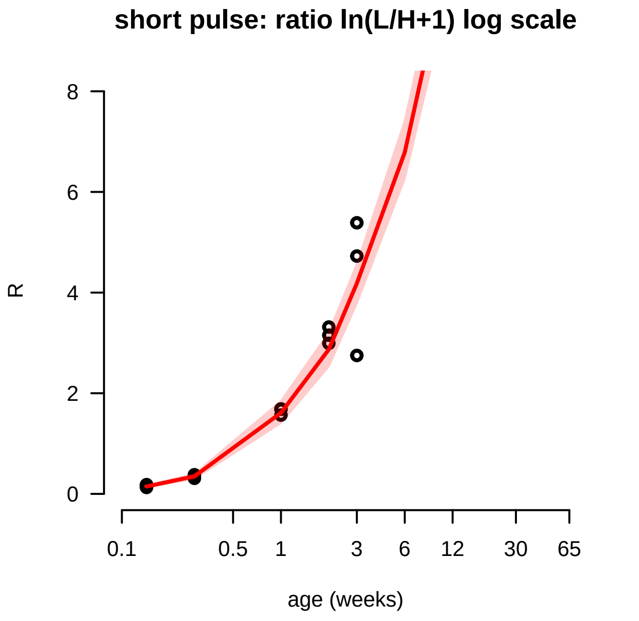

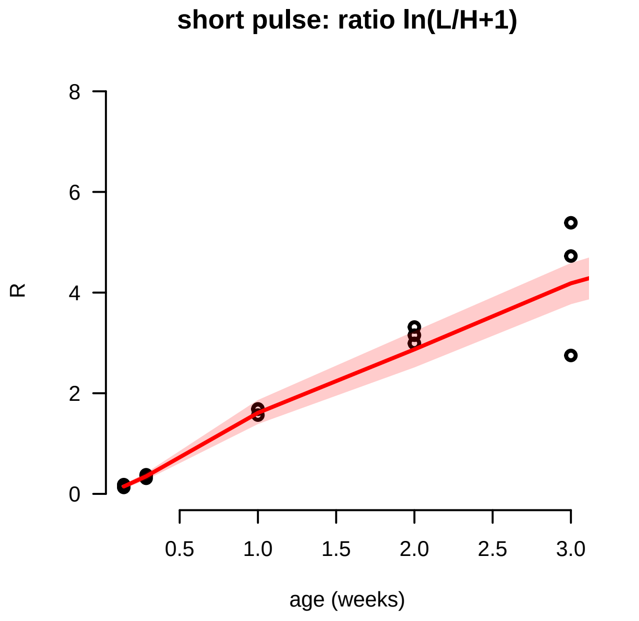

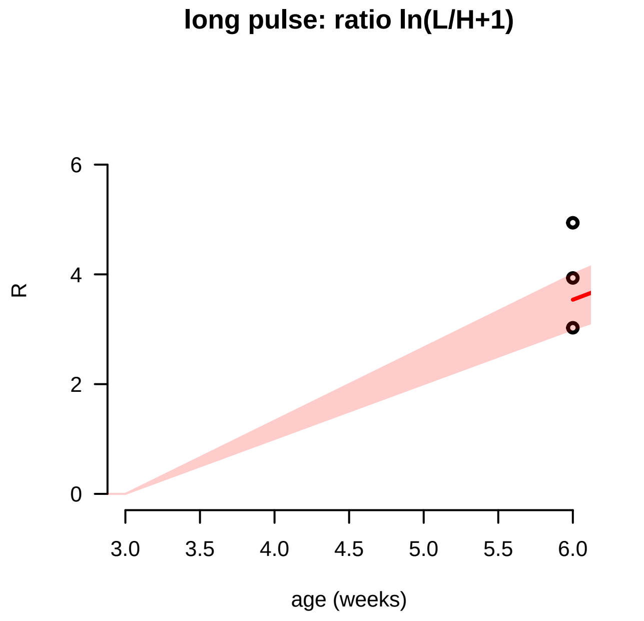

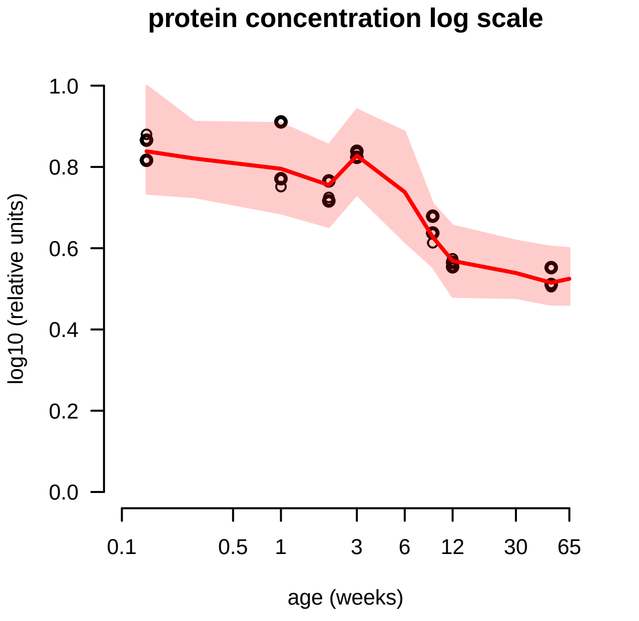

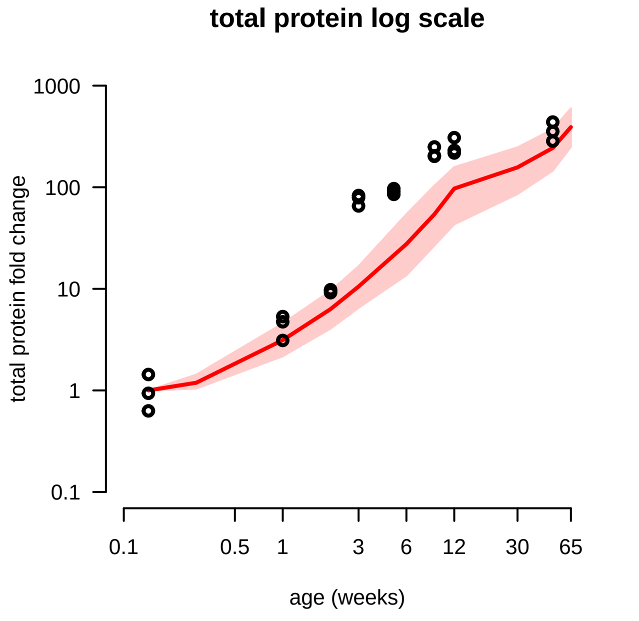

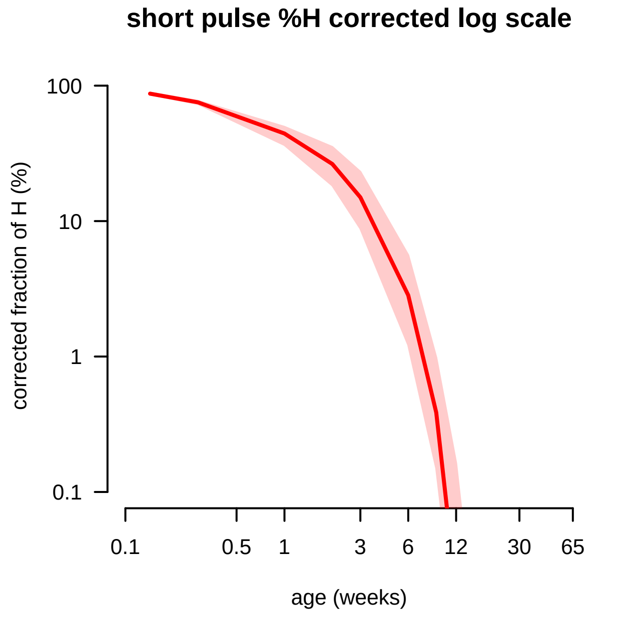

P35279−2

>sp|P35279−2|RAB6A_MOUSE

Isoform 2 of Ras−related protein Rab−6A

GN=Rab6a

Med H1/2: 4.7 days

Q5 H1/2: 4 days

Q95 H1/2: 6.1 days

reliable model fit: yes

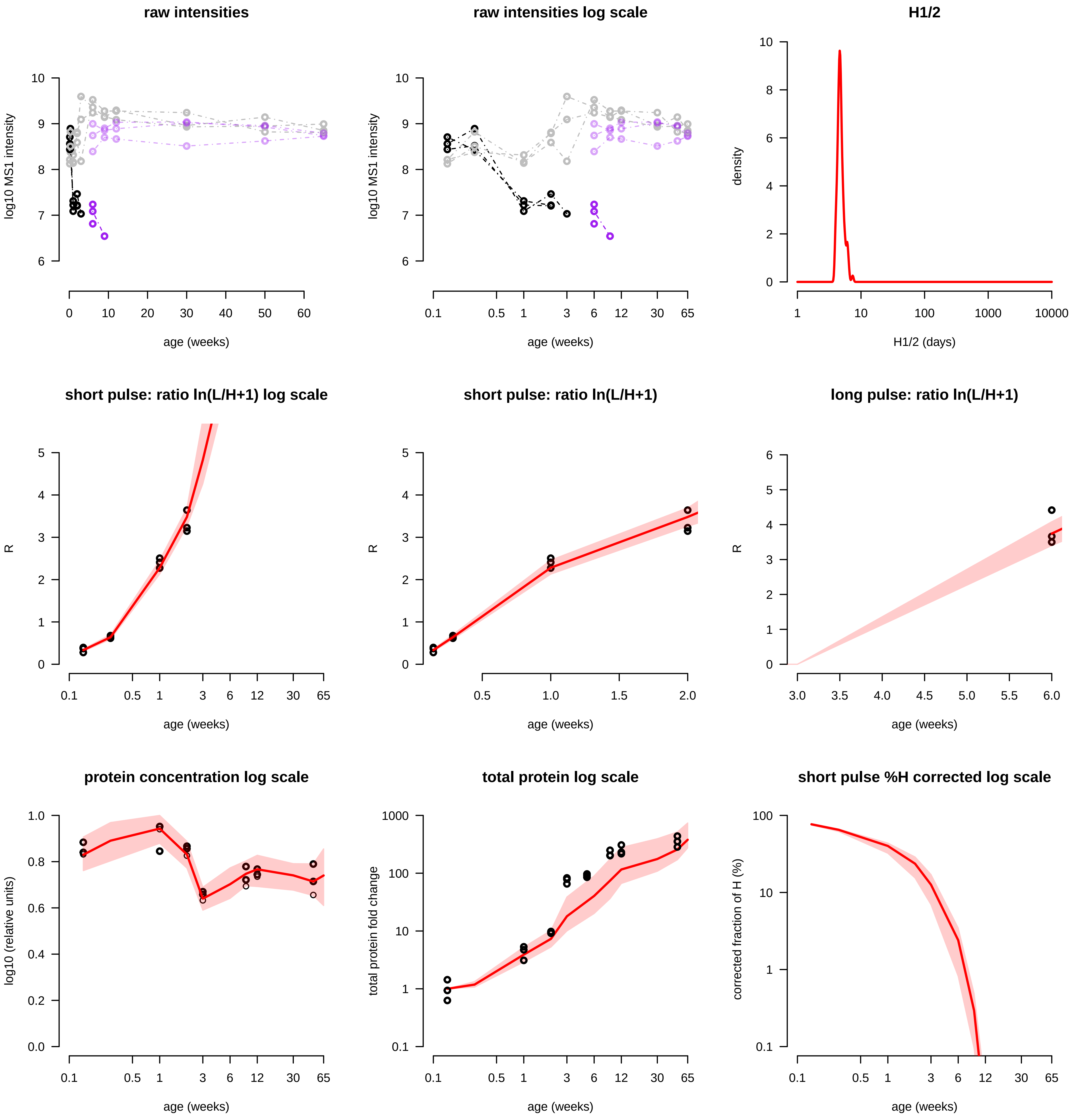

Q8BXZ1

>sp|Q8BXZ1|TMX3_MOUSE

Protein disulfide−isomerase TMX3

GN=Tmx3

Med H1/2: 158.7 days

Q5 H1/2: 72 days

Q95 H1/2: 2661.3 days

reliable model fit: no

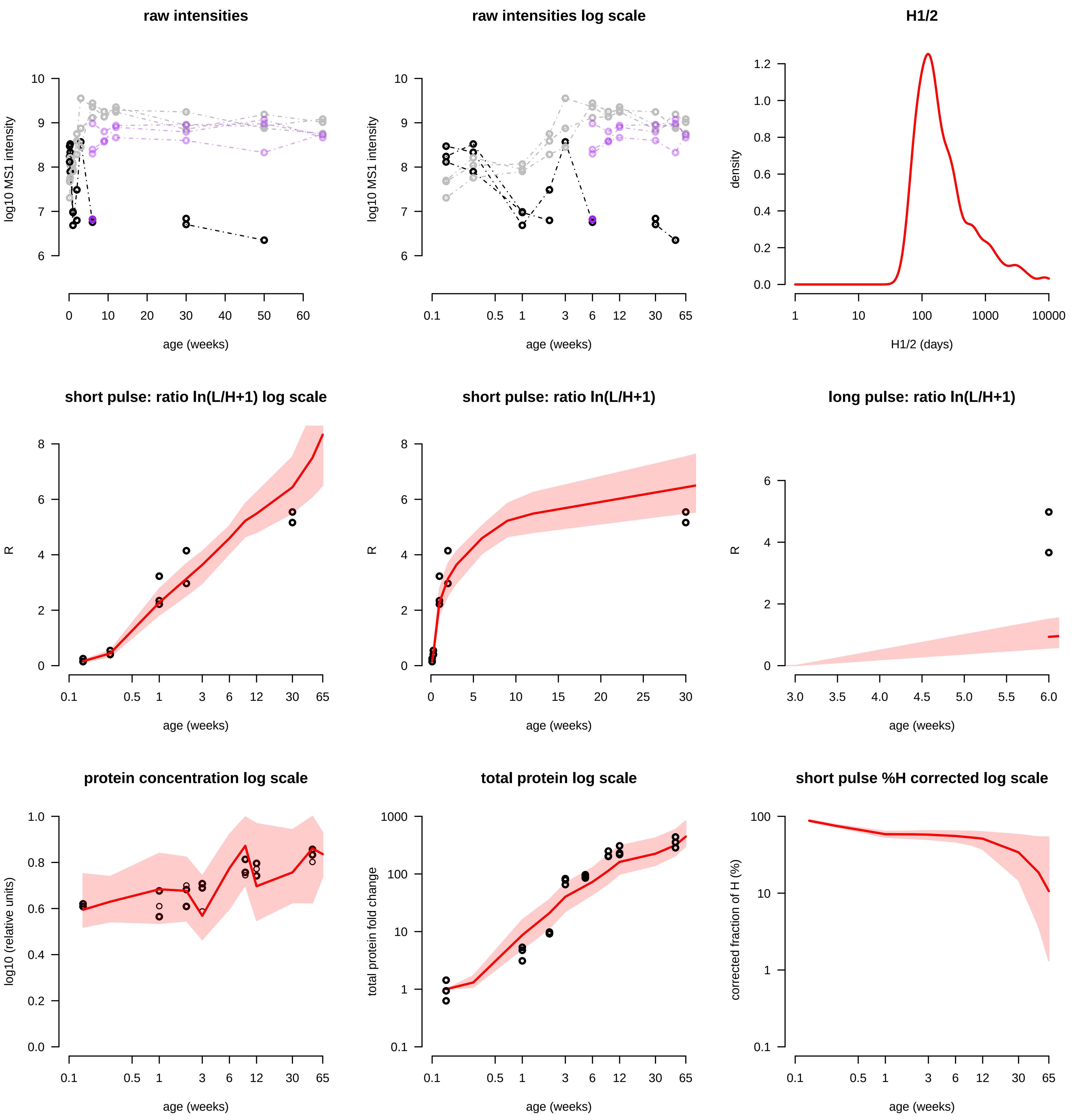

O35326

>sp|O35326|SRSF5_MOUSE

Serine/arginine−rich splicing factor 5

GN=Srsf5

Med H1/2: 7 days

Q5 H1/2: 6 days

Q95 H1/2: 9 days

reliable model fit: yes

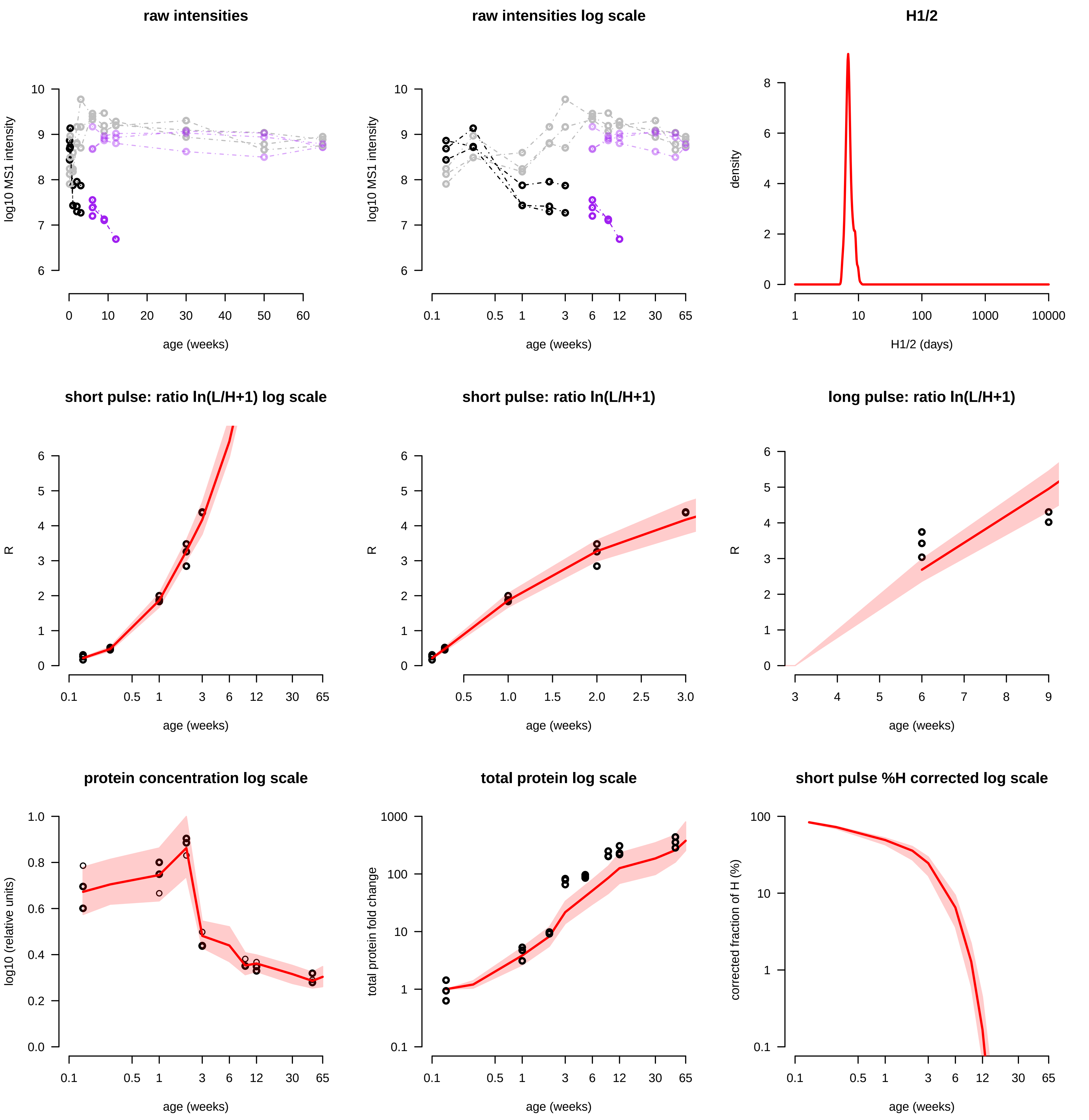

O35295

>sp|O35295|PURB_MOUSE

Transcriptional activator protein Pur−beta

GN=Purb

Med H1/2: 15.8 days

Q5 H1/2: 12.3 days

Q95 H1/2: 21.9 days

reliable model fit: yes

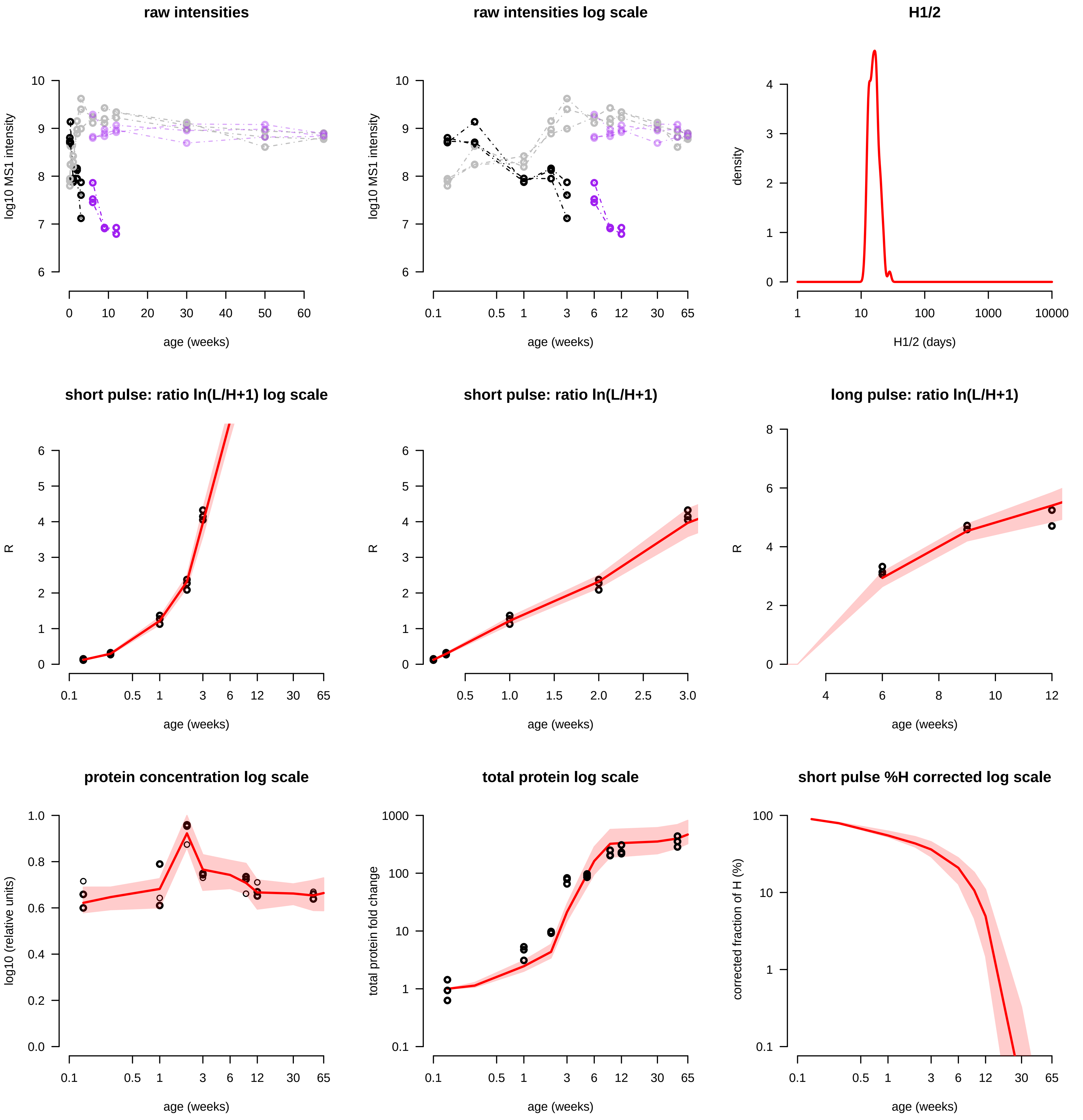

Q9CQU3

>sp|Q9CQU3|RER1_MOUSE

Protein RER1

GN=Rer1

Med H1/2: 3.8 days

Q5 H1/2: 3.1 days

Q95 H1/2: 4.8 days

reliable model fit: yes

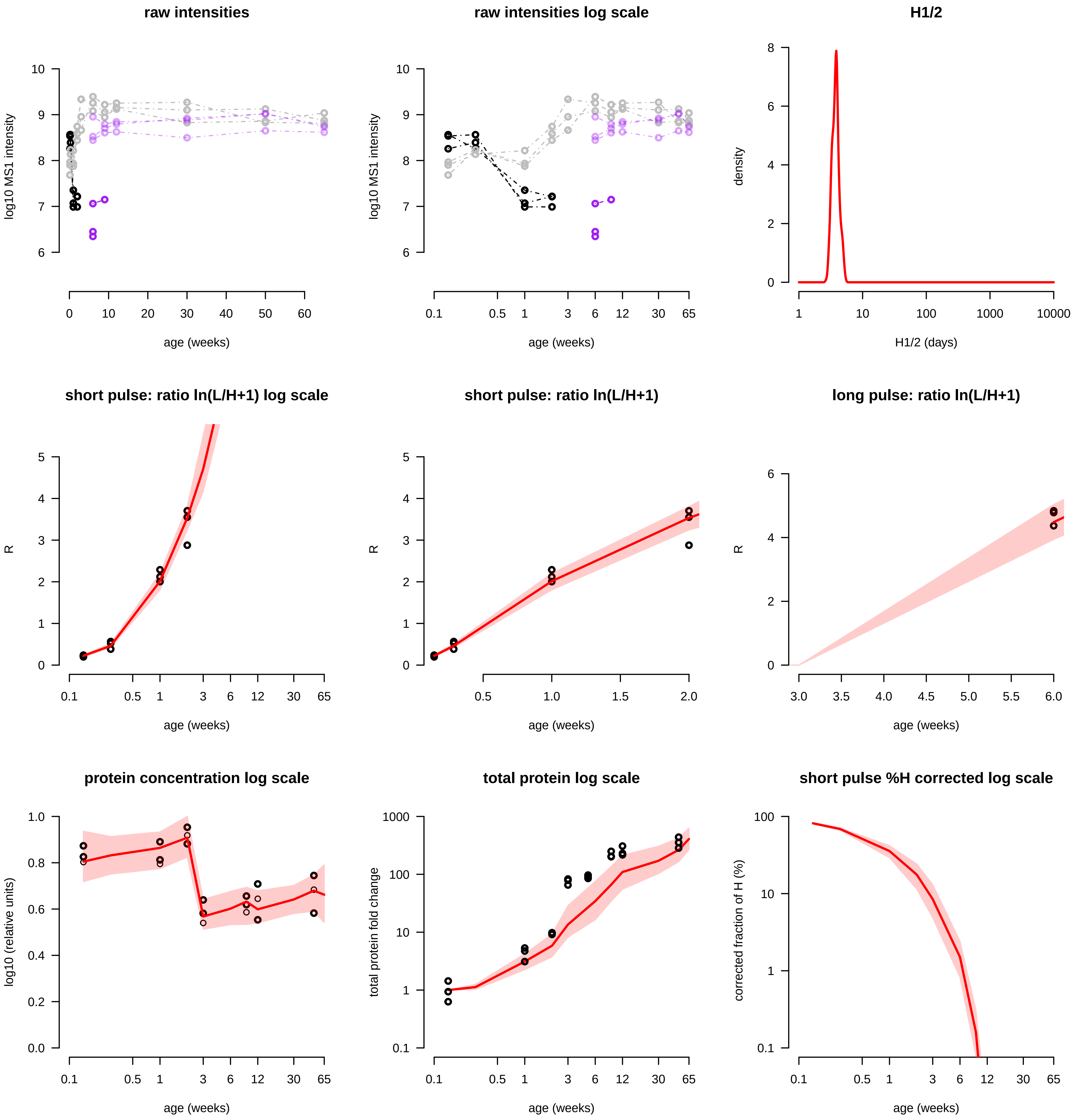

Q9CQ60

>sp|Q9CQ60|6PGL_MOUSE

6−phosphogluconolactonase

GN=Pgls

Med H1/2: 11.1 days

Q5 H1/2: 8.5 days

Q95 H1/2: 18.1 days

reliable model fit: yes

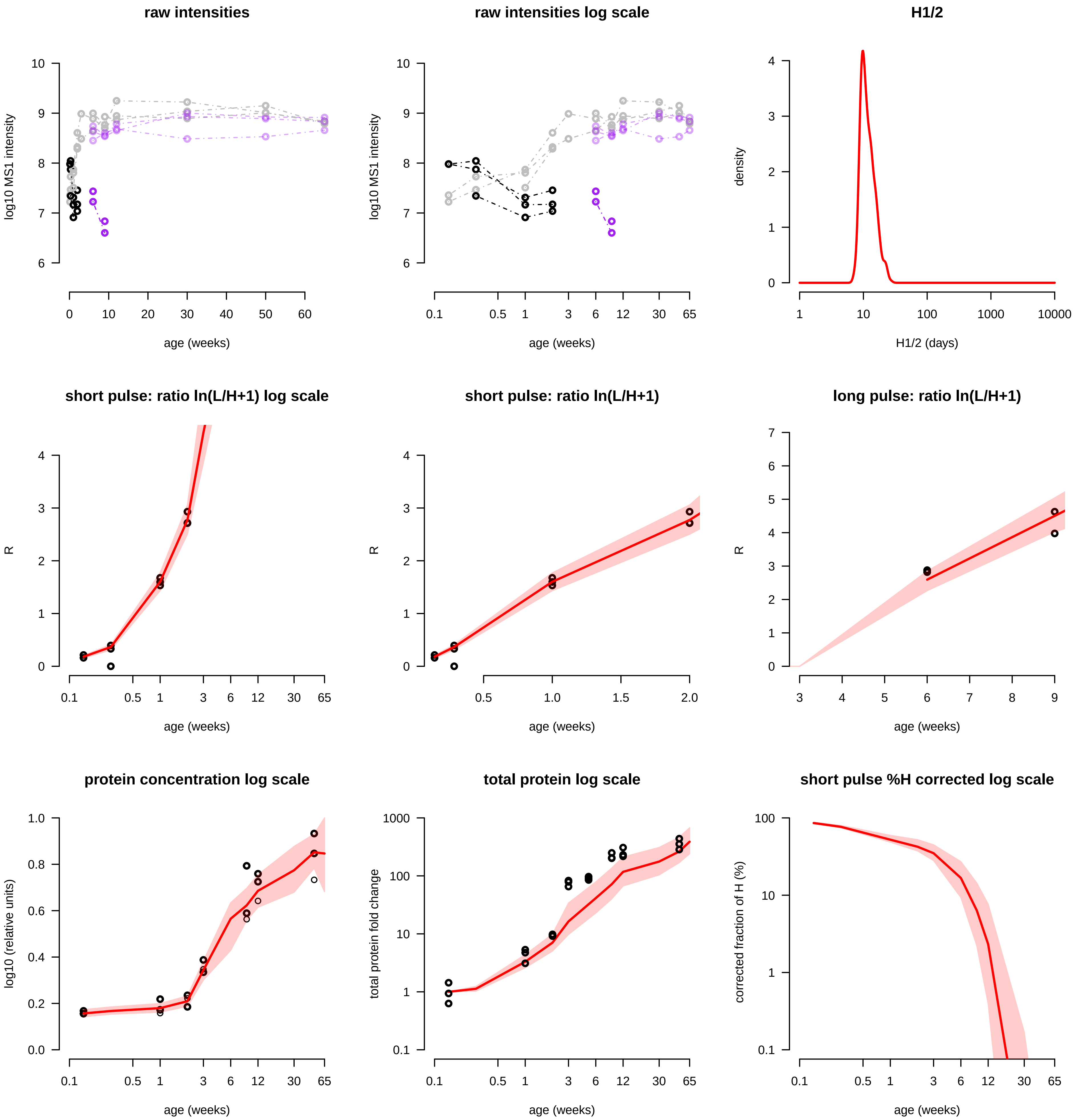

Q9DB73;Q9DB73−2

>sp|Q9DB73|NB5R1_MOUSE

NADH−cytochrome b5 reductase 1

GN=Cyb5r1

Med H1/2: 8.1 days

Q5 H1/2: 5.4 days

Q95 H1/2: 15.8 days

reliable model fit: yes

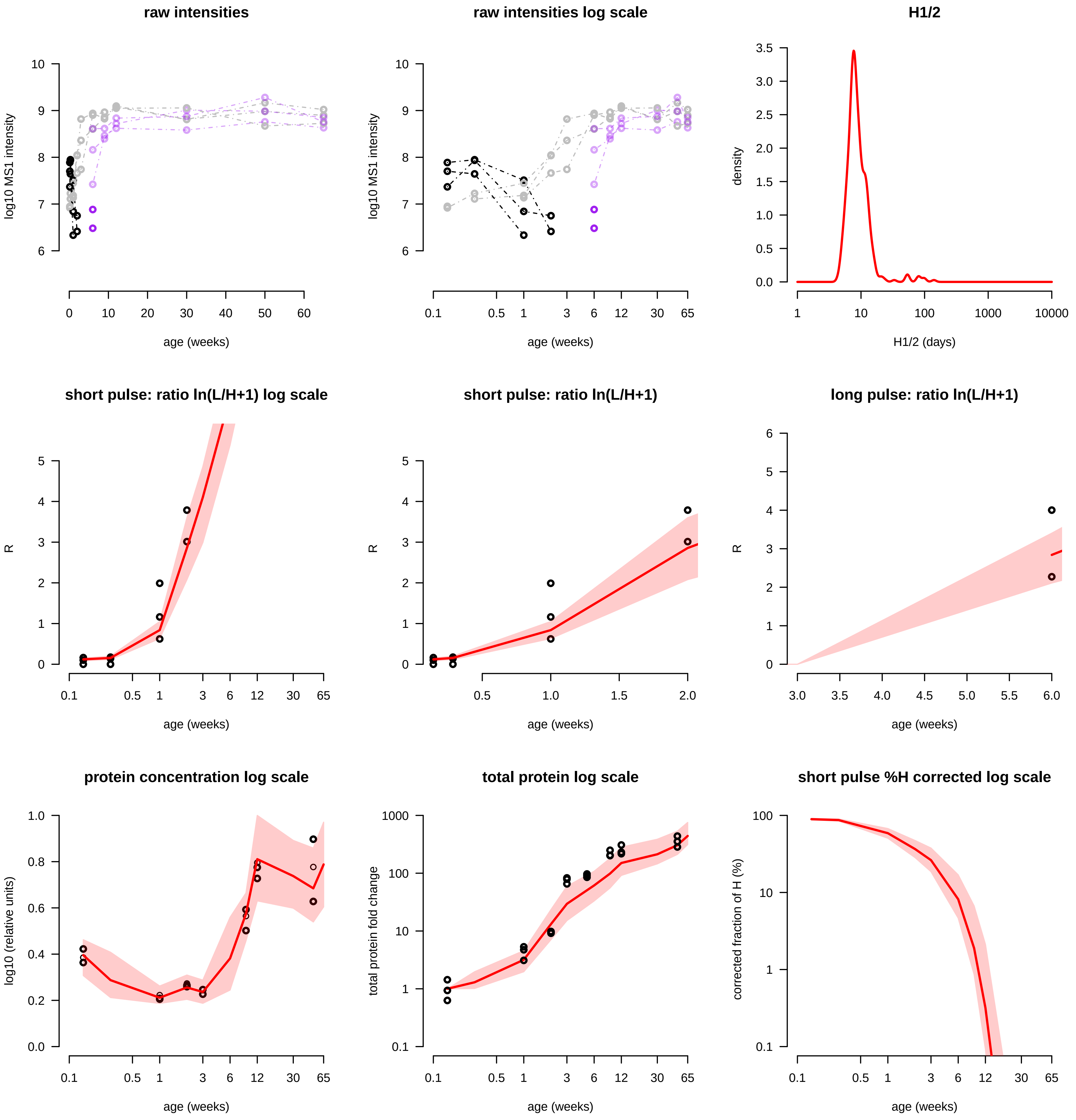

Q8BJ71;Q8BJ71−2

>sp|Q8BJ71|NUP93_MOUSE

Nuclear pore complex protein Nup93

GN=Nup93

Med H1/2: 10.5 days

Q5 H1/2: 7.9 days

Q95 H1/2: 16.1 days

reliable model fit: no

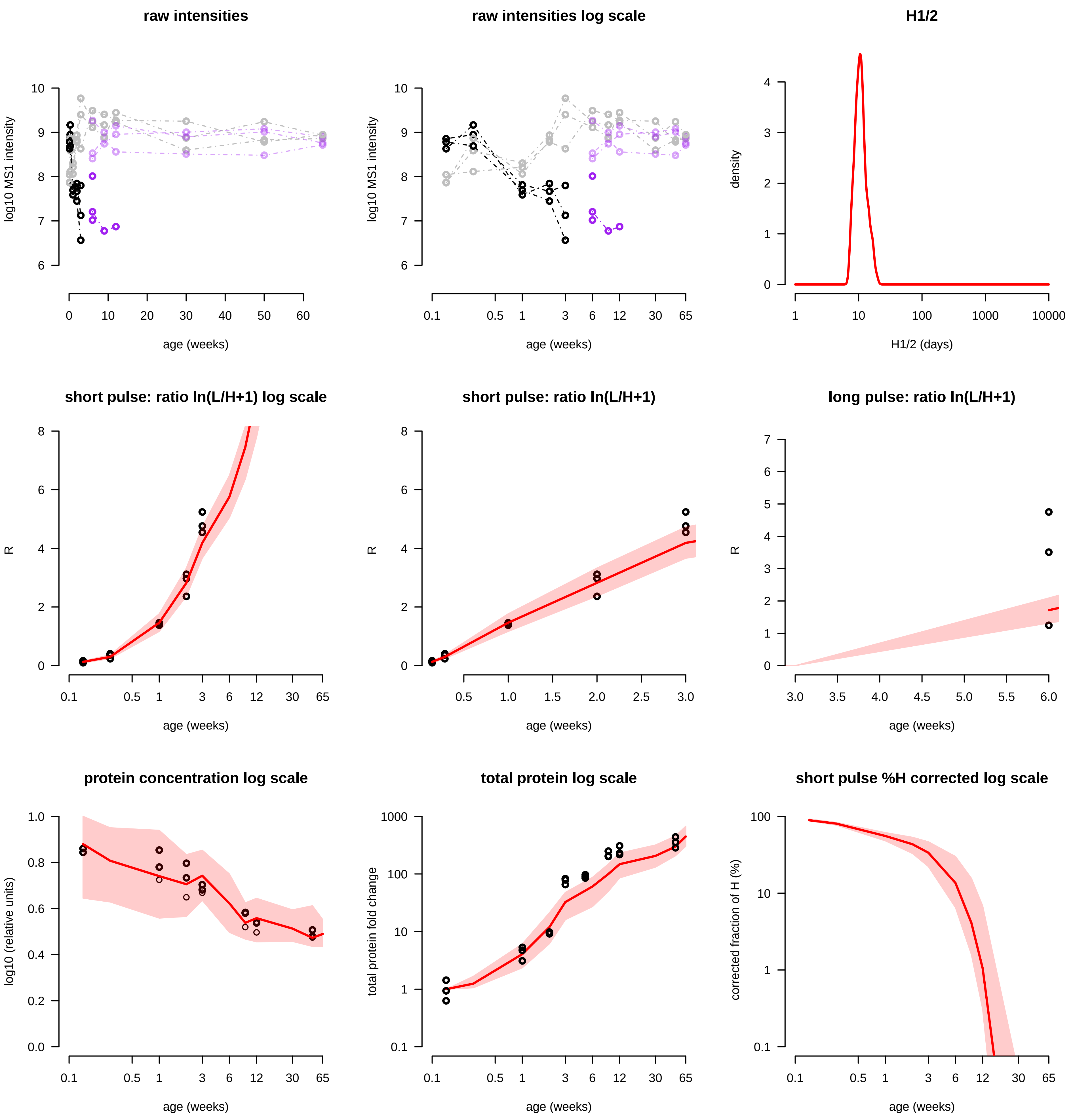

P28658

>sp|P28658|ATX10_MOUSE

Ataxin−10

GN=Atxn10

Med H1/2: 5.4 days

Q5 H1/2: 4.1 days

Q95 H1/2: 7.6 days

reliable model fit: yes

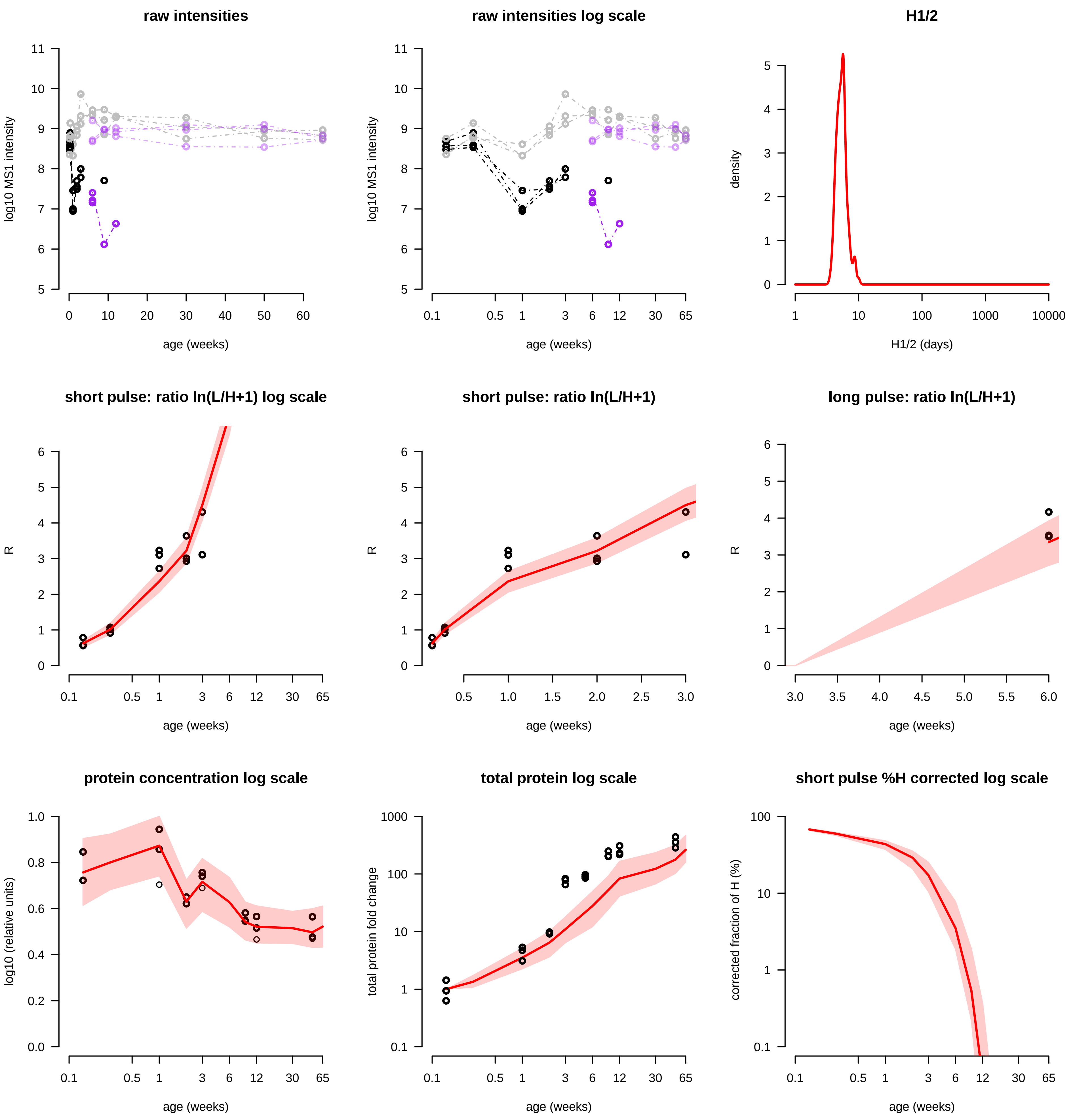

P14115

>sp|P14115|RL27A_MOUSE

60S ribosomal protein L27a

GN=Rpl27a

Med H1/2: 7.3 days

Q5 H1/2: 5.8 days

Q95 H1/2: 9.6 days

reliable model fit: yes

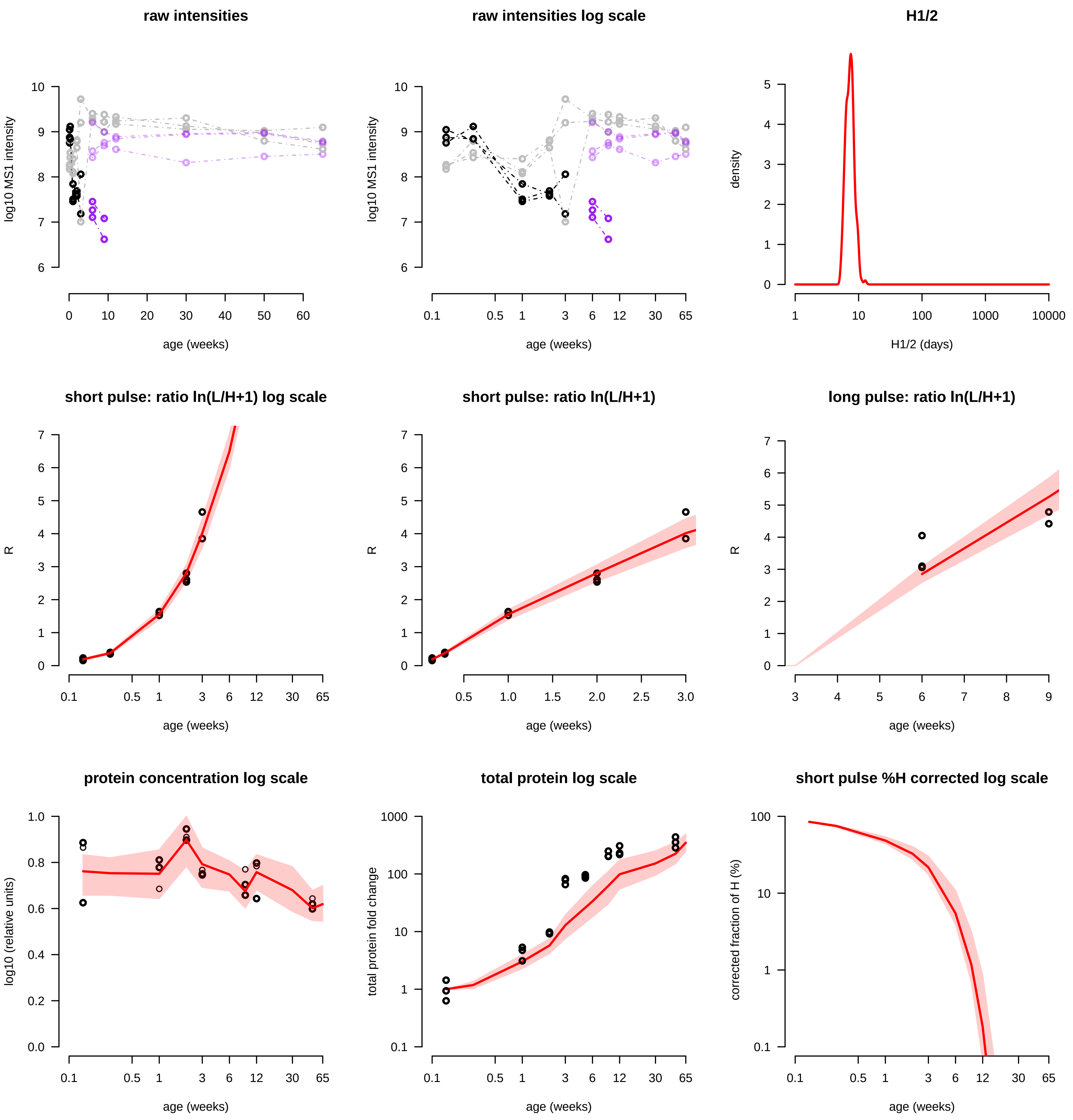

Q8K0D5

>sp|Q8K0D5|EFGM_MOUSE

Elongation factor G, mitochondrial

GN=Gfm1

Med H1/2: 4.4 days

Q5 H1/2: 3.6 days

Q95 H1/2: 5.6 days

reliable model fit: yes

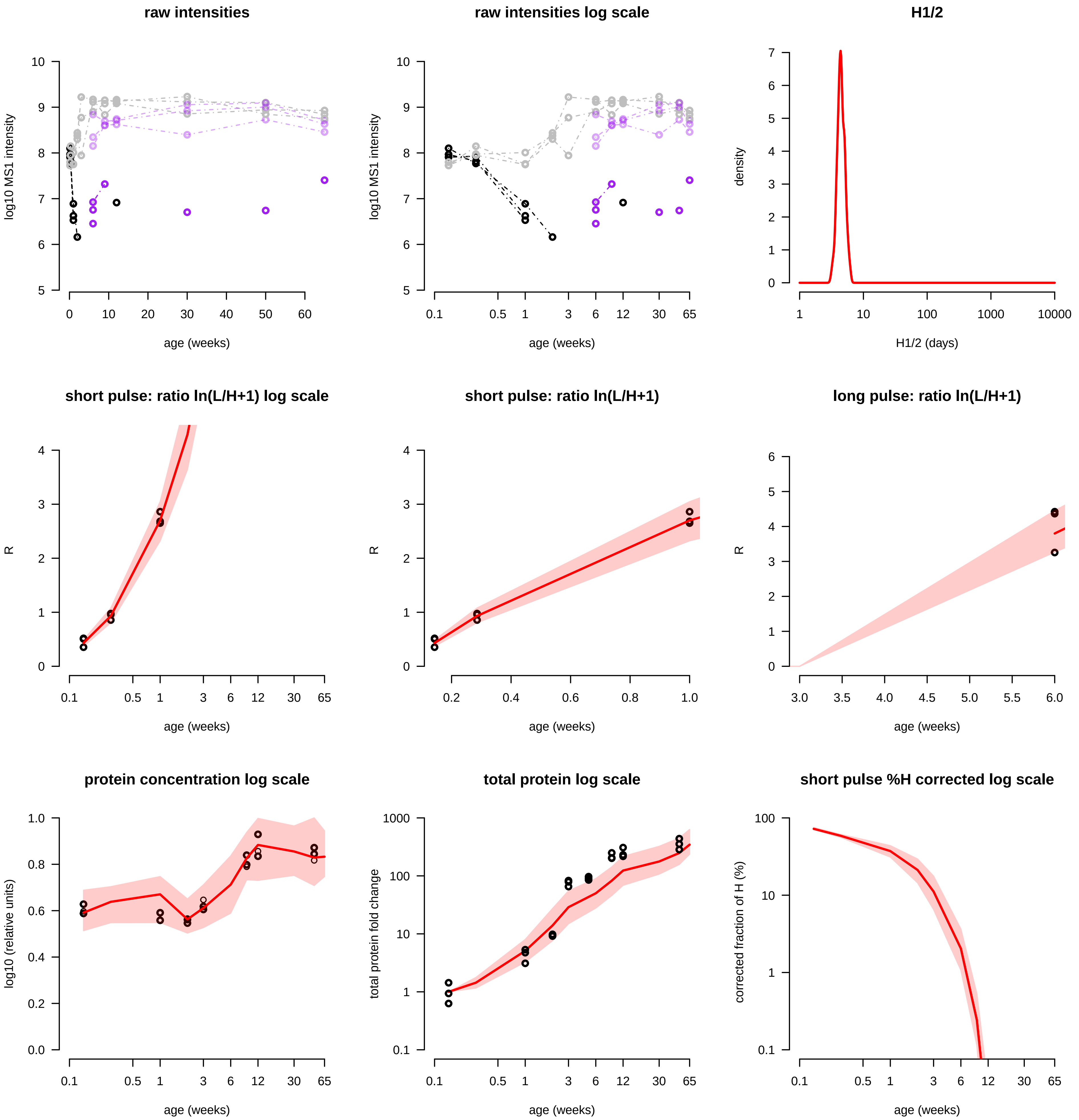

A2ASZ8−2;A2ASZ8−3;A2ASZ8;A2ASZ8−4;A2ASZ8−5

>sp|A2ASZ8−2|SCMC2_MOUSE

Isoform 2 of Calcium−binding mitochondrial carrier protein SCaMC−2

GN=Slc25a25

Med H1/2: 12.2 days

Q5 H1/2: 9 days

Q95 H1/2: 17.3 days

reliable model fit: yes

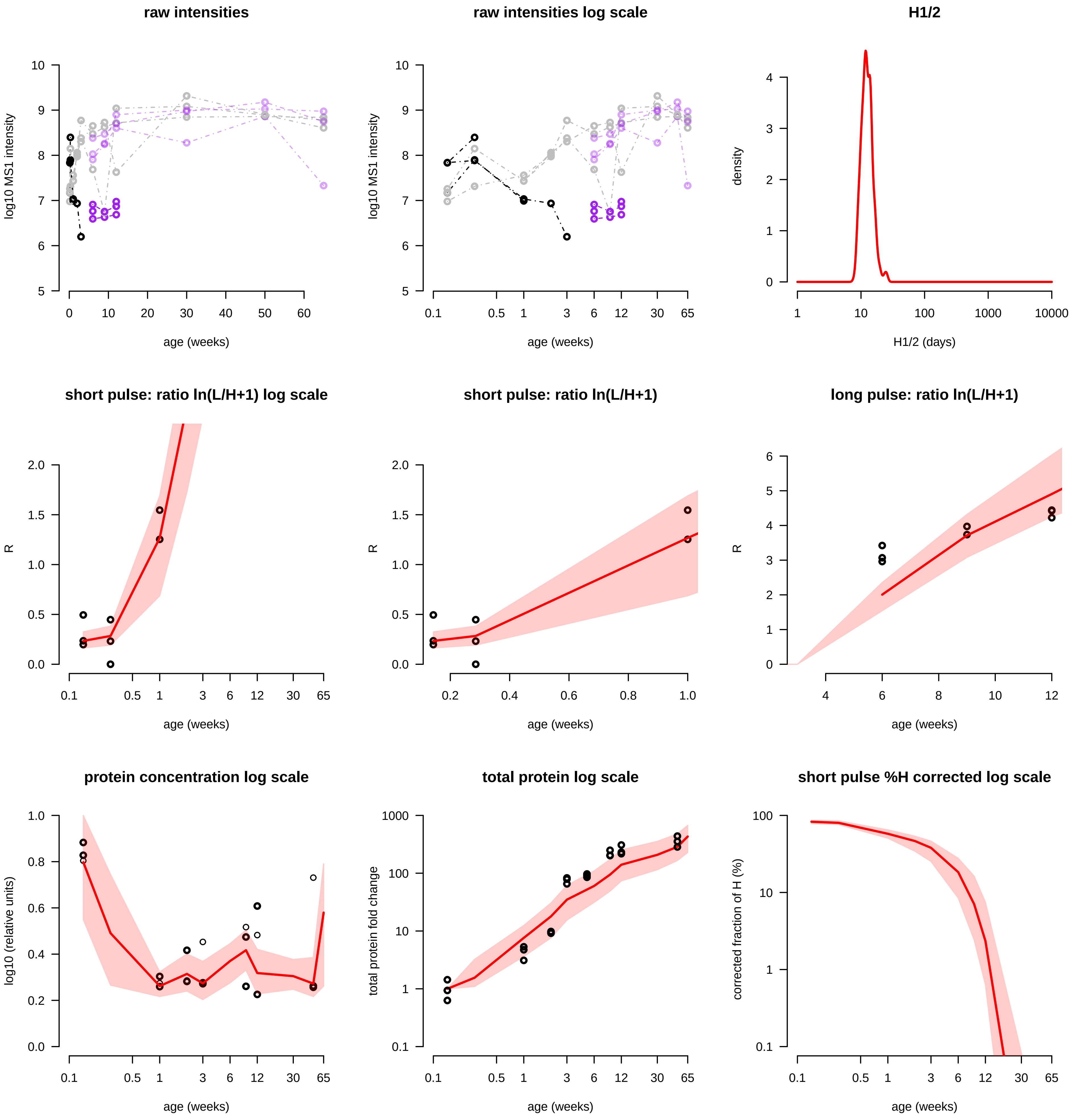

B2RY56

>sp|B2RY56|RBM25_MOUSE

RNA−binding protein 25

GN=Rbm25

Med H1/2: 3.9 days

Q5 H1/2: 3.3 days

Q95 H1/2: 4.7 days

reliable model fit: yes

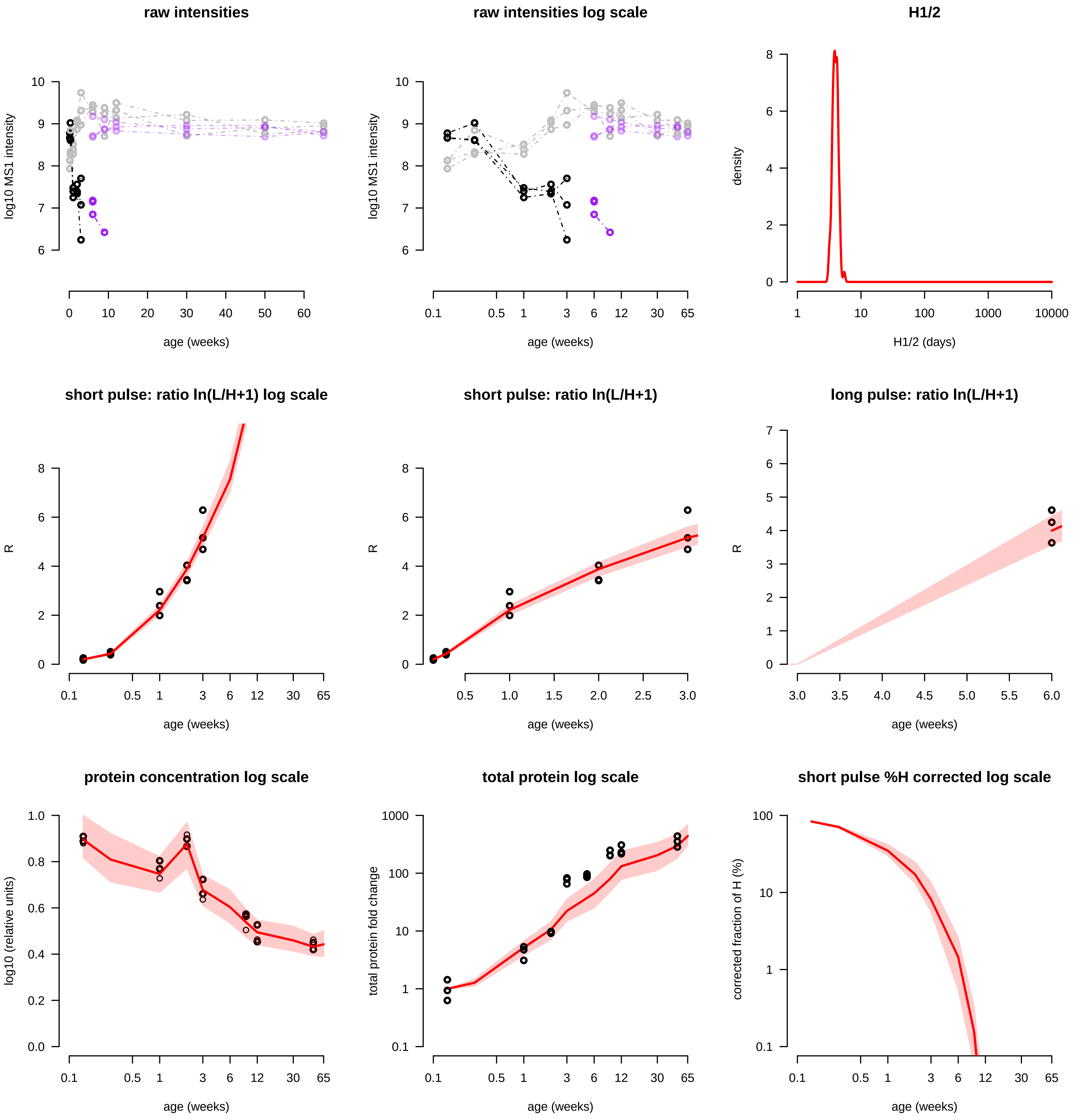

Q9CQ69

>sp|Q9CQ69|QCR8_MOUSE

Cytochrome b−c1 complex subunit 8

GN=Uqcrq

Med H1/2: 13.8 days

Q5 H1/2: 9.4 days

Q95 H1/2: 33.8 days

reliable model fit: yes

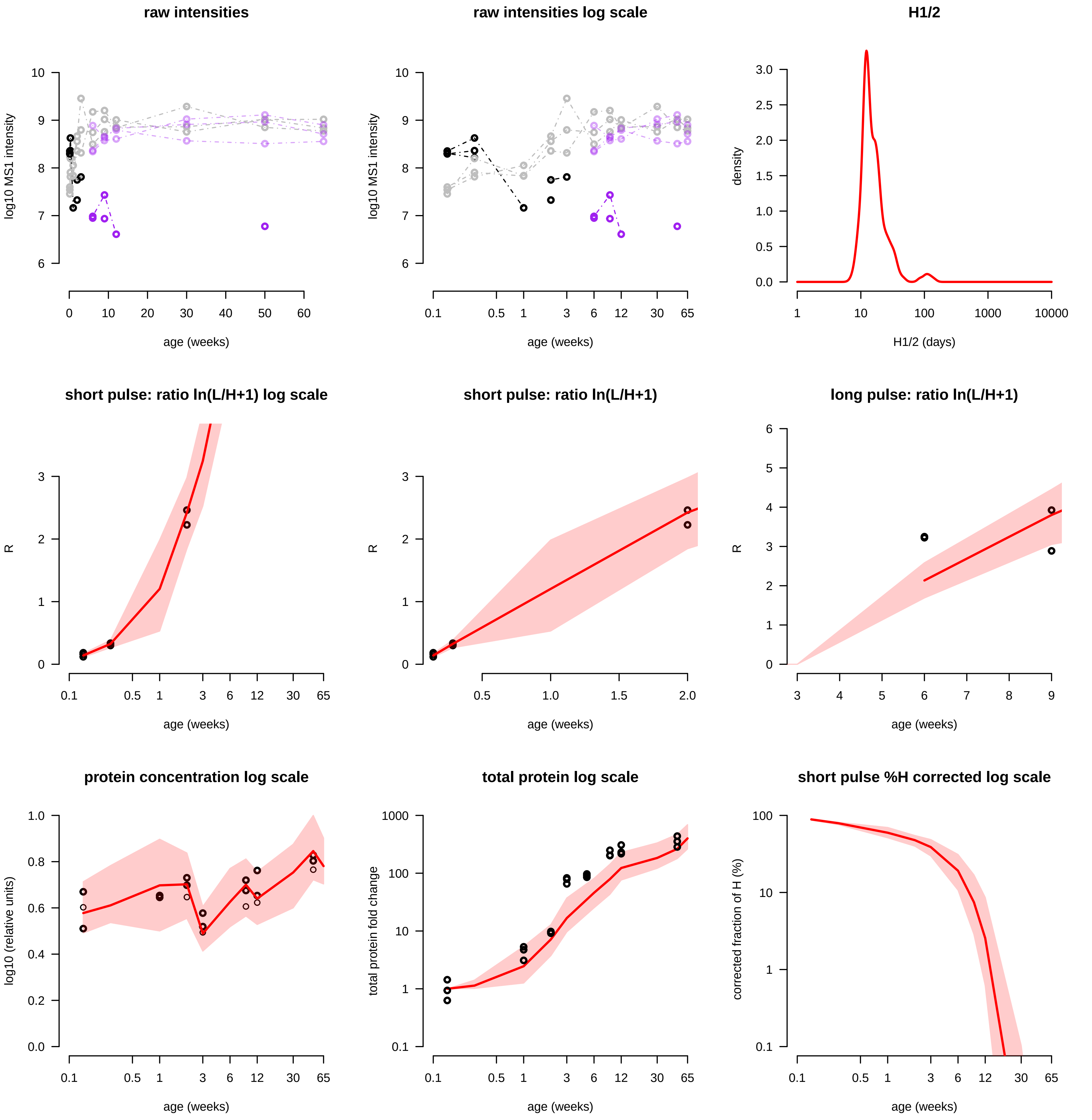

Q9WV54

>sp|Q9WV54|ASAH1_MOUSE

Acid ceramidase

GN=Asah1

Med H1/2: 5.6 days

Q5 H1/2: 4.1 days

Q95 H1/2: 7.9 days

reliable model fit: yes

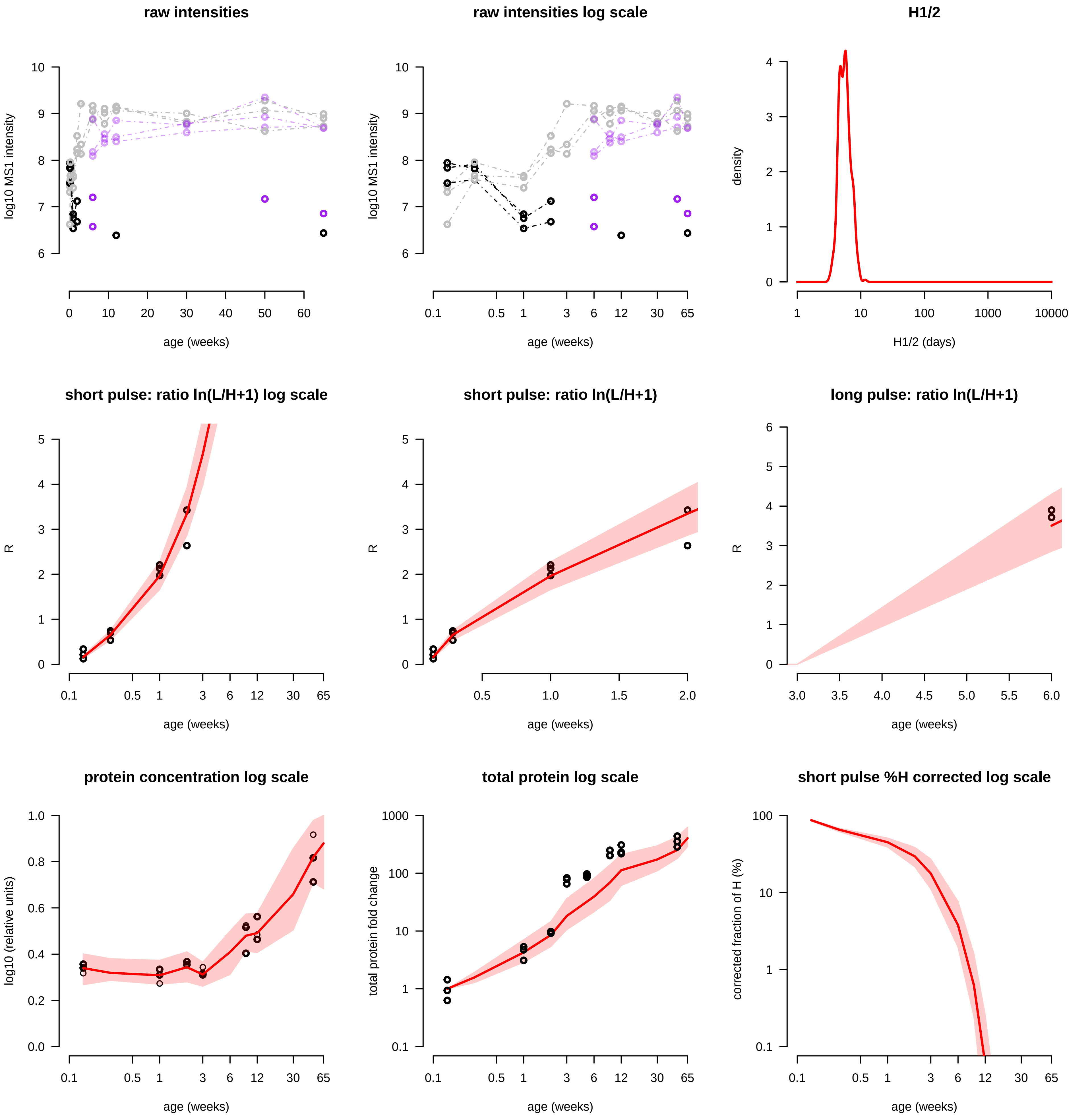

Q6A026

>sp|Q6A026|PDS5A_MOUSE

Sister chromatid cohesion protein PDS5 homolog A

GN=Pds5a

Med H1/2: 5 days

Q5 H1/2: 4.1 days

Q95 H1/2: 6.4 days

reliable model fit: yes

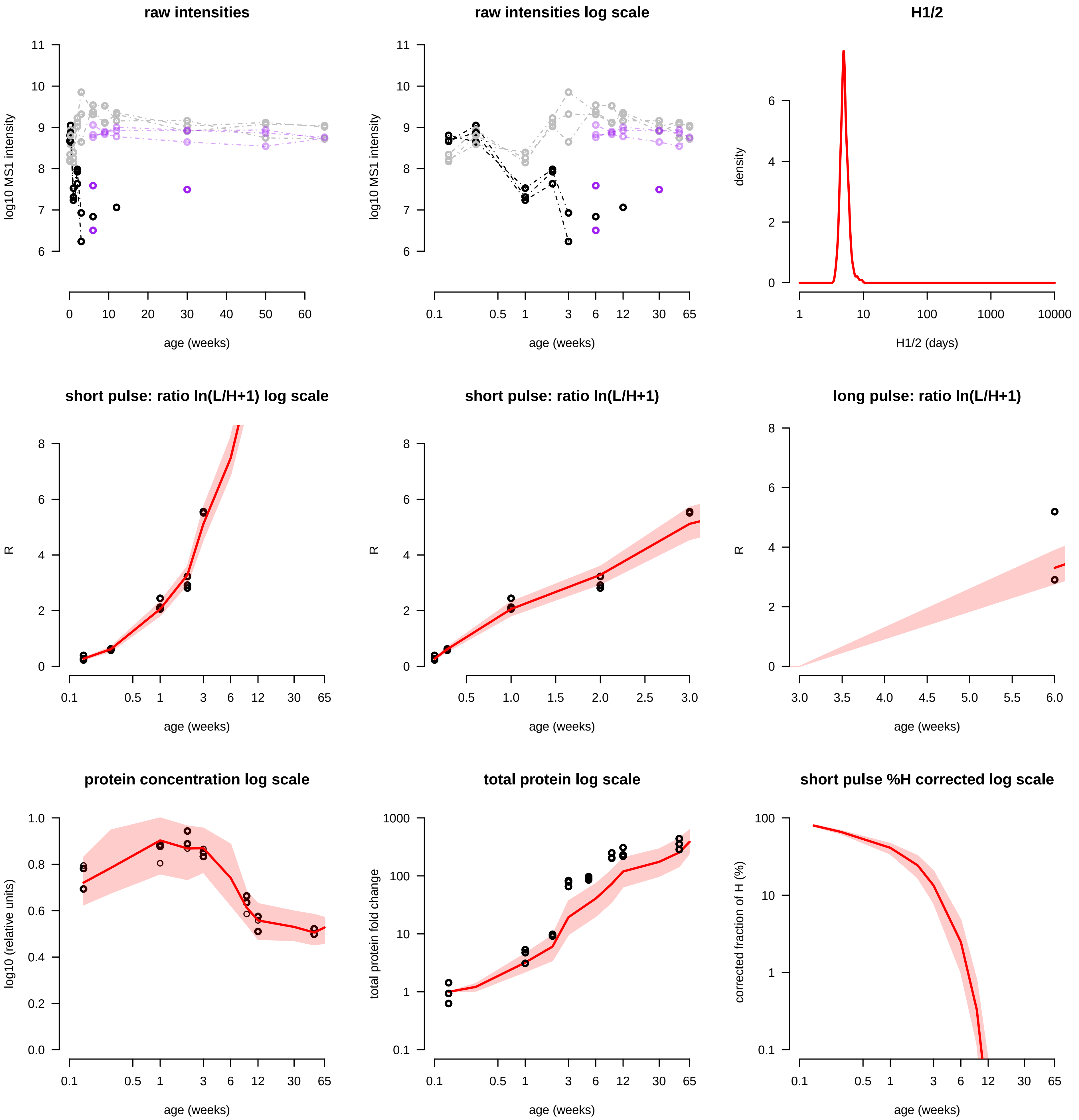

Q91WD5

>sp|Q91WD5|NDUS2_MOUSE

NADH dehydrogenase [ubiquinone] iron−sulfur protein 2, mitochondrial

GN=Ndufs2

Med H1/2: 7.4 days

Q5 H1/2: 5.6 days

Q95 H1/2: 10.7 days

reliable model fit: yes

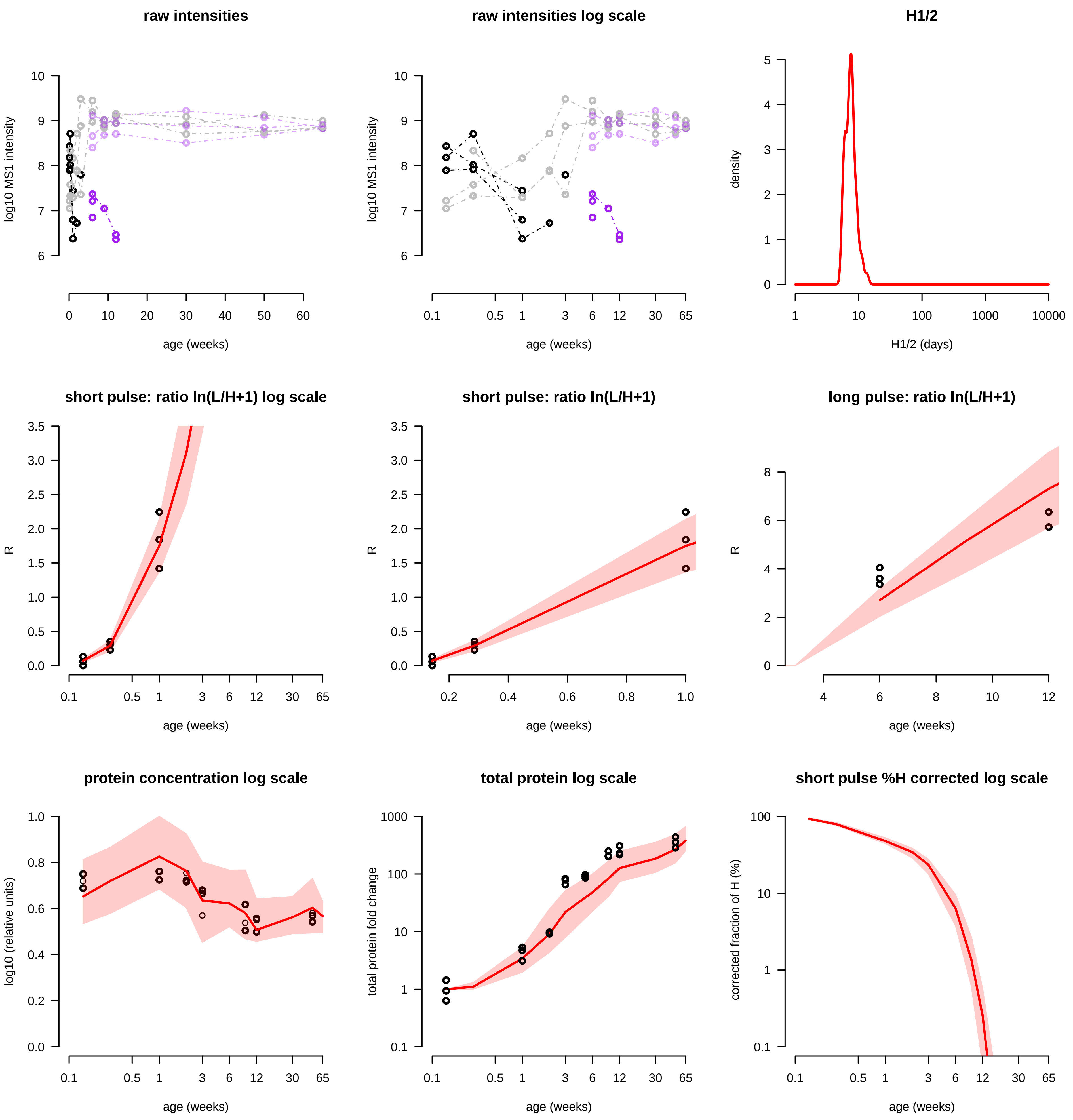

O35551;O35551−6;O35551−2;O35551−3;O35551−5

>sp|O35551|RABE1_MOUSE

Rab GTPase−binding effector protein 1

GN=Rabep1

Med H1/2: 17.9 days

Q5 H1/2: 10.6 days

Q95 H1/2: 43.4 days

reliable model fit: yes

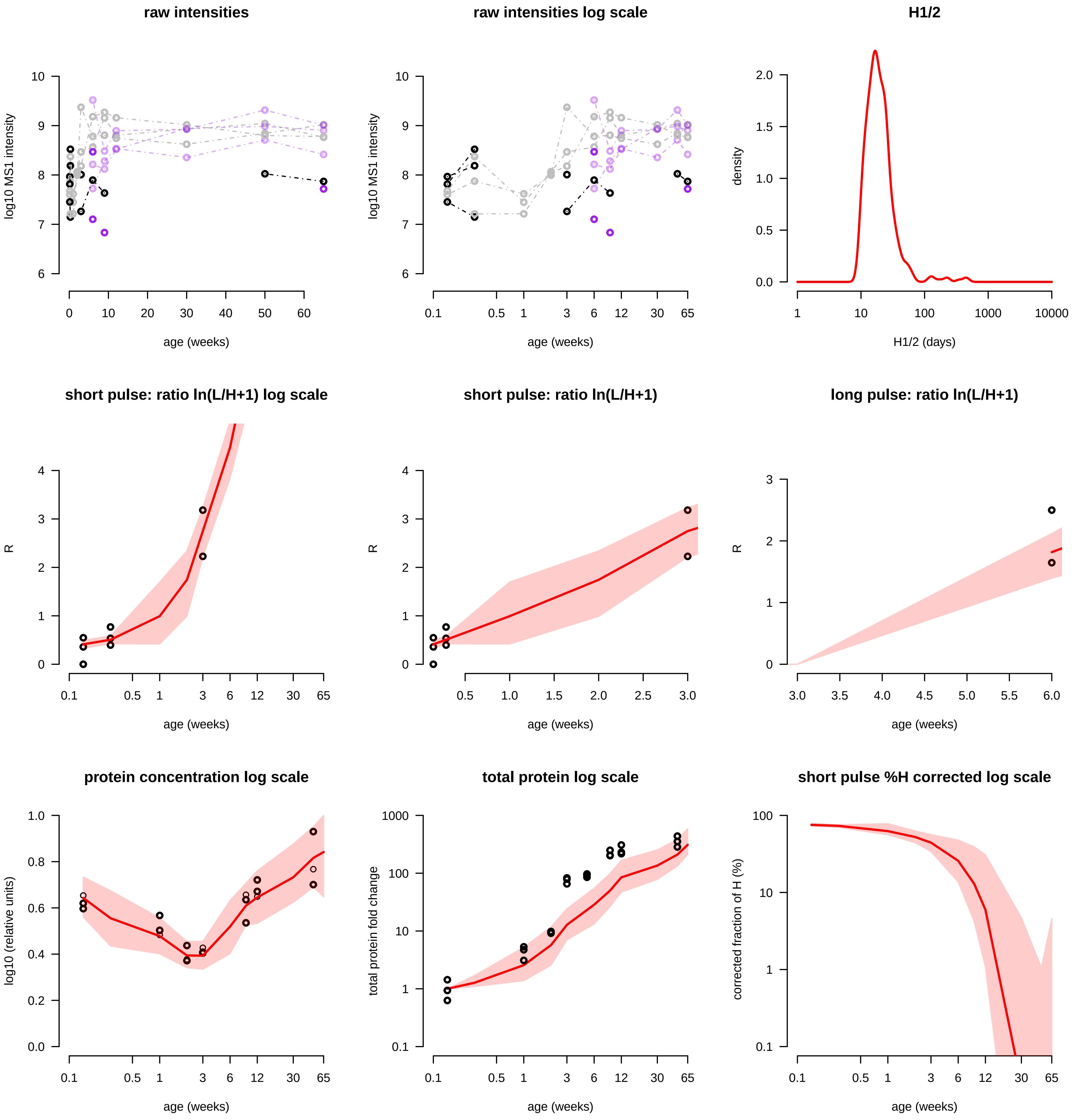

Q99KK2;Q99KK2−2

>sp|Q99KK2|NEUA_MOUSE

N−acylneuraminate cytidylyltransferase

GN=Cmas

Med H1/2: 5.7 days

Q5 H1/2: 4.7 days

Q95 H1/2: 7.2 days

reliable model fit: yes

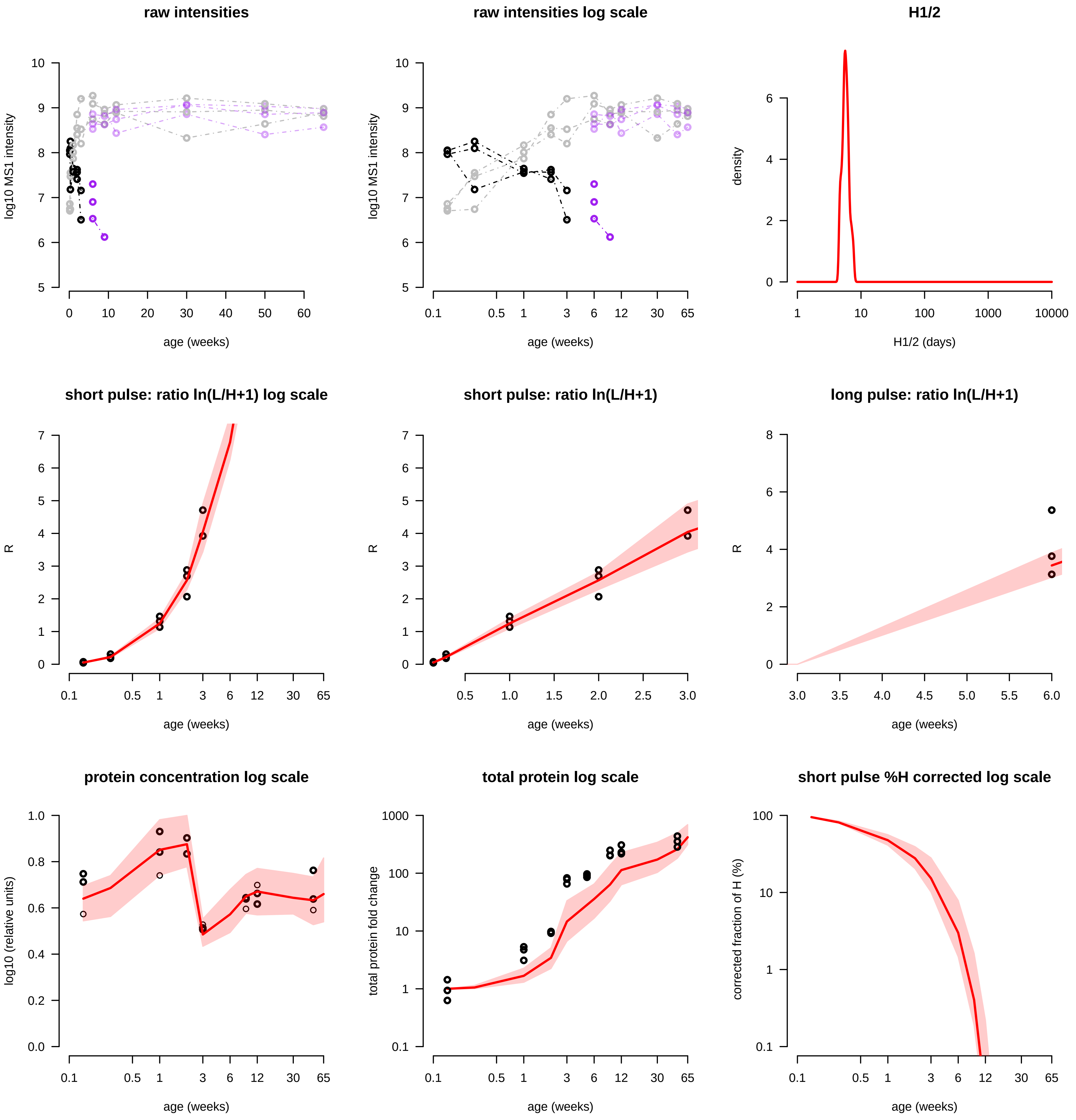

Q9Z2N8

>sp|Q9Z2N8|ACL6A_MOUSE

Actin−like protein 6A

GN=Actl6a

Med H1/2: 5.5 days

Q5 H1/2: 3.7 days

Q95 H1/2: 10.3 days

reliable model fit: yes

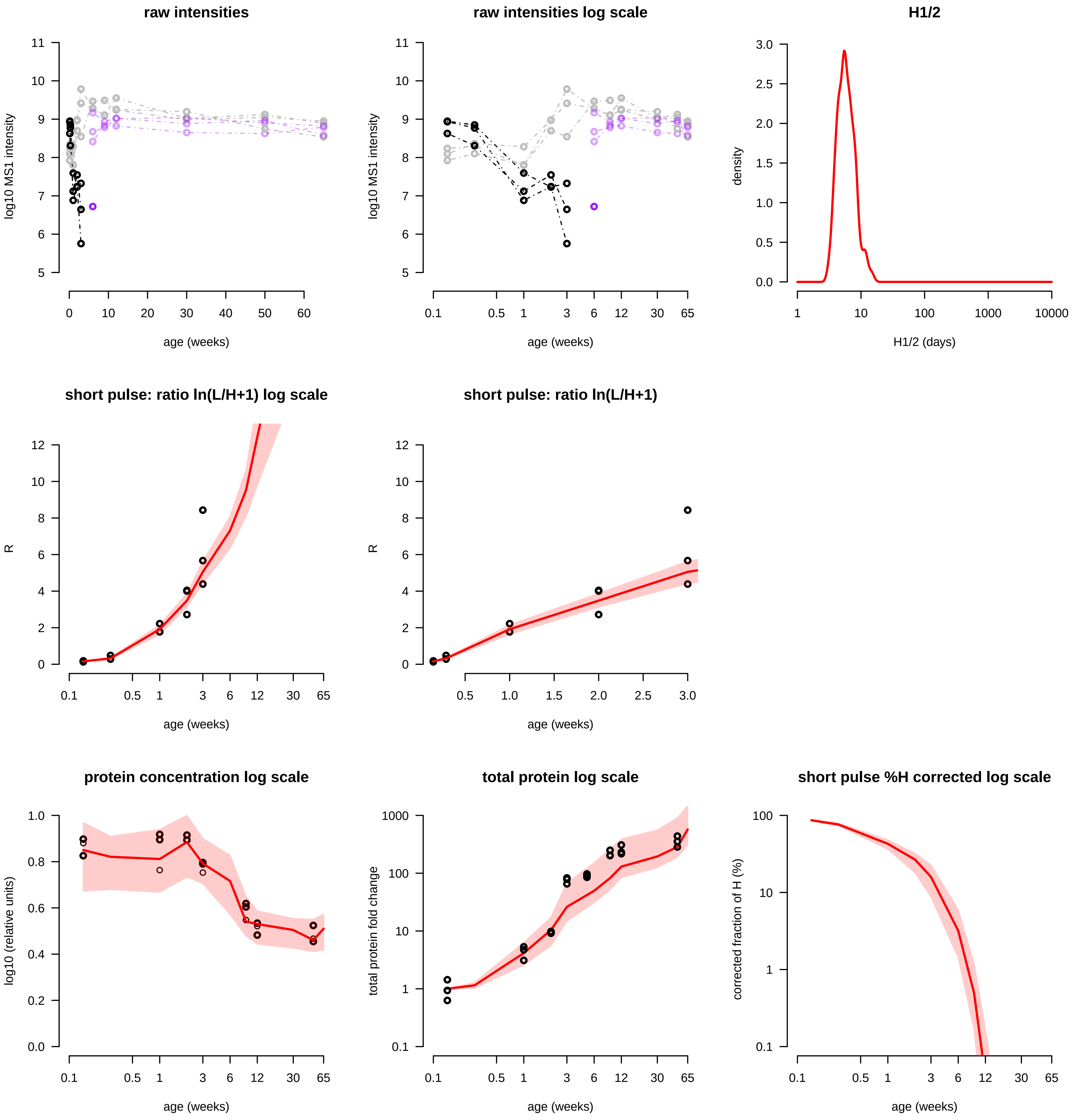

Q8C0E2;Q8C0E2−2

>sp|Q8C0E2|VP26B_MOUSE

Vacuolar protein sorting−associated protein 26B

GN=Vps26b

Med H1/2: 558.1 days

Q5 H1/2: 187.4 days

Q95 H1/2: 10036.3 days

reliable model fit: no

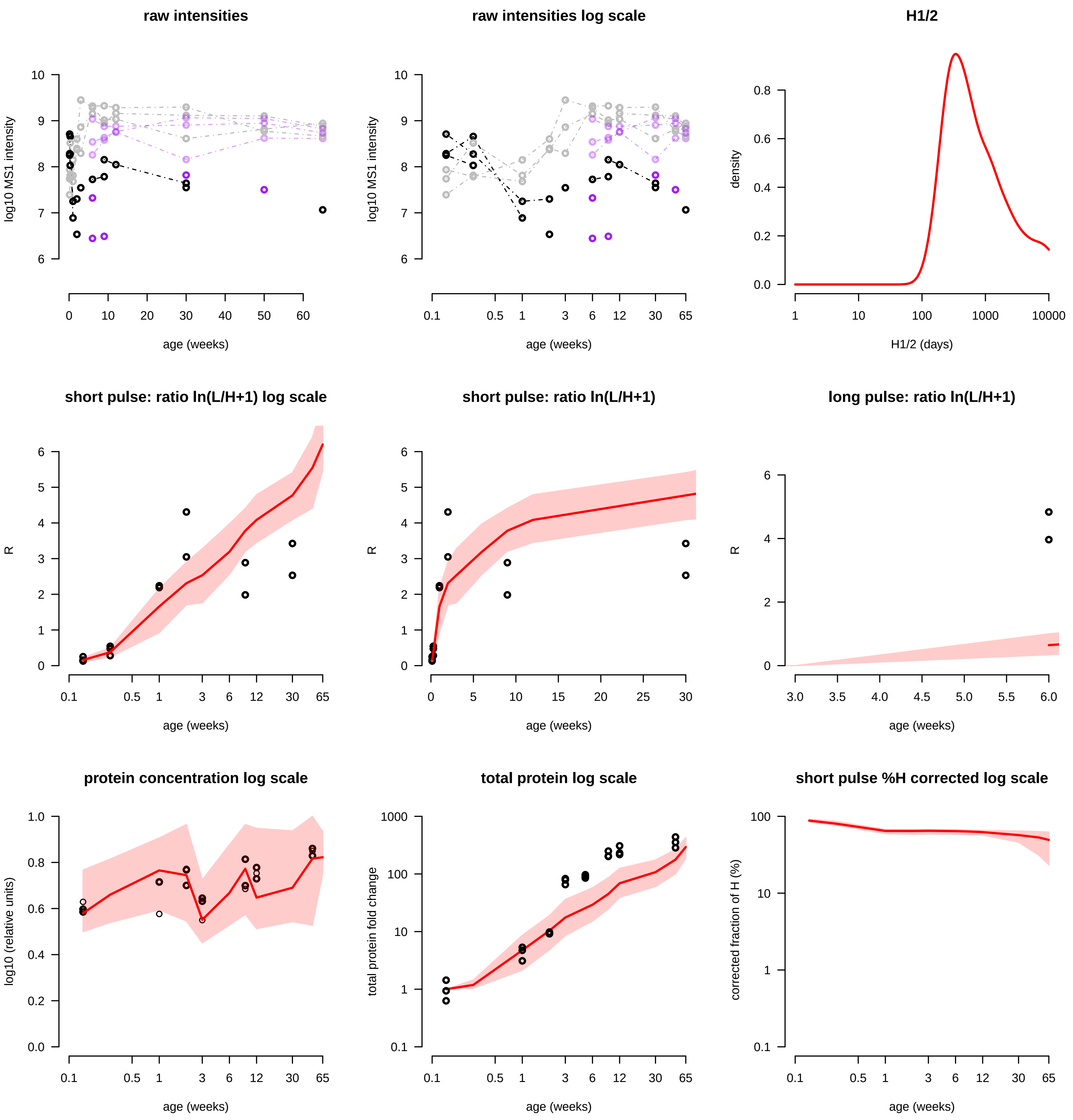

Q6ZQ08;Q6ZQ08−4

>sp|Q6ZQ08|CNOT1_MOUSE

CCR4−NOT transcription complex subunit 1

GN=Cnot1

Med H1/2: 484.8 days

Q5 H1/2: 171.2 days

Q95 H1/2: 4297.6 days

reliable model fit: no

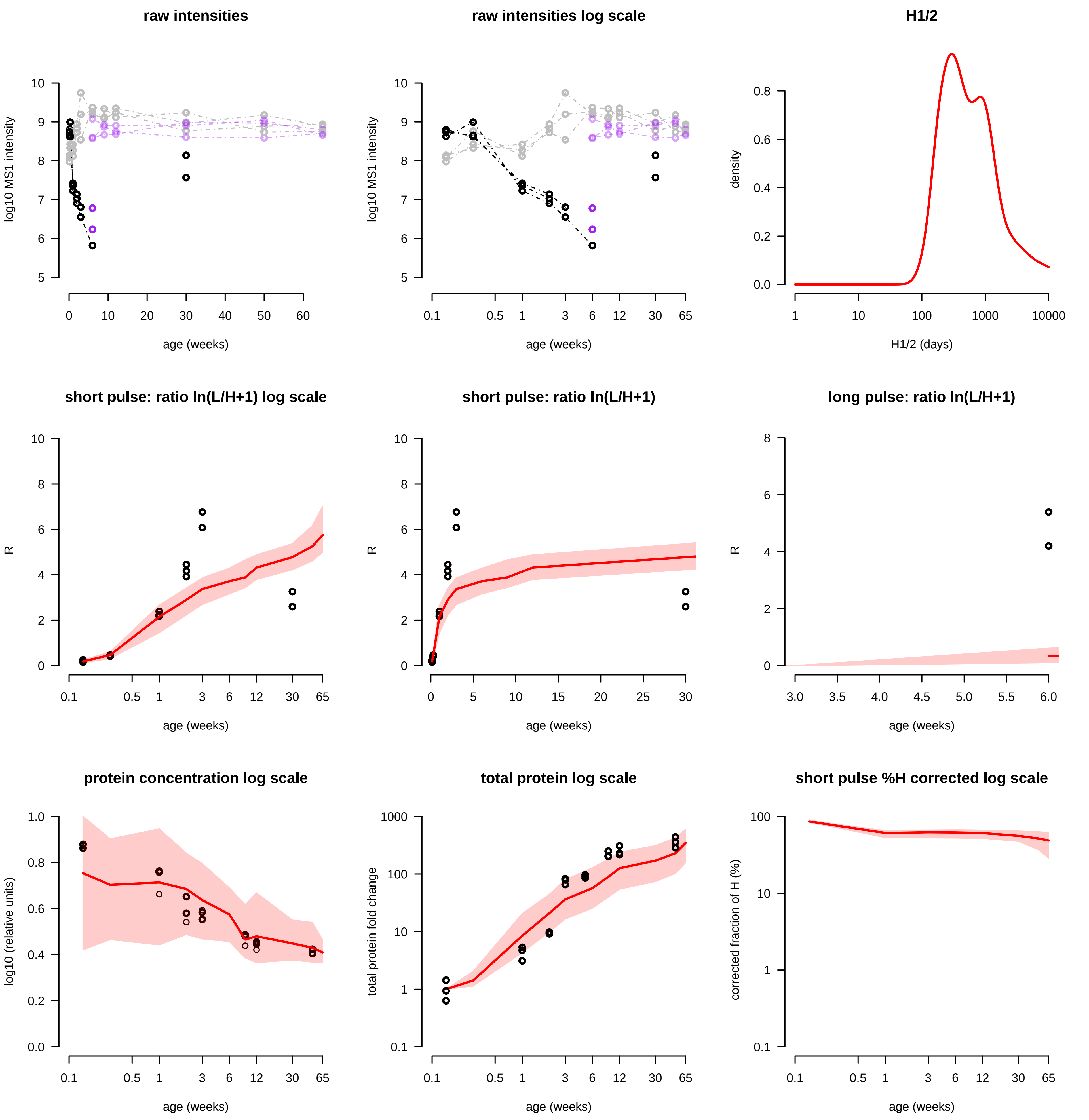

P61620

>sp|P61620|S61A1_MOUSE

Protein transport protein Sec61 subunit alpha isoform 1

GN=Sec61a1

Med H1/2: 7.2 days

Q5 H1/2: 5.6 days

Q95 H1/2: 9.6 days

reliable model fit: yes

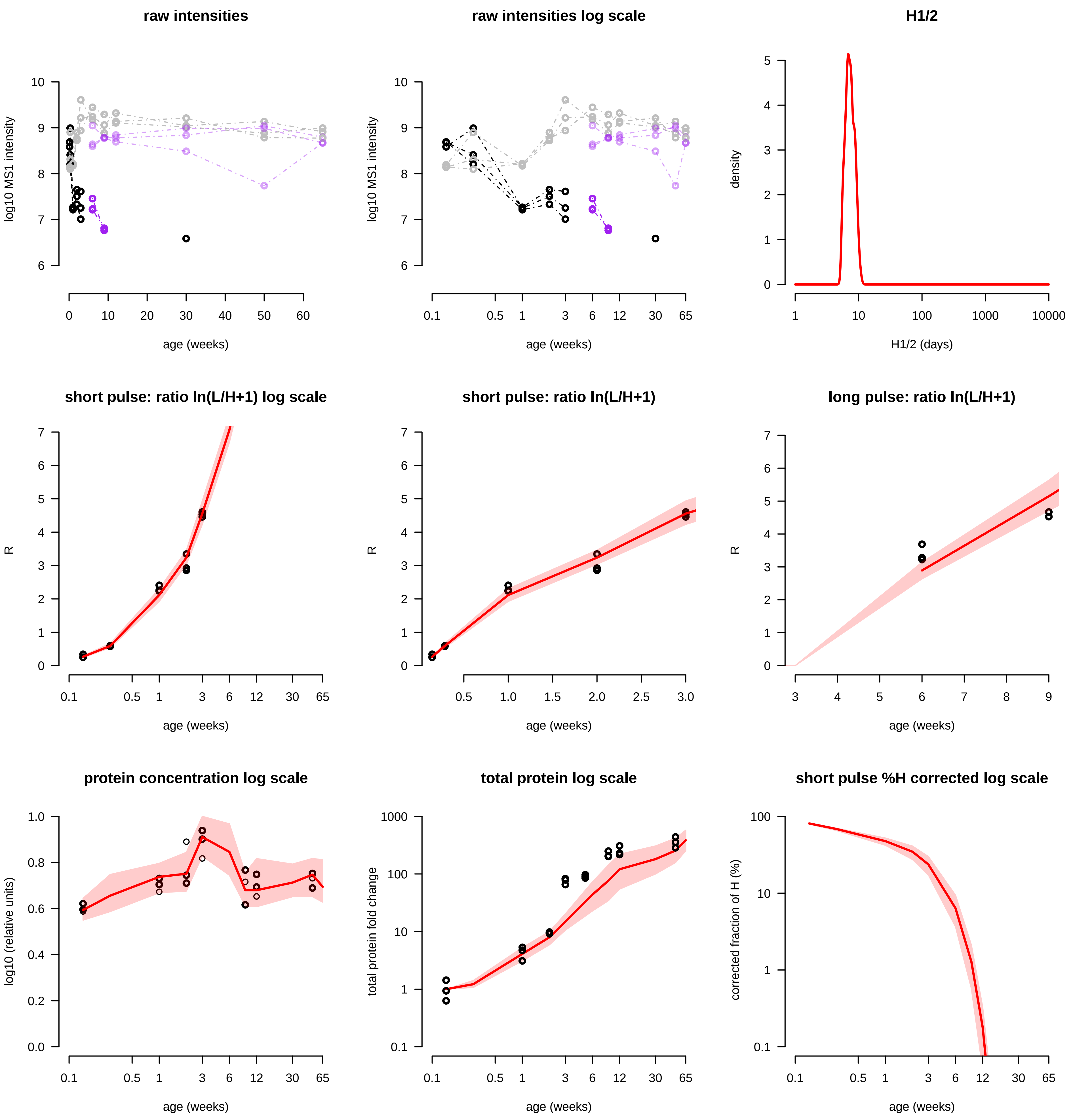

P84104−2;P84104

>sp|P84104−2|SRSF3_MOUSE

Isoform Short of Serine/arginine−rich splicing factor 3

GN=Srsf3

Med H1/2: 39.5 days

Q5 H1/2: 25.4 days

Q95 H1/2: 61.3 days

reliable model fit: no

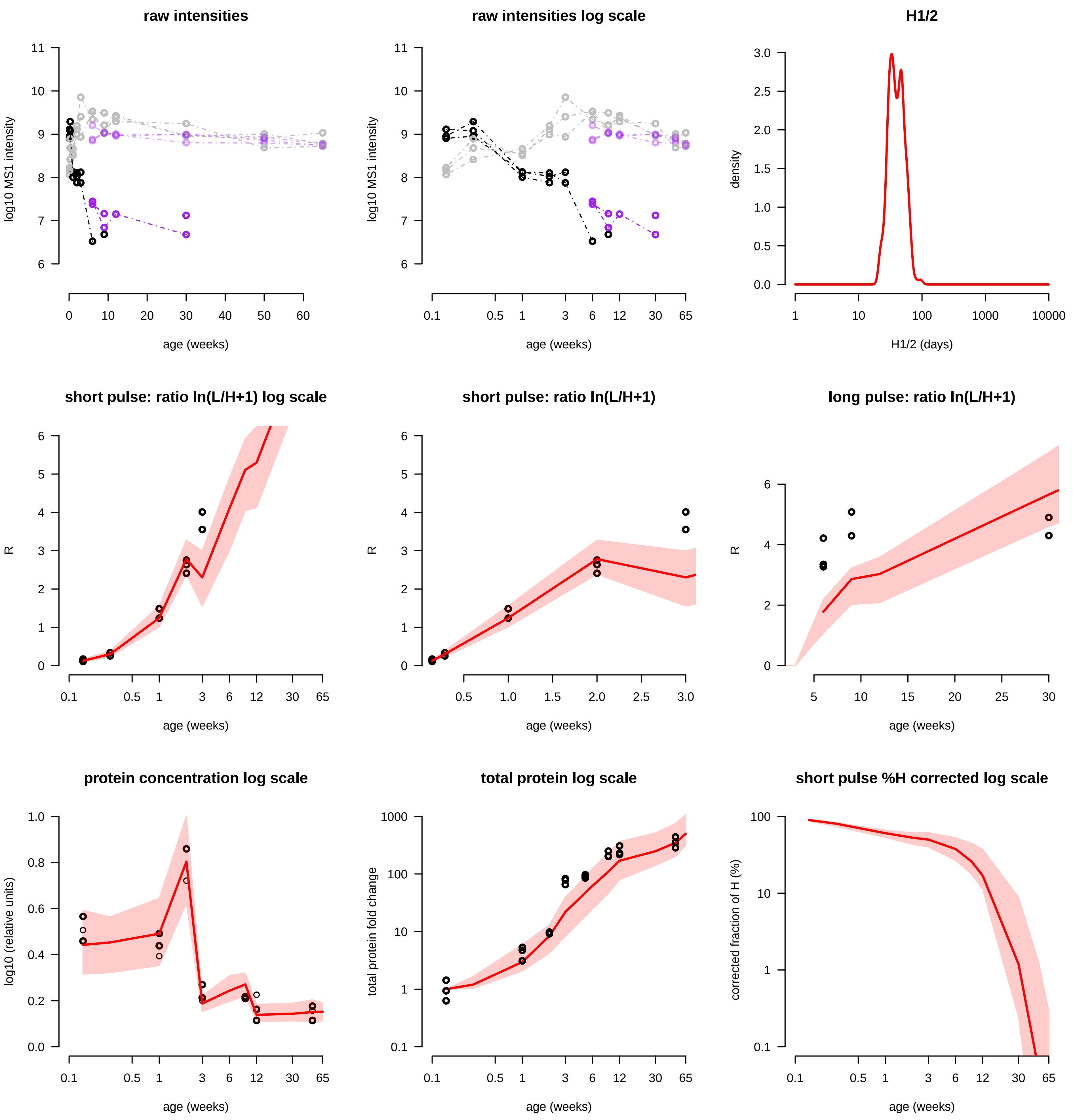

Q07076

>sp|Q07076|ANXA7_MOUSE

Annexin A7

GN=Anxa7

Med H1/2: 5.9 days

Q5 H1/2: 4.7 days

Q95 H1/2: 7.8 days

reliable model fit: yes

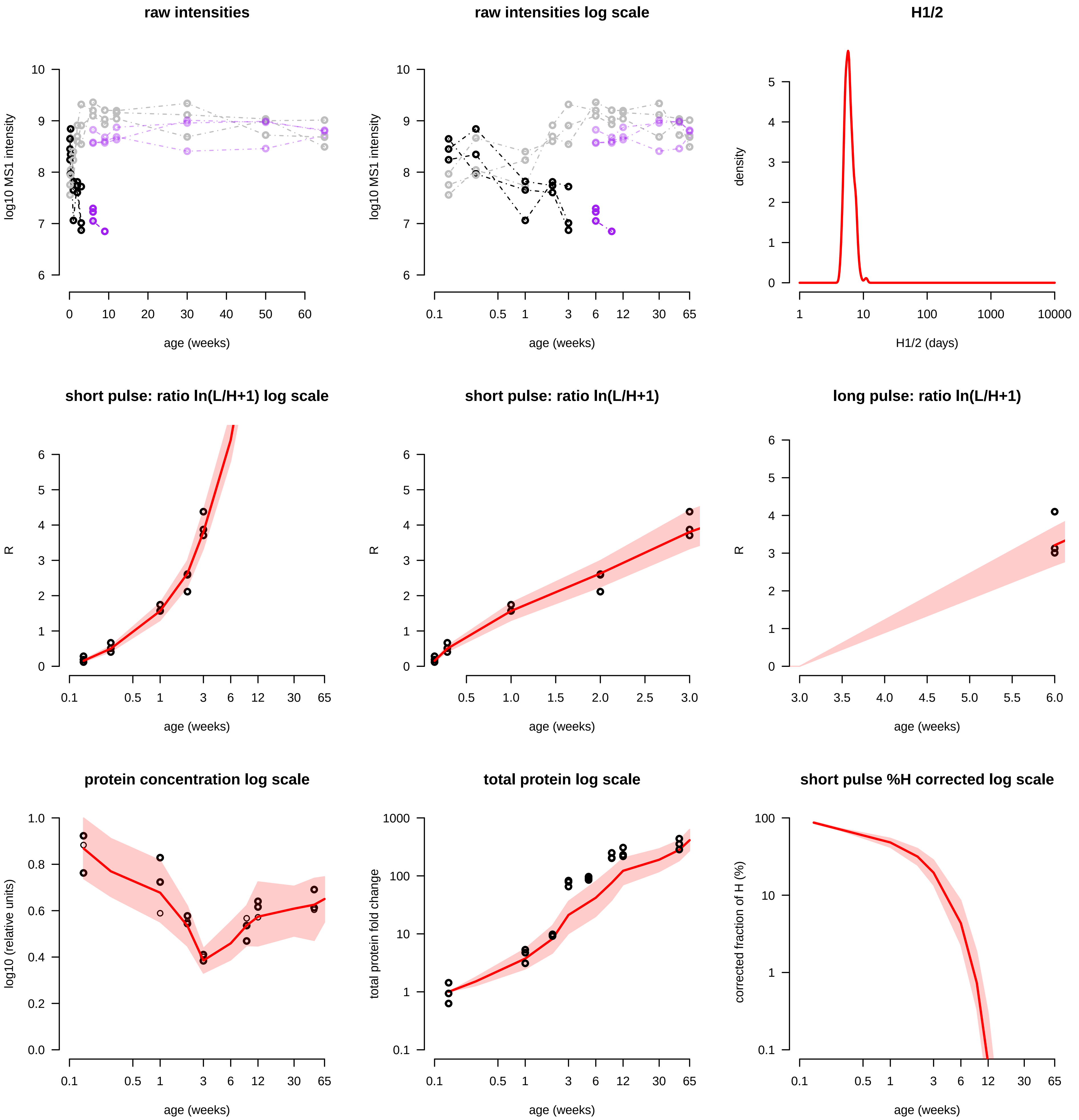

Q99P88

>sp|Q99P88|NU155_MOUSE

Nuclear pore complex protein Nup155

GN=Nup155

Med H1/2: 4.9 days

Q5 H1/2: 3.9 days

Q95 H1/2: 6 days

reliable model fit: yes

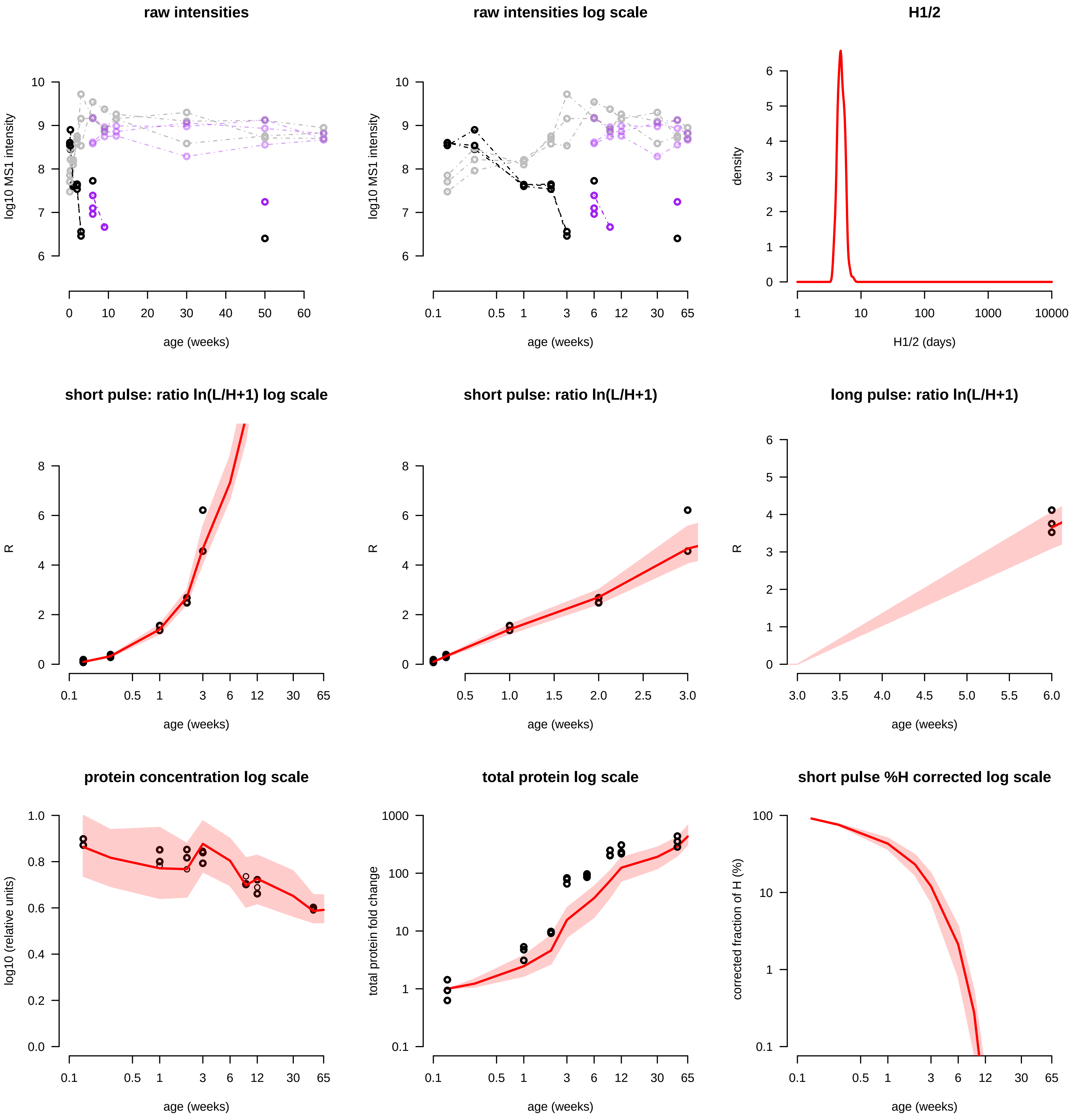

Q6ZWY3

>sp|Q6ZWY3|RS27L_MOUSE

40S ribosomal protein S27−like

GN=Rps27l

Med H1/2: 92.1 days

Q5 H1/2: 35.6 days

Q95 H1/2: 926.2 days

reliable model fit: no

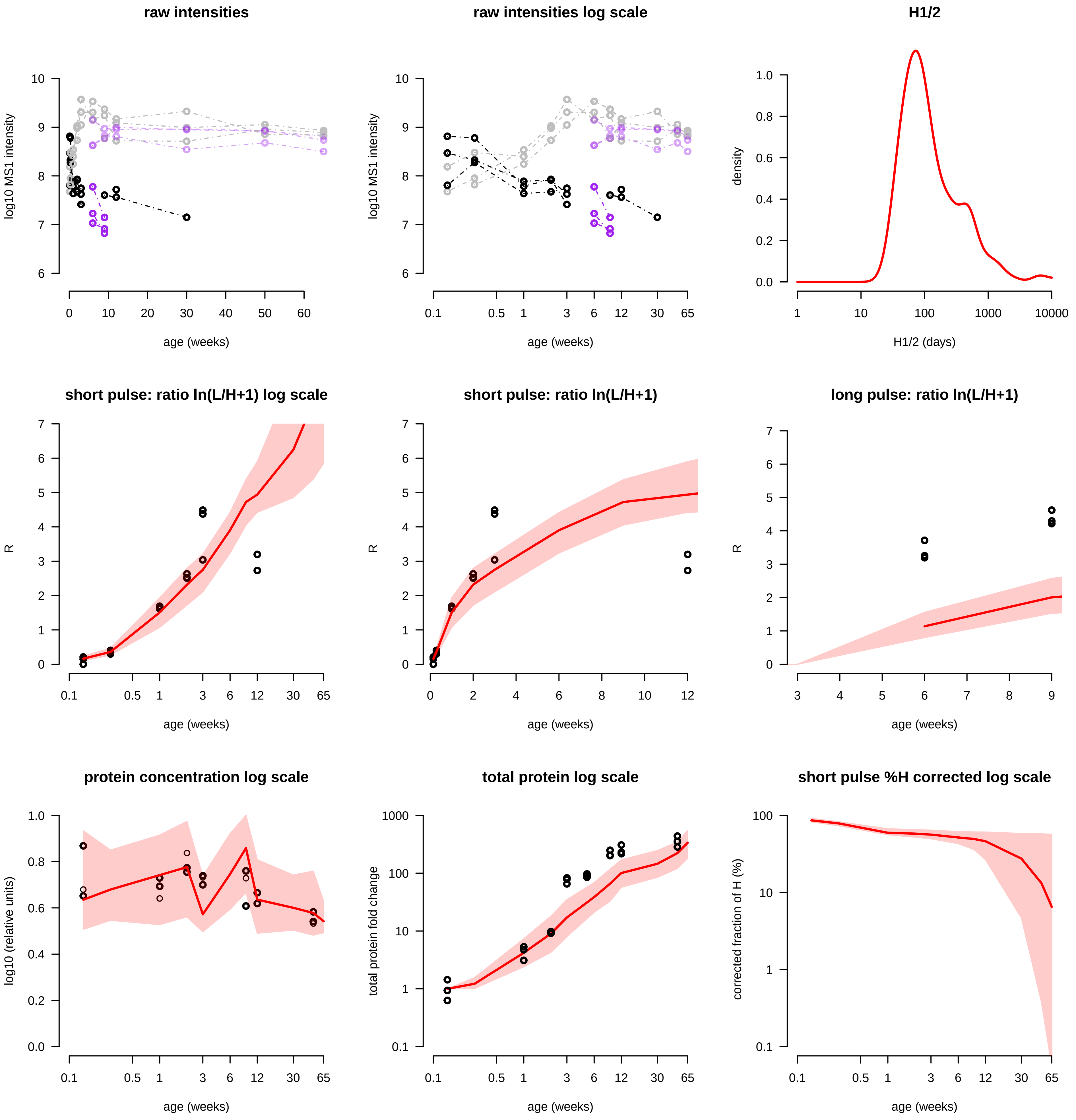

Q91VM9;Q91VM9−2

>sp|Q91VM9|IPYR2_MOUSE

Inorganic pyrophosphatase 2, mitochondrial

GN=Ppa2

Med H1/2: 9 days

Q5 H1/2: 3.3 days

Q95 H1/2: 89.1 days

reliable model fit: yes

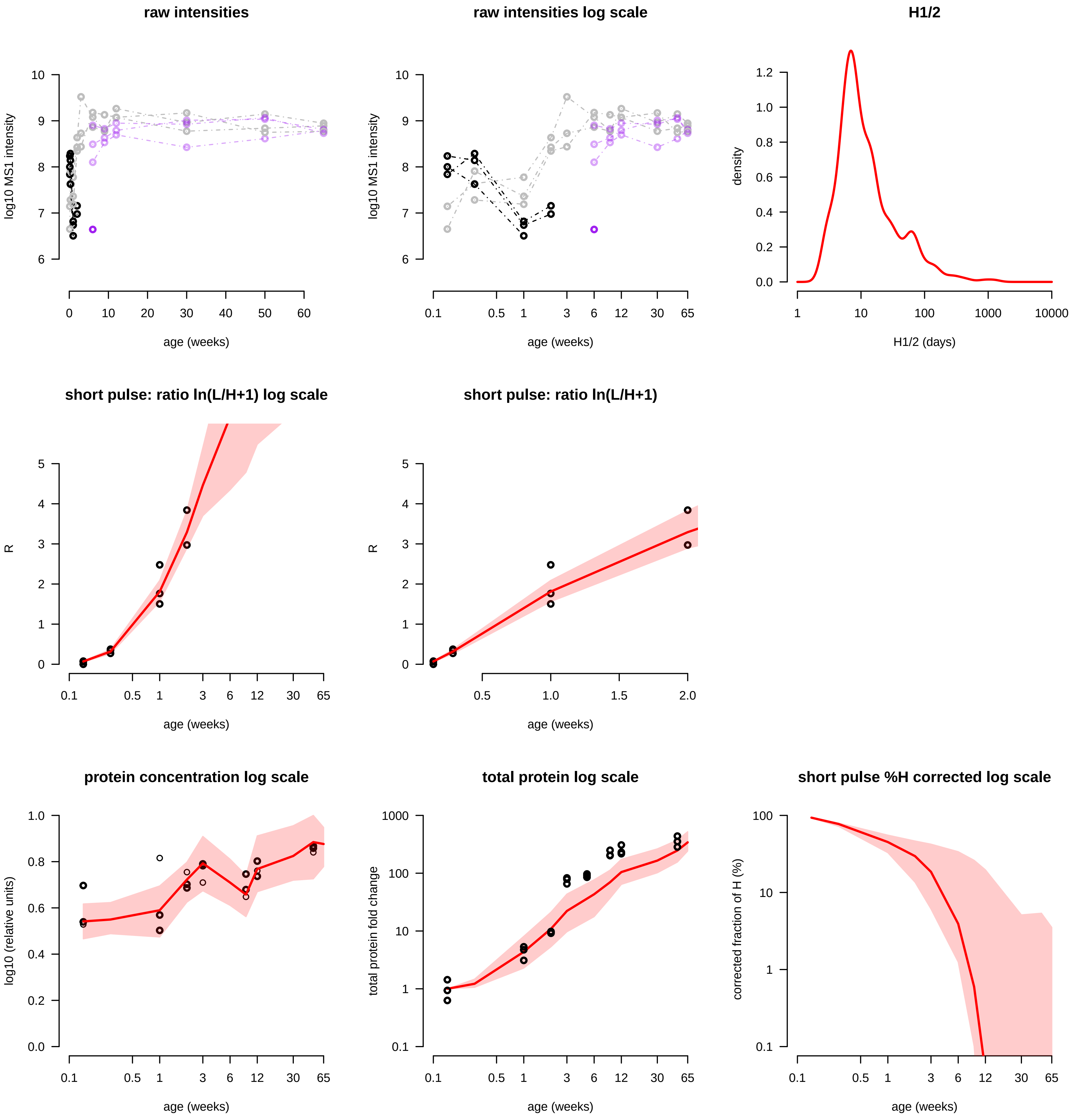

Q9CRA4

>sp|Q9CRA4|MSMO1_MOUSE

Methylsterol monooxygenase 1

GN=Msmo1

Med H1/2: 7.3 days

Q5 H1/2: 4.8 days

Q95 H1/2: 11.8 days

reliable model fit: yes

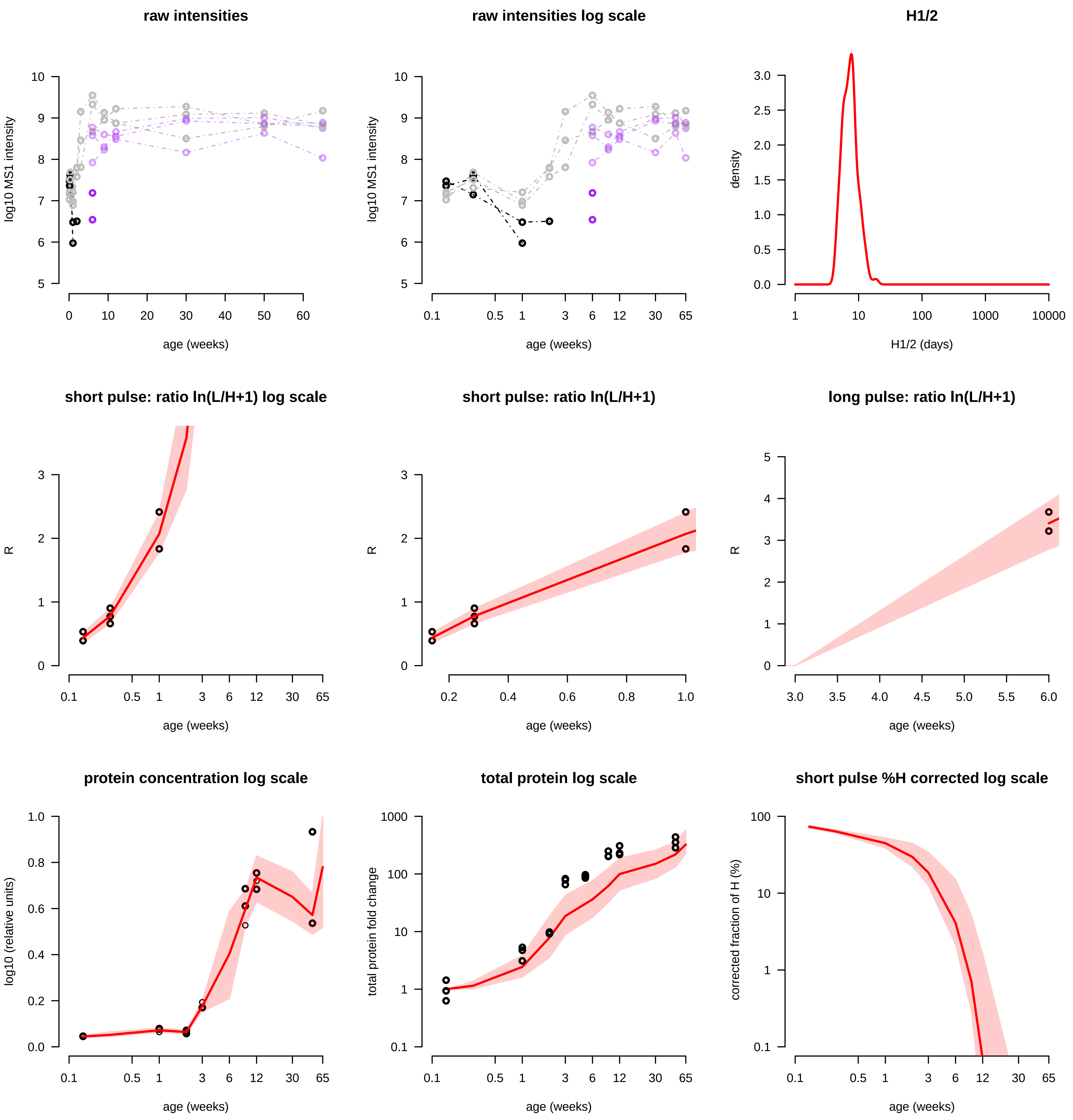

Q9CZU3

>sp|Q9CZU3|MTREX_MOUSE

Exosome RNA helicase MTR4

GN=Mtrex

Med H1/2: 5.1 days

Q5 H1/2: 2.8 days

Q95 H1/2: 12.5 days

reliable model fit: yes

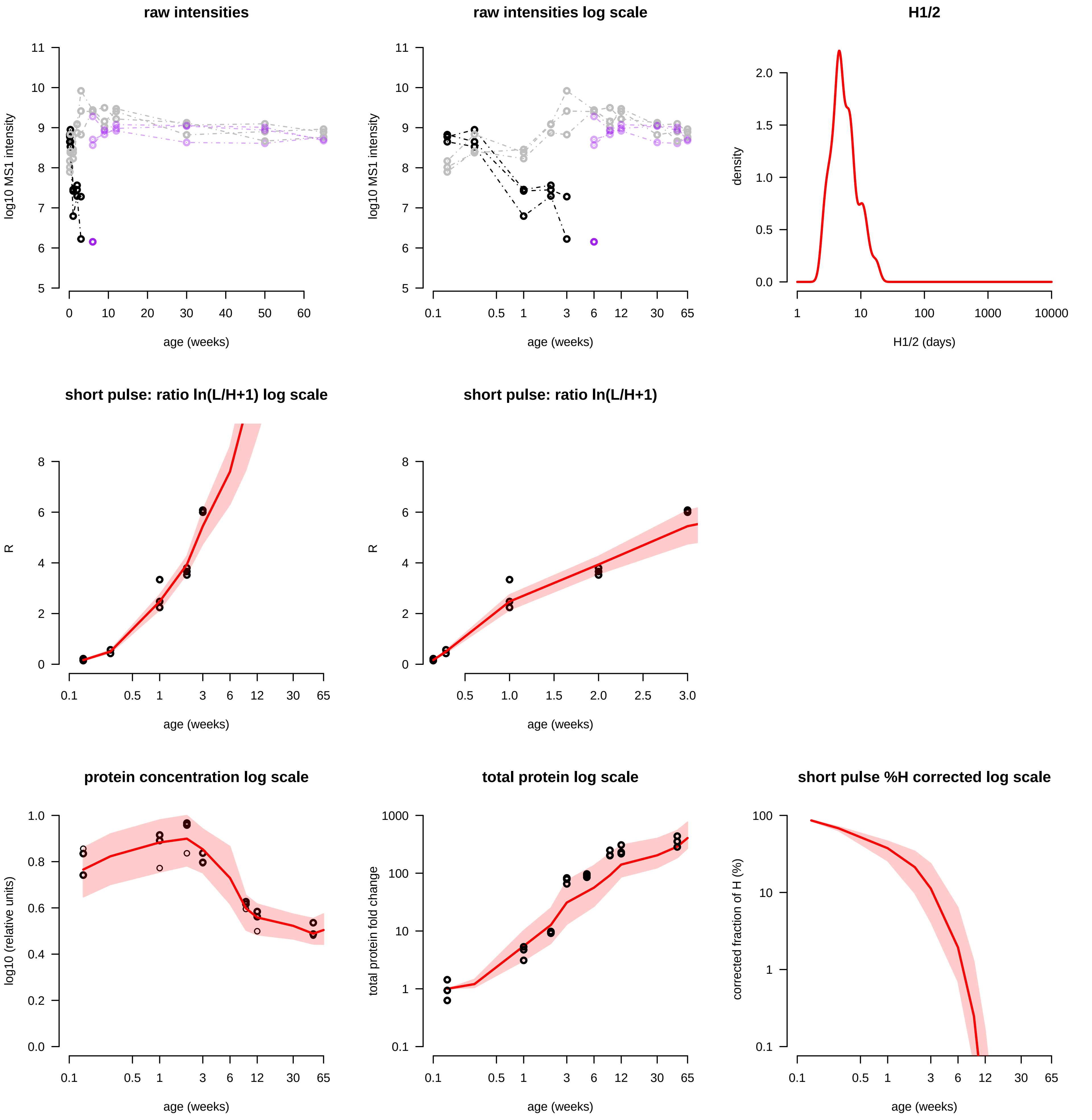

P70288

>sp|P70288|HDAC2_MOUSE

Histone deacetylase 2

GN=Hdac2

Med H1/2: 5.5 days

Q5 H1/2: 4.5 days

Q95 H1/2: 7 days

reliable model fit: yes

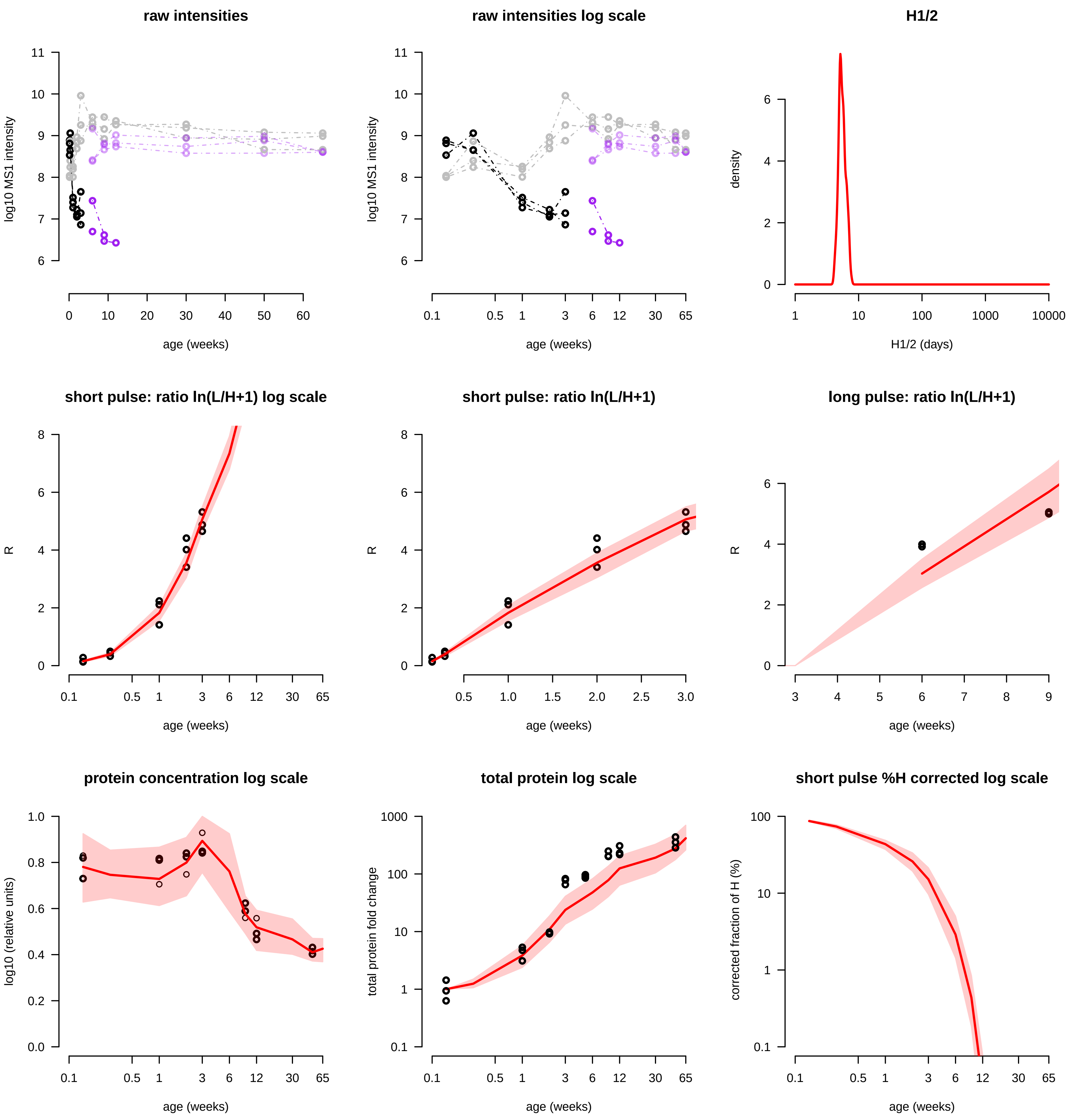

Q9JLQ0

>sp|Q9JLQ0|CD2AP_MOUSE

CD2−associated protein

GN=Cd2ap

Med H1/2: 3 days

Q5 H1/2: 2.5 days

Q95 H1/2: 4.3 days

reliable model fit: yes

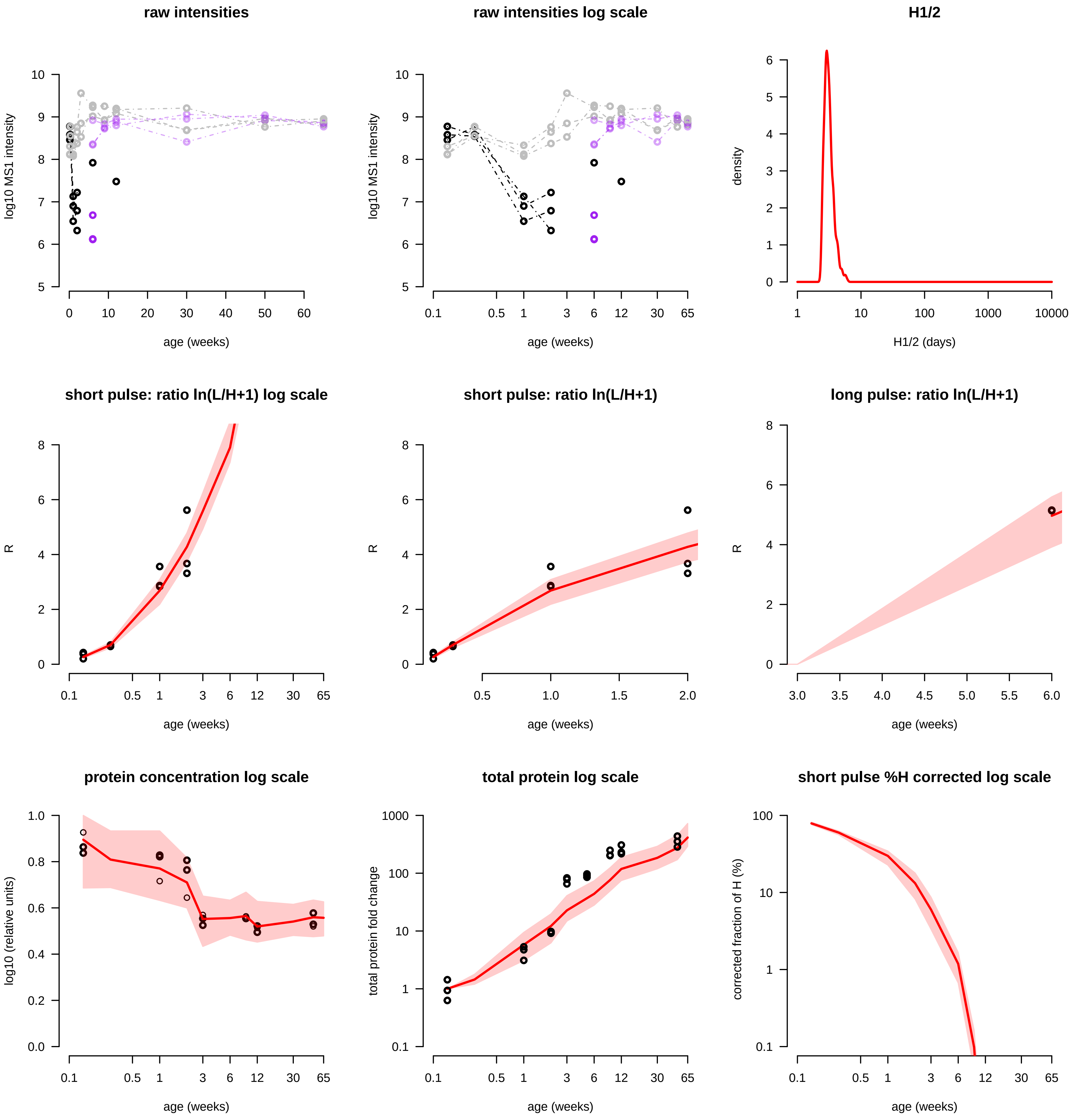

Q9CXT8

>sp|Q9CXT8|MPPB_MOUSE

Mitochondrial−processing peptidase subunit beta

GN=Pmpcb

Med H1/2: 3.6 days

Q5 H1/2: 2.8 days

Q95 H1/2: 4.7 days

reliable model fit: yes

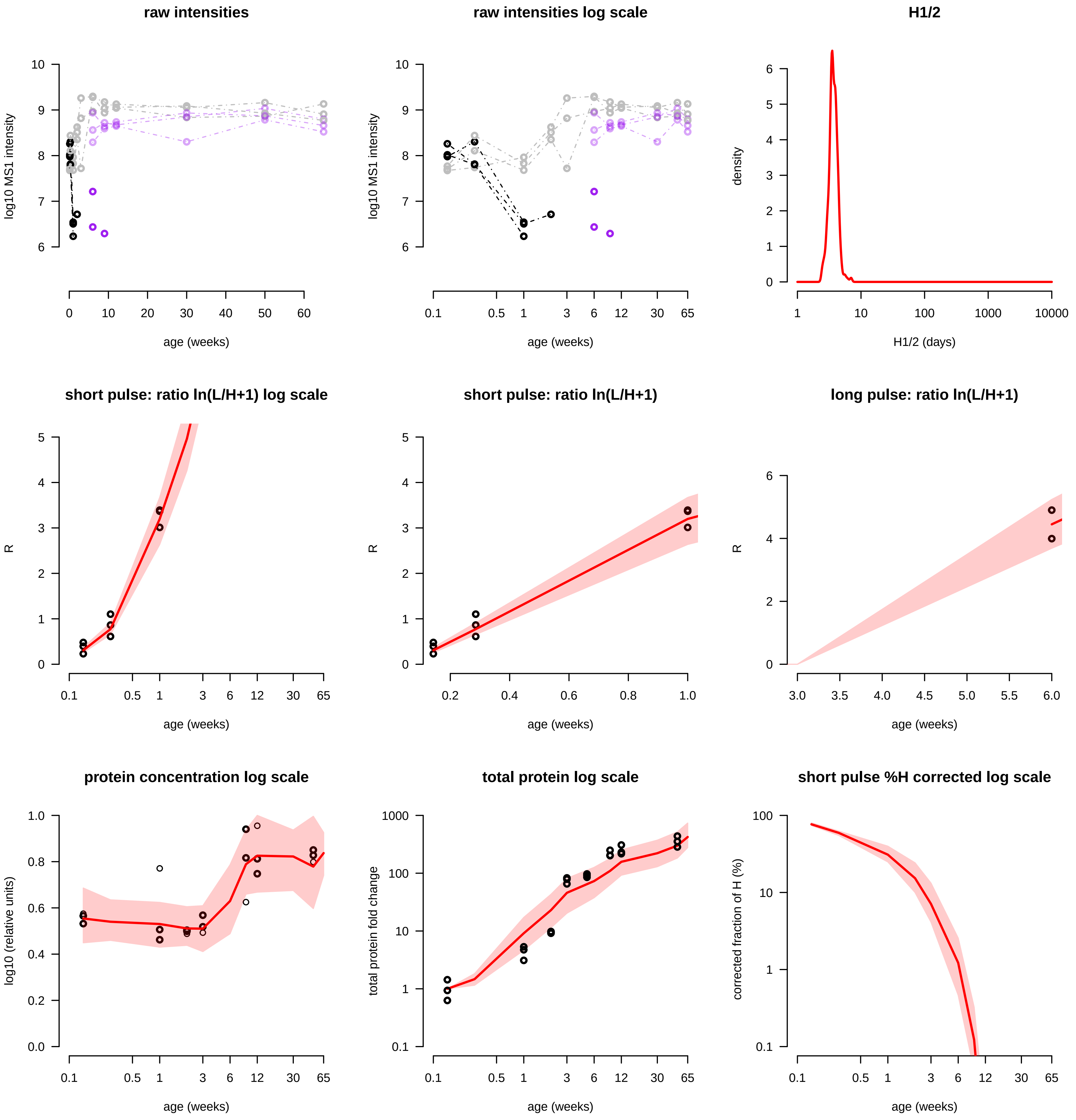

Q5SWU9;Q5SWU9−2

>sp|Q5SWU9|ACACA_MOUSE

Acetyl−CoA carboxylase 1

GN=Acaca

Med H1/2: 3 days

Q5 H1/2: 2.5 days

Q95 H1/2: 4.3 days

reliable model fit: yes

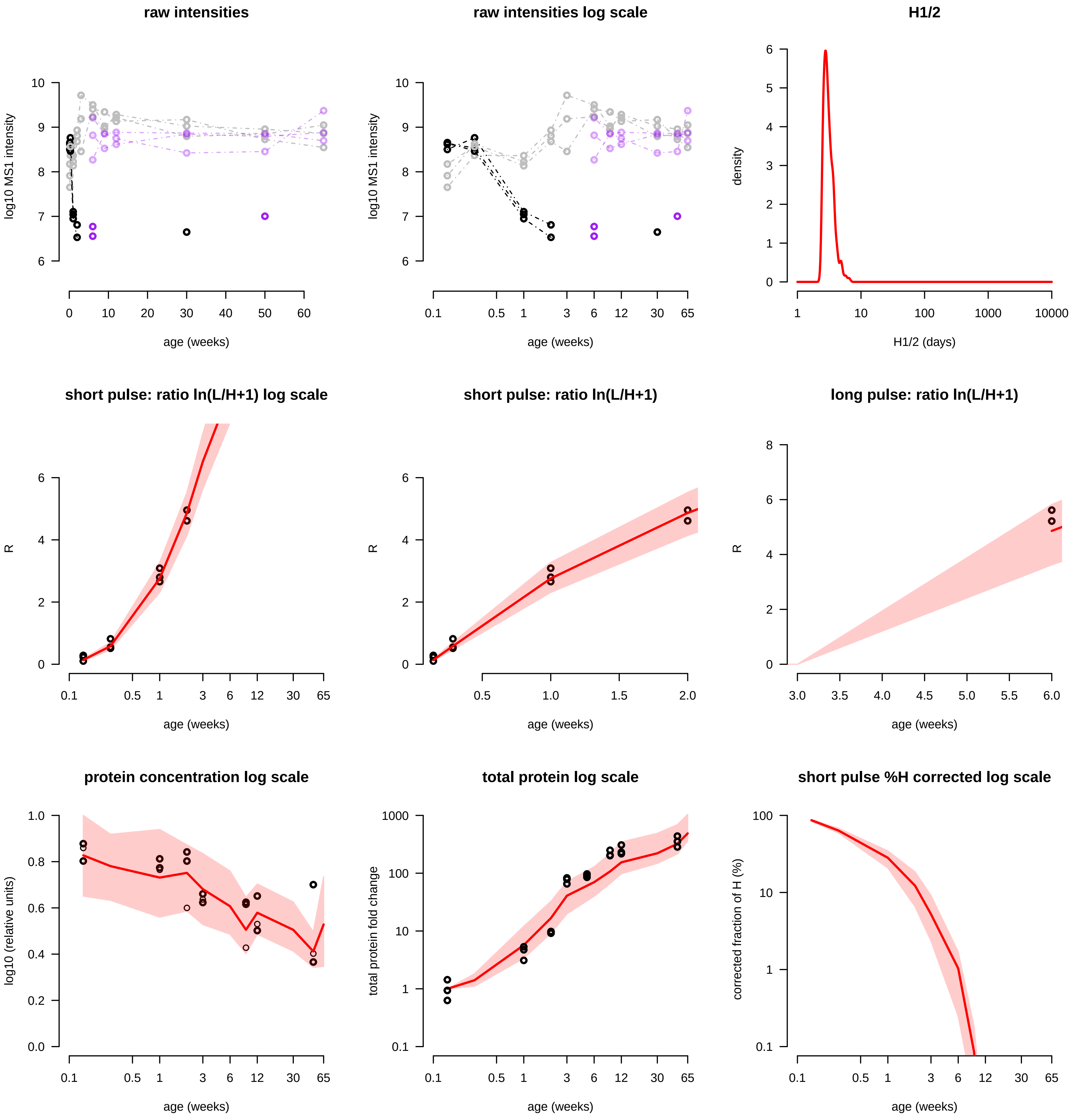

Q8BU14

>sp|Q8BU14|SEC62_MOUSE

Translocation protein SEC62

GN=Sec62

Med H1/2: 4.6 days

Q5 H1/2: 3 days

Q95 H1/2: 7.3 days

reliable model fit: yes

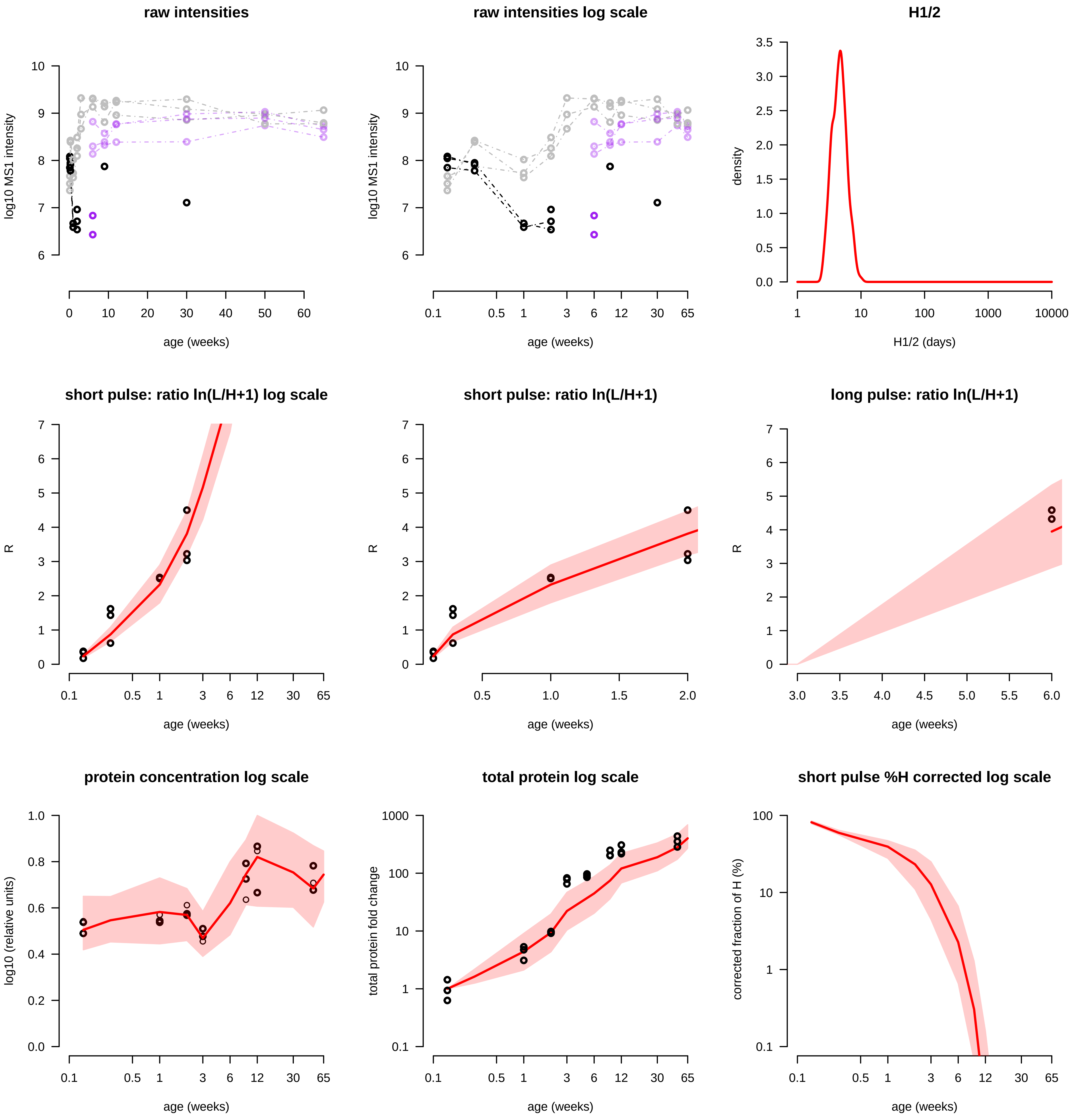

Q9DBE8

>sp|Q9DBE8|ALG2_MOUSE

Alpha−1,3/1,6−mannosyltransferase ALG2

GN=Alg2

Med H1/2: 5.2 days

Q5 H1/2: 4.3 days

Q95 H1/2: 7.1 days

reliable model fit: yes

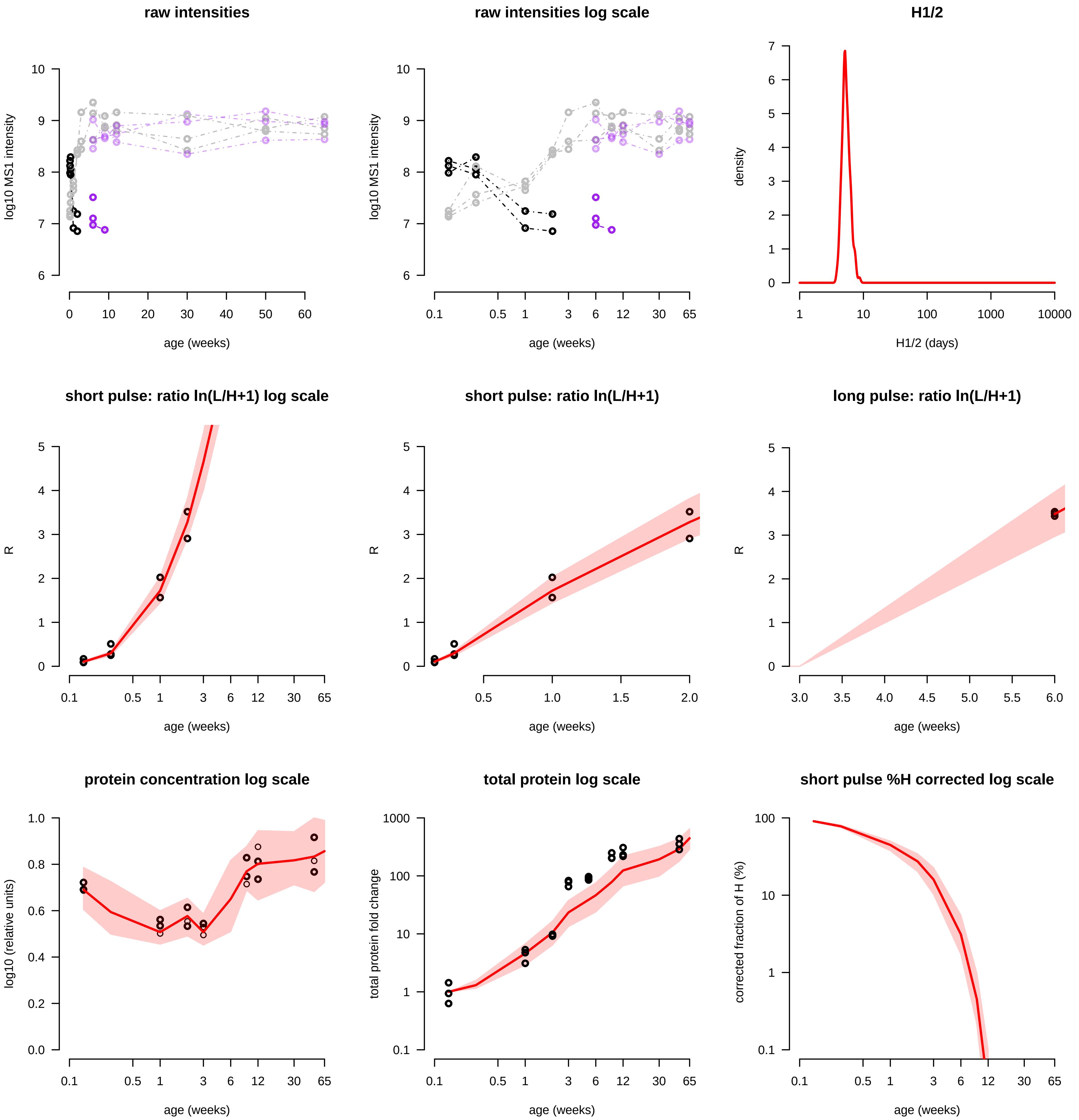

Q8VBT0

>sp|Q8VBT0|TMX1_MOUSE

Thioredoxin−related transmembrane protein 1

GN=Tmx1

Med H1/2: 4.8 days

Q5 H1/2: 4 days

Q95 H1/2: 5.6 days

reliable model fit: yes

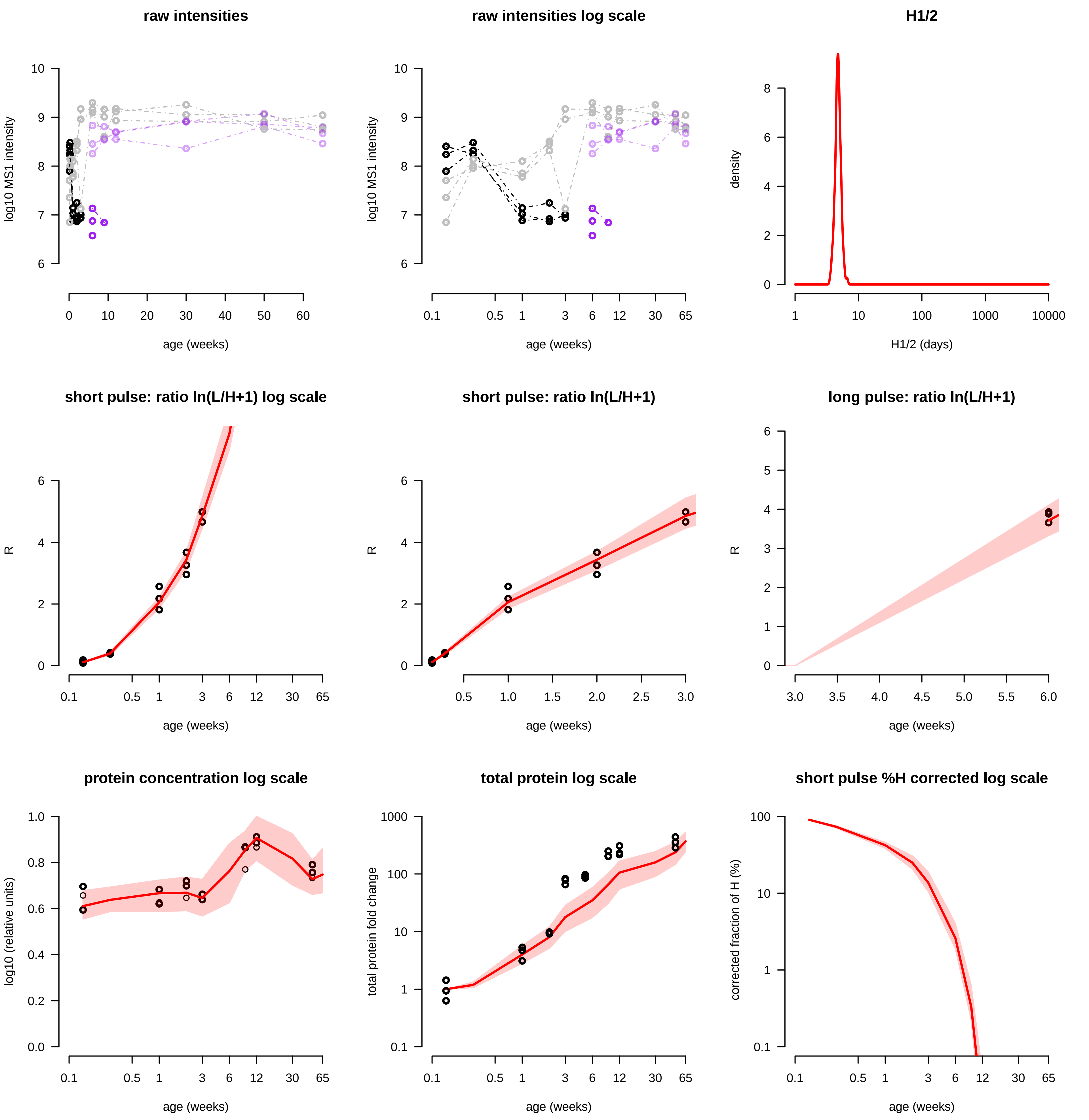

Q61584−3;Q61584;Q61584−6;Q61584−2;Q61584−7

>sp|Q61584−3|FXR1_MOUSE

Isoform B of Fragile X mental retardation syndrome−related protein 1

GN=Fxr1

Med H1/2: 3368 days

Q5 H1/2: 1279.8 days

Q95 H1/2: 66589.1 days

reliable model fit: yes

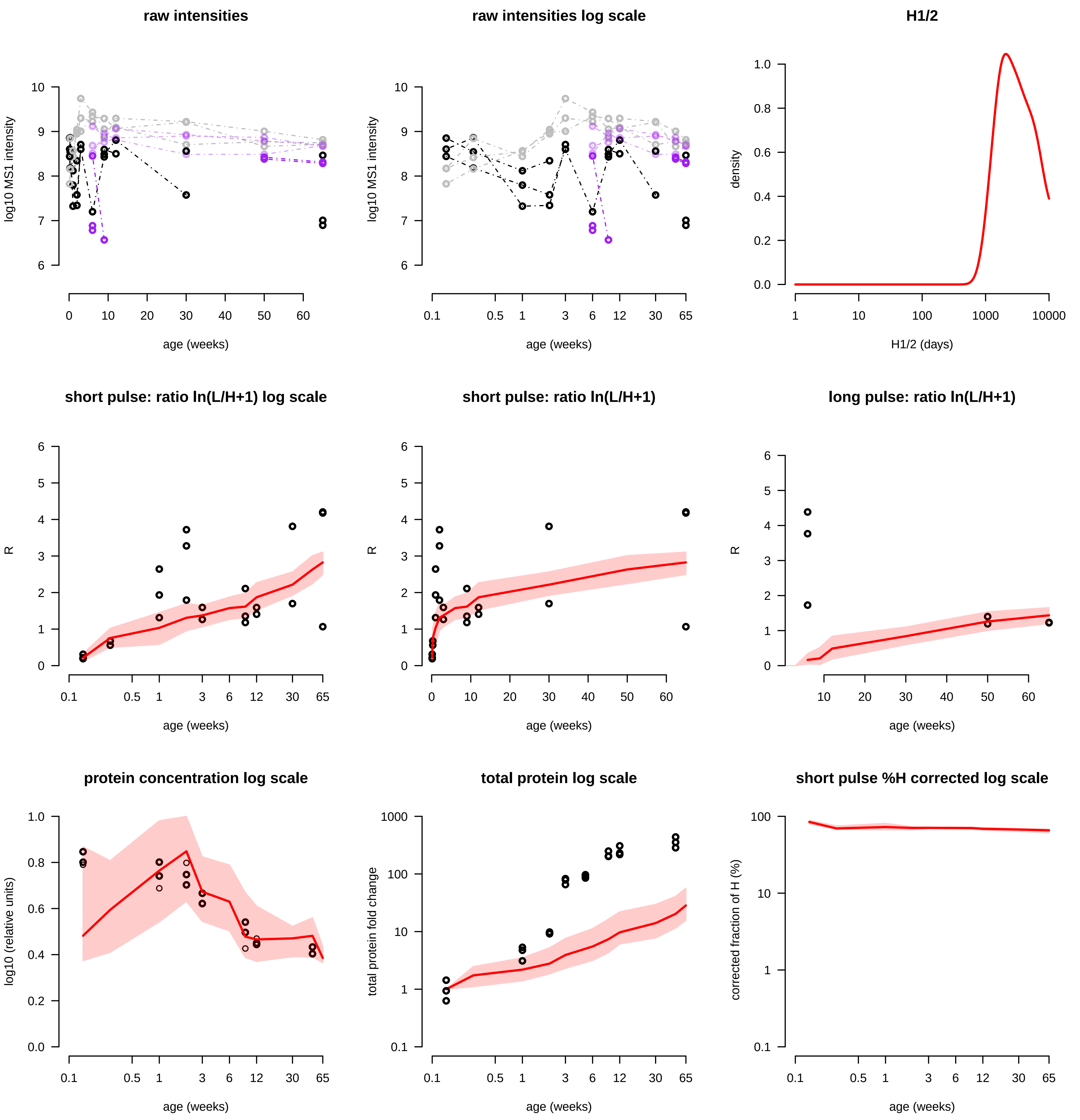

O88653

>sp|O88653|LTOR3_MOUSE

Ragulator complex protein LAMTOR3

GN=Lamtor3

Med H1/2: 4.3 days

Q5 H1/2: 2.7 days

Q95 H1/2: 8.7 days

reliable model fit: yes

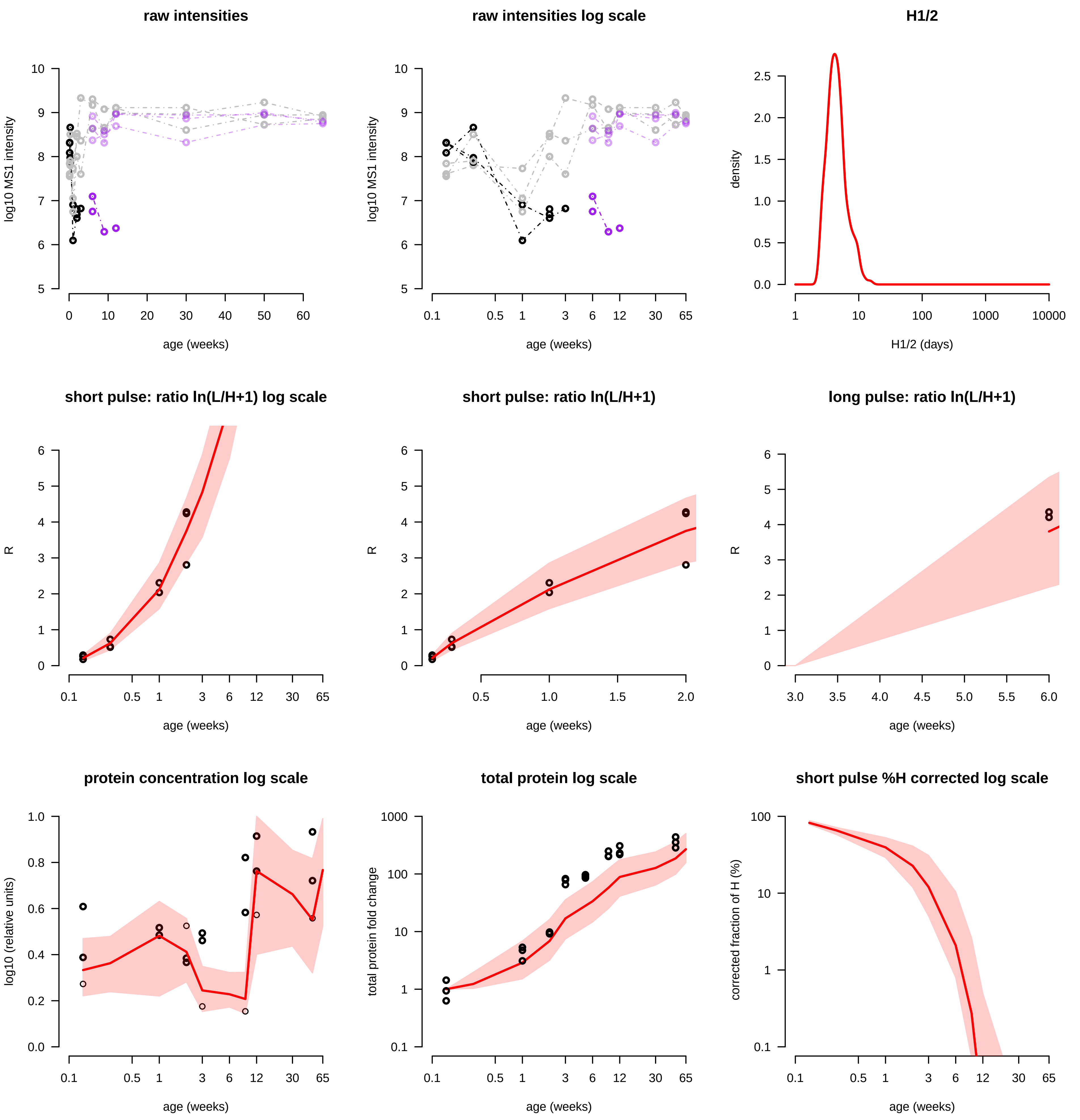

Q8BKG3

>sp|Q8BKG3|PTK7_MOUSE

Inactive tyrosine−protein kinase 7

GN=Ptk7

Med H1/2: 3.4 days

Q5 H1/2: 2.8 days

Q95 H1/2: 4.4 days

reliable model fit: yes

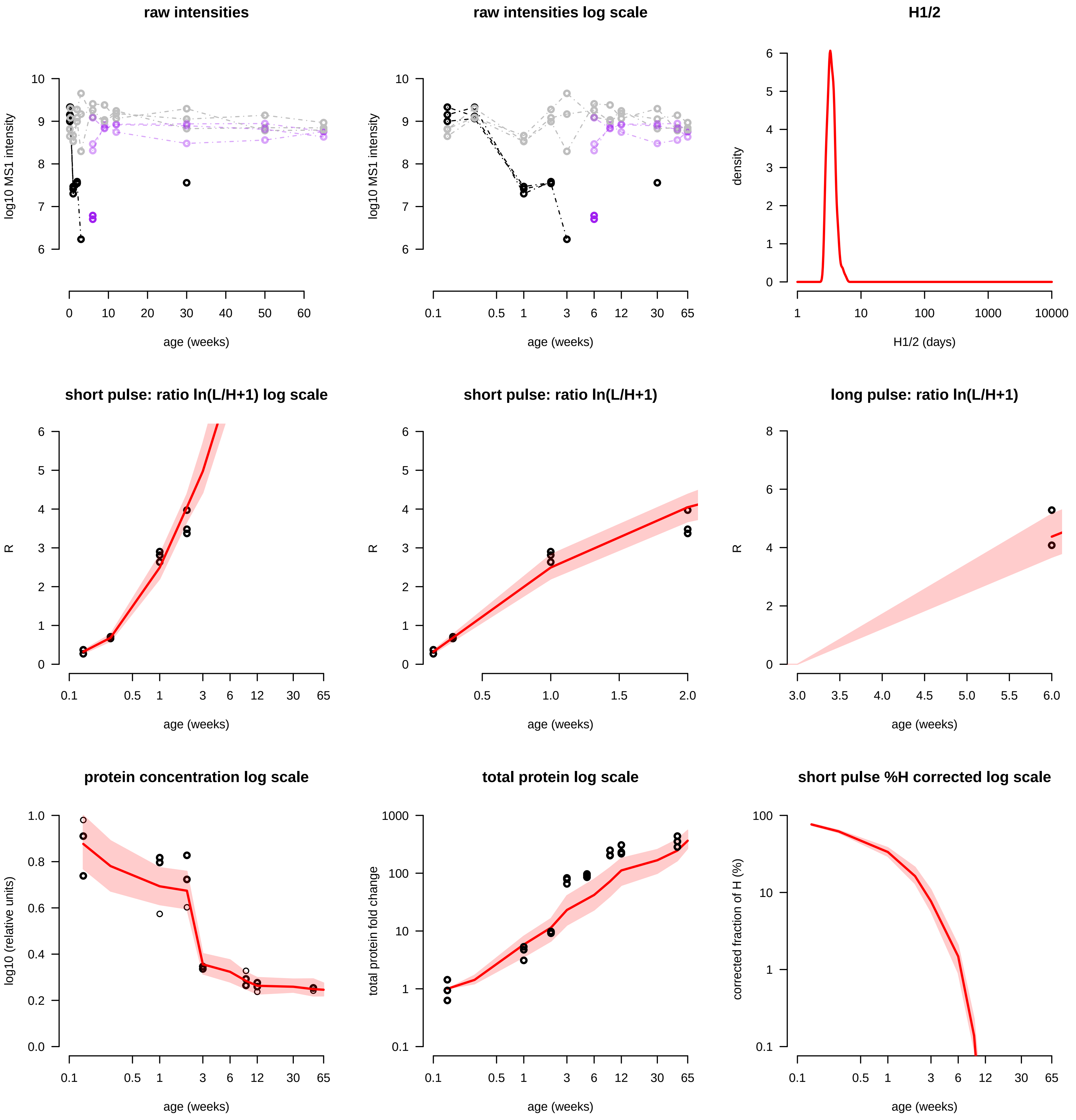

Q6A068

>sp|Q6A068|CDC5L_MOUSE

Cell division cycle 5−like protein

GN=Cdc5l

Med H1/2: 4.9 days

Q5 H1/2: 4 days

Q95 H1/2: 6.8 days

reliable model fit: no

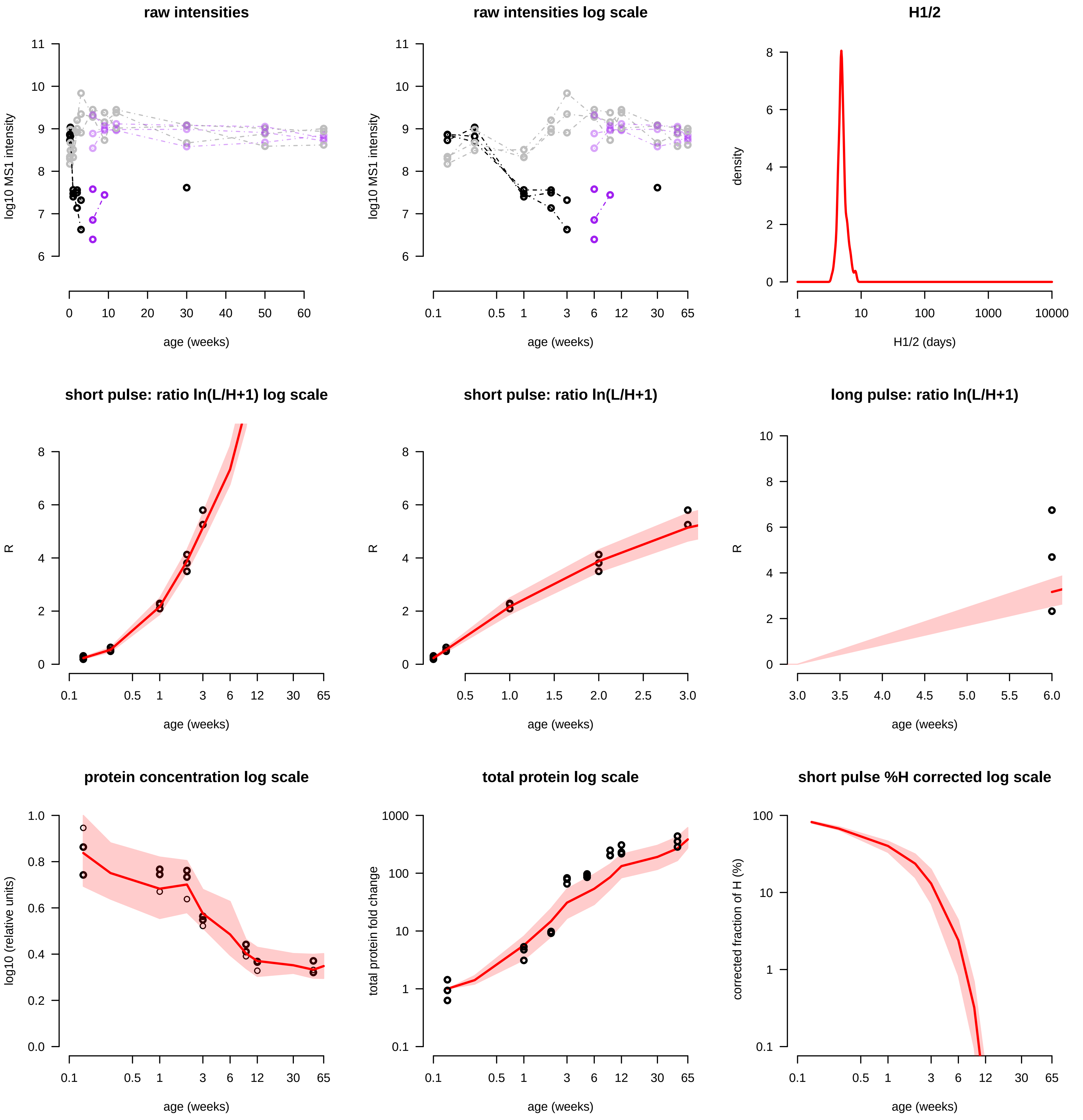

Q6PFD9

>sp|Q6PFD9|NUP98_MOUSE

Nuclear pore complex protein Nup98−Nup96

GN=Nup98

Med H1/2: 3.3 days

Q5 H1/2: 2.6 days

Q95 H1/2: 4.3 days

reliable model fit: yes

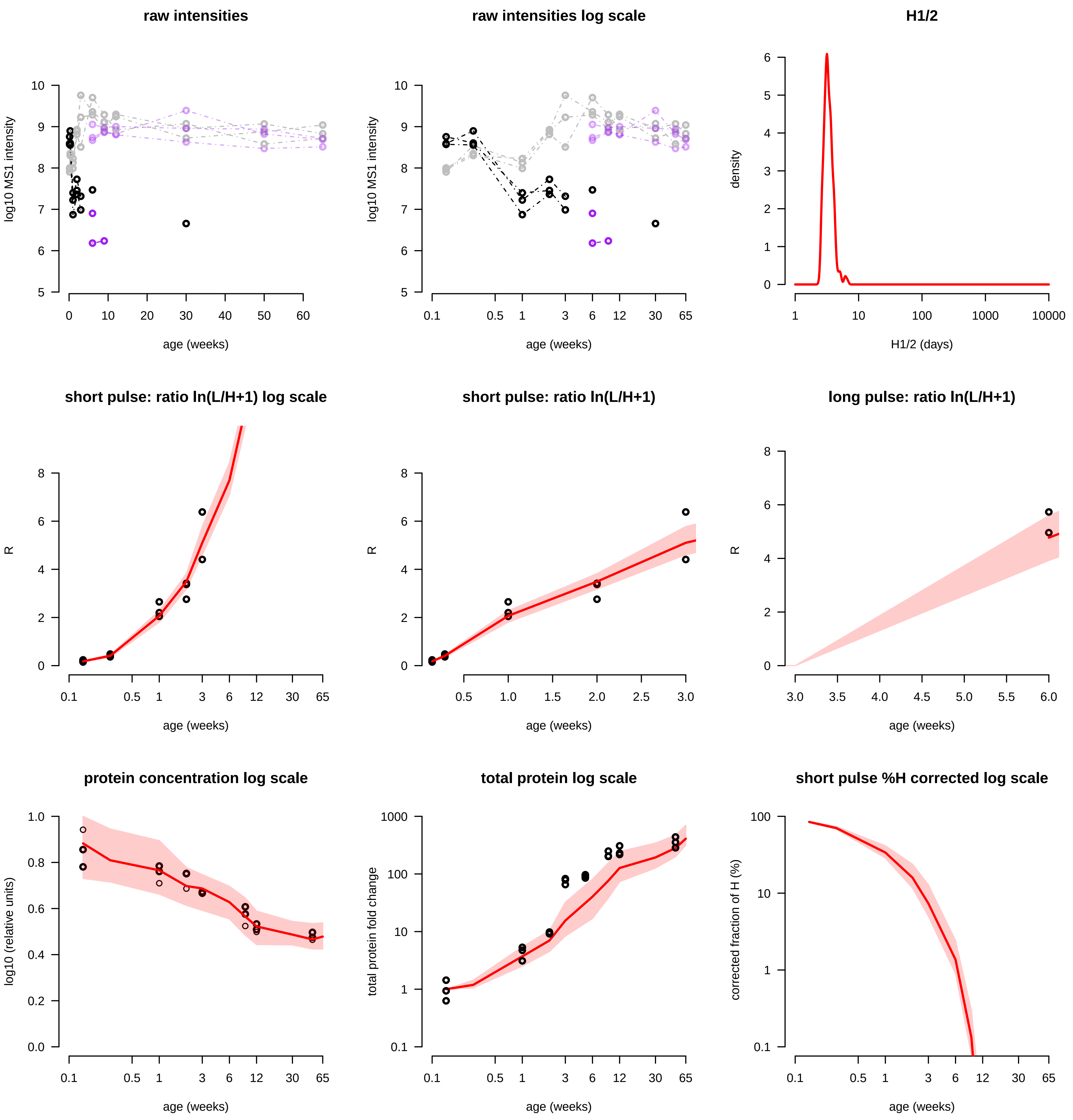

P62900

>sp|P62900|RL31_MOUSE

60S ribosomal protein L31

GN=Rpl31

Med H1/2: 8.2 days

Q5 H1/2: 6.2 days

Q95 H1/2: 12 days

reliable model fit: yes

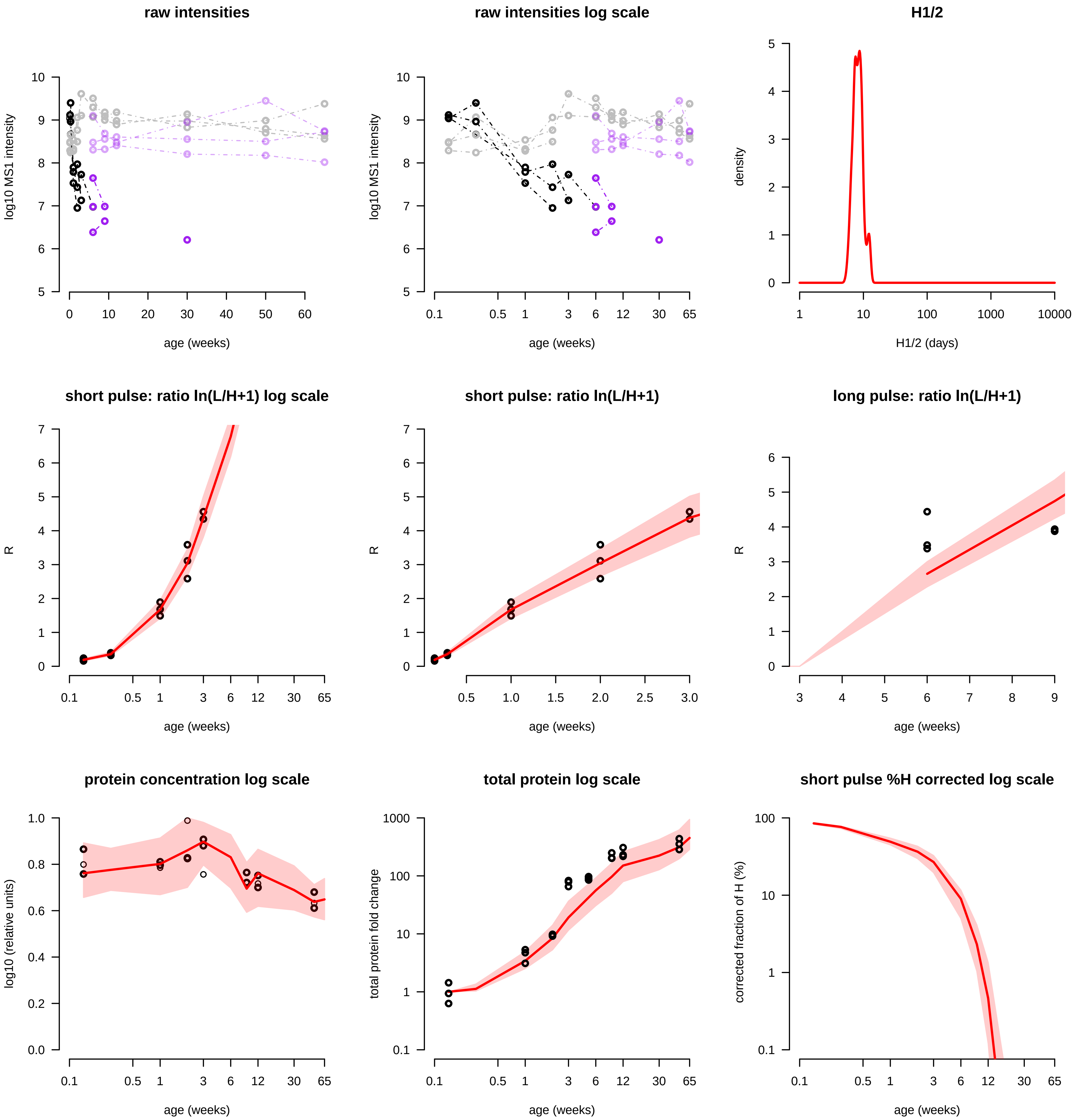

Q99LC3

>sp|Q99LC3|NDUAA_MOUSE

IADH dehydrogenase [ubiquinone] 1 alpha subcomplex subunit 10, mitochondria

GN=Ndufa10

Med H1/2: 7.6 days

Q5 H1/2: 5.6 days

Q95 H1/2: 10.7 days

reliable model fit: yes

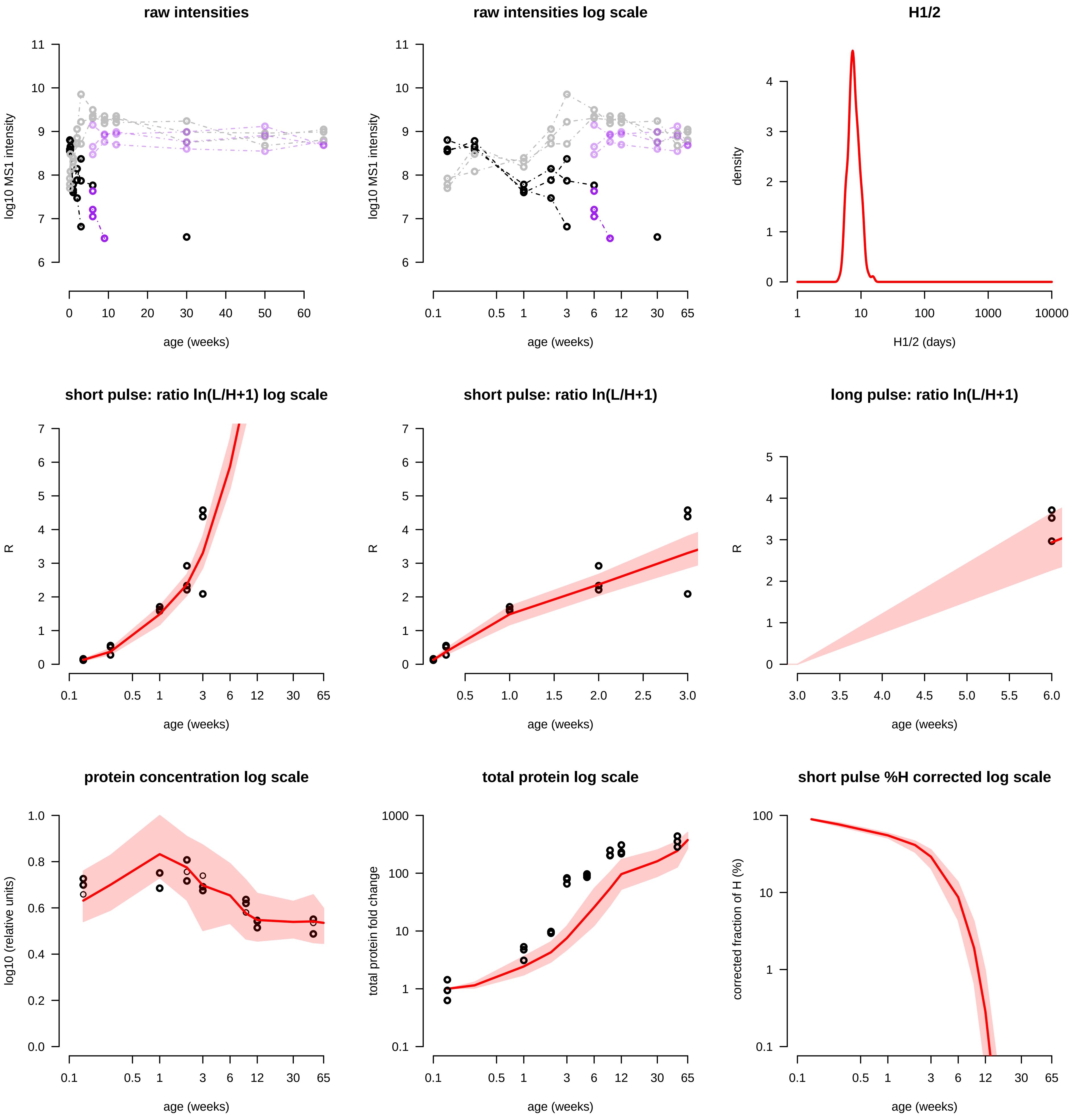

Q8BG51−3

>sp|Q8BG51−3|MIRO1_MOUSE

Isoform 3 of Mitochondrial Rho GTPase 1

GN=Rhot1

Med H1/2: 29.7 days

Q5 H1/2: 8 days

Q95 H1/2: 387.7 days

reliable model fit: yes

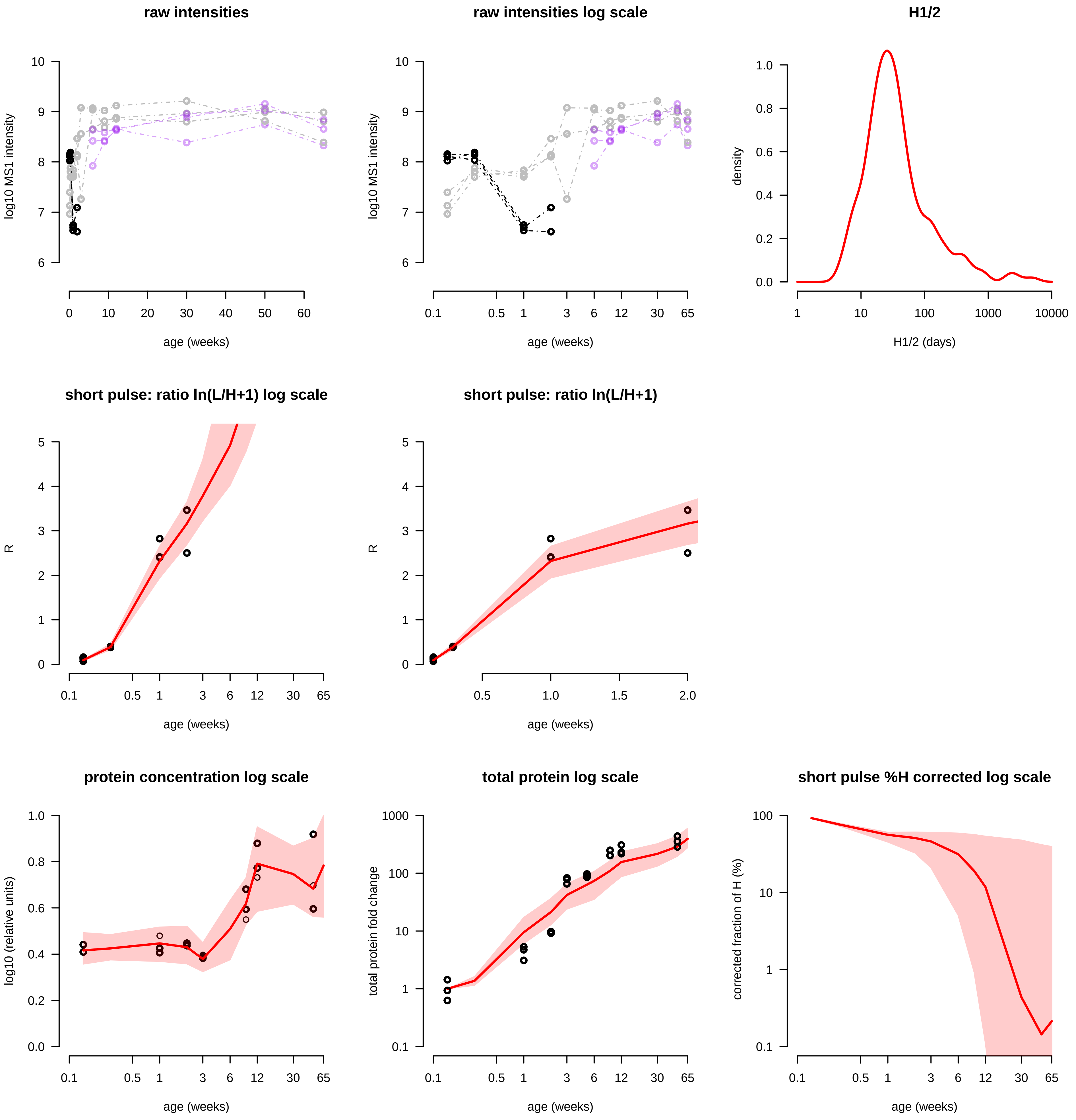

P46061

>sp|P46061|RAGP1_MOUSE

Ran GTPase−activating protein 1

GN=Rangap1

Med H1/2: 7.7 days

Q5 H1/2: 6.2 days

Q95 H1/2: 9.9 days

reliable model fit: yes

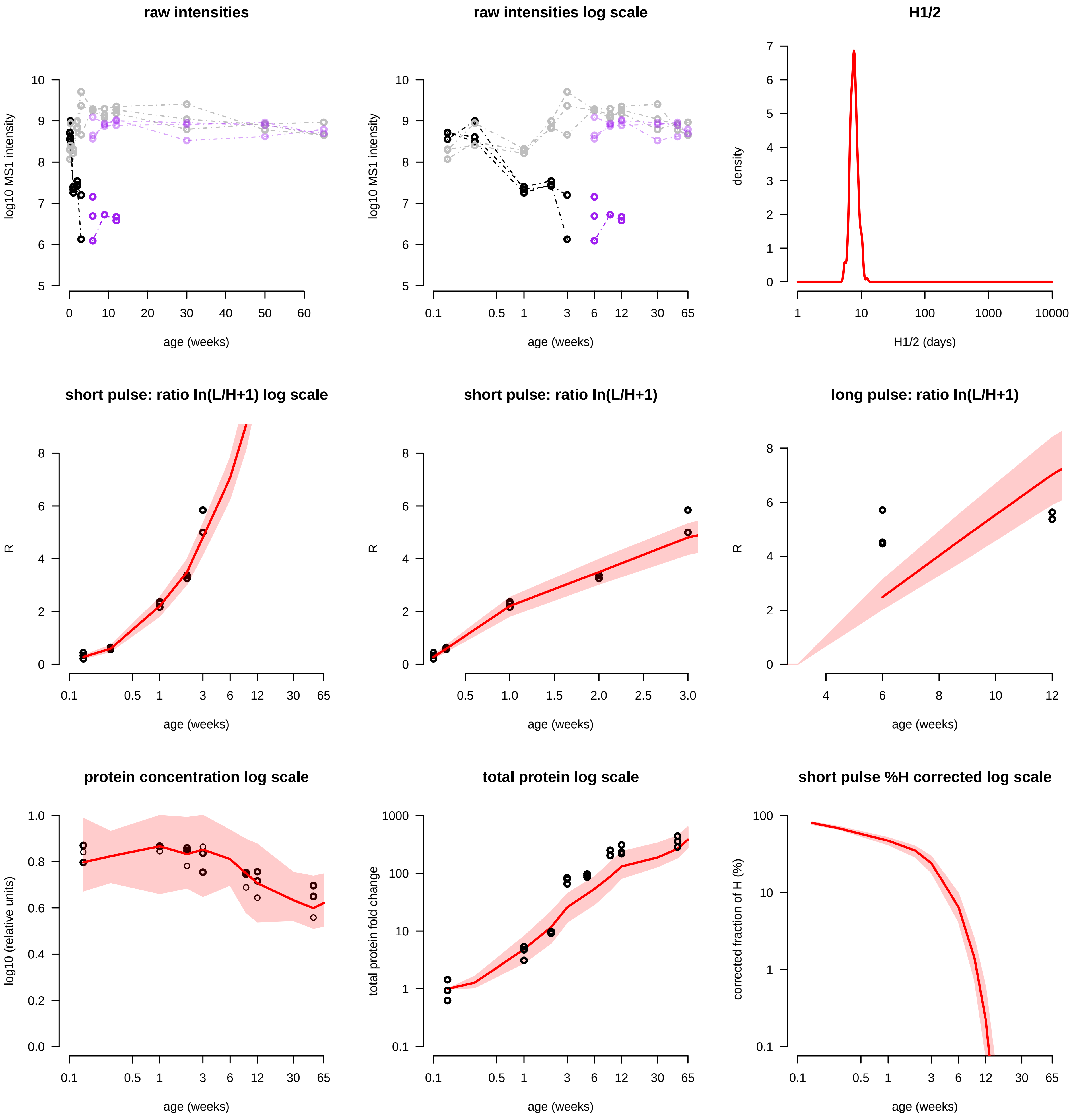

Q9CQR4

>sp|Q9CQR4|ACO13_MOUSE

Acyl−coenzyme A thioesterase 13

GN=Acot13

Med H1/2: 6.5 days

Q5 H1/2: 4.2 days

Q95 H1/2: 12.2 days

reliable model fit: yes

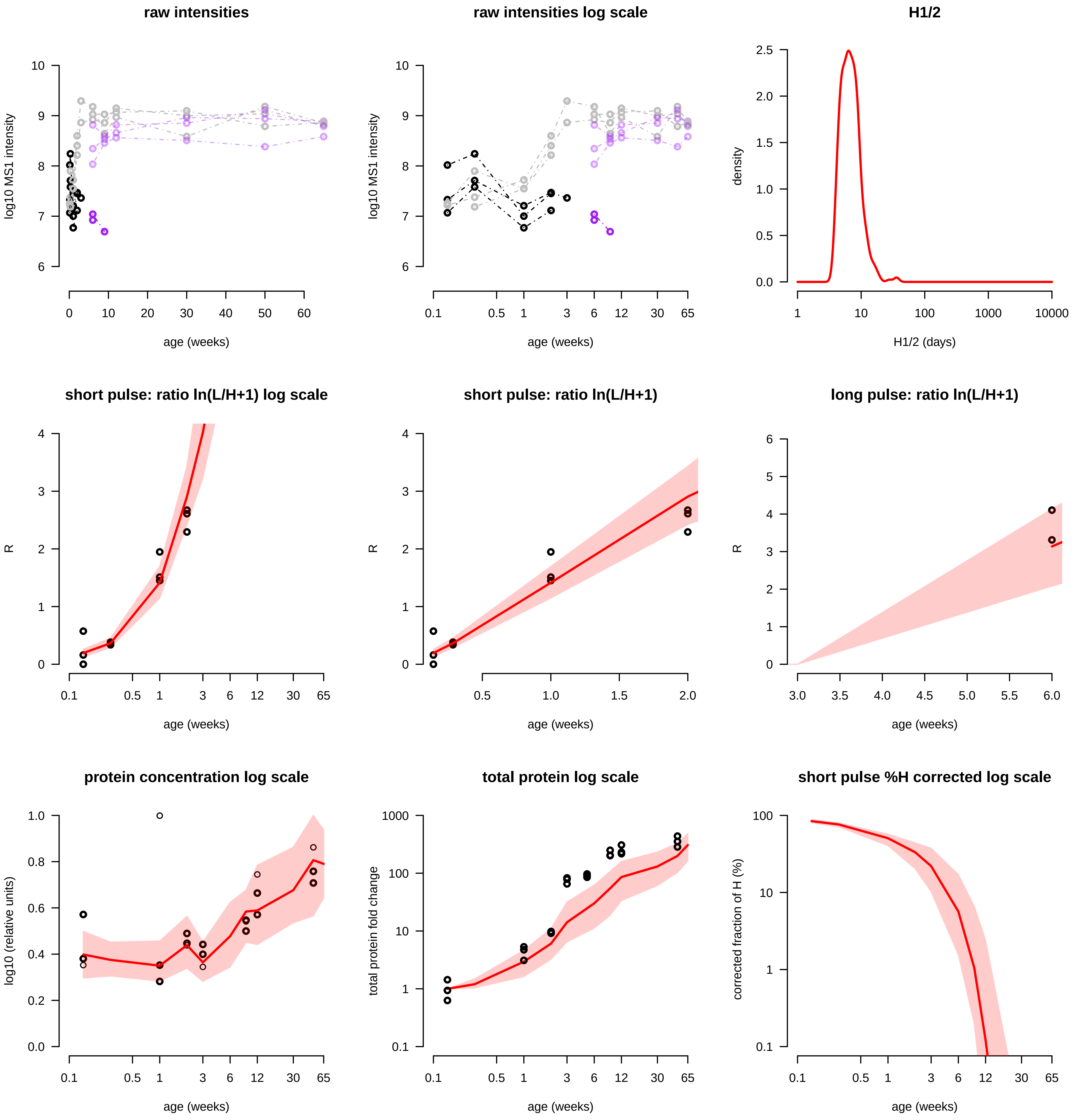

Q9JI78

>sp|Q9JI78|NGLY1_MOUSE

Peptide−N(4)−(N−acetyl−beta−glucosaminyl)asparagine amidase

GN=Ngly1

Med H1/2: 7.6 days

Q5 H1/2: 3 days

Q95 H1/2: 43.4 days

reliable model fit: yes

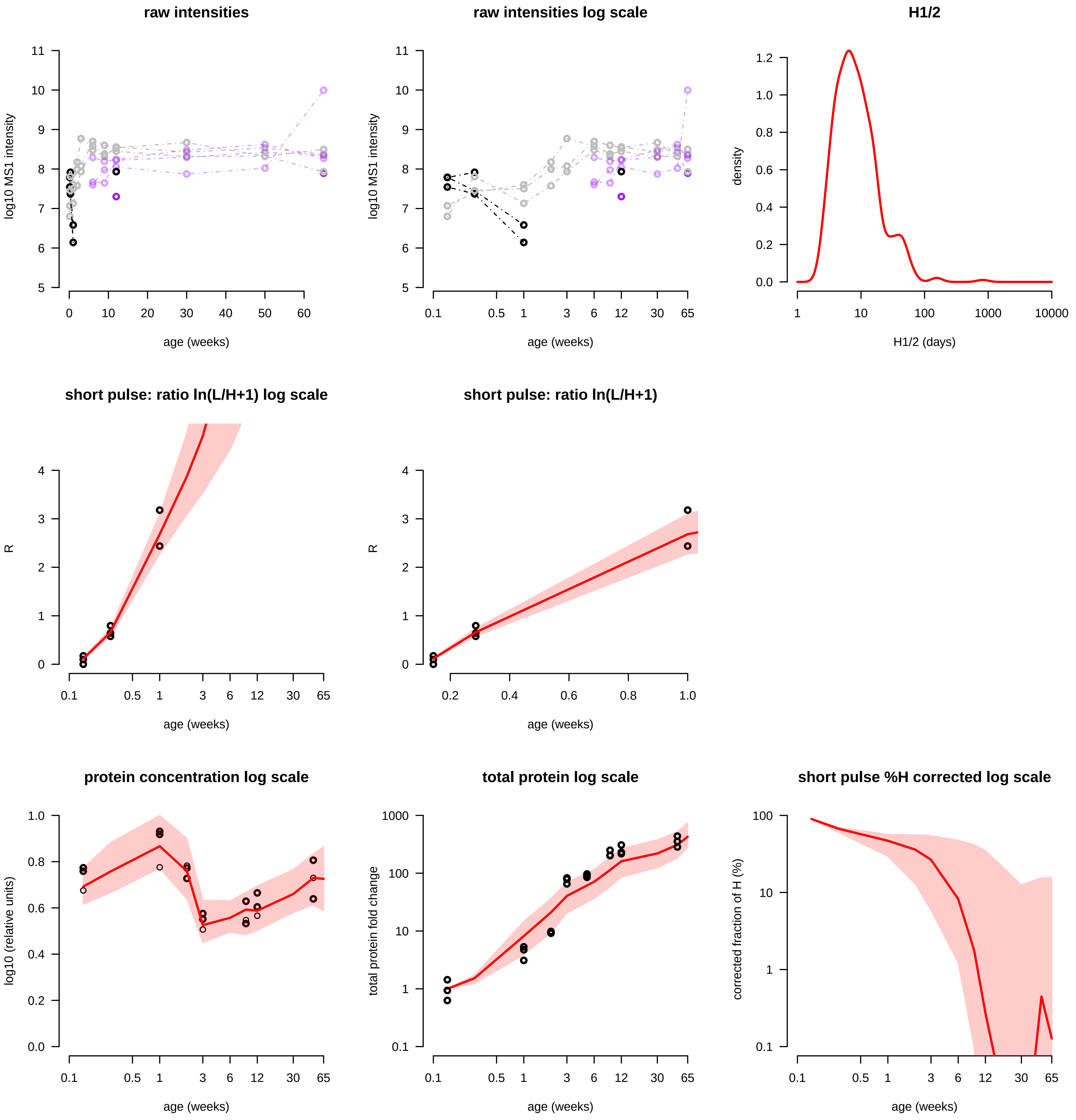

Q80XI3;Q80XI3−3

>sp|Q80XI3|IF4G3_MOUSE

Eukaryotic translation initiation factor 4 gamma 3

GN=Eif4g3

Med H1/2: 4.7 days

Q5 H1/2: 3.4 days

Q95 H1/2: 7.1 days

reliable model fit: yes

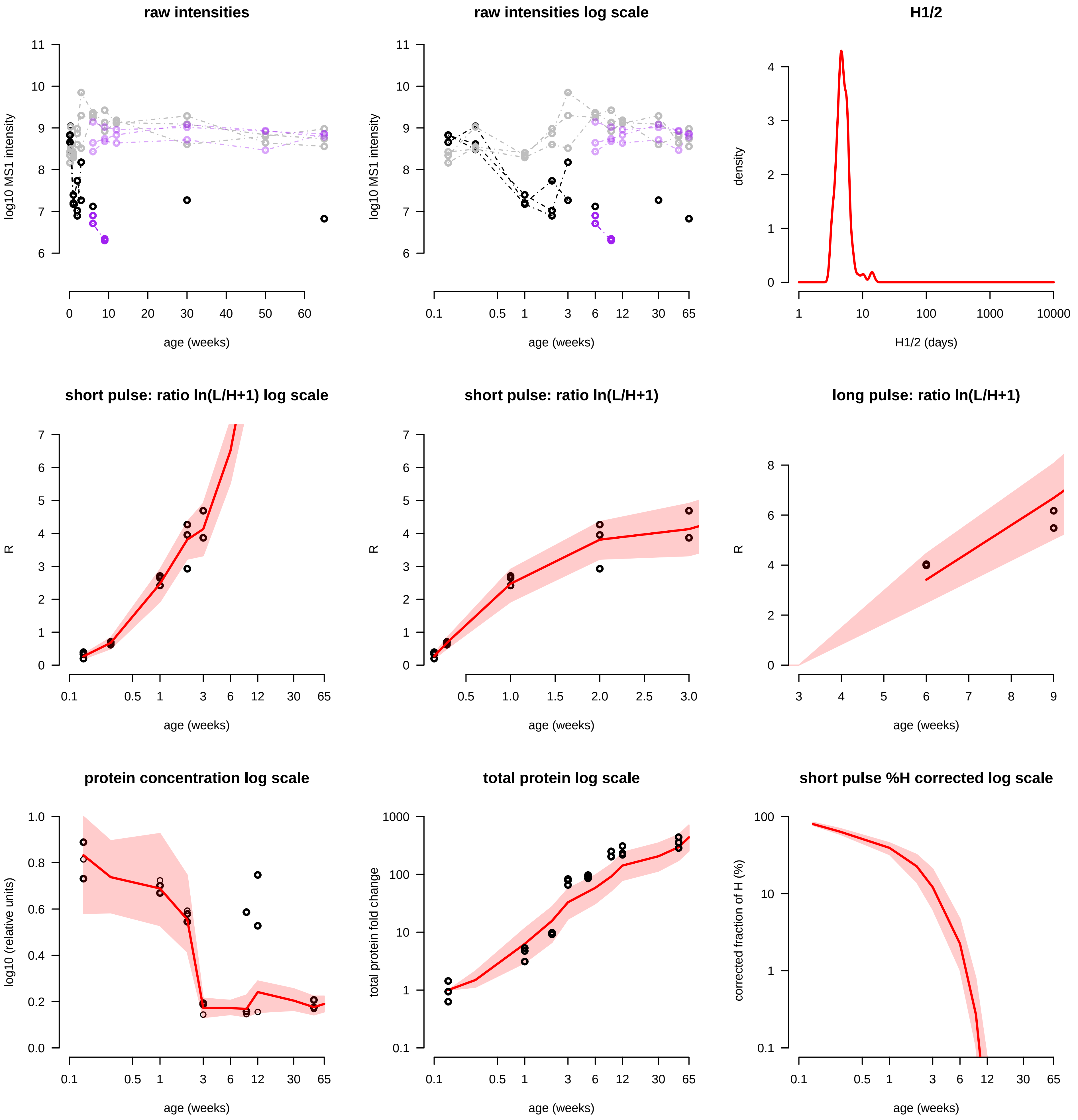

Q01405

>sp|Q01405|SC23A_MOUSE

Protein transport protein Sec23A

GN=Sec23a

Med H1/2: 4.9 days

Q5 H1/2: 3.9 days

Q95 H1/2: 6 days

reliable model fit: yes

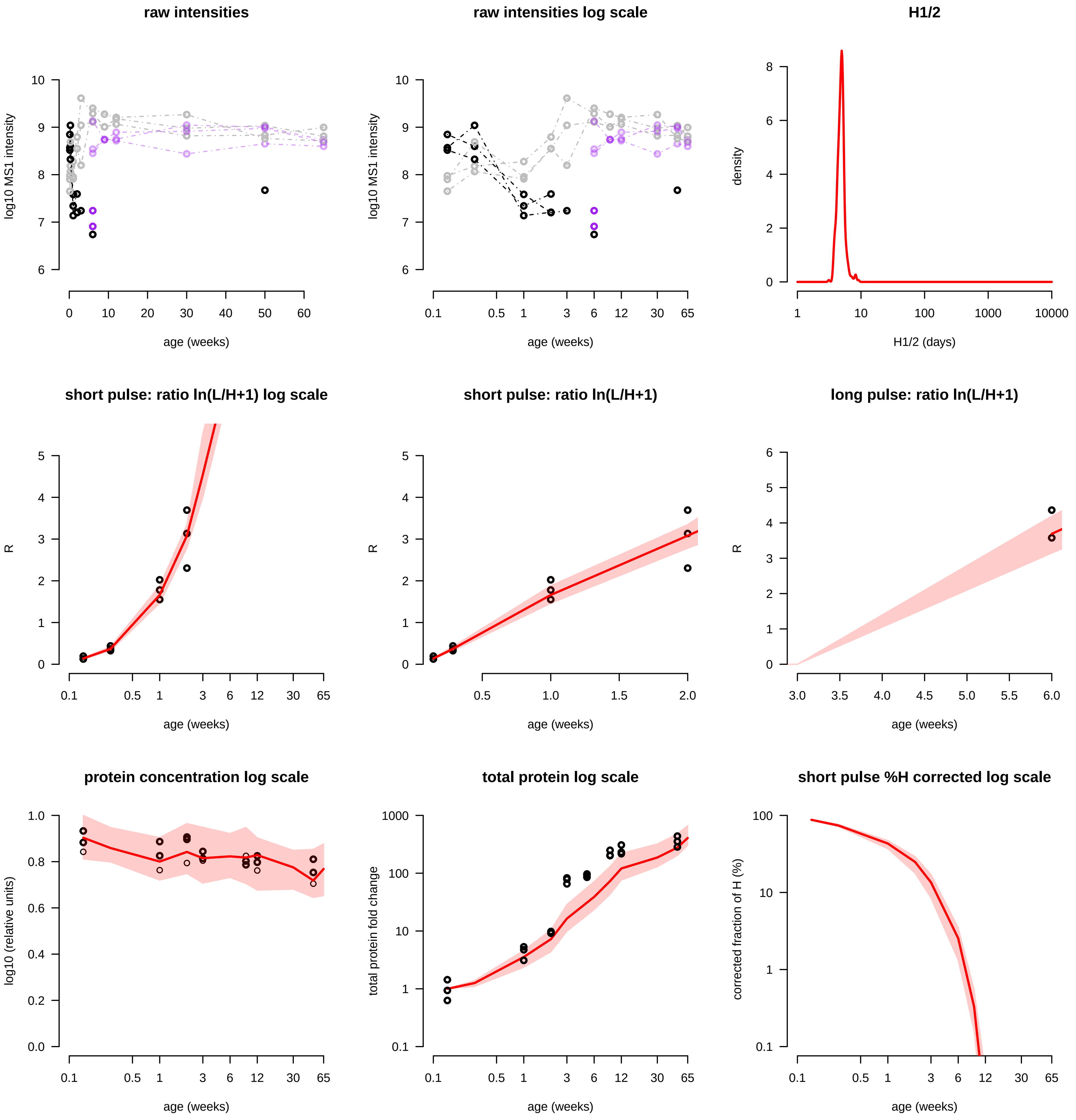

Q61655

>sp|Q61655|DD19A_MOUSE

ATP−dependent RNA helicase DDX19A

GN=Ddx19a

Med H1/2: 5.4 days

Q5 H1/2: 4.5 days

Q95 H1/2: 6.1 days

reliable model fit: yes

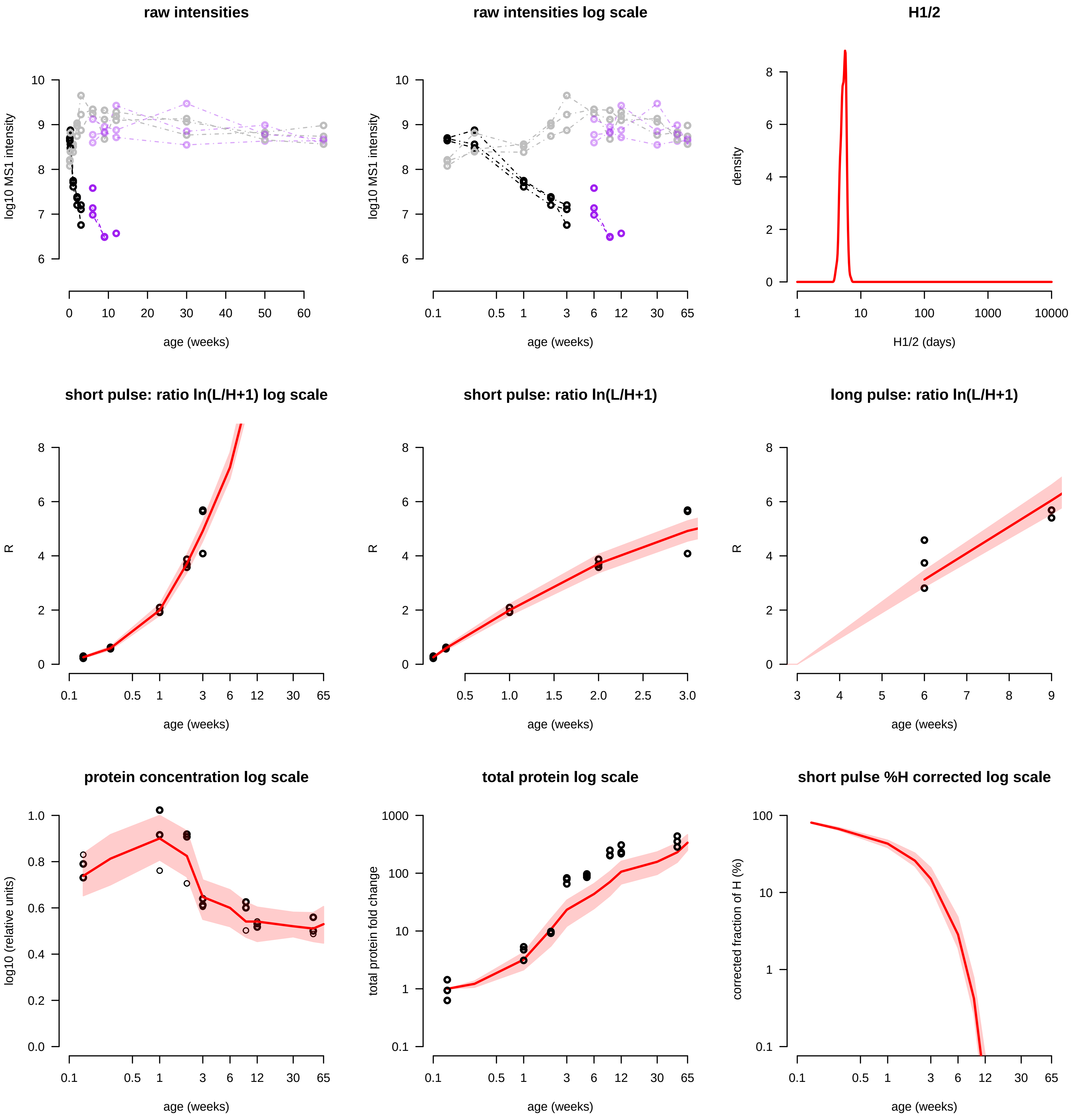

Q99KH8

>sp|Q99KH8|STK24_MOUSE

Serine/threonine−protein kinase 24

GN=Stk24

Med H1/2: 4 days

Q5 H1/2: 2.9 days

Q95 H1/2: 5 days

reliable model fit: yes

raw intensities

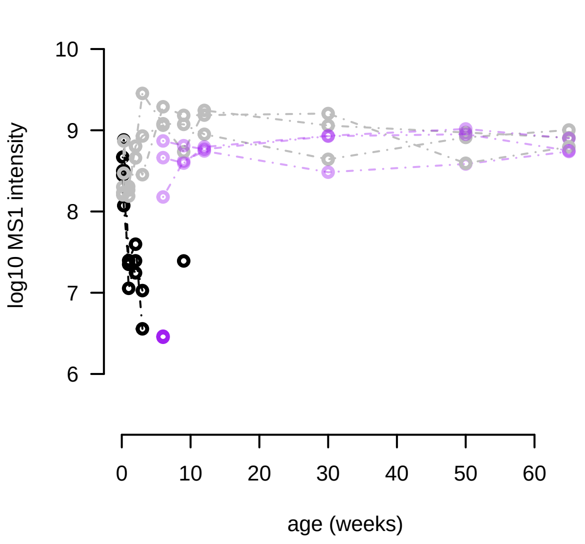

raw intensities log scale

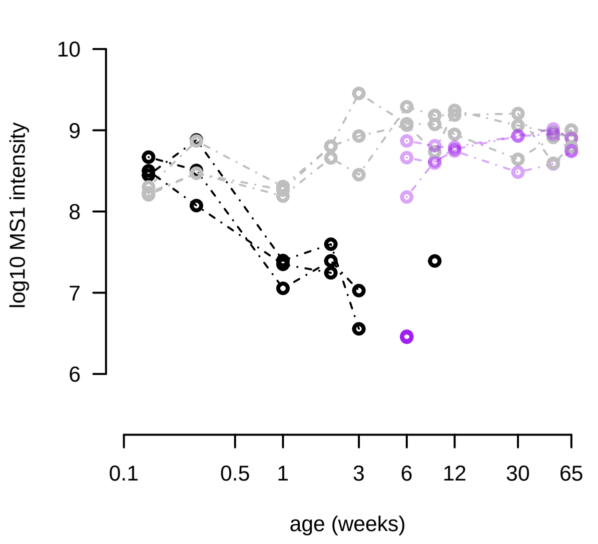

H1/2

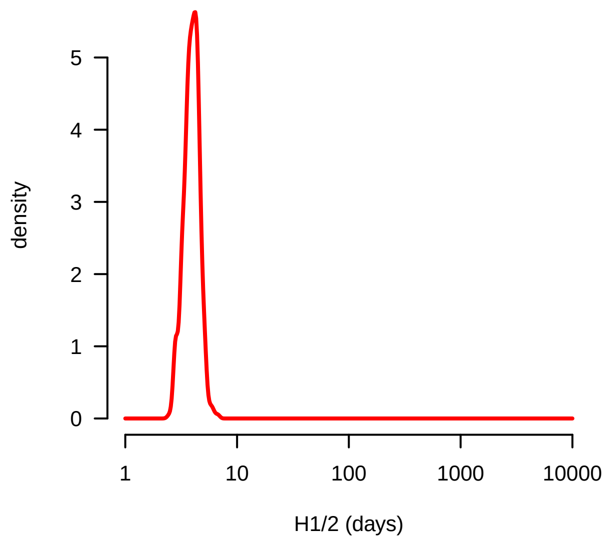

short pulse: ratio ln(L/H+1) log scale

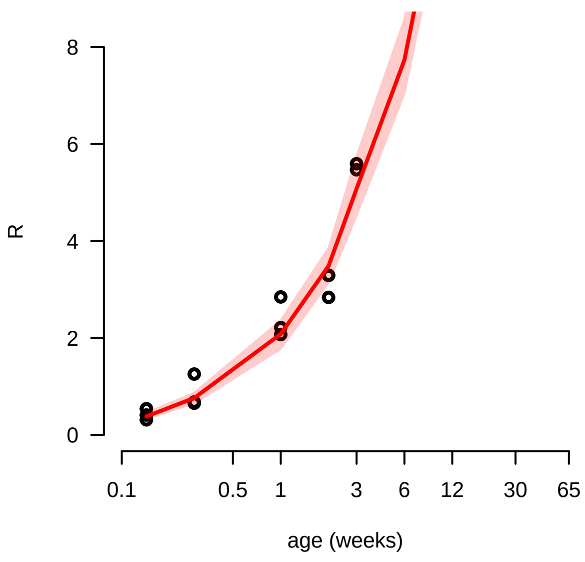

short pulse: ratio ln(L/H+1)

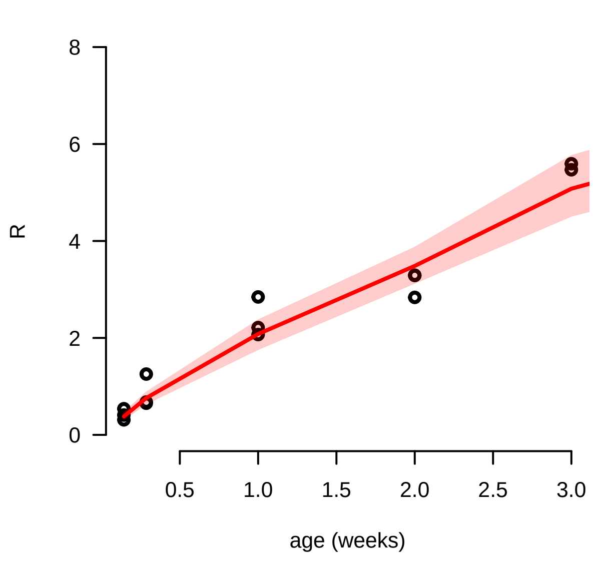

long pulse: ratio ln(L/H+1)

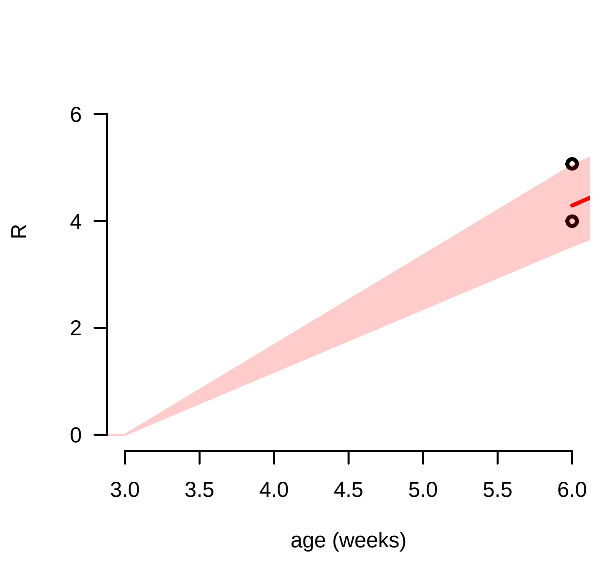

protein concentration log scale

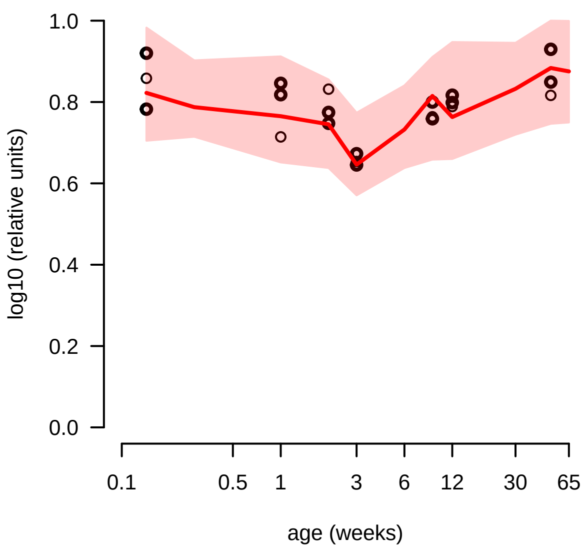

total protein log scale

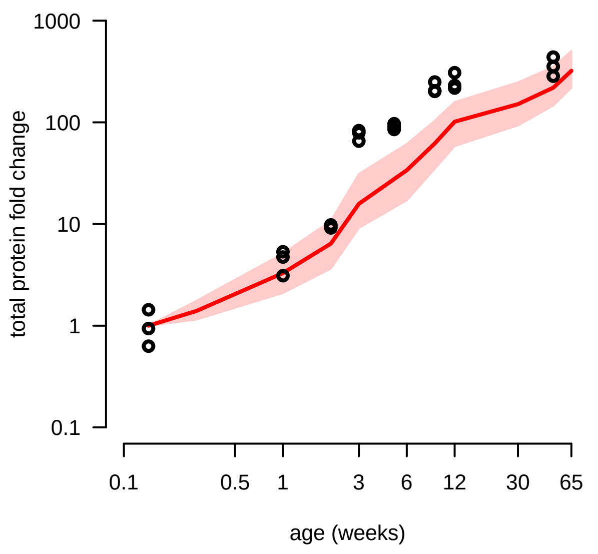

short pulse %H corrected log scale

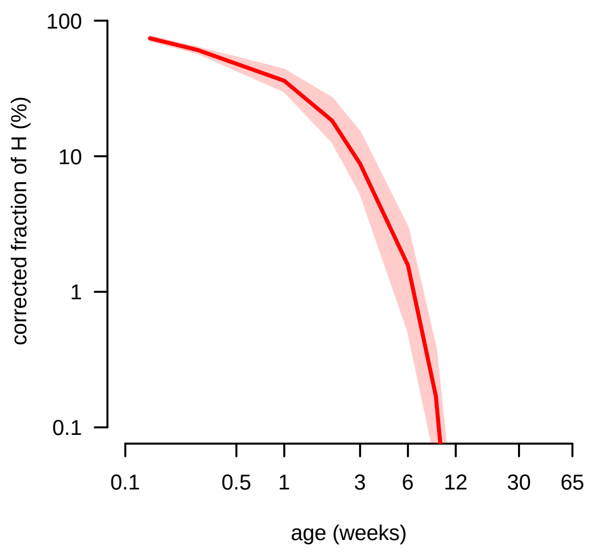

P97496−2;P97496

>sp|P97496−2|SMRC1_MOUSE

Isoform 2 of SWI/SNF complex subunit SMARCC1

GN=Smarcc1

Med H1/2: 3.9 days

Q5 H1/2: 3 days

Q95 H1/2: 4.9 days

reliable model fit: yes

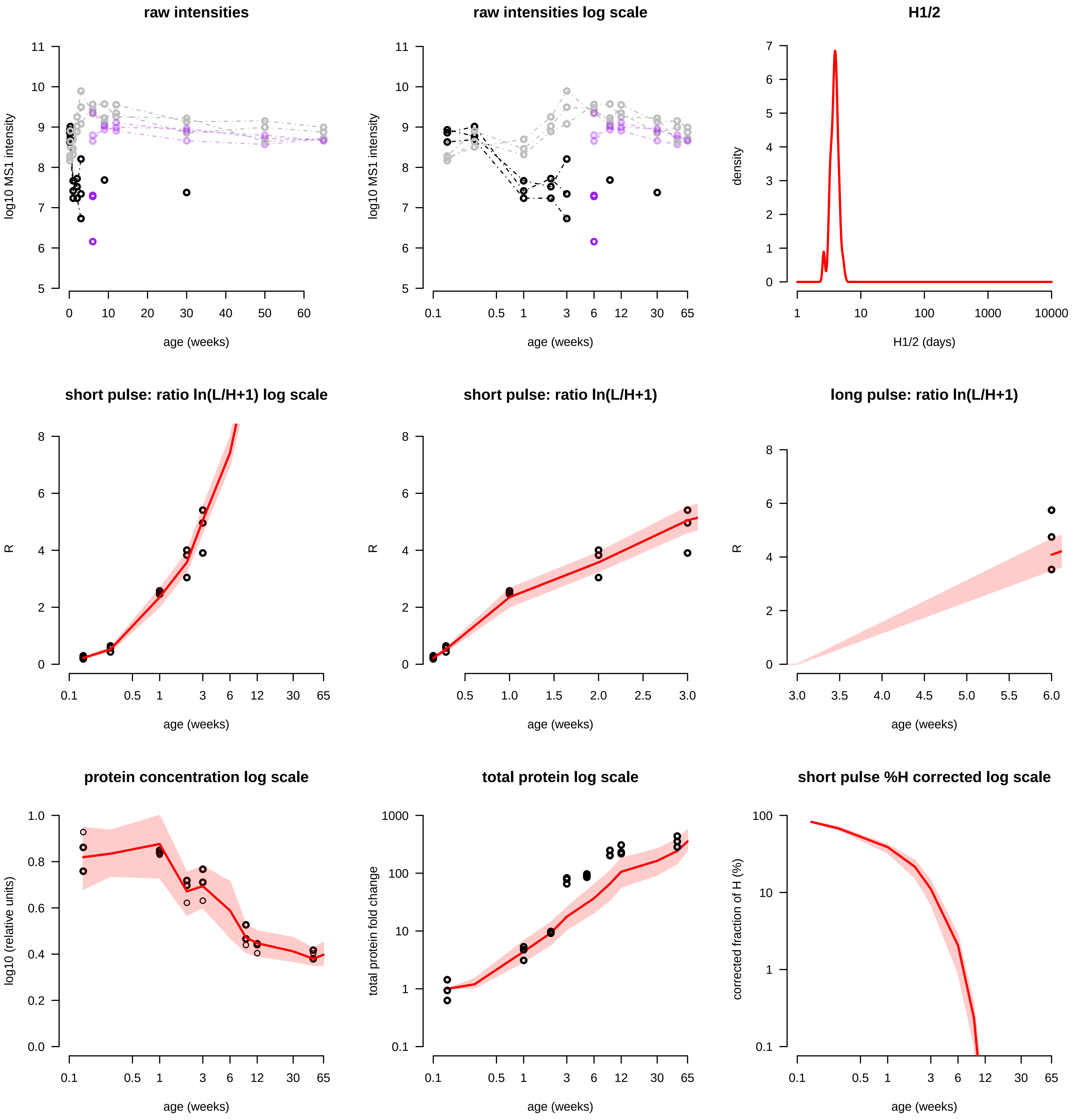

Q8R0F8

>sp|Q8R0F8|FAHD1_MOUSE

Acylpyruvase FAHD1, mitochondrial

GN=Fahd1

Med H1/2: 507.7 days

Q5 H1/2: 106.3 days

Q95 H1/2: 4361.3 days

reliable model fit: yes

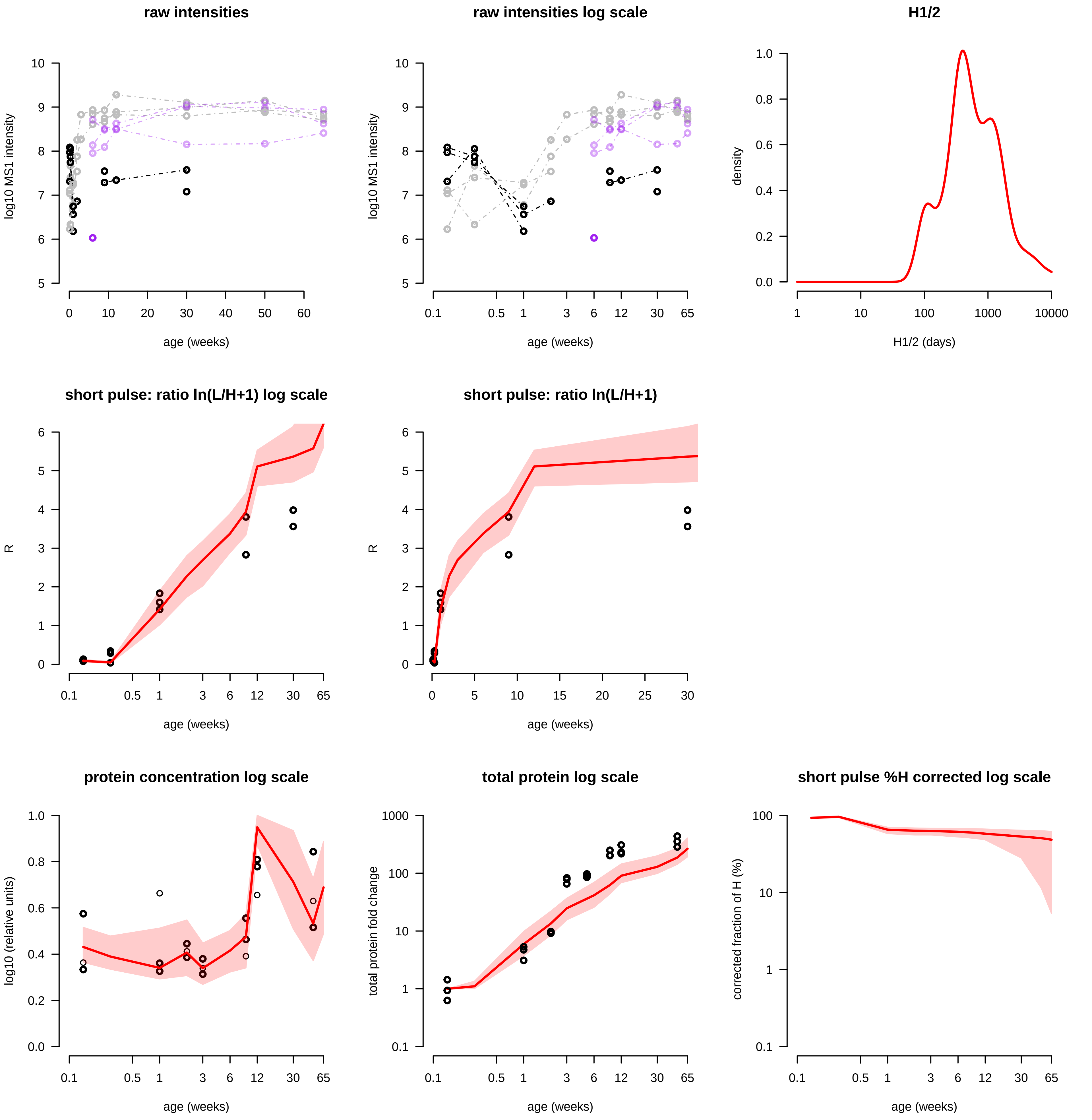

Q3UJU9

>sp|Q3UJU9|RMD3_MOUSE

Regulator of microtubule dynamics protein 3

GN=Rmdn3

Med H1/2: 6.7 days

Q5 H1/2: 2.7 days

Q95 H1/2: 37.9 days

reliable model fit: yes

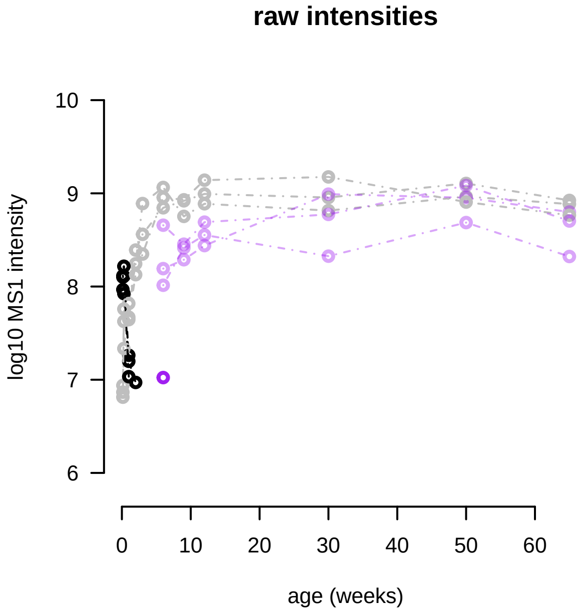

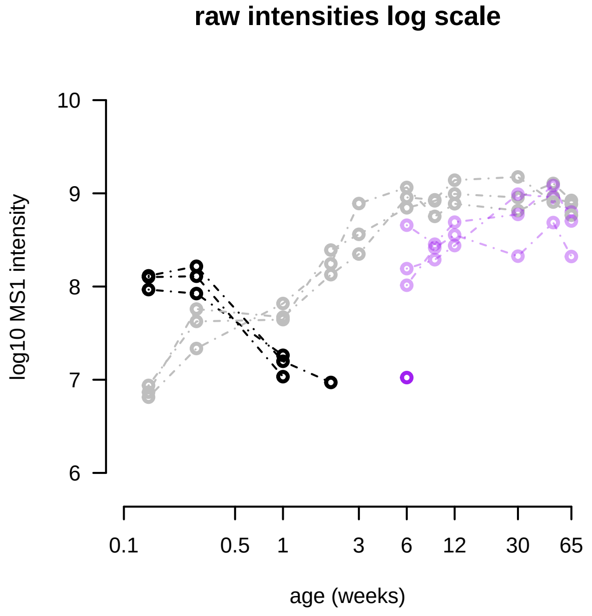

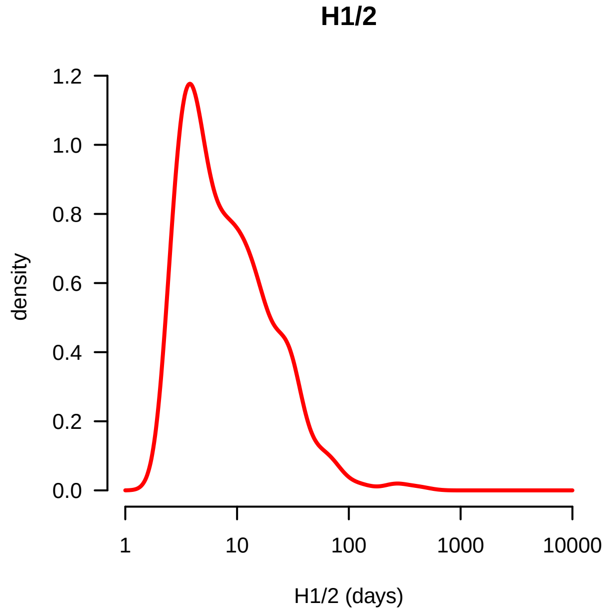

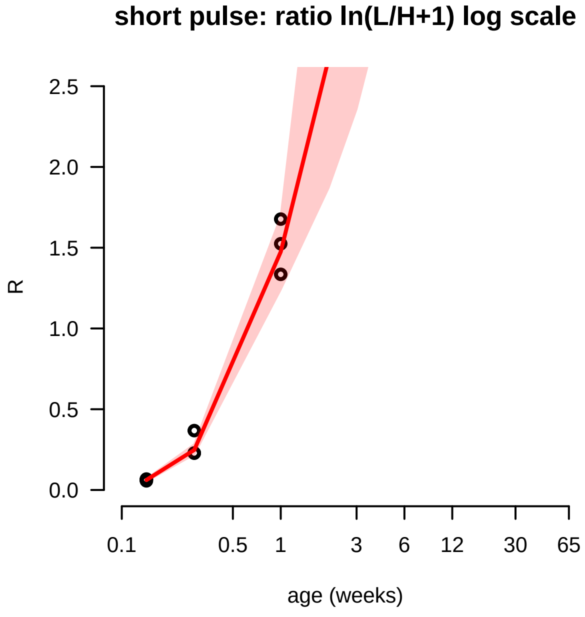

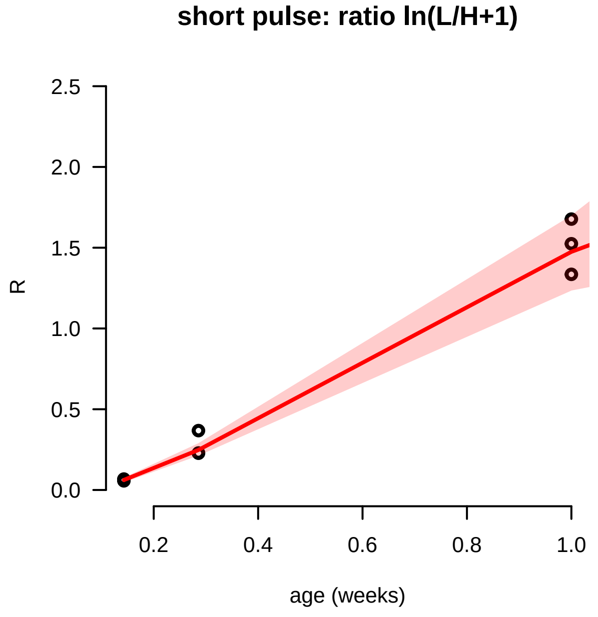

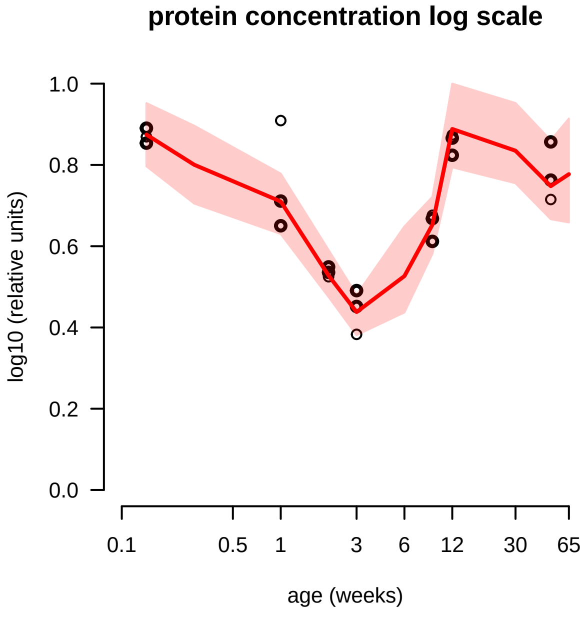

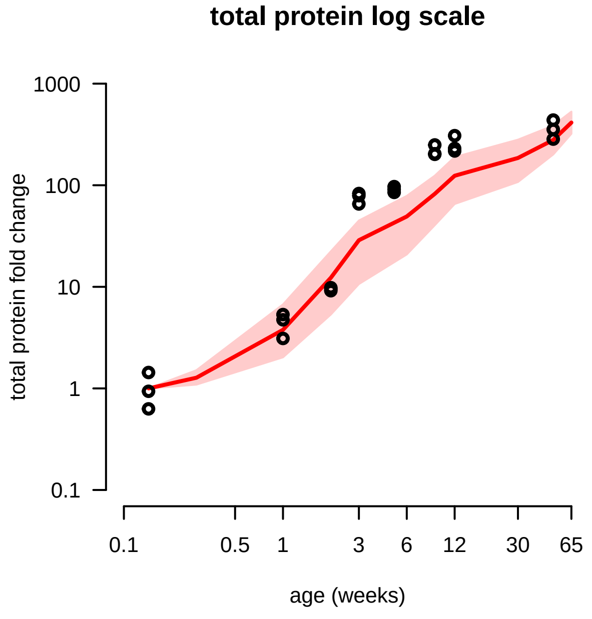

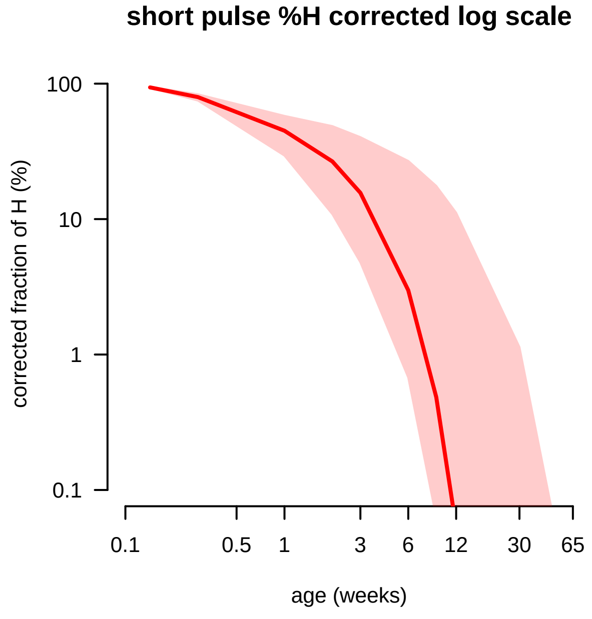

Q9R112

>sp|Q9R112|SQOR_MOUSE

Sulfide:quinone oxidoreductase, mitochondrial

GN=Sqor

Med H1/2: 10 days

Q5 H1/2: 3.5 days

Q95 H1/2: 78.5 days

reliable model fit: yes

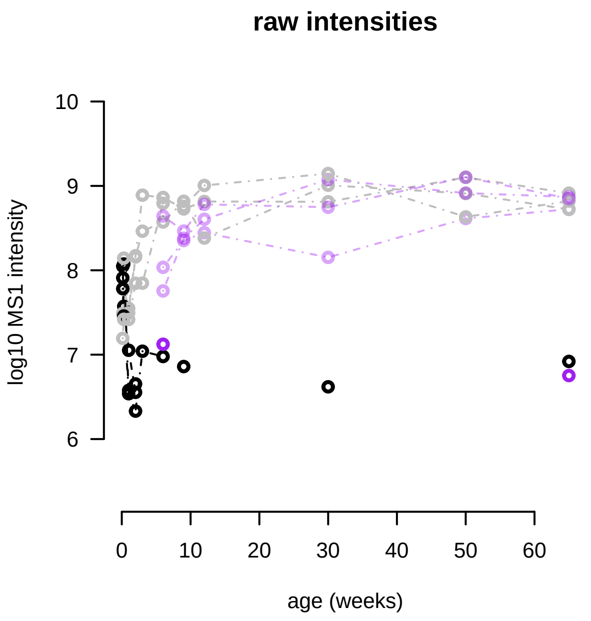

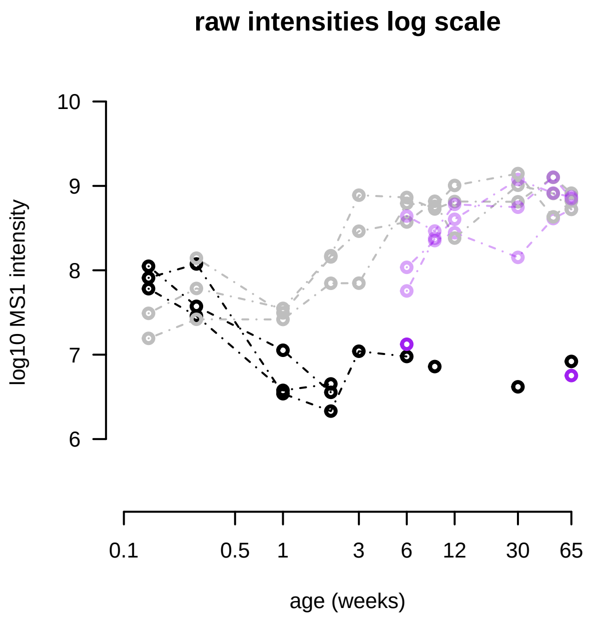

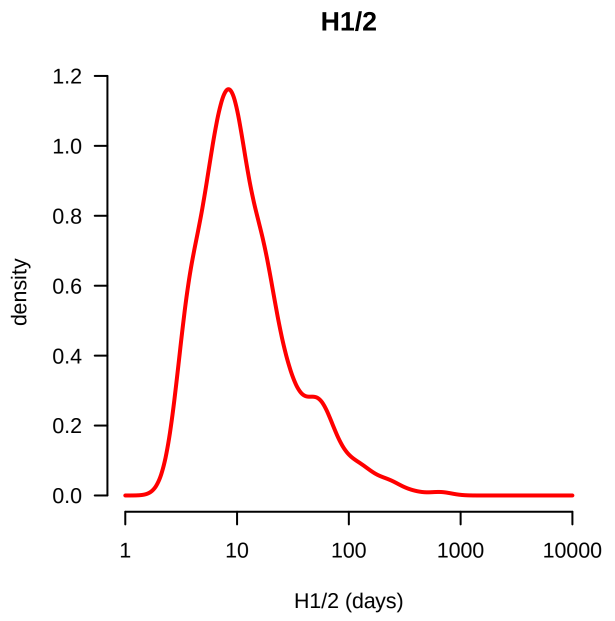

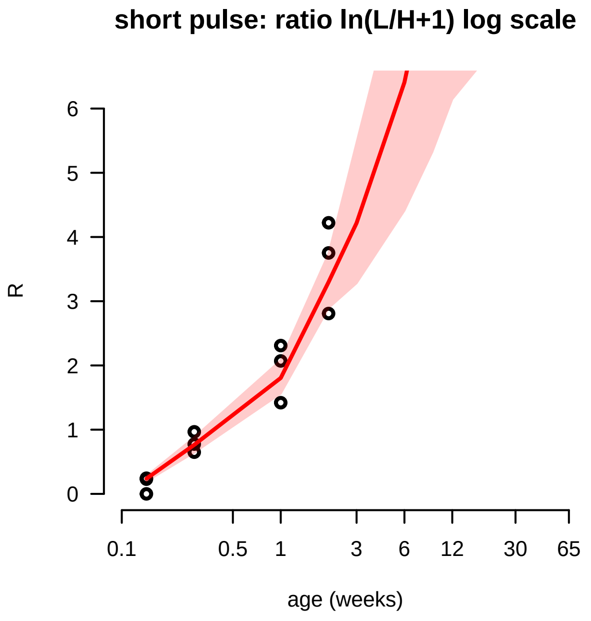

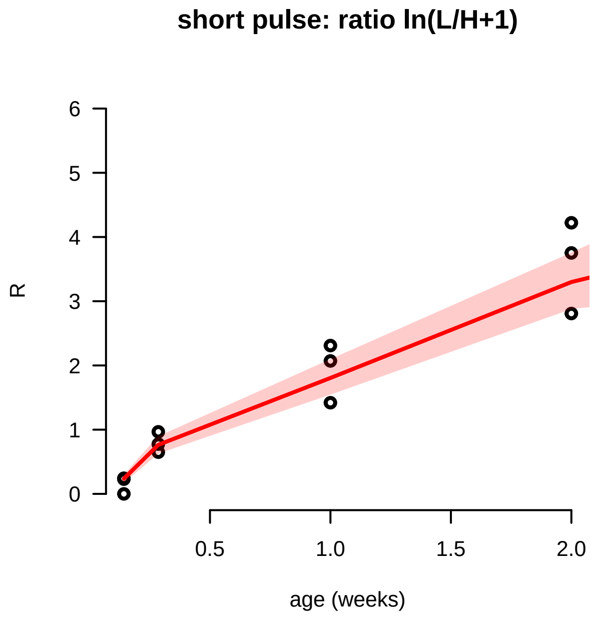

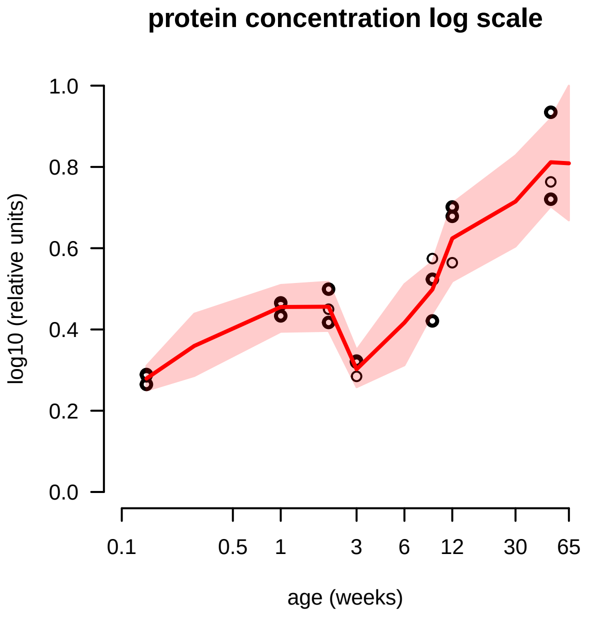

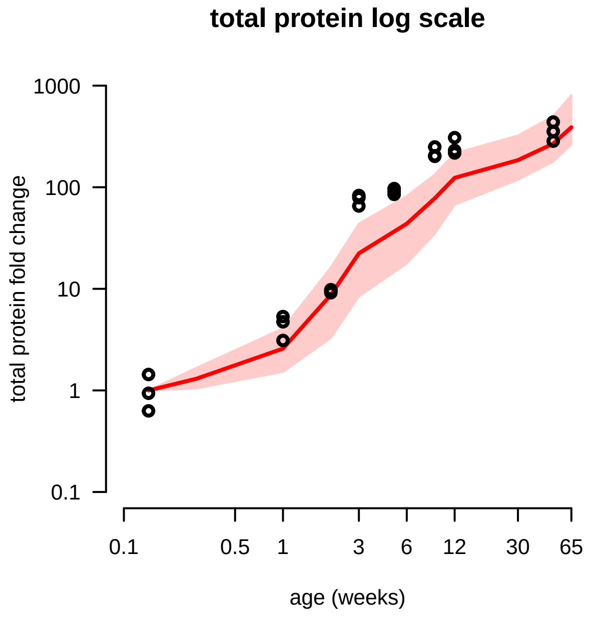

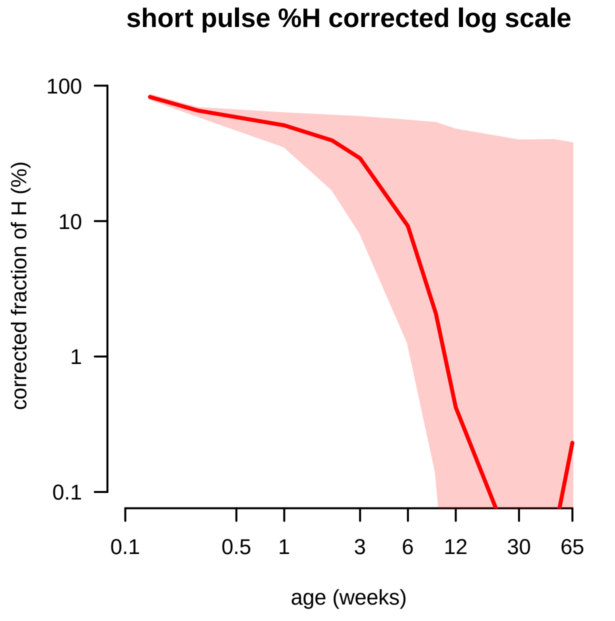

P15116

>sp|P15116|CADH2_MOUSE

Cadherin−2

GN=Cdh2

Med H1/2: 4.1 days

Q5 H1/2: 2.8 days

Q95 H1/2: 7.4 days

reliable model fit: yes

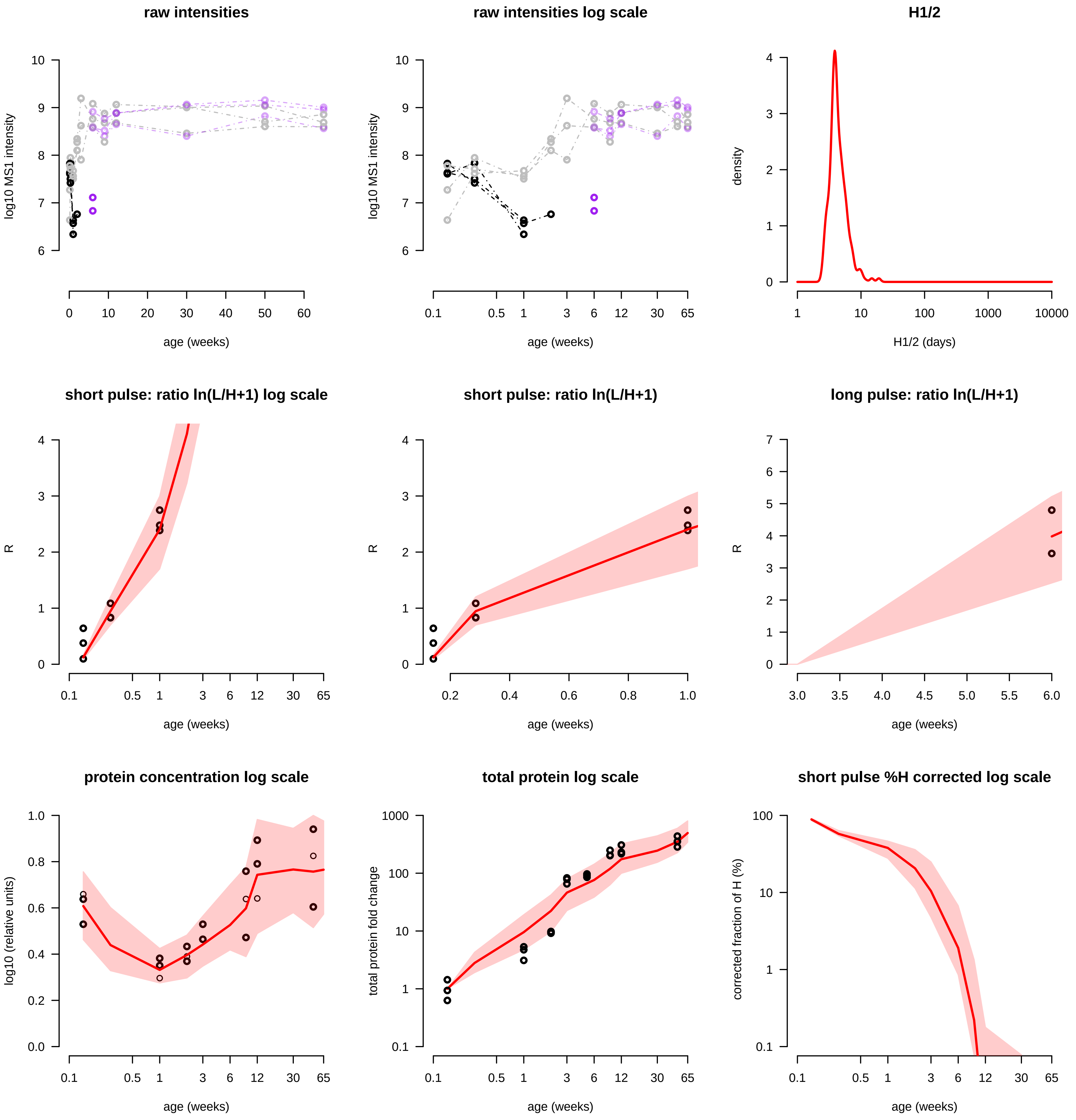

Q5SUR0

>sp|Q5SUR0|PUR4_MOUSE

Phosphoribosylformylglycinamidine synthase

GN=Pfas

Med H1/2: 62.6 days

Q5 H1/2: 36.4 days

Q95 H1/2: 151.1 days

reliable model fit: yes

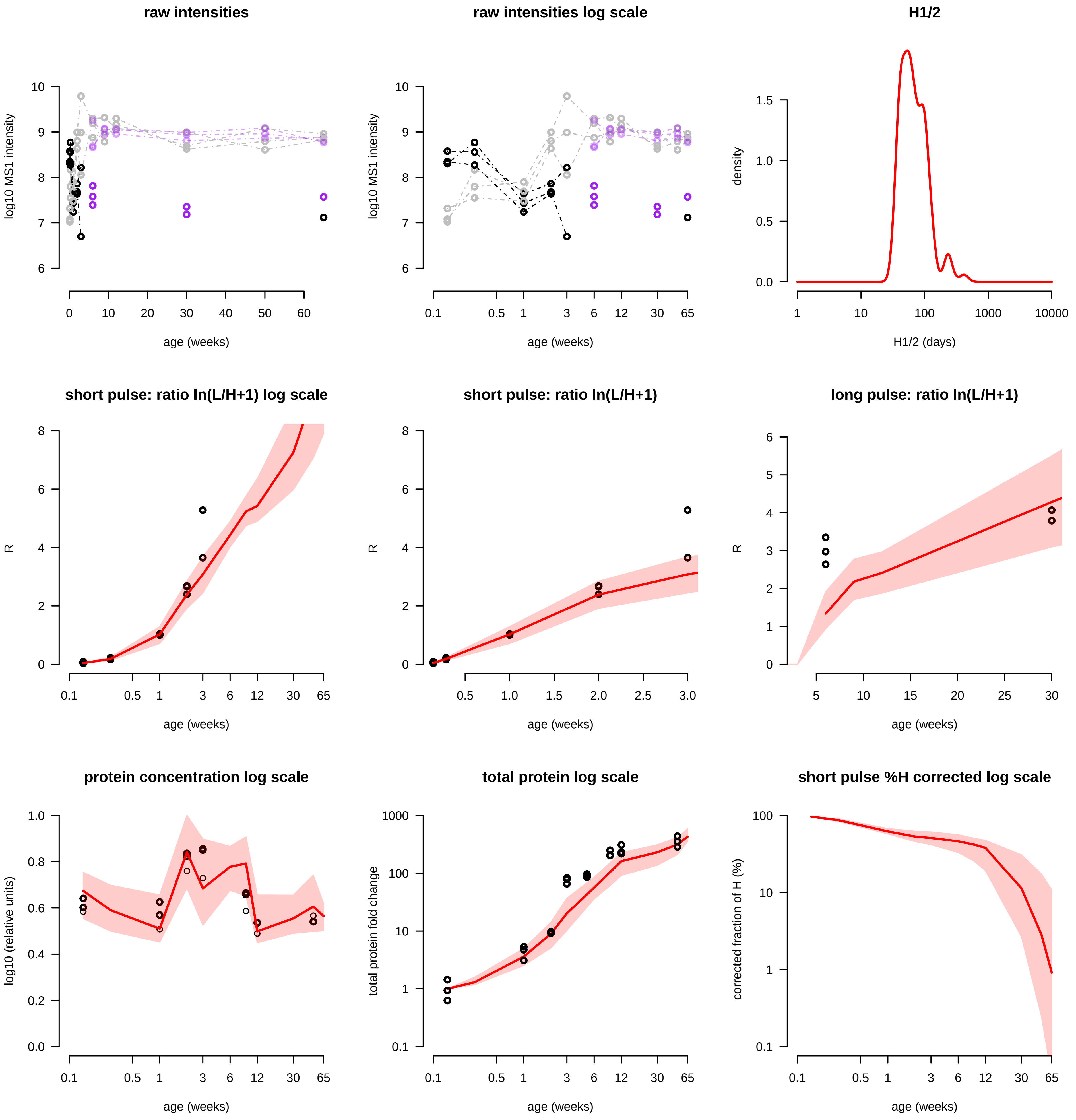

P43883

>sp|P43883|PLIN2_MOUSE

Perilipin−2

GN=Plin2

Med H1/2: 9.8 days

Q5 H1/2: 3.8 days

Q95 H1/2: 97.1 days

reliable model fit: yes

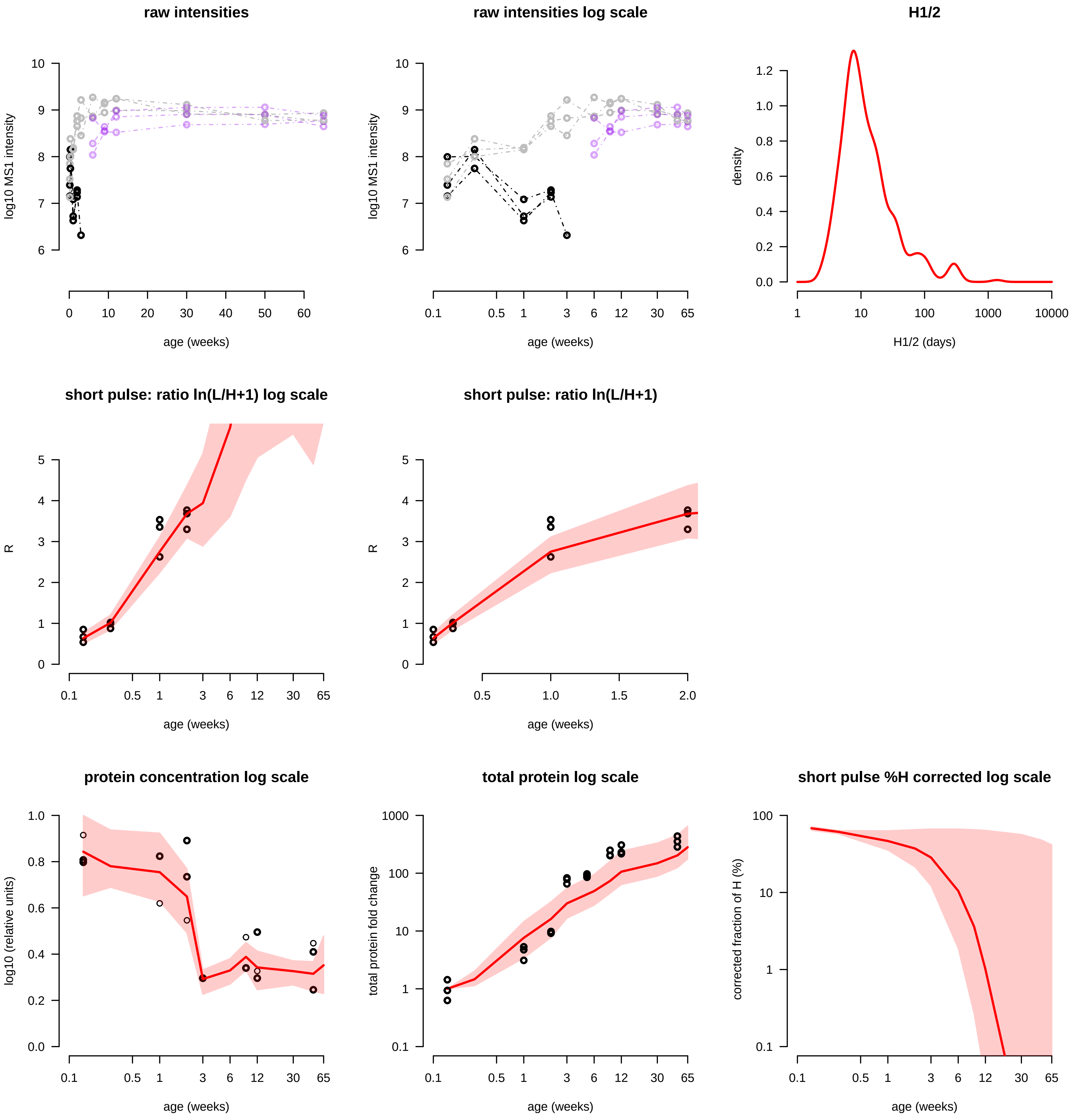

Q8C7X2−2

>sp|Q8C7X2−2|EMC1_MOUSE

Isoform 2 of ER membrane protein complex subunit 1

GN=Emc1

Med H1/2: 19.4 days

Q5 H1/2: 11.3 days

Q95 H1/2: 38.7 days

reliable model fit: no

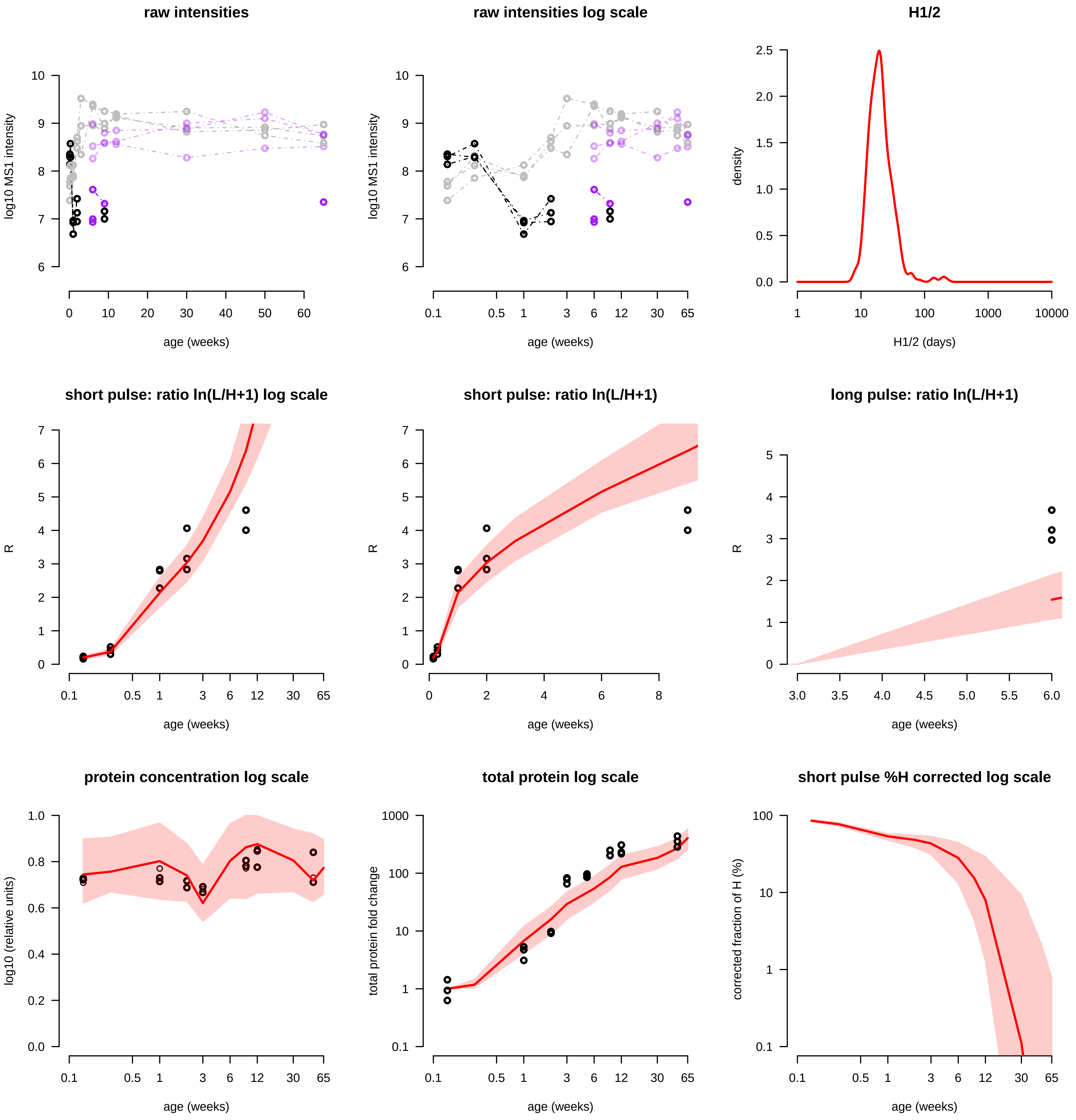

Q91V01

>sp|Q91V01|MBOA5_MOUSE

Lysophospholipid acyltransferase 5

GN=Lpcat3

Med H1/2: 6.1 days

Q5 H1/2: 4.4 days

Q95 H1/2: 9.6 days

reliable model fit: yes

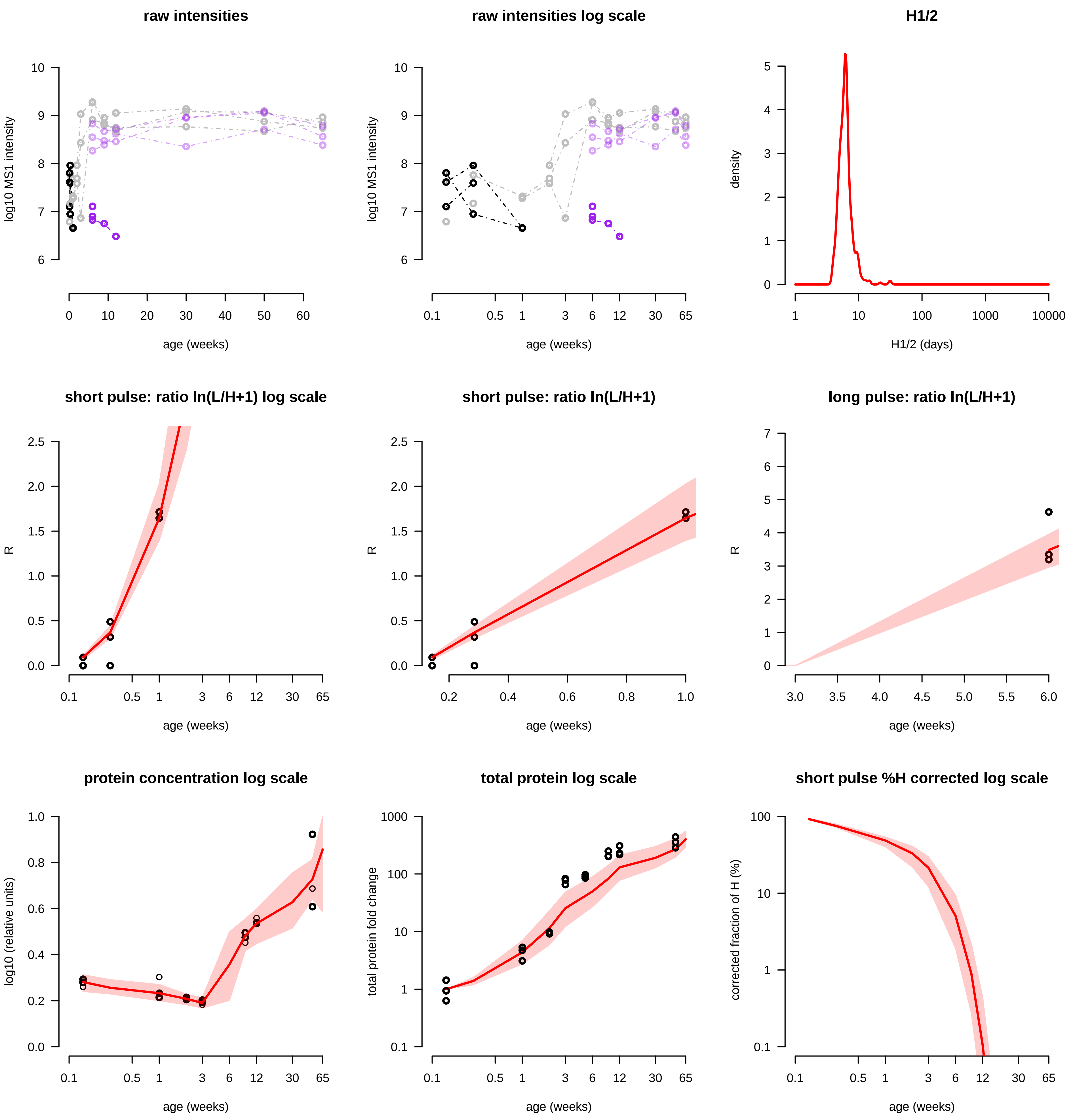

Q9CQZ5

>sp|Q9CQZ5|NDUA6_MOUSE

NADH dehydrogenase [ubiquinone] 1 alpha subcomplex subunit 6

GN=Ndufa6

Med H1/2: 6.3 days

Q5 H1/2: 5 days

Q95 H1/2: 9.3 days

reliable model fit: yes

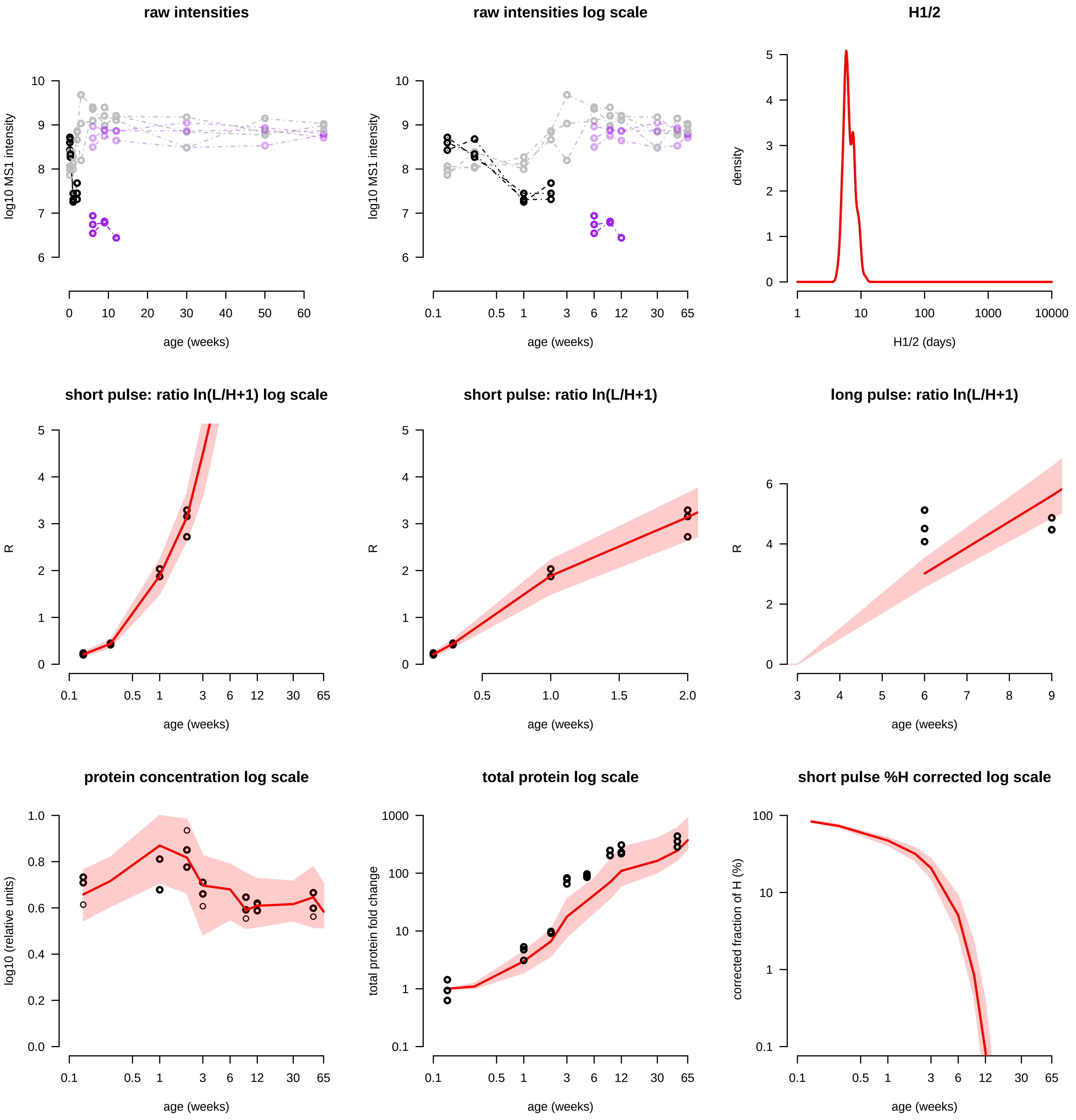

P13439

>sp|P13439|UMPS_MOUSE

Uridine 5'−monophosphate synthase

GN=Umps

Med H1/2: 4.6 days

Q5 H1/2: 3.7 days

Q95 H1/2: 6.1 days

reliable model fit: yes

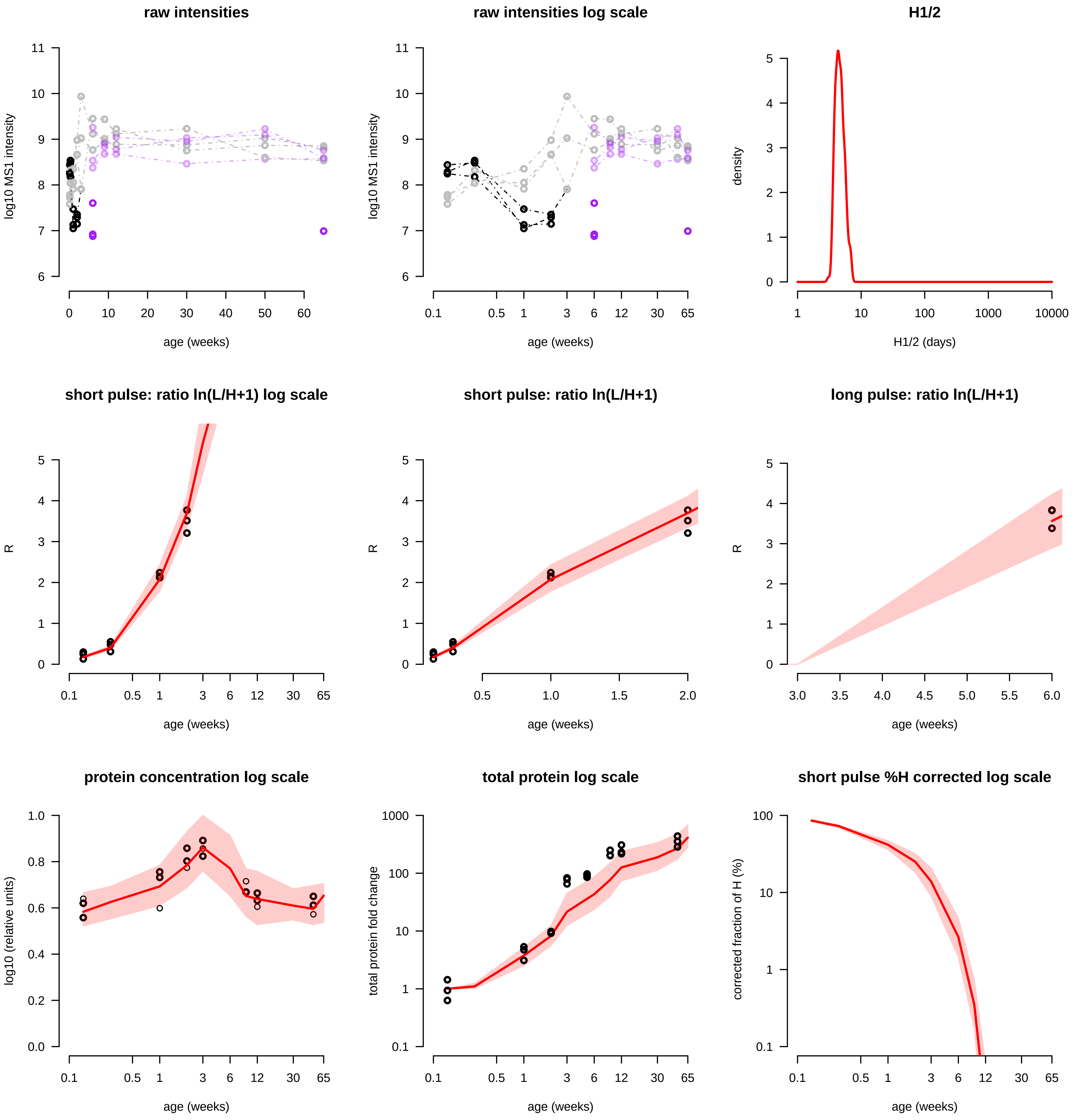

Q8R326;Q8R326−2

>sp|Q8R326|PSPC1_MOUSE

Paraspeckle component 1

GN=Pspc1

Med H1/2: 5 days

Q5 H1/2: 4.3 days

Q95 H1/2: 6.3 days

reliable model fit: yes

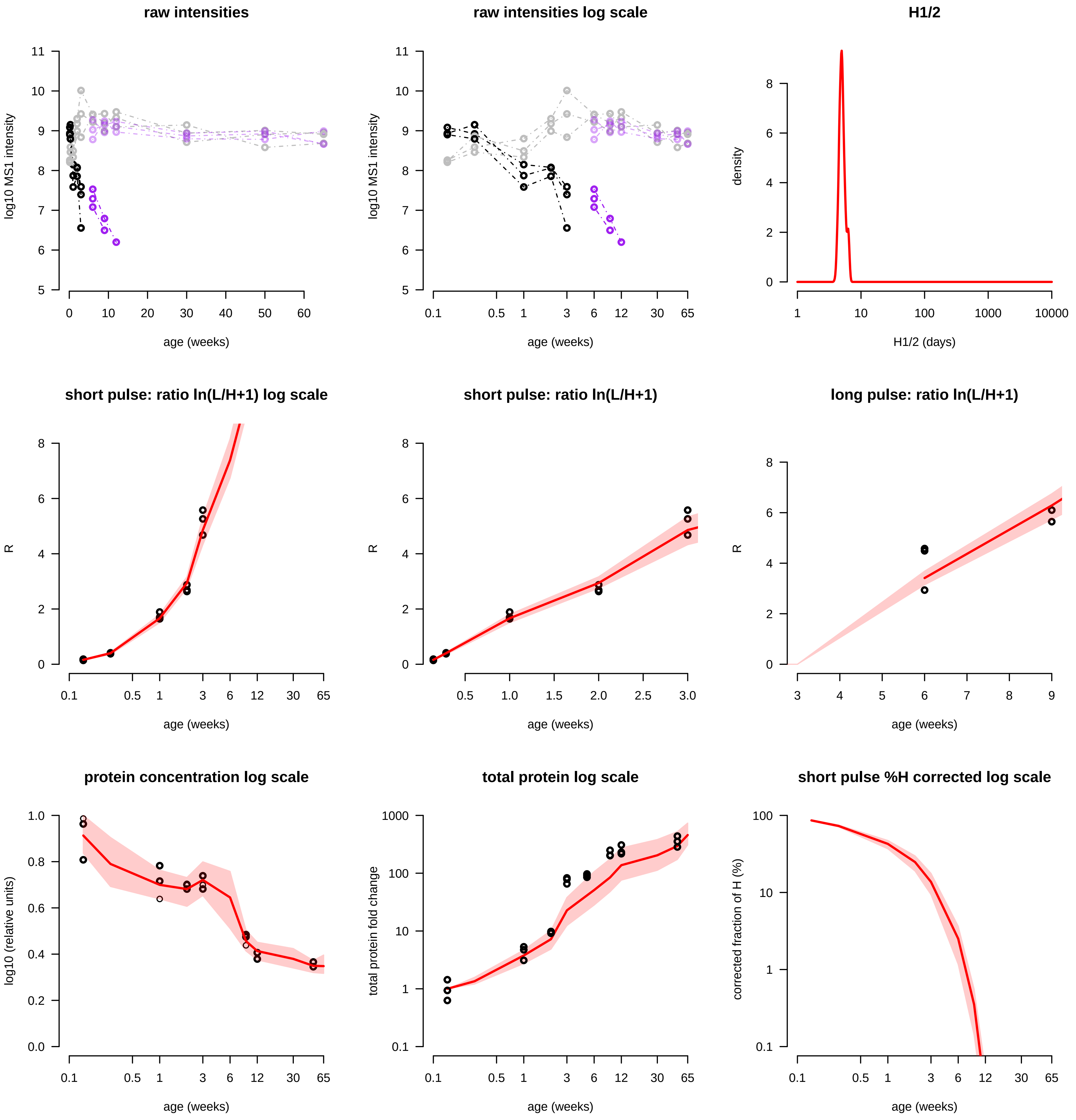

Q62048

>sp|Q62048|PEA15_MOUSE

Astrocytic phosphoprotein PEA−15

GN=Pea15

Med H1/2: 6.2 days

Q5 H1/2: 4.4 days

Q95 H1/2: 12.9 days

reliable model fit: yes

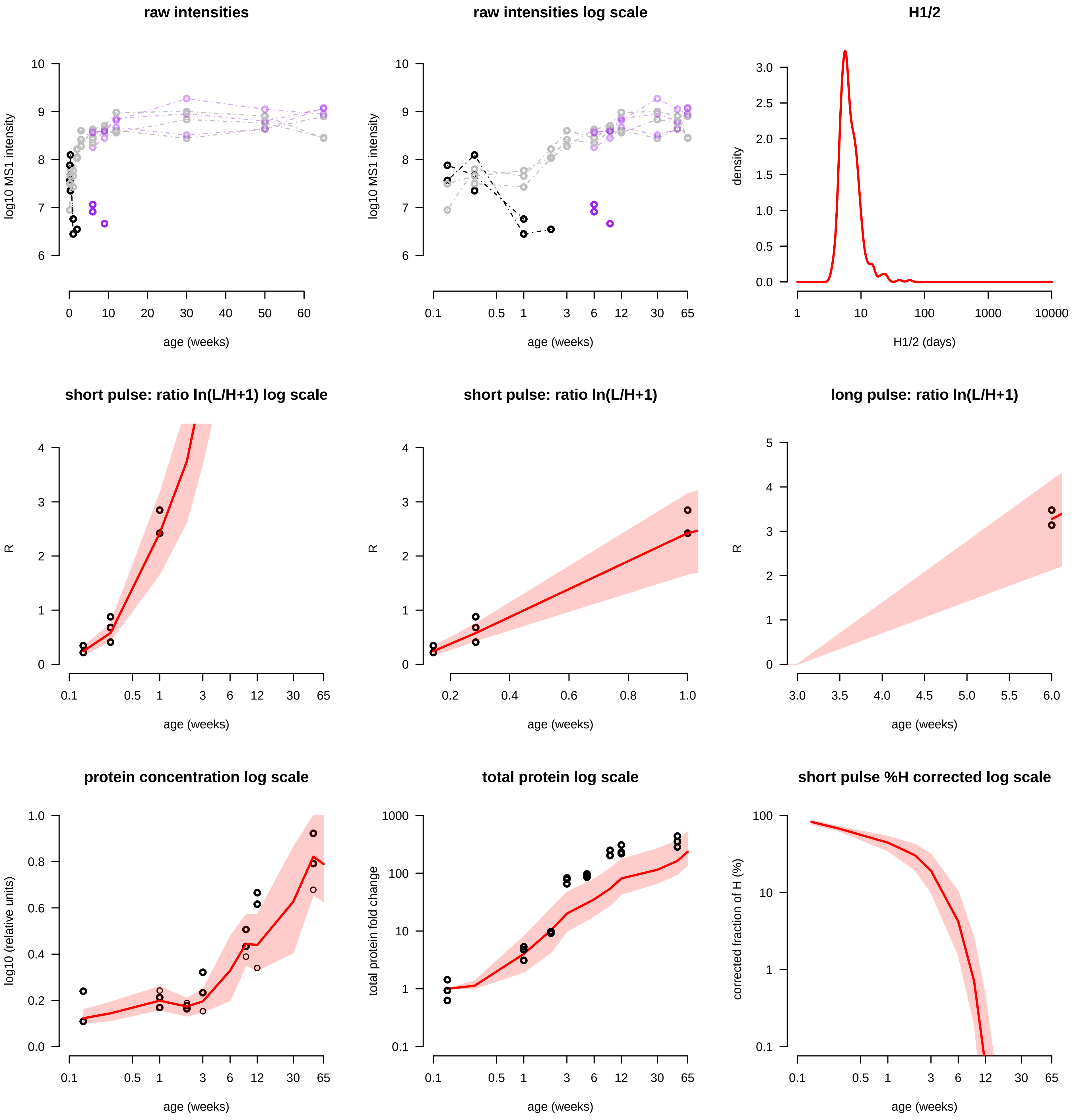

P70698

>sp|P70698|PYRG1_MOUSE

CTP synthase 1

GN=Ctps1

Med H1/2: 3.3 days

Q5 H1/2: 2.8 days

Q95 H1/2: 4.3 days

reliable model fit: yes

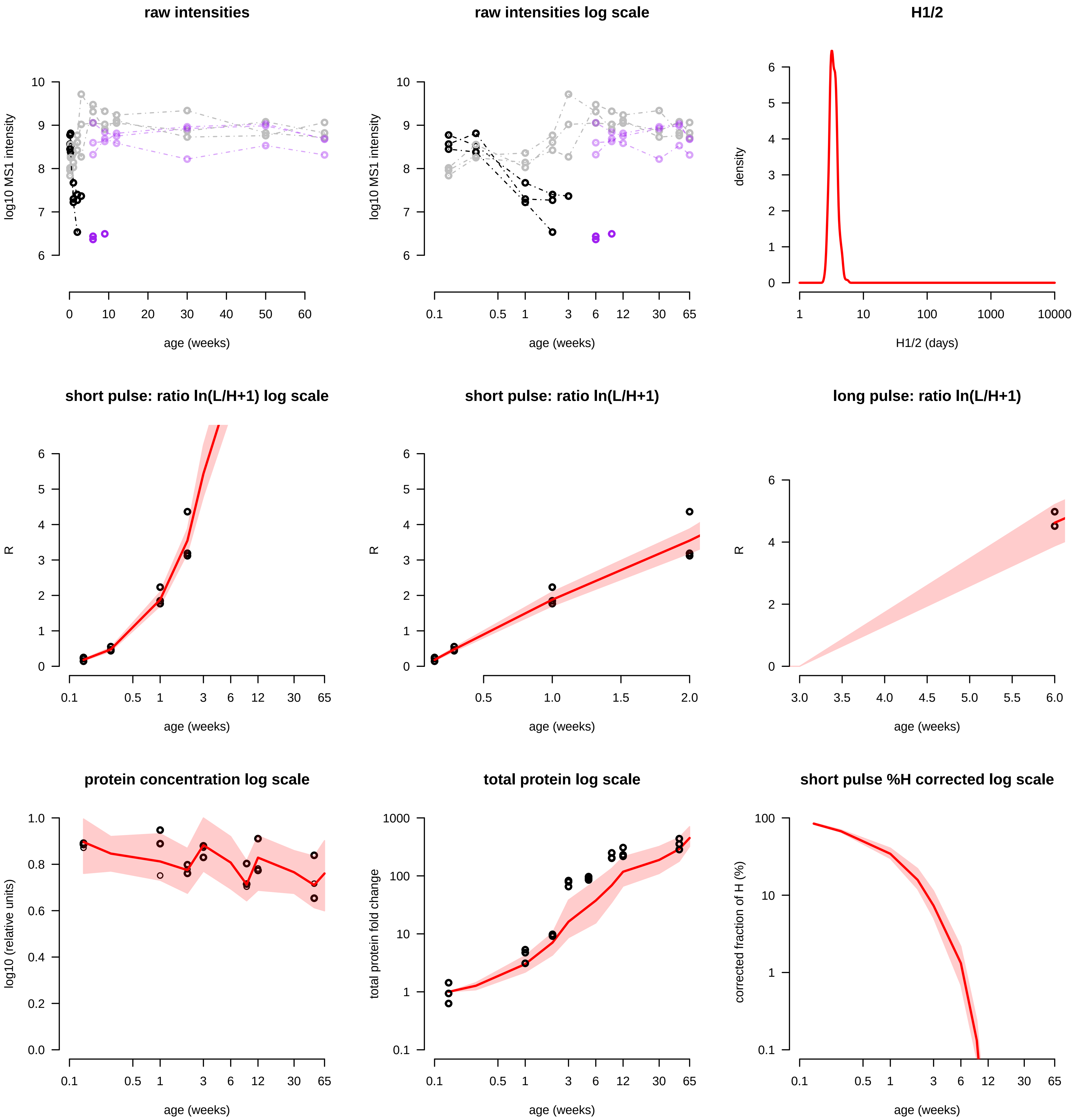

P20060

>sp|P20060|HEXB_MOUSE

Beta−hexosaminidase subunit beta

GN=Hexb

Med H1/2: 3.9 days

Q5 H1/2: 2.7 days

Q95 H1/2: 5.2 days

reliable model fit: yes

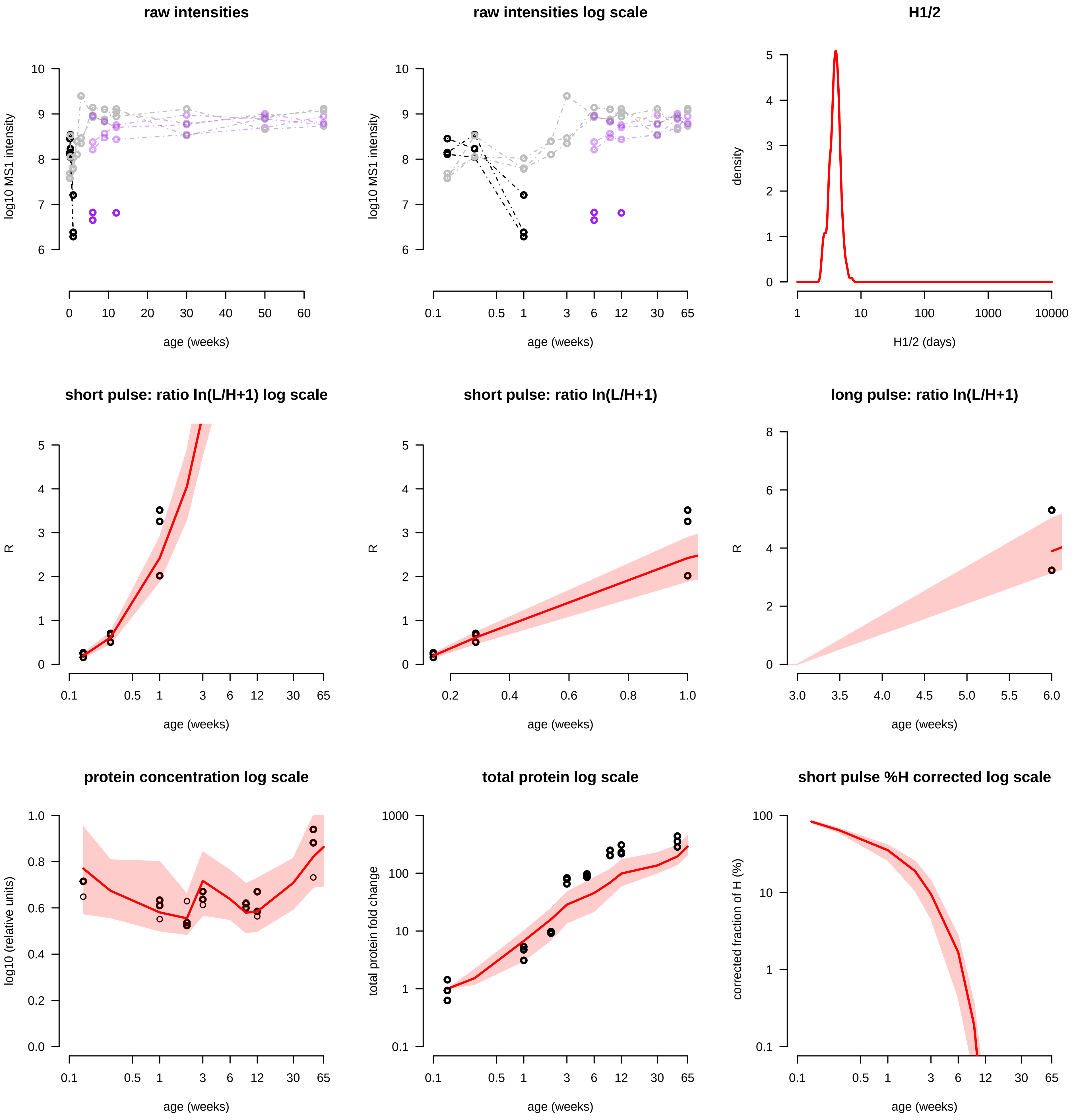

Q8K019−2;Q8K019;Q8K019−3

>sp|Q8K019−2|BCLF1_MOUSE

Isoform 2 of Bcl−2−associated transcription factor 1

GN=Bclaf1

Med H1/2: 4.2 days

Q5 H1/2: 3.5 days

Q95 H1/2: 5.3 days

reliable model fit: yes

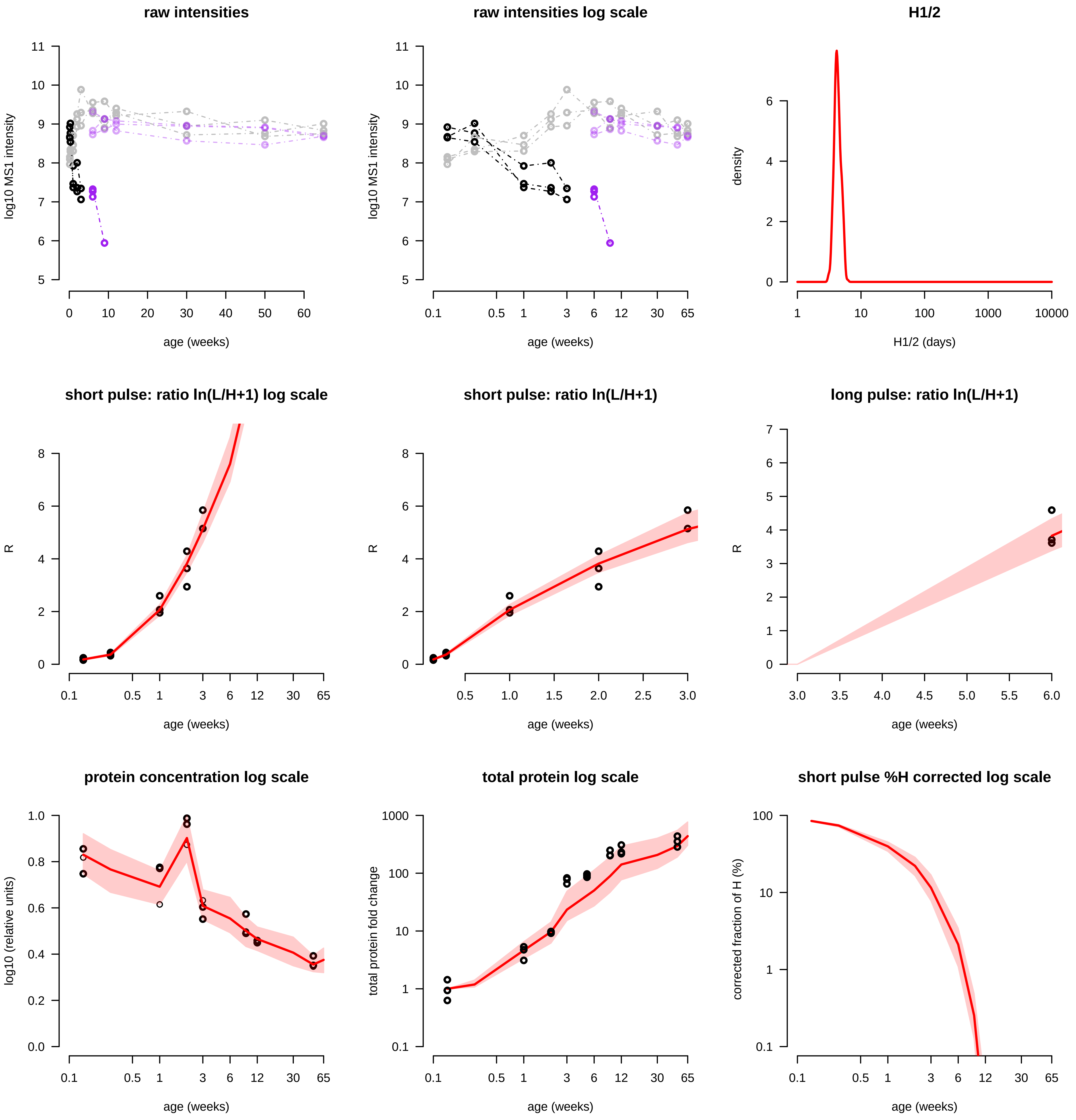

Q8K215

>sp|Q8K215|LYRM4_MOUSE

LYR motif−containing protein 4

GN=Lyrm4

Med H1/2: 26.1 days

Q5 H1/2: 8.7 days

Q95 H1/2: 221.3 days

reliable model fit: yes

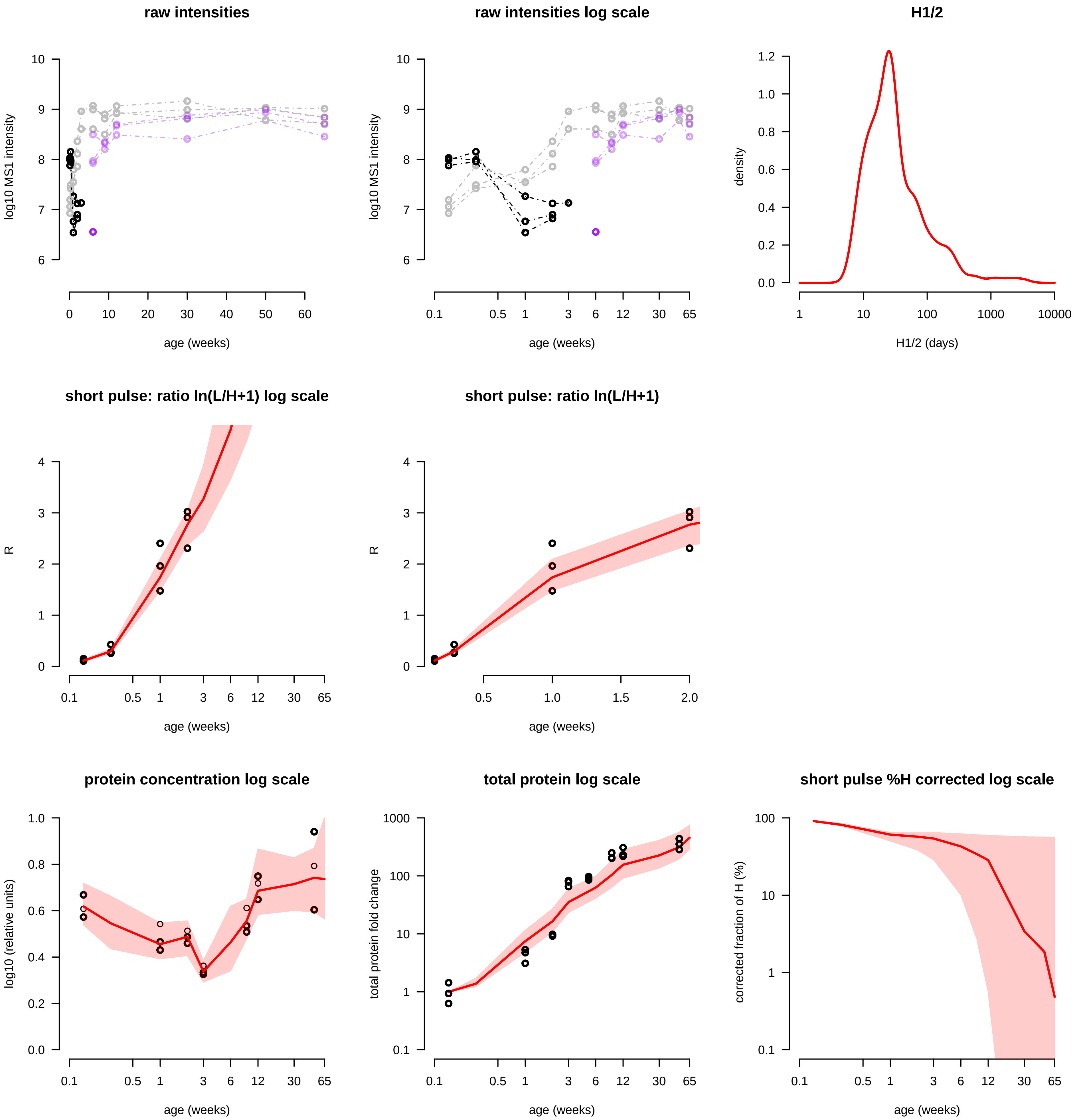

Q9CXZ1

>sp|Q9CXZ1|NDUS4_MOUSE

NADH dehydrogenase [ubiquinone] iron−sulfur protein 4, mitochondrial

GN=Ndufs4

Med H1/2: 7.1 days

Q5 H1/2: 5.6 days

Q95 H1/2: 9.9 days

reliable model fit: yes

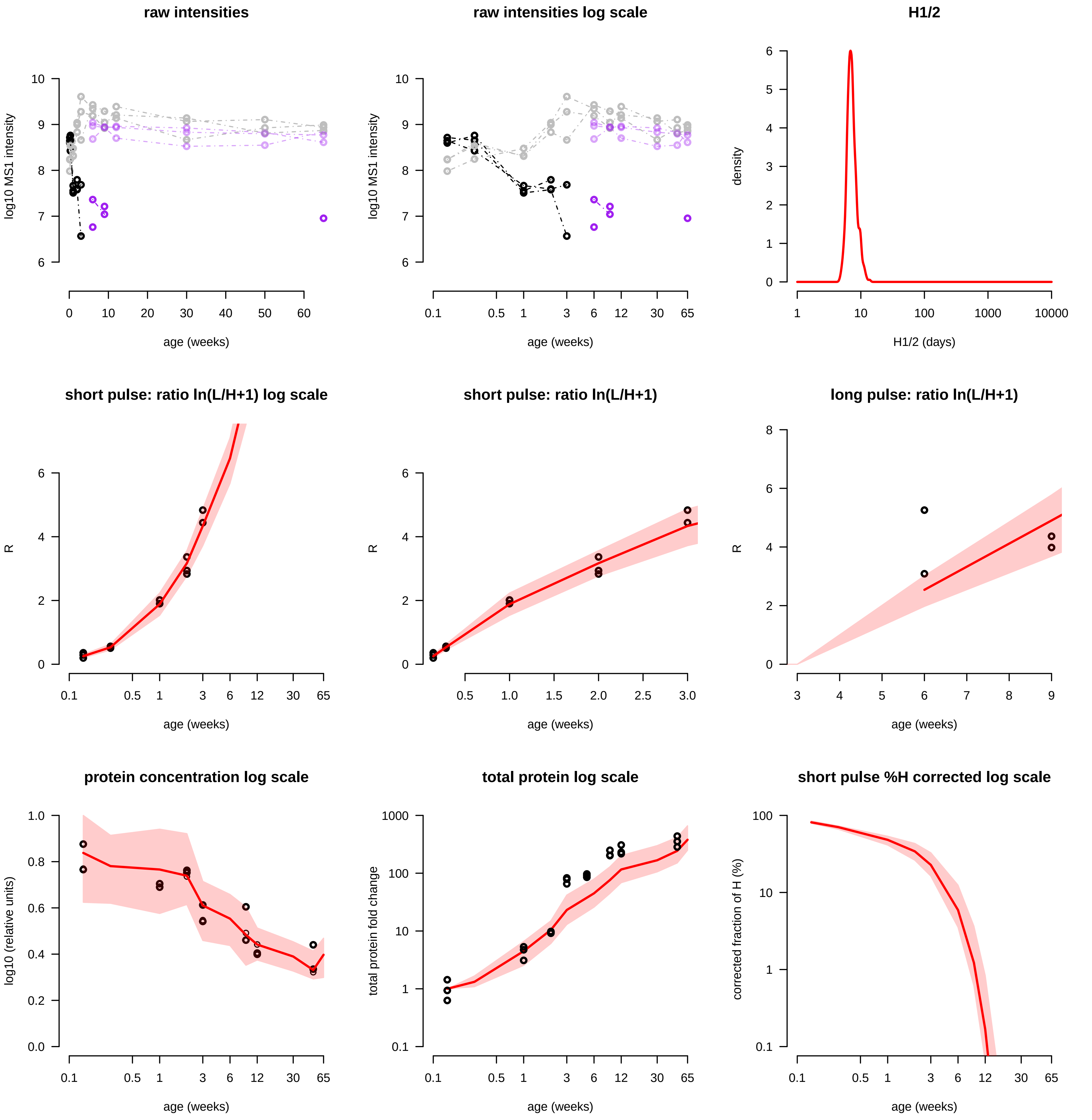

Q8VH51−2;Q8VH51;Q8VH51−3

>sp|Q8VH51−2|RBM39_MOUSE

Isoform 2 of RNA−binding protein 39

GN=Rbm39

Med H1/2: 6.6 days

Q5 H1/2: 5.5 days

Q95 H1/2: 8.1 days

reliable model fit: yes

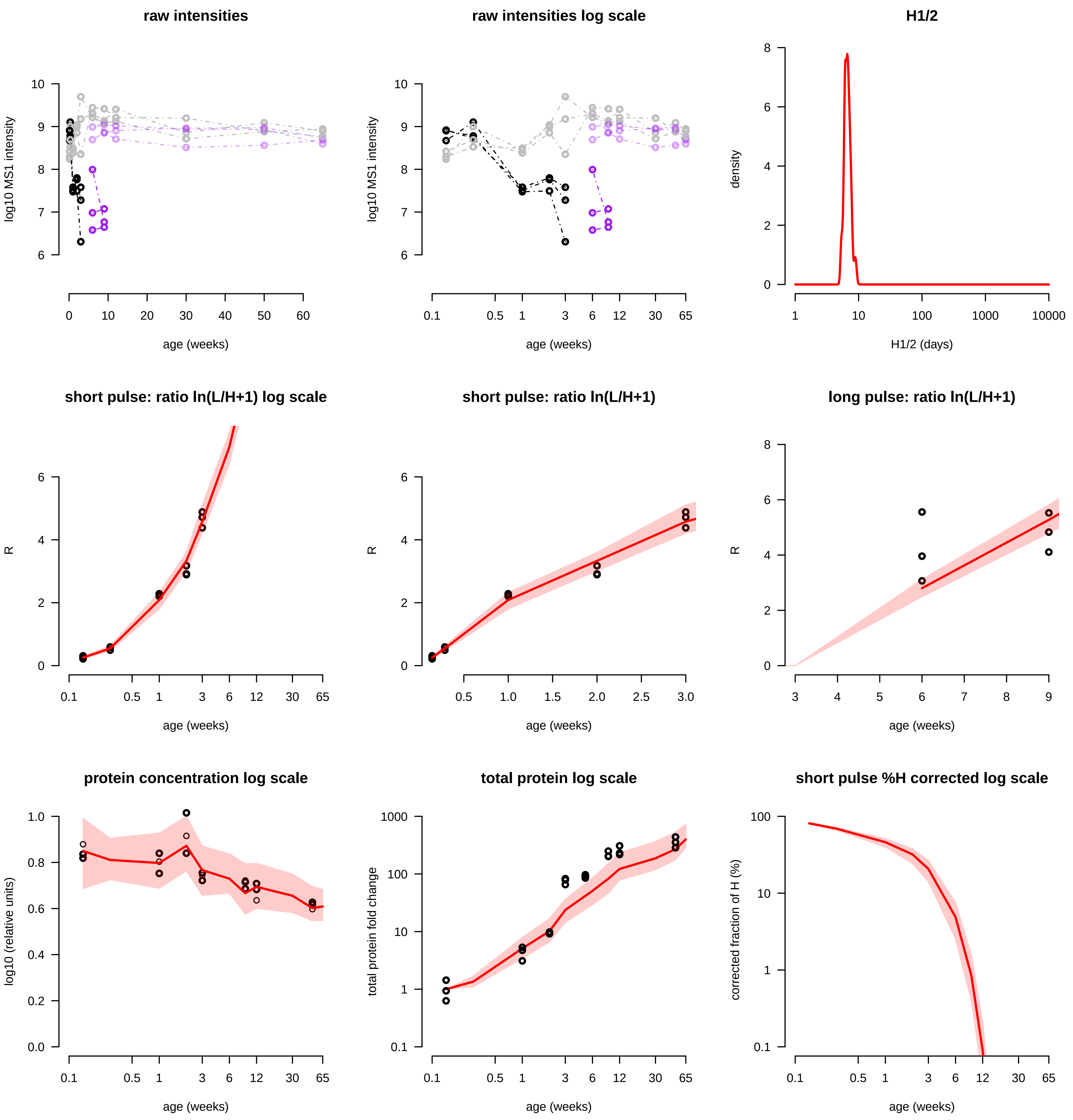

Q9QXX0

>sp|Q9QXX0|JAG1_MOUSE

Protein jagged−1

GN=Jag1

Med H1/2: 11.3 days

Q5 H1/2: 8.3 days

Q95 H1/2: 14.9 days

reliable model fit: yes

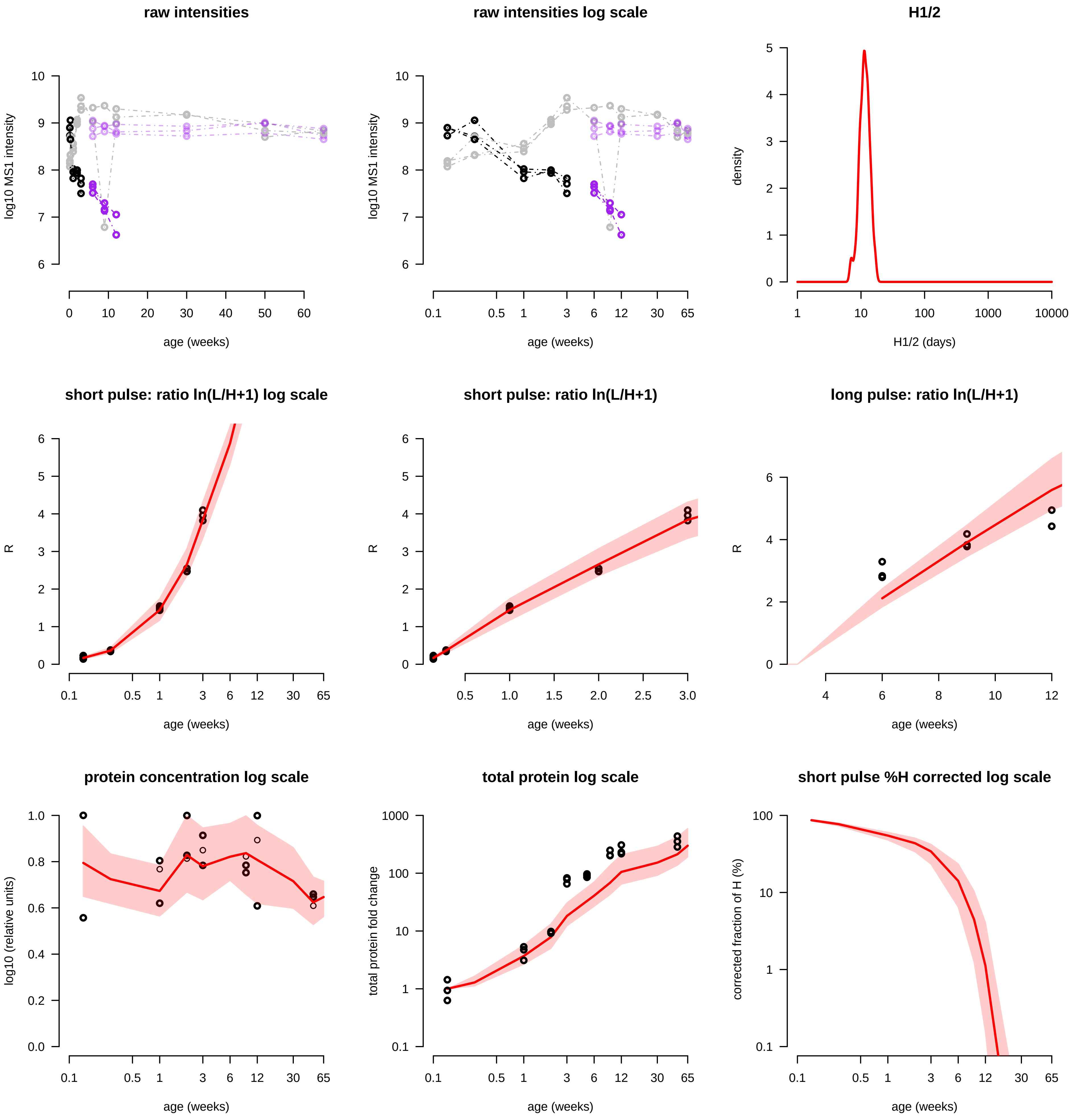

Q80WJ7

>sp|Q80WJ7|LYRIC_MOUSE

Protein LYRIC

GN=Mtdh

Med H1/2: 4.4 days

Q5 H1/2: 3 days

Q95 H1/2: 8.7 days

reliable model fit: yes

raw intensities

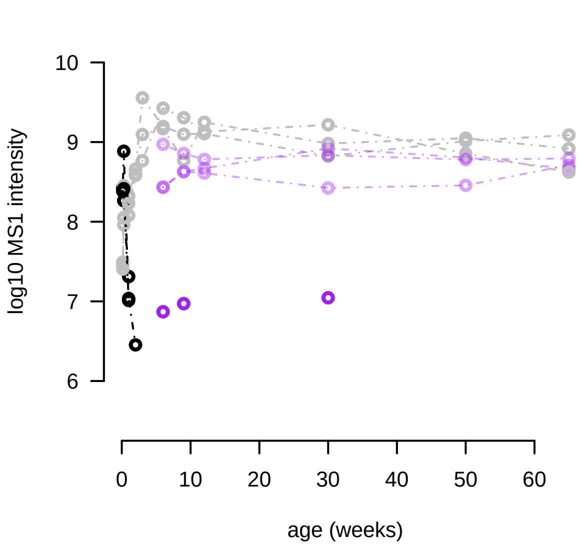

raw intensities log scale

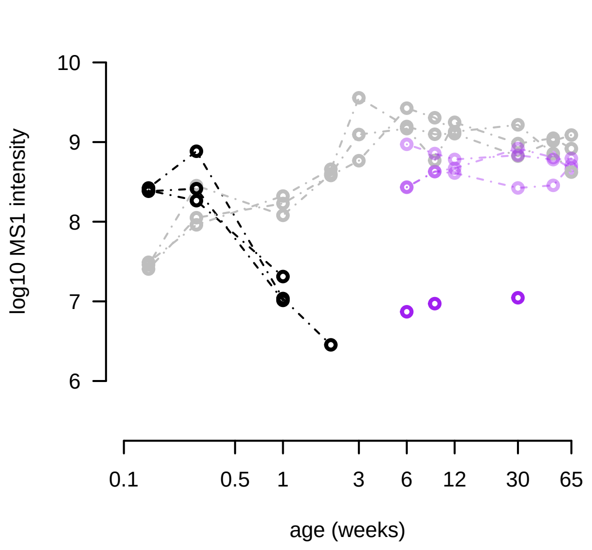

H1/2

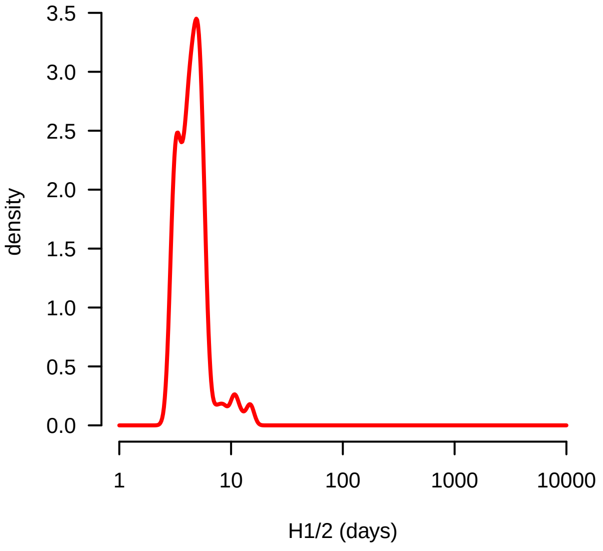

short pulse: ratio ln(L/H+1) log scale

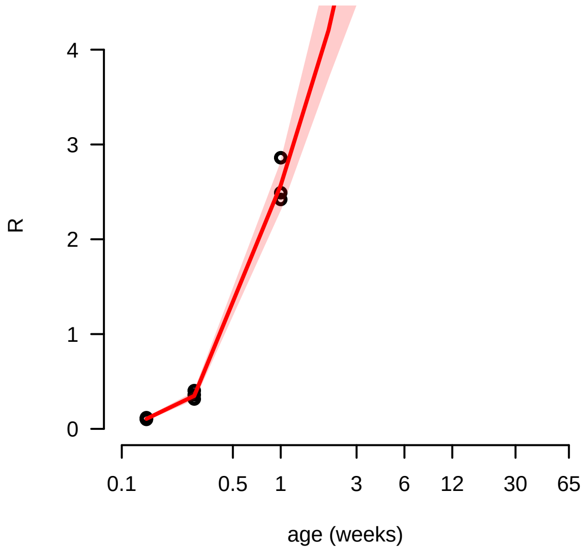

short pulse: ratio ln(L/H+1)

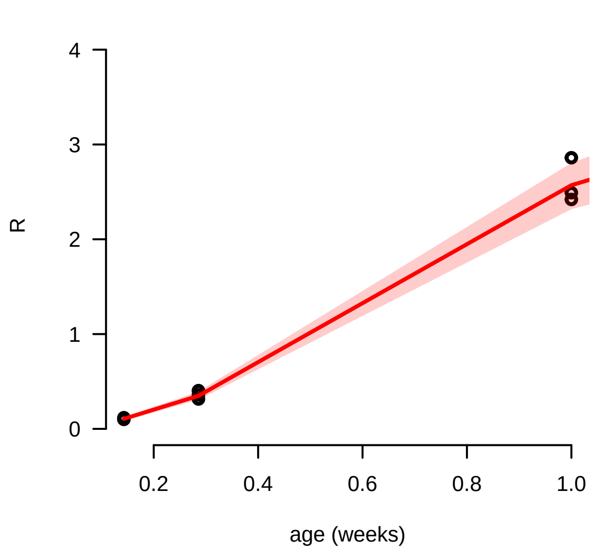

protein concentration log scale

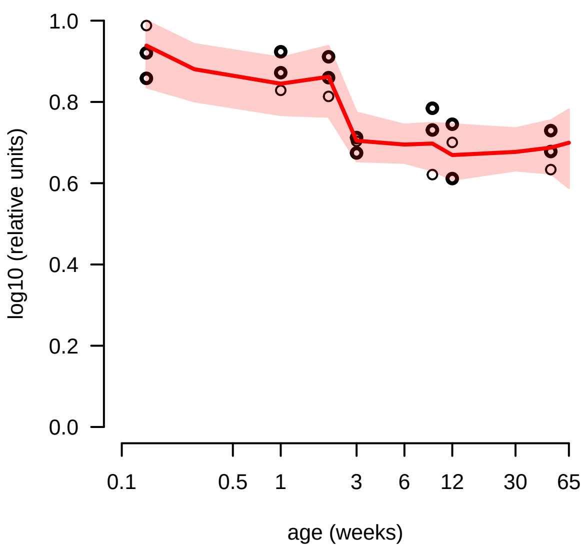

total protein log scale

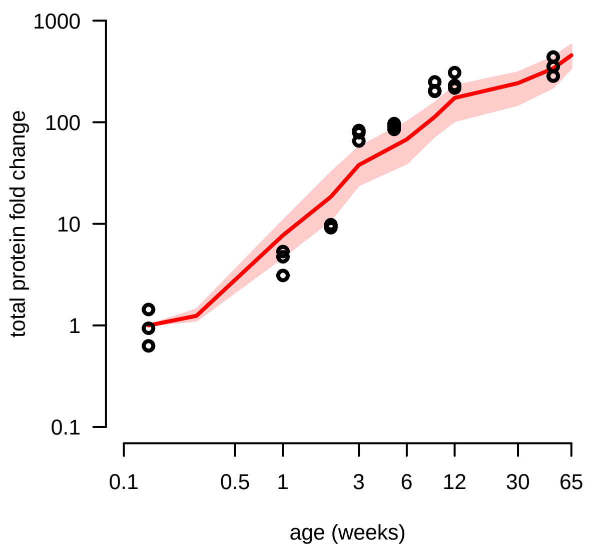

short pulse %H corrected log scale

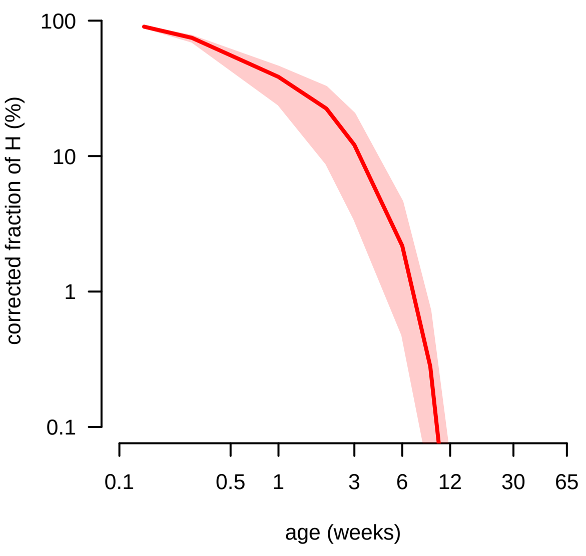

Q8K354

>sp|Q8K354|CBR3_MOUSE

Carbonyl reductase [NADPH] 3

GN=Cbr3

Med H1/2: 15.1 days

Q5 H1/2: 4.4 days

Q95 H1/2: 143.4 days

reliable model fit: yes

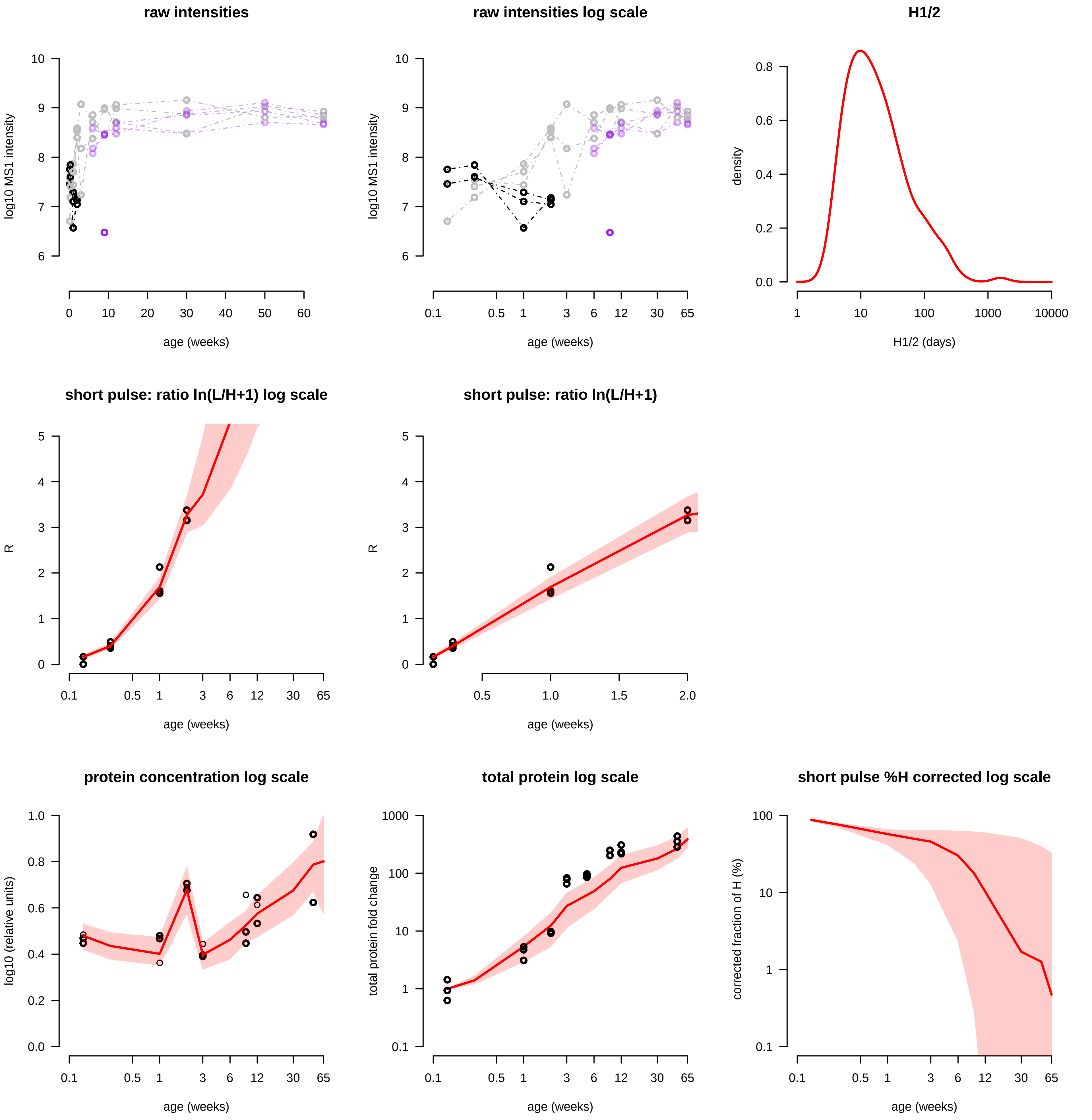

Q9CQW1

>sp|Q9CQW1|YKT6_MOUSE

Synaptobrevin homolog YKT6

GN=Ykt6

Med H1/2: 1408.6 days

Q5 H1/2: 359.7 days

Q95 H1/2: 16155.6 days

reliable model fit: yes

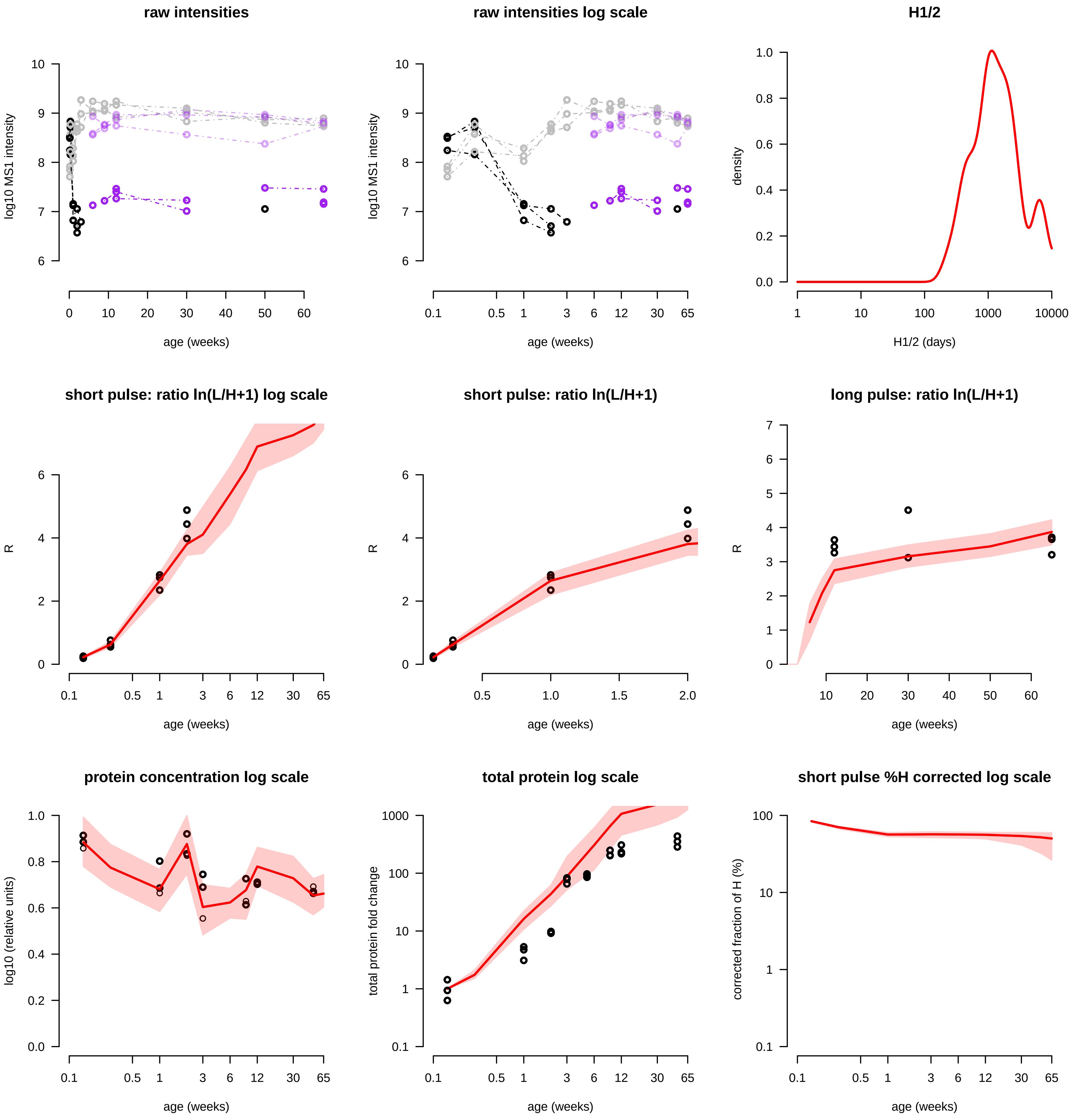

Q8BMP6

>sp|Q8BMP6|GCP60_MOUSE

Golgi resident protein GCP60

GN=Acbd3

Med H1/2: 6 days

Q5 H1/2: 4.7 days

Q95 H1/2: 8.2 days

reliable model fit: yes

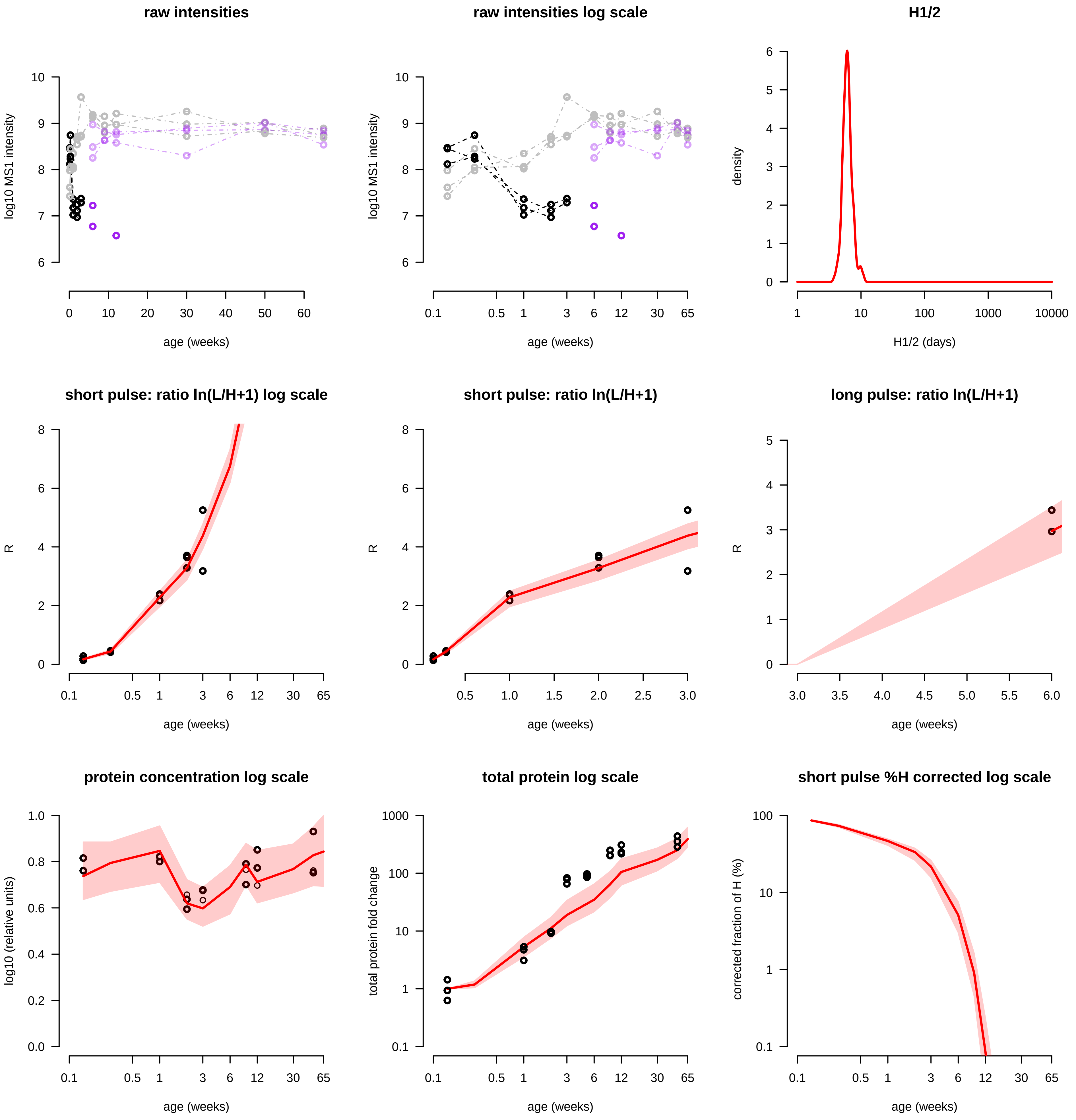

Q6PHZ2

>sp|Q6PHZ2|KCC2D_MOUSE

Calcium/calmodulin−dependent protein kinase type II subunit delta

GN=Camk2d

Med H1/2: 4.3 days

Q5 H1/2: 3.6 days

Q95 H1/2: 5.2 days

reliable model fit: yes

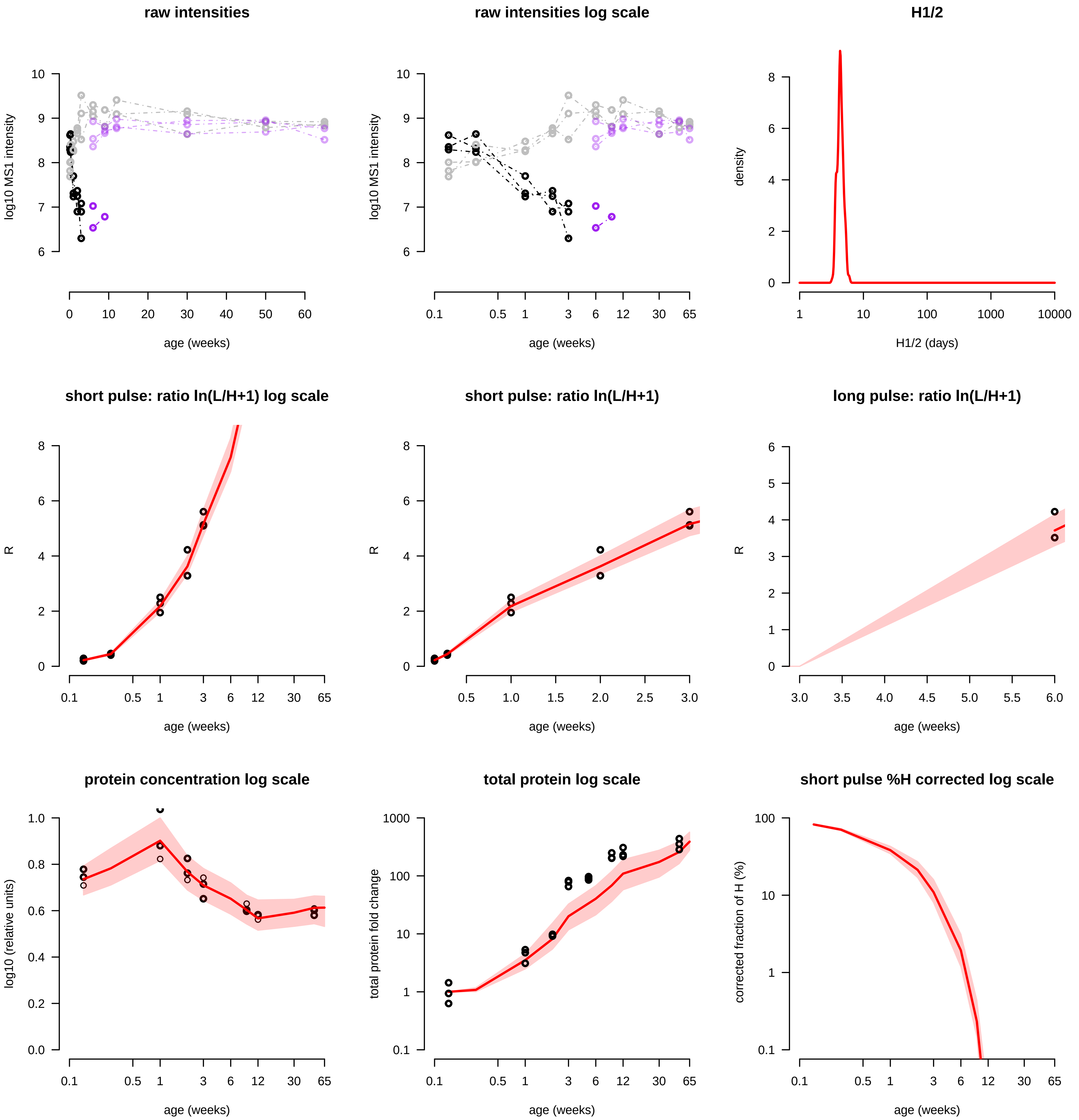

Q8BJY1

>sp|Q8BJY1|PSMD5_MOUSE

26S proteasome non−ATPase regulatory subunit 5

GN=Psmd5

Med H1/2: 4.9 days

Q5 H1/2: 3.8 days

Q95 H1/2: 7 days

reliable model fit: yes

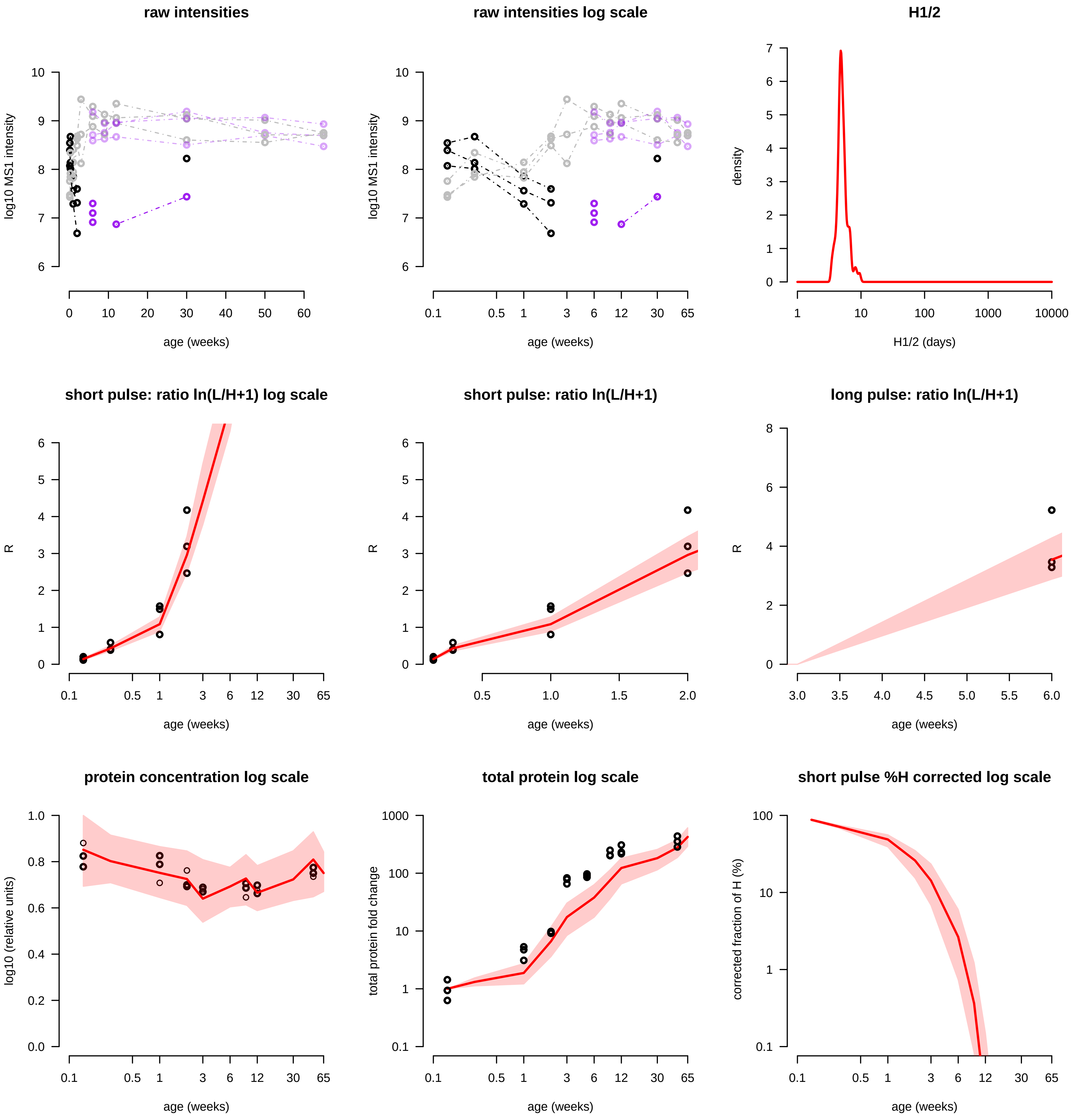

O70475

>sp|O70475|UGDH_MOUSE

UDP−glucose 6−dehydrogenase

GN=Ugdh

Med H1/2: 4 days

Q5 H1/2: 3.1 days

Q95 H1/2: 5.2 days

reliable model fit: yes

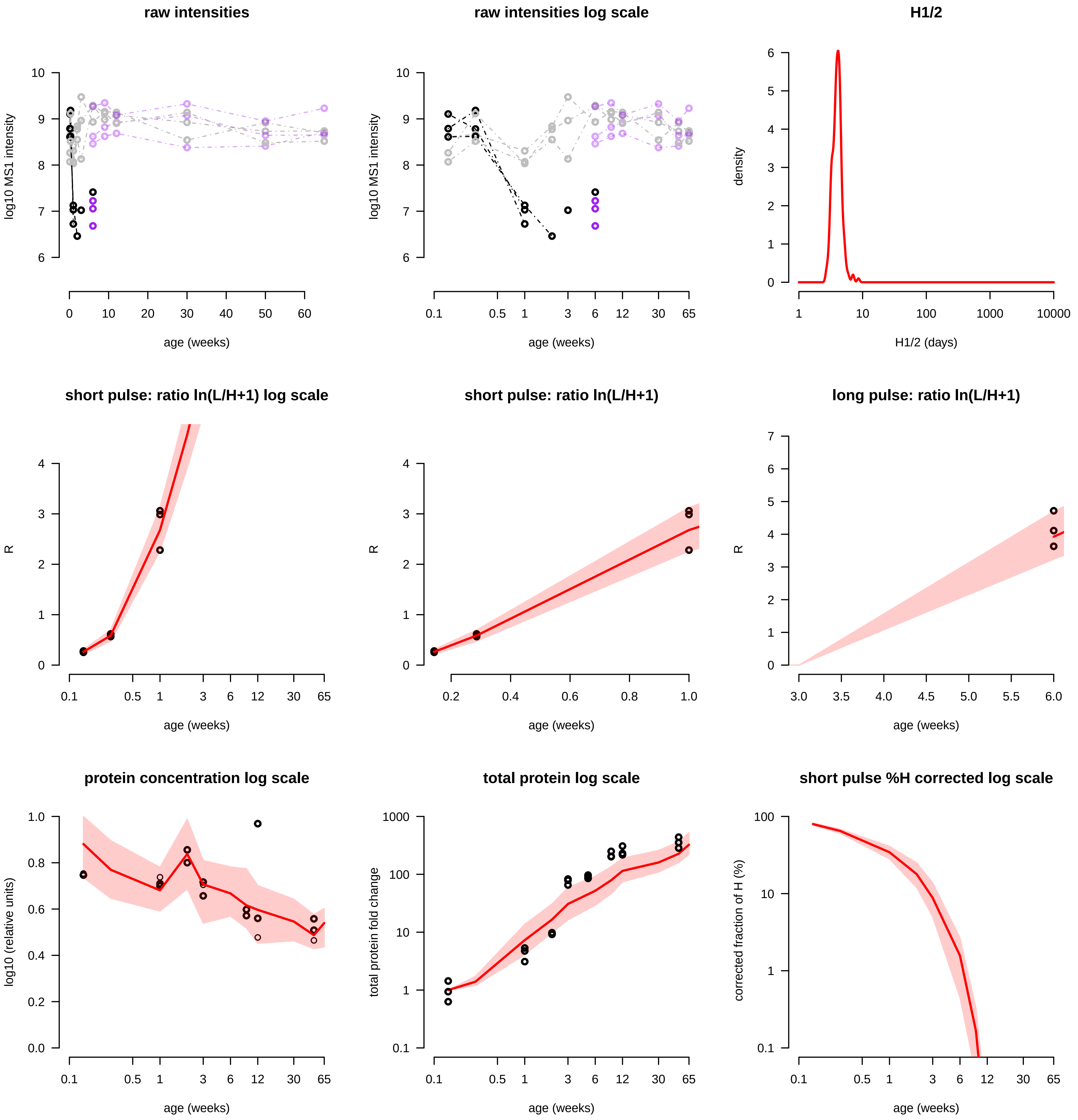

P18572−2;P18572

>sp|P18572−2|BASI_MOUSE

Isoform 2 of Basigin

GN=Bsg

Med H1/2: 7.9 days

Q5 H1/2: 6.5 days

Q95 H1/2: 10 days

reliable model fit: yes

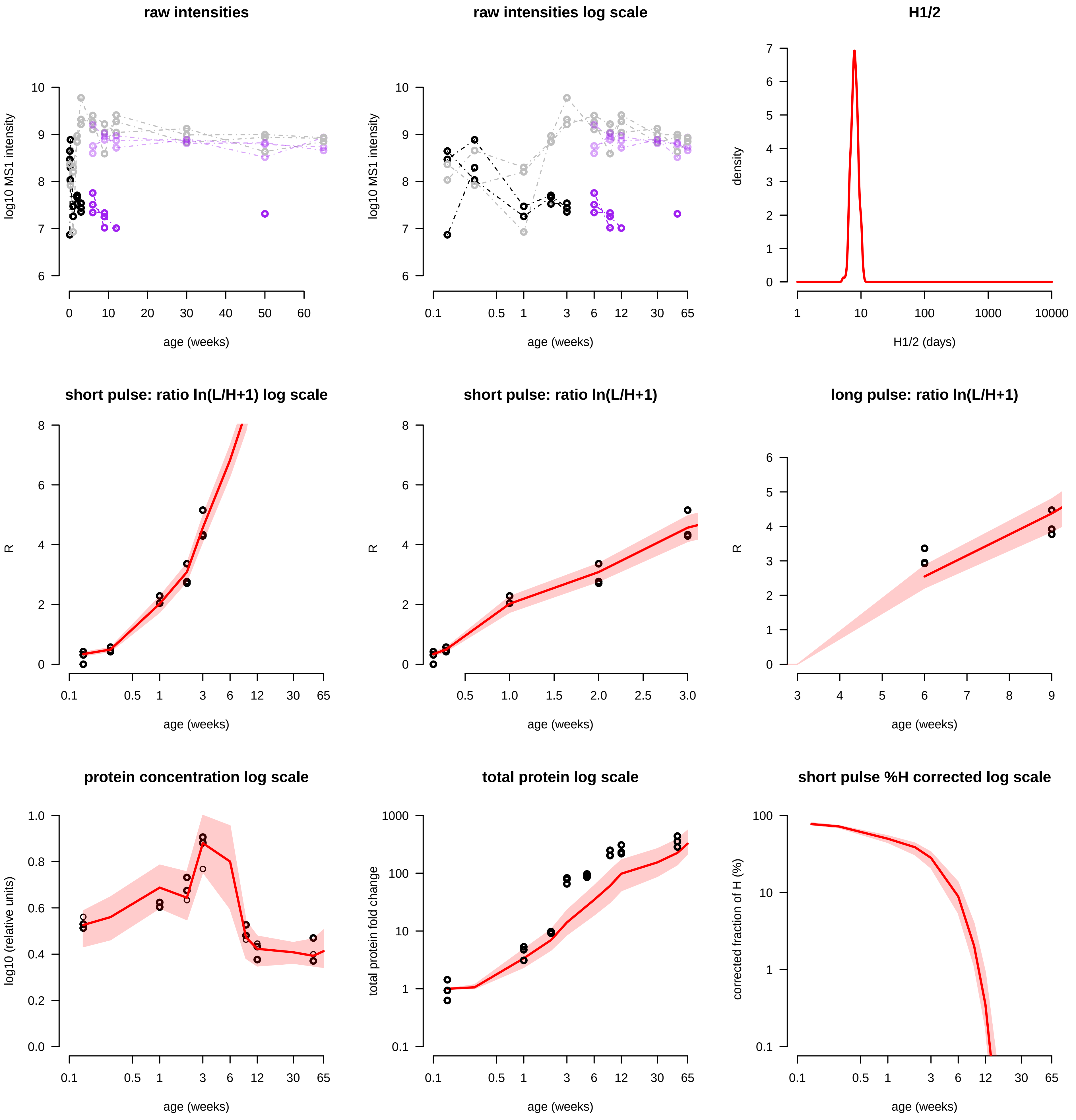

P11031

>sp|P11031|TCP4_MOUSE

Activated RNA polymerase II transcriptional coactivator p15

GN=Sub1

Med H1/2: 6.9 days

Q5 H1/2: 4.7 days

Q95 H1/2: 11.6 days

reliable model fit: no

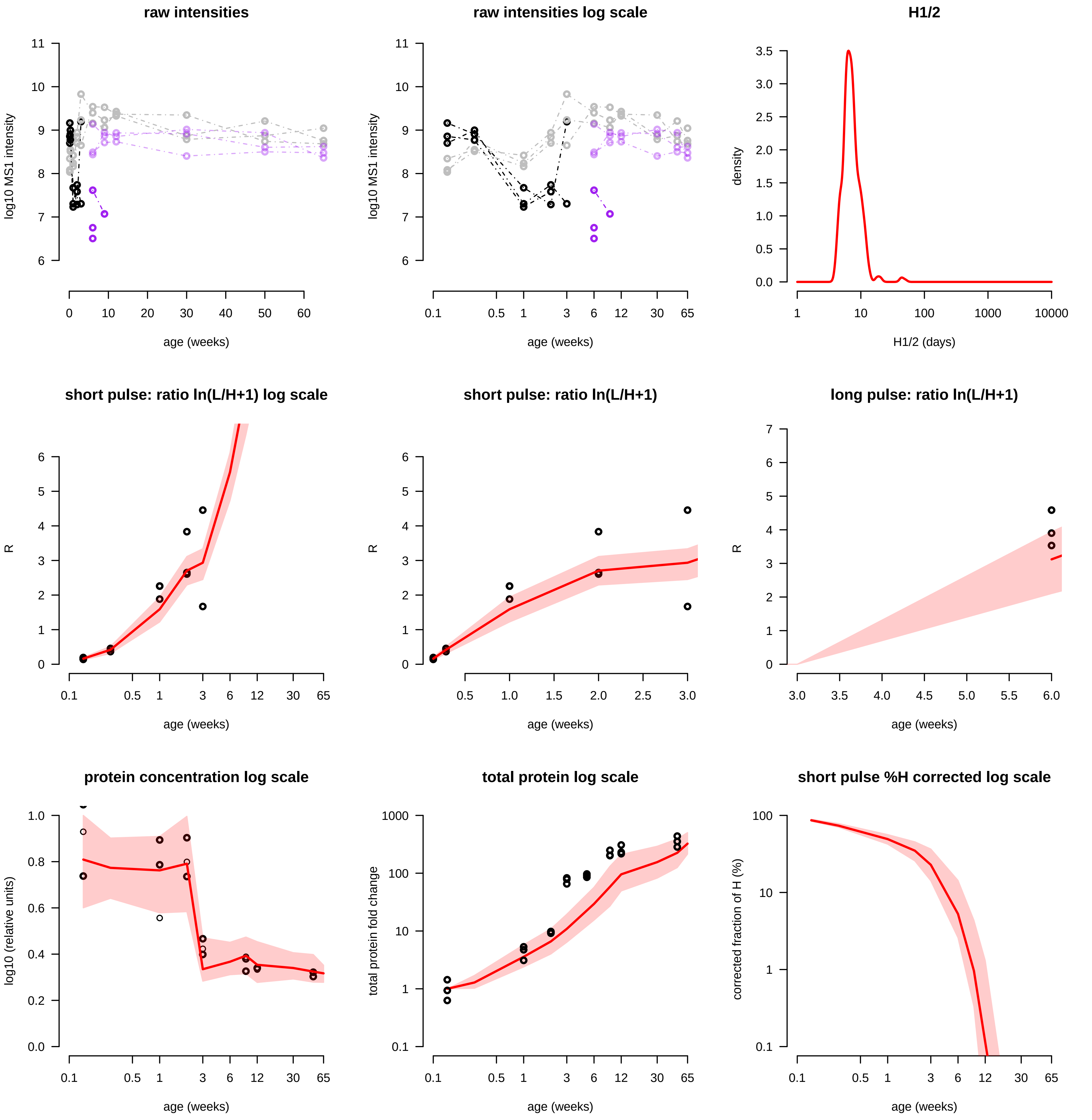

Q9QX47−3;Q9QX47

>sp|Q9QX47−3|SON_MOUSE

Isoform 3 of Protein SON

GN=Son

Med H1/2: 3.3 days

Q5 H1/2: 2.8 days

Q95 H1/2: 3.8 days

reliable model fit: yes

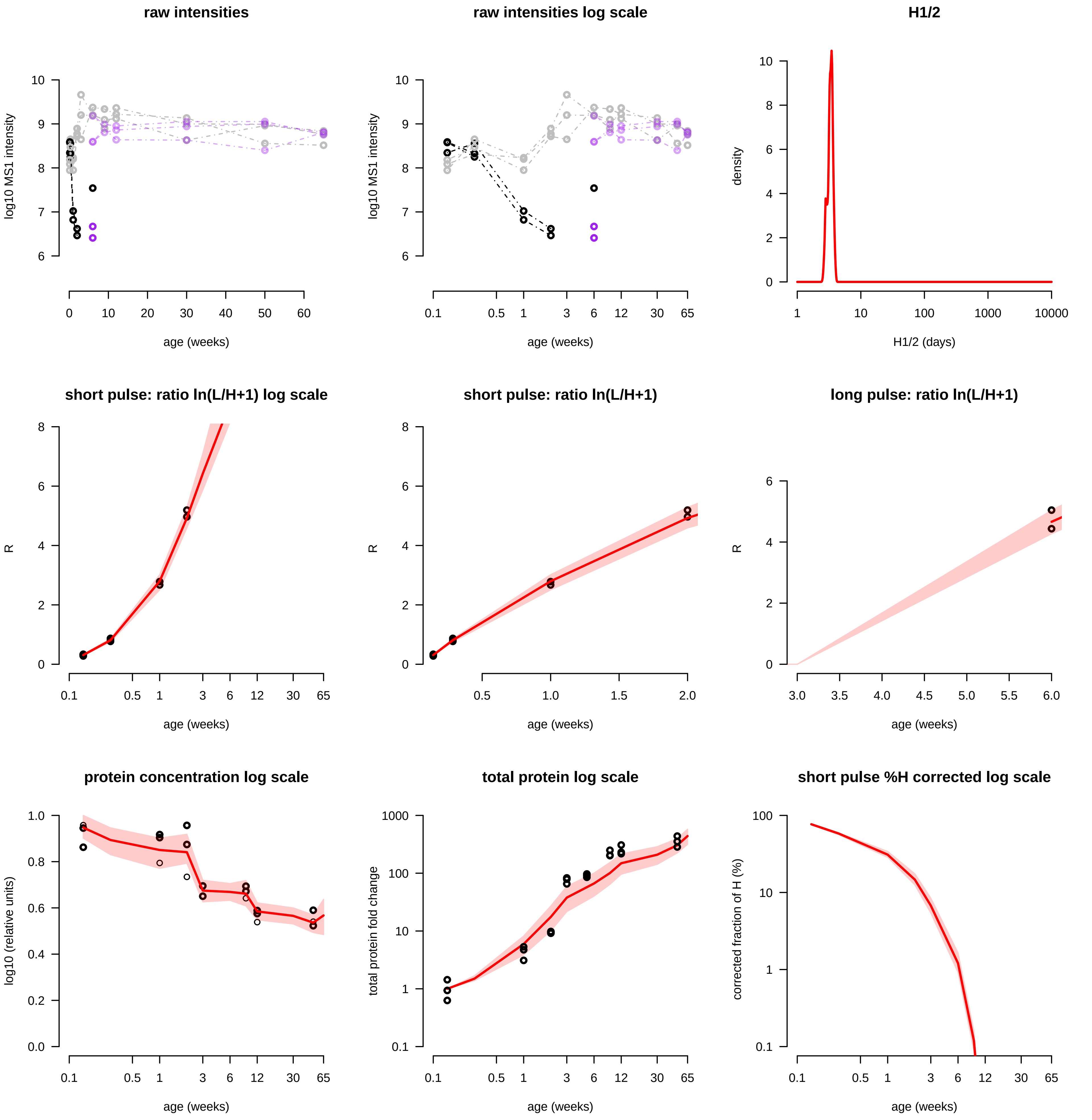

Q8BGD9

>sp|Q8BGD9|IF4B_MOUSE

Eukaryotic translation initiation factor 4B

GN=Eif4b

Med H1/2: 5.7 days

Q5 H1/2: 4.7 days

Q95 H1/2: 6.7 days

reliable model fit: yes

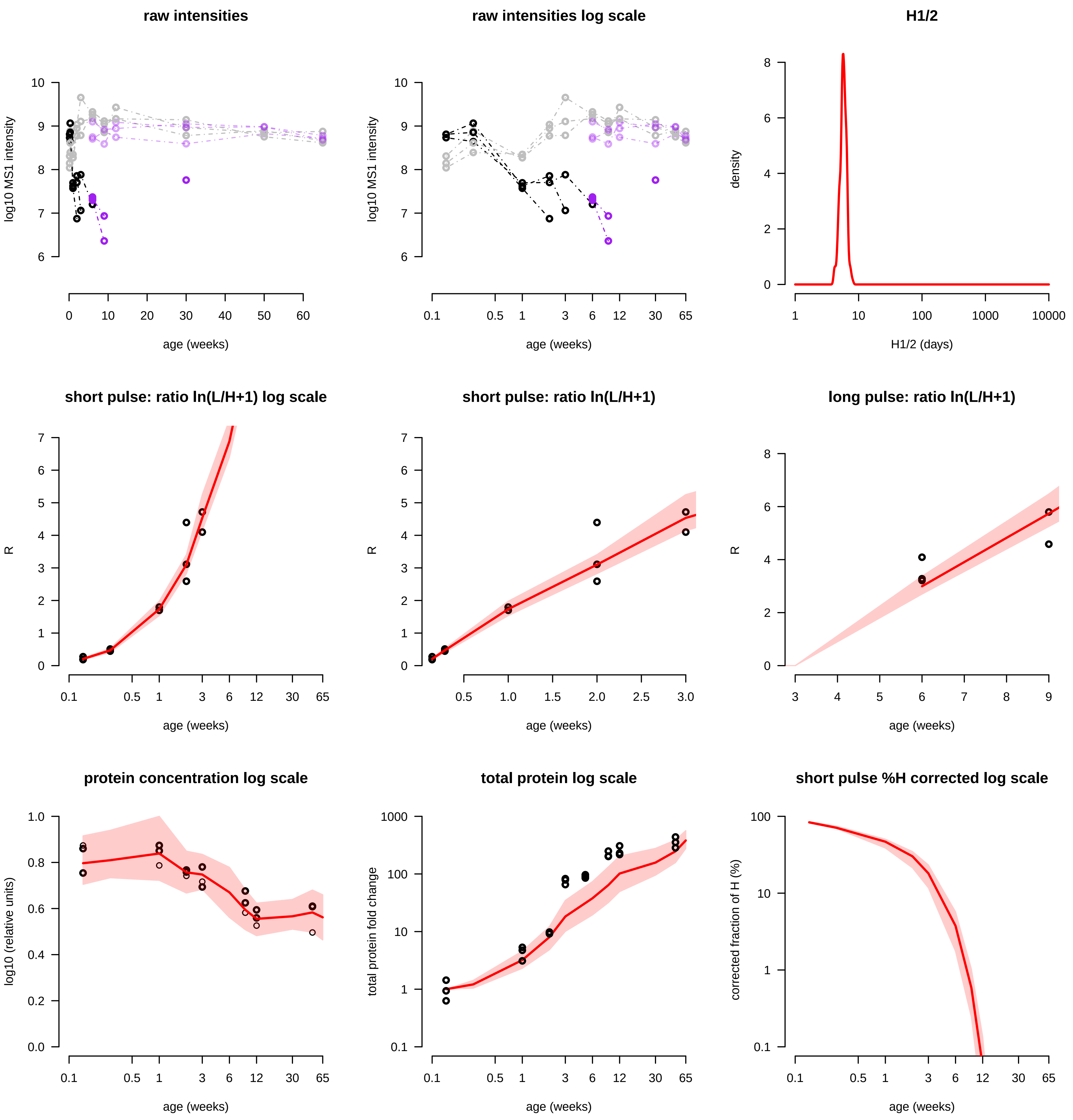

Q60902;Q60902−2;Q60902−3

>sp|Q60902|EP15R_MOUSE

Epidermal growth factor receptor substrate 15−like 1

GN=Eps15l1

Med H1/2: 1826.7 days

Q5 H1/2: 629.2 days

Q95 H1/2: 9155.9 days

reliable model fit: no

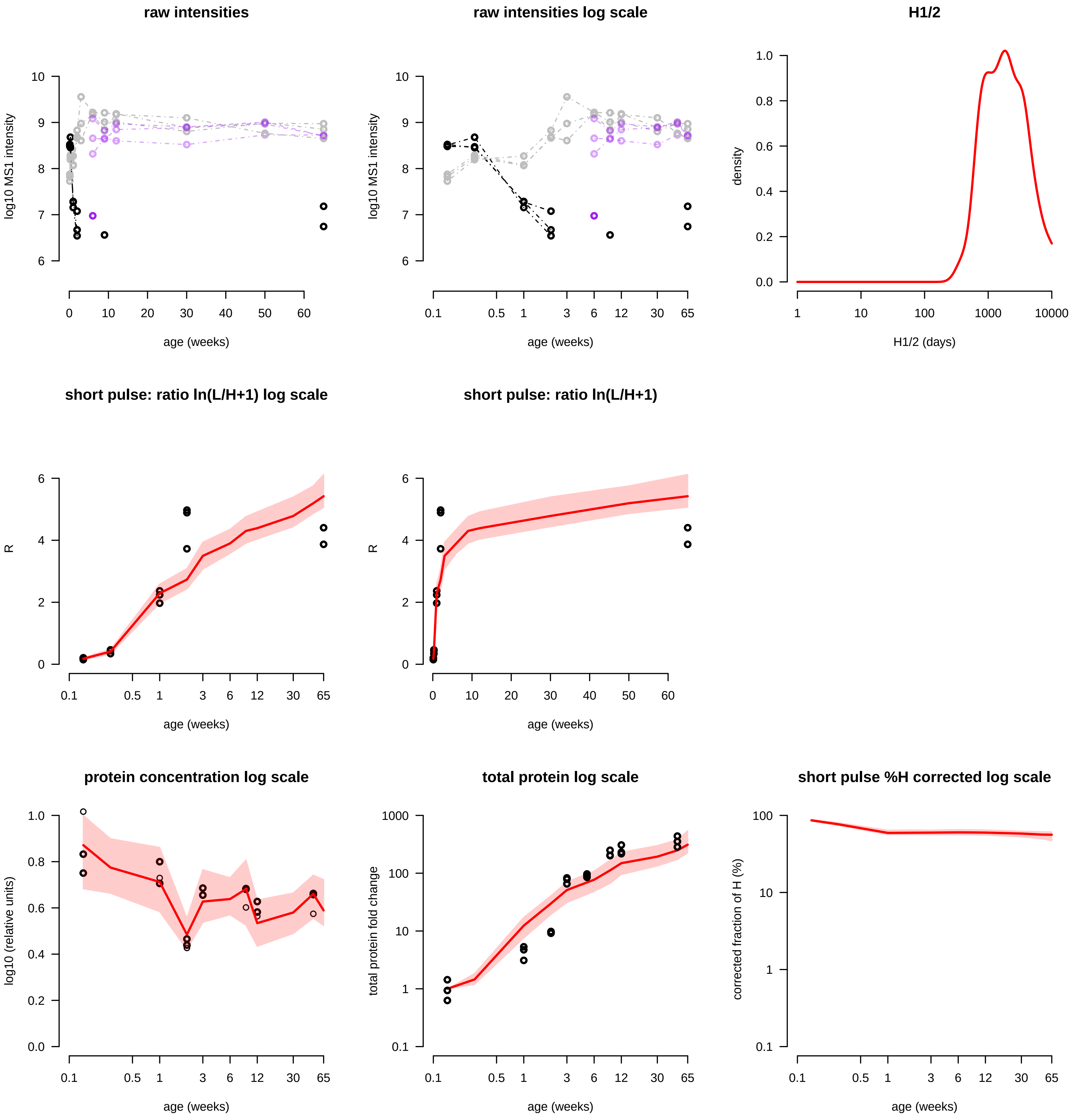

O88441

>sp|O88441|MTX2_MOUSE

Metaxin−2

GN=Mtx2

Med H1/2: 20.9 days

Q5 H1/2: 8.1 days

Q95 H1/2: 159.4 days

reliable model fit: yes

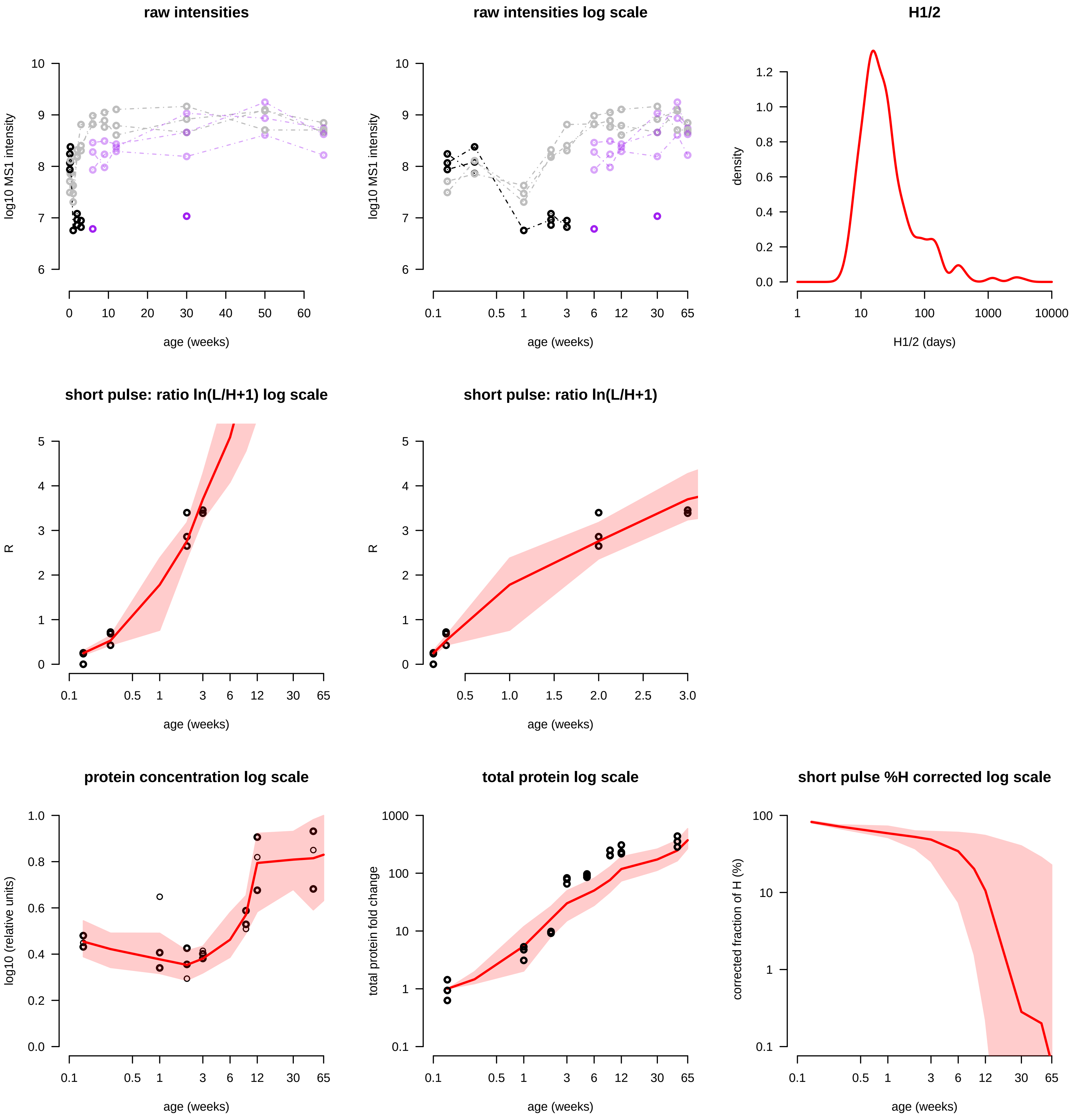

P51655

>sp|P51655|GPC4_MOUSE

Glypican−4

GN=Gpc4

Med H1/2: 4.7 days

Q5 H1/2: 3.6 days

Q95 H1/2: 6.2 days

reliable model fit: yes

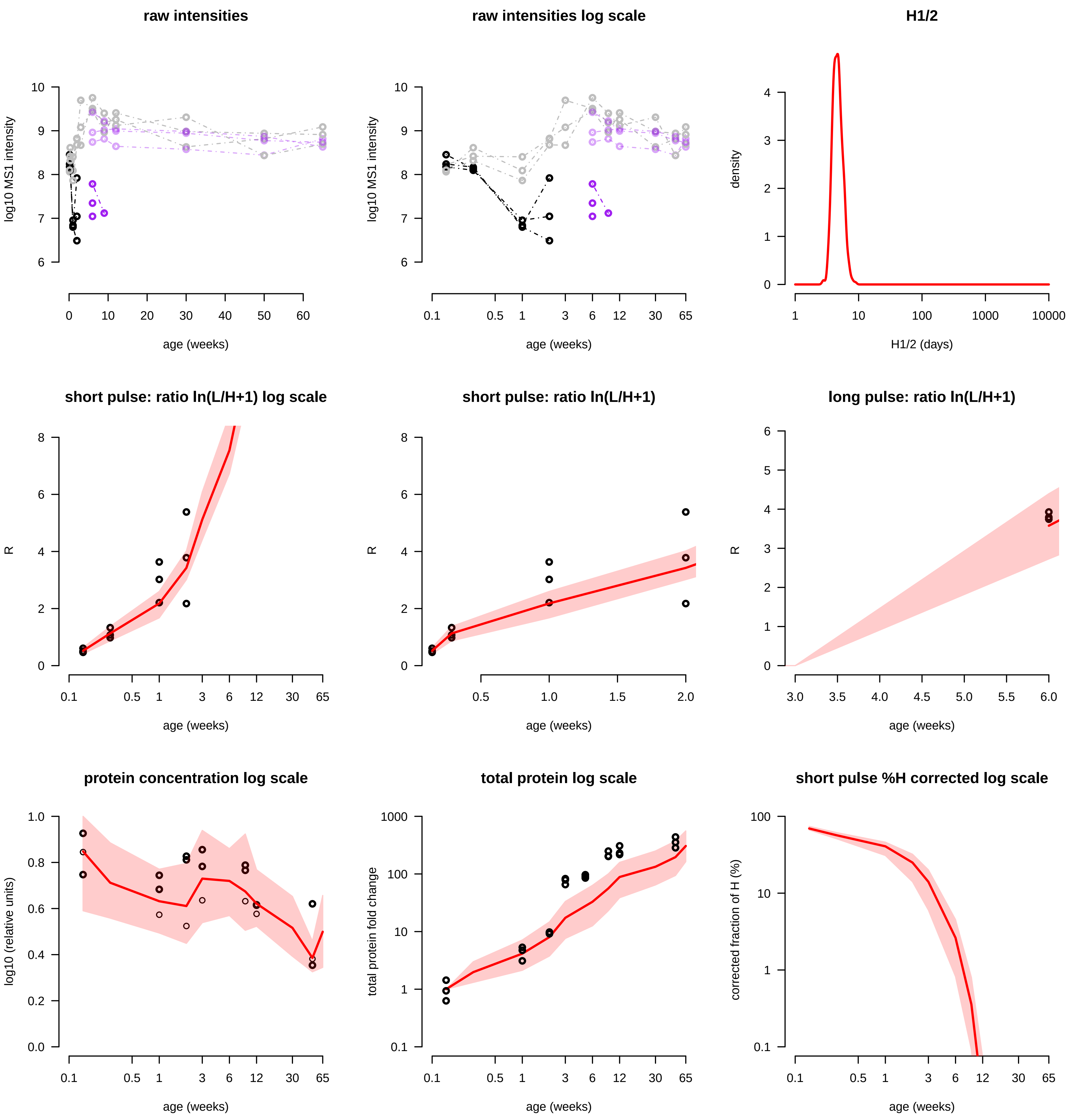

Q9JKC8

>sp|Q9JKC8|AP3M1_MOUSE

AP−3 complex subunit mu−1

GN=Ap3m1

Med H1/2: 6.3 days

Q5 H1/2: 3.1 days

Q95 H1/2: 33 days

reliable model fit: yes

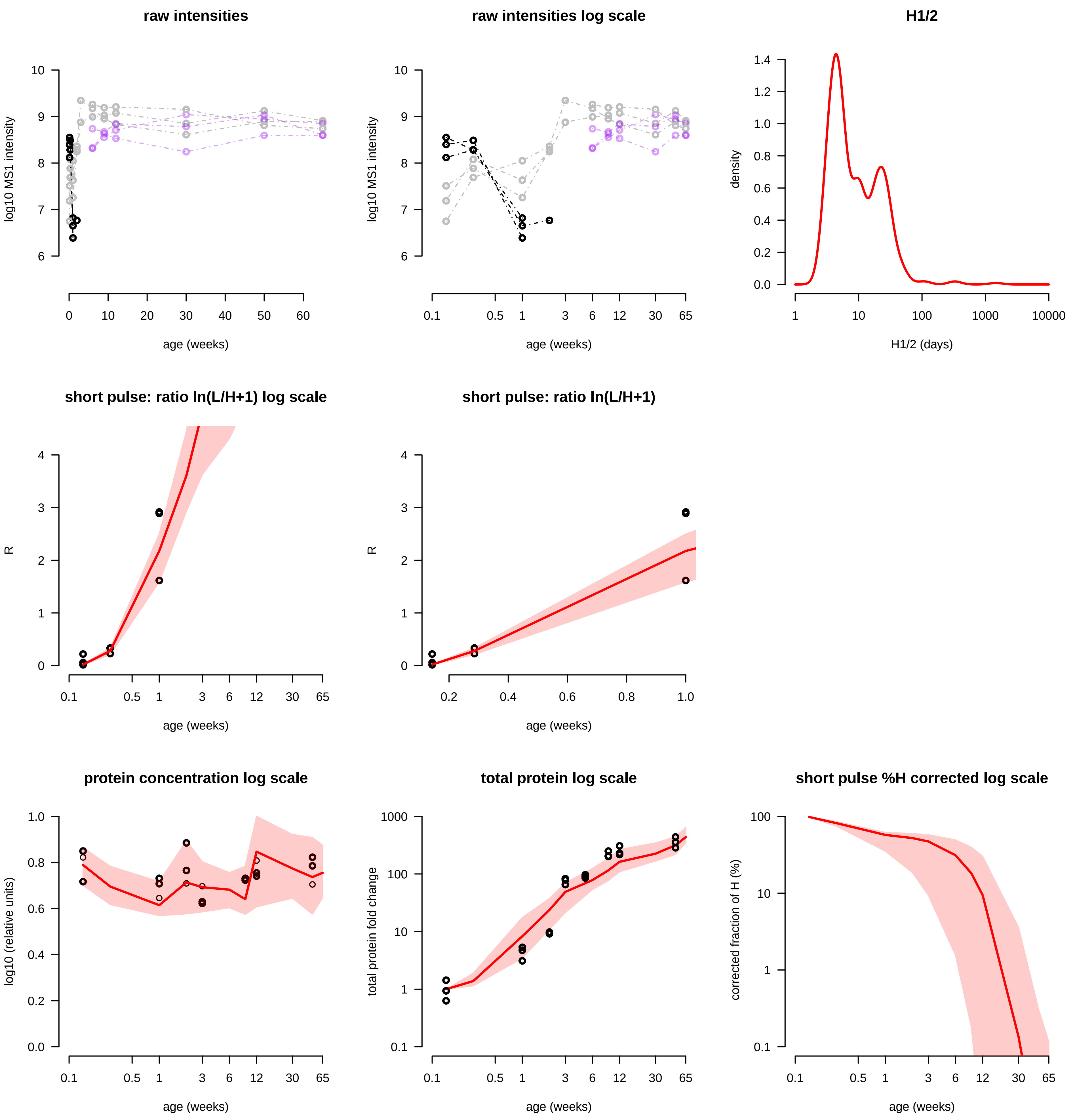

Q9CQI6

>sp|Q9CQI6|COTL1_MOUSE

Coactosin−like protein

GN=Cotl1

Med H1/2: 9.6 days

Q5 H1/2: 4.1 days

Q95 H1/2: 62.5 days

reliable model fit: yes

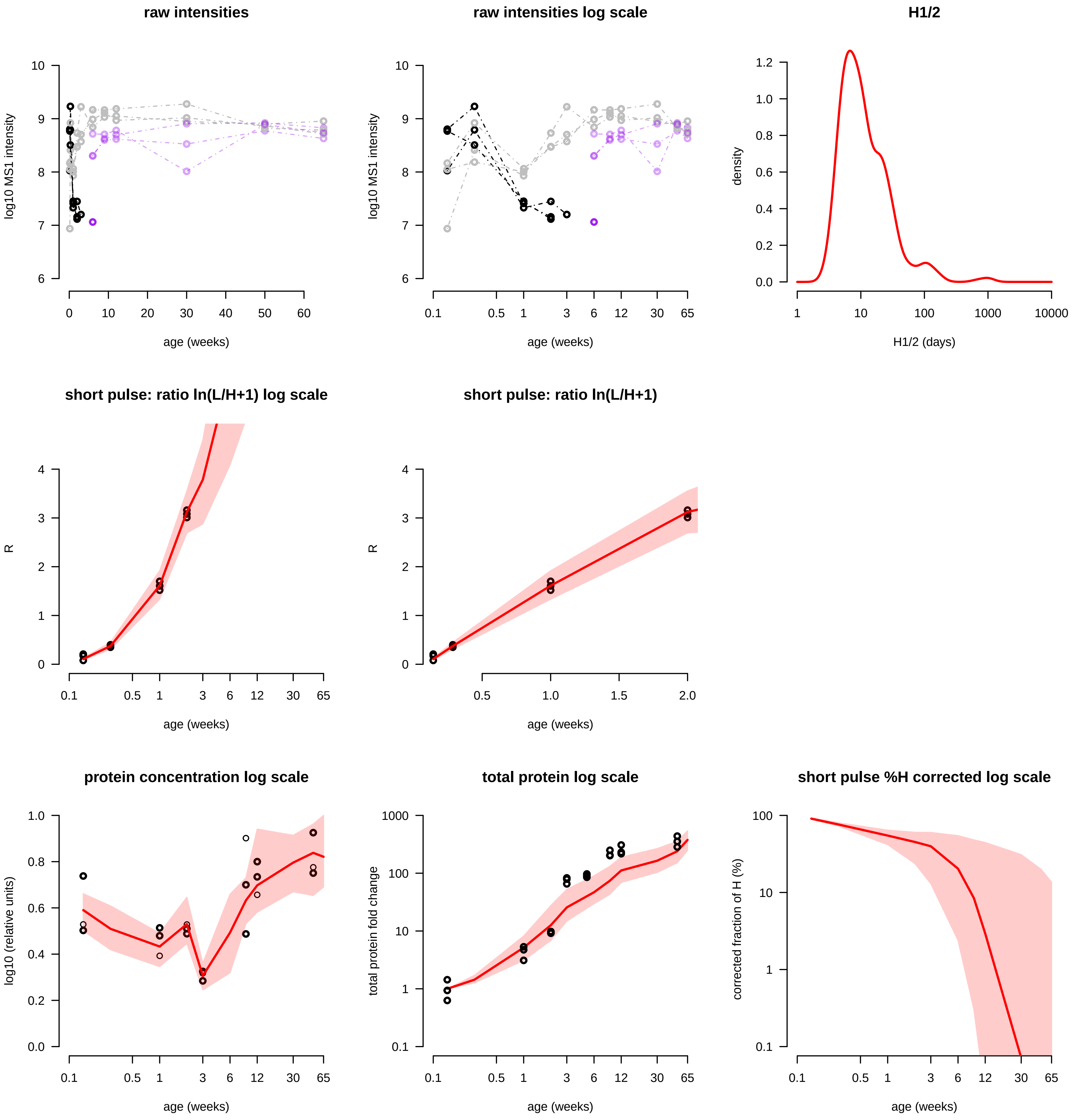

Q9ESD7−2;Q9ESD7−3

>sp|Q9ESD7−2|DYSF_MOUSE

Isoform 2 of Dysferlin

GN=Dysf

Med H1/2: 1203.4 days

Q5 H1/2: 260.3 days

Q95 H1/2: 15381 days

reliable model fit: no

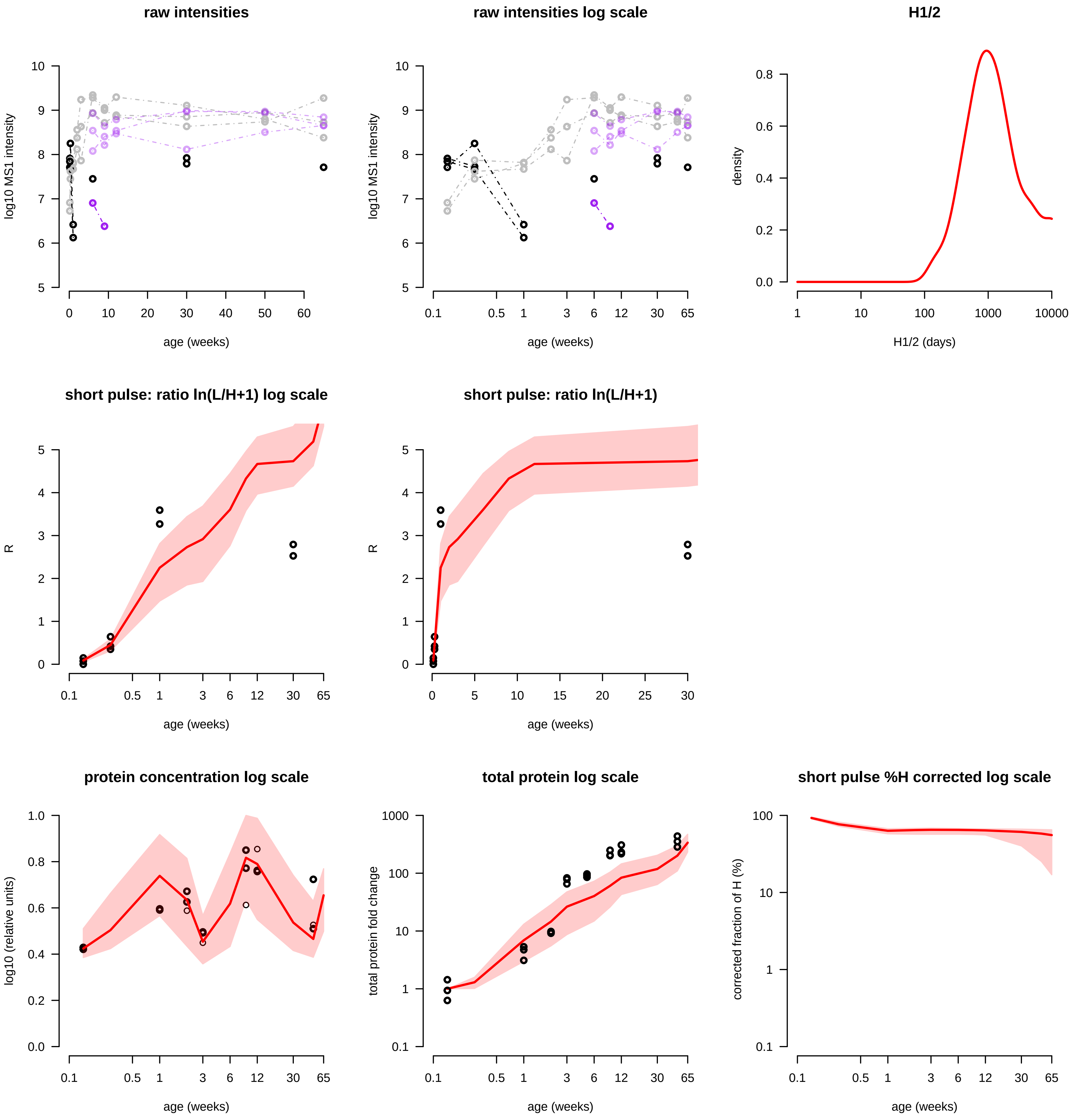

P51855

>sp|P51855|GSHB_MOUSE

Glutathione synthetase

GN=Gss

Med H1/2: 5.2 days

Q5 H1/2: 2.8 days

Q95 H1/2: 38.7 days

reliable model fit: yes

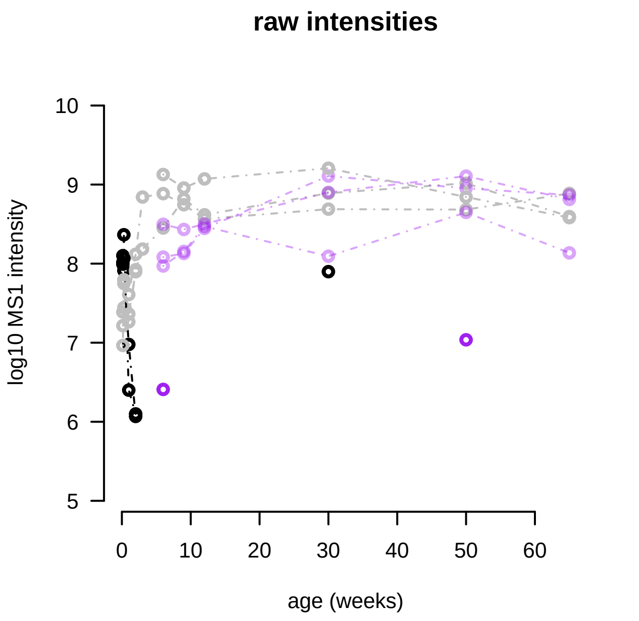

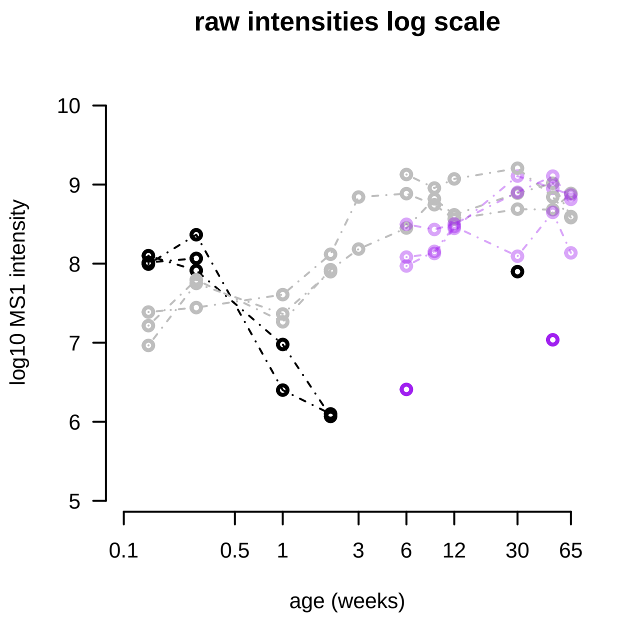

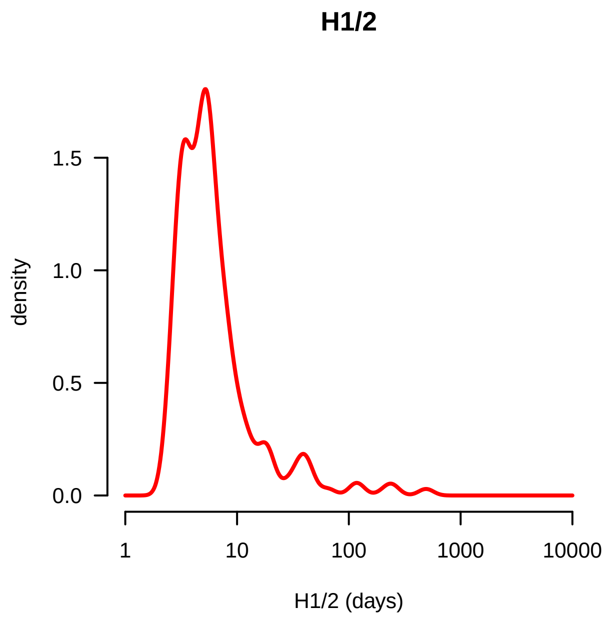

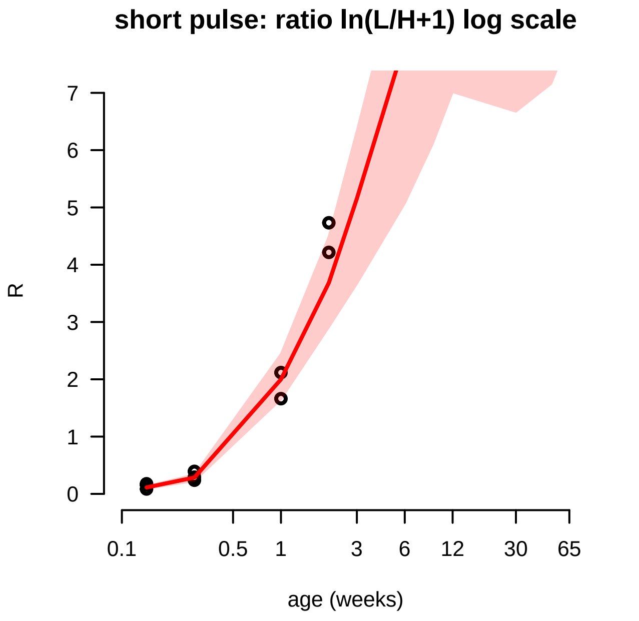

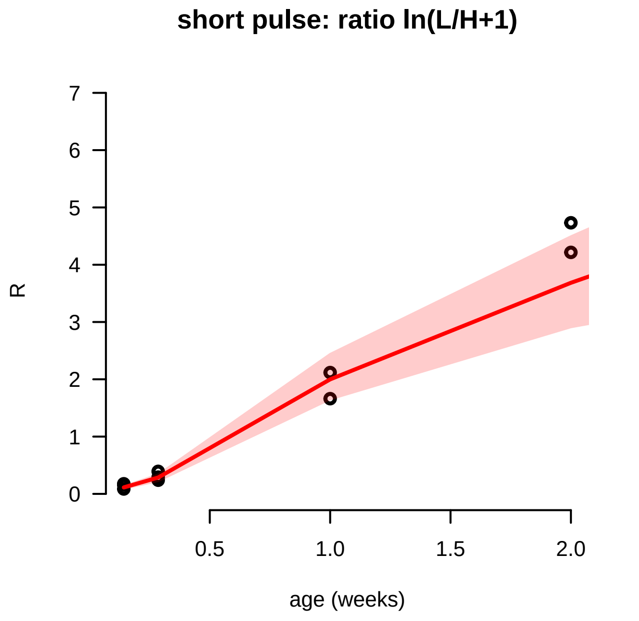

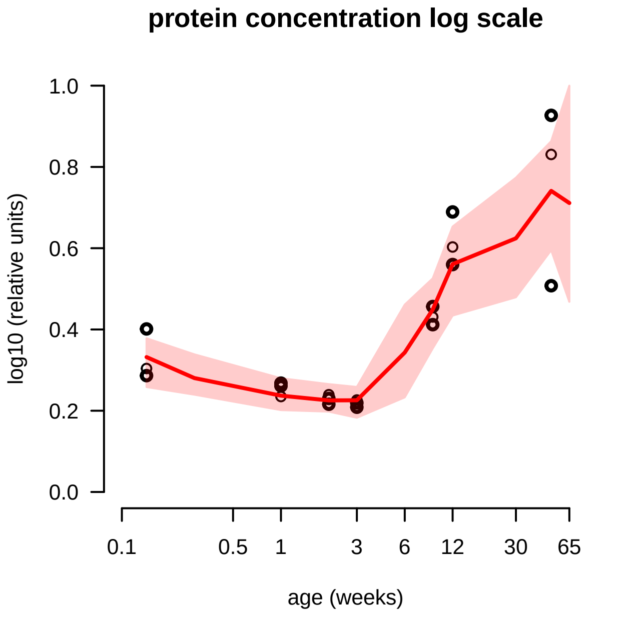

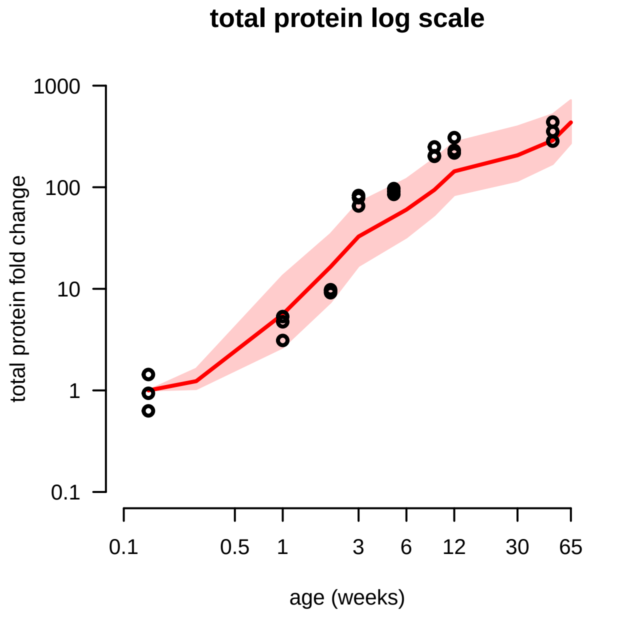

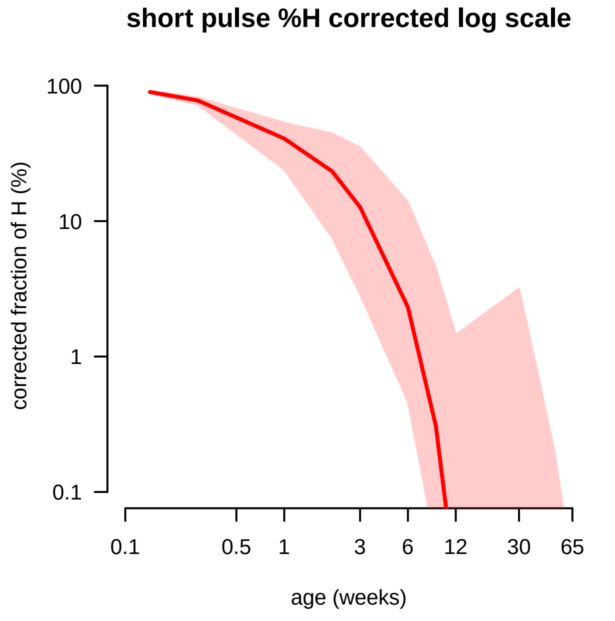

Q9D0L7;Q9D0L7−2

>sp|Q9D0L7|ARM10_MOUSE

Armadillo repeat−containing protein 10

GN=Armc10

Med H1/2: 4 days

Q5 H1/2: 3.4 days

Q95 H1/2: 4.9 days

reliable model fit: yes

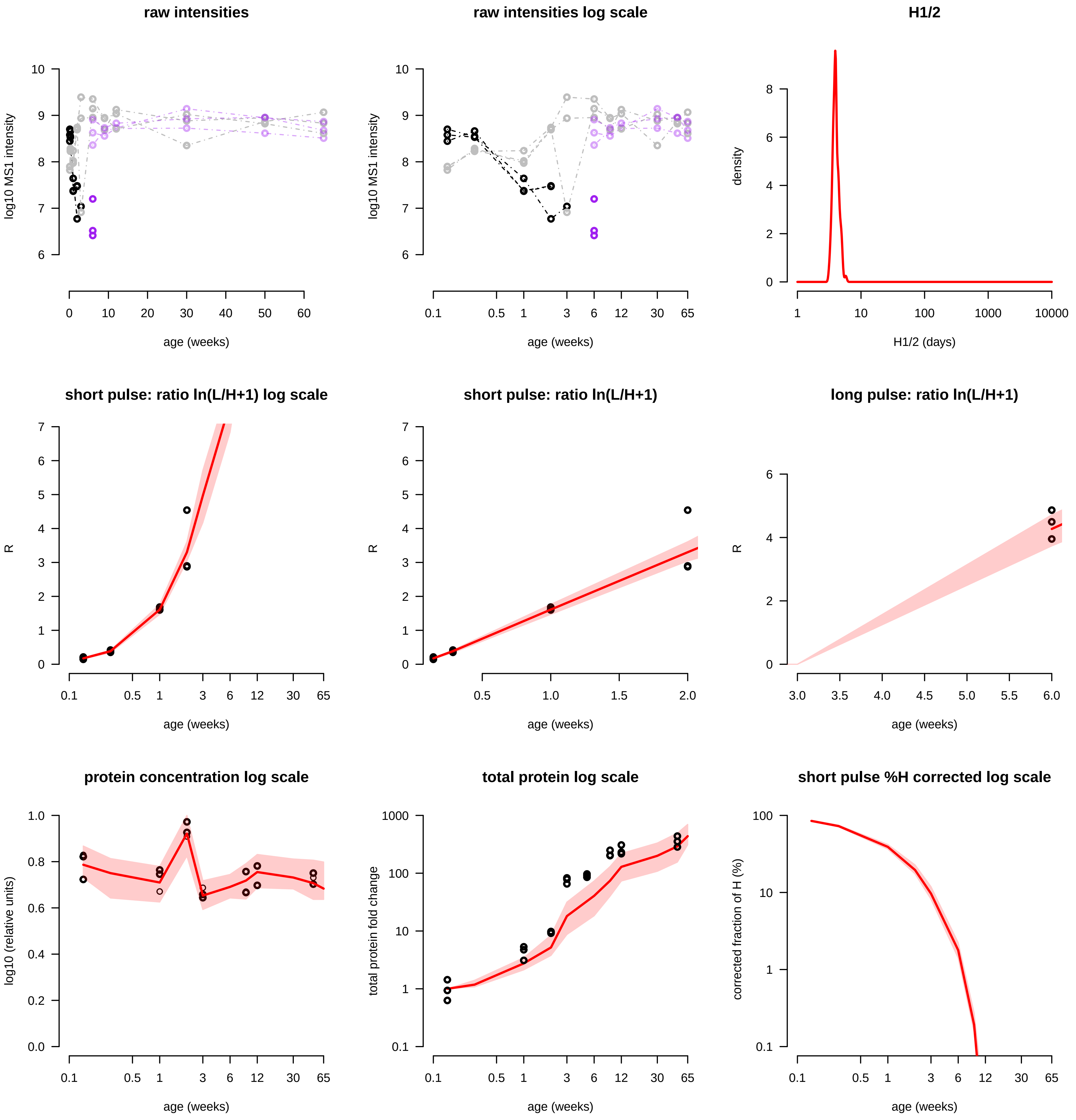

P08775

>sp|P08775|RPB1_MOUSE

DNA−directed RNA polymerase II subunit RPB1

GN=Polr2a

Med H1/2: 10.7 days

Q5 H1/2: 3.9 days

Q95 H1/2: 53.4 days

reliable model fit: yes

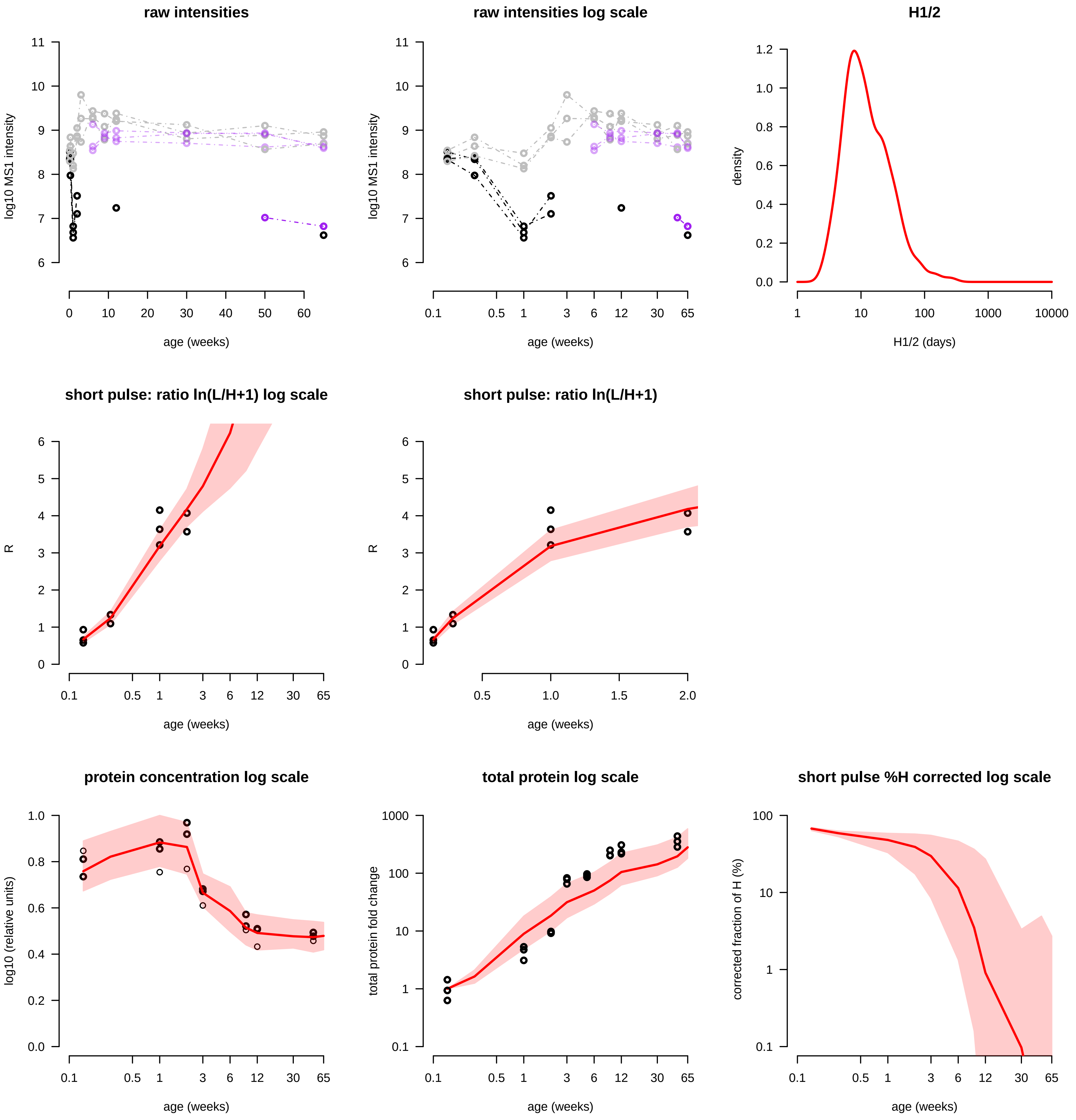

Q9D880

>sp|Q9D880|TIM50_MOUSE

Mitochondrial import inner membrane translocase subunit TIM50

GN=Timm50

Med H1/2: 4.9 days

Q5 H1/2: 3.8 days

Q95 H1/2: 6.8 days

reliable model fit: yes

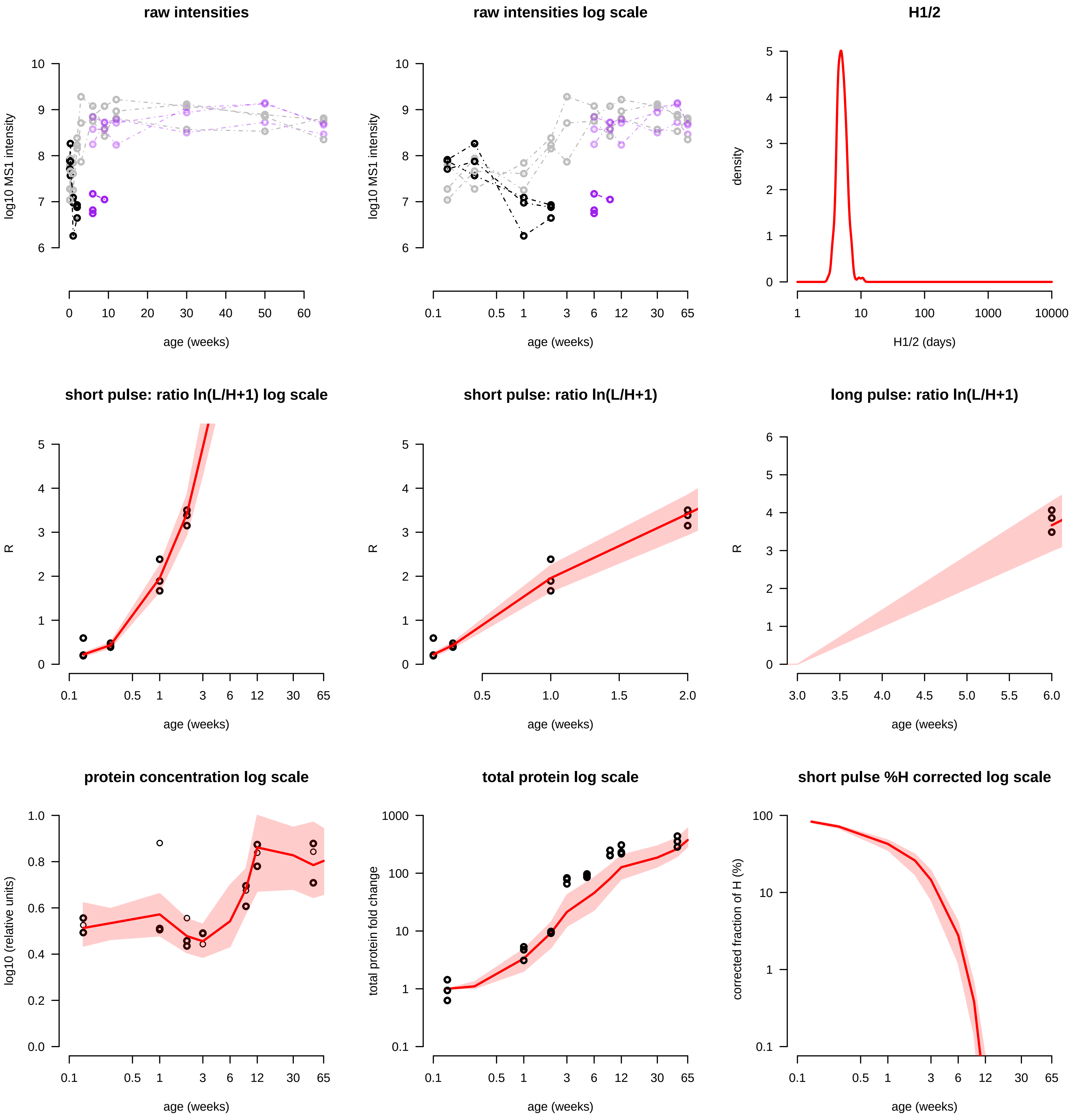

Q8CG76

>sp|Q8CG76|ARK72_MOUSE

Aflatoxin B1 aldehyde reductase member 2

GN=Akr7a2

Med H1/2: 5.6 days

Q5 H1/2: 5 days

Q95 H1/2: 7.4 days

reliable model fit: yes

raw intensities

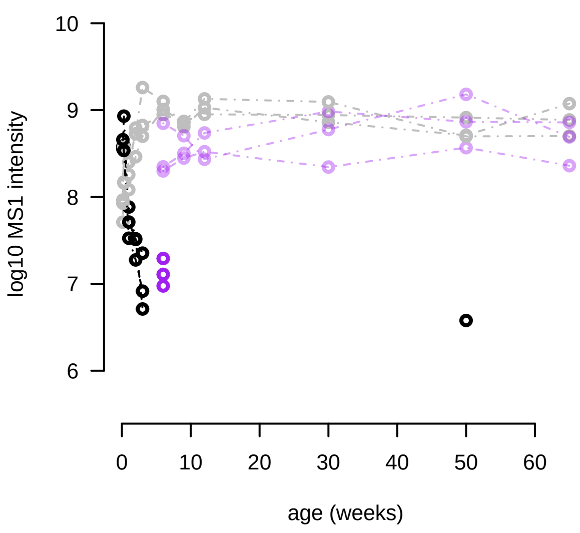

raw intensities log scale

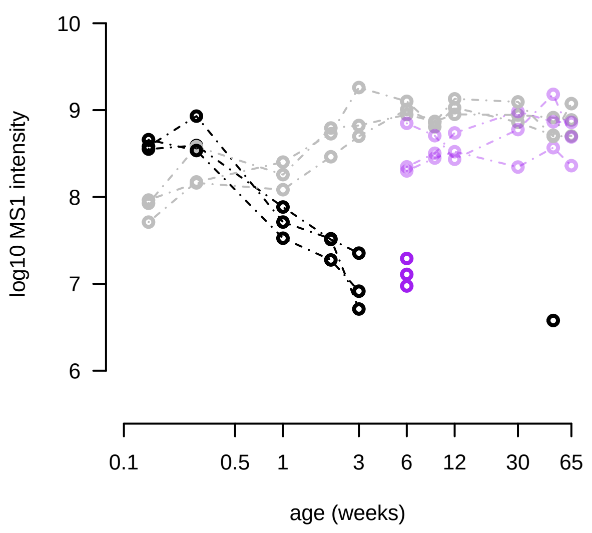

H1/2

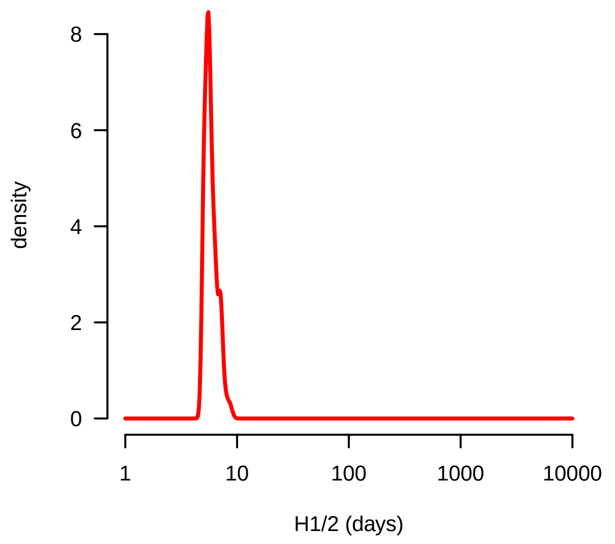

short pulse: ratio ln(L/H+1) log scale

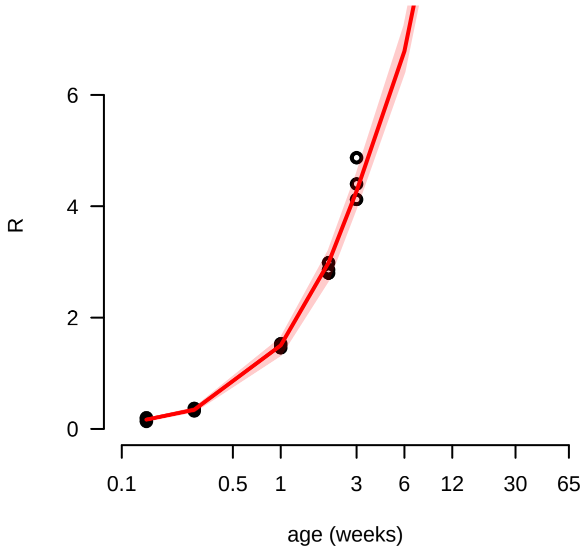

short pulse: ratio ln(L/H+1)

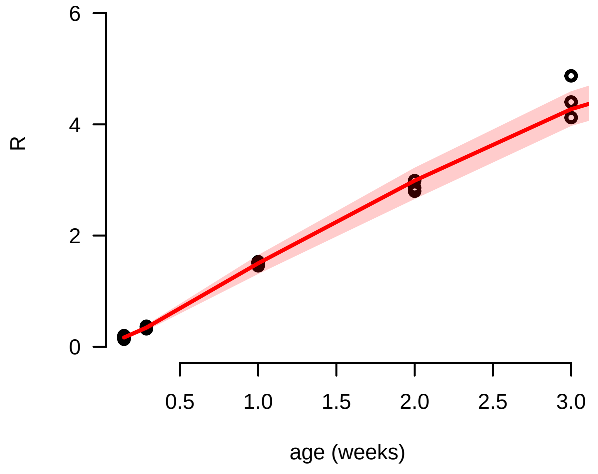

long pulse: ratio ln(L/H+1)

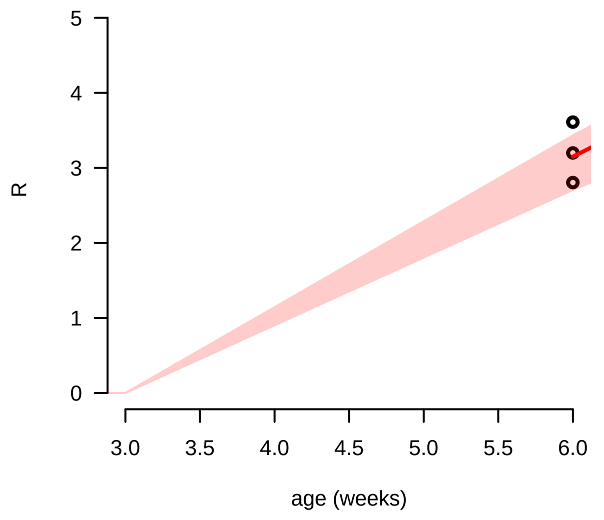

protein concentration log scale

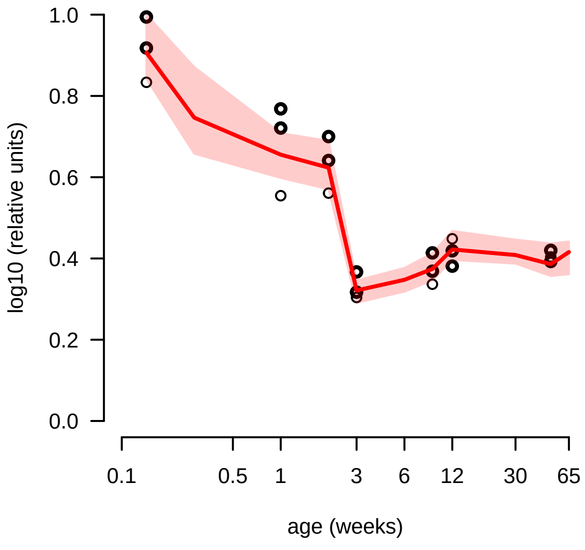

total protein log scale

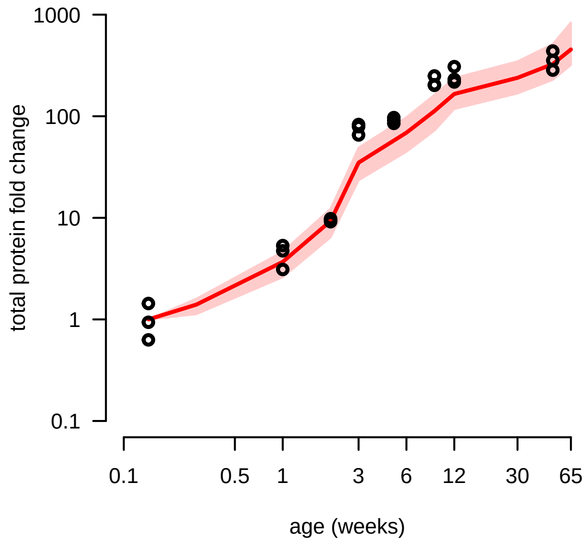

short pulse %H corrected log scale

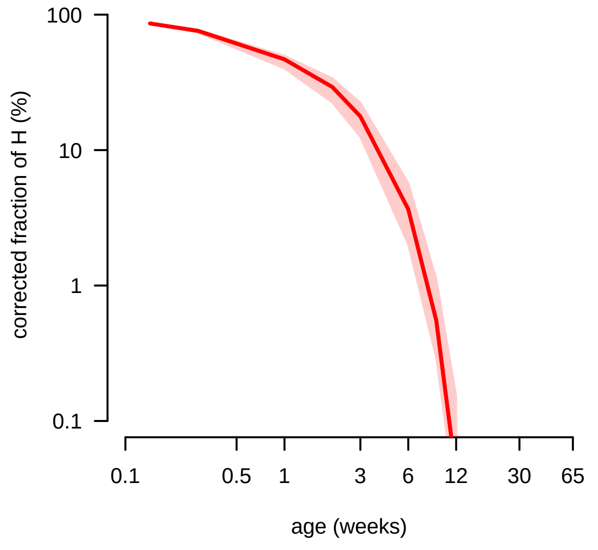

Q9QZQ1−2;Q9QZQ1

>sp|Q9QZQ1−2|AFAD_MOUSE

Isoform 1 of Afadin

GN=Afdn

Med H1/2: 3.7 days

Q5 H1/2: 2.6 days

Q95 H1/2: 10.3 days

reliable model fit: yes

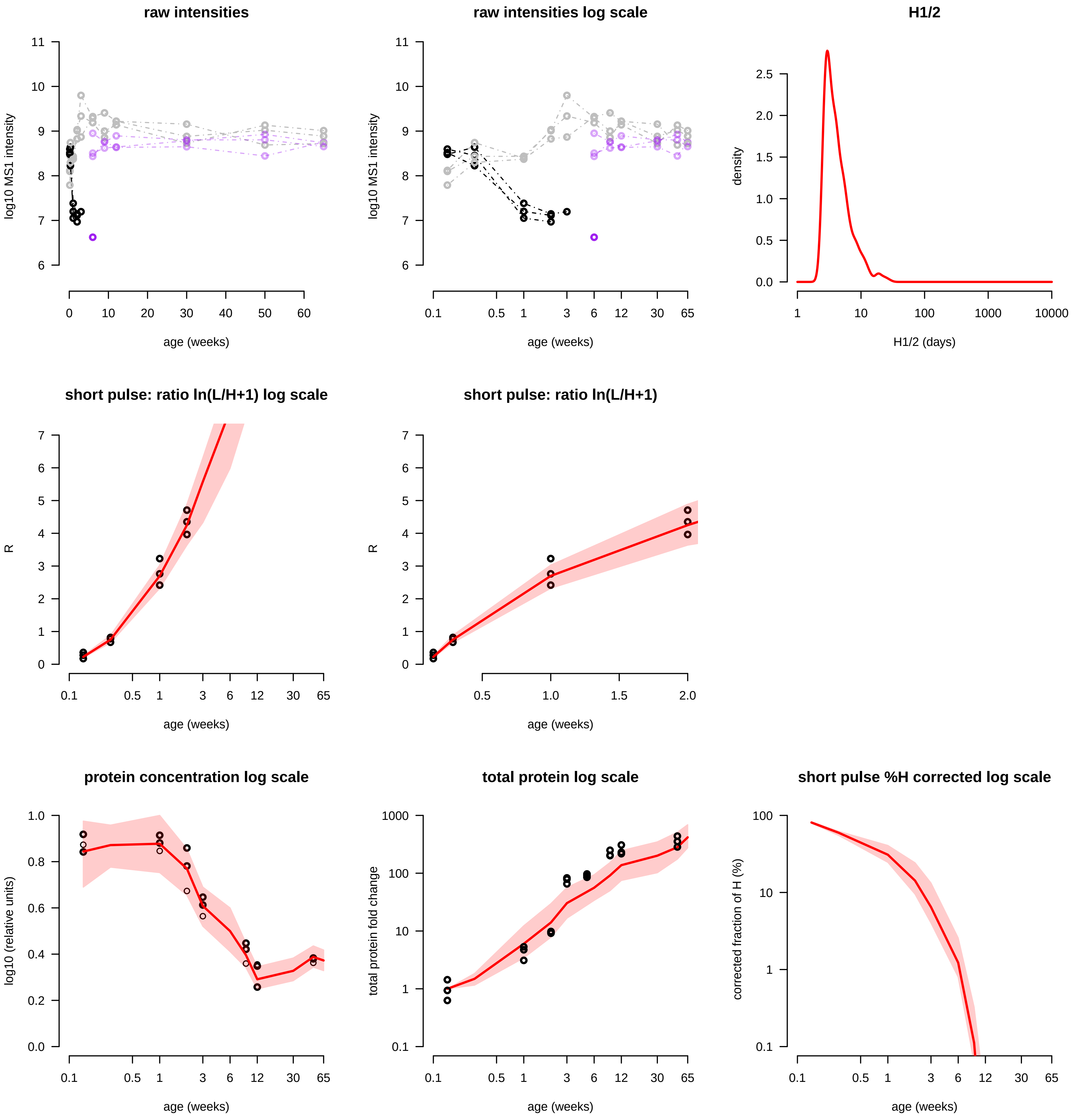

Q66JS6

>sp|Q66JS6|EI3JB_MOUSE

Eukaryotic translation initiation factor 3 subunit J−B

GN=Eif3j2

Med H1/2: 6.7 days

Q5 H1/2: 4.9 days

Q95 H1/2: 10.3 days

reliable model fit: yes

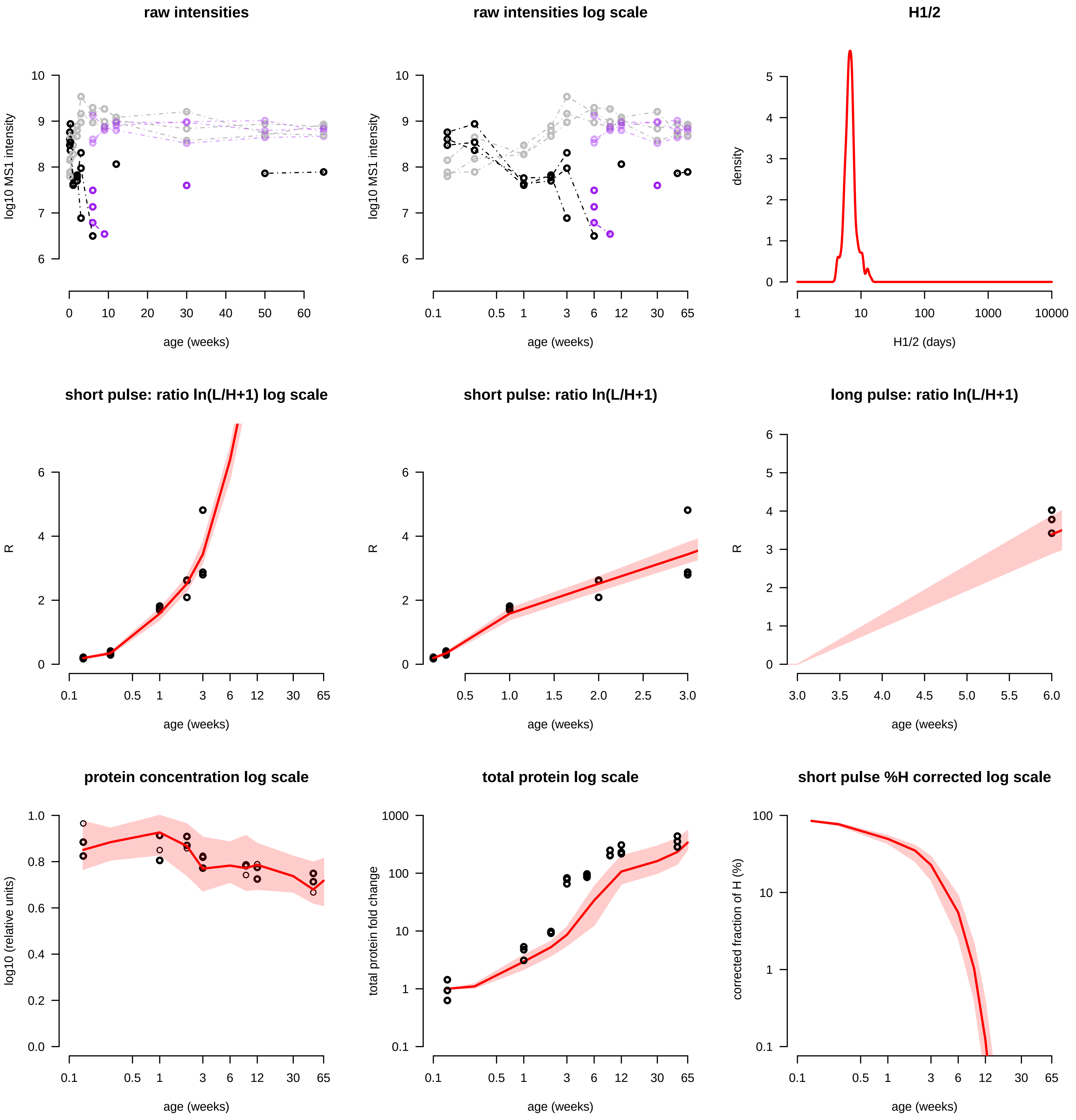

Q8BKF1

>sp|Q8BKF1|RPOM_MOUSE

DNA−directed RNA polymerase, mitochondrial

GN=Polrmt

Med H1/2: 18.2 days

Q5 H1/2: 13.4 days

Q95 H1/2: 30.4 days

reliable model fit: yes

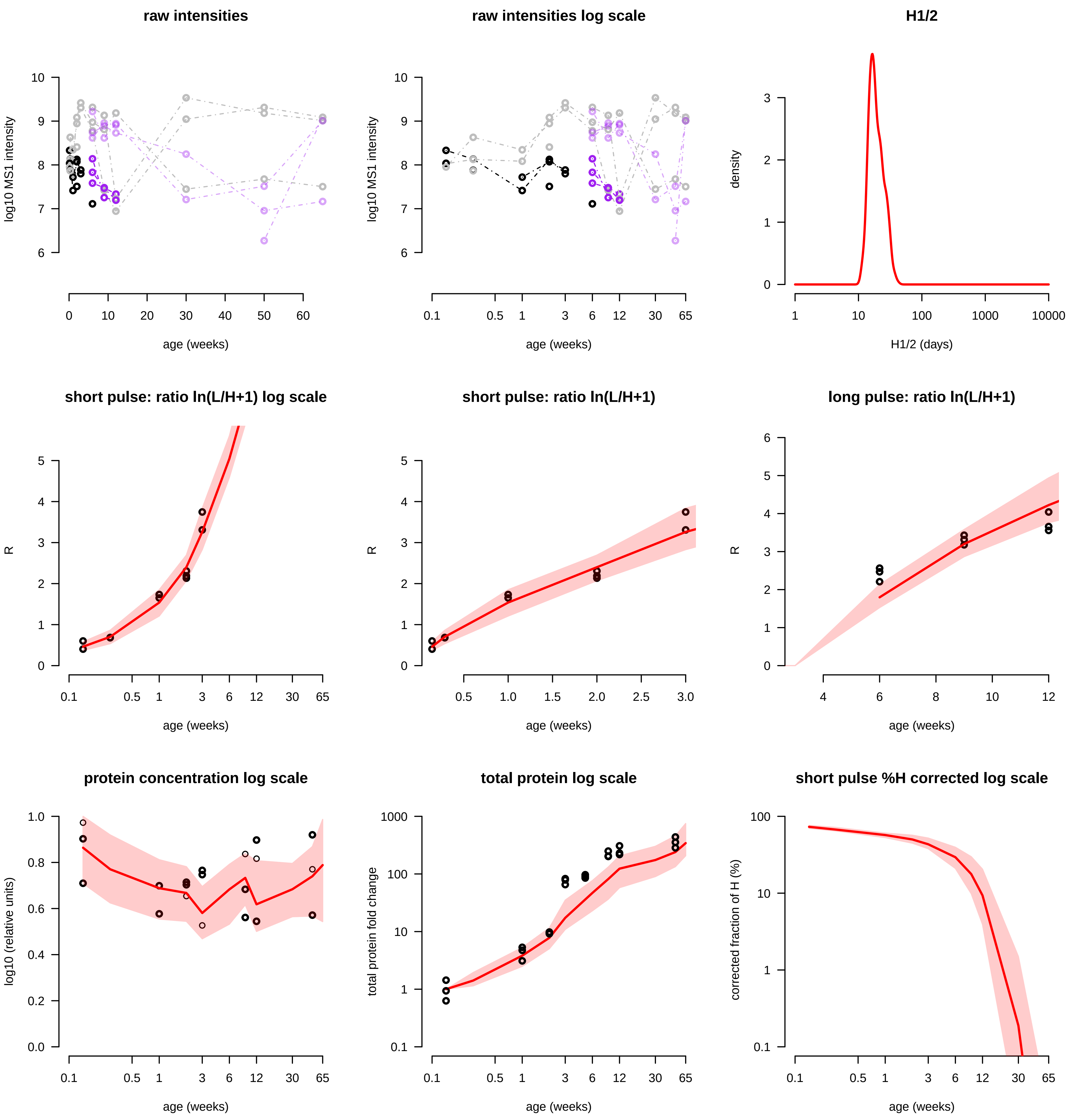

Q8BTI8;Q8BTI8−3;Q8BTI8−2

>sp|Q8BTI8|SRRM2_MOUSE

Serine/arginine repetitive matrix protein 2

GN=Srrm2

Med H1/2: 3.4 days

Q5 H1/2: 2.7 days

Q95 H1/2: 4.2 days

reliable model fit: yes

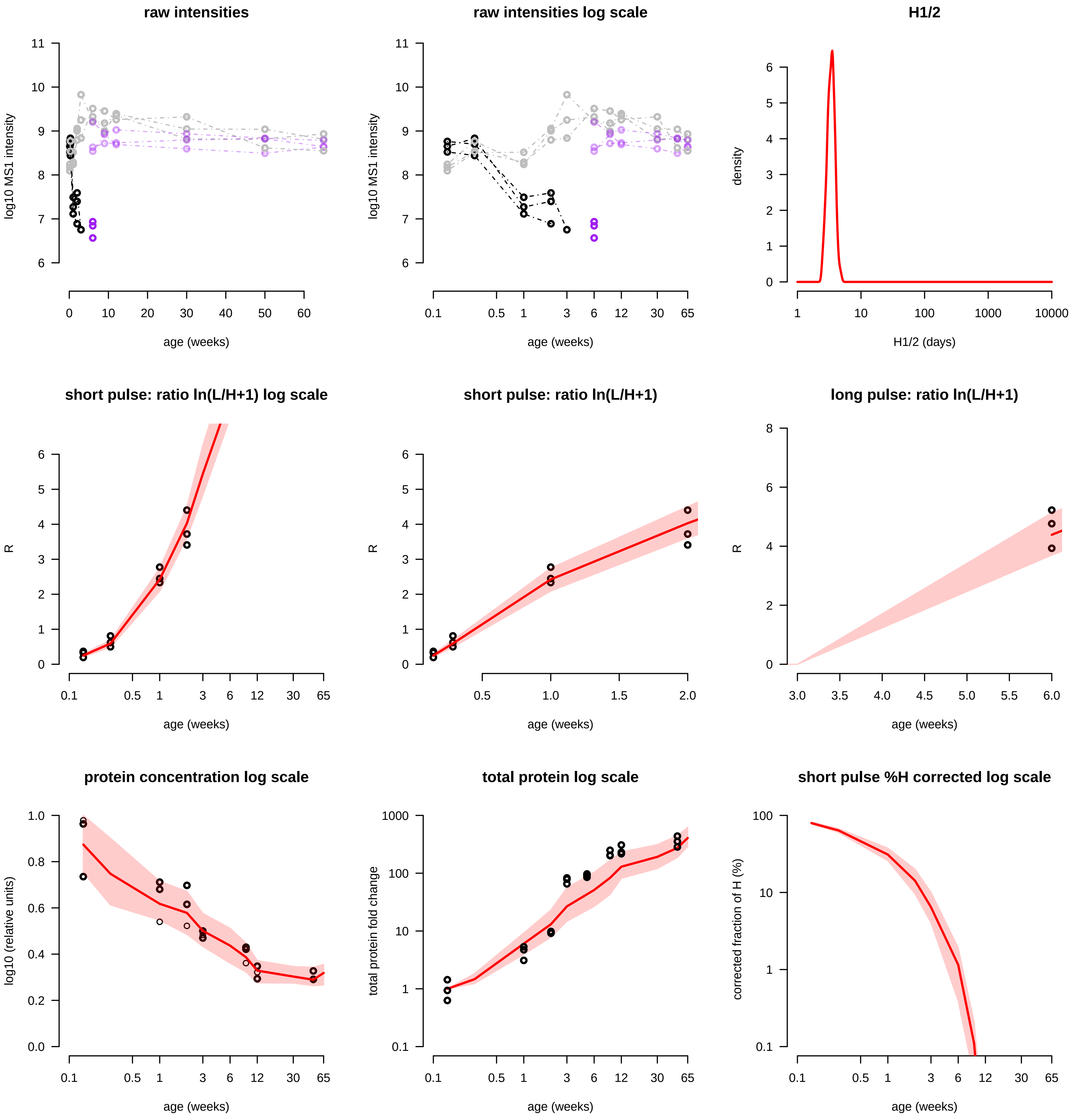

P62996

>sp|P62996|TRA2B_MOUSE

Transformer−2 protein homolog beta

GN=Tra2b

Med H1/2: 9.4 days

Q5 H1/2: 8 days

Q95 H1/2: 12.1 days

reliable model fit: yes

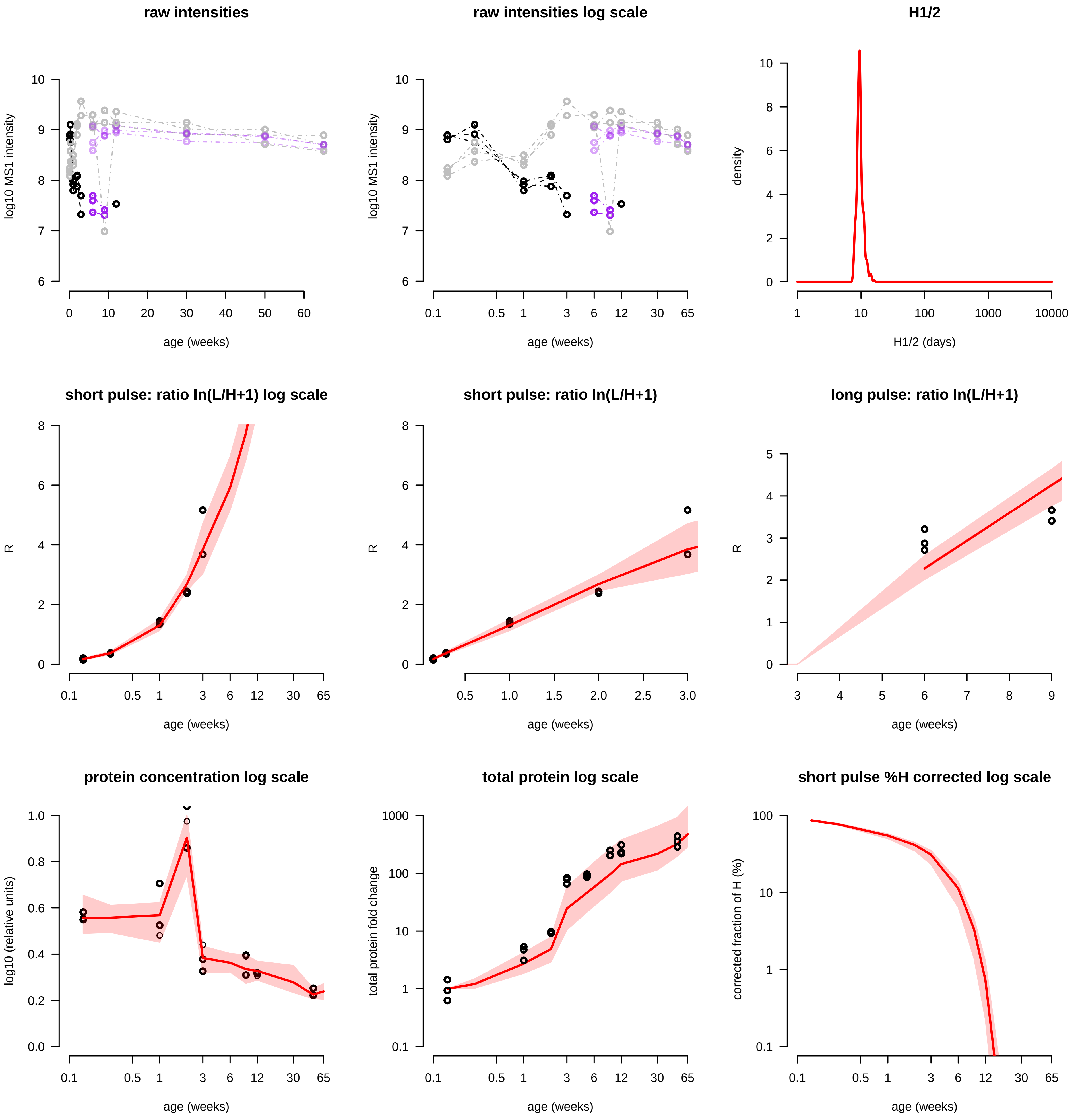

P97927

>sp|P97927|LAMA4_MOUSE

Laminin subunit alpha−4

GN=Lama4

Med H1/2: 3.6 days

Q5 H1/2: 2.8 days

Q95 H1/2: 4.5 days

reliable model fit: yes

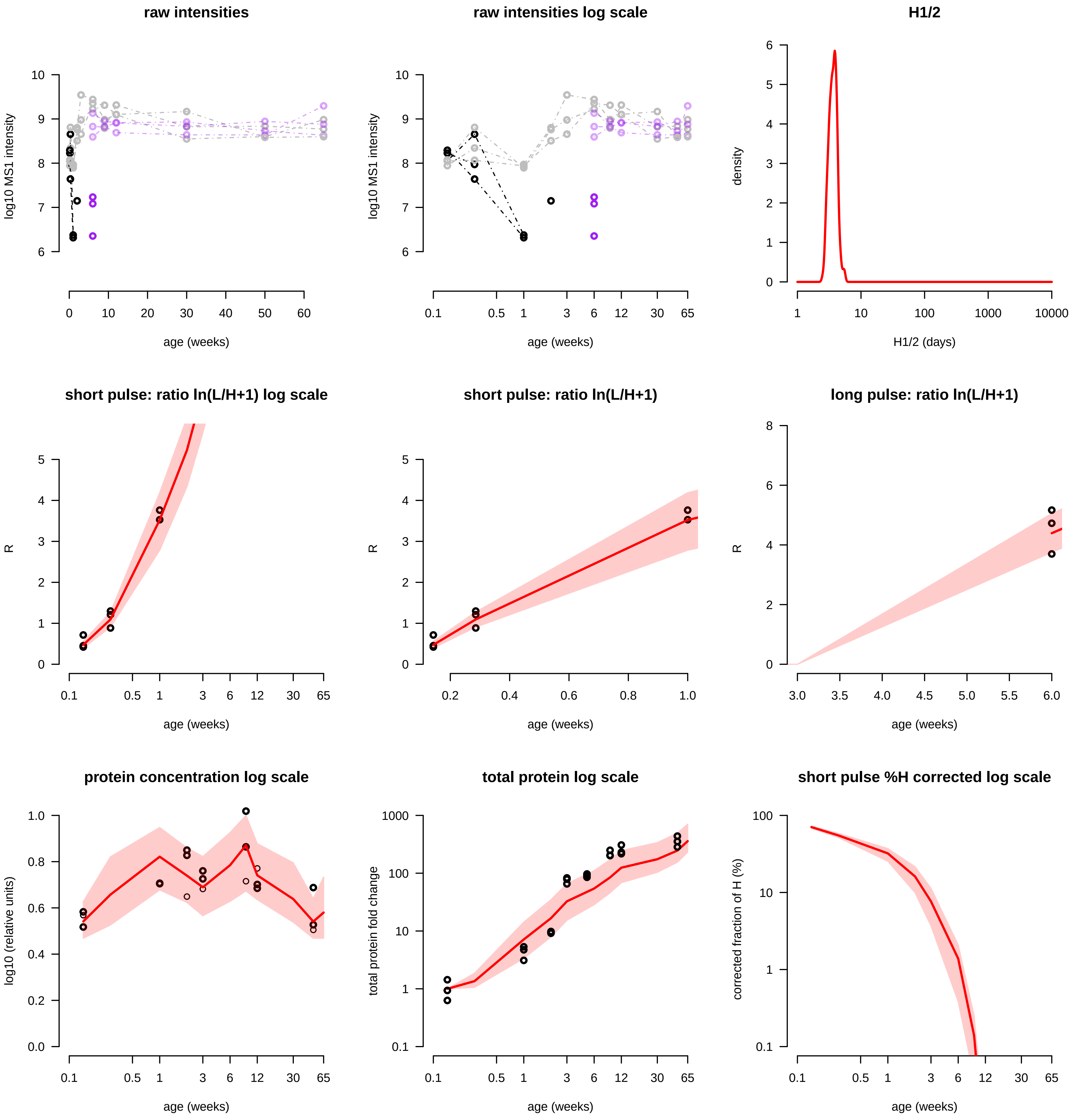

Q8CGY8

>sp|Q8CGY8|OGT1_MOUSE

’−N−acetylglucosamine−−peptide N−acetylglucosaminyltransferase 110 kDa sul

GN=Ogt

Med H1/2: 3.7 days

Q5 H1/2: 2.6 days

Q95 H1/2: 6.7 days

reliable model fit: yes

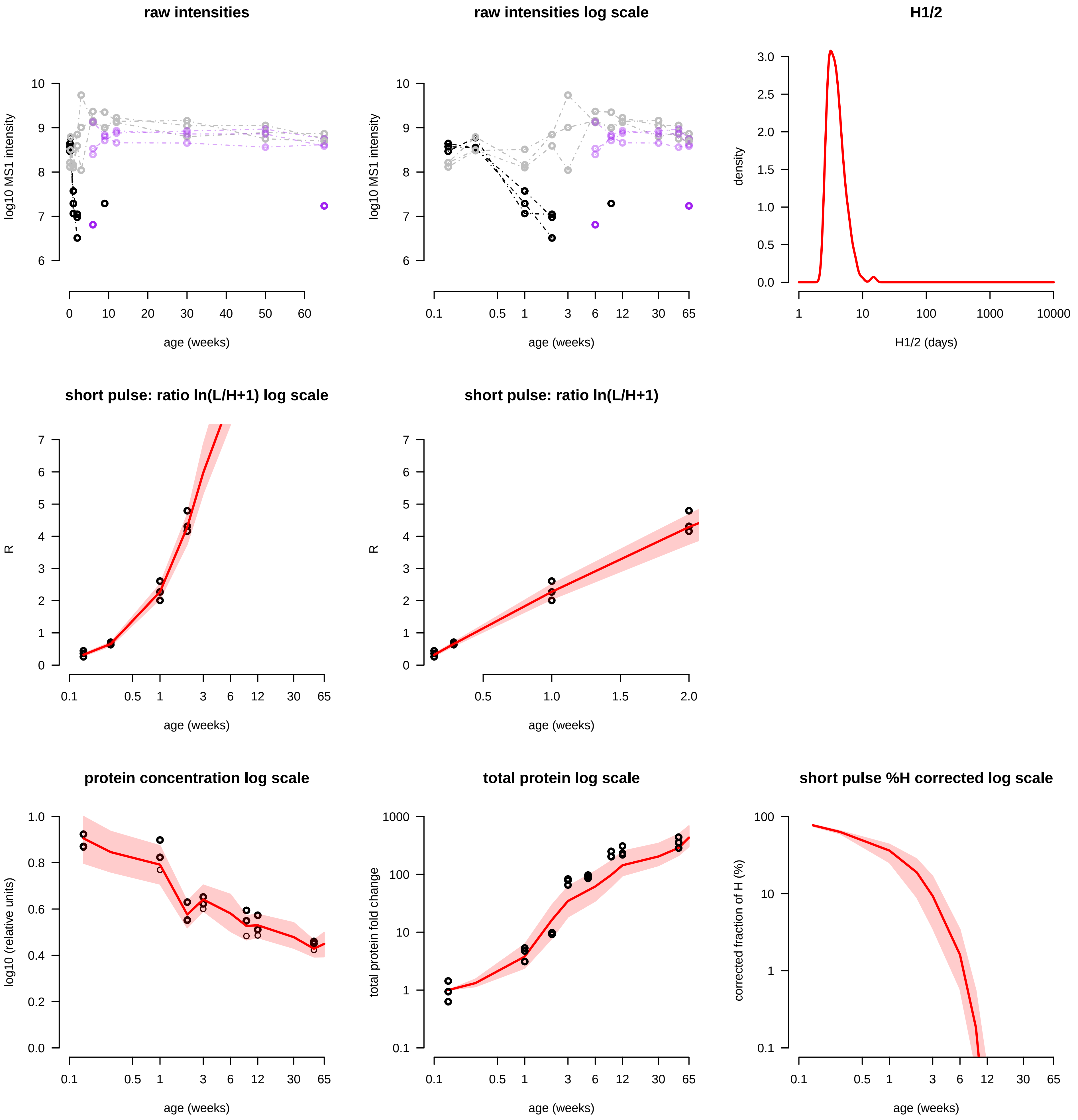

Q9D6U8

>sp|Q9D6U8|F162A_MOUSE

Protein FAM162A

GN=Fam162a

Med H1/2: 4.7 days

Q5 H1/2: 4 days

Q95 H1/2: 6 days

reliable model fit: yes

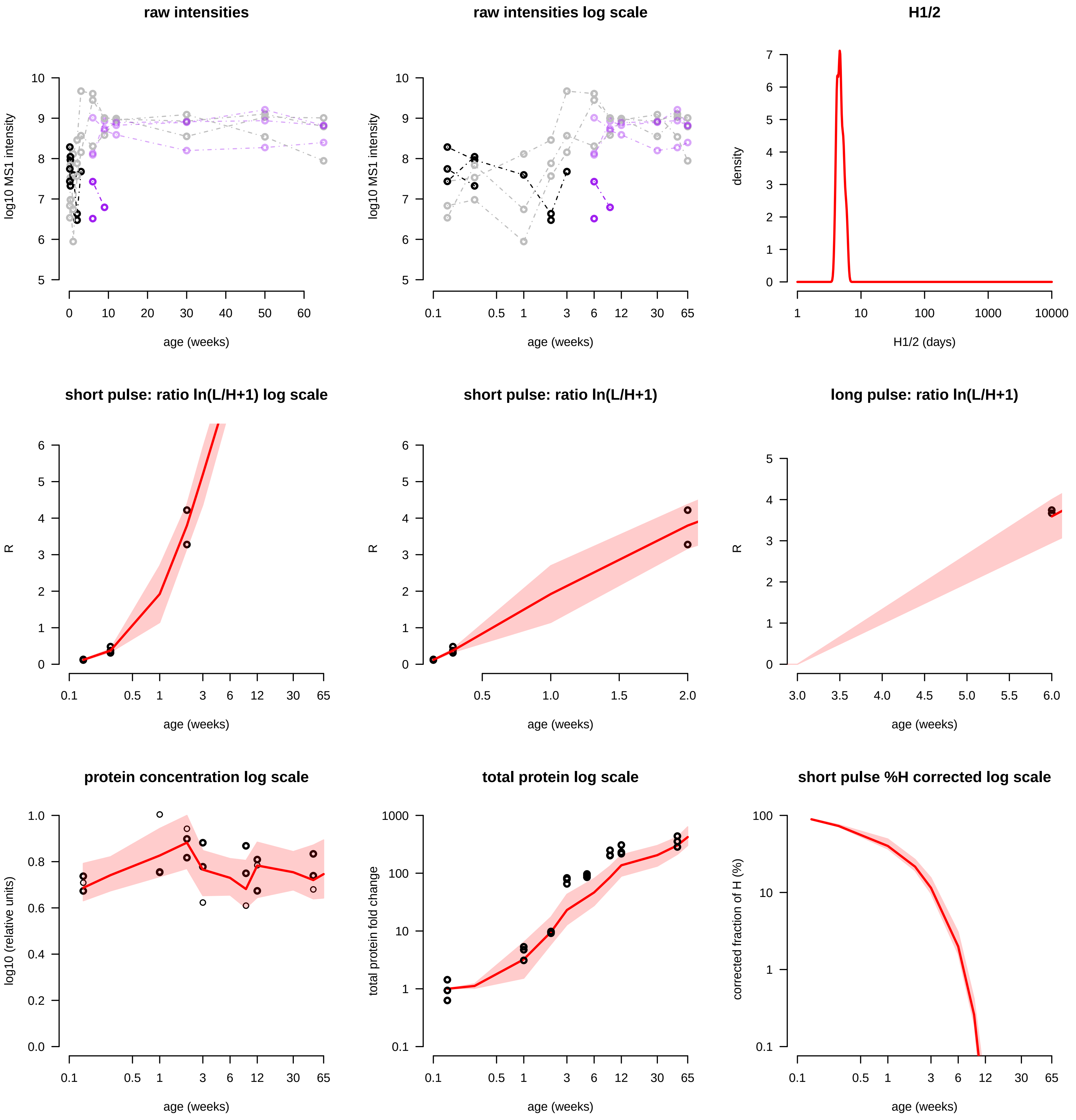

Q9CQ45

>sp|Q9CQ45|NENF_MOUSE

Neudesin

GN=Nenf

Med H1/2: 3.5 days

Q5   H1/2: 2.5 days

Q95 H1/2: 7.7 days

reliable model fit: yes

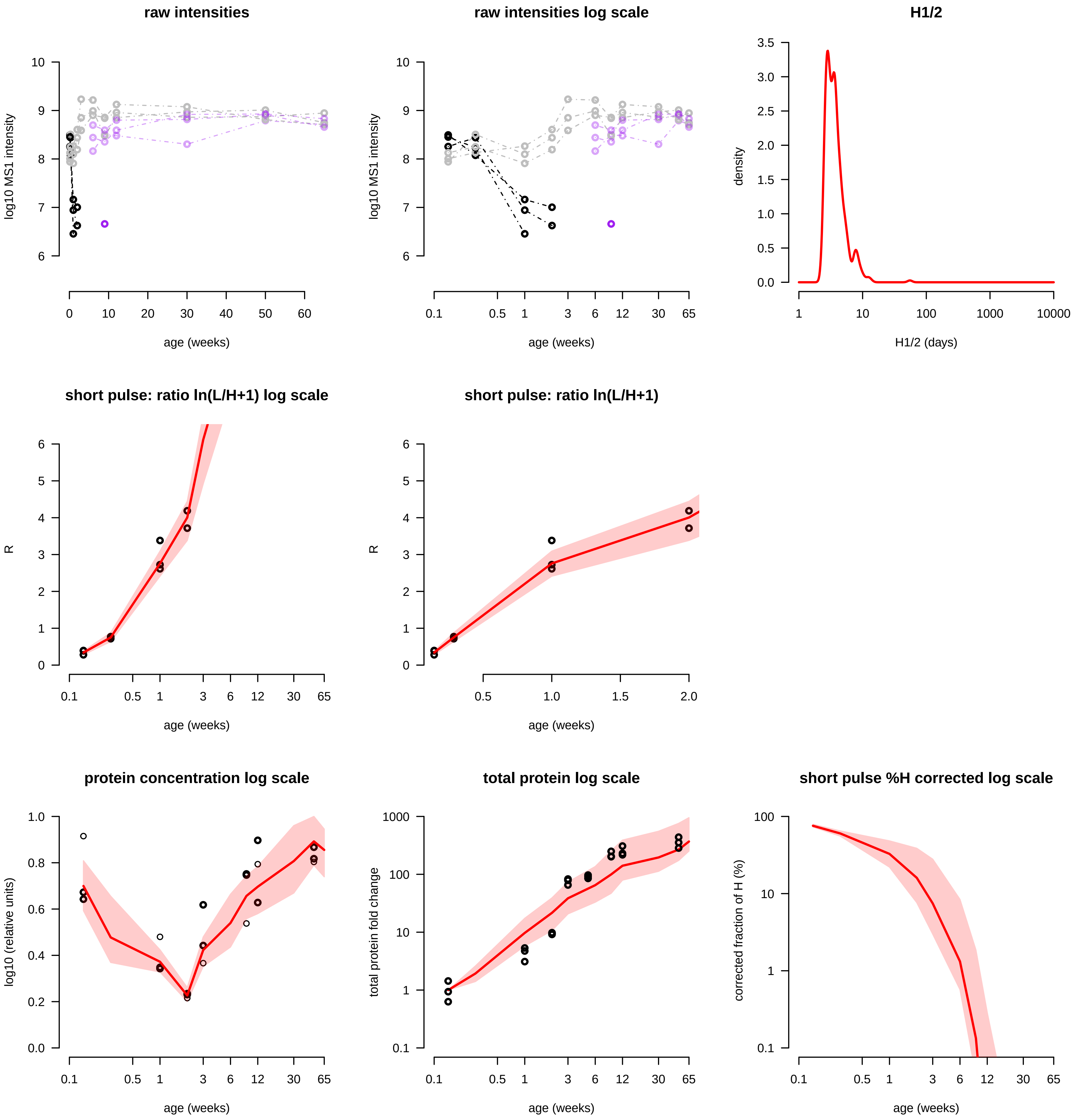

Q9CWZ3;Q9CWZ3−2

>sp|Q9CWZ3|RBM8A_MOUSE

RNA−binding protein 8A

GN=Rbm8a

Med H1/2: 6.8 days

Q5 H1/2: 5.7 days

Q95 H1/2: 8.9 days

reliable model fit: yes

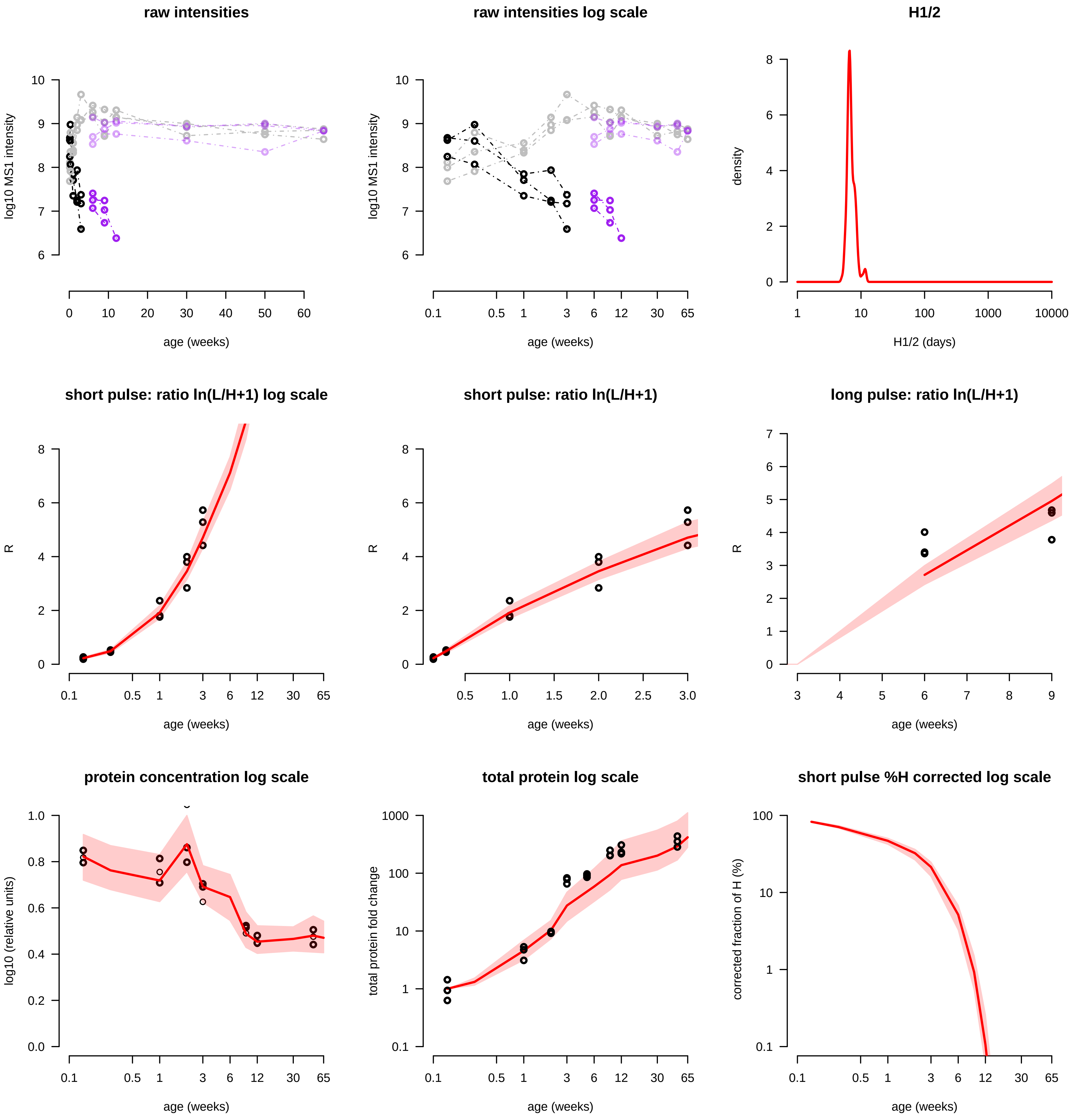

P62774

>sp|P62774|MTPN_MOUSE

Myotrophin

GN=Mtpn

Med H1/2: 124.5 days

Q5 H1/2: 33.1 days

Q95 H1/2: 1048.5 days

reliable model fit: yes

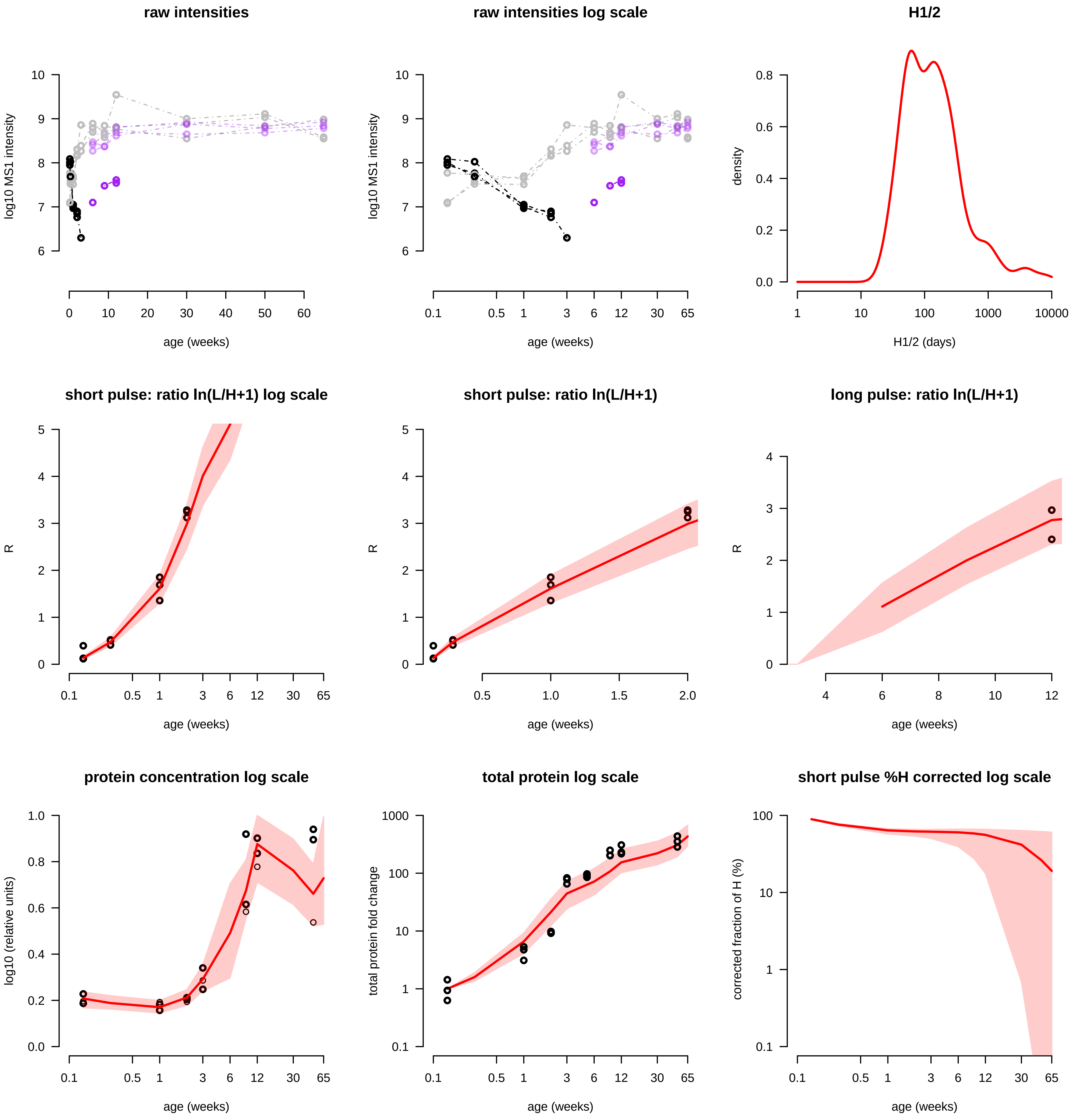

O35864

>sp|O35864|CSN5_MOUSE

COP9 signalosome complex subunit 5

GN=Cops5

Med H1/2: 7.1 days

Q5 H1/2: 5.2 days

Q95 H1/2: 9.4 days

reliable model fit: yes

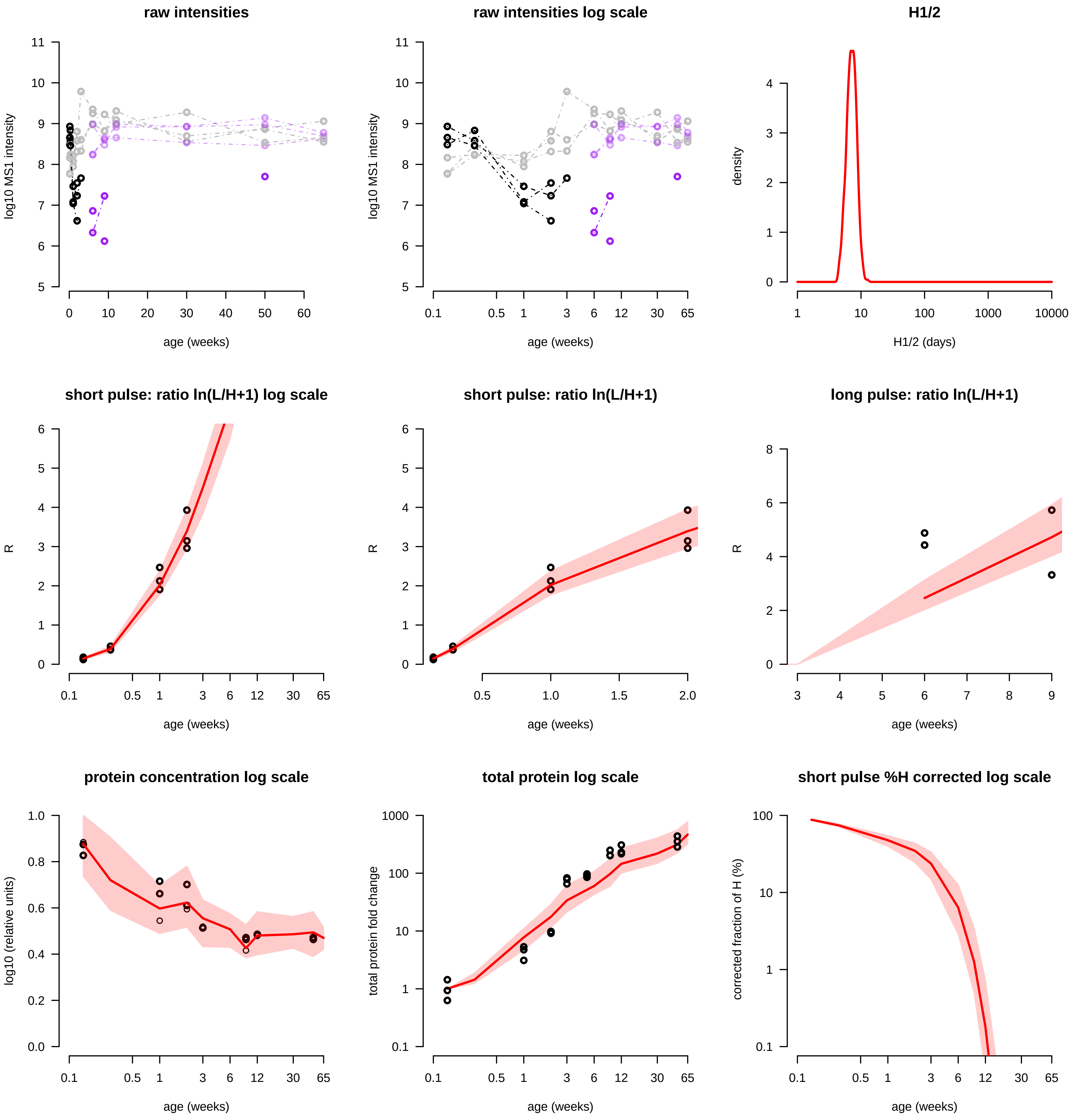

Q9D5V5

>sp|Q9D5V5|CUL5_MOUSE

Cullin−5

GN=Cul5

Med H1/2: 6.3 days

Q5 H1/2: 3.6 days

Q95 H1/2: 21.6 days

reliable model fit: yes

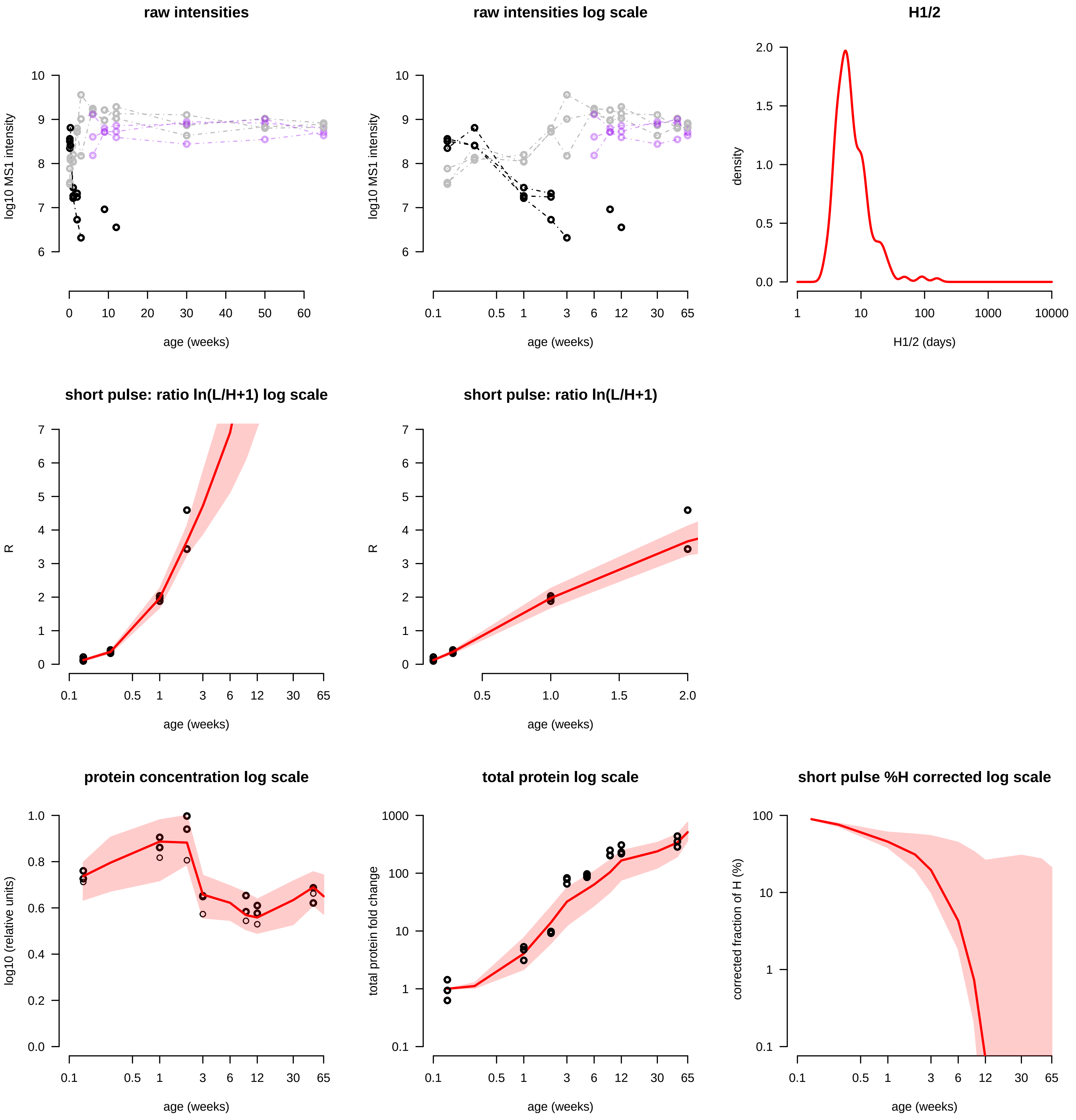

Q8CCJ3;Q8CCJ3−1

>sp|Q8CCJ3|UFL1_MOUSE

E3 UFM1−protein ligase 1

GN=Ufl1

Med H1/2: 5.2 days

Q5 H1/2: 4.2 days

Q95 H1/2: 7.1 days

reliable model fit: yes

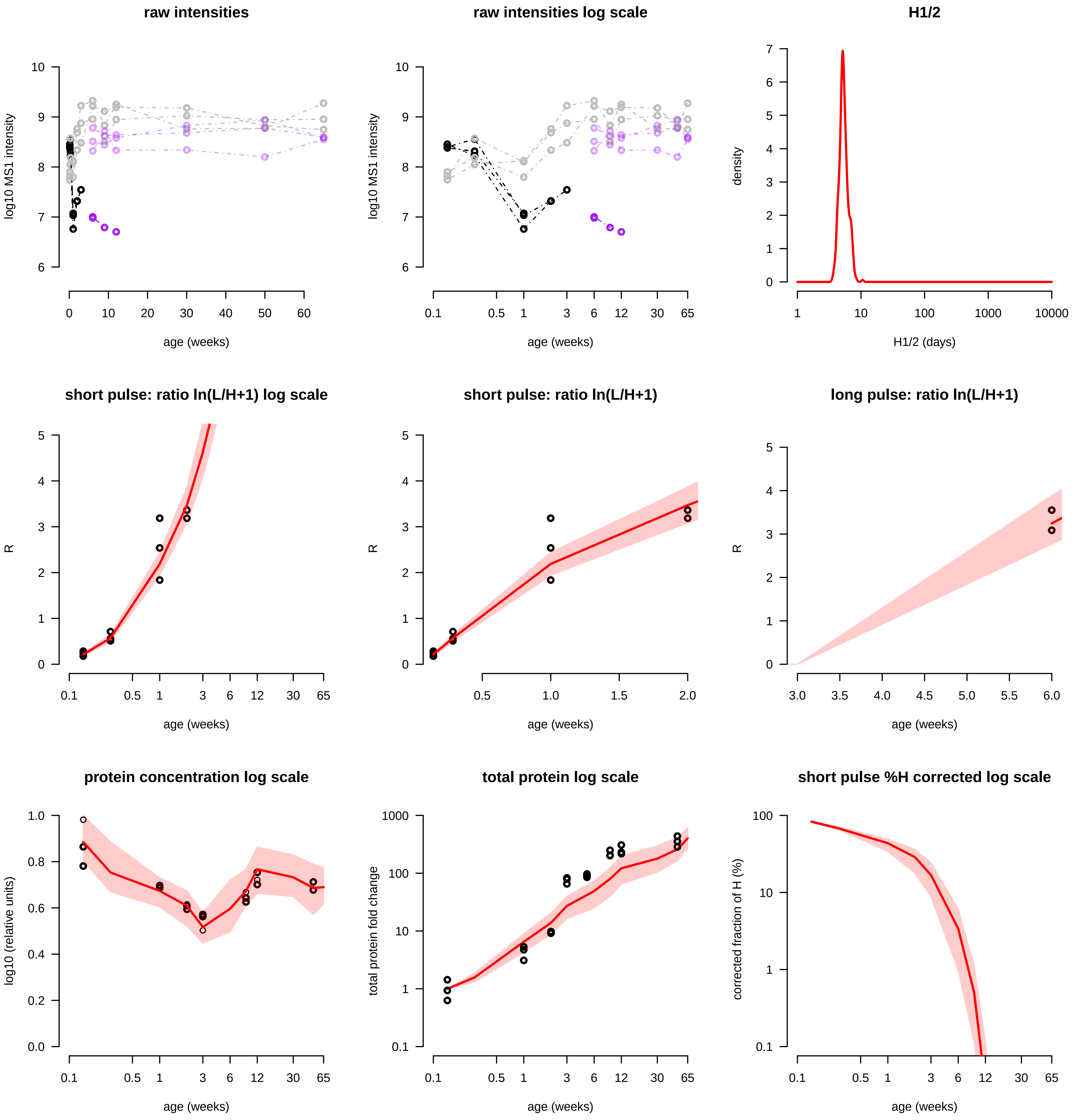

Q8BMJ3

>sp|Q8BMJ3|IF1AX_MOUSE

Eukaryotic translation initiation factor 1A, X−chromosomal

GN=Eif1ax

Med H1/2: 7.7 days

Q5 H1/2: 6.2 days

Q95 H1/2: 11.4 days

reliable model fit: yes

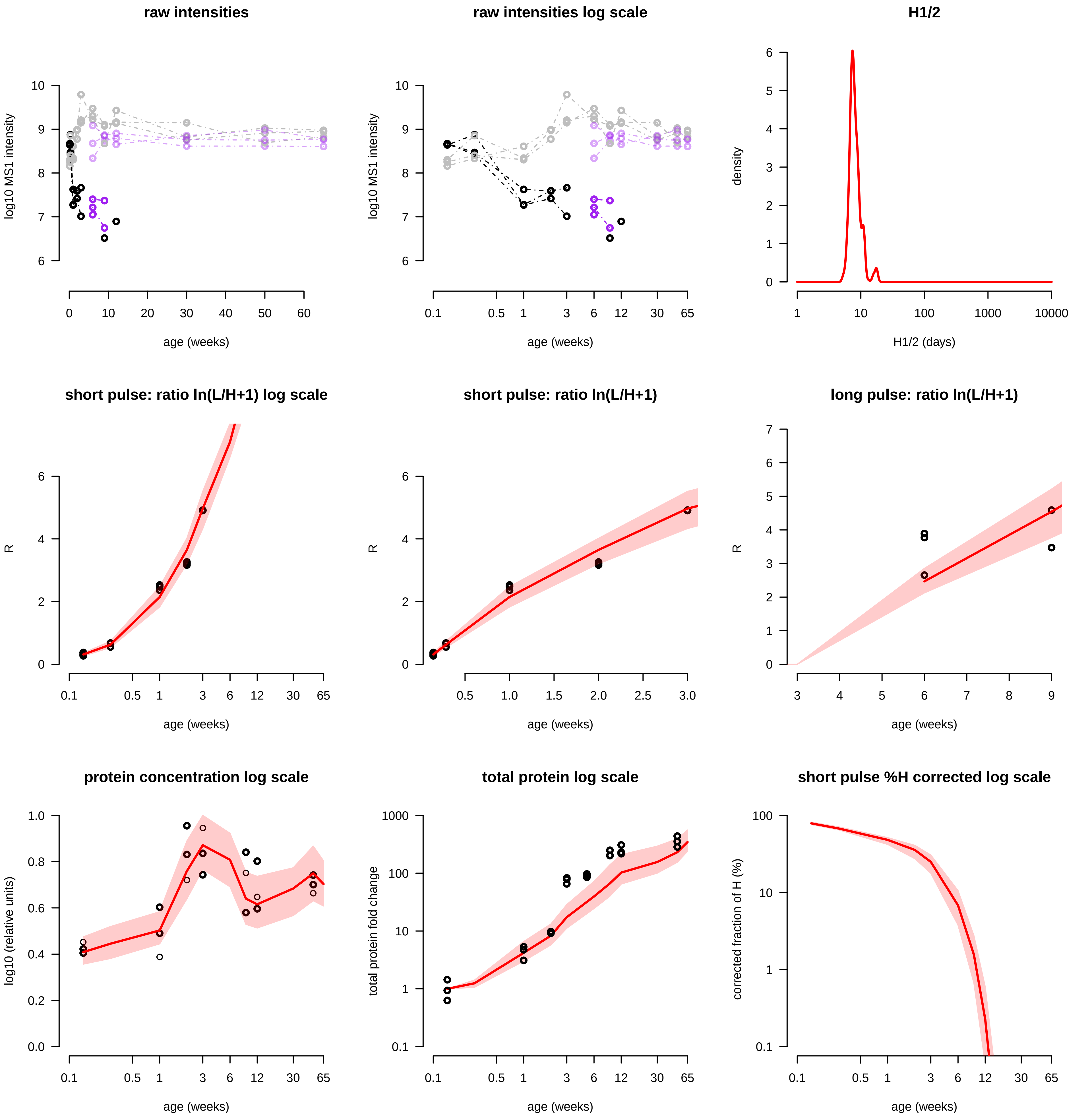

Q5SUF2;Q5SUF2−3;Q5SUF2−2

>sp|Q5SUF2|LC7L3_MOUSE

Luc7−like protein 3

GN=Luc7l3

Med H1/2: 920.6 days

Q5 H1/2: 198.7 days

Q95 H1/2: 7928.7 days

reliable model fit: yes

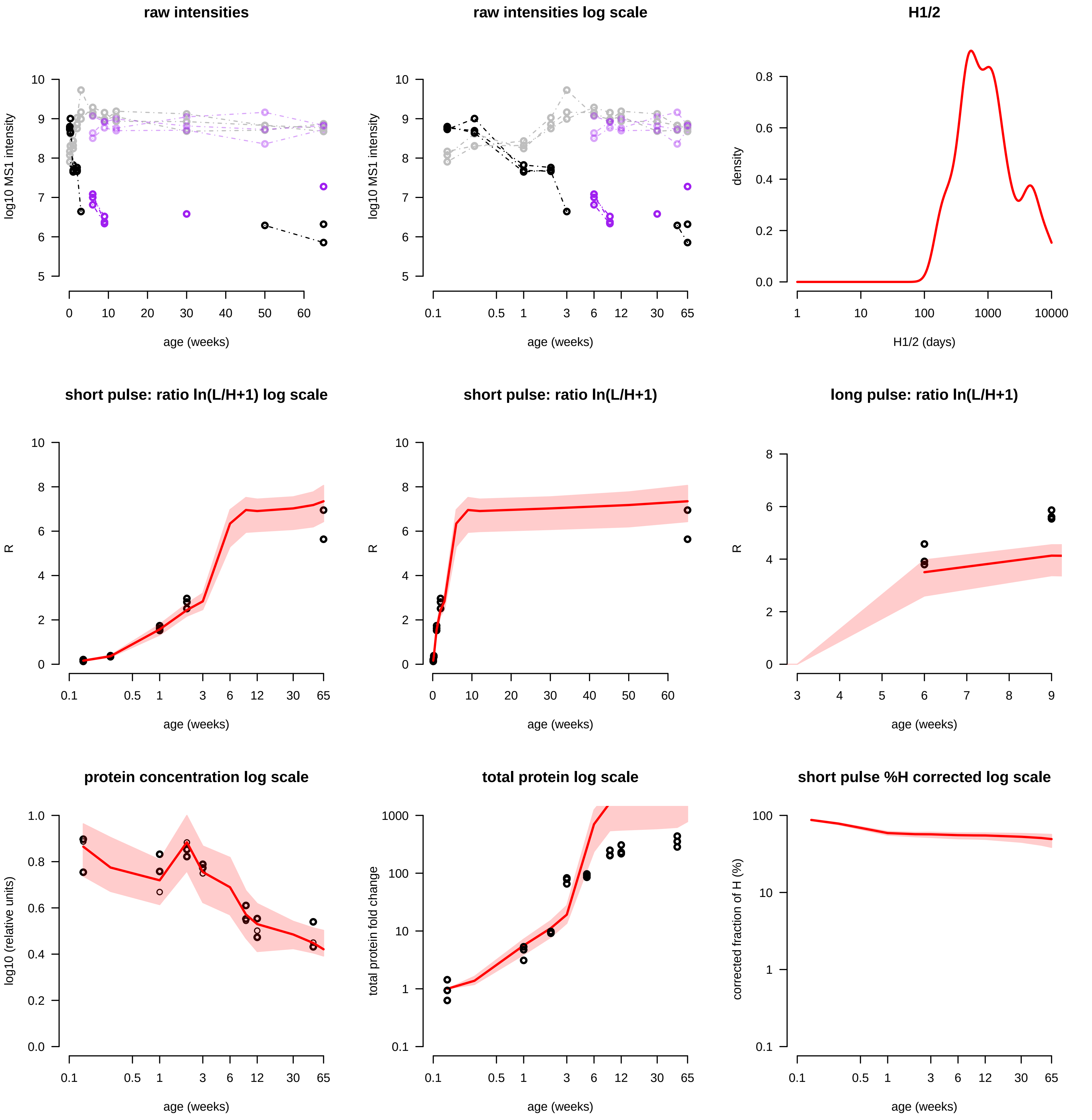

Q9CS42

>sp|Q9CS42|PRPS2_MOUSE

Ribose−phosphate pyrophosphokinase 2

GN=Prps2

Med H1/2: 8.3 days

Q5 H1/2: 5.8 days

Q95 H1/2: 15 days

reliable model fit: yes

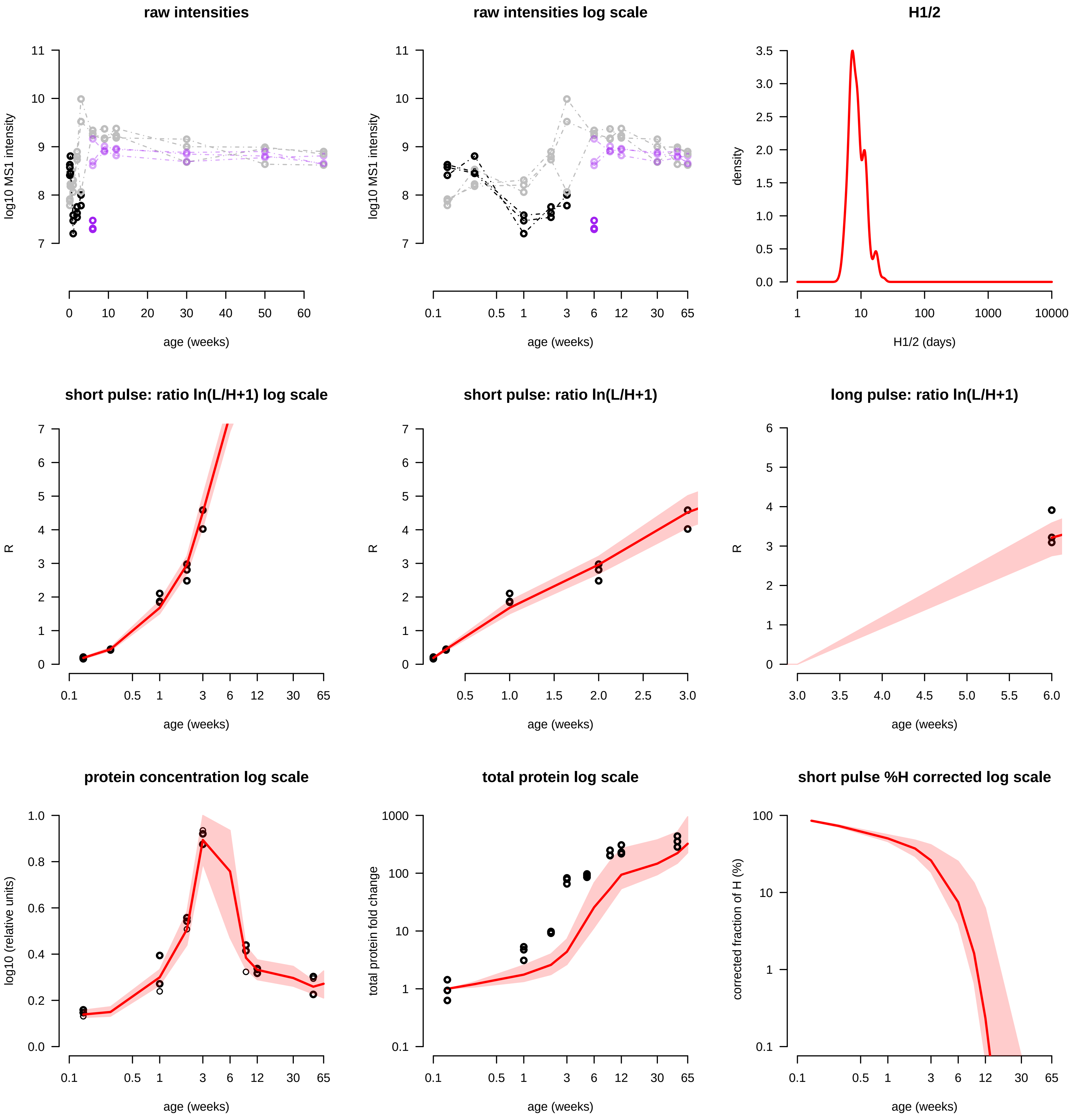

Q99KB8;Q99KB8−2

>sp|Q99KB8|GLO2_MOUSE

Hydroxyacylglutathione hydrolase, mitochondrial

GN=Hagh

Med H1/2: 7 days

Q5 H1/2: 5.3 days

Q95 H1/2: 12.3 days

reliable model fit: yes

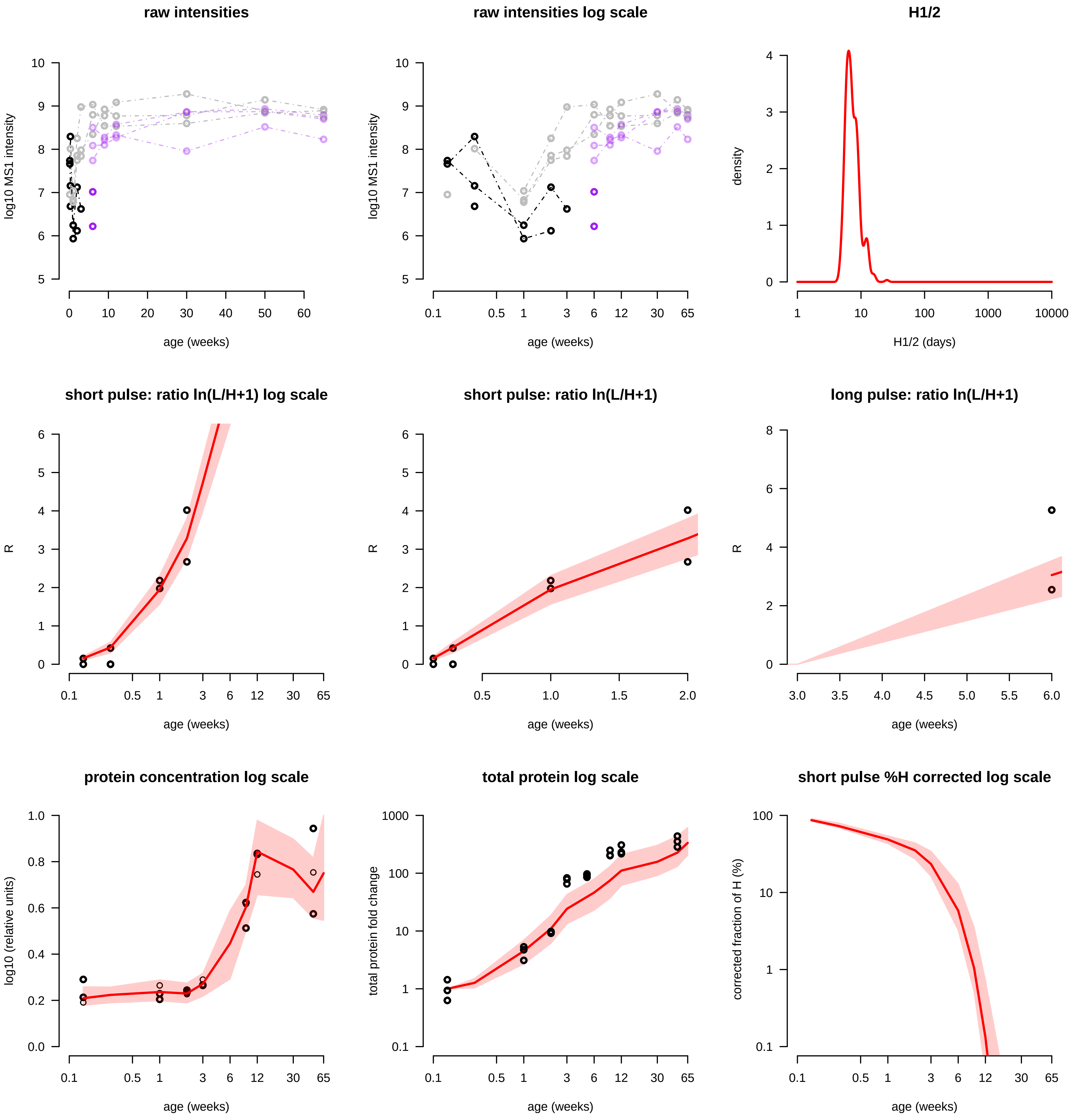

Q99JW4

>sp|Q99JW4|LIMS1_MOUSE

LIM and senescent cell antigen−like−containing domain protein 1

GN=Lims1

Med H1/2: 20.2 days

Q5 H1/2: 9 days

Q95 H1/2: 169.5 days

reliable model fit: no

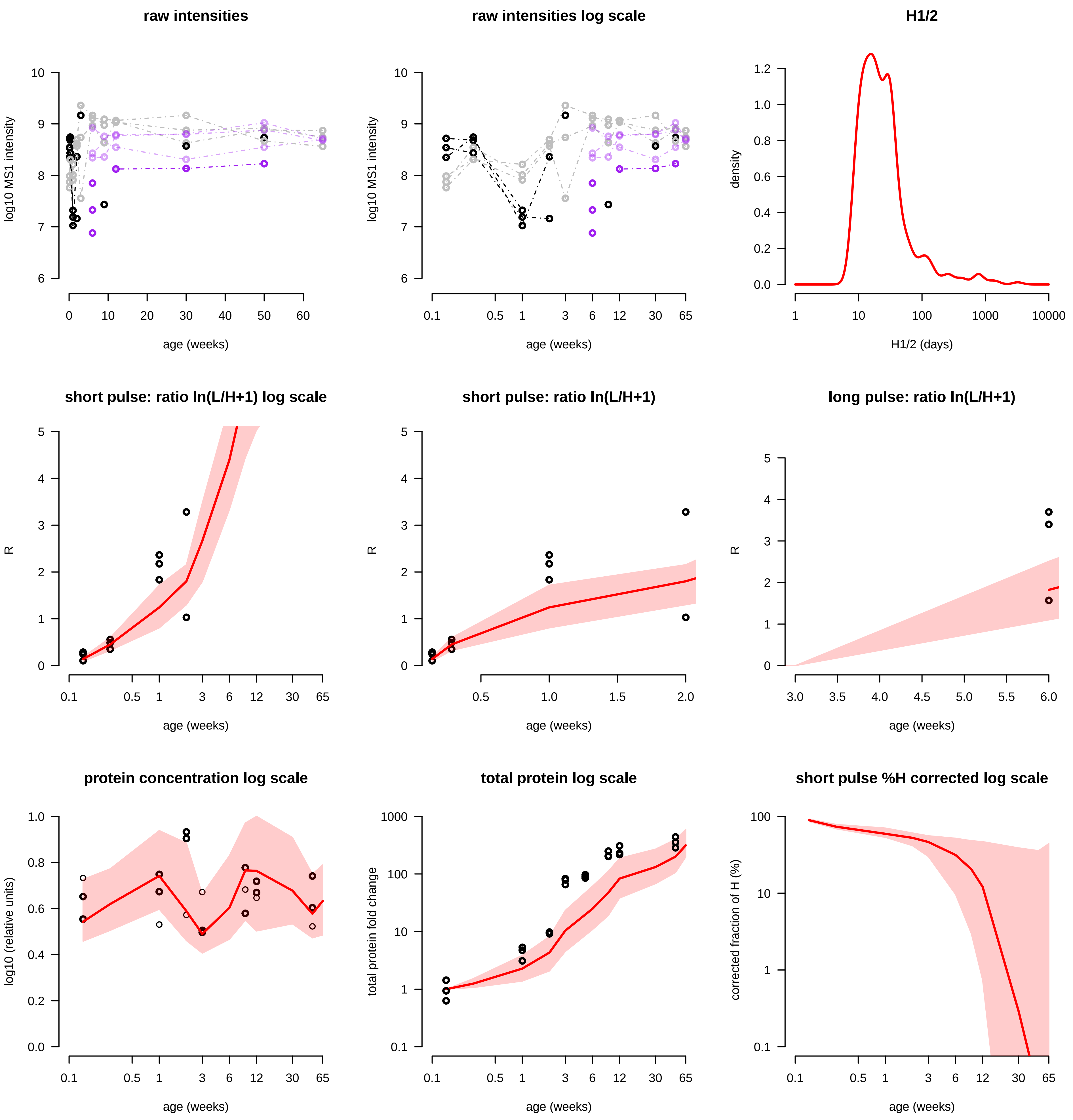

Q8K3C3

>sp|Q8K3C3|LZIC_MOUSE

Protein LZIC

GN=Lzic

Med H1/2: 11.4 days

Q5 H1/2: 4.3 days

Q95 H1/2: 103.2 days

reliable model fit: yes

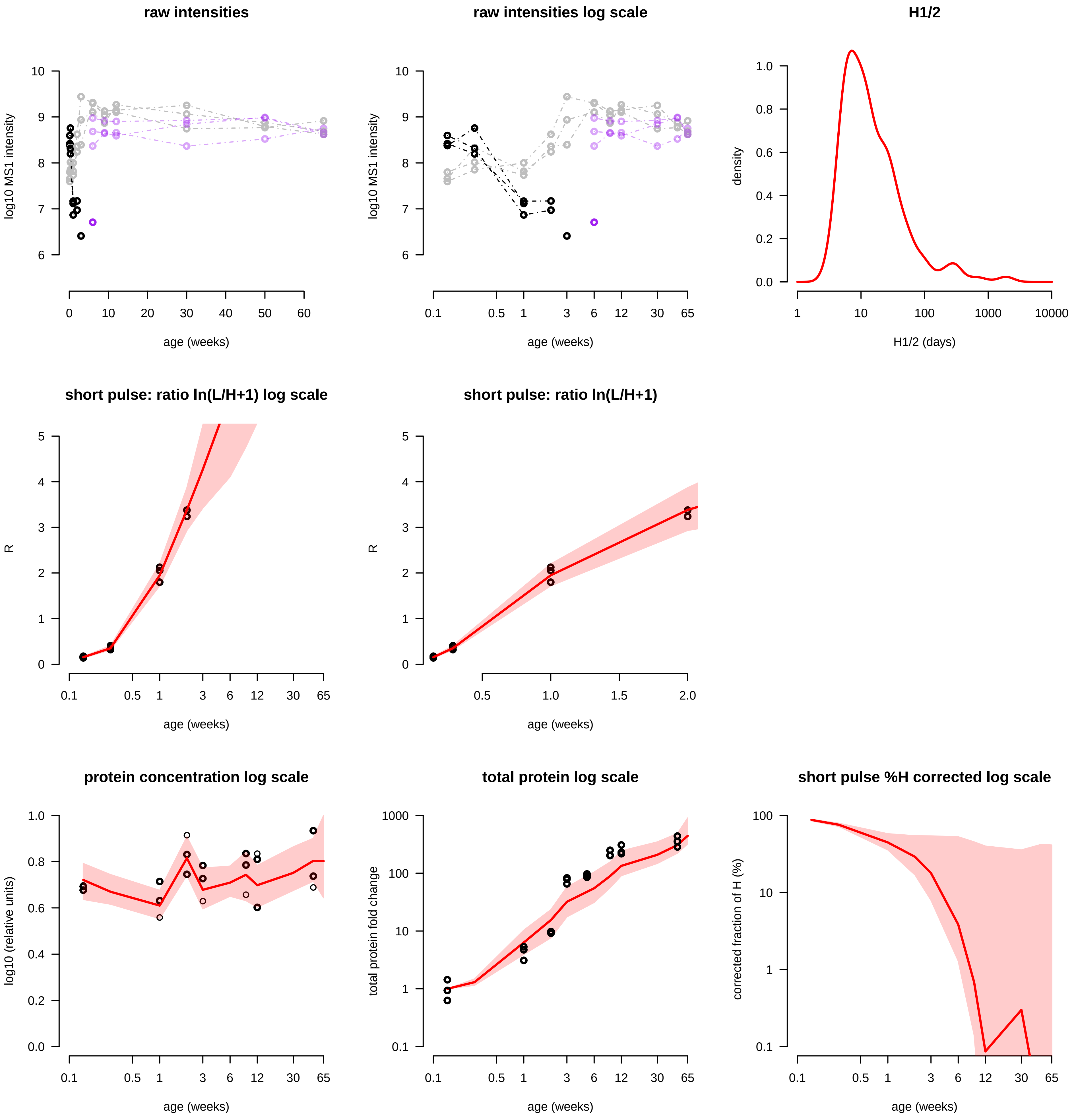

Q8K0T0

>sp|Q8K0T0|RTN1_MOUSE

Reticulon−1

GN=Rtn1

Med H1/2: 10.8 days

Q5 H1/2: 8.3 days

Q95 H1/2: 16 days

reliable model fit: yes

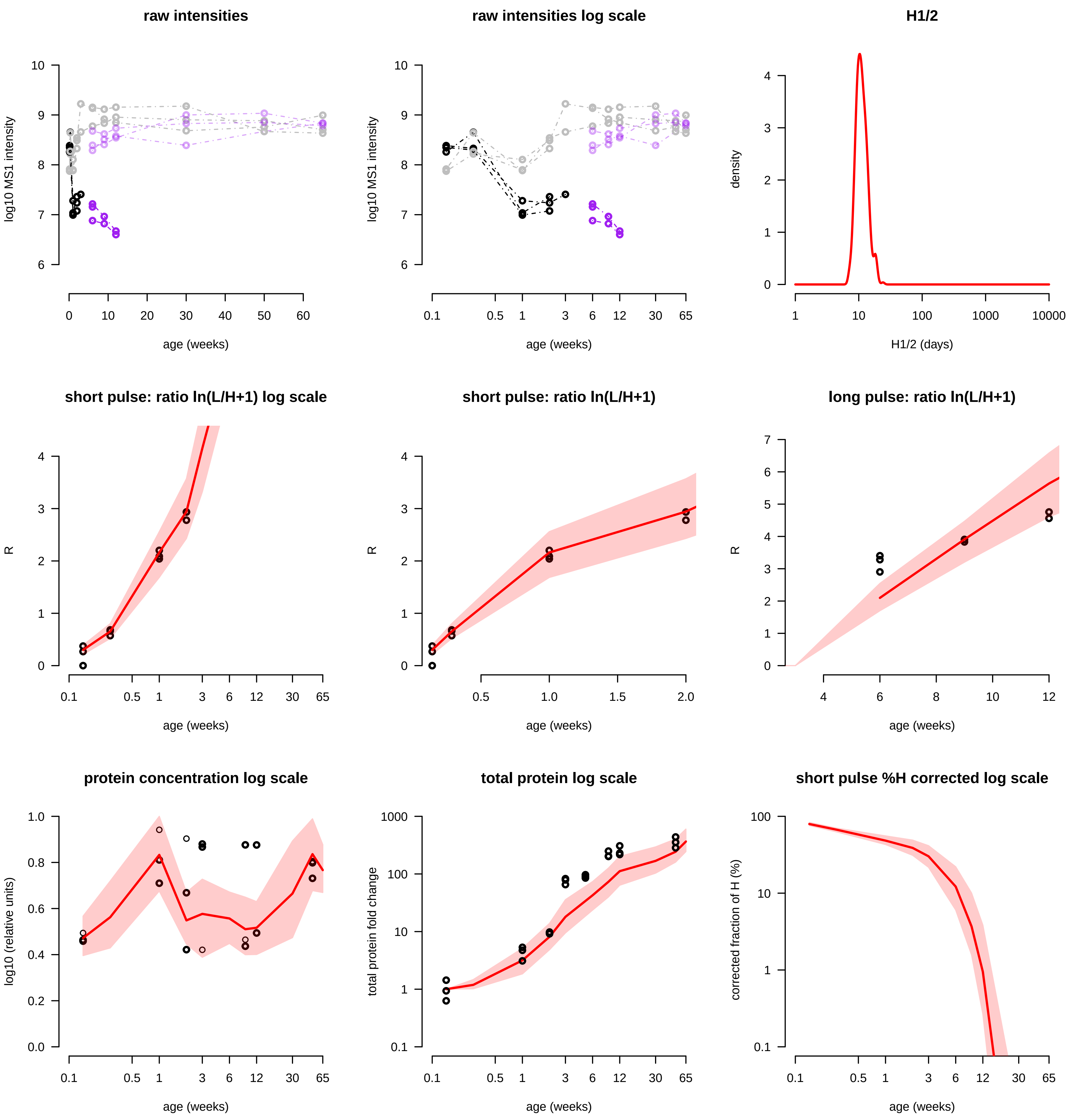

Q61235

>sp|Q61235|SNTB2_MOUSE

Beta−2−syntrophin

GN=Sntb2

Med H1/2: 9.4 days

Q5 H1/2: 4 days

Q95 H1/2: 82.5 days

reliable model fit: yes

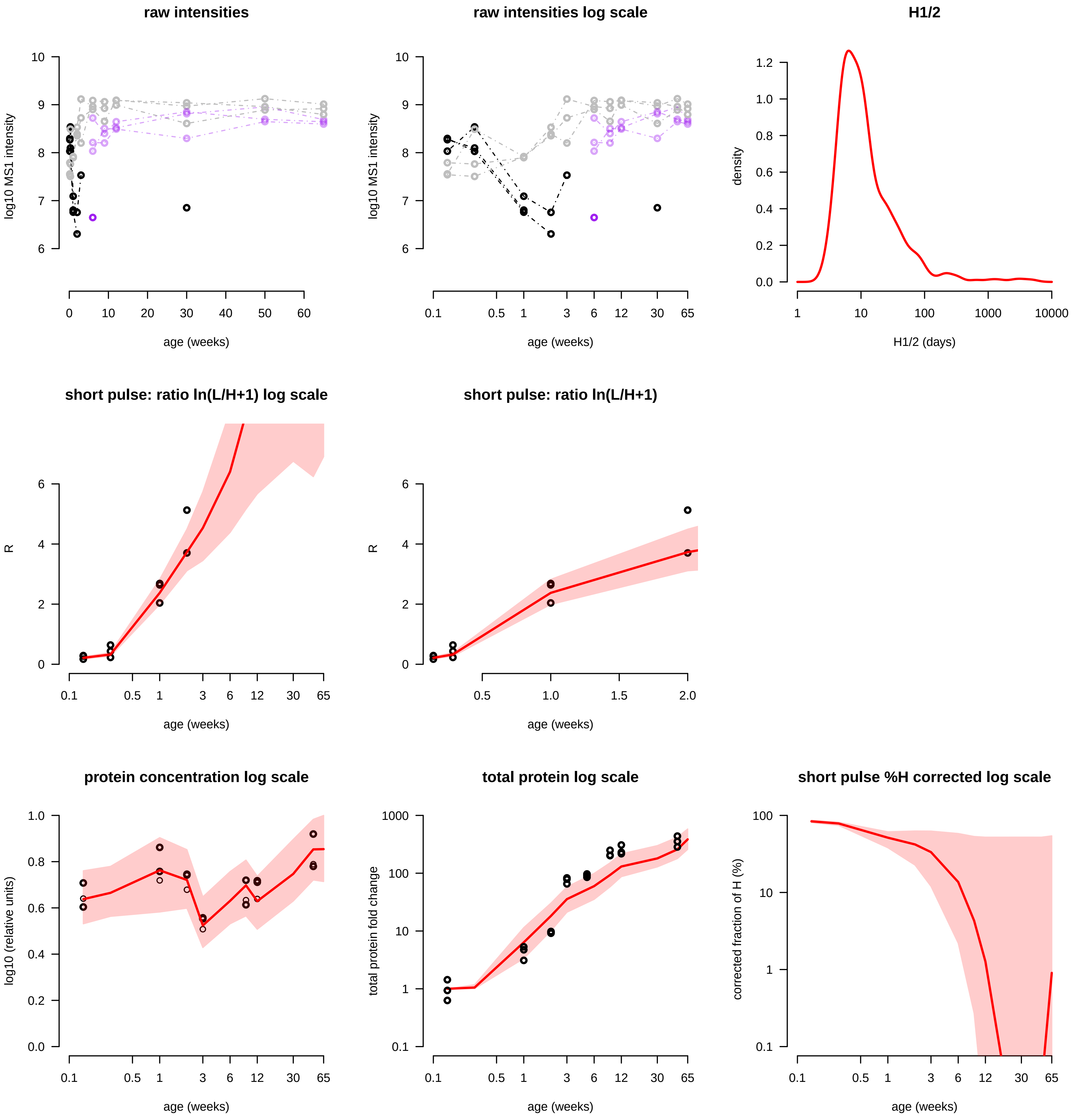

P54923

>sp|P54923|ADPRH_MOUSE

[Protein ADP−ribosylarginine] hydrolase

GN=Adprh

Med H1/2: 6.7 days

Q5 H1/2: 5.5 days

Q95 H1/2: 9.3 days

reliable model fit: yes

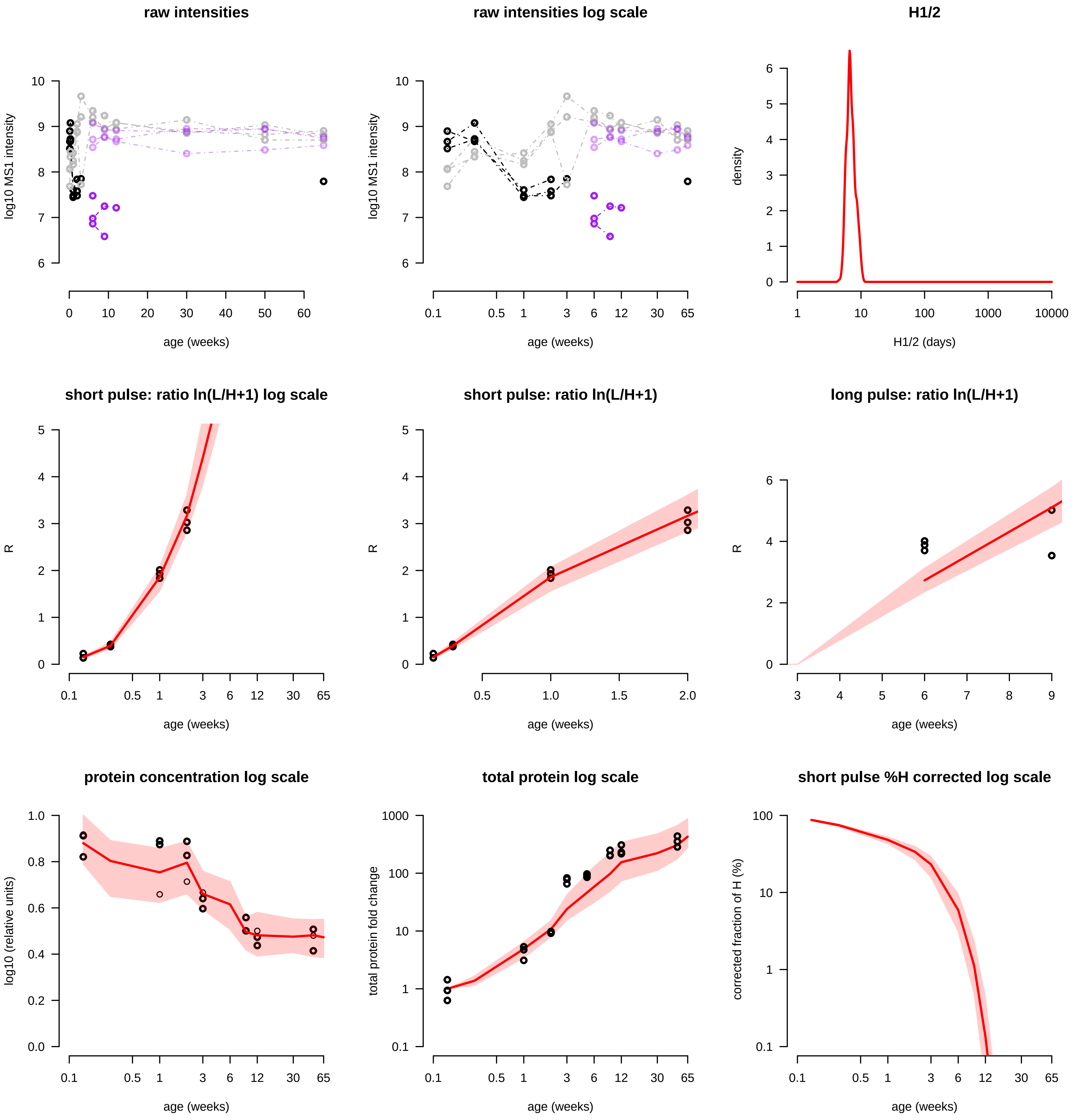

P14901

>sp|P14901|HMOX1_MOUSE

Heme oxygenase 1

GN=Hmox1

Med H1/2: 12.6 days

Q5 H1/2: 3.5 days

Q95 H1/2: 110 days

reliable model fit: yes

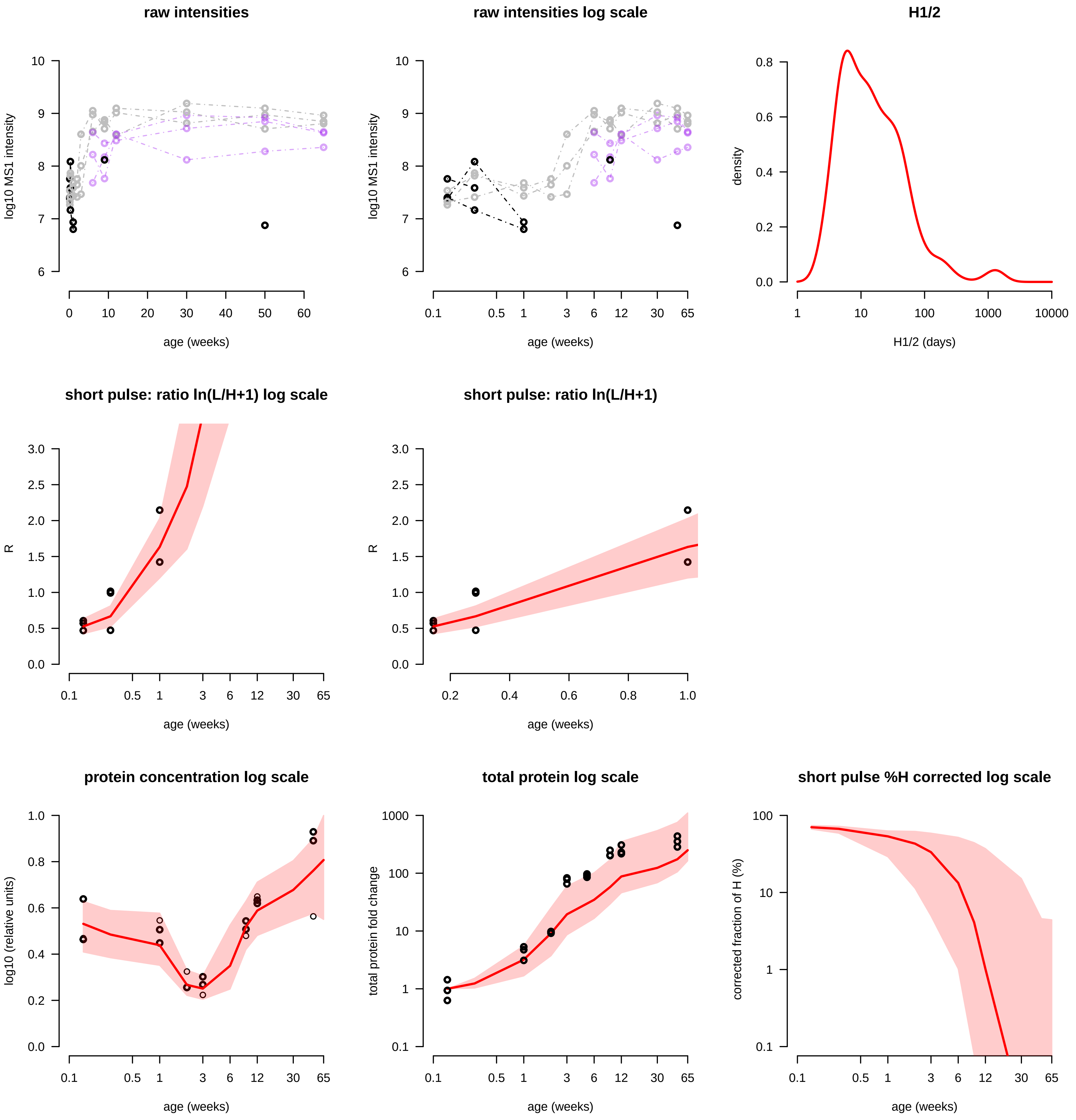

O54950

>sp|O54950|AAKG1_MOUSE

5'−AMP−activated protein kinase subunit gamma−1

GN=Prkag1

Med H1/2: 7.9 days

Q5 H1/2: 3.7 days

Q95 H1/2: 24.8 days

reliable model fit: yes

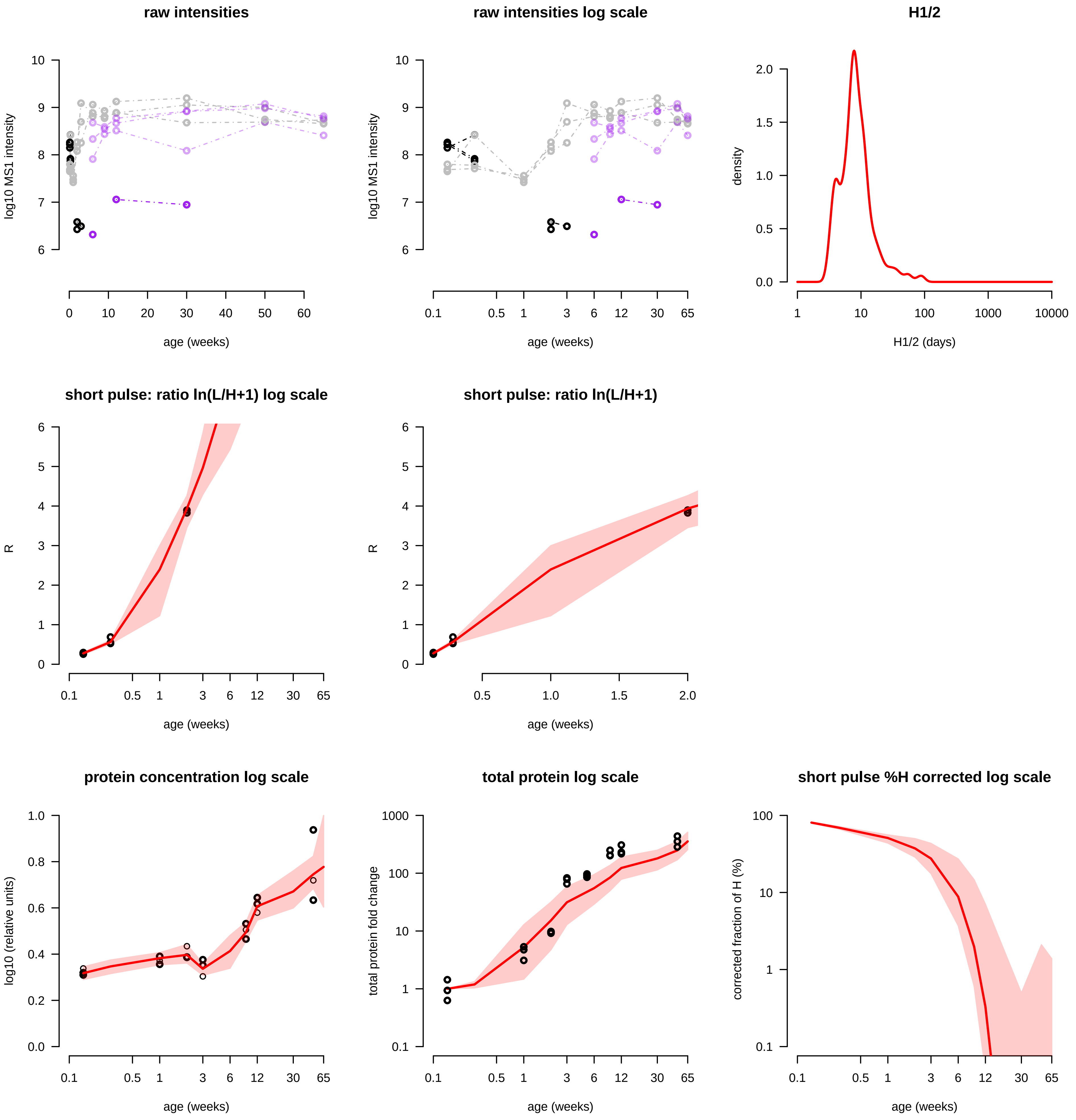

Q99JR1

>sp|Q99JR1|SFXN1_MOUSE

Sideroflexin−1

GN=Sfxn1

Med H1/2: 9.4 days

Q5 H1/2: 6.6 days

Q95 H1/2: 18.5 days

reliable model fit: no

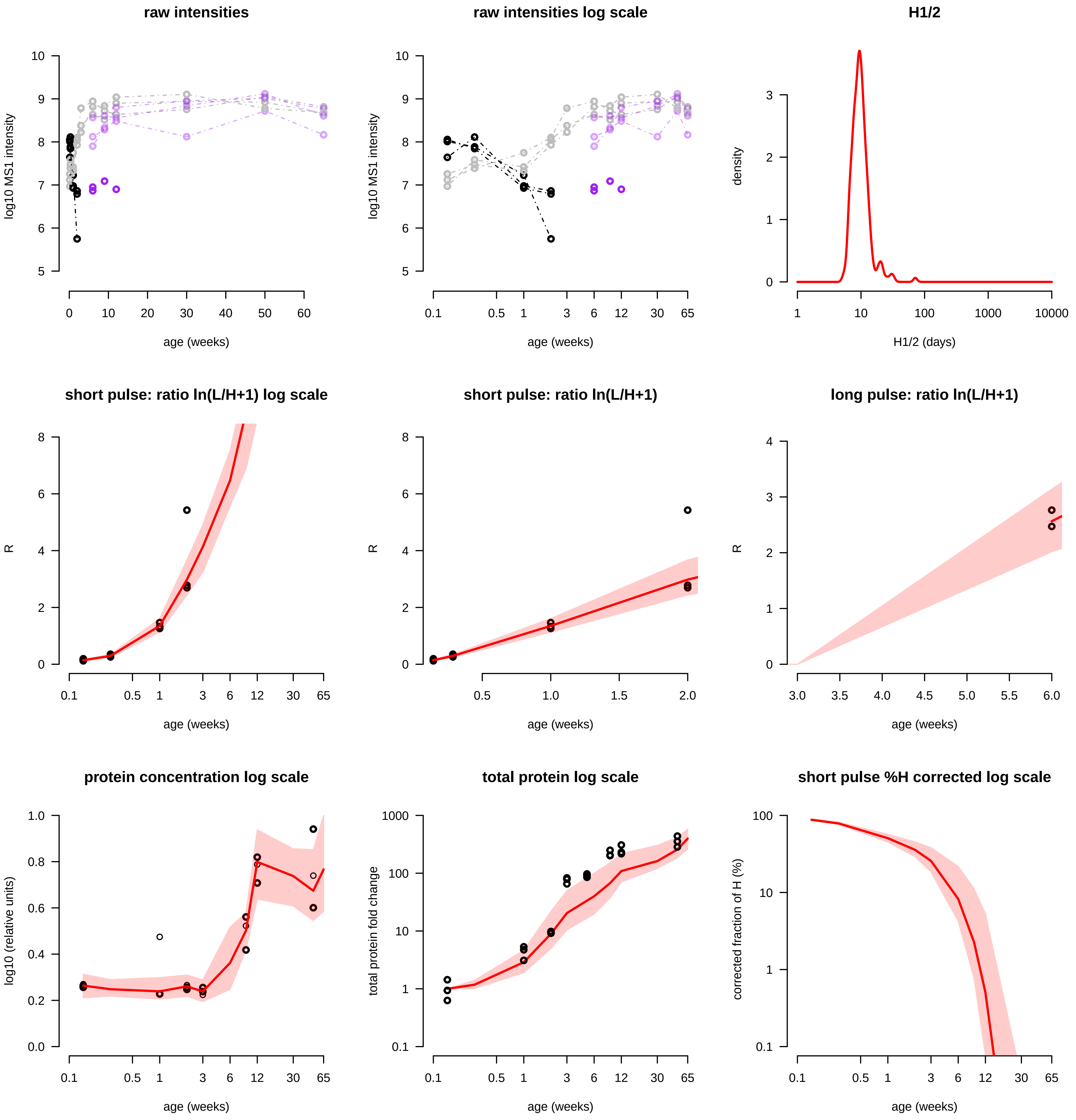

Q8R0W0

>sp|Q8R0W0|EPIPL_MOUSE

Epiplakin

GN=Eppk1

Med H1/2: 16.9 days

Q5 H1/2: 5.5 days

Q95 H1/2: 172.6 days

reliable model fit: yes

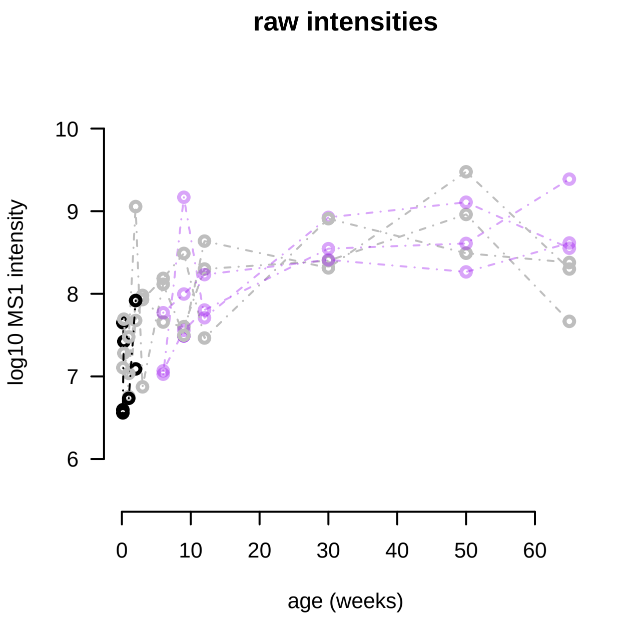

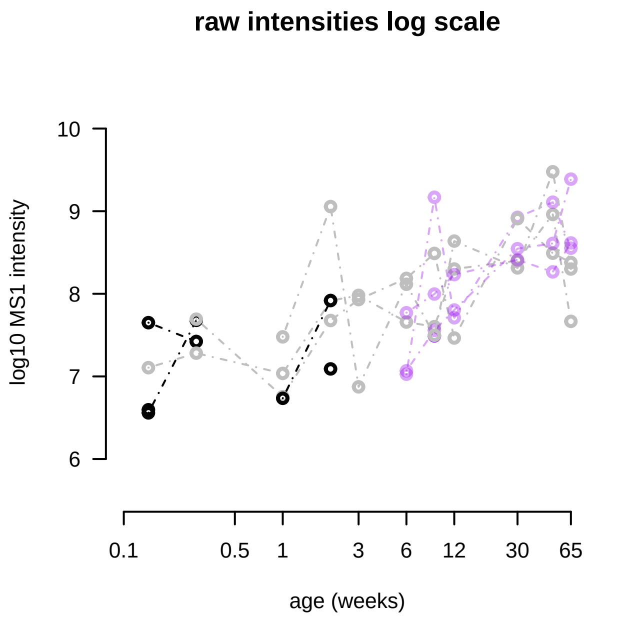

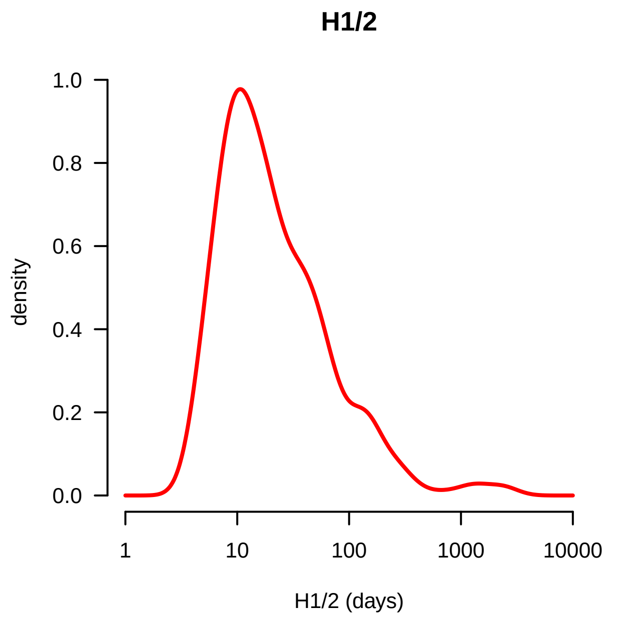

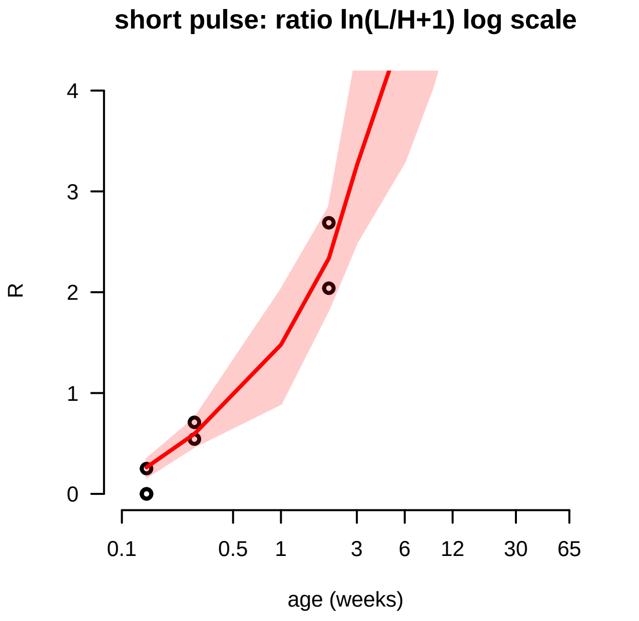

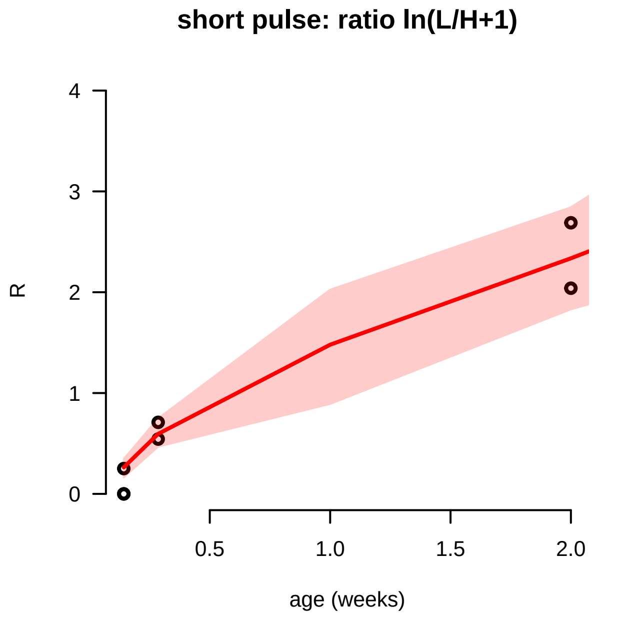

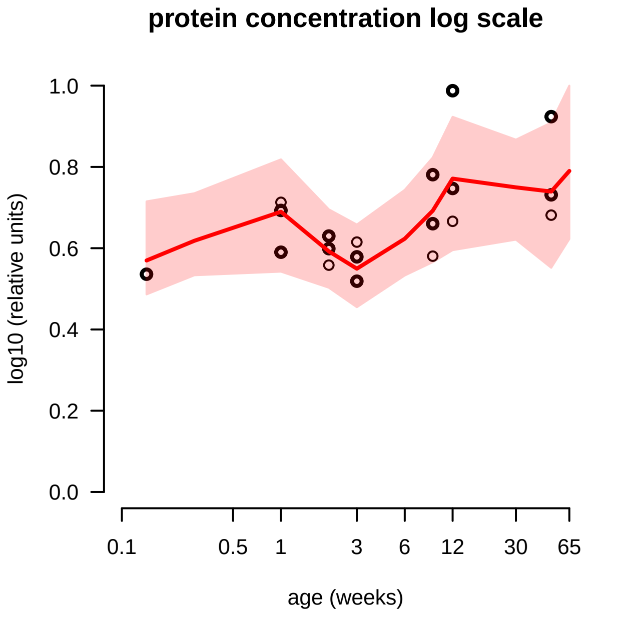

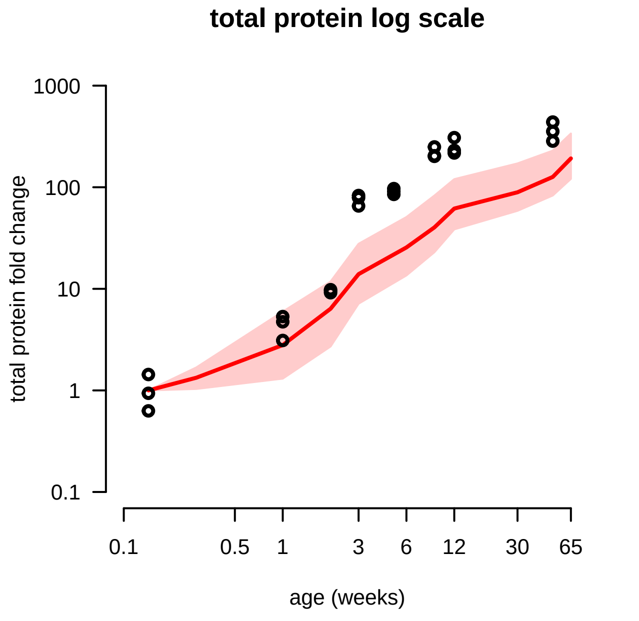

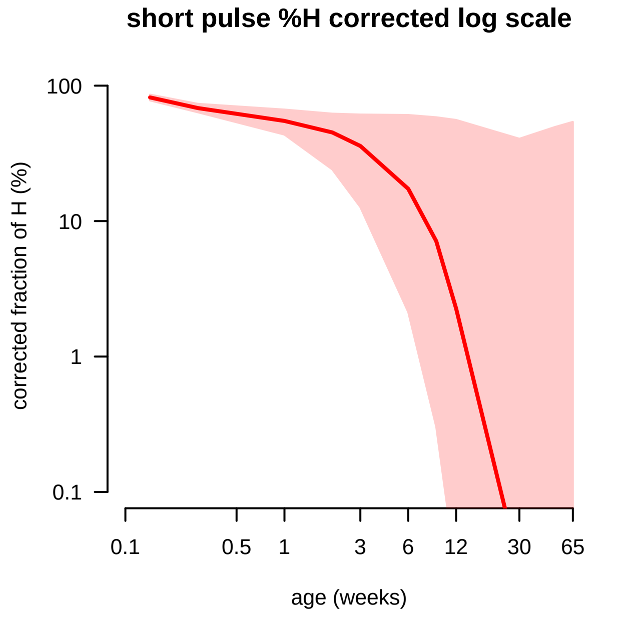

Q6PGB6−2;Q6PGB6;Q6PGB6−4;Q6PGB6−3

>sp|Q6PGB6−2|NAA50_MOUSE

Isoform 2 of N−alpha−acetyltransferase 50

GN=Naa50

Med H1/2: 4.9 days

Q5 H1/2: 4 days

Q95 H1/2: 6 days

reliable model fit: yes

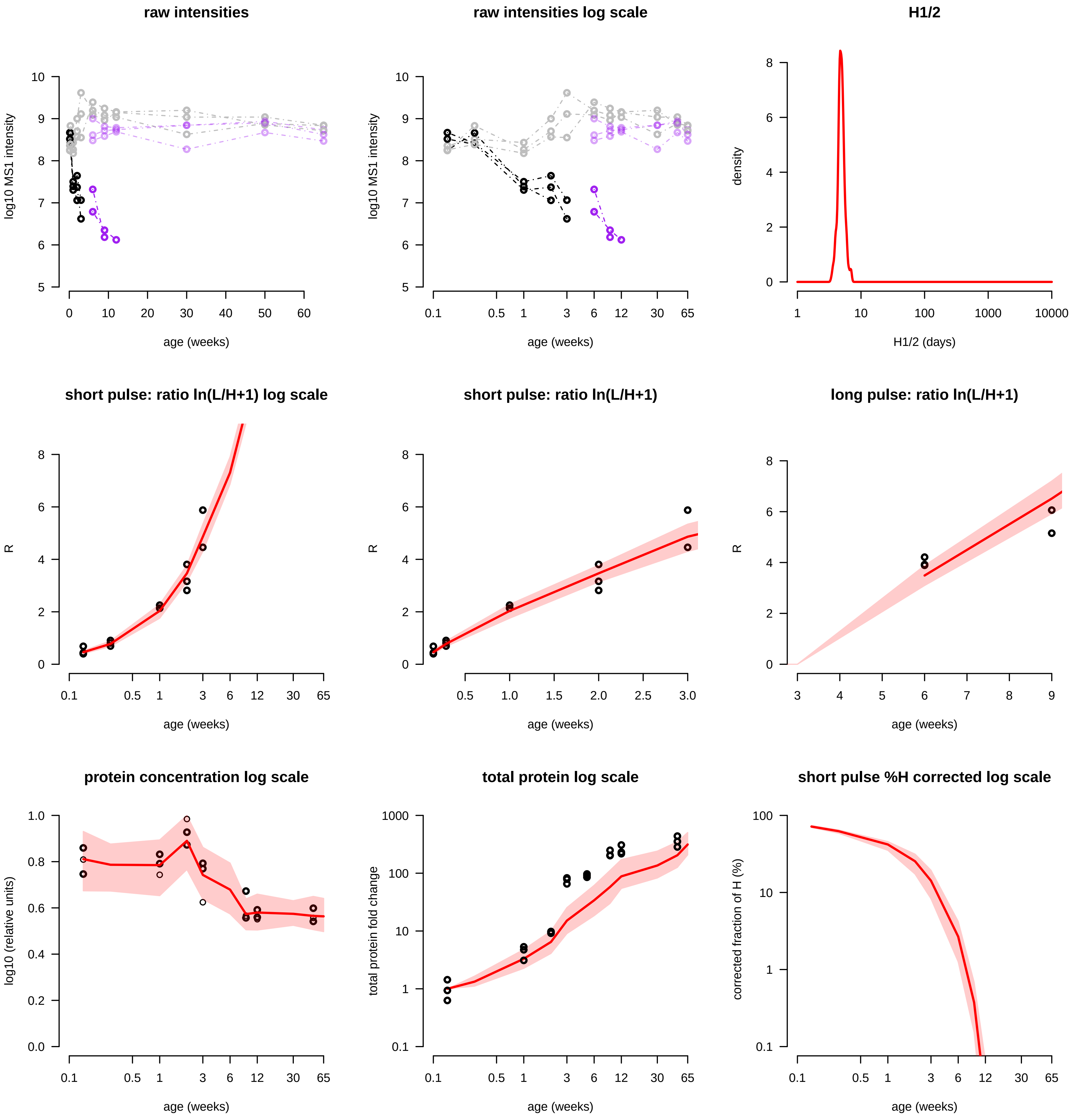

Q9CQM5

>sp|Q9CQM5|TXD17_MOUSE

Thioredoxin domain−containing protein 17

GN=Txndc17

Med H1/2: 9.6 days

Q5 H1/2: 7.2 days

Q95 H1/2: 20.5 days

reliable model fit: yes

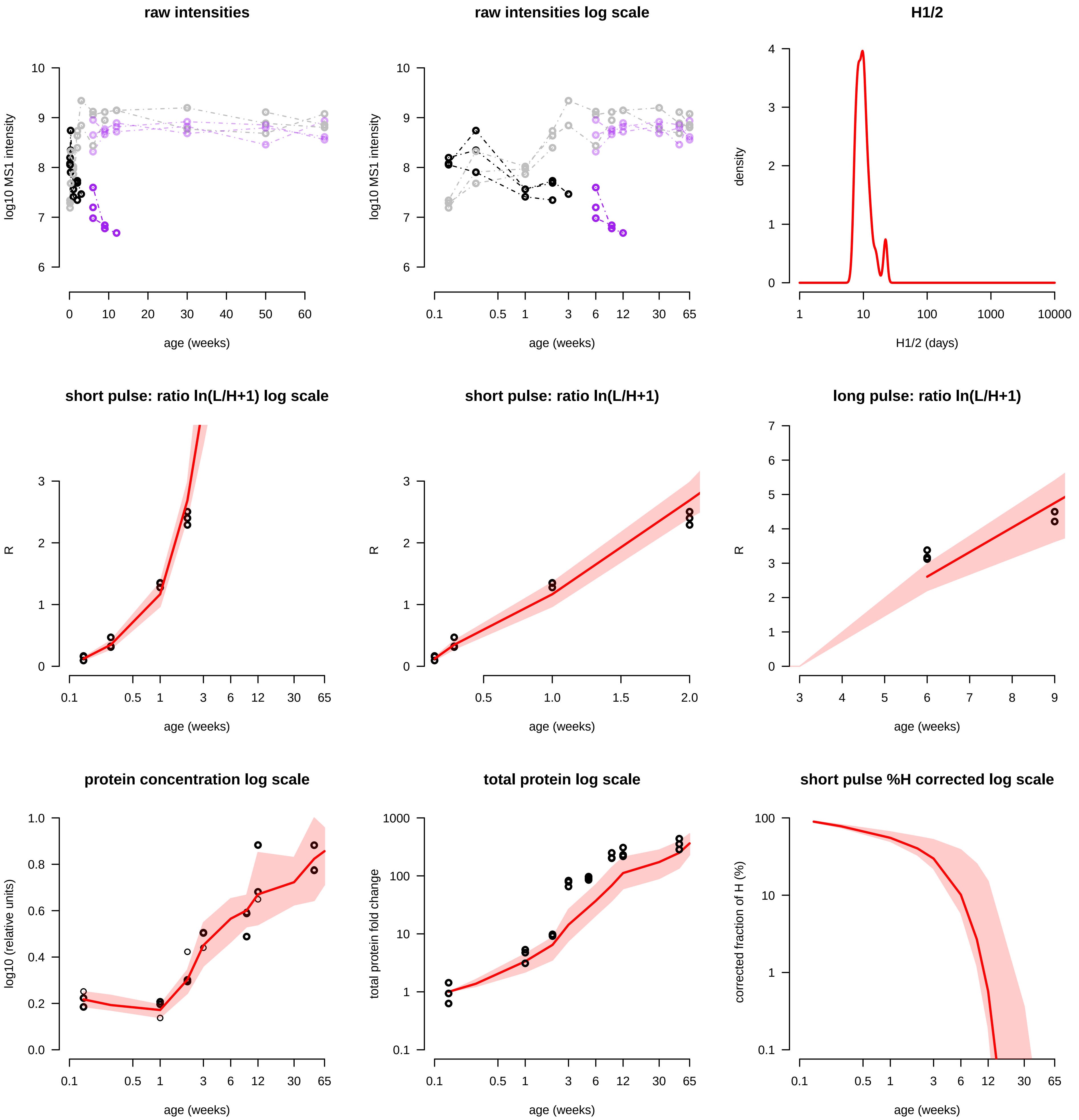

Q9D024;Q9D024−2;Q9D024−3

>sp|Q9D024|CCD47_MOUSE

PAT complex subunit CCDC47

GN=Ccdc47

Med H1/2: 6.5 days

Q5 H1/2: 3.1 days

Q95 H1/2: 67.3 days

reliable model fit: no

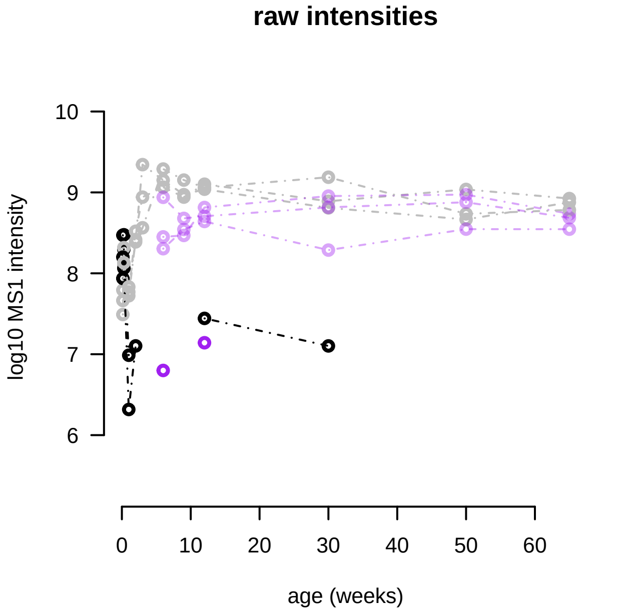

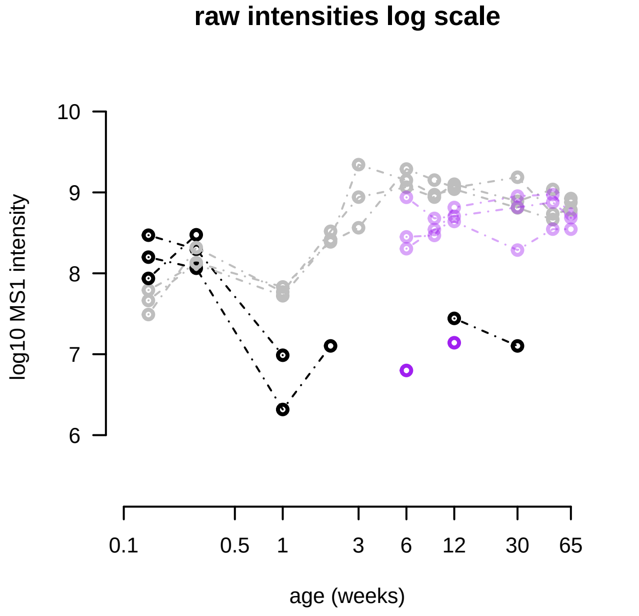

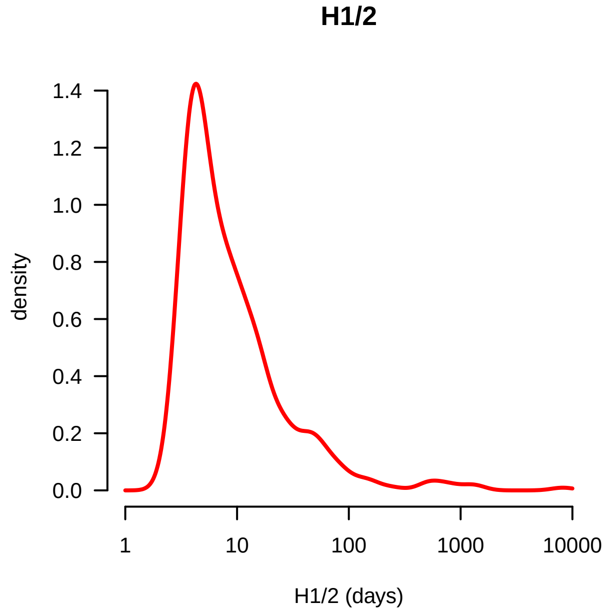

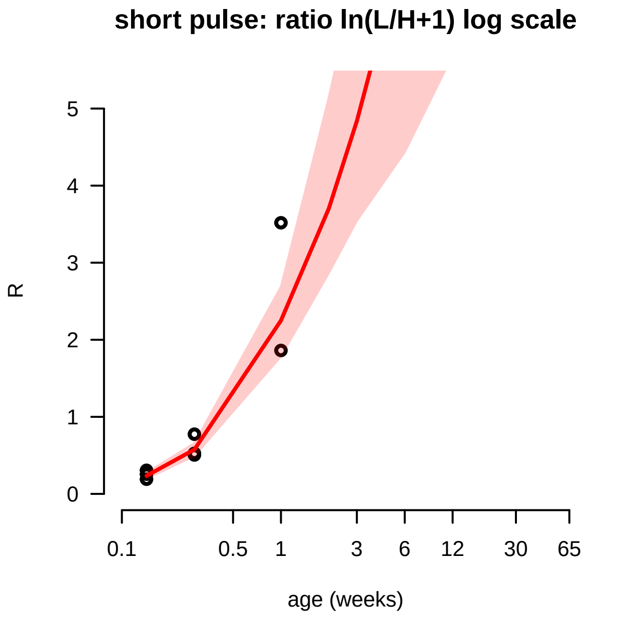

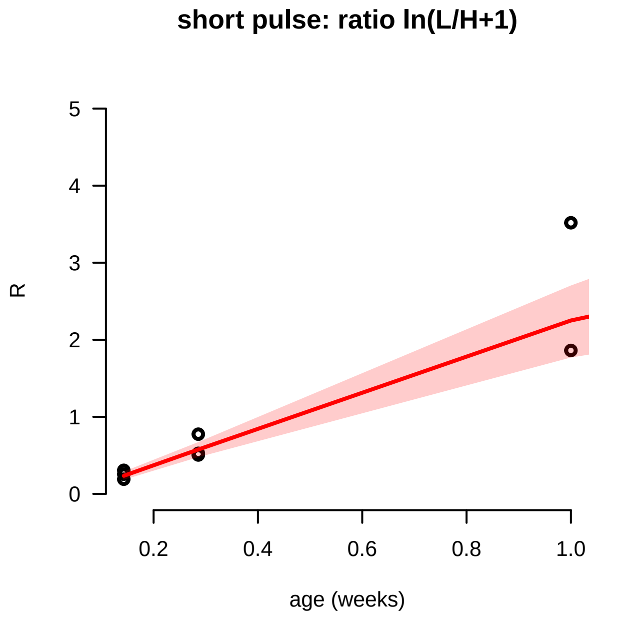

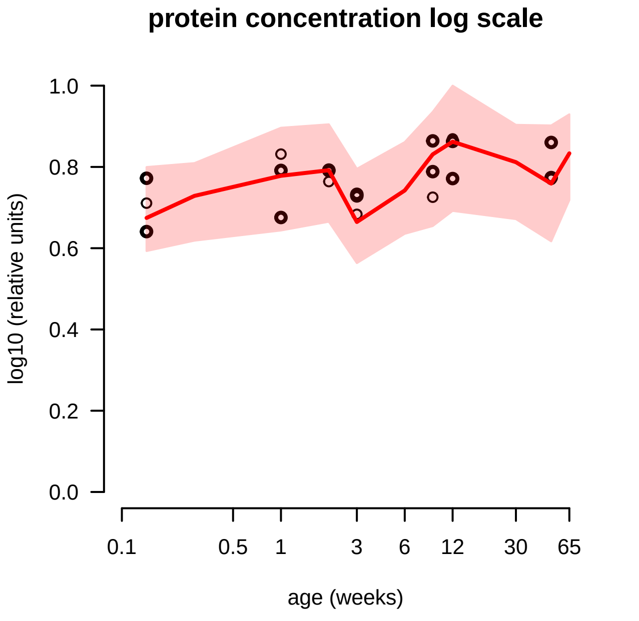

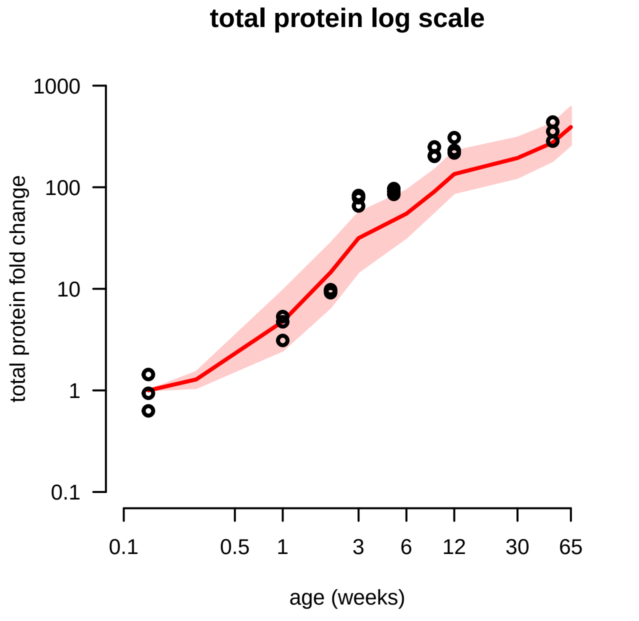

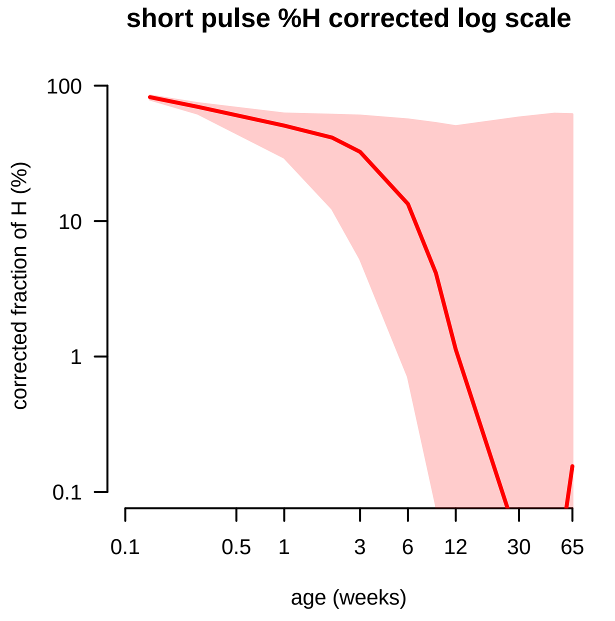

Q3UVK0

>sp|Q3UVK0|ERMP1_MOUSE

Endoplasmic reticulum metallopeptidase 1

GN=Ermp1

Med H1/2: 5.8 days

Q5 H1/2: 4.6 days

Q95 H1/2: 7.7 days

reliable model fit: yes

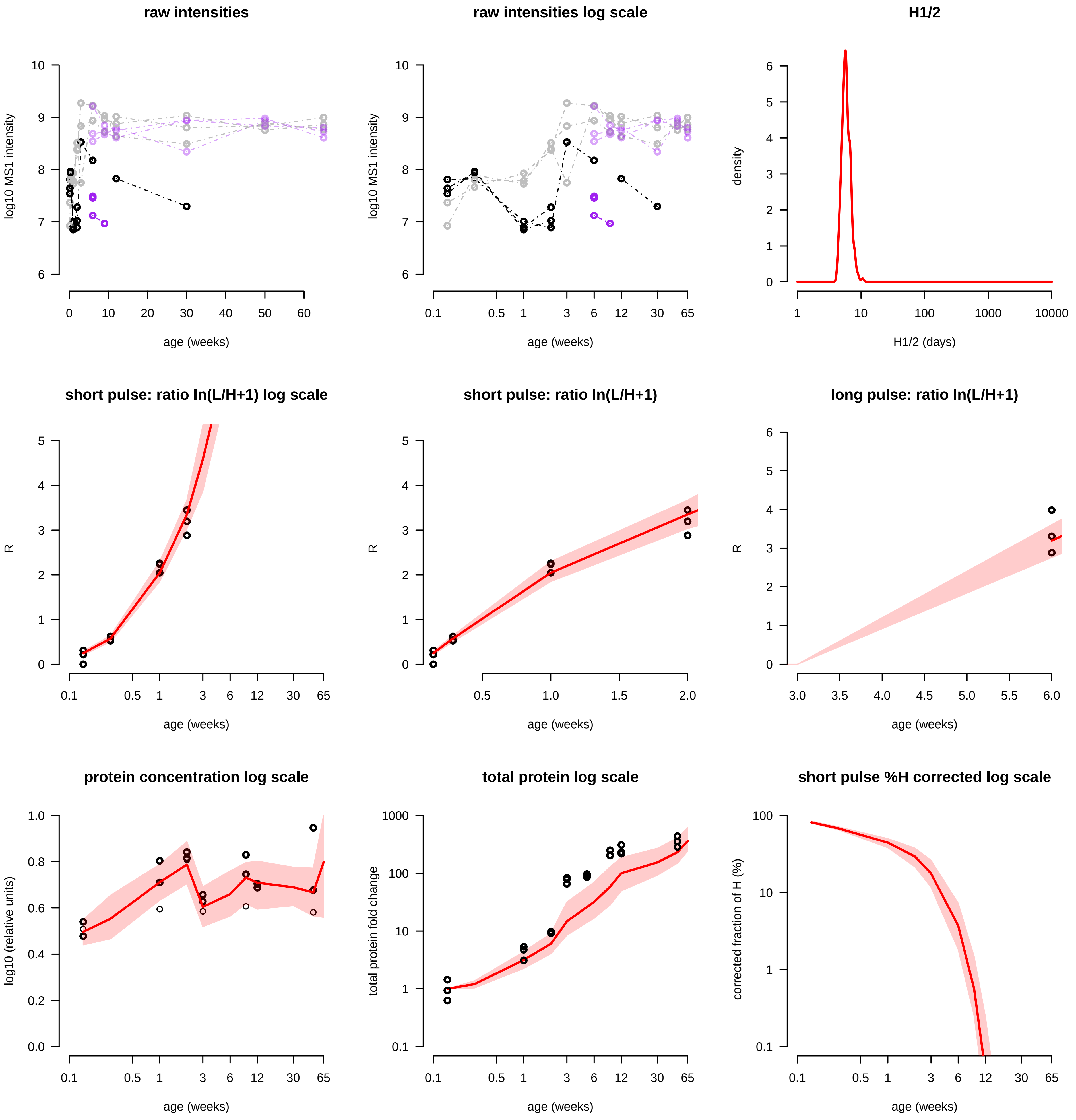

Q6PGL7;Q6PGL7−2

>sp|Q6PGL7|WASC2_MOUSE

WASH complex subunit 2

GN=Washc2

Med H1/2: 18.2 days

Q5 H1/2: 4.2 days

Q95 H1/2: 279.3 days

reliable model fit: no

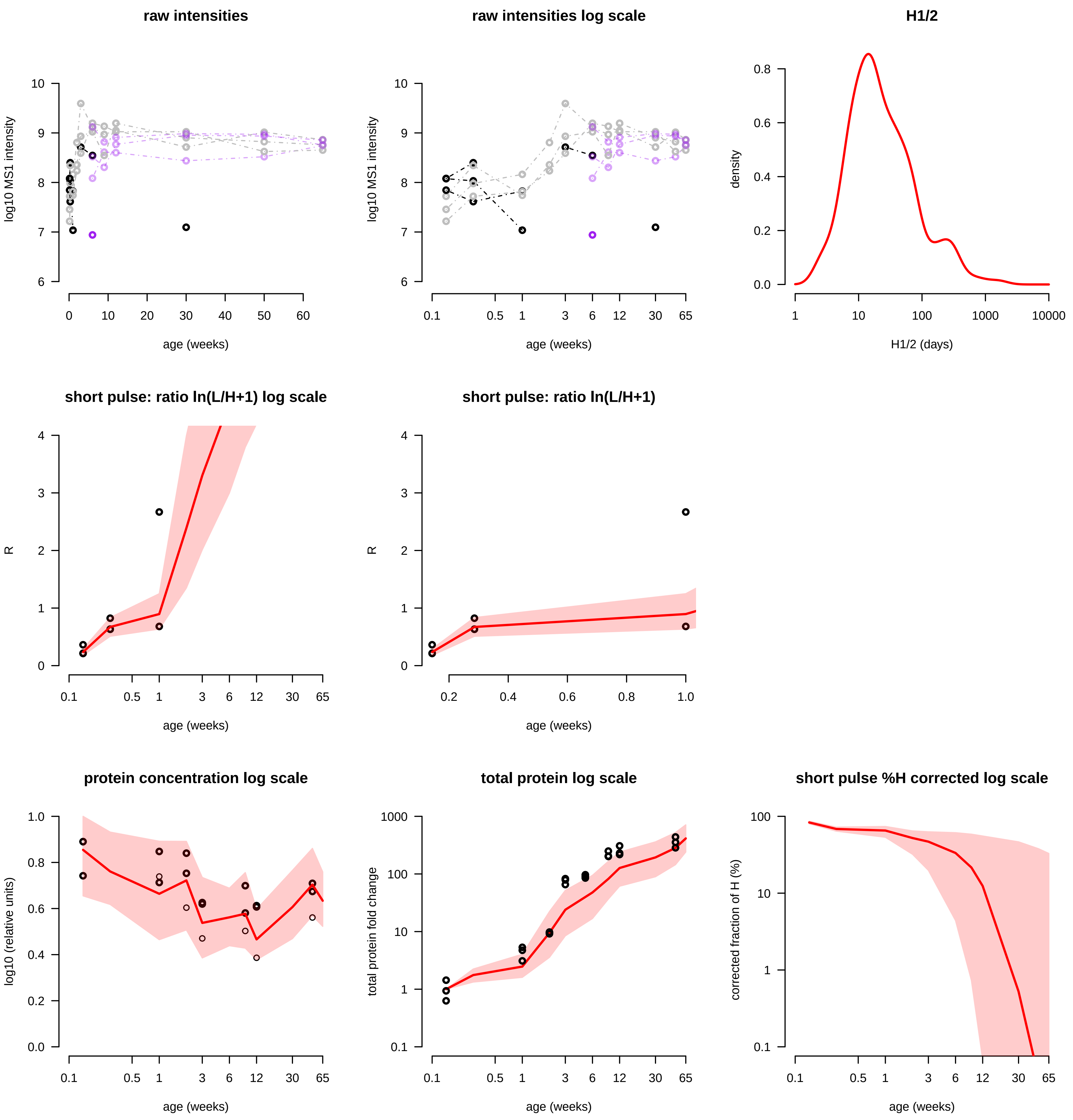

Q61687

>sp|Q61687|ATRX_MOUSE

Transcriptional regulator ATRX

GN=Atrx

Med H1/2: 2.7 days

Q5   H1/2: 2.4 days

Q95 H1/2: 3.4 days

reliable model fit: yes

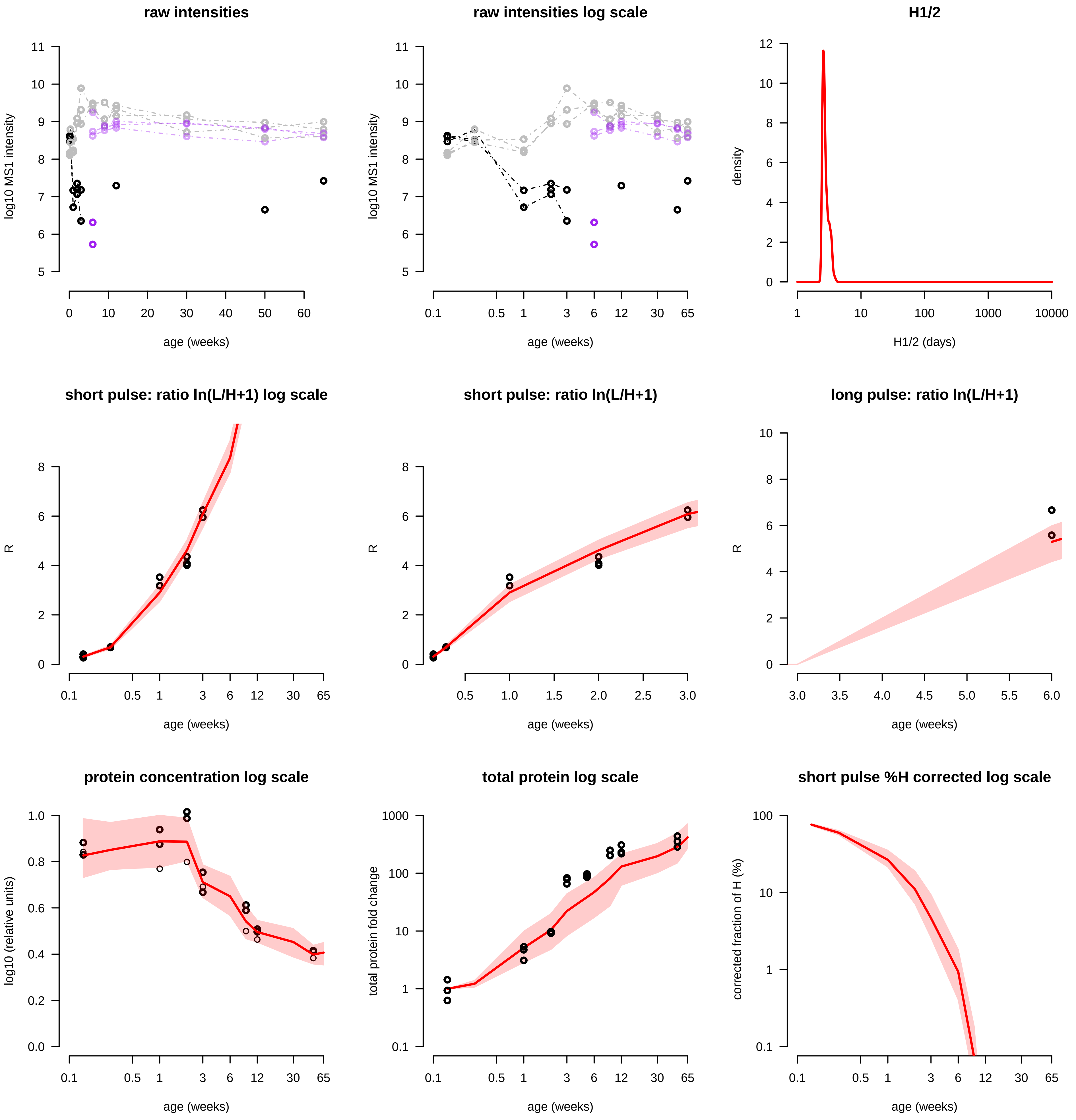

P16406

>sp|P16406|AMPE_MOUSE

Glutamyl aminopeptidase

GN=Enpep

Med H1/2: 150.5 days

Q5 H1/2: 76.6 days

Q95 H1/2: 1102 days

reliable model fit: no

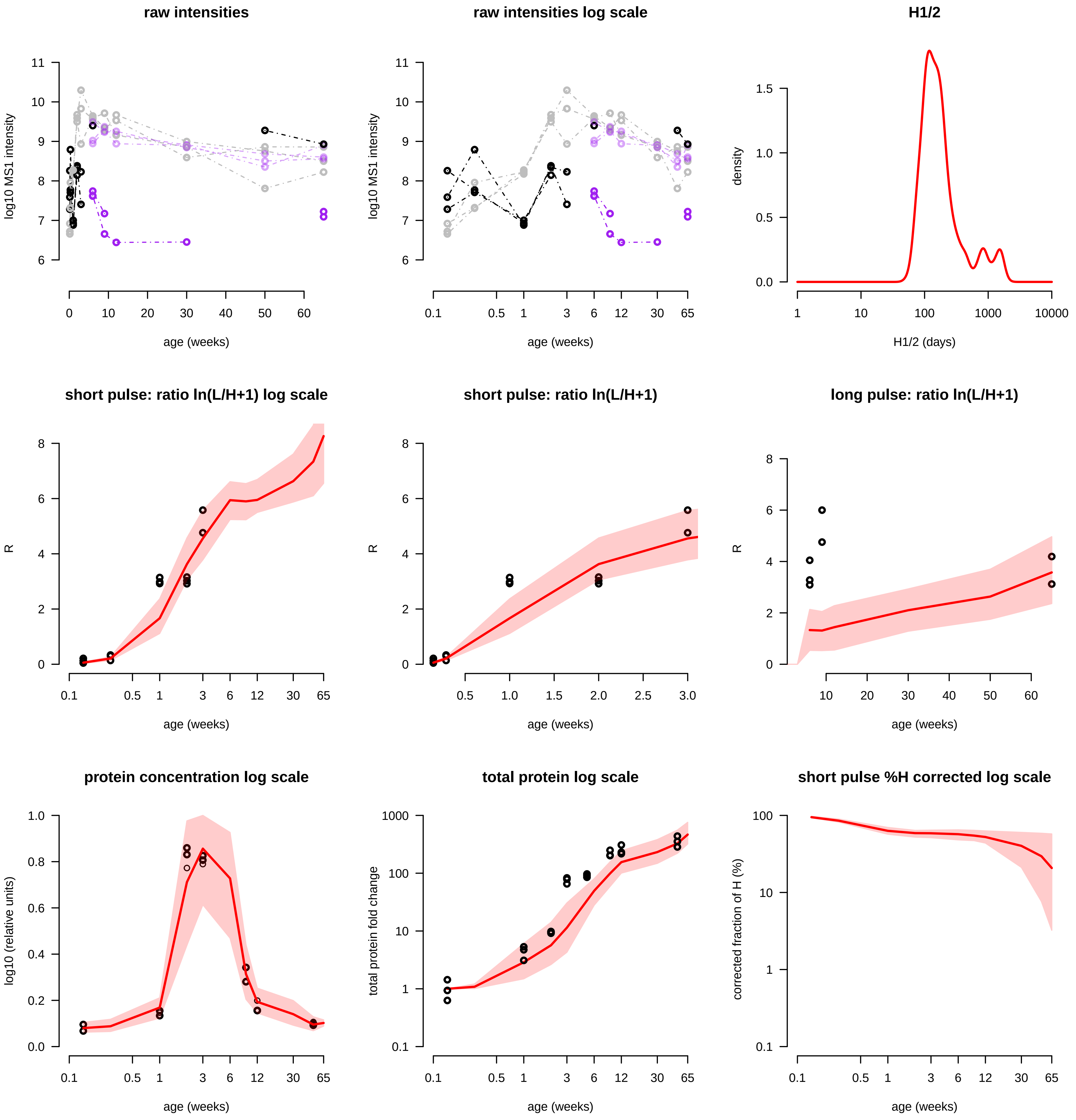

P97384

>sp|P97384|ANX11_MOUSE

Annexin A11

GN=Anxa11

Med H1/2: 4.7 days

Q5 H1/2: 3.8 days

Q95 H1/2: 5.8 days

reliable model fit: yes

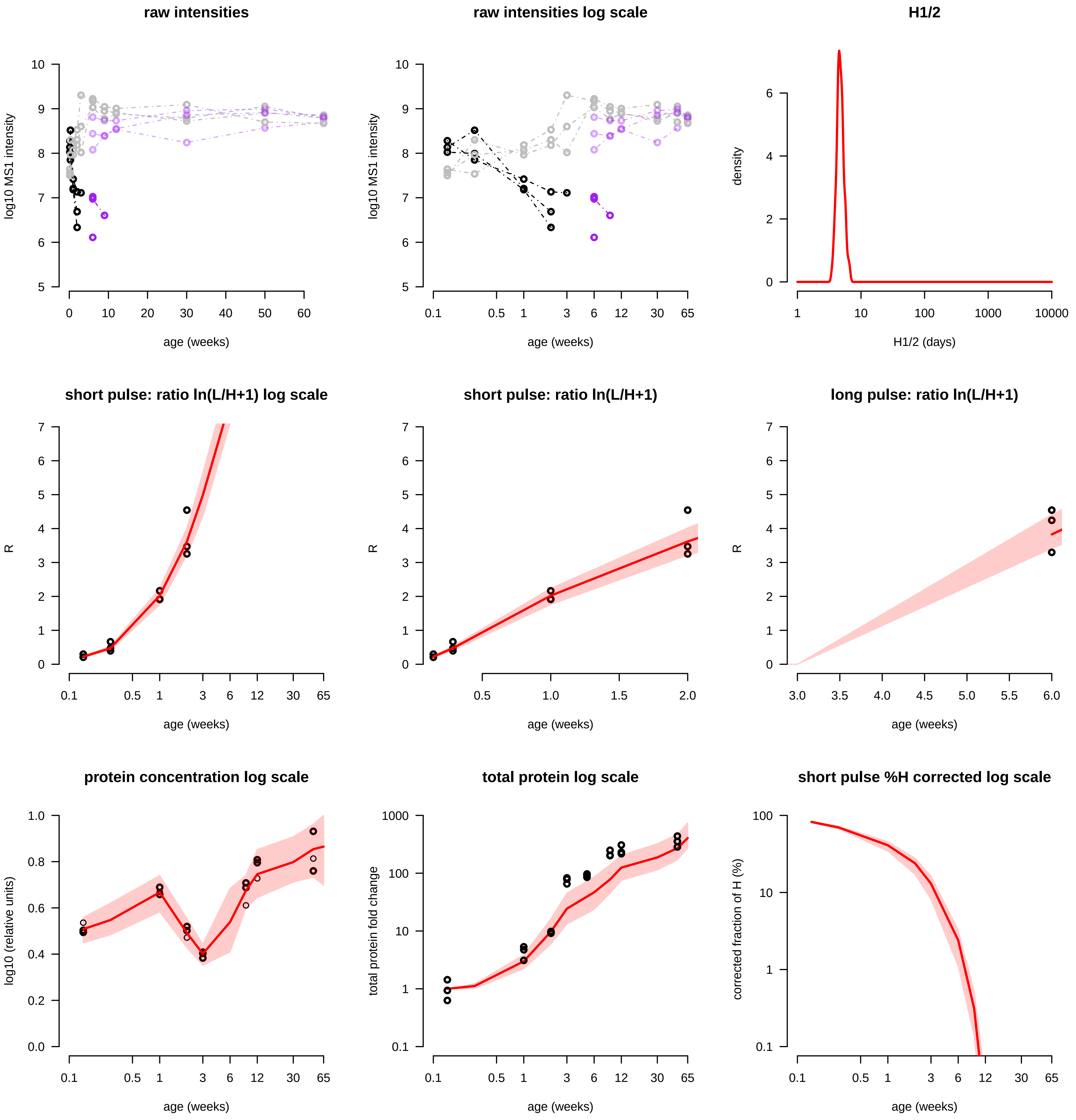

P10493

>sp|P10493|NID1_MOUSE

Nidogen−1

GN=Nid1

Med H1/2: 4.2 days

Q5 H1/2: 3.6 days

Q95 H1/2: 5.1 days

reliable model fit: yes

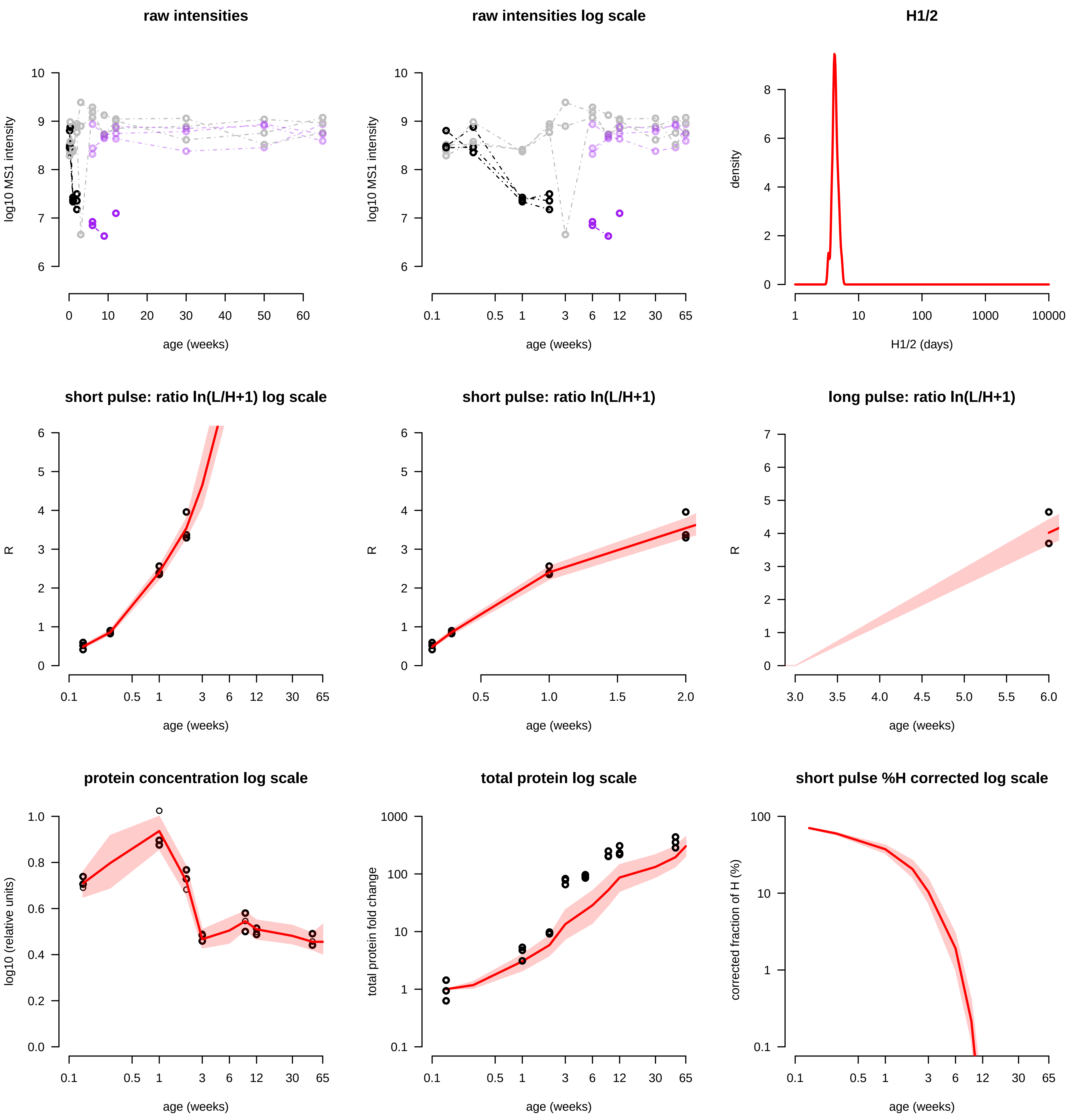

P35505

>sp|P35505|FAAA_MOUSE

Fumarylacetoacetase

GN=Fah

Med H1/2: 4.7 days

Q5 H1/2: 3.7 days

Q95 H1/2: 6.3 days

reliable model fit: yes

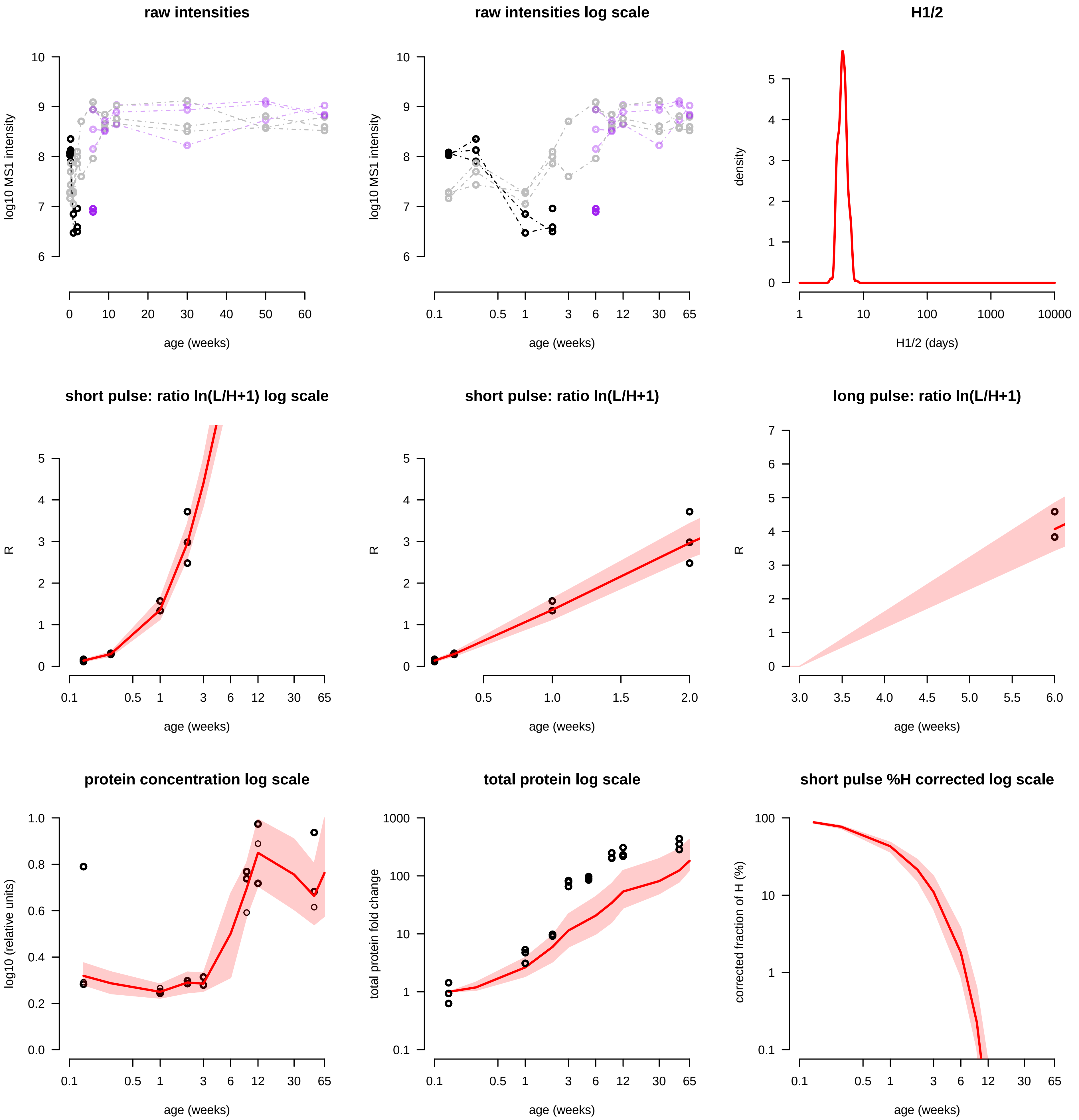

O35143

>sp|O35143|ATIF1_MOUSE

ATPase inhibitor, mitochondrial

GN=Atp5if1

Med H1/2: 11.1 days

Q5 H1/2: 8.2 days

Q95 H1/2: 16.7 days

reliable model fit: yes

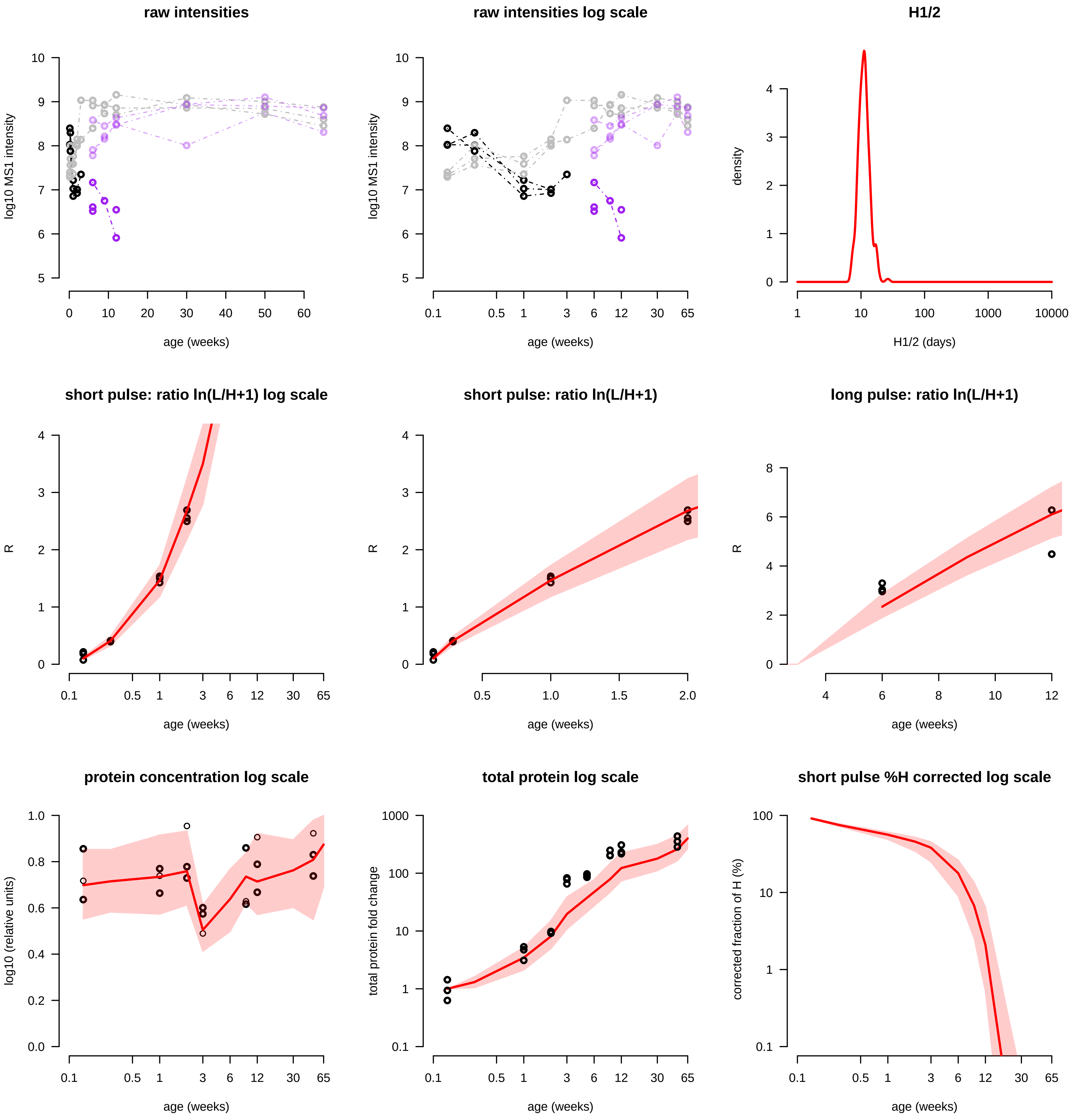

Q99MD9

>sp|Q99MD9|NASP_MOUSE

Nuclear autoantigenic sperm protein

GN=Nasp

Med H1/2: 3.8 days

Q5 H1/2: 3.1 days

Q95 H1/2: 4.9 days

reliable model fit: yes

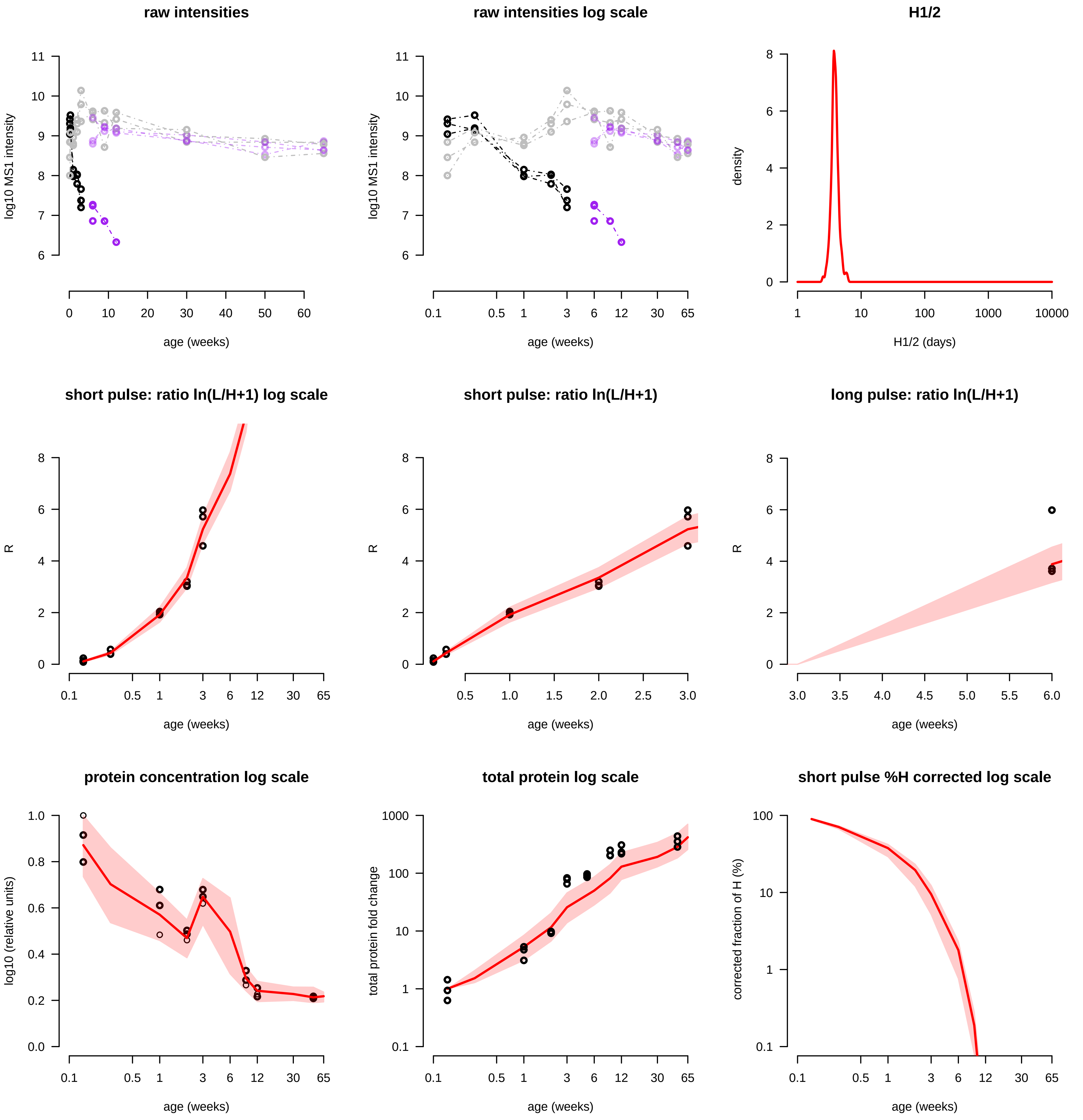

P10711

>sp|P10711|TCEA1_MOUSE

Transcription elongation factor A protein 1

GN=Tcea1

Med H1/2: 55 days

Q5 H1/2: 13.9 days

Q95 H1/2: 513.9 days

reliable model fit: yes

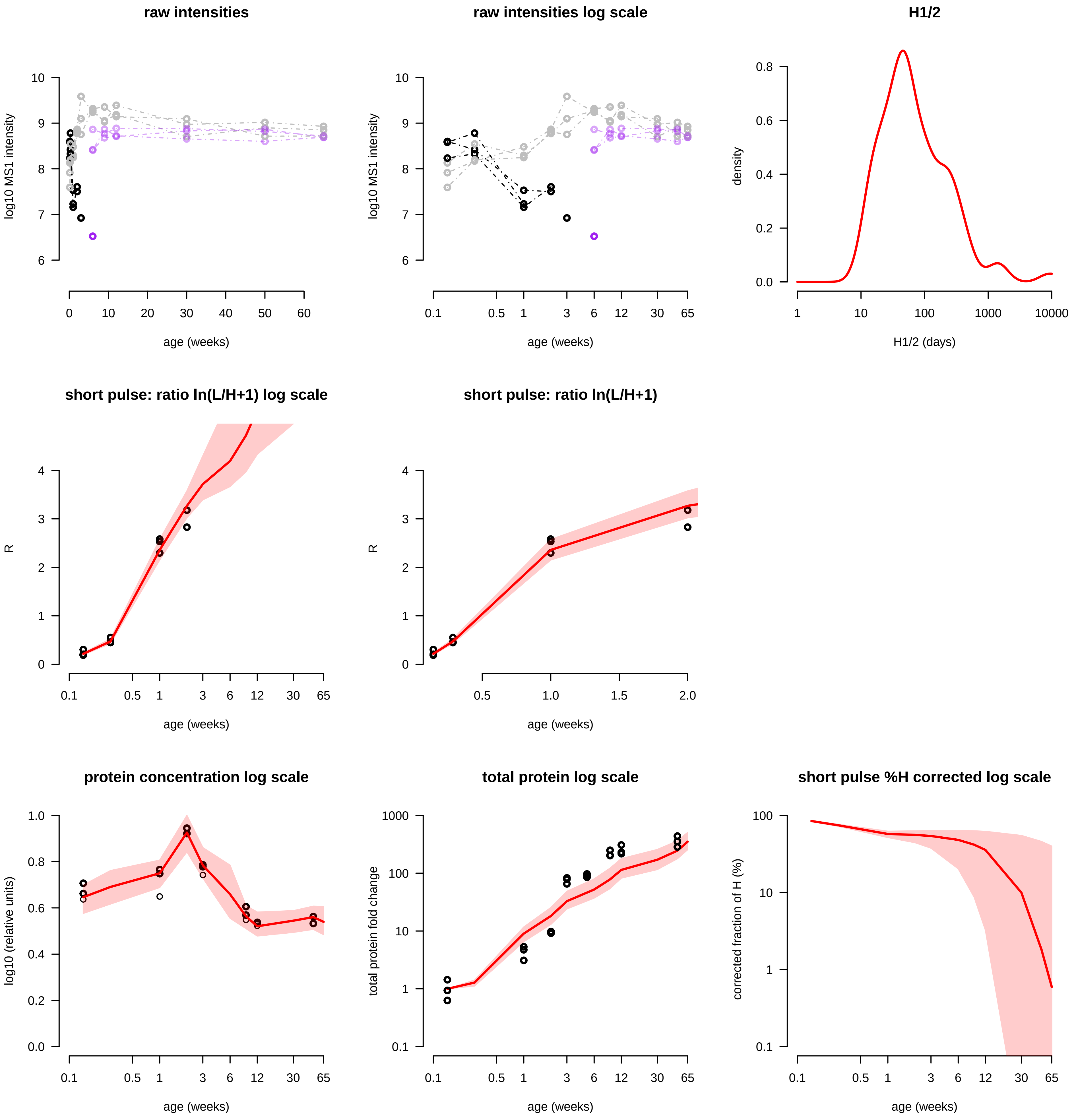

P24452

>sp|P24452|CAPG_MOUSE

Macrophage−capping protein

GN=Capg

Med H1/2: 7.2 days

Q5 H1/2: 4.7 days

Q95 H1/2: 16.4 days

reliable model fit: yes

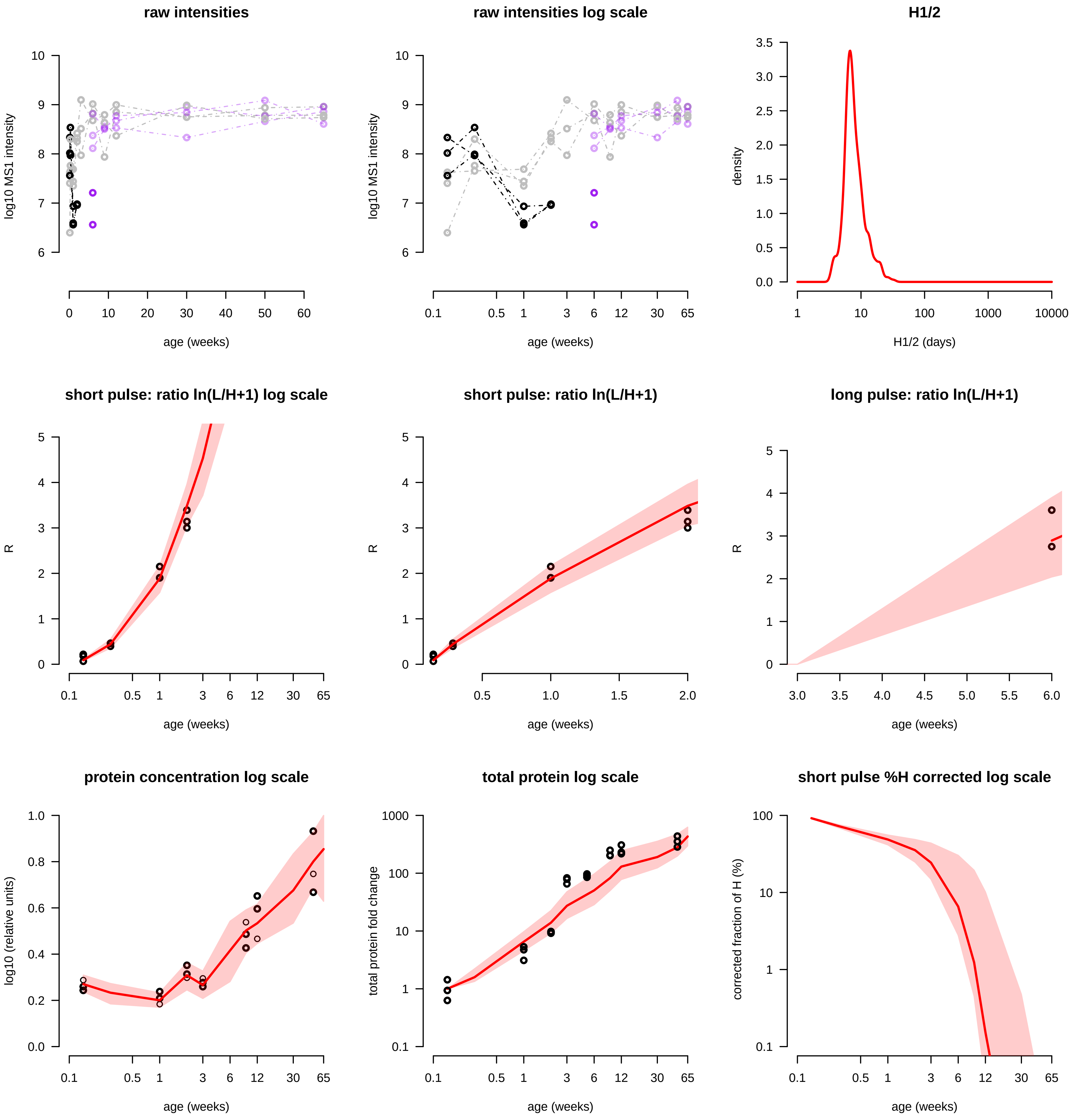

Q9QXK3;Q9QXK3−4

>sp|Q9QXK3|COPG2_MOUSE

Coatomer subunit gamma−2

GN=Copg2

Med H1/2: 7.4 days

Q5 H1/2: 6.1 days

Q95 H1/2: 9.6 days

reliable model fit: yes

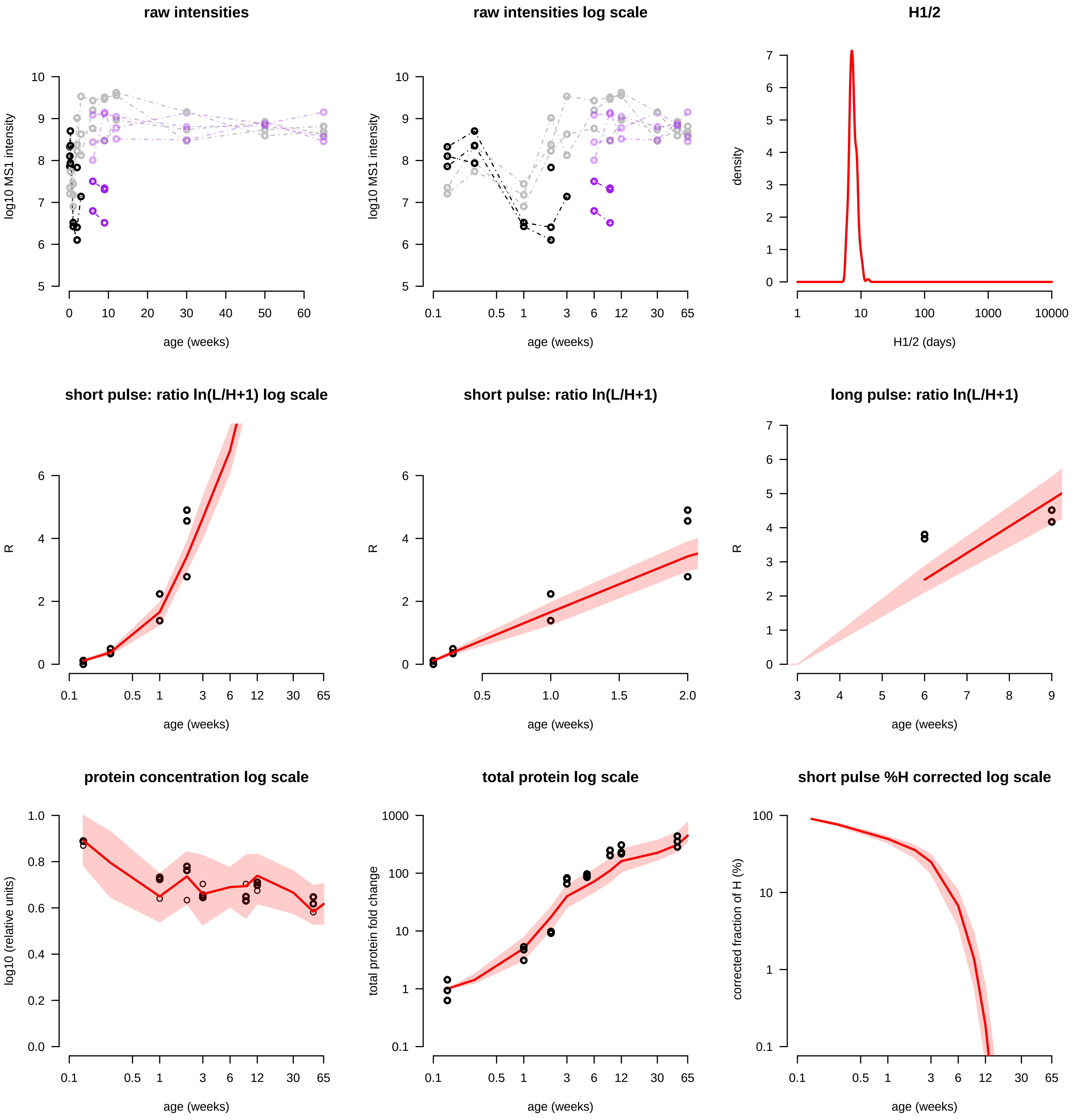

Q9JJ28

>sp|Q9JJ28|FLII_MOUSE

Protein flightless−1 homolog

GN=Flii

Med H1/2: 1869.9 days

Q5 H1/2: 559.4 days

Q95 H1/2: 27436.6 days

reliable model fit: no

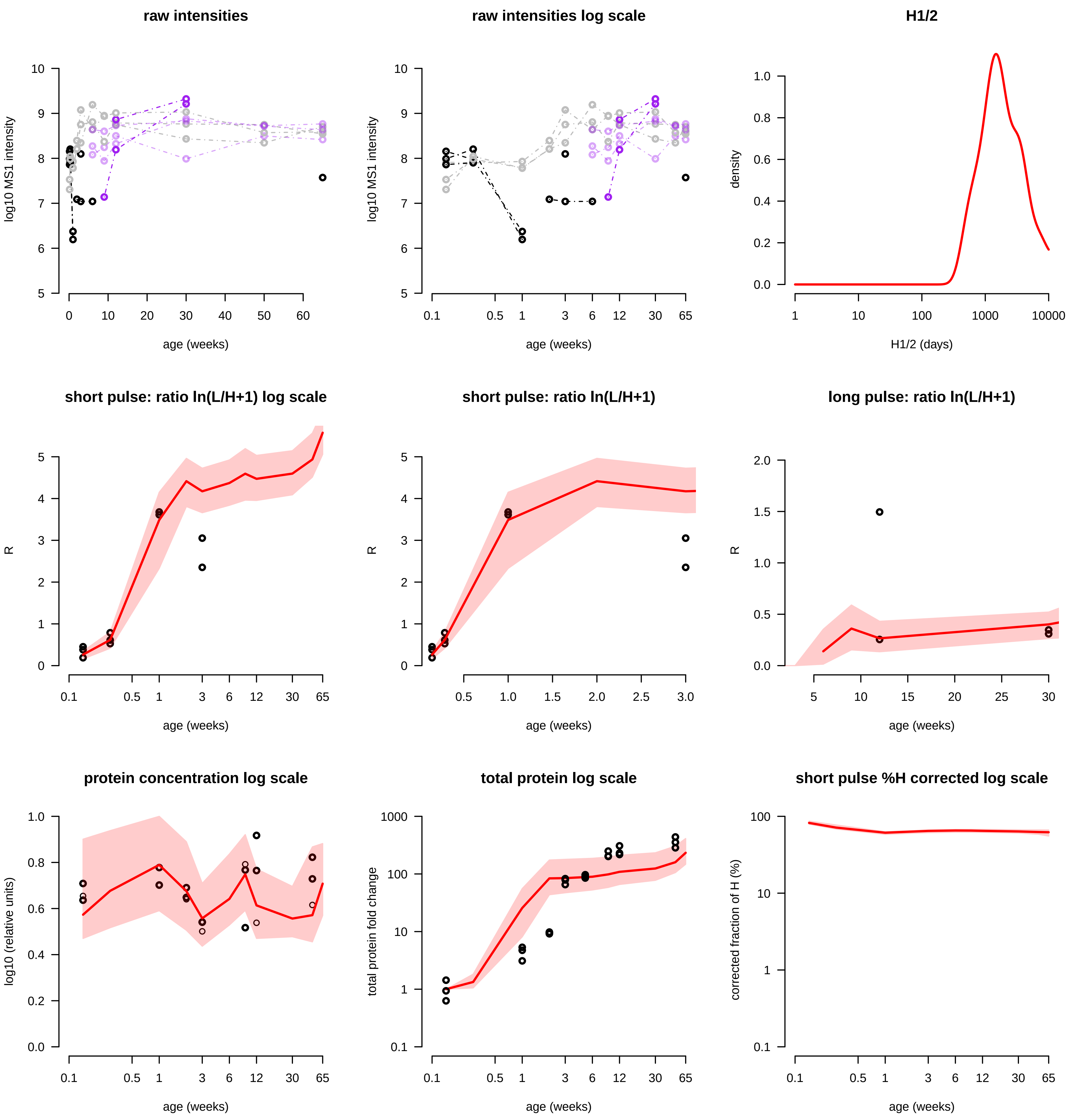

Q9DBS1

>sp|Q9DBS1|TMM43_MOUSE

Transmembrane protein 43

GN=Tmem43

Med H1/2: 27.9 days

Q5 H1/2: 9.5 days

Q95 H1/2: 147.5 days

reliable model fit: yes

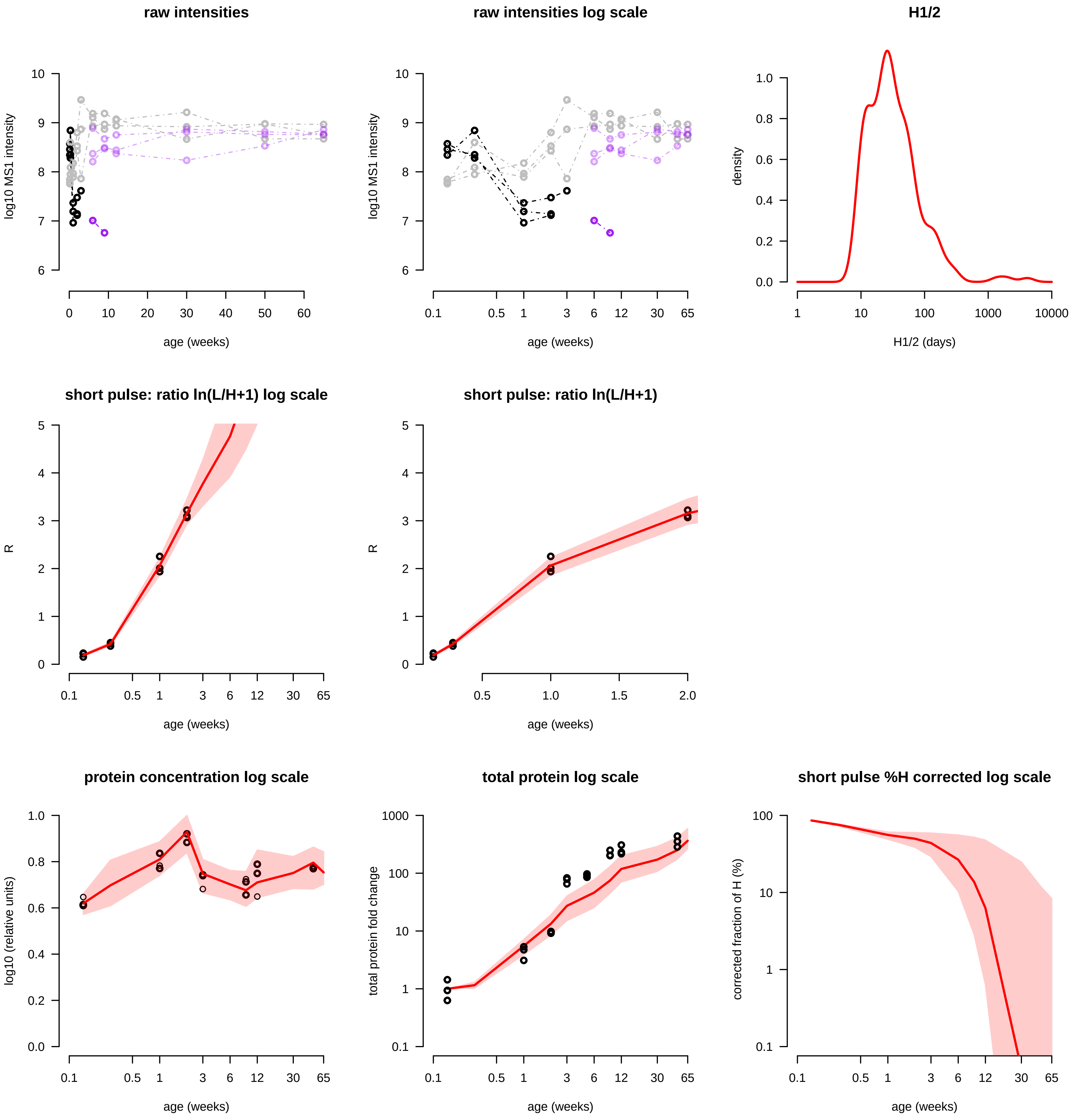

Q99LJ6

>sp|Q99LJ6|GPX7_MOUSE

Glutathione peroxidase 7

GN=Gpx7

Med H1/2: 72.4 days

Q5 H1/2: 44.6 days

Q95 H1/2: 338.2 days

reliable model fit: yes

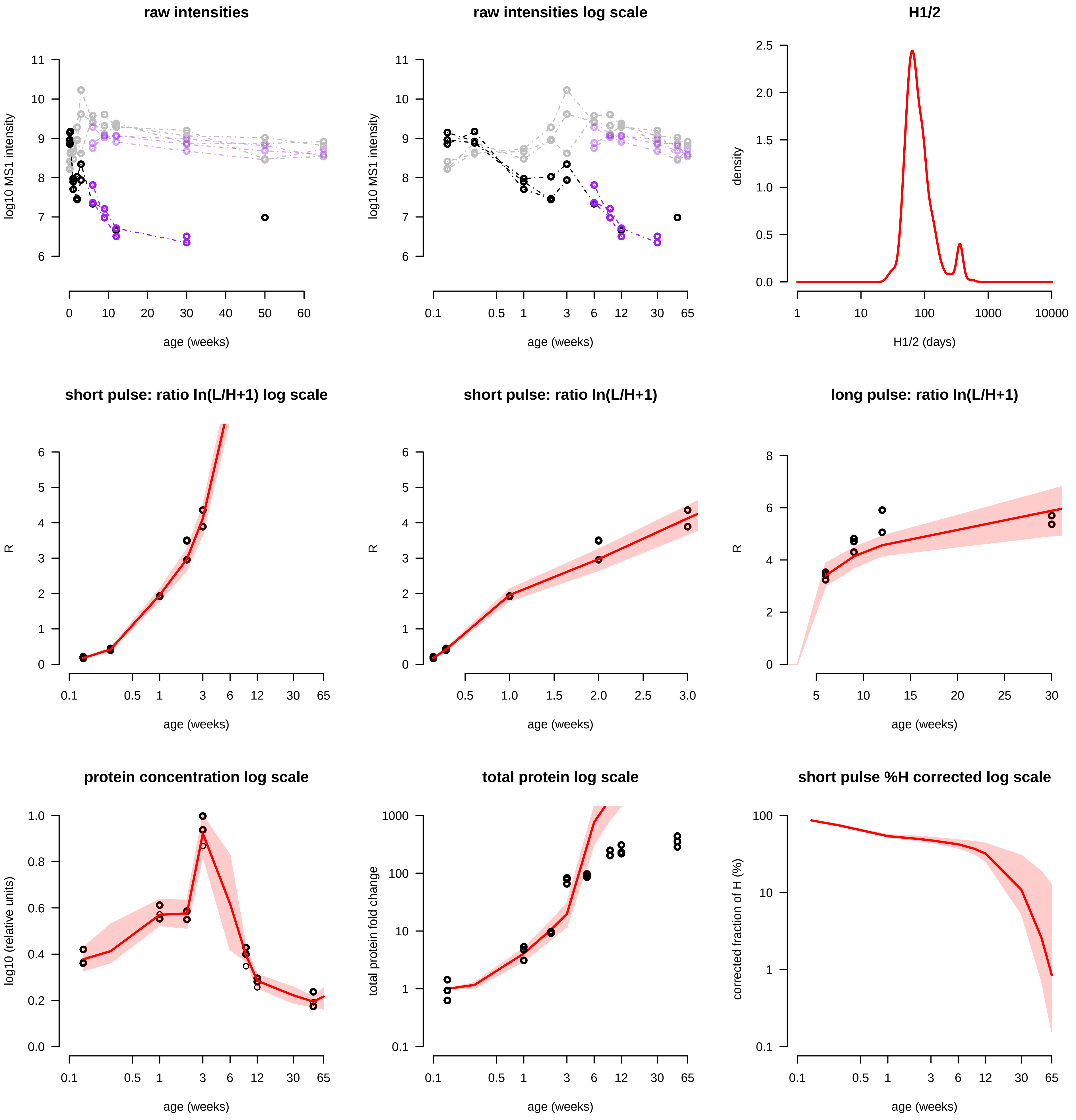

Q6PAV2−2;Q6PAV2

>sp|Q6PAV2−2|HERC4_MOUSE

Isoform 2 of Probable E3 ubiquitin−protein ligase HERC4

GN=Herc4

Med H1/2: 16.4 days

Q5 H1/2: 5 days

Q95 H1/2: 197 days

reliable model fit: yes

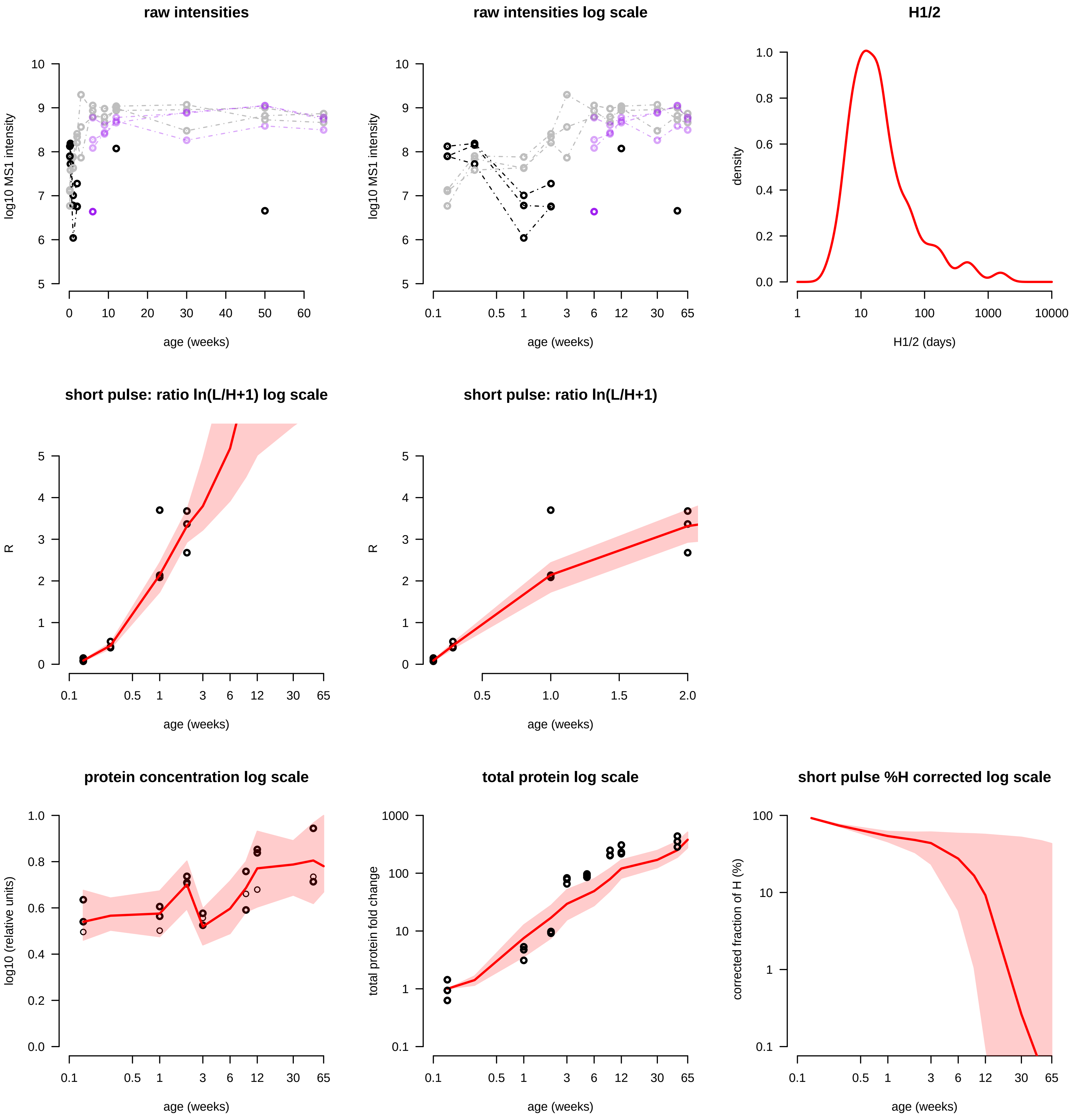

Q9Z0K8

>sp|Q9Z0K8|VNN1_MOUSE

Pantetheinase

GN=Vnn1

Med H1/2: 11.3 days

Q5 H1/2: 7.5 days

Q95 H1/2: 21.2 days

reliable model fit: yes

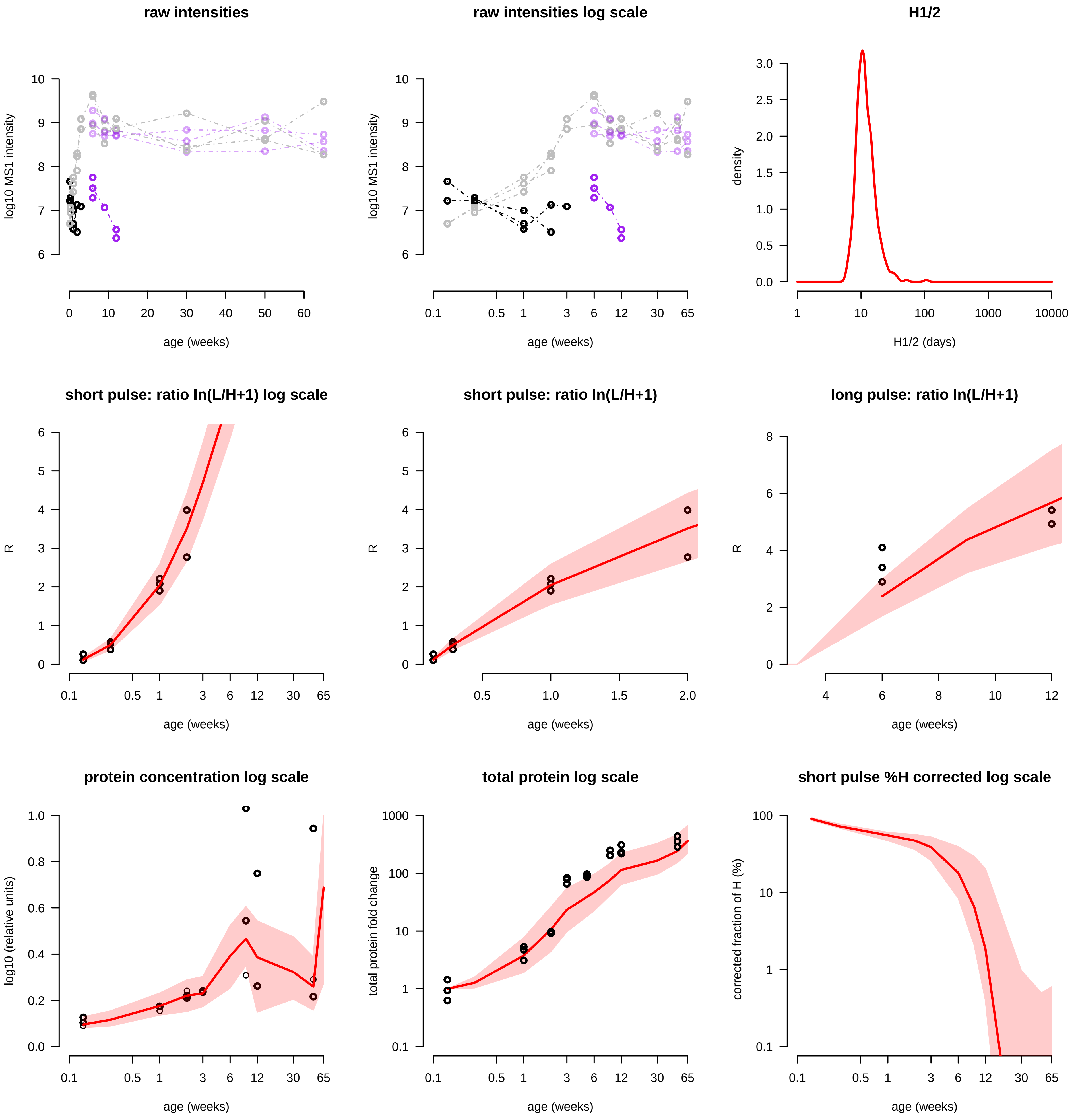

Q6PE01

>sp|Q6PE01|SNR40_MOUSE

U5 small nuclear ribonucleoprotein 40 kDa protein

GN=Snrnp40

Med H1/2: 5.2 days

Q5 H1/2: 4.2 days

Q95 H1/2: 6.6 days

reliable model fit: yes

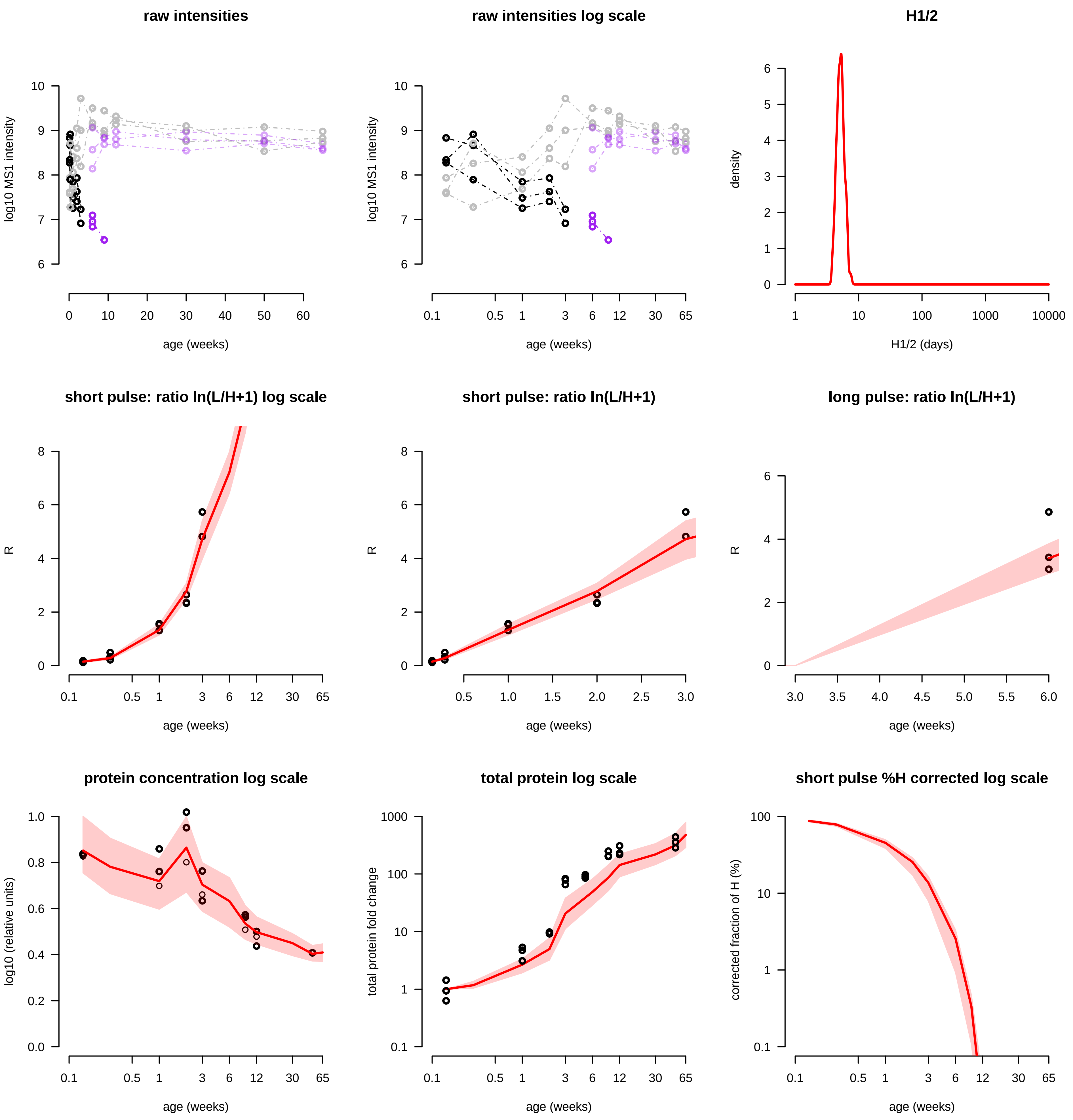

Q925I1;Q925I1−2

>sp|Q925I1|ATAD3_MOUSE

ATPase family AAA domain−containing protein 3

GN=Atad3

Med H1/2: 13 days

Q5 H1/2: 4.9 days

Q95 H1/2: 100.7 days

reliable model fit: yes

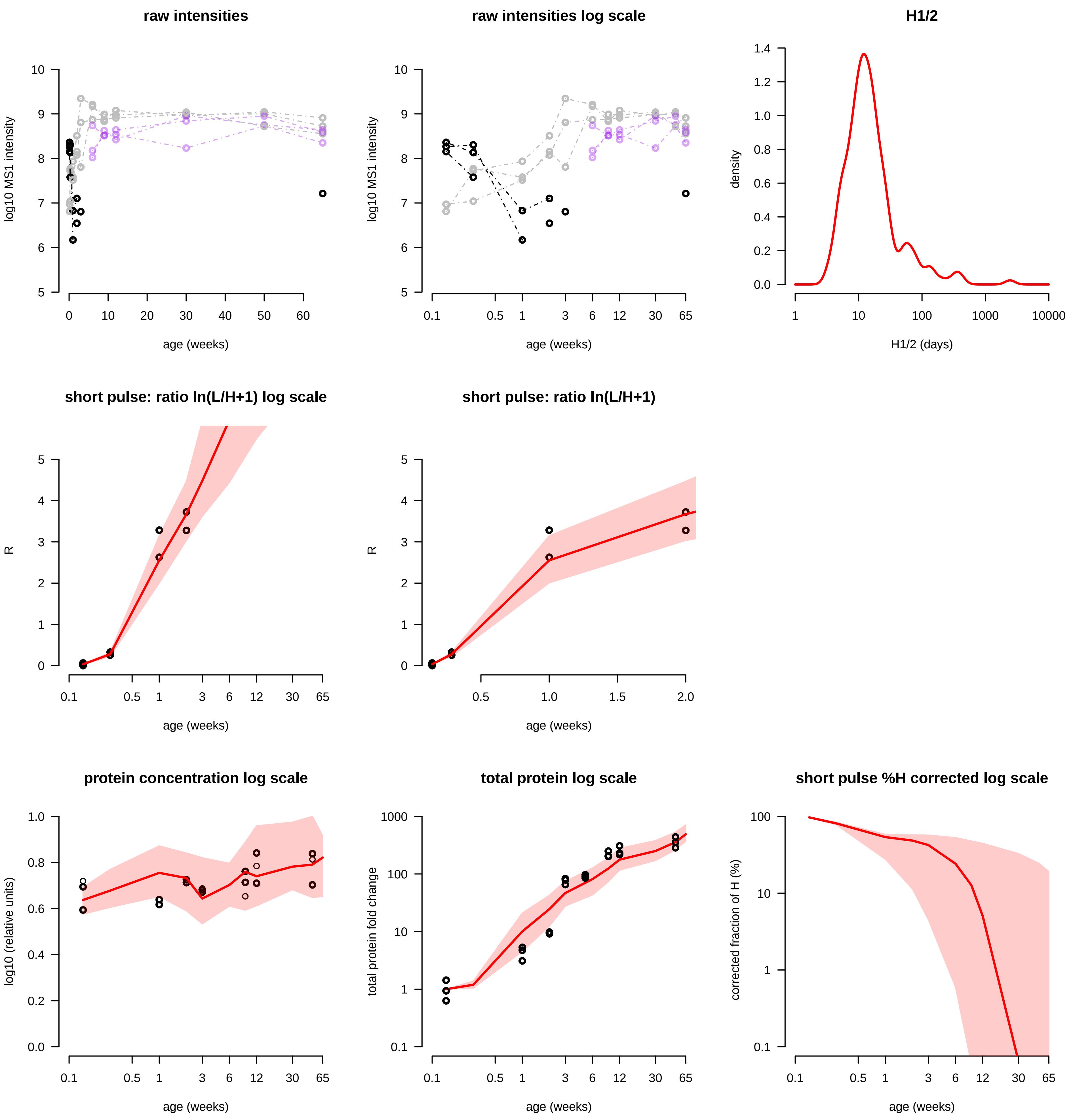

P15508

>sp|P15508|SPTB1_MOUSE

Spectrin beta chain, erythrocytic

GN=Sptb

Med H1/2: 1481.4 days

Q5 H1/2: 362 days

Q95 H1/2: 31874.6 days

reliable model fit: no

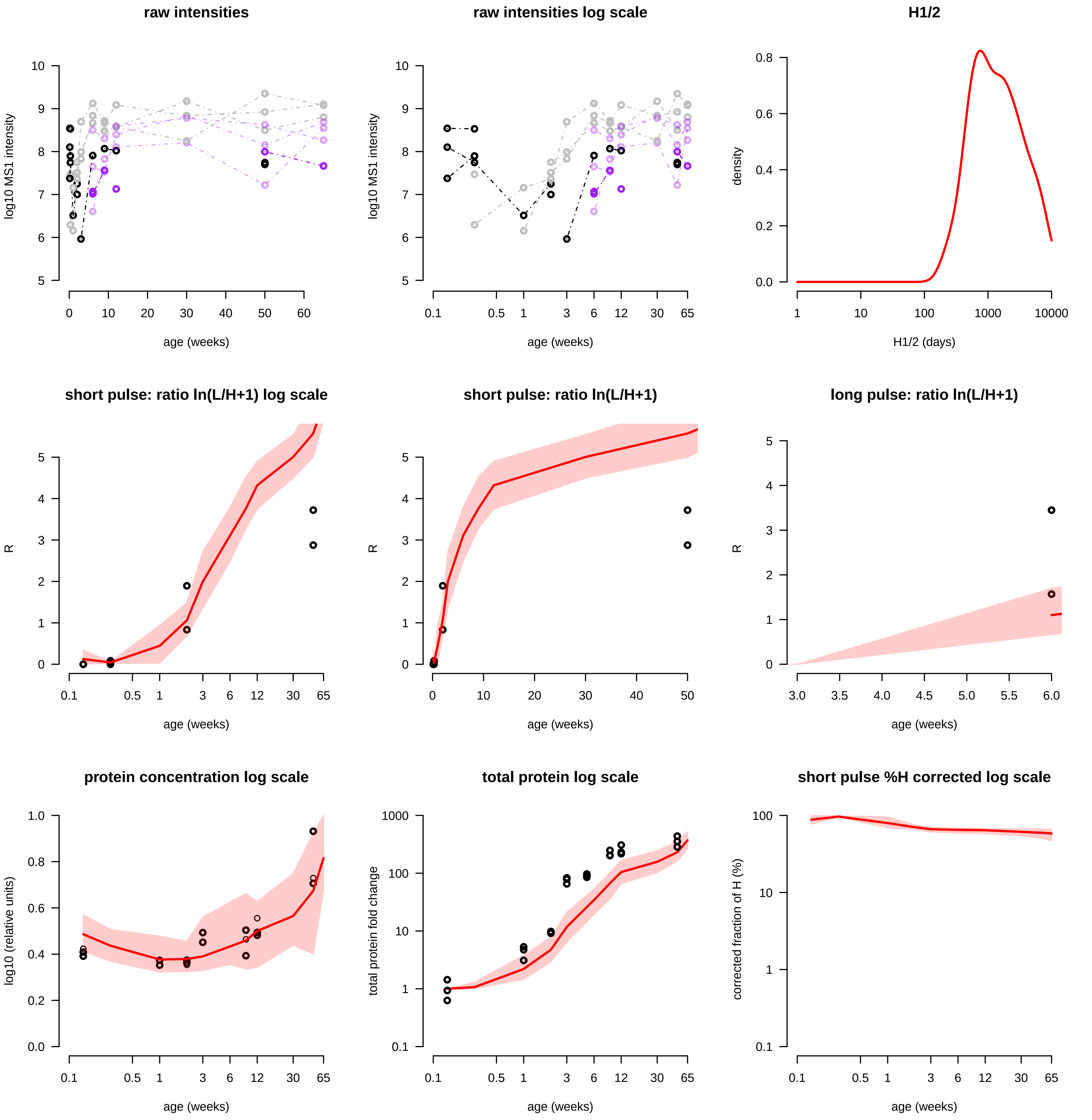

Q9JLN9

>sp|Q9JLN9|MTOR_MOUSE

Serine/threonine−protein kinase mTOR

GN=Mtor

Med H1/2: 4 days

Q5 H1/2: 2.6 days

Q95 H1/2: 10.2 days

reliable model fit: yes

raw intensities

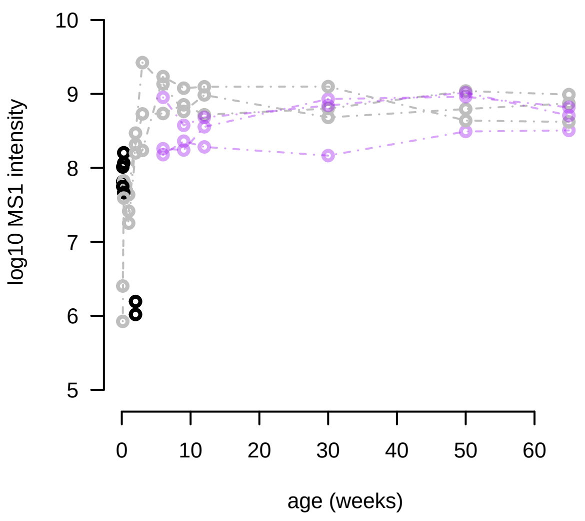

raw intensities log scale

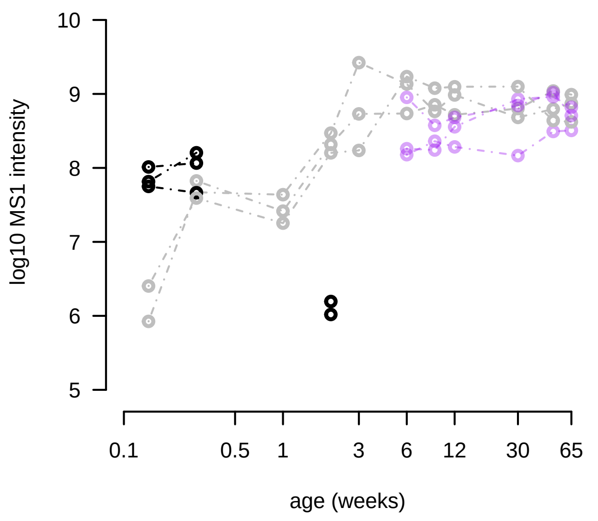

H1/2

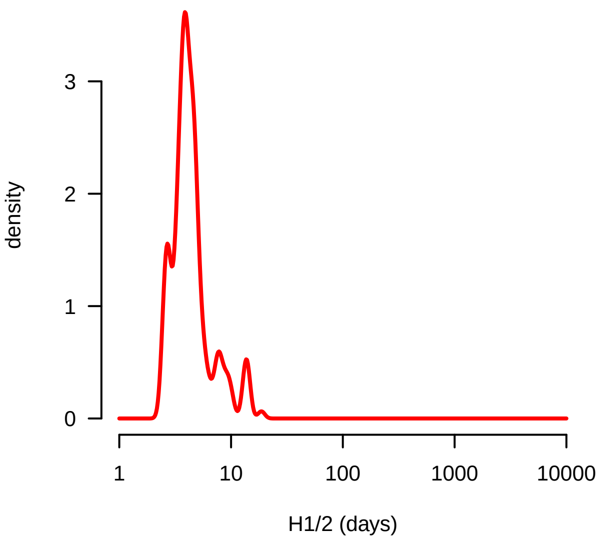

short pulse: ratio ln(L/H+1) log scale

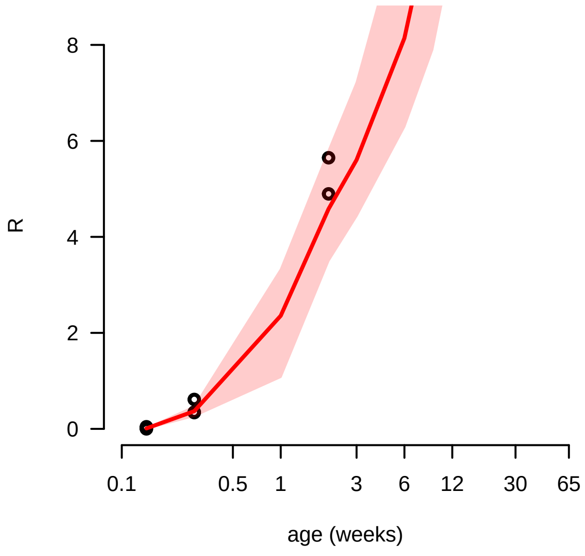

short pulse: ratio ln(L/H+1)

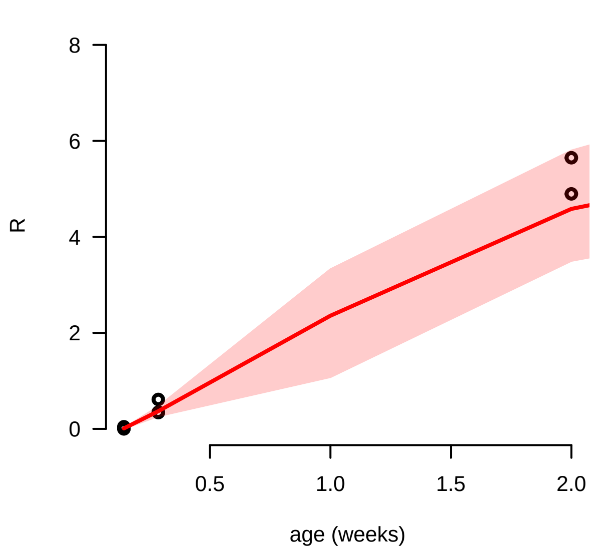

protein concentration log scale

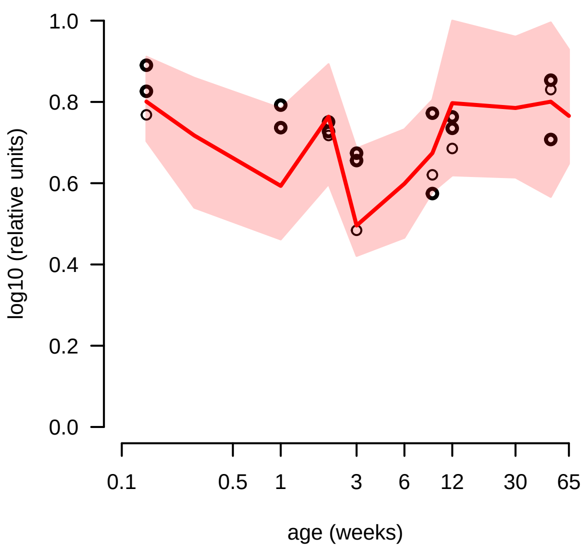

total protein log scale

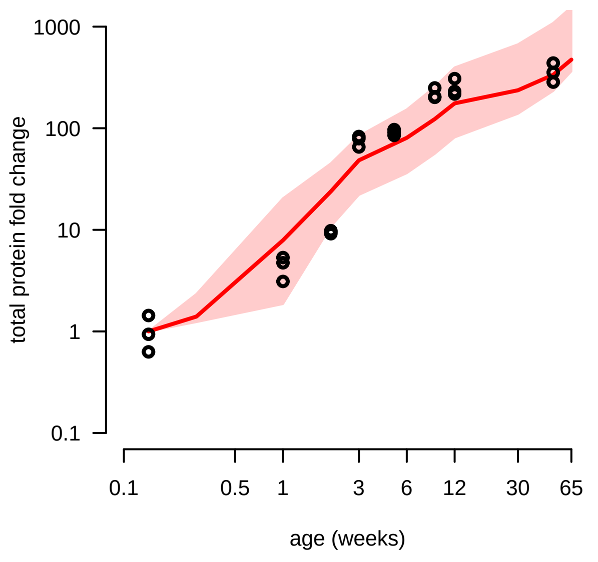

short pulse %H corrected log scale

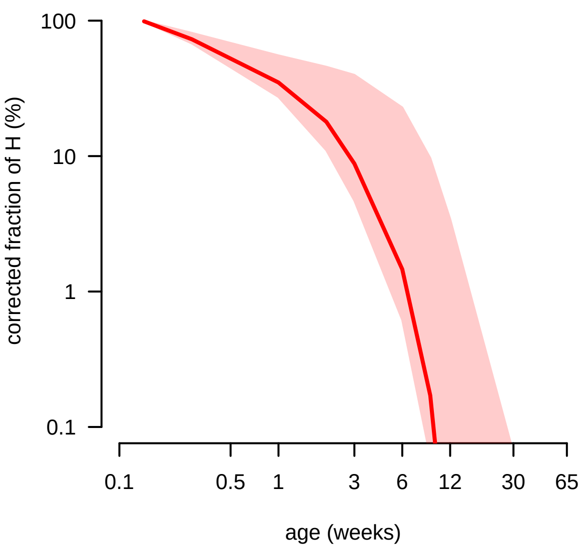

P23492

>sp|P23492|PNPH_MOUSE

Purine nucleoside phosphorylase

GN=Pnp

Med H1/2: 5.2 days

Q5 H1/2: 4.2 days

Q95 H1/2: 6.8 days

reliable model fit: yes

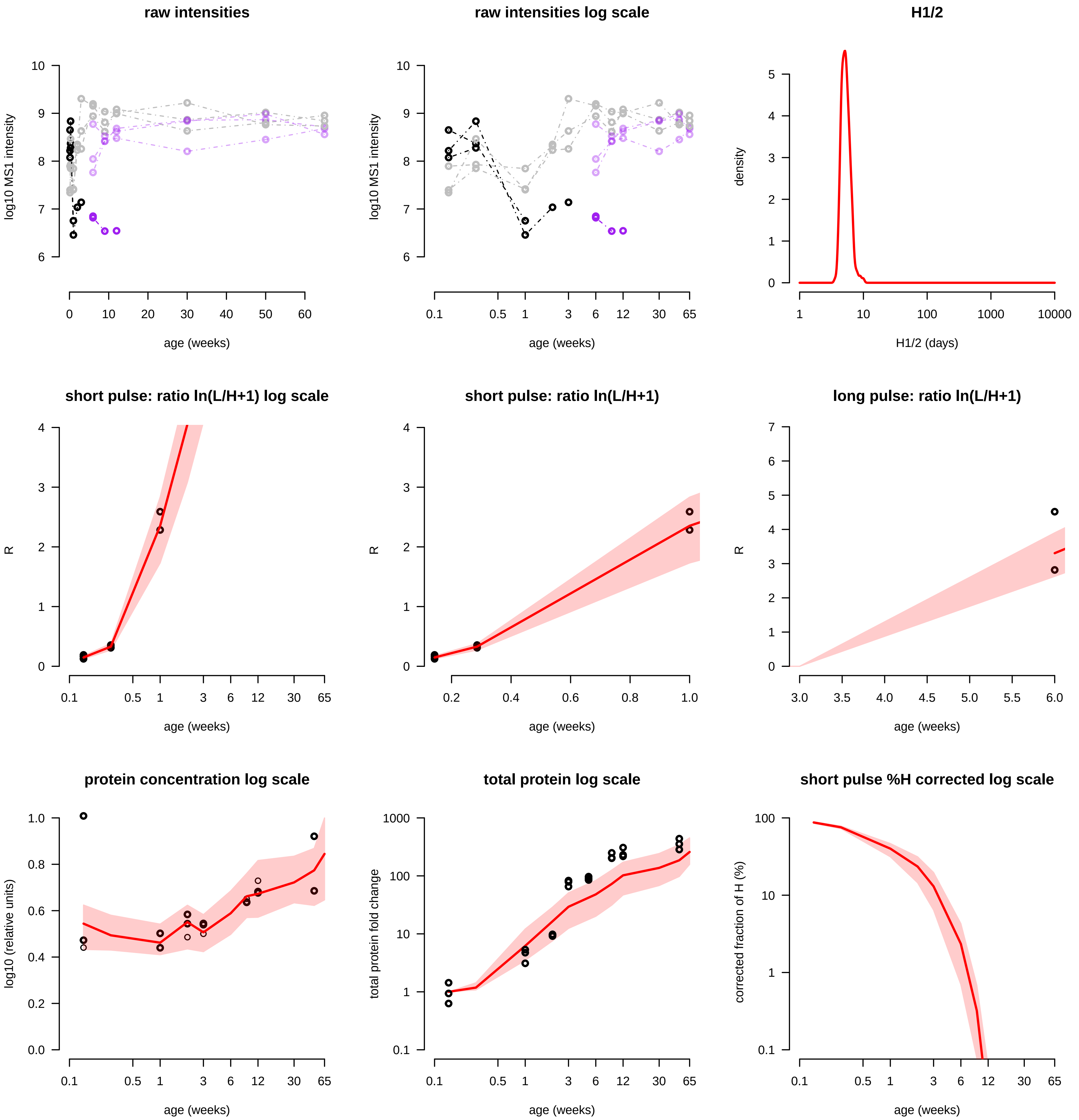

O88543

>sp|O88543|CSN3_MOUSE

COP9 signalosome complex subunit 3

GN=Cops3

Med H1/2: 3.7 days

Q5   H1/2: 3.2 days

Q95 H1/2: 4.7 days

reliable model fit: yes

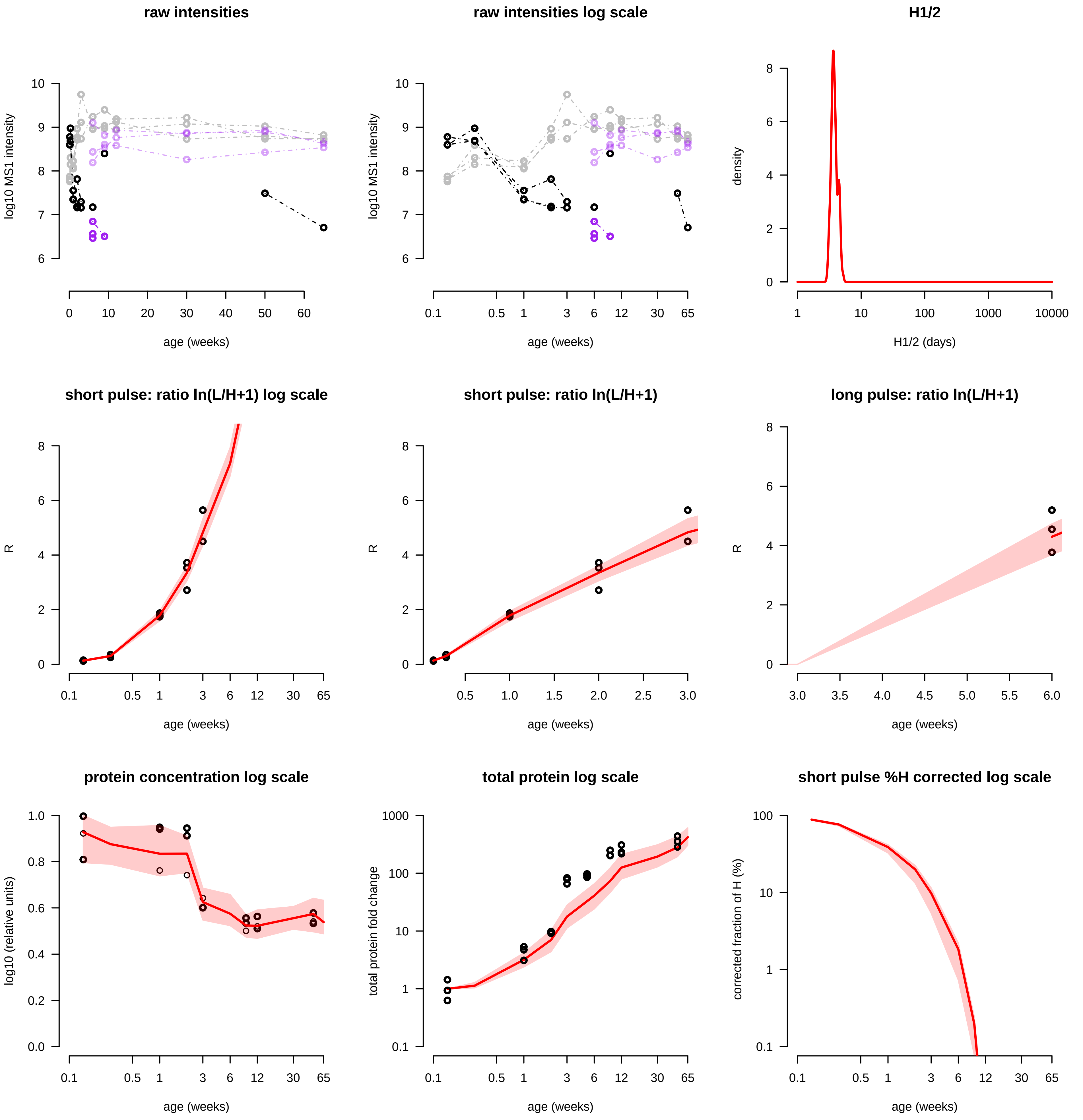

P48024

>sp|P48024|EIF1_MOUSE

Eukaryotic translation initiation factor 1

GN=Eif1

Med H1/2: 6 days

Q5 H1/2: 5 days

Q95 H1/2: 7.7 days

reliable model fit: yes

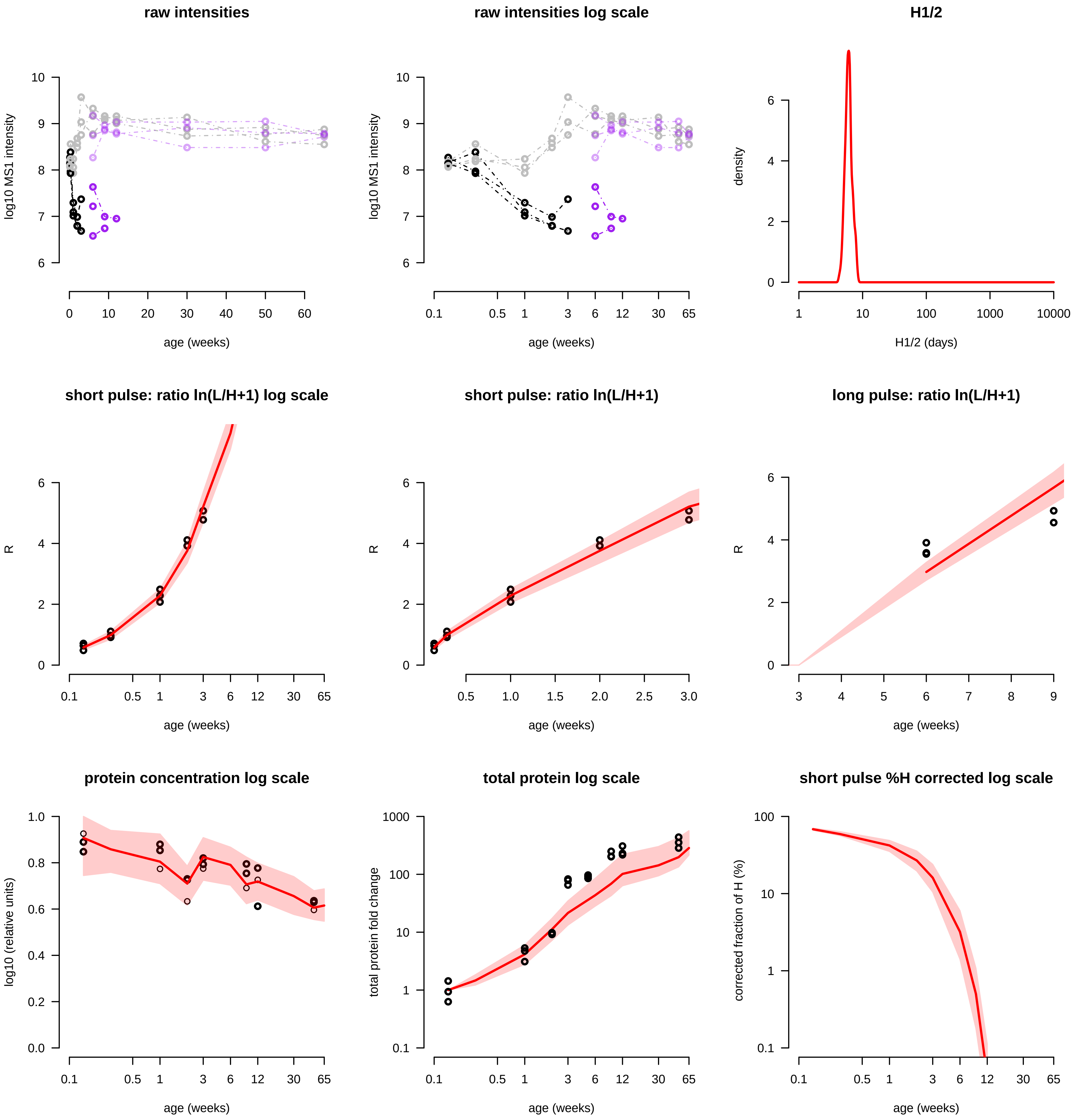

Q9DBL7

>sp|Q9DBL7|COASY_MOUSE

Bifunctional coenzyme A synthase

GN=Coasy

Med H1/2: 5.5 days

Q5 H1/2: 4.3 days

Q95 H1/2: 15.3 days

reliable model fit: yes

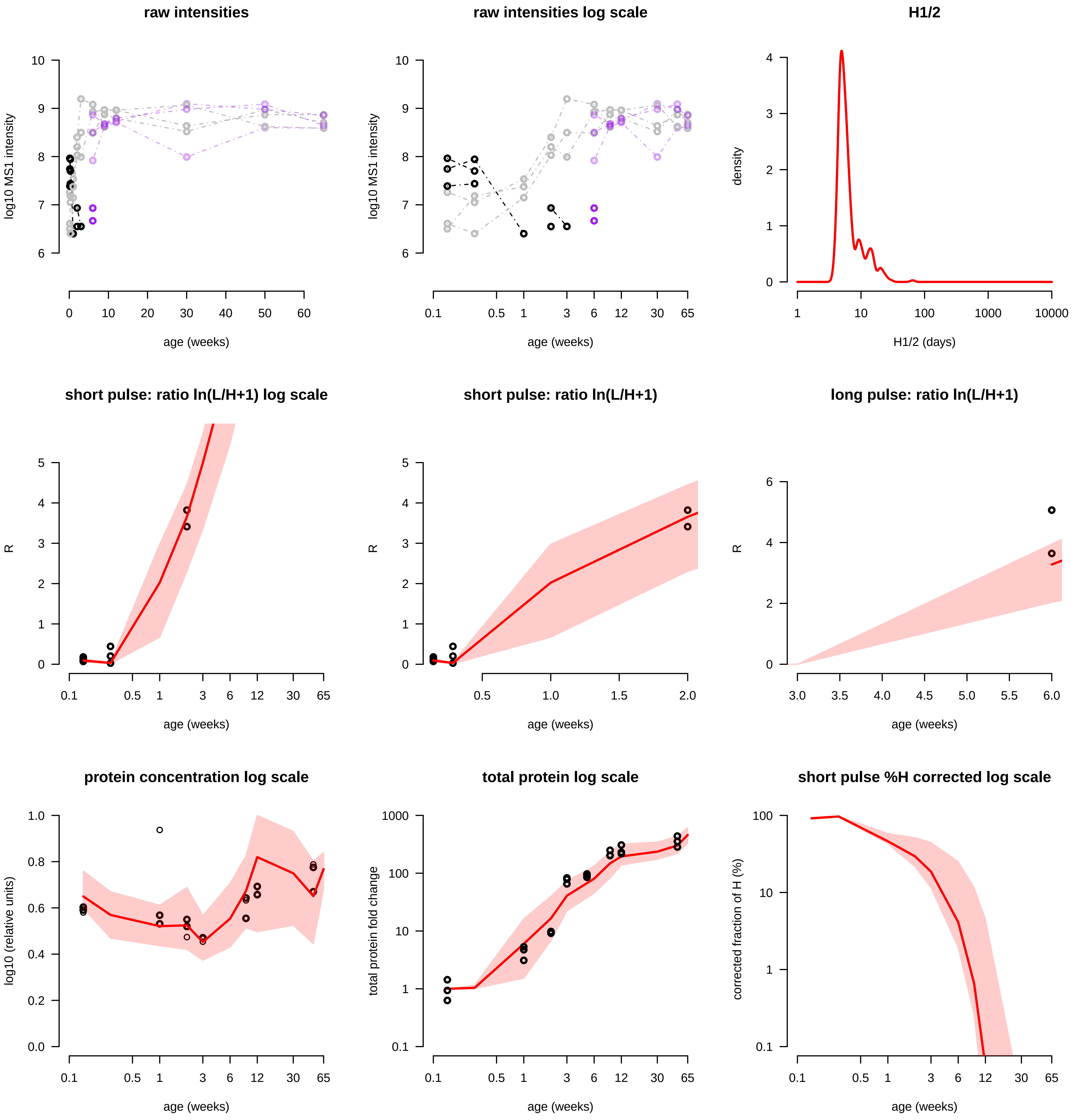

Q9JHS4

>sp|Q9JHS4|CLPX_MOUSE

ATP−dependent Clp protease ATP−binding subunit clpX−like, mitochondrial

GN=Clpx

Med H1/2: 387.2 days

Q5 H1/2: 163.2 days

Q95 H1/2: 3664.5 days

reliable model fit: yes

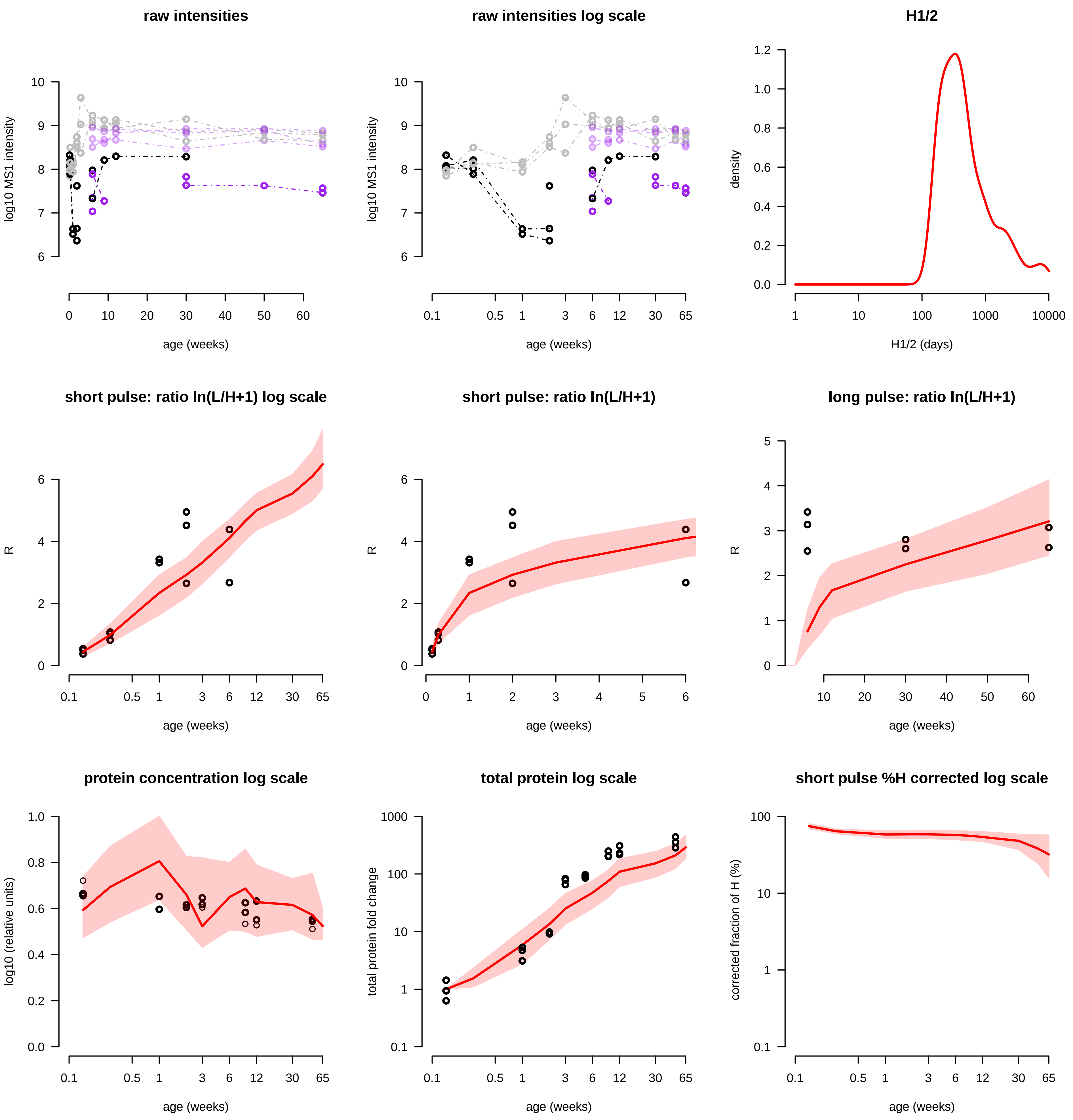

O08759;O08759−2;O08759−3

>sp|O08759|UBE3A_MOUSE

Ubiquitin−protein ligase E3A

GN=Ube3a

Med H1/2: 4.6 days

Q5 H1/2: 3.4 days

Q95 H1/2: 7.2 days

reliable model fit: yes

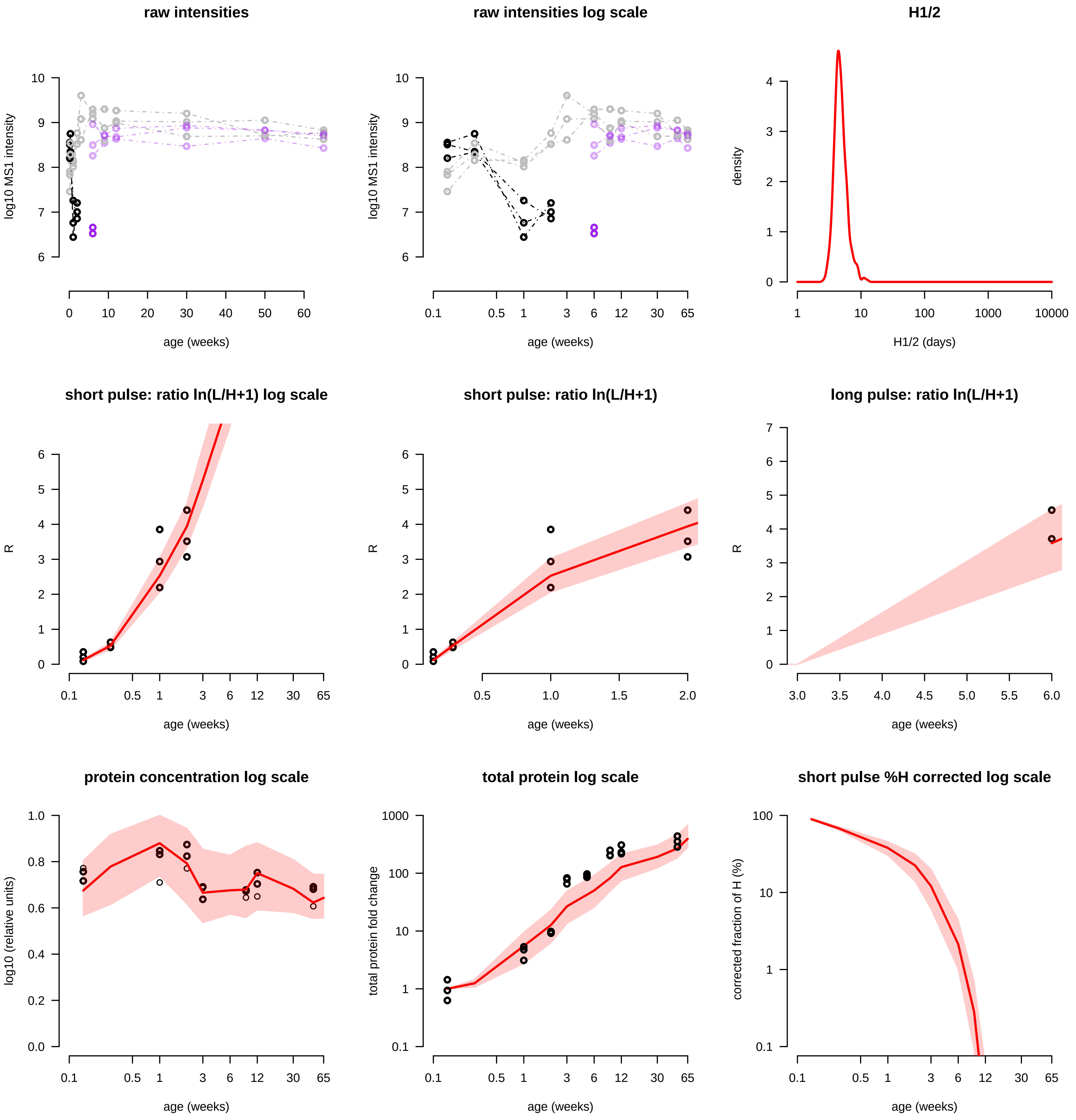

Q9JKX6

>sp|Q9JKX6|NUDT5_MOUSE

ADP−sugar pyrophosphatase

GN=Nudt5

Med H1/2: 6.7 days

Q5 H1/2: 4.7 days

Q95 H1/2: 10 days

reliable model fit: yes

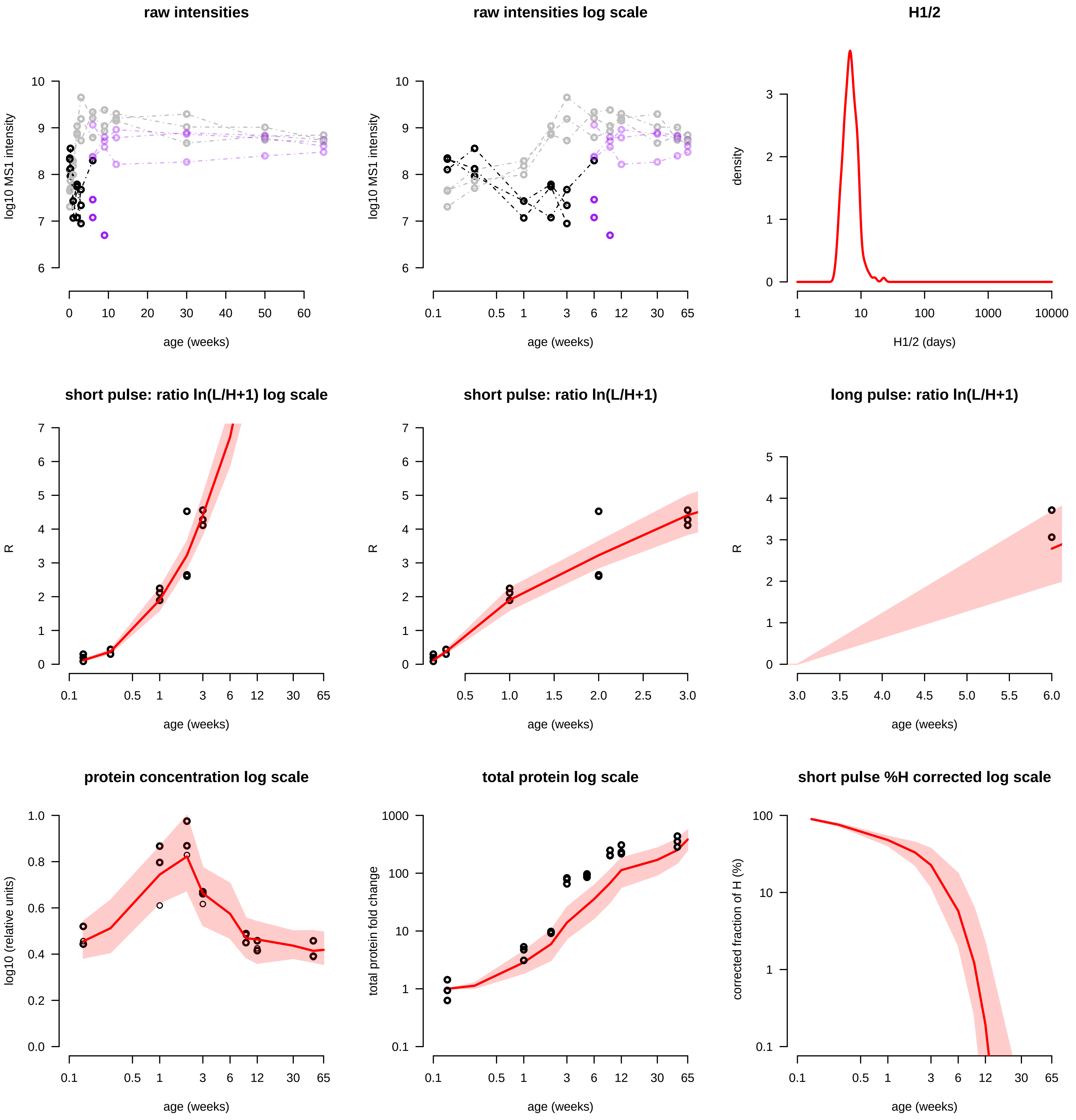

P47964

>sp|P47964|RL36_MOUSE

60S ribosomal protein L36

GN=Rpl36

Med H1/2: 7.8 days

Q5 H1/2: 6.3 days

Q95 H1/2: 10.2 days

reliable model fit: yes

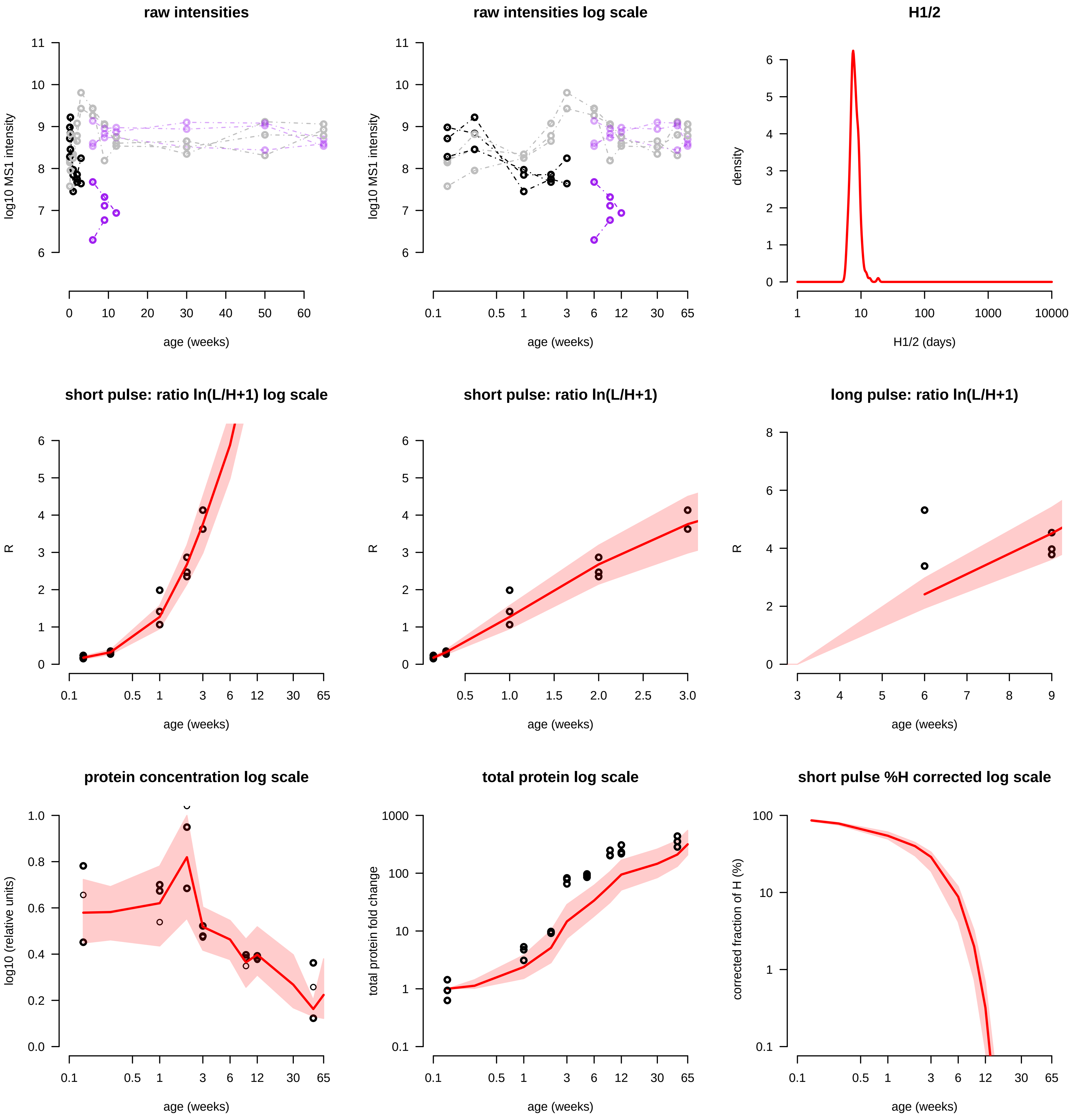

Q9JIW9

>sp|Q9JIW9|RALB_MOUSE

Ras−related protein Ral−B

GN=Ralb

Med H1/2: 4.8 days

Q5 H1/2: 3.8 days

Q95 H1/2: 6.6 days

reliable model fit: yes

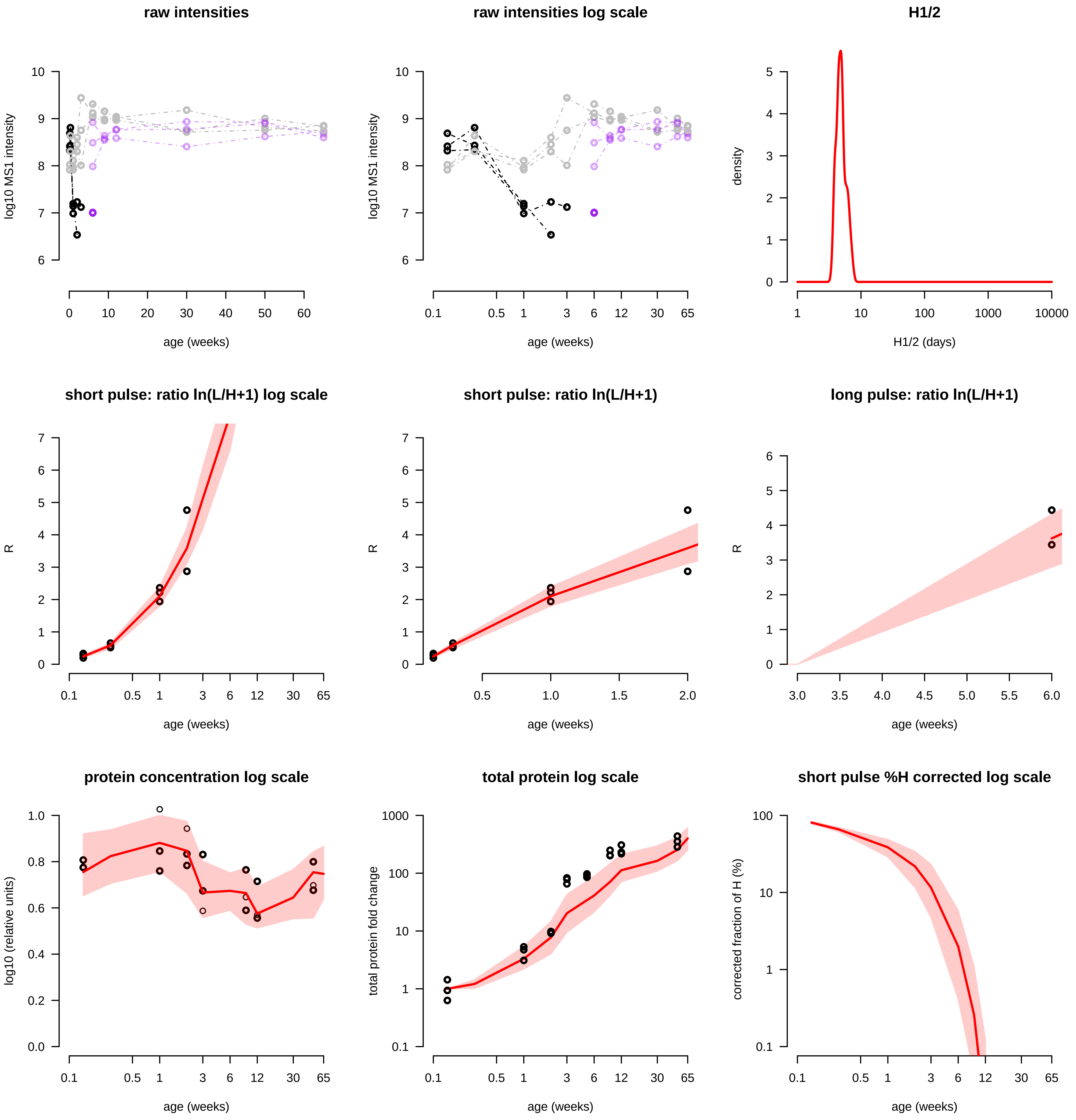

Q99J47;Q99J47−2

>sp|Q99J47|DRS7B_MOUSE

Dehydrogenase/reductase SDR family member 7B

GN=Dhrs7b

Med H1/2: 6.8 days

Q5 H1/2: 3.1 days

Q95 H1/2: 58.3 days

reliable model fit: yes

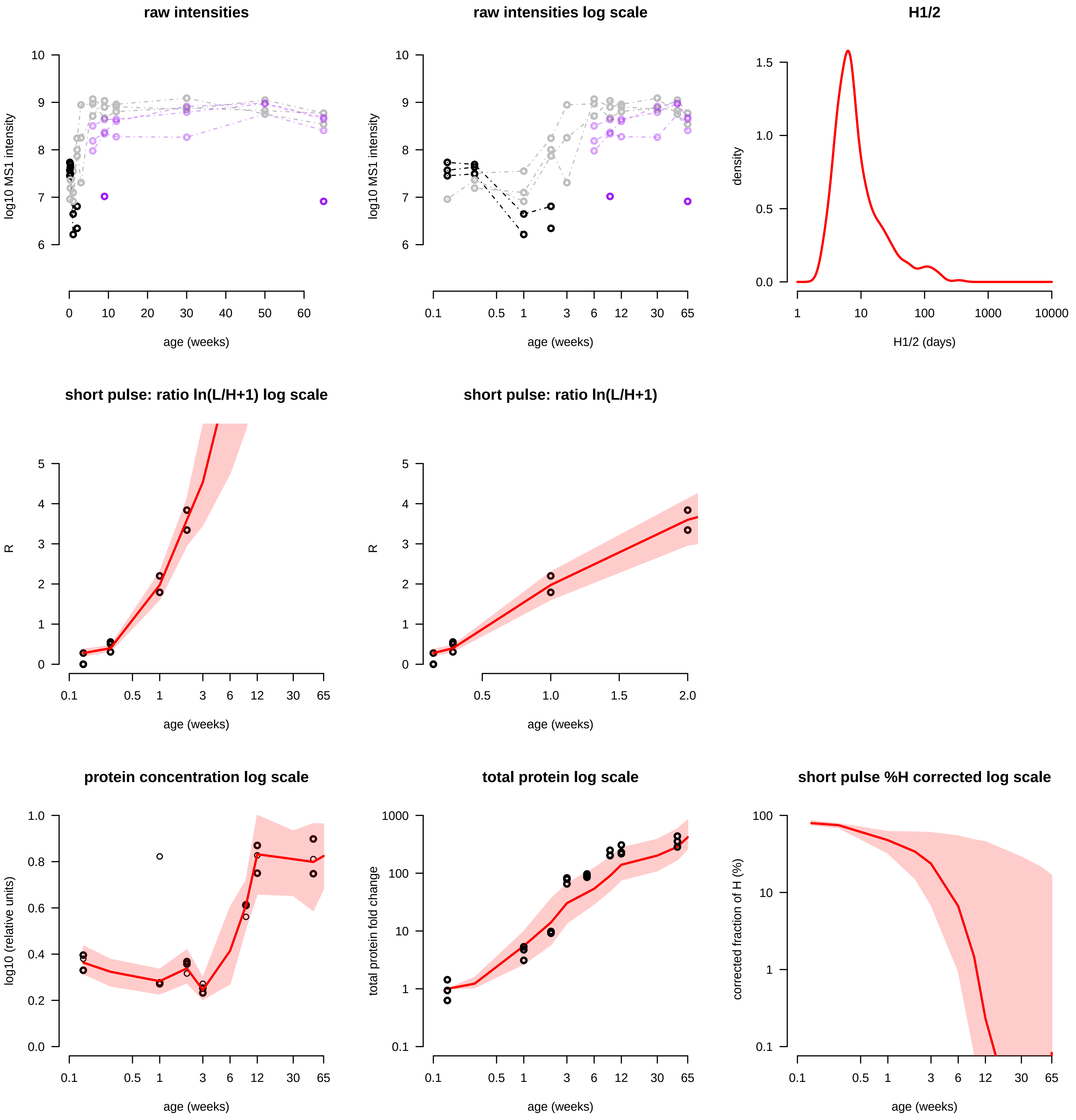

Q60649

>sp|Q60649|CLPB_MOUSE

Caseinolytic peptidase B protein homolog

GN=Clpb

Med H1/2: 4.8 days

Q5 H1/2: 3.7 days

Q95 H1/2: 8 days

reliable model fit: yes

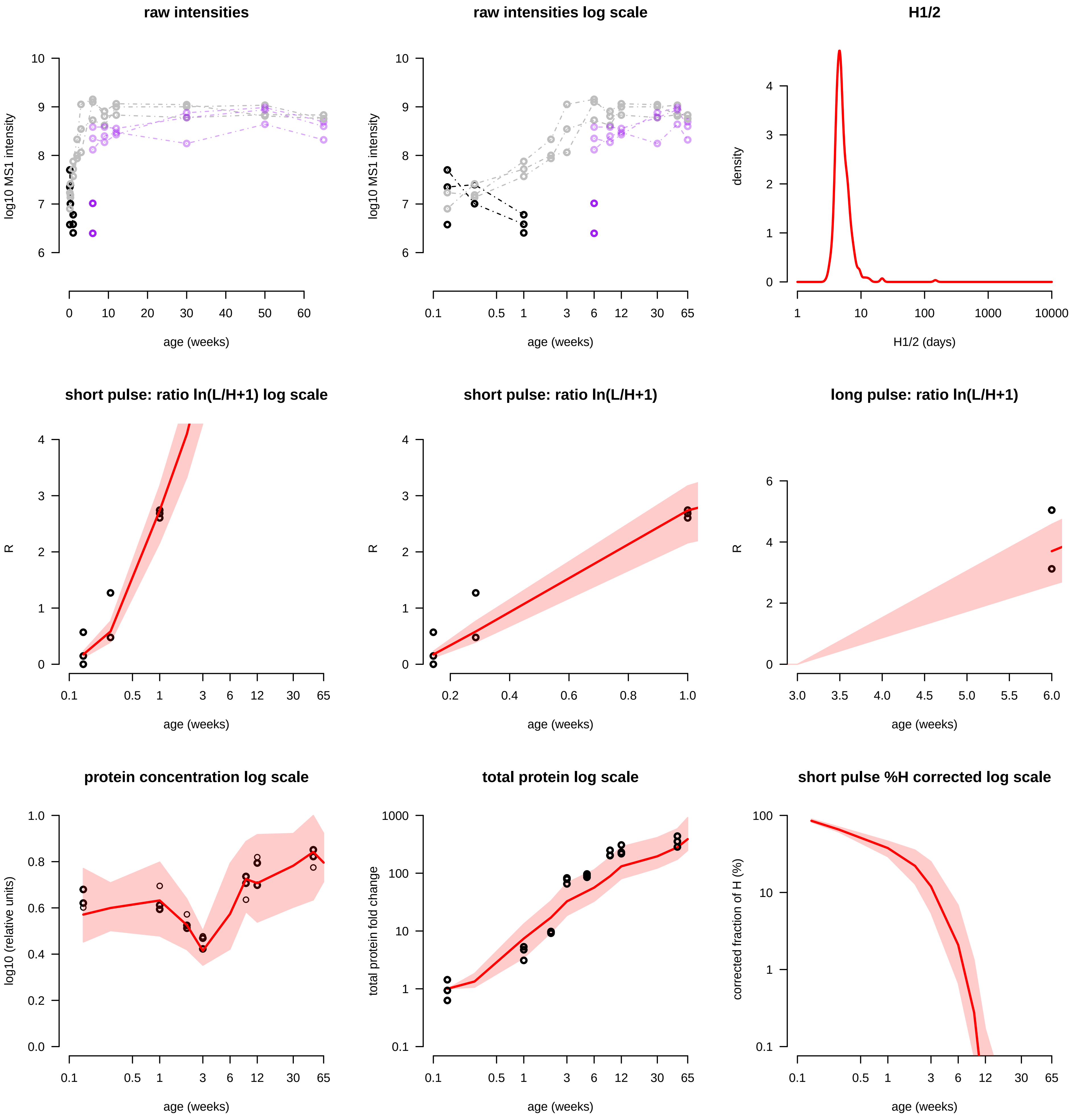

Q69Z99

>sp|Q69Z99|ZN512_MOUSE

Zinc finger protein 512

GN=Znf512

Med H1/2: 6 days

Q5 H1/2: 4.2 days

Q95 H1/2: 11.5 days

reliable model fit: yes

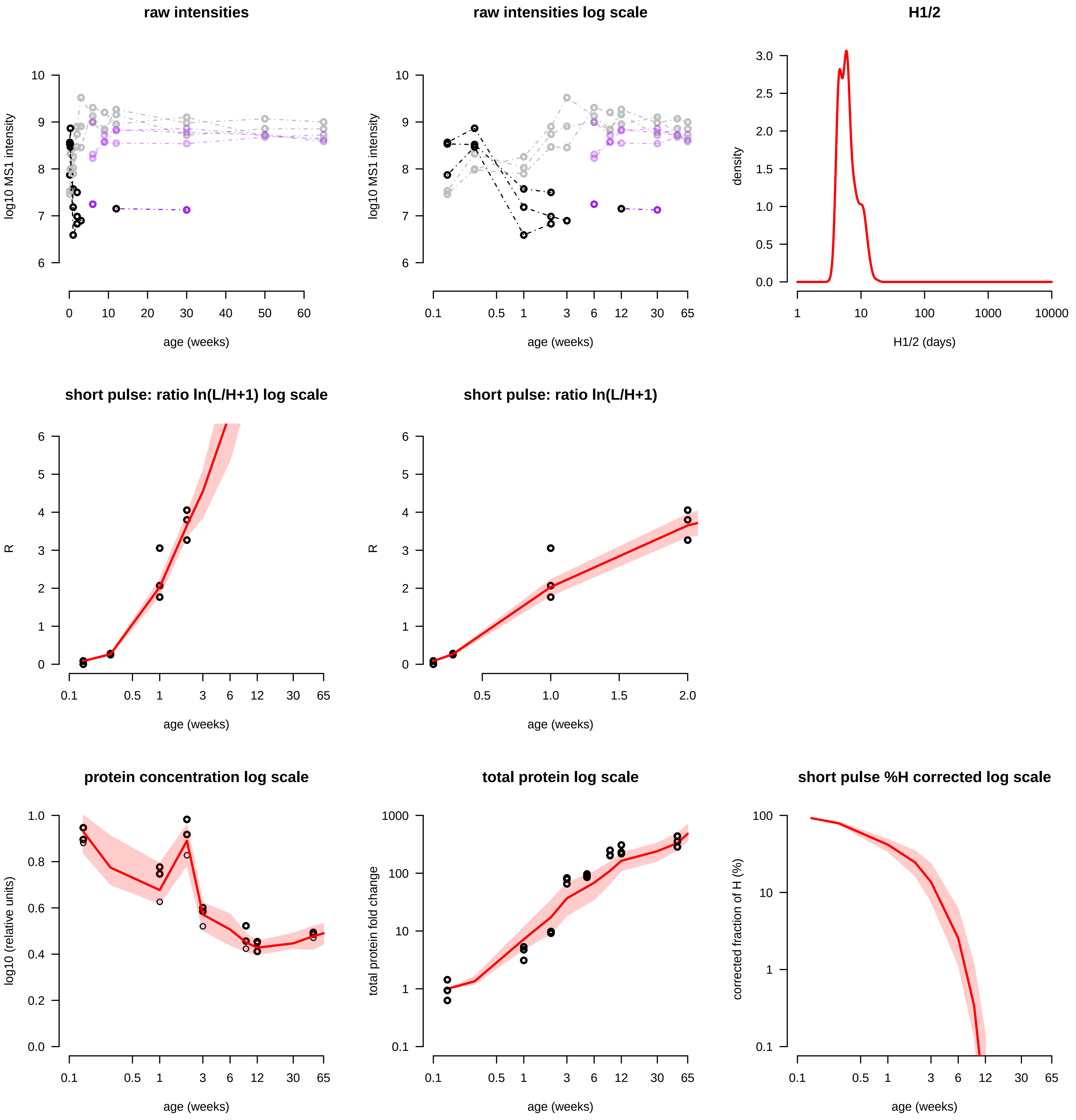

O70566

>sp|O70566|DIAP2_MOUSE

Protein diaphanous homolog 2

GN=Diaph2

Med H1/2: 114 days

Q5 H1/2: 40.5 days

Q95 H1/2: 1084.4 days

reliable model fit: yes

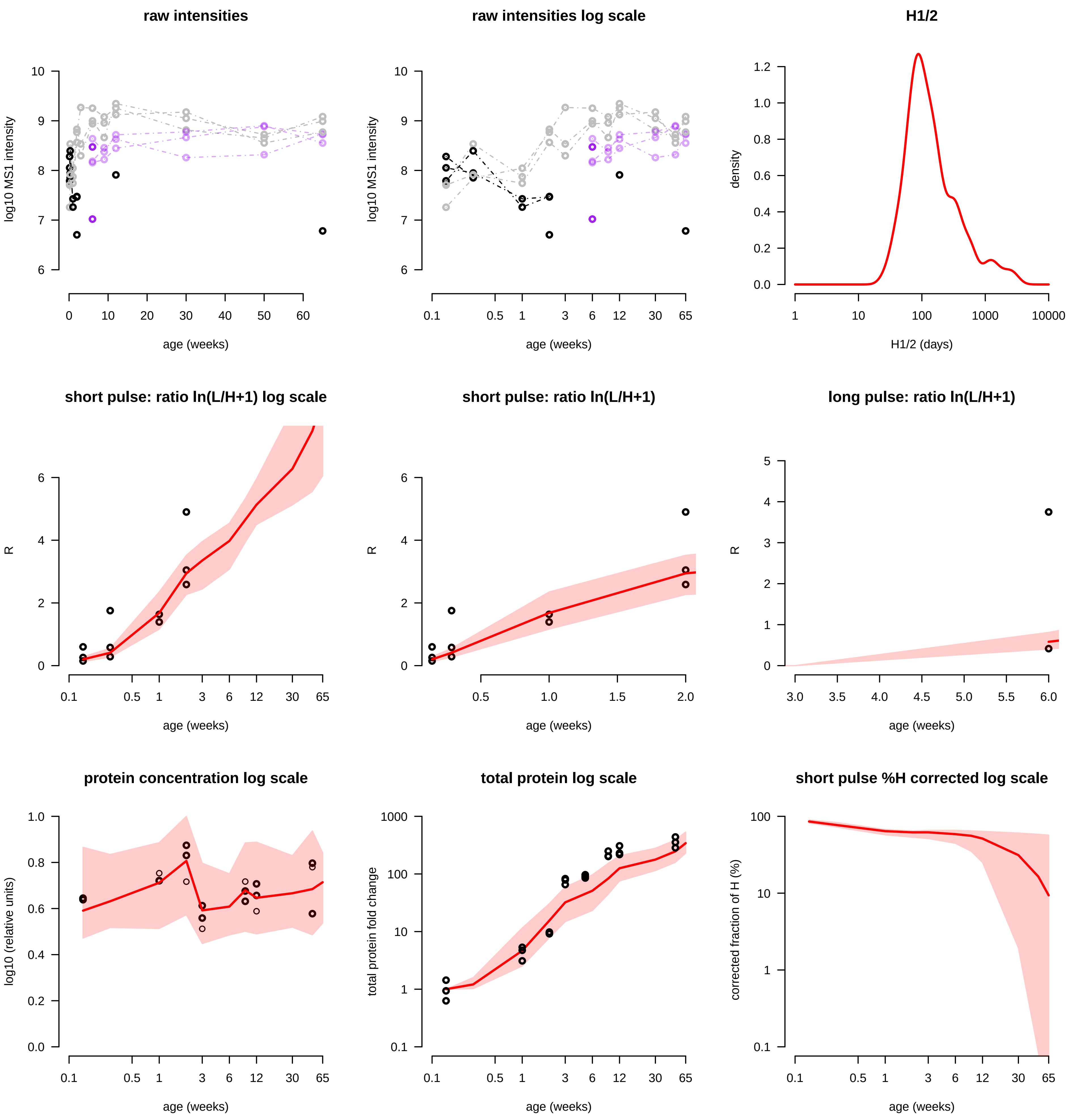

‌9JMH9−6;Q9JMH9−1;Q9JMH9−4;Q9JMH9−2;Q9JMH9−5;‌

>sp|Q9JMH9|MY18A_MOUSE

Unconventional myosin−XVIIIa

GN=Myo18a

Med H1/2: 3.5 days

Q5 H1/2: 2.6 days

Q95 H1/2: 4.9 days

reliable model fit: yes

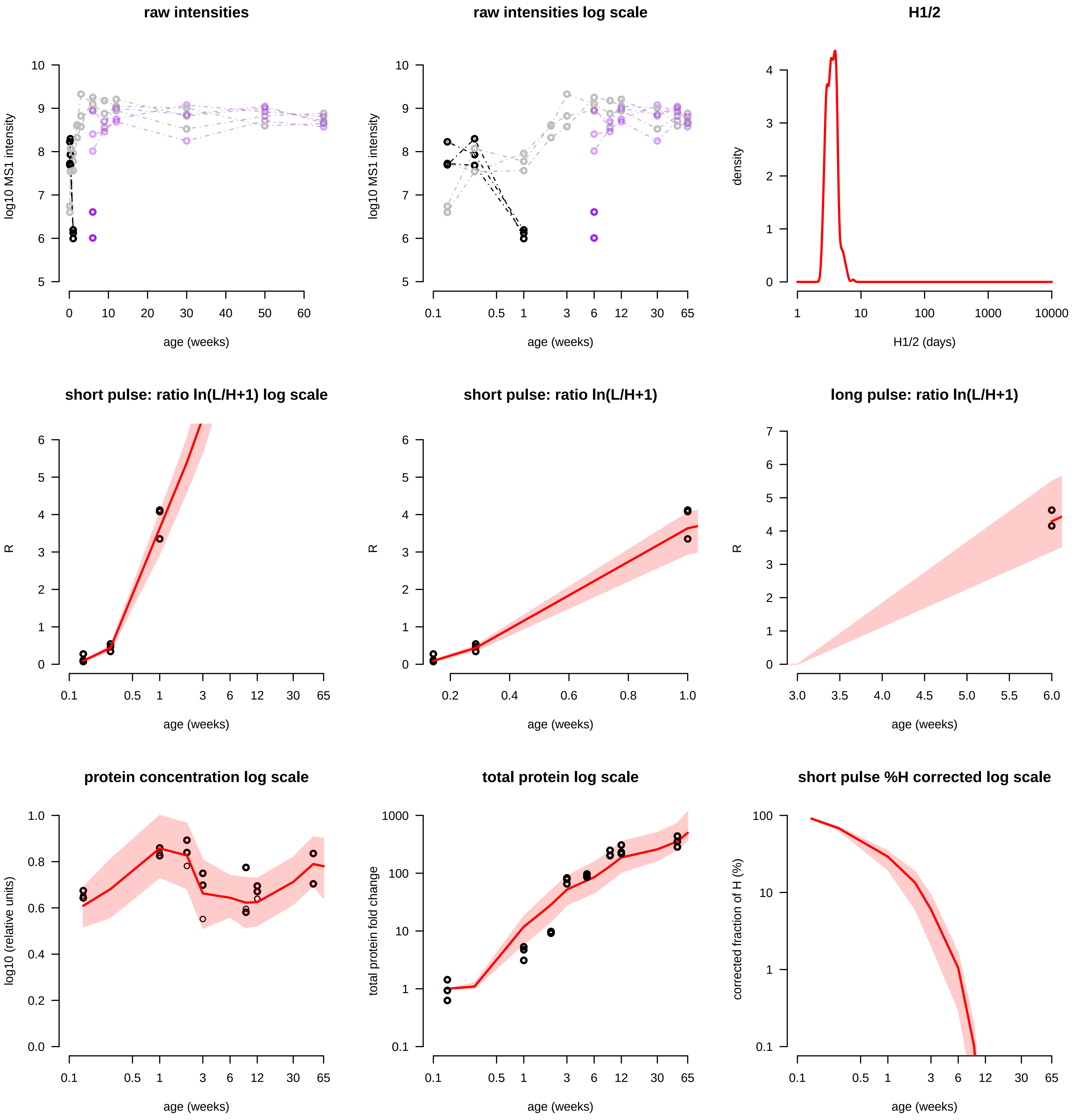

Q9JMH6;Q9JMH6−2

>sp|Q9JMH6|TRXR1_MOUSE

Thioredoxin reductase 1, cytoplasmic

GN=Txnrd1

Med H1/2: 4.8 days

Q5 H1/2: 4 days

Q95 H1/2: 5.8 days

reliable model fit: yes

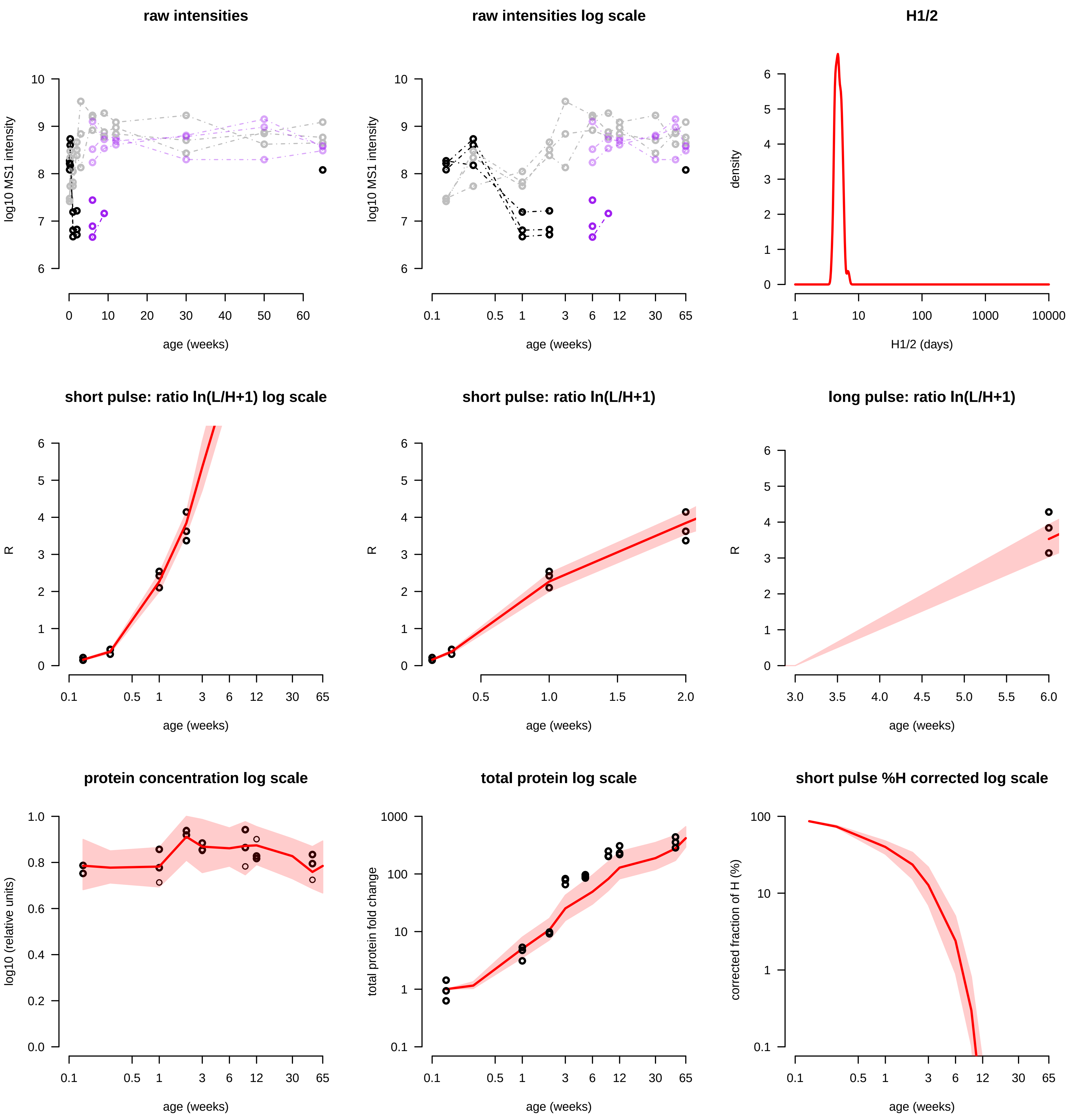

Q6ZQ58

>sp|Q6ZQ58|LARP1_MOUSE

La−related protein 1

GN=Larp1

Med H1/2: 3.6 days

Q5 H1/2: 2.8 days

Q95 H1/2: 4.9 days

reliable model fit: yes

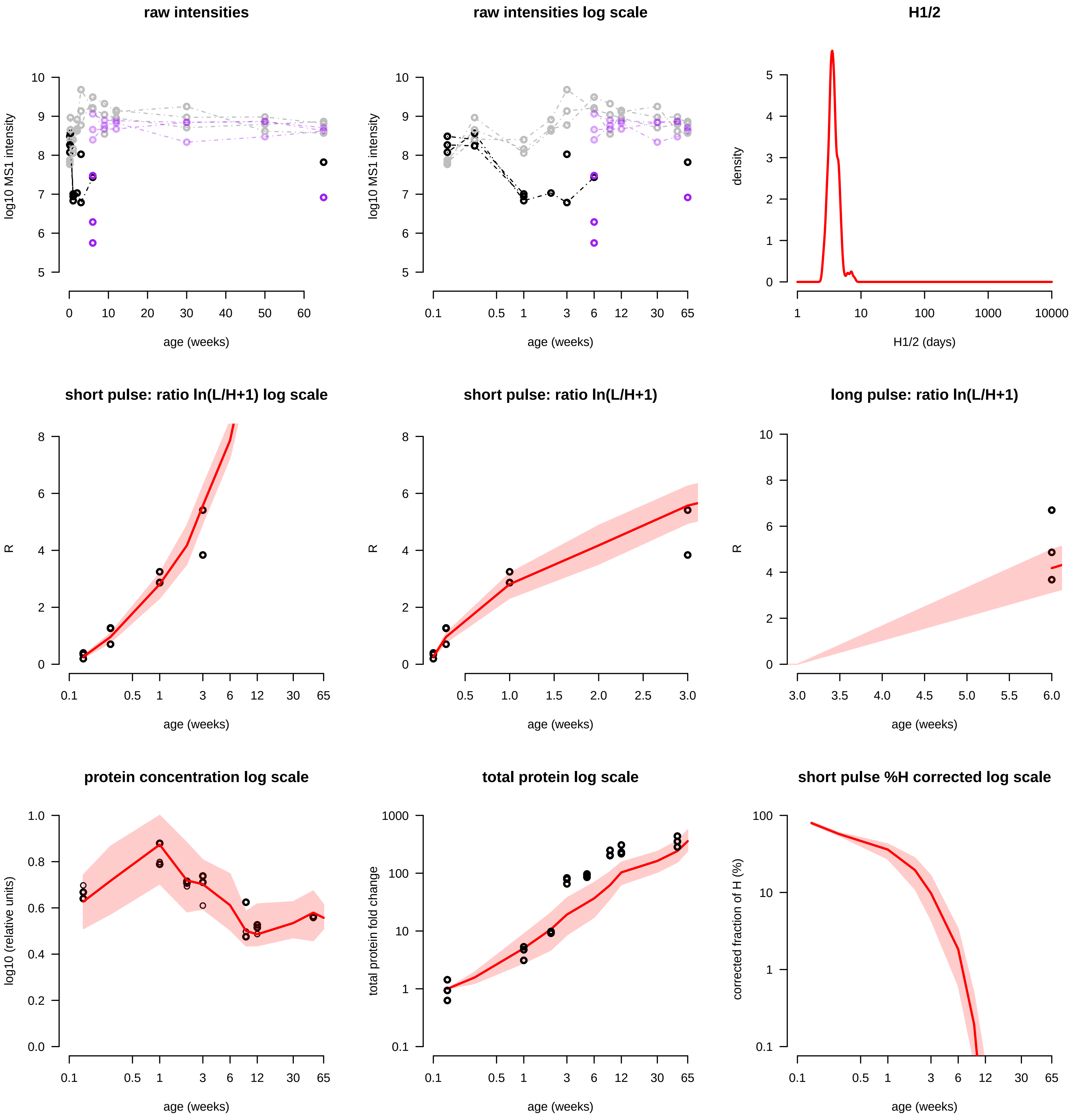

O55126

>sp|O55126|NIPS2_MOUSE

Protein NipSnap homolog 2

GN=Nipsnap2

Med H1/2: 8.5 days

Q5 H1/2: 6.5 days

Q95 H1/2: 14 days

reliable model fit: yes

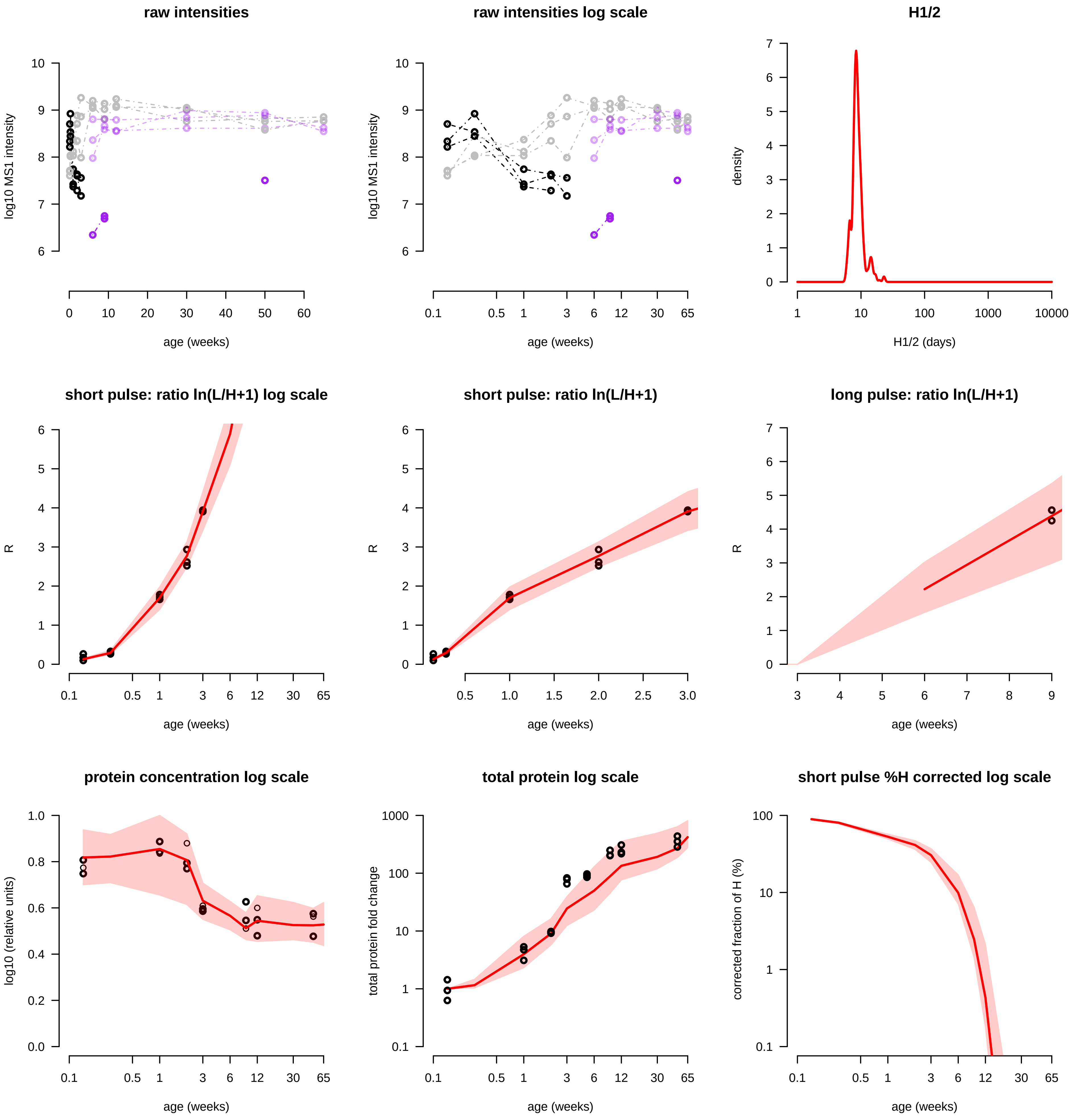

Q922P9

>sp|Q922P9|GLYR1_MOUSE

Putative oxidoreductase GLYR1

GN=Glyr1

Med H1/2: 5.6 days

Q5 H1/2: 4.6 days

Q95 H1/2: 7.5 days

reliable model fit: yes

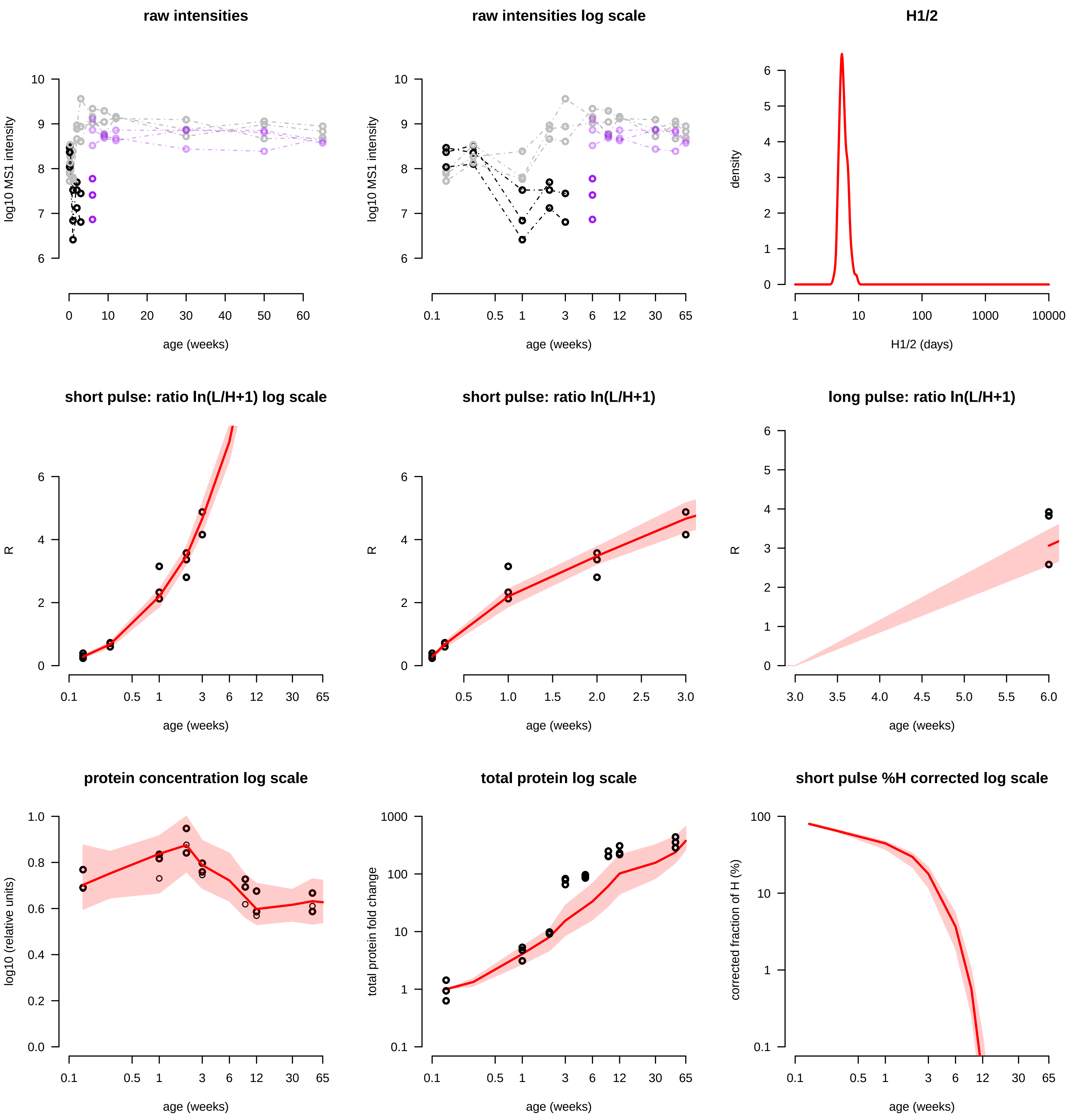

Q9QYB5−2

>sp|Q9QYB5−2|ADDG_MOUSE

Isoform 1 of Gamma−adducin

GN=Add3

Med H1/2: 4.4 days

Q5 H1/2: 3.4 days

Q95 H1/2: 6.3 days

reliable model fit: yes

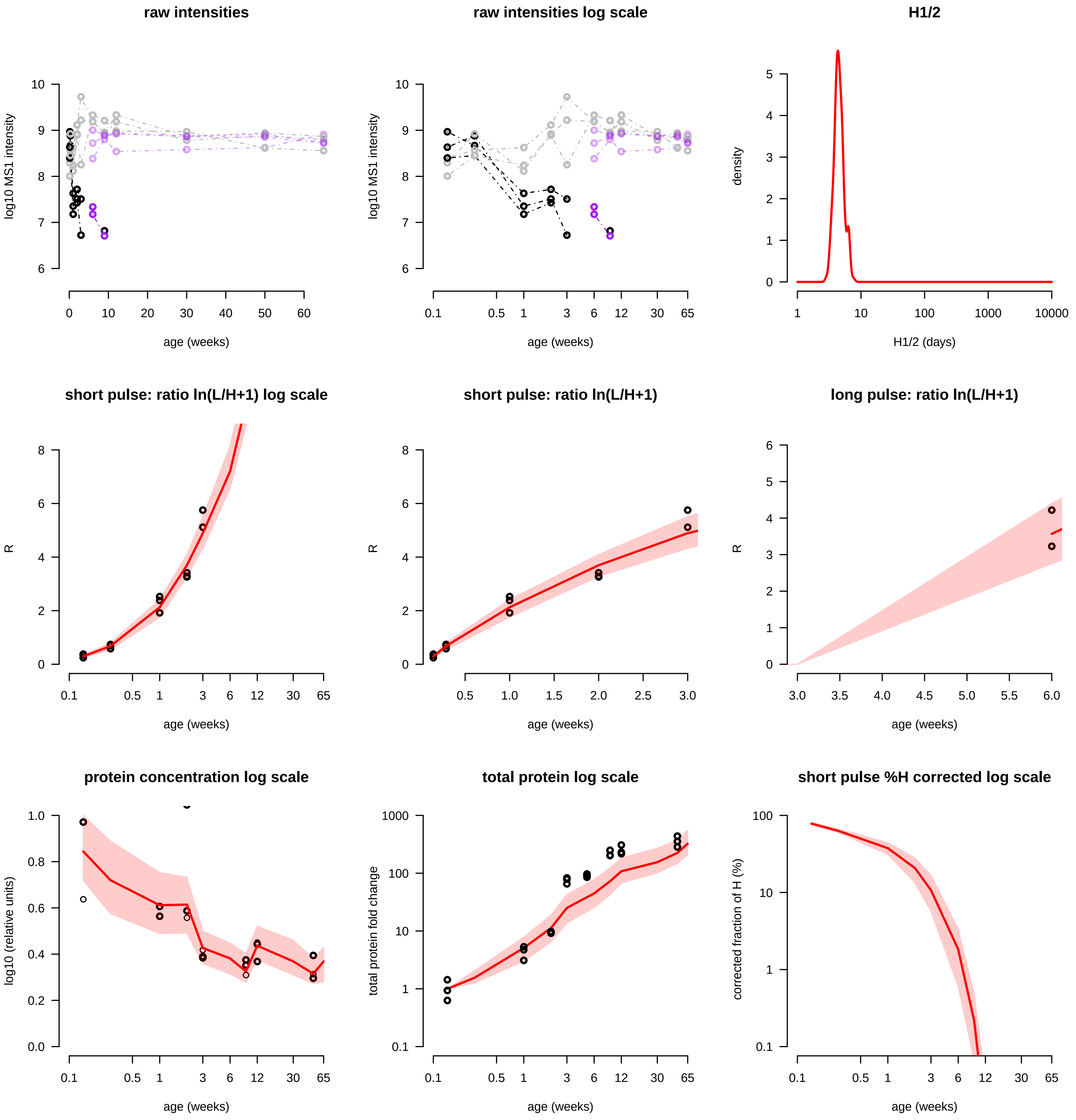

Q8BJ03−2;Q8BJ03

>sp|Q8BJ03−2|COX15_MOUSE

Isoform 2 of Cytochrome c oxidase assembly protein COX15 homolog

GN=Cox15

Med H1/2: 4.9 days

Q5 H1/2: 3.2 days

Q95 H1/2: 7.5 days

reliable model fit: yes

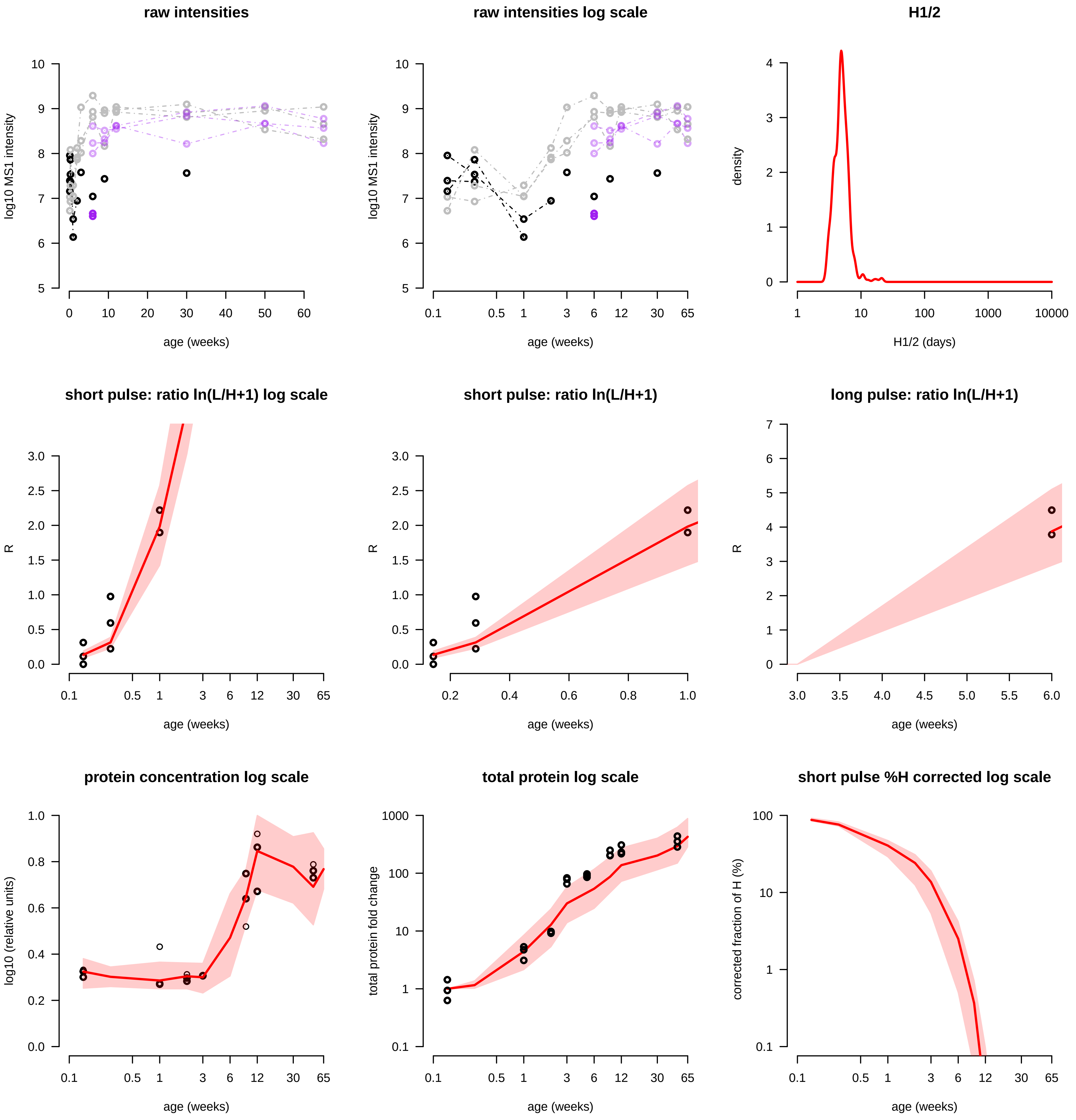

O09106

>sp|O09106|HDAC1_MOUSE

Histone deacetylase 1

GN=Hdac1

Med H1/2: 2642.3 days

Q5 H1/2: 731 days

Q95 H1/2: 19860.7 days

reliable model fit: yes

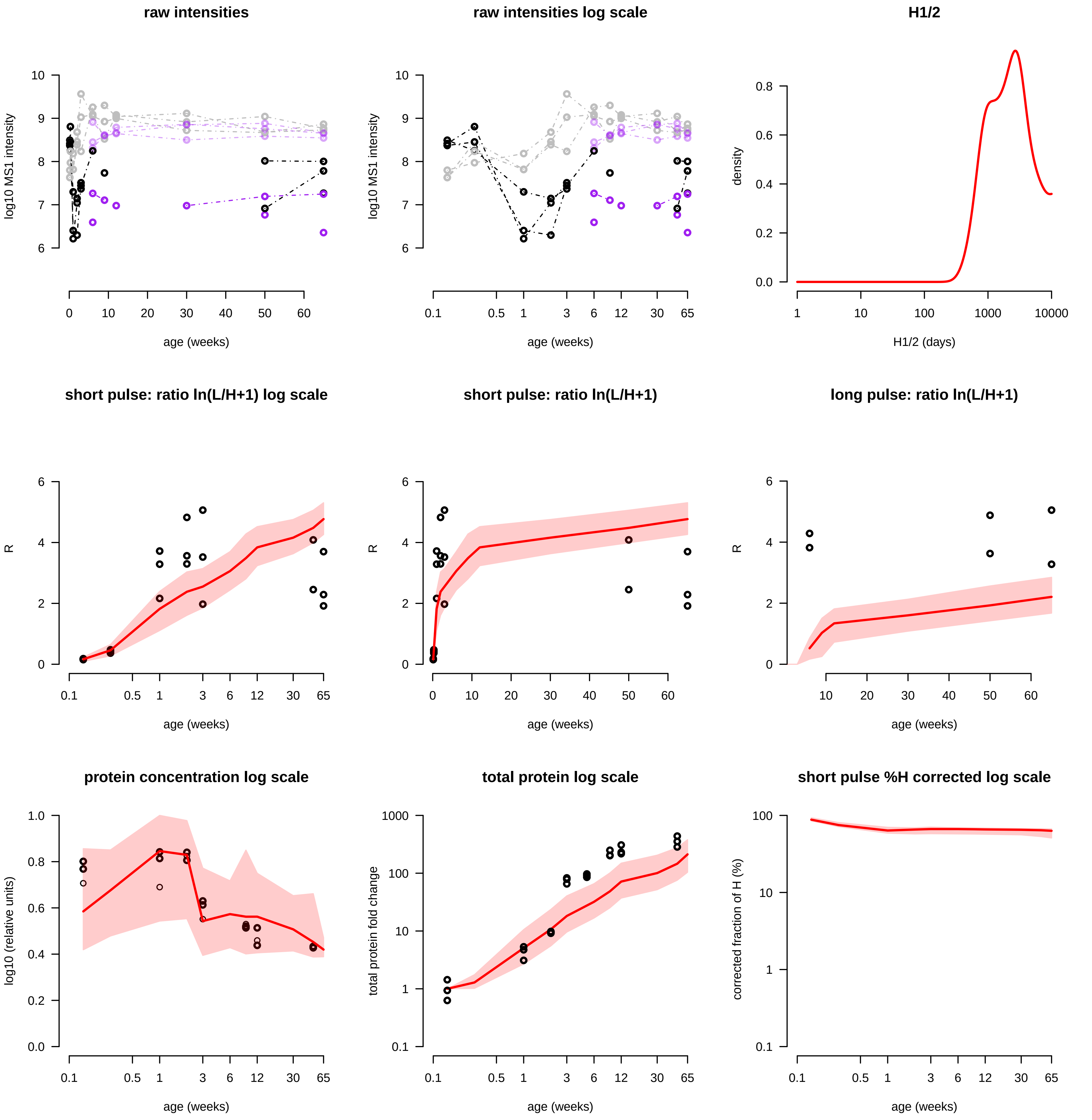

Q9R190

>sp|Q9R190|MTA2_MOUSE

Metastasis−associated protein MTA2

GN=Mta2

Med H1/2: 5.2 days

Q5 H1/2: 2.9 days

Q95 H1/2: 19.1 days

reliable model fit: yes

raw intensities

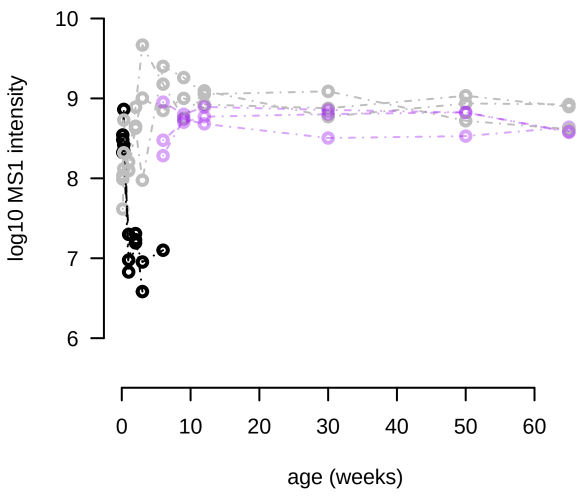

raw intensities log scale

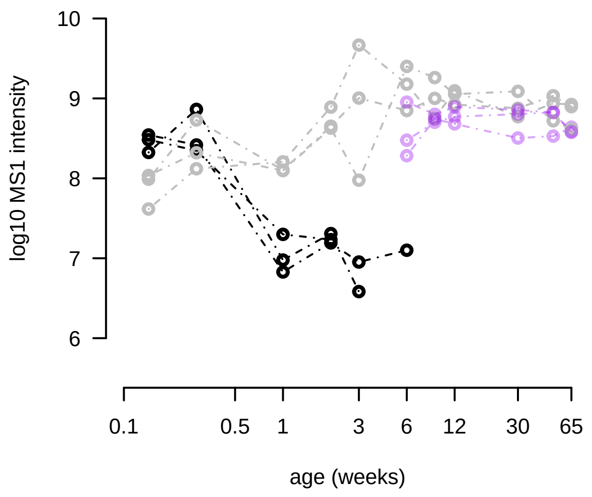

H1/2

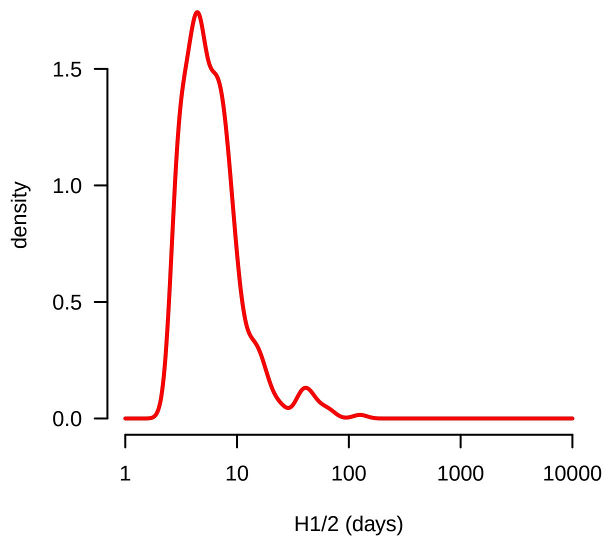

short pulse: ratio ln(L/H+1) log scale

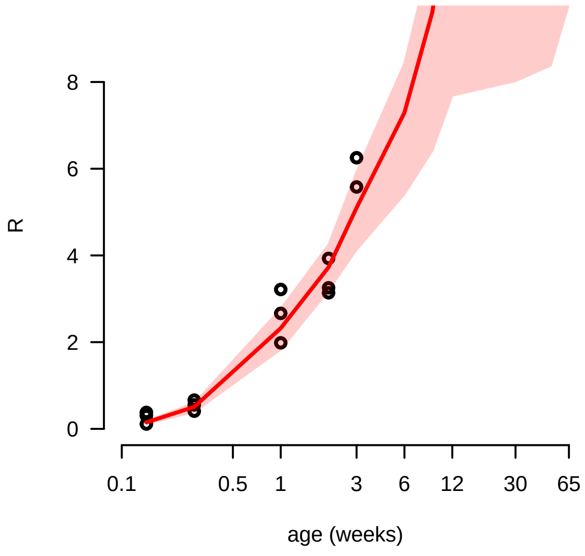

short pulse: ratio ln(L/H+1)

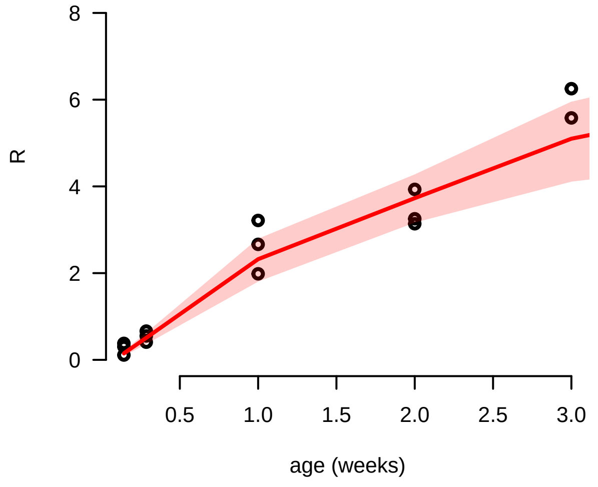

protein concentration log scale

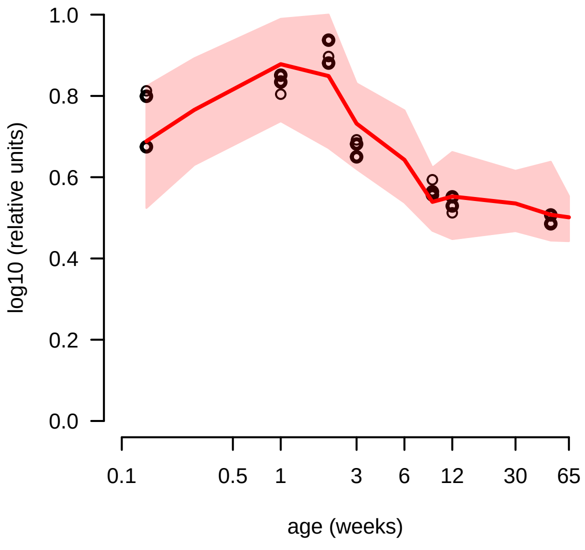

total protein log scale

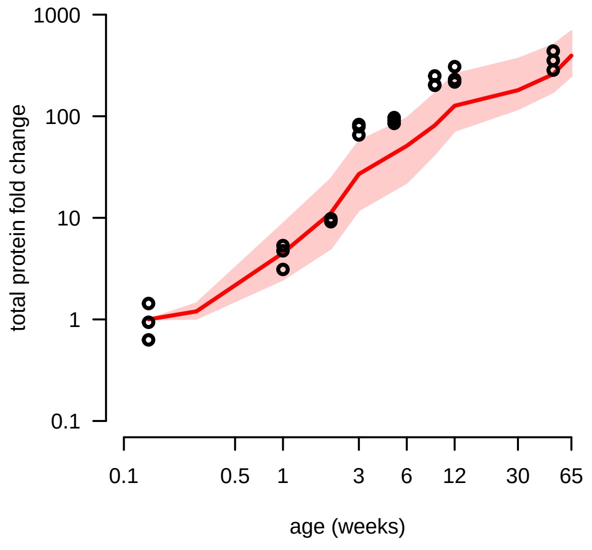

short pulse %H corrected log scale

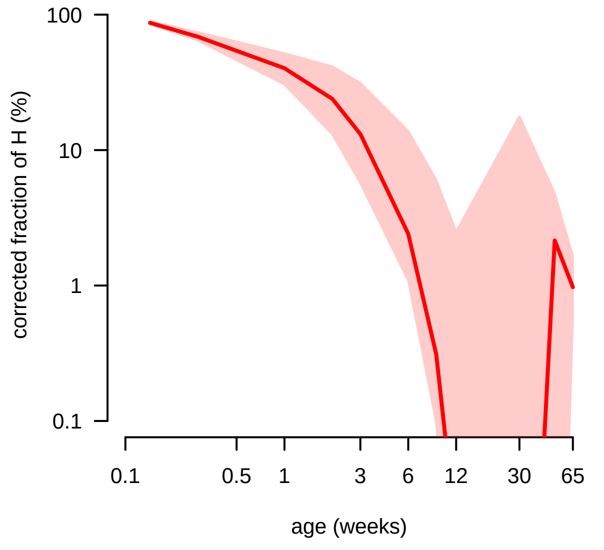

Q6IRU5−2;Q6IRU5;Q6IRU5−3

>sp|Q6IRU5−2|CLCB_MOUSE

Isoform 2 of Clathrin light chain B

GN=Cltb

Med H1/2: 35.3 days

Q5 H1/2: 15.5 days

Q95 H1/2: 249 days

reliable model fit: yes

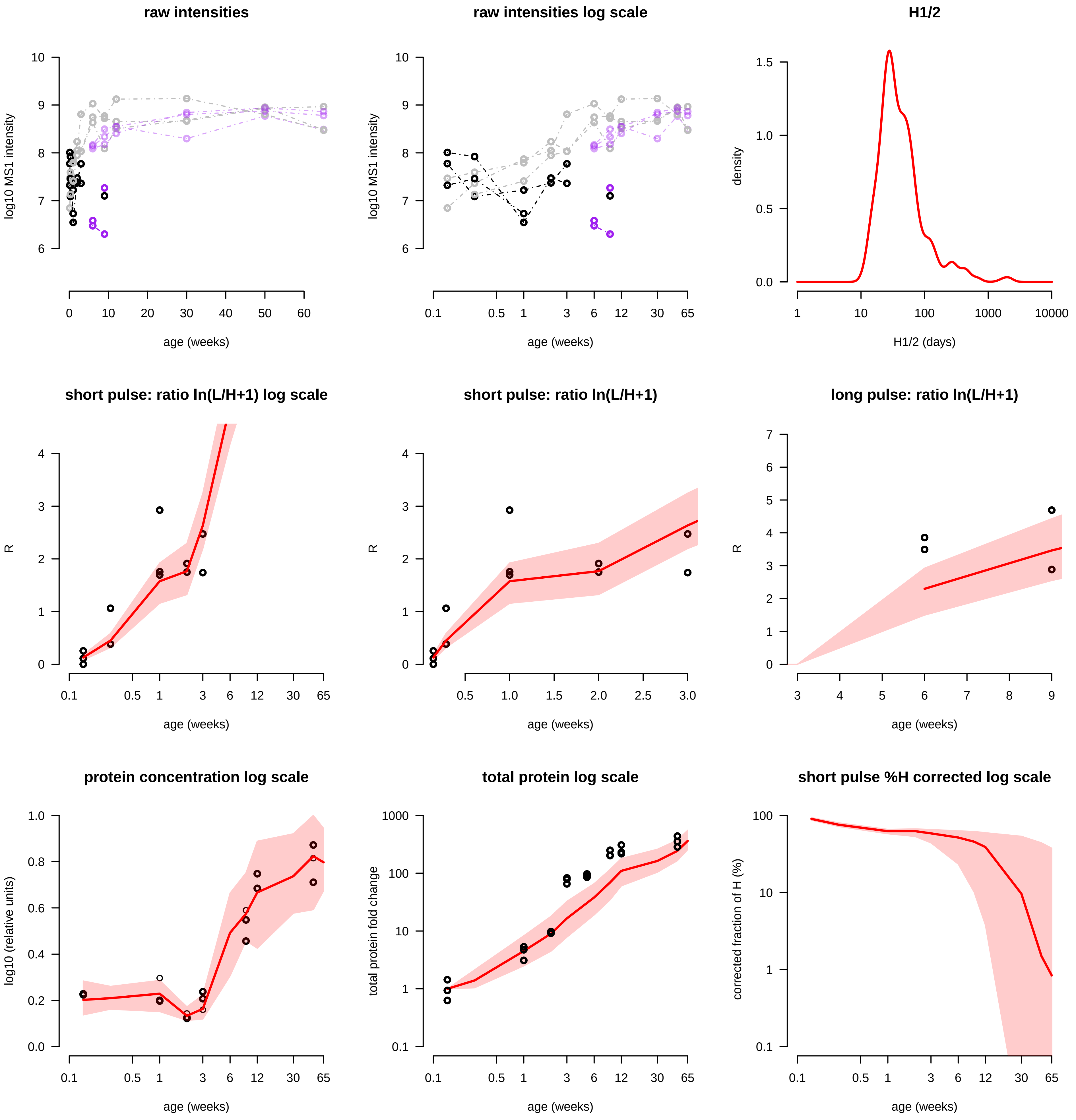

P46664

>sp|P46664|PURA2_MOUSE

Adenylosuccinate synthetase isozyme 2

GN=Adss2

Med H1/2: 4.9 days

Q5 H1/2: 4.1 days

Q95 H1/2: 6.2 days

reliable model fit: yes

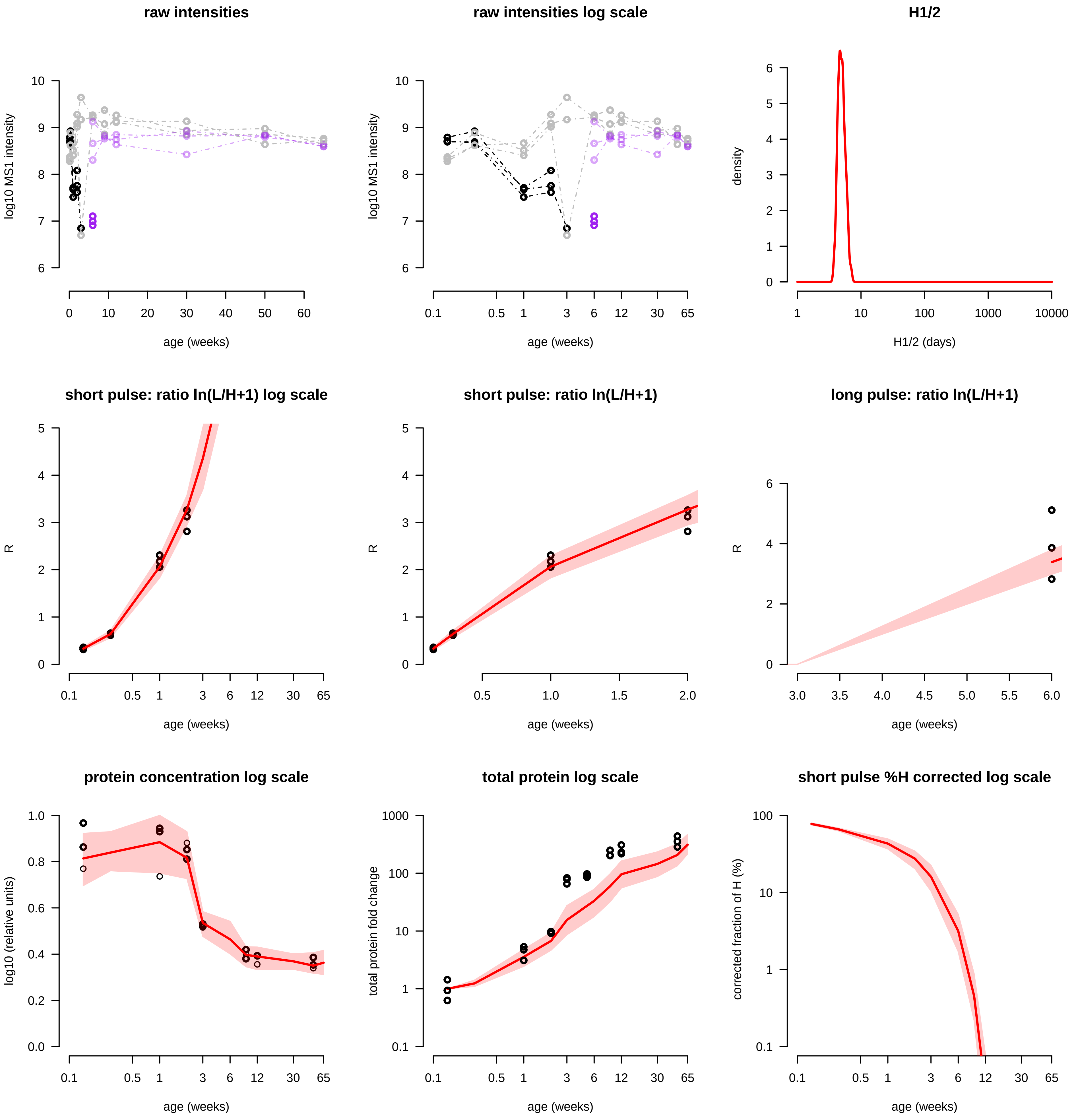

Q9D0L8;Q9D0L8−2;Q9D0L8−3

>sp|Q9D0L8|MCES_MOUSE

mRNA cap guanine−N7 methyltransferase

GN=Rnmt

Med H1/2: 3.9 days

Q5 H1/2: 3 days

Q95 H1/2: 5 days

reliable model fit: yes

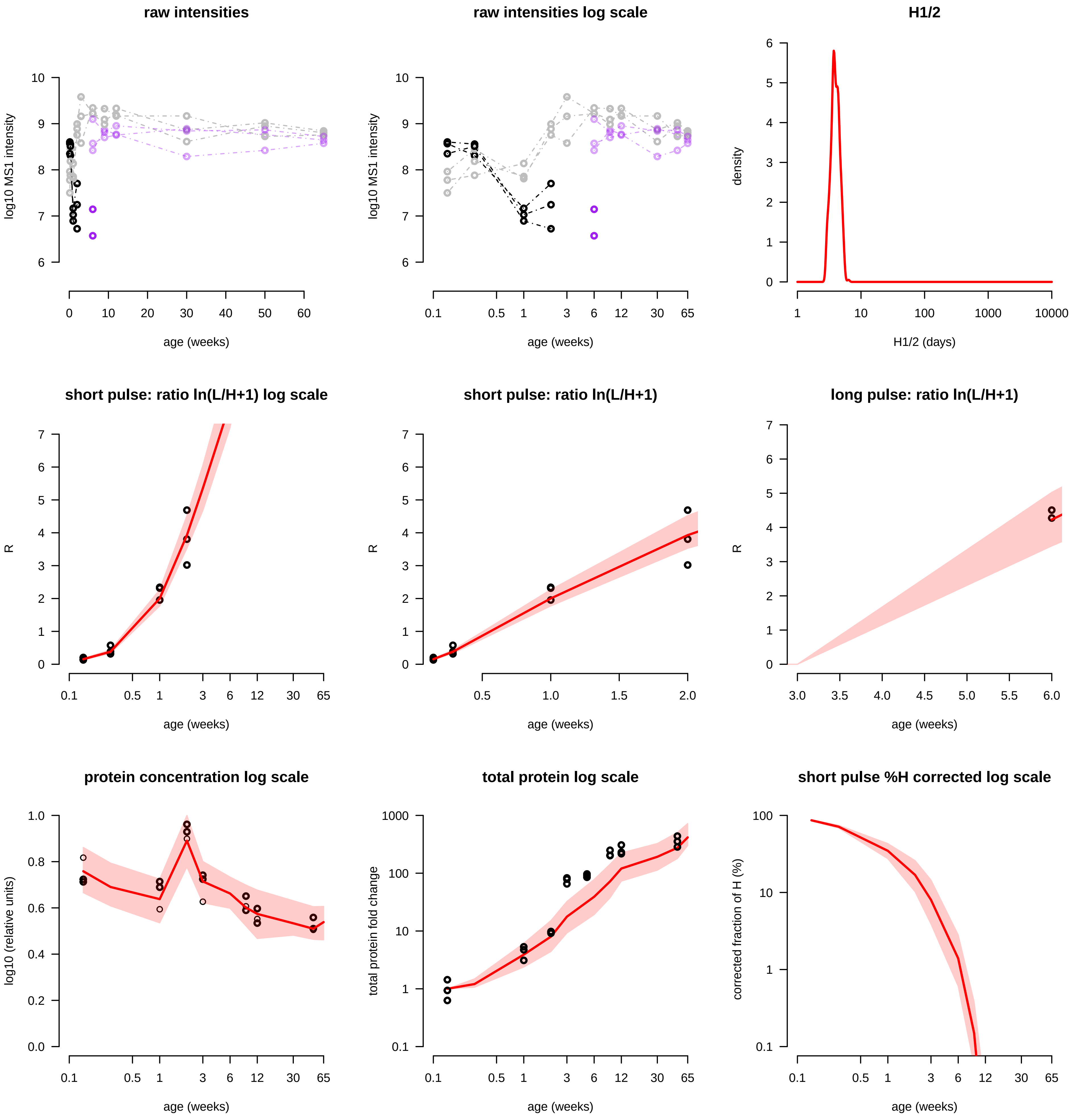

Q8BLN5

>sp|Q8BLN5|LSS_MOUSE

Lanosterol synthase

GN=Lss

Med H1/2: 1312 days

Q5 H1/2: 304.4 days

Q95 H1/2: 14605.6 days

reliable model fit: no

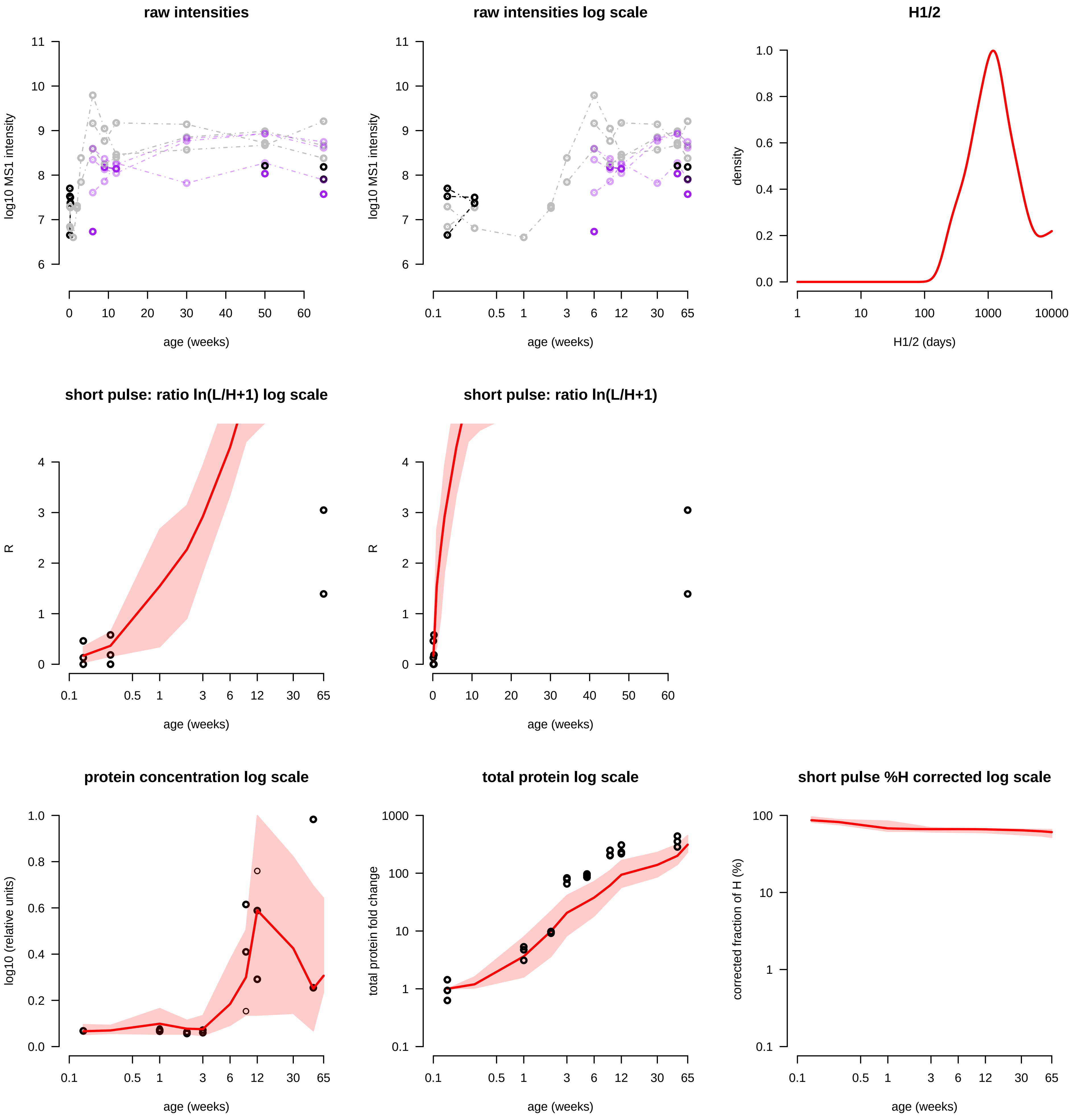

Q6NZC7

>sp|Q6NZC7|S23IP_MOUSE

SEC23−interacting protein

GN=Sec23ip

Med H1/2: 12.8 days

Q5 H1/2: 4.8 days

Q95 H1/2: 139 days

reliable model fit: yes

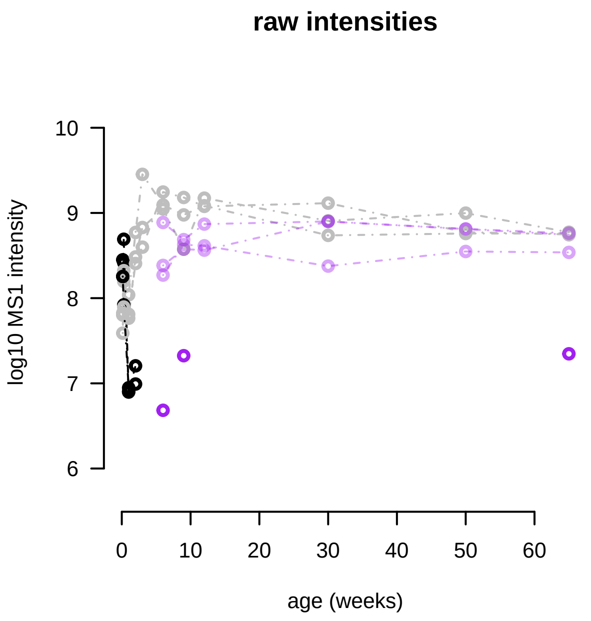

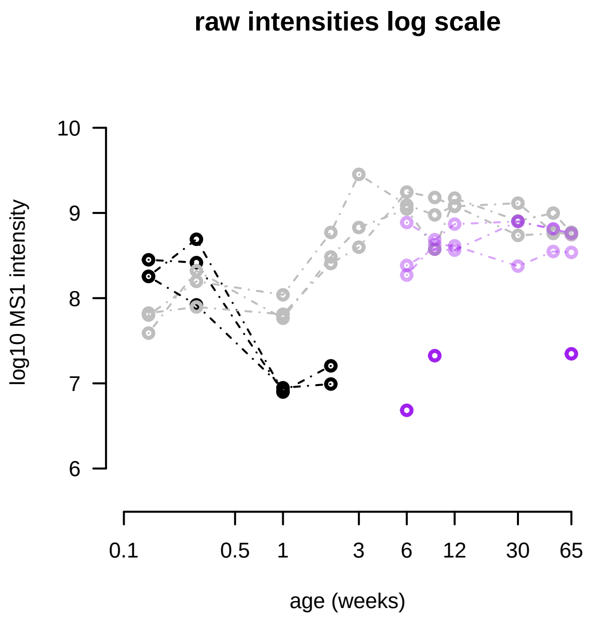

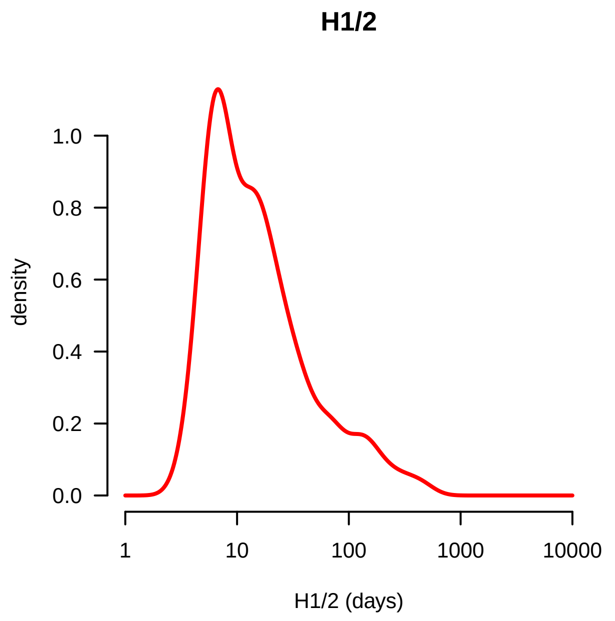

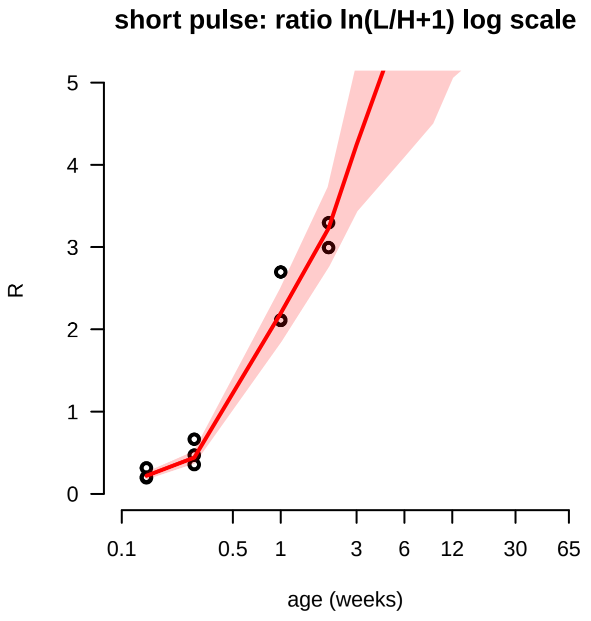

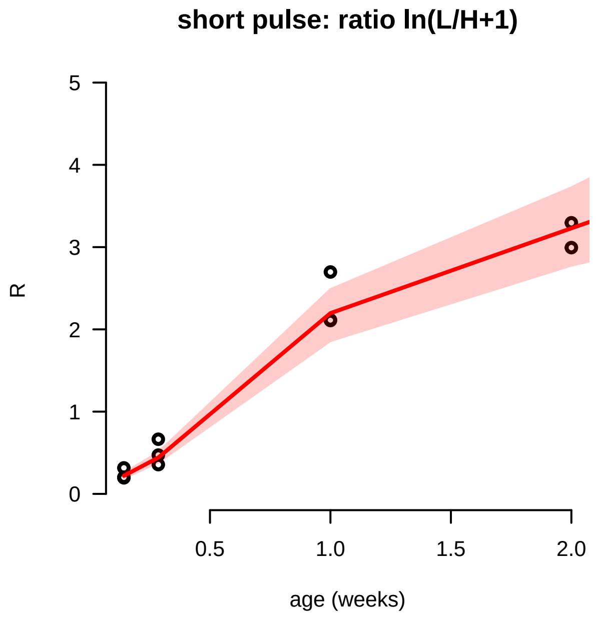

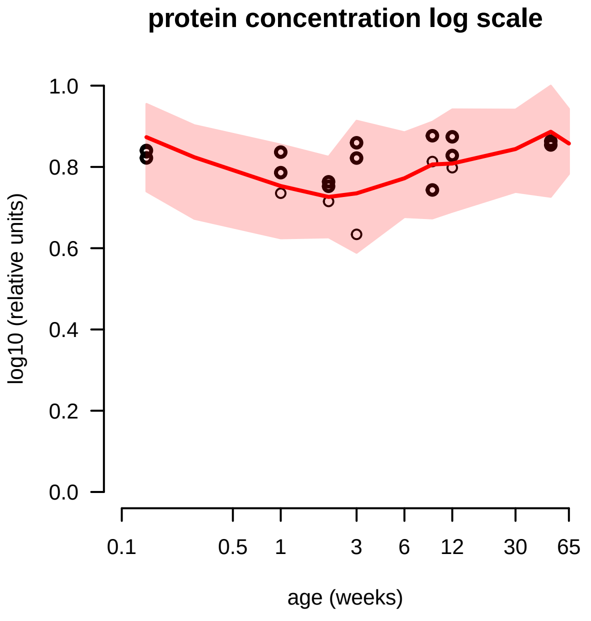

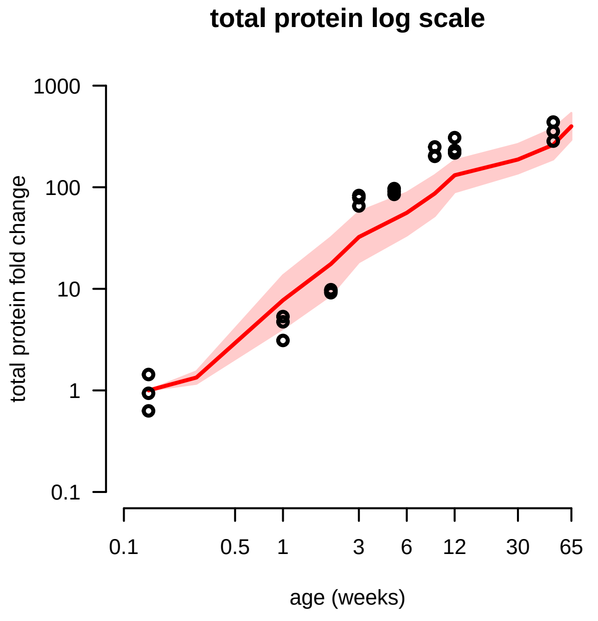

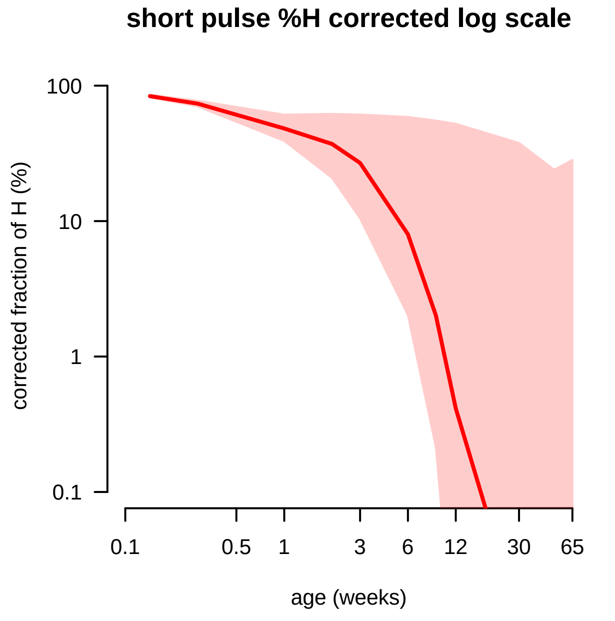

Q9DCS9

>sp|Q9DCS9|NDUBA_MOUSE

NADH dehydrogenase [ubiquinone] 1 beta subcomplex subunit 10

GN=Ndufb10

Med H1/2: 7.5 days

Q5 H1/2: 5.4 days

Q95 H1/2: 11.3 days

reliable model fit: yes

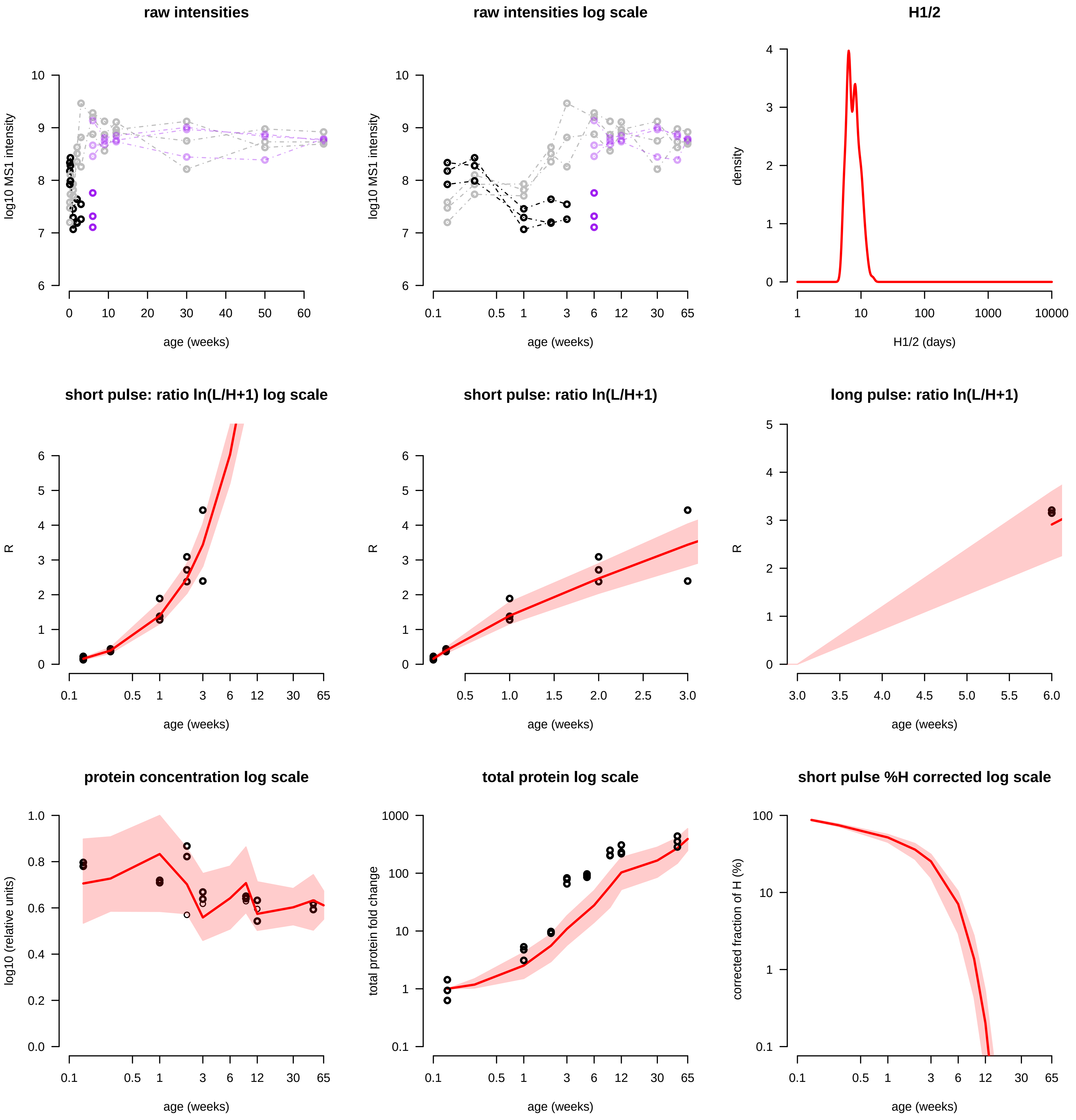

Q8BMA6

>sp|Q8BMA6|SRP68_MOUSE

Signal recognition particle subunit SRP68

GN=Srp68

Med H1/2: 268.4 days

Q5 H1/2: 126 days

Q95 H1/2: 1402.6 days

reliable model fit: no

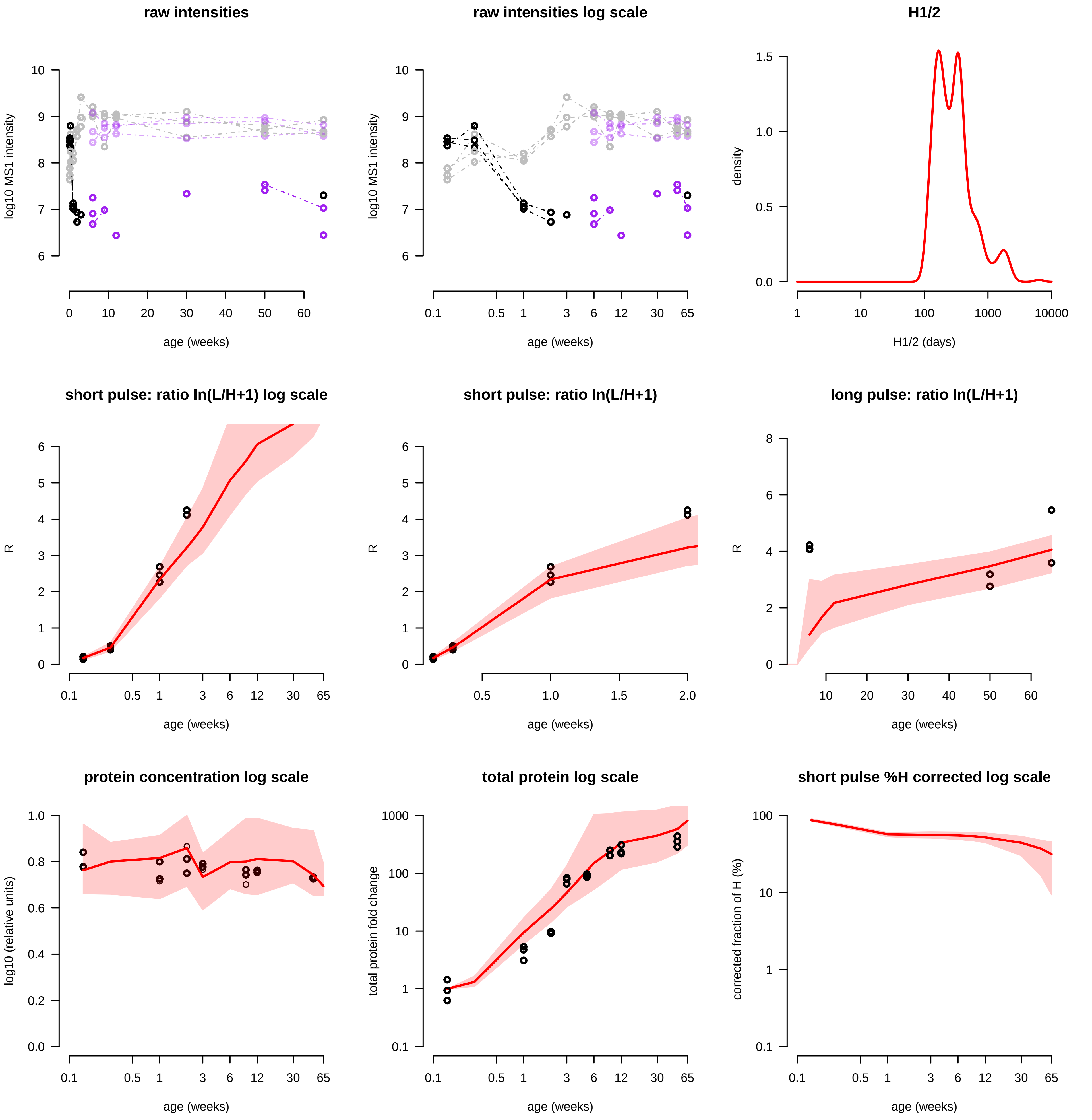

Q7TT37

>sp|Q7TT37|ELP1_MOUSE

Elongator complex protein 1

GN=Elp1

Med H1/2: 2.9 days

Q5 H1/2: 2.5 days

Q95 H1/2: 3.5 days

reliable model fit: yes

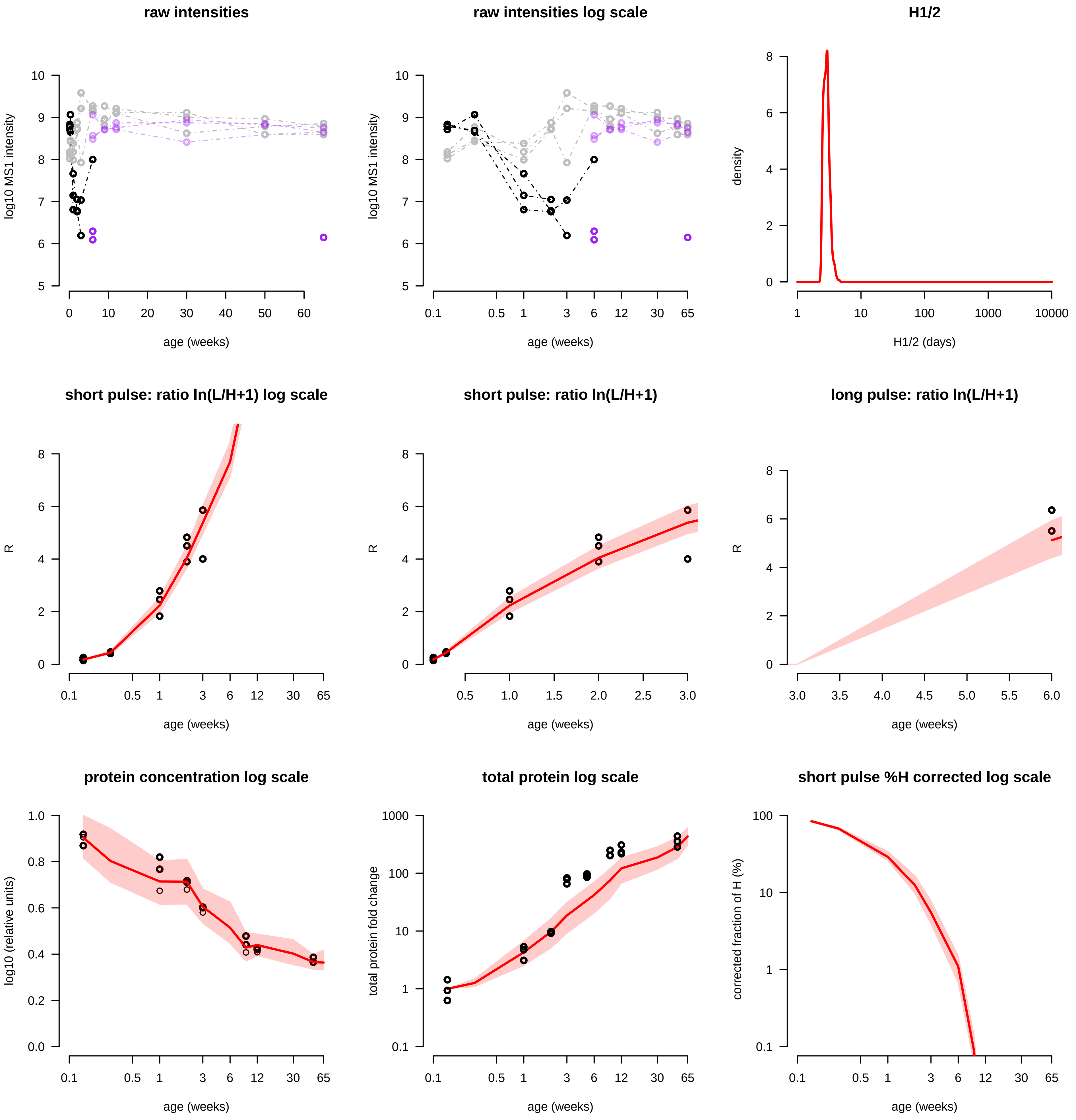

Q9DCZ4−2;Q9DCZ4;Q9DCZ4−3

>sp|Q9DCZ4−2|MIC26_MOUSE

Isoform 2 of MICOS complex subunit Mic26

GN=Apoo

Med H1/2: 6.5 days

Q5 H1/2: 3 days

Q95 H1/2: 77.2 days

reliable model fit: yes

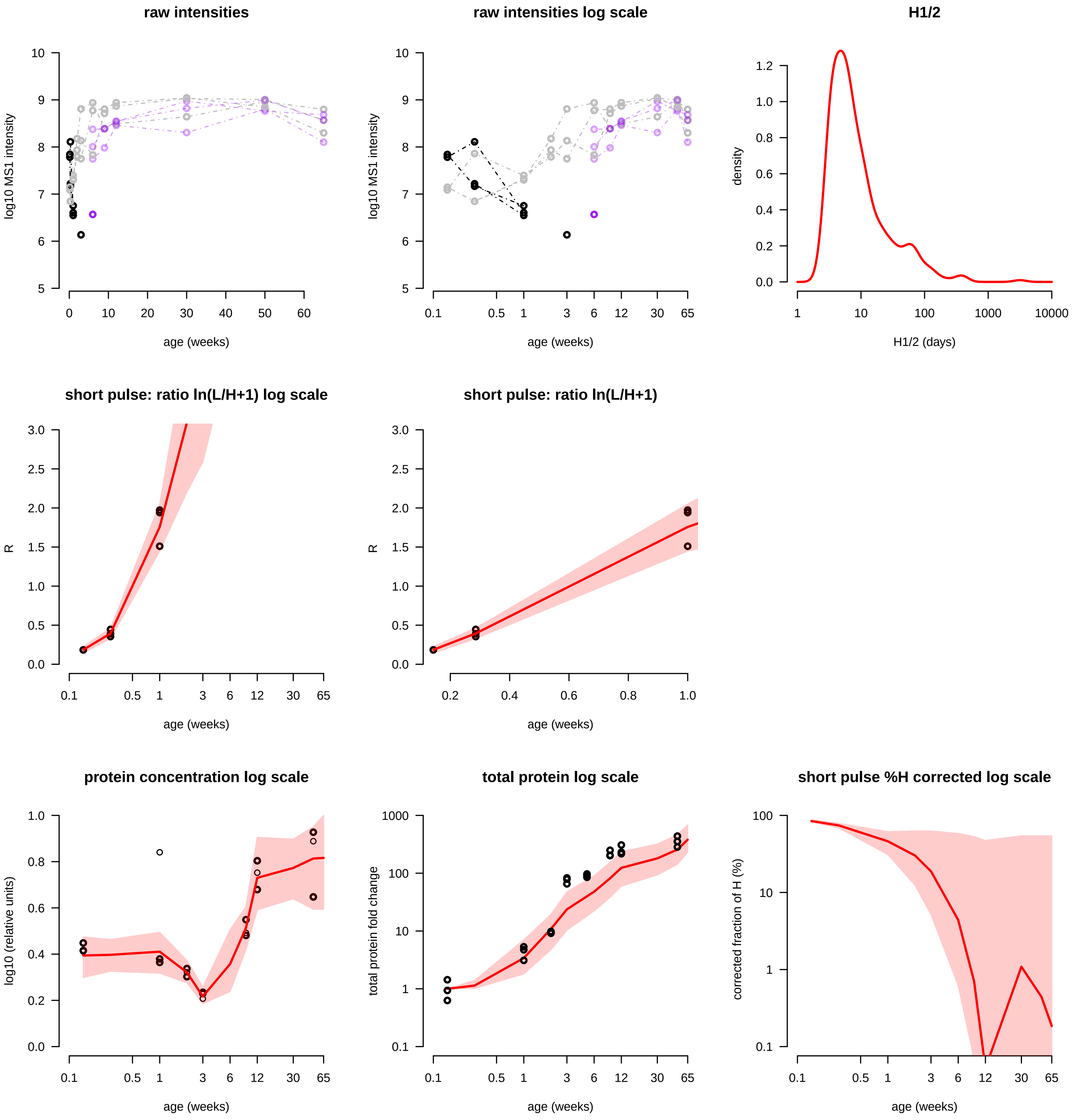

P35282

>sp|P35282|RAB21_MOUSE

Ras−related protein Rab−21

GN=Rab21

Med H1/2: 8.6 days

Q5 H1/2: 6.1 days

Q95 H1/2: 13.2 days

reliable model fit: yes

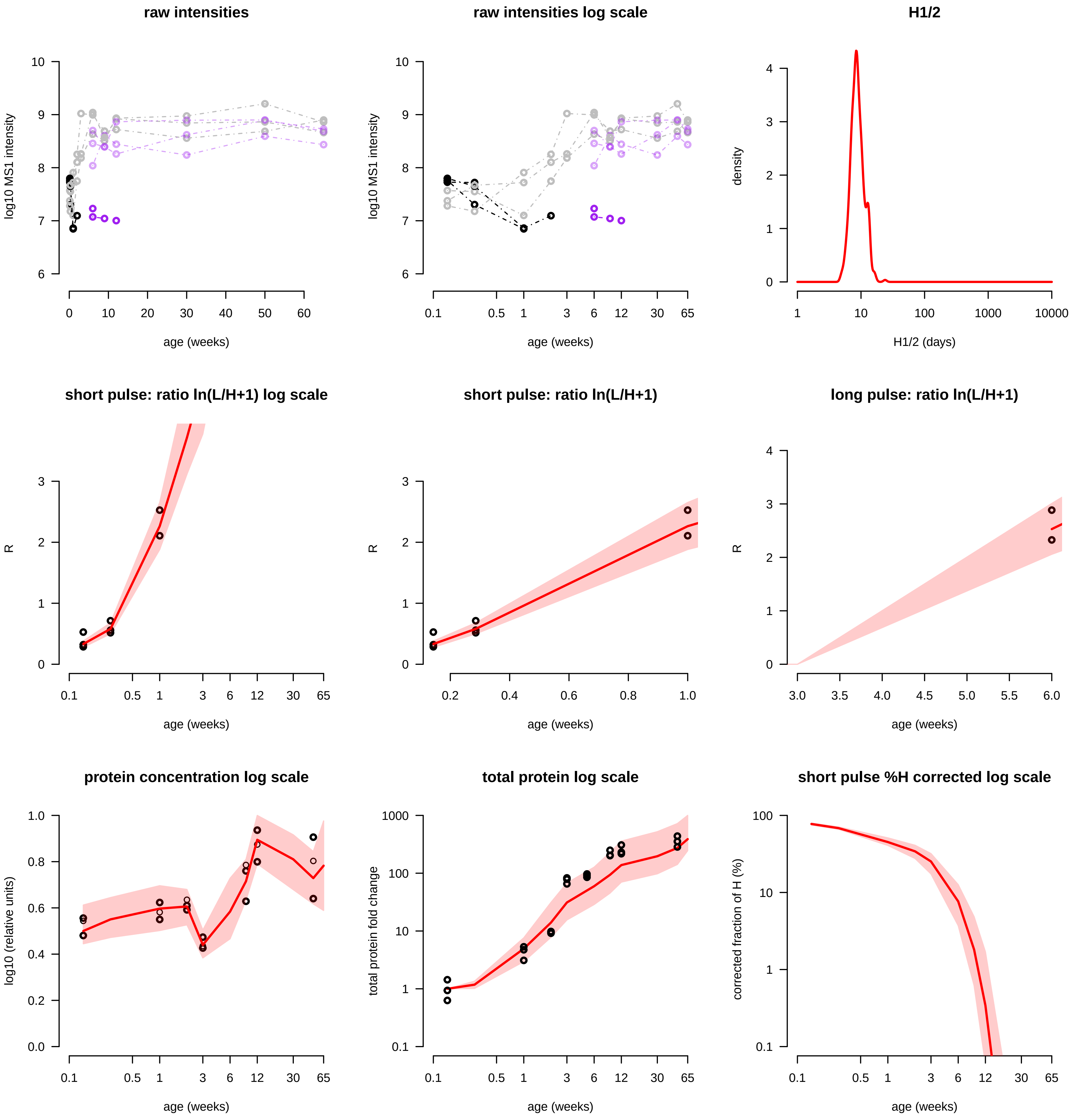

Q91YR9

>sp|Q91YR9|PTGR1_MOUSE

Prostaglandin reductase 1

GN=Ptgr1

Med H1/2: 178.6 days

Q5 H1/2: 95.7 days

Q95 H1/2: 807.5 days

reliable model fit: yes

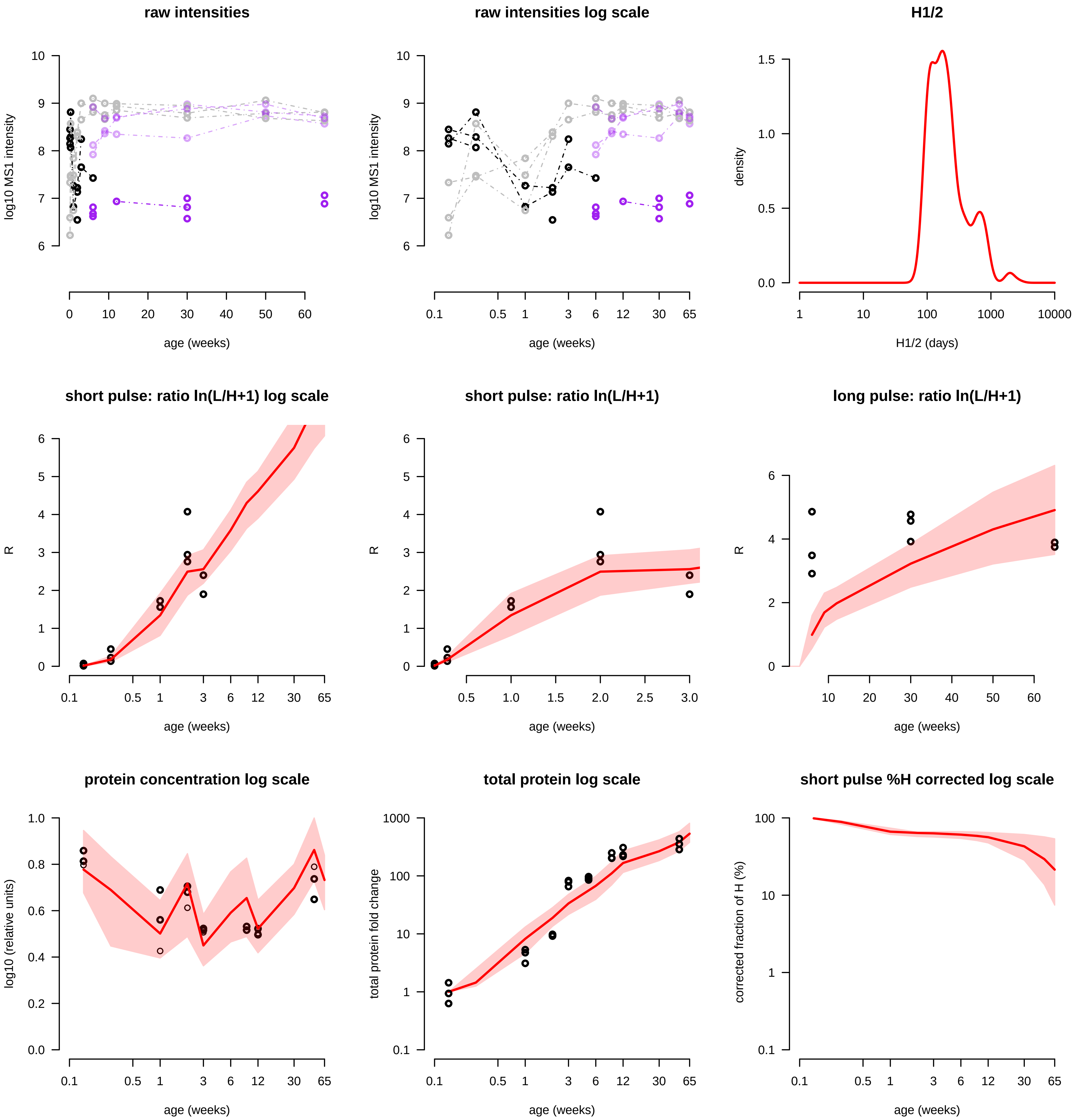

Q61753

>sp|Q61753|SERA_MOUSE

D−3−phosphoglycerate dehydrogenase

GN=Phgdh

Med H1/2: 4.3 days

Q5 H1/2: 3.4 days

Q95 H1/2: 5.4 days

reliable model fit: yes

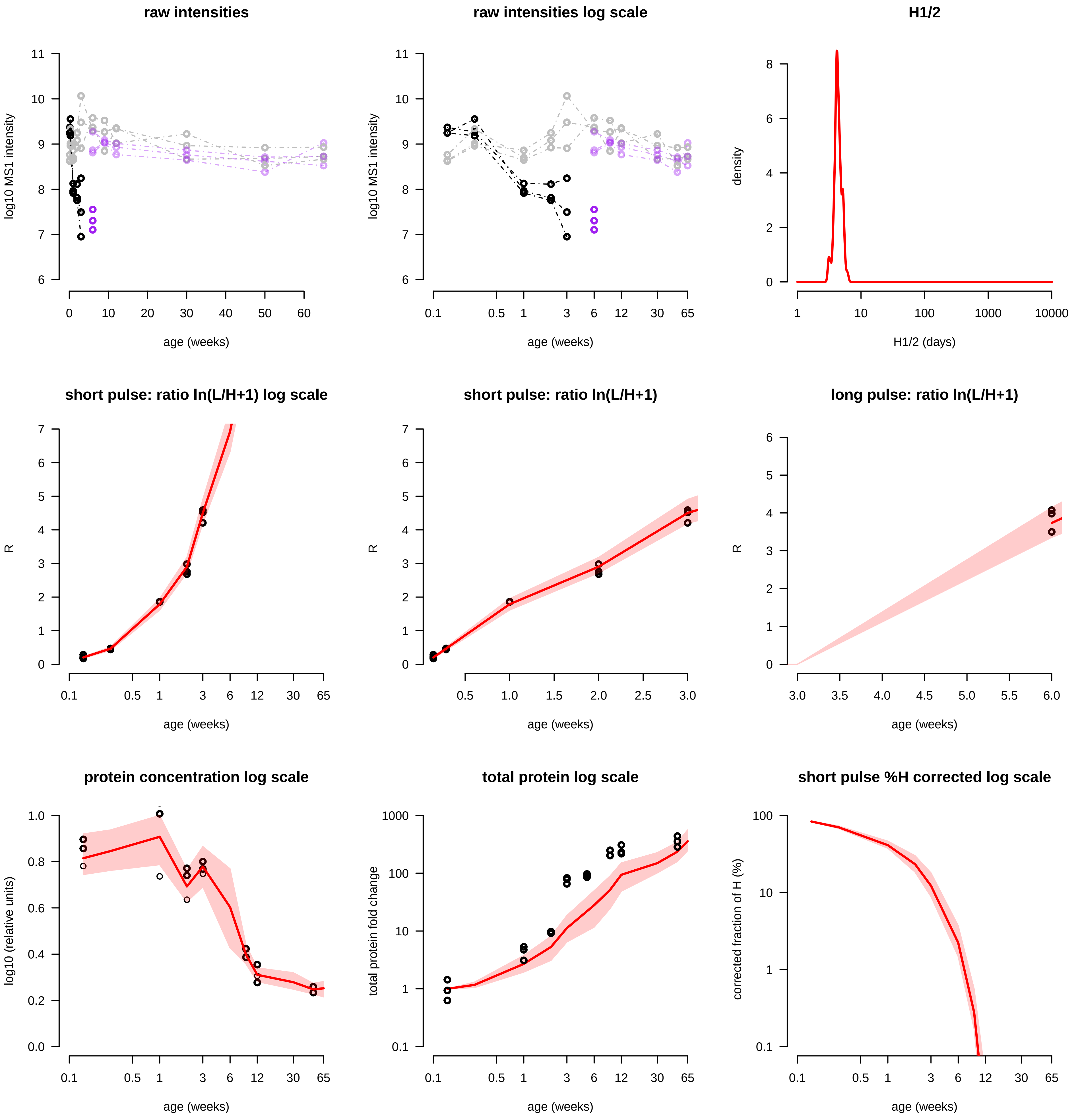

Q80XU3

>sp|Q80XU3|NUCKS_MOUSE

Nuclear ubiquitous casein and cyclin−dependent kinase substrate 1

GN=Nucks1

Med H1/2: 8.3 days

Q5 H1/2: 6.9 days

Q95 H1/2: 13.5 days

reliable model fit: yes

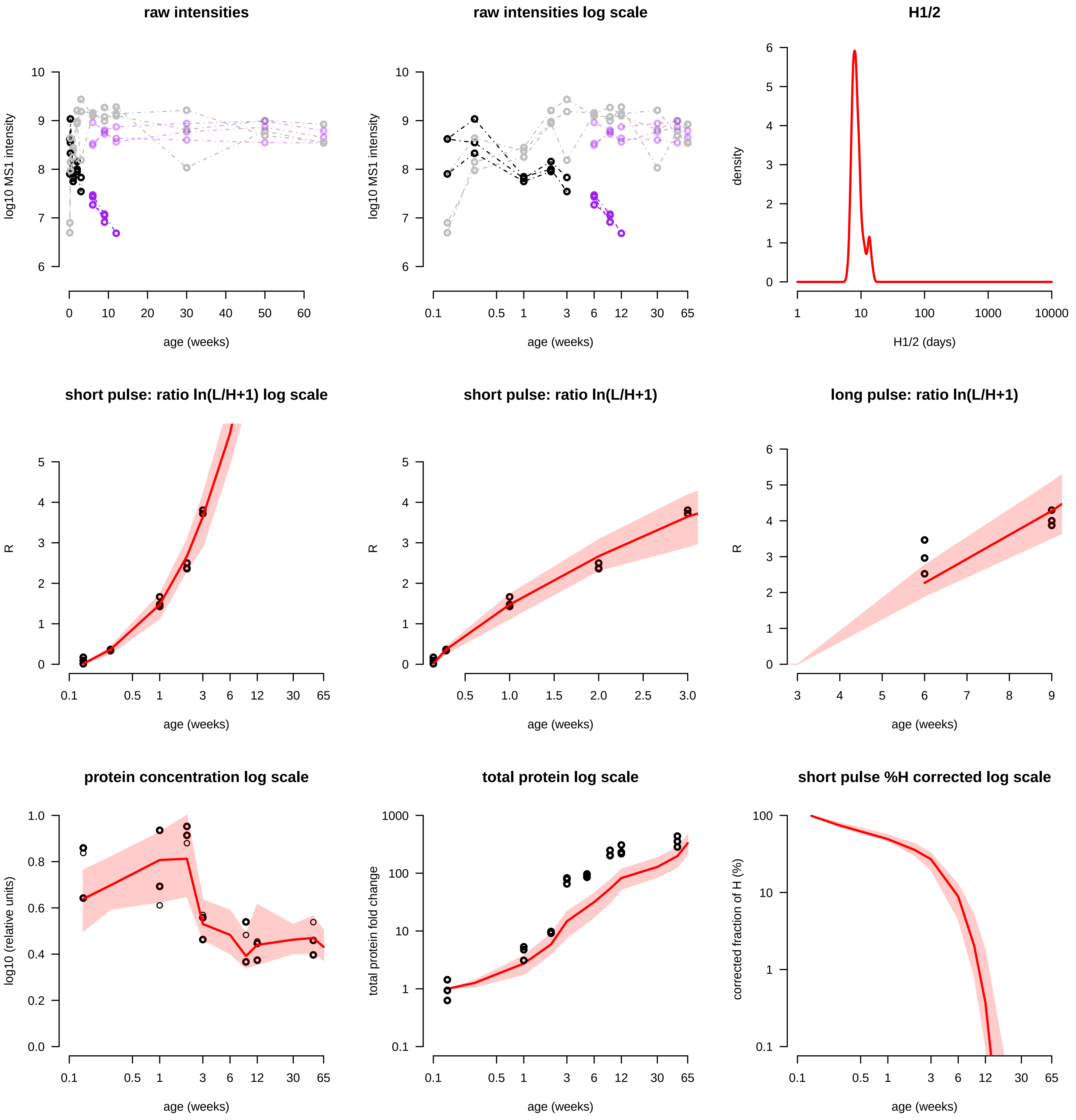

P55937;P55937−1

>sp|P55937|GOGA3_MOUSE

Golgin subfamily A member 3

GN=Golga3

Med H1/2: 4.1 days

Q5 H1/2: 2.5 days

Q95 H1/2: 14.5 days

reliable model fit: yes

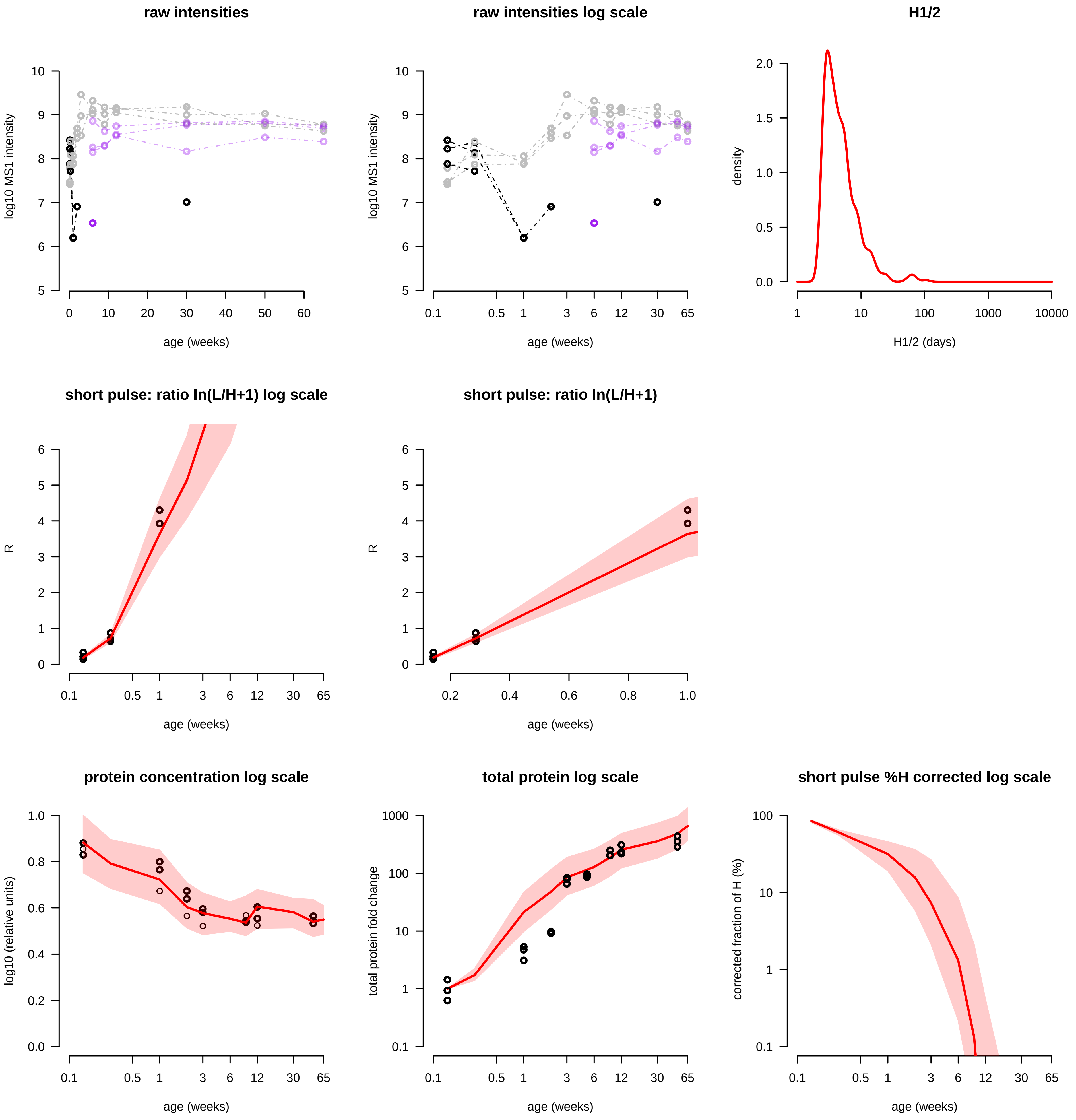

Q9D020−1

>sp|Q9D020−1|5NT3A_MOUSE

Isoform 1 of Cytosolic 5'−nucleotidase 3A

GN=Nt5c3a

Med H1/2: 5 days

Q5 H1/2: 2.5 days

Q95 H1/2: 51.8 days

reliable model fit: no

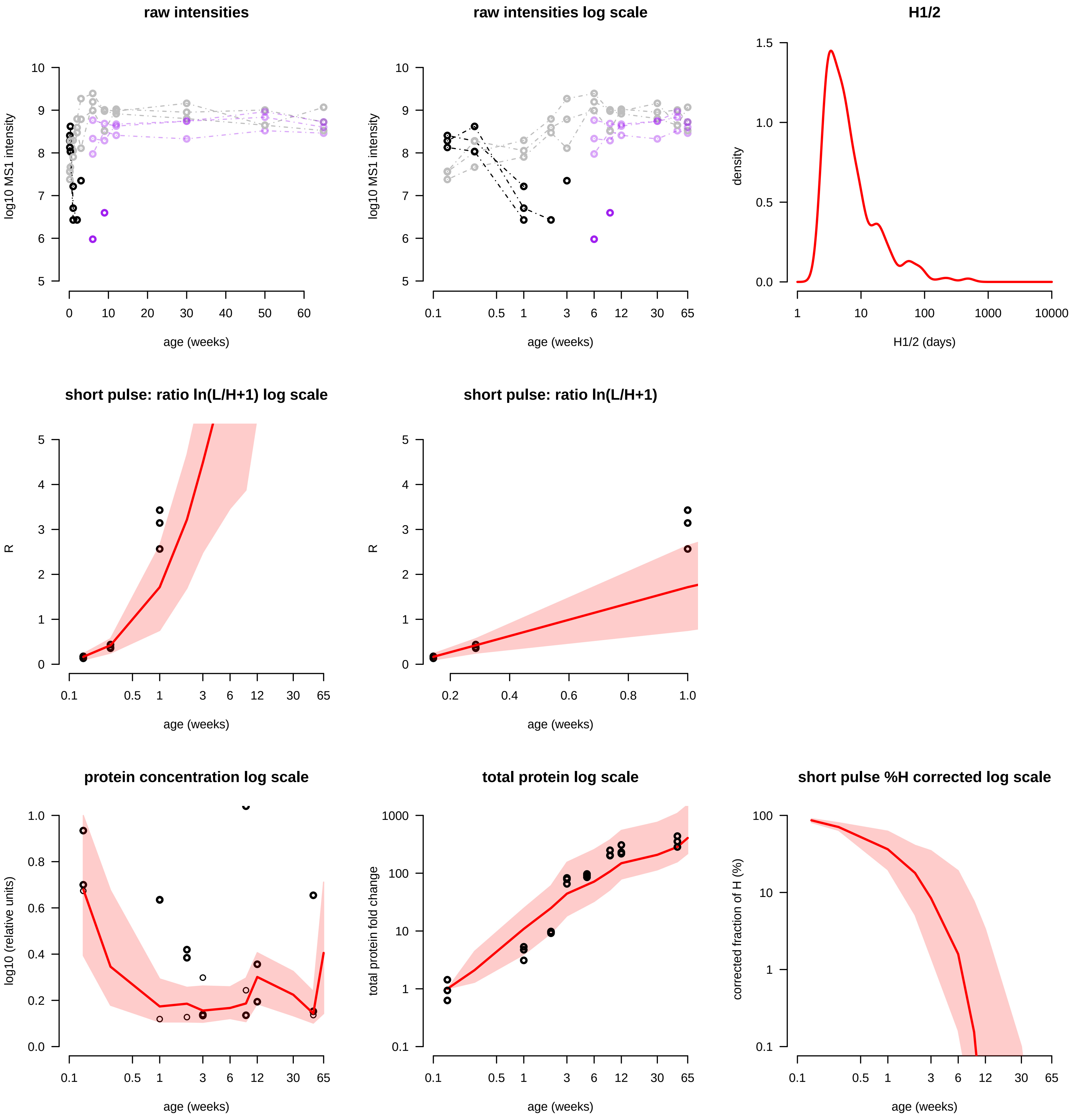

Q02257

>sp|Q02257|PLAK_MOUSE

Junction plakoglobin

GN=Jup

Med H1/2: 4.3 days

Q5 H1/2: 3.4 days

Q95 H1/2: 5.3 days

reliable model fit: yes

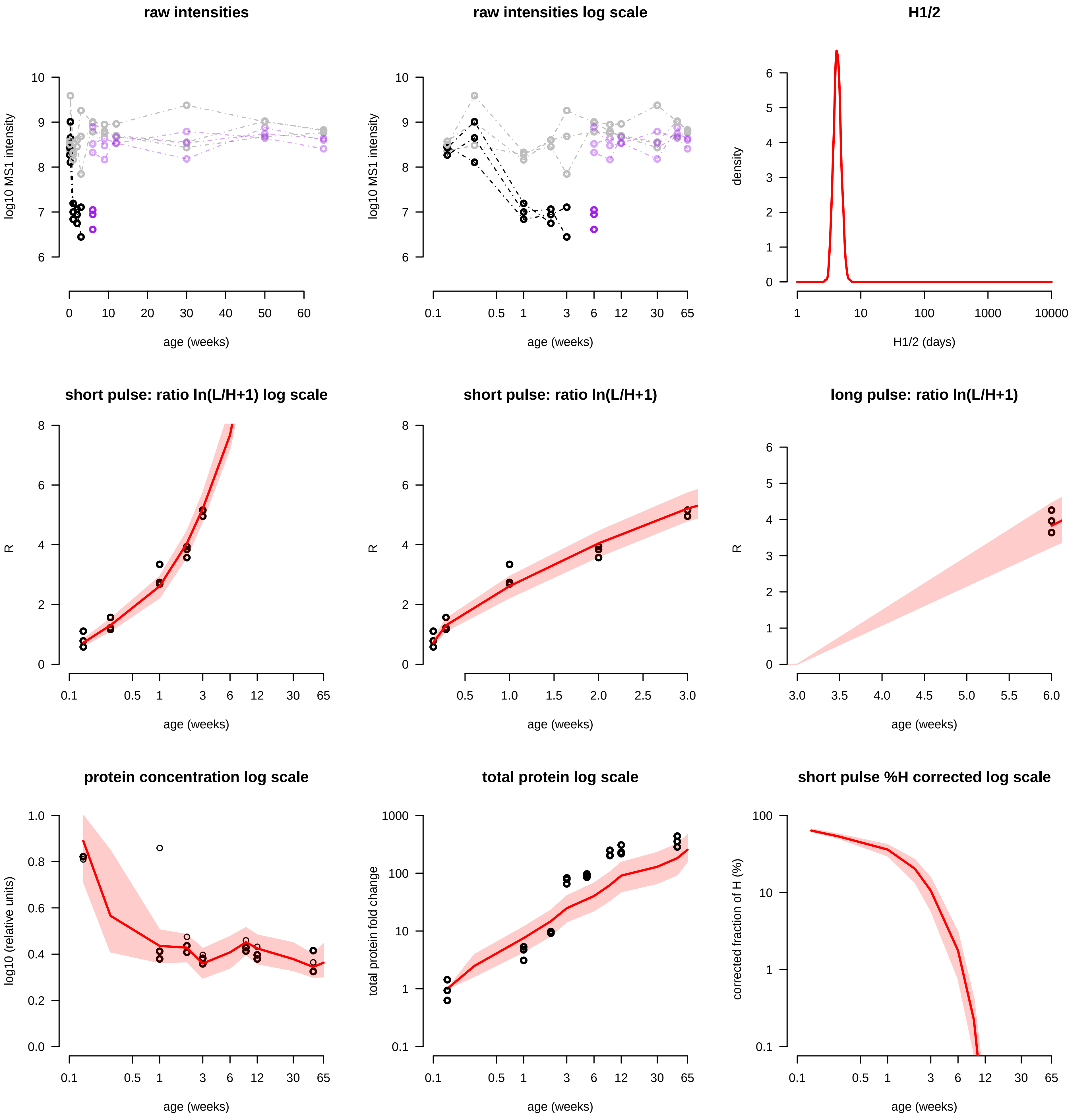

Q9CYR0

>sp|Q9CYR0|SSBP_MOUSE

Single−stranded DNA−binding protein, mitochondrial

GN=Ssbp1

Med H1/2: 453 days

Q5 H1/2: 164 days

Q95 H1/2: 5037.8 days

reliable model fit: no

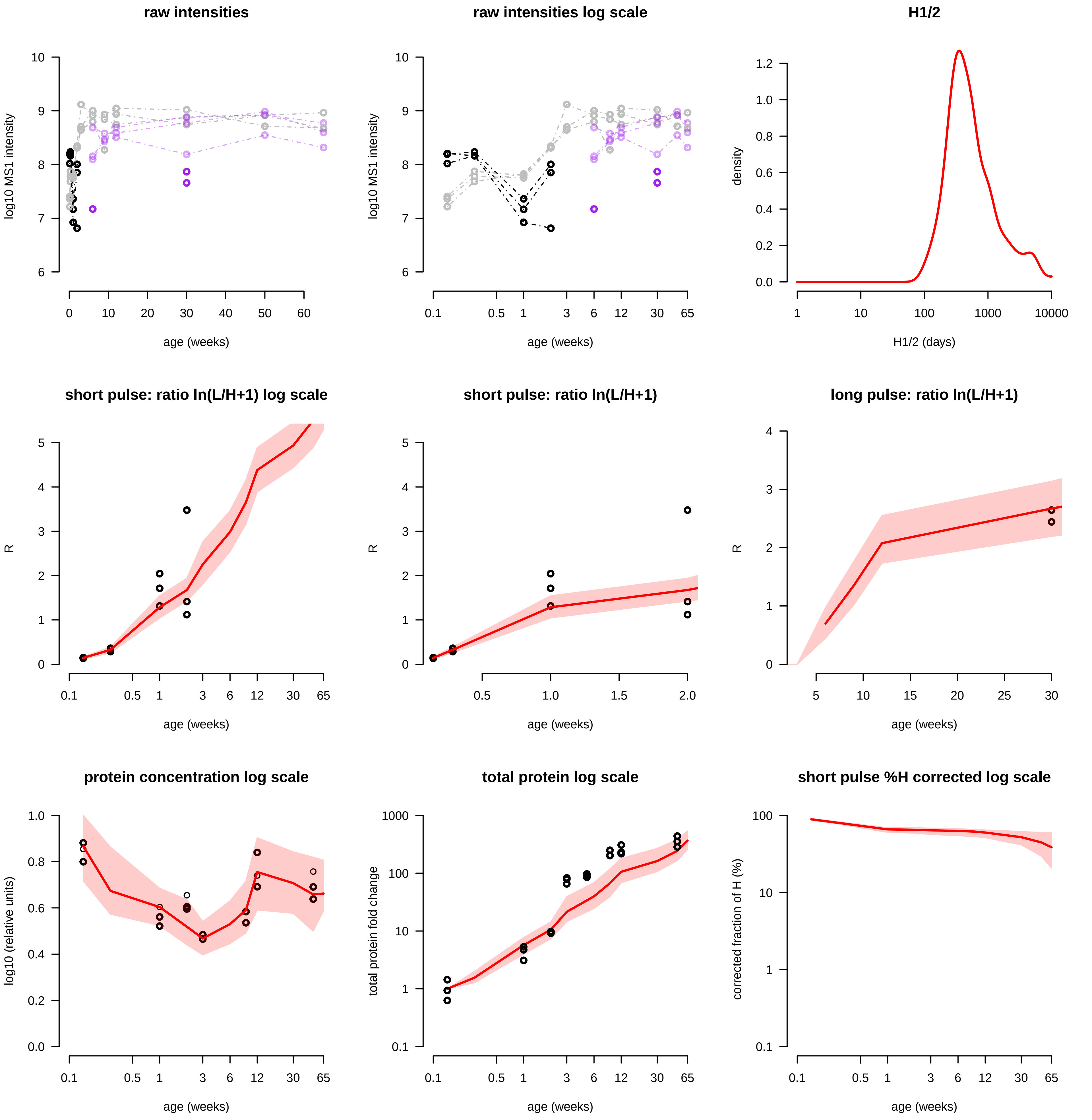

Q3TLH4

>sp|Q3TLH4|PRC2C_MOUSE

Protein PRRC2C

GN=Prrc2c

Med H1/2: 19.4 days

Q5 H1/2: 16.2 days

Q95 H1/2: 24.2 days

reliable model fit: yes

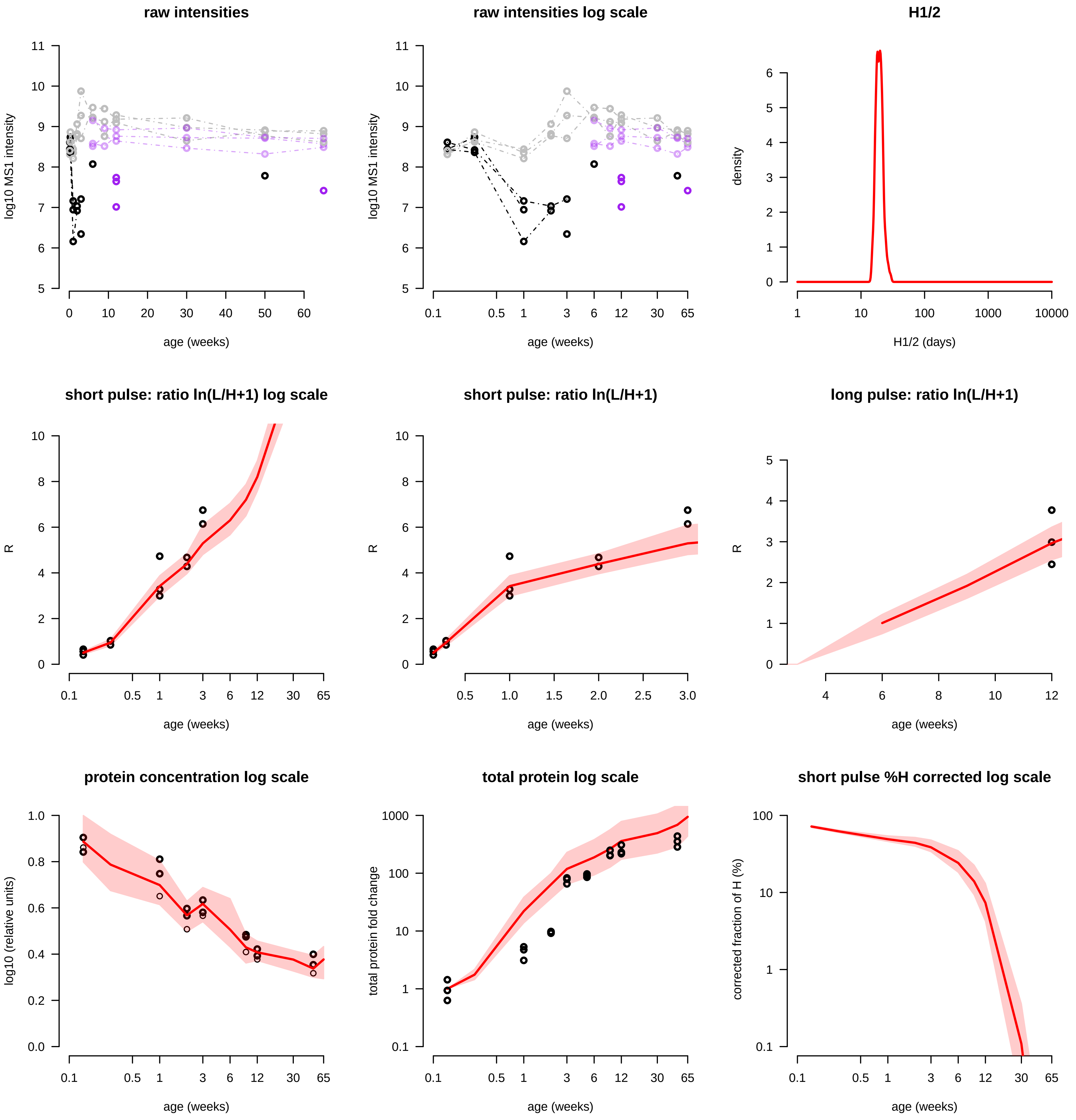

Q8BPU7

>sp|Q8BPU7|ELMO1_MOUSE

Engulfment and cell motility protein 1

GN=Elmo1

Med H1/2: 5.2 days

Q5 H1/2: 3.9 days

Q95 H1/2: 7.6 days

reliable model fit: yes

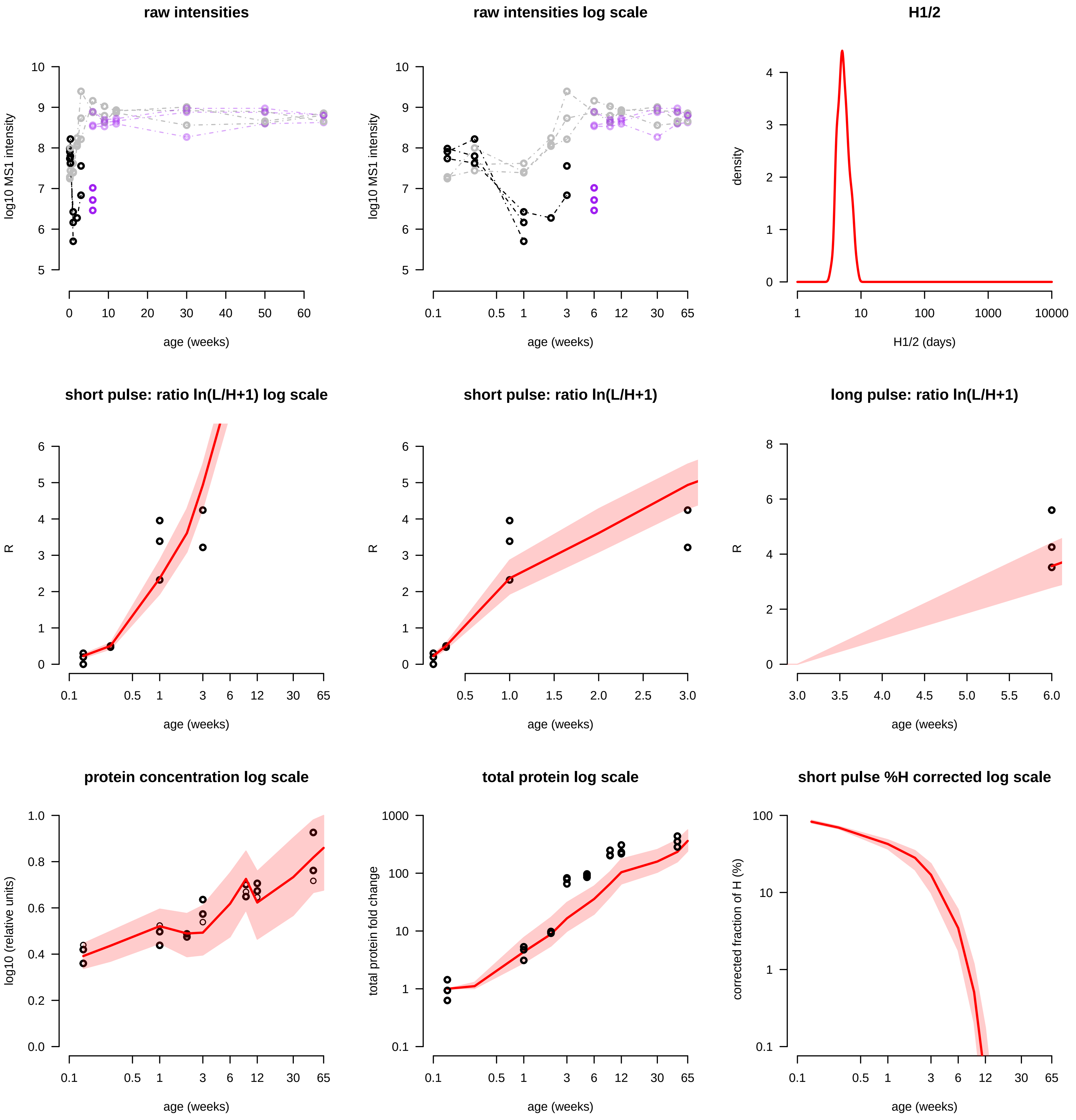

Q08943;Q08943−2

>sp|Q08943|SSRP1_MOUSE

FACT complex subunit SSRP1

GN=Ssrp1

Med H1/2: 3388.7 days

Q5 H1/2: 728.5 days

Q95 H1/2: 43130.6 days

reliable model fit: no

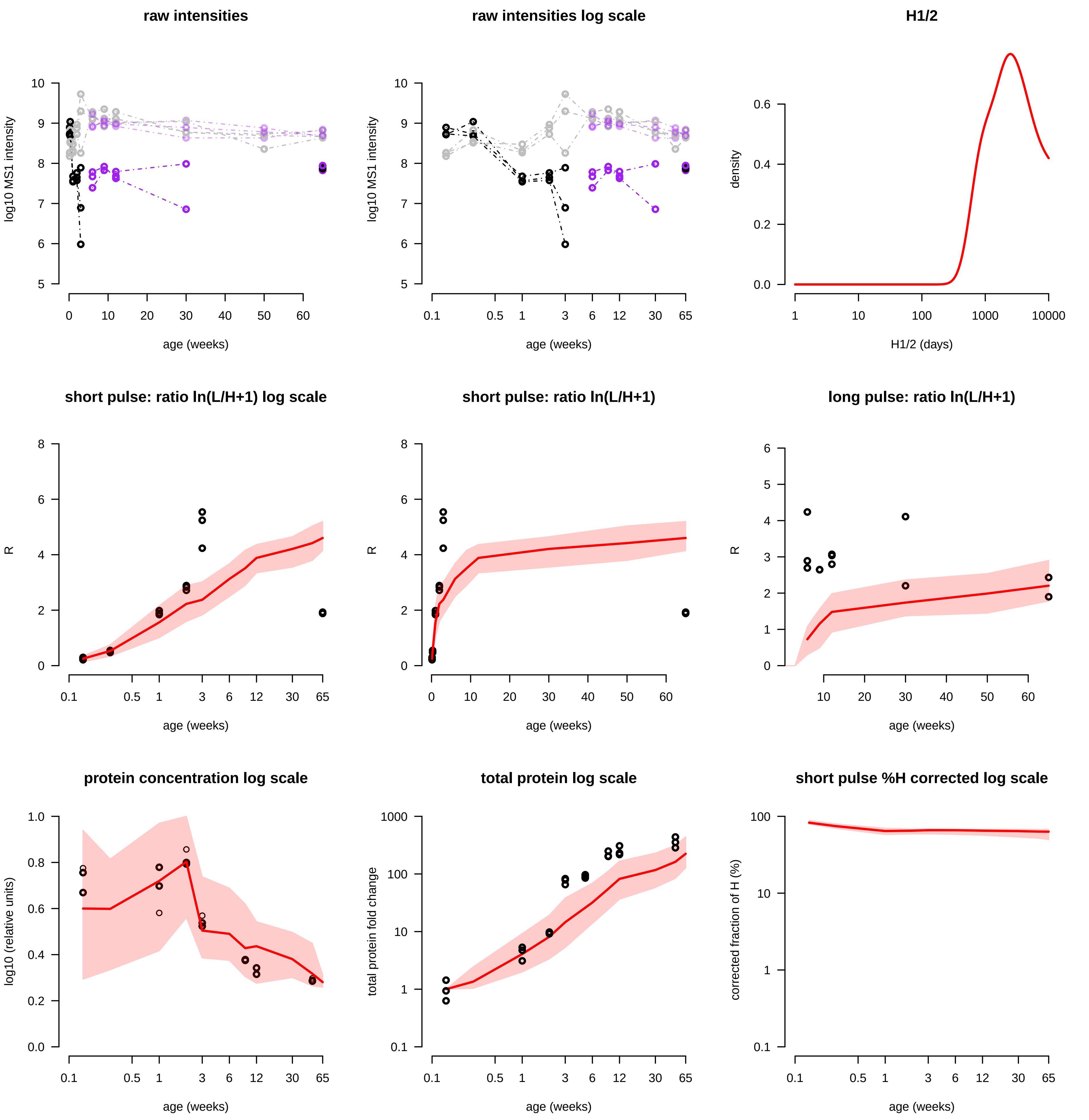

Q9WTQ5

>sp|Q9WTQ5|AKA12_MOUSE

A−kinase anchor protein 12

GN=Akap12

Med H1/2: 9.2 days

Q5 H1/2: 2.7 days

Q95 H1/2: 24.6 days

reliable model fit: yes

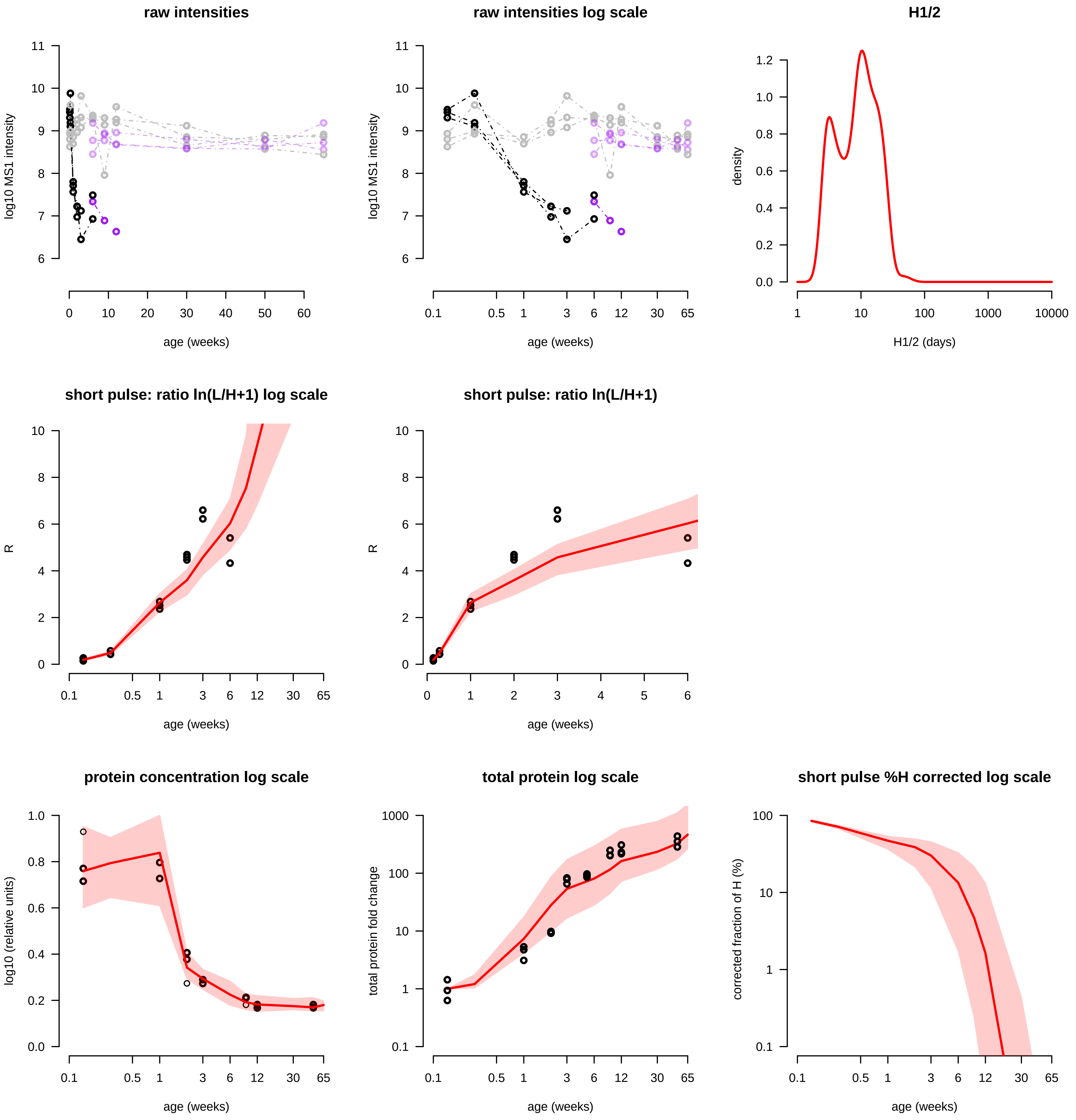

Q8BWQ6;Q8BWQ6−3

>sp|Q8BWQ6|VP35L_MOUSE

VPS35 endosomal protein−sorting factor−like

GN=Vps35l

Med H1/2: 2066.2 days

Q5 H1/2: 538.4 days

Q95 H1/2: 16201.7 days

reliable model fit: no

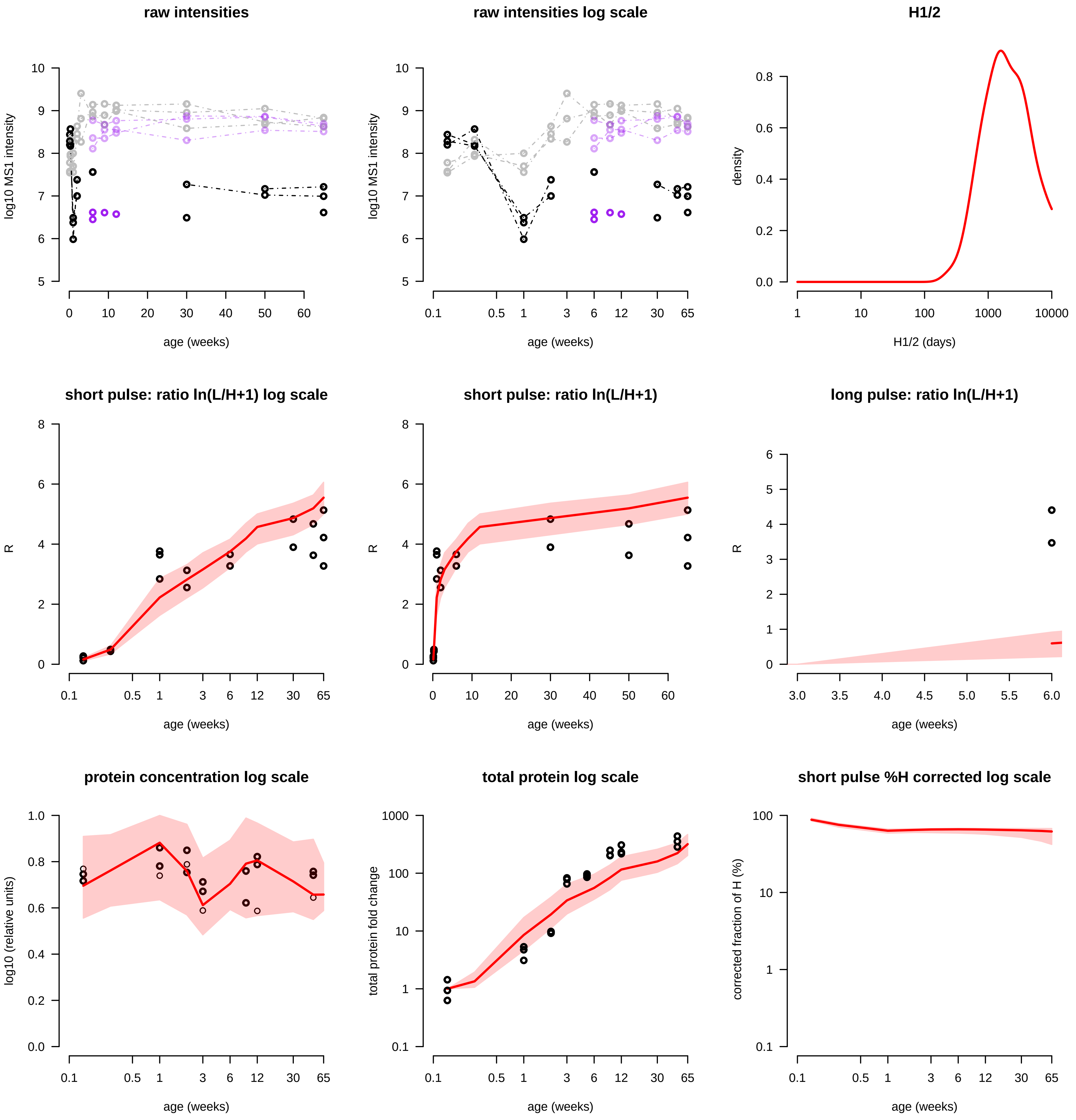

Q64669

>sp|Q64669|NQO1_MOUSE

NAD(P)H dehydrogenase [quinone] 1

GN=Nqo1

Med H1/2: 10.3 days

Q5 H1/2: 5.5 days

Q95 H1/2: 87.3 days

reliable model fit: yes

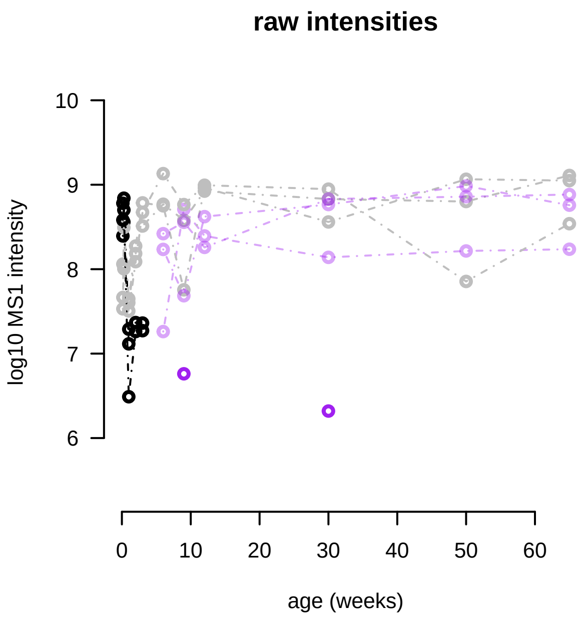

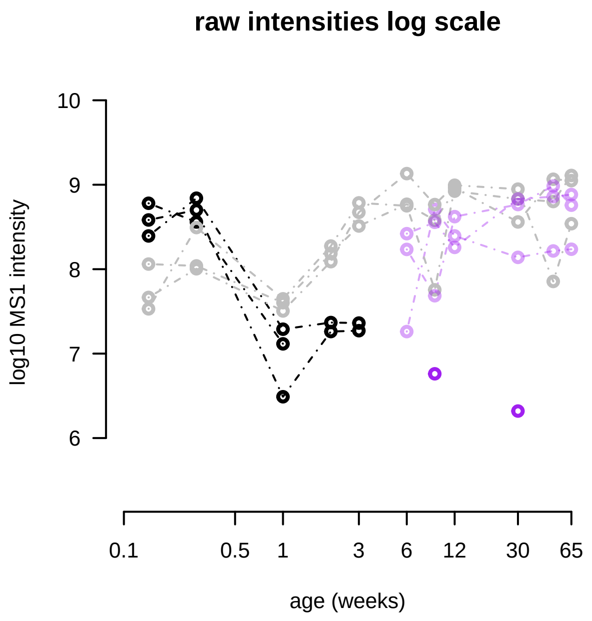

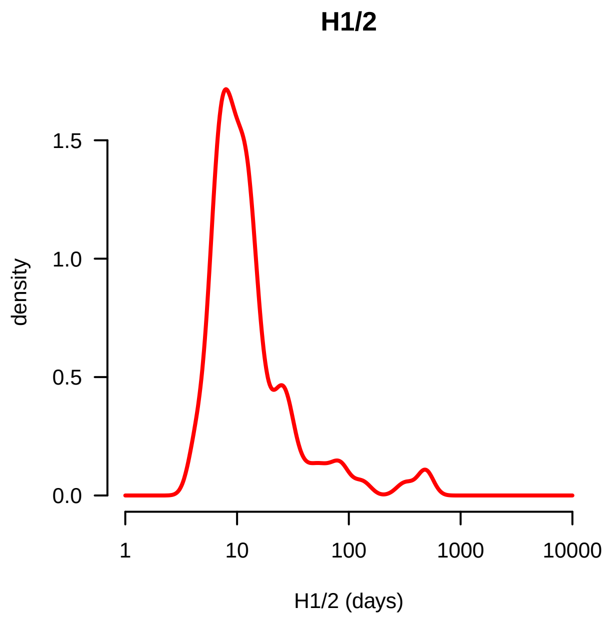

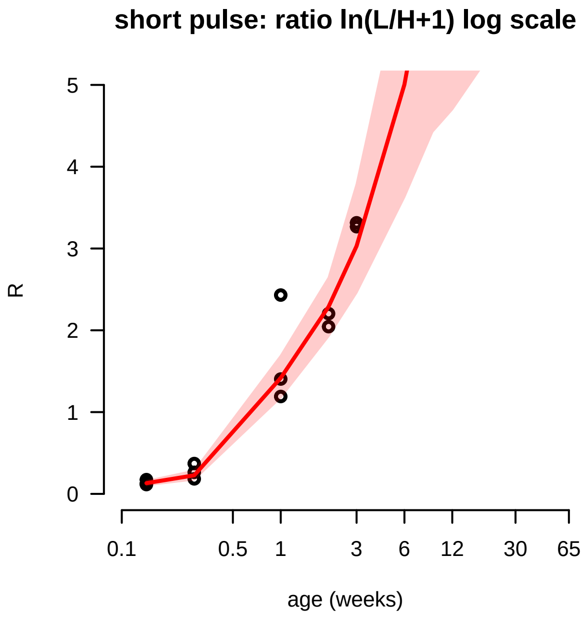

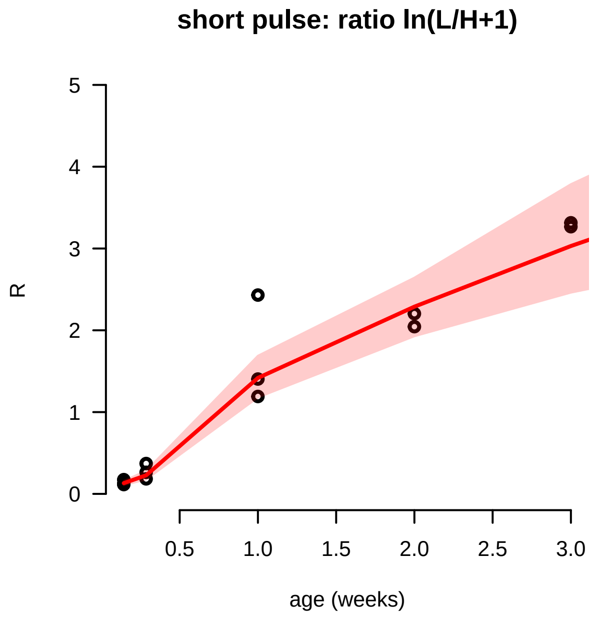

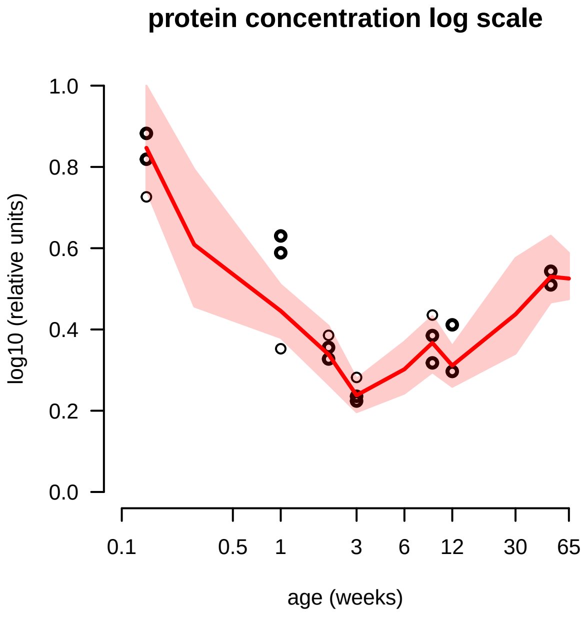

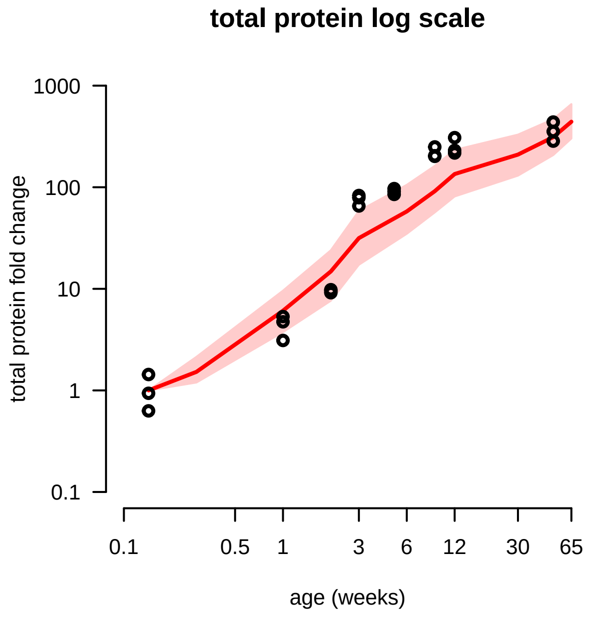

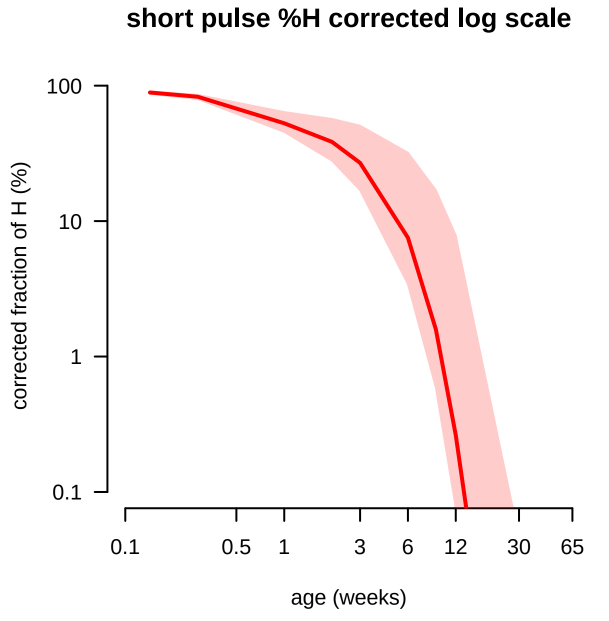

Q9EQU5−2

>sp|Q9EQU5−2|SET_MOUSE

Isoform 2 of Protein SET

GN=Set

Med H1/2: 158.4 days

Q5 H1/2: 59.3 days

Q95 H1/2: 1083.4 days

reliable model fit: no

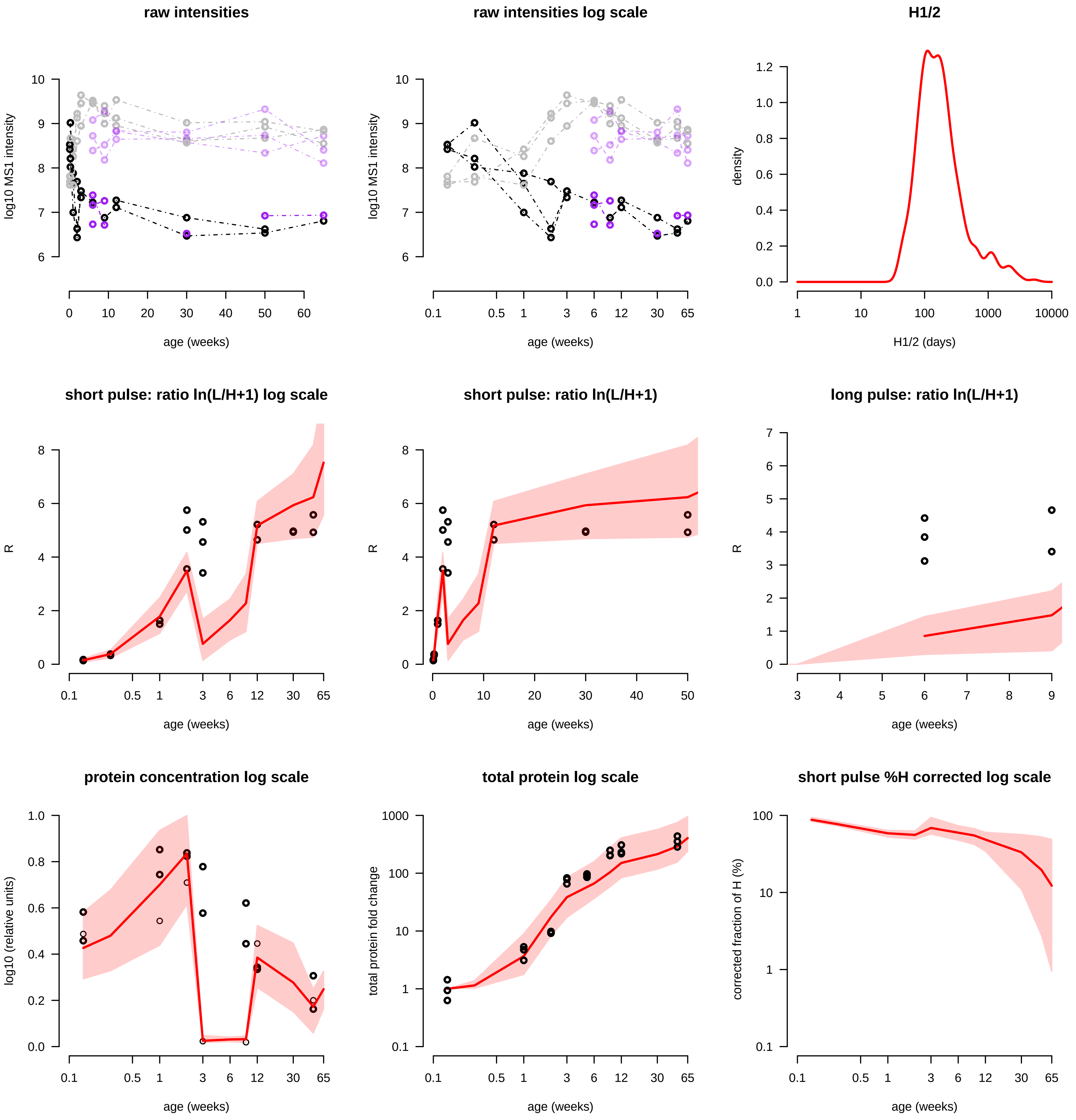

Q9QYC0;Q9QYC0−2

>sp|Q9QYC0|ADDA_MOUSE

Alpha−adducin

GN=Add1

Med H1/2: 4.8 days

Q5 H1/2: 4 days

Q95 H1/2: 6.1 days

reliable model fit: yes

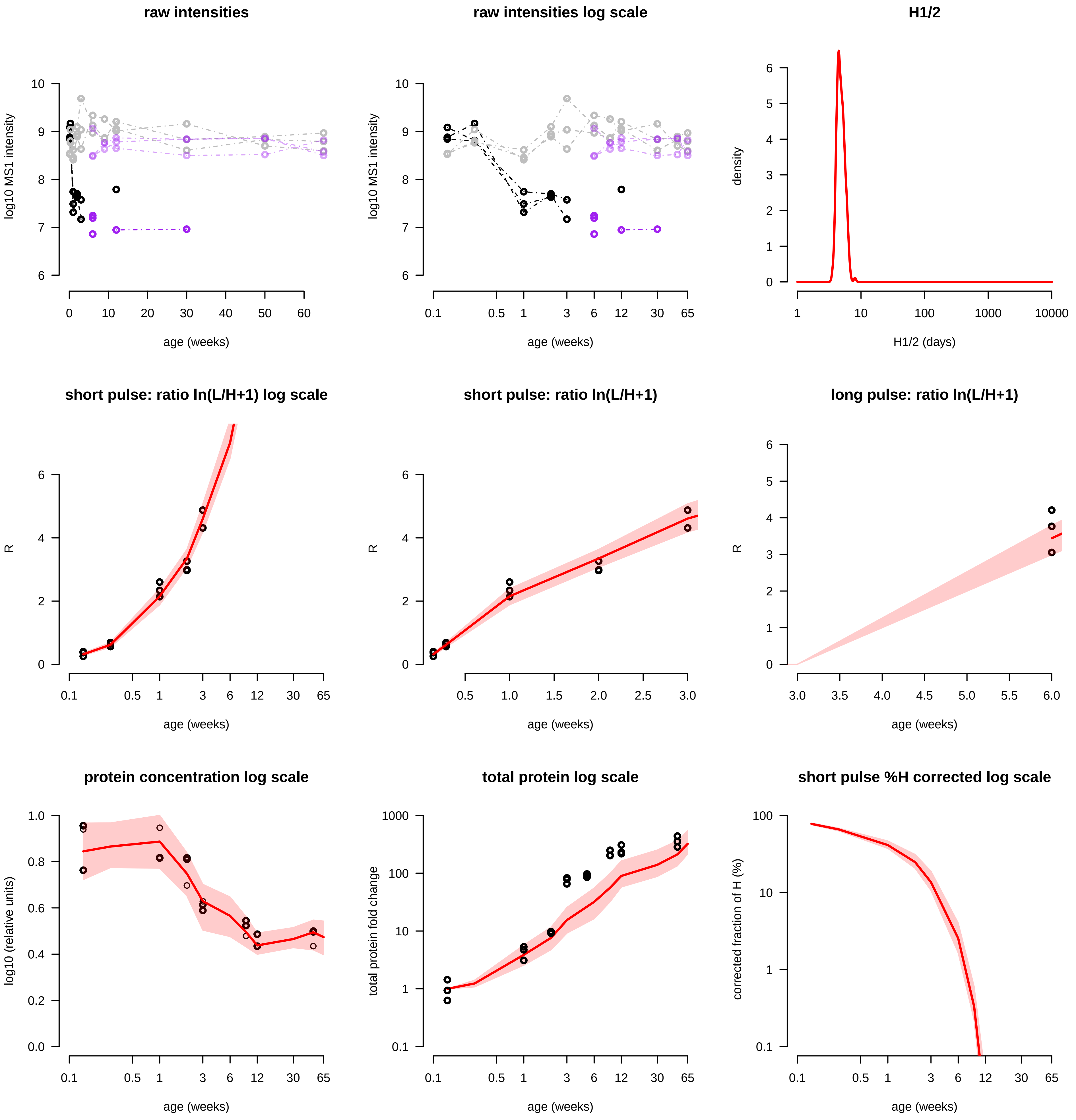

Q8R4H2

>sp|Q8R4H2|ARHGC_MOUSE

Rho guanine nucleotide exchange factor 12

GN=Arhgef12

Med H1/2: 4.1 days

Q5 H1/2: 2.5 days

Q95 H1/2: 16.5 days

reliable model fit: yes

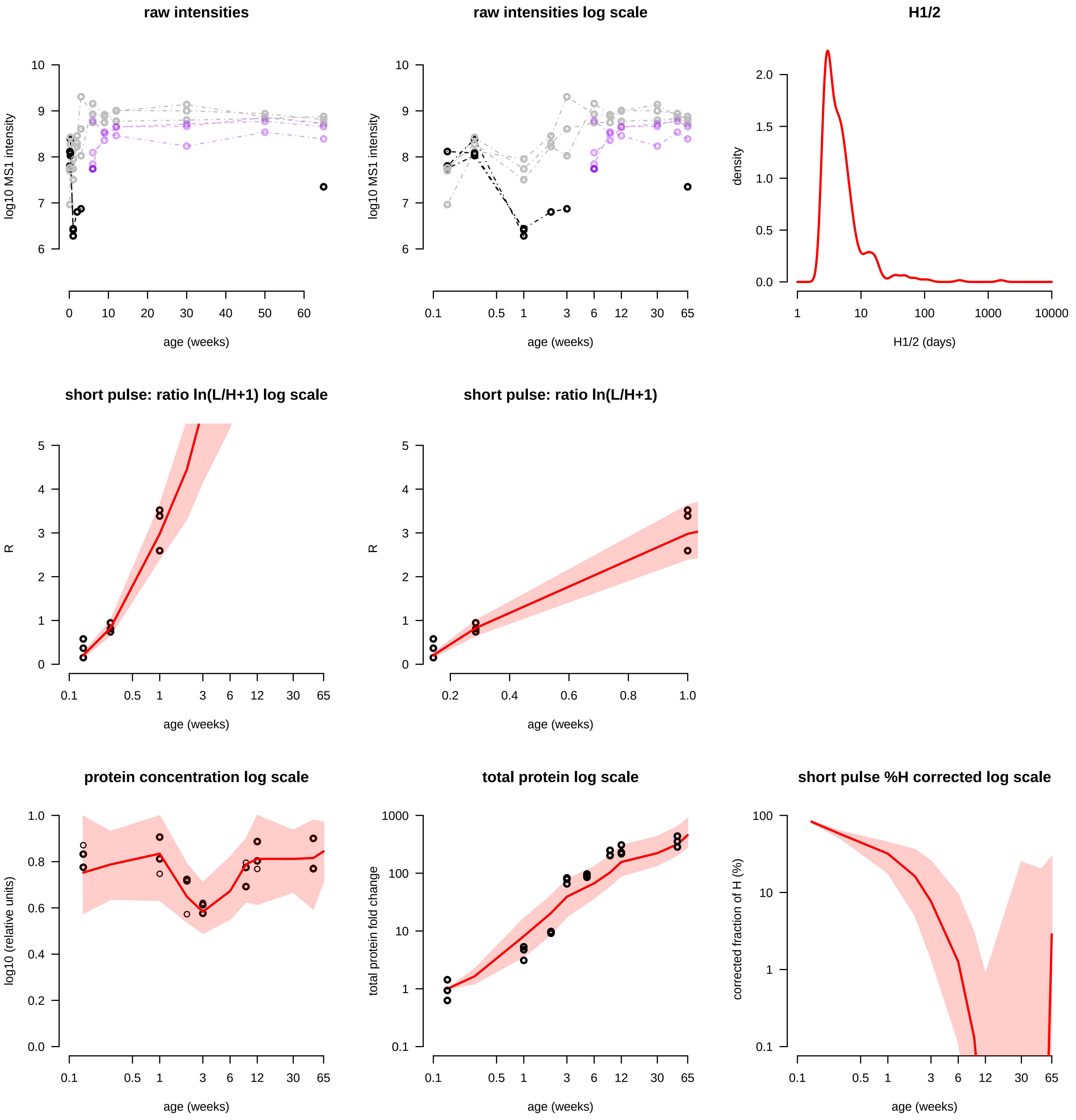

P63030

>sp|P63030|MPC1_MOUSE

Mitochondrial pyruvate carrier 1

GN=Mpc1

Med H1/2: 21.1 days

Q5 H1/2: 12.5 days

Q95 H1/2: 64.2 days

reliable model fit: yes

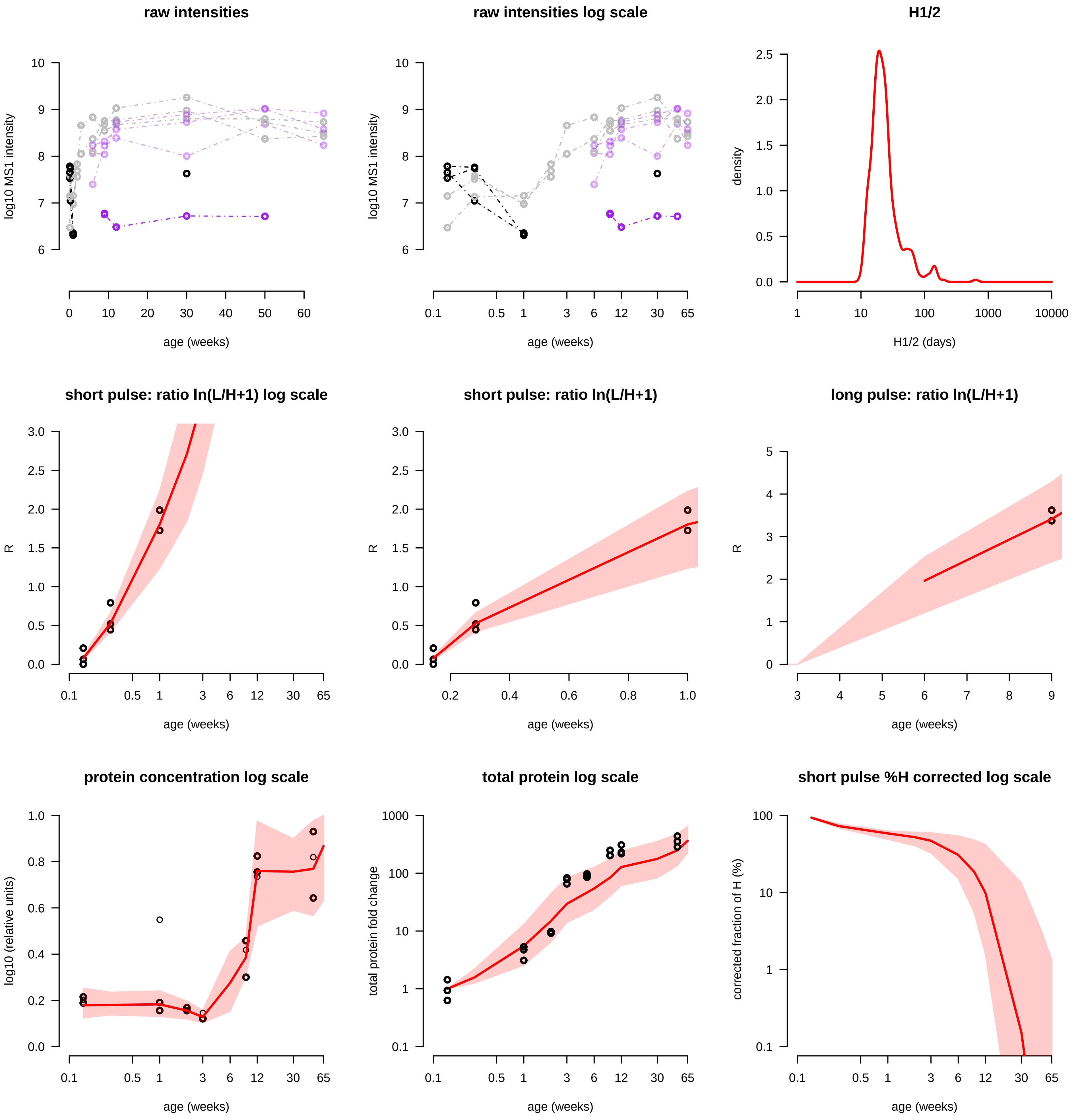

O88447

>sp|O88447|KLC1_MOUSE

Kinesin light chain 1

GN=Klc1

Med H1/2: 14.9 days

Q5 H1/2: 10.6 days

Q95 H1/2: 21.7 days

reliable model fit: yes

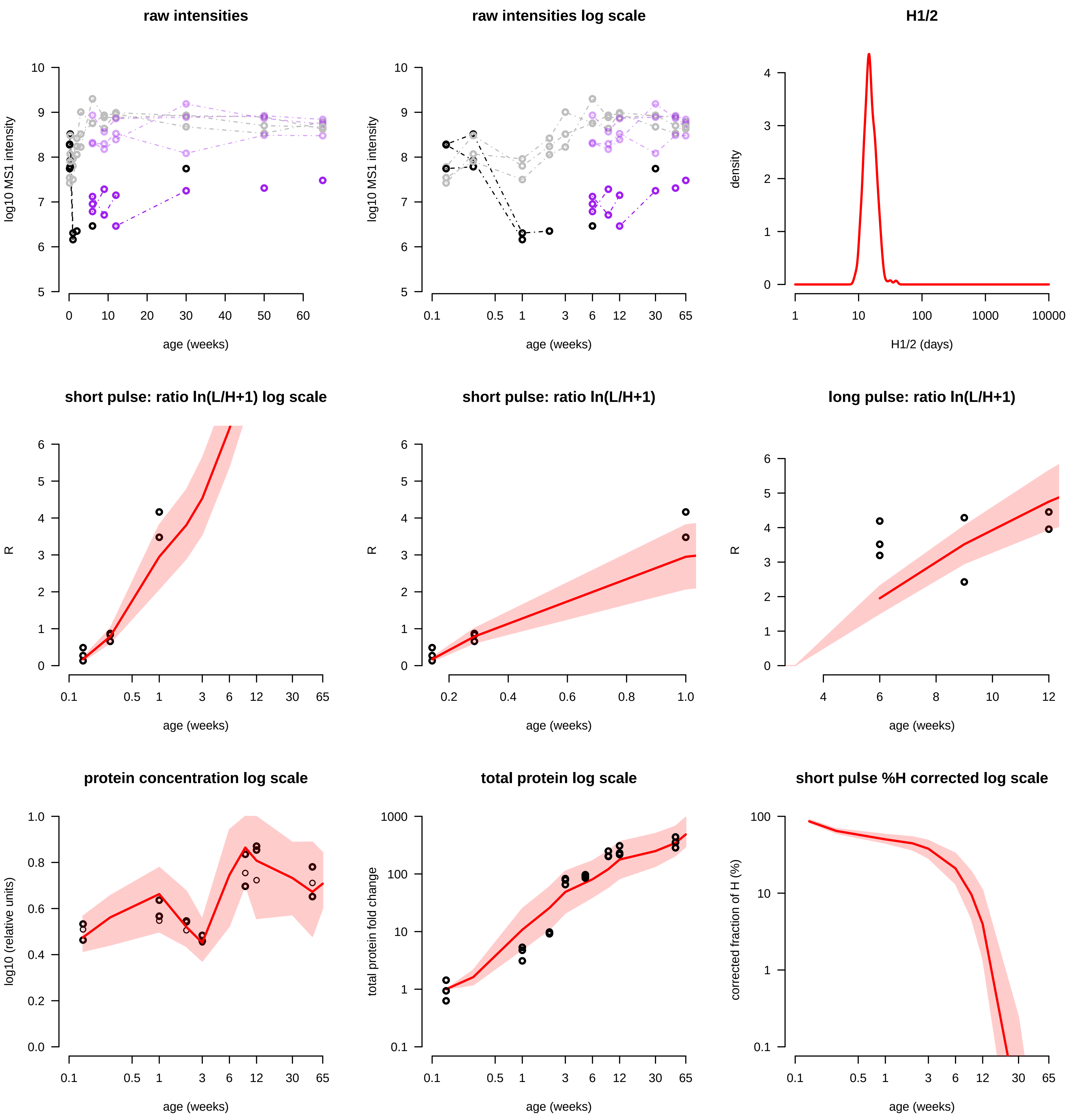

Q61205

>sp|Q61205|PA1B3_MOUSE

Platelet−activating factor acetylhydrolase IB subunit alpha1

GN=Pafah1b3

Med H1/2: 5.3 days

Q5 H1/2: 4.4 days

Q95 H1/2: 6.4 days

reliable model fit: yes

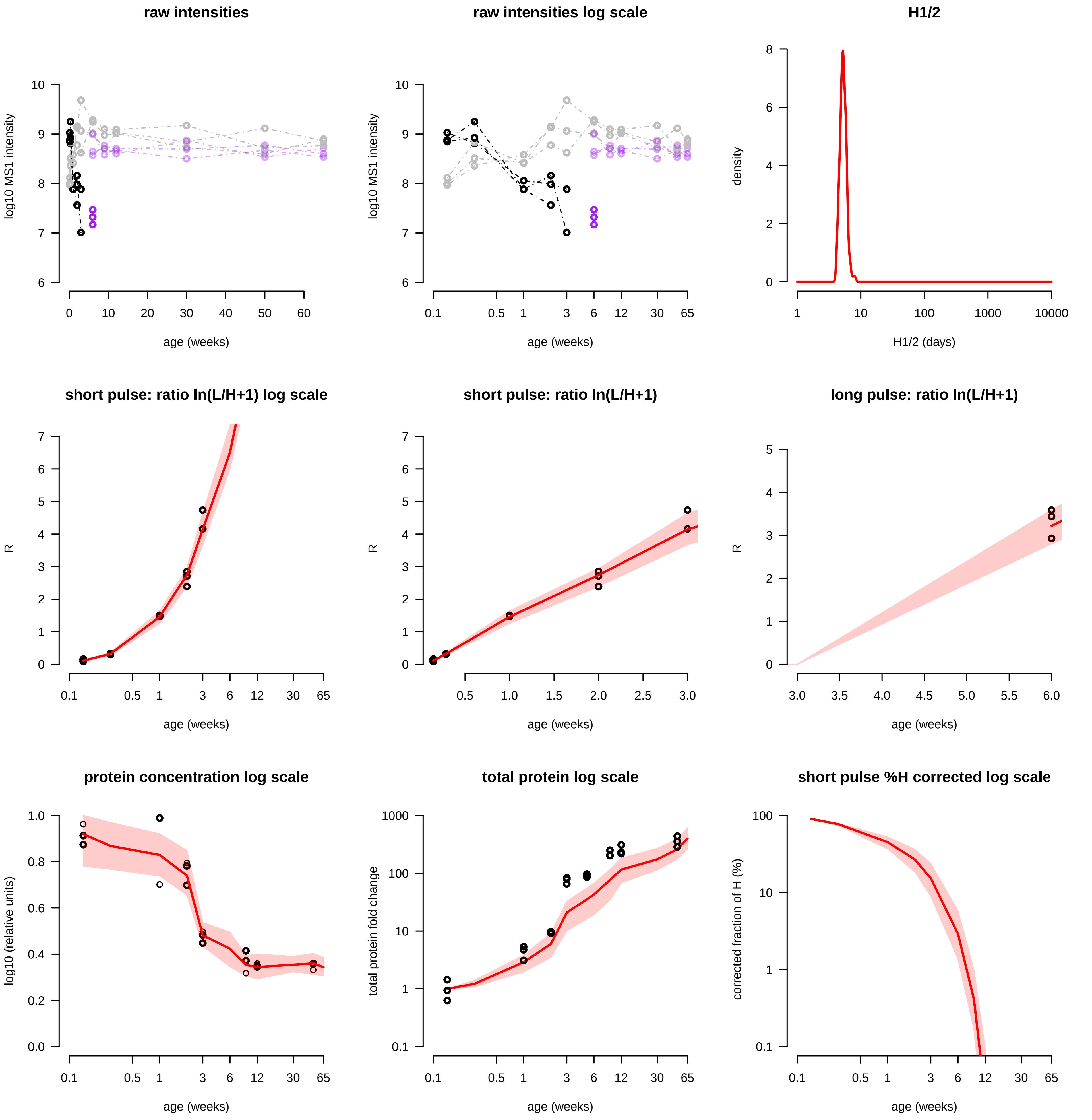

Q8C854;Q8C854−3;Q8C854−2

>sp|Q8C854|MYEF2_MOUSE

Myelin expression factor 2

GN=Myef2

Med H1/2: 66.2 days

Q5 H1/2: 43.9 days

Q95 H1/2: 149.7 days

reliable model fit: no

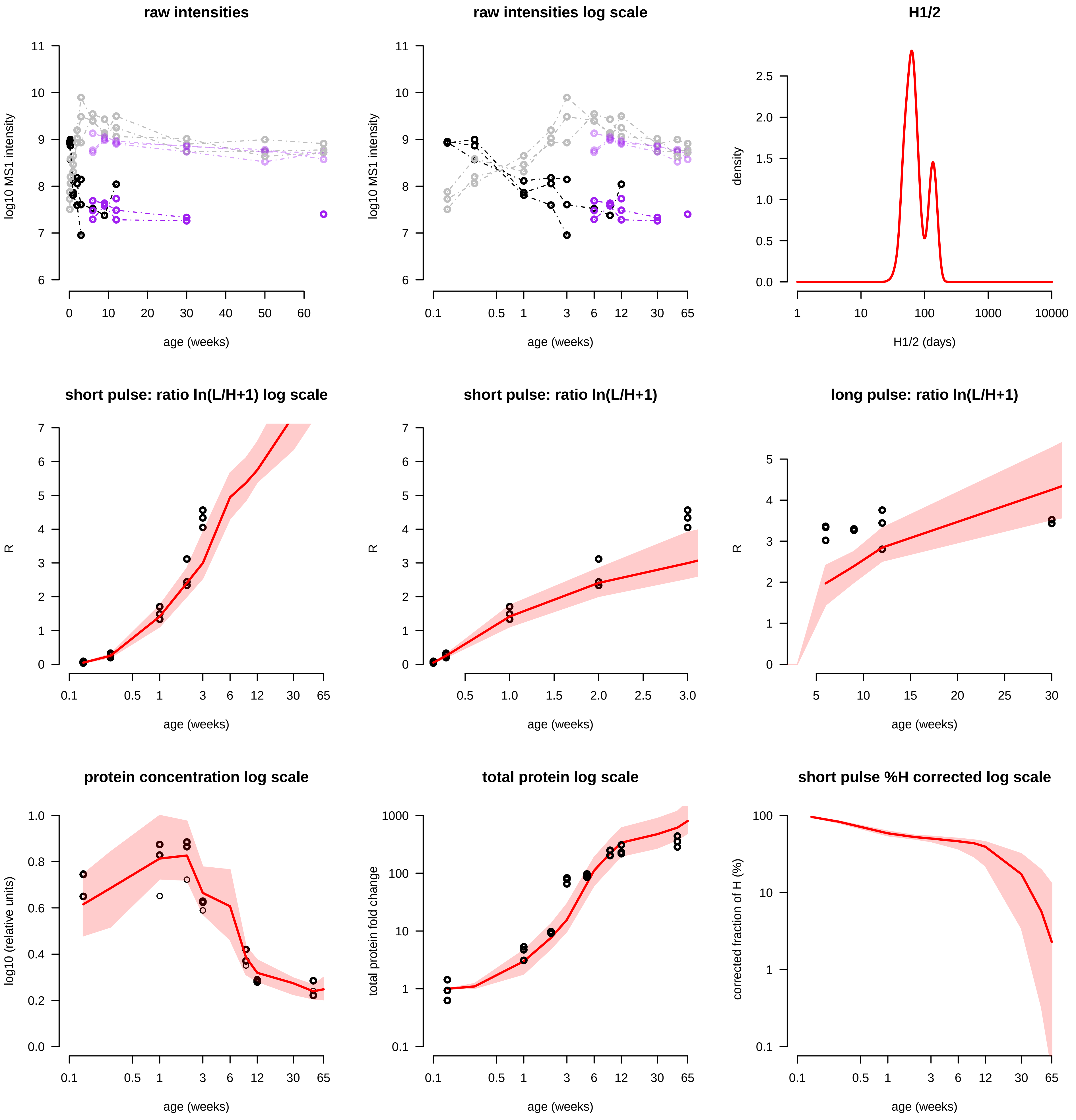

Q99JX3;Q99JX3−2

>sp|Q99JX3|GORS2_MOUSE

Golgi reassembly−stacking protein 2

GN=Gorasp2

Med H1/2: 7.6 days

Q5 H1/2: 3.4 days

Q95 H1/2: 26.8 days

reliable model fit: yes

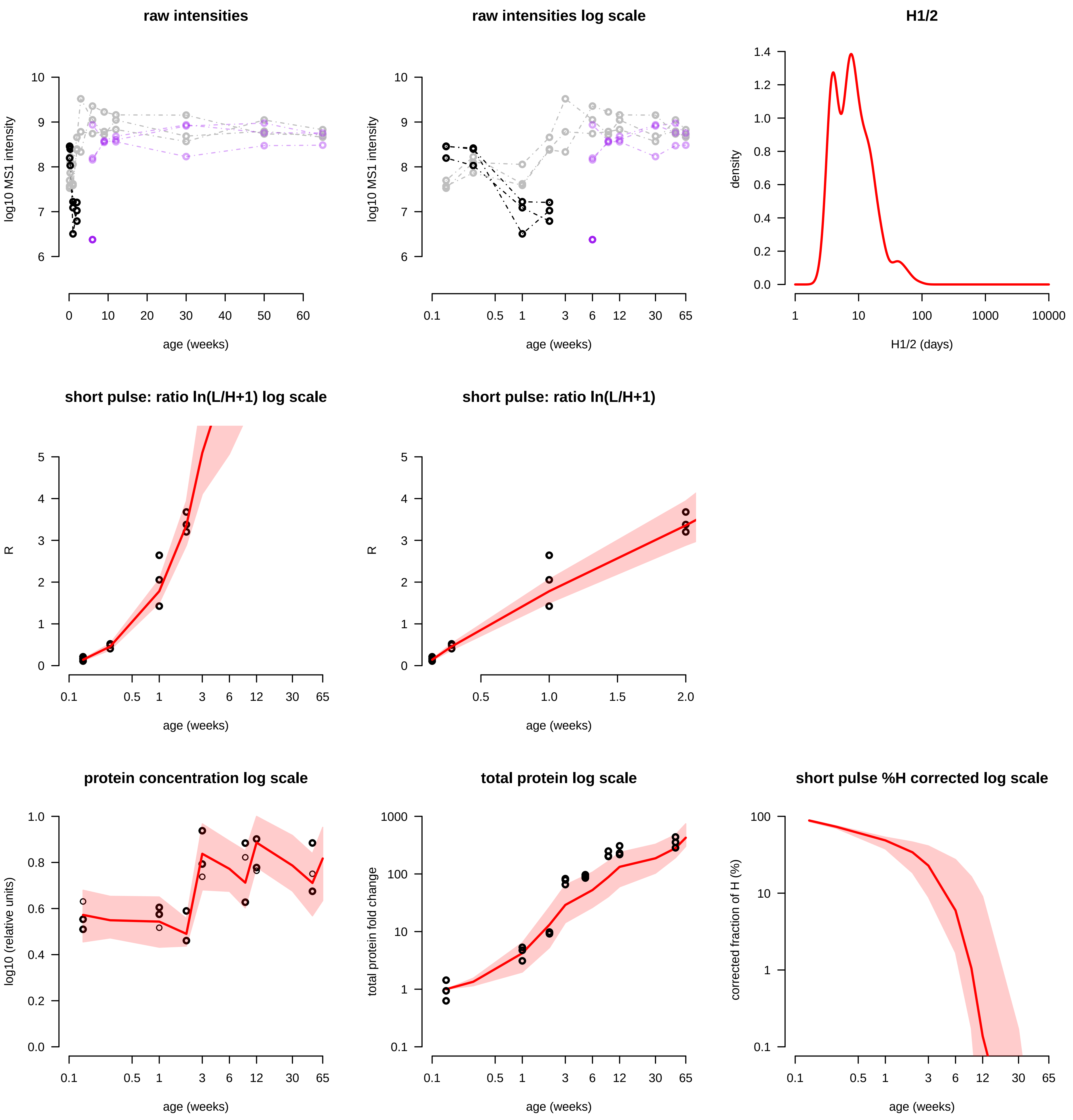

8A65−2;Q58A65;Q58A65−5;Q58A65−6;Q58A65−3;Q58A65

>sp|Q58A65−2|JIP4_MOUSE

Isoform 2 of C−Jun−amino−terminal kinase−interacting protein 4

GN=Spag9

Med H1/2: 3.6 days

Q5 H1/2: 2.6 days

Q95 H1/2: 11.7 days

reliable model fit: yes

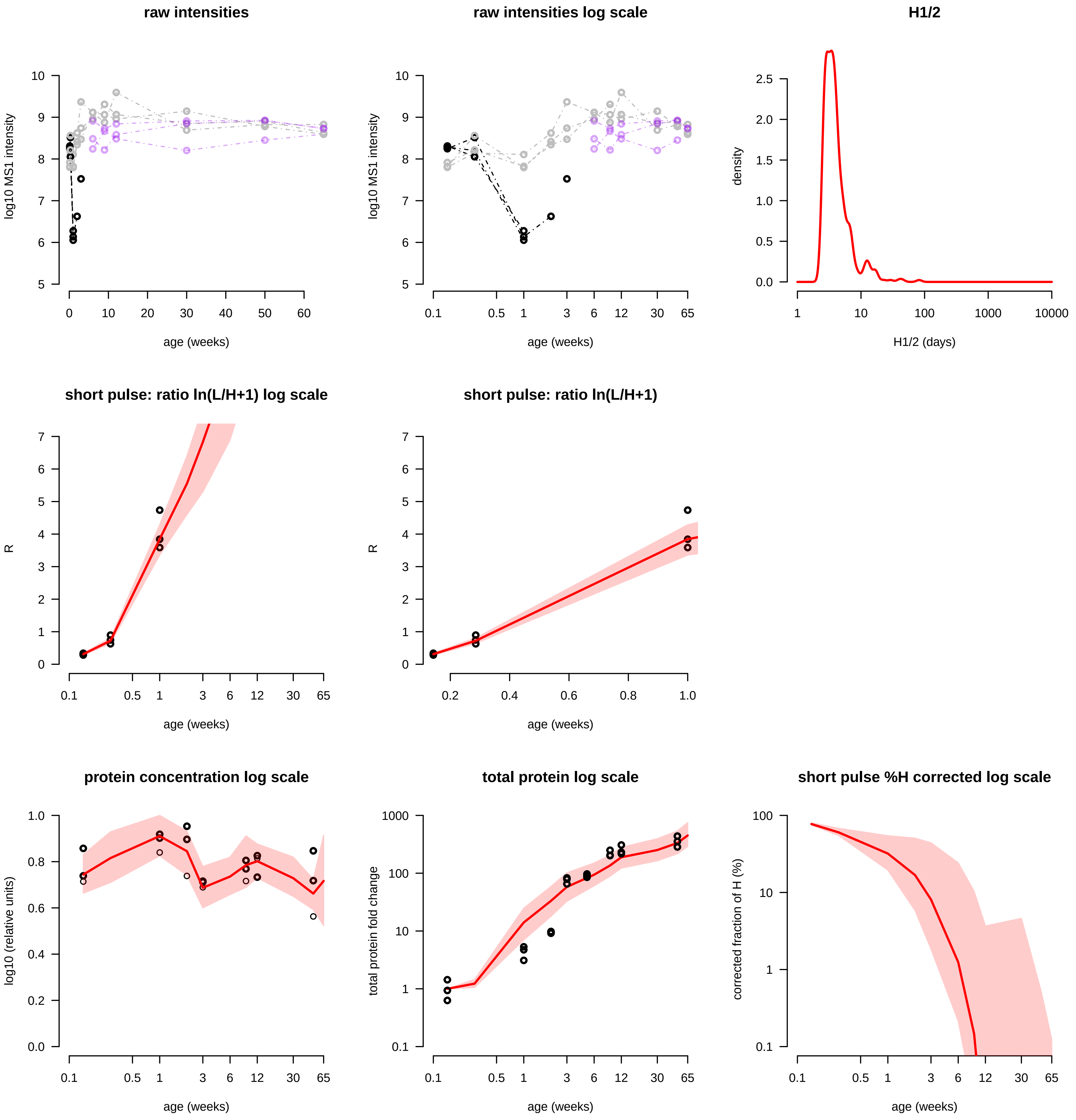

Q9ERG2

>sp|Q9ERG2|STRN3_MOUSE

Striatin−3

GN=Strn3

Med H1/2: 4.3 days

Q5 H1/2: 3.5 days

Q95 H1/2: 5.6 days

reliable model fit: yes

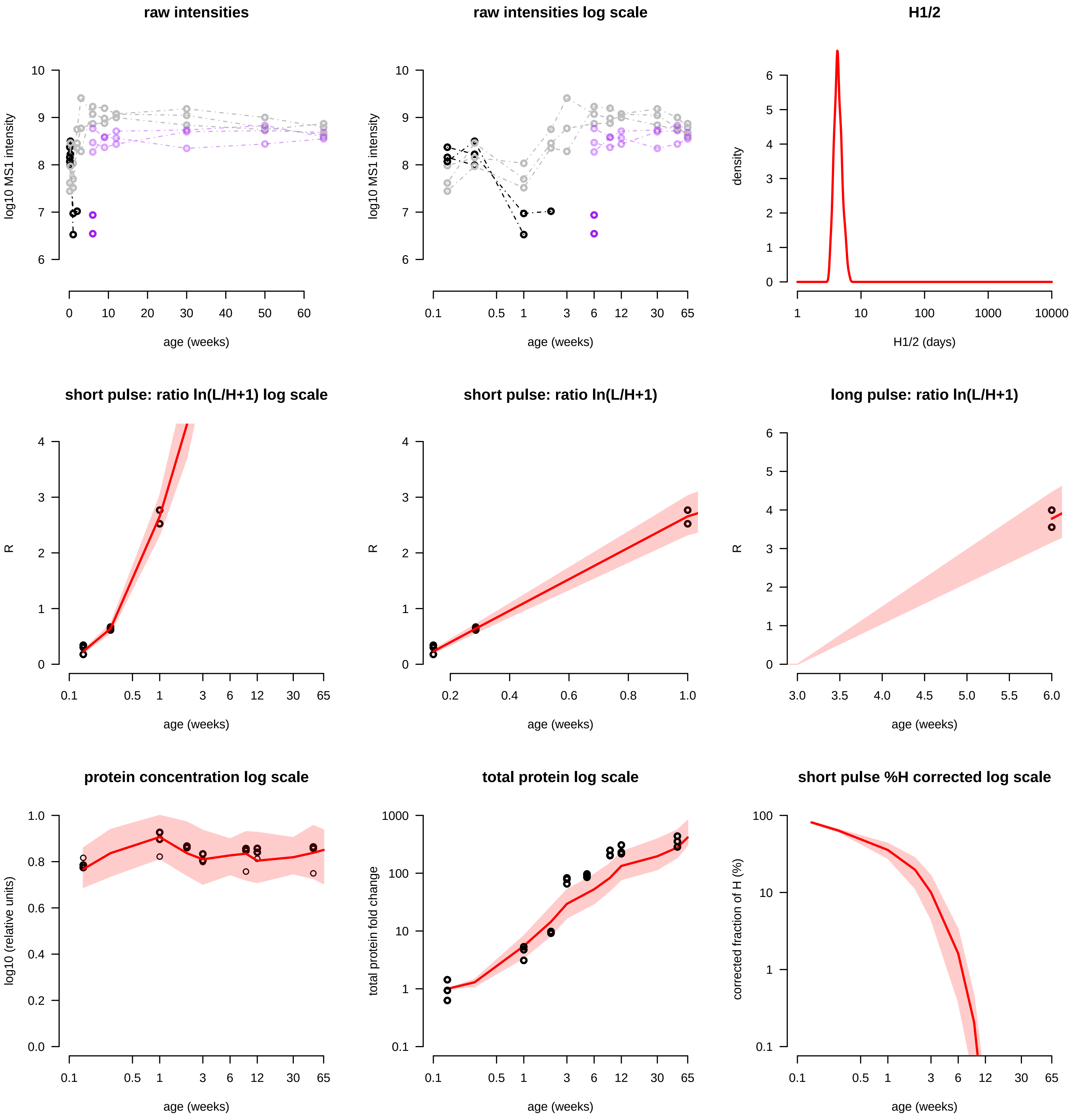

Q9WVL3;Q9WVL3−2

>sp|Q9WVL3|S12A7_MOUSE

Solute carrier family 12 member 7

GN=Slc12a7

Med H1/2: 5 days

Q5 H1/2: 2.6 days

Q95 H1/2: 29.6 days

reliable model fit: yes

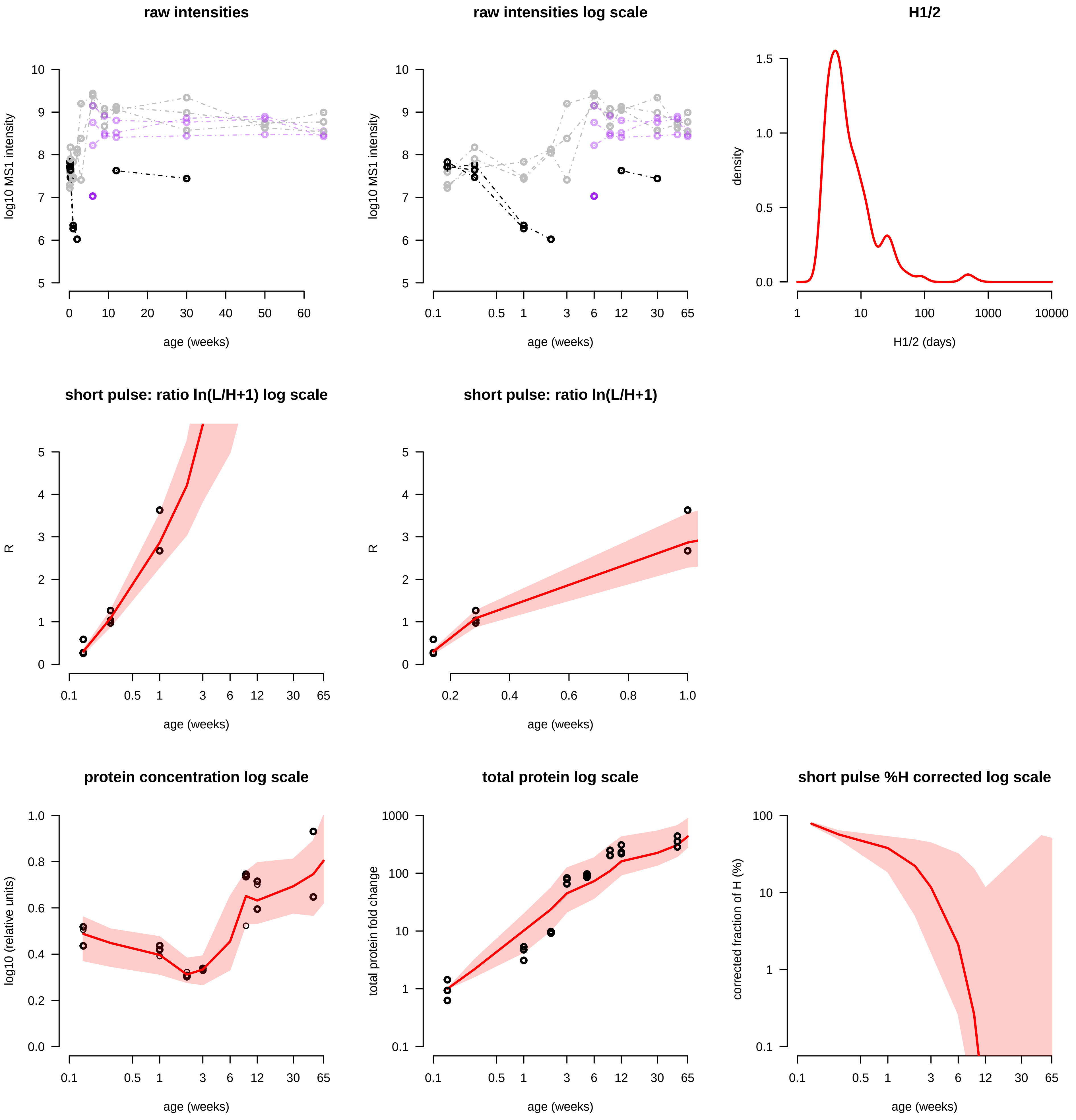

Q8BKZ9

>sp|Q8BKZ9|ODPX_MOUSE

Pyruvate dehydrogenase protein X component, mitochondrial

GN=Pdhx

Med H1/2: 4.5 days

Q5 H1/2: 3.3 days

Q95 H1/2: 6 days

reliable model fit: yes

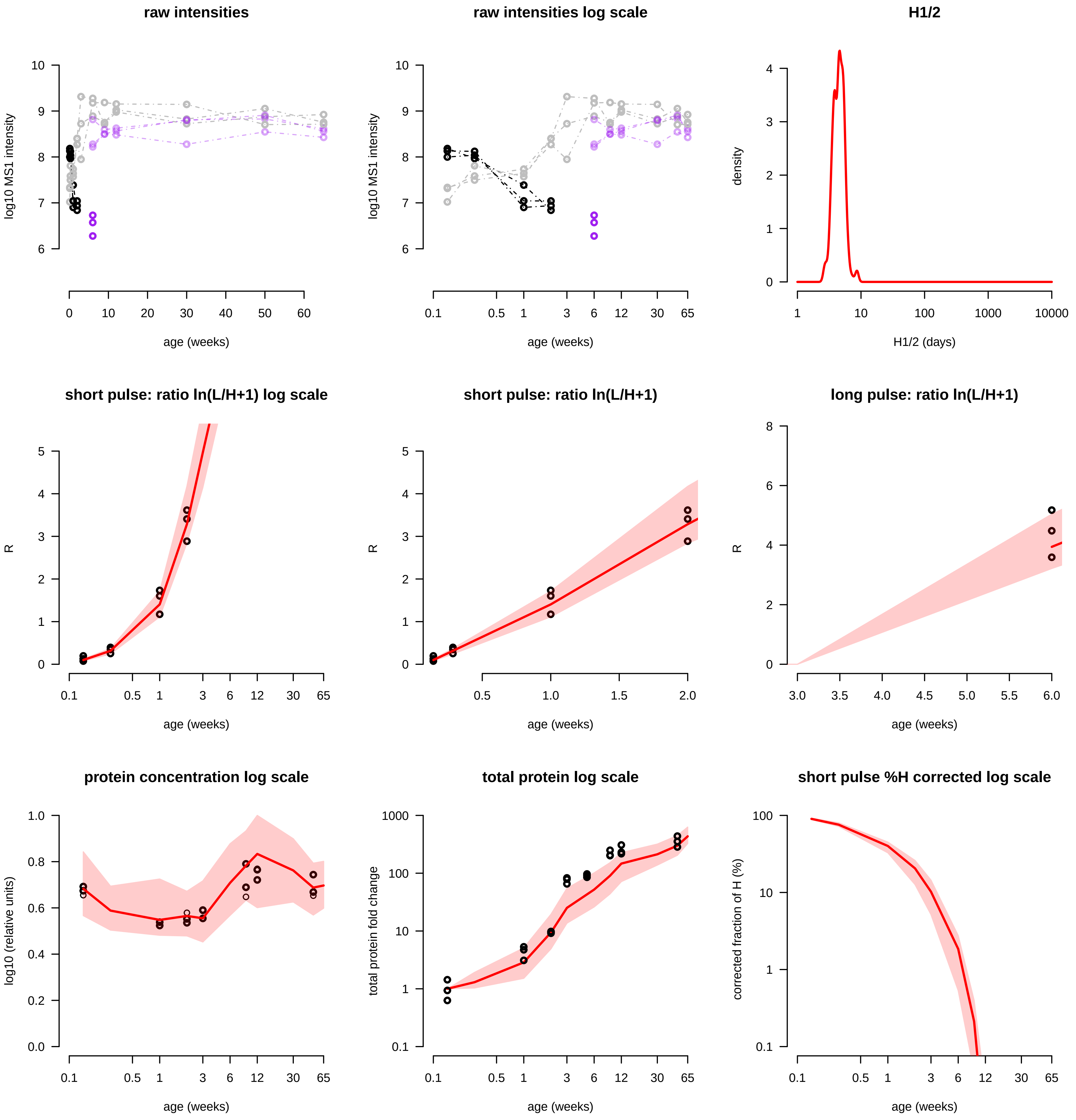

Q9CQB5

>sp|Q9CQB5|CISD2_MOUSE

CDGSH iron−sulfur domain−containing protein 2

GN=Cisd2

Med H1/2: 5.8 days

Q5 H1/2: 4.6 days

Q95 H1/2: 9.4 days

reliable model fit: yes

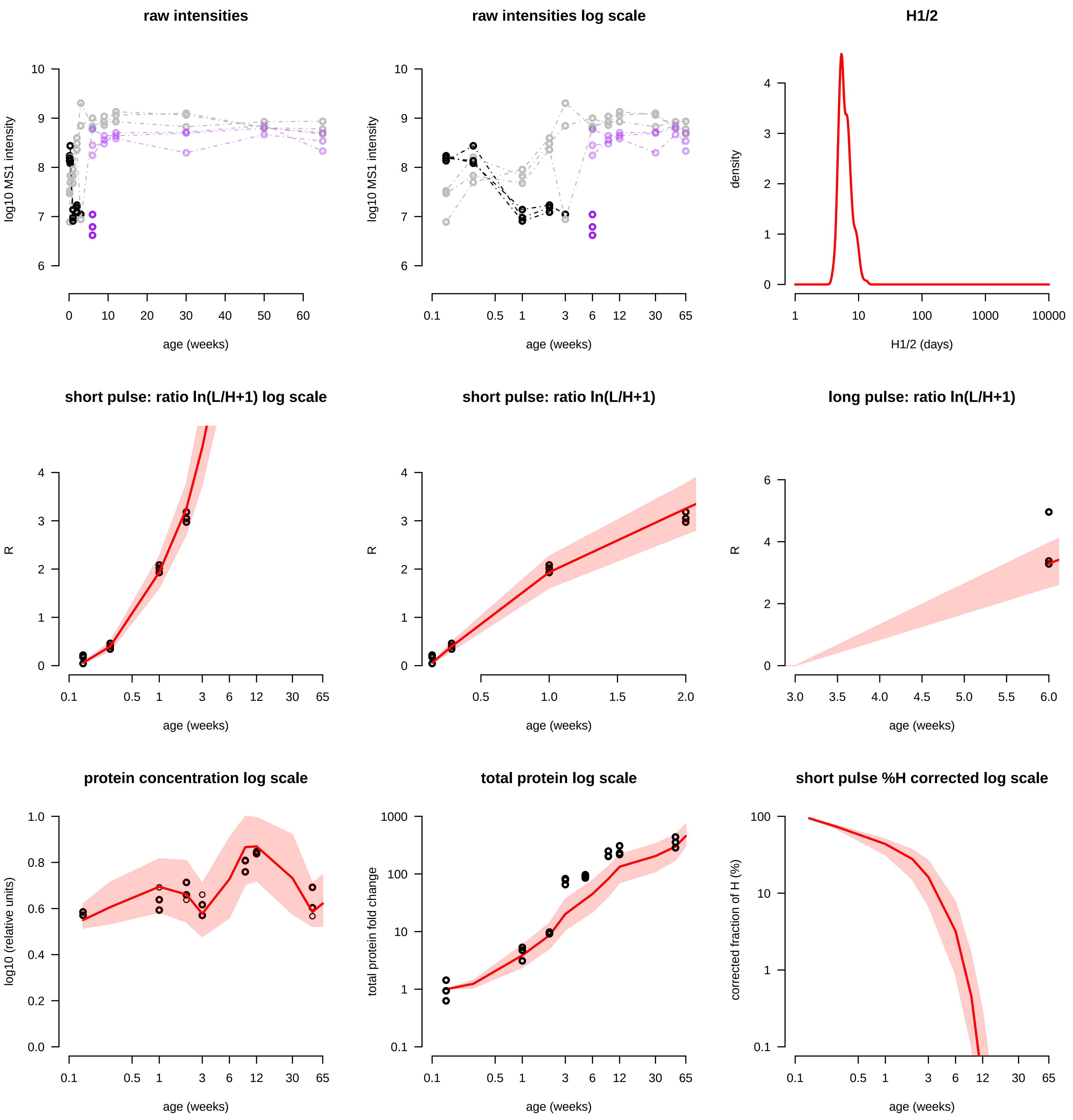

O88712

>sp|O88712|CTBP1_MOUSE

C−terminal−binding protein 1

GN=Ctbp1

Med H1/2: 5.1 days

Q5 H1/2: 3.9 days

Q95 H1/2: 6.7 days

reliable model fit: yes

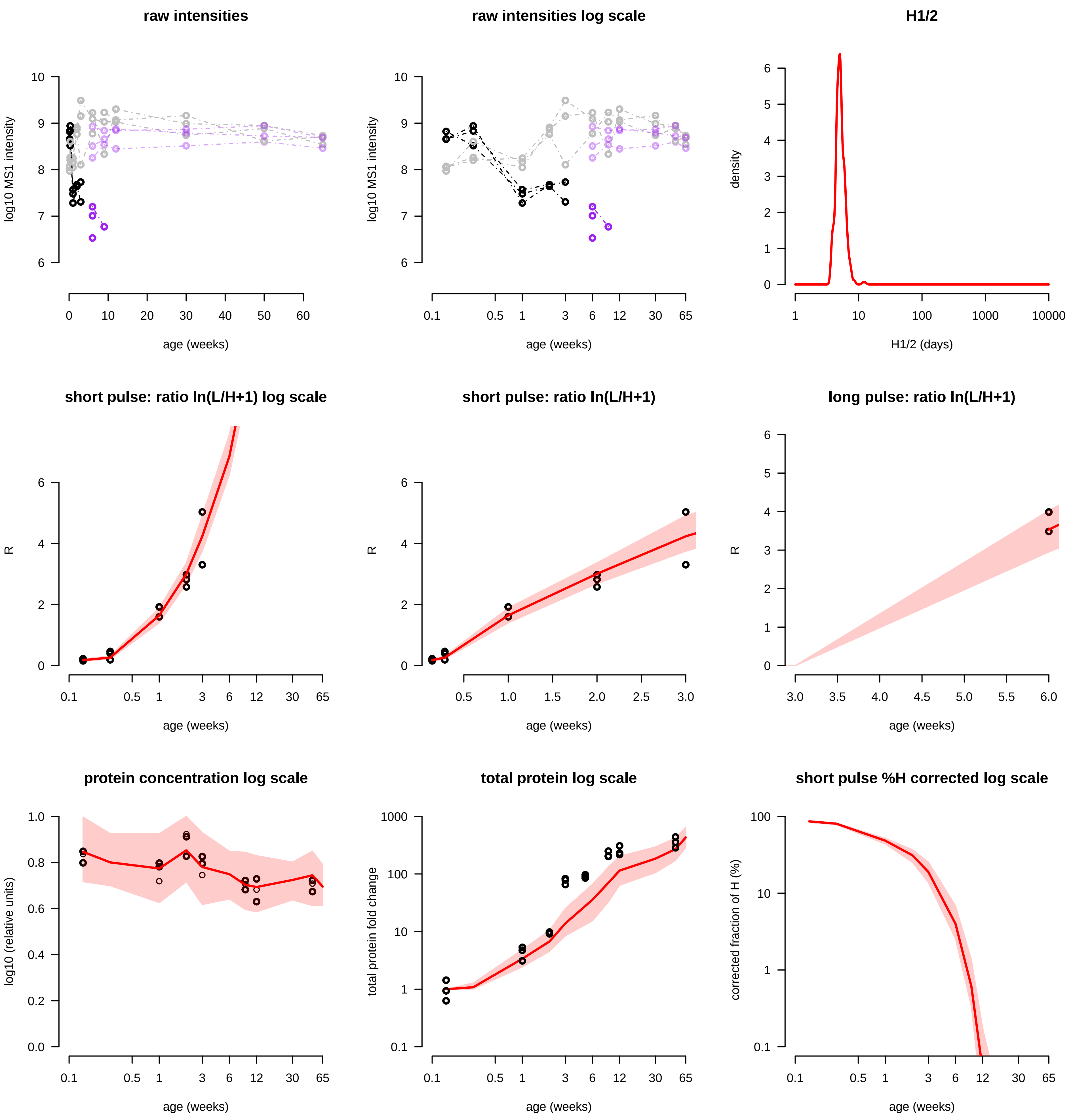

O70194

>sp|O70194|EIF3D_MOUSE

Eukaryotic translation initiation factor 3 subunit D

GN=Eif3d

Med H1/2: 46.2 days

Q5 H1/2: 29 days

Q95 H1/2: 96.5 days

reliable model fit: yes

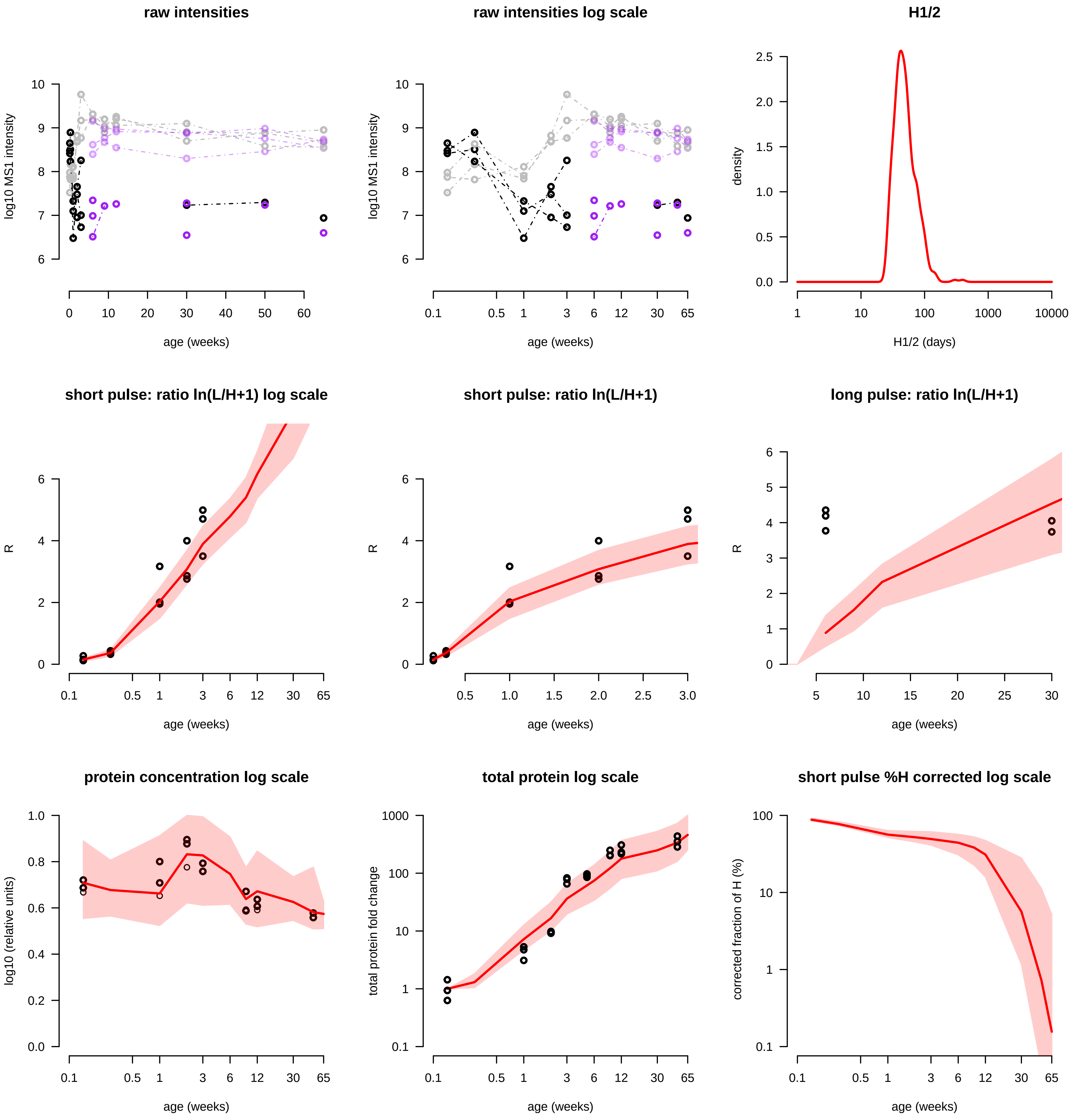

P61021

>sp|P61021|RAB5B_MOUSE

Ras−related protein Rab−5B

GN=Rab5b

Med H1/2: 6.5 days

Q5 H1/2: 4.4 days

Q95 H1/2: 10 days

reliable model fit: yes

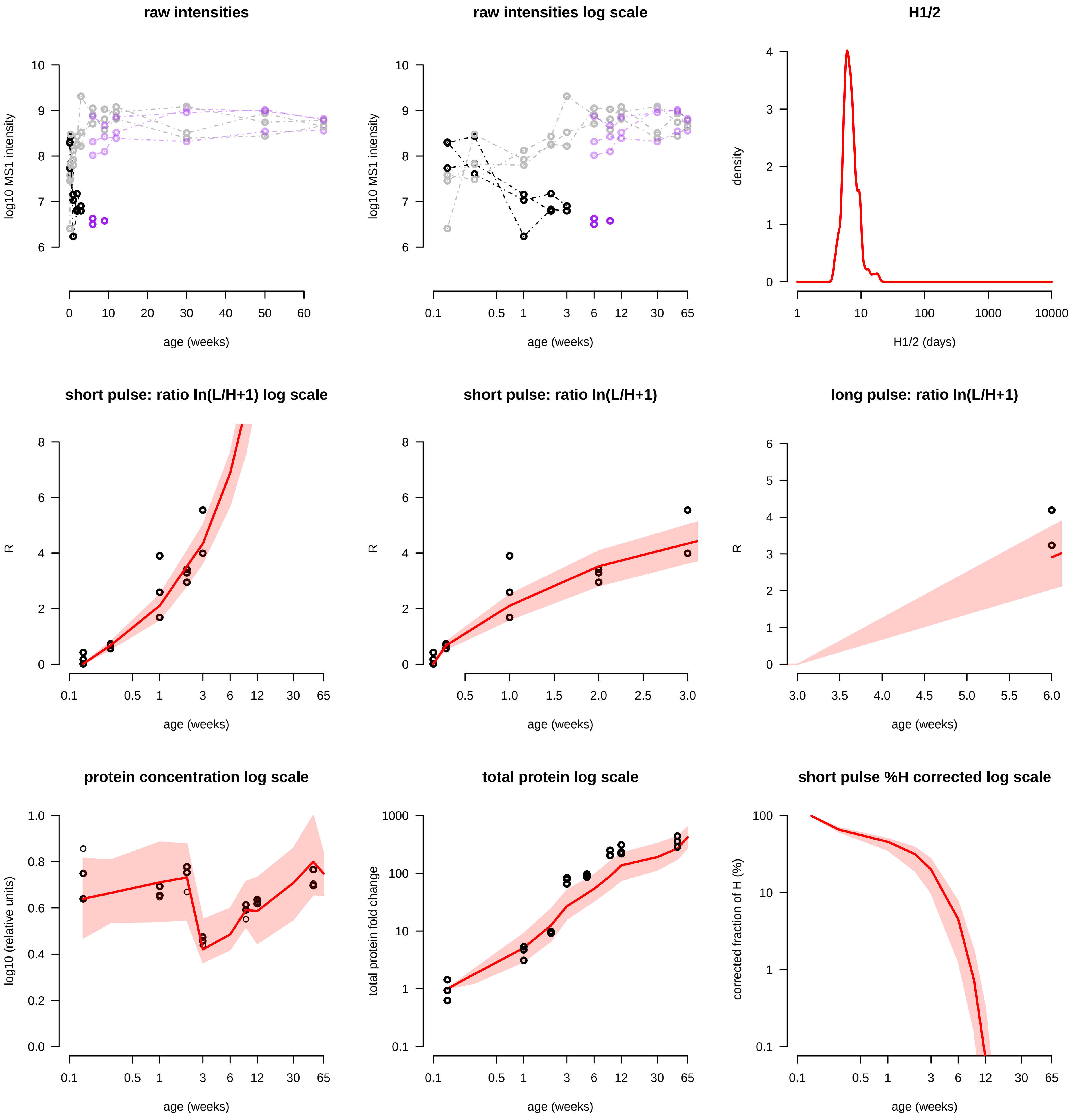

Q9JIY5

>sp|Q9JIY5|HTRA2_MOUSE

Serine protease HTRA2, mitochondrial

GN=Htra2

Med H1/2: 6.3 days

Q5 H1/2: 4.3 days

Q95 H1/2: 10.2 days

reliable model fit: yes

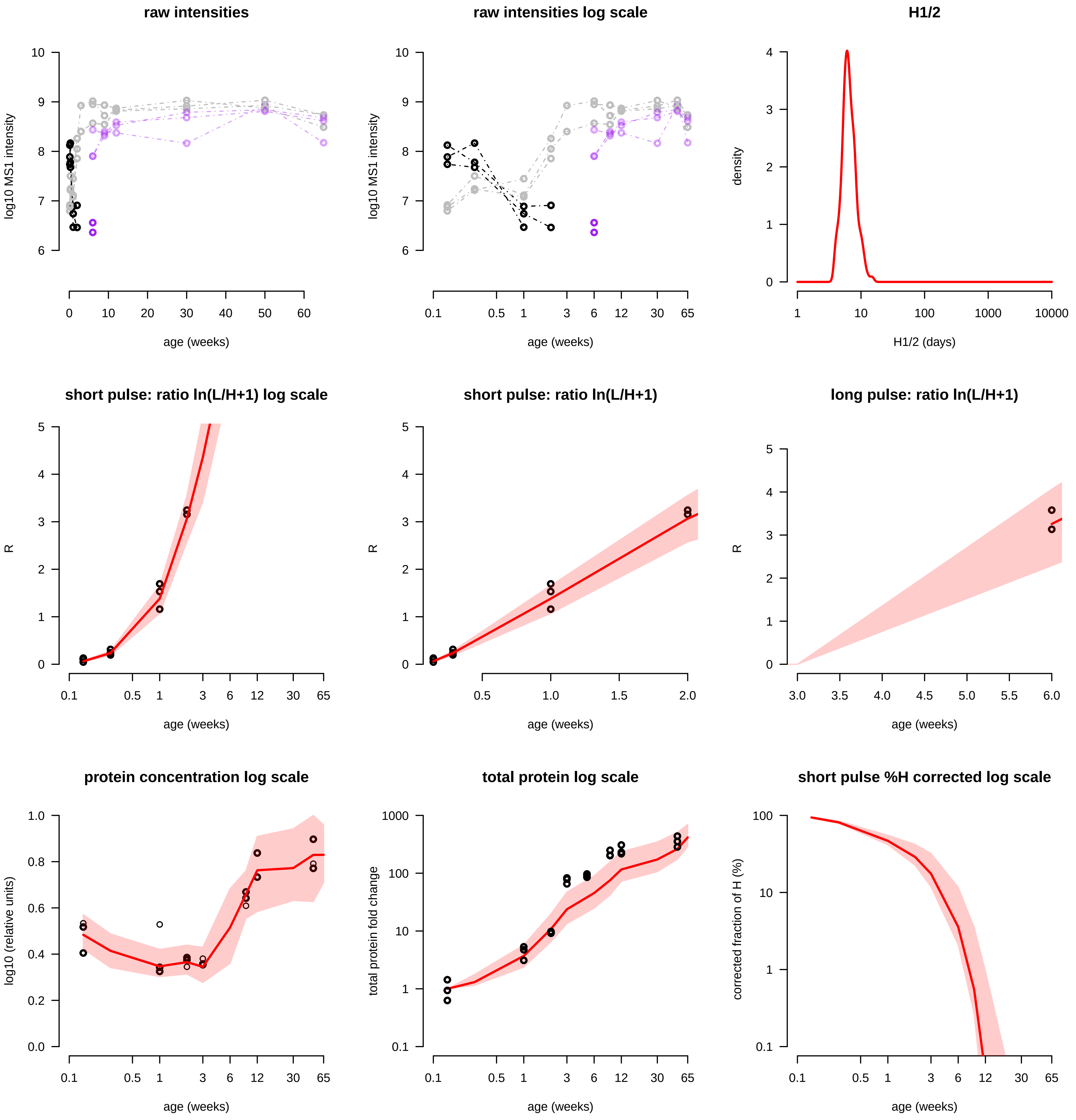

Q9DB15

>sp|Q9DB15|RM12_MOUSE

39S ribosomal protein L12, mitochondrial

GN=Mrpl12

Med H1/2: 5 days

Q5 H1/2: 2.6 days

Q95 H1/2: 37.6 days

reliable model fit: no

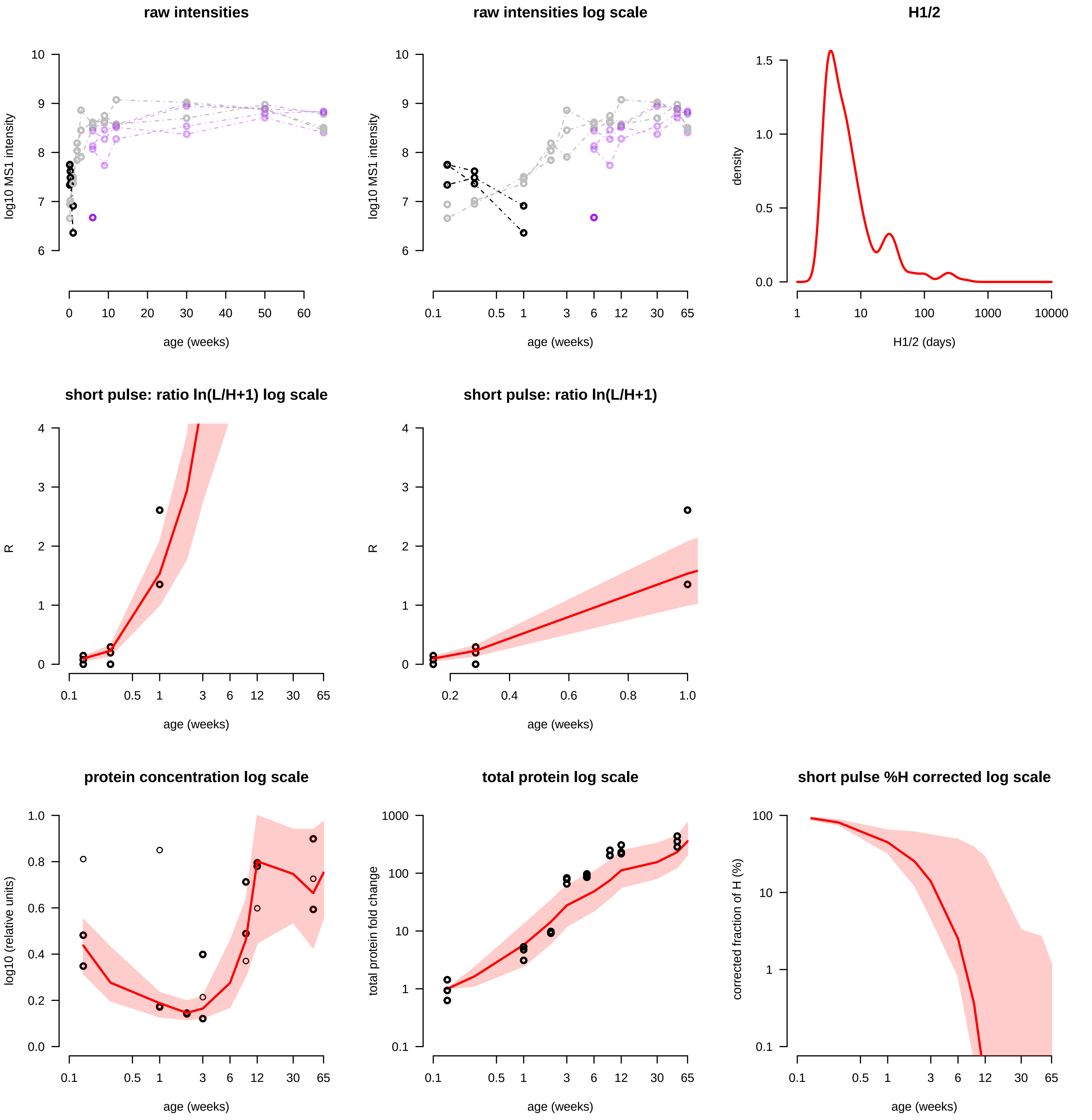

Q3UKJ7;Q3UKJ7−2

>sp|Q3UKJ7|SMU1_MOUSE

WD40 repeat−containing protein SMU1

GN=Smu1

Med H1/2: 3.5 days

Q5 H1/2: 2.8 days

Q95 H1/2: 4.3 days

reliable model fit: yes

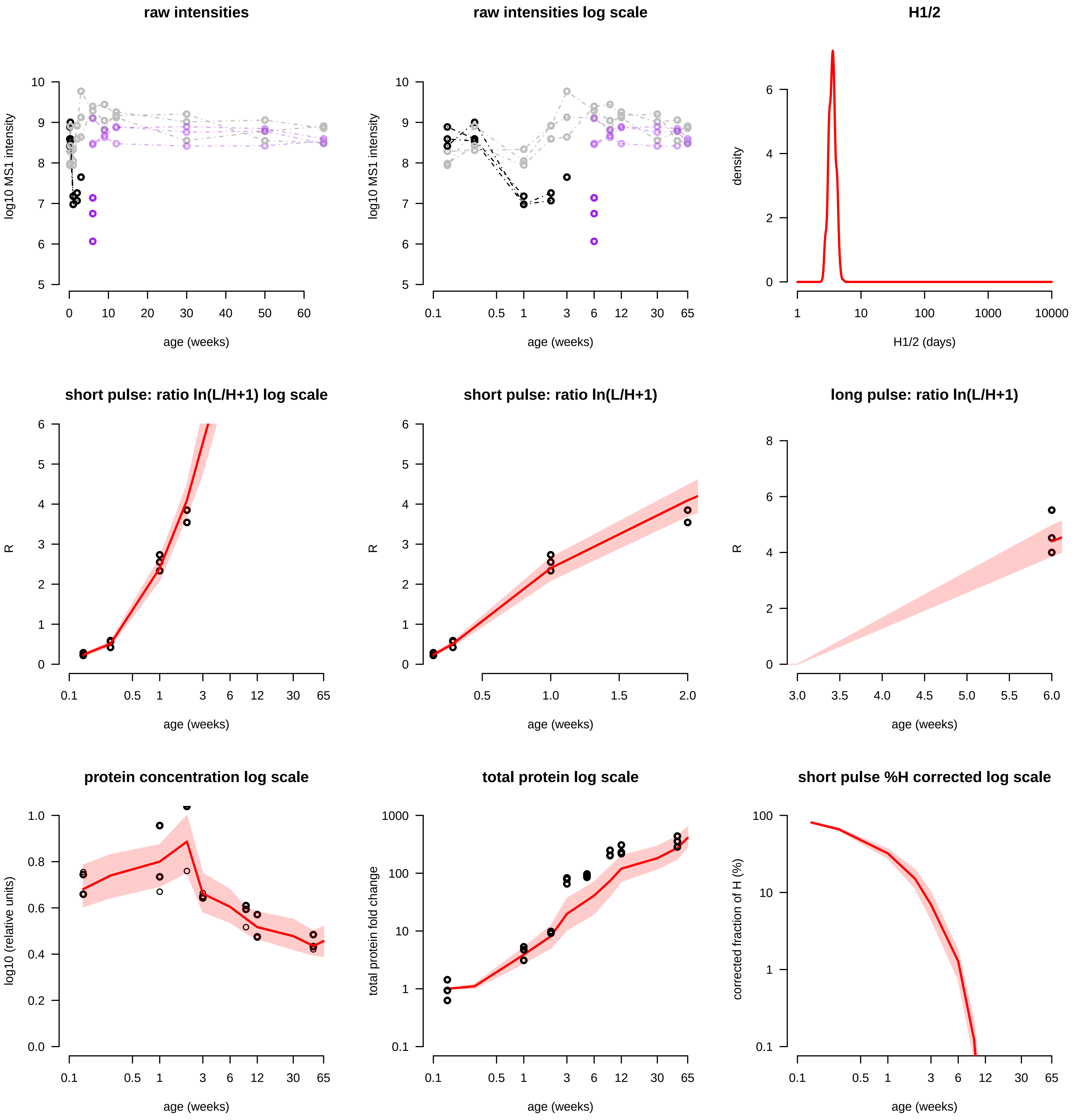

Q8R1A4;Q8R1A4−2

>sp|Q8R1A4|DOCK7_MOUSE

Dedicator of cytokinesis protein 7

GN=Dock7

Med H1/2: 6.2 days

Q5 H1/2: 2.9 days

Q95 H1/2: 24.6 days

reliable model fit: yes

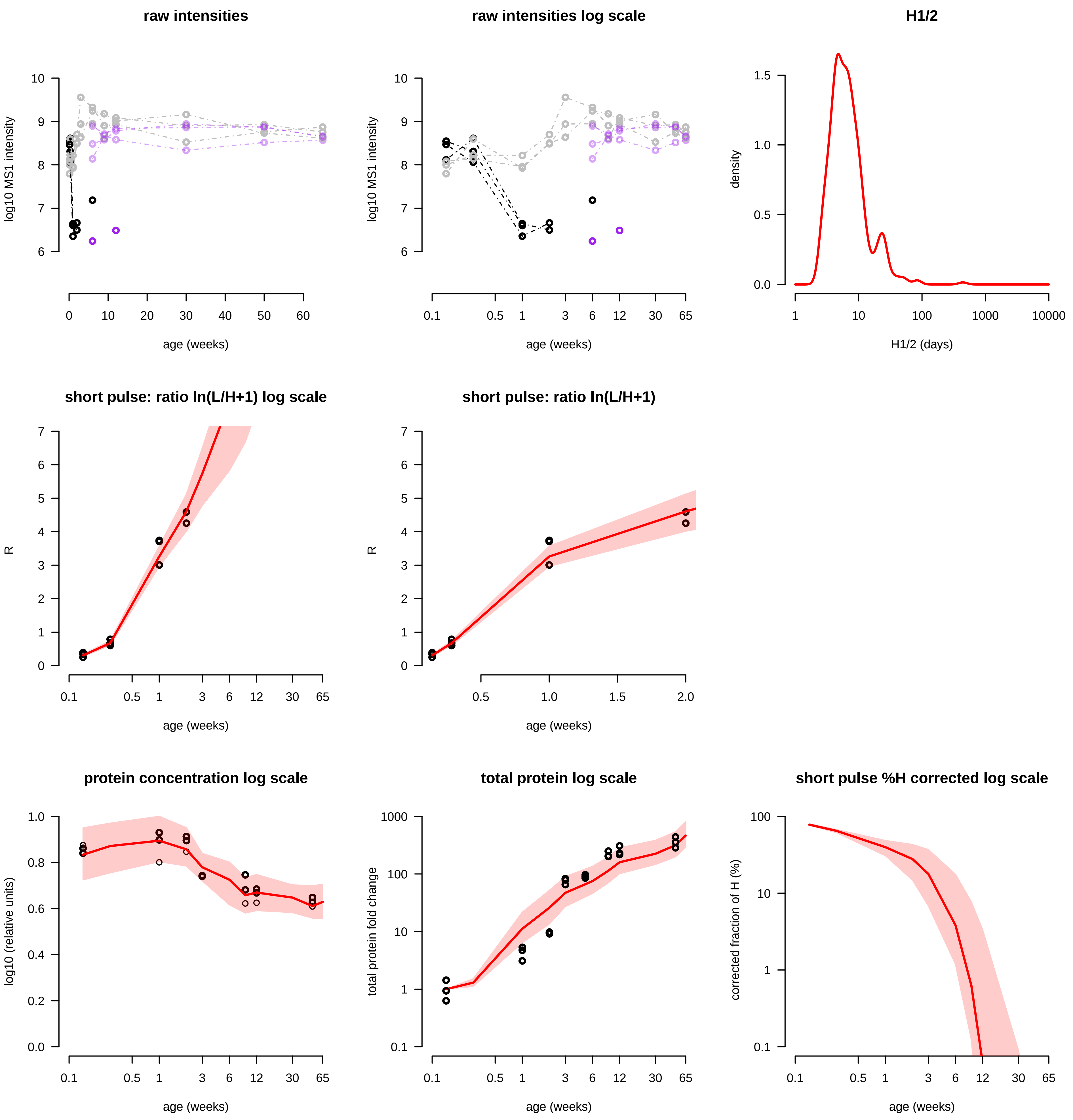

P06330;P01757;P01756

>sp|P06330|HVM51_MOUSE

Ig heavy chain V region AC38 205.12

Med H1/2: 43.4 days

Q5 H1/2: 17.4 days

Q95 H1/2: 228 days

reliable model fit: yes

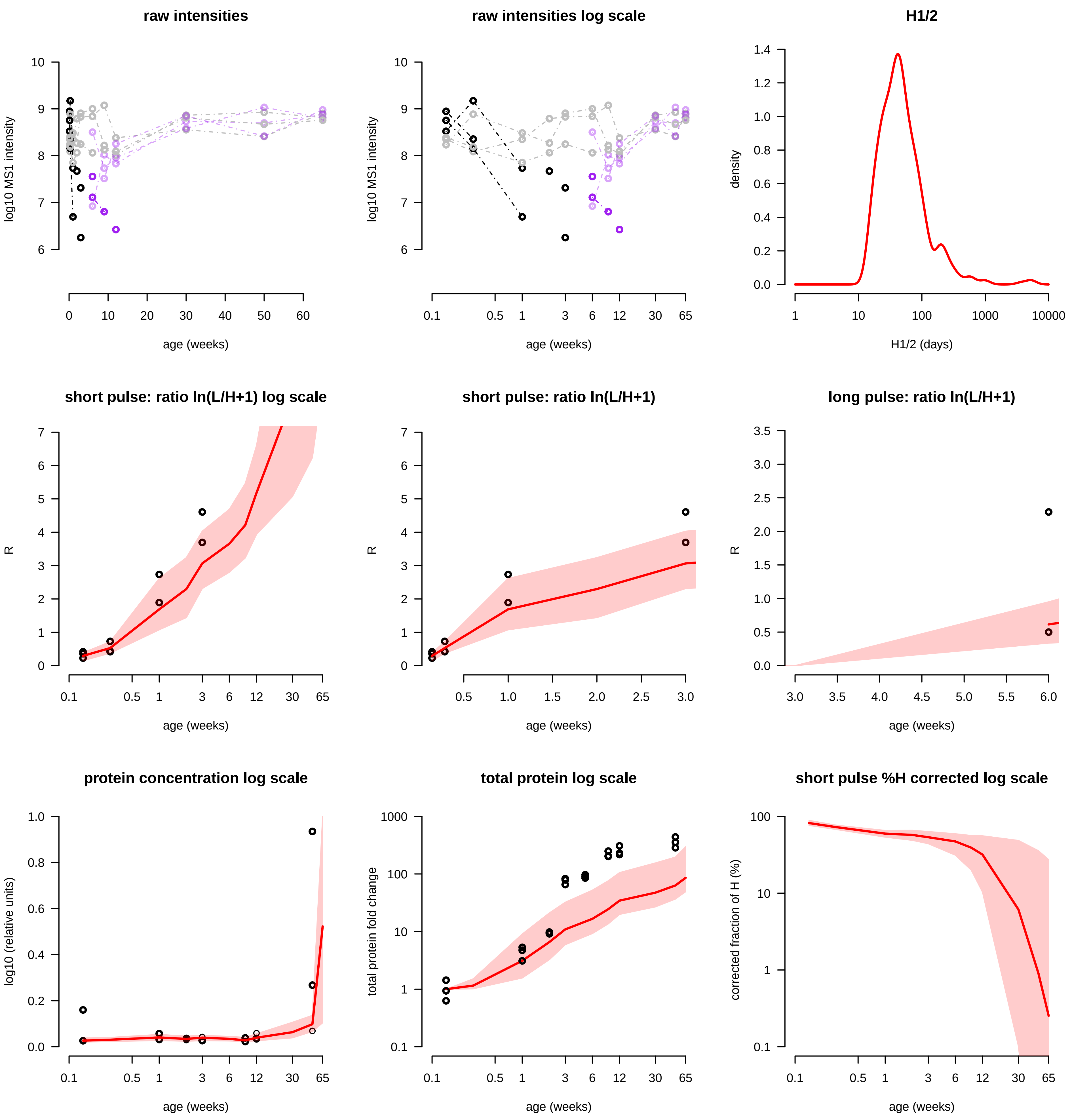

P70333

>sp|P70333|HNRH2_MOUSE

Heterogeneous nuclear ribonucleoprotein H2

GN=Hnrnph2

Med H1/2: 5.9 days

Q5 H1/2: 5 days

Q95 H1/2: 7.3 days

reliable model fit: yes

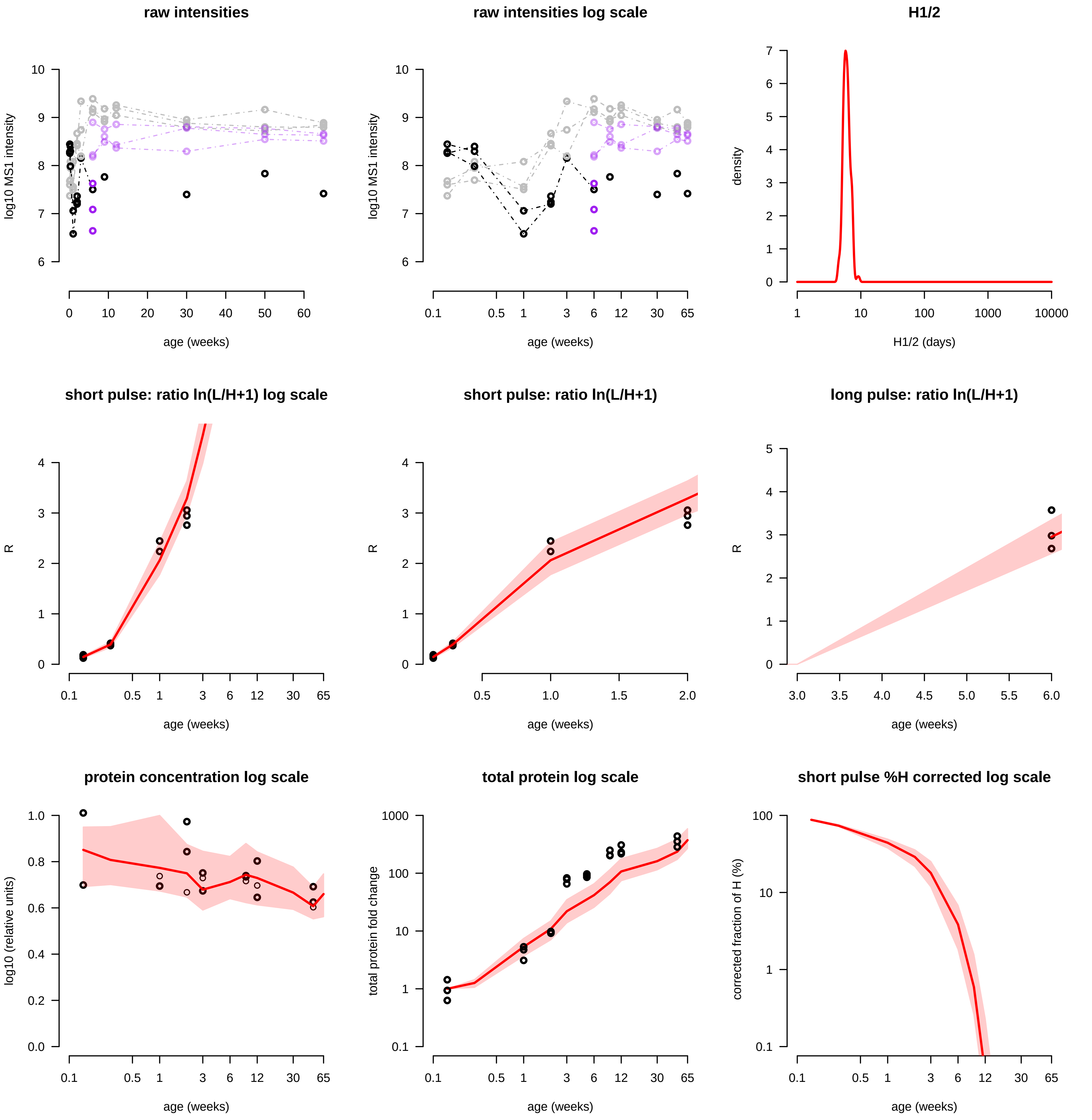

Q9CR51

>sp|Q9CR51|VATG1_MOUSE

V−type proton ATPase subunit G 1

GN=Atp6v1g1

Med H1/2: 4.3 days

Q5 H1/2: 3.7 days

Q95 H1/2: 5.2 days

reliable model fit: yes

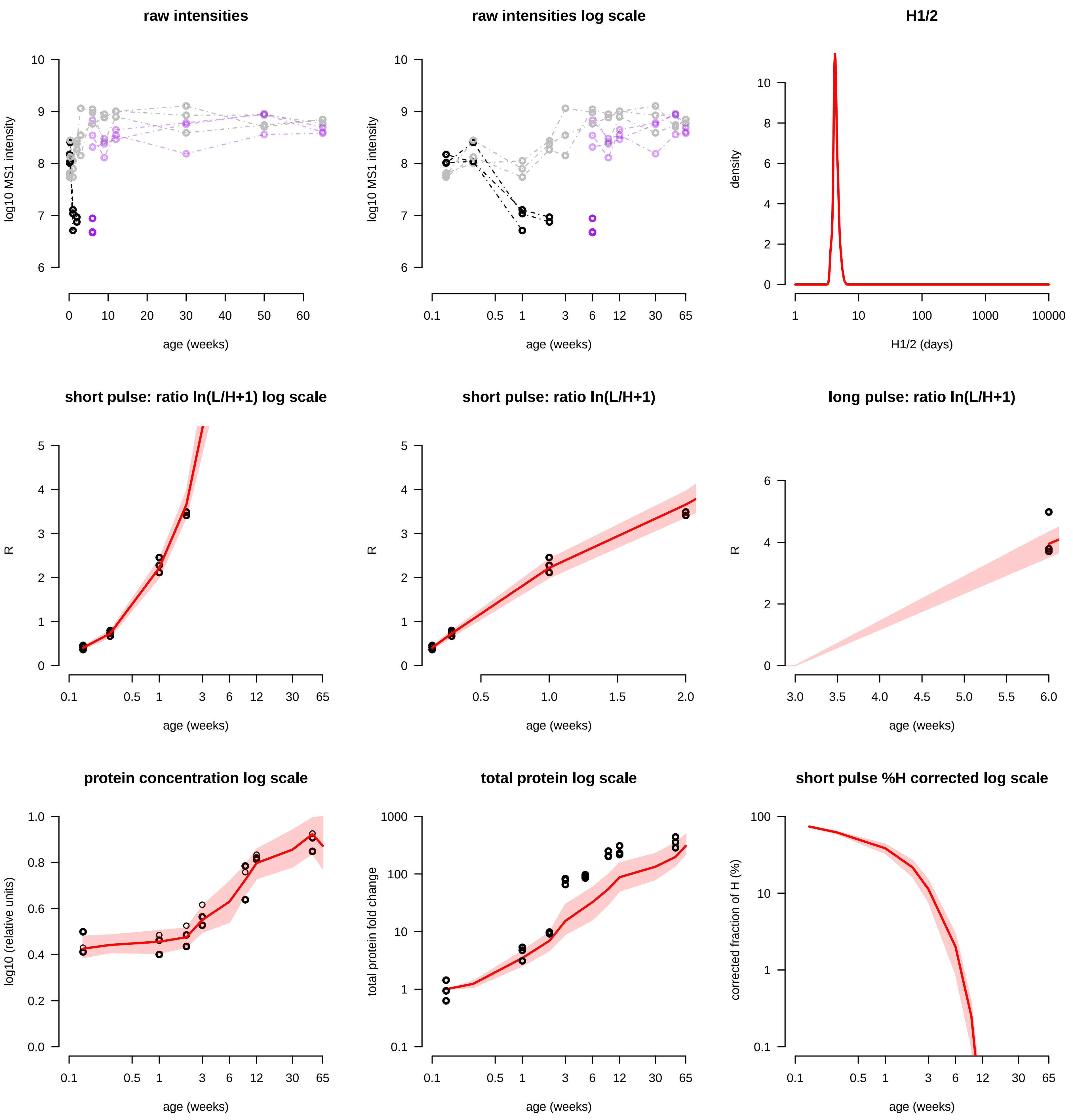

Q61686

>sp|Q61686|CBX5_MOUSE

Chromobox protein homolog 5

GN=Cbx5

Med H1/2: 21.5 days

Q5 H1/2: 13.8 days

Q95 H1/2: 43.2 days

reliable model fit: yes

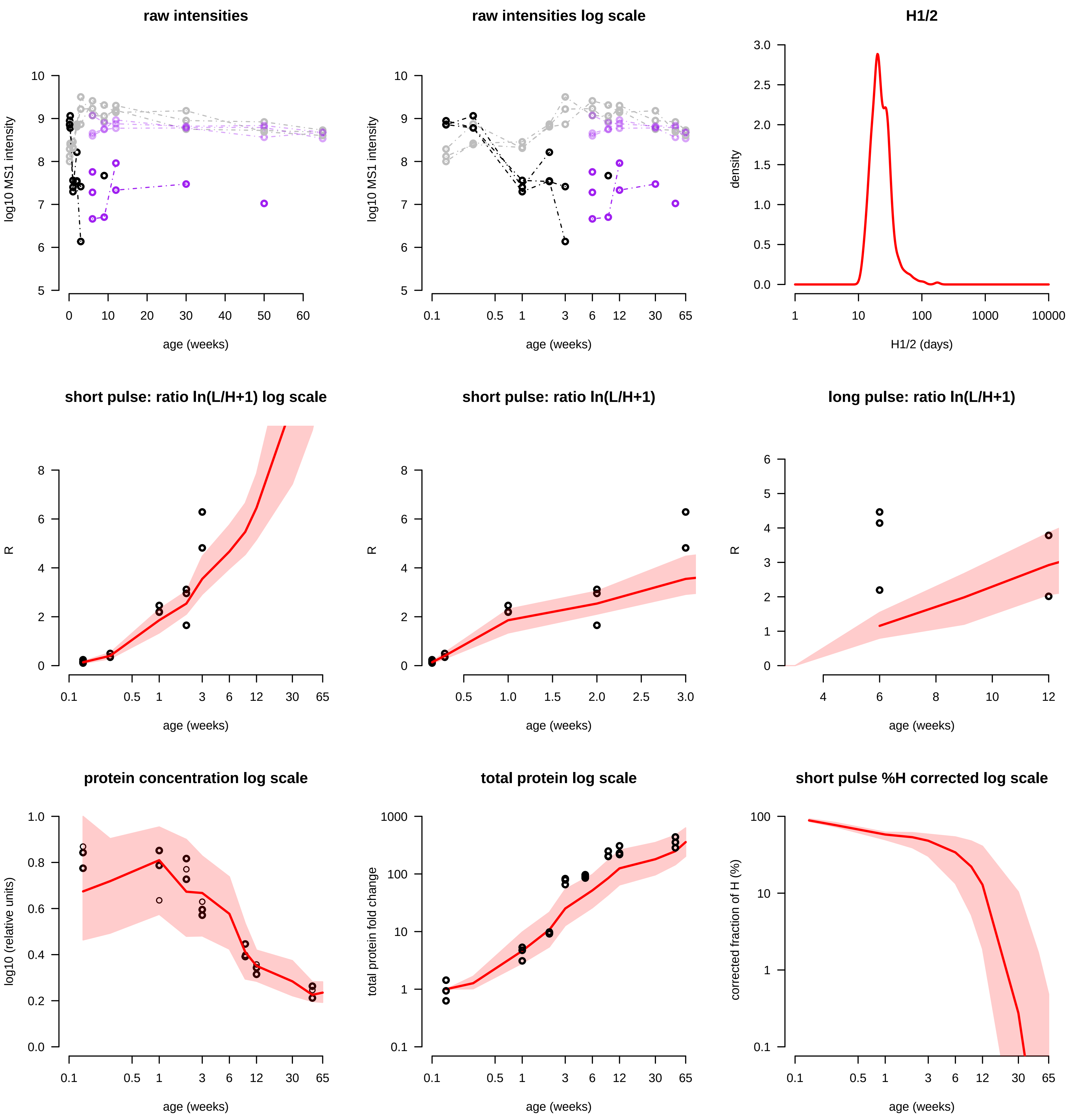

Q9CQH3

>sp|Q9CQH3|NDUB5_MOUSE

NADH dehydrogenase [ubiquinone] 1 beta subcomplex subunit 5, mitochondrial

GN=Ndufb5

Med H1/2: 6 days

Q5 H1/2: 4.5 days

Q95 H1/2: 10.2 days

reliable model fit: yes

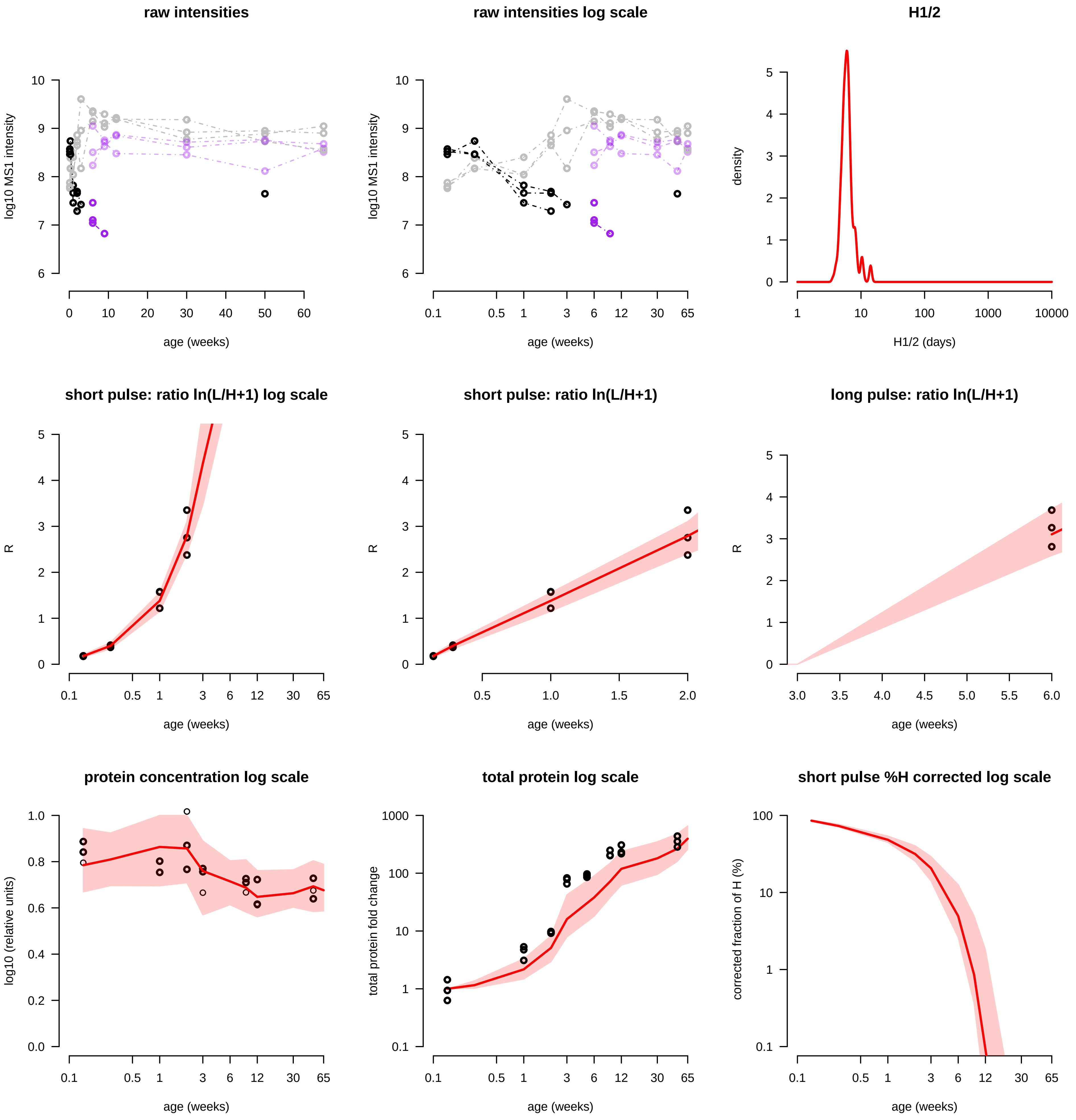

D3YZP9

>sp|D3YZP9|CCDC6_MOUSE

Coiled−coil domain−containing protein 6

GN=Ccdc6

Med H1/2: 3.8 days

Q5 H1/2: 3.1 days

Q95 H1/2: 4.9 days

reliable model fit: yes

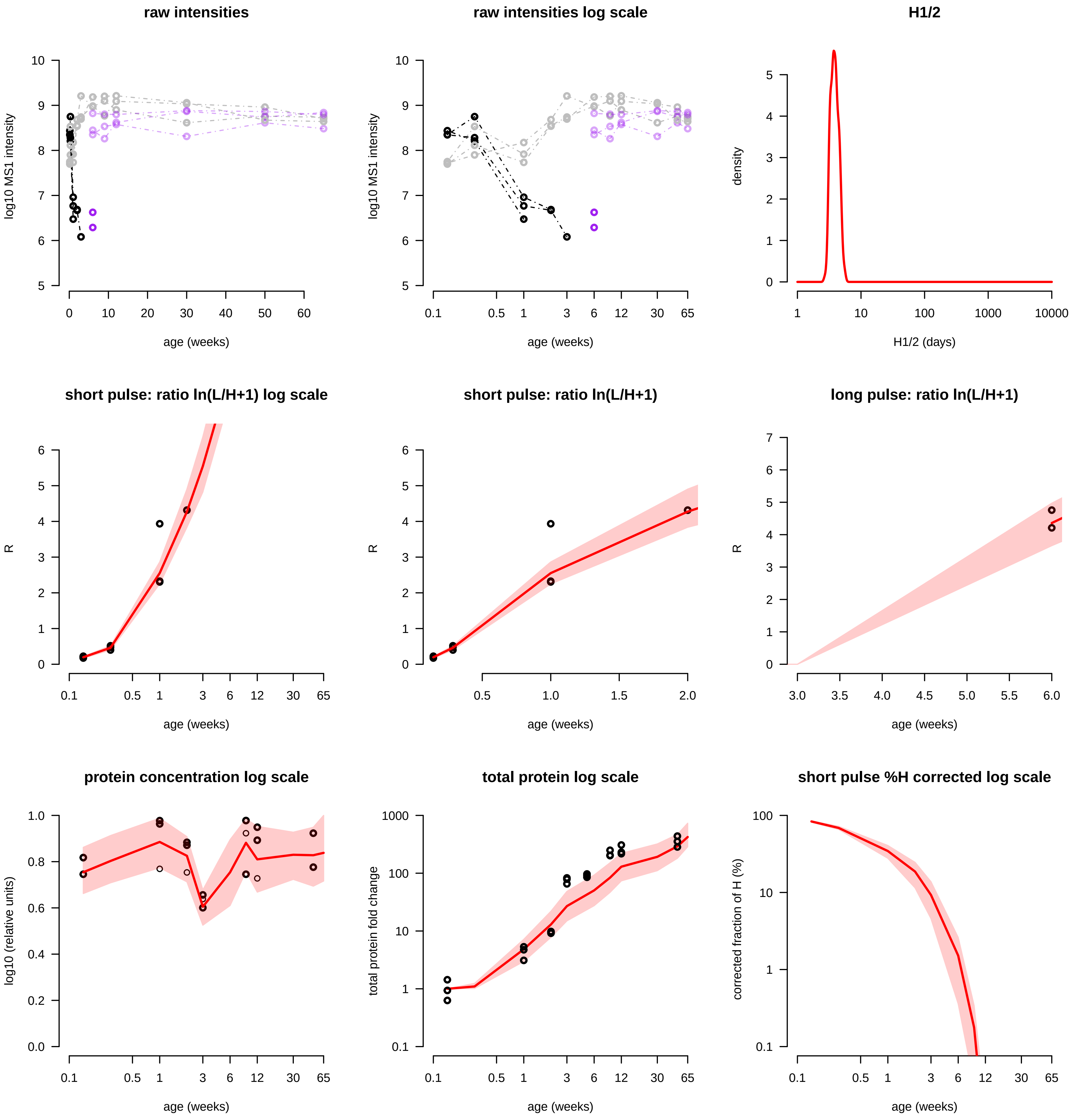

O08585

>sp|O08585|CLCA_MOUSE

Clathrin light chain A

GN=Clta

Med H1/2: 4.9 days

Q5 H1/2: 4.2 days

Q95 H1/2: 6.2 days

reliable model fit: yes

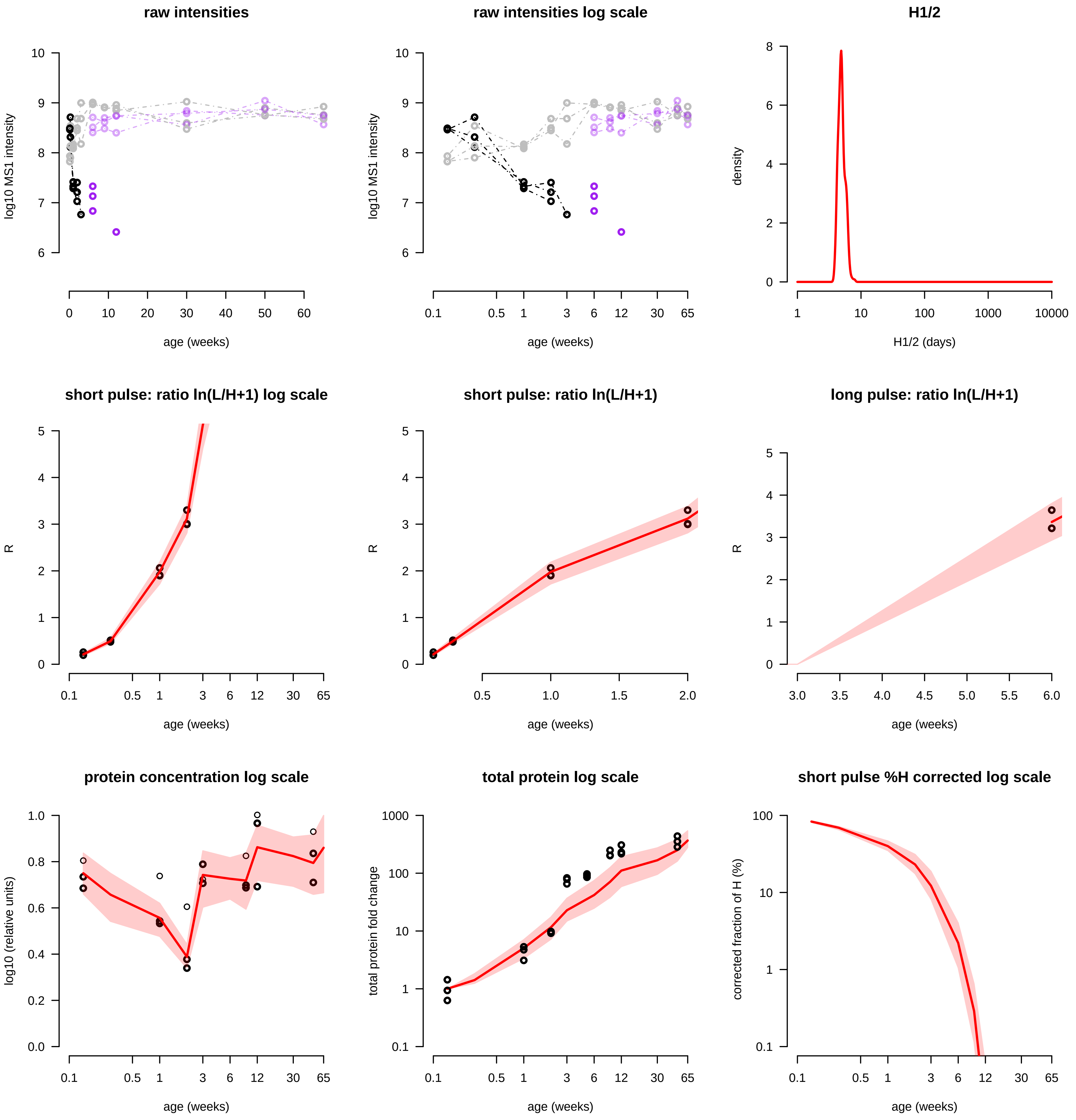

P51863

>sp|P51863|VA0D1_MOUSE

V−type proton ATPase subunit d 1

GN=Atp6v0d1

Med H1/2: 681.4 days

Q5 H1/2: 174.3 days

Q95 H1/2: 8744.2 days

reliable model fit: yes

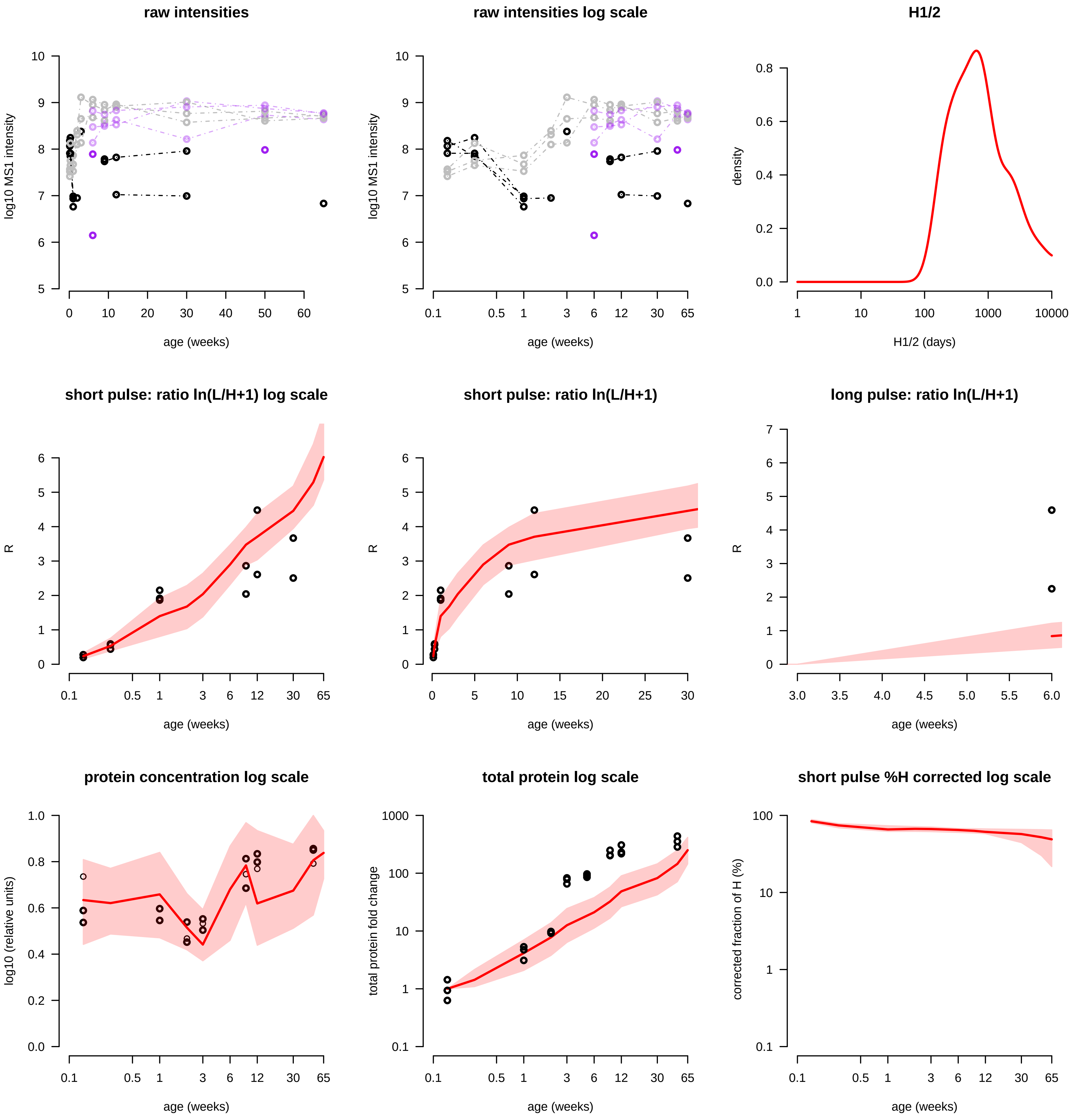

Q9ER00

>sp|Q9ER00|STX12_MOUSE

Syntaxin−12

GN=Stx12

Med H1/2: 3.4 days

Q5 H1/2: 2.9 days

Q95 H1/2: 4.2 days

reliable model fit: yes

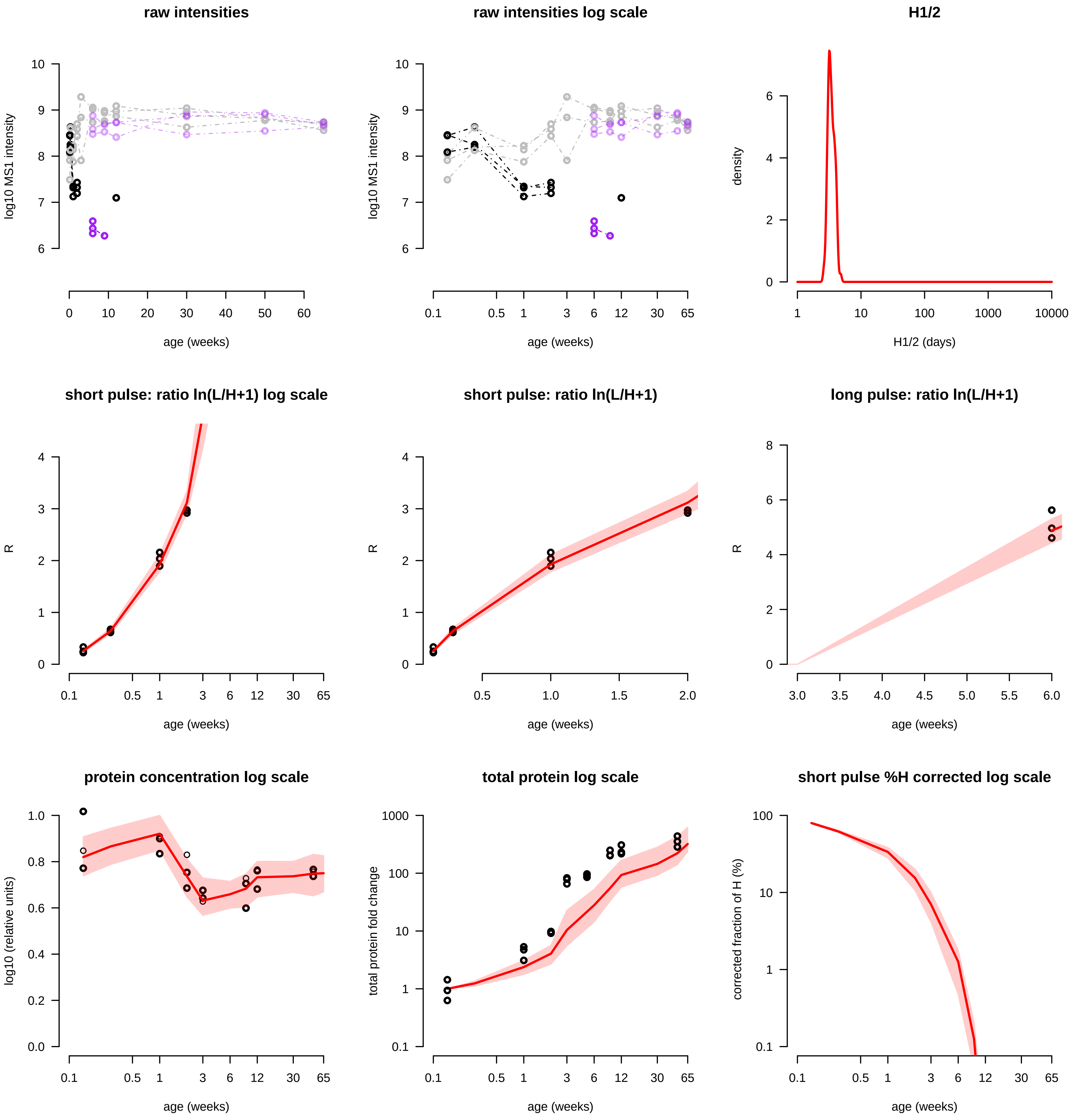

Q8R3B1

>sp|Q8R3B1|PLCD1_MOUSE

1−phosphatidylinositol 4,5−bisphosphate phosphodiesterase delta−1

GN=Plcd1

Med H1/2: 8.7 days

Q5 H1/2: 3.5 days

Q95 H1/2: 53.3 days

reliable model fit: yes

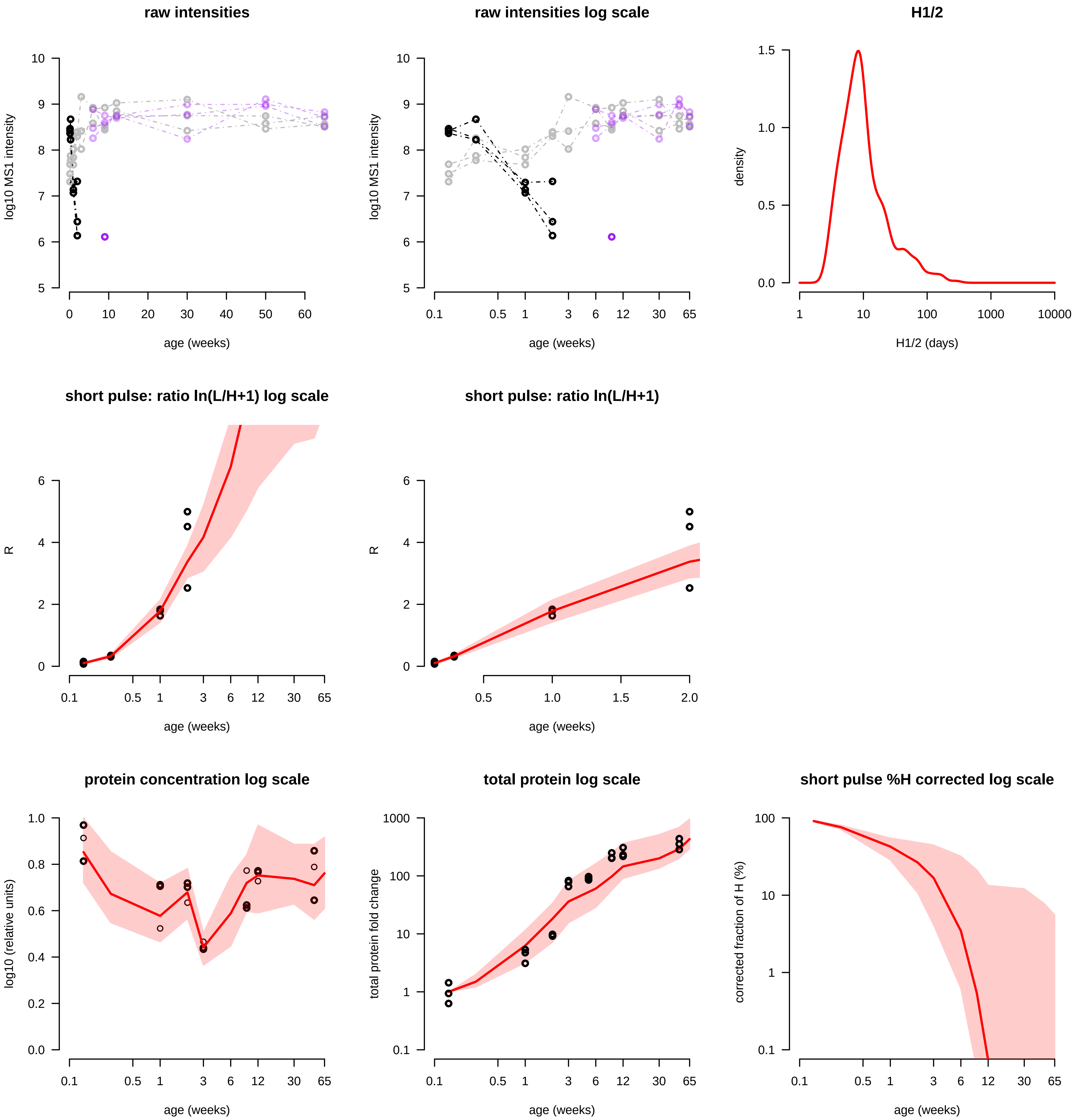

Q9DC69

>sp|Q9DC69|NDUA9_MOUSE

NADH dehydrogenase [ubiquinone] 1 alpha subcomplex subunit 9, mitochondria

GN=Ndufa9

Med H1/2: 6.7 days

Q5 H1/2: 3.1 days

Q95 H1/2: 19 days

reliable model fit: yes

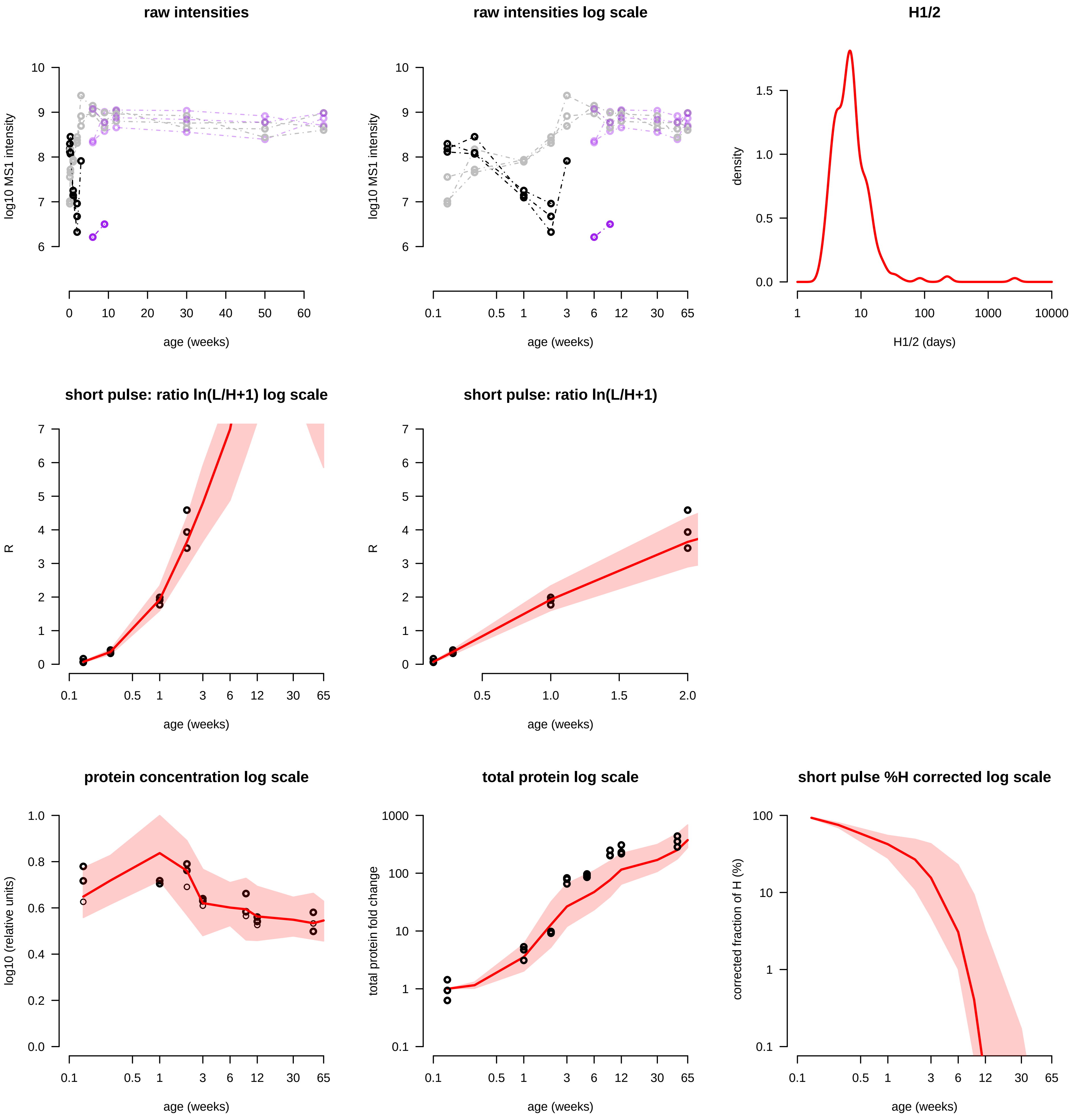

P18608

>sp|P18608|HMGN1_MOUSE

Non−histone chromosomal protein HMG−14

GN=Hmgn1

Med H1/2: 7.1 days

Q5 H1/2: 5.1 days

Q95 H1/2: 11.5 days

reliable model fit: no

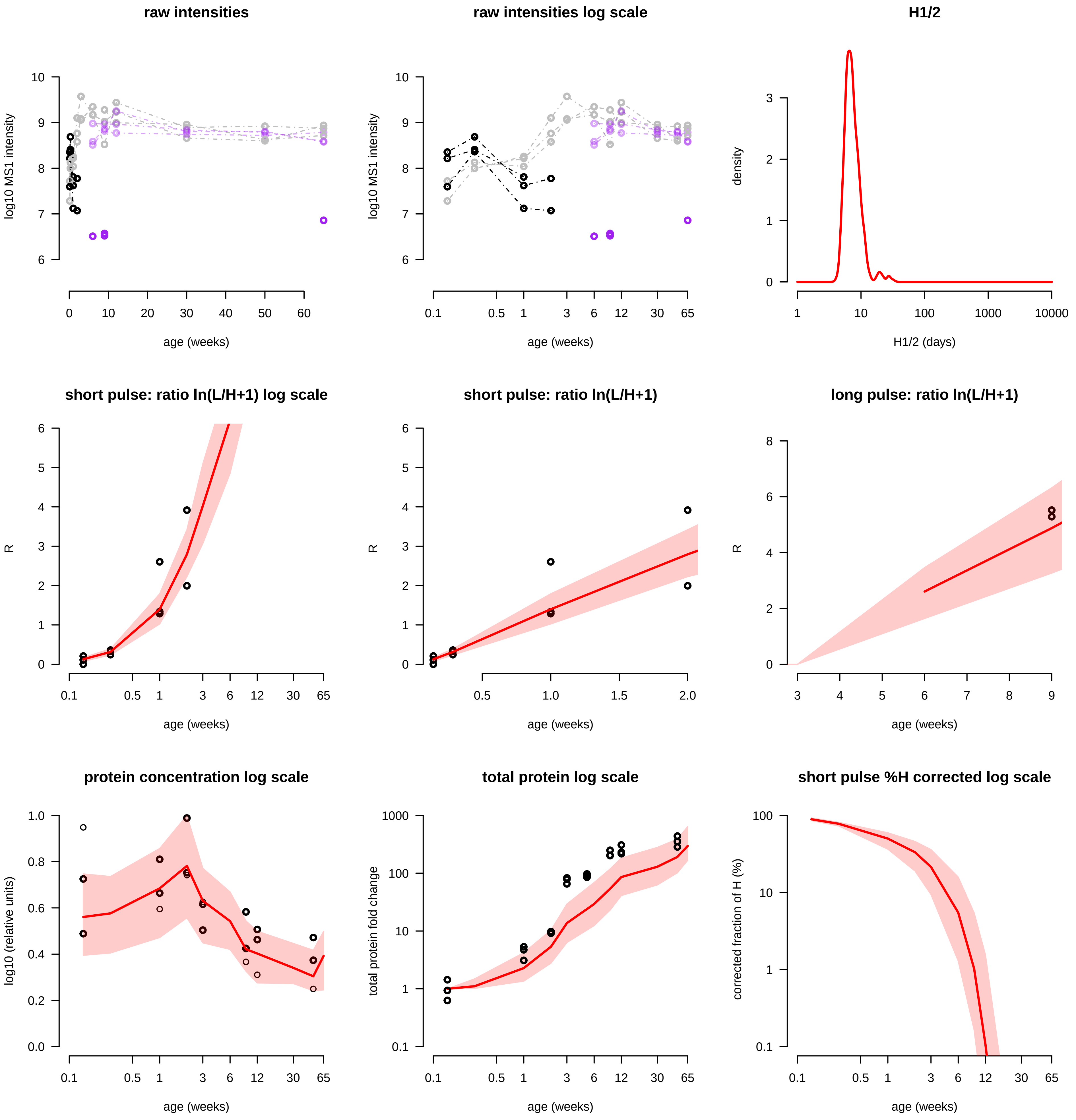

C0HKG6;C0HKG5

>sp|C0HKG6|RNT2B_MOUSE

Ribonuclease T2−B

GN=Rnaset2b

Med H1/2: 4.4 days

Q5 H1/2: 3.6 days

Q95 H1/2: 5.4 days

reliable model fit: yes

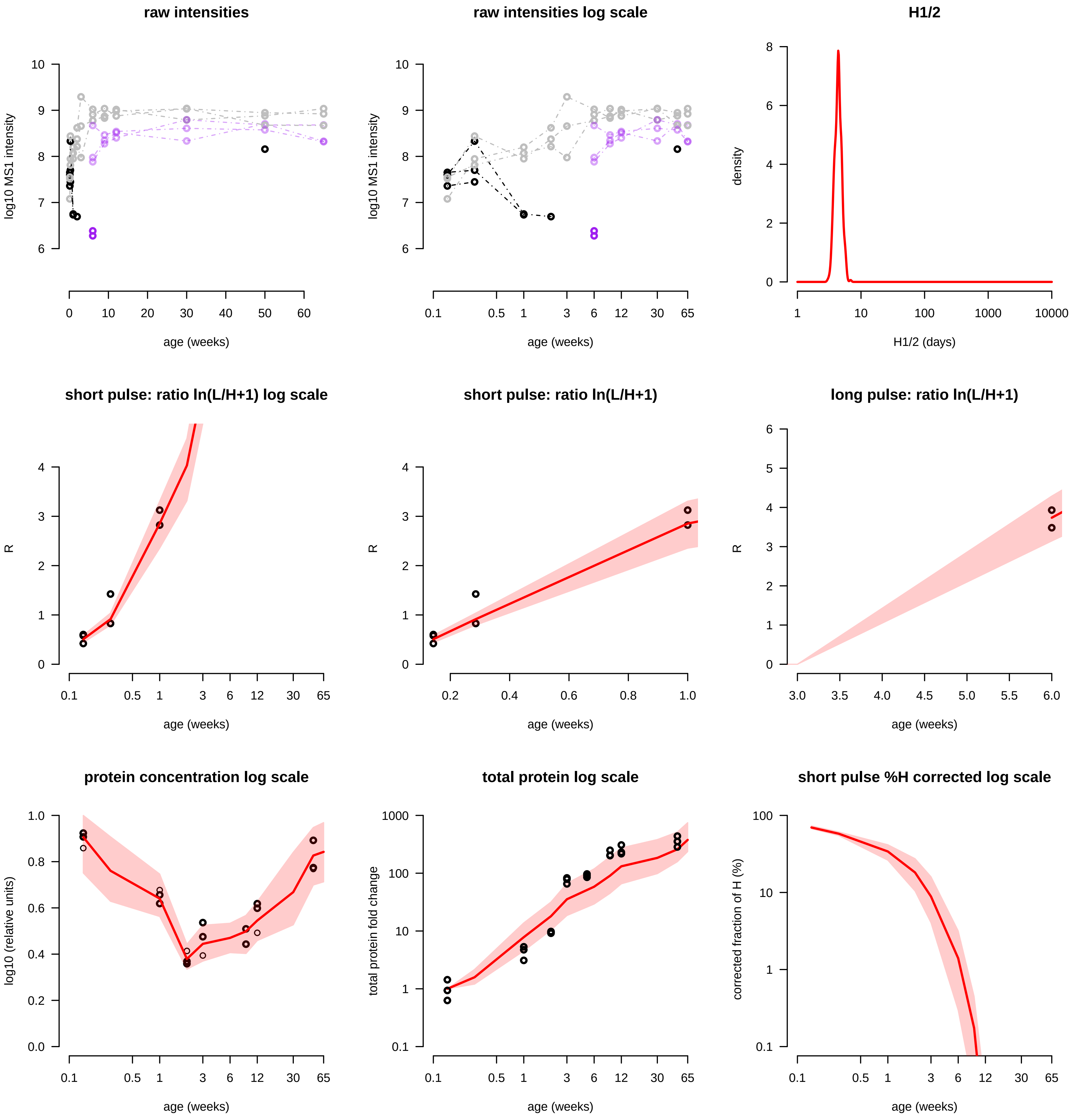

Q9D6S7

>sp|Q9D6S7|RRFM_MOUSE

Ribosome−recycling factor, mitochondrial

GN=Mrrf

Med H1/2: 6.9 days

Q5 H1/2: 3 days

Q95 H1/2: 61.3 days

reliable model fit: yes

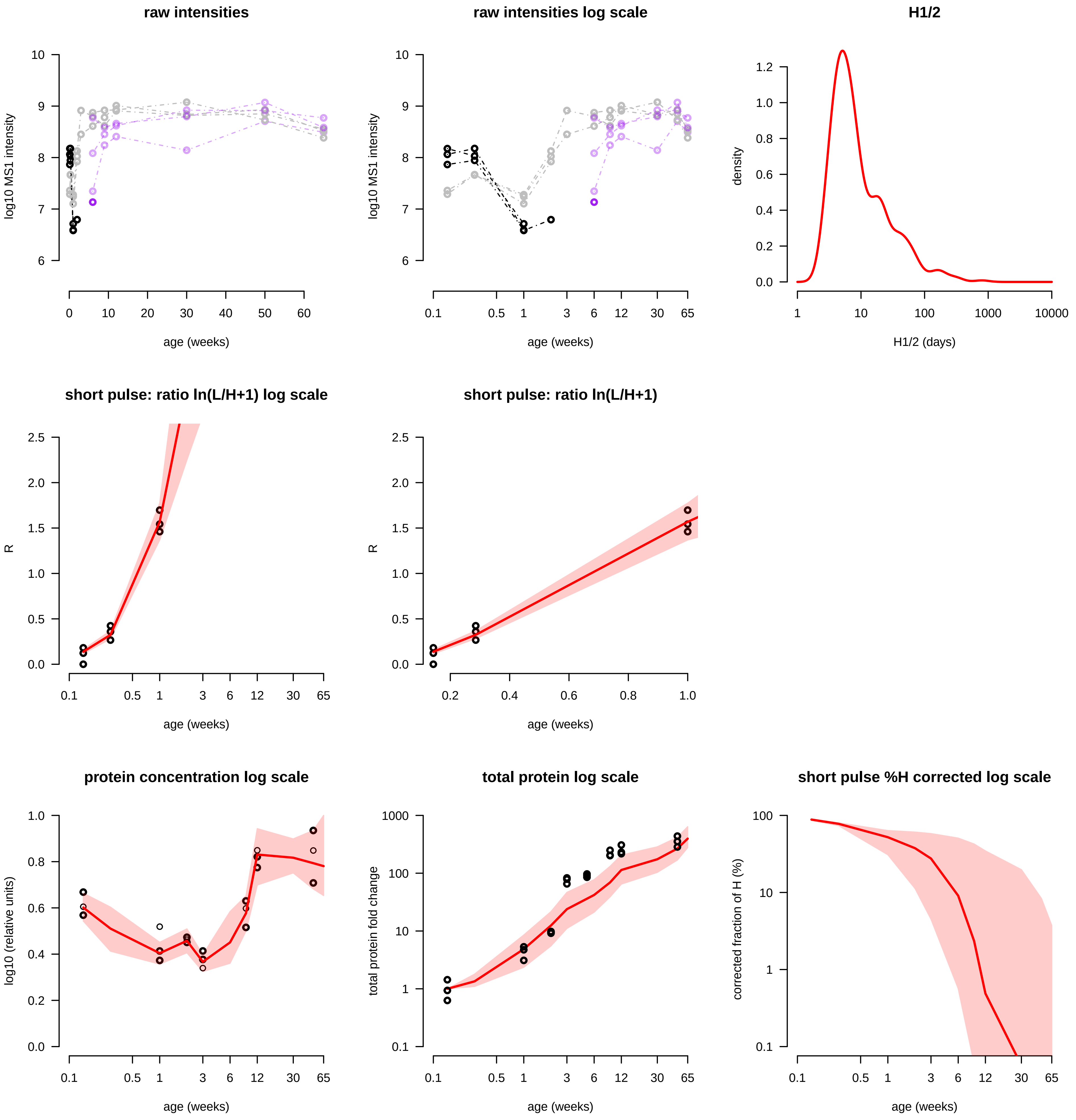

P30999

>sp|P30999|CTND1_MOUSE

Catenin delta−1

GN=Ctnnd1

Med H1/2: 15.1 days

Q5 H1/2: 5.6 days

Q95 H1/2: 215.6 days

reliable model fit: yes

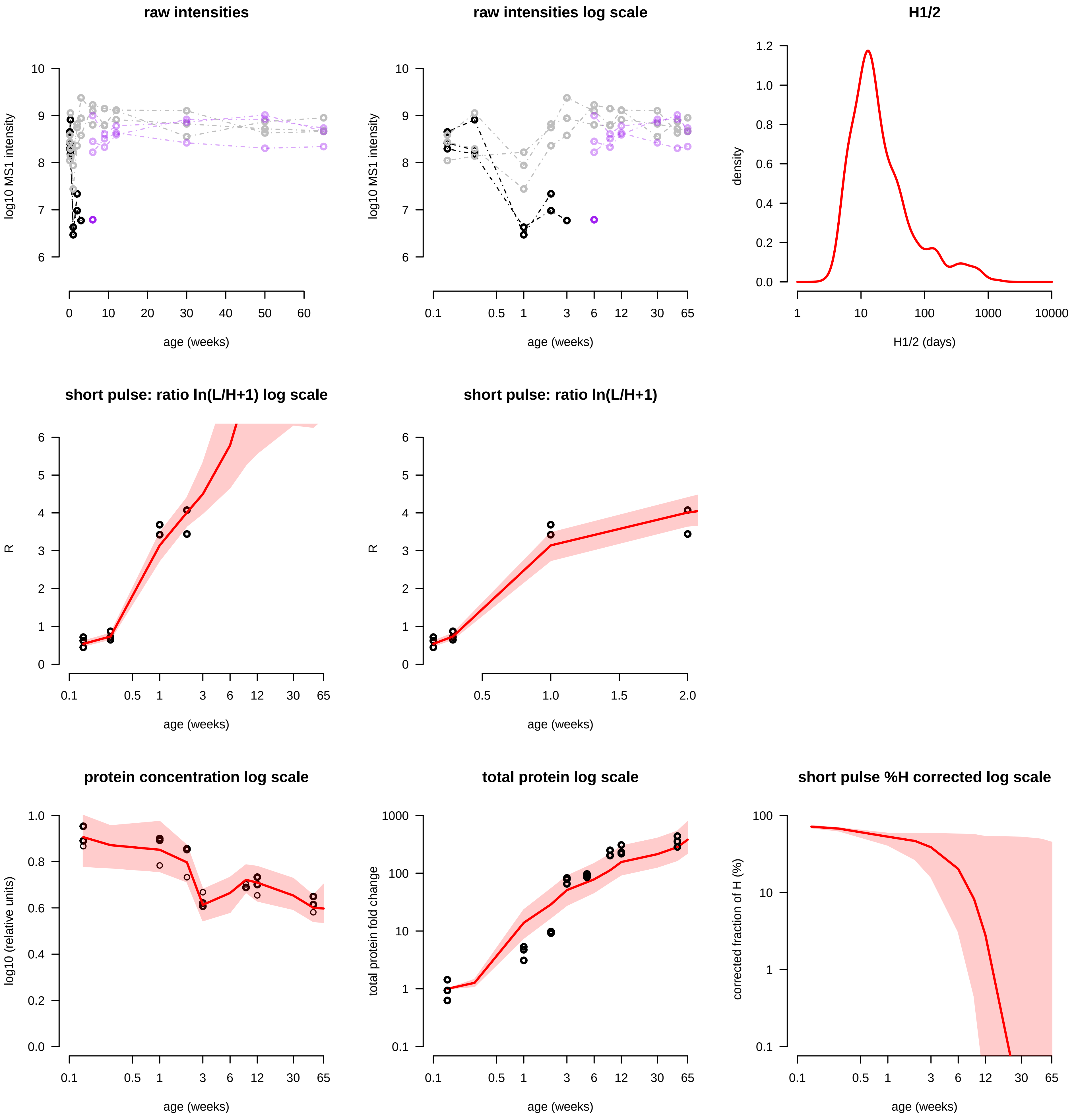

Q8CHH9;Q8CHH9−2

>sp|Q8CHH9|SEPT8_MOUSE

Septin−8

GN=Septin8

Med H1/2: 5.7 days

Q5 H1/2: 4.1 days

Q95 H1/2: 7.6 days

reliable model fit: yes

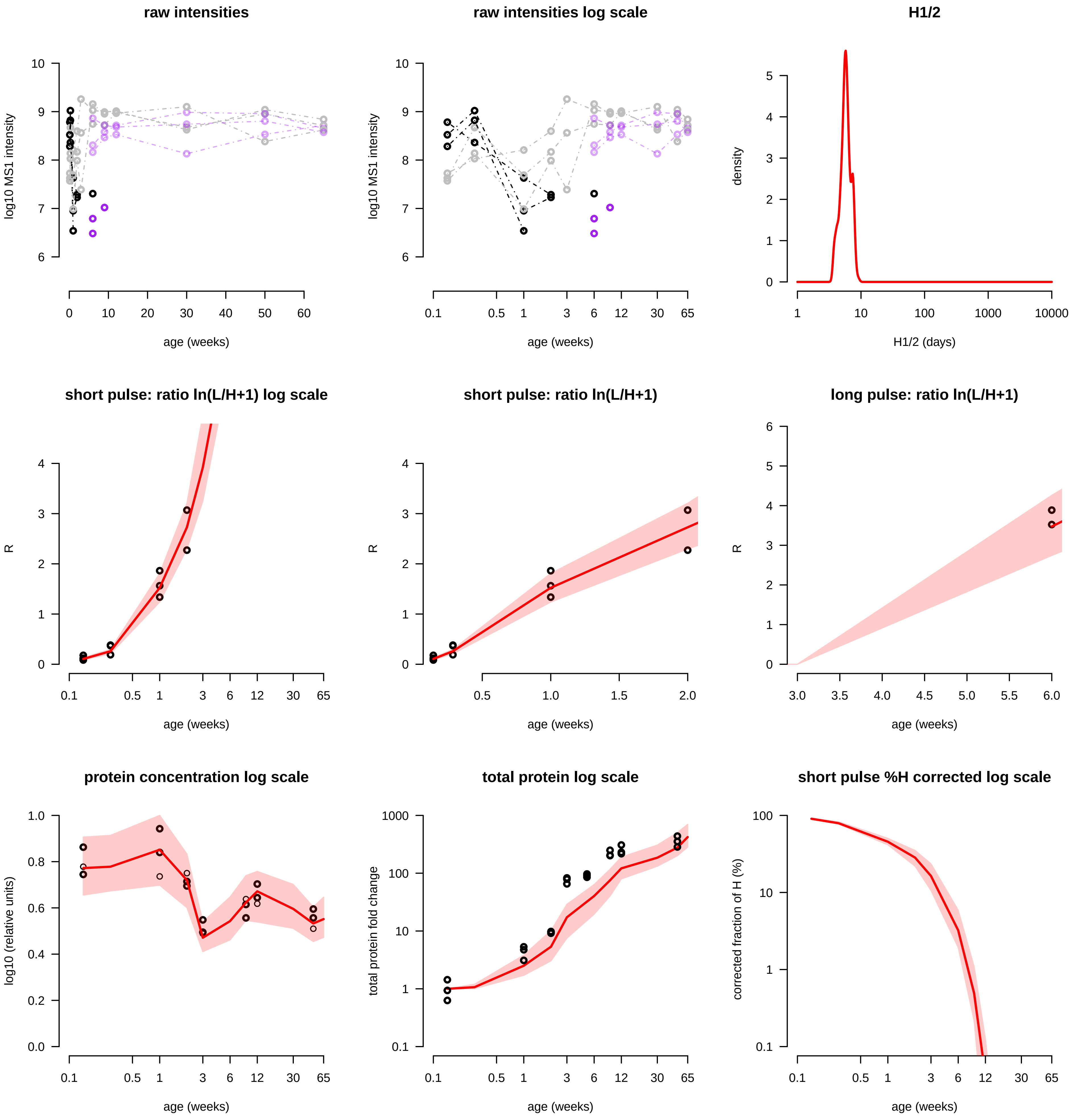

Q8WTY4

>sp|Q8WTY4|CPIN1_MOUSE

Anamorsin

GN=Ciapin1

Med H1/2: 15.9 days

Q5 H1/2: 7.3 days

Q95 H1/2: 97.2 days

reliable model fit: yes

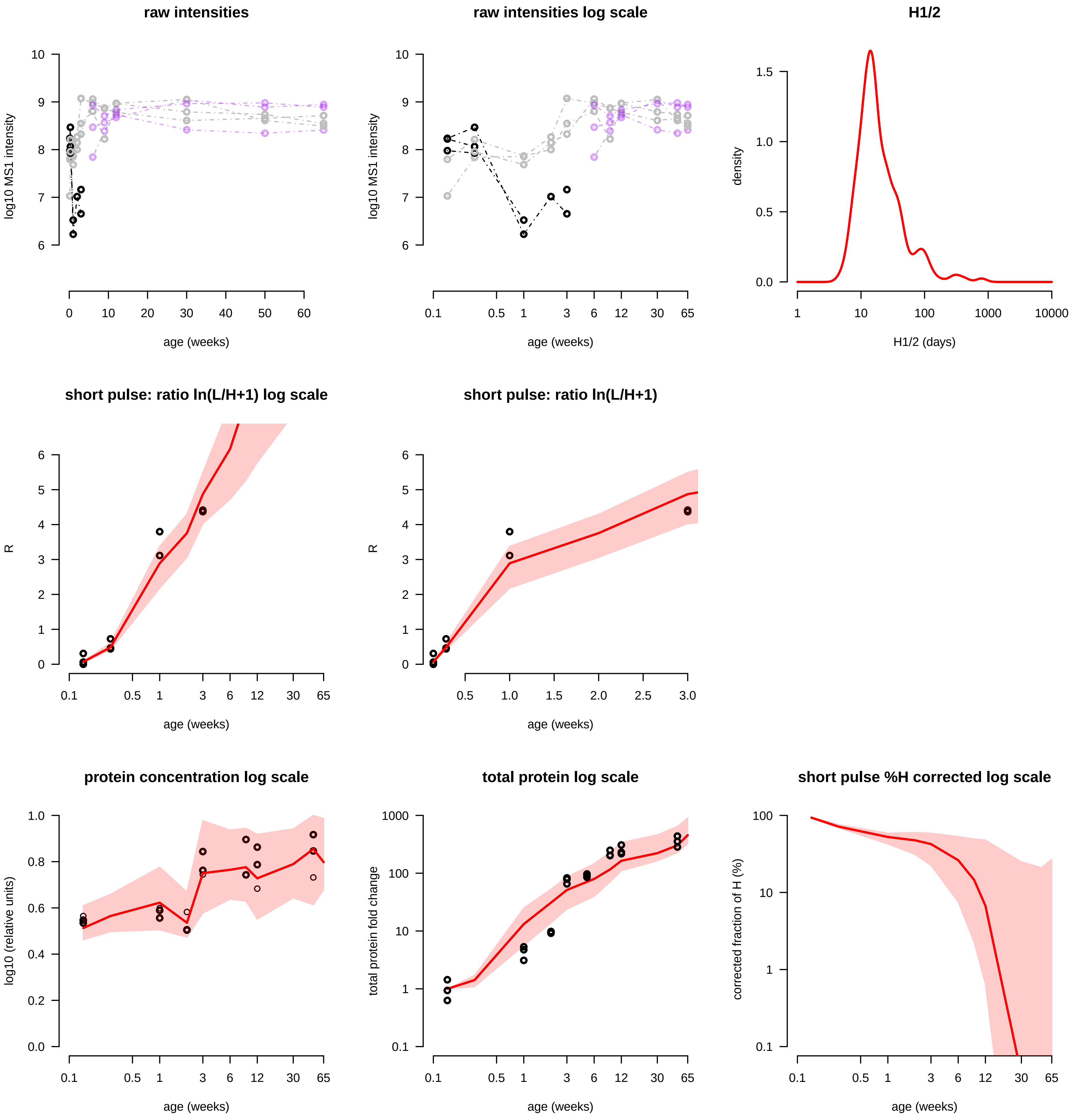

Q07113

>sp|Q07113|MPRI_MOUSE

Cation−independent mannose−6−phosphate receptor

GN=Igf2r

Med H1/2: 9.7 days

Q5 H1/2: 7.9 days

Q95 H1/2: 13.8 days

reliable model fit: yes

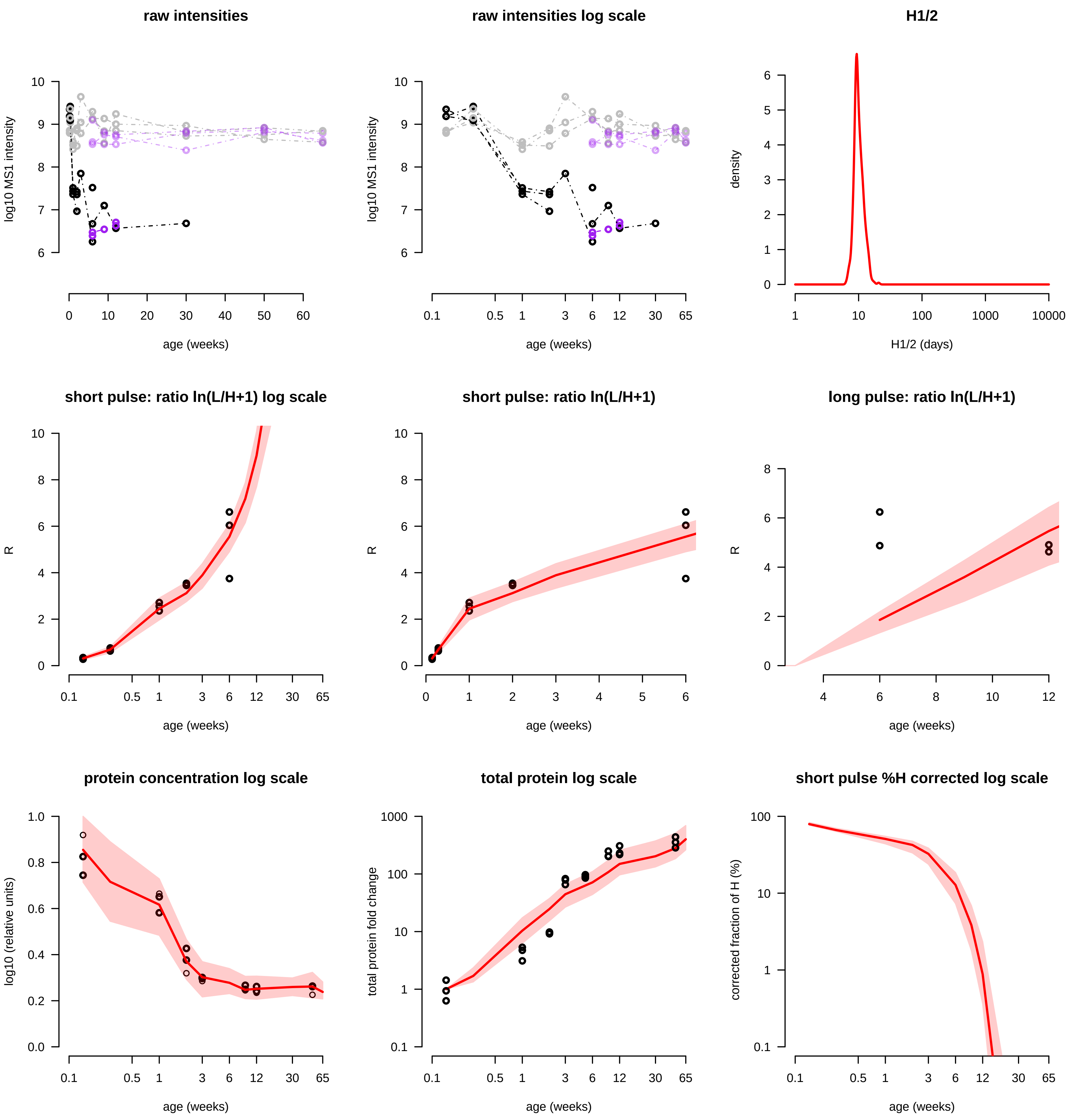

Q91WU5

>sp|Q91WU5|AS3MT_MOUSE

Arsenite methyltransferase

GN=As3mt

Med H1/2: 332.3 days

Q5 H1/2: 161.3 days

Q95 H1/2: 1372.2 days

reliable model fit: yes

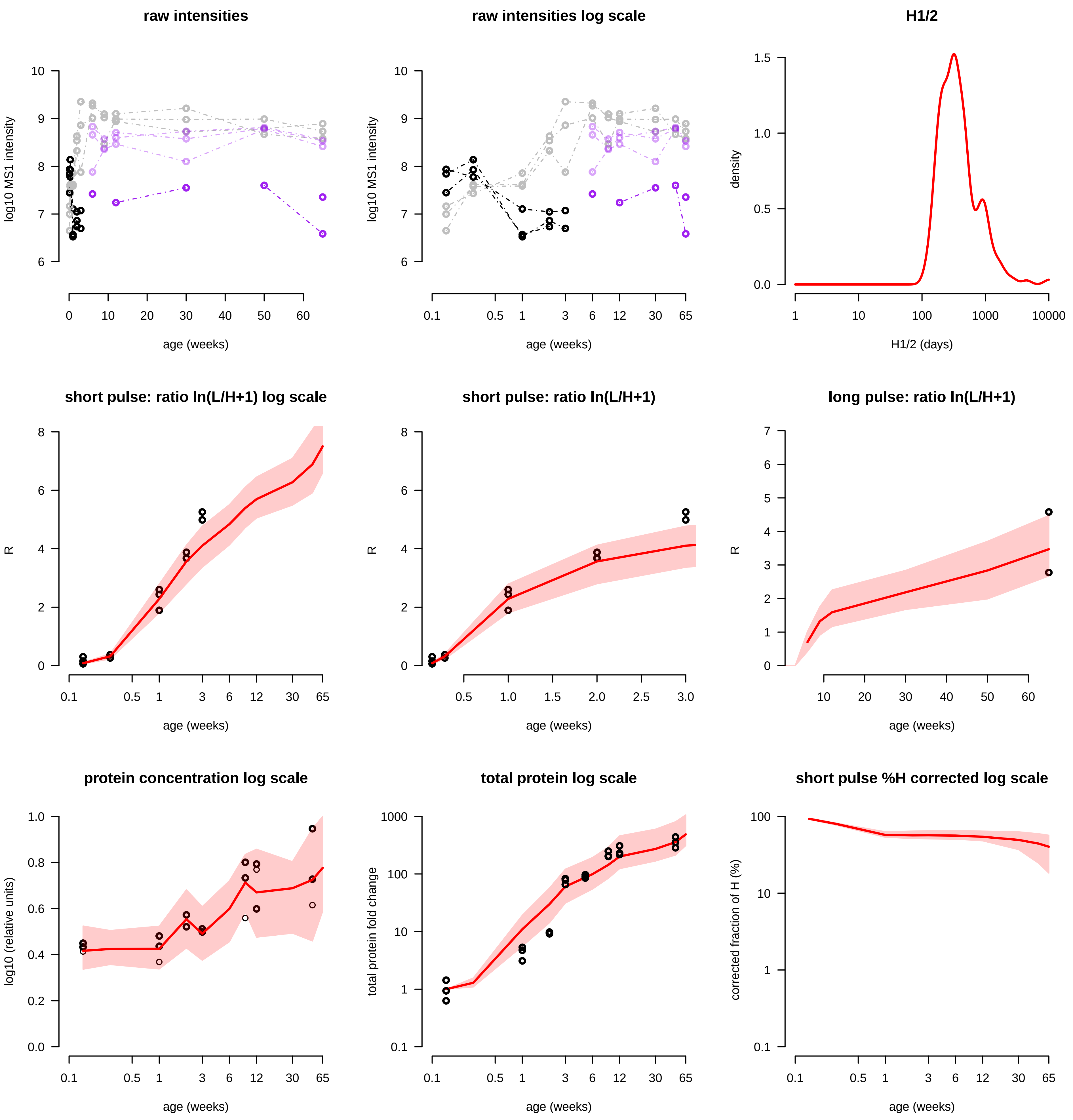

Q63918

>sp|Q63918|CAVN2_MOUSE

Caveolae−associated protein 2

GN=Cavin2

Med H1/2: 4.7 days

Q5 H1/2: 3.1 days

Q95 H1/2: 7.5 days

reliable model fit: yes

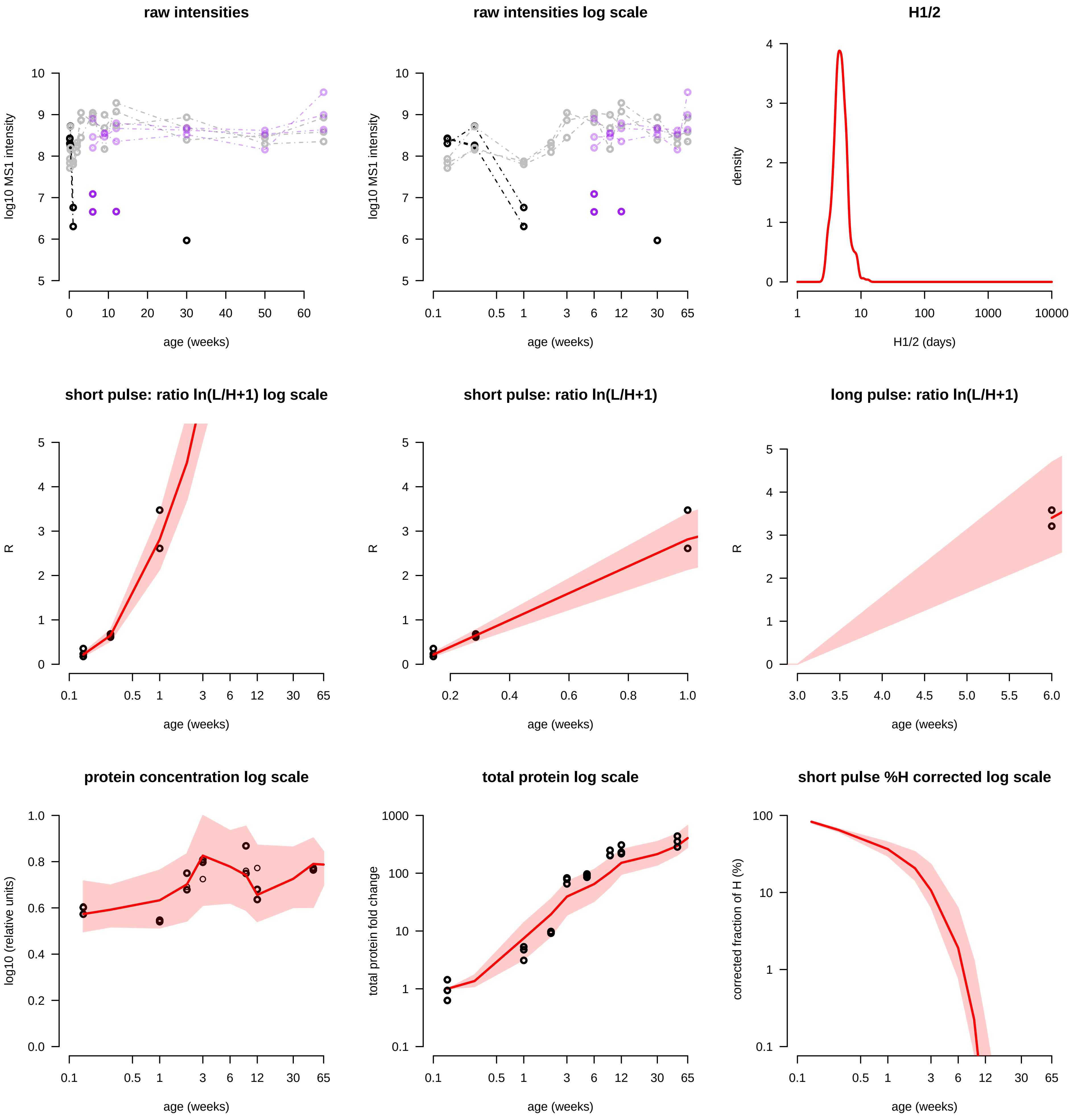

Q923D2

>sp|Q923D2|BLVRB_MOUSE

Flavin reductase (NADPH)

GN=Blvrb

Med H1/2: 11 days

Q5 H1/2: 8.6 days

Q95 H1/2: 15.6 days

reliable model fit: yes

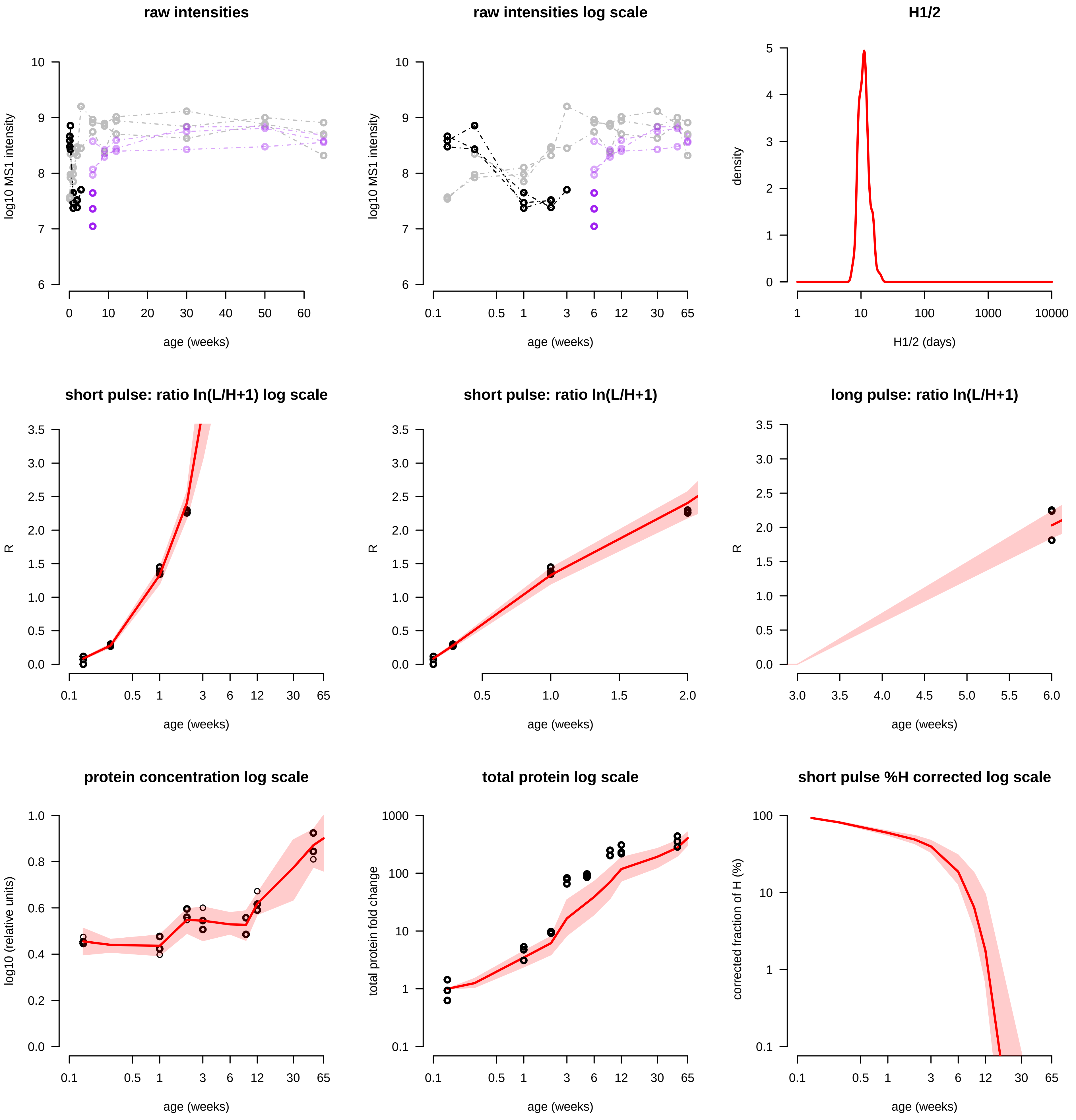

Q9R099

>sp|Q9R099|TBL2_MOUSE

Transducin beta−like protein 2

GN=Tbl2

Med H1/2: 9.1 days

Q5 H1/2: 4.1 days

Q95 H1/2: 41.9 days

reliable model fit: yes

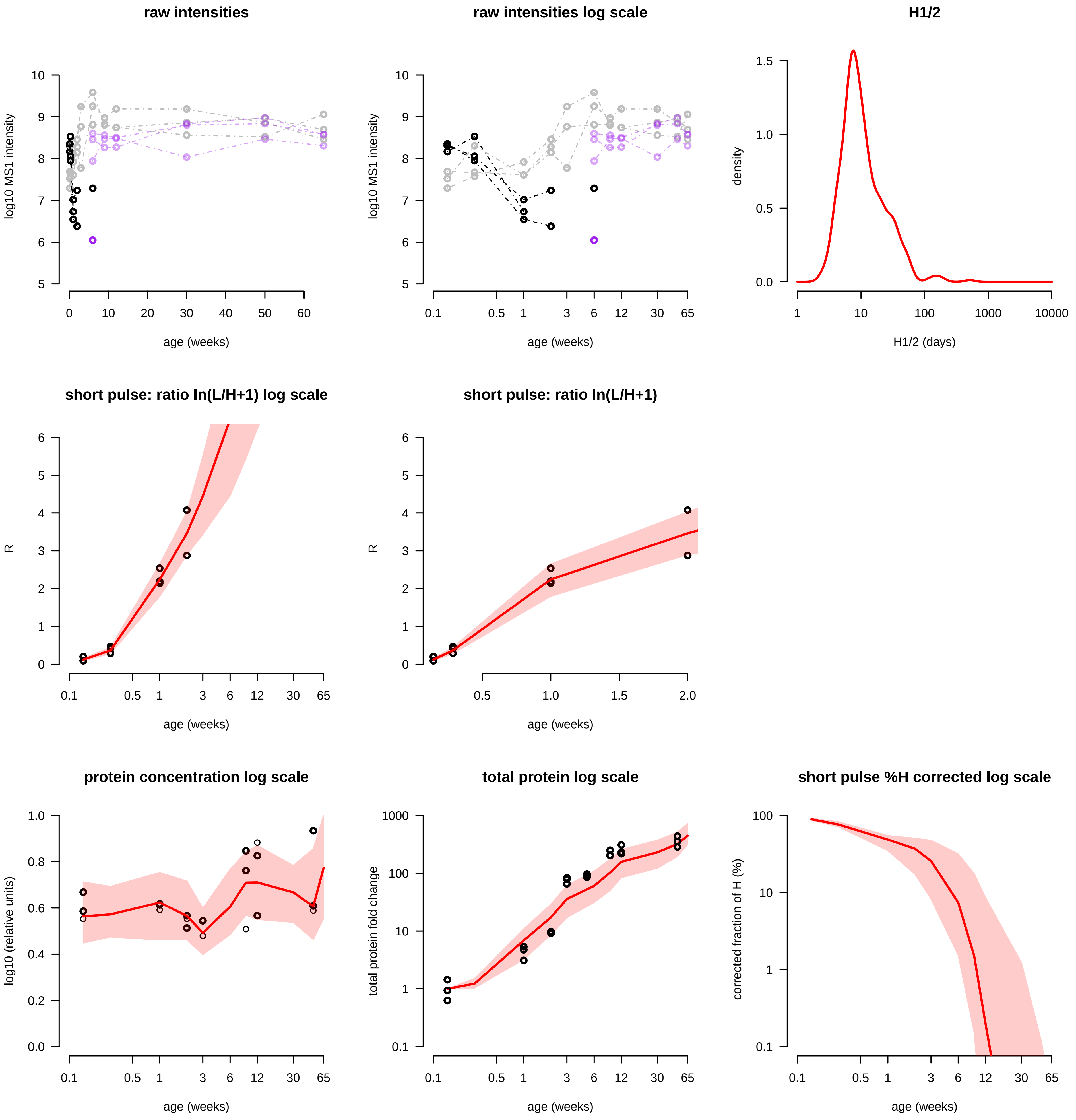

Q9CZL5

>sp|Q9CZL5|PHS2_MOUSE

Pterin−4−alpha−carbinolamine dehydratase 2

GN=Pcbd2

Med H1/2: 745.5 days

Q5 H1/2: 213.1 days

Q95 H1/2: 6438.2 days

reliable model fit: yes

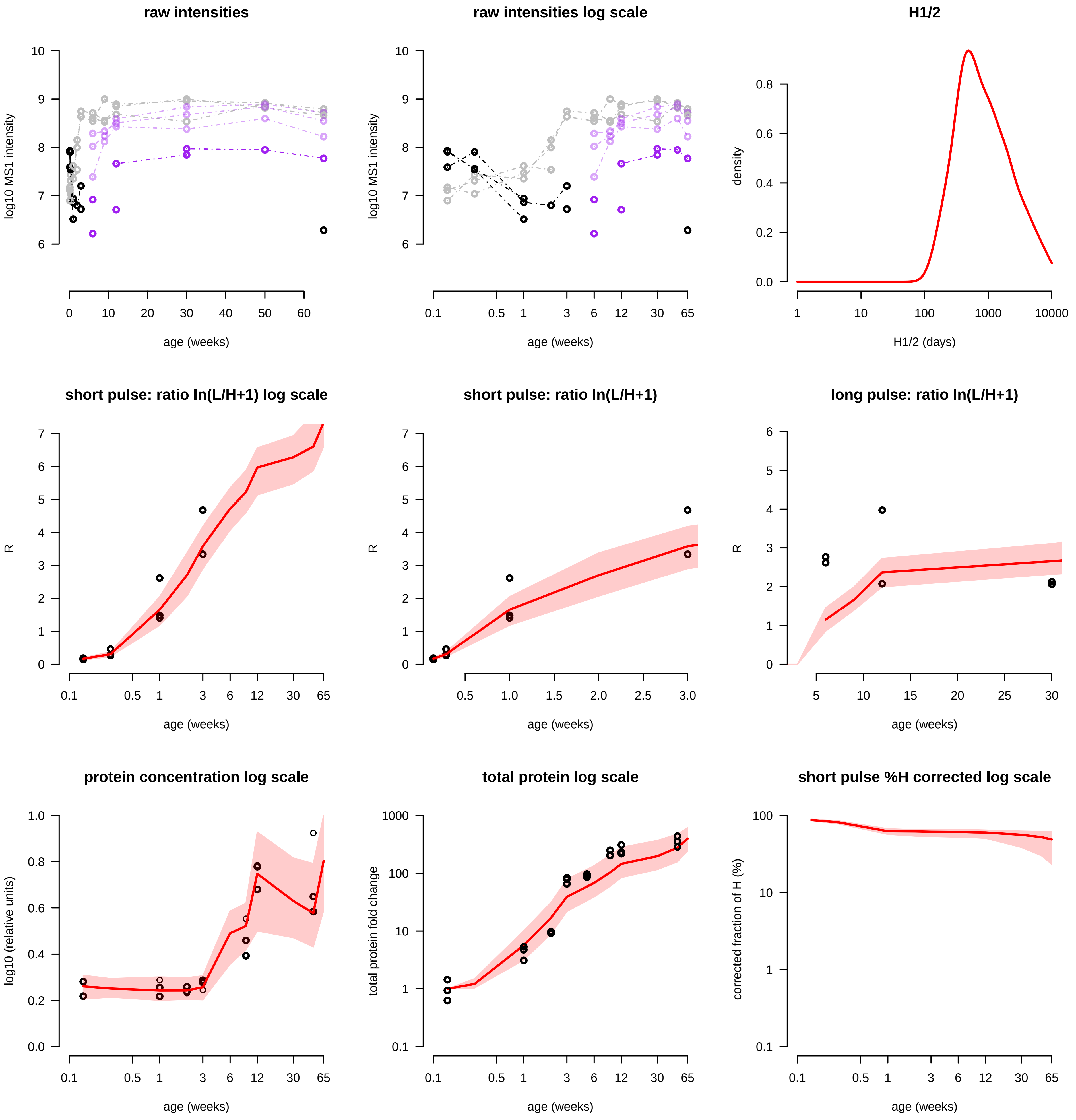

Q99L04

>sp|Q99L04|DHRS1_MOUSE

Dehydrogenase/reductase SDR family member 1

GN=Dhrs1

Med H1/2: 7.3 days

Q5 H1/2: 6.2 days

Q95 H1/2: 9.1 days

reliable model fit: yes

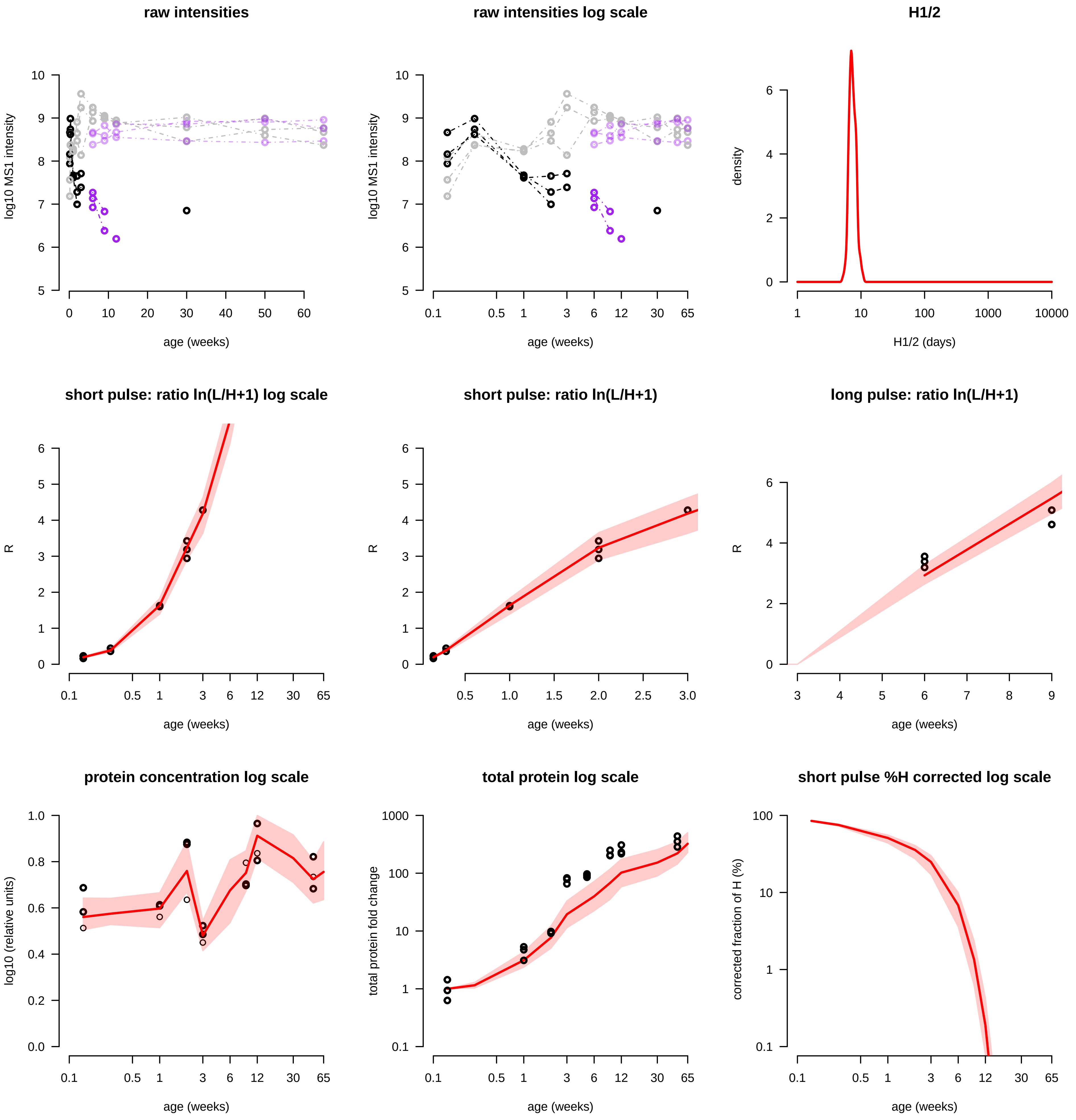

O89020;O89020−3;O89020−2

>sp|O89020|AFAM_MOUSE

Afamin

GN=Afm

Med H1/2: 10.1 days

Q5 H1/2: 7.9 days

Q95 H1/2: 13.4 days

reliable model fit: yes

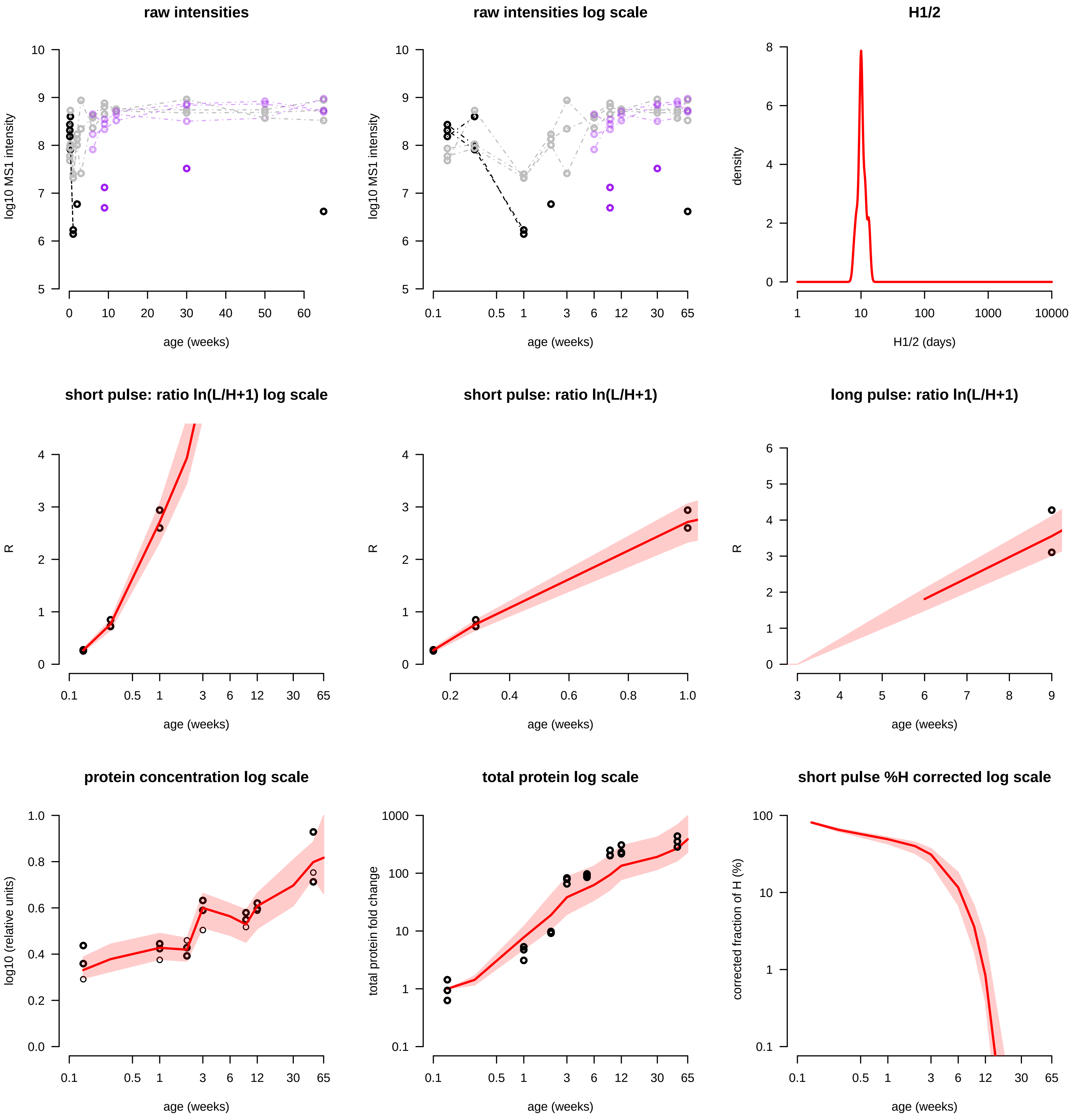

Q8VBZ3

>sp|Q8VBZ3|CLPT1_MOUSE

Cleft lip and palate transmembrane protein 1 homolog

GN=Clptm1

Med H1/2: 7 days

Q5 H1/2: 5.4 days

Q95 H1/2: 10.5 days

reliable model fit: yes

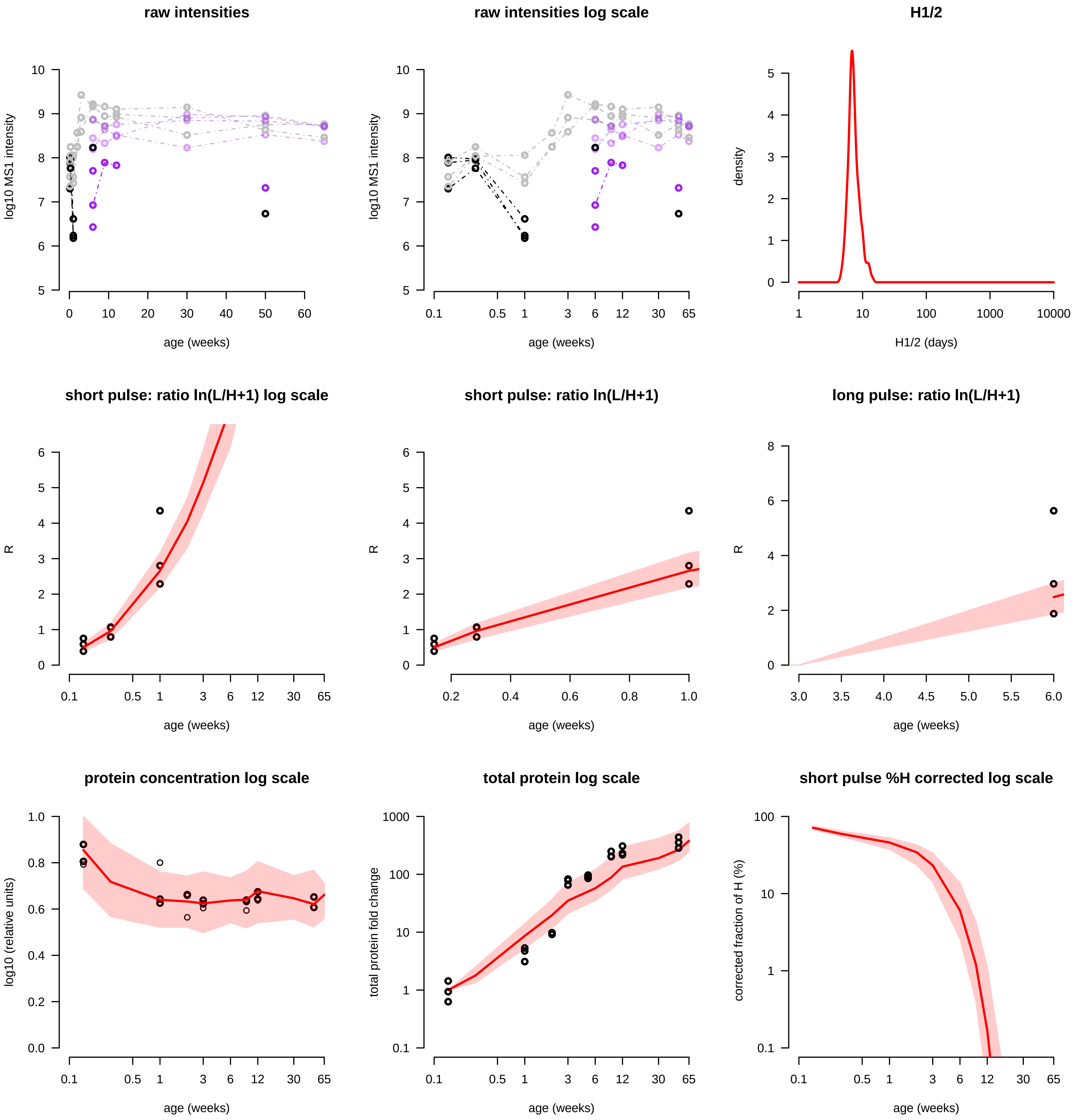

Q9D0S9

>sp|Q9D0S9|HINT2_MOUSE

Histidine triad nucleotide−binding protein 2, mitochondrial

GN=Hint2

Med H1/2: 7.3 days

Q5 H1/2: 5 days

Q95 H1/2: 15 days

reliable model fit: yes

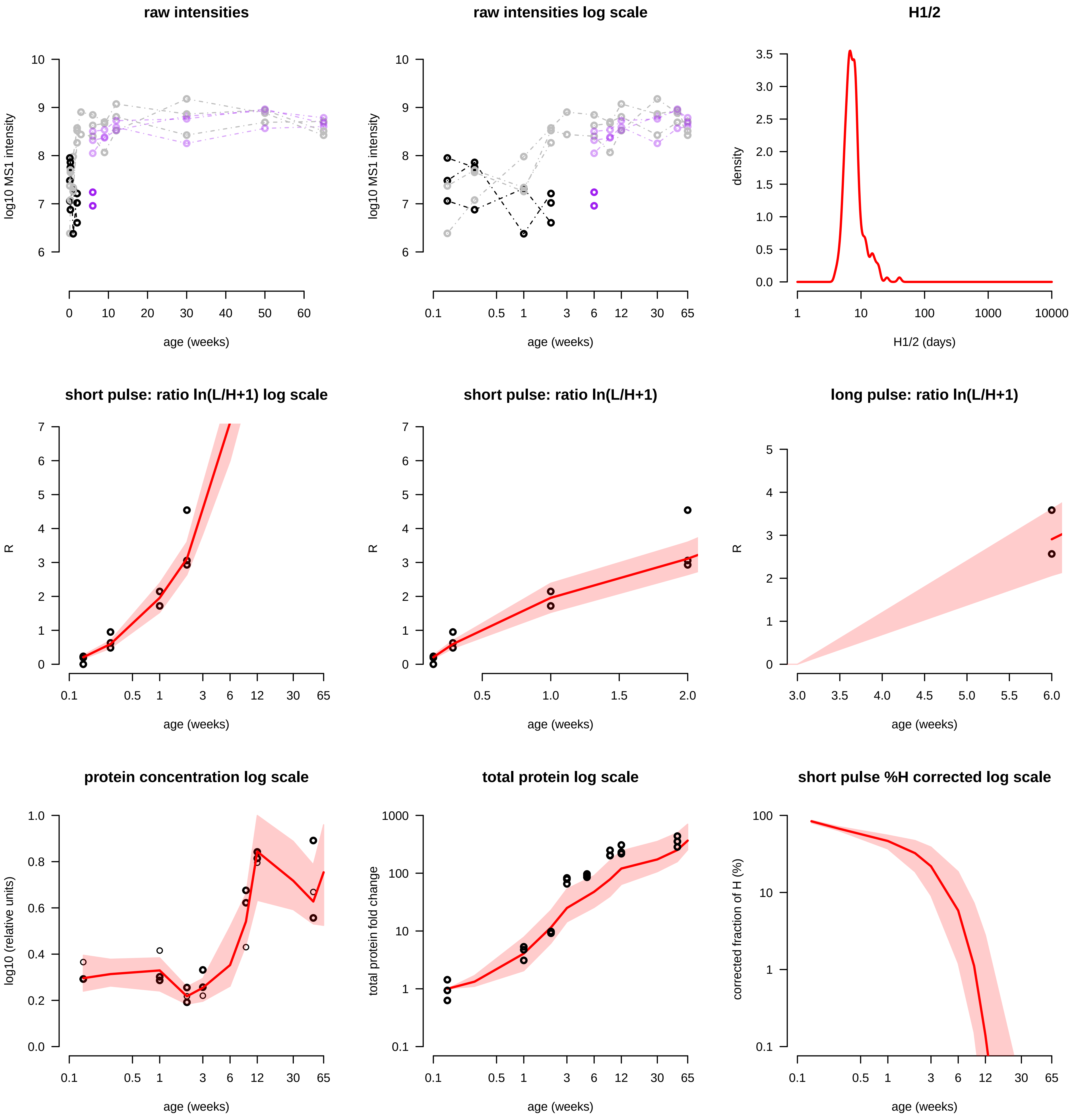

Q91VH2

>sp|Q91VH2|SNX9_MOUSE

Sorting nexin−9

GN=Snx9

Med H1/2: 6.8 days

Q5 H1/2: 3.3 days

Q95 H1/2: 30.8 days

reliable model fit: yes

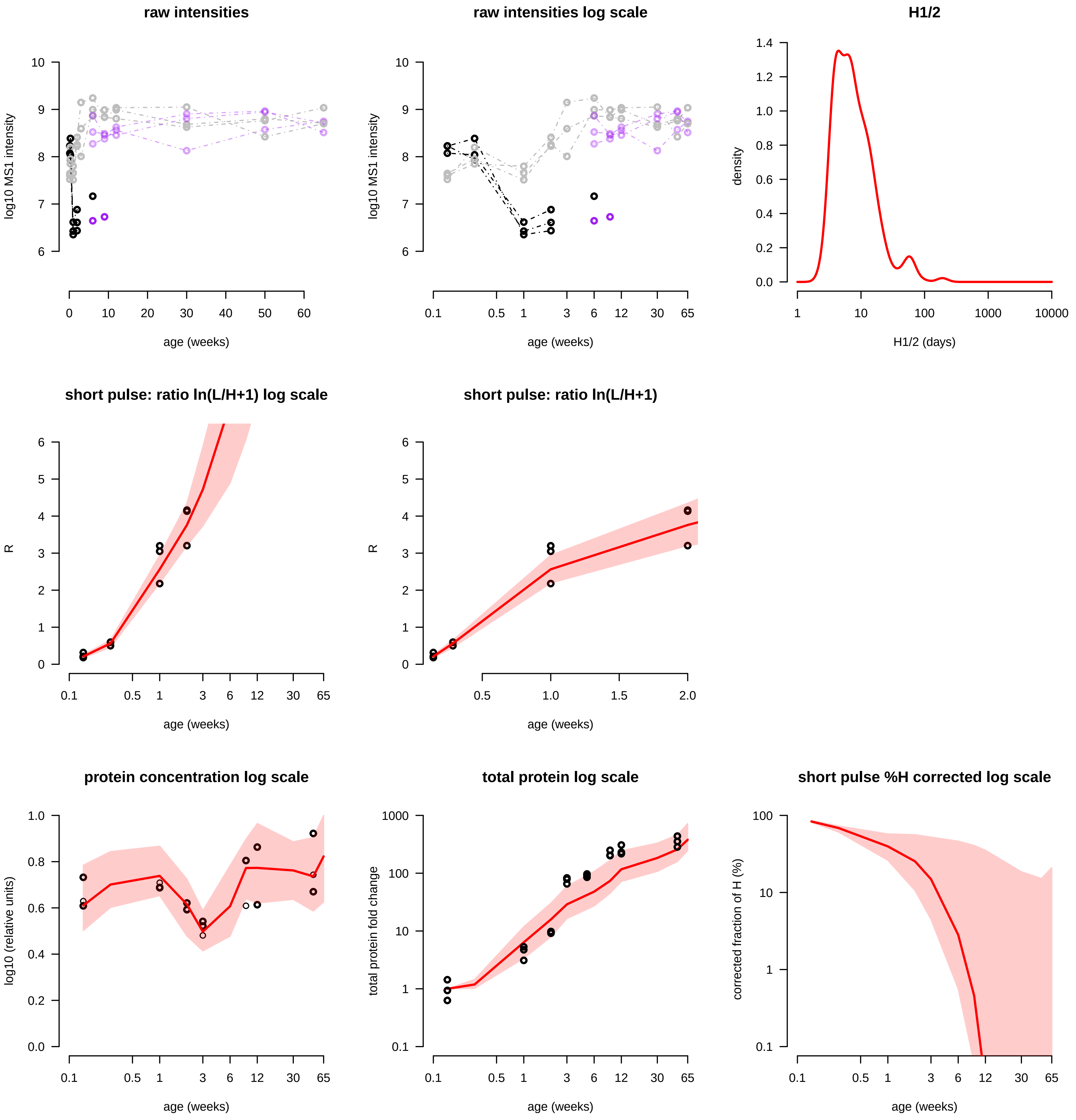

Q9Z1G3

>sp|Q9Z1G3|VATC1_MOUSE

V−type proton ATPase subunit C 1

GN=Atp6v1c1

Med H1/2: 5.6 days

Q5 H1/2: 2.8 days

Q95 H1/2: 41.1 days

reliable model fit: yes

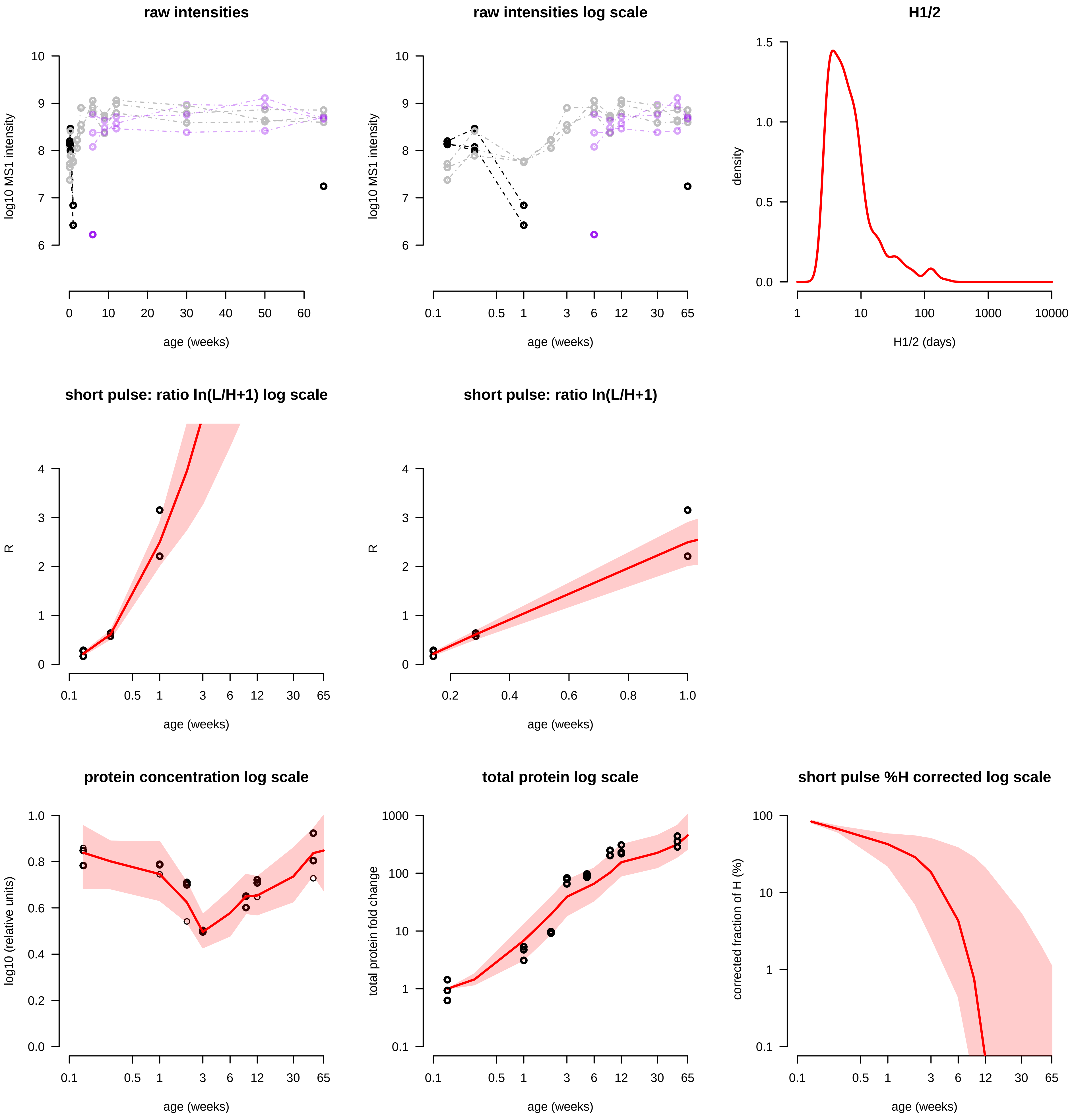

Q64737

>sp|Q64737|PUR2_MOUSE

Trifunctional purine biosynthetic protein adenosine−3

GN=Gart

Med H1/2: 3.5 days

Q5 H1/2: 2.9 days

Q95 H1/2: 4.5 days

reliable model fit: yes

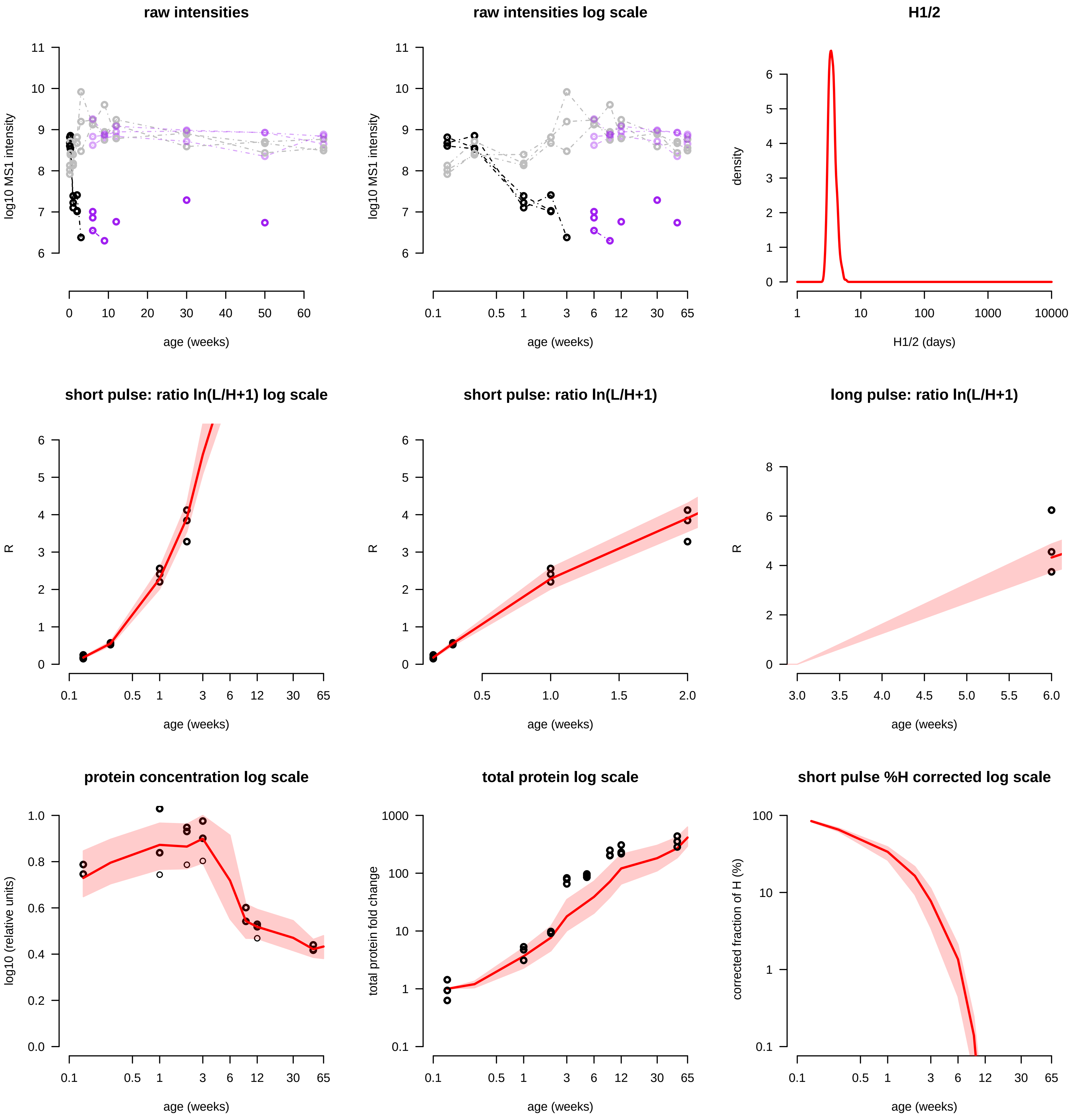

Q924M7

>sp|Q924M7|MPI_MOUSE

Mannose−6−phosphate isomerase

GN=Mpi

Med H1/2: 8.5 days

Q5 H1/2: 6.1 days

Q95 H1/2: 14.2 days

reliable model fit: yes

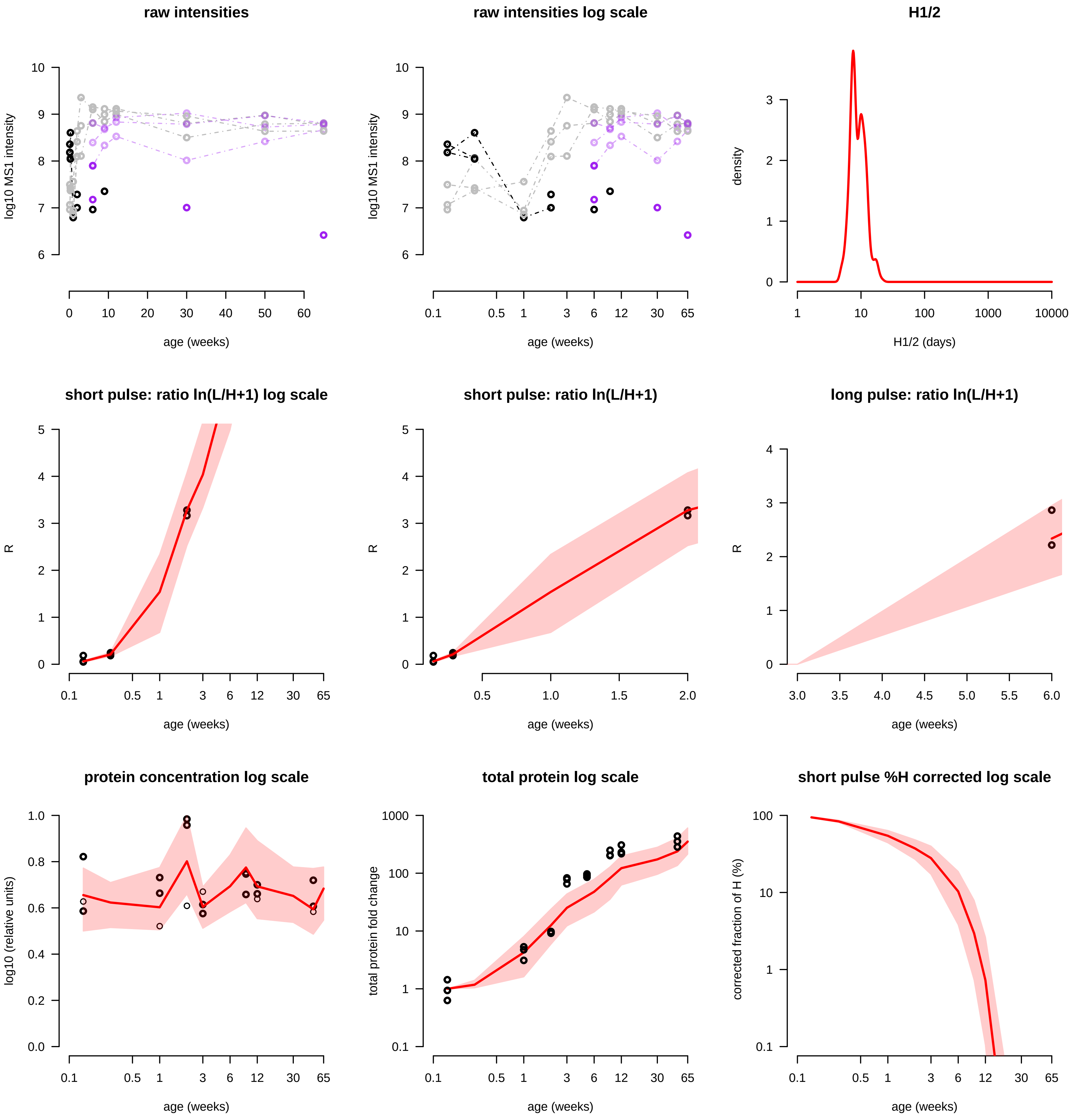

O70252

>sp|O70252|HMOX2_MOUSE

Heme oxygenase 2

GN=Hmox2

Med H1/2: 5 days

Q5 H1/2: 4.3 days

Q95 H1/2: 6 days

reliable model fit: yes

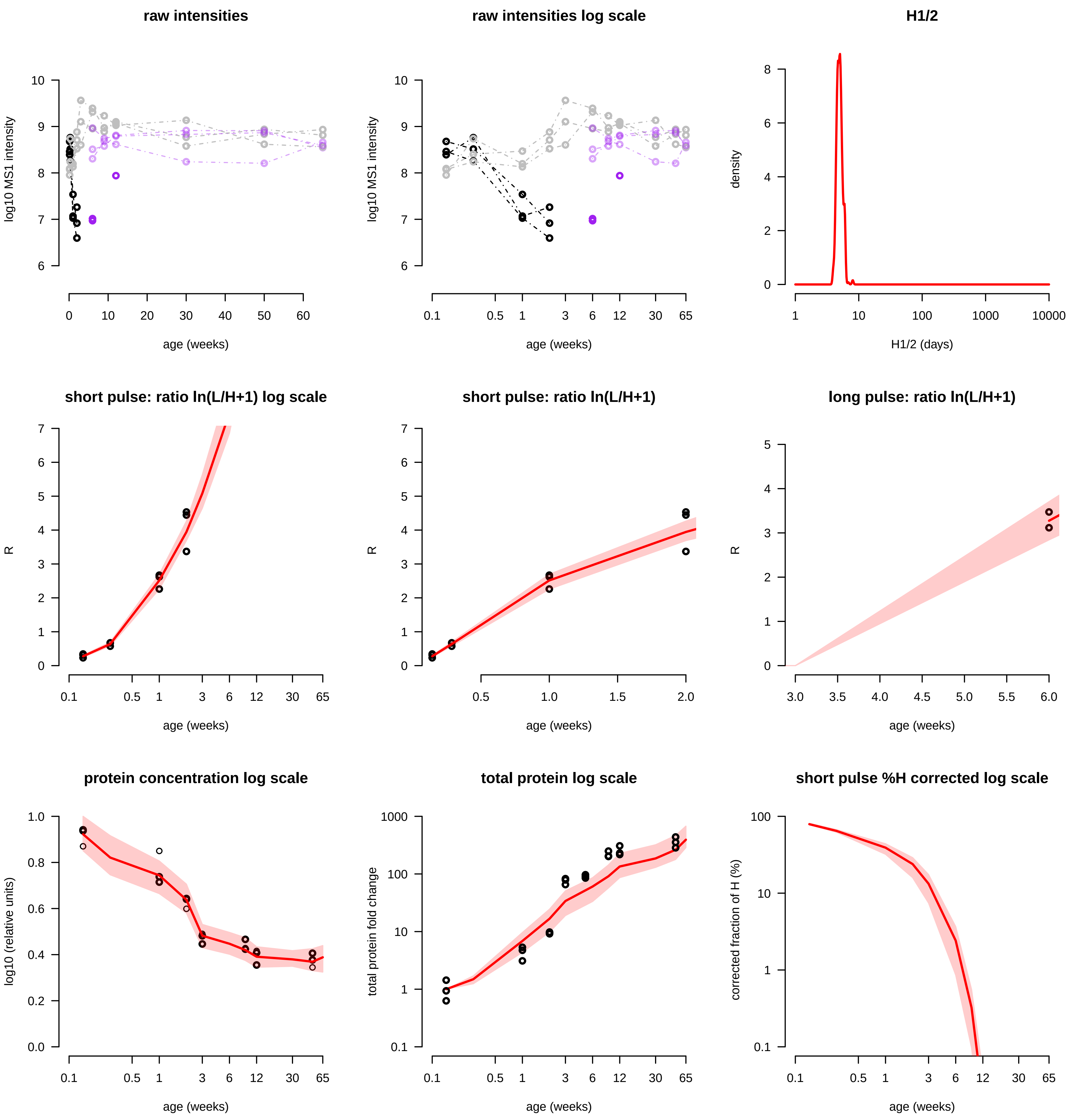

Q8CI32

>sp|Q8CI32|BAG5_MOUSE

BAG family molecular chaperone regulator 5

GN=Bag5

Med H1/2: 5.2 days

Q5 H1/2: 2.7 days

Q95 H1/2: 14.4 days

reliable model fit: yes

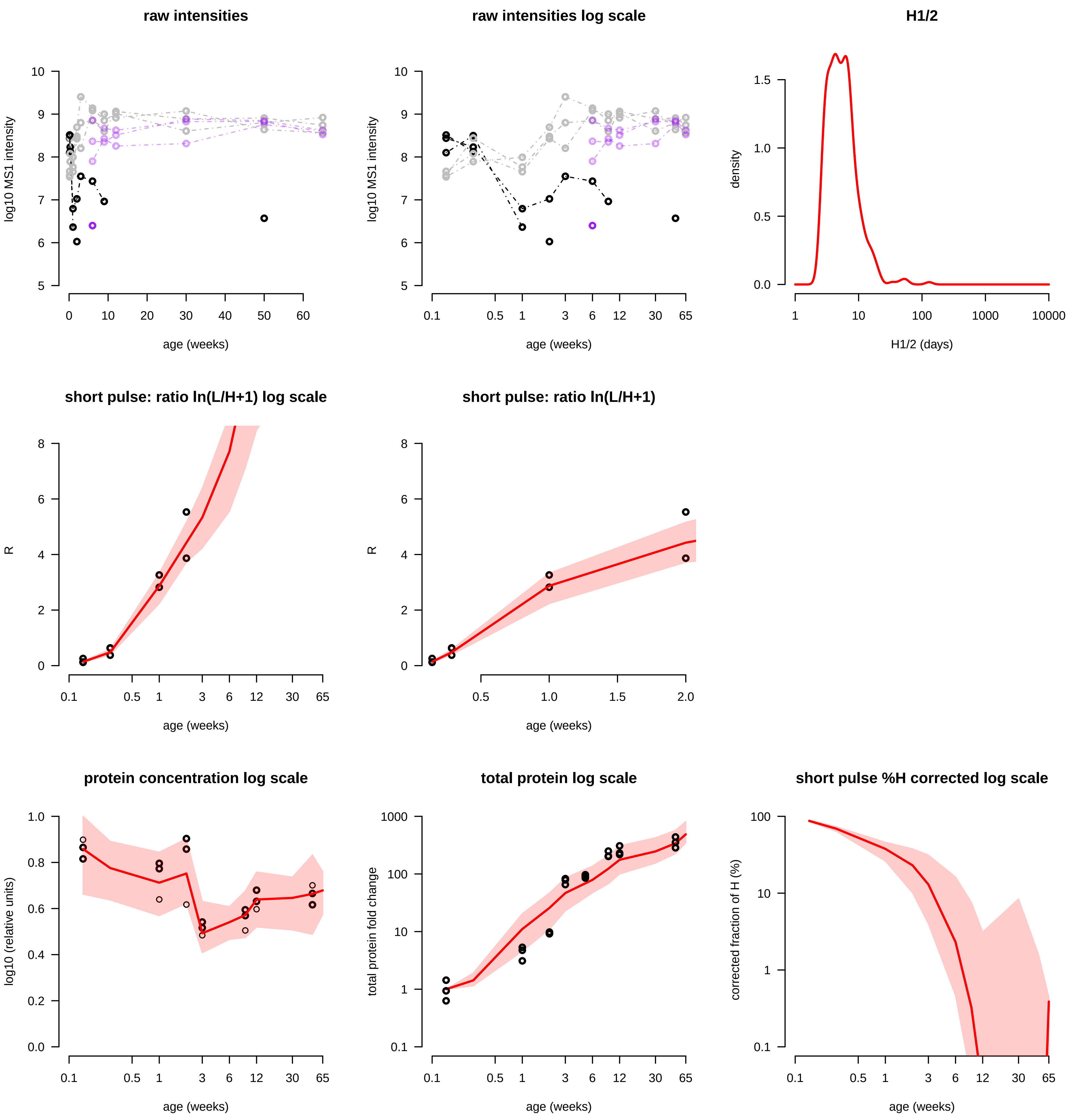

Q61490

>sp|Q61490|CD166_MOUSE

CD166 antigen

GN=Alcam

Med H1/2: 3.4 days

Q5 H1/2: 2.5 days

Q95 H1/2: 11.9 days

reliable model fit: yes

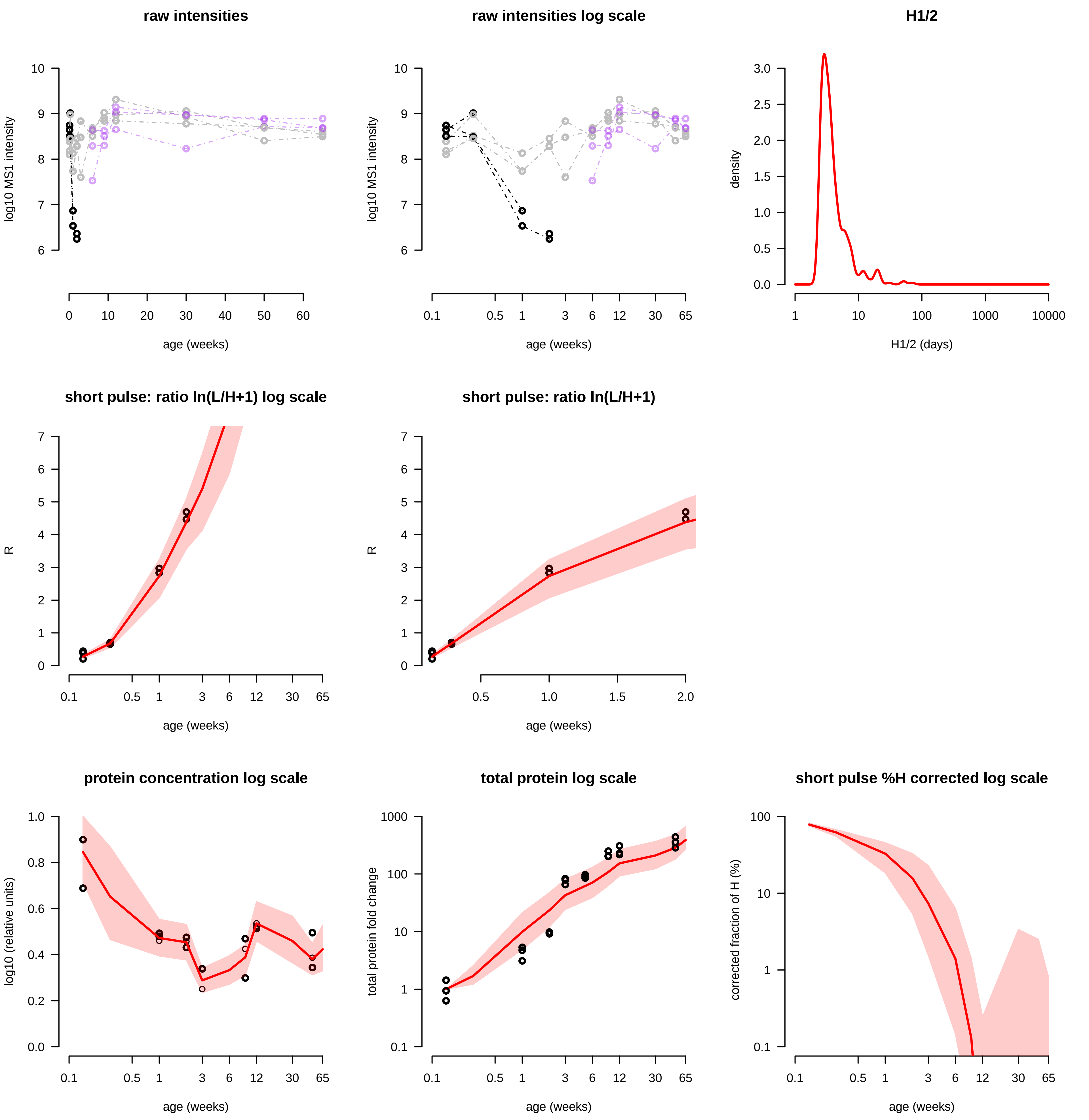

Q8VBW6

>sp|Q8VBW6|ULA1_MOUSE

NEDD8−activating enzyme E1 regulatory subunit

GN=Nae1

Med H1/2: 4.3 days

Q5 H1/2: 3.4 days

Q95 H1/2: 5.9 days

reliable model fit: yes

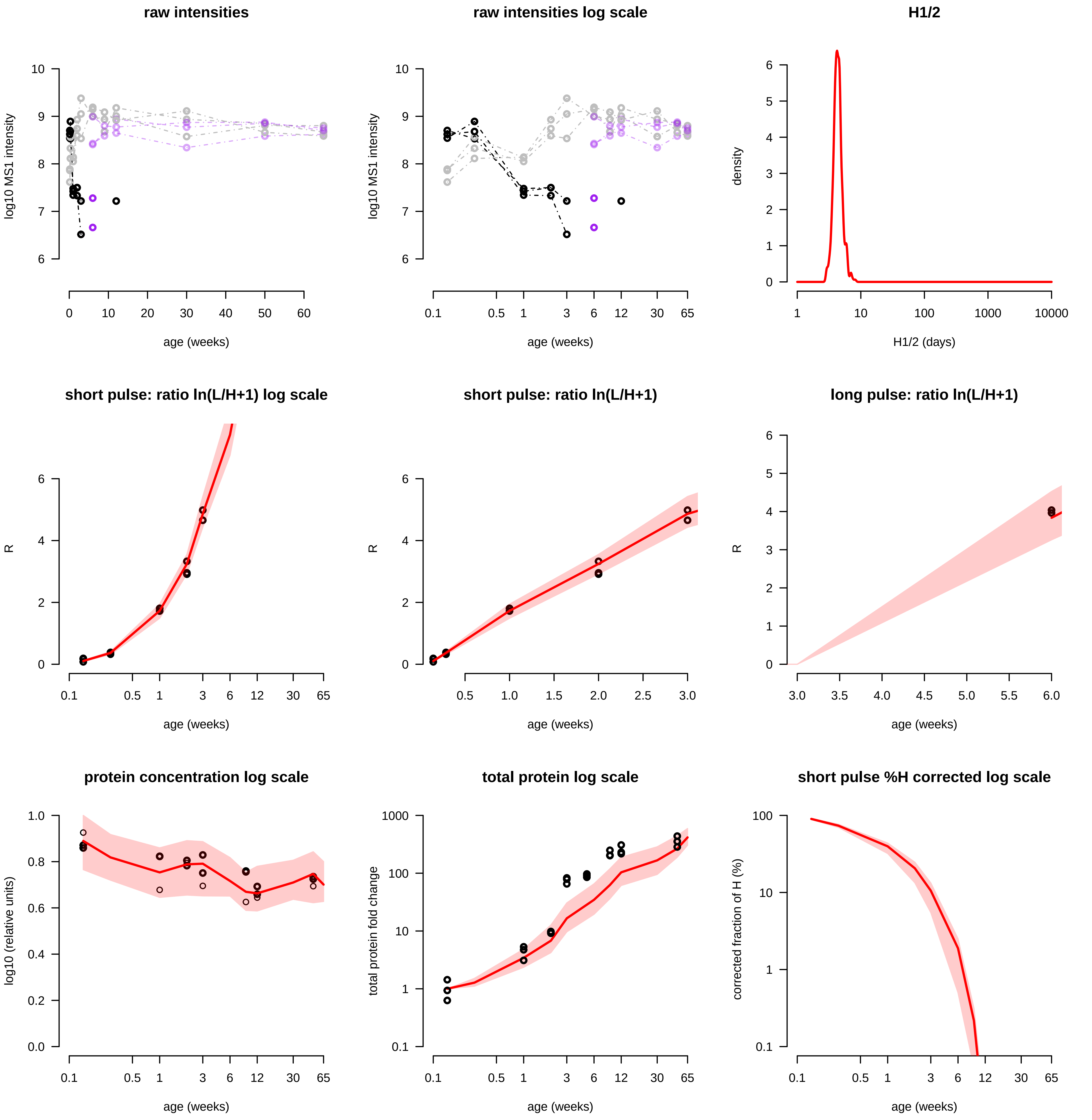

Q9CQE1

>sp|Q9CQE1|NPS3B_MOUSE

Protein NipSnap homolog 3B

GN=Nipsnap3b

Med H1/2: 5.5 days

Q5 H1/2: 4.5 days

Q95 H1/2: 6.5 days

reliable model fit: yes

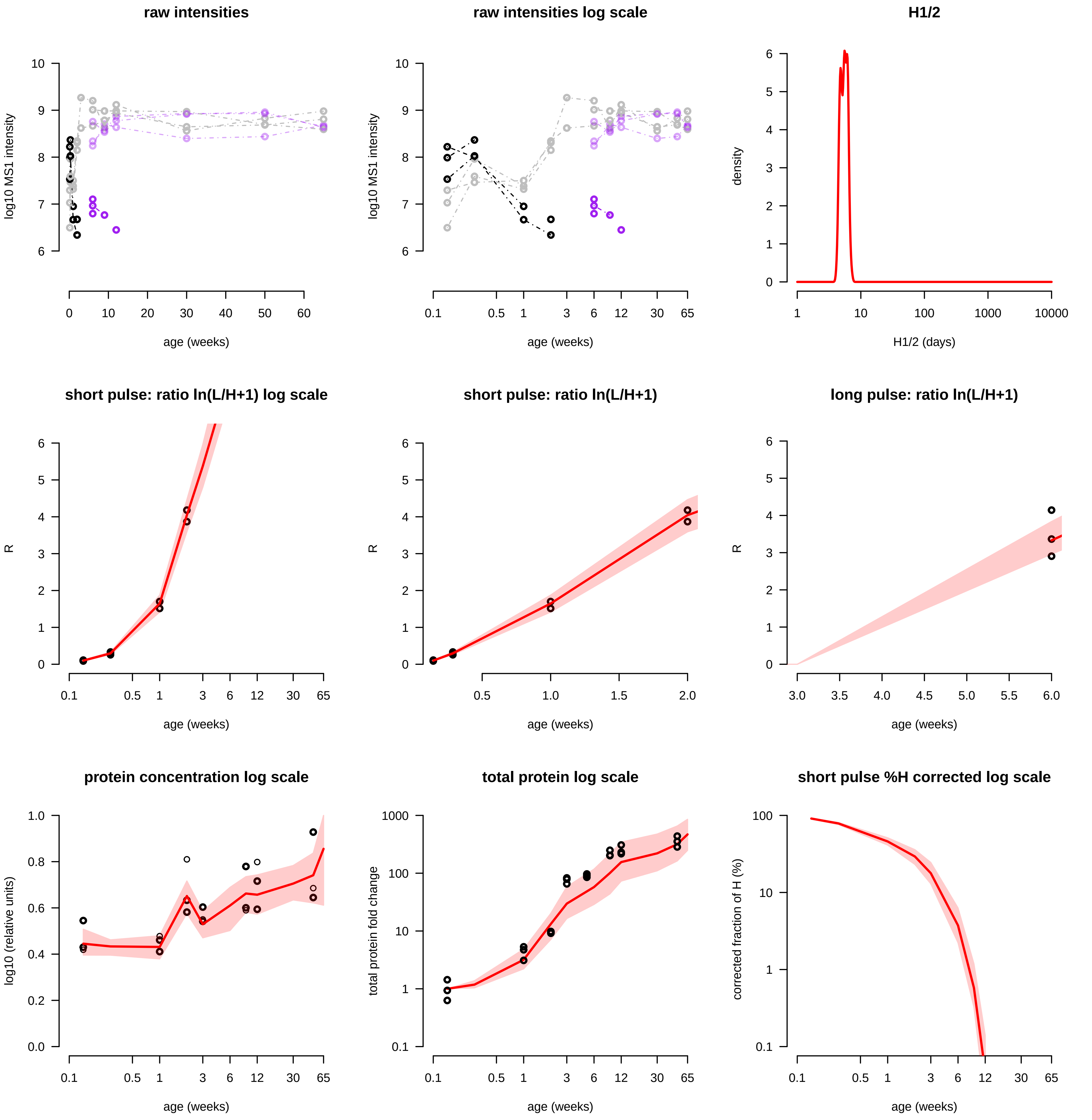

Q91VW5

>sp|Q91VW5|GOGA4_MOUSE

Golgin subfamily A member 4

GN=Golga4

Med H1/2: 3.3 days

Q5 H1/2: 2.5 days

Q95 H1/2: 14.1 days

reliable model fit: yes

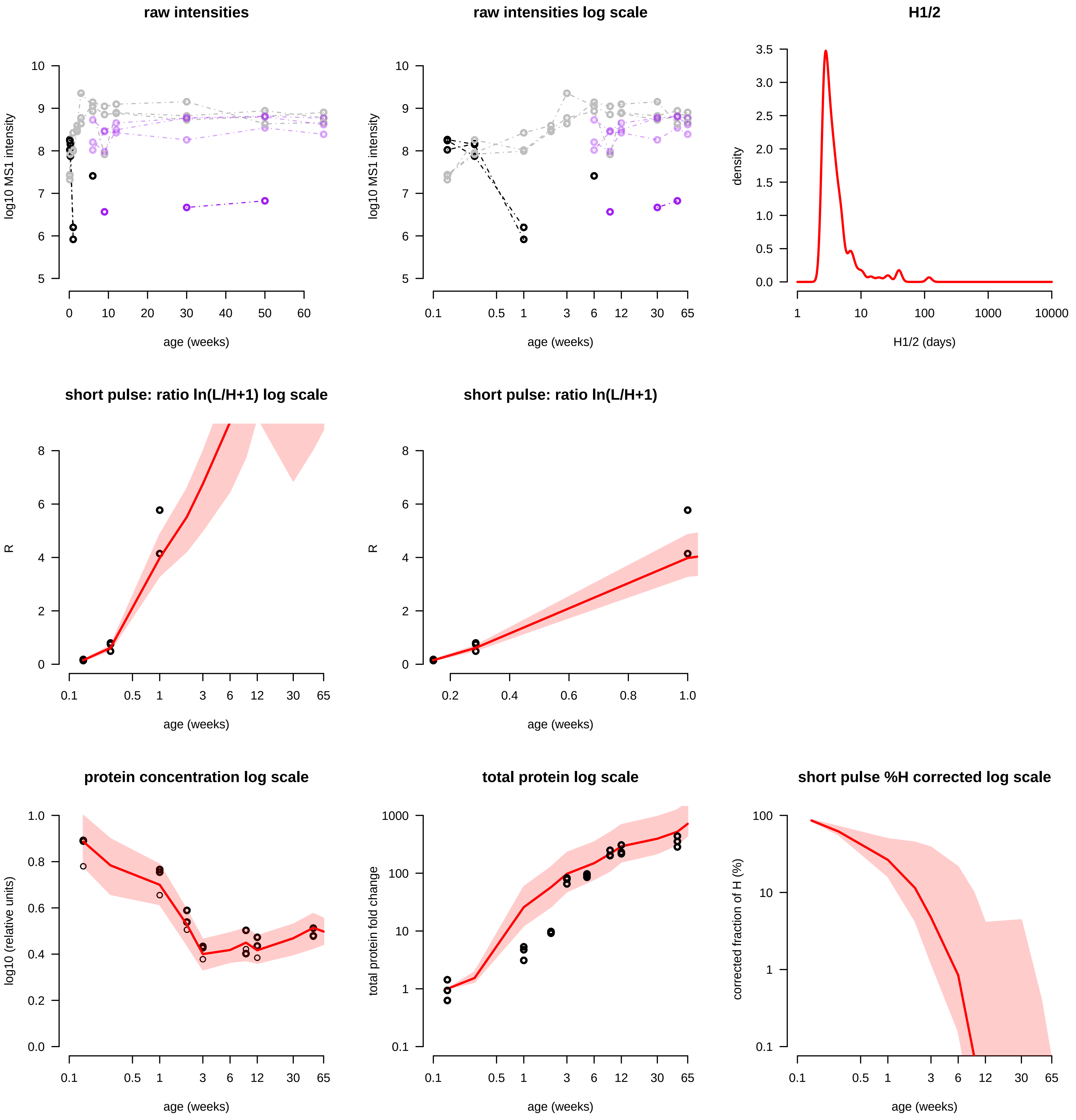

P52503

>sp|P52503|NDUS6_MOUSE

NADH dehydrogenase [ubiquinone] iron−sulfur protein 6, mitochondrial

GN=Ndufs6

Med H1/2: 5.3 days

Q5 H1/2: 4.3 days

Q95 H1/2: 7 days

reliable model fit: yes

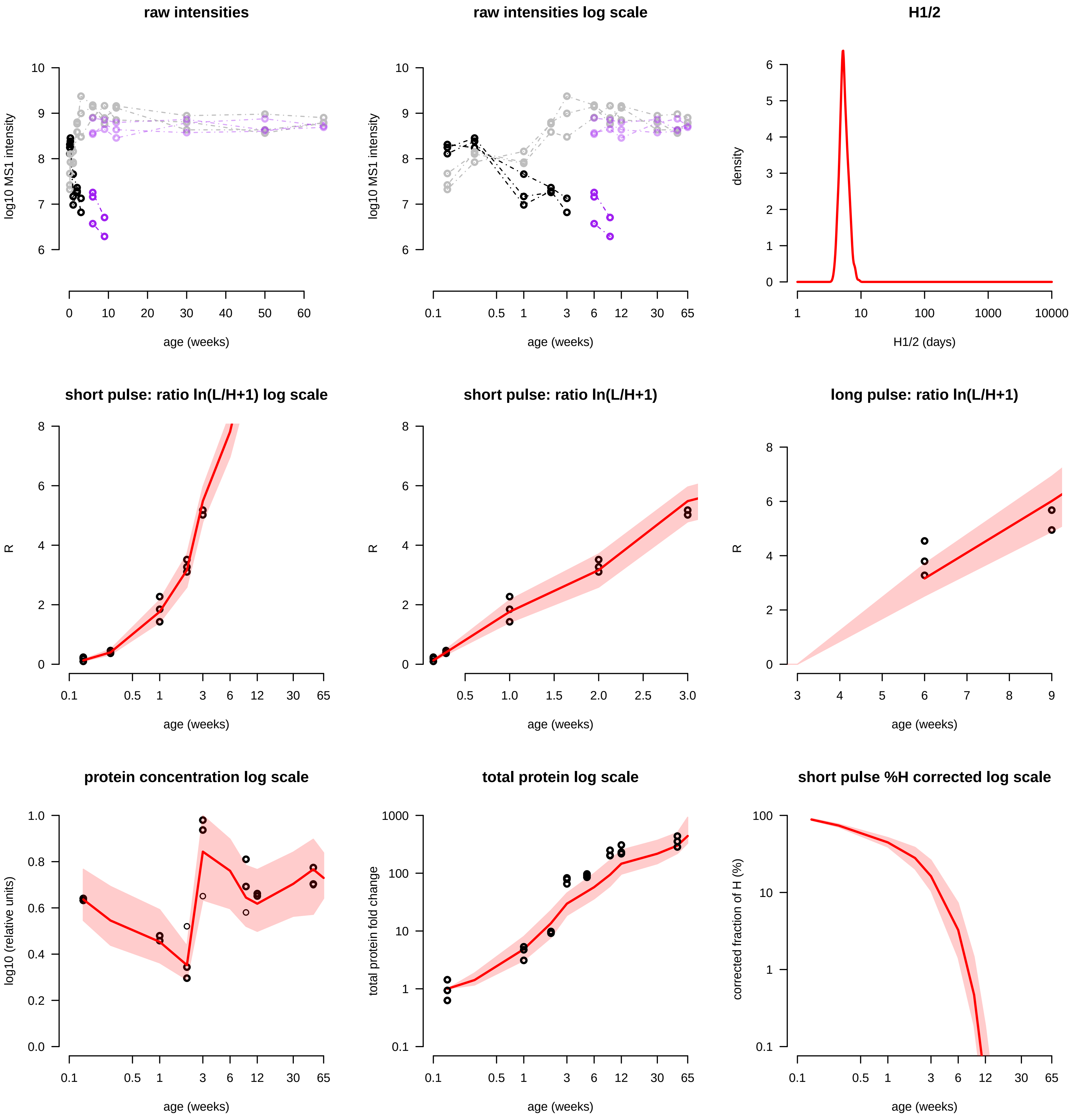

Q6PDL0

>sp|Q6PDL0|DC1L2_MOUSE

Cytoplasmic dynein 1 light intermediate chain 2

GN=Dync1li2

Med H1/2: 22.1 days

Q5 H1/2: 8.4 days

Q95 H1/2: 227.1 days

reliable model fit: no

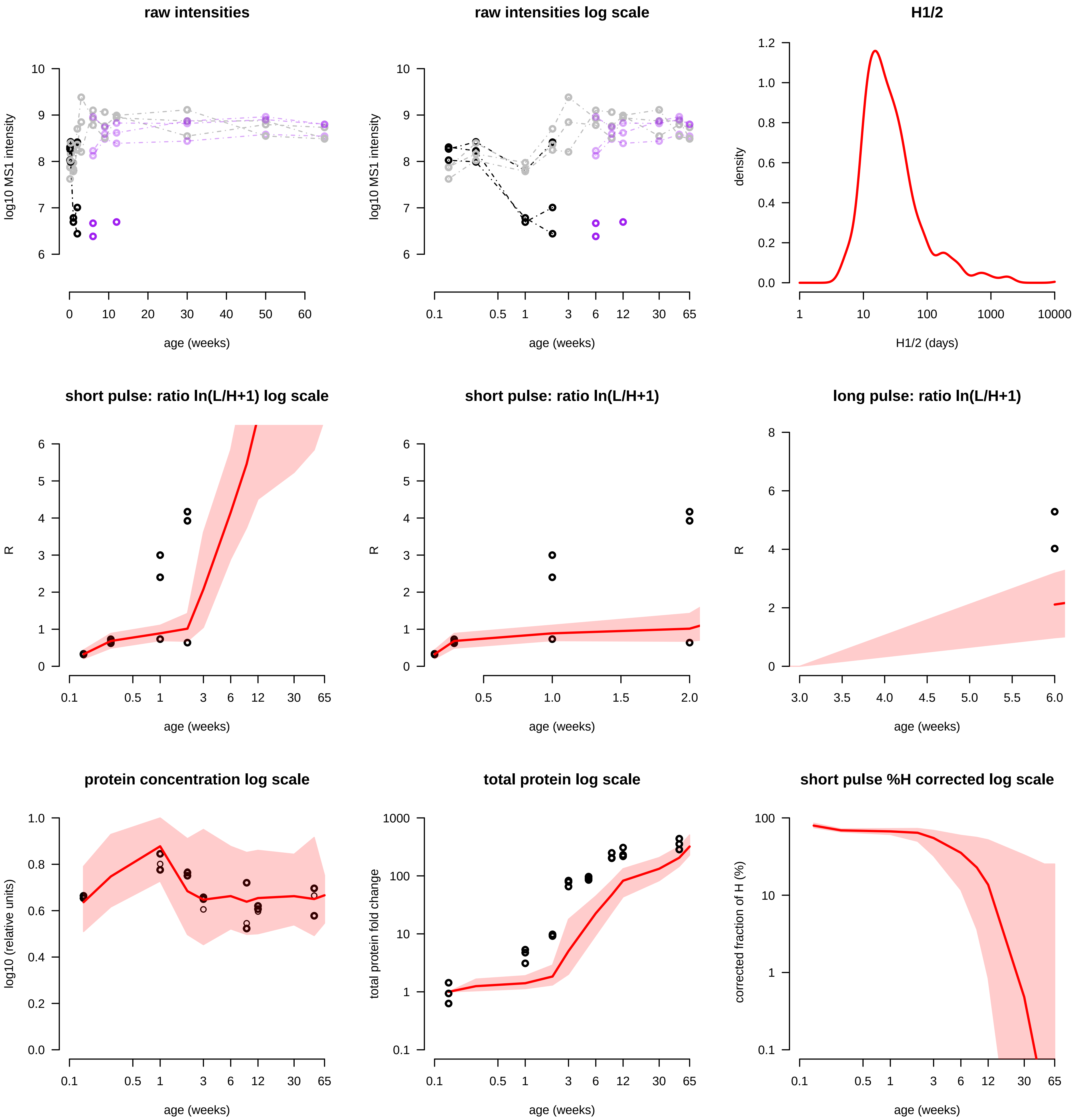

Q3THS6

>sp|Q3THS6|METK2_MOUSE

S−adenosylmethionine synthase isoform type−2

GN=Mat2a

Med H1/2: 3.9 days

Q5 H1/2: 3.2 days

Q95 H1/2: 5.1 days

reliable model fit: yes

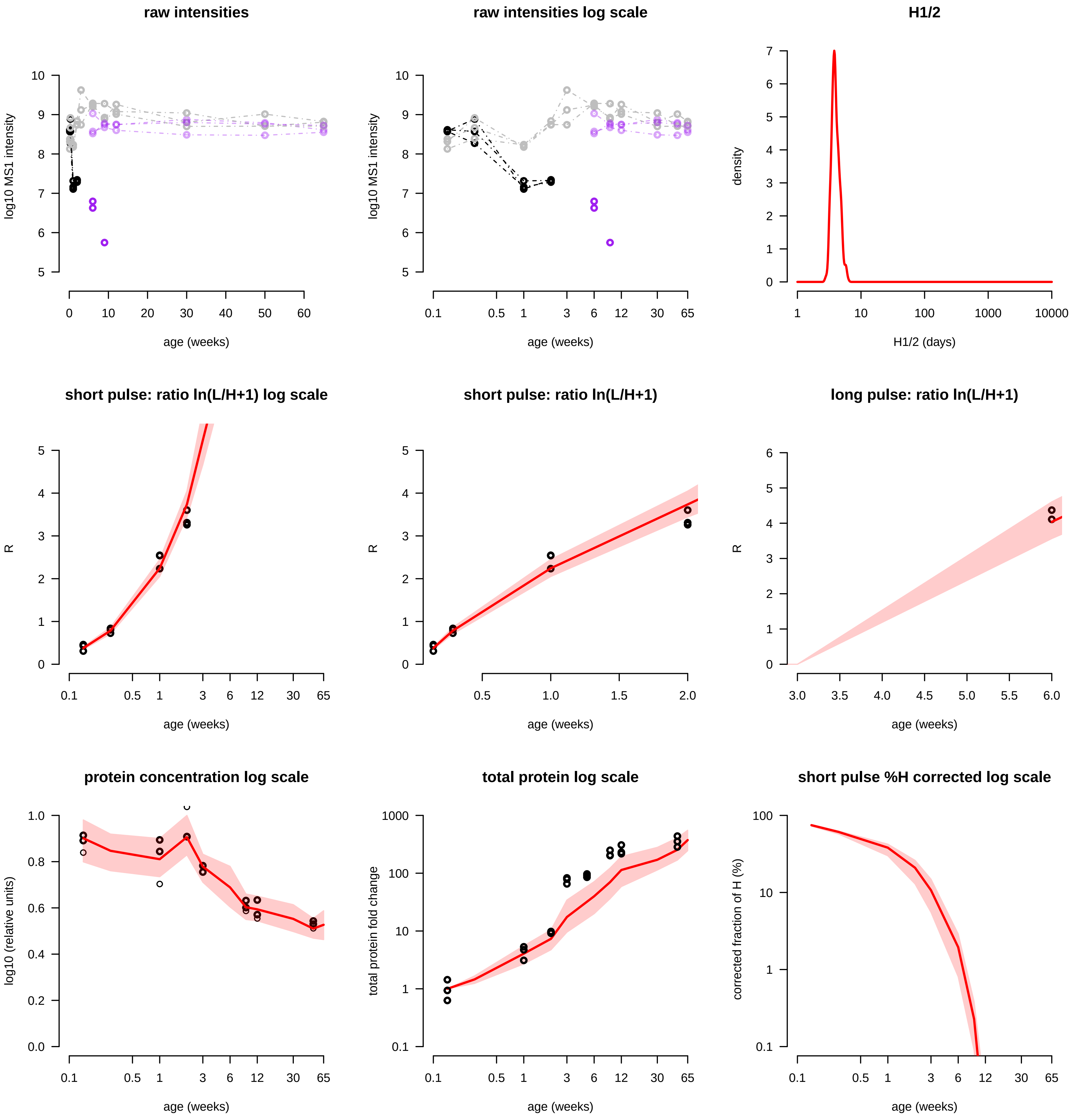

Q61739−2

>sp|Q61739−2|ITA6_MOUSE

Isoform Alpha−6X1A of Integrin alpha−6

GN=Itga6

Med H1/2: 3.7 days

Q5 H1/2: 2.7 days

Q95 H1/2: 5.5 days

reliable model fit: yes

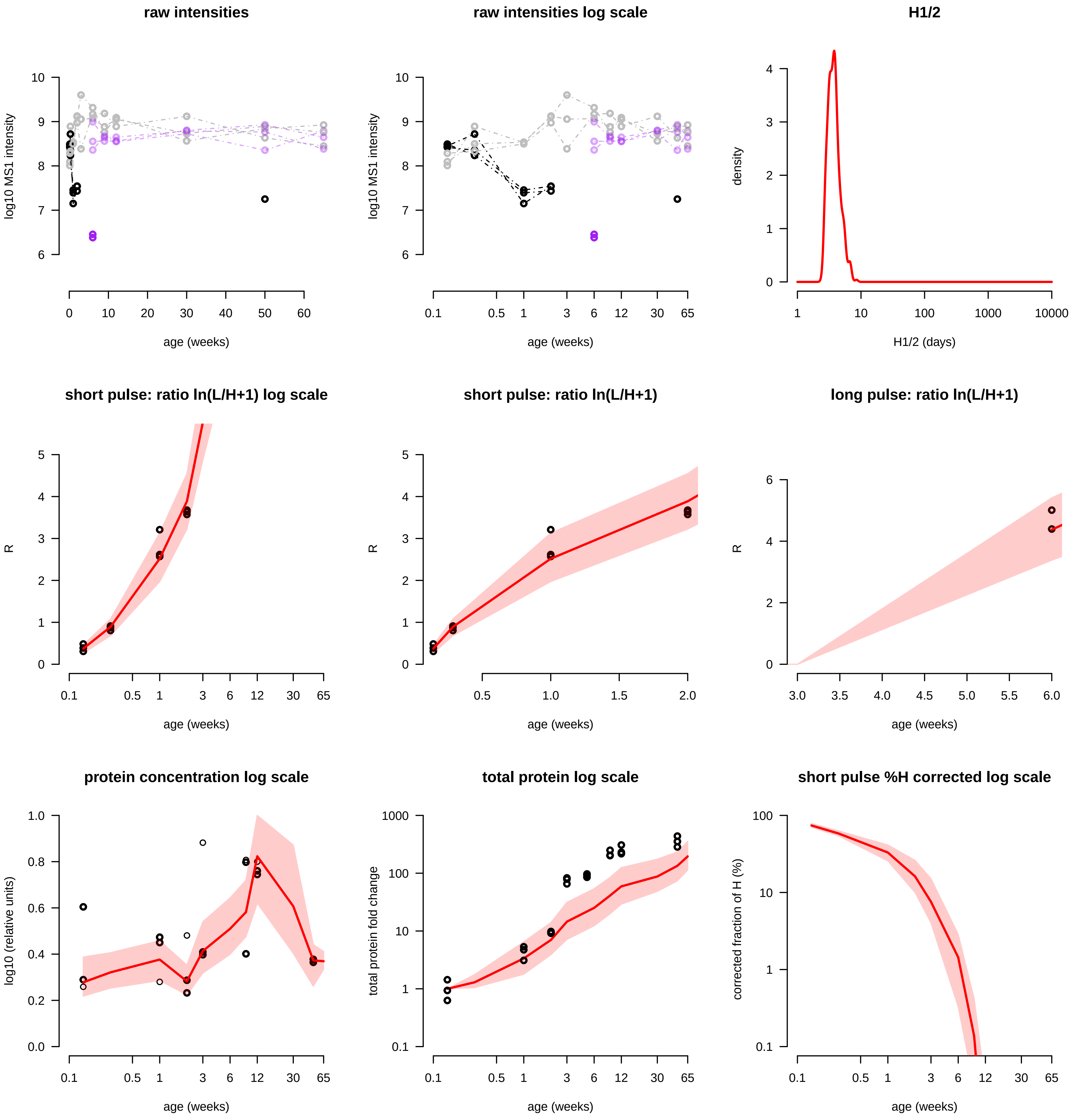

Q6P069−2;Q6P069

>sp|Q6P069−2|SORCN_MOUSE

Isoform 2 of Sorcin

GN=Sri

Med H1/2: 14.7 days

Q5 H1/2: 4.5 days

Q95 H1/2: 167.3 days

reliable model fit: yes

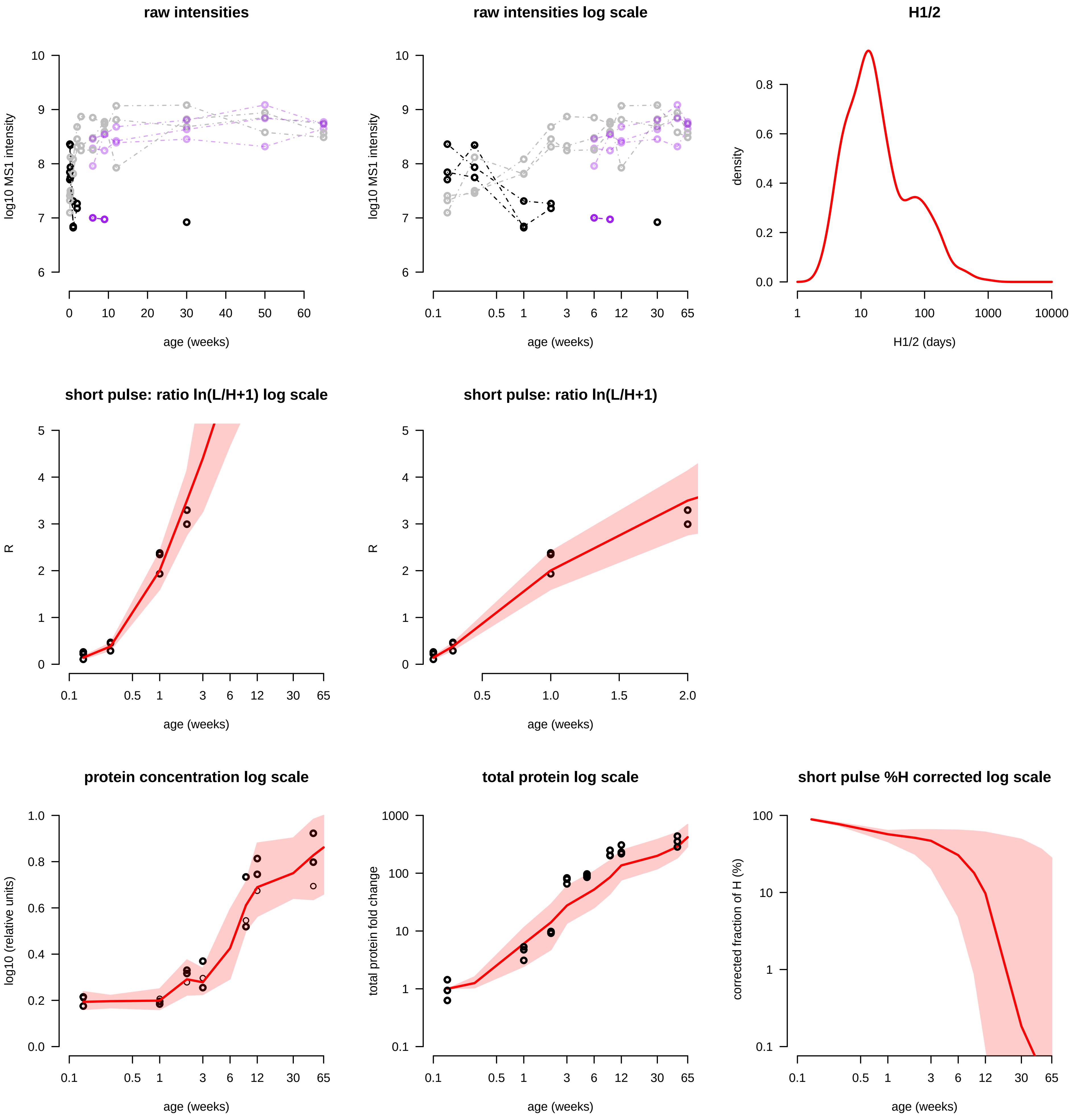

Q9D1P2

>sp|Q9D1P2|KAT8_MOUSE

Histone acetyltransferase KAT8

GN=Kat8

Med H1/2: 10.3 days

Q5 H1/2: 8 days

Q95 H1/2: 13.7 days

reliable model fit: yes

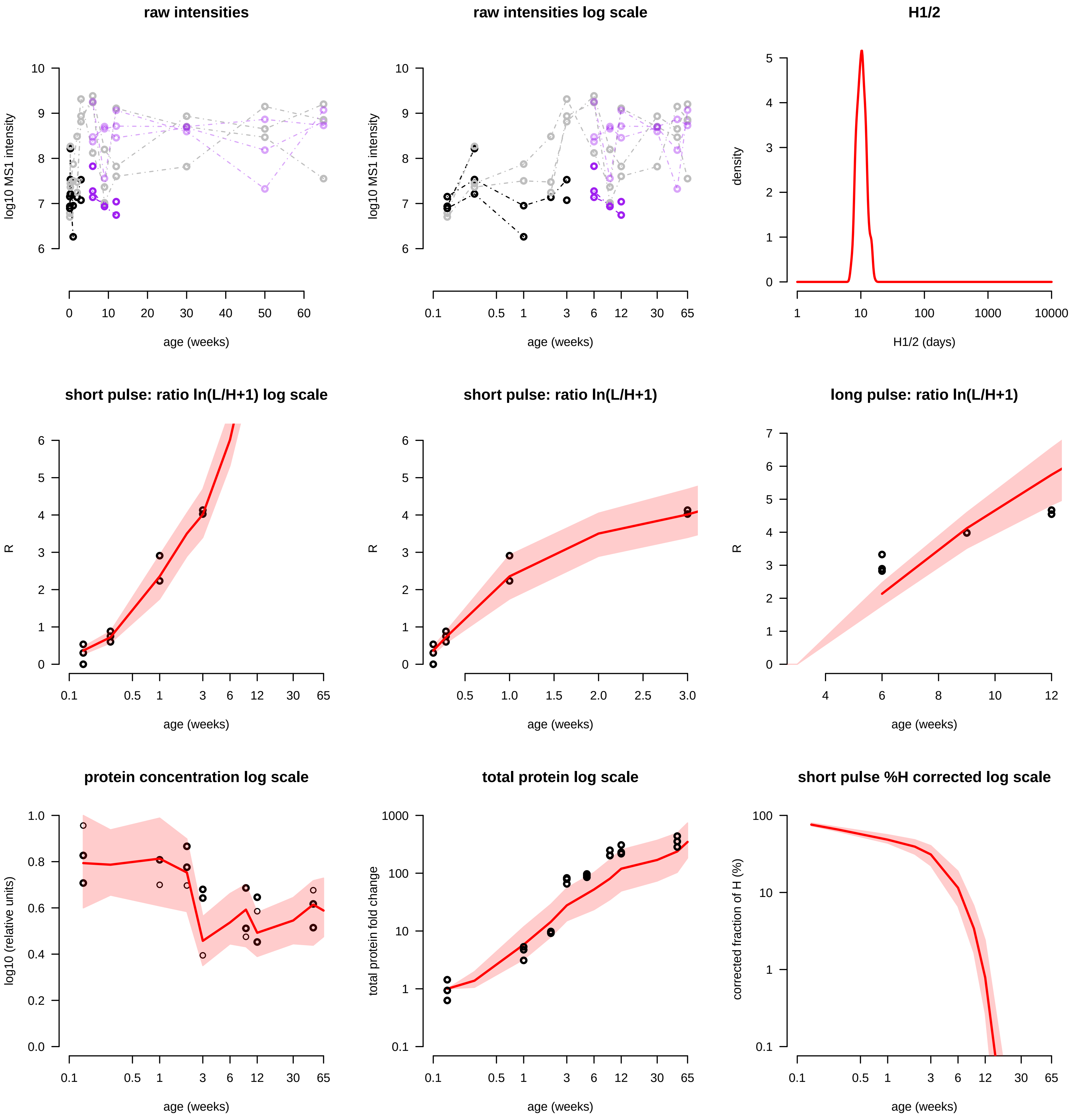

Q99M87;Q99M87−3

>sp|Q99M87|DNJA3_MOUSE

DnaJ homolog subfamily A member 3, mitochondrial

GN=Dnaja3

Med H1/2: 14.1 days

Q5 H1/2: 5.1 days

Q95 H1/2: 130.5 days

reliable model fit: no

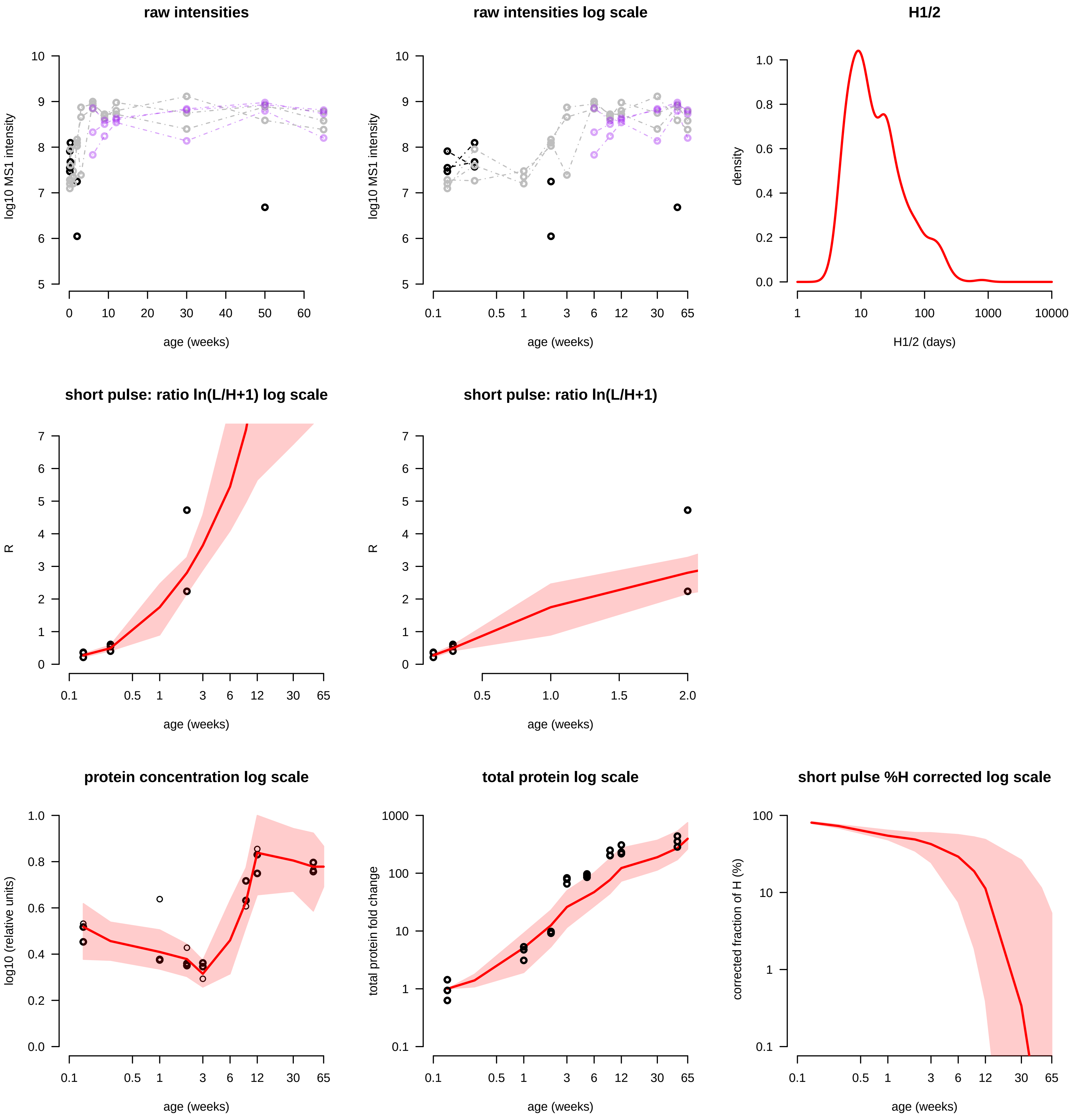

E9PVX6;E9PVX6−2

>sp|E9PVX6|KI67_MOUSE

Proliferation marker protein Ki−67

GN=Mki67

Med H1/2: 10.1 days

Q5 H1/2: 8.3 days

Q95 H1/2: 12.9 days

reliable model fit: yes

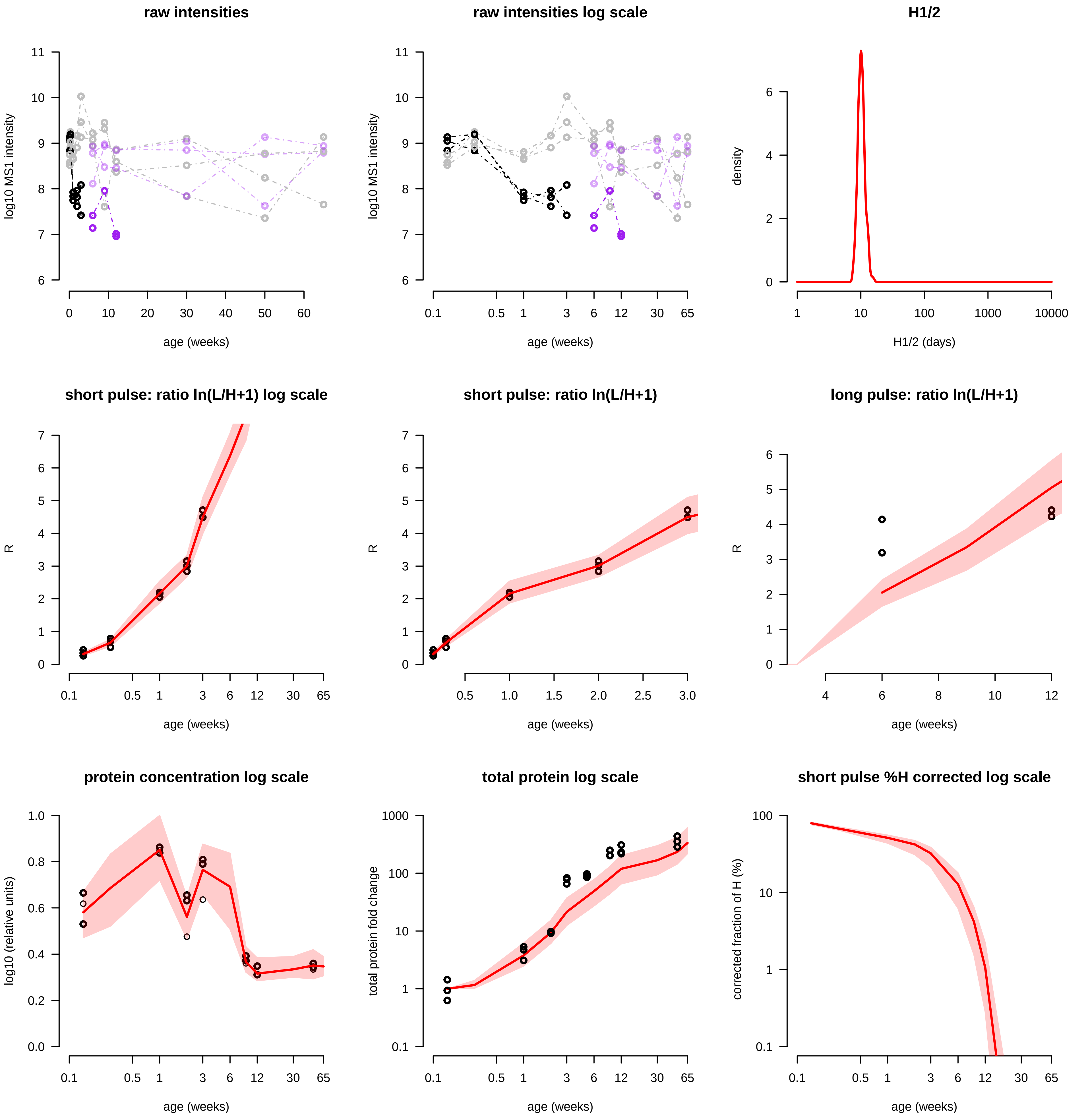

Q63844

>sp|Q63844|MK03_MOUSE

Mitogen−activated protein kinase 3

GN=Mapk3

Med H1/2: 5.6 days

Q5   H1/2: 4.5 days

Q95 H1/2: 7.1 days

reliable model fit: yes

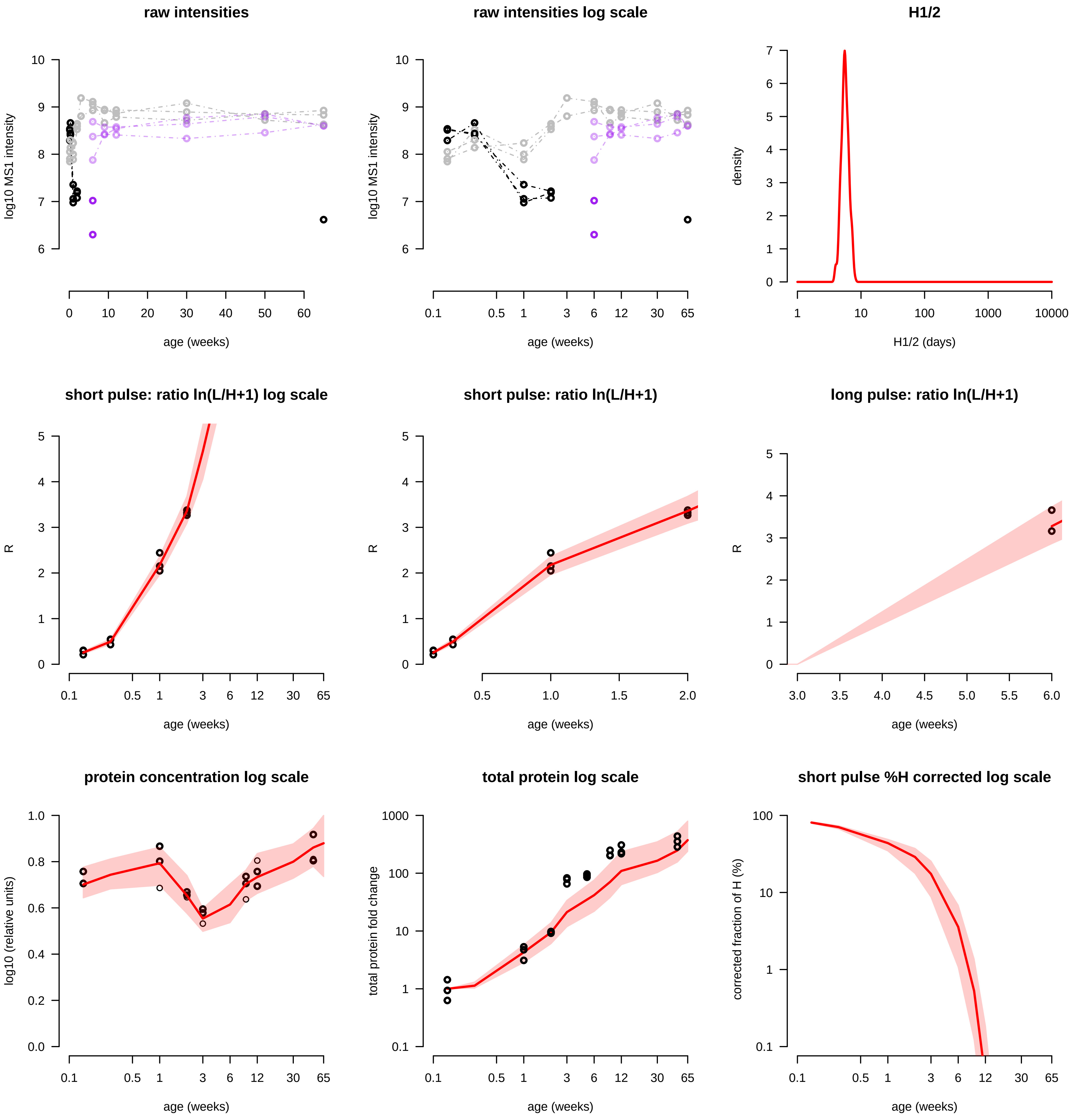

Q3URD3−2;Q3URD3;Q3URD3−5

>sp|Q3URD3−2|SLMAP_MOUSE

Isoform 2 of Sarcolemmal membrane−associated protein

GN=Slmap

Med H1/2: 1159.7 days

Q5 H1/2: 205.2 days

Q95 H1/2: 10732.5 days

reliable model fit: yes

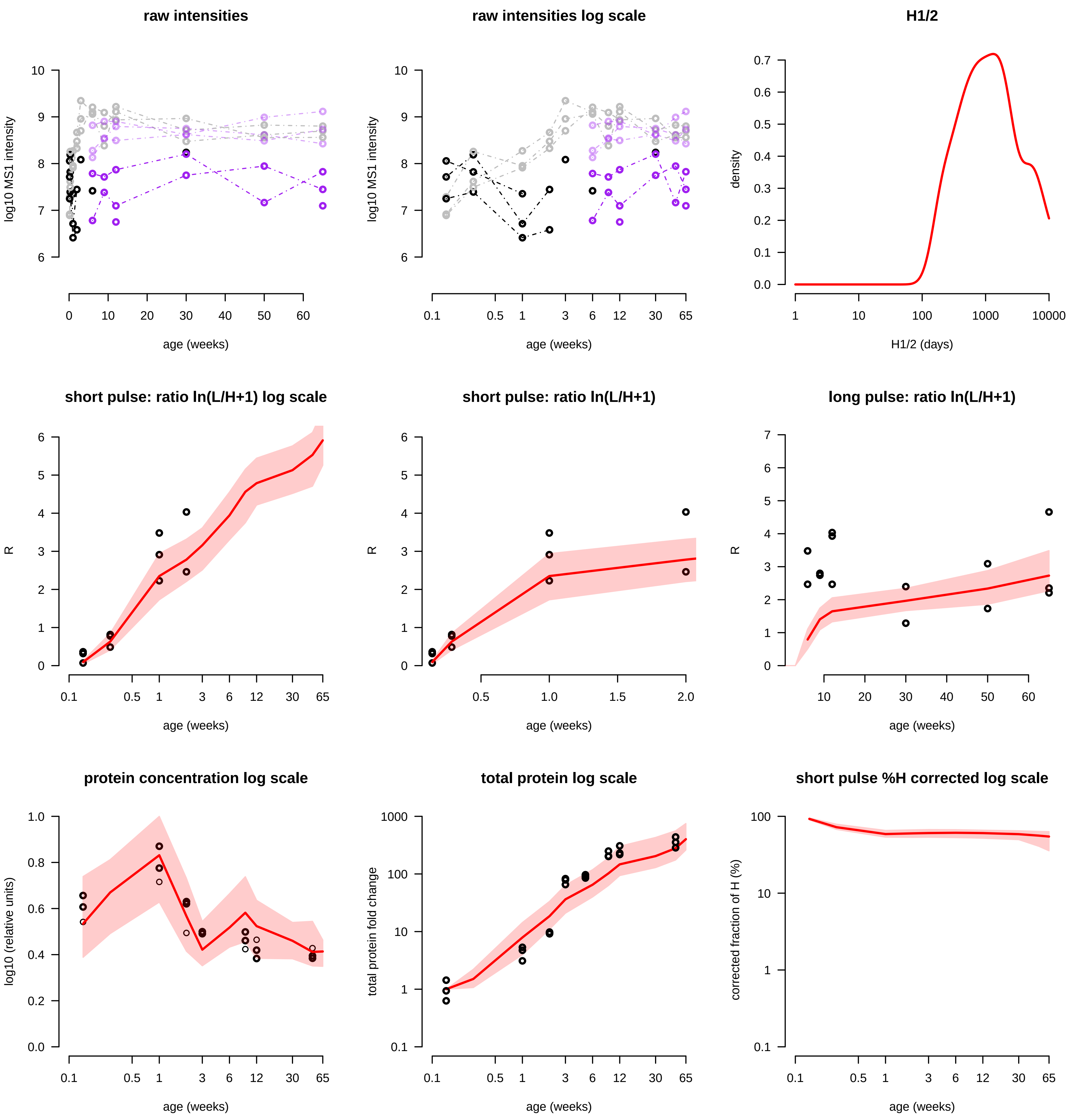

P60670;P60670−2

>sp|P60670|NPL4_MOUSE

Nuclear protein localization protein 4 homolog

GN=Nploc4

Med H1/2: 18.5 days

Q5 H1/2: 13.4 days

Q95 H1/2: 43.3 days

reliable model fit: yes

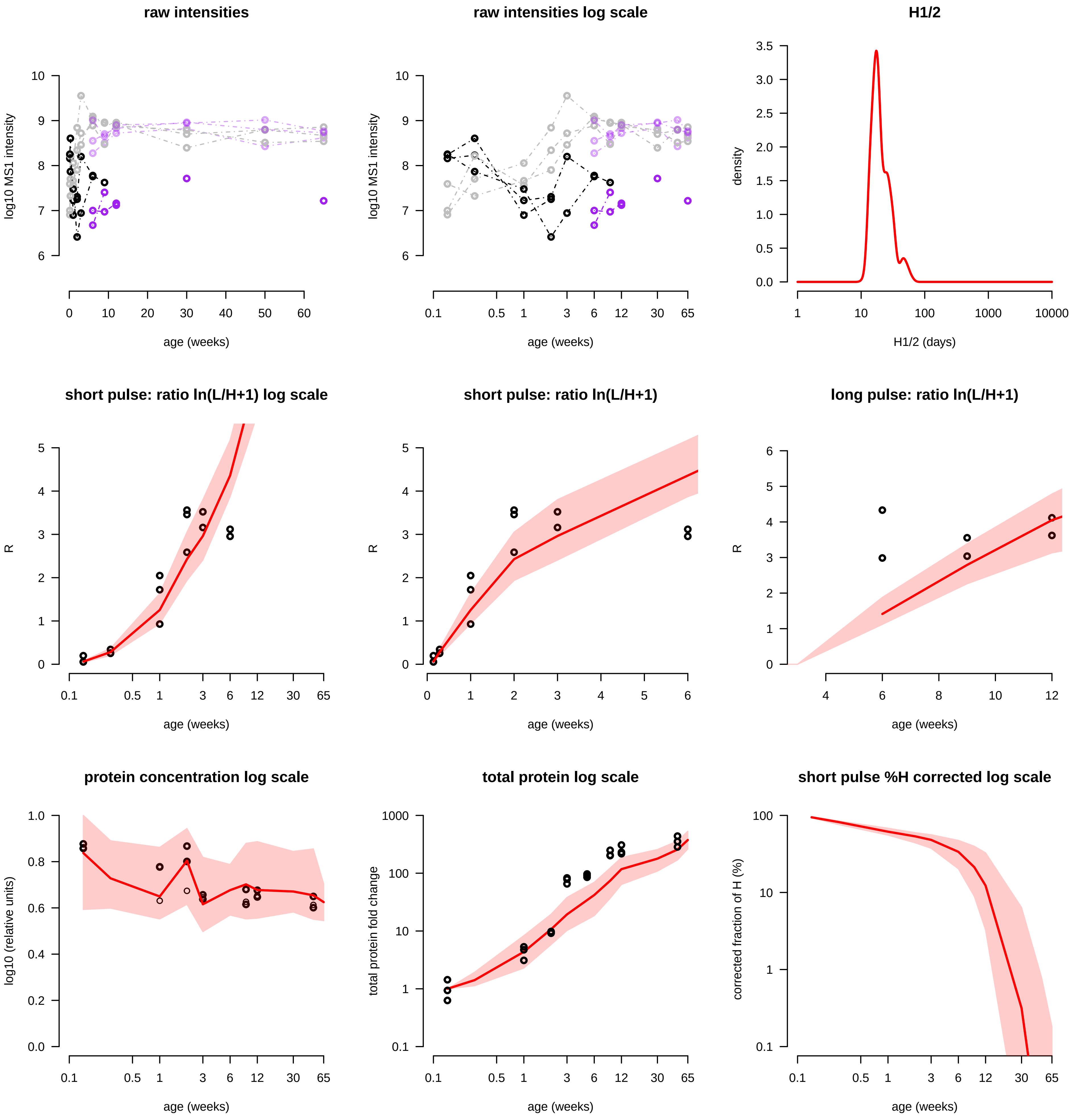

E9Q555

>sp|E9Q555|RN213_MOUSE

E3 ubiquitin−protein ligase RNF213

GN=Rnf213

Med H1/2: 8.4 days

Q5 H1/2: 3.9 days

Q95 H1/2: 19.3 days

reliable model fit: yes

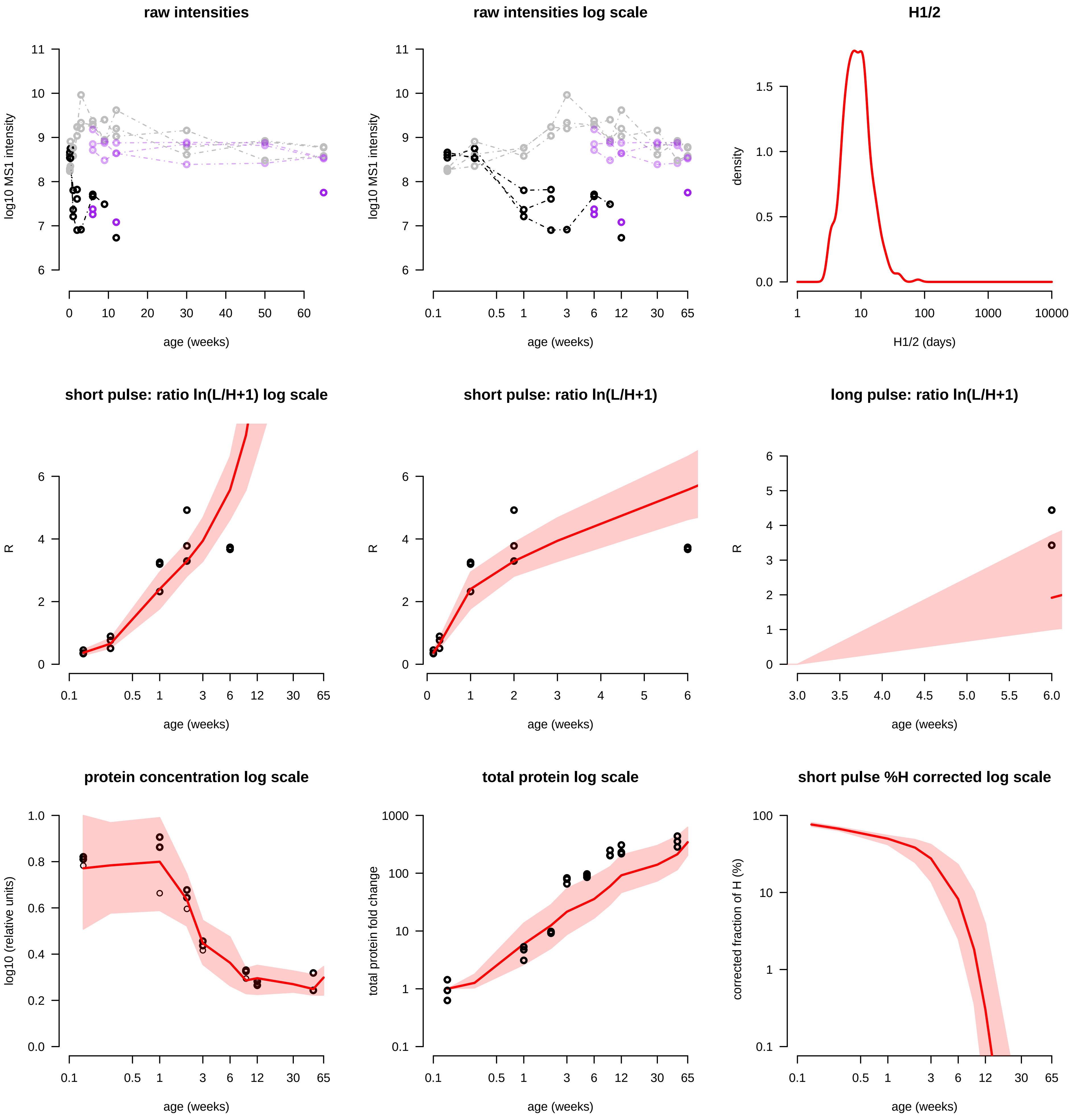

Q9D1G5

>sp|Q9D1G5|LRC57_MOUSE

Leucine−rich repeat−containing protein 57

GN=Lrrc57

Med H1/2: 13 days

Q5 H1/2: 5.2 days

Q95 H1/2: 144.8 days

reliable model fit: yes

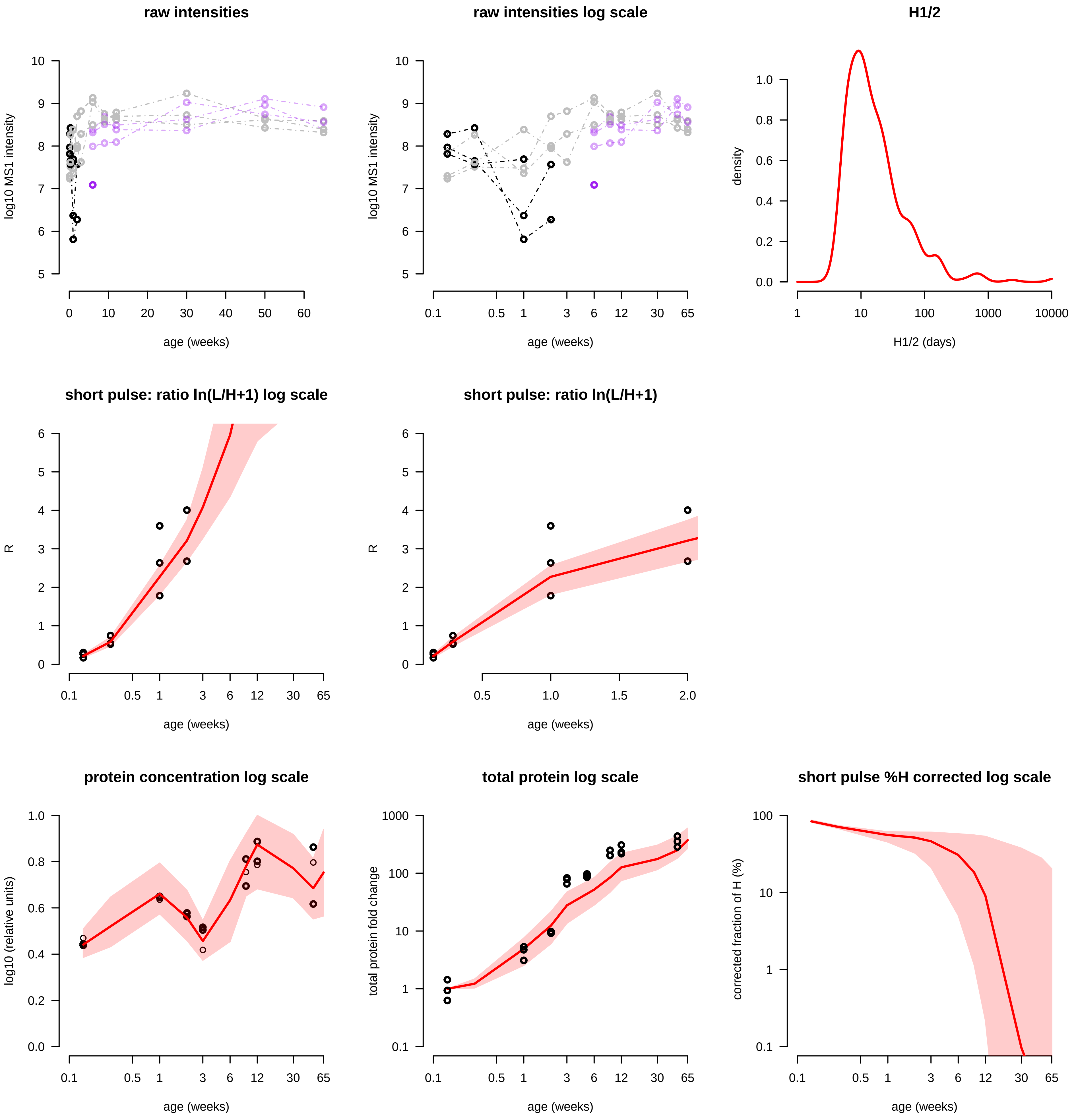

Q9Z0R4

>sp|Q9Z0R4|ITSN1_MOUSE

Intersectin−1

GN=Itsn1

Med H1/2: 818.9 days

Q5 H1/2: 252.6 days

Q95 H1/2: 7546.7 days

reliable model fit: yes

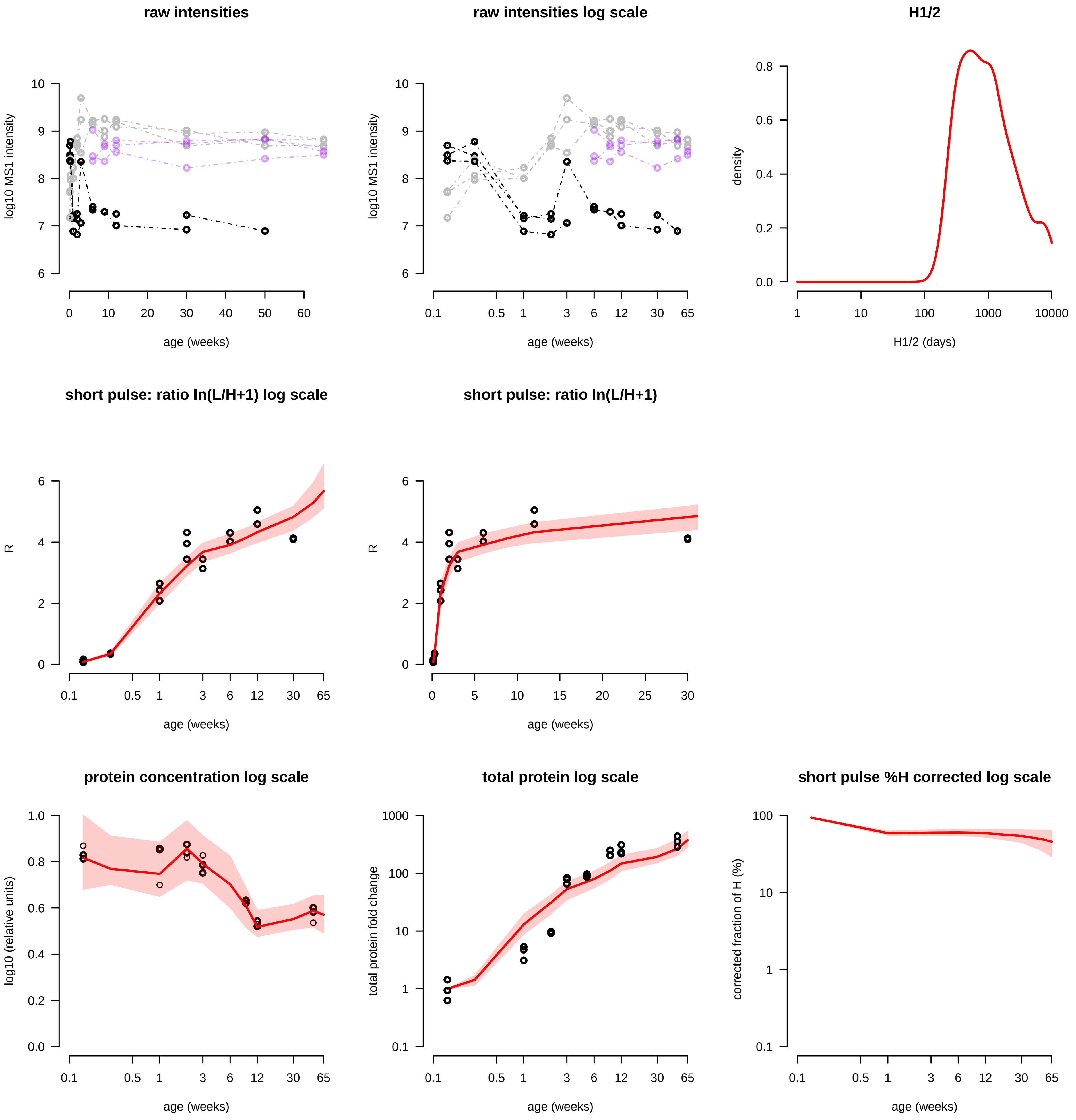

Q9QZE7

>sp|Q9QZE7|TSNAX_MOUSE

Translin−associated protein X

GN=Tsnax

Med H1/2: 3.5 days

Q5 H1/2: 2.8 days

Q95 H1/2: 4.8 days

reliable model fit: yes

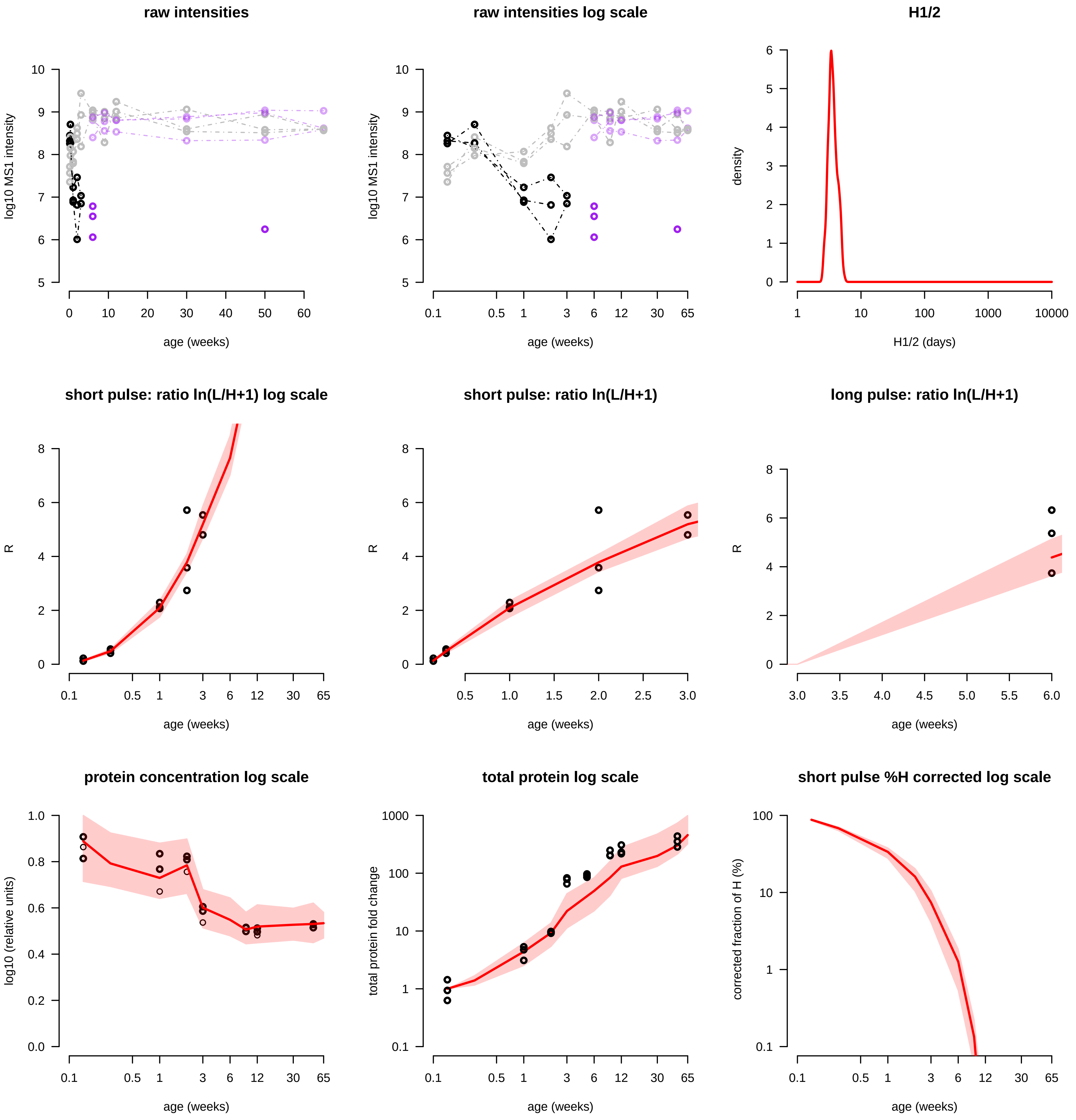

Q8K4B0

>sp|Q8K4B0|MTA1_MOUSE

Metastasis−associated protein MTA1

GN=Mta1

Med H1/2: 3.9 days

Q5 H1/2: 3.2 days

Q95 H1/2: 4.7 days

reliable model fit: yes

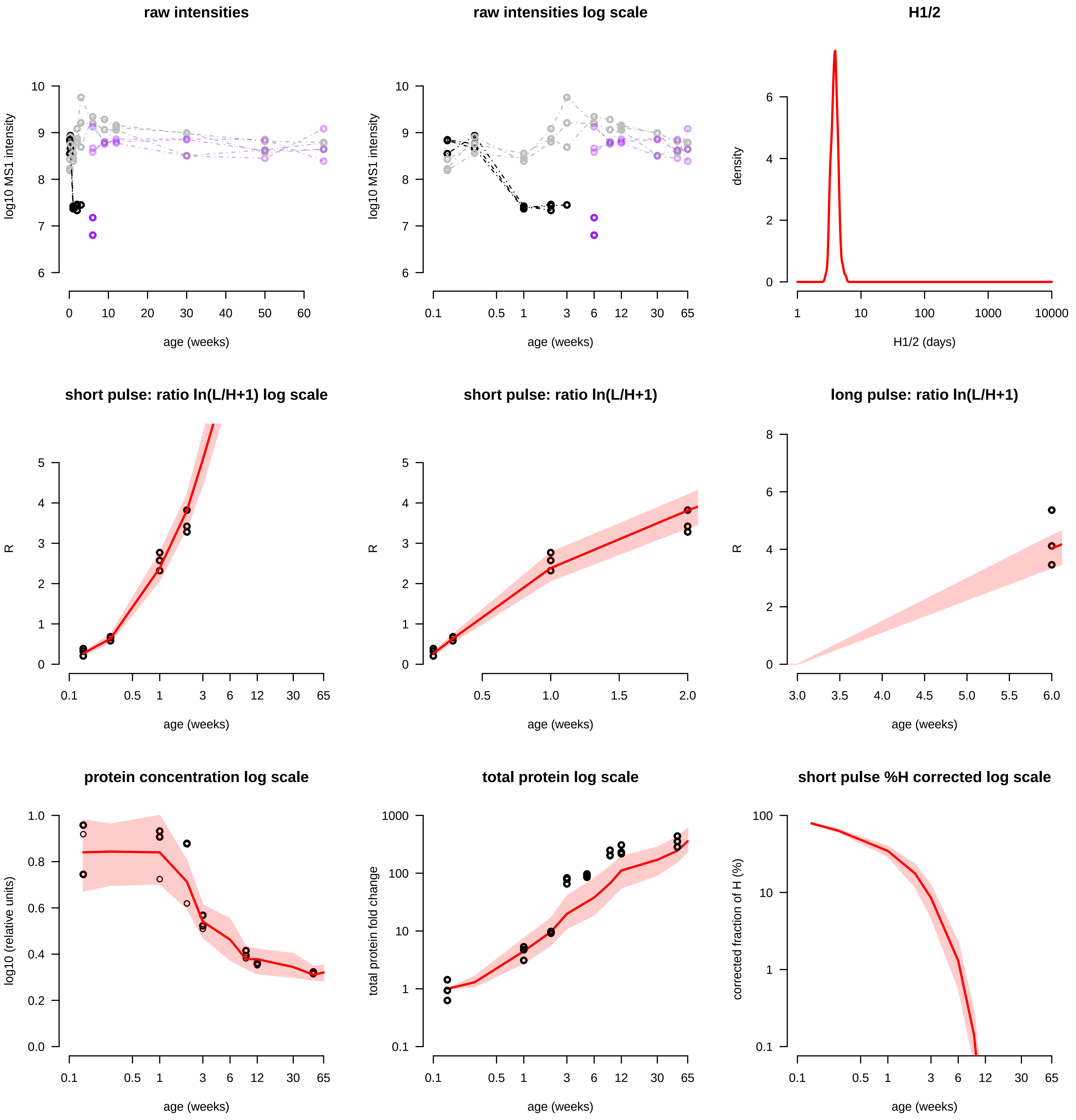

B2RQC6;B2RQC6−2

>sp|B2RQC6|PYR1_MOUSE

CAD protein

GN=Cad

Med H1/2: 20.4 days

Q5 H1/2: 9.1 days

Q95 H1/2: 99.3 days

reliable model fit: no

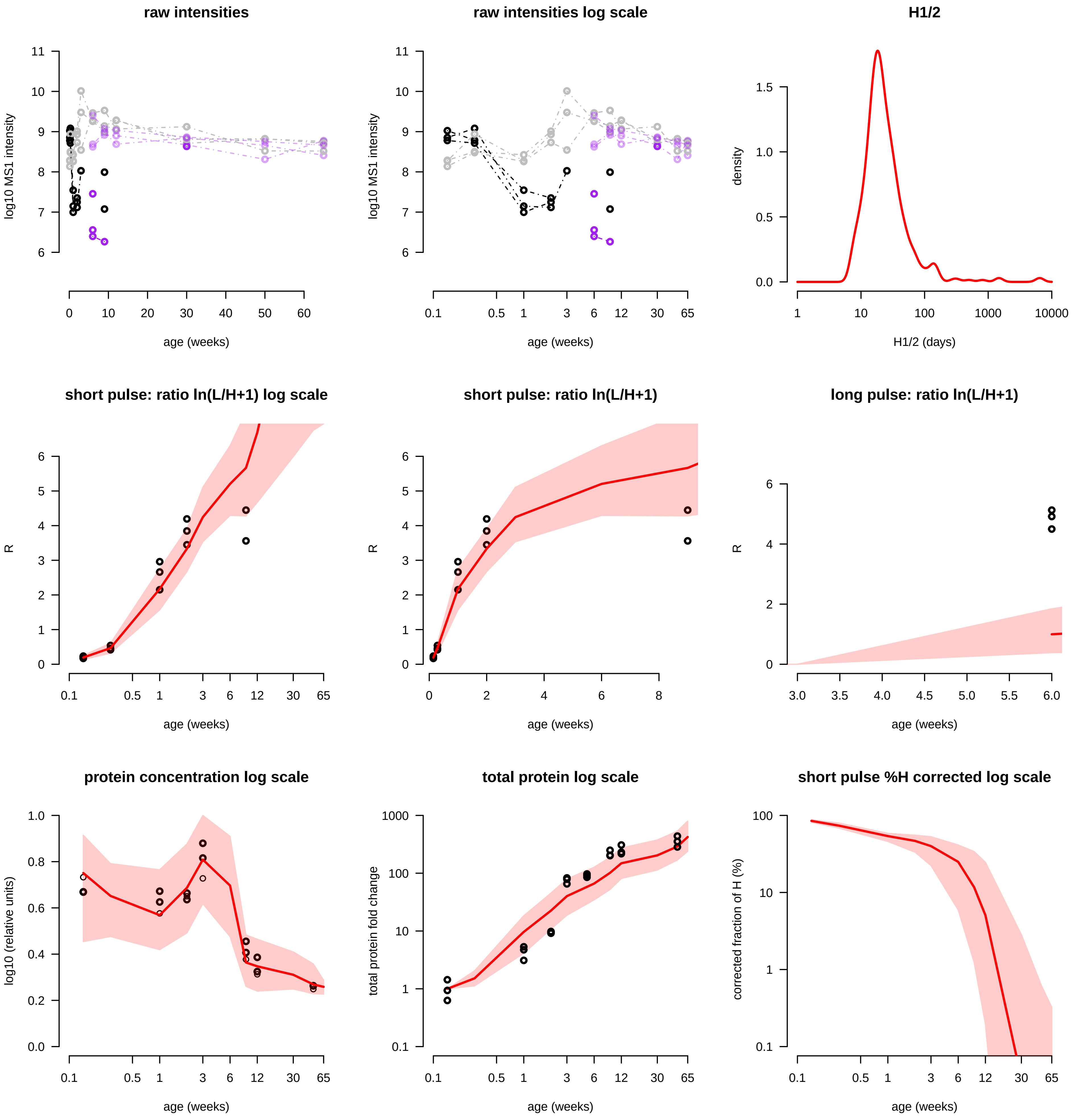

P41731

>sp|P41731|CD63_MOUSE

CD63 antigen

GN=Cd63

Med H1/2: 5 days

Q5 H1/2: 3.9 days

Q95 H1/2: 7.5 days

reliable model fit: yes

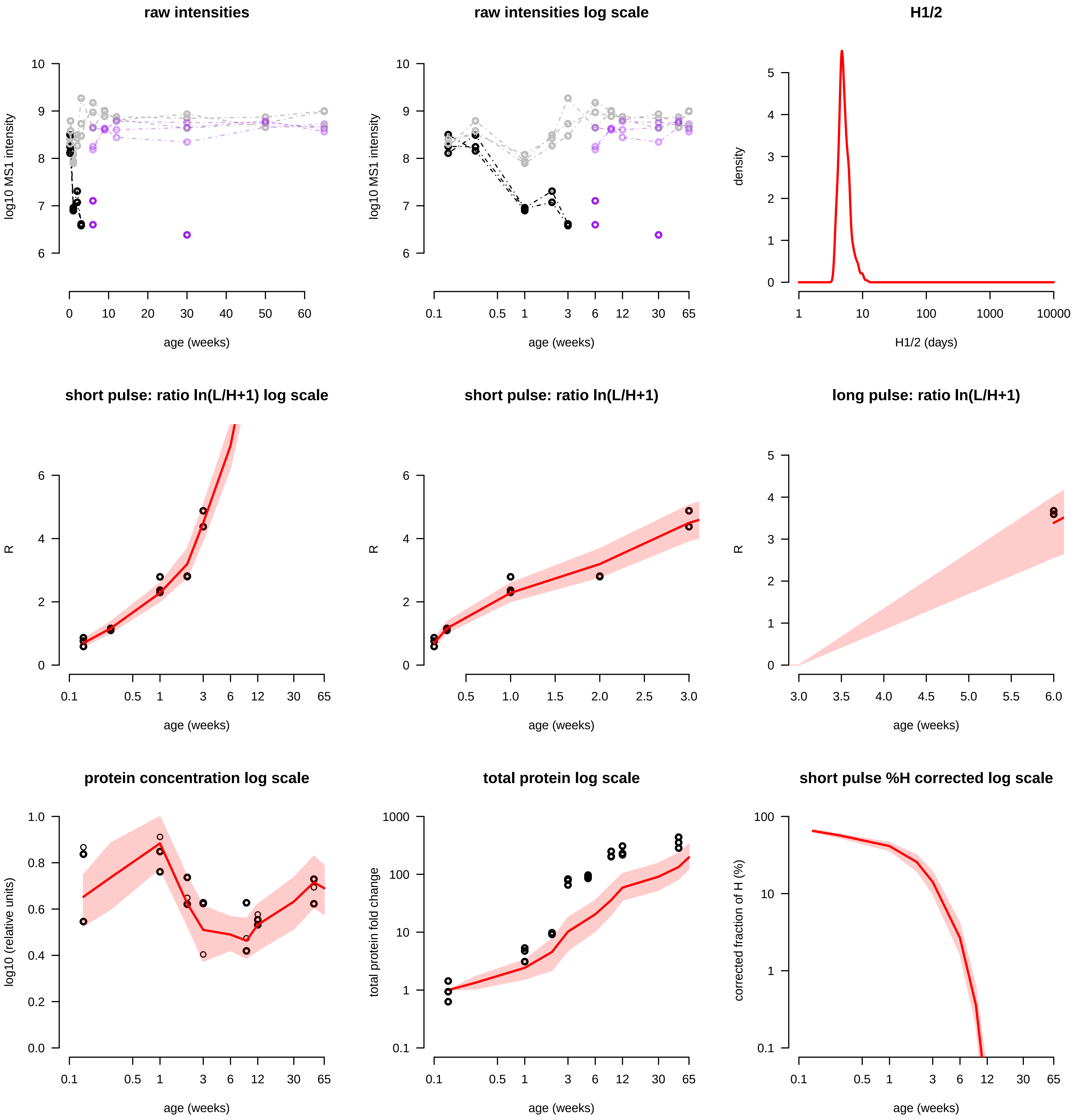

P17156

>sp|P17156|HSP72_MOUSE

Heat shock−related 70 kDa protein 2

GN=Hspa2

Med H1/2: 1764.9 days

Q5 H1/2: 500.8 days

Q95 H1/2: 25613.4 days

reliable model fit: yes

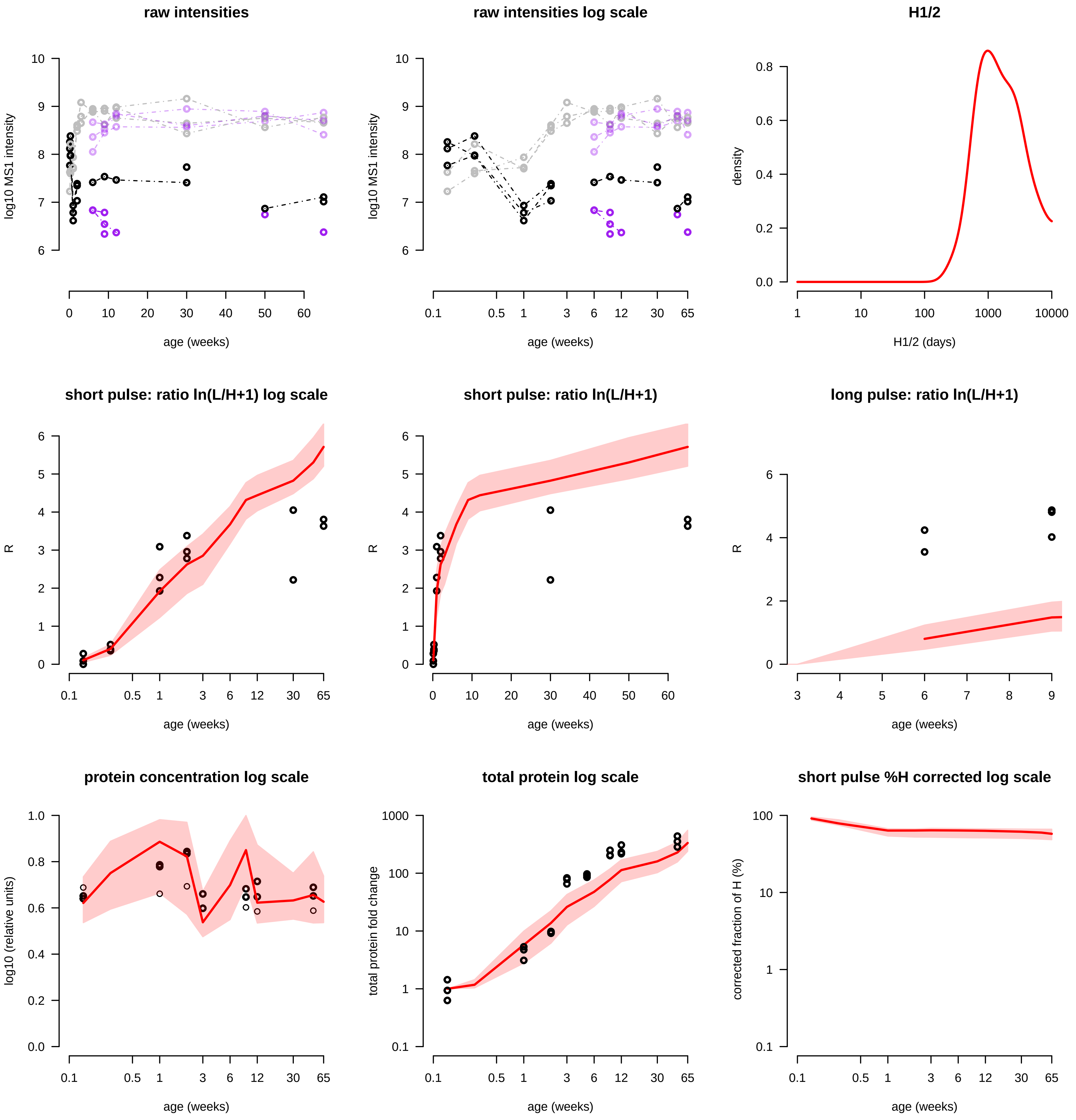

Q8BUR4;Q8BUR4−2

>sp|Q8BUR4|DOCK1_MOUSE

Dedicator of cytokinesis protein 1

GN=Dock1

Med H1/2: 149.8 days

Q5 H1/2: 60.6 days

Q95 H1/2: 1692.8 days

reliable model fit: no

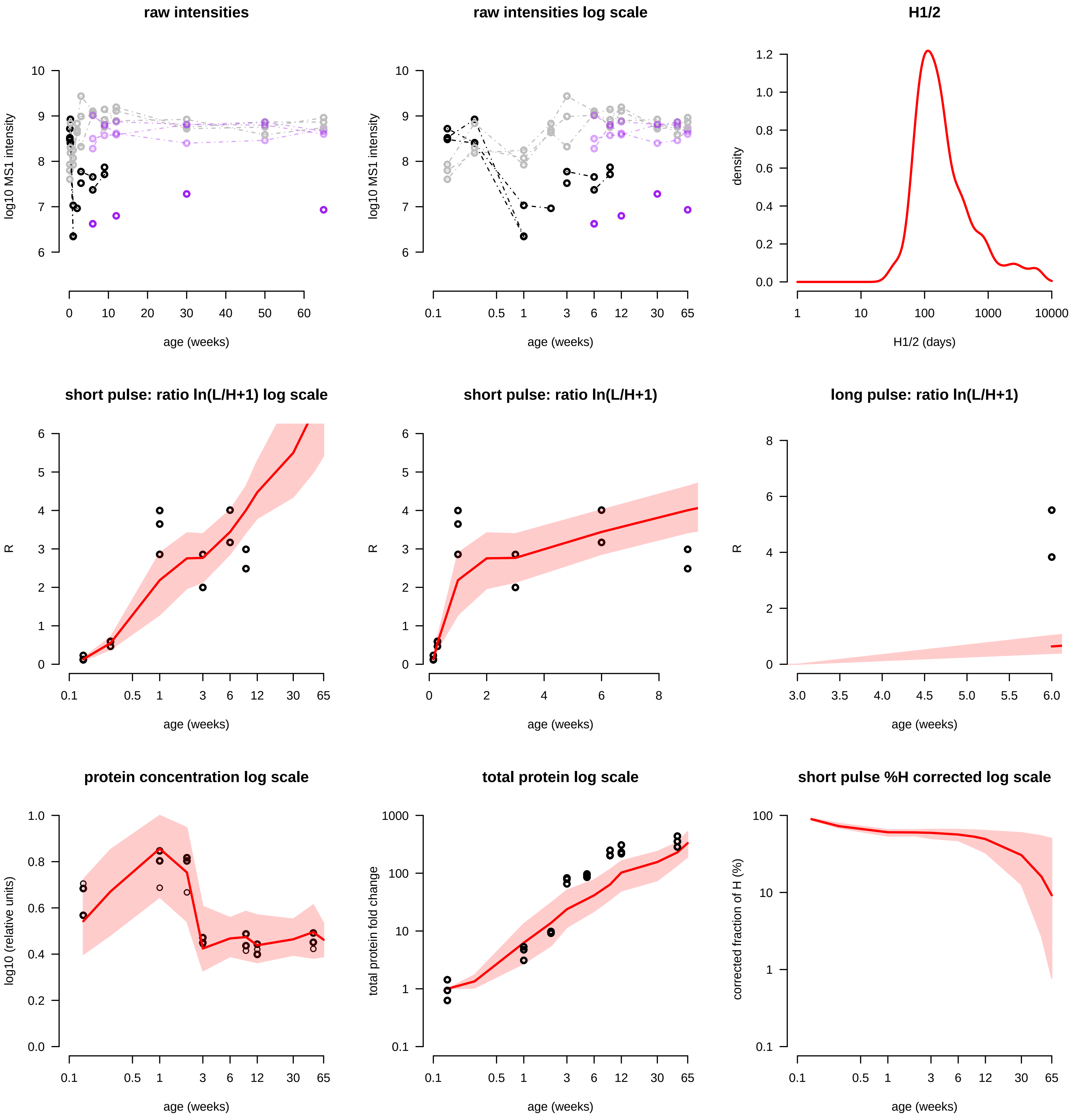

Q8CBW3−2;Q8CBW3−4

>sp|Q8CBW3−2|ABI1_MOUSE

Isoform 2 of Abl interactor 1

GN=Abi1

Med H1/2: 4 days

Q5 H1/2: 3.3 days

Q95 H1/2: 5 days

reliable model fit: yes

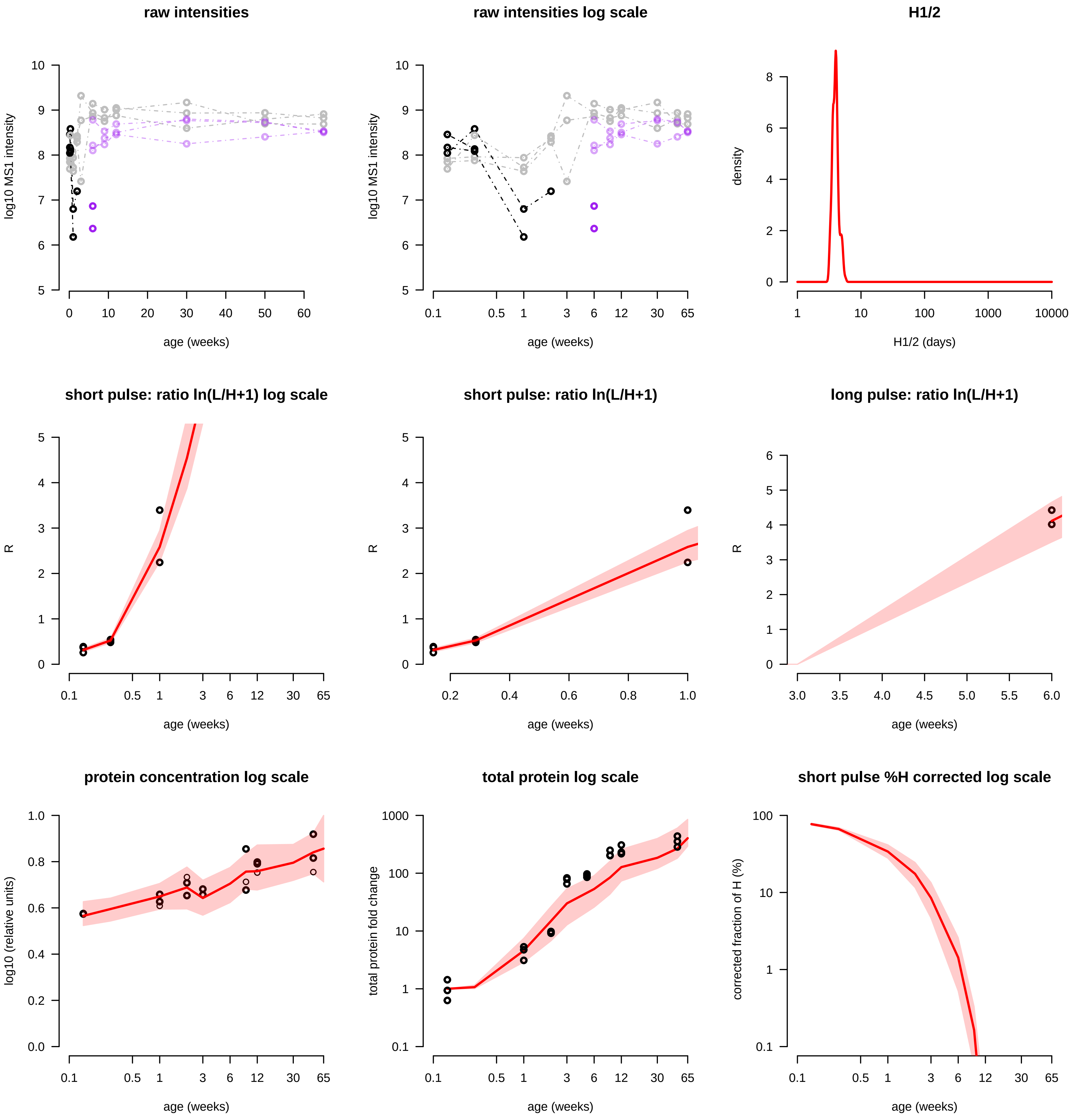

Q9QYF1

>sp|Q9QYF1|RDH11_MOUSE

Retinol dehydrogenase 11

GN=Rdh11

Med H1/2: 10.5 days

Q5 H1/2: 3.3 days

Q95 H1/2: 79 days

reliable model fit: yes

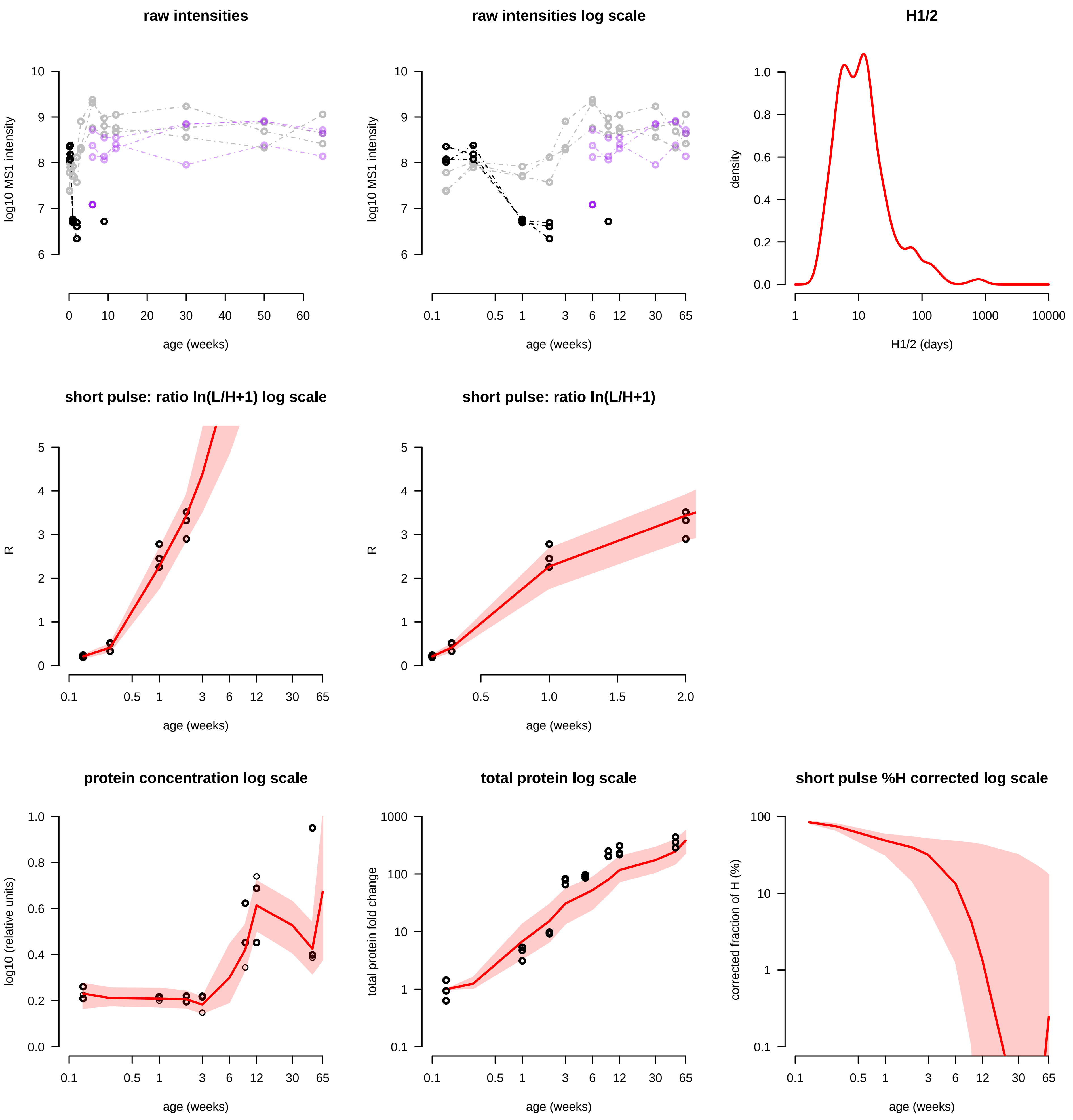

P61290

>sp|P61290|PSME3_MOUSE

Proteasome activator complex subunit 3

GN=Psme3

Med H1/2: 32.4 days

Q5 H1/2: 15.9 days

Q95 H1/2: 330.7 days

reliable model fit: no

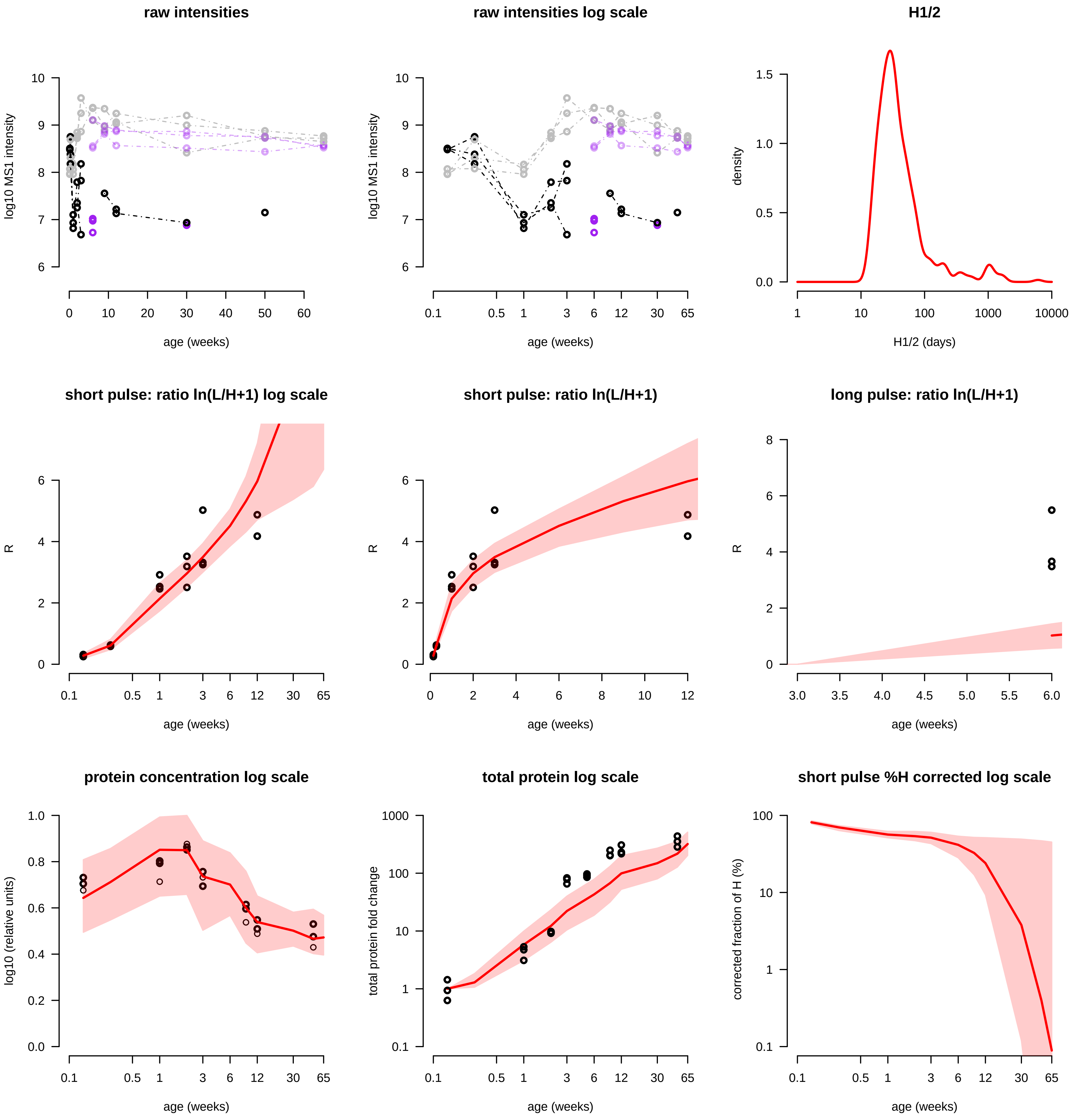

O88967

>sp|O88967|YMEL1_MOUSE

ATP−dependent zinc metalloprotease YME1L1

GN=Yme1l1

Med H1/2: 660.2 days

Q5 H1/2: 148.3 days

Q95 H1/2: 9891 days

reliable model fit: yes

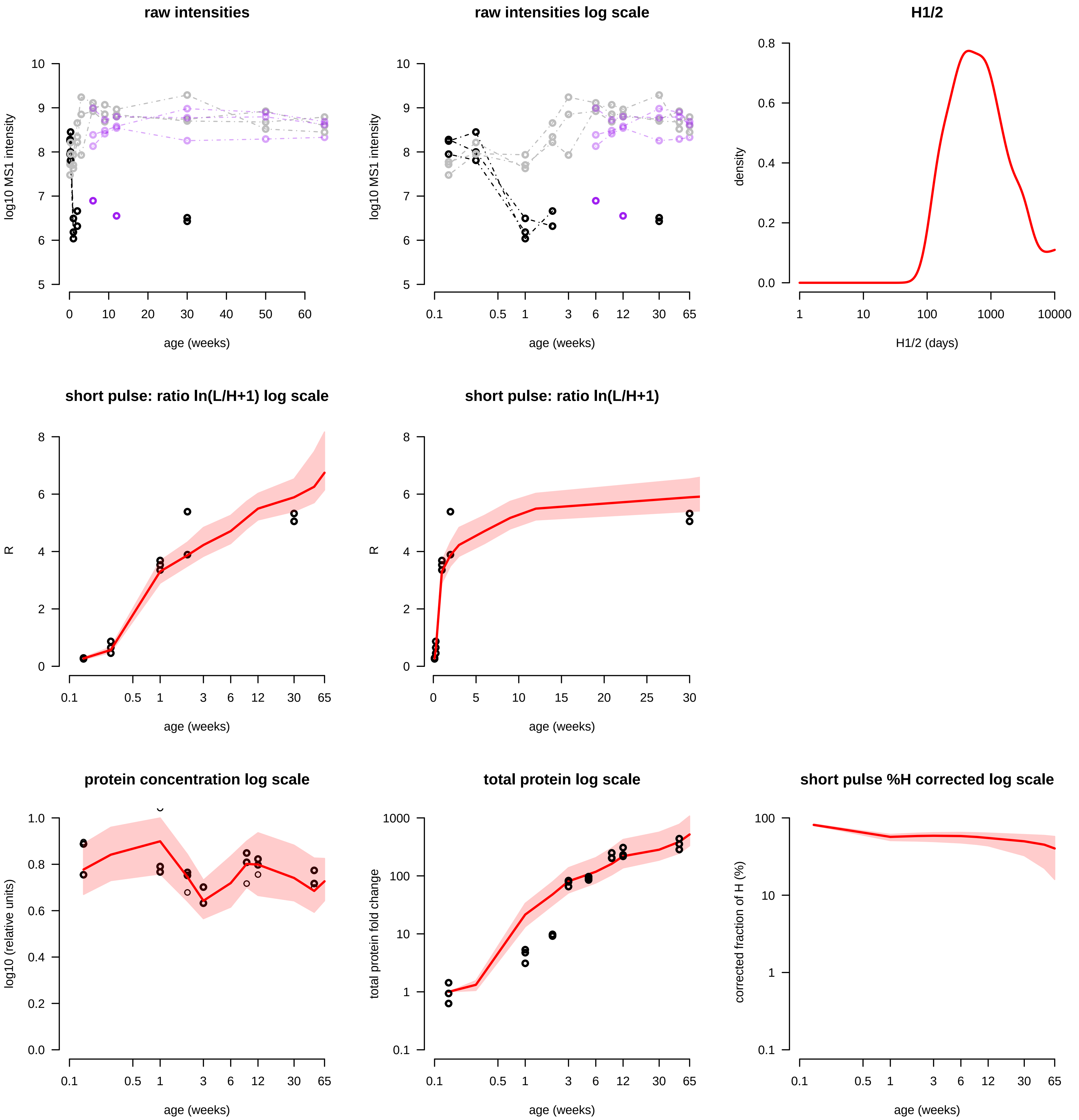

Q9CPQ3

>sp|Q9CPQ3|TOM22_MOUSE

Mitochondrial import receptor subunit TOM22 homolog

GN=Tomm22

Med H1/2: 7.8 days

Q5 H1/2: 6.3 days

Q95 H1/2: 10.2 days

reliable model fit: yes

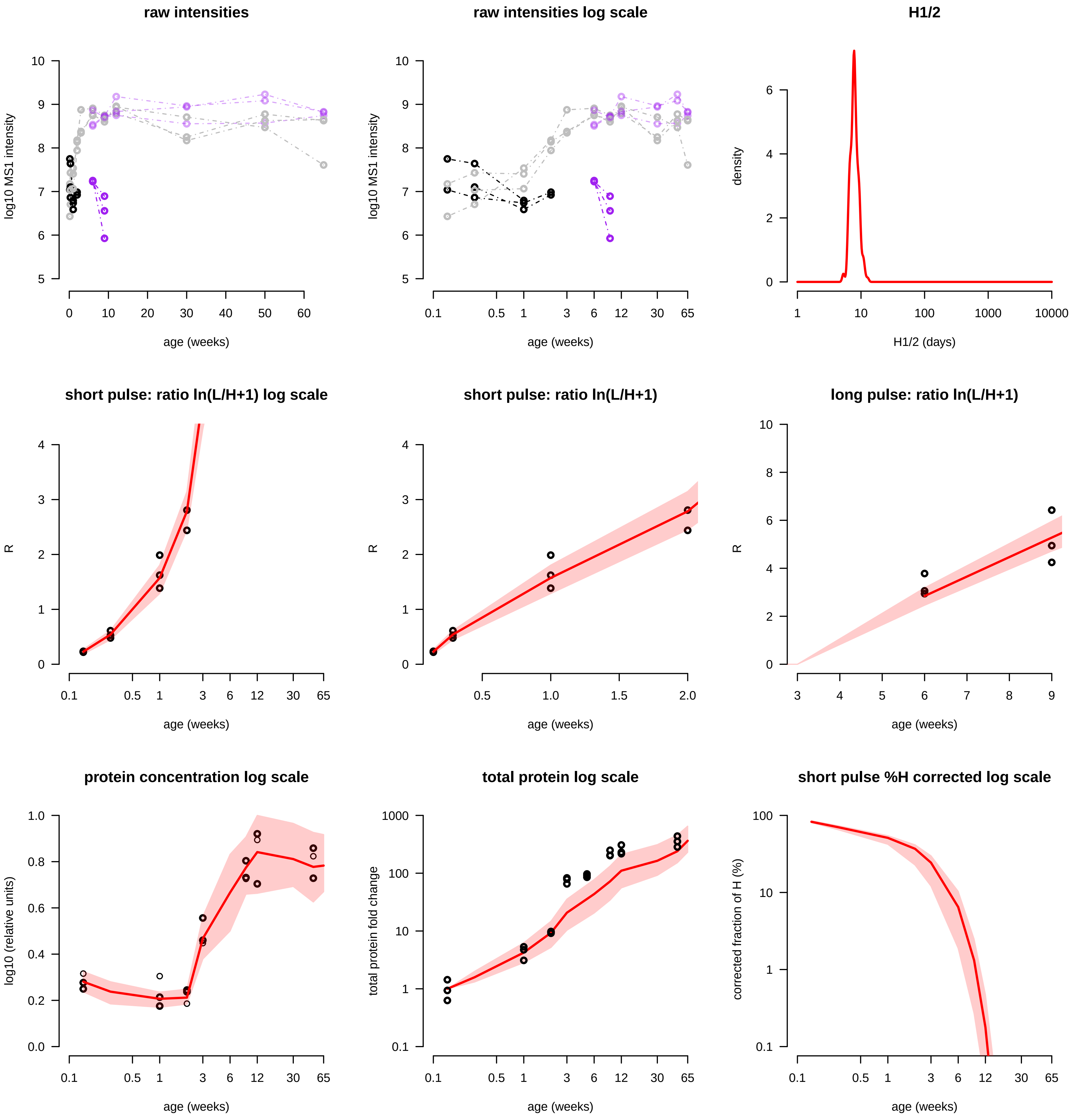

Q7TMM9

>sp|Q7TMM9|TBB2A_MOUSE

Tubulin beta−2A chain

GN=Tubb2a

Med H1/2: 6.3 days

Q5 H1/2: 4.8 days

Q95 H1/2: 8.1 days

reliable model fit: yes

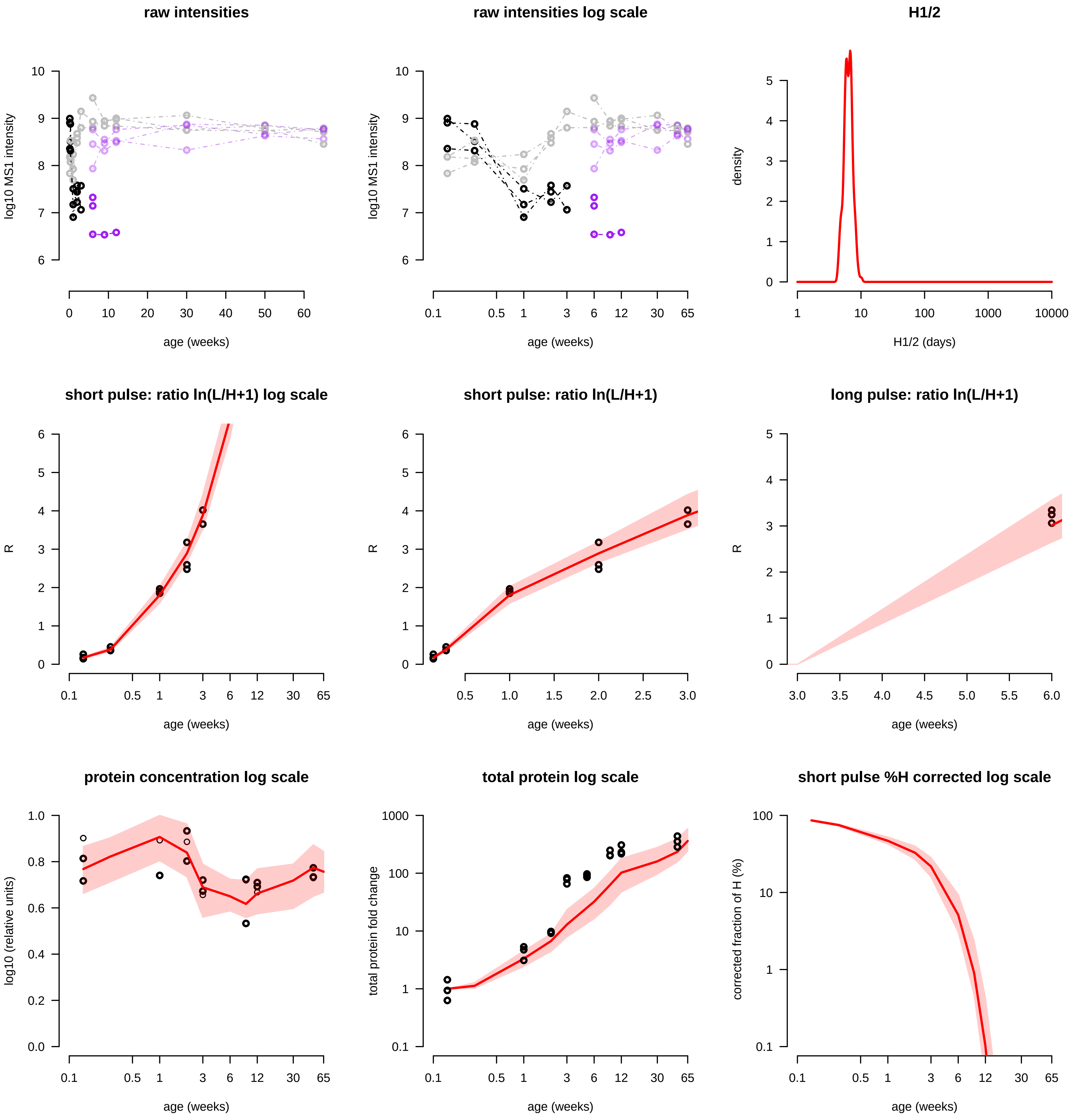

Q5SW19;Q5SW19−3;Q5SW19−2

>sp|Q5SW19|CLU_MOUSE

Clustered mitochondria protein homolog

GN=Cluh

Med H1/2: 3.5 days

Q5 H1/2: 2.6 days

Q95 H1/2: 4.7 days

reliable model fit: yes

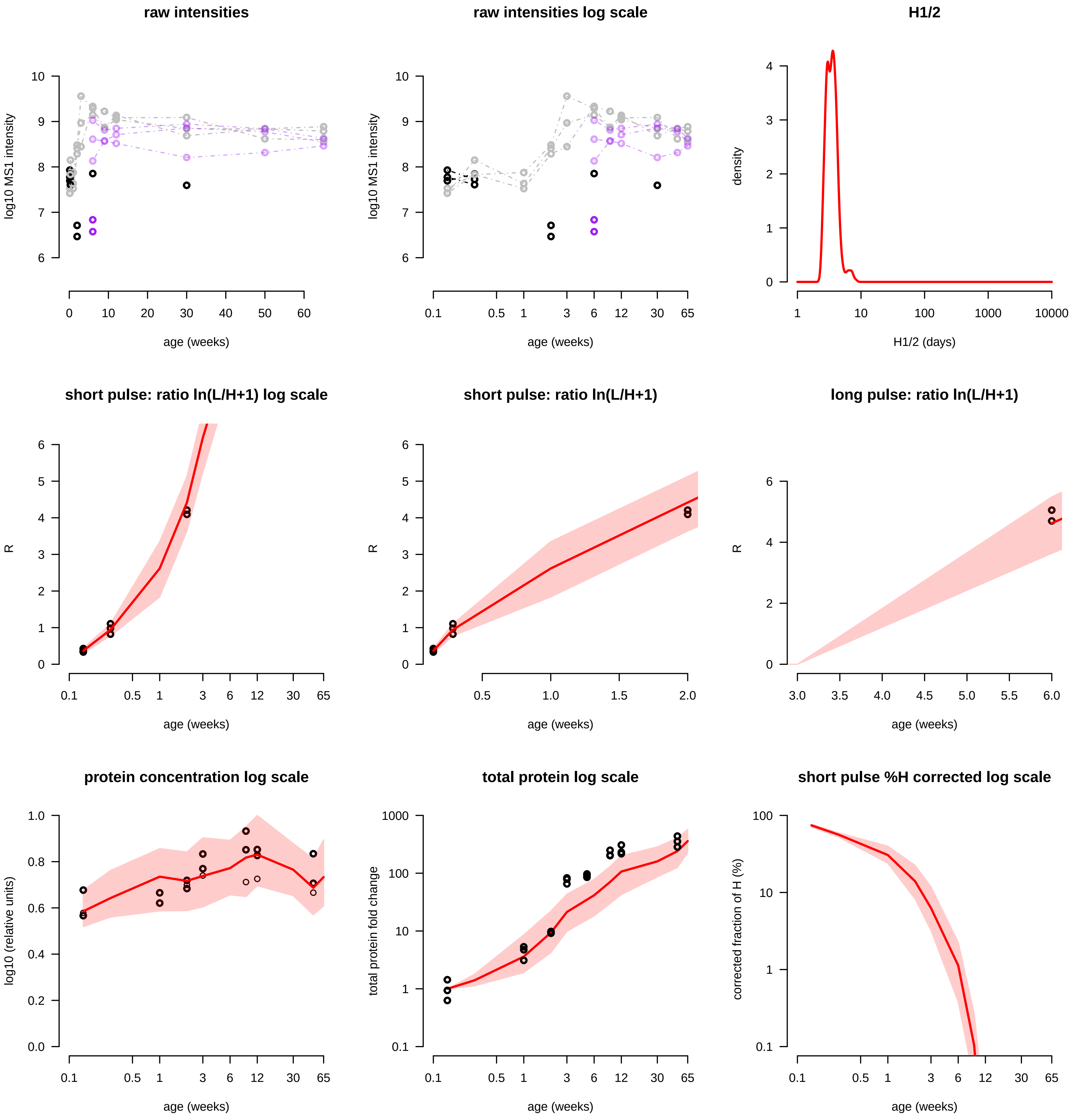

P97823;P97823−2

>sp|P97823|LYPA1_MOUSE

Acyl−protein thioesterase 1

GN=Lypla1

Med H1/2: 19.3 days

Q5 H1/2: 11.8 days

Q95 H1/2: 41.5 days

reliable model fit: yes

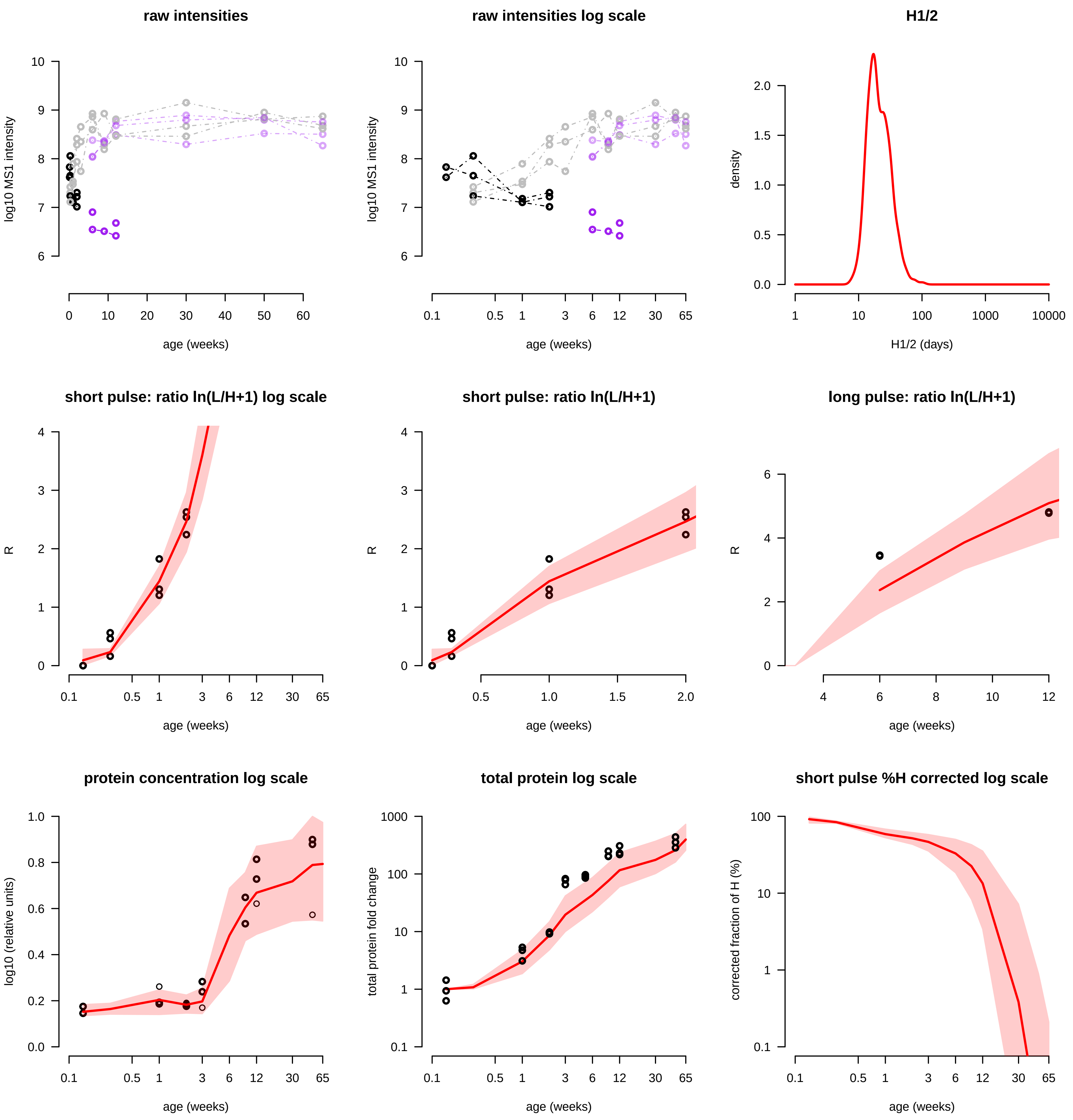

P10833

>sp|P10833|RRAS_MOUSE

Ras−related protein R−Ras

GN=Rras

Med H1/2: 13.2 days

Q5 H1/2: 4.3 days

Q95 H1/2: 159.3 days

reliable model fit: yes

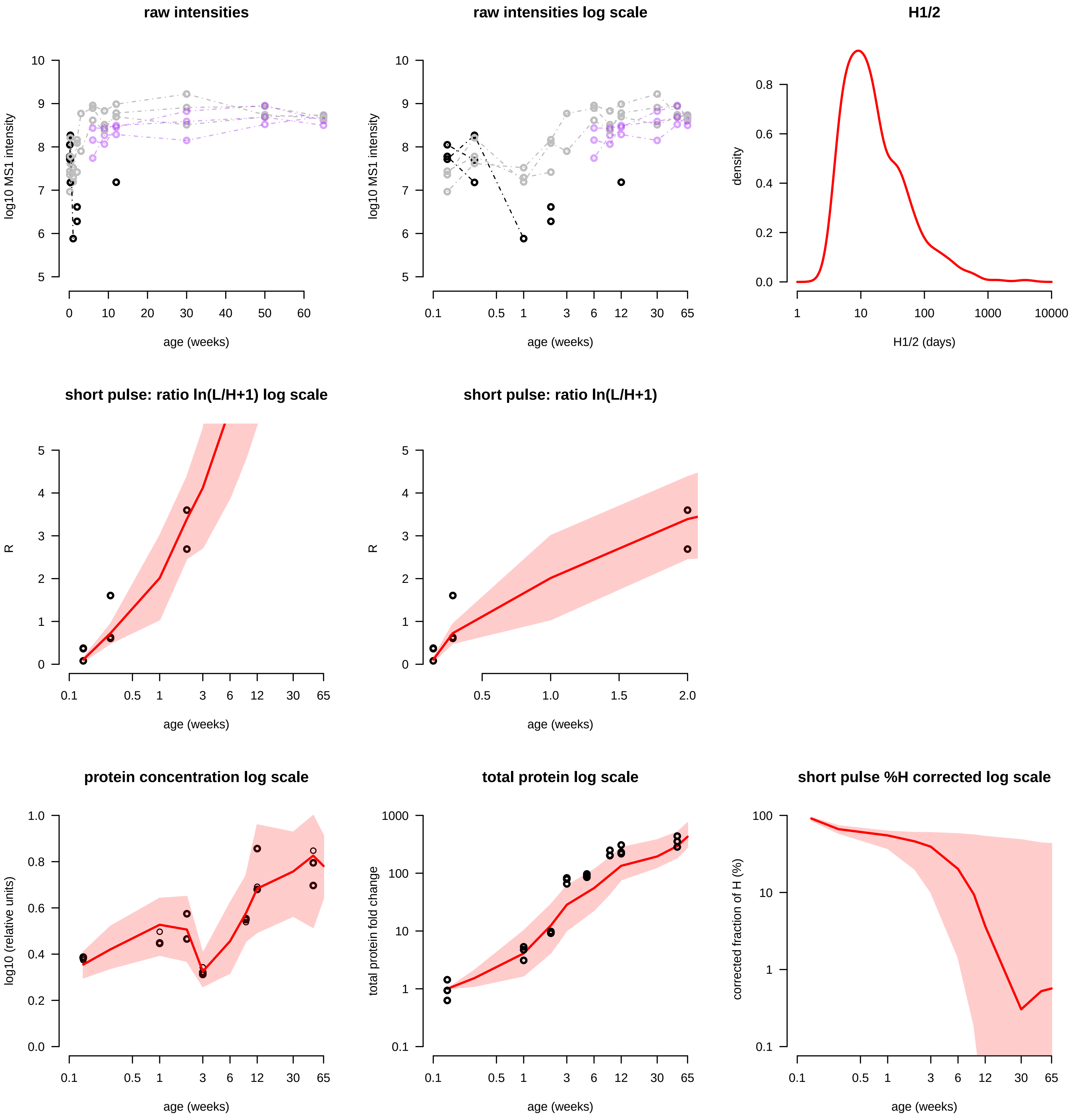

Q9Z110

>sp|Q9Z110|P5CS_MOUSE

Delta−1−pyrroline−5−carboxylate synthase

GN=Aldh18a1

Med H1/2: 4.3 days

Q5 H1/2: 2.9 days

Q95 H1/2: 5.6 days

reliable model fit: yes

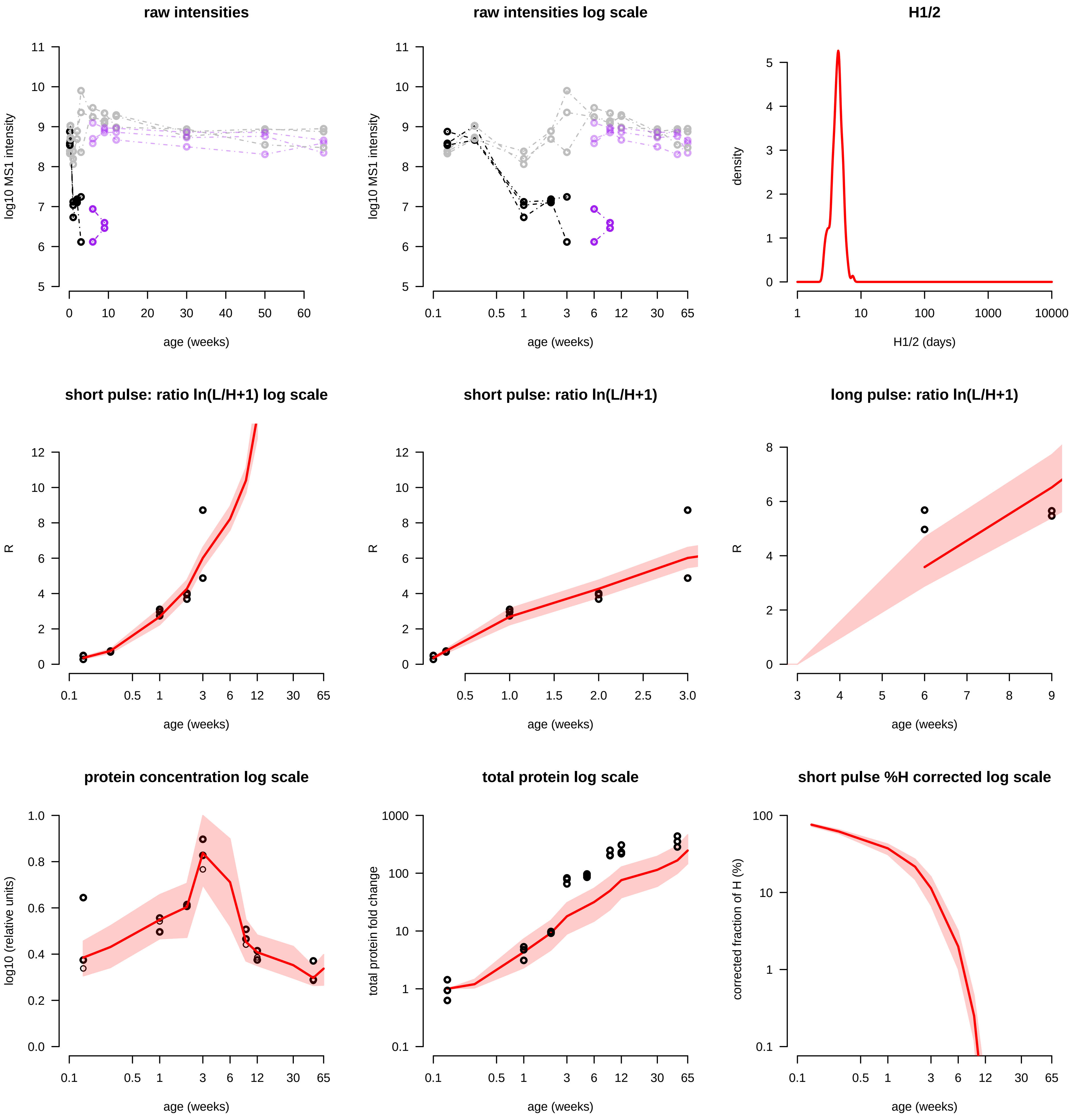

YS9−4;Q9QYS9−3;Q9QYS9−2;Q9QYS9;Q9QYS9−7;Q9QY

>sp|Q9QYS9−8|QKI_MOUSE

Isoform 8 of Protein quaking

GN=Qki

Med H1/2: 25.7 days

Q5 H1/2: 9.9 days

Q95 H1/2: 315.8 days

reliable model fit: yes

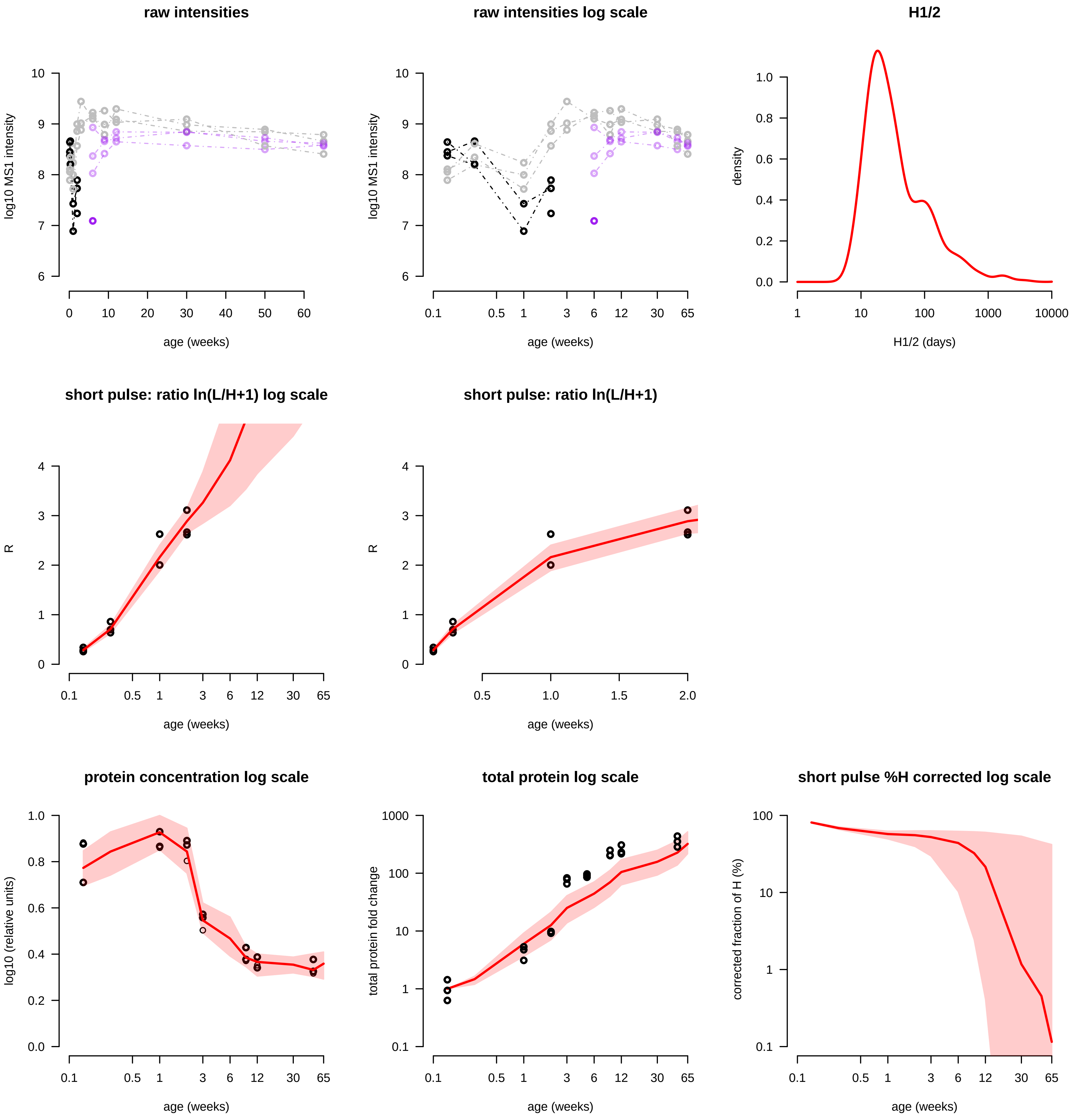

P61922;P61922−2

>sp|P61922|GABT_MOUSE

4−aminobutyrate aminotransferase, mitochondrial

GN=Abat

Med H1/2: 5.9 days

Q5 H1/2: 2.8 days

Q95 H1/2: 51.4 days

reliable model fit: yes

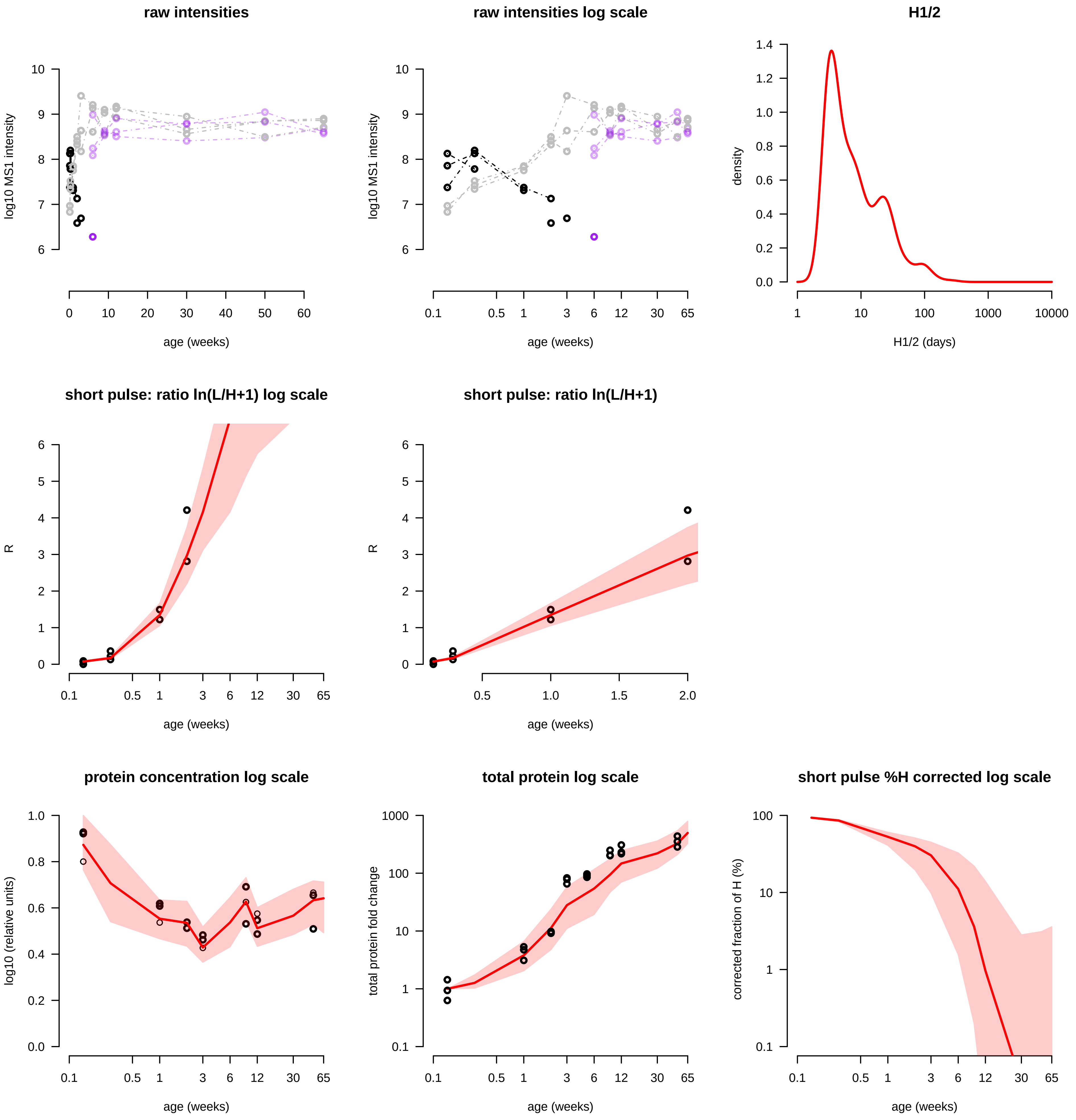

P42567;P42567−2

>sp|P42567|EPS15_MOUSE

Epidermal growth factor receptor substrate 15

GN=Eps15

Med H1/2: 282.3 days

Q5 H1/2: 109.1 days

Q95 H1/2: 2587.3 days

reliable model fit: yes

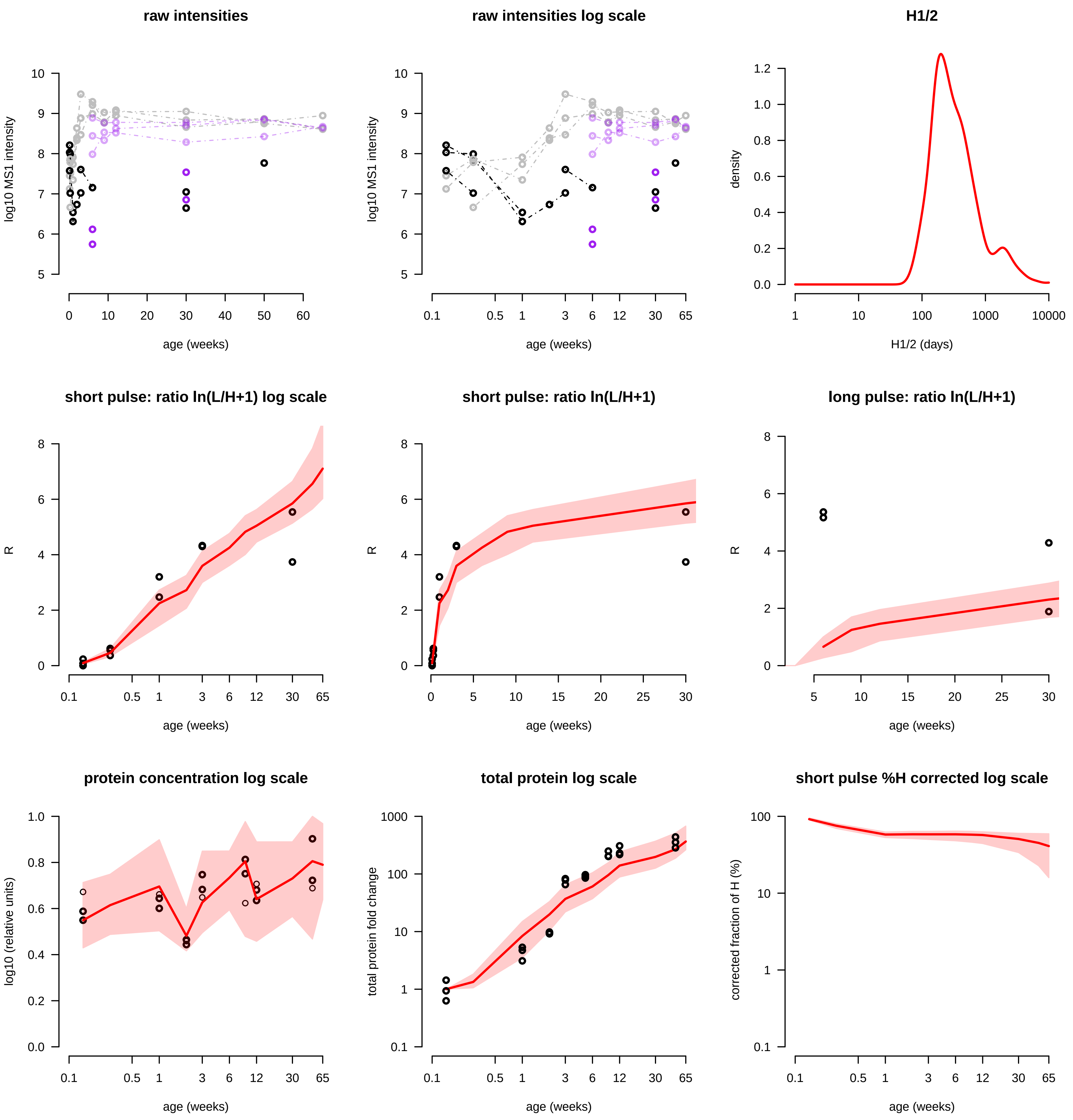

A2BH40;A2BH40−3

>sp|A2BH40|ARI1A_MOUSE

AT−rich interactive domain−containing protein 1A

GN=Arid1a

Med H1/2: 29.4 days

Q5 H1/2: 20.5 days

Q95 H1/2: 44.5 days

reliable model fit: no

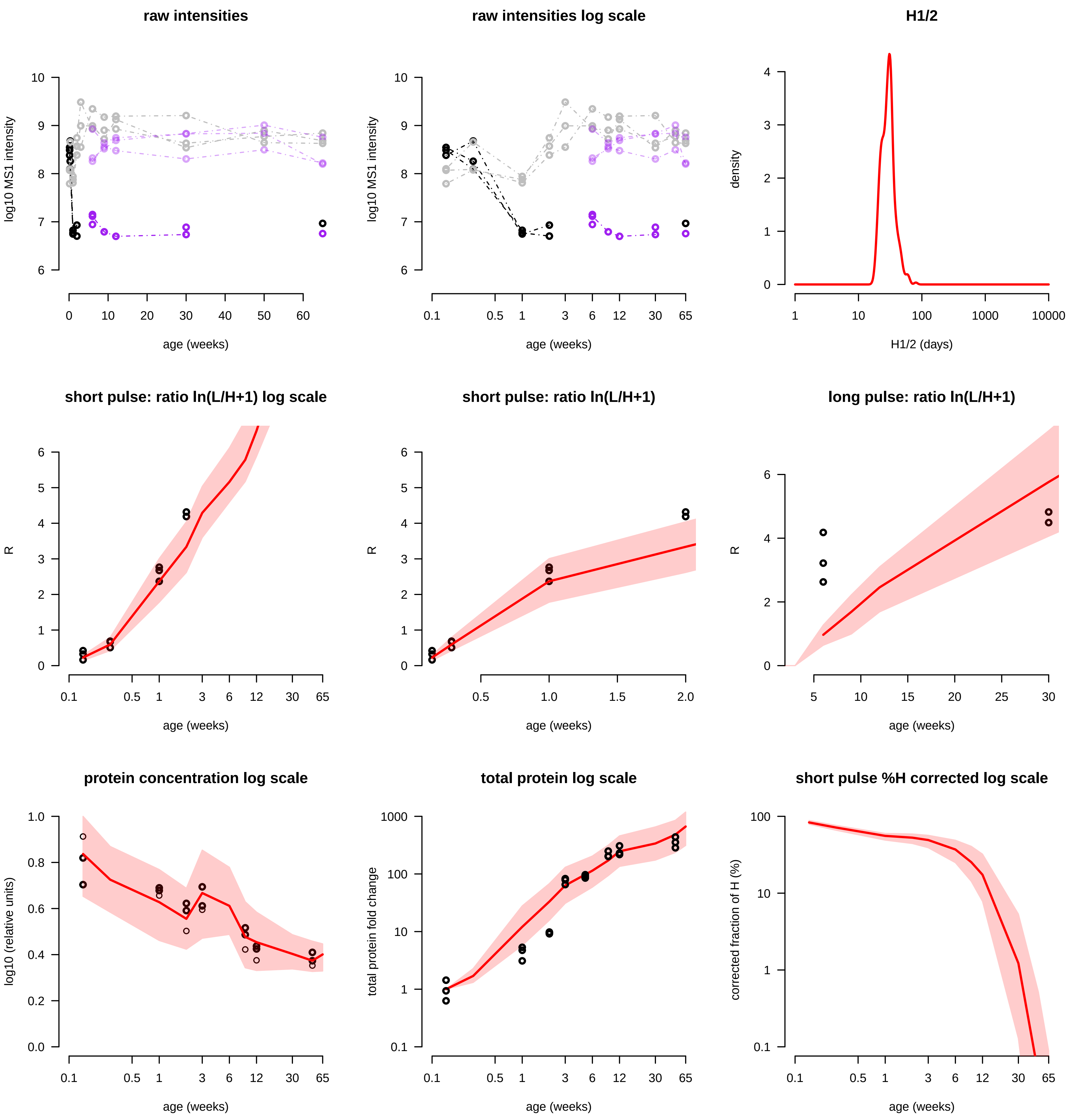

B2RXS4

>sp|B2RXS4|PLXB2_MOUSE

Plexin−B2

GN=Plxnb2

Med H1/2: 20.7 days

Q5 H1/2: 7.2 days

Q95 H1/2: 216.7 days

reliable model fit: yes

raw intensities

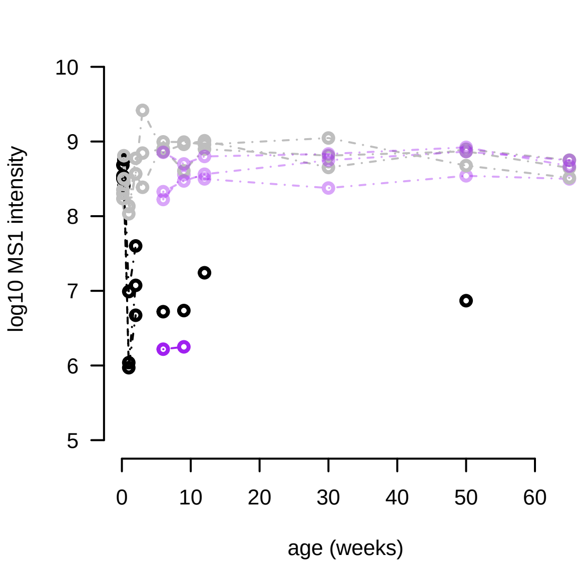

raw intensities log scale

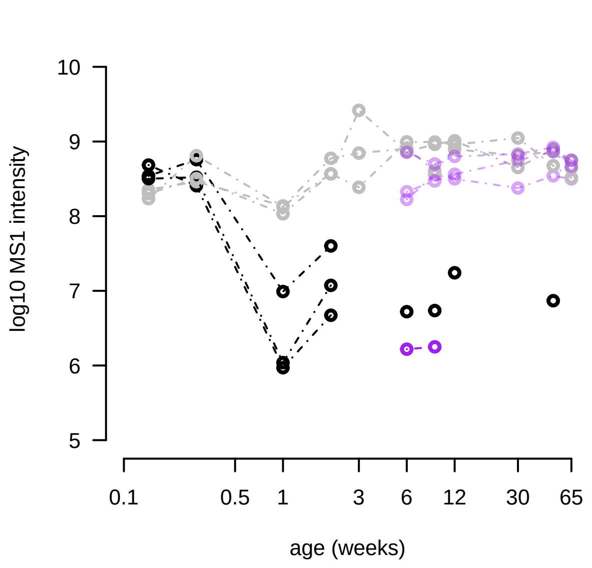

H1/2

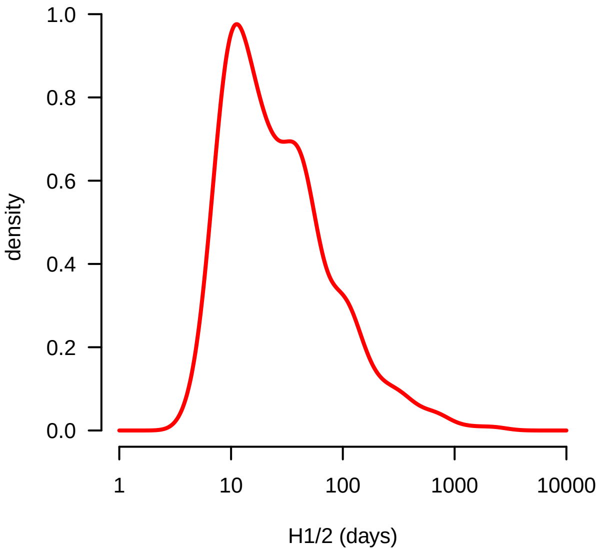

short pulse: ratio ln(L/H+1) log scale

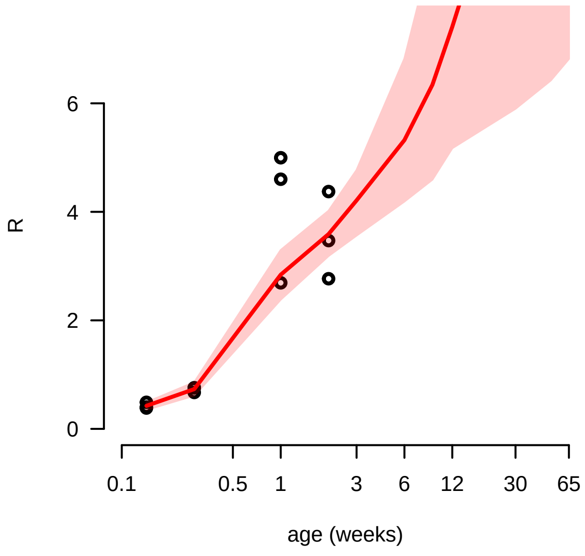

short pulse: ratio ln(L/H+1)

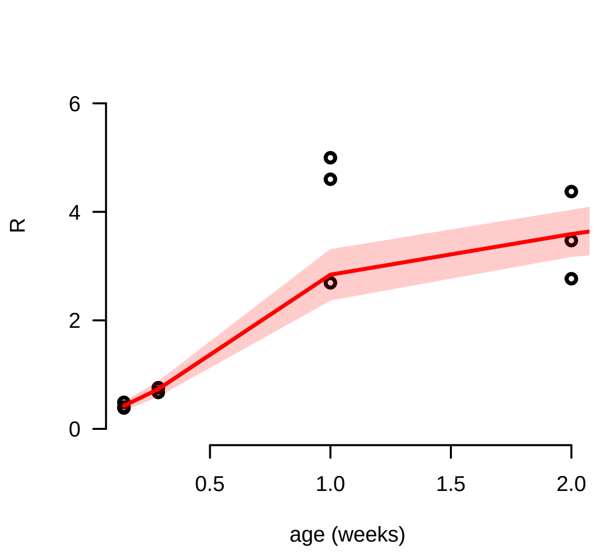

protein concentration log scale

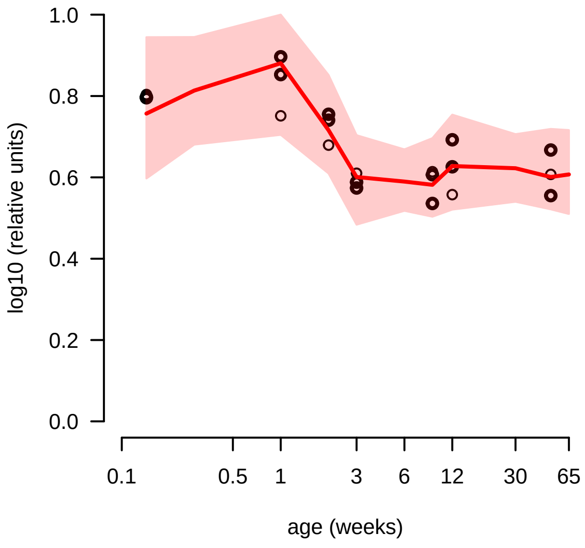

total protein log scale

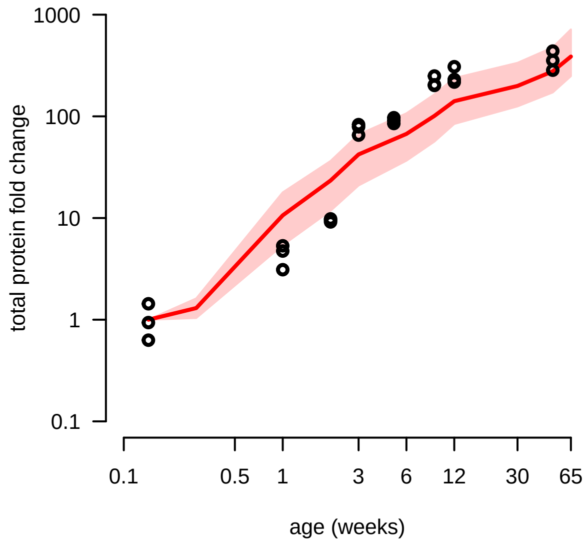

short pulse %H corrected log scale

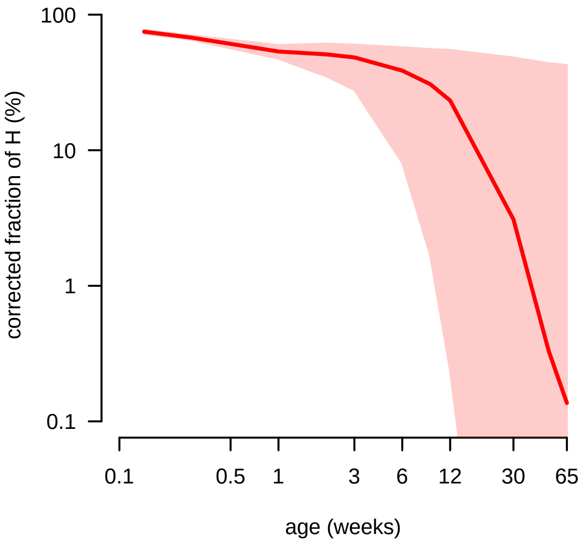

Q8BVY0

>sp|Q8BVY0|RL1D1_MOUSE

Ribosomal L1 domain−containing protein 1

GN=Rsl1d1

Med H1/2: 4.2 days

Q5 H1/2: 3.4 days

Q95 H1/2: 5.4 days

reliable model fit: yes

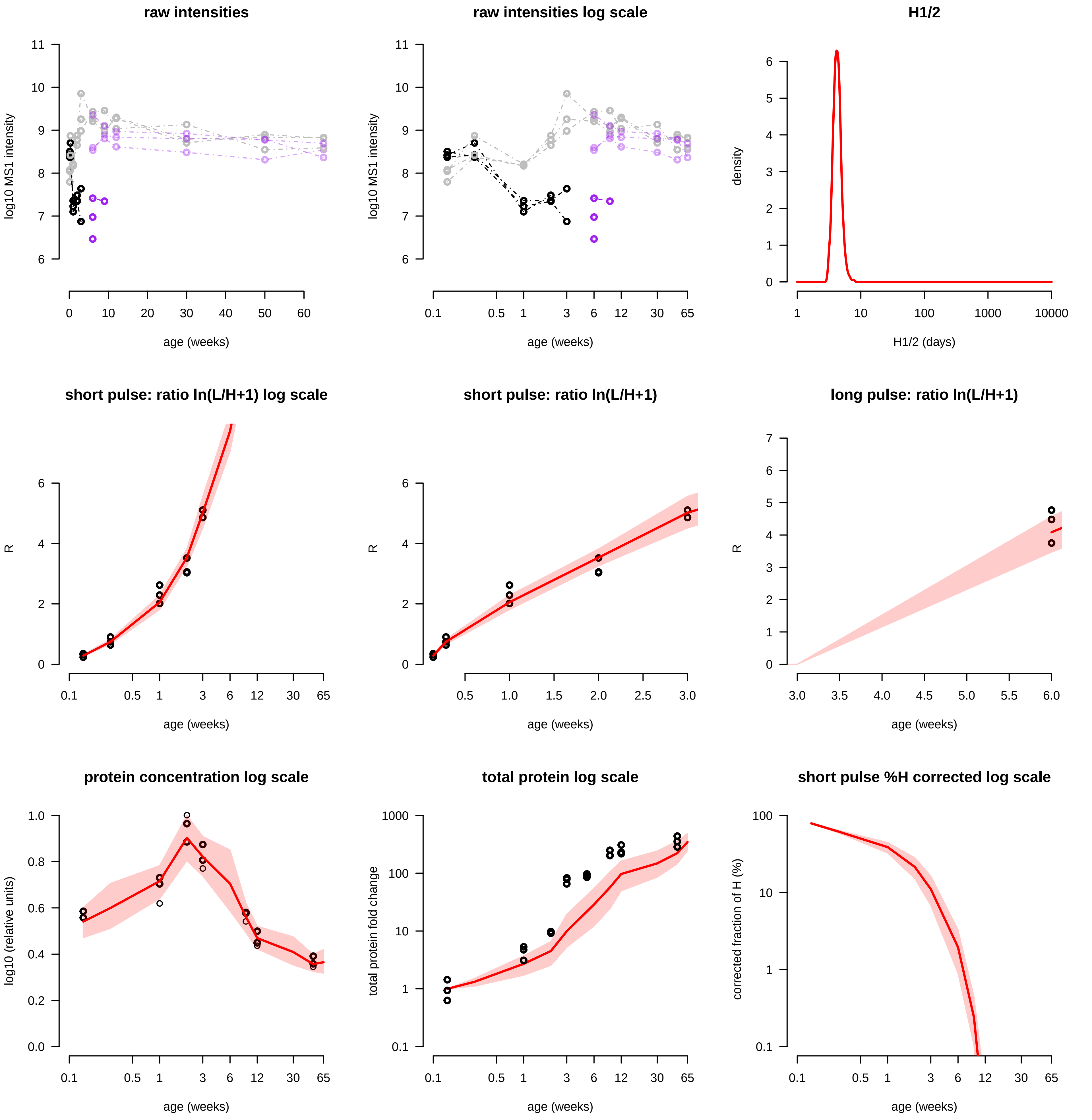

Q9Z2M7

>sp|Q9Z2M7|PMM2_MOUSE

Phosphomannomutase 2

GN=Pmm2

Med H1/2: 8.5 days

Q5 H1/2: 6.7 days

Q95 H1/2: 11.9 days

reliable model fit: yes

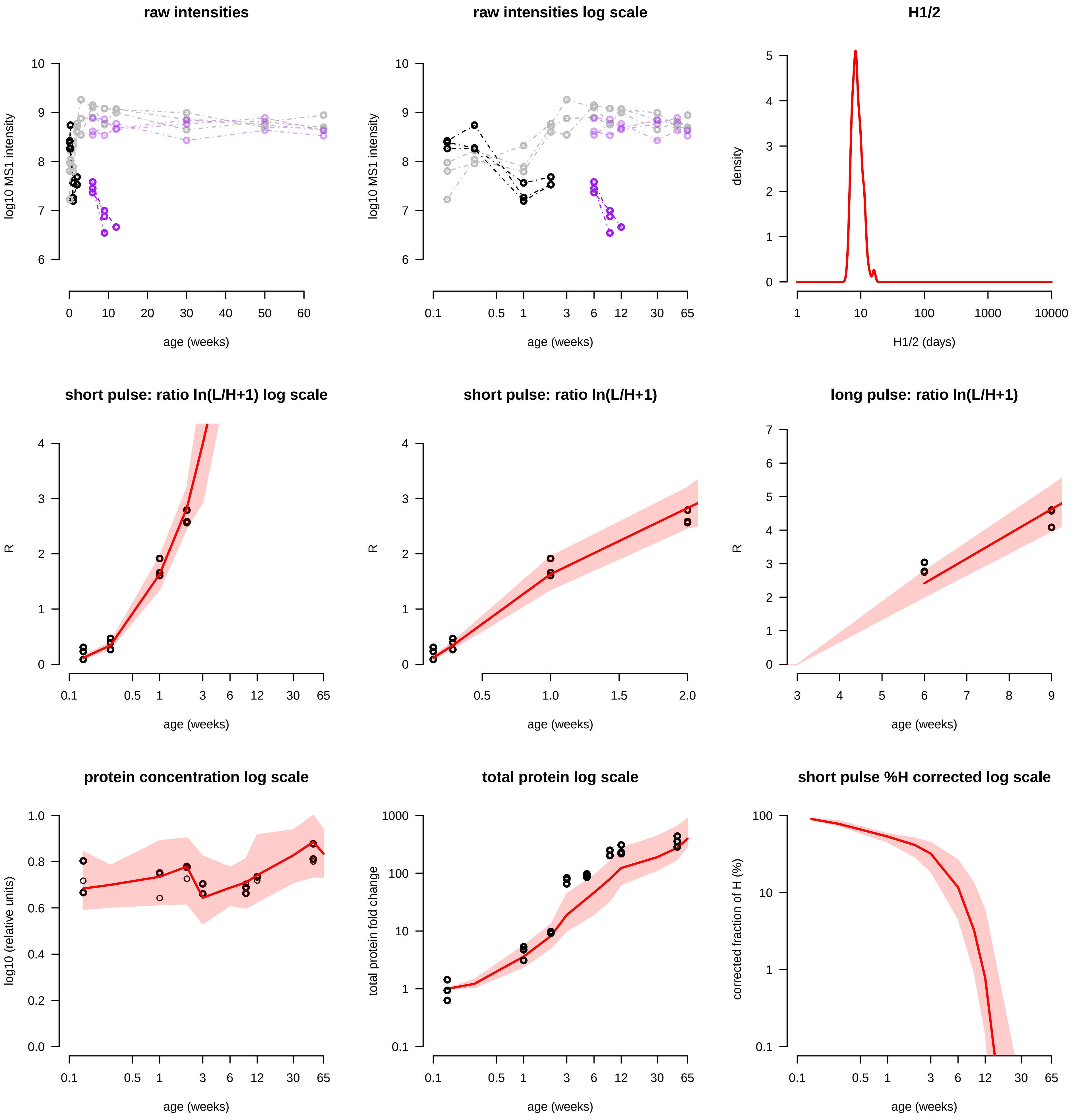

O09061

>sp|O09061|PSB1_MOUSE

Proteasome subunit beta type−1

GN=Psmb1

Med H1/2: 7.6 days

Q5 H1/2: 5.9 days

Q95 H1/2: 10.9 days

reliable model fit: yes

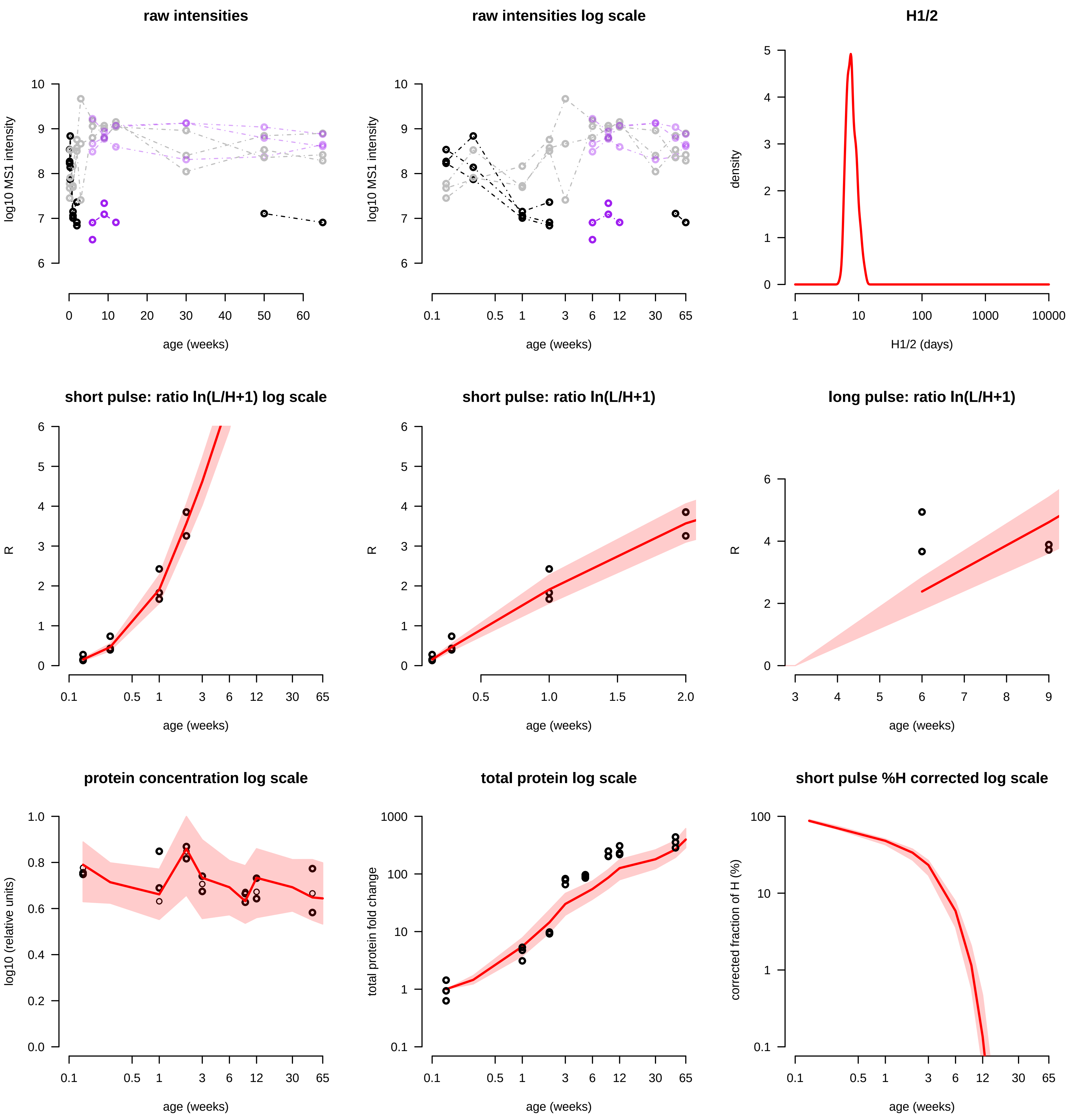

Q63932

>sp|Q63932|MP2K2_MOUSE

Dual specificity mitogen−activated protein kinase kinase 2

GN=Map2k2

Med H1/2: 6.2 days

Q5 H1/2: 3 days

Q95 H1/2: 66.5 days

reliable model fit: yes

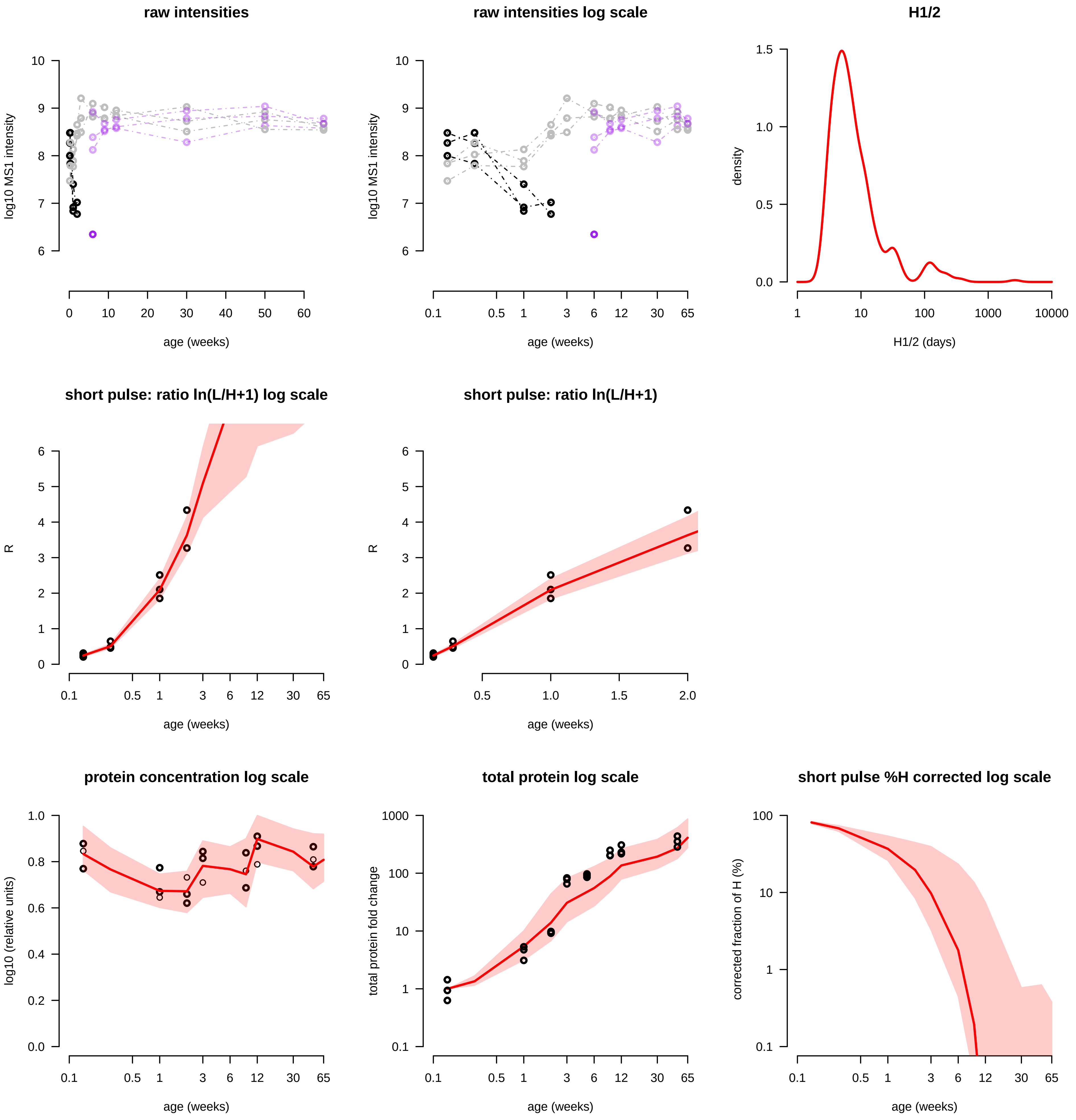

Q8C2Q3

>sp|Q8C2Q3|RBM14_MOUSE

RNA−binding protein 14

GN=Rbm14

Med H1/2: 4.4 days

Q5 H1/2: 3.8 days

Q95 H1/2: 5.9 days

reliable model fit: yes

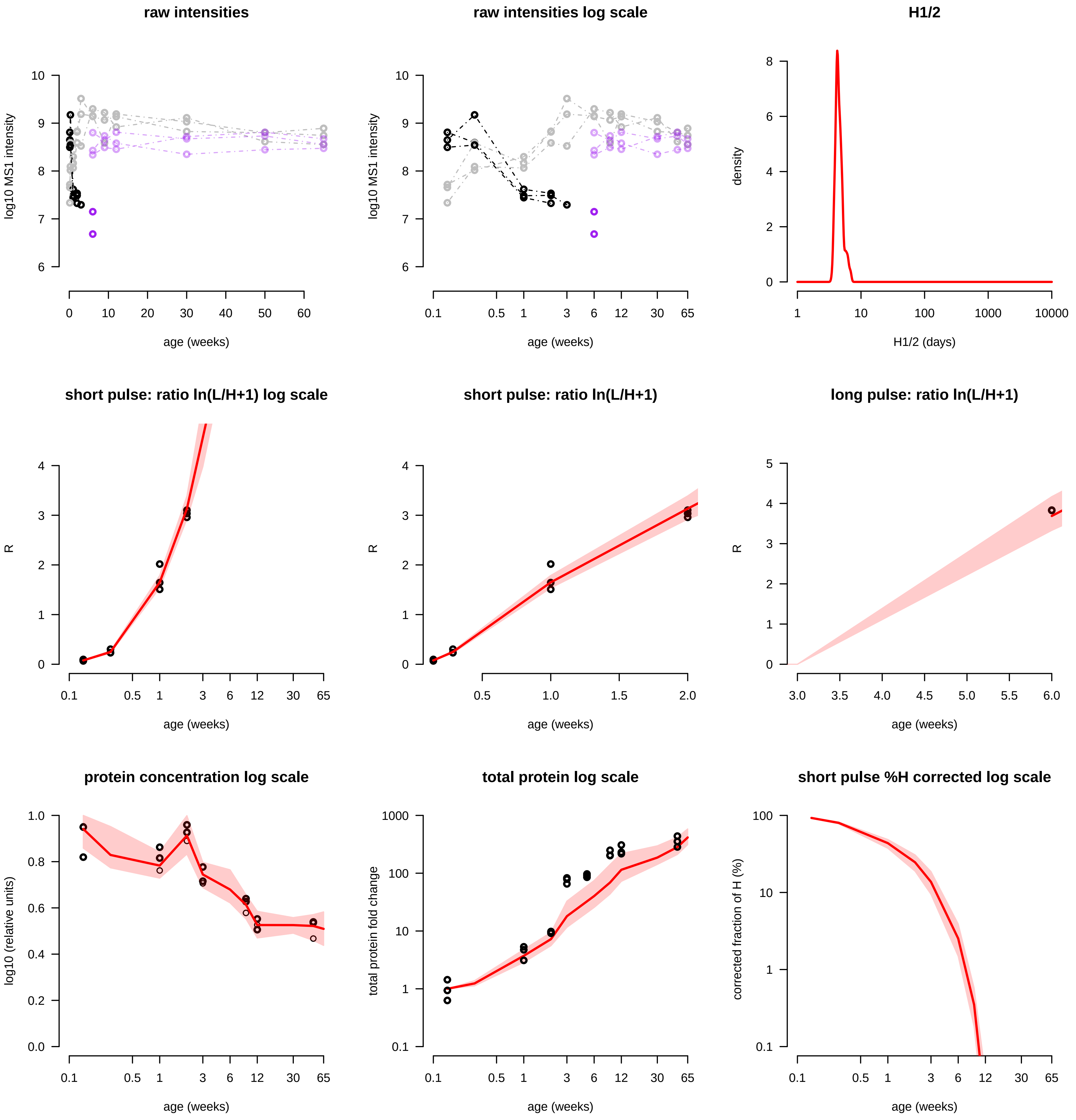

Q9CZY3;Q9CZY3−2

>sp|Q9CZY3|UB2V1_MOUSE

Ubiquitin−conjugating enzyme E2 variant 1

GN=Ube2v1

Med H1/2: 15 days

Q5 H1/2: 11.6 days

Q95 H1/2: 20.9 days

reliable model fit: yes

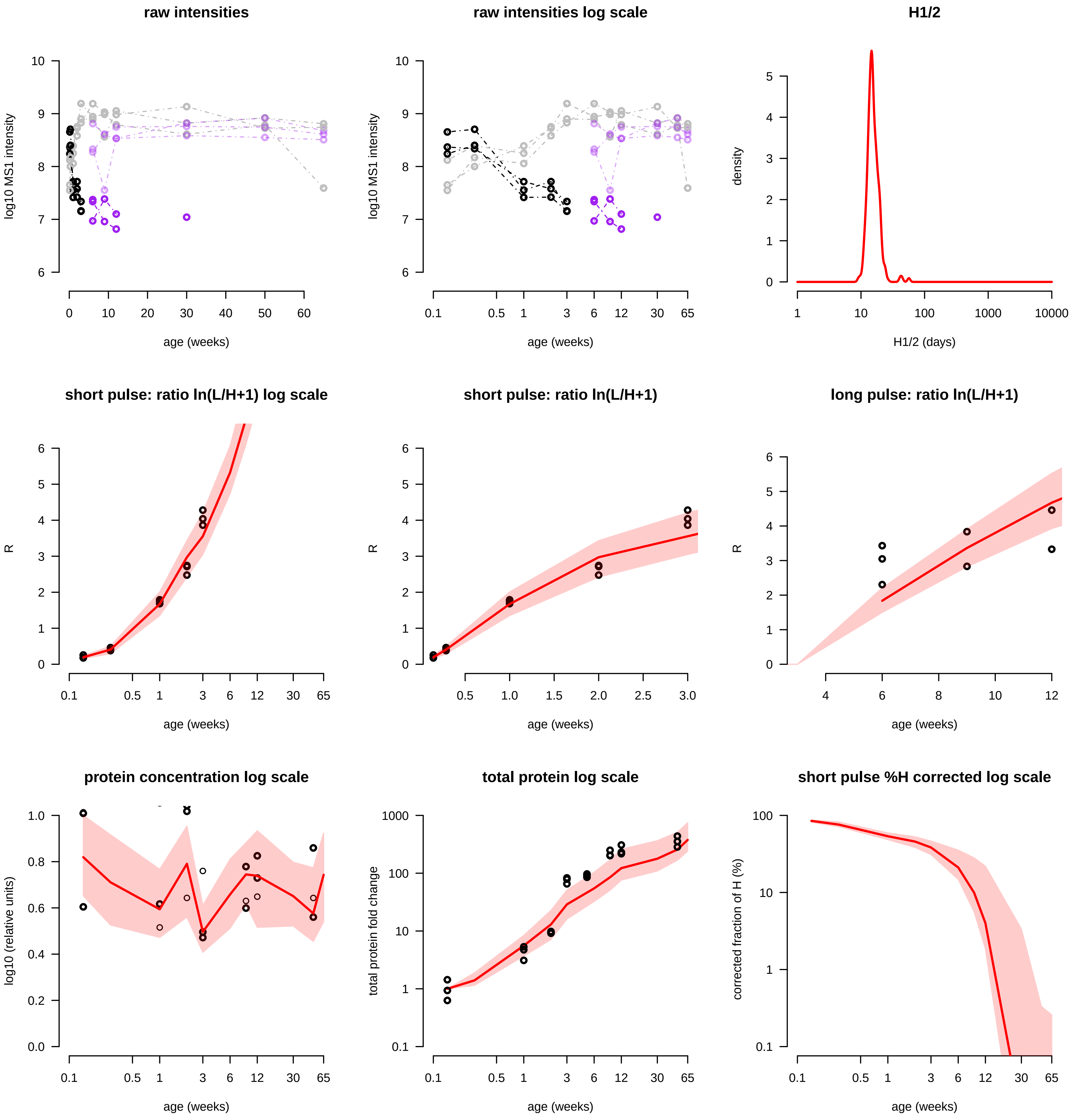

Q91YP2

>sp|Q91YP2|NEUL_MOUSE

Neurolysin, mitochondrial

GN=Nln

Med H1/2: 9.4 days

Q5 H1/2: 3.9 days

Q95 H1/2: 44.6 days

reliable model fit: yes

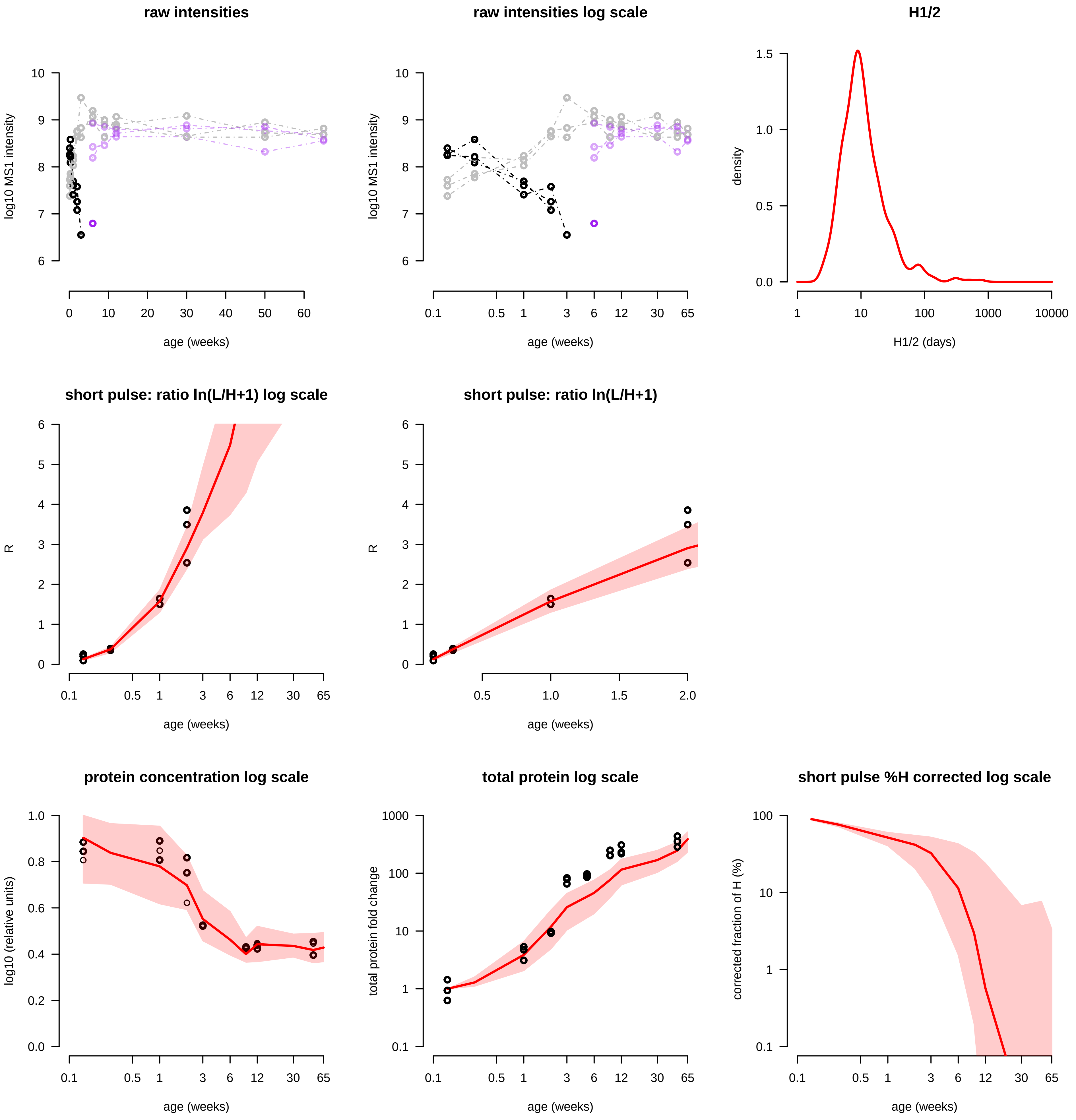

P11679

>sp|P11679|K2C8_MOUSE

Keratin, type II cytoskeletal 8

GN=Krt8

Med H1/2: 4.6 days

Q5 H1/2: 3.8 days

Q95 H1/2: 5.6 days

reliable model fit: yes

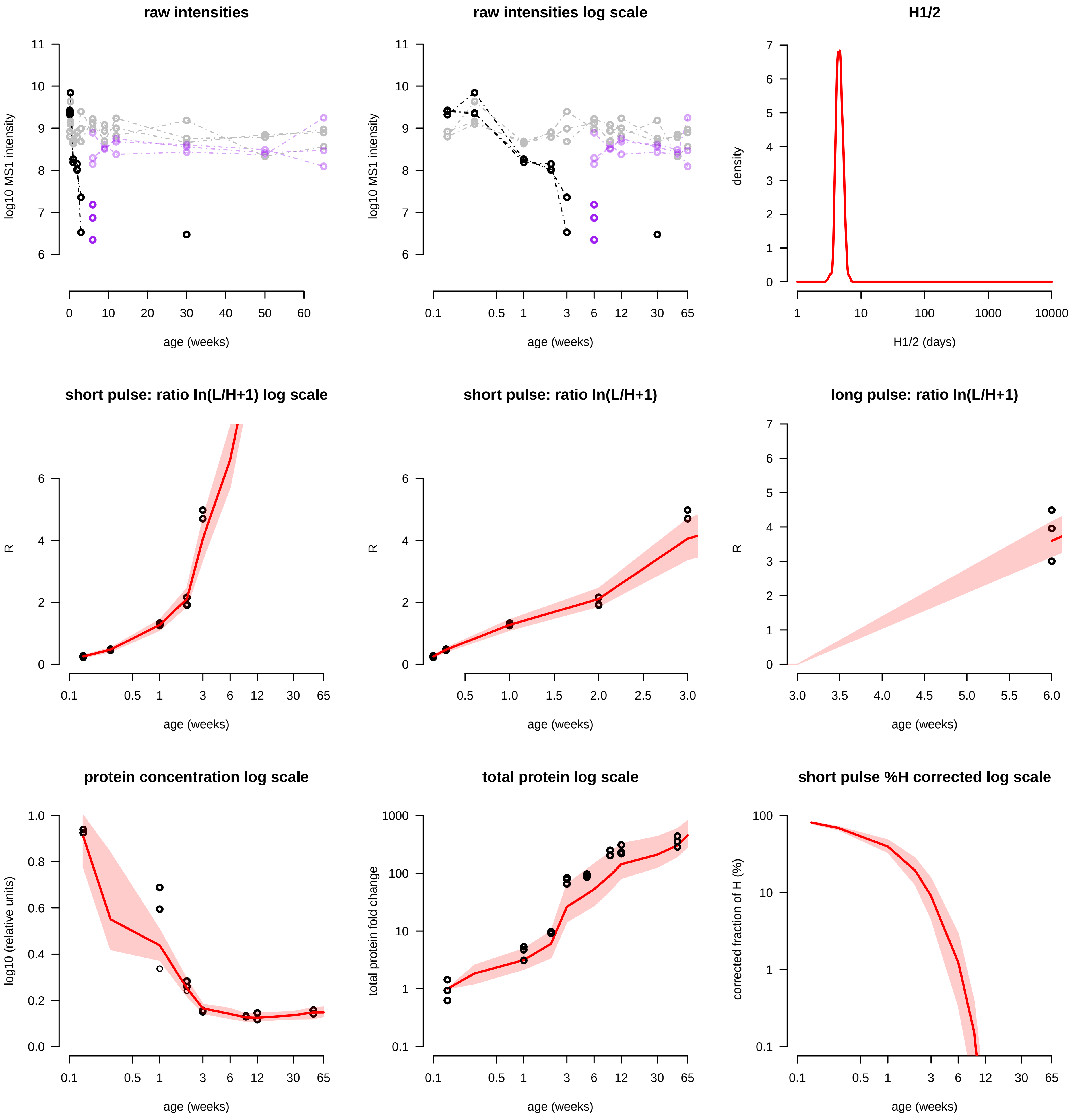

Q60739;Q60739−2

>sp|Q60739|BAG1_MOUSE

BAG family molecular chaperone regulator 1

GN=Bag1

Med H1/2: 58.4 days

Q5 H1/2: 24 days

Q95 H1/2: 203 days

reliable model fit: no

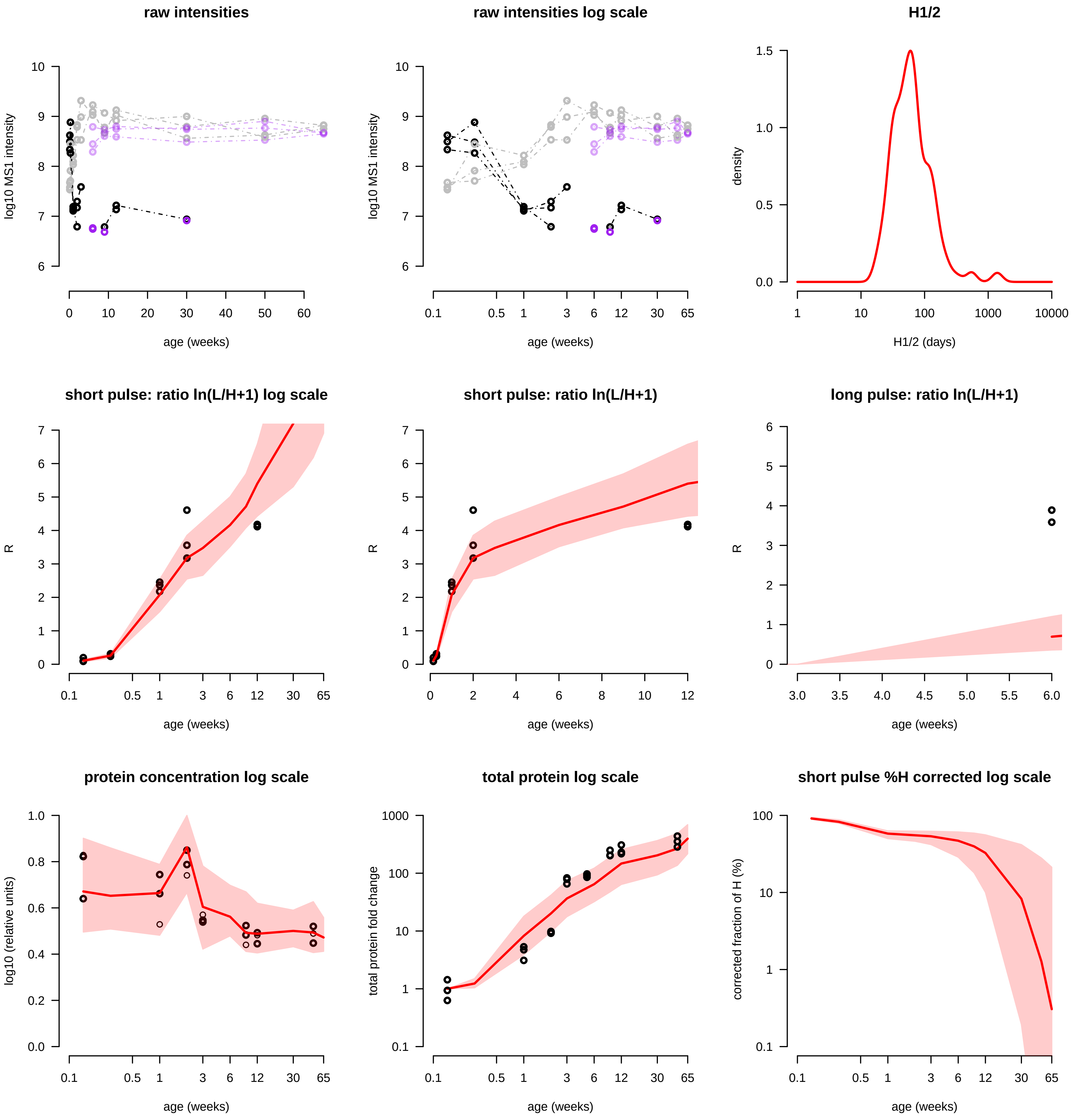

P50136

>sp|P50136|ODBA_MOUSE

2−oxoisovalerate dehydrogenase subunit alpha, mitochondrial

GN=Bckdha

Med H1/2: 11 days

Q5 H1/2: 3 days

Q95 H1/2: 106 days

reliable model fit: yes

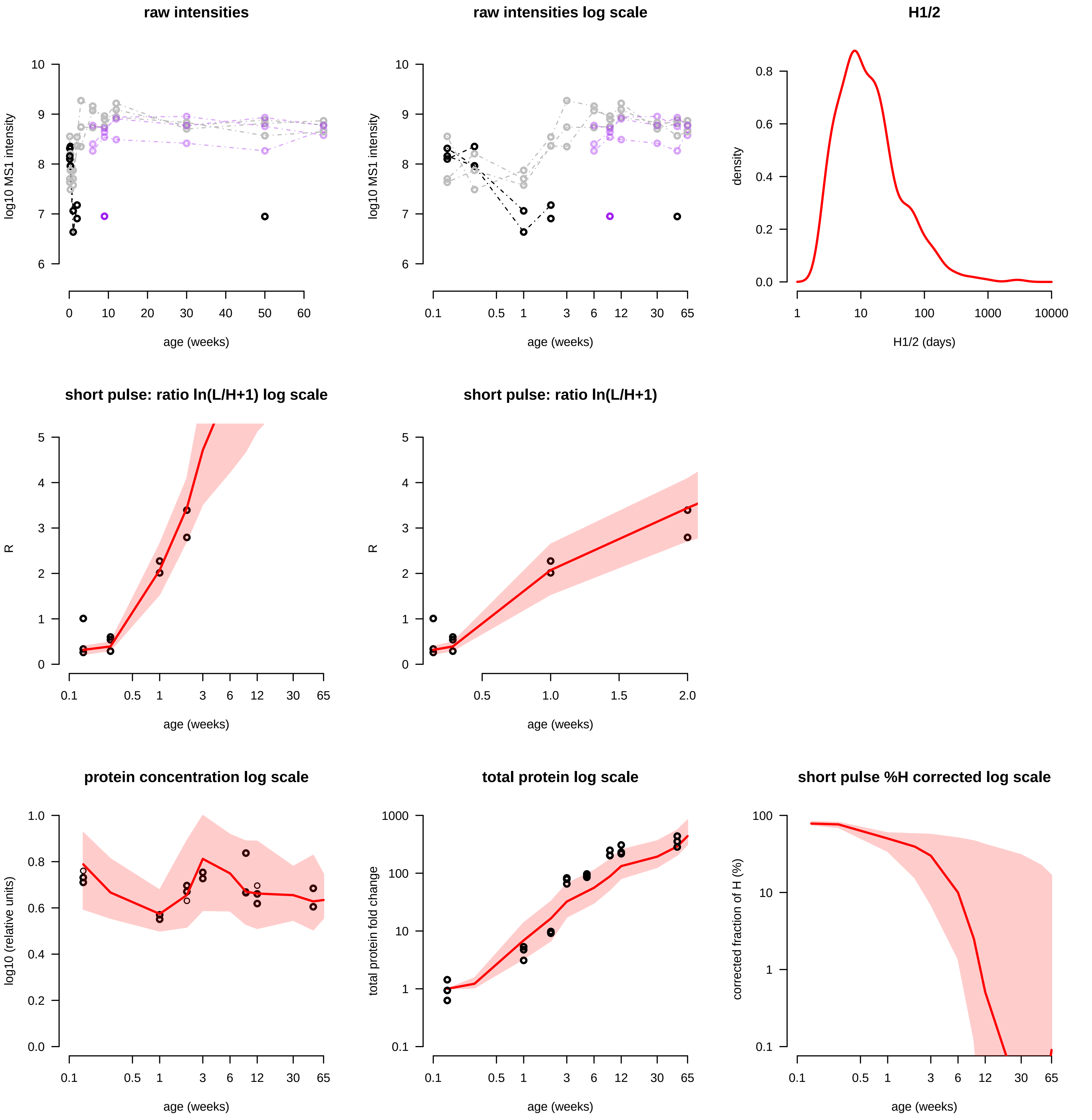

Q8CCF0;Q8CCF0−2

>sp|Q8CCF0|PRP31_MOUSE

U4/U6 small nuclear ribonucleoprotein Prp31

GN=Prpf31

Med H1/2: 1206.4 days

Q5 H1/2: 361.8 days

Q95 H1/2: 11420.4 days

reliable model fit: yes

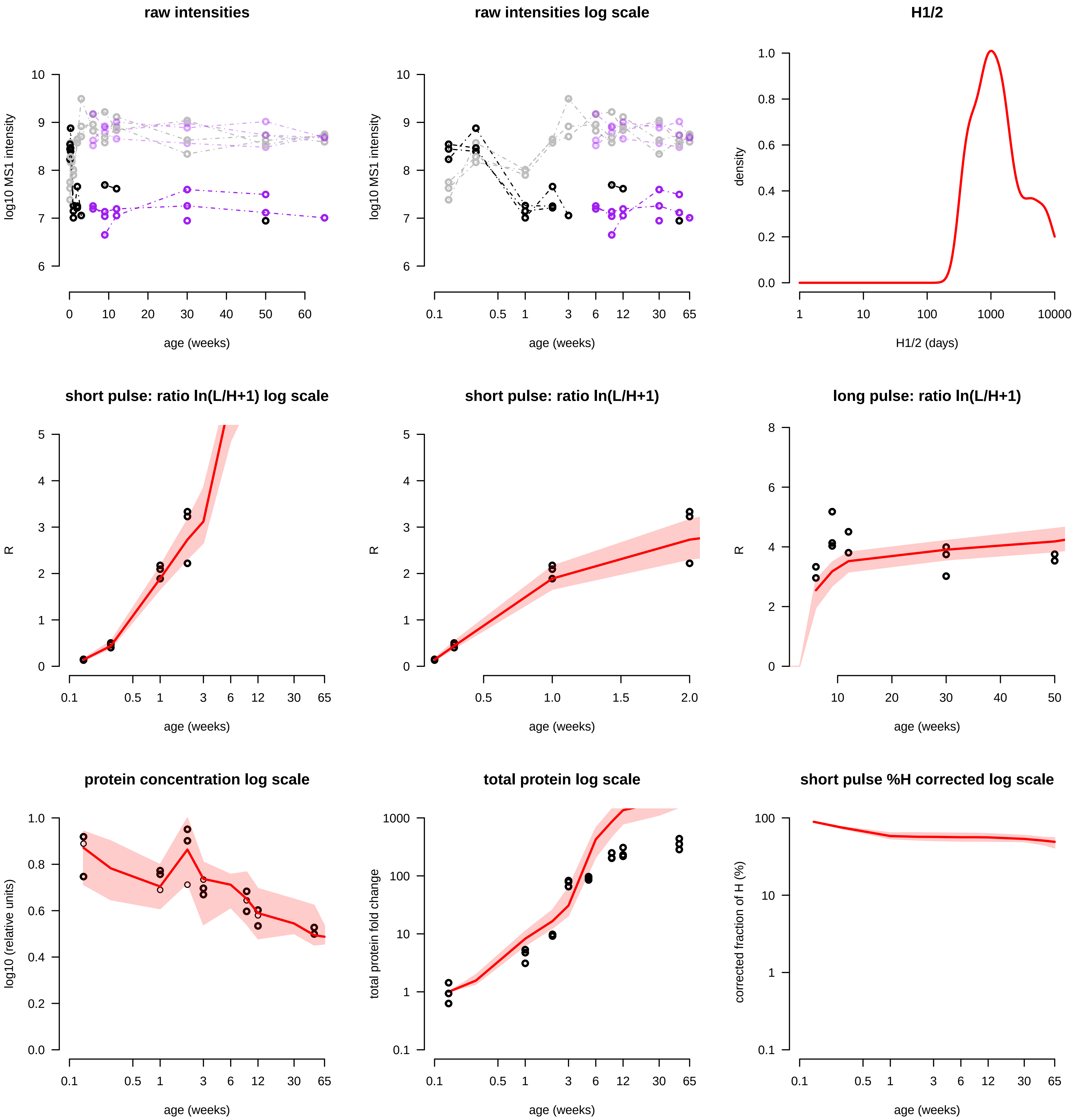

Q9ER72;Q9ER72−2

>sp|Q9ER72|SYCC_MOUSE

Cysteine−−tRNA ligase, cytoplasmic

GN=Cars1

Med H1/2: 3.2 days

Q5 H1/2: 2.6 days

Q95 H1/2: 4 days

reliable model fit: yes

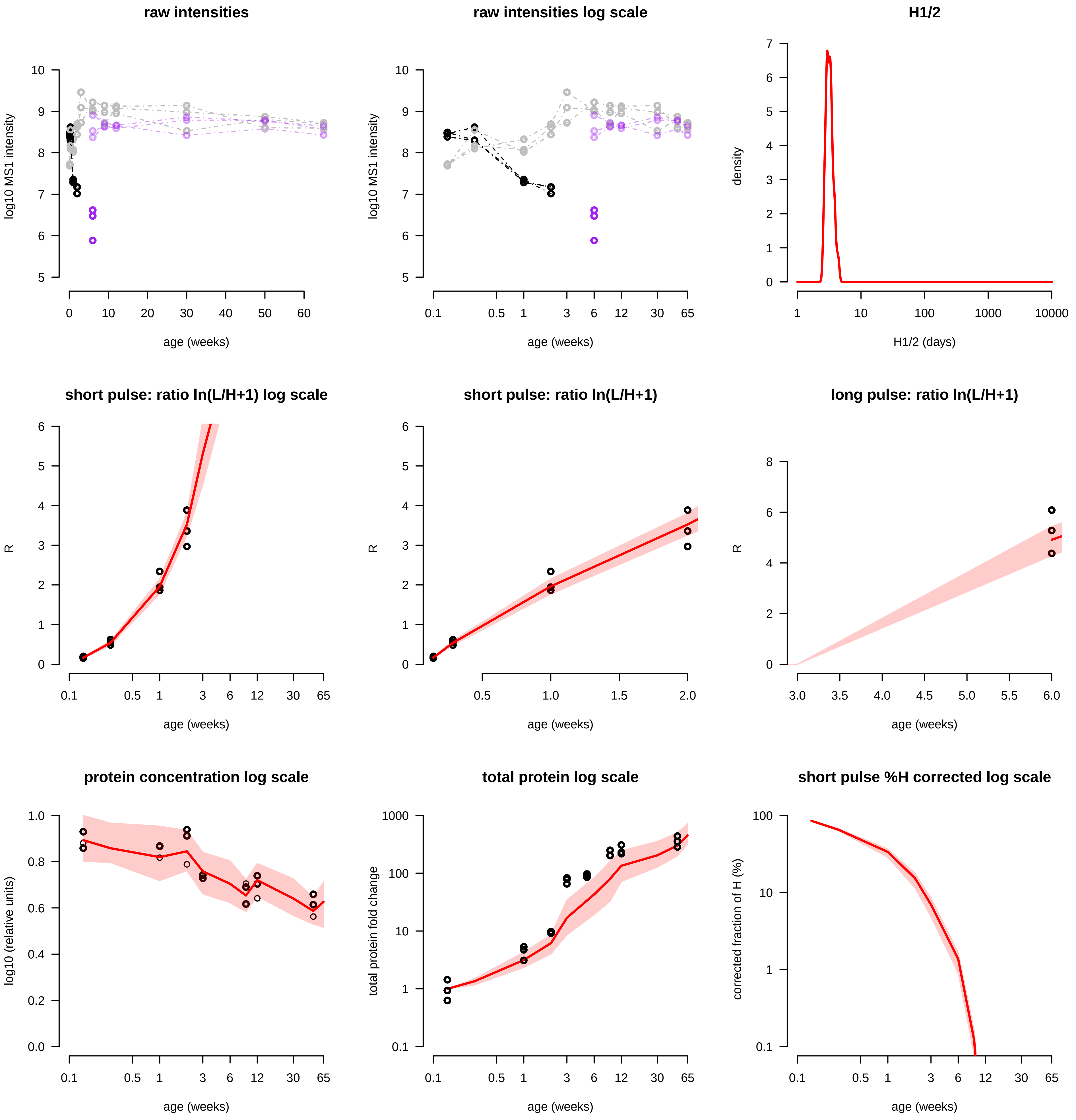

Q7TNG5;Q7TNG5−2

>sp|Q7TNG5|EMAL2_MOUSE

Echinoderm microtubule−associated protein−like 2

GN=Eml2

Med H1/2: 20 days

Q5 H1/2: 6.2 days

Q95 H1/2: 293.4 days

reliable model fit: yes

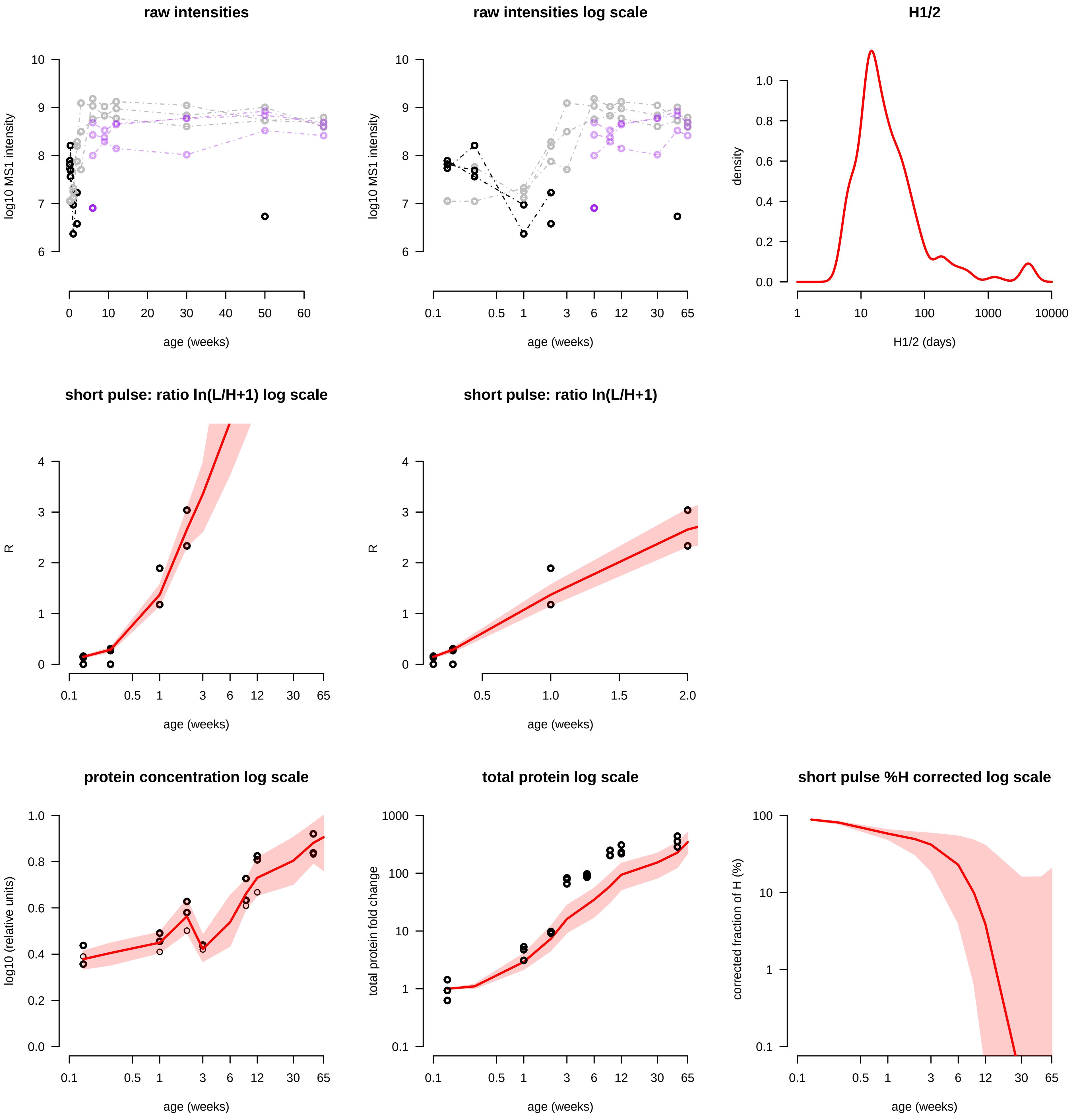

Q9JI10;Q9JI10−2

>sp|Q9JI10|STK3_MOUSE

Serine/threonine−protein kinase 3

GN=Stk3

Med H1/2: 3.5 days

Q5 H1/2: 3 days

Q95 H1/2: 4.4 days

reliable model fit: yes

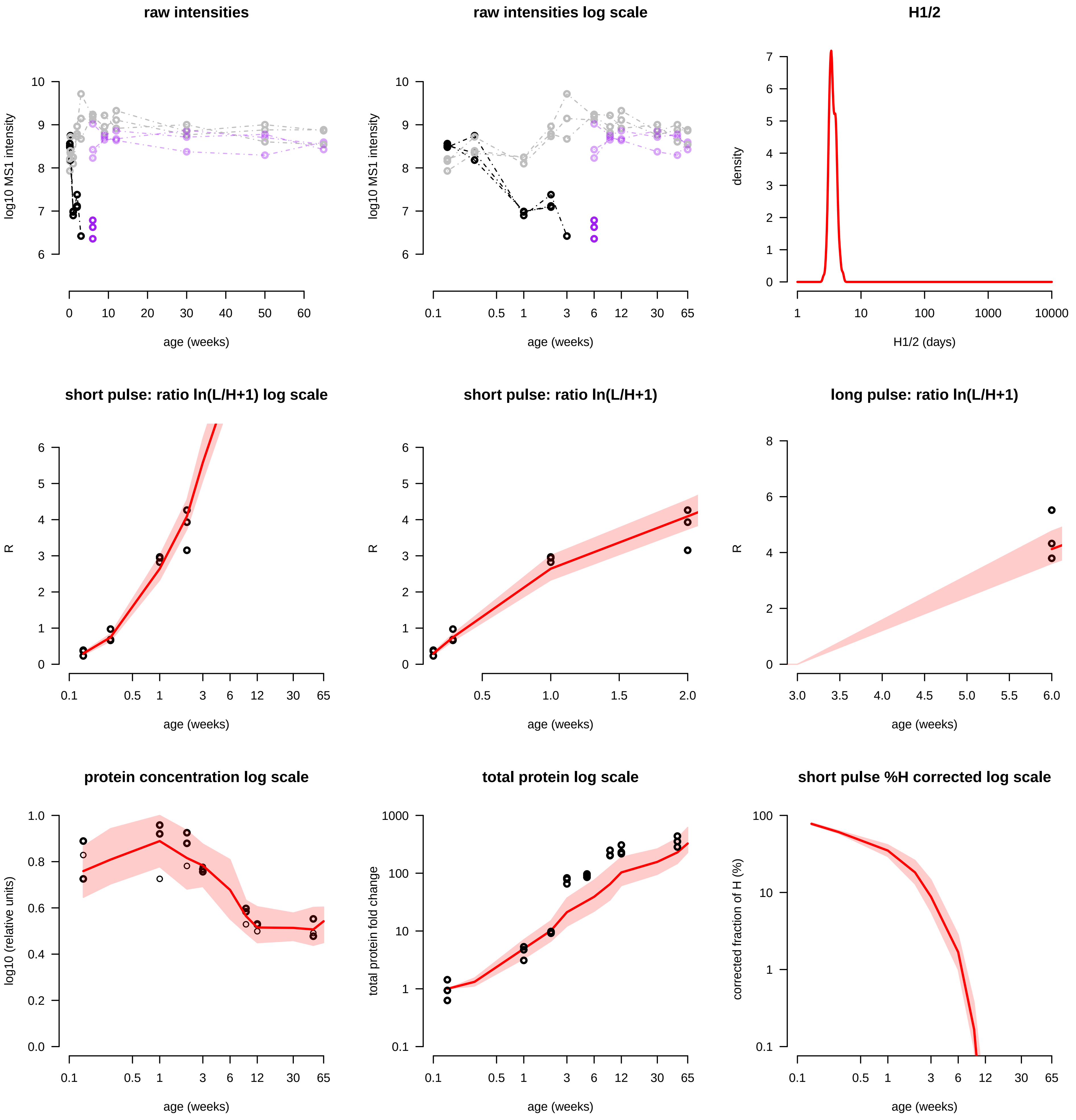

Q8CCS6−2;Q8CCS6

>sp|Q8CCS6−2|PABP2_MOUSE

Isoform 2 of Polyadenylate−binding protein 2

GN=Pabpn1

Med H1/2: 12.1 days

Q5 H1/2: 8.9 days

Q95 H1/2: 17.9 days

reliable model fit: yes

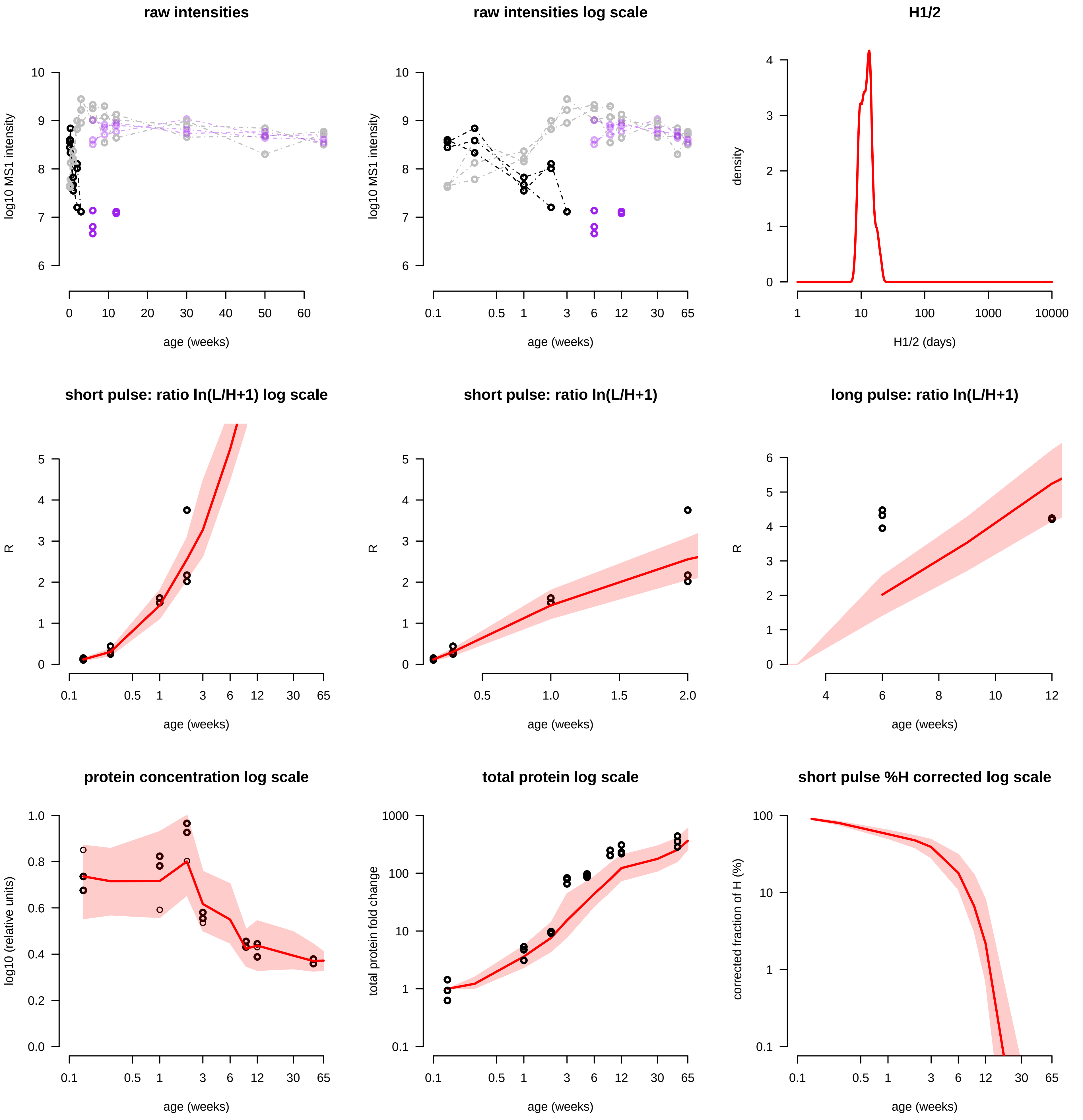

Q8BKE6

>sp|Q8BKE6|CP20A_MOUSE

Cytochrome P450 20A1

GN=Cyp20a1

Med H1/2: 4.4 days

Q5 H1/2: 2.9 days

Q95 H1/2: 8 days

reliable model fit: yes

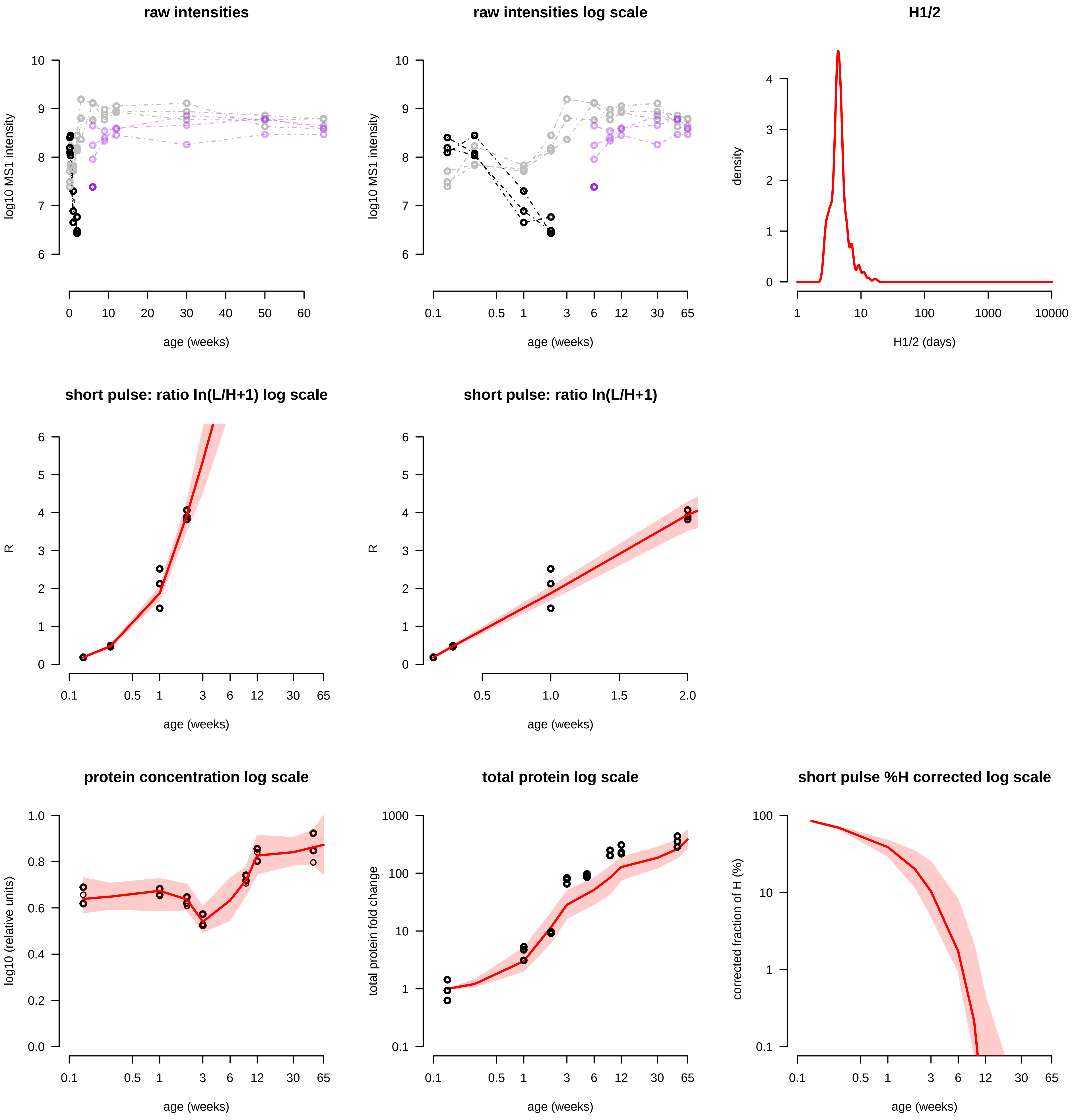

Q4VAA2;Q4VAA2−2

>sp|Q4VAA2|CDV3_MOUSE

Protein CDV3

GN=Cdv3

Med H1/2: 4.6 days

Q5 H1/2: 3.6 days

Q95 H1/2: 6.6 days

reliable model fit: yes

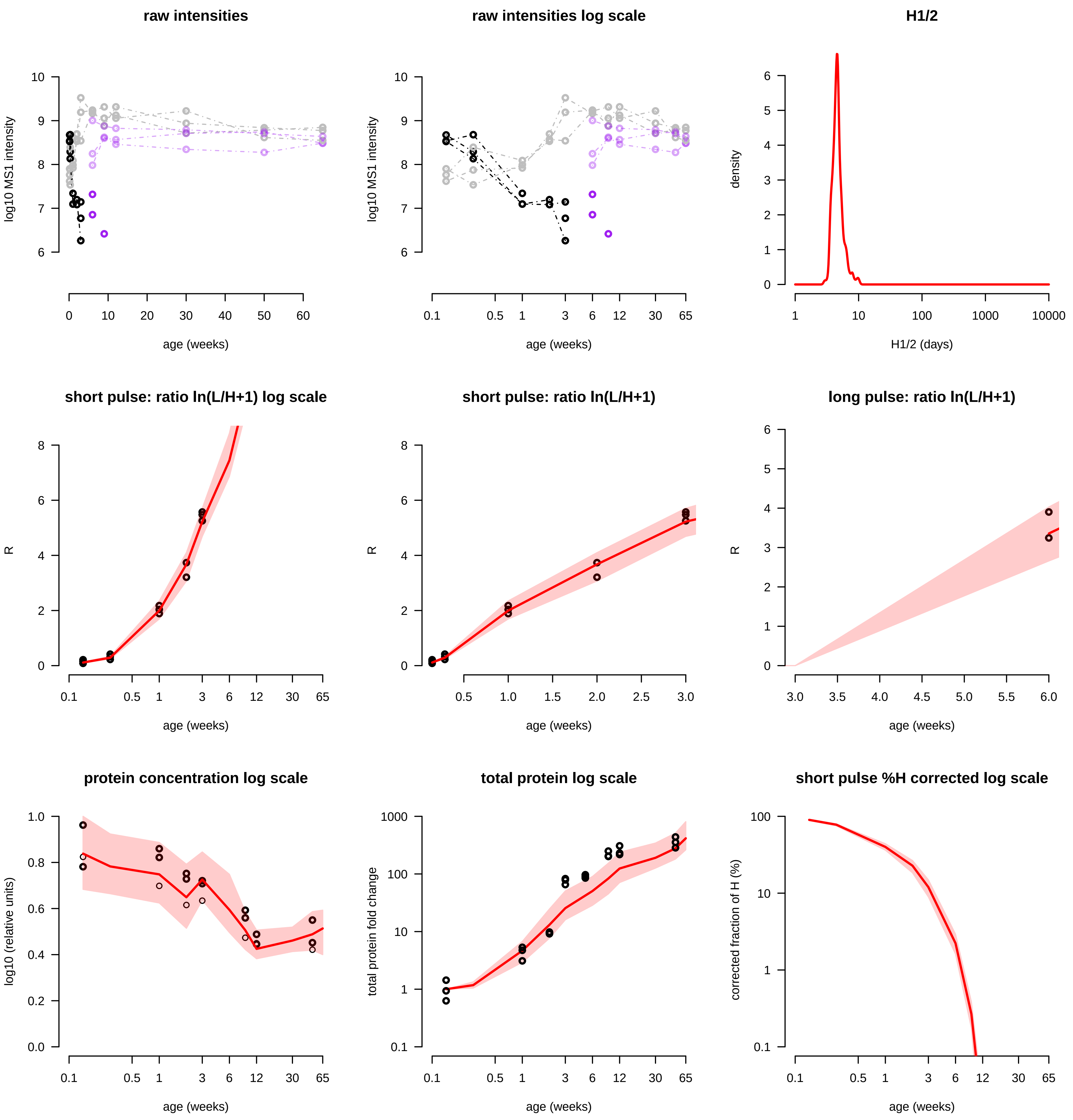

Q9ES28;Q9ES28−2;Q9ES28−7;Q9ES28−4;Q9ES28−6

>sp|Q9ES28|ARHG7_MOUSE

Rho guanine nucleotide exchange factor 7

GN=Arhgef7

Med H1/2: 3.1 days

Q5 H1/2: 2.6 days

Q95 H1/2: 4 days

reliable model fit: yes

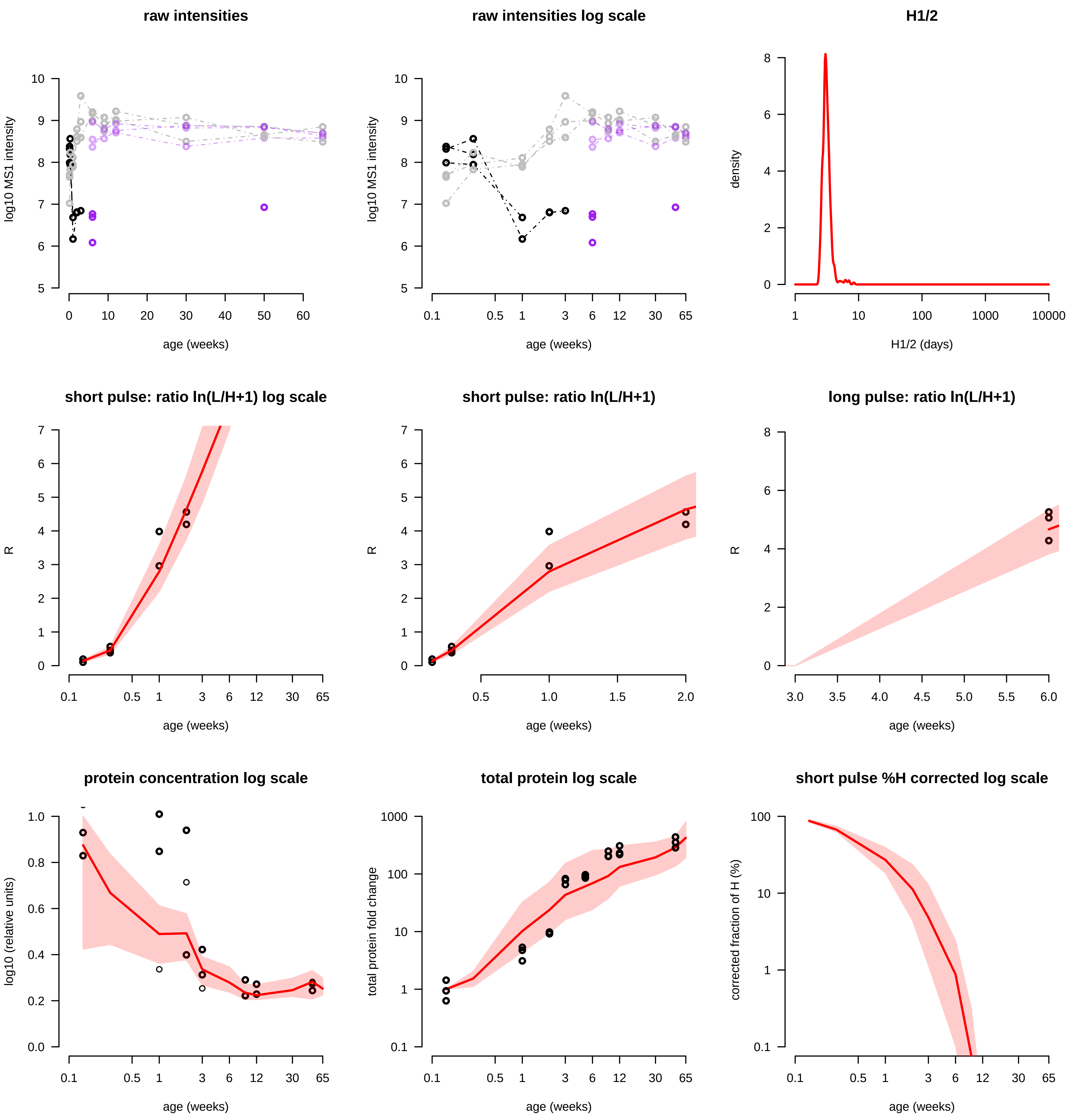

Q9Z130

>sp|Q9Z130|HNRDL_MOUSE

Heterogeneous nuclear ribonucleoprotein D−like

GN=Hnrnpdl

Med H1/2: 5.9 days

Q5 H1/2: 4.8 days

Q95 H1/2: 7.1 days

reliable model fit: yes

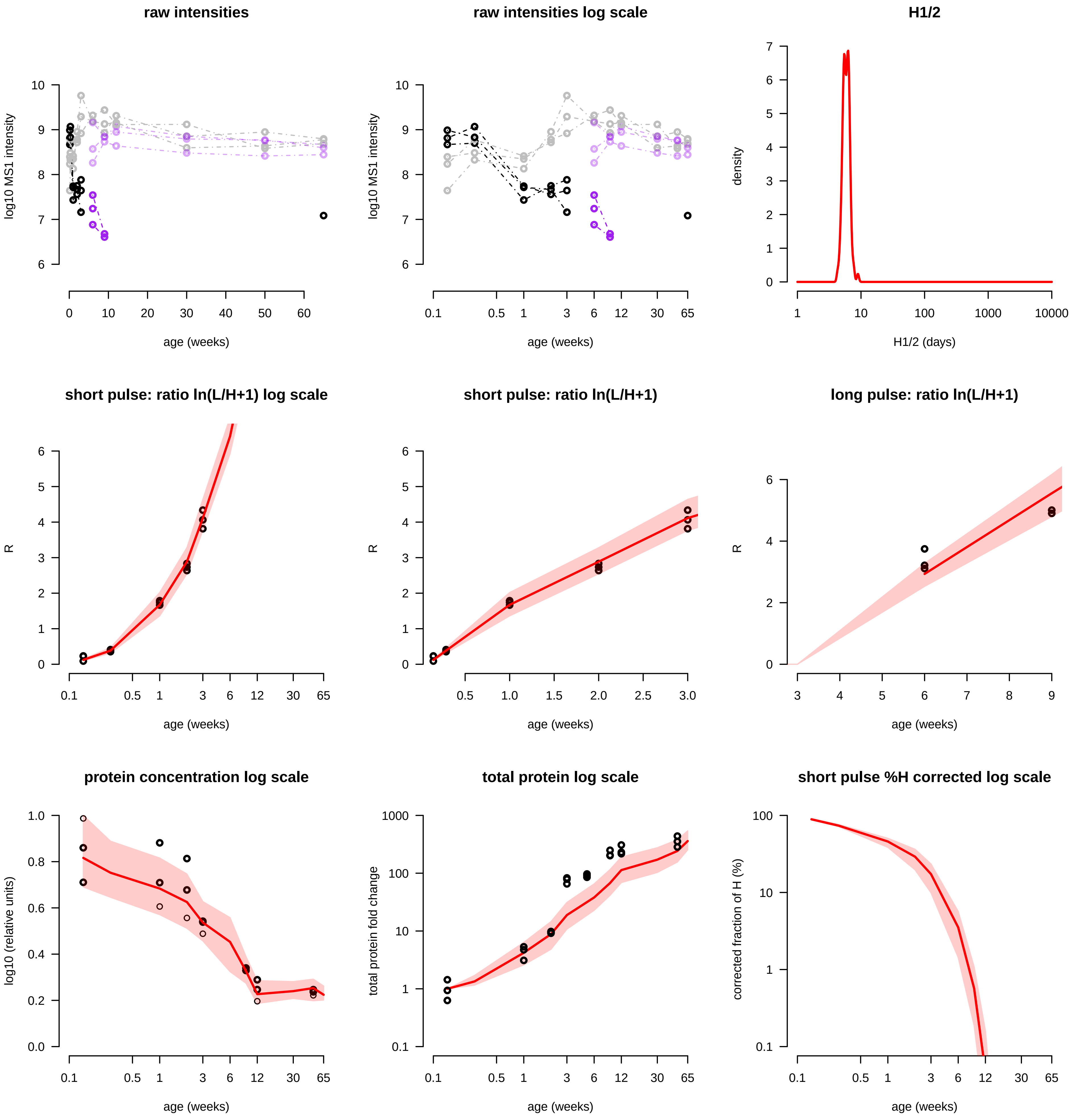

Q6P4S8

>sp|Q6P4S8|INT1_MOUSE

Integrator complex subunit 1

GN=Ints1

Med H1/2: 12.1 days

Q5 H1/2: 8.7 days

Q95 H1/2: 24.3 days

reliable model fit: yes

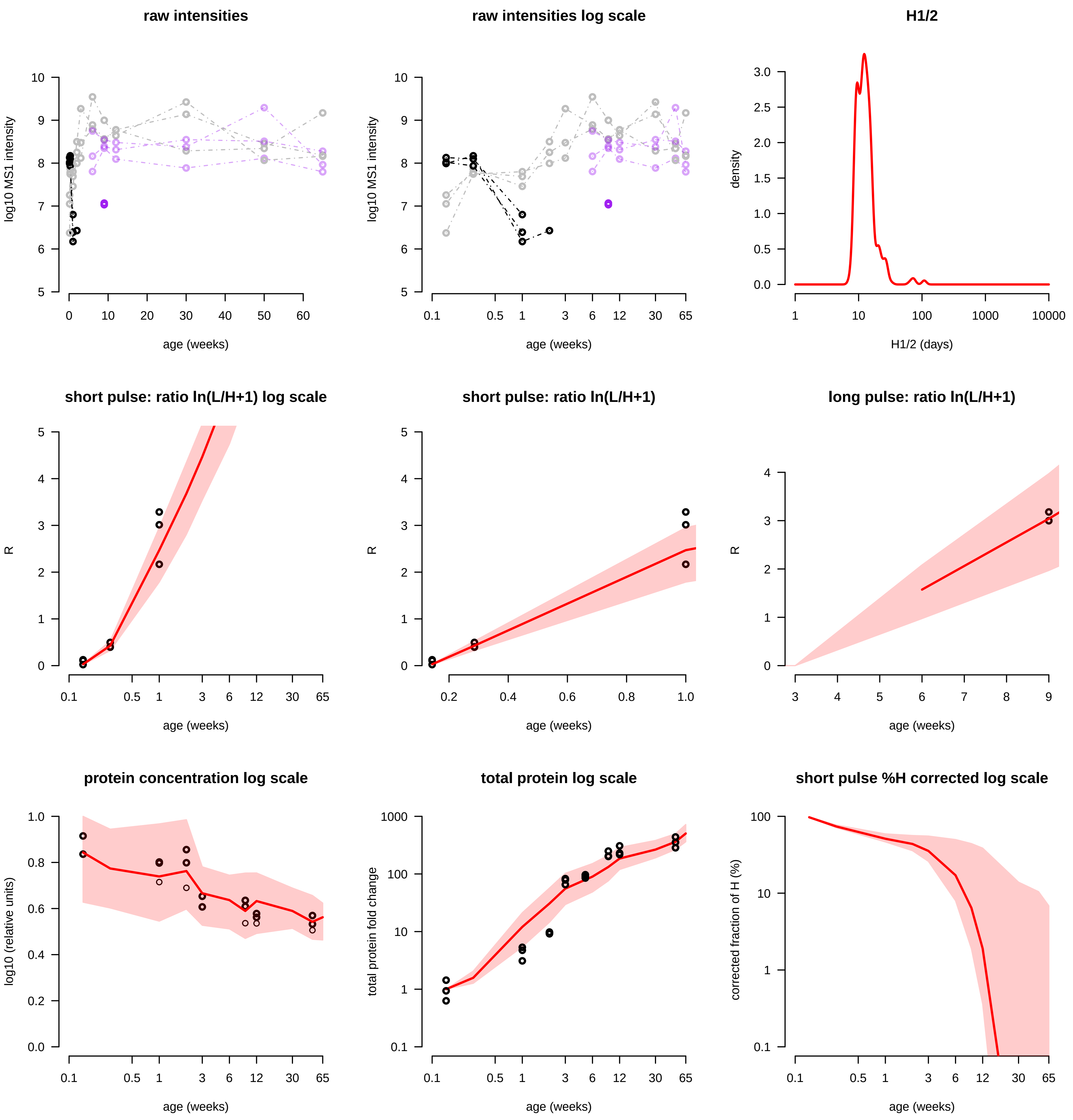

Q62418−3;Q62418−2;Q62418

>sp|Q62418−3|DBNL_MOUSE

Isoform 3 of Drebrin−like protein

GN=Dbnl

Med H1/2: 6.2 days

Q5 H1/2: 3.3 days

Q95 H1/2: 39.8 days

reliable model fit: yes

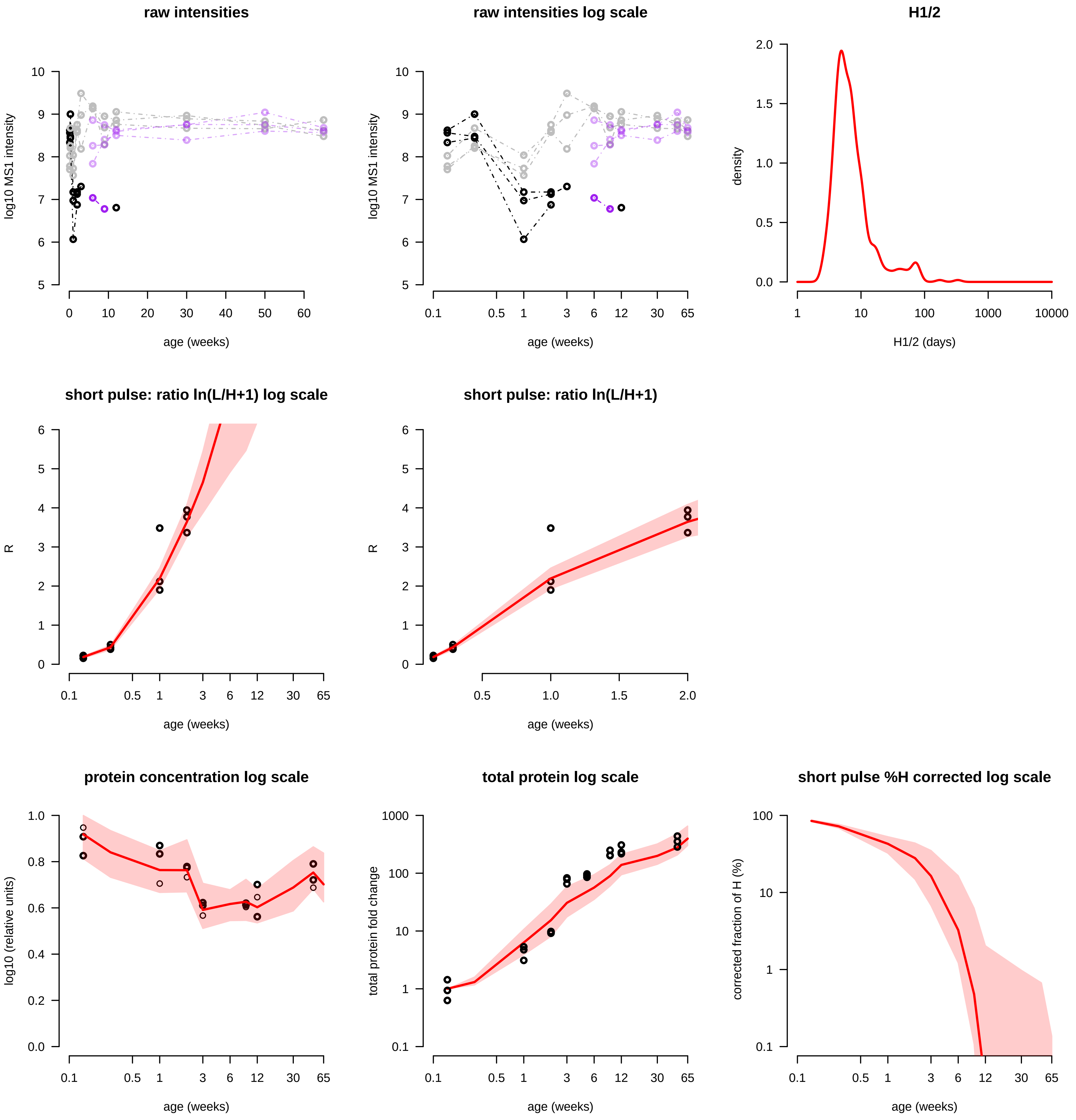

Q9ERF3

>sp|Q9ERF3|WDR61_MOUSE

WD repeat−containing protein 61

GN=Wdr61

Med H1/2: 3.8 days

Q5 H1/2: 3.1 days

Q95 H1/2: 4.7 days

reliable model fit: yes

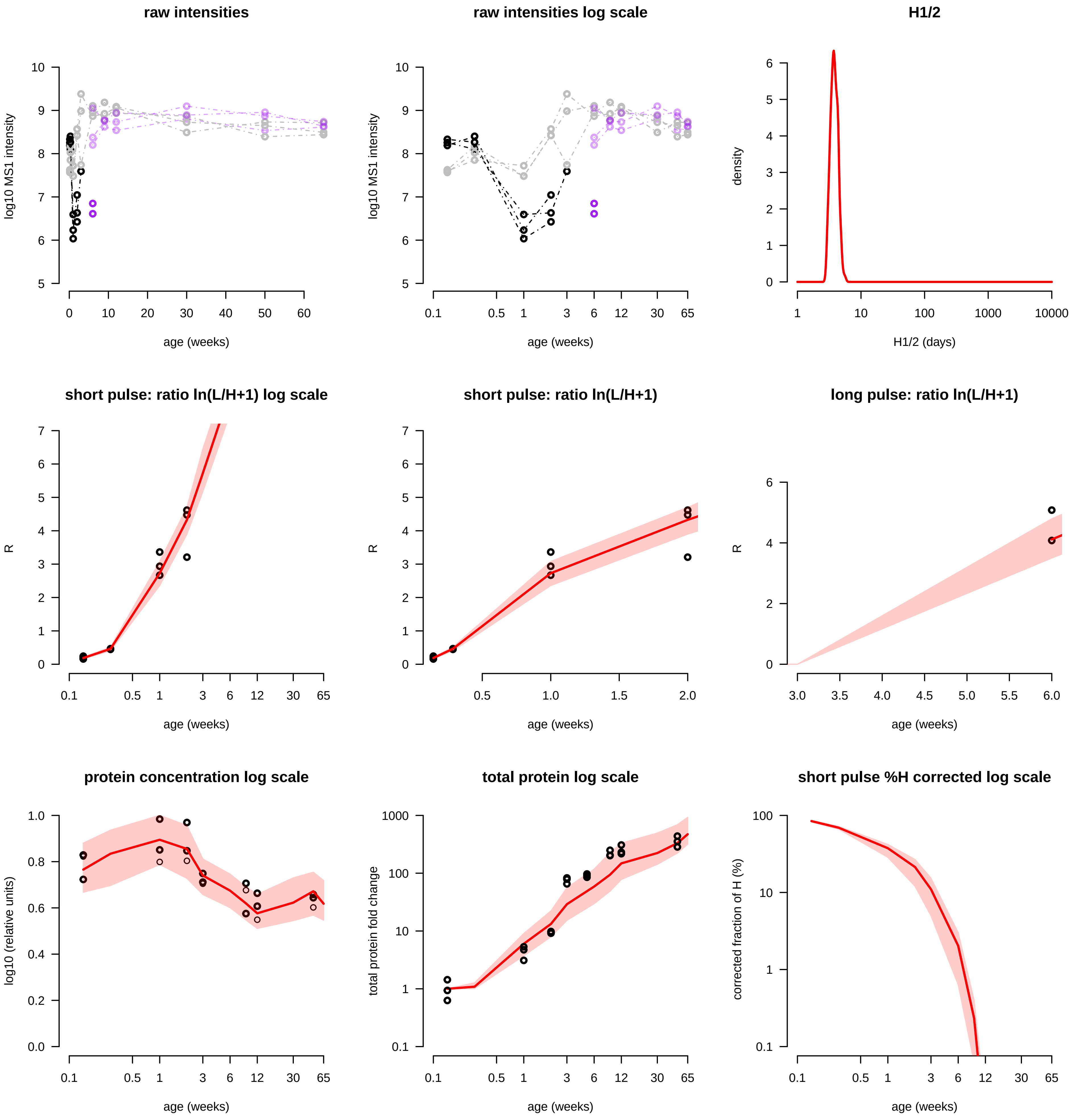

Q3TDN2;Q3TDN2−2

>sp|Q3TDN2|FAF2_MOUSE

FAS−associated factor 2

GN=Faf2

Med H1/2: 3.9 days

Q5 H1/2: 3.1 days

Q95 H1/2: 5.1 days

reliable model fit: yes

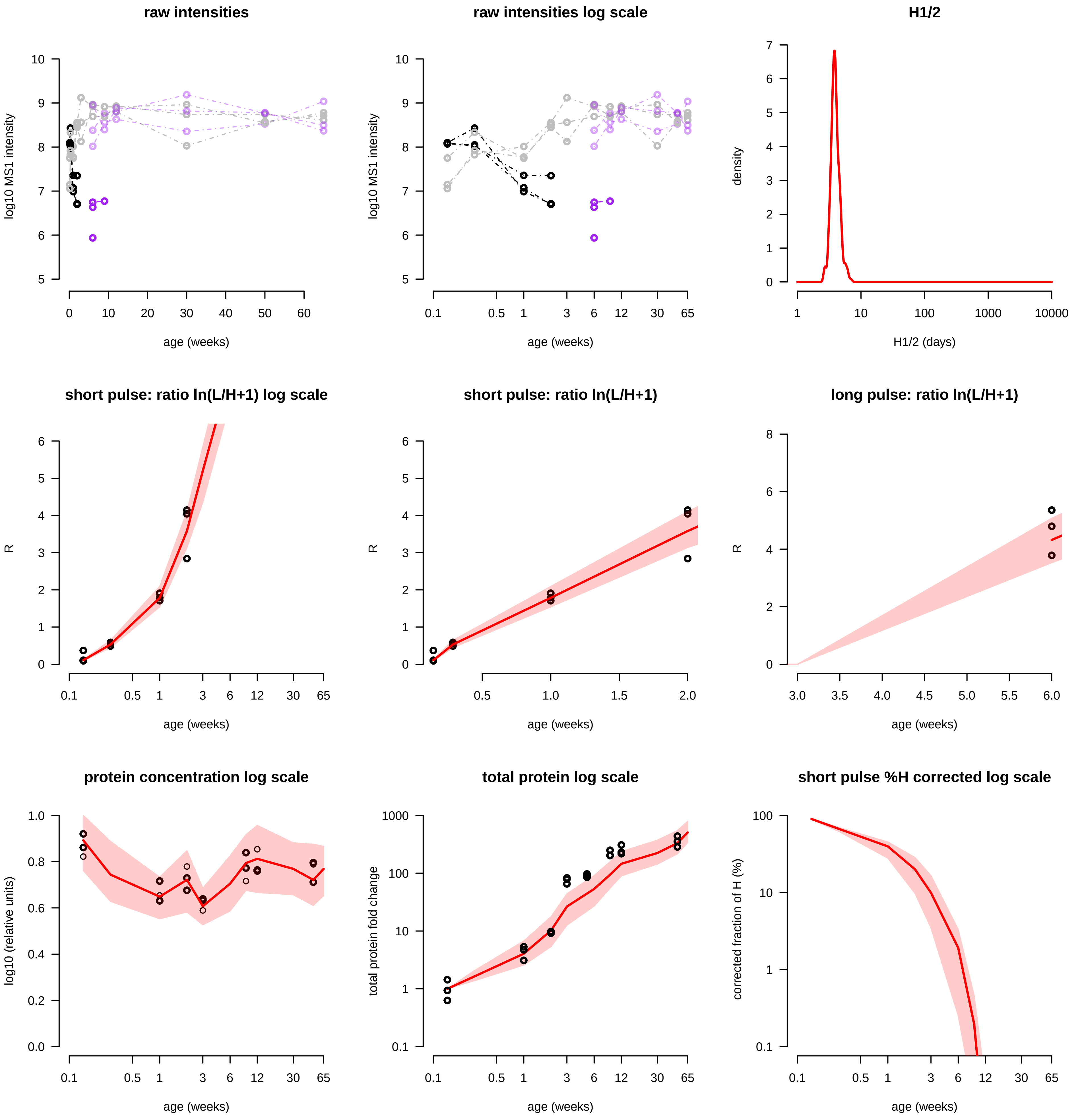

P35821

>sp|P35821|PTN1_MOUSE

Tyrosine−protein phosphatase non−receptor type 1

GN=Ptpn1

Med H1/2: 10.5 days

Q5 H1/2: 4.3 days

Q95 H1/2: 58.4 days

reliable model fit: yes

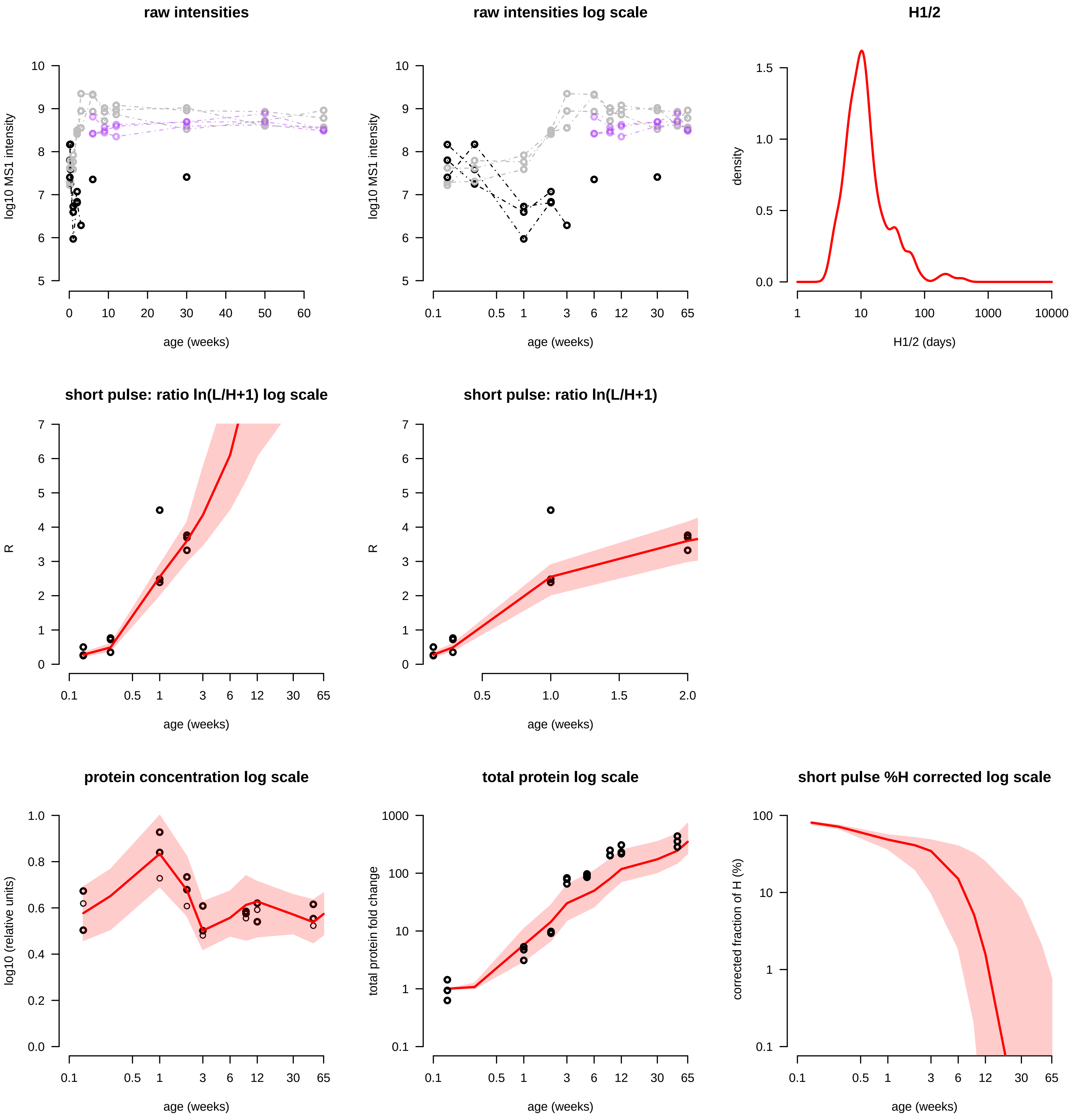

A3KMP2;A3KMP2−2

>sp|A3KMP2|TTC38_MOUSE

Tetratricopeptide repeat protein 38

GN=Ttc38

Med H1/2: 3.8 days

Q5 H1/2: 3.1 days

Q95 H1/2: 5.4 days

reliable model fit: yes

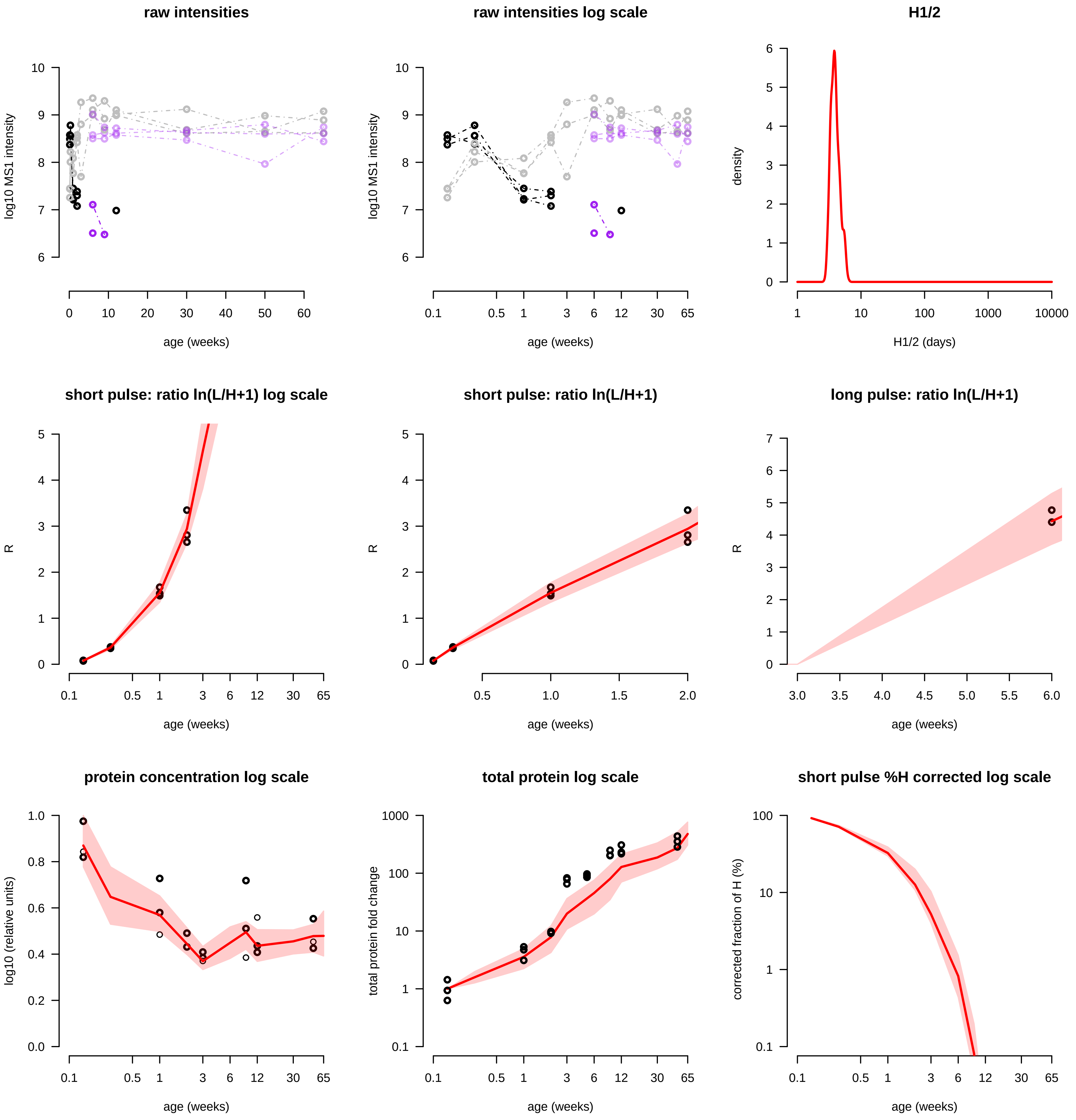

P11531

>sp|P11531|DMD_MOUSE

Dystrophin

GN=Dmd

Med H1/2: 2358 days

Q5 H1/2: 773.6 days

Q95 H1/2: 33173.4 days

reliable model fit: yes

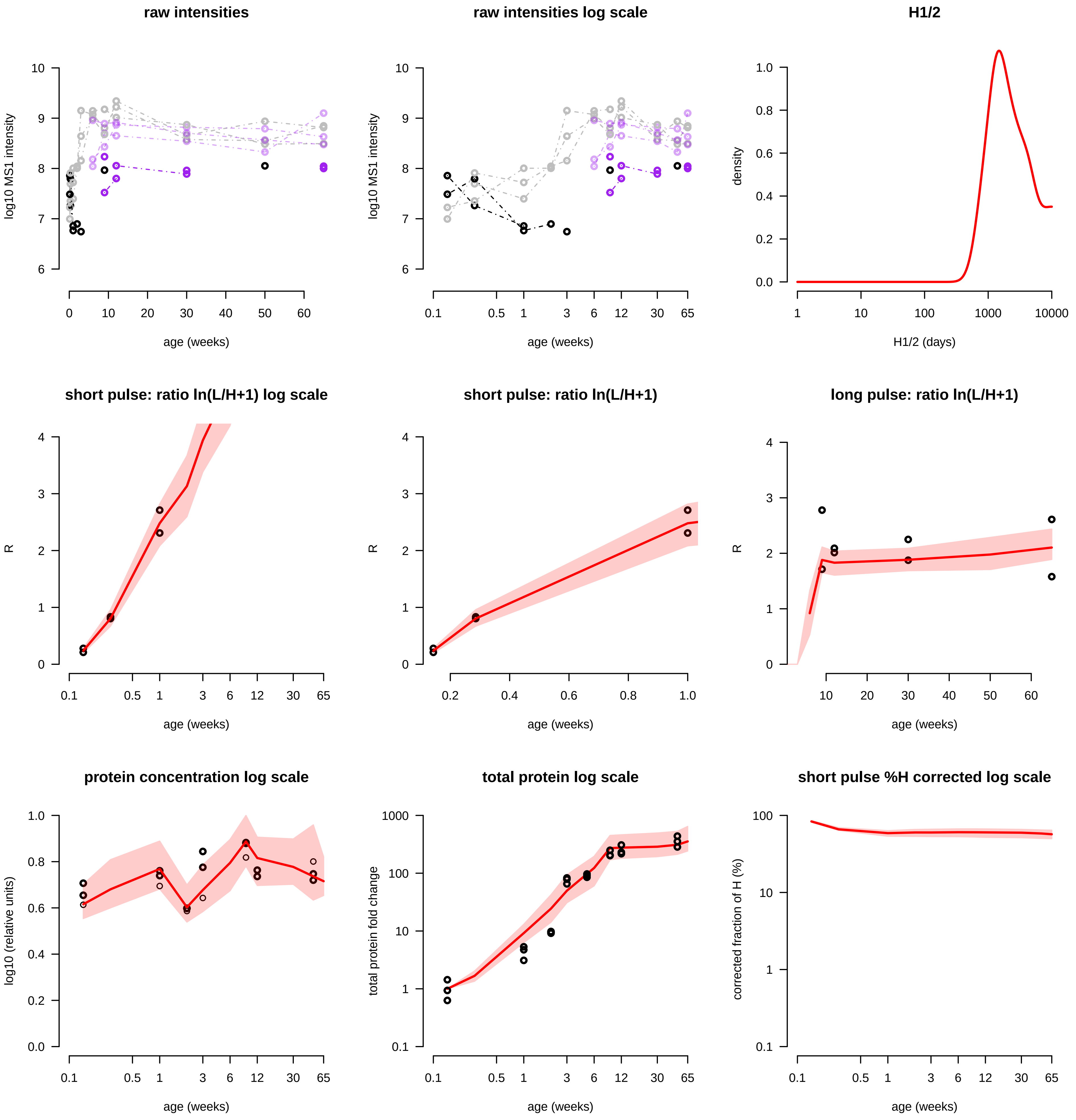

P35235−1;P35235

>sp|P35235−1|PTN11_MOUSE

Isoform 2 of Tyrosine−protein phosphatase non−receptor type 11

GN=Ptpn11

Med H1/2: 6.7 days

Q5 H1/2: 5.3 days

Q95 H1/2: 9.3 days

reliable model fit: yes

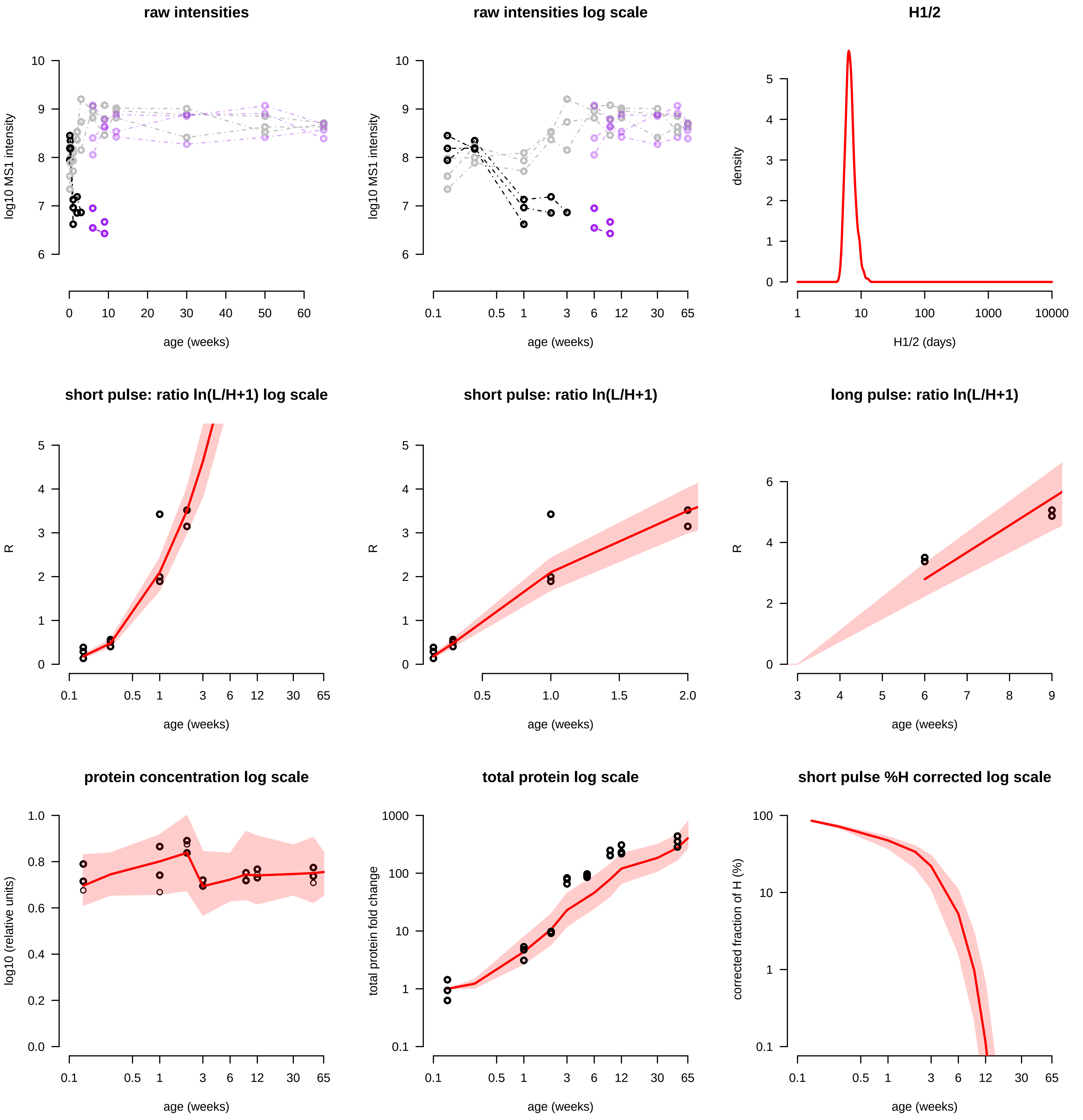

Q9WUQ2

>sp|Q9WUQ2|PREB_MOUSE

Prolactin regulatory element−binding protein

GN=Preb

Med H1/2: 4.3 days

Q5 H1/2: 3.5 days

Q95 H1/2: 5.8 days

reliable model fit: yes

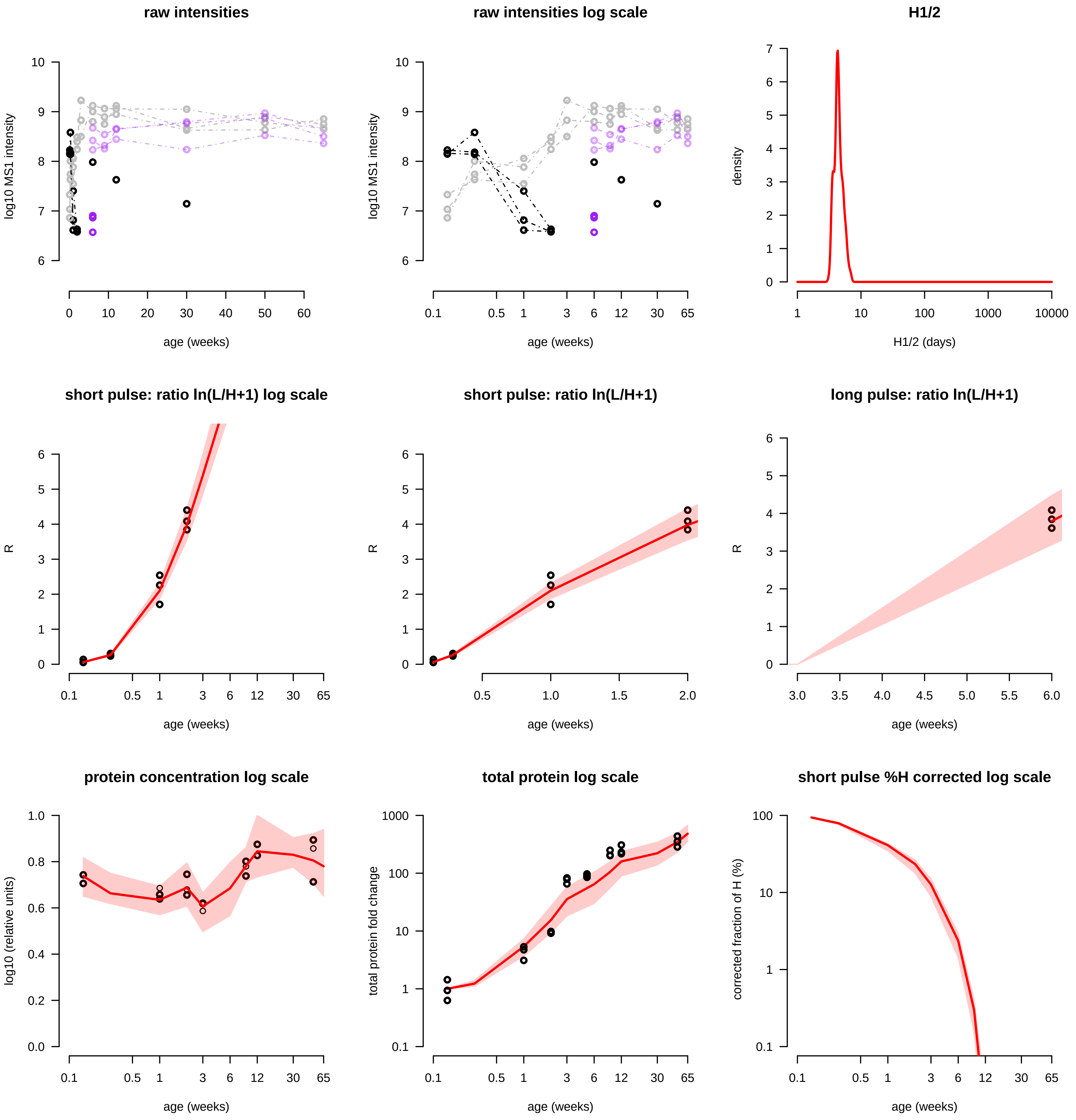

Q921M4−2;Q921M4

>sp|Q921M4−2|GOGA2_MOUSE

Isoform 2 of Golgin subfamily A member 2

GN=Golga2

Med H1/2: 3.6 days

Q5 H1/2: 2.6 days

Q95 H1/2: 12.5 days

reliable model fit: yes

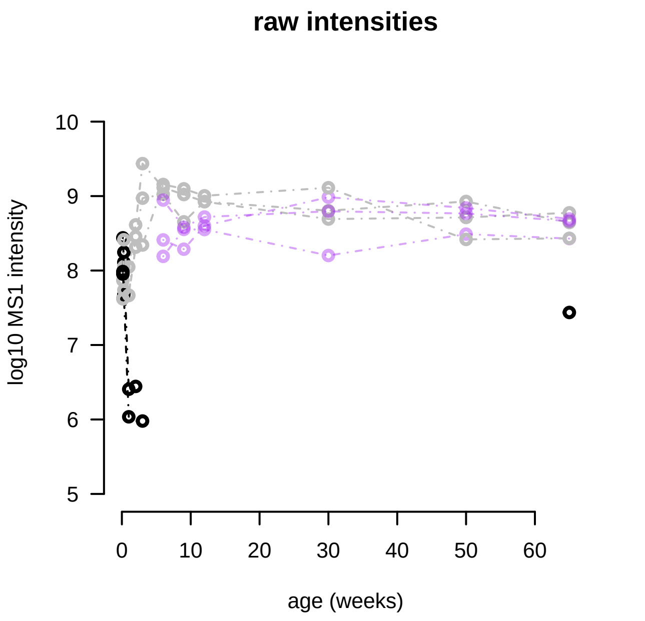

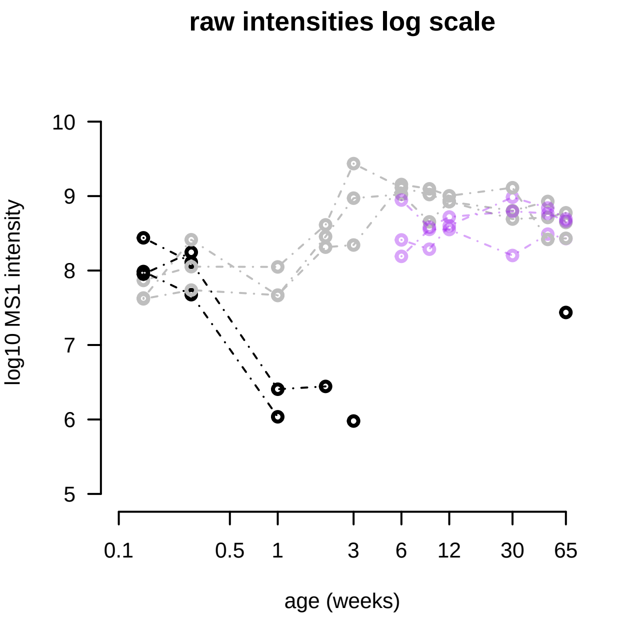

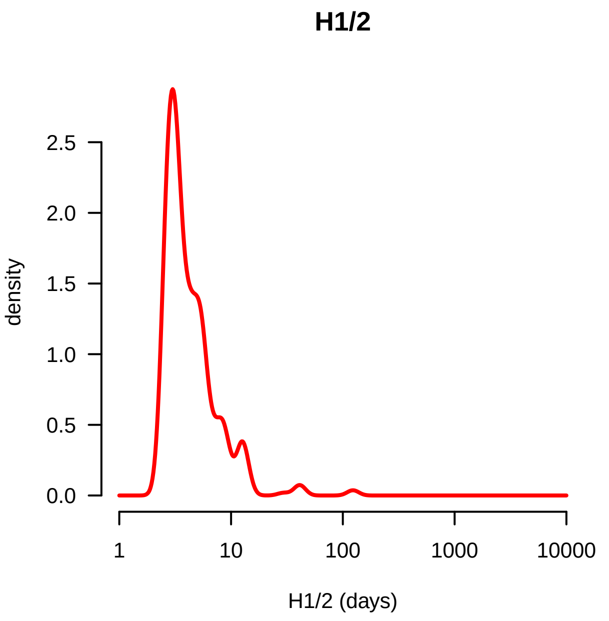

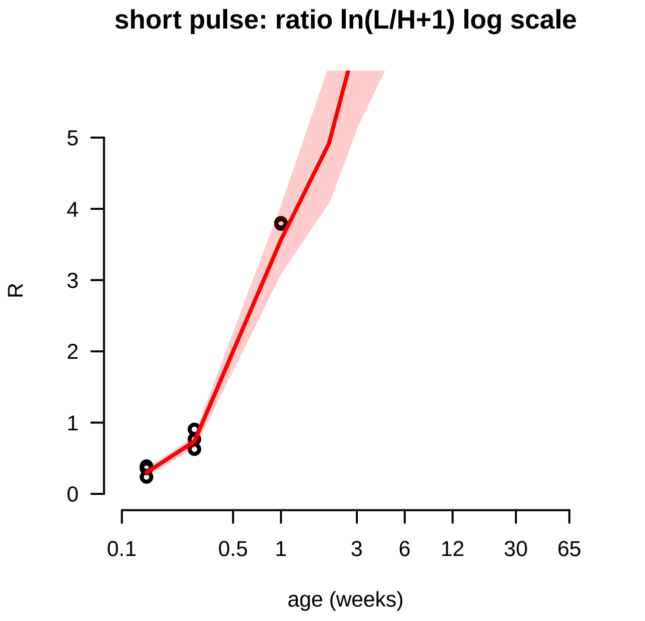

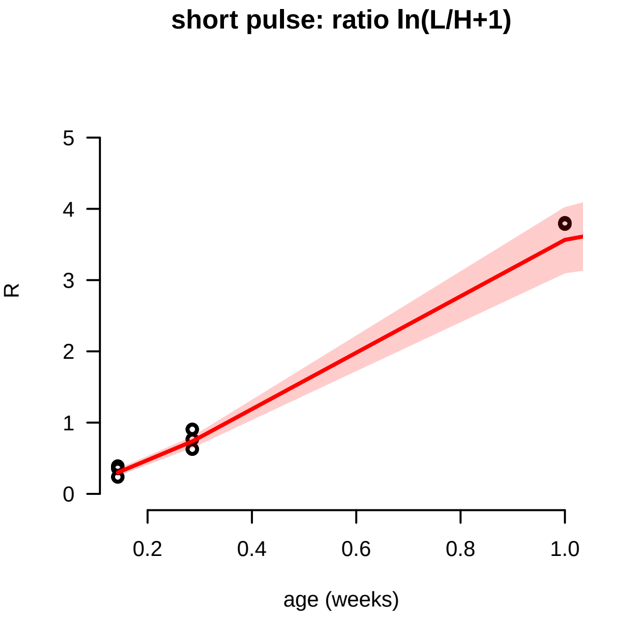

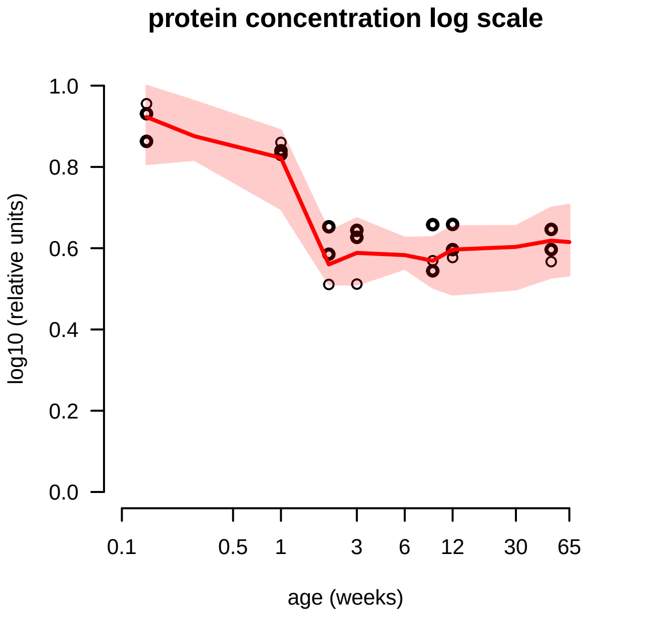

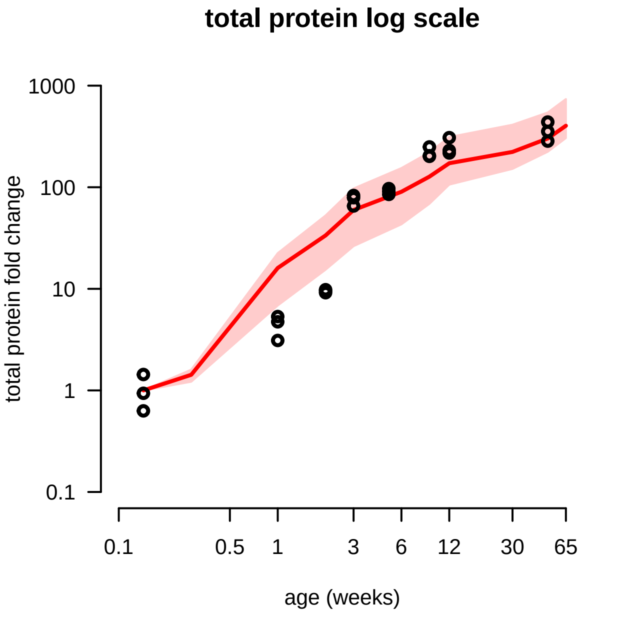

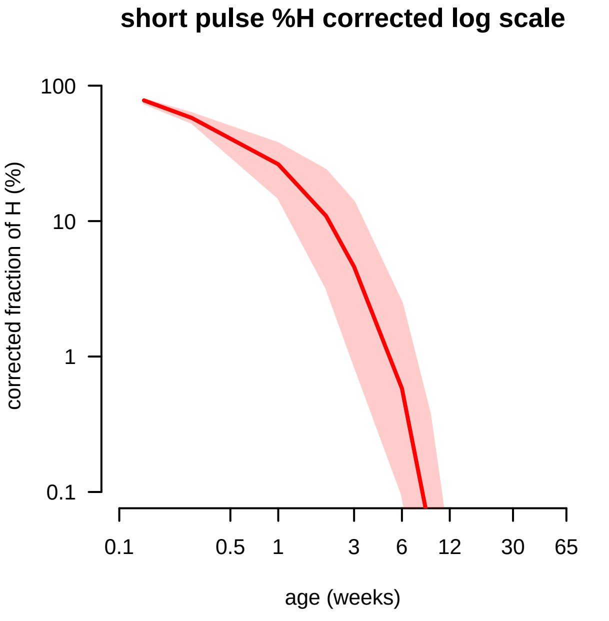

Q9QXE7

>sp|Q9QXE7|TBL1X_MOUSE

F−box−like/WD repeat−containing protein TBL1X

GN=Tbl1x

Med H1/2: 4.2 days

Q5 H1/2: 3.5 days

Q95 H1/2: 5.7 days

reliable model fit: yes

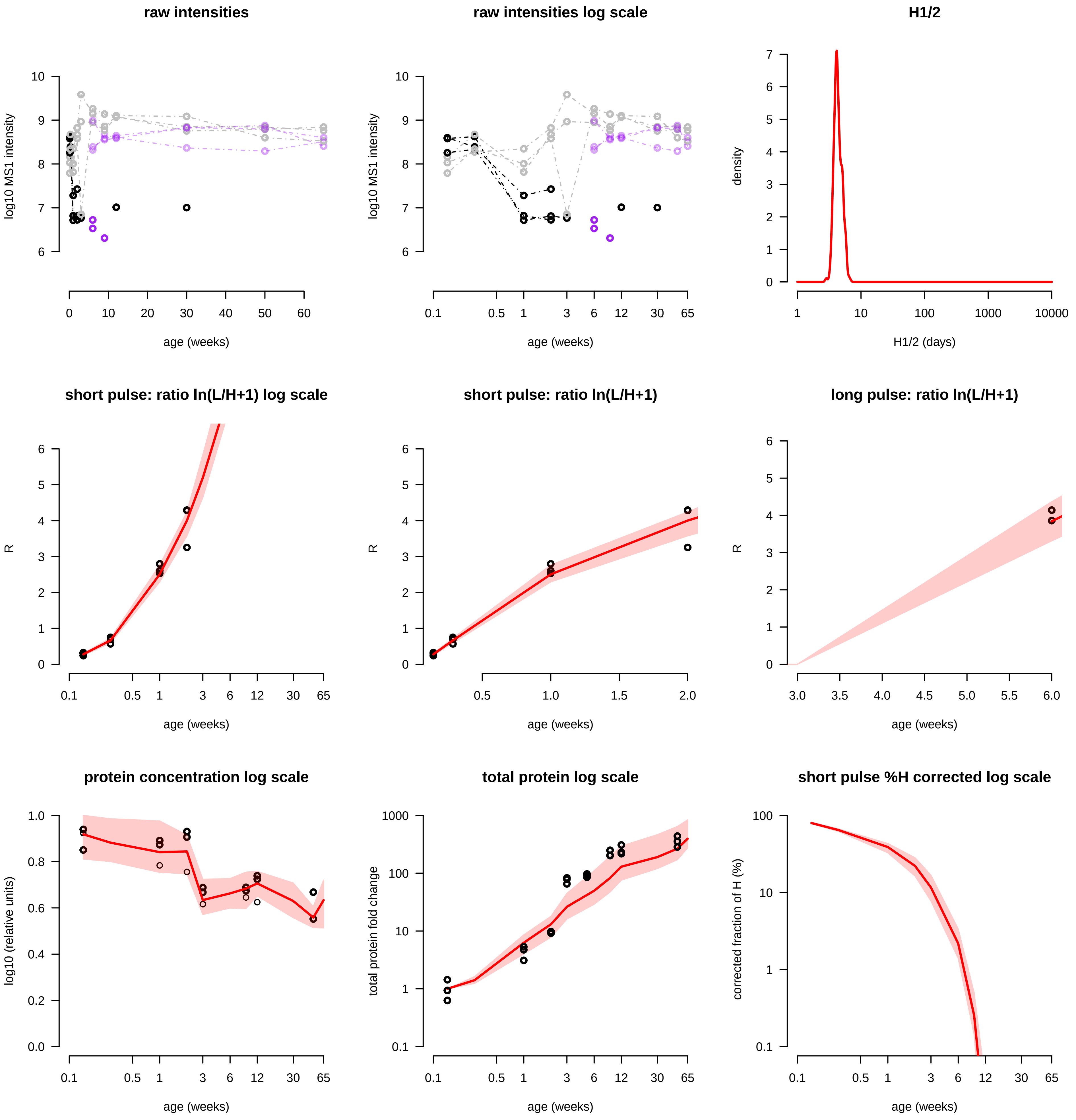

O08784

>sp|O08784|TCOF_MOUSE

Treacle protein

GN=Tcof1

Med H1/2: 3.4 days

Q5 H1/2: 2.9 days

Q95 H1/2: 3.8 days

reliable model fit: yes

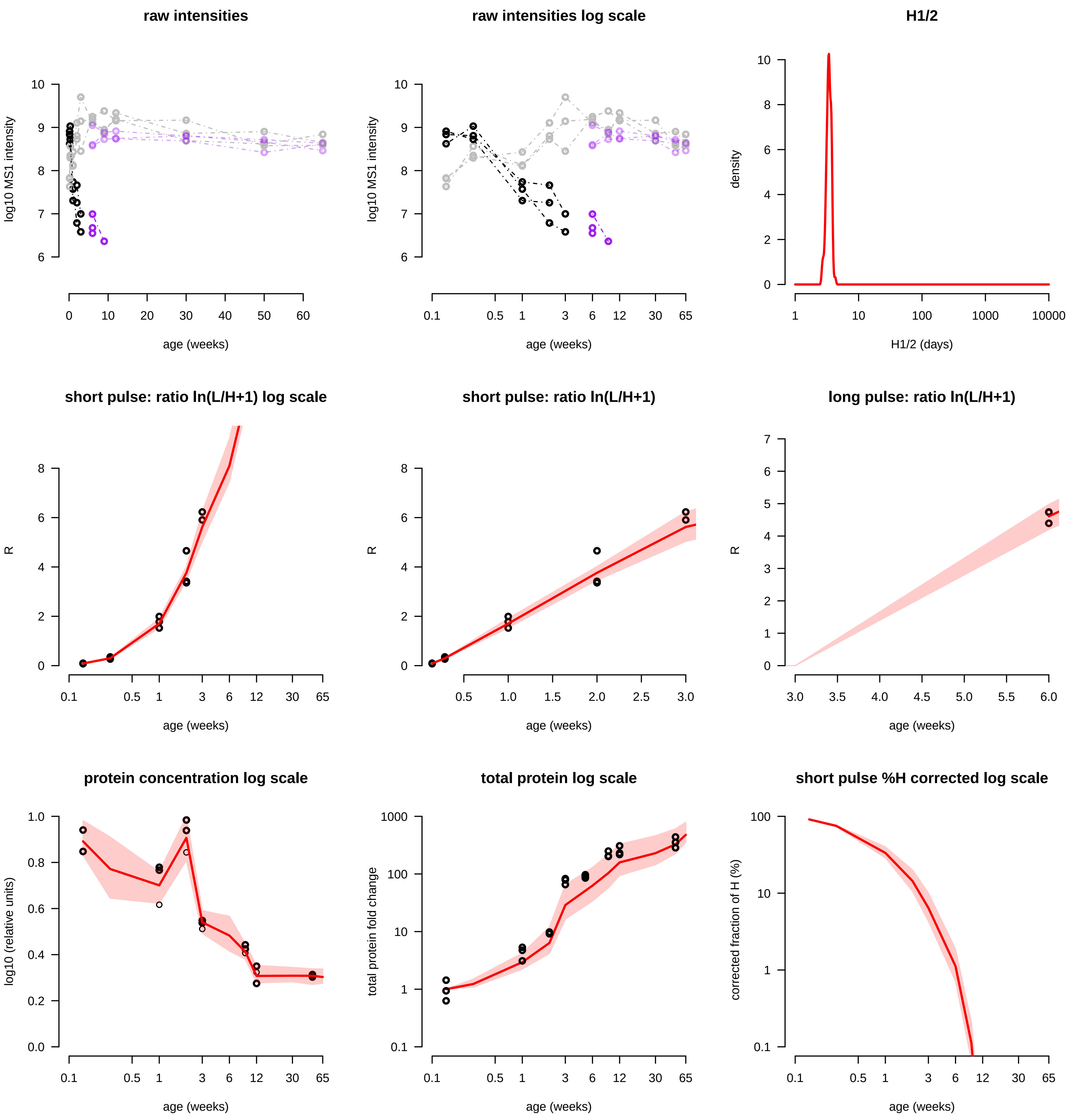

Q9CPP6

>sp|Q9CPP6|NDUA5_MOUSE

NADH dehydrogenase [ubiquinone] 1 alpha subcomplex subunit 5

GN=Ndufa5

Med H1/2: 4.4 days

Q5 H1/2: 3.3 days

Q95 H1/2: 6.5 days

reliable model fit: yes

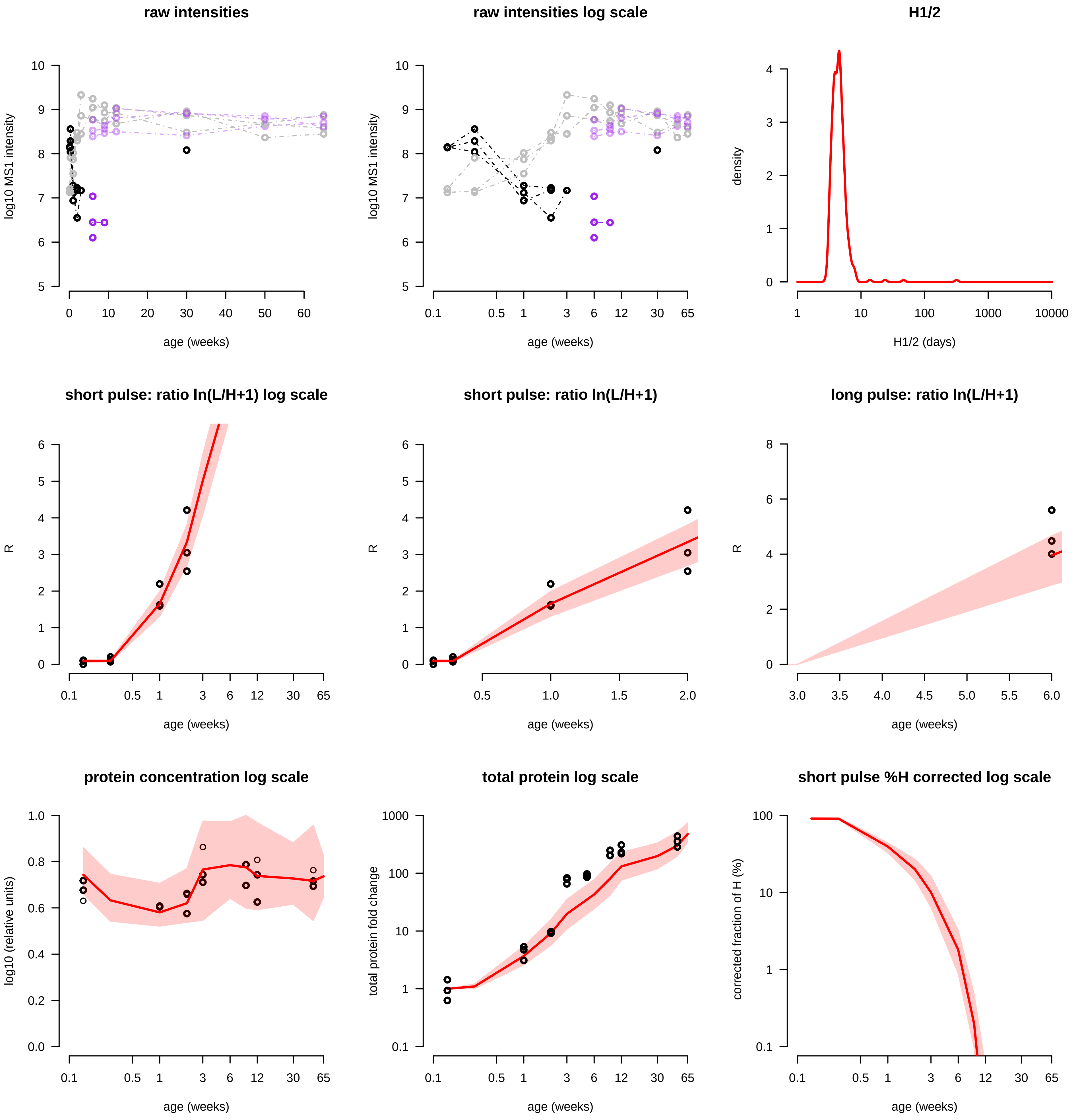

P47758

>sp|P47758|SRPRB_MOUSE

Signal recognition particle receptor subunit beta

GN=Srprb

Med H1/2: 3.7 days

Q5 H1/2: 3.1 days

Q95 H1/2: 5 days

reliable model fit: yes

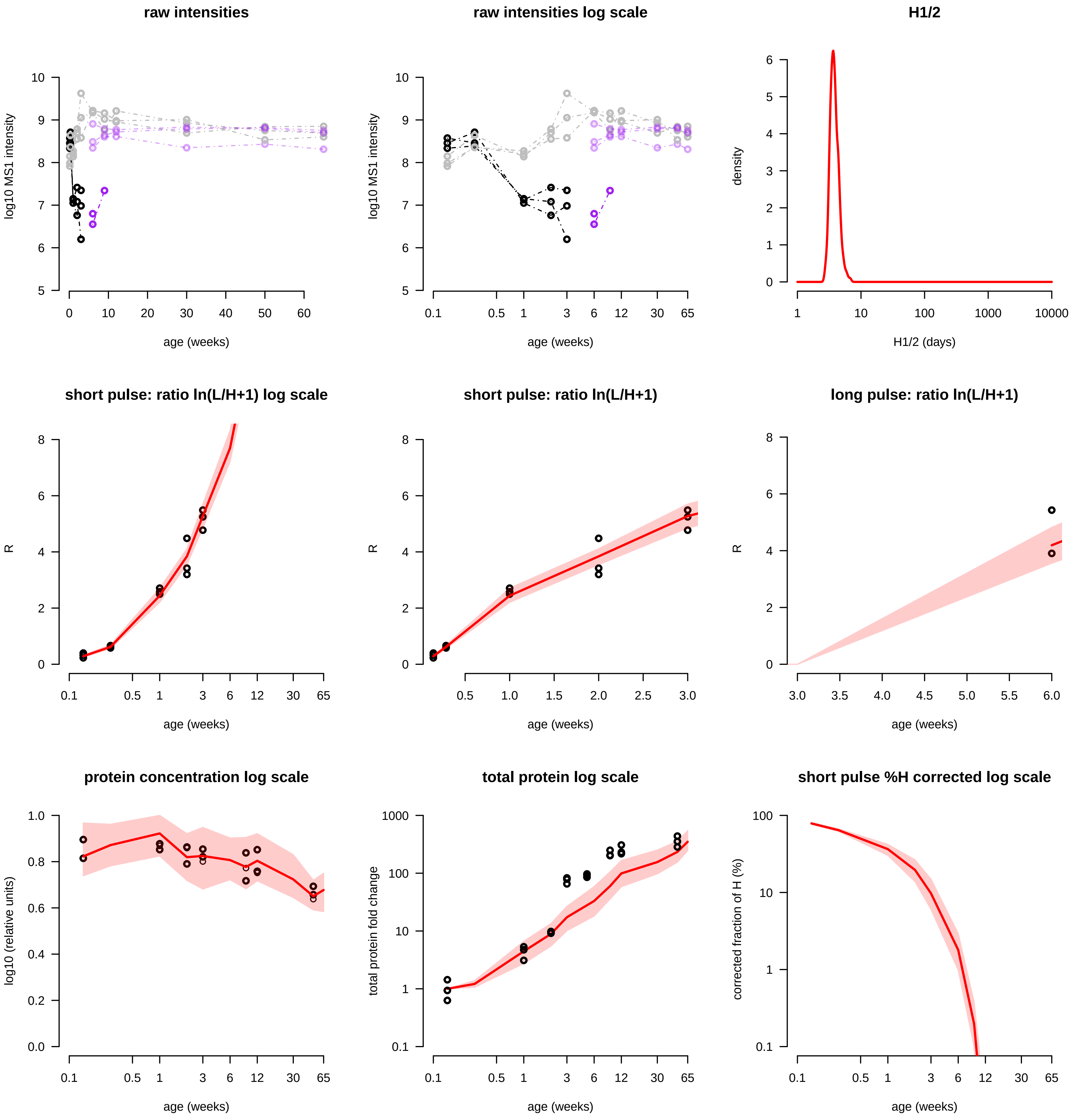

Q9WTX2

>sp|Q9WTX2|PRKRA_MOUSE

nterferon−inducible double−stranded RNA−dependent protein kinase activator A

GN=Prkra

Med H1/2: 4.6 days

Q5 H1/2: 3.9 days

Q95 H1/2: 5.5 days

reliable model fit: yes

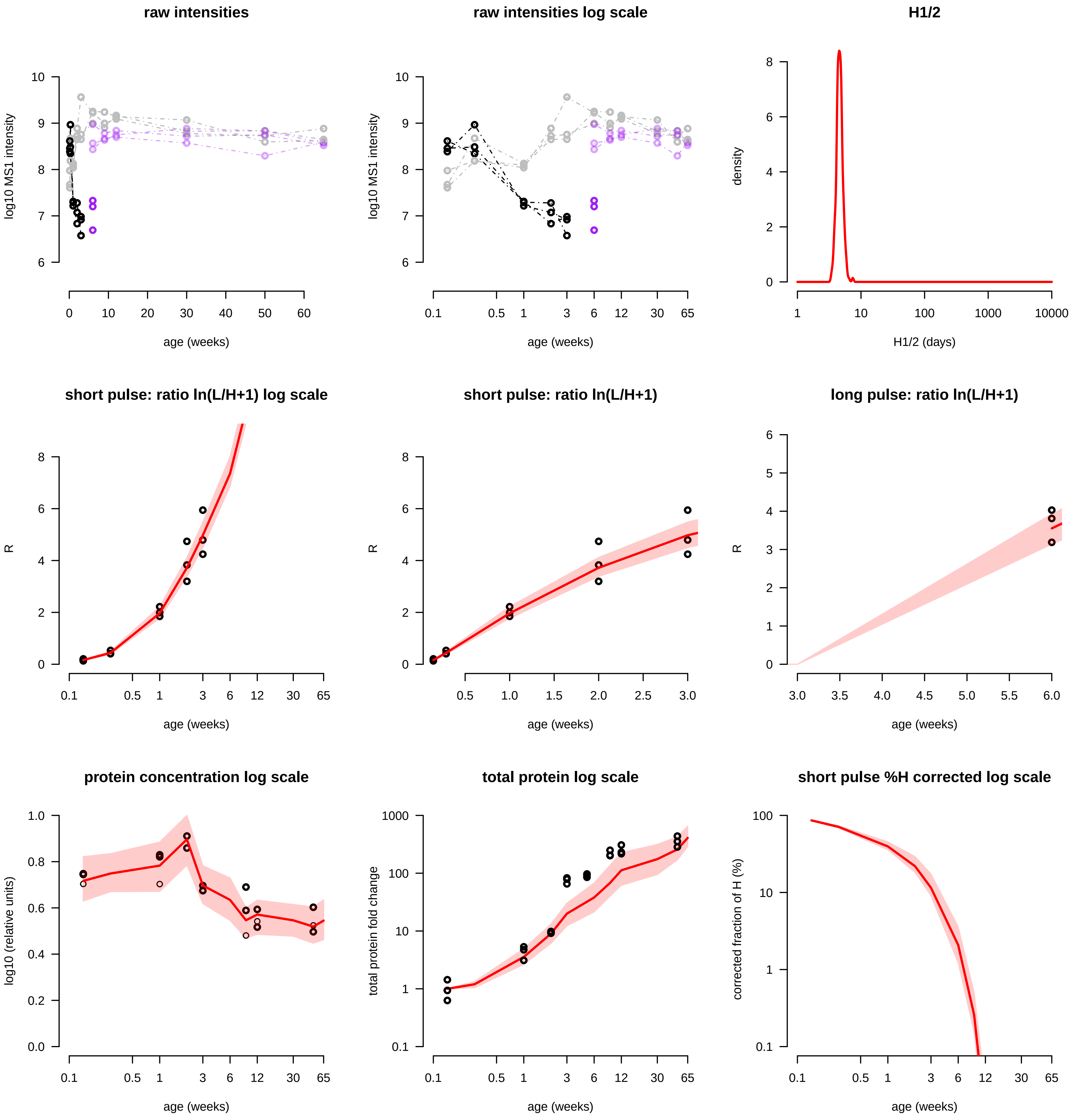

O54988

>sp|O54988|SLK_MOUSE

STE20−like serine/threonine−protein kinase

GN=Slk

Med H1/2: 29.3 days

Q5 H1/2: 19.5 days

Q95 H1/2: 62.2 days

reliable model fit: yes

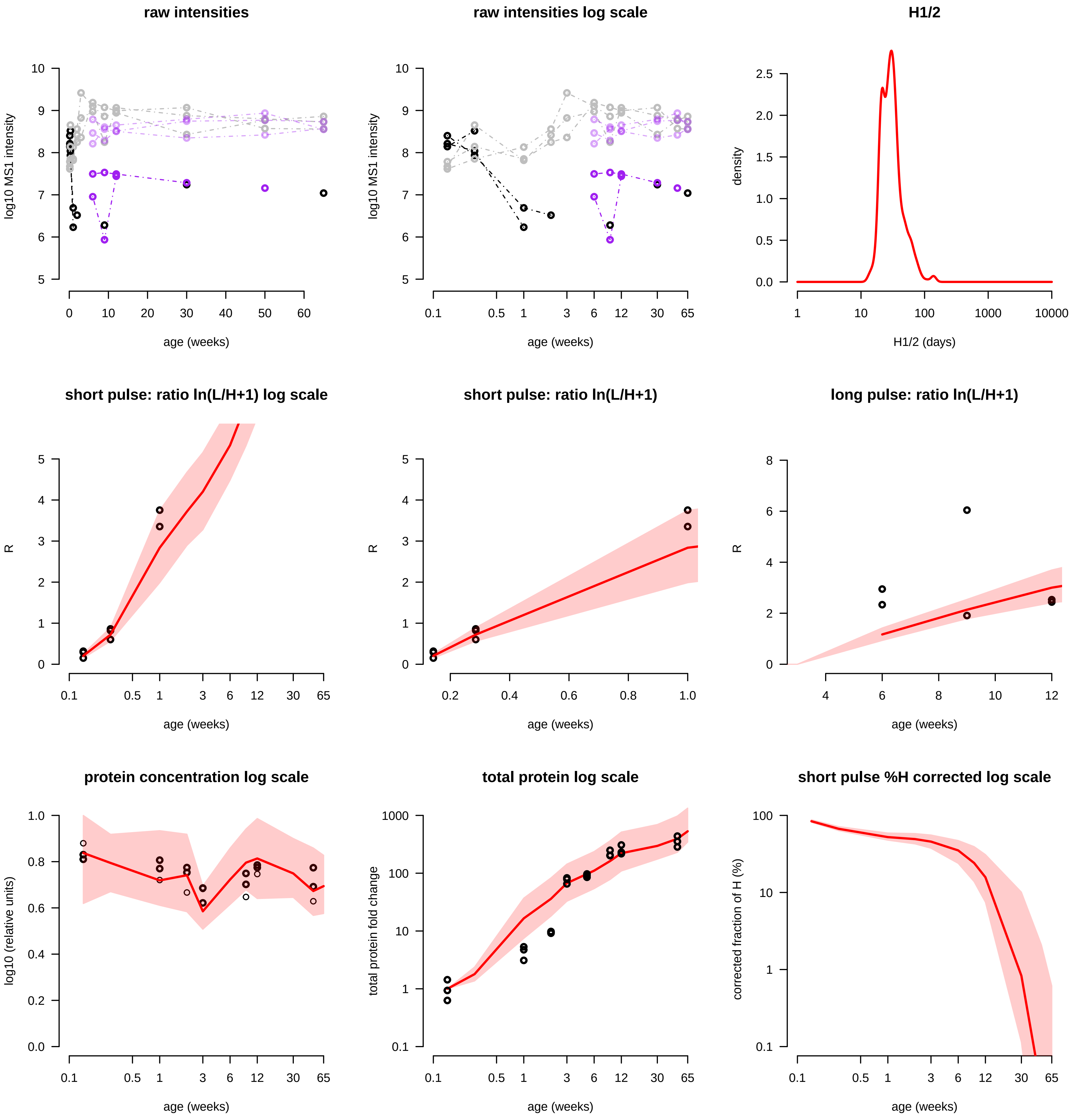

Q810A7

>sp|Q810A7|DDX42_MOUSE

ATP−dependent RNA helicase DDX42

GN=Ddx42

Med H1/2: 5.1 days

Q5 H1/2: 4 days

Q95 H1/2: 6.3 days

reliable model fit: yes

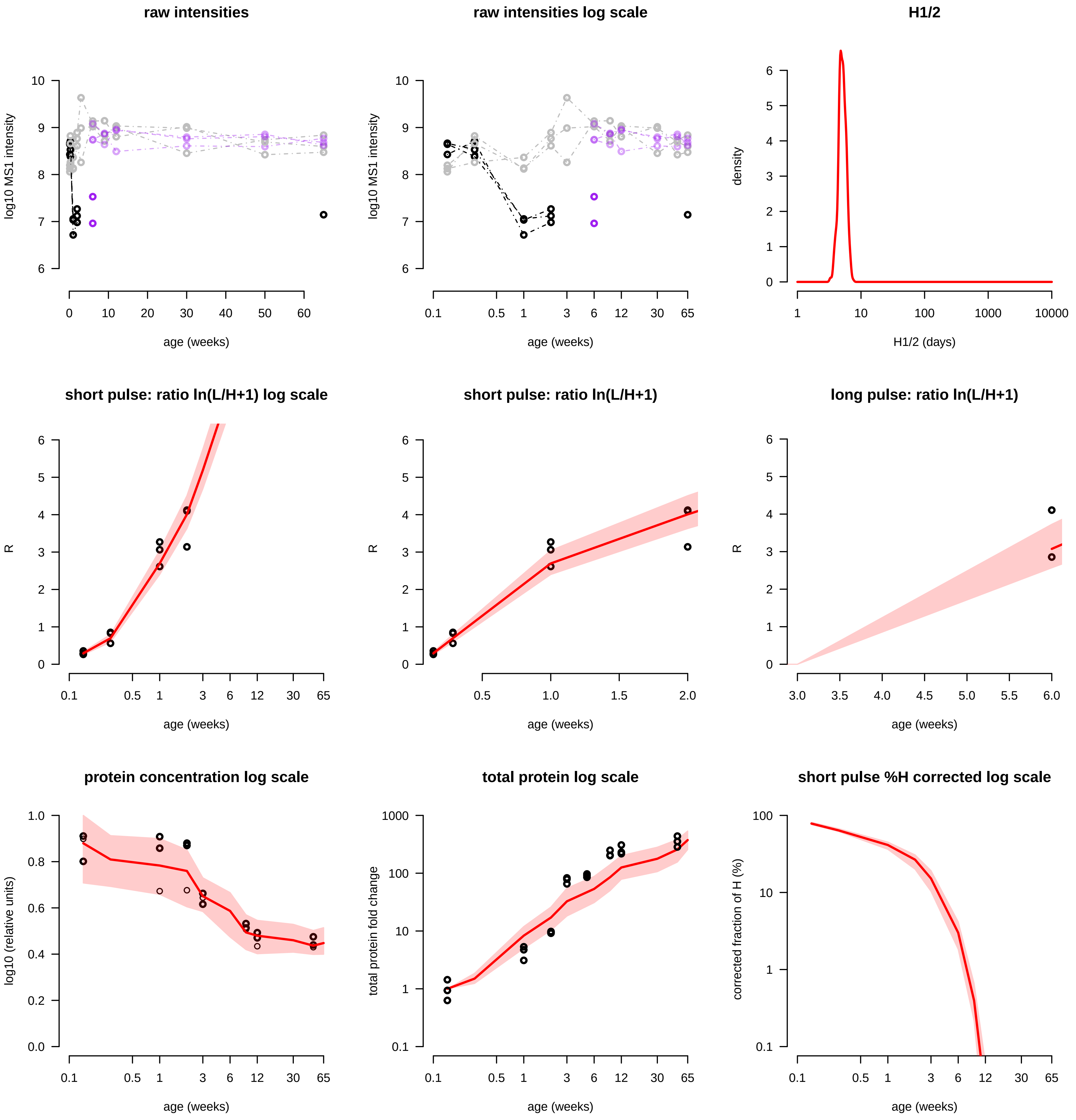

Q60692

>sp|Q60692|PSB6_MOUSE

Proteasome subunit beta type−6

GN=Psmb6

Med H1/2: 19.8 days

Q5 H1/2: 7.3 days

Q95 H1/2: 112.5 days

reliable model fit: yes

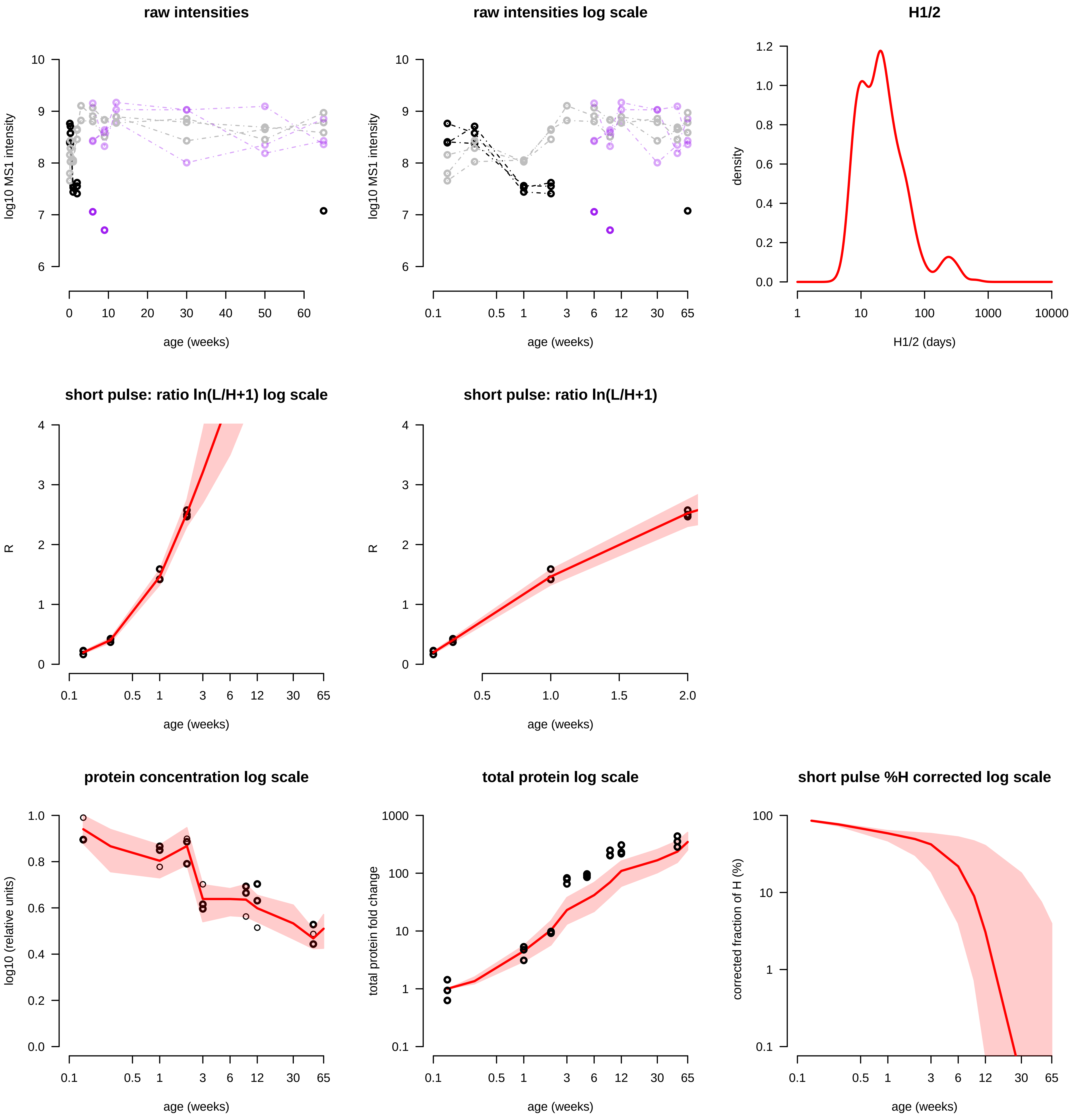

P97930

>sp|P97930|KTHY_MOUSE

Thymidylate kinase

GN=Dtymk

Med H1/2: 10.7 days

Q5 H1/2: 3.3 days

Q95 H1/2: 48.8 days

reliable model fit: yes

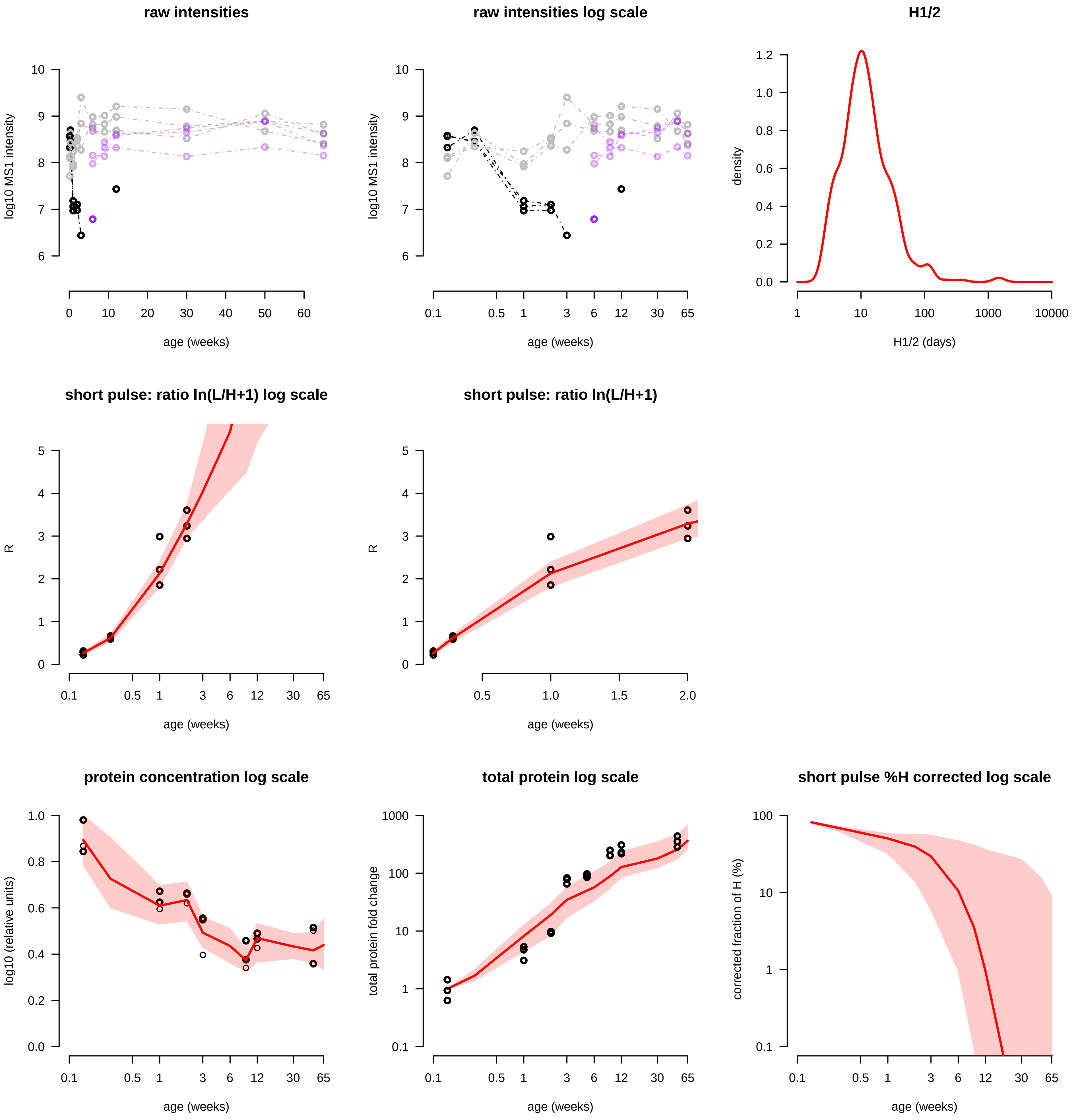

Q6DFV7

>sp|Q6DFV7|NCOA7_MOUSE

Nuclear receptor coactivator 7

GN=Ncoa7

Med H1/2: 8.3 days

Q5 H1/2: 6.3 days

Q95 H1/2: 12.8 days

reliable model fit: yes

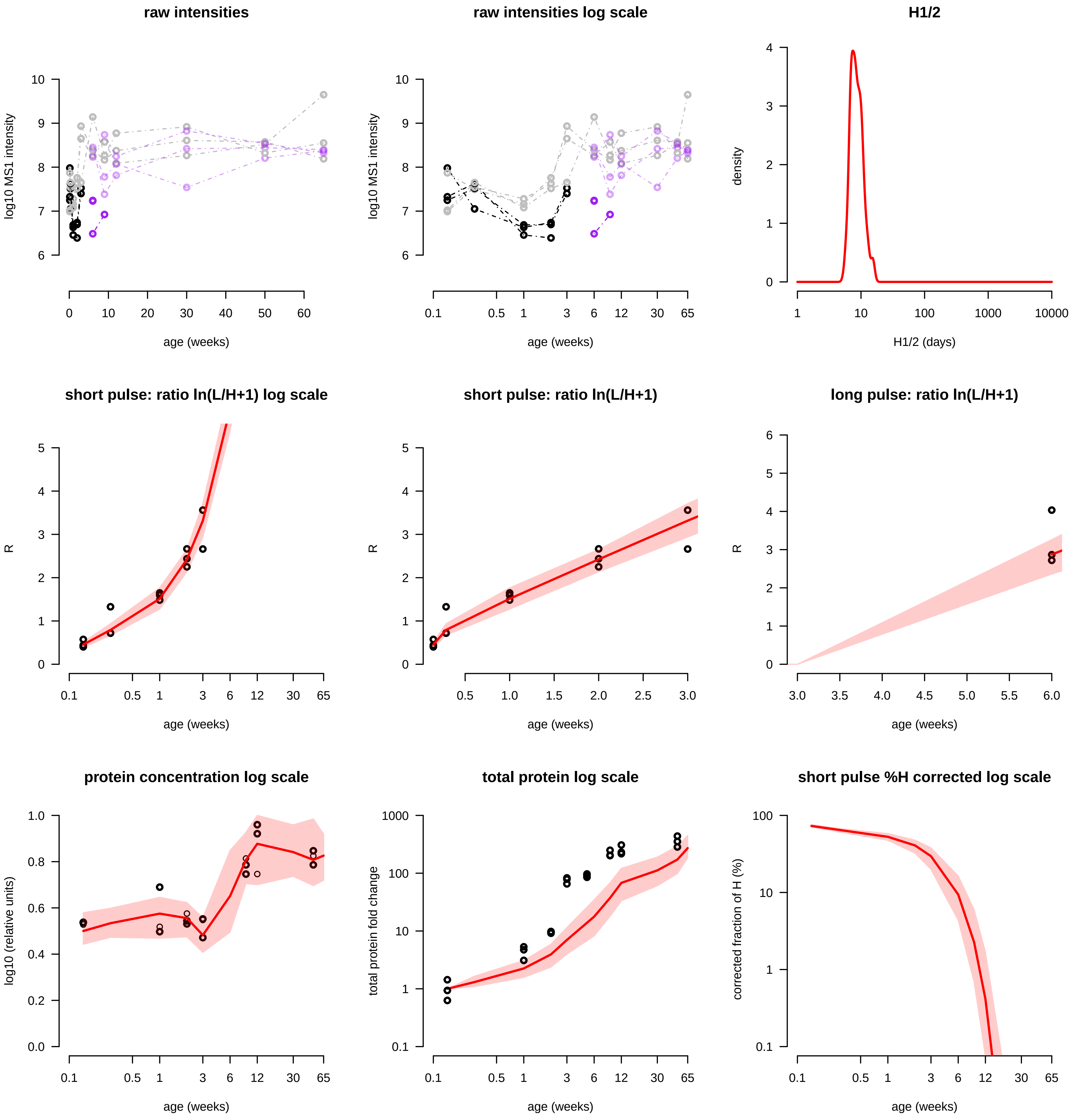

P47802

>sp|P47802|MTX1_MOUSE

Metaxin−1

GN=Mtx1

Med H1/2: 6.6 days

Q5 H1/2: 4.4 days

Q95 H1/2: 14.5 days

reliable model fit: yes

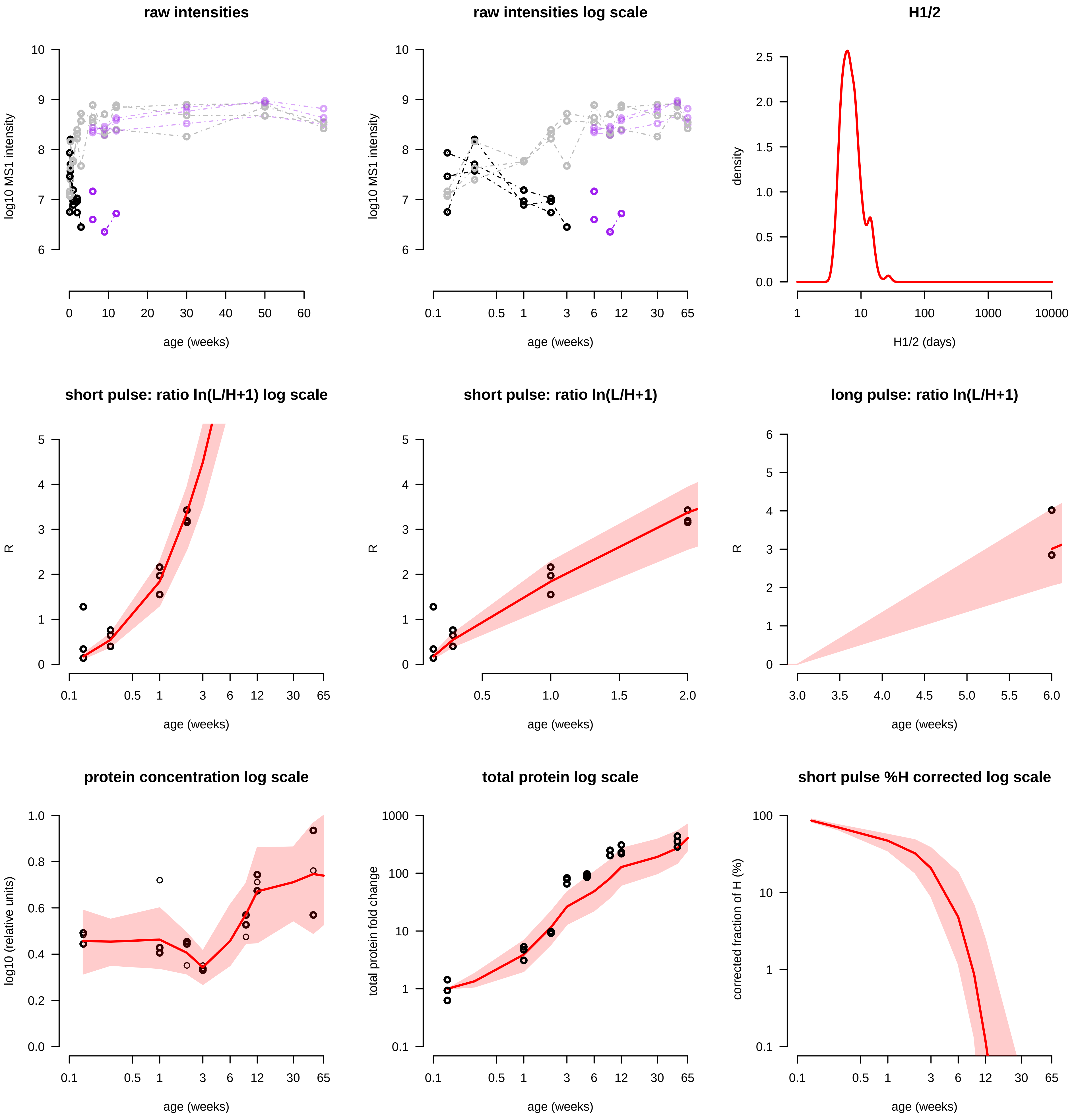

P14094

>sp|P14094|AT1B1_MOUSE

Sodium/potassium−transporting ATPase subunit beta−1

GN=Atp1b1

Med H1/2: 4 days

Q5 H1/2: 2.8 days

Q95 H1/2: 14.2 days

reliable model fit: yes

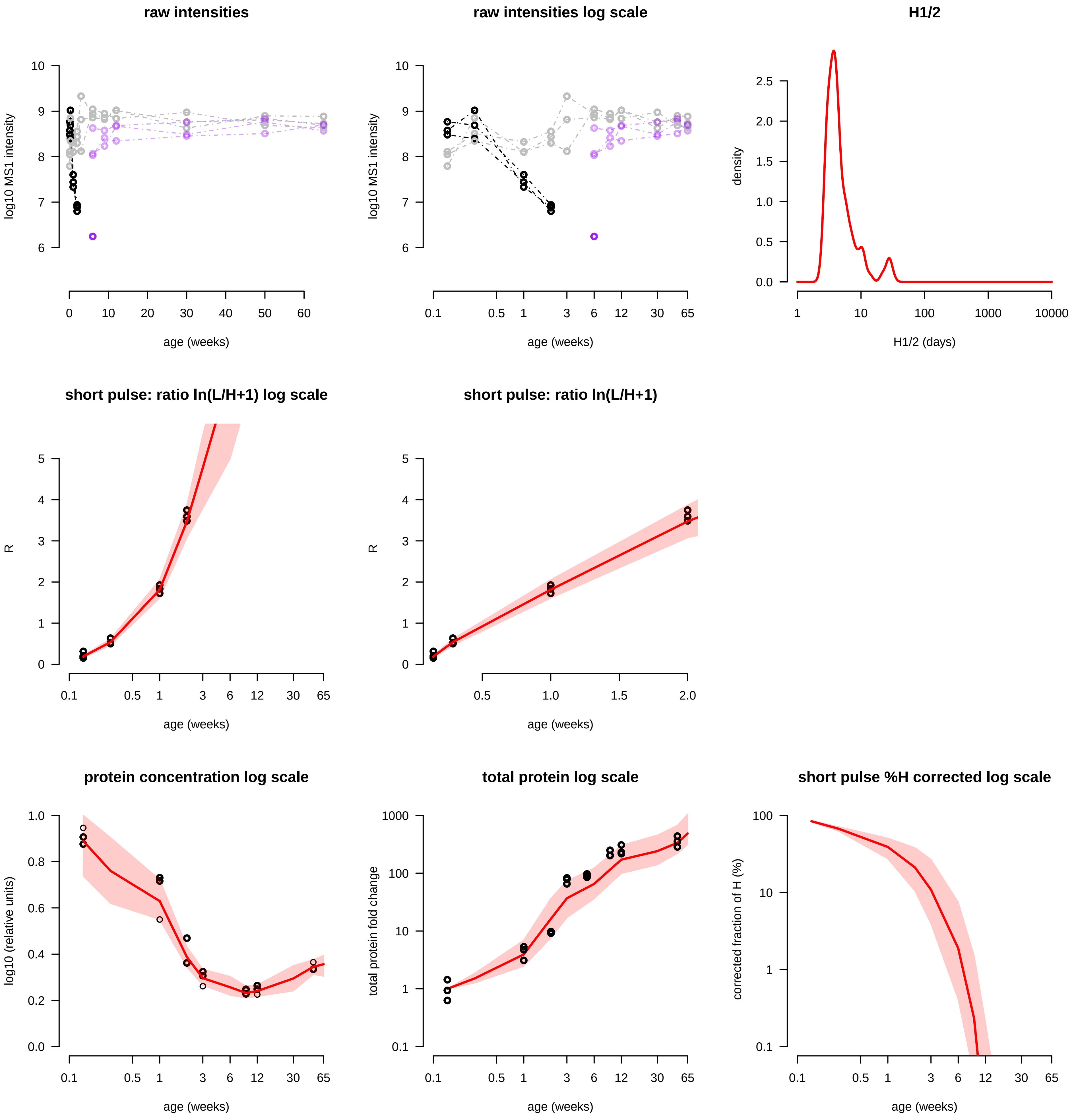

Q3UDE2

>sp|Q3UDE2|TTL12_MOUSE

Tubulin−−tyrosine ligase−like protein 12

GN=Ttll12

Med H1/2: 5.4 days

Q5 H1/2: 4.4 days

Q95 H1/2: 7.2 days

reliable model fit: yes

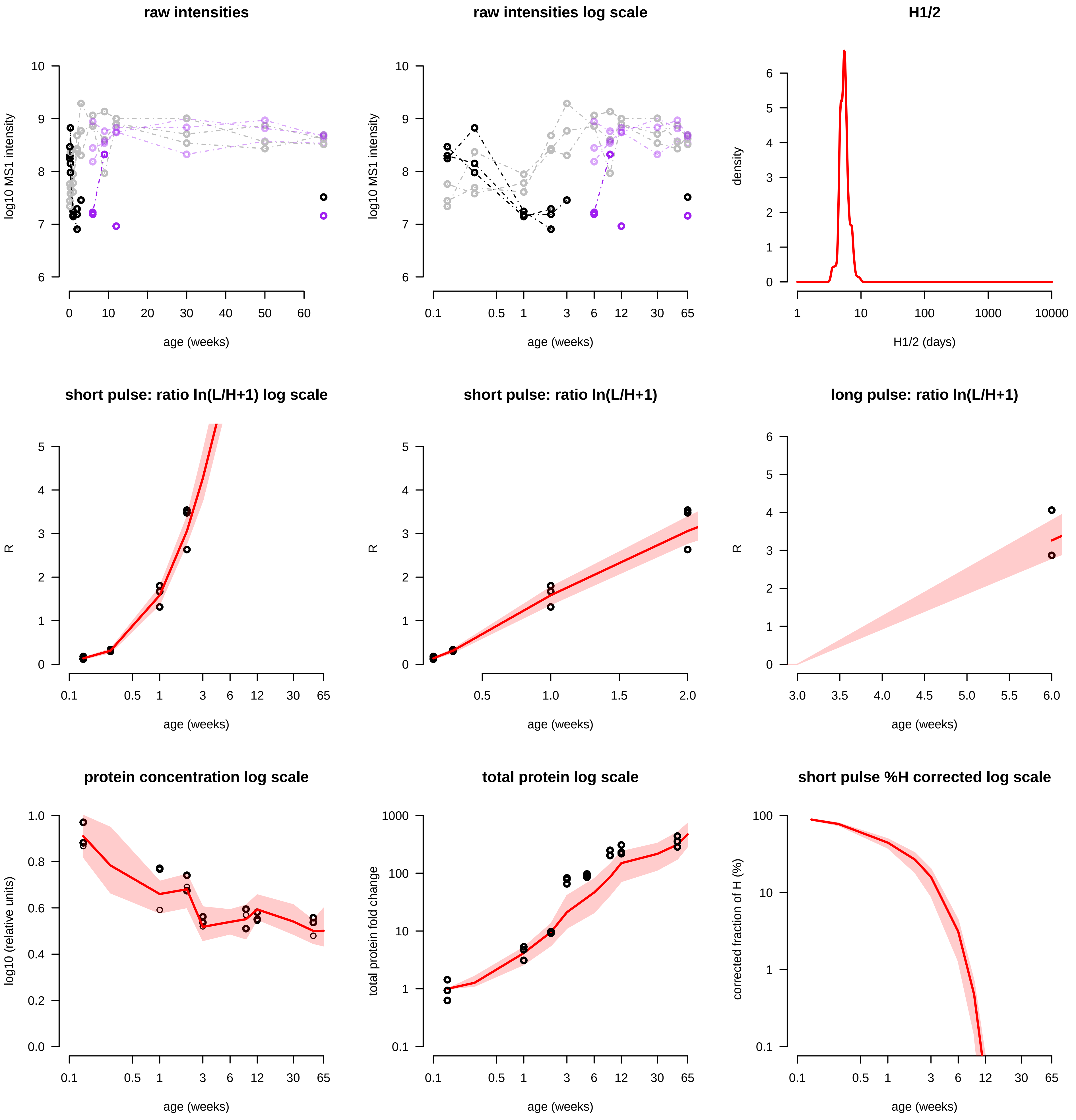

Q9WUL7

>sp|Q9WUL7|ARL3_MOUSE

ADP−ribosylation factor−like protein 3

GN=Arl3

Med H1/2: 14.3 days

Q5 H1/2: 6.7 days

Q95 H1/2: 74.6 days

reliable model fit: yes

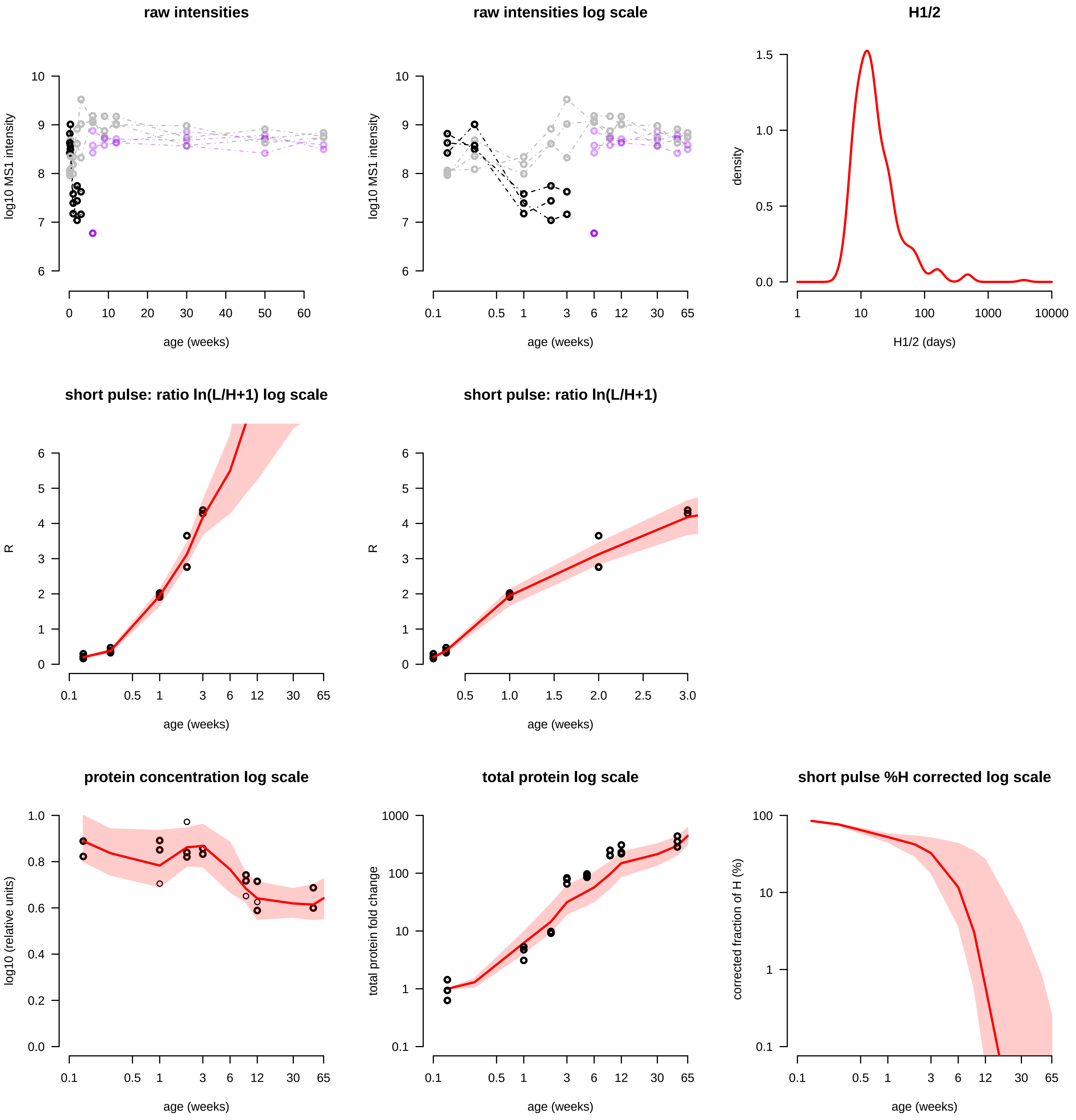

Q9QZ06

>sp|Q9QZ06|TOLIP_MOUSE

Toll−interacting protein

GN=Tollip

Med H1/2: 5.1 days

Q5 H1/2: 4 days

Q95 H1/2: 6.9 days

reliable model fit: yes

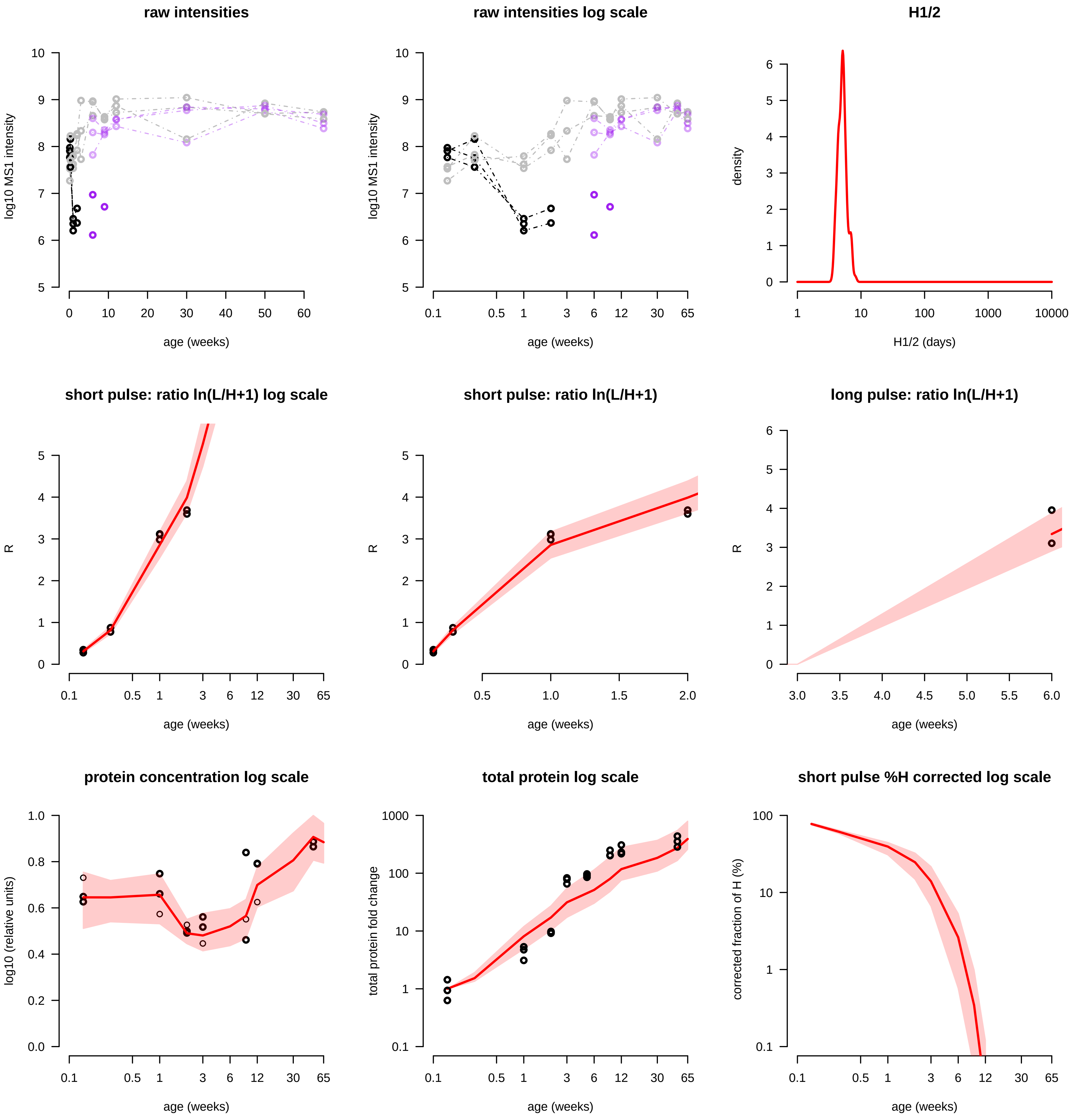

Q3TCH7

>sp|Q3TCH7|CUL4A_MOUSE

Cullin−4A

GN=Cul4a

Med H1/2: 3369.3 days

Q5 H1/2: 959.1 days

Q95 H1/2: 65272 days

reliable model fit: no

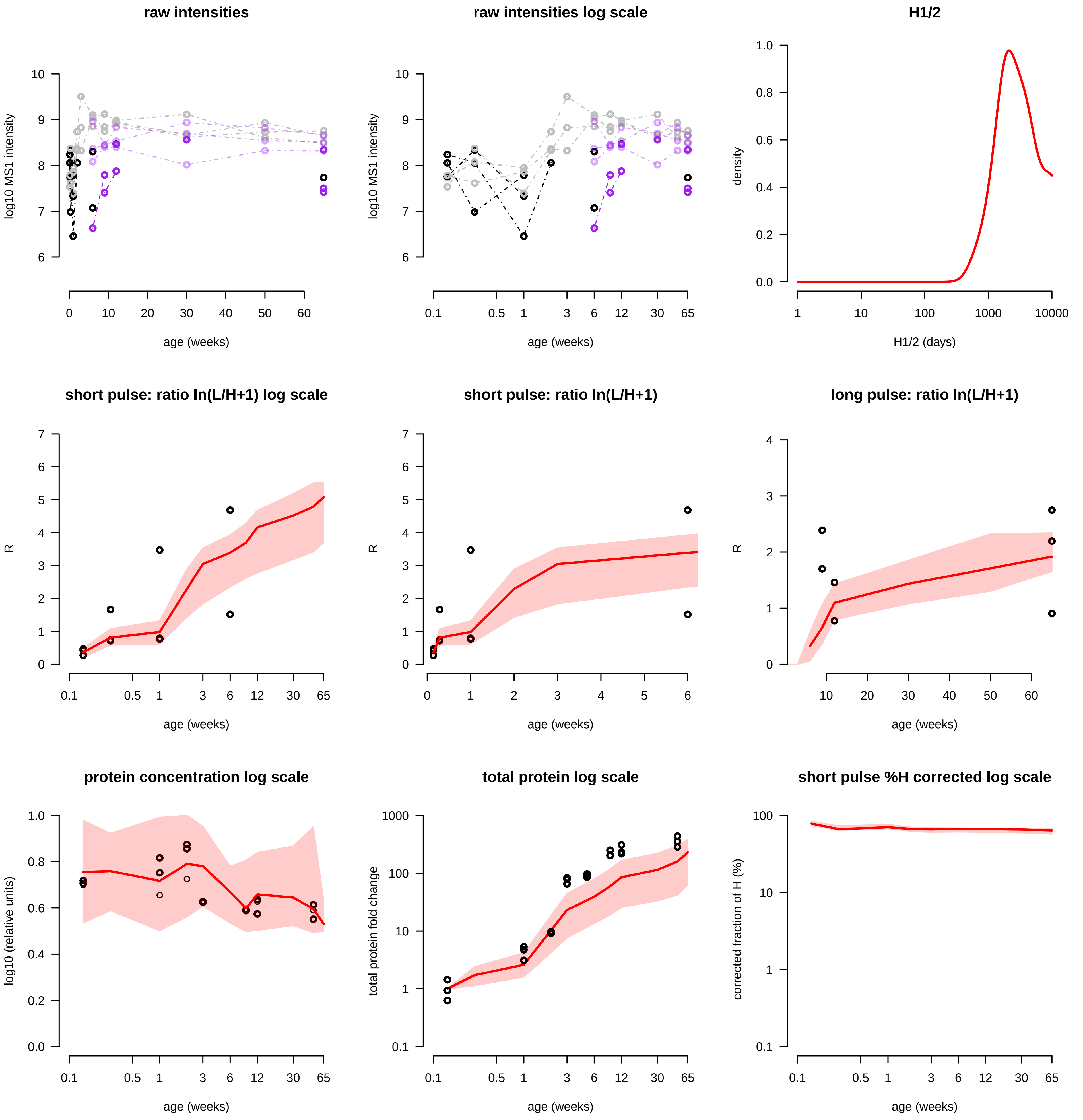

E9PZJ8

>sp|E9PZJ8|ASCC3_MOUSE

Activating signal cointegrator 1 complex subunit 3

GN=Ascc3

Med H1/2: 626.1 days

Q5 H1/2: 209 days

Q95 H1/2: 4718.6 days

reliable model fit: yes

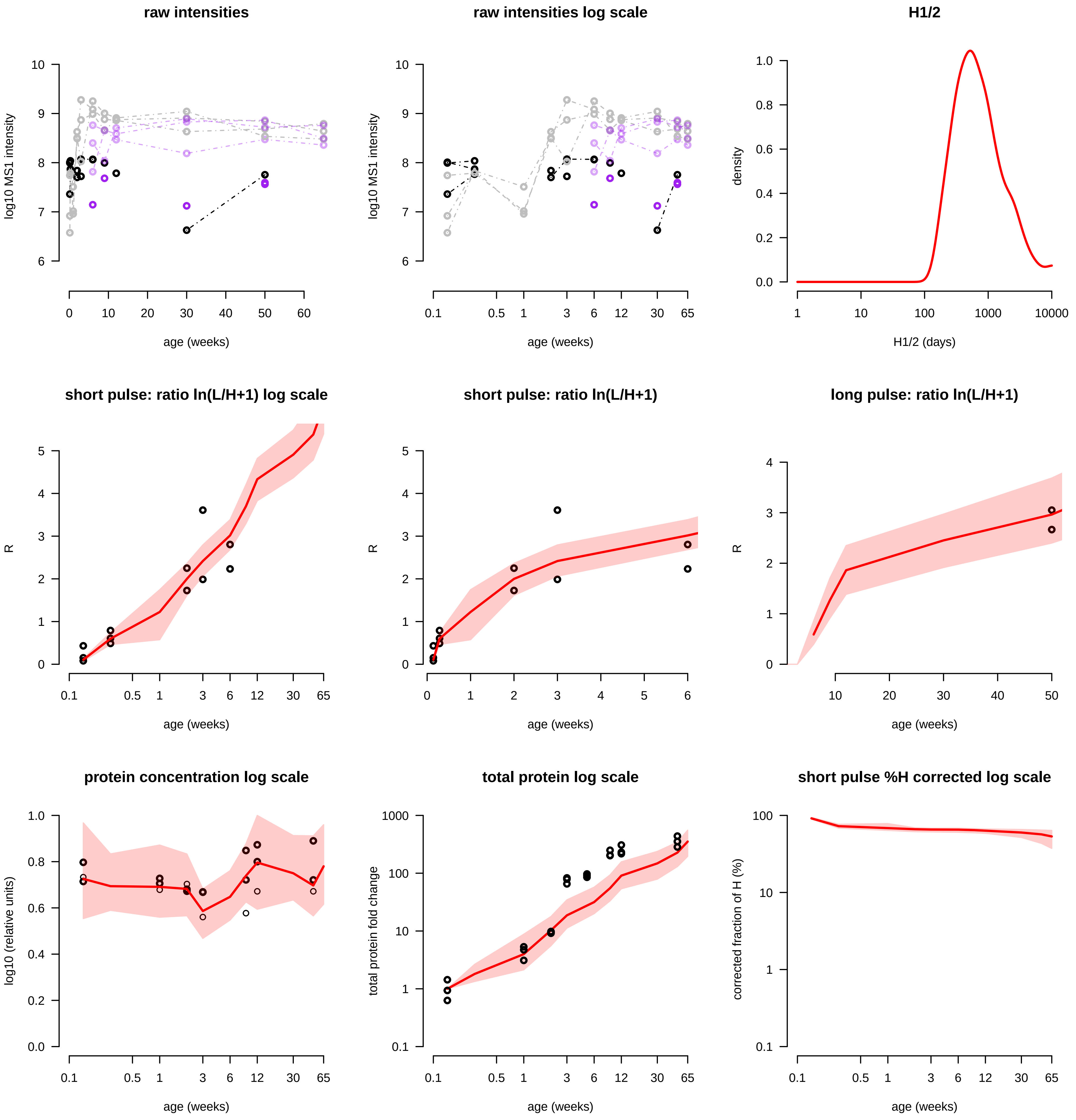

O88456

>sp|O88456|CPNS1_MOUSE

Calpain small subunit 1

GN=Capns1

Med H1/2: 5.9 days

Q5 H1/2: 4.7 days

Q95 H1/2: 8.9 days

reliable model fit: yes

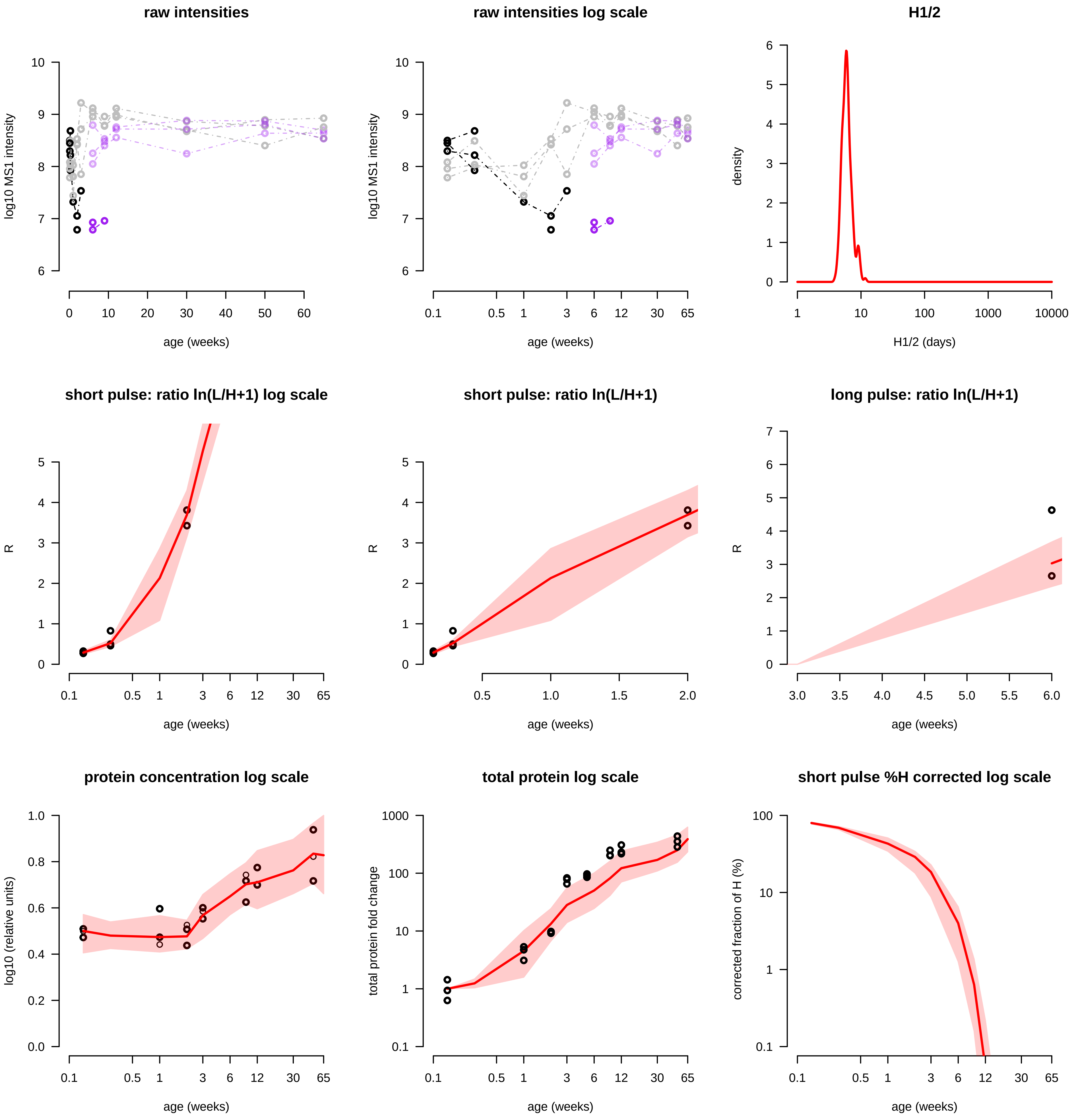

Q80W93

>sp|Q80W93|HYDIN_MOUSE

Hydrocephalus−inducing protein

GN=Hydin

Med H1/2: 685.3 days

Q5 H1/2: 244.8 days

Q95 H1/2: 7352.9 days

reliable model fit: no

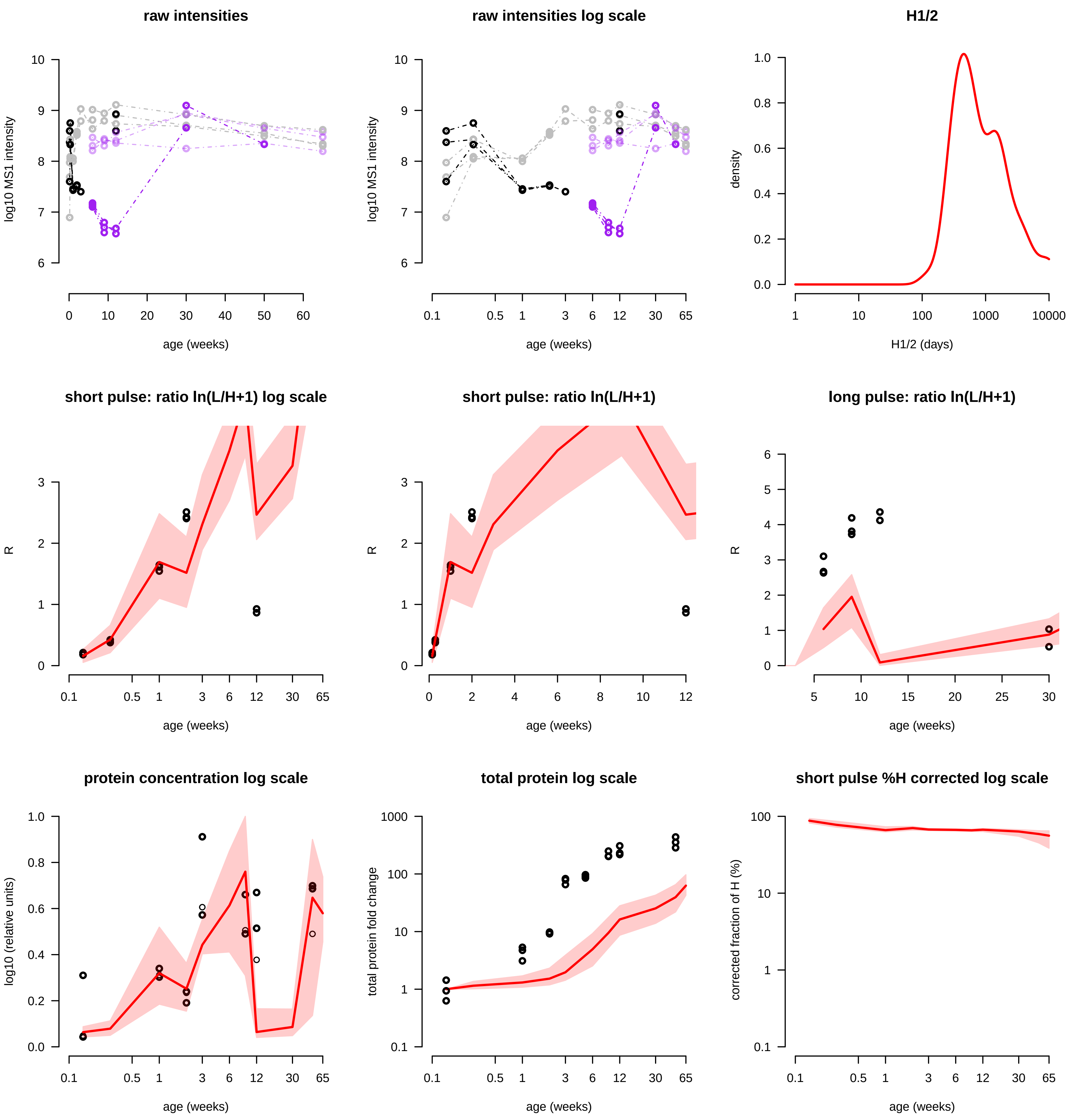

Q99J36

>sp|Q99J36|THUM1_MOUSE

THUMP domain−containing protein 1

GN=Thumpd1

Med H1/2: 4 days

Q5 H1/2: 3.3 days

Q95 H1/2: 5 days

reliable model fit: yes

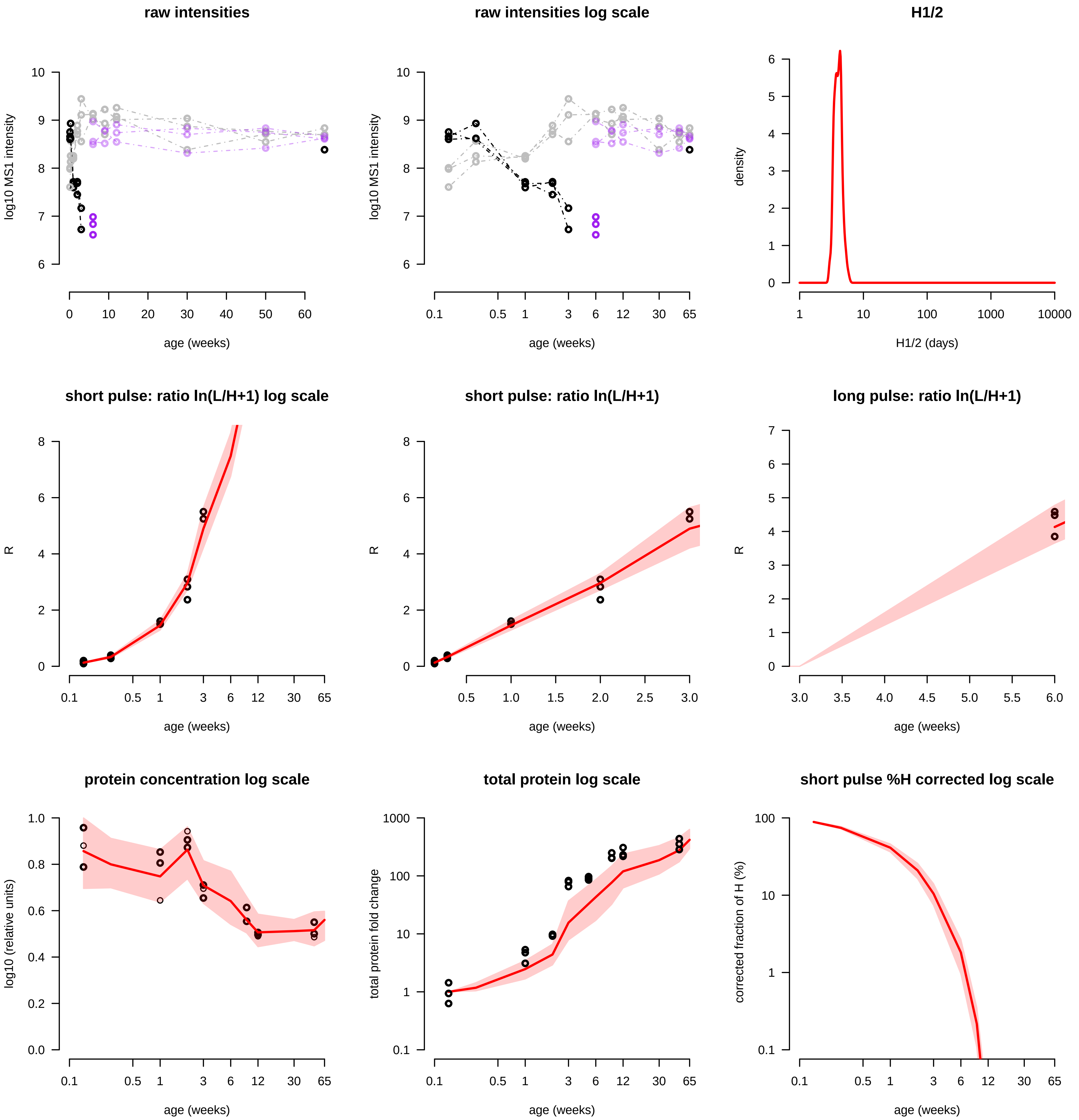

Q9ET01

>sp|Q9ET01|PYGL_MOUSE

Glycogen phosphorylase, liver form

GN=Pygl

Med H1/2: 6.5 days

Q5 H1/2: 3.2 days

Q95 H1/2: 20.1 days

reliable model fit: yes

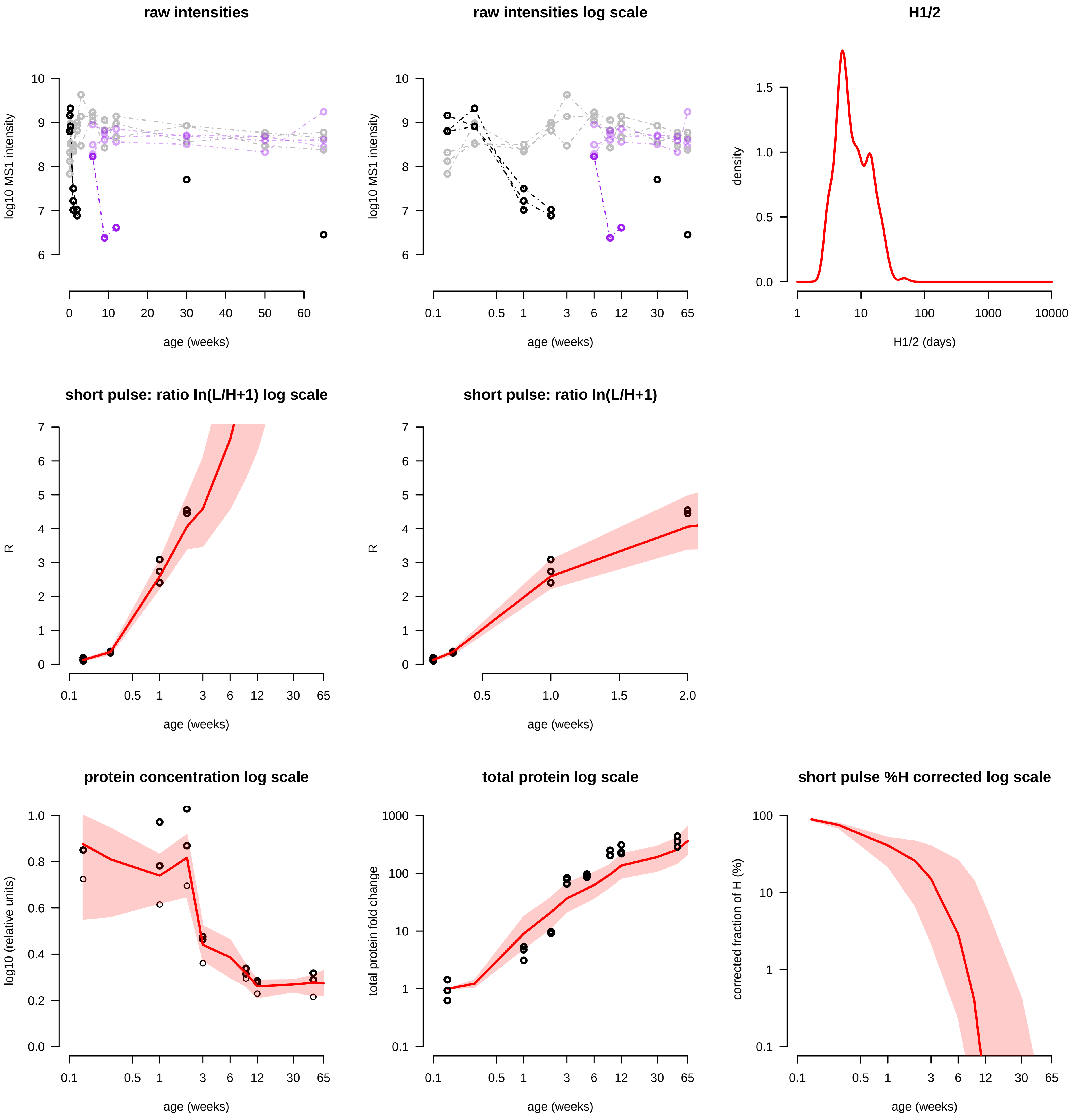

Q91WP6

>sp|Q91WP6|SPA3N_MOUSE

Serine protease inhibitor A3N

GN=Serpina3n

Med H1/2: 3.1 days

Q5 H1/2: 2.5 days

Q95 H1/2: 5.3 days

reliable model fit: yes

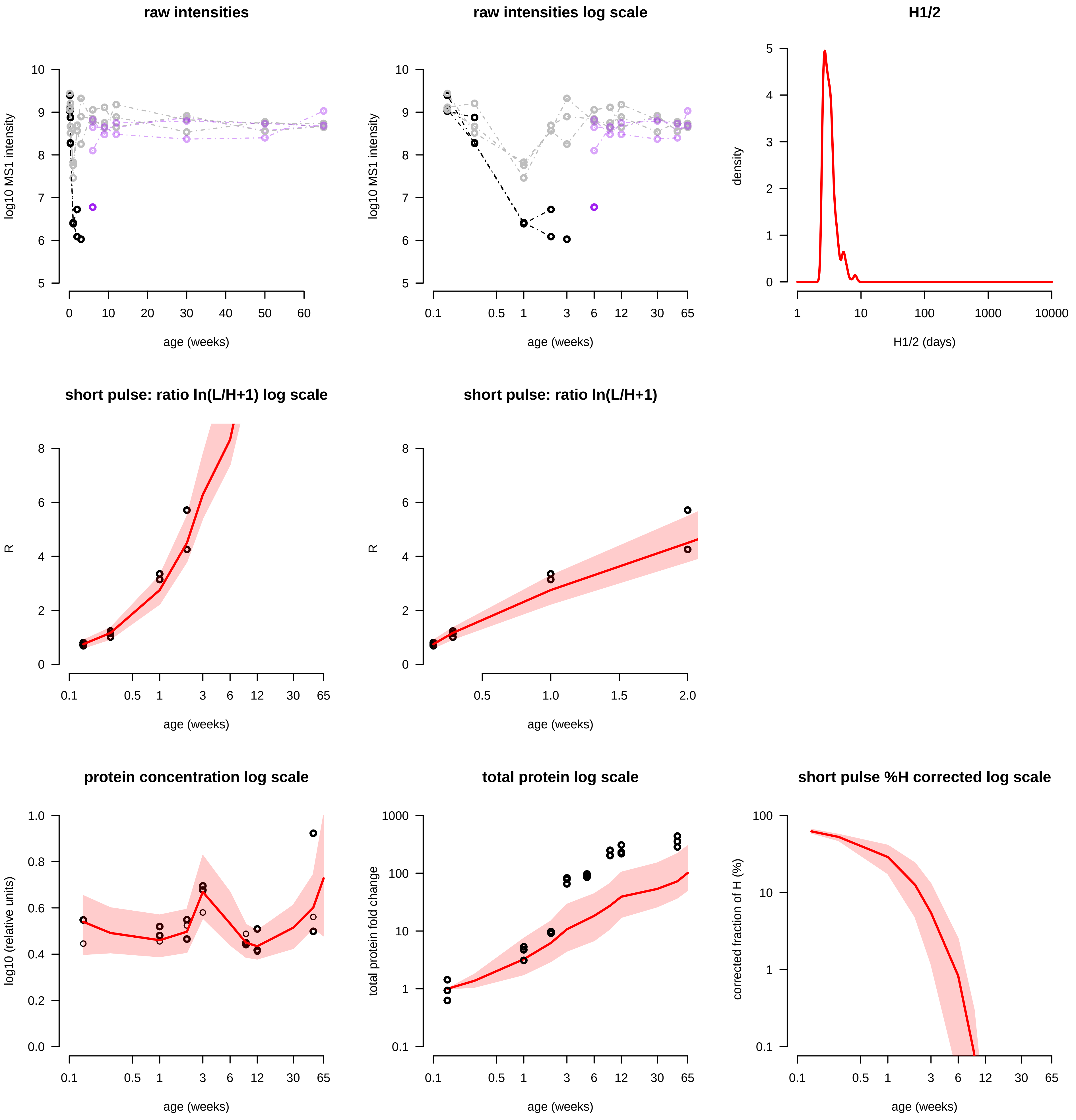

Q9EPL8

>sp|Q9EPL8|IPO7_MOUSE

Importin−7

GN=Ipo7

Med H1/2: 3.9 days

Q5 H1/2: 3.2 days

Q95 H1/2: 4.9 days

reliable model fit: yes

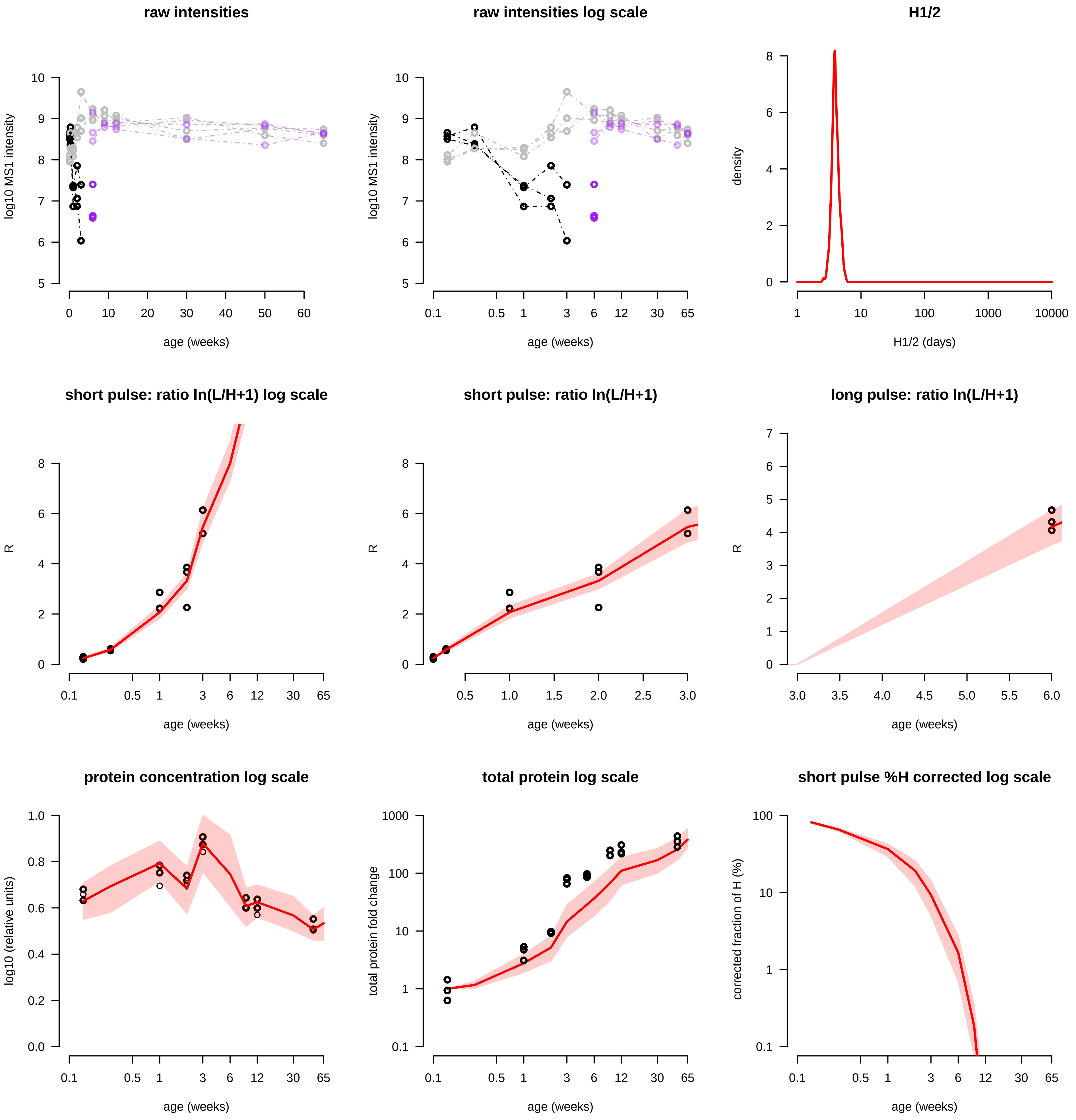

Q6NV83−2;Q6NV83

>sp|Q6NV83−2|SR140_MOUSE

Isoform 2 of U2 snRNP−associated SURP motif−containing protein

GN=U2surp

Med H1/2: 70.2 days

Q5 H1/2: 27.6 days

Q95 H1/2: 284.5 days

reliable model fit: no

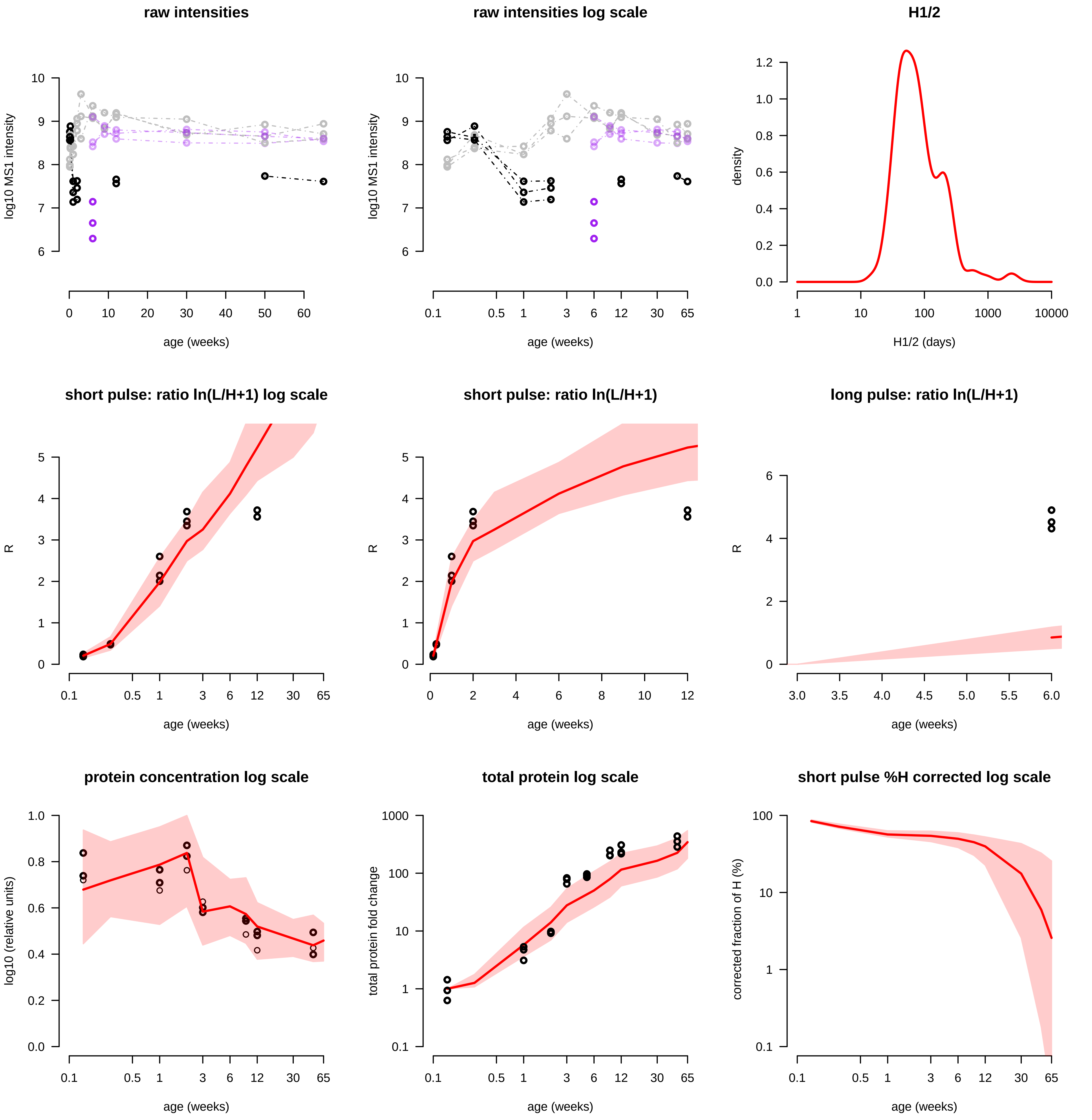

P12265

>sp|P12265|BGLR_MOUSE

Beta−glucuronidase

GN=Gusb

Med H1/2: 4.1 days

Q5 H1/2: 3.2 days

Q95 H1/2: 5.5 days

reliable model fit: yes

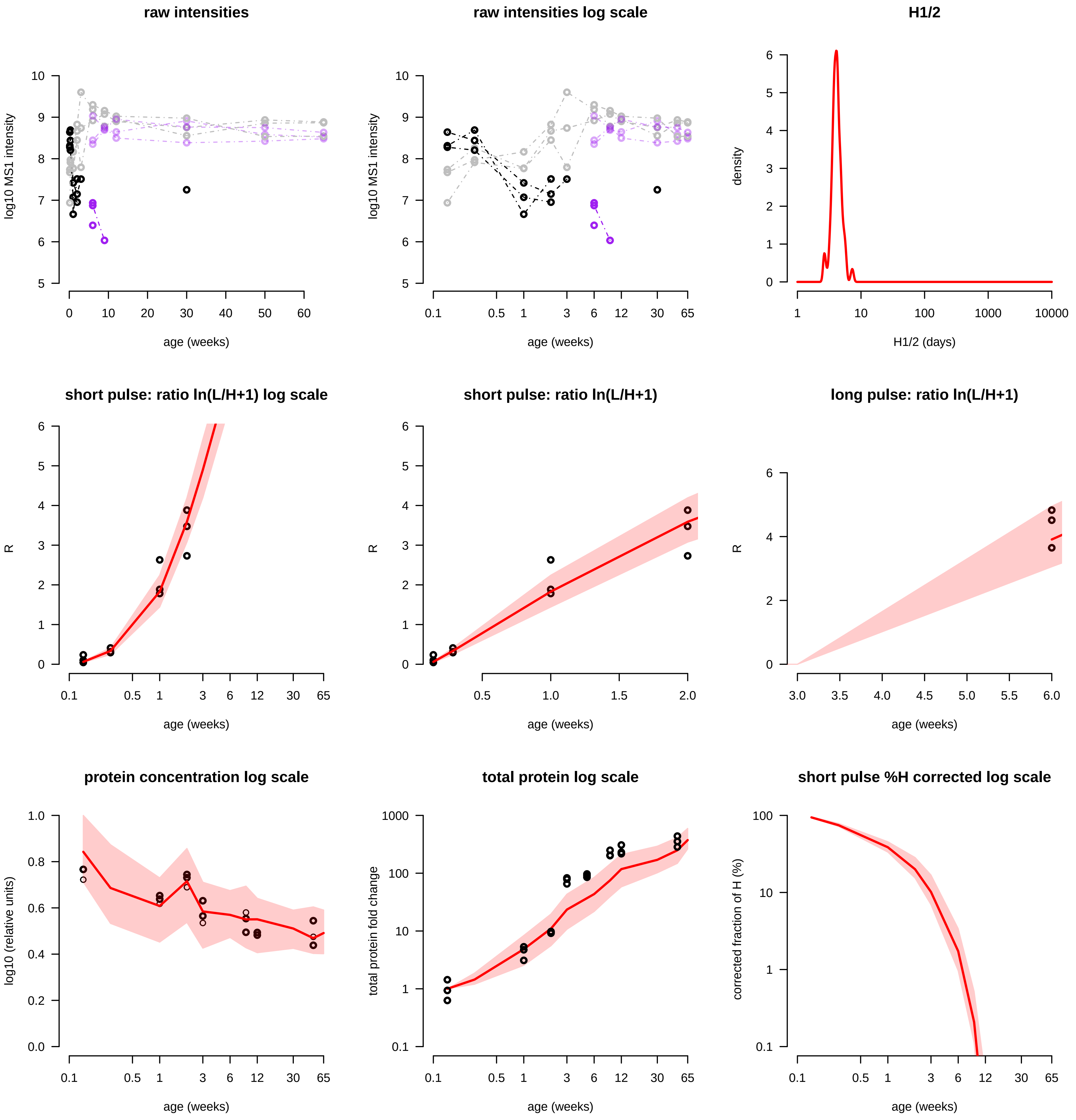

Q9EP72

>sp|Q9EP72|EMC7_MOUSE

ER membrane protein complex subunit 7

GN=Emc7

Med H1/2: 15.5 days

Q5 H1/2: 6.1 days

Q95 H1/2: 122 days

reliable model fit: yes

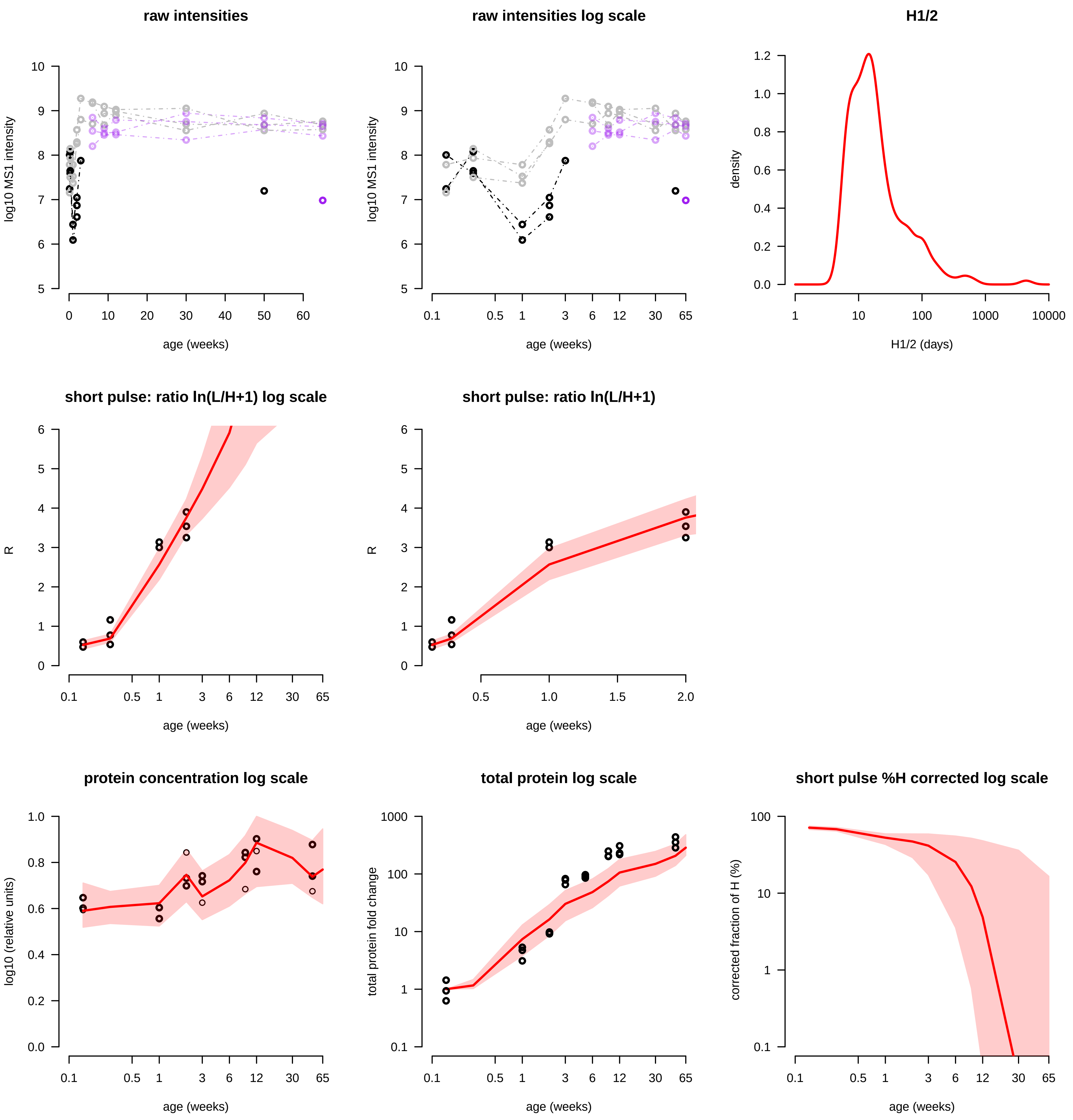

Q3UJB9

>sp|Q3UJB9|EDC4_MOUSE

Enhancer of mRNA−decapping protein 4

GN=Edc4

Med H1/2: 3.1 days

Q5 H1/2: 2.6 days

Q95 H1/2: 3.9 days

reliable model fit: yes

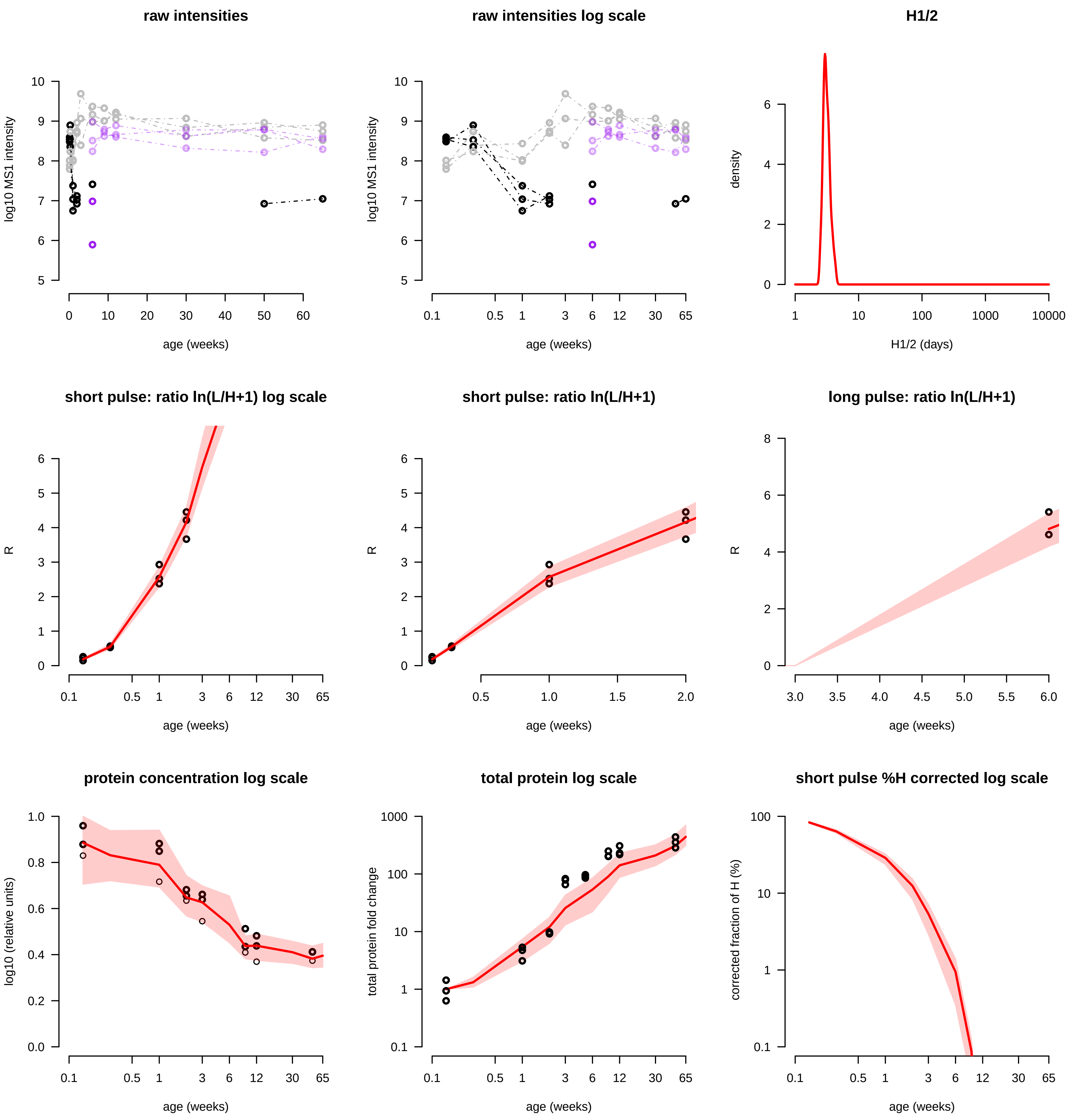

Q62186

>sp|Q62186|SSRD_MOUSE

Translocon−associated protein subunit delta

GN=Ssr4

Med H1/2: 5.1 days

Q5 H1/2: 4.4 days

Q95 H1/2: 6 days

reliable model fit: yes

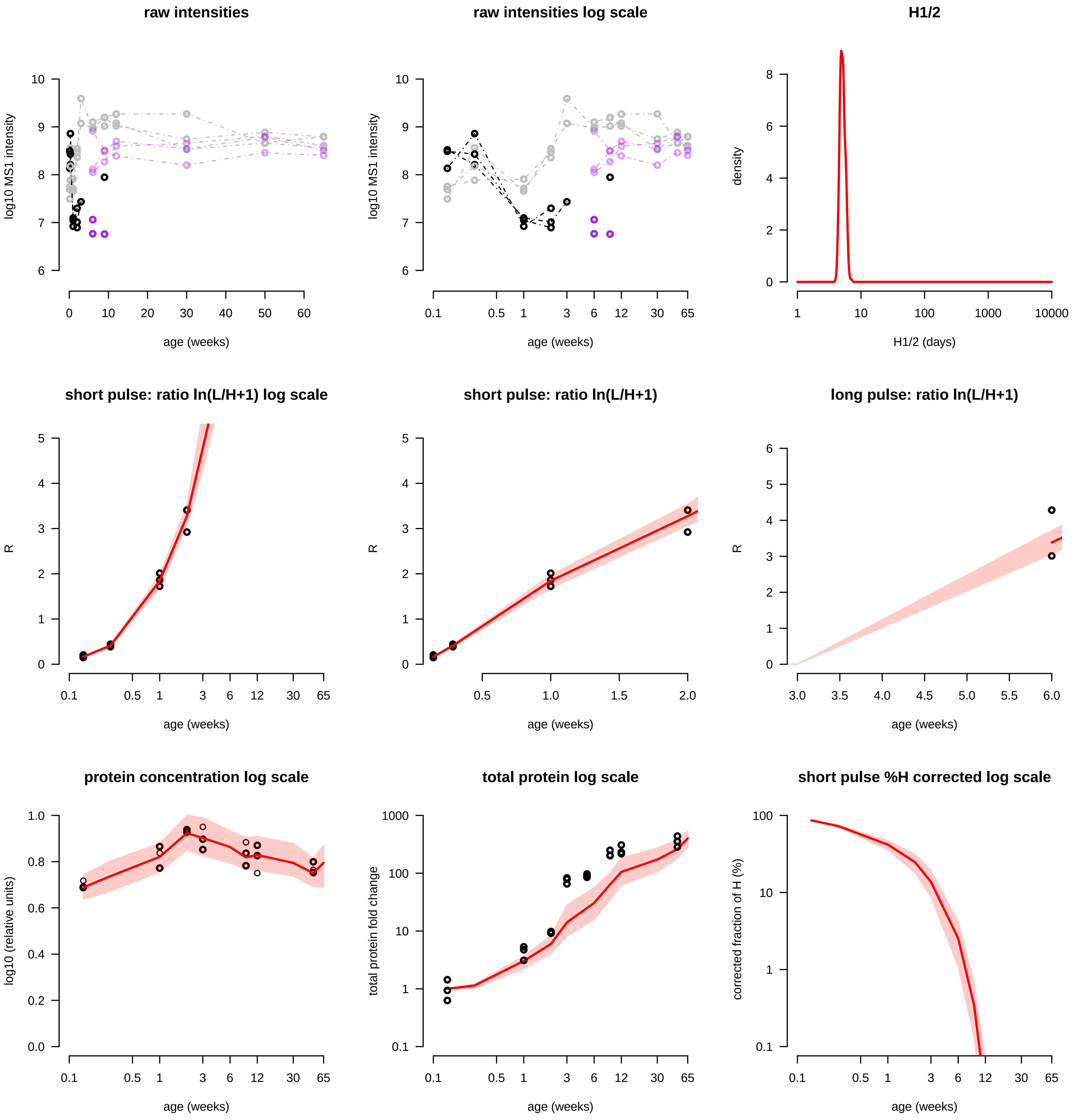

P56812

>sp|P56812|PDCD5_MOUSE

Programmed cell death protein 5

GN=Pdcd5

Med H1/2: 9.7 days

Q5 H1/2: 8.1 days

Q95 H1/2: 11.3 days

reliable model fit: yes

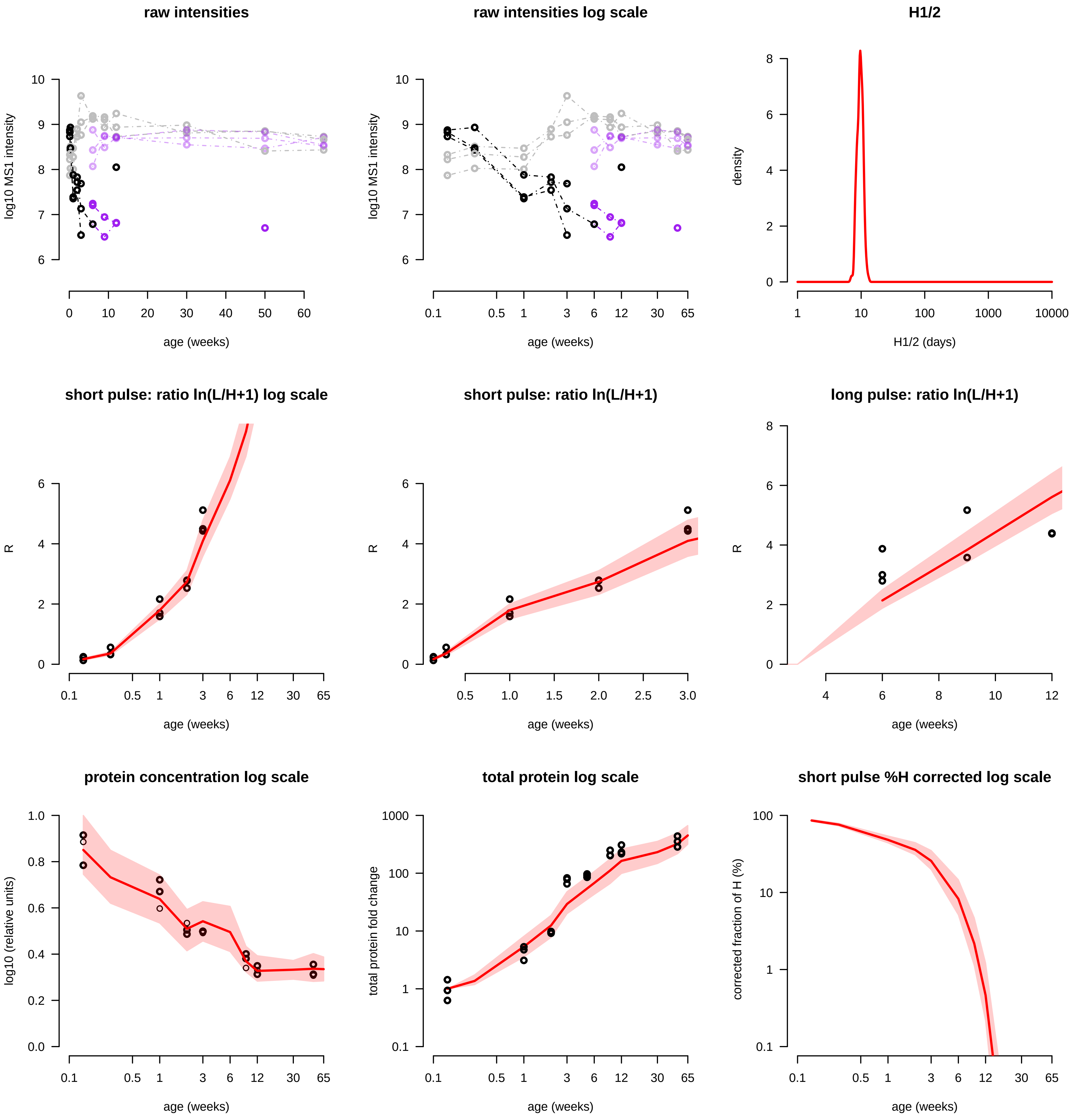

O08539

>sp|O08539|BIN1_MOUSE

Myc box−dependent−interacting protein 1

GN=Bin1

Med H1/2: 23.1 days

Q5 H1/2: 11 days

Q95 H1/2: 183.4 days

reliable model fit: no

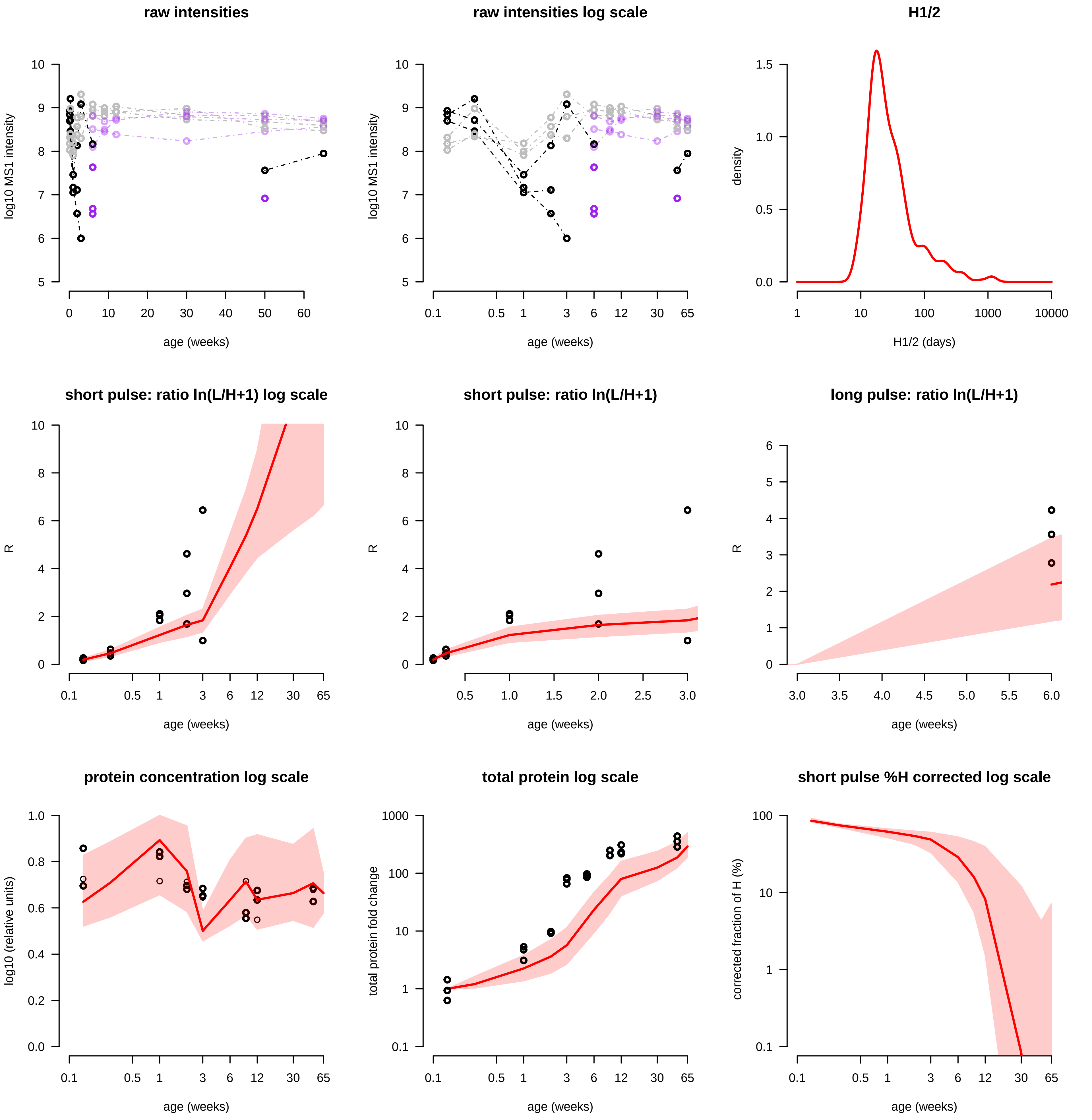

Q3UNZ8;Q3UNZ8−2

>sp|Q3UNZ8|QORL2_MOUSE

Quinone oxidoreductase−like protein 2

GN=Cryzl2

Med H1/2: 15.3 days

Q5 H1/2: 5.4 days

Q95 H1/2: 233.2 days

reliable model fit: yes

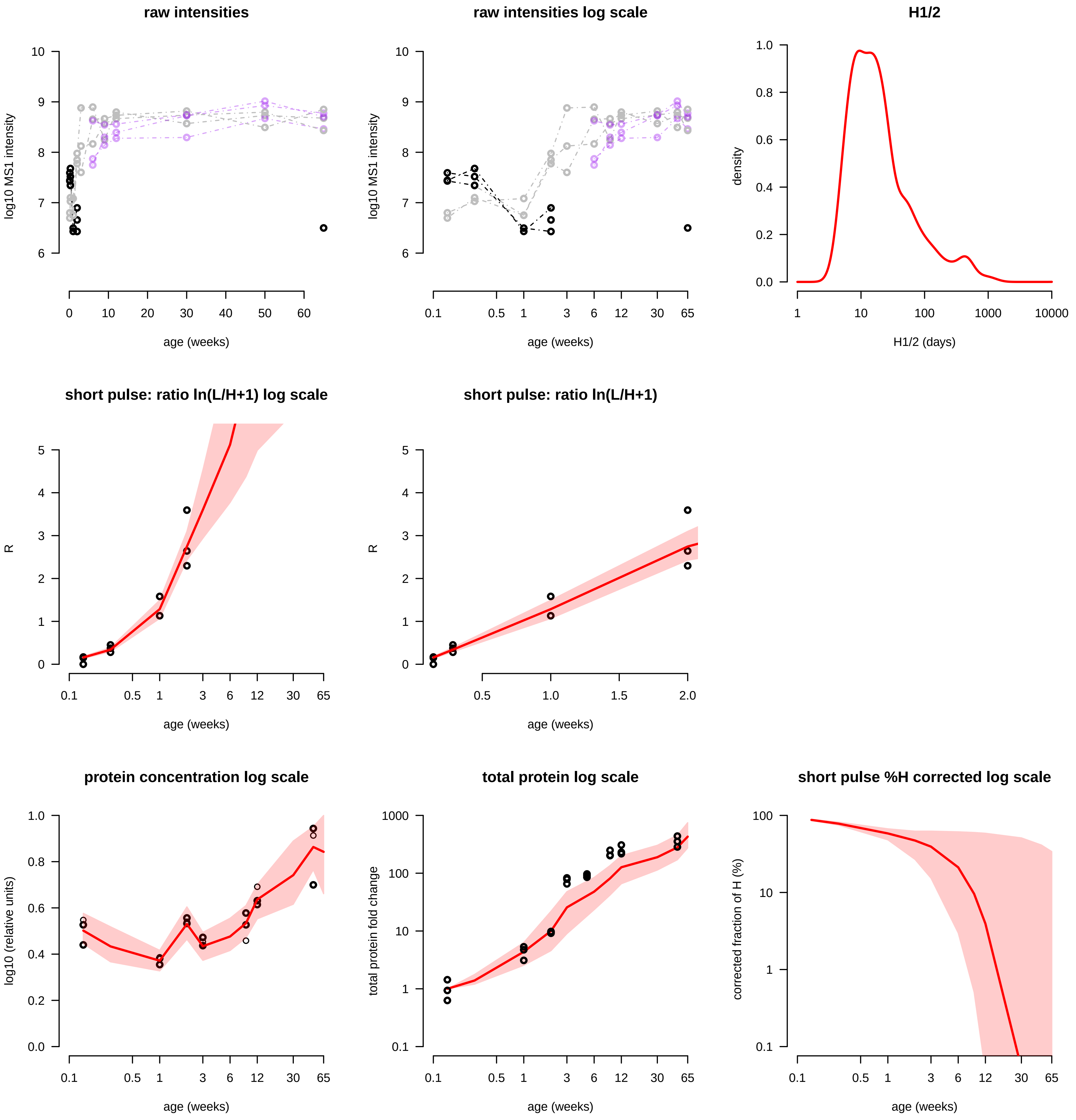

Q9EPK7;Q9EPK7−2

>sp|Q9EPK7|XPO7_MOUSE

Exportin−7

GN=Xpo7

Med H1/2: 186.6 days

Q5 H1/2: 53.2 days

Q95 H1/2: 2743.5 days

reliable model fit: no

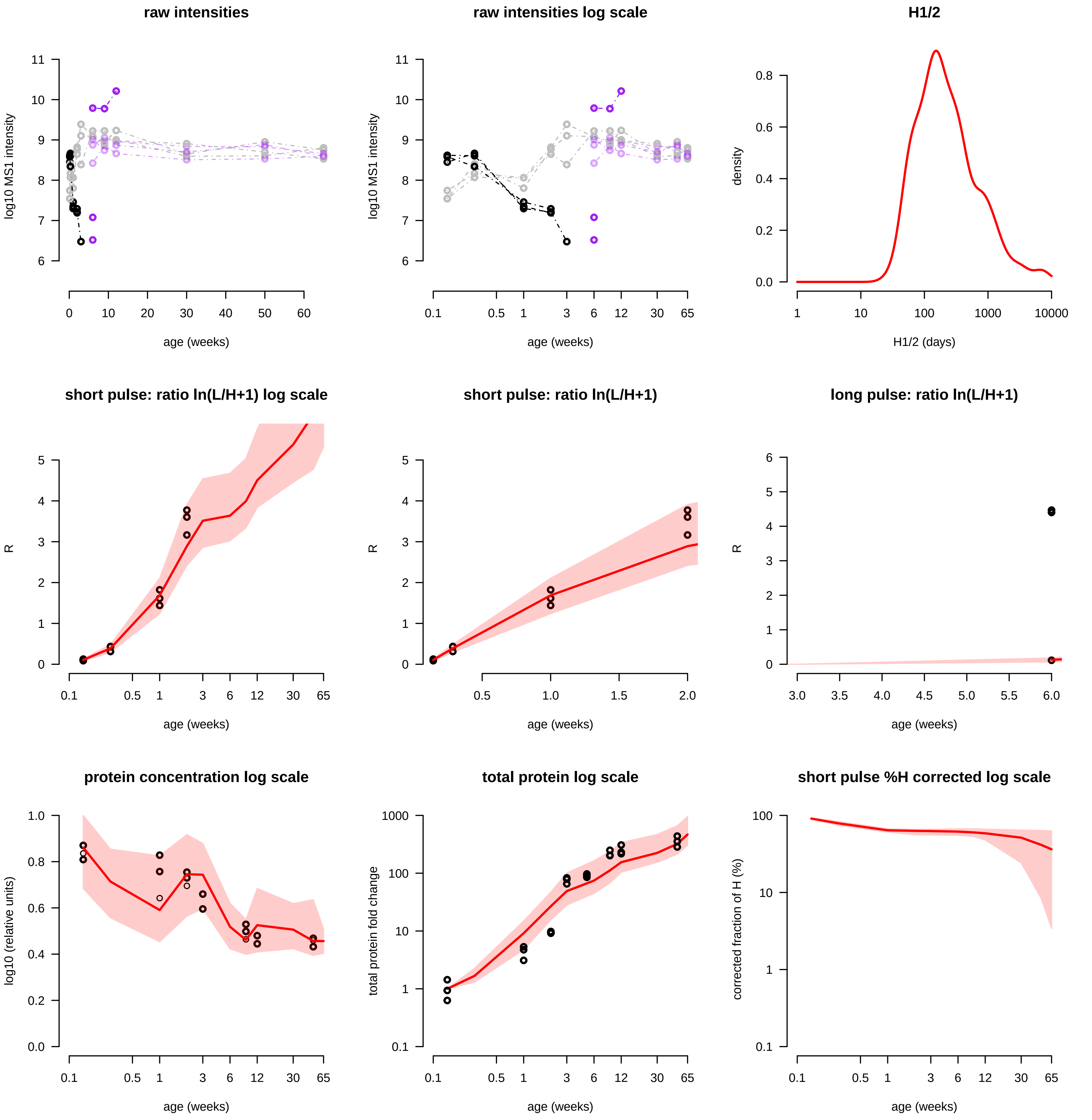

Q3UMR5;Q3UMR5−2

>sp|Q3UMR5|MCU_MOUSE

Calcium uniporter protein, mitochondrial

GN=Mcu

Med H1/2: 10 days

Q5 H1/2: 4.1 days

Q95 H1/2: 40.3 days

reliable model fit: yes

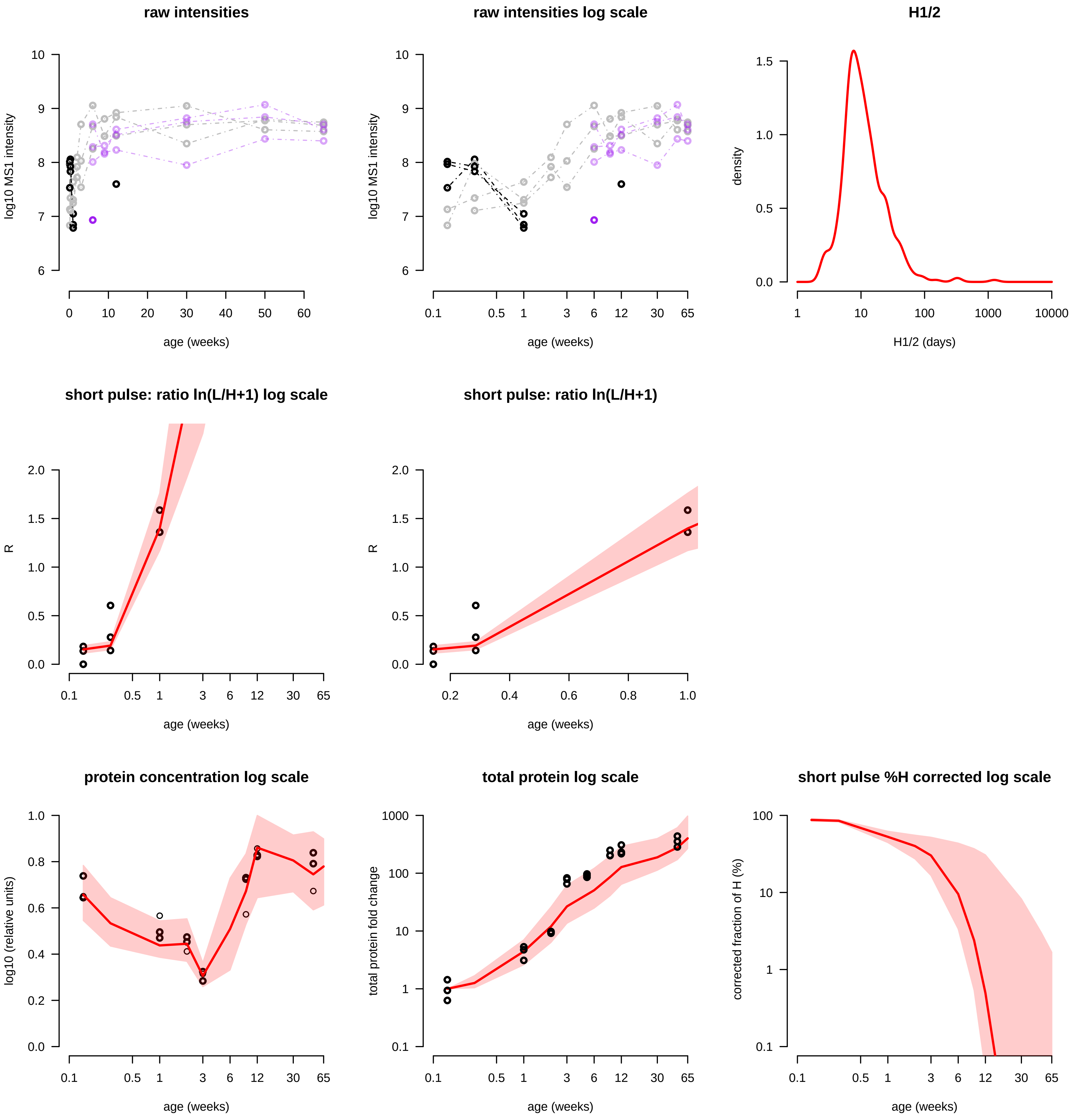

P28667

>sp|P28667|MRP_MOUSE

MARCKS−related protein

GN=Marcksl1

Med H1/2: 4.6 days

Q5 H1/2: 3.7 days

Q95 H1/2: 5.7 days

reliable model fit: yes

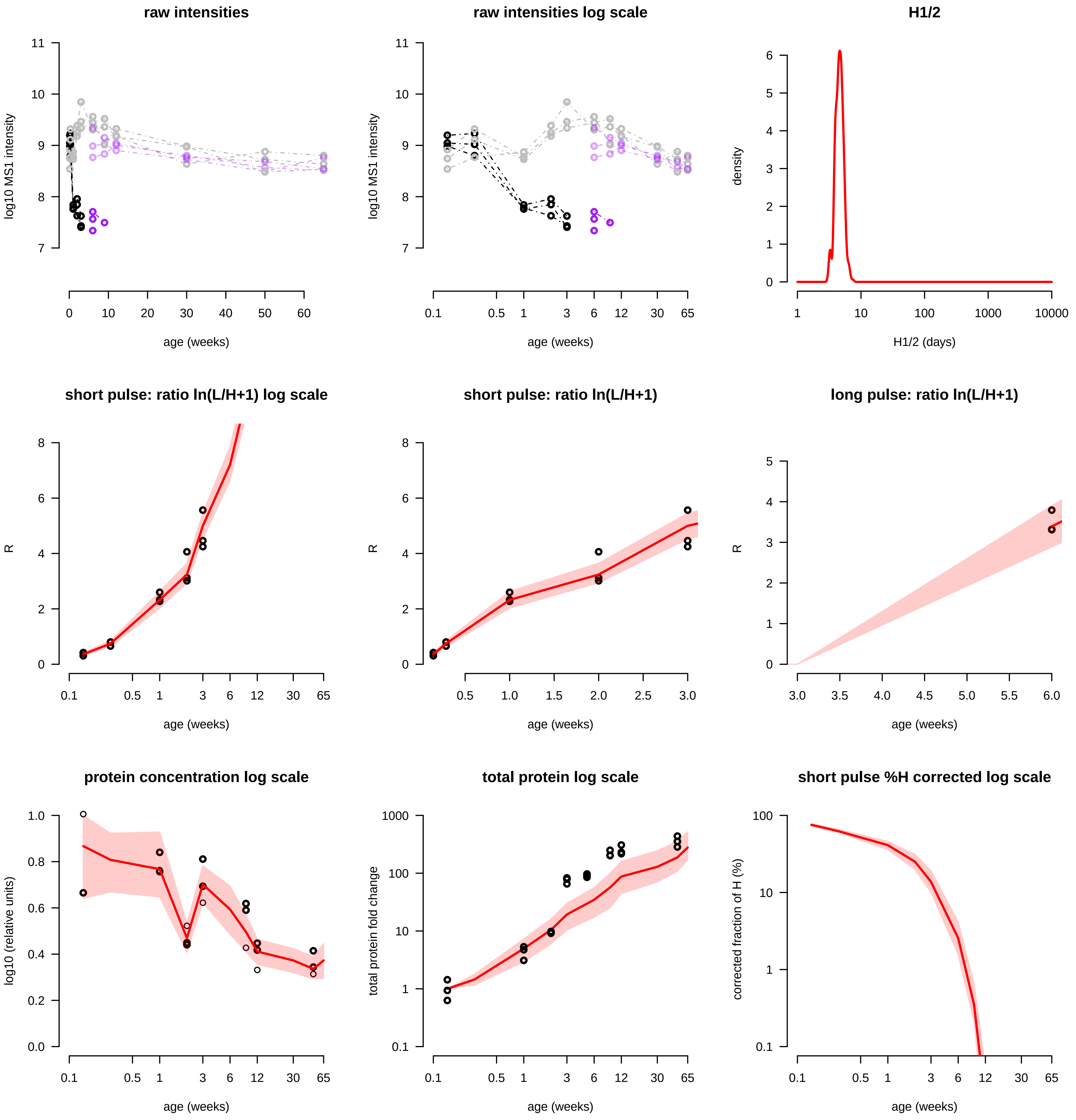

Q9R1C7;Q9R1C7−2

>sp|Q9R1C7|PR40A_MOUSE

Pre−mRNA−processing factor 40 homolog A

GN=Prpf40a

Med H1/2: 4.7 days

Q5 H1/2: 3.9 days

Q95 H1/2: 6 days

reliable model fit: yes

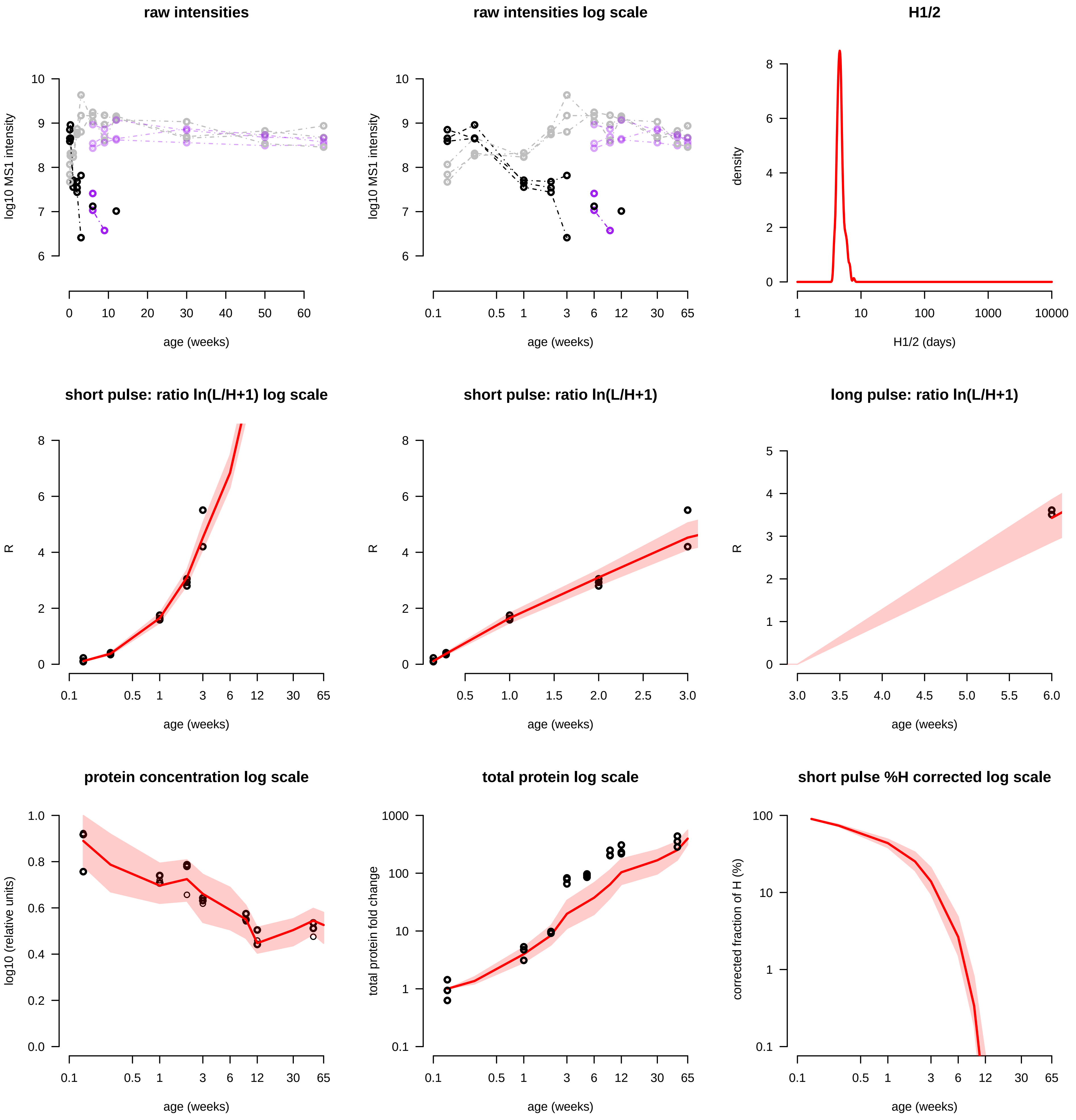

O35691

>sp|O35691|PININ_MOUSE

Pinin

GN=Pnn

Med H1/2: 4.9 days

Q5 H1/2: 4.3 days

Q95 H1/2: 5.9 days

reliable model fit: yes

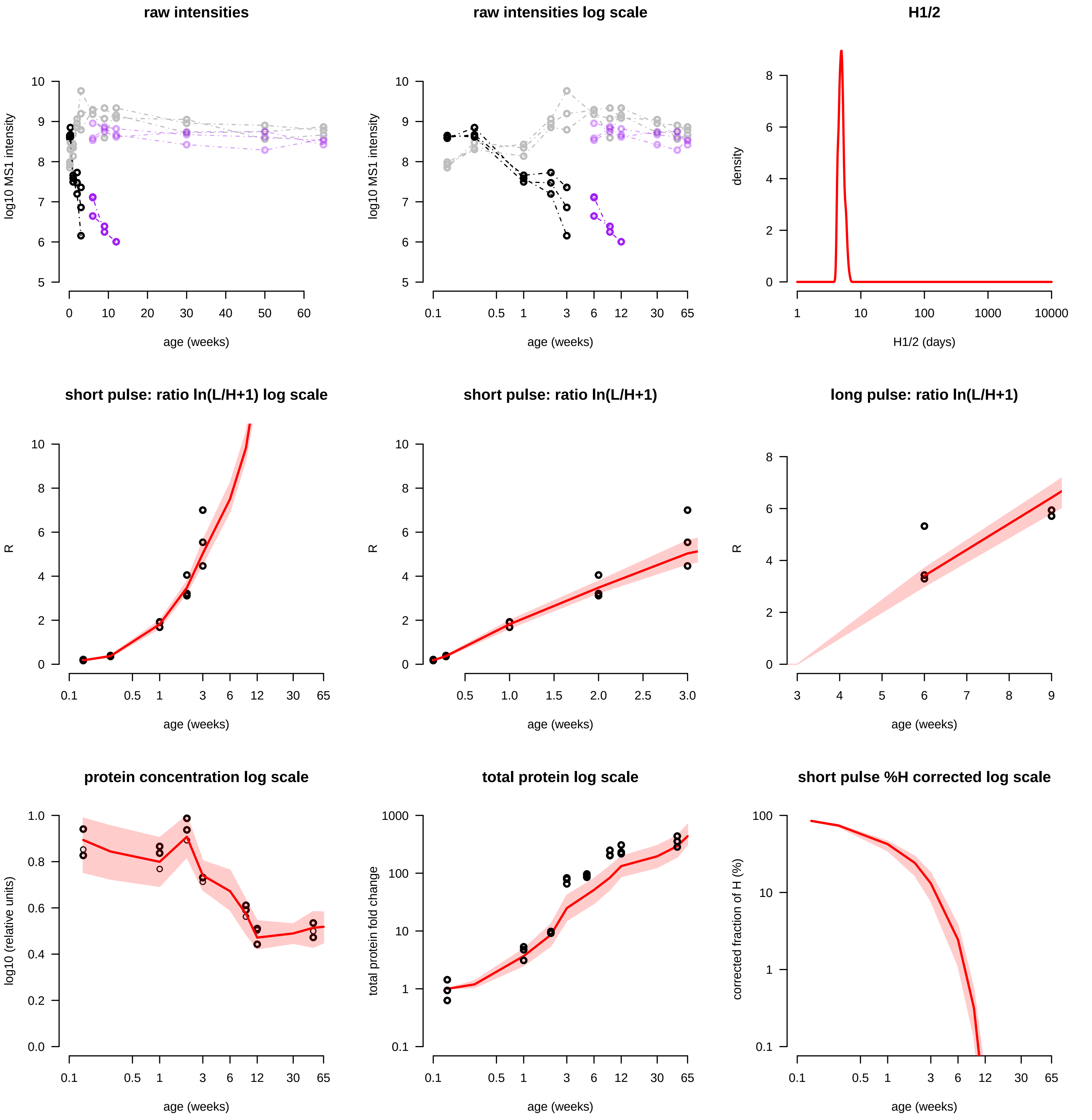

O88322

>sp|O88322|NID2_MOUSE

Nidogen−2

GN=Nid2

Med H1/2: 7.2 days

Q5 H1/2: 3.5 days

Q95 H1/2: 32.3 days

reliable model fit: yes

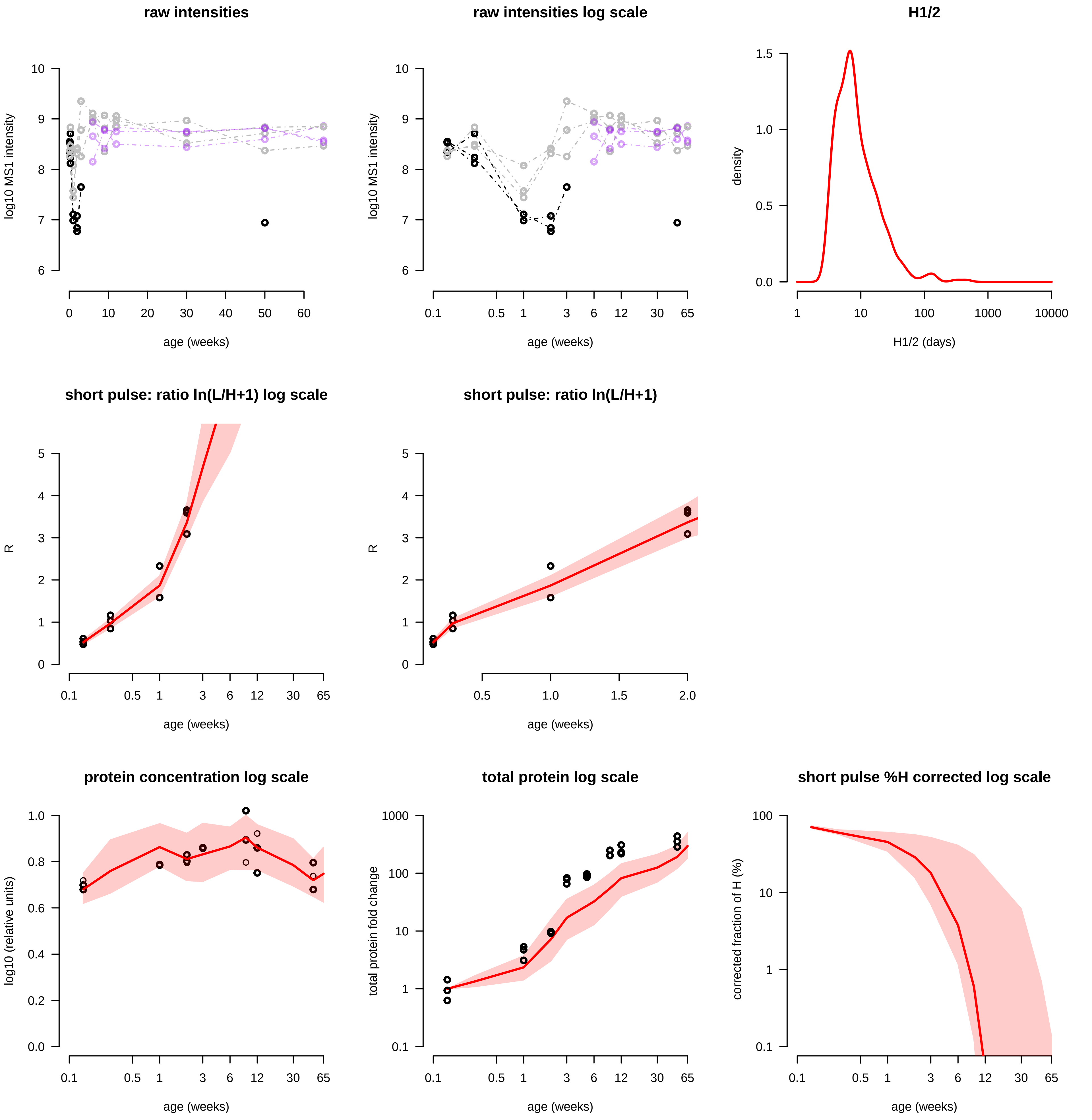

Q8BH69

>sp|Q8BH69|SPS1_MOUSE

Selenide, water dikinase 1

GN=Sephs1

Med H1/2: 9.4 days

Q5 H1/2: 7 days

Q95 H1/2: 15.5 days

reliable model fit: yes

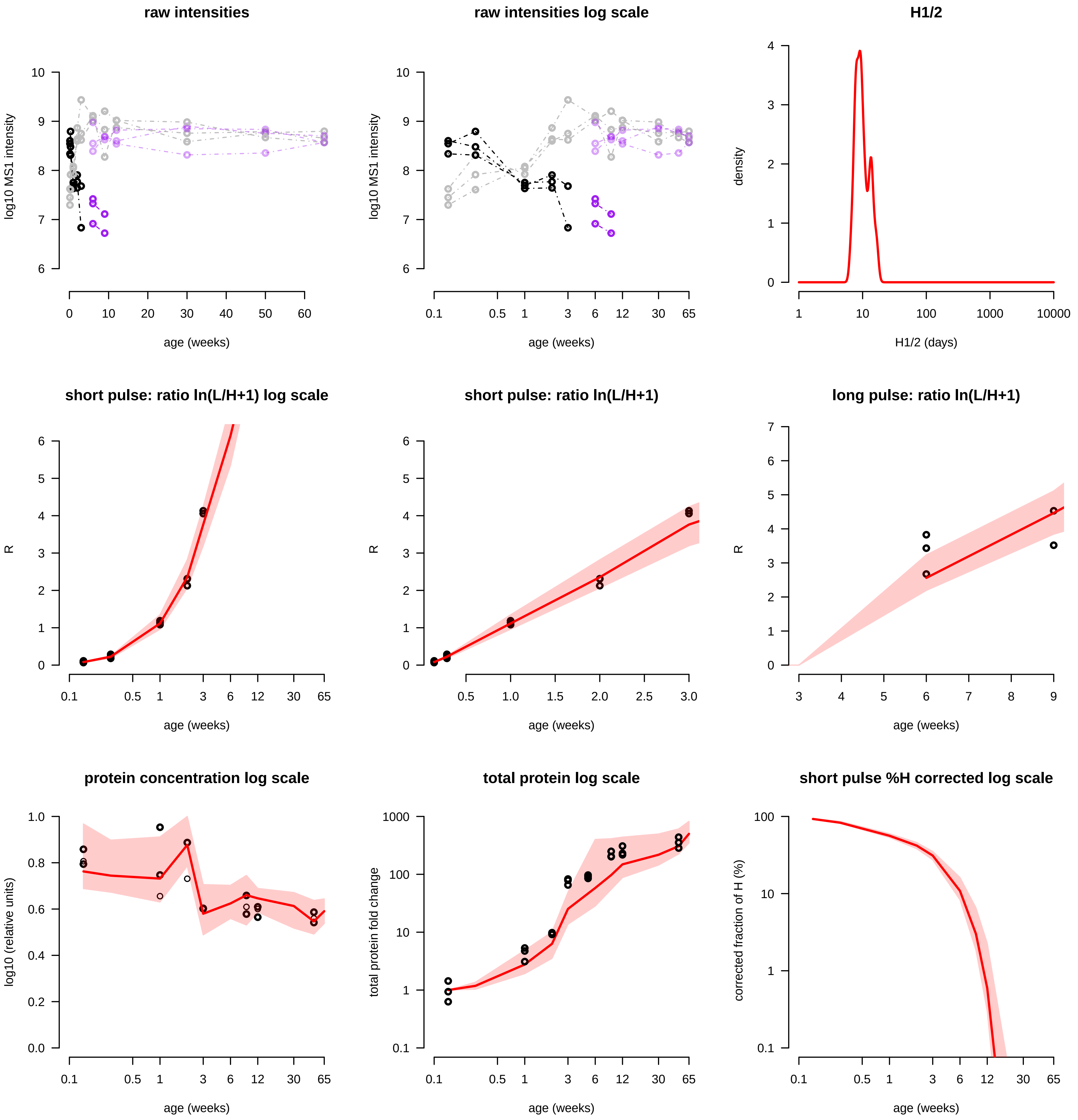

Q8VE37

>sp|Q8VE37|RCC1_MOUSE

Regulator of chromosome condensation

GN=Rcc1

Med H1/2: 431.6 days

Q5 H1/2: 150.3 days

Q95 H1/2: 7745.3 days

reliable model fit: yes

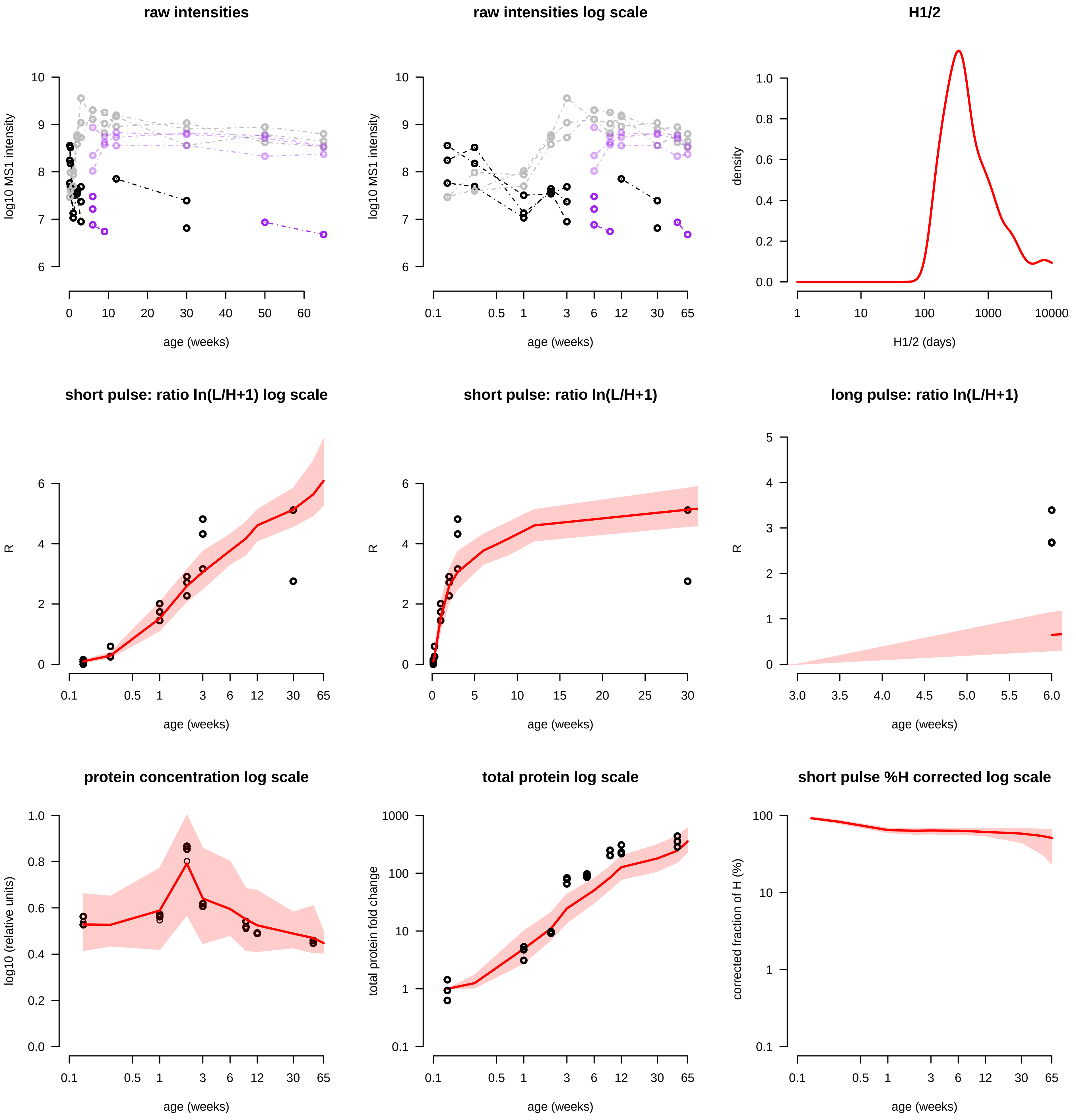

Q9DCH4

>sp|Q9DCH4|EIF3F_MOUSE

Eukaryotic translation initiation factor 3 subunit F

GN=Eif3f

Med H1/2: 831.4 days

Q5 H1/2: 265.6 days

Q95 H1/2: 5592 days

reliable model fit: no

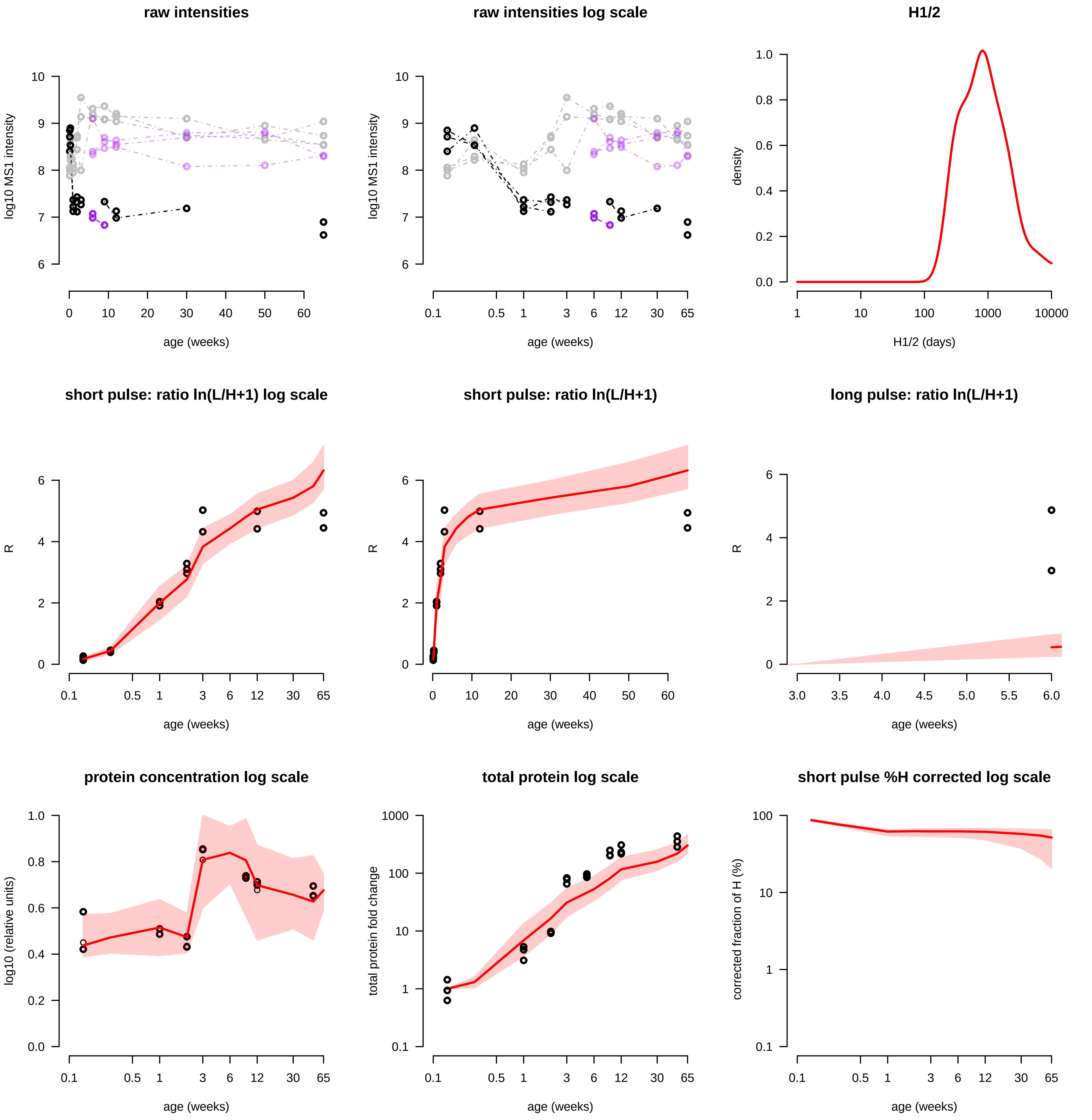

Q8BIJ7

>sp|Q8BIJ7|RUFY1_MOUSE

RUN and FYVE domain−containing protein 1

GN=Rufy1

Med H1/2: 18.9 days

Q5 H1/2: 8.9 days

Q95 H1/2: 101.3 days

reliable model fit: yes

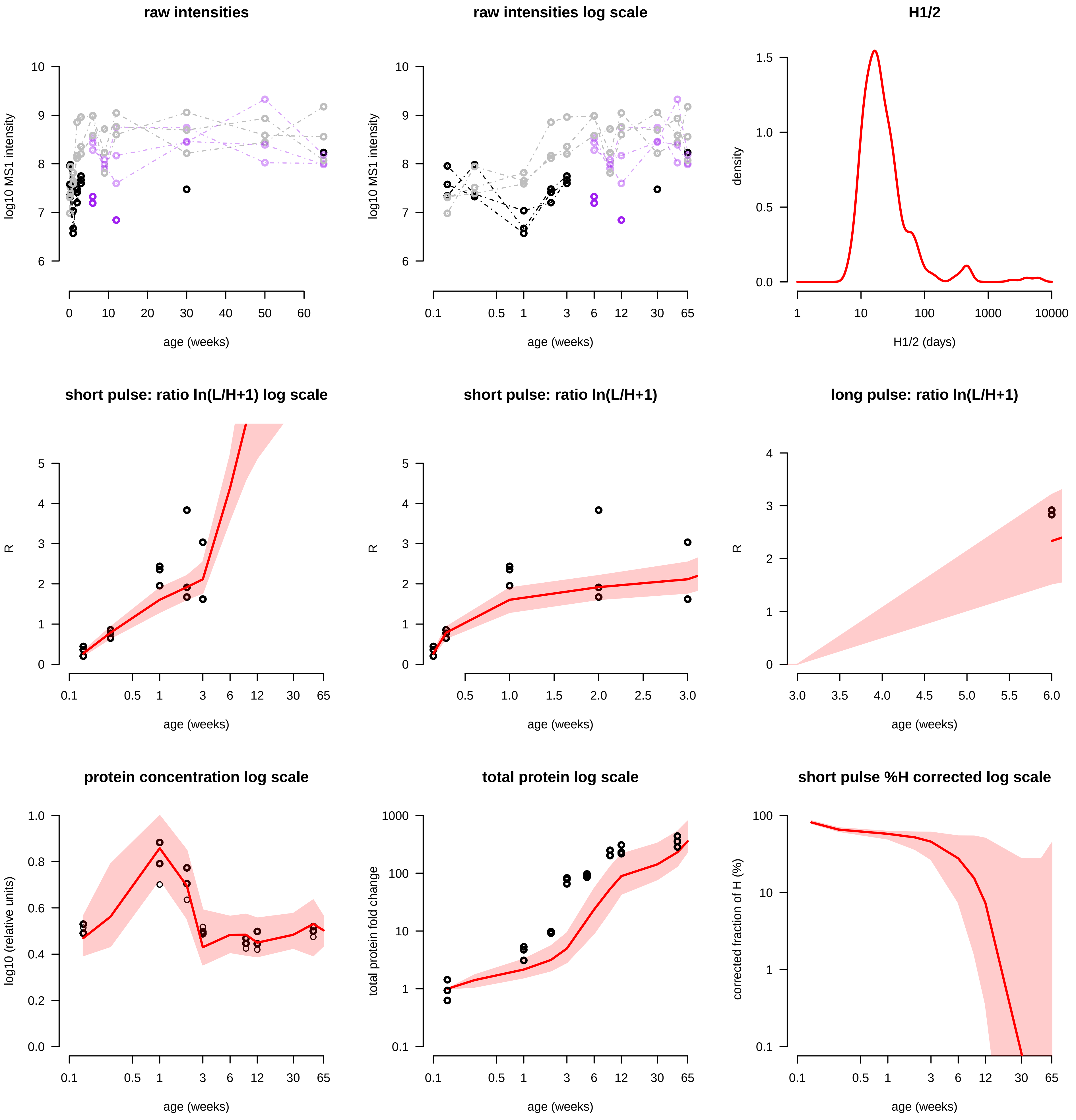

P58021

>sp|P58021|TM9S2_MOUSE

Transmembrane 9 superfamily member 2

GN=Tm9sf2

Med H1/2: 16.2 days

Q5 H1/2: 4.9 days

Q95 H1/2: 146.4 days

reliable model fit: yes

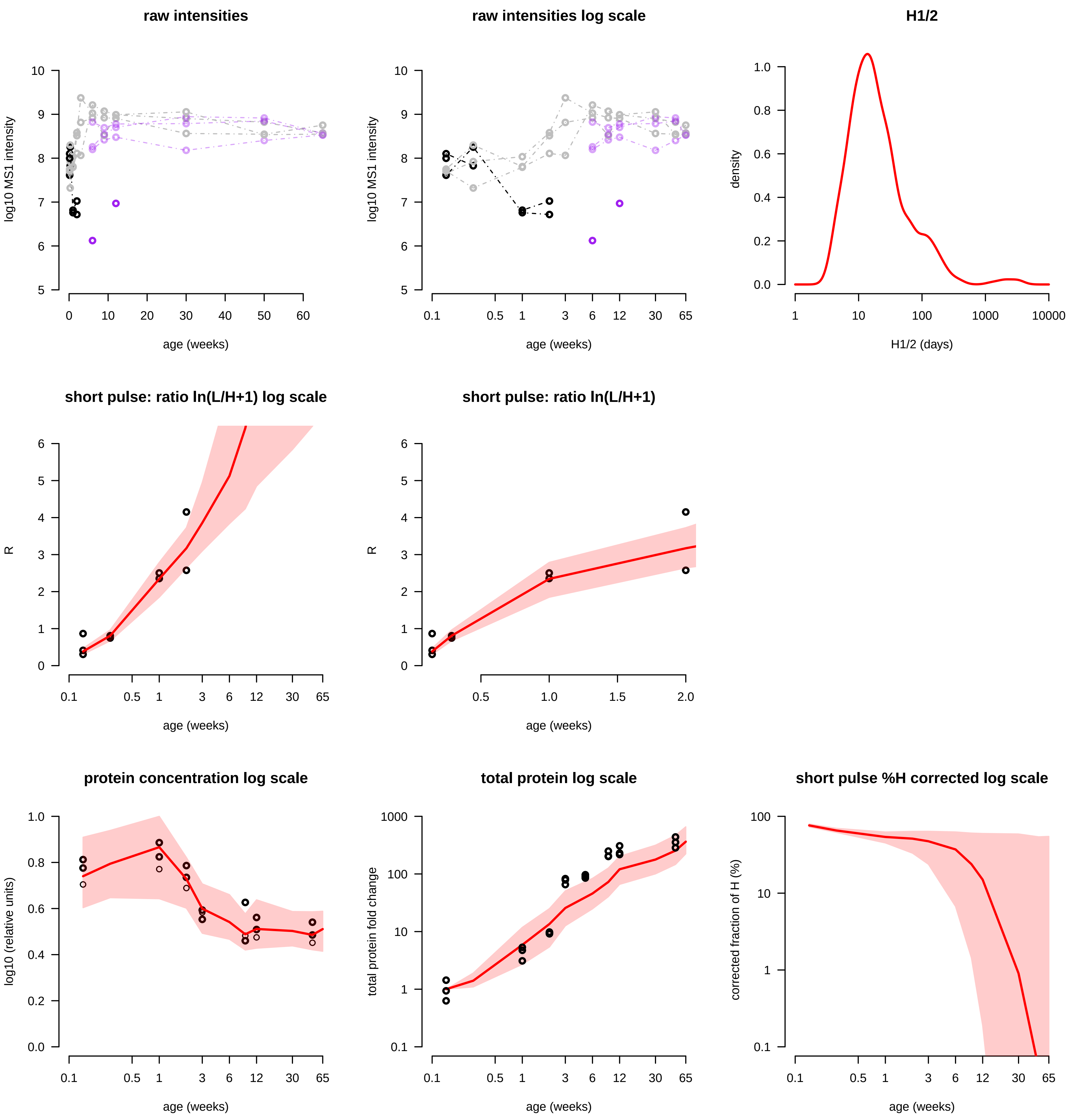

Q99LD8

>sp|Q99LD8|DDAH2_MOUSE

N(G),N(G)−dimethylarginine dimethylaminohydrolase 2

GN=Ddah2

Med H1/2: 99.5 days

Q5 H1/2: 50.9 days

Q95 H1/2: 289.4 days

reliable model fit: no

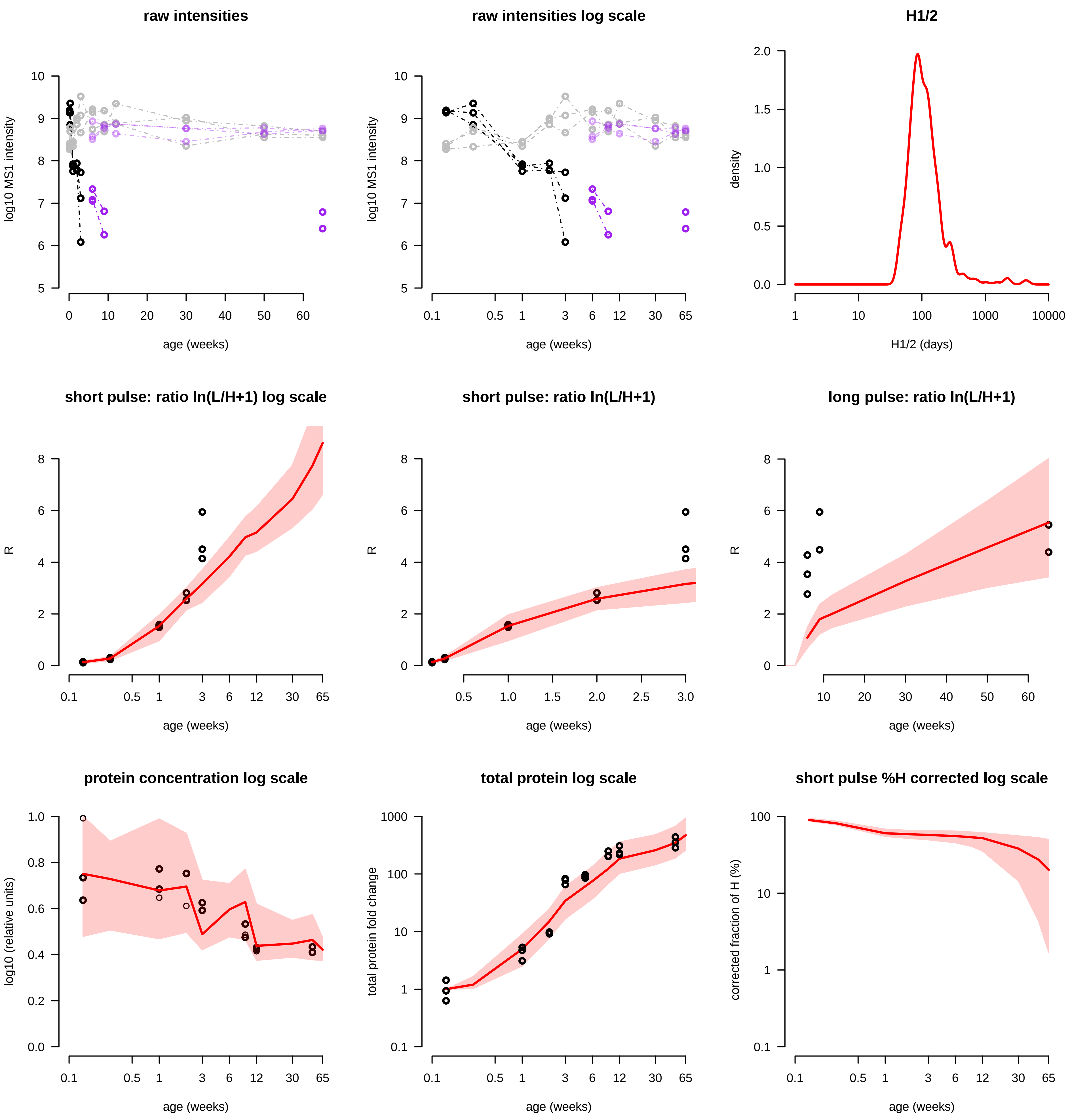

Q60715−2

>sp|Q60715−2|P4HA1_MOUSE

Isoform 2 of Prolyl 4−hydroxylase subunit alpha−1

GN=P4ha1

Med H1/2: 4.9 days

Q5 H1/2: 2.7 days

Q95 H1/2: 11.9 days

reliable model fit: yes

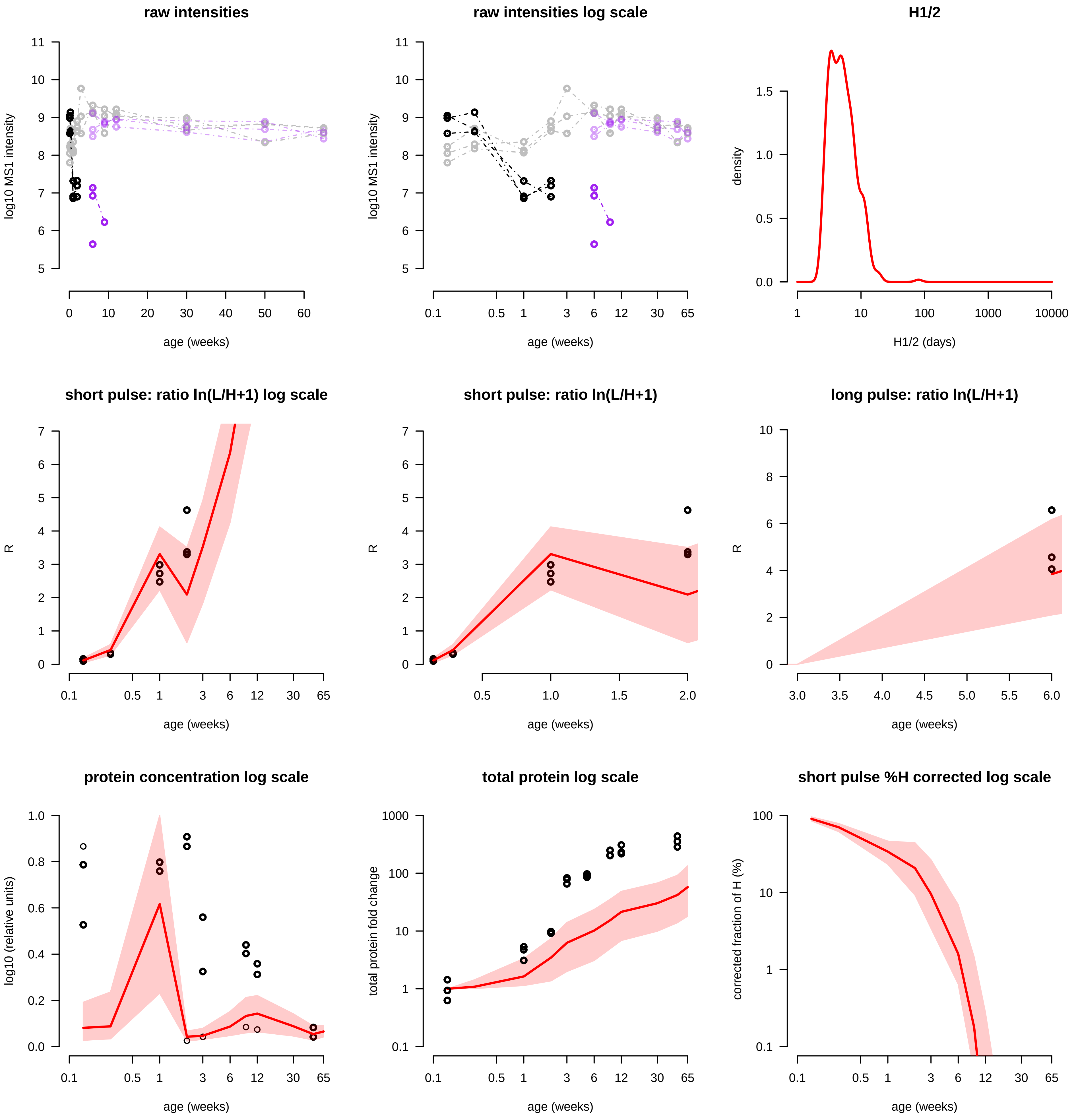

Q99K70

>sp|Q99K70|RRAGC_MOUSE

Ras−related GTP−binding protein C

GN=Rragc

Med H1/2: 6.8 days

Q5 H1/2: 3.5 days

Q95 H1/2: 26.9 days

reliable model fit: yes

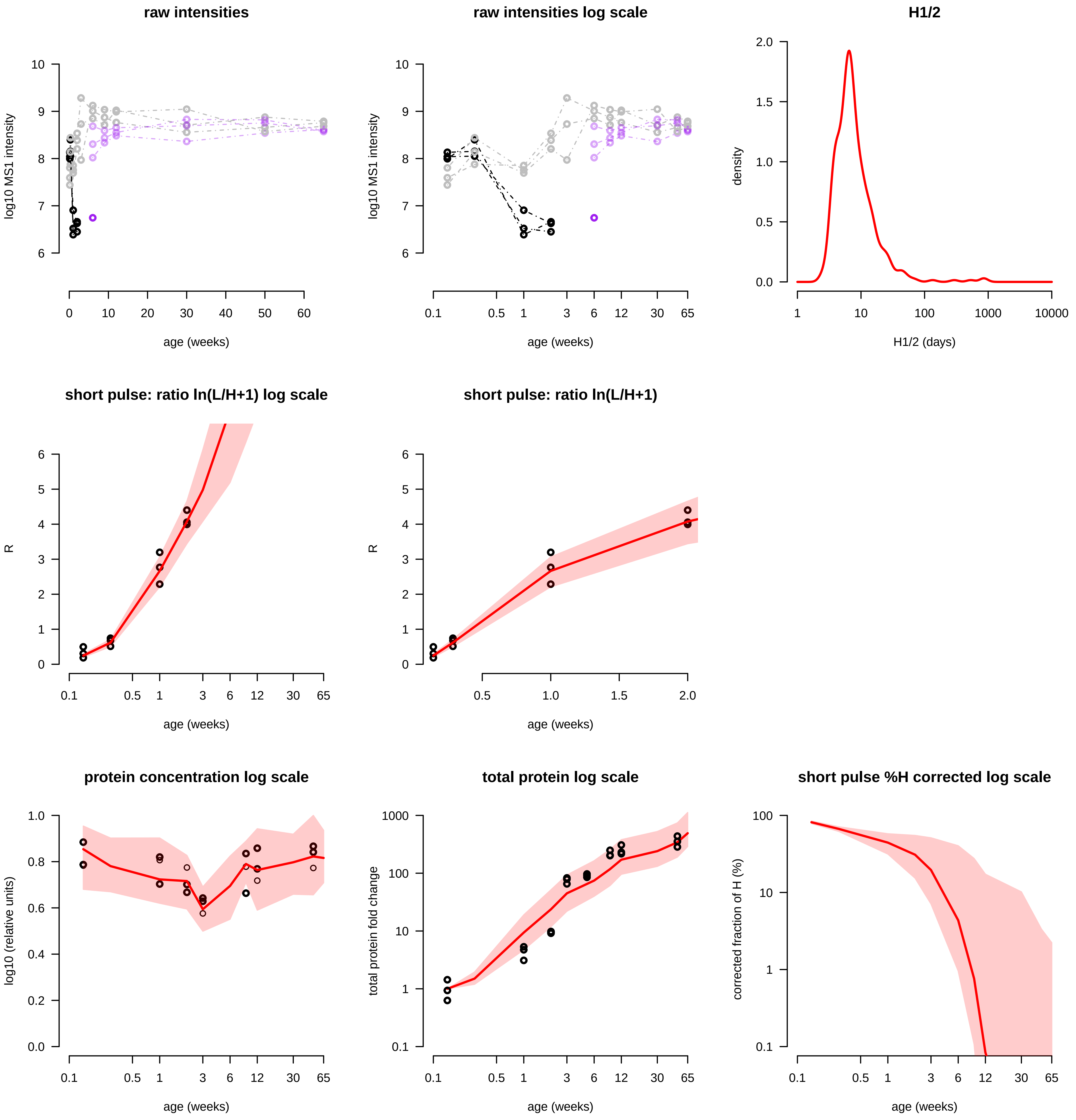

Q61387

>sp|Q61387|COX7R_MOUSE

Cytochrome c oxidase subunit 7A−related protein, mitochondrial

GN=Cox7a2l

Med H1/2: 5.3 days

Q5 H1/2: 4 days

Q95 H1/2: 7.7 days

reliable model fit: yes

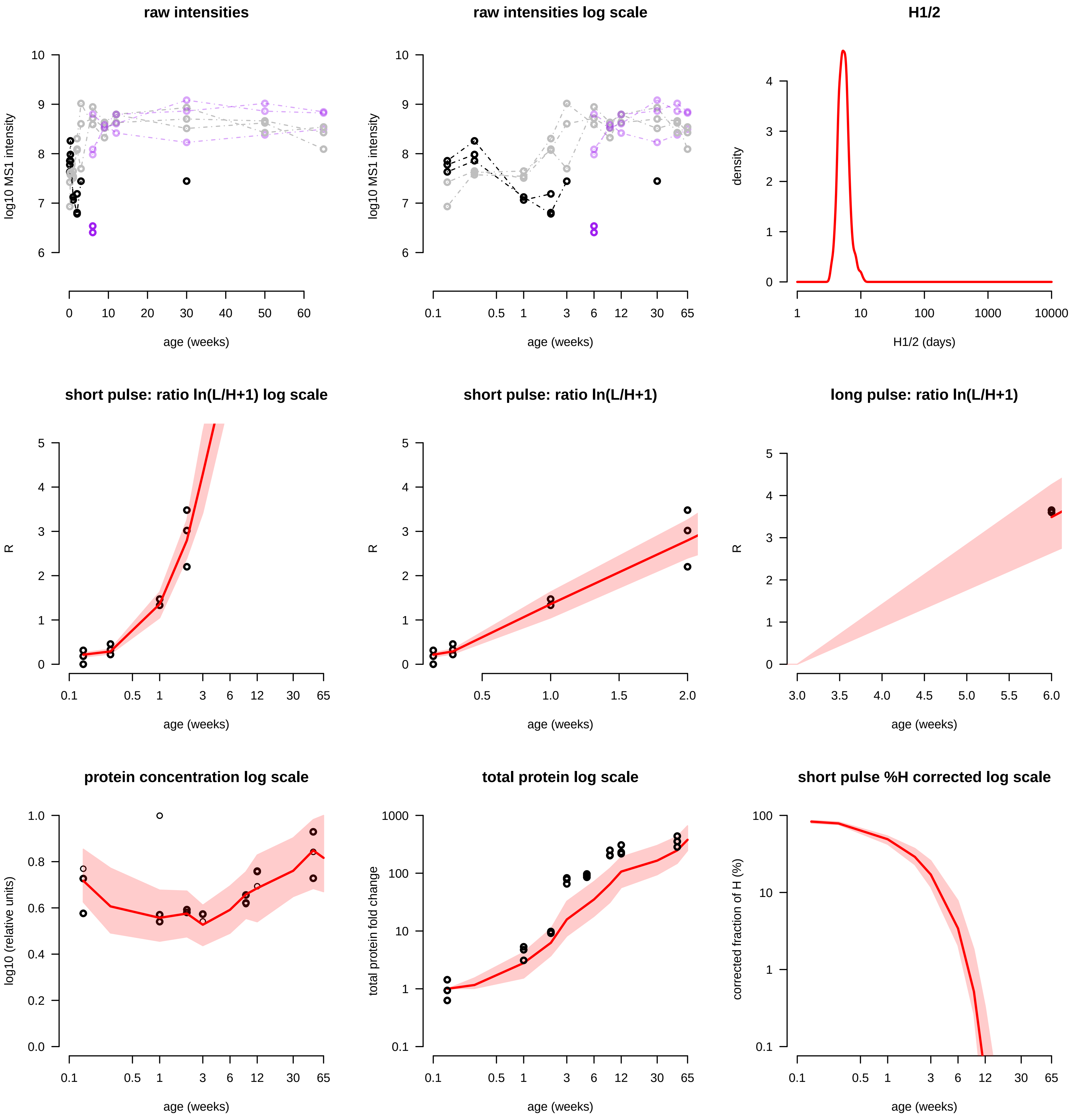

P56546−2;P56546

>sp|P56546−2|CTBP2_MOUSE

Isoform 2 of C−terminal−binding protein 2

GN=Ctbp2

Med H1/2: 4.3 days

Q5 H1/2: 3.4 days

Q95 H1/2: 6 days

reliable model fit: yes

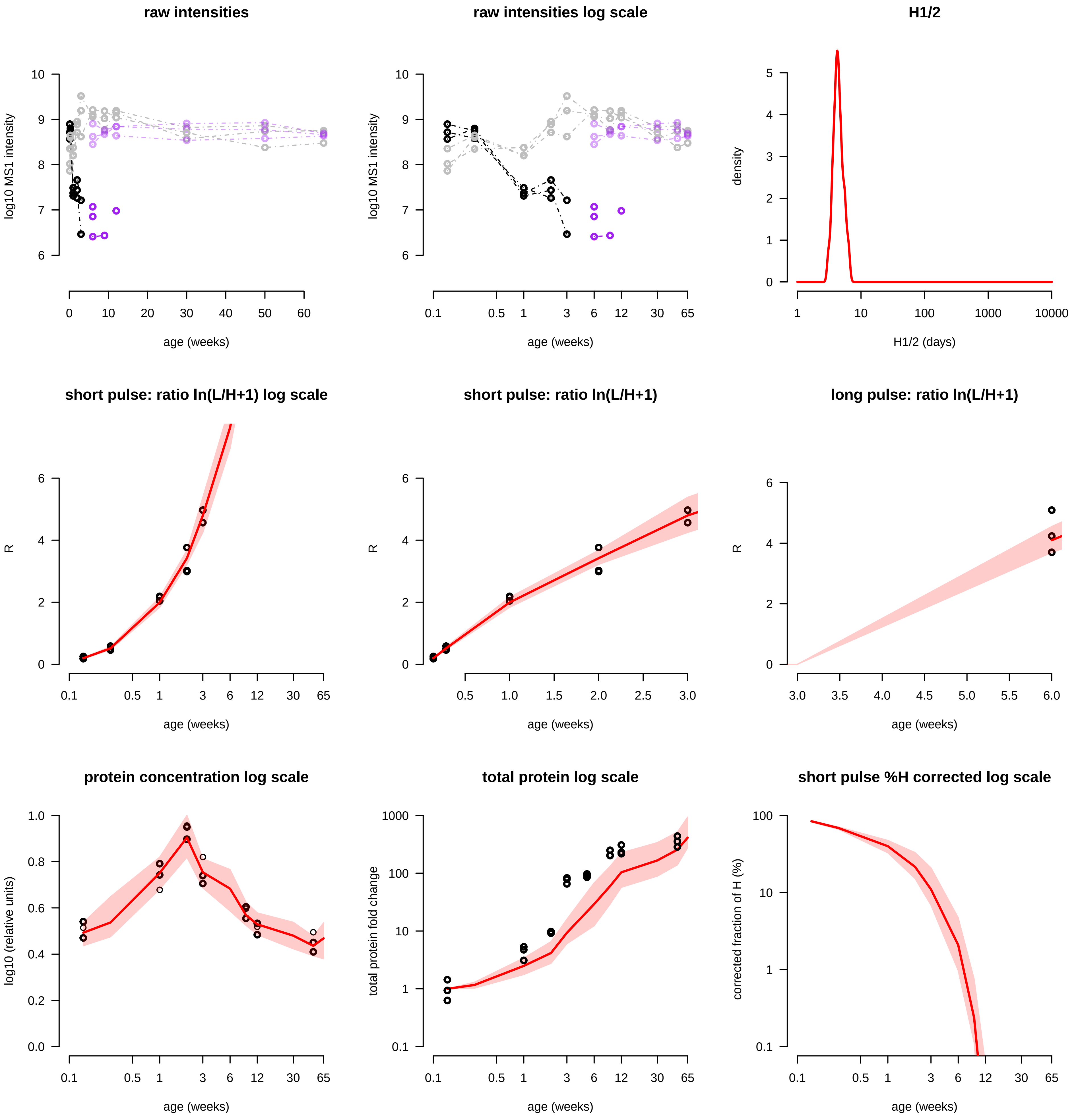

Q9R0E2

>sp|Q9R0E2|PLOD1_MOUSE

Procollagen−lysine,2−oxoglutarate 5−dioxygenase 1

GN=Plod1

Med H1/2: 3.4 days

Q5 H1/2: 2.8 days

Q95 H1/2: 4.2 days

reliable model fit: yes

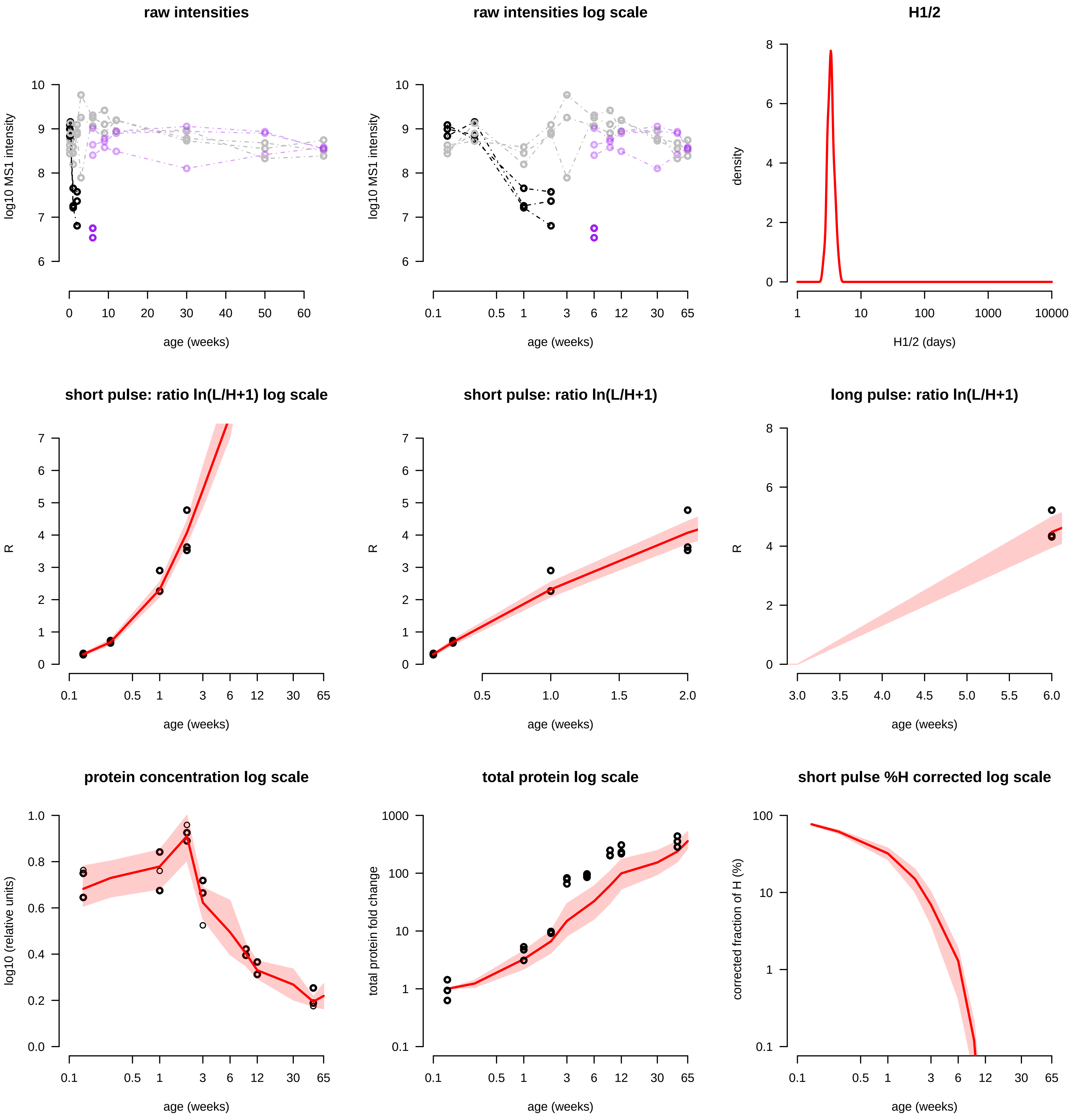

Q99LI8

>sp|Q99LI8|HGS_MOUSE

Hepatocyte growth factor−regulated tyrosine kinase substrate

GN=Hgs

Med H1/2: 1119.2 days

Q5 H1/2: 341.3 days

Q95 H1/2: 9440.5 days

reliable model fit: yes

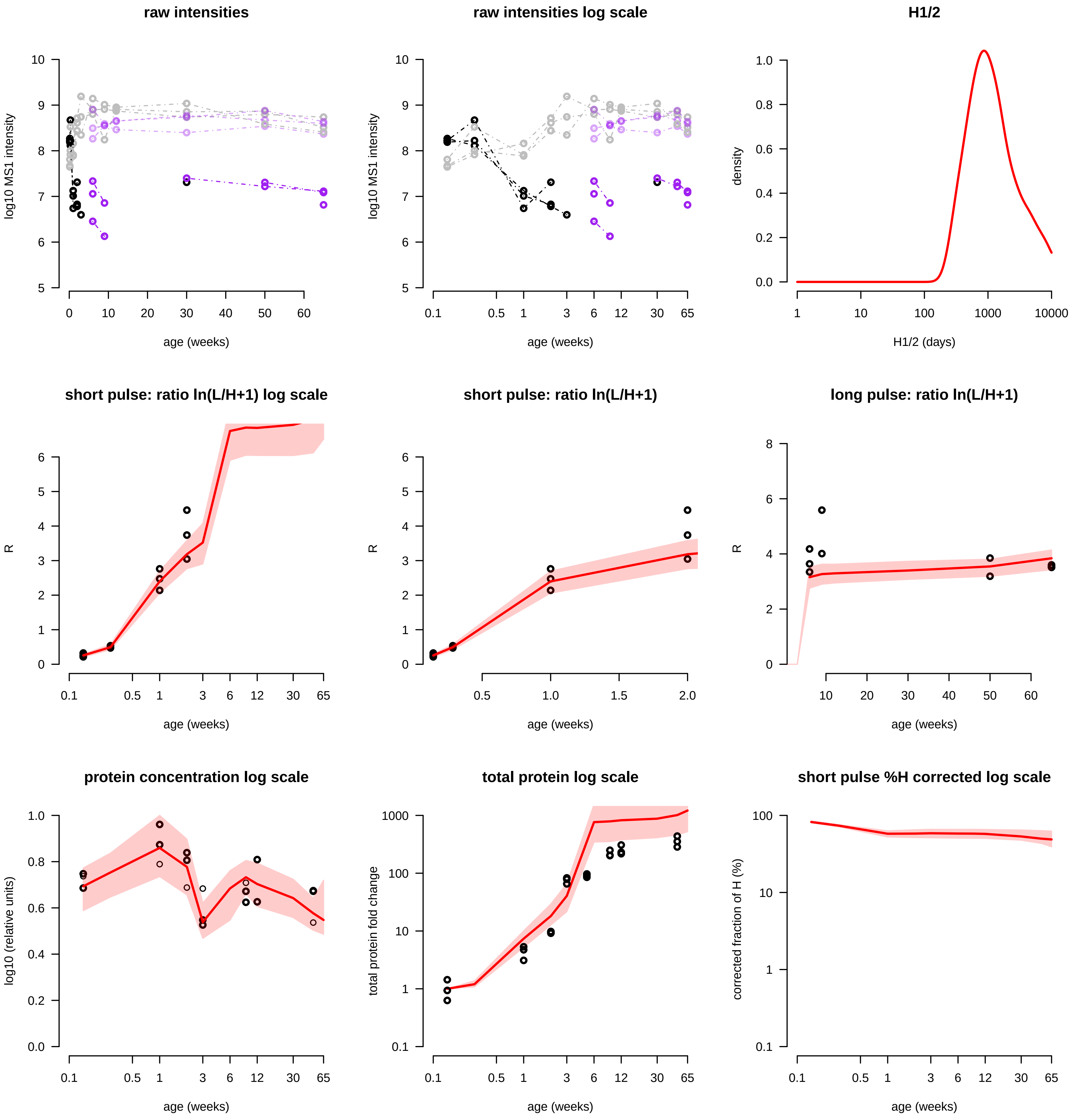

Q9WVA3

>sp|Q9WVA3|BUB3_MOUSE

Mitotic checkpoint protein BUB3

GN=Bub3

Med H1/2: 6 days

Q5 H1/2: 5 days

Q95 H1/2: 7.2 days

reliable model fit: yes

raw intensities

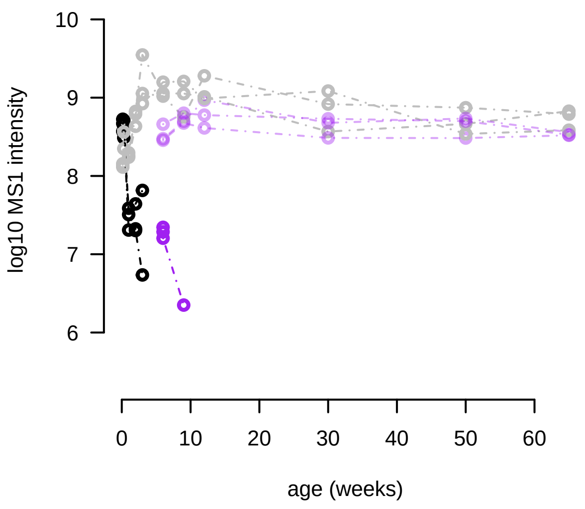

raw intensities log scale

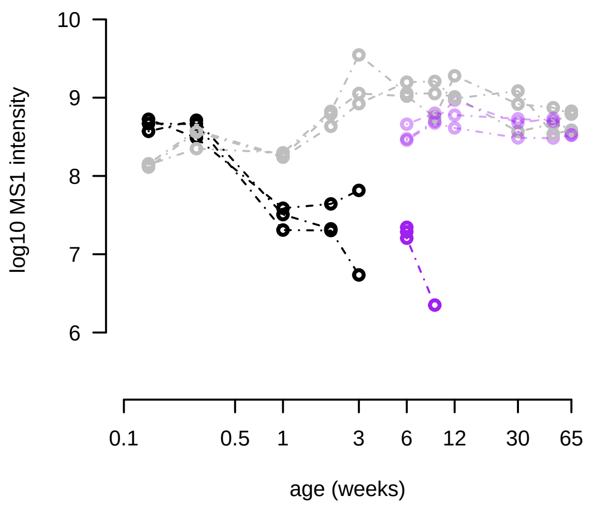

H1/2

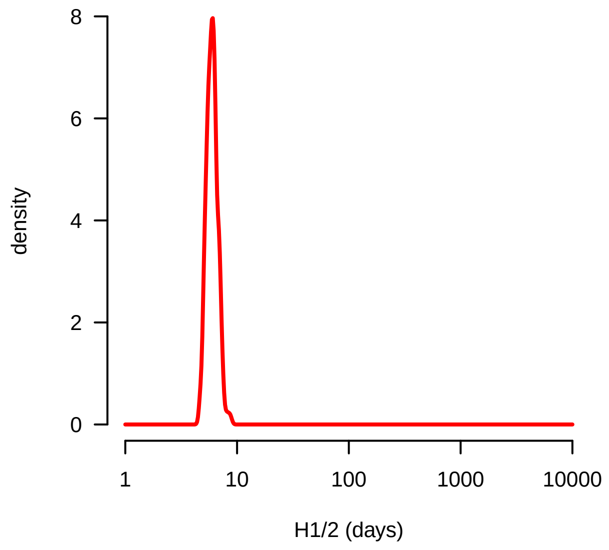

short pulse: ratio ln(L/H+1) log scale

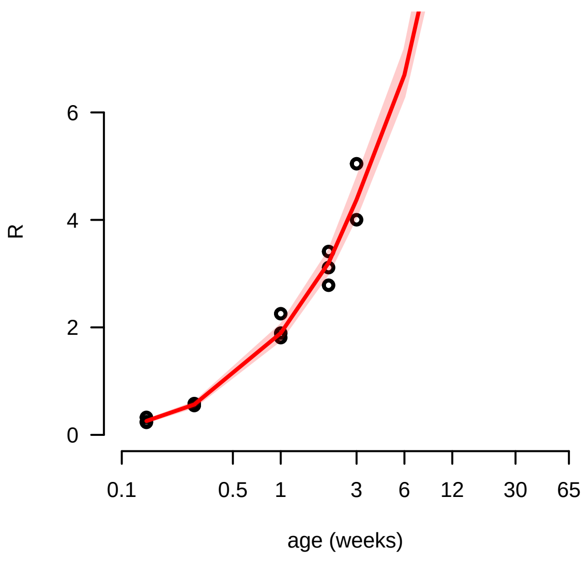

short pulse: ratio ln(L/H+1)

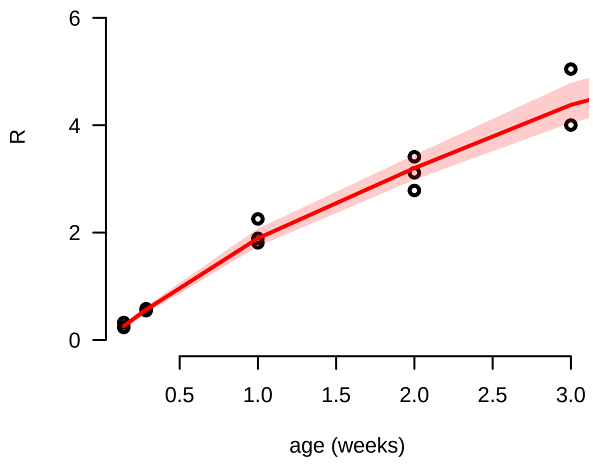

long pulse: ratio ln(L/H+1)

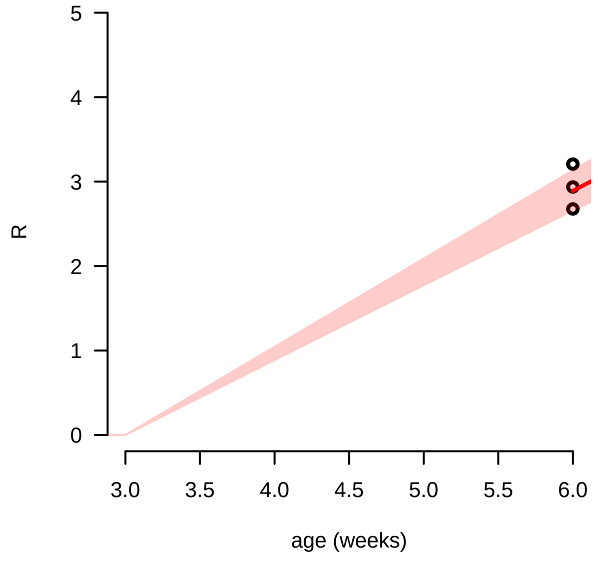

protein concentration log scale

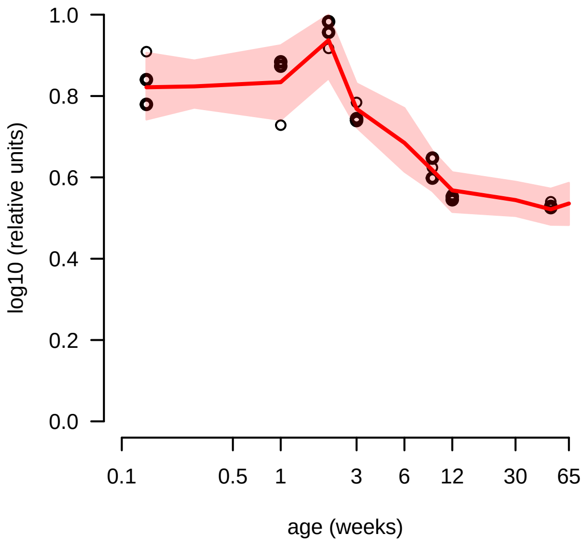

total protein log scale

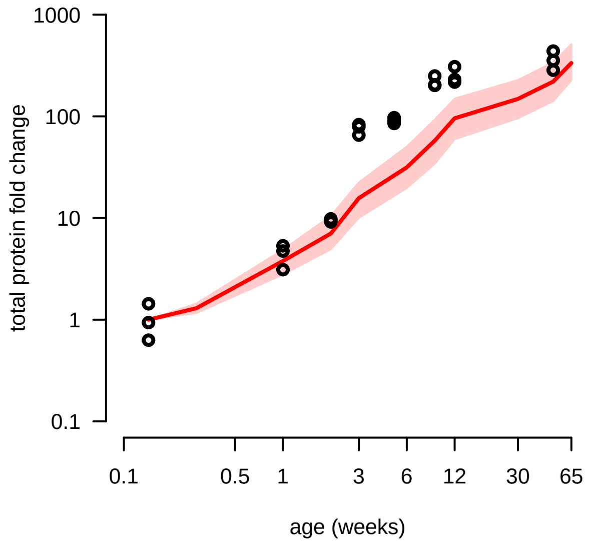

short pulse %H corrected log scale

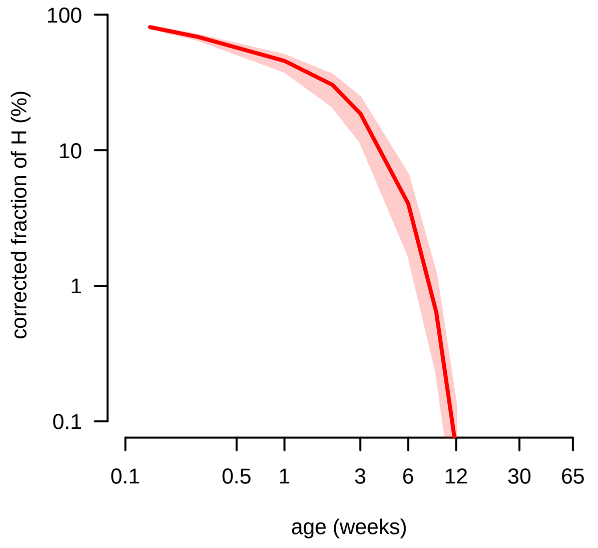

Q60714

>sp|Q60714|S27A1_MOUSE

Long−chain fatty acid transport protein 1

GN=Slc27a1

Med H1/2: 14.5 days

Q5 H1/2: 4.3 days

Q95 H1/2: 158 days

reliable model fit: yes

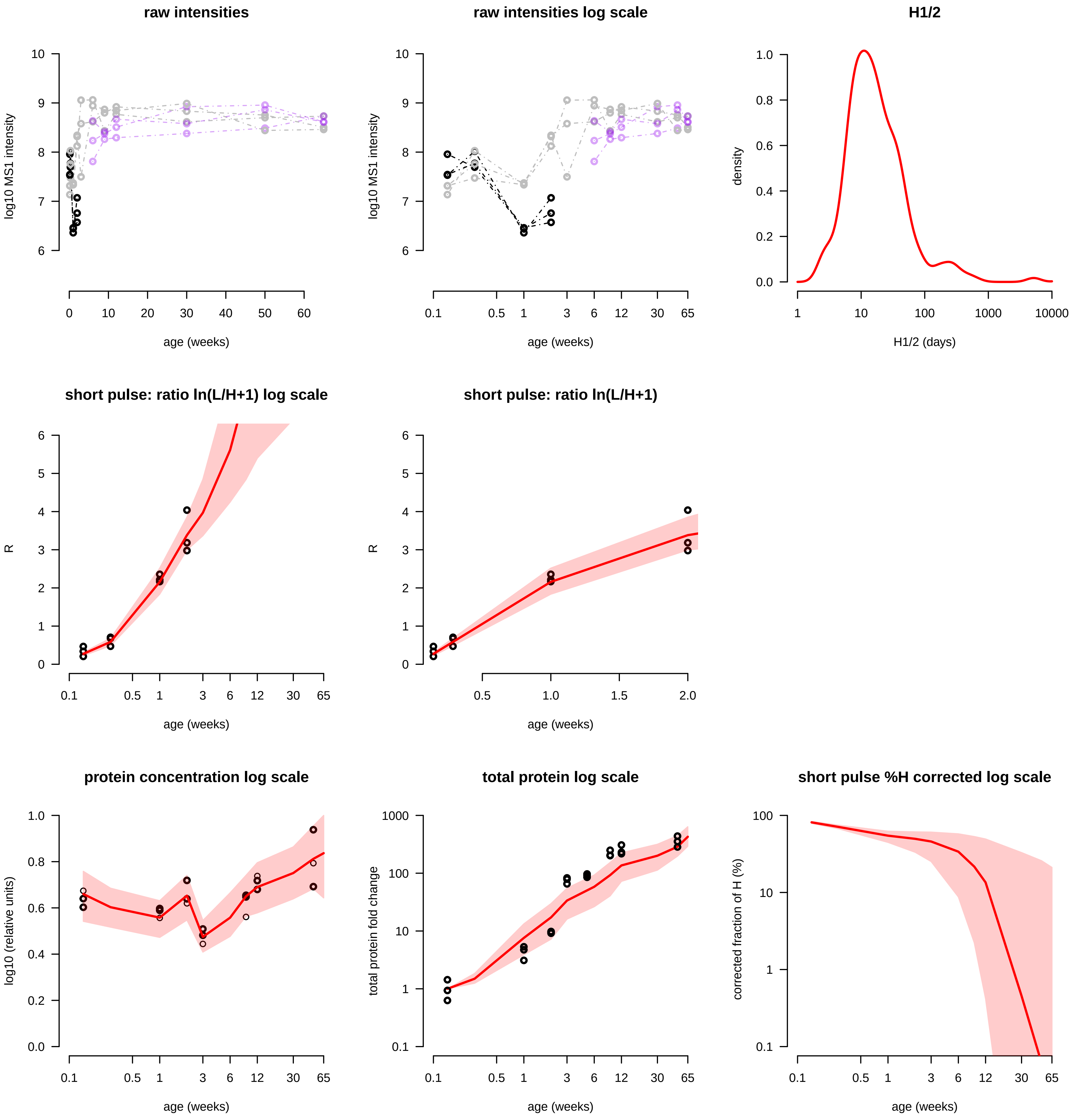

Q64487−11;Q64487−10

>sp|Q64487−11|PTPRD_MOUSE

Isoform K of Receptor−type tyrosine−protein phosphatase delta

GN=Ptprd

Med H1/2: 2511.8 days

Q5 H1/2: 742 days

Q95 H1/2: 35845.1 days

reliable model fit: yes

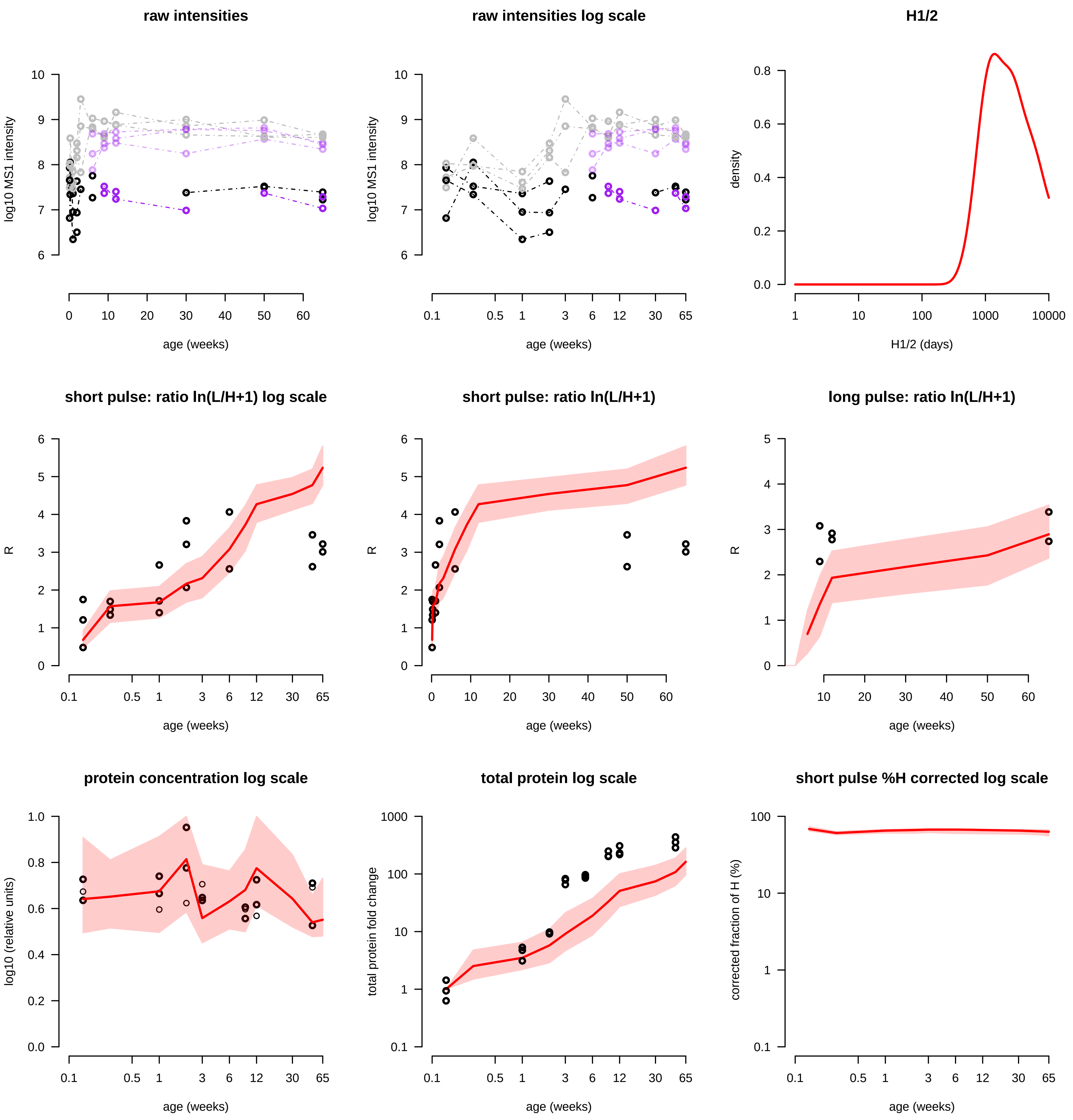

Q62093

>sp|Q62093|SRSF2_MOUSE

Serine/arginine−rich splicing factor 2

GN=Srsf2

Med H1/2: 8 days

Q5 H1/2: 6.5 days

Q95 H1/2: 10.8 days

reliable model fit: yes

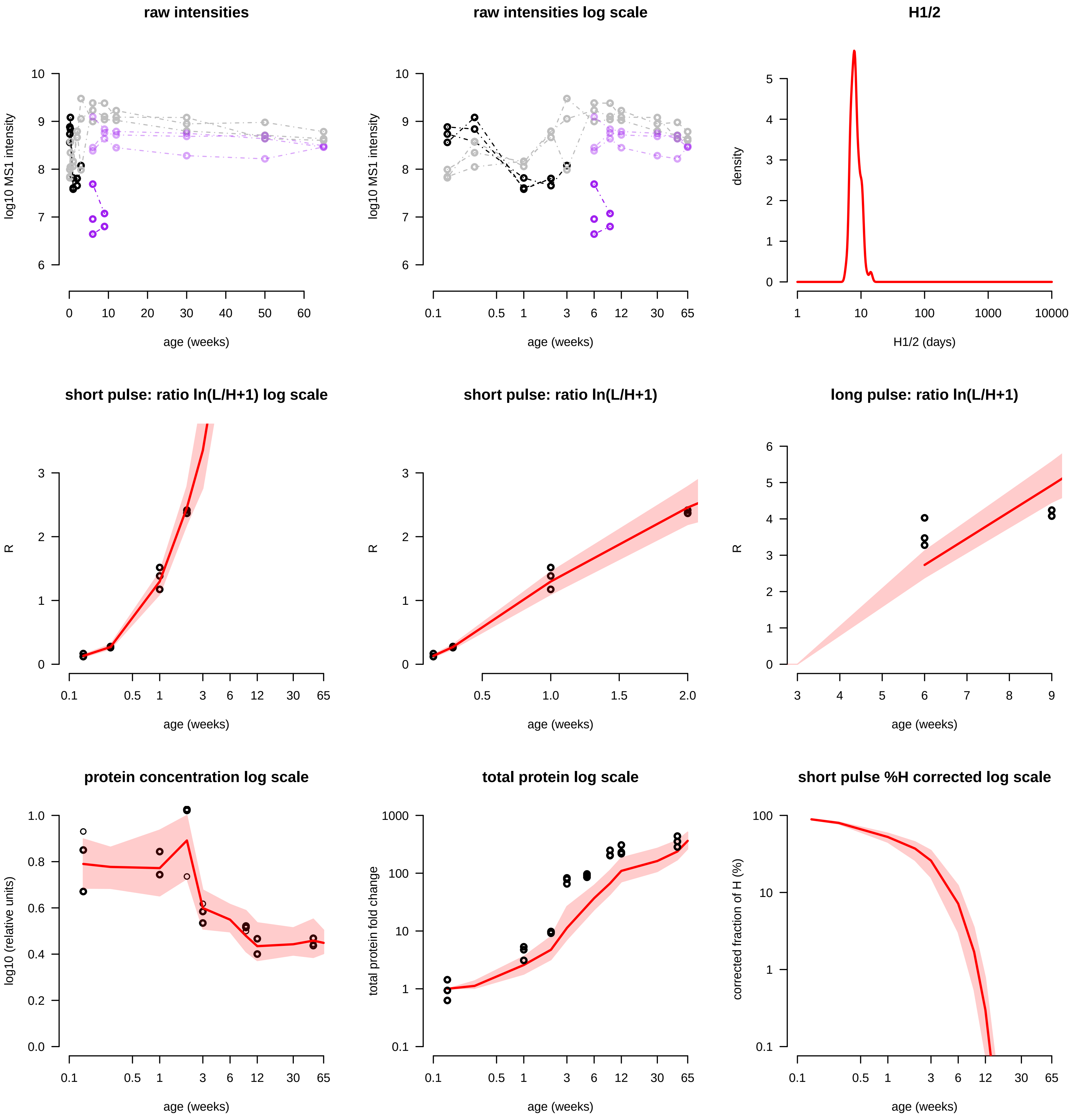

P48774

>sp|P48774|GSTM5_MOUSE

Glutathione S−transferase Mu 5

GN=Gstm5

Med H1/2: 4.1 days

Q5 H1/2: 3.4 days

Q95 H1/2: 5.1 days

reliable model fit: yes

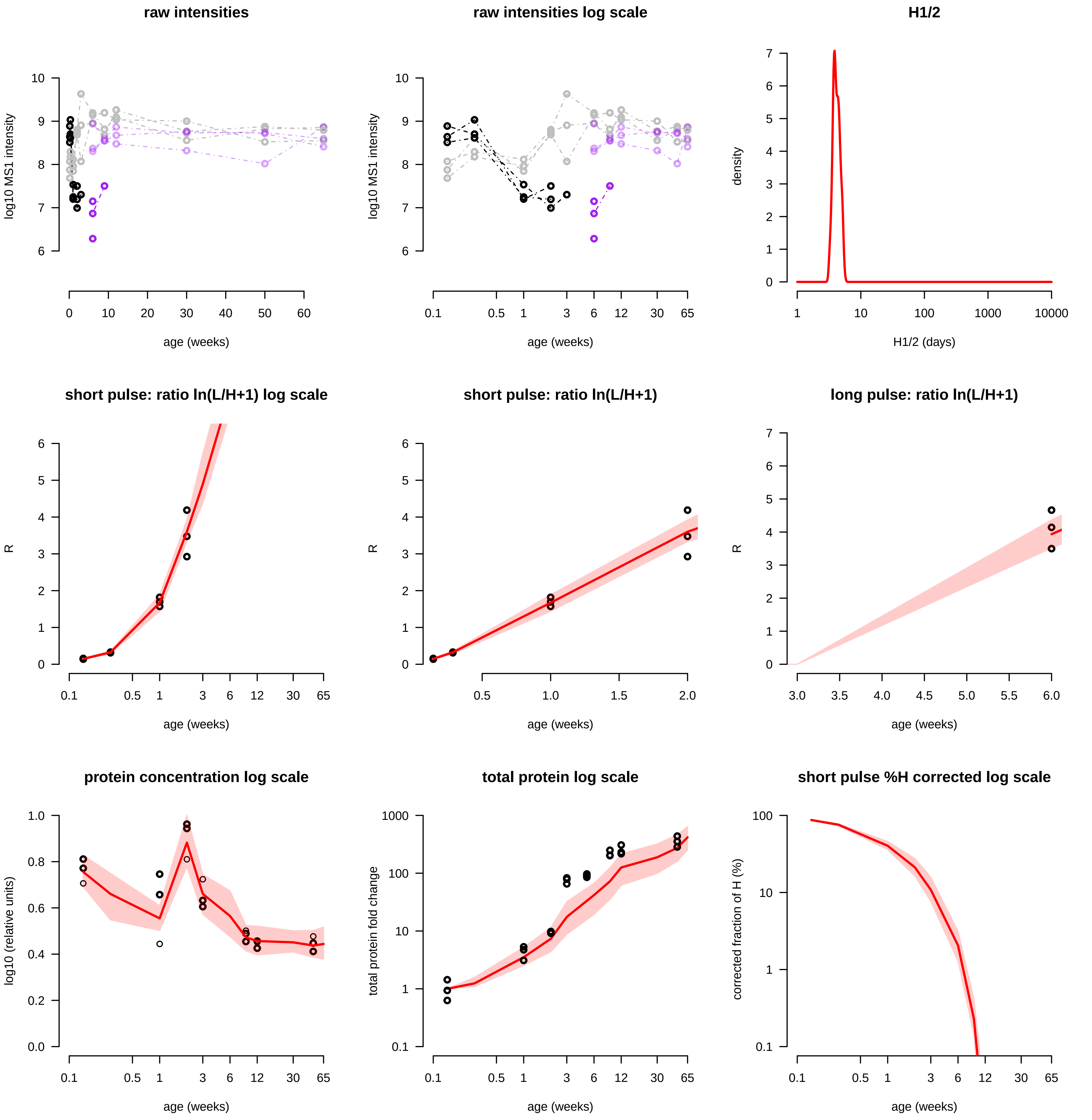

Q6P542

>sp|Q6P542|ABCF1_MOUSE

ATP−binding cassette sub−family F member 1

GN=Abcf1

Med H1/2: 5.2 days

Q5 H1/2: 3.7 days

Q95 H1/2: 7.8 days

reliable model fit: no

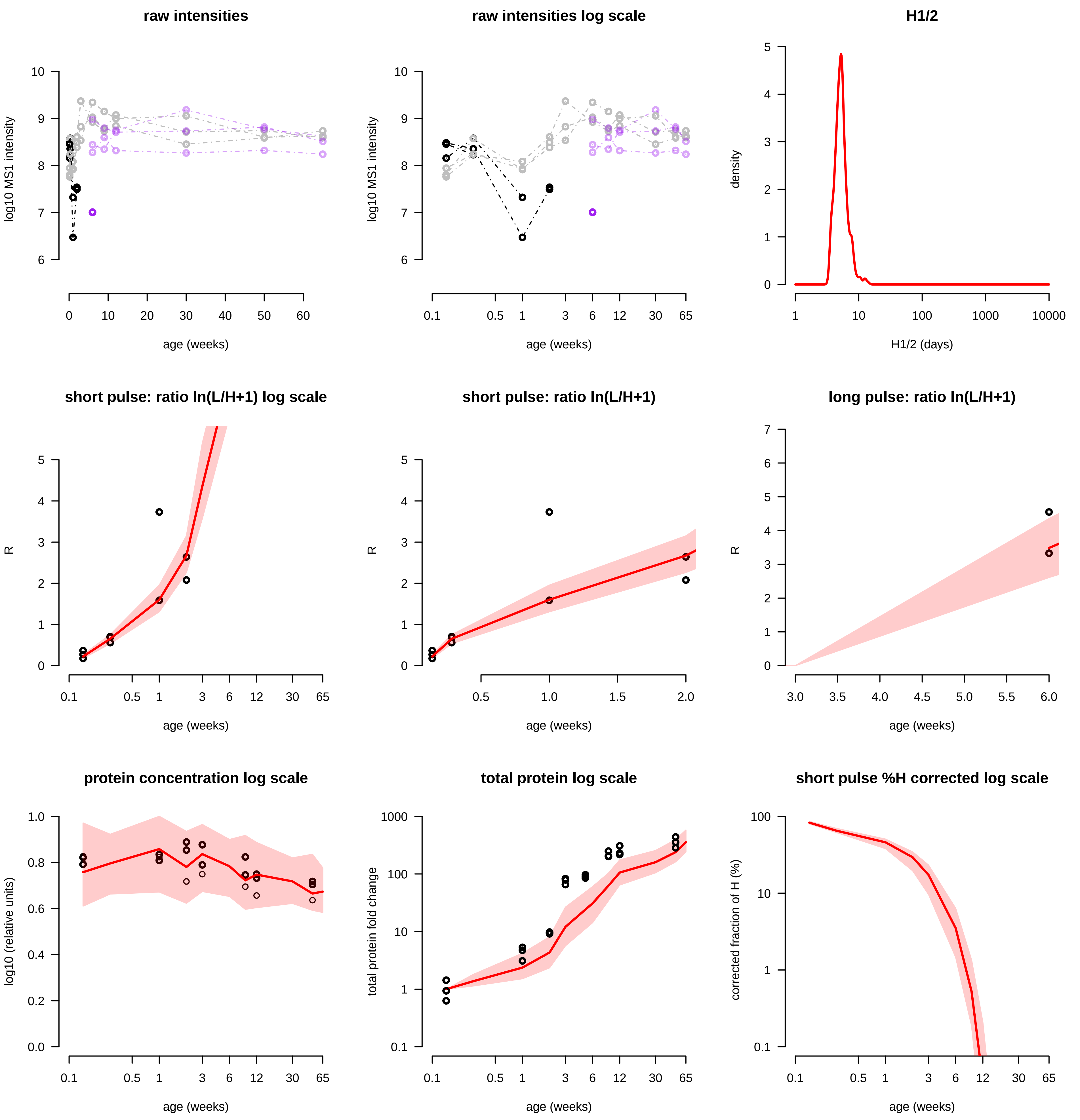

Q70FJ1−2;Q70FJ1;Q70FJ1−3

>sp|Q70FJ1−2|AKAP9_MOUSE

Isoform 2 of A−kinase anchor protein 9

GN=Akap9

Med H1/2: 15.5 days

Q5 H1/2: 6.5 days

Q95 H1/2: 253.5 days

reliable model fit: yes

raw intensities

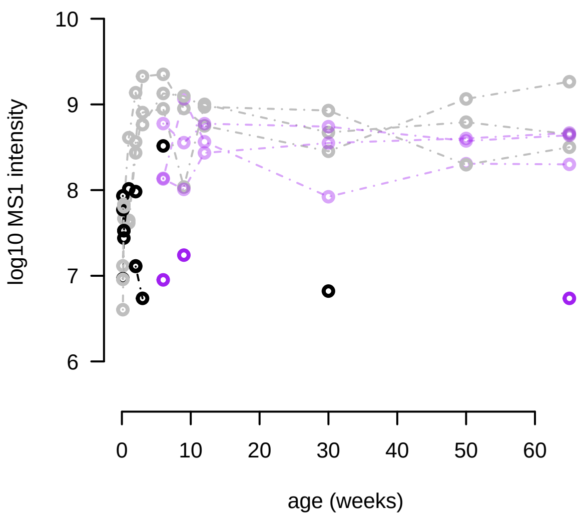

raw intensities log scale

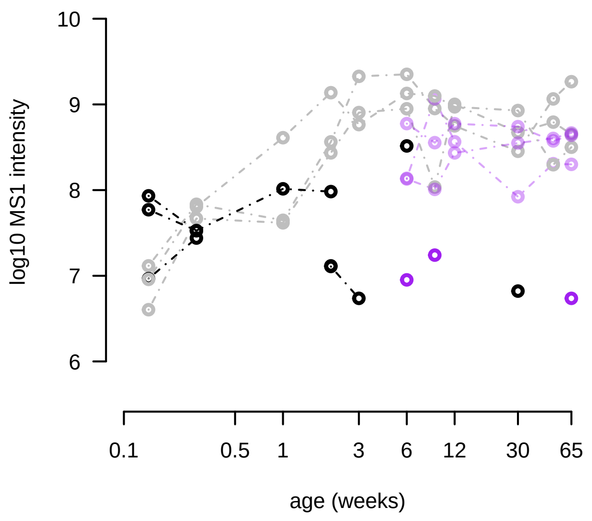

H1/2

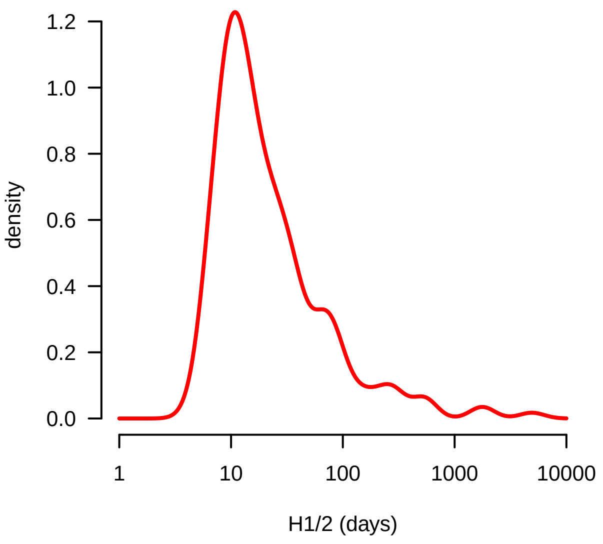

short pulse: ratio ln(L/H+1) log scale

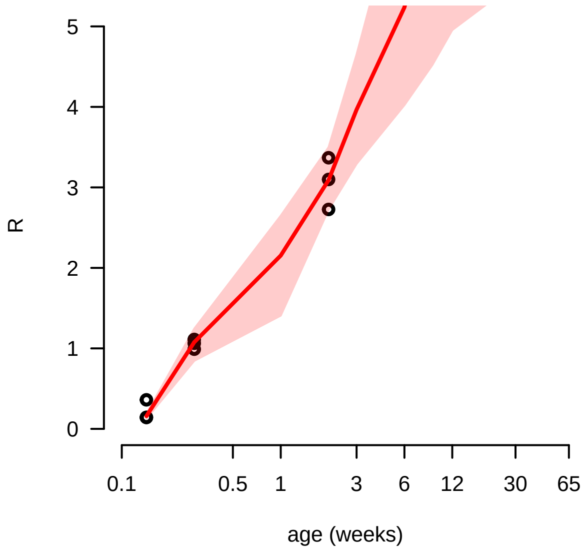

short pulse: ratio ln(L/H+1)

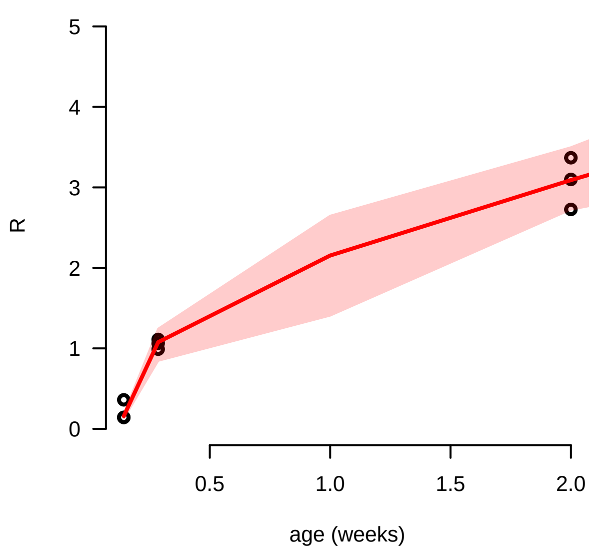

protein concentration log scale

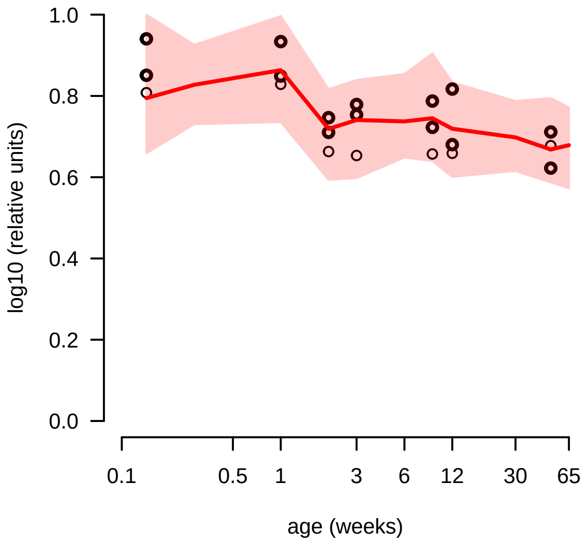

total protein log scale

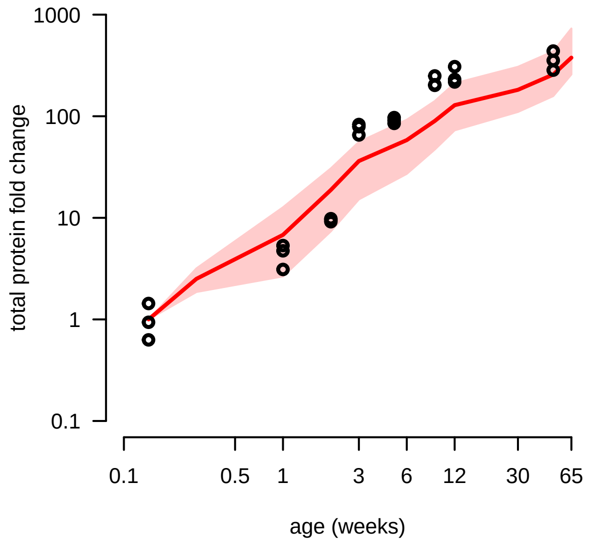

short pulse %H corrected log scale

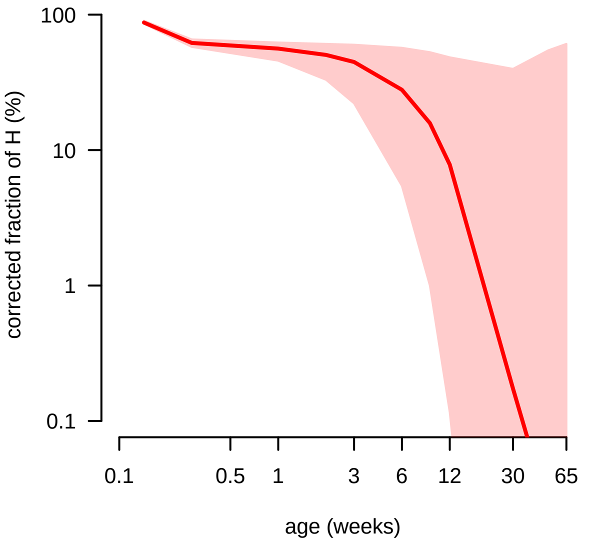

Q9JMG1

>sp|Q9JMG1|EDF1_MOUSE

Endothelial differentiation−related factor 1

GN=Edf1

Med H1/2: 5.7 days

Q5 H1/2: 4.2 days

Q95 H1/2: 7.8 days

reliable model fit: yes

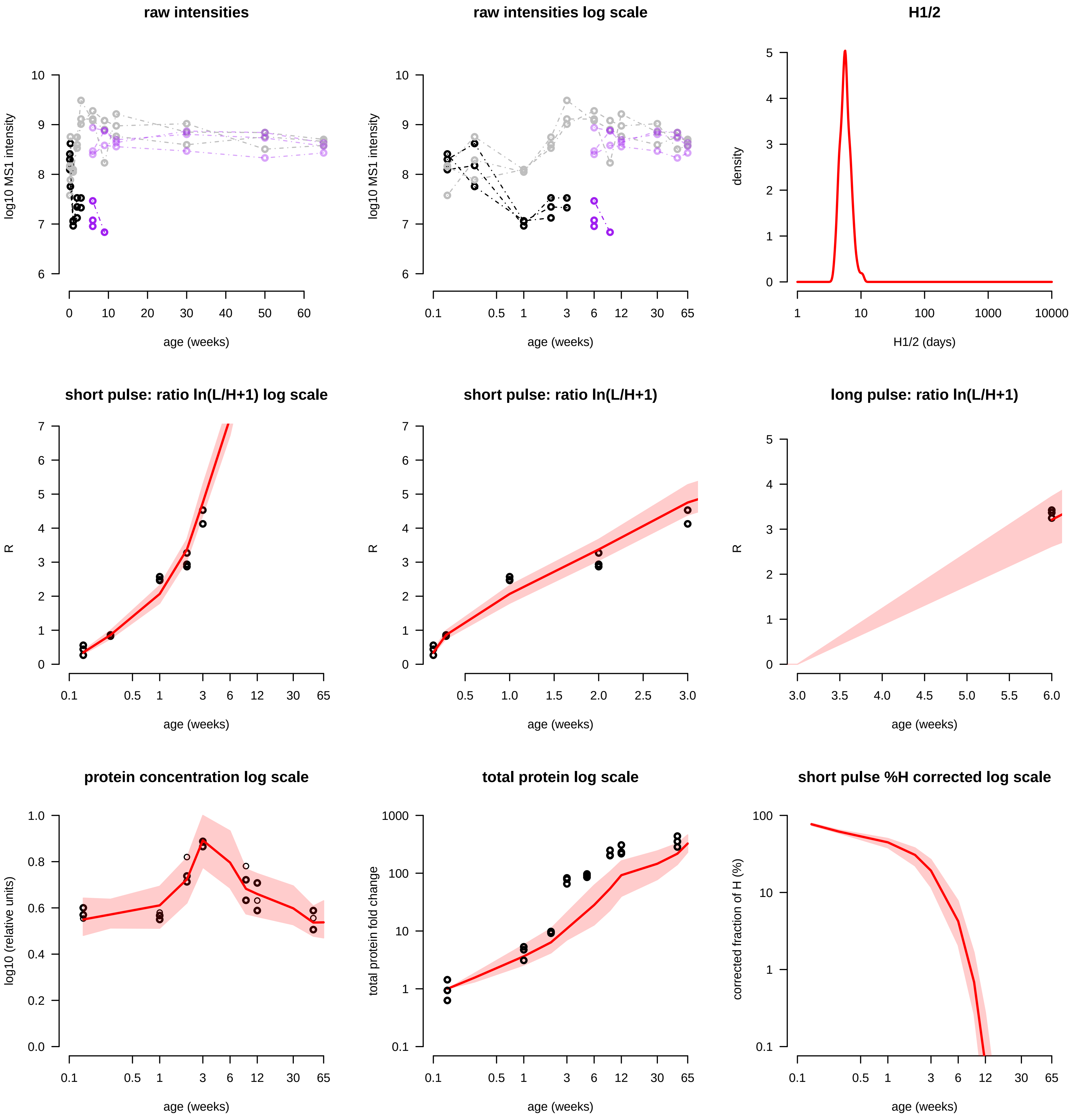

P61082

>sp|P61082|UBC12_MOUSE

NEDD8−conjugating enzyme Ubc12

GN=Ube2m

Med H1/2: 7.5 days

Q5 H1/2: 6.3 days

Q95 H1/2: 9.7 days

reliable model fit: yes

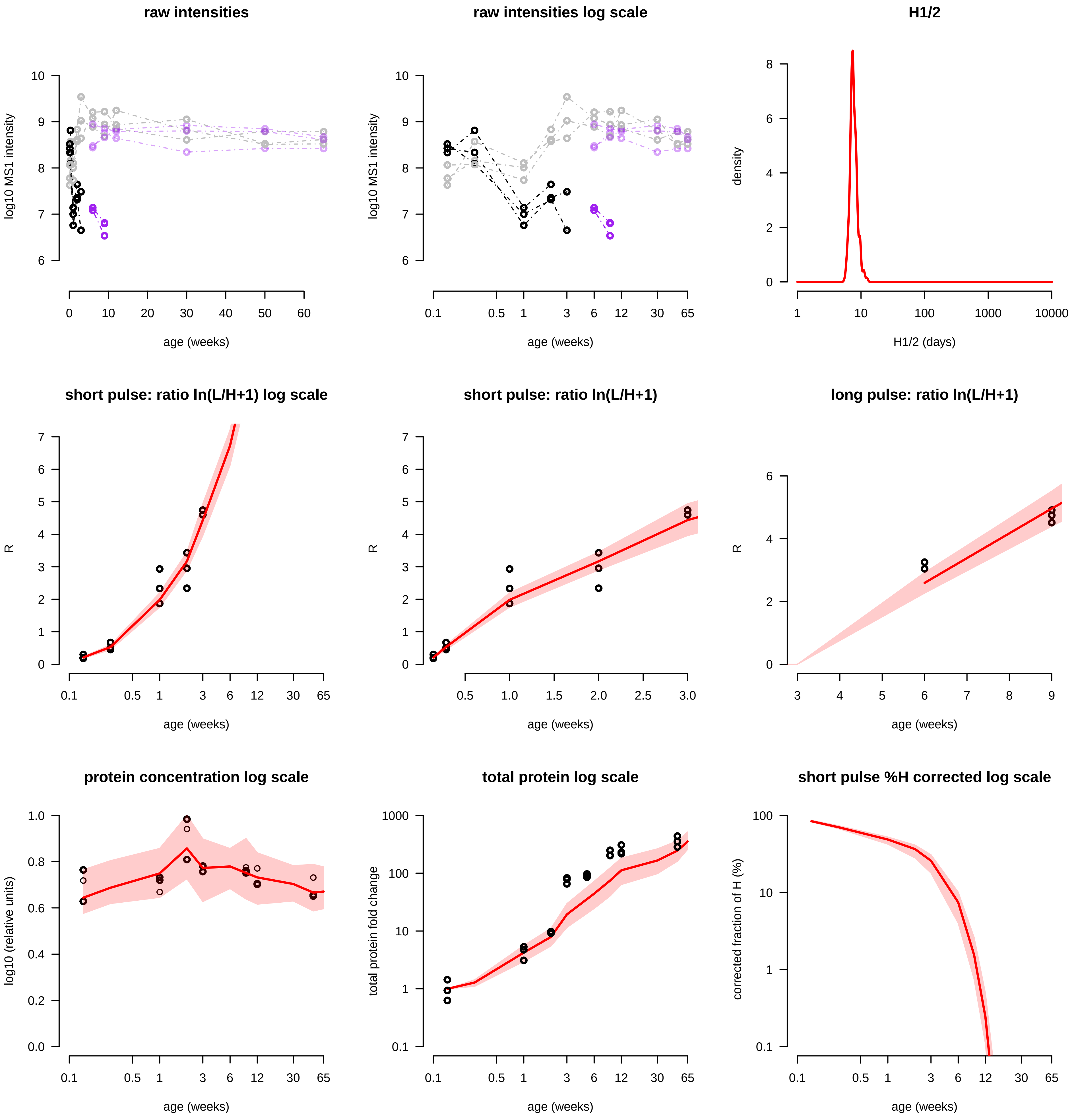

P84084

>sp|P84084|ARF5_MOUSE

ADP−ribosylation factor 5

GN=Arf5

Med H1/2: 6.9 days

Q5 H1/2: 5.3 days

Q95 H1/2: 9.8 days

reliable model fit: yes

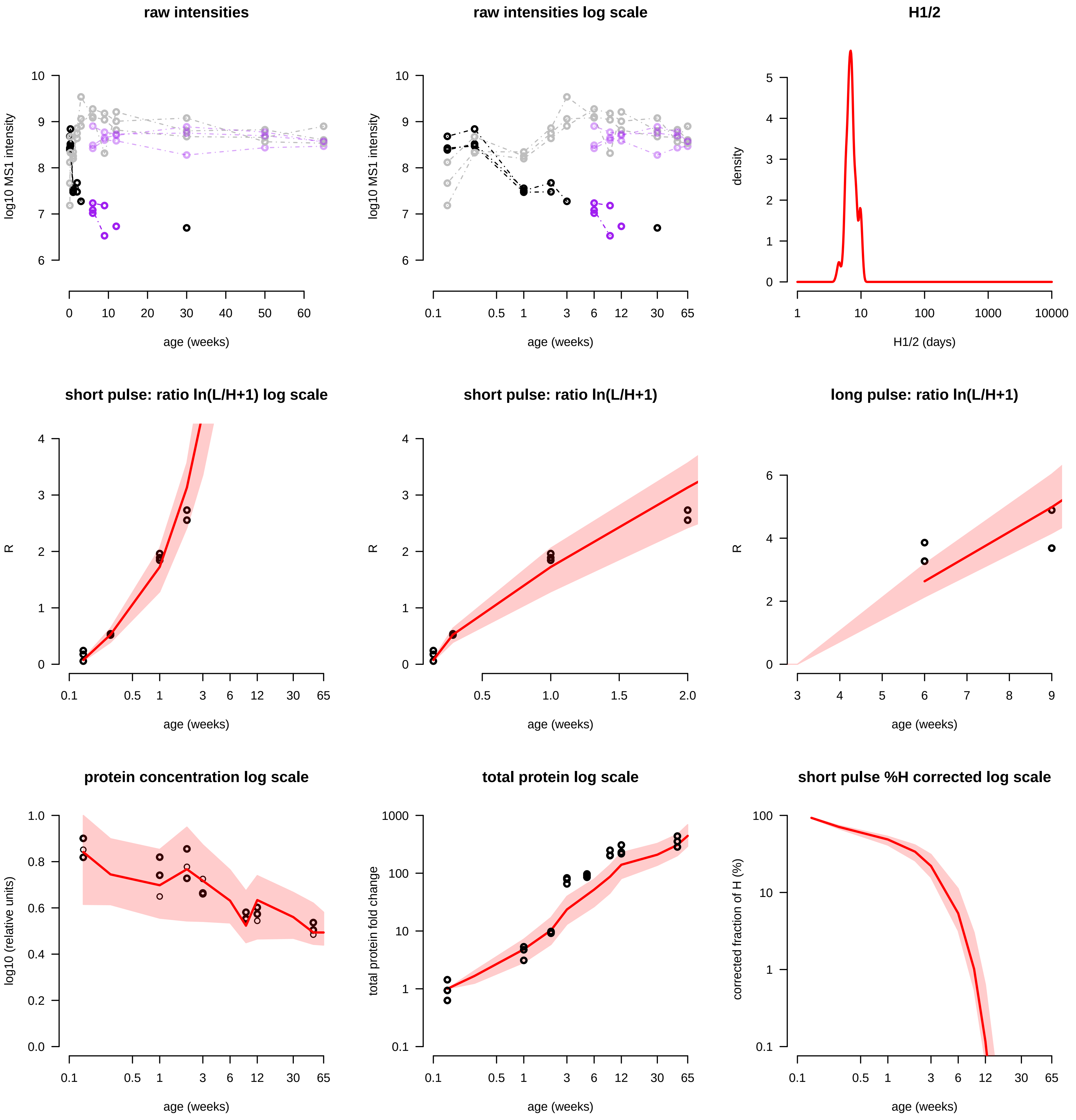

Q8C0M9

>sp|Q8C0M9|ASGL1_MOUSE

Isoaspartyl peptidase/L−asparaginase

GN=Asrgl1

Med H1/2: 26.2 days

Q5 H1/2: 7.9 days

Q95 H1/2: 477.2 days

reliable model fit: yes

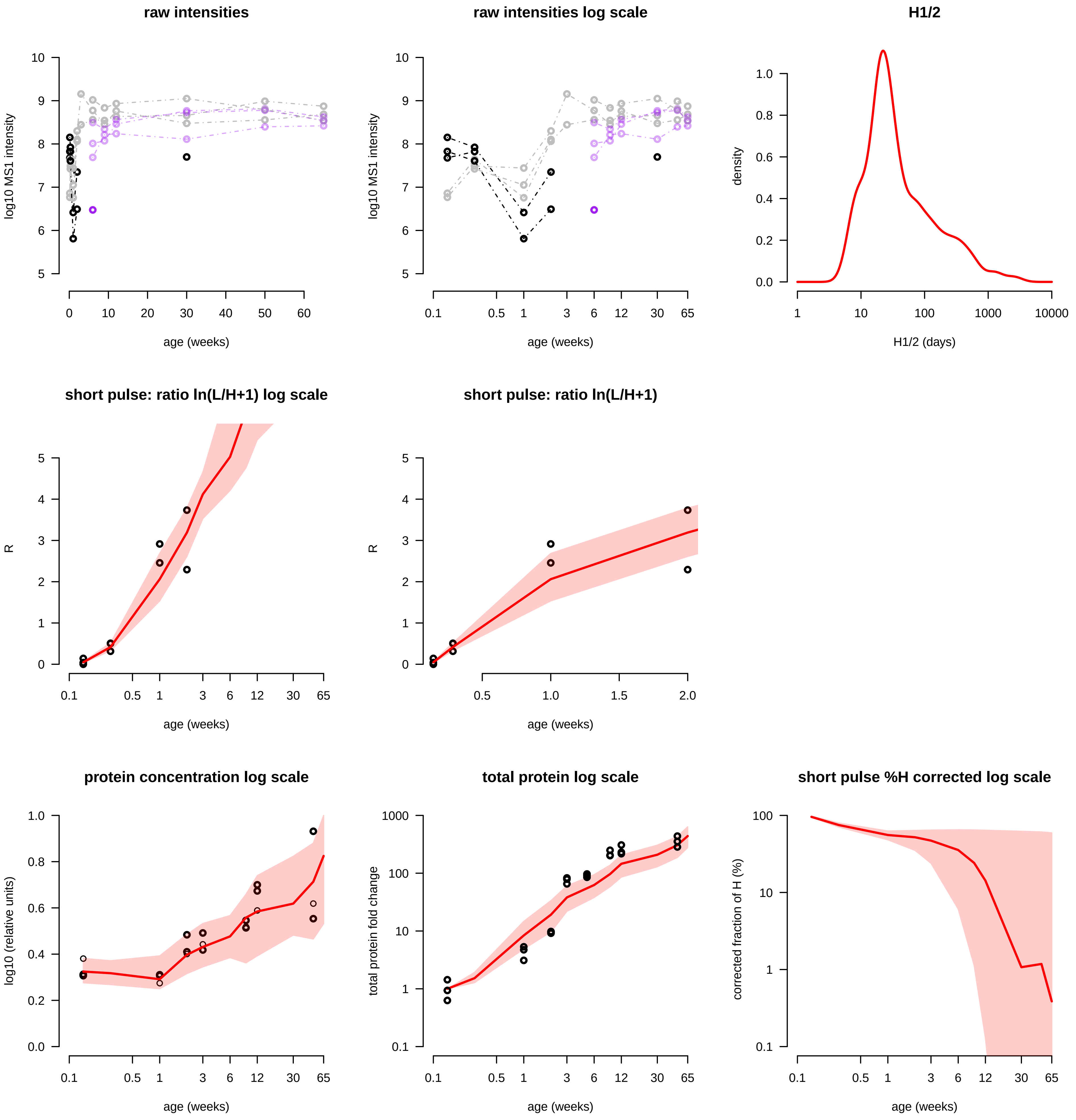

P07742

>sp|P07742|RIR1_MOUSE

Ribonucleoside−diphosphate reductase large subunit

GN=Rrm1

Med H1/2: 3.8 days

Q5 H1/2: 2.7 days

Q95 H1/2: 8.1 days

reliable model fit: yes

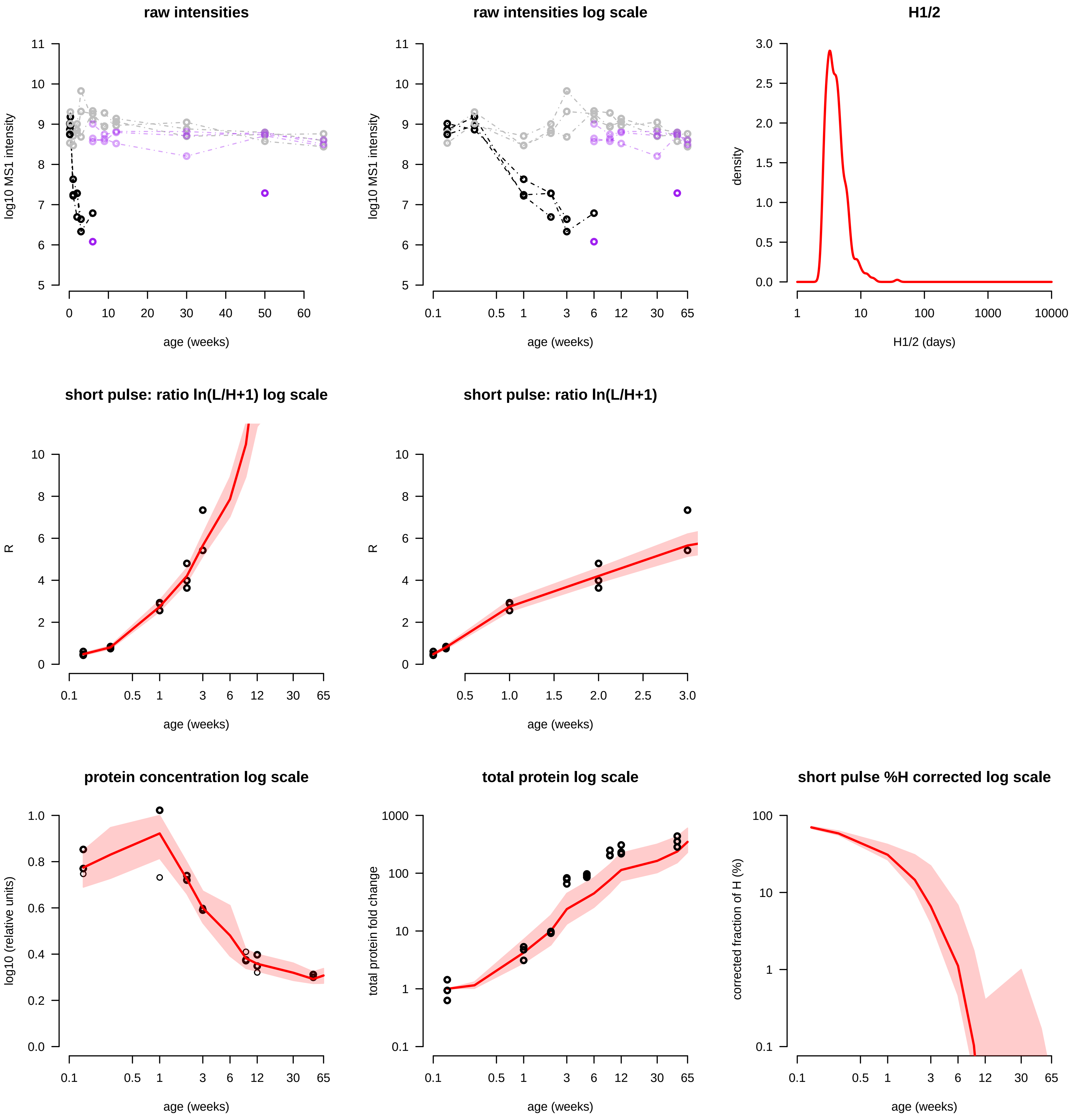

E9Q414

>sp|E9Q414|APOB_MOUSE

Apolipoprotein B−100

GN=Apob

Med H1/2: 3225.3 days

Q5 H1/2: 1049.6 days

Q95 H1/2: 117115.2 days

reliable model fit: no

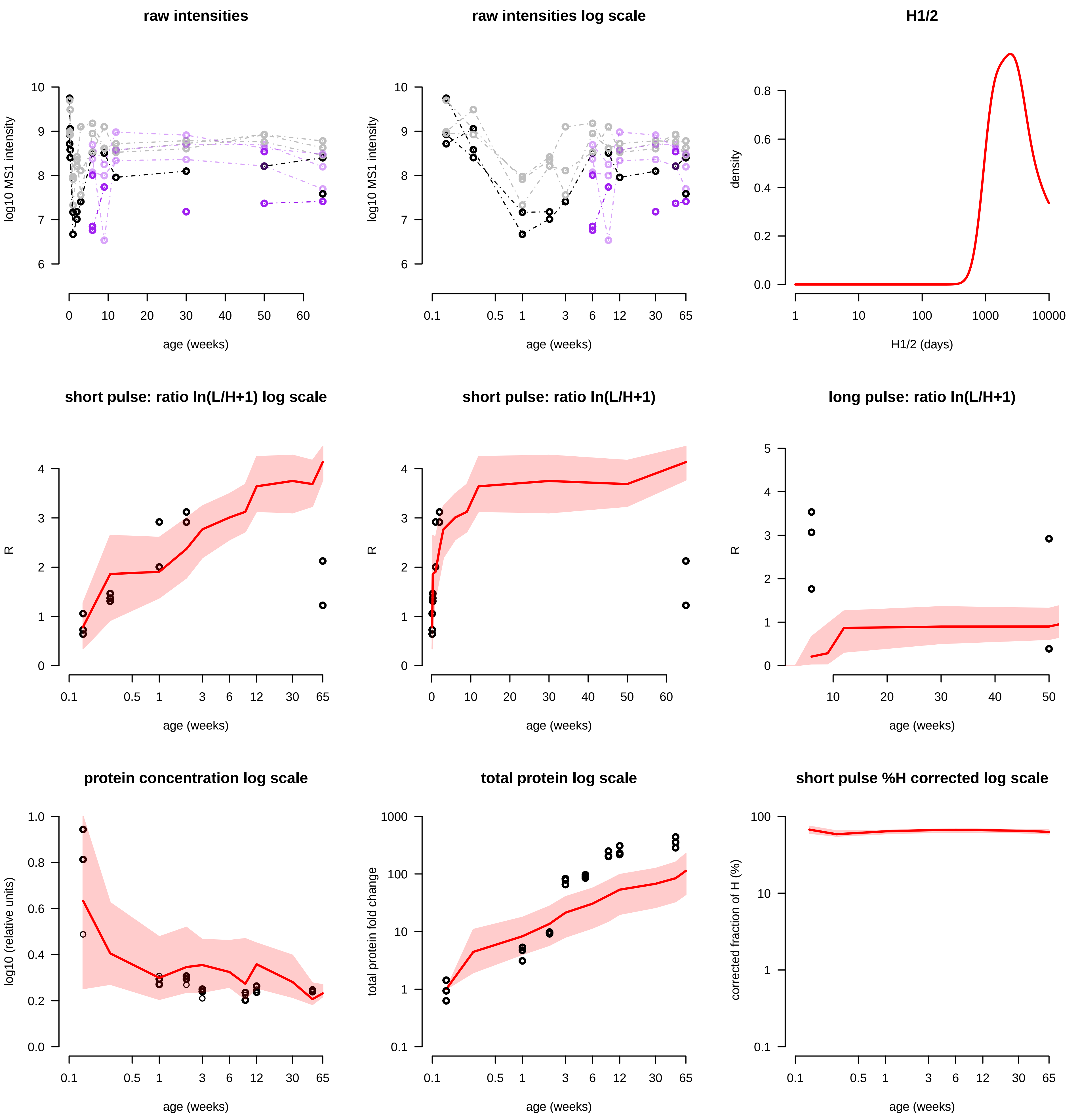

Q9D6Y9

>sp|Q9D6Y9|GLGB_MOUSE

1,4−alpha−glucan−branching enzyme

GN=Gbe1

Med H1/2: 555.8 days

Q5 H1/2: 185.2 days

Q95 H1/2: 4427.2 days

reliable model fit: no

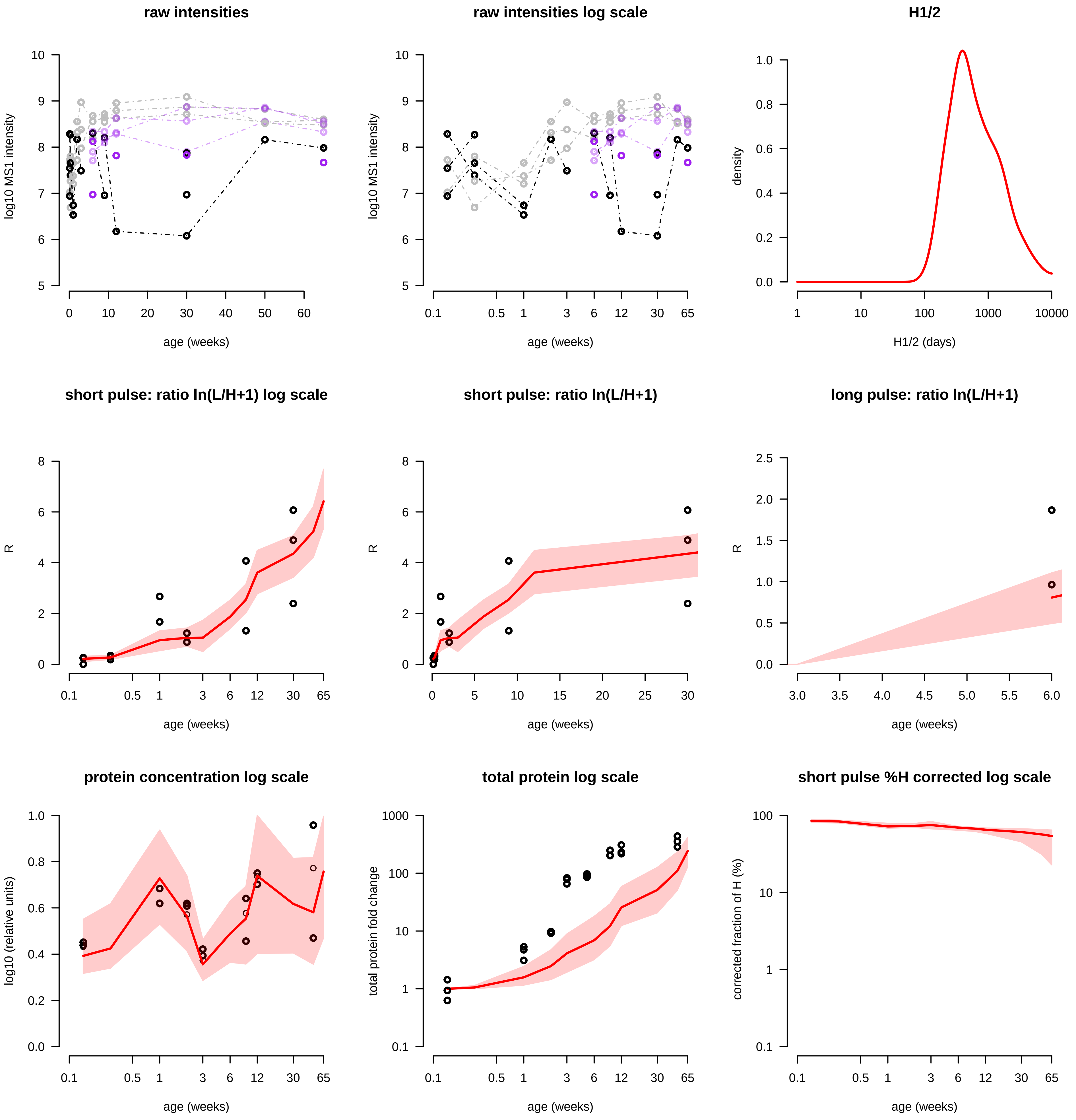

O35074

>sp|O35074|PTGIS_MOUSE

Prostacyclin synthase

GN=Ptgis

Med H1/2: 9.1 days

Q5 H1/2: 4.1 days

Q95 H1/2: 43.2 days

reliable model fit: yes

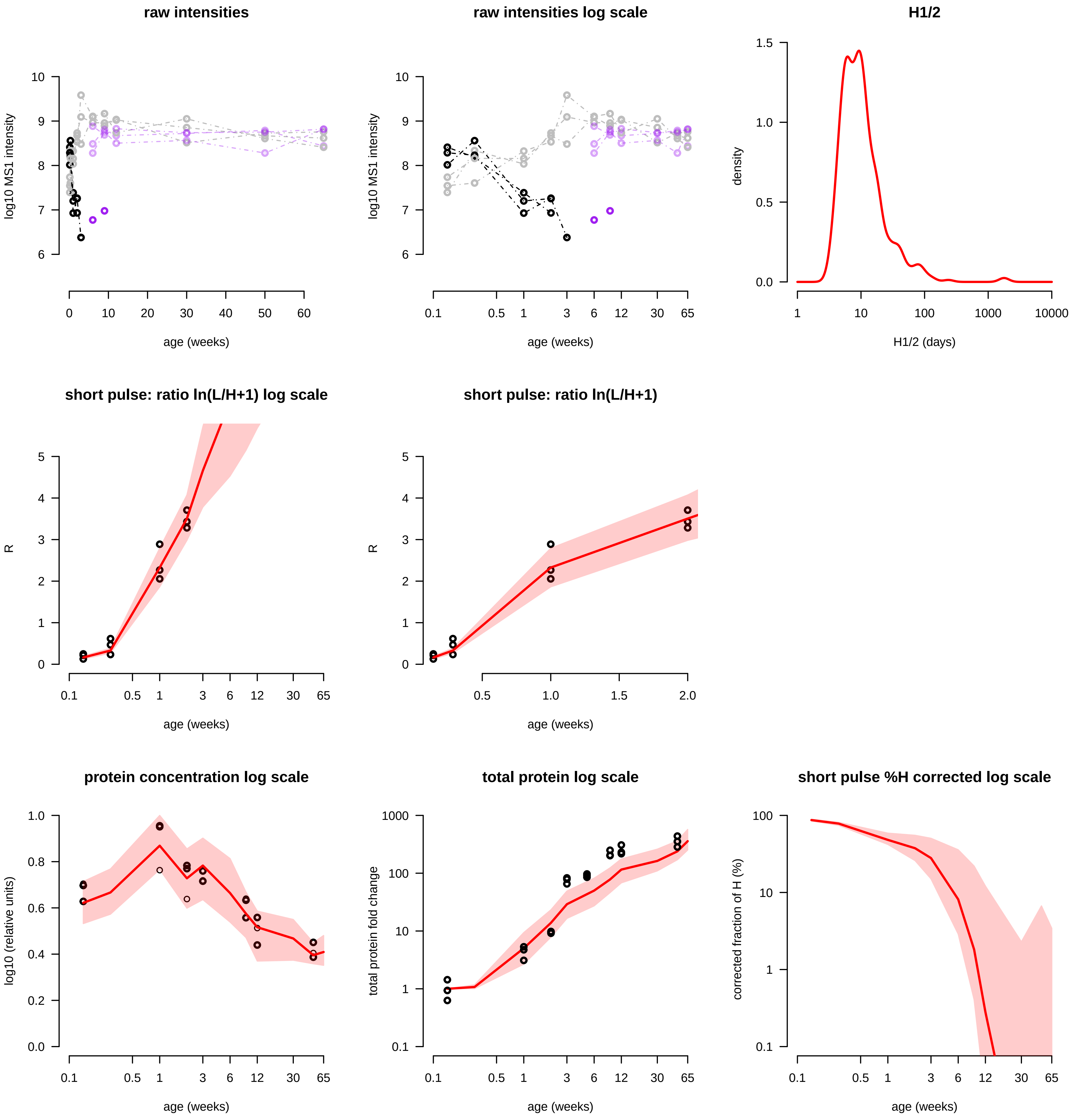

P62748

>sp|P62748|HPCL1_MOUSE

Hippocalcin−like protein 1

GN=Hpcal1

Med H1/2: 6.4 days

Q5 H1/2: 4.8 days

Q95 H1/2: 8.1 days

reliable model fit: yes

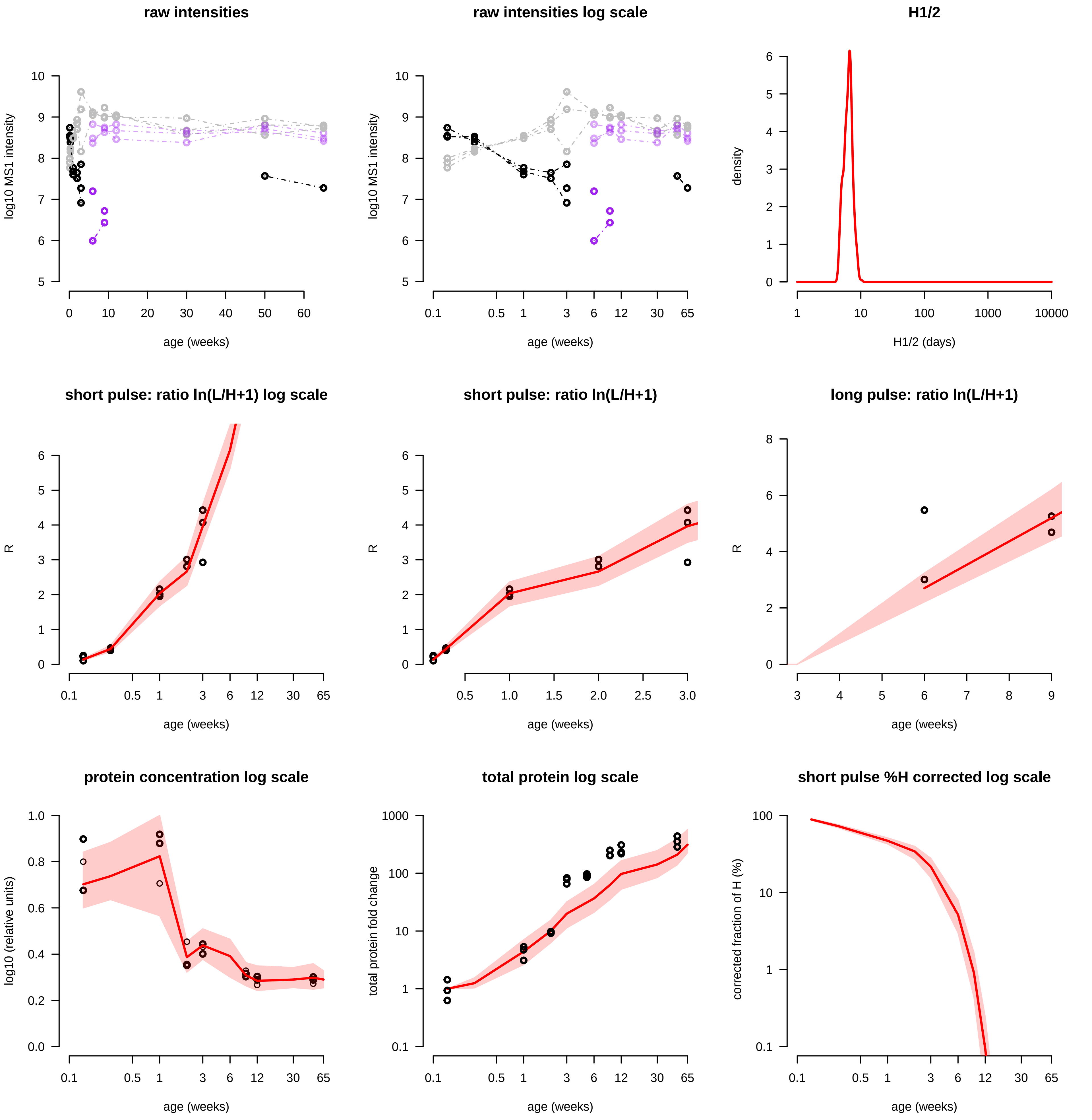

Q9DB27;Q9DB27−2

>sp|Q9DB27|MCTS1_MOUSE

Malignant T−cell−amplified sequence 1

GN=Mcts1

Med H1/2: 139.2 days

Q5 H1/2: 50.8 days

Q95 H1/2: 717.6 days

reliable model fit: yes

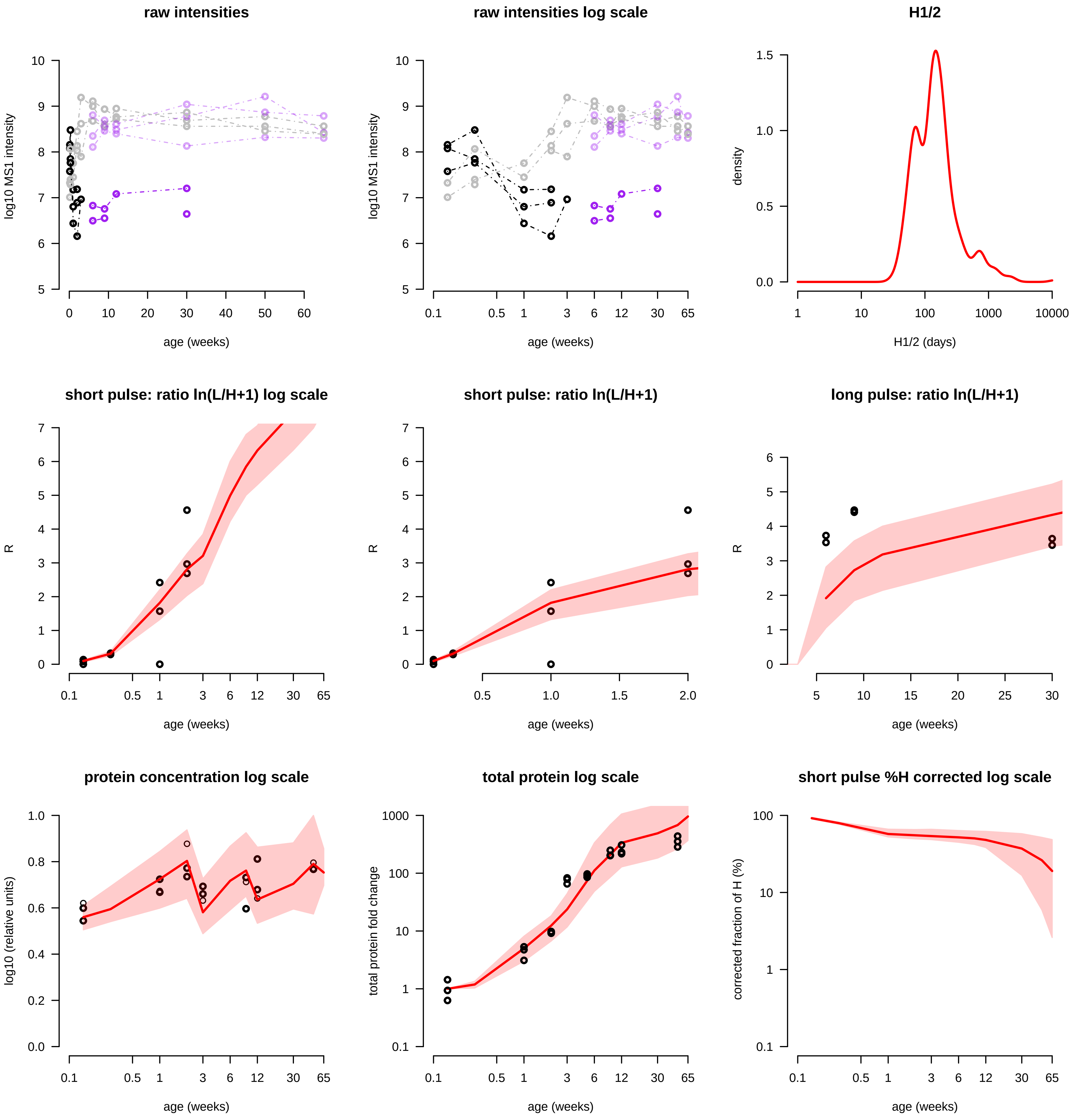

O54833

>sp|O54833|CSK22_MOUSE

Casein kinase II subunit alpha'

GN=Csnk2a2

Med H1/2: 7.9 days

Q5 H1/2: 3.1 days

Q95 H1/2: 35.9 days

reliable model fit: yes

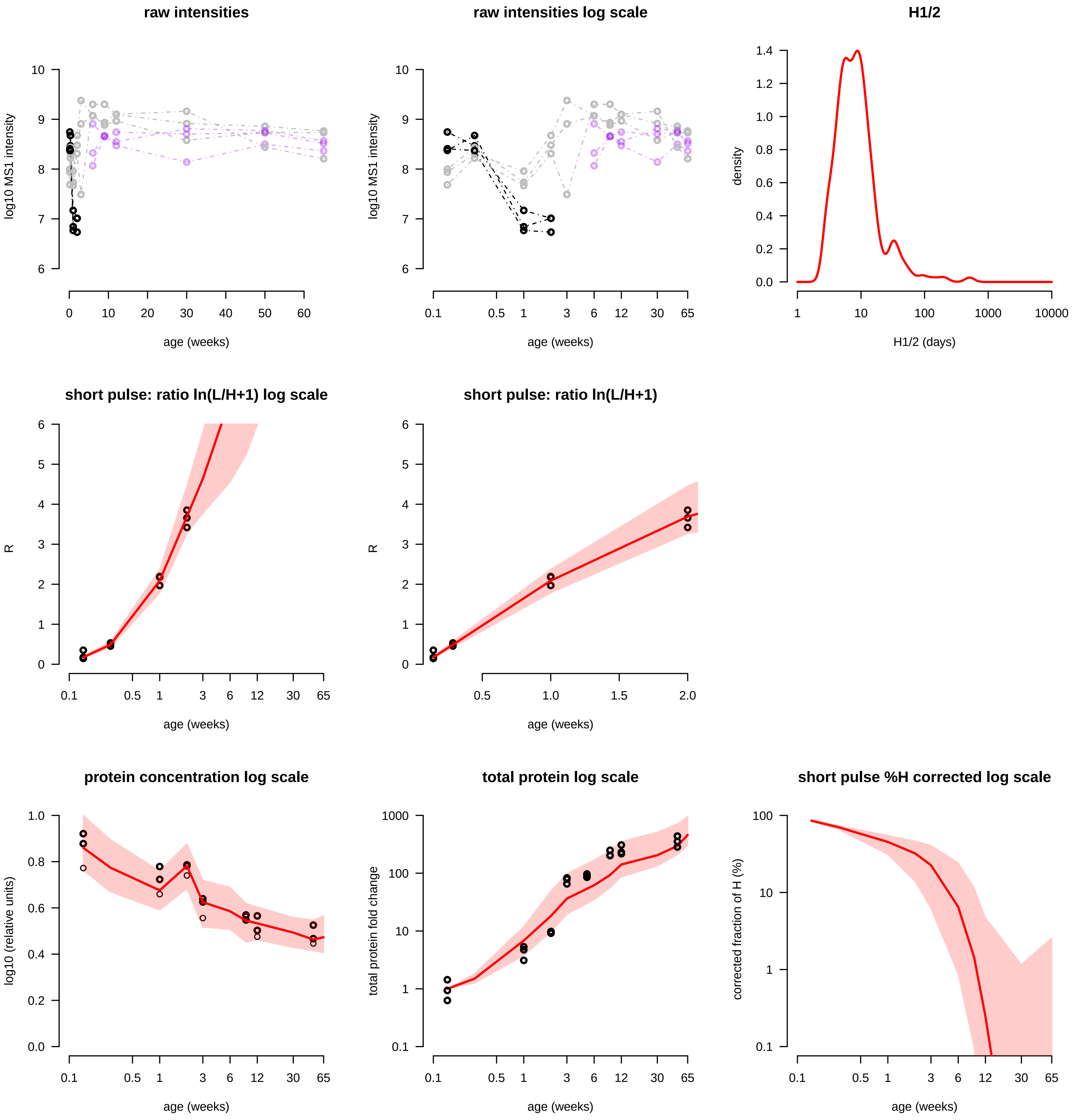

Q9CXA2

>sp|Q9CXA2|T3HPD_MOUSE

Trans−L−3−hydroxyproline dehydratase

GN=L3hypdh

Med H1/2: 6.3 days

Q5 H1/2: 5 days

Q95 H1/2: 9.2 days

reliable model fit: yes

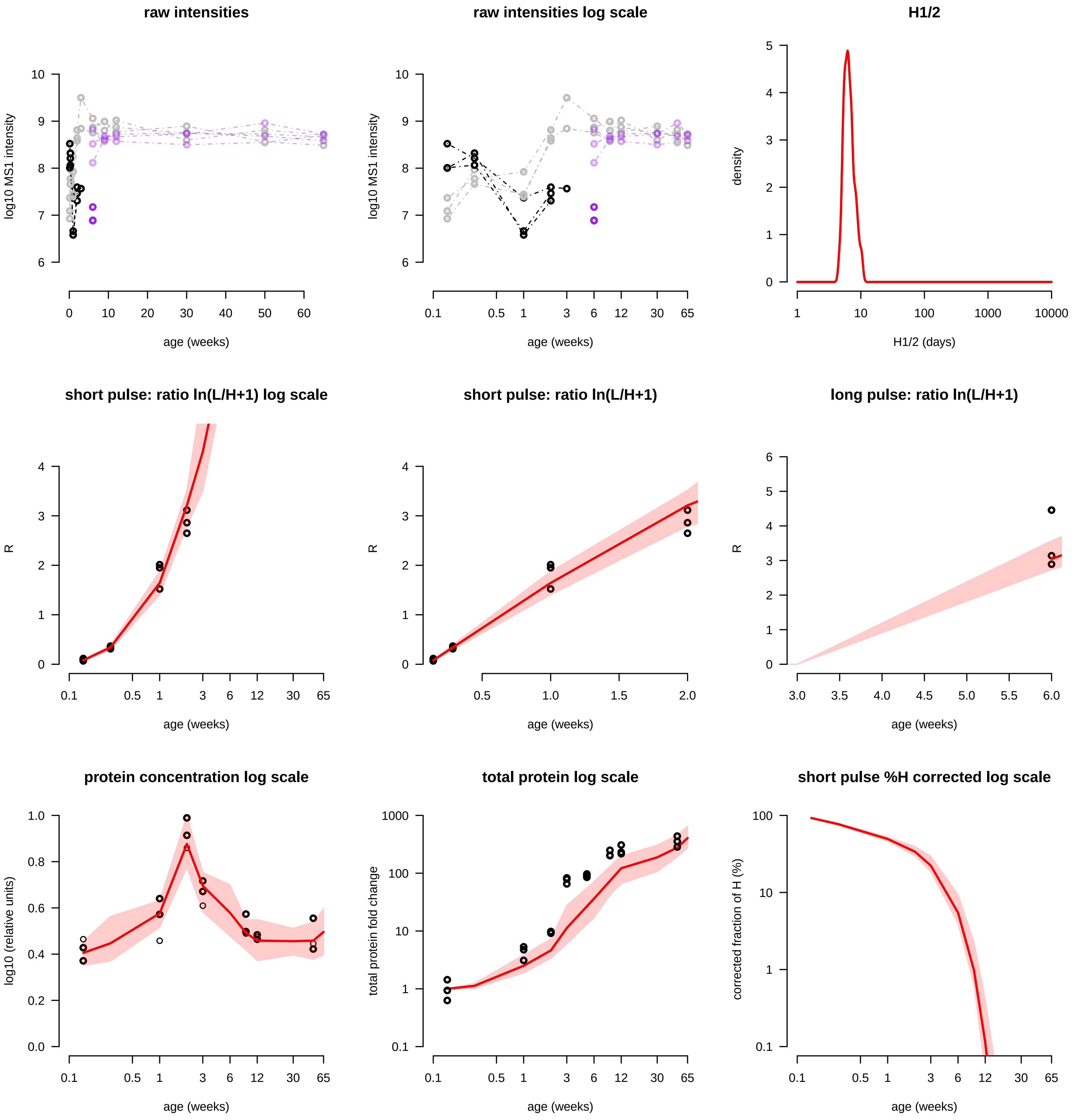

Q9QUH0

>sp|Q9QUH0|GLRX1_MOUSE

Glutaredoxin−1

GN=Glrx

Med H1/2: 36.8 days

Q5 H1/2: 10.4 days

Q95 H1/2: 901.5 days

reliable model fit: yes

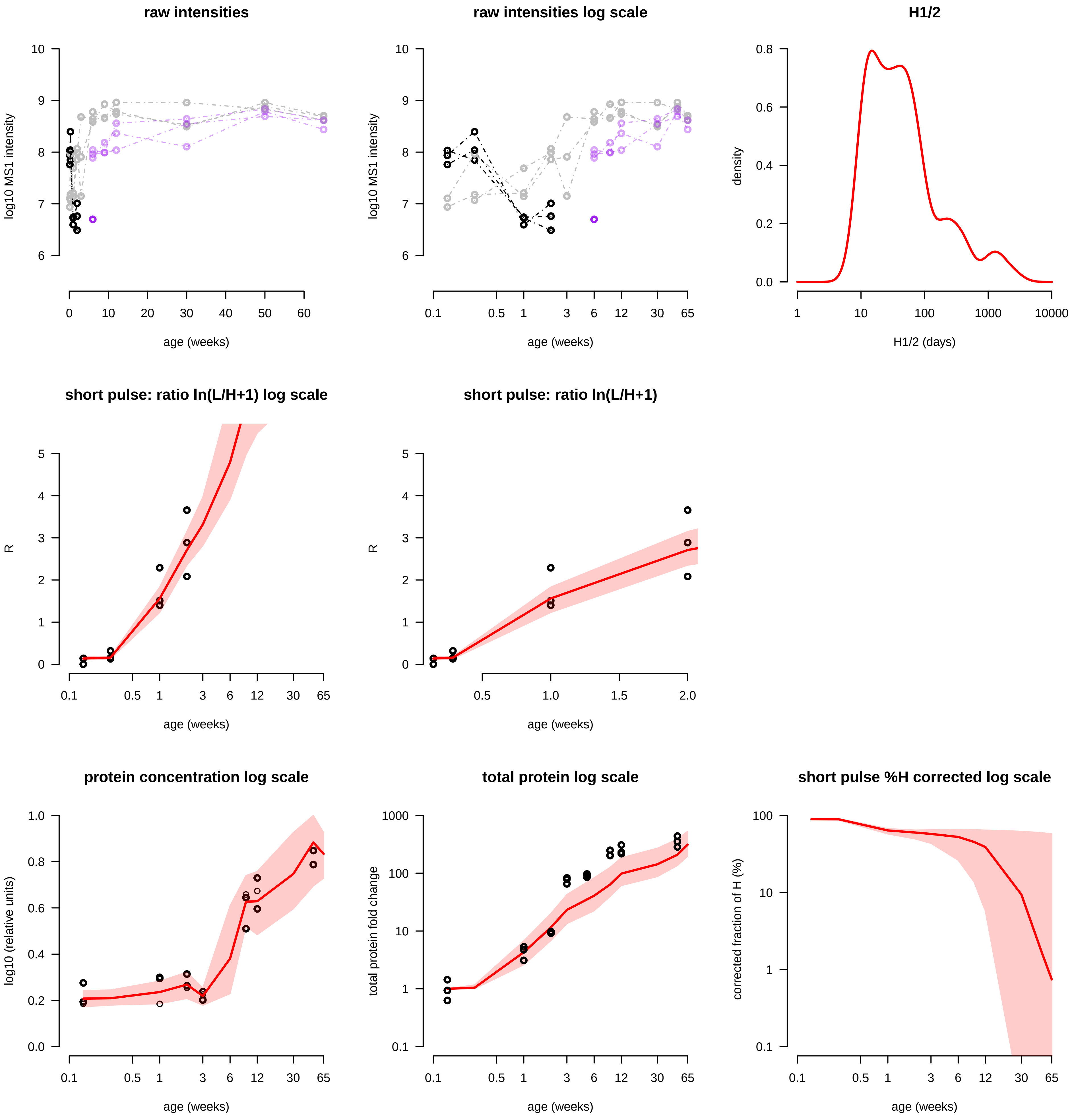

Q9CZ04−2;Q9CZ04

>sp|Q9CZ04−2|CSN7A_MOUSE

Isoform 2 of COP9 signalosome complex subunit 7a

GN=Cops7a

Med H1/2: 4.2 days

Q5 H1/2: 3.4 days

Q95 H1/2: 5.7 days

reliable model fit: yes

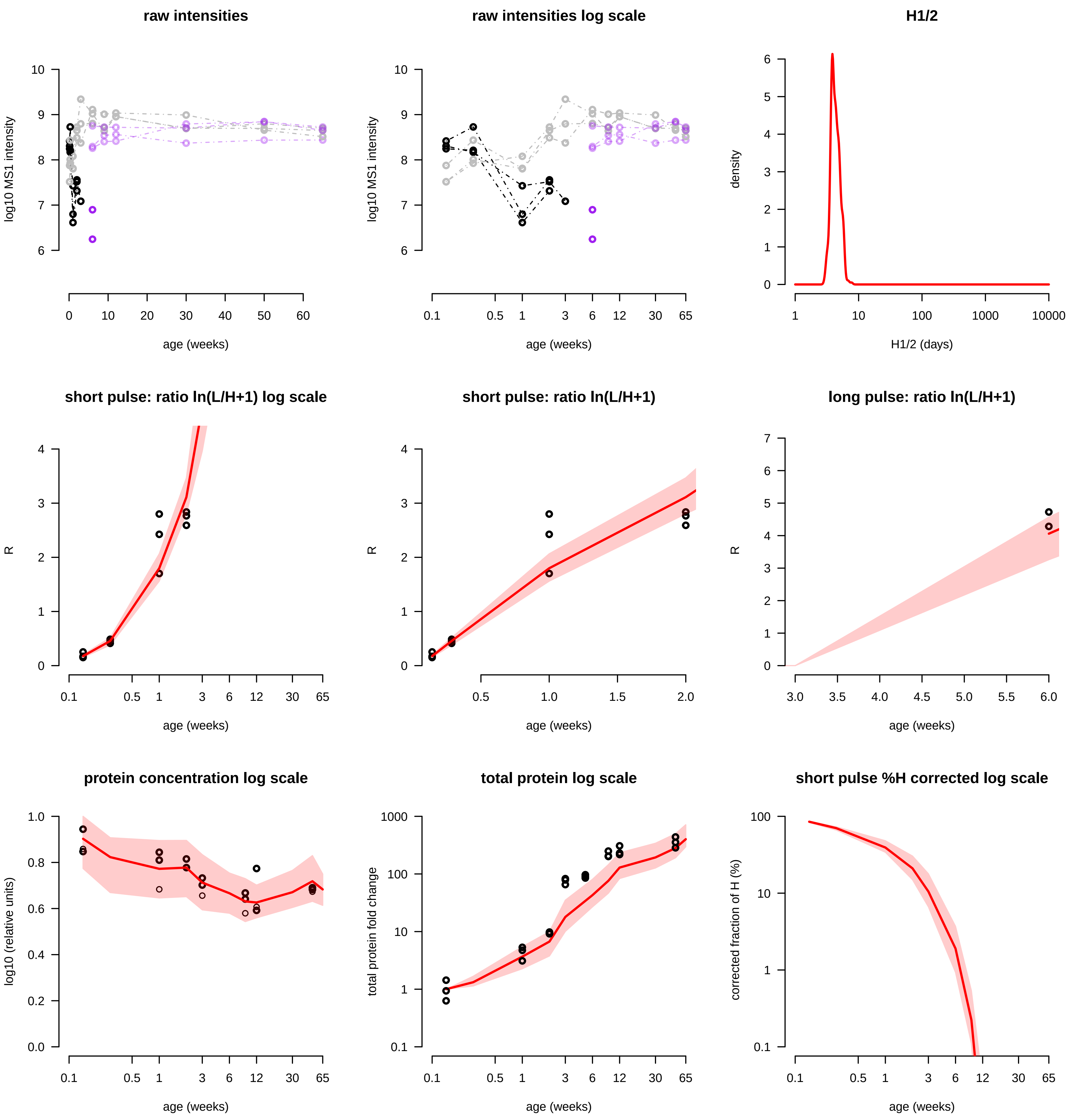

Q64010;Q64010−2

>sp|Q64010|CRK_MOUSE

Adapter molecule crk

GN=Crk

Med H1/2: 4.7 days

Q5 H1/2: 3.7 days

Q95 H1/2: 5.7 days

reliable model fit: yes

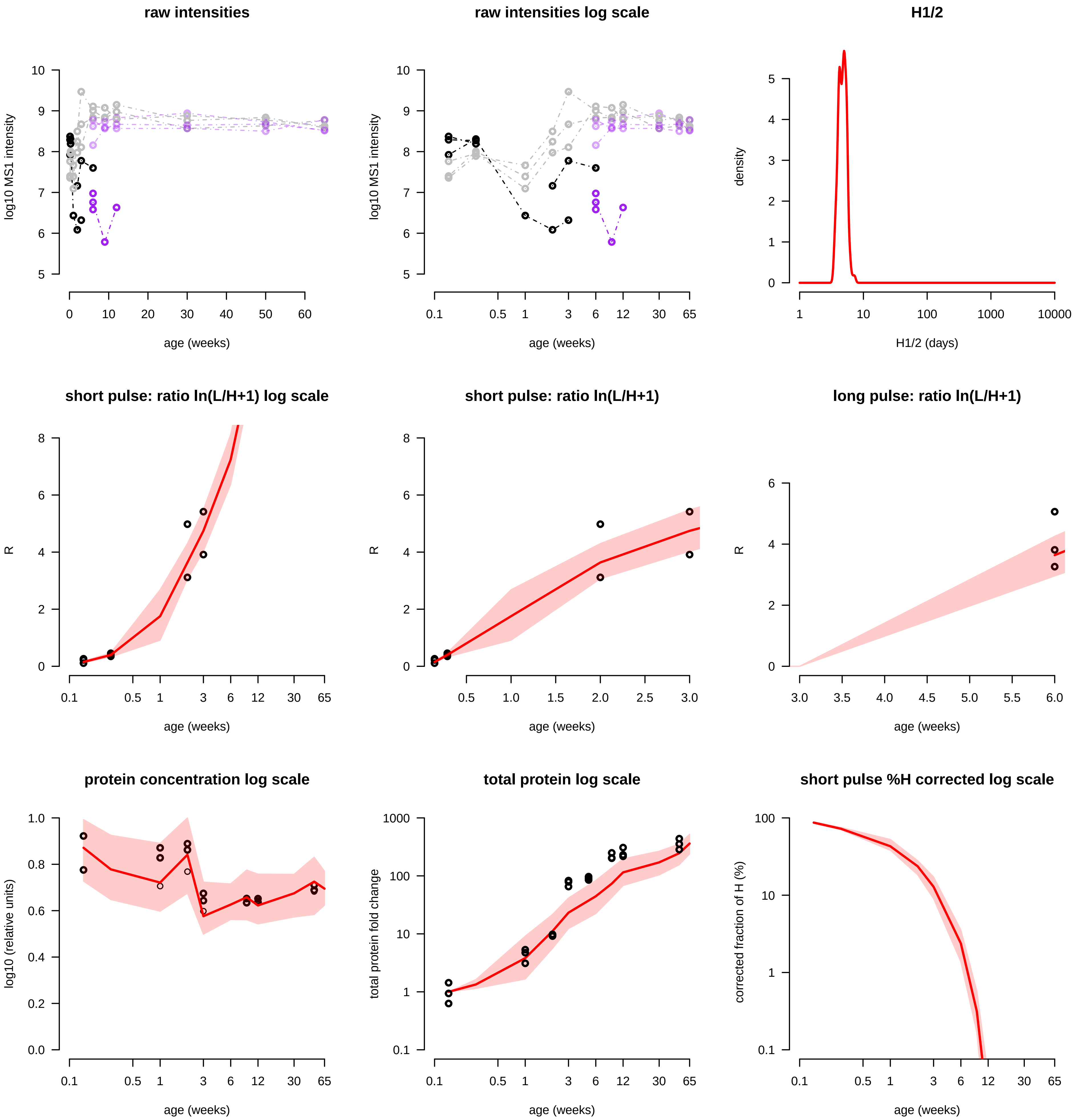

P81117

>sp|P81117|NUCB2_MOUSE

Nucleobindin−2

GN=Nucb2

Med H1/2: 4.4 days

Q5 H1/2: 3.2 days

Q95 H1/2: 6.1 days

reliable model fit: yes

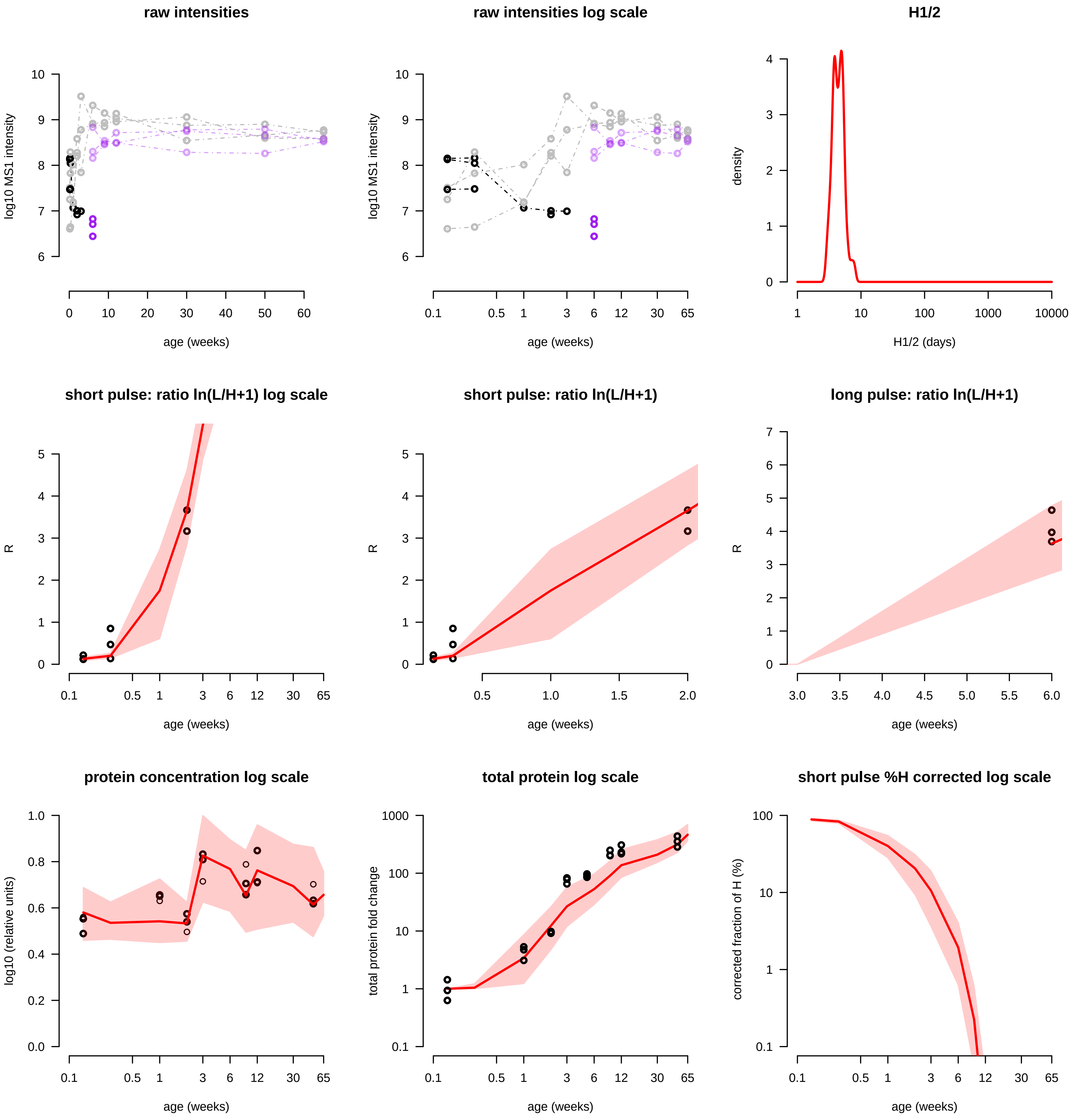

Q3UHX2

>sp|Q3UHX2|HAP28_MOUSE

28 kDa heat− and acid−stable phosphoprotein

GN=Pdap1

Med H1/2: 4.1 days

Q5 H1/2: 3.3 days

Q95 H1/2: 5.8 days

reliable model fit: yes

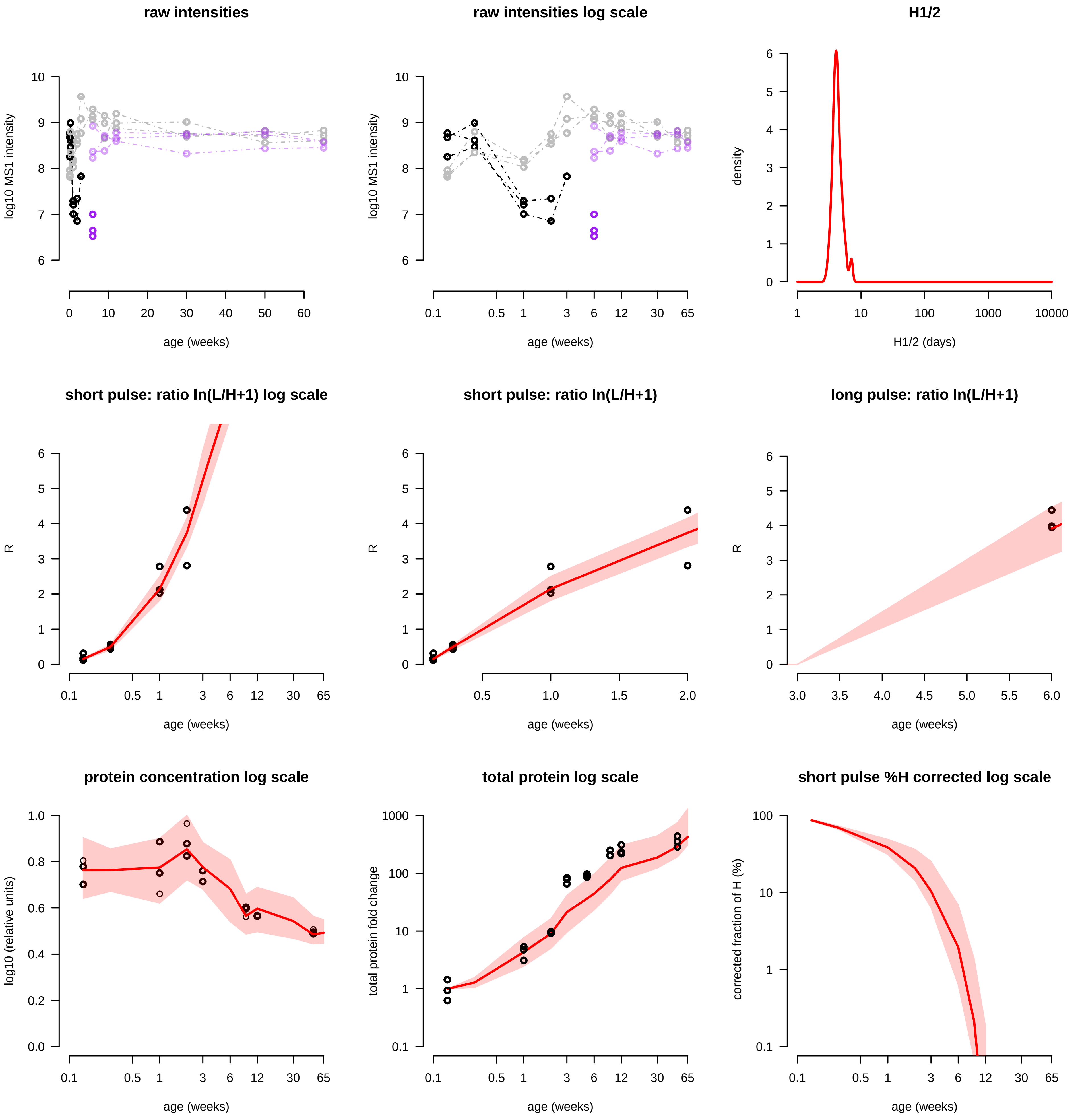

Q9CX56

>sp|Q9CX56|PSMD8_MOUSE

26S proteasome non−ATPase regulatory subunit 8

GN=Psmd8

Med H1/2: 4.7 days

Q5 H1/2: 3.9 days

Q95 H1/2: 5.7 days

reliable model fit: yes

raw intensities

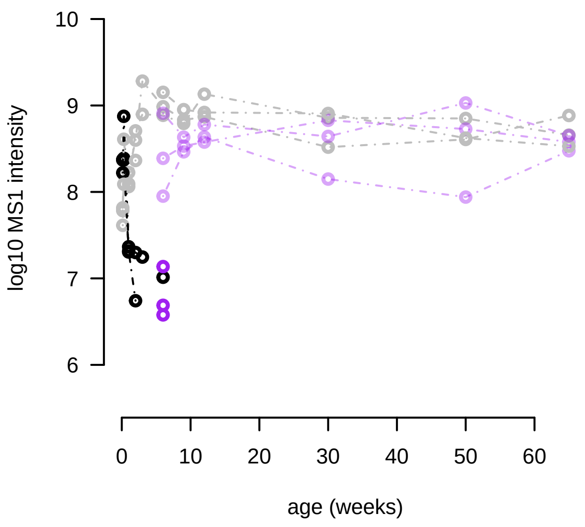

raw intensities log scale

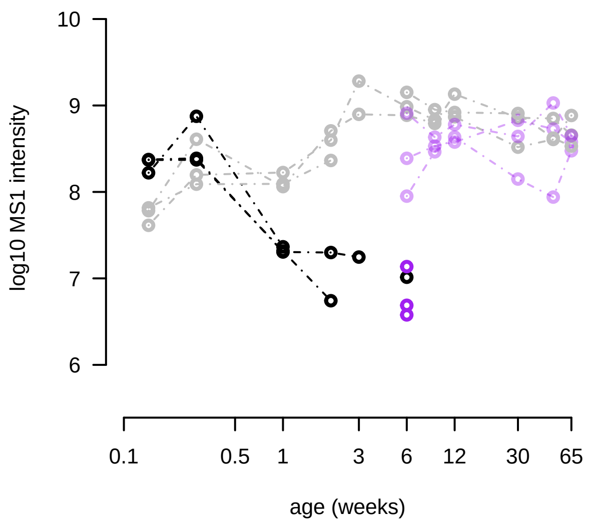

H1/2

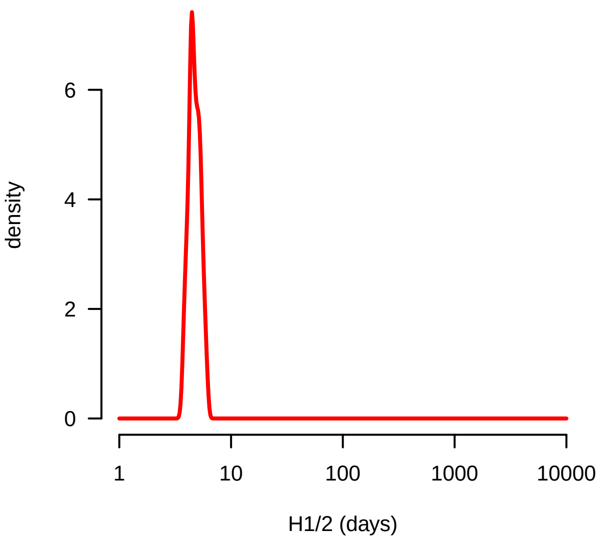

short pulse: ratio ln(L/H+1) log scale

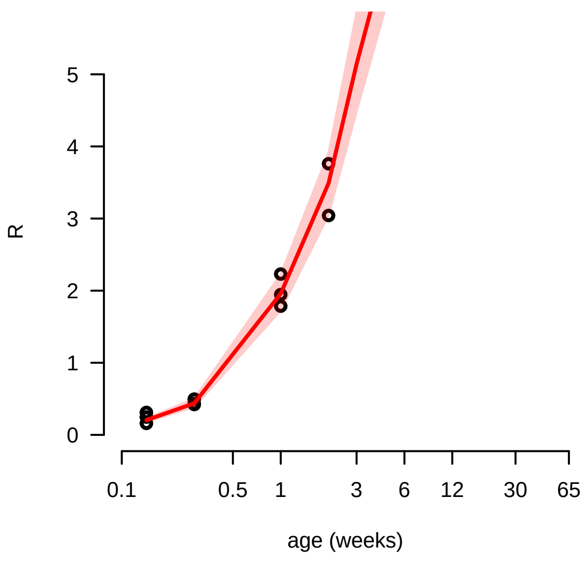

short pulse: ratio ln(L/H+1)

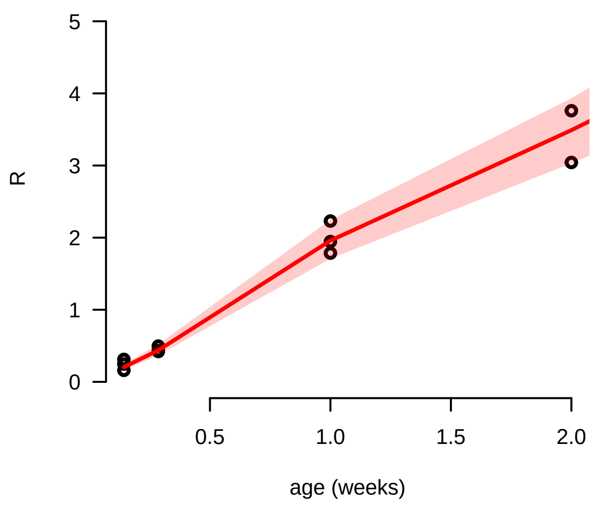

long pulse: ratio ln(L/H+1)

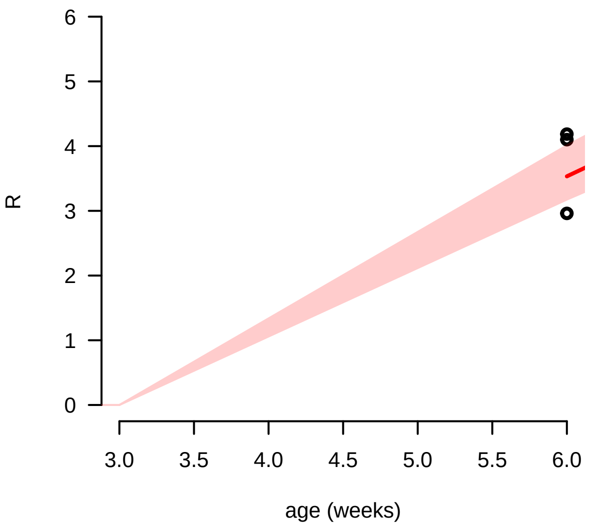

protein concentration log scale

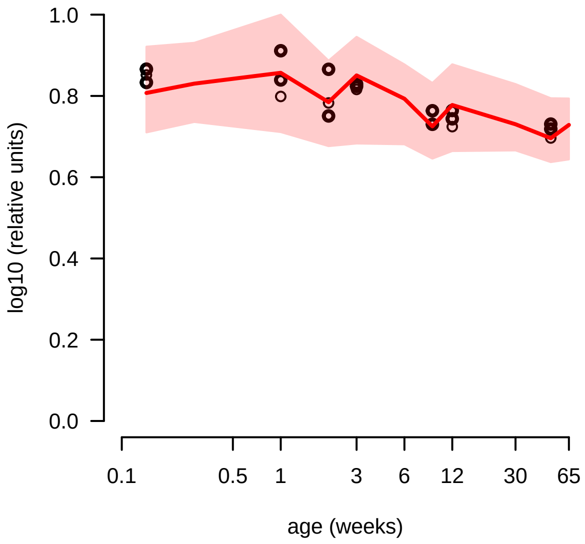

total protein log scale

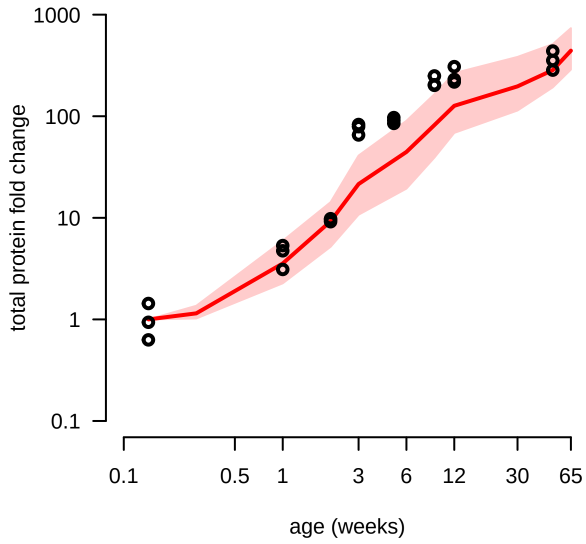

short pulse %H corrected log scale

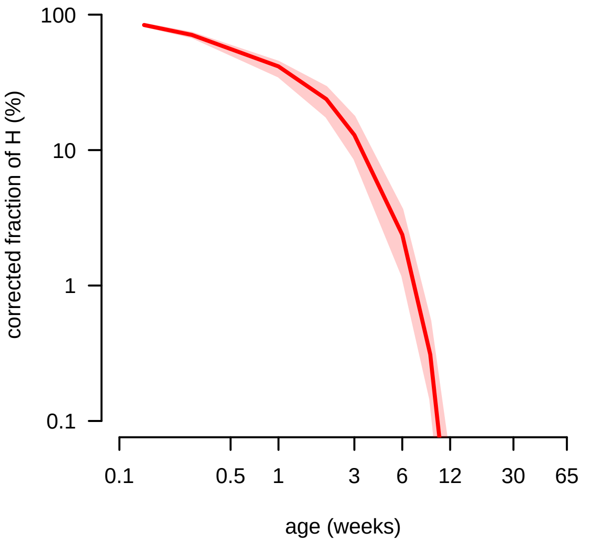

Q8R3Q6

>sp|Q8R3Q6|MIX23_MOUSE

Protein MIX23

GN=Mix23

Med H1/2: 6.2 days

Q5 H1/2: 4.6 days

Q95 H1/2: 8.8 days

reliable model fit: yes

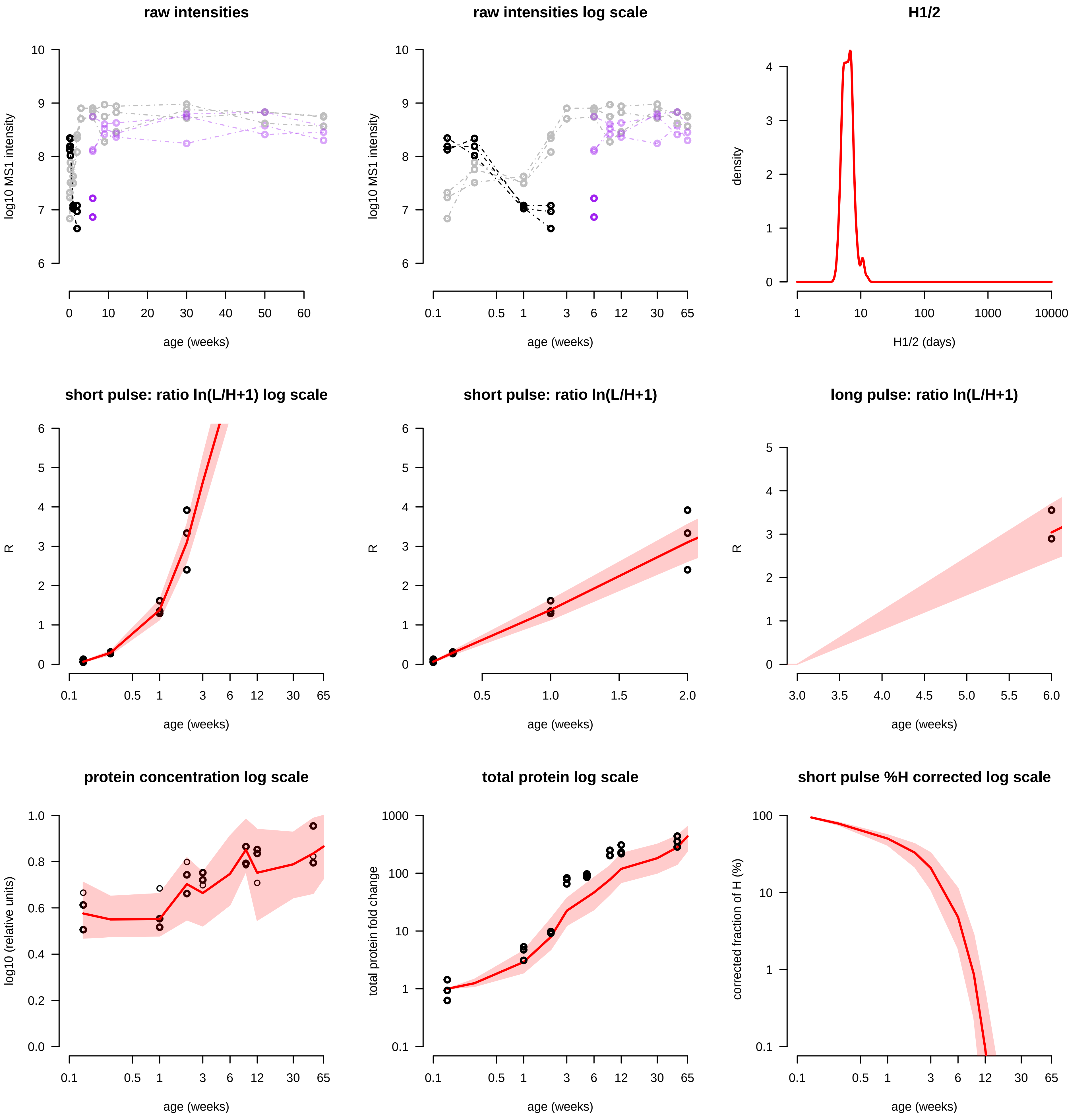

Q9R0B9

>sp|Q9R0B9|PLOD2_MOUSE

Procollagen−lysine,2−oxoglutarate 5−dioxygenase 2

GN=Plod2

Med H1/2: 3.7 days

Q5 H1/2: 2.9 days

Q95 H1/2: 4.8 days

reliable model fit: yes

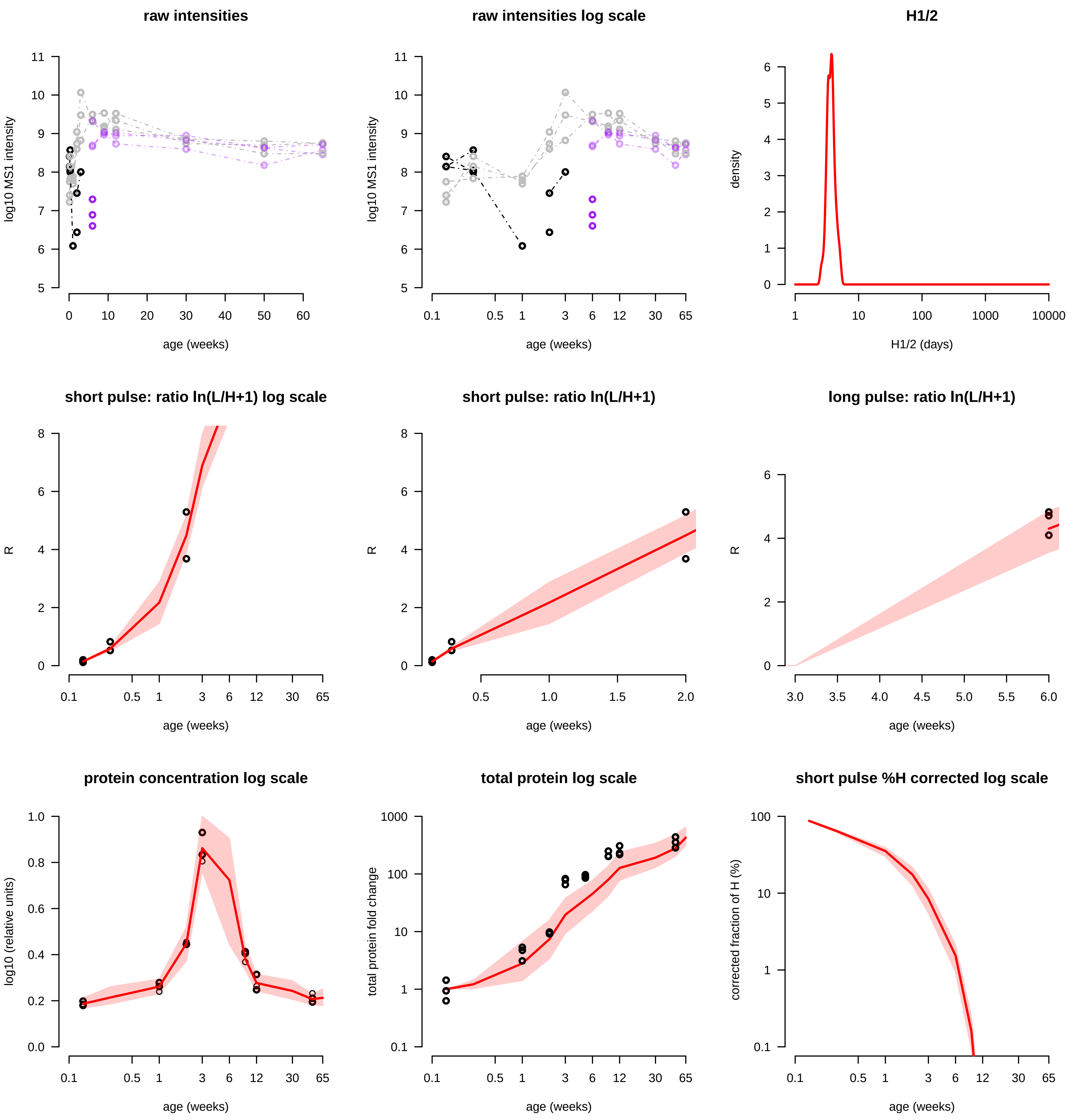

Q8BMG7

>sp|Q8BMG7|RBGPR_MOUSE

Rab3 GTPase−activating protein non−catalytic subunit

GN=Rab3gap2

Med H1/2: 4.5 days

Q5 H1/2: 2.7 days

Q95 H1/2: 26.7 days

reliable model fit: yes

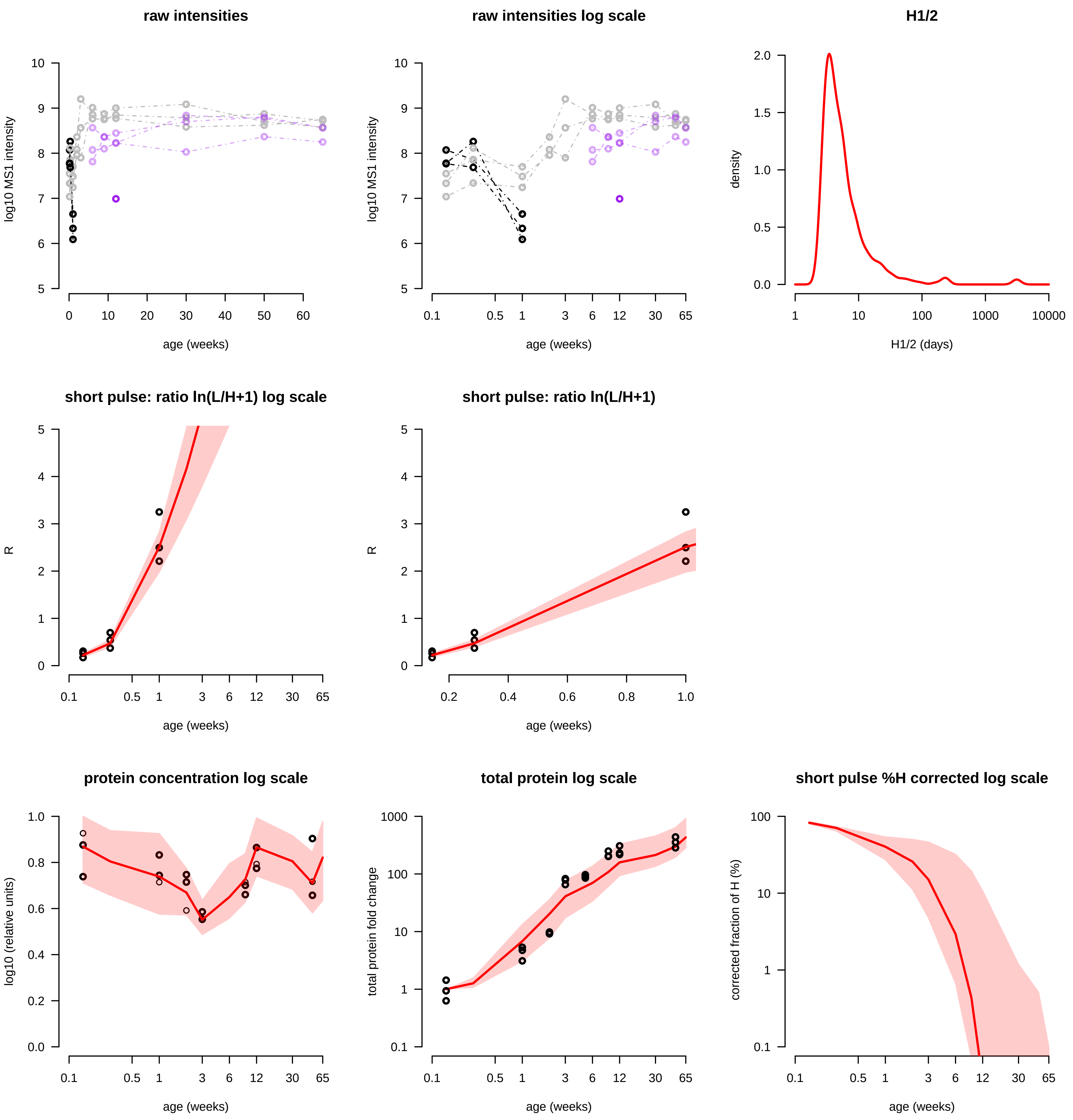

Q8CJG0

>sp|Q8CJG0|AGO2_MOUSE

Protein argonaute−2

GN=Ago2

Med H1/2: 12.2 days

Q5 H1/2: 4.5 days

Q95 H1/2: 198.2 days

reliable model fit: yes

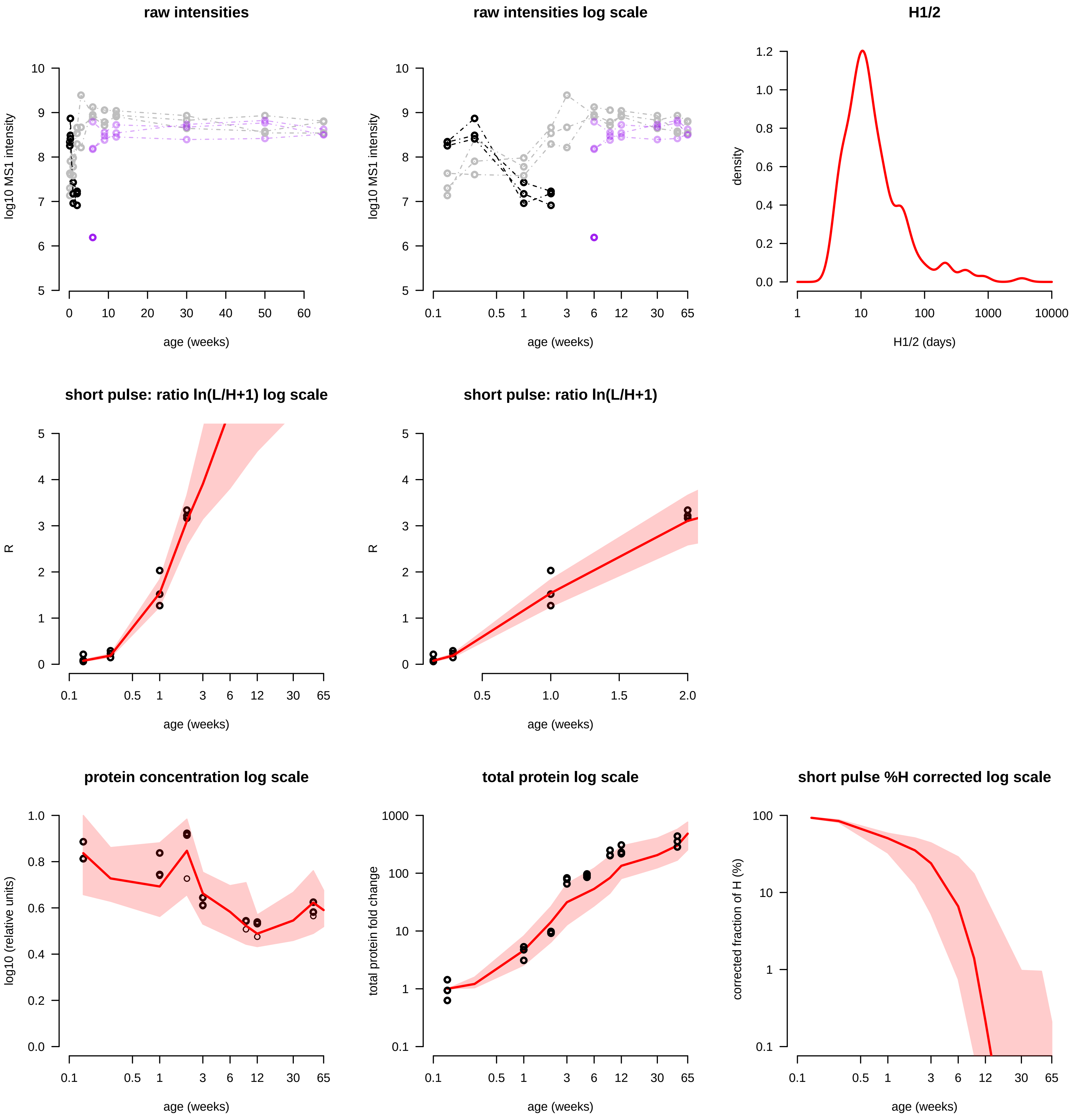

Q9WUA3;Q9WUA3−2

>sp|Q9WUA3|PFKAP_MOUSE

ATP−dependent 6−phosphofructokinase, platelet type

GN=Pfkp

Med H1/2: 886.2 days

Q5 H1/2: 283.7 days

Q95 H1/2: 7705 days

reliable model fit: yes

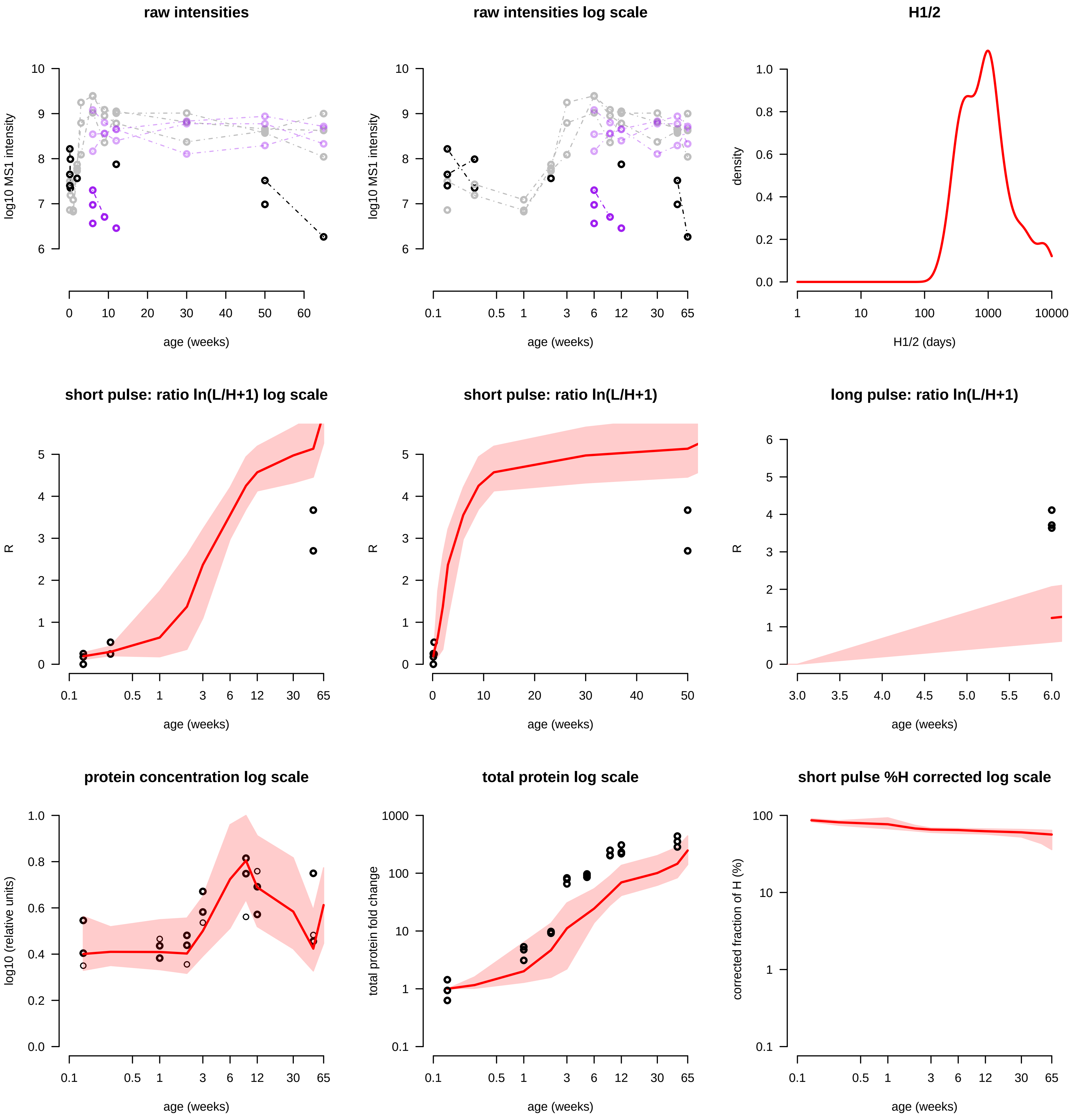

Q8BHG2;Q8BHG2−3;Q8BHG2−2

>sp|Q8BHG2|CZIB_MOUSE

CXXC motif containing zinc binding protein

GN=Czib

Med H1/2: 5 days

Q5 H1/2: 4 days

Q95 H1/2: 6.7 days

reliable model fit: yes

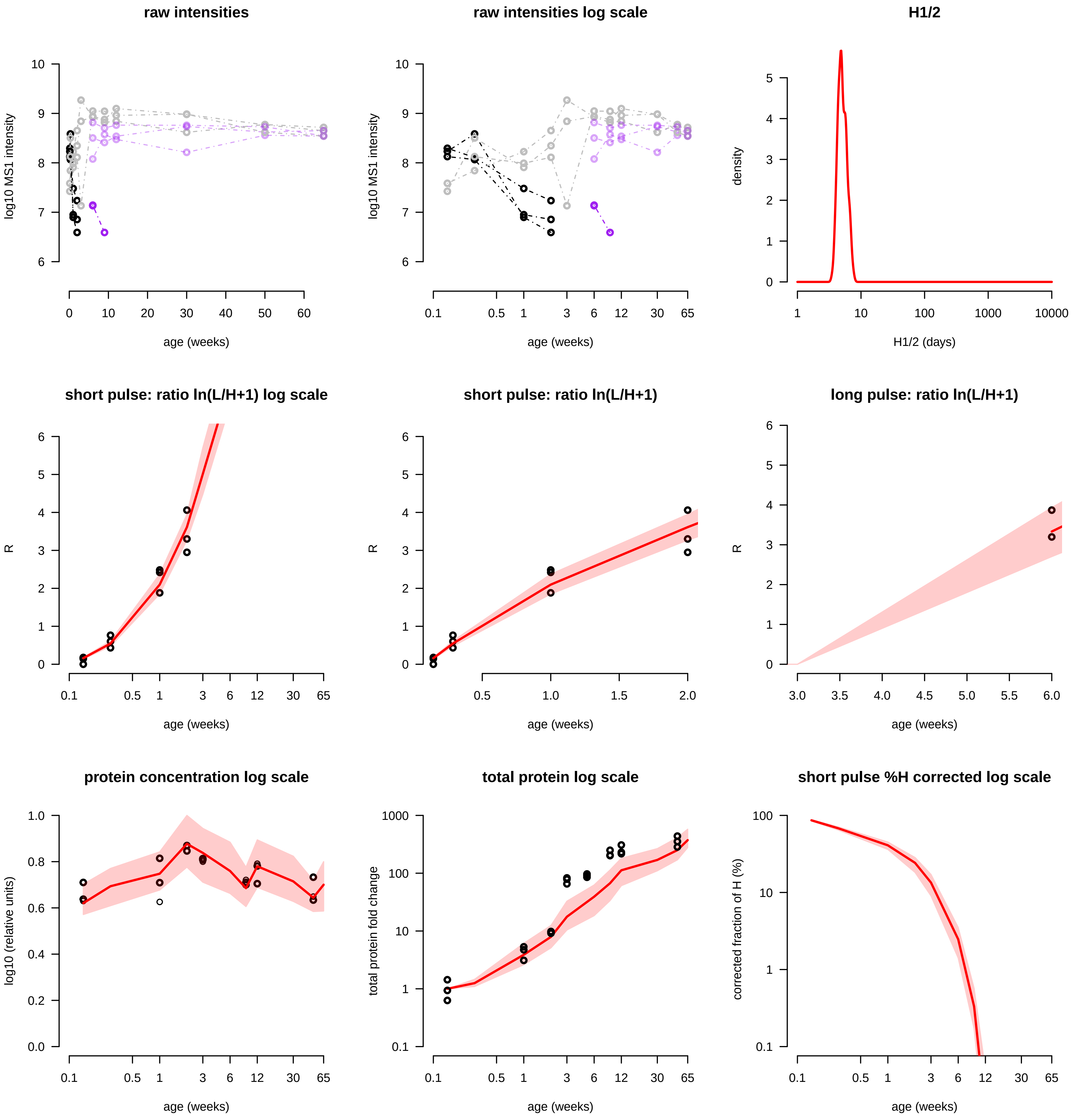

Q9CQV7;Q9CQV7−3

>sp|Q9CQV7|TIM14_MOUSE

Mitochondrial import inner membrane translocase subunit TIM14

GN=Dnajc19

Med H1/2: 4.9 days

Q5 H1/2: 2.8 days

Q95 H1/2: 25.8 days

reliable model fit: yes

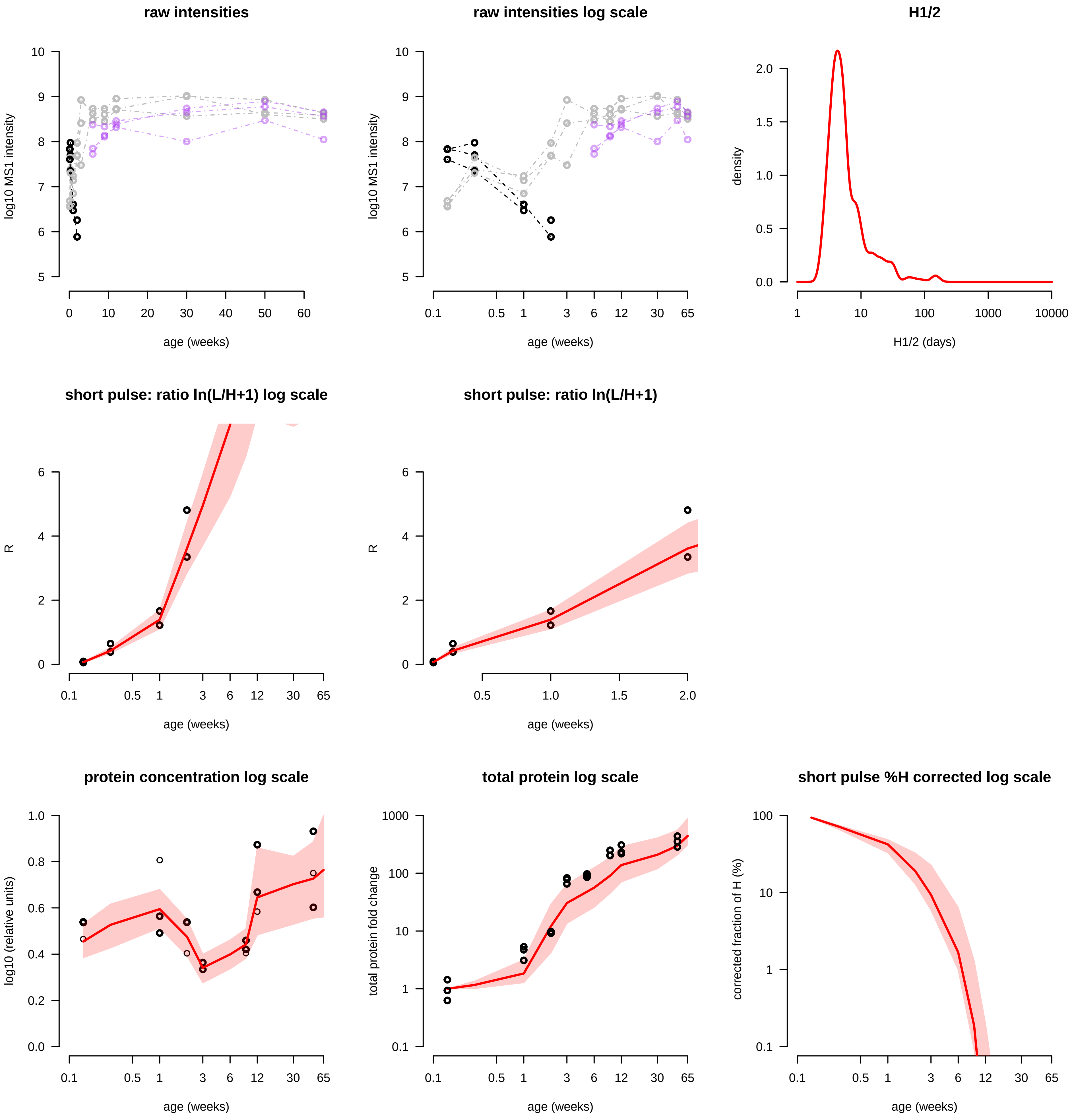

Q3ULD5

>sp|Q3ULD5|MCCB_MOUSE

Methylcrotonoyl−CoA carboxylase beta chain, mitochondrial

GN=Mccc2

Med H1/2: 10 days

Q5 H1/2: 3.9 days

Q95 H1/2: 80.9 days

reliable model fit: yes

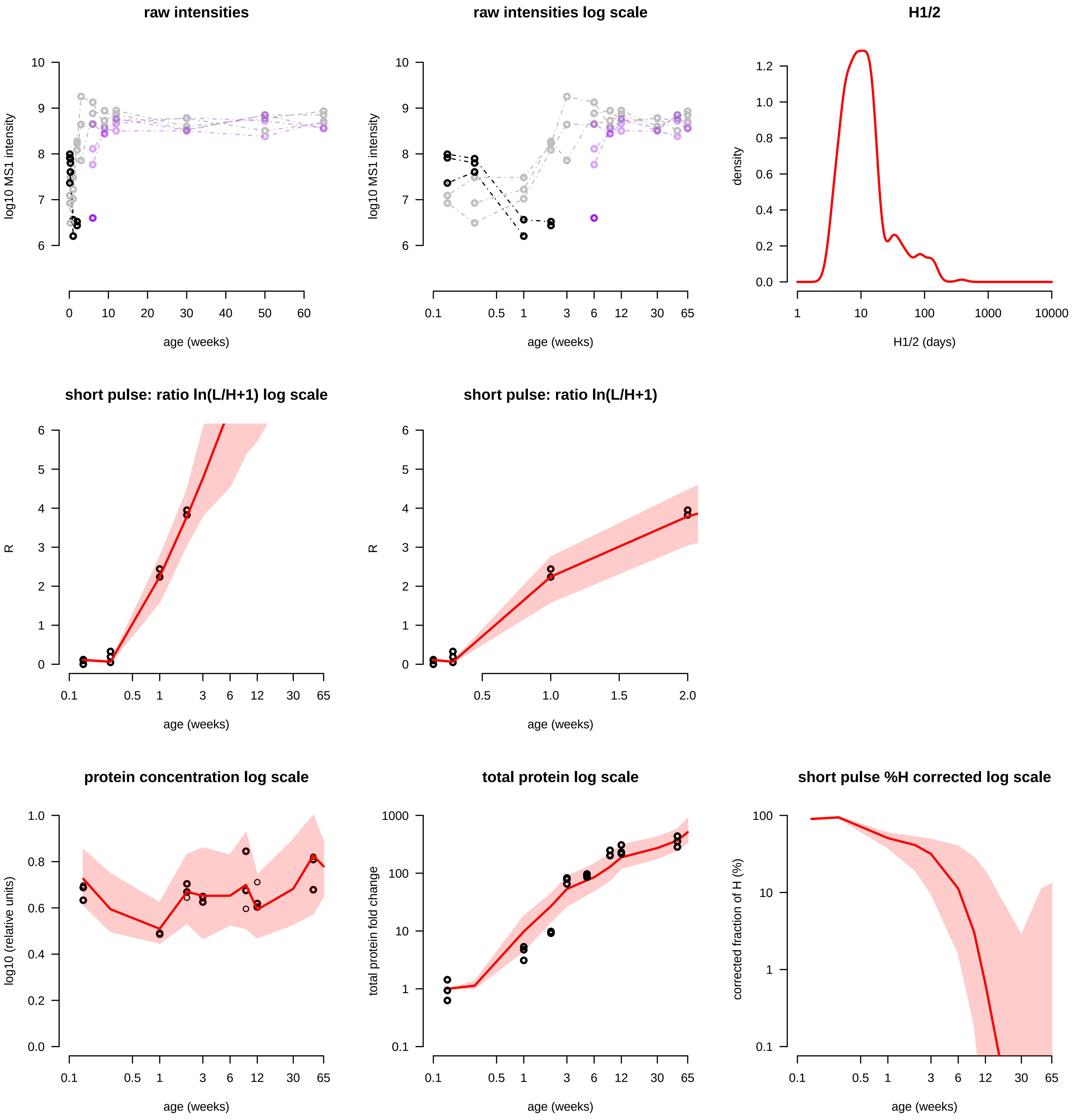

Q91YE6

>sp|Q91YE6|IPO9_MOUSE

Importin−9

GN=Ipo9

Med H1/2: 3.6 days

Q5 H1/2: 2.9 days

Q95 H1/2: 4.6 days

reliable model fit: yes

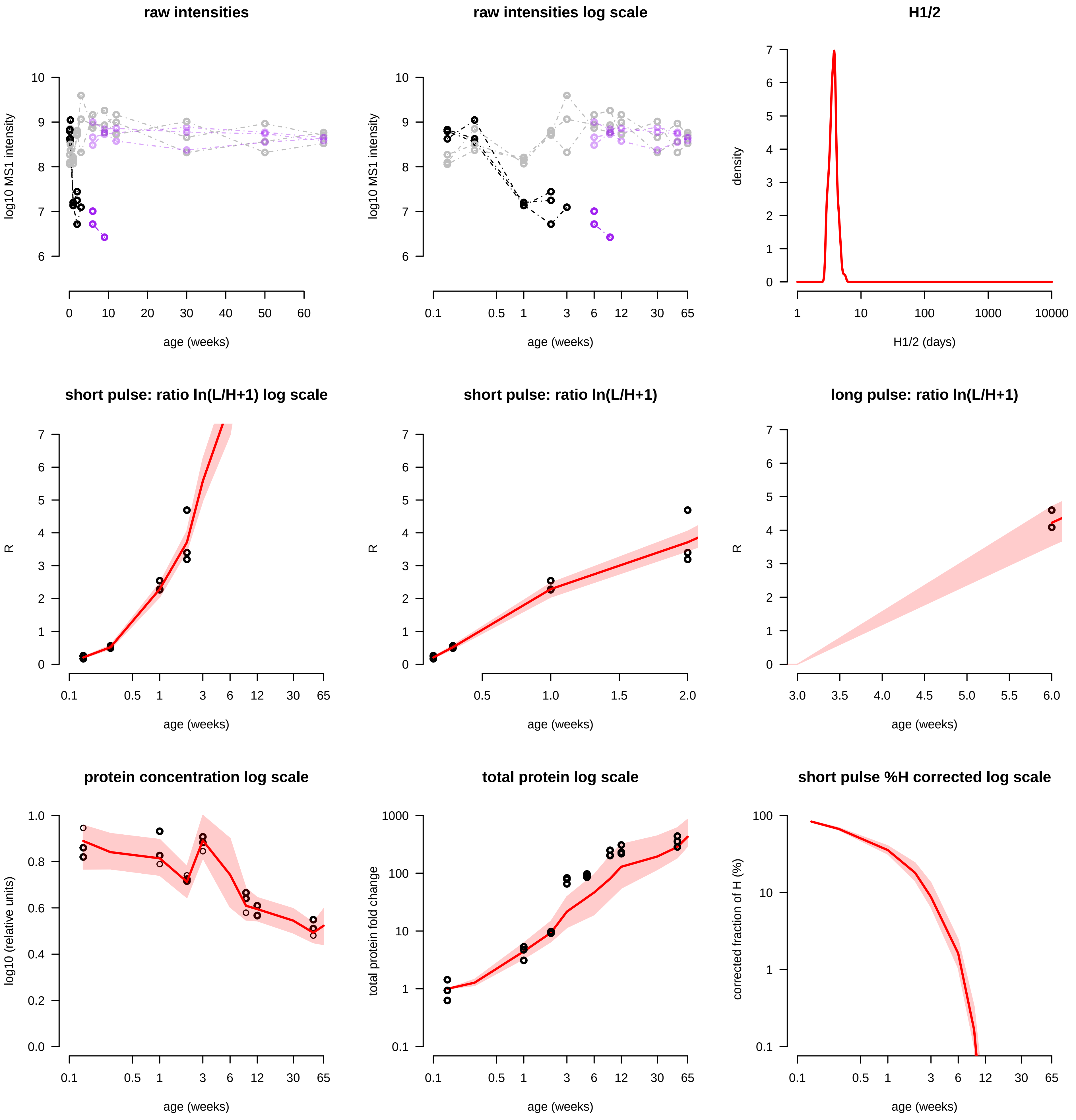

P21278

>sp|P21278|GNA11_MOUSE

Guanine nucleotide−binding protein subunit alpha−11

GN=Gna11

Med H1/2: 5.2 days

Q5 H1/2: 4.5 days

Q95 H1/2: 6.3 days

reliable model fit: yes

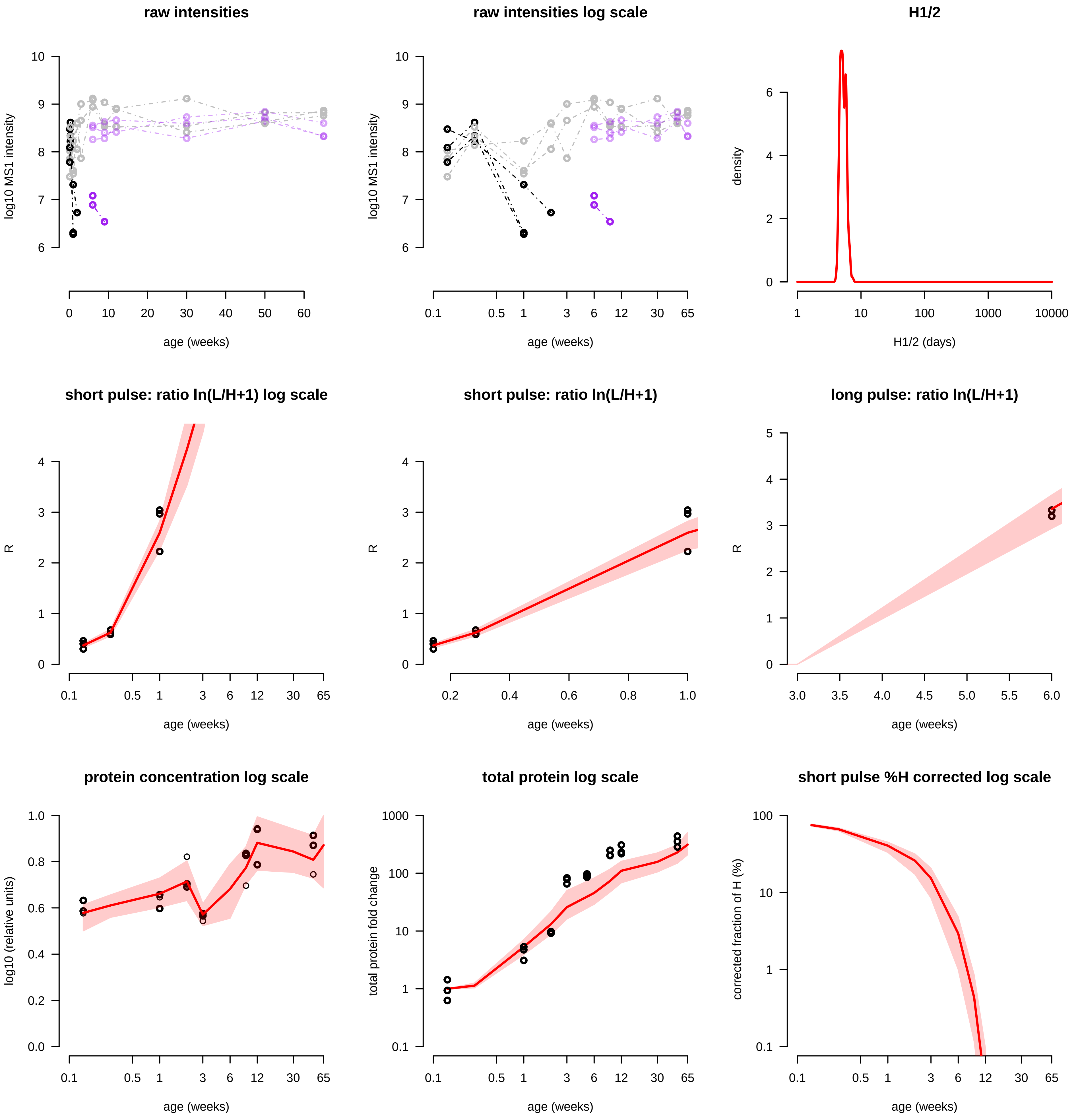

Q9Z1G4−3;Q9Z1G4−2

>sp|Q9Z1G4−3|VPP1_MOUSE

Isoform A1−III of V−type proton ATPase 116 kDa subunit a1

GN=Atp6v0a1

Med H1/2: 6.5 days

Q5 H1/2: 3 days

Q95 H1/2: 28.7 days

reliable model fit: yes

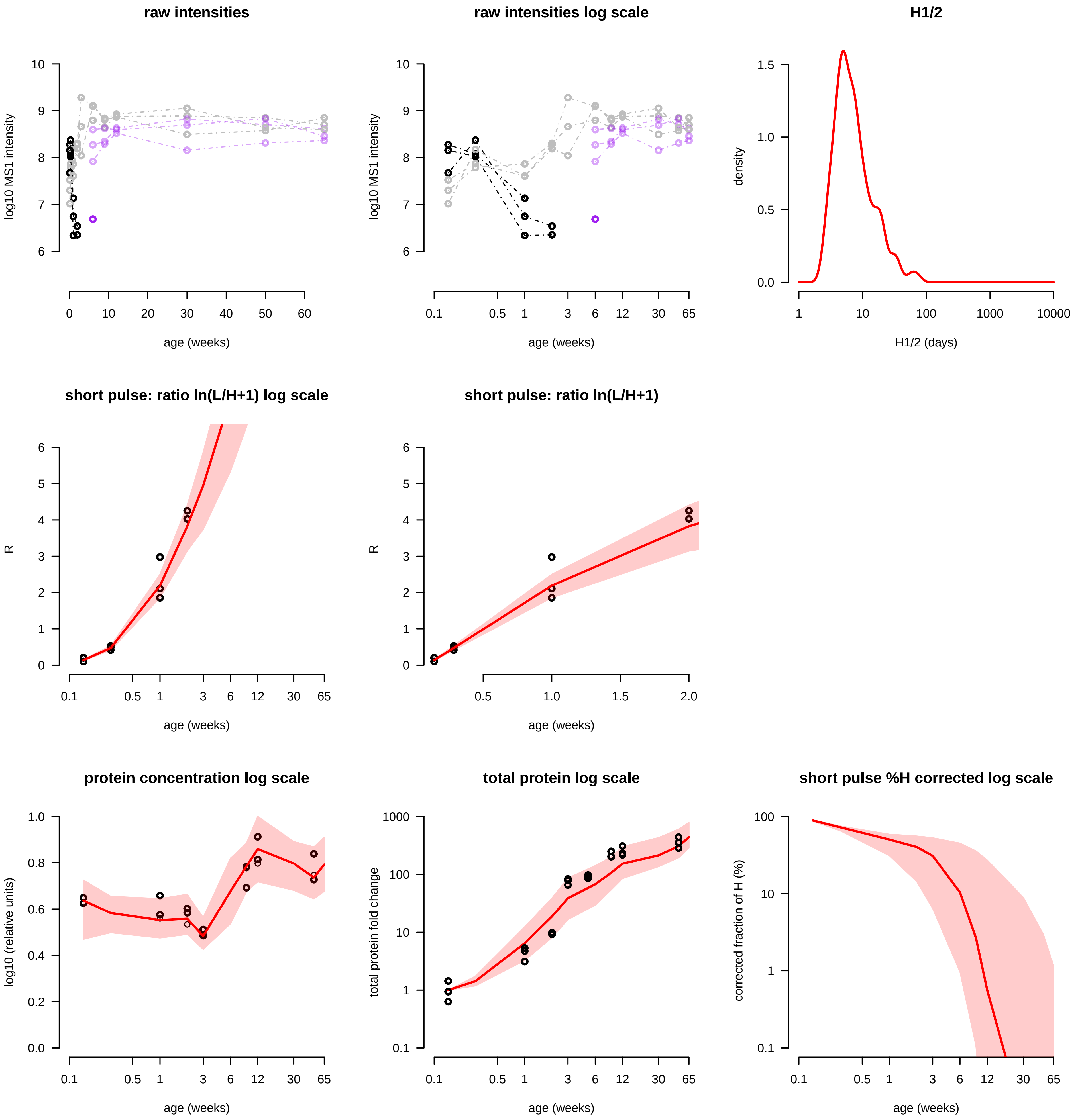

Q9CQR6

>sp|Q9CQR6|PPP6_MOUSE

Serine/threonine−protein phosphatase 6 catalytic subunit

GN=Ppp6c

Med H1/2: 4.6 days

Q5 H1/2: 3.8 days

Q95 H1/2: 6 days

reliable model fit: yes

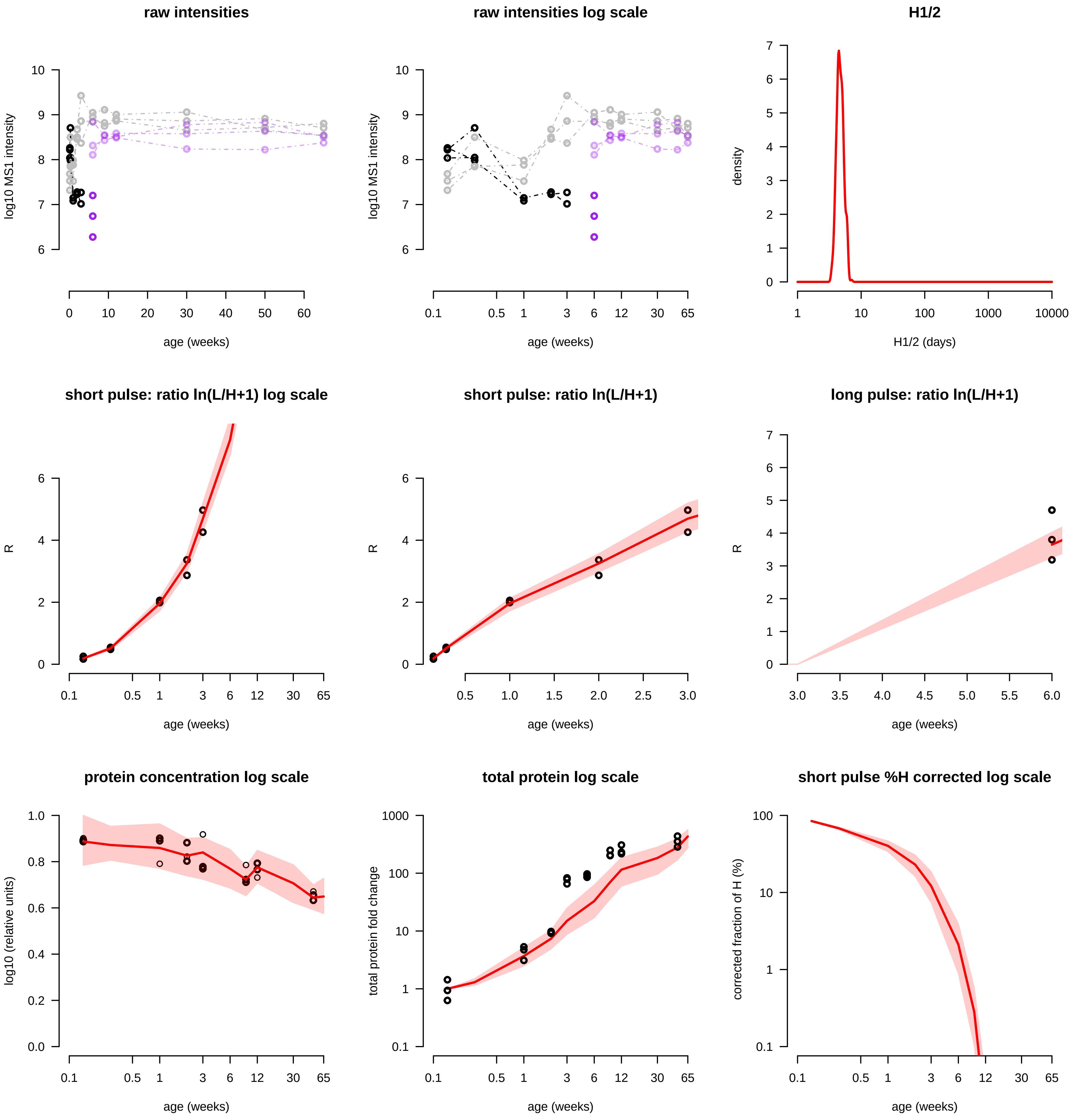

Q922D4−2;Q922D4;Q922D4−3;Q922D4−4

>sp|Q922D4−2|PP6R3_MOUSE

Isoform 2 of Serine/threonine−protein phosphatase 6 regulatory subunit 3

GN=Ppp6r3

Med H1/2: 19.5 days

Q5 H1/2: 6.4 days

Q95 H1/2: 206.5 days

reliable model fit: yes

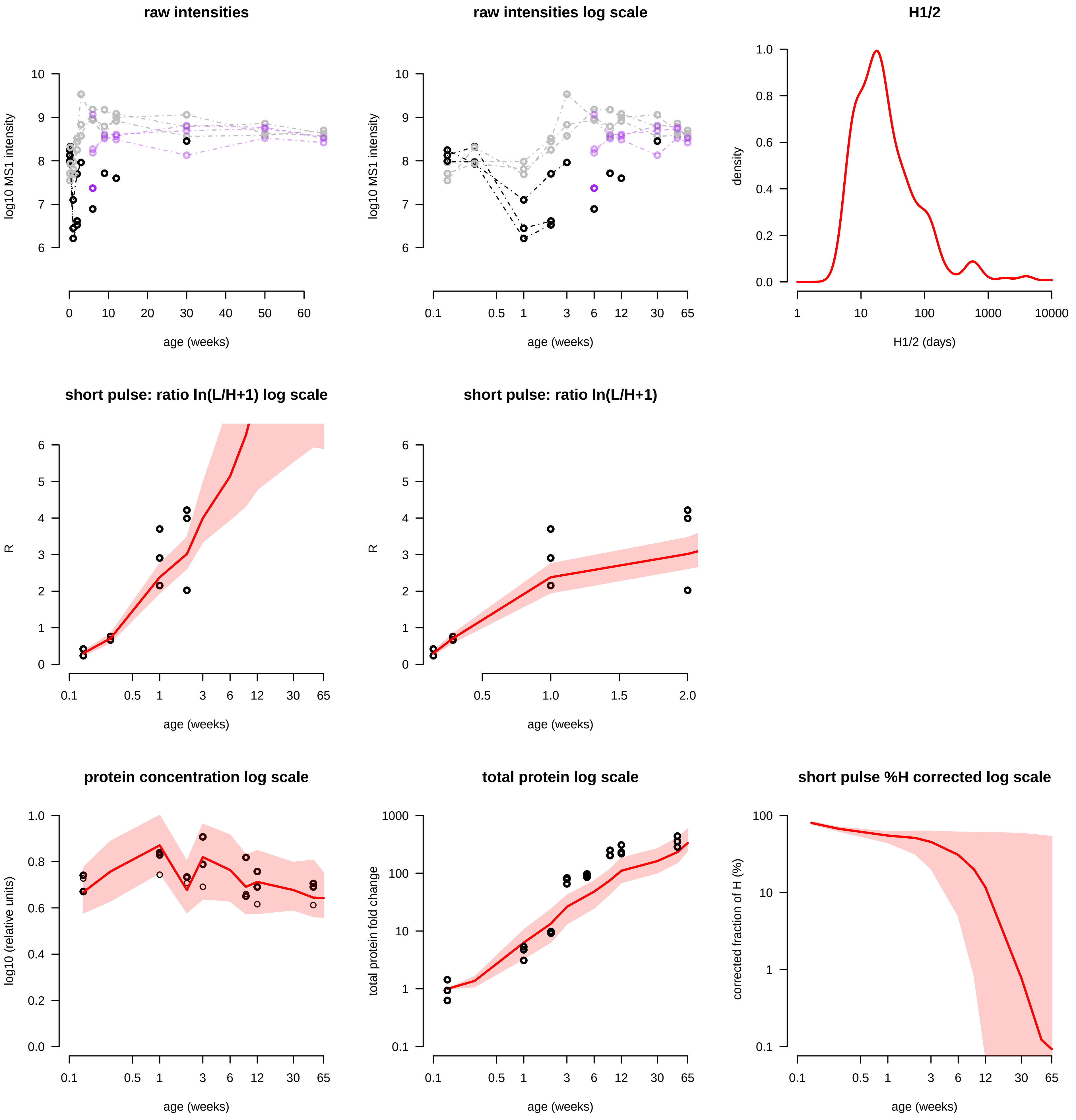

Q3TKT4;Q3TKT4−2

>sp|Q3TKT4|SMCA4_MOUSE

Transcription activator BRG1

GN=Smarca4

Med H1/2: 3 days

Q5 H1/2: 2.6 days

Q95 H1/2: 3.5 days

reliable model fit: yes

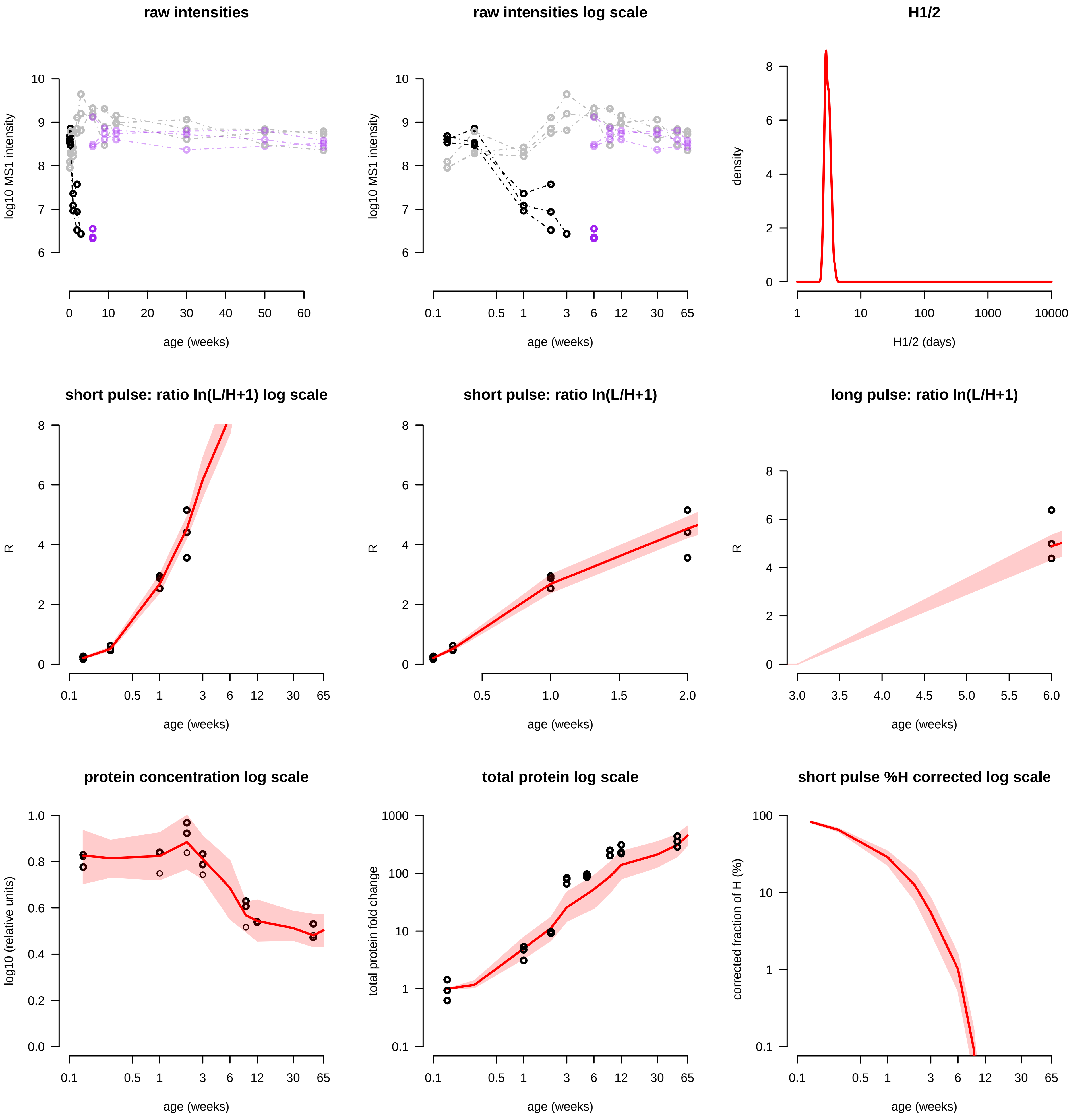

Q9EPE9

>sp|Q9EPE9|AT131_MOUSE

Endoplasmic reticulum transmembrane helix translocase

GN=Atp13a1

Med H1/2: 502 days

Q5 H1/2: 163.4 days

Q95 H1/2: 12010.5 days

reliable model fit: yes

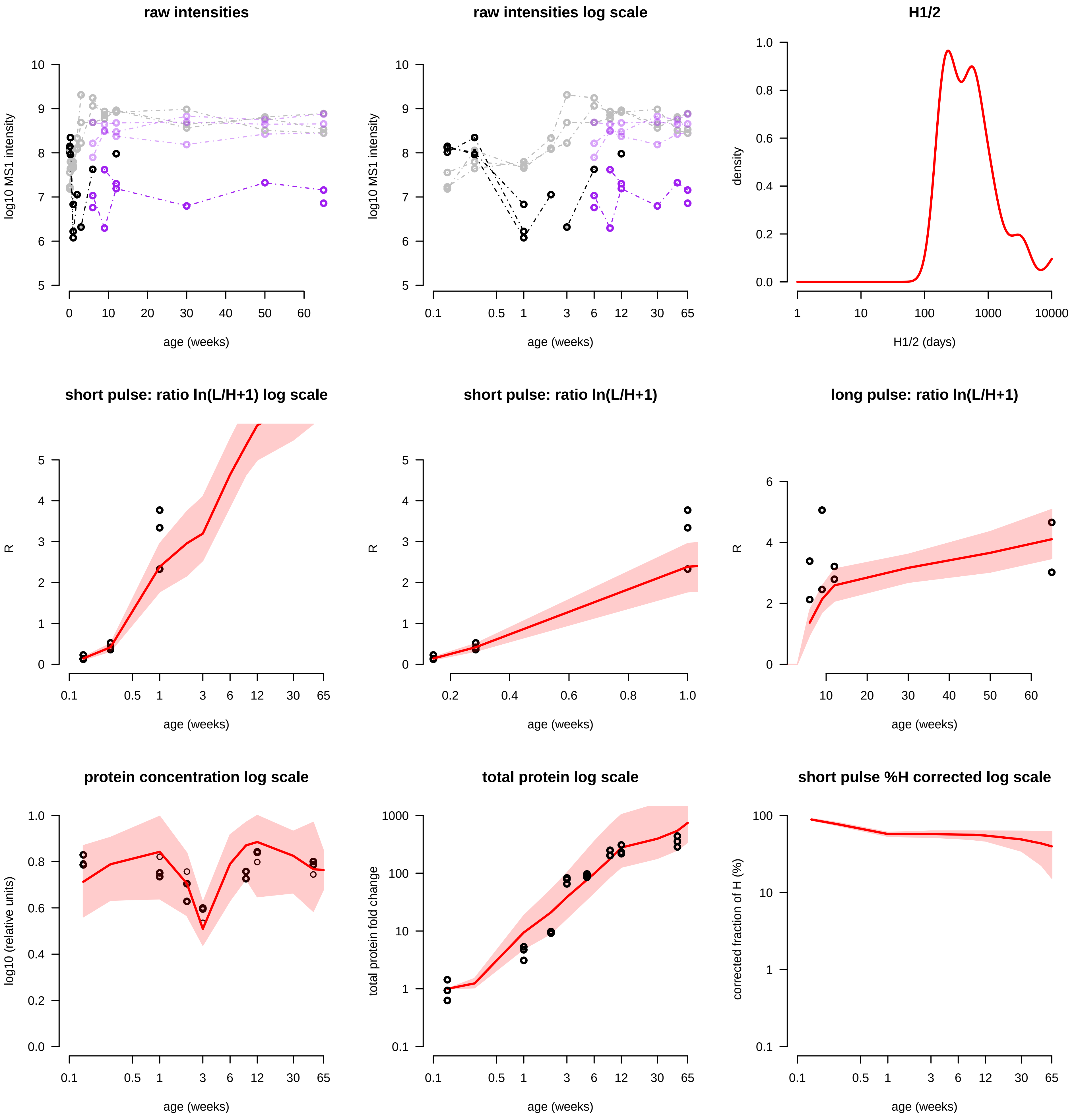

Q9CX00

>sp|Q9CX00|IST1_MOUSE

IST1 homolog

GN=Ist1

Med H1/2: 4.4 days

Q5 H1/2: 3.4 days

Q95 H1/2: 6.1 days

reliable model fit: yes

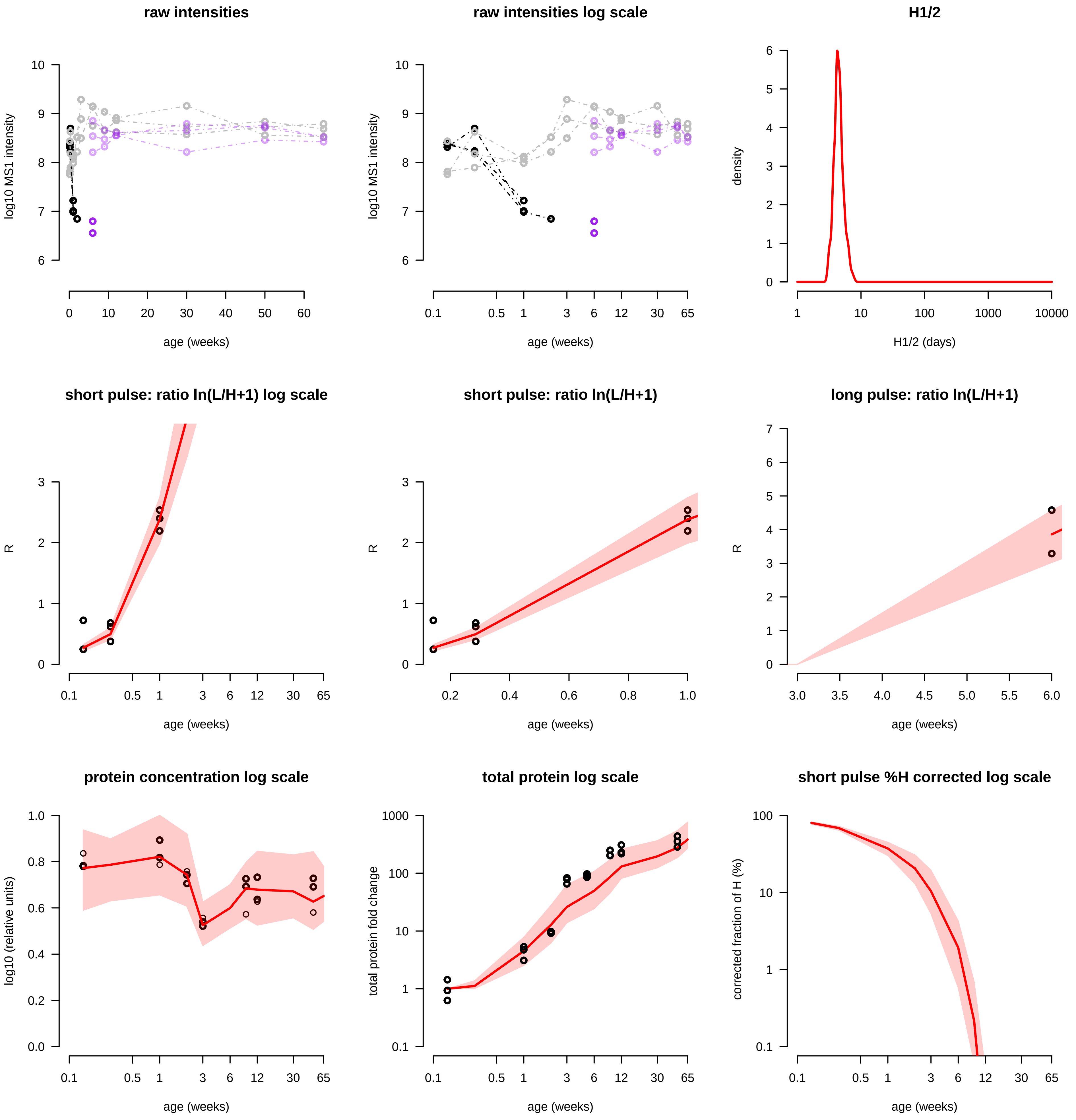

Q9D0T1

>sp|Q9D0T1|NH2L1_MOUSE

NHP2−like protein 1

GN=Snu13

Med H1/2: 7 days

Q5 H1/2: 5.5 days

Q95 H1/2: 9.1 days

reliable model fit: yes

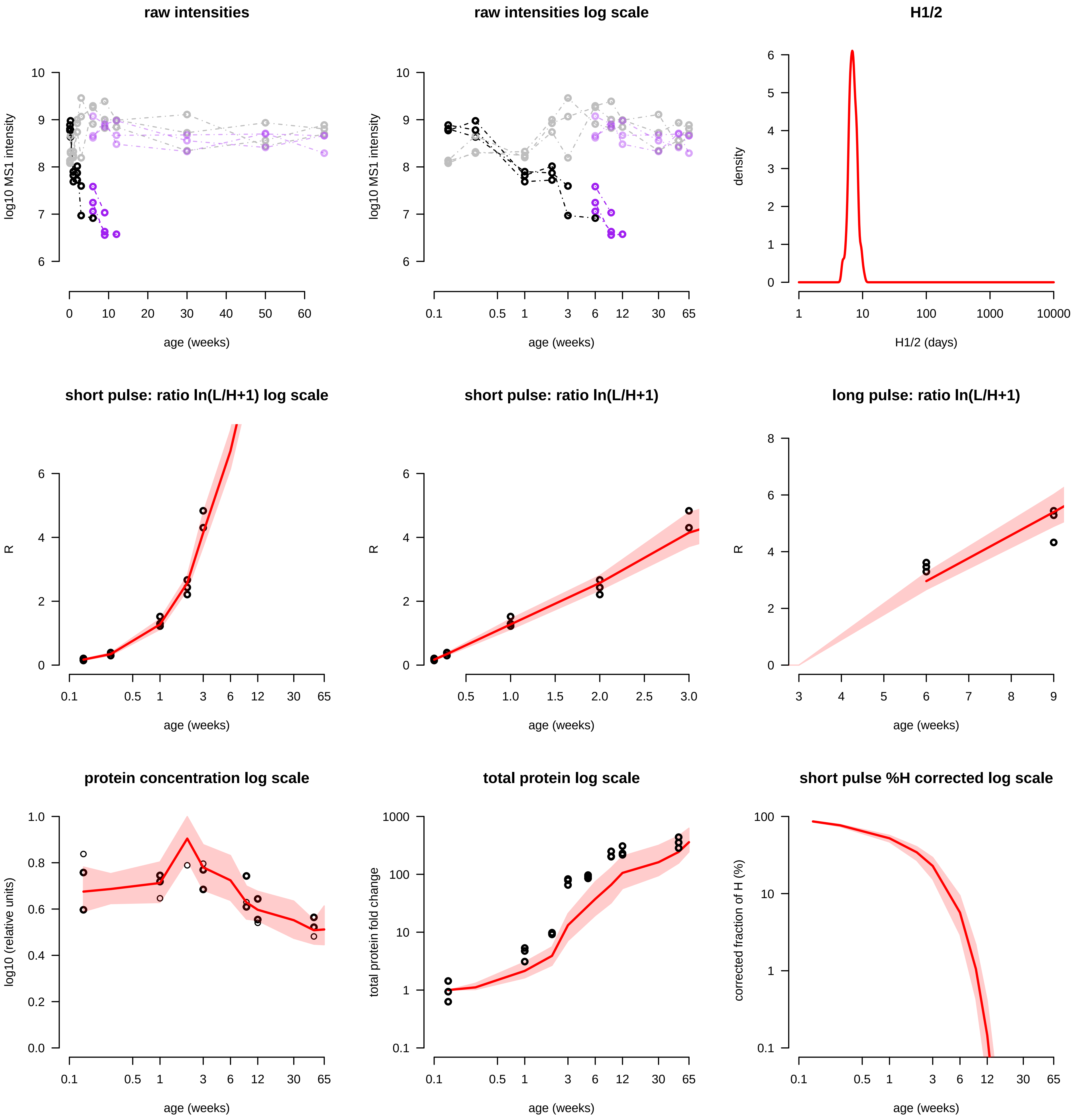

Q64674

>sp|Q64674|SPEE_MOUSE

Spermidine synthase

GN=Srm

Med H1/2: 4.5 days

Q5 H1/2: 3.7 days

Q95 H1/2: 6 days

reliable model fit: yes

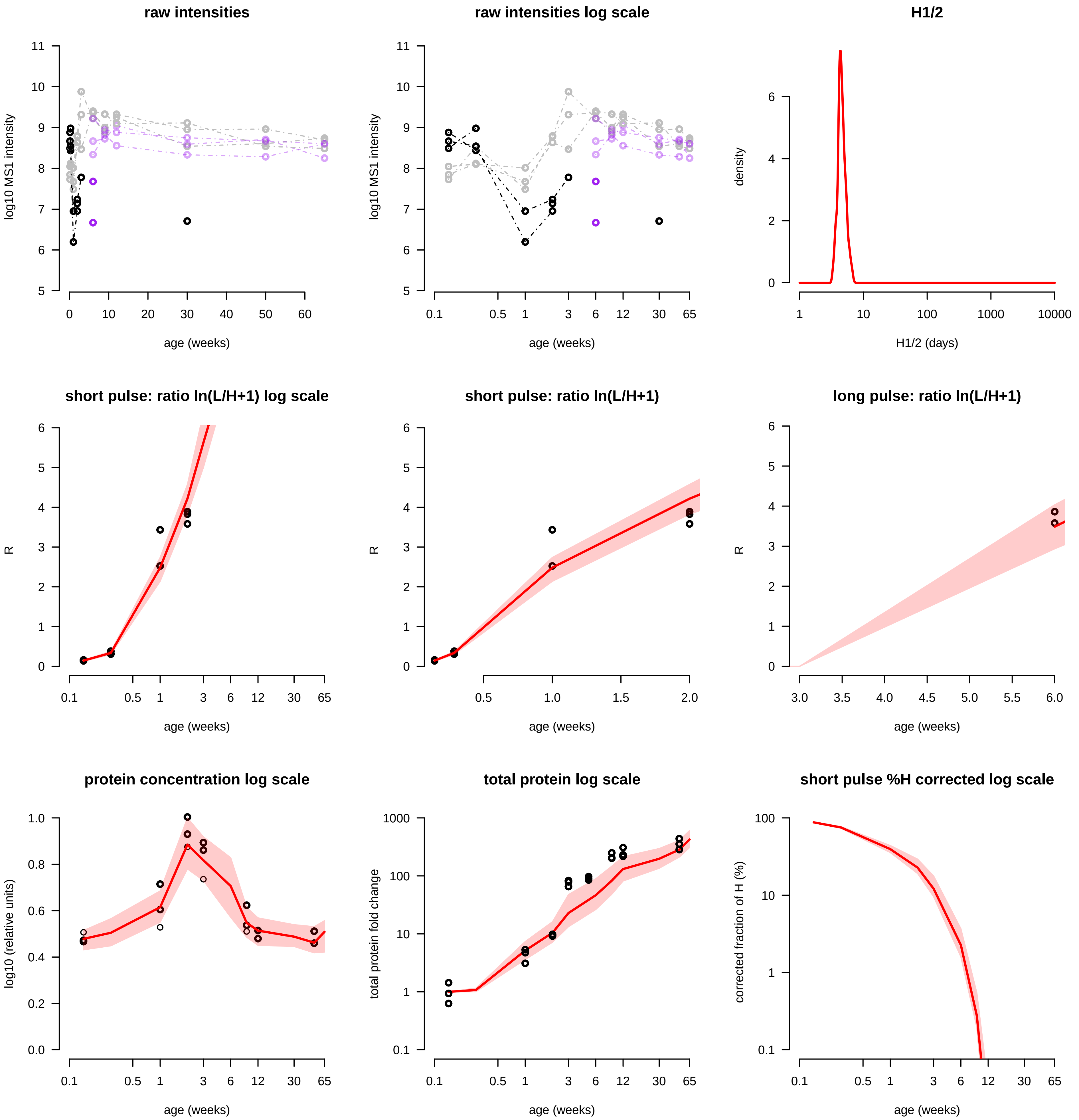

O88811;O88811−2

>sp|O88811|STAM2_MOUSE

Signal transducing adapter molecule 2

GN=Stam2

Med H1/2: 9.5 days

Q5 H1/2: 3.7 days

Q95 H1/2: 95.7 days

reliable model fit: yes

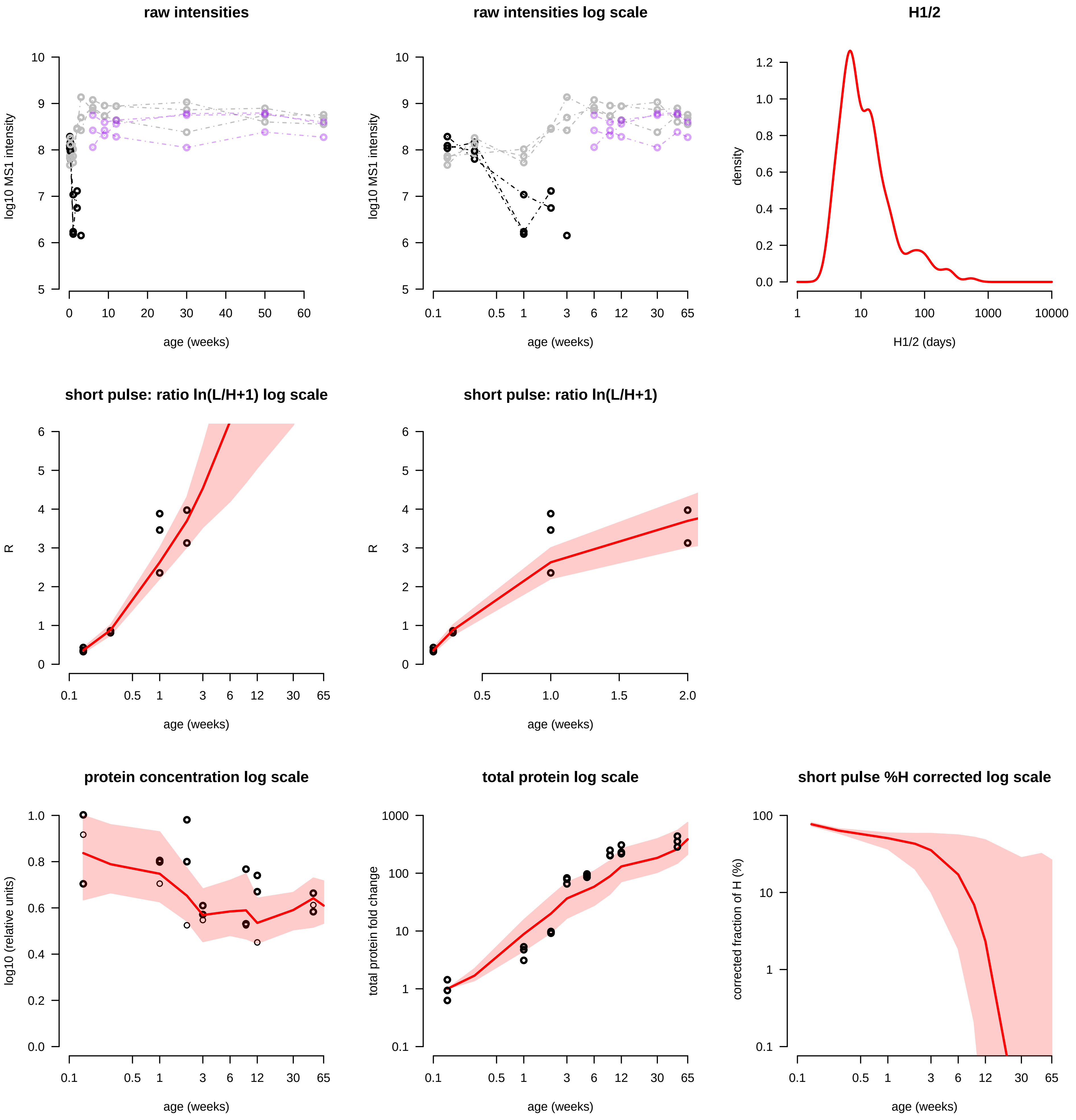

Q2TBE6

>sp|Q2TBE6|P4K2A_MOUSE

Phosphatidylinositol 4−kinase type 2−alpha

GN=Pi4k2a

Med H1/2: 4.3 days

Q5 H1/2: 2.6 days

Q95 H1/2: 37.9 days

reliable model fit: yes

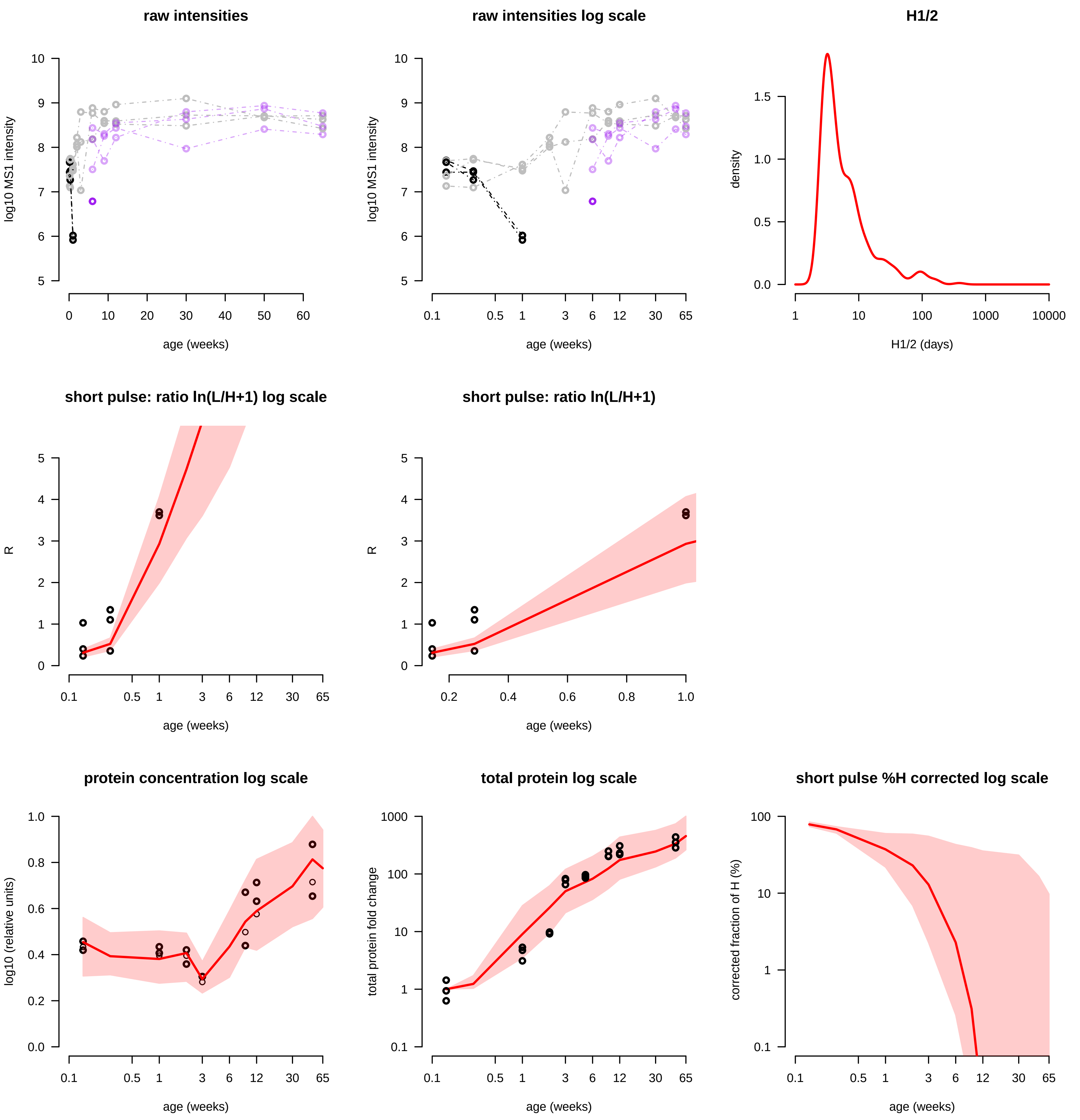

Q9CR86

>sp|Q9CR86|CHSP1_MOUSE

Calcium−regulated heat stable protein 1

GN=Carhsp1

Med H1/2: 4.8 days

Q5 H1/2: 3.4 days

Q95 H1/2: 6.8 days

reliable model fit: yes

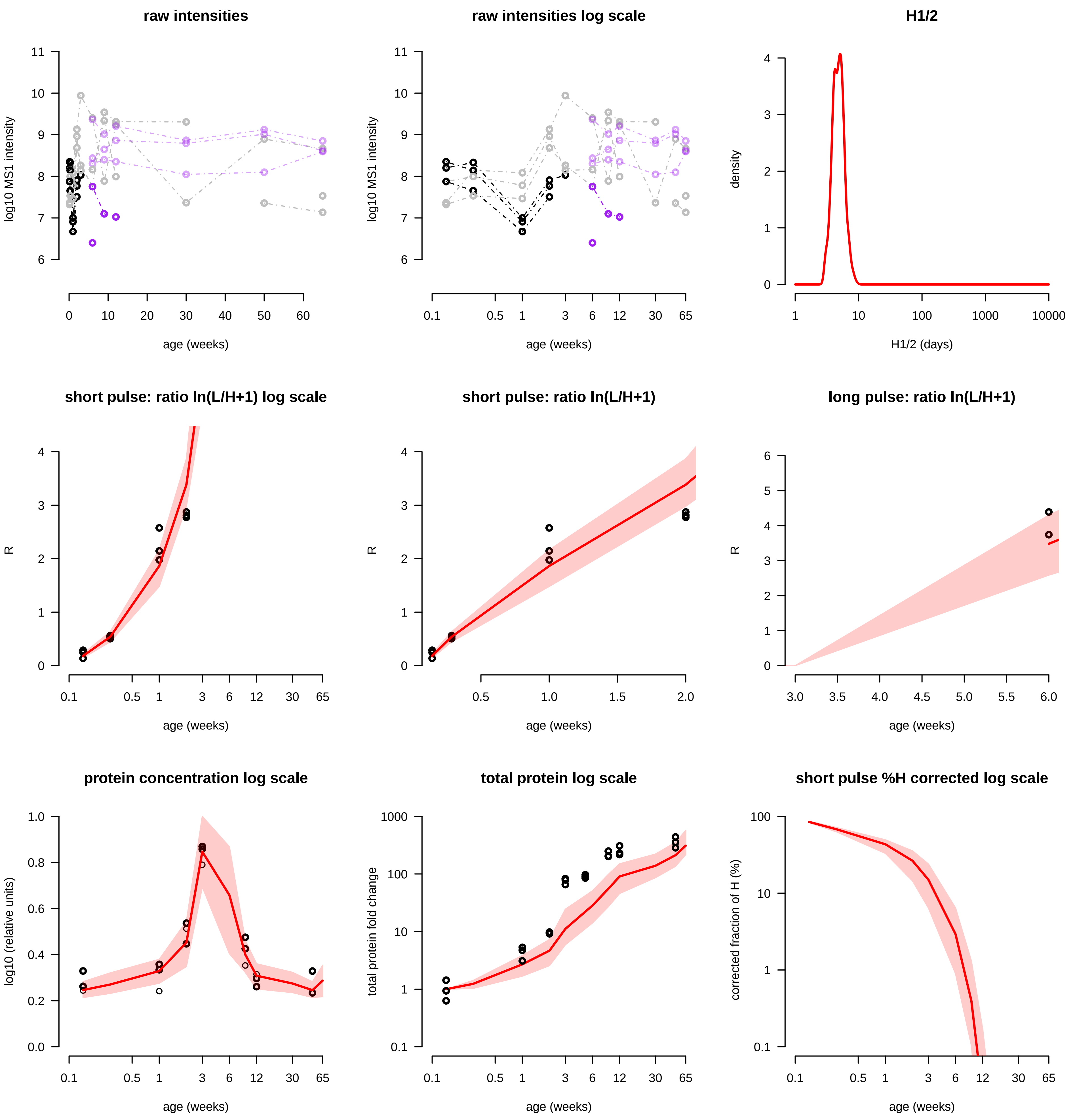

Q91YR7;Q91YR7−2

>sp|Q91YR7|PRP6_MOUSE

Pre−mRNA−processing factor 6

GN=Prpf6

Med H1/2: 2202.3 days

Q5 H1/2: 500.7 days

Q95 H1/2: 22227 days

reliable model fit: yes

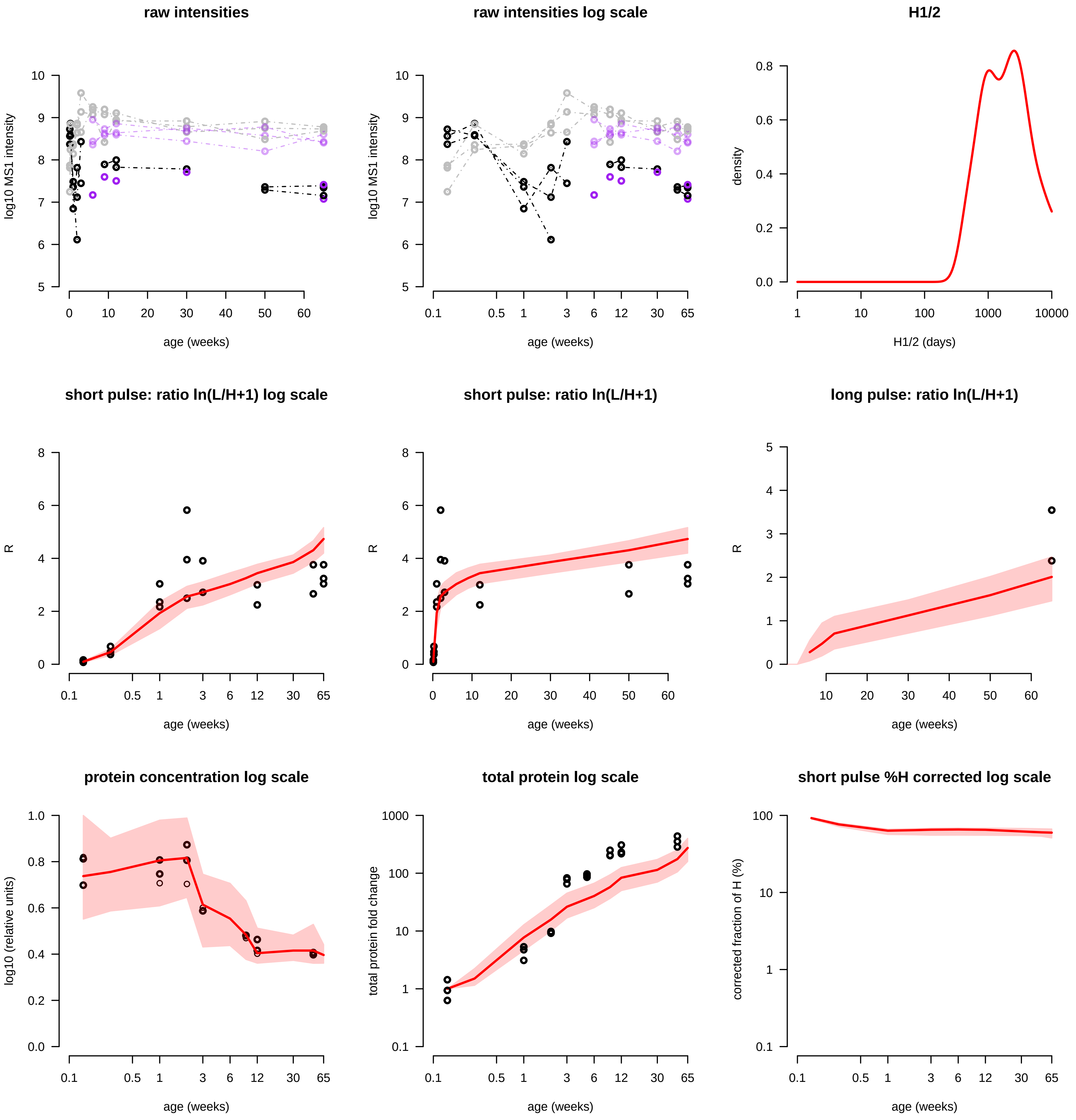

A2ADY9

>sp|A2ADY9|DDI2_MOUSE

Protein DDI1 homolog 2

GN=Ddi2

Med H1/2: 4.9 days

Q5 H1/2: 4.1 days

Q95 H1/2: 6.9 days

reliable model fit: yes

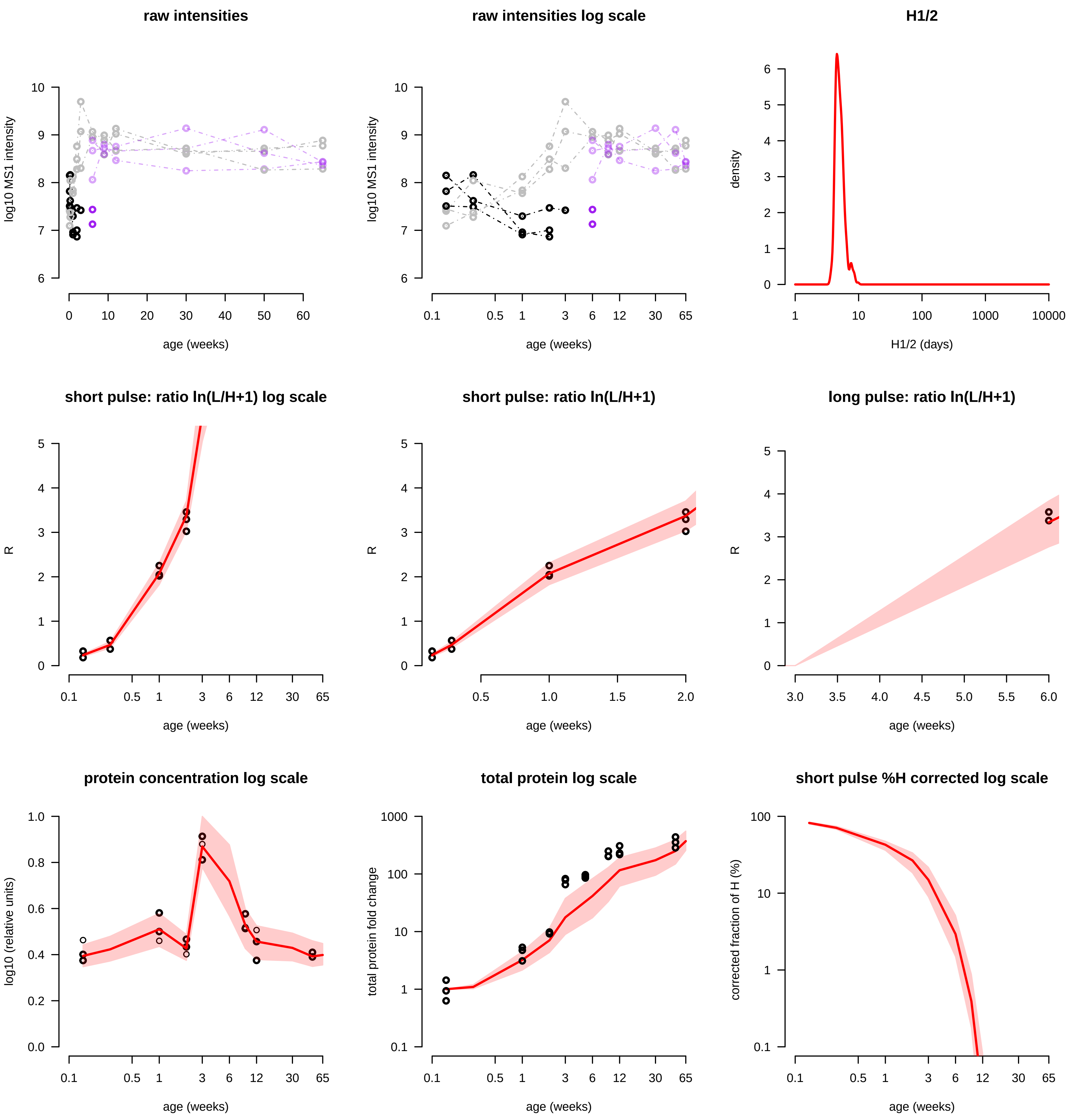

Q6DIC0

>sp|Q6DIC0|SMCA2_MOUSE

Probable global transcription activator SNF2L2

GN=Smarca2

Med H1/2: 6.5 days

Q5 H1/2: 3.7 days

Q95 H1/2: 20.6 days

reliable model fit: yes

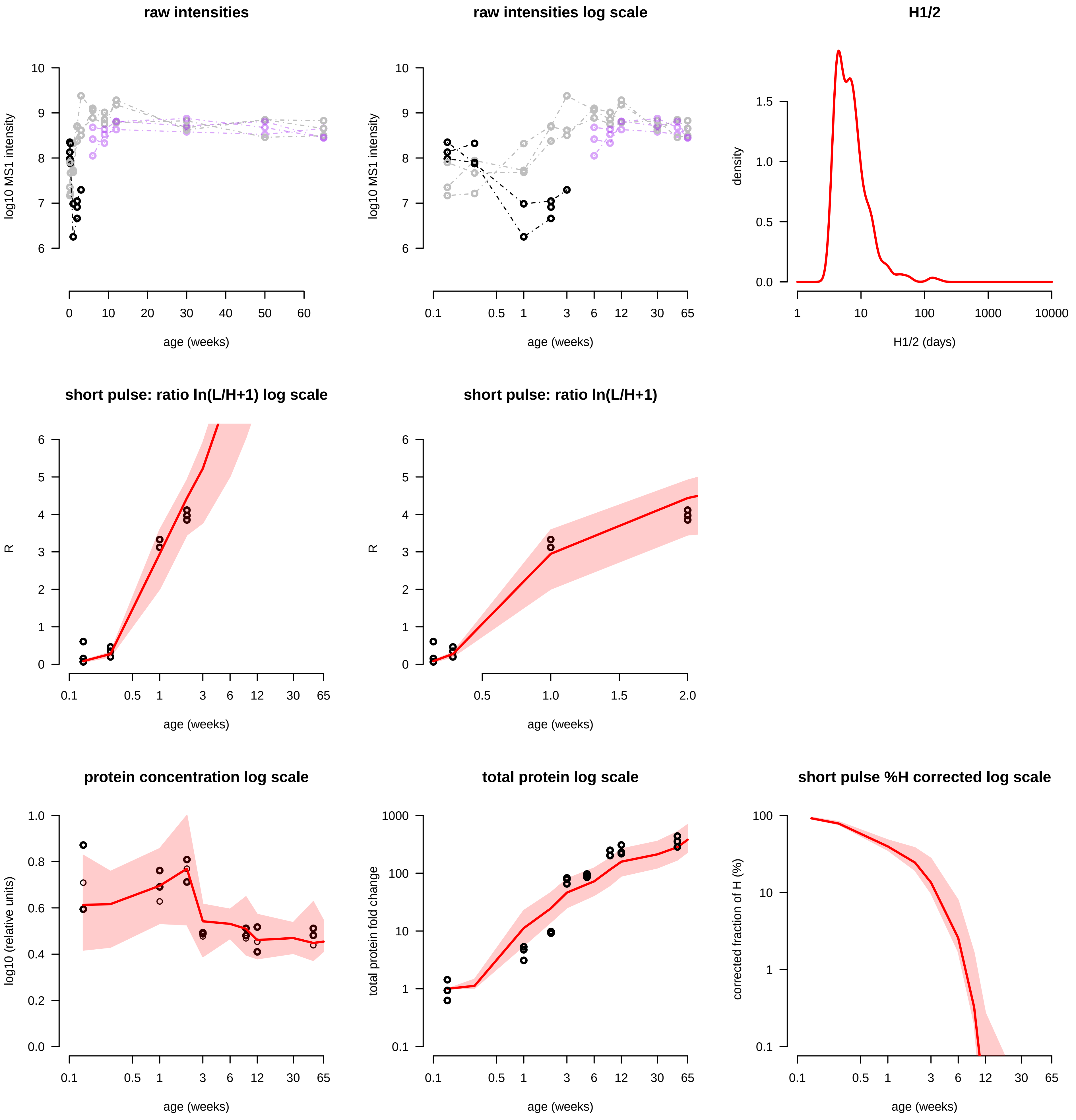

Q9EQS3

>sp|Q9EQS3|MYCBP_MOUSE

c−Myc−binding protein

GN=Mycbp

Med H1/2: 5.2 days

Q5 H1/2: 2.6 days

Q95 H1/2: 11.3 days

reliable model fit: yes

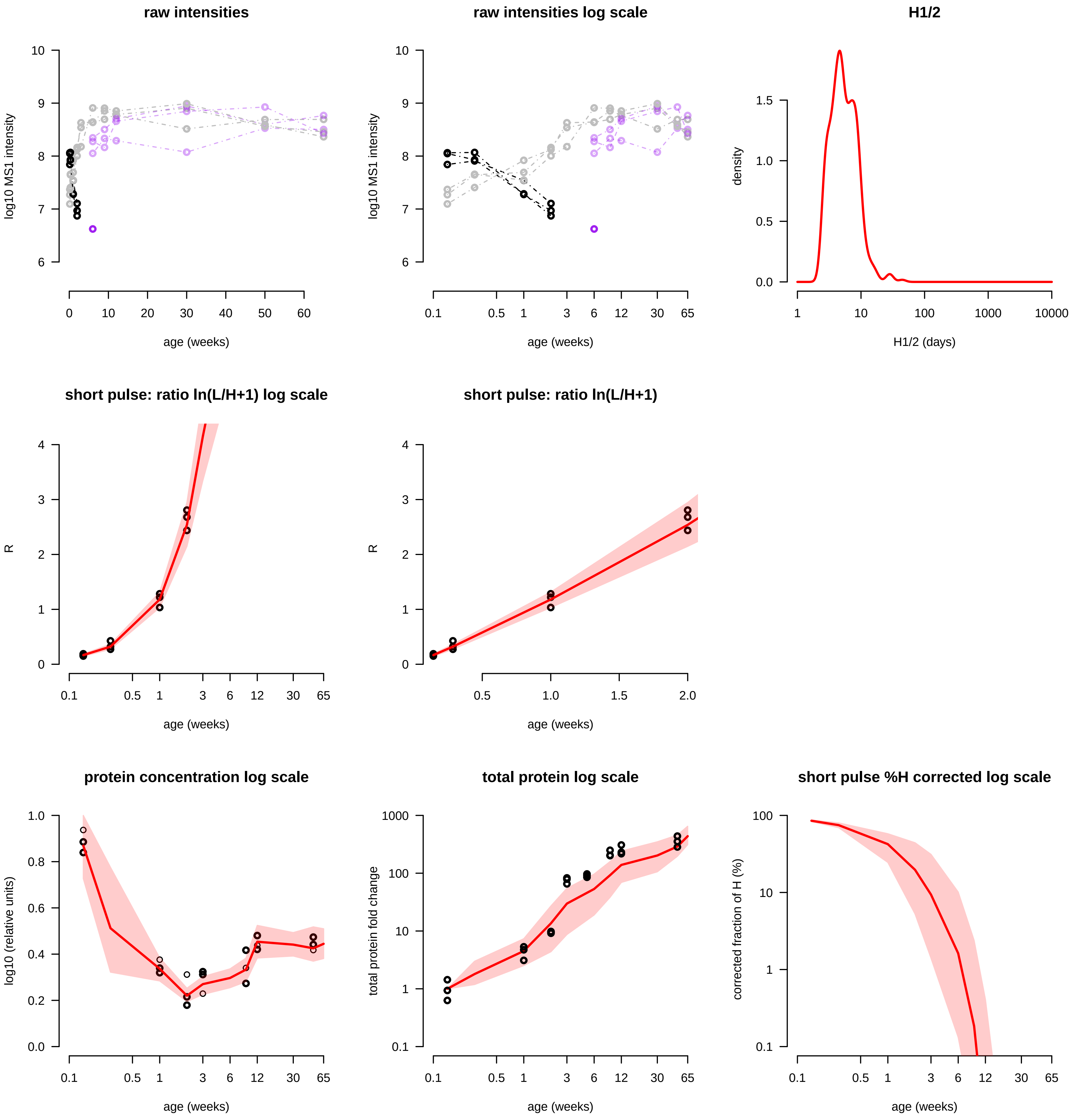

Q8BK72

>sp|Q8BK72|RT27_MOUSE

28S ribosomal protein S27, mitochondrial

GN=Mrps27

Med H1/2: 10.2 days

Q5 H1/2: 3.8 days

Q95 H1/2: 72.3 days

reliable model fit: yes

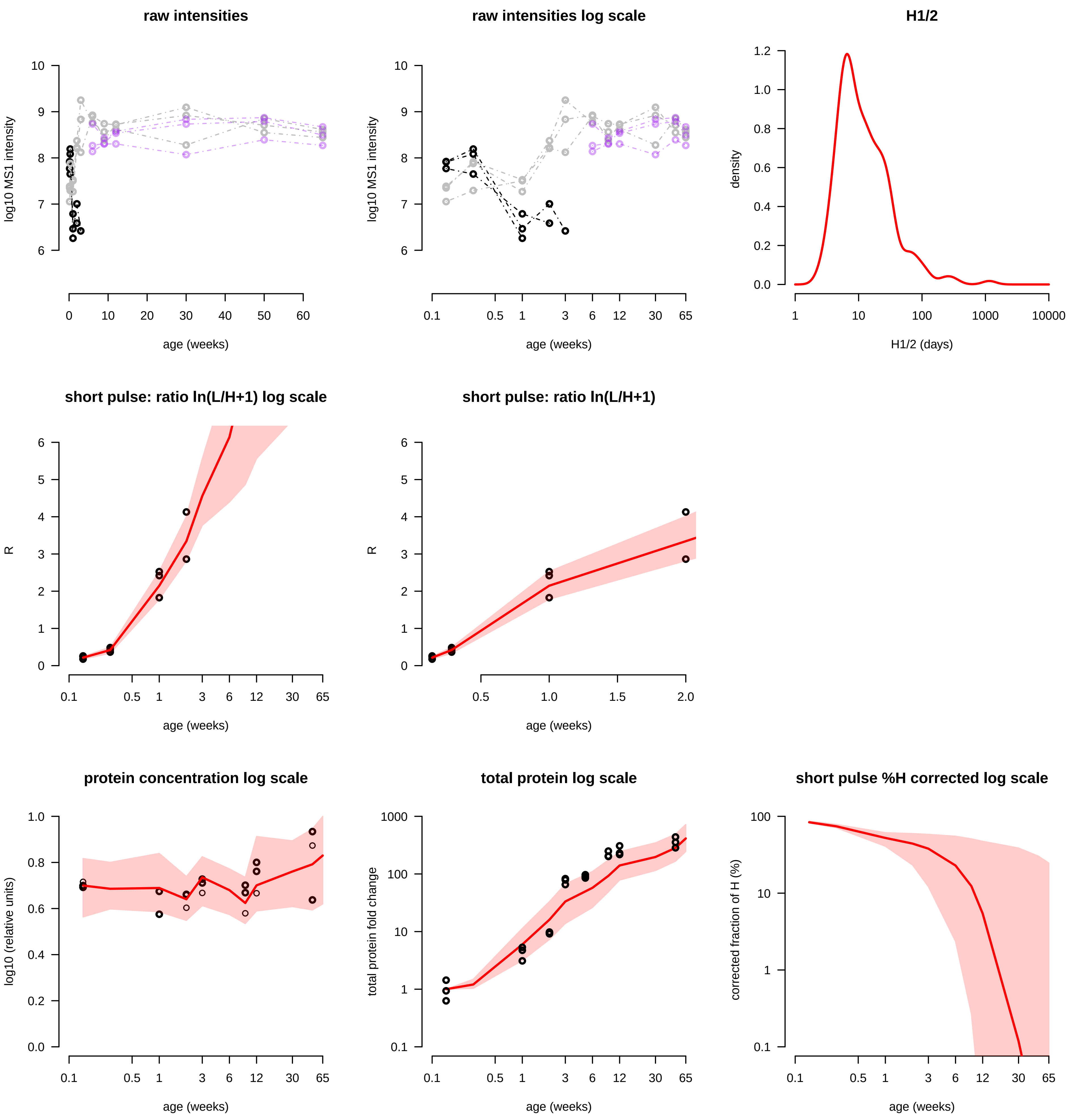

Q6P5D8

>sp|Q6P5D8|SMHD1_MOUSE

ructural maintenance of chromosomes flexible hinge domain−containing protein

GN=Smchd1

Med H1/2: 144.2 days

Q5 H1/2: 67.2 days

Q95 H1/2: 300.1 days

reliable model fit: yes

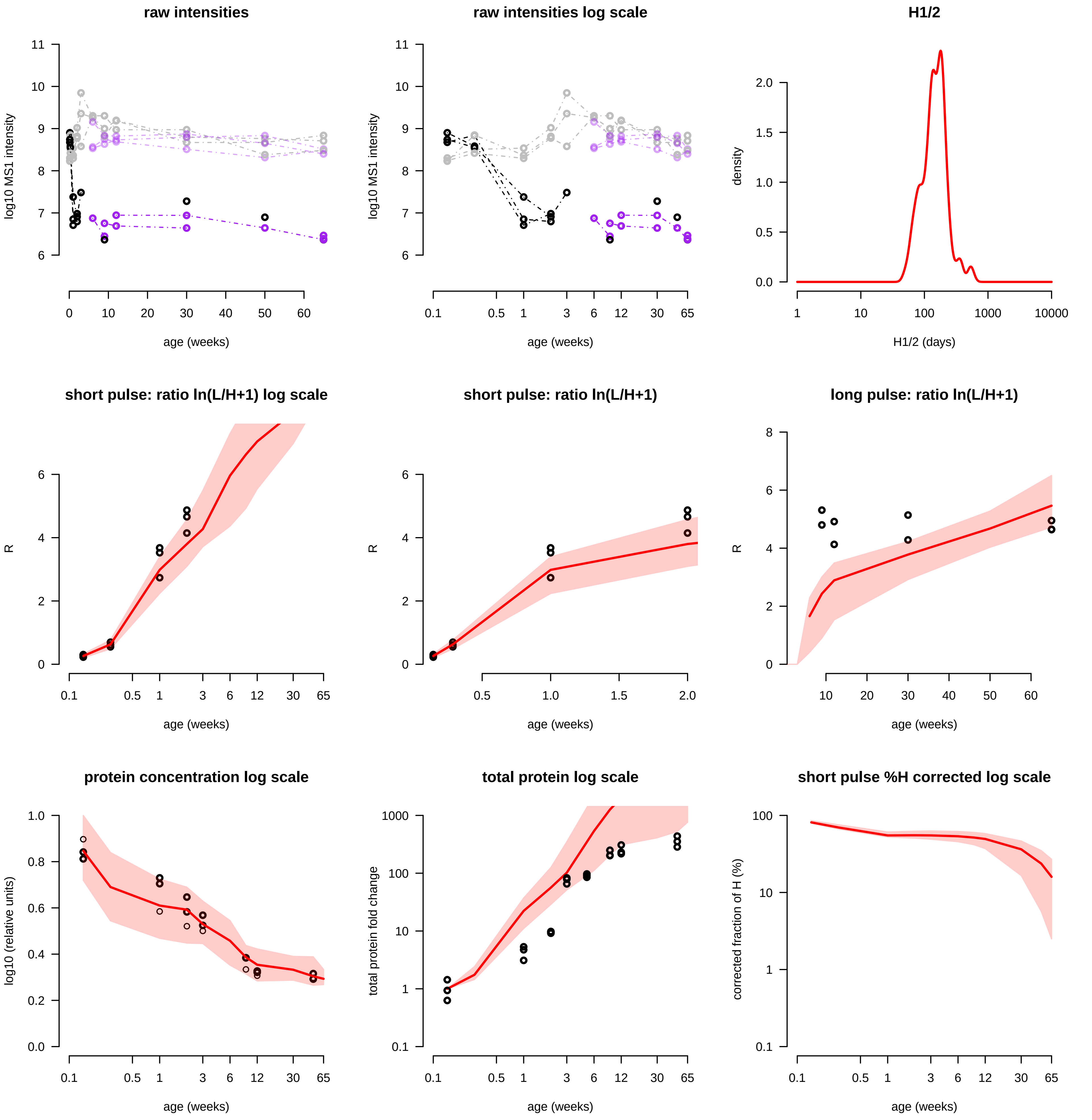

Q61187

>sp|Q61187|TS101_MOUSE

Tumor susceptibility gene 101 protein

GN=Tsg101

Med H1/2: 3.7 days

Q5 H1/2: 2.5 days

Q95 H1/2: 12.4 days

reliable model fit: yes

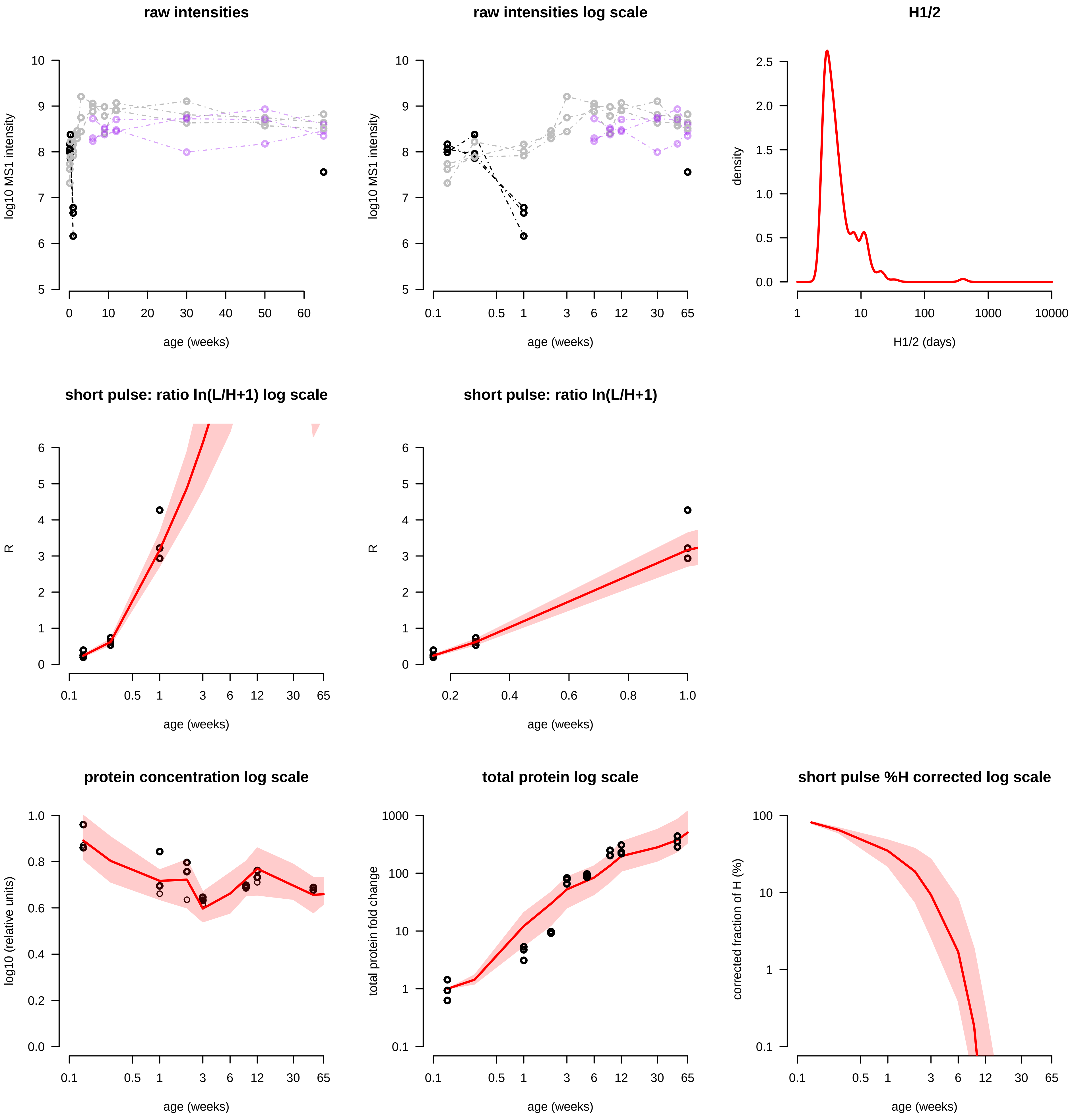

Q6PD03

>sp|Q6PD03|2A5A_MOUSE

erine/threonine−protein phosphatase 2A 56 kDa regulatory subunit alpha isofor

GN=Ppp2r5a

Med H1/2: 5.2 days

Q5 H1/2: 3.8 days

Q95 H1/2: 7.3 days

reliable model fit: yes

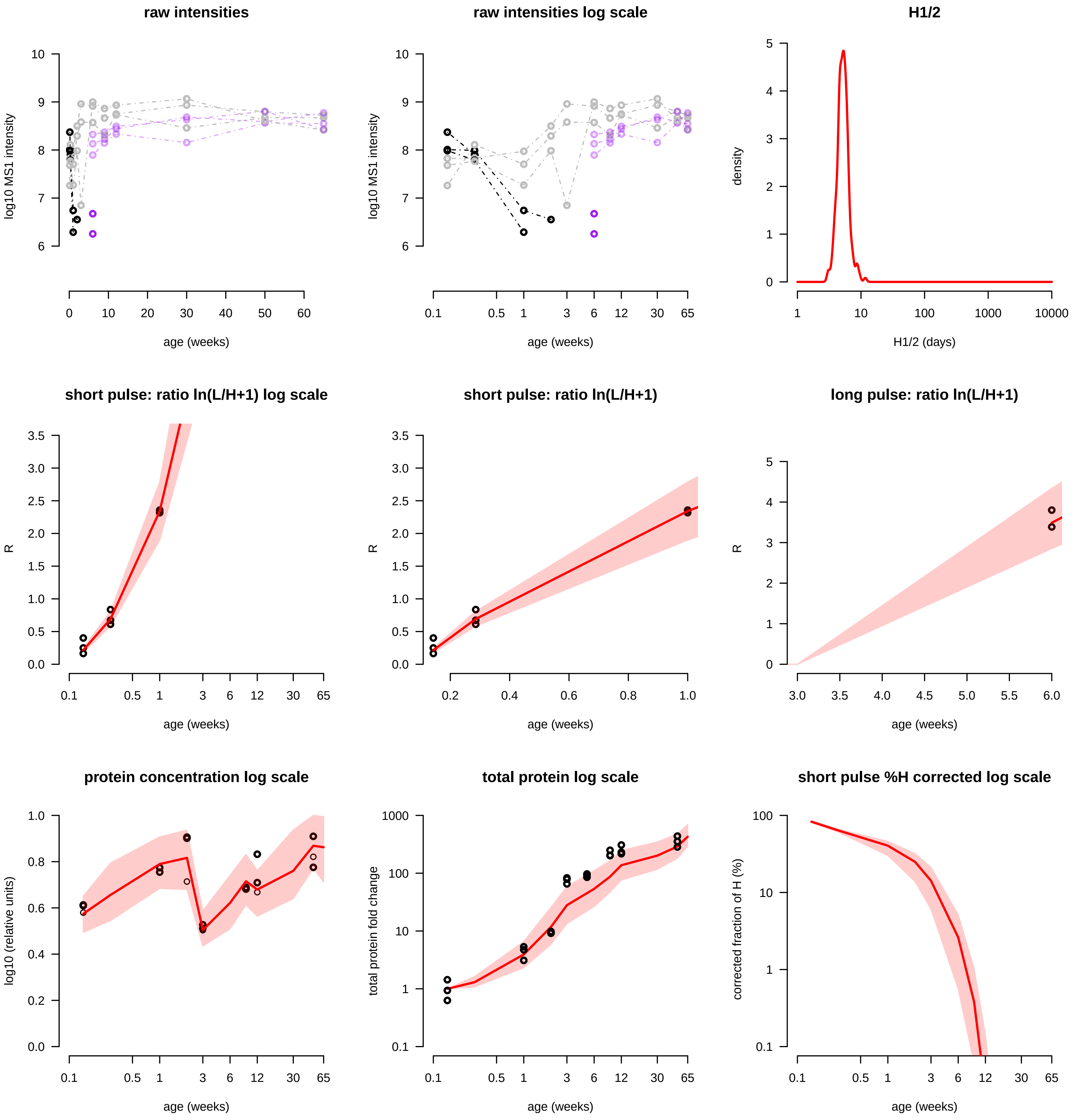

Q3TDQ1

>sp|Q3TDQ1|STT3B_MOUSE

Dolichyl−diphosphooligosaccharide−−protein glycosyltransferase subunit STT3B

GN=Stt3b

Med H1/2: 4.5 days

Q5 H1/2: 3.8 days

Q95 H1/2: 5.4 days

reliable model fit: yes

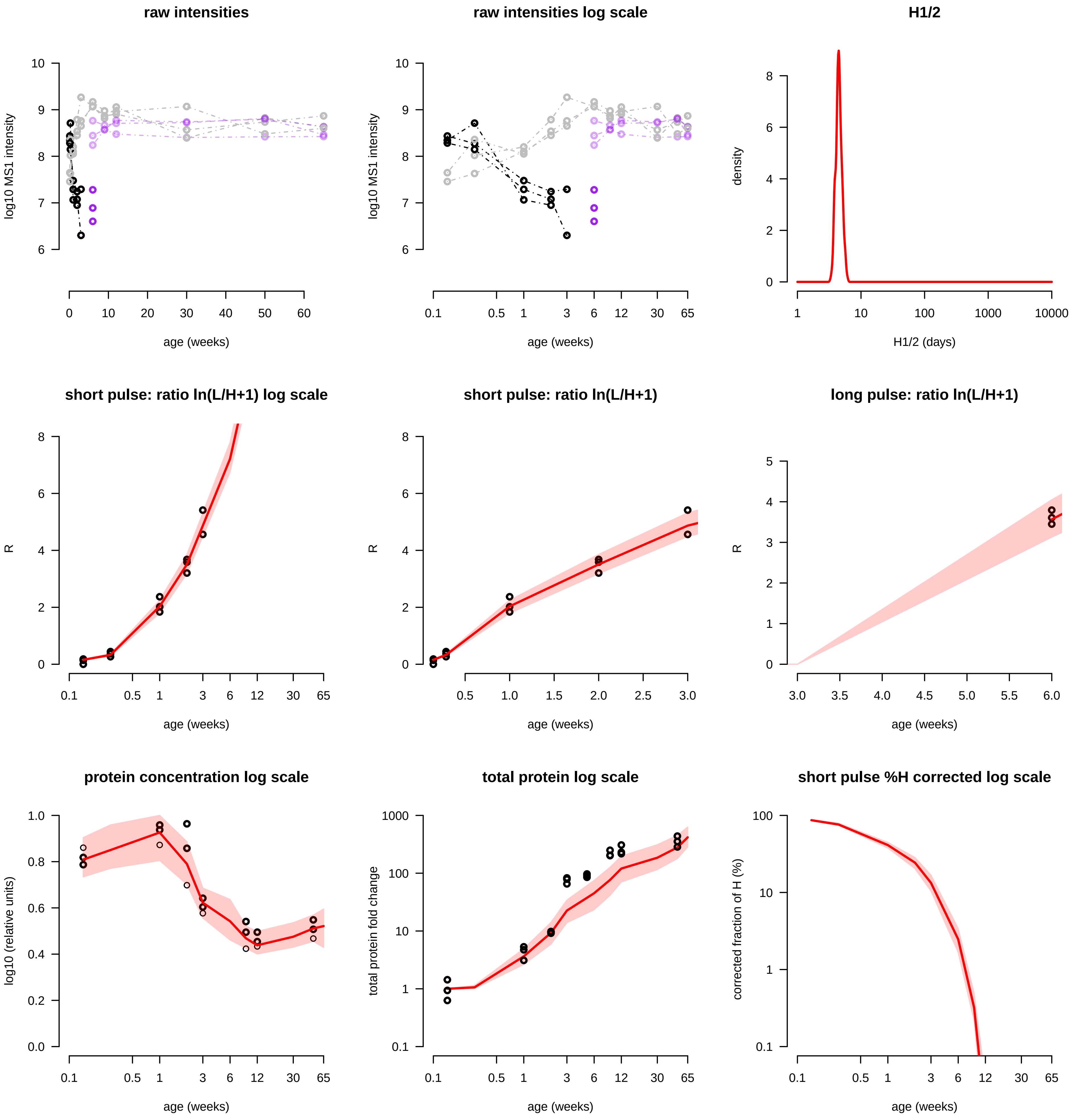

P22892

>sp|P22892|AP1G1_MOUSE

AP−1 complex subunit gamma−1

GN=Ap1g1

Med H1/2: 6.8 days

Q5 H1/2: 3 days

Q95 H1/2: 99.4 days

reliable model fit: yes

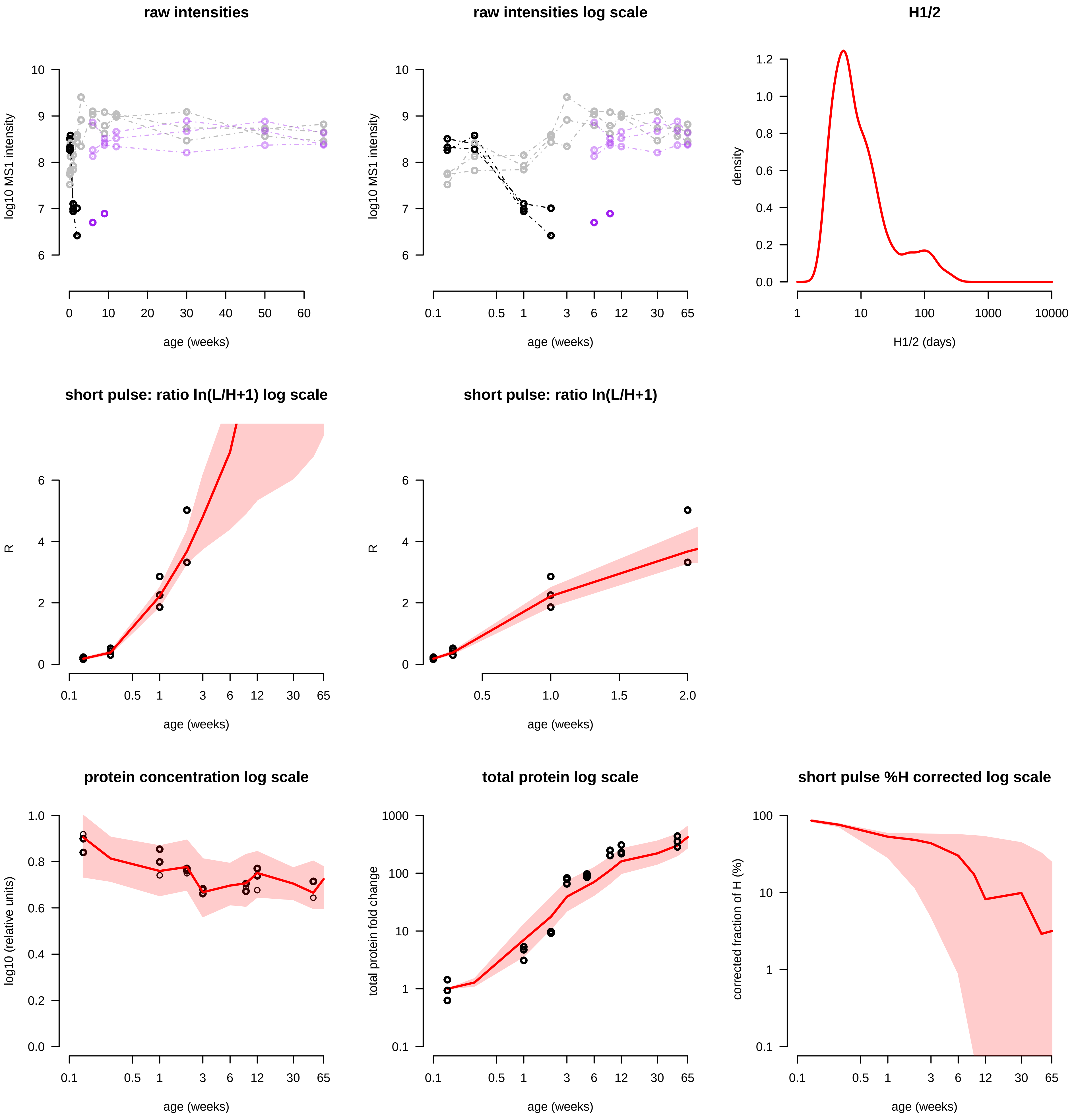

P27601

>sp|P27601|GNA13_MOUSE

Guanine nucleotide−binding protein subunit alpha−13

GN=Gna13

Med H1/2: 4.7 days

Q5 H1/2: 3.8 days

Q95 H1/2: 5.9 days

reliable model fit: yes

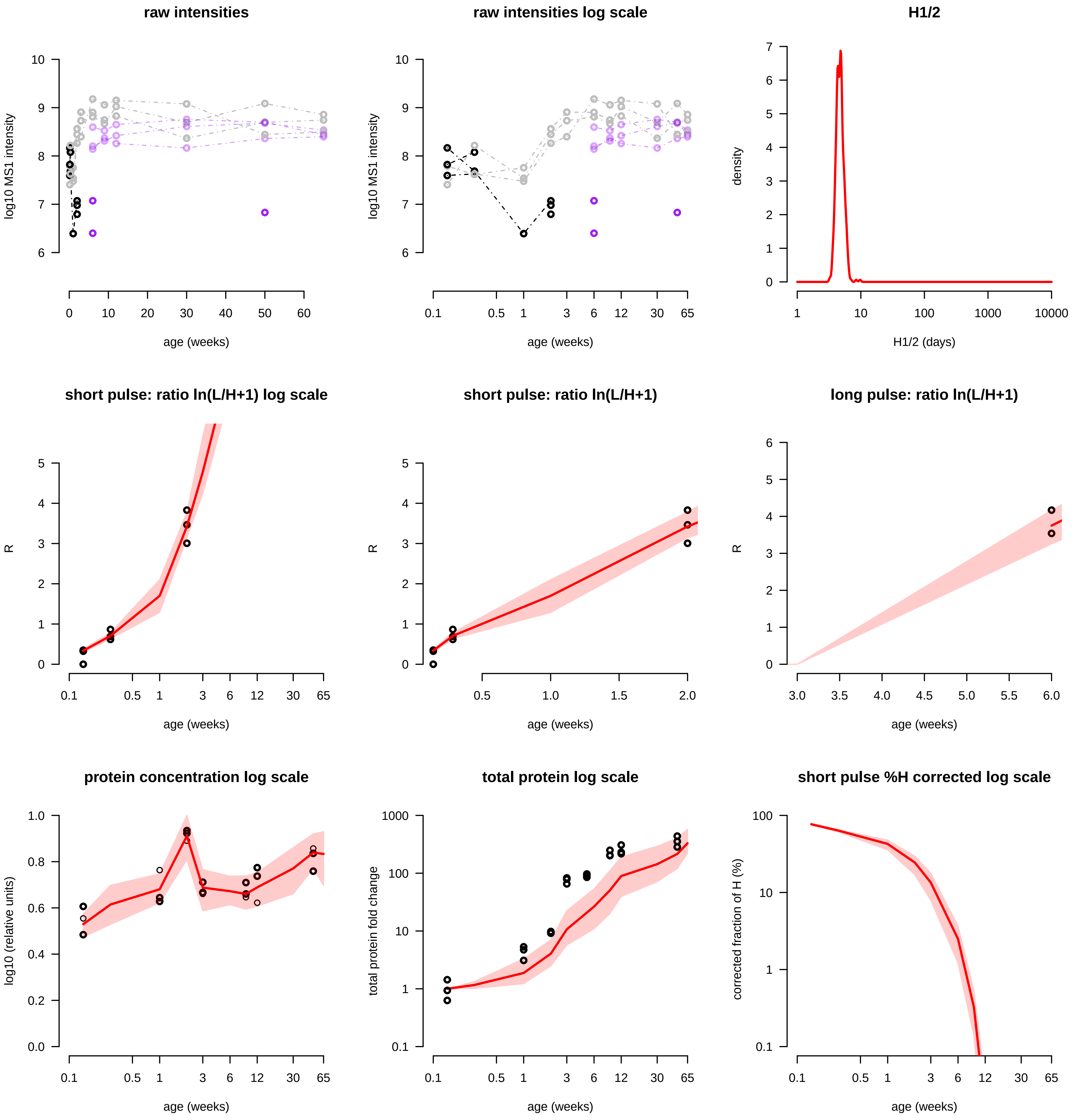

Q9Z1M8

>sp|Q9Z1M8|RED_MOUSE

Protein Red

GN=Ik

Med H1/2: 15.9 days

Q5 H1/2: 5.7 days

Q95 H1/2: 89.3 days

reliable model fit: yes

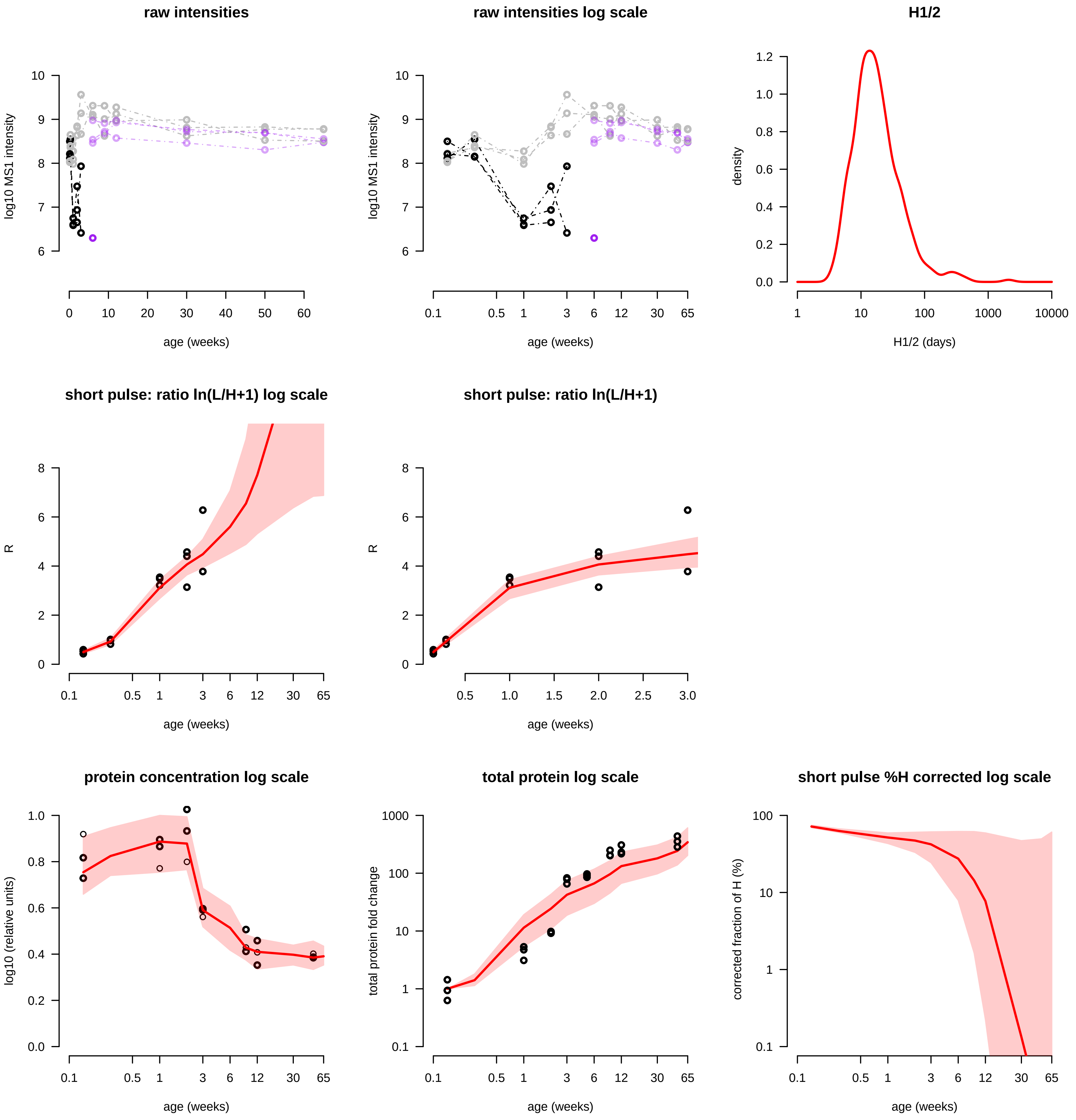

Q5U458

>sp|Q5U458|DJC11_MOUSE

DnaJ homolog subfamily C member 11

GN=Dnajc11

Med H1/2: 8.8 days

Q5 H1/2: 4.6 days

Q95 H1/2: 132.2 days

reliable model fit: yes

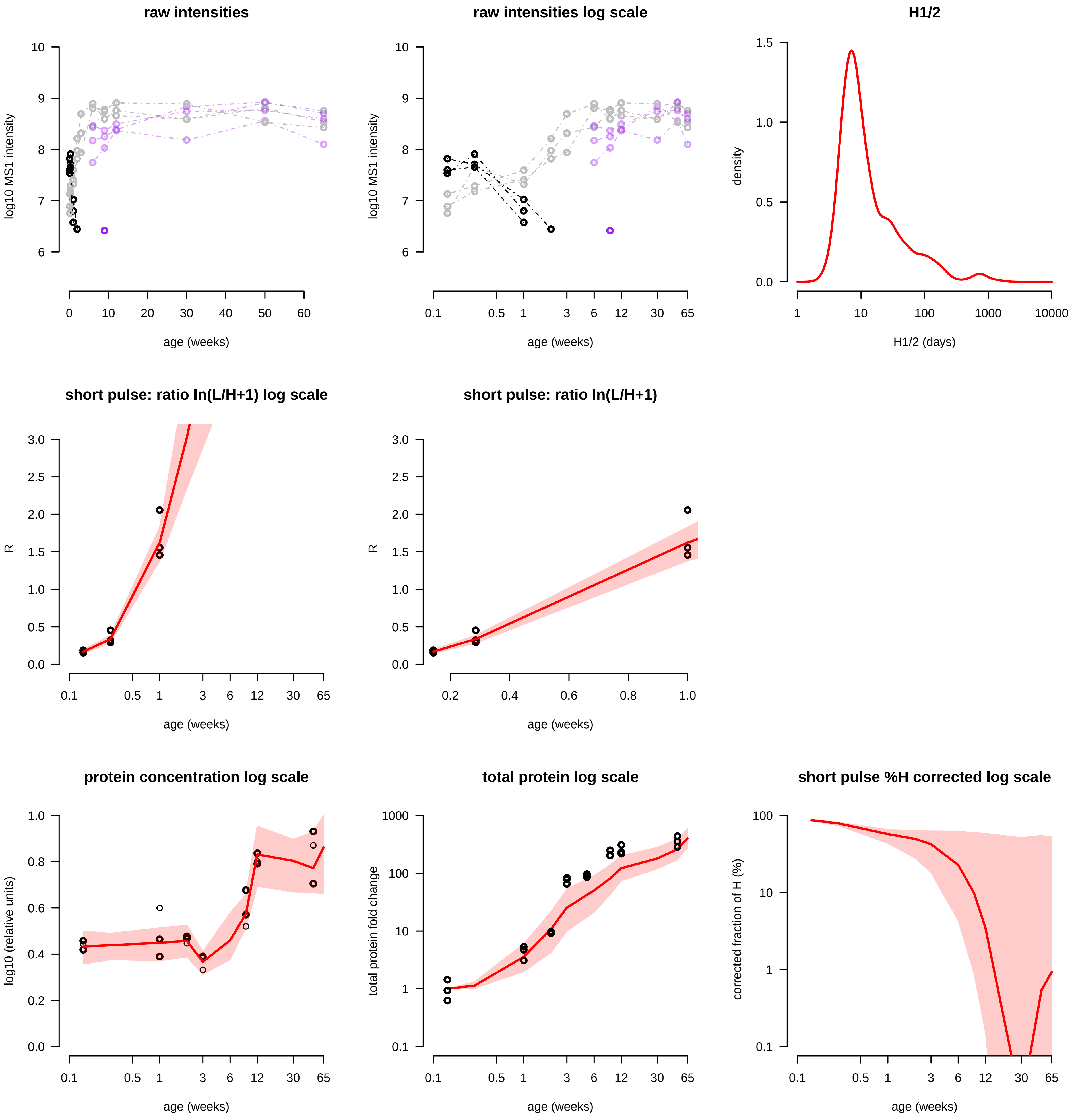

Q8K183

>sp|Q8K183|PDXK_MOUSE

Pyridoxal kinase

GN=Pdxk

Med H1/2: 5 days

Q5 H1/2: 4 days

Q95 H1/2: 6.2 days

reliable model fit: yes

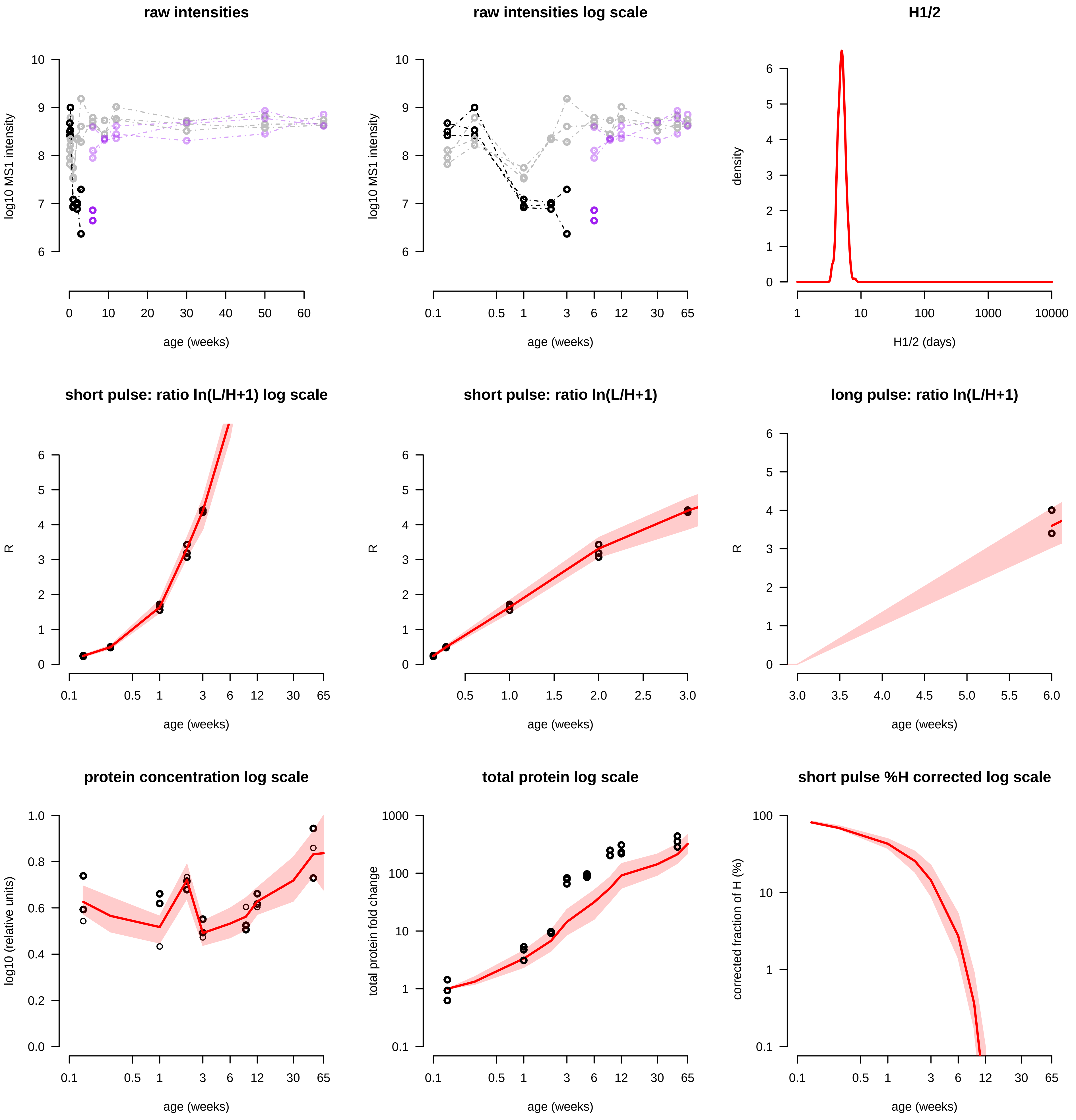

Q8K2Q7

>sp|Q8K2Q7|BROX_MOUSE

BRO1 domain−containing protein BROX

GN=Brox

Med H1/2: 3.8 days

Q5   H1/2: 2.5 days

Q95 H1/2: 20 days

reliable model fit: yes

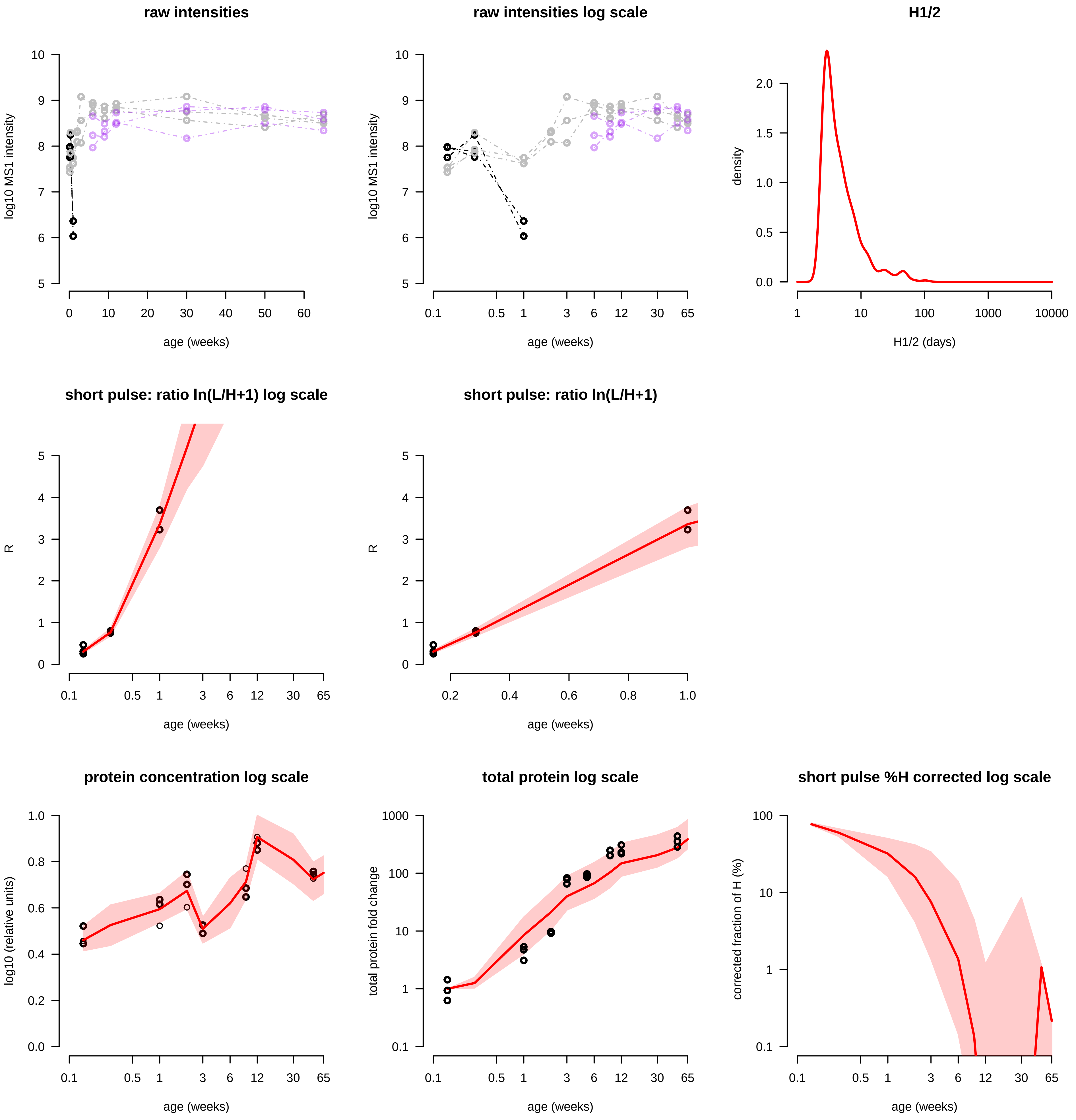

Q9CQY6

>sp|Q9CQY6|UQCC2_MOUSE

Ubiquinol−cytochrome−c reductase complex assembly factor 2

GN=Uqcc2

Med H1/2: 6.9 days

Q5 H1/2: 4.5 days

Q95 H1/2: 12.1 days

reliable model fit: yes

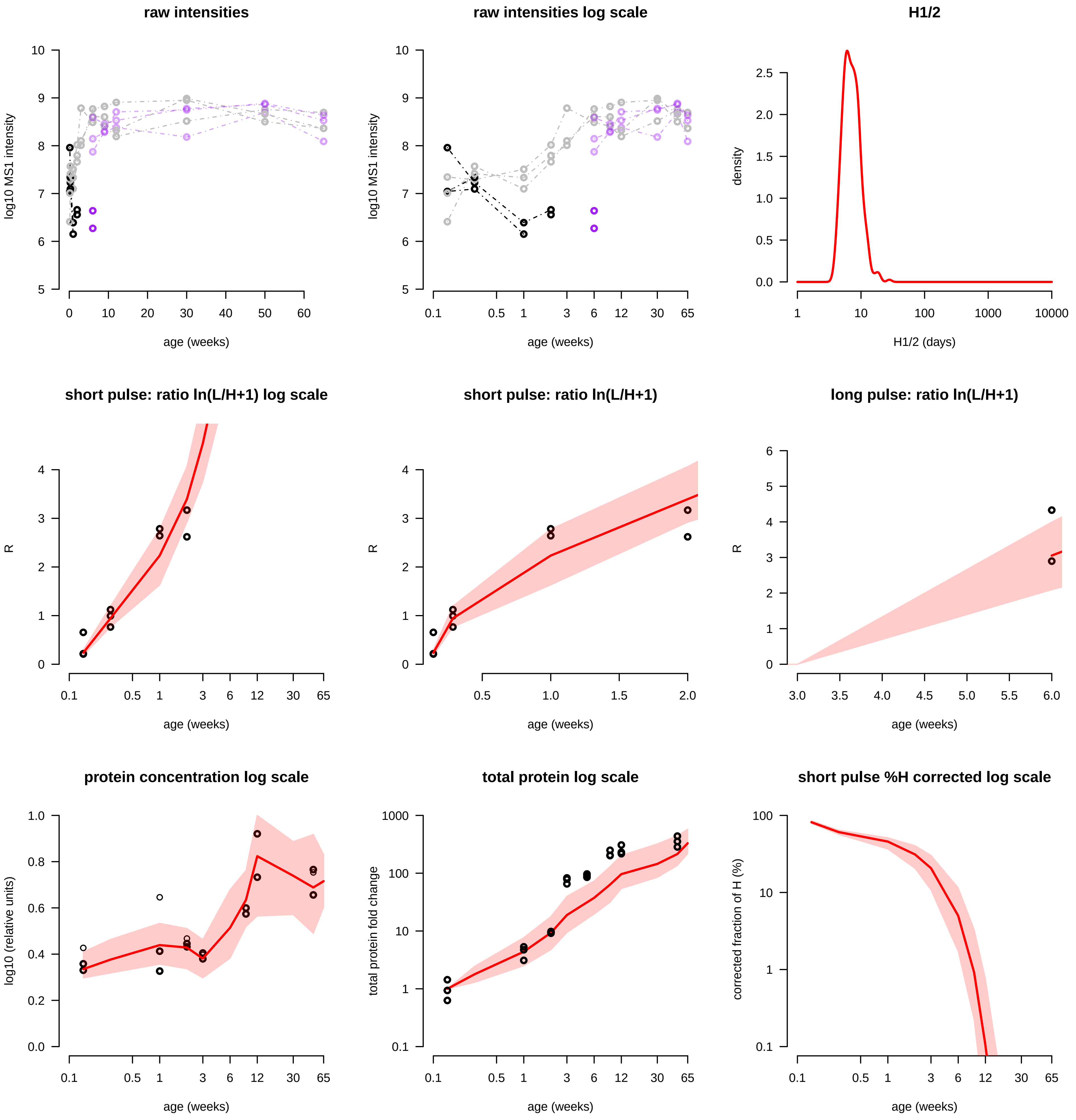

Q9CWZ7

>sp|Q9CWZ7|SNAG_MOUSE

Gamma−soluble NSF attachment protein

GN=Napg

Med H1/2: 5.2 days

Q5 H1/2: 3 days

Q95 H1/2: 23.4 days

reliable model fit: yes

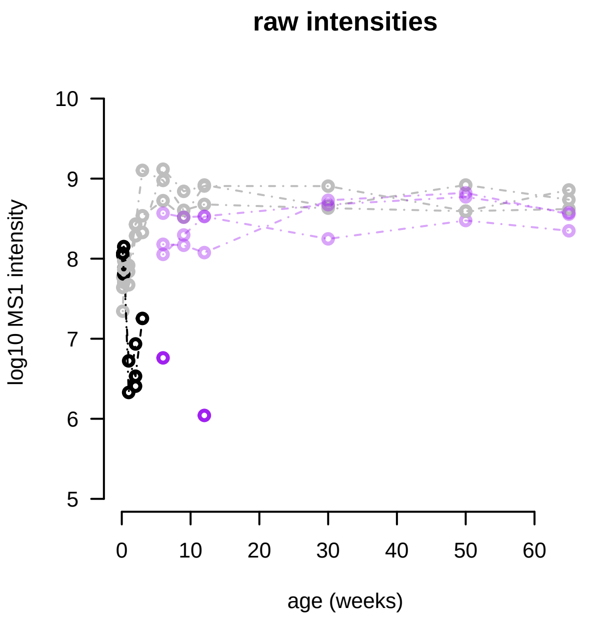

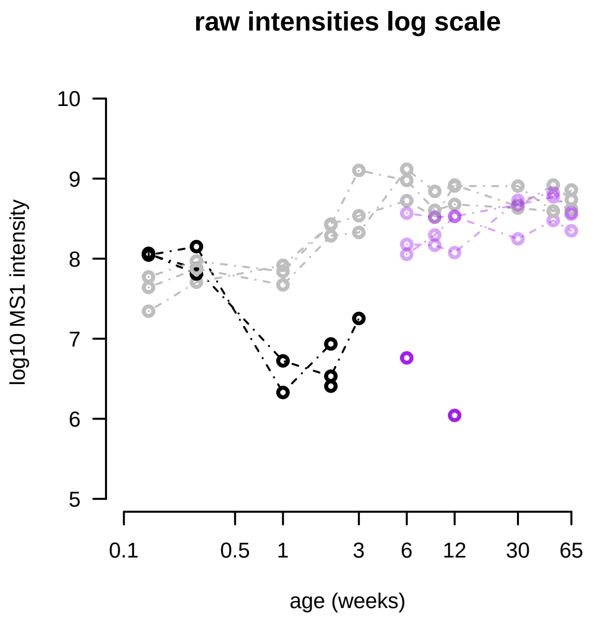

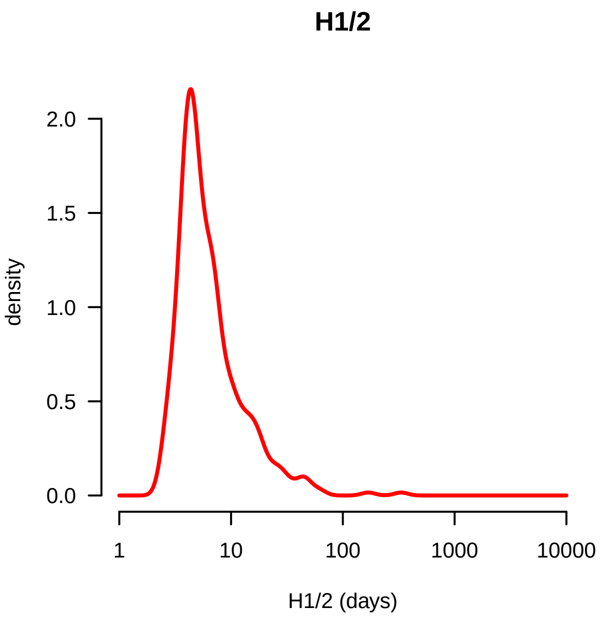

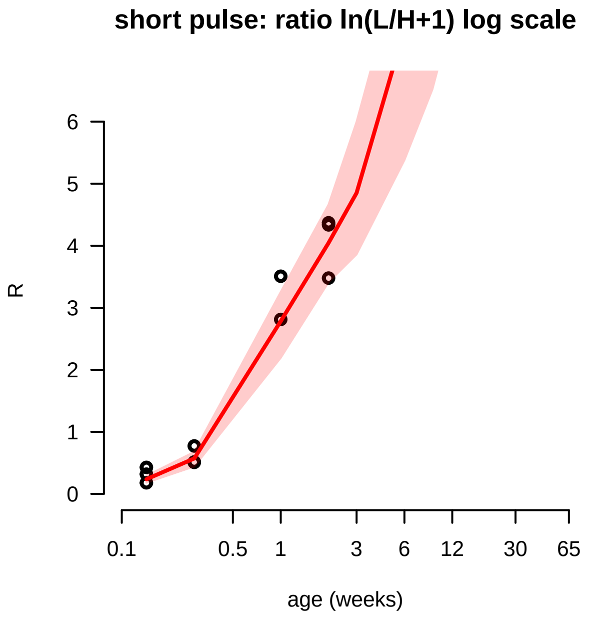

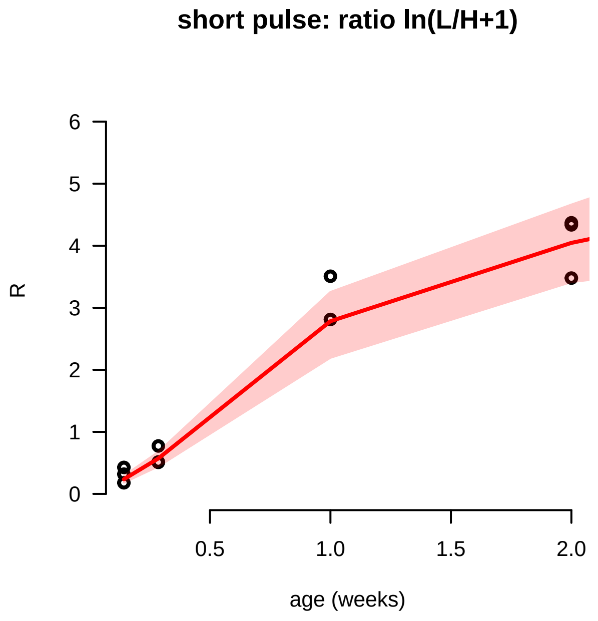

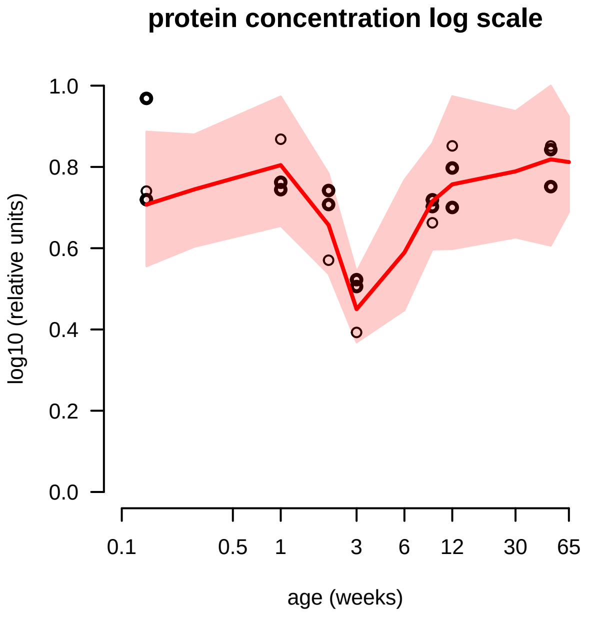

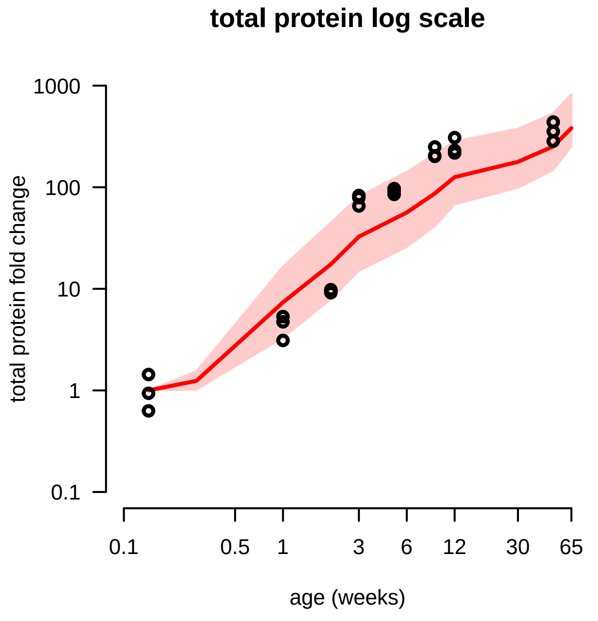

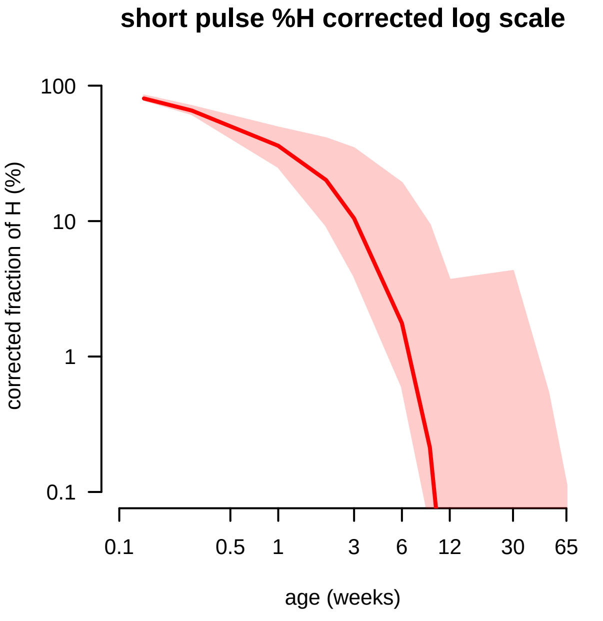

Q8BFQ4

>sp|Q8BFQ4|WDR82_MOUSE

WD repeat−containing protein 82

GN=Wdr82

Med H1/2: 4.9 days

Q5 H1/2: 4.1 days

Q95 H1/2: 6.3 days

reliable model fit: yes

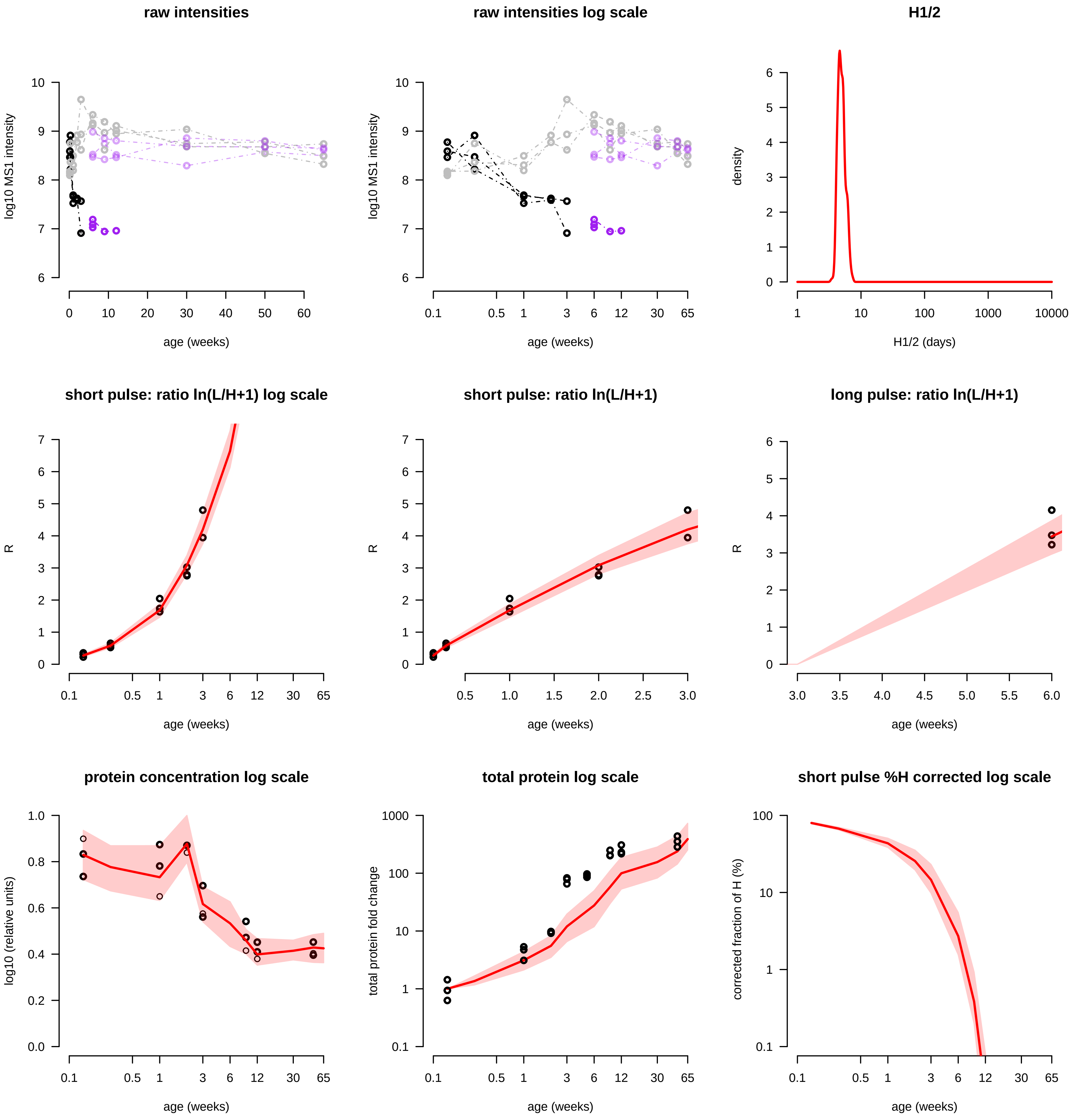

Q6ZPF4

>sp|Q6ZPF4|FMNL3_MOUSE

Formin−like protein 3

GN=Fmnl3

Med H1/2: 6 days

Q5 H1/2: 2.9 days

Q95 H1/2: 50.8 days

reliable model fit: yes

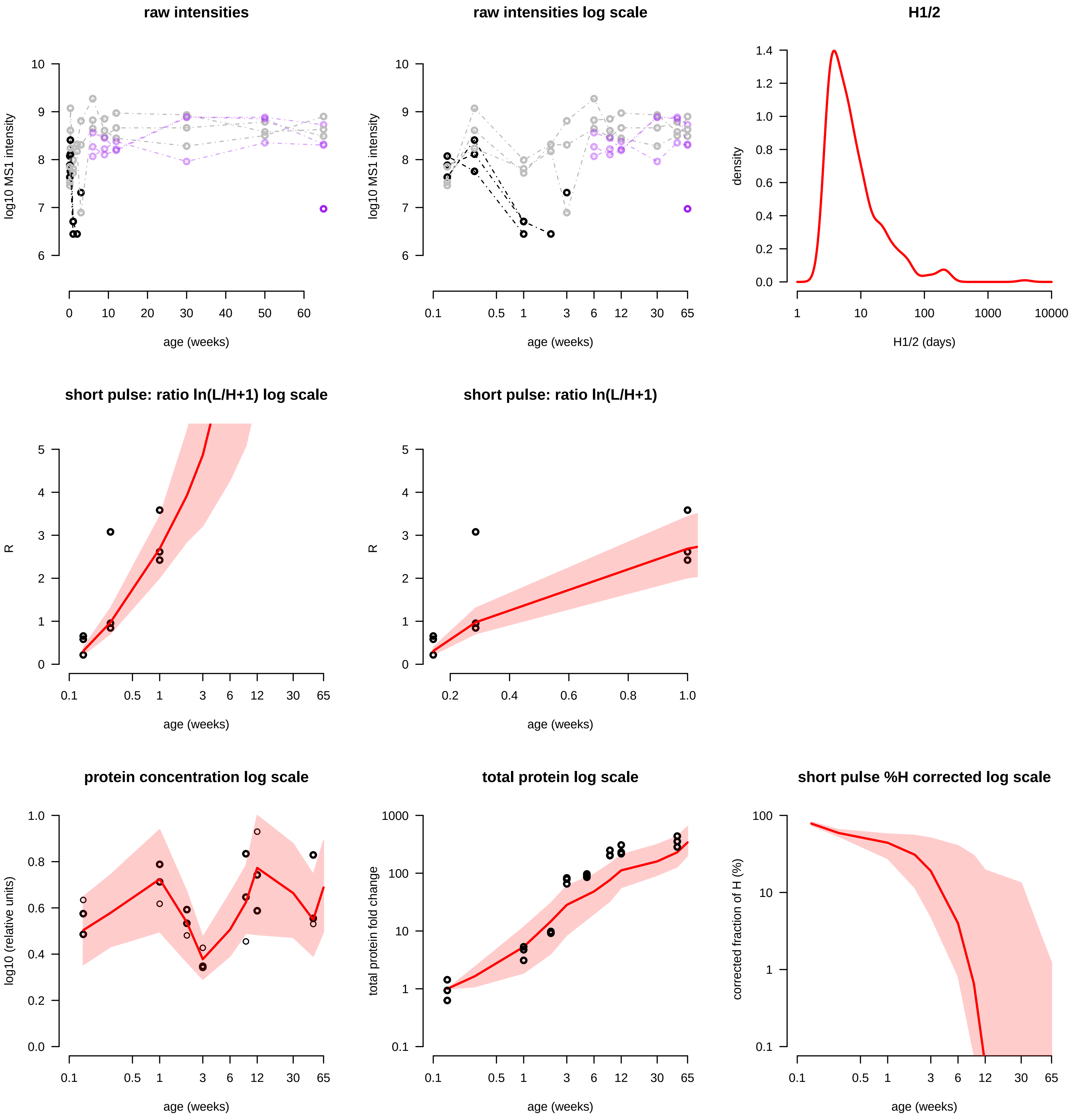

Q5SUC9

>sp|Q5SUC9|SCO1_MOUSE

Protein SCO1 homolog, mitochondrial

GN=Sco1

Med H1/2: 9.7 days

Q5 H1/2: 3.3 days

Q95 H1/2: 110.1 days

reliable model fit: yes

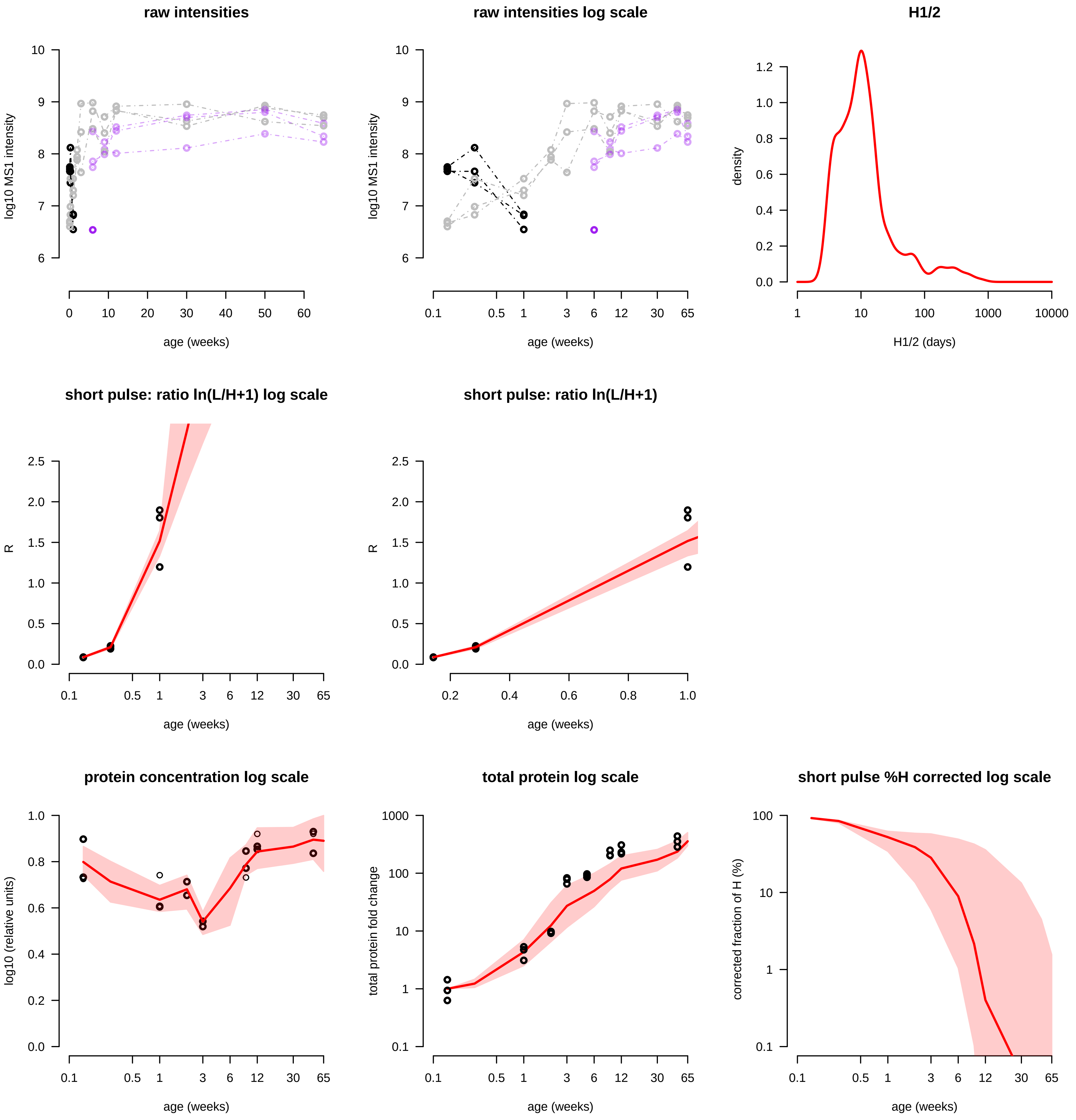

Q9D7P6

>sp|Q9D7P6|ISCU_MOUSE

Iron−sulfur cluster assembly enzyme ISCU, mitochondrial

GN=Iscu

Med H1/2: 5.8 days

Q5 H1/2: 2.6 days

Q95 H1/2: 37.7 days

reliable model fit: yes

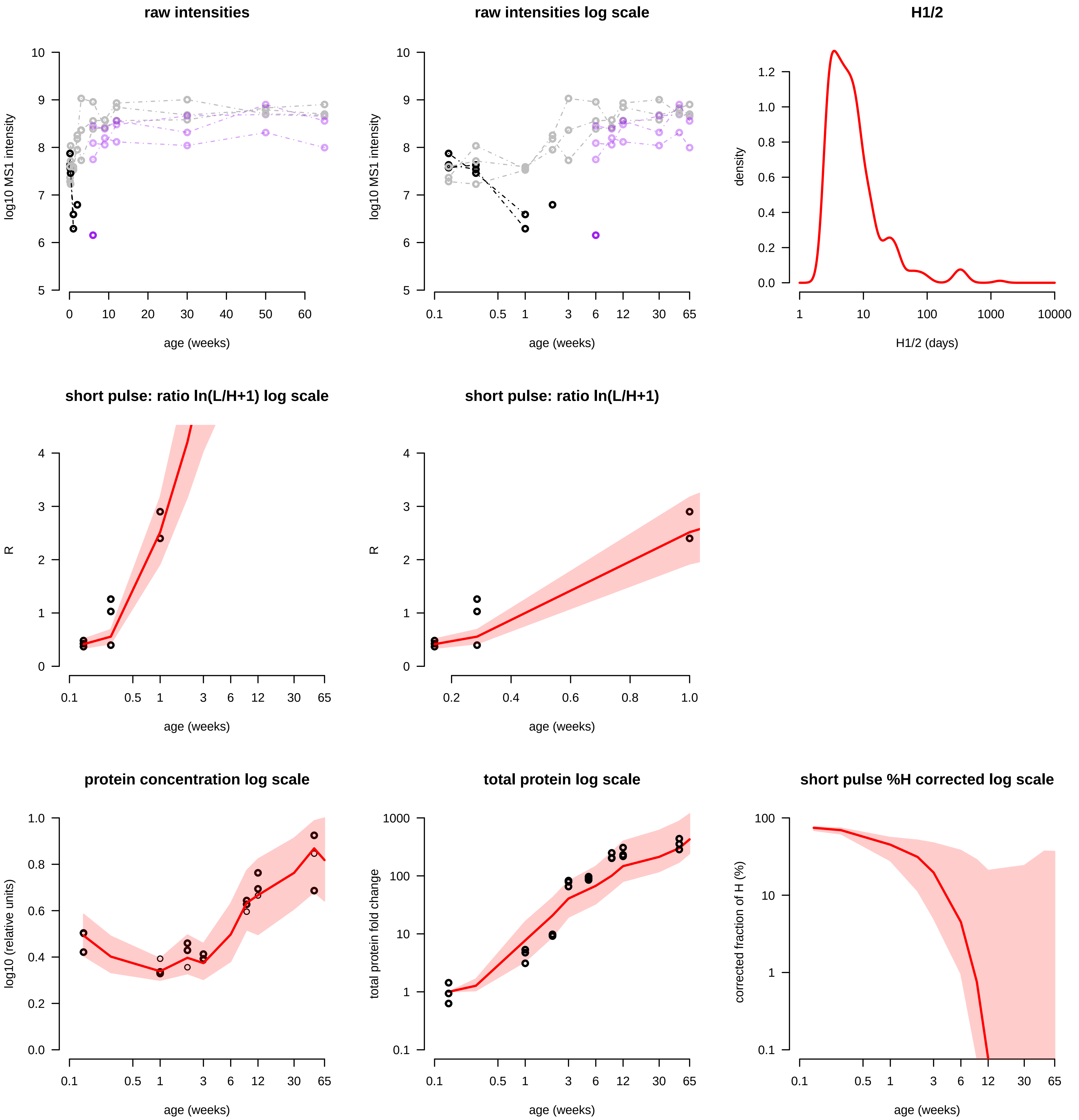

Q9D114

>sp|Q9D114|MESH1_MOUSE

Guanosine−3',5'−bis(diphosphate) 3'−pyrophosphohydrolase MESH1

GN=Hddc3

Med H1/2: 4.4 days

Q5 H1/2: 3.6 days

Q95 H1/2: 6.2 days

reliable model fit: yes

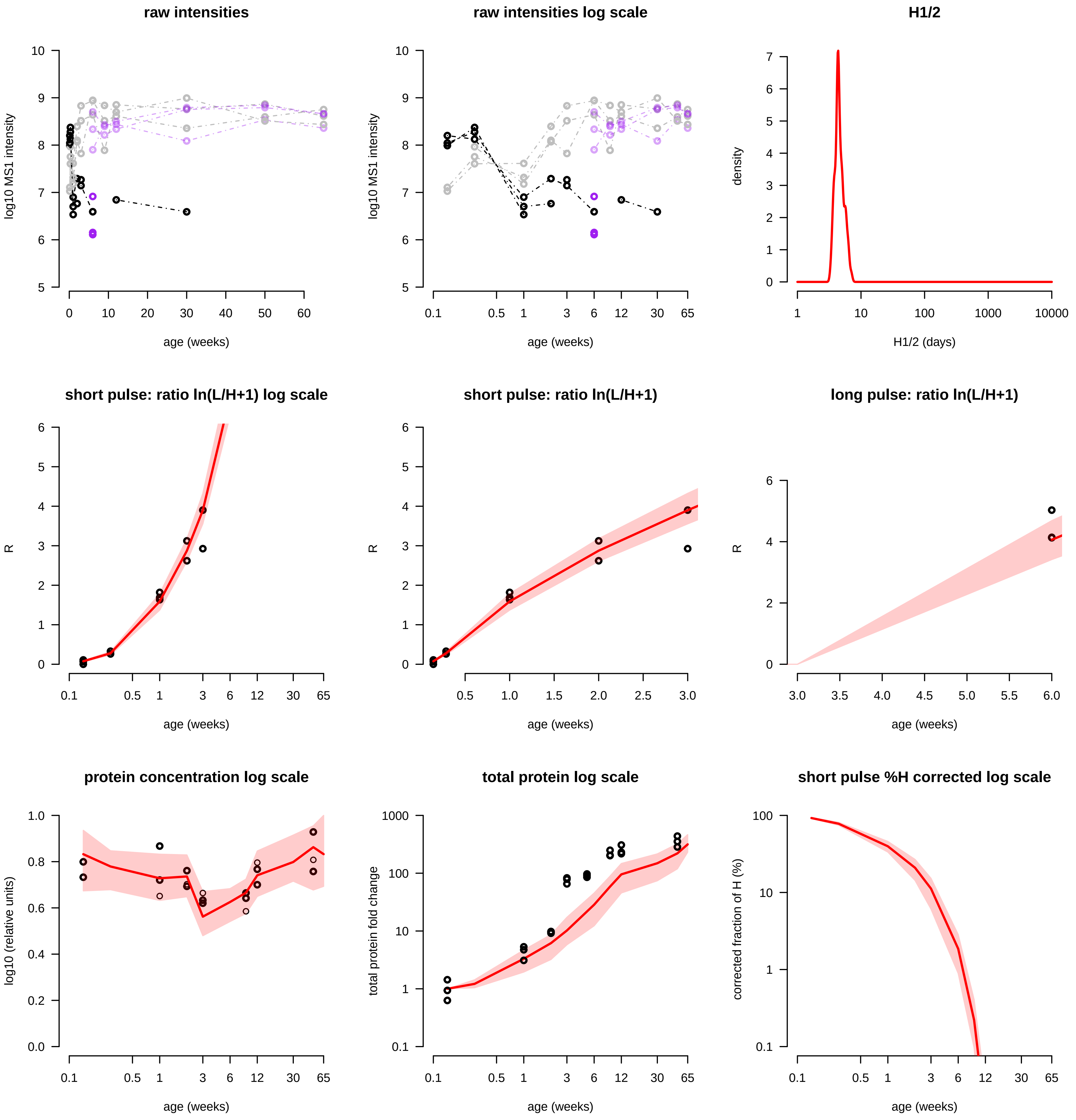

G5E870

>sp|G5E870|TRIPC_MOUSE

E3 ubiquitin−protein ligase TRIP12

GN=Trip12

Med H1/2: 12 days

Q5 H1/2: 9.7 days

Q95 H1/2: 16.3 days

reliable model fit: yes

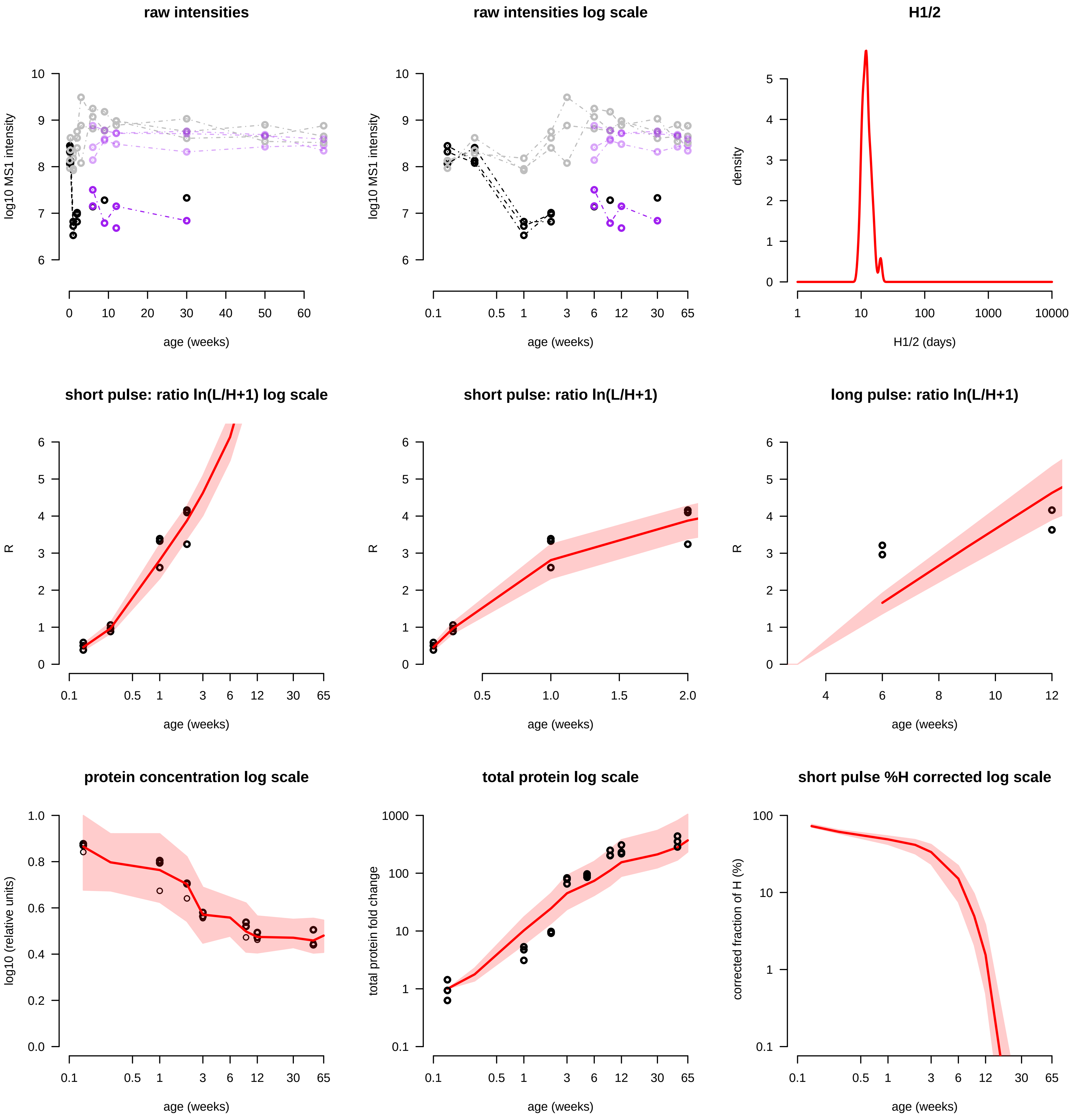

Q62203

>sp|Q62203|SF3A2_MOUSE

Splicing factor 3A subunit 2

GN=Sf3a2

Med H1/2: 5.7 days

Q5 H1/2: 4.5 days

Q95 H1/2: 8.4 days

reliable model fit: no

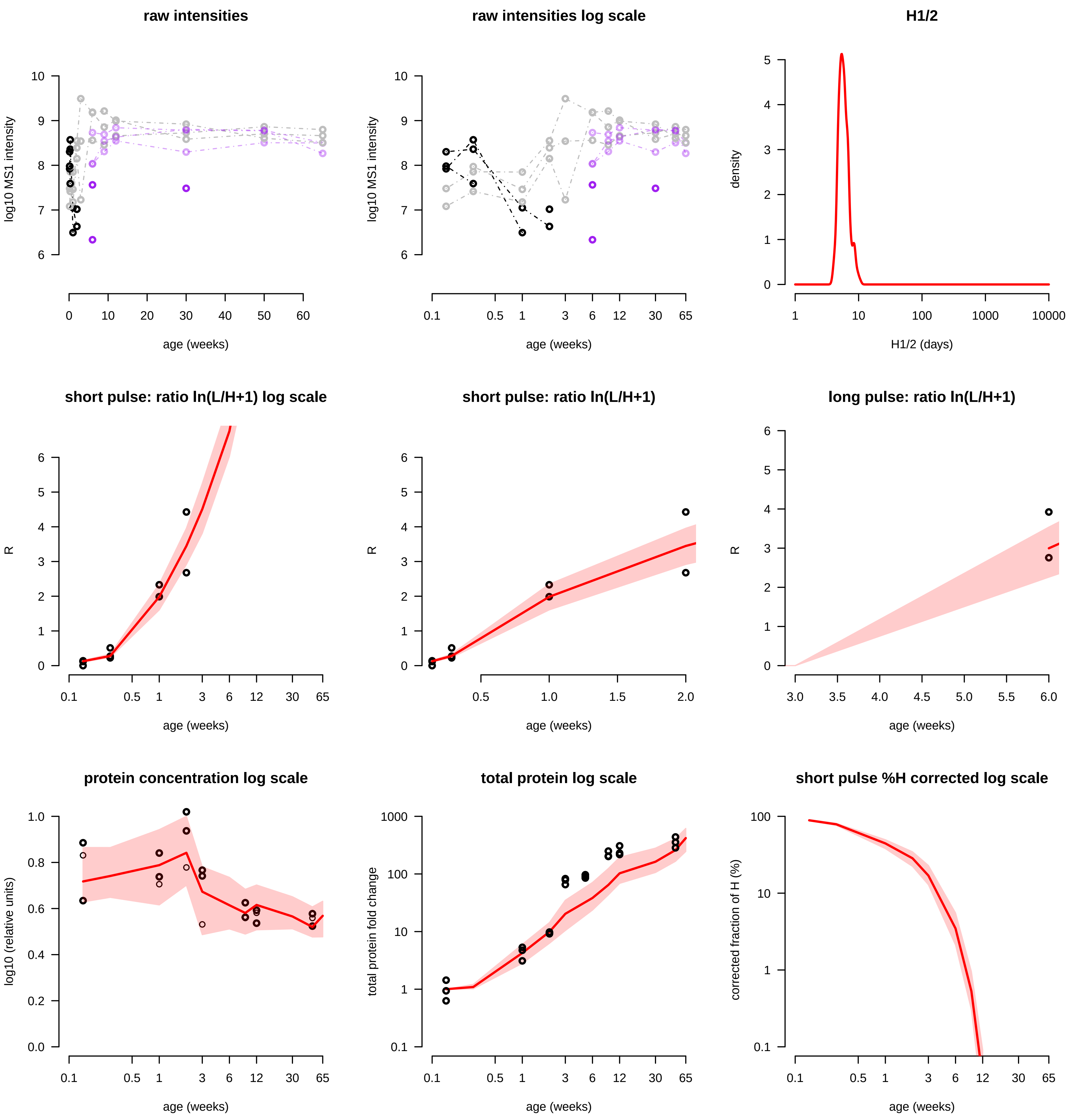

Q8C570

>sp|Q8C570|RAE1L_MOUSE

mRNA export factor

GN=Rae1

Med H1/2: 3.9 days

Q5 H1/2: 2.8 days

Q95 H1/2: 5.4 days

reliable model fit: yes

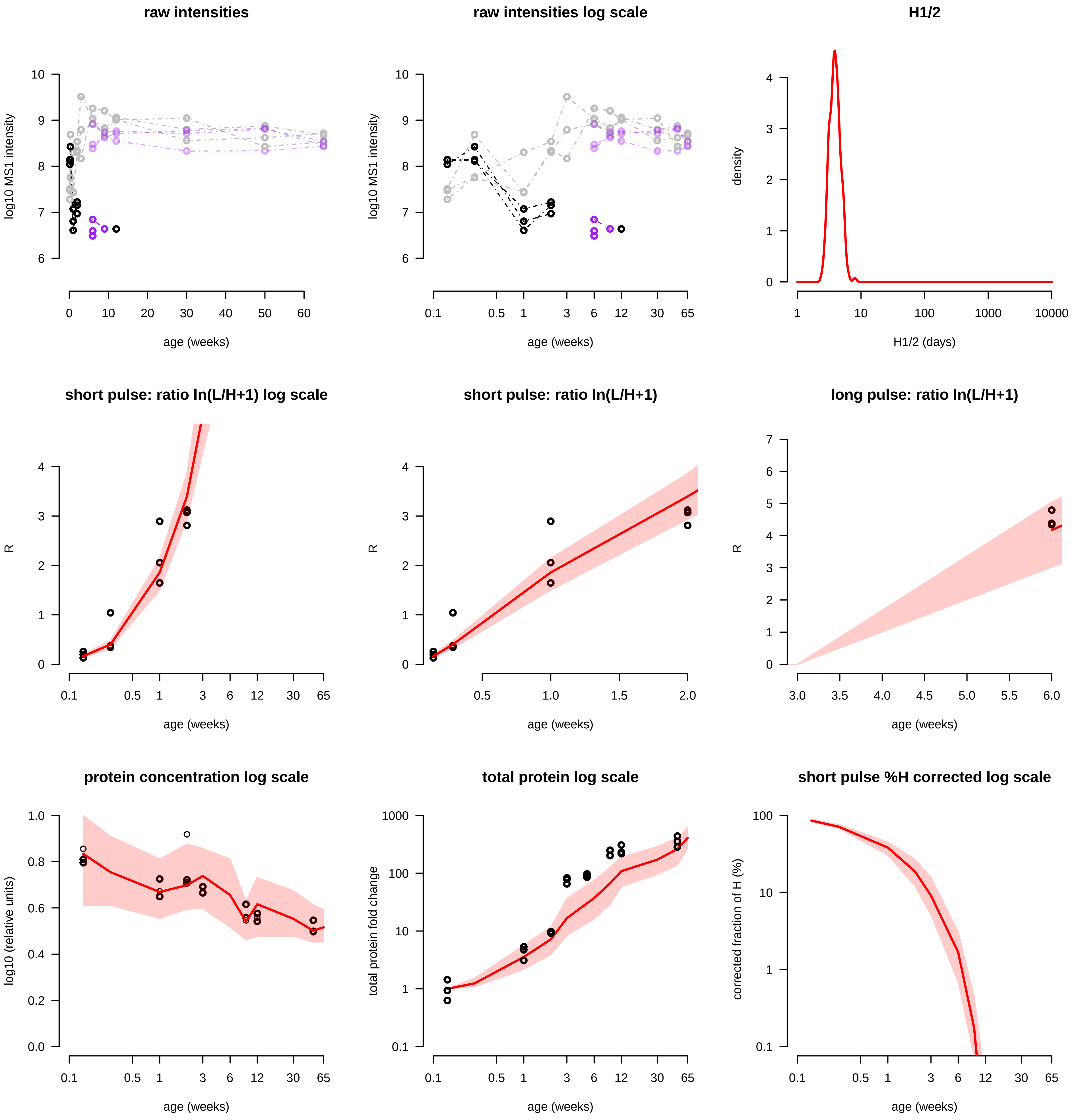

P03987−2;P03987

>sp|P03987−2|IGHG3_MOUSE

Isoform 2 of Ig gamma−3 chain C region

Med H1/2: 33.9 days

Q5 H1/2: 10.5 days

Q95 H1/2: 1007.9 days

reliable model fit: no

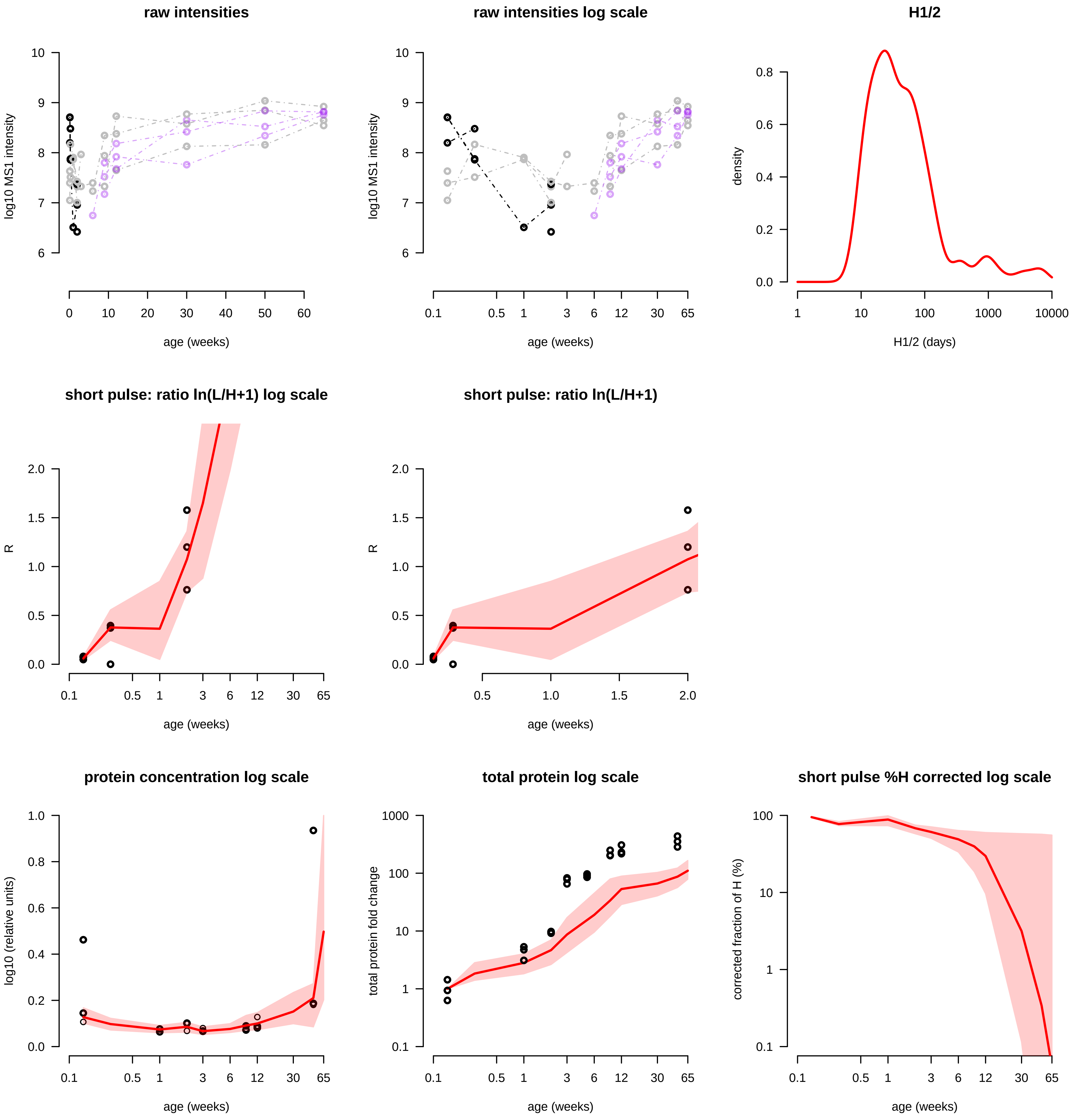

O88532

>sp|O88532|ZFR_MOUSE

Zinc finger RNA−binding protein

GN=Zfr

Med H1/2: 11.6 days

Q5 H1/2: 5.4 days

Q95 H1/2: 149.8 days

reliable model fit: yes

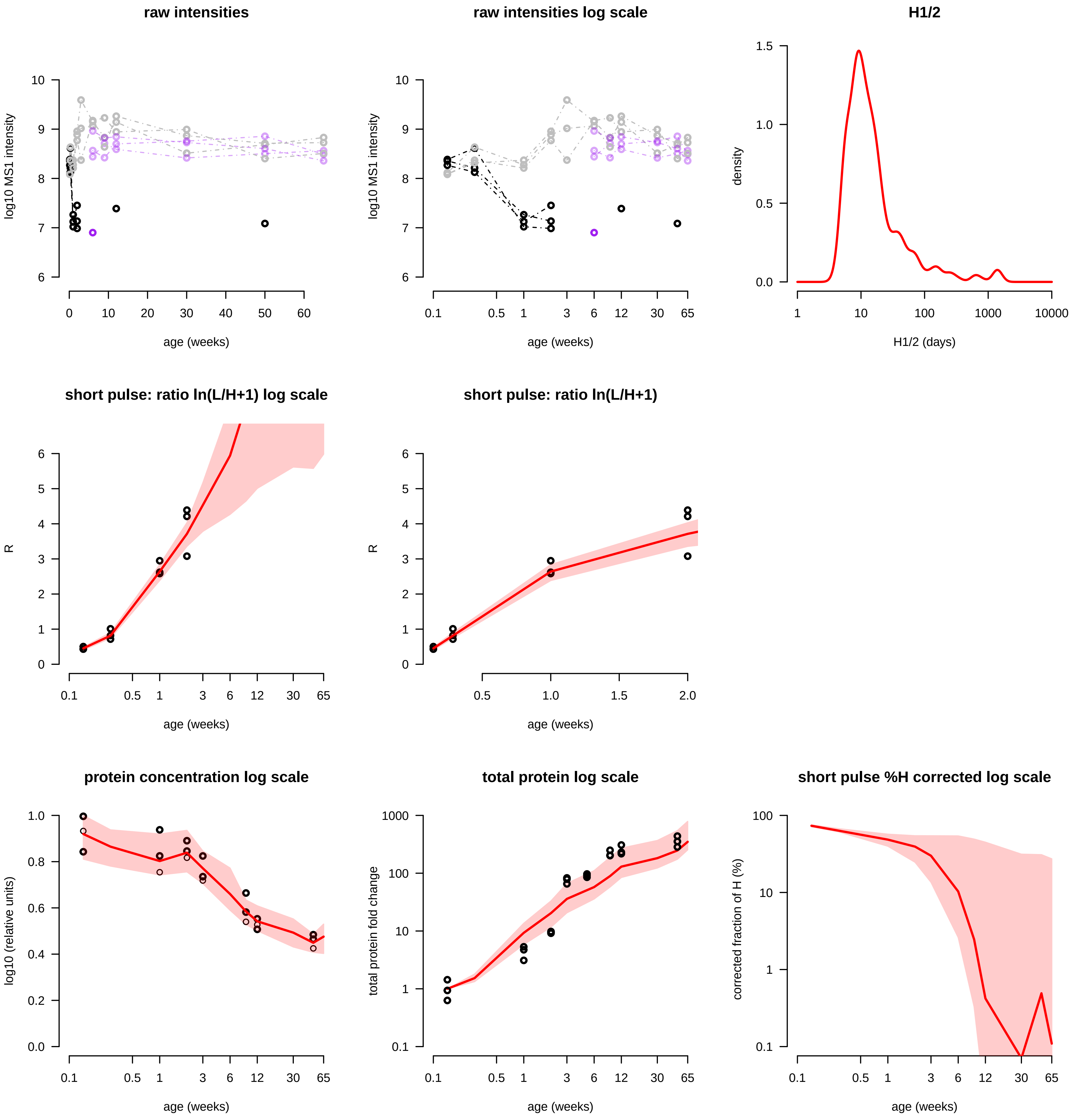

Q9CQJ6

>sp|Q9CQJ6|DENR_MOUSE

Density−regulated protein

GN=Denr

Med H1/2: 4.1 days

Q5 H1/2: 3 days

Q95 H1/2: 7.6 days

reliable model fit: yes

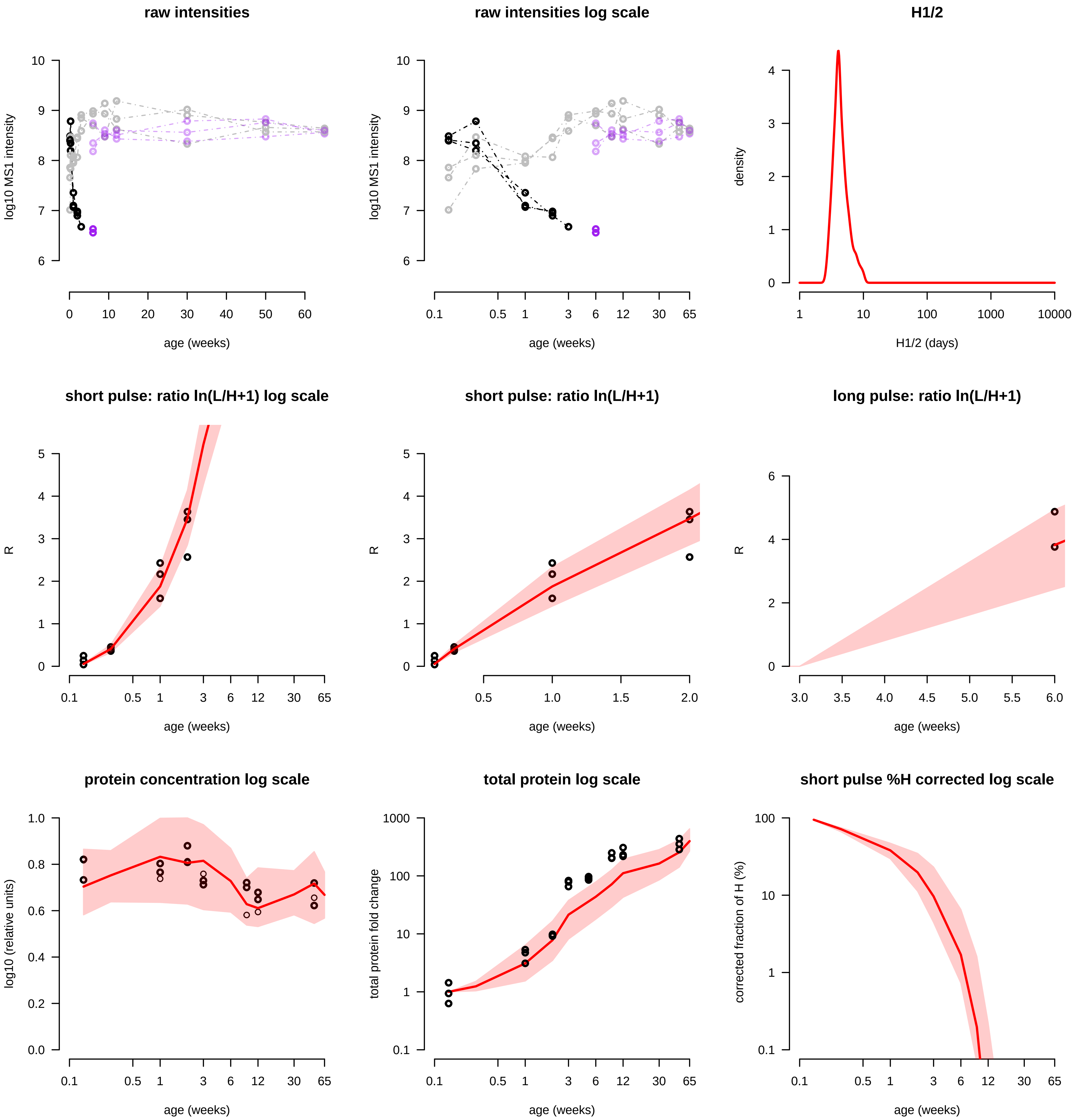

O55106

>sp|O55106|STRN_MOUSE

Striatin

GN=Strn

Med H1/2: 4.2 days

Q5 H1/2: 3.3 days

Q95 H1/2: 5.7 days

reliable model fit: yes

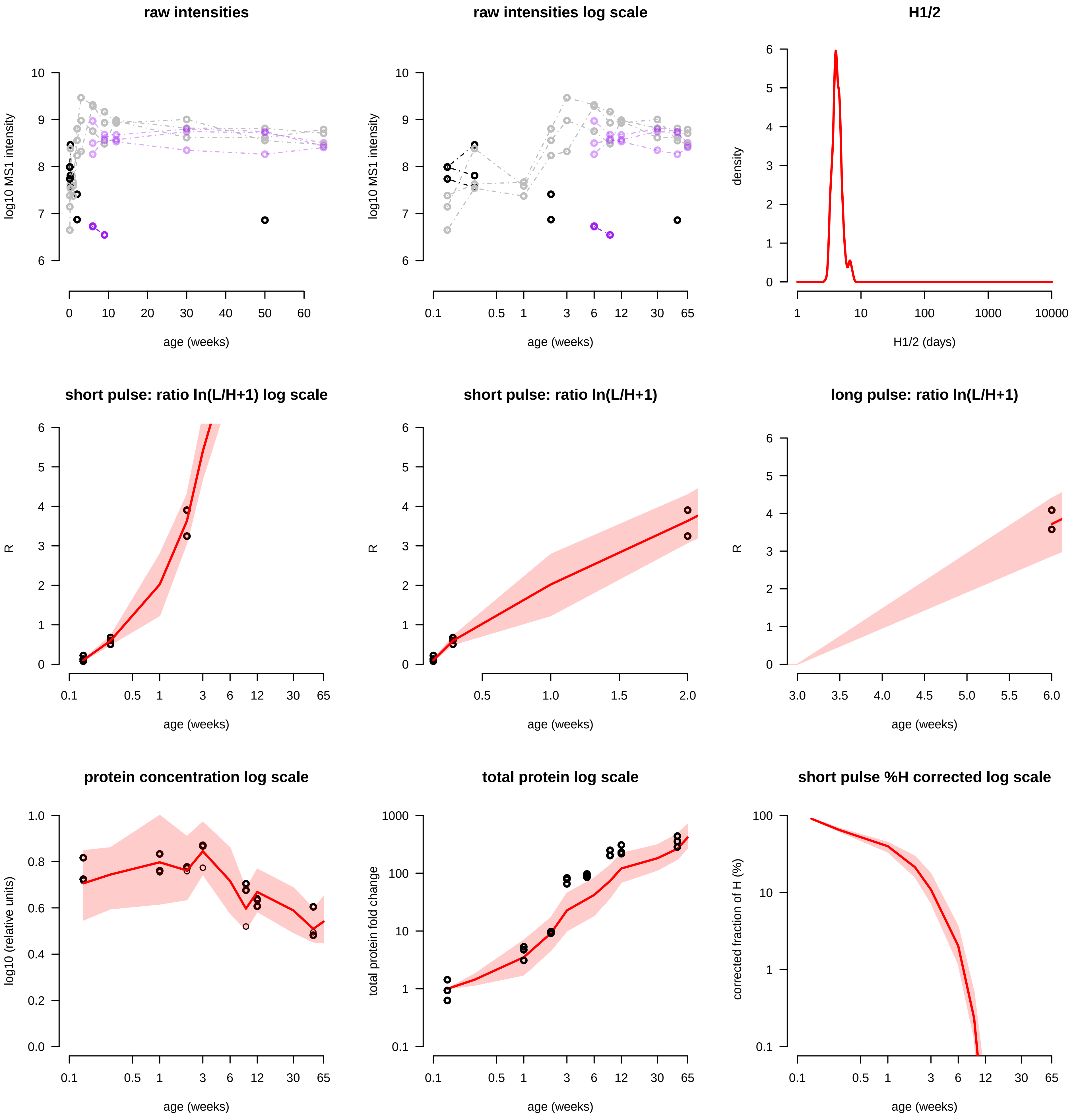

Q80X82

>sp|Q80X82|SYMPK_MOUSE

Symplekin

GN=Sympk

Med H1/2: 3.5 days

Q5 H1/2: 2.9 days

Q95 H1/2: 4.6 days

reliable model fit: yes

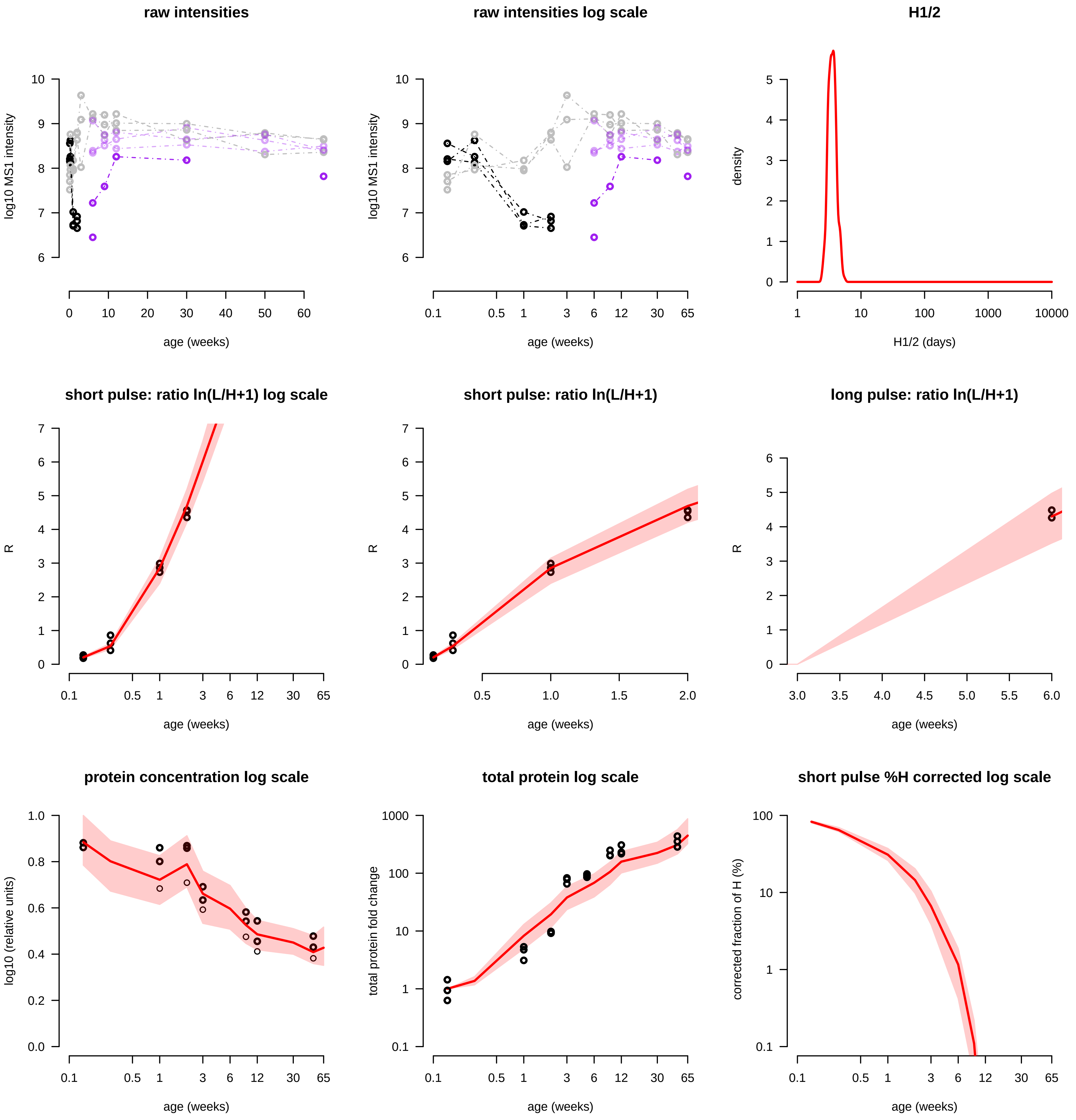

Q8K1Z0

>sp|Q8K1Z0|COQ9_MOUSE

Ubiquinone biosynthesis protein COQ9, mitochondrial

GN=Coq9

Med H1/2: 6.2 days

Q5 H1/2: 3.7 days

Q95 H1/2: 10.7 days

reliable model fit: yes

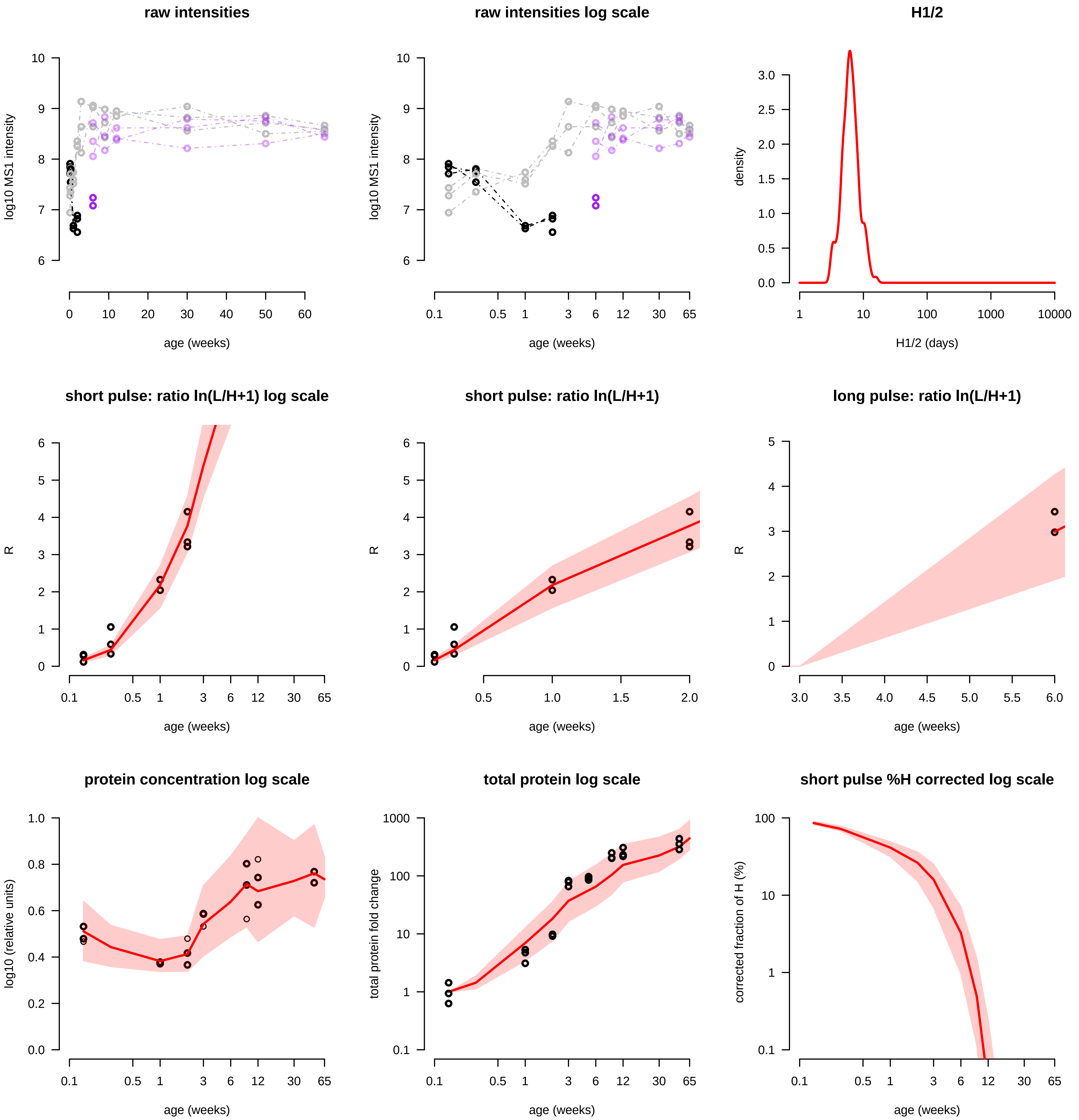

Q9D6K8

>sp|Q9D6K8|FUND2_MOUSE

FUN14 domain−containing protein 2

GN=Fundc2

Med H1/2: 23.6 days

Q5 H1/2: 9.9 days

Q95 H1/2: 172.7 days

reliable model fit: no

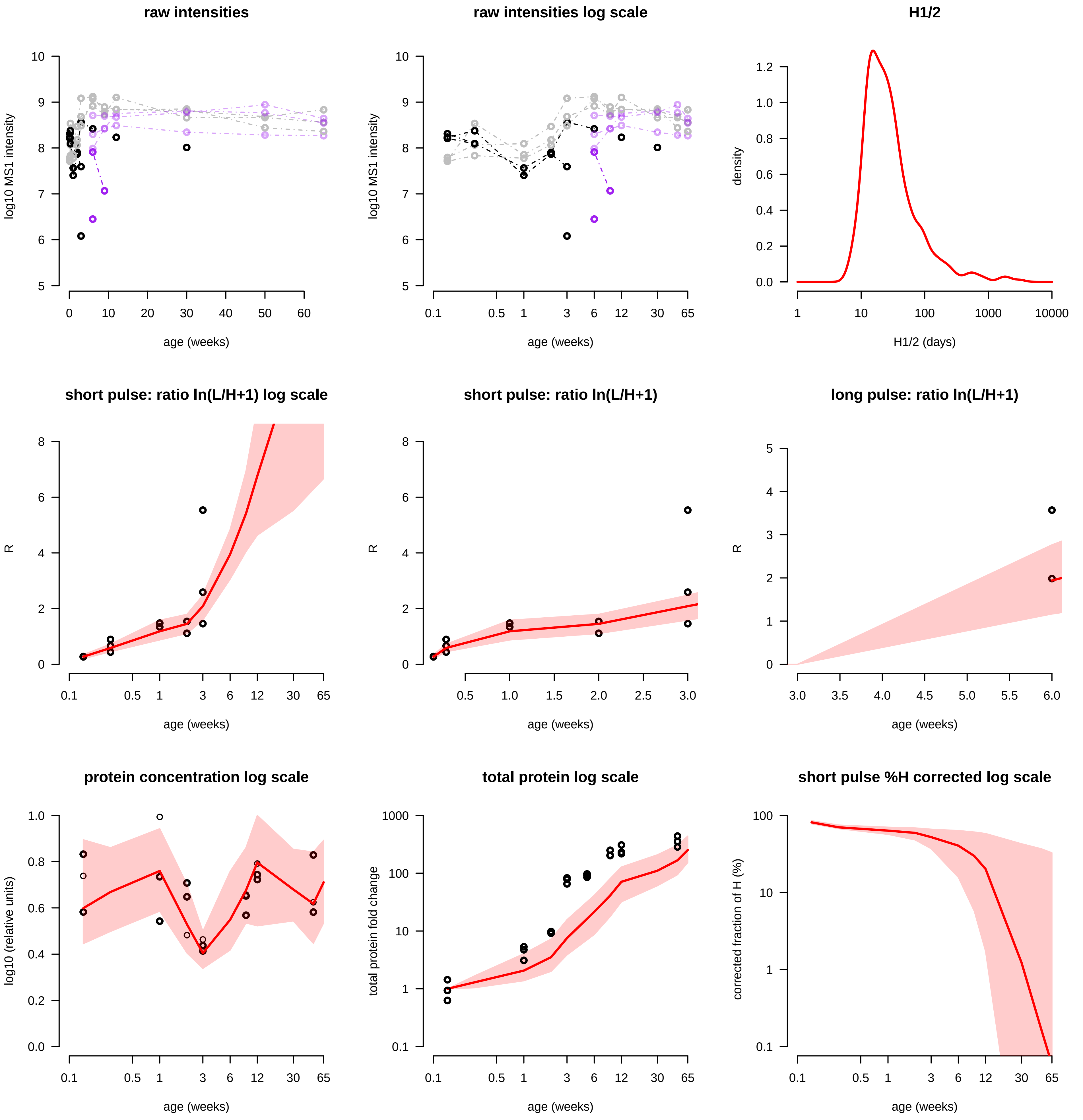

Q9CQS8

>sp|Q9CQS8|SC61B_MOUSE

Protein transport protein Sec61 subunit beta

GN=Sec61b

Med H1/2: 8.5 days

Q5 H1/2: 7 days

Q95 H1/2: 11.7 days

reliable model fit: yes

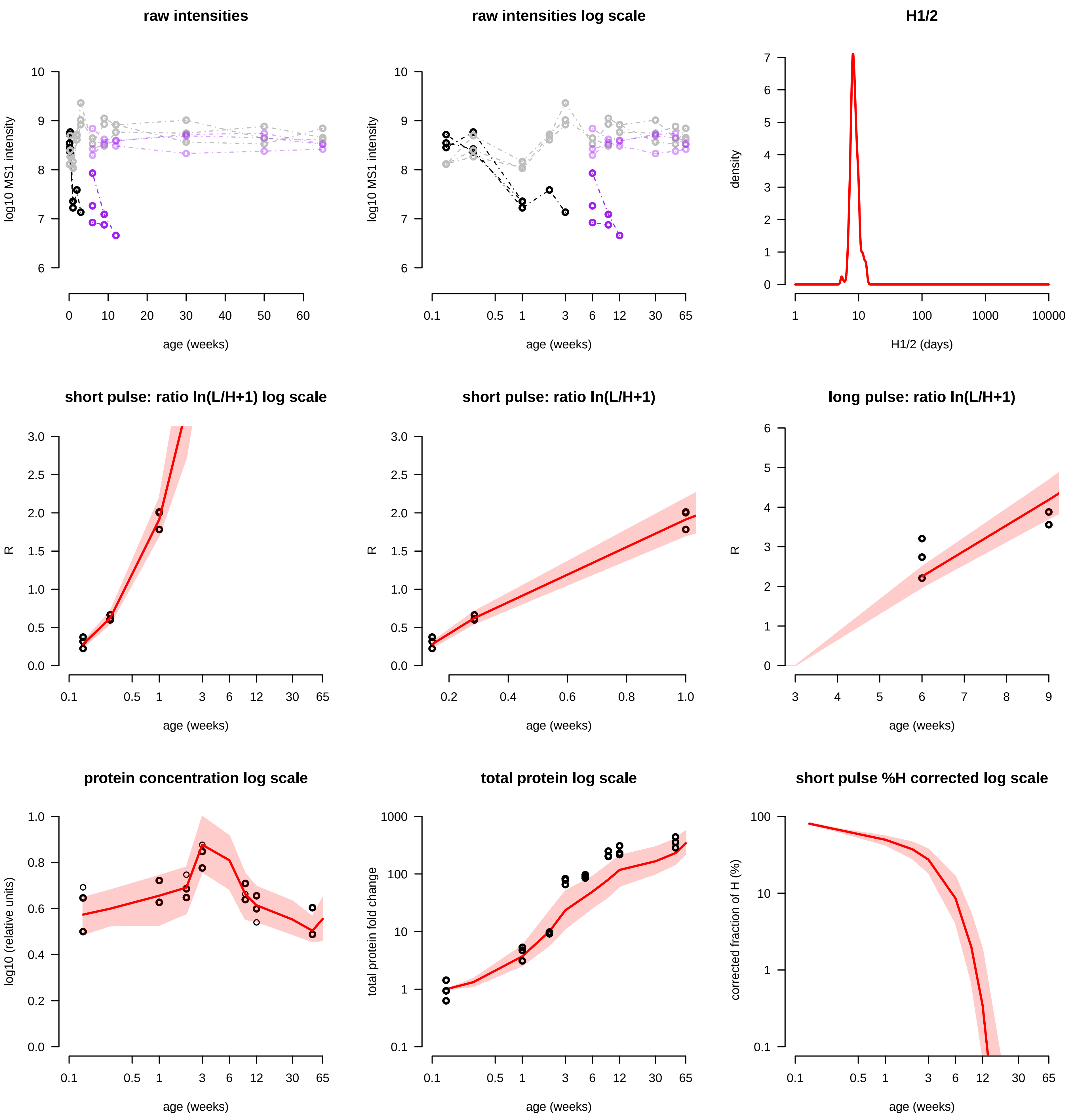

Q9Z277;Q9Z277−2

>sp|Q9Z277|BAZ1B_MOUSE

Tyrosine−protein kinase BAZ1B

GN=Baz1b

Med H1/2: 258.6 days

Q5 H1/2: 106 days

Q95 H1/2: 1653.6 days

reliable model fit: no

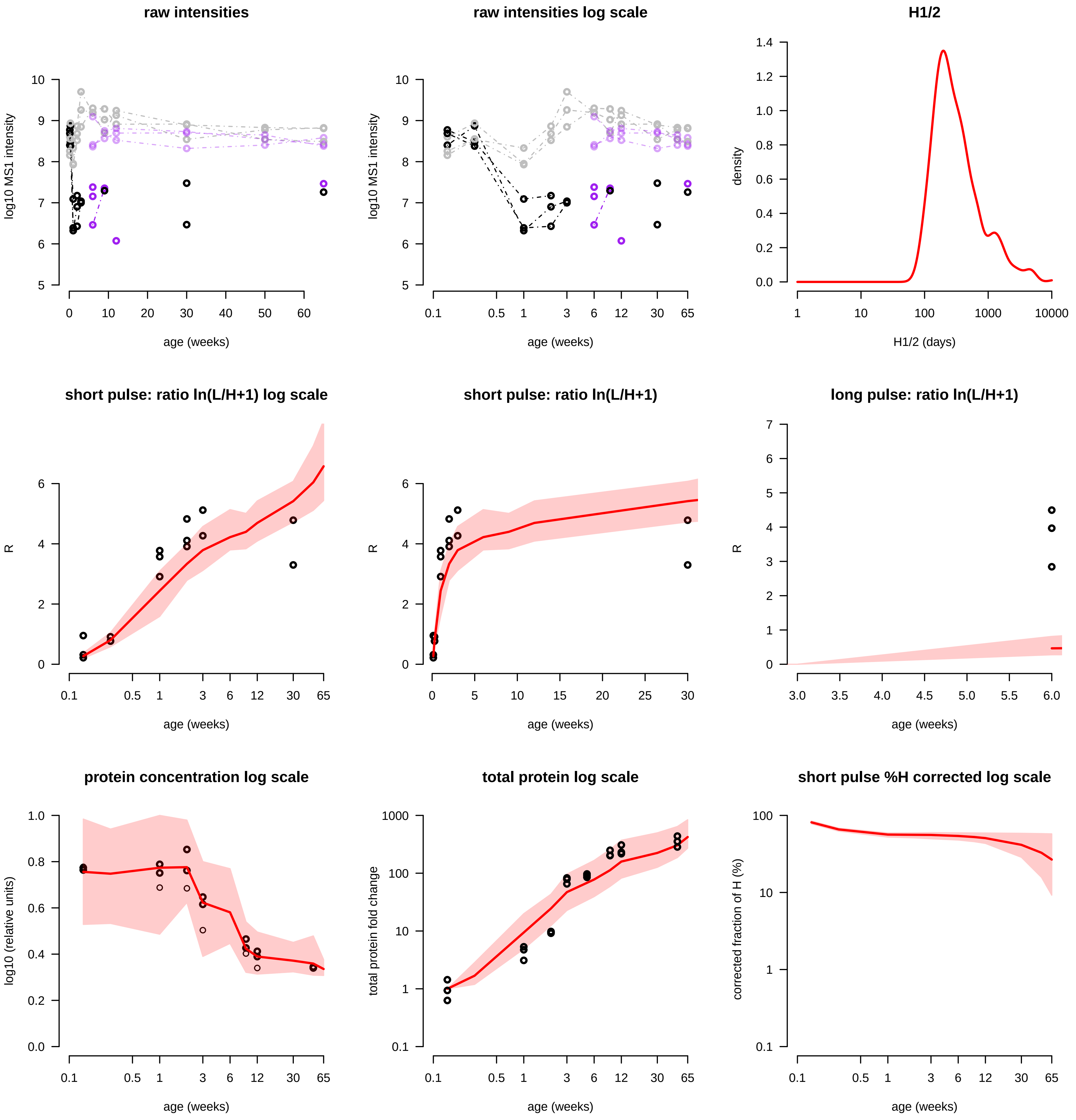

Q8K1X1

>sp|Q8K1X1|WDR11_MOUSE

WD repeat−containing protein 11

GN=Wdr11

Med H1/2: 23.5 days

Q5 H1/2: 18.4 days

Q95 H1/2: 38 days

reliable model fit: yes

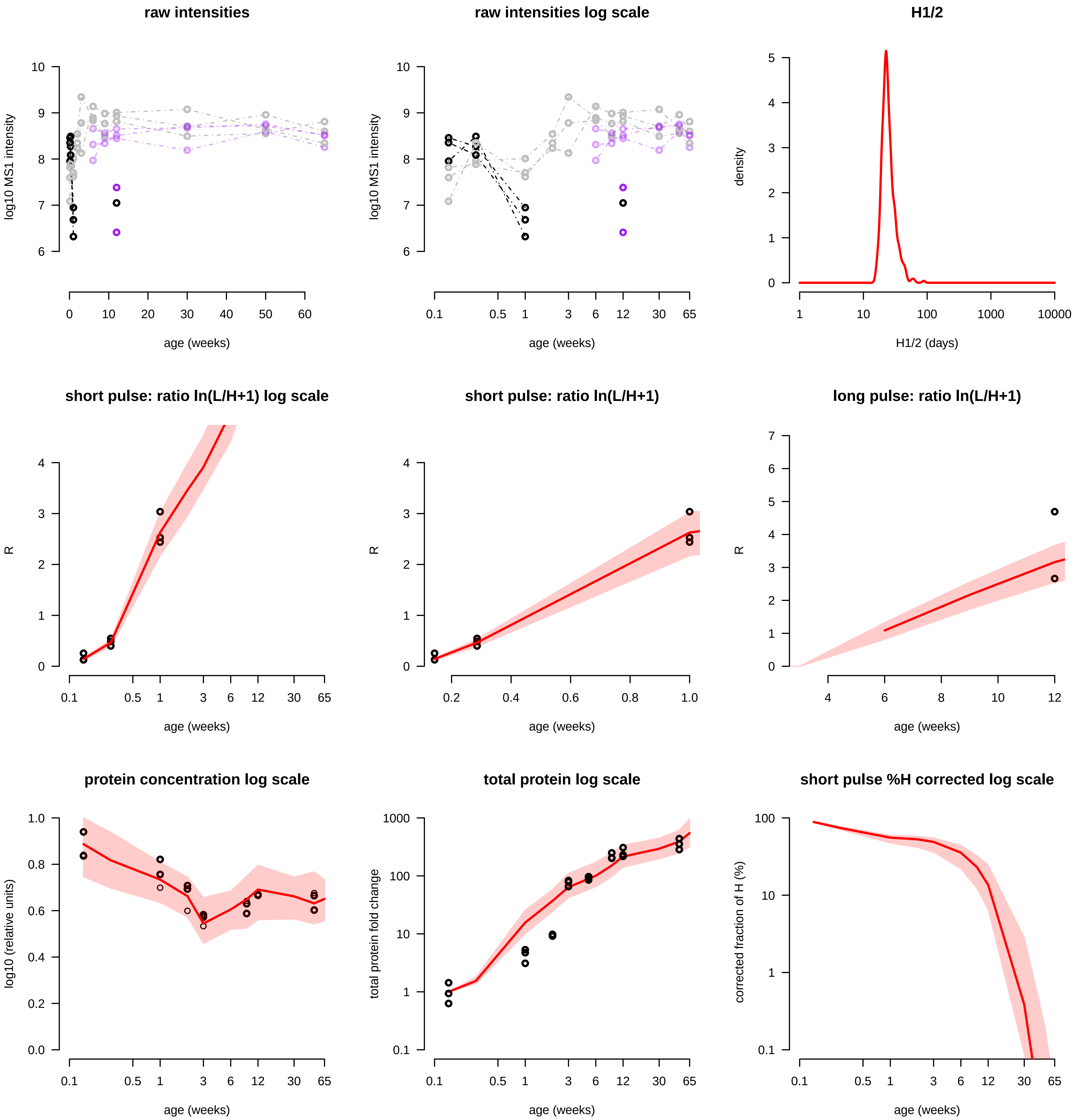

Q9CSH3

>sp|Q9CSH3|RRP44_MOUSE

Exosome complex exonuclease RRP44

GN=Dis3

Med H1/2: 6.4 days

Q5 H1/2: 4.9 days

Q95 H1/2: 8.9 days

reliable model fit: no

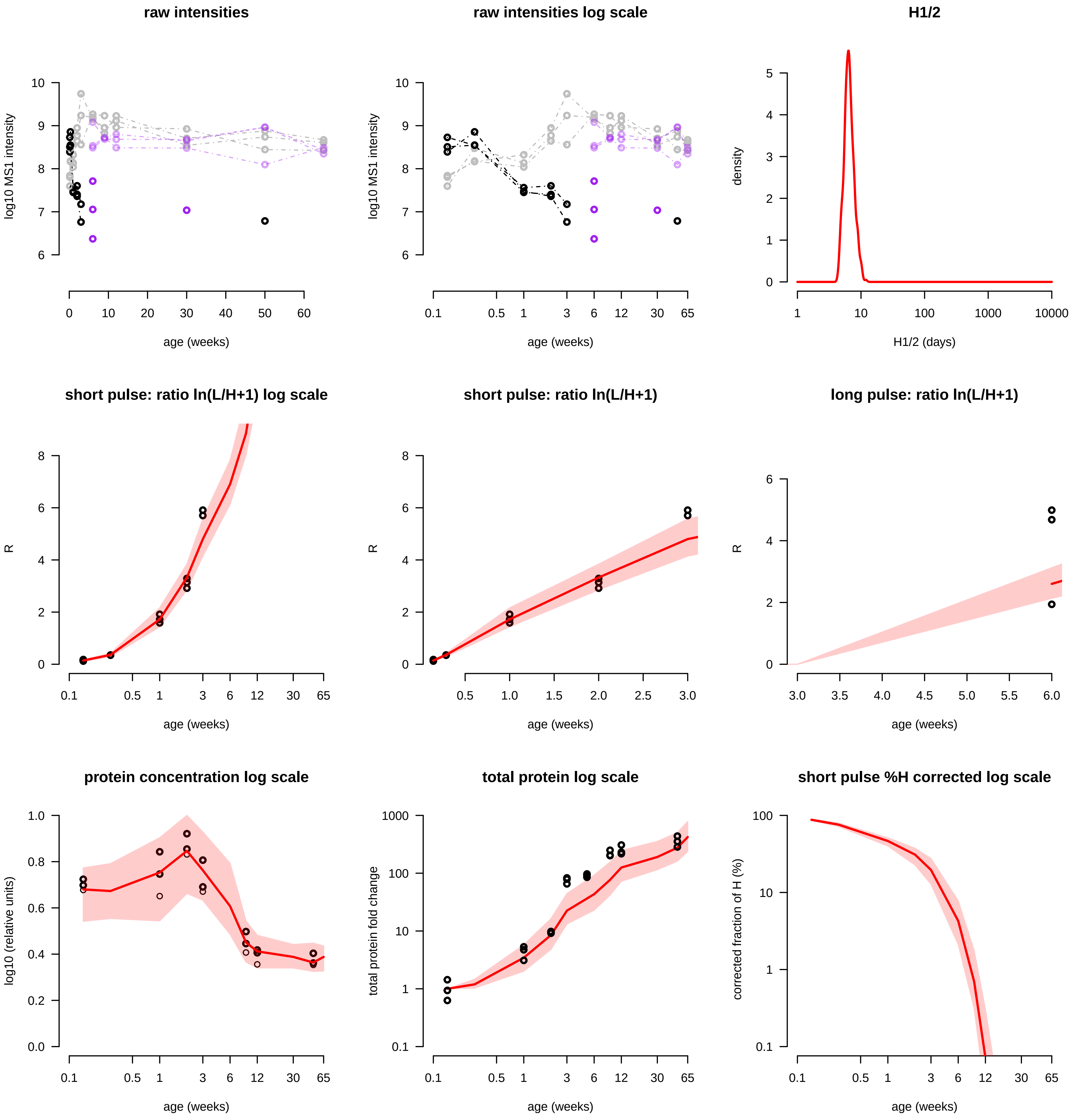

Q60631;Q60631−2

>sp|Q60631|GRB2_MOUSE

Growth factor receptor−bound protein 2

GN=Grb2

Med H1/2: 19 days

Q5 H1/2: 4.7 days

Q95 H1/2: 150.3 days

reliable model fit: yes

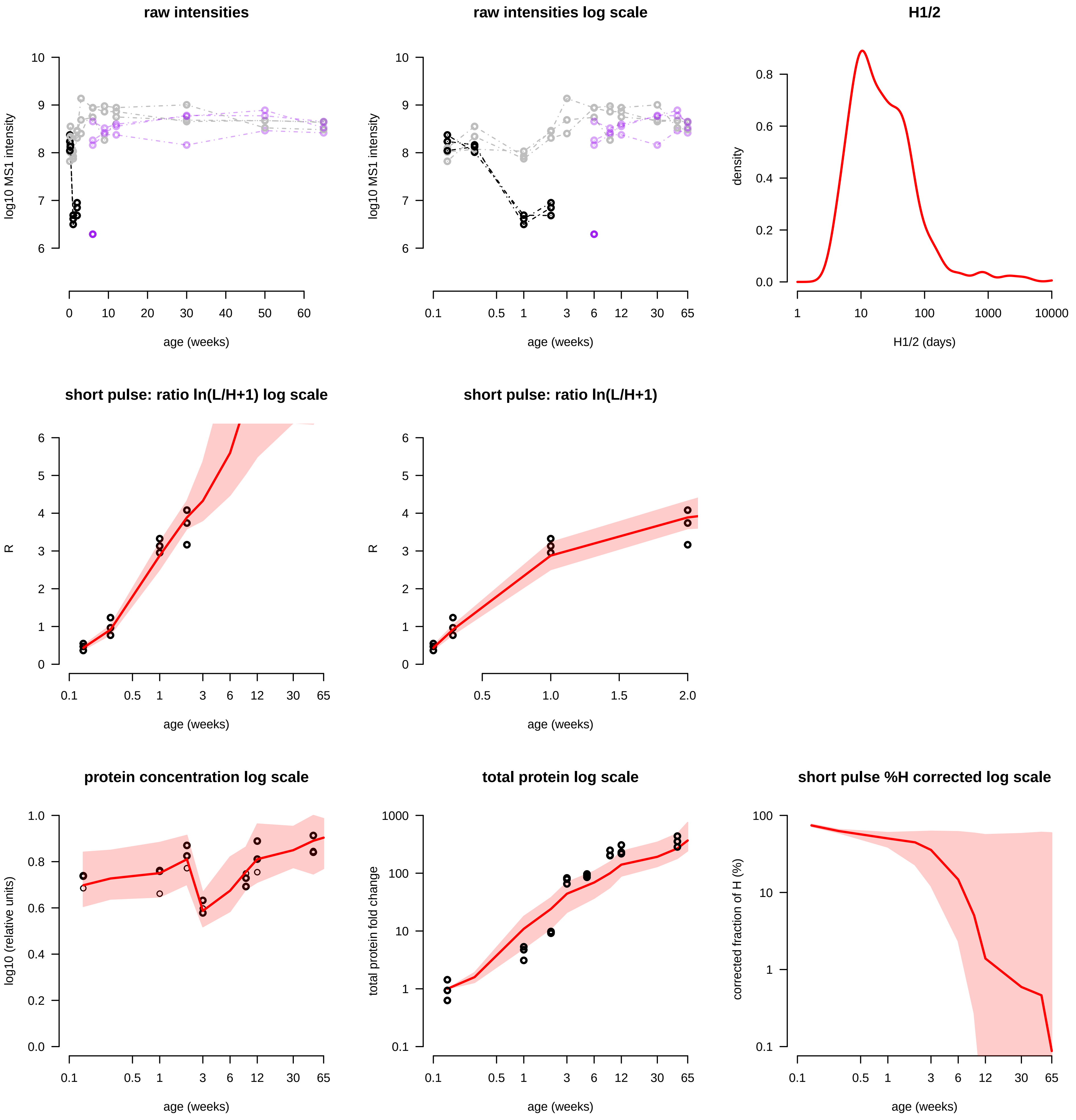

P57784

>sp|P57784|RU2A_MOUSE

U2 small nuclear ribonucleoprotein A'

GN=Snrpa1

Med H1/2: 5.1 days

Q5 H1/2: 4.2 days

Q95 H1/2: 7.8 days

reliable model fit: yes

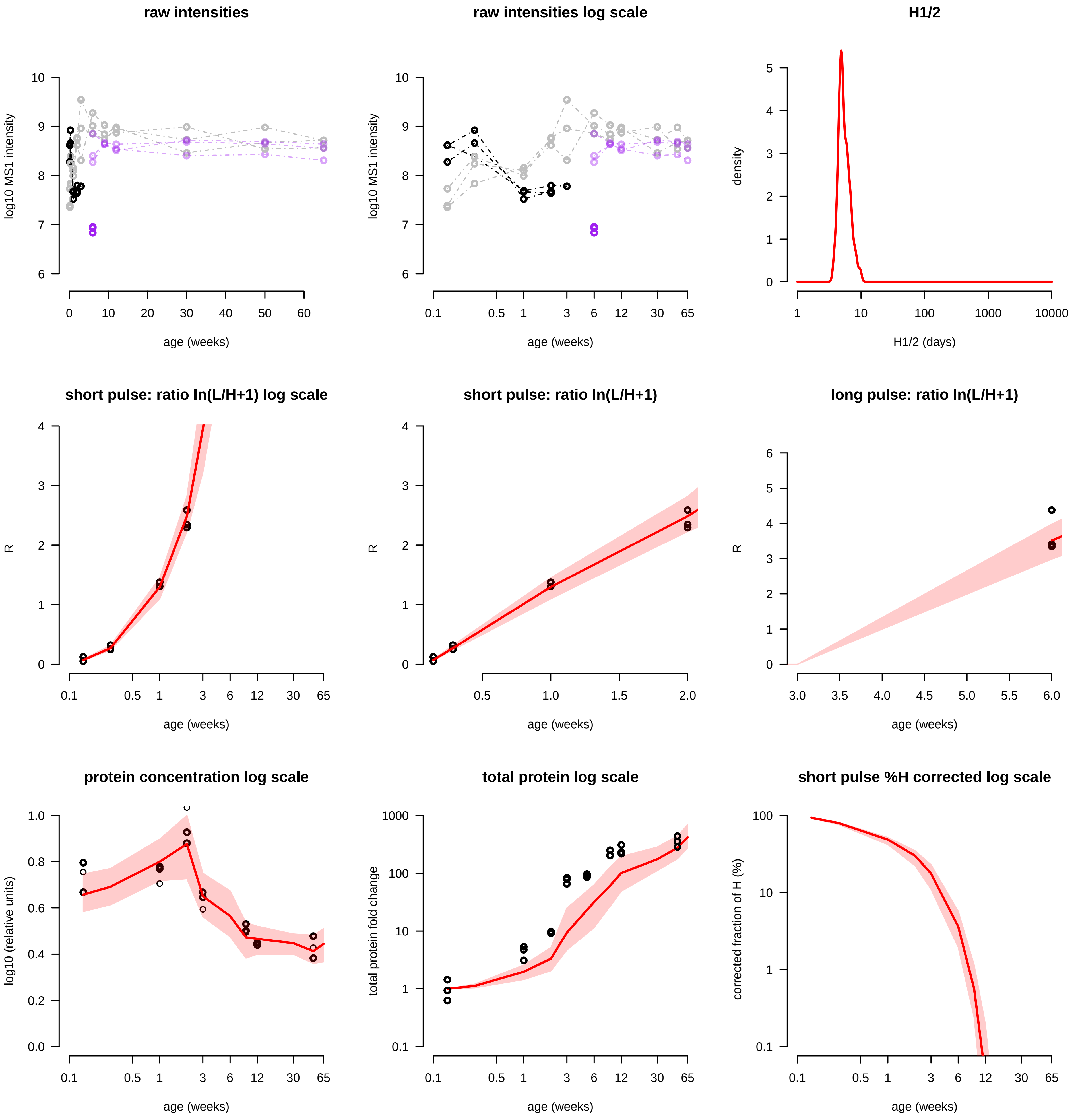

Q6PHN9

>sp|Q6PHN9|RAB35_MOUSE

Ras−related protein Rab−35

GN=Rab35

Med H1/2: 9.8 days

Q5 H1/2: 4 days

Q95 H1/2: 44.4 days

reliable model fit: yes

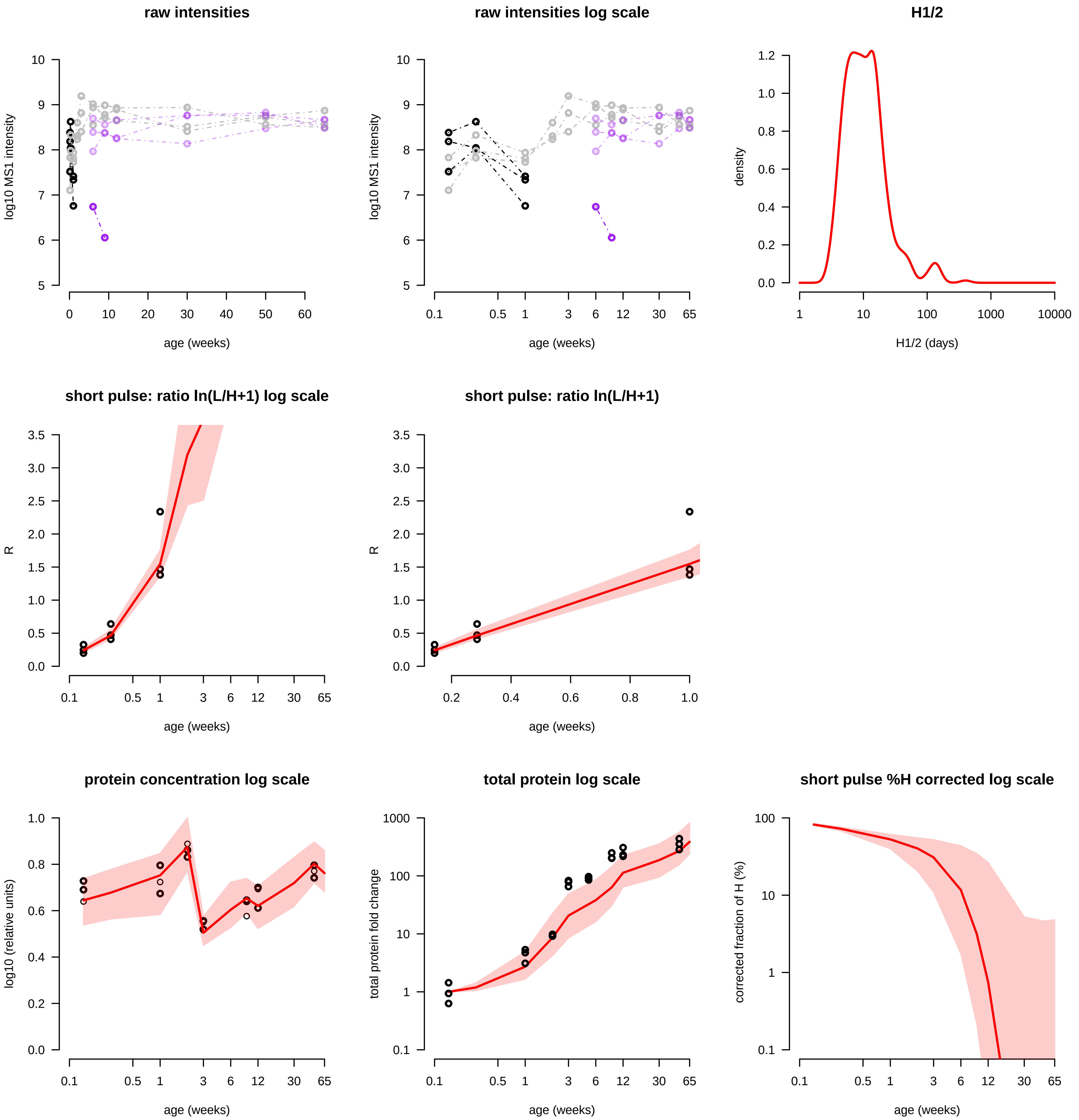

P70227

>sp|P70227|ITPR3_MOUSE

Inositol 1,4,5−trisphosphate receptor type 3

GN=Itpr3

Med H1/2: 8.7 days

Q5 H1/2: 4.7 days

Q95 H1/2: 78.8 days

reliable model fit: no

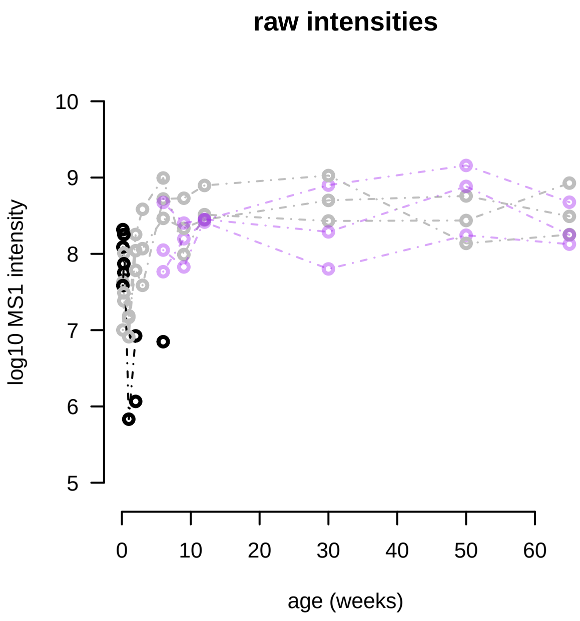

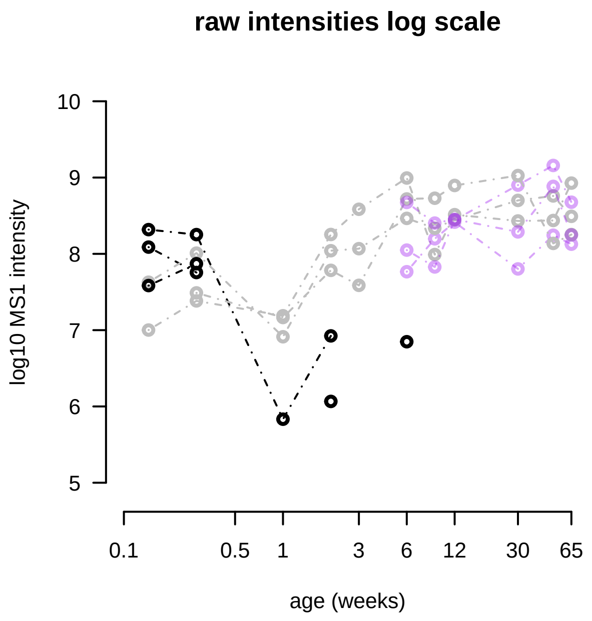

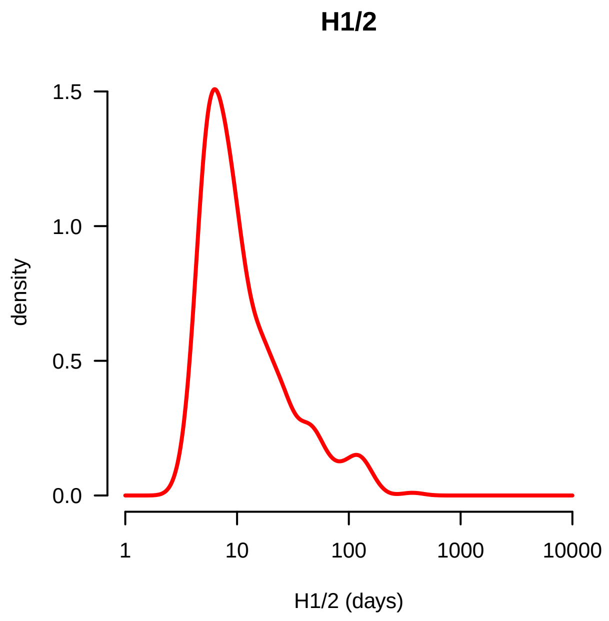

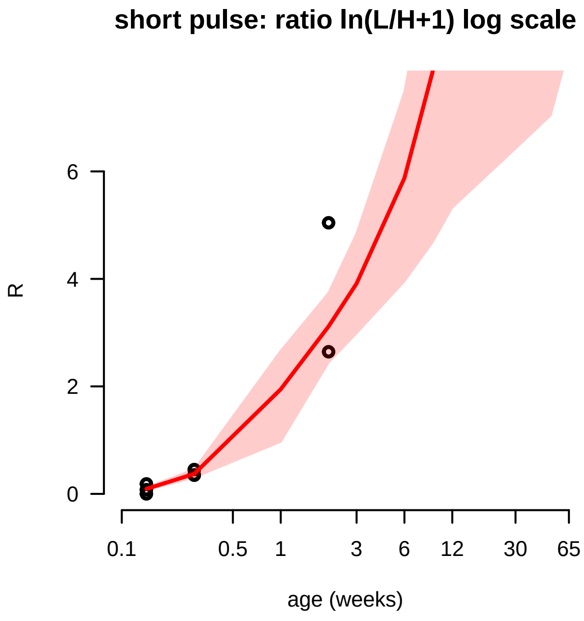

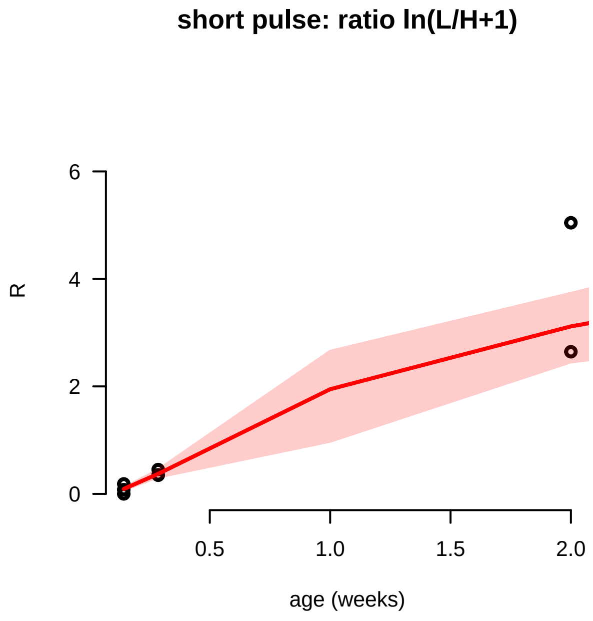

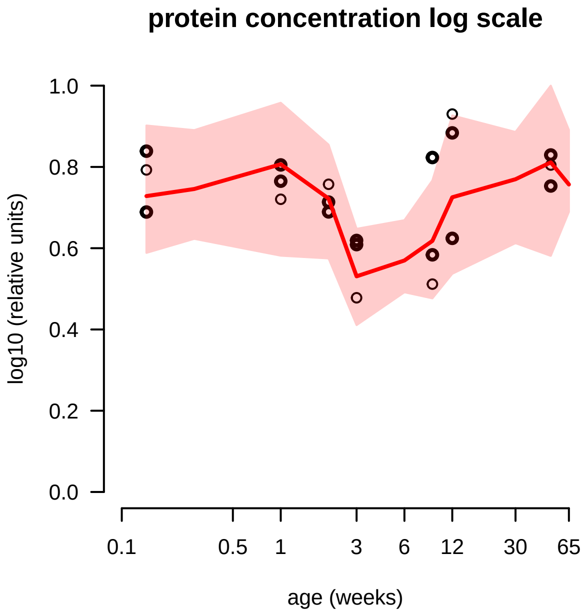

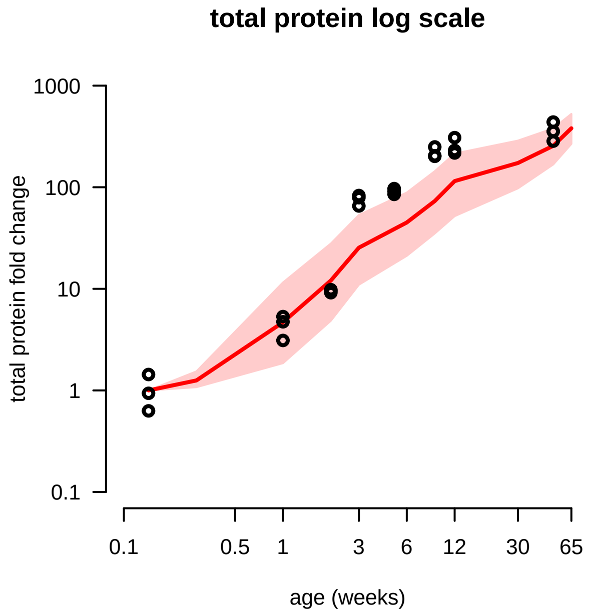

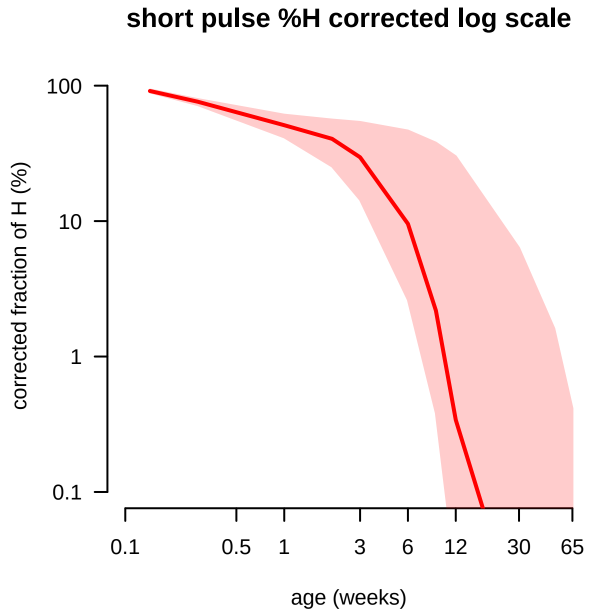

P15105

>sp|P15105|GLNA_MOUSE

Glutamine synthetase

GN=Glul

Med H1/2: 13.9 days

Q5 H1/2: 4.7 days

Q95 H1/2: 99.4 days

reliable model fit: yes

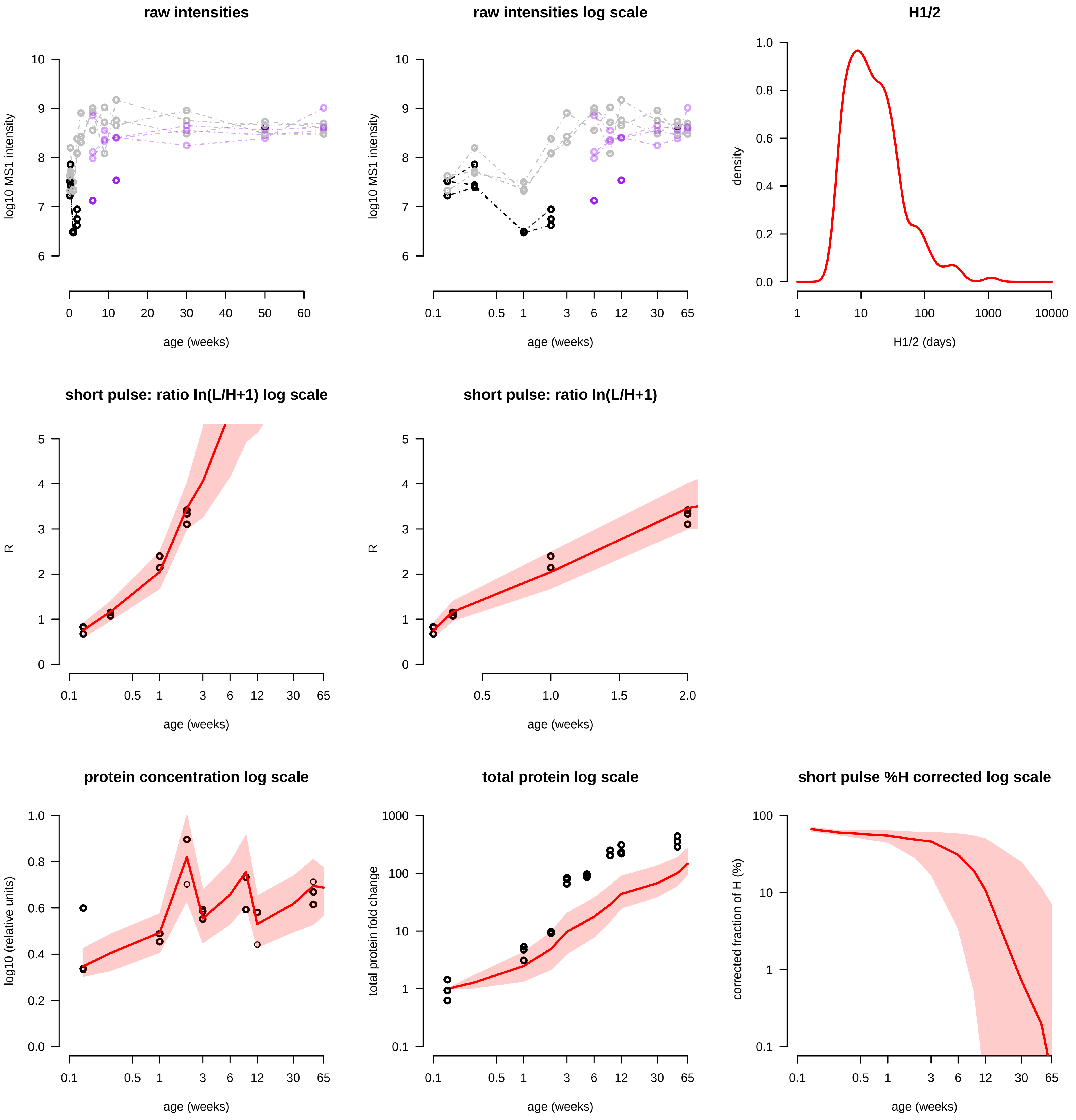

Q8R1F1

>sp|Q8R1F1|NIBA2_MOUSE

Protein Niban 2

GN=Niban2

Med H1/2: 5.1 days

Q5 H1/2: 2.5 days

Q95 H1/2: 34.1 days

reliable model fit: yes

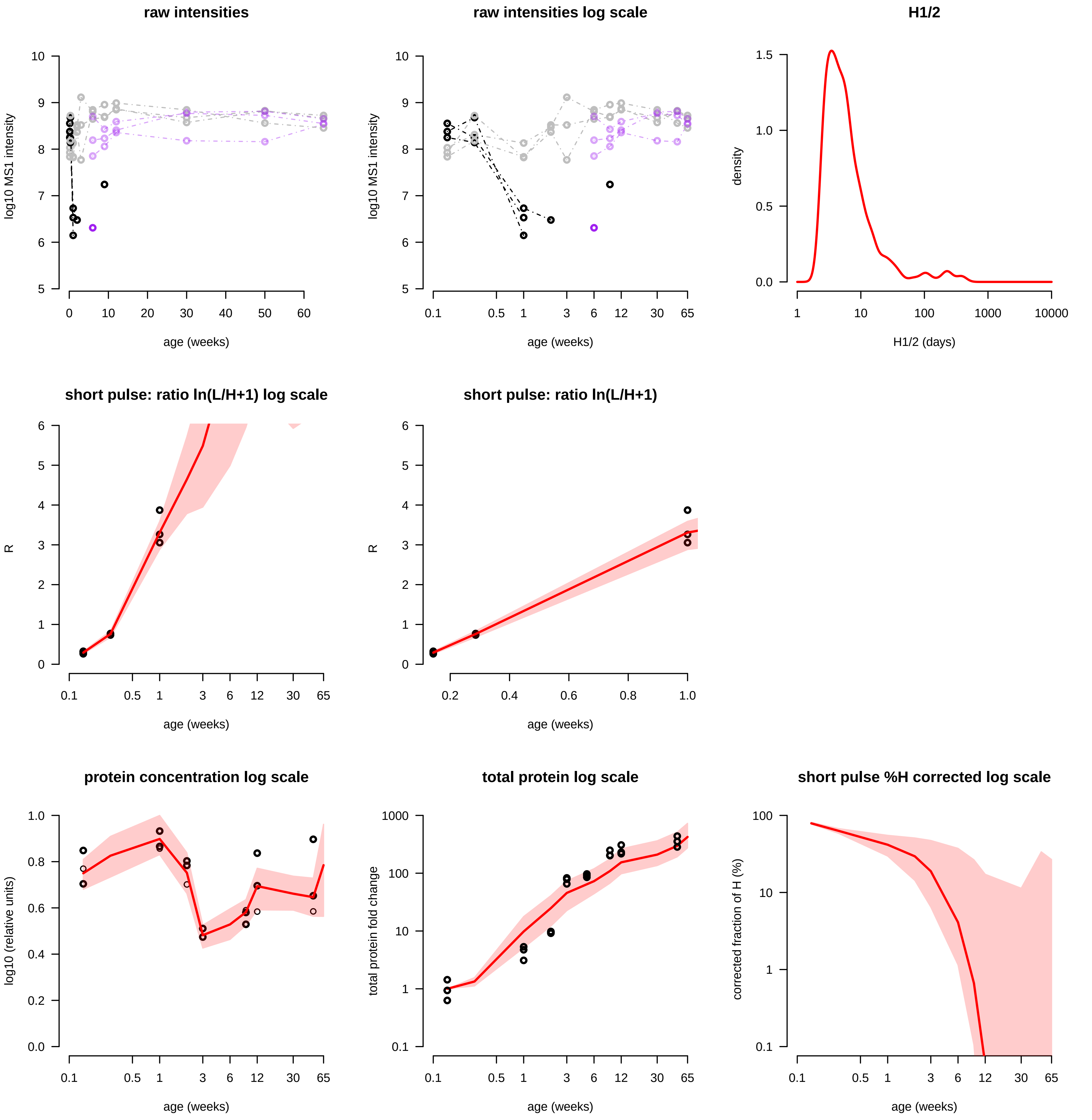

Q9D7X3

>sp|Q9D7X3|DUS3_MOUSE

Dual specificity protein phosphatase 3

GN=Dusp3

Med H1/2: 6.5 days

Q5 H1/2: 4.9 days

Q95 H1/2: 9.5 days

reliable model fit: yes

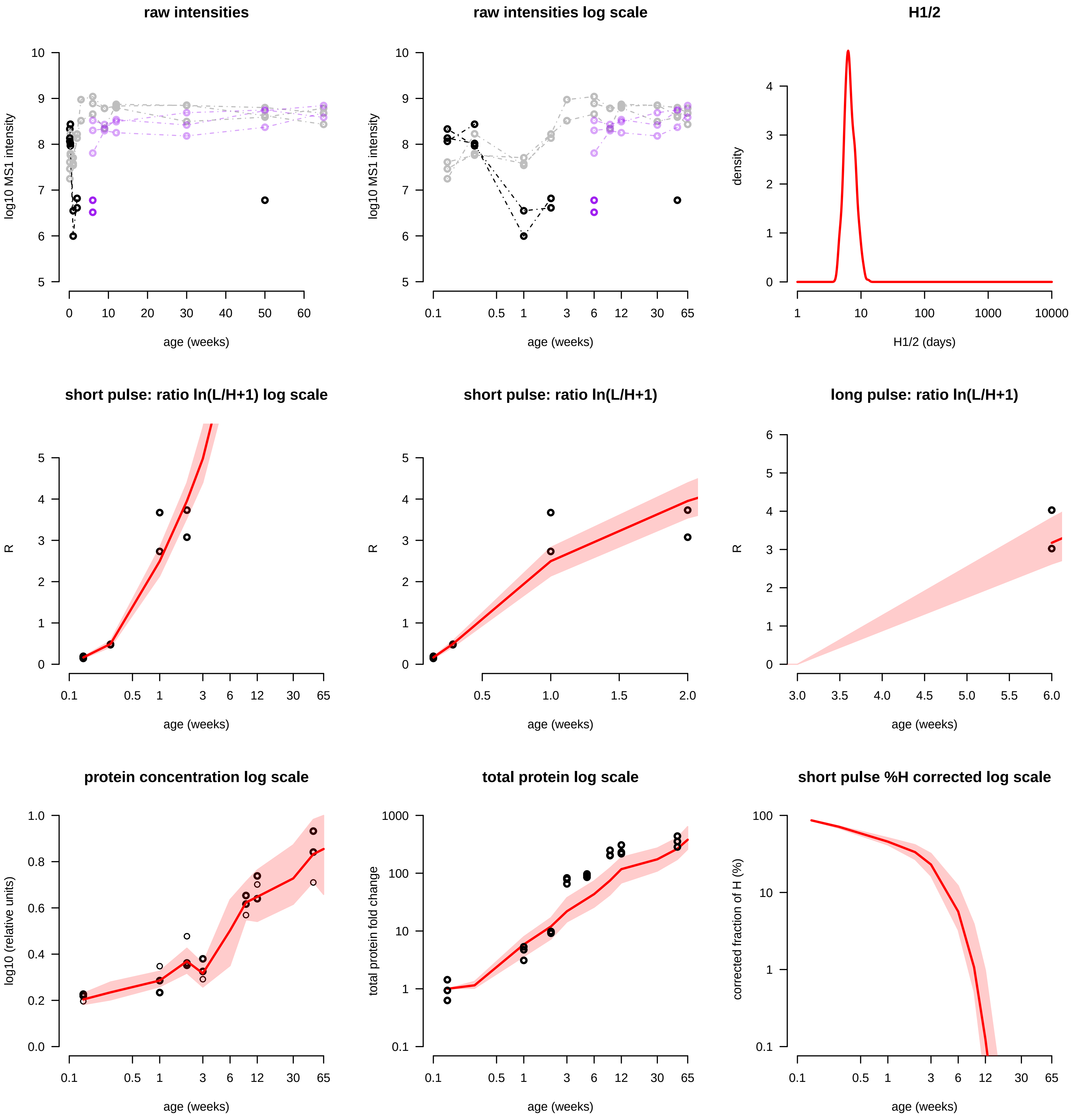

P22907;P22907−2

>sp|P22907|HEM3_MOUSE

Porphobilinogen deaminase

GN=Hmbs

Med H1/2: 4.2 days

Q5 H1/2: 3.3 days

Q95 H1/2: 5.4 days

reliable model fit: yes

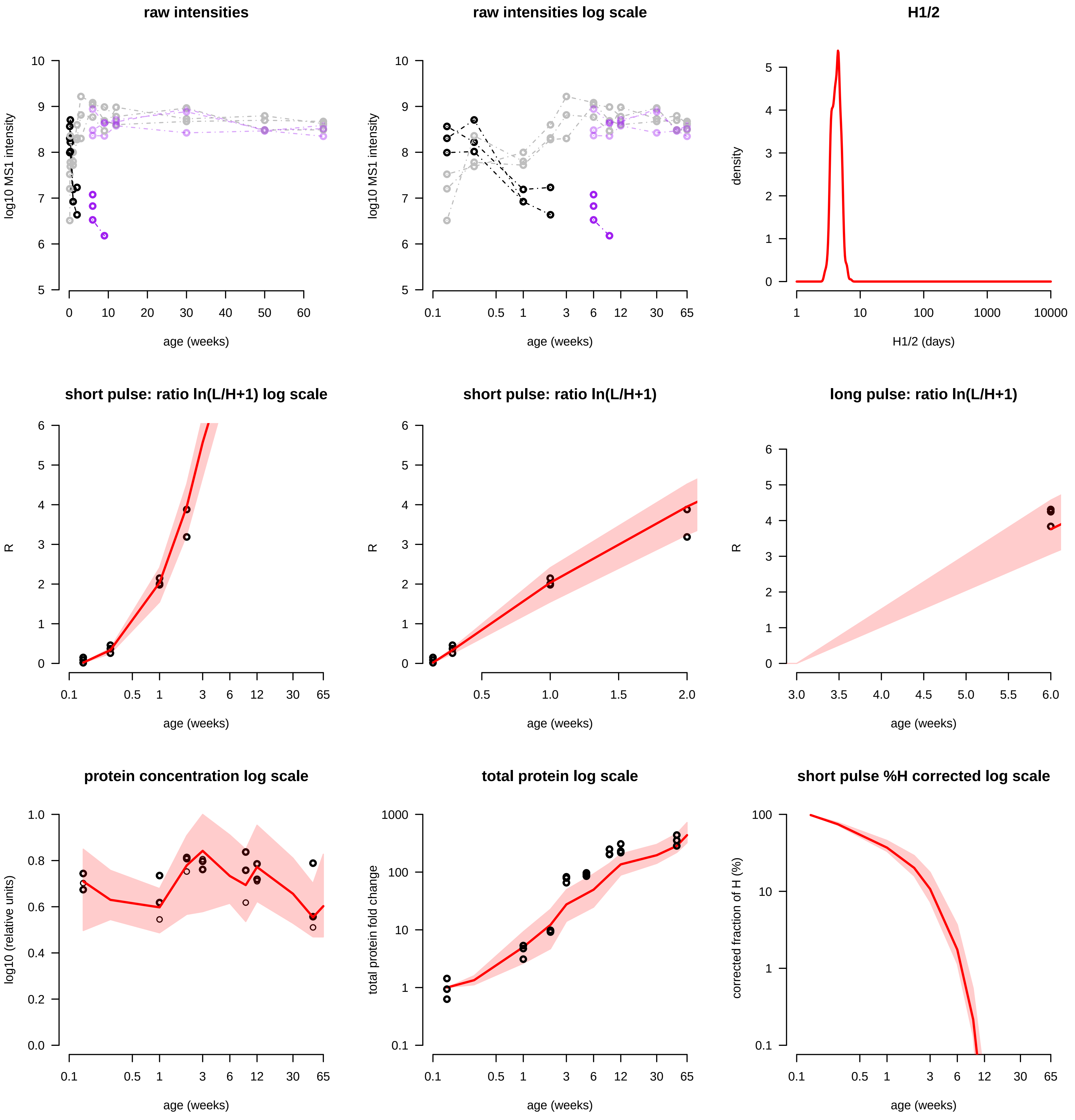

P49182

>sp|P49182|HEP2_MOUSE

Heparin cofactor 2

GN=Serpind1

Med H1/2: 9.7 days

Q5 H1/2: 3.7 days

Q95 H1/2: 63 days

reliable model fit: yes

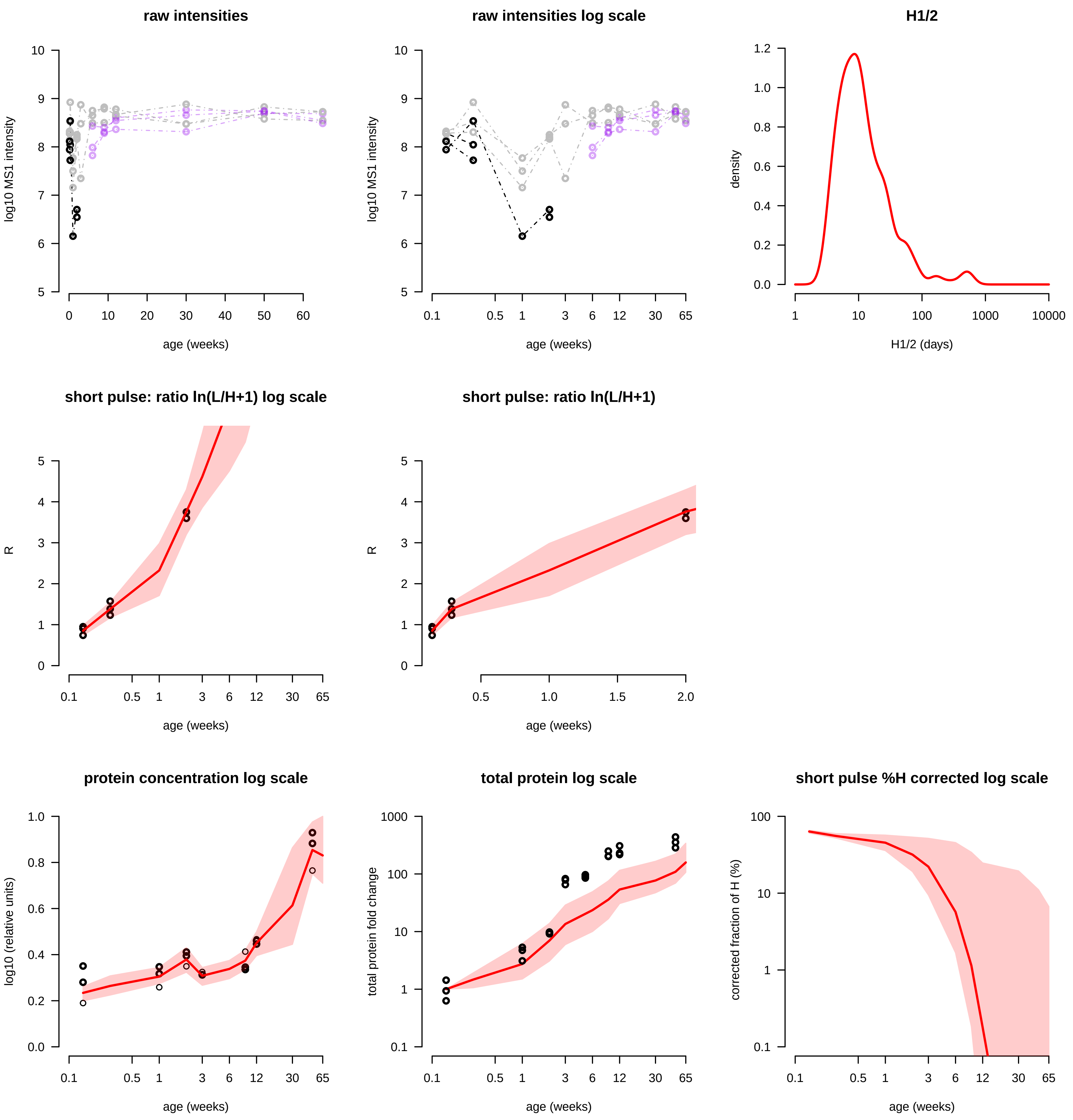

Q9WV85

>sp|Q9WV85|NDK3_MOUSE

Nucleoside diphosphate kinase 3

GN=Nme3

Med H1/2: 8.6 days

Q5 H1/2: 6.2 days

Q95 H1/2: 14 days

reliable model fit: yes

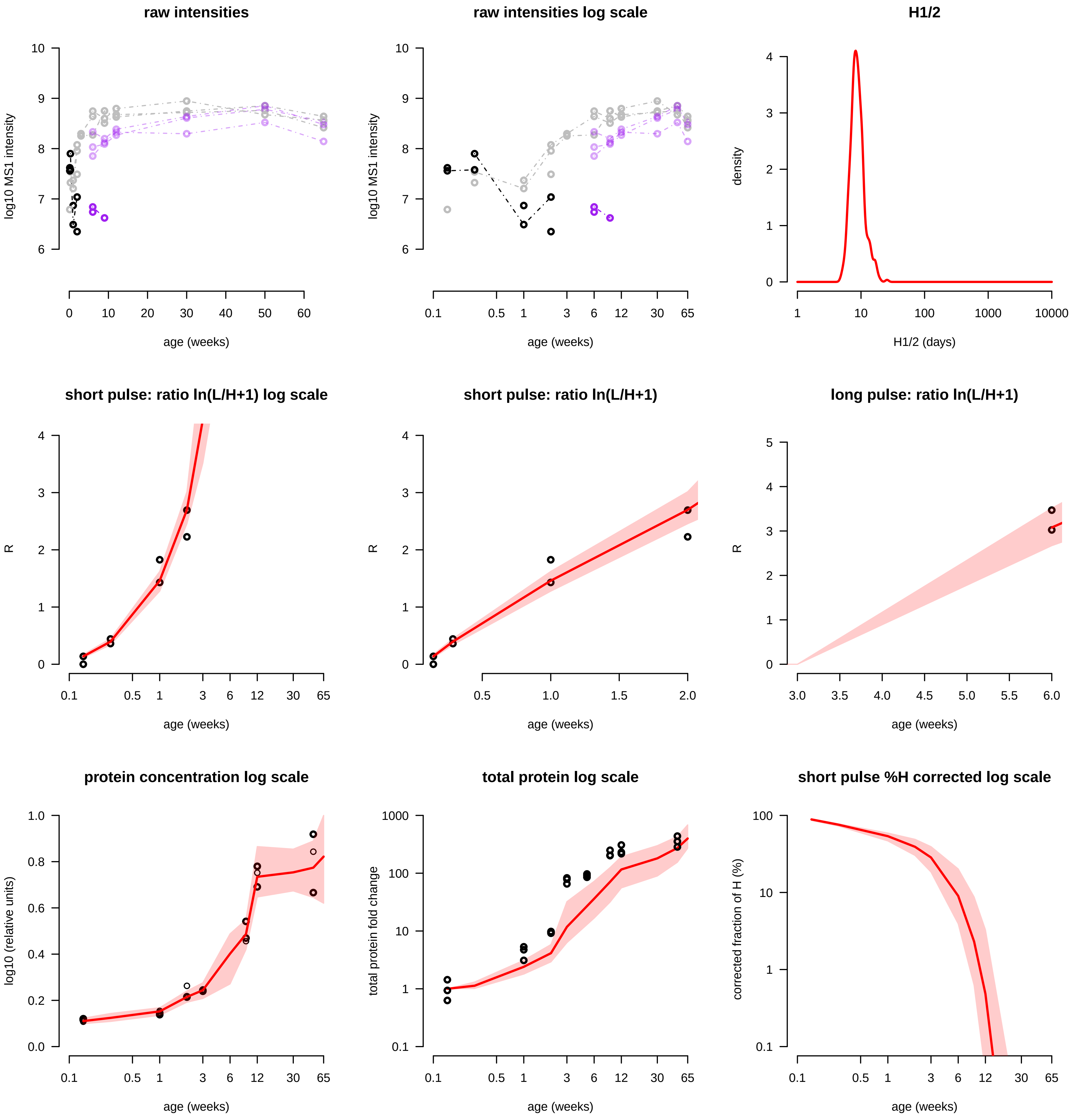

P49615

>sp|P49615|CDK5_MOUSE

Cyclin−dependent−like kinase 5

GN=Cdk5

Med H1/2: 3.2 days

Q5 H1/2: 2.7 days

Q95 H1/2: 3.9 days

reliable model fit: yes

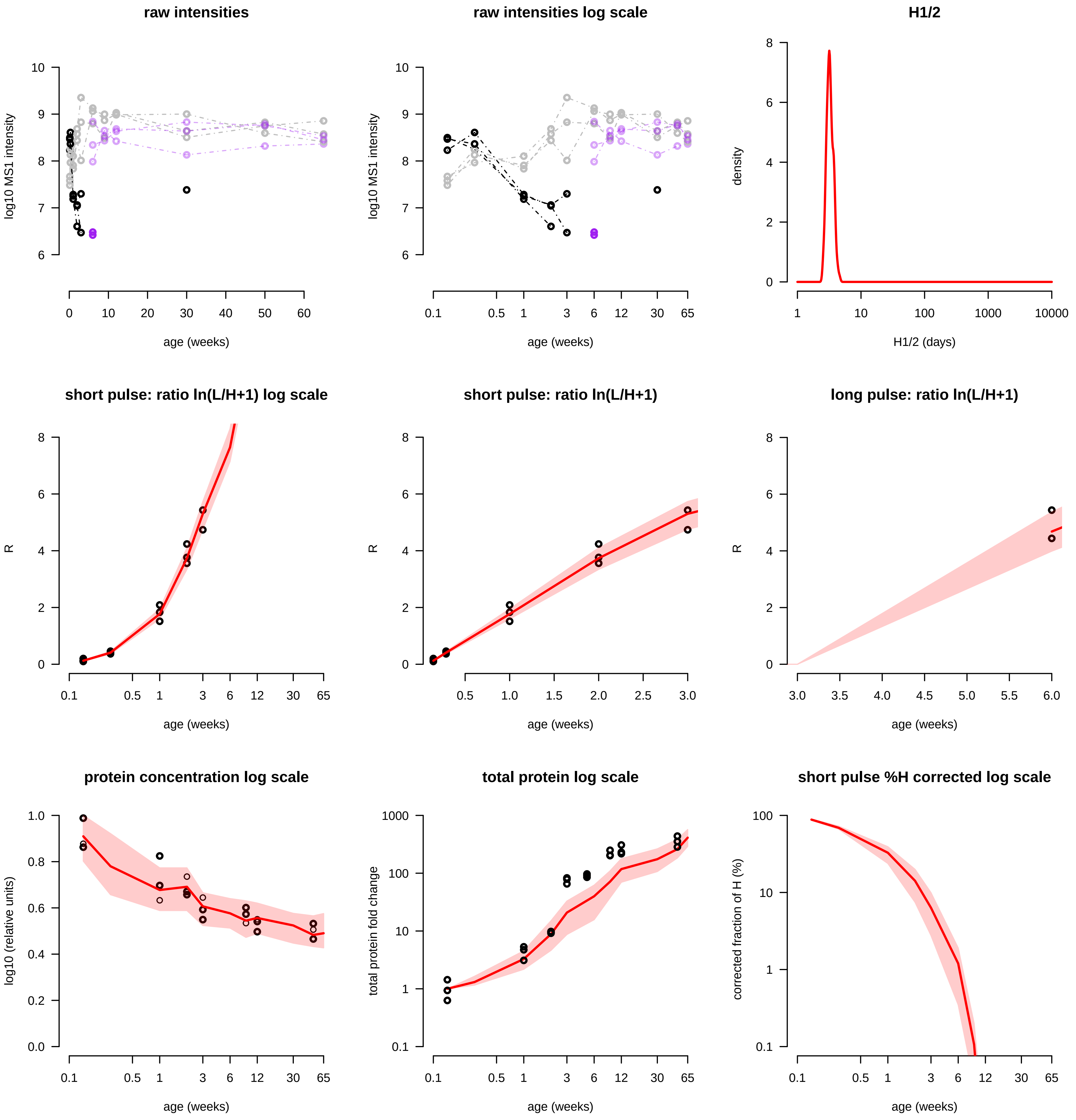

P58871

>sp|P58871|TB182_MOUSE

182 kDa tankyrase−1−binding protein

GN=Tnks1bp1

Med H1/2: 3.6 days

Q5 H1/2: 2.5 days

Q95 H1/2: 32.4 days

reliable model fit: yes

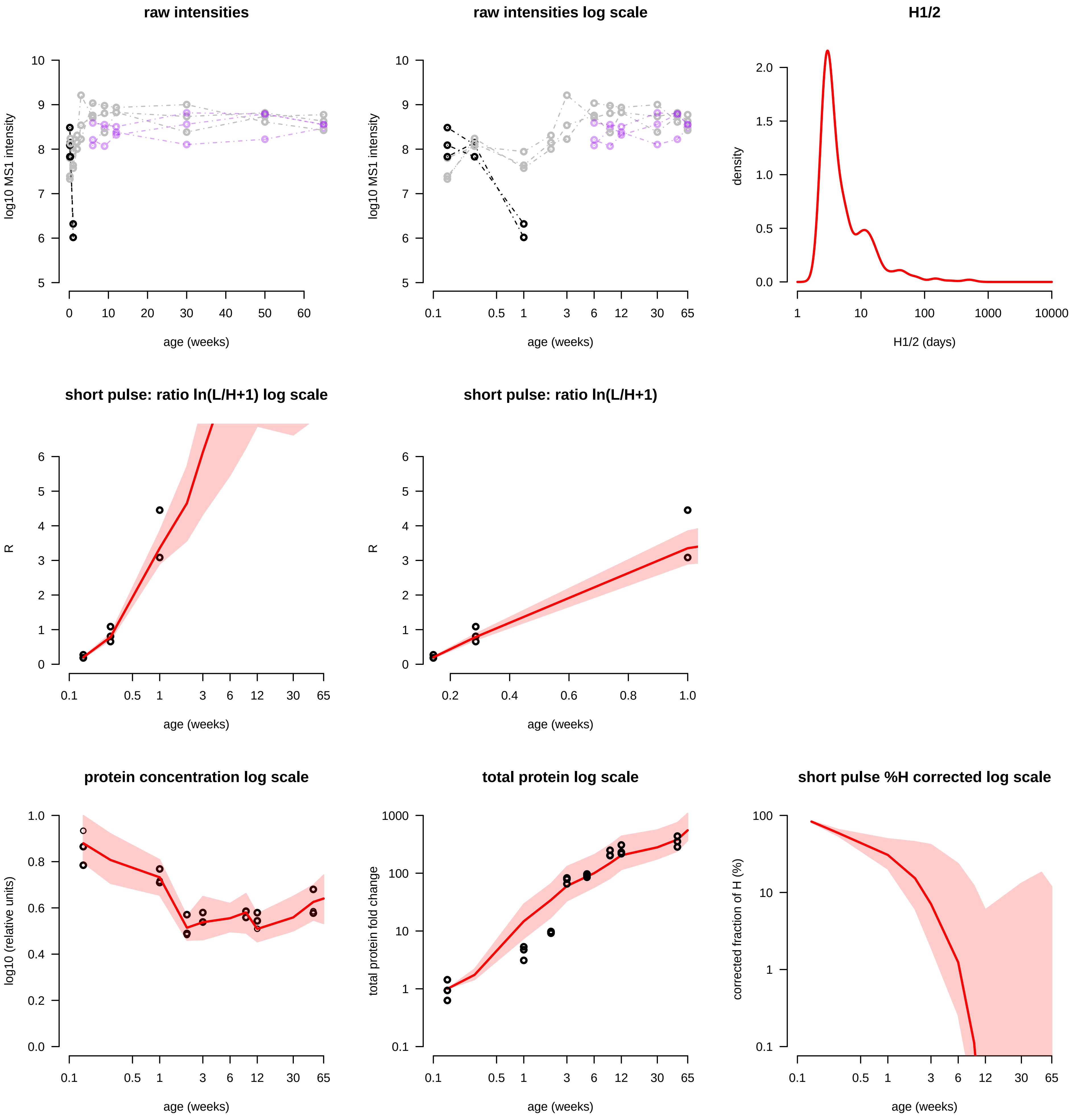

Q99JX4

>sp|Q99JX4|EIF3M_MOUSE

Eukaryotic translation initiation factor 3 subunit M

GN=Eif3m

Med H1/2: 36.8 days

Q5 H1/2: 10.5 days

Q95 H1/2: 364.6 days

reliable model fit: yes

raw intensities

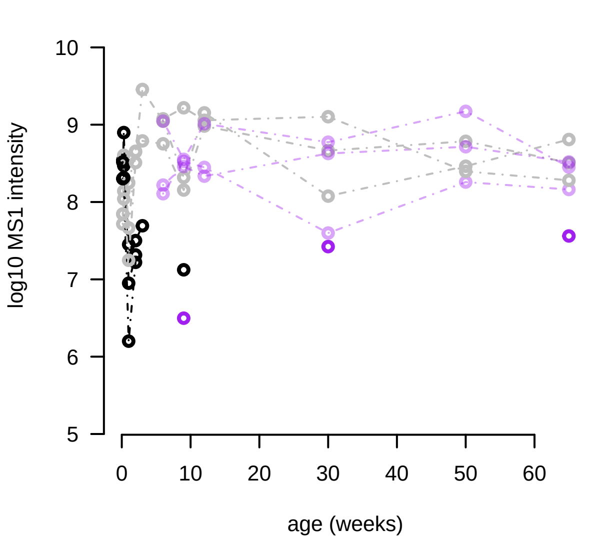

raw intensities log scale

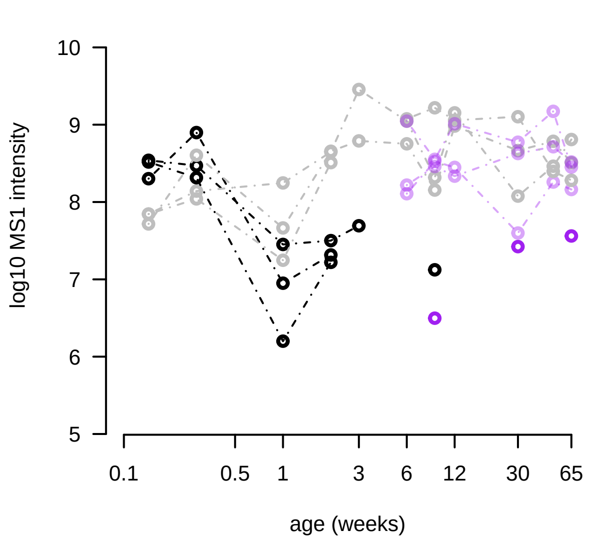

H1/2

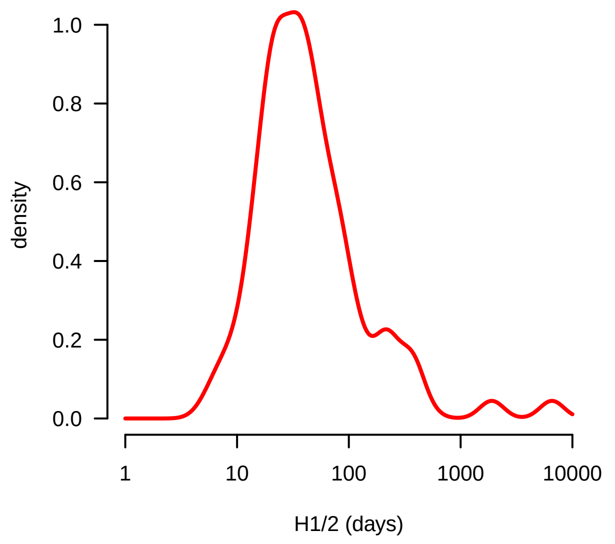

short pulse: ratio ln(L/H+1) log scale

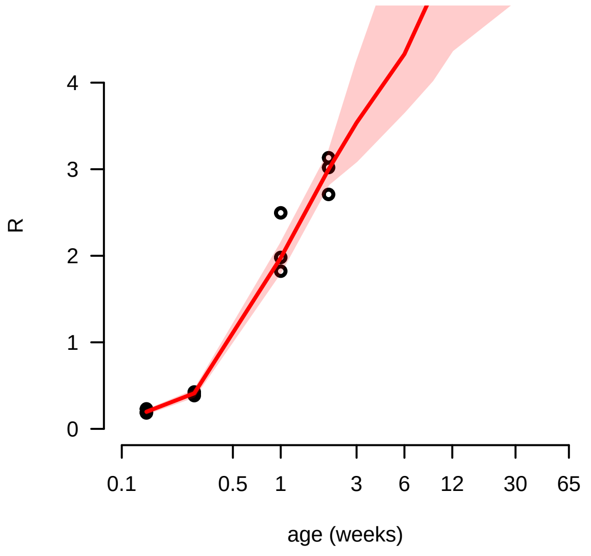

short pulse: ratio ln(L/H+1)

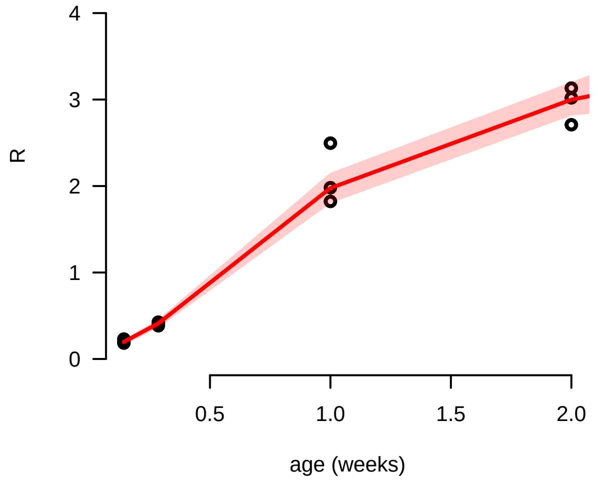

protein concentration log scale

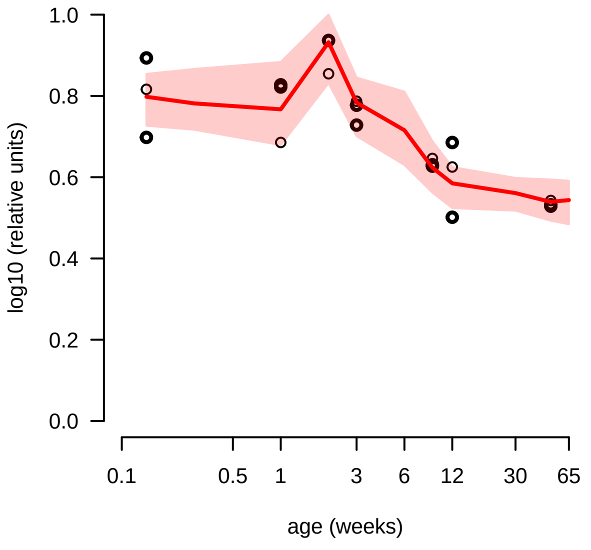

total protein log scale

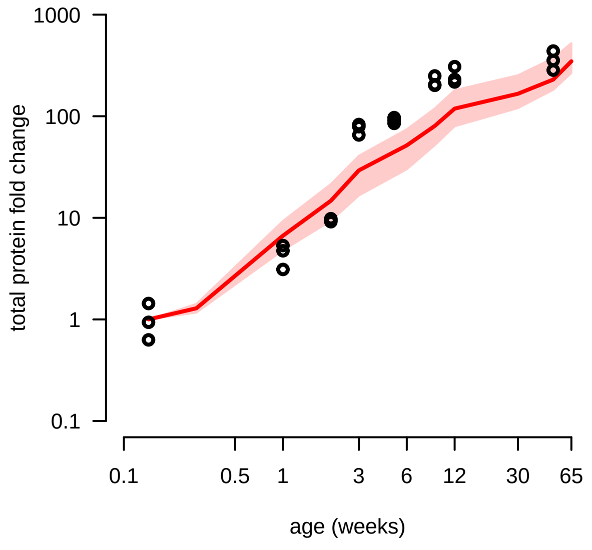

short pulse %H corrected log scale

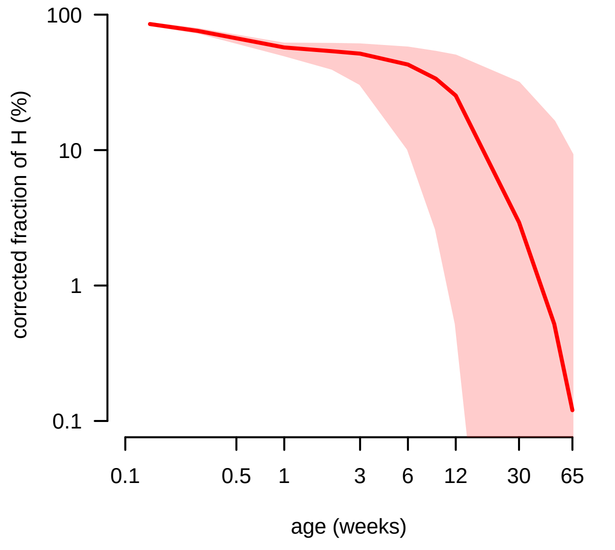

P63254

>sp|P63254|CRIP1_MOUSE

Cysteine−rich protein 1

GN=Crip1

Med H1/2: 8.7 days

Q5 H1/2: 4.5 days

Q95 H1/2: 23 days

reliable model fit: yes

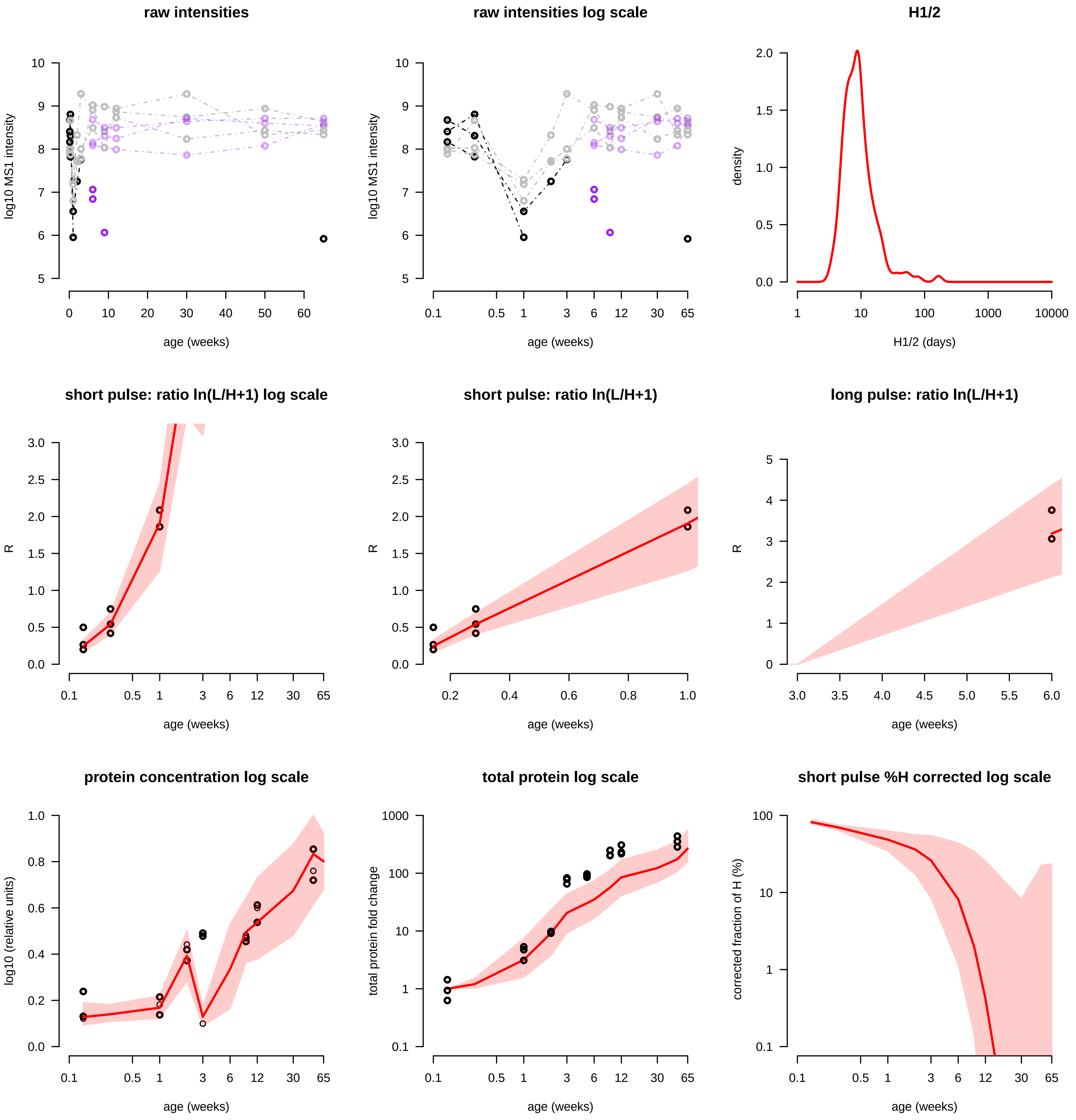

Q8BH04

>sp|Q8BH04|PCKGM_MOUSE

Phosphoenolpyruvate carboxykinase [GTP], mitochondrial

GN=Pck2

Med H1/2: 10.7 days

Q5 H1/2: 4.2 days

Q95 H1/2: 26.7 days

reliable model fit: yes

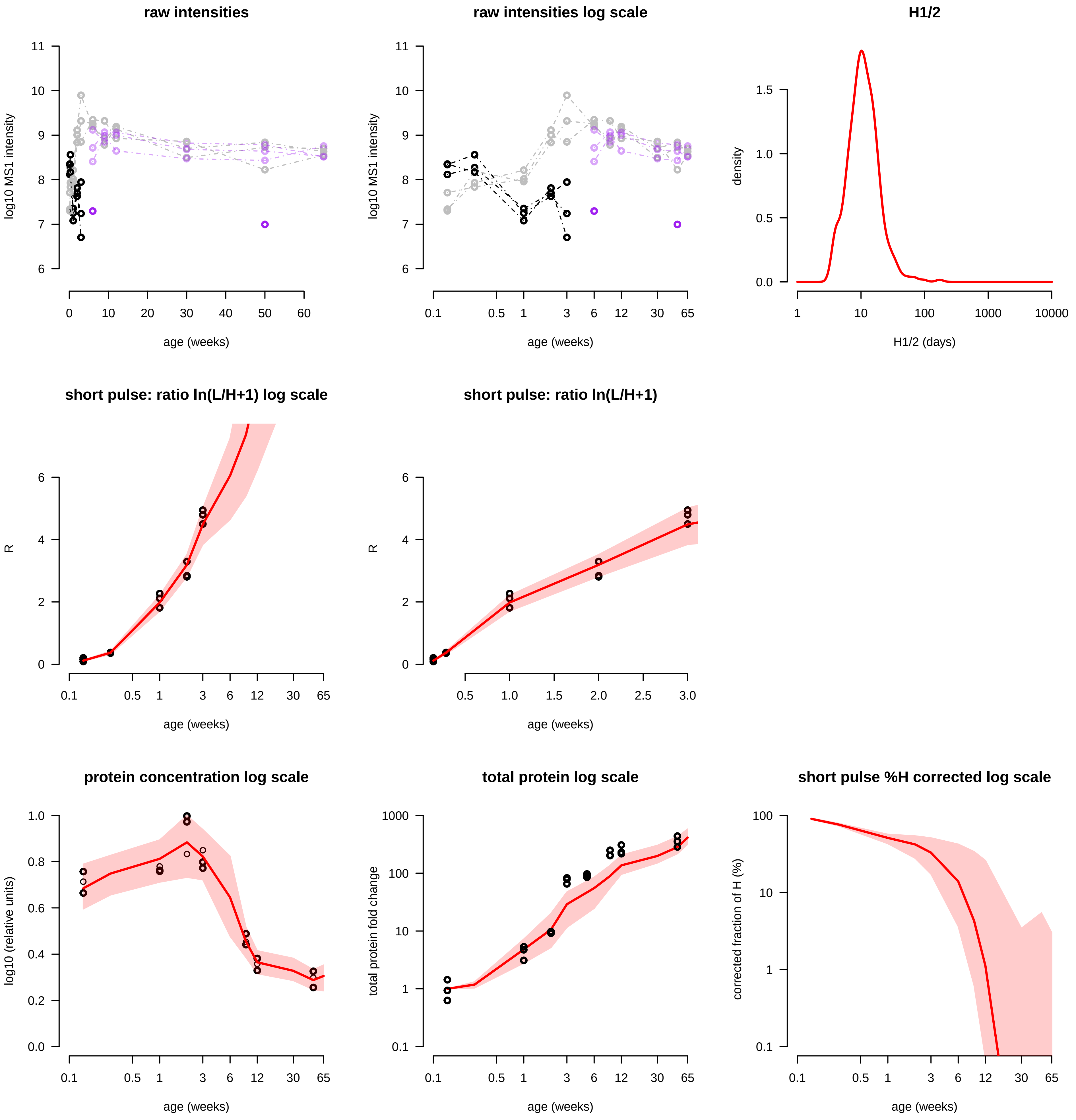

O08528

>sp|O08528|HXK2_MOUSE

Hexokinase−2

GN=Hk2

Med H1/2: 7.2 days

Q5 H1/2: 2.8 days

Q95 H1/2: 70.5 days

reliable model fit: yes

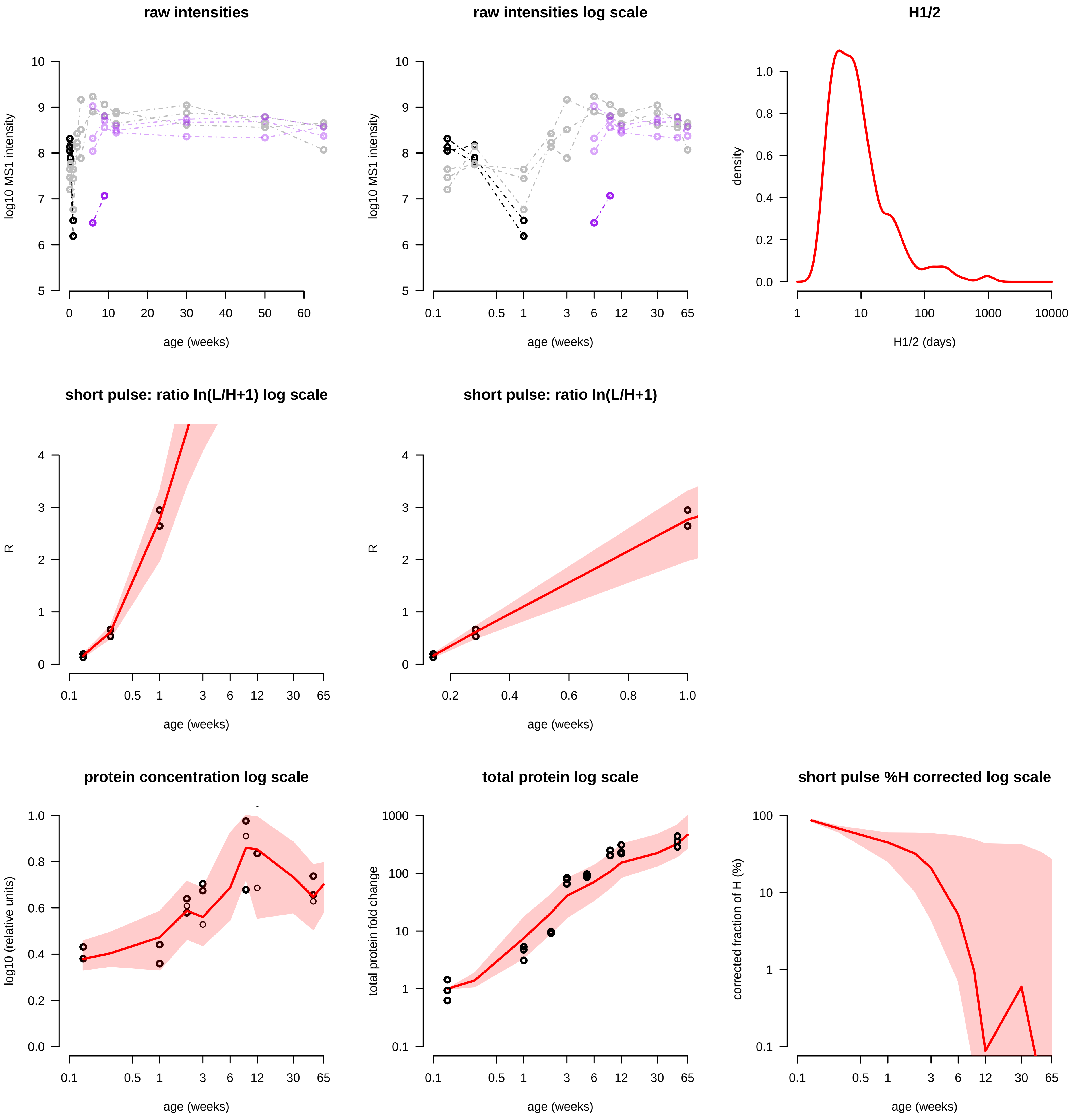

P62071

>sp|P62071|RRAS2_MOUSE

Ras−related protein R−Ras2

GN=Rras2

Med H1/2: 5.1 days

Q5 H1/2: 4.3 days

Q95 H1/2: 6.6 days

reliable model fit: yes

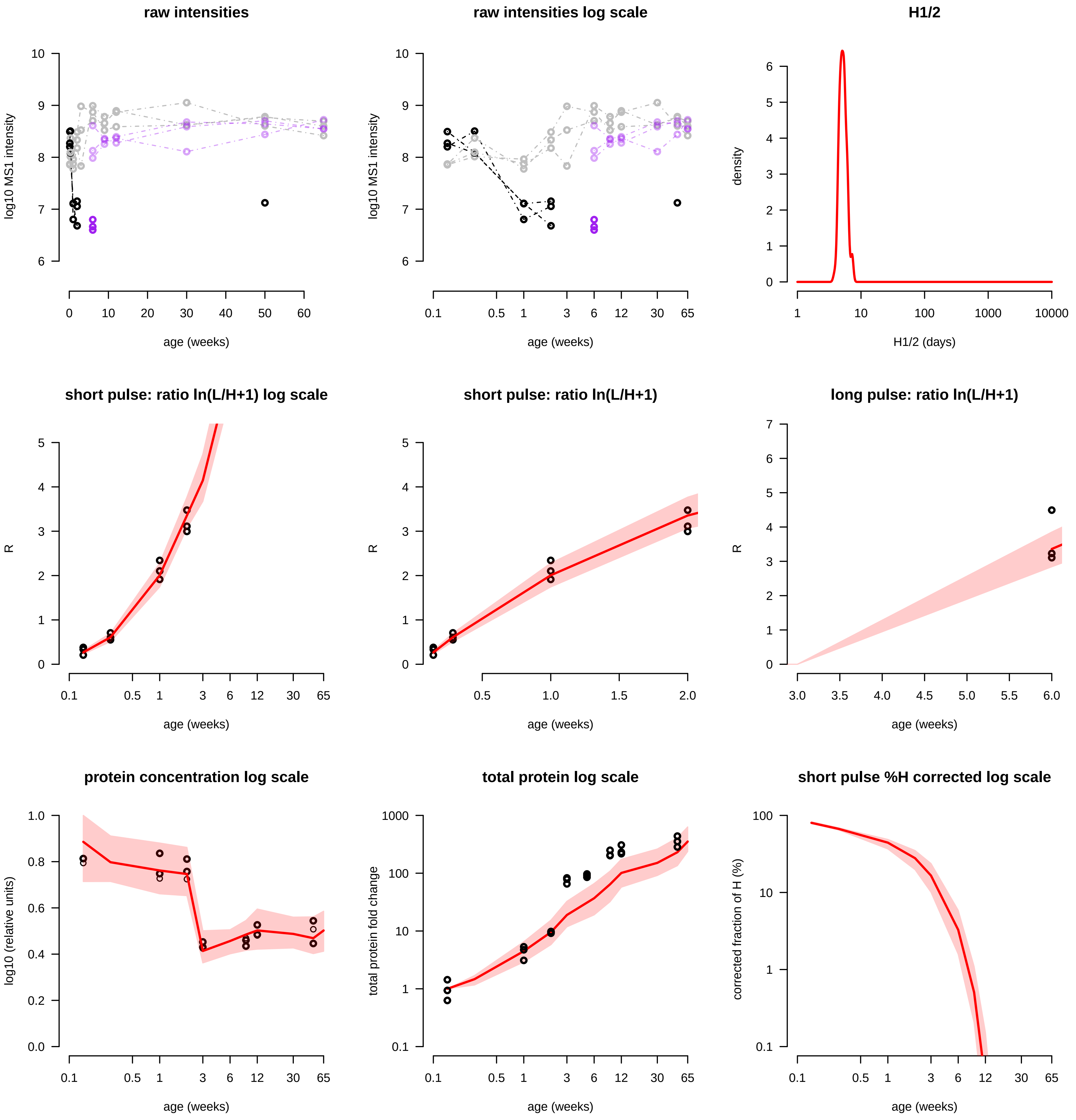

Q9Z2A9;Q9Z2A9−2

>sp|Q9Z2A9|GGT5_MOUSE

Glutathione hydrolase 5 proenzyme

GN=Ggt5

Med H1/2: 10.9 days

Q5 H1/2: 3.4 days

Q95 H1/2: 99 days

reliable model fit: yes

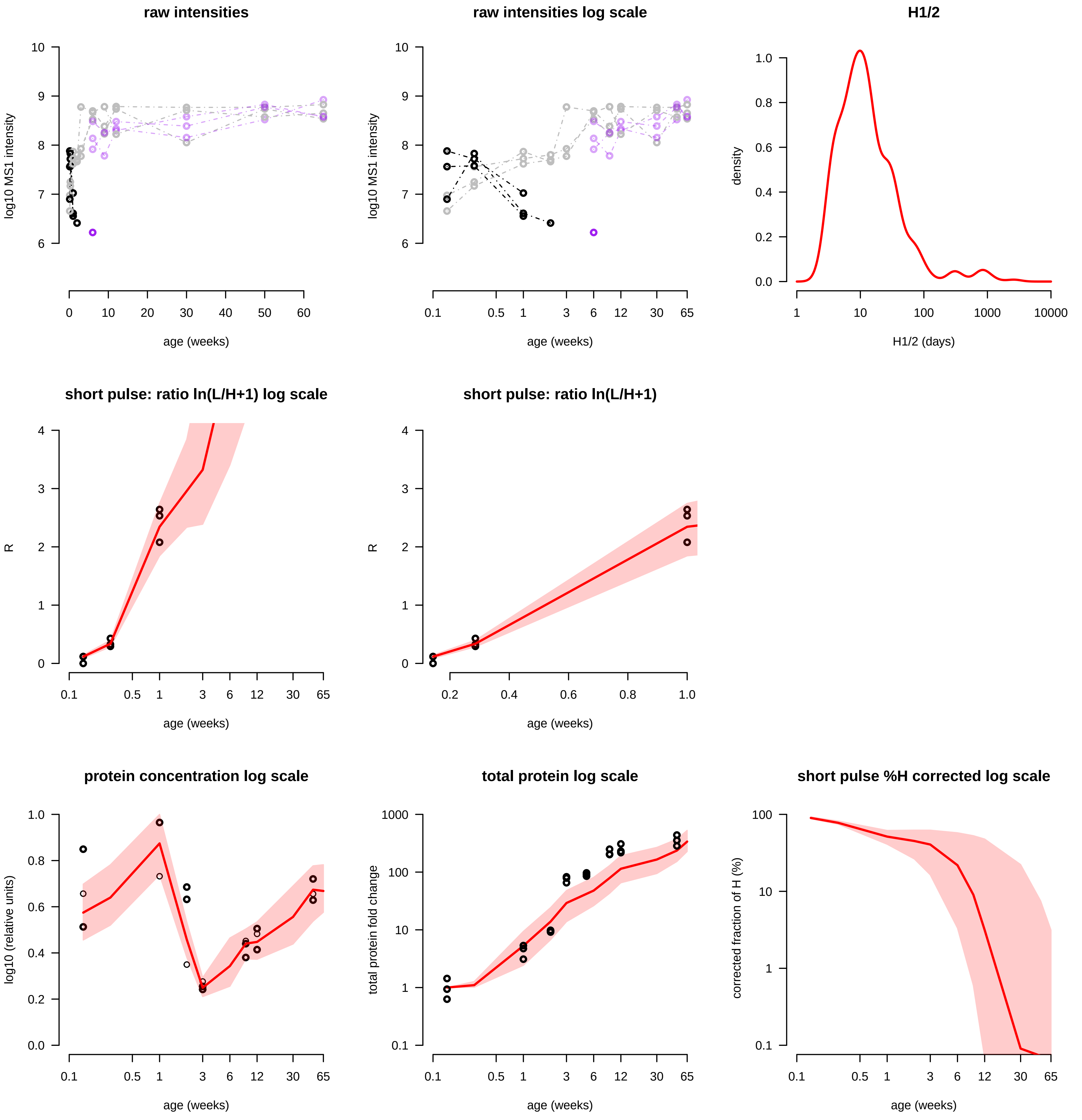

Q80SU7

>sp|Q80SU7|GVIN1_MOUSE

Interferon−induced very large GTPase 1

GN=Gvin1

Med H1/2: 2457.6 days

Q5 H1/2: 555 days

Q95 H1/2: 14281.8 days

reliable model fit: no

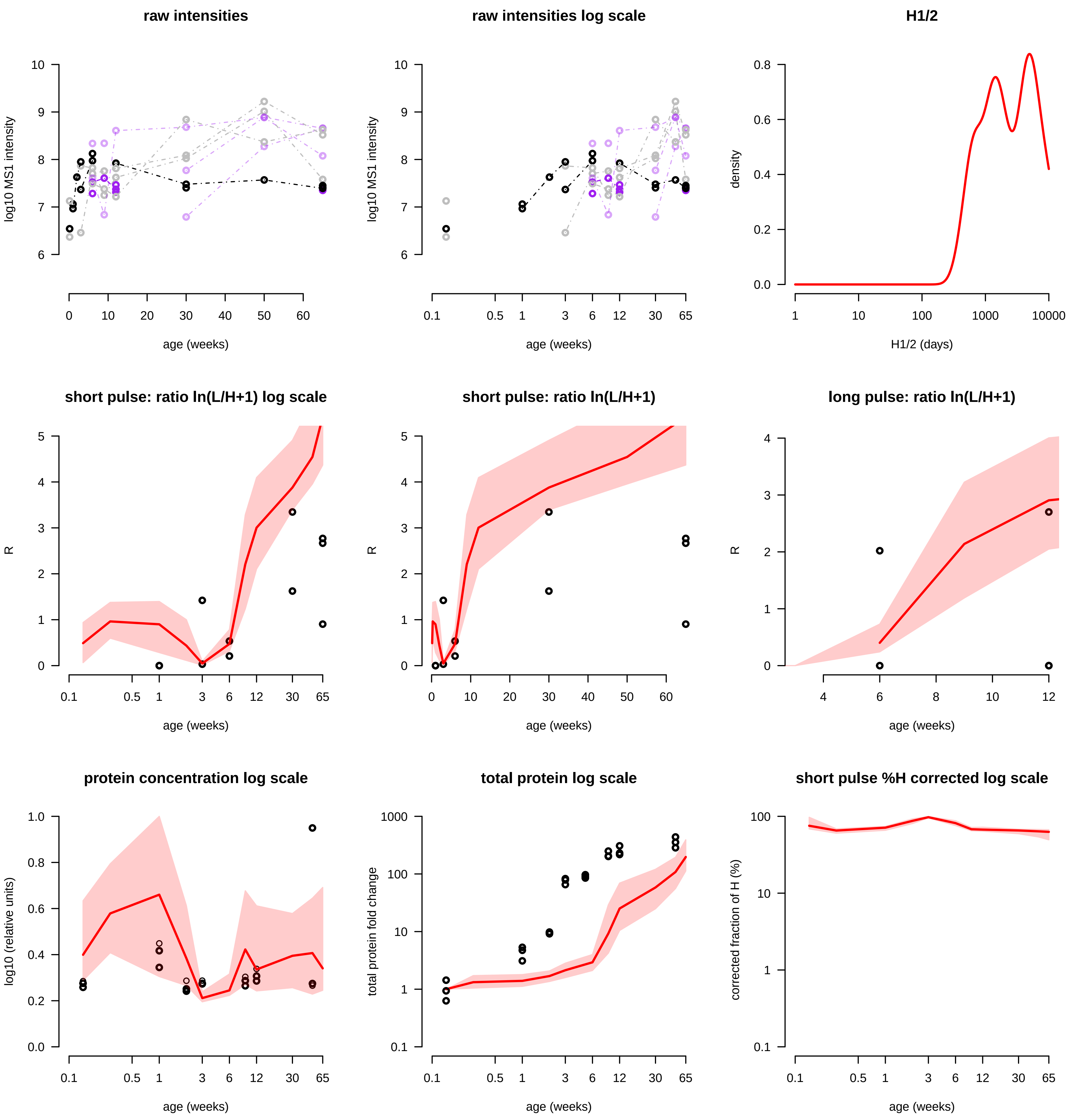

P84096

>sp|P84096|RHOG_MOUSE

Rho−related GTP−binding protein RhoG

GN=Rhog

Med H1/2: 4.4 days

Q5 H1/2: 3.1 days

Q95 H1/2: 5.6 days

reliable model fit: yes

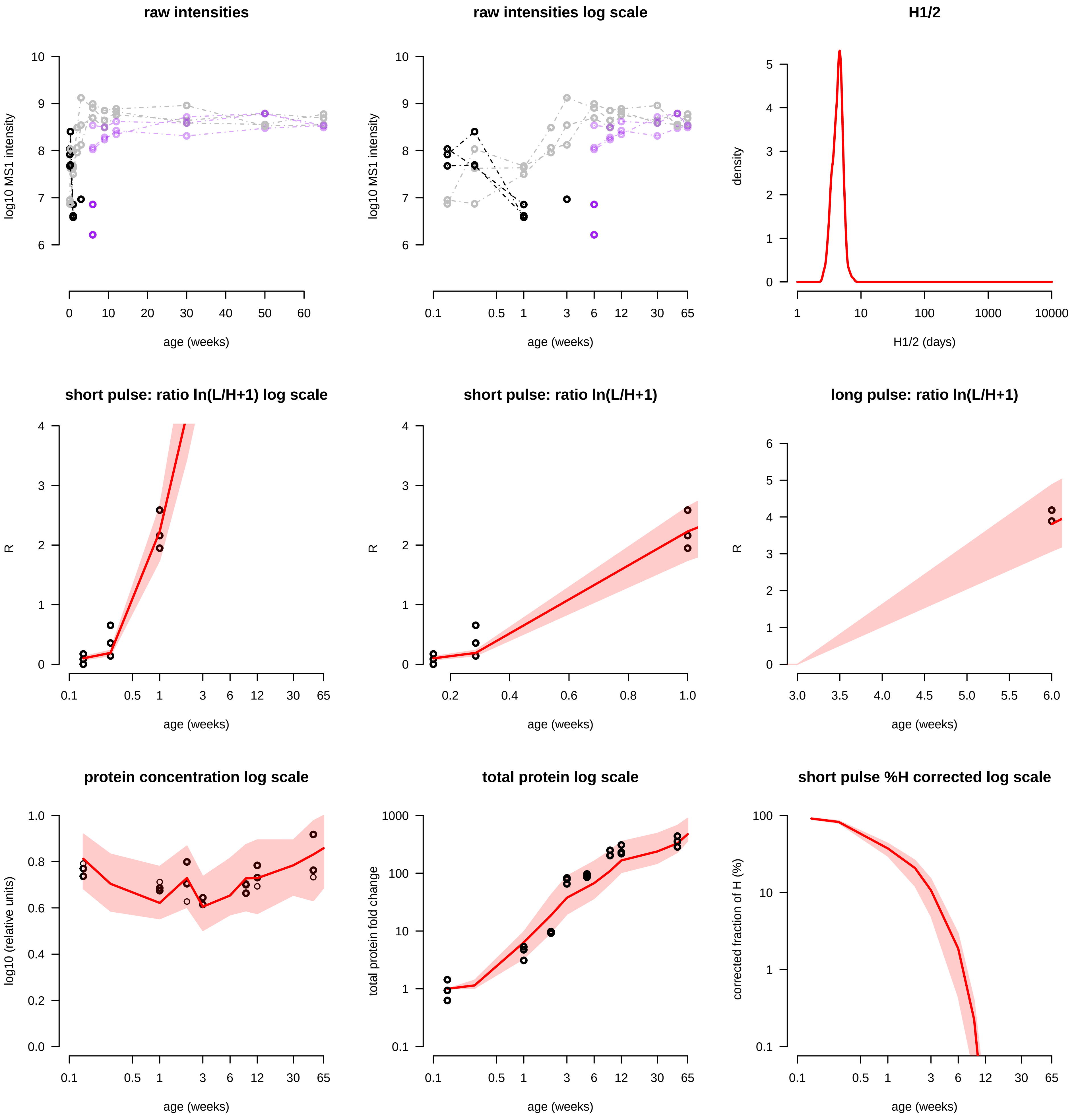

Q3UGR5;Q3UGR5−2

>sp|Q3UGR5|HDHD2_MOUSE

Haloacid dehalogenase−like hydrolase domain−containing protein 2

GN=Hdhd2

Med H1/2: 2315 days

Q5 H1/2: 625.4 days

Q95 H1/2: 22506.5 days

reliable model fit: no

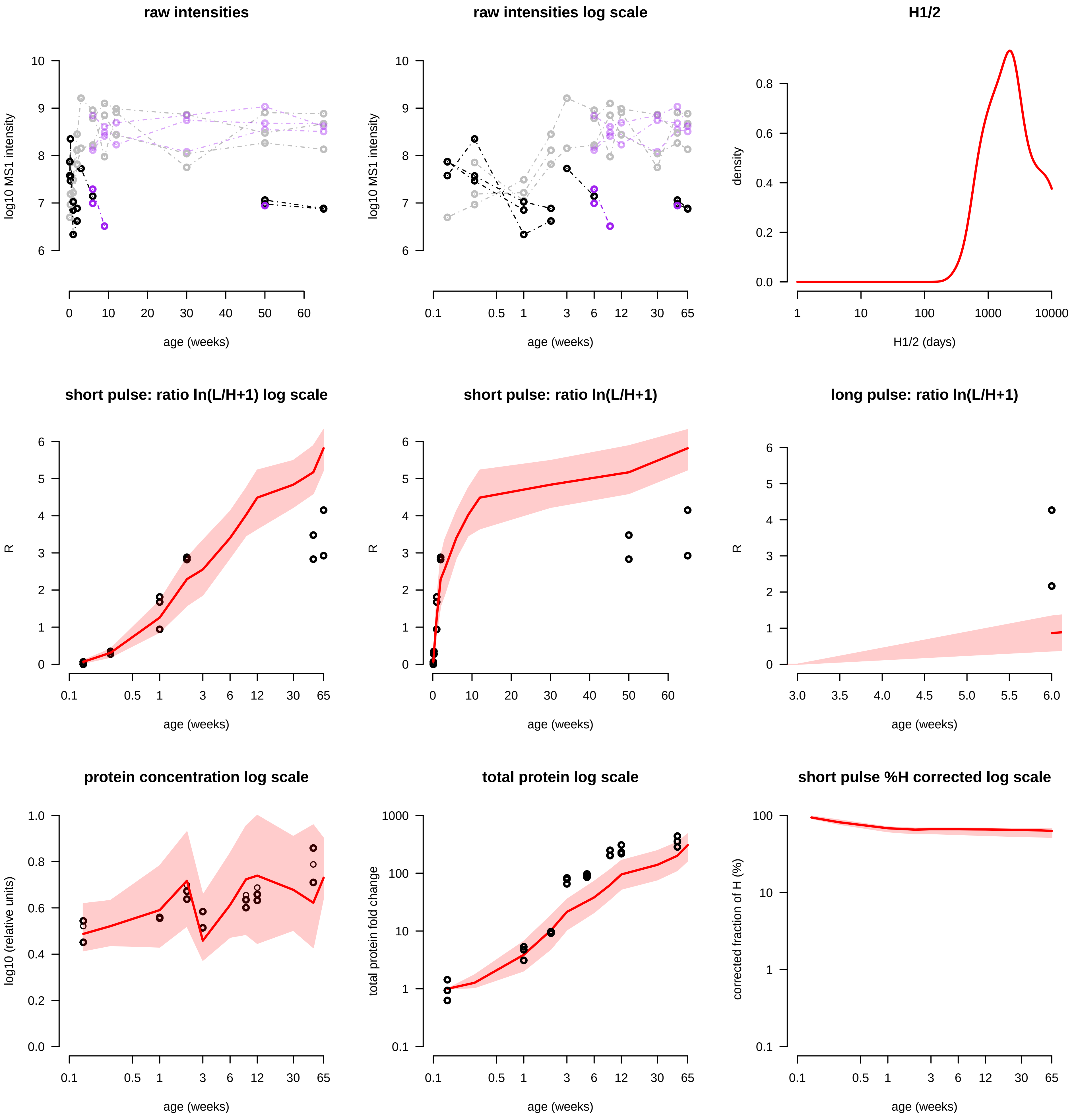

Q80VD1

>sp|Q80VD1|FA98B_MOUSE

Protein FAM98B

GN=Fam98b

Med H1/2: 6.2 days

Q5 H1/2: 4.1 days

Q95 H1/2: 14.1 days

reliable model fit: yes

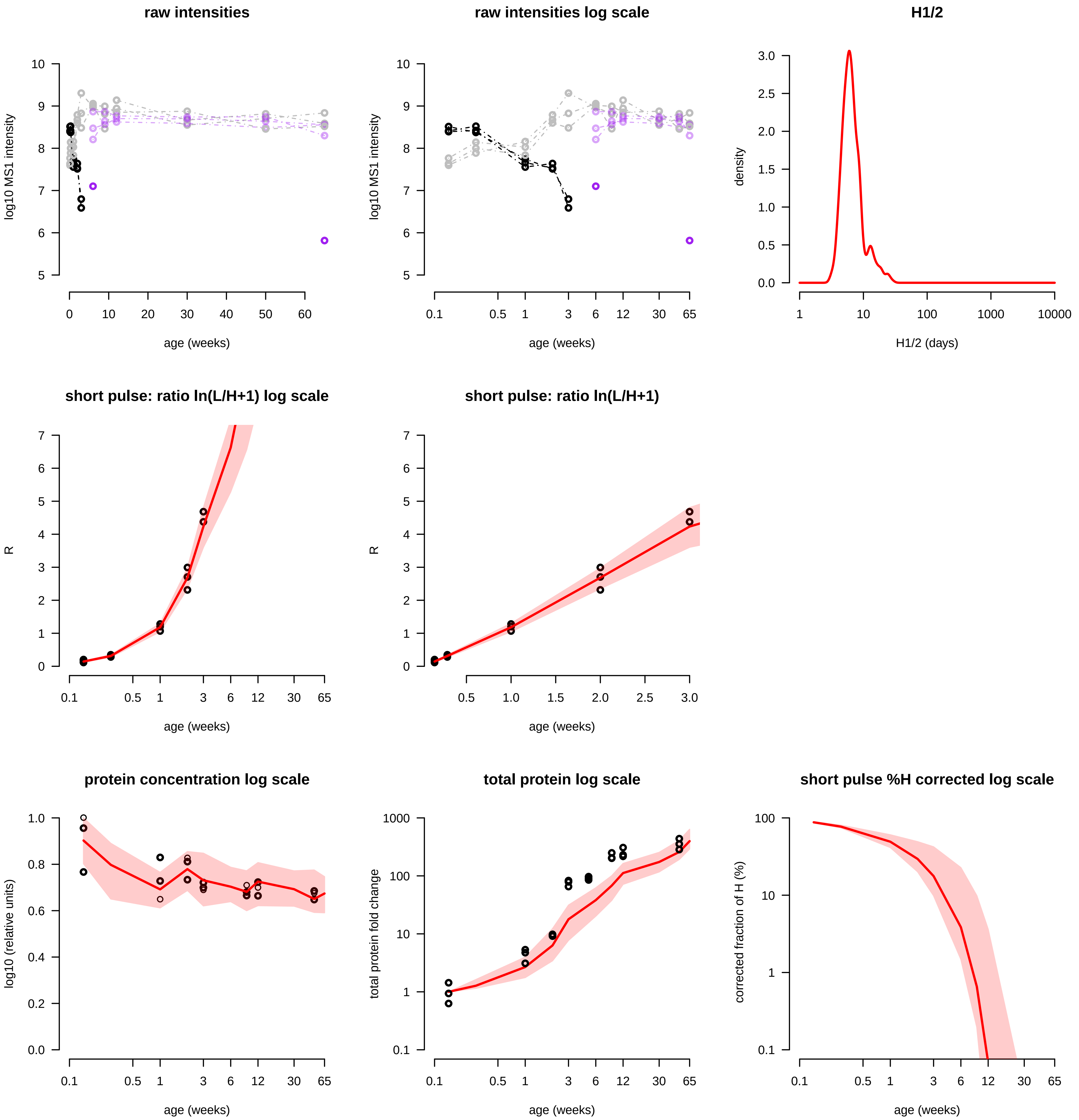

P11404

>sp|P11404|FABPH_MOUSE

Fatty acid−binding protein, heart

GN=Fabp3

Med H1/2: 4.9 days

Q5 H1/2: 3.5 days

Q95 H1/2: 7.4 days

reliable model fit: yes

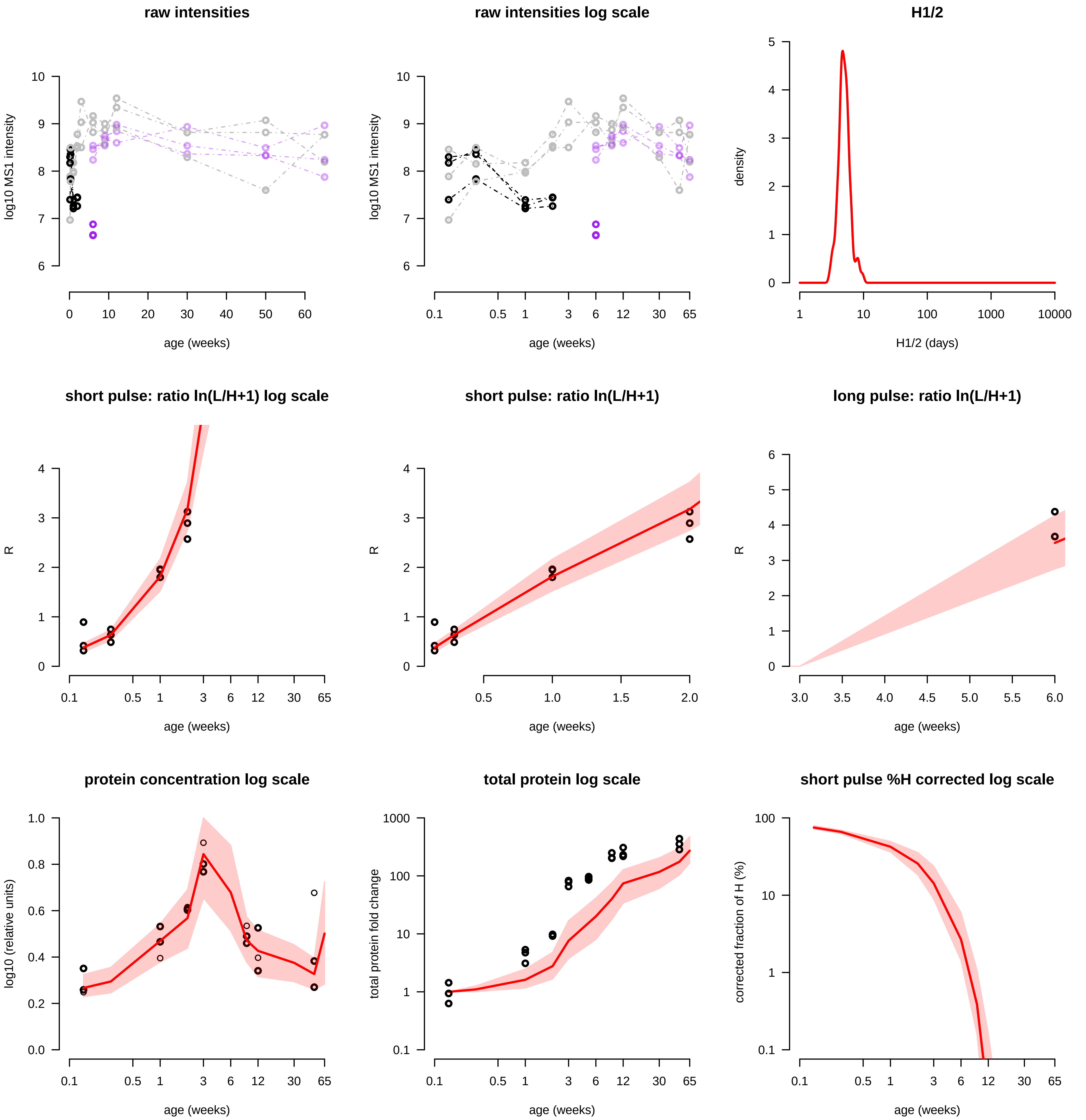

E9Q634

>sp|E9Q634|MYO1E_MOUSE

Unconventional myosin−Ie

GN=Myo1e

Med H1/2: 1122.6 days

Q5 H1/2: 363.4 days

Q95 H1/2: 15459.6 days

reliable model fit: yes

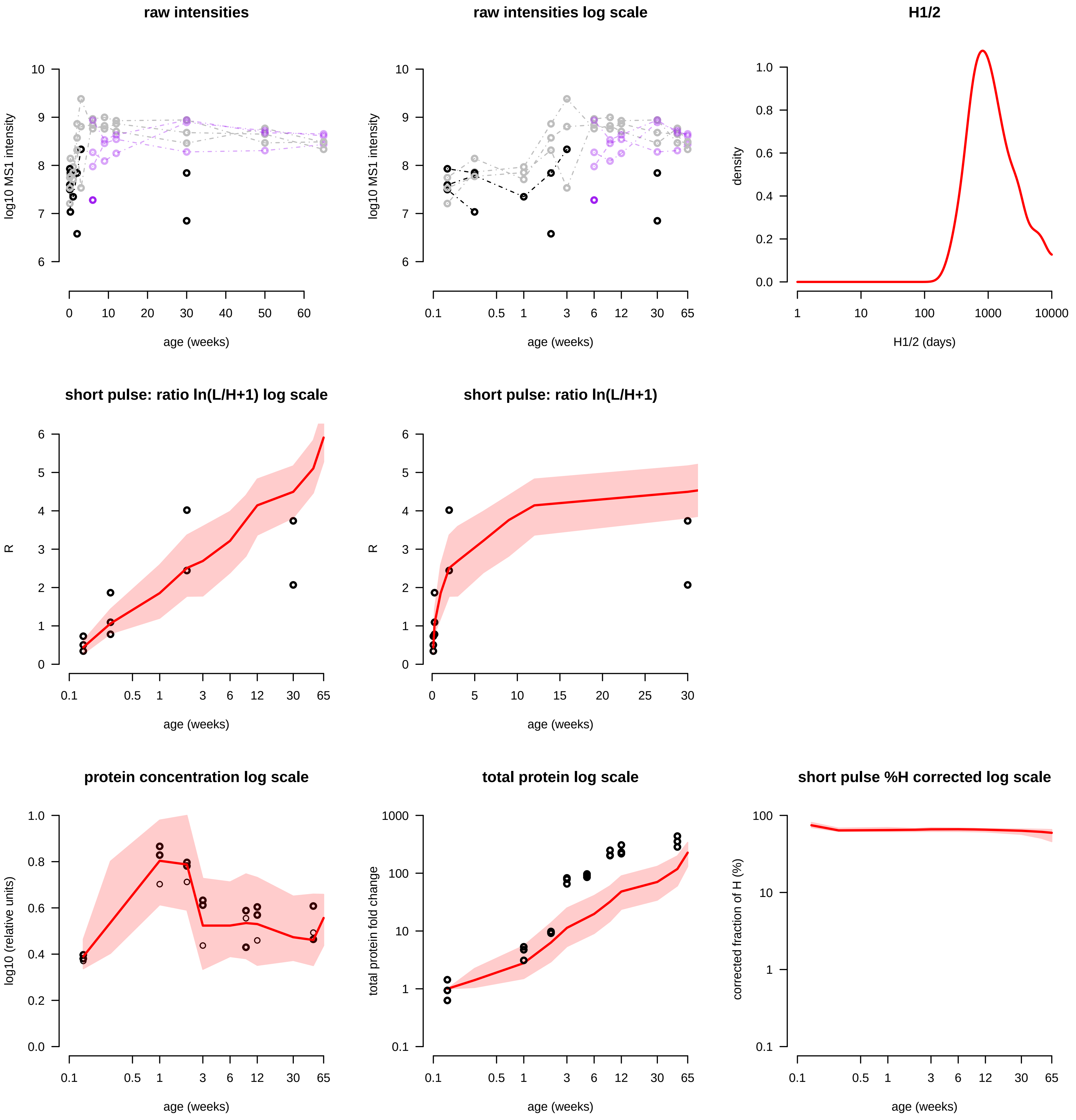

Q8K4F5

>sp|Q8K4F5|ABHDB_MOUSE

Protein ABHD11

GN=Abhd11

Med H1/2: 22.4 days

Q5 H1/2: 16.3 days

Q95 H1/2: 40.3 days

reliable model fit: yes

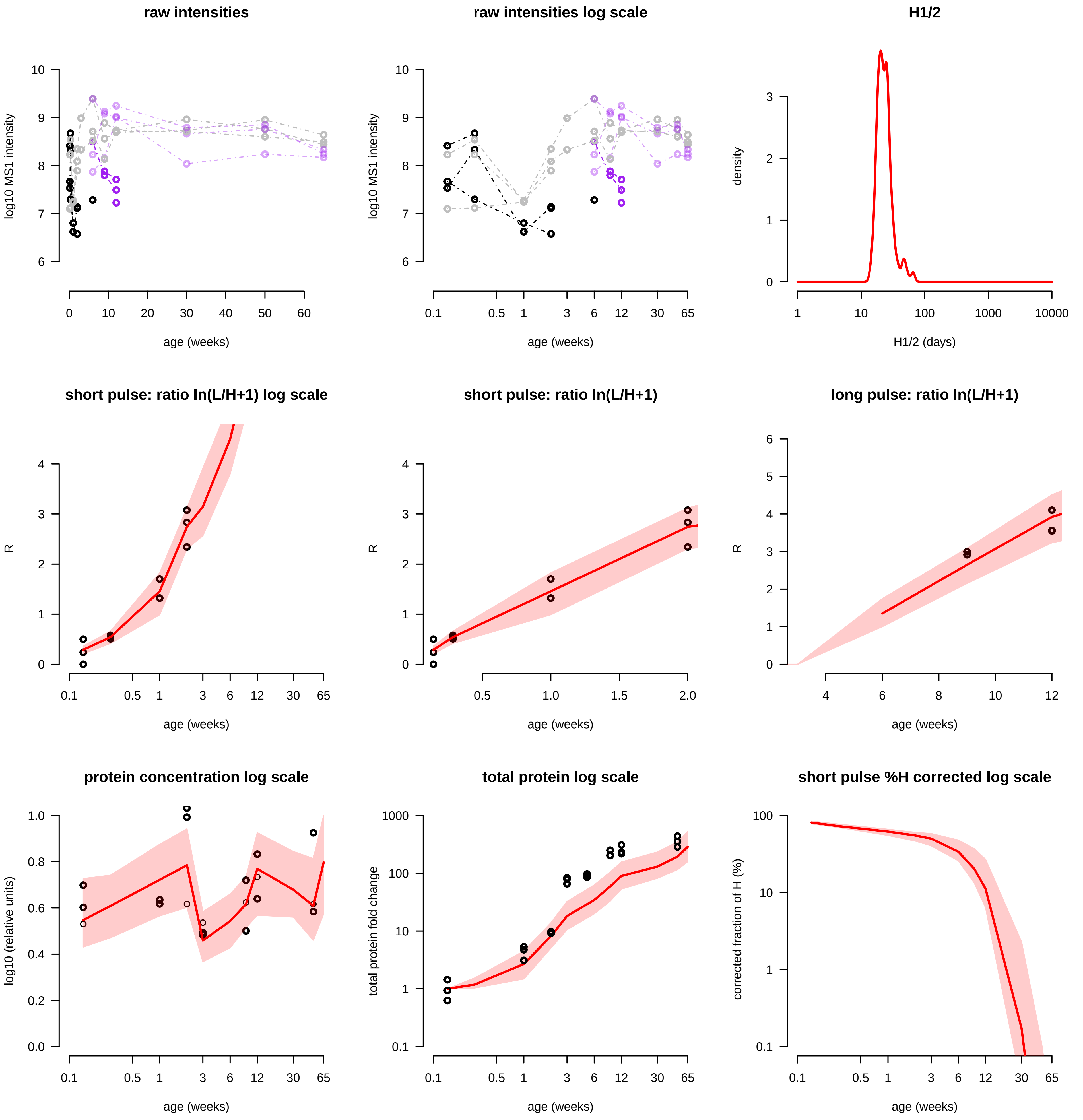

Q61464−4

>sp|Q61464−4|ZN638_MOUSE

Isoform 4 of Zinc finger protein 638

GN=Znf638

Med H1/2: 3.3 days

Q5 H1/2: 2.6 days

Q95 H1/2: 4.5 days

reliable model fit: yes

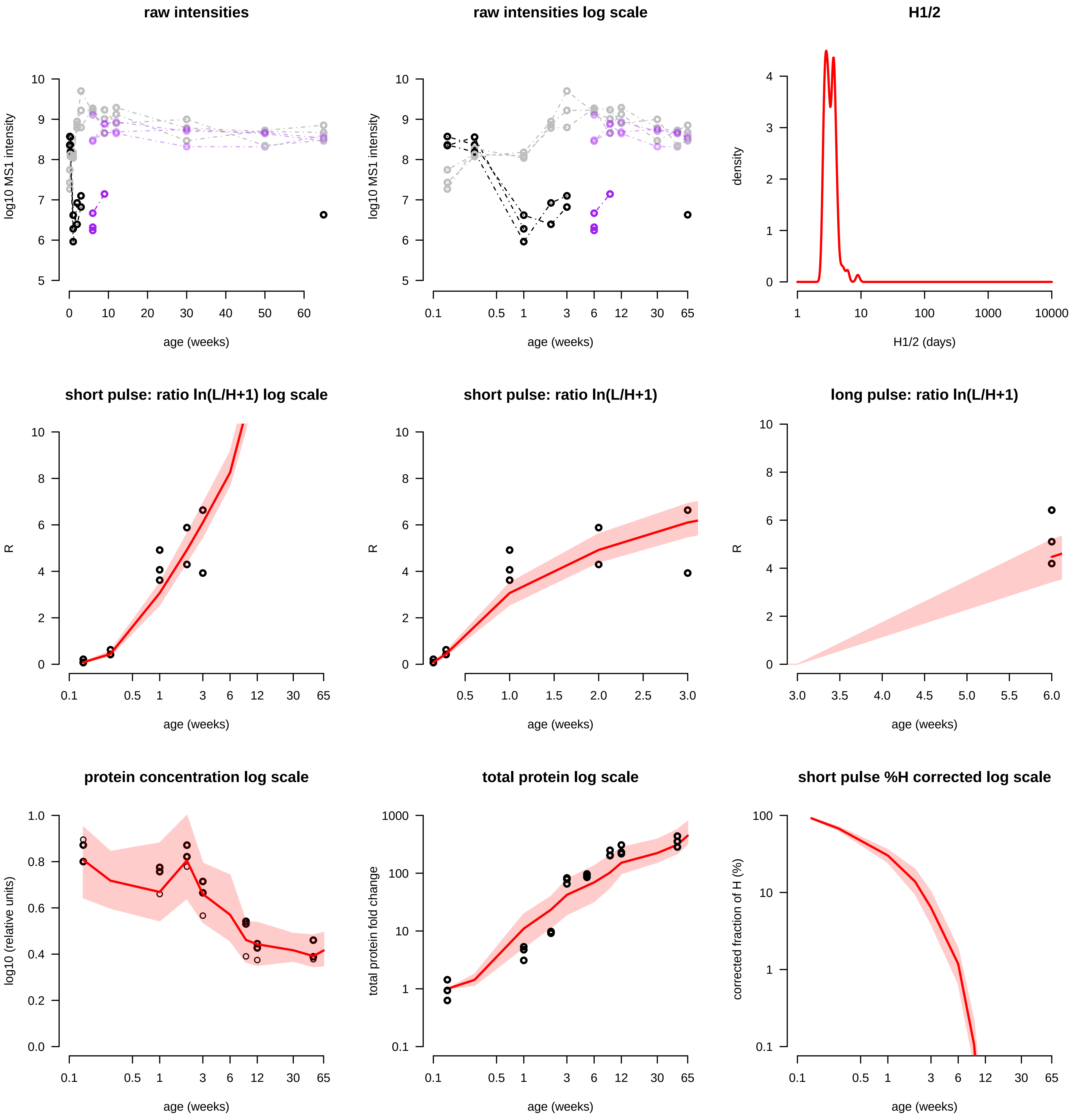

Q6P8I4

>sp|Q6P8I4|PCNP_MOUSE

PEST proteolytic signal−containing nuclear protein

GN=Pcnp

Med H1/2: 7.8 days

Q5 H1/2: 6.2 days

Q95 H1/2: 9.9 days

reliable model fit: yes

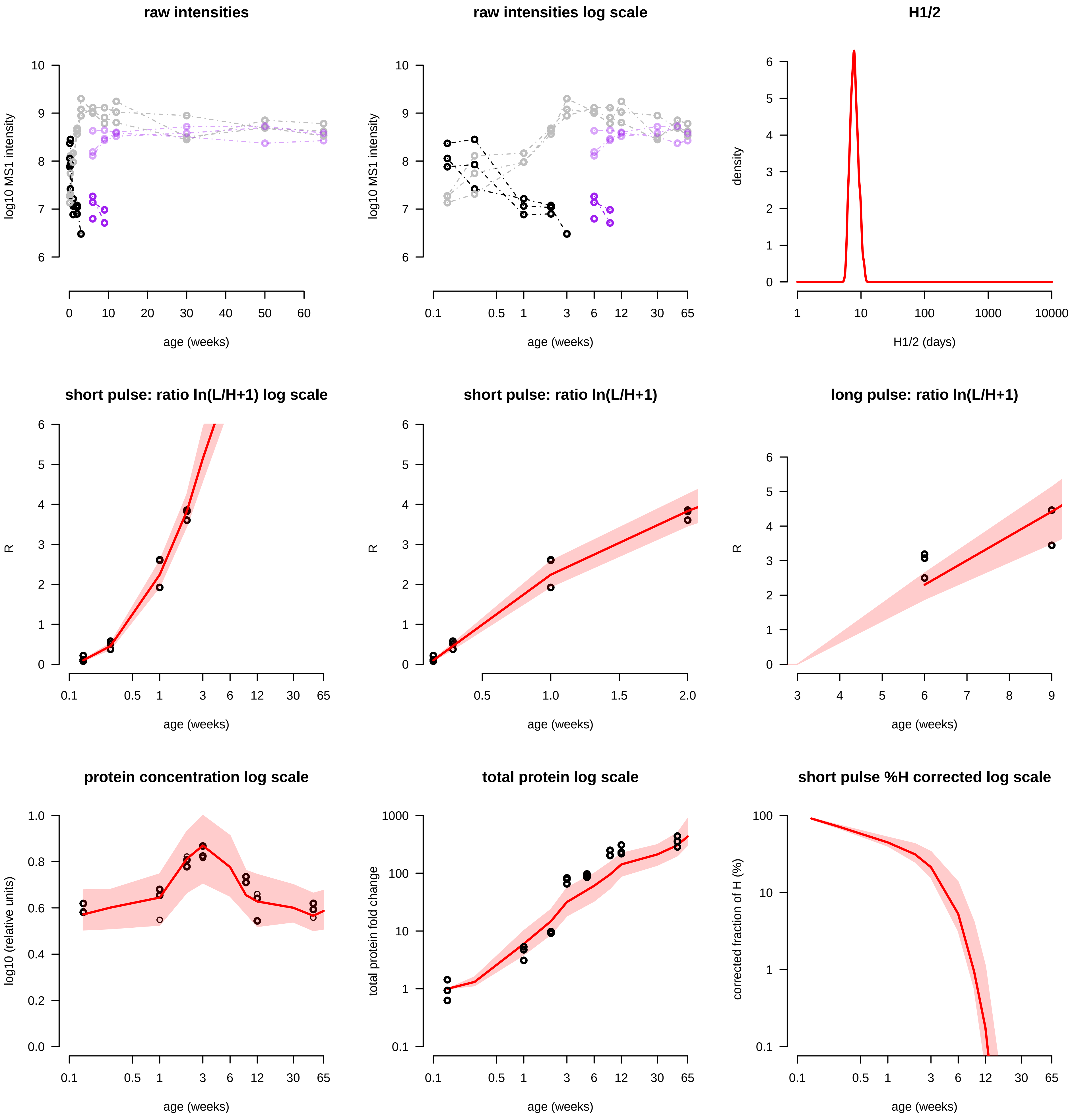

P70362

>sp|P70362|UFD1_MOUSE

Ubiquitin recognition factor in ER−associated degradation protein 1

GN=Ufd1

Med H1/2: 13.4 days

Q5 H1/2: 4.4 days

Q95 H1/2: 73.2 days

reliable model fit: yes

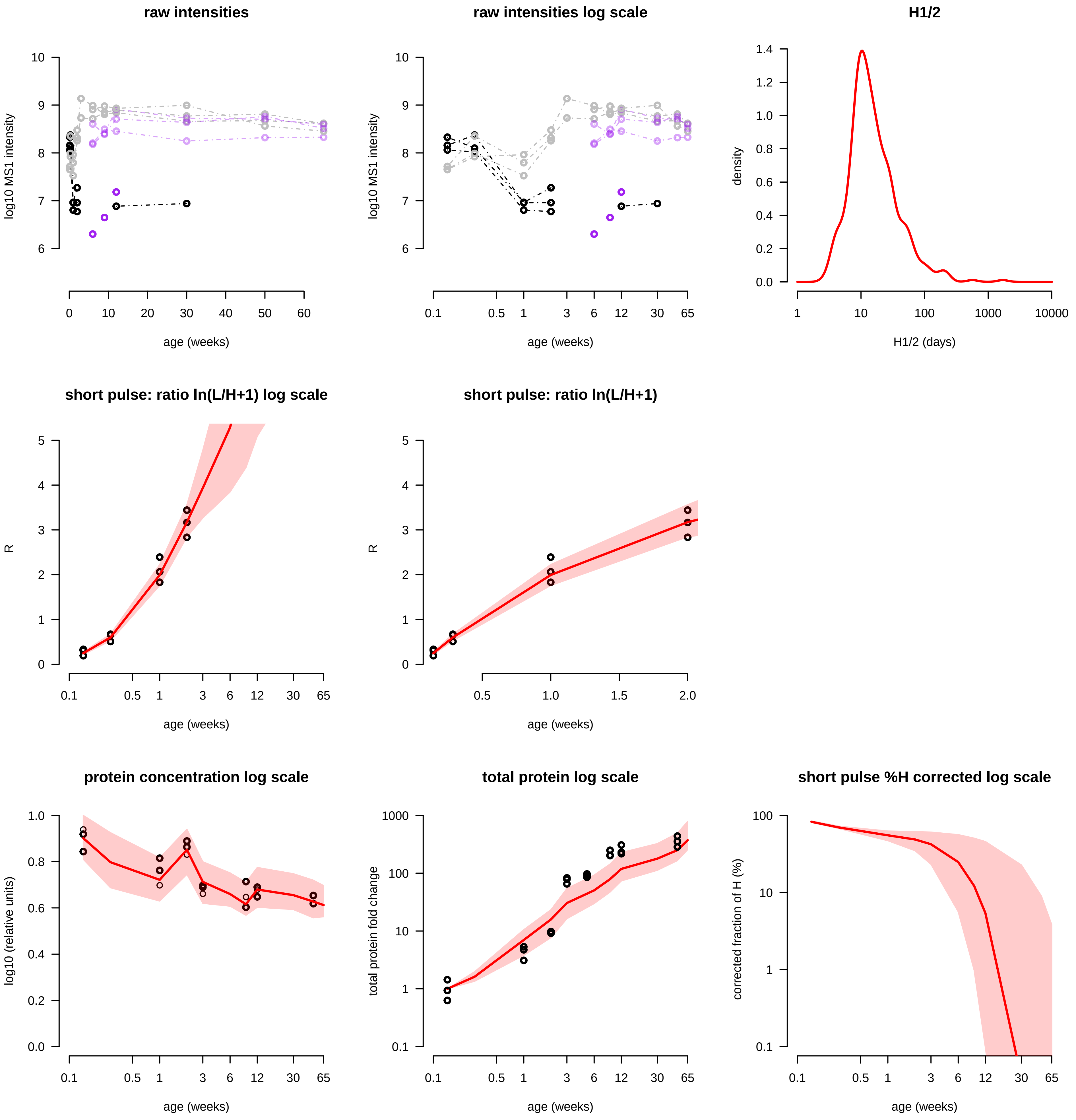

Q9JKB1

>sp|Q9JKB1|UCHL3_MOUSE

Ubiquitin carboxyl−terminal hydrolase isozyme L3

GN=Uchl3

Med H1/2: 5.5 days

Q5 H1/2: 4.4 days

Q95 H1/2: 7.2 days

reliable model fit: yes

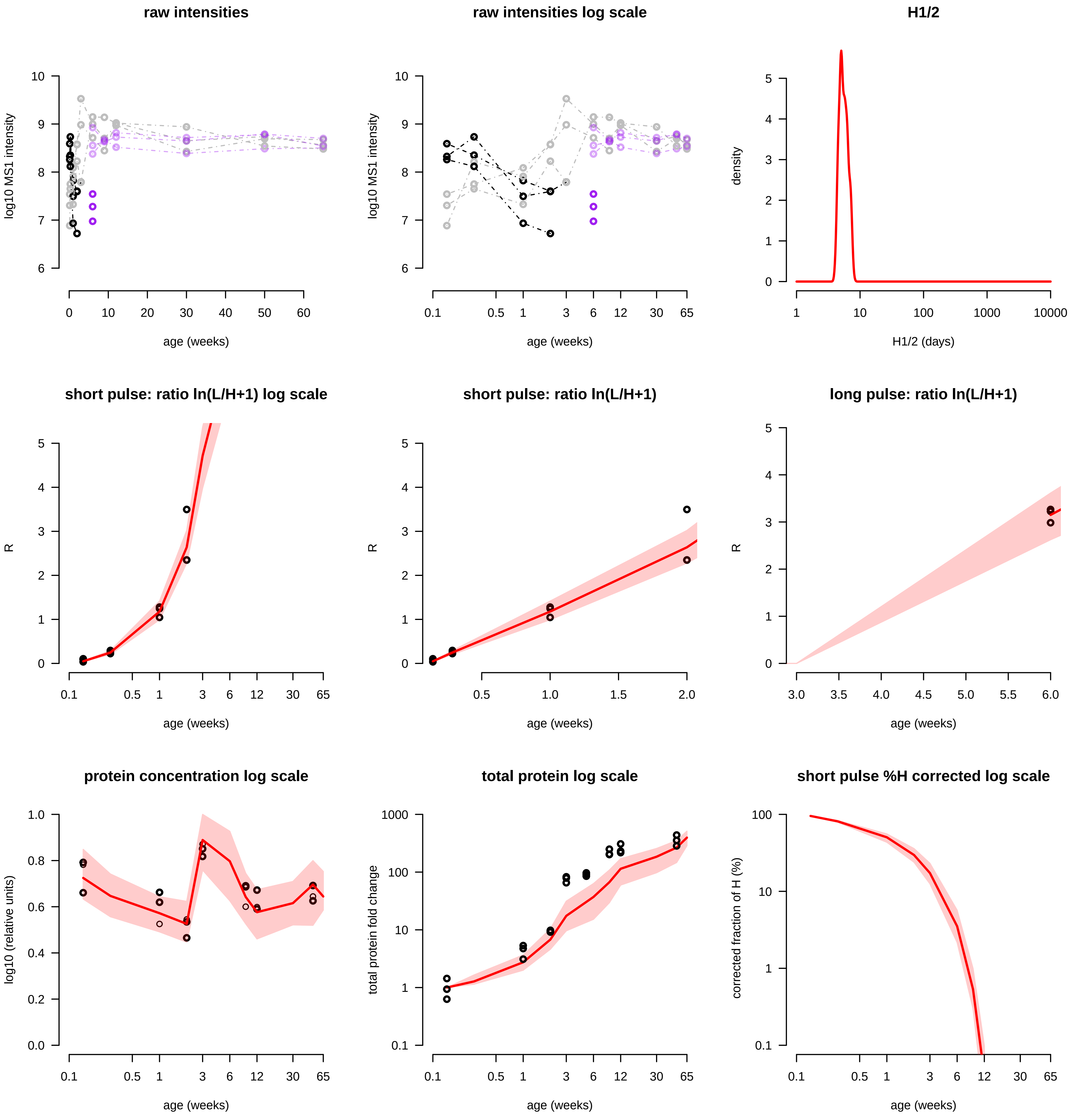

Q8VEJ9

>sp|Q8VEJ9|VPS4A_MOUSE

Vacuolar protein sorting−associated protein 4A

GN=Vps4a

Med H1/2: 1553.1 days

Q5 H1/2: 355.4 days

Q95 H1/2: 16495.5 days

reliable model fit: no

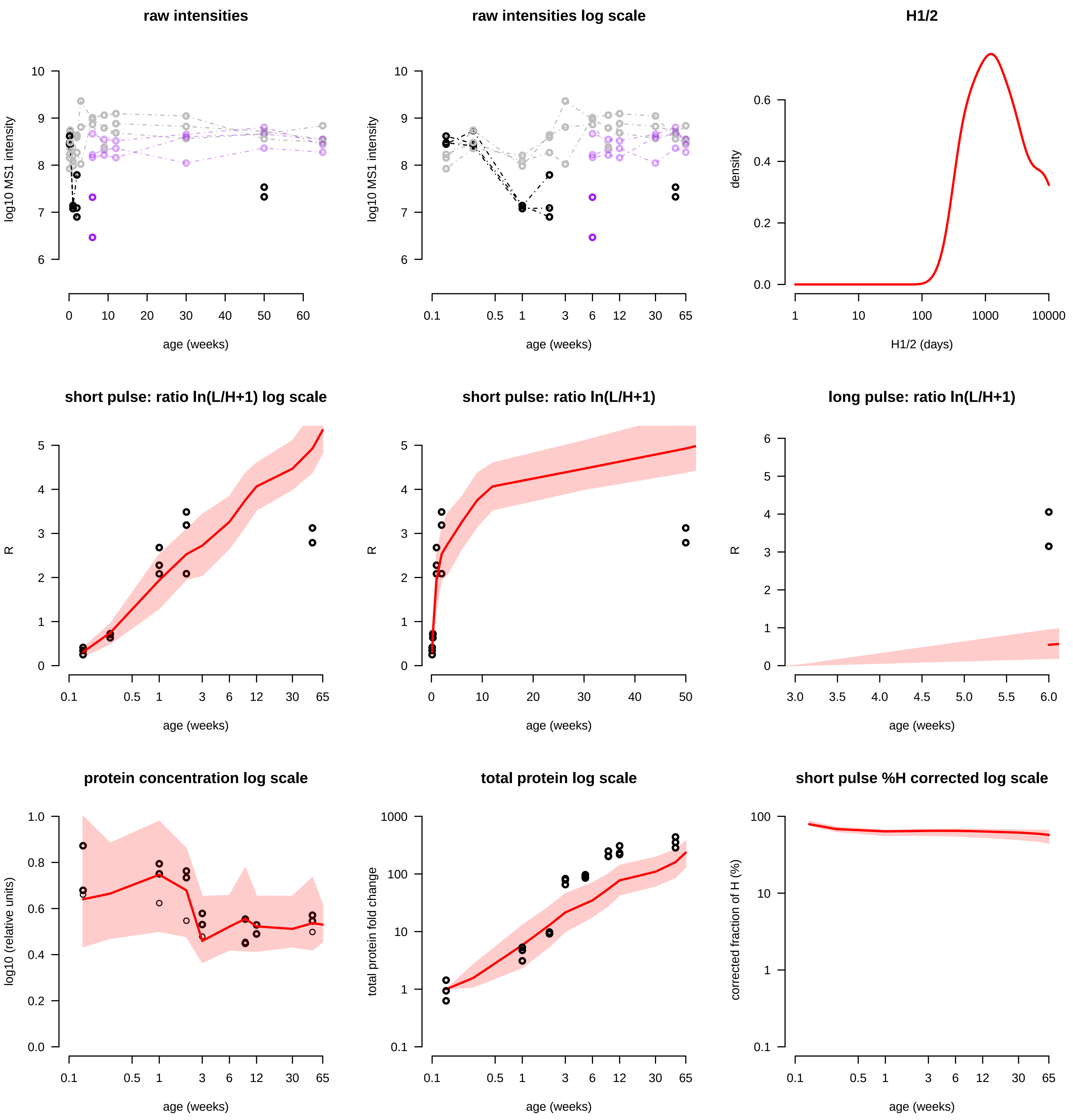

Q8VC30

>sp|Q8VC30|TKFC_MOUSE

Triokinase/FMN cyclase

GN=Tkfc

Med H1/2: 4.1 days

Q5 H1/2: 2.8 days

Q95 H1/2: 14.2 days

reliable model fit: yes

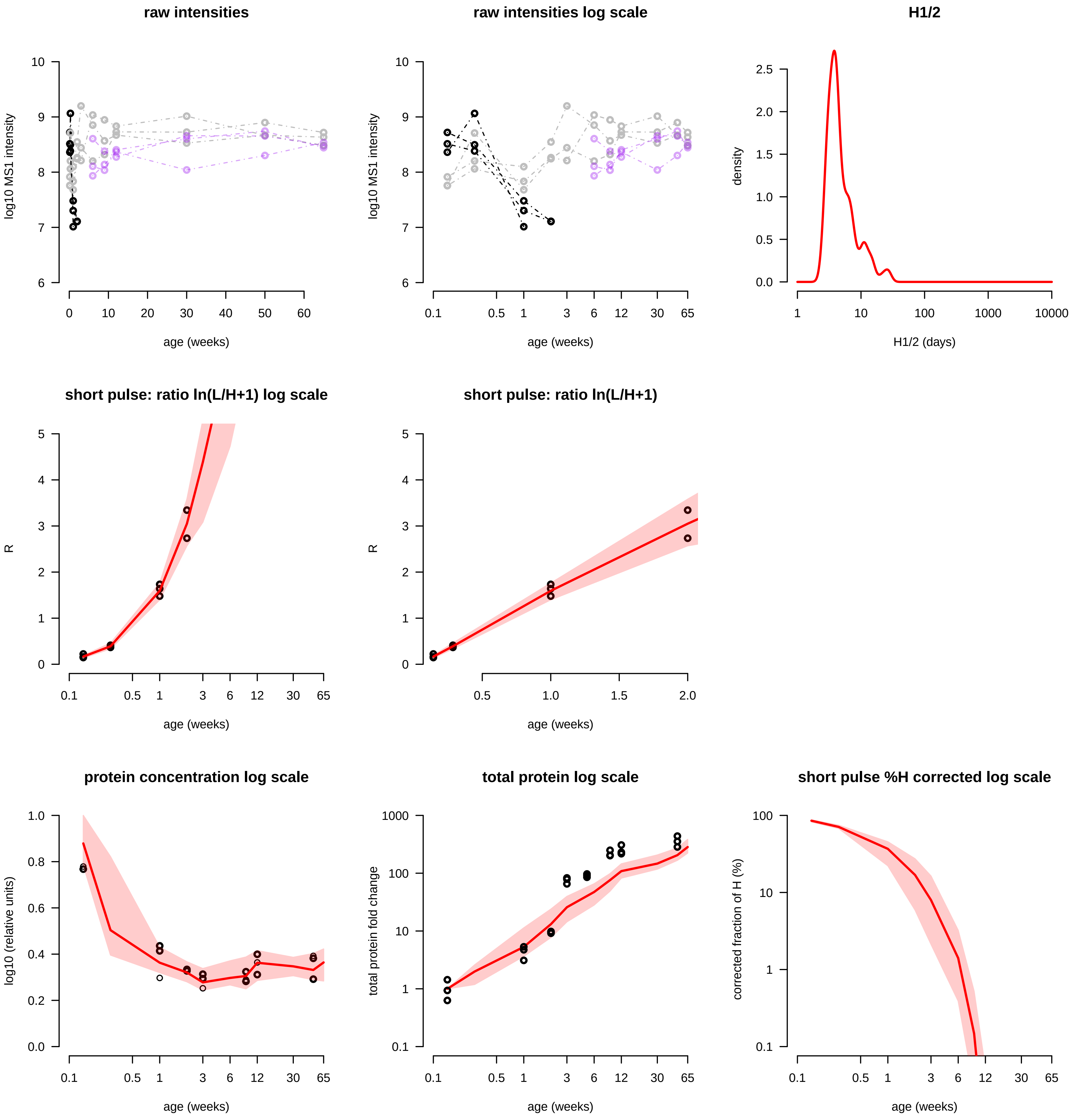

Q59J78

>sp|Q59J78|NDUF2_MOUSE

NADH dehydrogenase [ubiquinone] 1 alpha subcomplex assembly factor 2

GN=Ndufaf2

Med H1/2: 4.4 days

Q5 H1/2: 3.3 days

Q95 H1/2: 5.5 days

reliable model fit: yes

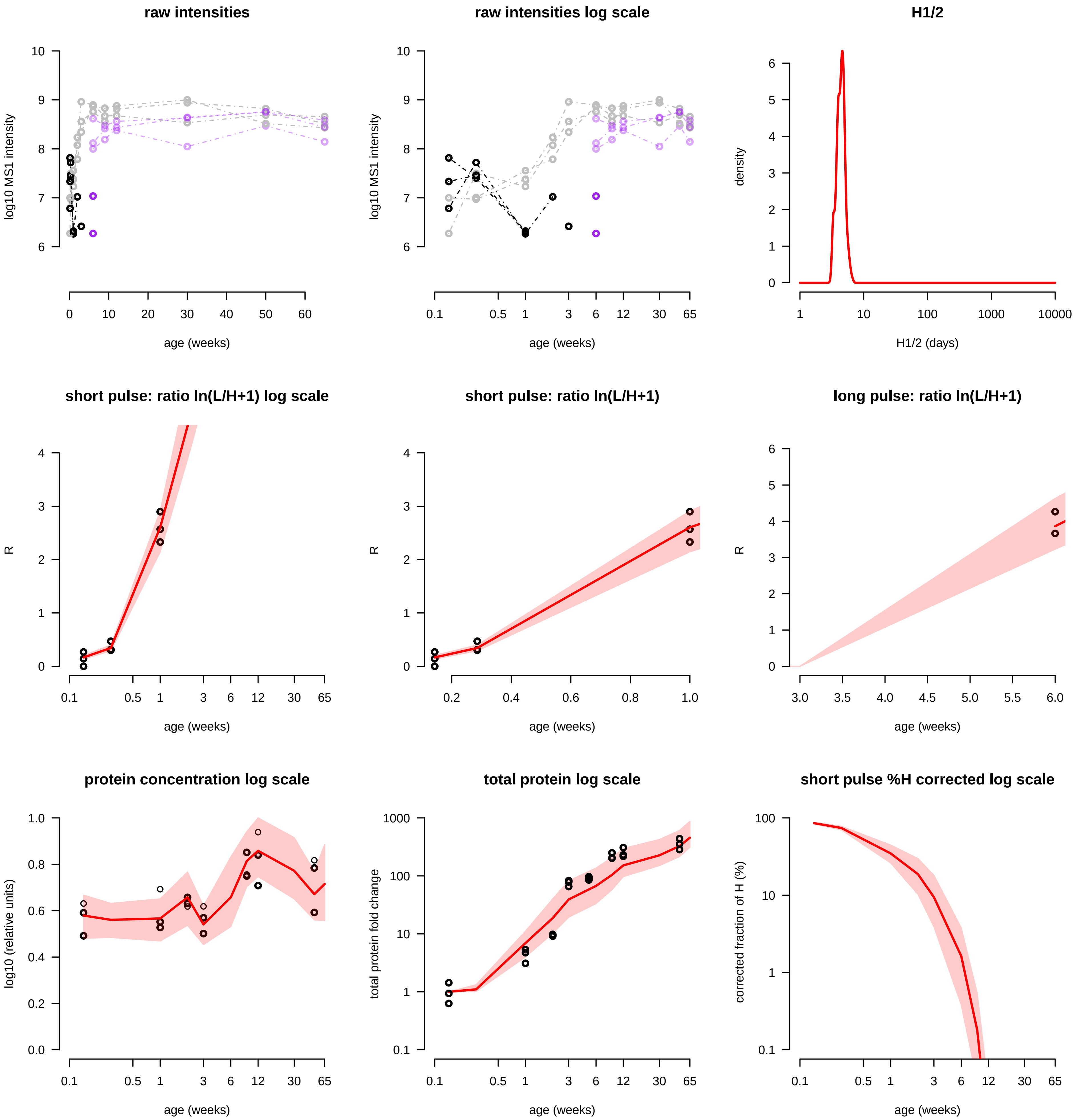

Q1HFZ0;Q1HFZ0−2

>sp|Q1HFZ0|NSUN2_MOUSE

RNA cytosine C(5)−methyltransferase NSUN2

GN=Nsun2

Med H1/2: 4.7 days

Q5 H1/2: 3.1 days

Q95 H1/2: 6.7 days

reliable model fit: yes

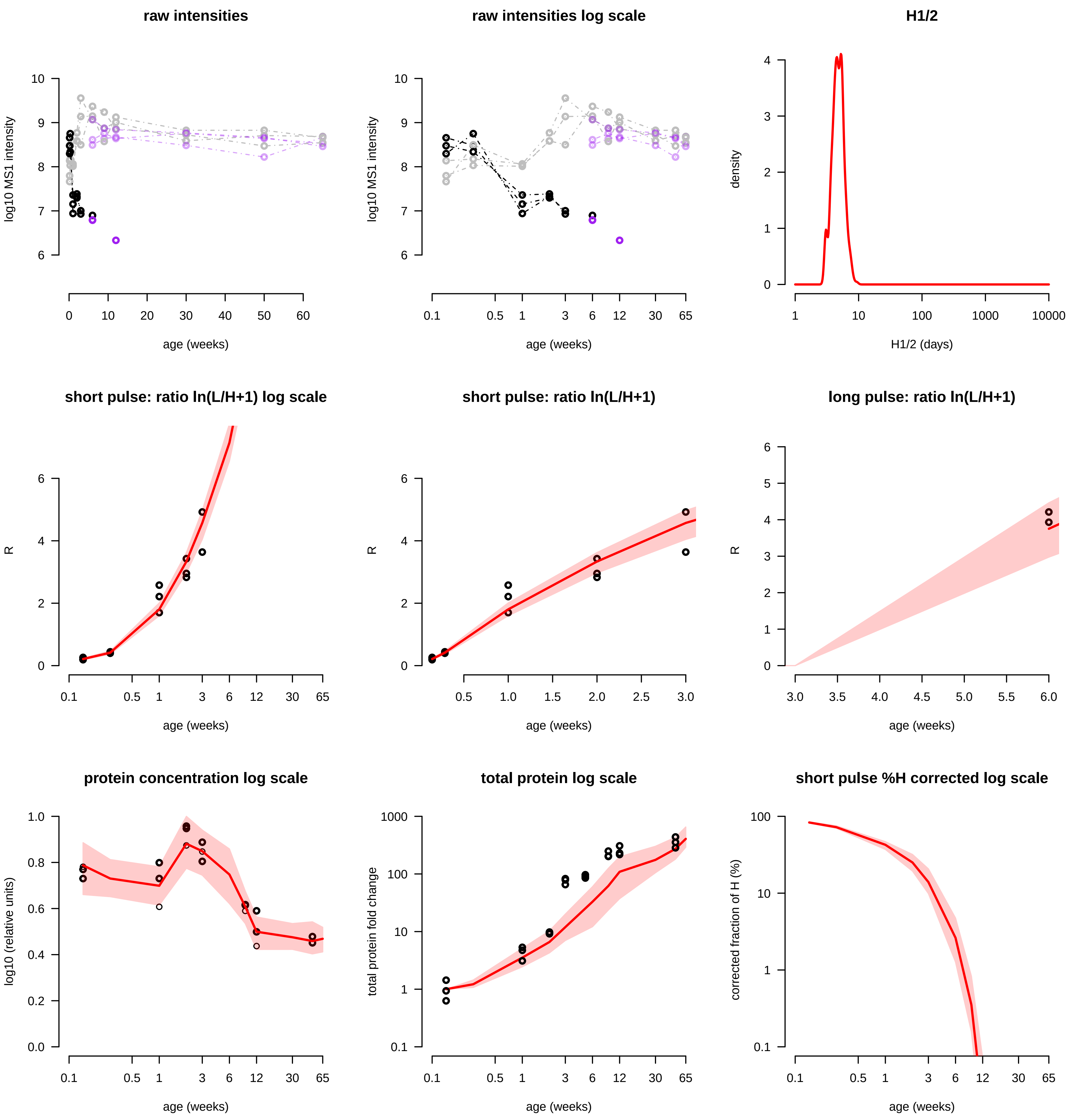

Q8VCM8

>sp|Q8VCM8|NCLN_MOUSE

Nicalin

GN=Ncln

Med H1/2: 7 days

Q5 H1/2: 3.4 days

Q95 H1/2: 32.3 days

reliable model fit: yes

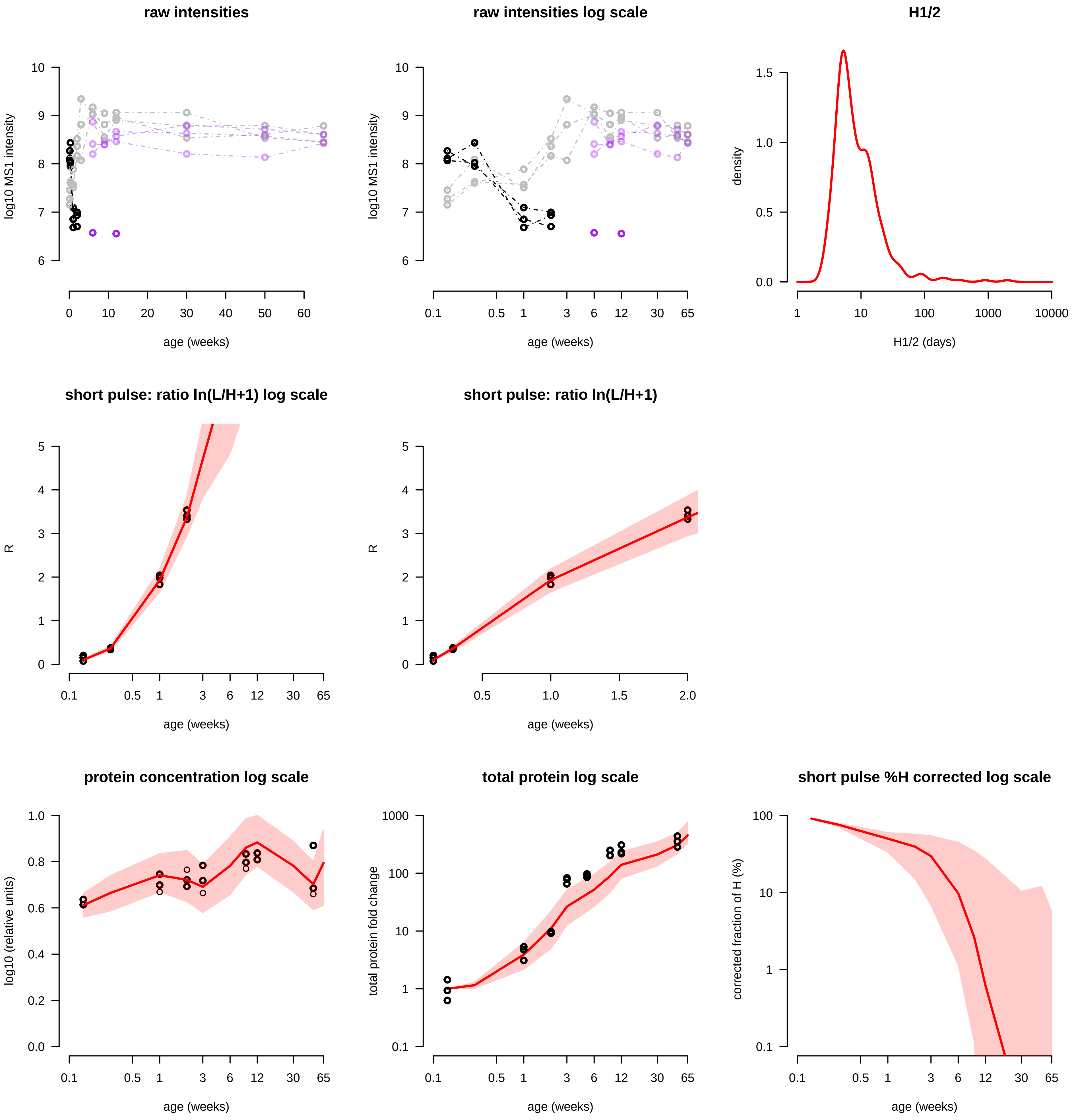

Q9D958

>sp|Q9D958|SPCS1_MOUSE

Signal peptidase complex subunit 1

GN=Spcs1

Med H1/2: 13.3 days

Q5 H1/2: 5.7 days

Q95 H1/2: 269.2 days

reliable model fit: no

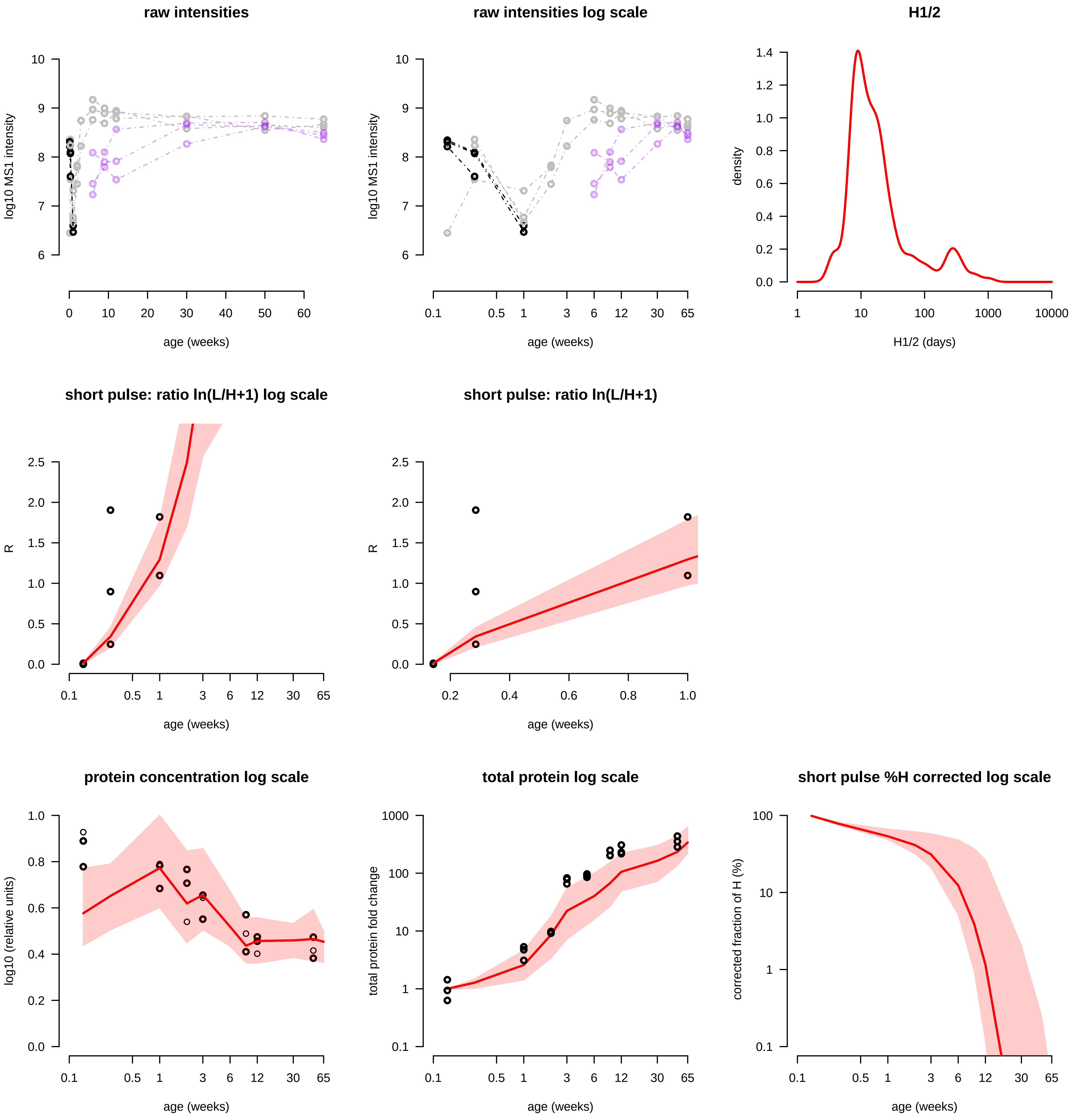

O08738

>sp|O08738|CASP6_MOUSE

Caspase−6

GN=Casp6

Med H1/2: 5.4 days

Q5 H1/2: 4 days

Q95 H1/2: 7.4 days

reliable model fit: yes

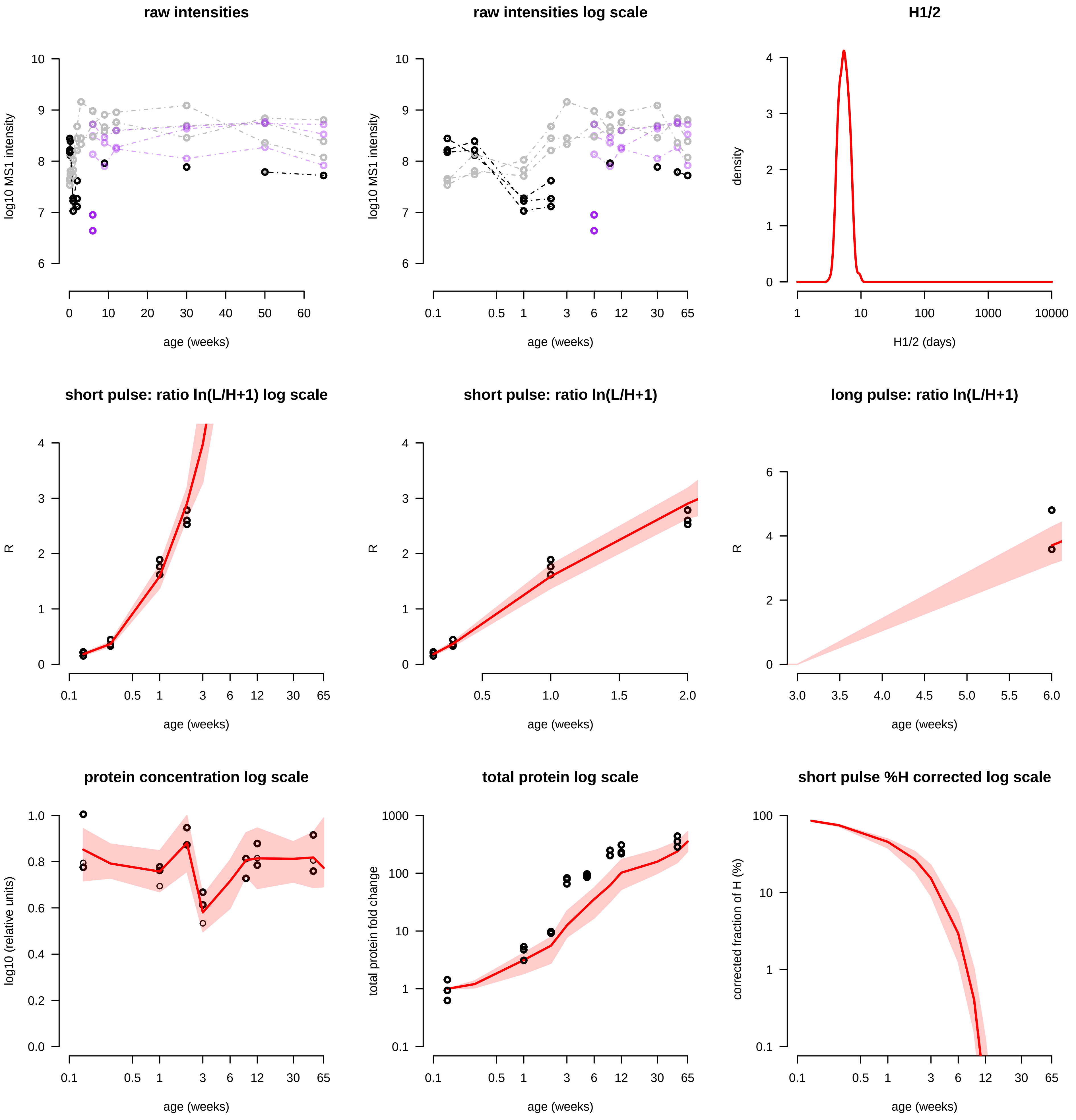

P62880

>sp|P62880|GBB2_MOUSE

Guanine nucleotide−binding protein G(I)/G(S)/G(T) subunit beta−2

GN=Gnb2

Med H1/2: 27 days

Q5 H1/2: 15.8 days

Q95 H1/2: 71.3 days

reliable model fit: yes

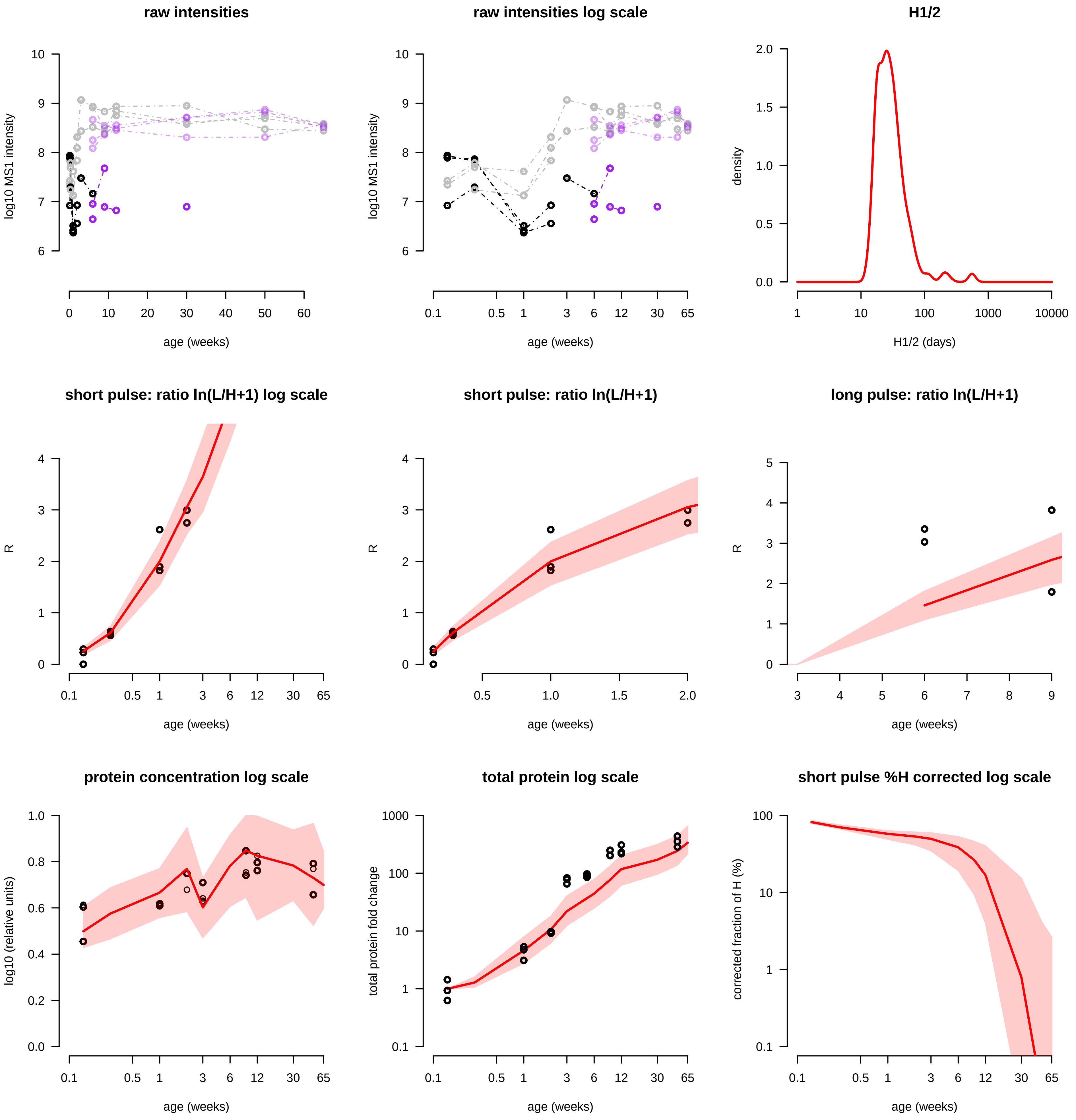

P06802−2;P06802

>sp|P06802−2|ENPP1_MOUSE

soform 1 of Ectonucleotide pyrophosphatase/phosphodiesterase family member

GN=Enpp1

Med H1/2: 8 days

Q5 H1/2: 3.4 days

Q95 H1/2: 82.1 days

reliable model fit: yes

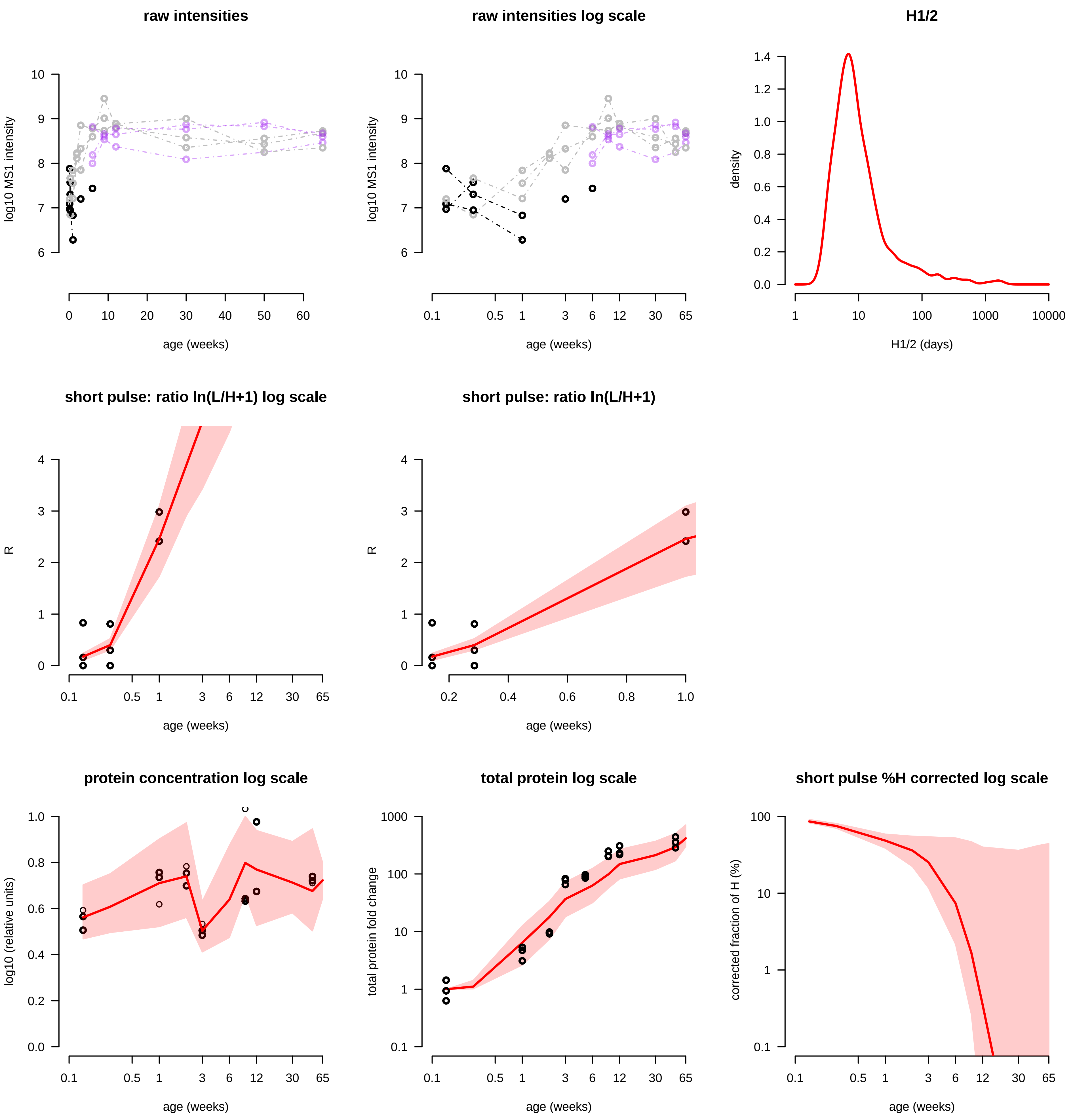

P61079

>sp|P61079|UB2D3_MOUSE

Ubiquitin−conjugating enzyme E2 D3

GN=Ube2d3

Med H1/2: 7.9 days

Q5 H1/2: 5.9 days

Q95 H1/2: 11.4 days

reliable model fit: yes

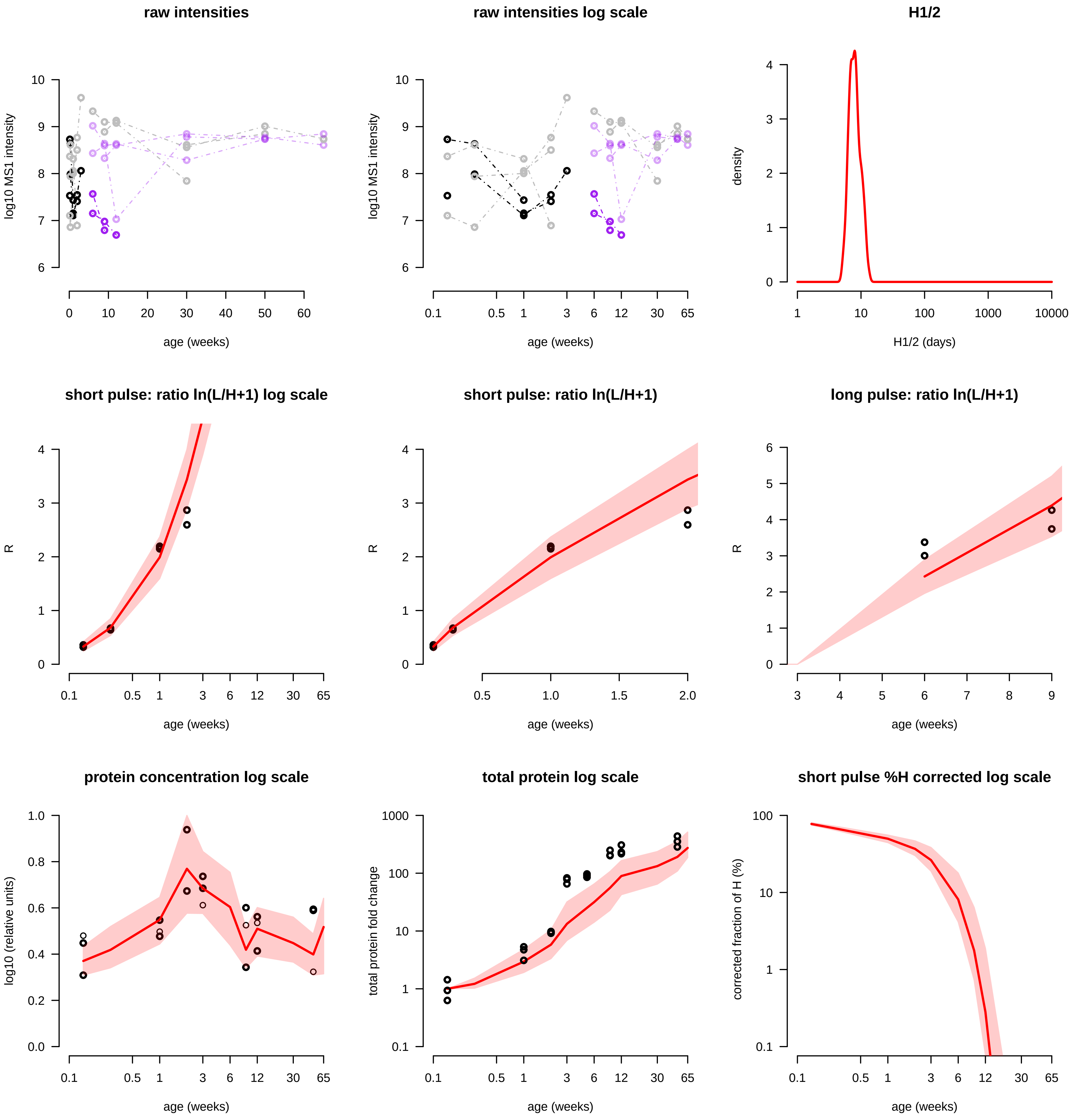

Q6P9J9

>sp|Q6P9J9|ANO6_MOUSE

Anoctamin−6

GN=Ano6

Med H1/2: 10.5 days

Q5 H1/2: 4 days

Q95 H1/2: 76.4 days

reliable model fit: no

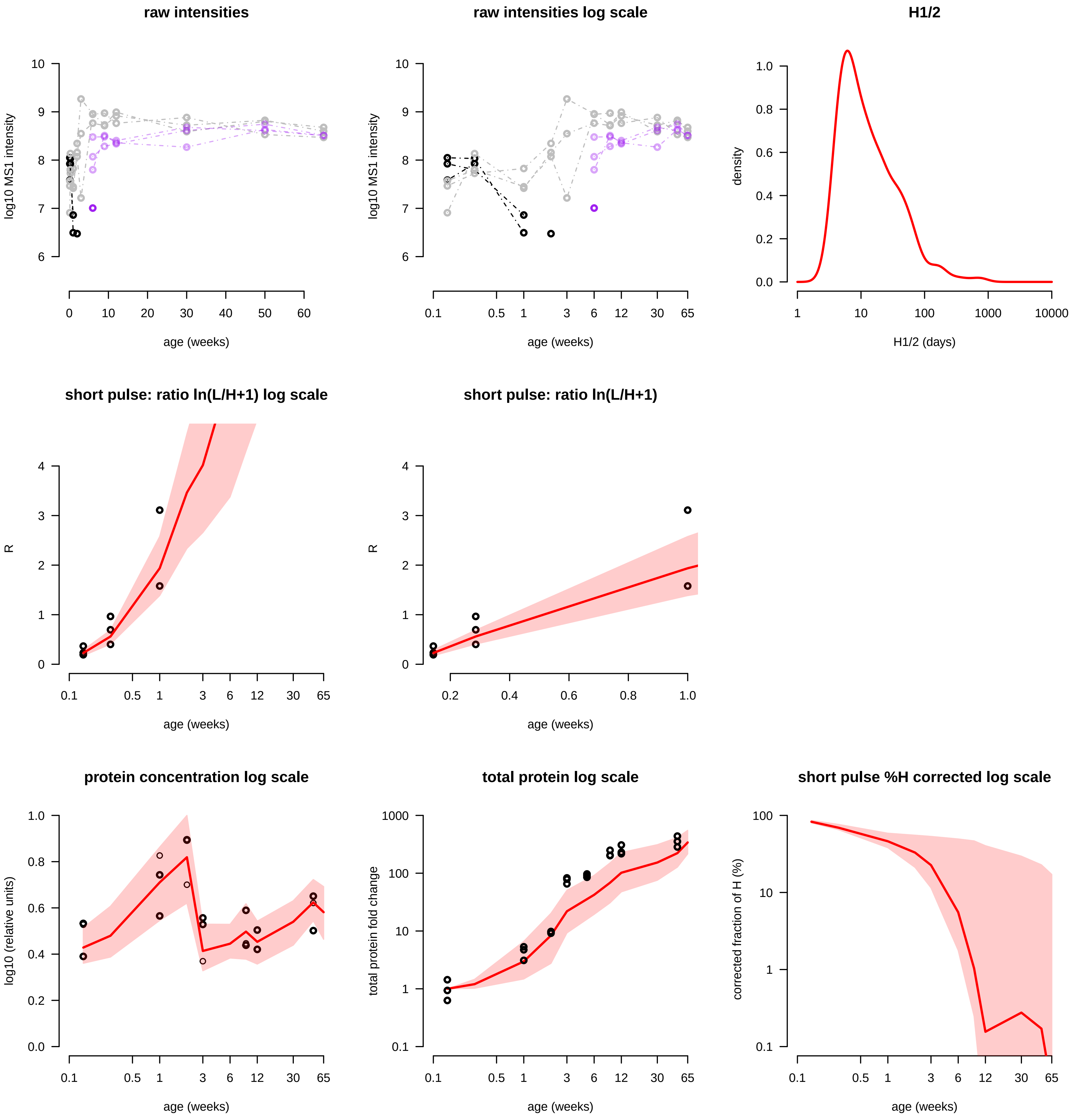

Q8R574

>sp|Q8R574|KPRB_MOUSE

Phosphoribosyl pyrophosphate synthase−associated protein 2

GN=Prpsap2

Med H1/2: 12.2 days

Q5 H1/2: 5.6 days

Q95 H1/2: 108.6 days

reliable model fit: yes

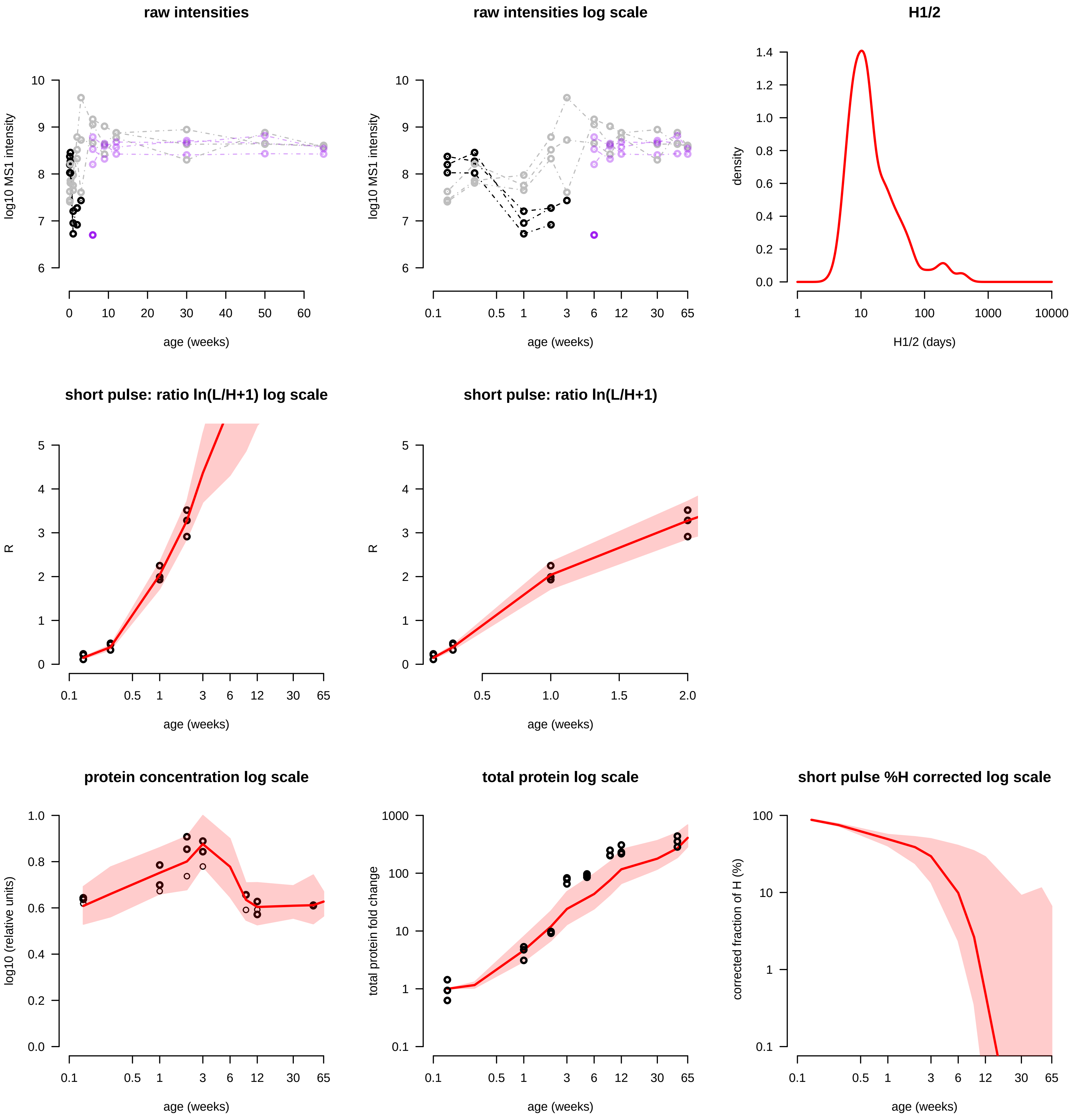

P21126

>sp|P21126|UBL4A_MOUSE

Ubiquitin−like protein 4A

GN=Ubl4a

Med H1/2: 9.1 days

Q5 H1/2: 3.1 days

Q95 H1/2: 134.5 days

reliable model fit: yes

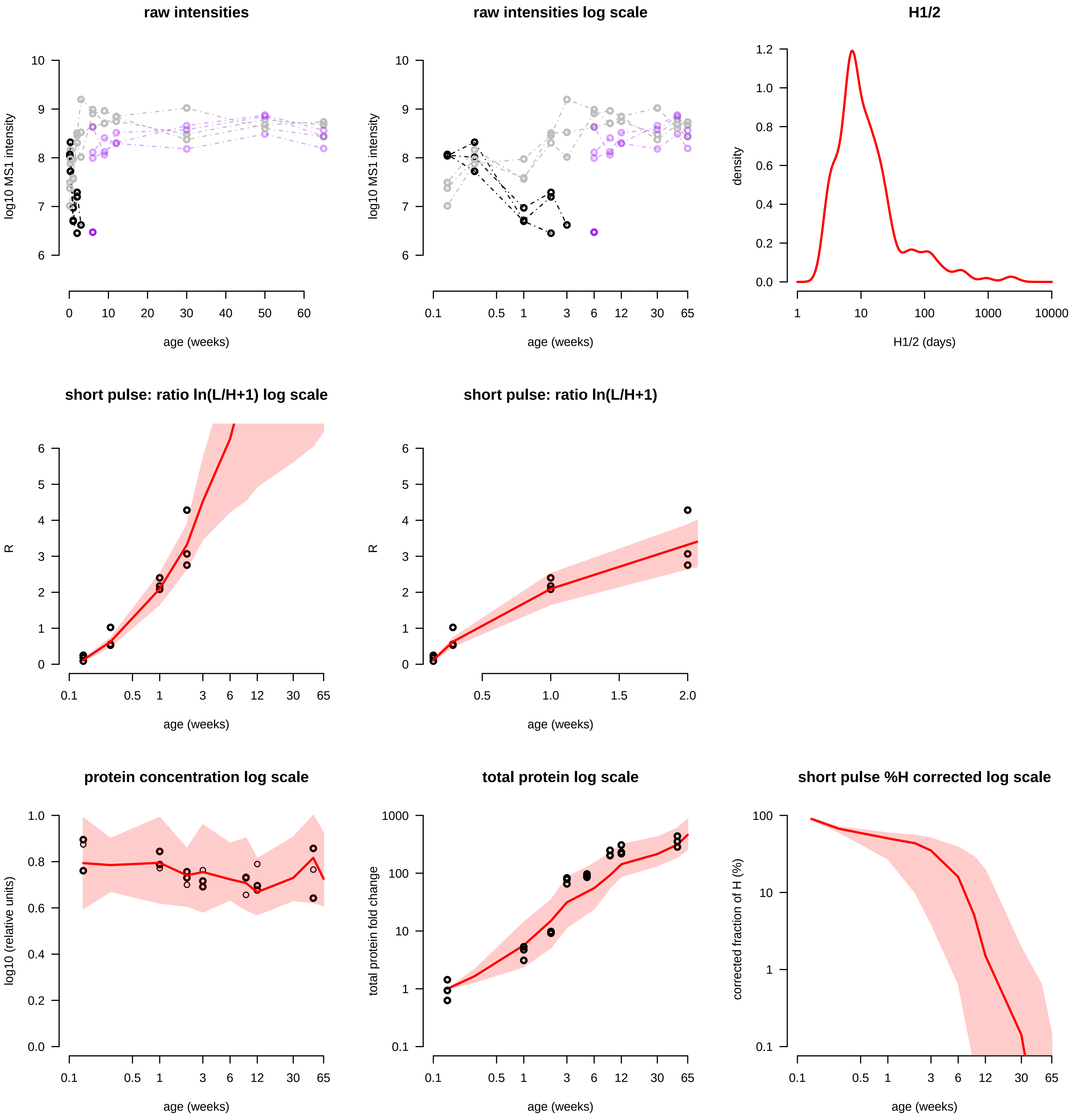

Q99KP3

>sp|Q99KP3|CRYL1_MOUSE

Lambda−crystallin homolog

GN=Cryl1

Med H1/2: 10.4 days

Q5 H1/2: 7.5 days

Q95 H1/2: 15.9 days

reliable model fit: yes

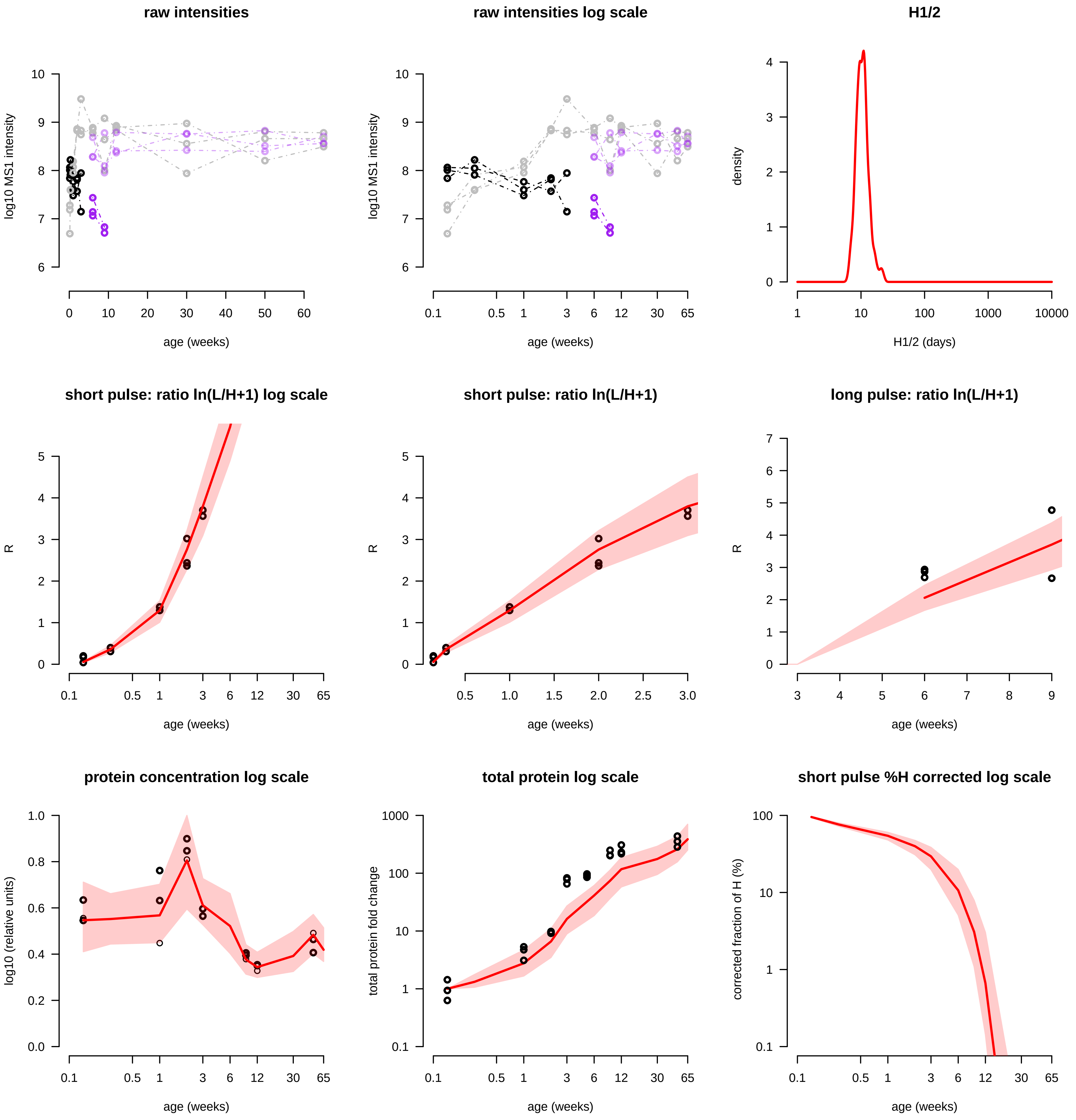

Q8BPB0

>sp|Q8BPB0|MOB1B_MOUSE

MOB kinase activator 1B

GN=Mob1b

Med H1/2: 6 days

Q5 H1/2: 4.9 days

Q95 H1/2: 7.9 days

reliable model fit: yes

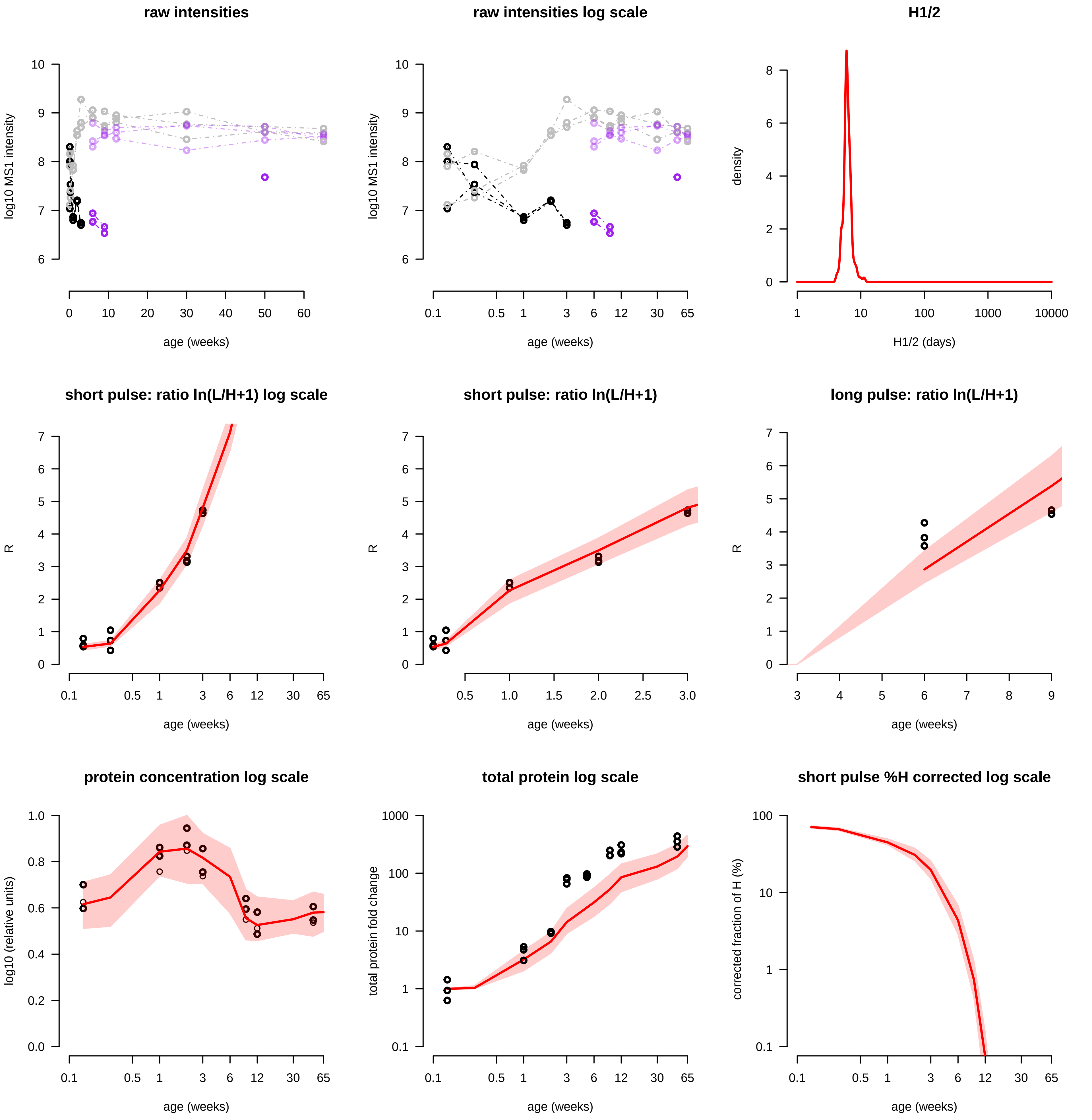

Q8BHC4

>sp|Q8BHC4|DCAKD_MOUSE

Dephospho−CoA kinase domain−containing protein

GN=Dcakd

Med H1/2: 28.5 days

Q5 H1/2: 10.7 days

Q95 H1/2: 218 days

reliable model fit: yes

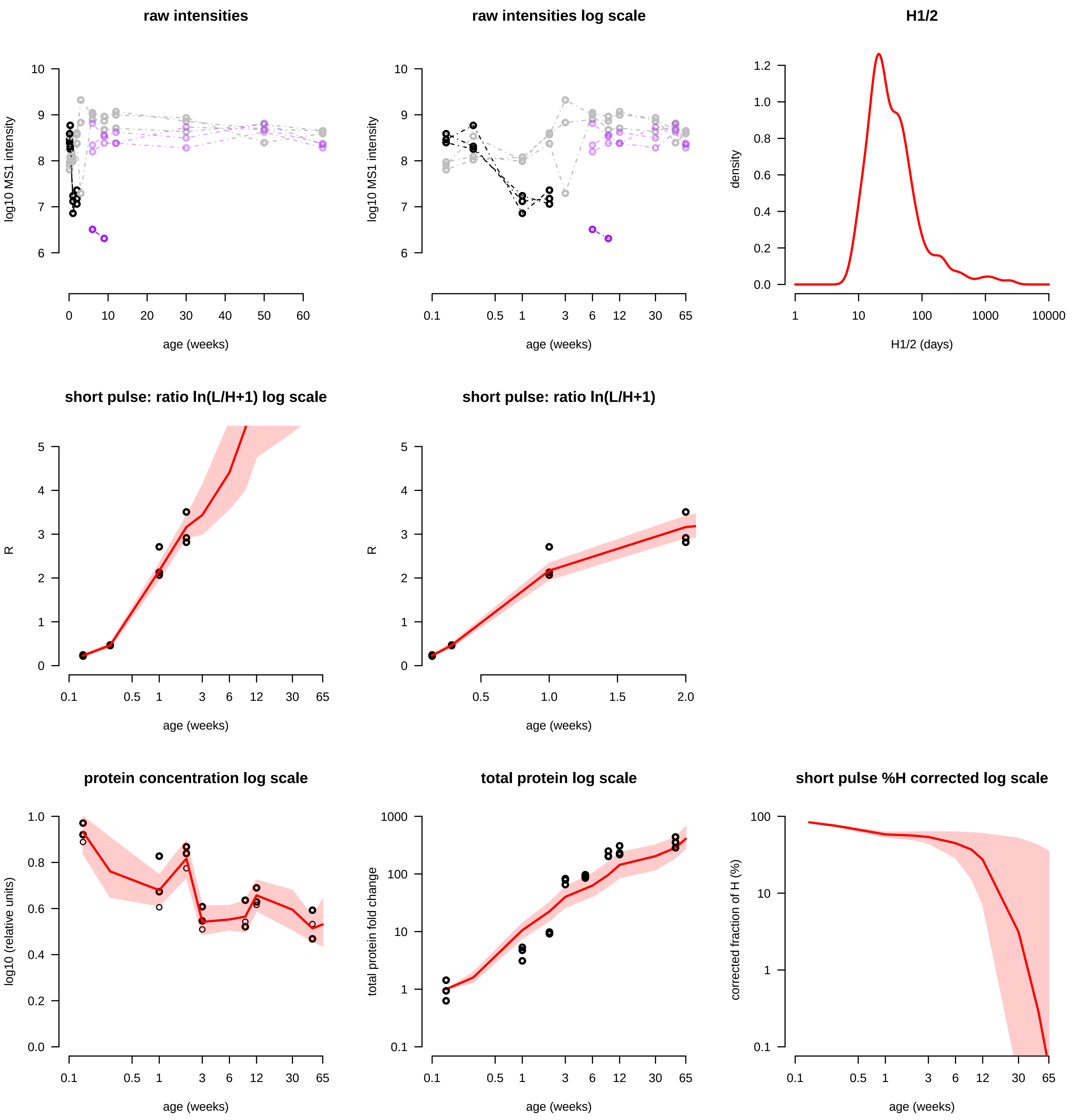

Q8C8R3−4;Q8C8R3;Q8C8R3−5

>sp|Q8C8R3−4|ANK2_MOUSE

Isoform 4 of Ankyrin−2

GN=Ank2

Med H1/2: 6.9 days

Q5 H1/2: 5.9 days

Q95 H1/2: 8.5 days

reliable model fit: yes

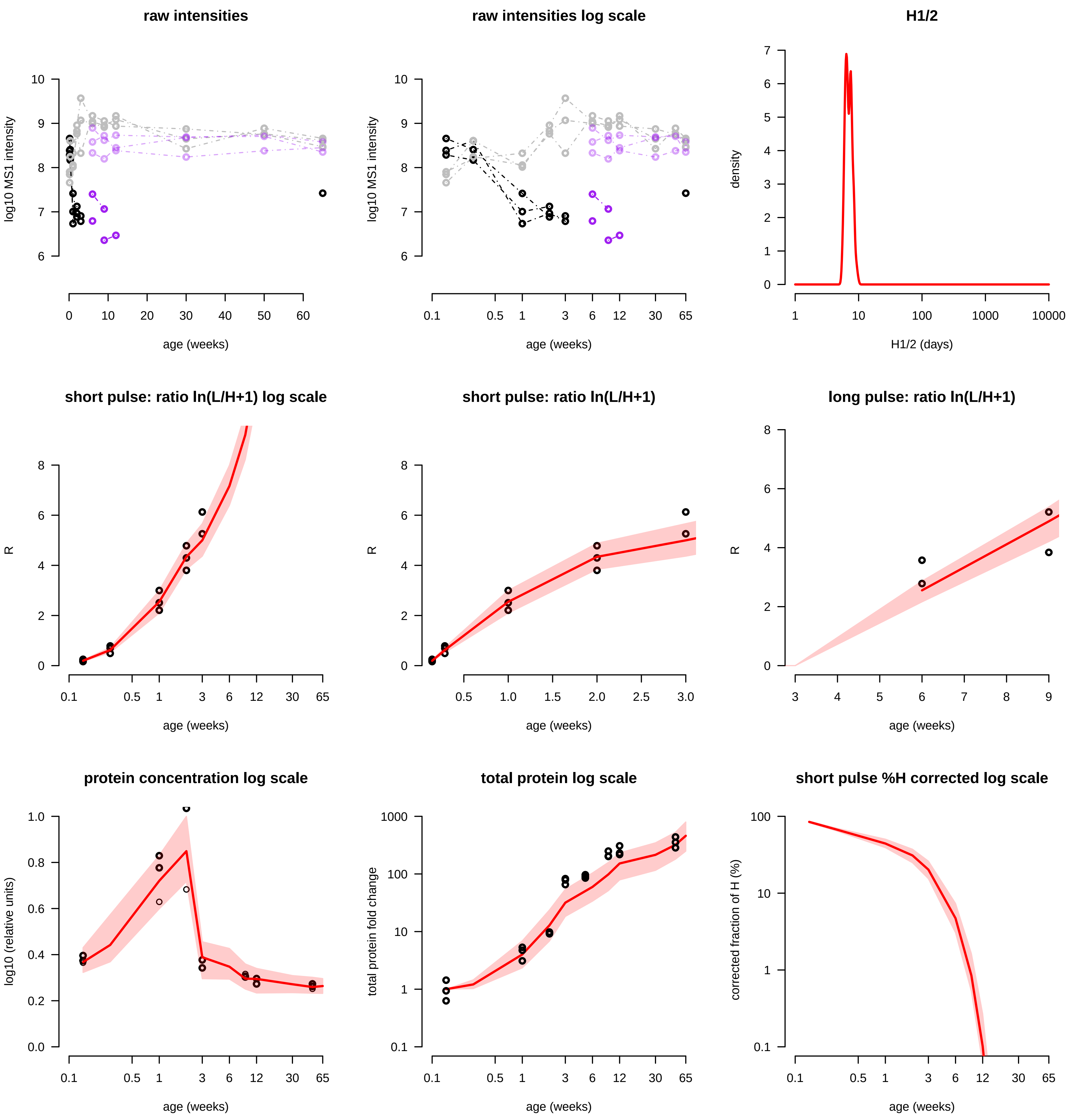

P63024

>sp|P63024|VAMP3_MOUSE

Vesicle−associated membrane protein 3

GN=Vamp3

Med H1/2: 5.8 days

Q5 H1/2: 4.6 days

Q95 H1/2: 7.4 days

reliable model fit: yes

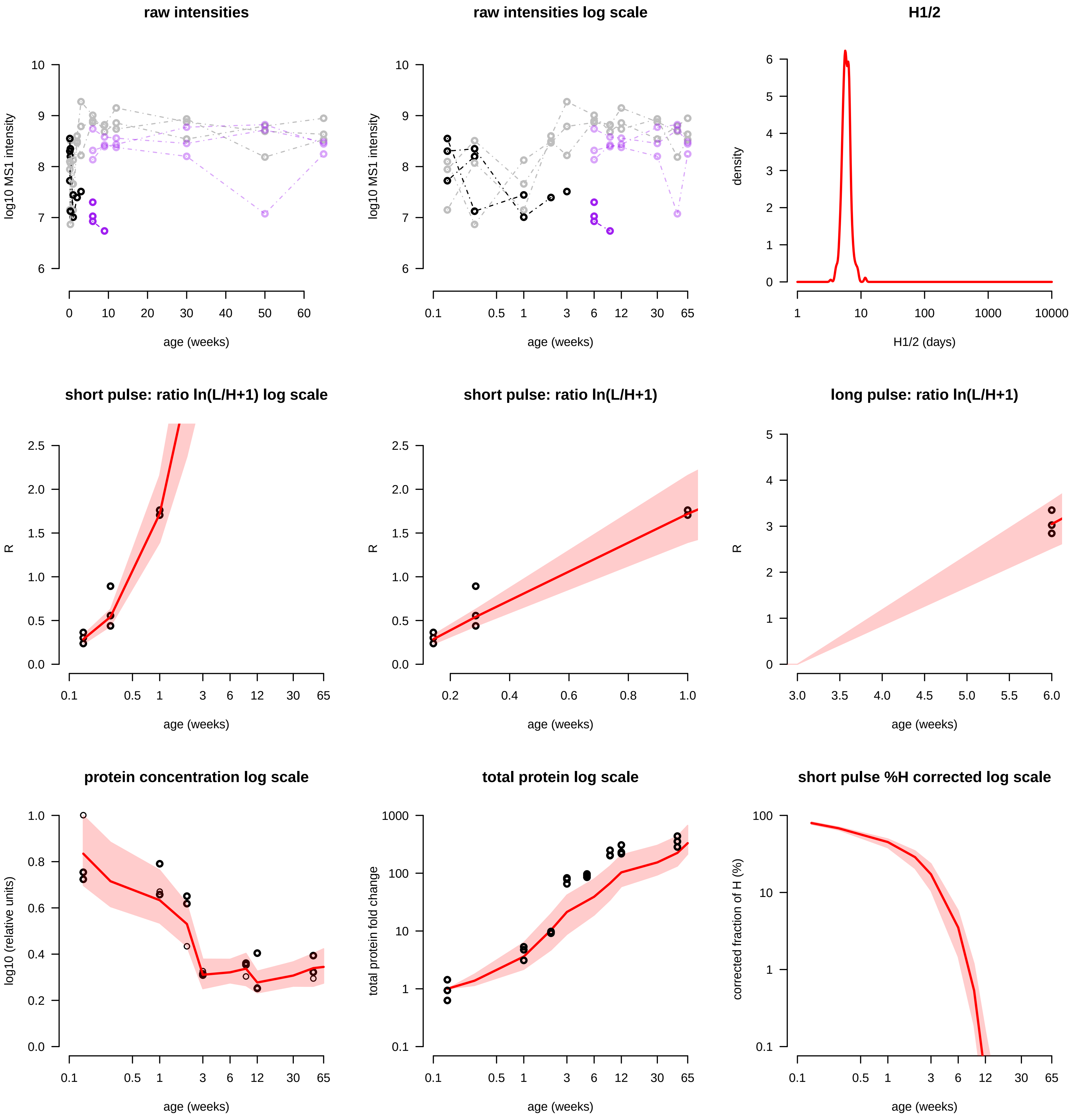

Q9JHW4

>sp|Q9JHW4|SELB_MOUSE

Selenocysteine−specific elongation factor

GN=Eefsec

Med H1/2: 4.2 days

Q5 H1/2: 3.4 days

Q95 H1/2: 5.4 days

reliable model fit: yes

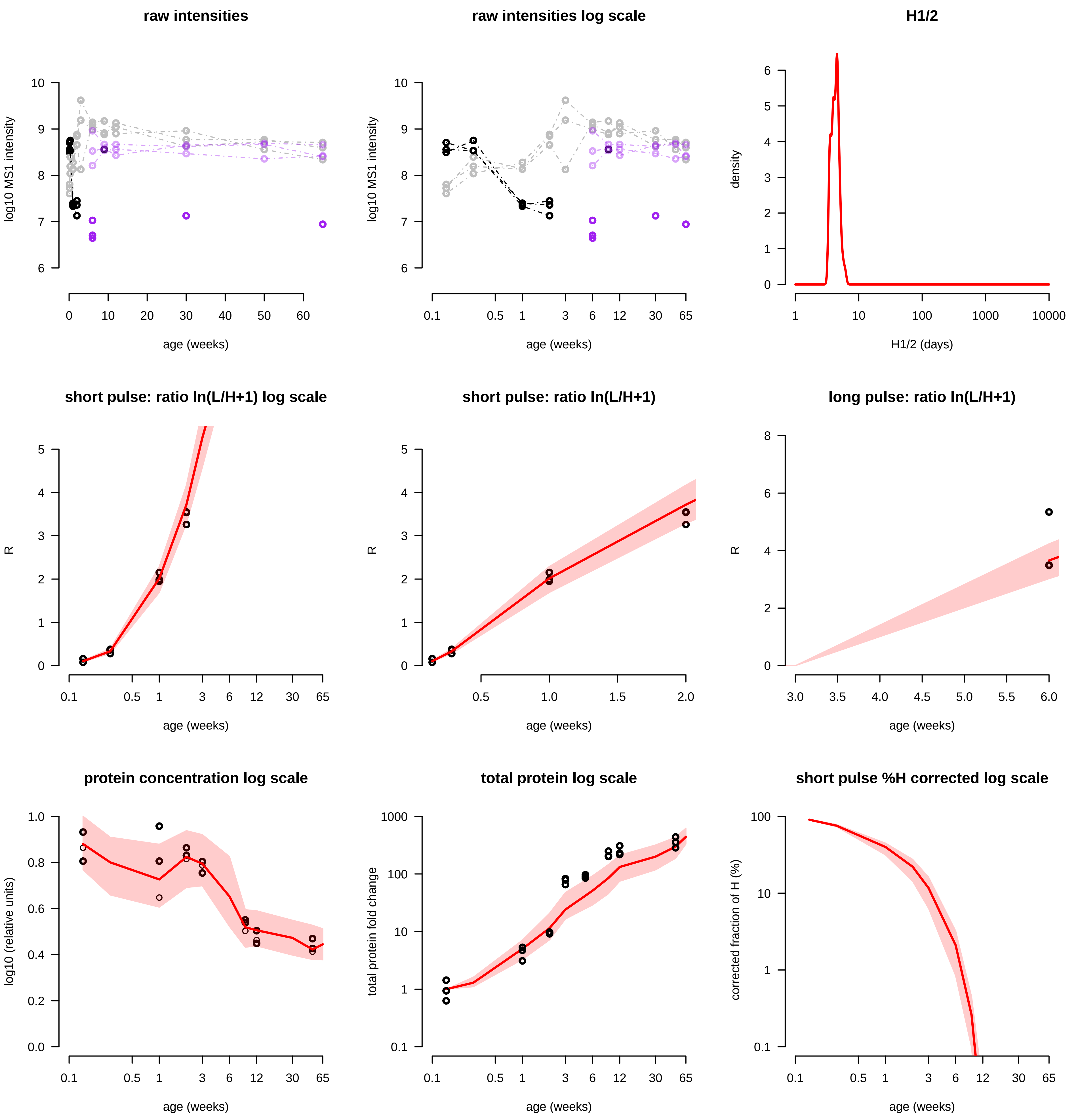

Q8K2K6−3;Q8K2K6−1;Q8K2K6

>sp|Q8K2K6−3|AGFG1_MOUSE

Isoform 3 of Arf−GAP domain and FG repeat−containing protein 1

GN=Agfg1

Med H1/2: 14.4 days

Q5 H1/2: 6.4 days

Q95 H1/2: 88.3 days

reliable model fit: yes

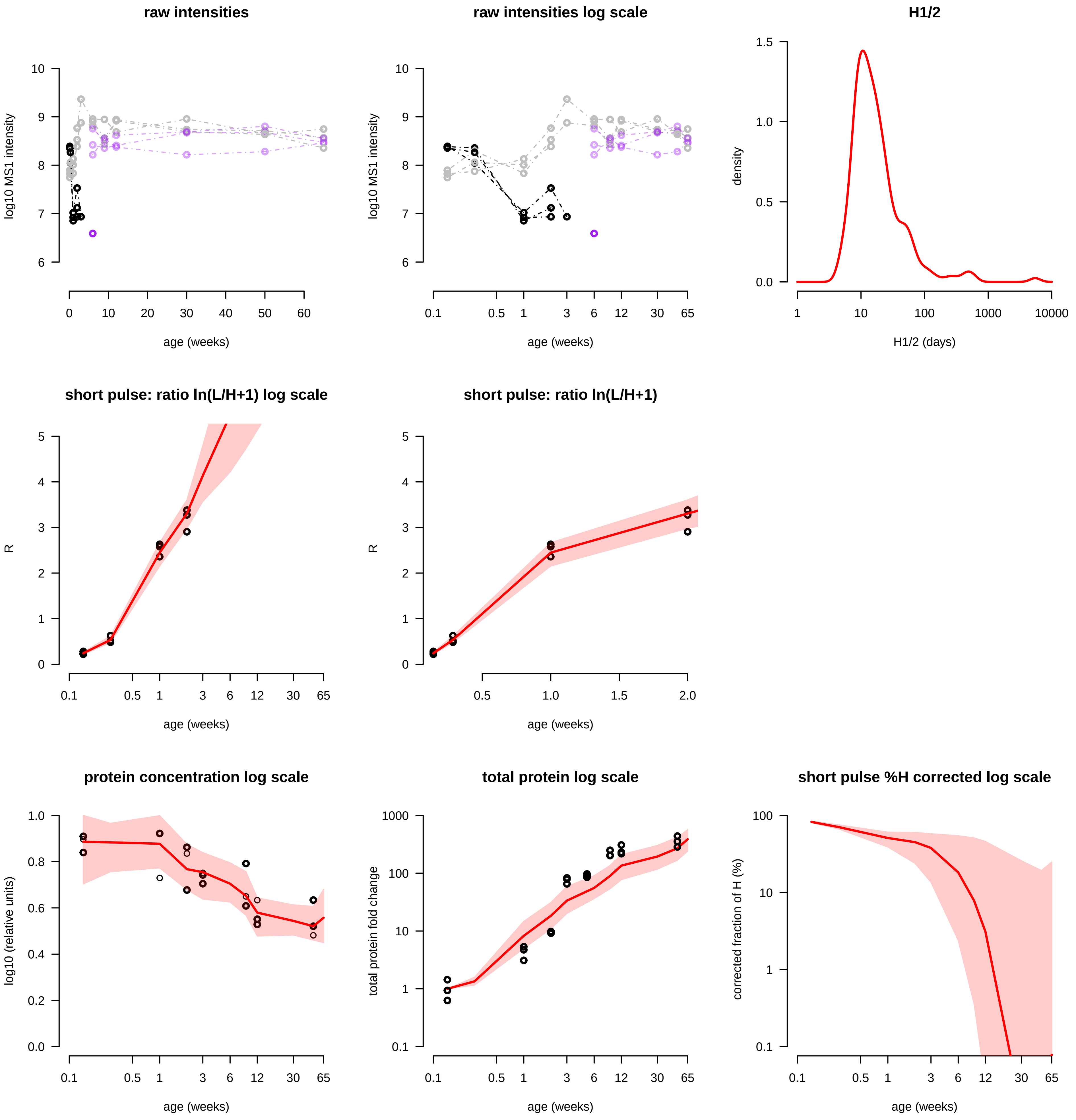

P46467

>sp|P46467|VPS4B_MOUSE

Vacuolar protein sorting−associated protein 4B

GN=Vps4b

Med H1/2: 3.6 days

Q5   H1/2: 3.5 days

Q95 H1/2: 5.1 days

reliable model fit: no

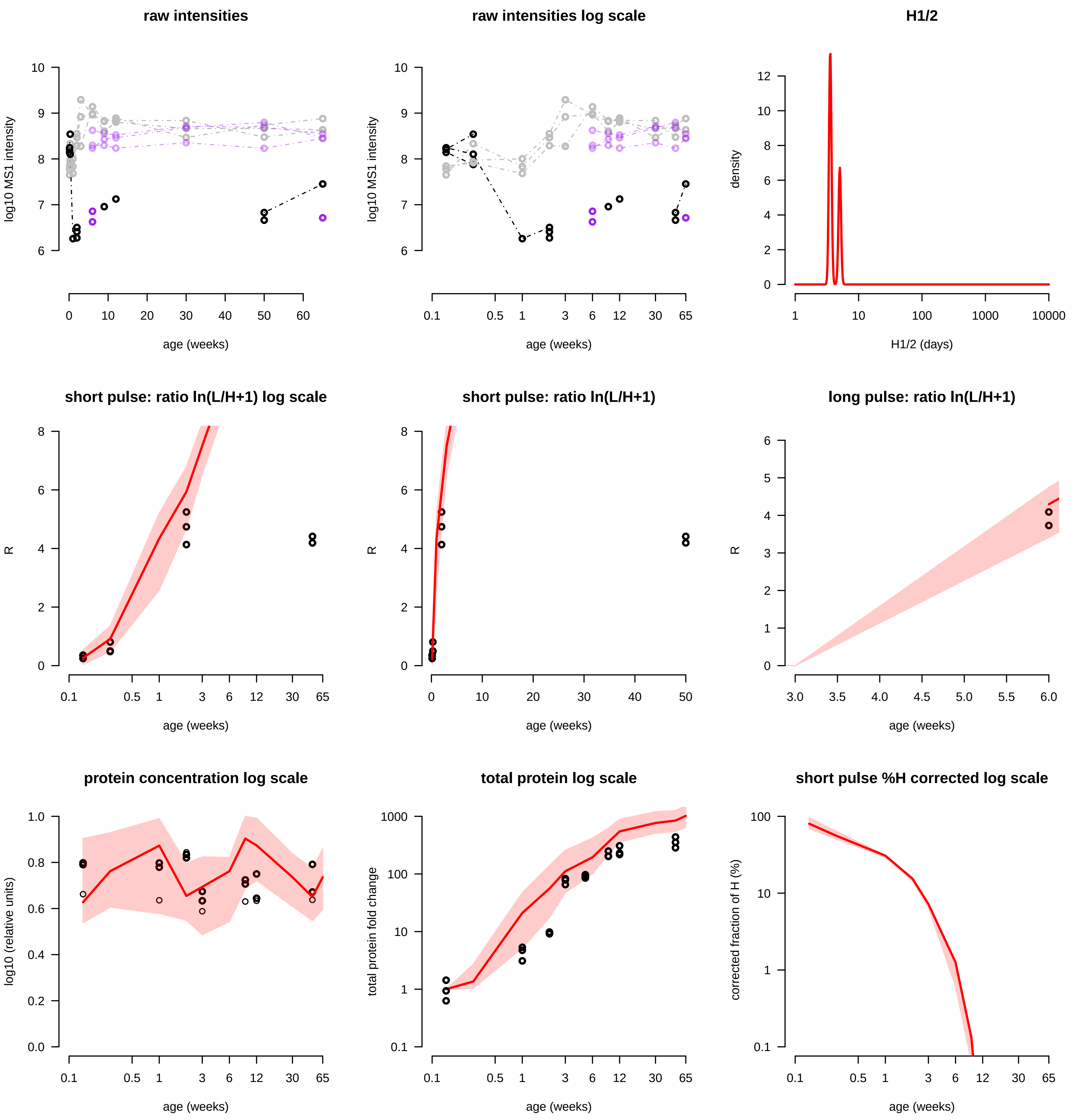

P61087

>sp|P61087|UBE2K_MOUSE

Ubiquitin−conjugating enzyme E2 K

GN=Ube2k

Med H1/2: 35.6 days

Q5 H1/2: 11.8 days

Q95 H1/2: 312.6 days

reliable model fit: yes

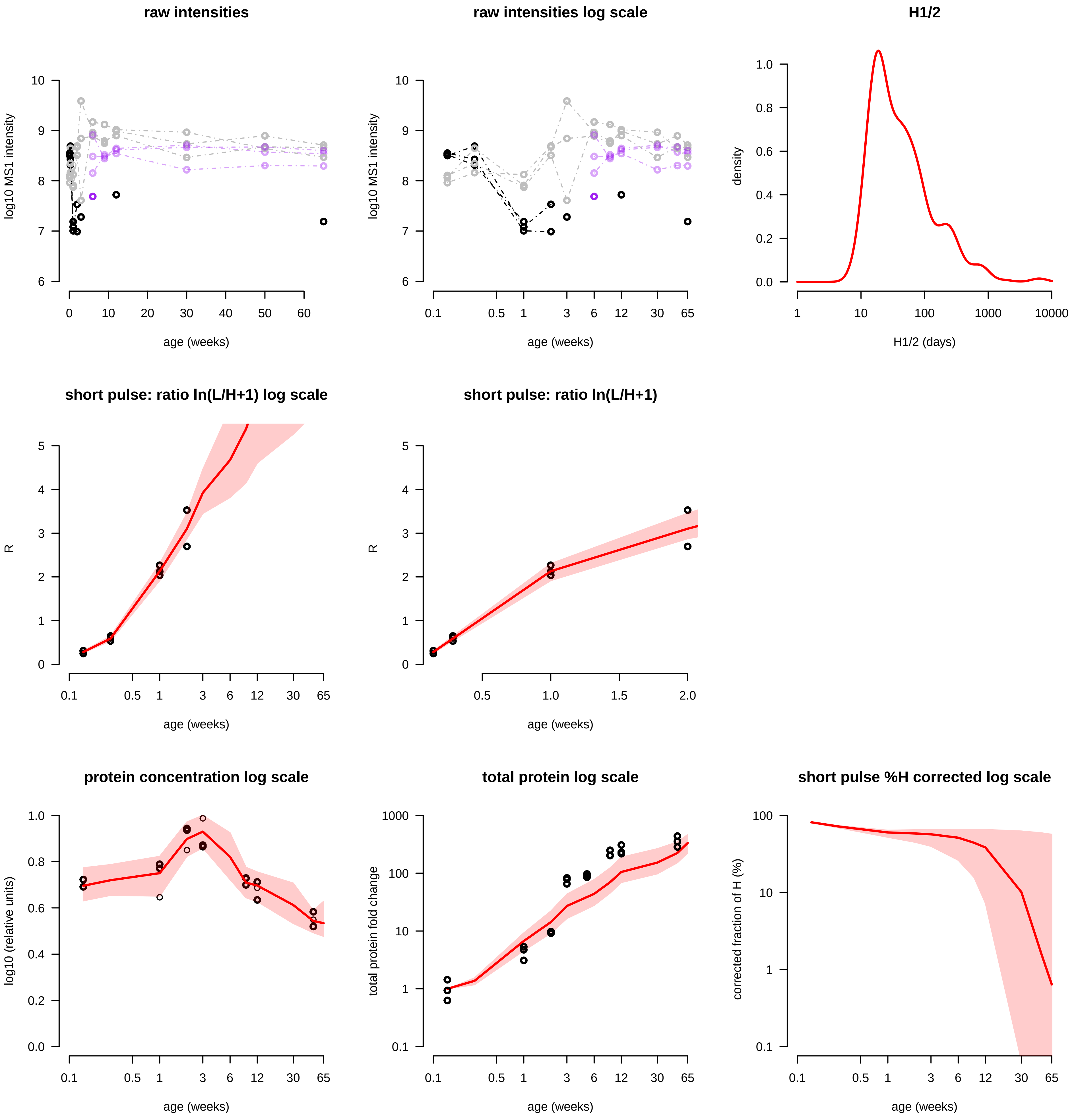

O35638

>sp|O35638|STAG2_MOUSE

Cohesin subunit SA−2

GN=Stag2

Med H1/2: 3 days

Q5 H1/2: 2.5 days

Q95 H1/2: 3.6 days

reliable model fit: yes

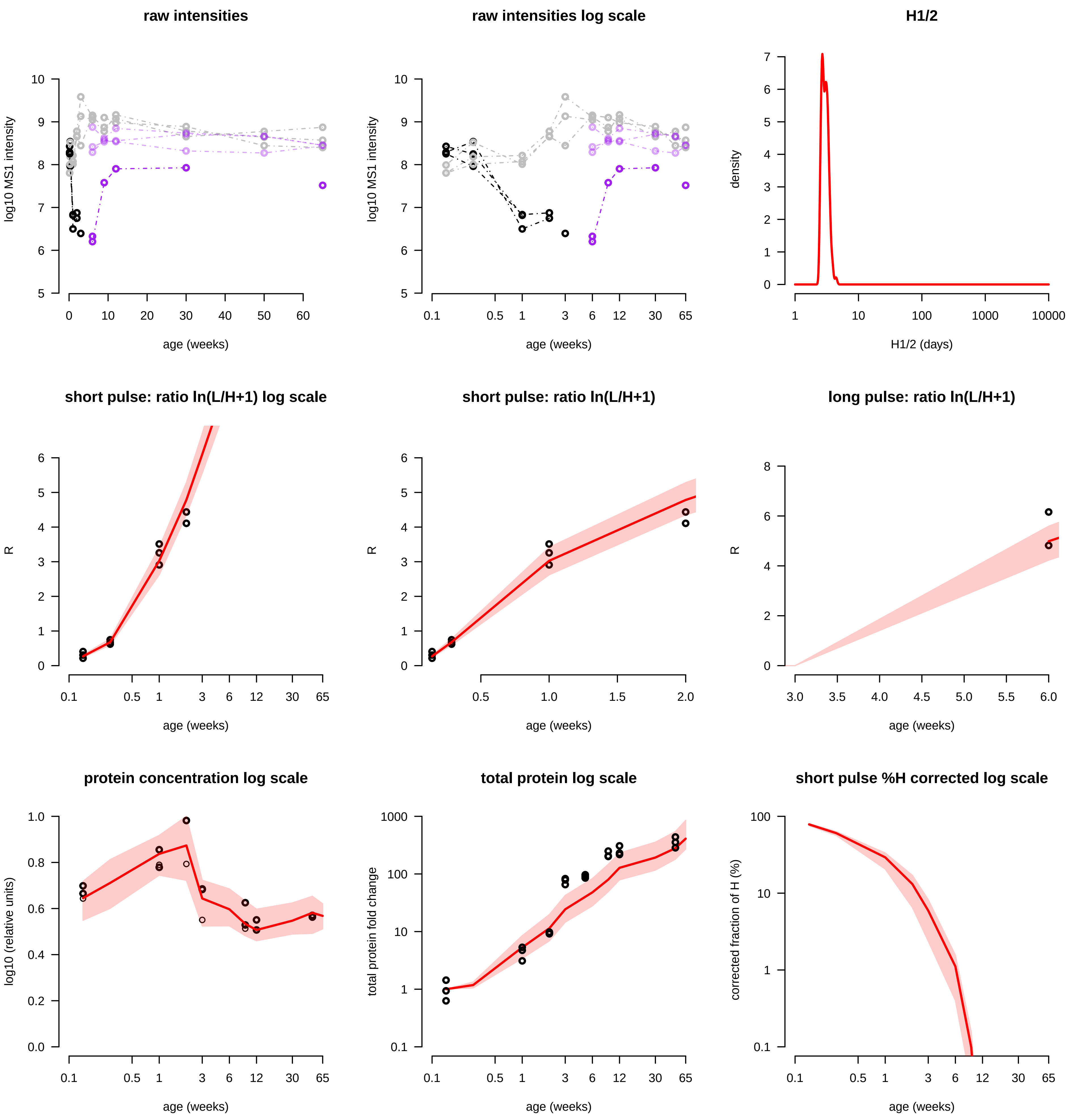

P61965

>sp|P61965|WDR5_MOUSE

WD repeat−containing protein 5

GN=Wdr5

Med H1/2: 9.4 days

Q5 H1/2: 4.2 days

Q95 H1/2: 70.5 days

reliable model fit: yes

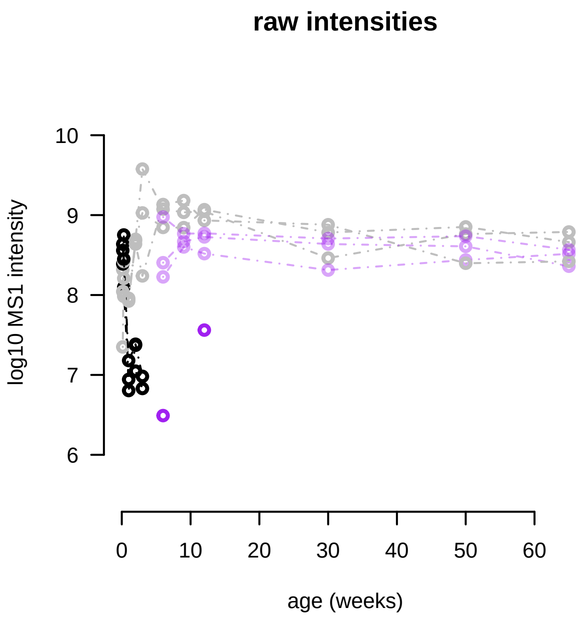

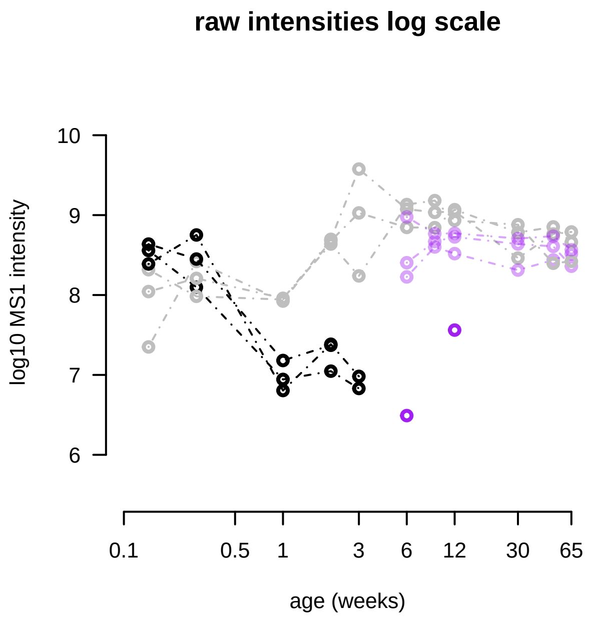

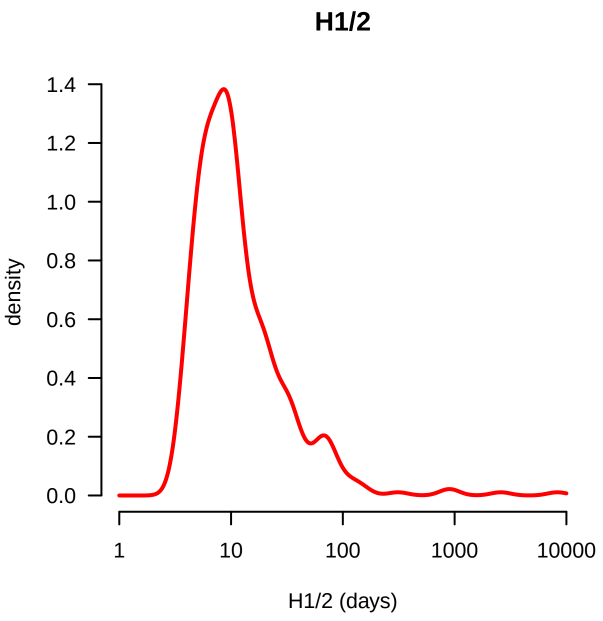

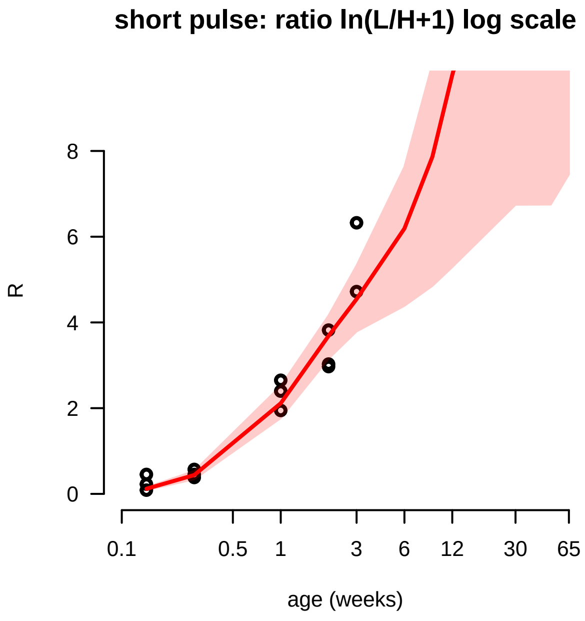

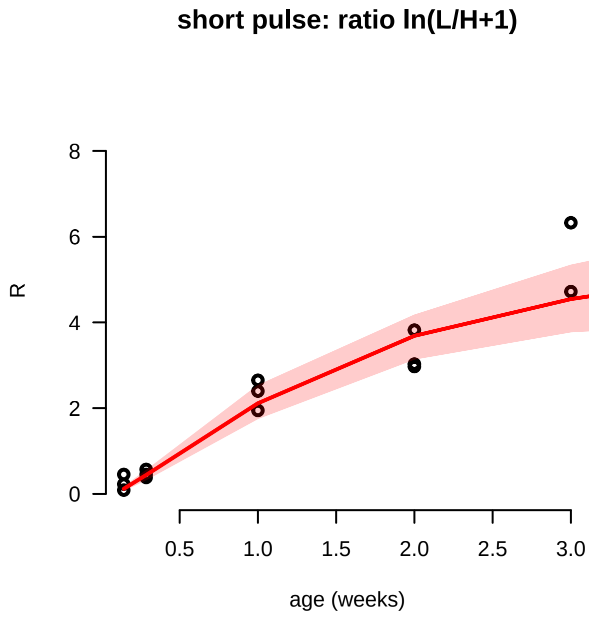

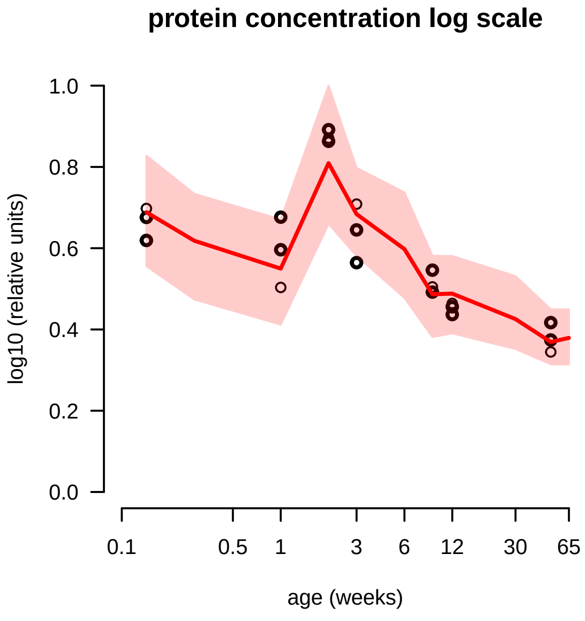

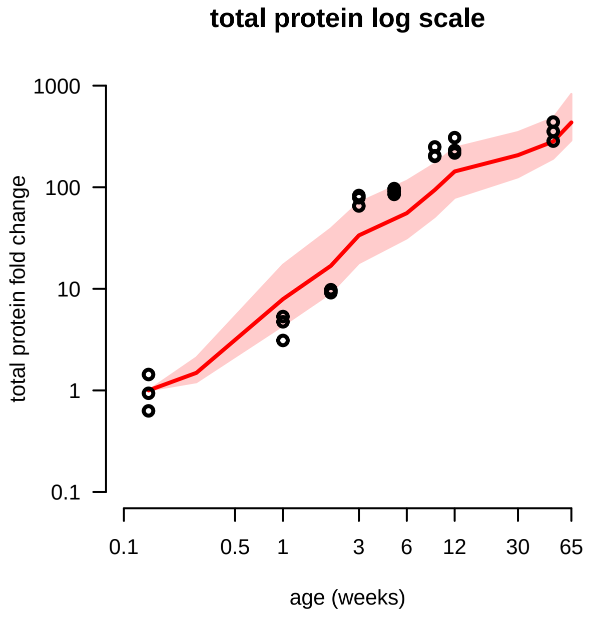

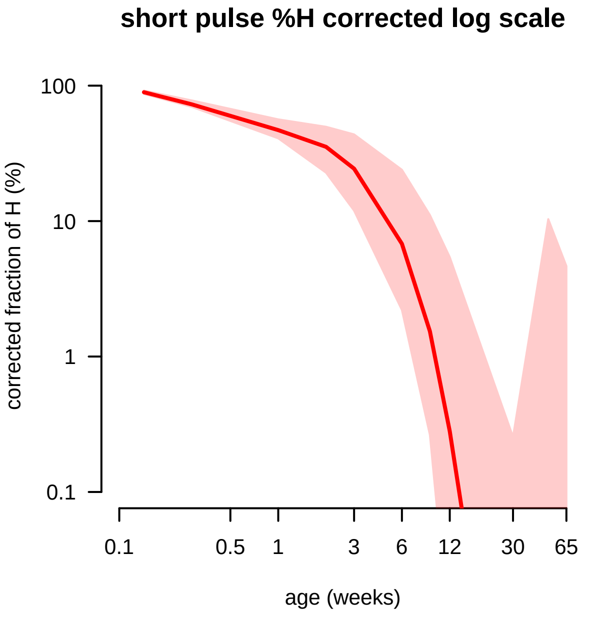

Q9CQD1

>sp|Q9CQD1|RAB5A_MOUSE

Ras−related protein Rab−5A

GN=Rab5a

Med H1/2: 4.8 days

Q5 H1/2: 3.5 days

Q95 H1/2: 7.2 days

reliable model fit: no

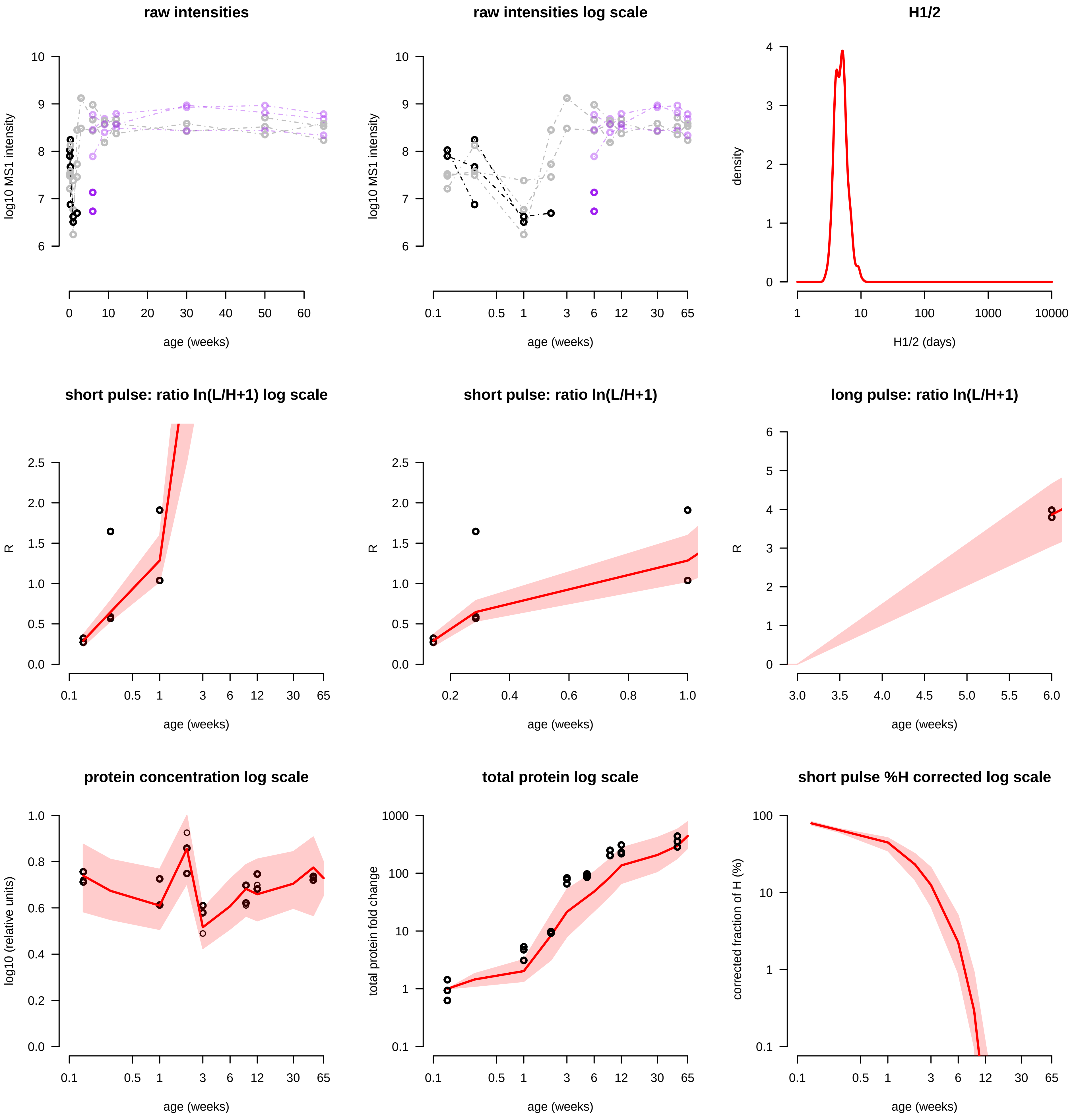

Q9CSN1

>sp|Q9CSN1|SNW1_MOUSE

SNW domain−containing protein 1

GN=Snw1

Med H1/2: 3.3 days

Q5 H1/2: 2.7 days

Q95 H1/2: 4.3 days

reliable model fit: yes

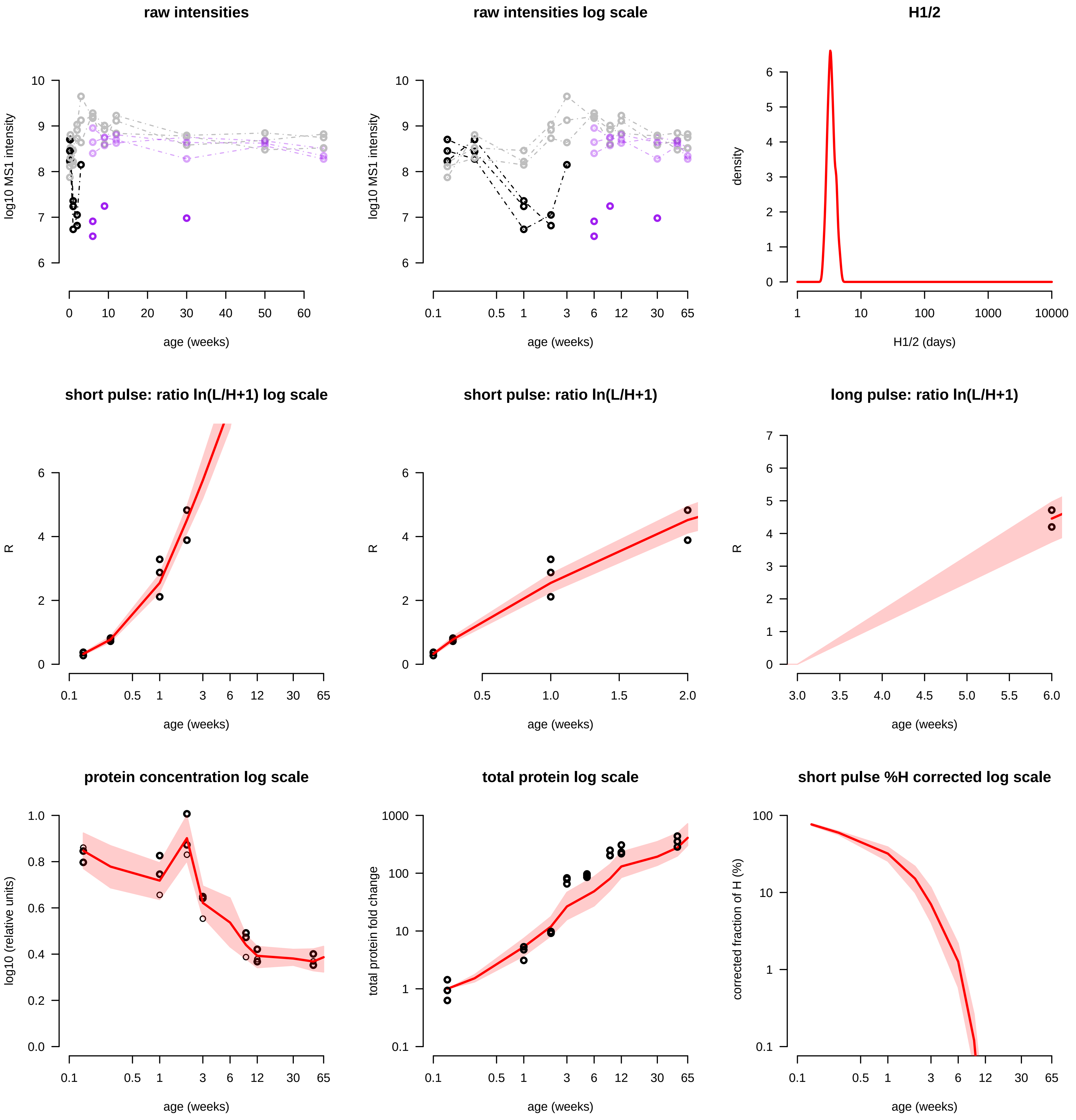

Q9CQV6

>sp|Q9CQV6|MLP3B_MOUSE

Microtubule−associated proteins 1A/1B light chain 3B

GN=Map1lc3b

Med H1/2: 13.8 days

Q5 H1/2: 5.3 days

Q95 H1/2: 137.3 days

reliable model fit: yes

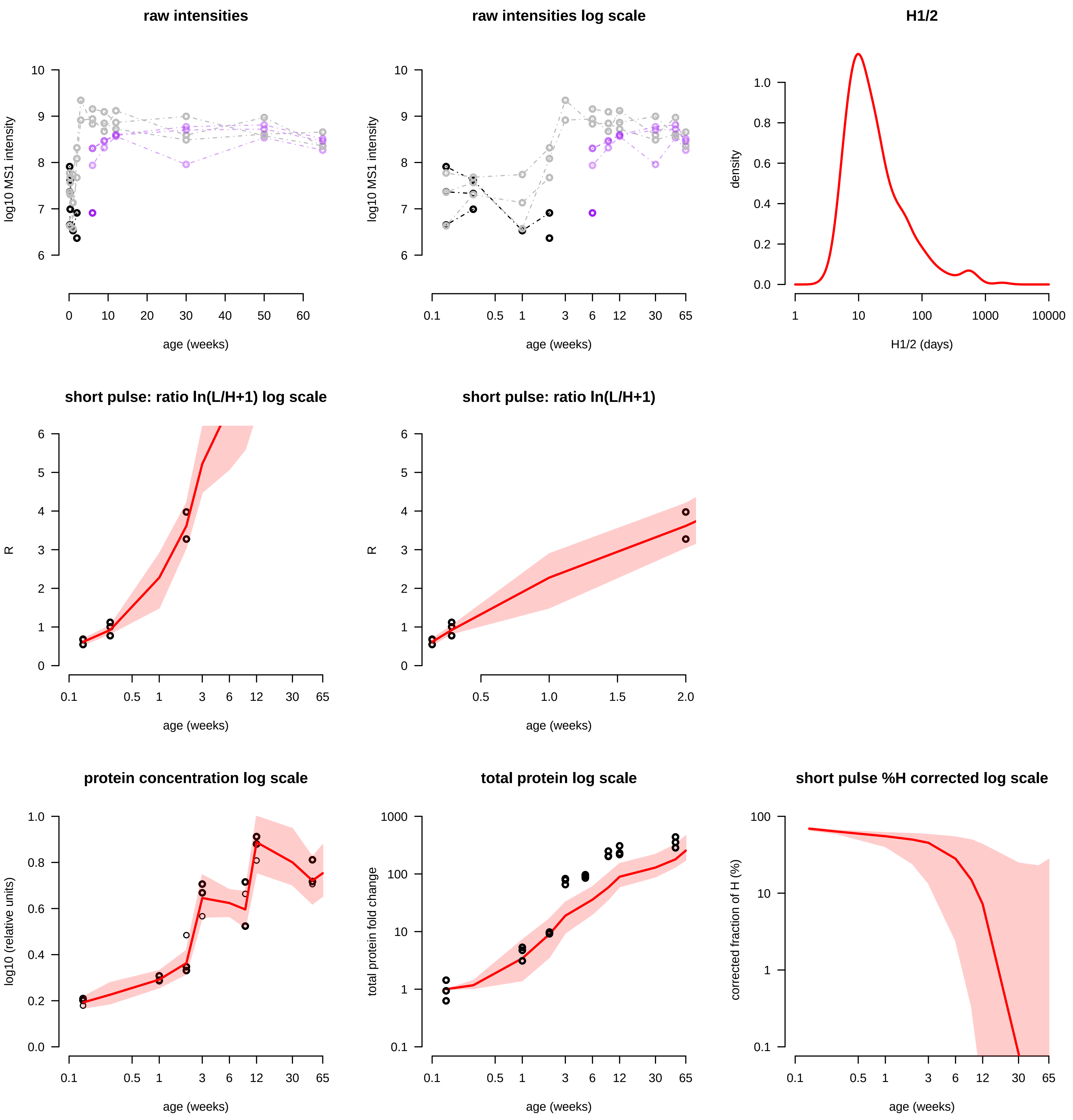

Q9CXW2

>sp|Q9CXW2|RT22_MOUSE

28S ribosomal protein S22, mitochondrial

GN=Mrps22

Med H1/2: 9.3 days

Q5 H1/2: 3.6 days

Q95 H1/2: 578.2 days

reliable model fit: yes

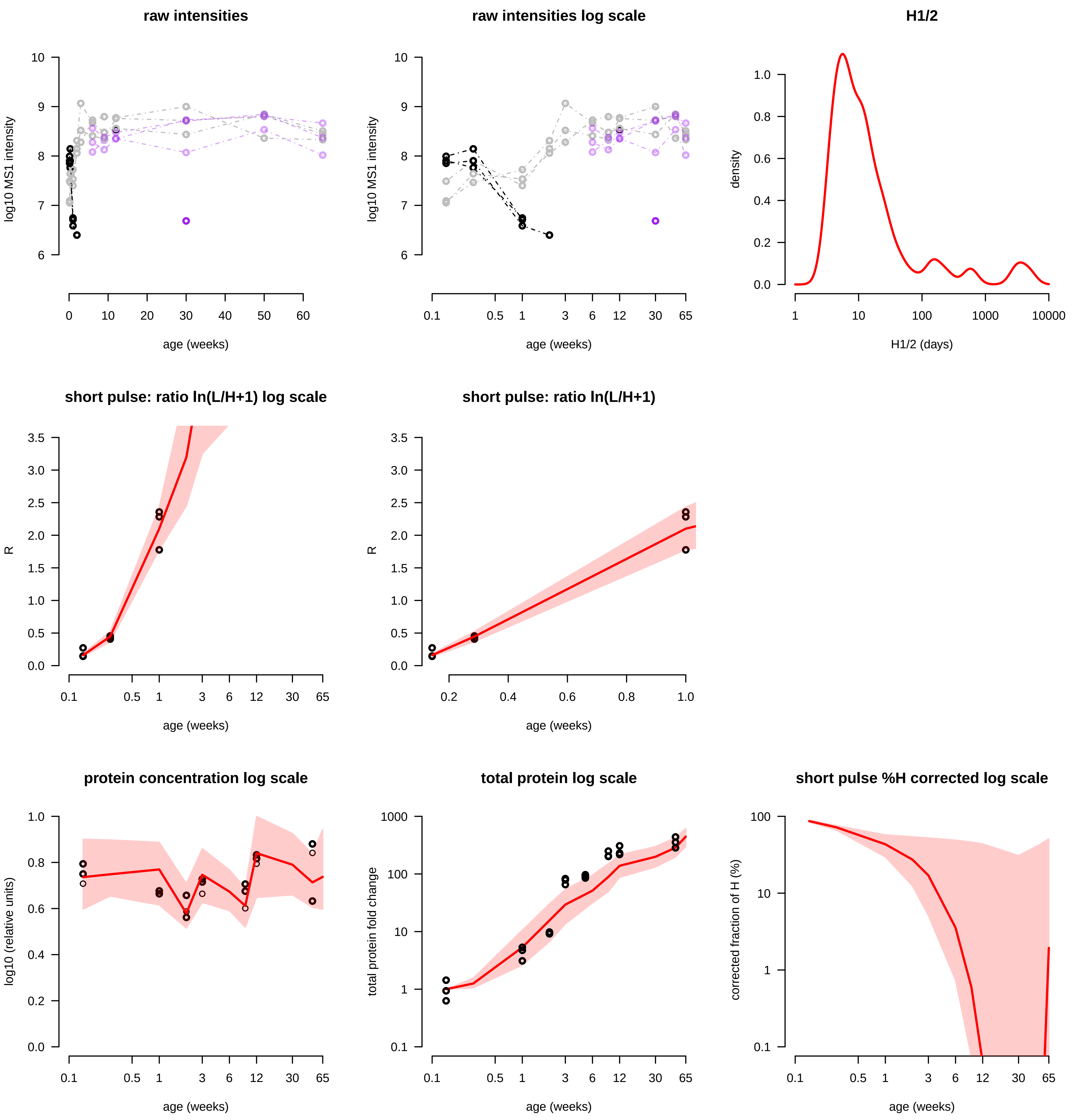

P09242

>sp|P09242|PPBT_MOUSE

Alkaline phosphatase, tissue−nonspecific isozyme

GN=Alpl

Med H1/2: 5.6 days

Q5 H1/2: 3.8 days

Q95 H1/2: 10.7 days

reliable model fit: yes

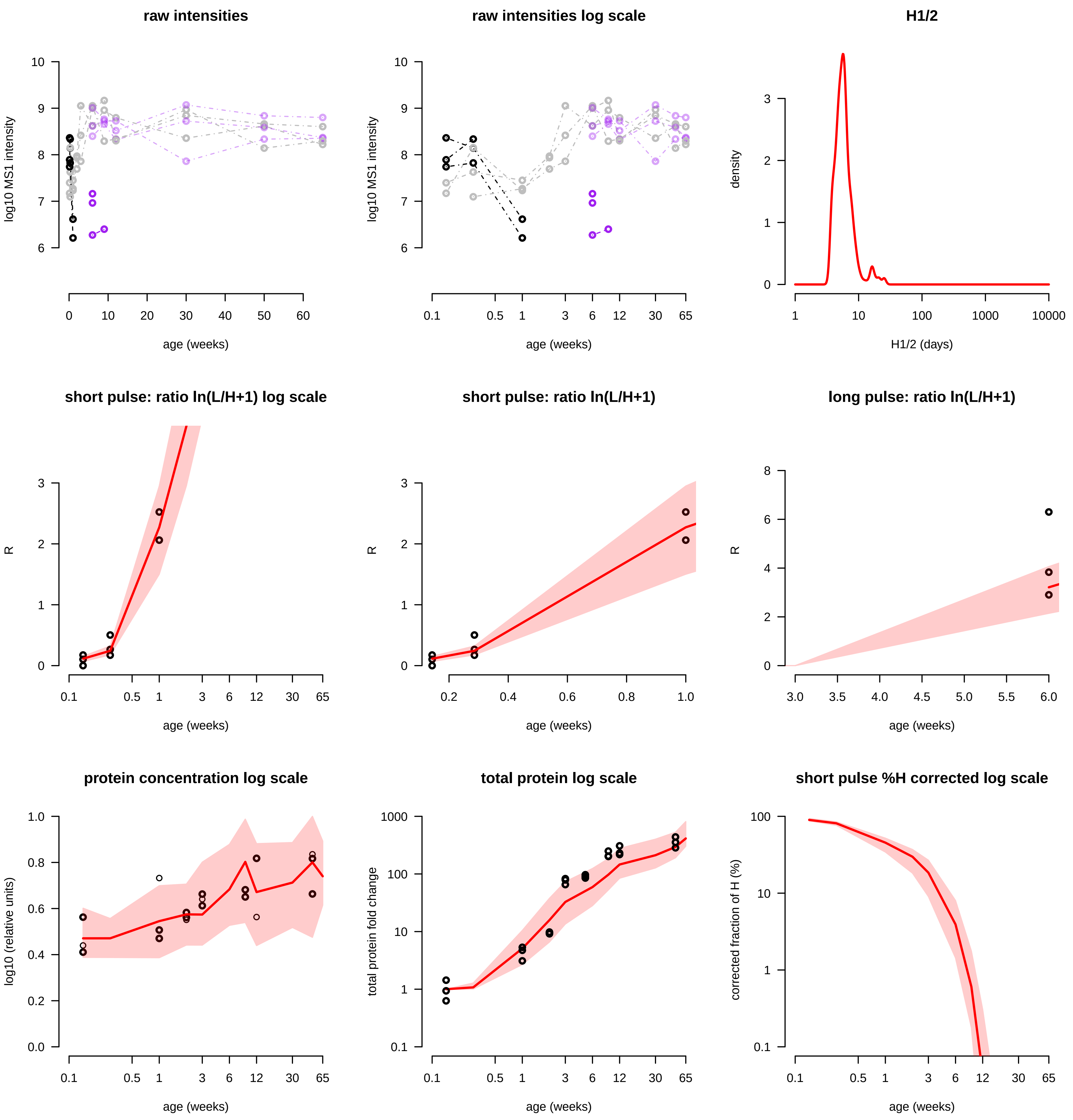

P58242

>sp|P58242|ASM3B_MOUSE

Acid sphingomyelinase−like phosphodiesterase 3b

GN=Smpdl3b

Med H1/2: 4.9 days

Q5 H1/2: 3.6 days

Q95 H1/2: 8 days

reliable model fit: yes

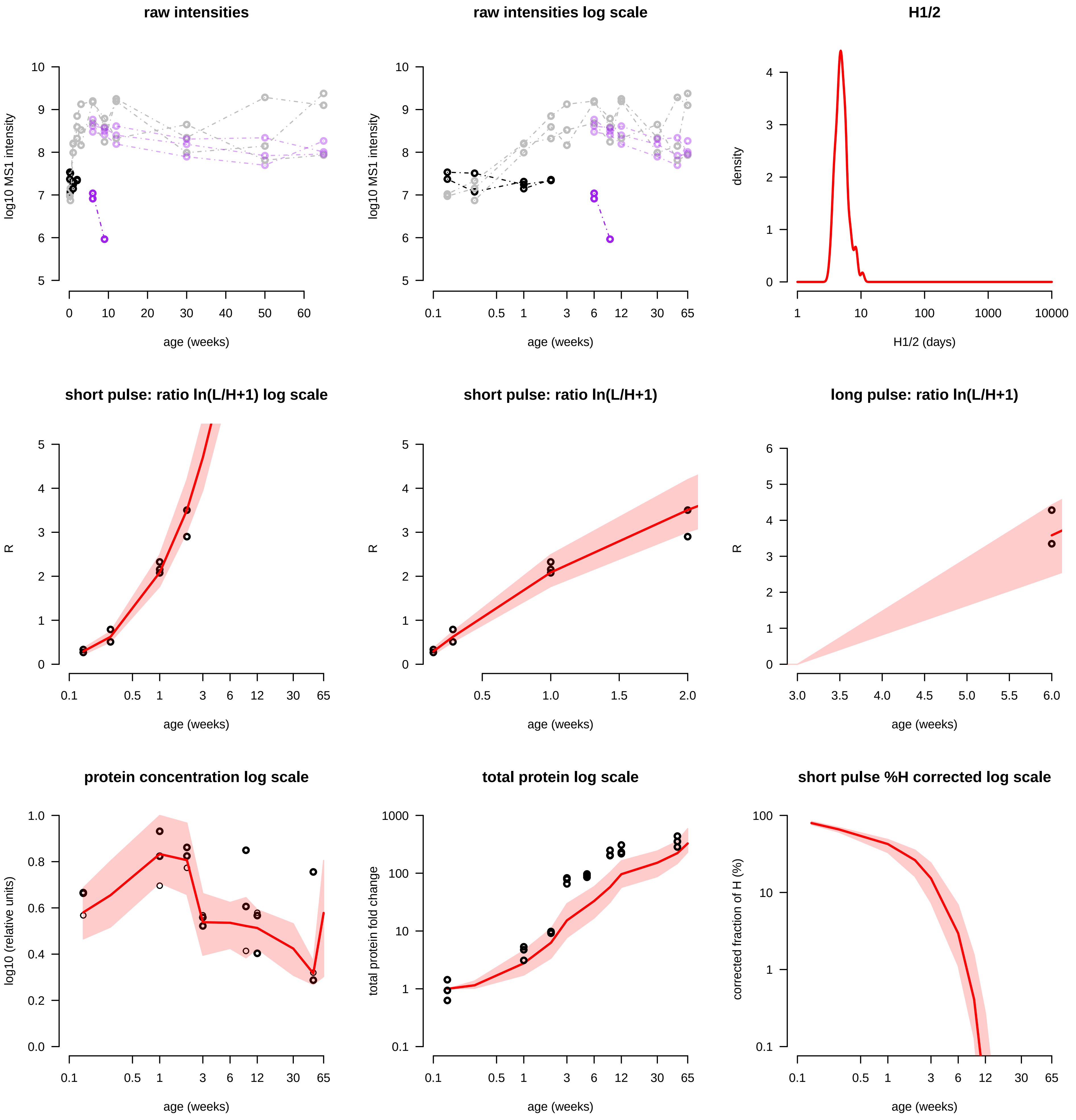

Q99104

>sp|Q99104|MYO5A_MOUSE

Unconventional myosin−Va

GN=Myo5a

Med H1/2: 880.8 days

Q5 H1/2: 315.5 days

Q95 H1/2: 8363 days

reliable model fit: no

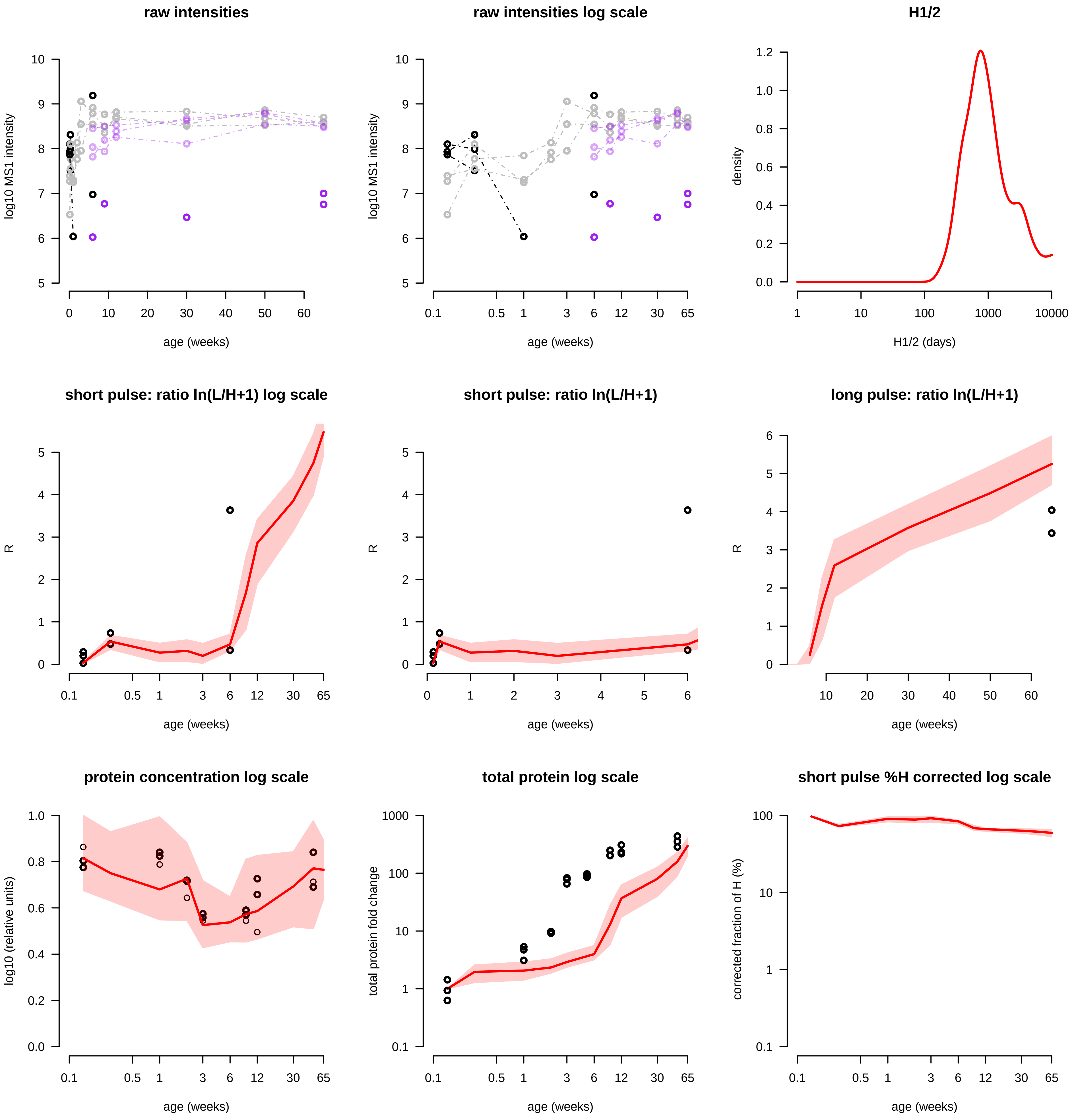

Q9R257

>sp|Q9R257|HEBP1_MOUSE

Heme−binding protein 1

GN=Hebp1

Med H1/2: 7.4 days

Q5 H1/2: 4 days

Q95 H1/2: 41 days

reliable model fit: yes

raw intensities

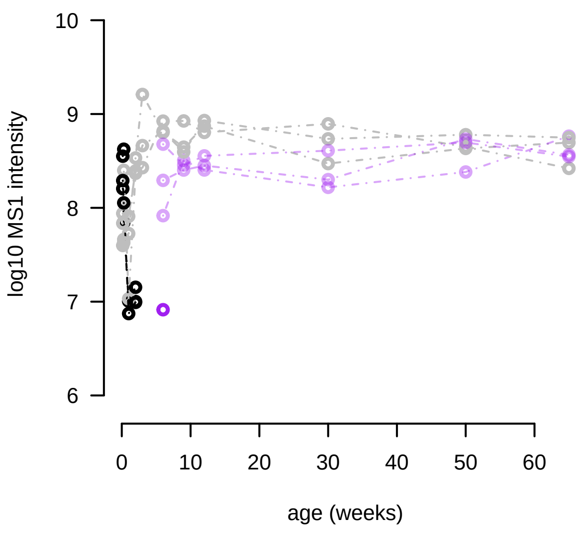

raw intensities log scale

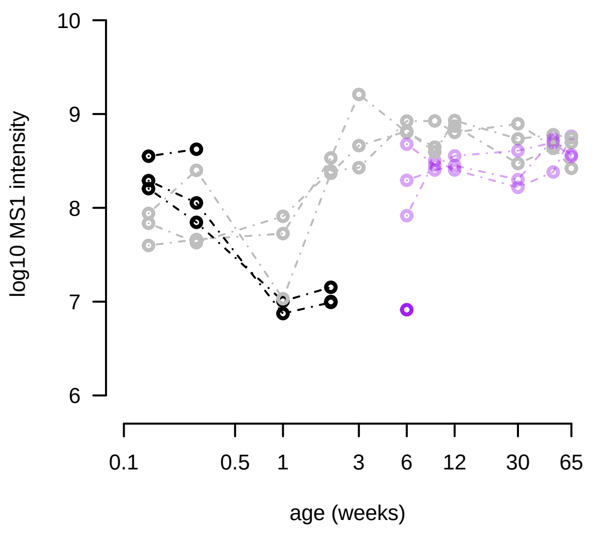

H1/2

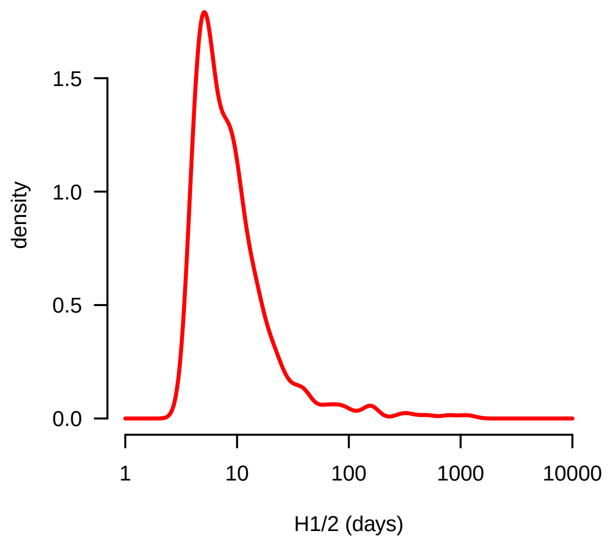

short pulse: ratio ln(L/H+1) log scale

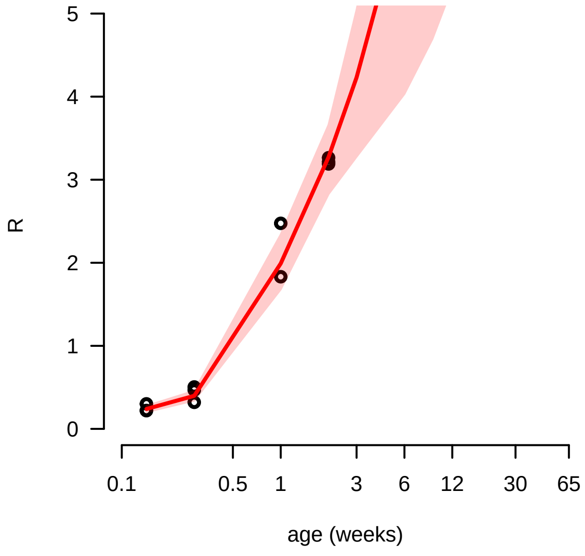

short pulse: ratio ln(L/H+1)

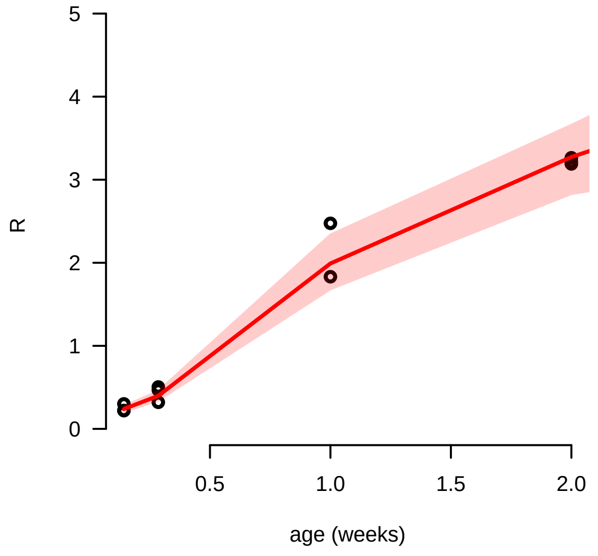

protein concentration log scale

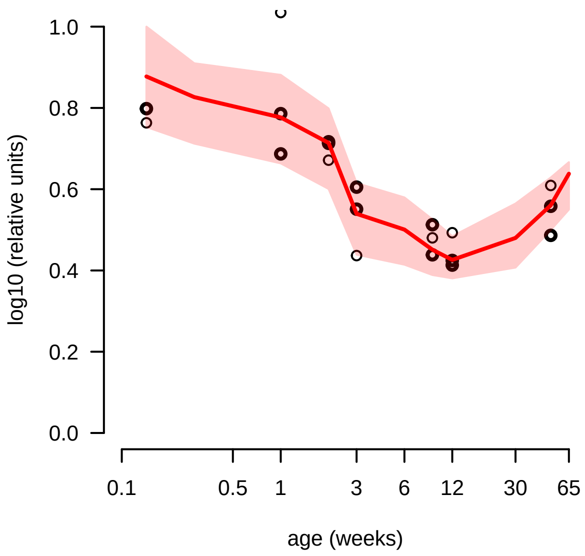

total protein log scale

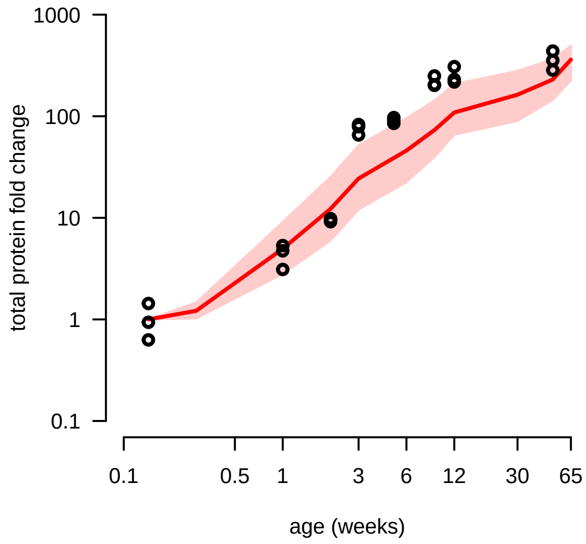

short pulse %H corrected log scale

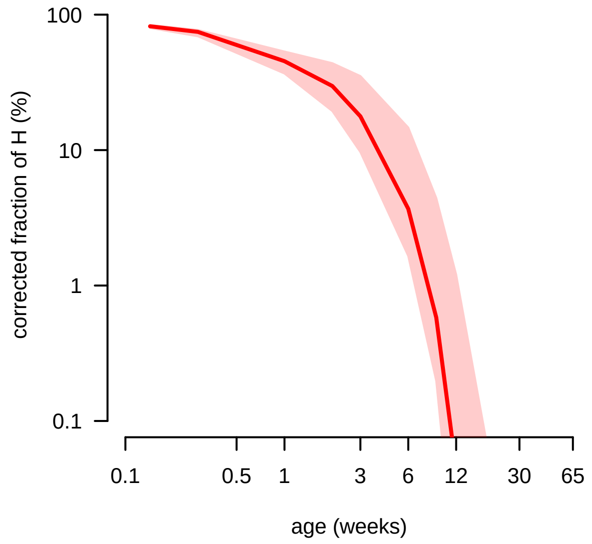

Q8VEH8

>sp|Q8VEH8|ERLEC_MOUSE

Endoplasmic reticulum lectin 1

GN=Erlec1

Med H1/2: 9.5 days

Q5 H1/2: 3.1 days

Q95 H1/2: 78.6 days

reliable model fit: yes

raw intensities

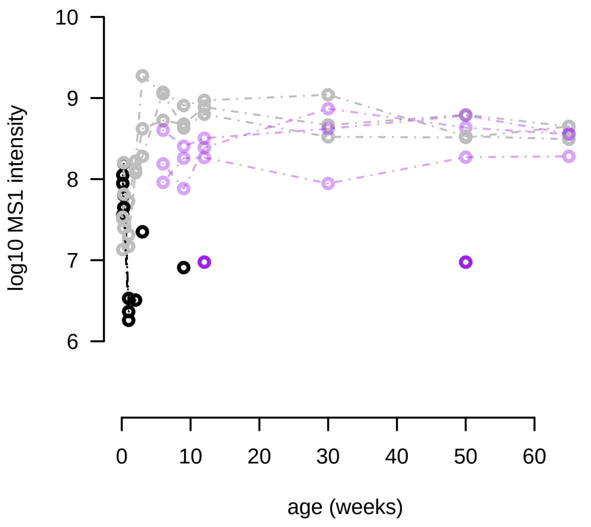

raw intensities log scale

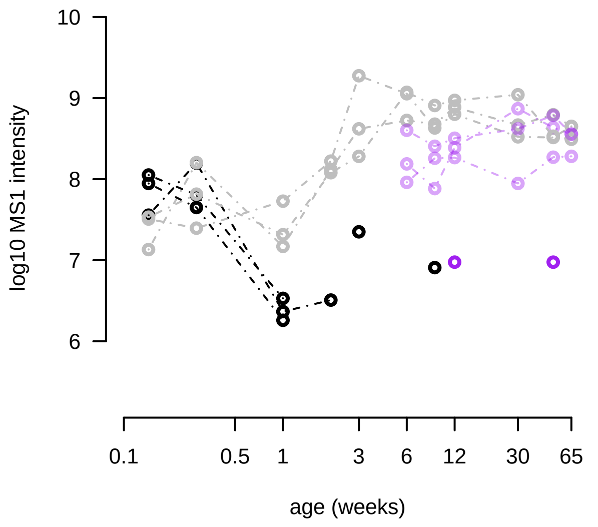

H1/2

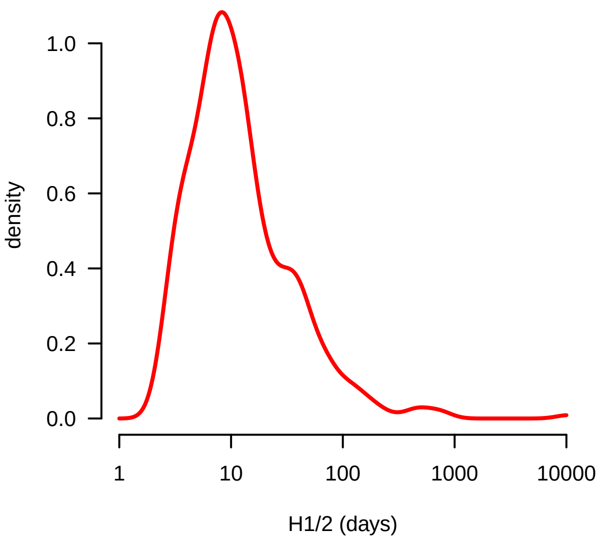

short pulse: ratio ln(L/H+1) log scale

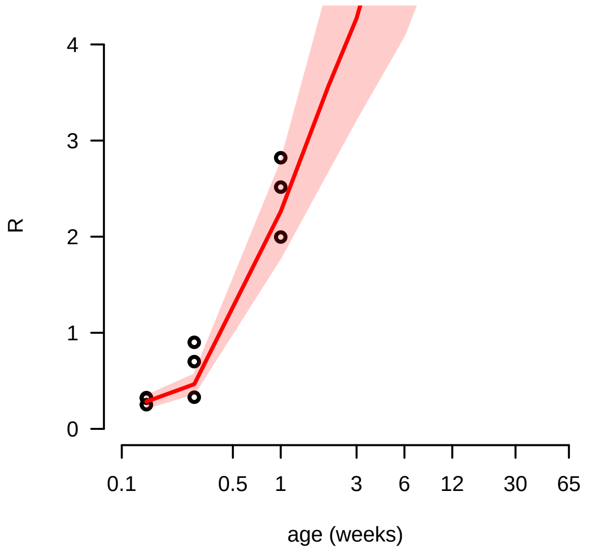

short pulse: ratio ln(L/H+1)

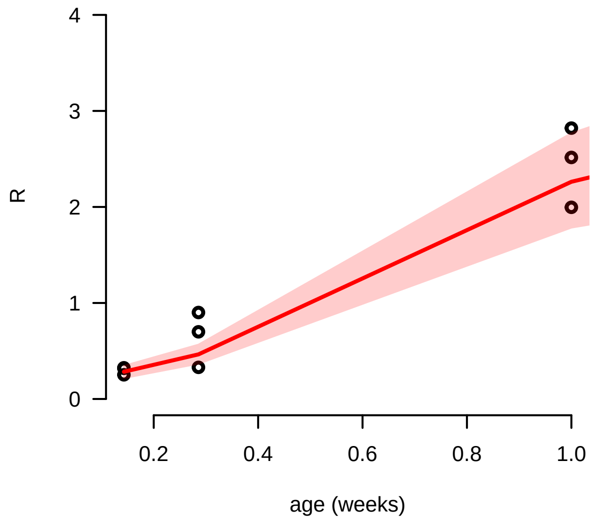

protein concentration log scale

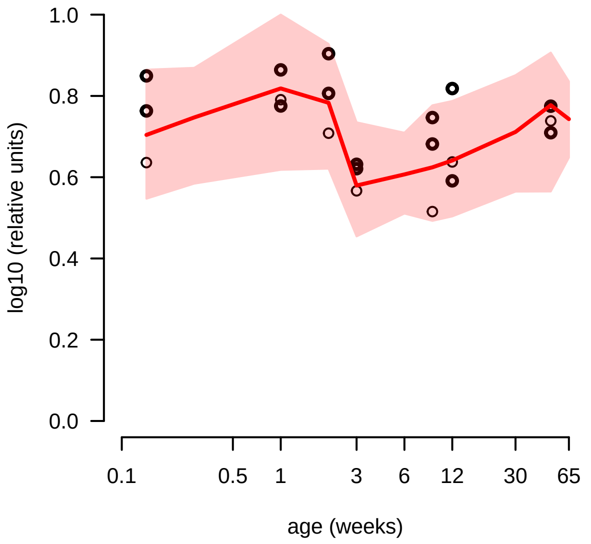

total protein log scale

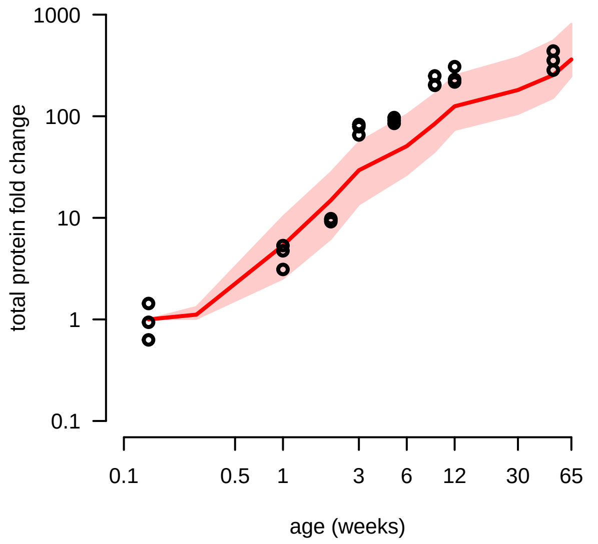

short pulse %H corrected log scale

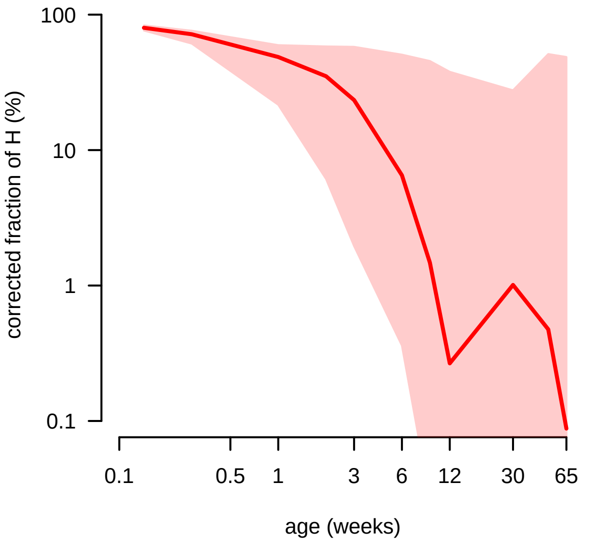

Q8BH97

>sp|Q8BH97|RCN3_MOUSE

Reticulocalbin−3

GN=Rcn3

Med H1/2: 50.4 days

Q5 H1/2: 17.4 days

Q95 H1/2: 452.9 days

reliable model fit: no

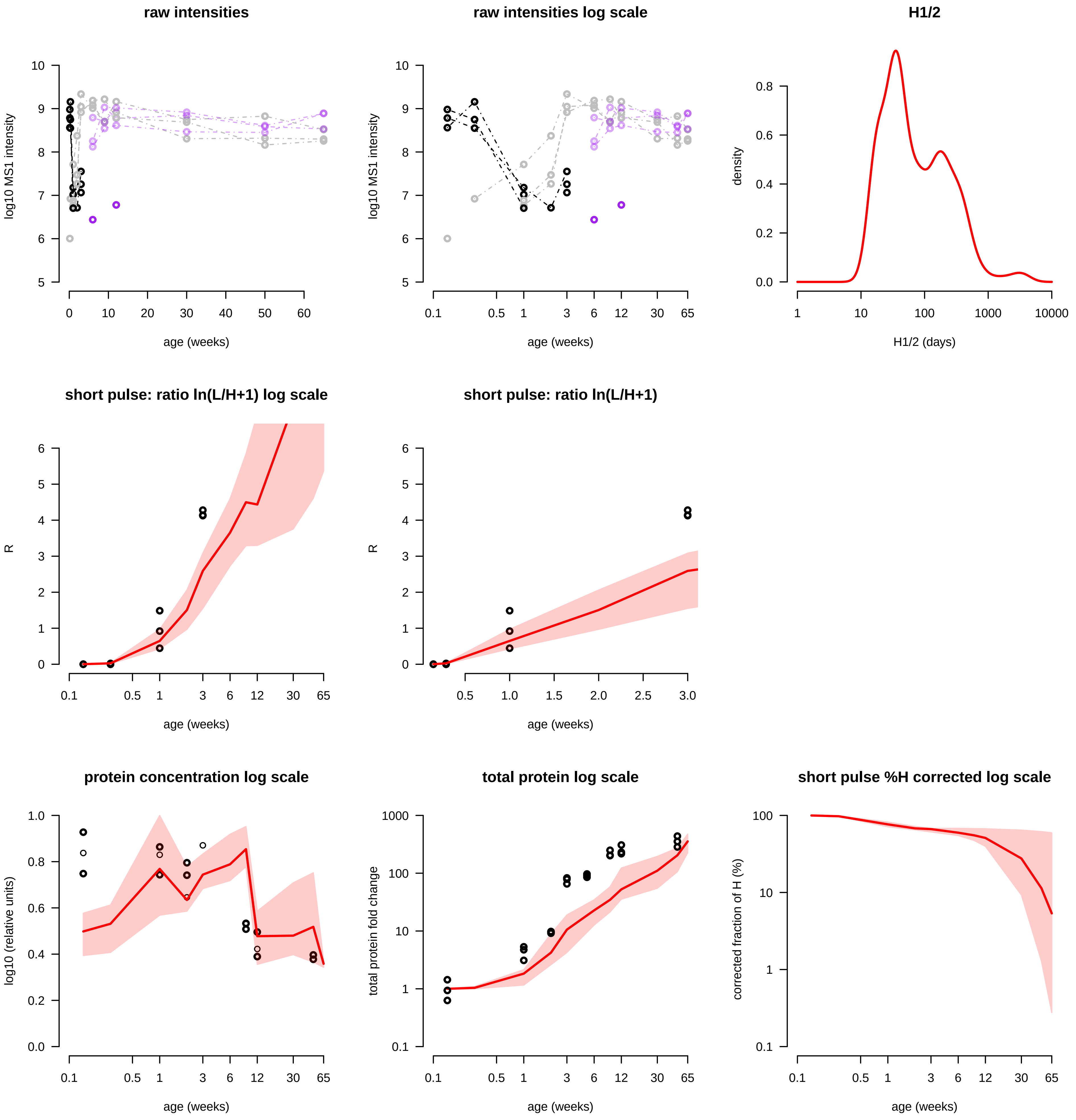

Q9R0N0

>sp|Q9R0N0|GALK1_MOUSE

Galactokinase

GN=Galk1

Med H1/2: 4.8 days

Q5 H1/2: 4 days

Q95 H1/2: 6 days

reliable model fit: yes

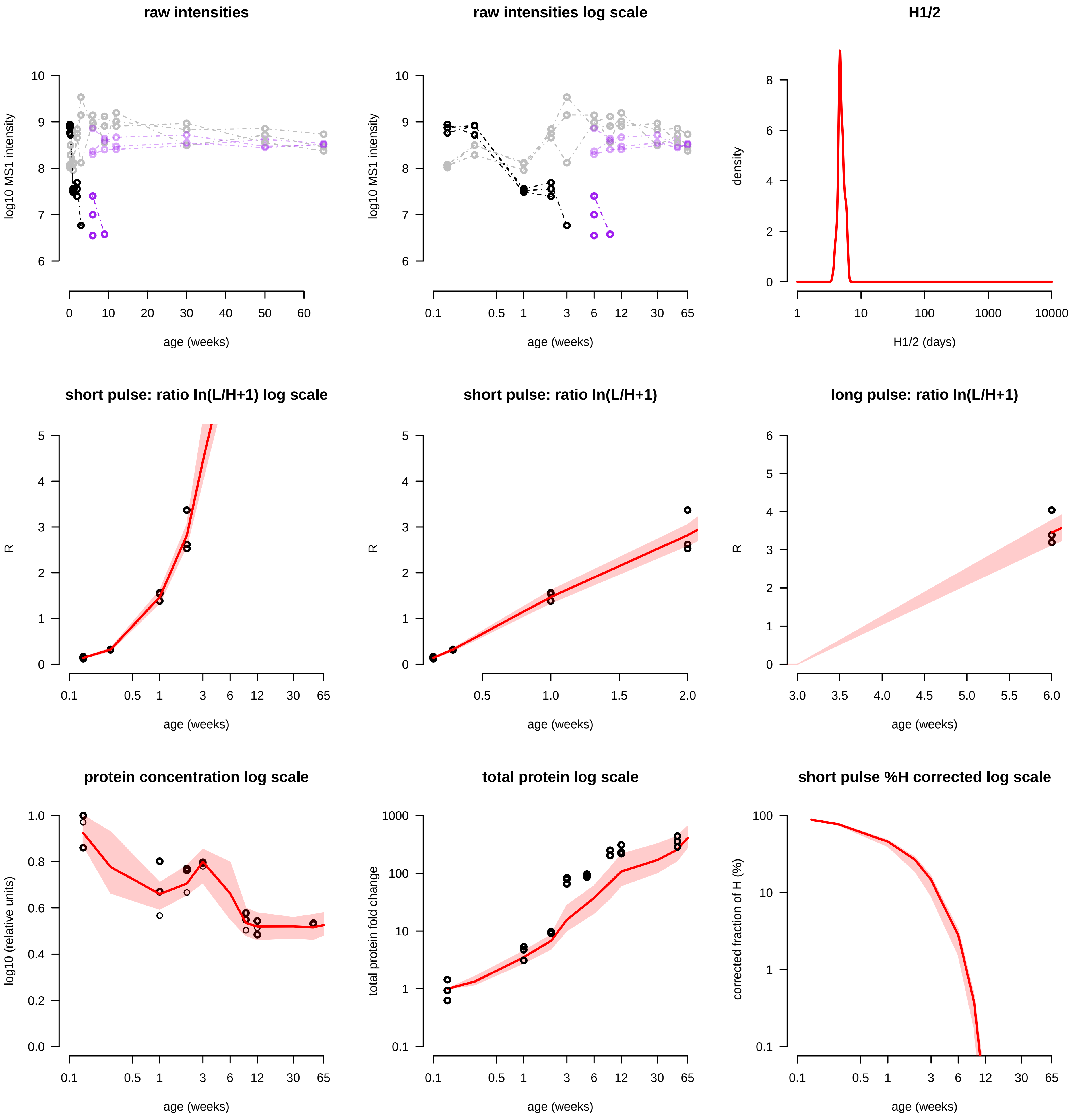

Q8VEE1

>sp|Q8VEE1|LMCD1_MOUSE

LIM and cysteine−rich domains protein 1

GN=Lmcd1

Med H1/2: 3.7 days

Q5 H1/2: 3 days

Q95 H1/2: 4.8 days

reliable model fit: yes

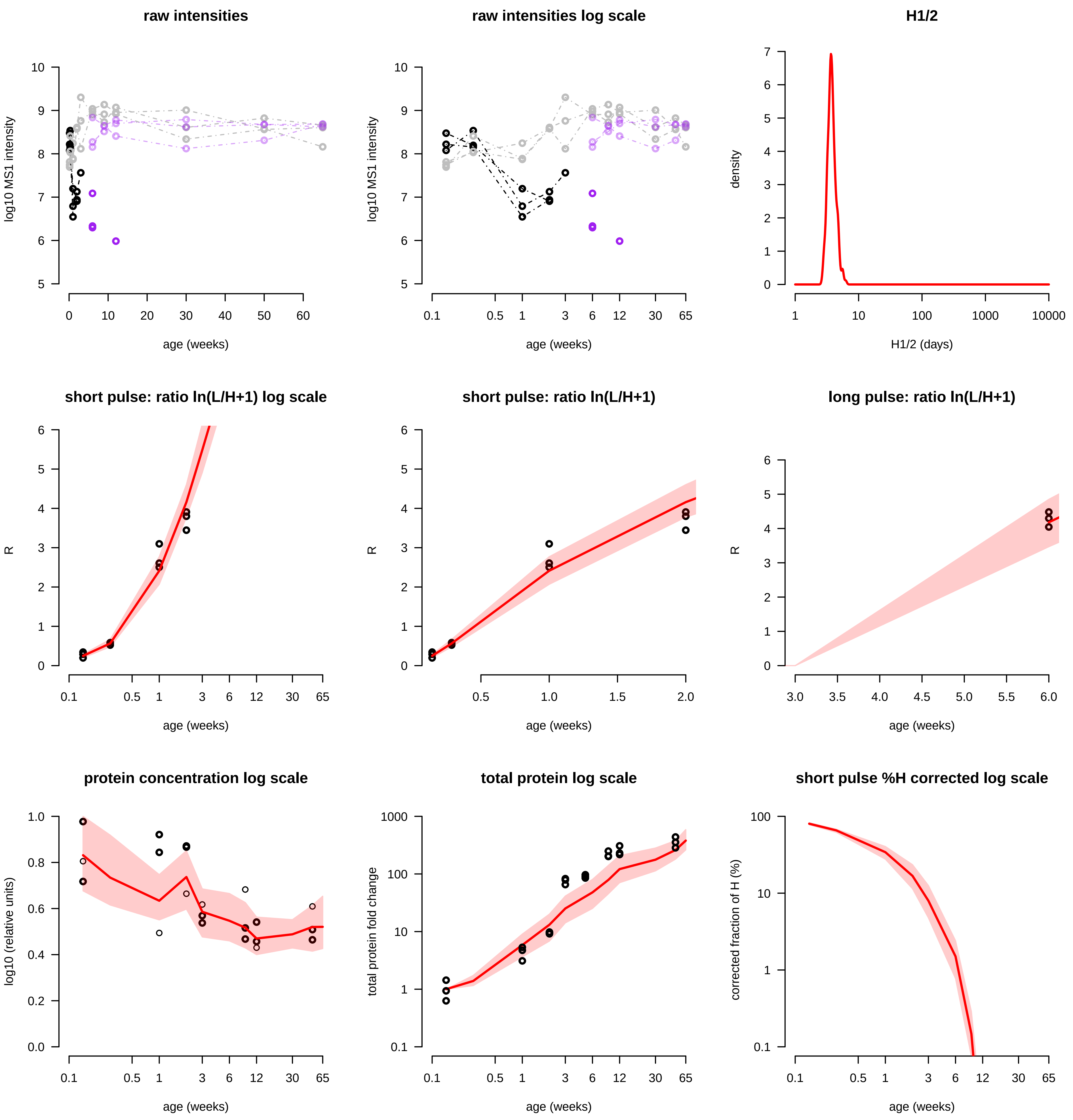

Q9WVQ5

>sp|Q9WVQ5|MTNB_MOUSE

Methylthioribulose−1−phosphate dehydratase

GN=Apip

Med H1/2: 10.1 days

Q5 H1/2: 4.4 days

Q95 H1/2: 108.7 days

reliable model fit: yes

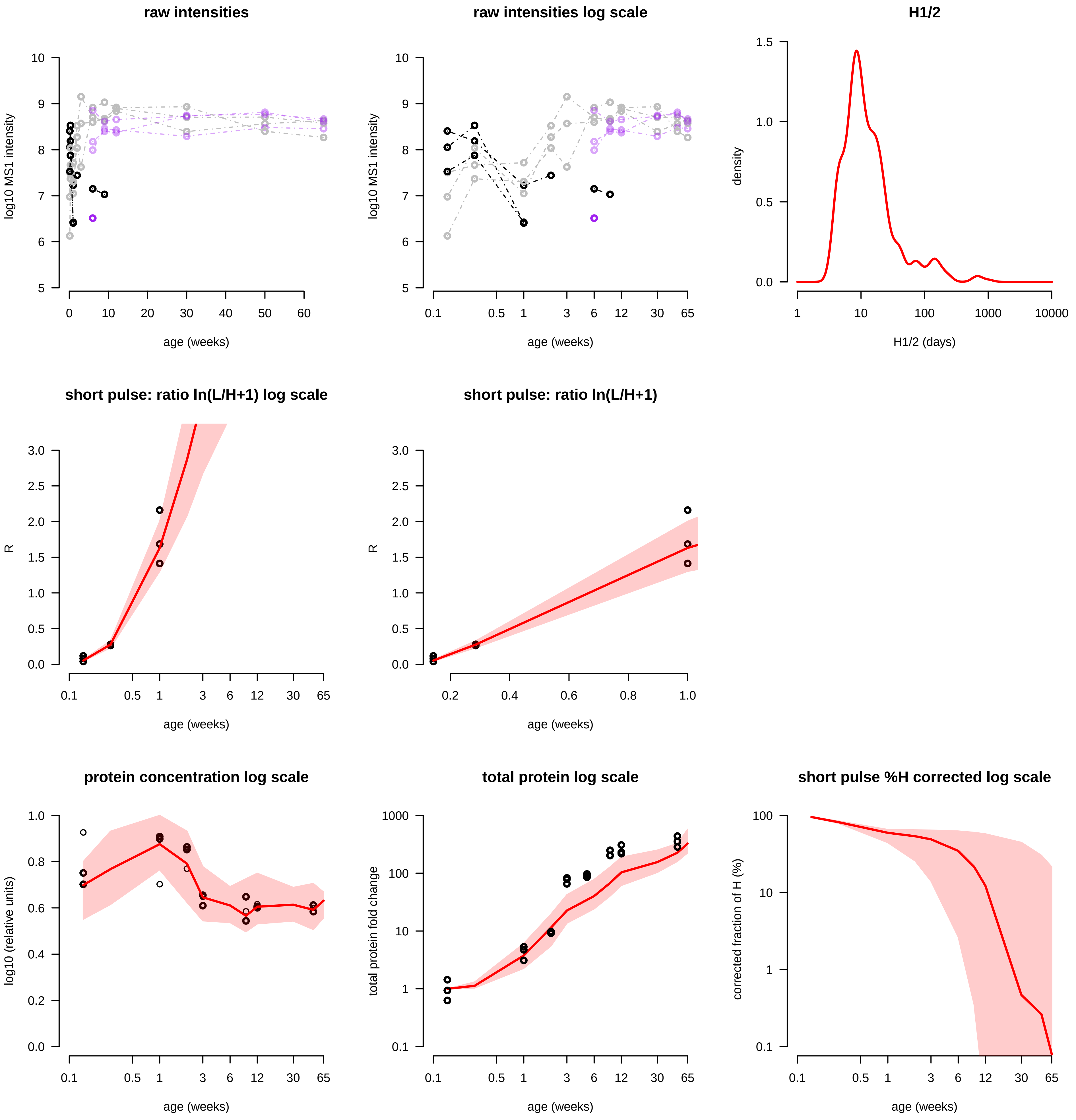

O35215

>sp|O35215|DOPD_MOUSE

D−dopachrome decarboxylase

GN=Ddt

Med H1/2: 7 days

Q5 H1/2: 4.6 days

Q95 H1/2: 12.1 days

reliable model fit: yes

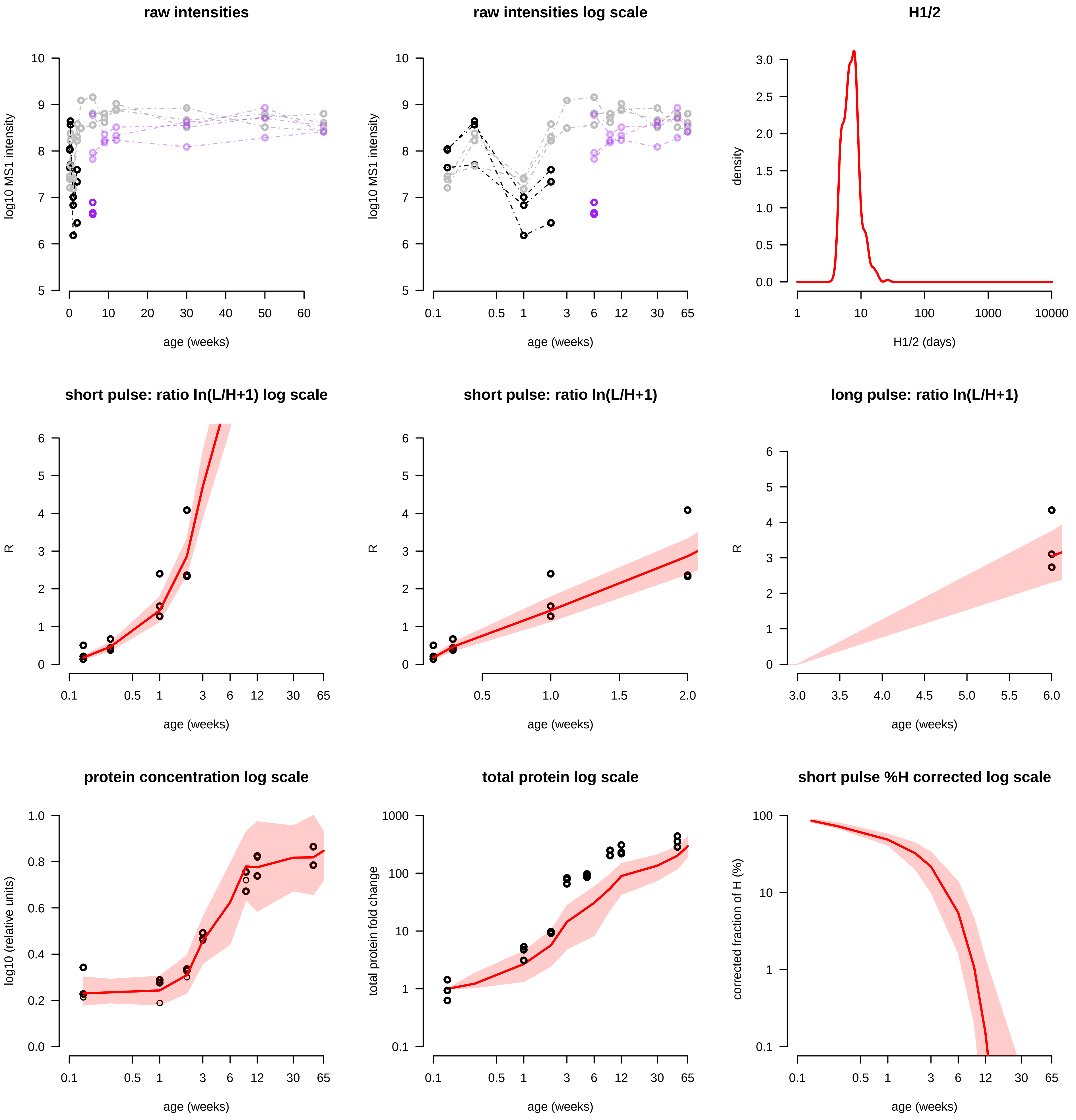

Q03173;Q03173−5

>sp|Q03173|ENAH_MOUSE

Protein enabled homolog

GN=Enah

Med H1/2: 5.9 days

Q5 H1/2: 3.1 days

Q95 H1/2: 32.2 days

reliable model fit: yes

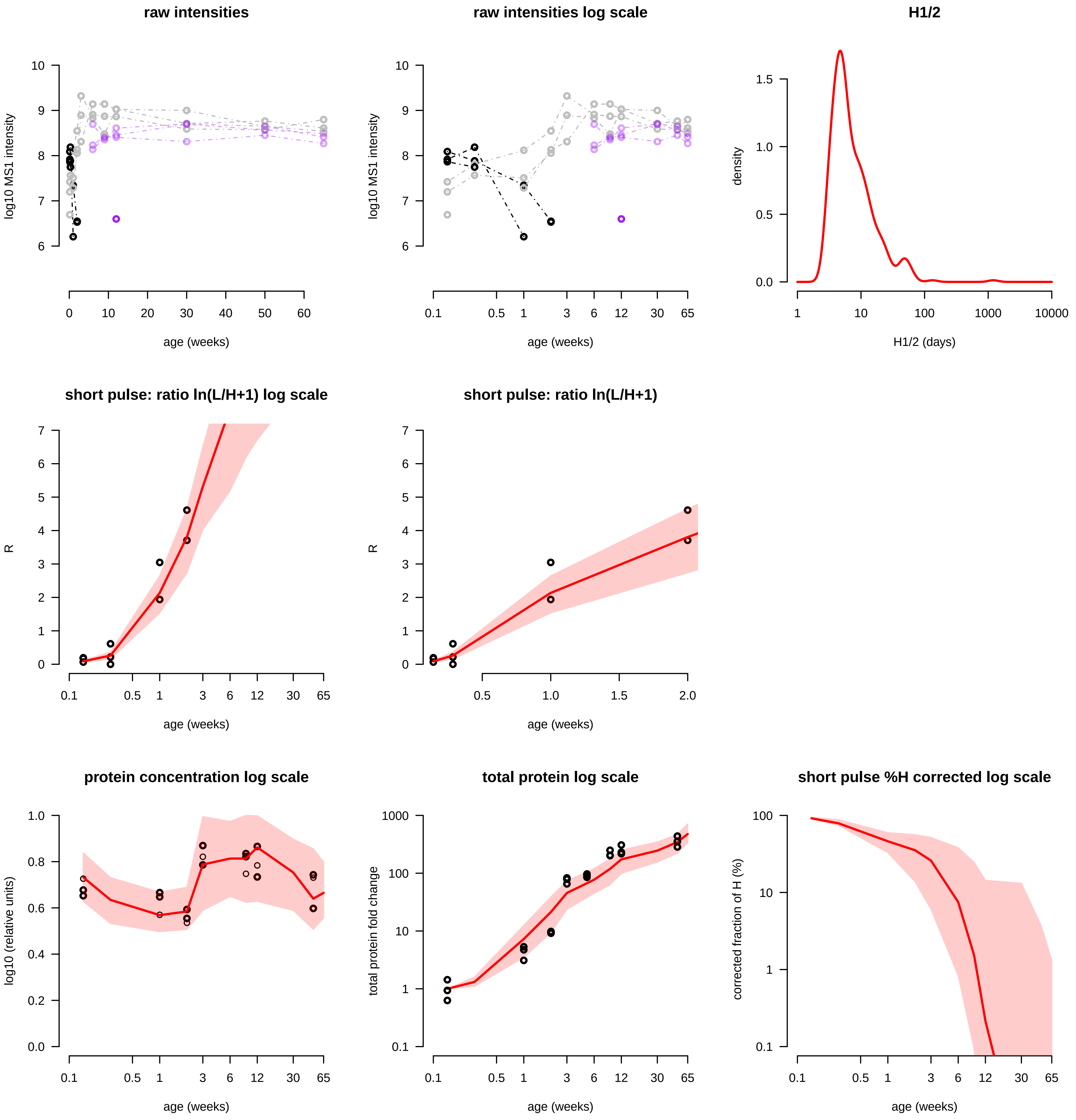

Q9JK48−2;Q9JK48;Q9JK48−3

>sp|Q9JK48−2|SHLB1_MOUSE

Isoform 2 of Endophilin−B1

GN=Sh3glb1

Med H1/2: 5.6 days

Q5 H1/2: 4.1 days

Q95 H1/2: 7.8 days

reliable model fit: yes

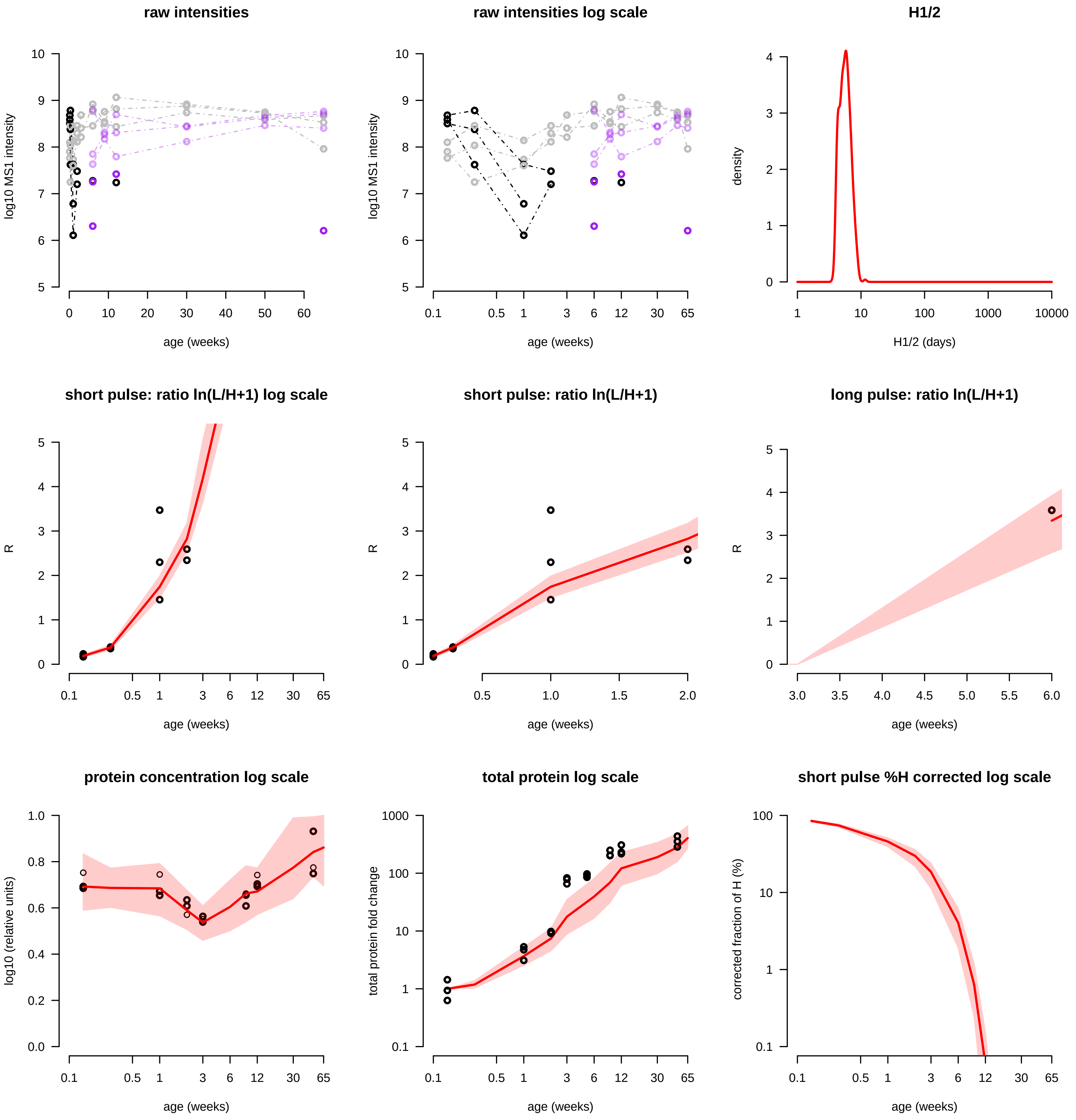

P56376

>sp|P56376|ACYP1_MOUSE

Acylphosphatase−1

GN=Acyp1

Med H1/2: 6.4 days

Q5 H1/2: 5.3 days

Q95 H1/2: 8.8 days

reliable model fit: yes

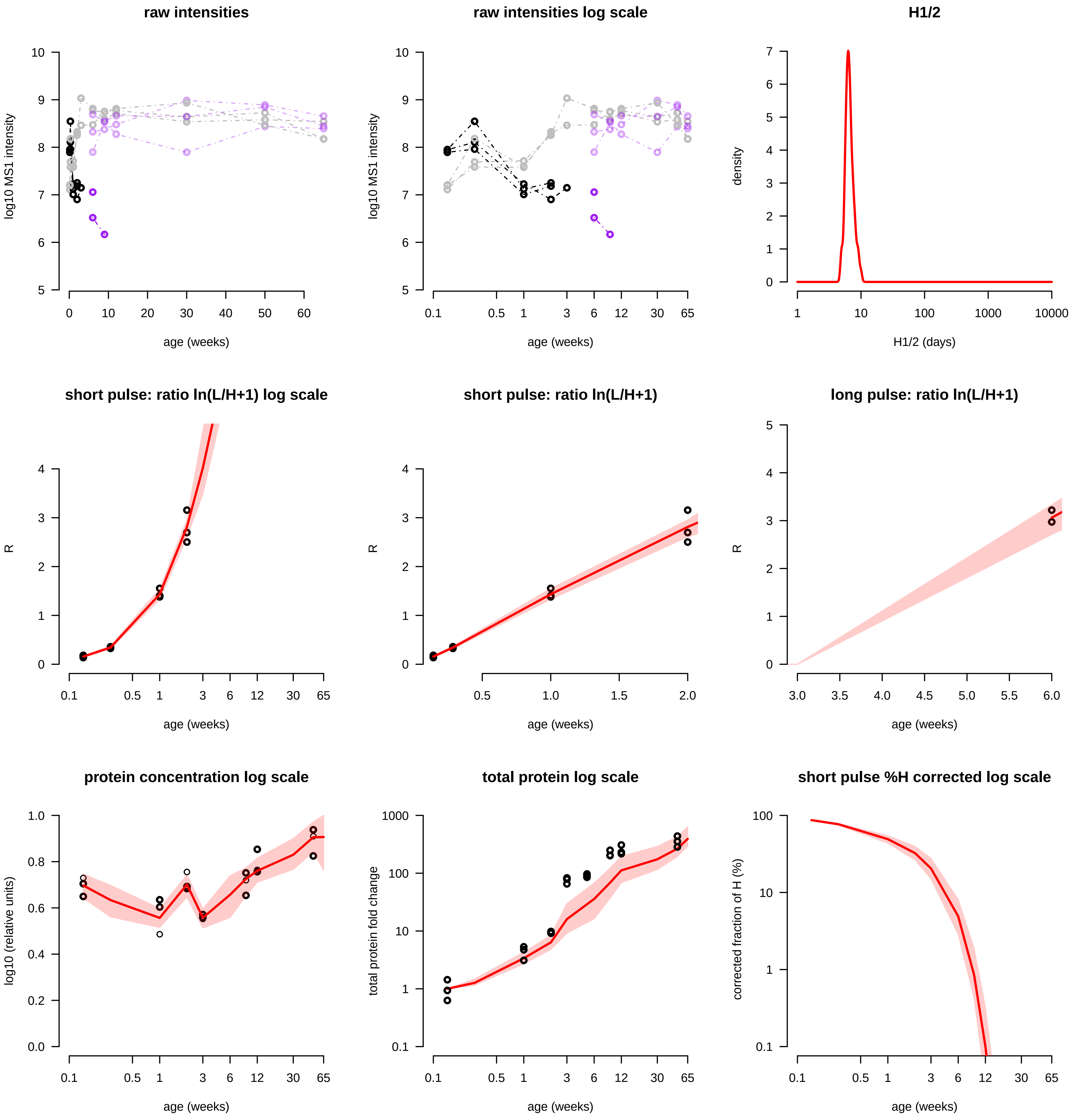

Q8BP92

>sp|Q8BP92|RCN2_MOUSE

Reticulocalbin−2

GN=Rcn2

Med H1/2: 5 days

Q5   H1/2: 4.3 days

Q95 H1/2: 6 days

reliable model fit: yes

raw intensities

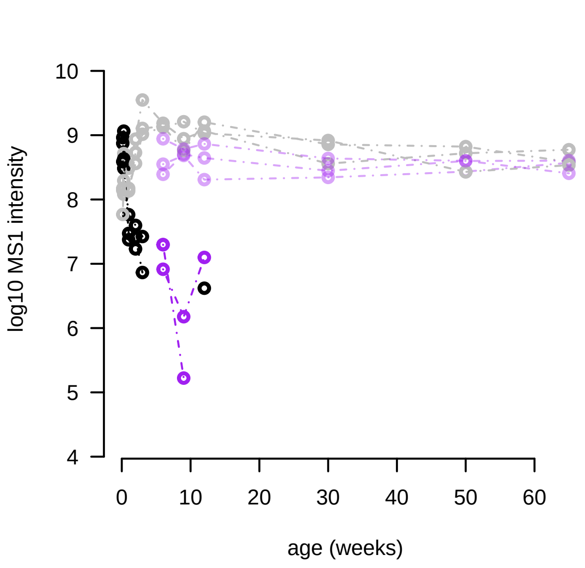

raw intensities log scale

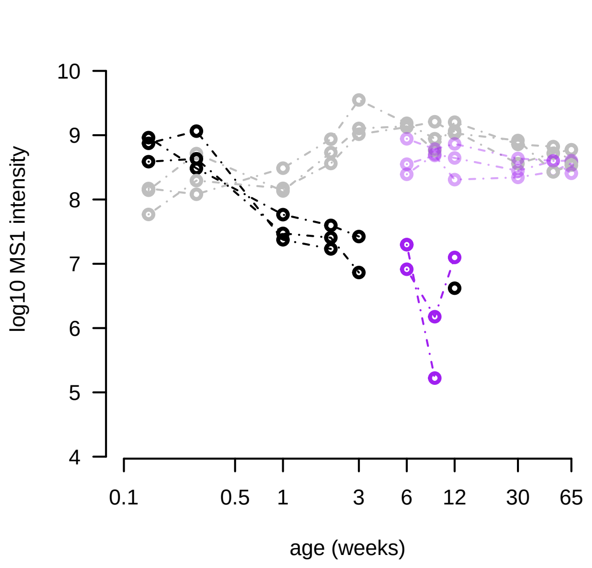

H1/2

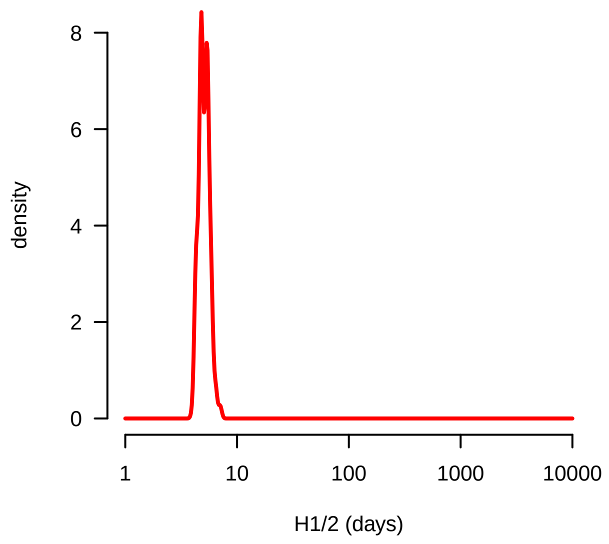

short pulse: ratio ln(L/H+1) log scale

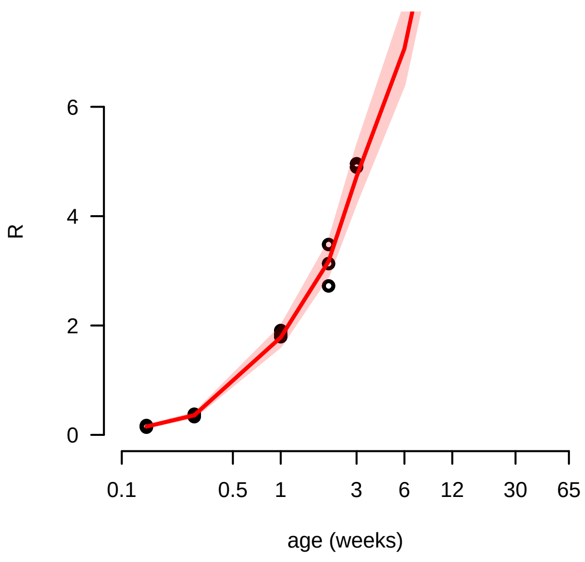

short pulse: ratio ln(L/H+1)

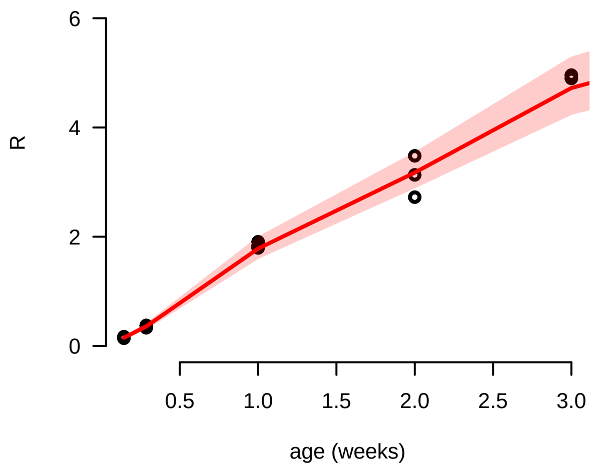

long pulse: ratio ln(L/H+1)

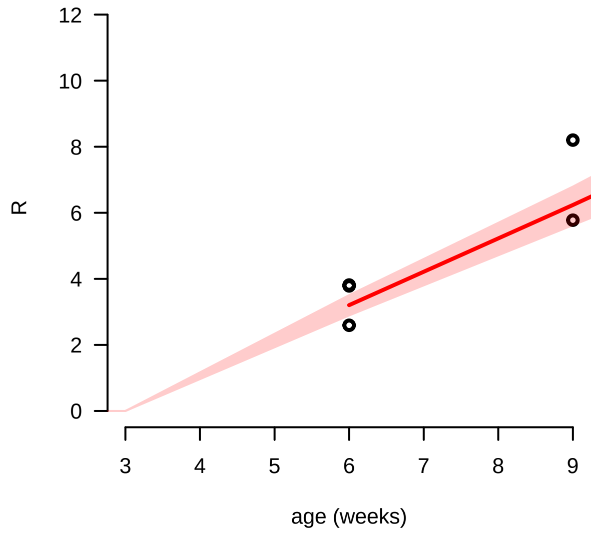

protein concentration log scale

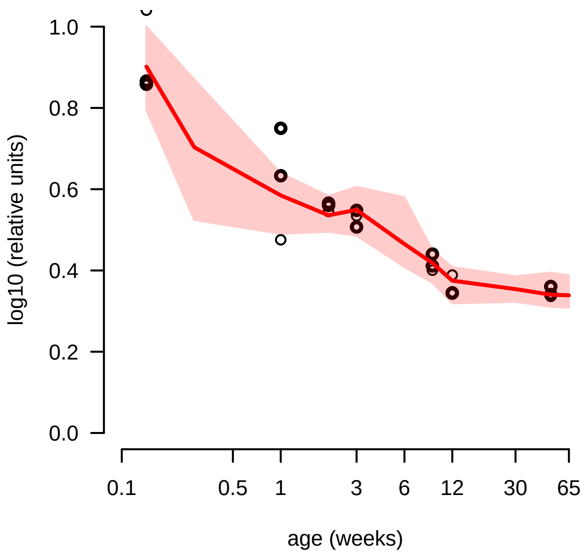

total protein log scale

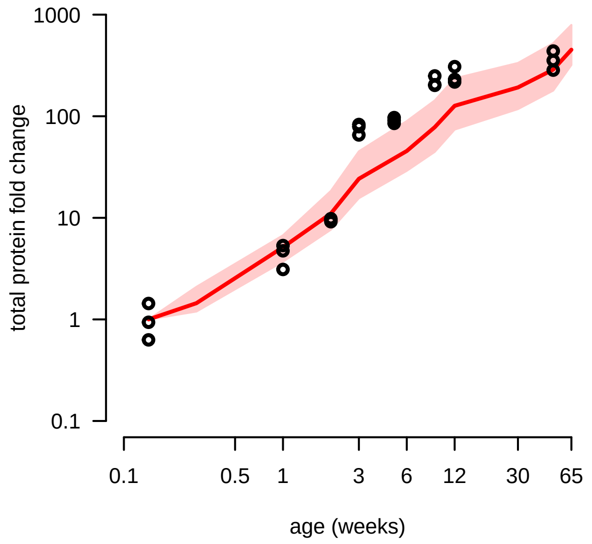

short pulse %H corrected log scale

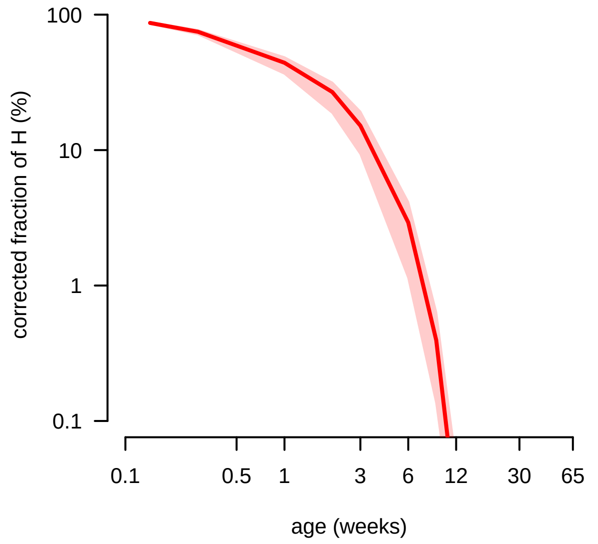

P19137

>sp|P19137|LAMA1_MOUSE

Laminin subunit alpha−1

GN=Lama1

Med H1/2: 12.4 days

Q5 H1/2: 3.9 days

Q95 H1/2: 102.1 days

reliable model fit: yes

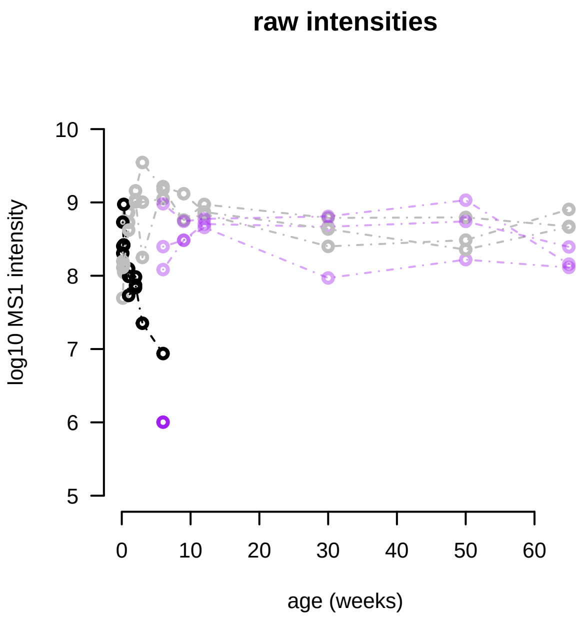

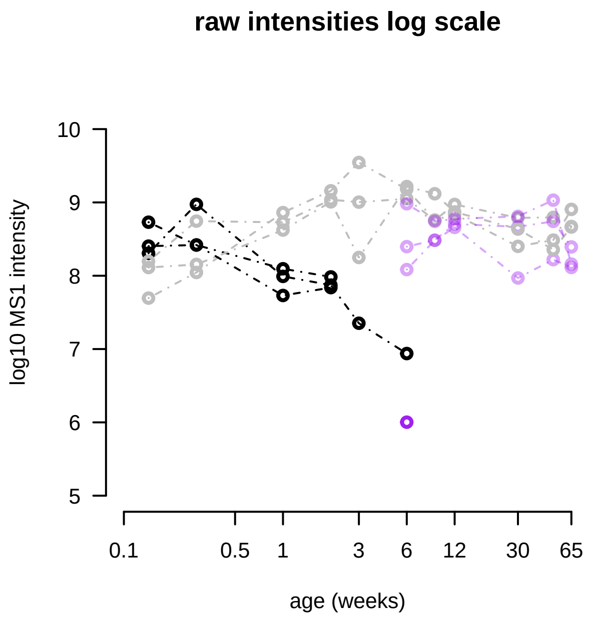

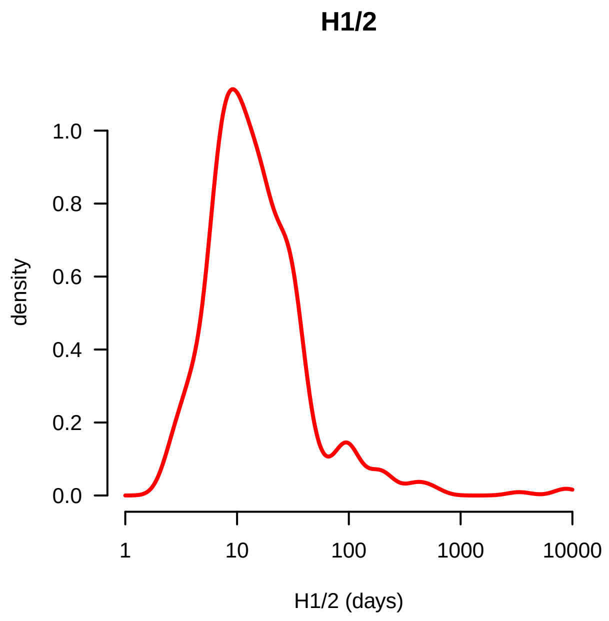

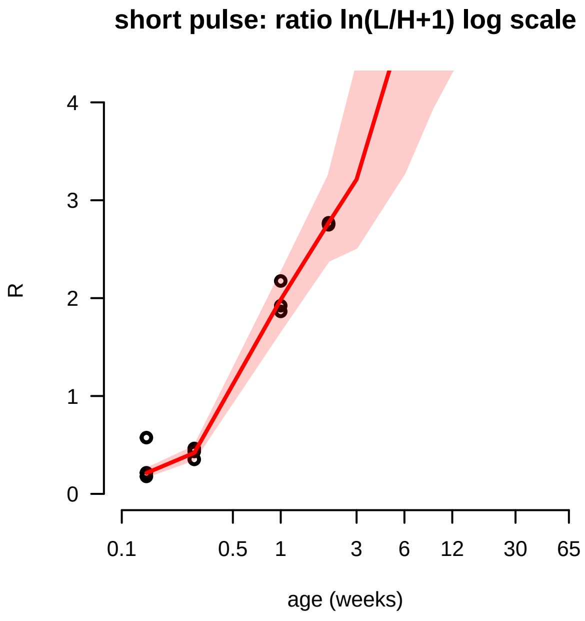

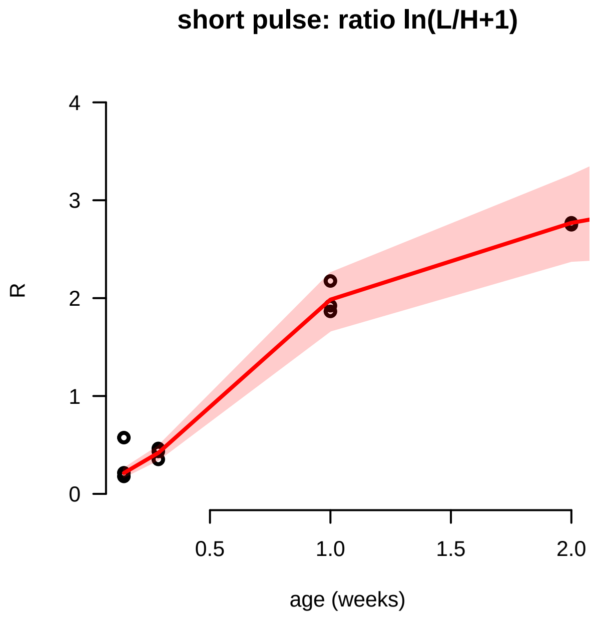

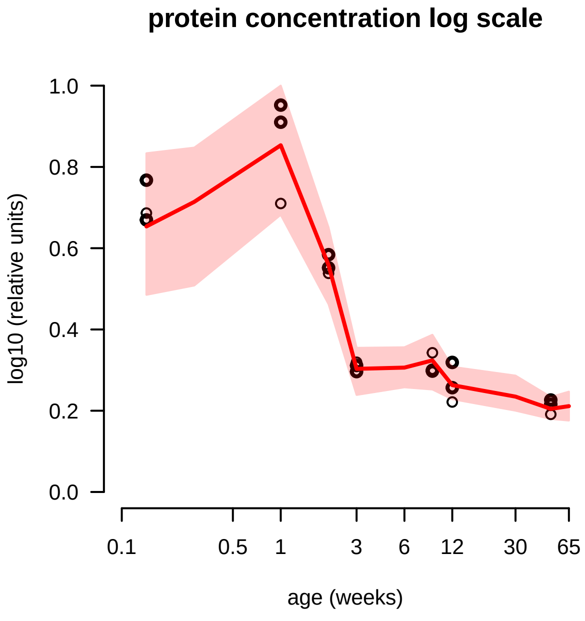

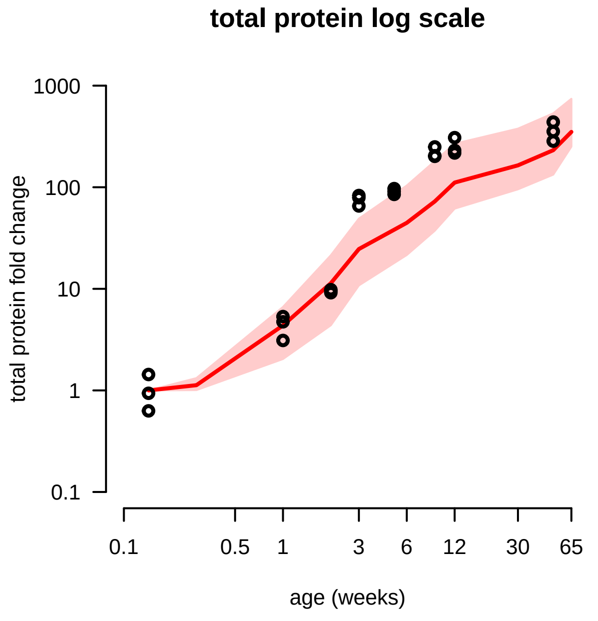

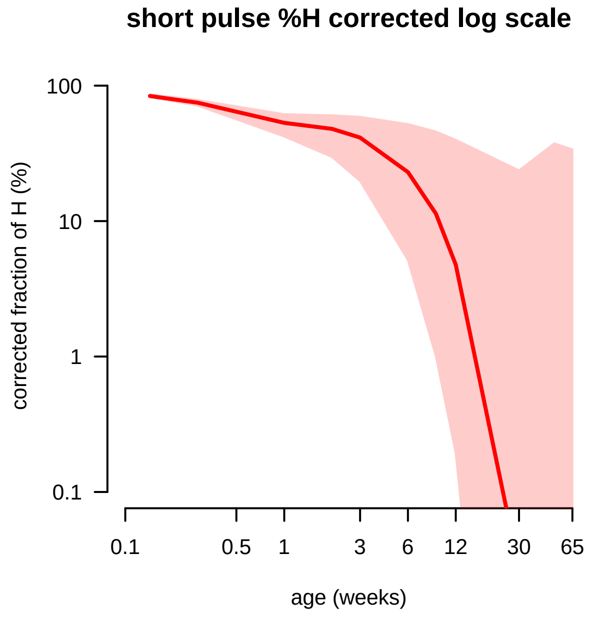

Q99MZ7

>sp|Q99MZ7|PECR_MOUSE

Peroxisomal trans−2−enoyl−CoA reductase

GN=Pecr

Med H1/2: 7.1 days

Q5 H1/2: 5.1 days

Q95 H1/2: 10.4 days

reliable model fit: yes

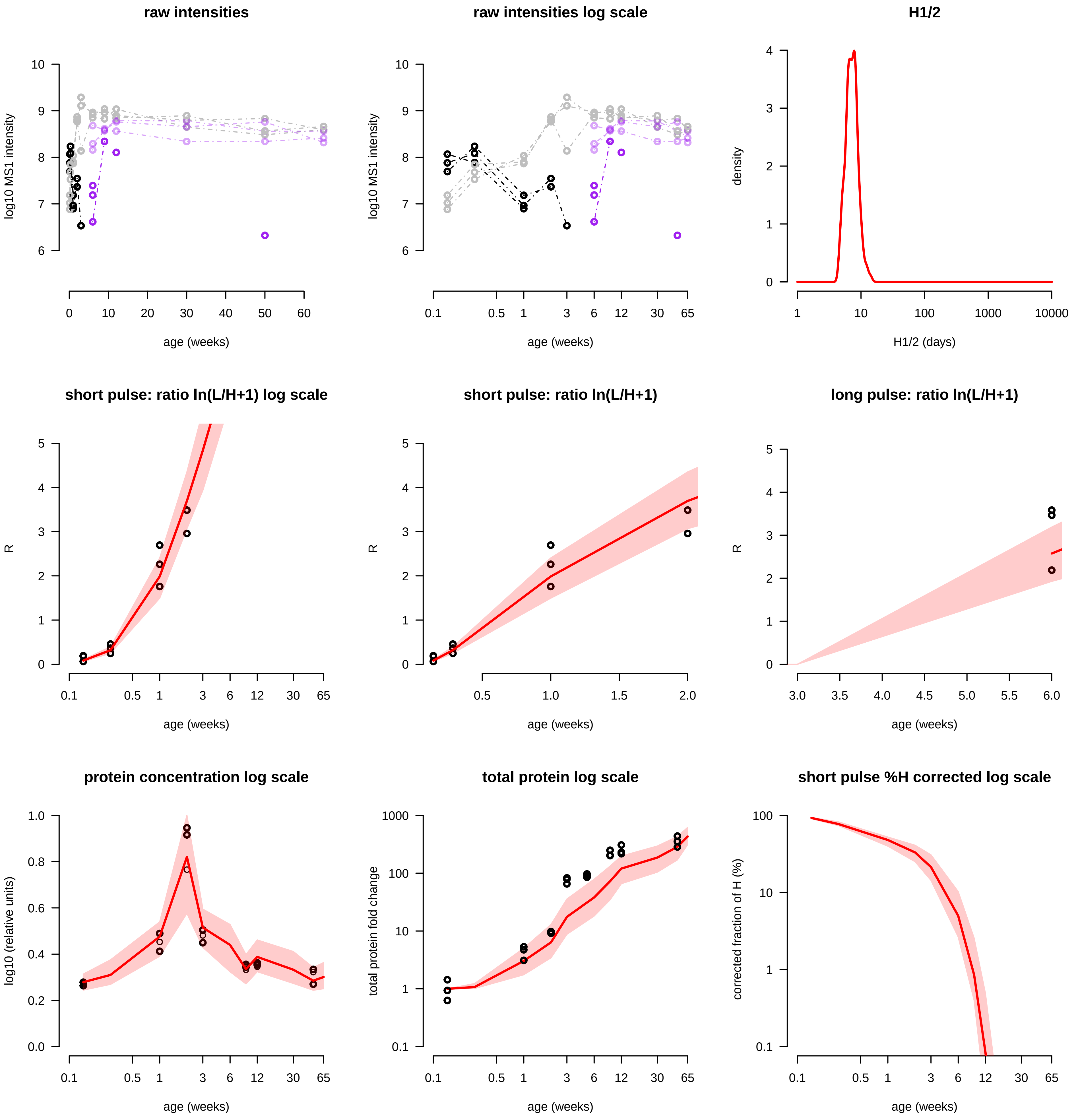

Q8R2Y2;Q8R2Y2−2

>sp|Q8R2Y2|MUC18_MOUSE

Cell surface glycoprotein MUC18

GN=Mcam

Med H1/2: 4.4 days

Q5 H1/2: 3.4 days

Q95 H1/2: 6.1 days

reliable model fit: yes

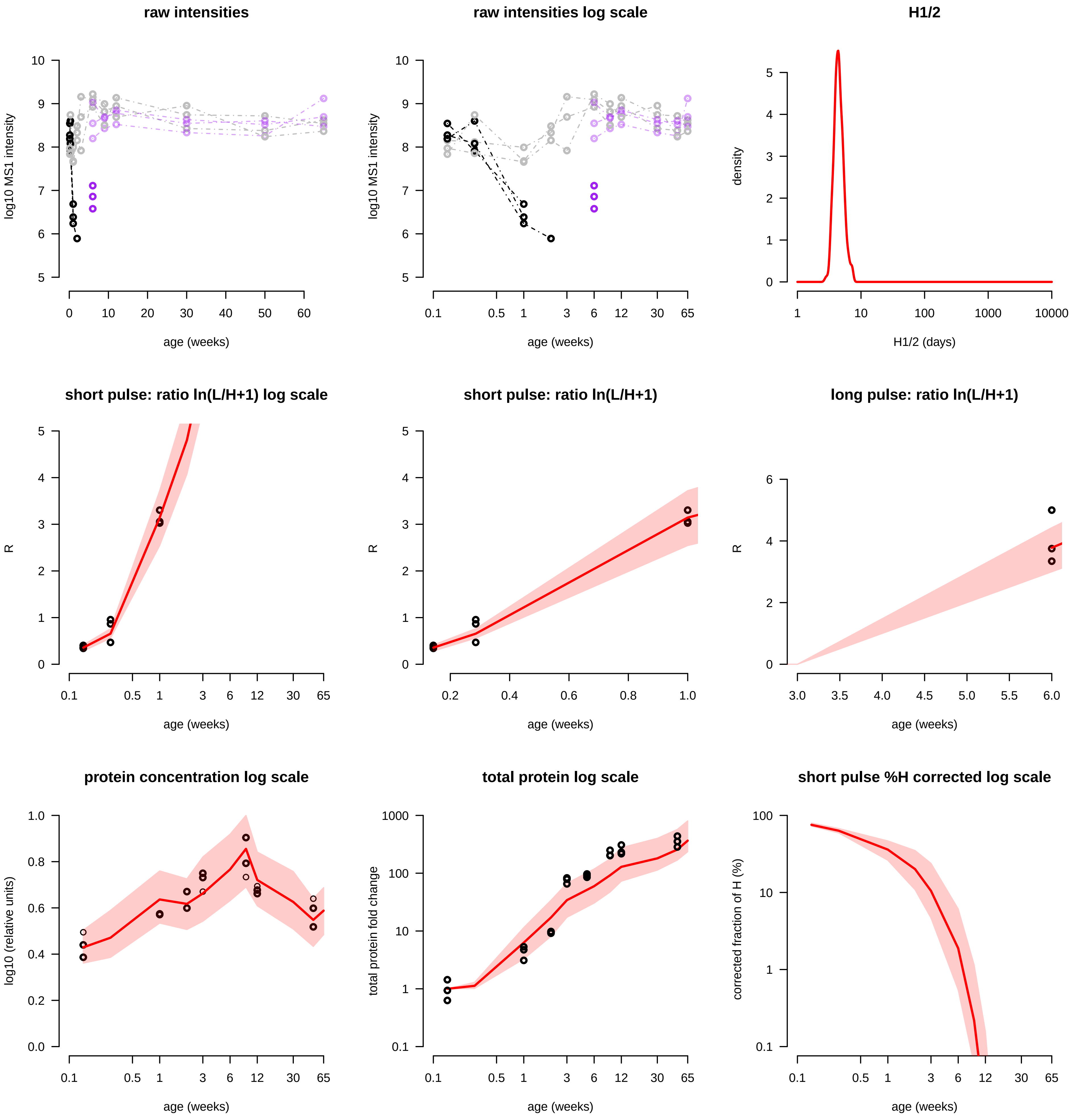

Q91VK1

>sp|Q91VK1|BZW2_MOUSE

Basic leucine zipper and W2 domain−containing protein 2

GN=Bzw2

Med H1/2: 6.9 days

Q5 H1/2: 3 days

Q95 H1/2: 61.5 days

reliable model fit: yes

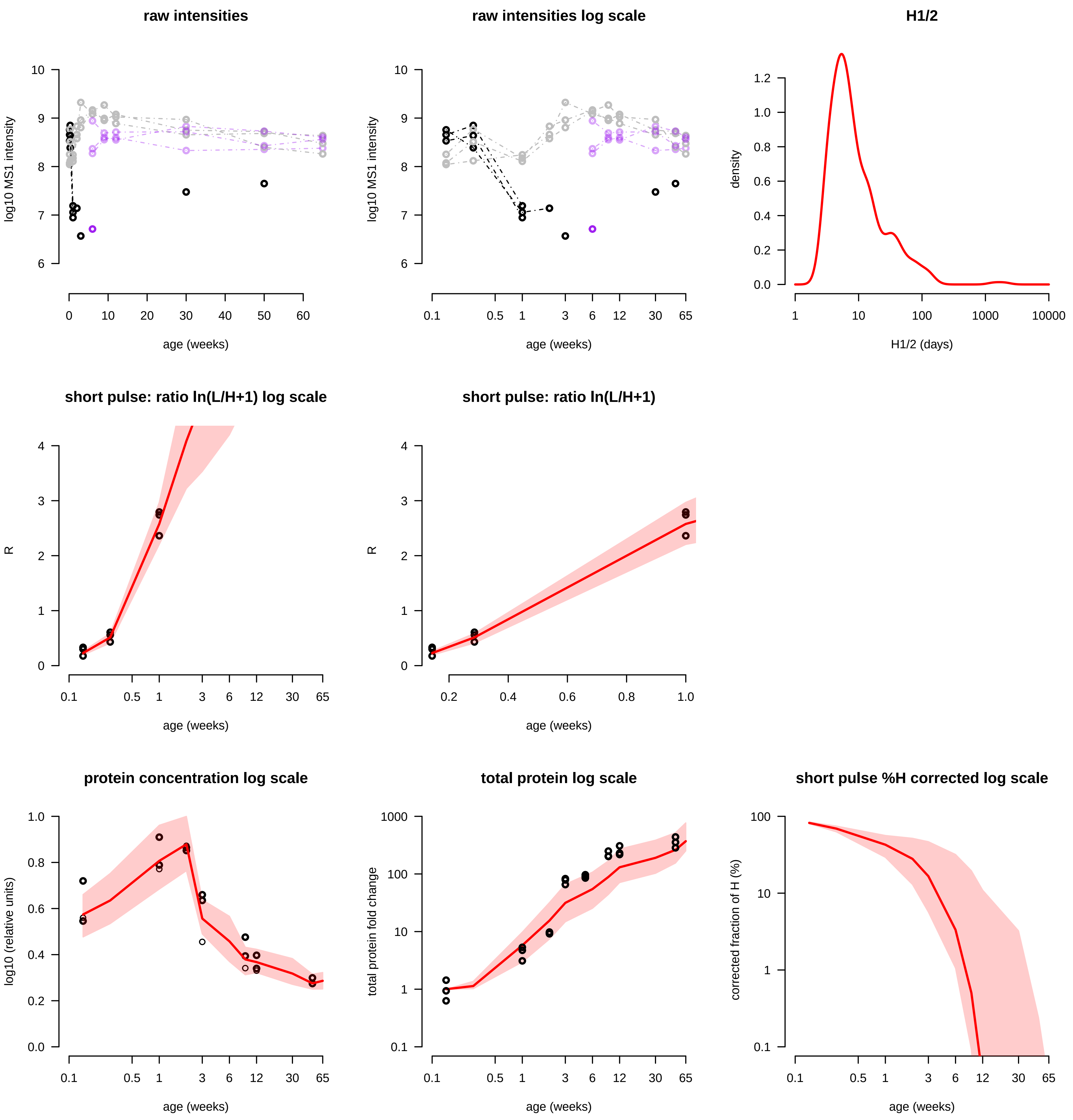

P29788

>sp|P29788|VTNC_MOUSE

Vitronectin

GN=Vtn

Med H1/2: 11.7 days

Q5 H1/2: 4.7 days

Q95 H1/2: 63.5 days

reliable model fit: yes

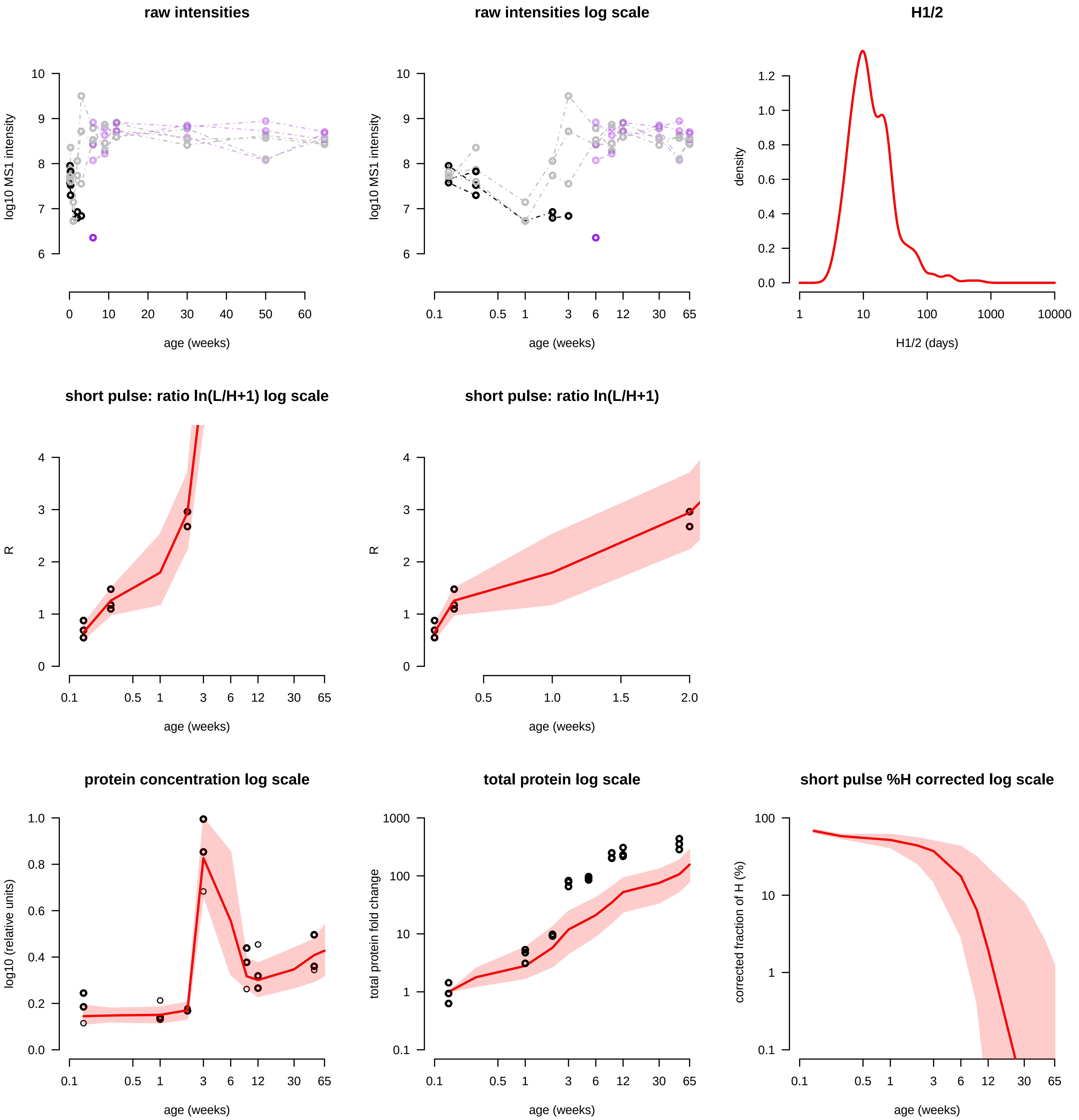

Q8VE97

>sp|Q8VE97|SRSF4_MOUSE

Serine/arginine−rich splicing factor 4

GN=Srsf4

Med H1/2: 669 days

Q5 H1/2: 276.3 days

Q95 H1/2: 10464.3 days

reliable model fit: yes

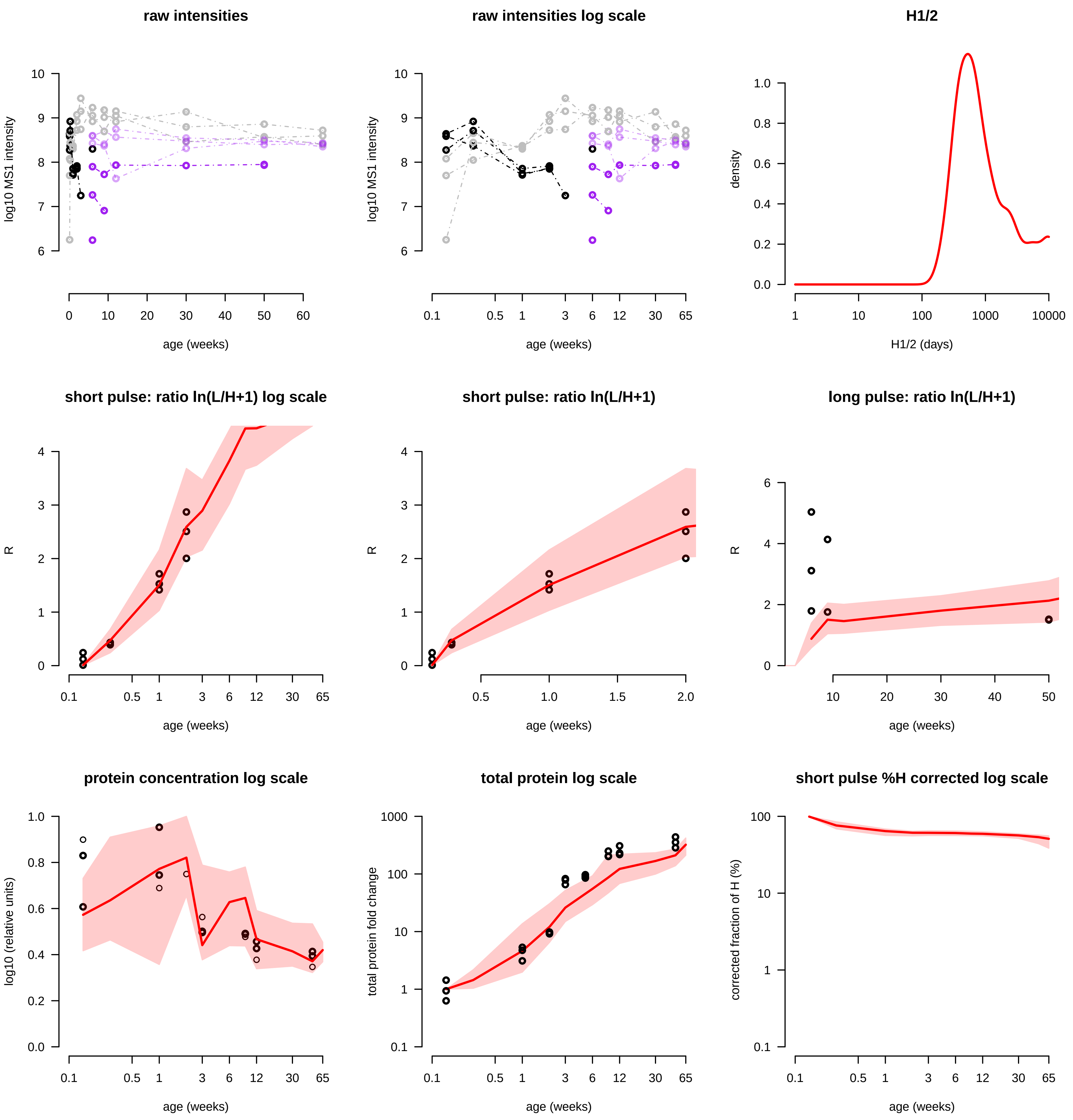

Q62433

>sp|Q62433|NDRG1_MOUSE

Protein NDRG1

GN=Ndrg1

Med H1/2: 37.3 days

Q5 H1/2: 15.9 days

Q95 H1/2: 308.1 days

reliable model fit: yes

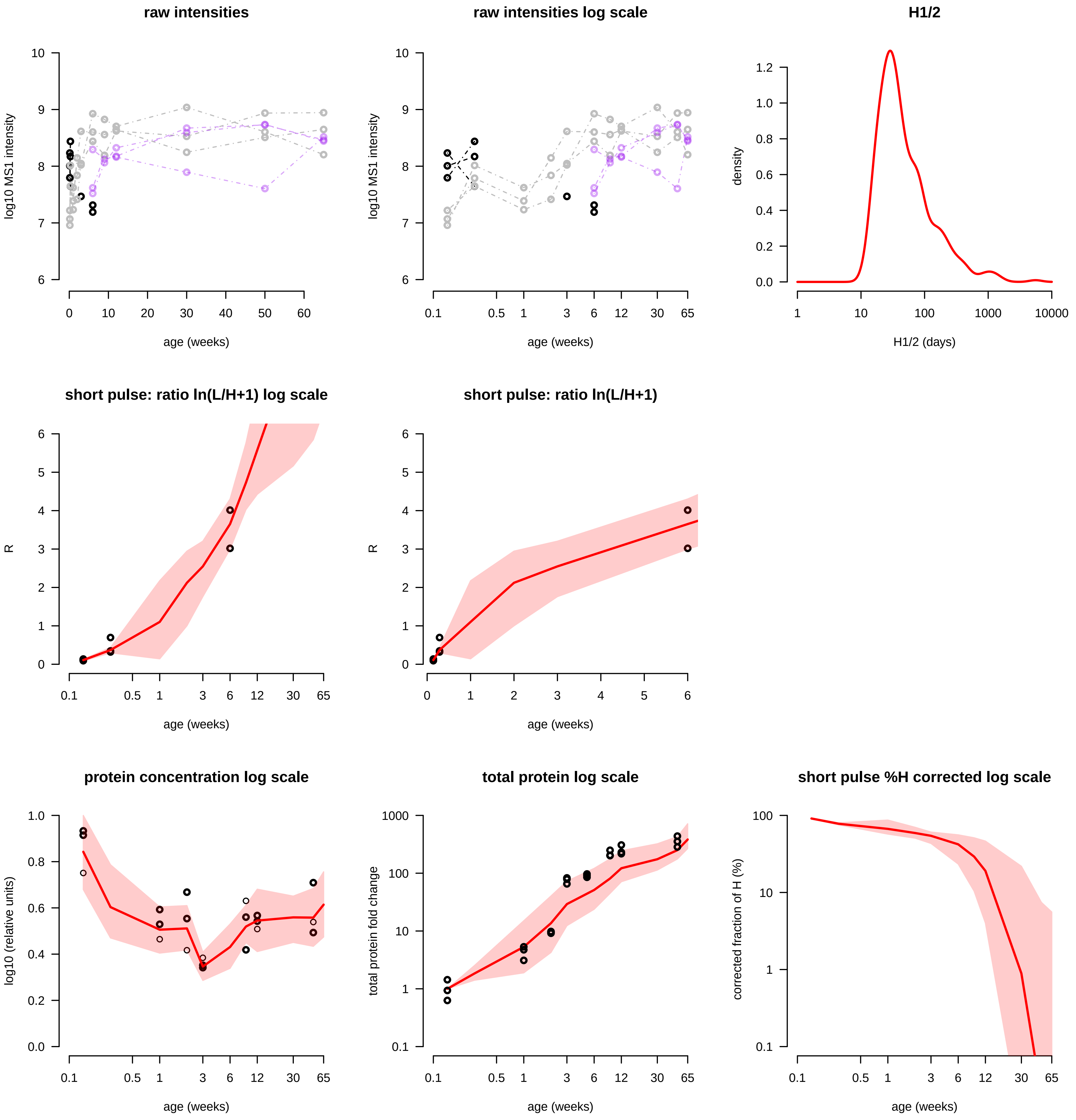

Q9CRC9

>sp|Q9CRC9|GNPI2_MOUSE

Glucosamine−6−phosphate isomerase 2

GN=Gnpda2

Med H1/2: 560.3 days

Q5 H1/2: 160.5 days

Q95 H1/2: 5706.9 days

reliable model fit: yes

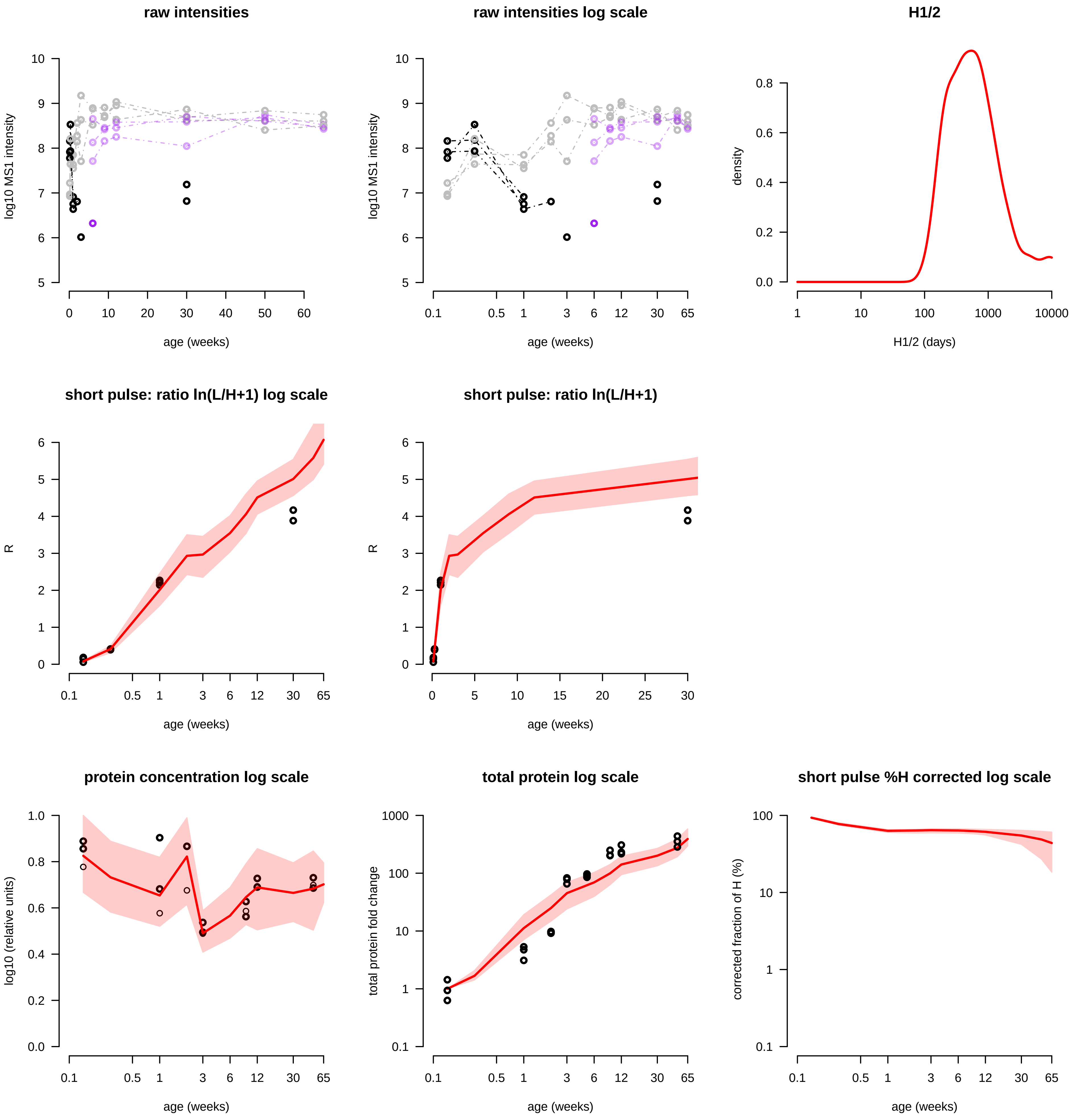

Q9DBS5

>sp|Q9DBS5|KLC4_MOUSE

Kinesin light chain 4

GN=Klc4

Med H1/2: 7 days

Q5 H1/2: 3.8 days

Q95 H1/2: 31.2 days

reliable model fit: yes

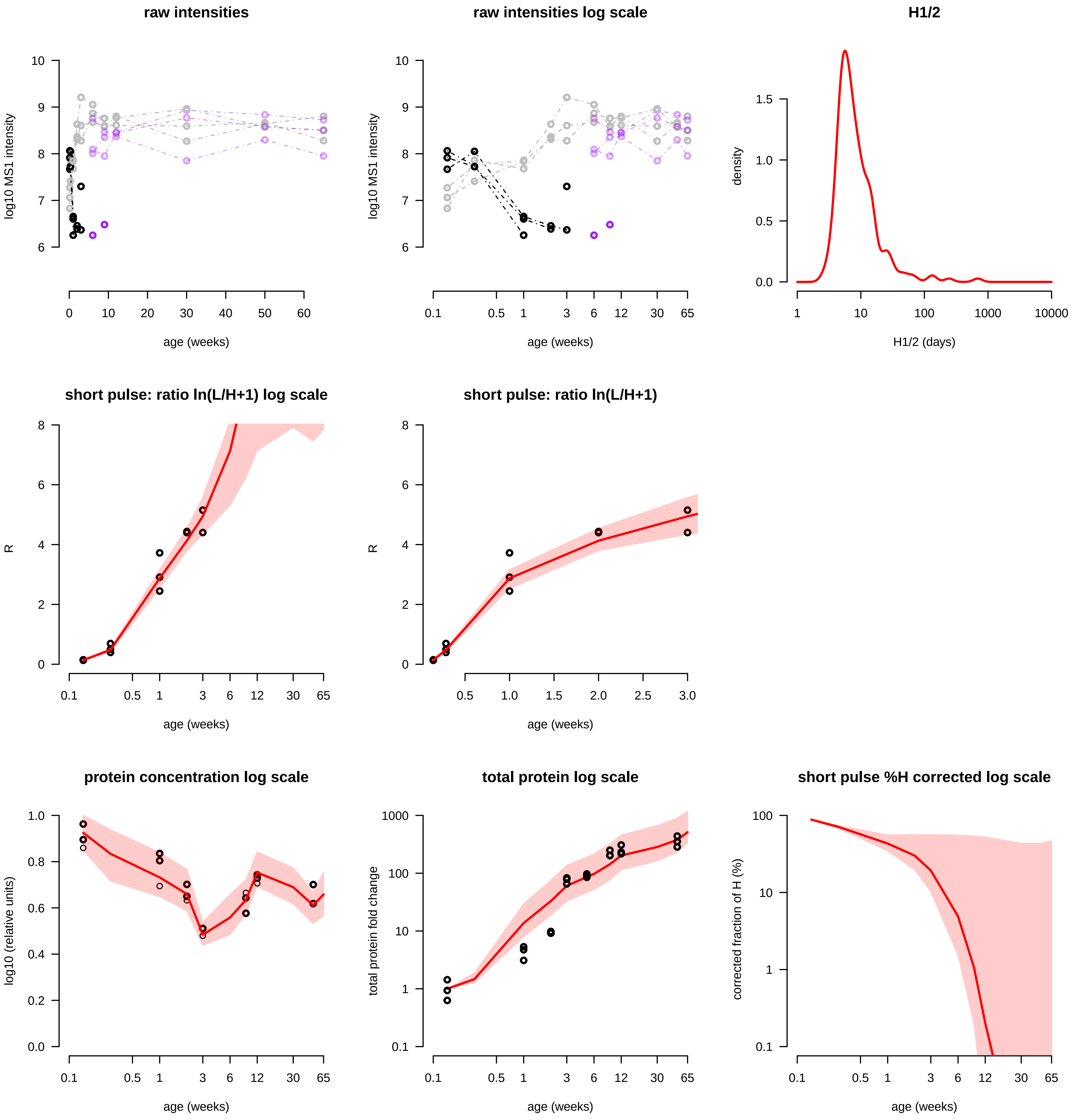

Q9D7G0

>sp|Q9D7G0|PRPS1_MOUSE

Ribose−phosphate pyrophosphokinase 1

GN=Prps1

Med H1/2: 8.7 days

Q5 H1/2: 7 days

Q95 H1/2: 11.2 days

reliable model fit: yes

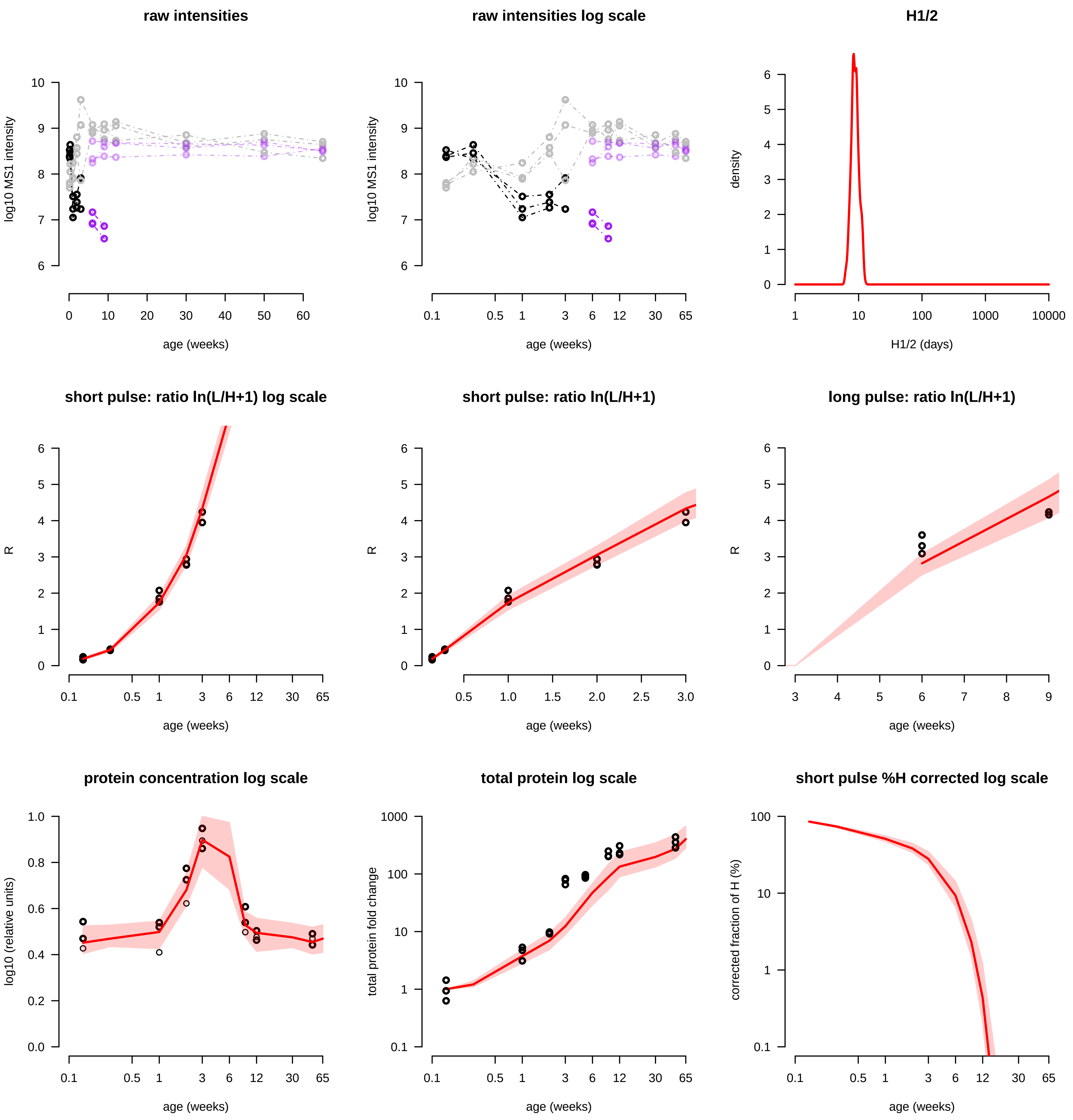

Q9D8S3

>sp|Q9D8S3|ARFG3_MOUSE

ADP−ribosylation factor GTPase−activating protein 3

GN=Arfgap3

Med H1/2: 5.6 days

Q5 H1/2: 3 days

Q95 H1/2: 36.4 days

reliable model fit: yes

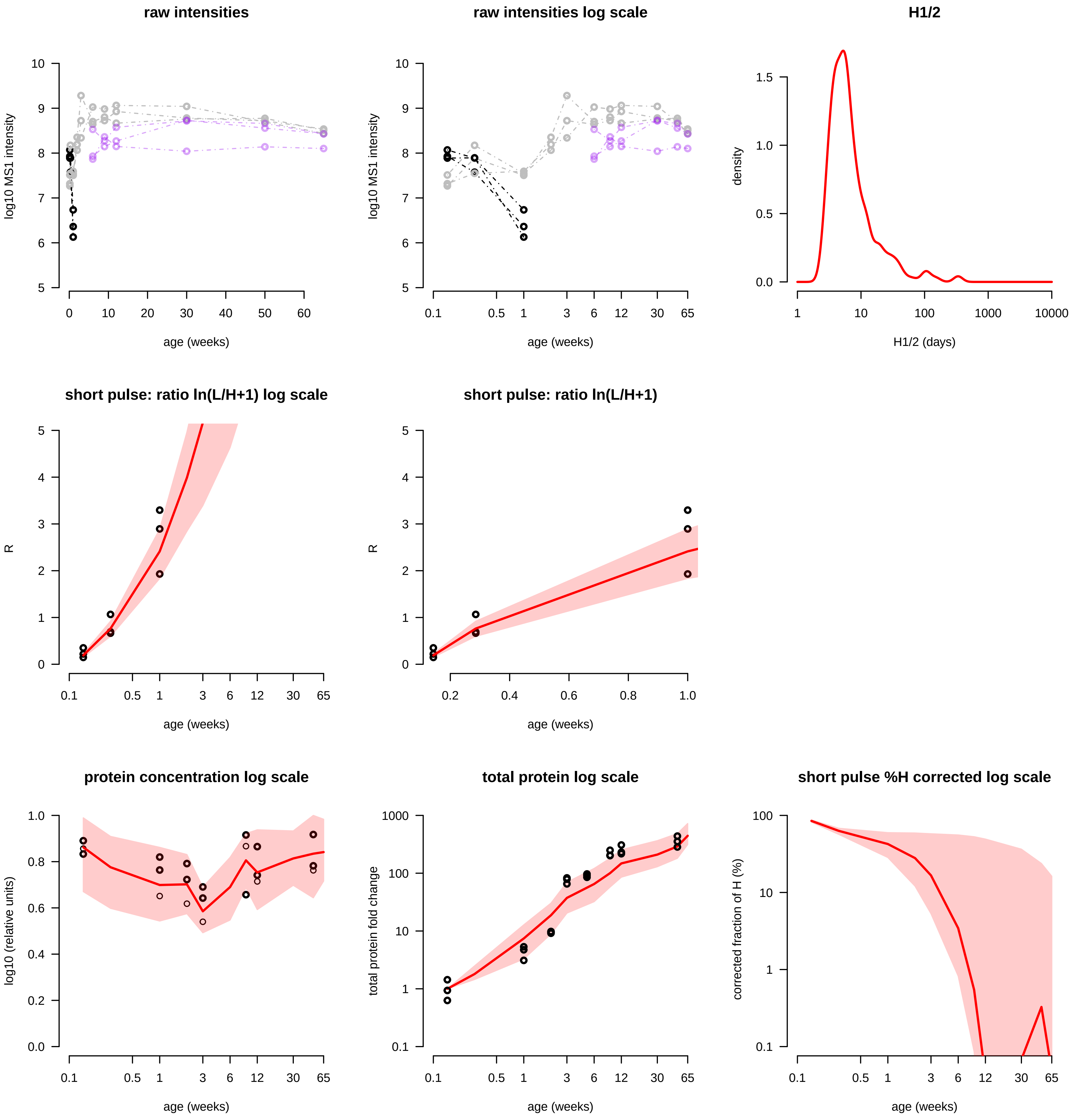

Q9CR09

>sp|Q9CR09|UFC1_MOUSE

Ubiquitin−fold modifier−conjugating enzyme 1

GN=Ufc1

Med H1/2: 10.7 days

Q5 H1/2: 5.4 days

Q95 H1/2: 42.6 days

reliable model fit: yes

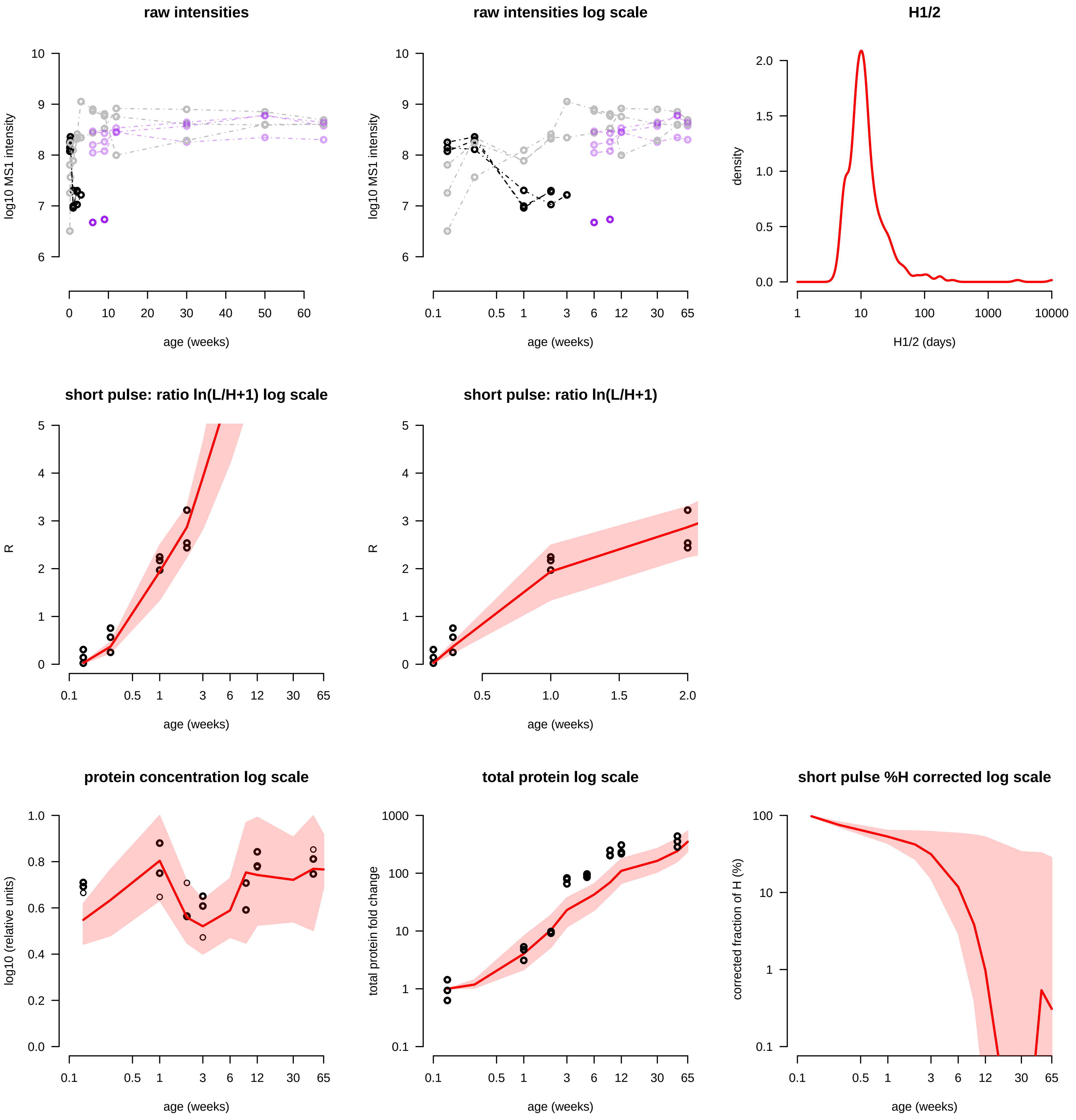

Q9WU42;Q9WU42−2

>sp|Q9WU42|NCOR2_MOUSE

Nuclear receptor corepressor 2

GN=Ncor2

Med H1/2: 20.3 days

Q5 H1/2: 8.5 days

Q95 H1/2: 92.7 days

reliable model fit: yes

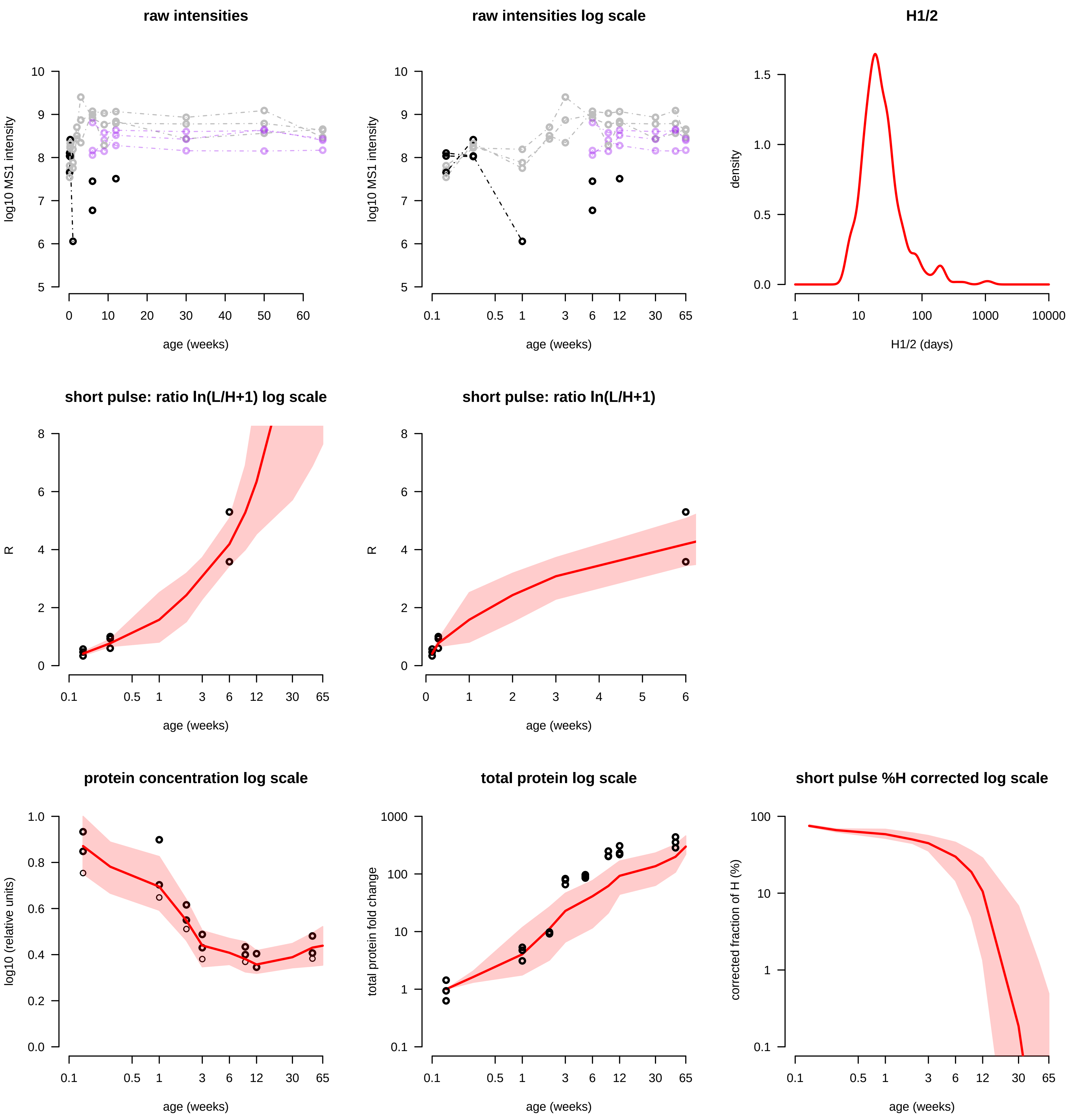

Q7TQH0−2;Q7TQH0−3;Q7TQH0

>sp|Q7TQH0−2|ATX2L_MOUSE

Isoform 2 of Ataxin−2−like protein

GN=Atxn2l

Med H1/2: 393.6 days

Q5 H1/2: 145.6 days

Q95 H1/2: 1534.5 days

reliable model fit: yes

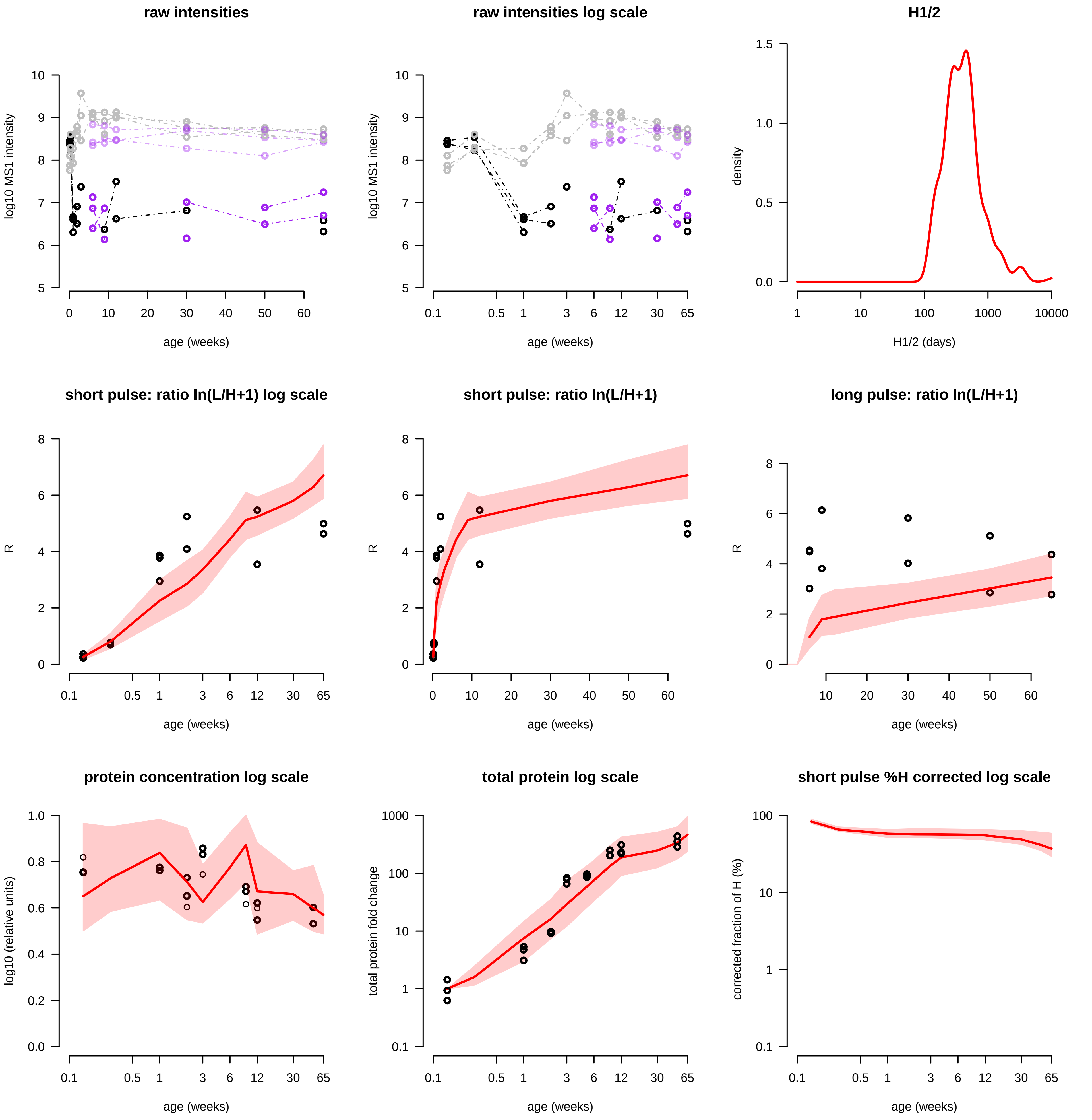

P61924

>sp|P61924|COPZ1_MOUSE

Coatomer subunit zeta−1

GN=Copz1

Med H1/2: 7 days

Q5 H1/2: 5.8 days

Q95 H1/2: 10.5 days

reliable model fit: yes

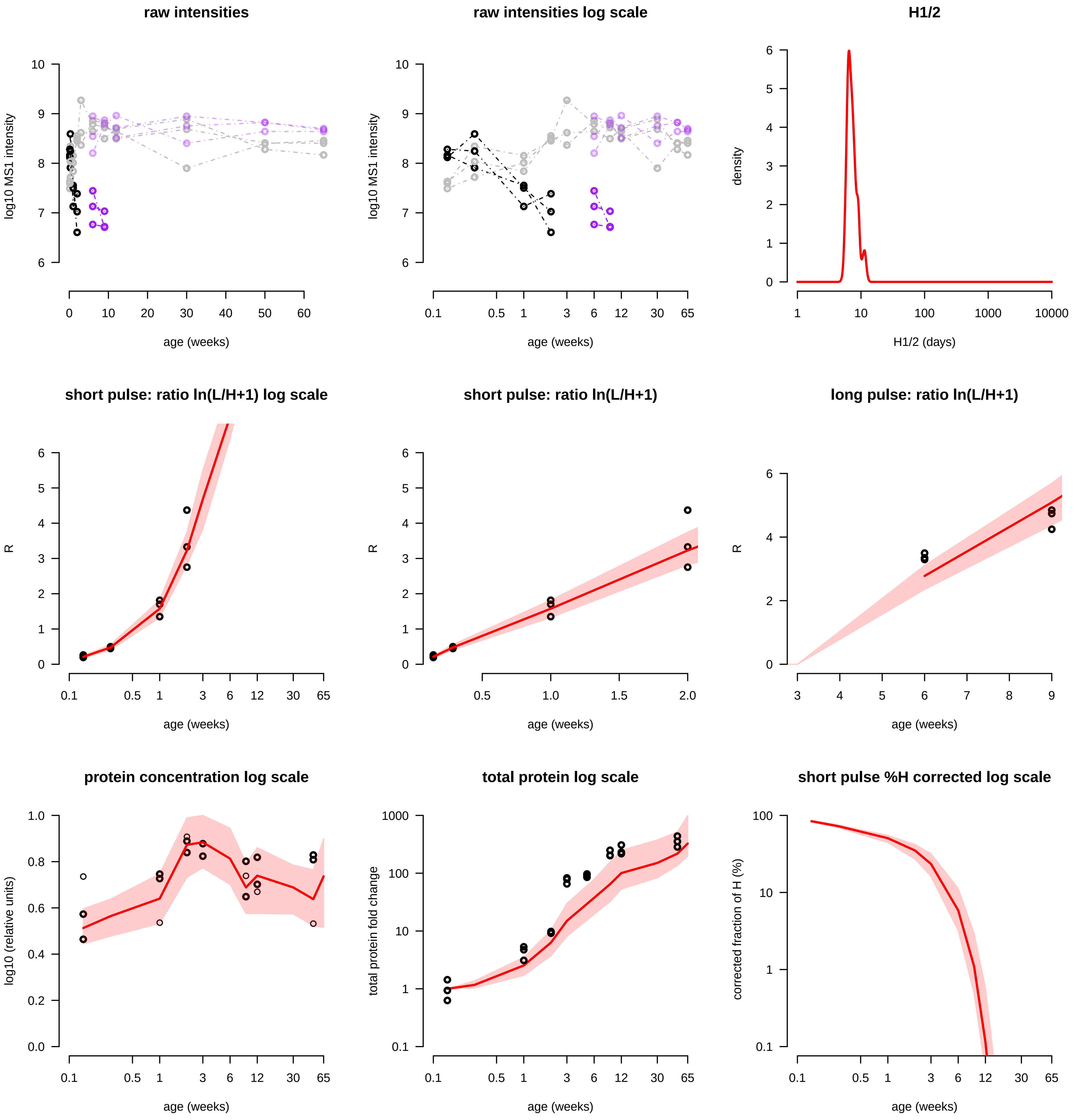

P70303;P70303−2

>sp|P70303|PYRG2_MOUSE

CTP synthase 2

GN=Ctps2

Med H1/2: 3.4 days

Q5 H1/2: 2.8 days

Q95 H1/2: 4.3 days

reliable model fit: yes

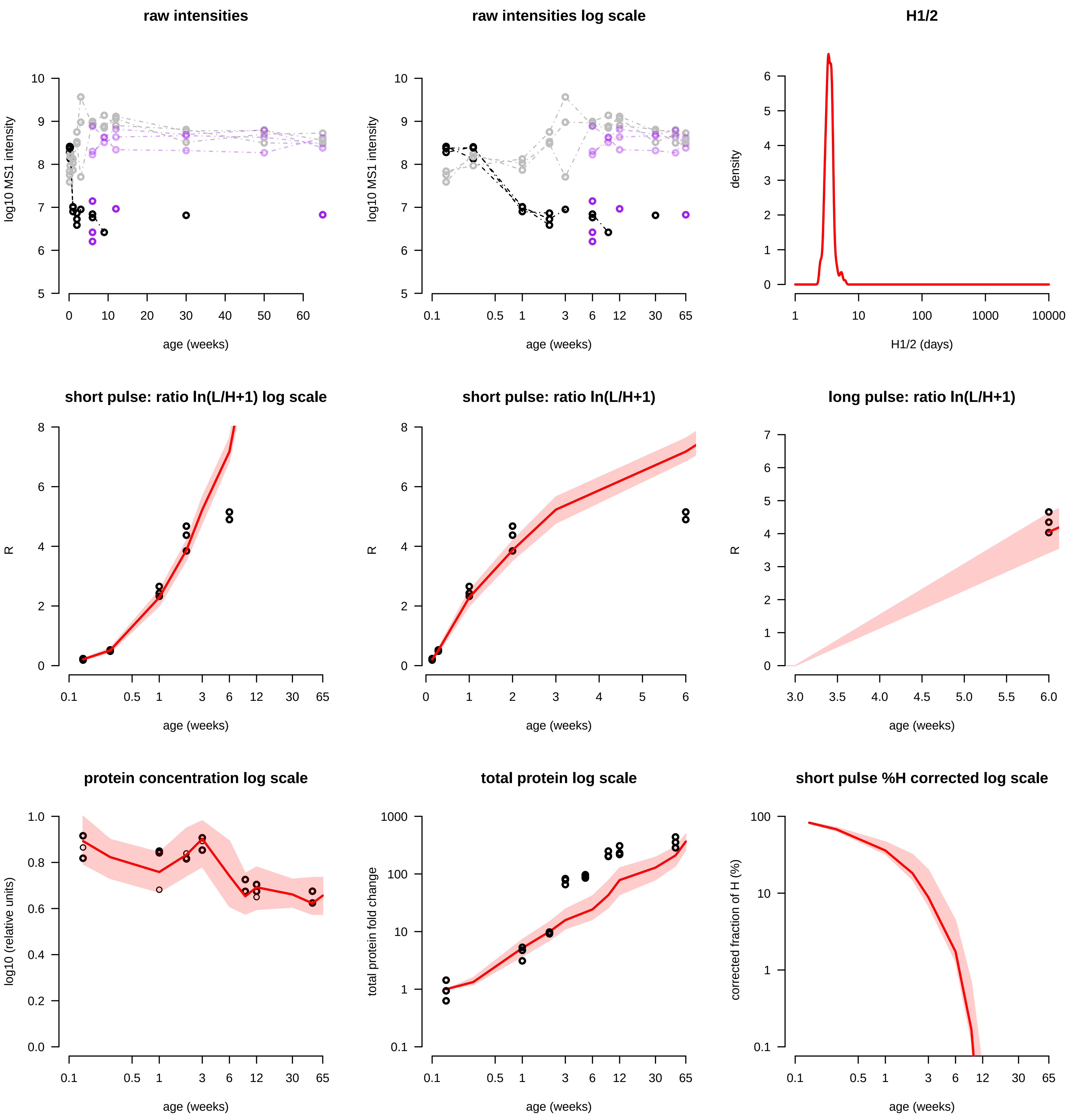

Q8CFI7

>sp|Q8CFI7|RPB2_MOUSE

DNA−directed RNA polymerase II subunit RPB2

GN=Polr2b

Med H1/2: 9.4 days

Q5 H1/2: 7.6 days

Q95 H1/2: 12.2 days

reliable model fit: no

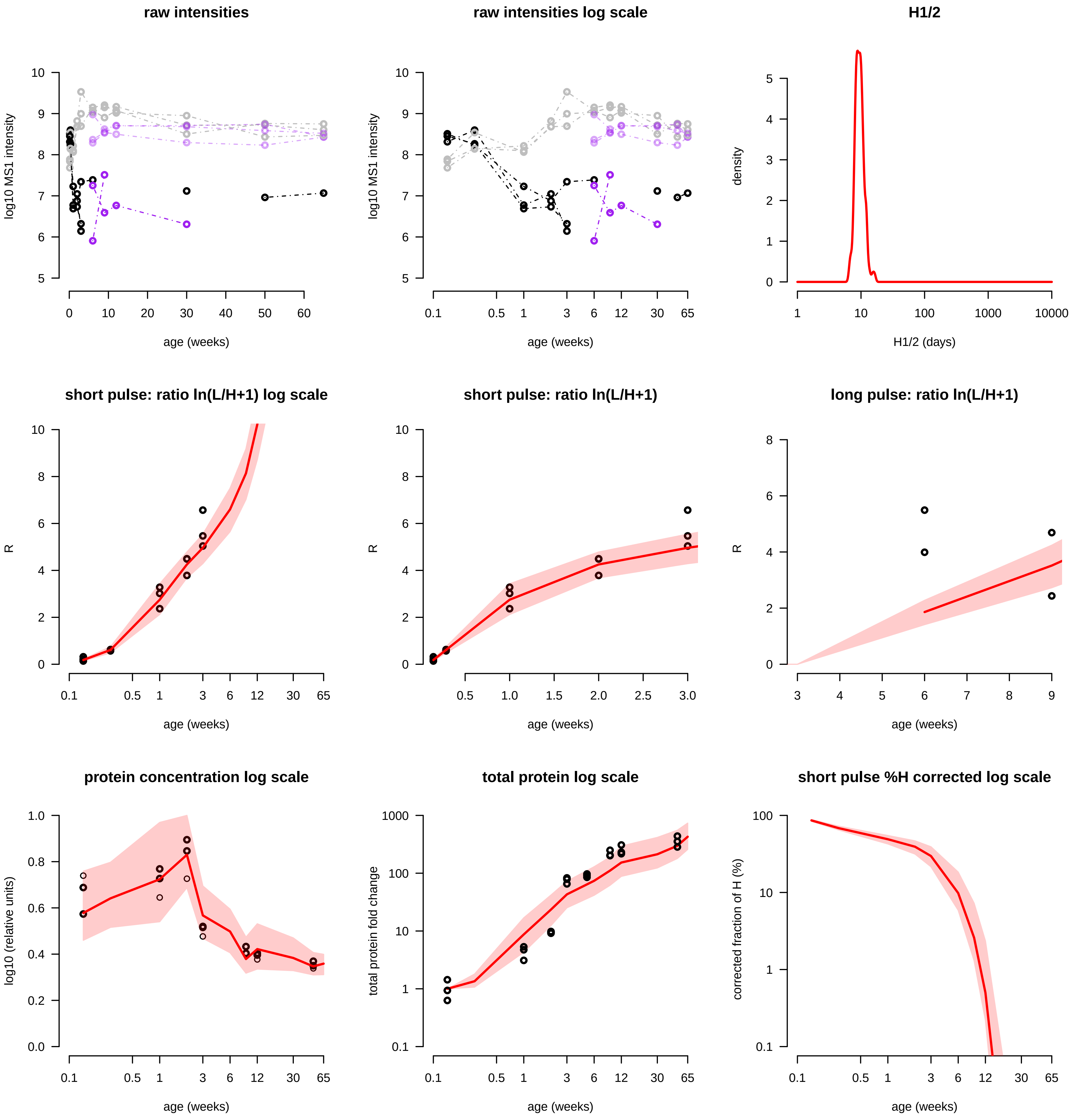

P08030

>sp|P08030|APT_MOUSE

Adenine phosphoribosyltransferase

GN=Aprt

Med H1/2: 4.4 days

Q5 H1/2: 3.6 days

Q95 H1/2: 5.7 days

reliable model fit: yes

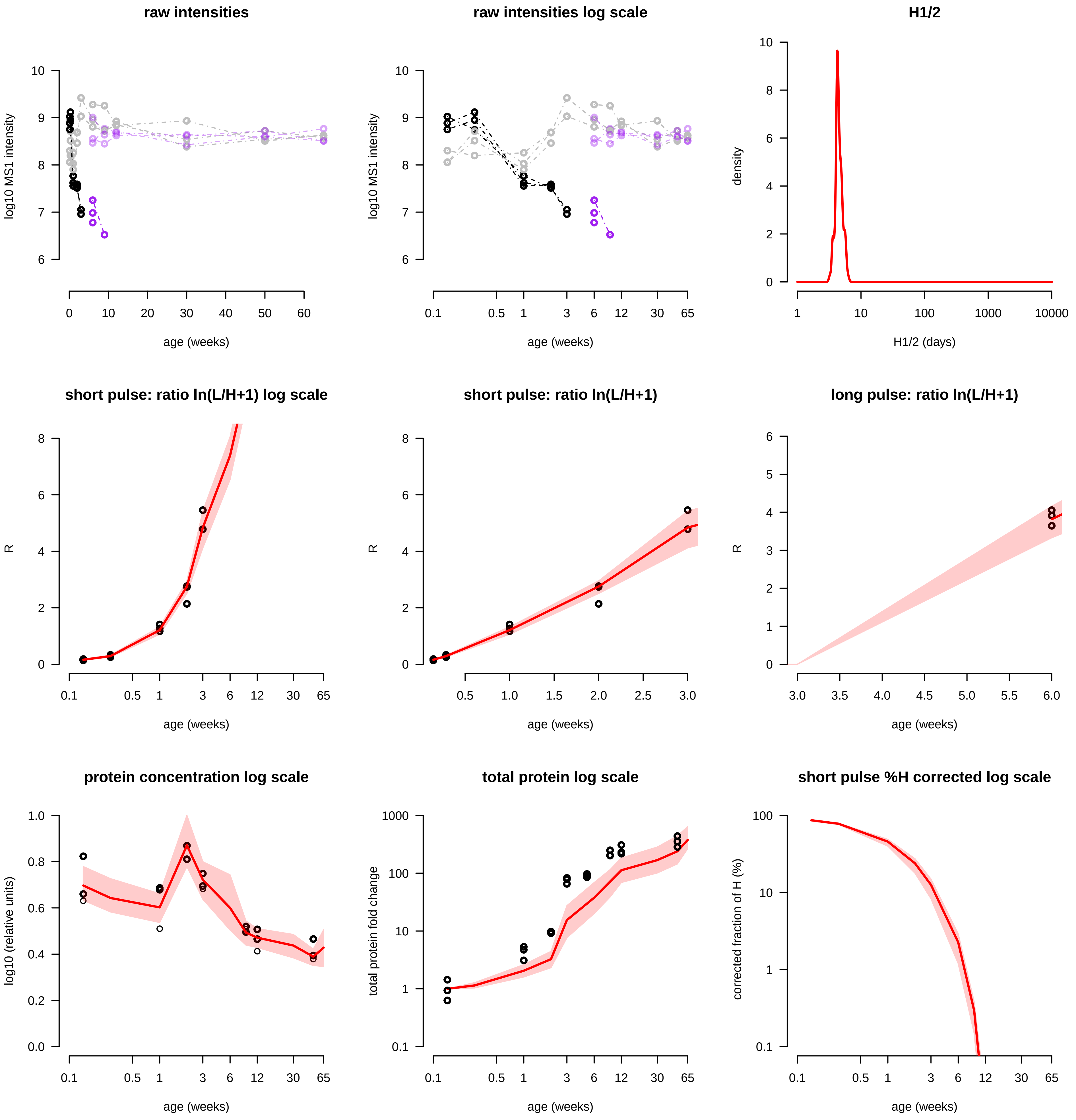

Q9QY36

>sp|Q9QY36|NAA10_MOUSE

N−alpha−acetyltransferase 10

GN=Naa10

Med H1/2: 5 days

Q5 H1/2: 4.1 days

Q95 H1/2: 6.7 days

reliable model fit: yes

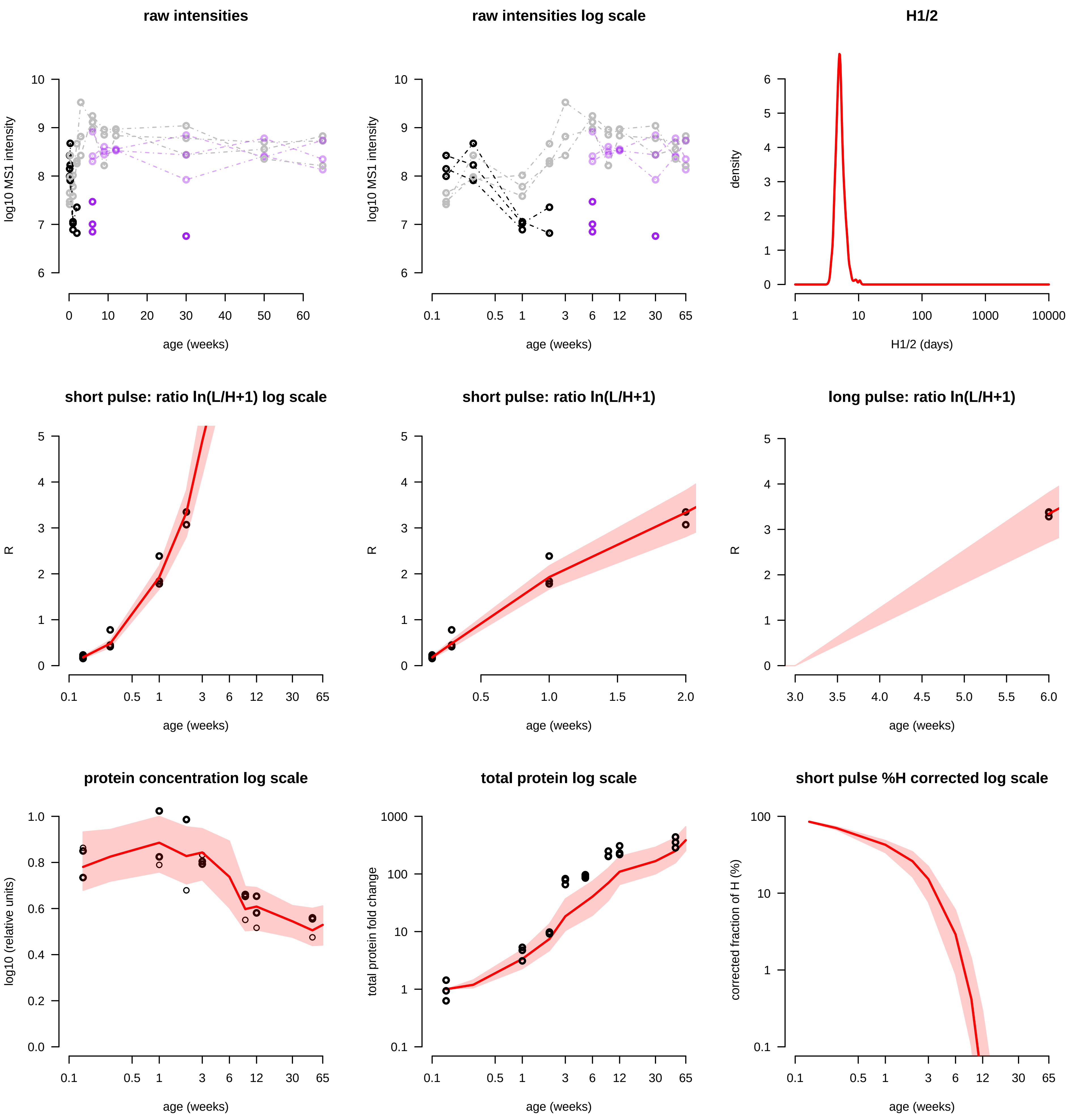

P13864;P13864−2

>sp|P13864|DNMT1_MOUSE

DNA (cytosine−5)−methyltransferase 1

GN=Dnmt1

Med H1/2: 3.3 days

Q5 H1/2: 2.6 days

Q95 H1/2: 4.4 days

reliable model fit: yes

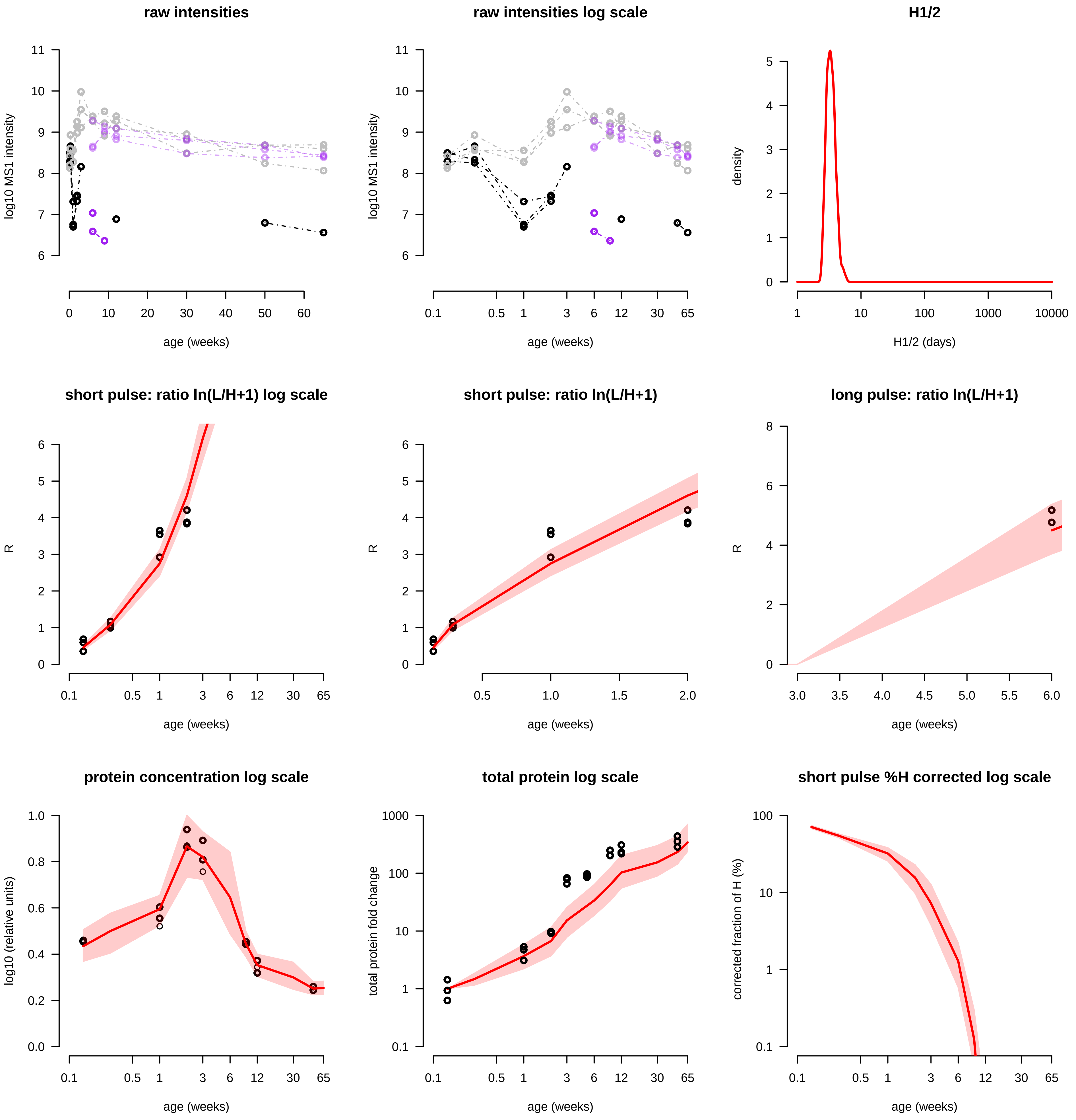

Q80SZ7

>sp|Q80SZ7|GBG5_MOUSE

Guanine nucleotide−binding protein G(I)/G(S)/G(O) subunit gamma−5

GN=Gng5

Med H1/2: 9.2 days

Q5 H1/2: 6.7 days

Q95 H1/2: 16.6 days

reliable model fit: yes

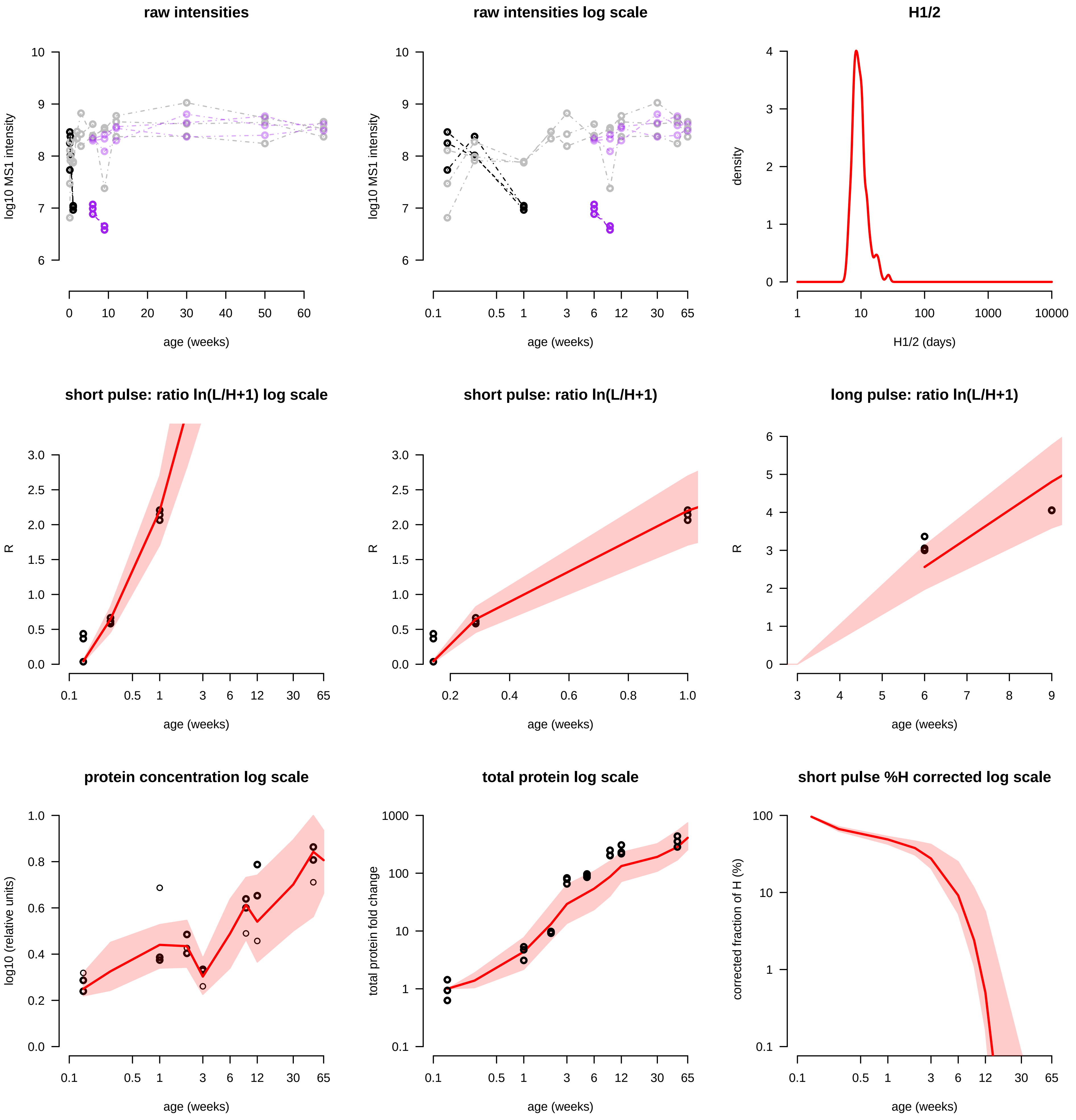

B1AZI6

>sp|B1AZI6|THOC2_MOUSE

THO complex subunit 2

GN=Thoc2

Med H1/2: 4.2 days

Q5 H1/2: 3.5 days

Q95 H1/2: 5.1 days

reliable model fit: yes

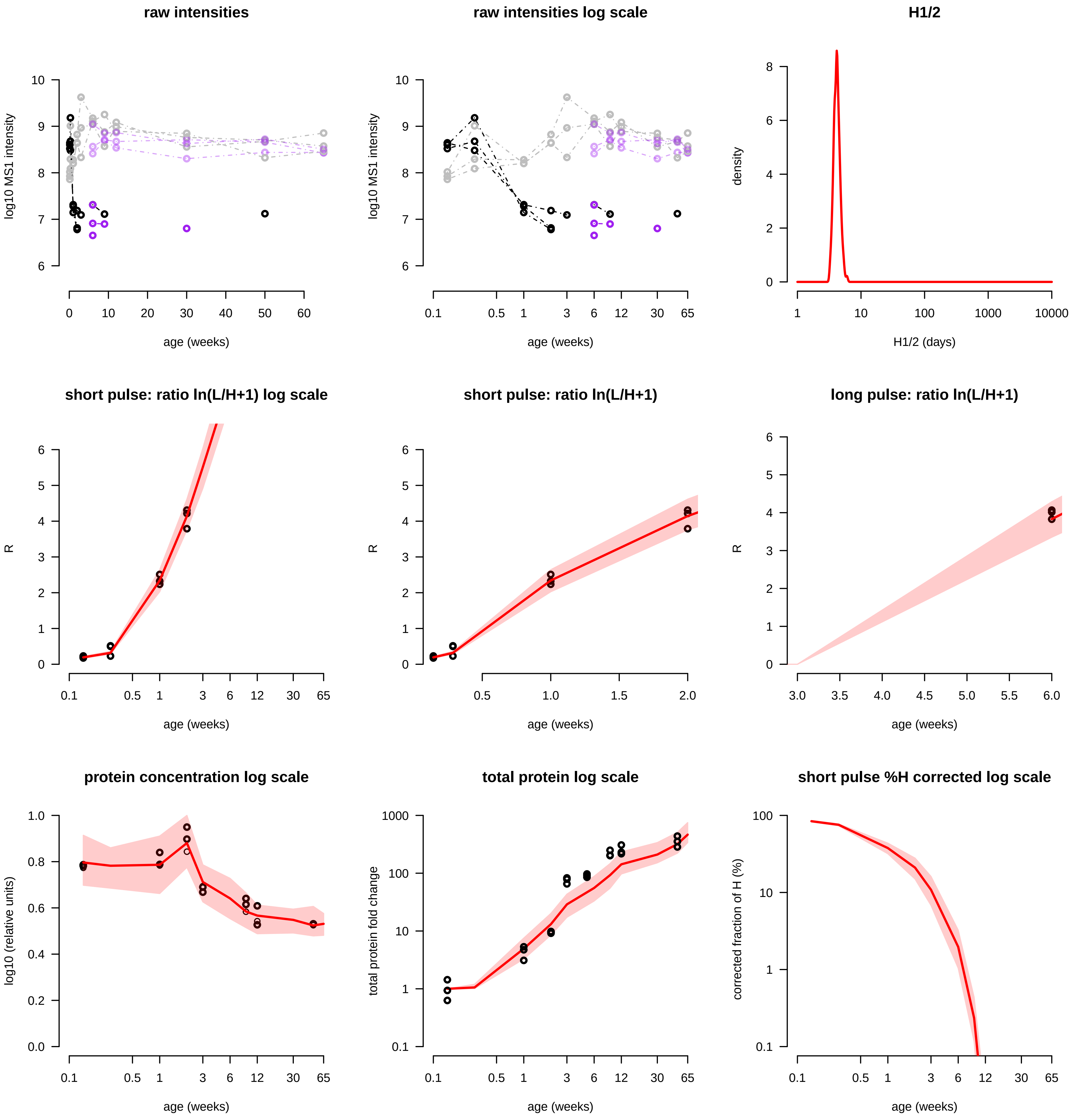

Q3UMU9−3;Q3UMU9

>sp|Q3UMU9−3|HDGR2_MOUSE

Isoform 3 of Hepatoma−derived growth factor−related protein 2

GN=Hdgfl2

Med H1/2: 4.8 days

Q5 H1/2: 3.5 days

Q95 H1/2: 7.7 days

reliable model fit: yes

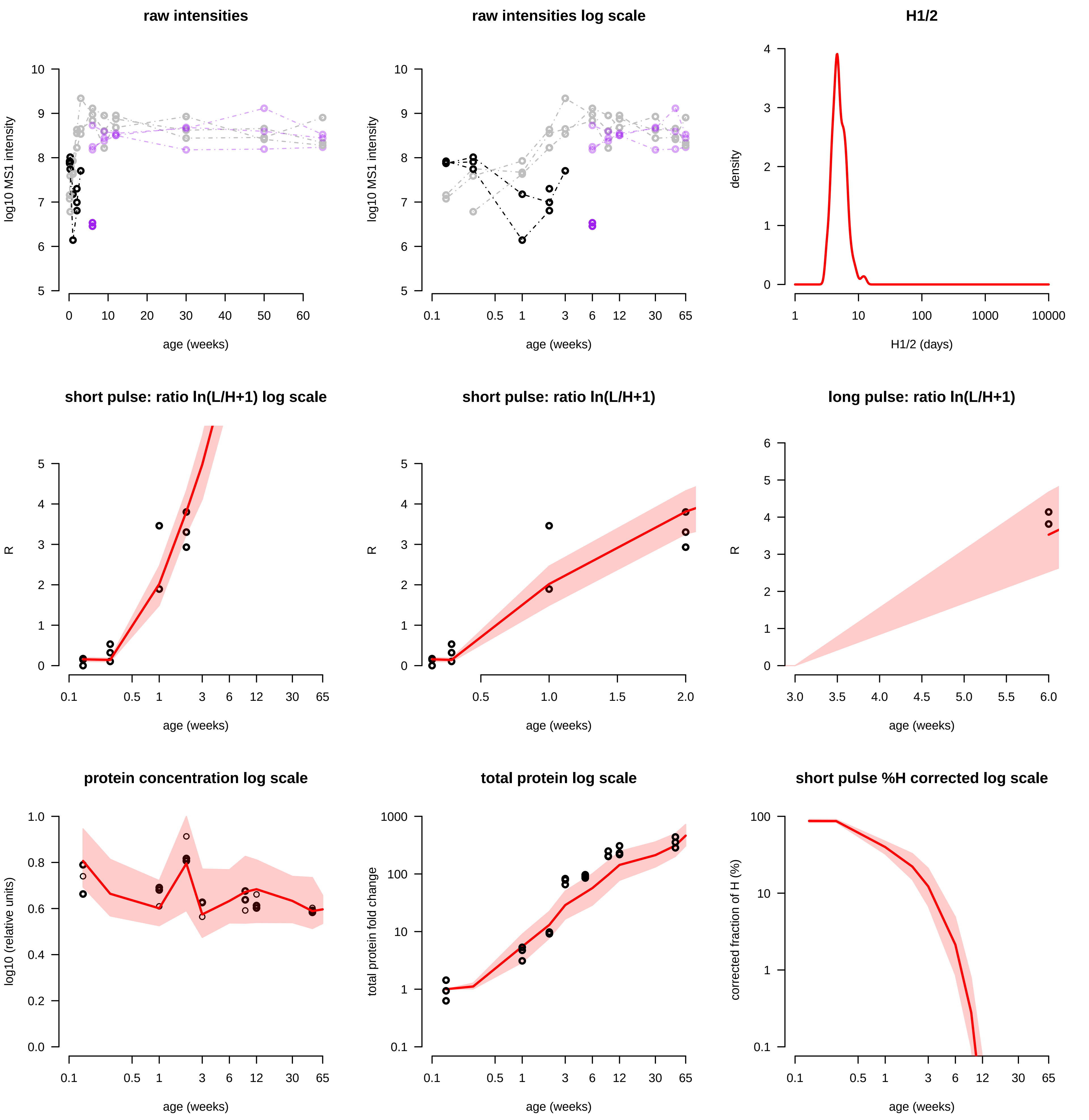

Q8BHE8

>sp|Q8BHE8|MAIP1_MOUSE

m−AAA protease−interacting protein 1, mitochondrial

GN=Maip1

Med H1/2: 5.5 days

Q5 H1/2: 3.7 days

Q95 H1/2: 10.7 days

reliable model fit: no

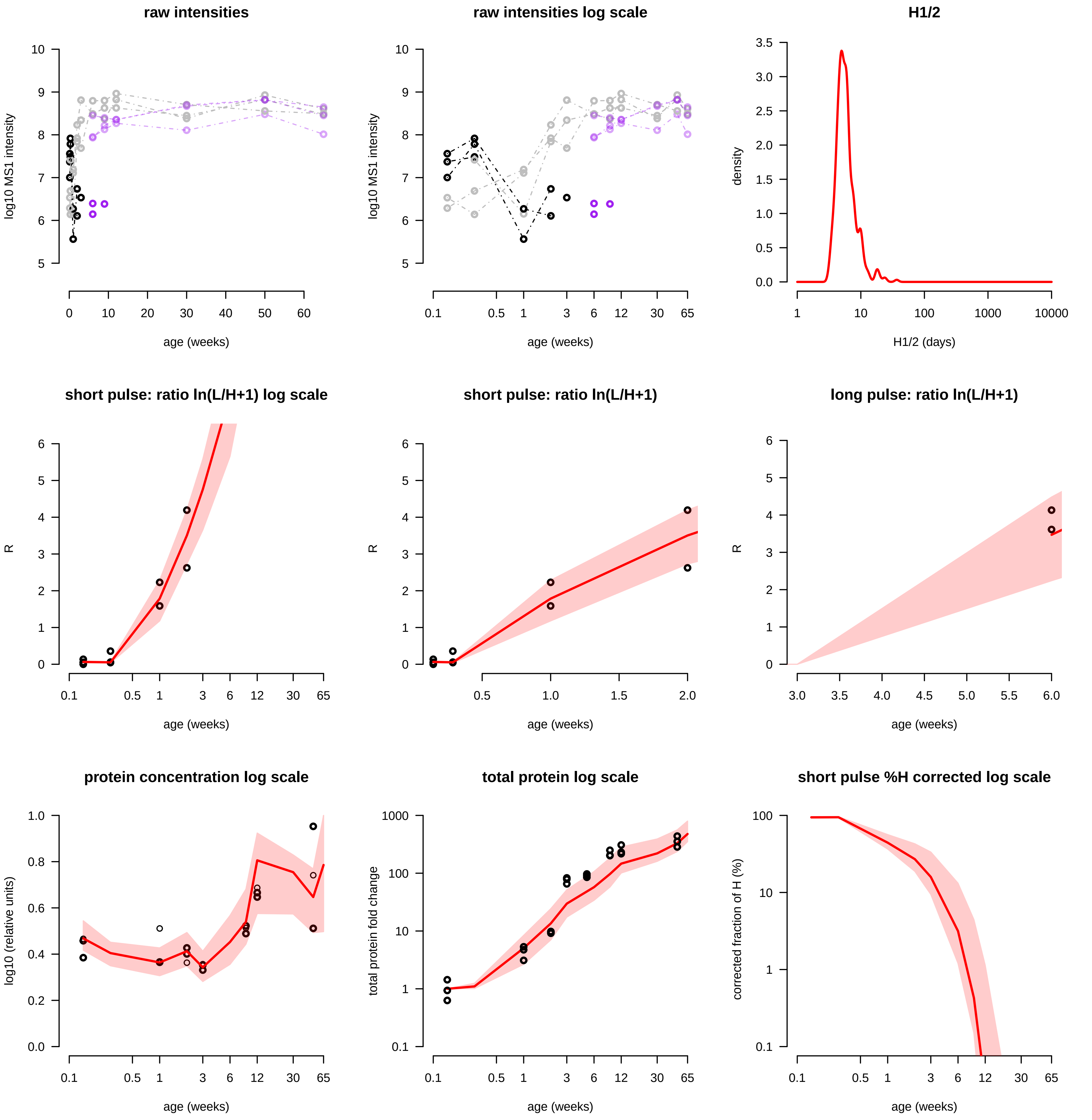

P60521

>sp|P60521|GBRL2_MOUSE

Gamma−aminobutyric acid receptor−associated protein−like 2

GN=Gabarapl2

Med H1/2: 5.5 days

Q5   H1/2: 3.8 days

Q95 H1/2: 9.6 days

reliable model fit: yes

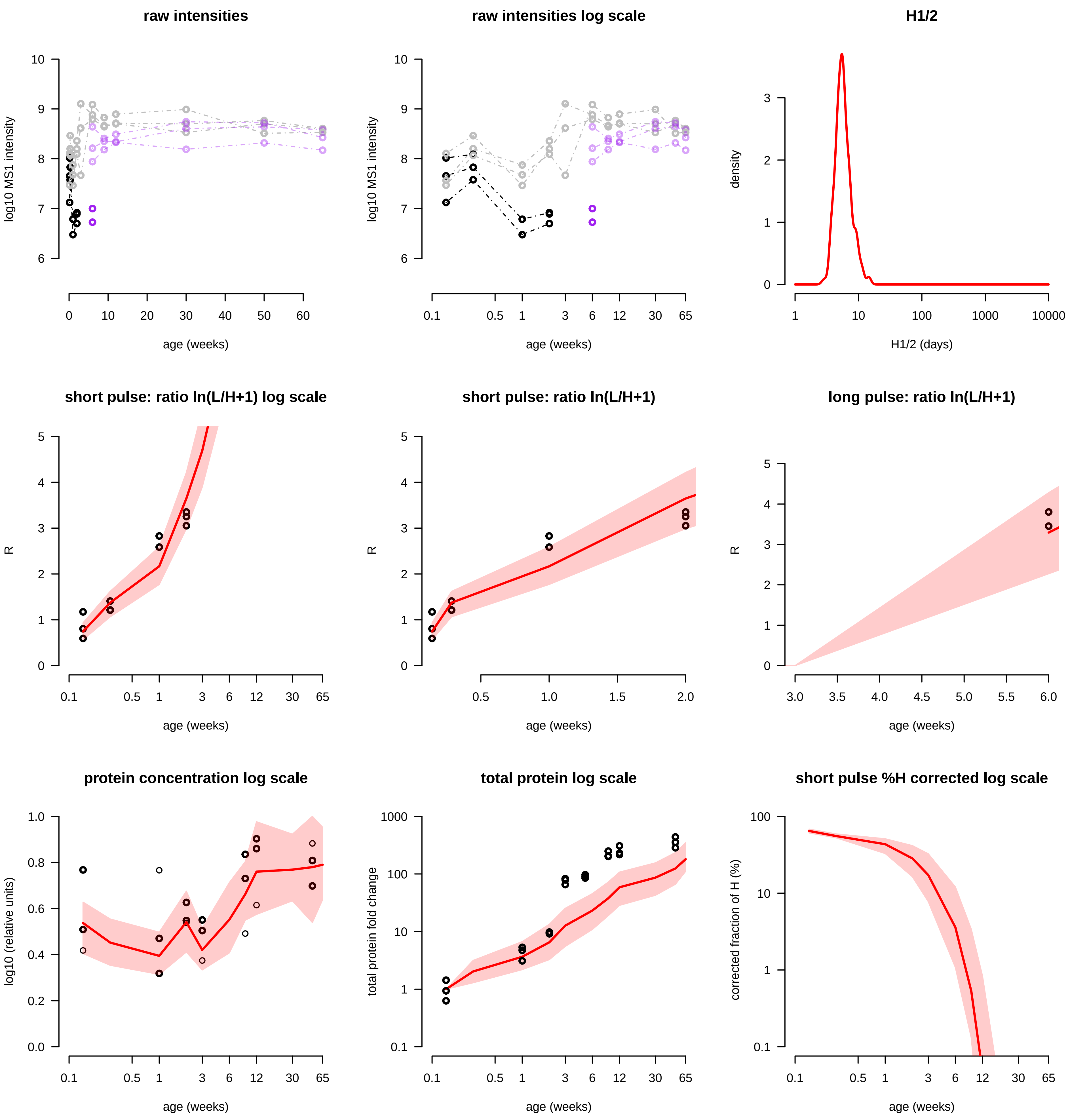

P63330

>sp|P63330|PP2AA_MOUSE

Serine/threonine−protein phosphatase 2A catalytic subunit alpha isoform

GN=Ppp2ca

Med H1/2: 7.6 days

Q5 H1/2: 3.4 days

Q95 H1/2: 100 days

reliable model fit: yes

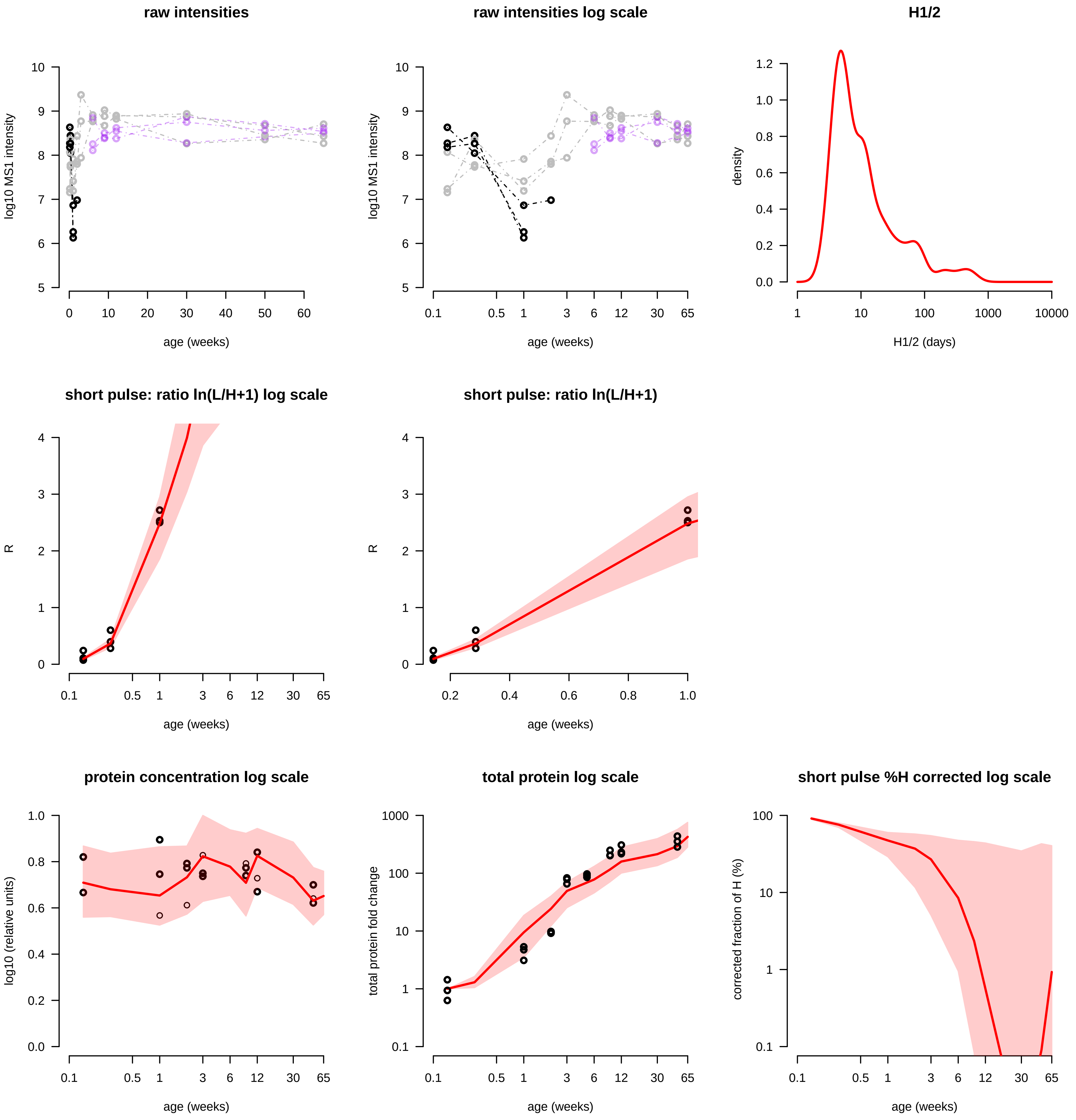

Q99LD4

>sp|Q99LD4|CSN1_MOUSE

COP9 signalosome complex subunit 1

GN=Gps1

Med H1/2: 5 days

Q5 H1/2: 4 days

Q95 H1/2: 6.6 days

reliable model fit: yes

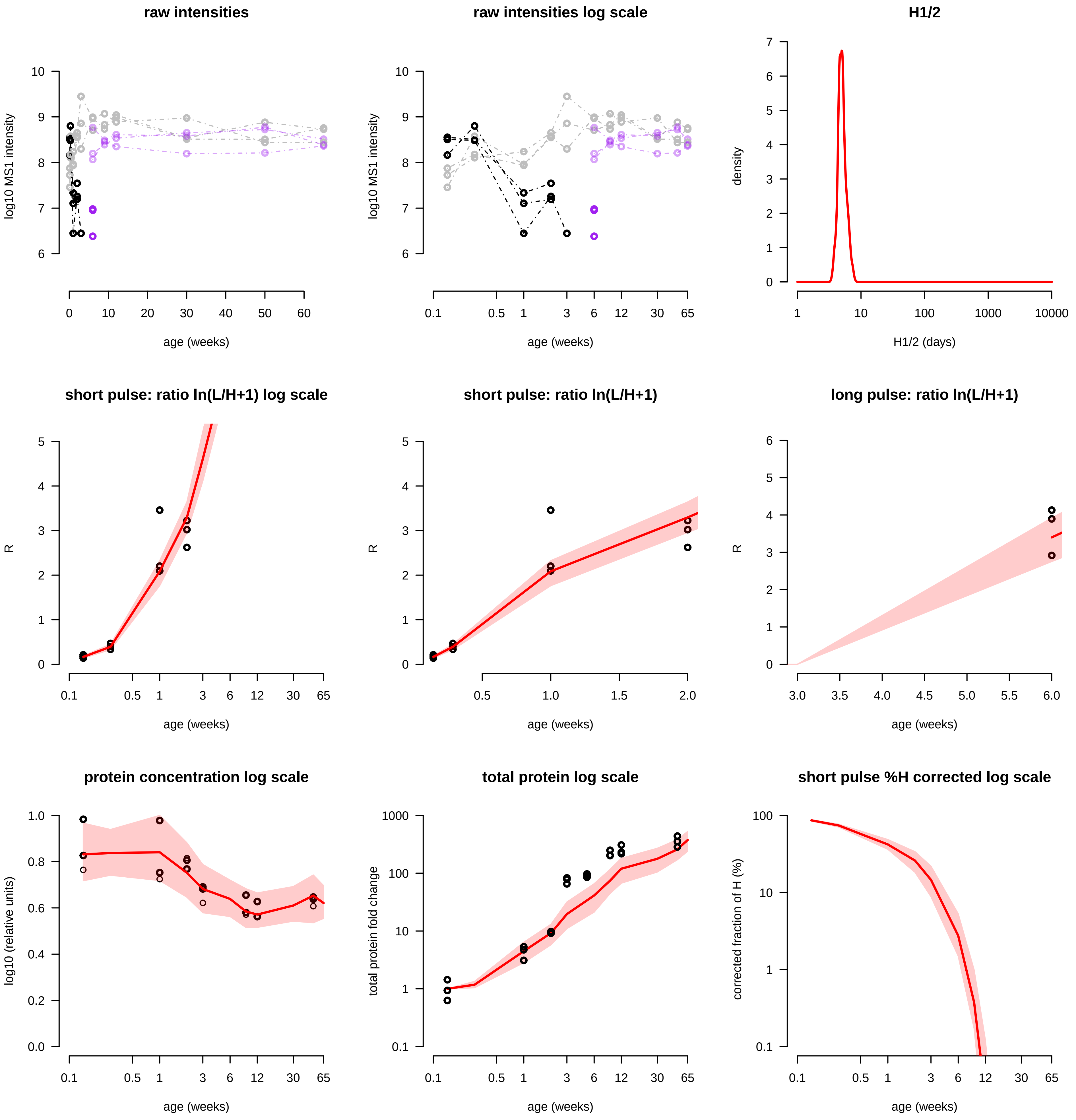

P28867

>sp|P28867|KPCD_MOUSE

Protein kinase C delta type

GN=Prkcd

Med H1/2: 4.7 days

Q5 H1/2: 3.5 days

Q95 H1/2: 7.3 days

reliable model fit: yes

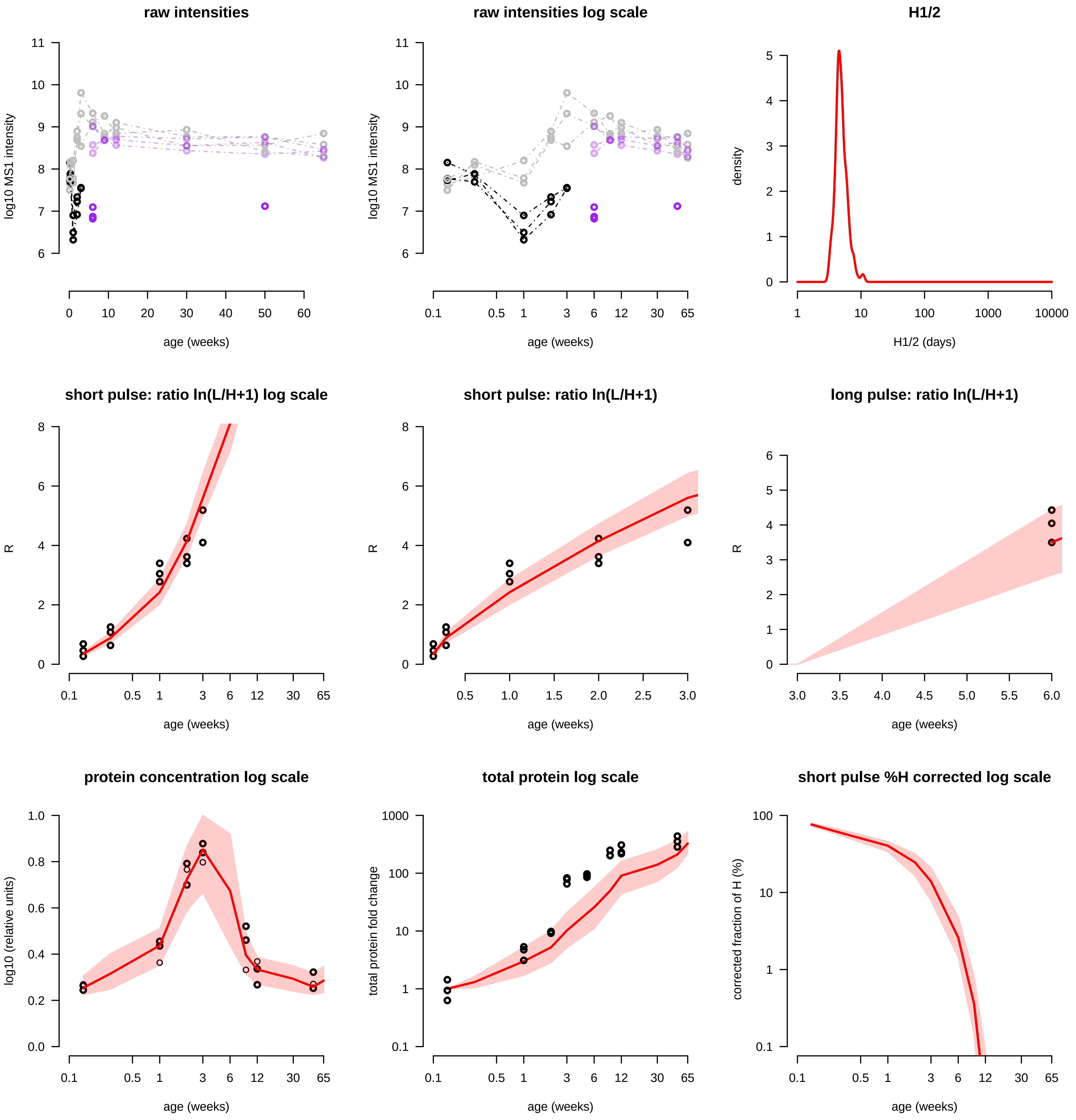

Q8BIW1

>sp|Q8BIW1|PRUN1_MOUSE

Exopolyphosphatase PRUNE1

GN=Prune1

Med H1/2: 4.8 days

Q5 H1/2: 3.7 days

Q95 H1/2: 6.6 days

reliable model fit: yes

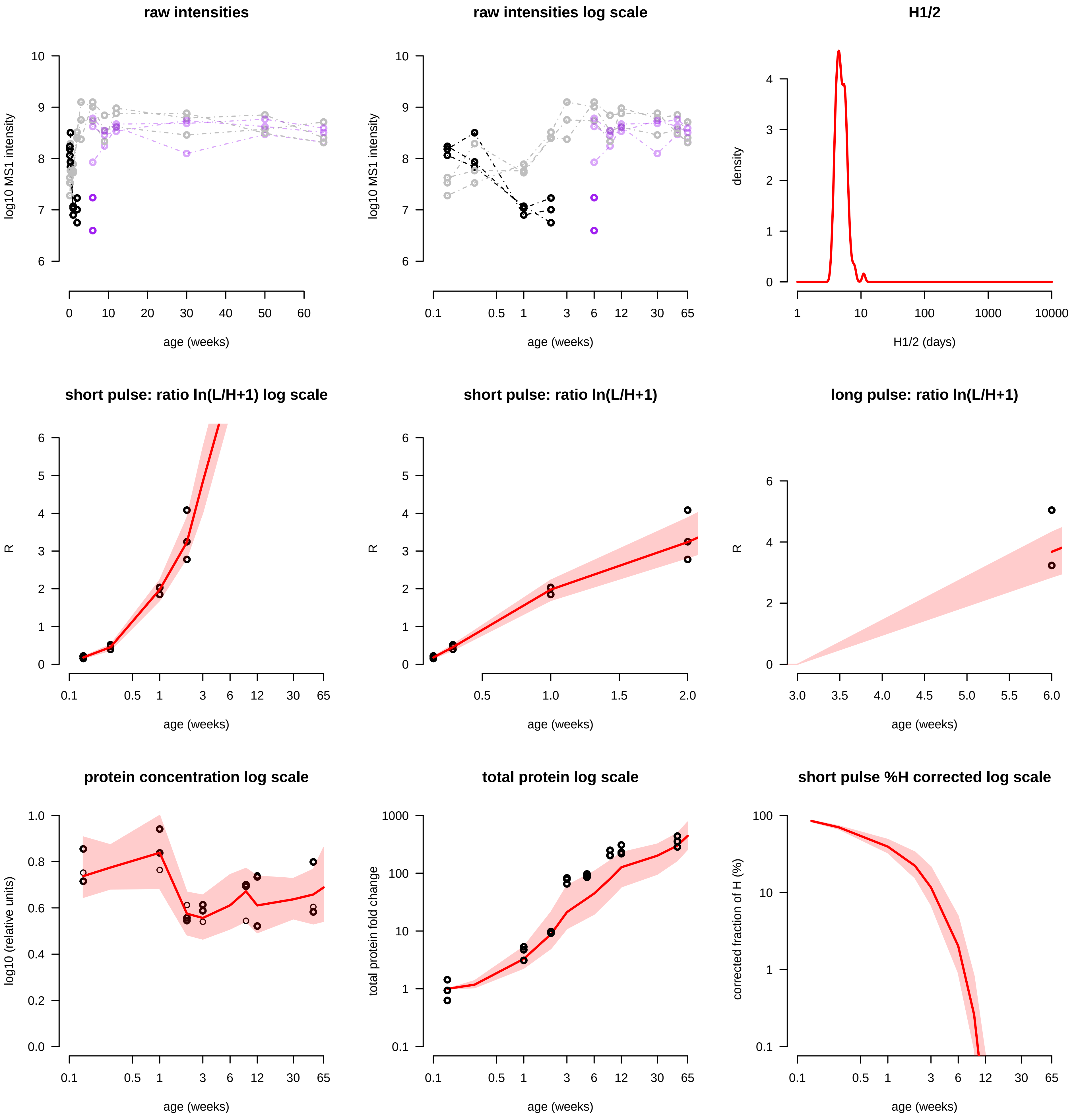

O70152

>sp|O70152|DPM1_MOUSE

Dolichol−phosphate mannosyltransferase subunit 1

GN=Dpm1

Med H1/2: 5.6 days

Q5 H1/2: 4.5 days

Q95 H1/2: 7.5 days

reliable model fit: yes

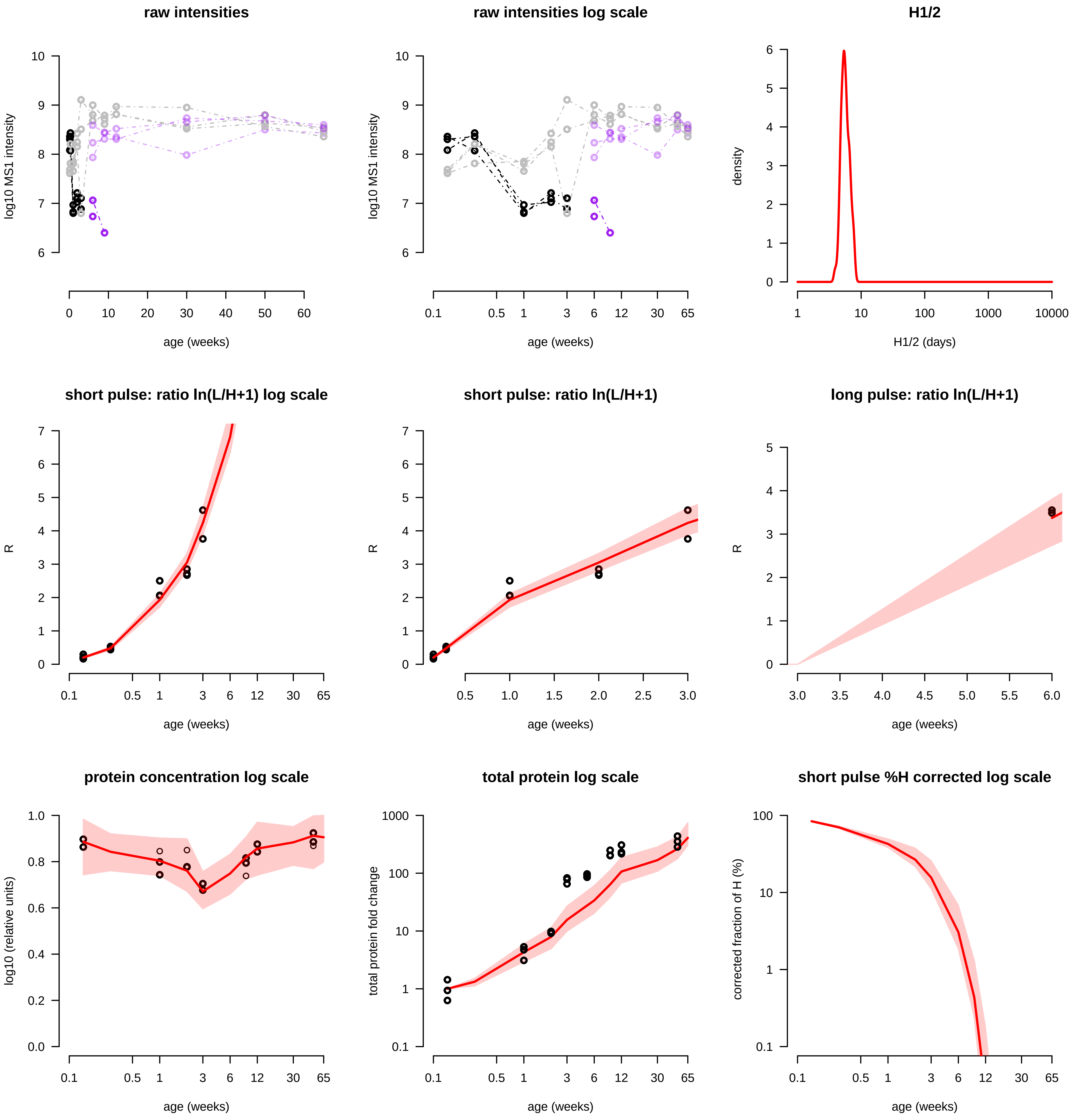

Q8BSQ9;Q8BSQ9−2

>sp|Q8BSQ9|PB1_MOUSE

Protein polybromo−1

GN=Pbrm1

Med H1/2: 9.5 days

Q5 H1/2: 8.2 days

Q95 H1/2: 11.9 days

reliable model fit: yes

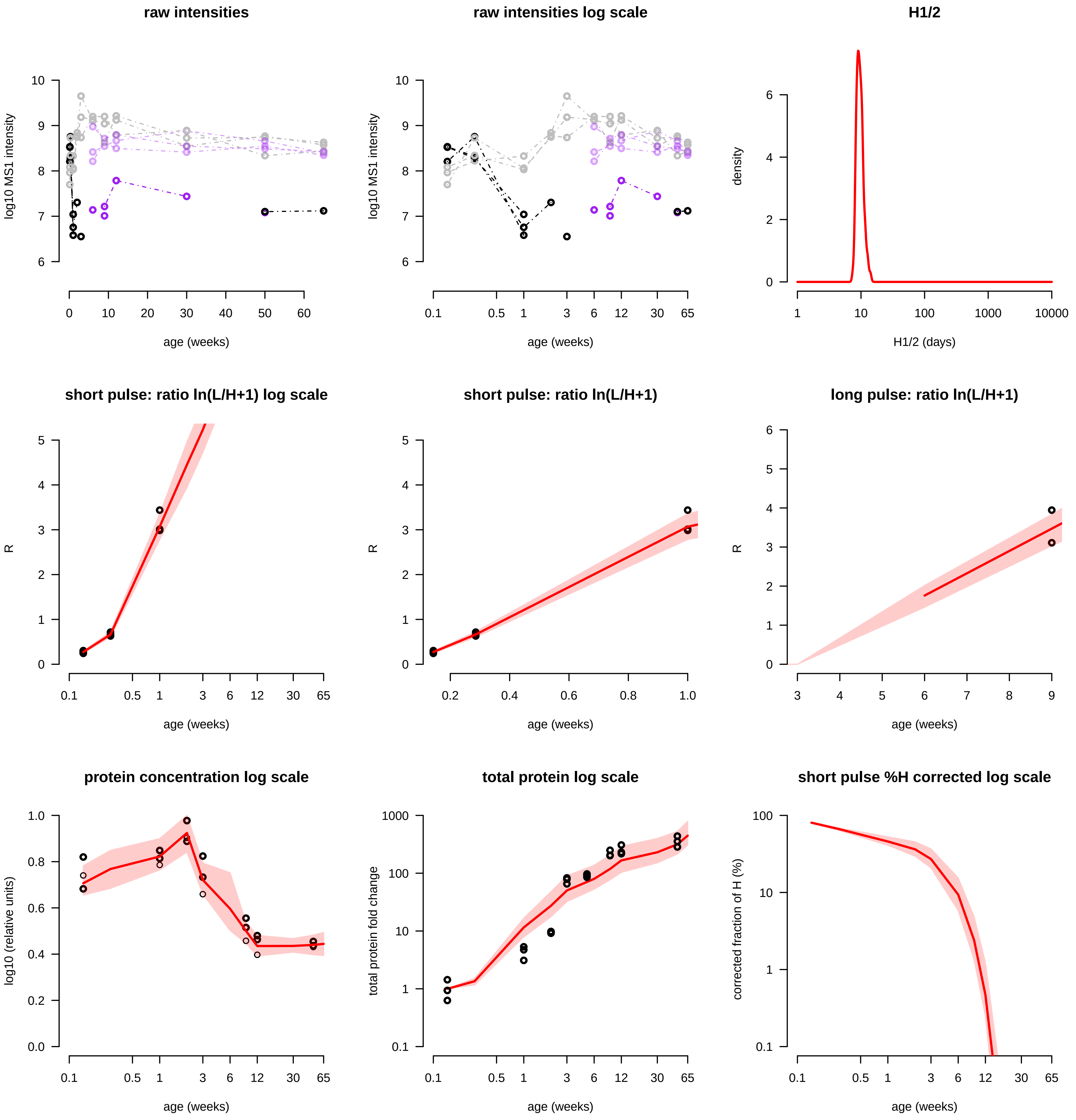

Q9CT10

>sp|Q9CT10|RANB3_MOUSE

Ran−binding protein 3

GN=Ranbp3

Med H1/2: 4.1 days

Q5 H1/2: 2.9 days

Q95 H1/2: 7.2 days

reliable model fit: yes

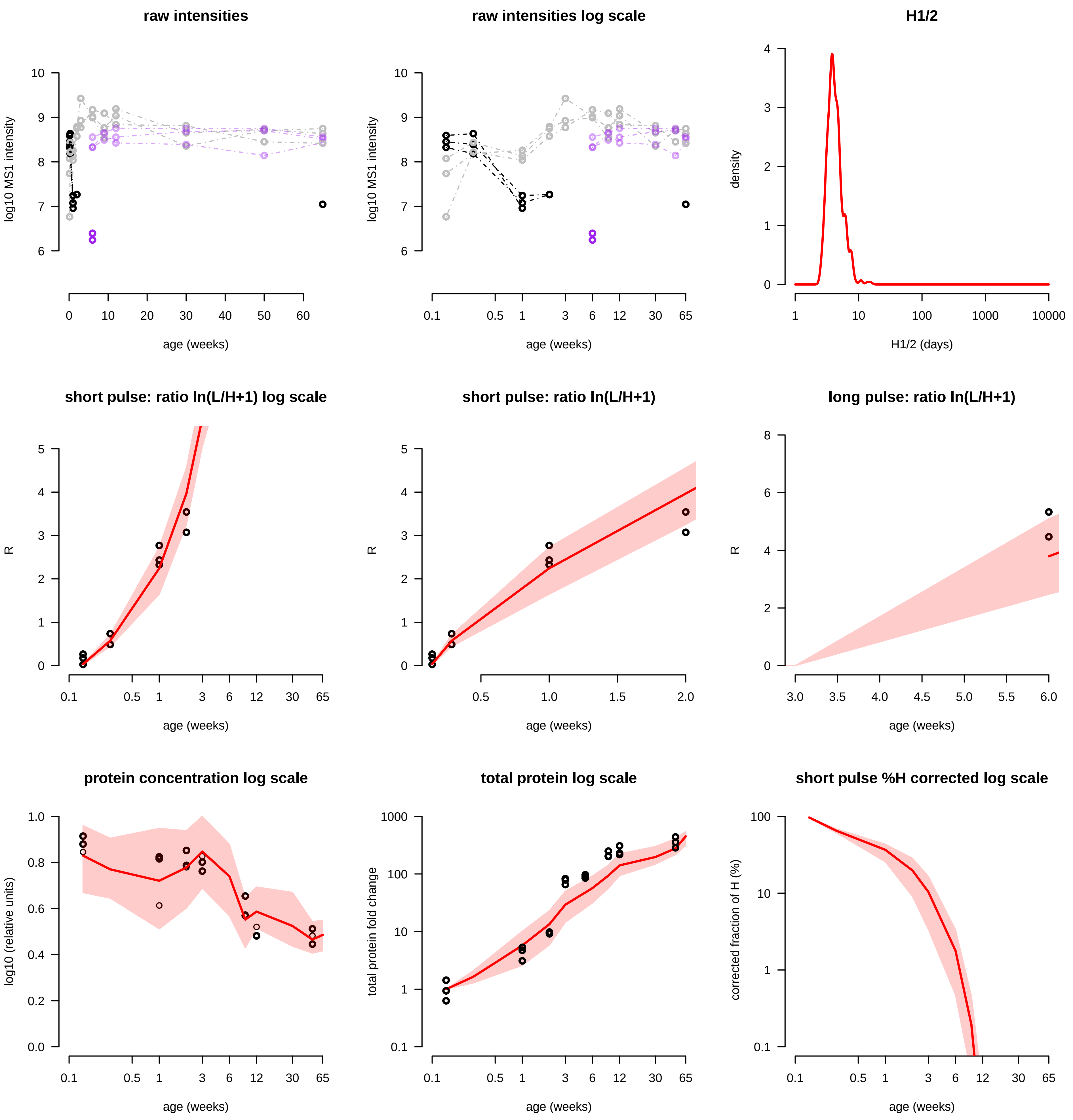

Q8BY87−2;Q8BY87

>sp|Q8BY87−2|UBP47_MOUSE

Isoform 2 of Ubiquitin carboxyl−terminal hydrolase 47

GN=Usp47

Med H1/2: 5.3 days

Q5 H1/2: 2.7 days

Q95 H1/2: 15.8 days

reliable model fit: yes

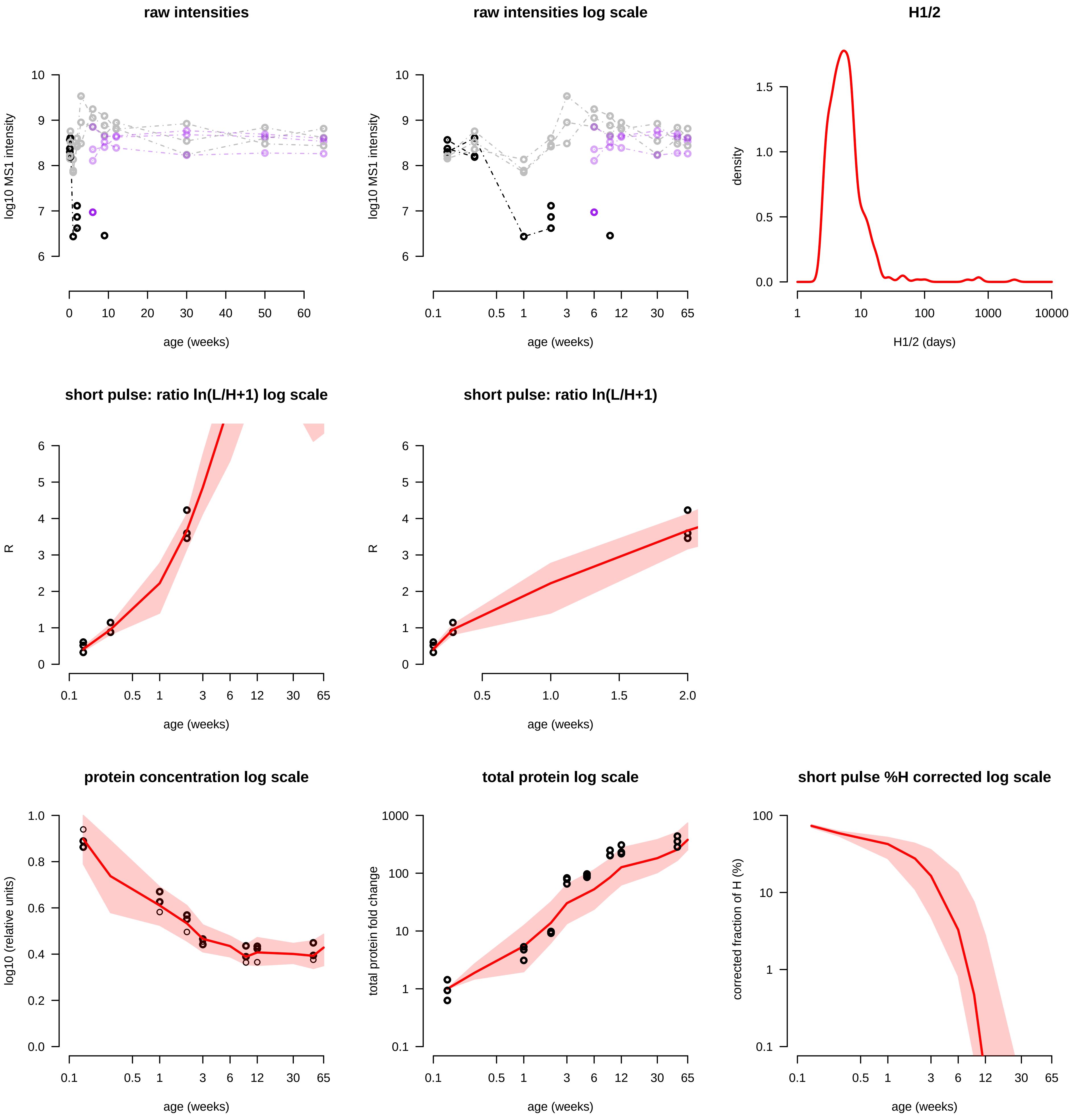

Q9DBG7

>sp|Q9DBG7|SRPRA_MOUSE

Signal recognition particle receptor subunit alpha

GN=Srpra

Med H1/2: 54 days

Q5 H1/2: 32.3 days

Q95 H1/2: 107.2 days

reliable model fit: yes

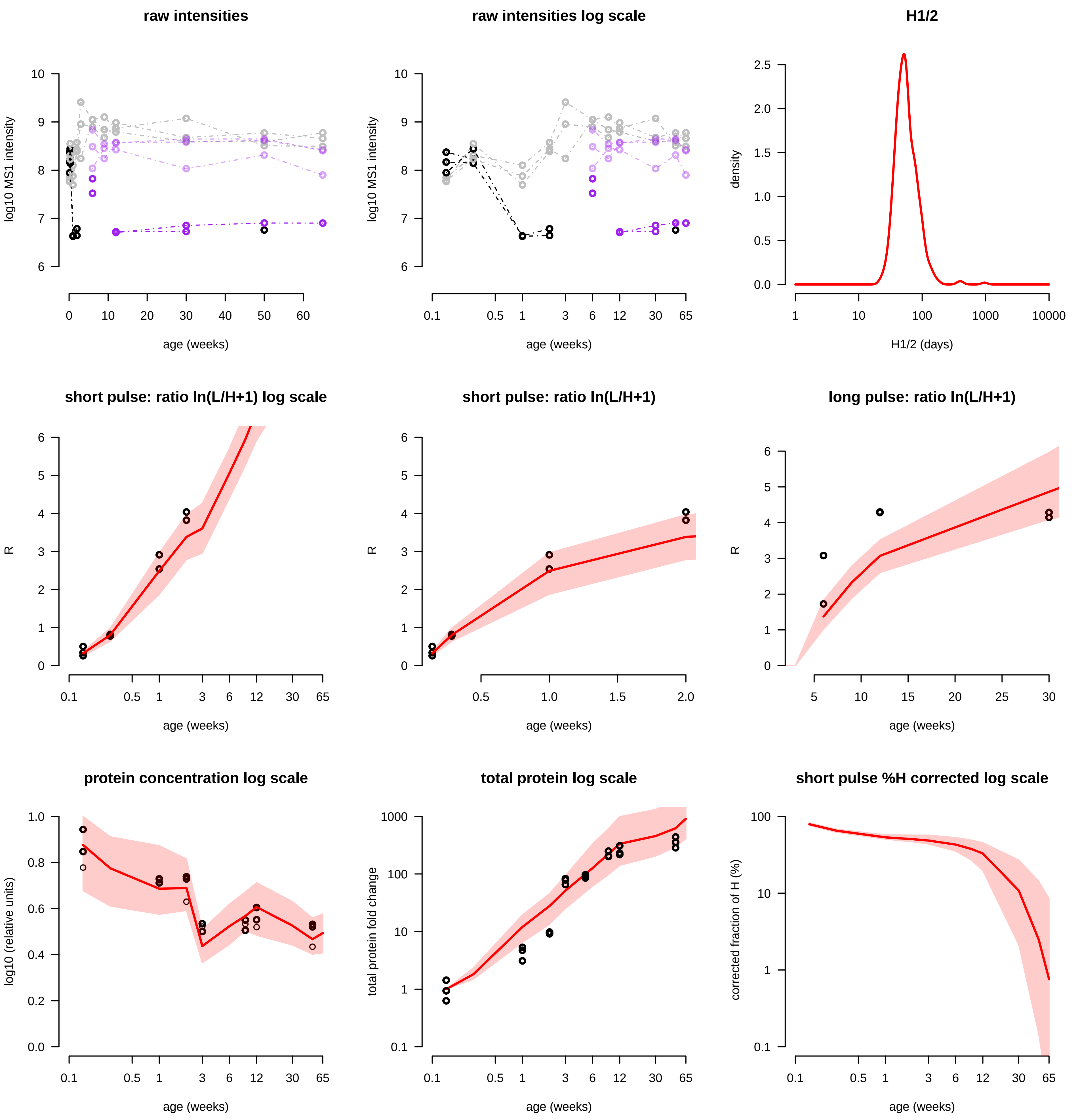

Q9CYG7

>sp|Q9CYG7|TOM34_MOUSE

Mitochondrial import receptor subunit TOM34

GN=Tomm34

Med H1/2: 12.2 days

Q5 H1/2: 4.3 days

Q95 H1/2: 78.8 days

reliable model fit: yes

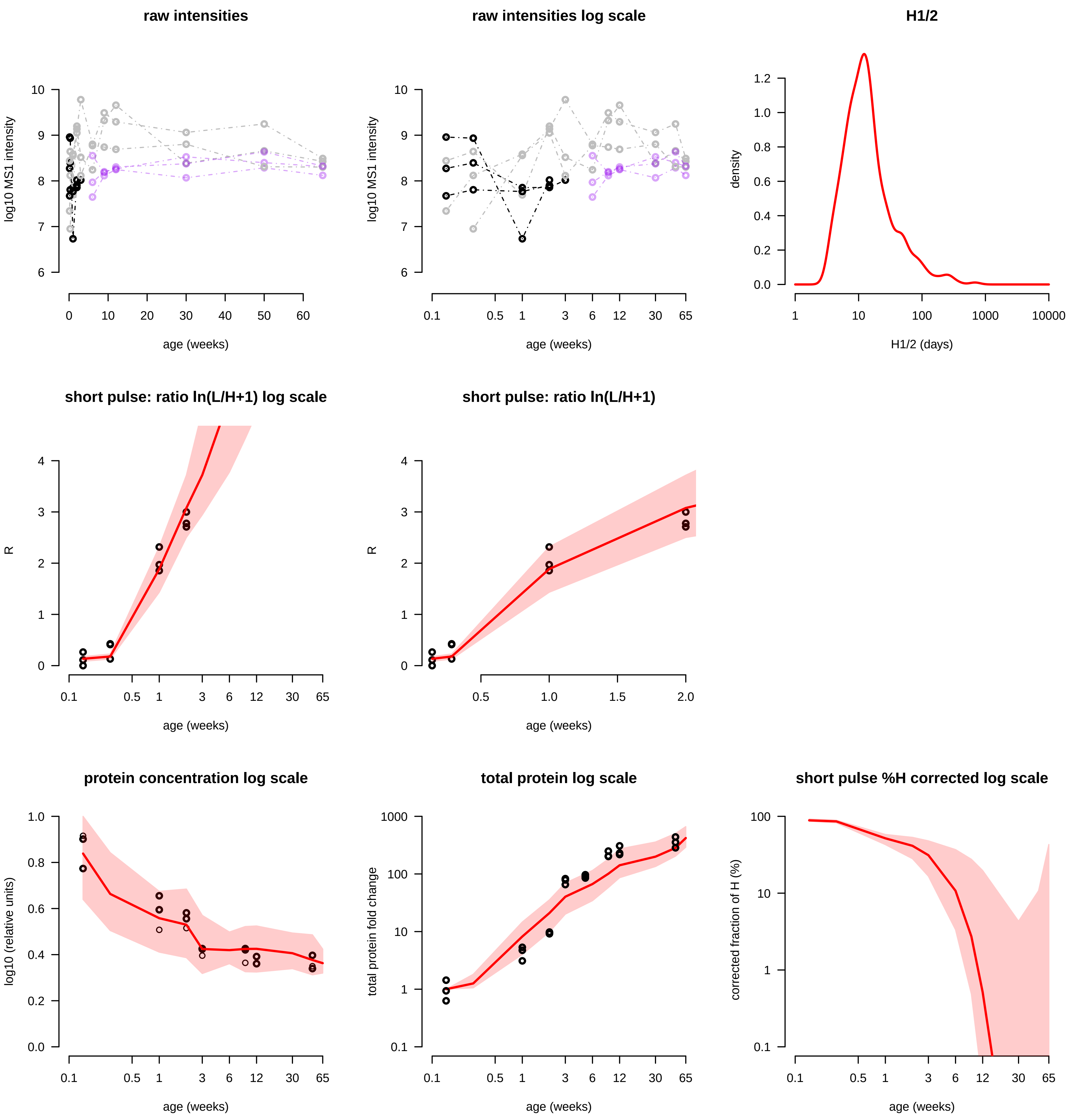

Q8BJD1

>sp|Q8BJD1|ITIH5_MOUSE

Inter−alpha−trypsin inhibitor heavy chain H5

GN=Itih5

Med H1/2: 19.9 days

Q5 H1/2: 6.6 days

Q95 H1/2: 218 days

reliable model fit: yes

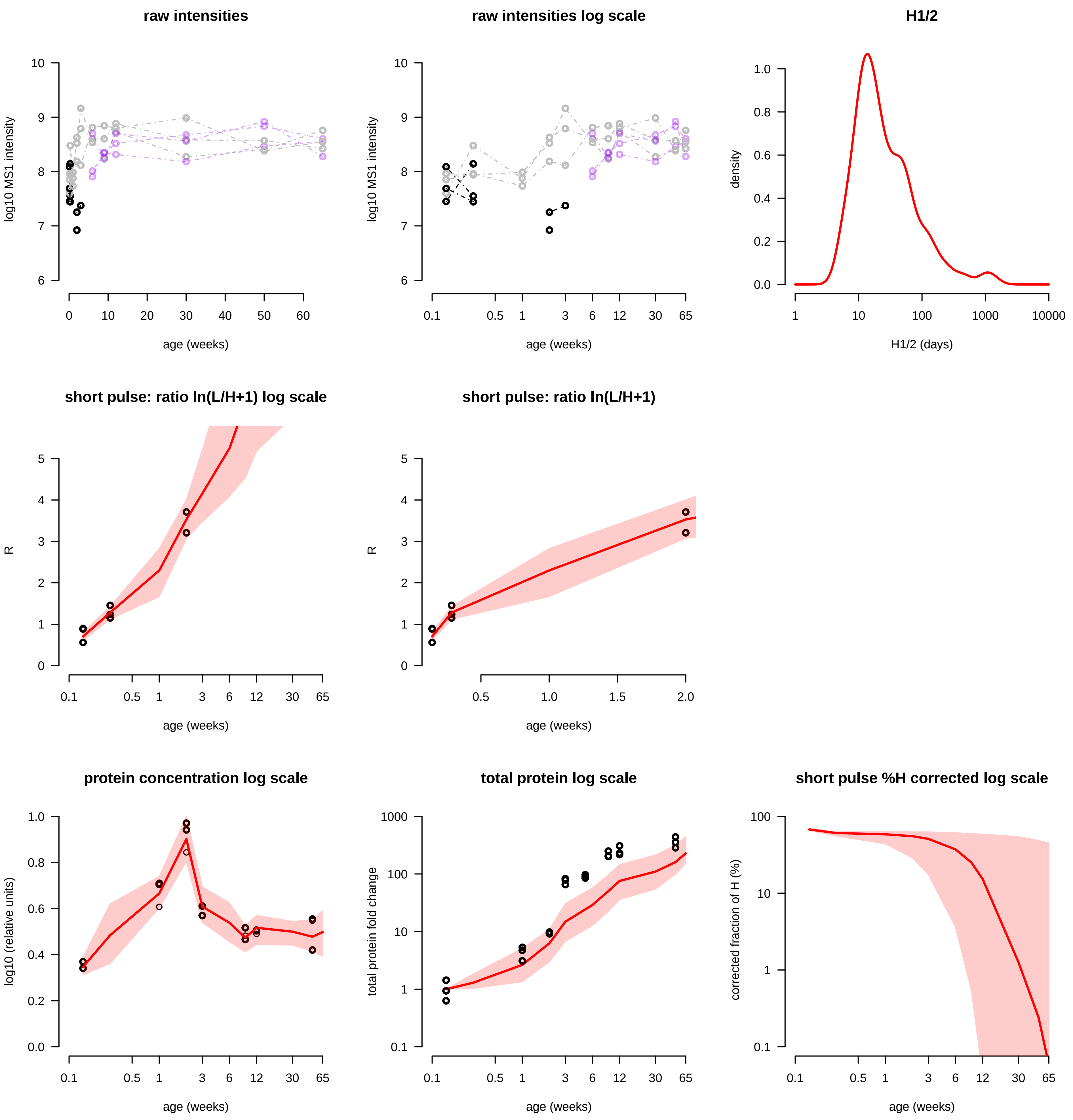

Q9ER88;Q9ER88−2

>sp|Q9ER88|RT29_MOUSE

28S ribosomal protein S29, mitochondrial

GN=Dap3

Med H1/2: 7.1 days

Q5 H1/2: 2.9 days

Q95 H1/2: 41.6 days

reliable model fit: yes

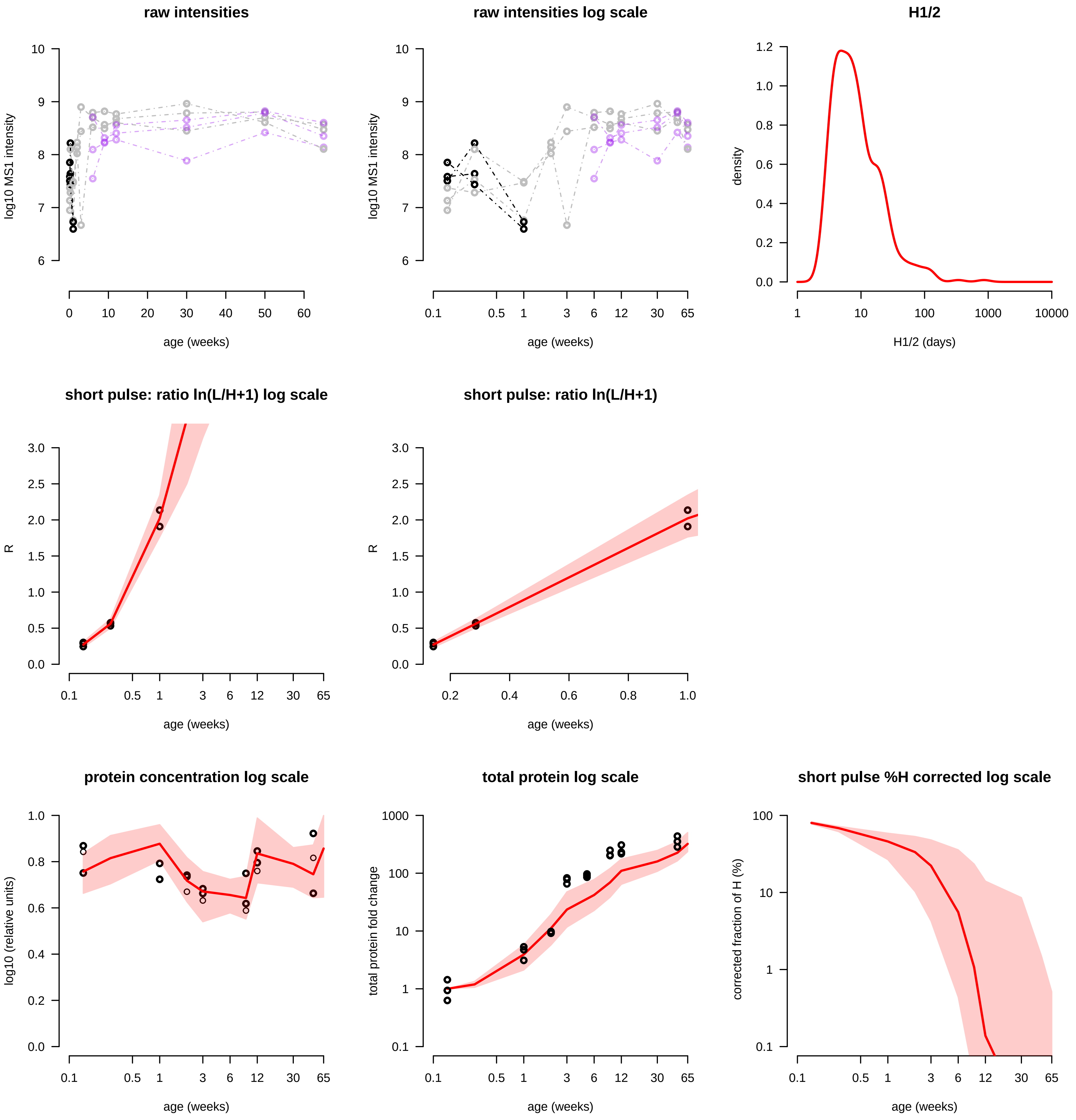

Q9DCL8

>sp|Q9DCL8|IPP2_MOUSE

Protein phosphatase inhibitor 2

GN=Ppp1r2

Med H1/2: 3.8 days

Q5 H1/2: 3.1 days

Q95 H1/2: 4.8 days

reliable model fit: yes

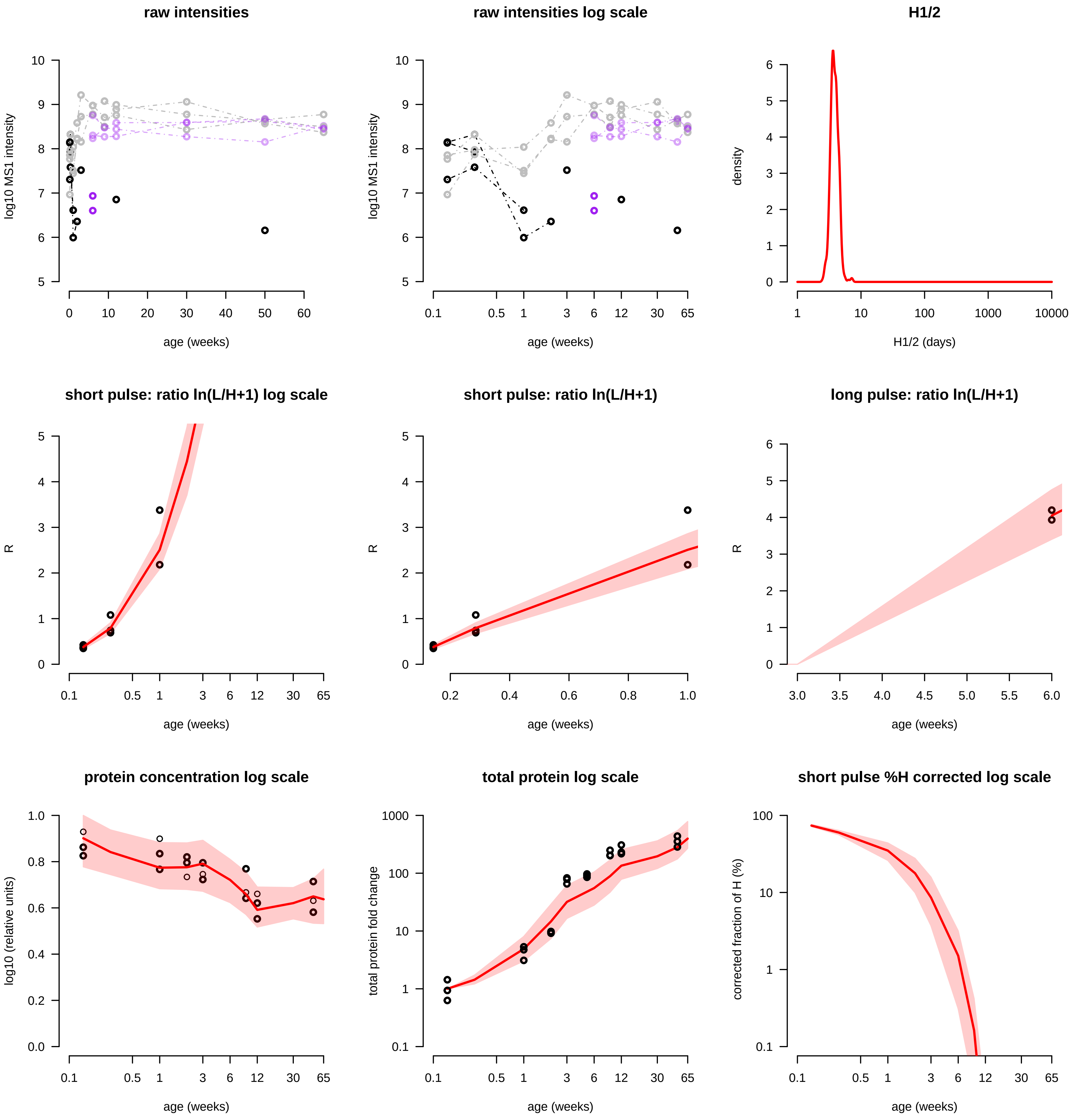

Q99MI1;Q99MI1−2

>sp|Q99MI1|RB6I2_MOUSE

ELKS/Rab6−interacting/CAST family member 1

GN=Erc1

Med H1/2: 3.9 days

Q5 H1/2: 2.6 days

Q95 H1/2: 12.7 days

reliable model fit: yes

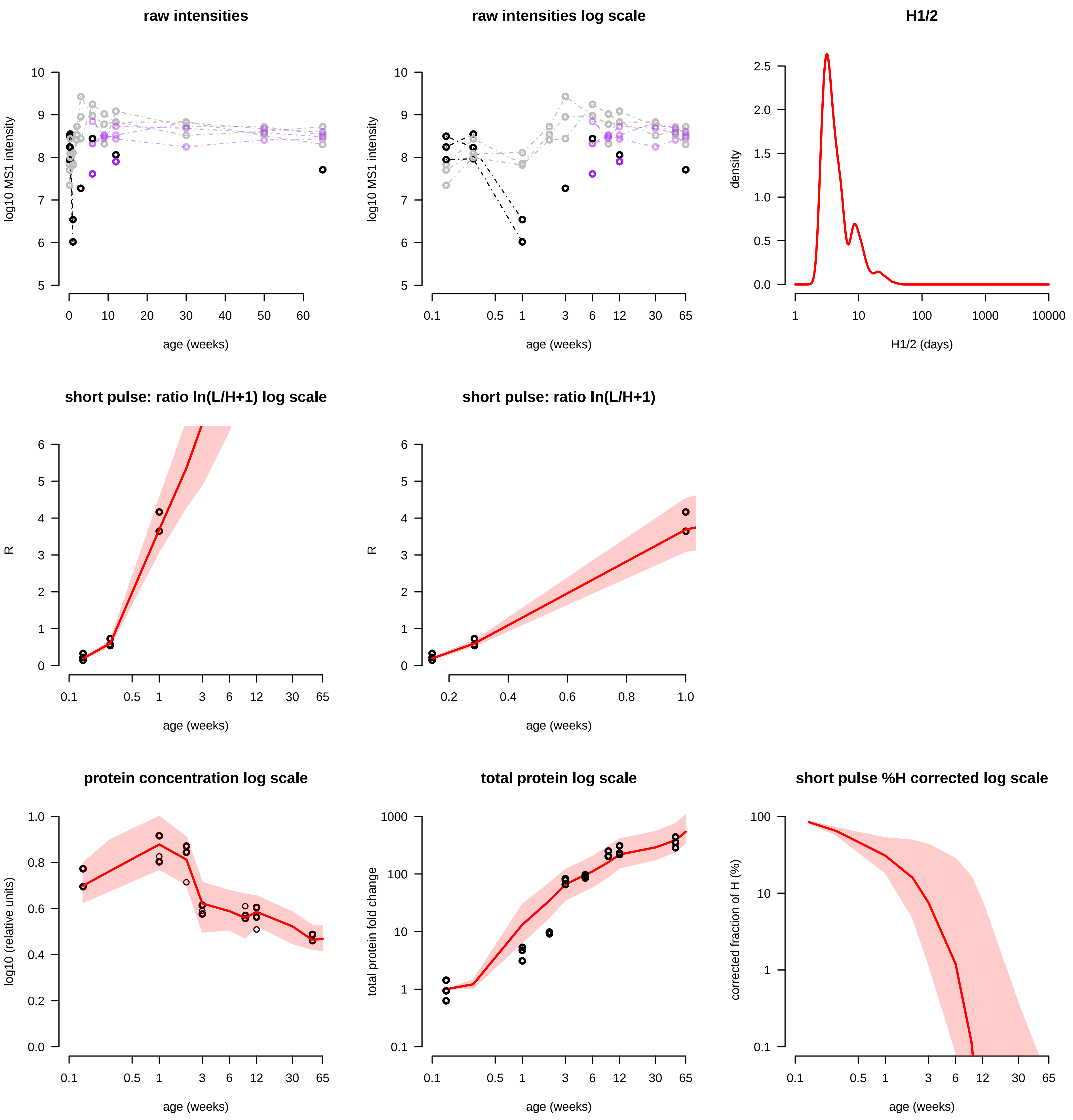

Q8R0G9

>sp|Q8R0G9|NU133_MOUSE

Nuclear pore complex protein Nup133

GN=Nup133

Med H1/2: 5.1 days

Q5 H1/2: 3.6 days

Q95 H1/2: 9.5 days

reliable model fit: no

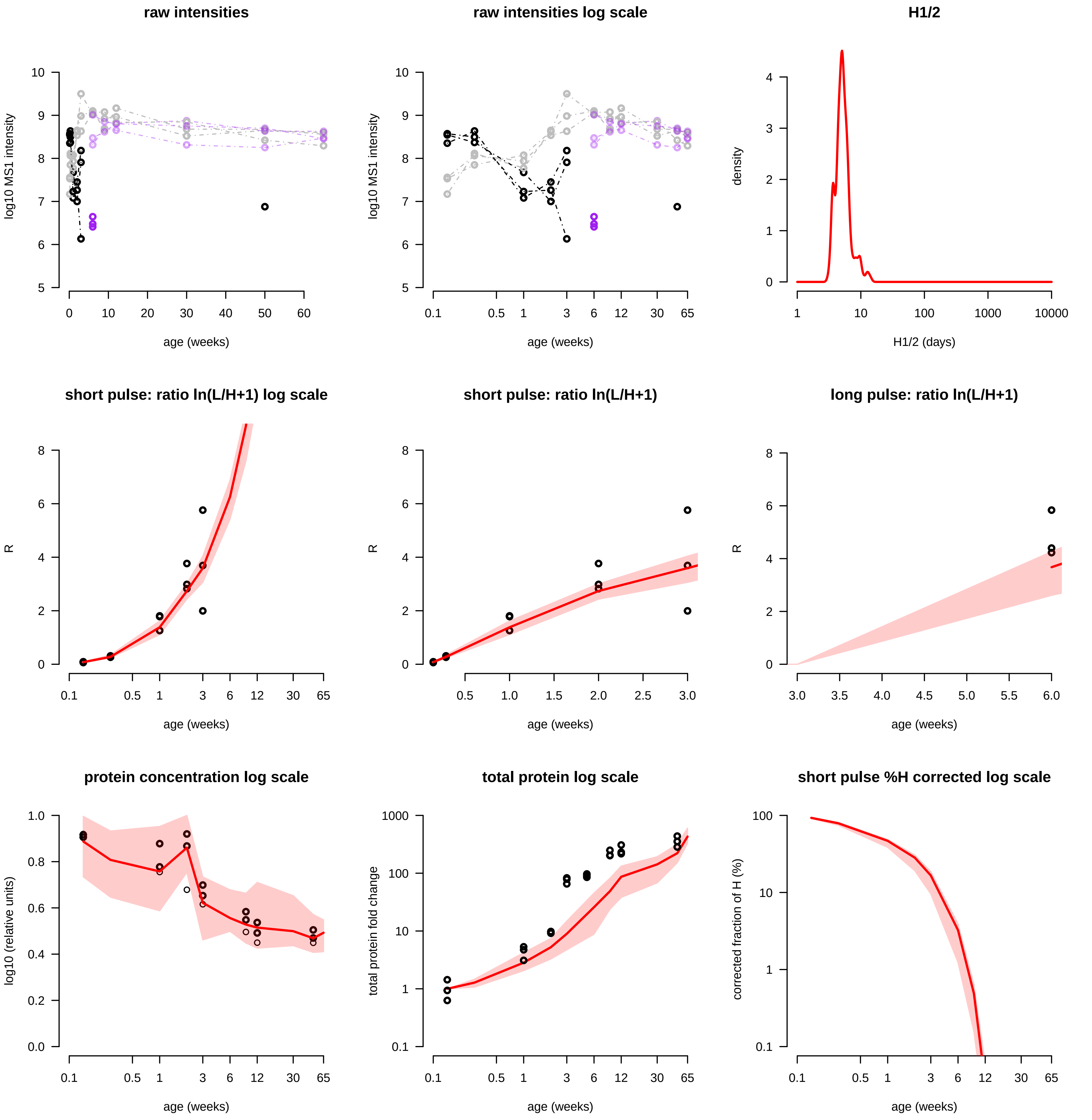

Q8BGS2;Q8BGS2−2

>sp|Q8BGS2|BOLA2_MOUSE

BolA−like protein 2

GN=Bola2

Med H1/2: 13.1 days

Q5 H1/2: 6.4 days

Q95 H1/2: 67.2 days

reliable model fit: yes

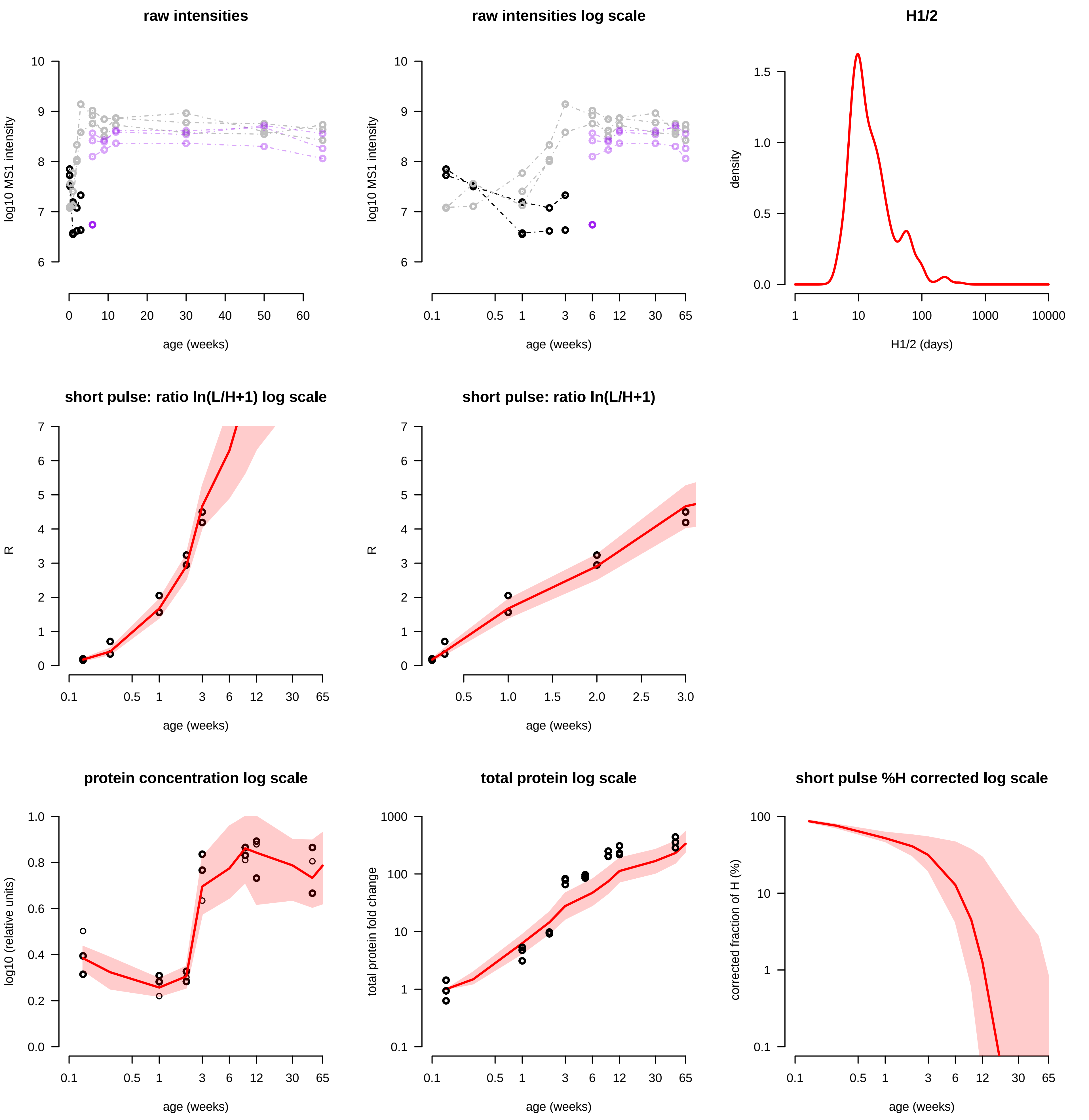

A2ASQ1−3;A2ASQ1−2;A2ASQ1

>sp|A2ASQ1−3|AGRIN_MOUSE

Isoform 3 of Agrin

GN=Agrn

Med H1/2: 4.8 days

Q5 H1/2: 2.7 days

Q95 H1/2: 12.6 days

reliable model fit: yes

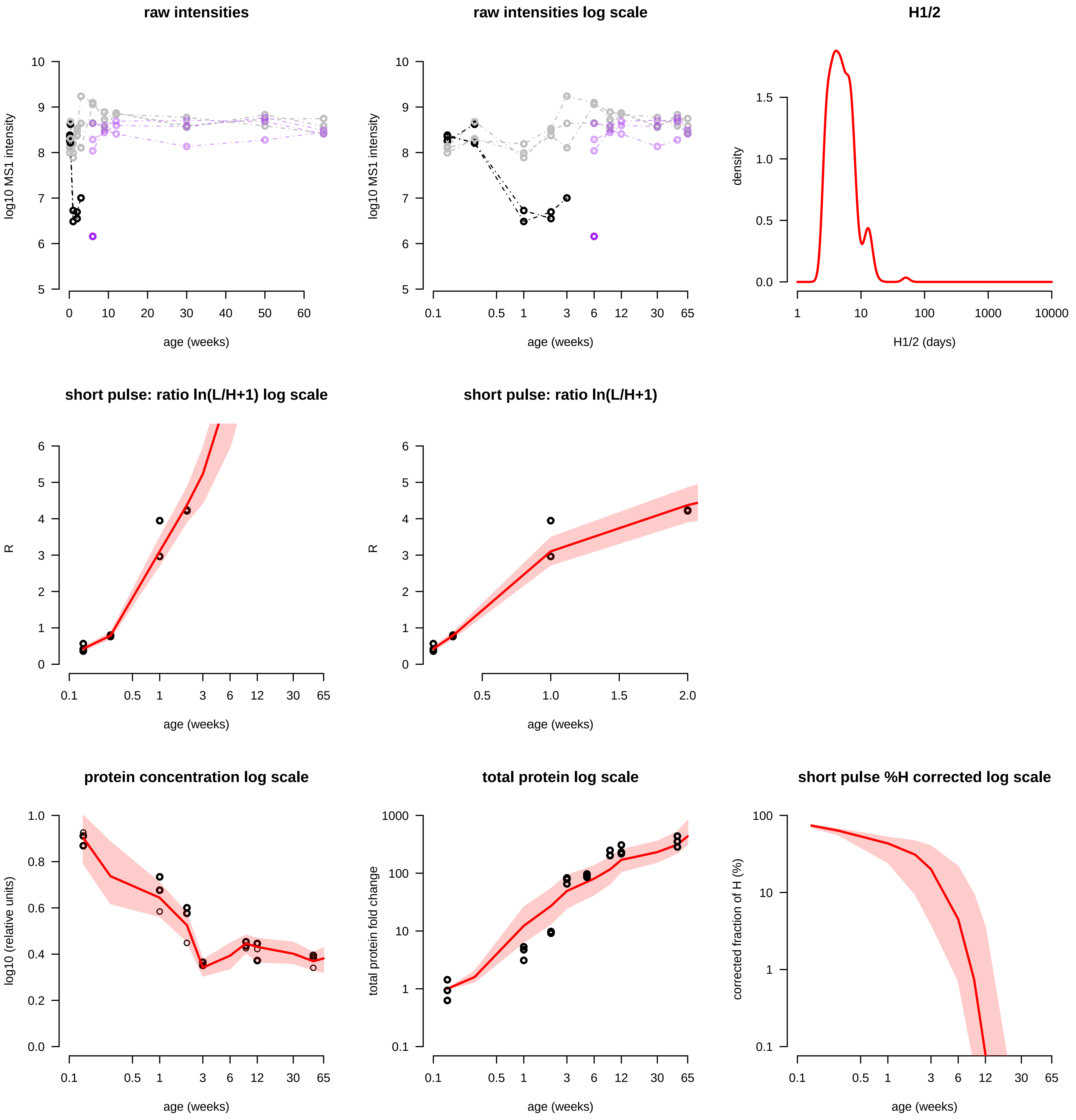

O08808

>sp|O08808|DIAP1_MOUSE

Protein diaphanous homolog 1

GN=Diaph1

Med H1/2: 5.1 days

Q5 H1/2: 2.6 days

Q95 H1/2: 27.6 days

reliable model fit: yes

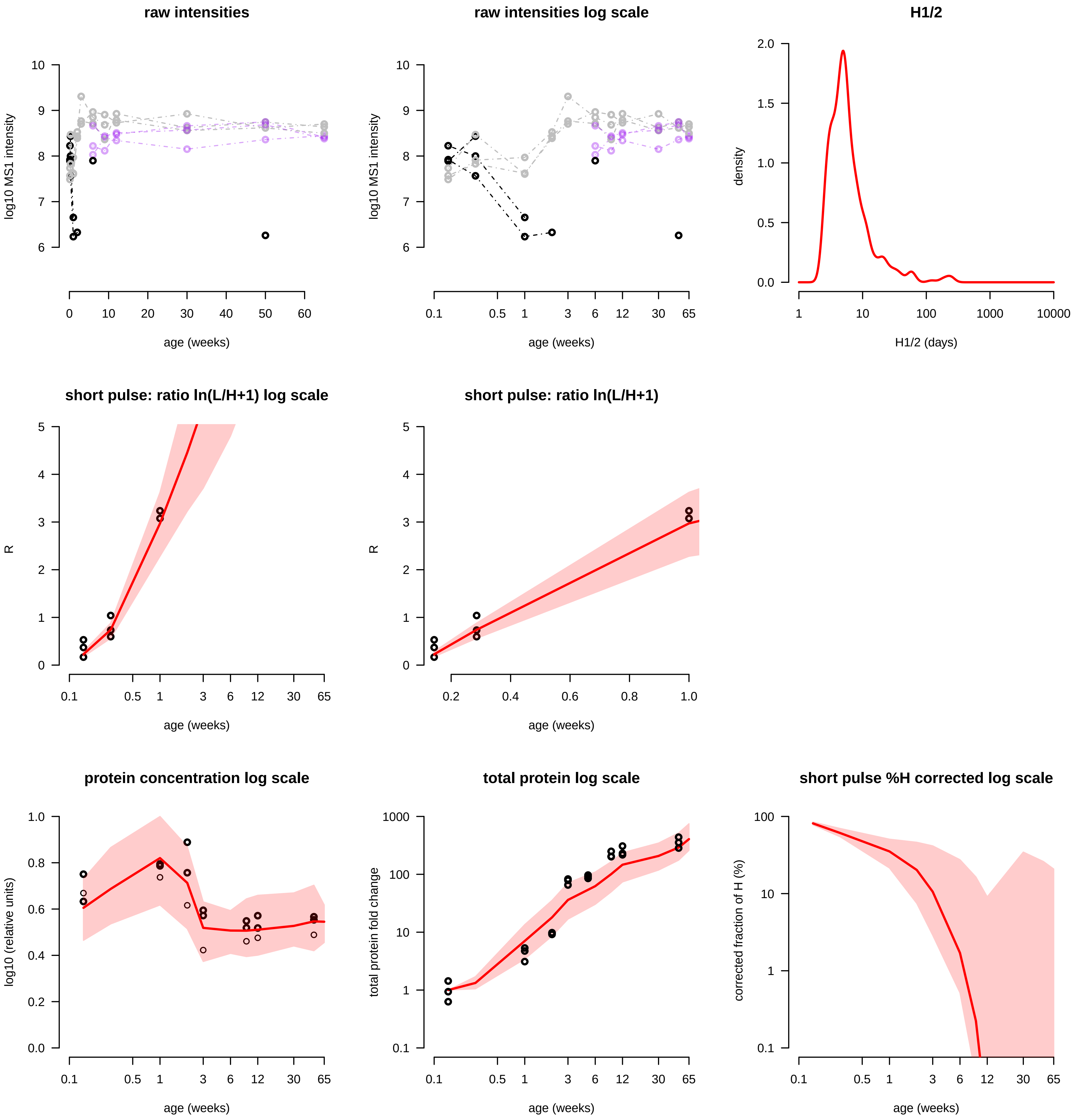

Q8BH66

>sp|Q8BH66|ATLA1_MOUSE

Atlastin−1

GN=Atl1

Med H1/2: 7.8 days

Q5 H1/2: 6.2 days

Q95 H1/2: 10.8 days

reliable model fit: yes

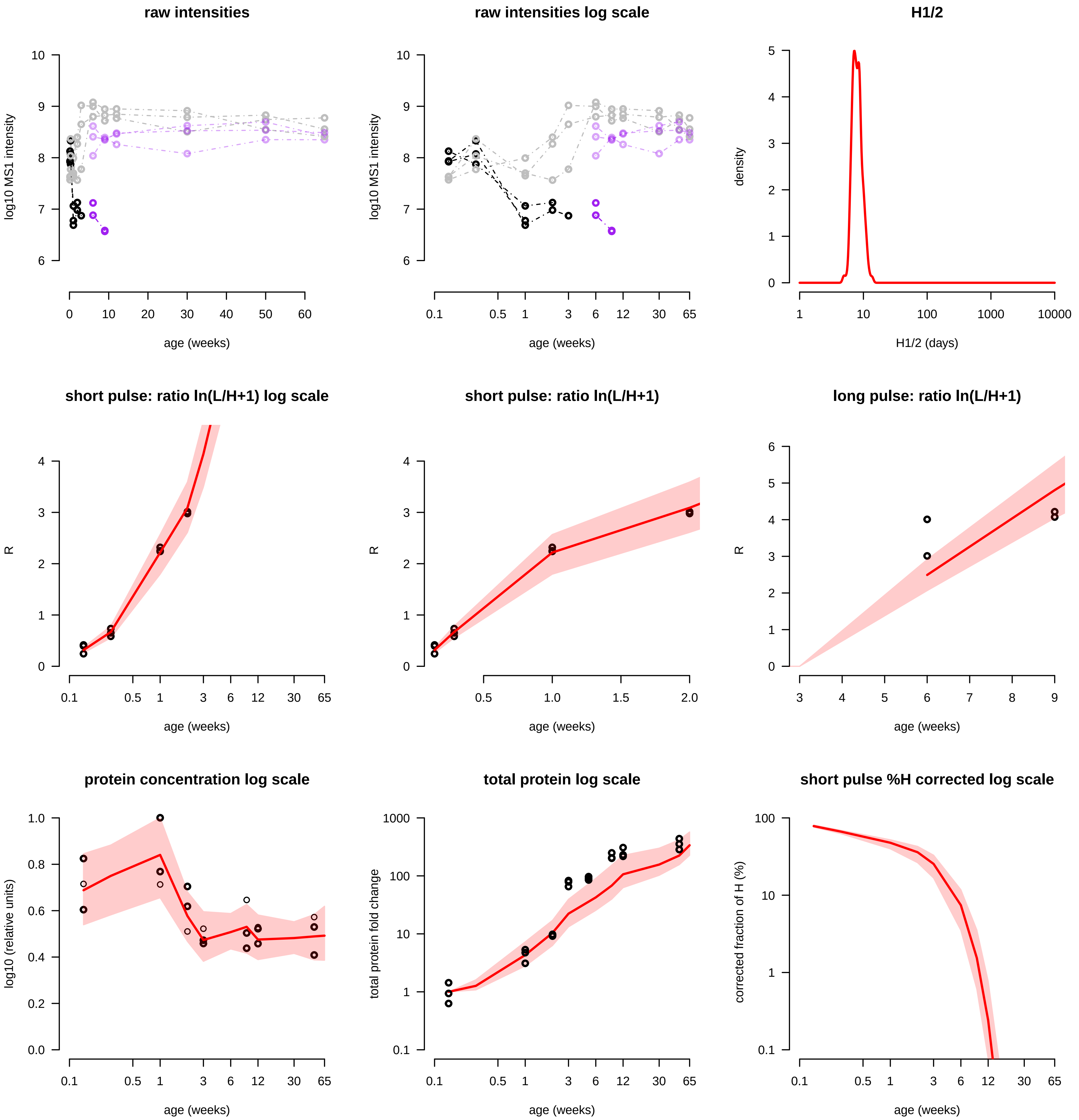

P60824

>sp|P60824|CIRBP_MOUSE

Cold−inducible RNA−binding protein

GN=Cirbp

Med H1/2: 9.4 days

Q5 H1/2: 7.7 days

Q95 H1/2: 12.2 days

reliable model fit: yes

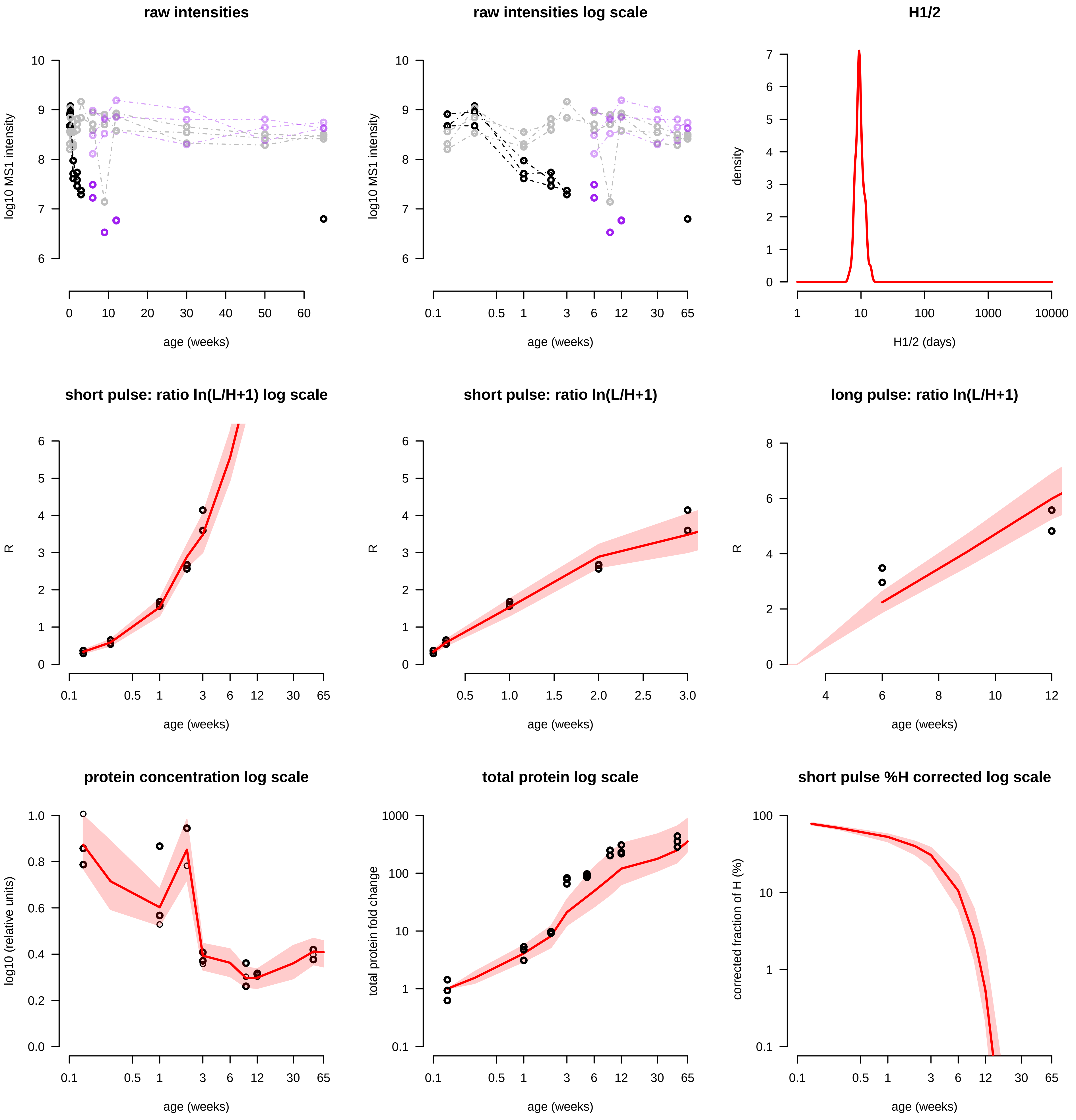

Q62384

>sp|Q62384|ZPR1_MOUSE

Zinc finger protein ZPR1

GN=Zpr1

Med H1/2: 4.8 days

Q5 H1/2: 2.9 days

Q95 H1/2: 19.3 days

reliable model fit: yes

raw intensities

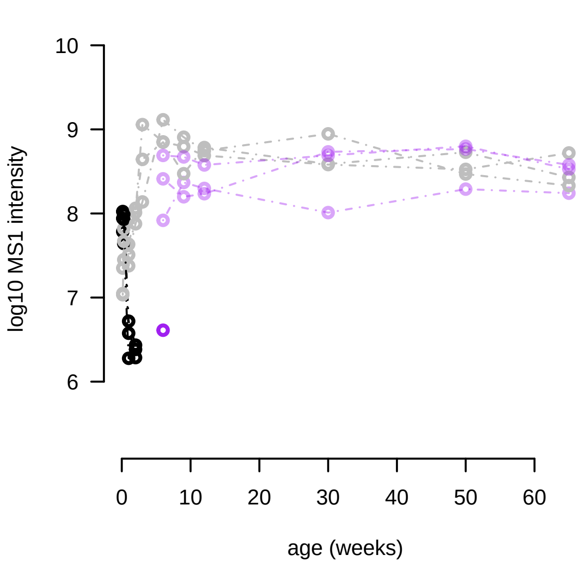

raw intensities log scale

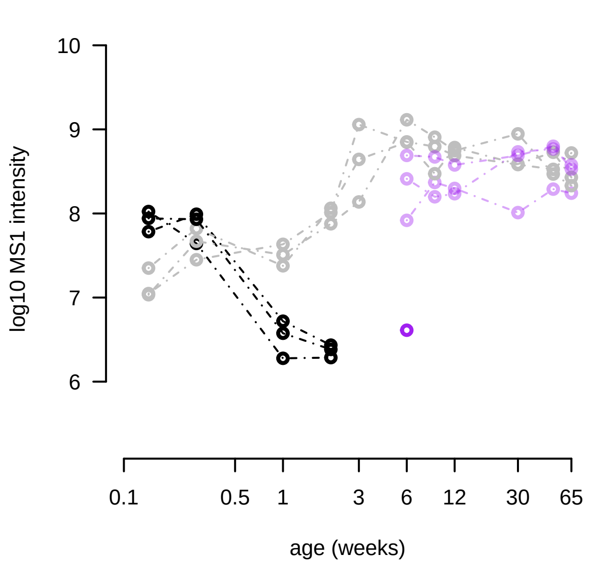

H1/2

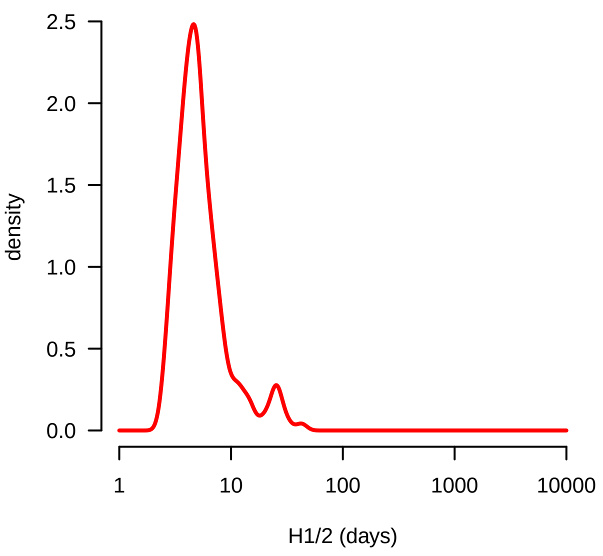

short pulse: ratio ln(L/H+1) log scale

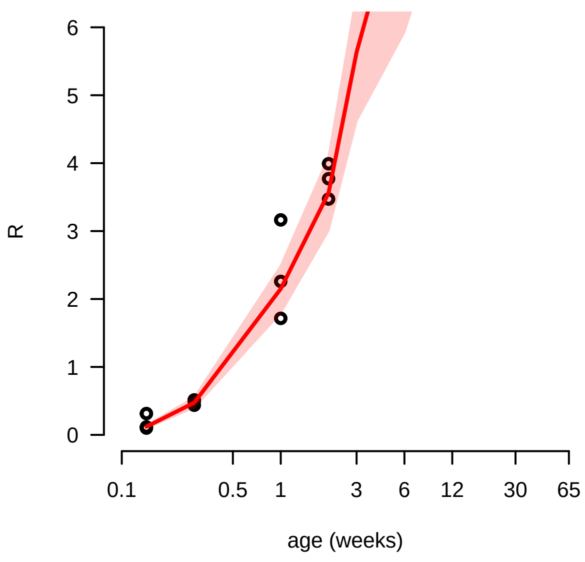

short pulse: ratio ln(L/H+1)

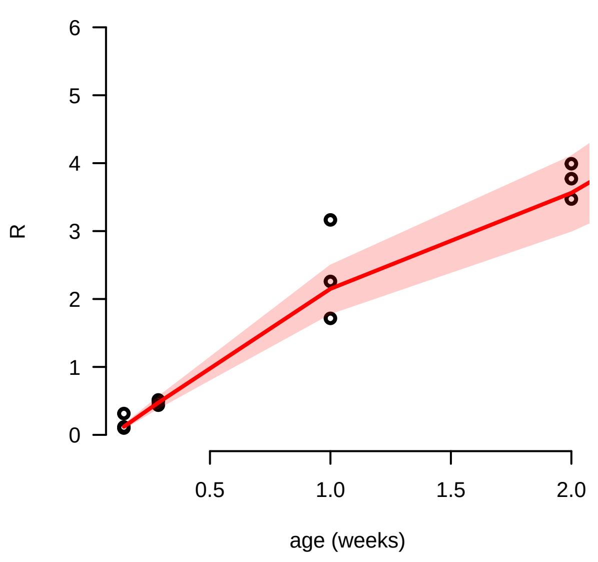

protein concentration log scale

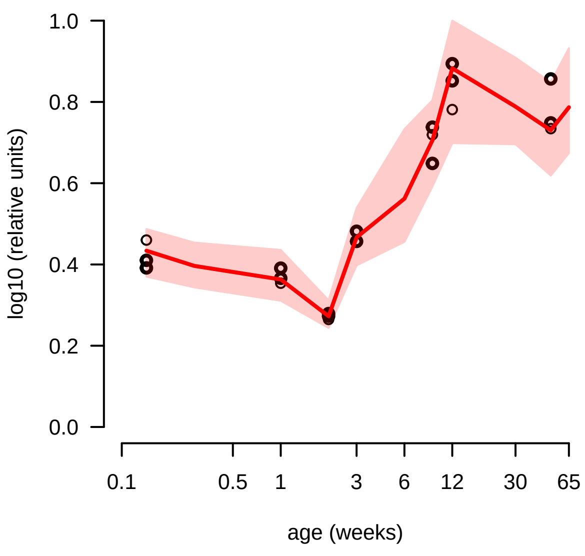

total protein log scale

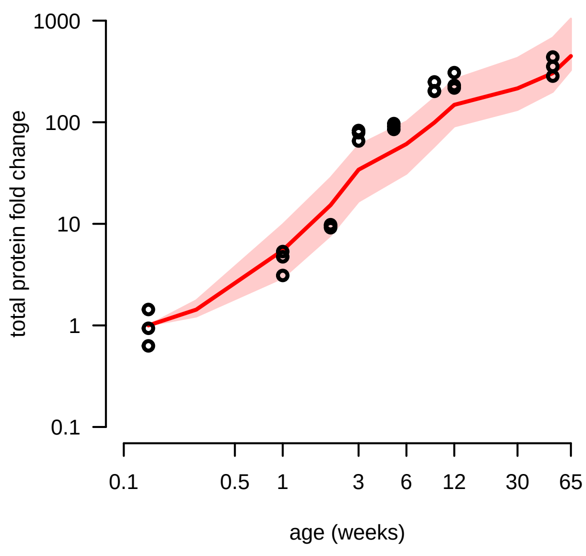

short pulse %H corrected log scale

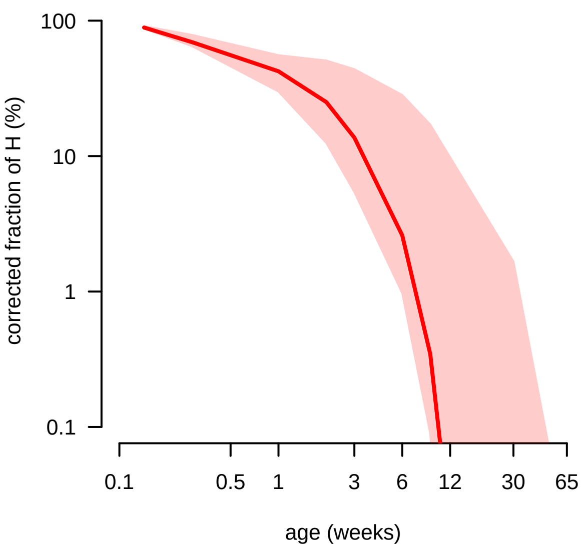

Q8BG95

>sp|Q8BG95|MYPT2_MOUSE

Protein phosphatase 1 regulatory subunit 12B

GN=Ppp1r12b

Med H1/2: 9.2 days

Q5 H1/2: 6.8 days

Q95 H1/2: 14.2 days

reliable model fit: yes

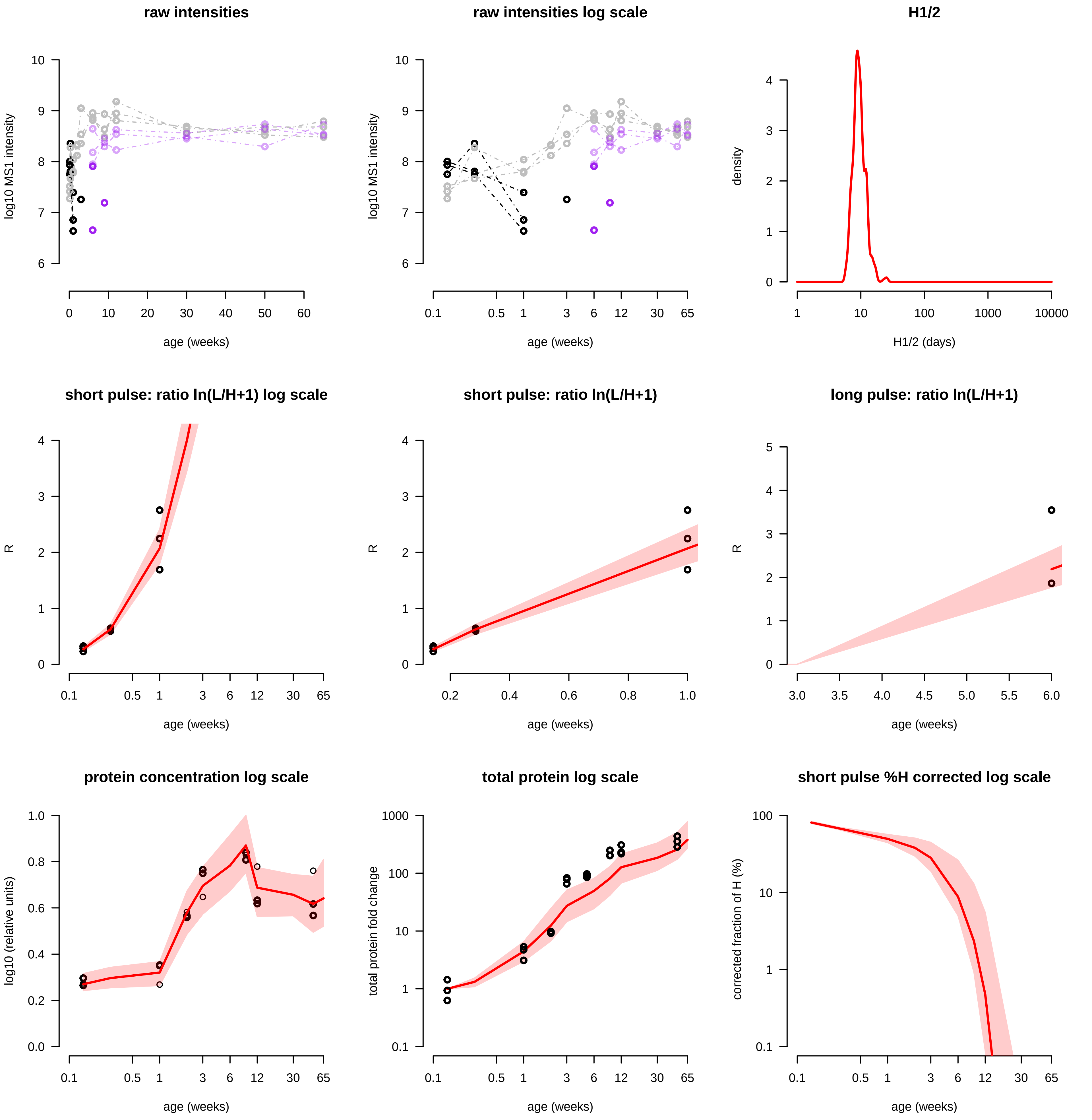

Q8R180

>sp|Q8R180|ERO1A_MOUSE

ERO1−like protein alpha

GN=Ero1a

Med H1/2: 9.6 days

Q5 H1/2: 4 days

Q95 H1/2: 30.5 days

reliable model fit: yes

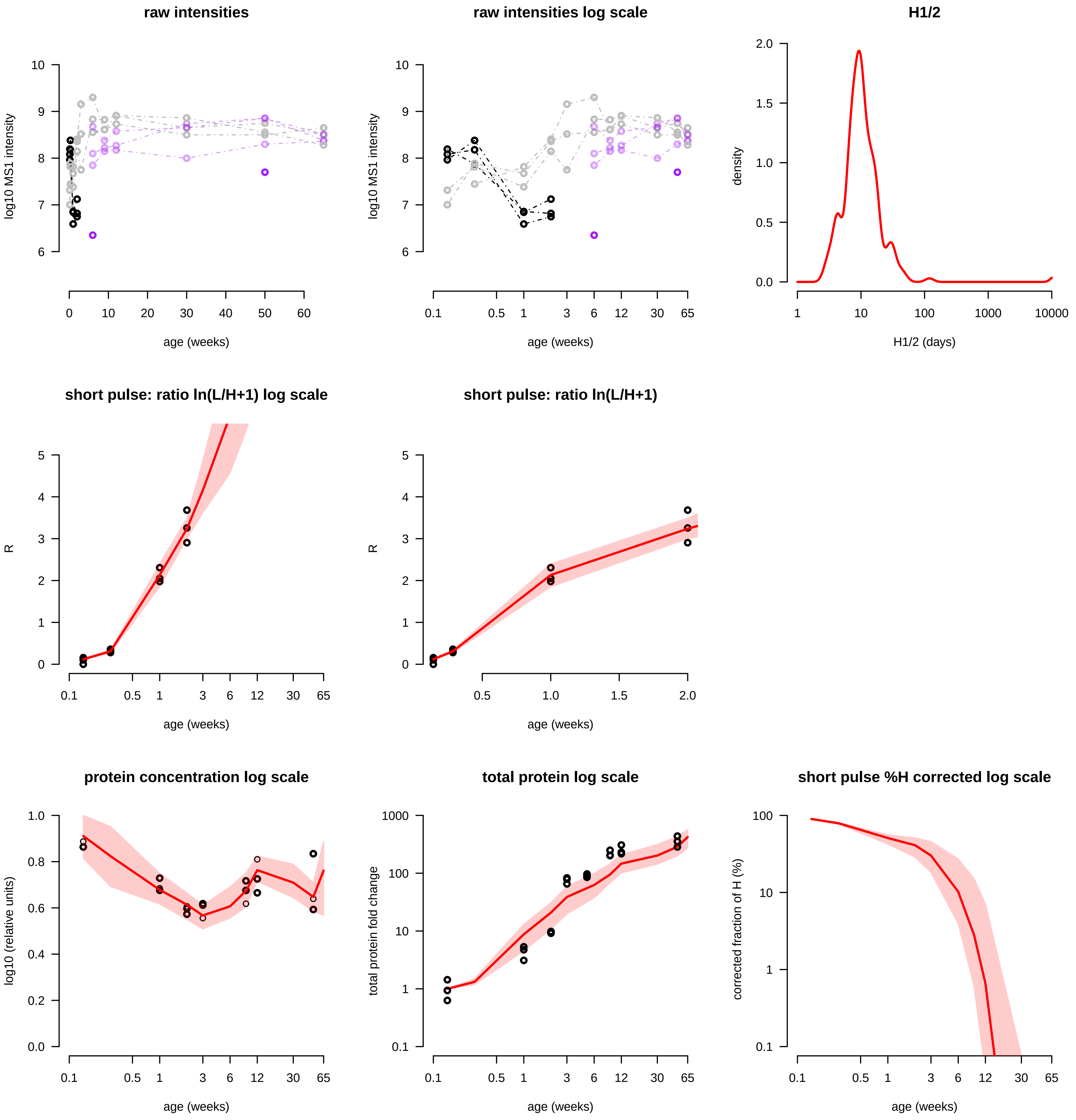

Q99K28;Q99K28−2

>sp|Q99K28|ARFG2_MOUSE

ADP−ribosylation factor GTPase−activating protein 2

GN=Arfgap2

Med H1/2: 7.4 days

Q5 H1/2: 3.6 days

Q95 H1/2: 40 days

reliable model fit: yes

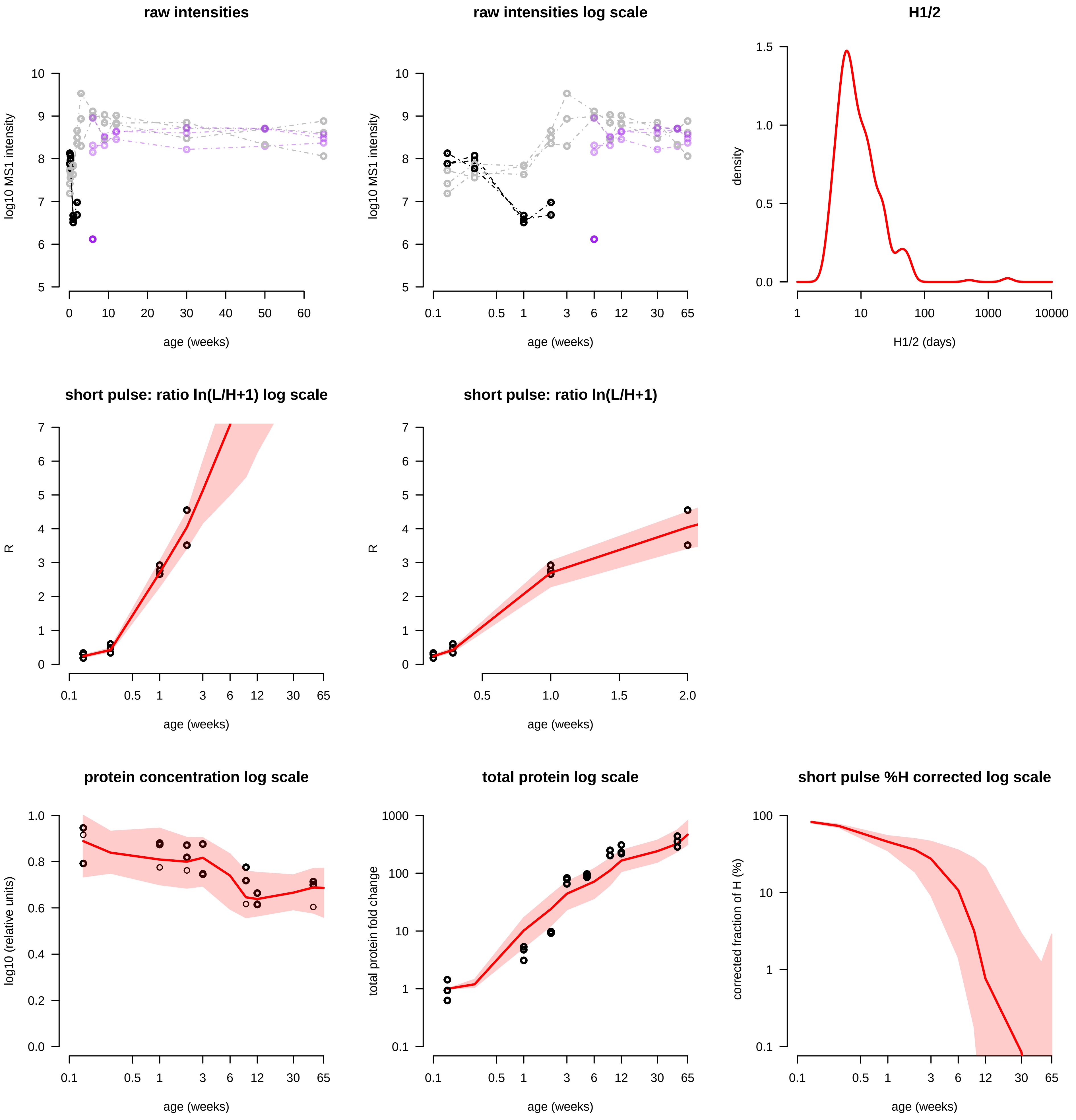

Q9Z0V7

>sp|Q9Z0V7|TI17B_MOUSE

Mitochondrial import inner membrane translocase subunit Tim17−B

GN=Timm17b

Med H1/2: 14.2 days

Q5 H1/2: 4.4 days

Q95 H1/2: 100.4 days

reliable model fit: yes

raw intensities

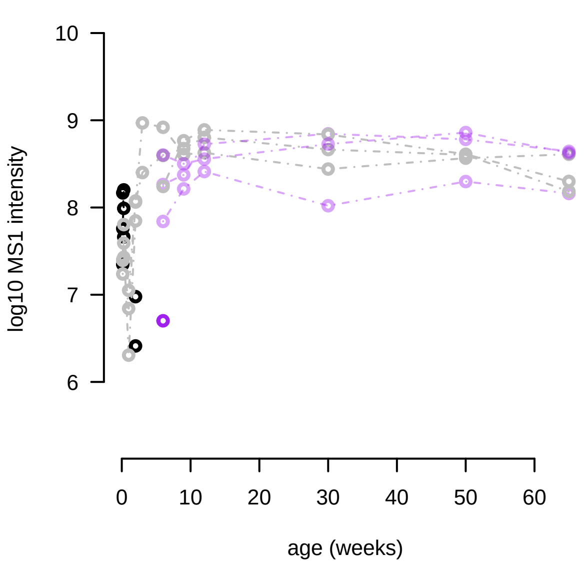

raw intensities log scale

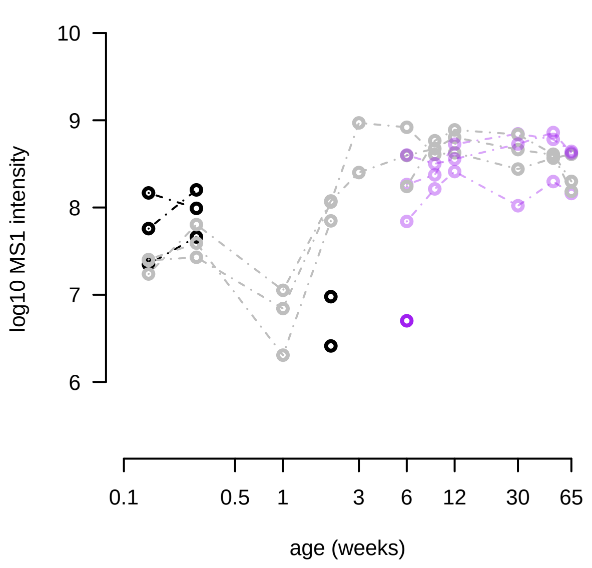

H1/2

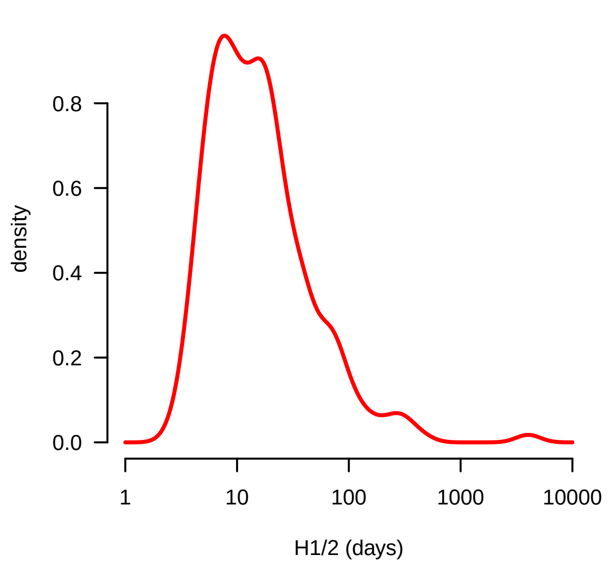

short pulse: ratio ln(L/H+1) log scale

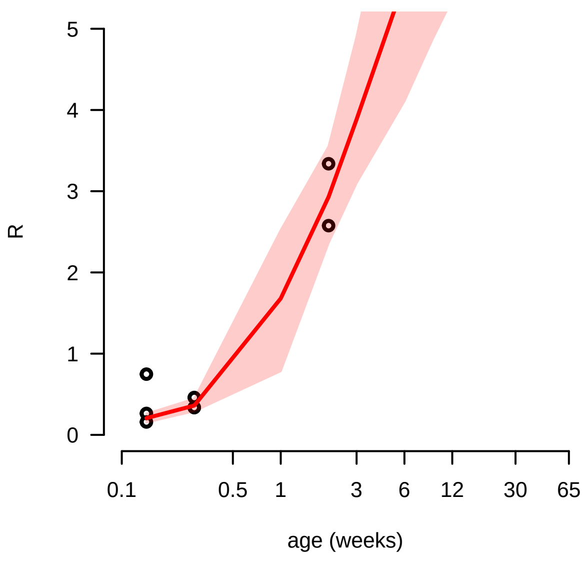

short pulse: ratio ln(L/H+1)

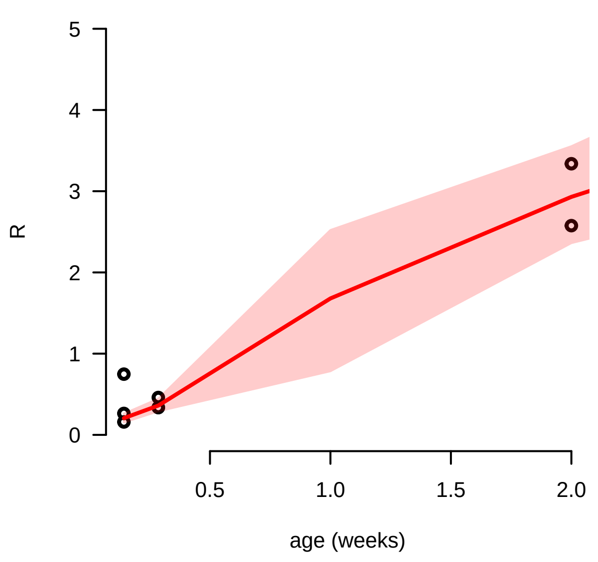

protein concentration log scale

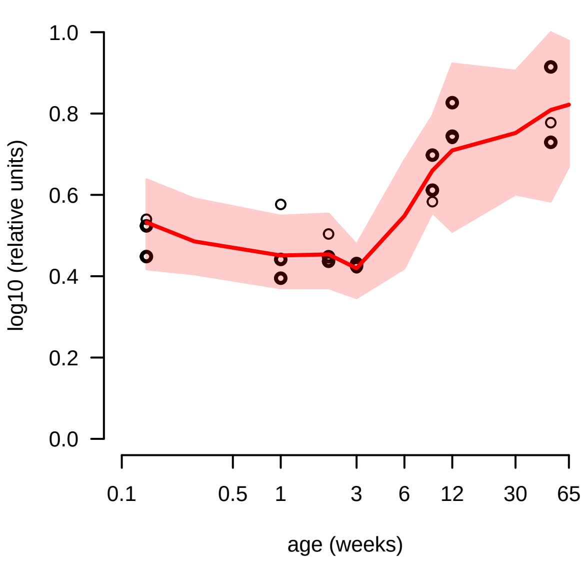

total protein log scale

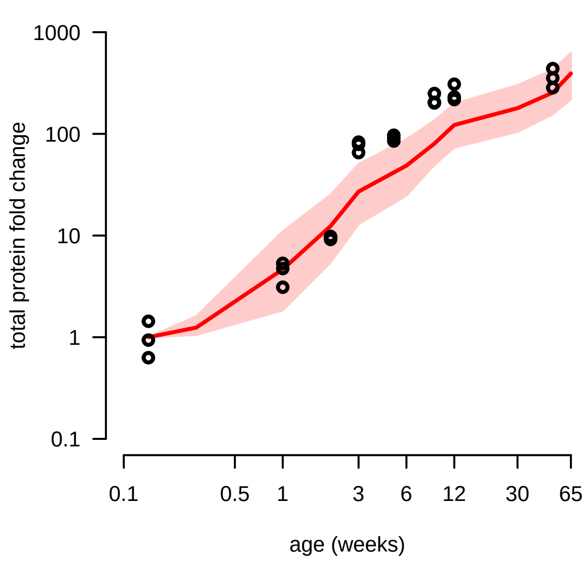

short pulse %H corrected log scale

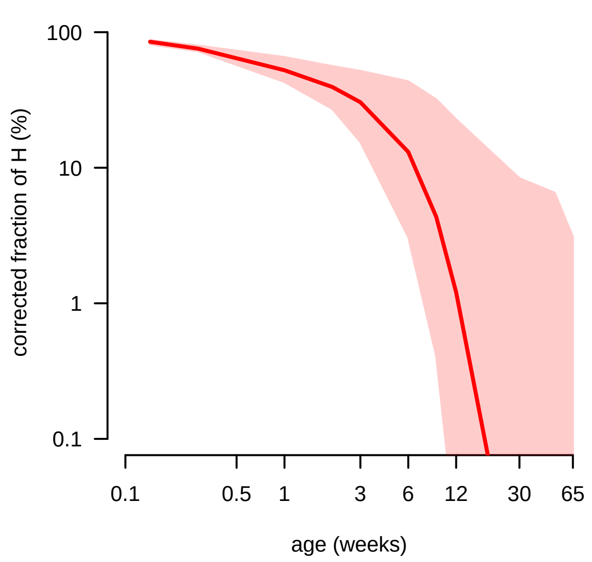

P53986

>sp|P53986|MOT1_MOUSE

Monocarboxylate transporter 1

GN=Slc16a1

Med H1/2: 13.7 days

Q5 H1/2: 10.3 days

Q95 H1/2: 17.9 days

reliable model fit: yes

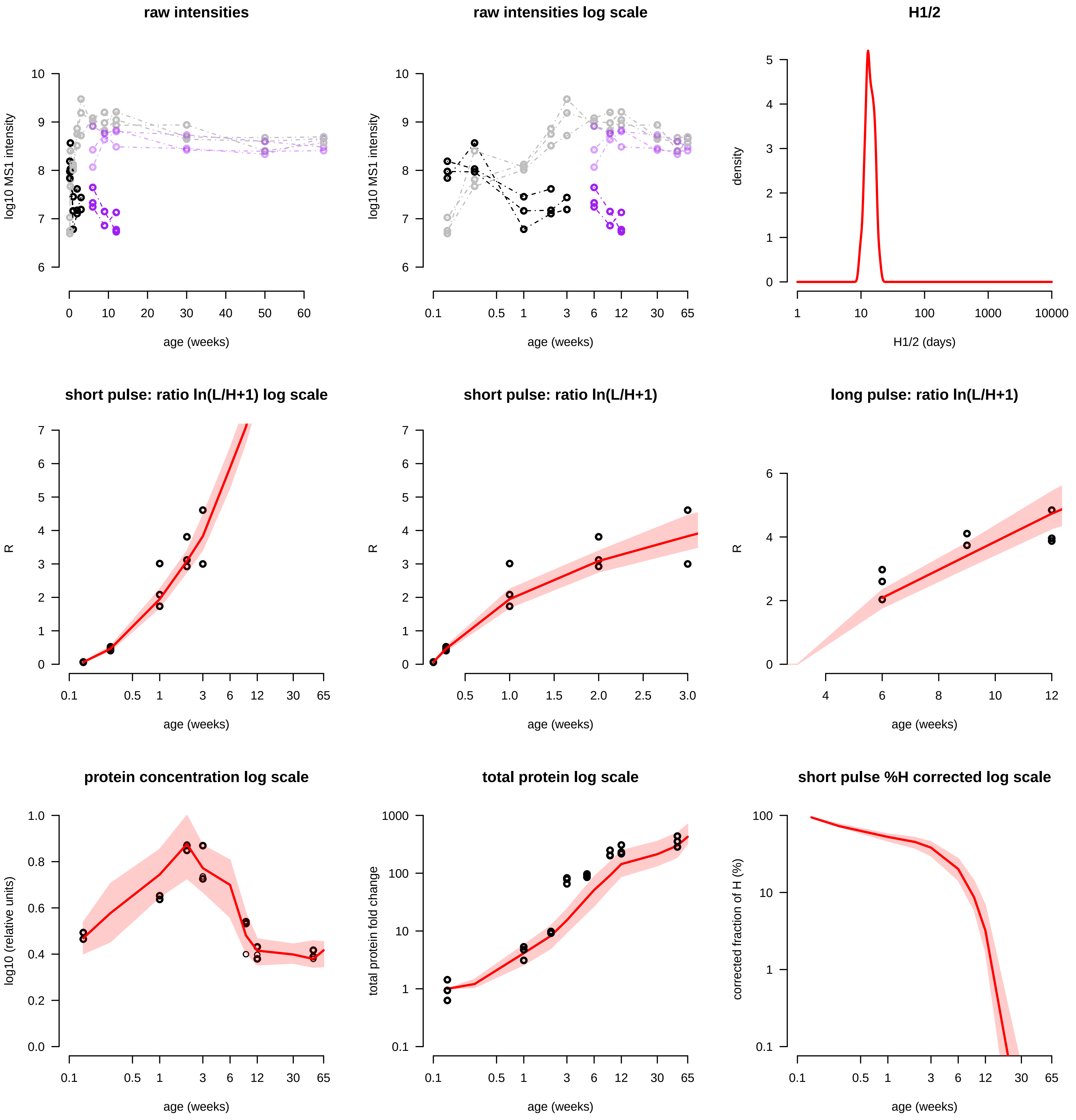

Q9D4H8;Q9D4H8−2

>sp|Q9D4H8|CUL2_MOUSE

Cullin−2

GN=Cul2

Med H1/2: 4 days

Q5 H1/2: 2.7 days

Q95 H1/2: 5.6 days

reliable model fit: yes

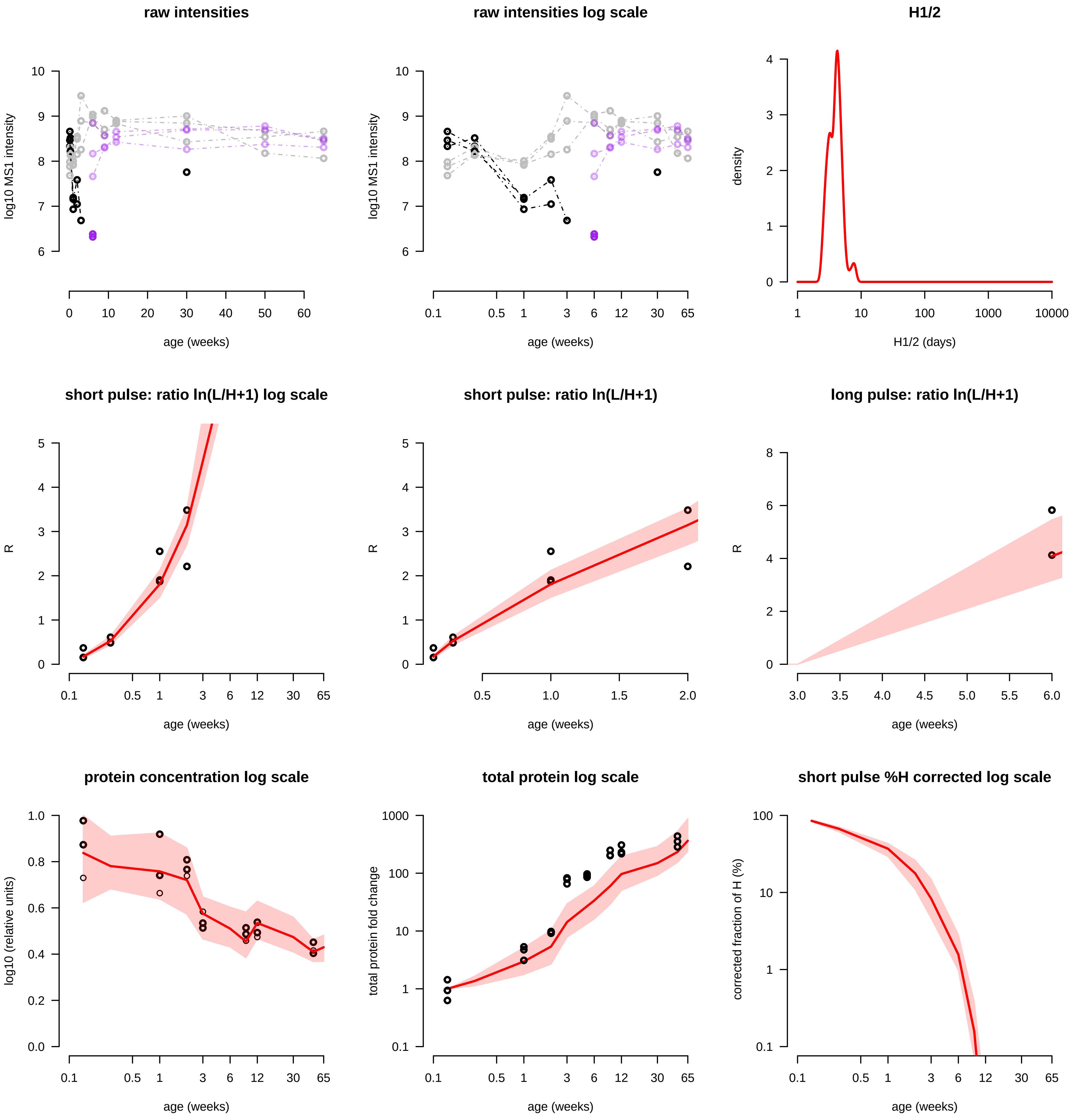

O09111

>sp|O09111|NDUBB_MOUSE

NADH dehydrogenase [ubiquinone] 1 beta subcomplex subunit 11, mitochondria

GN=Ndufb11

Med H1/2: 5.2 days

Q5 H1/2: 4.1 days

Q95 H1/2: 6.6 days

reliable model fit: yes

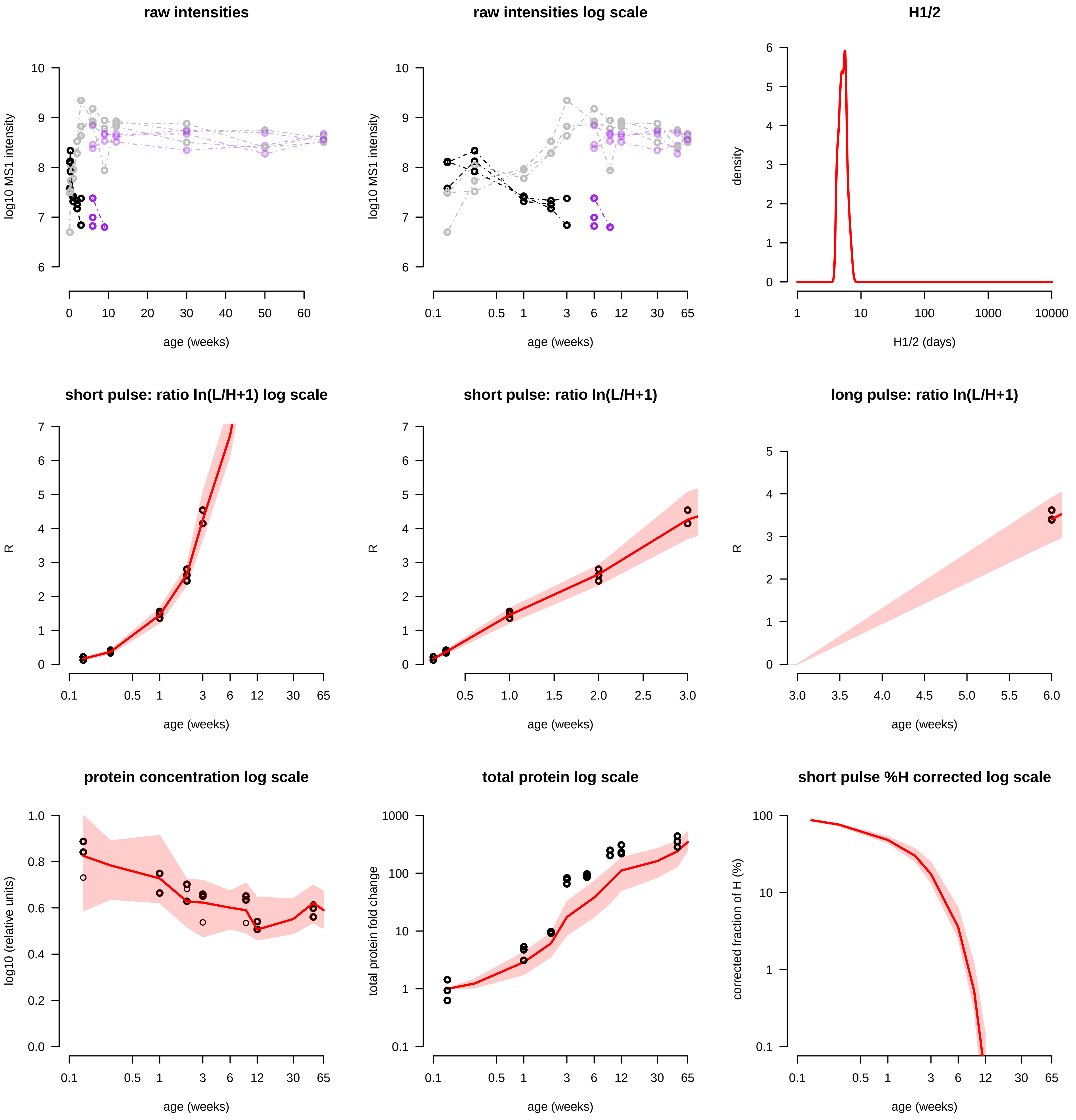

A2AWA9;A2AWA9−2

>sp|A2AWA9|RBGP1_MOUSE

Rab GTPase−activating protein 1

GN=Rabgap1

Med H1/2: 12.5 days

Q5 H1/2: 4.8 days

Q95 H1/2: 248.4 days

reliable model fit: yes

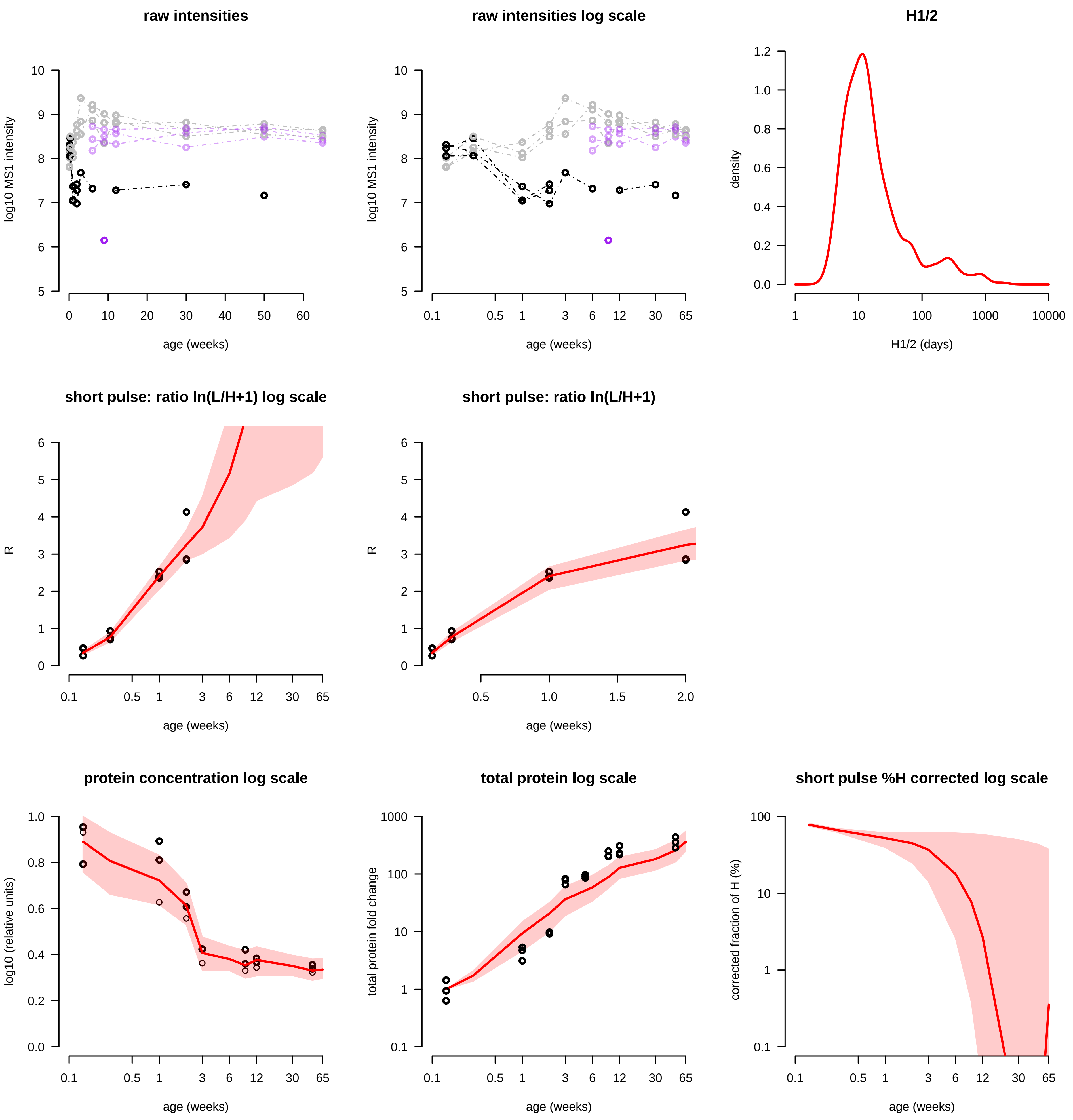

Q9Z0P5;Q9Z0P5−2

>sp|Q9Z0P5|TWF2_MOUSE

Twinfilin−2

GN=Twf2

Med H1/2: 5.5 days

Q5 H1/2: 2.6 days

Q95 H1/2: 30.5 days

reliable model fit: yes

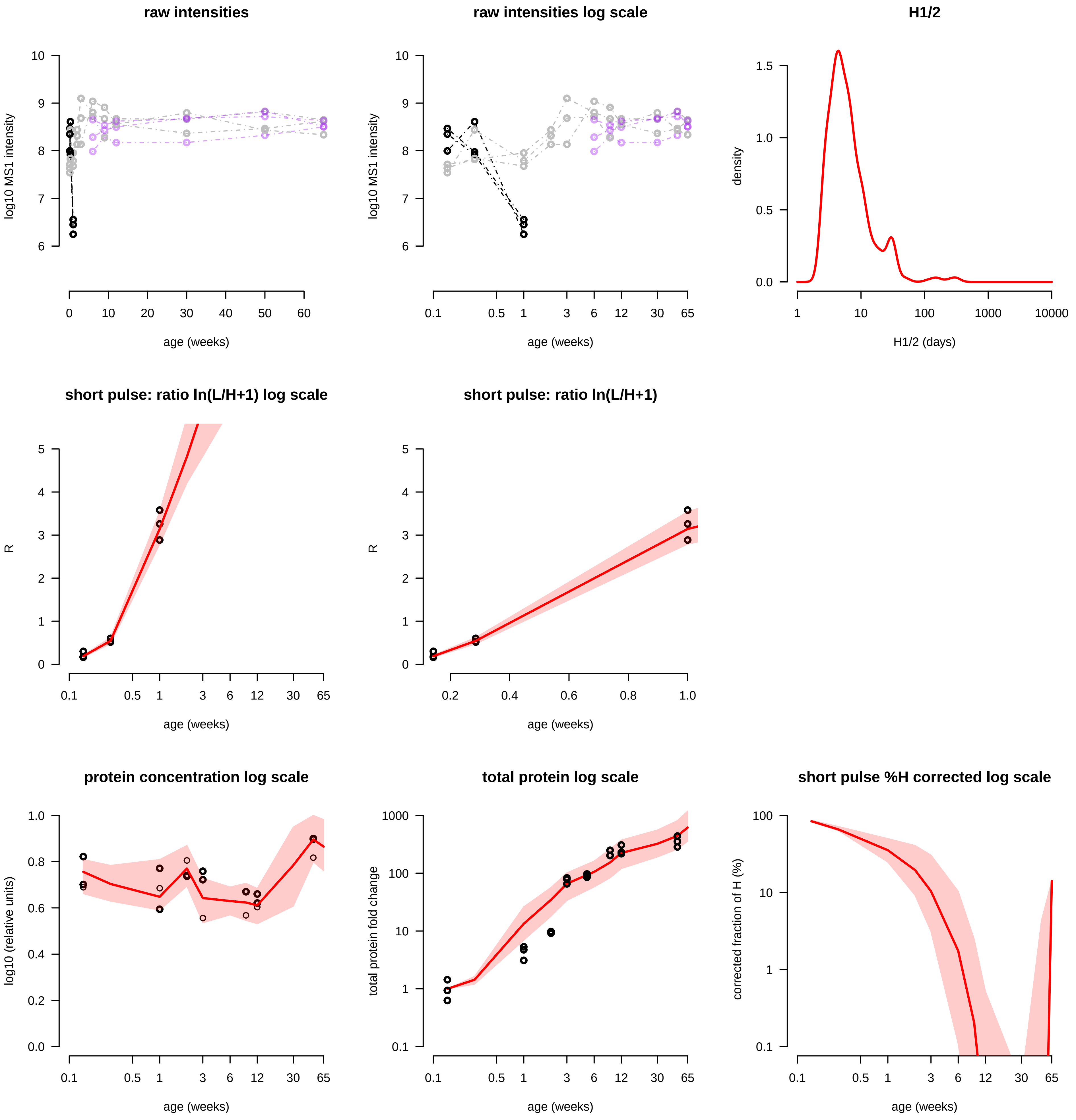

Q3UPH1

>sp|Q3UPH1|PRRC1_MOUSE

Protein PRRC1

GN=Prrc1

Med H1/2: 4.5 days

Q5 H1/2: 3.5 days

Q95 H1/2: 6.2 days

reliable model fit: yes

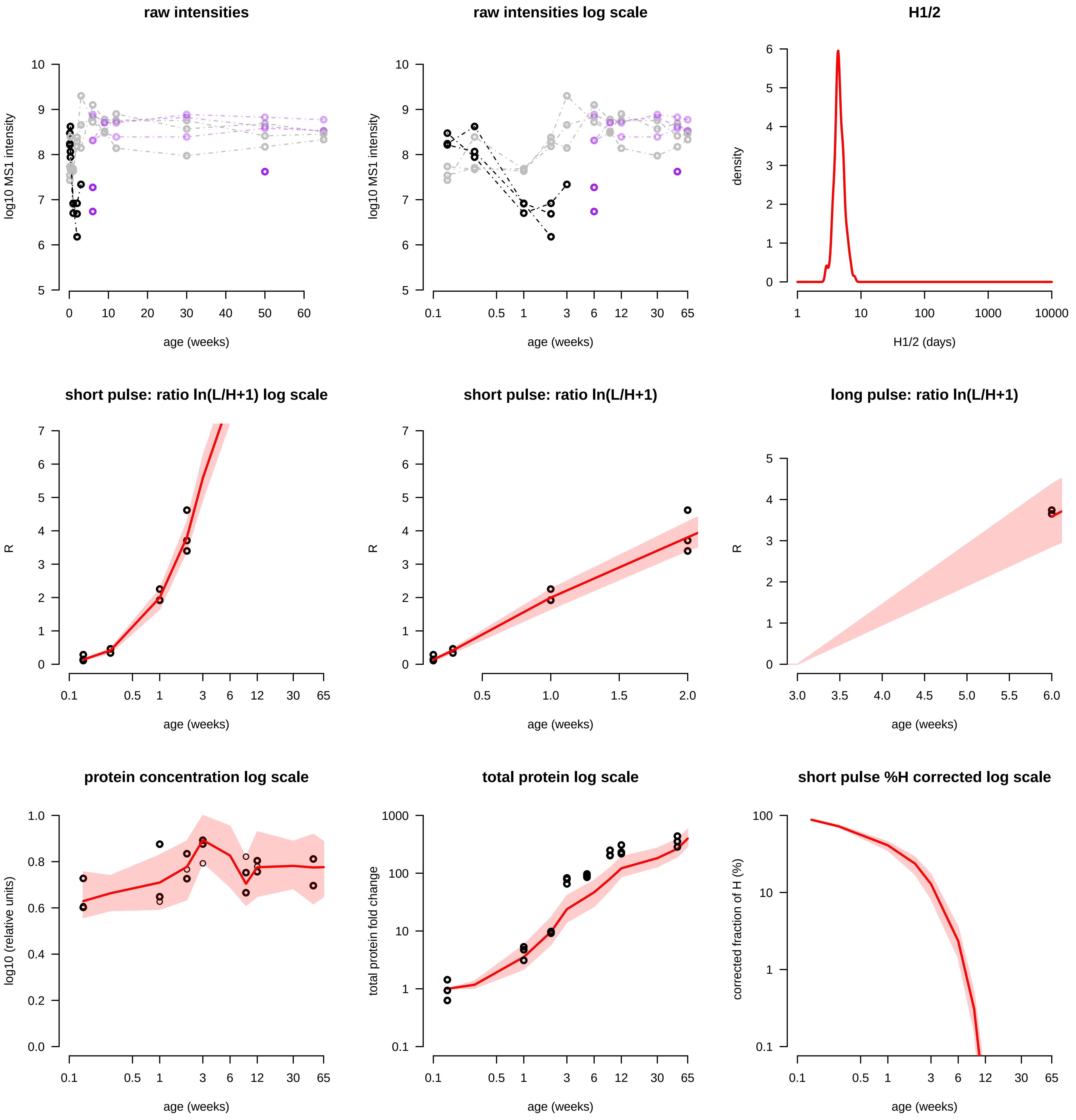

Q91VJ4

>sp|Q91VJ4|STK38_MOUSE

Serine/threonine−protein kinase 38

GN=Stk38

Med H1/2: 478.8 days

Q5 H1/2: 146.3 days

Q95 H1/2: 8757.6 days

reliable model fit: yes

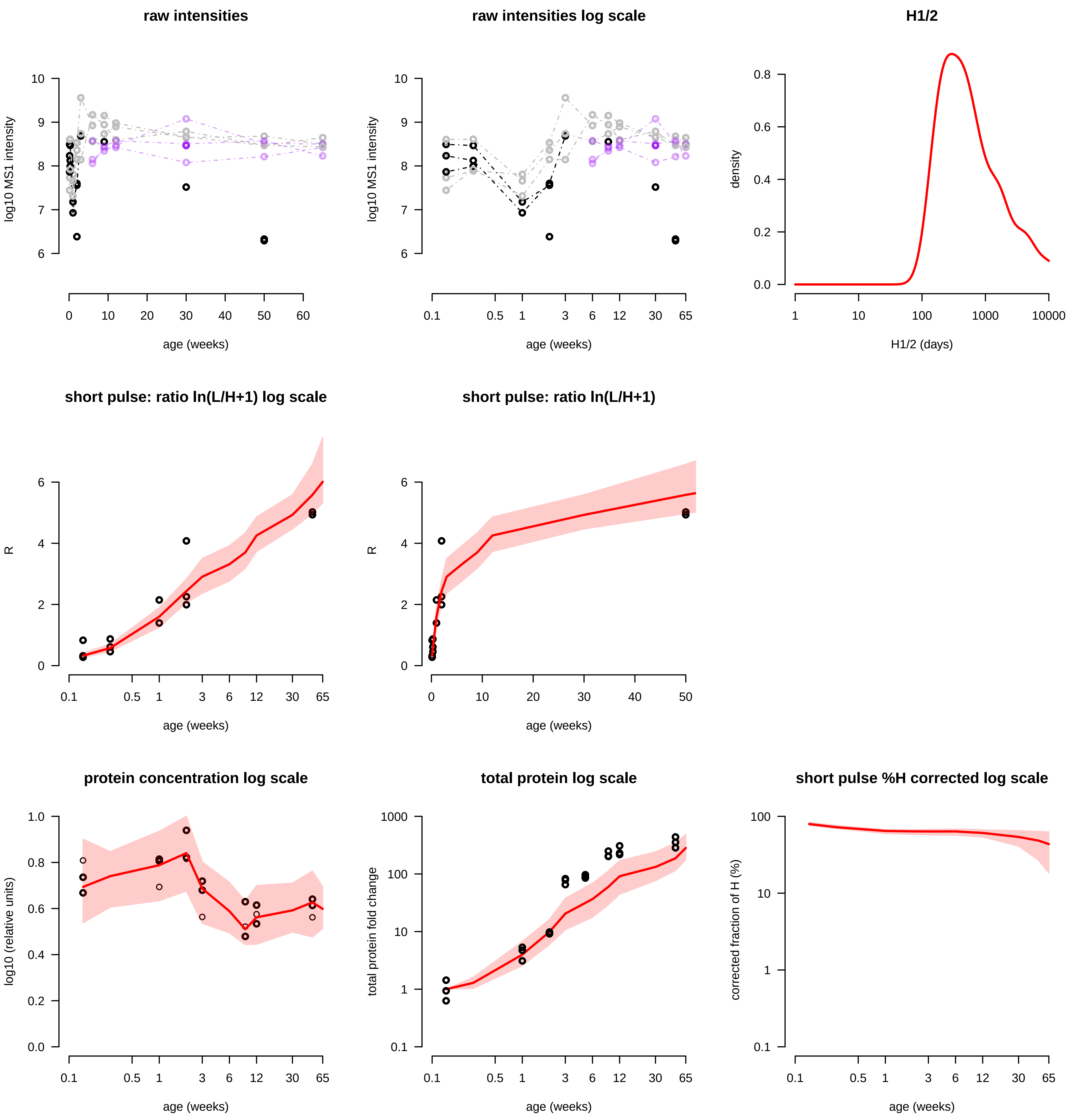

Q3U9G9

>sp|Q3U9G9|LBR_MOUSE

Delta(14)−sterol reductase LBR

GN=Lbr

Med H1/2: 4.5 days

Q5 H1/2: 3.5 days

Q95 H1/2: 8.1 days

reliable model fit: yes

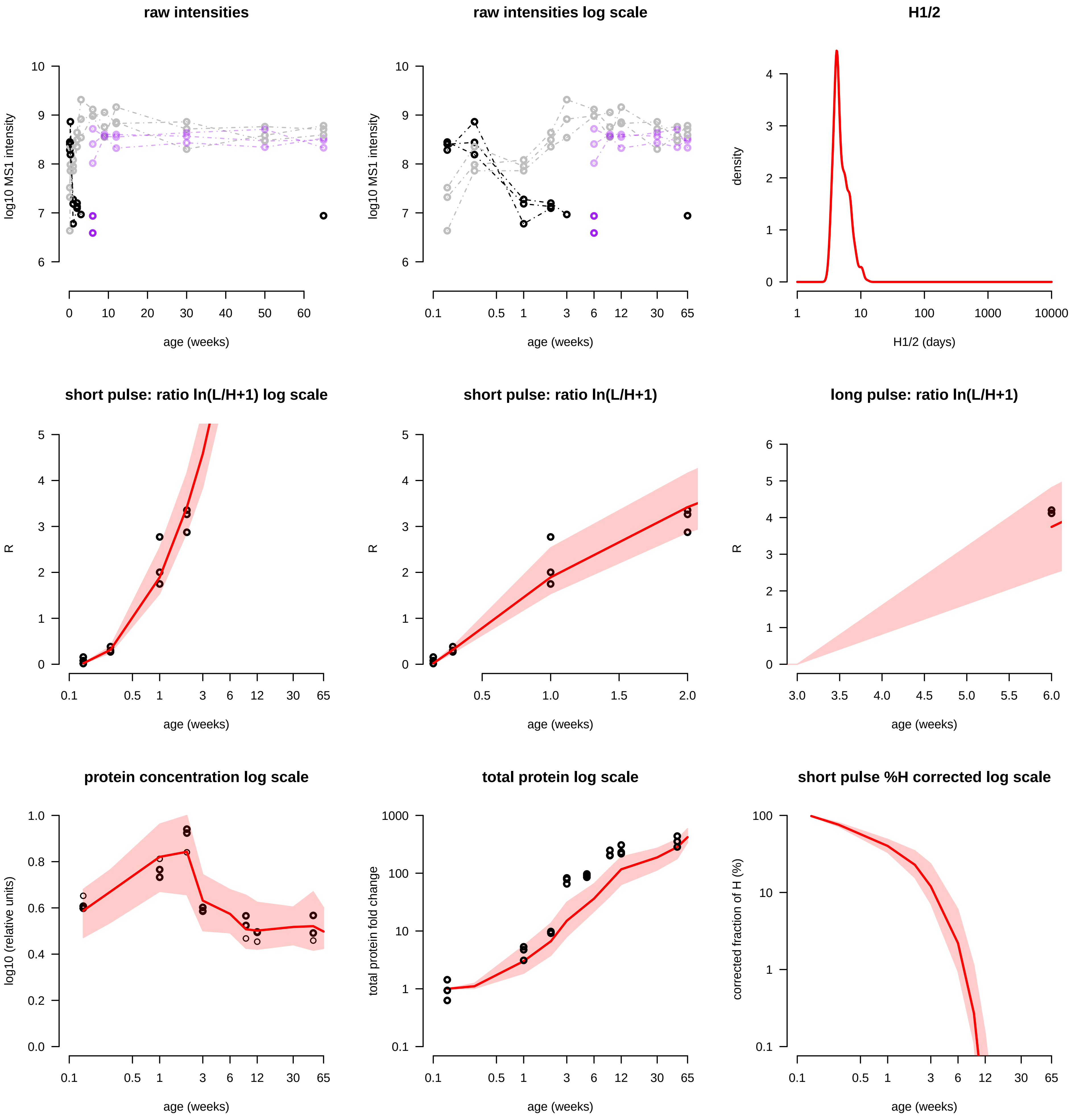

Q921J2

>sp|Q921J2|RHEB_MOUSE

GTP−binding protein Rheb

GN=Rheb

Med H1/2: 10.3 days

Q5 H1/2: 8 days

Q95 H1/2: 15.5 days

reliable model fit: yes

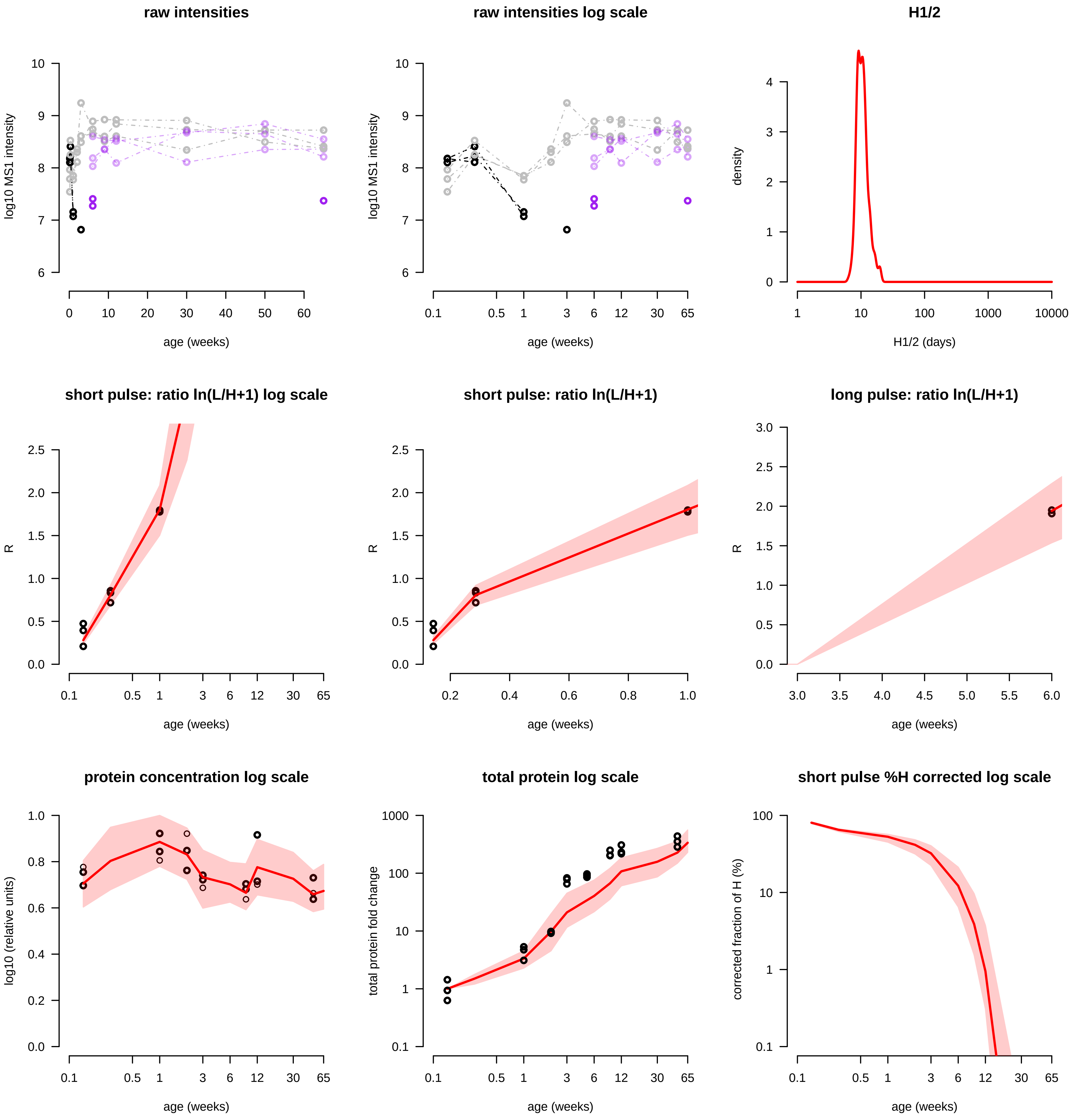

Q9QXB9

>sp|Q9QXB9|DRG2_MOUSE

Developmentally−regulated GTP−binding protein 2

GN=Drg2

Med H1/2: 3015.2 days

Q5 H1/2: 831.2 days

Q95 H1/2: 32214.9 days

reliable model fit: yes

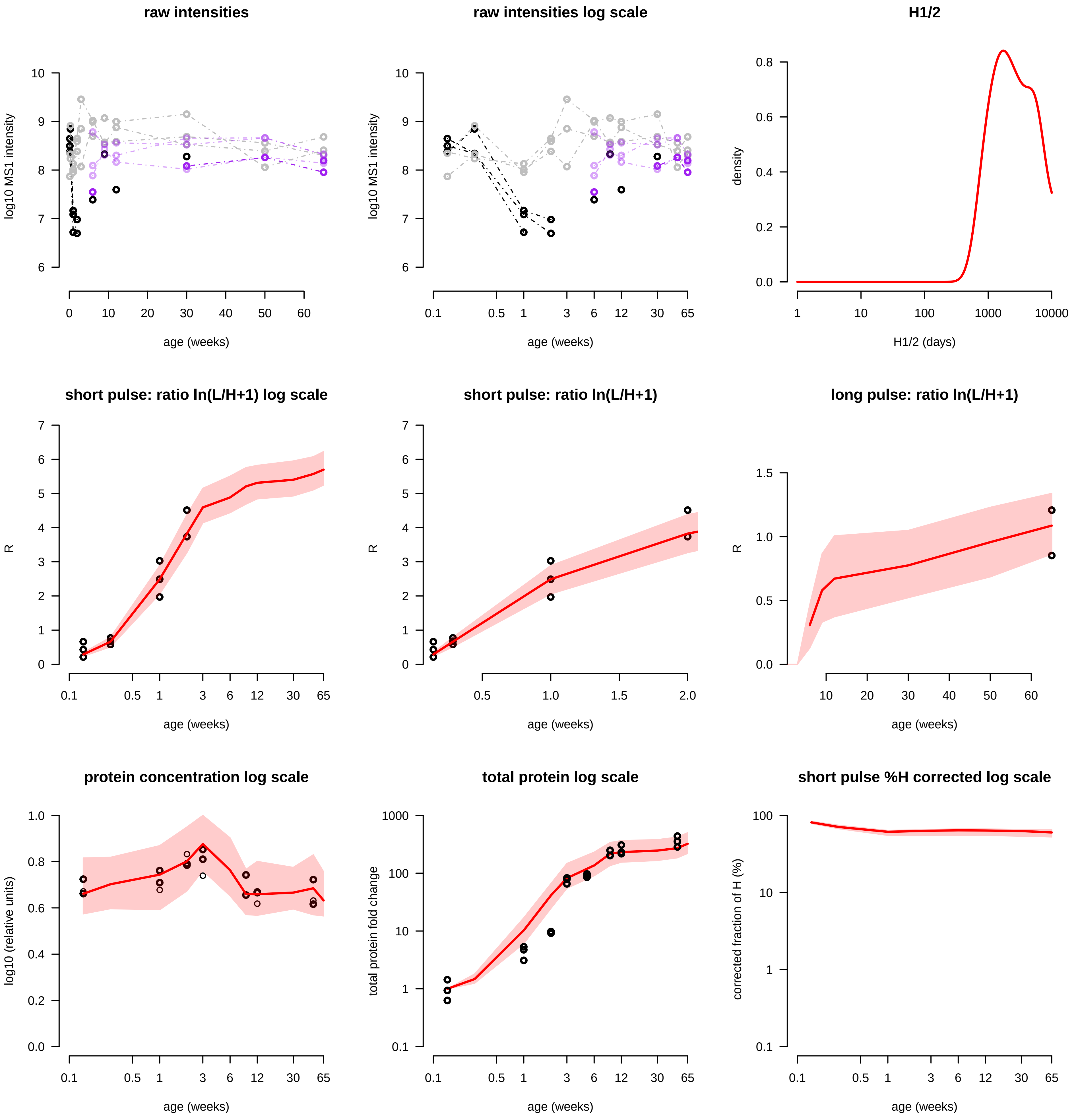

Q921X9

>sp|Q921X9|PDIA5_MOUSE

Protein disulfide−isomerase A5

GN=Pdia5

Med H1/2: 4.1 days

Q5 H1/2: 3.3 days

Q95 H1/2: 4.9 days

reliable model fit: yes

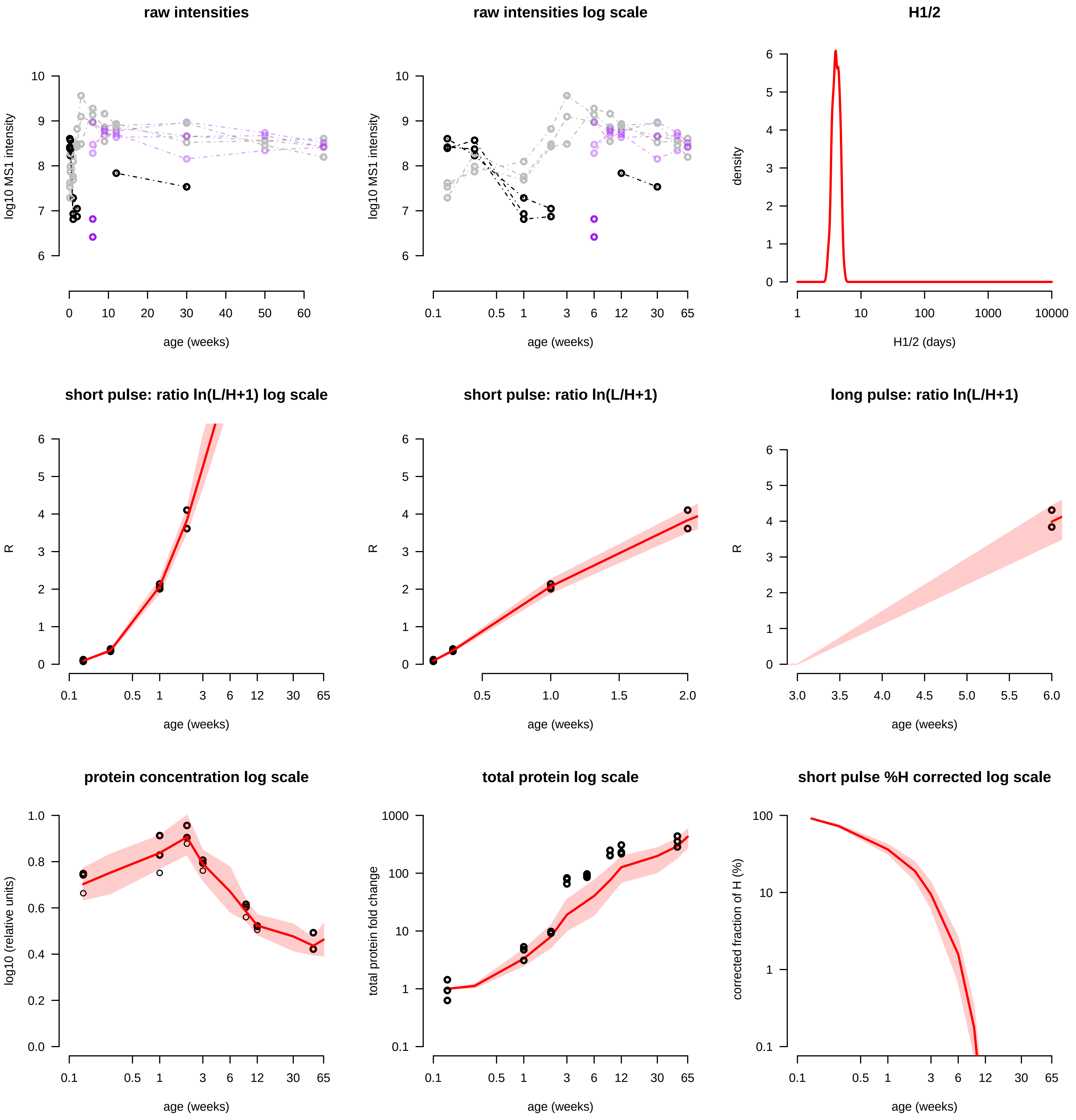

P32883

>sp|P32883|RASK_MOUSE

GTPase KRas

GN=Kras

Med H1/2: 5.8 days

Q5 H1/2: 3 days

Q95 H1/2: 21.4 days

reliable model fit: yes

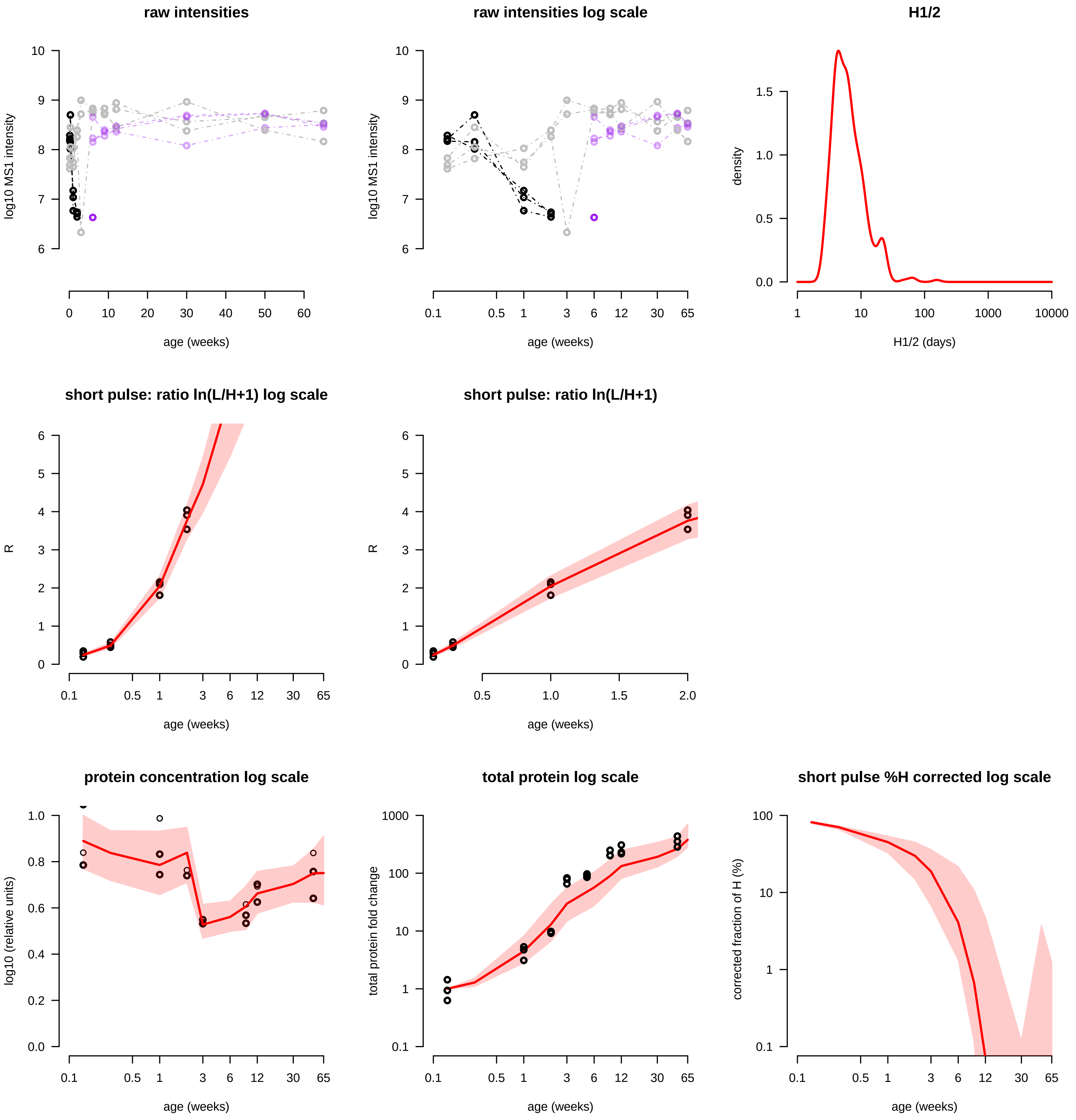

Q8K3H0

>sp|Q8K3H0|DP13A_MOUSE

DCC−interacting protein 13−alpha

GN=Appl1

Med H1/2: 43 days

Q5 H1/2: 9.9 days

Q95 H1/2: 1142.3 days

reliable model fit: yes

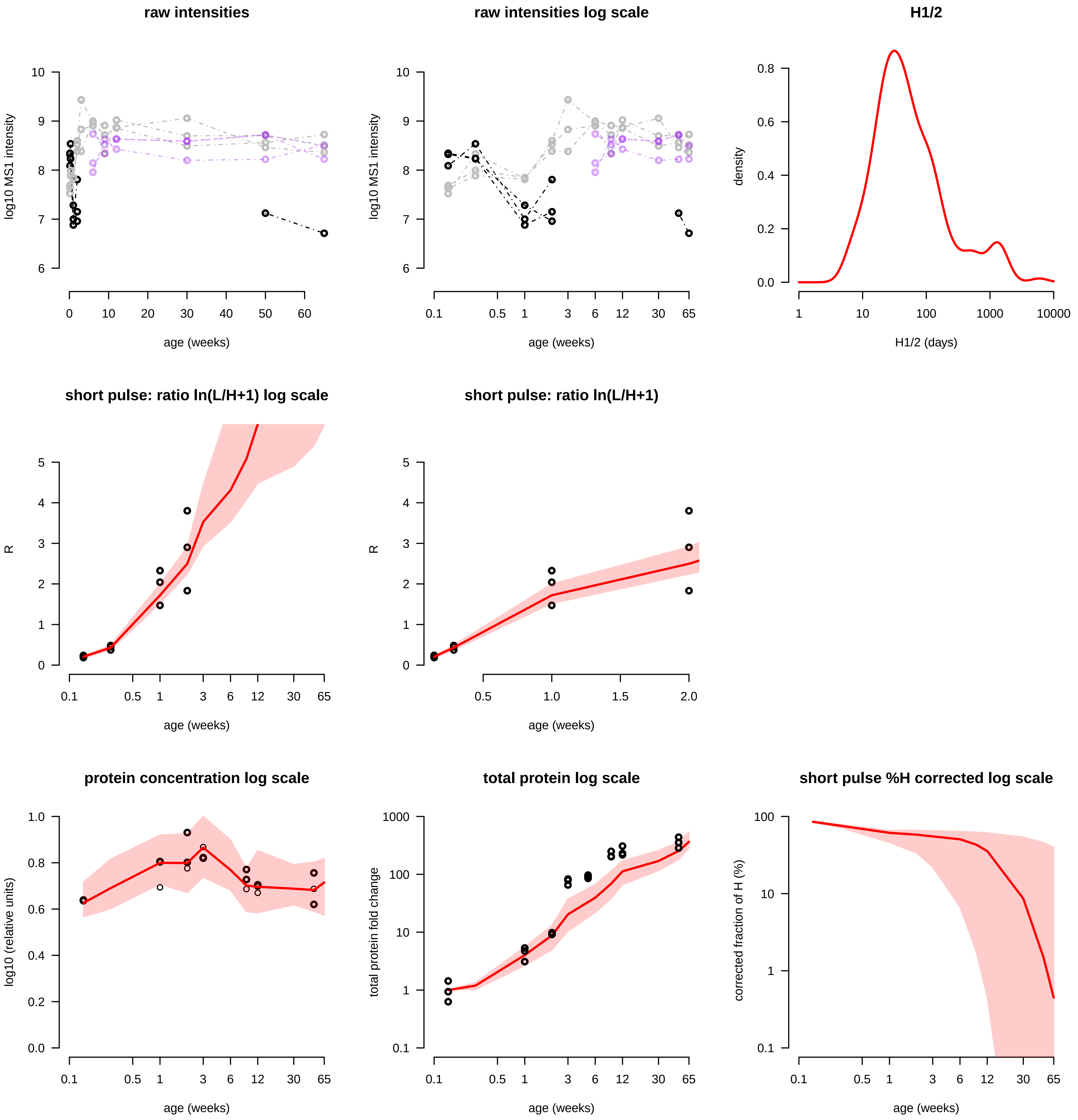

Q9CWU6

>sp|Q9CWU6|UQCC1_MOUSE

Ubiquinol−cytochrome−c reductase complex assembly factor 1

GN=Uqcc1

Med H1/2: 12.5 days

Q5 H1/2: 3.9 days

Q95 H1/2: 124 days

reliable model fit: yes

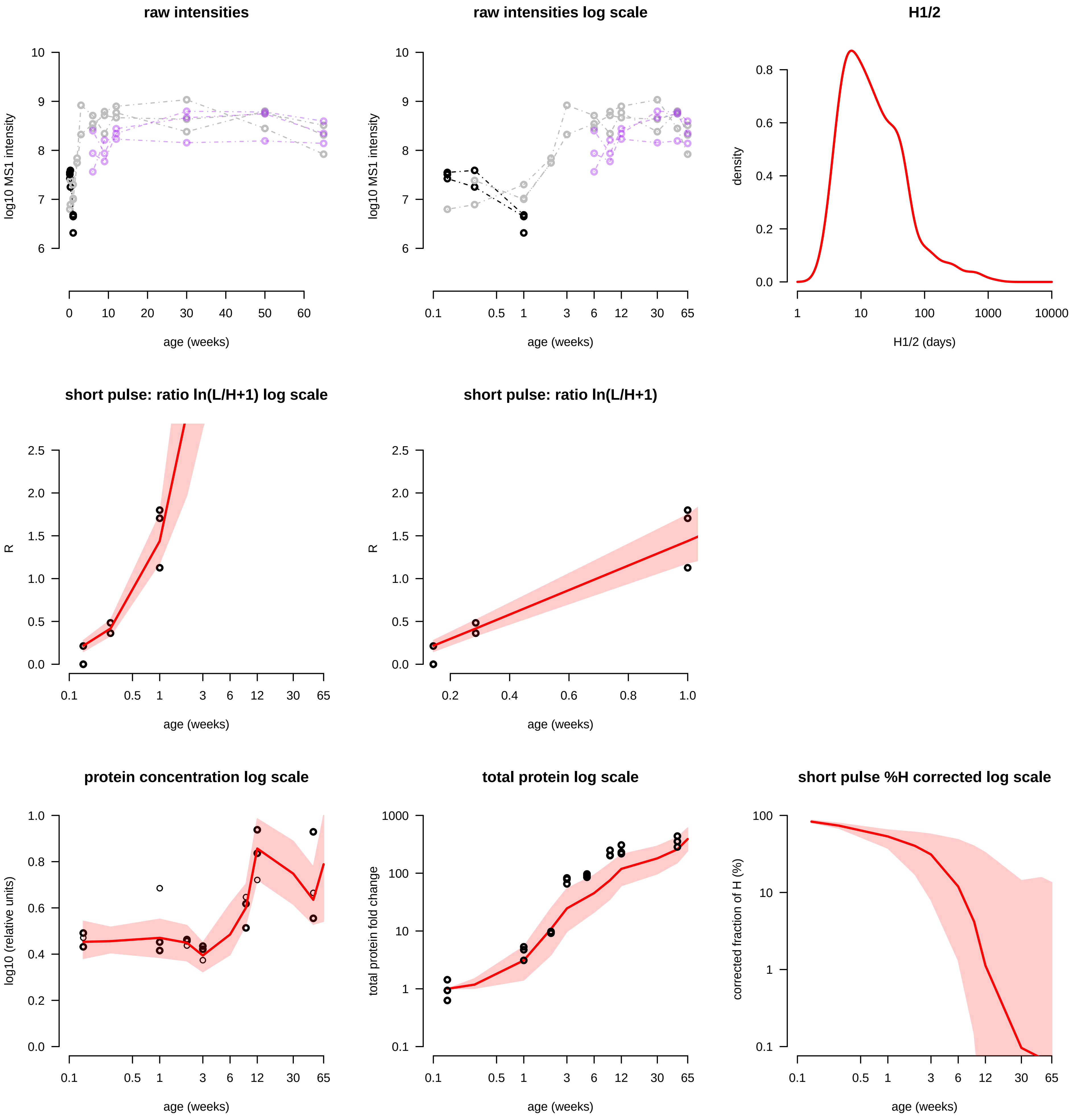

Q3TW96;Q3TW96−2

>sp|Q3TW96|UAP1L_MOUSE

UDP−N−acetylhexosamine pyrophosphorylase−like protein 1

GN=Uap1l1

Med H1/2: 4.6 days

Q5 H1/2: 2.7 days

Q95 H1/2: 14 days

reliable model fit: yes

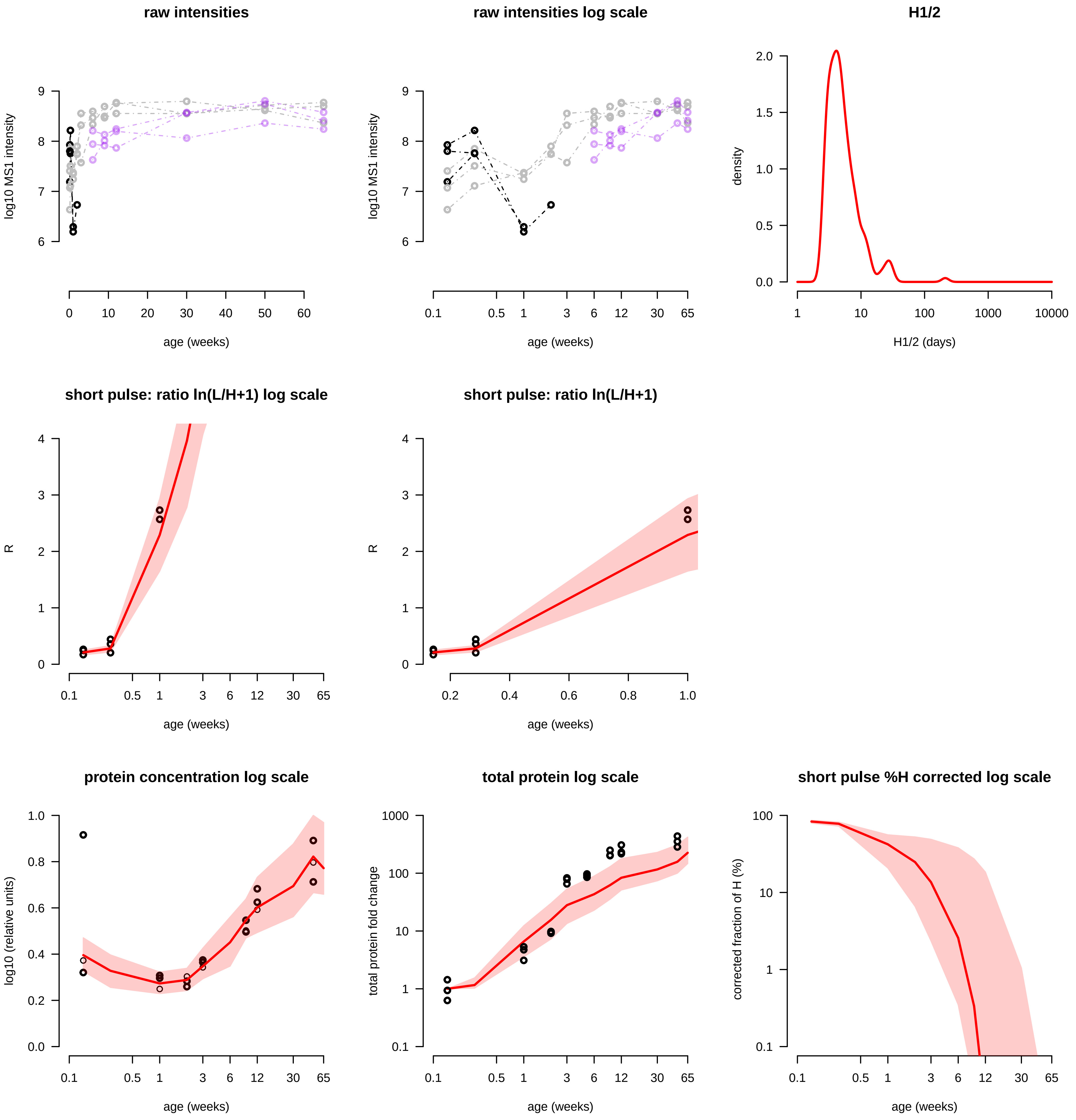

P48722;P48722−2

>sp|P48722|HS74L_MOUSE

Heat shock 70 kDa protein 4L

GN=Hspa4l

Med H1/2: 12.3 days

Q5 H1/2: 4.9 days

Q95 H1/2: 186 days

reliable model fit: yes

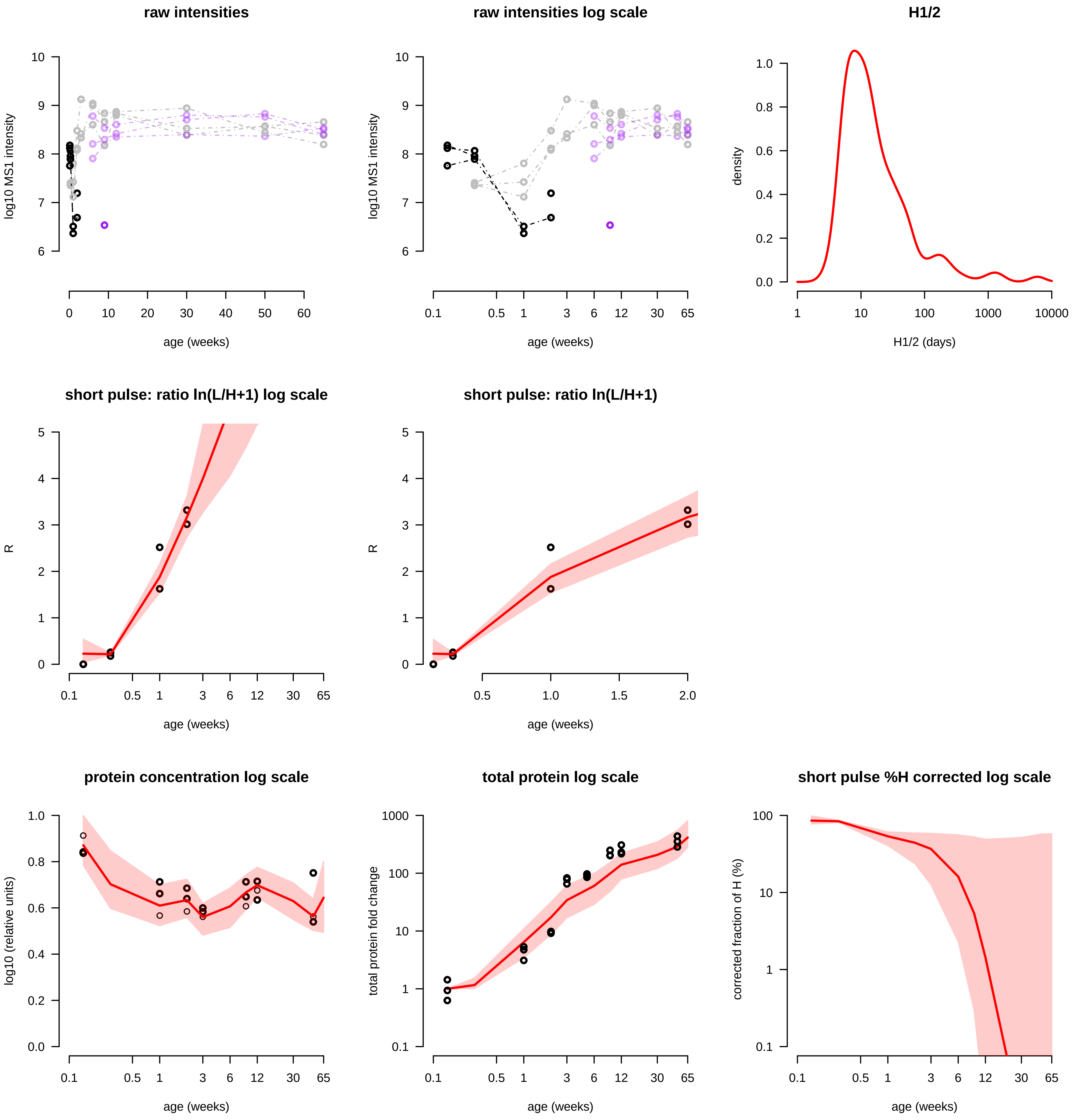

O70493

>sp|O70493|SNX12_MOUSE

Sorting nexin−12

GN=Snx12

Med H1/2: 32.5 days

Q5 H1/2: 9.4 days

Q95 H1/2: 273.7 days

reliable model fit: yes

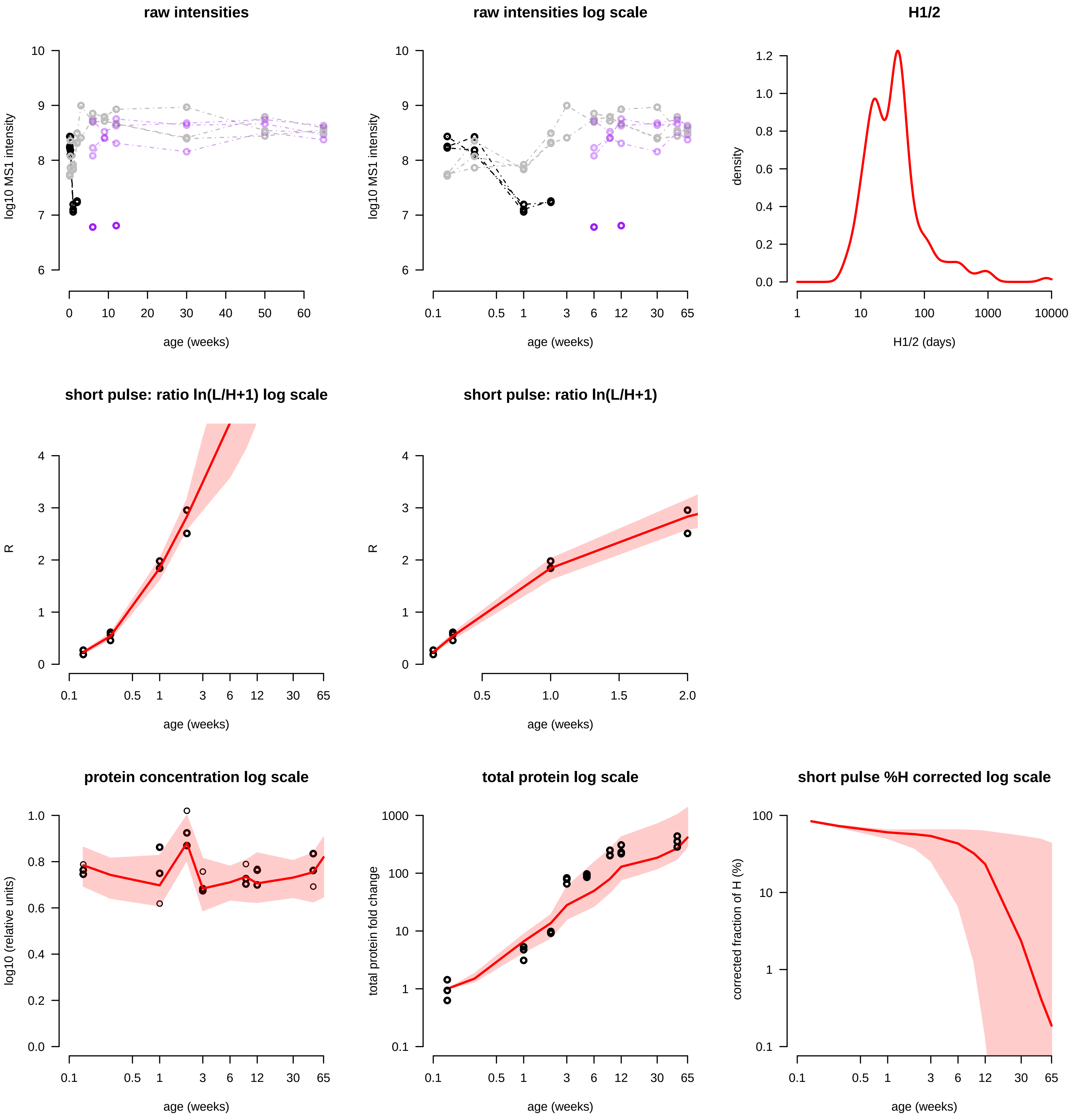

Q9Z0Y1

>sp|Q9Z0Y1|DCTN3_MOUSE

Dynactin subunit 3

GN=Dctn3

Med H1/2: 5.2 days

Q5 H1/2: 3.9 days

Q95 H1/2: 7.5 days

reliable model fit: yes

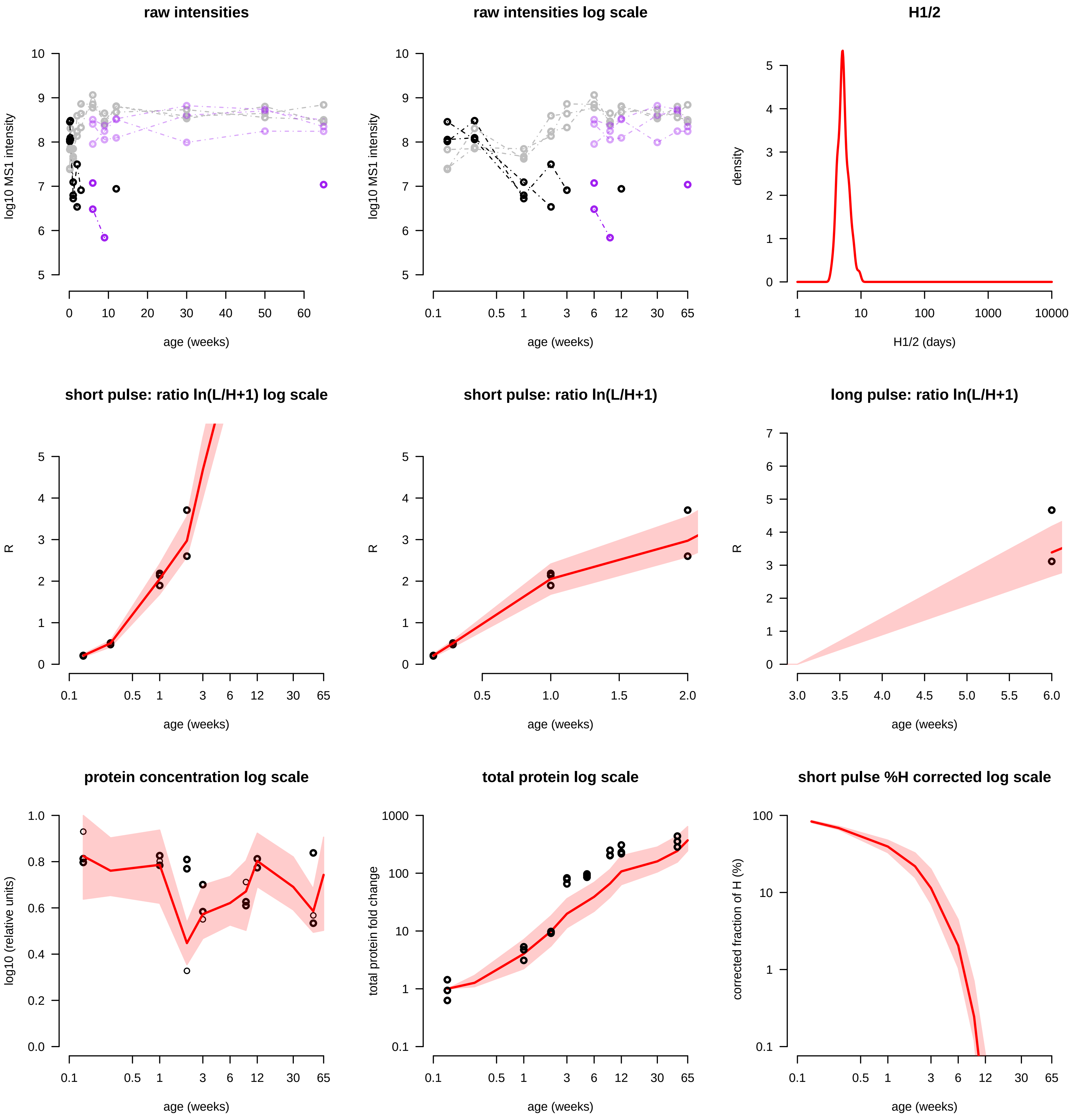

O09044

>sp|O09044|SNP23_MOUSE

Synaptosomal−associated protein 23

GN=Snap23

Med H1/2: 6.2 days

Q5 H1/2: 3 days

Q95 H1/2: 61.8 days

reliable model fit: yes

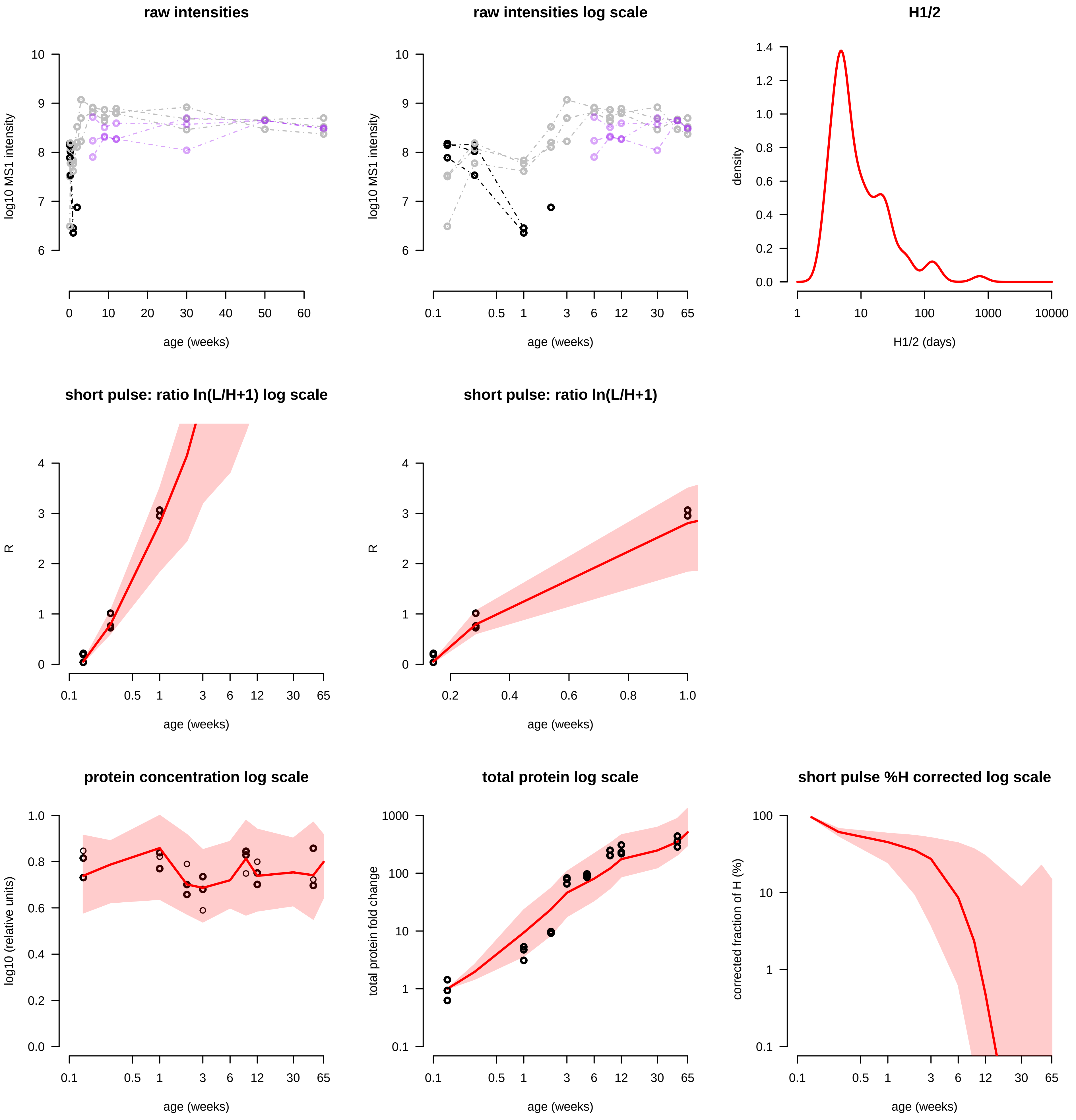

Q99LB6−2

>sp|Q99LB6−2|MAT2B_MOUSE

Isoform 2 of Methionine adenosyltransferase 2 subunit beta

GN=Mat2b

Med H1/2: 4.6 days

Q5 H1/2: 3.7 days

Q95 H1/2: 6.2 days

reliable model fit: yes

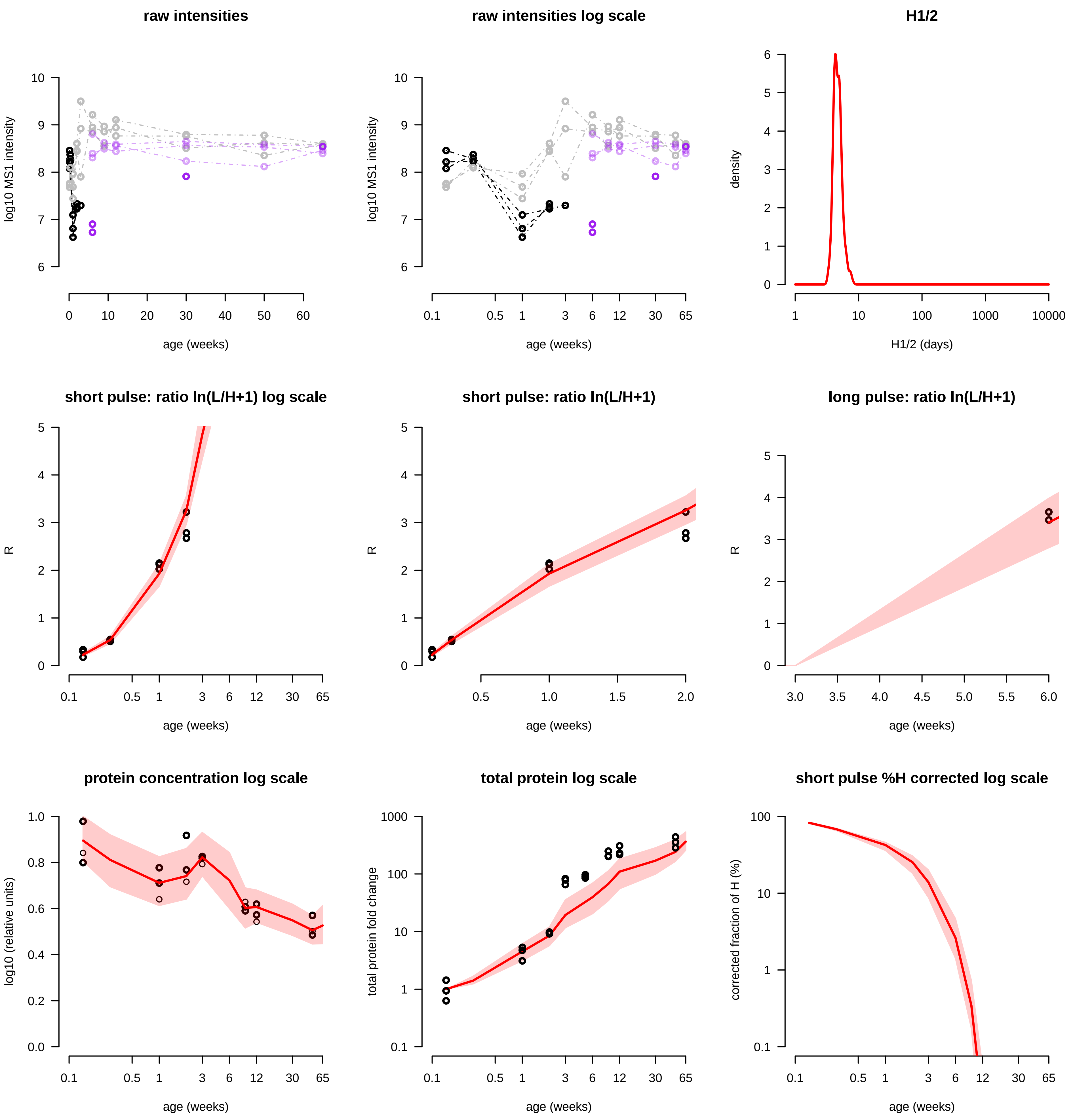

P52332

>sp|P52332|JAK1_MOUSE

Tyrosine−protein kinase JAK1

GN=Jak1

Med H1/2: 3.7 days

Q5 H1/2: 2.6 days

Q95 H1/2: 12.5 days

reliable model fit: yes

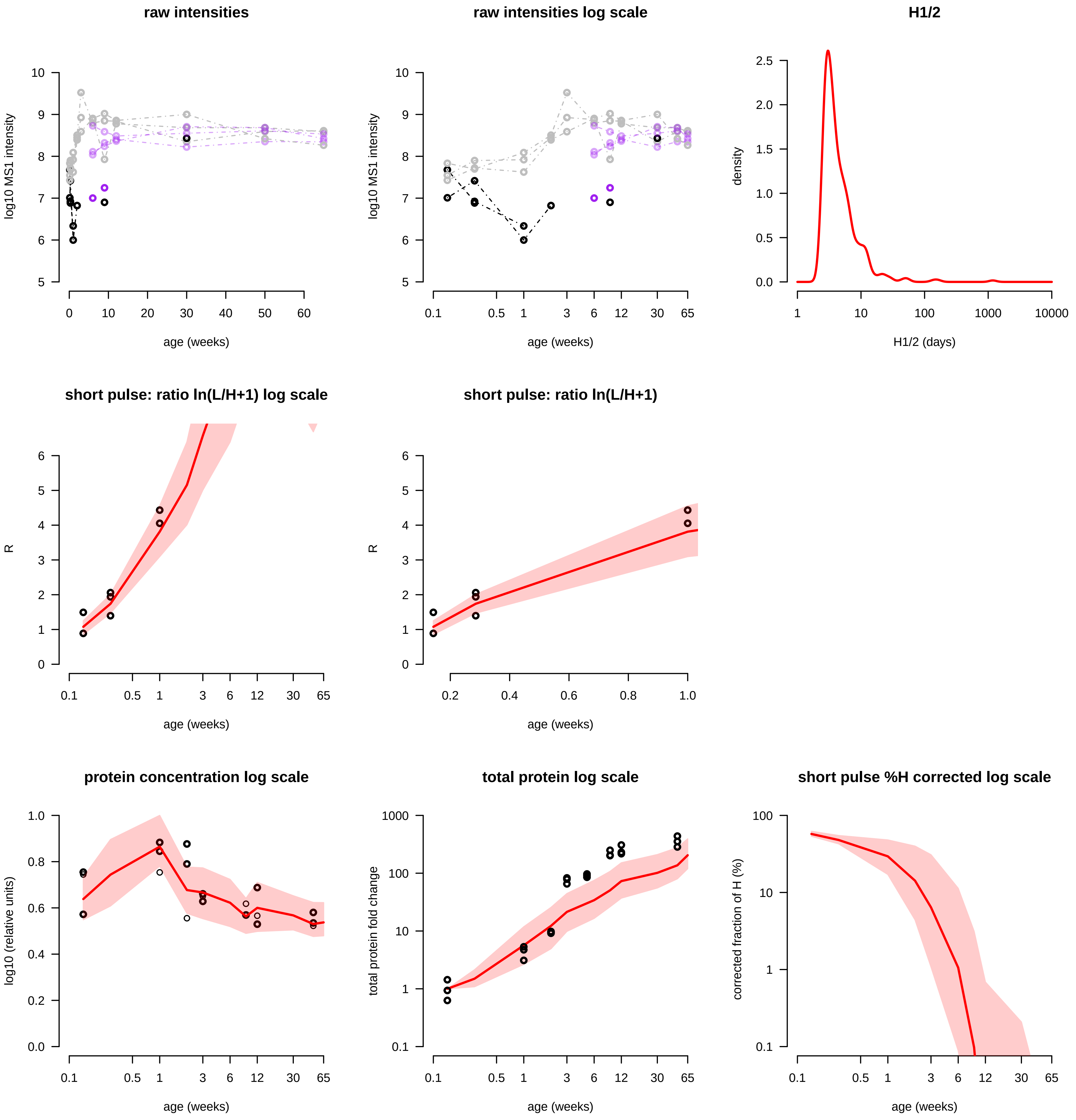

Q3UYV9

>sp|Q3UYV9|NCBP1_MOUSE

Nuclear cap−binding protein subunit 1

GN=Ncbp1

Med H1/2: 4.9 days

Q5 H1/2: 3.8 days

Q95 H1/2: 7.8 days

reliable model fit: no

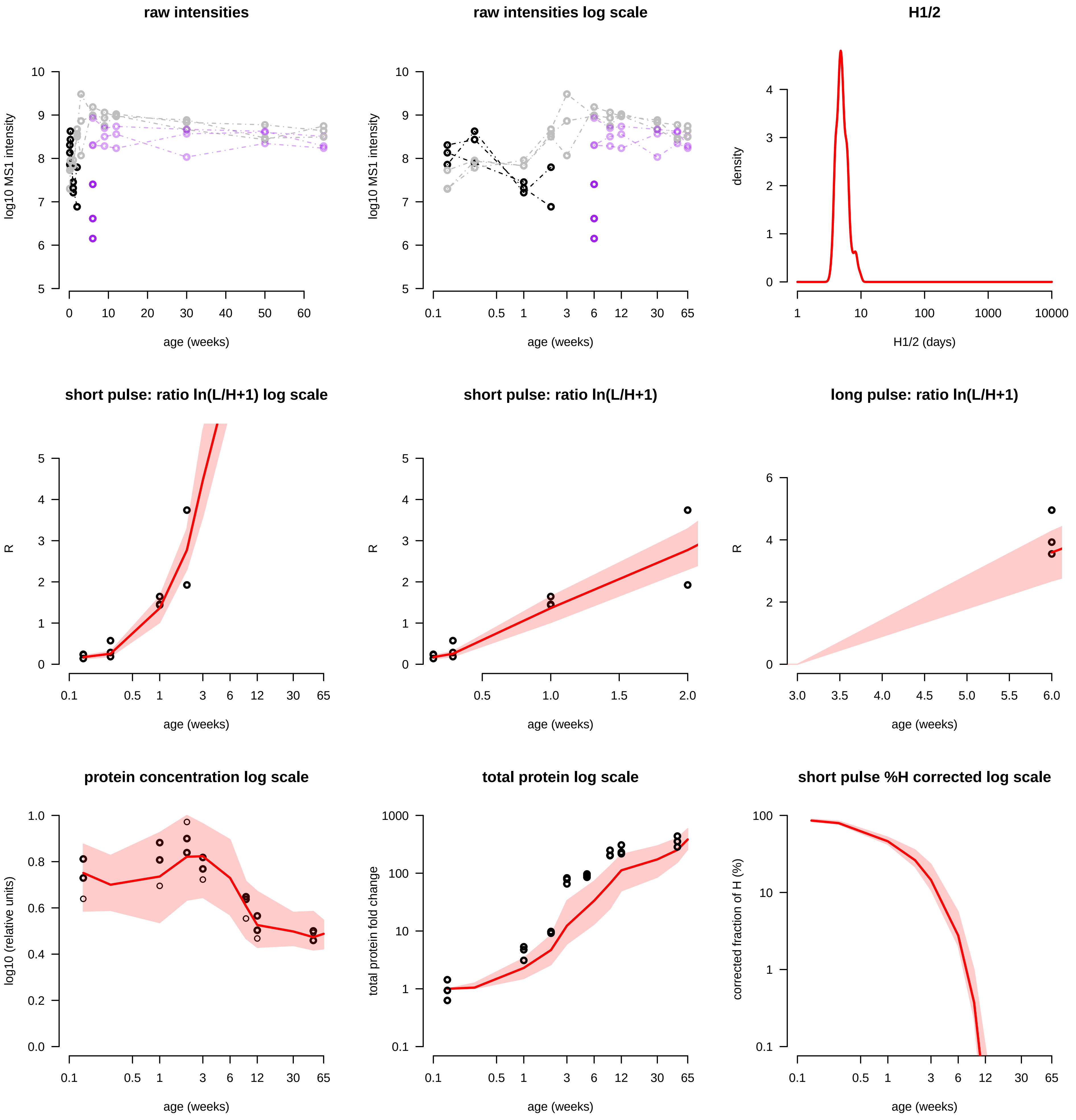

Q61830

>sp|Q61830|MRC1_MOUSE

Macrophage mannose receptor 1

GN=Mrc1

Med H1/2: 6.9 days

Q5 H1/2: 5 days

Q95 H1/2: 10.8 days

reliable model fit: yes

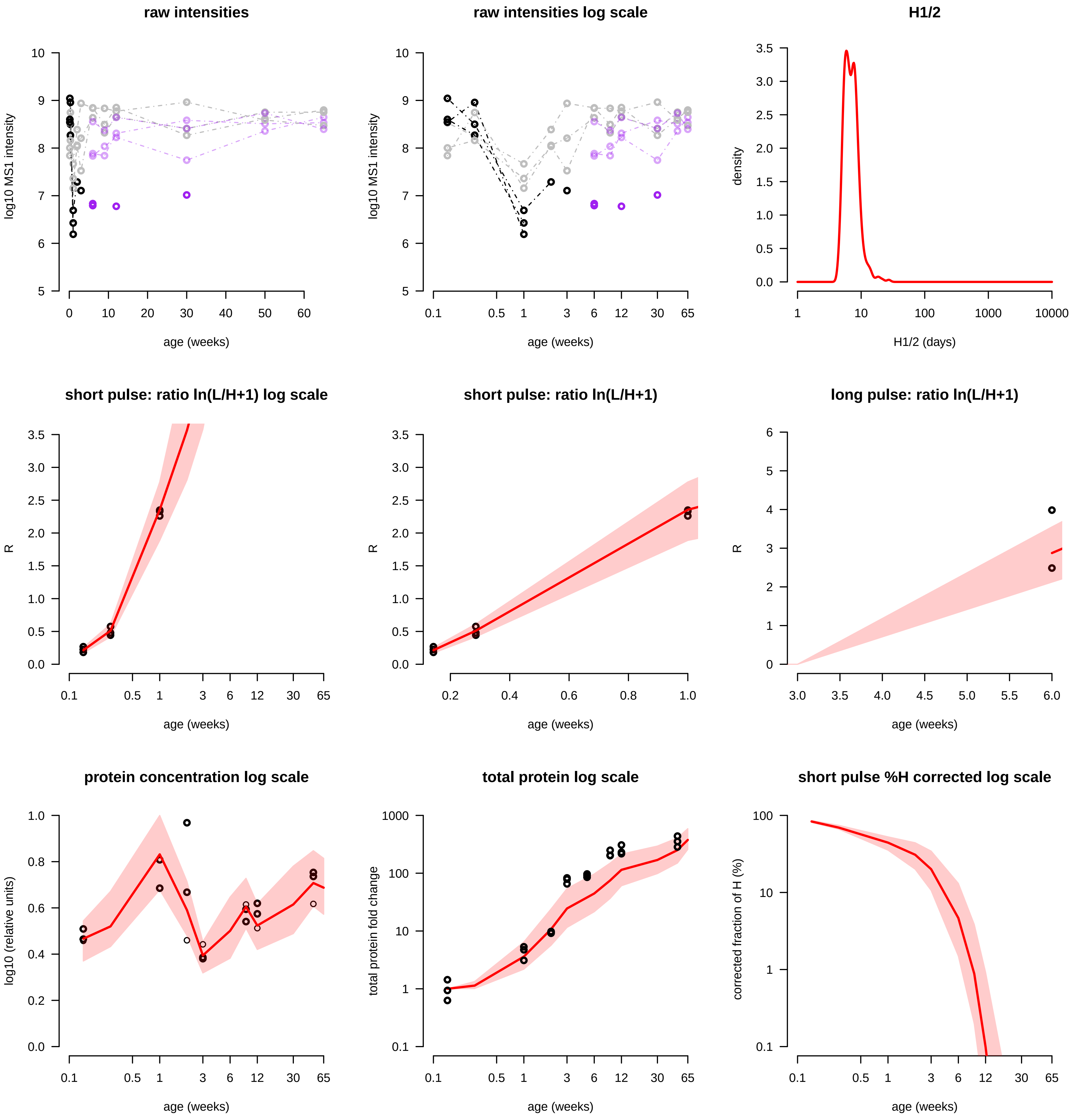

Q08093

>sp|Q08093|CNN2_MOUSE

Calponin−2

GN=Cnn2

Med H1/2: 4.7 days

Q5 H1/2: 3.3 days

Q95 H1/2: 6.8 days

reliable model fit: yes

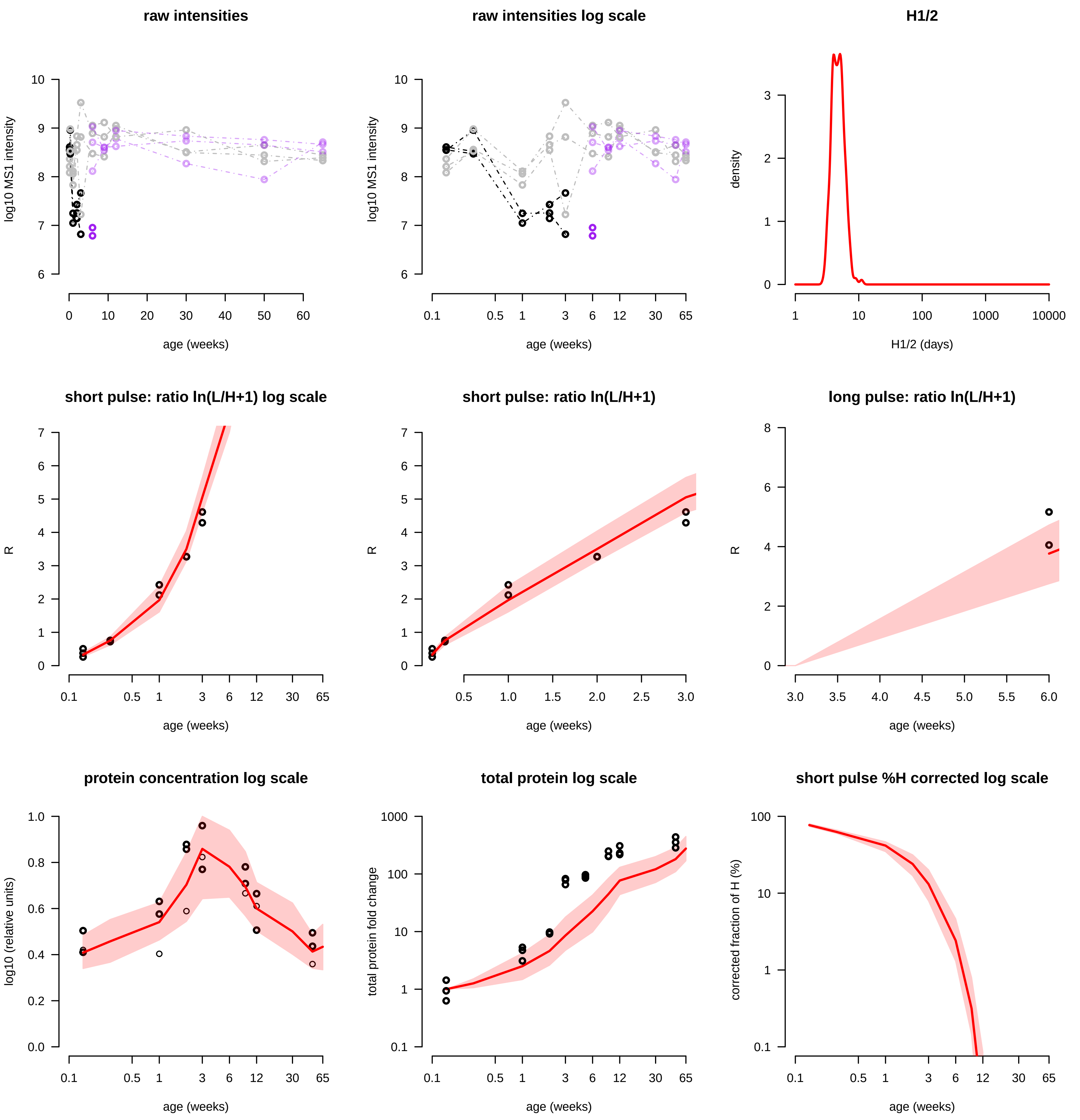

Q8K0U4

>sp|Q8K0U4|HS12A_MOUSE

Heat shock 70 kDa protein 12A

GN=Hspa12a

Med H1/2: 8.1 days

Q5 H1/2: 5.6 days

Q95 H1/2: 13.4 days

reliable model fit: yes

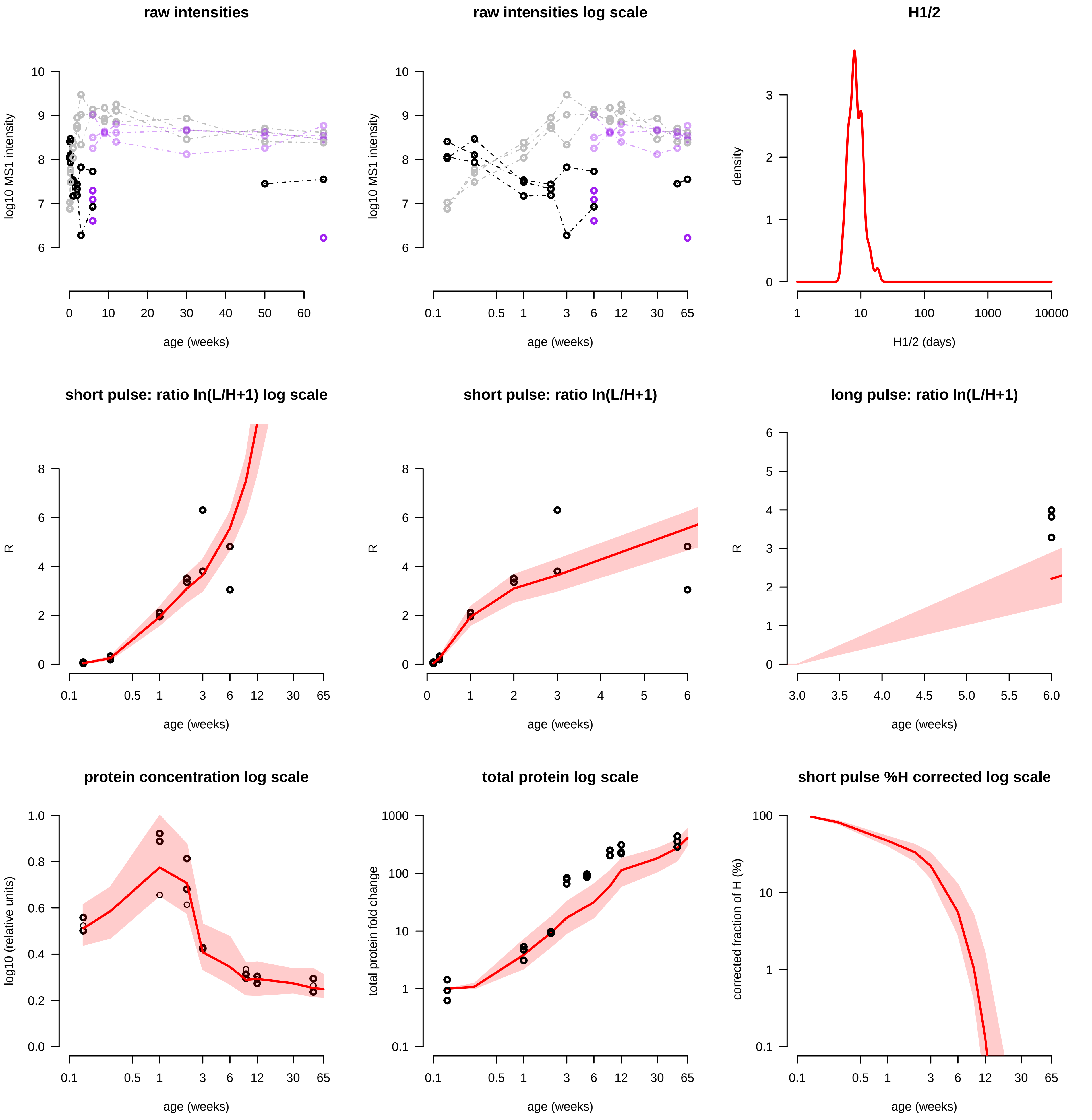

Q6P3A8;Q6P3A8−2

>sp|Q6P3A8|ODBB_MOUSE

2−oxoisovalerate dehydrogenase subunit beta, mitochondrial

GN=Bckdhb

Med H1/2: 5.9 days

Q5 H1/2: 4.1 days

Q95 H1/2: 9.8 days

reliable model fit: no

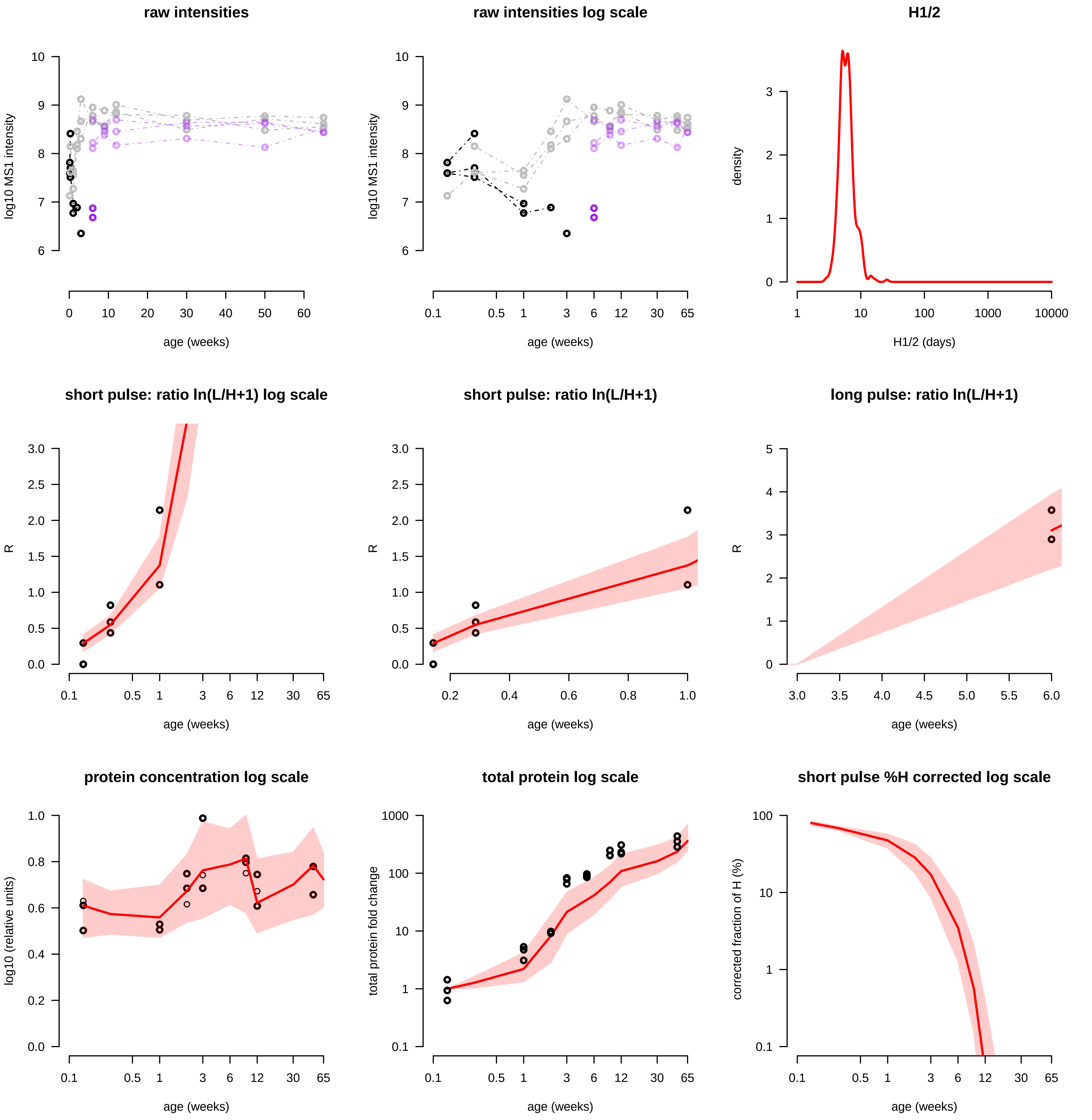

O70400

>sp|O70400|PDLI1_MOUSE

PDZ and LIM domain protein 1

GN=Pdlim1

Med H1/2: 5.9 days

Q5 H1/2: 2.9 days

Q95 H1/2: 40.4 days

reliable model fit: yes

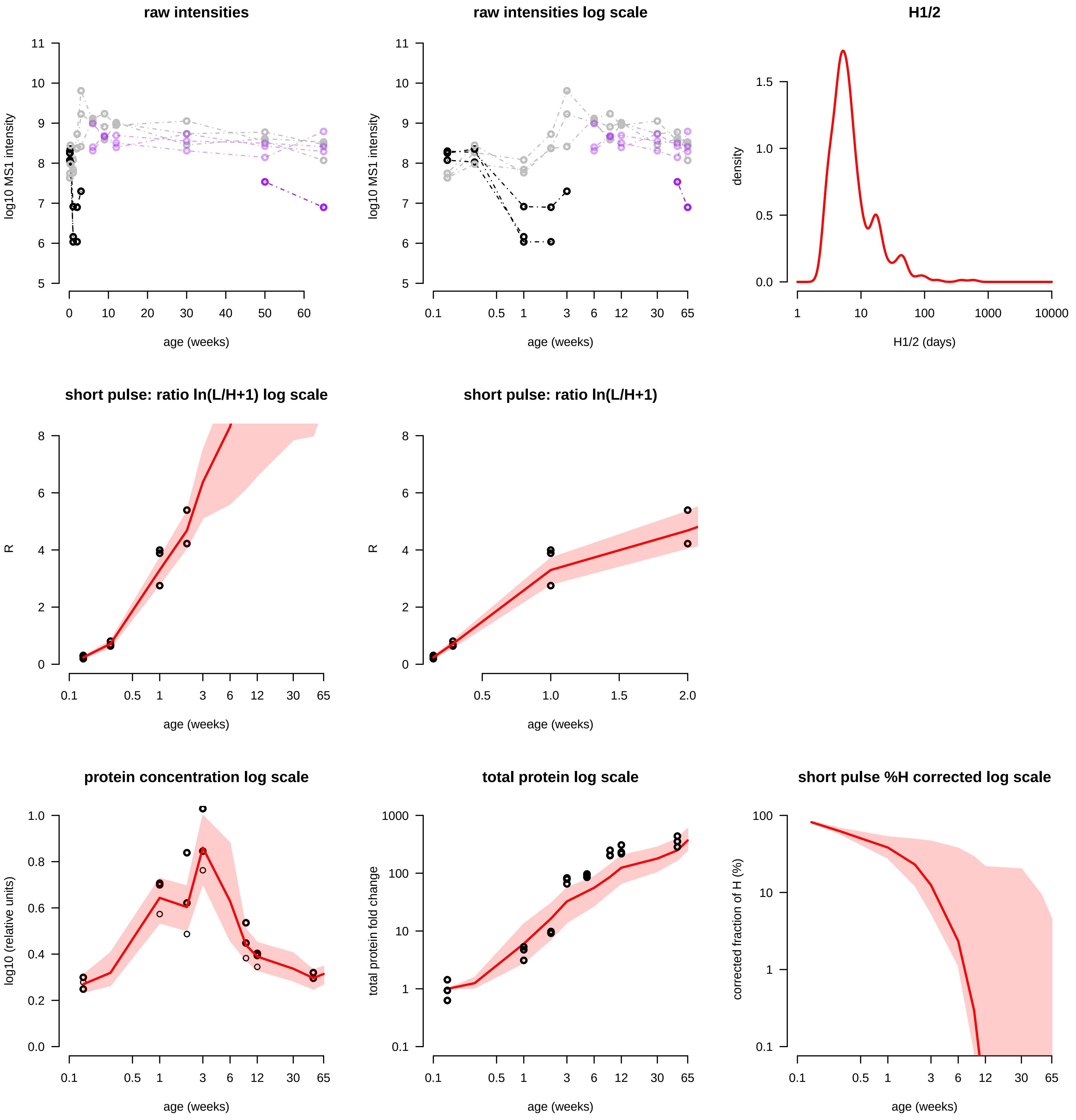

Q8BUV3

>sp|Q8BUV3|GEPH_MOUSE

Gephyrin

GN=Gphn

Med H1/2: 4 days

Q5 H1/2: 2.6 days

Q95 H1/2: 37.7 days

reliable model fit: yes

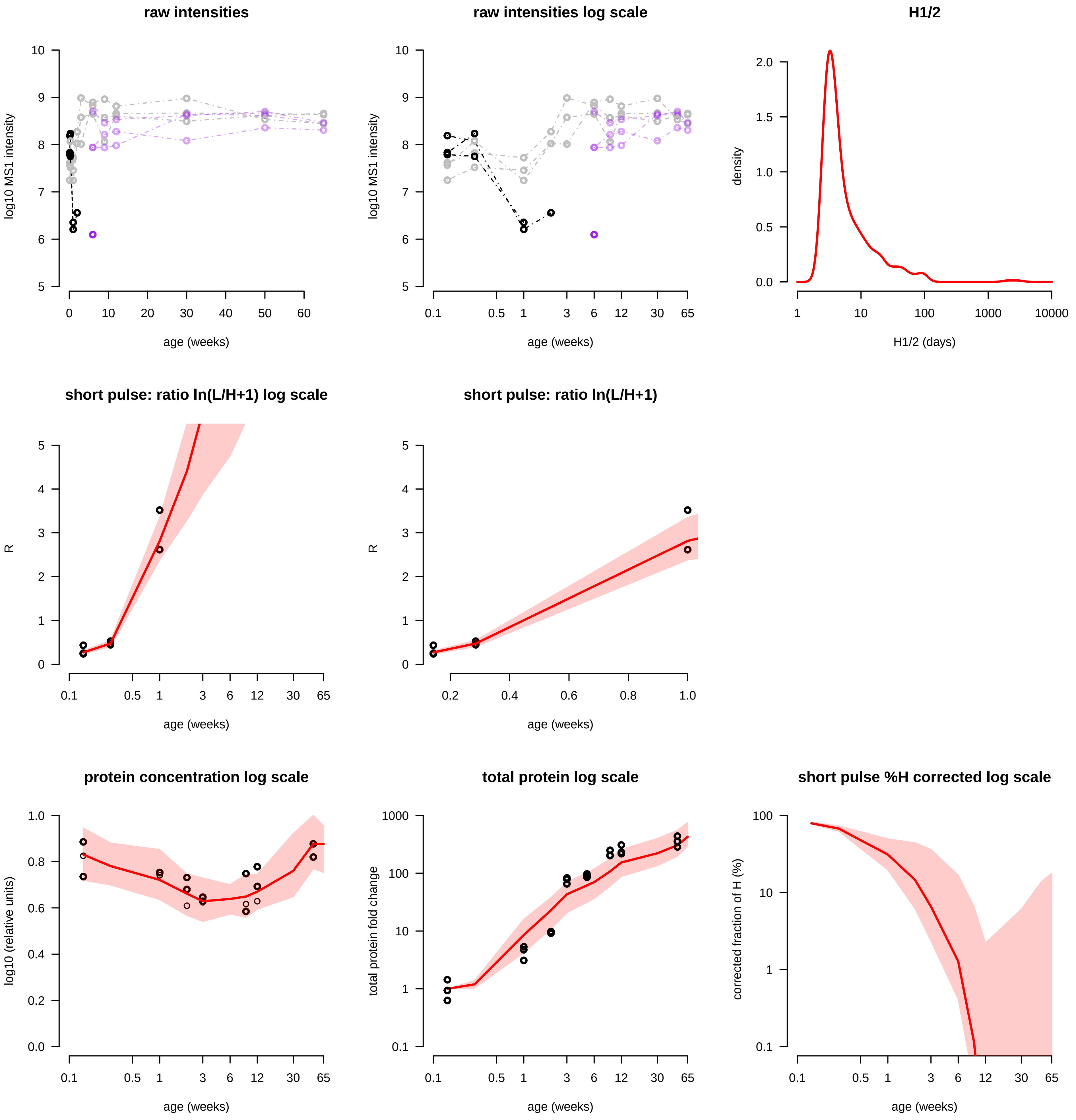

Q8K021

>sp|Q8K021|SCAM1_MOUSE

Secretory carrier−associated membrane protein 1

GN=Scamp1

Med H1/2: 18.4 days

Q5 H1/2: 7 days

Q95 H1/2: 179.5 days

reliable model fit: yes

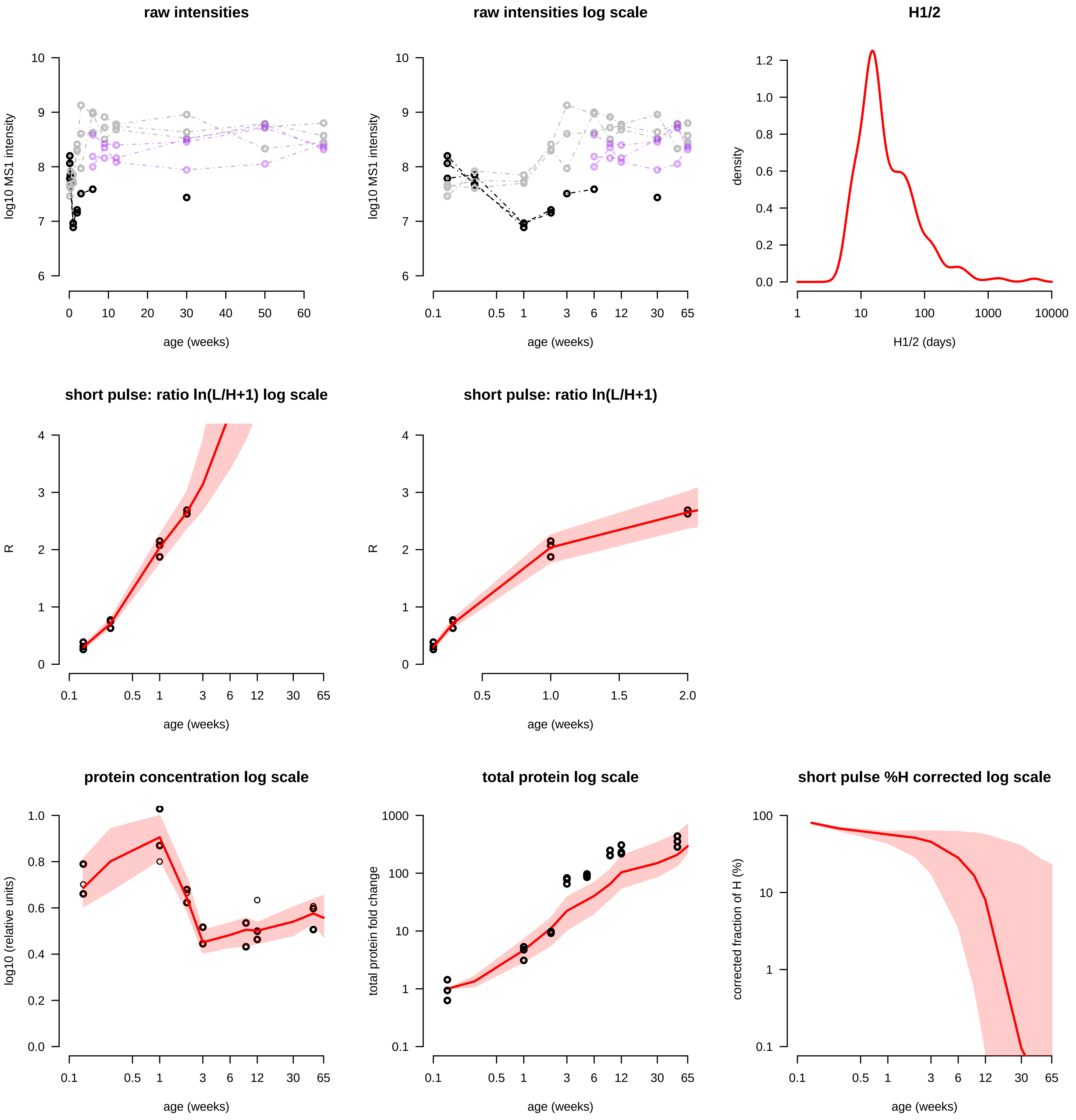

P61979−3

>sp|P61979−3|HNRPK_MOUSE

Isoform 3 of Heterogeneous nuclear ribonucleoprotein K

GN=Hnrnpk

Med H1/2: 16.1 days

Q5 H1/2: 12.1 days

Q95 H1/2: 27 days

reliable model fit: yes

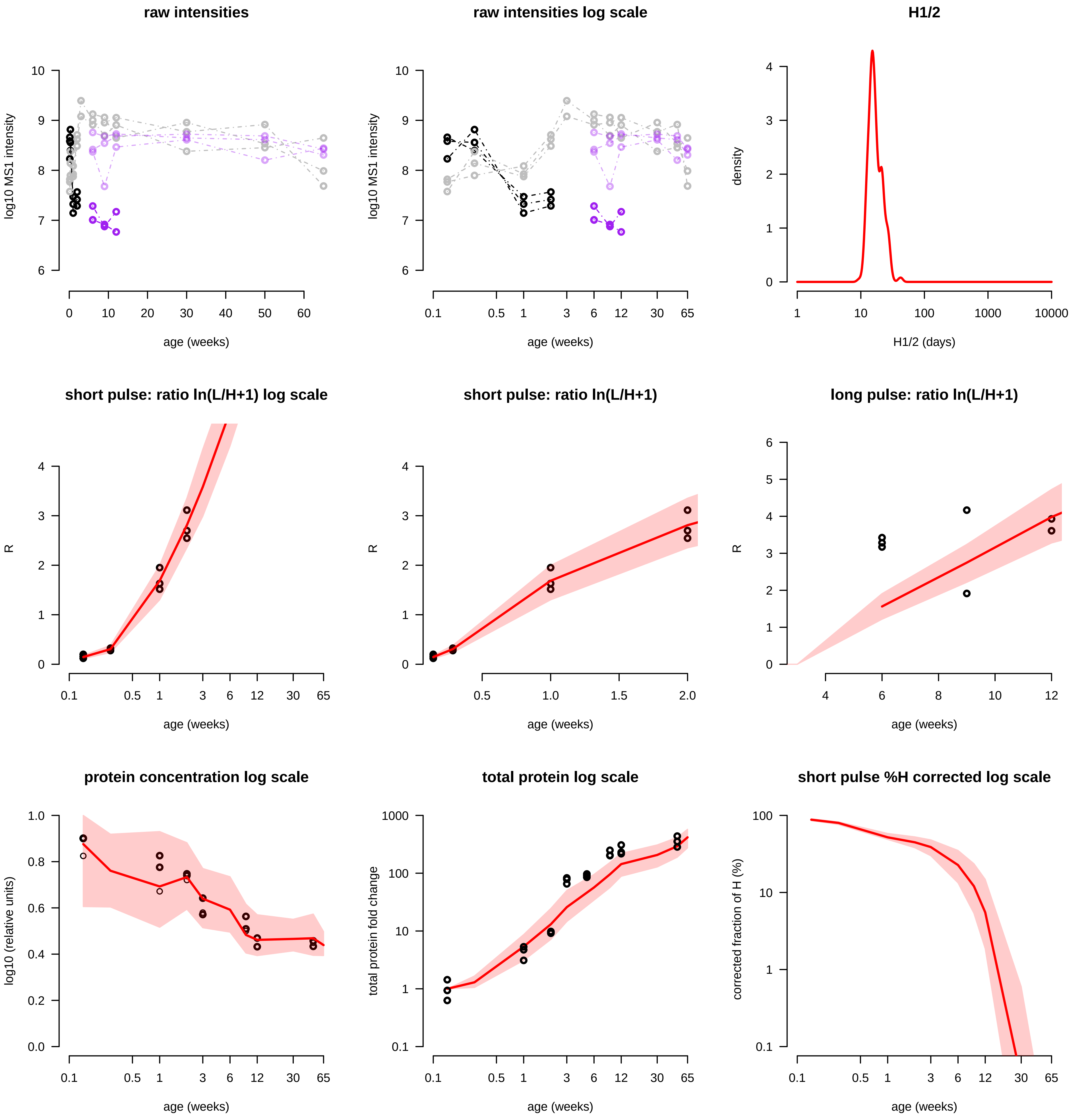

Q505F5

>sp|Q505F5|LRC47_MOUSE

Leucine−rich repeat−containing protein 47

GN=Lrrc47

Med H1/2: 4.2 days

Q5 H1/2: 3.7 days

Q95 H1/2: 5.2 days

reliable model fit: yes

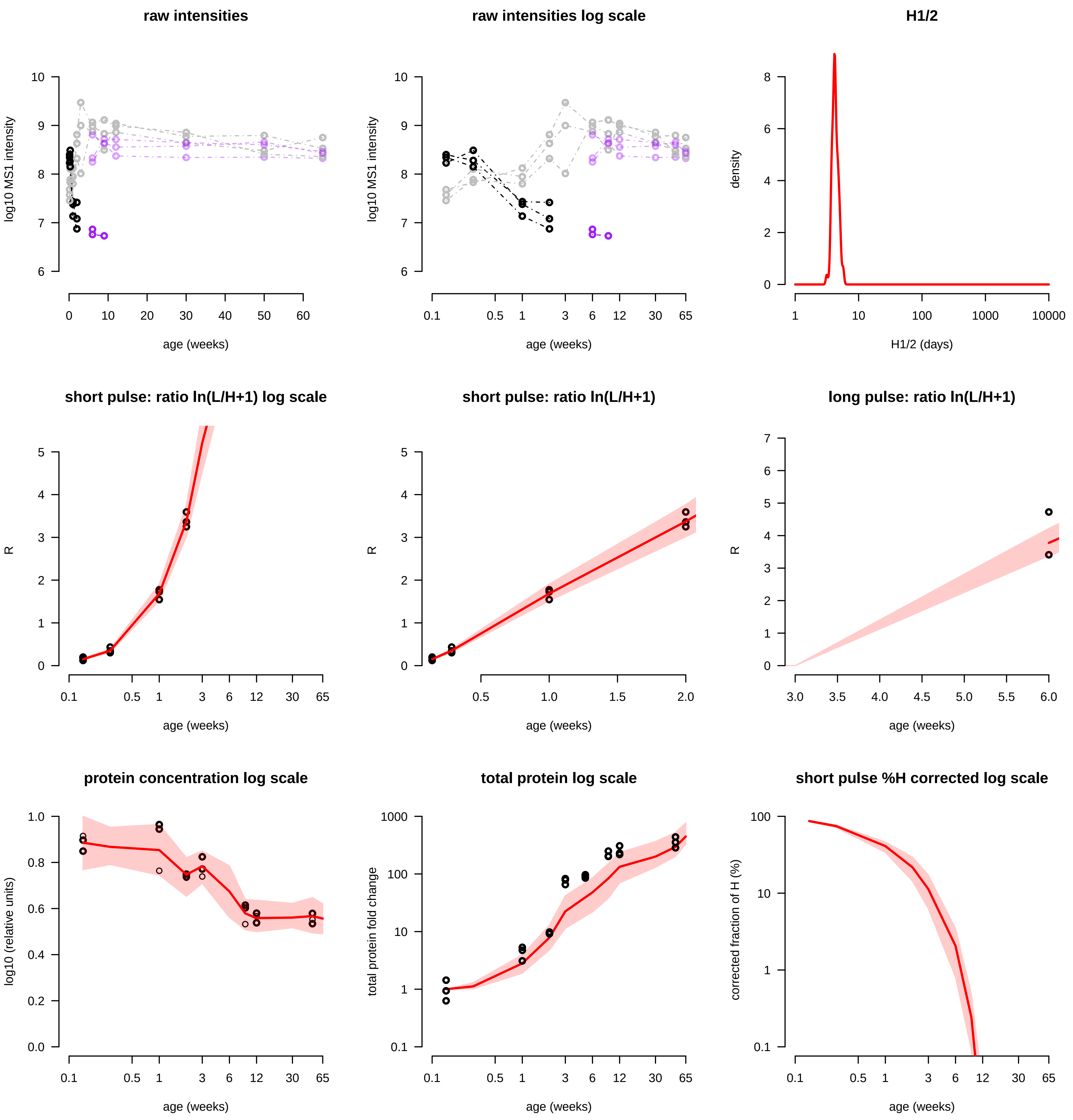

Q6P2K6

>sp|Q6P2K6|P4R3A_MOUSE

Serine/threonine−protein phosphatase 4 regulatory subunit 3A

GN=Ppp4r3a

Med H1/2: 4.6 days

Q5 H1/2: 2.8 days

Q95 H1/2: 10.1 days

reliable model fit: yes

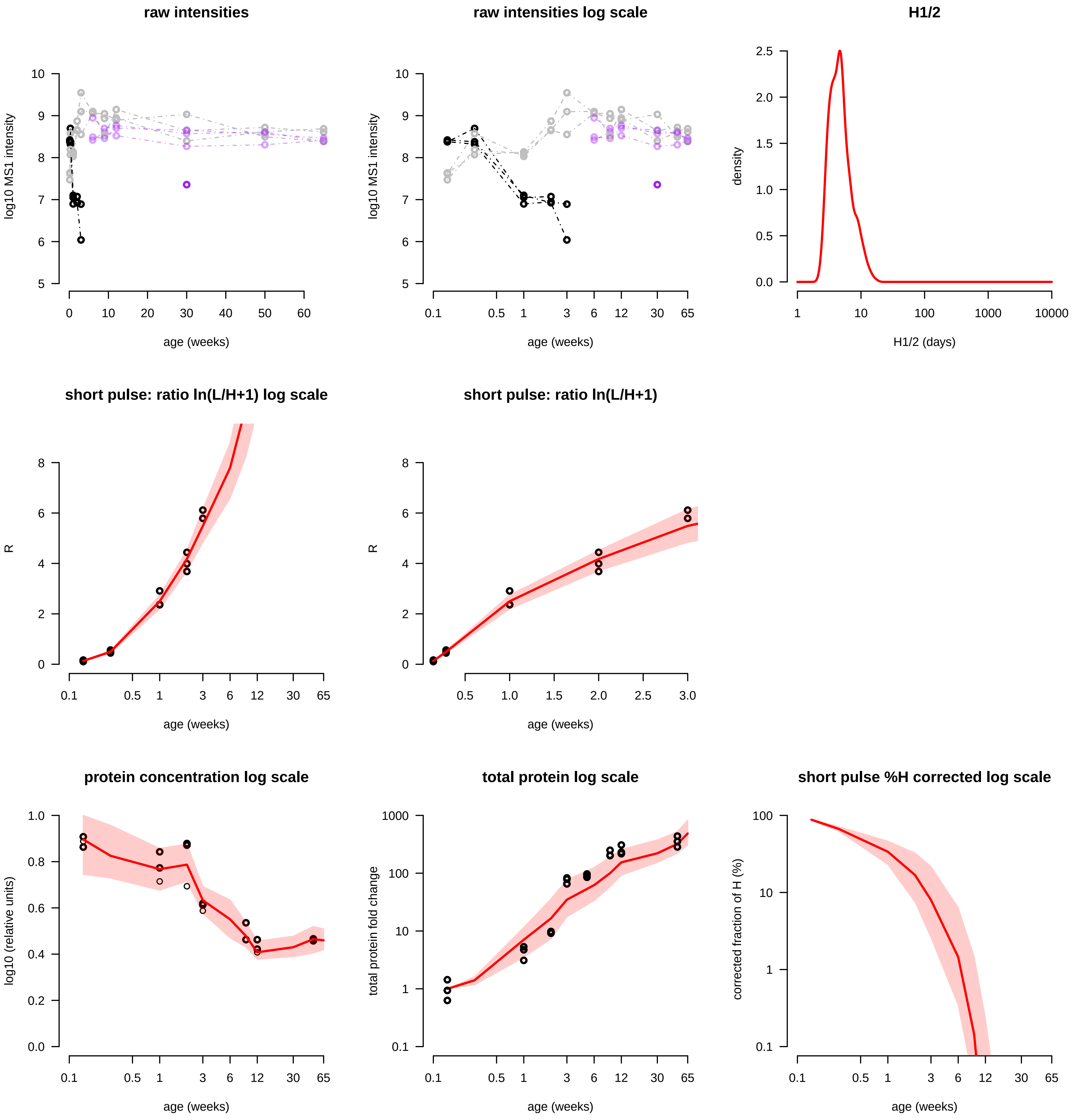

Q9CQC7

>sp|Q9CQC7|NDUB4_MOUSE

NADH dehydrogenase [ubiquinone] 1 beta subcomplex subunit 4

GN=Ndufb4

Med H1/2: 8.7 days

Q5 H1/2: 5.1 days

Q95 H1/2: 16.4 days

reliable model fit: yes

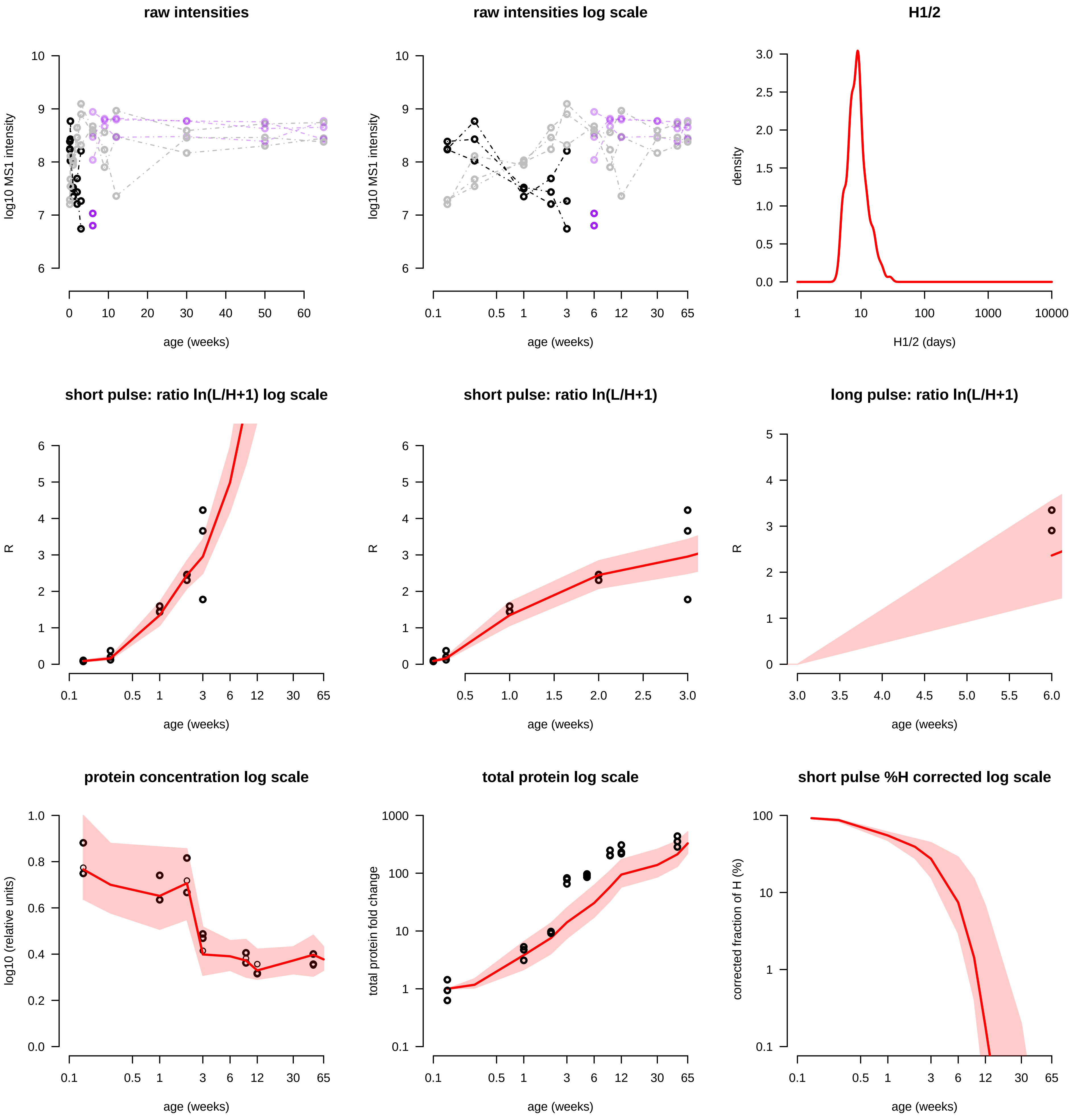

Q80U93

>sp|Q80U93|NU214_MOUSE

Nuclear pore complex protein Nup214

GN=Nup214

Med H1/2: 3.8 days

Q5 H1/2: 3.1 days

Q95 H1/2: 4.7 days

reliable model fit: yes

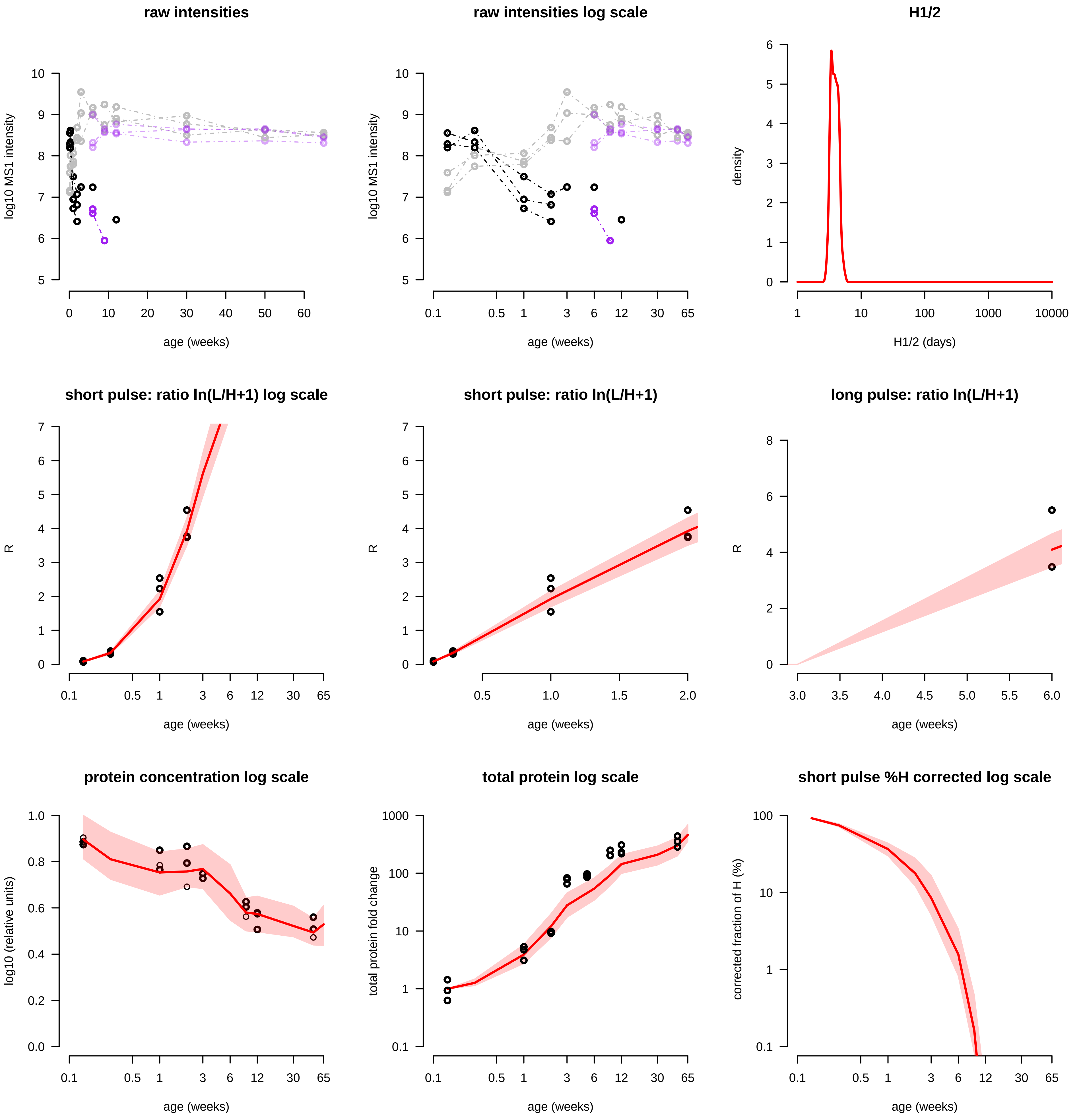

Q8BH74

>sp|Q8BH74|NU107_MOUSE

Nuclear pore complex protein Nup107

GN=Nup107

Med H1/2: 11.4 days

Q5 H1/2: 4.2 days

Q95 H1/2: 78.9 days

reliable model fit: yes

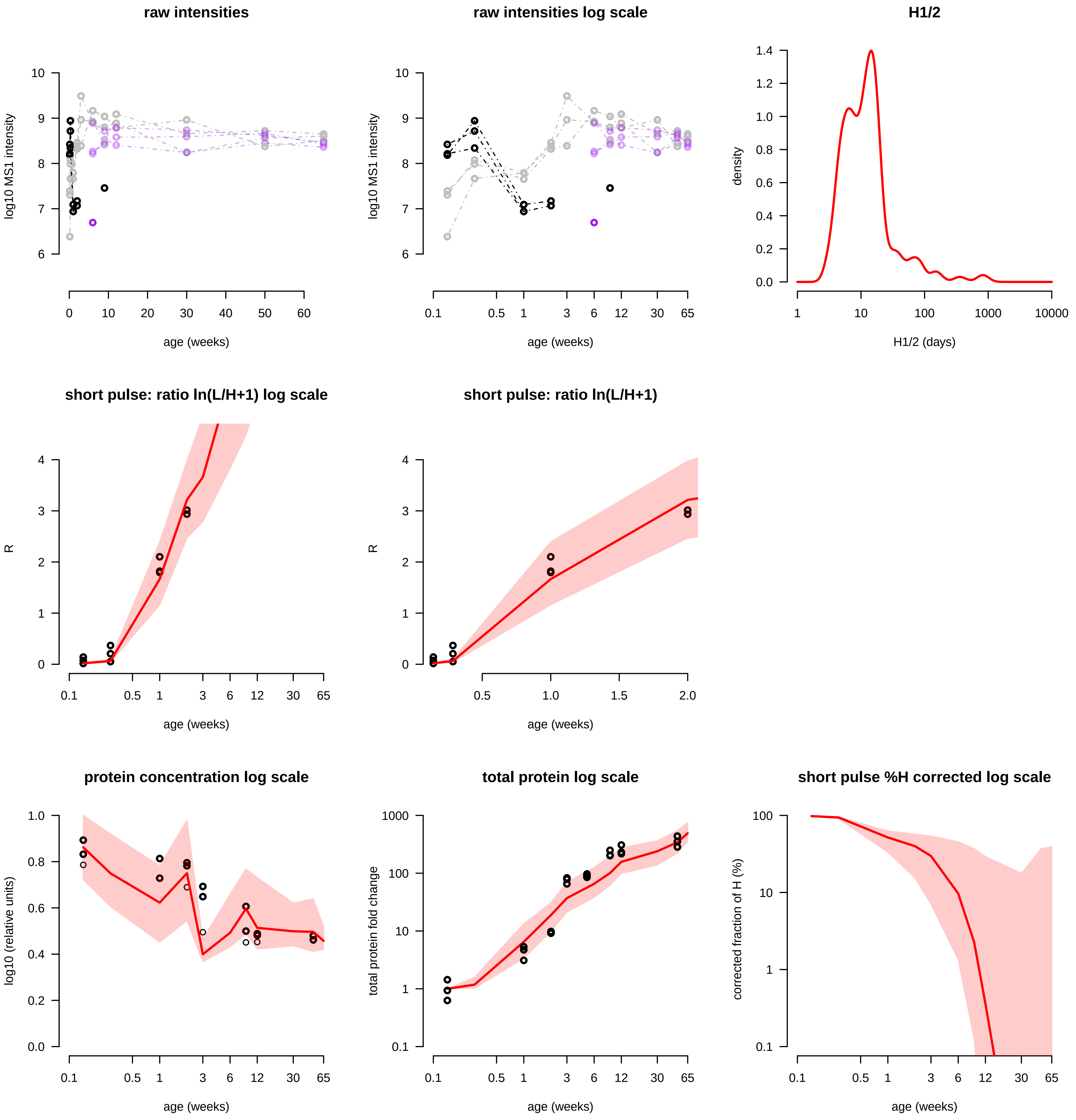

Q62351

>sp|Q62351|TFR1_MOUSE

Transferrin receptor protein 1

GN=Tfrc

Med H1/2: 4.5 days

Q5 H1/2: 2.7 days

Q95 H1/2: 11.7 days

reliable model fit: yes

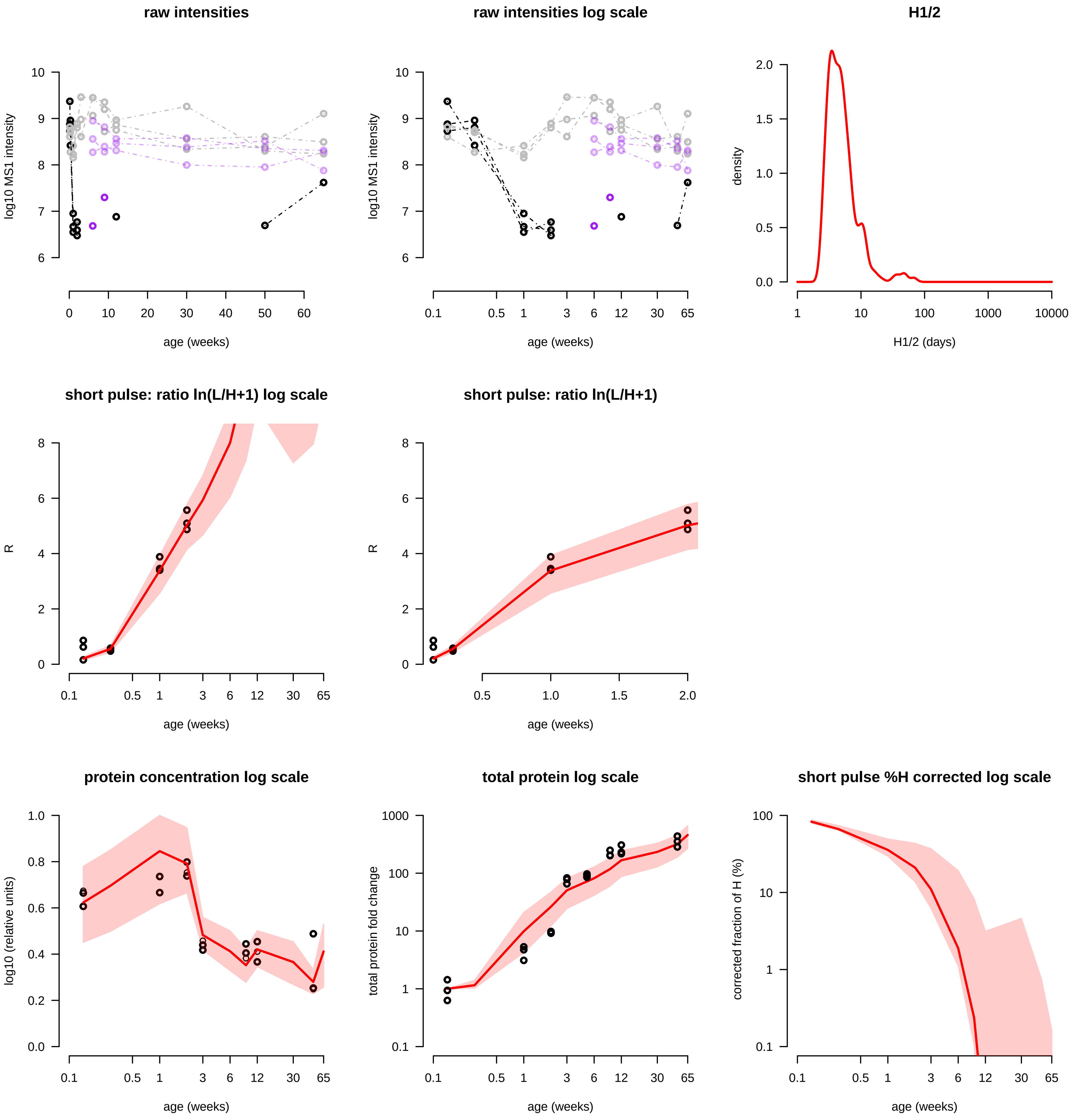

P08122

>sp|P08122|CO4A2_MOUSE

Collagen alpha−2(IV) chain

GN=Col4a2

Med H1/2: 18.5 days

Q5 H1/2: 5.1 days

Q95 H1/2: 131.7 days

reliable model fit: yes

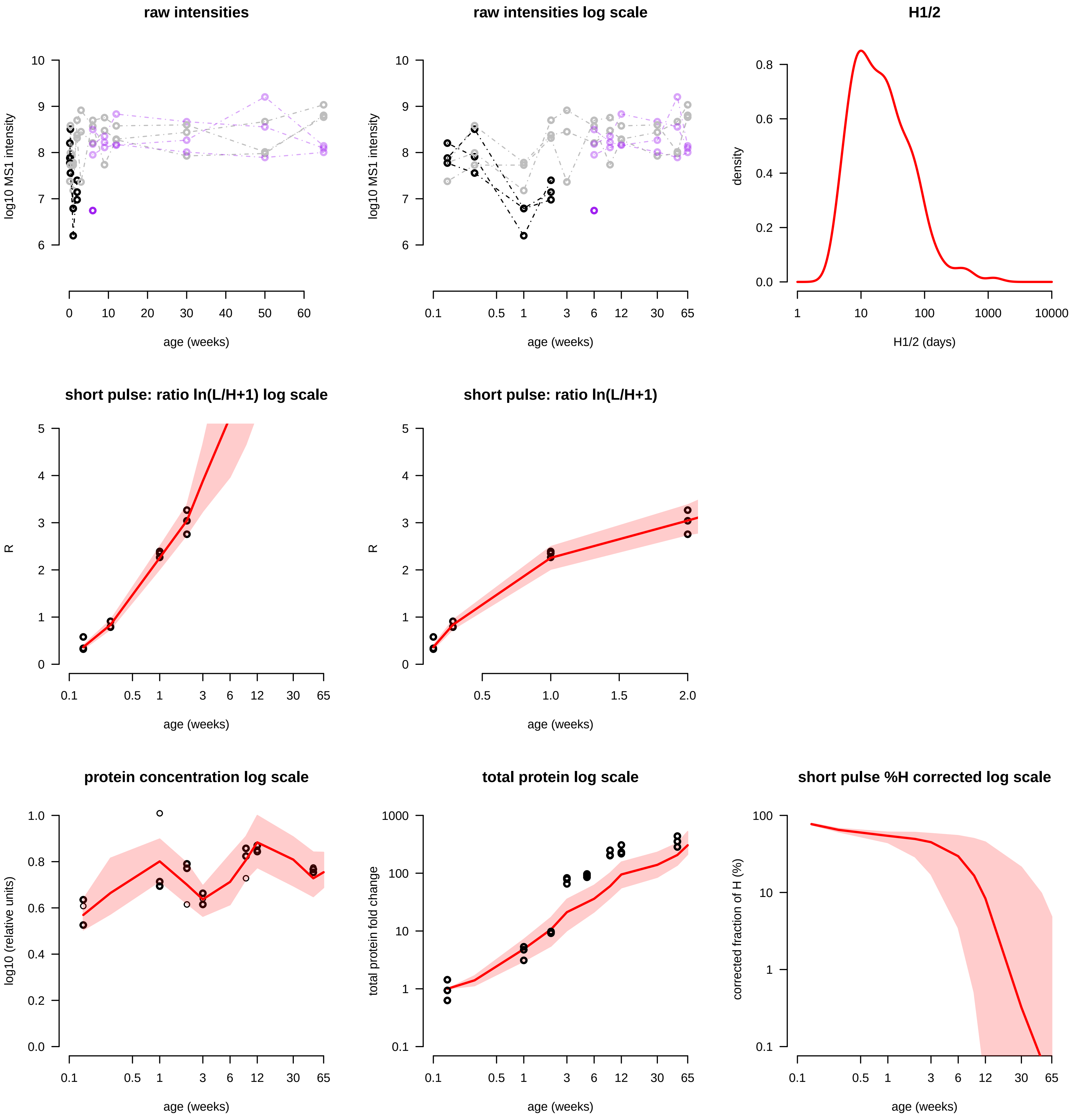

E9Q912

>sp|E9Q912|GDS1_MOUSE

Rap1 GTPase−GDP dissociation stimulator 1

GN=Rap1gds1

Med H1/2: 4.1 days

Q5 H1/2: 3.5 days

Q95 H1/2: 4.8 days

reliable model fit: yes

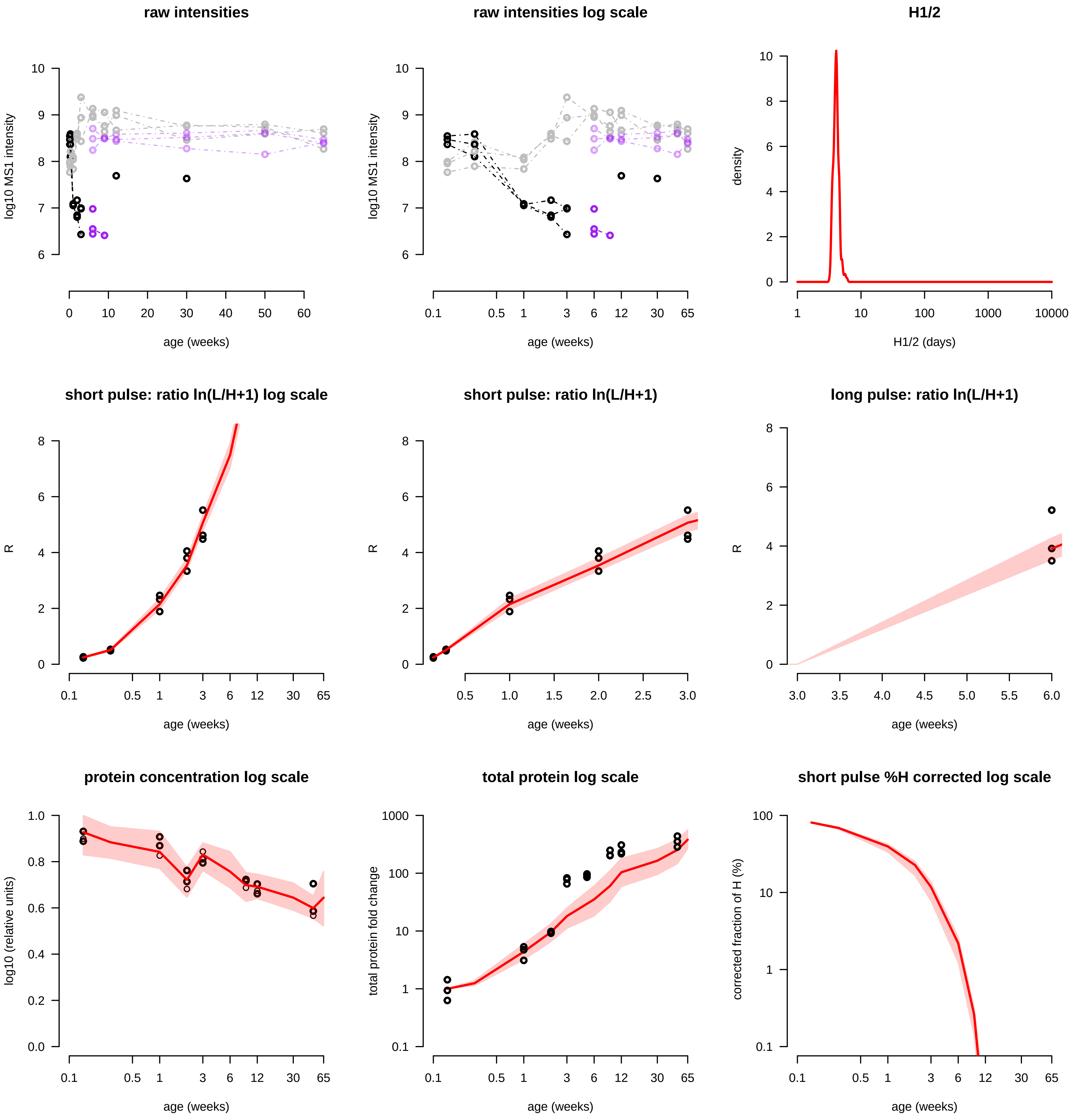

P70318

>sp|P70318|TIAR_MOUSE

Nucleolysin TIAR

GN=Tial1

Med H1/2: 1498.3 days

Q5 H1/2: 461.6 days

Q95 H1/2: 13735.4 days

reliable model fit: yes

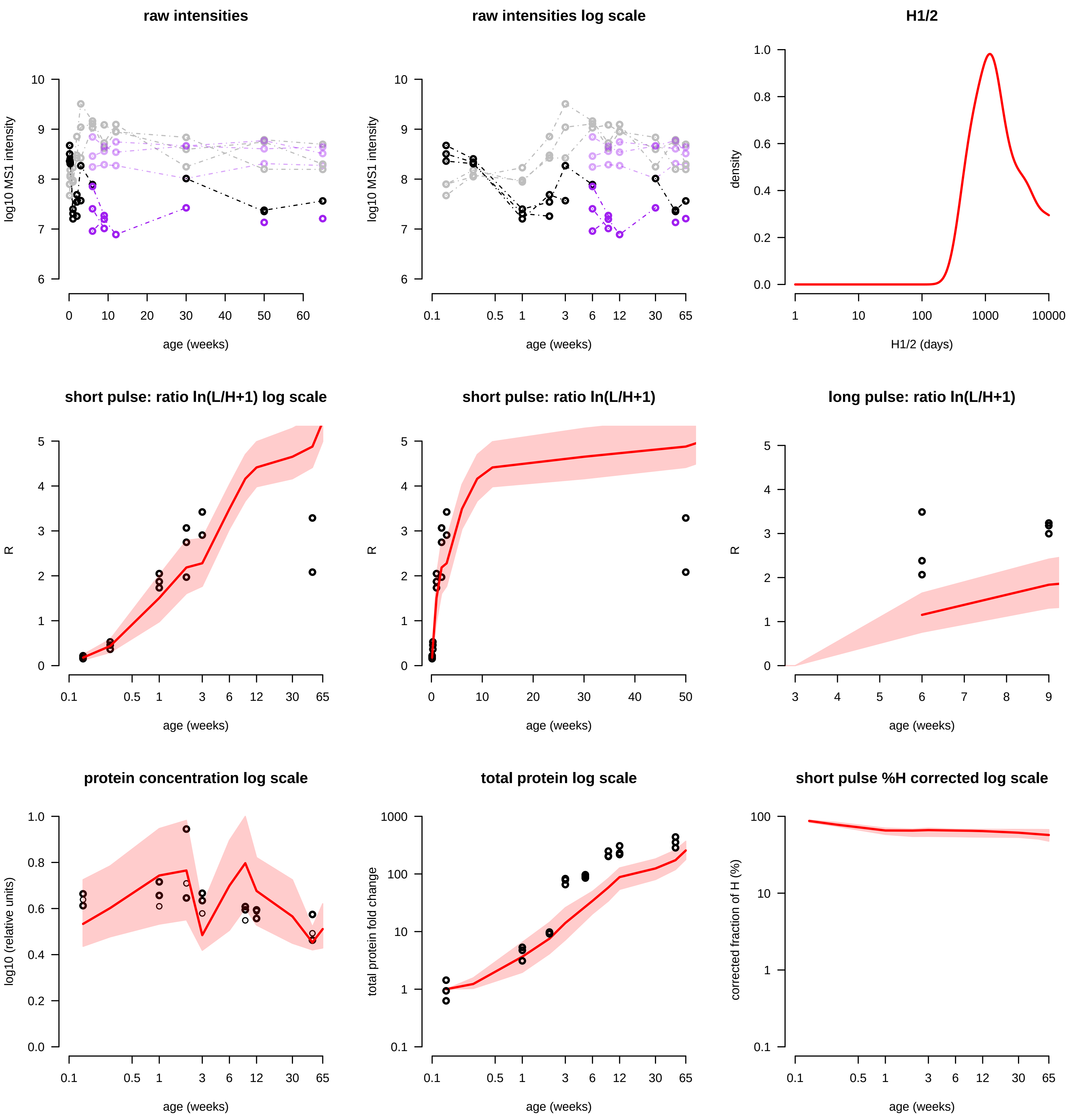

P56212;P56212−2

>sp|P56212|ARP19_MOUSE

cAMP−regulated phosphoprotein 19

GN=Arpp19

Med H1/2: 4.2 days

Q5 H1/2: 3.5 days

Q95 H1/2: 5.3 days

reliable model fit: yes

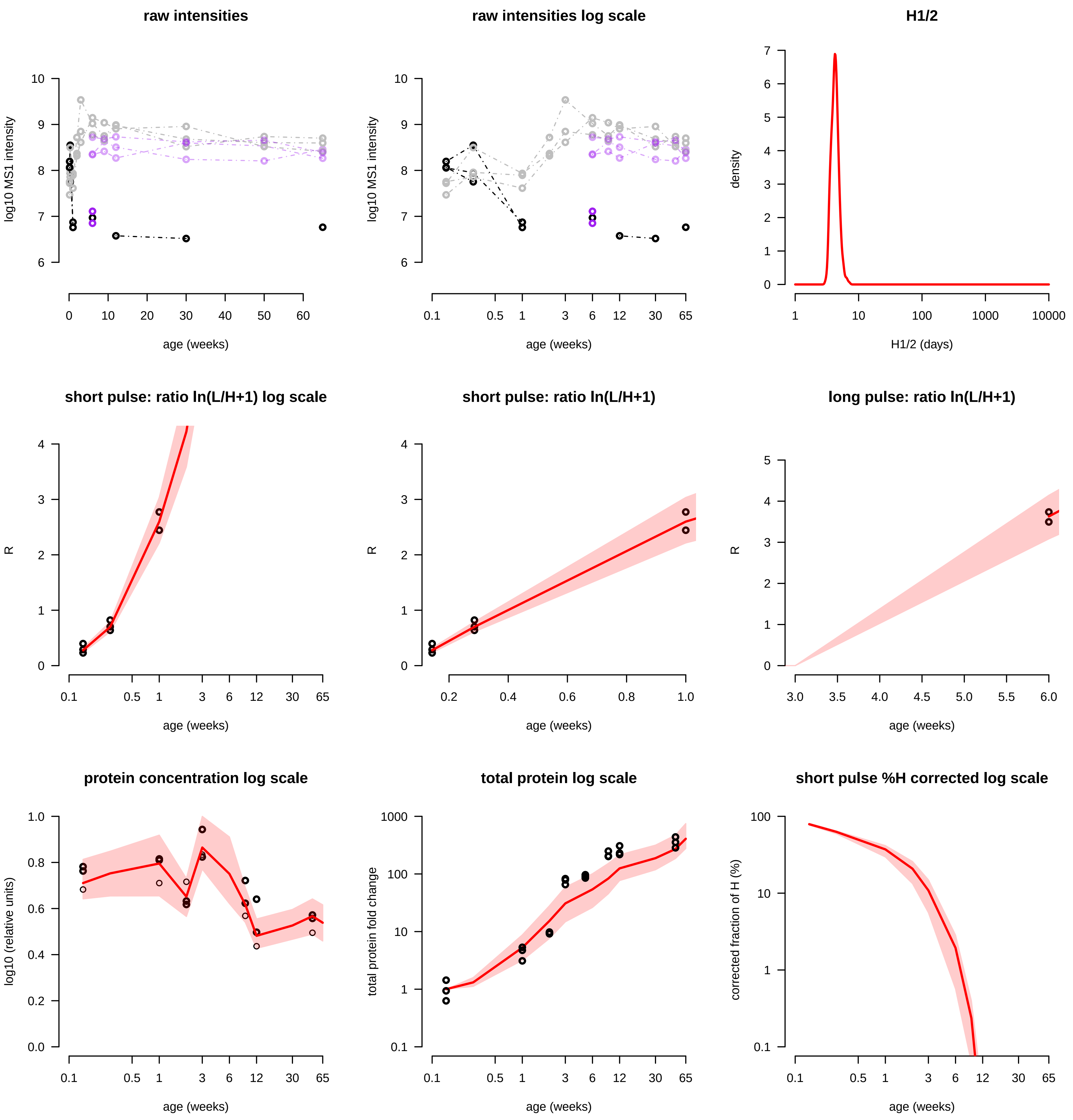

P70399−5;P70399;P70399−6

>sp|P70399−5|TP53B_MOUSE

Isoform 2 of TP53−binding protein 1

GN=Tp53bp1

Med H1/2: 4.4 days

Q5 H1/2: 2.9 days

Q95 H1/2: 13.5 days

reliable model fit: yes

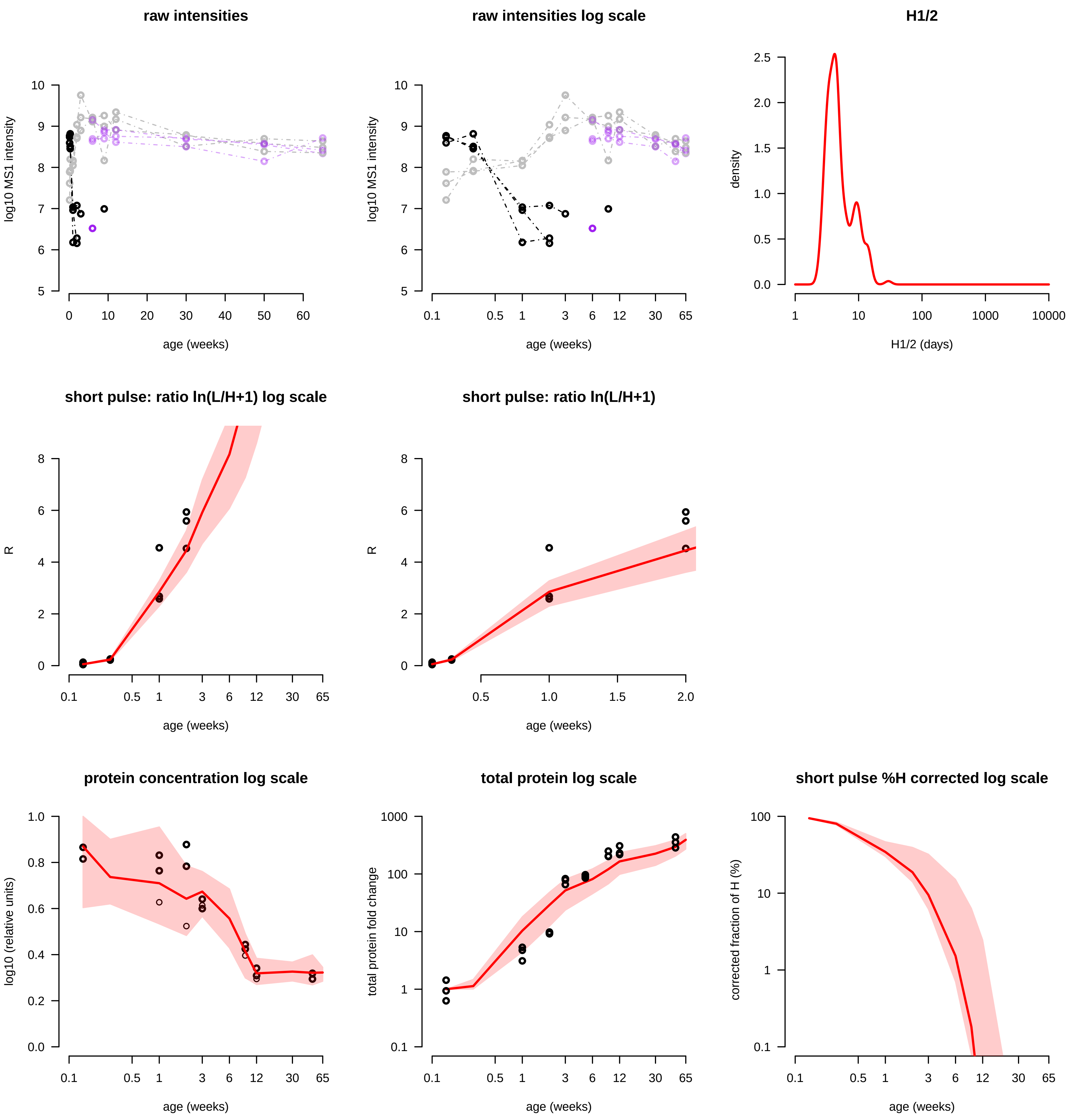

Q80UU9

>sp|Q80UU9|PGRC2_MOUSE

Membrane−associated progesterone receptor component 2

GN=Pgrmc2

Med H1/2: 4.6 days

Q5 H1/2: 2.8 days

Q95 H1/2: 30.9 days

reliable model fit: yes

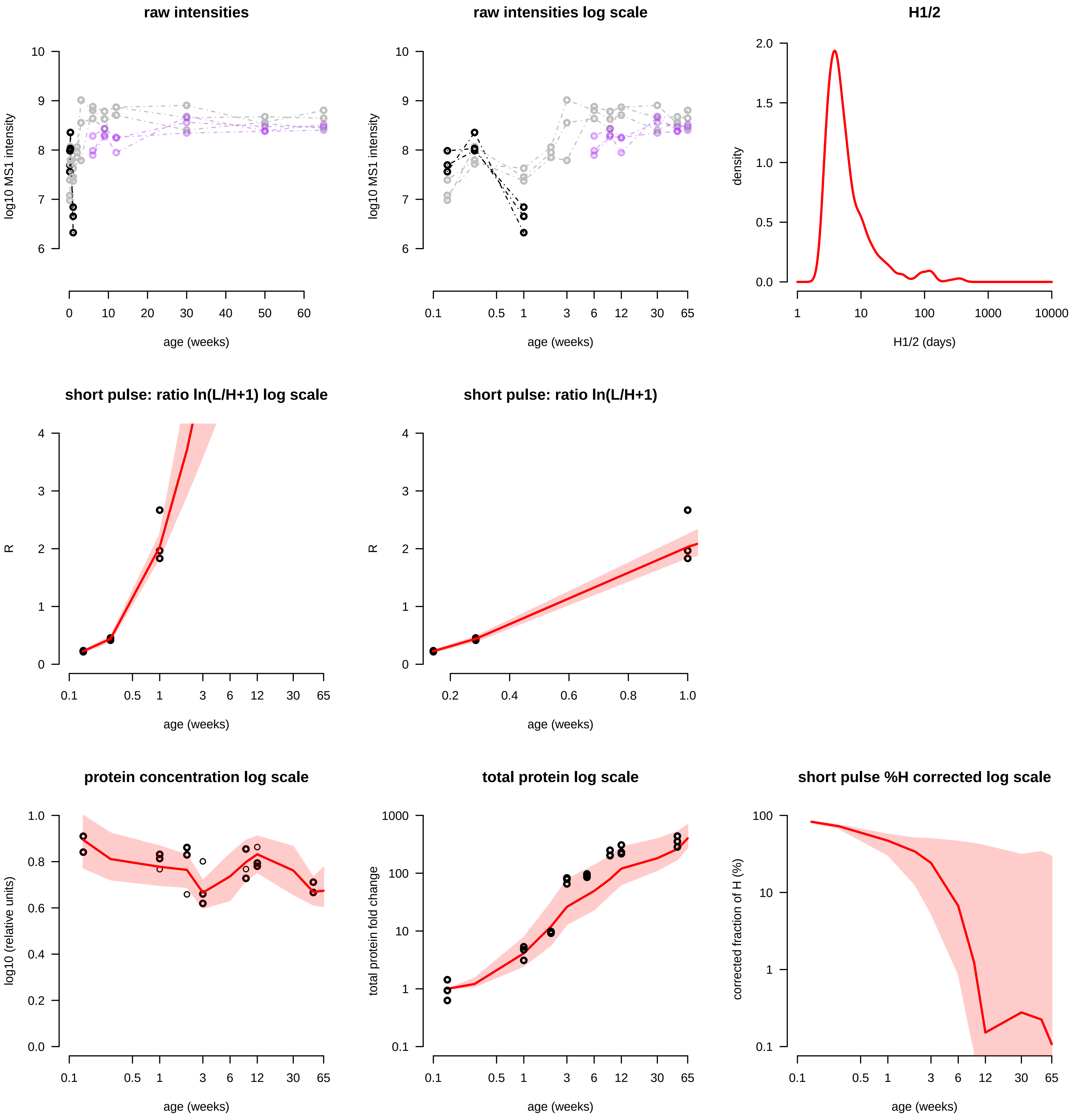

Q6P9R2

>sp|Q6P9R2|OXSR1_MOUSE

Serine/threonine−protein kinase OSR1

GN=Oxsr1

Med H1/2: 5 days

Q5 H1/2: 3.9 days

Q95 H1/2: 6.5 days

reliable model fit: yes

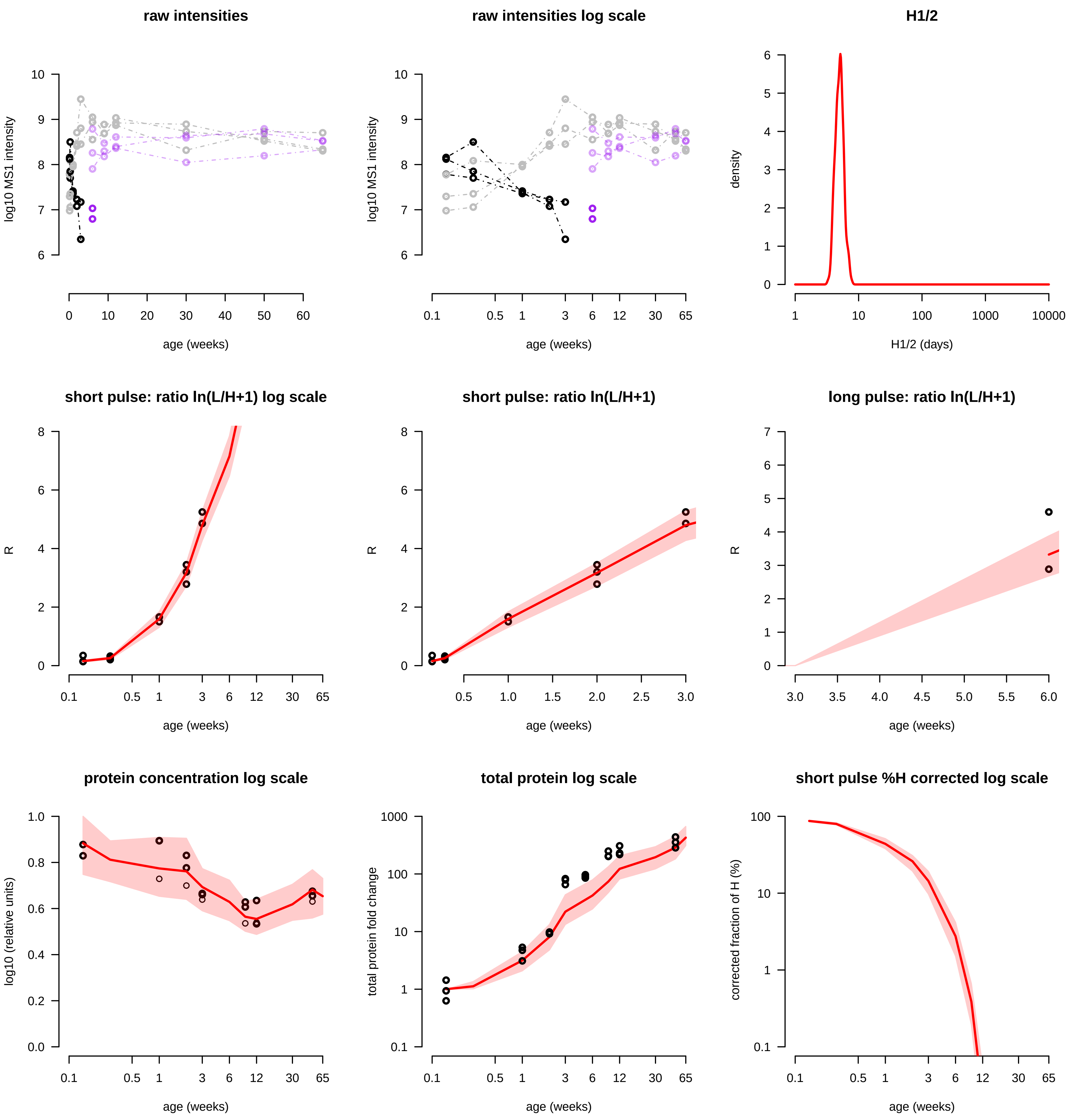

Q91ZU6−4;Q91ZU6;Q91ZU6−2

>sp|Q91ZU6−4|DYST_MOUSE

Isoform 4 of Dystonin

GN=Dst

Med H1/2: 12.6 days

Q5 H1/2: 4.5 days

Q95 H1/2: 156.1 days

reliable model fit: yes

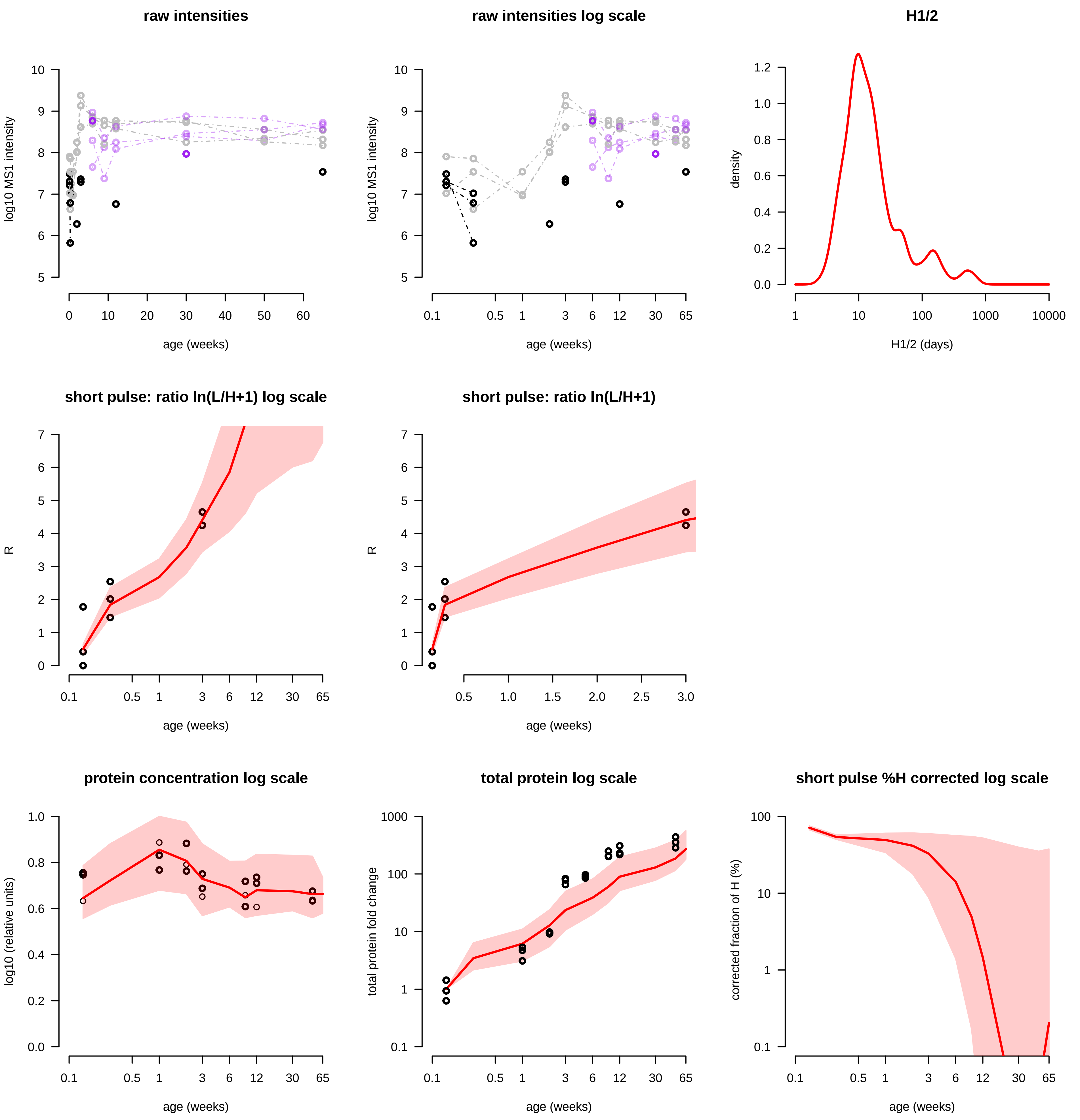

Q9Z315

>sp|Q9Z315|SNUT1_MOUSE

U4/U6.U5 tri−snRNP−associated protein 1

GN=Sart1

Med H1/2: 4.7 days

Q5 H1/2: 2.6 days

Q95 H1/2: 15.7 days

reliable model fit: yes

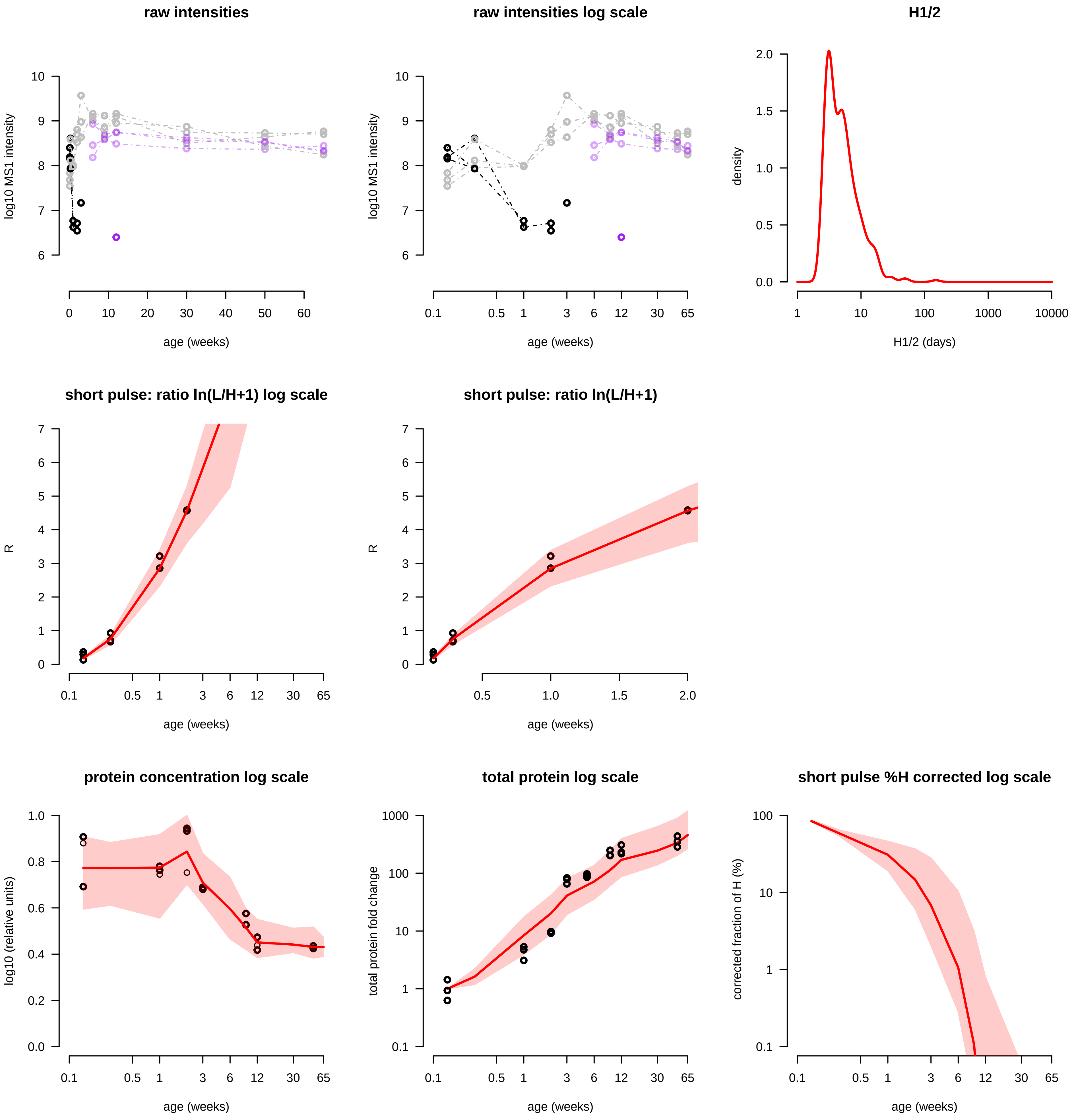

P35441

>sp|P35441|TSP1_MOUSE

Thrombospondin−1

GN=Thbs1

Med H1/2: 32.7 days

Q5 H1/2: 22.9 days

Q95 H1/2: 68.4 days

reliable model fit: no

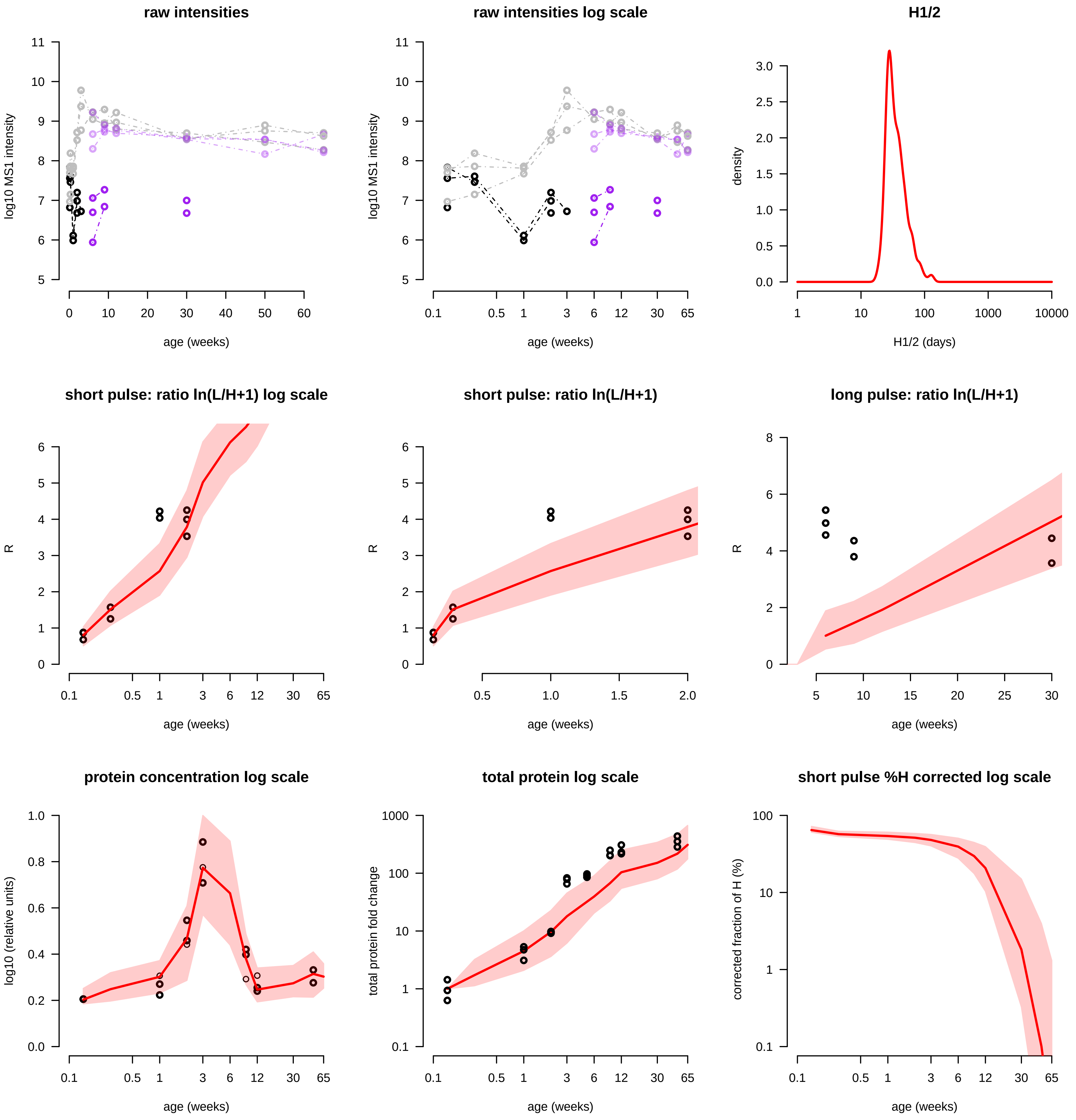

Q8BL97−4;Q8BL97−2;Q8BL97;Q8BL97−3

>sp|Q8BL97−4|SRSF7_MOUSE

Isoform 4 of Serine/arginine−rich splicing factor 7

GN=Srsf7

Med H1/2: 8.5 days

Q5 H1/2: 6.7 days

Q95 H1/2: 9.8 days

reliable model fit: yes

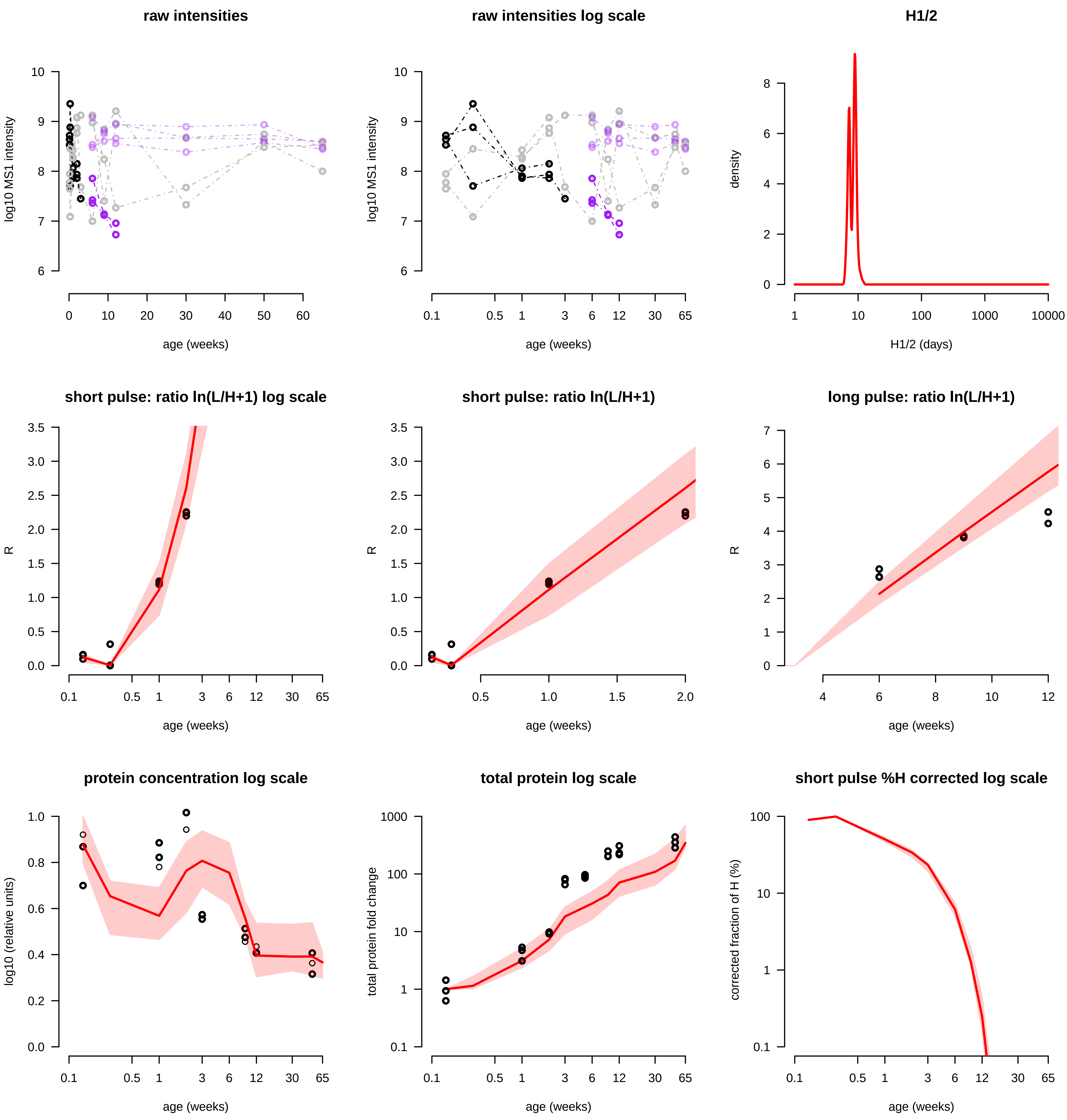

Q8VEE4

>sp|Q8VEE4|RFA1_MOUSE

Replication protein A 70 kDa DNA−binding subunit

GN=Rpa1

Med H1/2: 3.8 days

Q5 H1/2: 3.1 days

Q95 H1/2: 4.7 days

reliable model fit: yes

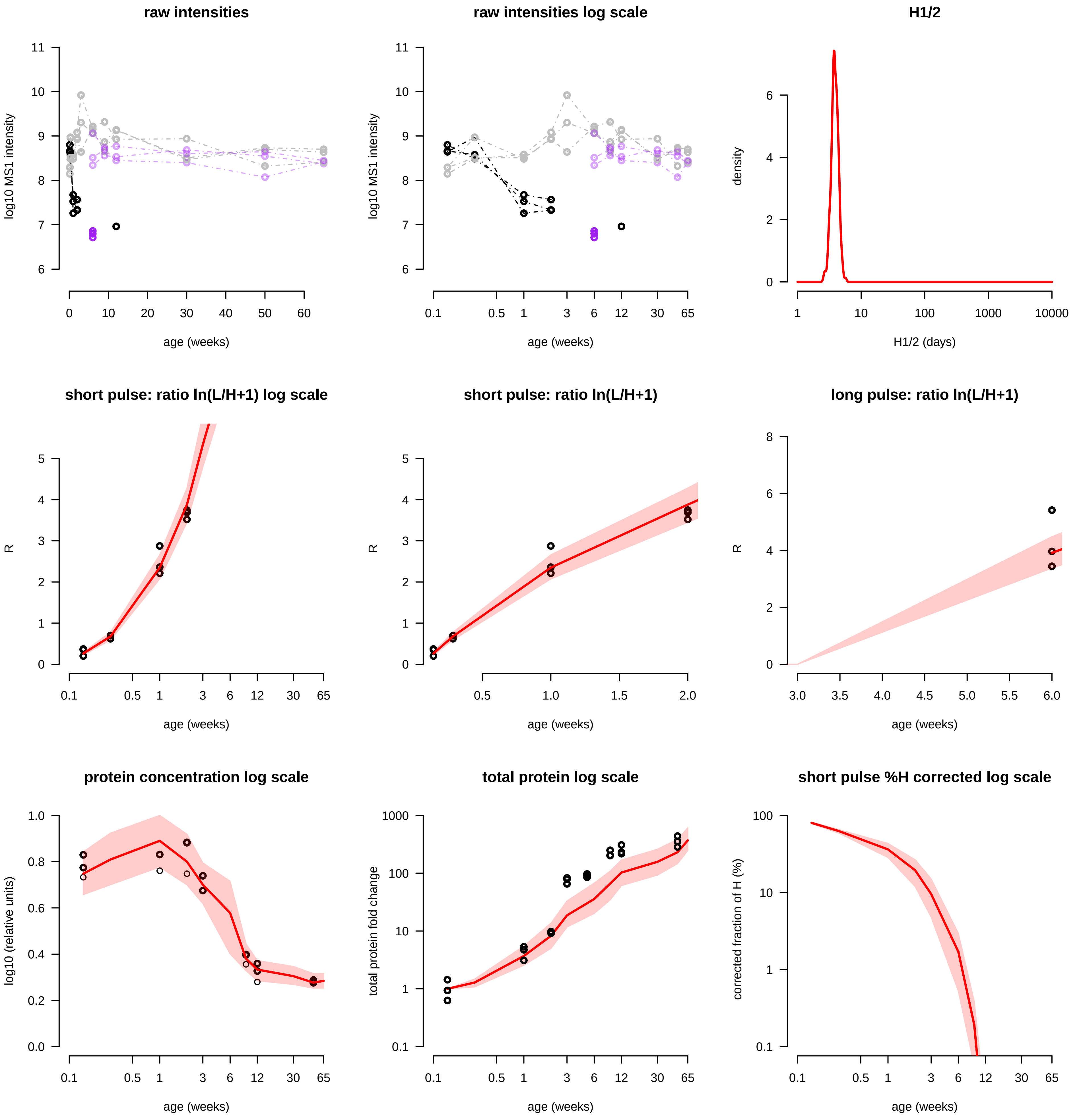

Q62383

>sp|Q62383|SPT6H_MOUSE

Transcription elongation factor SPT6

GN=Supt6h

Med H1/2: 3.9 days

Q5 H1/2: 2.5 days

Q95 H1/2: 15.1 days

reliable model fit: yes

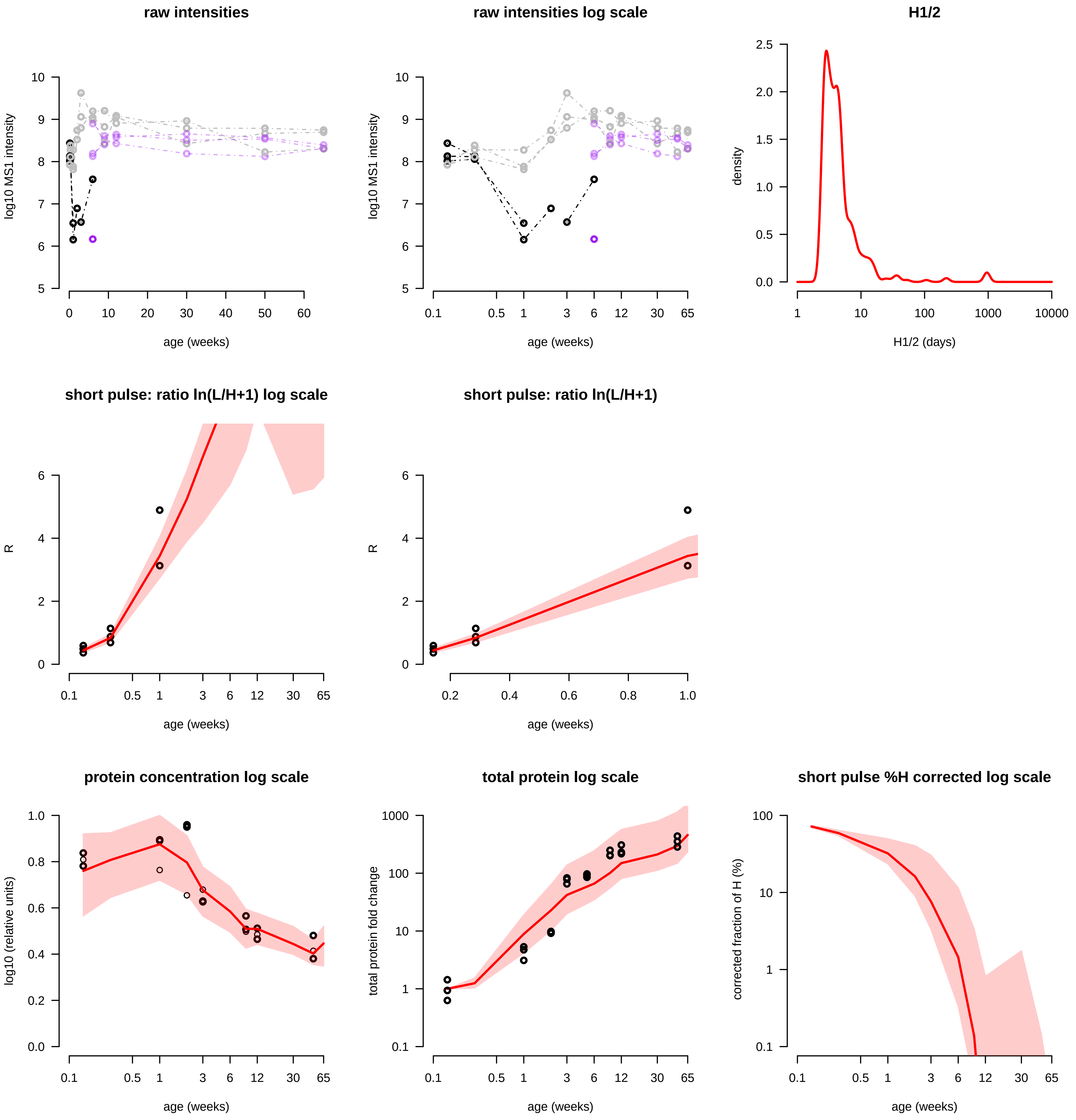

Q8CG72;Q8CG72−2

>sp|Q8CG72|ADPRS_MOUSE

ADP−ribose glycohydrolase ARH3

GN=Adprs

Med H1/2: 6.4 days

Q5 H1/2: 4.4 days

Q95 H1/2: 20 days

reliable model fit: yes

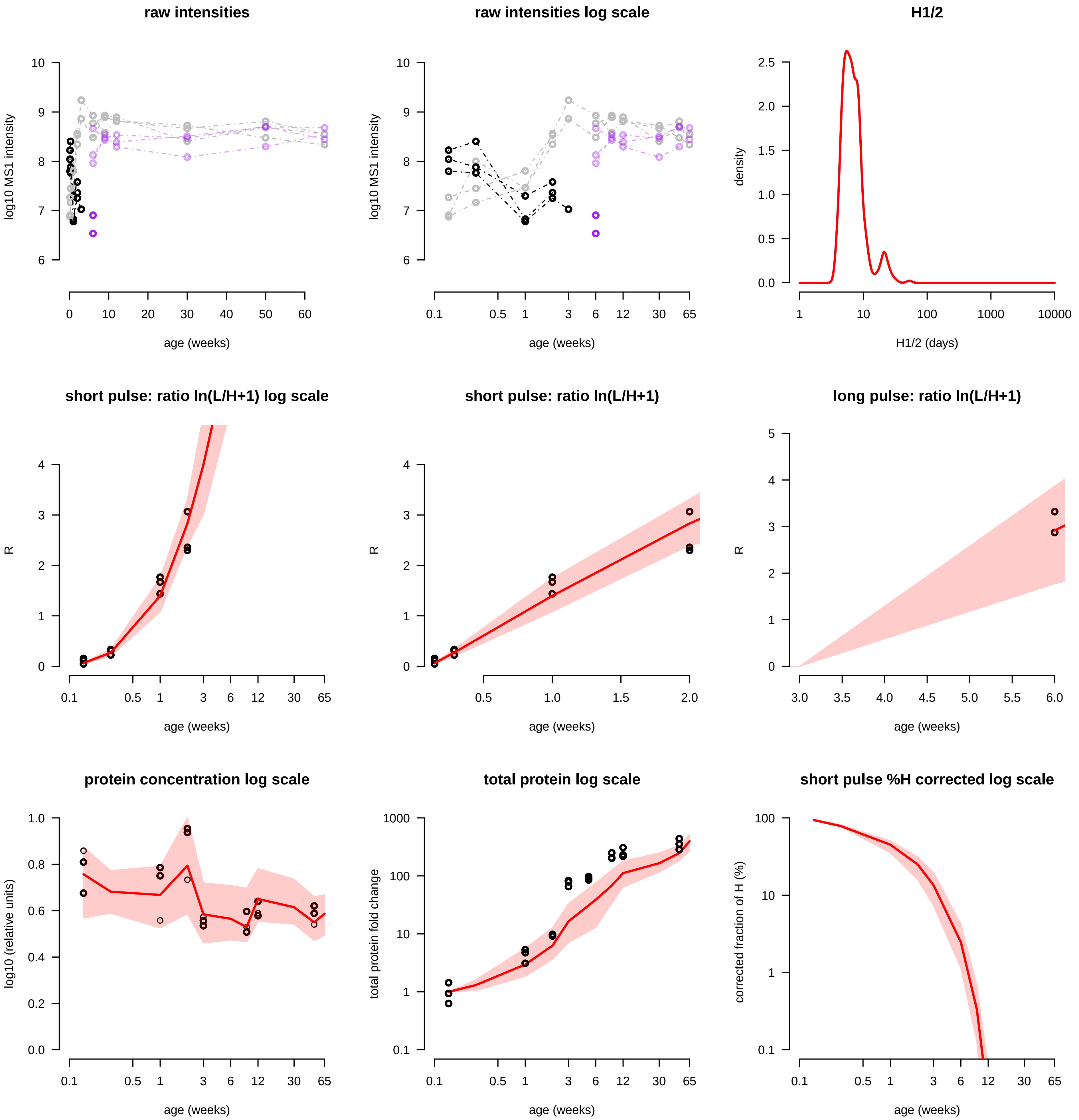

Q6ZQ88

>sp|Q6ZQ88|KDM1A_MOUSE

Lysine−specific histone demethylase 1A

GN=Kdm1a

Med H1/2: 18.5 days

Q5 H1/2: 13.6 days

Q95 H1/2: 36.6 days

reliable model fit: no

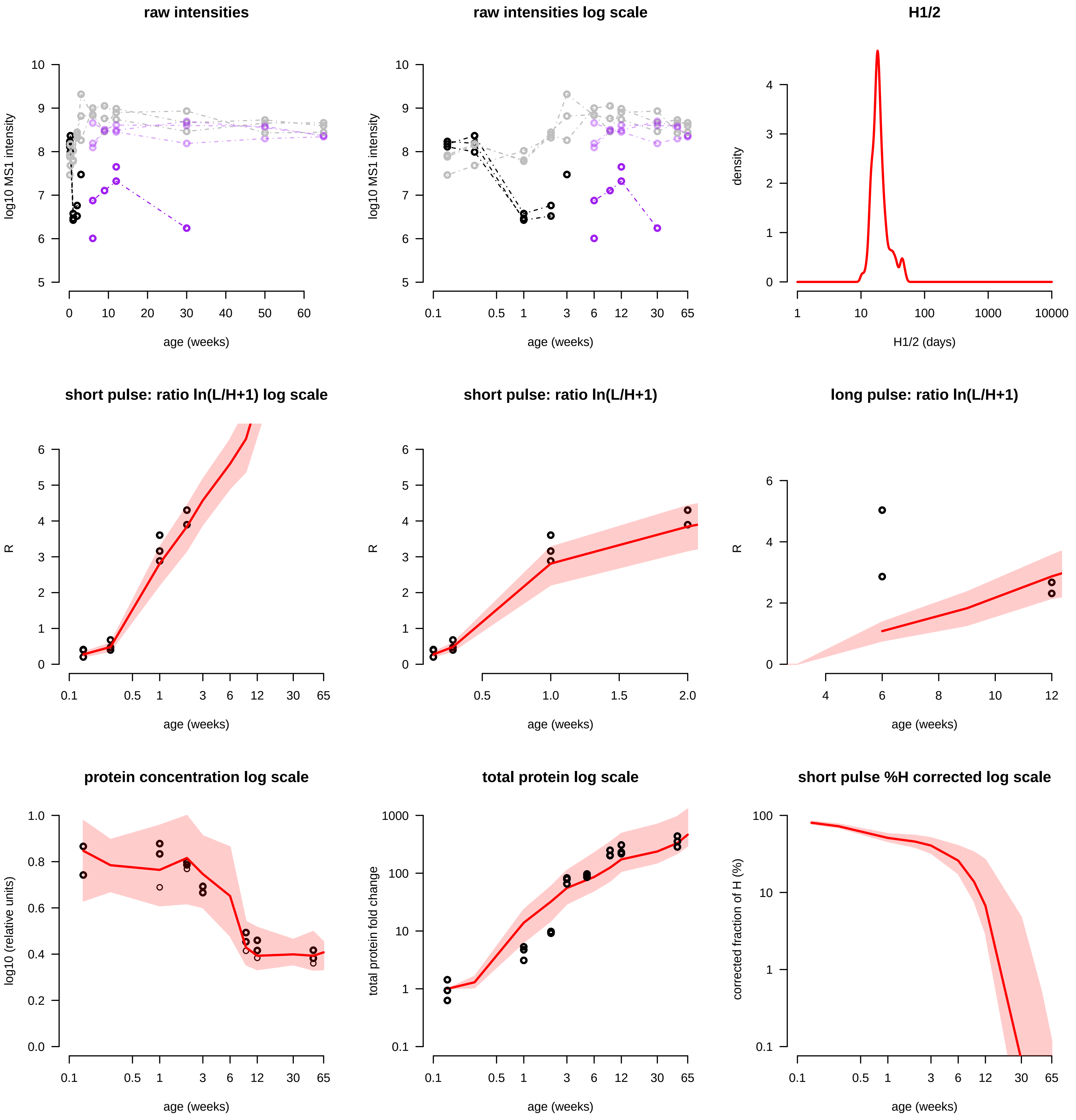

P47811;P47811−4;P47811−2

>sp|P47811|MK14_MOUSE

Mitogen−activated protein kinase 14

GN=Mapk14

Med H1/2: 1621.9 days

Q5 H1/2: 425 days

Q95 H1/2: 18930.7 days

reliable model fit: yes

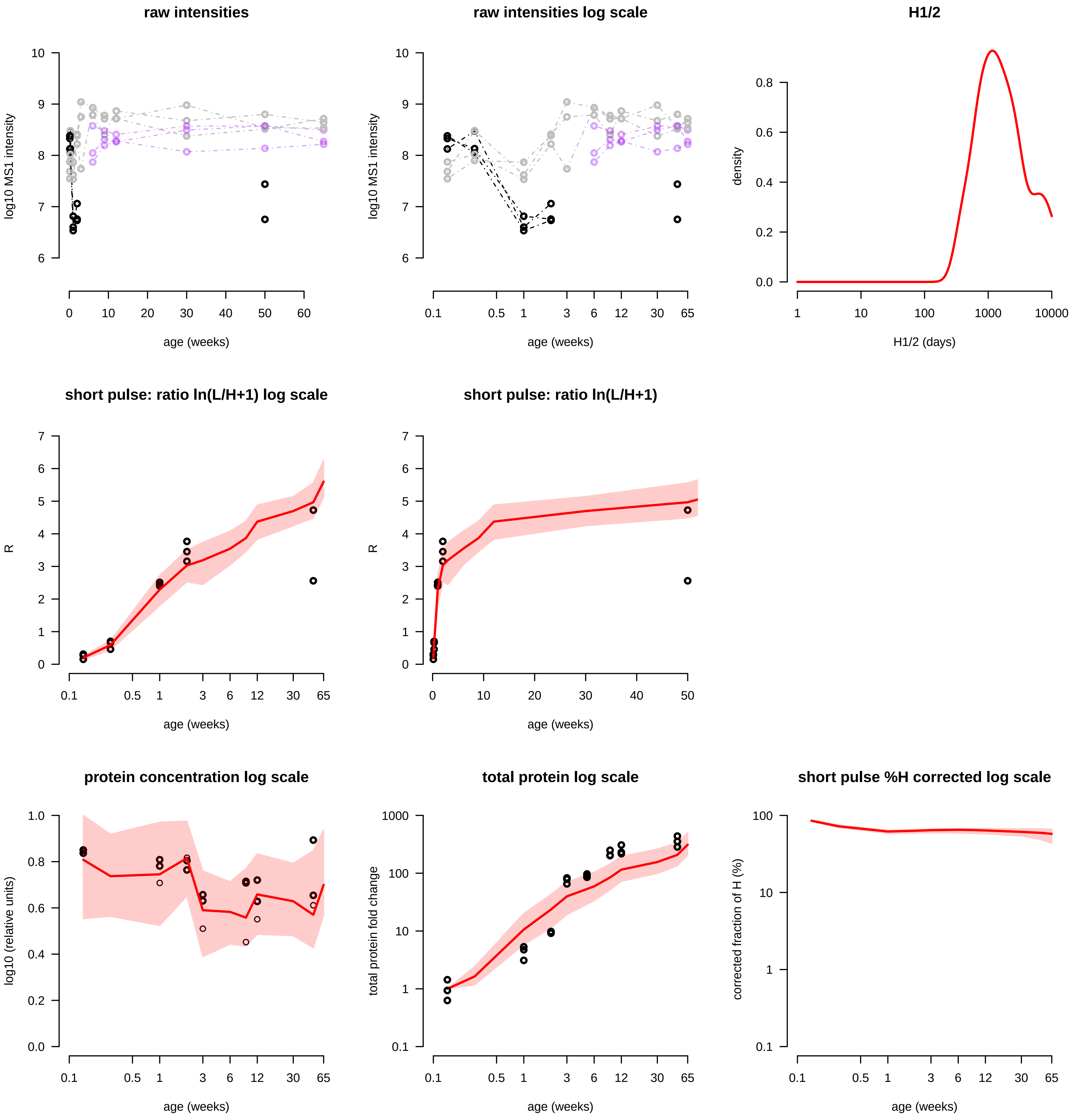

Q8CI04

>sp|Q8CI04|COG3_MOUSE

Conserved oligomeric Golgi complex subunit 3

GN=Cog3

Med H1/2: 7.7 days

Q5 H1/2: 5.7 days

Q95 H1/2: 11.4 days

reliable model fit: yes

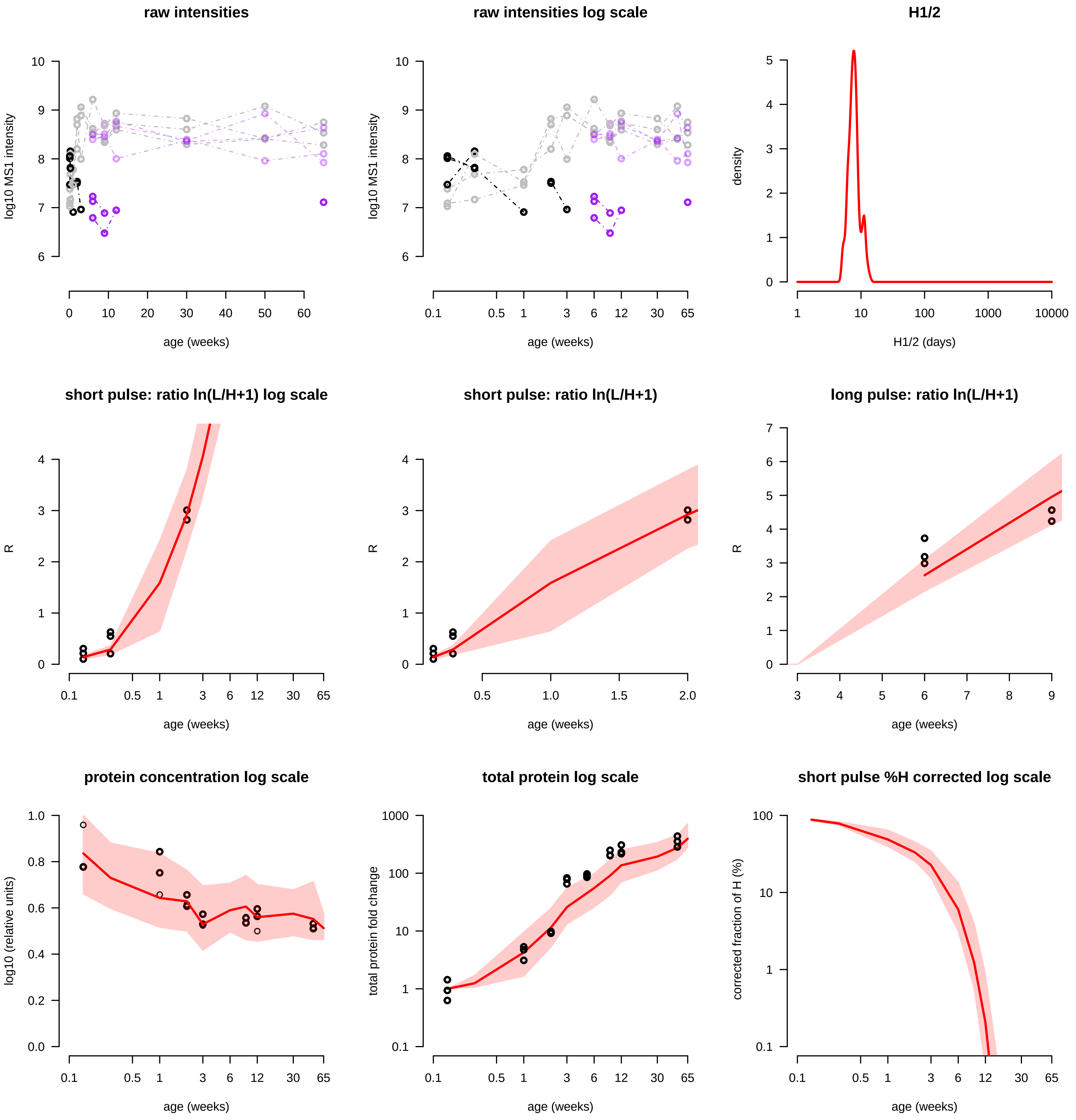

Q8CG71

>sp|Q8CG71|P3H2_MOUSE

Prolyl 3−hydroxylase 2

GN=P3h2

Med H1/2: 8.1 days

Q5 H1/2: 5.5 days

Q95 H1/2: 14.5 days

reliable model fit: yes

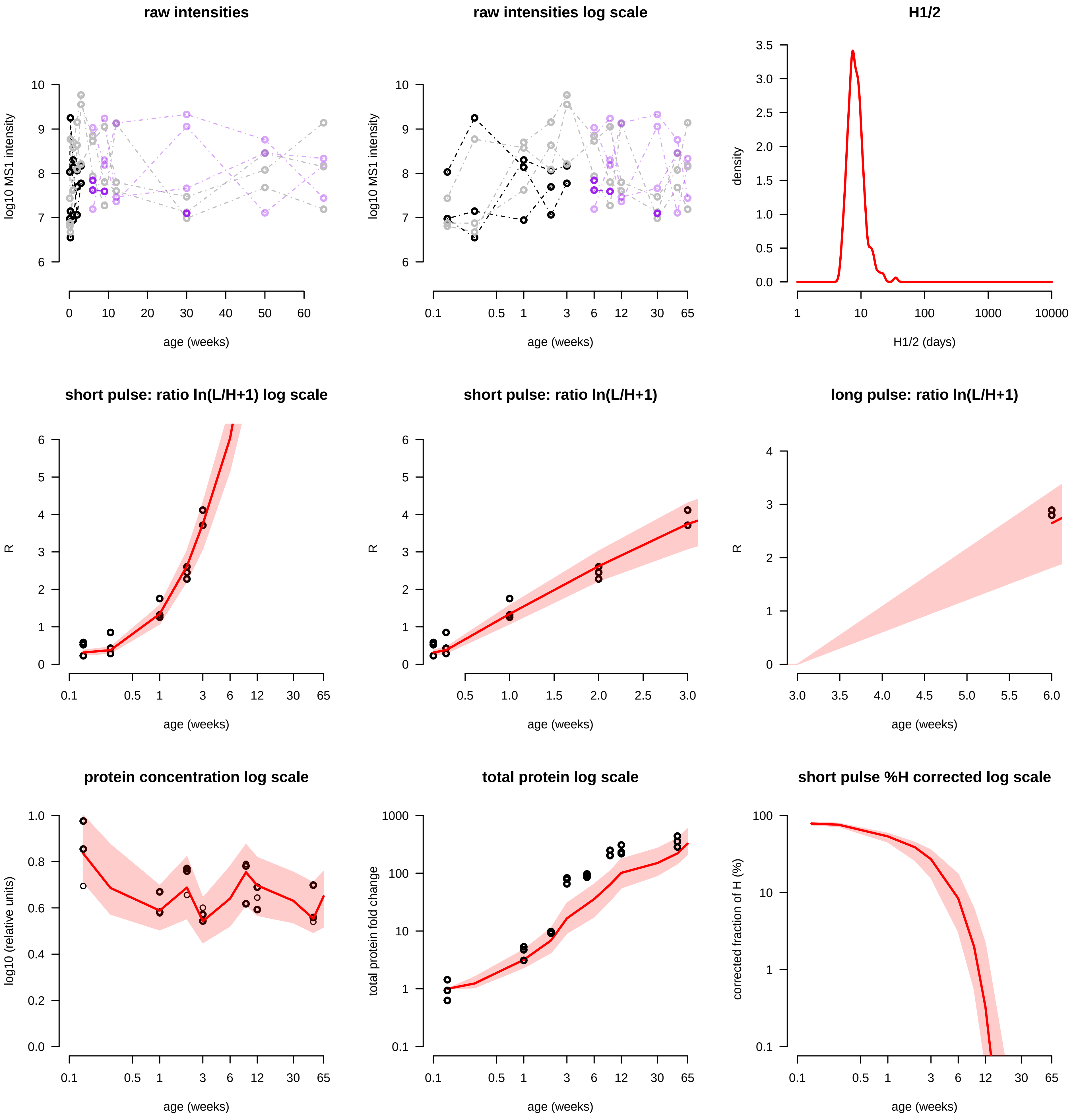

Q6P549

>sp|Q6P549|SHIP2_MOUSE

Phosphatidylinositol 3,4,5−trisphosphate 5−phosphatase 2

GN=Inppl1

Med H1/2: 5.5 days

Q5 H1/2: 4.4 days

Q95 H1/2: 7.5 days

reliable model fit: yes

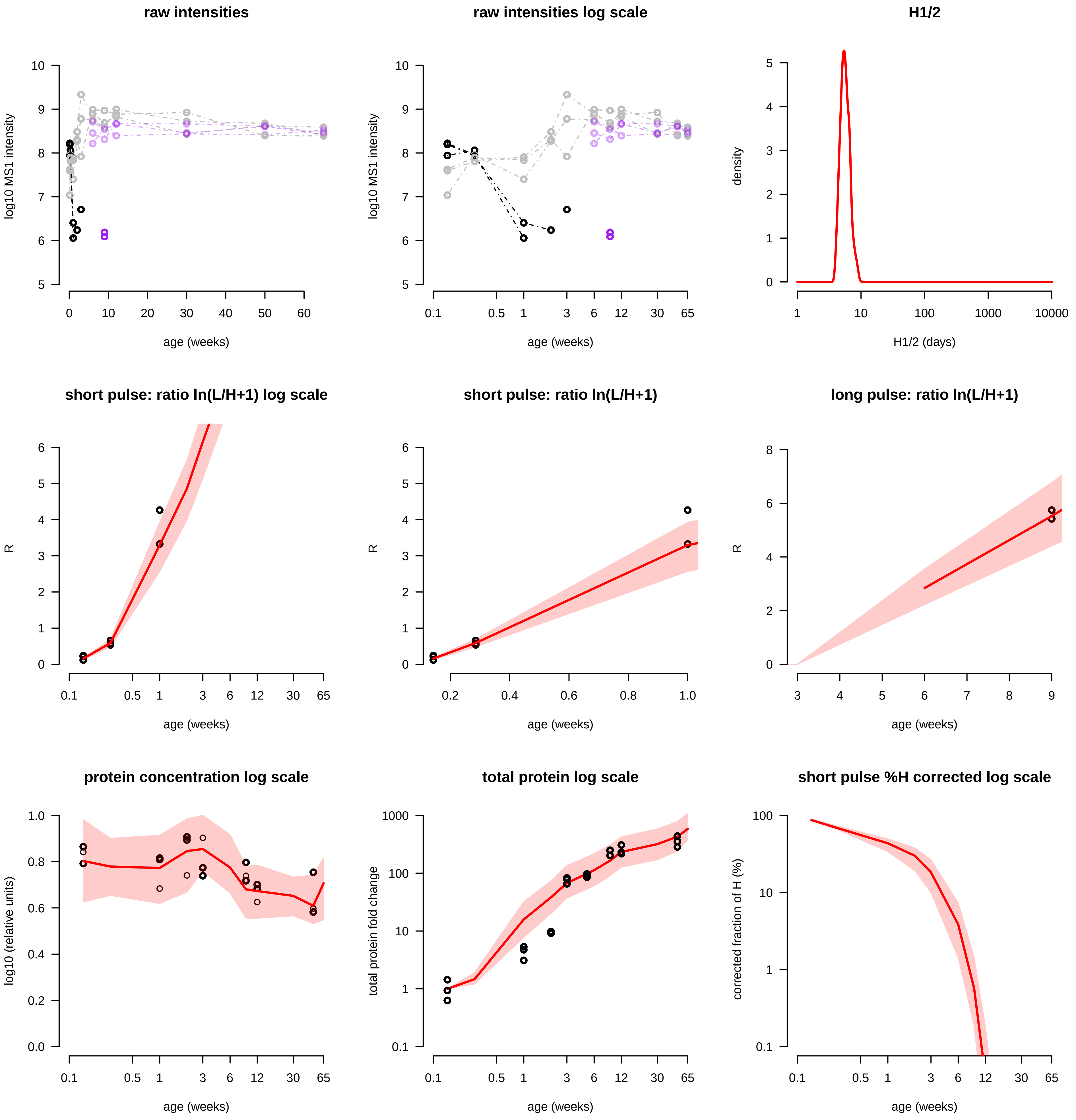

Q3TIX9

>sp|Q3TIX9|SNUT2_MOUSE

U4/U6.U5 tri−snRNP−associated protein 2

GN=Usp39

Med H1/2: 5.8 days

Q5 H1/2: 4.8 days

Q95 H1/2: 7.1 days

reliable model fit: yes

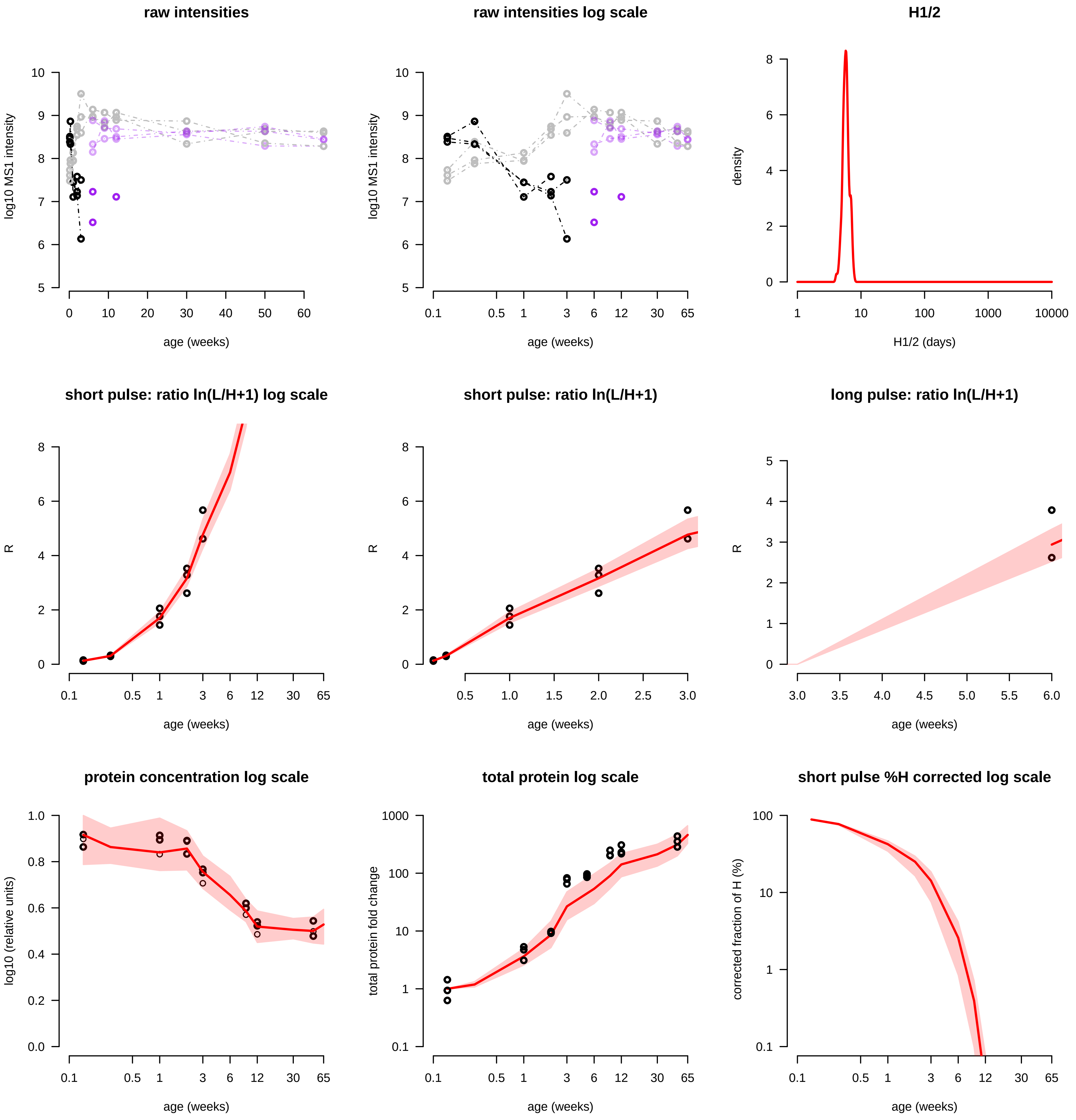

Q9DCT8

>sp|Q9DCT8|CRIP2_MOUSE

Cysteine−rich protein 2

GN=Crip2

Med H1/2: 5.4 days

Q5 H1/2: 4.2 days

Q95 H1/2: 7.3 days

reliable model fit: yes

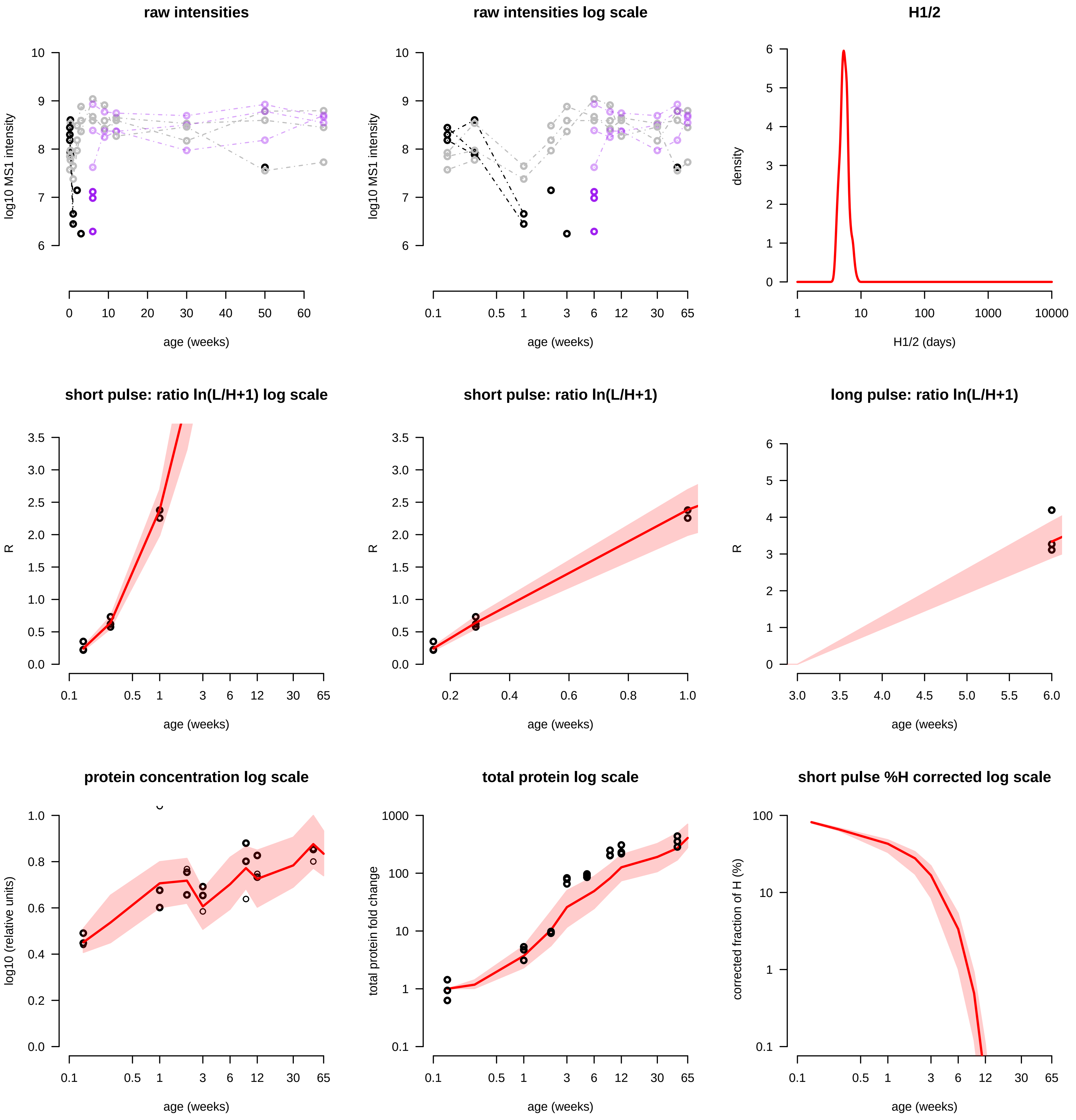

P62307

>sp|P62307|RUXF_MOUSE

Small nuclear ribonucleoprotein F

GN=Snrpf

Med H1/2: 24.5 days

Q5 H1/2: 11.5 days

Q95 H1/2: 79.8 days

reliable model fit: yes

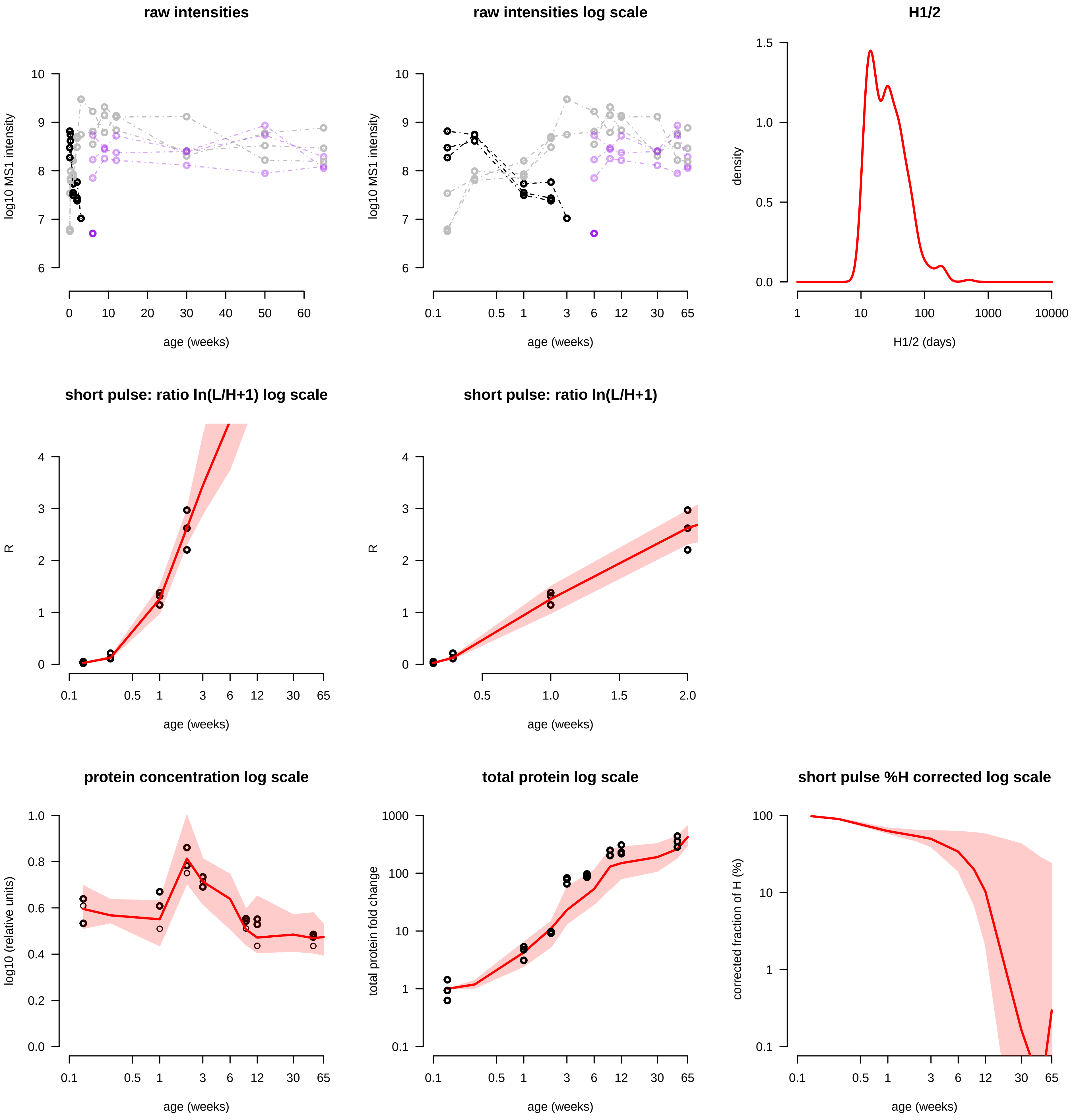

Q9DB25

>sp|Q9DB25|ALG5_MOUSE

Dolichyl−phosphate beta−glucosyltransferase

GN=Alg5

Med H1/2: 7.1 days

Q5 H1/2: 3 days

Q95 H1/2: 52.1 days

reliable model fit: yes

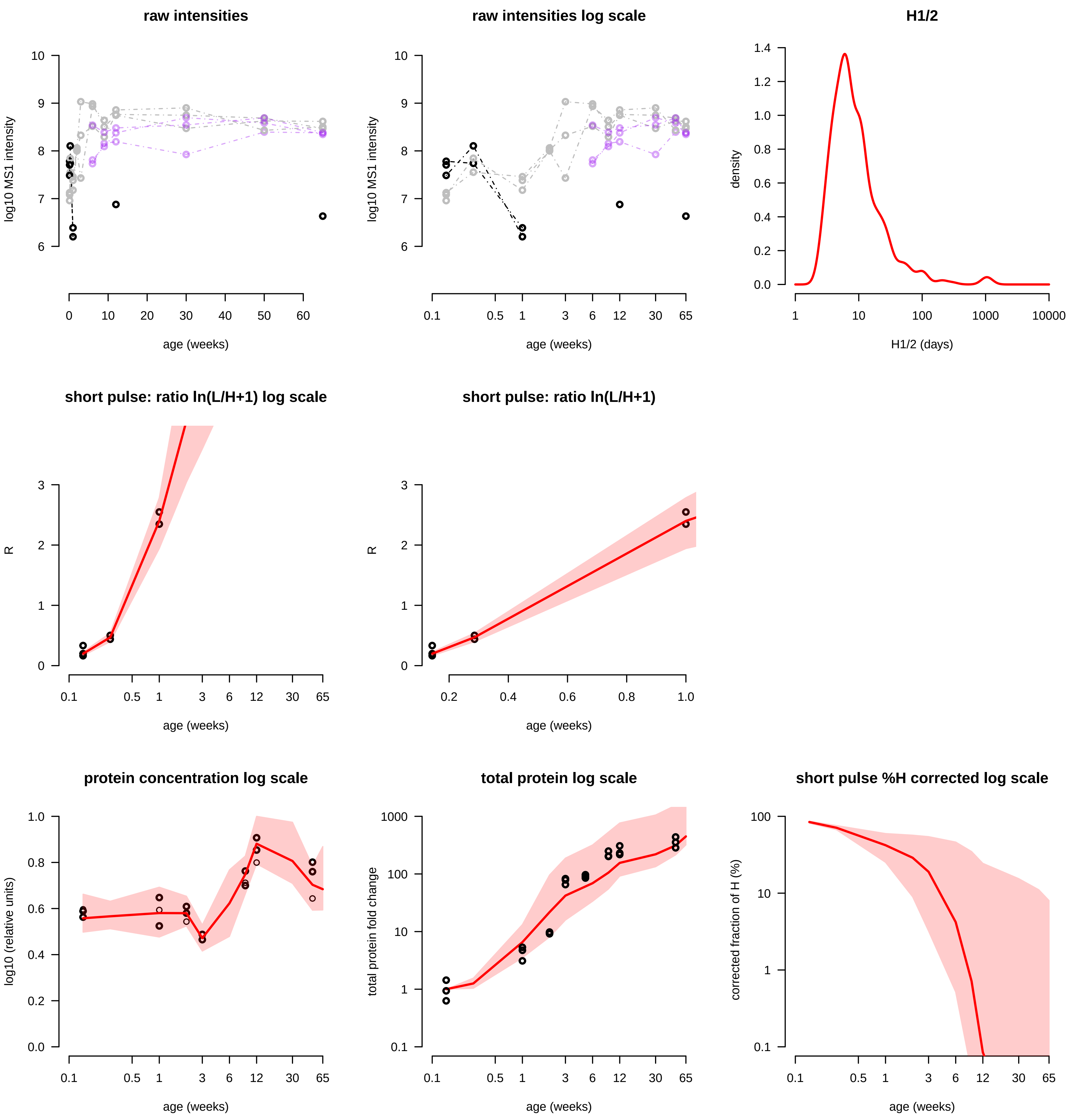

Q9DBZ5;Q9DBZ5−2

>sp|Q9DBZ5|EIF3K_MOUSE

Eukaryotic translation initiation factor 3 subunit K

GN=Eif3k

Med H1/2: 5.8 days

Q5 H1/2: 4.5 days

Q95 H1/2: 8.2 days

reliable model fit: yes

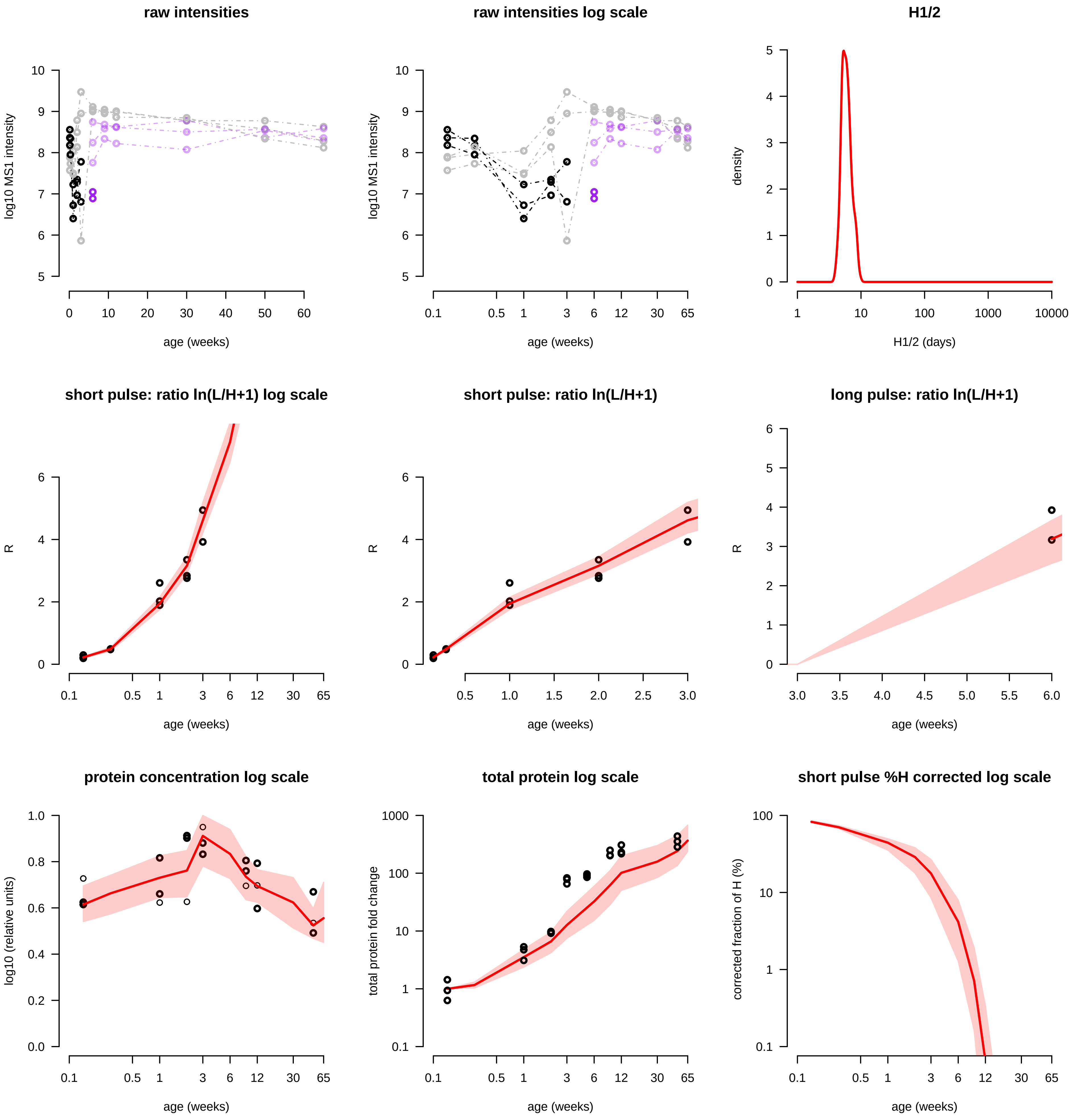

Q8C181;Q8C181−4;Q8C181−3;Q8C181−2

>sp|Q8C181|MBNL2_MOUSE

Muscleblind−like protein 2

GN=Mbnl2

Med H1/2: 6.5 days

Q5 H1/2: 4.2 days

Q95 H1/2: 17.4 days

reliable model fit: yes

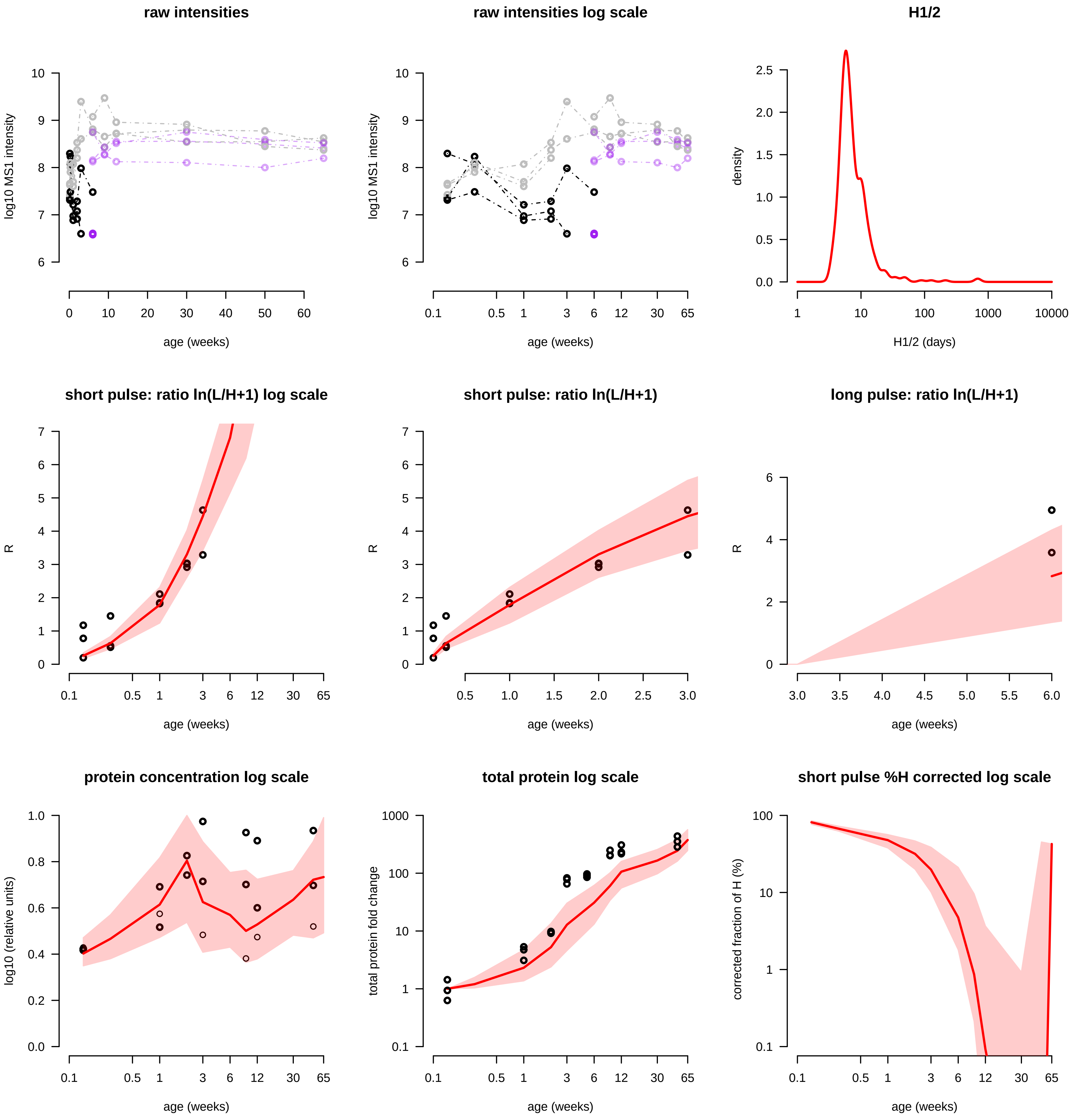

P63094;P63094−3

>sp|P63094|GNAS2_MOUSE

Guanine nucleotide−binding protein G(s) subunit alpha isoforms short

GN=Gnas

Med H1/2: 6.4 days

Q5 H1/2: 4.8 days

Q95 H1/2: 9.5 days

reliable model fit: yes

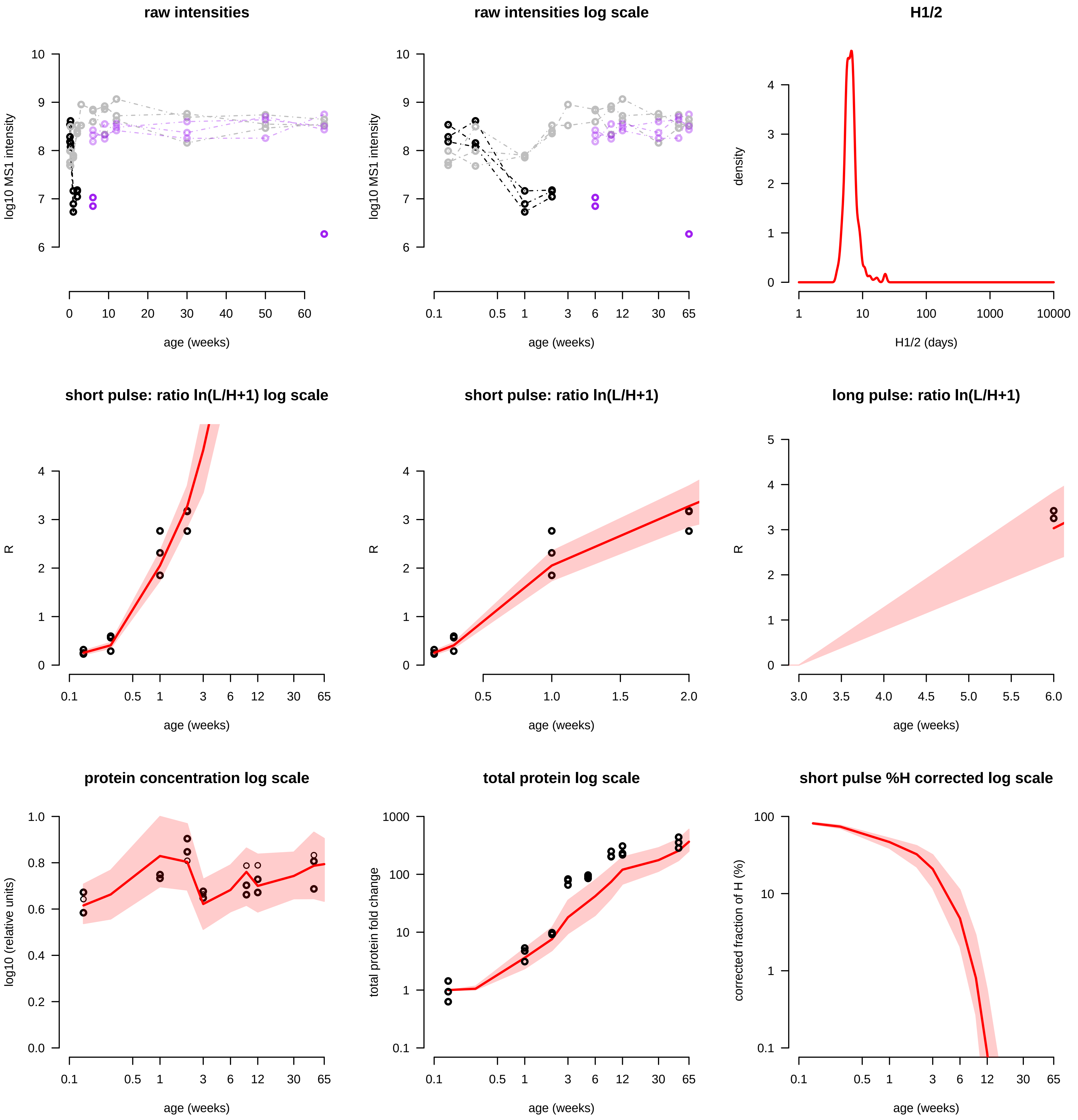

Q9Z0Z4−2;Q9Z0Z4

>sp|Q9Z0Z4−2|HEPH_MOUSE

Isoform 2 of Hephaestin

GN=Heph

Med H1/2: 6 days

Q5 H1/2: 4.1 days

Q95 H1/2: 11.5 days

reliable model fit: yes

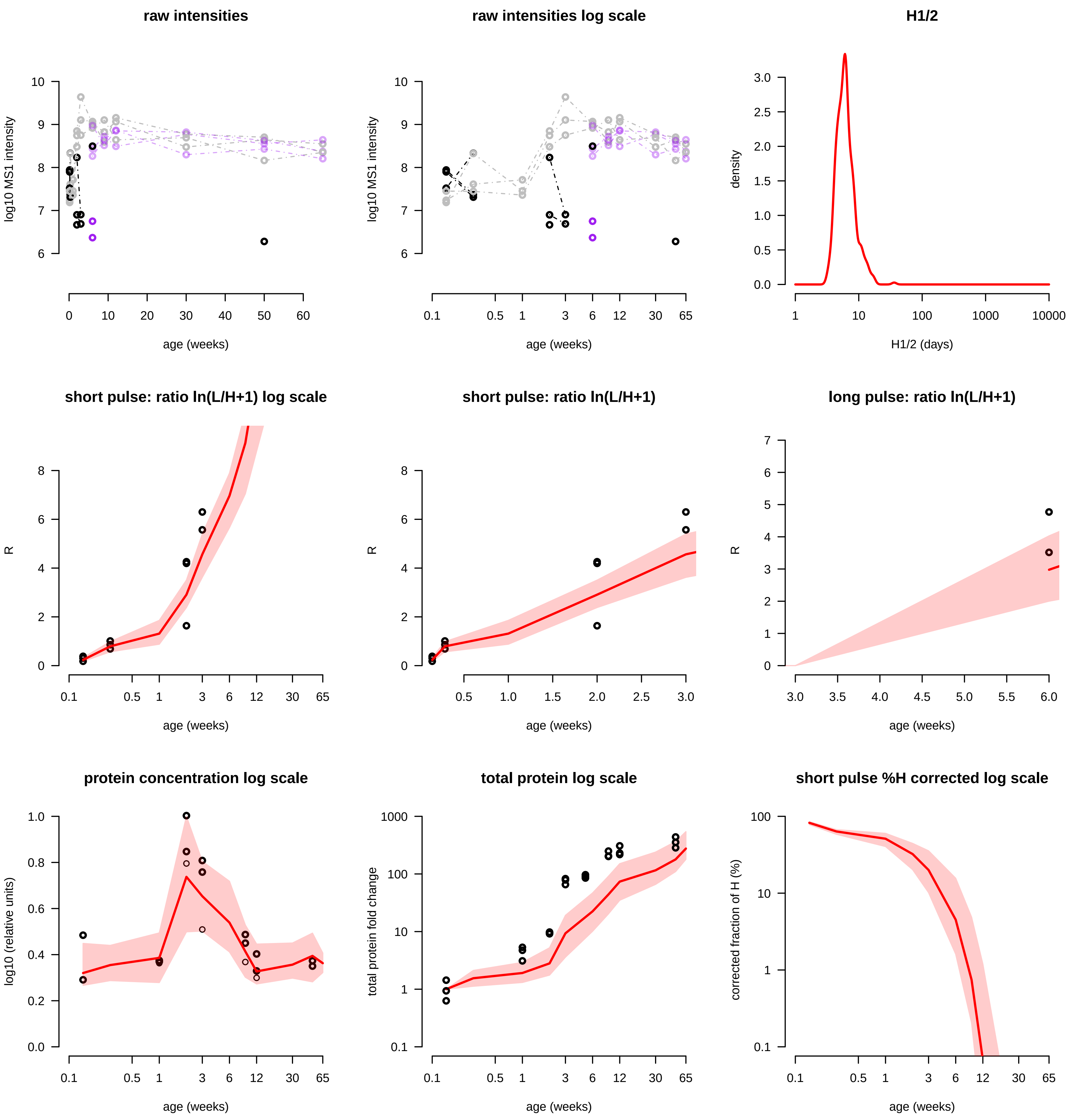

Q920L1

>sp|Q920L1|FADS1_MOUSE

Acyl−CoA (8−3)−desaturase

GN=Fads1

Med H1/2: 19.7 days

Q5 H1/2: 6.4 days

Q95 H1/2: 168 days

reliable model fit: yes

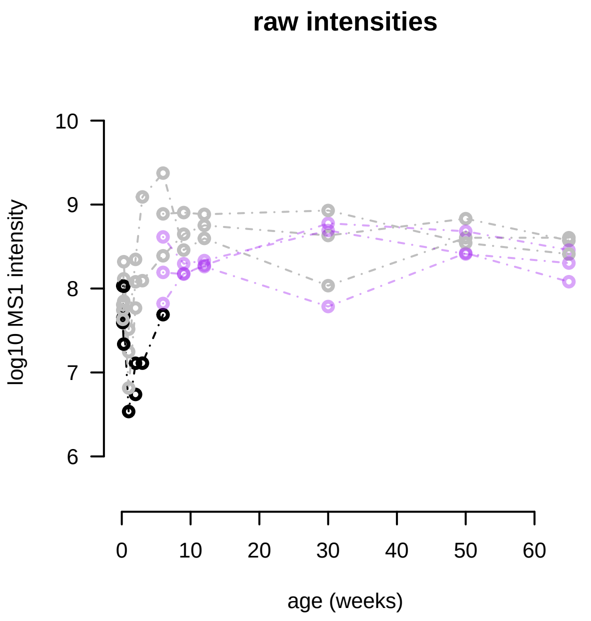

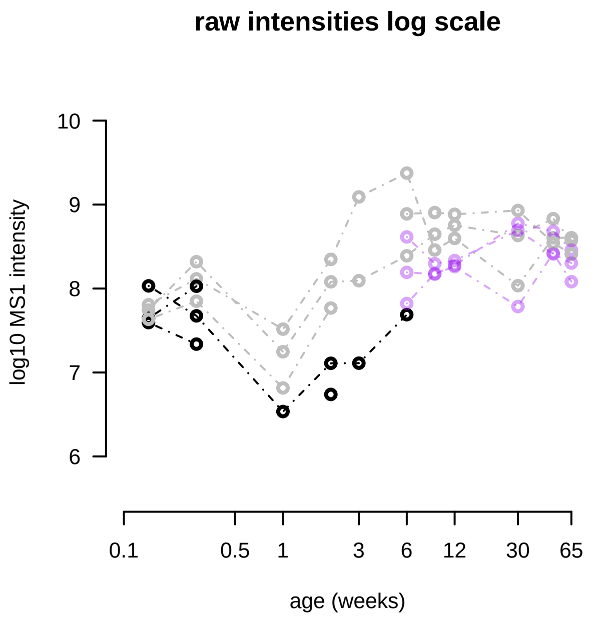

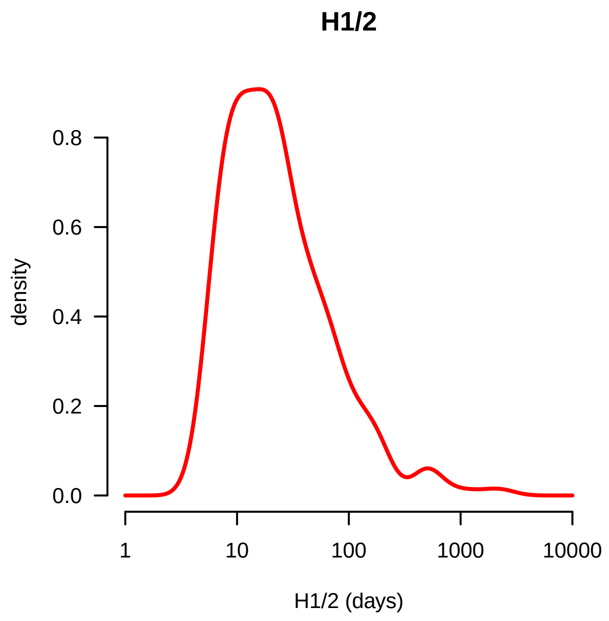

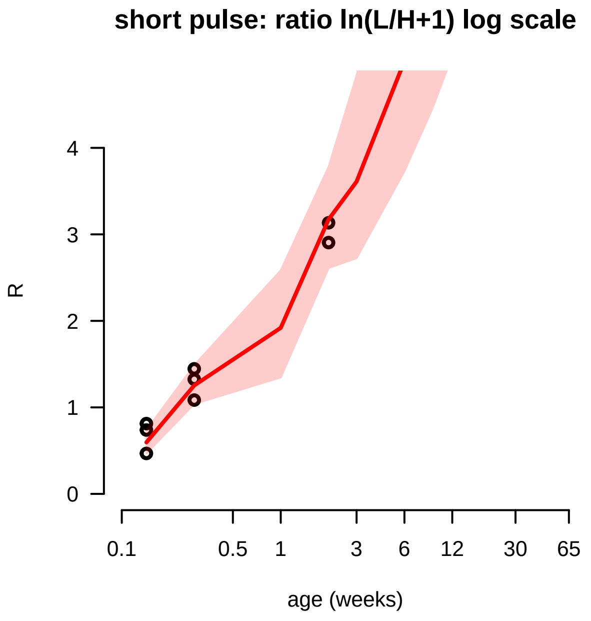

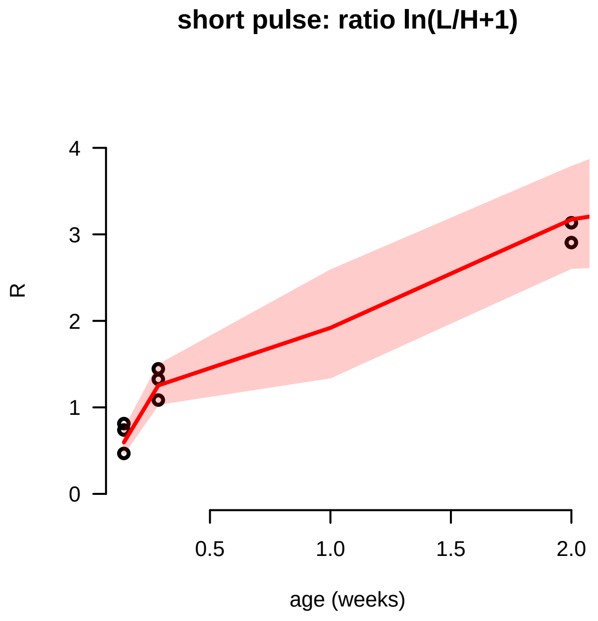

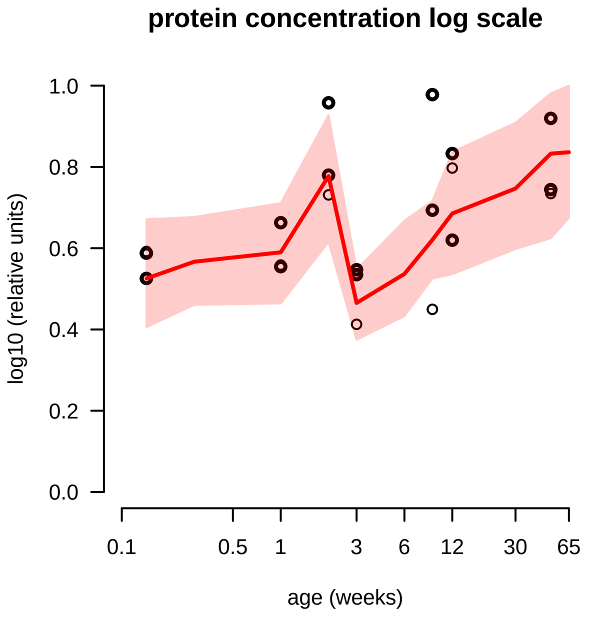

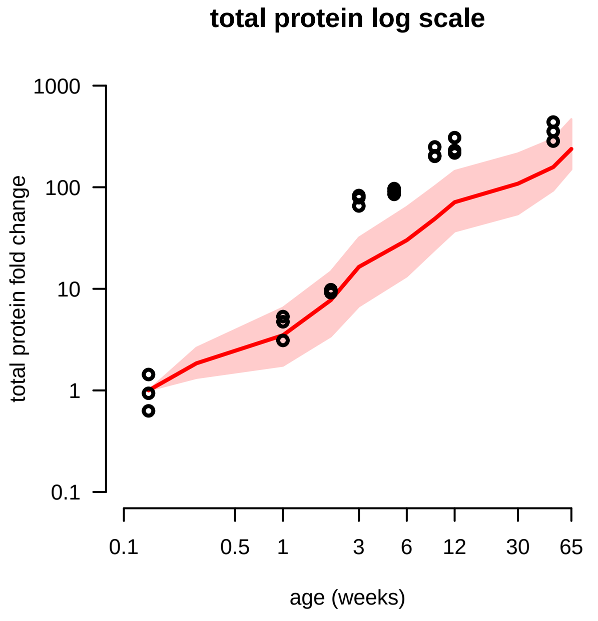

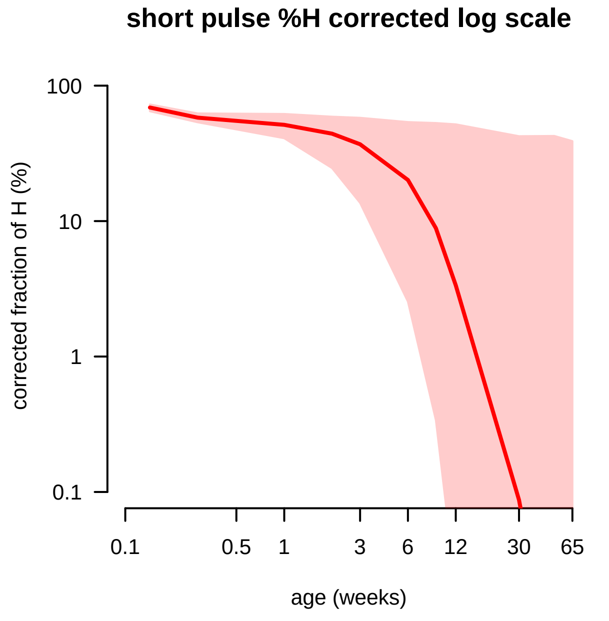

P32233

>sp|P32233|DRG1_MOUSE

Developmentally−regulated GTP−binding protein 1

GN=Drg1

Med H1/2: 4.1 days

Q5 H1/2: 2.9 days

Q95 H1/2: 5.9 days

reliable model fit: yes

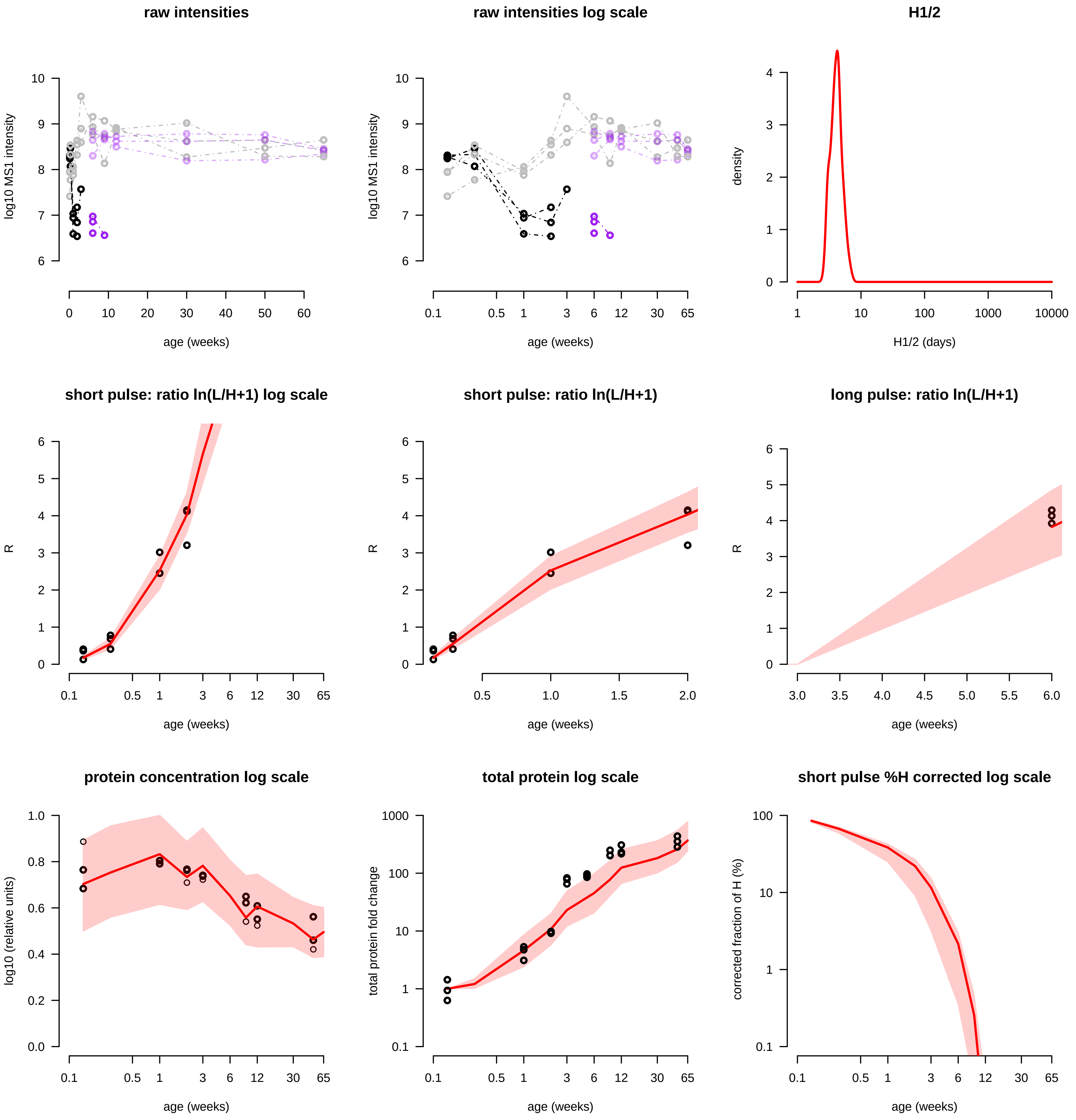

Q91VN4

>sp|Q91VN4|MIC25_MOUSE

MICOS complex subunit Mic25

GN=Chchd6

Med H1/2: 17.5 days

Q5 H1/2: 8.4 days

Q95 H1/2: 99.2 days

reliable model fit: yes

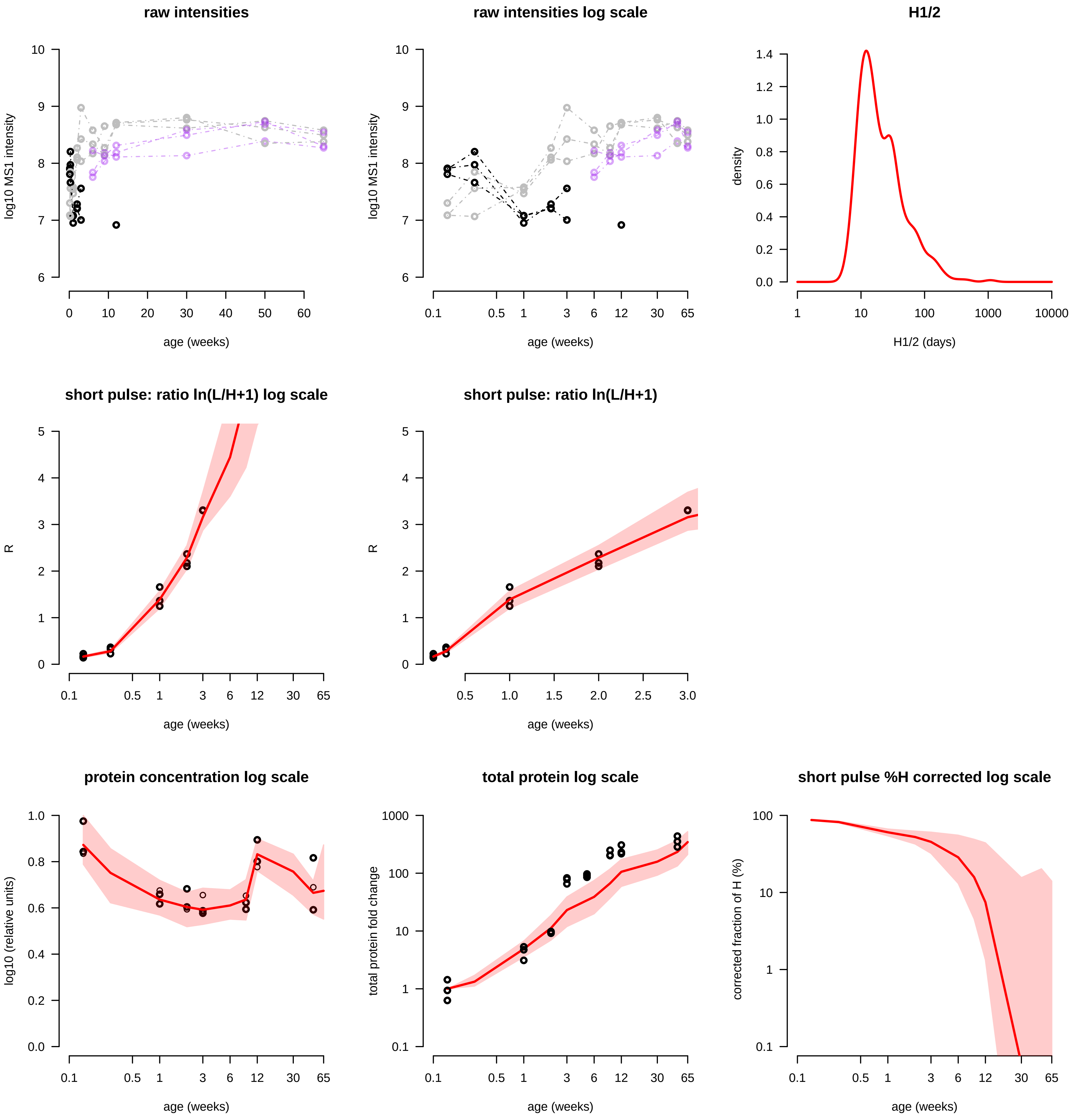

Q3TJD7

>sp|Q3TJD7|PDLI7_MOUSE

PDZ and LIM domain protein 7

GN=Pdlim7

Med H1/2: 102.5 days

Q5 H1/2: 19.4 days

Q95 H1/2: 906.1 days

reliable model fit: no

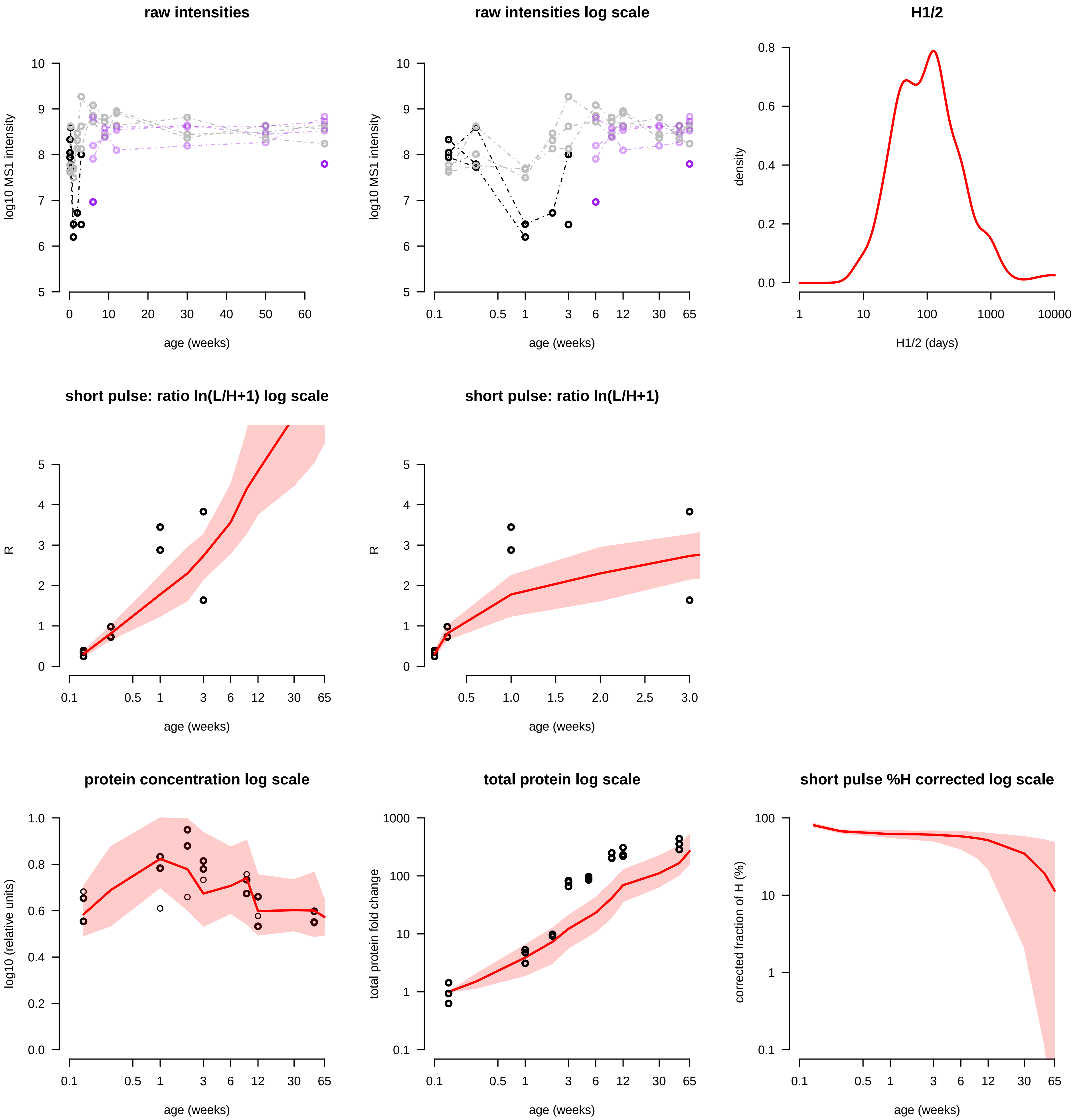

Q3TIV5;Q3TIV5−2

>sp|Q3TIV5|ZC3HF_MOUSE

Zinc finger CCCH domain−containing protein 15

GN=Zc3h15

Med H1/2: 6.4 days

Q5 H1/2: 3.1 days

Q95 H1/2: 51 days

reliable model fit: yes

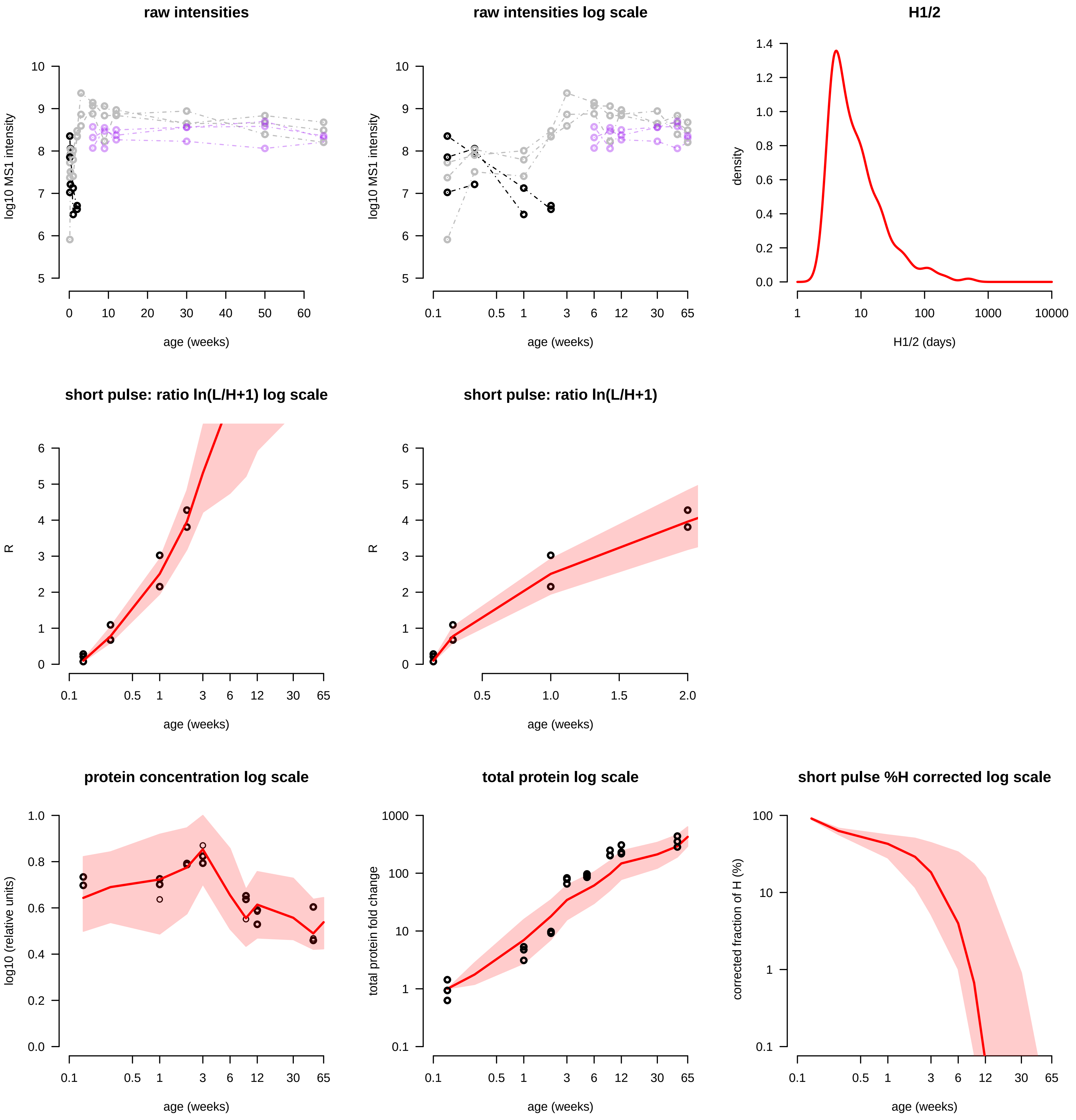

Q9D358

>sp|Q9D358|PPAC_MOUSE

Low molecular weight phosphotyrosine protein phosphatase

GN=Acp1

Med H1/2: 6.4 days

Q5 H1/2: 5.3 days

Q95 H1/2: 8 days

reliable model fit: yes

raw intensities

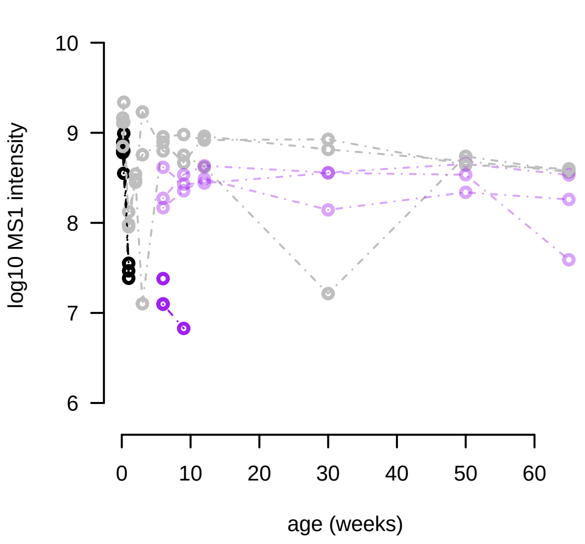

raw intensities log scale

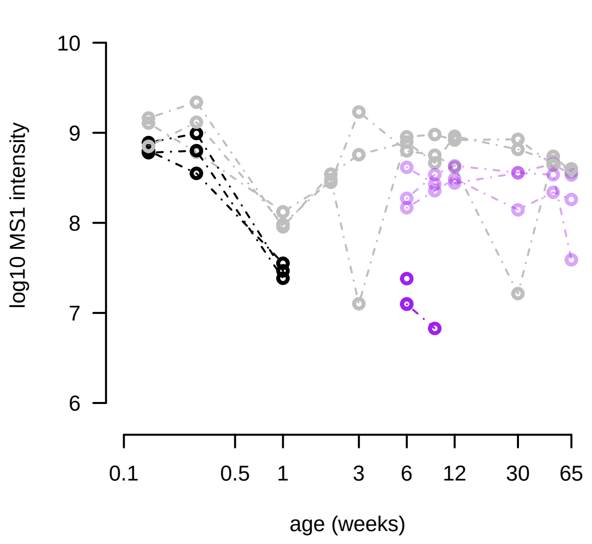

H1/2

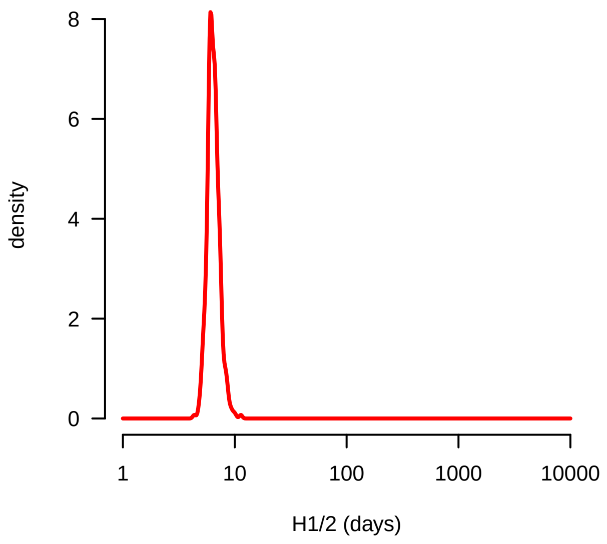

short pulse: ratio ln(L/H+1) log scale

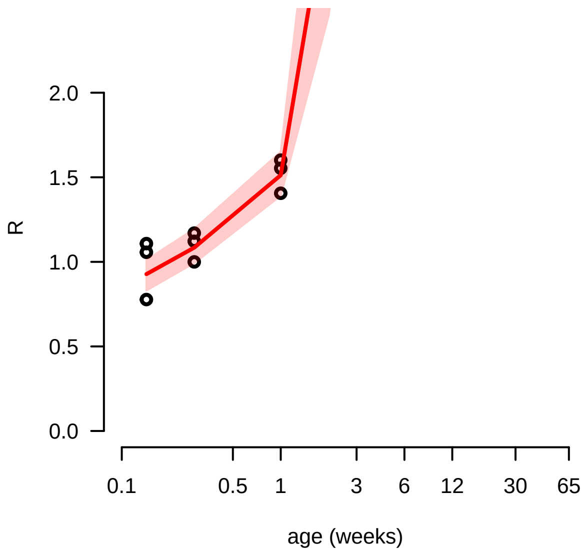

short pulse: ratio ln(L/H+1)

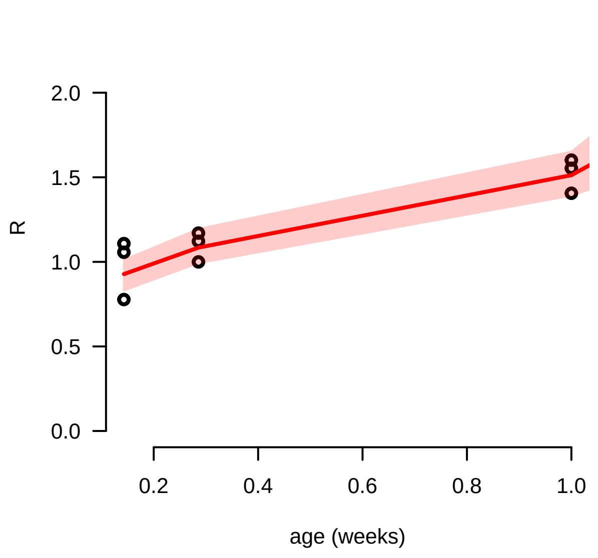

long pulse: ratio ln(L/H+1)

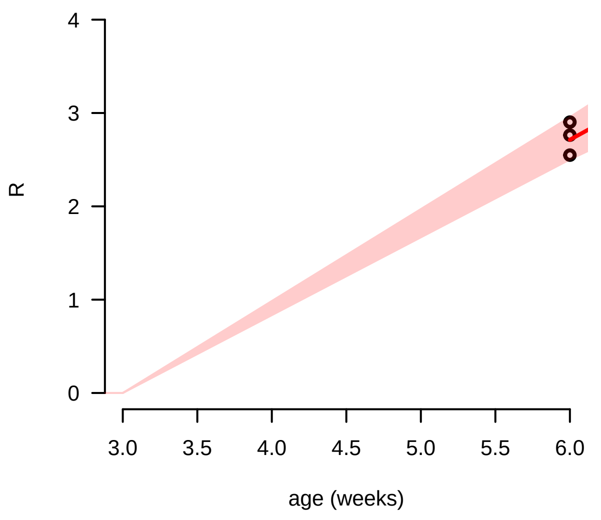

protein concentration log scale

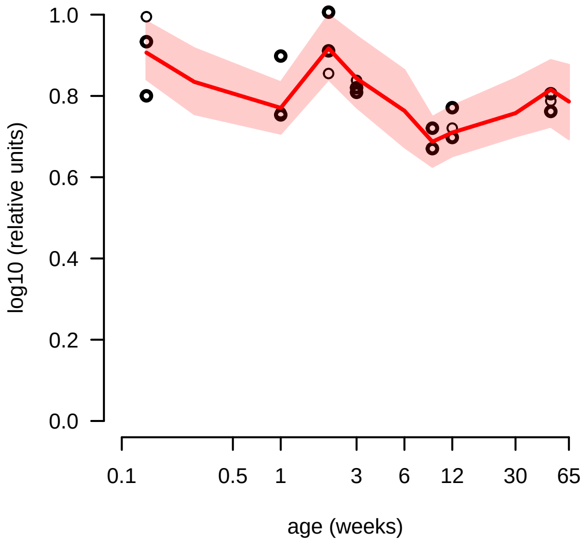

total protein log scale

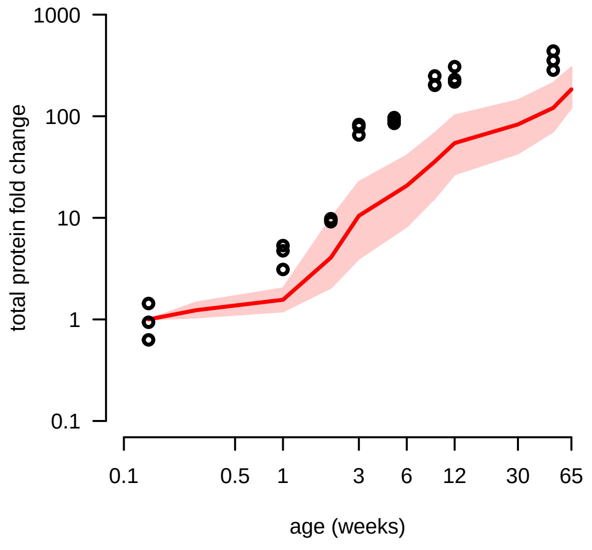

short pulse %H corrected log scale

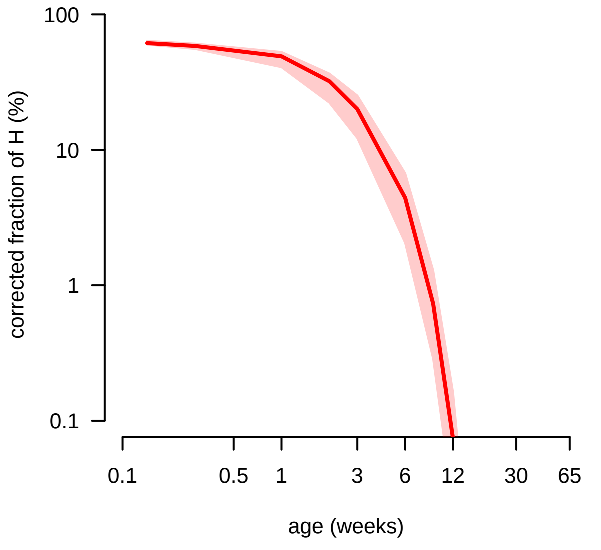

Q8BP48

>sp|Q8BP48|MAP11_MOUSE

Methionine aminopeptidase 1

GN=Metap1

Med H1/2: 8.9 days

Q5 H1/2: 2.8 days

Q95 H1/2: 164.8 days

reliable model fit: yes

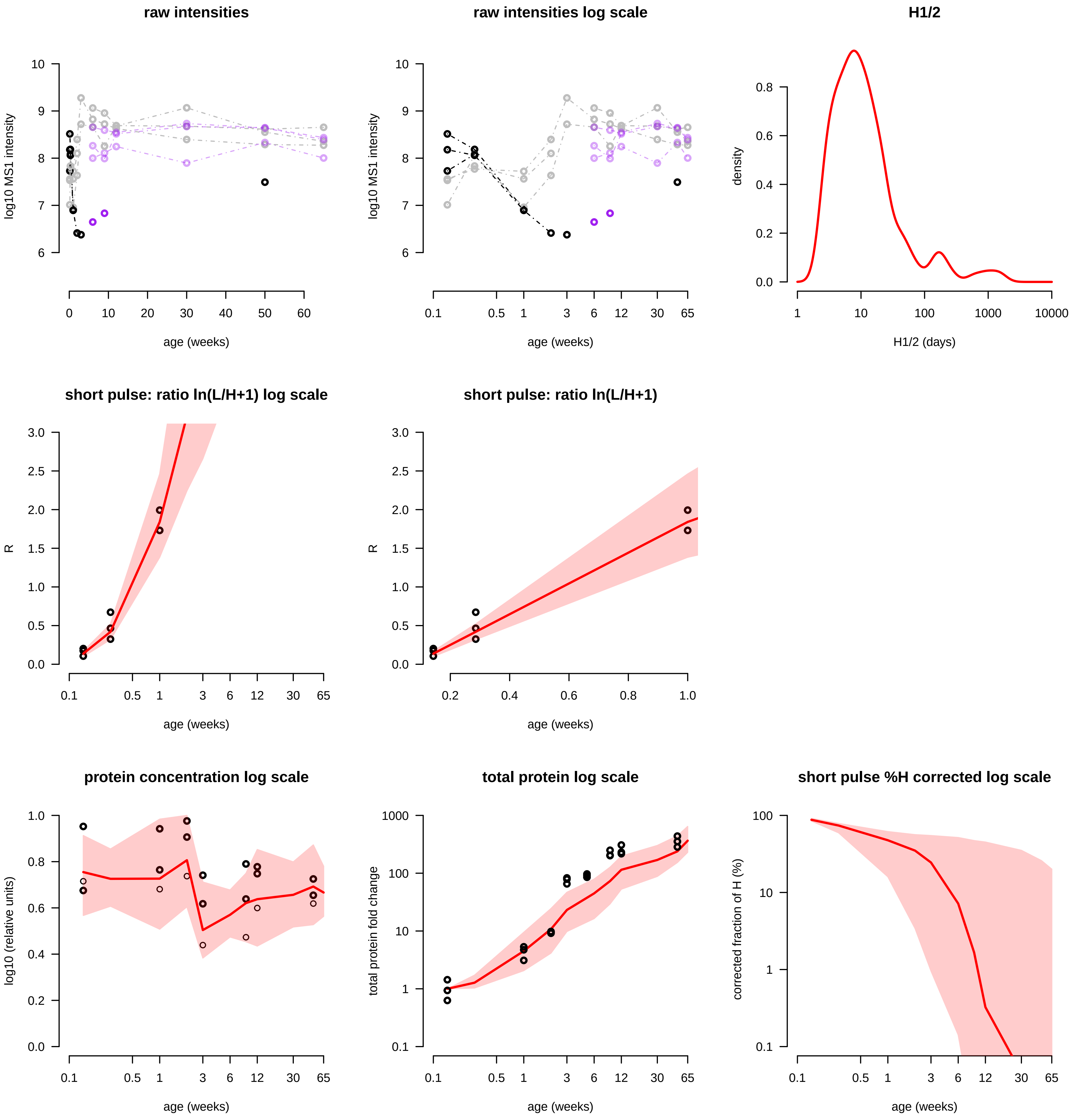

Q9QZ23

>sp|Q9QZ23|NFU1_MOUSE

NFU1 iron−sulfur cluster scaffold homolog, mitochondrial

GN=Nfu1

Med H1/2: 8.5 days

Q5 H1/2: 6.2 days

Q95 H1/2: 13.7 days

reliable model fit: yes

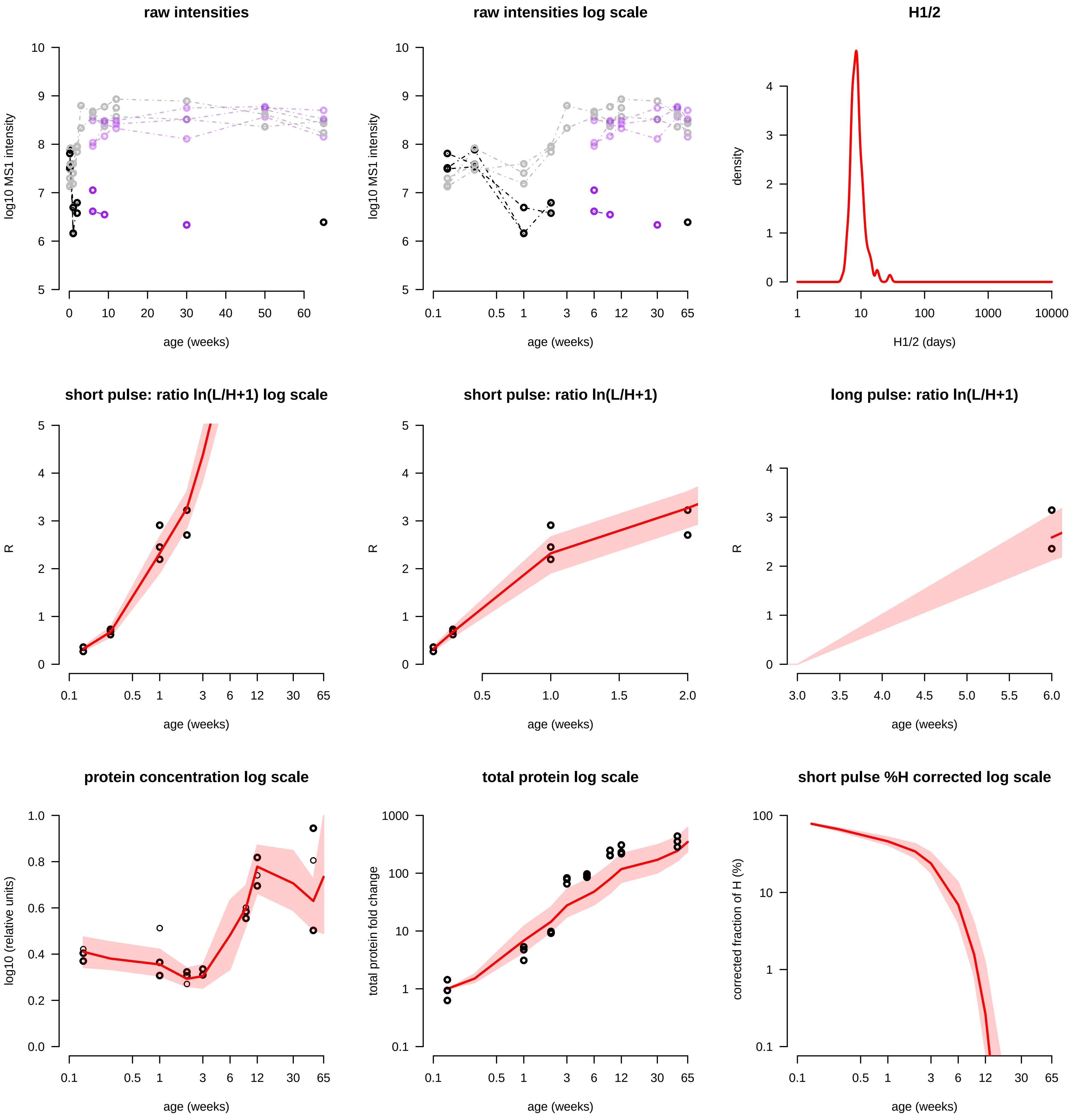

P97390

>sp|P97390|VPS45_MOUSE

Vacuolar protein sorting−associated protein 45

GN=Vps45

Med H1/2: 5.6 days

Q5 H1/2: 3 days

Q95 H1/2: 13.4 days

reliable model fit: yes

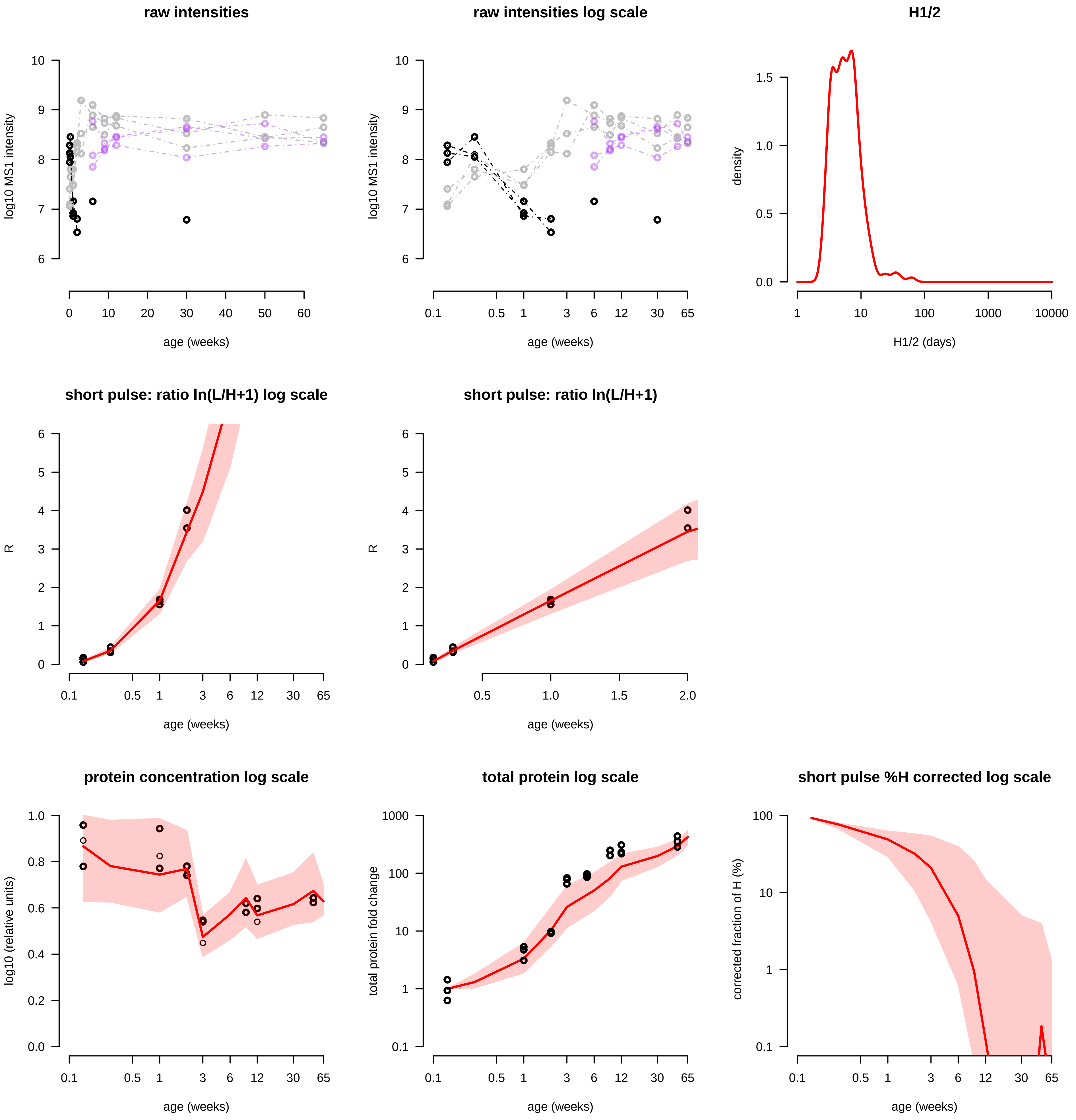

Q8R3N6

>sp|Q8R3N6|THOC1_MOUSE

THO complex subunit 1

GN=Thoc1

Med H1/2: 5.1 days

Q5 H1/2: 4 days

Q95 H1/2: 6.7 days

reliable model fit: yes

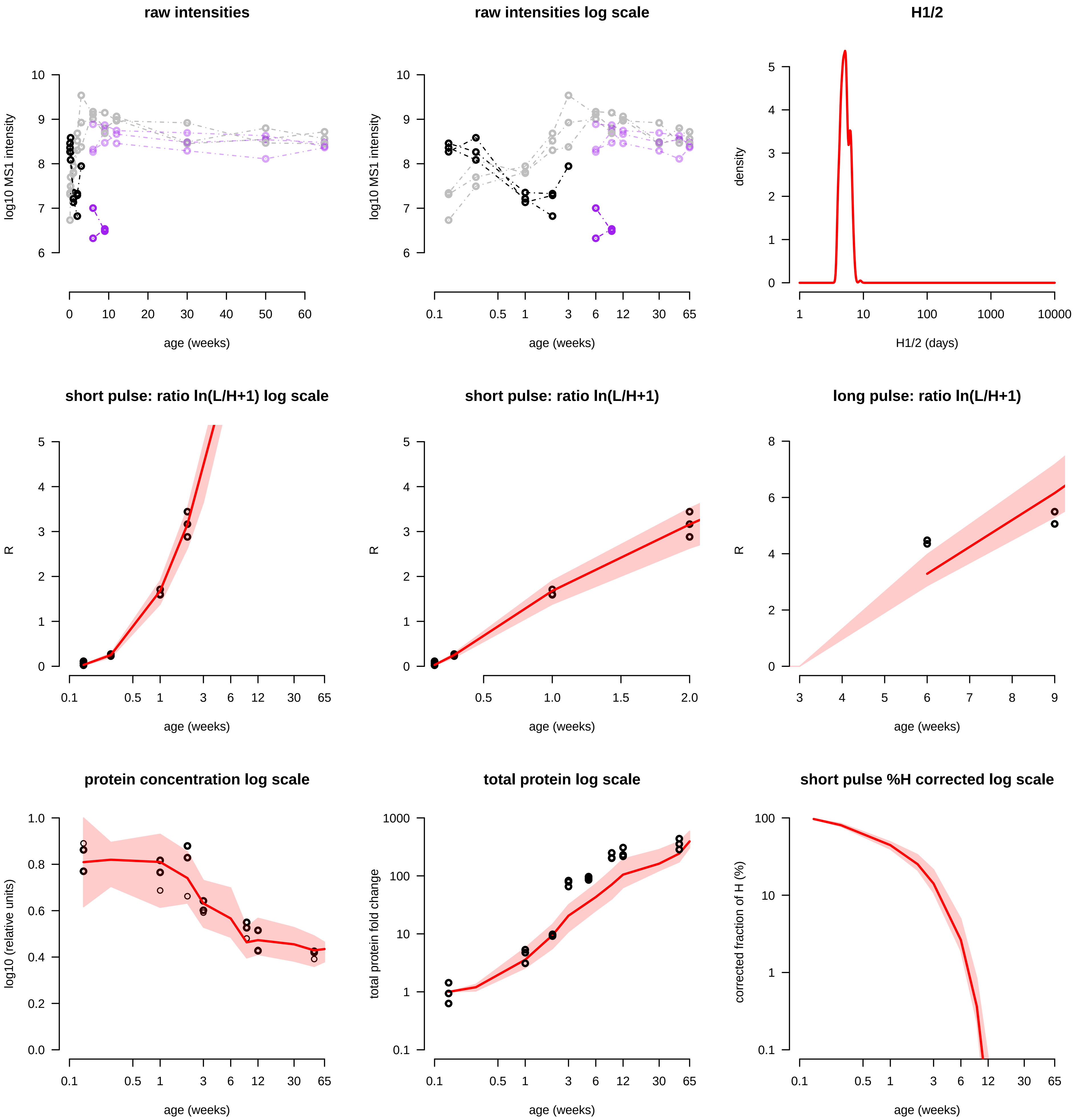

Q8BTX9

>sp|Q8BTX9|HSDL1_MOUSE

Inactive hydroxysteroid dehydrogenase−like protein 1

GN=Hsdl1

Med H1/2: 5.4 days

Q5 H1/2: 4 days

Q95 H1/2: 7.8 days

reliable model fit: yes

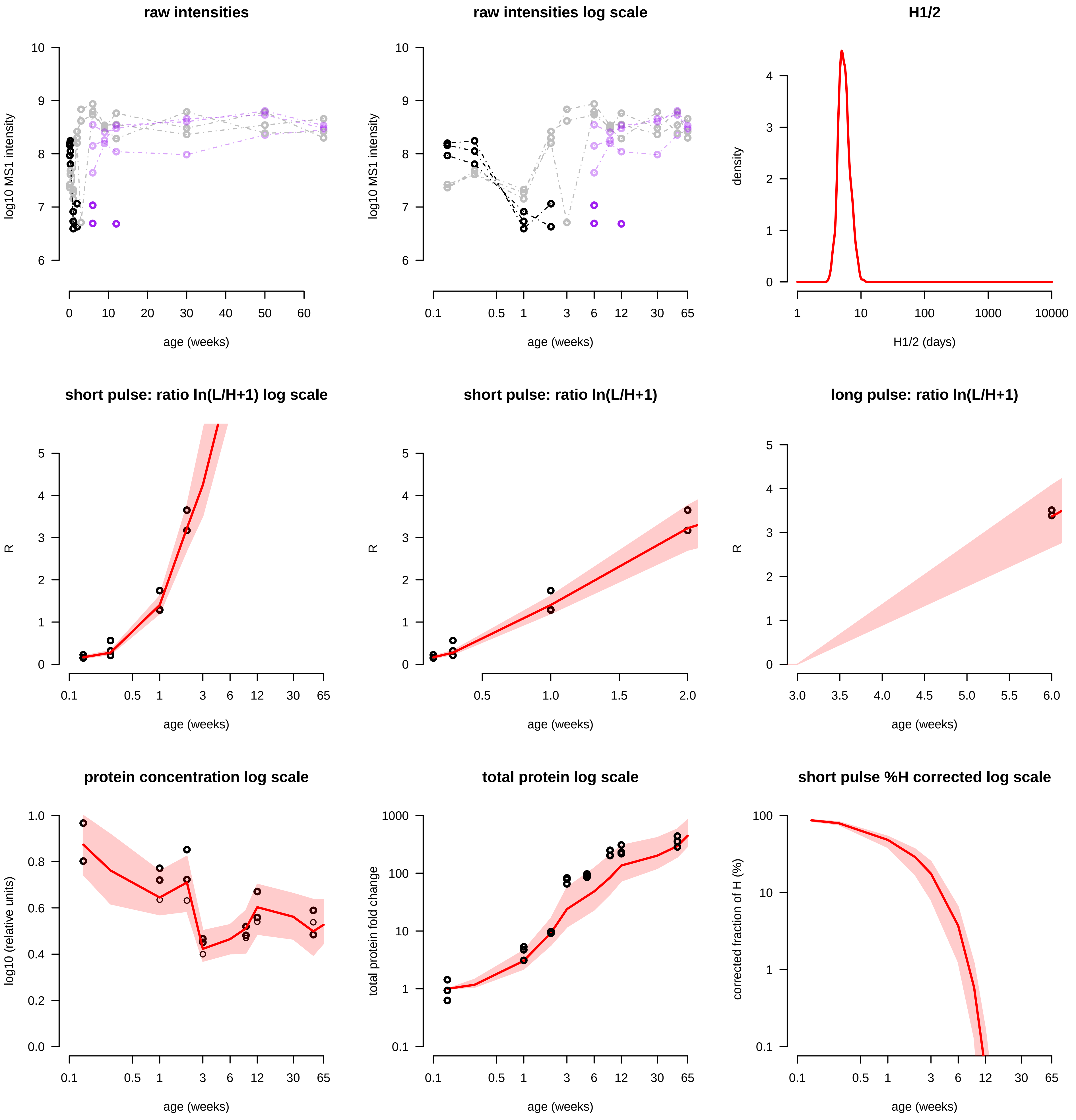

Q6P9Q6;Q6P9Q6−2

>sp|Q6P9Q6|FKB15_MOUSE

FK506−binding protein 15

GN=Fkbp15

Med H1/2: 1909 days

Q5 H1/2: 568.8 days

Q95 H1/2: 12166.1 days

reliable model fit: yes

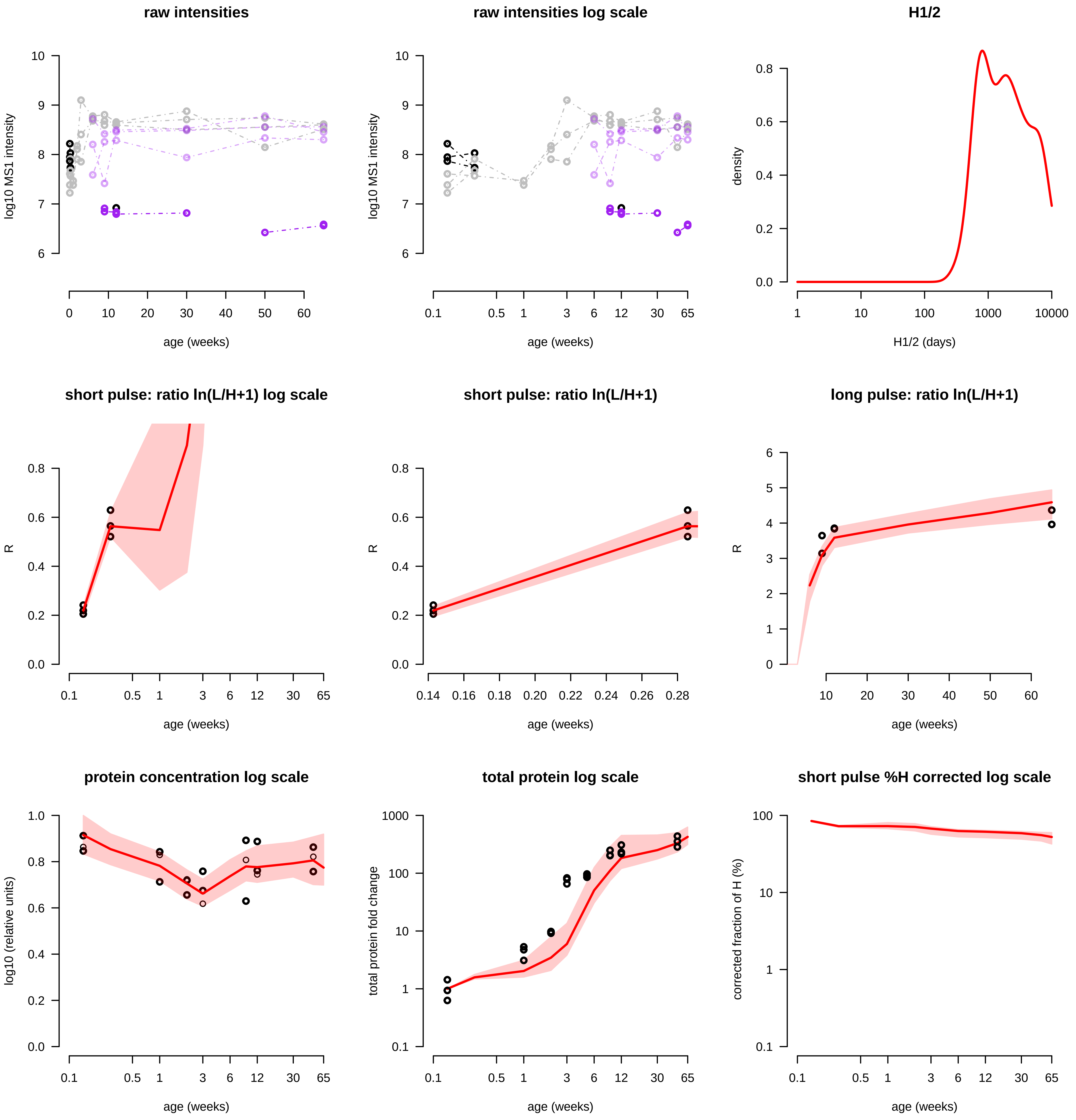

Q9CWU9

>sp|Q9CWU9|NUP37_MOUSE

Nucleoporin Nup37

GN=Nup37

Med H1/2: 16 days

Q5 H1/2: 5 days

Q95 H1/2: 149.3 days

reliable model fit: yes

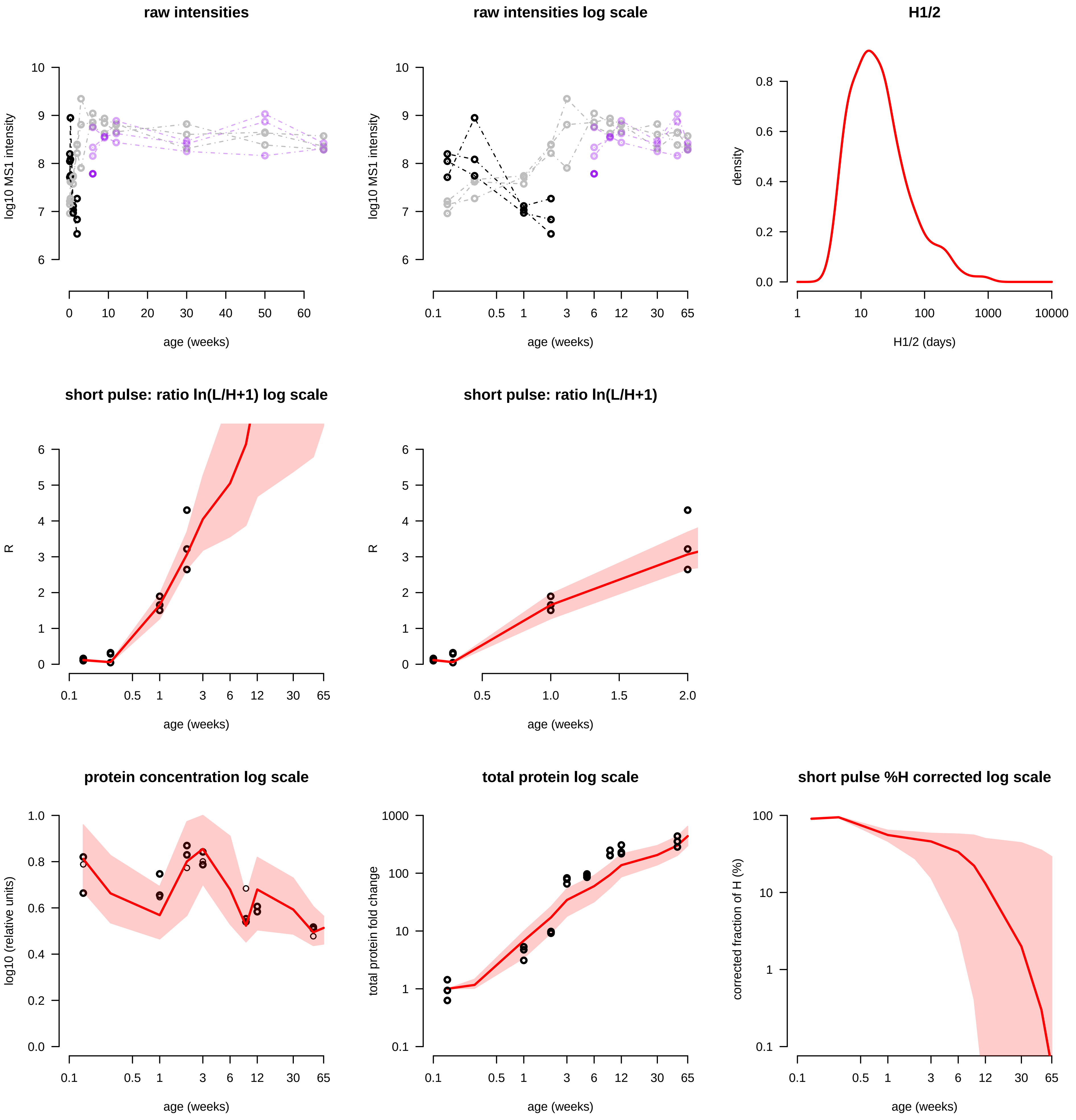

Q6NS46

>sp|Q6NS46|RRP5_MOUSE

Protein RRP5 homolog

GN=Pdcd11

Med H1/2: 3.8 days

Q5 H1/2: 3 days

Q95 H1/2: 4.8 days

reliable model fit: yes

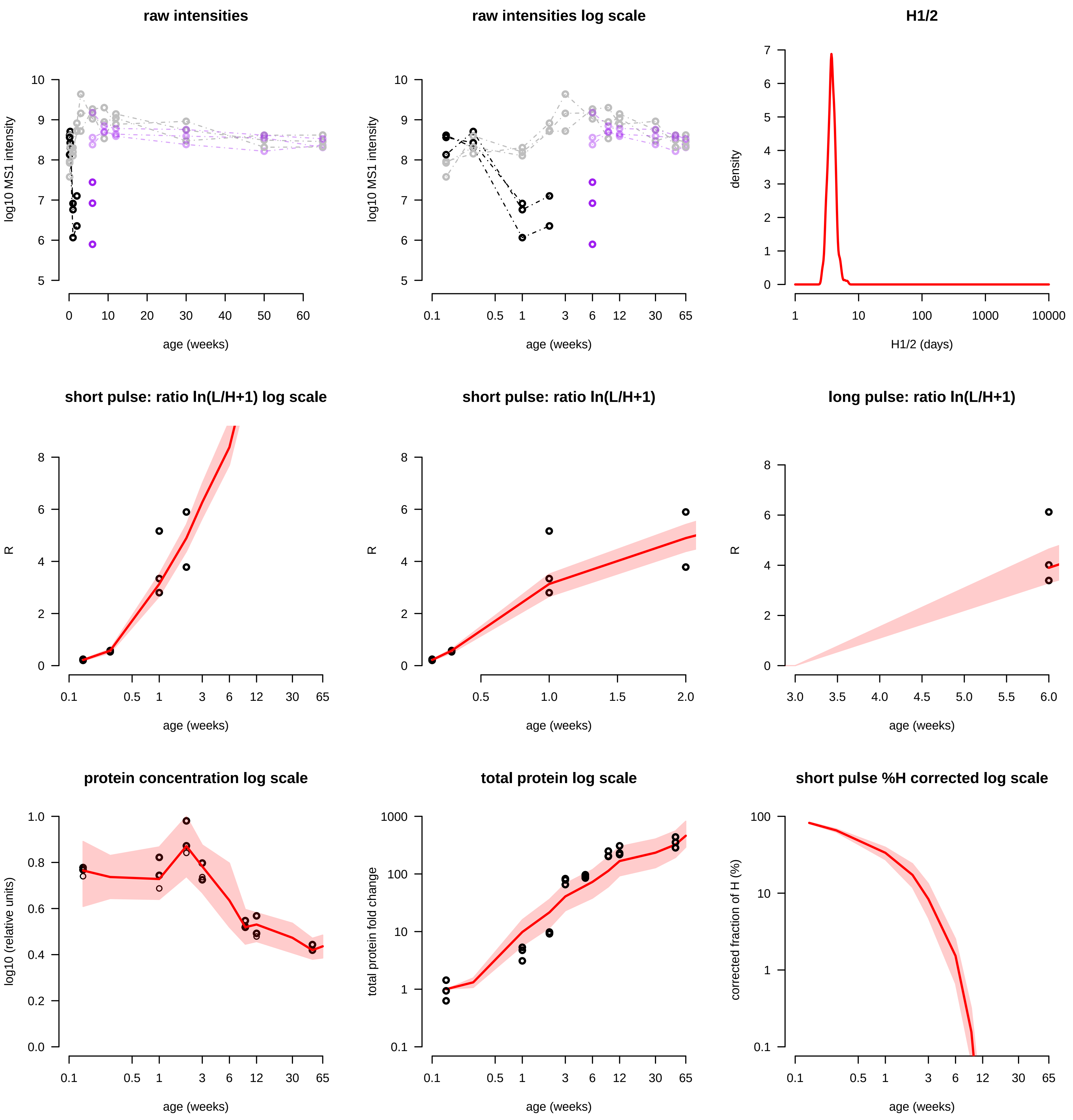

P56135

>sp|P56135|ATPK_MOUSE

ATP synthase subunit f, mitochondrial

GN=Atp5mf

Med H1/2: 6.2 days

Q5 H1/2: 5 days

Q95 H1/2: 8.5 days

reliable model fit: yes

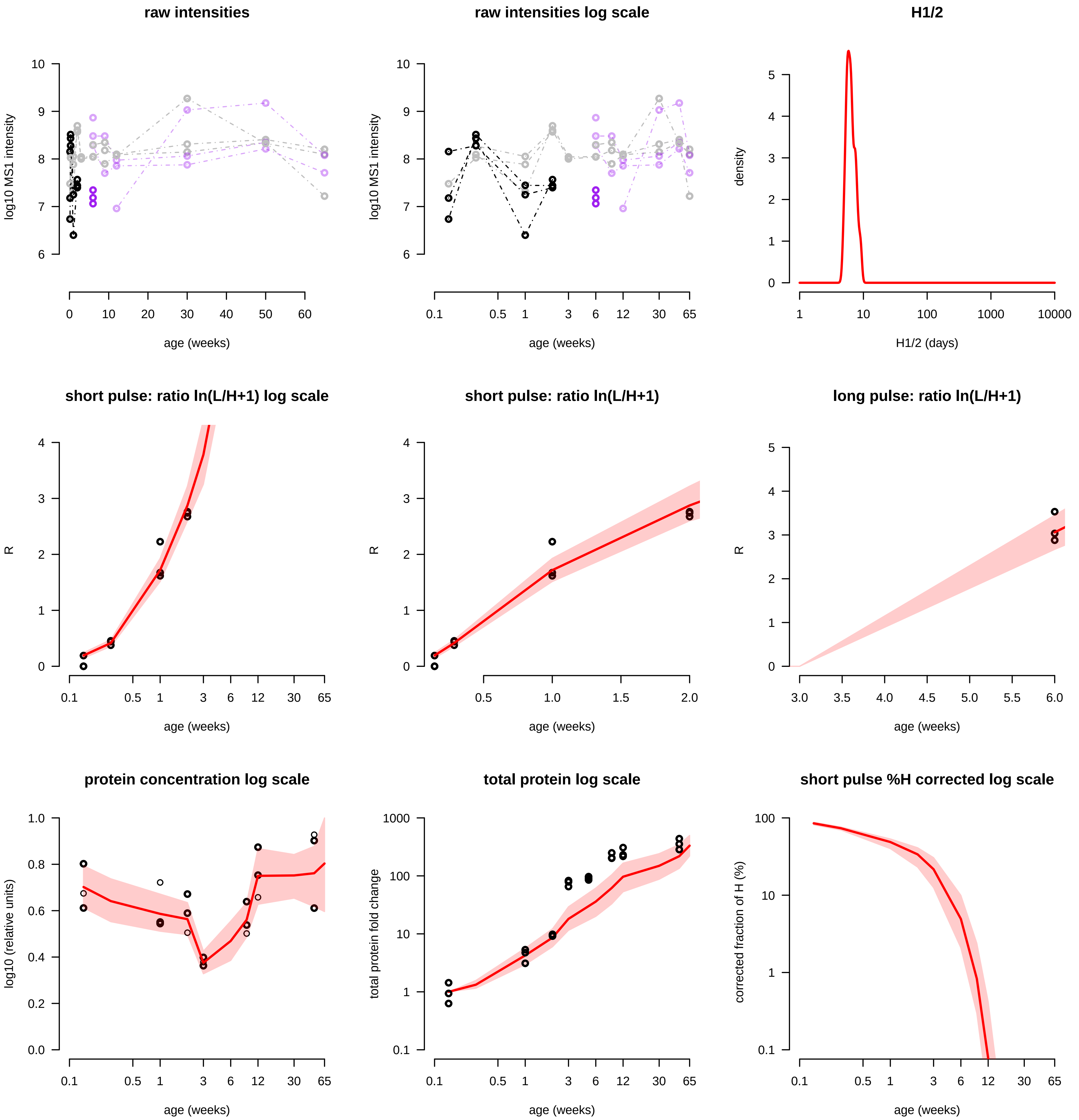

P60904

>sp|P60904|DNJC5_MOUSE

DnaJ homolog subfamily C member 5

GN=Dnajc5

Med H1/2: 11.4 days

Q5 H1/2: 7.8 days

Q95 H1/2: 22.9 days

reliable model fit: yes

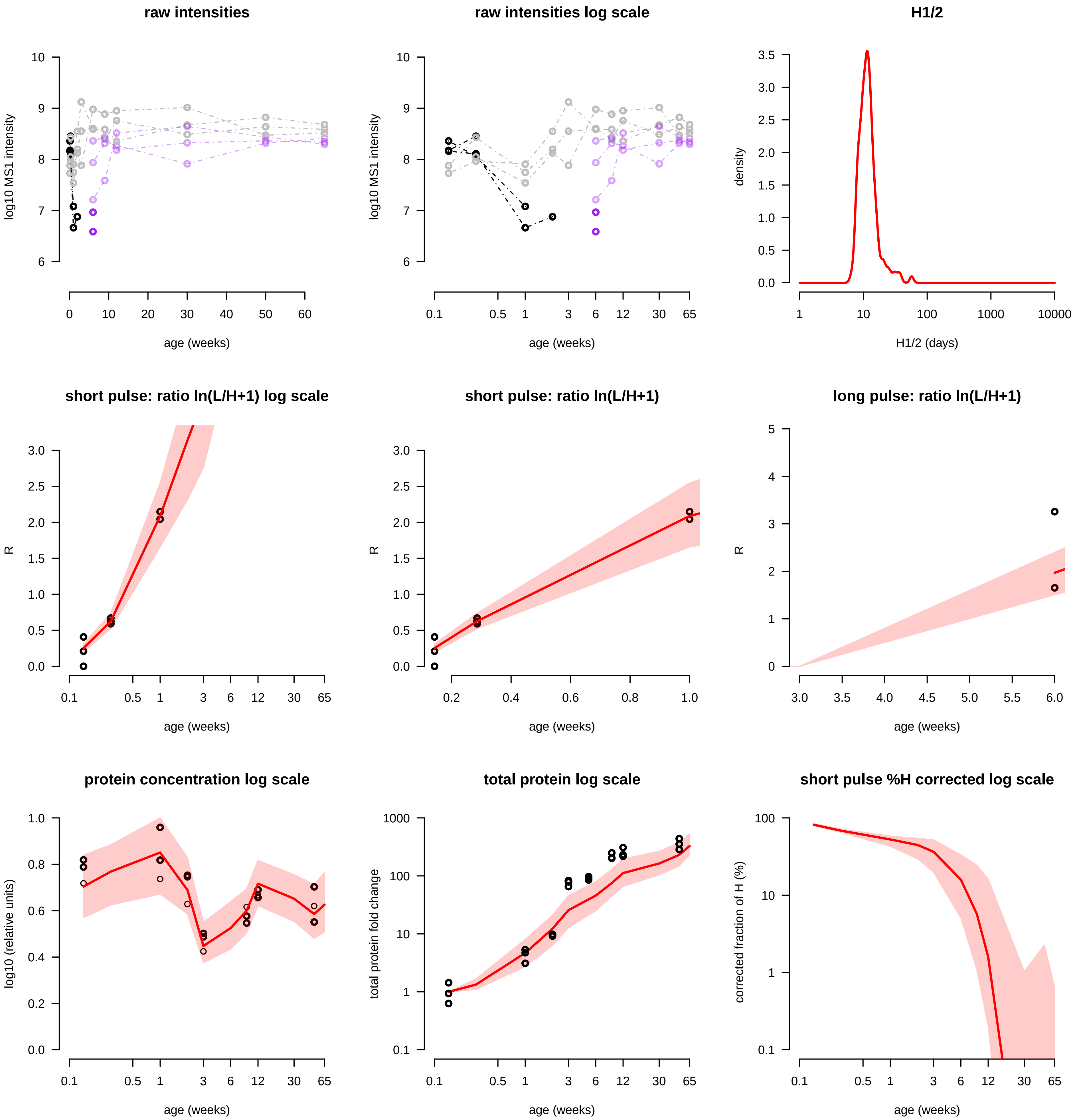

A6H8H2−2

>sp|A6H8H2−2|DEN4C_MOUSE

Isoform 2 of DENN domain−containing protein 4C

GN=Dennd4c

Med H1/2: 8.4 days

Q5 H1/2: 3.2 days

Q95 H1/2: 44 days

reliable model fit: yes

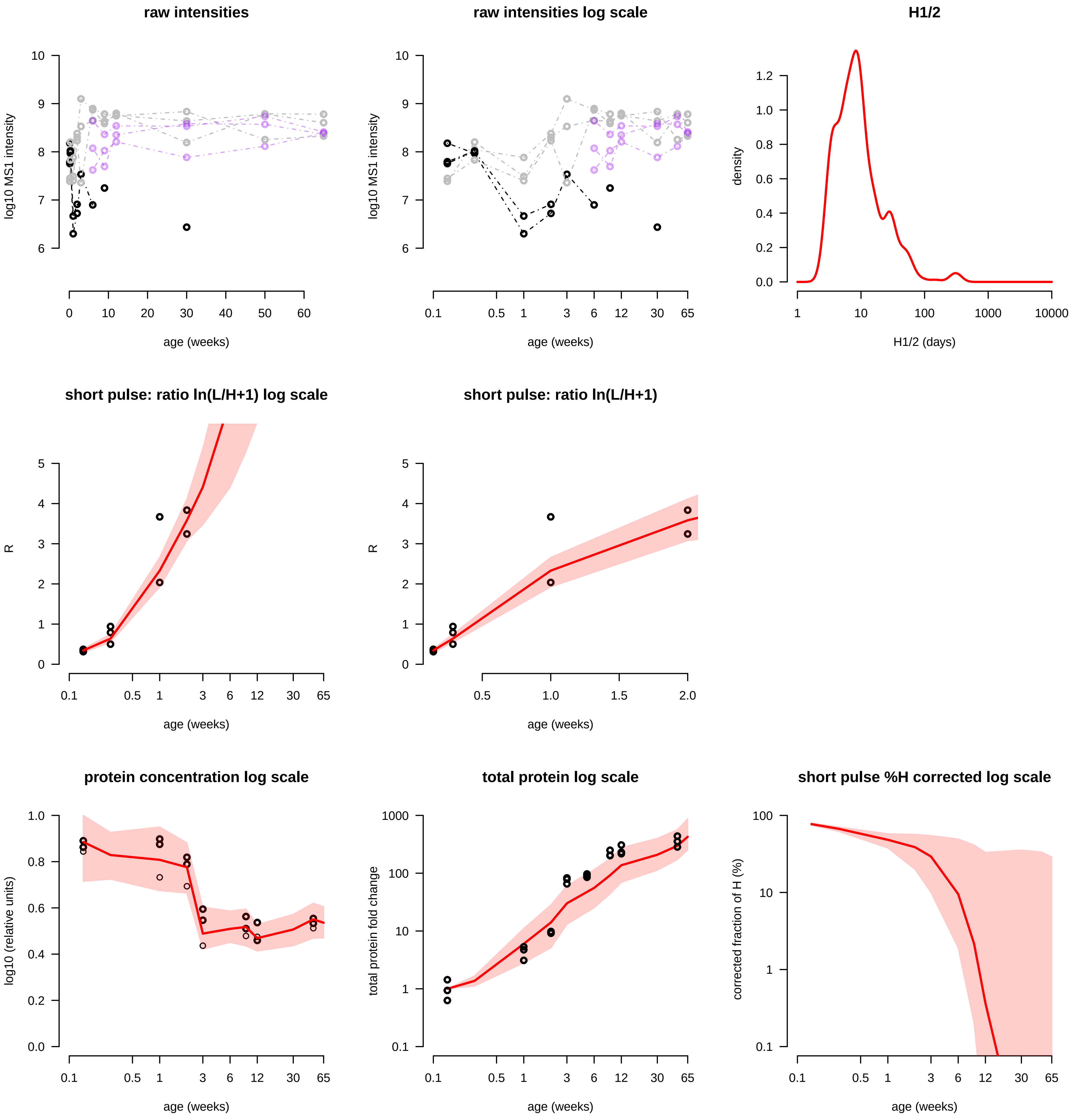

O35343

>sp|O35343|IMA3_MOUSE

Importin subunit alpha−3

GN=Kpna4

Med H1/2: 4.4 days

Q5 H1/2: 3.7 days

Q95 H1/2: 5.4 days

reliable model fit: yes

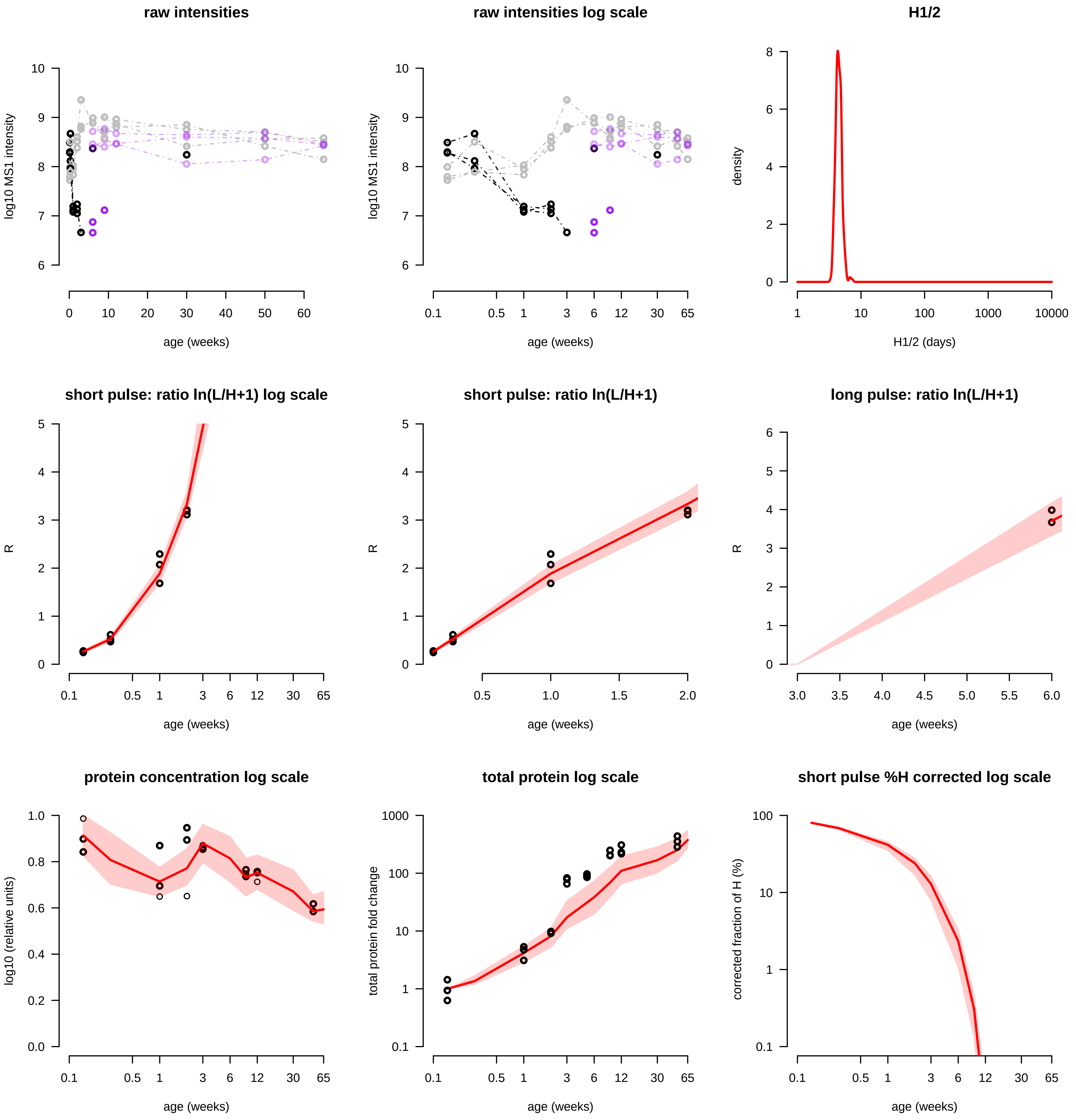

P35123

>sp|P35123|UBP4_MOUSE

Ubiquitin carboxyl−terminal hydrolase 4

GN=Usp4

Med H1/2: 5.8 days

Q5 H1/2: 3 days

Q95 H1/2: 24.2 days

reliable model fit: yes

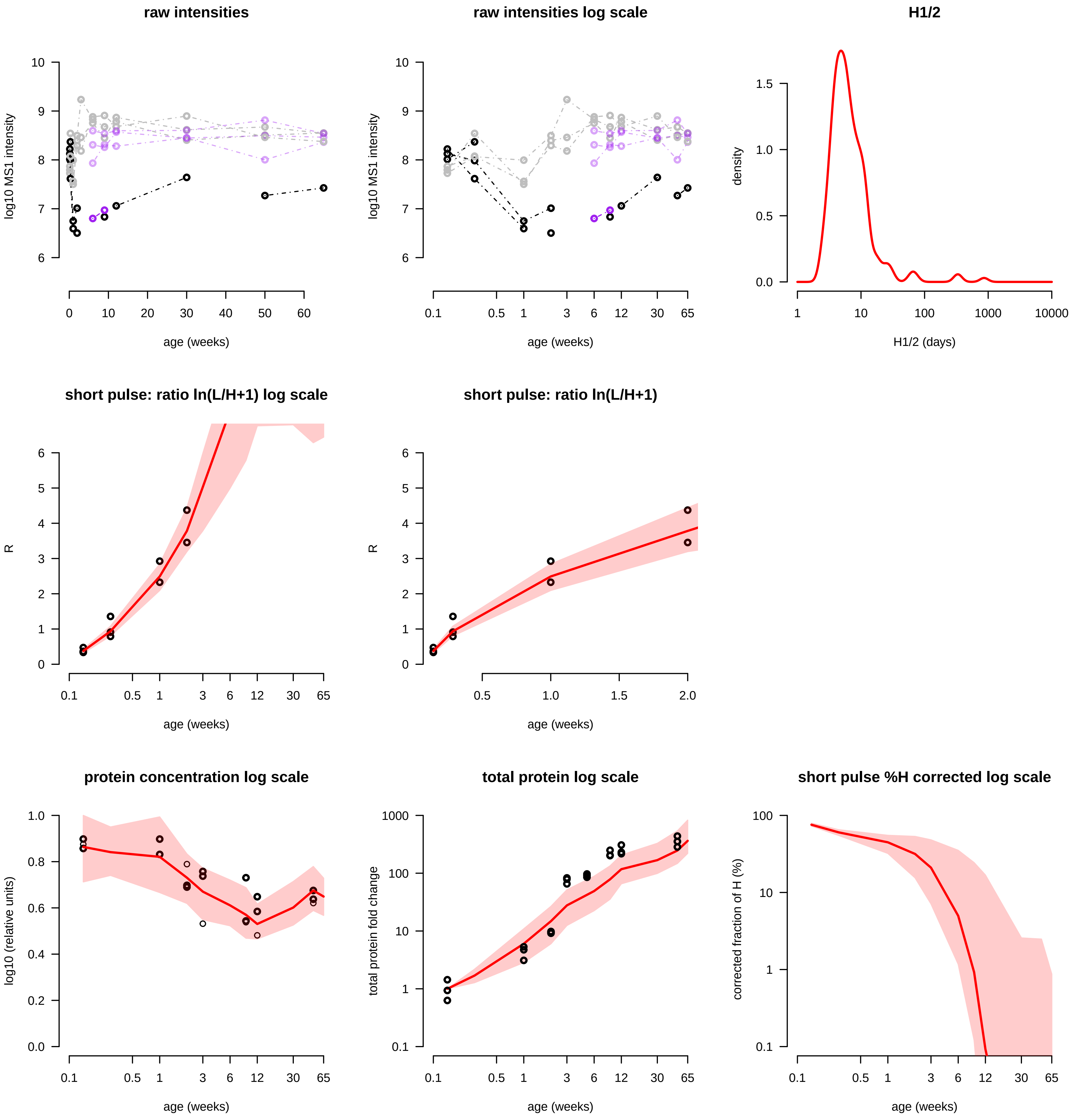

Q9DCC4

>sp|Q9DCC4|P5CR3_MOUSE

Pyrroline−5−carboxylate reductase 3

GN=Pycr3

Med H1/2: 8.3 days

Q5 H1/2: 6.2 days

Q95 H1/2: 11.1 days

reliable model fit: no

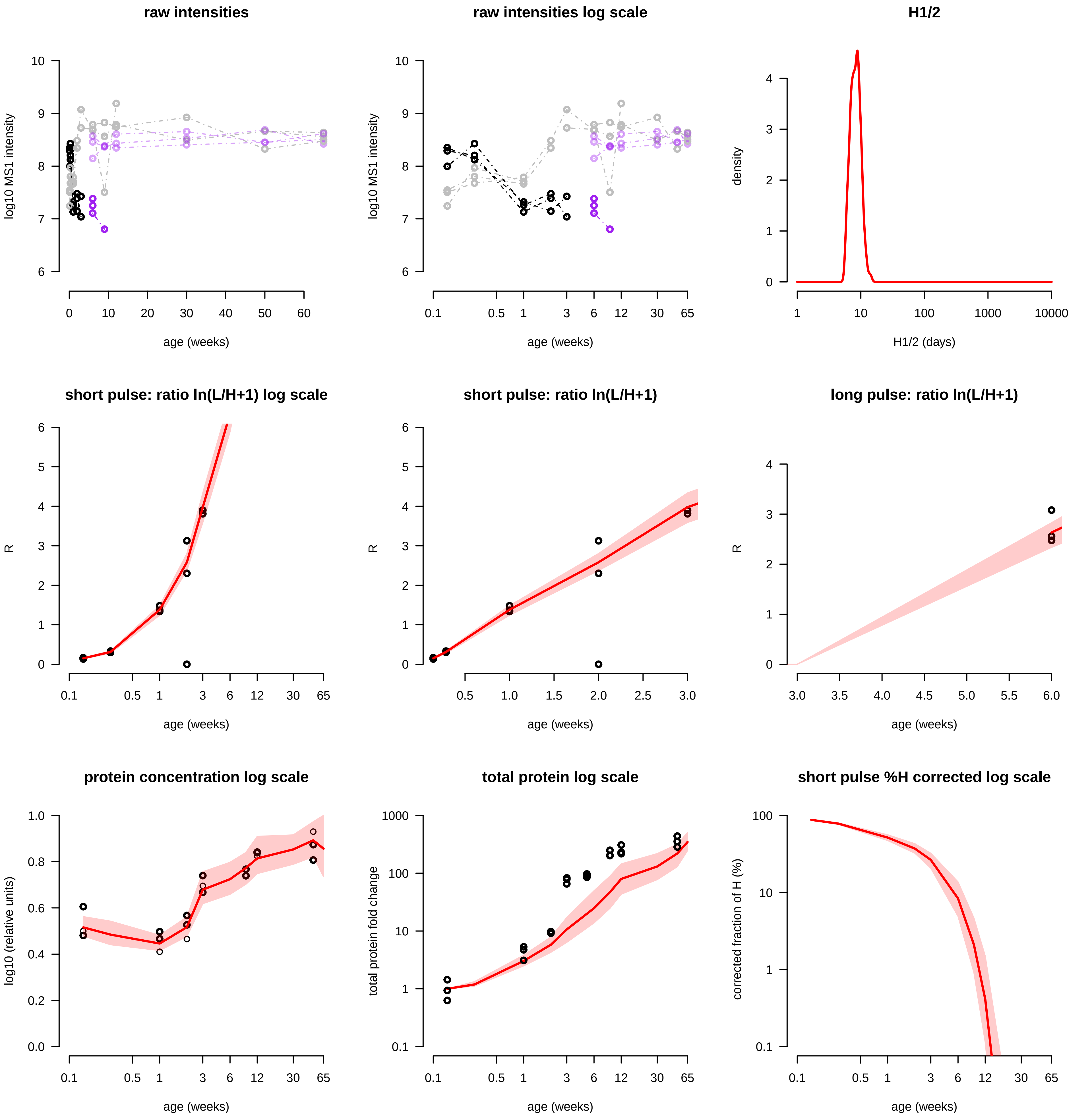

Q60676

>sp|Q60676|PPP5_MOUSE

Serine/threonine−protein phosphatase 5

GN=Ppp5c

Med H1/2: 16.4 days

Q5 H1/2: 5.5 days

Q95 H1/2: 195.2 days

reliable model fit: yes

raw intensities

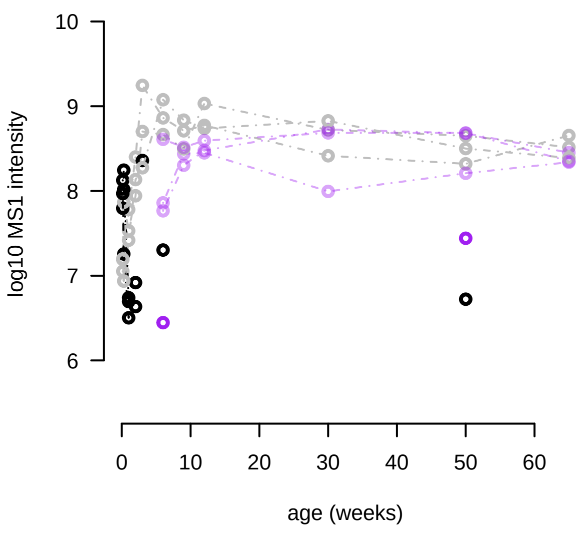

raw intensities log scale

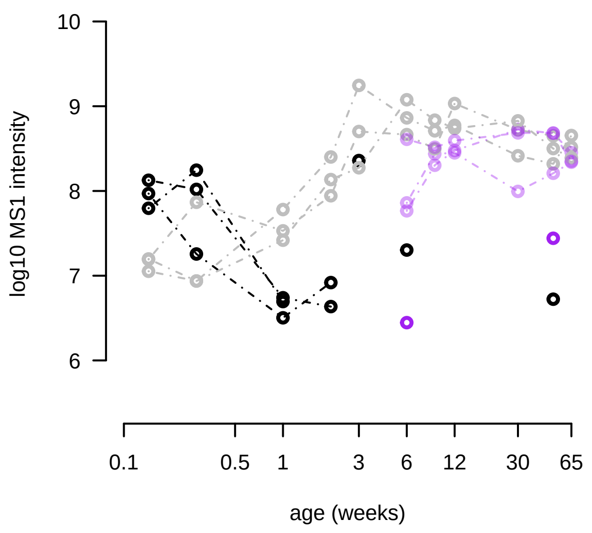

H1/2

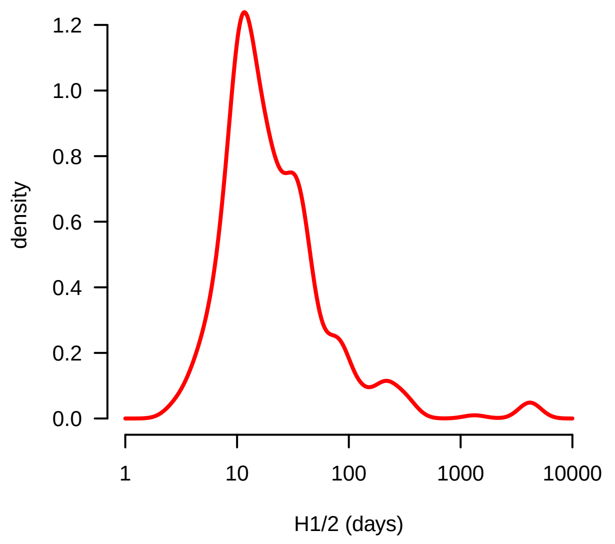

short pulse: ratio ln(L/H+1) log scale

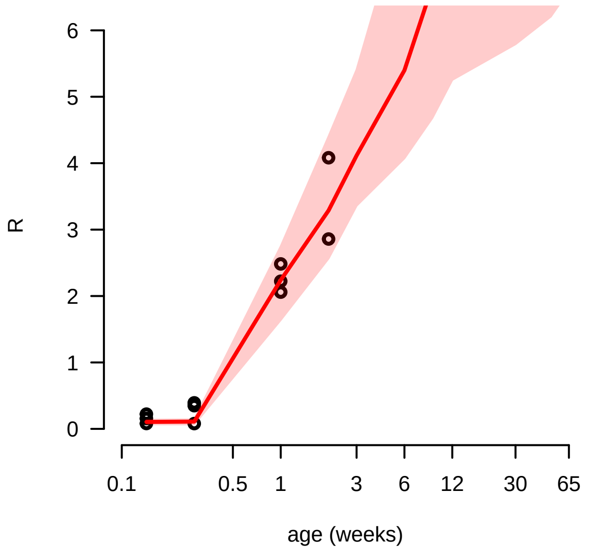

short pulse: ratio ln(L/H+1)

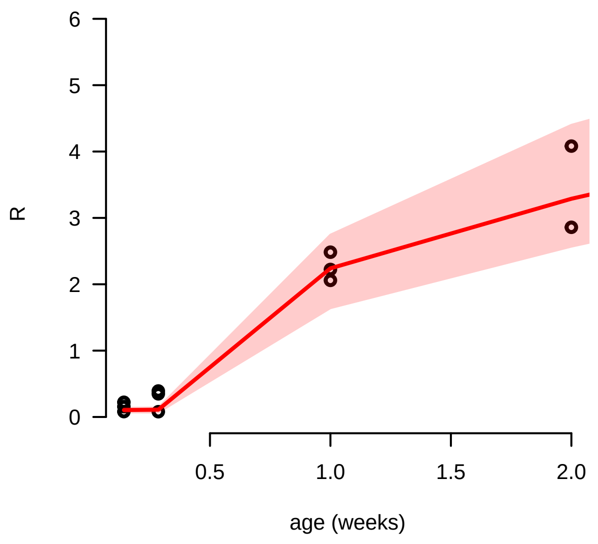

protein concentration log scale

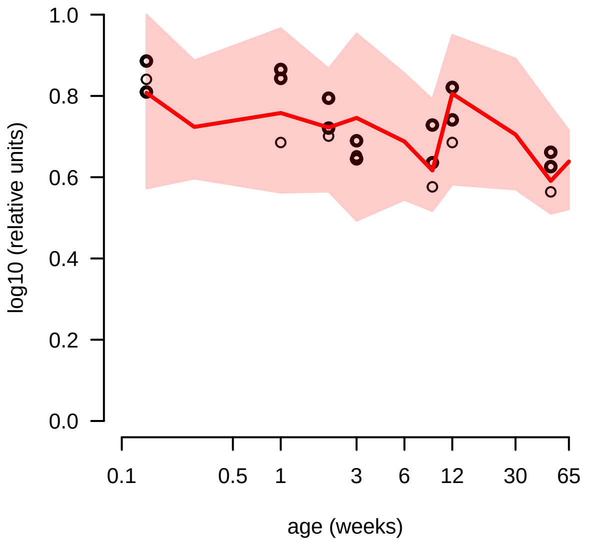

total protein log scale

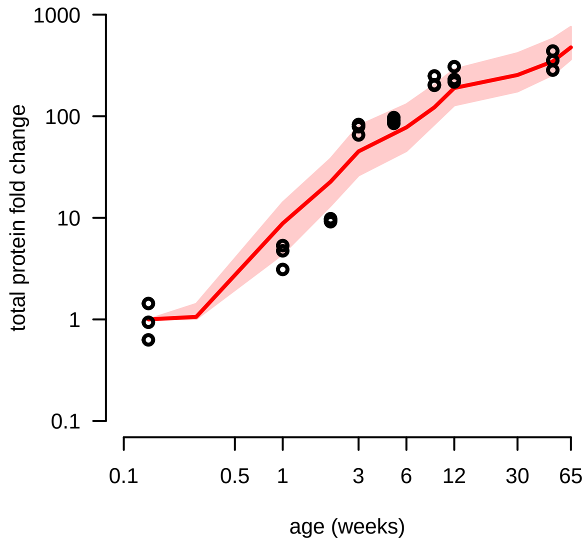

short pulse %H corrected log scale

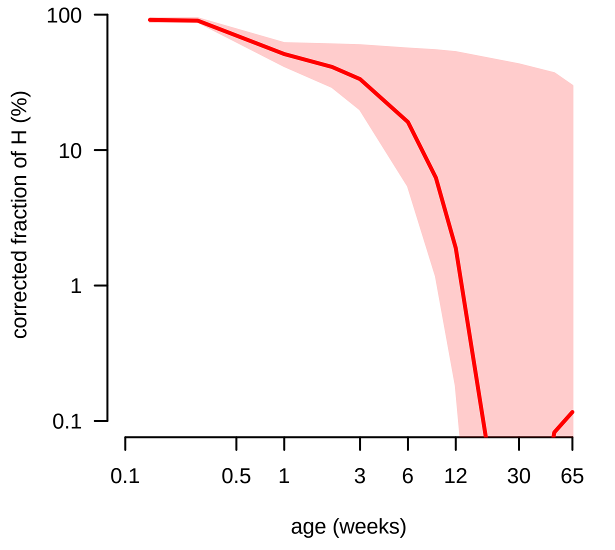

Q62419

>sp|Q62419|SH3G1_MOUSE

Endophilin−A2

GN=Sh3gl1

Med H1/2: 3.8 days

Q5 H1/2: 3.1 days

Q95 H1/2: 4.6 days

reliable model fit: yes

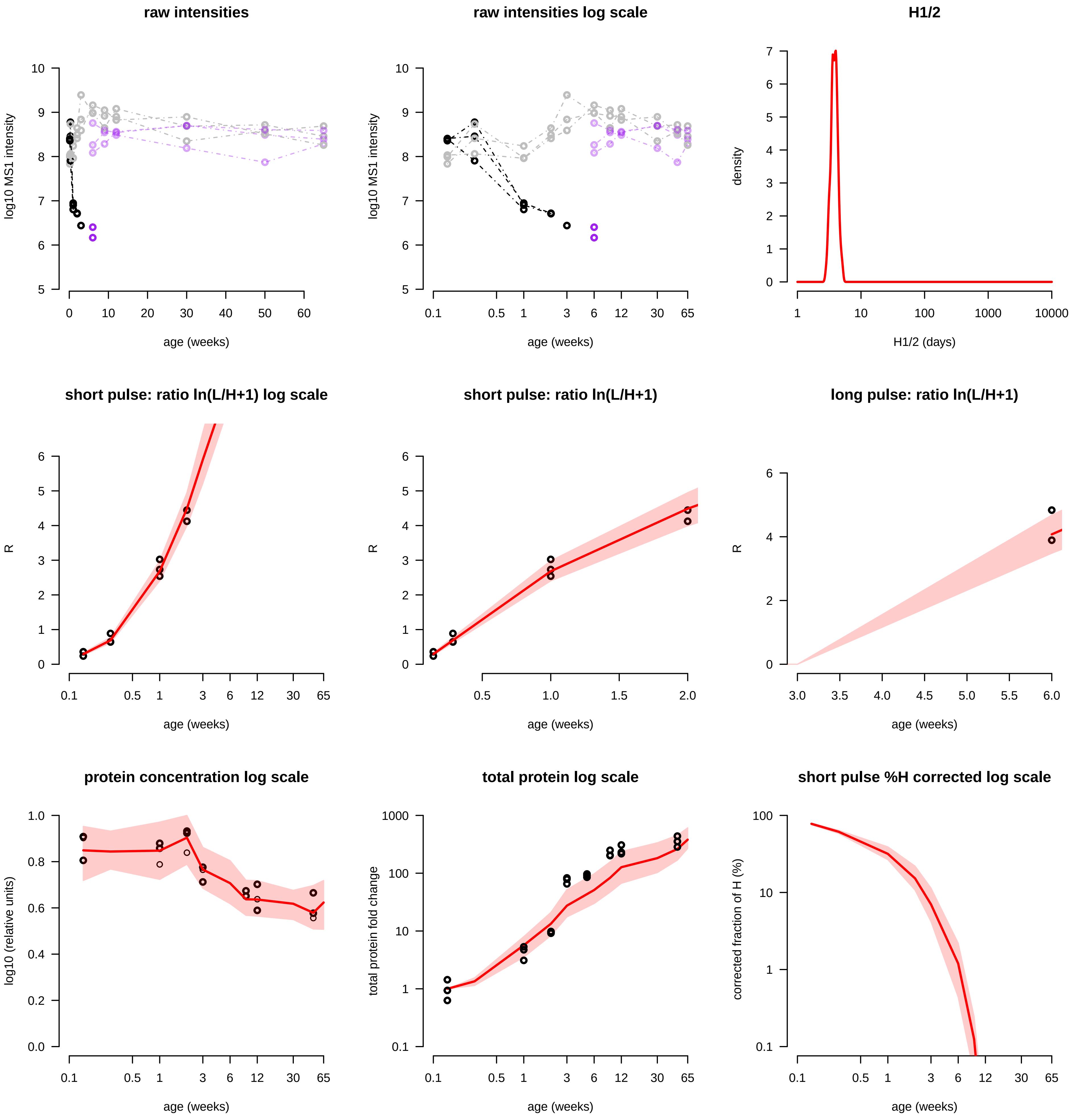

Q8BTS4

>sp|Q8BTS4|NUP54_MOUSE

Nuclear pore complex protein Nup54

GN=Nup54

Med H1/2: 4.2 days

Q5 H1/2: 3.5 days

Q95 H1/2: 5 days

reliable model fit: yes

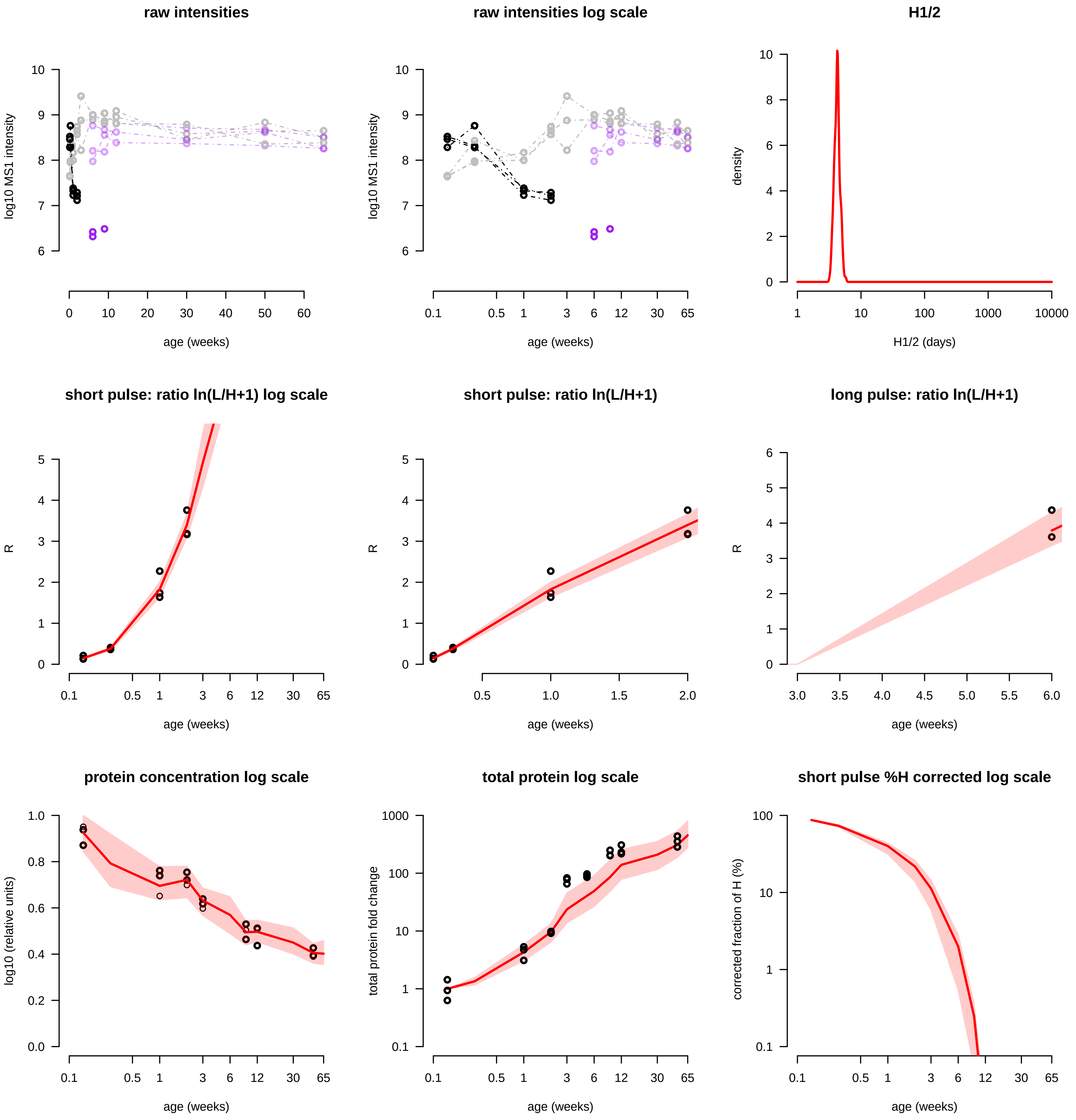

Q61183;Q61183−3;Q61183−4;Q61183−2

>sp|Q61183|PAPOA_MOUSE

Poly(A) polymerase alpha

GN=Papola

Med H1/2: 15.5 days

Q5 H1/2: 6.9 days

Q95 H1/2: 111.1 days

reliable model fit: yes

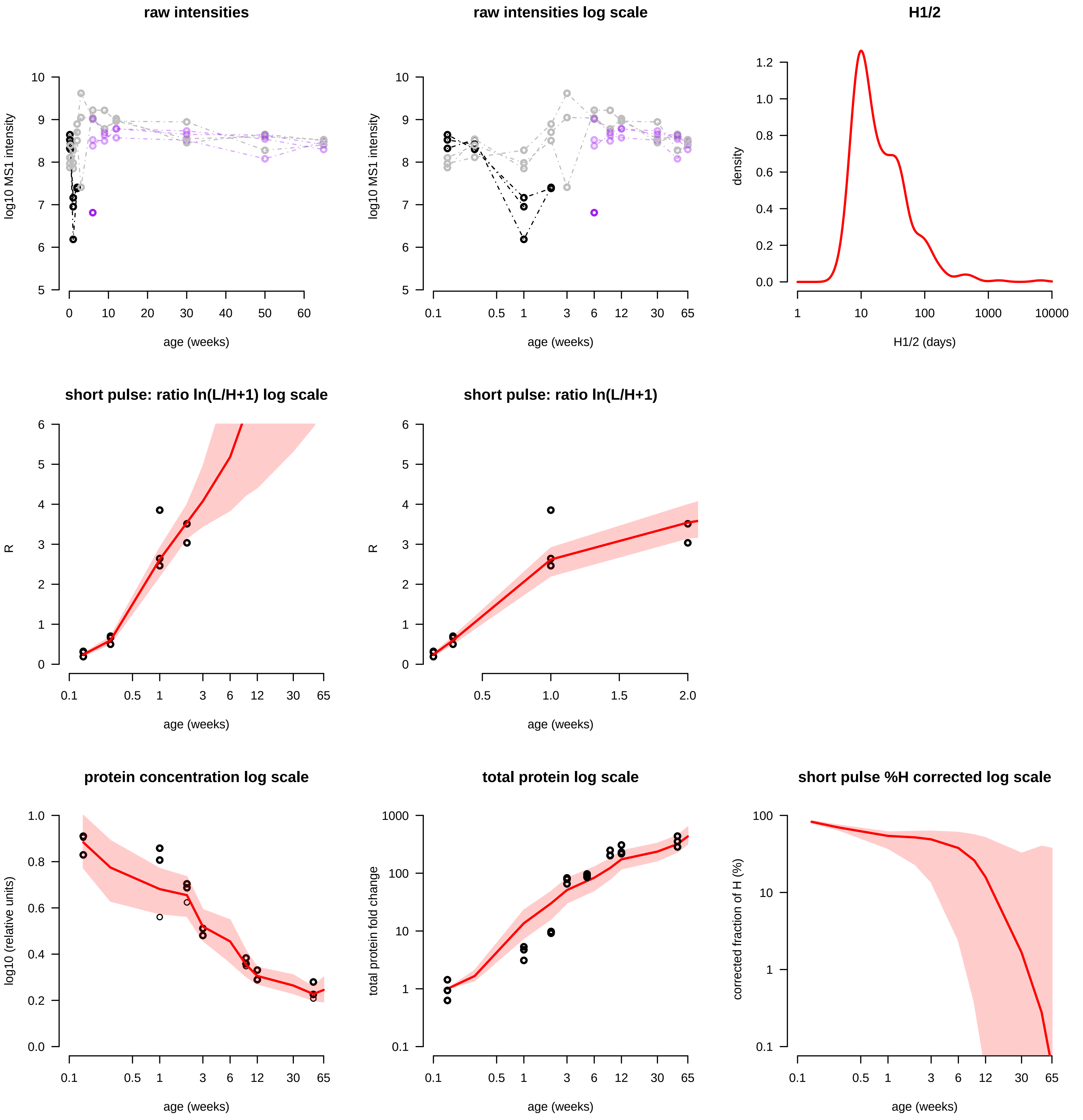

B8JK39

>sp|B8JK39|ITA9_MOUSE

Integrin alpha−9

GN=Itga9

Med H1/2: 532 days

Q5 H1/2: 144.1 days

Q95 H1/2: 5383 days

reliable model fit: yes

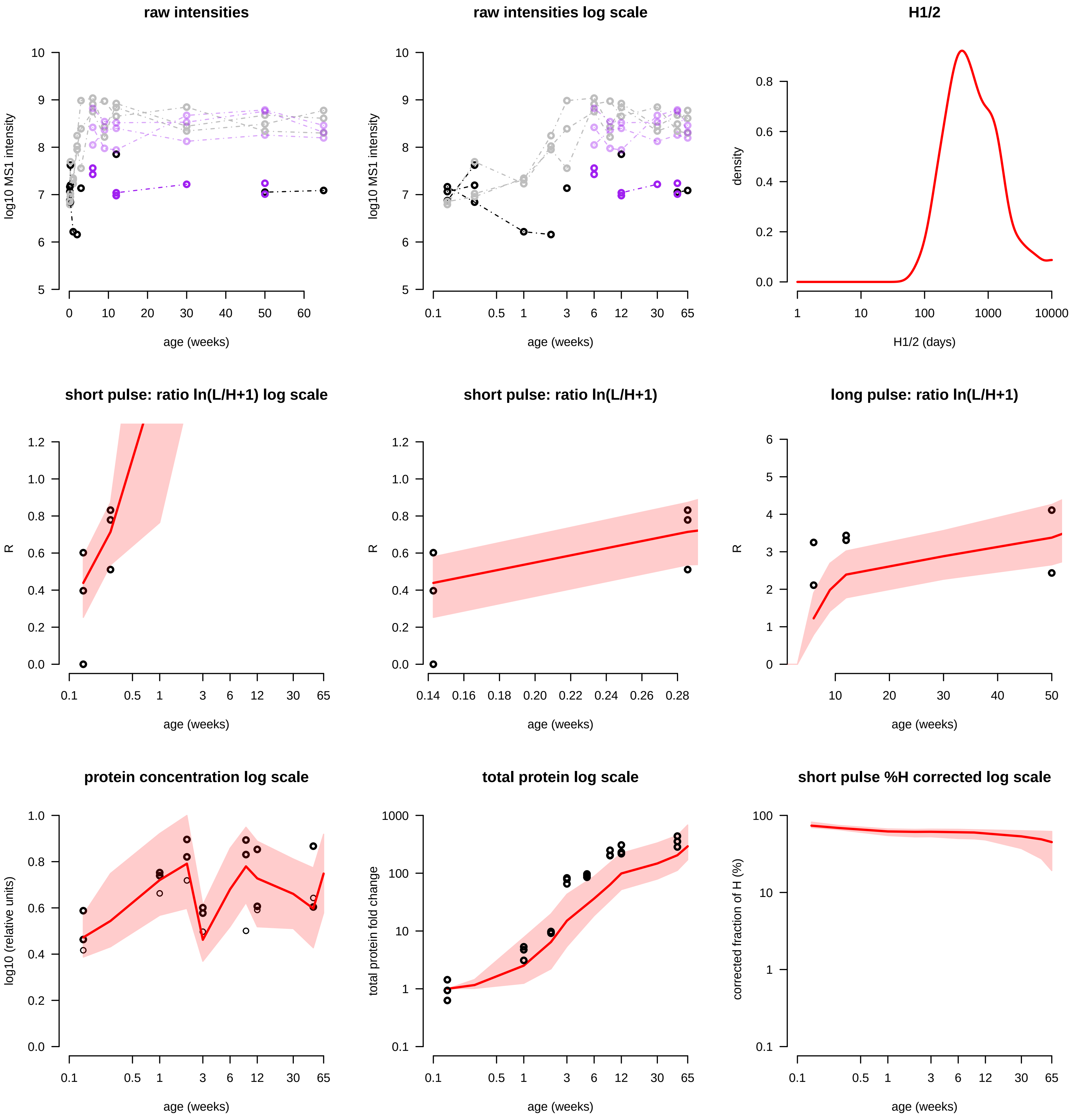

Q64337

>sp|Q64337|SQSTM_MOUSE

Sequestosome−1

GN=Sqstm1

Med H1/2: 6.7 days

Q5 H1/2: 2.6 days

Q95 H1/2: 48 days

reliable model fit: yes

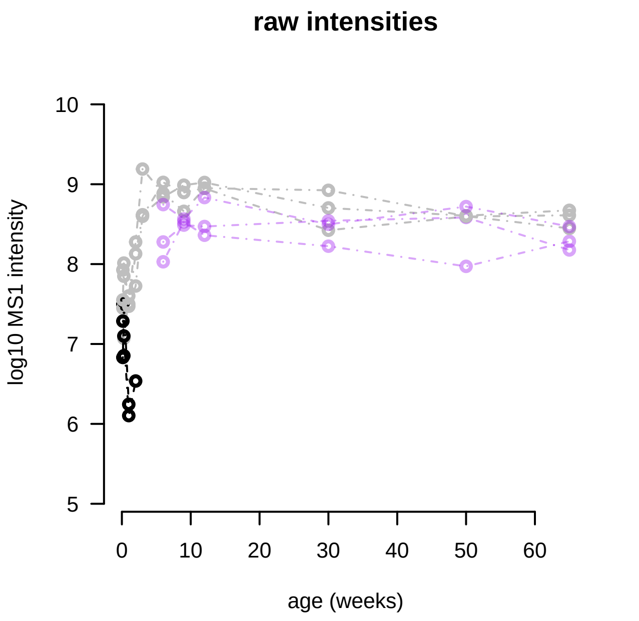

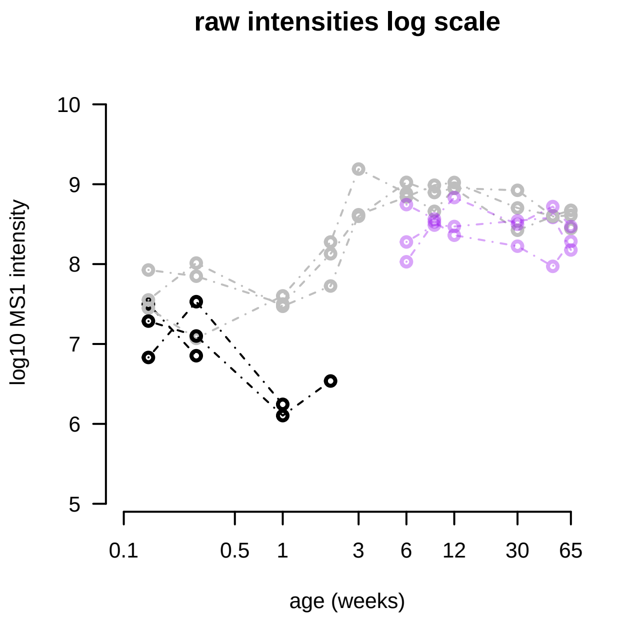

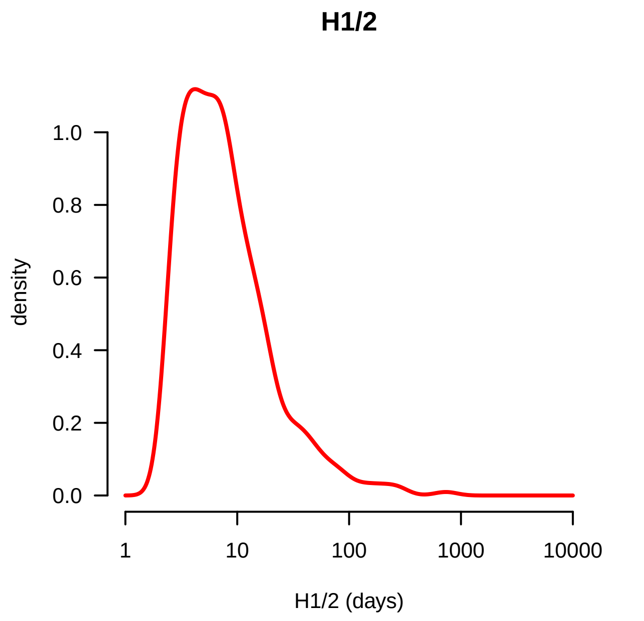

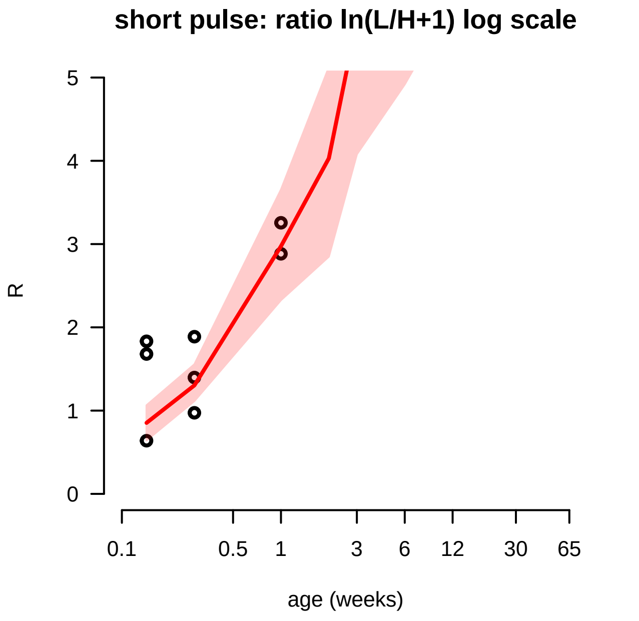

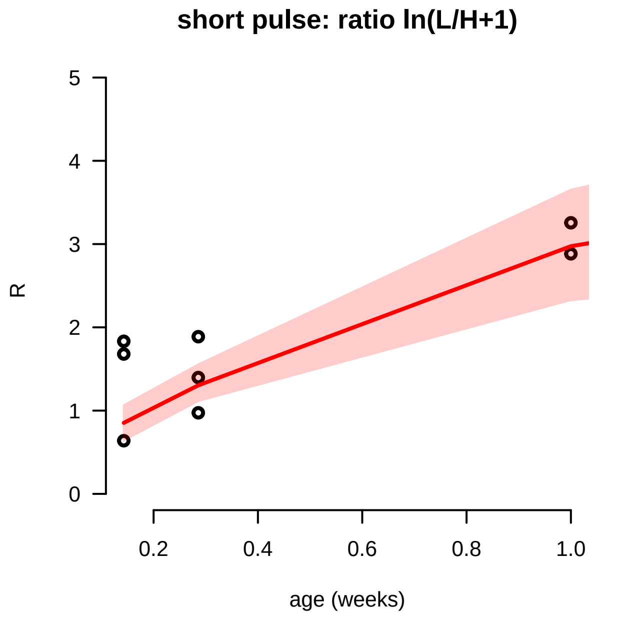

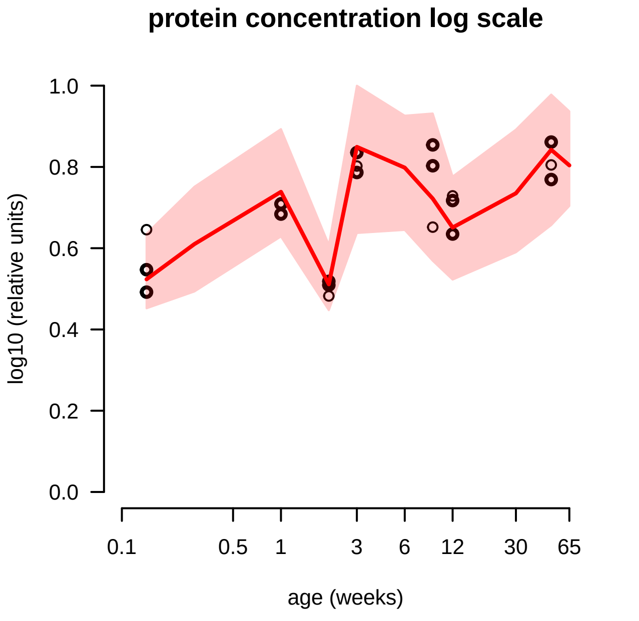

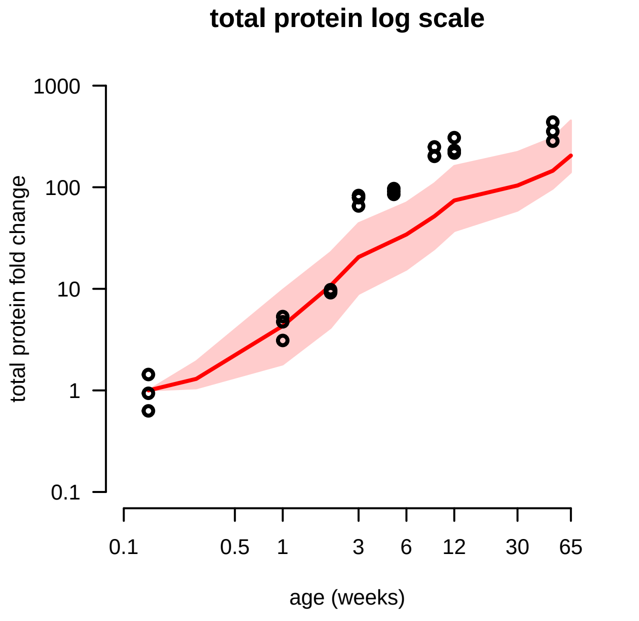

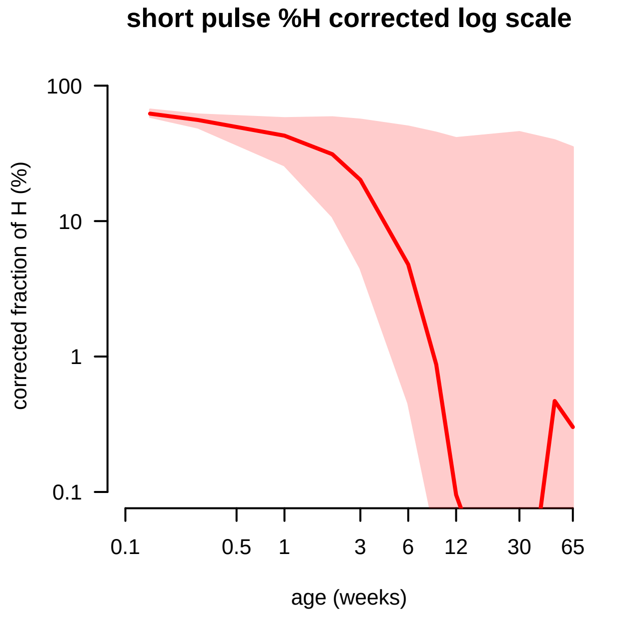

Q8K003

>sp|Q8K003|TMA7_MOUSE

Translation machinery−associated protein 7

GN=Tma7

Med H1/2: 8 days

Q5 H1/2: 6.3 days

Q95 H1/2: 10.2 days

reliable model fit: yes

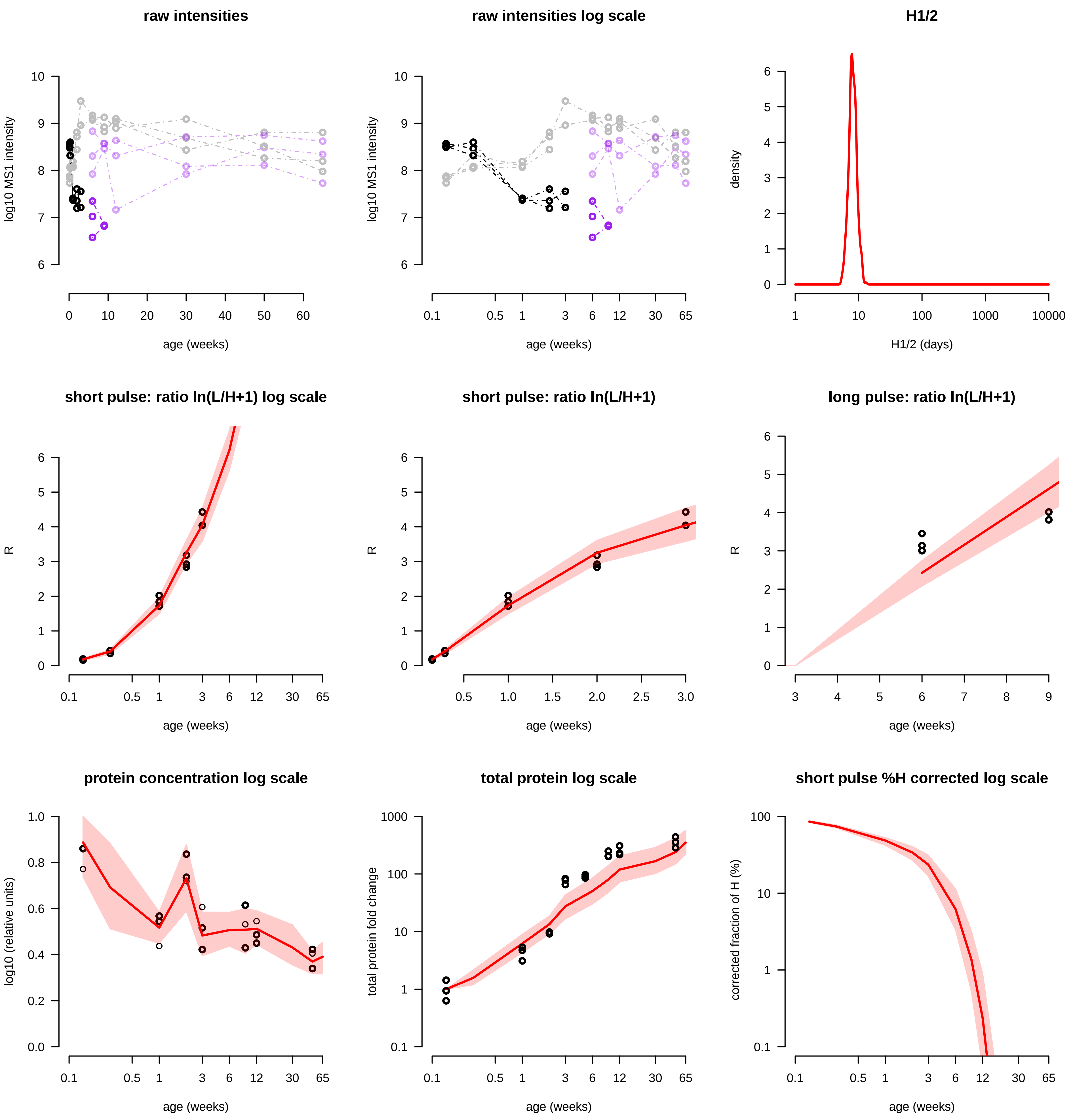

Q9D906

>sp|Q9D906|ATG7_MOUSE

Ubiquitin−like modifier−activating enzyme ATG7

GN=Atg7

Med H1/2: 5.1 days

Q5 H1/2: 2.7 days

Q95 H1/2: 28 days

reliable model fit: yes

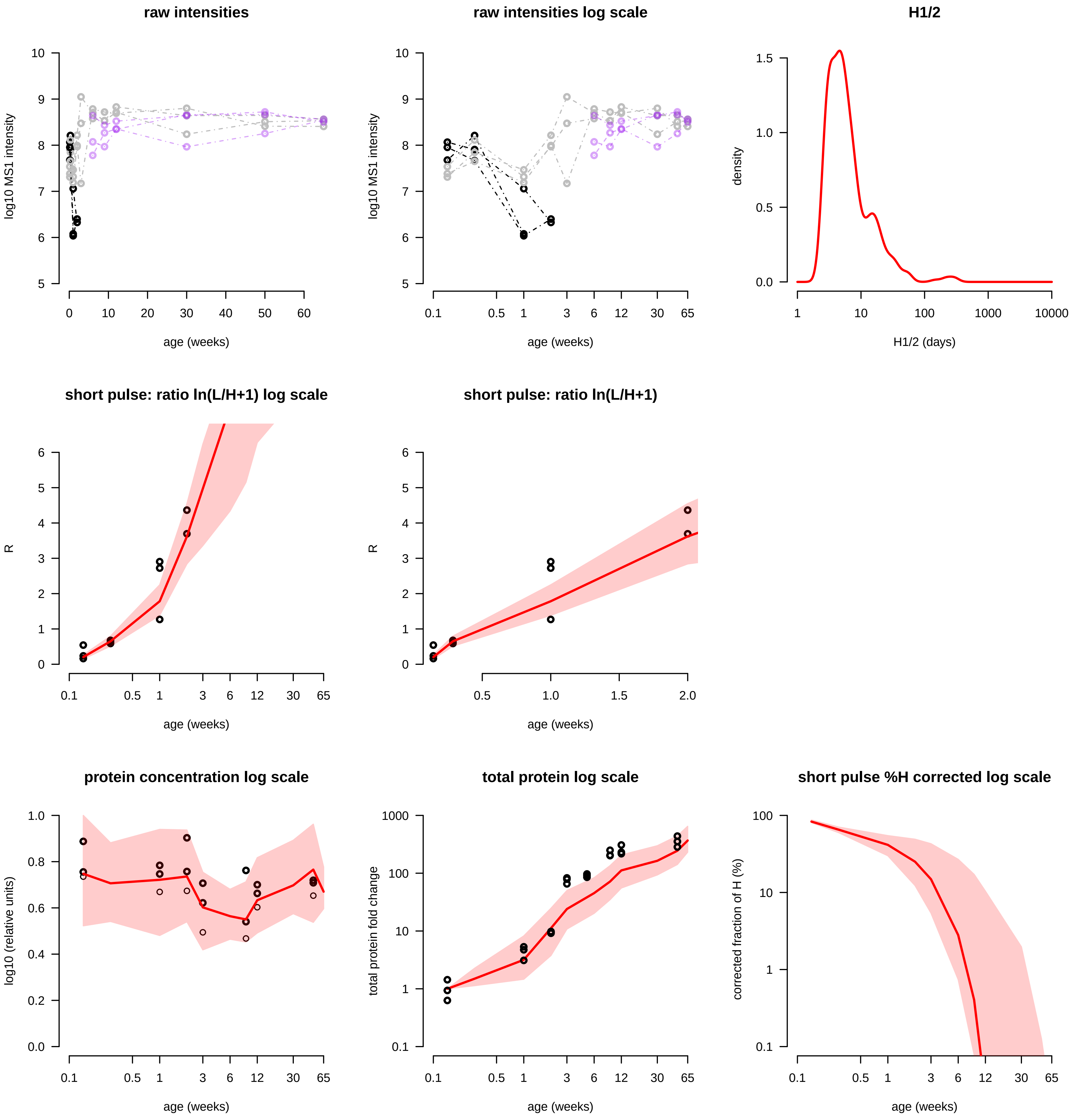

Q9D4H1

>sp|Q9D4H1|EXOC2_MOUSE

Exocyst complex component 2

GN=Exoc2

Med H1/2: 27.3 days

Q5 H1/2: 7.1 days

Q95 H1/2: 410.5 days

reliable model fit: no

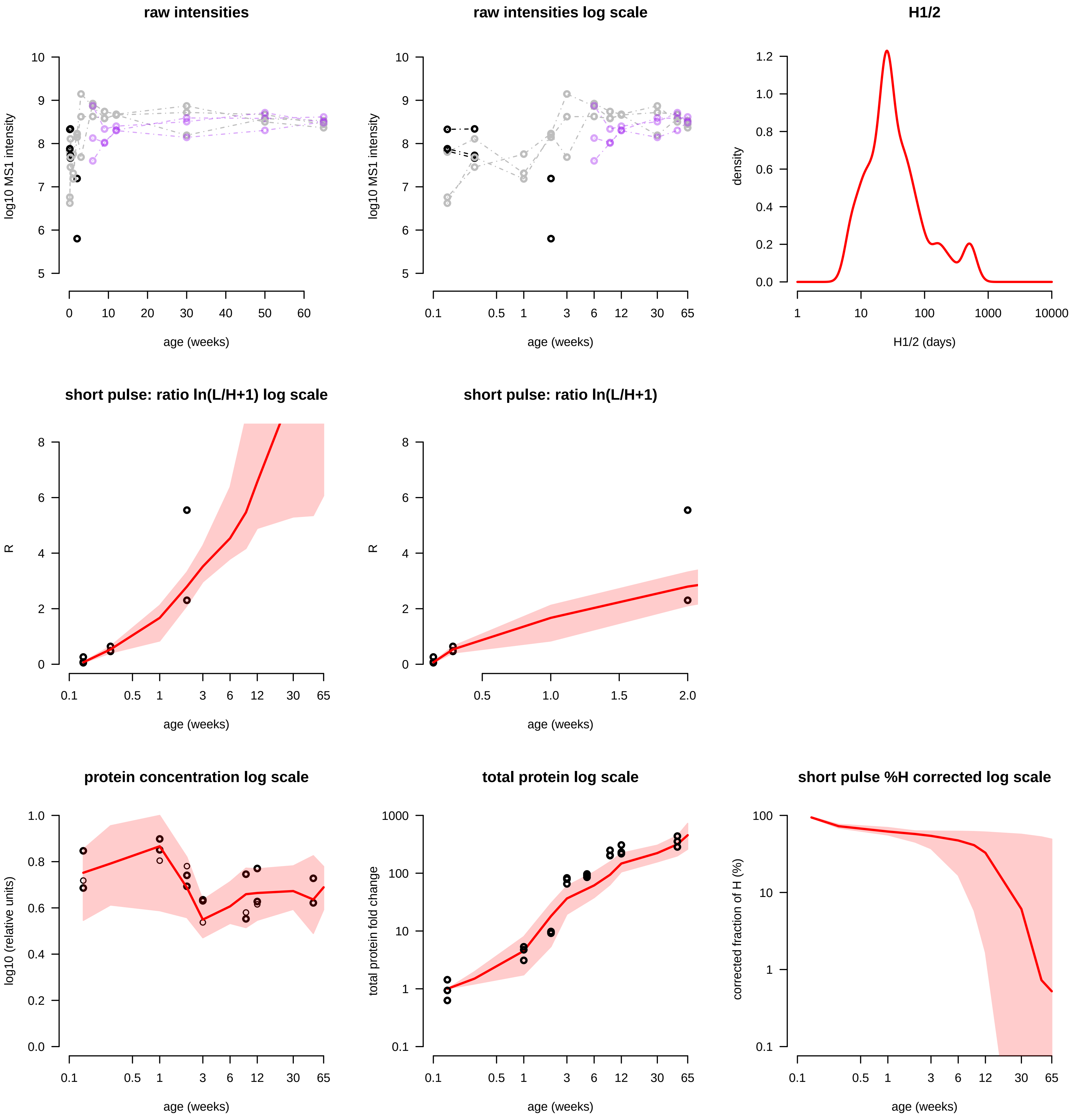

Q0P678

>sp|Q0P678|ZCH18_MOUSE

Zinc finger CCCH domain−containing protein 18

GN=Zc3h18

Med H1/2: 5.8 days

Q5 H1/2: 2.7 days

Q95 H1/2: 25.8 days

reliable model fit: yes

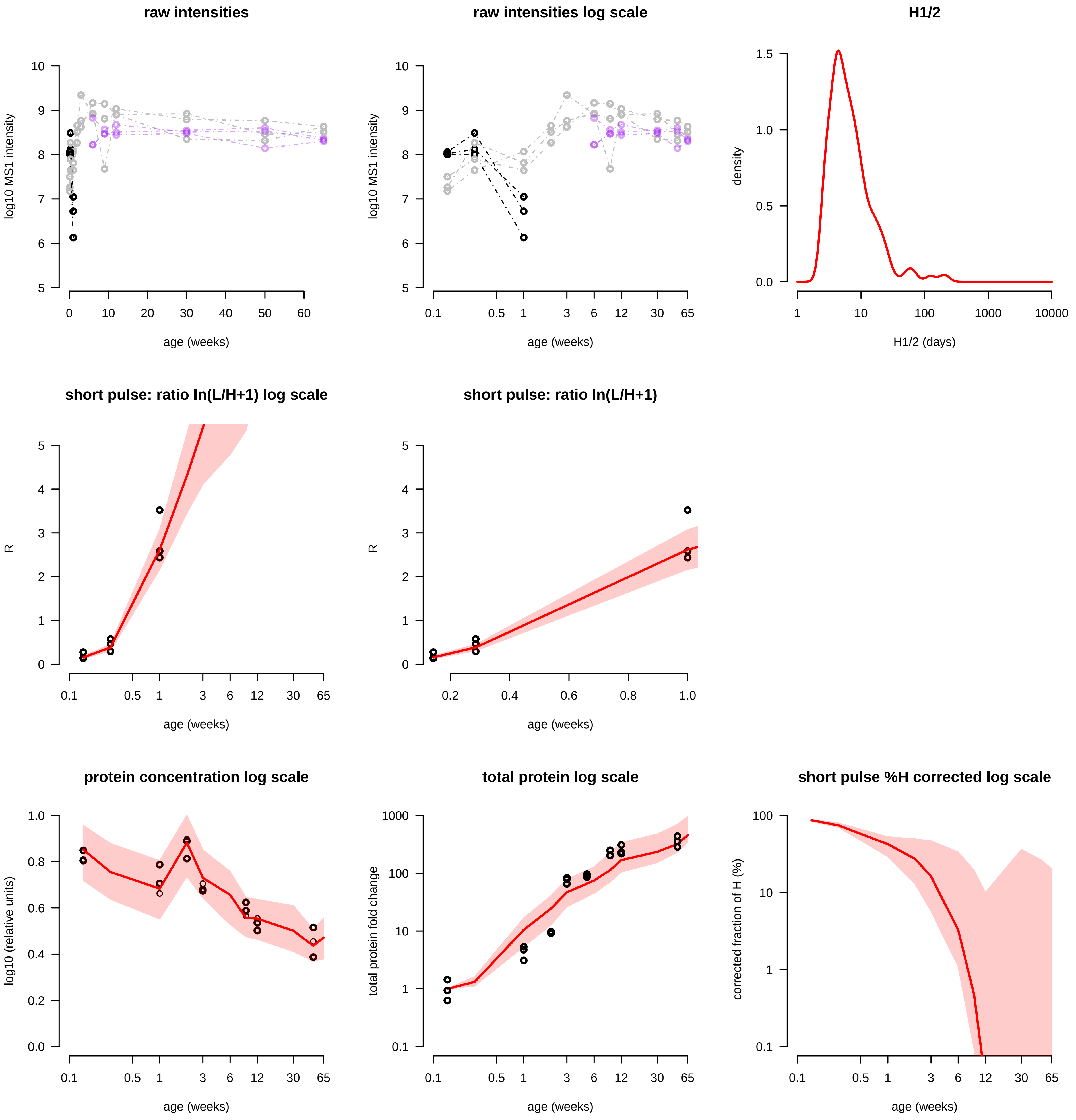

P62862

>sp|P62862|RS30_MOUSE

40S ribosomal protein S30

GN=Fau

Med H1/2: 9.2 days

Q5 H1/2: 3.9 days

Q95 H1/2: 104.6 days

reliable model fit: yes

raw intensities

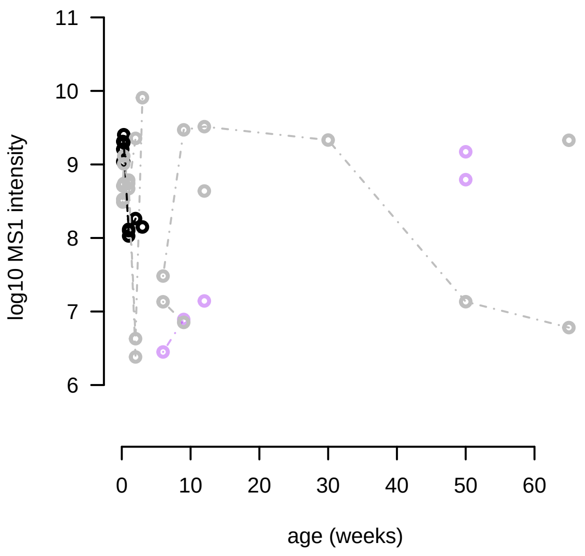

raw intensities log scale

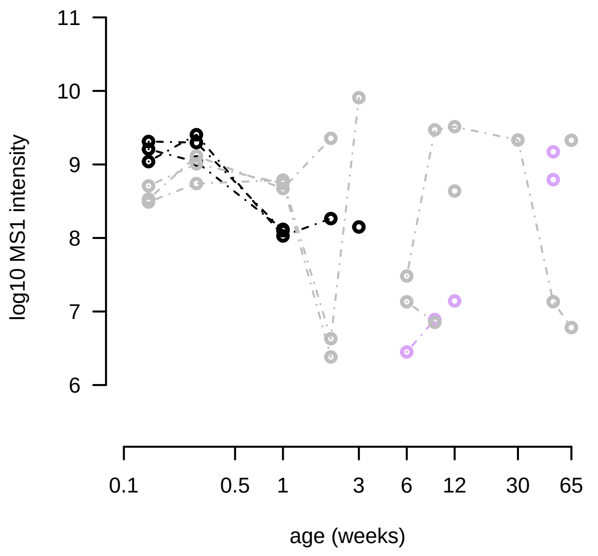

H1/2

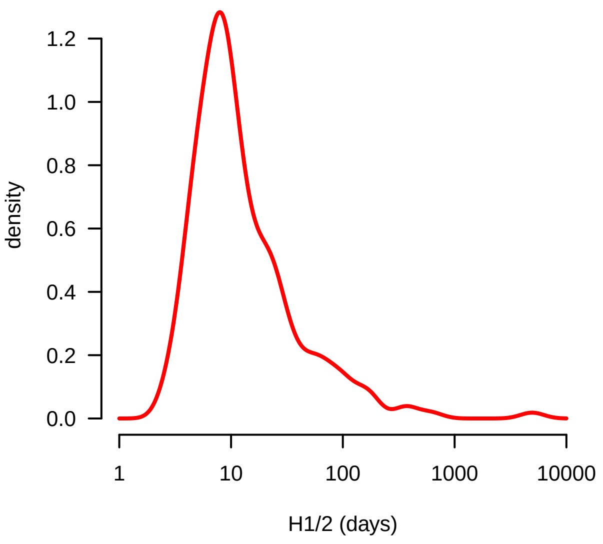

short pulse: ratio ln(L/H+1) log scale

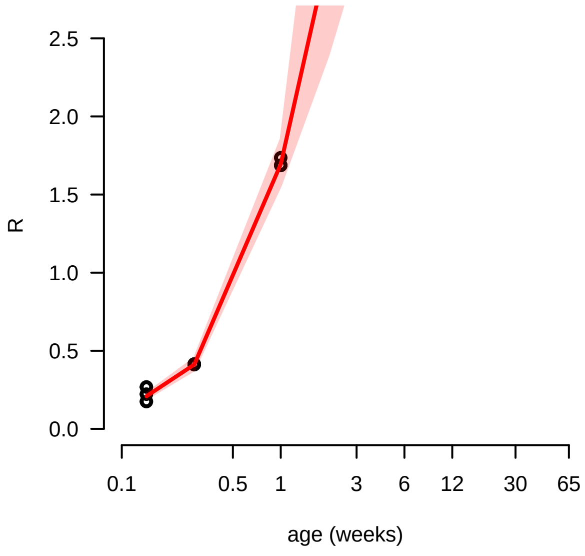

short pulse: ratio ln(L/H+1)

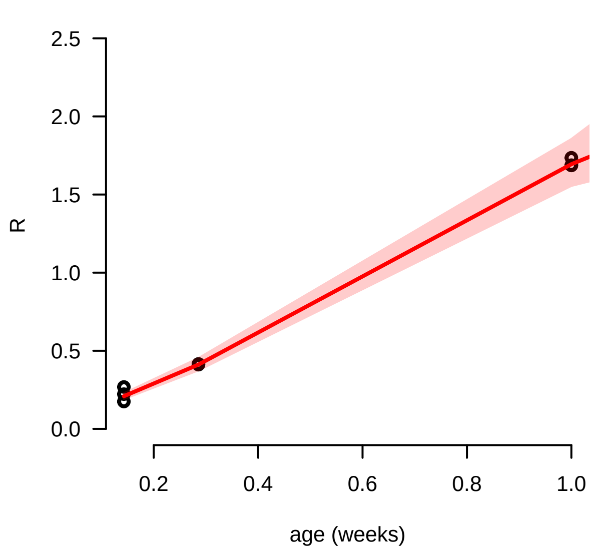

protein concentration log scale

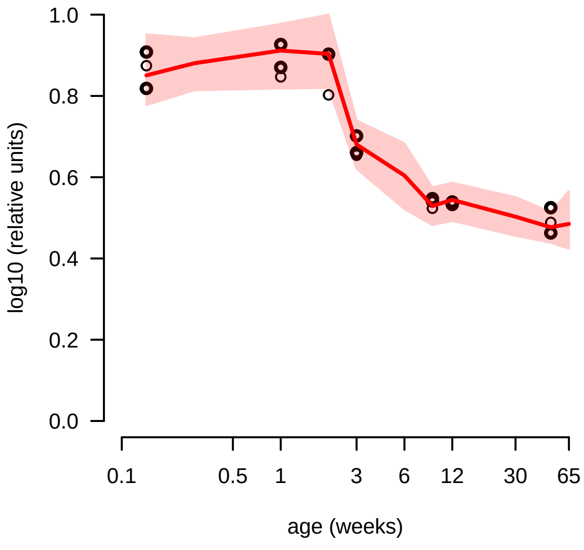

total protein log scale

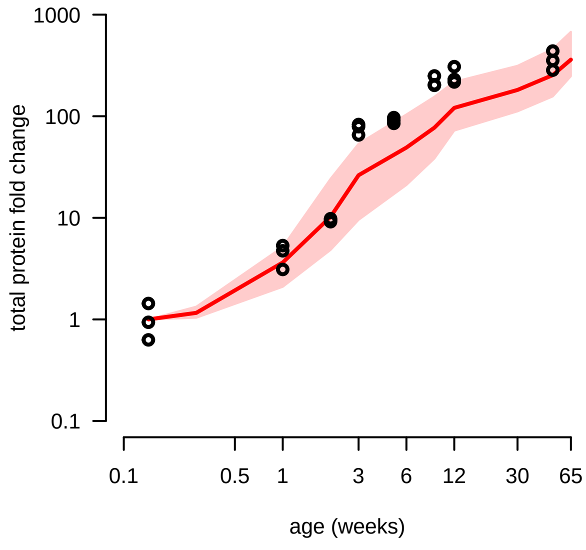

short pulse %H corrected log scale

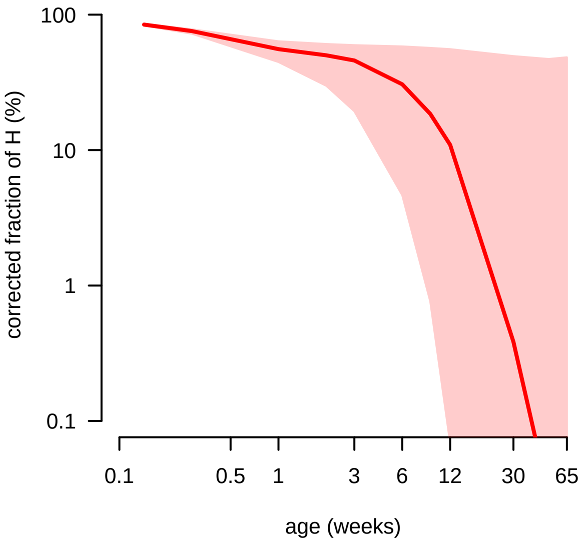

Q9CW46

>sp|Q9CW46|RAVR1_MOUSE

Ribonucleoprotein PTB−binding 1

GN=Raver1

Med H1/2: 4.4 days

Q5 H1/2: 2.6 days

Q95 H1/2: 25.4 days

reliable model fit: yes

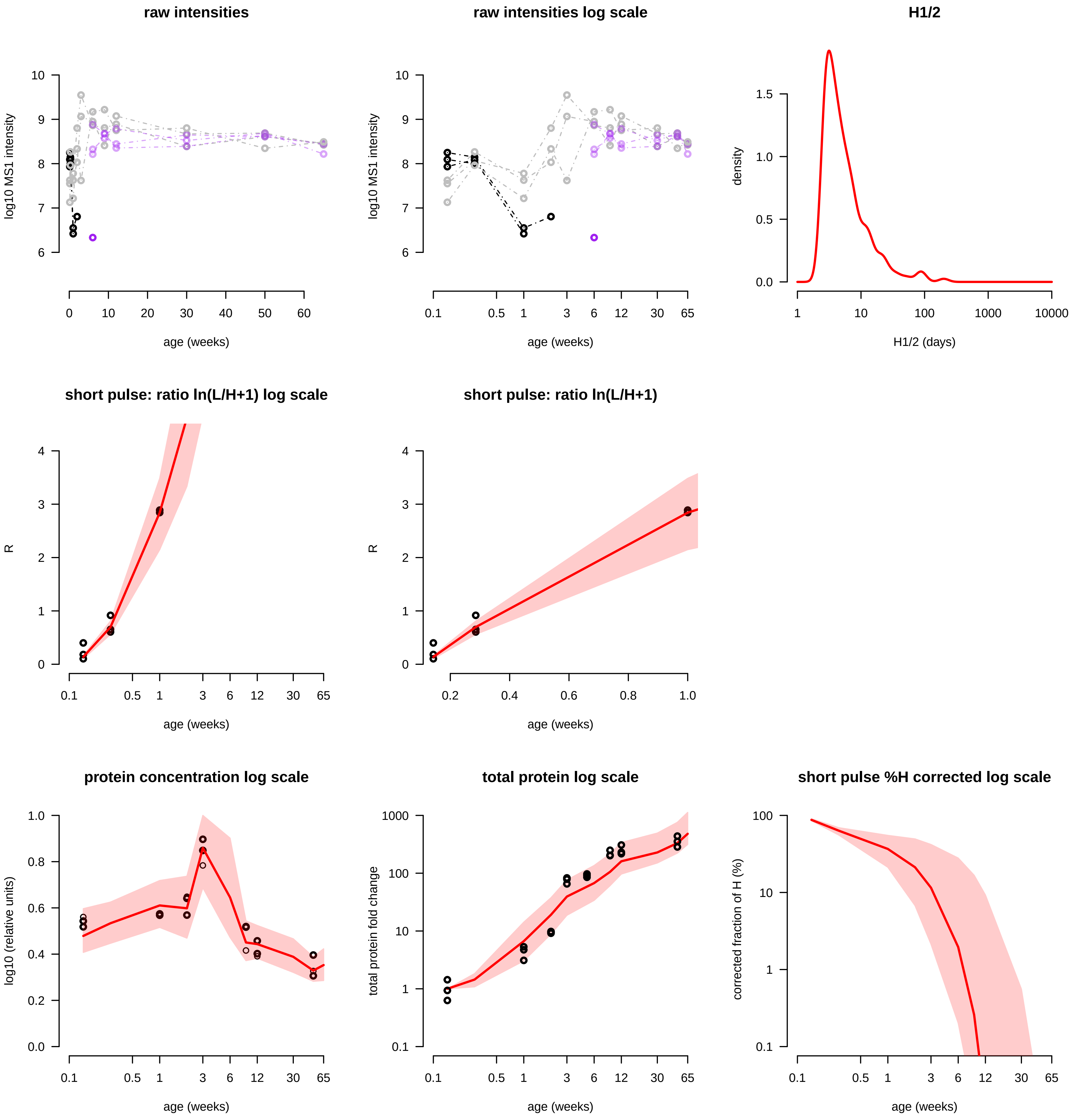

P84099

>sp|P84099|RL19_MOUSE

60S ribosomal protein L19

GN=Rpl19

Med H1/2: 270.8 days

Q5 H1/2: 127.6 days

Q95 H1/2: 1051.7 days

reliable model fit: yes

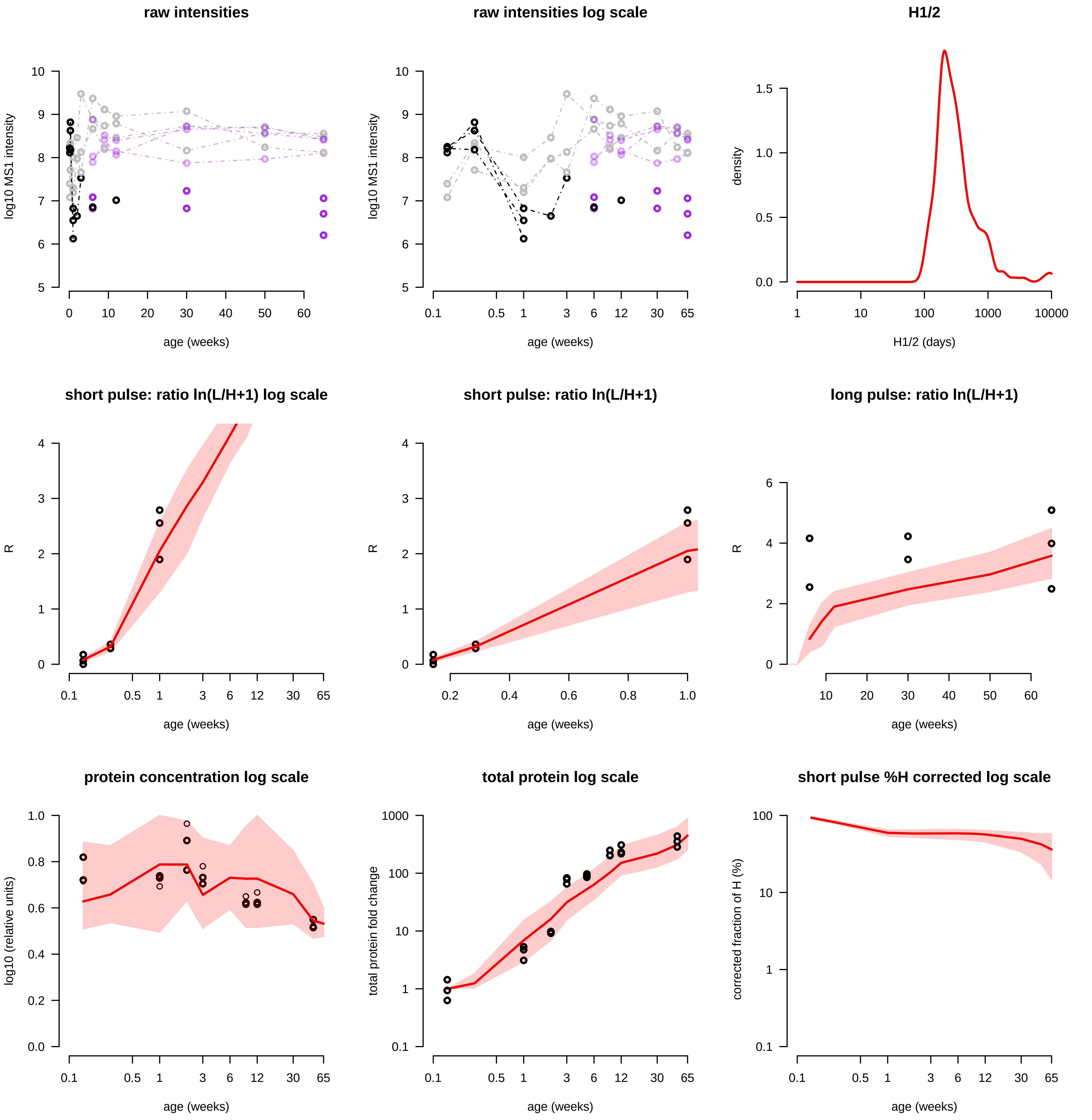

Q8CDG3;Q8CDG3−2

>sp|Q8CDG3|VCIP1_MOUSE

Deubiquitinating protein VCPIP1

GN=Vcpip1

Med H1/2: 1512.3 days

Q5 H1/2: 469 days

Q95 H1/2: 14942.1 days

reliable model fit: yes

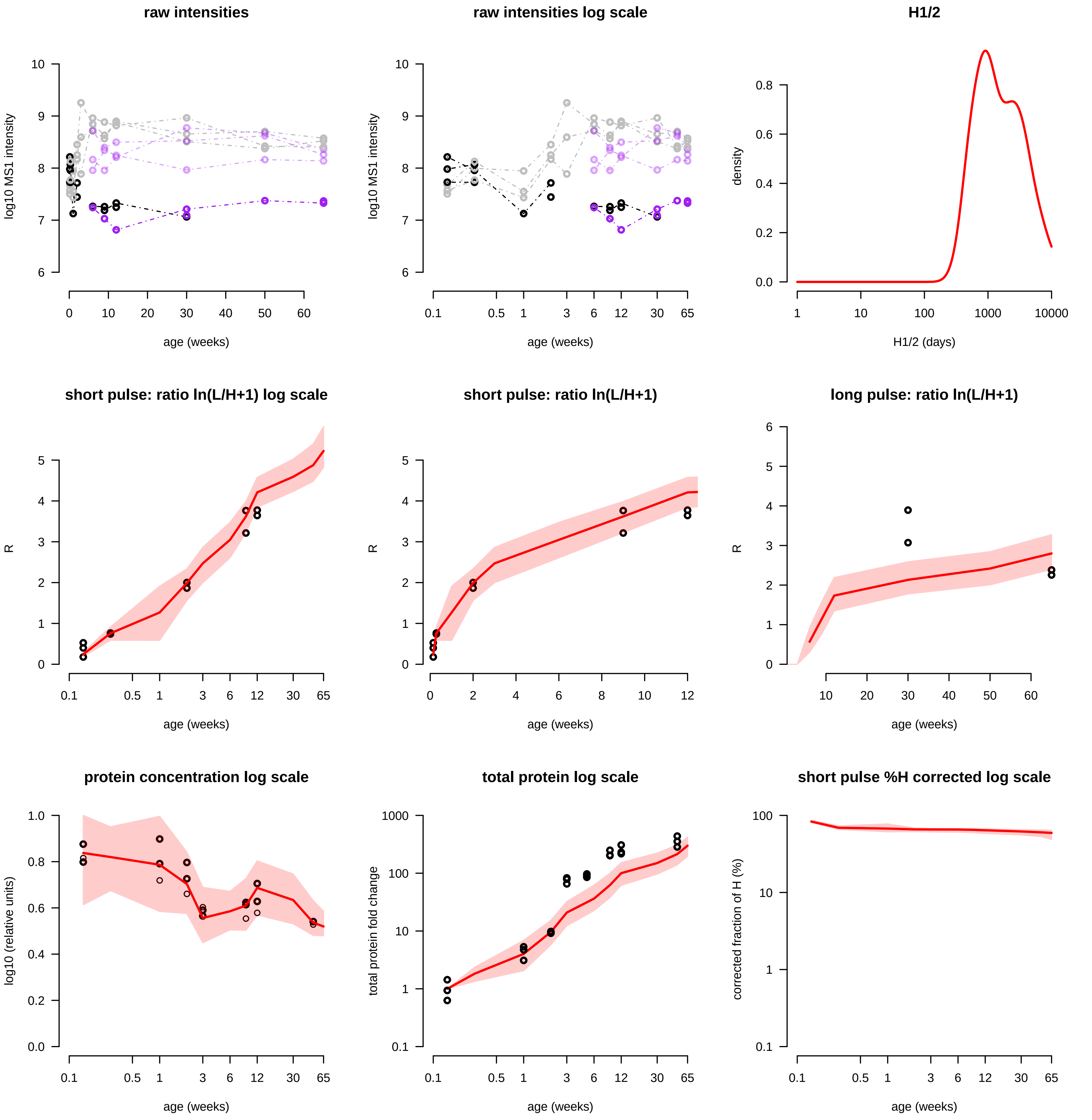

Q8BH58

>sp|Q8BH58|TIPRL_MOUSE

TIP41−like protein

GN=Tiprl

Med H1/2: 18.1 days

Q5 H1/2: 6.7 days

Q95 H1/2: 168 days

reliable model fit: yes

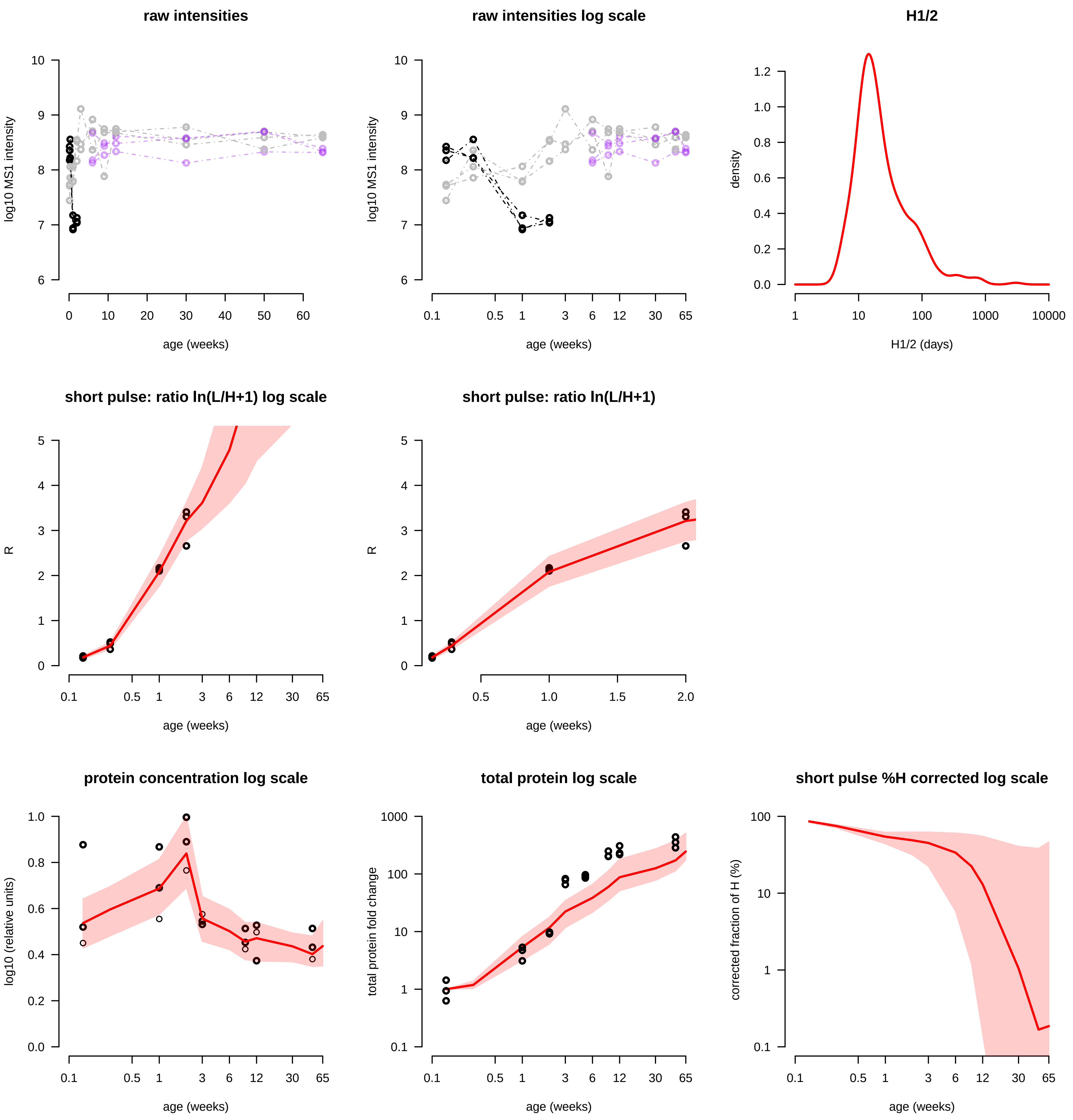

Q6NVF9

>sp|Q6NVF9|CPSF6_MOUSE

Cleavage and polyadenylation specificity factor subunit 6

GN=Cpsf6

Med H1/2: 6.3 days

Q5 H1/2: 5.1 days

Q95 H1/2: 9.3 days

reliable model fit: yes

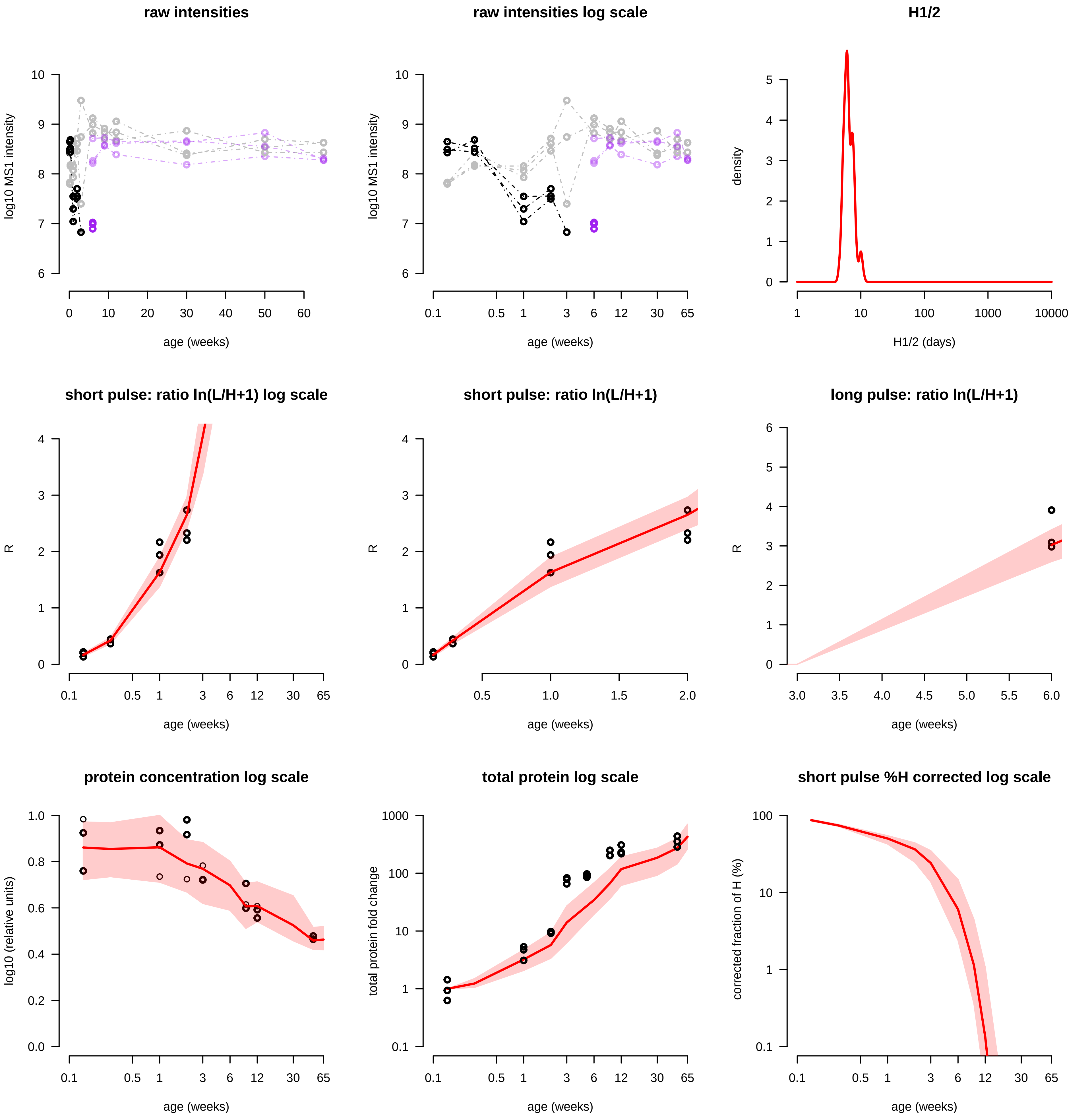

Q78IS1

>sp|Q78IS1|TMED3_MOUSE

Transmembrane emp24 domain−containing protein 3

GN=Tmed3

Med H1/2: 6 days

Q5 H1/2: 4.9 days

Q95 H1/2: 8.3 days

reliable model fit: yes

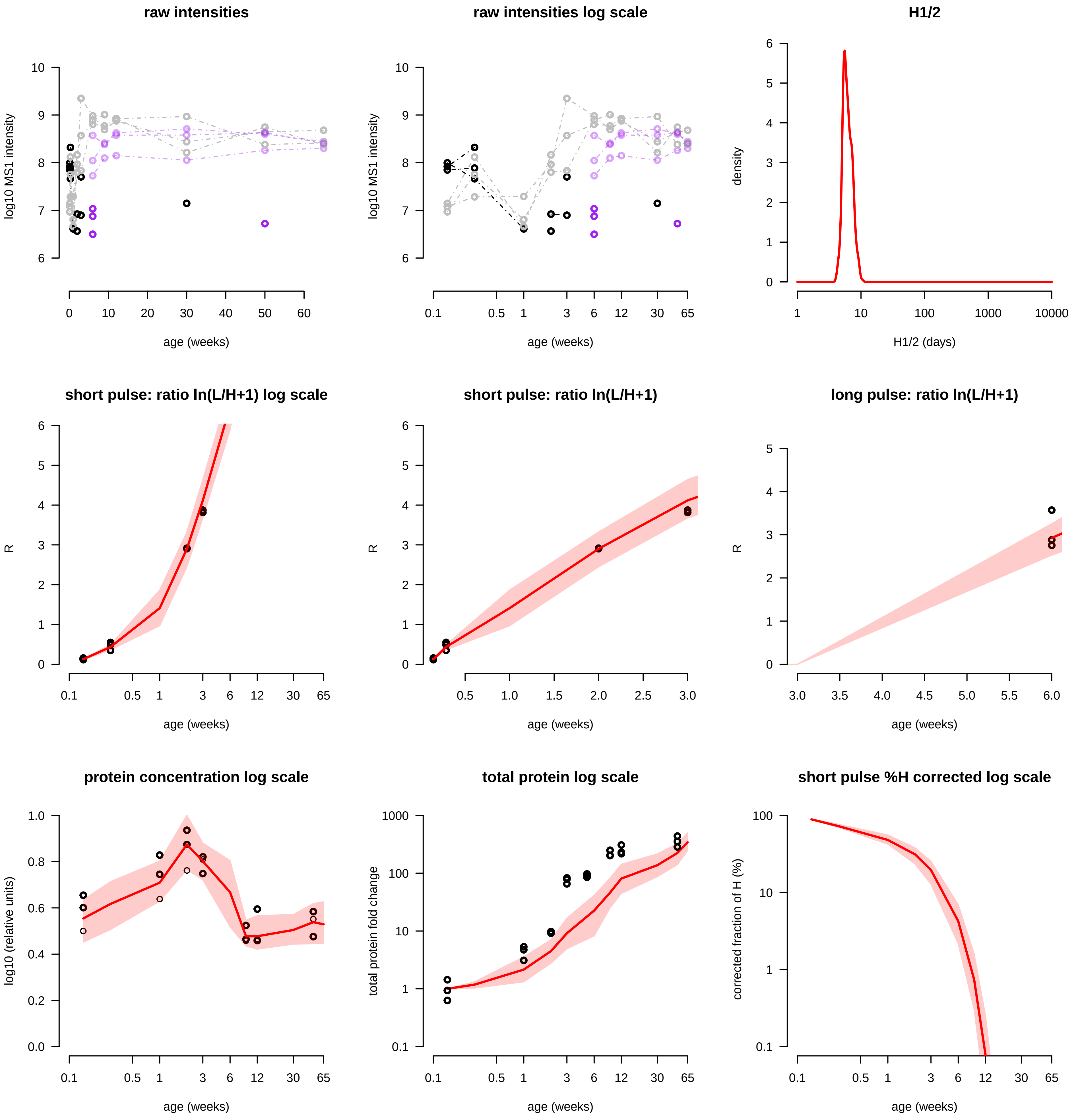

O70591

>sp|O70591|PFD2_MOUSE

Prefoldin subunit 2

GN=Pfdn2

Med H1/2: 6.7 days

Q5 H1/2: 2.8 days

Q95 H1/2: 110.4 days

reliable model fit: yes

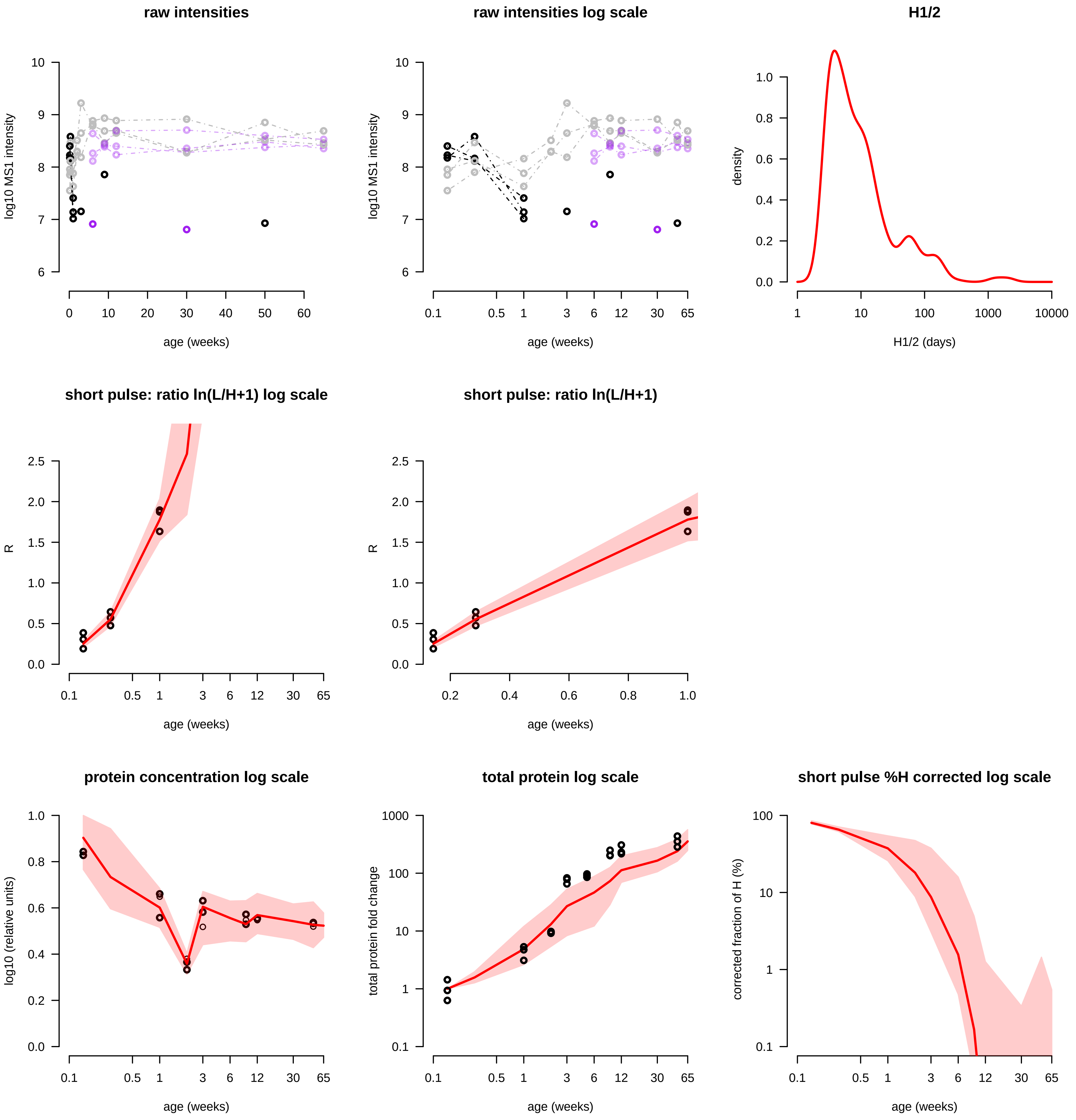

Q99M51

>sp|Q99M51|NCK1_MOUSE

Cytoplasmic protein NCK1

GN=Nck1

Med H1/2: 4.2 days

Q5 H1/2: 2.6 days

Q95 H1/2: 22.8 days

reliable model fit: yes

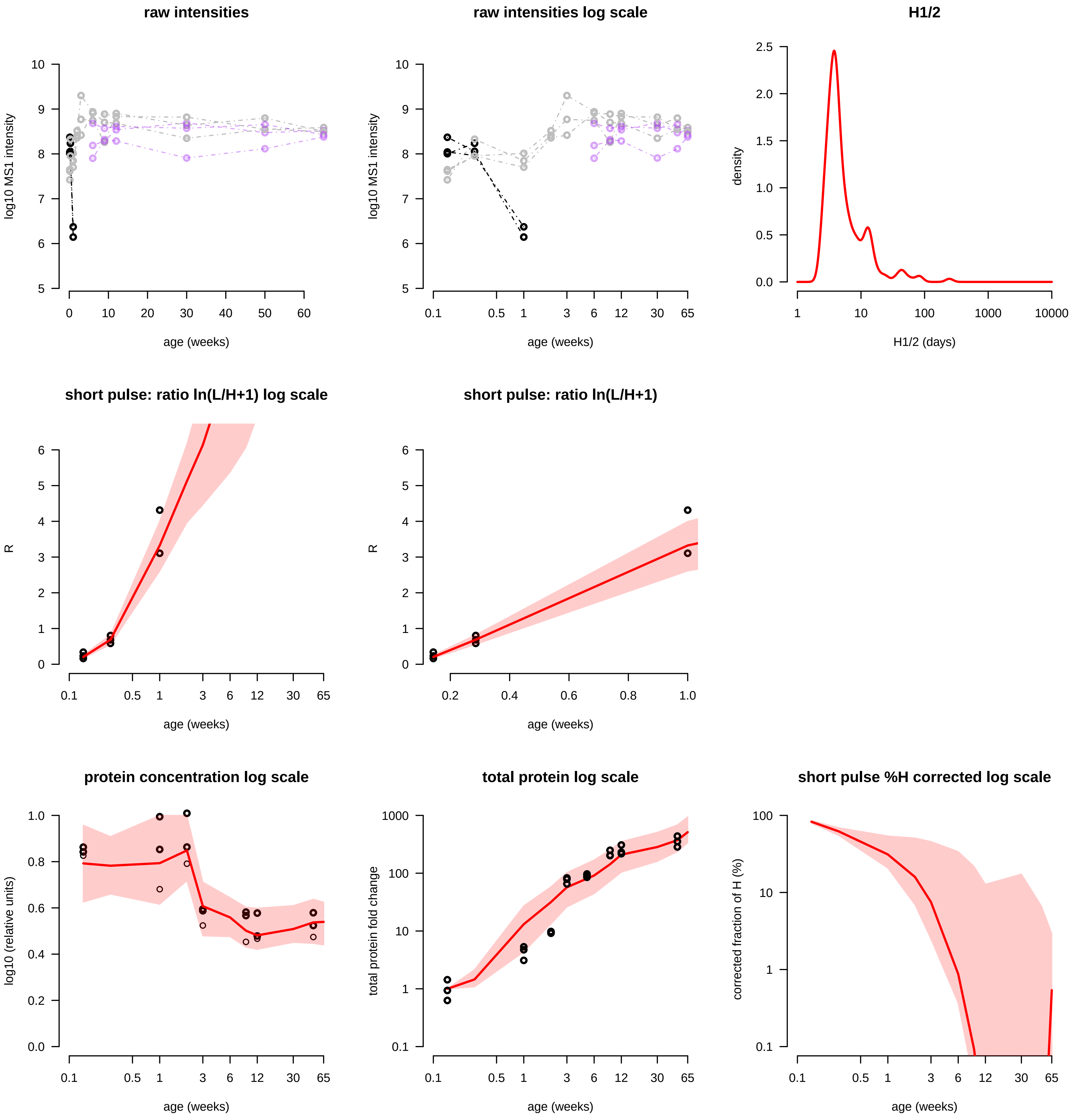

P61759

>sp|P61759|PFD3_MOUSE

Prefoldin subunit 3

GN=Vbp1

Med H1/2: 11.5 days

Q5 H1/2: 5.4 days

Q95 H1/2: 92.6 days

reliable model fit: yes

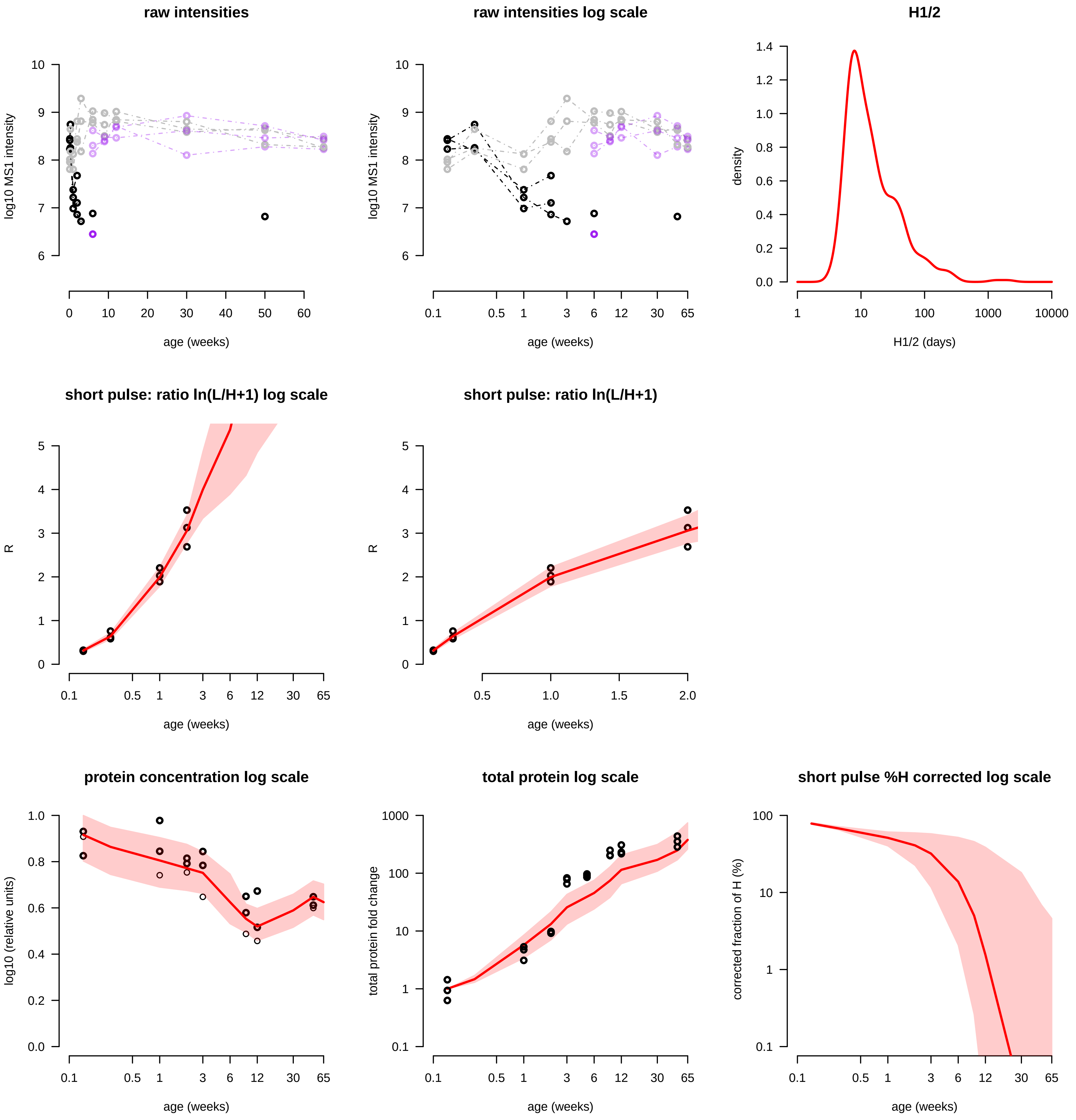

P15327

>sp|P15327|PMGE_MOUSE

Bisphosphoglycerate mutase

GN=Bpgm

Med H1/2: 13.1 days

Q5 H1/2: 8.6 days

Q95 H1/2: 20.5 days

reliable model fit: yes

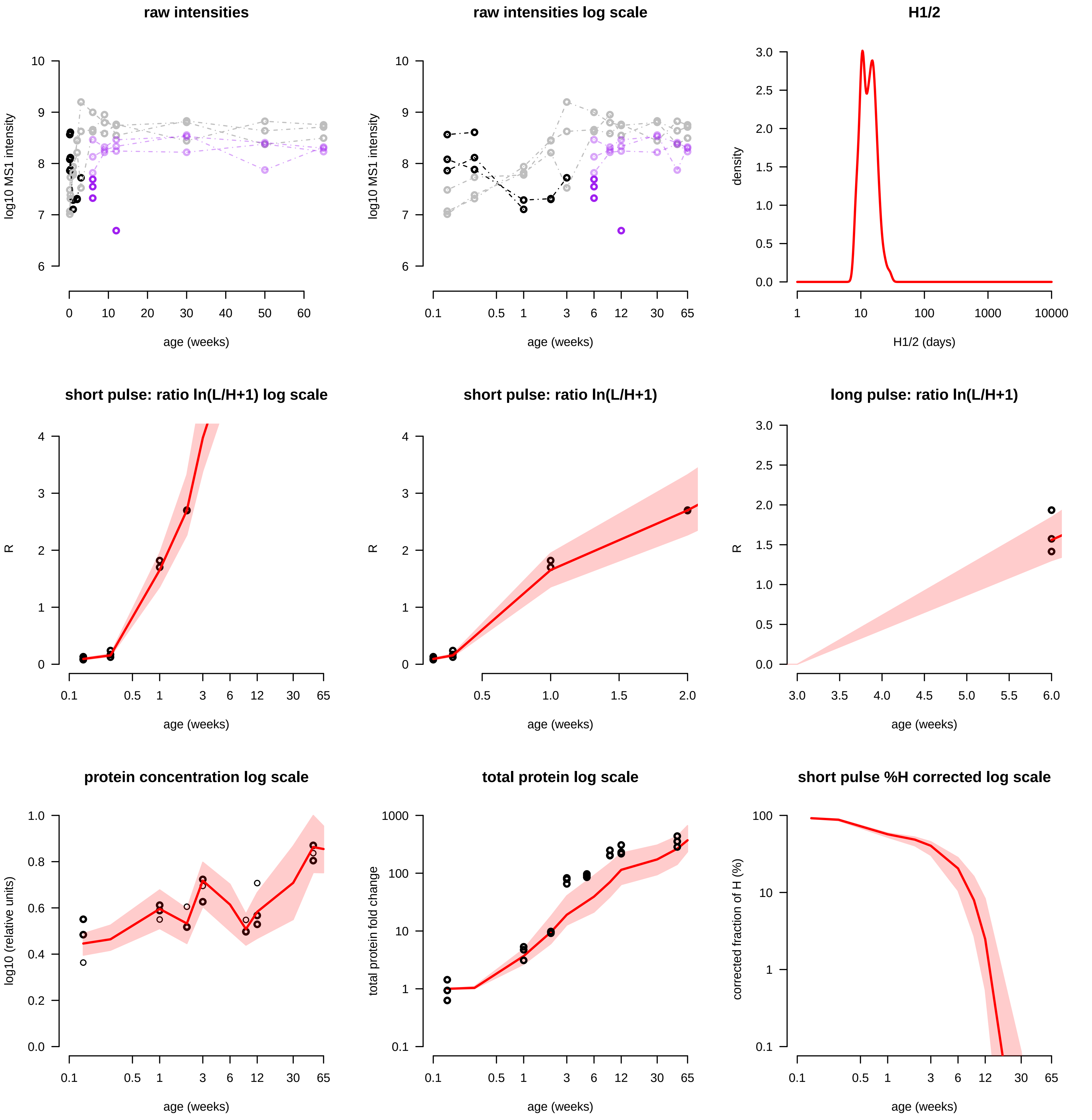

P38060

>sp|P38060|HMGCL_MOUSE

Hydroxymethylglutaryl−CoA lyase, mitochondrial

GN=Hmgcl

Med H1/2: 10.8 days

Q5 H1/2: 7.7 days

Q95 H1/2: 17.3 days

reliable model fit: yes

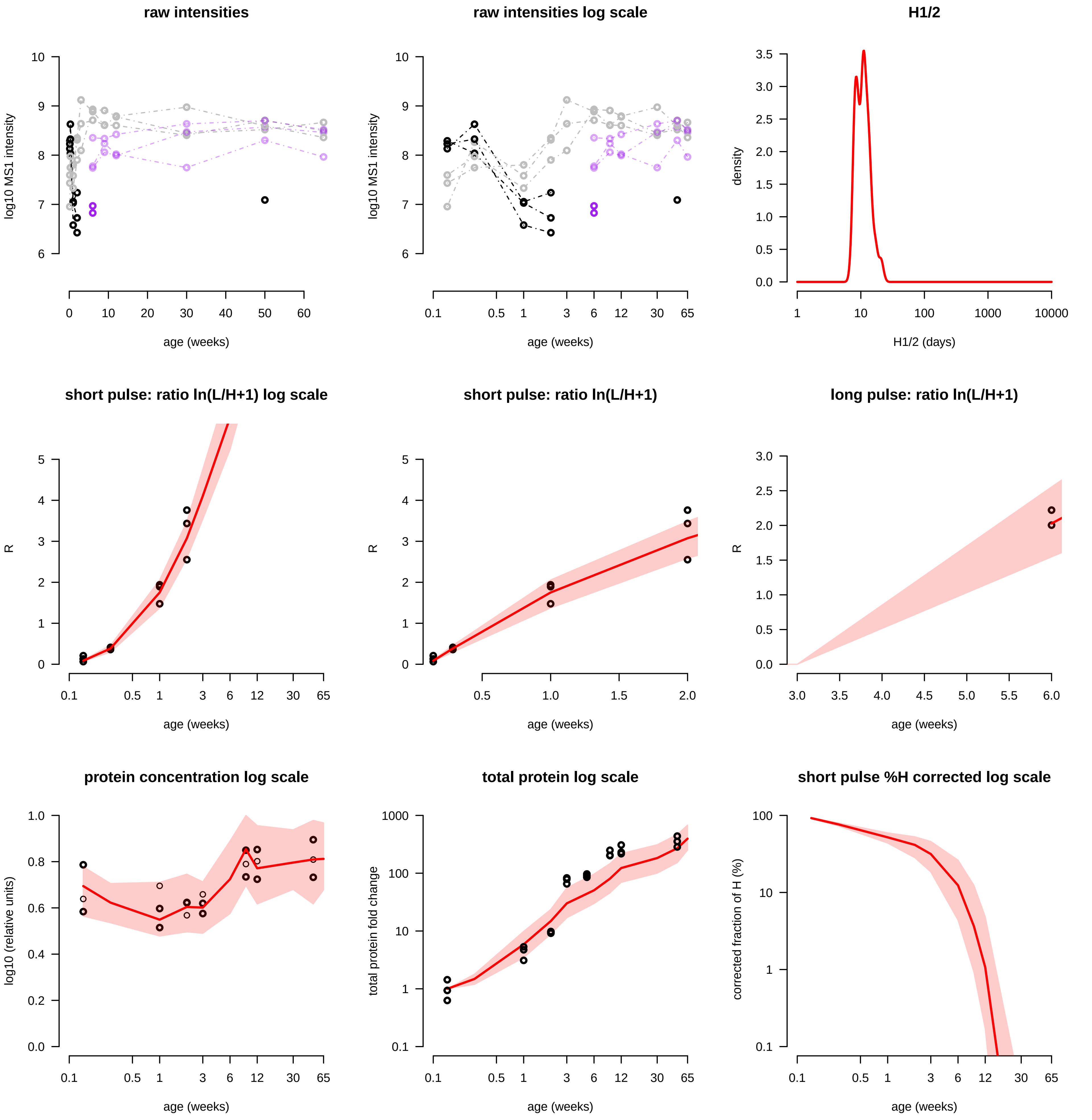

P70279

>sp|P70279|SURF6_MOUSE

Surfeit locus protein 6

GN=Surf6

Med H1/2: 681.5 days

Q5 H1/2: 152.3 days

Q95 H1/2: 10406.8 days

reliable model fit: yes

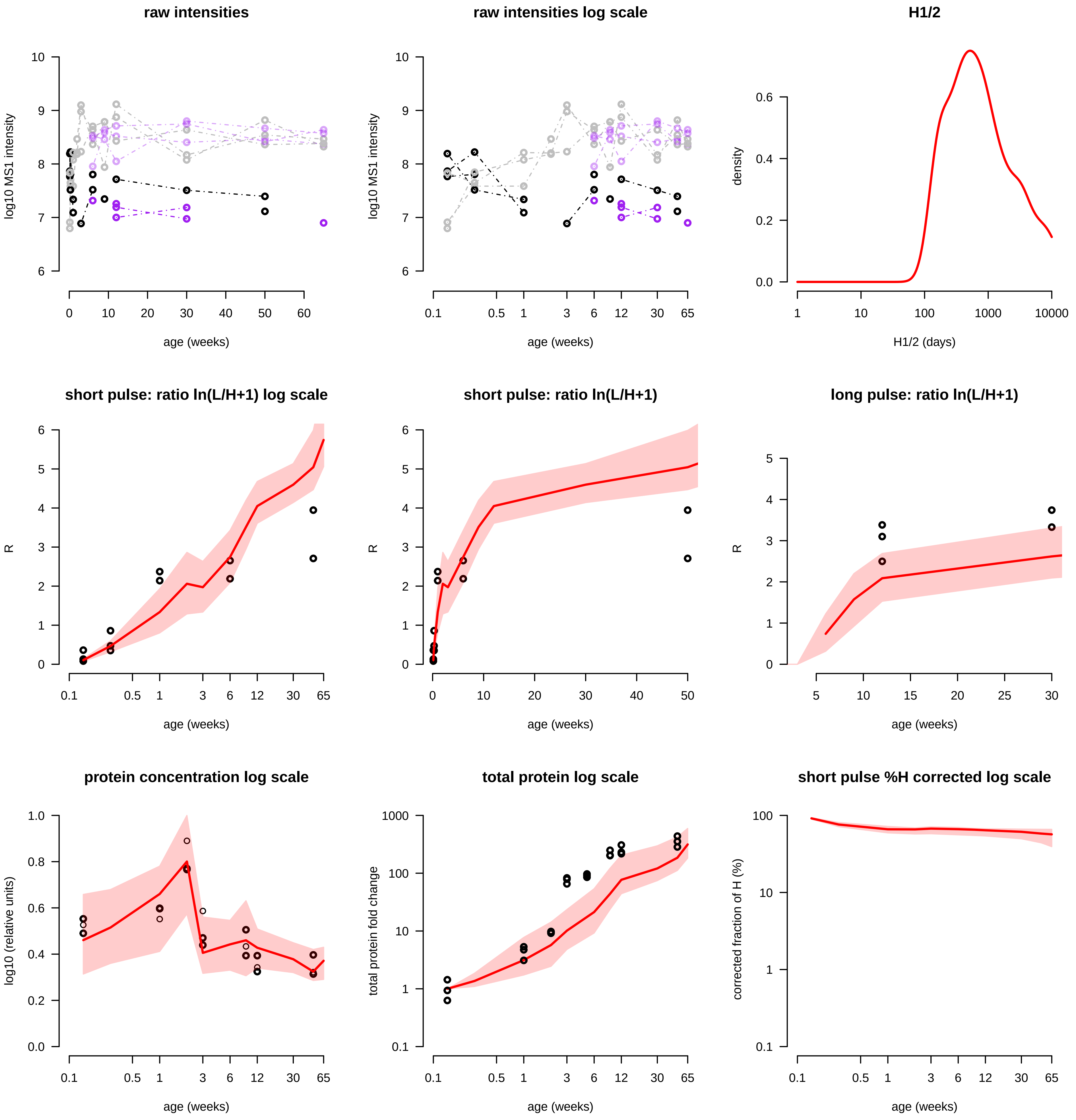

O70309;O70309−2

>sp|O70309|ITB5_MOUSE

Integrin beta−5

GN=Itgb5

Med H1/2: 2452.6 days

Q5 H1/2: 676.2 days

Q95 H1/2: 26466.7 days

reliable model fit: no

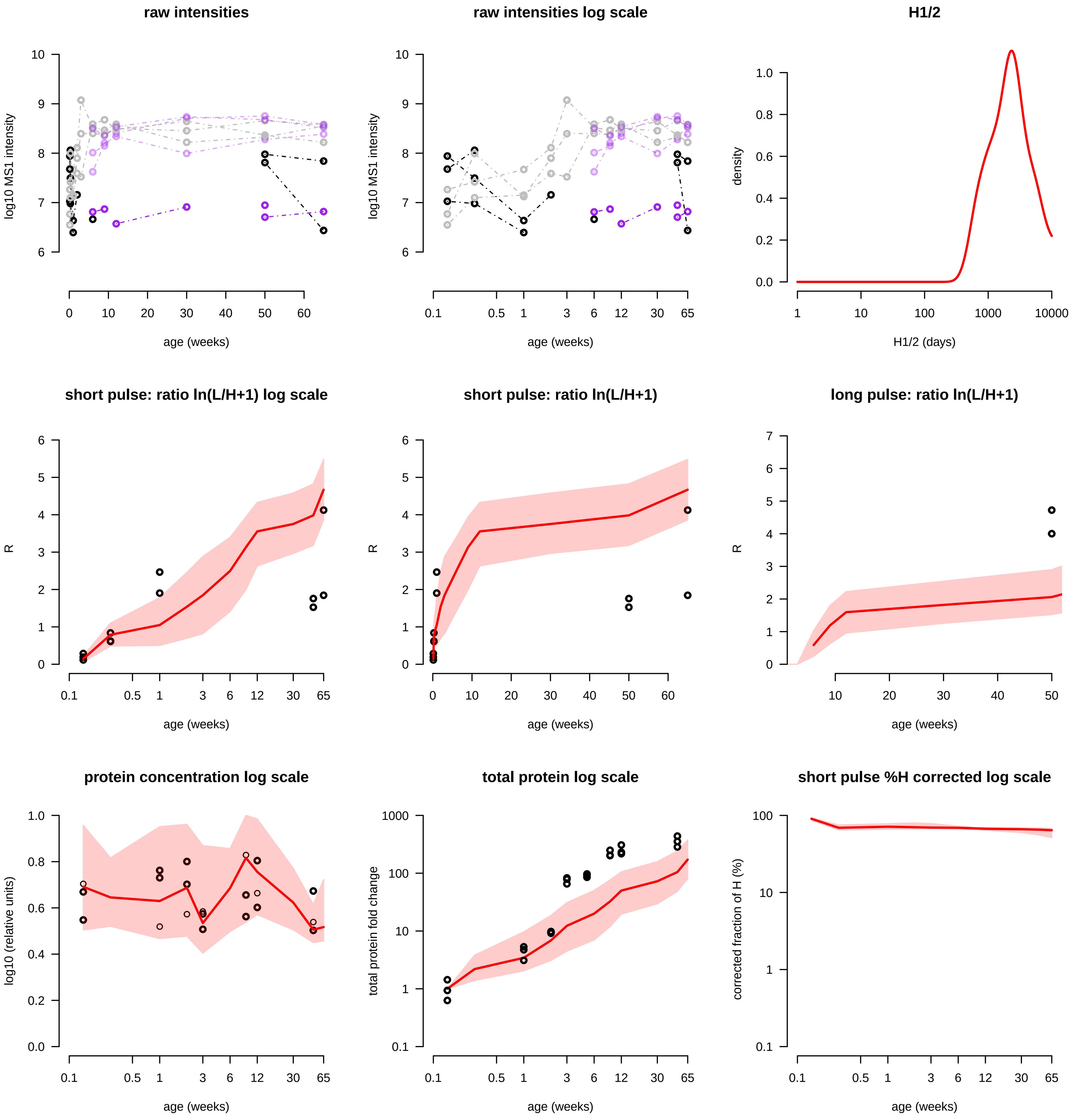

P30412

>sp|P30412|PPIC_MOUSE

Peptidyl−prolyl cis−trans isomerase C

GN=Ppic

Med H1/2: 4.9 days

Q5 H1/2: 4.1 days

Q95 H1/2: 6 days

reliable model fit: yes

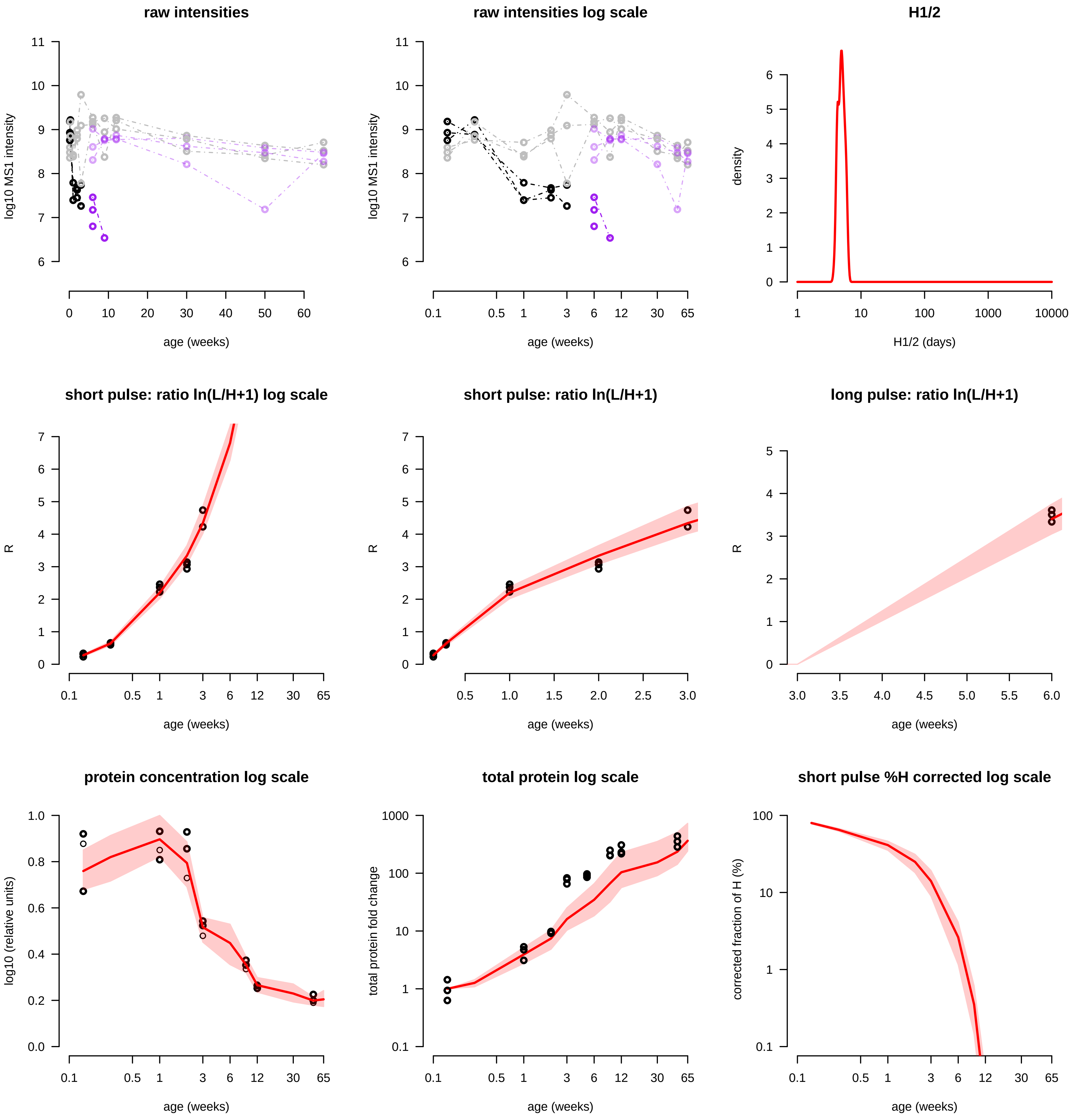

P59708

>sp|P59708|SF3B6_MOUSE

Splicing factor 3B subunit 6

GN=Sf3b6

Med H1/2: 5.2 days

Q5 H1/2: 3.9 days

Q95 H1/2: 7.7 days

reliable model fit: yes

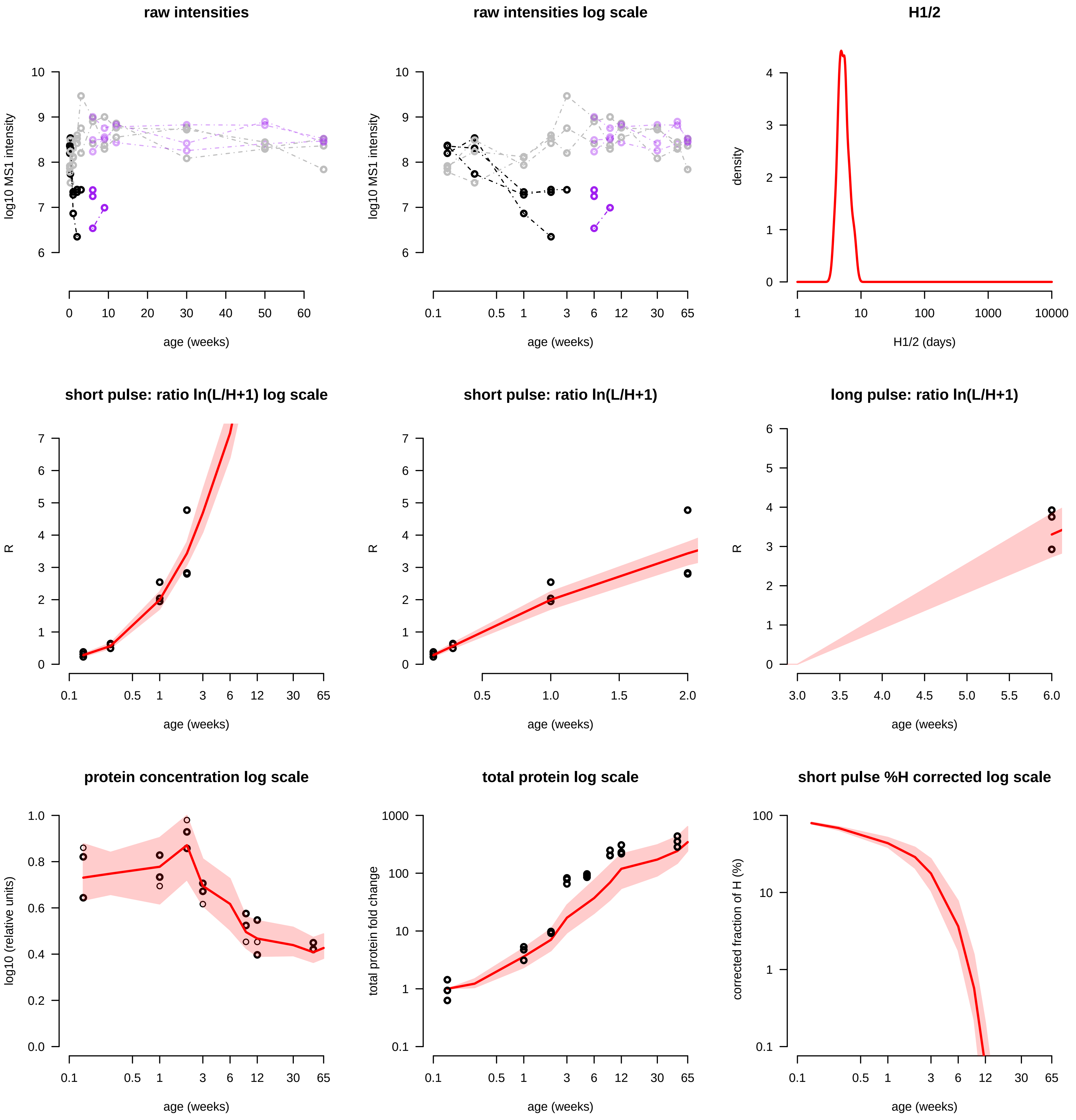

P51432

>sp|P51432|PLCB3_MOUSE

1−phosphatidylinositol 4,5−bisphosphate phosphodiesterase beta−3

GN=Plcb3

Med H1/2: 3.9 days

Q5 H1/2: 2.5 days

Q95 H1/2: 15 days

reliable model fit: yes

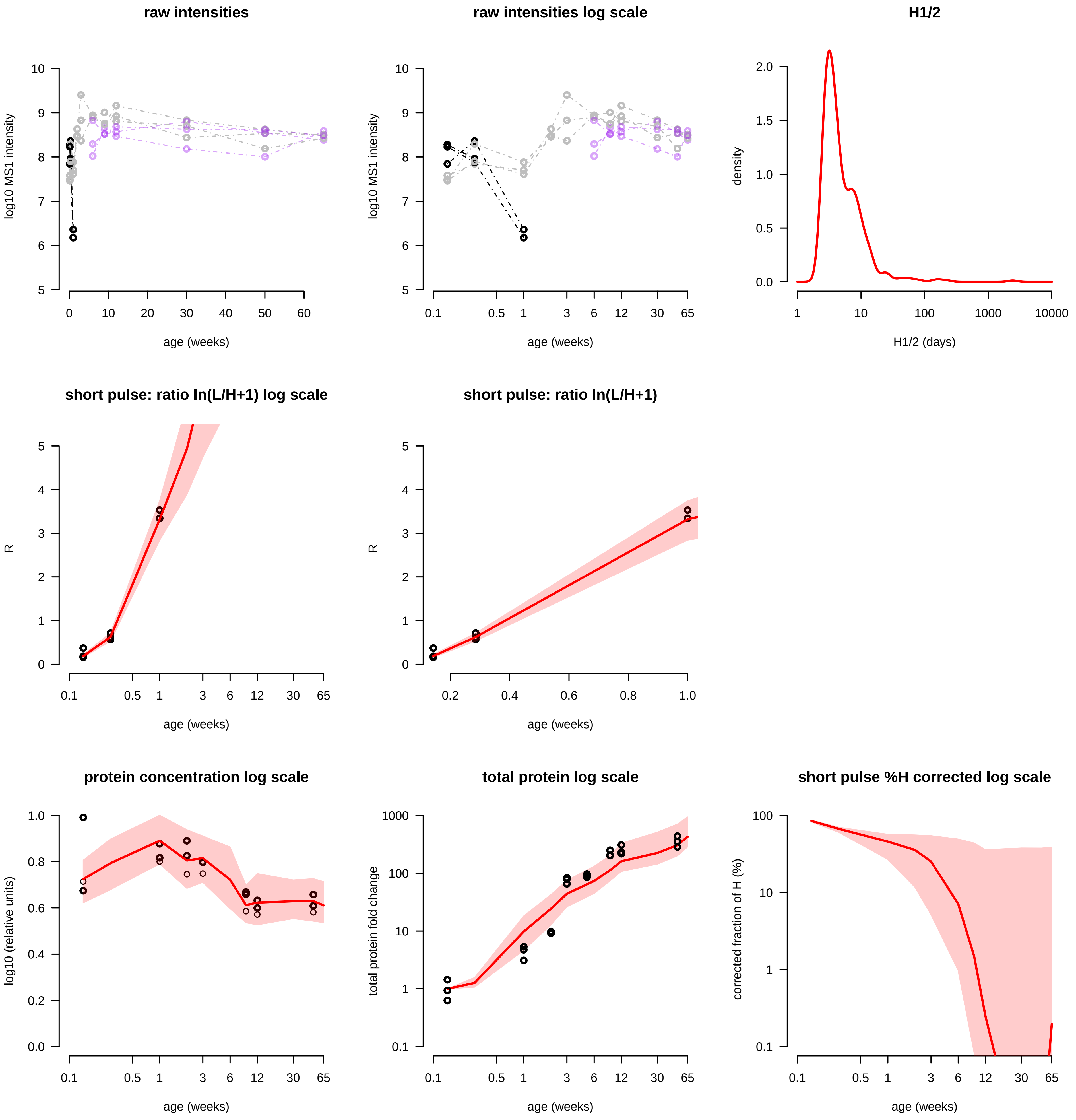

Q7TPD0

>sp|Q7TPD0|INT3_MOUSE

Integrator complex subunit 3

GN=Ints3

Med H1/2: 7.5 days

Q5 H1/2: 3.3 days

Q95 H1/2: 55.5 days

reliable model fit: yes

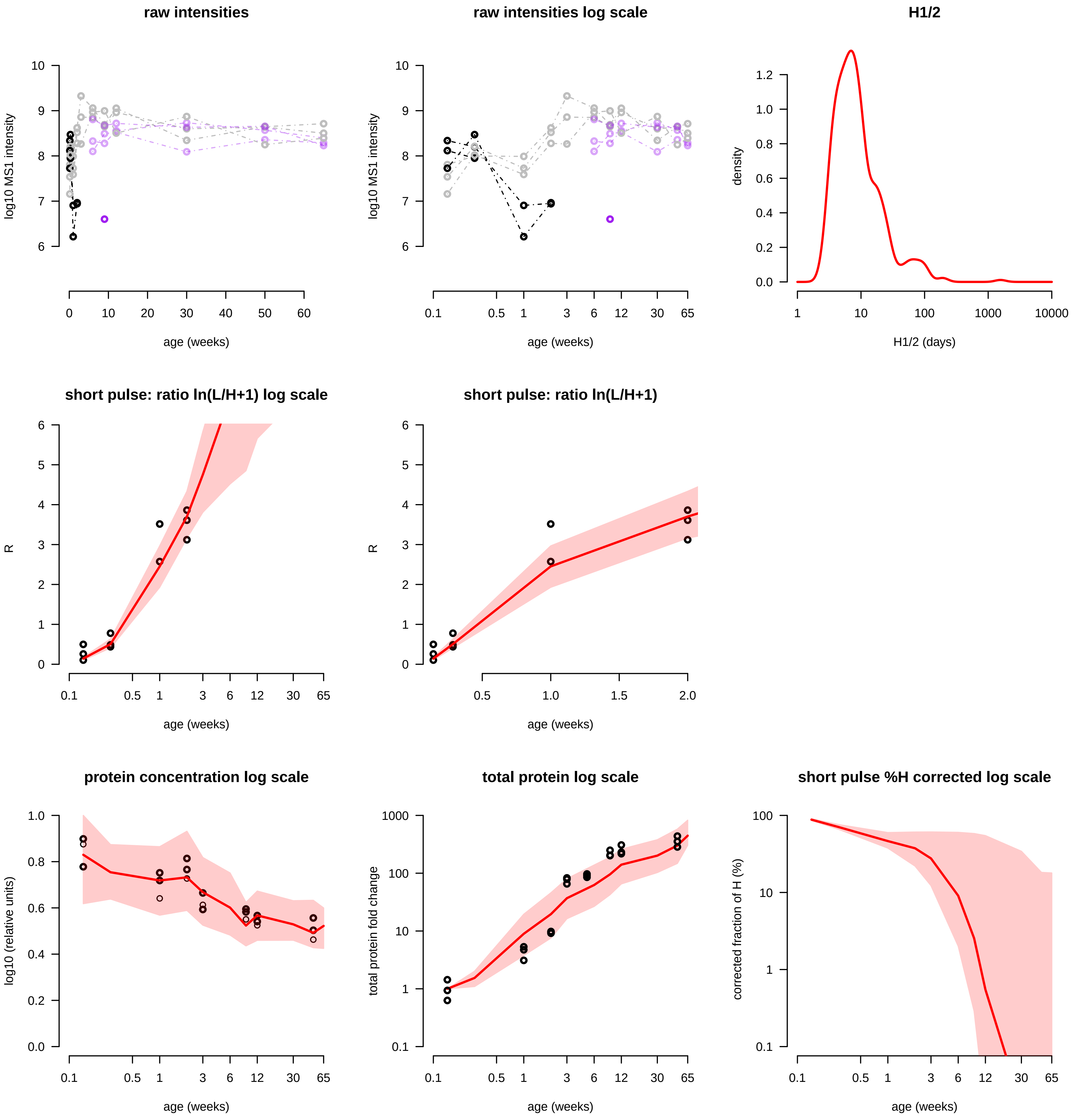

P23475

>sp|P23475|XRCC6_MOUSE

X−ray repair cross−complementing protein 6

GN=Xrcc6

Med H1/2: 3.3 days

Q5 H1/2: 2.6 days

Q95 H1/2: 4.7 days

reliable model fit: yes

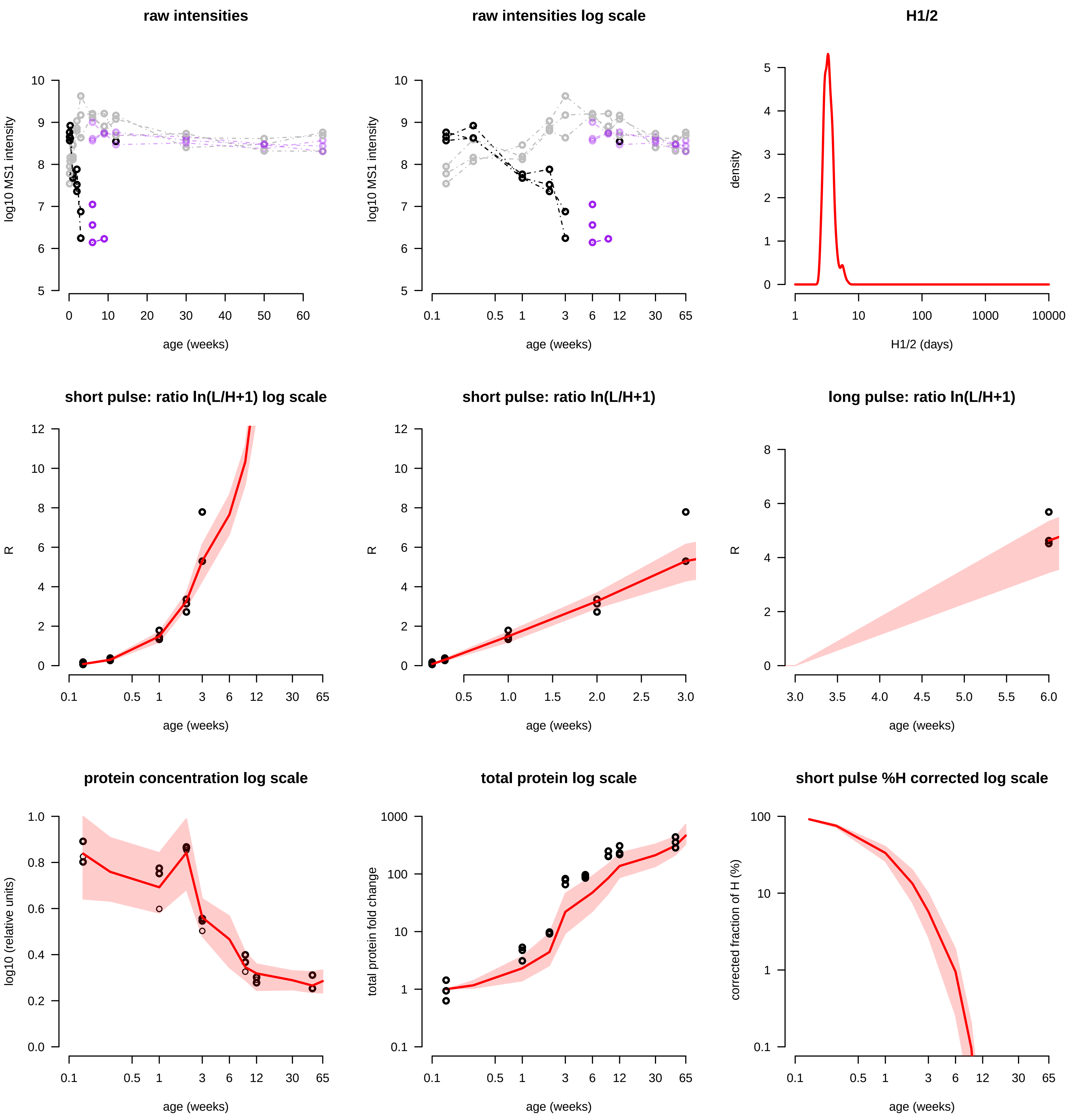

Q8C1Y8

>sp|Q8C1Y8|CCZ1_MOUSE

Vacuolar fusion protein CCZ1 homolog

GN=Ccz1

Med H1/2: 8.4 days

Q5 H1/2: 3.5 days

Q95 H1/2: 42.4 days

reliable model fit: yes

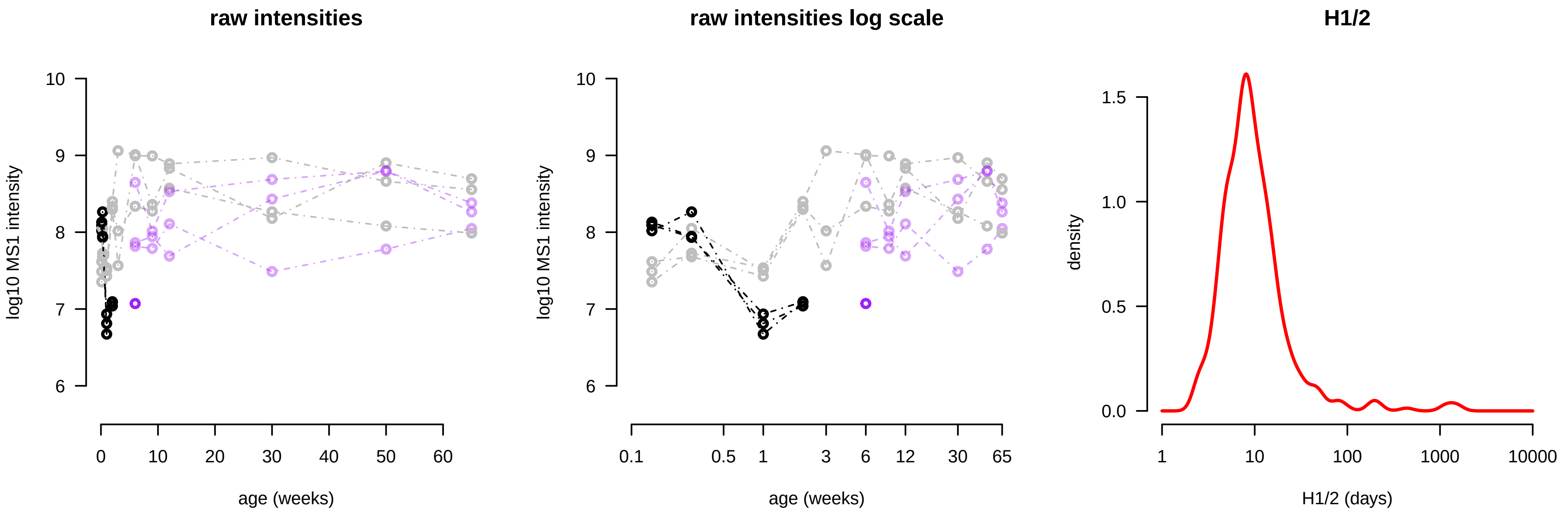

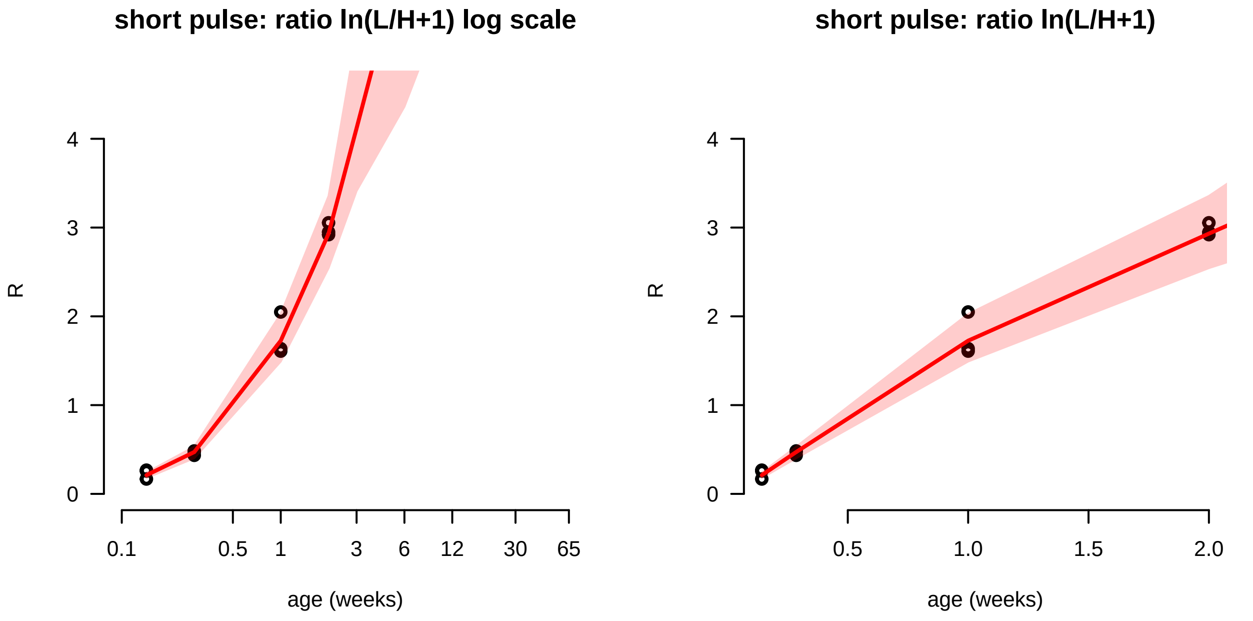

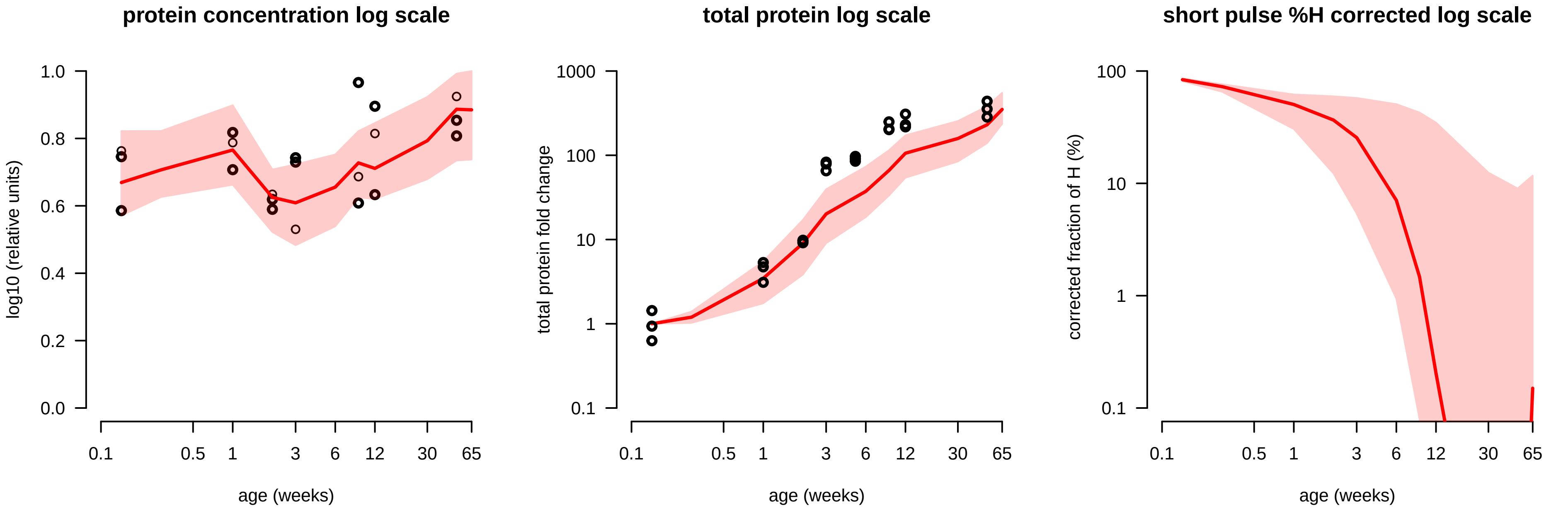

Q922Q4

>sp|Q922Q4|P5CR2_MOUSE

Pyrroline−5−carboxylate reductase 2

GN=Pycr2

Med H1/2: 4.4 days

Q5 H1/2: 3.6 days

Q95 H1/2: 5.3 days

reliable model fit: yes

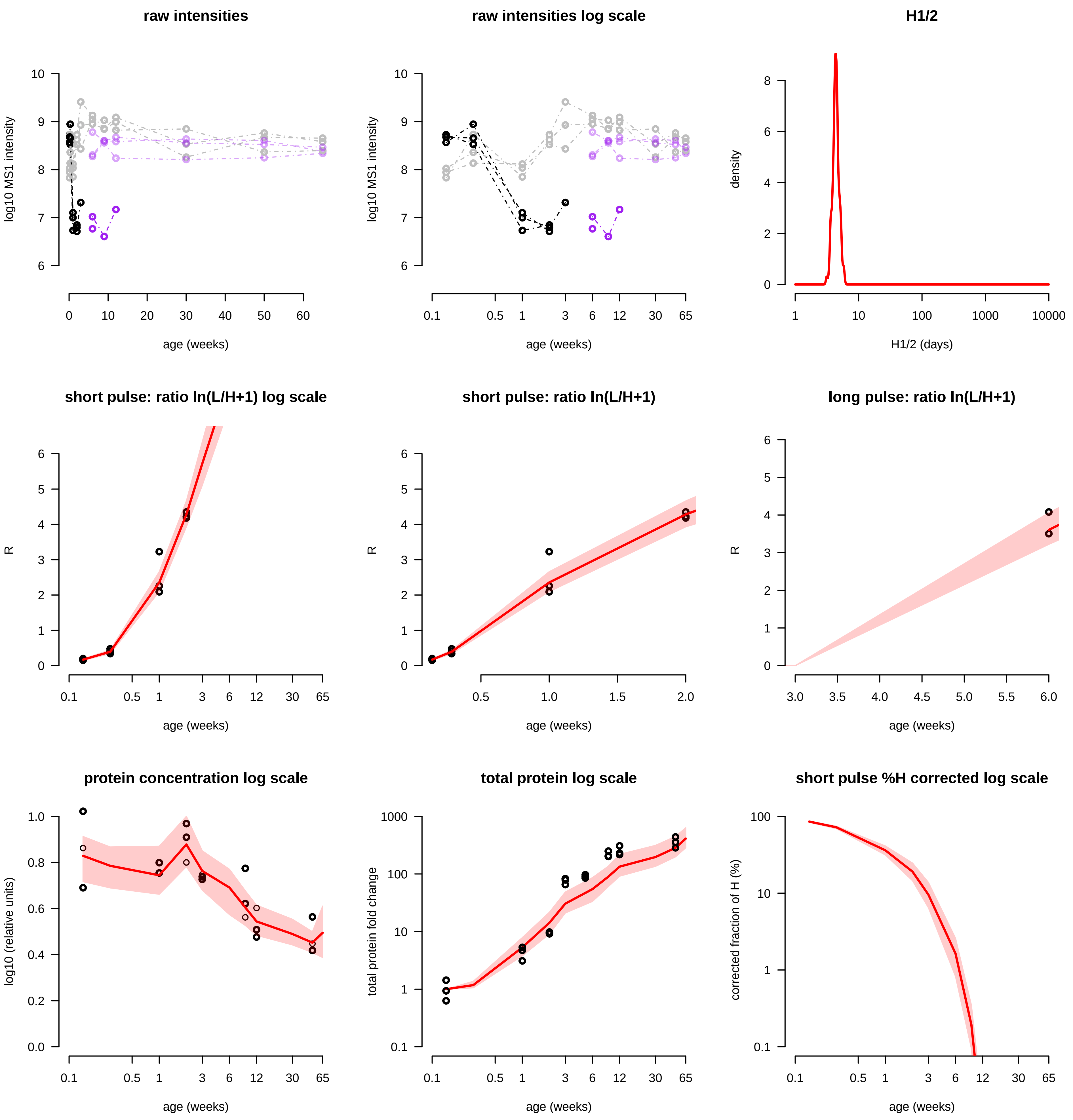

Q922V4

>sp|Q922V4|PLRG1_MOUSE

Pleiotropic regulator 1

GN=Plrg1

Med H1/2: 3.4 days

Q5 H1/2: 2.9 days

Q95 H1/2: 4.3 days

reliable model fit: yes

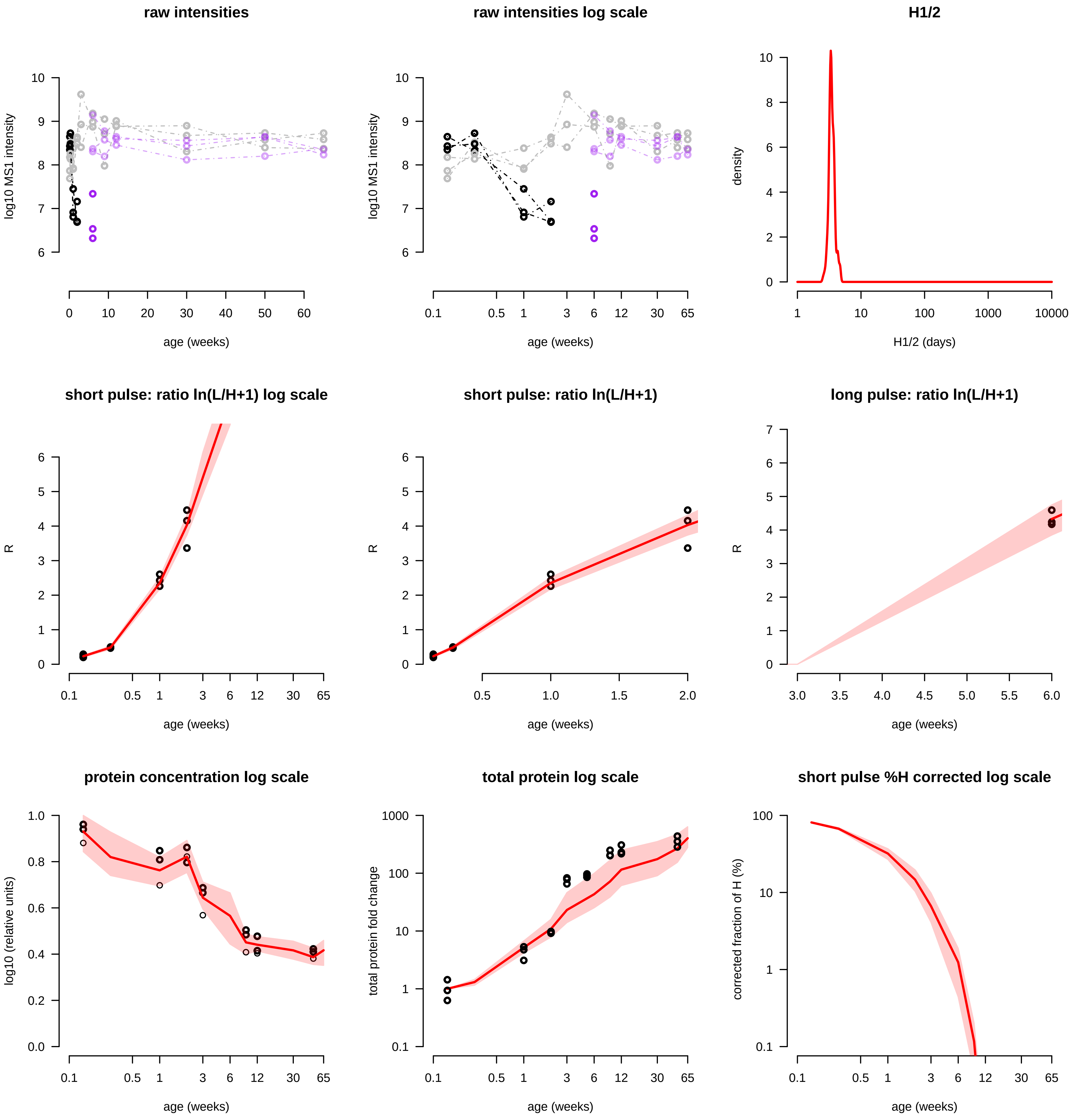

Q9CY57−5;Q9CY57−4;Q9CY57−3;Q9CY57−2;Q9CY57

>sp|Q9CY57−5|CHTOP_MOUSE

Isoform 5 of Chromatin target of PRMT1 protein

GN=Chtop

Med H1/2: 6.3 days

Q5 H1/2: 5.5 days

Q95 H1/2: 7.7 days

reliable model fit: yes

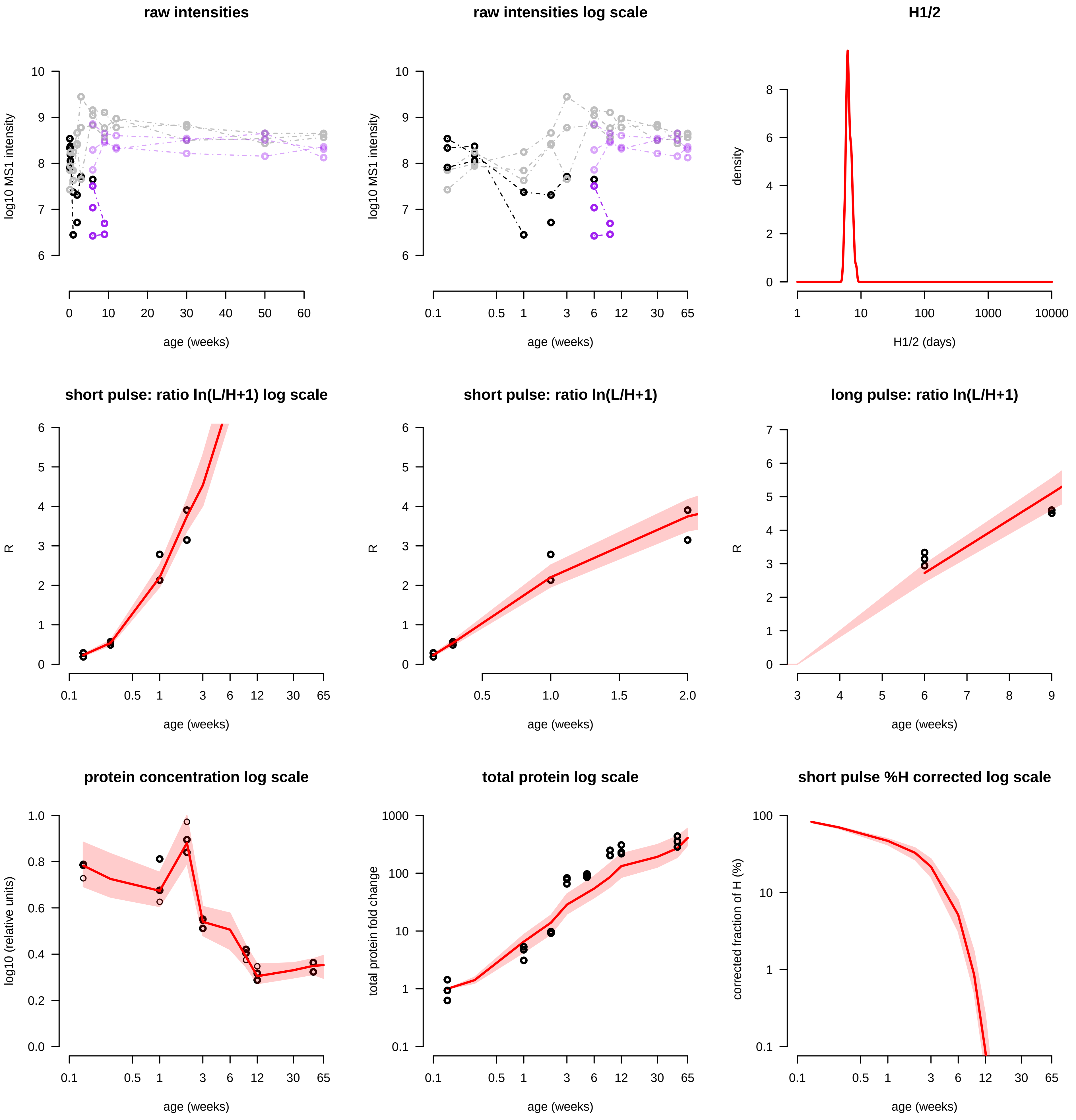

Q8BJS4−2;Q8BJS4;Q8BJS4−3

>sp|Q8BJS4−2|SUN2_MOUSE

Isoform 2 of SUN domain−containing protein 2

GN=Sun2

Med H1/2: 3.8 days

Q5 H1/2: 2.9 days

Q95 H1/2: 4.9 days

reliable model fit: yes

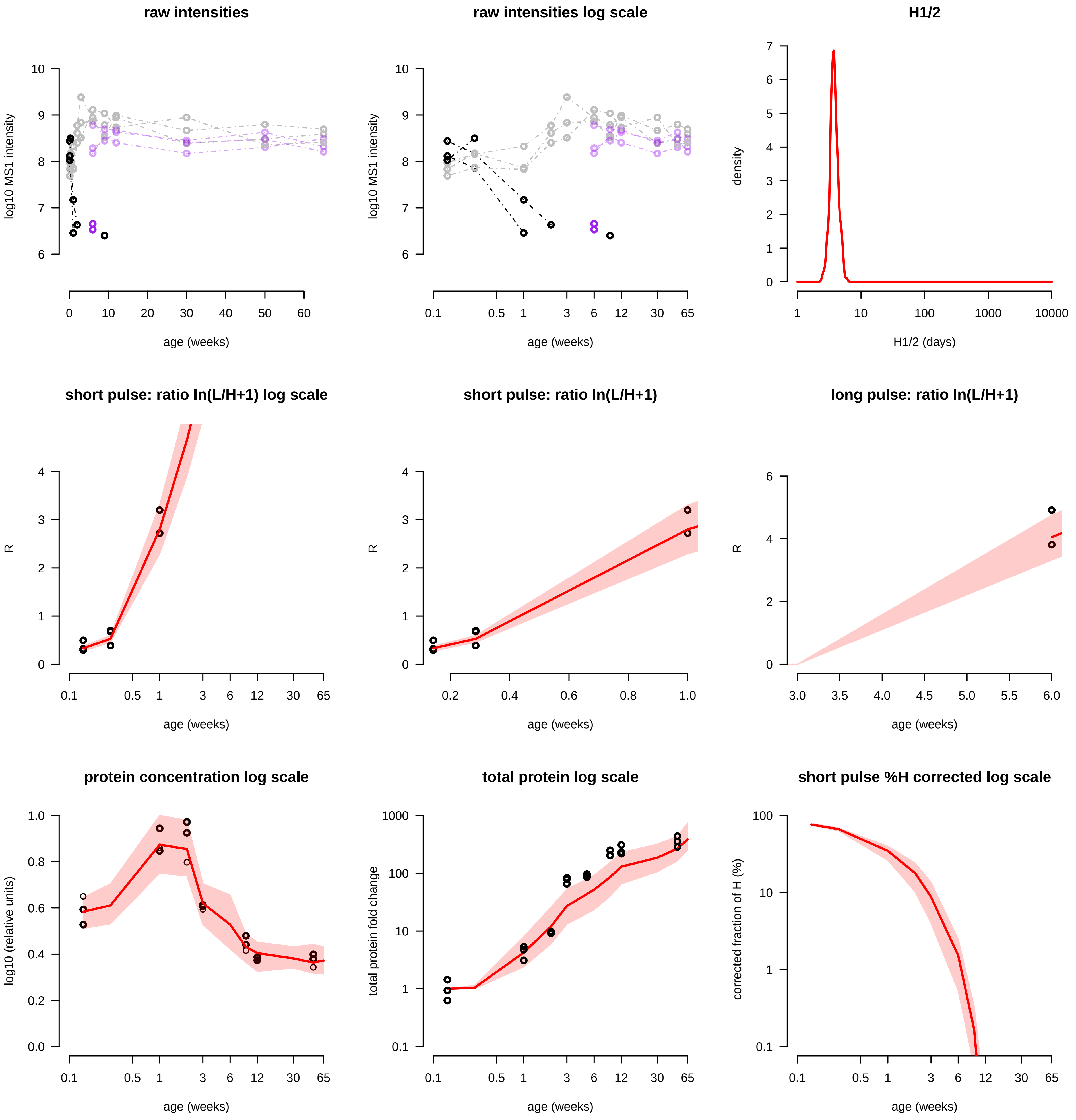

P56960

>sp|P56960|EXOSX_MOUSE

Exosome component 10

GN=Exosc10

Med H1/2: 5.2 days

Q5 H1/2: 2.9 days

Q95 H1/2: 14.8 days

reliable model fit: yes

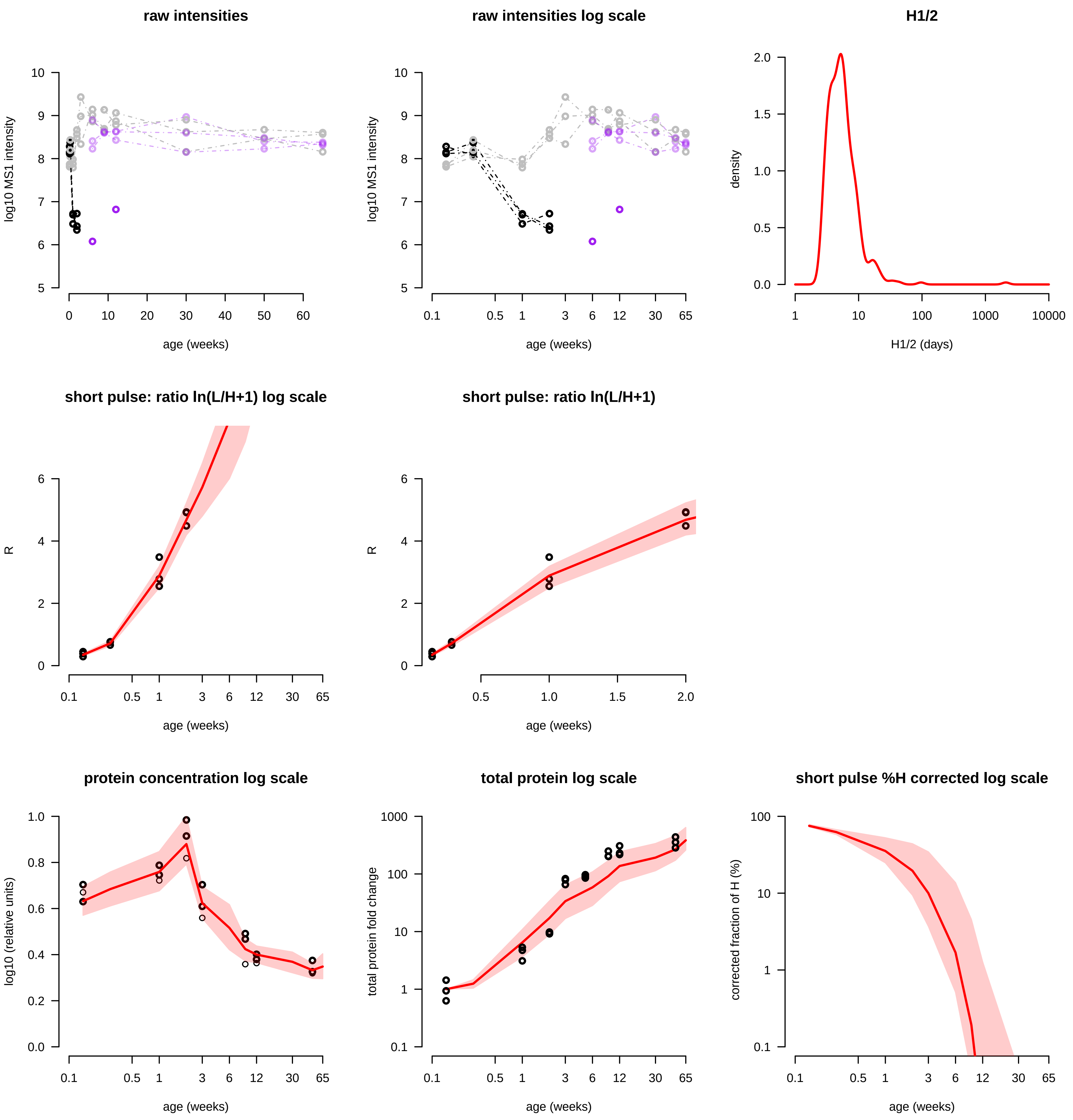

Q9ERR7

>sp|Q9ERR7|SEP15_MOUSE

Selenoprotein F

GN=Selenof

Med H1/2: 5.6 days

Q5   H1/2: 4.5 days

Q95 H1/2: 7.9 days

reliable model fit: yes

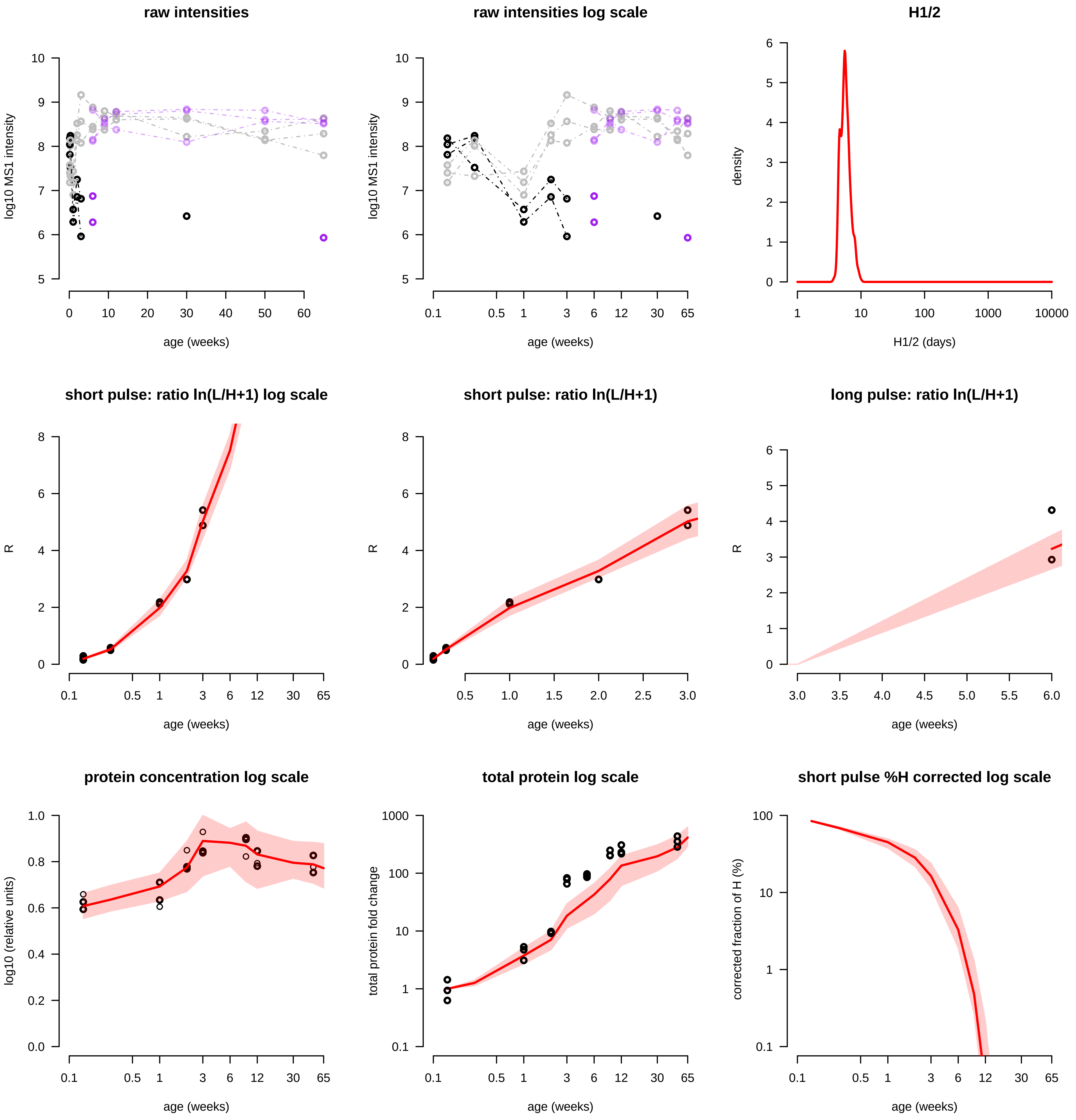

Q80X41;Q80X41−5;Q80X41−3;Q80X41−2

>sp|Q80X41|VRK1_MOUSE

Serine/threonine−protein kinase VRK1

GN=Vrk1

Med H1/2: 25.4 days

Q5 H1/2: 6.2 days

Q95 H1/2: 374.8 days

reliable model fit: yes

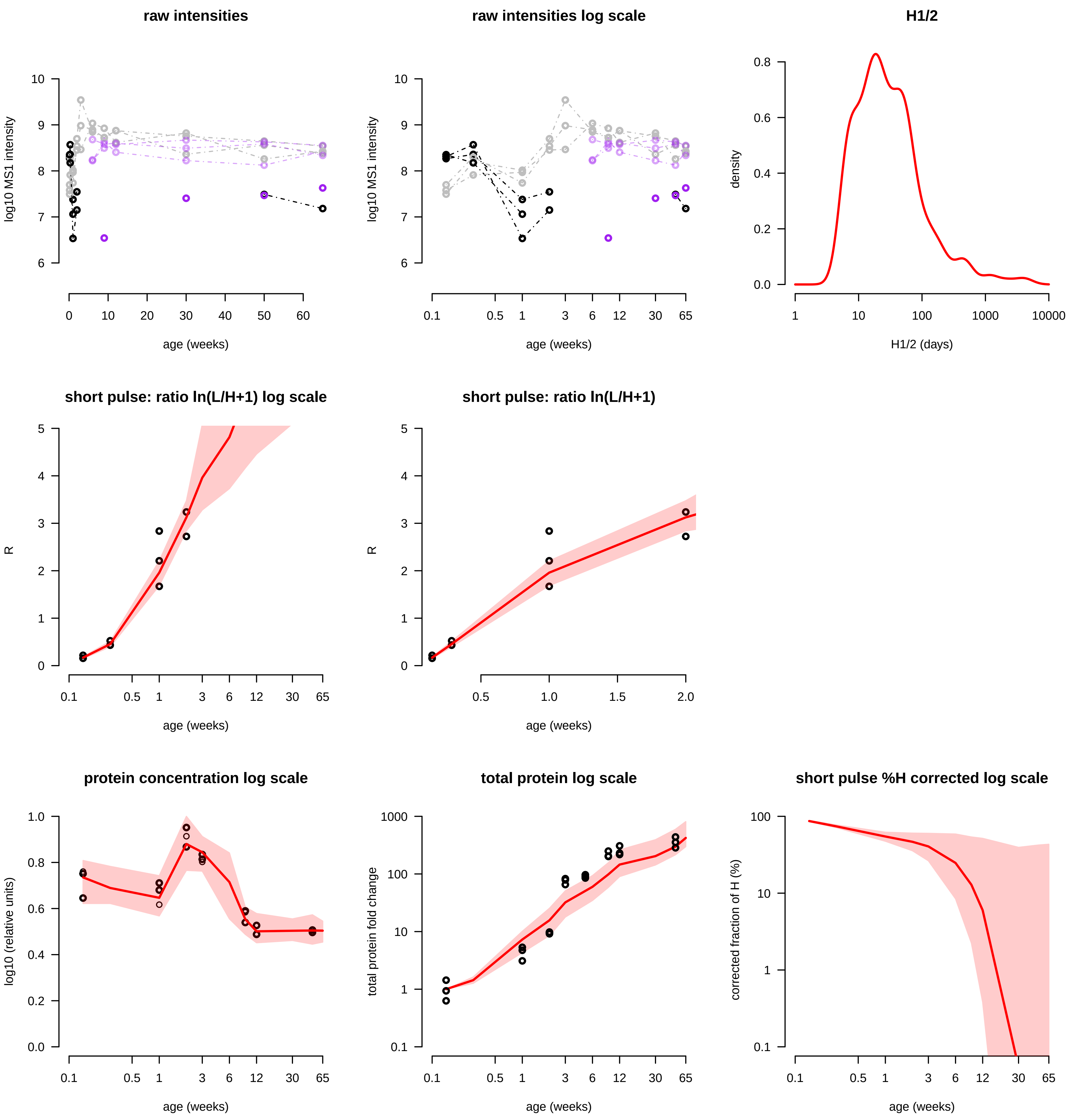

Q8BUK6

>sp|Q8BUK6|HOOK3_MOUSE

Protein Hook homolog 3

GN=Hook3

Med H1/2: 5.4 days

Q5 H1/2: 2.8 days

Q95 H1/2: 72.4 days

reliable model fit: yes

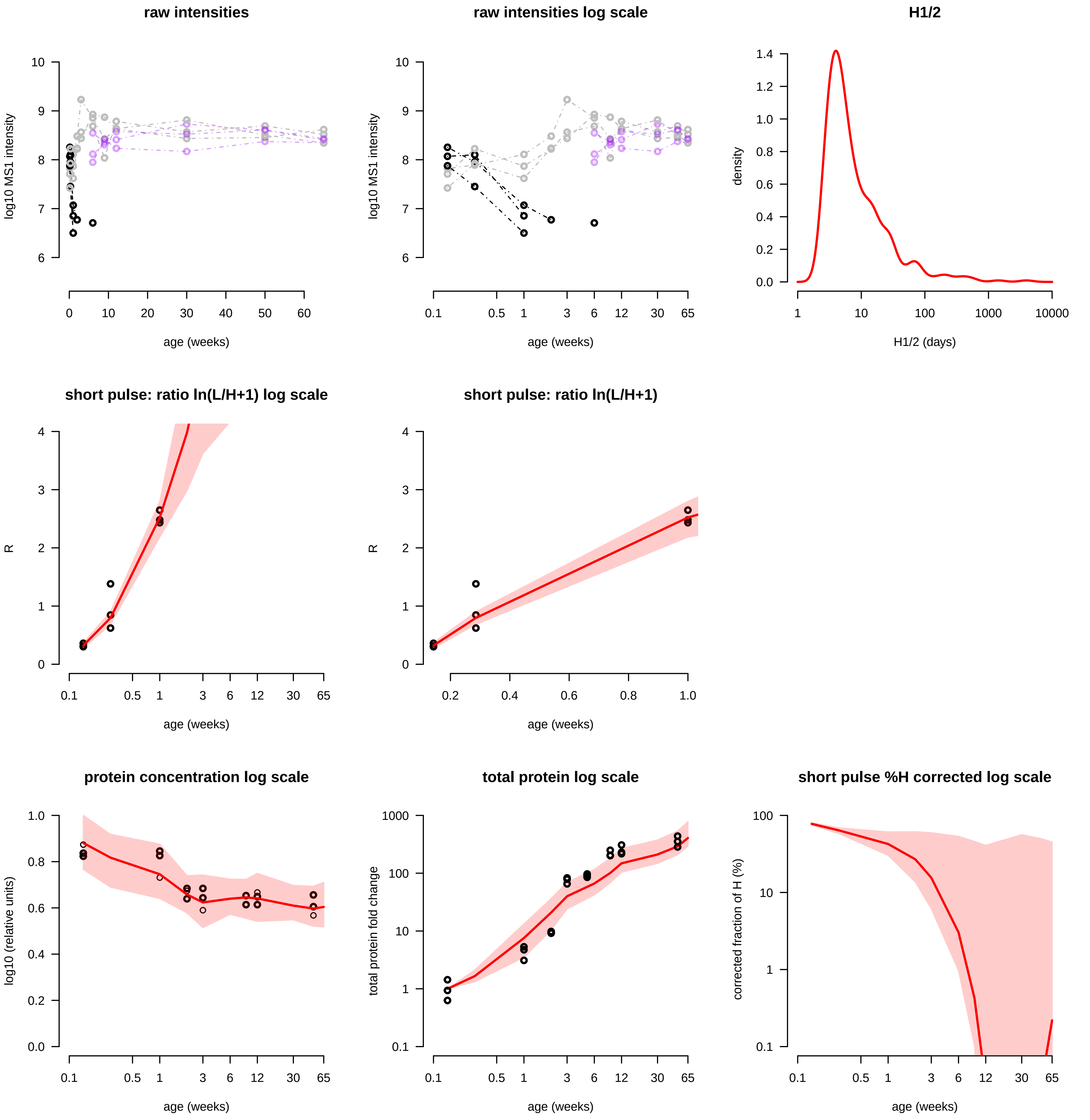

Q91YS8

>sp|Q91YS8|KCC1A_MOUSE

Calcium/calmodulin−dependent protein kinase type 1

GN=Camk1

Med H1/2: 4.7 days

Q5 H1/2: 3.7 days

Q95 H1/2: 6.3 days

reliable model fit: yes

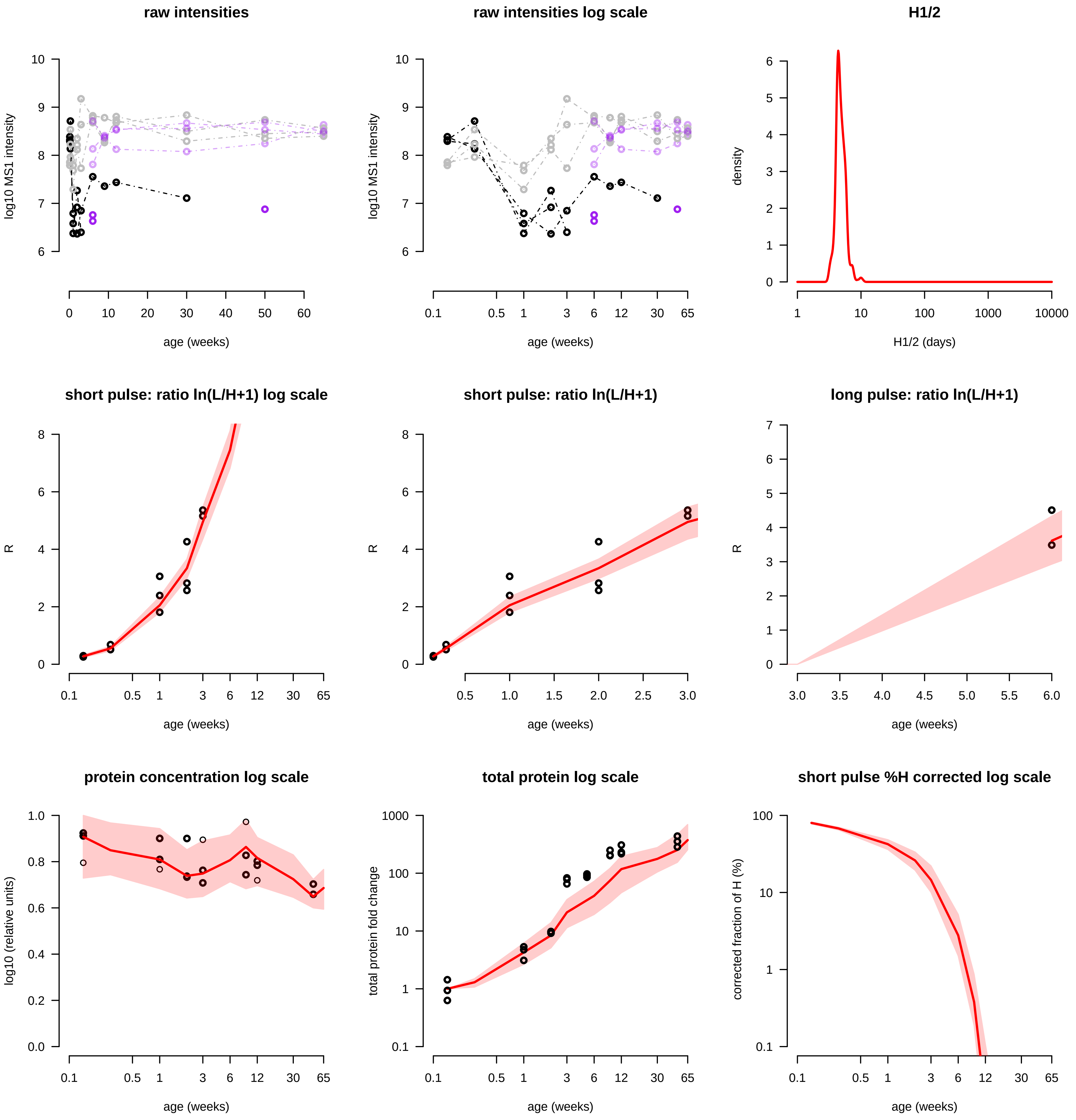

Q9DBR0

>sp|Q9DBR0|AKAP8_MOUSE

A−kinase anchor protein 8

GN=Akap8

Med H1/2: 10.9 days

Q5 H1/2: 4 days

Q95 H1/2: 124.1 days

reliable model fit: yes

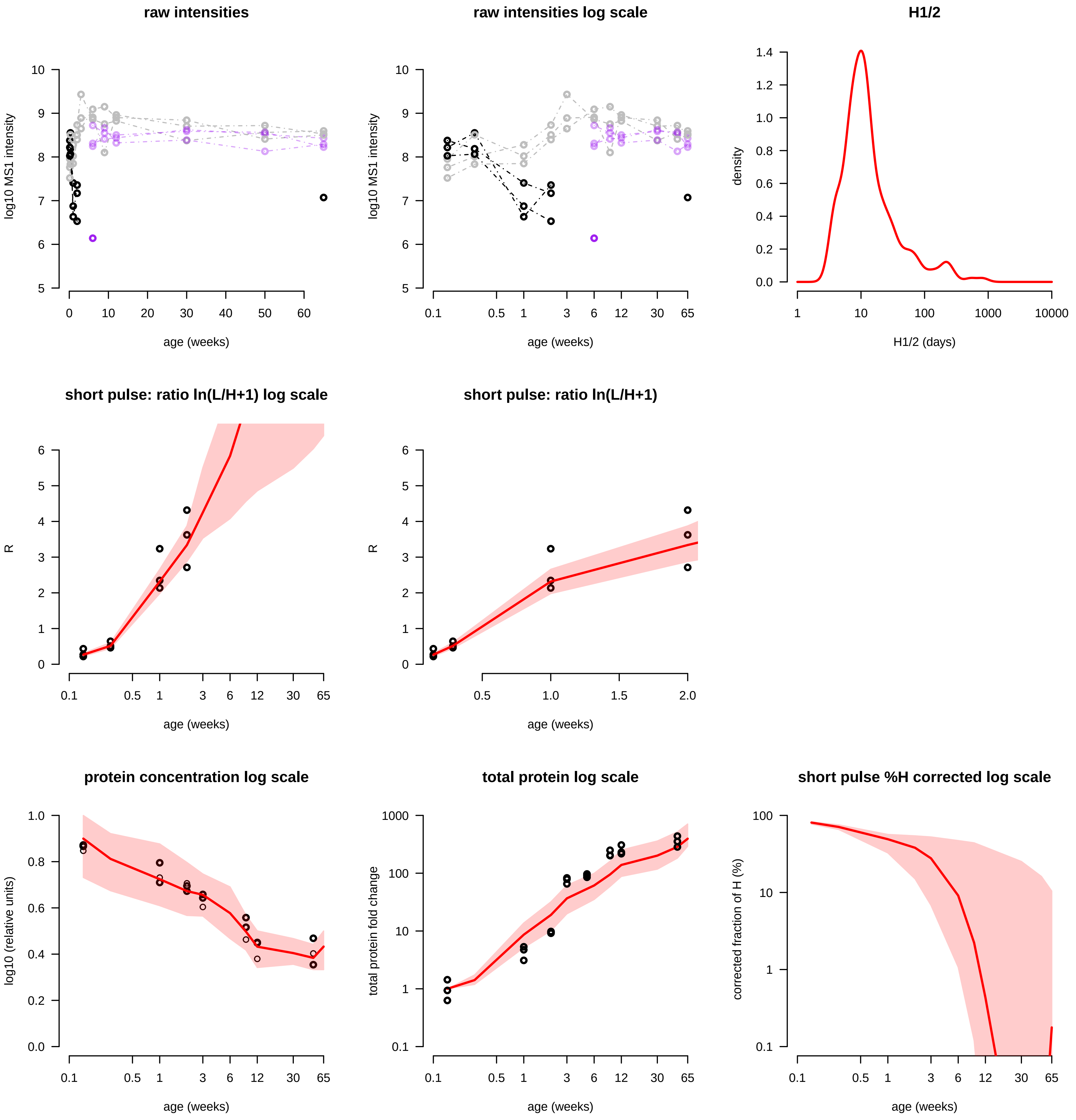

O35435

>sp|O35435|PYRD_MOUSE

Dihydroorotate dehydrogenase (quinone), mitochondrial

GN=Dhodh

Med H1/2: 11 days

Q5 H1/2: 4.8 days

Q95 H1/2: 80 days

reliable model fit: yes

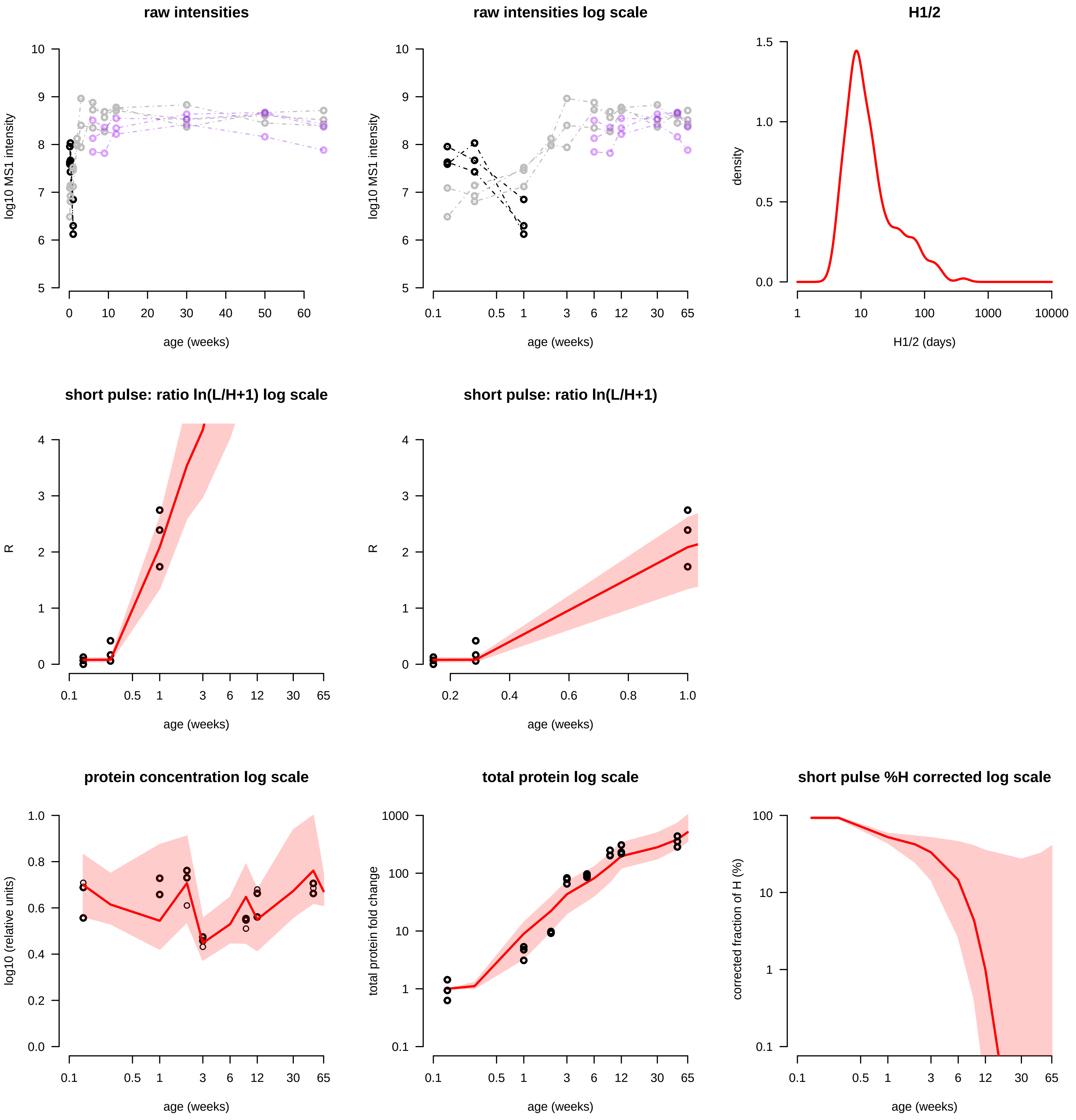

Q920A7;Q920A7−2

>sp|Q920A7|AFG31_MOUSE

AFG3−like protein 1

GN=Afg3l1

Med H1/2: 1188 days

Q5 H1/2: 237.9 days

Q95 H1/2: 17624.8 days

reliable model fit: yes

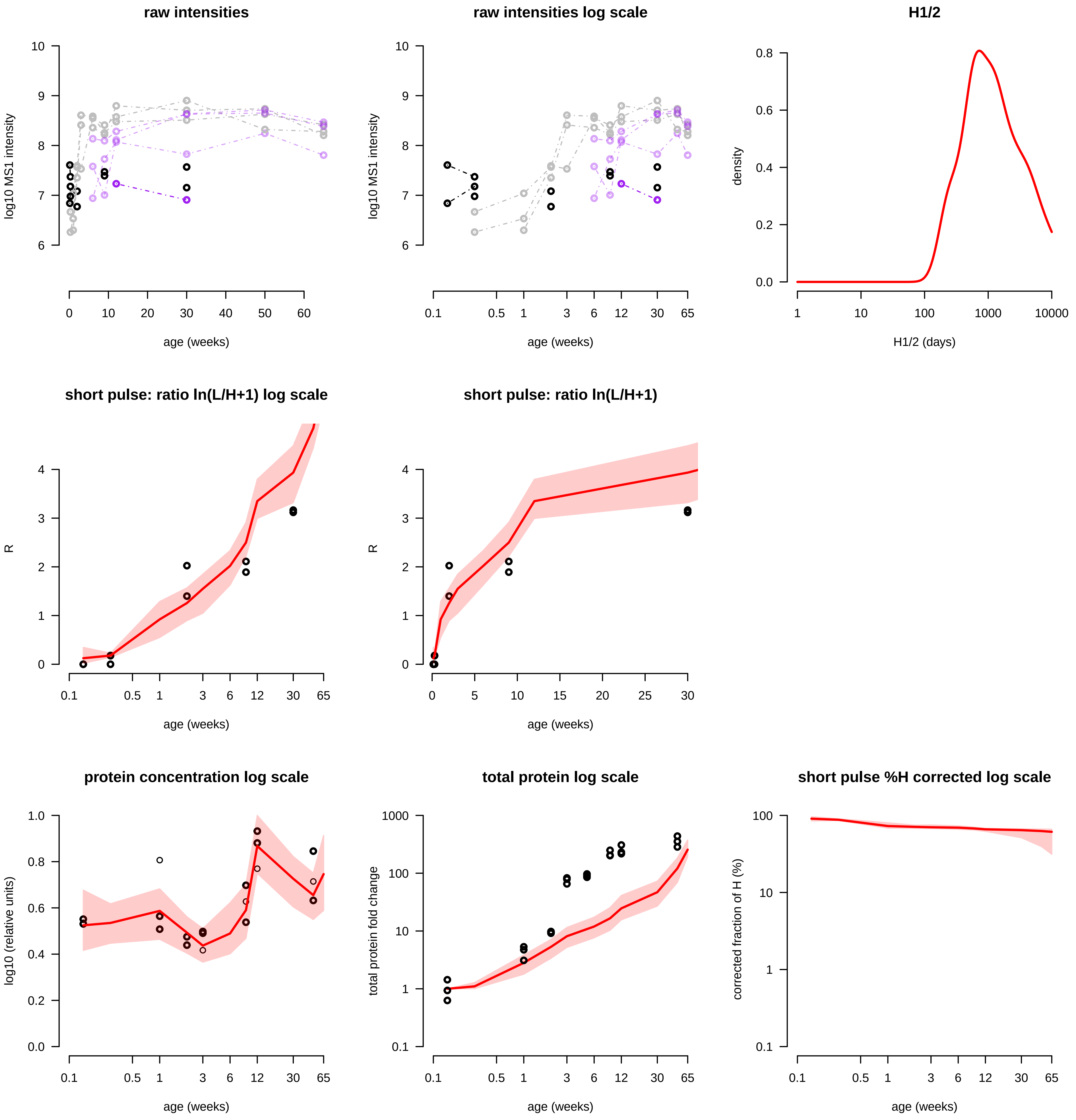

Q91WN1

>sp|Q91WN1|DNJC9_MOUSE

DnaJ homolog subfamily C member 9

GN=Dnajc9

Med H1/2: 15.3 days

Q5 H1/2: 5.8 days

Q95 H1/2: 149.7 days

reliable model fit: yes

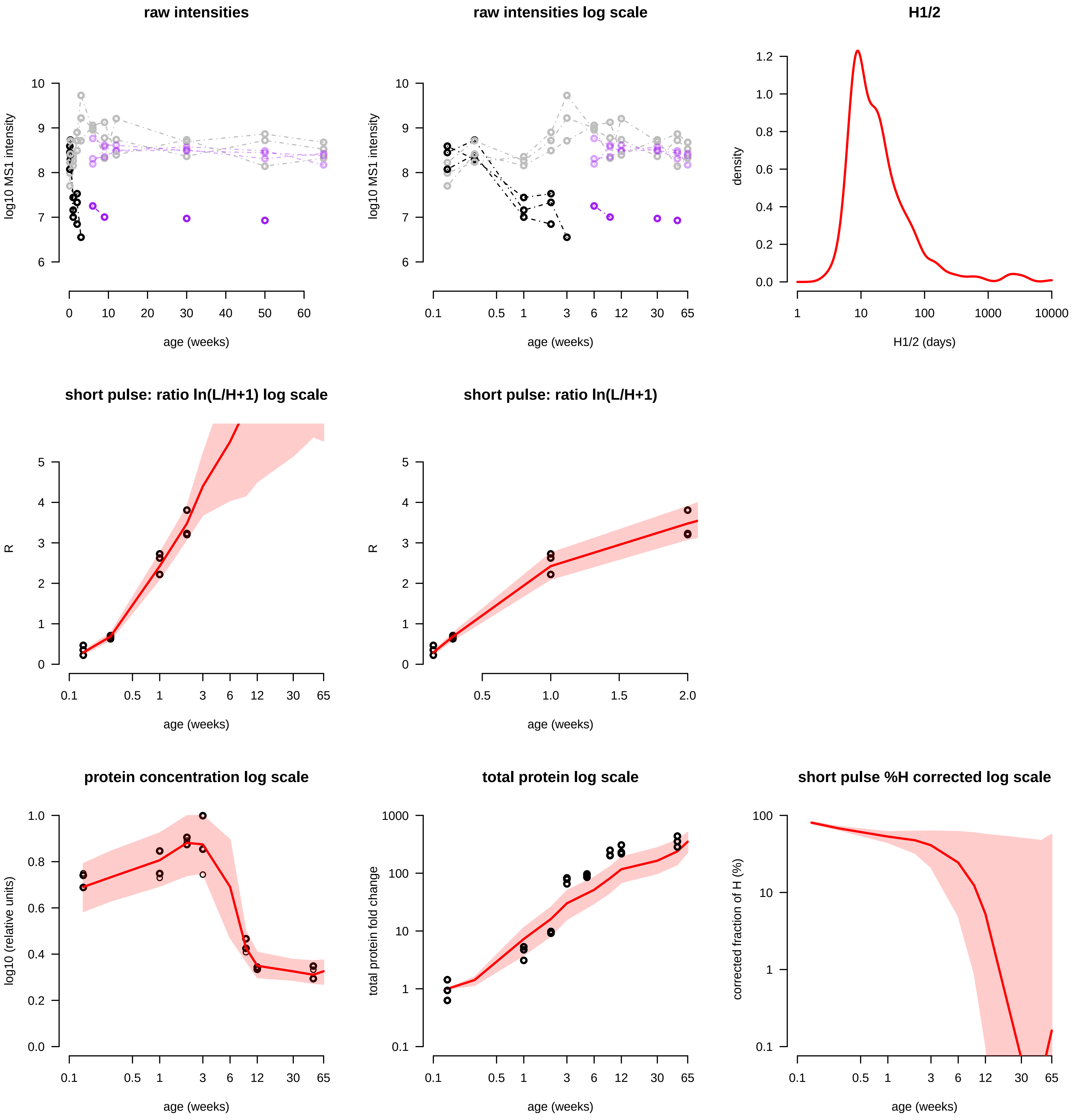

Q9QXK2

>sp|Q9QXK2|RAD18_MOUSE

E3 ubiquitin−protein ligase RAD18

GN=Rad18

Med H1/2: 22.8 days

Q5 H1/2: 7 days

Q95 H1/2: 230.4 days

reliable model fit: yes

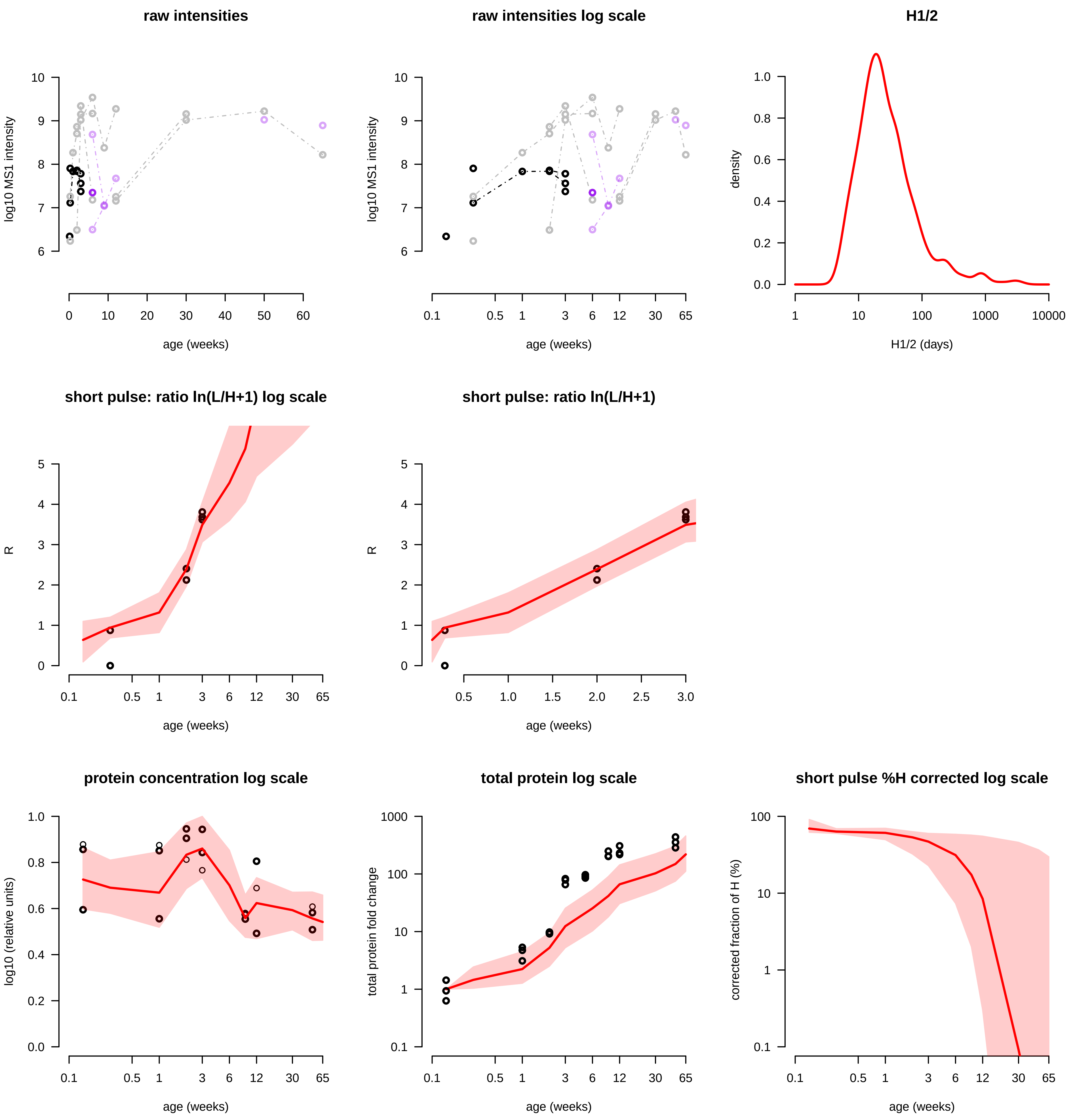

Q9Z103

>sp|Q9Z103|ADNP_MOUSE

Activity−dependent neuroprotector homeobox protein

GN=Adnp

Med H1/2: 1551.1 days

Q5 H1/2: 480 days

Q95 H1/2: 16615.5 days

reliable model fit: no

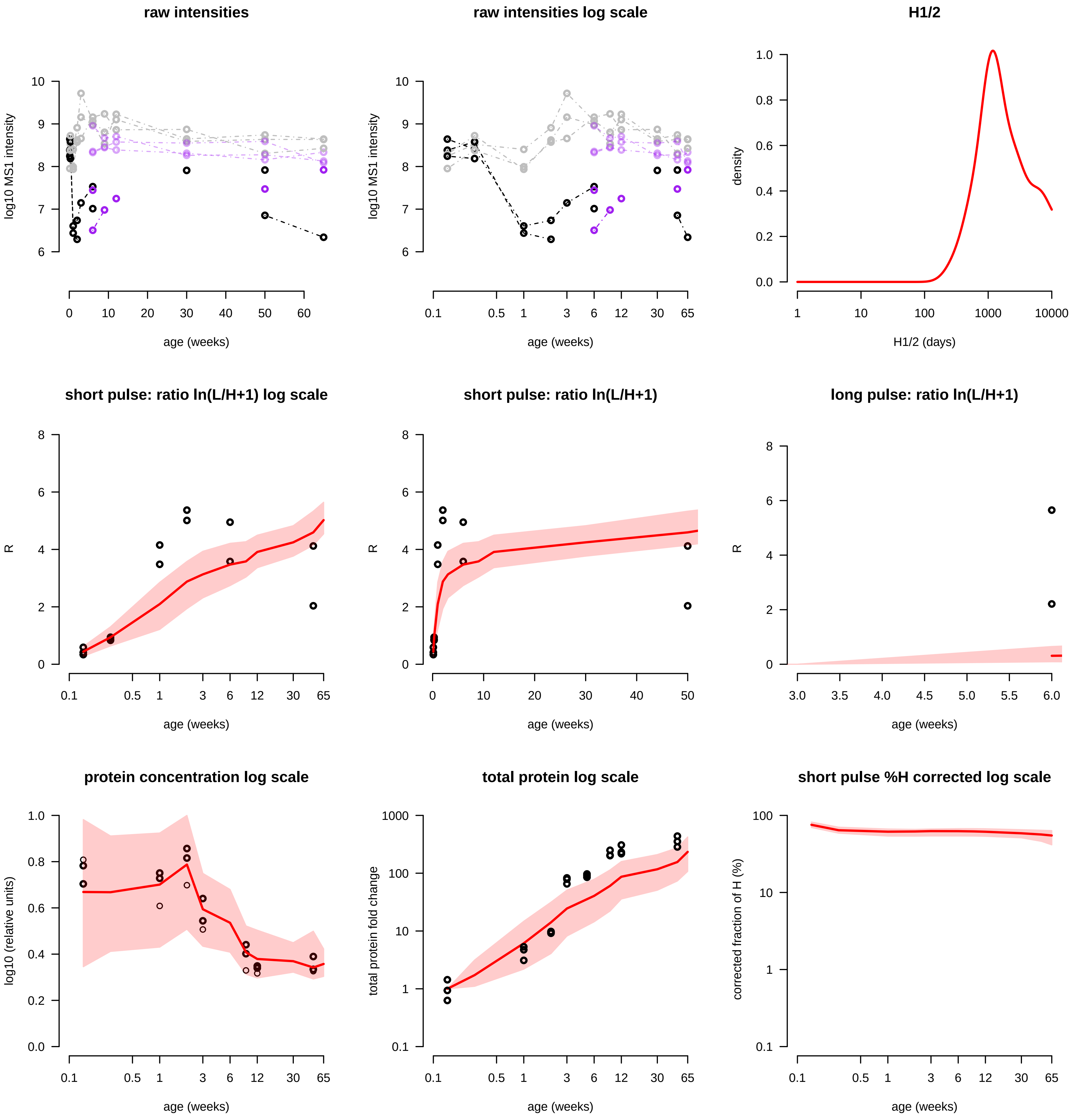

Q9JII5−2;Q9JII5

>sp|Q9JII5−2|DAZP1_MOUSE

Isoform 2 of DAZ−associated protein 1

GN=Dazap1

Med H1/2: 5.1 days

Q5 H1/2: 3.5 days

Q95 H1/2: 7.7 days

reliable model fit: no

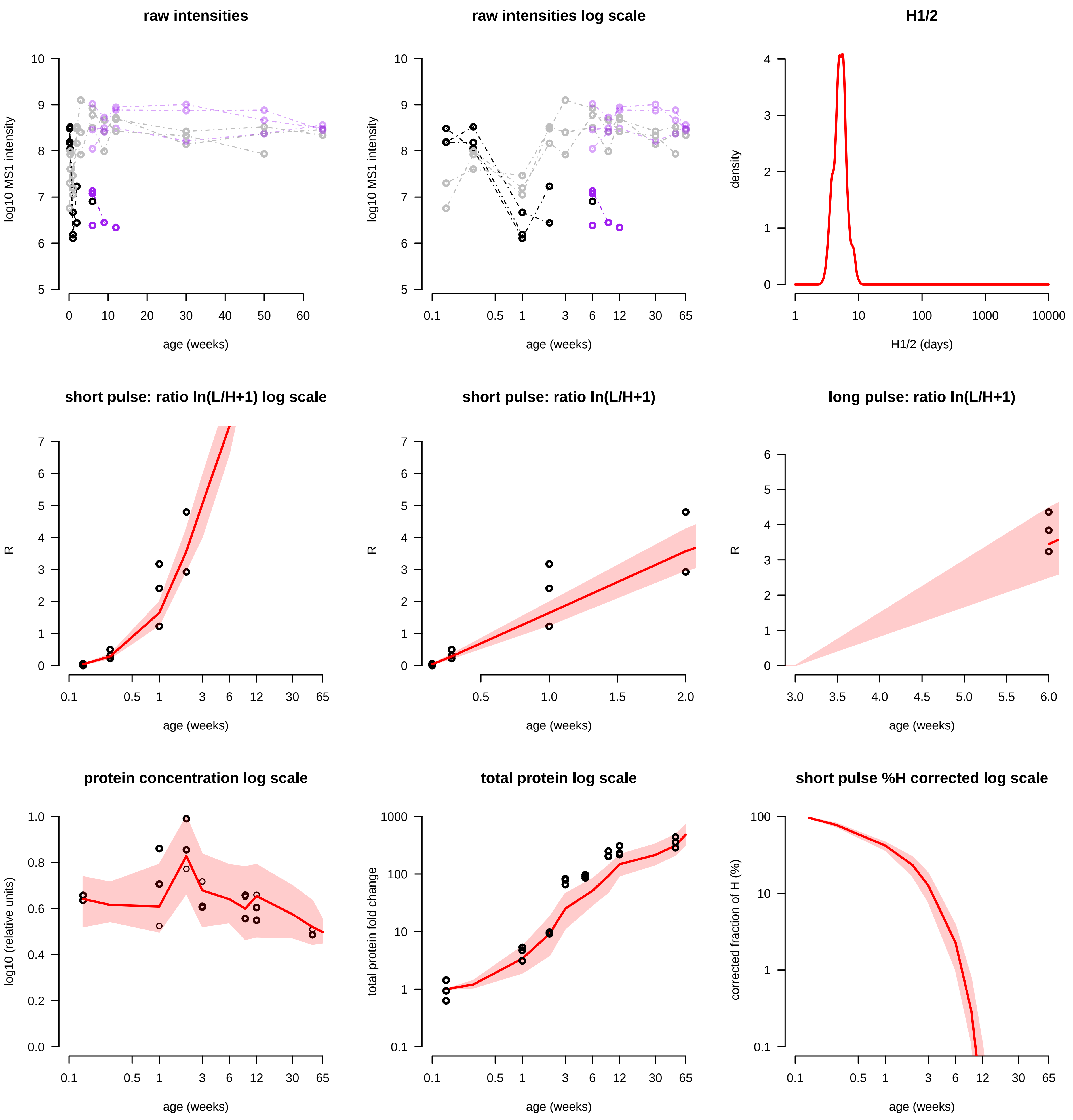

P70297

>sp|P70297|STAM1_MOUSE

Signal transducing adapter molecule 1

GN=Stam

Med H1/2: 5.3 days

Q5 H1/2: 3 days

Q95 H1/2: 25.1 days

reliable model fit: yes

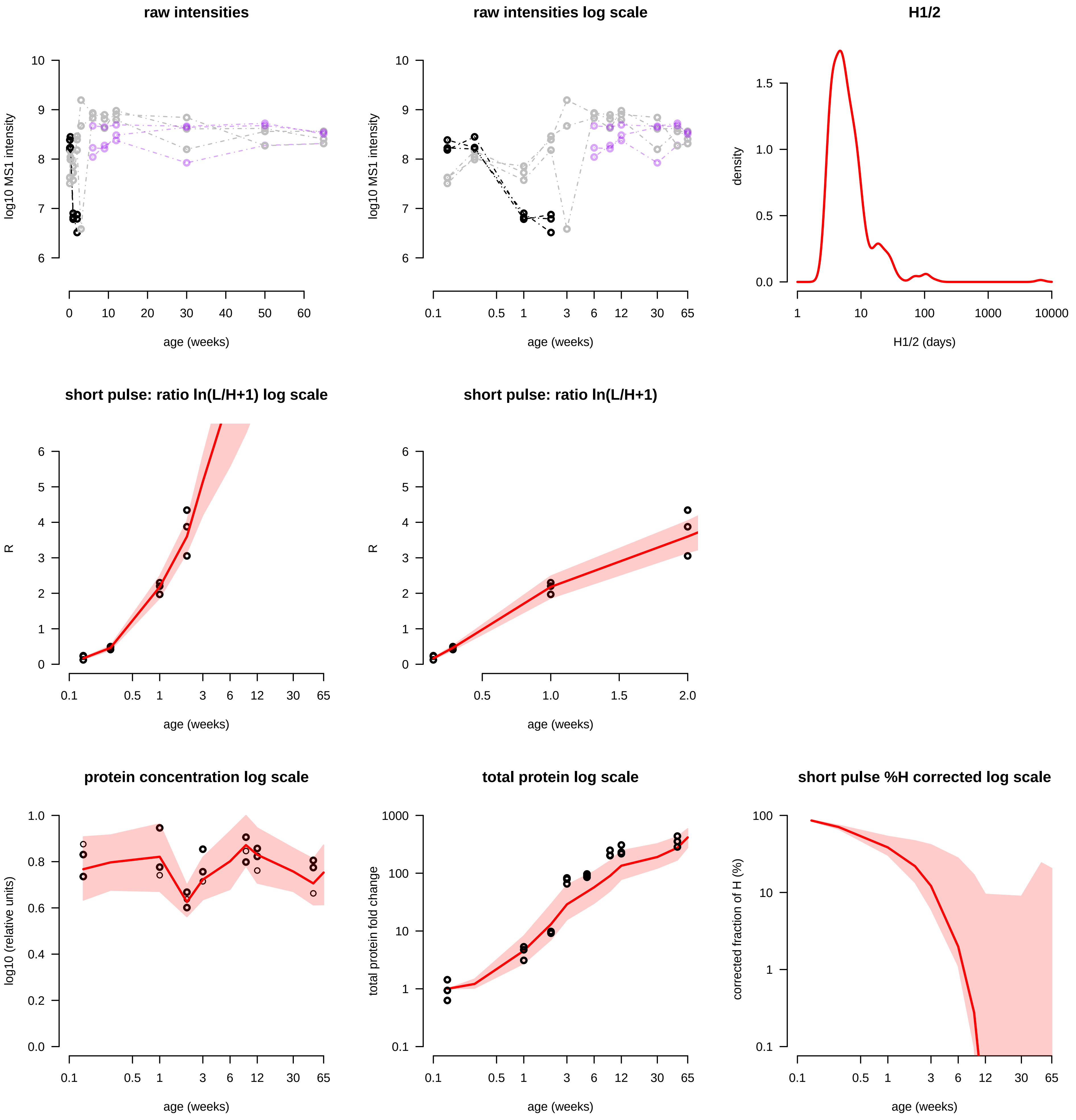

Q9EQ80

>sp|Q9EQ80|NIF3L_MOUSE

NIF3−like protein 1

GN=Nif3l1

Med H1/2: 5.2 days

Q5 H1/2: 3.6 days

Q95 H1/2: 8.4 days

reliable model fit: yes

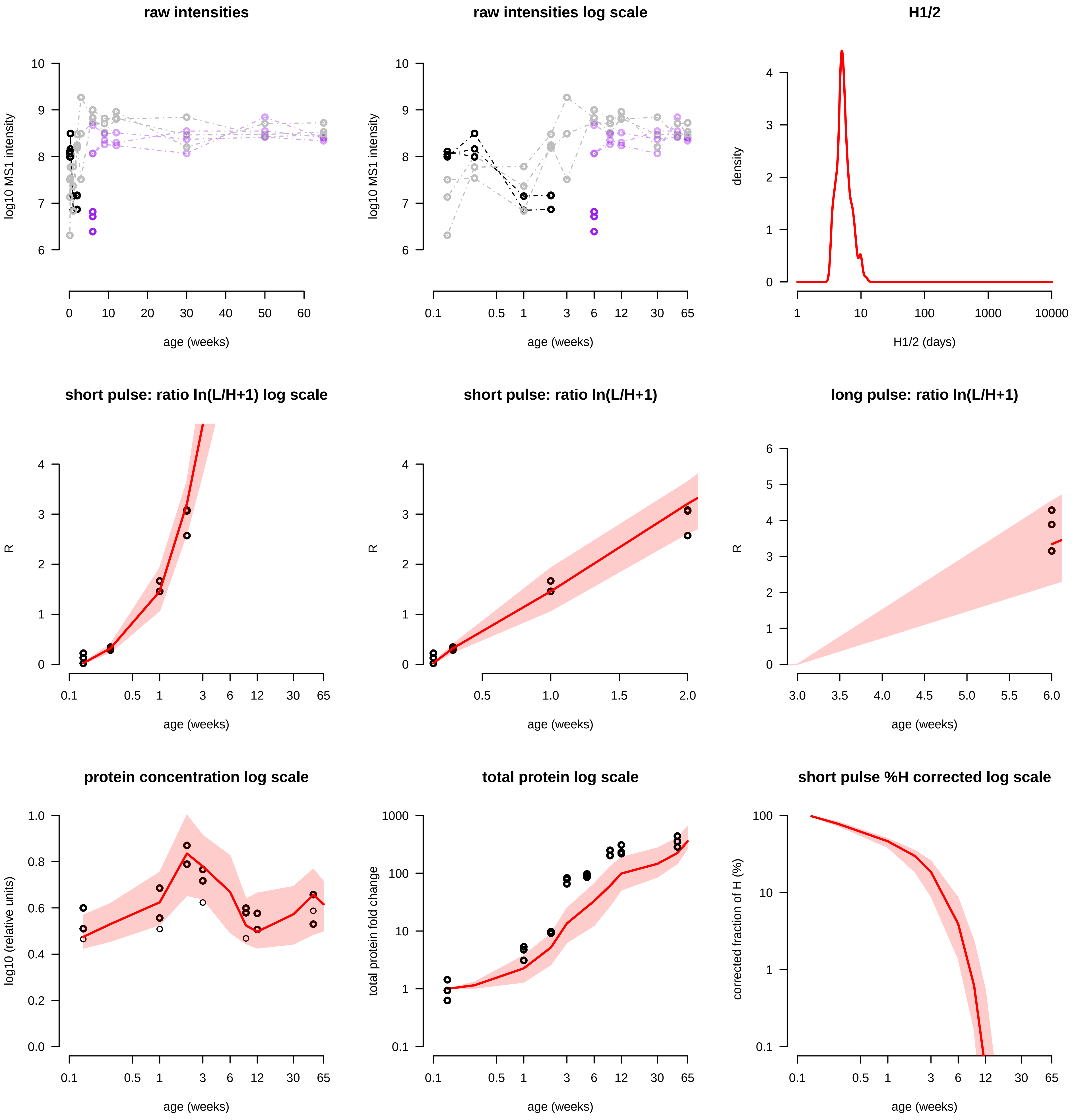

P98192

>sp|P98192|GNPAT_MOUSE

Dihydroxyacetone phosphate acyltransferase

GN=Gnpat

Med H1/2: 10.8 days

Q5 H1/2: 4.1 days

Q95 H1/2: 77.3 days

reliable model fit: yes

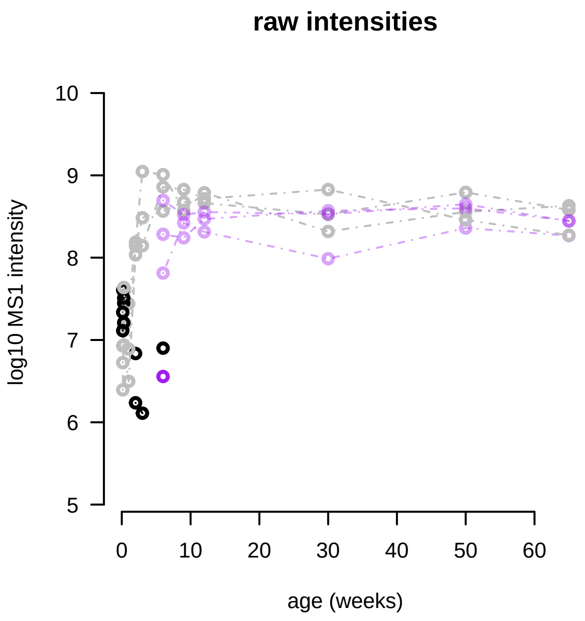

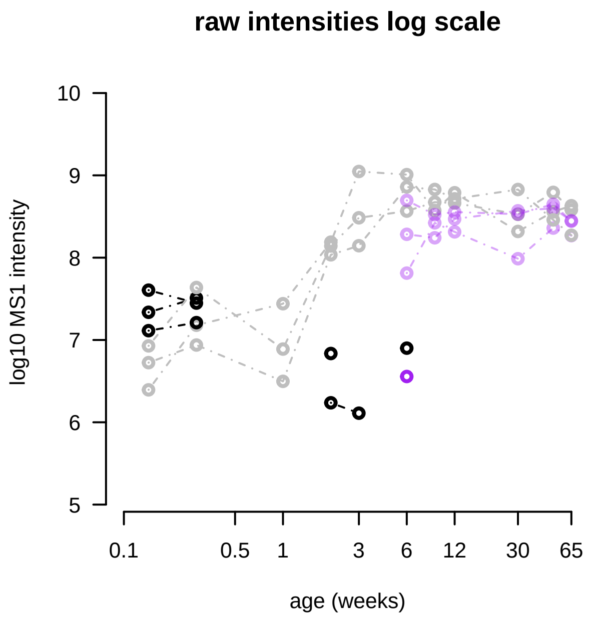

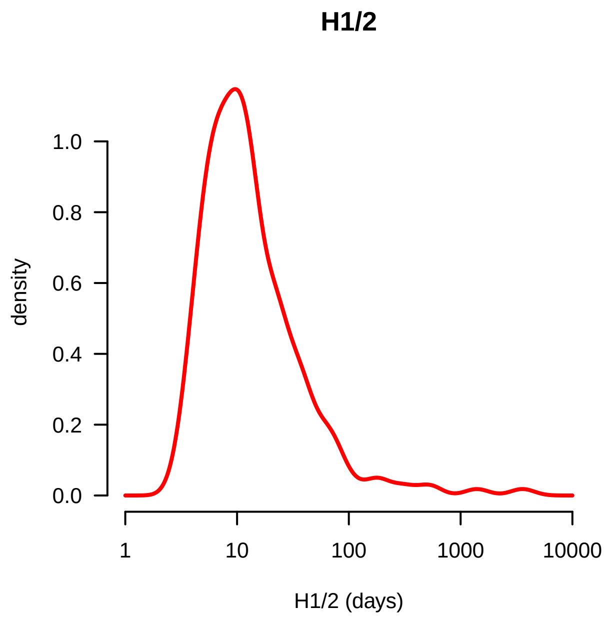

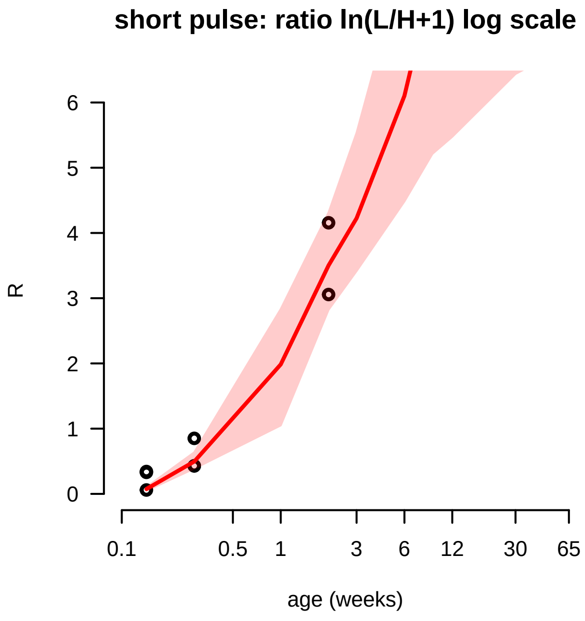

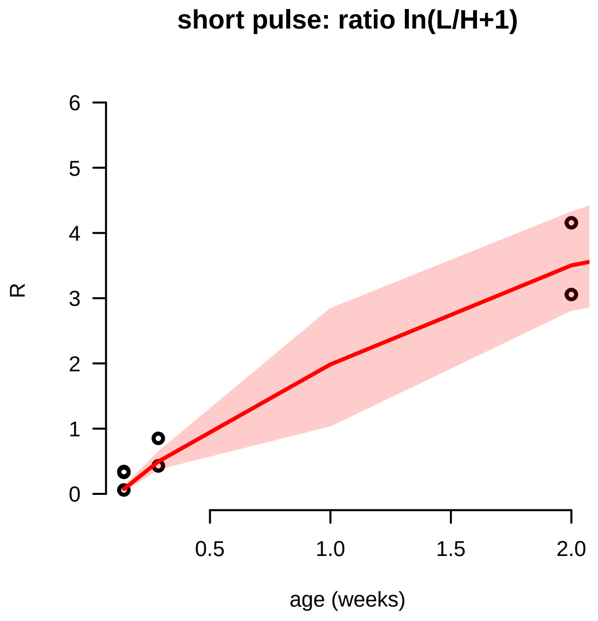

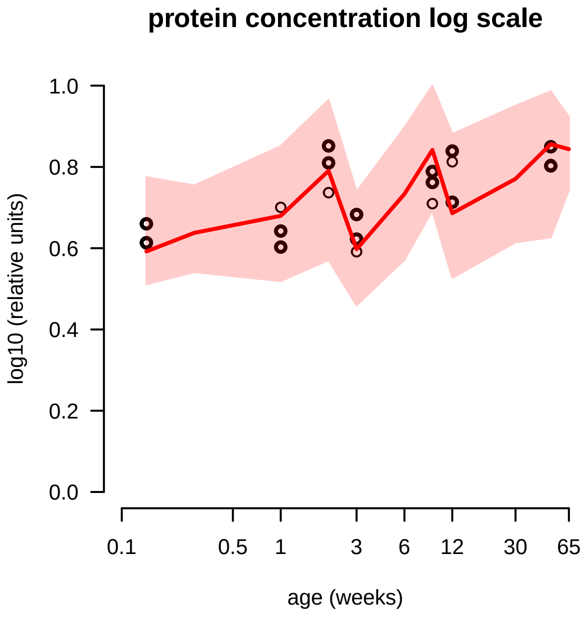

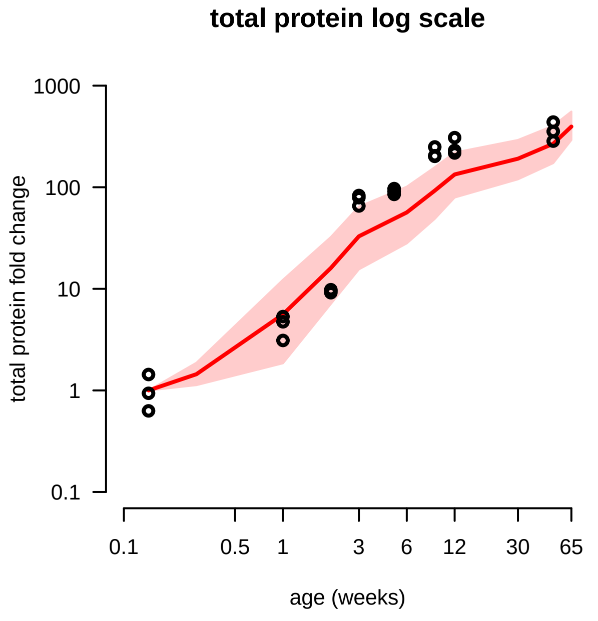

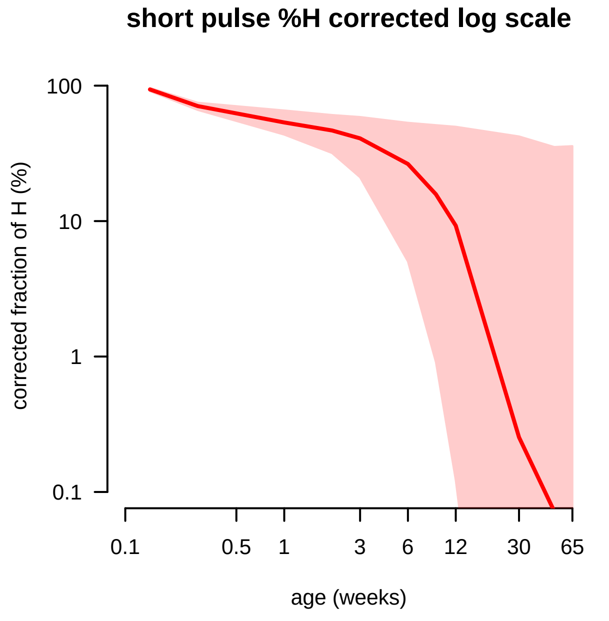

Q922U1

>sp|Q922U1|PRPF3_MOUSE

U4/U6 small nuclear ribonucleoprotein Prp3

GN=Prpf3

Med H1/2: 345.5 days

Q5 H1/2: 65 days

Q95 H1/2: 3801.4 days

reliable model fit: yes

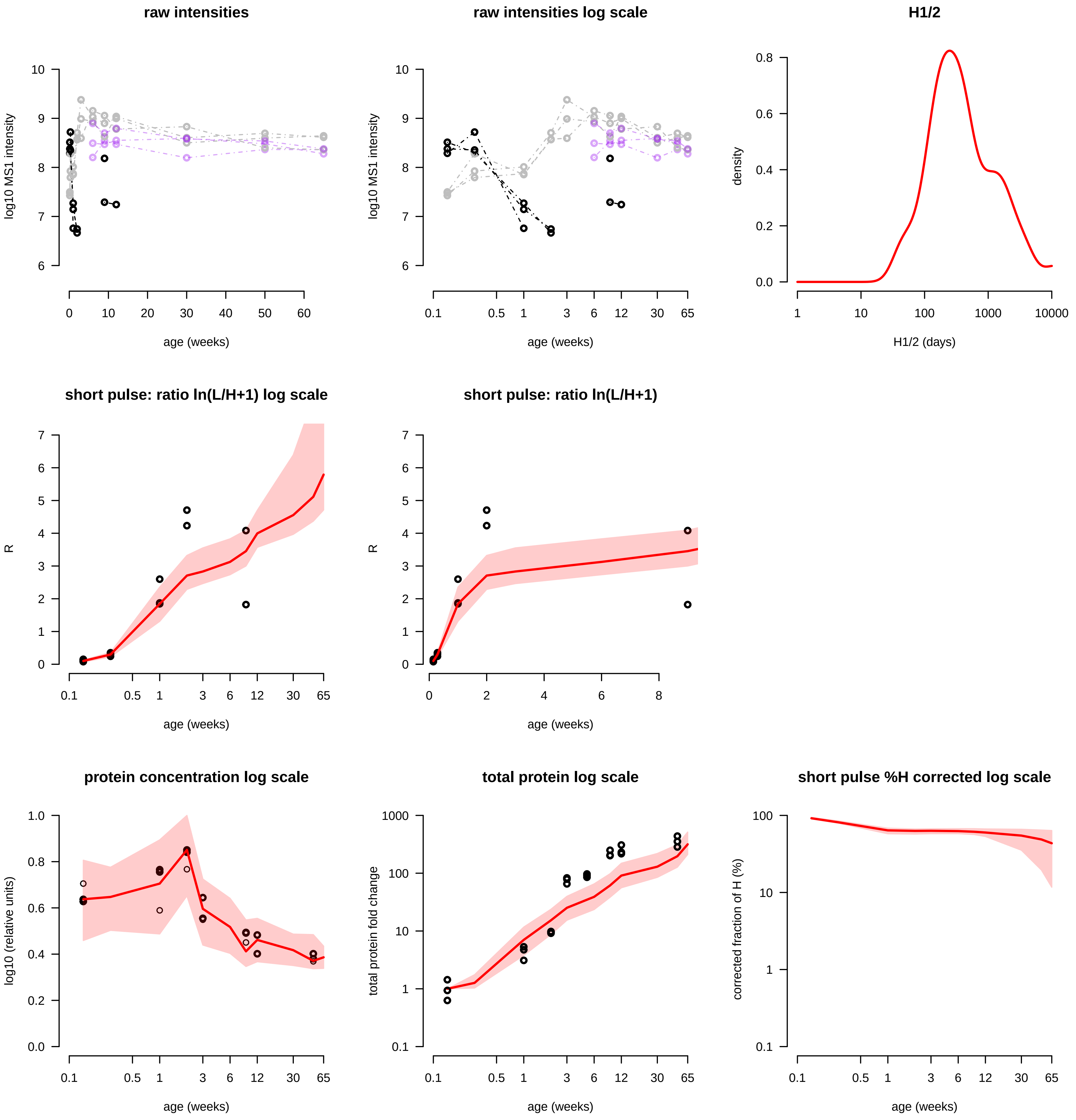

P29595

>sp|P29595|NEDD8_MOUSE

NEDD8

GN=Nedd8

Med H1/2: 18.8 days

Q5 H1/2: 5.5 days

Q95 H1/2: 173.2 days

reliable model fit: yes

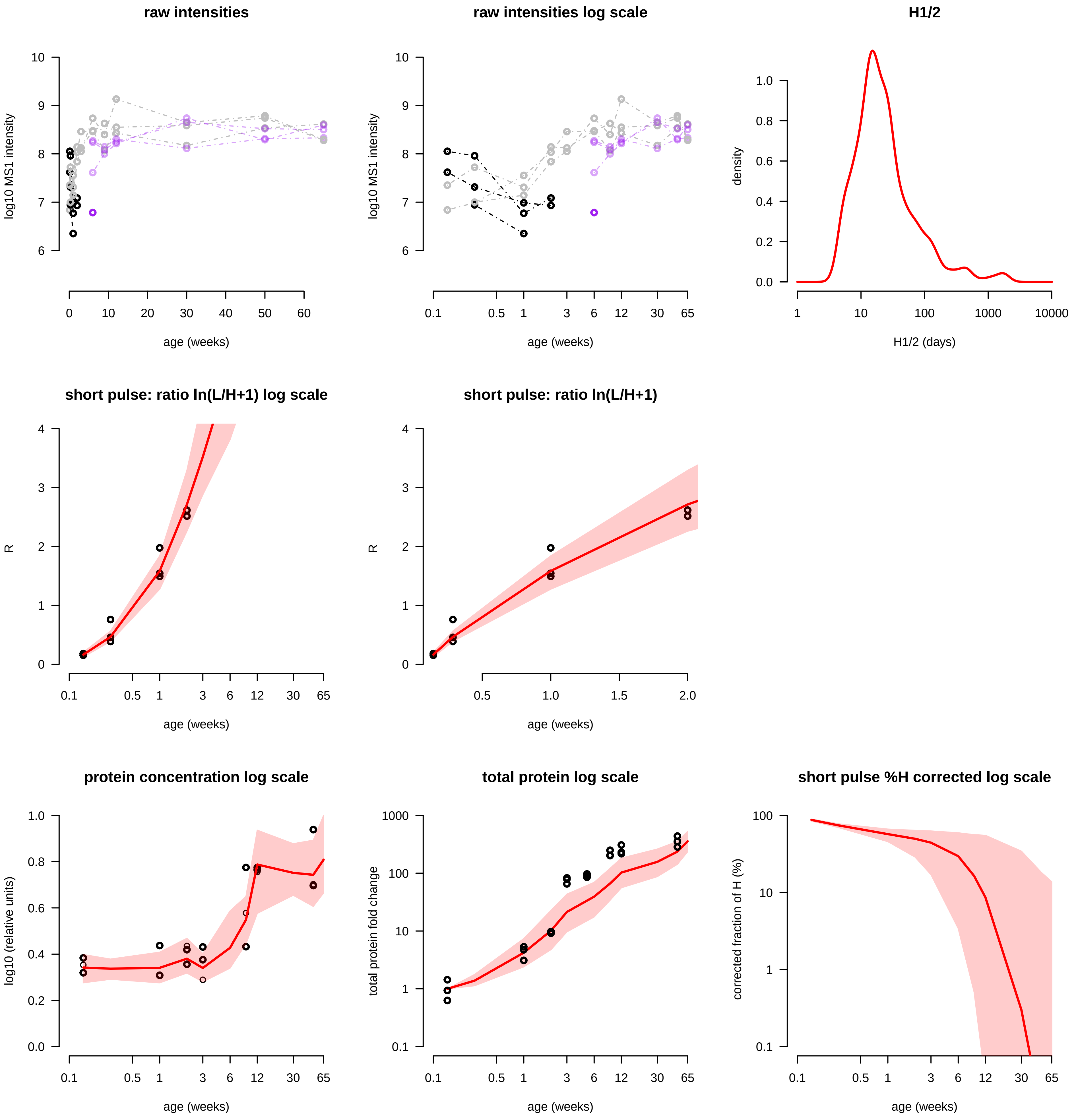

Q9JIQ3

>sp|Q9JIQ3|DBLOH_MOUSE

Diablo homolog, mitochondrial

GN=Diablo

Med H1/2: 7.3 days

Q5 H1/2: 2.9 days

Q95 H1/2: 76.1 days

reliable model fit: yes

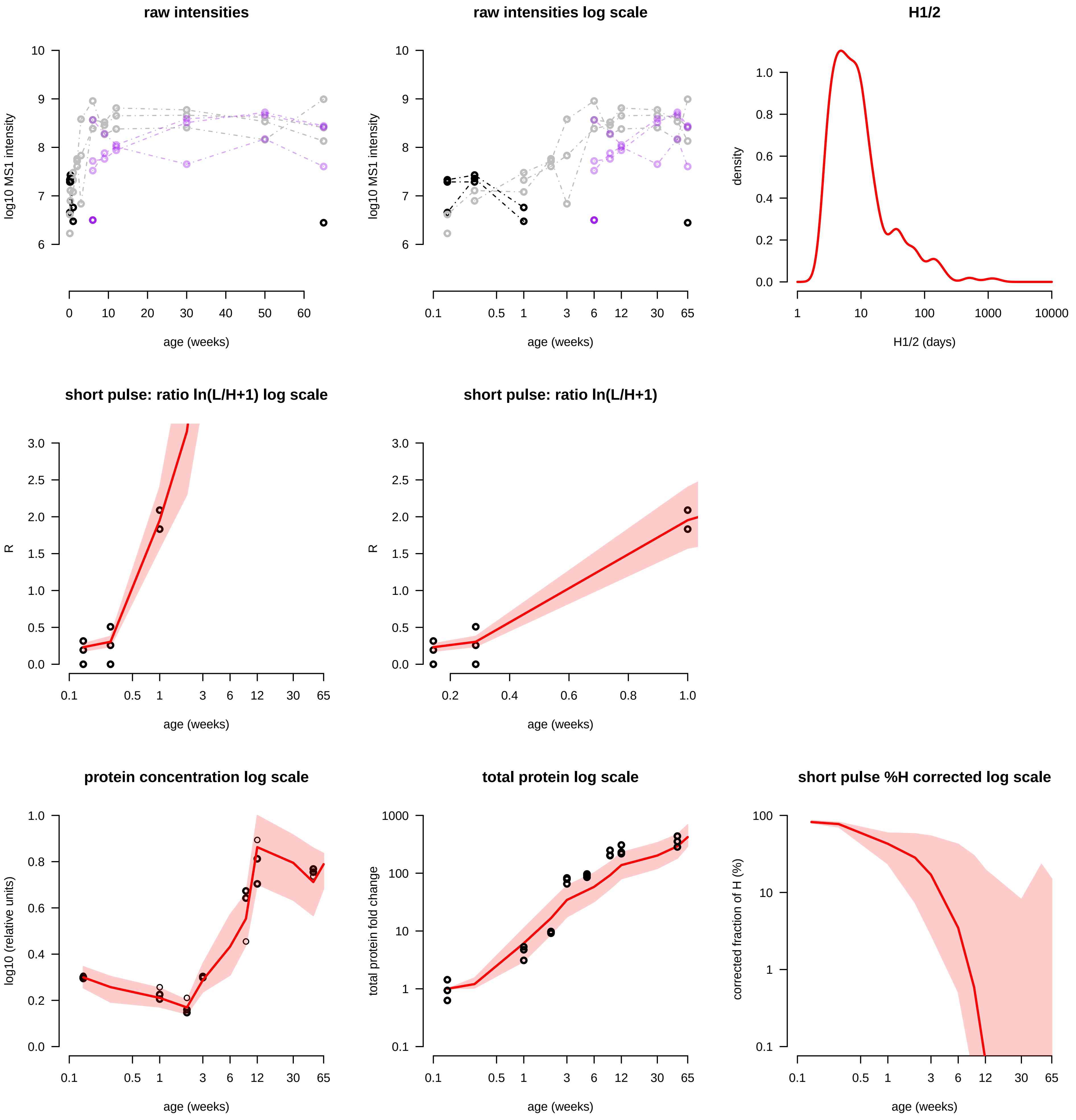

P98078;P98078−2;P98078−3

>sp|P98078|DAB2_MOUSE

Disabled homolog 2

GN=Dab2

Med H1/2: 3.7 days

Q5 H1/2: 3.1 days

Q95 H1/2: 4.7 days

reliable model fit: yes

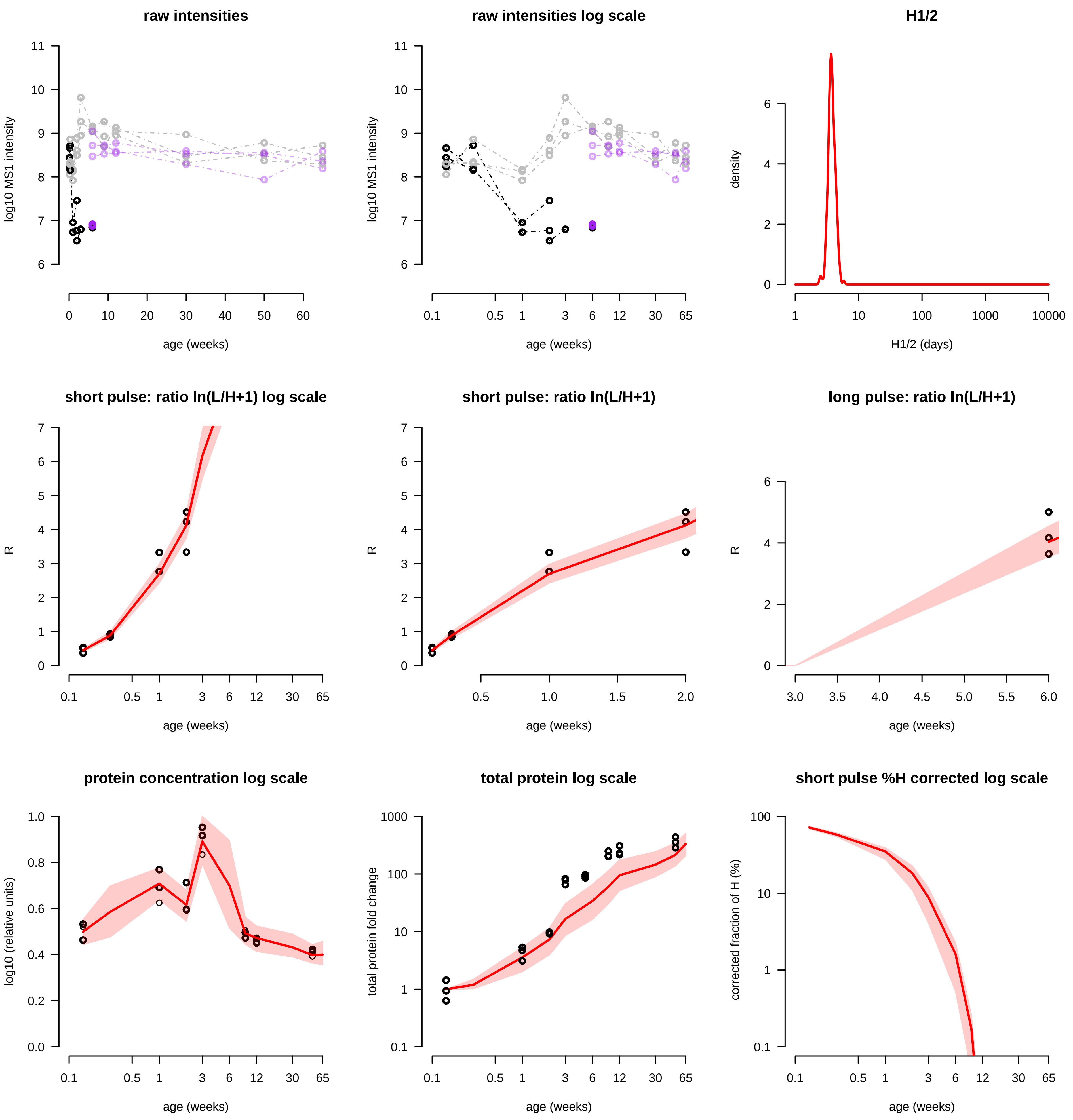

Q9CY66−2;Q9CY66;Q9CY66−3

>sp|Q9CY66−2|GAR1_MOUSE

Isoform 2 of H/ACA ribonucleoprotein complex subunit 1

GN=Gar1

Med H1/2: 6 days

Q5 H1/2: 4.3 days

Q95 H1/2: 8.9 days

reliable model fit: yes

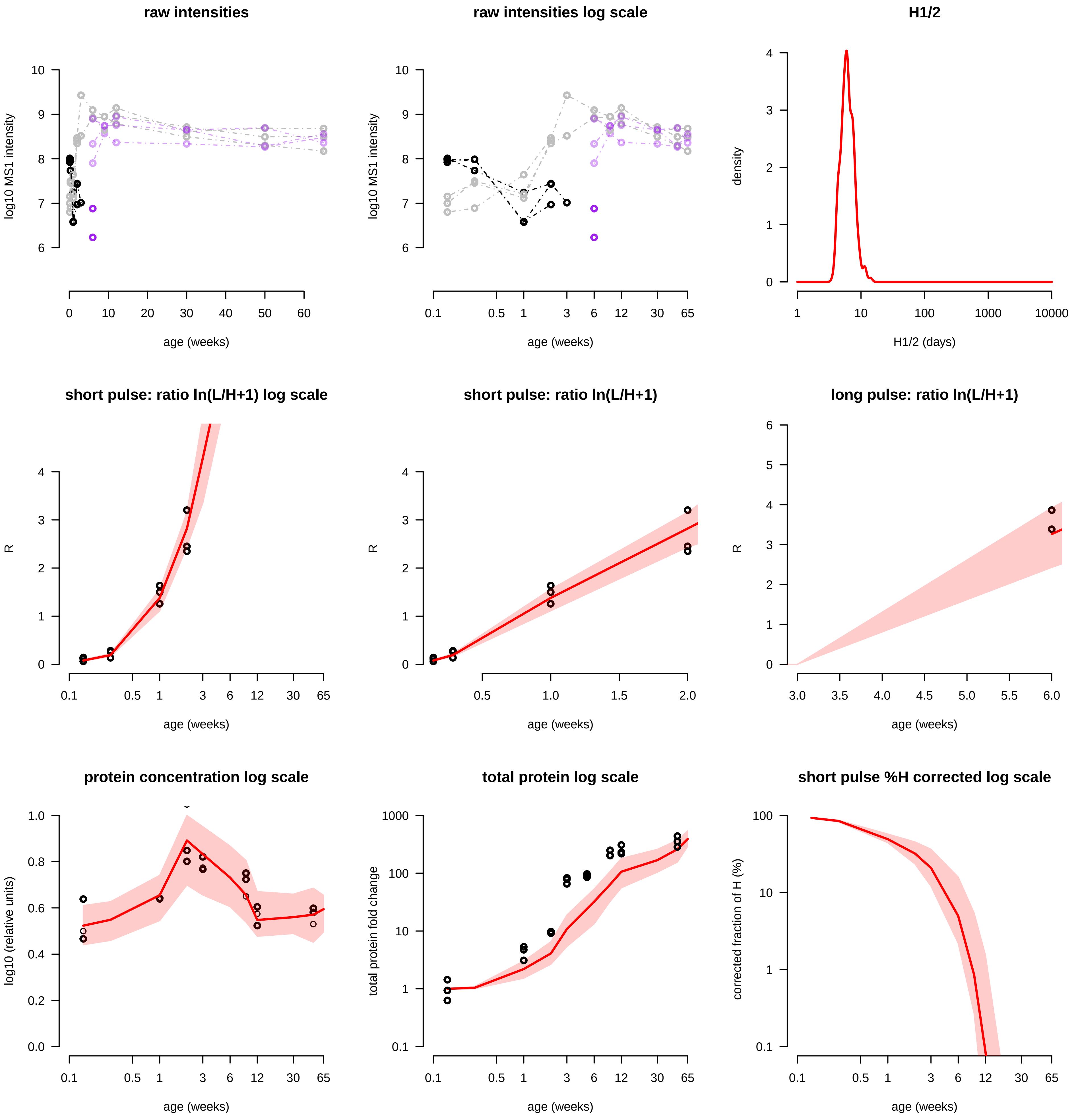

Q9QXT0

>sp|Q9QXT0|CNPY2_MOUSE

Protein canopy homolog 2

GN=Cnpy2

Med H1/2: 7.7 days

Q5 H1/2: 6 days

Q95 H1/2: 11.8 days

reliable model fit: yes

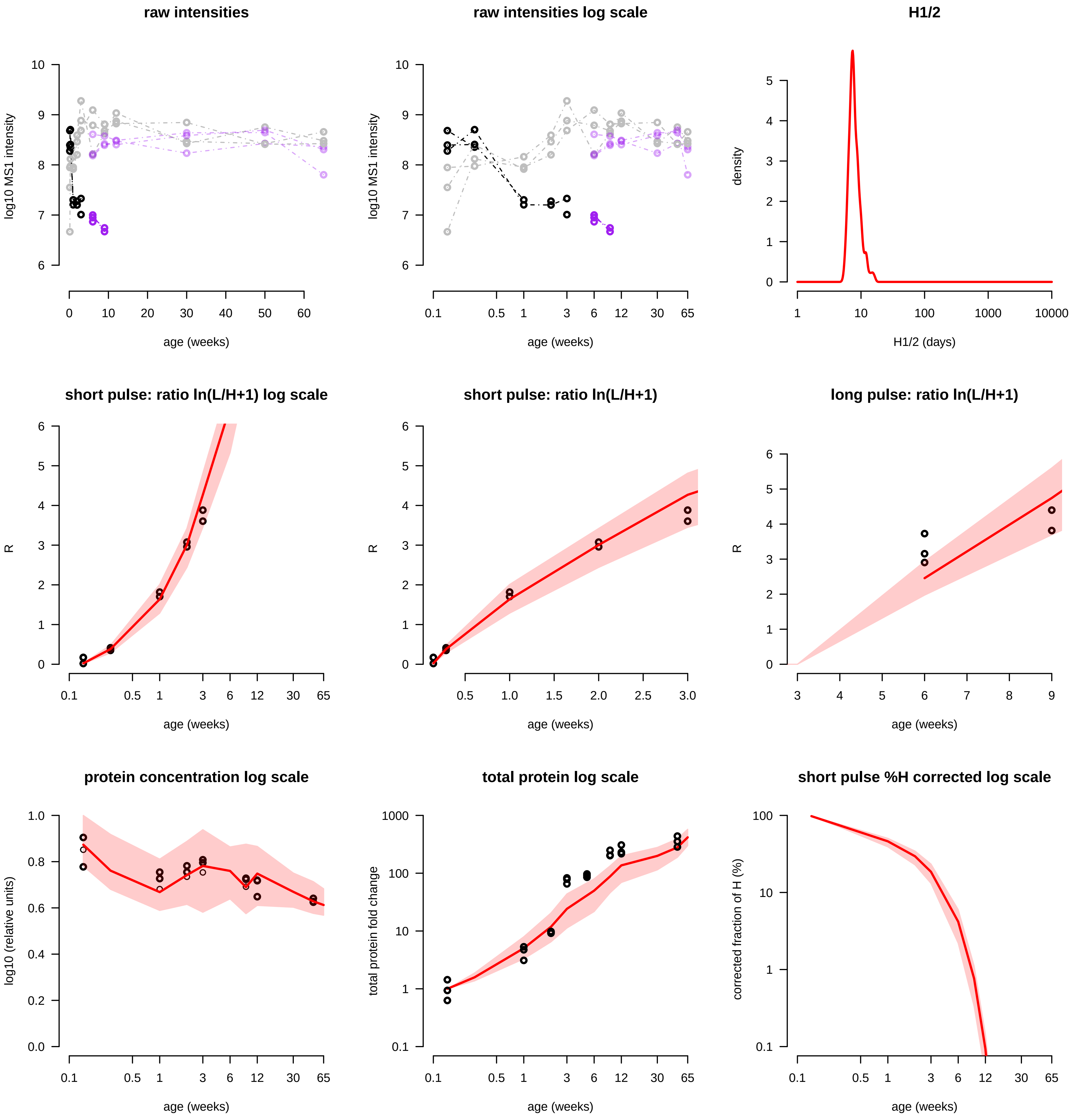

Q9Z1P6

>sp|Q9Z1P6|NDUA7_MOUSE

NADH dehydrogenase [ubiquinone] 1 alpha subcomplex subunit 7

GN=Ndufa7

Med H1/2: 4.3 days

Q5 H1/2: 2.7 days

Q95 H1/2: 6.1 days

reliable model fit: no

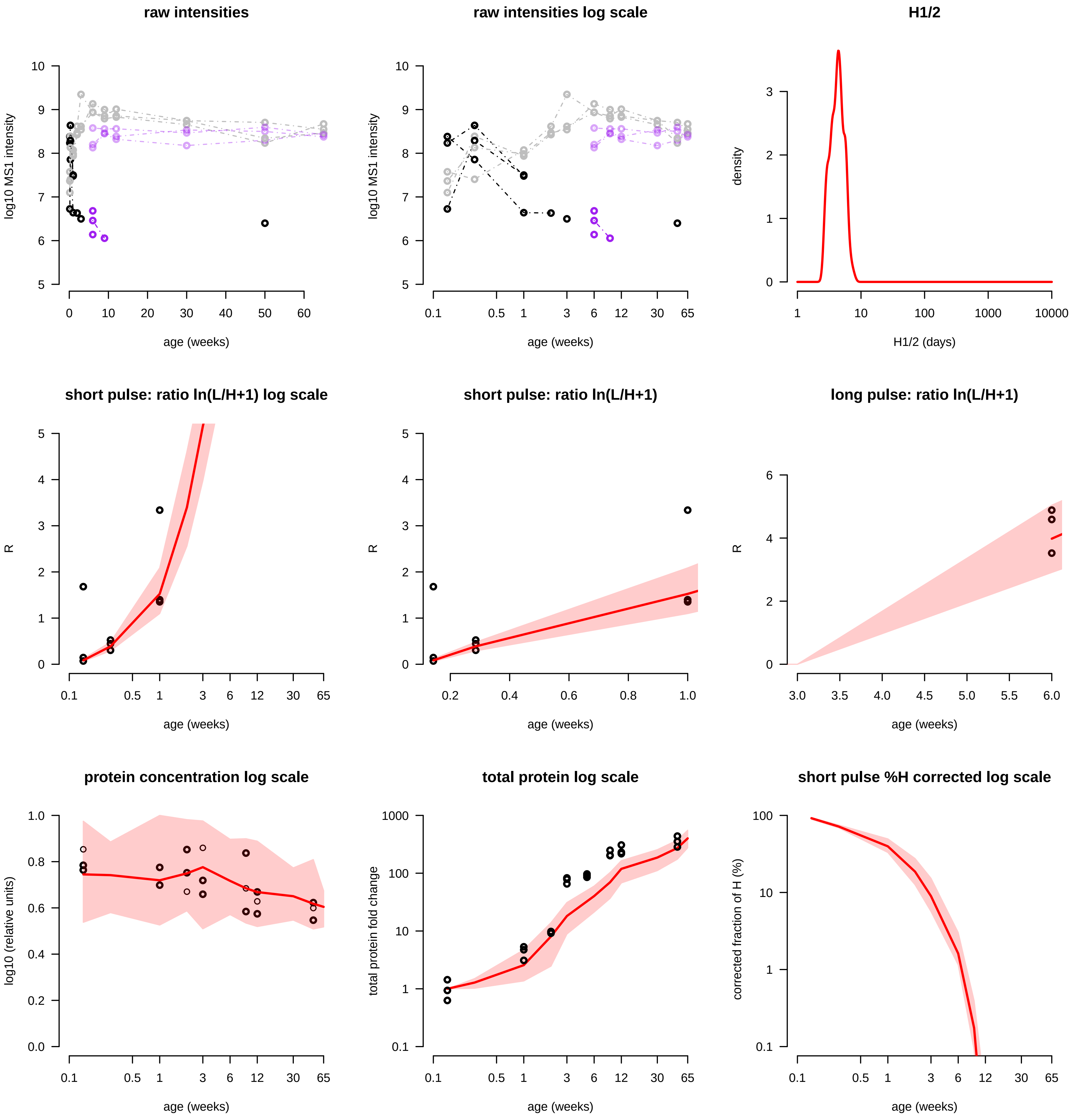

Q8BMB0−3;Q8BMB0

>sp|Q8BMB0−3|EMSY_MOUSE

Isoform 3 of BRCA2−interacting transcriptional repressor EMSY

GN=Emsy

Med H1/2: 13.6 days

Q5 H1/2: 4.3 days

Q95 H1/2: 134.1 days

reliable model fit: yes

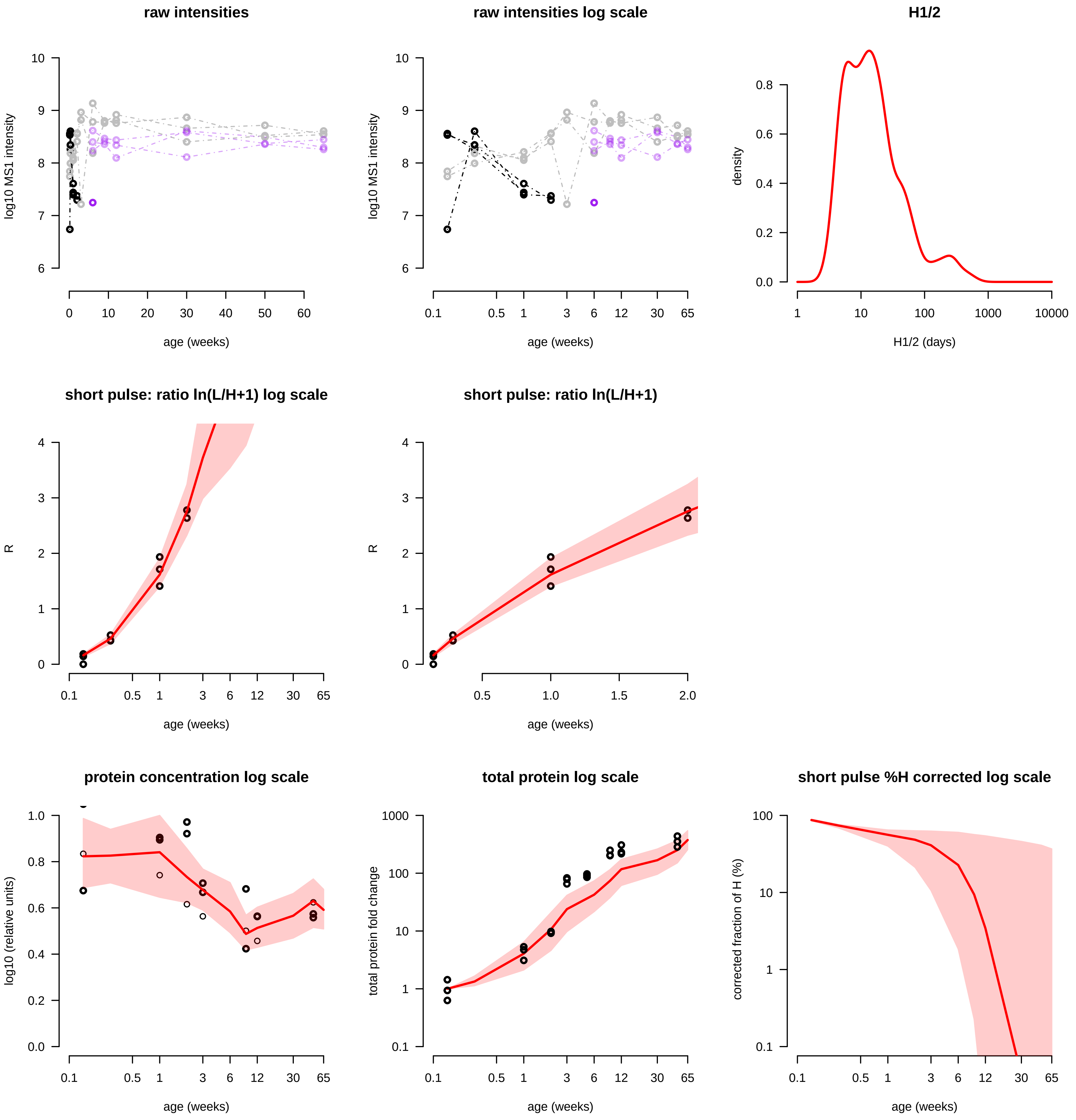

P25799;P25799−3;P25799−2;P25799−4

>sp|P25799|NFKB1_MOUSE

Nuclear factor NF−kappa−B p105 subunit

GN=Nfkb1

Med H1/2: 1701.7 days

Q5 H1/2: 427.9 days

Q95 H1/2: 29473.9 days

reliable model fit: no

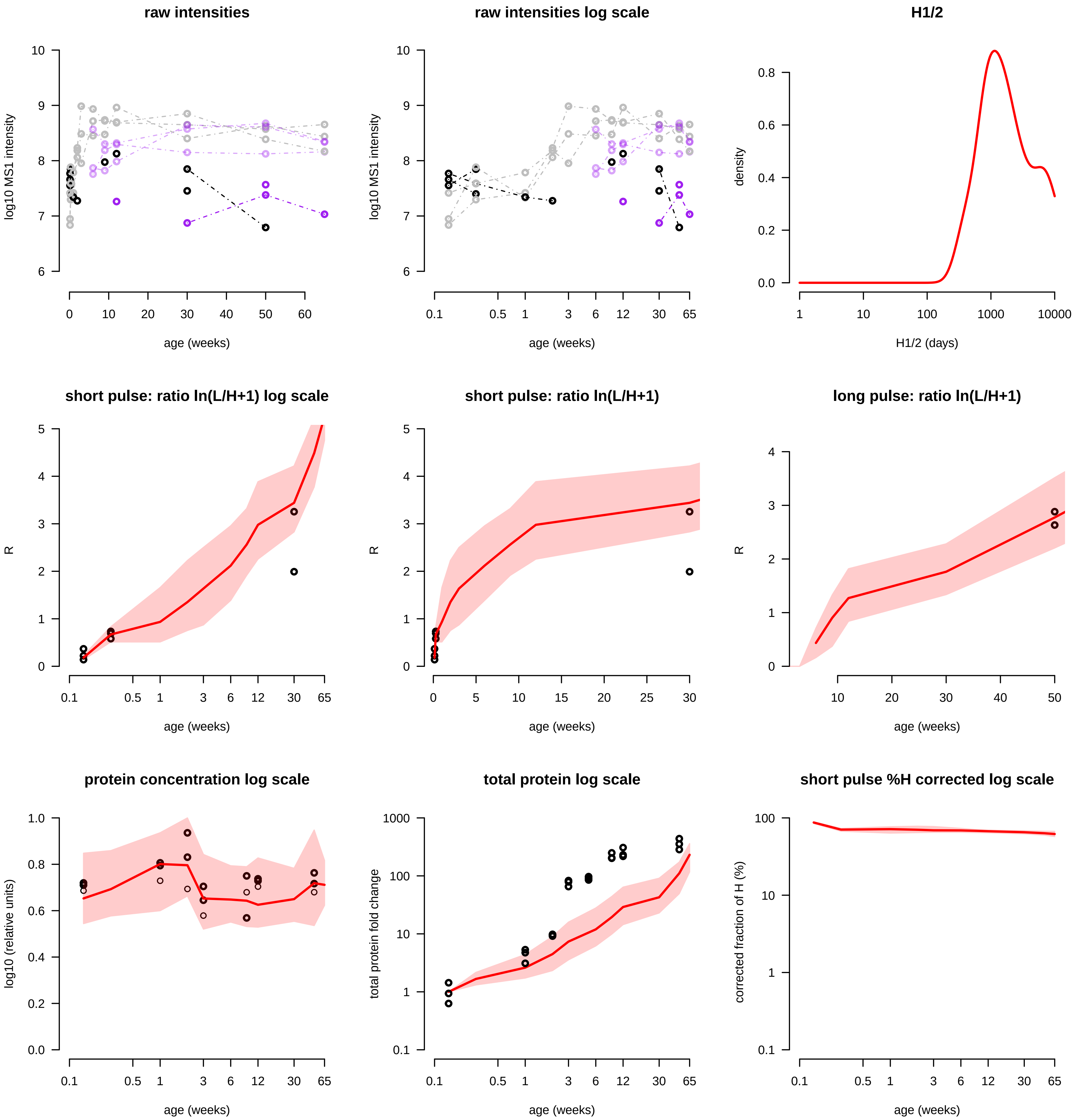

Q9D1J1

>sp|Q9D1J1|NECP2_MOUSE

Adaptin ear−binding coat−associated protein 2

GN=Necap2

Med H1/2: 10.4 days

Q5 H1/2: 3.7 days

Q95 H1/2: 87.9 days

reliable model fit: yes

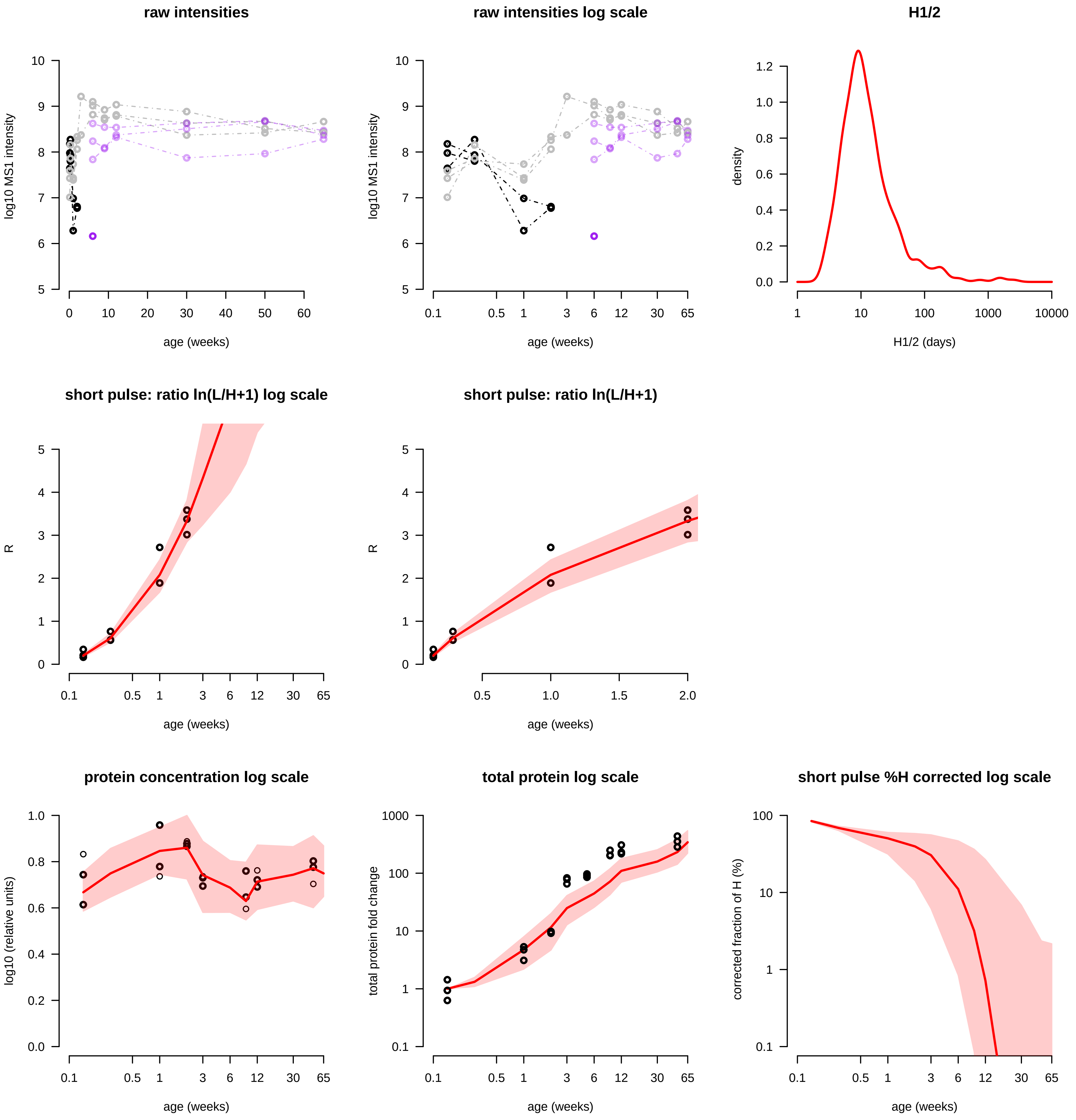

Q99LE6

>sp|Q99LE6|ABCF2_MOUSE

ATP−binding cassette sub−family F member 2

GN=Abcf2

Med H1/2: 3.6 days

Q5 H1/2: 2.5 days

Q95 H1/2: 14.6 days

reliable model fit: yes

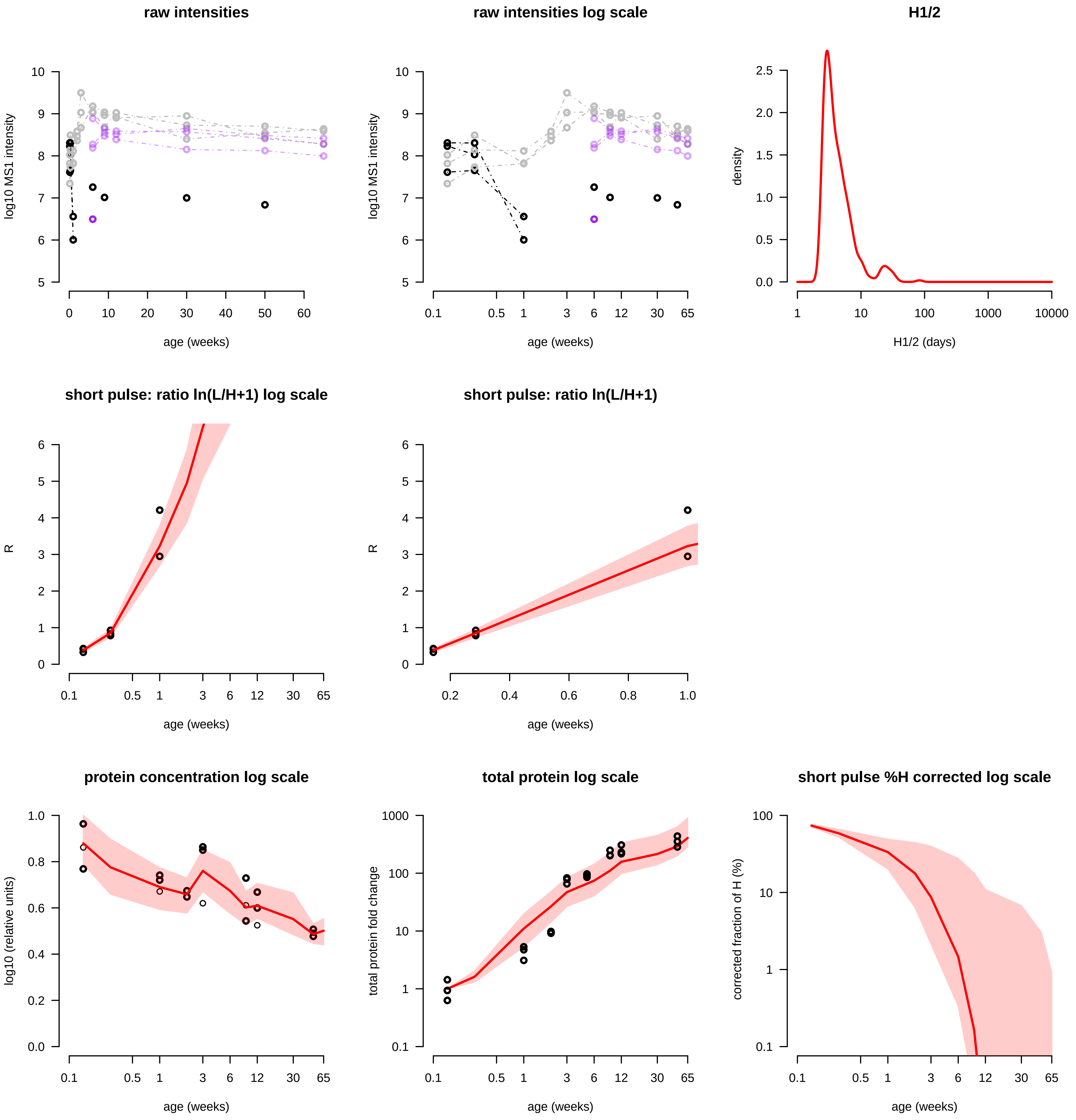

Q62077

>sp|Q62077|PLCG1_MOUSE

1−phosphatidylinositol 4,5−bisphosphate phosphodiesterase gamma−1

GN=Plcg1

Med H1/2: 4.7 days

Q5 H1/2: 2.6 days

Q95 H1/2: 17.6 days

reliable model fit: yes

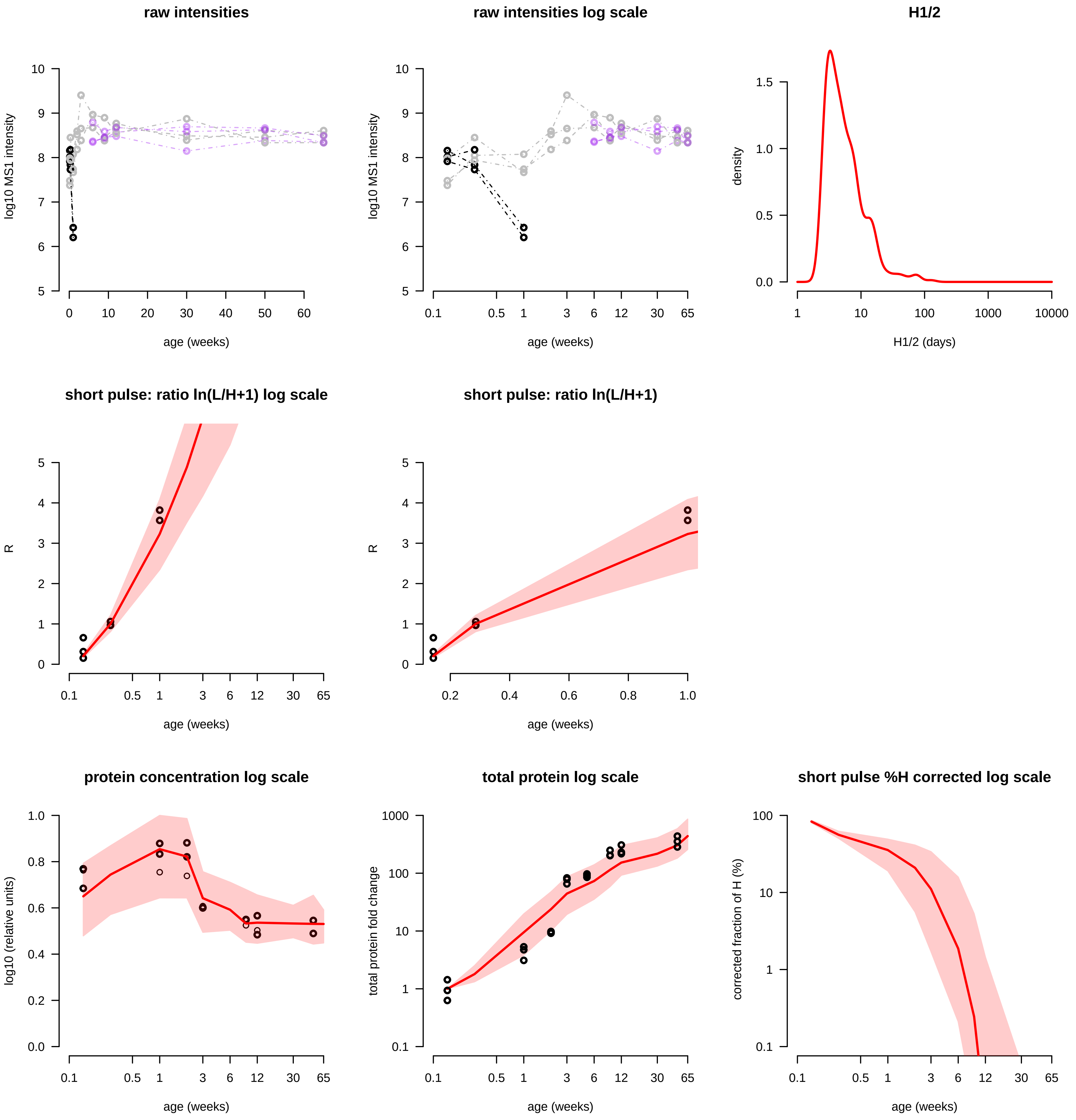

Q62523

>sp|Q62523|ZYX_MOUSE

Zyxin

GN=Zyx

Med H1/2: 4.8 days

Q5 H1/2: 2.7 days

Q95 H1/2: 14.7 days

reliable model fit: yes

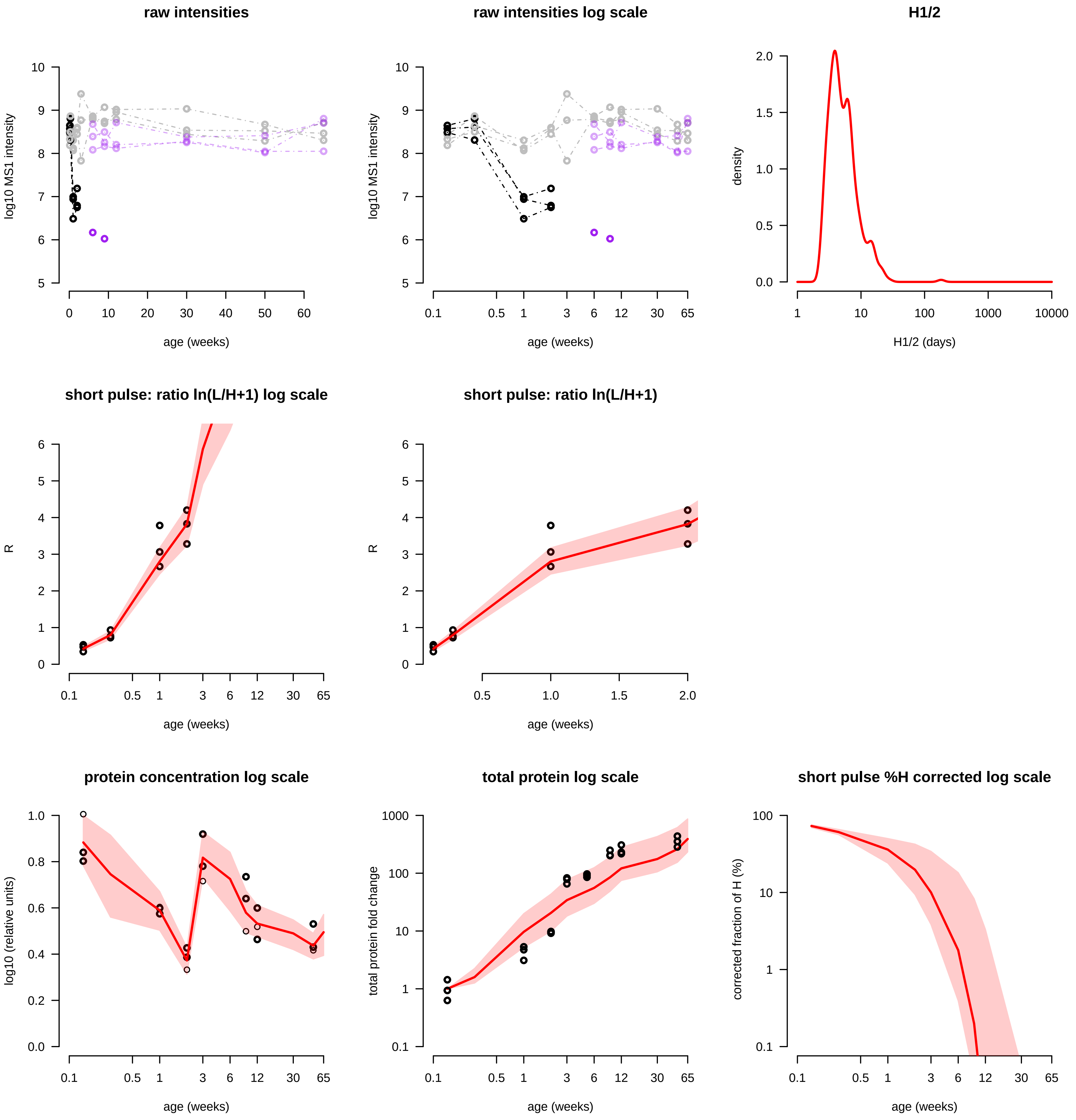

Q9DAW6

>sp|Q9DAW6|PRP4_MOUSE

U4/U6 small nuclear ribonucleoprotein Prp4

GN=Prpf4

Med H1/2: 5.7 days

Q5 H1/2: 3.2 days

Q95 H1/2: 18.5 days

reliable model fit: yes

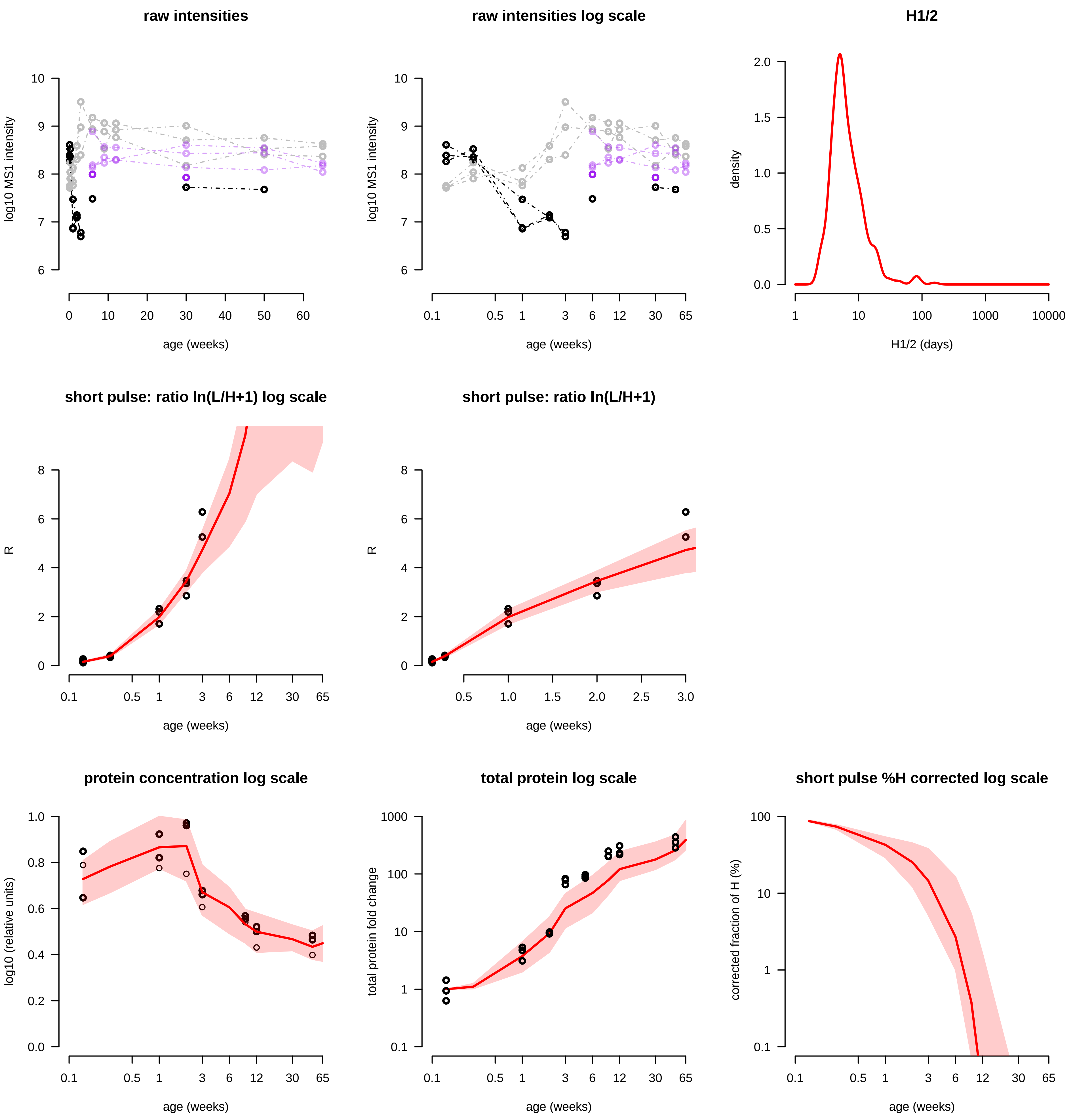

Q8BG81

>sp|Q8BG81|PDIP3_MOUSE

Polymerase delta−interacting protein 3

GN=Poldip3

Med H1/2: 3.9 days

Q5 H1/2: 3.1 days

Q95 H1/2: 5.3 days

reliable model fit: yes

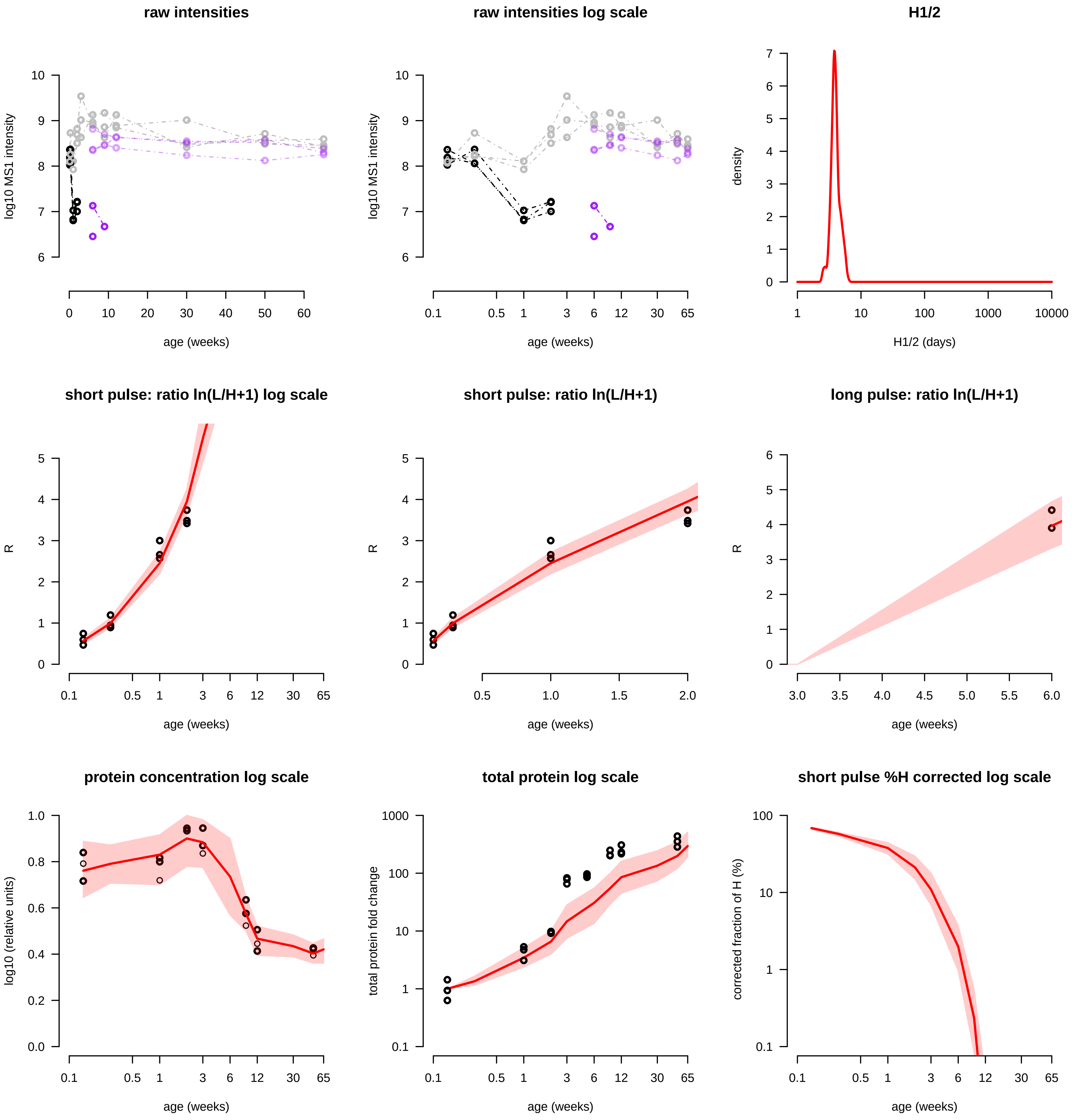

P16460

>sp|P16460|ASSY_MOUSE

Argininosuccinate synthase

GN=Ass1

Med H1/2: 3.8 days

Q5 H1/2: 3 days

Q95 H1/2: 5.8 days

reliable model fit: yes

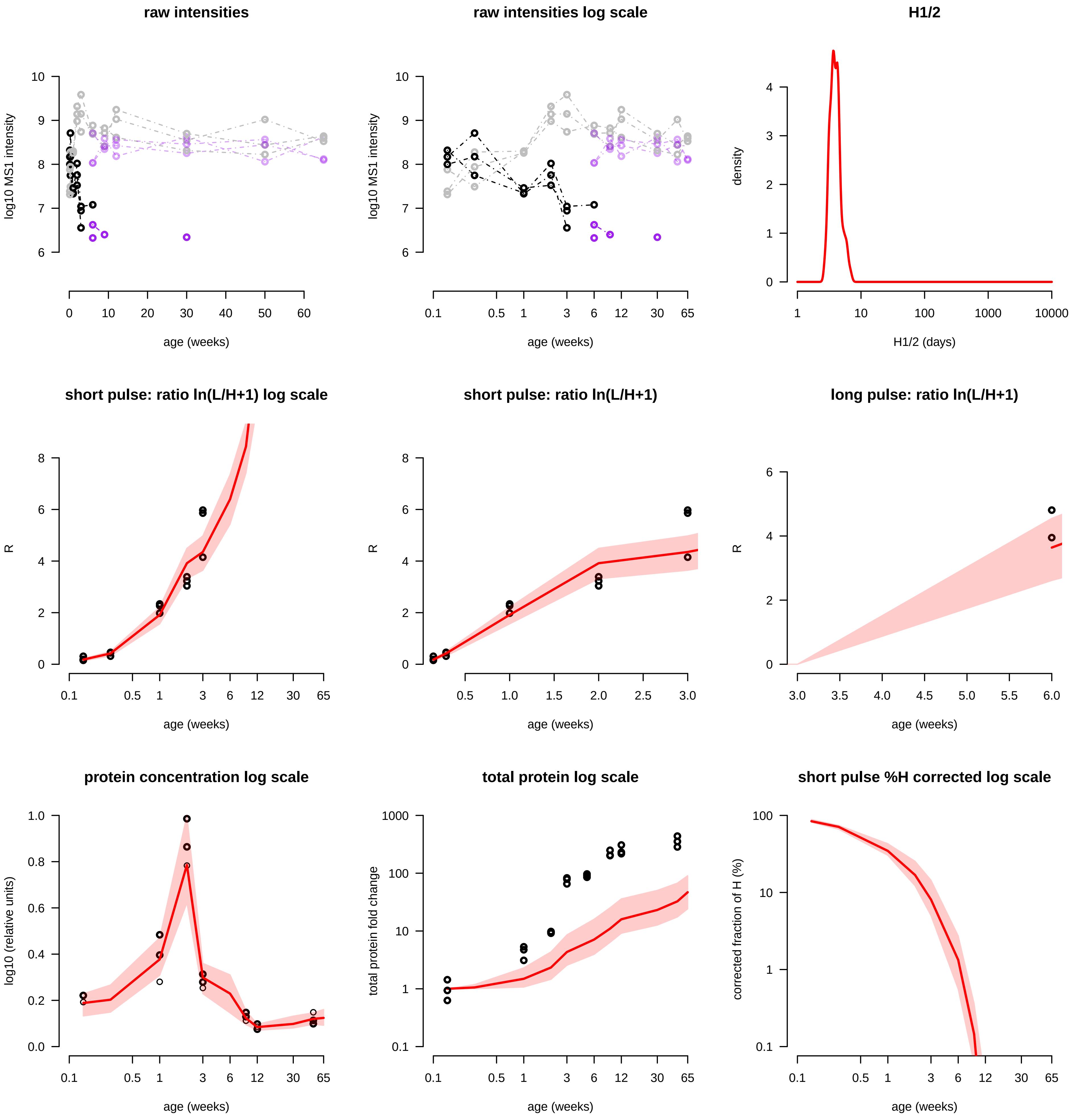

Q8VE47

>sp|Q8VE47|UBA5_MOUSE

Ubiquitin−like modifier−activating enzyme 5

GN=Uba5

Med H1/2: 5.7 days

Q5 H1/2: 2.6 days

Q95 H1/2: 21.2 days

reliable model fit: yes

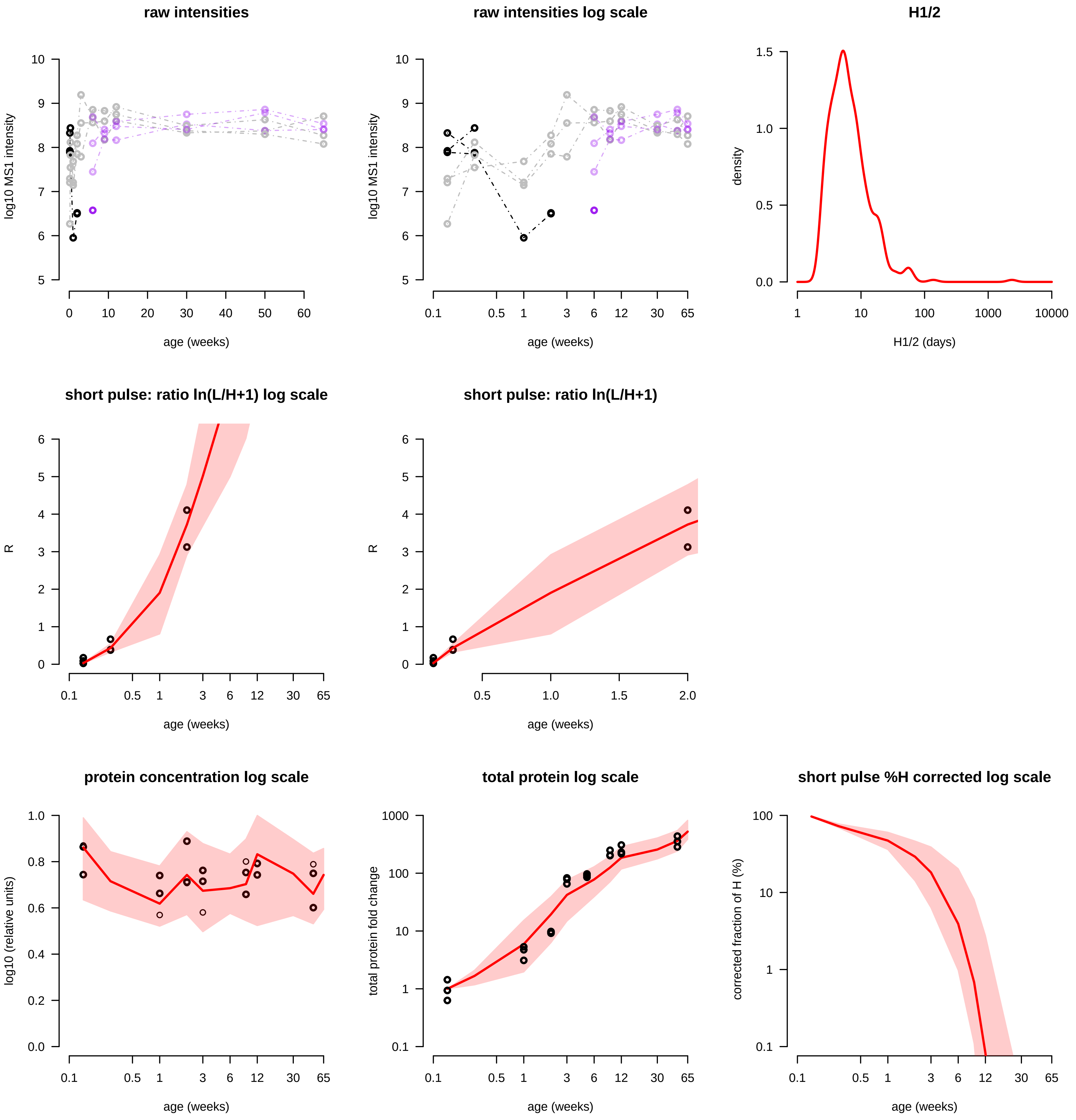

Q78JW9

>sp|Q78JW9|UBFD1_MOUSE

Ubiquitin domain−containing protein UBFD1

GN=Ubfd1

Med H1/2: 9.9 days

Q5 H1/2: 5.1 days

Q95 H1/2: 30.1 days

reliable model fit: yes

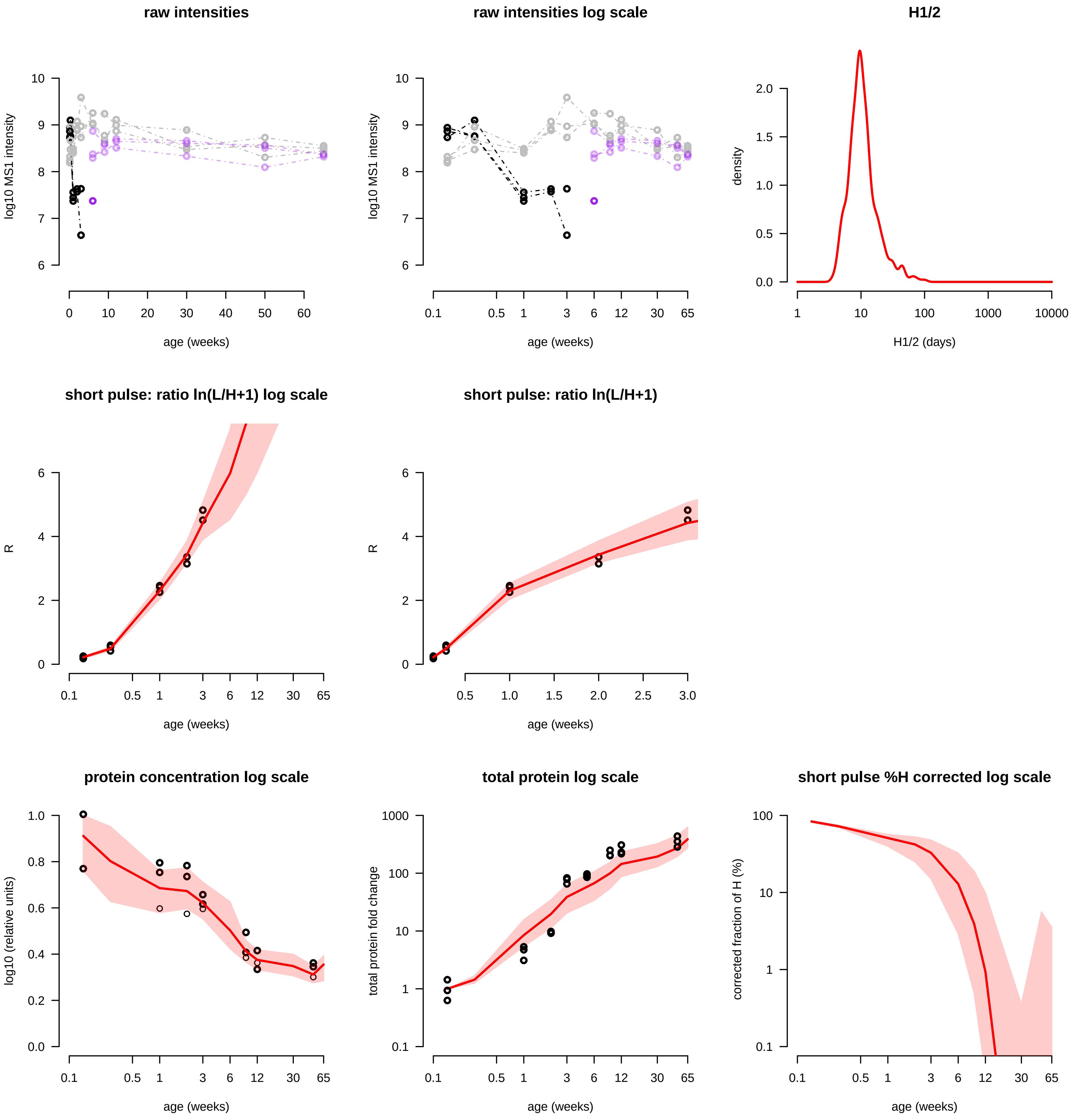

Q3UMB9

>sp|Q3UMB9|WASC4_MOUSE

WASH complex subunit 4

GN=Washc4

Med H1/2: 427.4 days

Q5 H1/2: 104.5 days

Q95 H1/2: 6478.8 days

reliable model fit: no

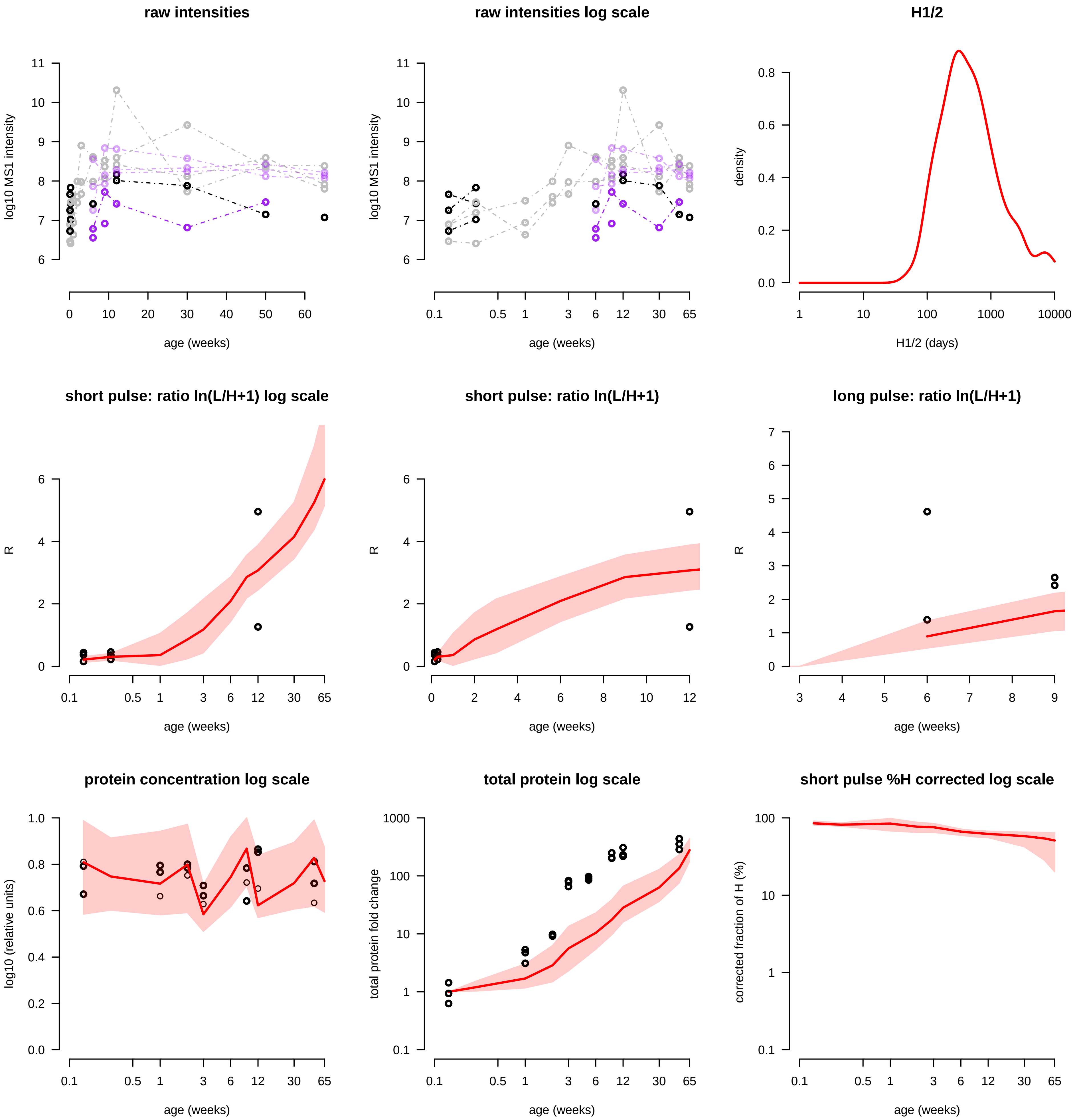

Q99KD5

>sp|Q99KD5|UN45A_MOUSE

Protein unc−45 homolog A

GN=Unc45a

Med H1/2: 12 days

Q5 H1/2: 8.7 days

Q95 H1/2: 19.7 days

reliable model fit: no

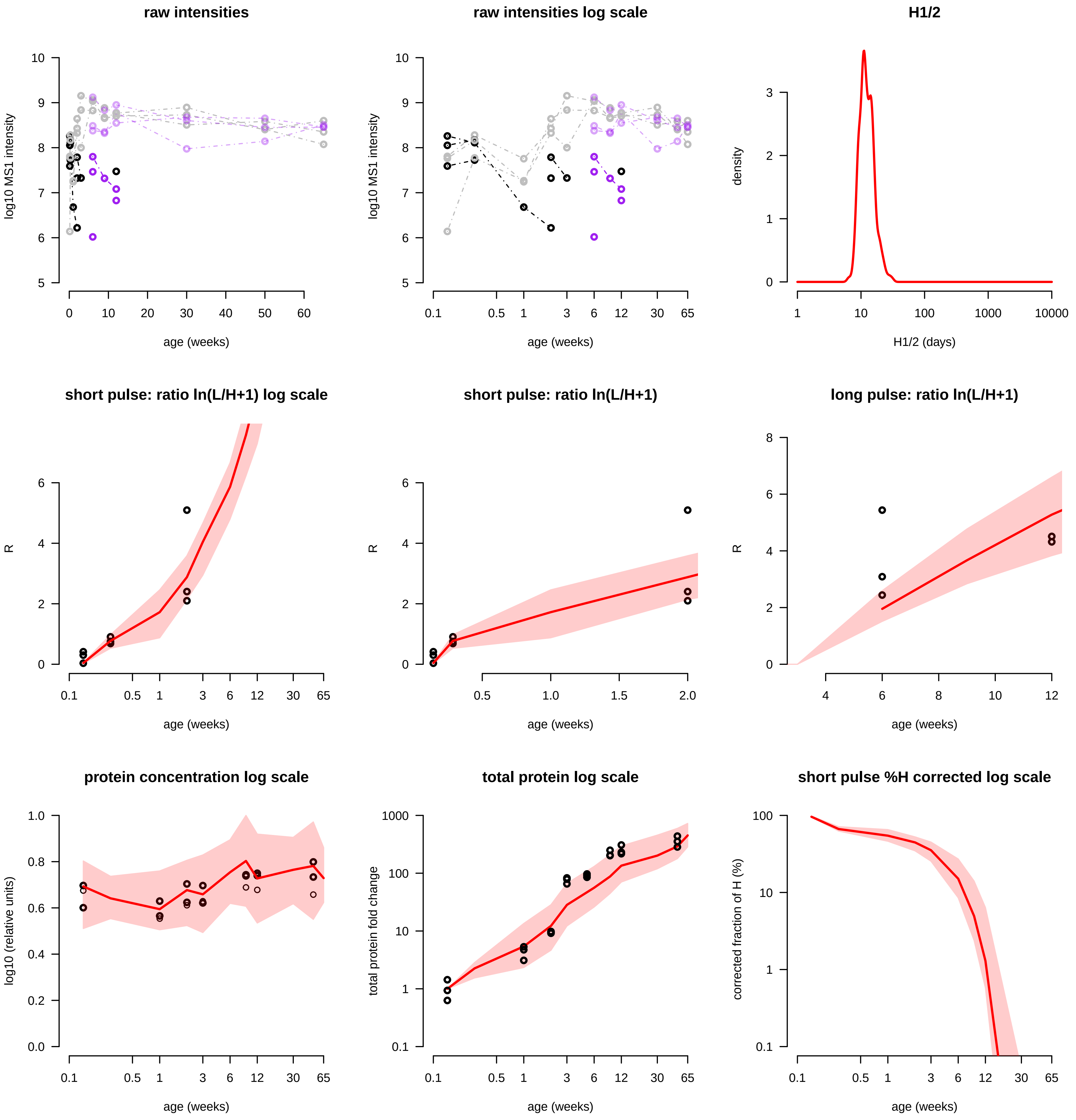

P27048;P63163

>sp|P27048|RSMB_MOUSE

Small nuclear ribonucleoprotein−associated protein B

GN=Snrpb

Med H1/2: 5.9 days

Q5 H1/2: 4.9 days

Q95 H1/2: 7.6 days

reliable model fit: yes

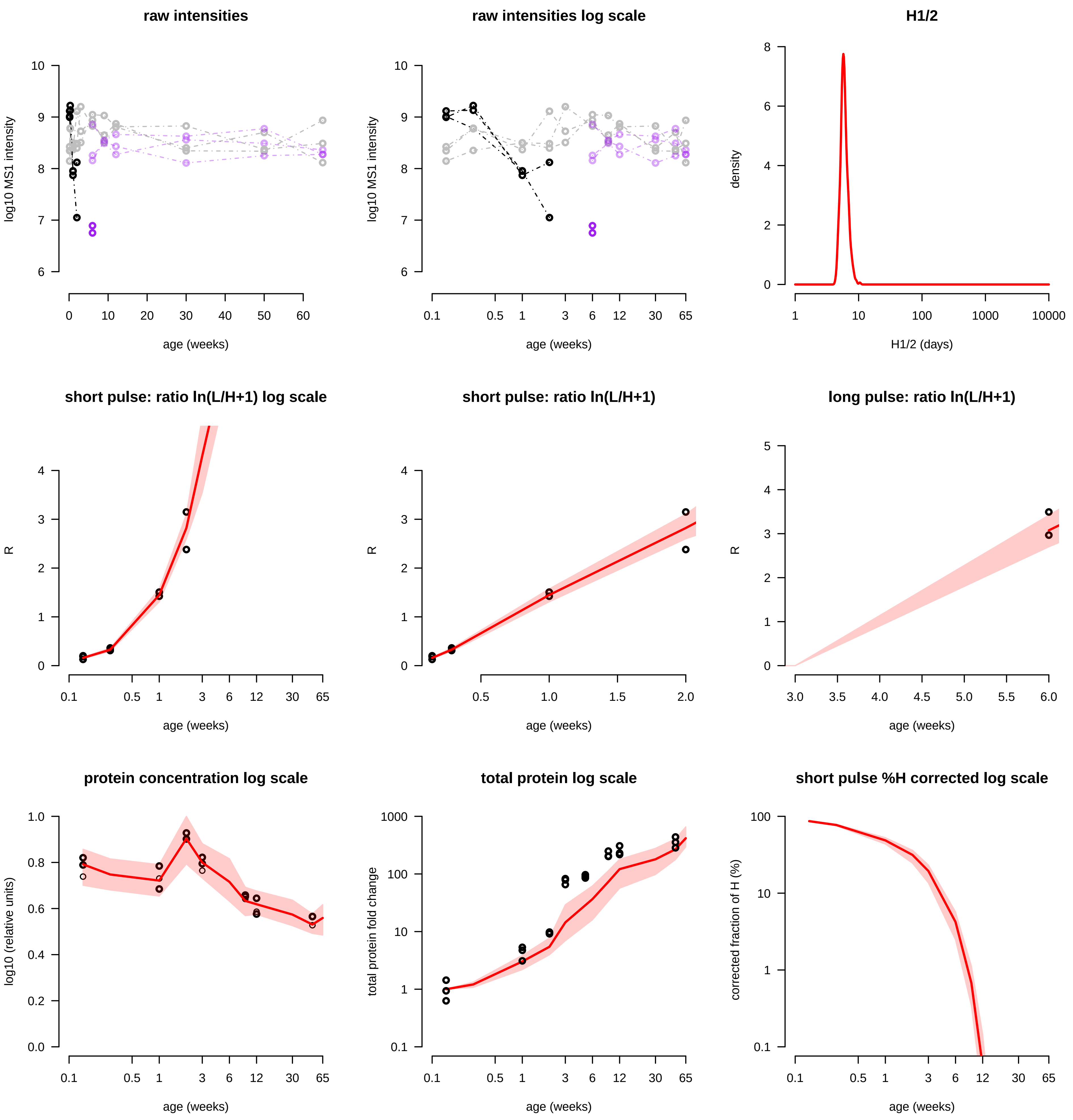

Q80ZK0

>sp|Q80ZK0|RT10_MOUSE

28S ribosomal protein S10, mitochondrial

GN=Mrps10

Med H1/2: 6.6 days

Q5 H1/2: 3.1 days

Q95 H1/2: 69.1 days

reliable model fit: yes

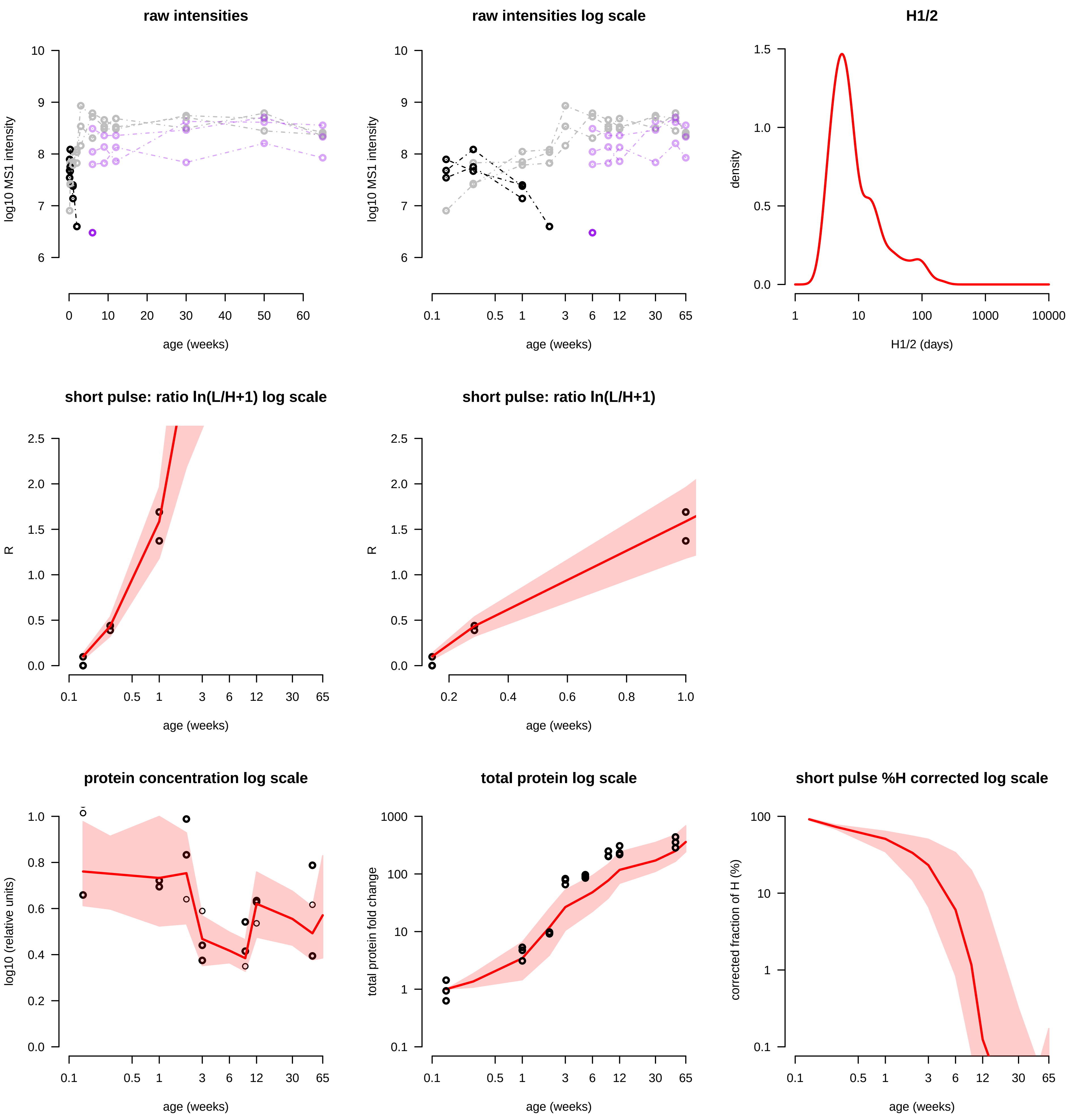

Q61576

>sp|Q61576|FKB10_MOUSE

Peptidyl−prolyl cis−trans isomerase FKBP10

GN=Fkbp10

Med H1/2: 4.8 days

Q5 H1/2: 3.8 days

Q95 H1/2: 6.2 days

reliable model fit: yes

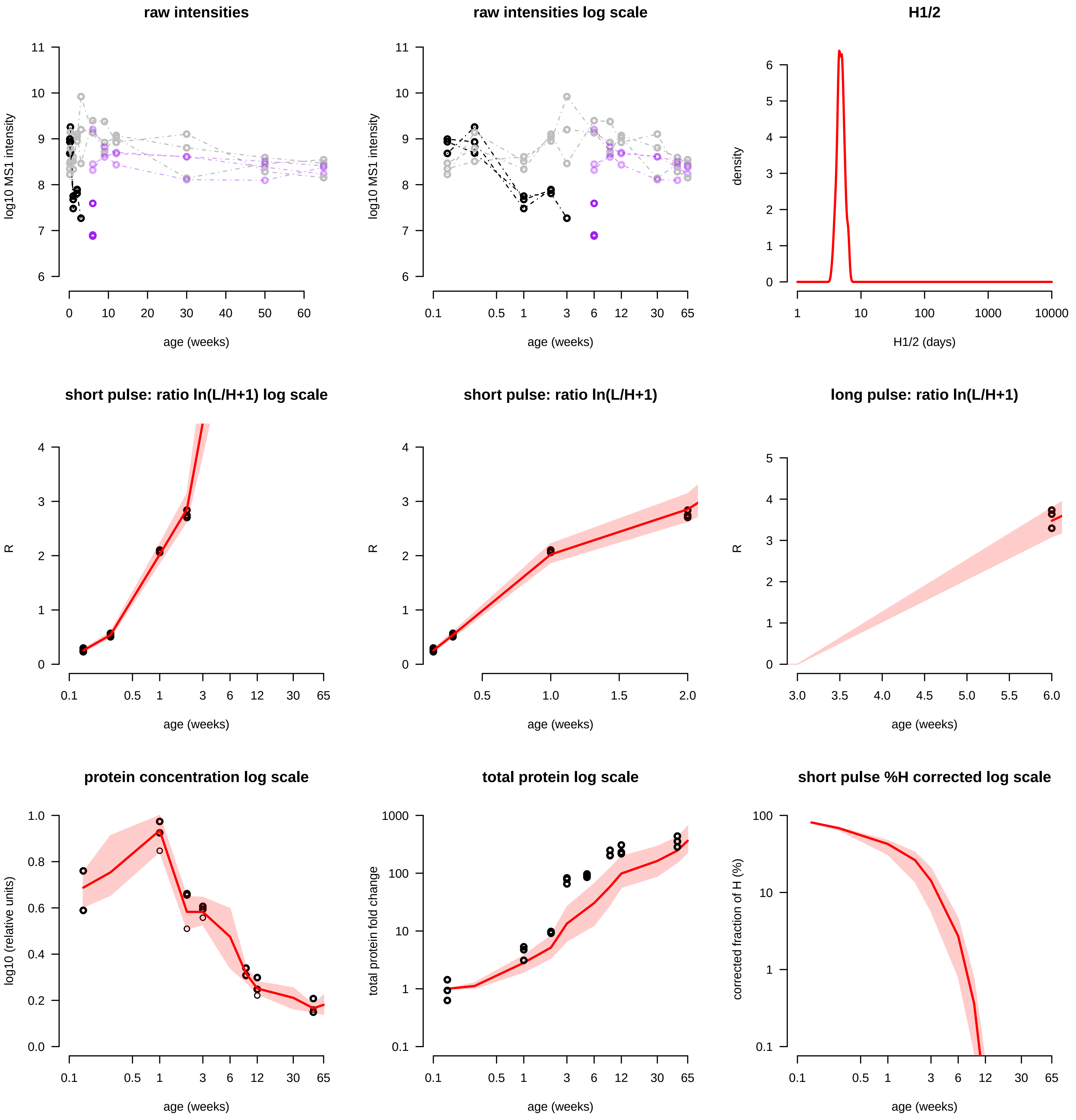

Q8BK63

>sp|Q8BK63|KC1A_MOUSE

Casein kinase I isoform alpha

GN=Csnk1a1

Med H1/2: 11.2 days

Q5 H1/2: 3.2 days

Q95 H1/2: 62.9 days

reliable model fit: yes

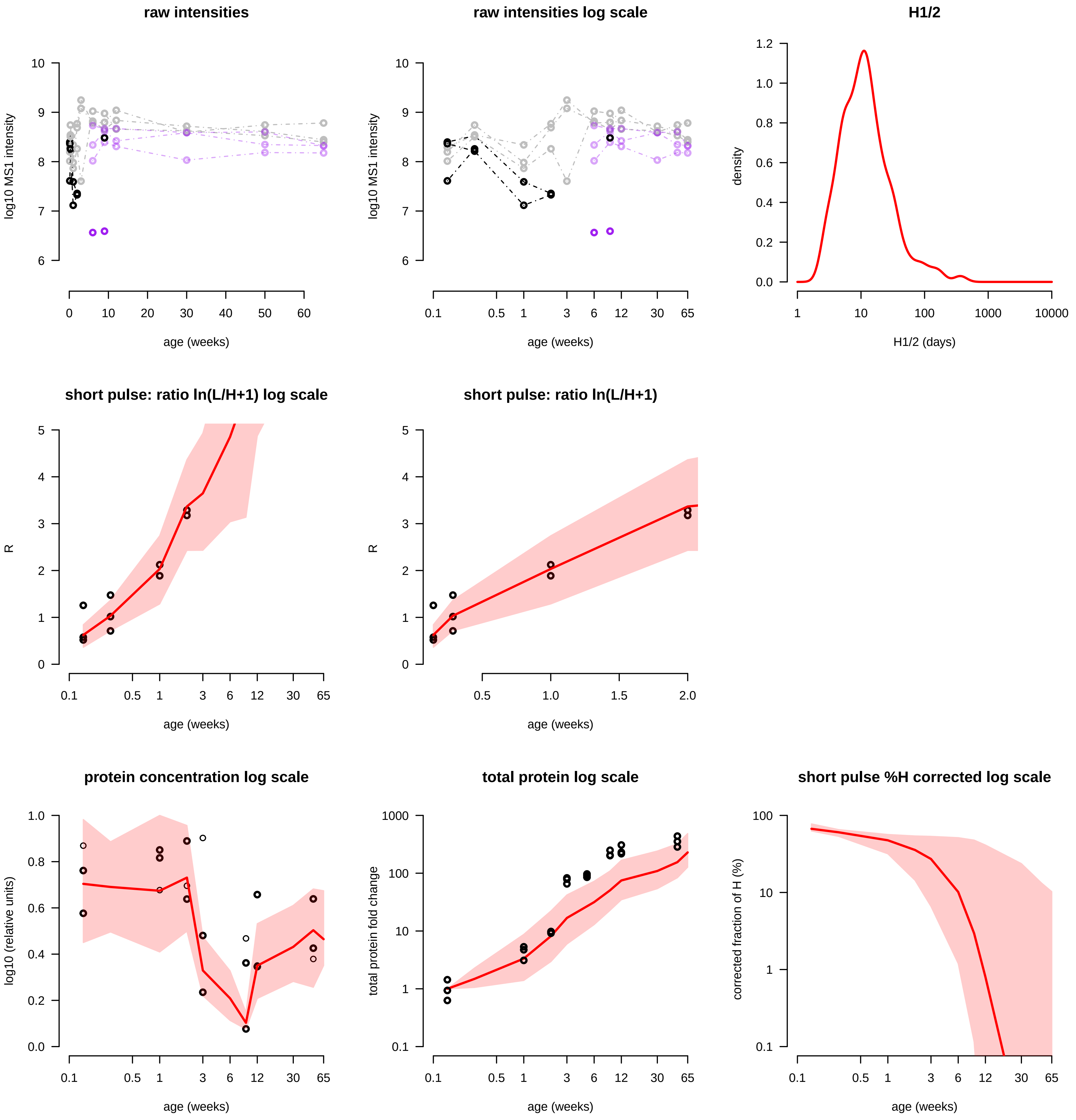

P70302

>sp|P70302|STIM1_MOUSE

Stromal interaction molecule 1

GN=Stim1

Med H1/2: 4.2 days

Q5   H1/2: 2.7 days

Q95 H1/2: 9.8 days

reliable model fit: yes

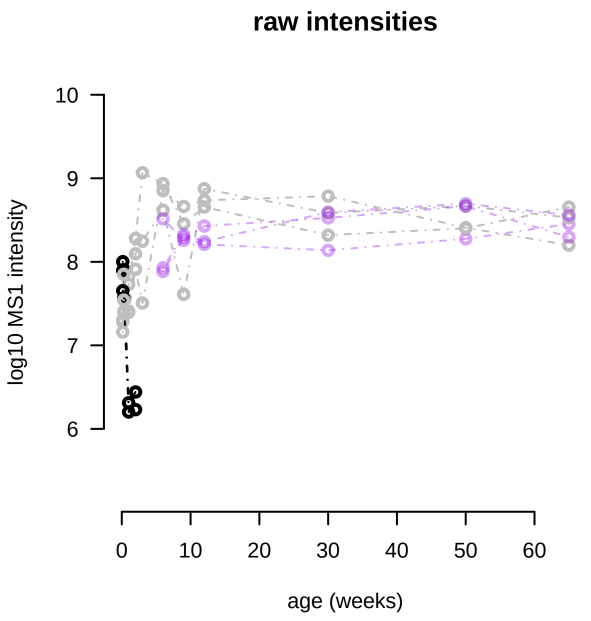

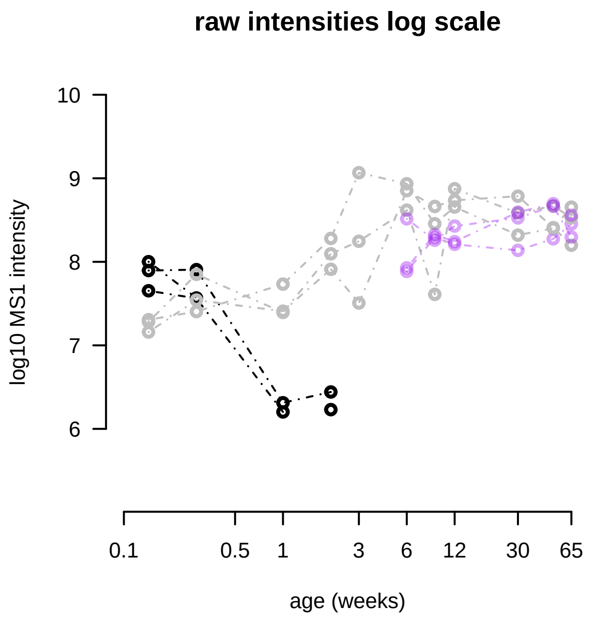

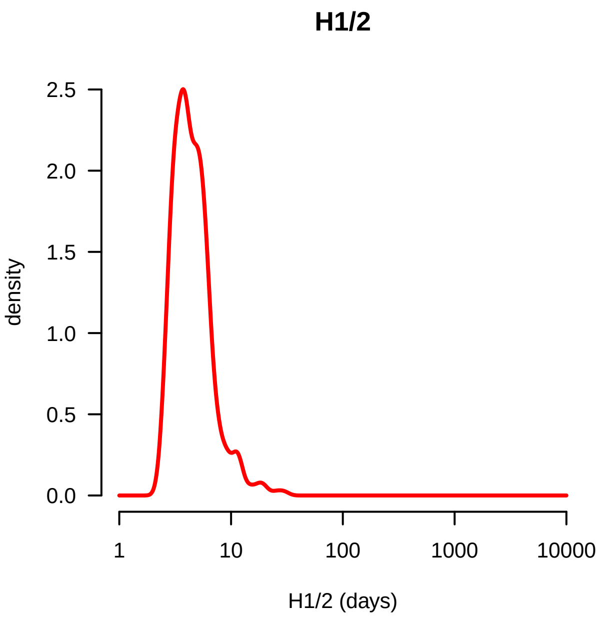

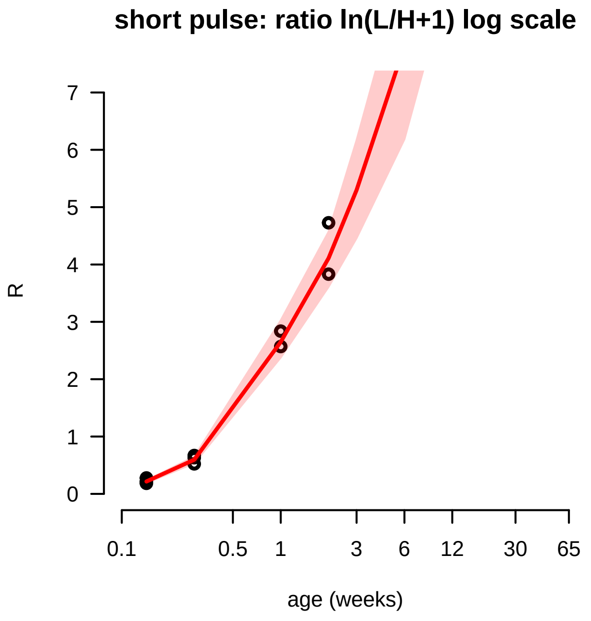

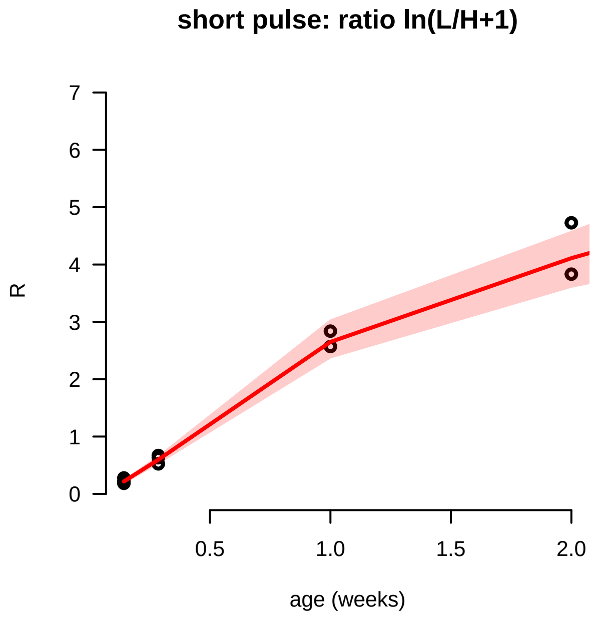

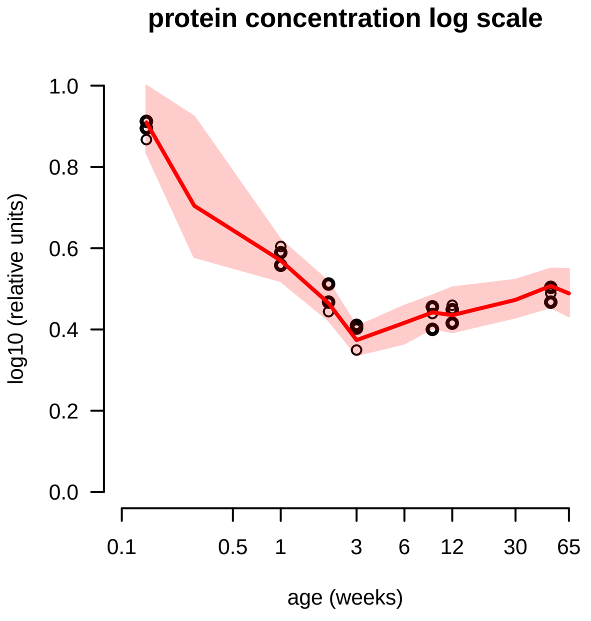

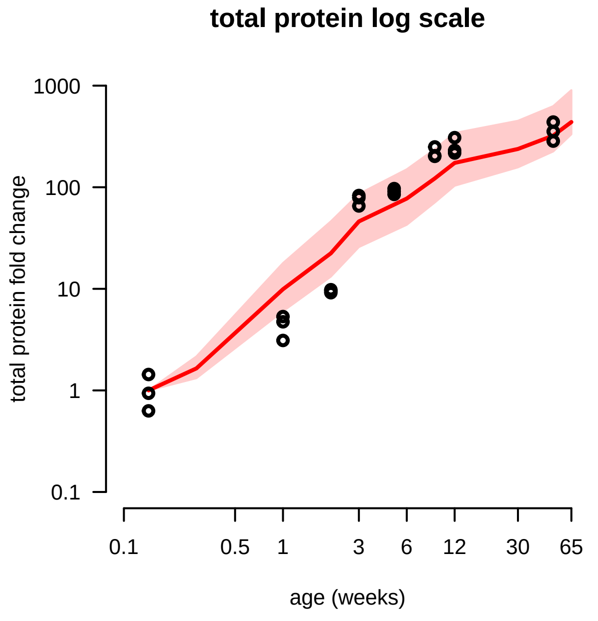

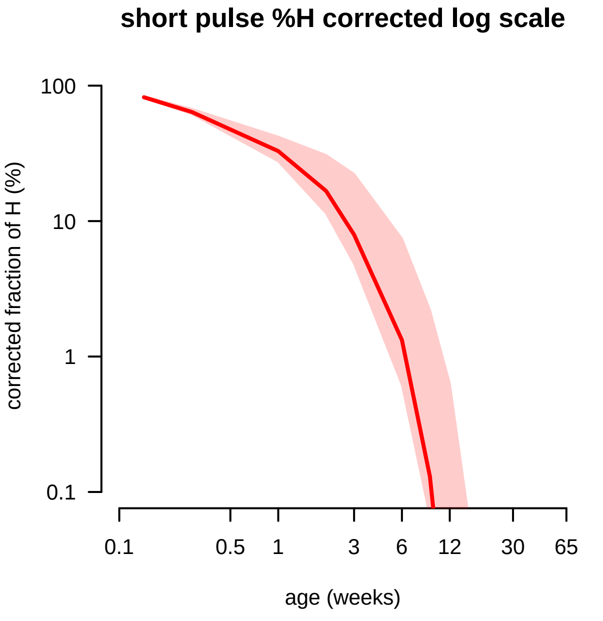

Q8R127

>sp|Q8R127|SCPDL_MOUSE

Saccharopine dehydrogenase−like oxidoreductase

GN=Sccpdh

Med H1/2: 13.1 days

Q5 H1/2: 6 days

Q95 H1/2: 104 days

reliable model fit: yes

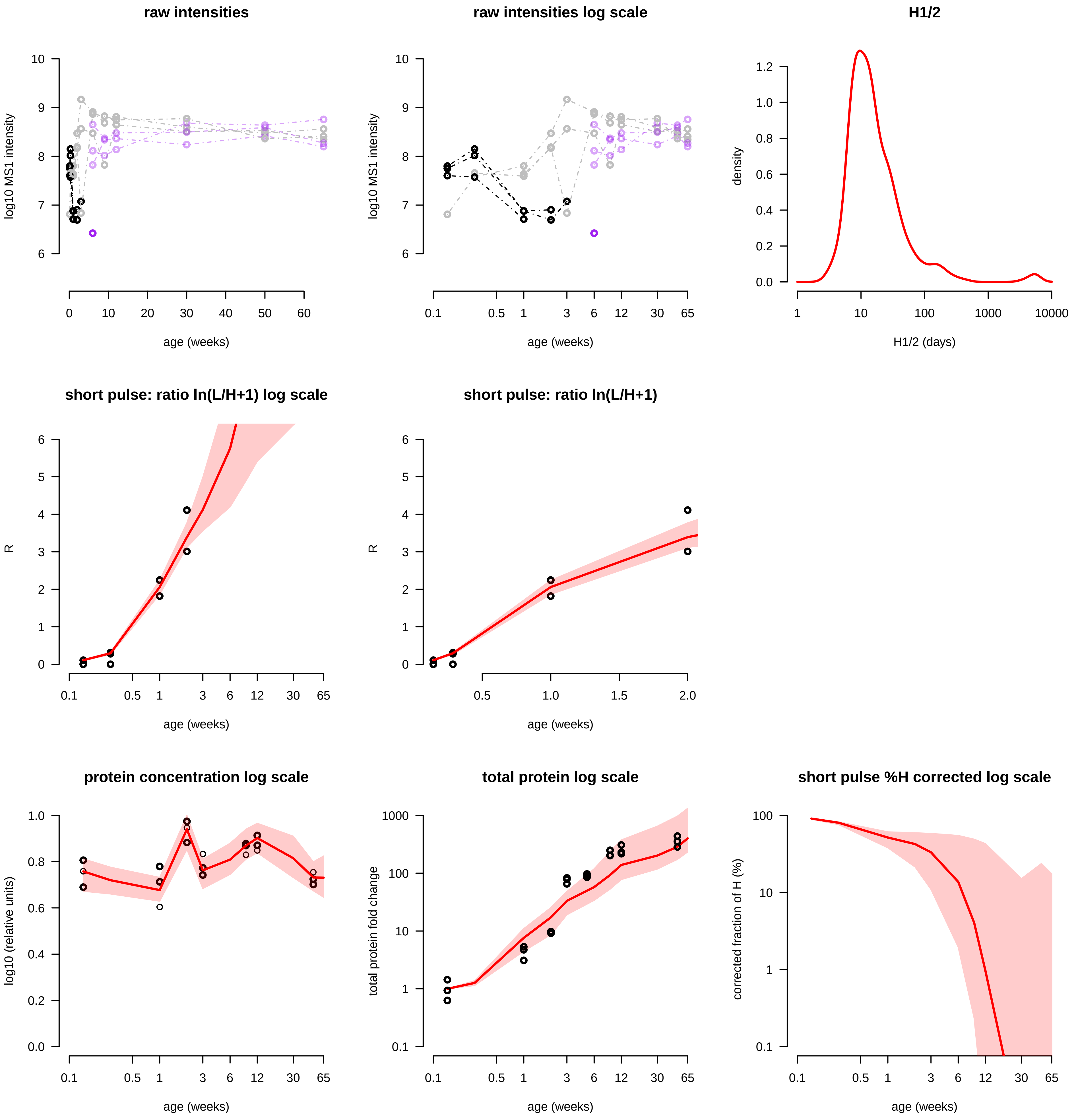

Q99JR8−2;Q99JR8

>sp|Q99JR8−2|SMRD2_MOUSE

I/SNF−related matrix−associated actin−dependent regulator of chromatin subfar

GN=Smarcd2

Med H1/2: 5.2 days

Q5 H1/2: 2.6 days

Q95 H1/2: 28.3 days

reliable model fit: yes

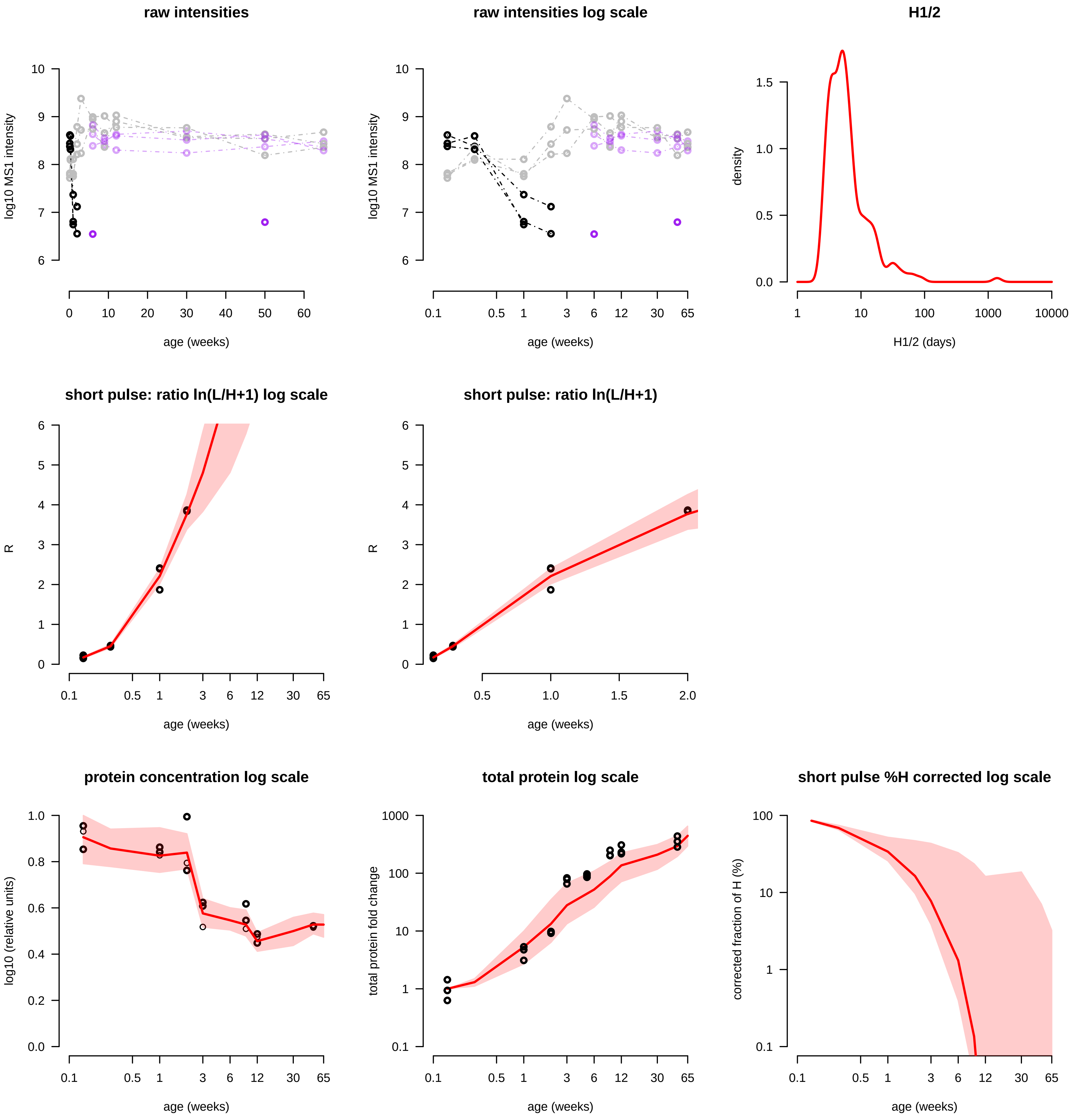

Q9D8S9

>sp|Q9D8S9|BOLA1_MOUSE

BolA−like protein 1

GN=Bola1

Med H1/2: 4.7 days

Q5   H1/2: 2.7 days

Q95 H1/2: 23 days

reliable model fit: yes

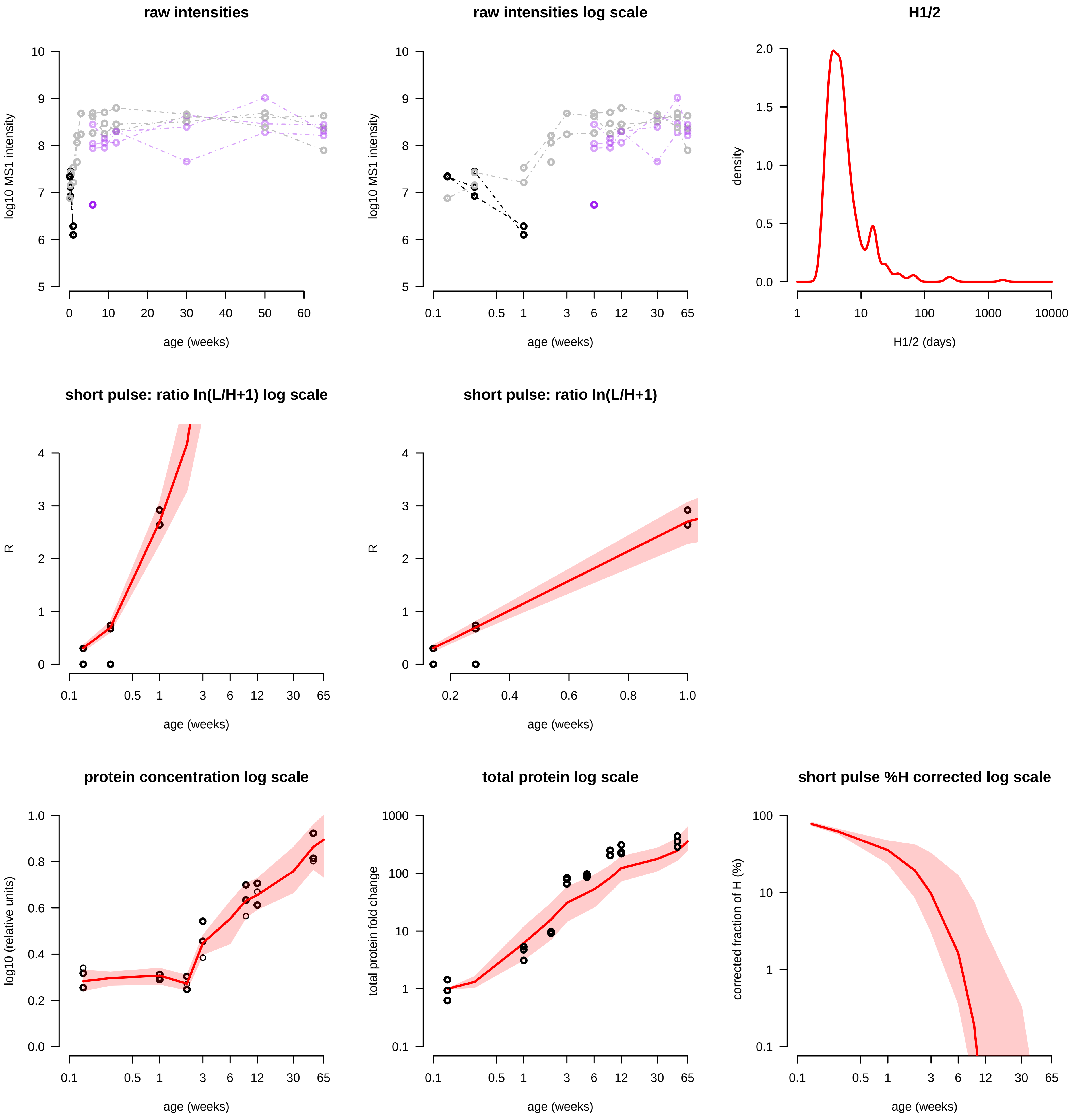

Q60520;Q60520−1

>sp|Q60520|SIN3A_MOUSE

Paired amphipathic helix protein Sin3a

GN=Sin3a

Med H1/2: 10.1 days

Q5 H1/2: 3.6 days

Q95 H1/2: 68.9 days

reliable model fit: yes

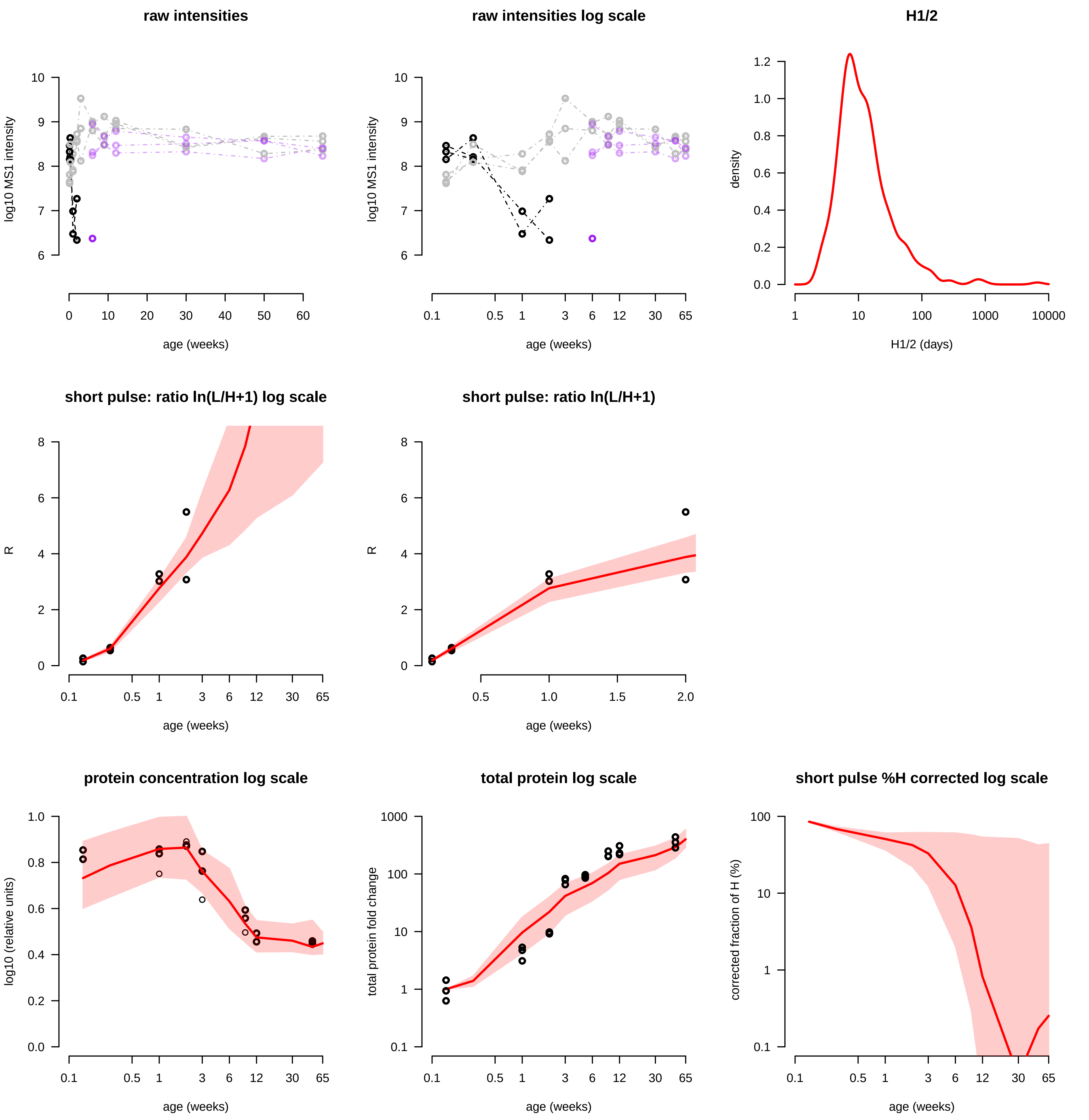

Q9QXS6−3;Q9QXS6;Q9QXS6−2

>sp|Q9QXS6−3|DREB_MOUSE

Isoform E2 of Drebrin

GN=Dbn1

Med H1/2: 6.8 days

Q5 H1/2: 2.8 days

Q95 H1/2: 21.5 days

reliable model fit: yes

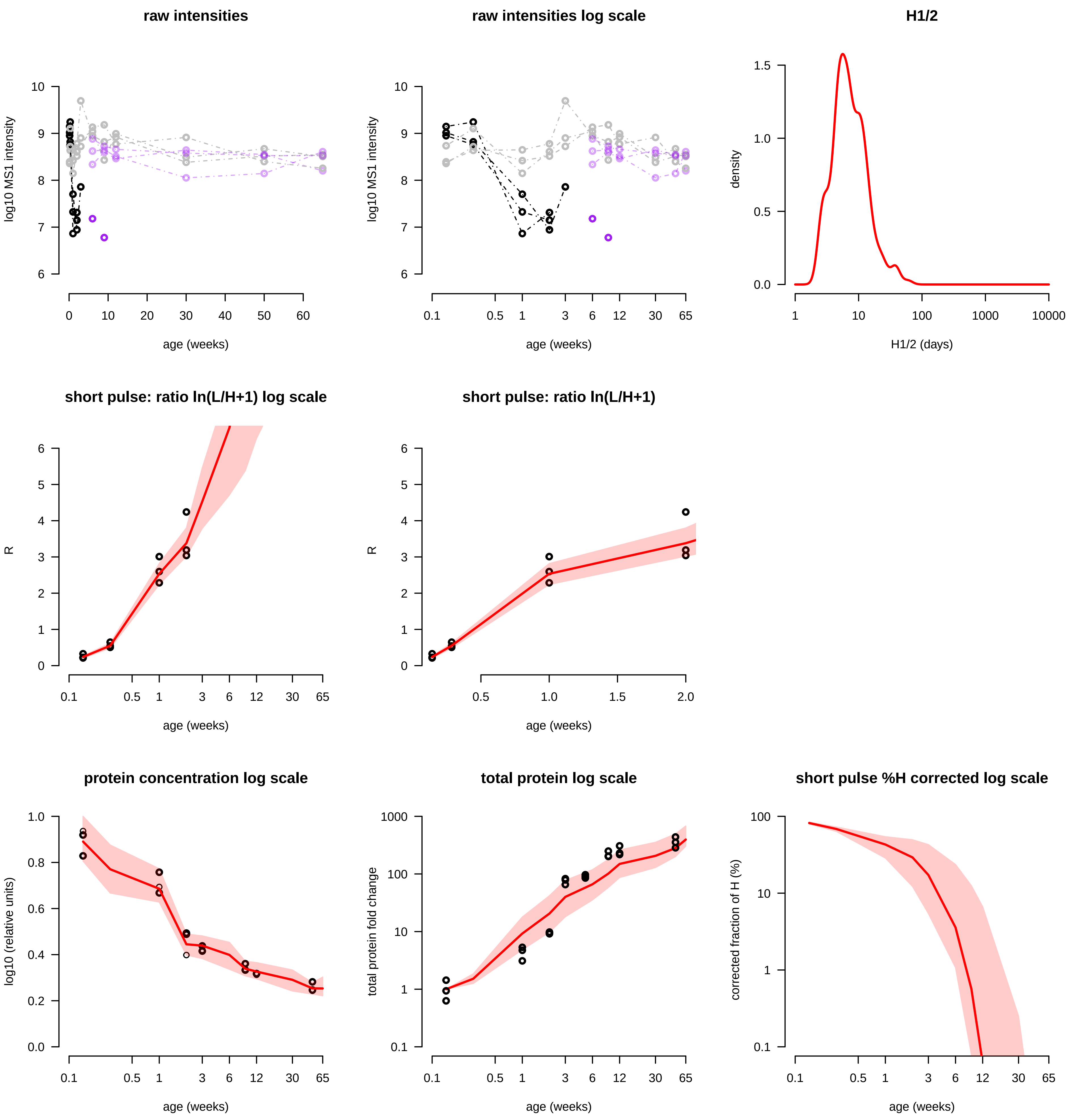

O35900

>sp|O35900|LSM2_MOUSE

U6 snRNA−associated Sm−like protein LSm2

GN=Lsm2

Med H1/2: 10.5 days

Q5 H1/2: 7.6 days

Q95 H1/2: 16.4 days

reliable model fit: yes

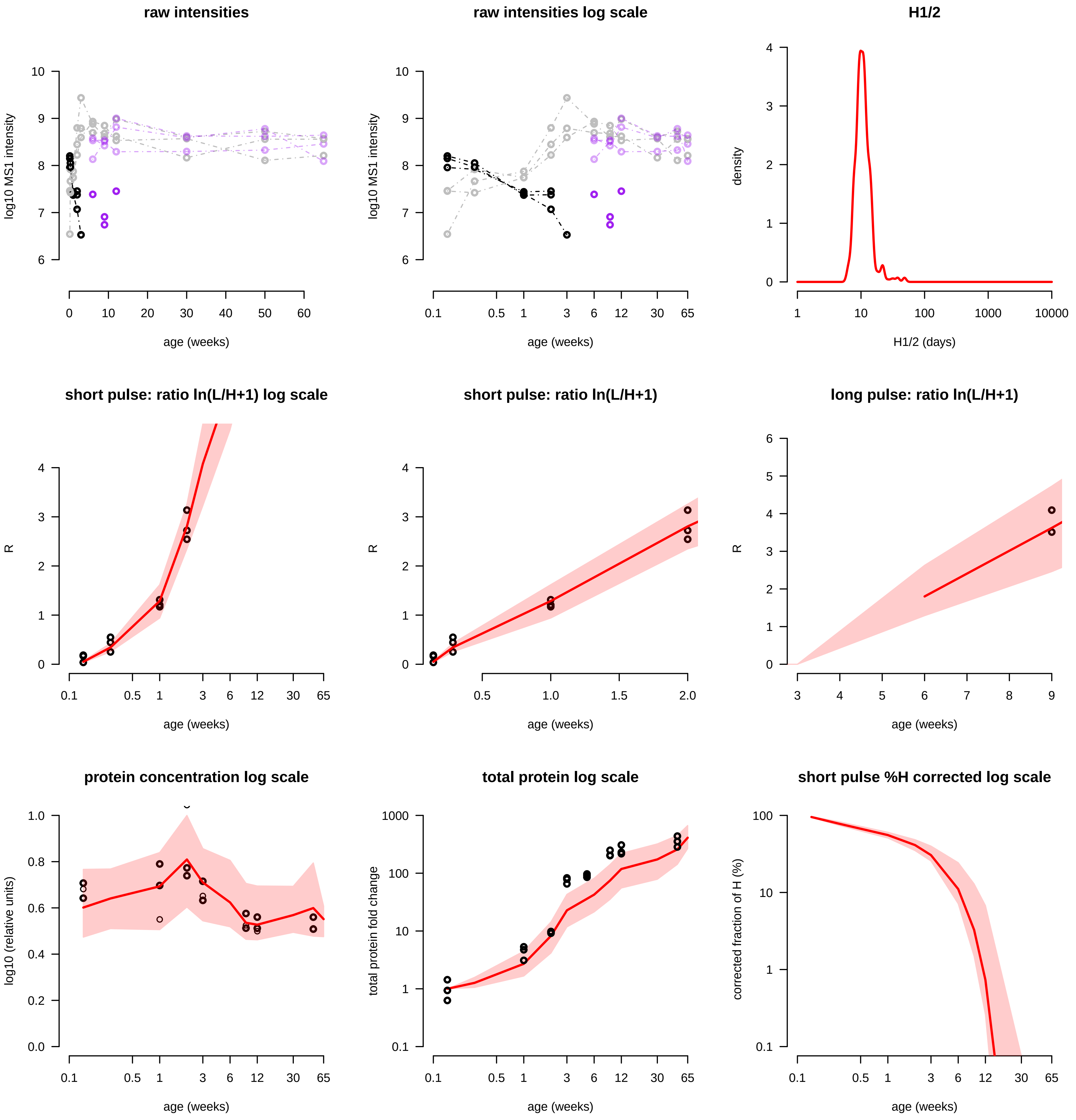

Q5HZI9

>sp|Q5HZI9|S2551_MOUSE

Mitochondrial nicotinamide adenine dinucleotide transporter SLC25A51

GN=Slc25a51

Med H1/2: 14.5 days

Q5 H1/2: 4.8 days

Q95 H1/2: 111.3 days

reliable model fit: no

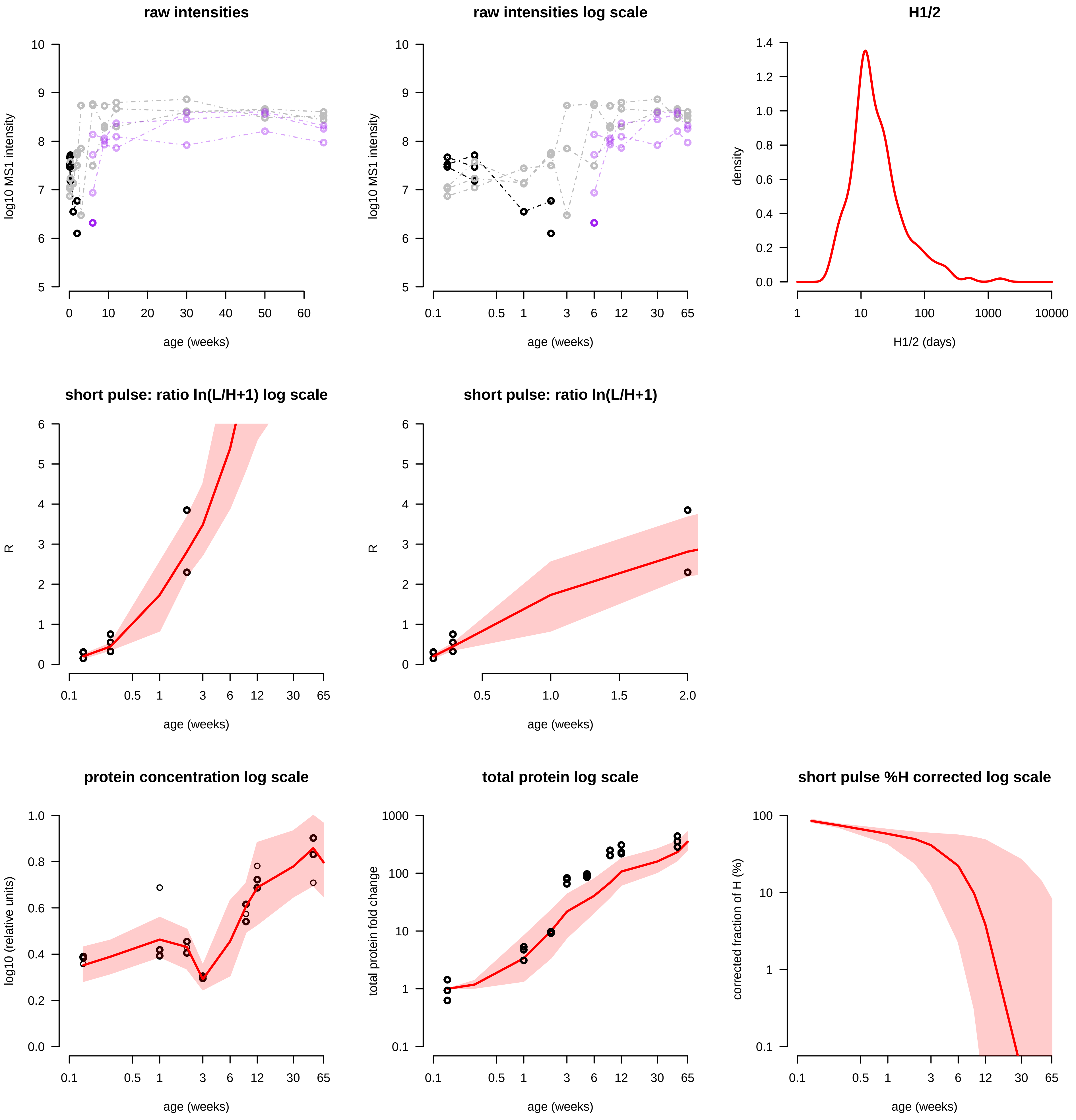

Q8BX09

>sp|Q8BX09|RBBP5_MOUSE

Retinoblastoma−binding protein 5

GN=Rbbp5

Med H1/2: 6 days

Q5 H1/2: 4.9 days

Q95 H1/2: 7.8 days

reliable model fit: yes

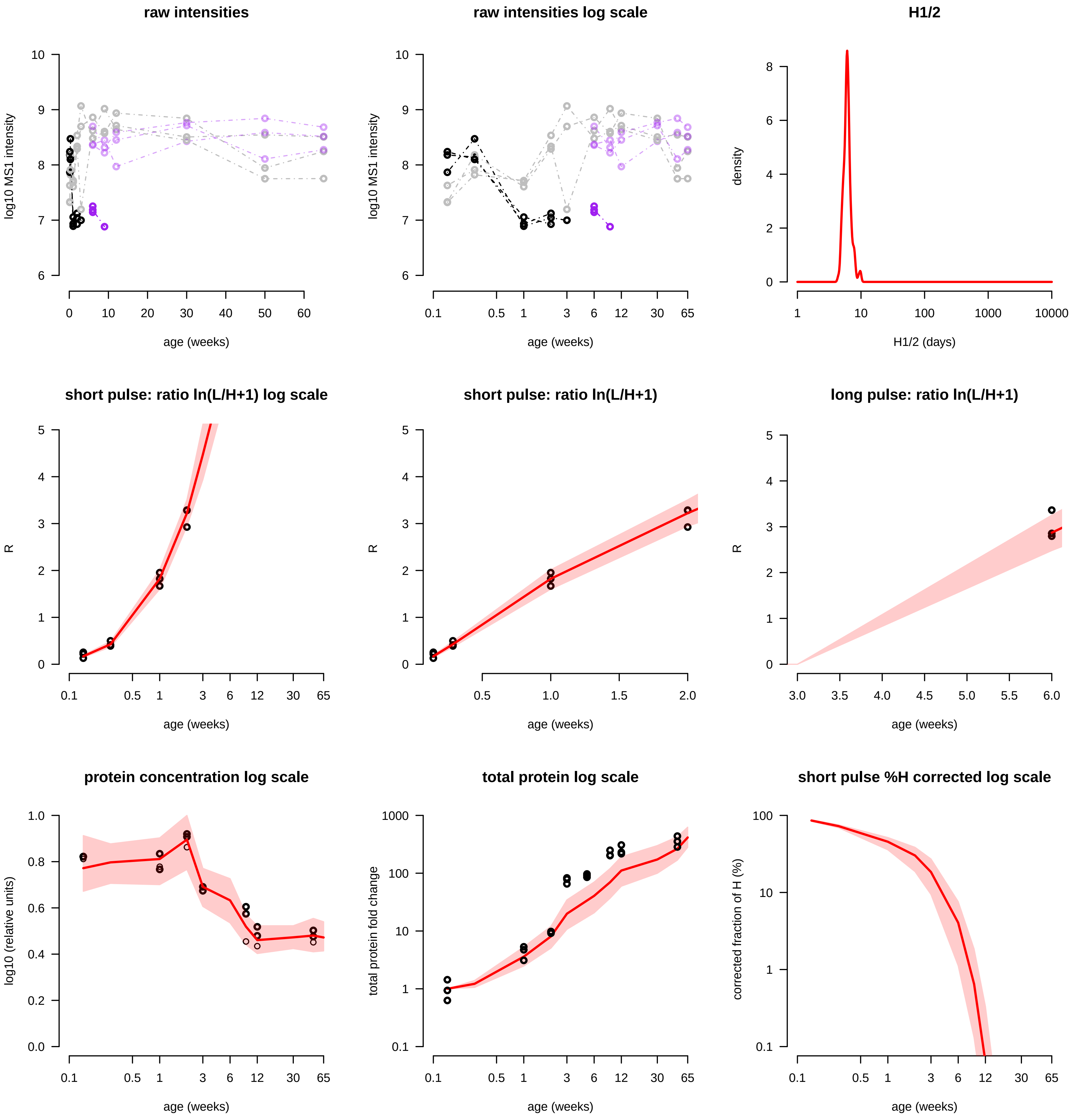

P97314

>sp|P97314|CSRP2_MOUSE

Cysteine and glycine−rich protein 2

GN=Csrp2

Med H1/2: 4.7 days

Q5 H1/2: 3.7 days

Q95 H1/2: 7.2 days

reliable model fit: yes

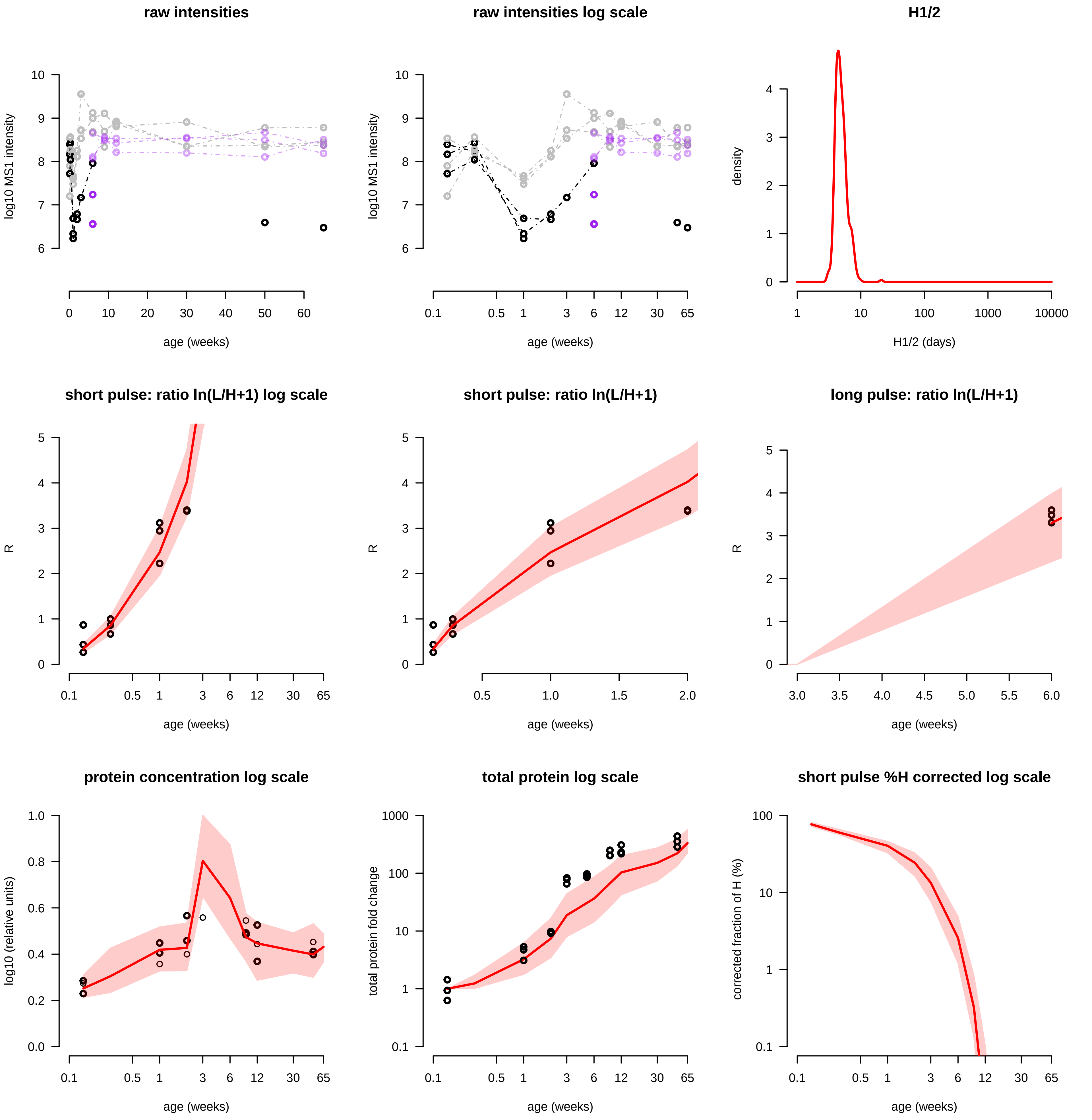

P47809

>sp|P47809|MP2K4_MOUSE

Dual specificity mitogen−activated protein kinase kinase 4

GN=Map2k4

Med H1/2: 4.2 days

Q5 H1/2: 3.5 days

Q95 H1/2: 5.2 days

reliable model fit: yes

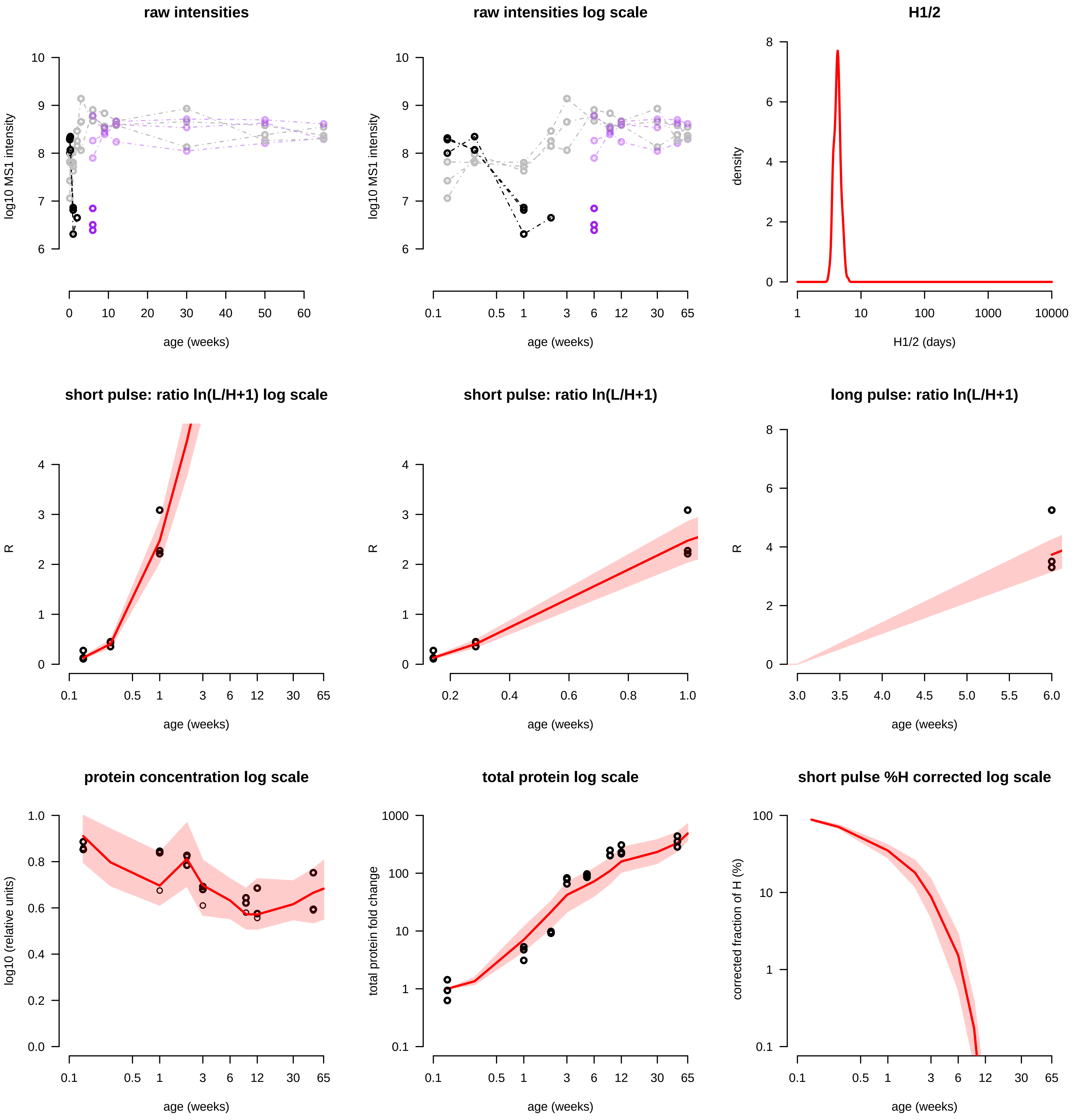

Q9WVJ3

>sp|Q9WVJ3|CBPQ_MOUSE

Carboxypeptidase Q

GN=Cpq

Med H1/2: 507.8 days

Q5 H1/2: 216.4 days

Q95 H1/2: 6359.6 days

reliable model fit: yes

raw intensities

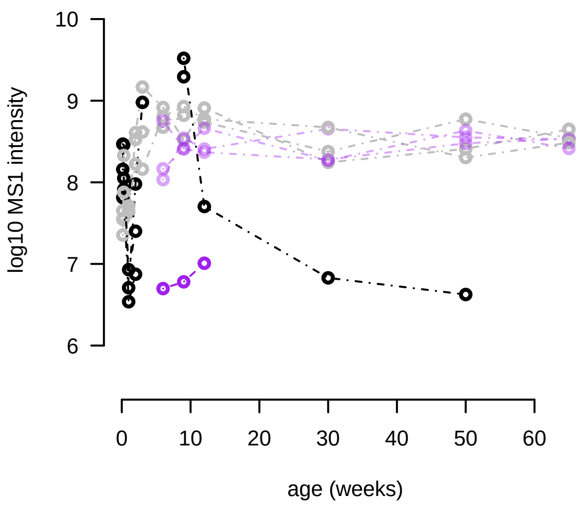

raw intensities log scale

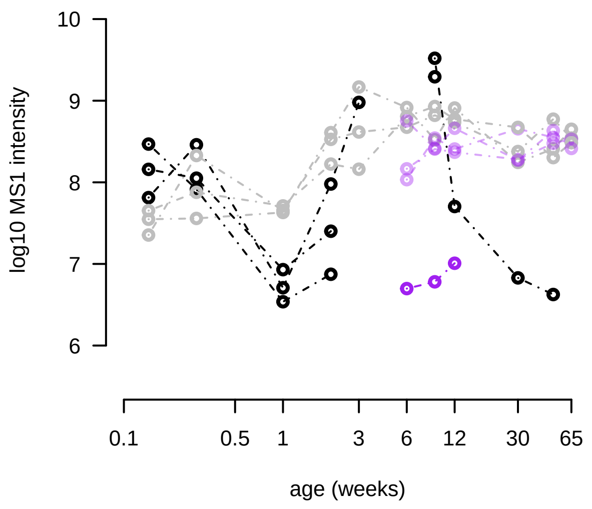

H1/2

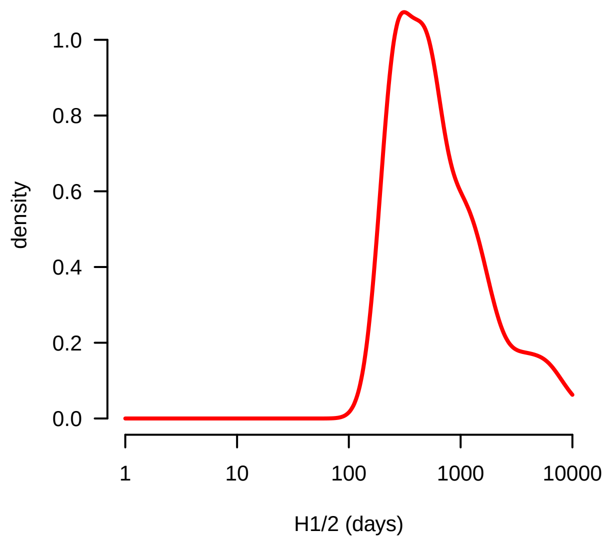

short pulse: ratio ln(L/H+1) log scale

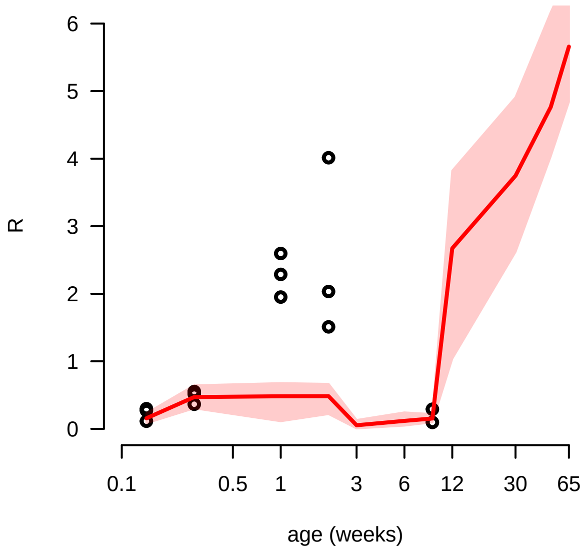

short pulse: ratio ln(L/H+1)

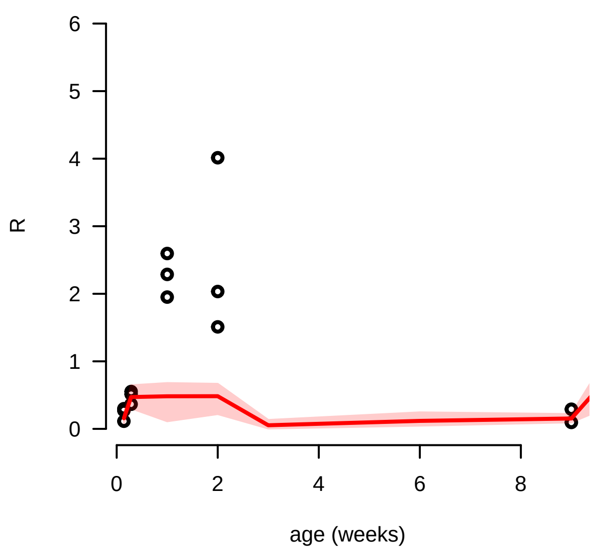

protein concentration log scale

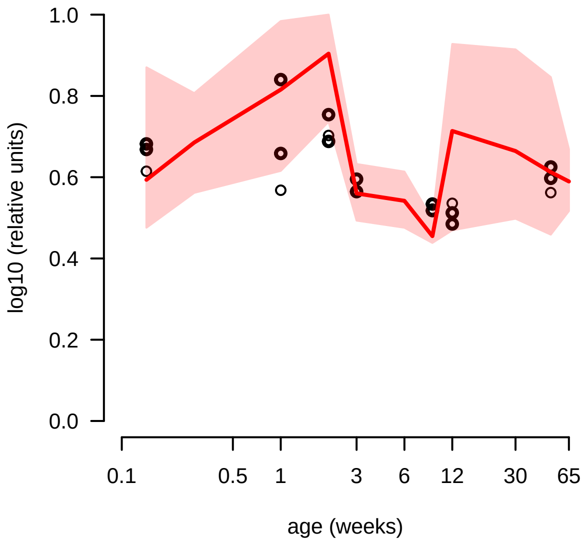

total protein log scale

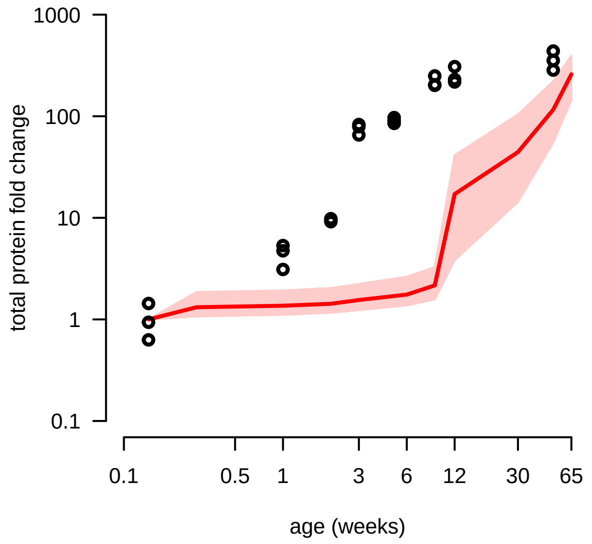

short pulse %H corrected log scale

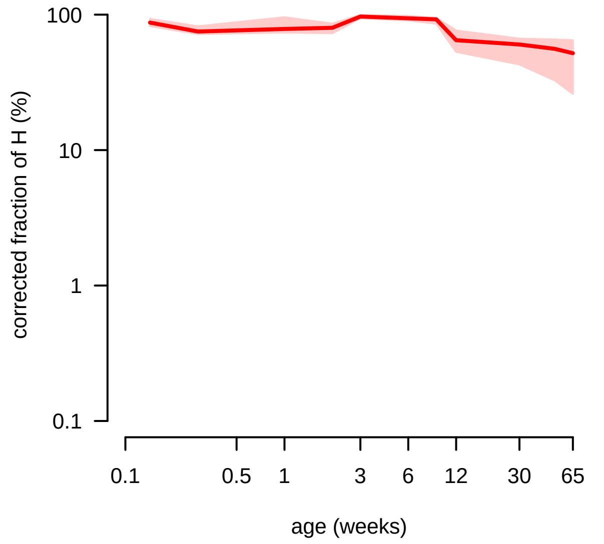

Q3UHD6;Q3UHD6−2

>sp|Q3UHD6|SNX27_MOUSE

Sorting nexin−27

GN=Snx27

Med H1/2: 5.3 days

Q5 H1/2: 2.7 days

Q95 H1/2: 28.3 days

reliable model fit: yes

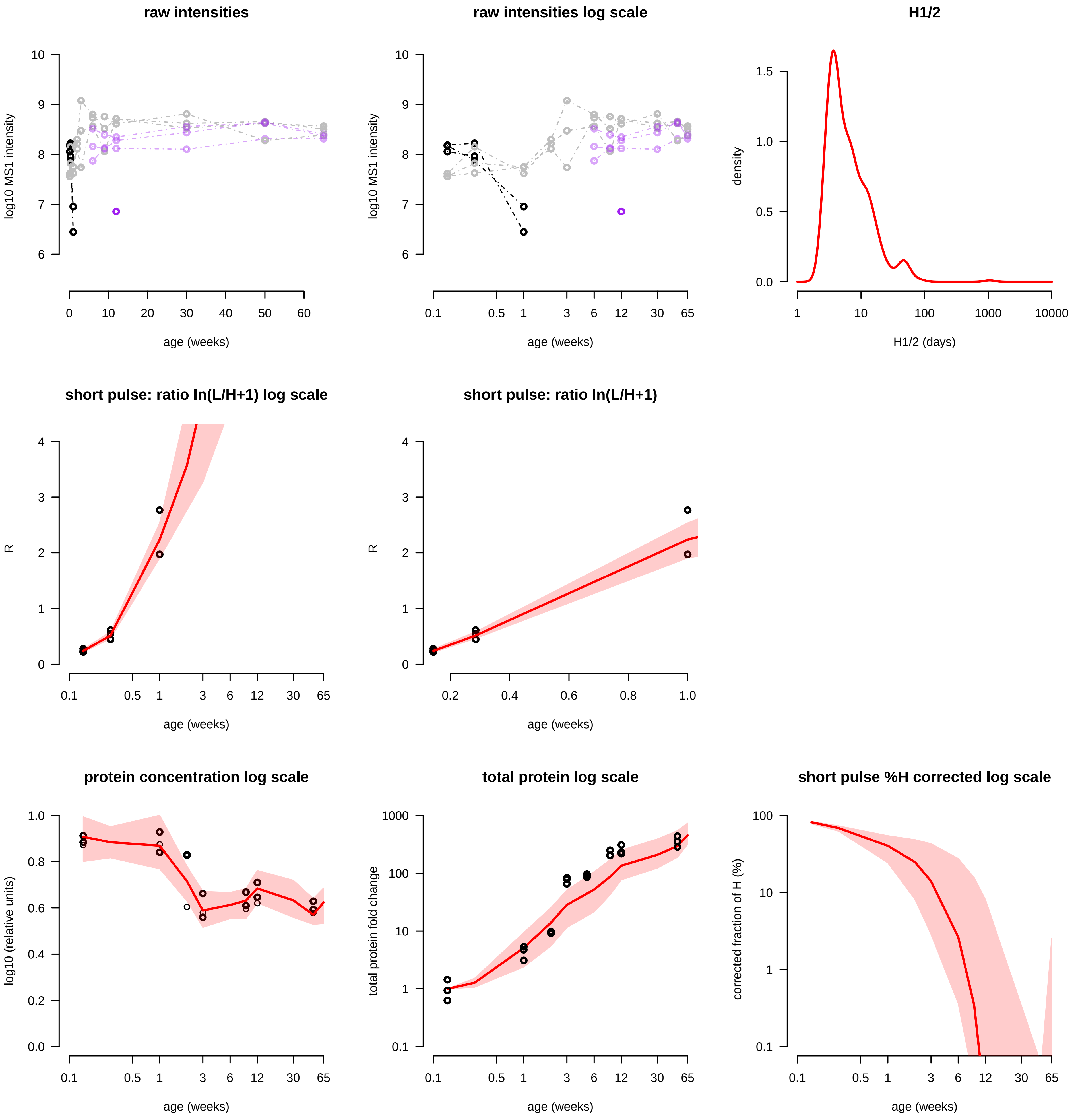

Q9Z0P4

>sp|Q9Z0P4|PALM_MOUSE

Paralemmin−1

GN=Palm

Med H1/2: 4.7 days

Q5 H1/2: 2.7 days

Q95 H1/2: 39 days

reliable model fit: yes

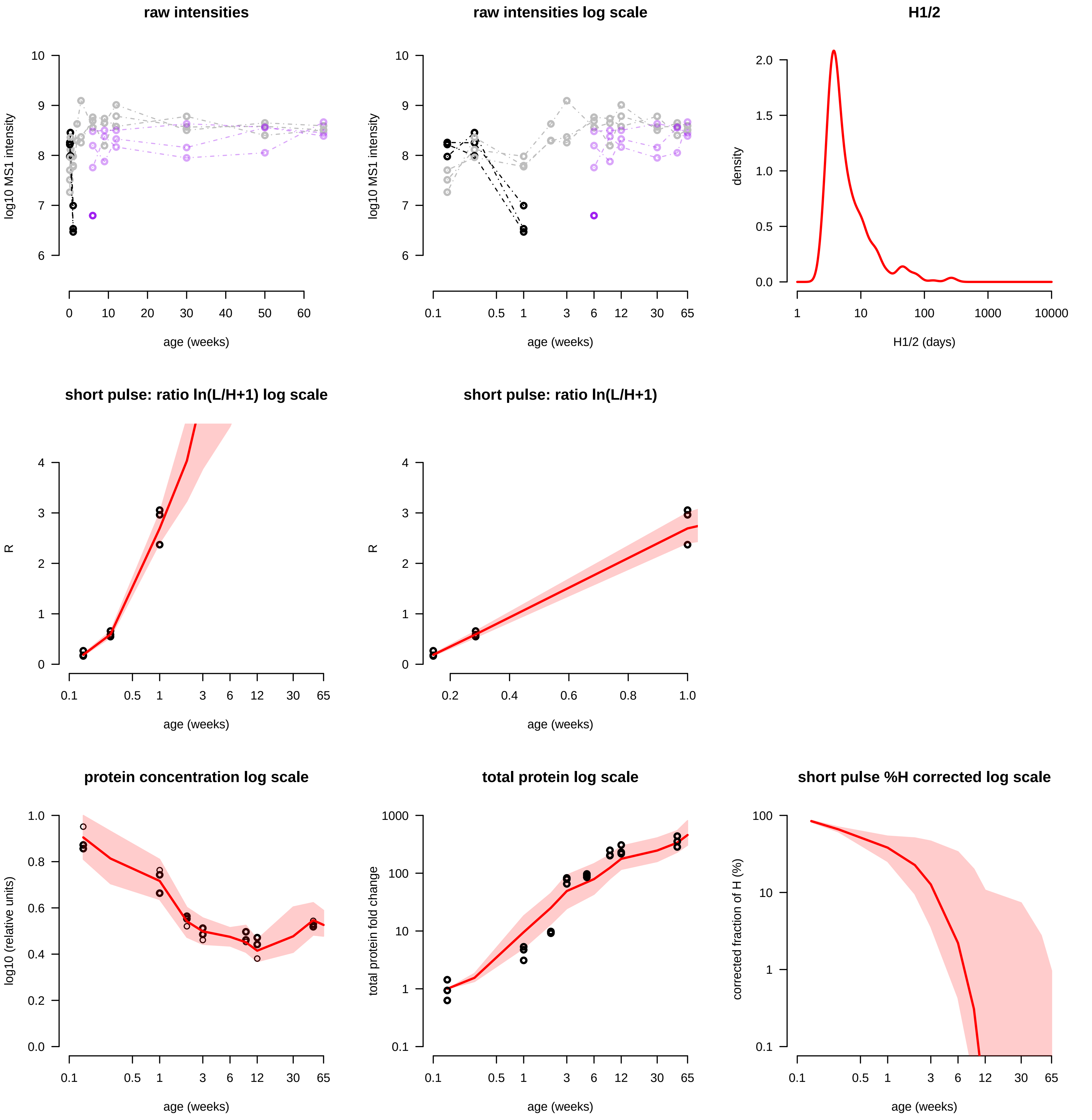

Q91YT7

>sp|Q91YT7|YTHD2_MOUSE

YTH domain−containing family protein 2

GN=Ythdf2

Med H1/2: 381.7 days

Q5 H1/2: 154.2 days

Q95 H1/2: 2273.2 days

reliable model fit: yes

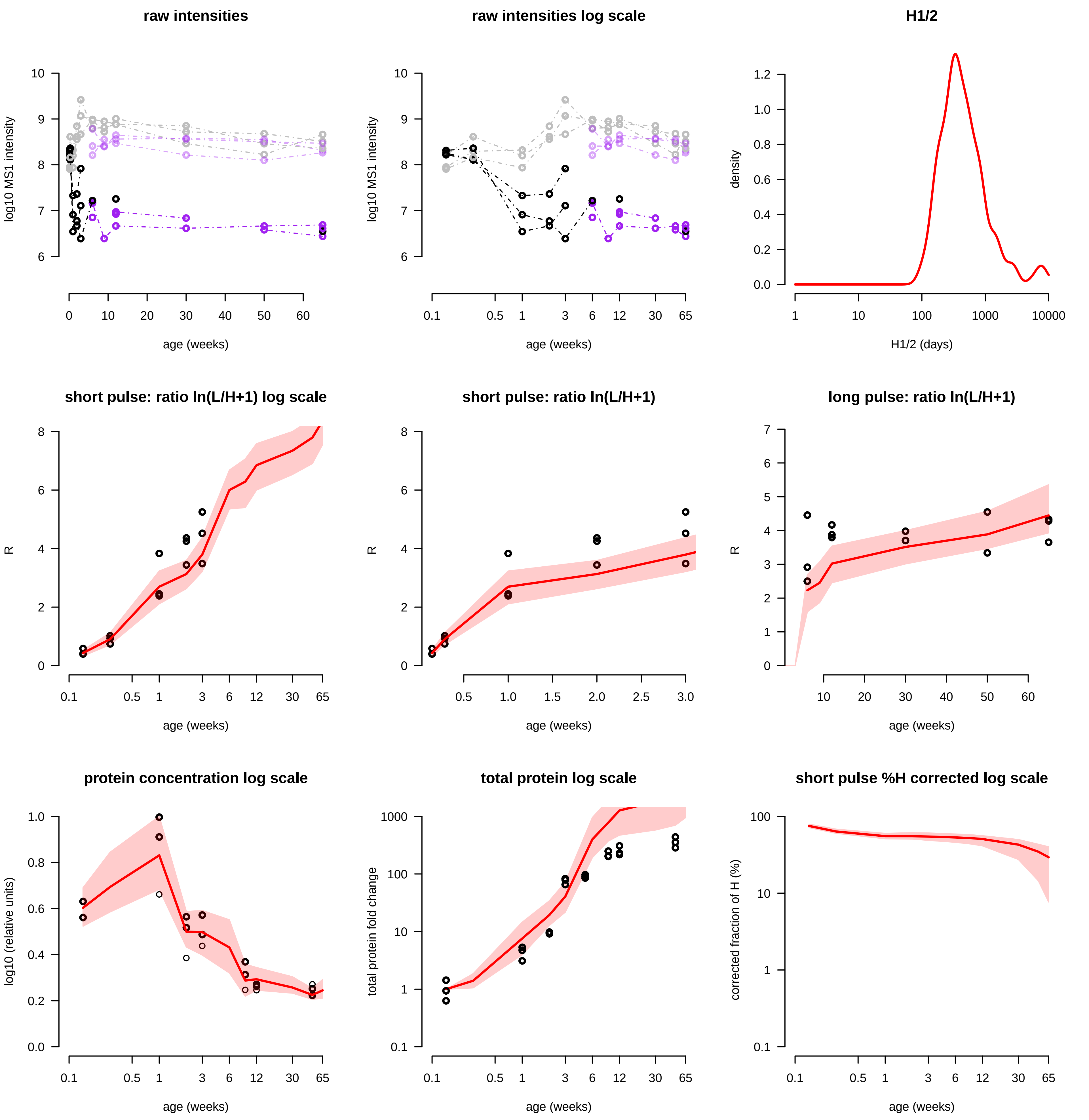

Q99LI2

>sp|Q99LI2|CLCC1_MOUSE

Chloride channel CLIC−like protein 1

GN=Clcc1

Med H1/2: 4.8 days

Q5 H1/2: 2.6 days

Q95 H1/2: 28.4 days

reliable model fit: yes

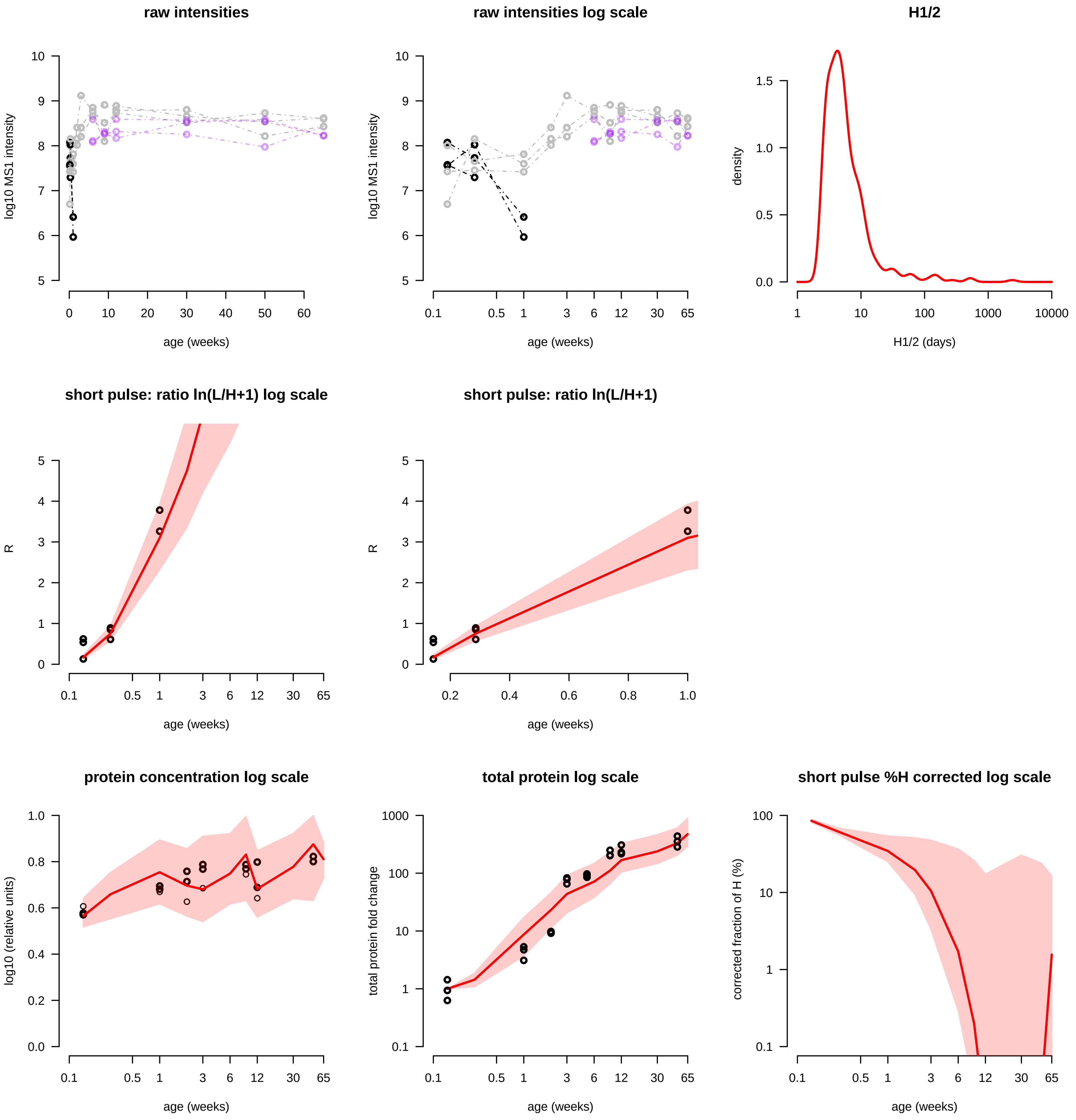

Q11136

>sp|Q11136|PEPD_MOUSE

Xaa−Pro dipeptidase

GN=Pepd

Med H1/2: 9 days

Q5 H1/2: 3.4 days

Q95 H1/2: 70.5 days

reliable model fit: yes

raw intensities

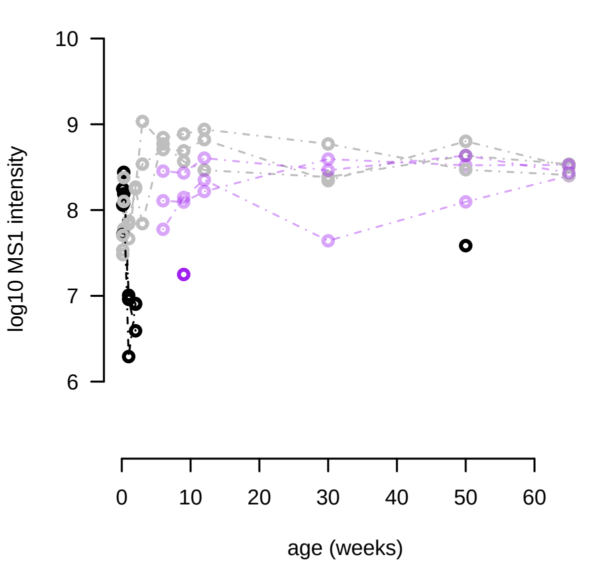

raw intensities log scale

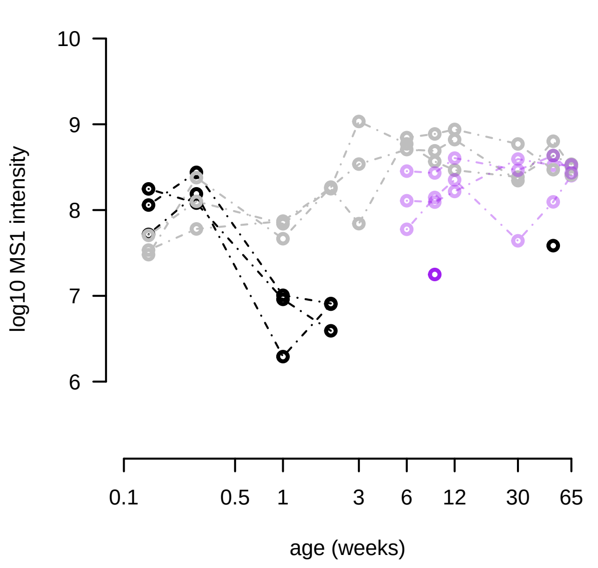

H1/2

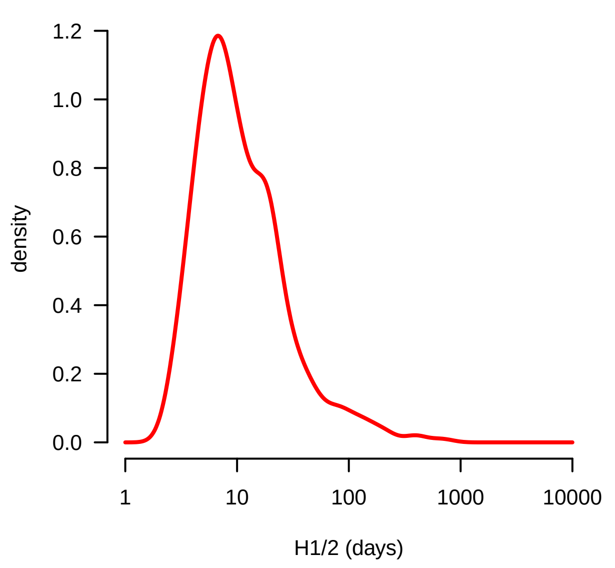

short pulse: ratio ln(L/H+1) log scale

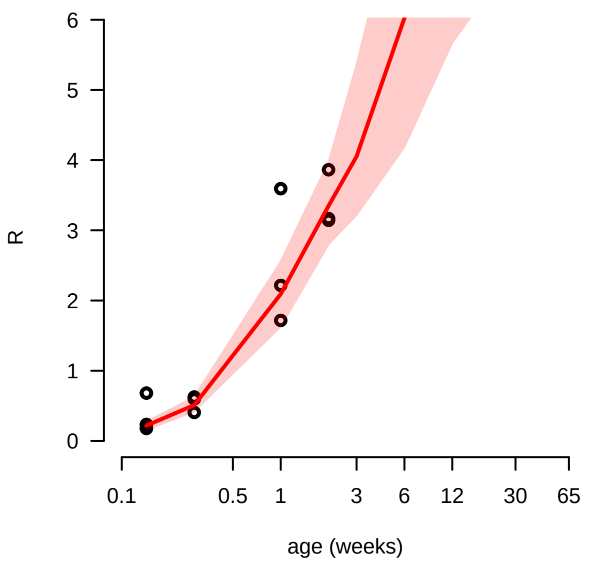

short pulse: ratio ln(L/H+1)

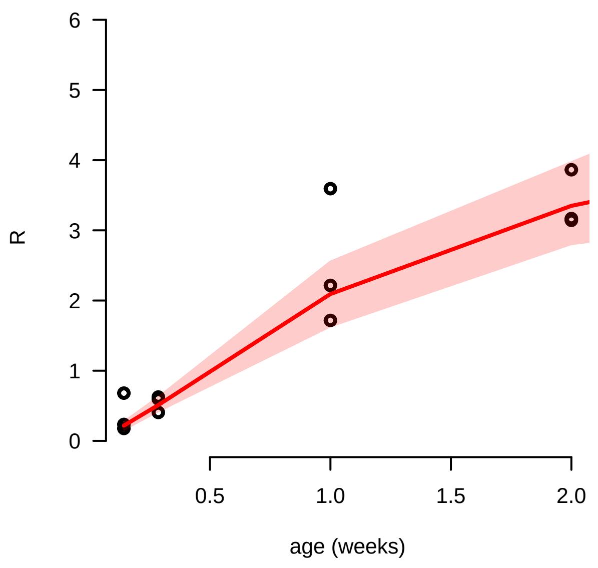

protein concentration log scale

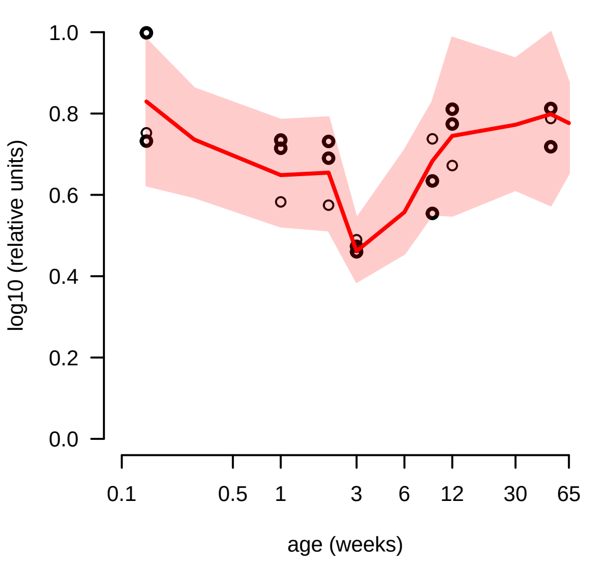

total protein log scale

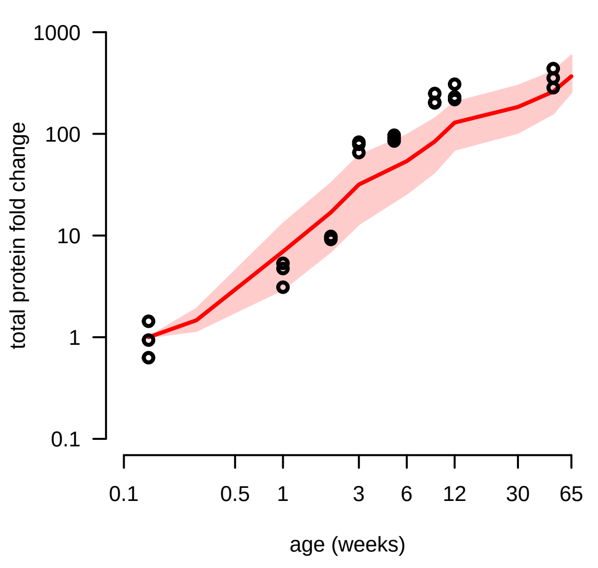

short pulse %H corrected log scale

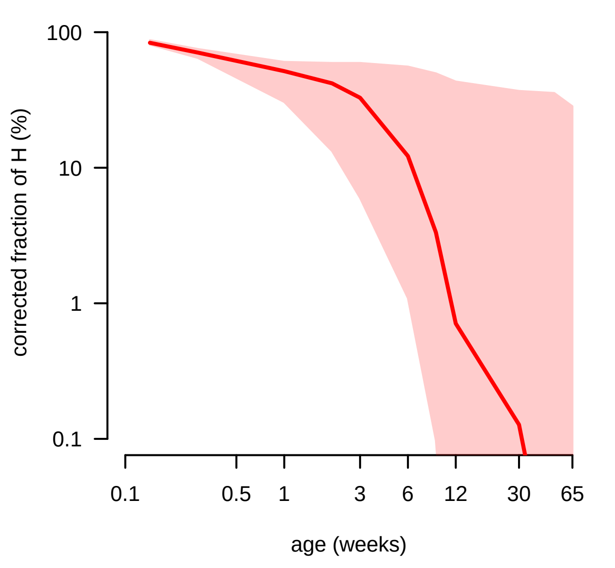

Q8N7N5;Q8N7N5−2

>sp|Q8N7N5|DCAF8_MOUSE

DDB1− and CUL4−associated factor 8

GN=Dcaf8

Med H1/2: 3.4 days

Q5 H1/2: 2.8 days

Q95 H1/2: 4.3 days

reliable model fit: yes

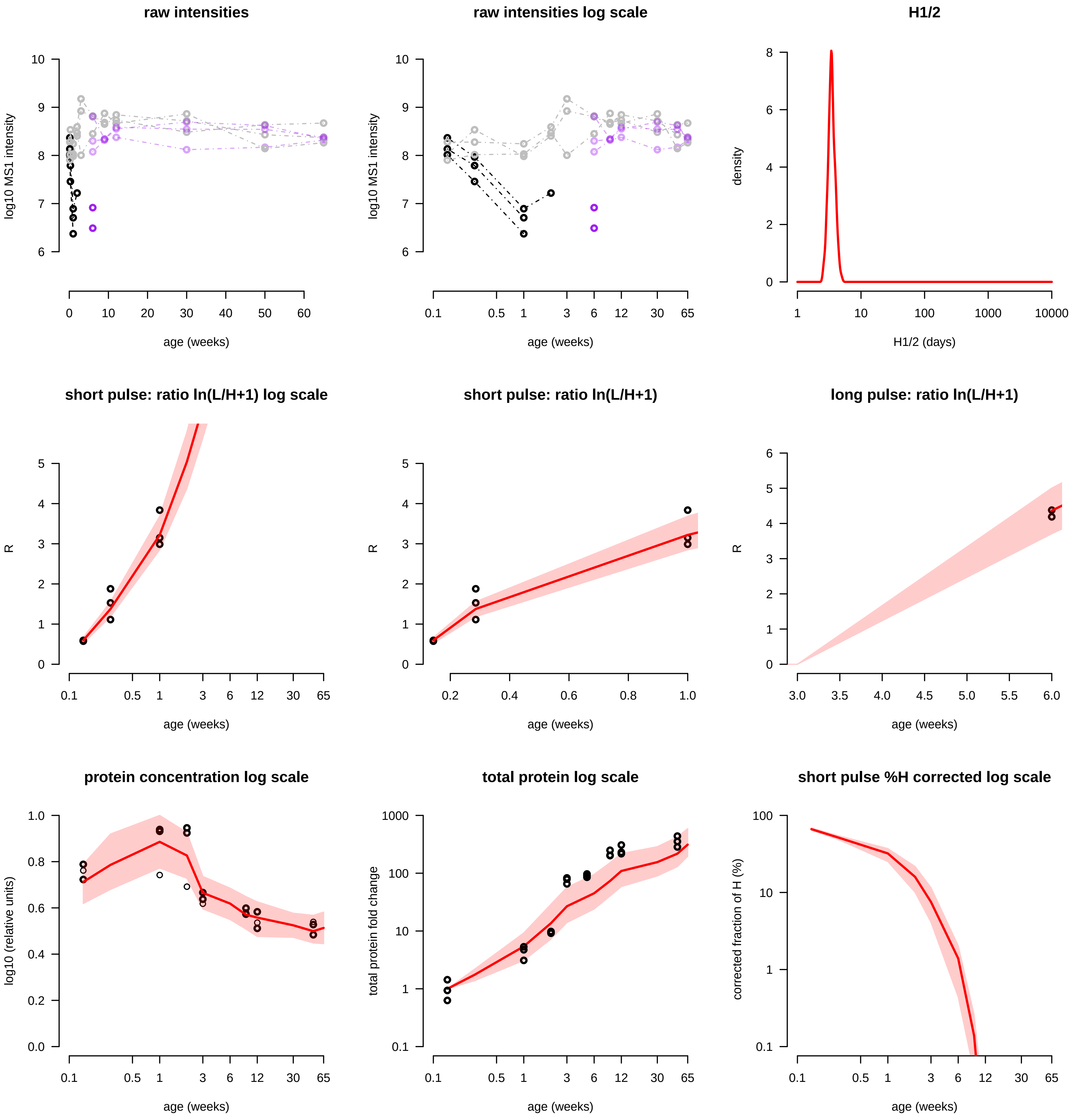

Q8C878

>sp|Q8C878|UBA3_MOUSE

NEDD8−activating enzyme E1 catalytic subunit

GN=Uba3

Med H1/2: 4.9 days

Q5 H1/2: 3.4 days

Q95 H1/2: 8.5 days

reliable model fit: yes

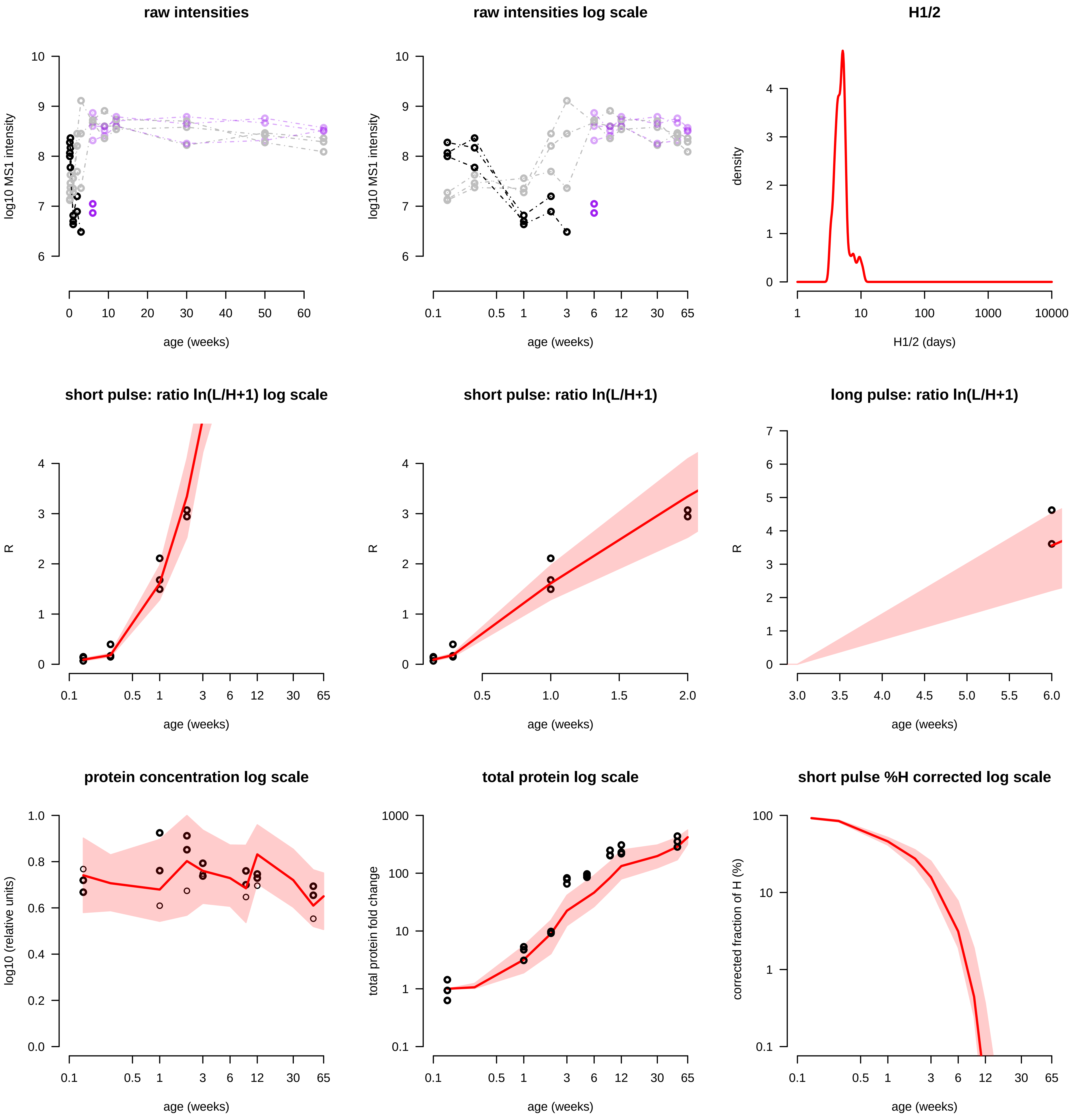

Q9D6J6;Q9D6J6−2

>sp|Q9D6J6|NDUV2_MOUSE

NADH dehydrogenase [ubiquinone] flavoprotein 2, mitochondrial

GN=Ndufv2

Med H1/2: 454.1 days

Q5 H1/2: 173.5 days

Q95 H1/2: 2227.1 days

reliable model fit: no

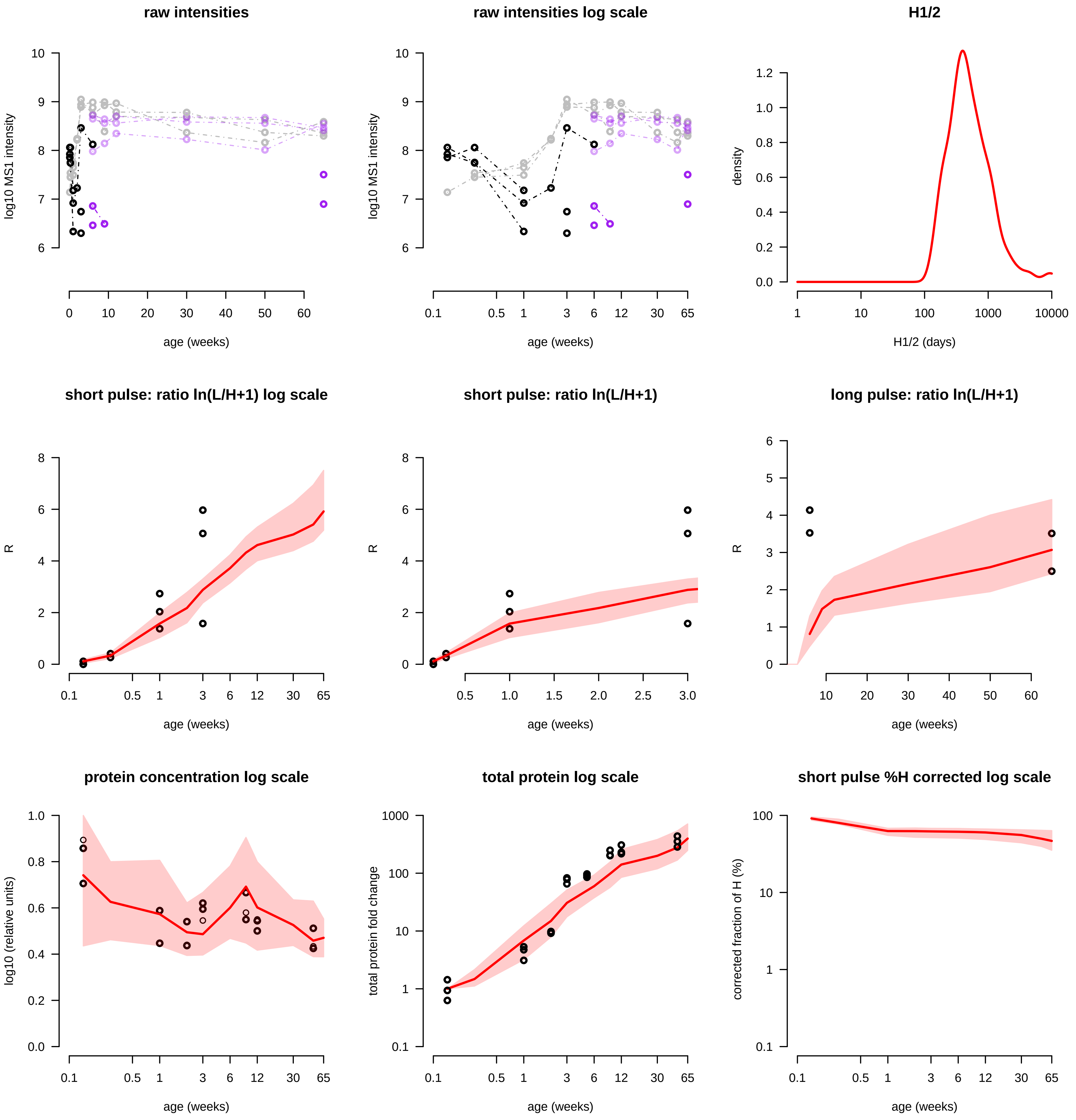

O54941

>sp|O54941|SMCE1_MOUSE

-related matrix−associated actin−dependent regulator of chromatin subfamily E

GN=Smarce1

Med H1/2: 4.1 days

Q5 H1/2: 2.7 days

Q95 H1/2: 7.8 days

reliable model fit: yes

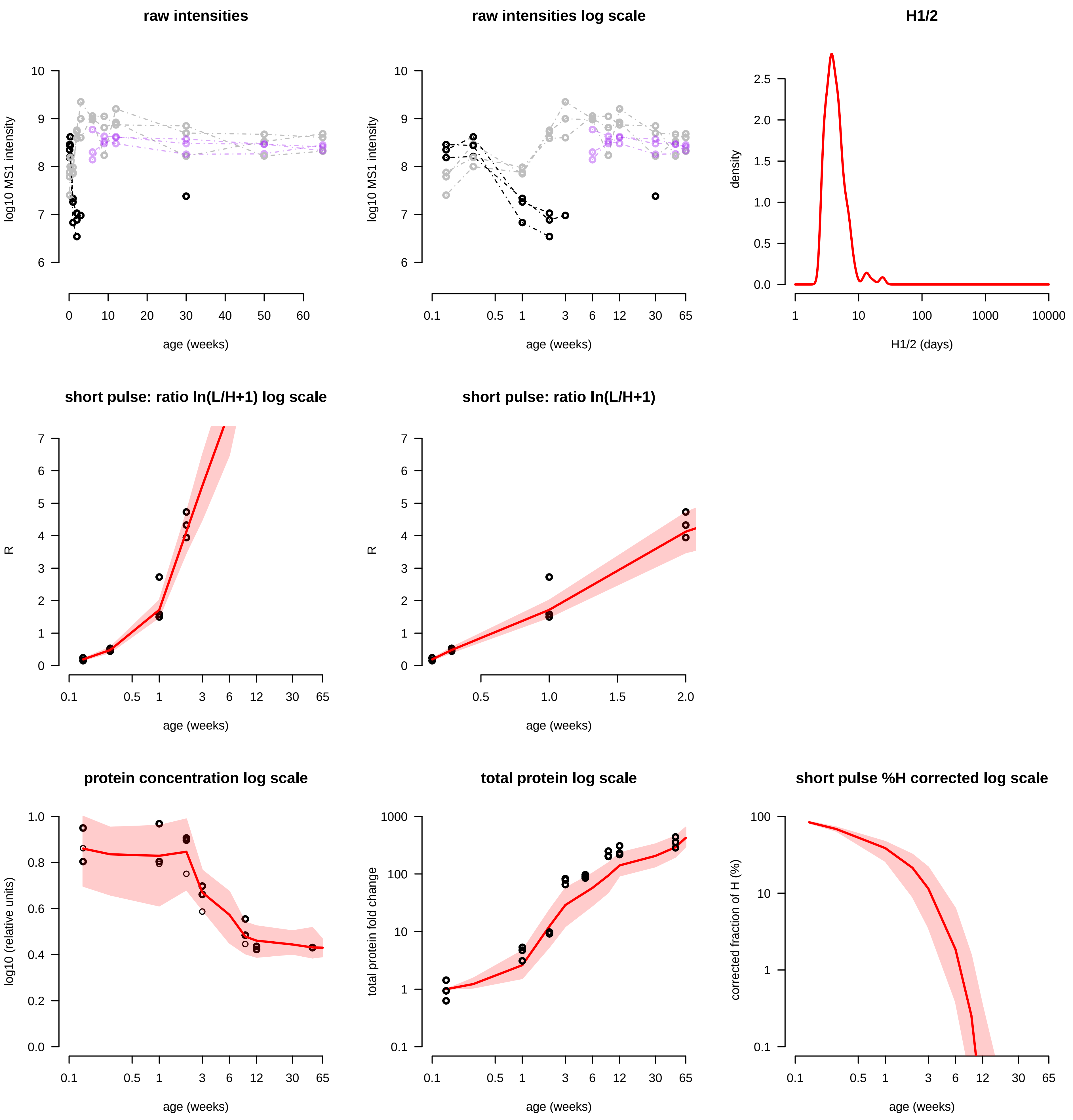

Q9QXY6

>sp|Q9QXY6|EHD3_MOUSE

EH domain−containing protein 3

GN=Ehd3

Med H1/2: 11.7 days

Q5 H1/2: 2.9 days

Q95 H1/2: 163.4 days

reliable model fit: yes

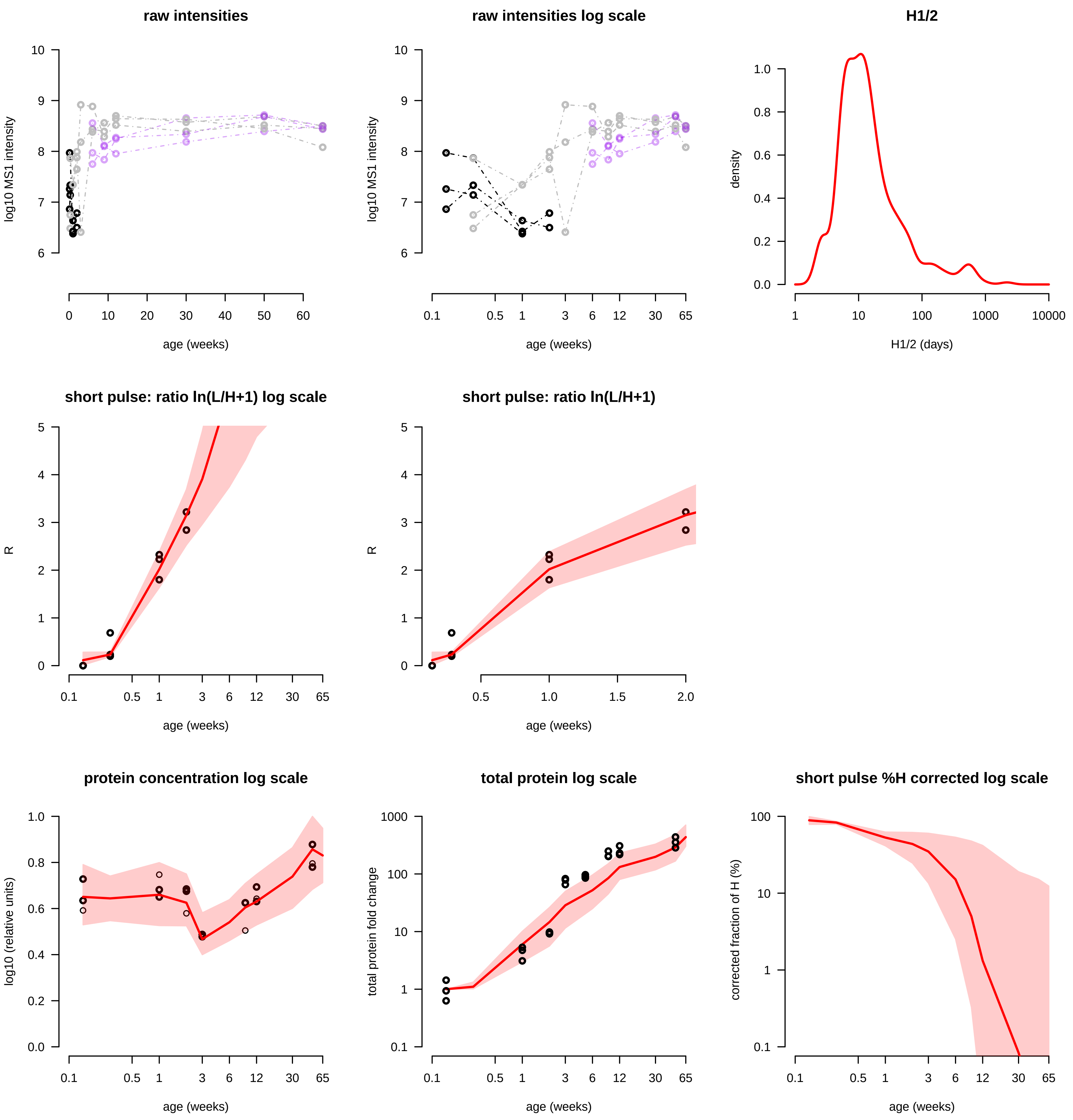

Q9ER41

>sp|Q9ER41|TOR1B_MOUSE

Torsin−1B

GN=Tor1b

Med H1/2: 15.1 days

Q5 H1/2: 5.3 days

Q95 H1/2: 94 days

reliable model fit: yes

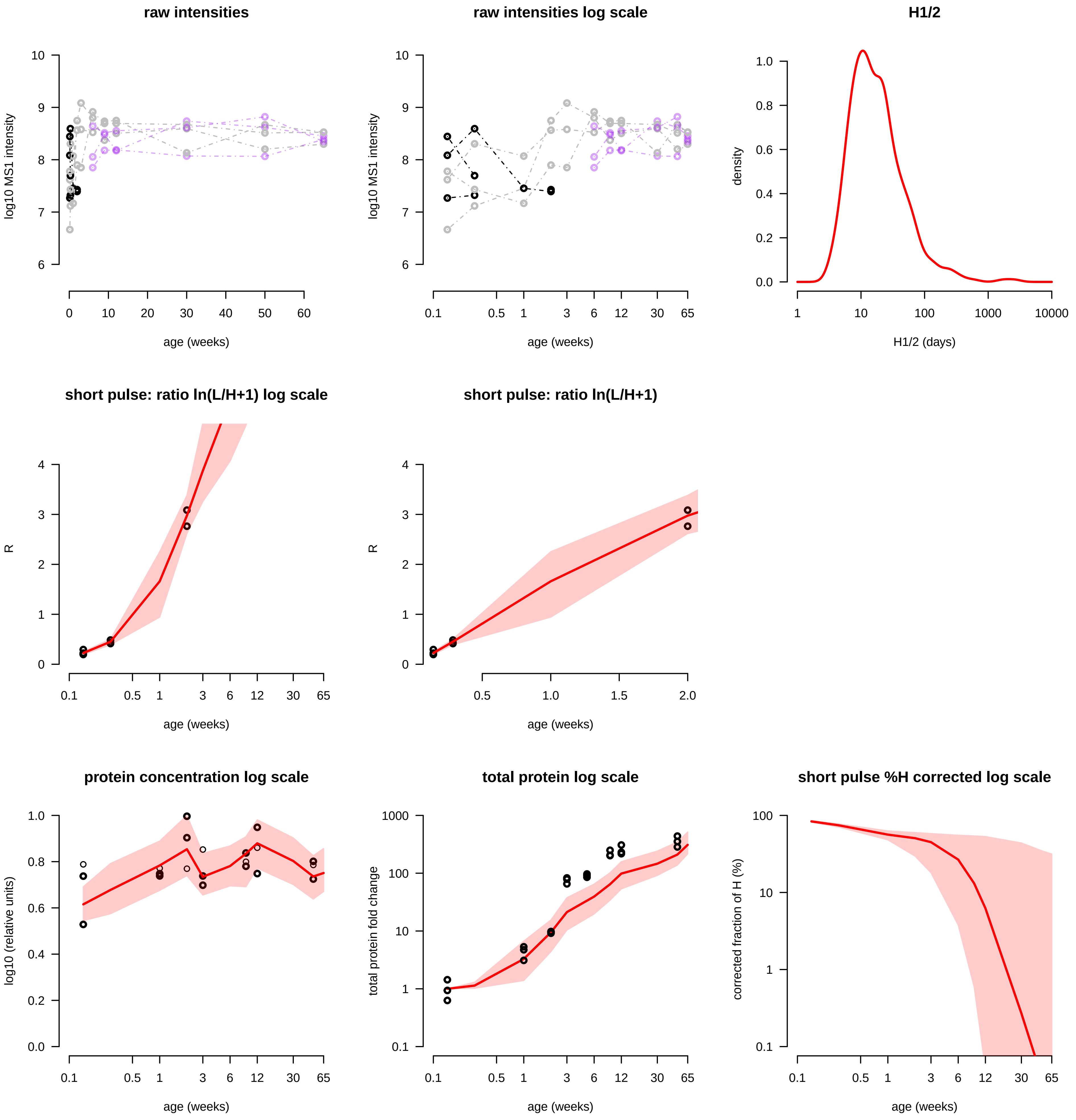

Q9R0P4

>sp|Q9R0P4|SMAP_MOUSE

Small acidic protein

GN=Smap

Med H1/2: 9.8 days

Q5 H1/2: 4.5 days

Q95 H1/2: 54.6 days

reliable model fit: yes

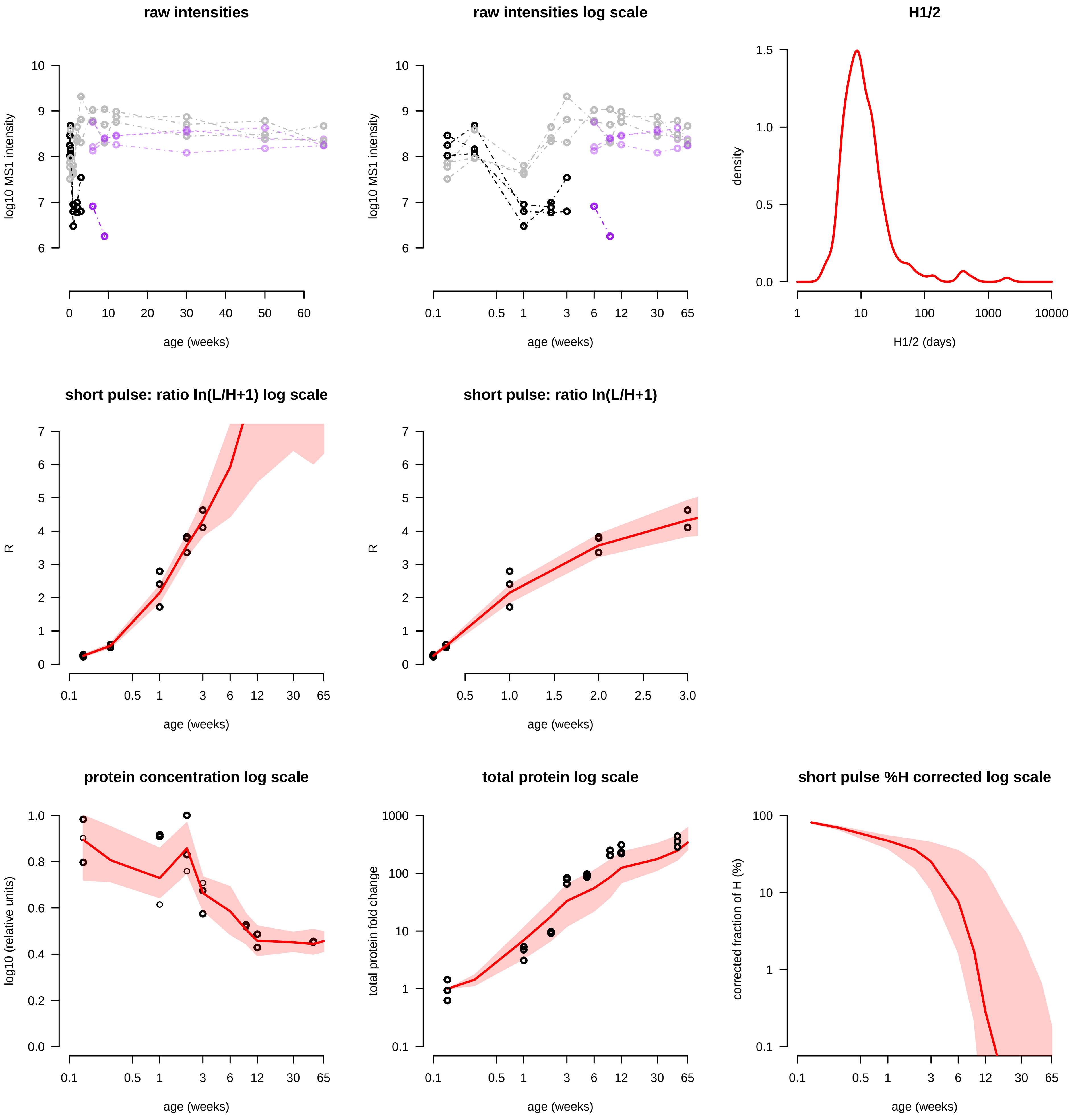

P17095

>sp|P17095|HMGA1_MOUSE

High mobility group protein HMG−I/HMG−Y

GN=Hmga1

Med H1/2: 4 days

Q5 H1/2: 3.1 days

Q95 H1/2: 11 days

reliable model fit: yes

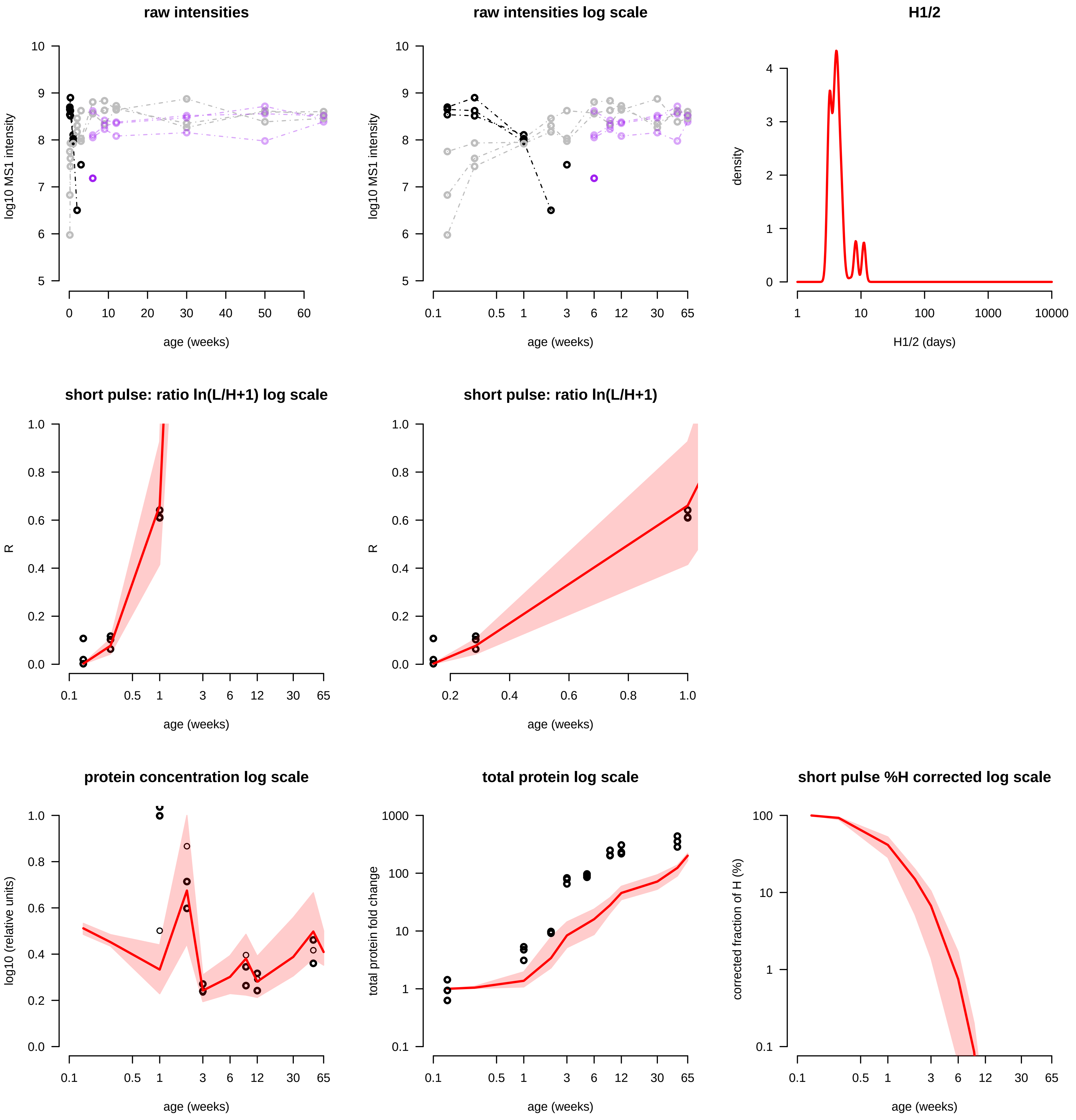

Q8BVQ5

>sp|Q8BVQ5|PPME1_MOUSE

Protein phosphatase methylesterase 1

GN=Ppme1

Med H1/2: 11 days

Q5   H1/2: 5.4 days

Q95 H1/2: 93.1 days

reliable model fit: yes

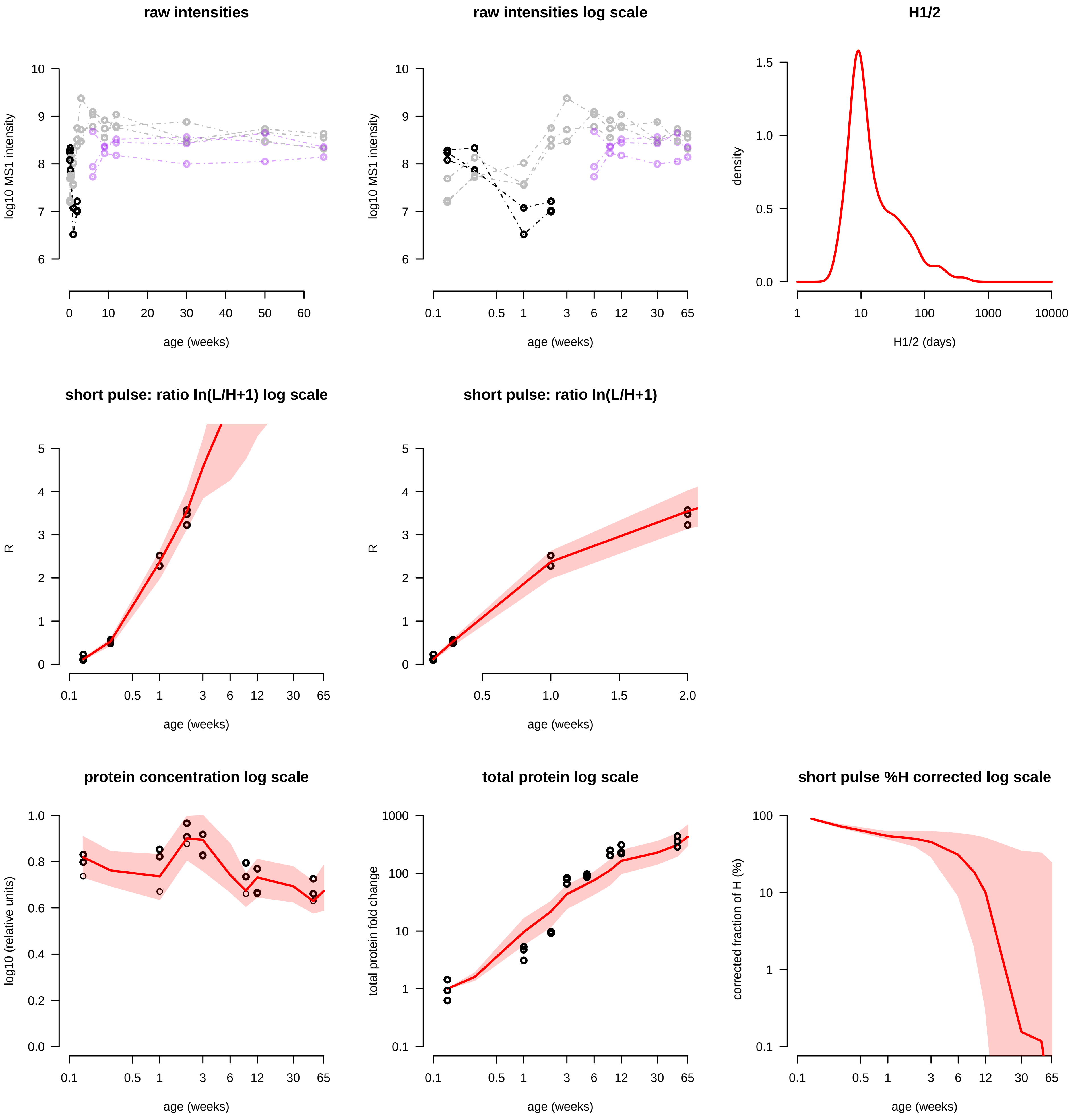

Q9D0W5

>sp|Q9D0W5|PPIL1_MOUSE

Peptidyl−prolyl cis−trans isomerase−like 1

GN=Ppil1

Med H1/2: 6 days

Q5 H1/2: 4.8 days

Q95 H1/2: 7.6 days

reliable model fit: yes

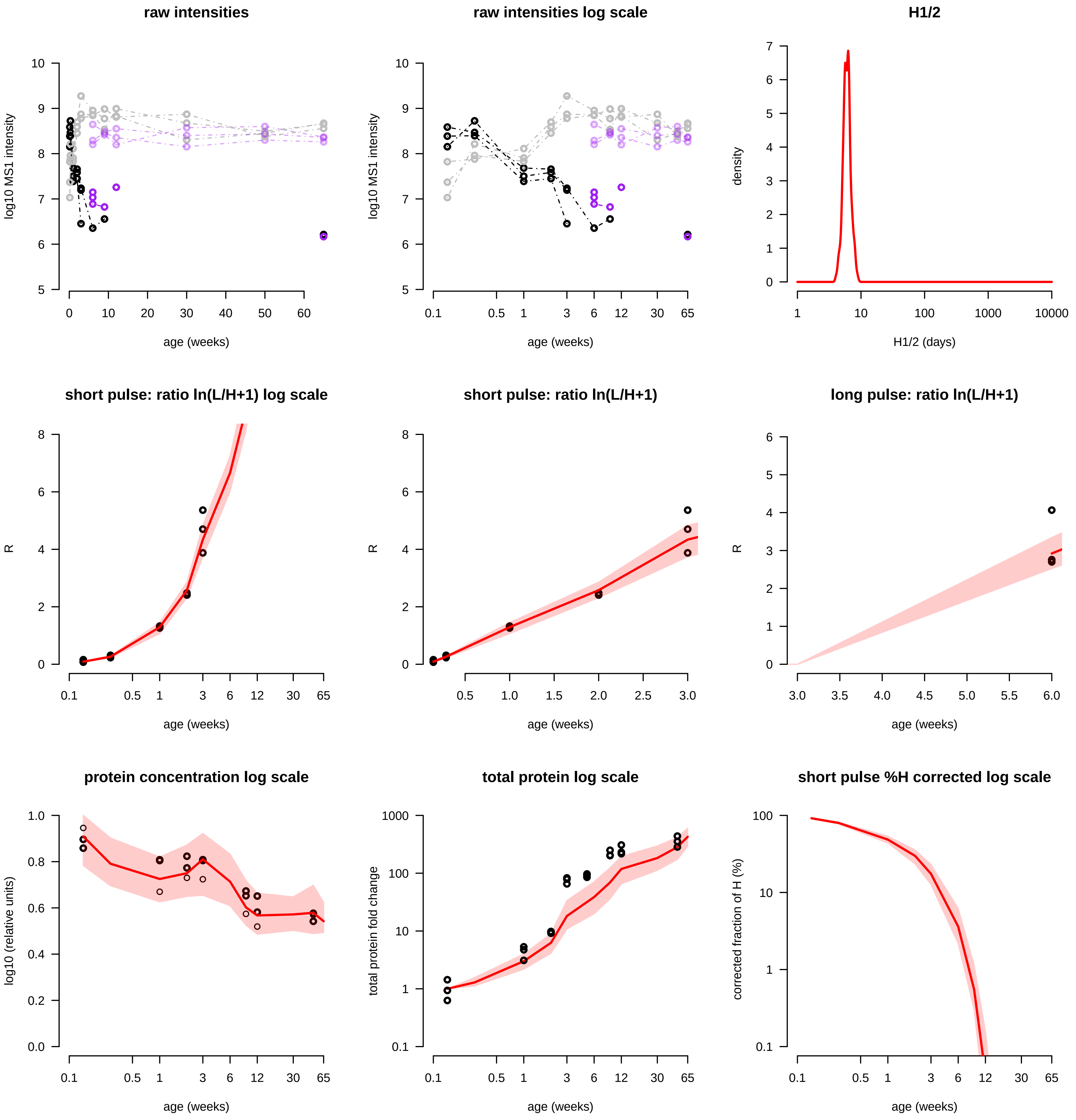

Q3UIA2;Q3UIA2−2;Q3UIA2−4

>sp|Q3UIA2|RHG17_MOUSE

Rho GTPase−activating protein 17

GN=Arhgap17

Med H1/2: 4.2 days

Q5 H1/2: 3.1 days

Q95 H1/2: 11.3 days

reliable model fit: yes

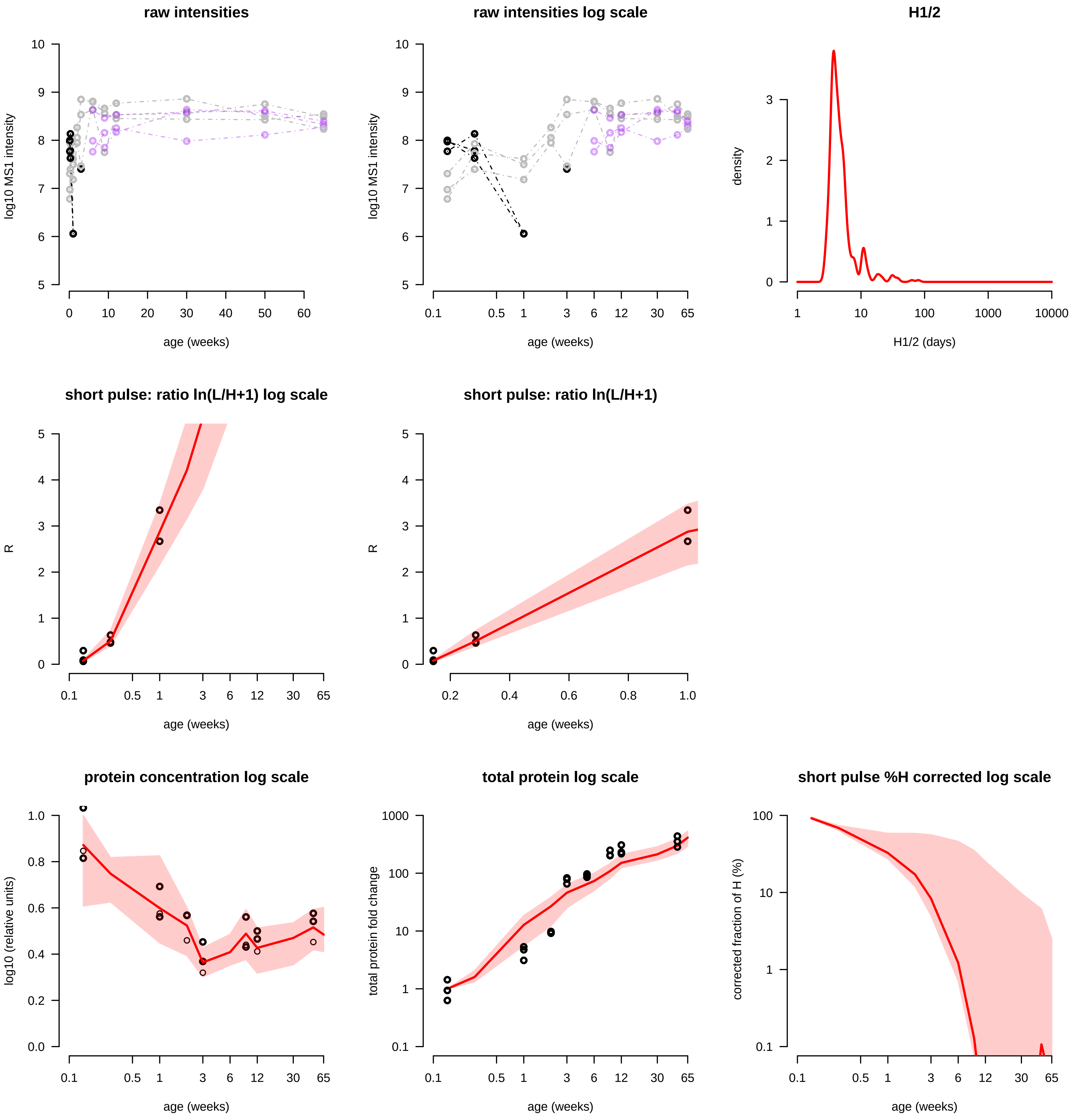

Q8JZX4

>sp|Q8JZX4|SPF45_MOUSE

Splicing factor 45

GN=Rbm17

Med H1/2: 97.2 days

Q5 H1/2: 26.9 days

Q95 H1/2: 1119.7 days

reliable model fit: no

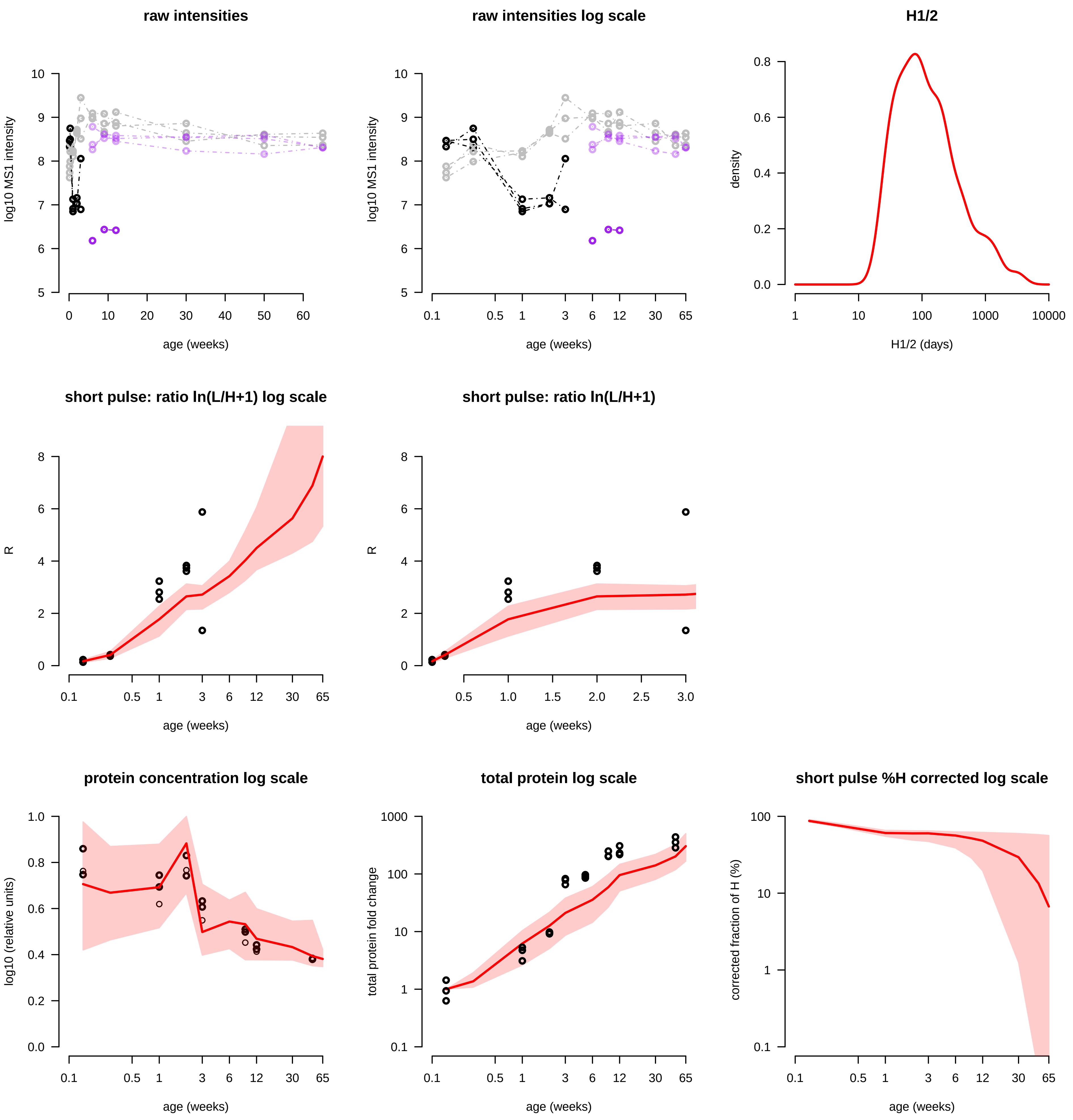

Q9CY50

>sp|Q9CY50|SSRA_MOUSE

Translocon−associated protein subunit alpha

GN=Ssr1

Med H1/2: 20.8 days

Q5 H1/2: 9.5 days

Q95 H1/2: 148.9 days

reliable model fit: yes

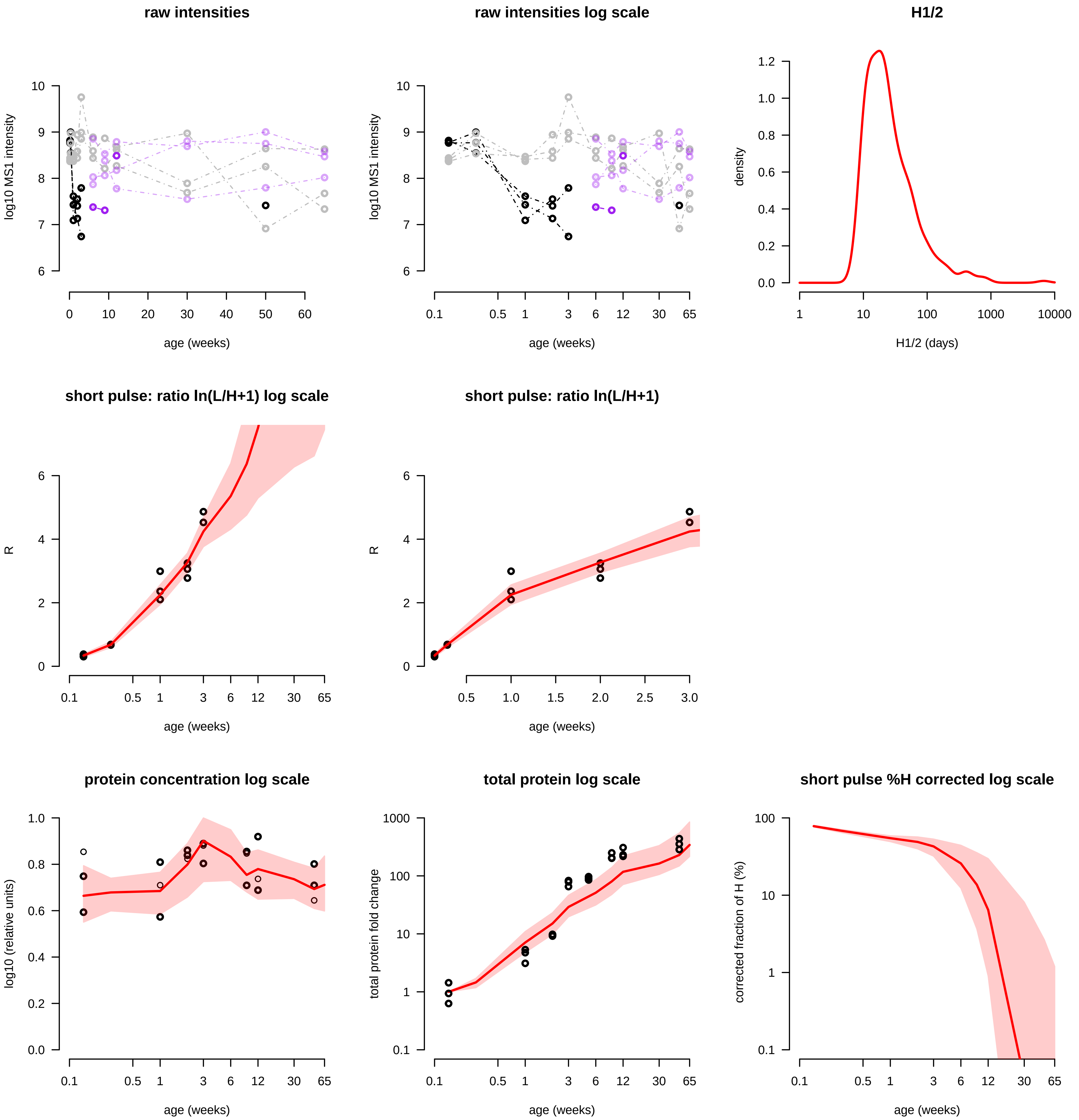

Q9JL62

>sp|Q9JL62|GLTP_MOUSE

Glycolipid transfer protein

GN=Gltp

Med H1/2: 6.5 days

Q5 H1/2: 5.1 days

Q95 H1/2: 9.2 days

reliable model fit: yes

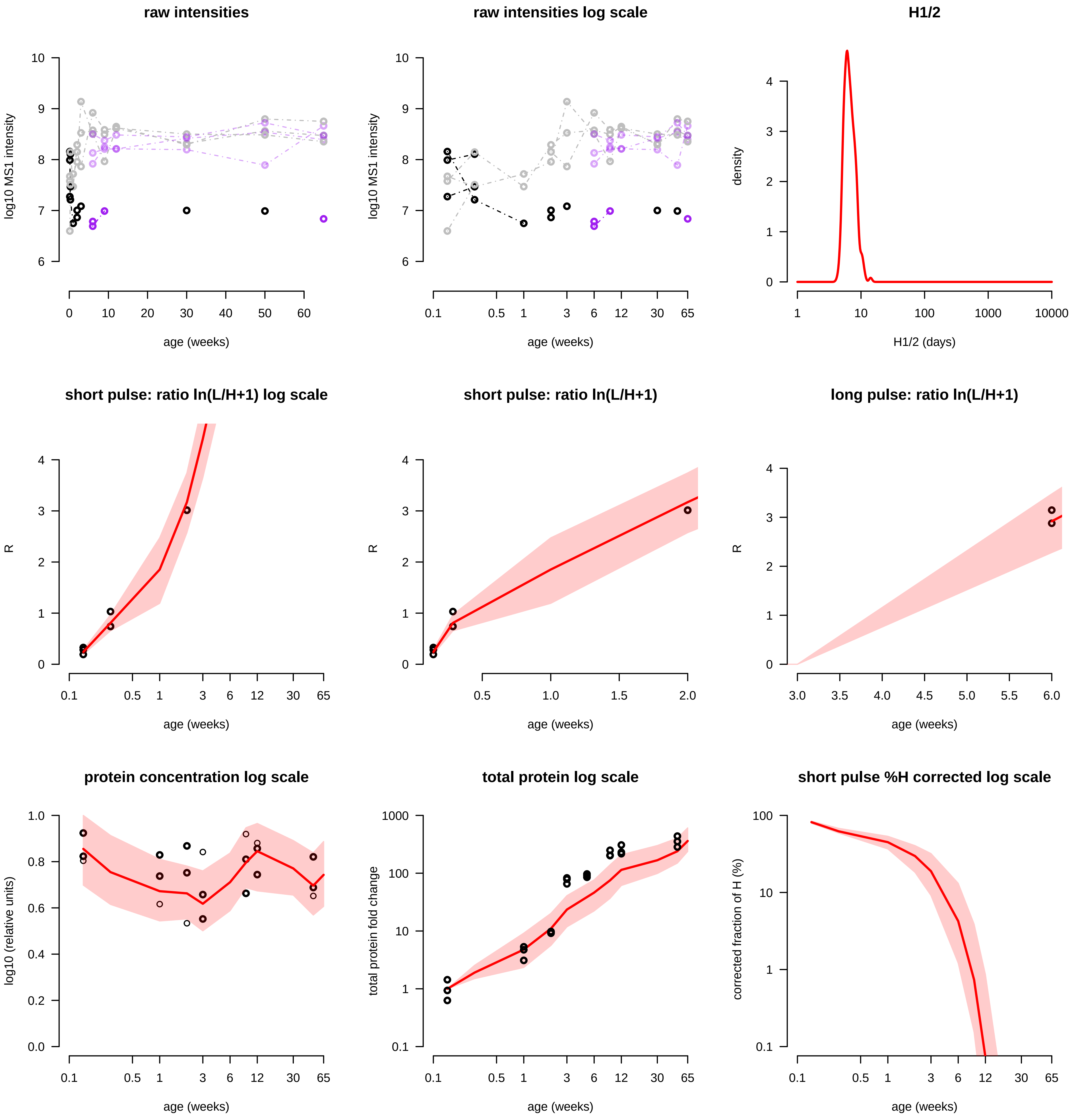

Q9EPK6

>sp|Q9EPK6|SIL1_MOUSE

Nucleotide exchange factor SIL1

GN=Sil1

Med H1/2: 5.7 days

Q5 H1/2: 2.9 days

Q95 H1/2: 17.1 days

reliable model fit: yes

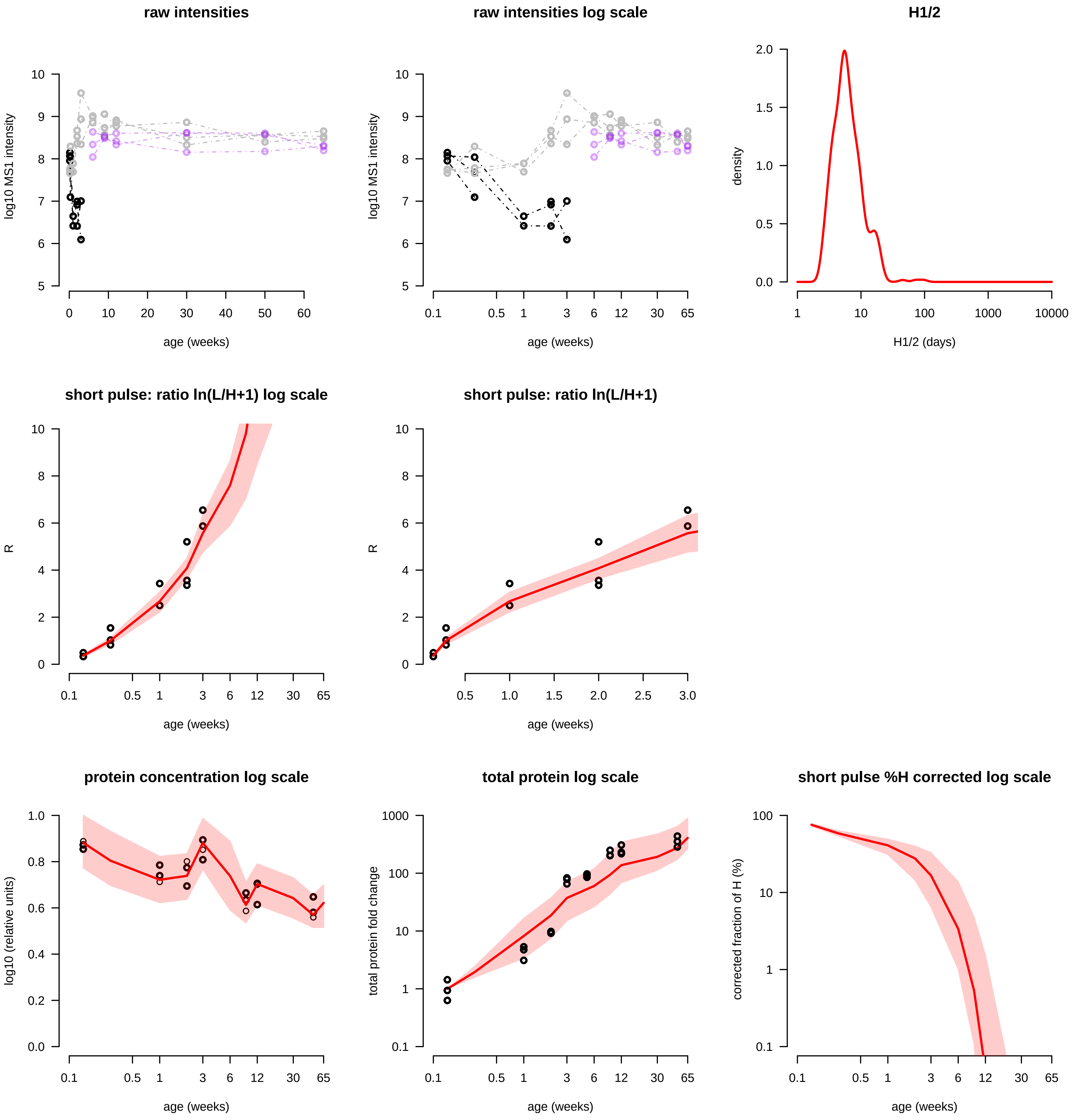

Q8R010

>sp|Q8R010|AIMP2_MOUSE

Aminoacyl tRNA synthase complex−interacting multifunctional protein 2

GN=Aimp2

Med H1/2: 4.8 days

Q5 H1/2: 4 days

Q95 H1/2: 5.7 days

reliable model fit: yes

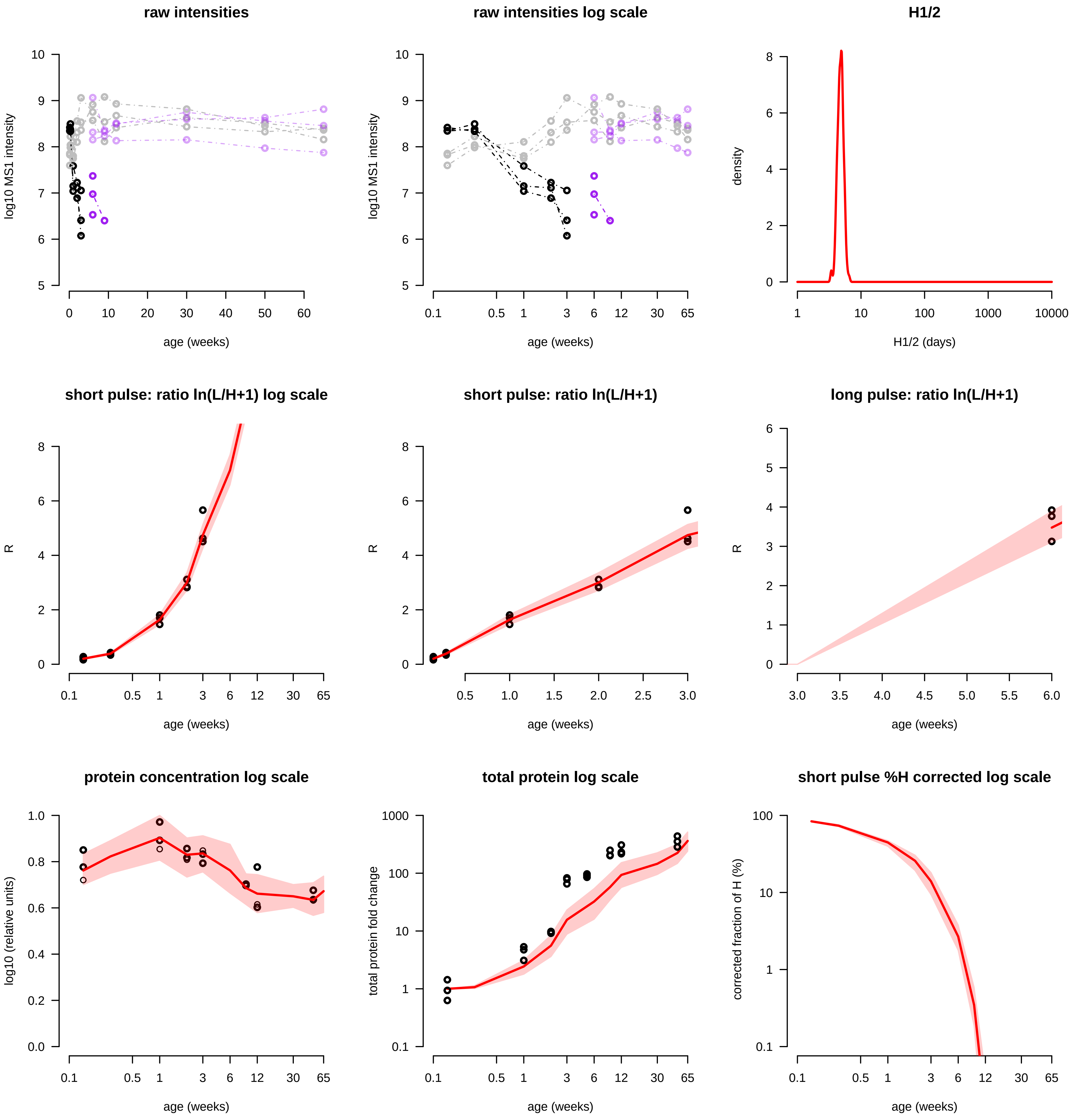

Q9DC23

>sp|Q9DC23|DJC10_MOUSE

DnaJ homolog subfamily C member 10

GN=Dnajc10

Med H1/2: 10 days

Q5 H1/2: 4 days

Q95 H1/2: 88.8 days

reliable model fit: yes

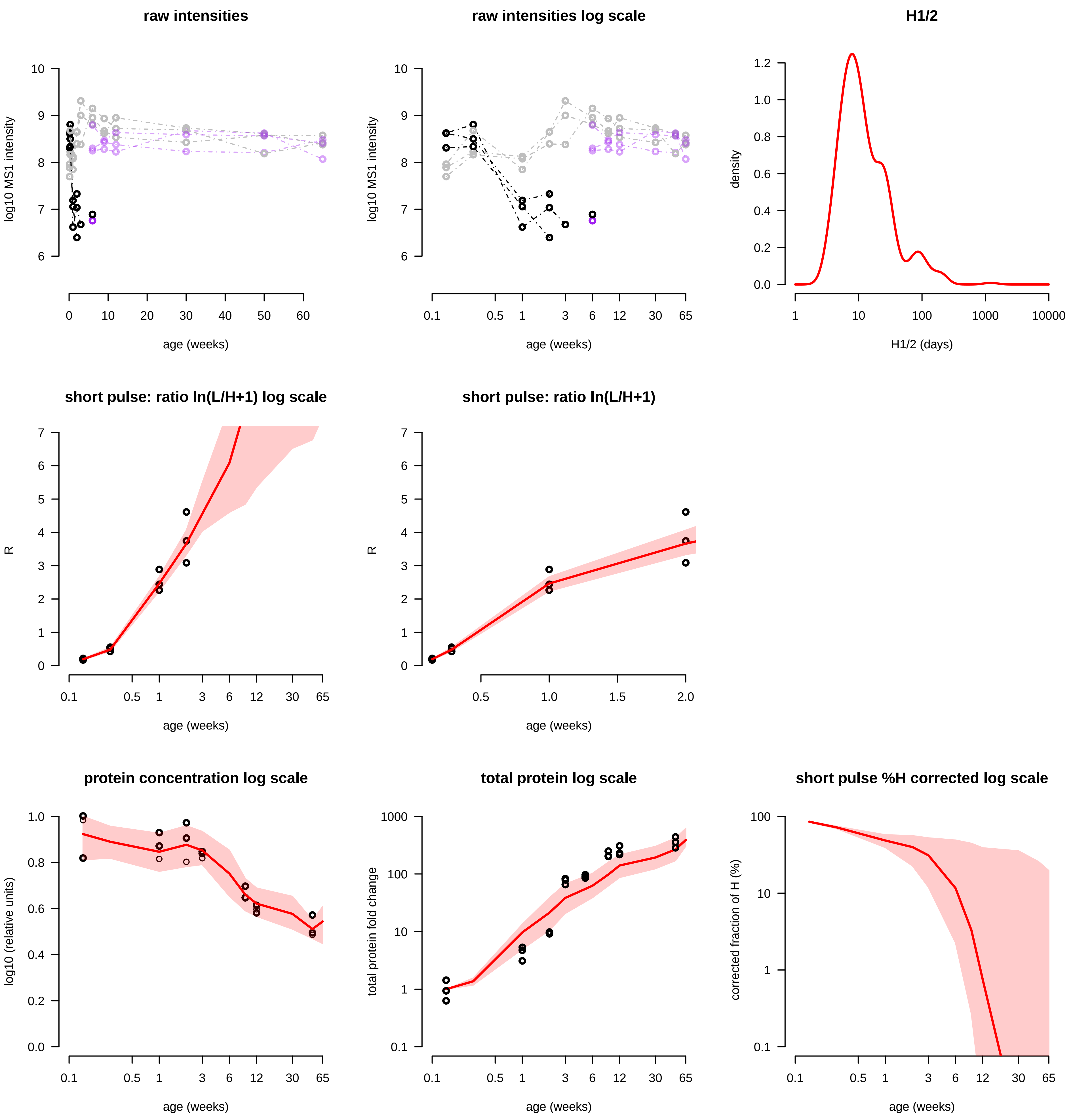

Q9D7S7−2;Q9D7S7

>sp|Q9D7S7−2|RL22L_MOUSE

Isoform 2 of 60S ribosomal protein L22−like 1

GN=Rpl22l1

Med H1/2: 6.3 days

Q5 H1/2: 4.9 days

Q95 H1/2: 9.4 days

reliable model fit: yes

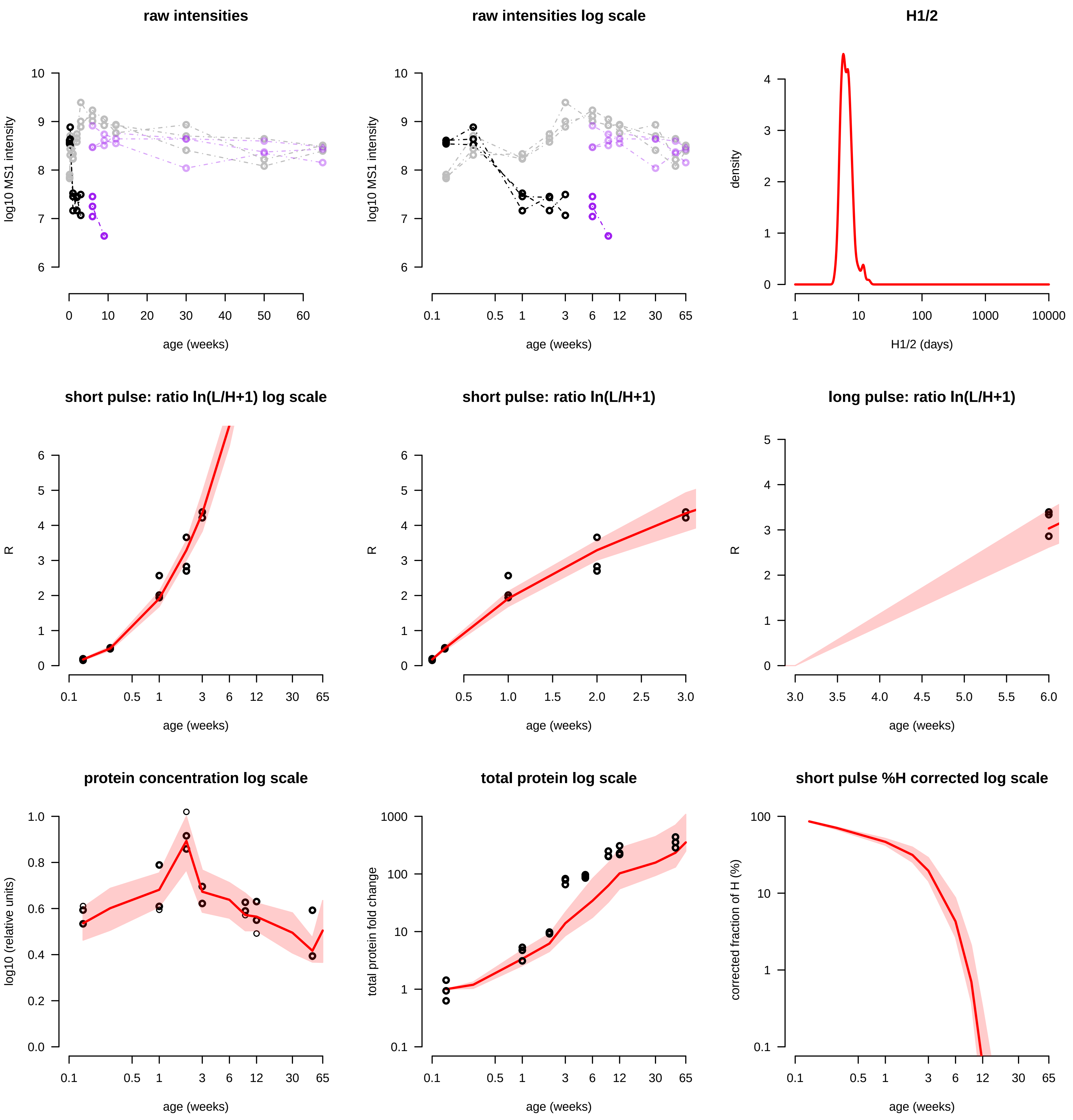

Q91XE8

>sp|Q91XE8|TM205_MOUSE

Transmembrane protein 205

GN=Tmem205

Med H1/2: 8.2 days

Q5 H1/2: 5.6 days

Q95 H1/2: 20.6 days

reliable model fit: no

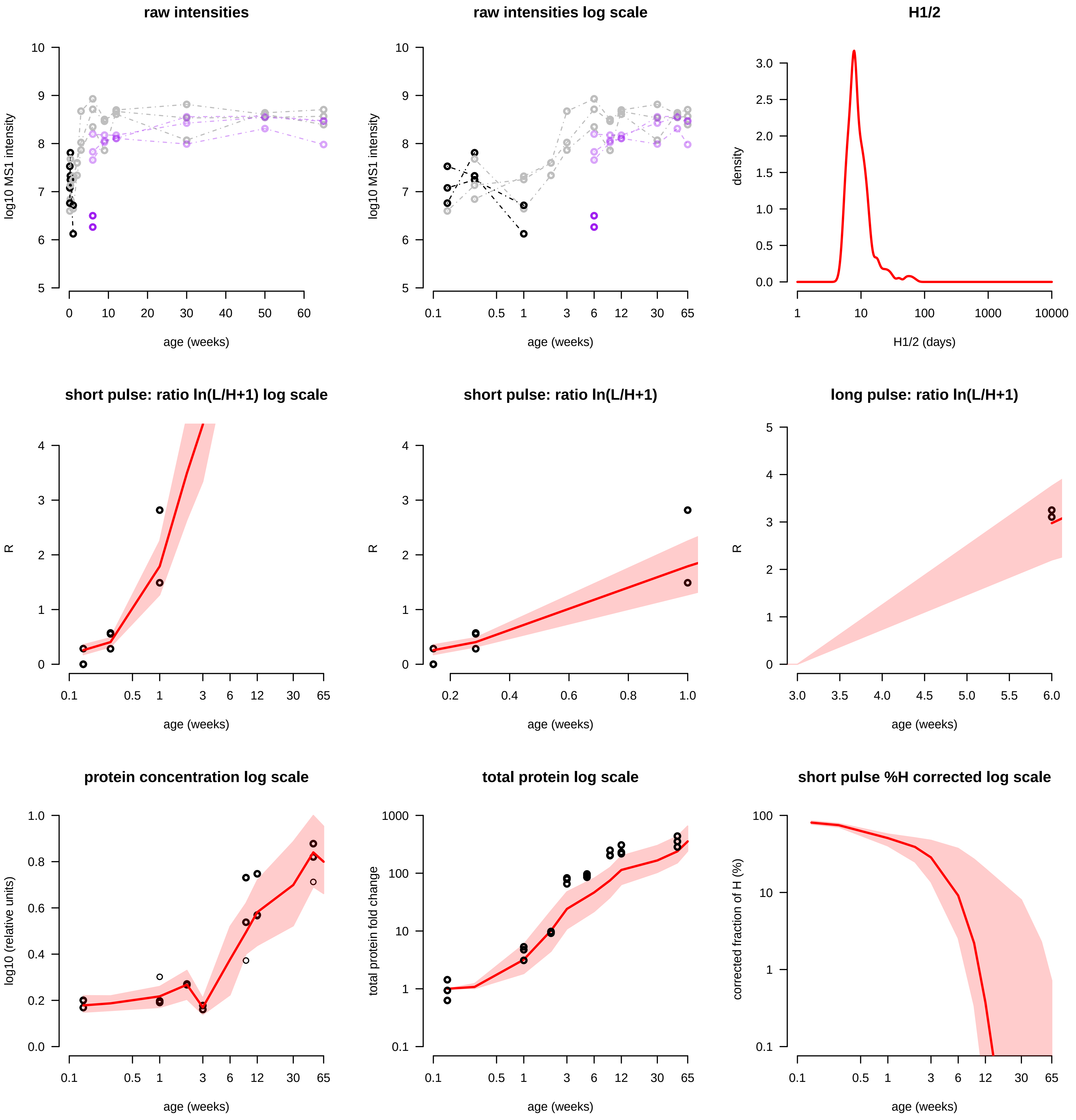

Q99ME9

>sp|Q99ME9|GTPB4_MOUSE

GTP−binding protein 4

GN=Gtpbp4

Med H1/2: 8.9 days

Q5 H1/2: 4 days

Q95 H1/2: 48.4 days

reliable model fit: yes

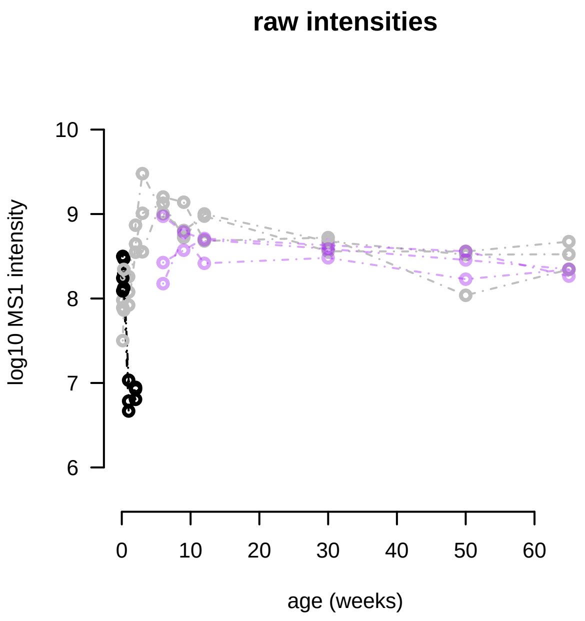

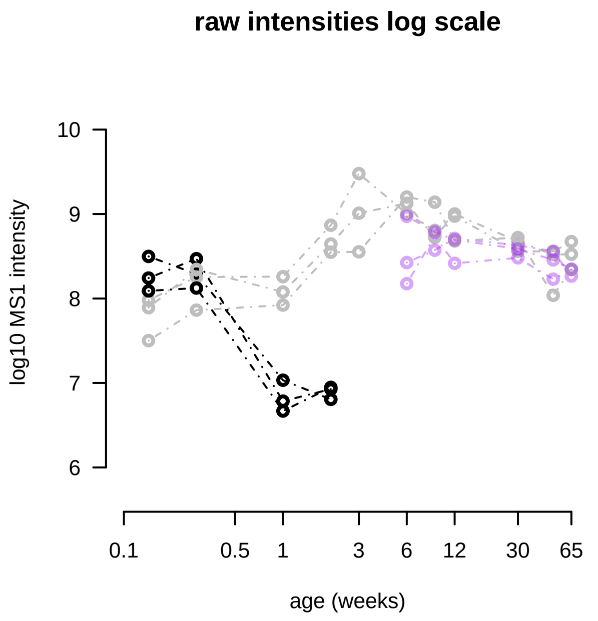

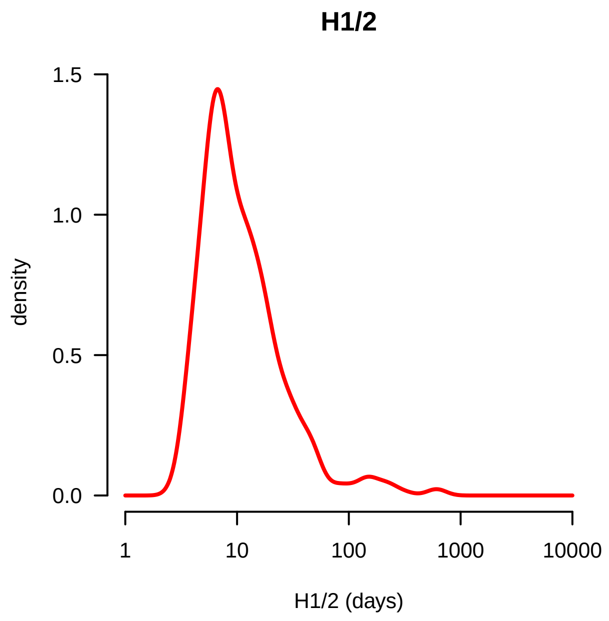

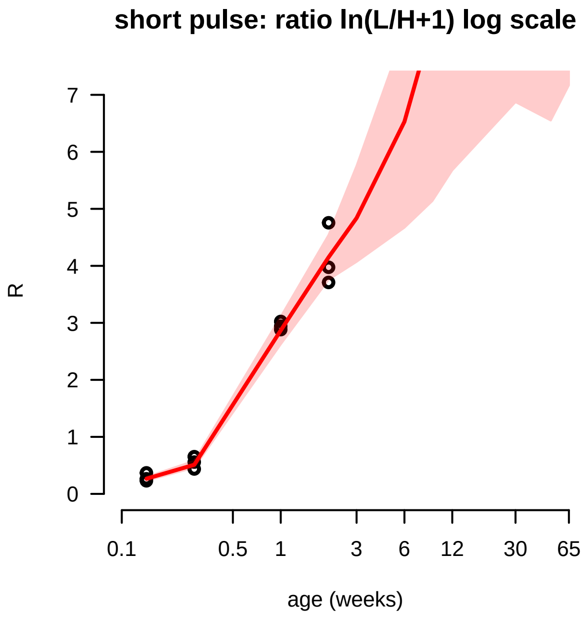

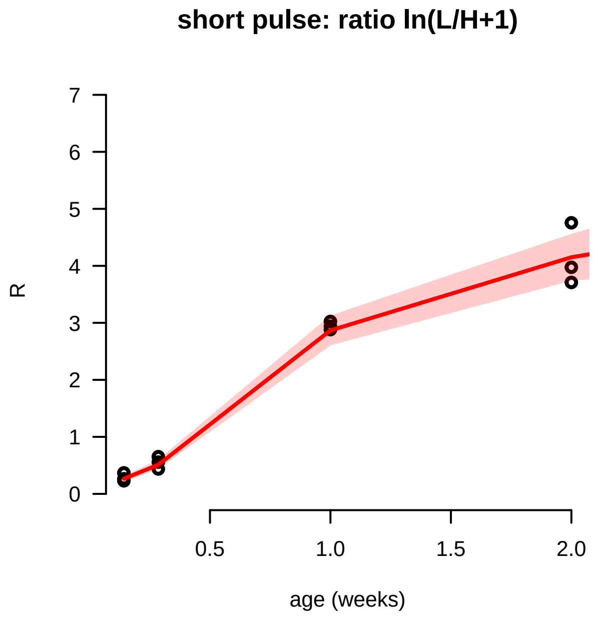

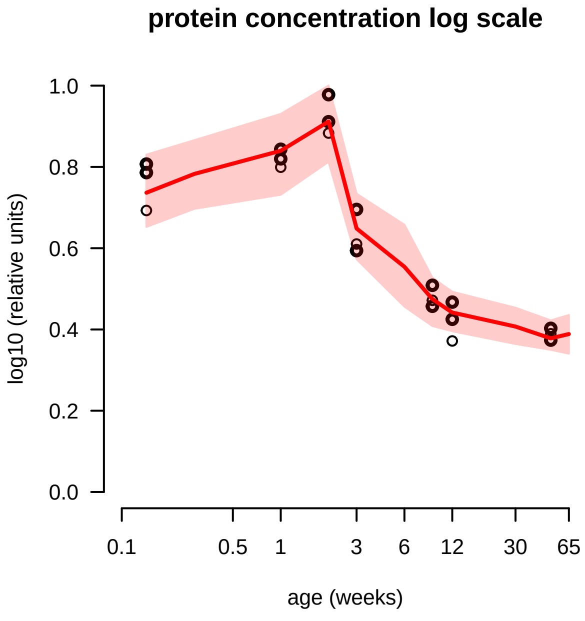

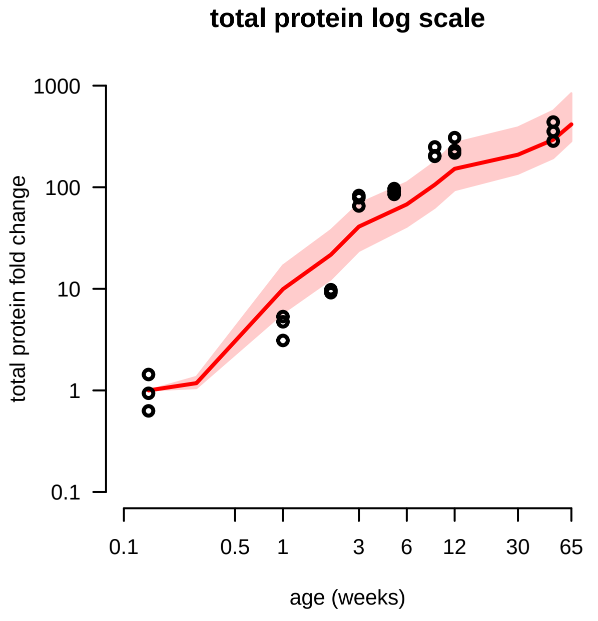

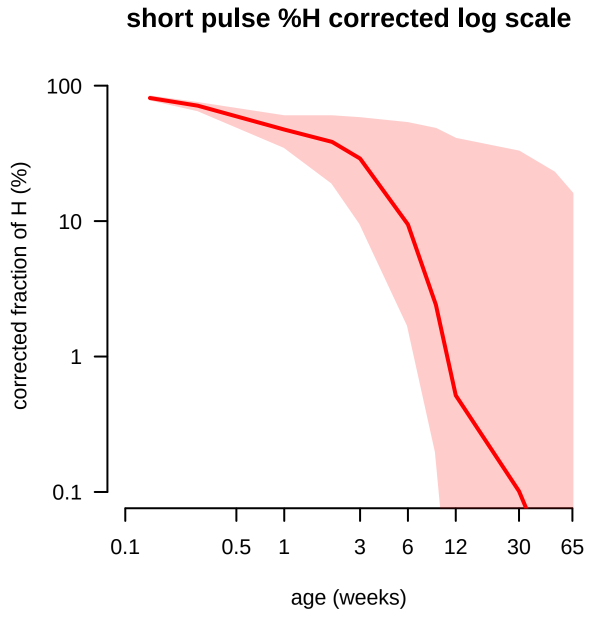

Q7TMF3

>sp|Q7TMF3|NDUAC_MOUSE

NADH dehydrogenase [ubiquinone] 1 alpha subcomplex subunit 12

GN=Ndufa12

Med H1/2: 5.3 days

Q5 H1/2: 4.1 days

Q95 H1/2: 8.7 days

reliable model fit: yes

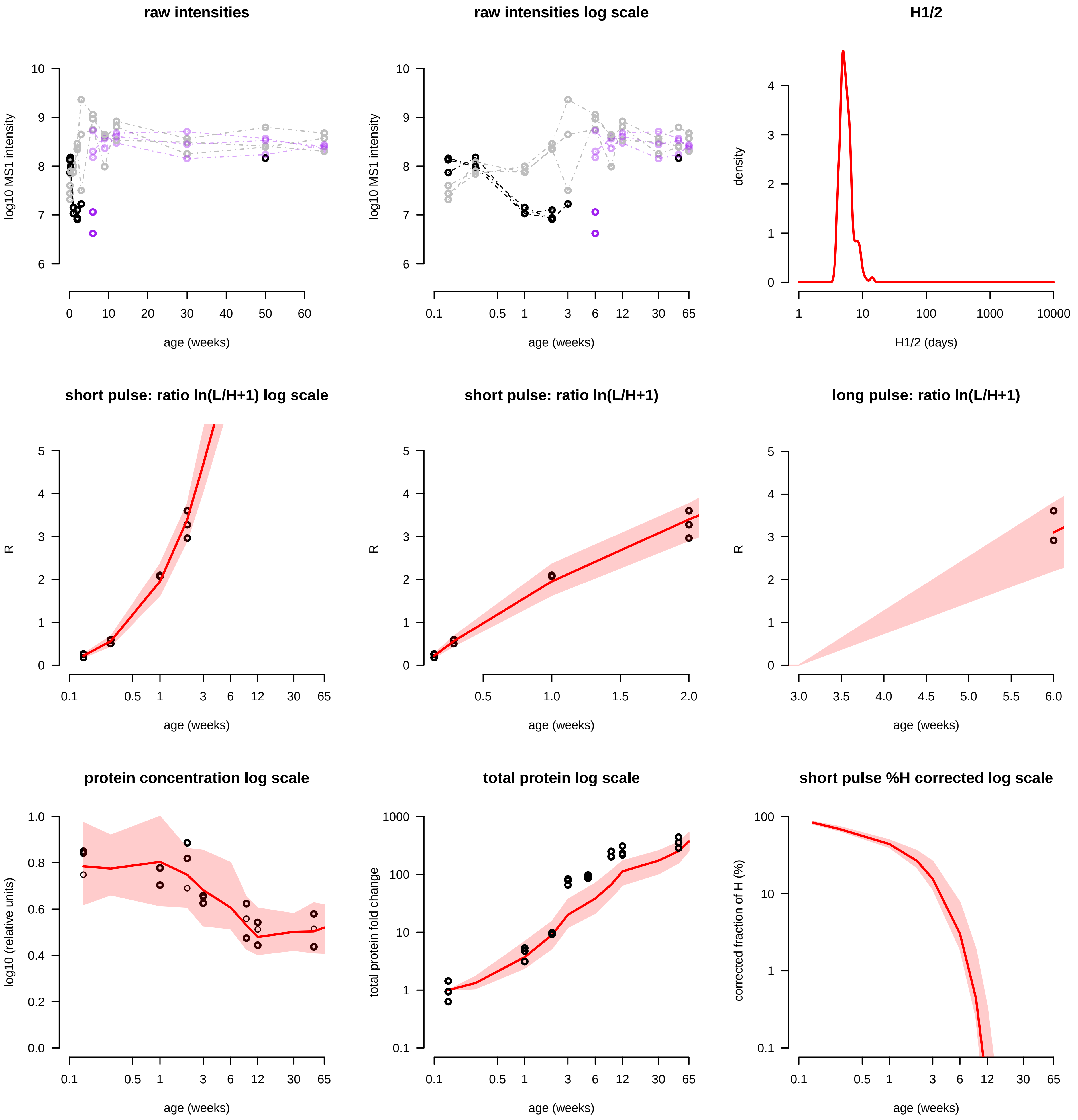

P63168

>sp|P63168|DYL1_MOUSE

Dynein light chain 1, cytoplasmic

GN=Dynll1

Med H1/2: 18.7 days

Q5 H1/2: 7.4 days

Q95 H1/2: 77.7 days

reliable model fit: yes

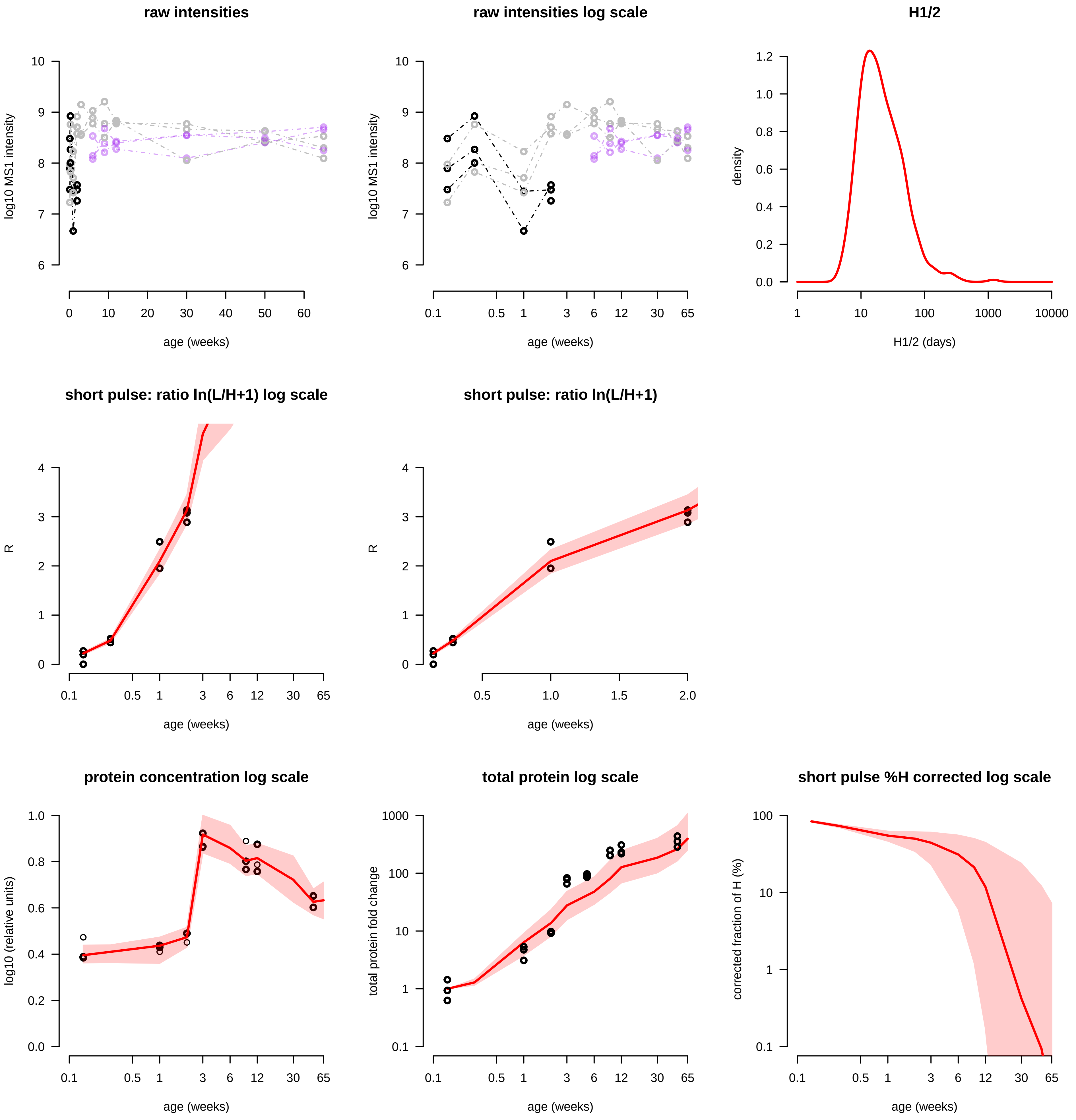

Q9Z2Q6

>sp|Q9Z2Q6|SEPT5_MOUSE

Septin−5

GN=Septin5

Med H1/2: 4.5 days

Q5 H1/2: 3.3 days

Q95 H1/2: 7 days

reliable model fit: yes

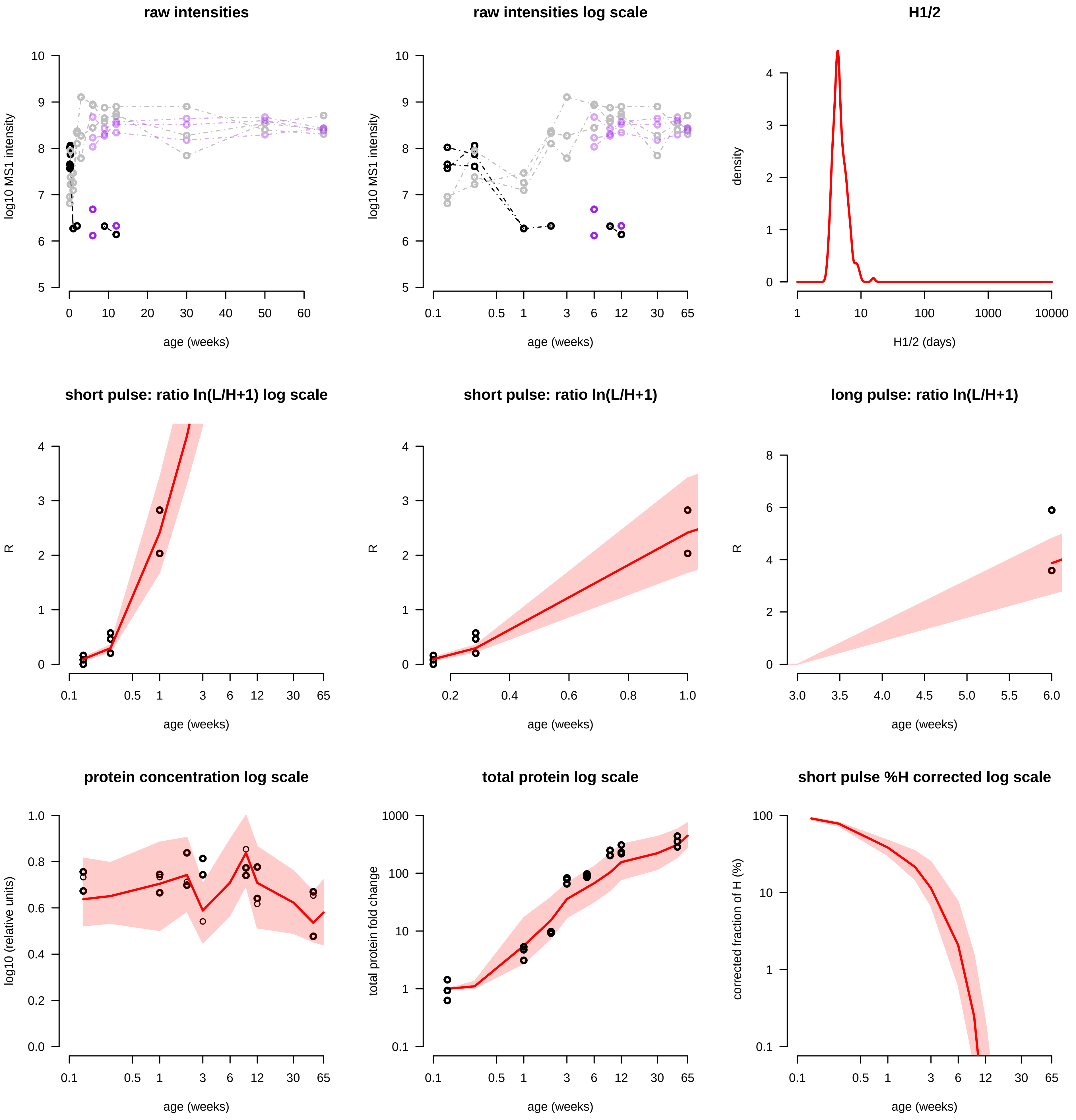

Q61206

>sp|Q61206|PA1B2_MOUSE

Platelet−activating factor acetylhydrolase IB subunit alpha2

GN=Pafah1b2

Med H1/2: 5.9 days

Q5 H1/2: 2.8 days

Q95 H1/2: 51.4 days

reliable model fit: yes

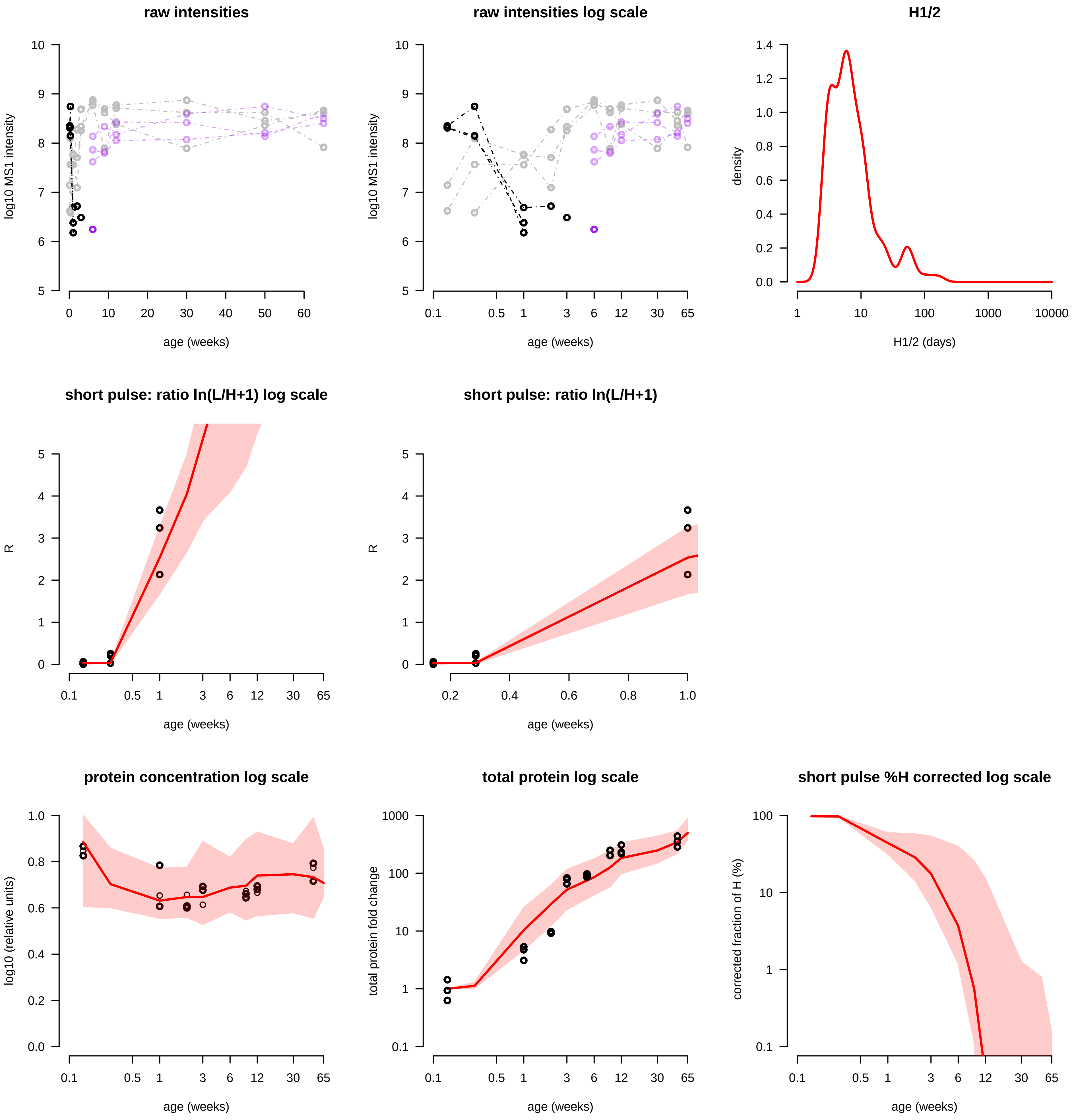

Q9WTL7

>sp|Q9WTL7|LYPA2_MOUSE

Acyl−protein thioesterase 2

GN=Lypla2

Med H1/2: 6.8 days

Q5 H1/2: 4.9 days

Q95 H1/2: 11.1 days

reliable model fit: yes

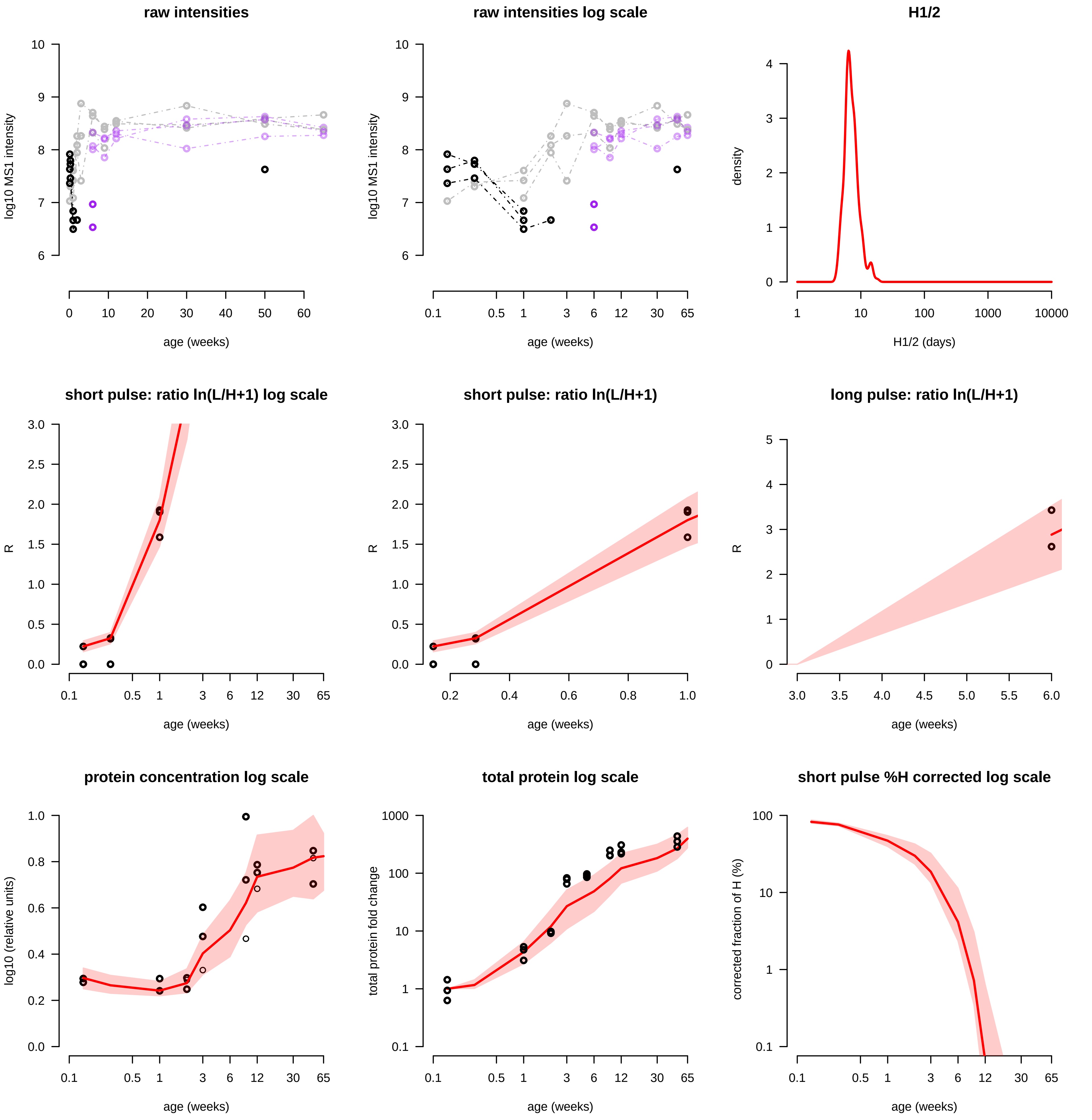

Q3TCN2;Q3TCN2−2

>sp|Q3TCN2|PLBL2_MOUSE

Putative phospholipase B−like 2

GN=Plbd2

Med H1/2: 5 days

Q5 H1/2: 2.7 days

Q95 H1/2: 36.5 days

reliable model fit: no

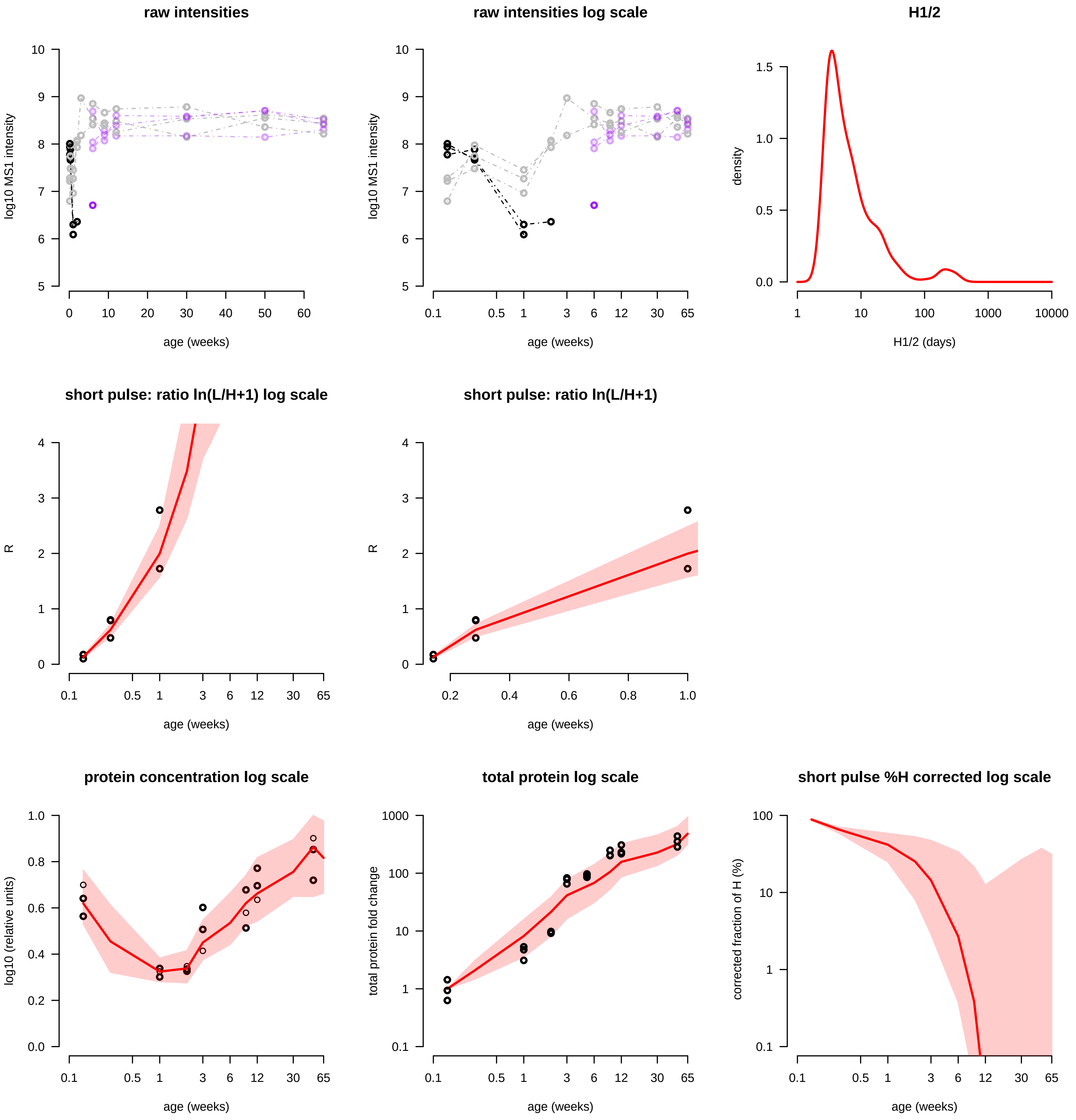

Q68FH4

>sp|Q68FH4|GALK2_MOUSE

N−acetylgalactosamine kinase

GN=Galk2

Med H1/2: 4.9 days

Q5 H1/2: 3.7 days

Q95 H1/2: 6.9 days

reliable model fit: no

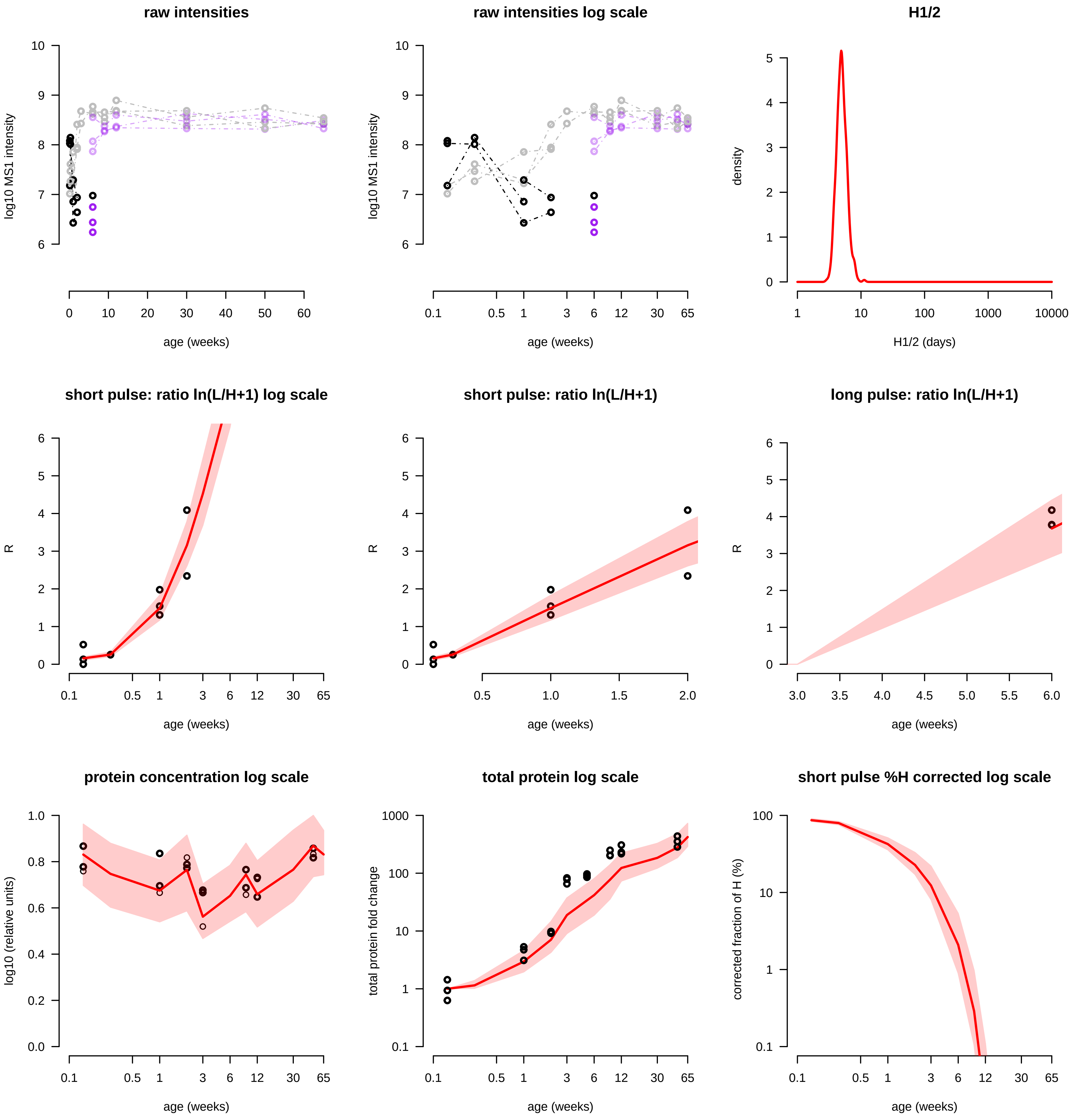

Q62241

>sp|Q62241|RU1C_MOUSE

U1 small nuclear ribonucleoprotein C

GN=Snrpc

Med H1/2: 1114.8 days

Q5 H1/2: 325.8 days

Q95 H1/2: 15924.7 days

reliable model fit: yes

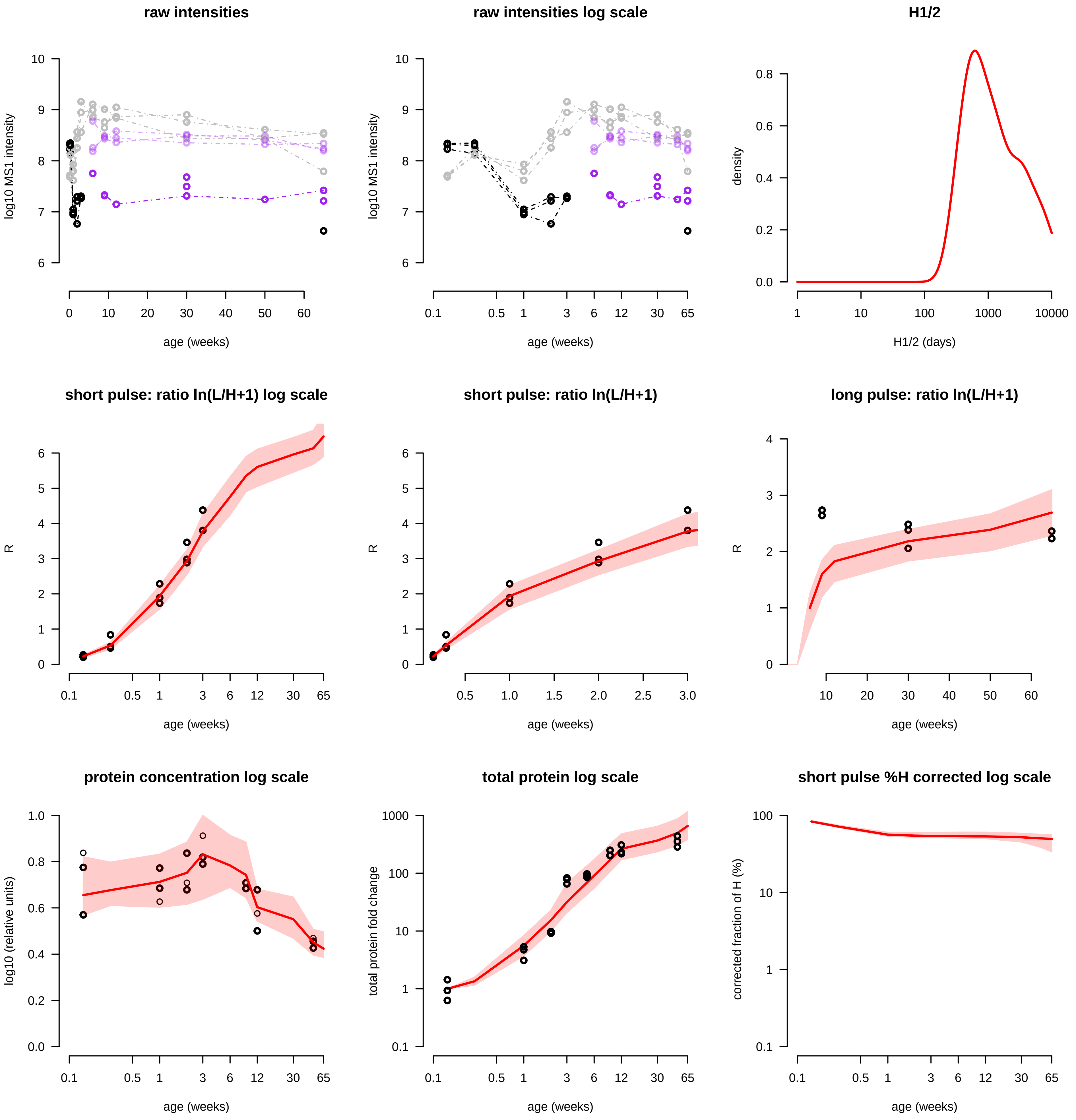

Q61239

>sp|Q61239|FNTA_MOUSE

Protein farnesyltransferase/geranylgeranyltransferase type−1 subunit alpha

GN=Fnta

Med H1/2: 8.3 days

Q5 H1/2: 3.2 days

Q95 H1/2: 133 days

reliable model fit: yes

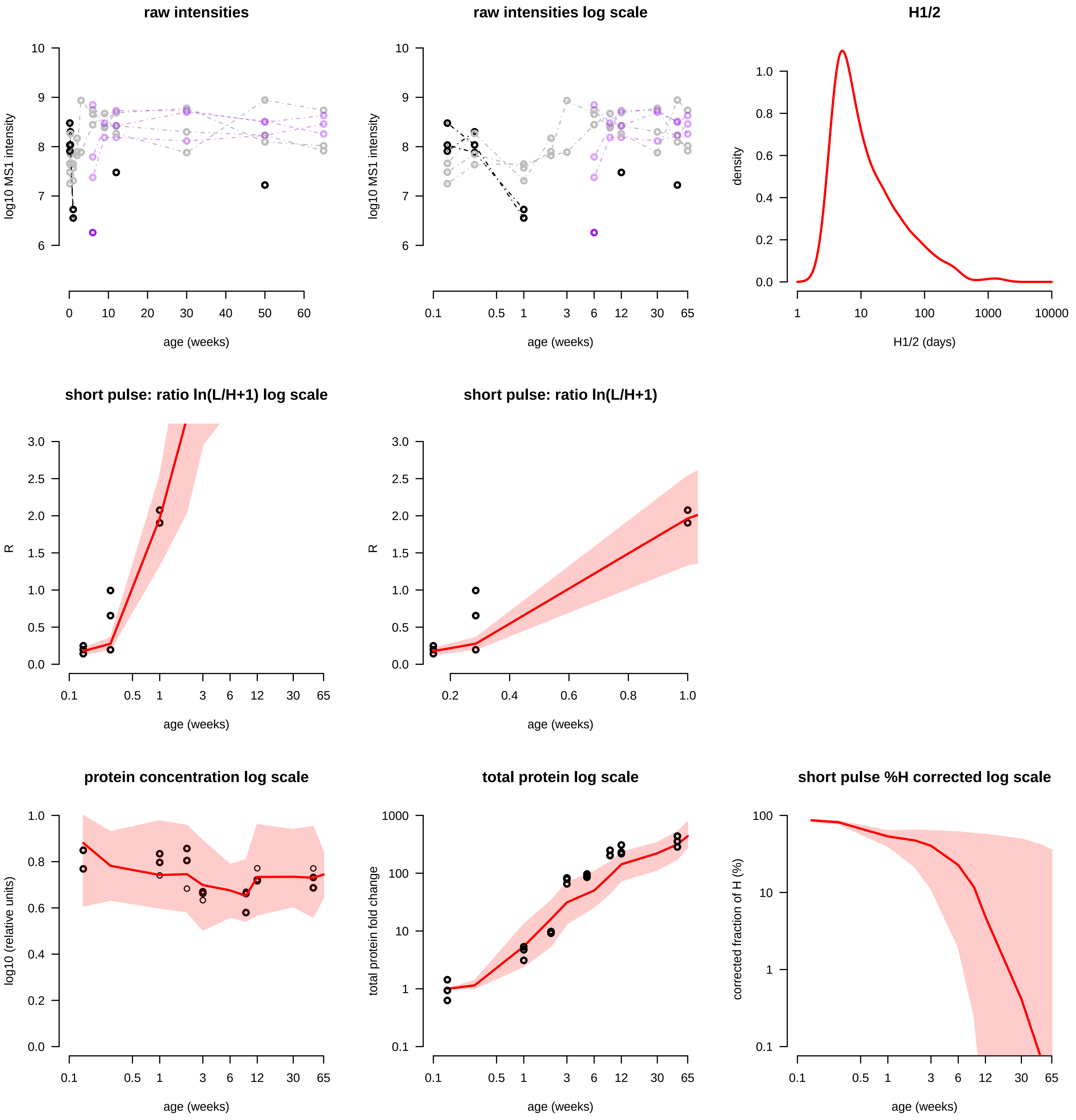

Q3V1L4

>sp|Q3V1L4|5NTC_MOUSE

Cytosolic purine 5'−nucleotidase

GN=Nt5c2

Med H1/2: 5 days

Q5   H1/2: 2.5 days

Q95 H1/2: 20.5 days

reliable model fit: yes

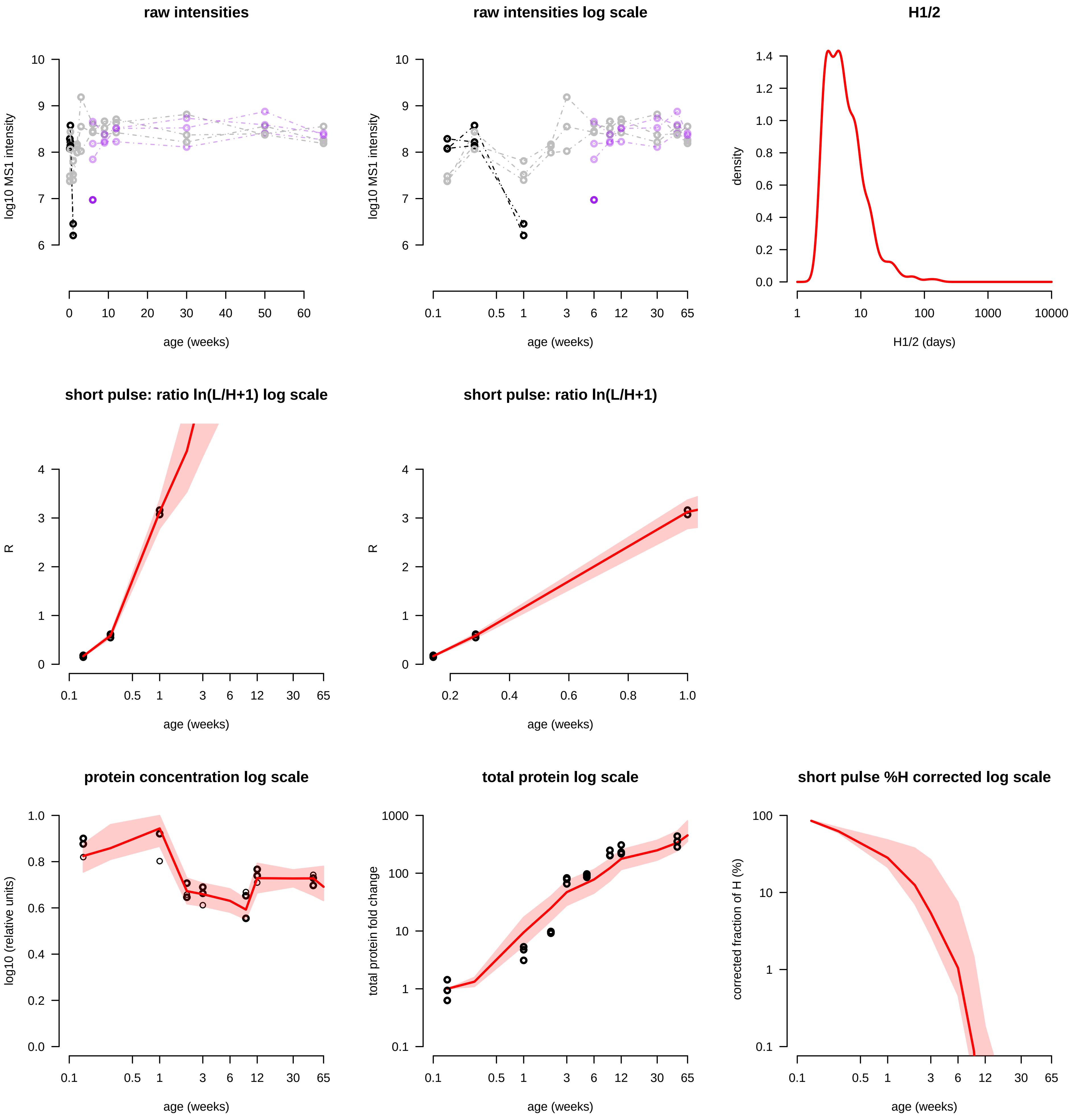

Q921H9

>sp|Q921H9|COA7_MOUSE

Cytochrome c oxidase assembly factor 7

GN=Coa7

Med H1/2: 7.9 days

Q5 H1/2: 4.2 days

Q95 H1/2: 30.4 days

reliable model fit: yes

raw intensities

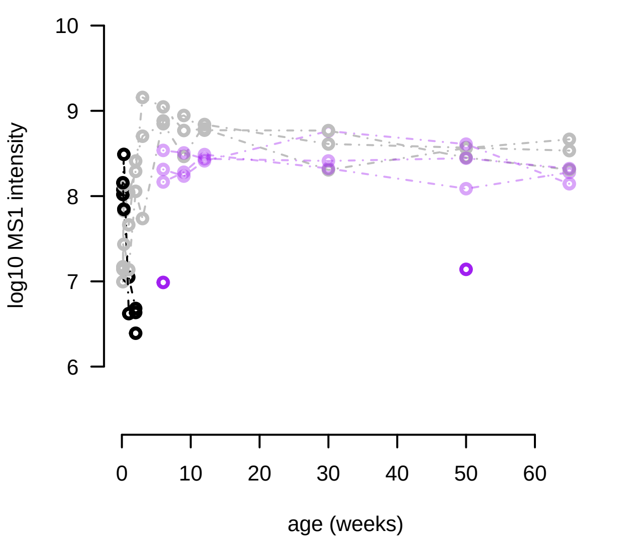

raw intensities log scale

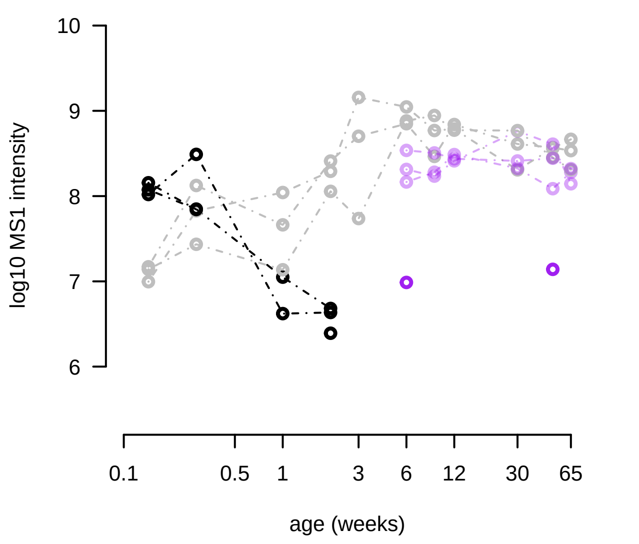

H1/2

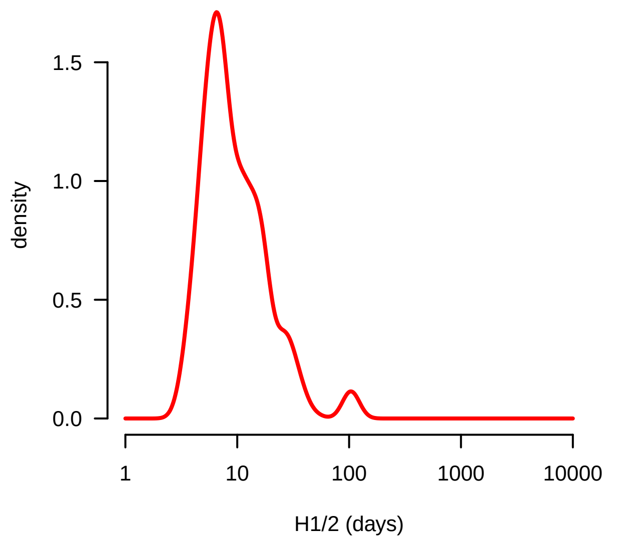

short pulse: ratio ln(L/H+1) log scale

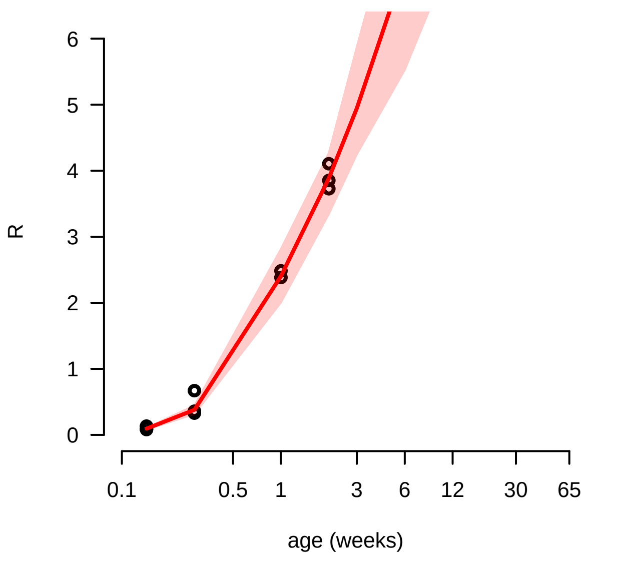

short pulse: ratio ln(L/H+1)

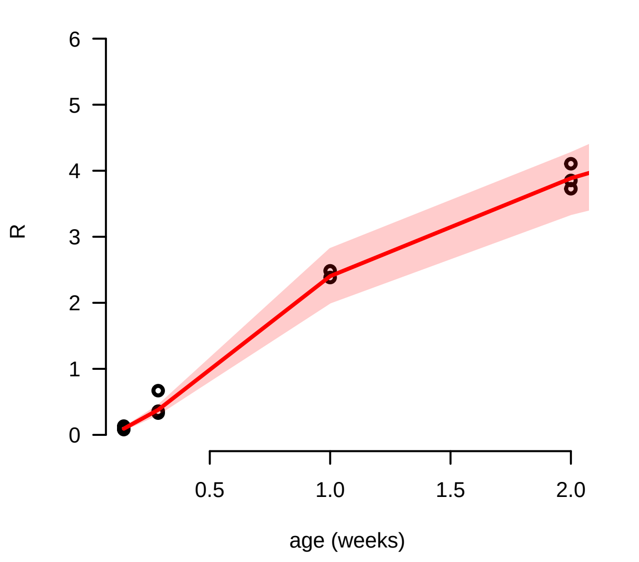

protein concentration log scale

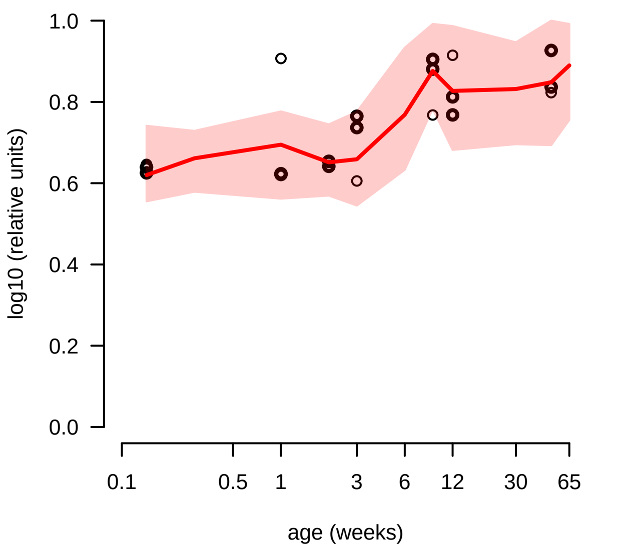

total protein log scale

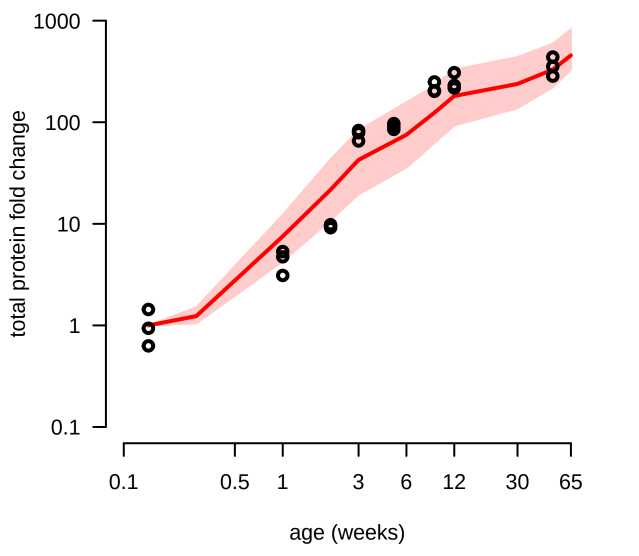

short pulse %H corrected log scale

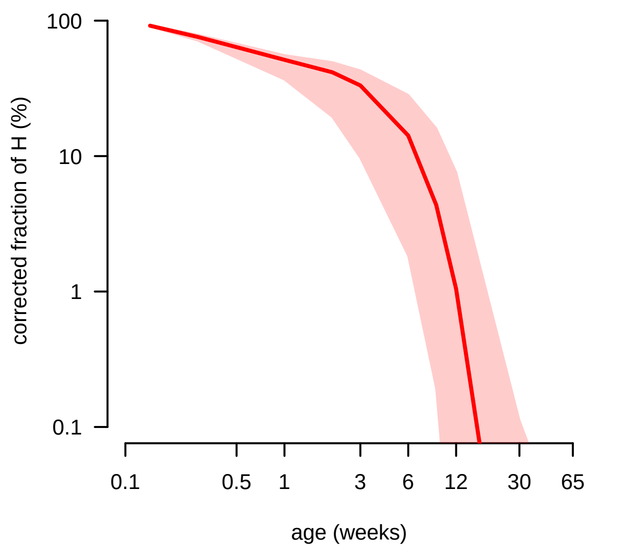

Q9ESU6;Q9ESU6−2

>sp|Q9ESU6|BRD4_MOUSE

Bromodomain−containing protein 4

GN=Brd4

Med H1/2: 11 days

Q5 H1/2: 3.5 days

Q95 H1/2: 93 days

reliable model fit: yes

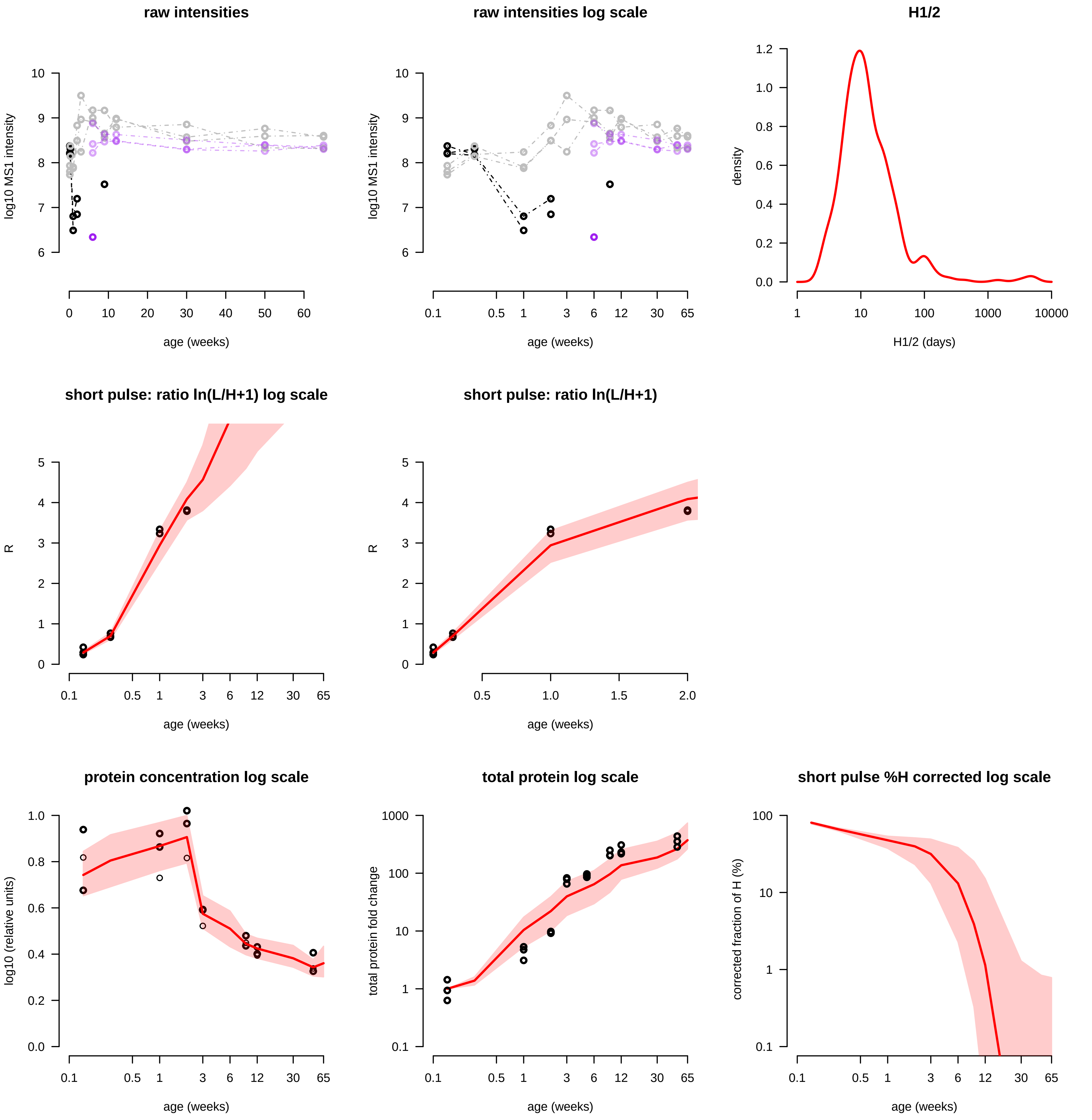

Q9Z1R2

>sp|Q9Z1R2|BAG6_MOUSE

Large proline−rich protein BAG6

GN=Bag6

Med H1/2: 6 days

Q5 H1/2: 3.1 days

Q95 H1/2: 18.4 days

reliable model fit: yes

raw intensities

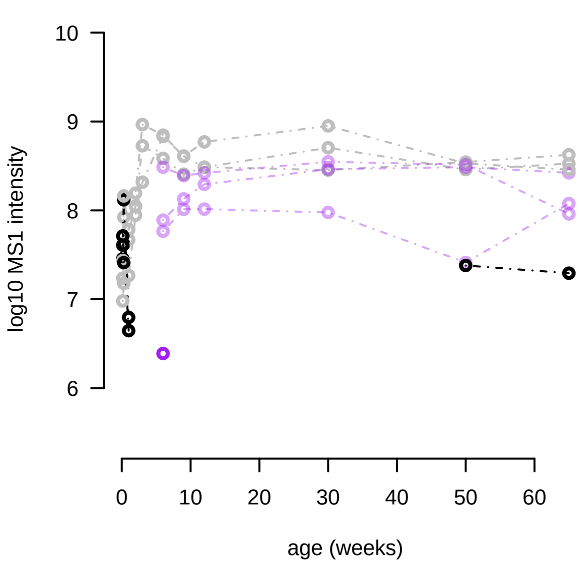

raw intensities log scale

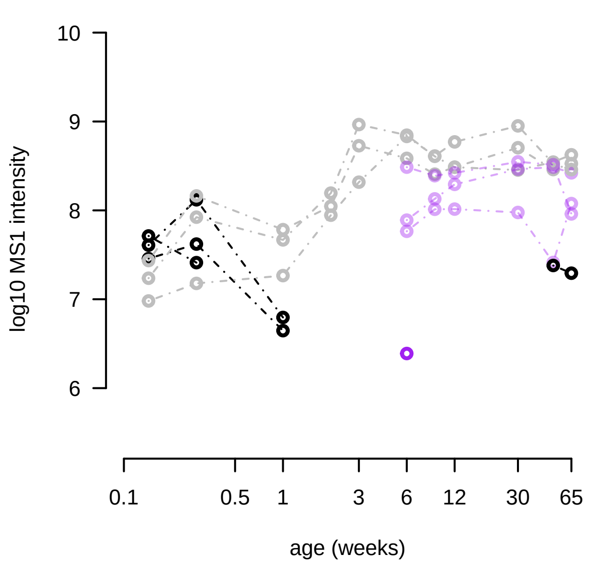

H1/2

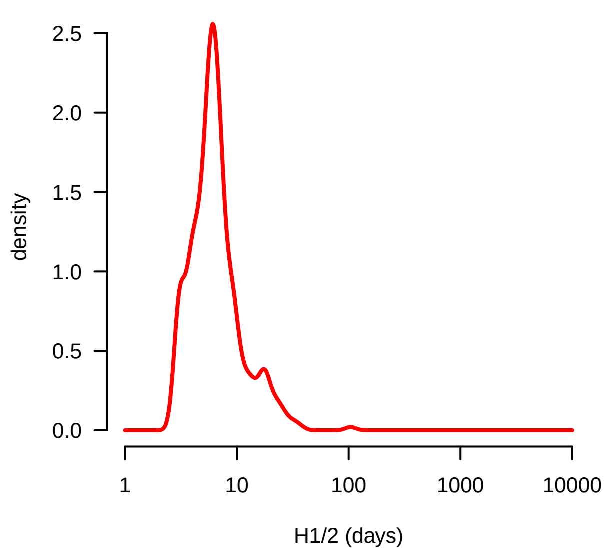

short pulse: ratio ln(L/H+1) log scale

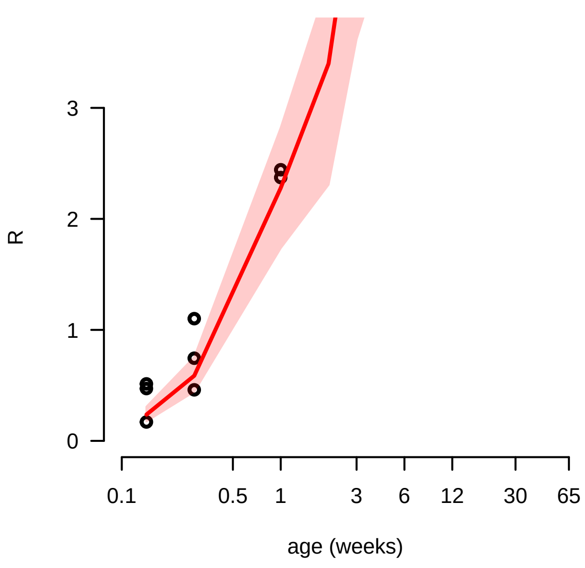

short pulse: ratio ln(L/H+1)

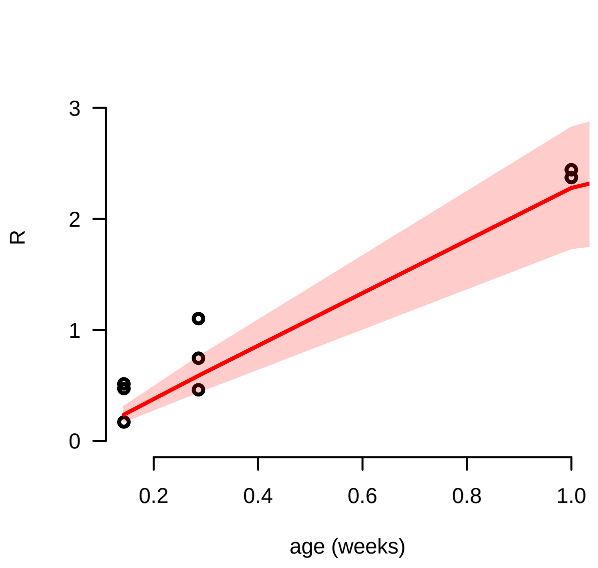

protein concentration log scale

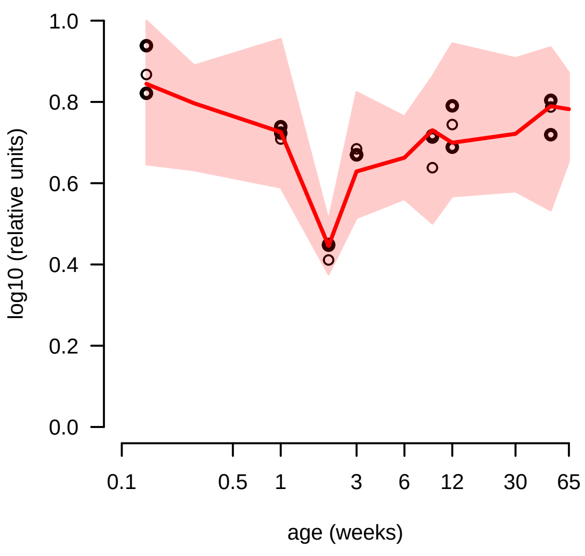

total protein log scale

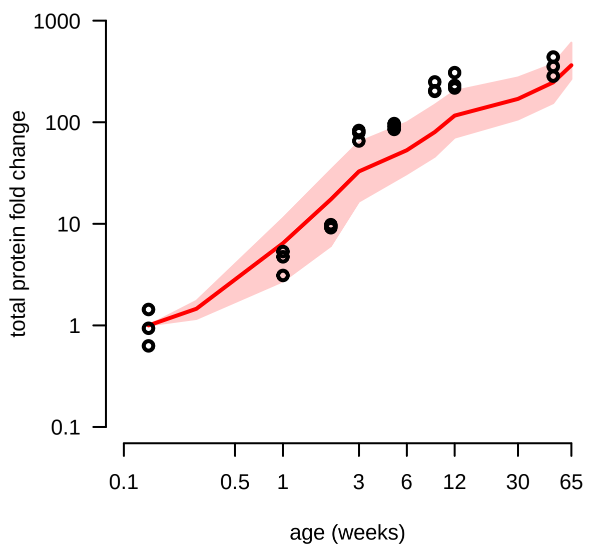

short pulse %H corrected log scale

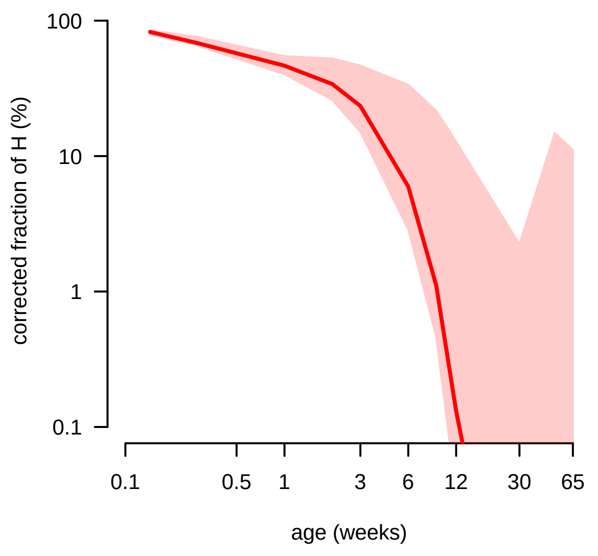

Q99PU8−2;Q99PU8

>sp|Q99PU8−2|DHX30_MOUSE

Isoform 2 of ATP−dependent RNA helicase DHX30

GN=Dhx30

Med H1/2: 7.1 days

Q5 H1/2: 3.5 days

Q95 H1/2: 50.4 days

reliable model fit: yes

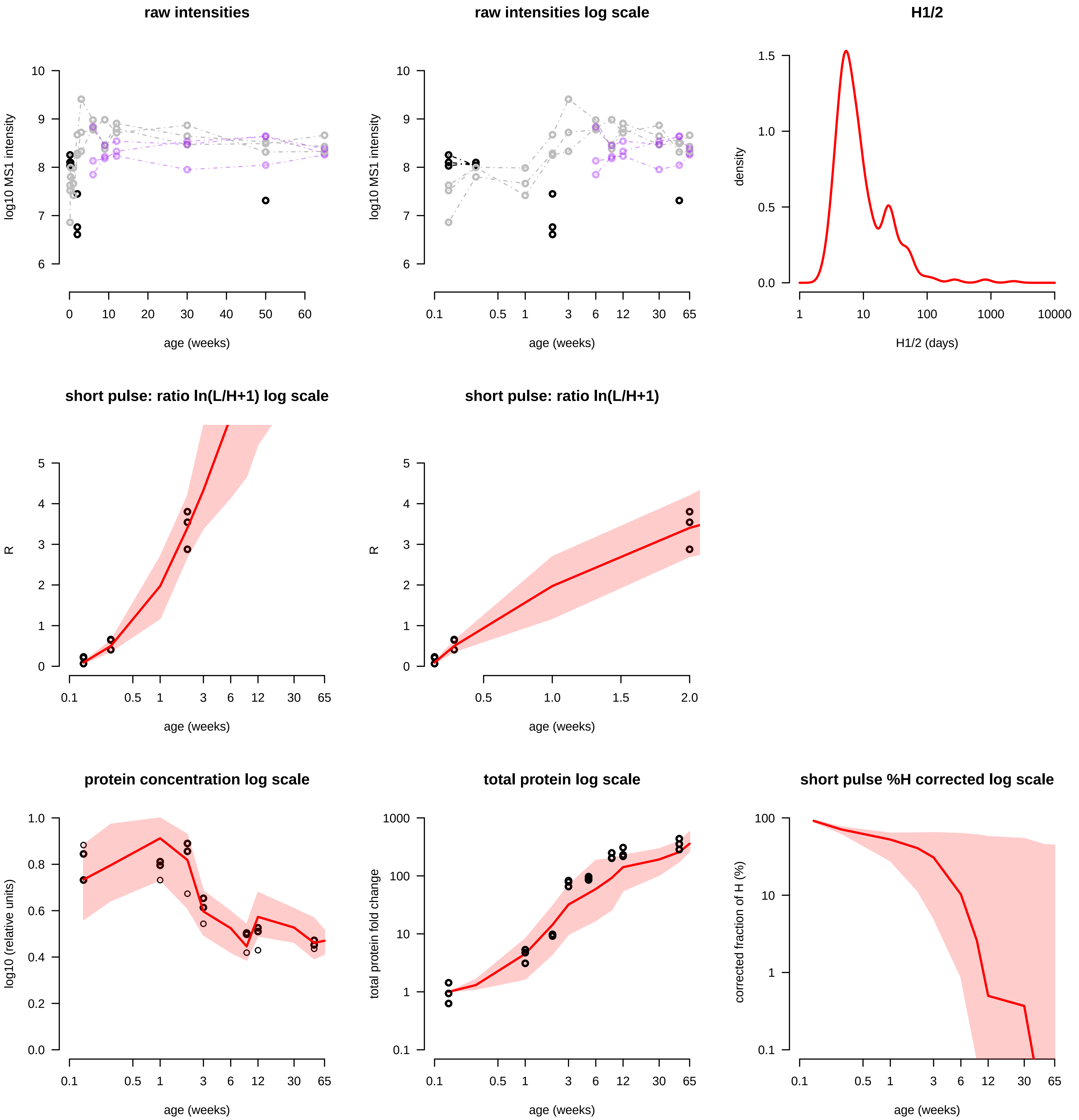

Q8VDL4−3;Q8VDL4

>sp|Q8VDL4−3|ADPGK_MOUSE

Isoform 3 of ADP−dependent glucokinase

GN=Adpgk

Med H1/2: 393.2 days

Q5 H1/2: 138.9 days

Q95 H1/2: 4477.9 days

reliable model fit: no

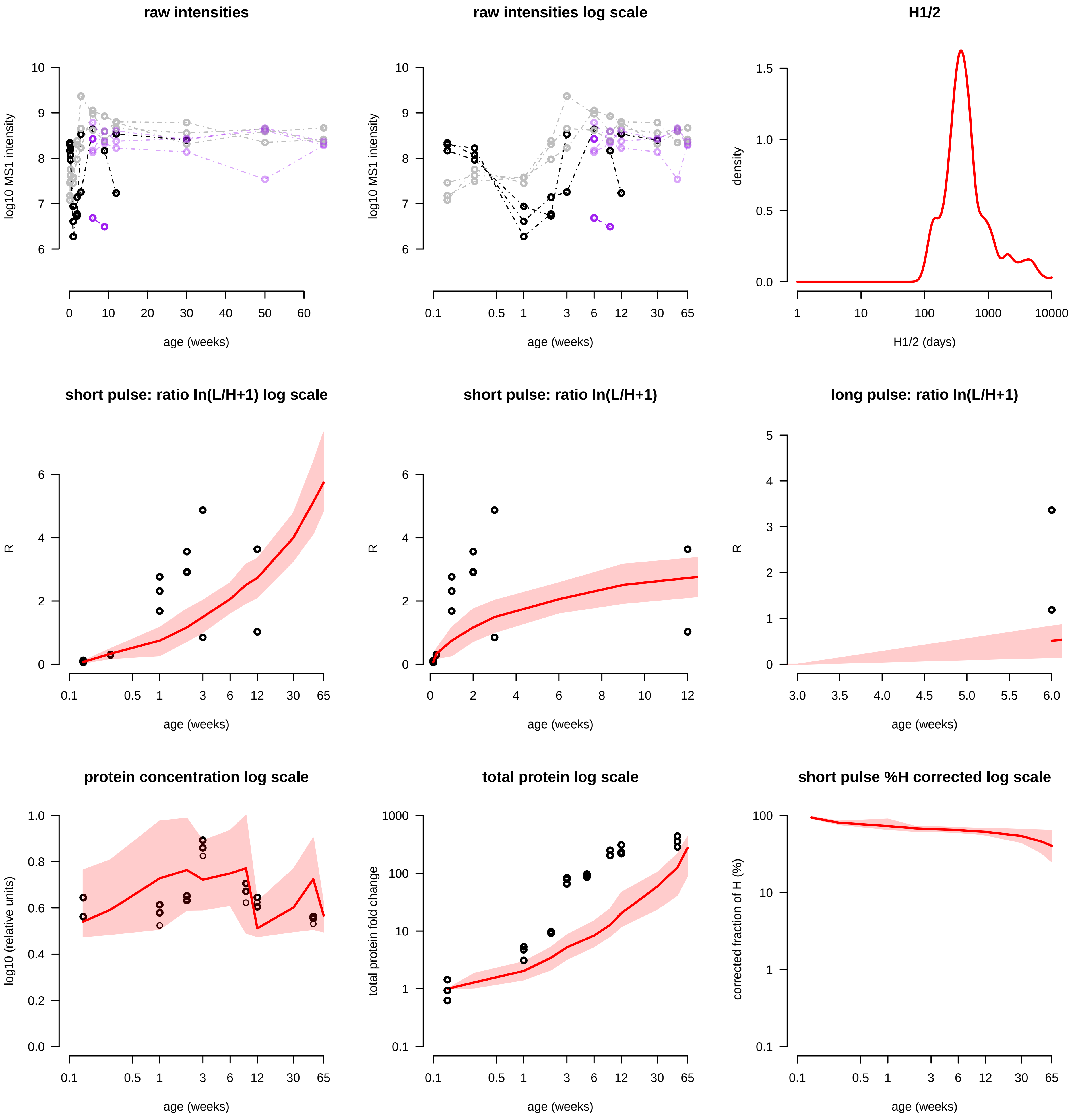

Q9D868−2;Q9D868

>sp|Q9D868−2|PPIH_MOUSE

Isoform 2 of Peptidyl−prolyl cis−trans isomerase H

GN=Ppih

Med H1/2: 4.5 days

Q5 H1/2: 3.5 days

Q95 H1/2: 6.8 days

reliable model fit: yes

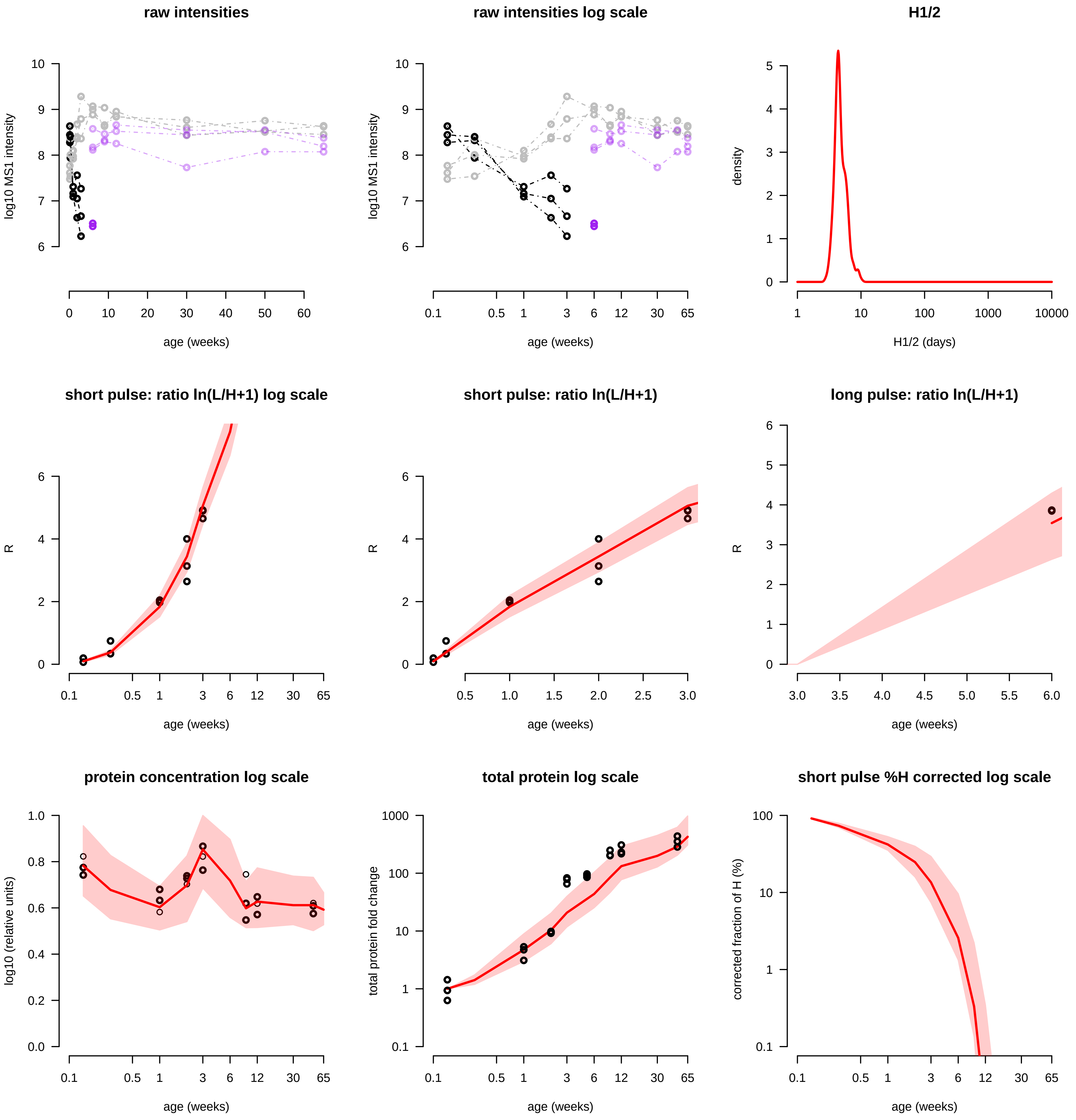

O35344

>sp|O35344|IMA4_MOUSE

Importin subunit alpha−4

GN=Kpna3

Med H1/2: 5.4 days

Q5 H1/2: 4.3 days

Q95 H1/2: 7.1 days

reliable model fit: yes

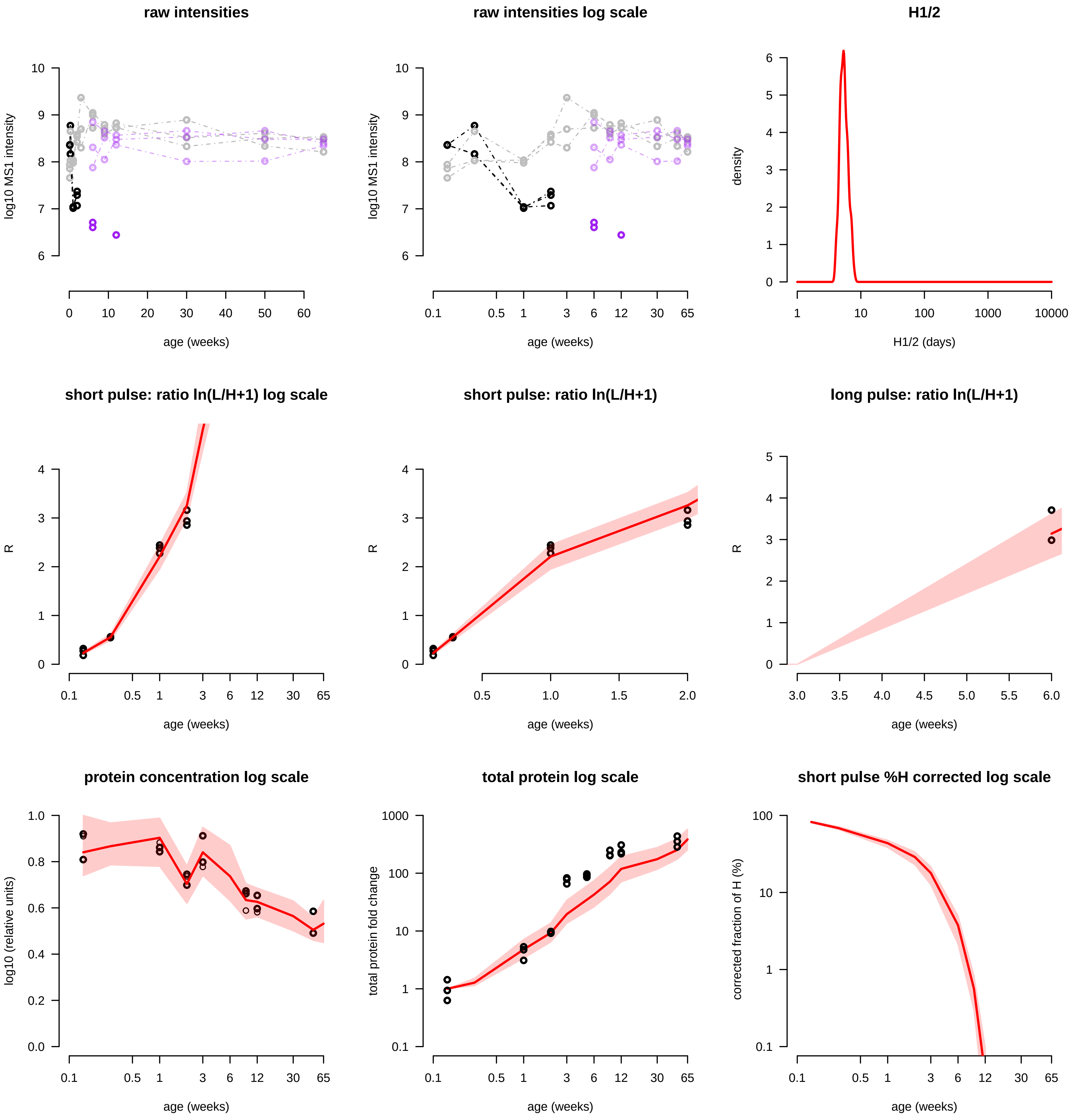

P53995

>sp|P53995|APC1_MOUSE

Anaphase−promoting complex subunit 1

GN=Anapc1

Med H1/2: 4.5 days

Q5 H1/2: 3.5 days

Q95 H1/2: 6.5 days

reliable model fit: yes

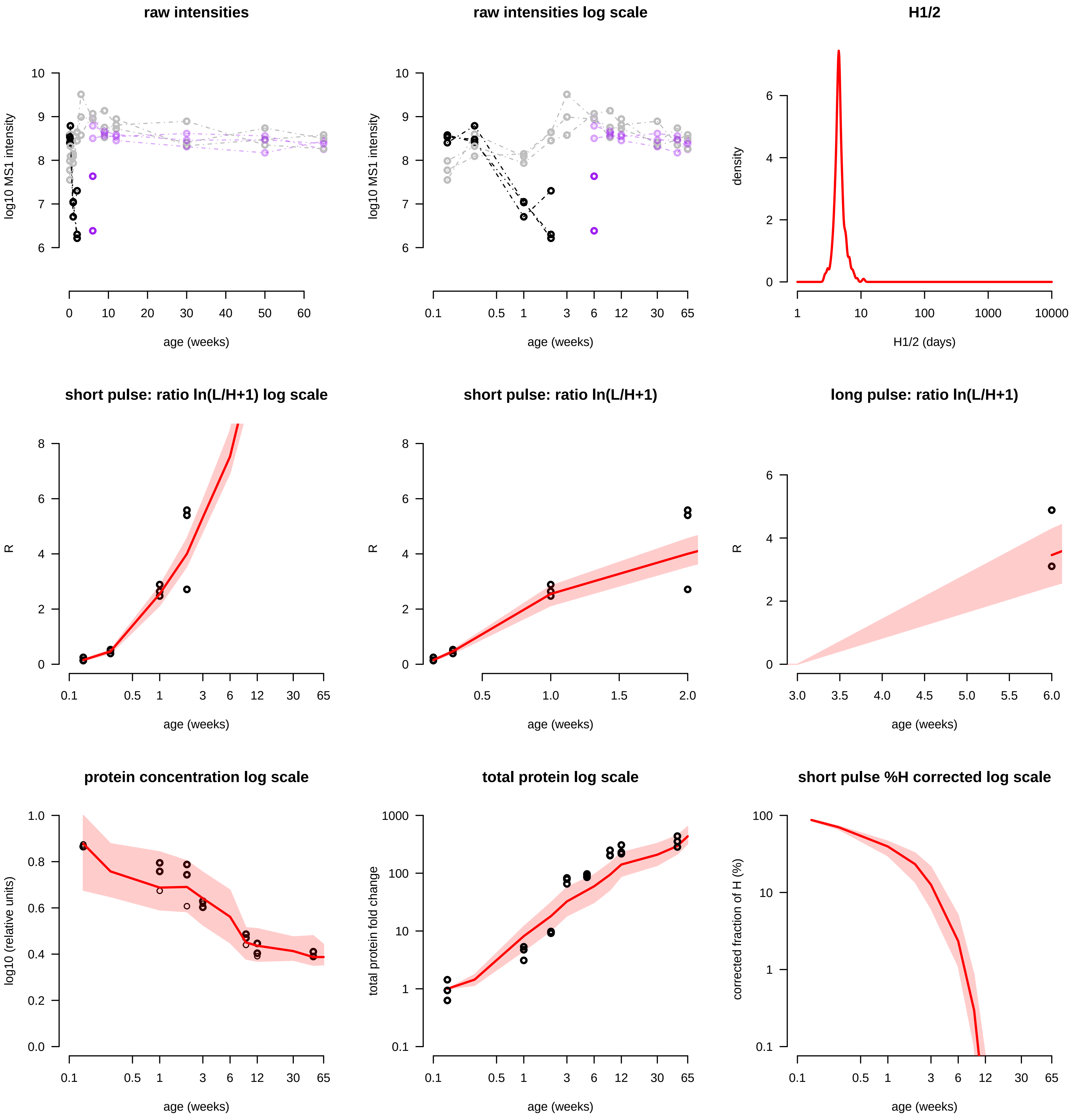

P61961

>sp|P61961|UFM1_MOUSE

Ubiquitin−fold modifier 1

GN=Ufm1

Med H1/2: 8.4 days

Q5 H1/2: 3.2 days

Q95 H1/2: 76.6 days

reliable model fit: yes

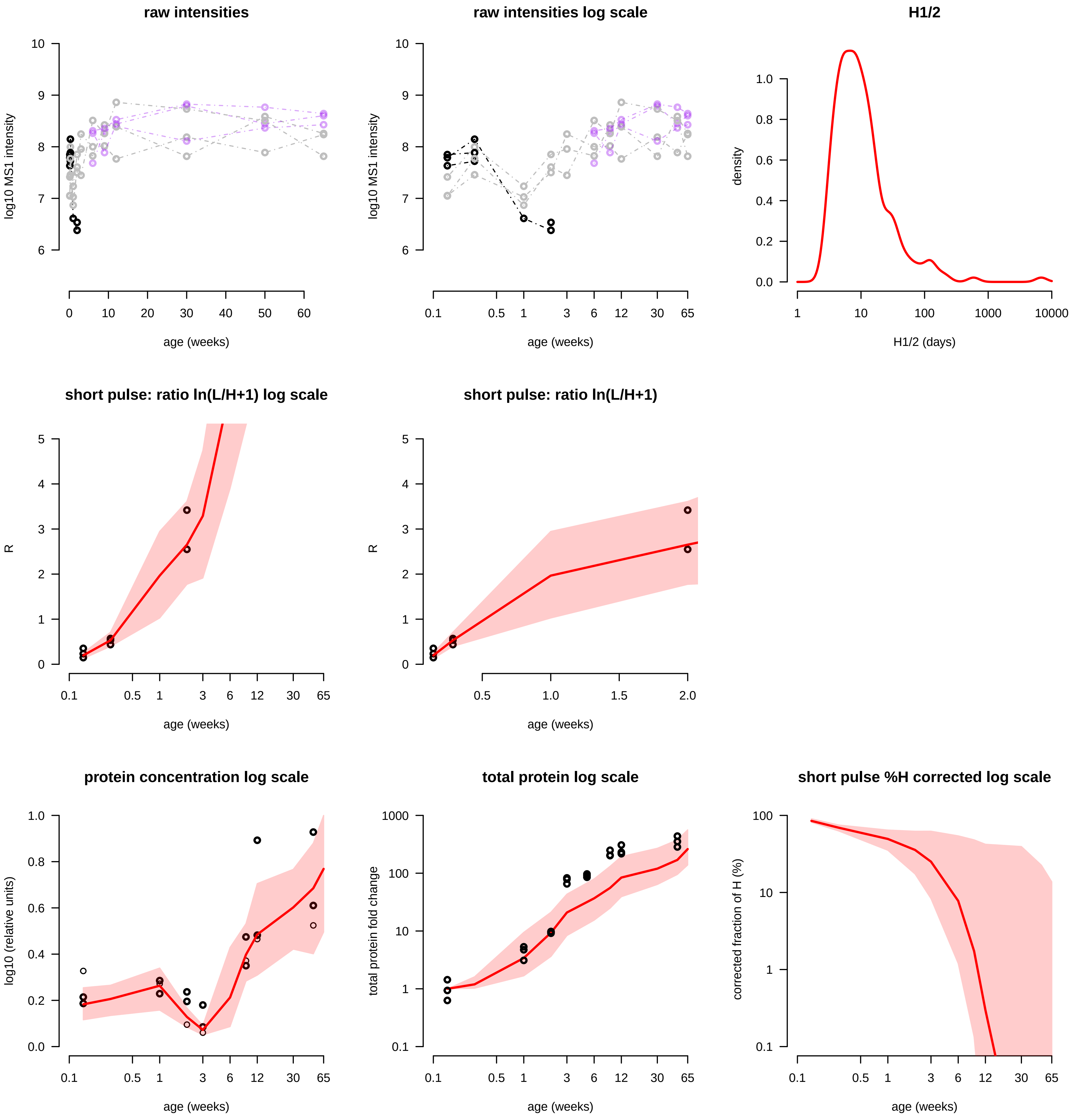

O08579

>sp|O08579|EMD_MOUSE

Emerin

GN=Emd

Med H1/2: 17 days

Q5 H1/2: 6.3 days

Q95 H1/2: 218.5 days

reliable model fit: yes

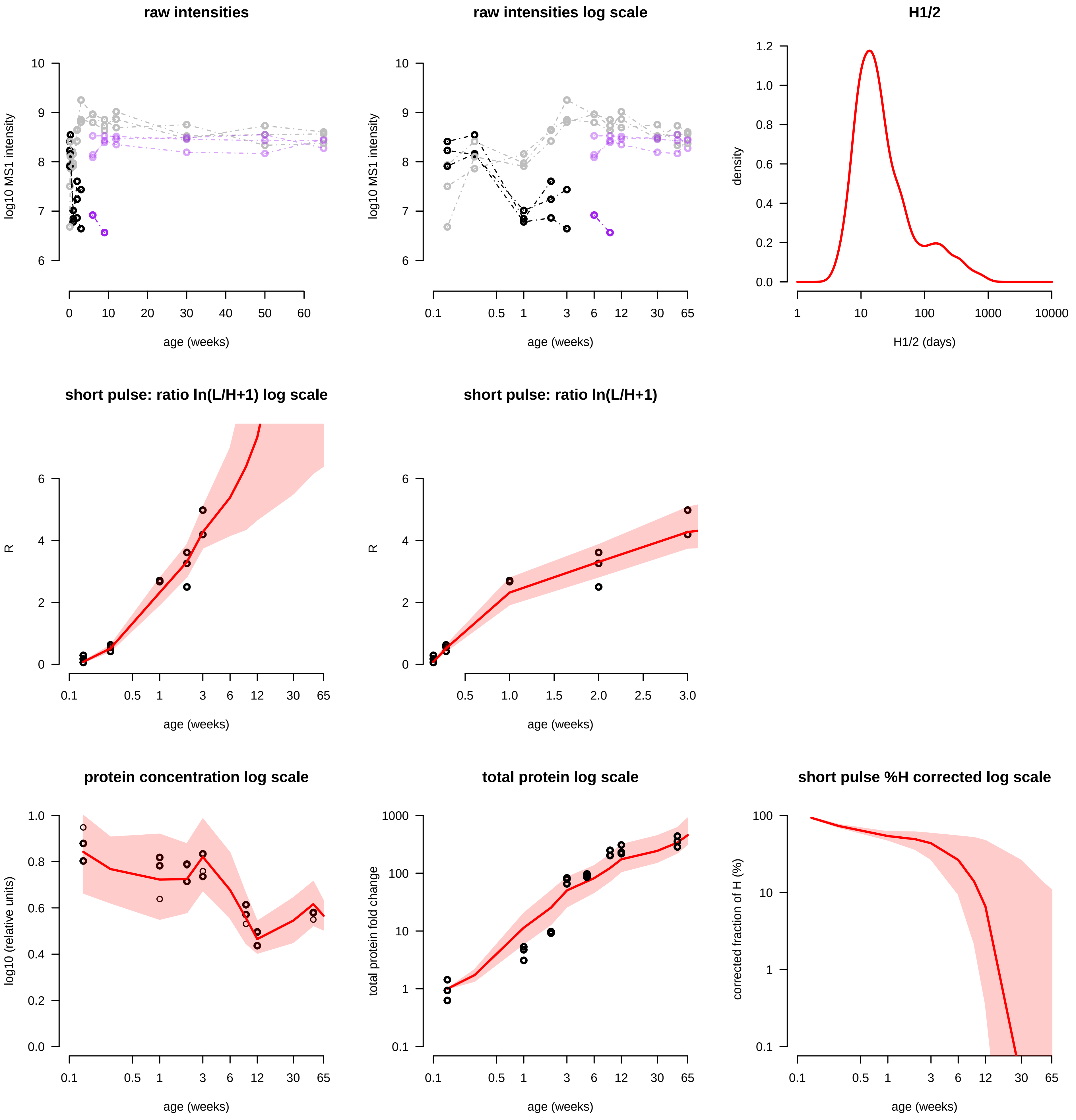

O08915

>sp|O08915|AIP_MOUSE

AH receptor−interacting protein

GN=Aip

Med H1/2: 7.8 days

Q5 H1/2: 3.7 days

Q95 H1/2: 60.2 days

reliable model fit: yes

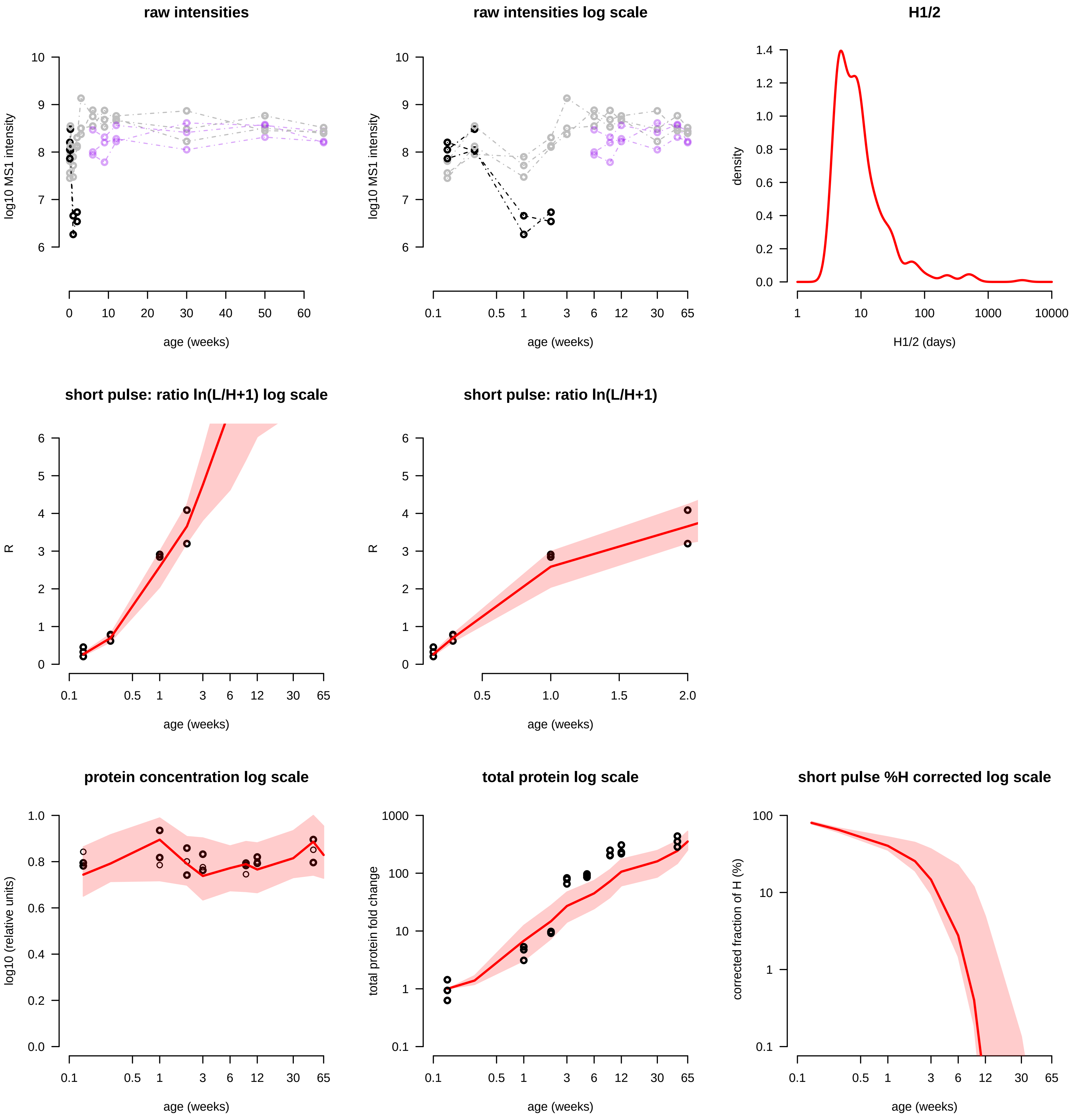

Q80V26

>sp|Q80V26|IMPA3_MOUSE

Golgi−resident adenosine 3',5'−bisphosphate 3'−phosphatase

GN=Bpnt2

Med H1/2: 3.9 days

Q5 H1/2: 3.3 days

Q95 H1/2: 4.9 days

reliable model fit: yes

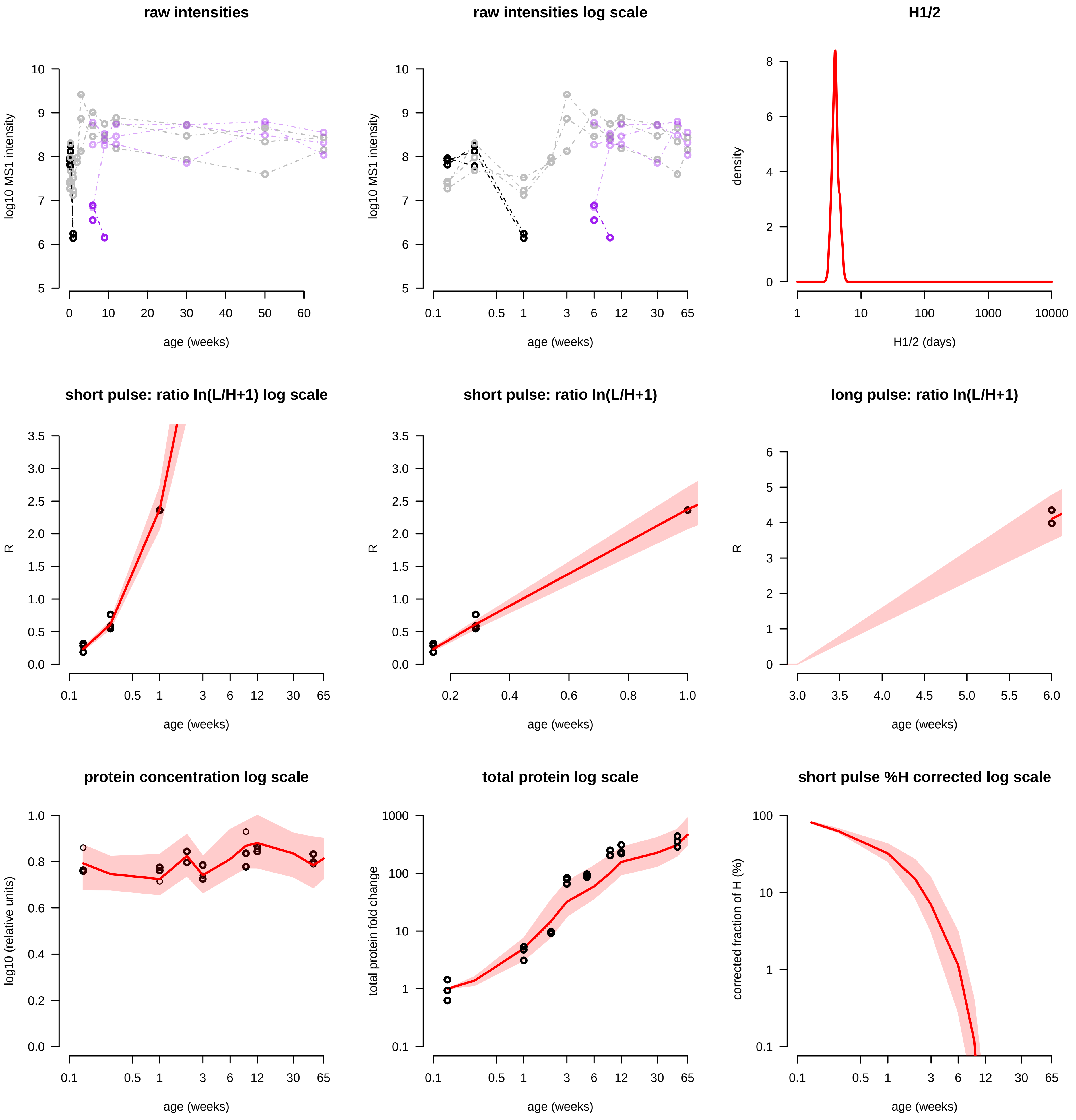

Q8R550−2;Q8R550;Q8R550−5;Q8R550−4

>sp|Q8R550−2|SH3K1_MOUSE

Isoform 2 of SH3 domain−containing kinase−binding protein 1

GN=Sh3kbp1

Med H1/2: 6 days

Q5 H1/2: 2.9 days

Q95 H1/2: 35 days

reliable model fit: yes

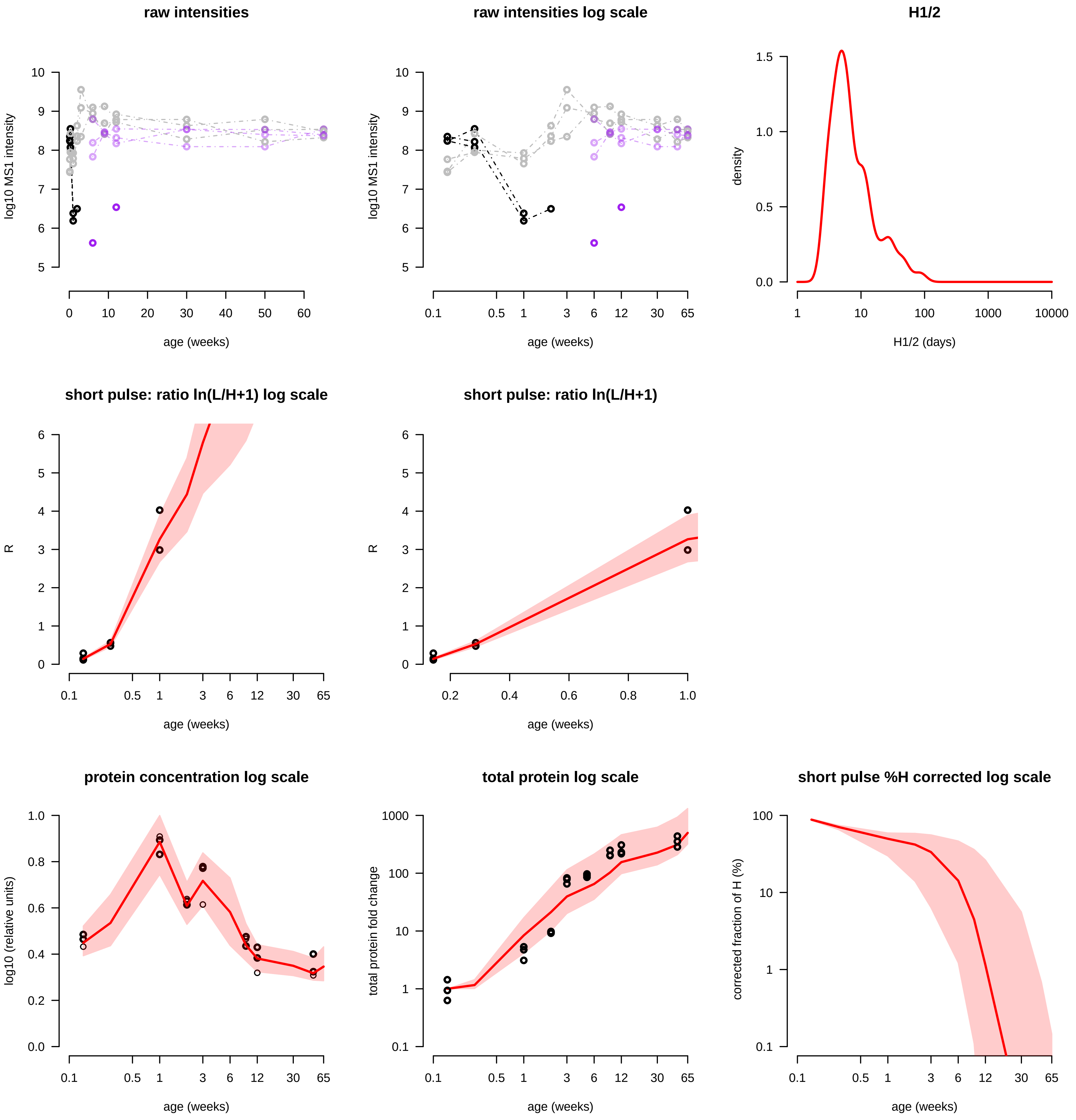

Q8CJF7

>sp|Q8CJF7|ELYS_MOUSE

Protein ELYS

GN=Ahctf1

Med H1/2: 4.8 days

Q5 H1/2: 2.9 days

Q95 H1/2: 47.3 days

reliable model fit: yes

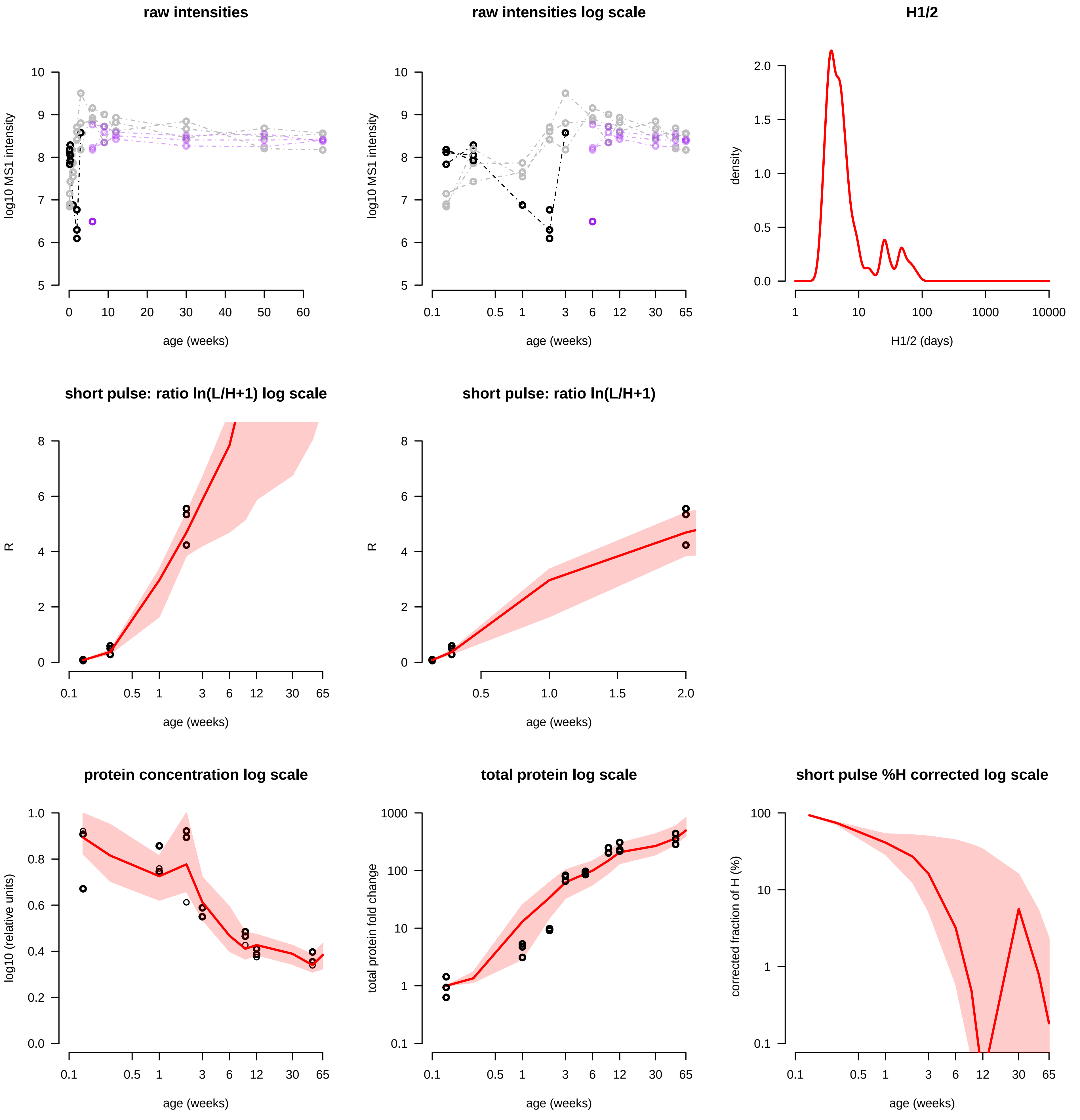

Q8CC35

>sp|Q8CC35|SYNPO_MOUSE

Synaptopodin

GN=Synpo

Med H1/2: 8.2 days

Q5 H1/2: 3.3 days

Q95 H1/2: 58.1 days

reliable model fit: yes

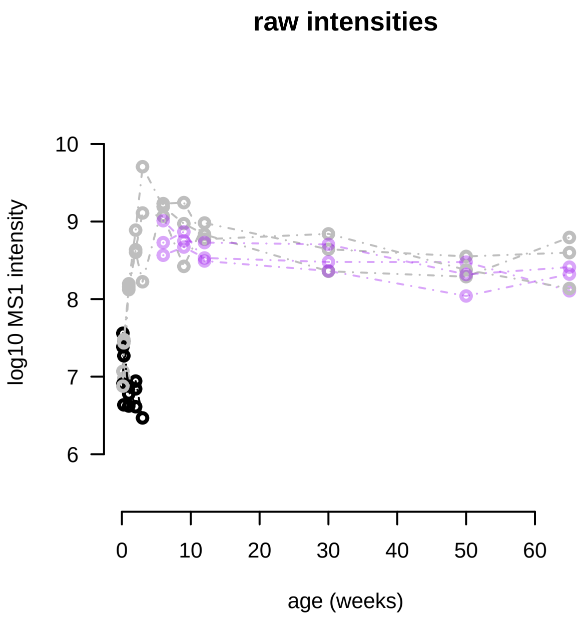

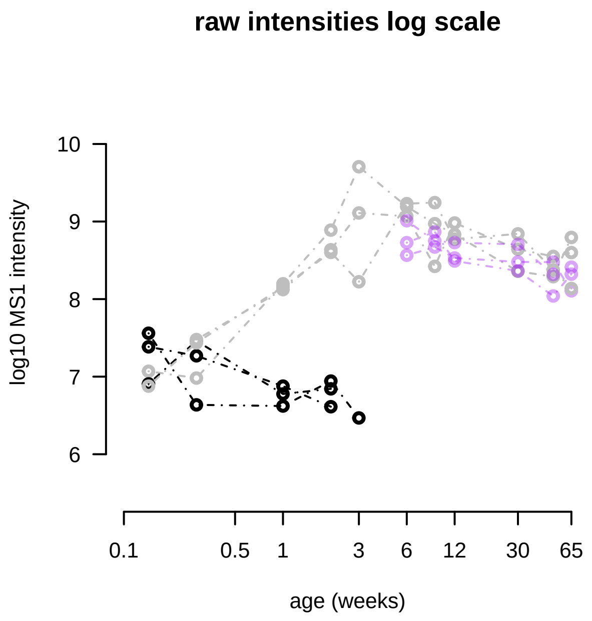

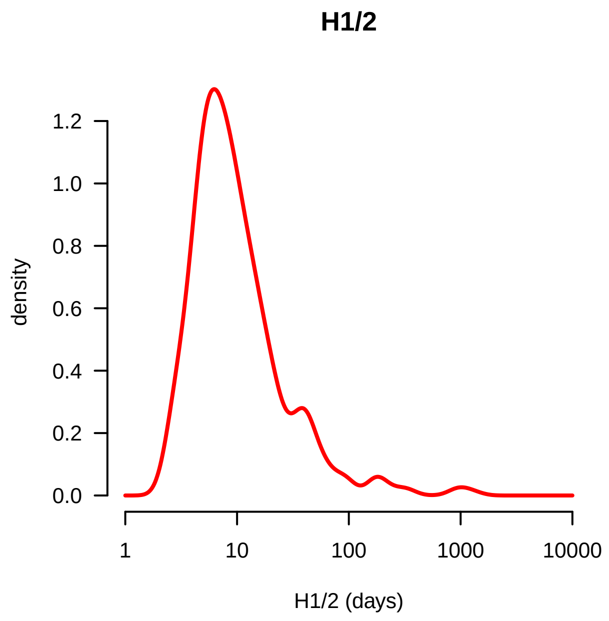

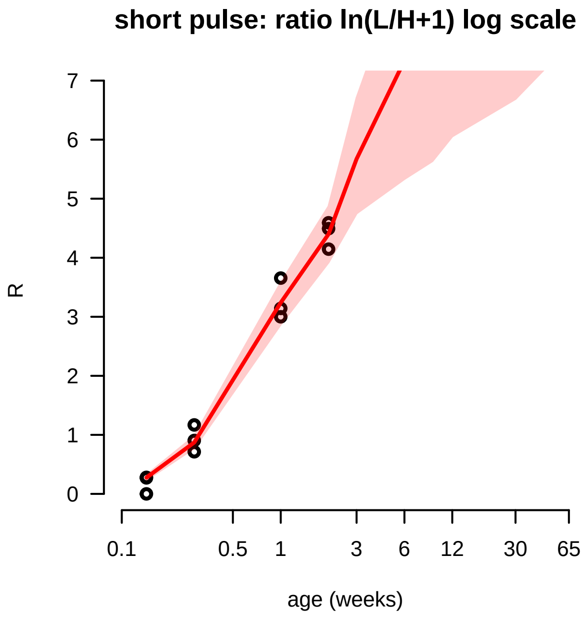

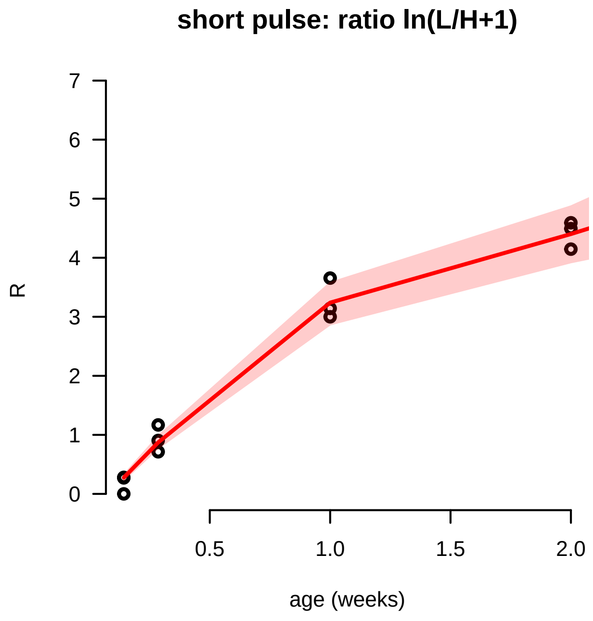

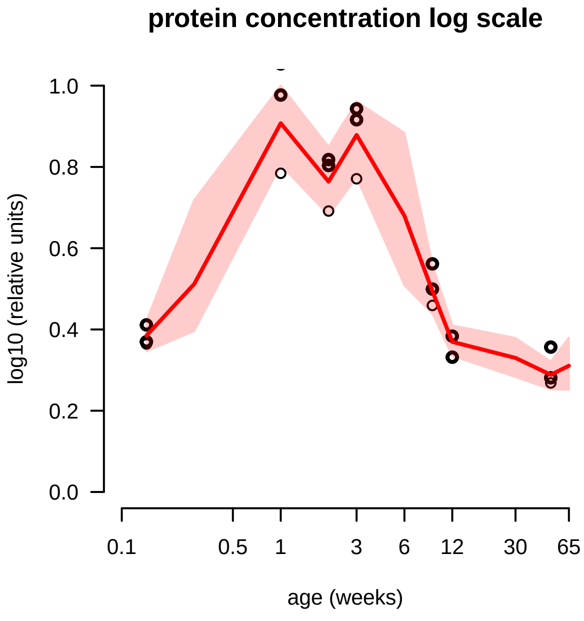

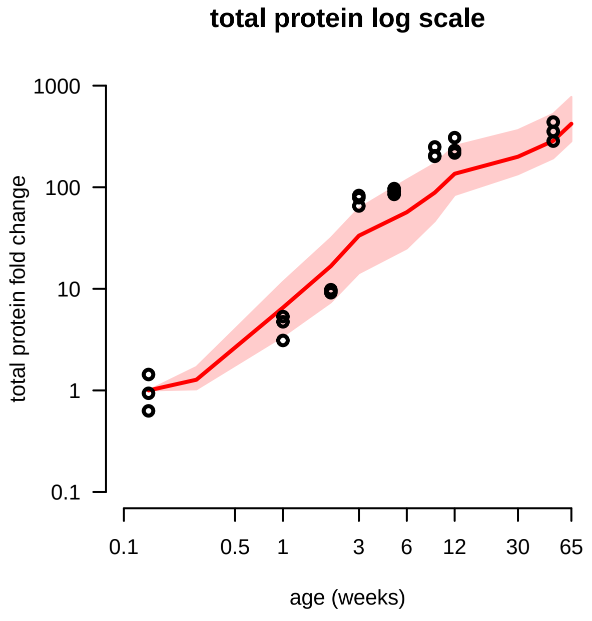

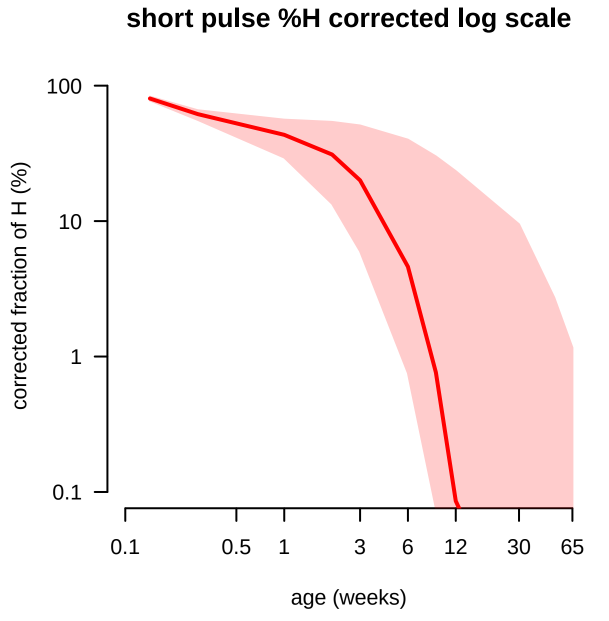

Q9D287

>sp|Q9D287|SPF27_MOUSE

Pre−mRNA−splicing factor SPF27

GN=Bcas2

Med H1/2: 6.6 days

Q5 H1/2: 2.5 days

Q95 H1/2: 33.7 days

reliable model fit: yes

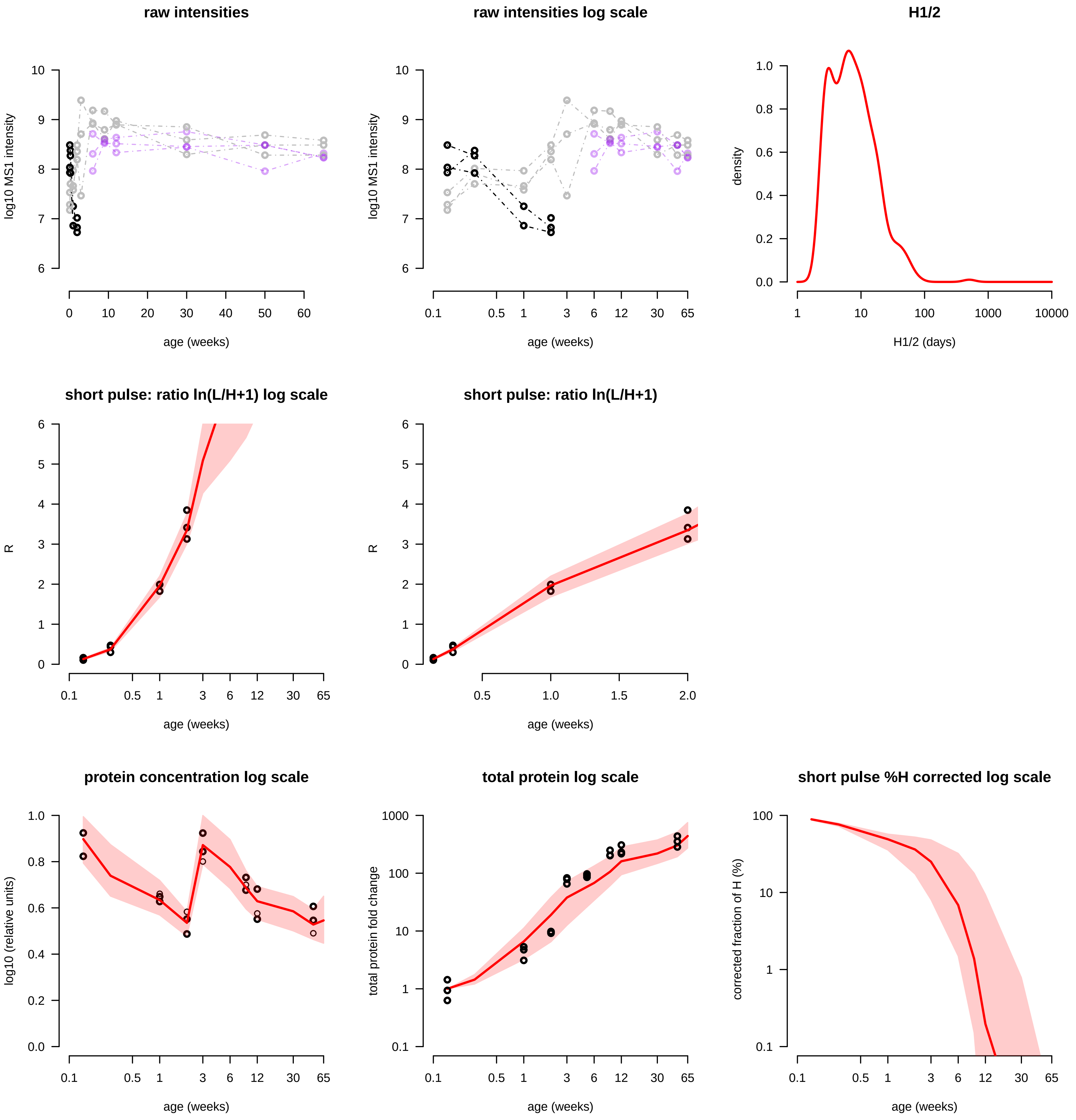

Q8BH61

>sp|Q8BH61|F13A_MOUSE

Coagulation factor XIII A chain

GN=F13a1

Med H1/2: 7.3 days

Q5 H1/2: 3.3 days

Q95 H1/2: 63.2 days

reliable model fit: yes

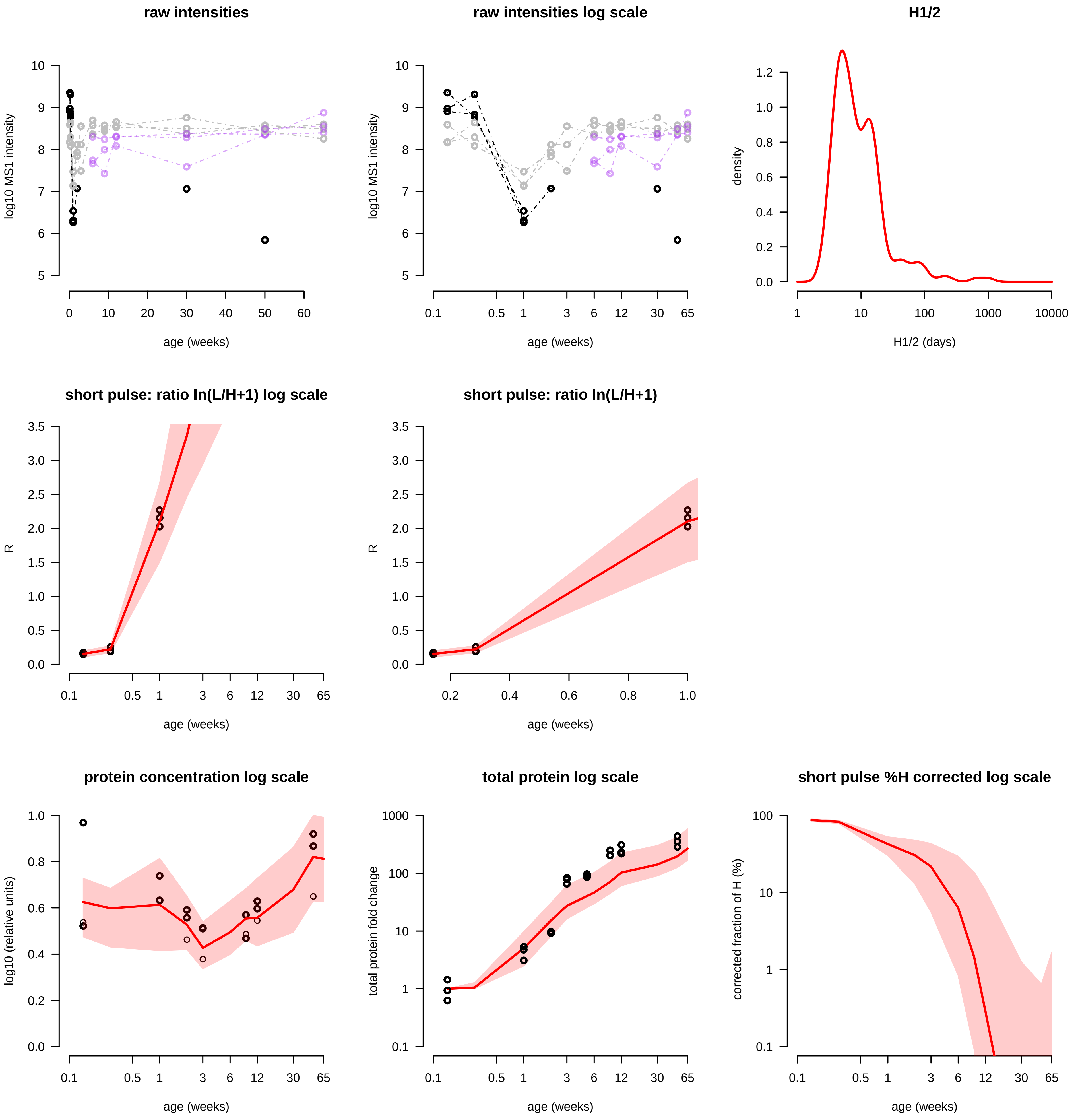

Q8R4N0

>sp|Q8R4N0|CLYBL_MOUSE

Citramalyl−CoA lyase, mitochondrial

GN=Clybl

Med H1/2: 11.2 days

Q5 H1/2: 4 days

Q95 H1/2: 125.1 days

reliable model fit: yes

raw intensities

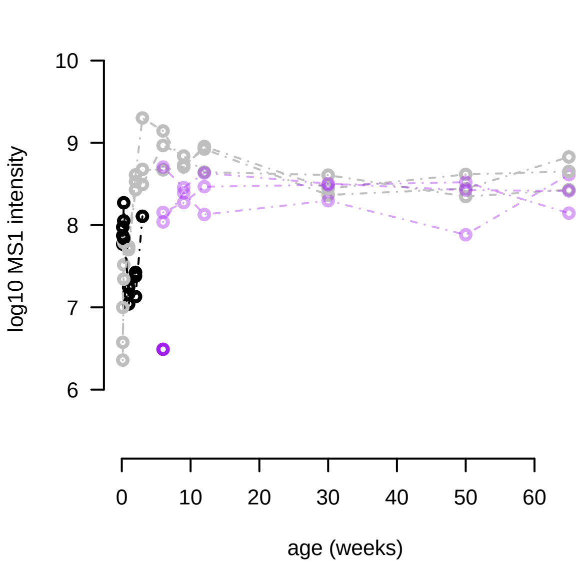

raw intensities log scale

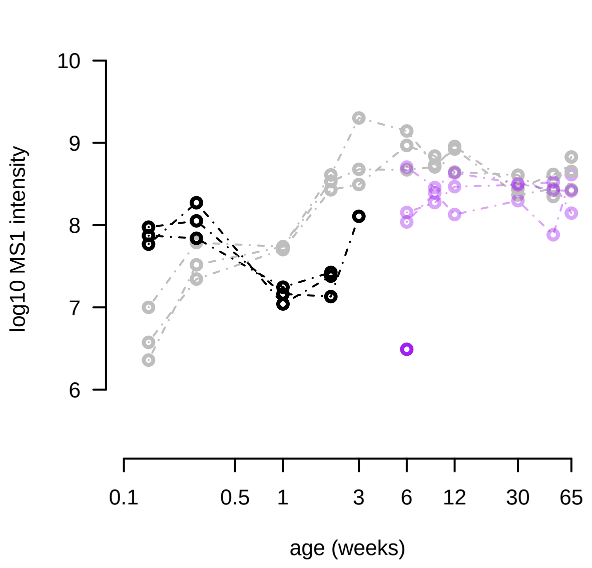

H1/2

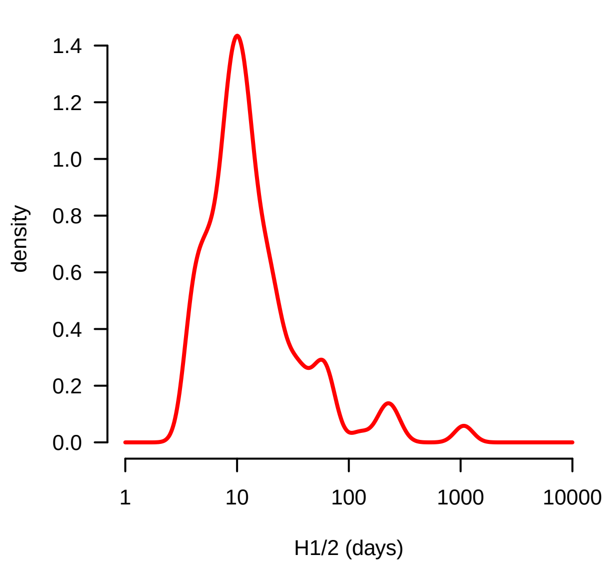

short pulse: ratio ln(L/H+1) log scale

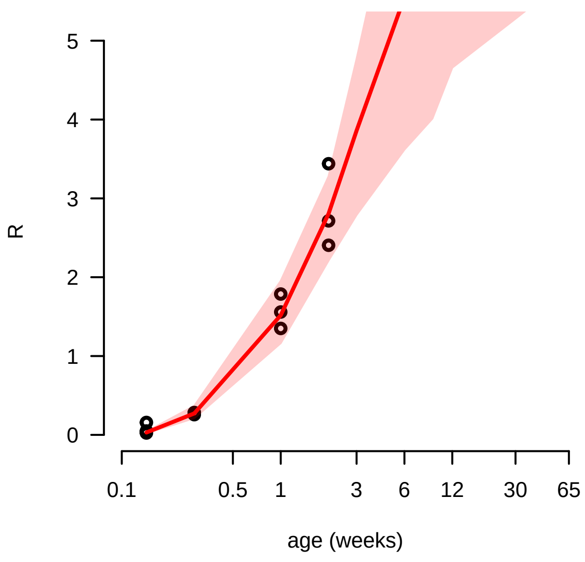

short pulse: ratio ln(L/H+1)

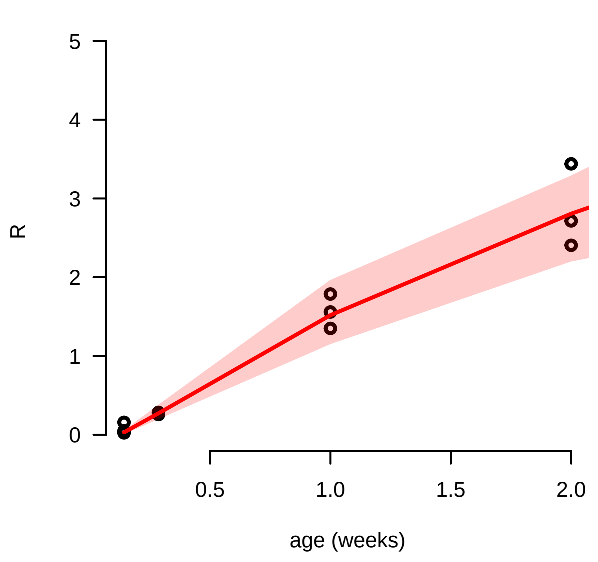

protein concentration log scale

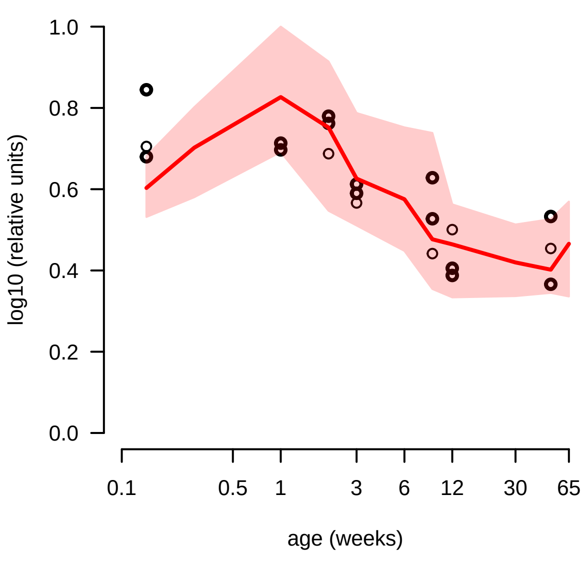

total protein log scale

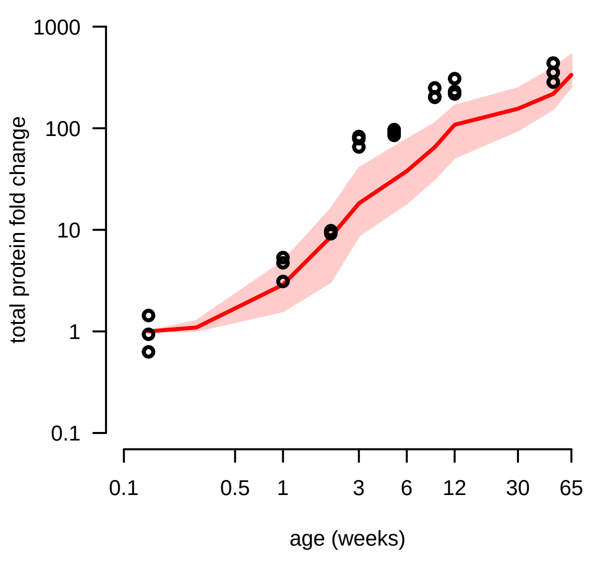

short pulse %H corrected log scale

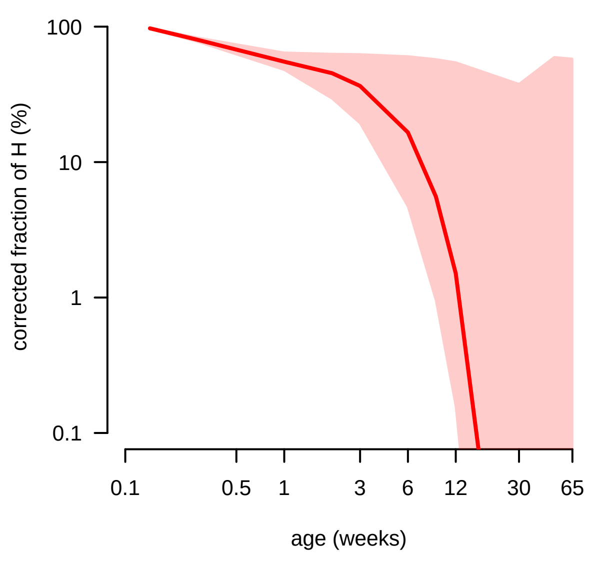

Q9CQI7

>sp|Q9CQI7|RU2B_MOUSE

U2 small nuclear ribonucleoprotein B"

GN=Snrpb2

Med H1/2: 7 days

Q5 H1/2: 4.7 days

Q95 H1/2: 16.4 days

reliable model fit: yes

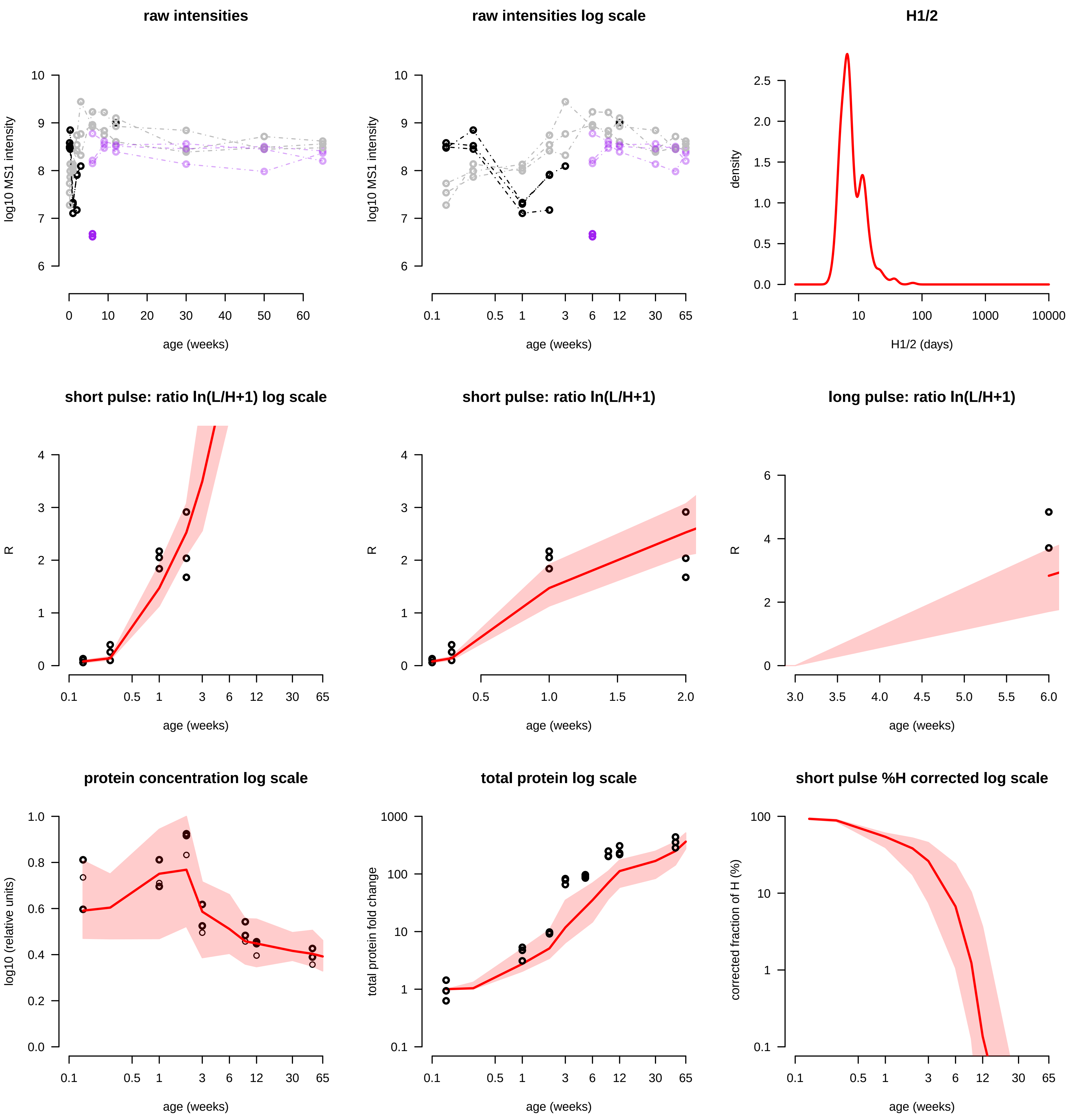

Q924C1

>sp|Q924C1|XPO5_MOUSE

Exportin−5

GN=Xpo5

Med H1/2: 22.1 days

Q5 H1/2: 13.1 days

Q95 H1/2: 82.7 days

reliable model fit: yes

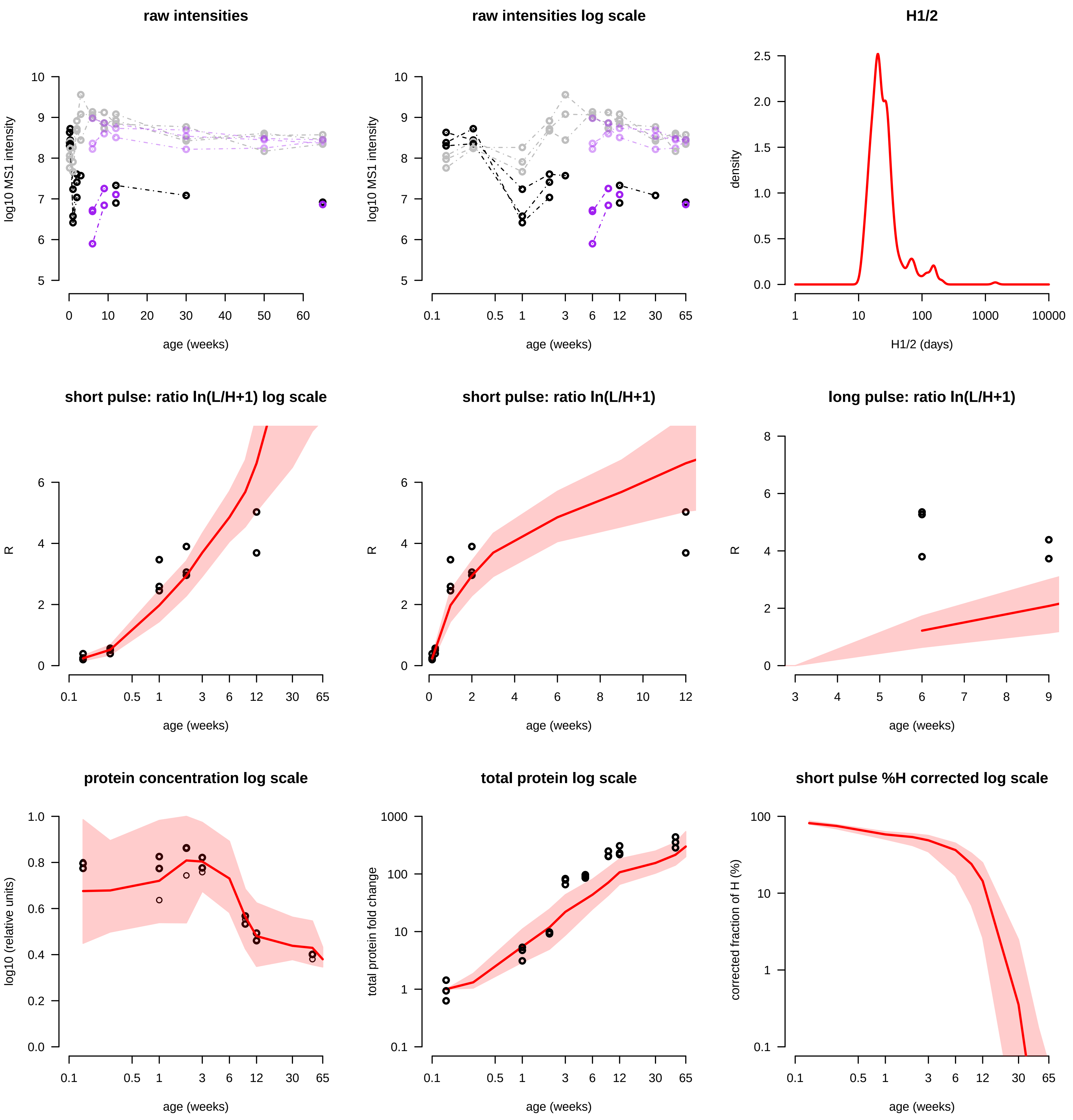

Q9D898

>sp|Q9D898|ARP5L_MOUSE

Actin−related protein 2/3 complex subunit 5−like protein

GN=Arpc5l

Med H1/2: 24.5 days

Q5 H1/2: 6.2 days

Q95 H1/2: 272.8 days

reliable model fit: no

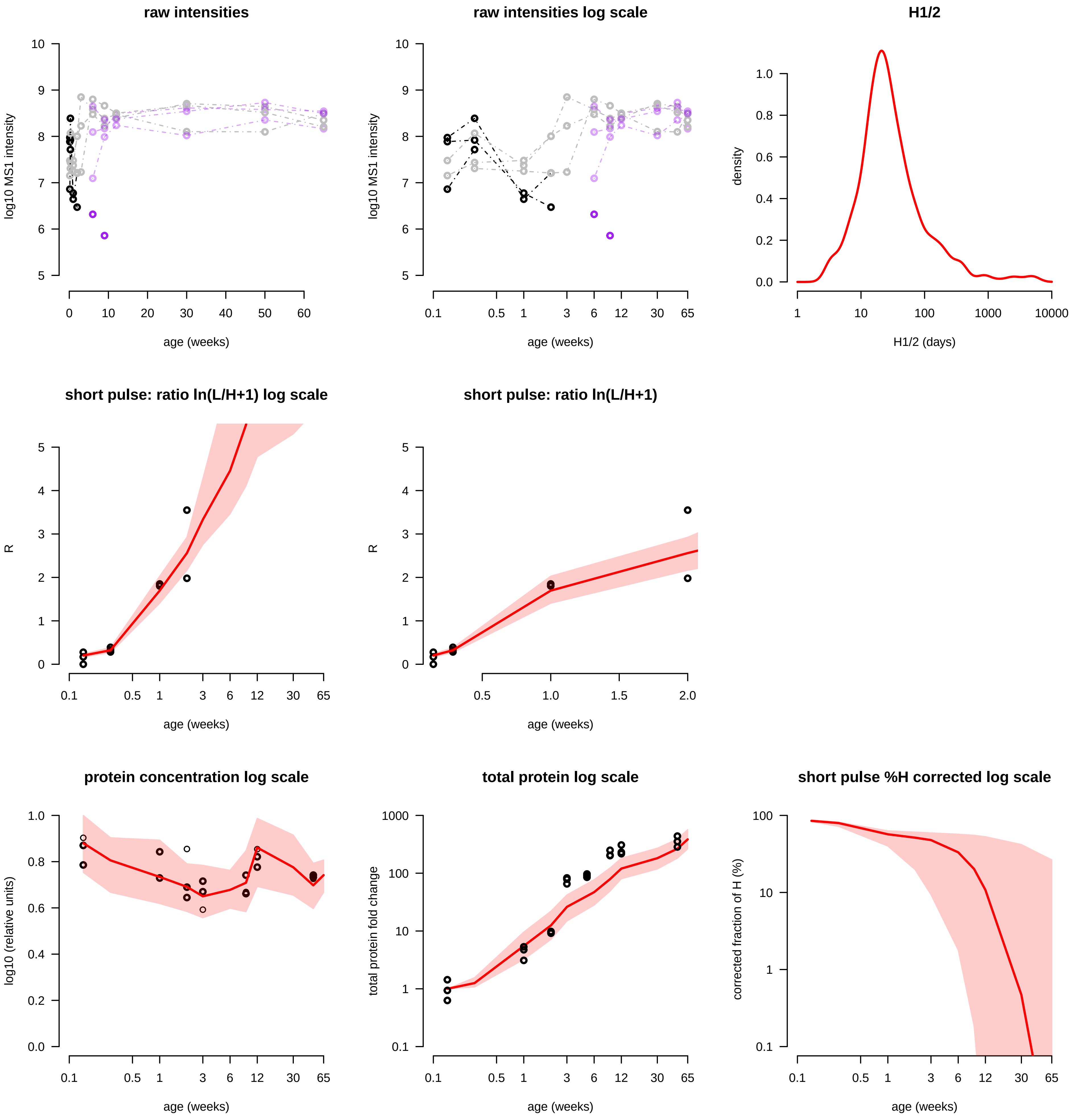

P03930

>sp|P03930|ATP8_MOUSE

ATP synthase protein 8

GN=Mtatp8

Med H1/2: 16.8 days

Q5 H1/2: 6 days

Q95 H1/2: 240.9 days

reliable model fit: yes

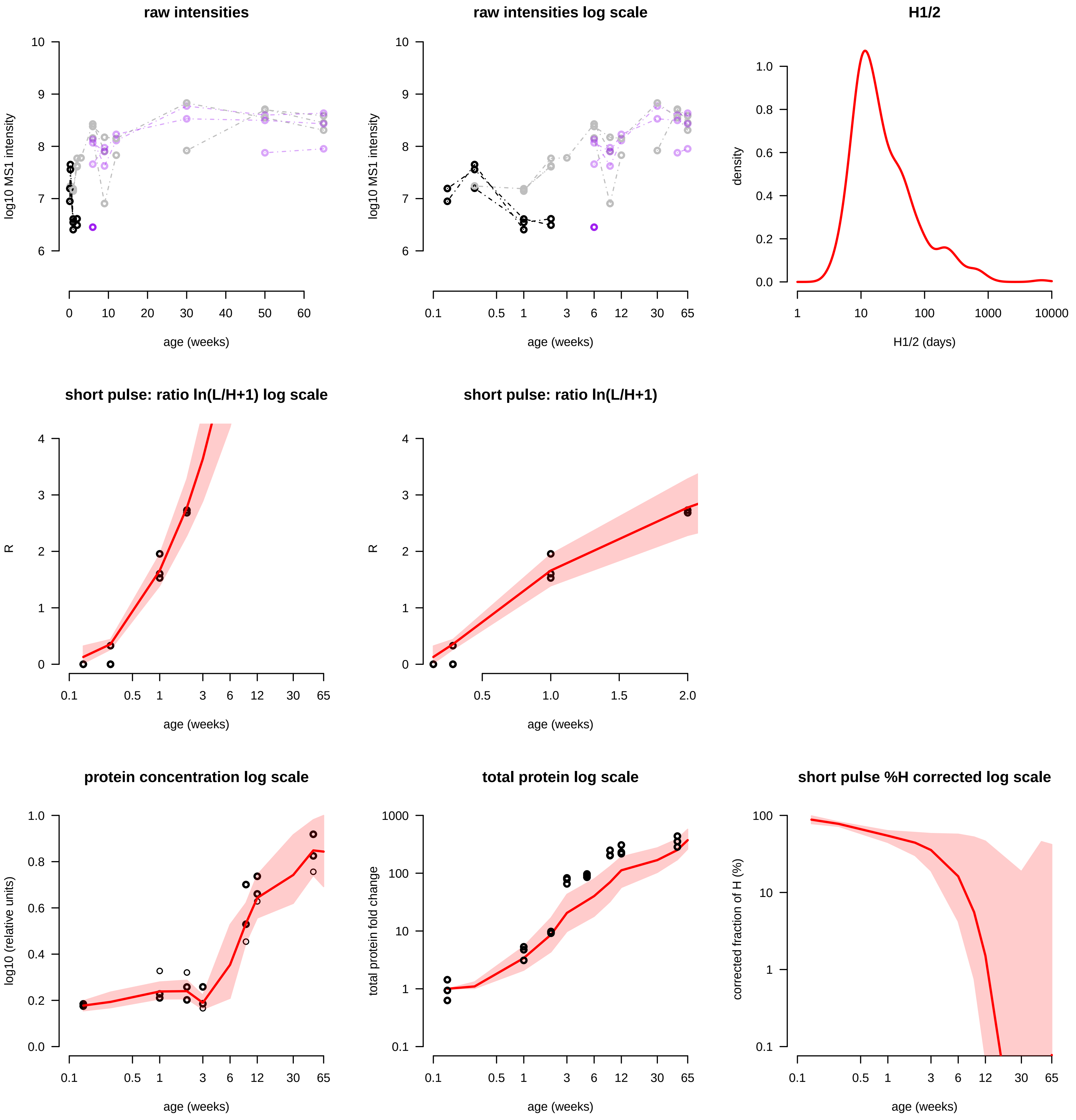

P52430

>sp|P52430|PON1_MOUSE

Serum paraoxonase/arylesterase 1

GN=Pon1

Med H1/2: 9.1 days

Q5 H1/2: 3.9 days

Q95 H1/2: 94.3 days

reliable model fit: yes

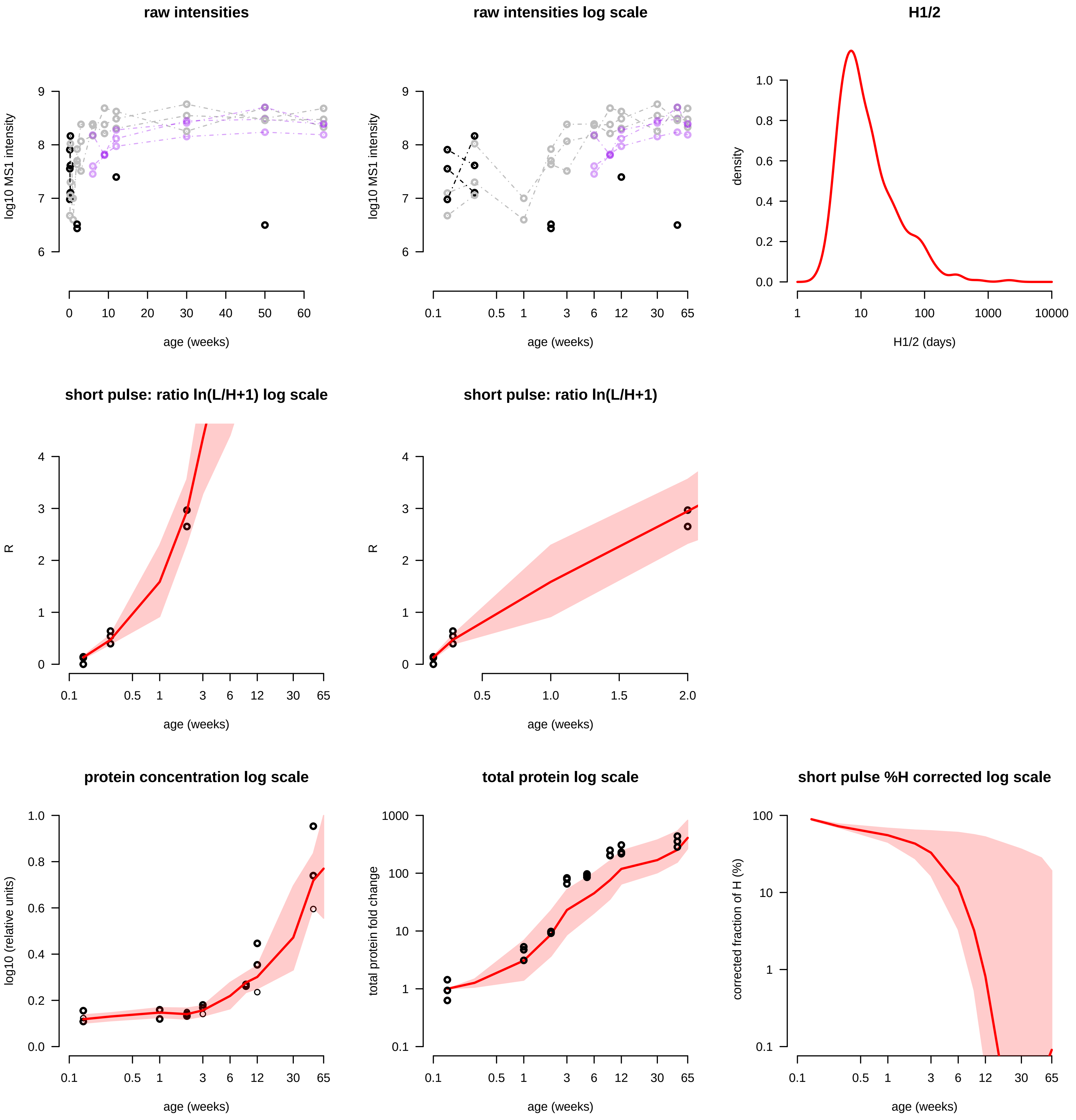

Q80WQ2

>sp|Q80WQ2|VAC14_MOUSE

Protein VAC14 homolog

GN=Vac14

Med H1/2: 13.1 days

Q5 H1/2: 4.8 days

Q95 H1/2: 88.6 days

reliable model fit: yes

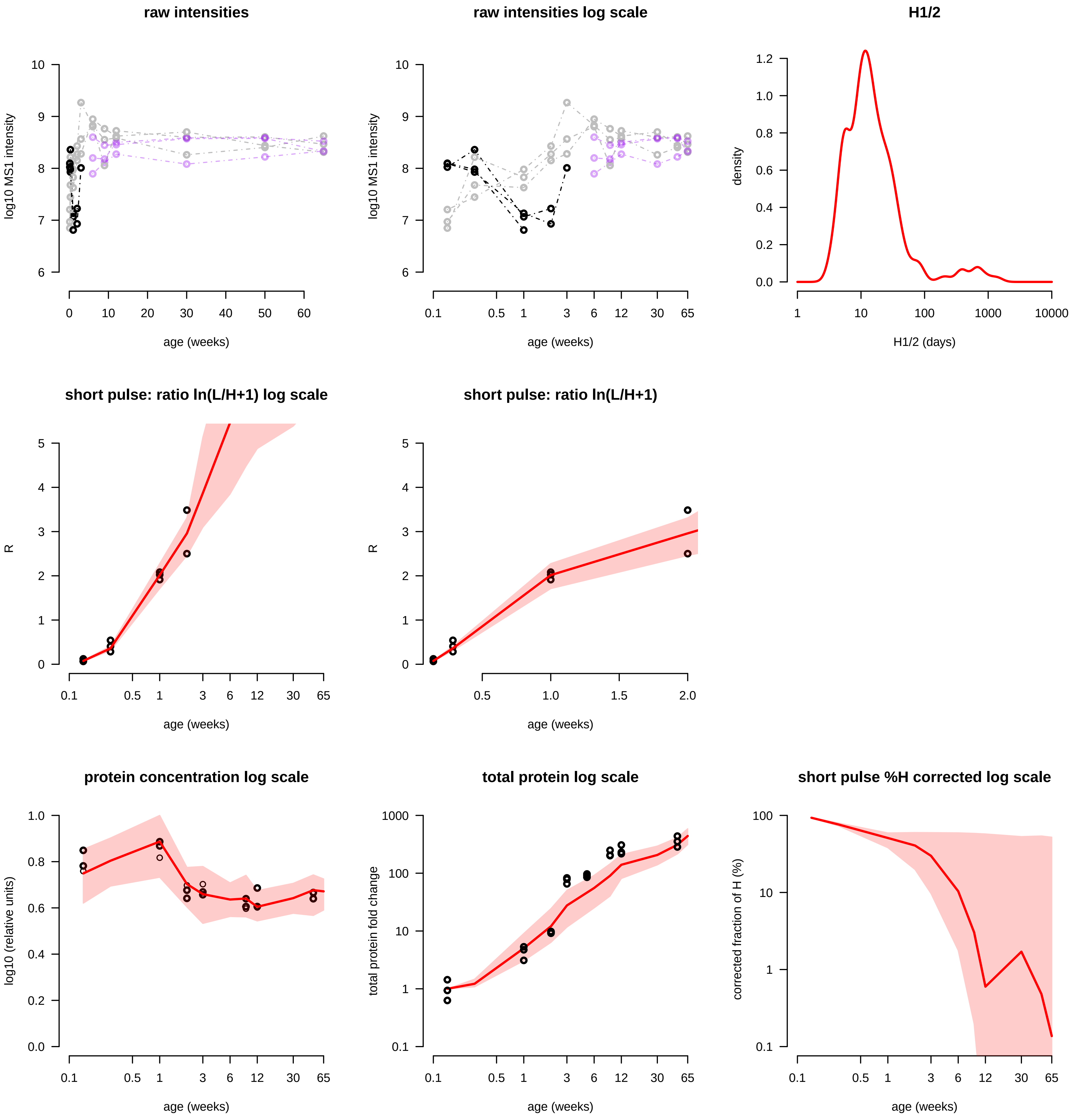

Q71RI9−2;Q71RI9

>sp|Q71RI9−2|KAT3_MOUSE

Isoform 2 of Kynurenine−−oxoglutarate transaminase 3

GN=Kyat3

Med H1/2: 8.4 days

Q5 H1/2: 6.5 days

Q95 H1/2: 10.6 days

reliable model fit: yes

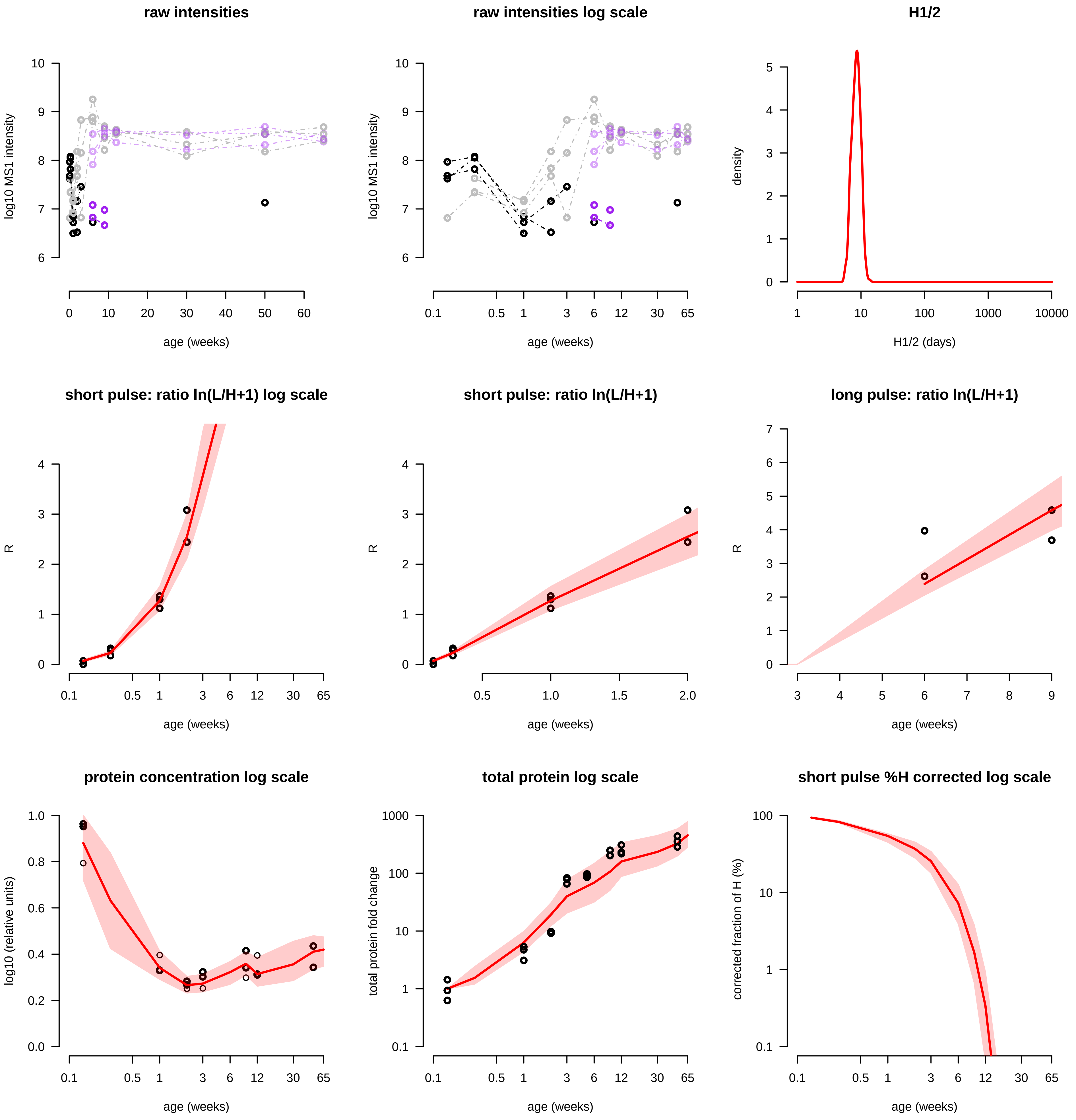

Q9D6M3

>sp|Q9D6M3|GHC1_MOUSE

Mitochondrial glutamate carrier 1

GN=Slc25a22

Med H1/2: 113.2 days

Q5 H1/2: 44.6 days

Q95 H1/2: 644.5 days

reliable model fit: yes

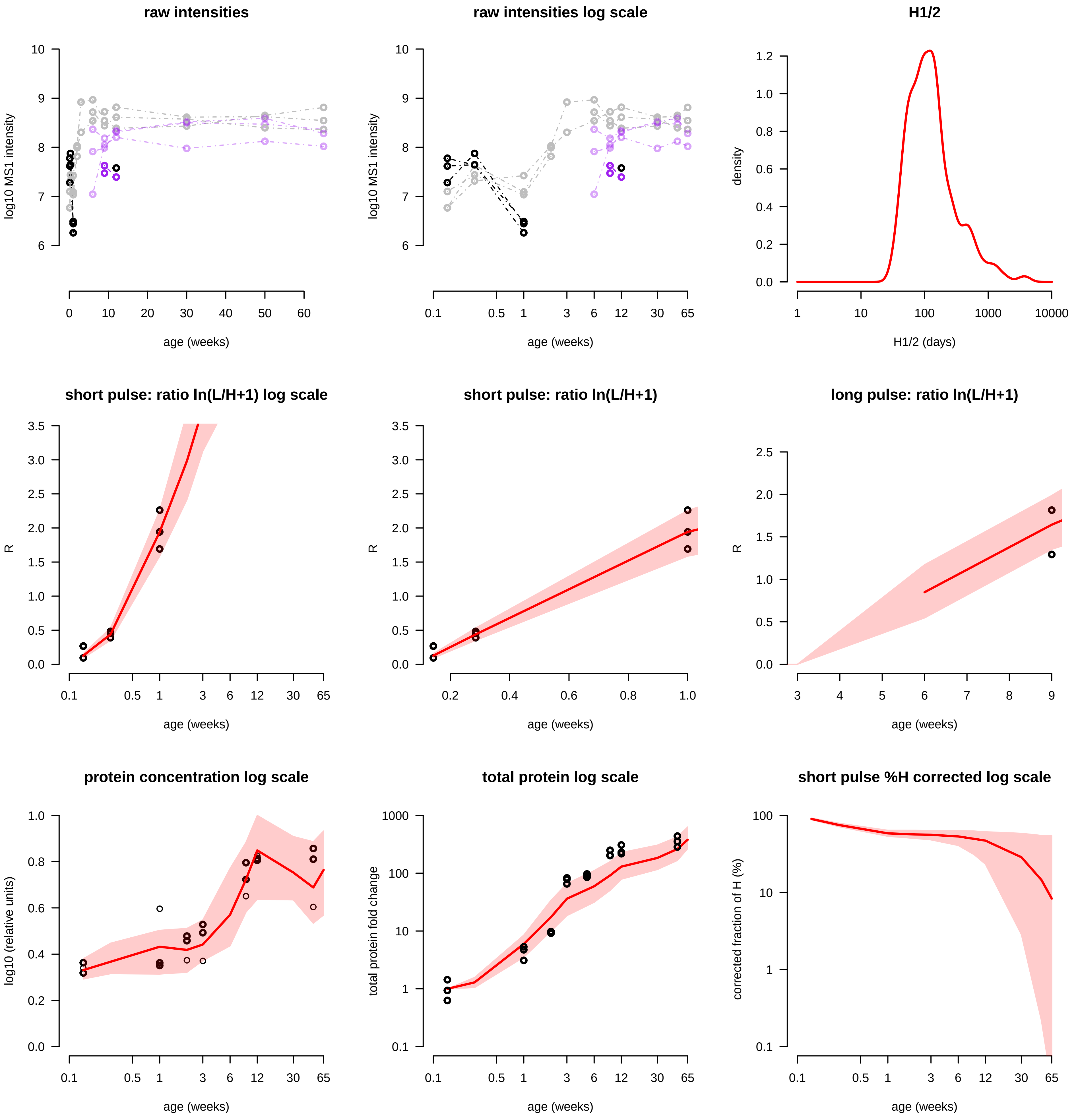

O08582

>sp|O08582|GTPB1_MOUSE

GTP−binding protein 1

GN=Gtpbp1

Med H1/2: 13.9 days

Q5 H1/2: 4.6 days

Q95 H1/2: 236.2 days

reliable model fit: yes

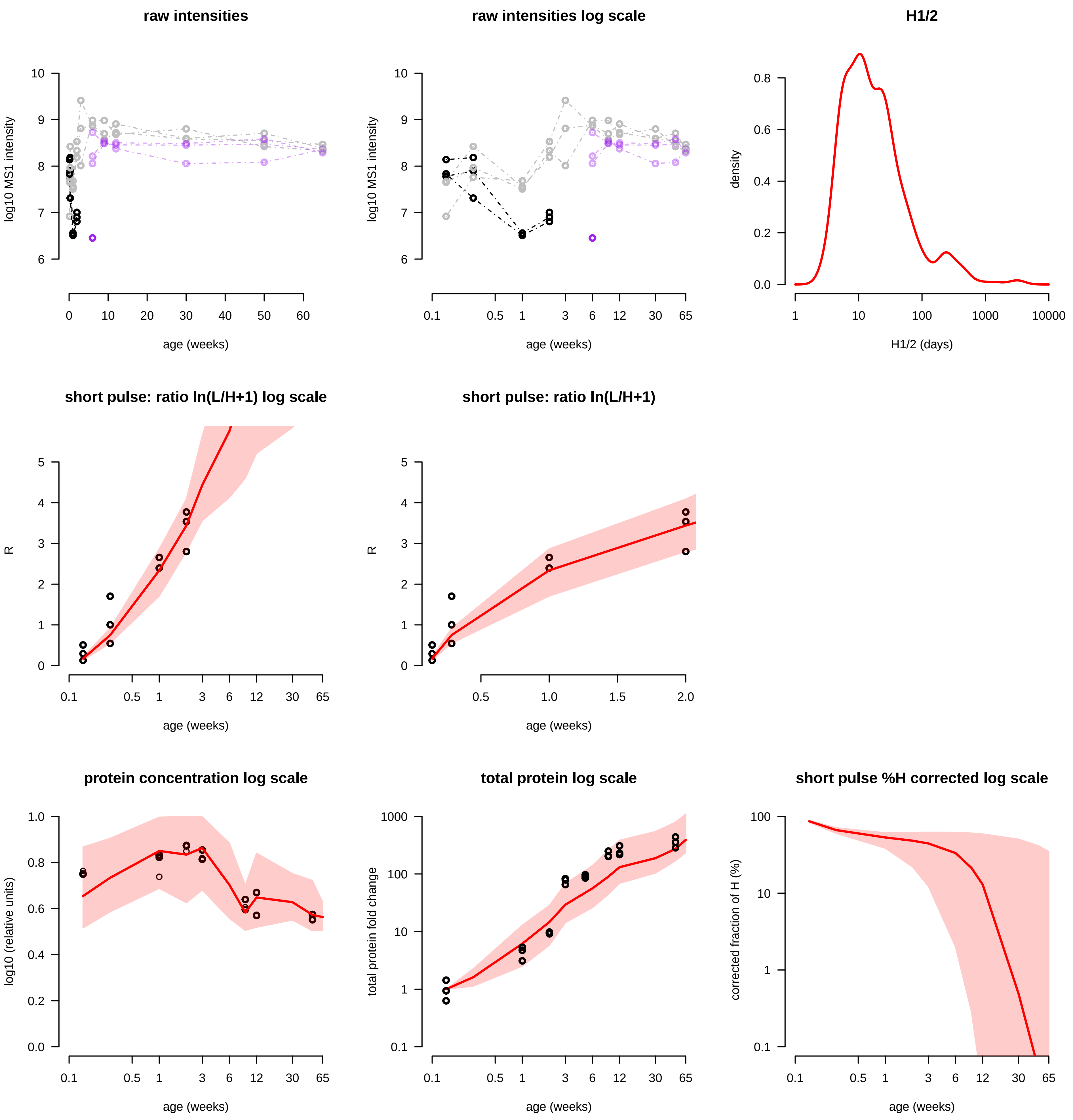

Q9QZ08

>sp|Q9QZ08|NAGK_MOUSE

N−acetyl−D−glucosamine kinase

GN=Nagk

Med H1/2: 8.1 days

Q5 H1/2: 6.3 days

Q95 H1/2: 10.9 days

reliable model fit: yes

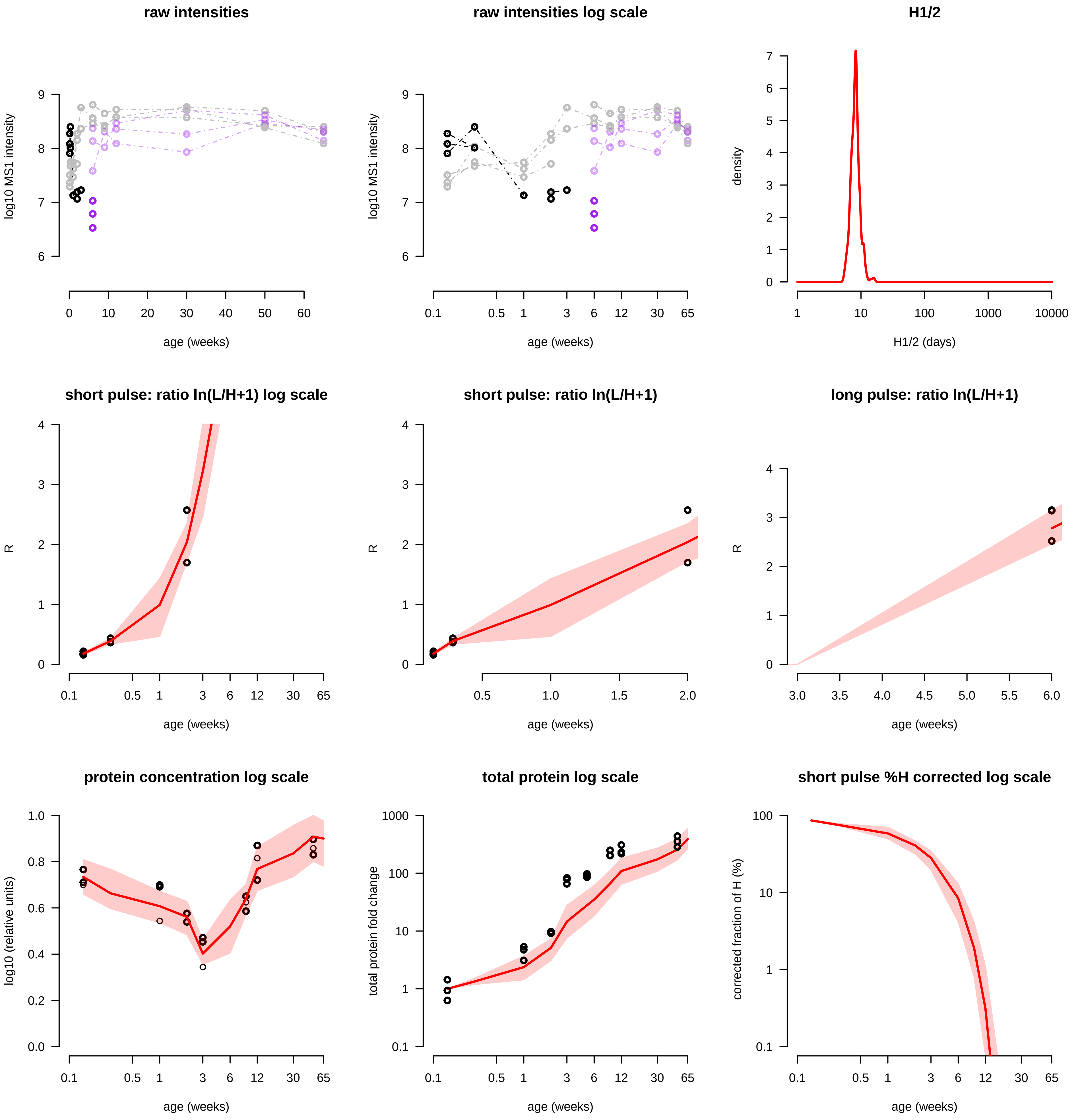

Q9QYI3

>sp|Q9QYI3|DNJC7_MOUSE

DnaJ homolog subfamily C member 7

GN=Dnajc7

Med H1/2: 488.5 days

Q5 H1/2: 87.3 days

Q95 H1/2: 8012.1 days

reliable model fit: yes

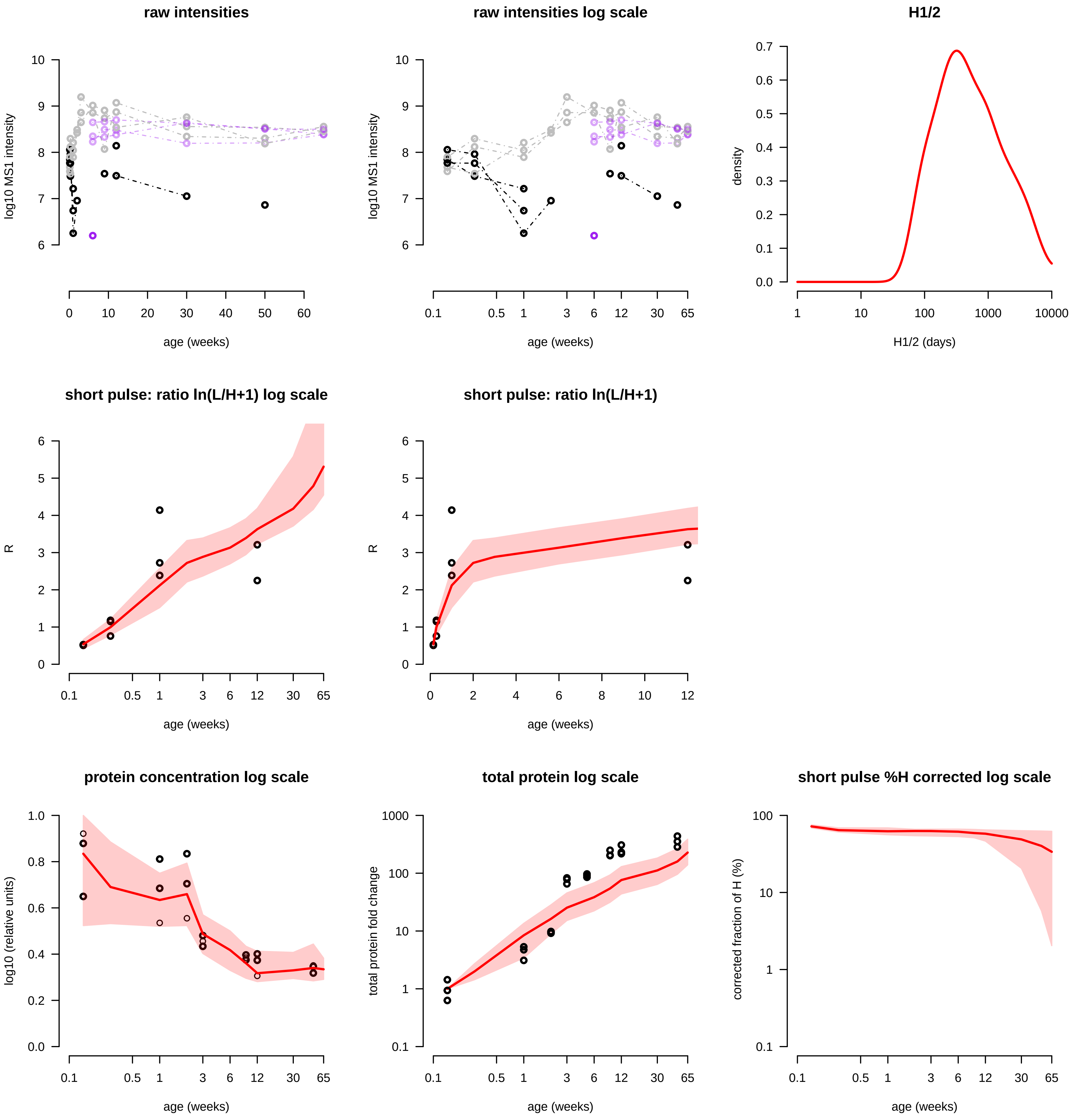

Q99LG2

>sp|Q99LG2|TNPO2_MOUSE

Transportin−2

GN=Tnpo2

Med H1/2: 3.4 days

Q5 H1/2: 2.7 days

Q95 H1/2: 4.4 days

reliable model fit: yes

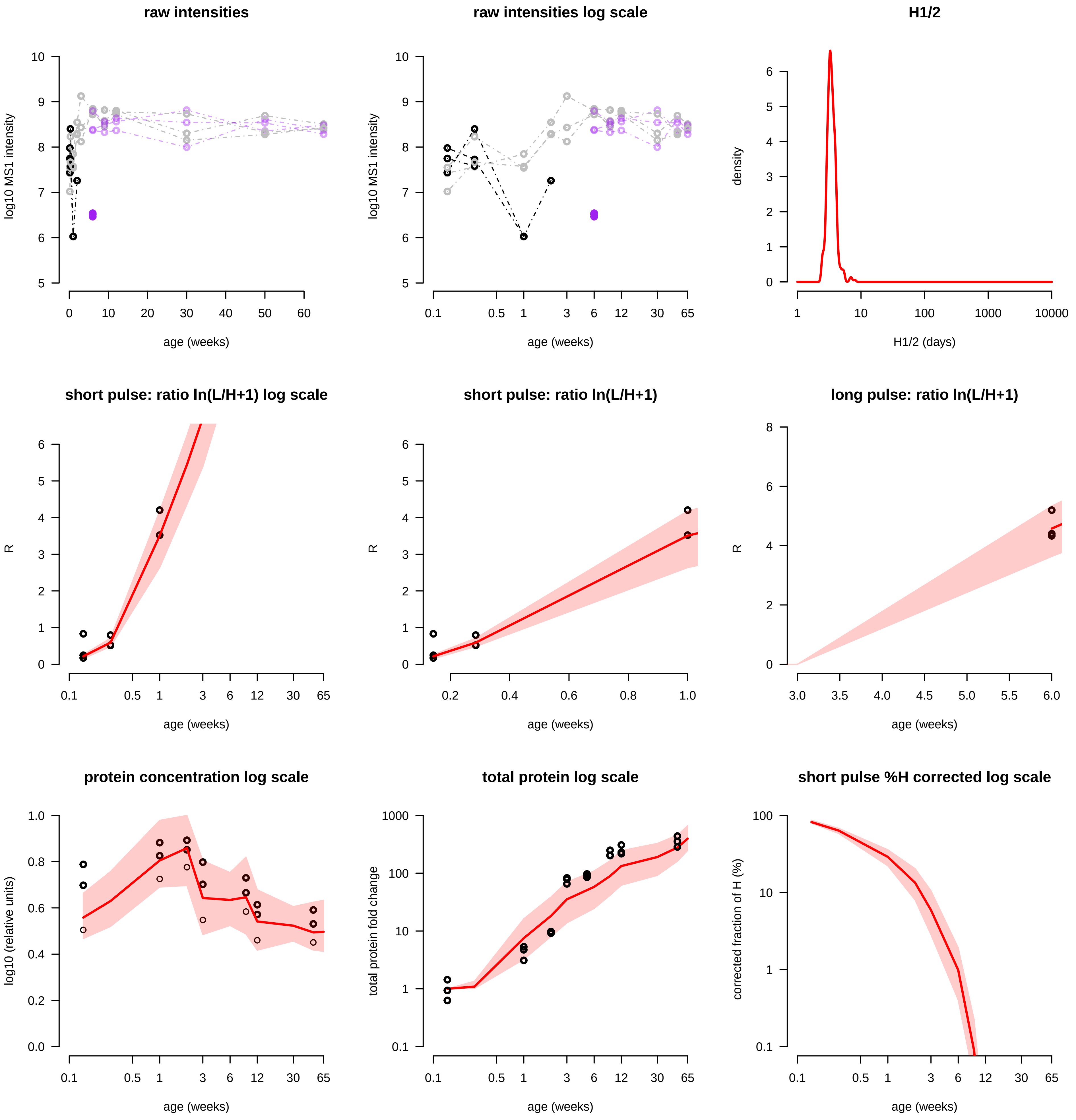

P27641

>sp|P27641|XRCC5_MOUSE

X−ray repair cross−complementing protein 5

GN=Xrcc5

Med H1/2: 10.1 days

Q5 H1/2: 3.6 days

Q95 H1/2: 54.8 days

reliable model fit: yes

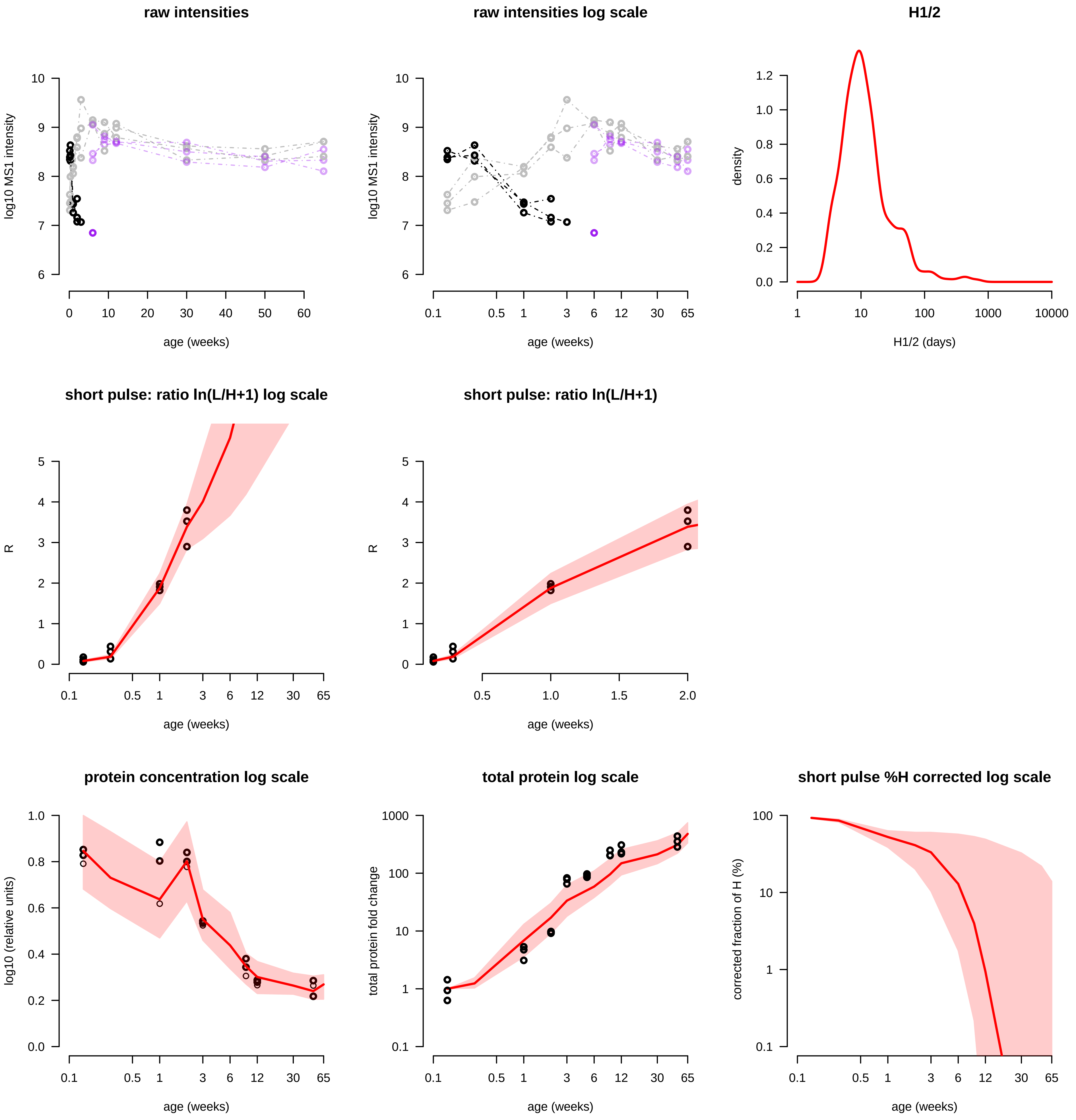

P63328−2;P63328

>sp|P63328−2|PP2BA_MOUSE

form 2 of Serine/threonine−protein phosphatase 2B catalytic subunit alpha isofo

GN=Ppp3ca

Med H1/2: 17.1 days

Q5 H1/2: 5.9 days

Q95 H1/2: 155.9 days

reliable model fit: yes

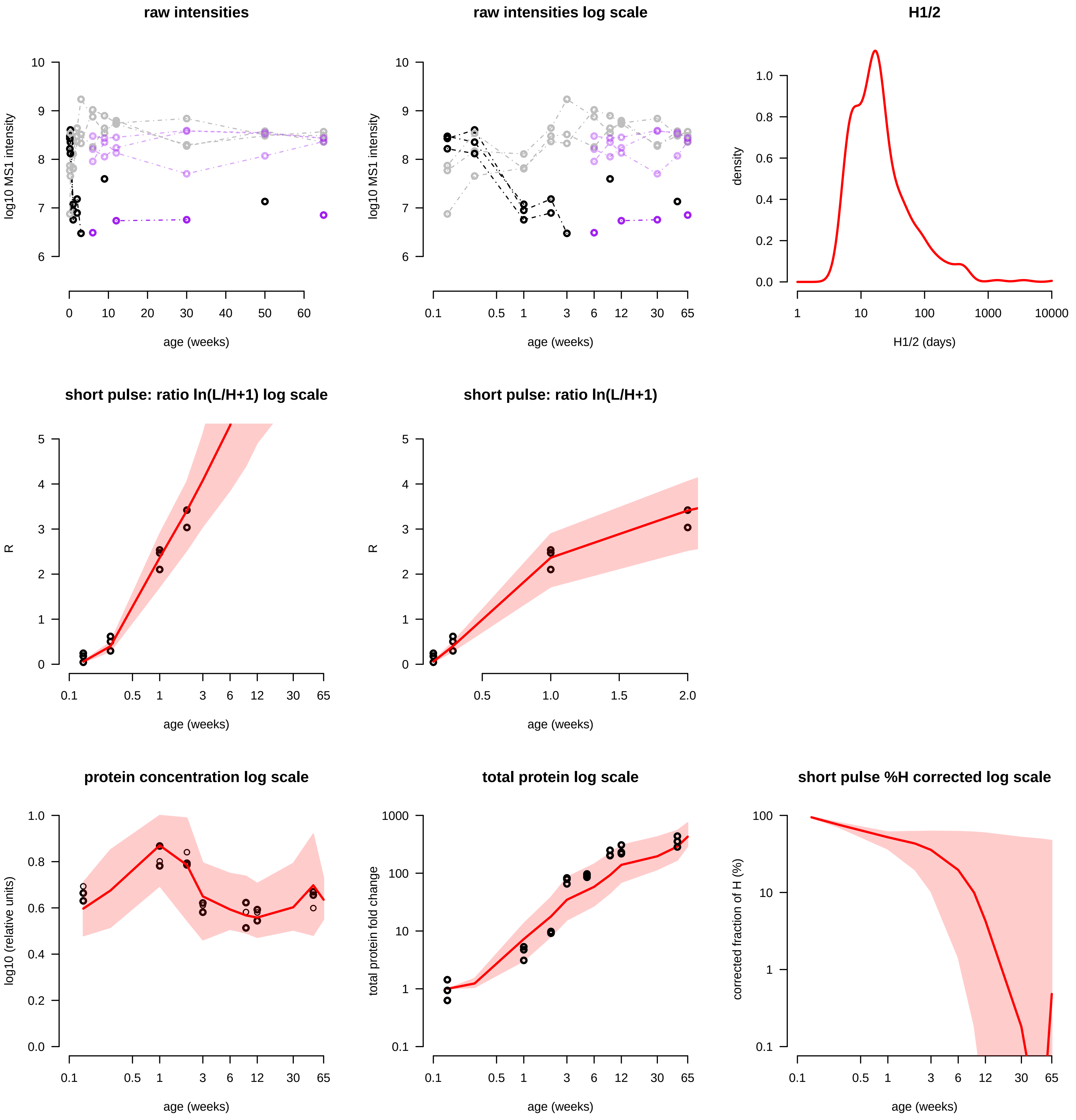

Q62393

>sp|Q62393|TPD52_MOUSE

Tumor protein D52

GN=Tpd52

Med H1/2: 5.4 days

Q5 H1/2: 3.9 days

Q95 H1/2: 8.3 days

reliable model fit: yes

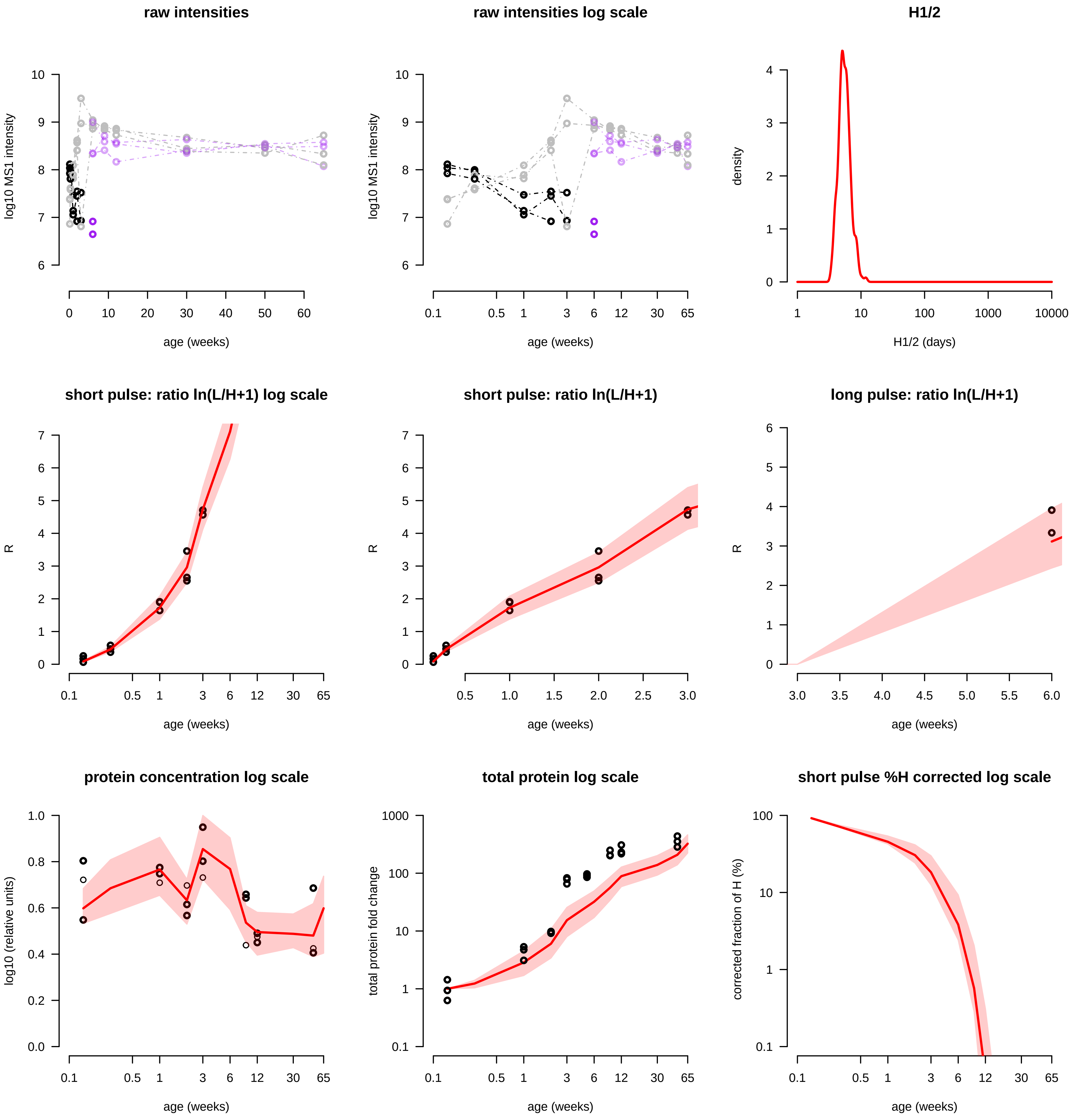

Q61292

>sp|Q61292|LAMB2_MOUSE

Laminin subunit beta−2

GN=Lamb2

Med H1/2: 18.8 days

Q5 H1/2: 5.8 days

Q95 H1/2: 215 days

reliable model fit: yes

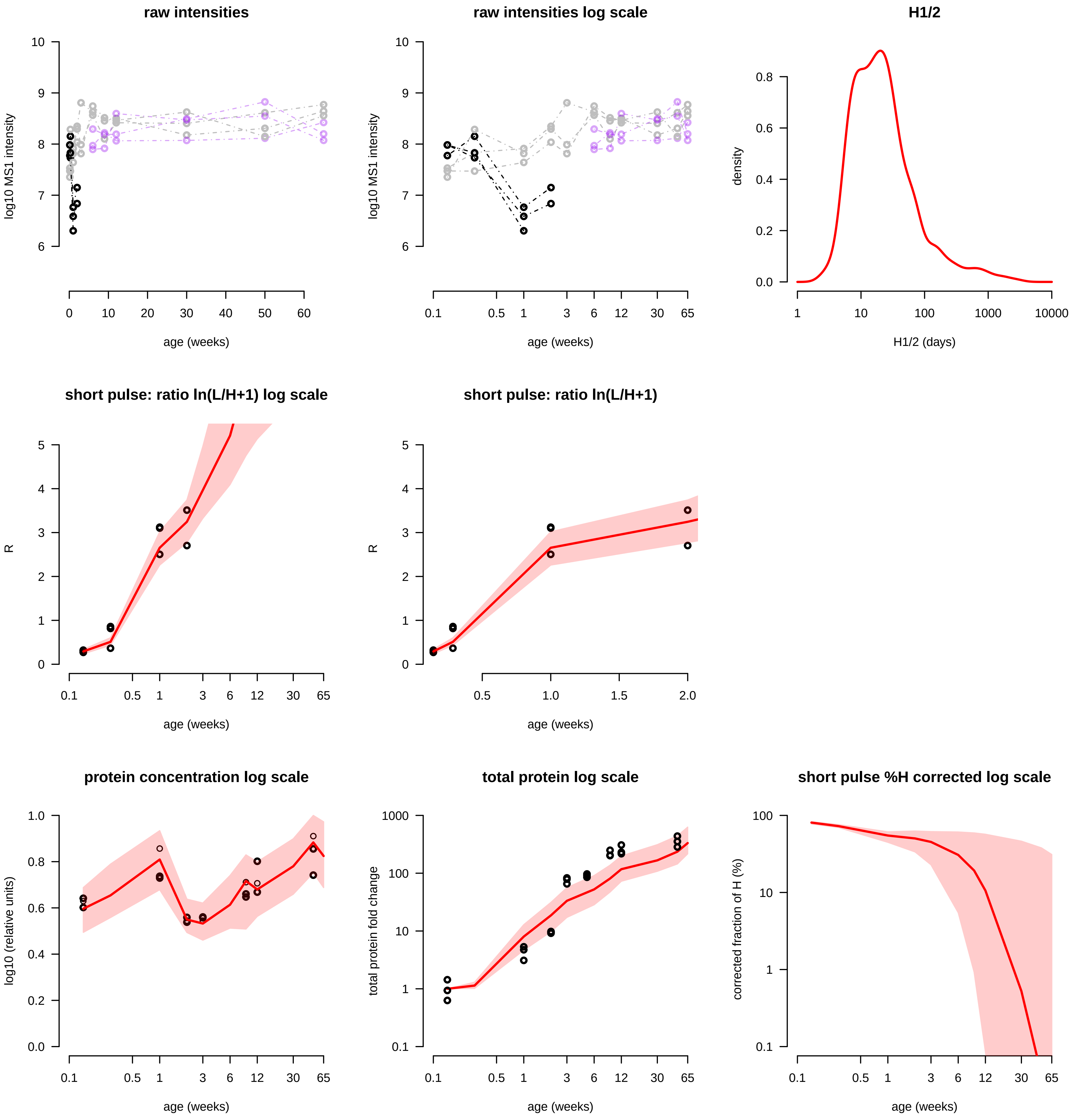

Q91VU7;Q91VU7−2

>sp|Q91VU7|PUS7_MOUSE

Pseudouridylate synthase 7 homolog

GN=Pus7

Med H1/2: 16.9 days

Q5 H1/2: 5.9 days

Q95 H1/2: 185.2 days

reliable model fit: yes

raw intensities

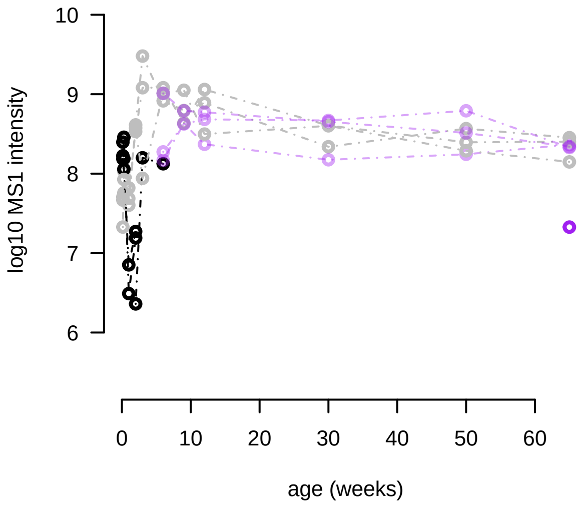

raw intensities log scale

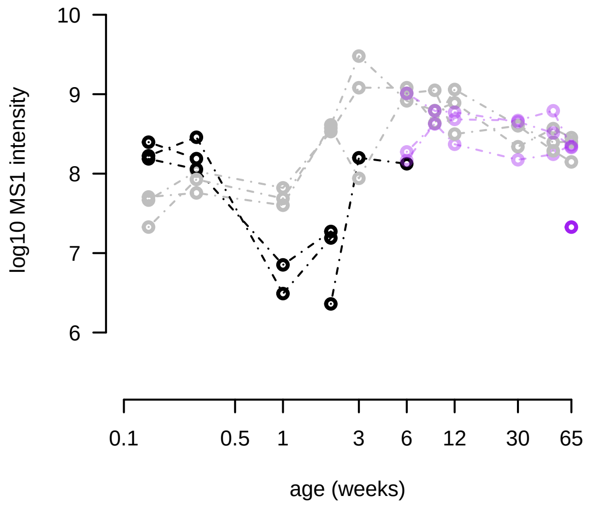

H1/2

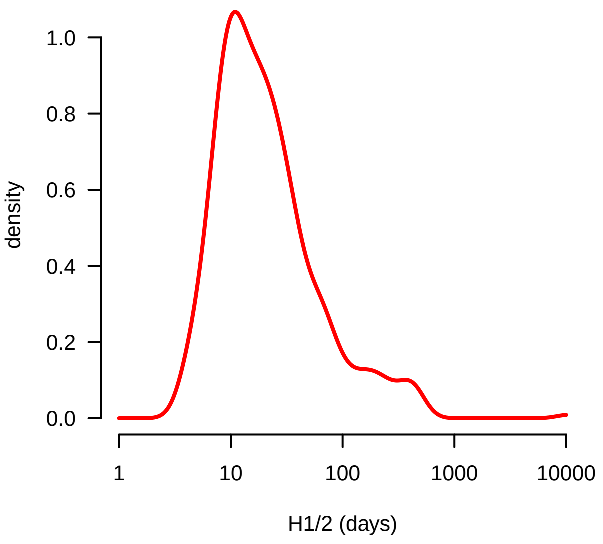

short pulse: ratio ln(L/H+1) log scale

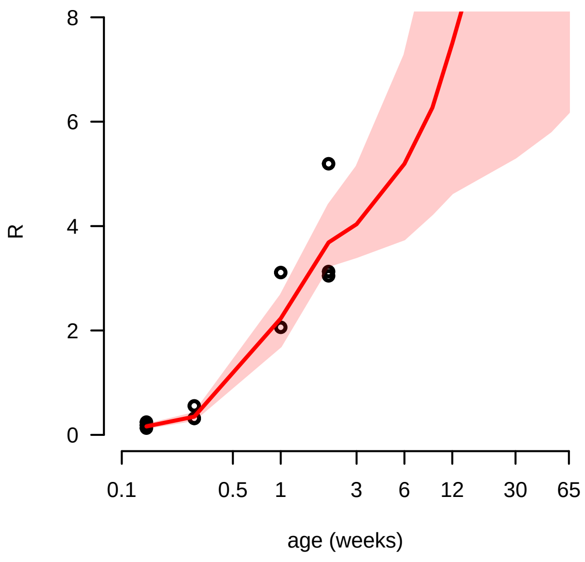

short pulse: ratio ln(L/H+1)

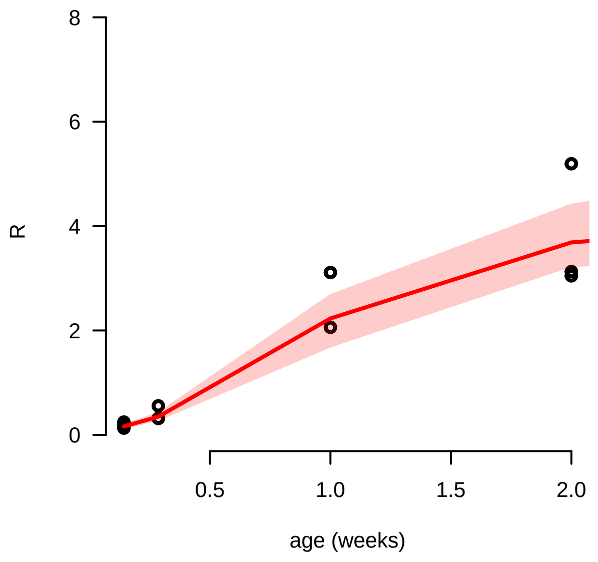

protein concentration log scale

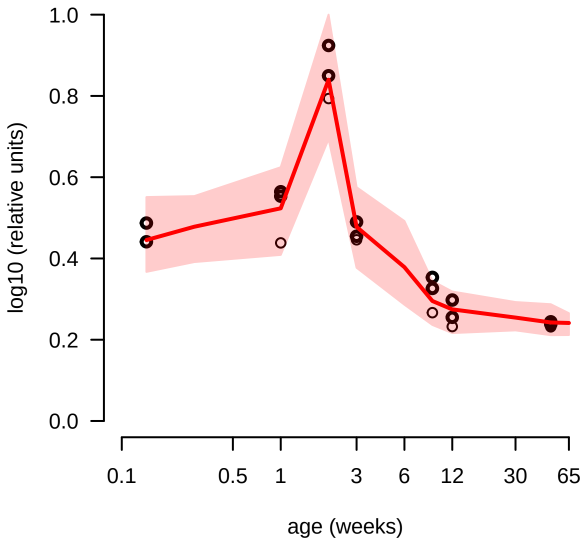

total protein log scale

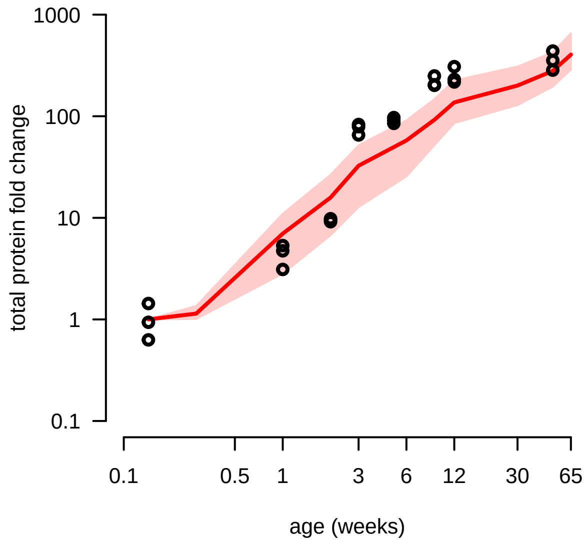

short pulse %H corrected log scale

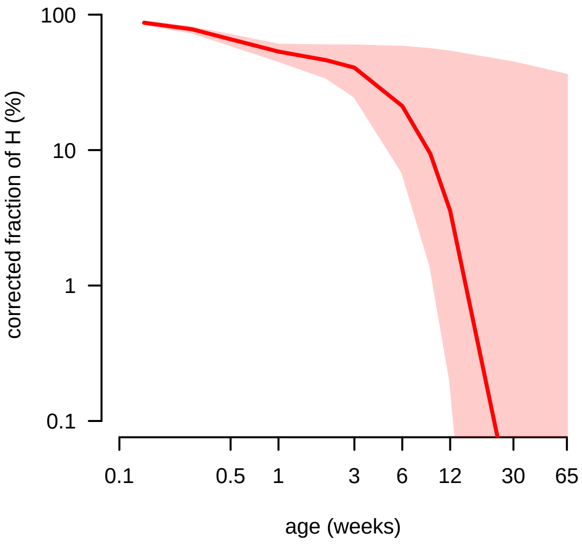

Q8R3G1

>sp|Q8R3G1|PP1R8_MOUSE

Nuclear inhibitor of protein phosphatase 1

GN=Ppp1r8

Med H1/2: 5.3 days

Q5 H1/2: 4.1 days

Q95 H1/2: 7.2 days

reliable model fit: yes

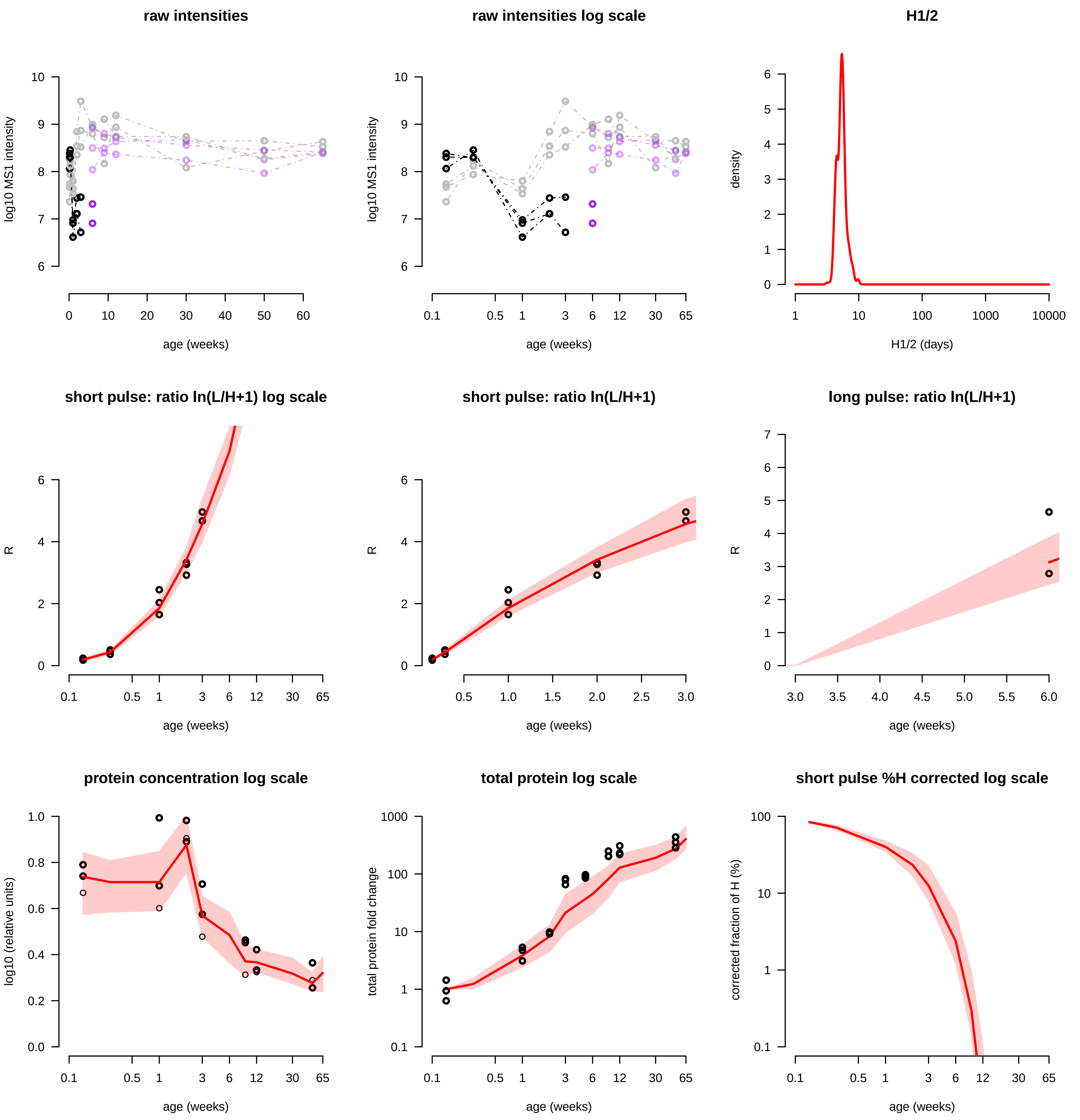

Q8VDQ1;Q8VDQ1−2

>sp|Q8VDQ1|PTGR2_MOUSE

Prostaglandin reductase 2

GN=Ptgr2

Med H1/2: 10.7 days

Q5 H1/2: 4.7 days

Q95 H1/2: 115.3 days

reliable model fit: yes

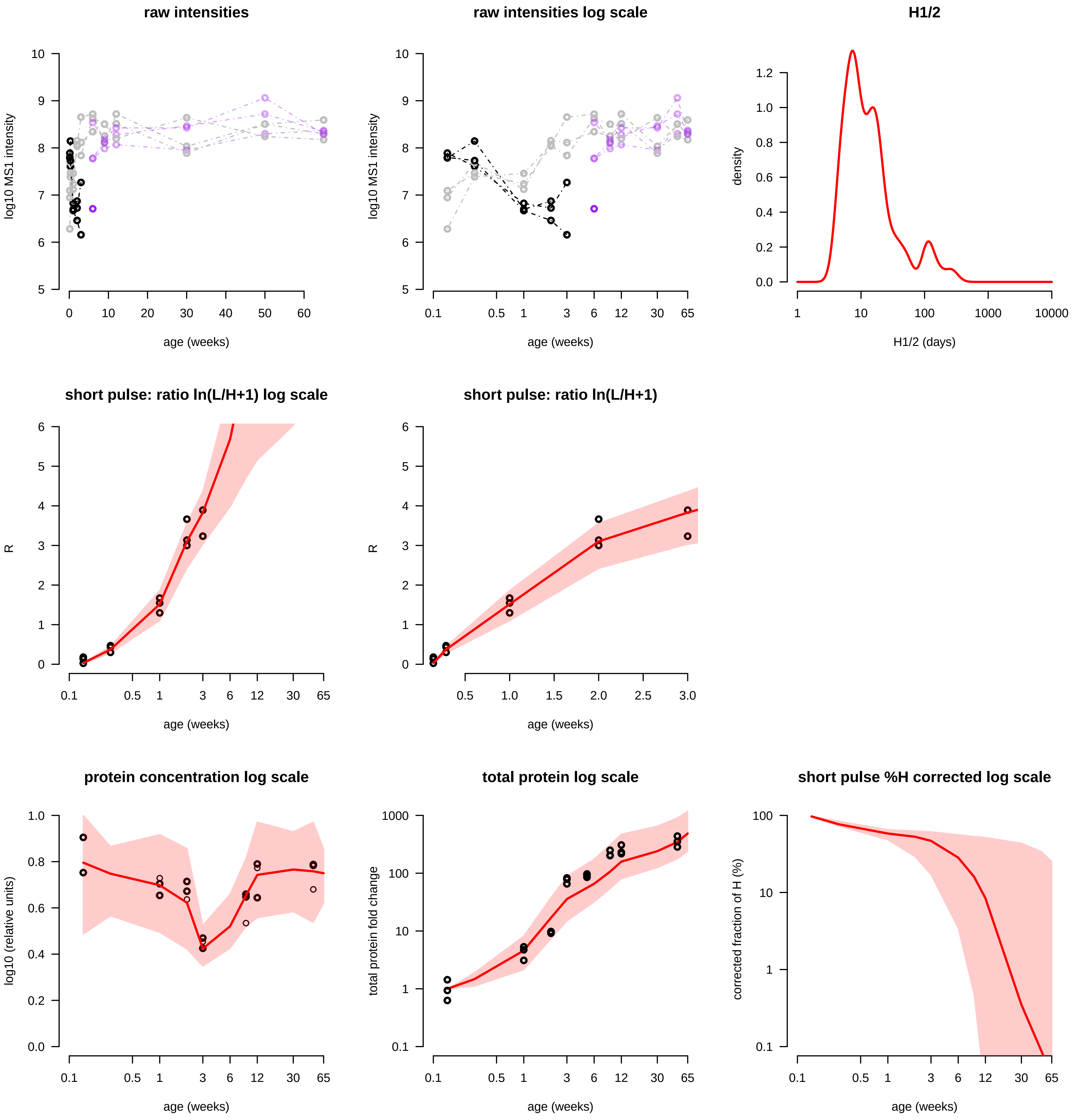

Q62059;Q62059−2

>sp|Q62059|CSPG2_MOUSE

Versican core protein

GN=Vcan

Med H1/2: 4 days

Q5 H1/2: 3.2 days

Q95 H1/2: 5.1 days

reliable model fit: yes

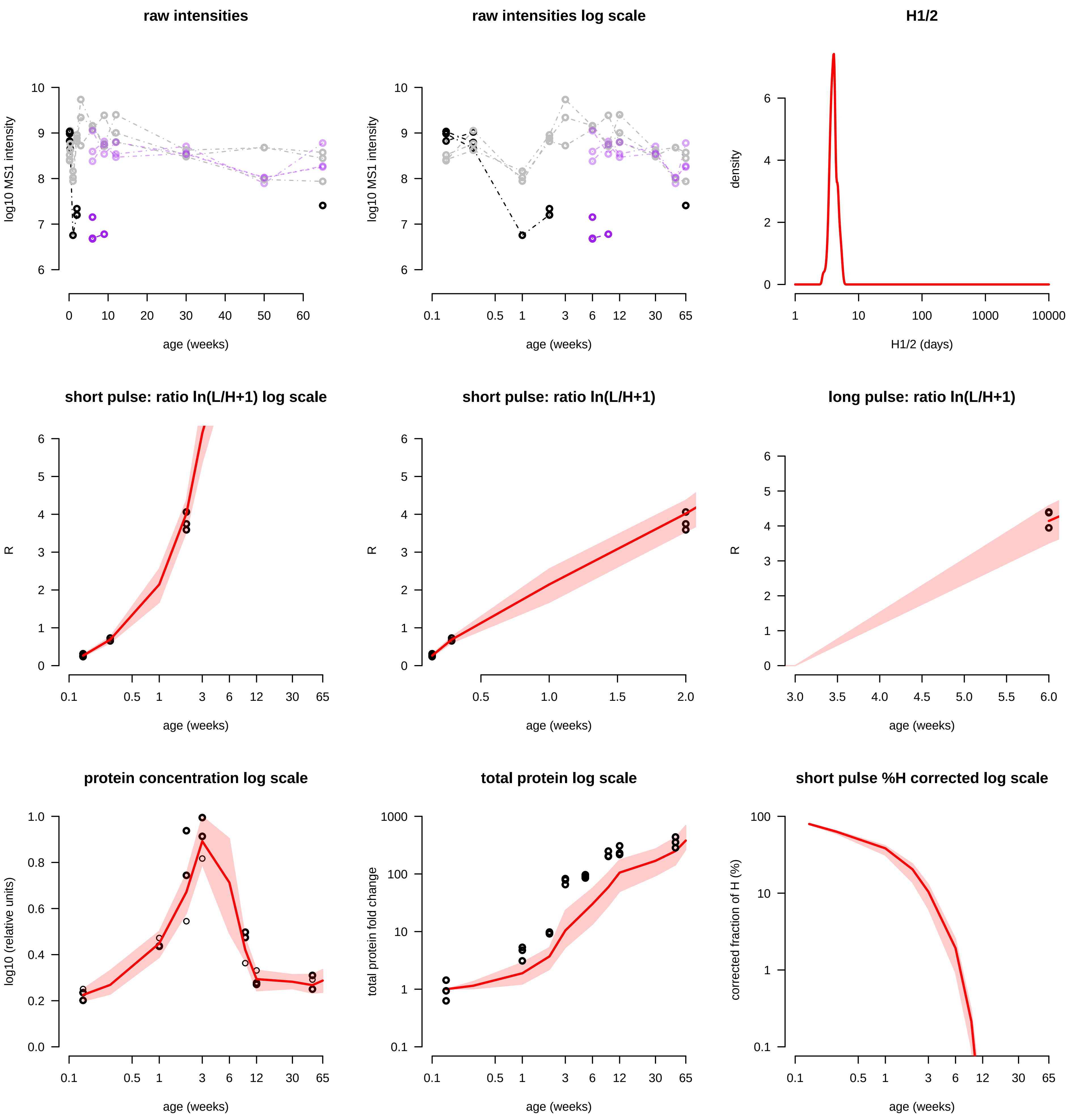

Q8BYK6−3;Q8BYK6;Q8BYK6−2

>sp|Q8BYK6−3|YTHD3_MOUSE

Isoform 3 of YTH domain−containing family protein 3

GN=Ythdf3

Med H1/2: 1669.6 days

Q5 H1/2: 546.5 days

Q95 H1/2: 17043.9 days

reliable model fit: yes

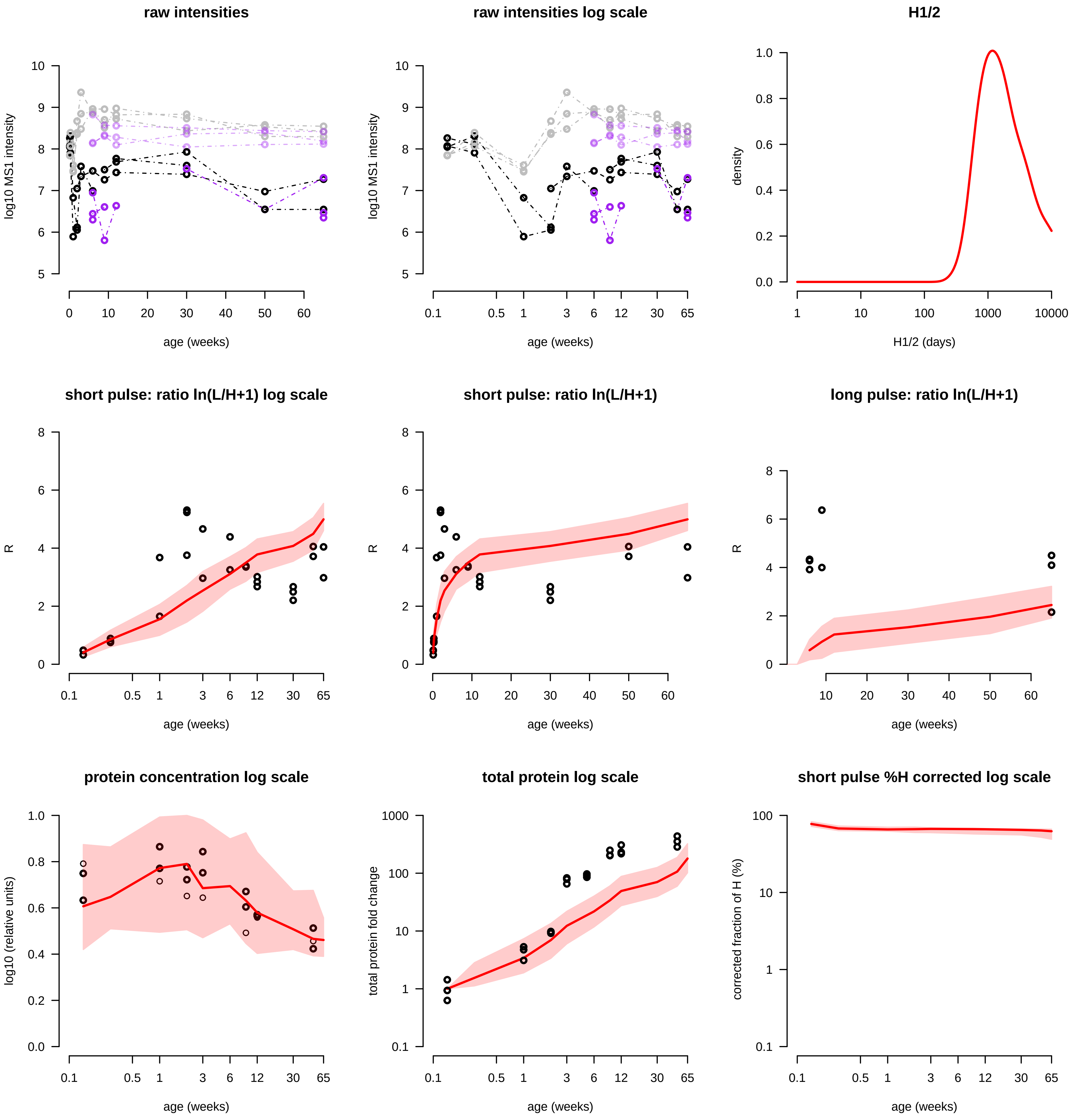

Q8BH79;Q8BH79−2;Q8BH79−4;Q8BH79−3

>sp|Q8BH79|ANO10_MOUSE

Anoctamin−10

GN=Ano10

Med H1/2: 6 days

Q5 H1/2: 2.8 days

Q95 H1/2: 56.1 days

reliable model fit: yes

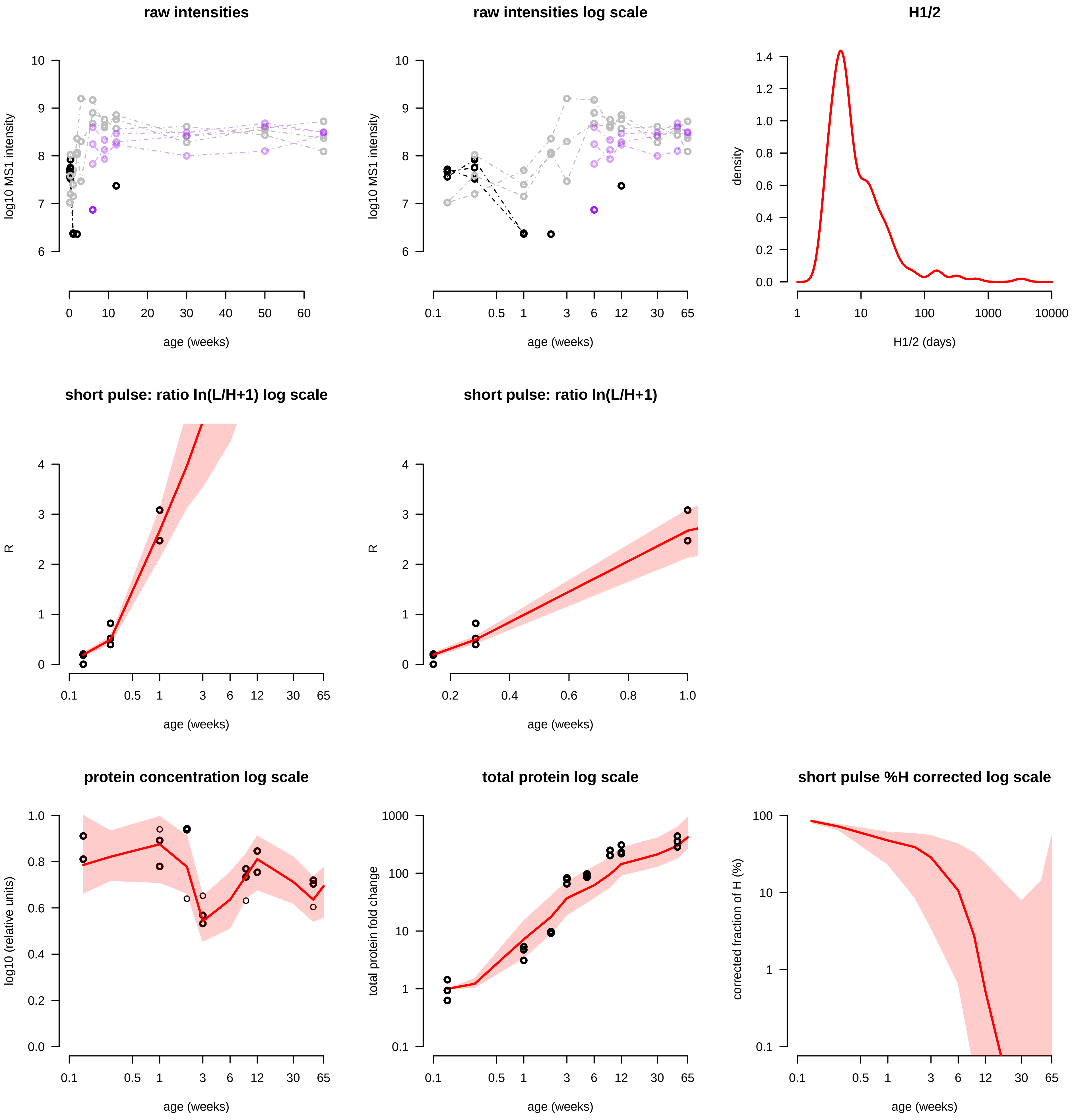

Q6ZWQ7

>sp|Q6ZWQ7|SPCS3_MOUSE

Signal peptidase complex subunit 3

GN=Spcs3

Med H1/2: 7.5 days

Q5 H1/2: 3.2 days

Q95 H1/2: 35.6 days

reliable model fit: yes

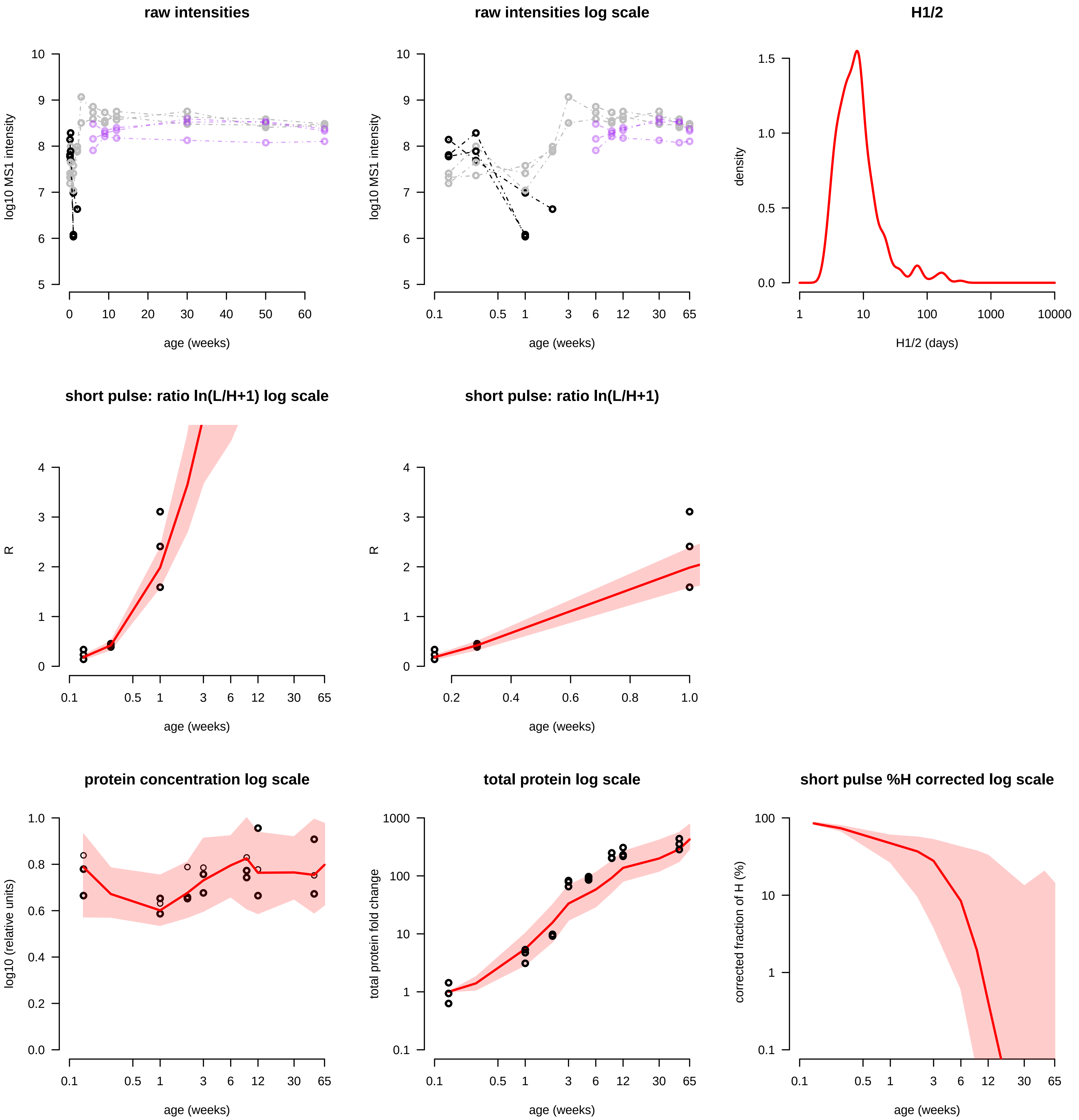

Q99K23

>sp|Q99K23|UFSP2_MOUSE

Ufm1−specific protease 2

GN=Ufsp2

Med H1/2: 5.6 days

Q5 H1/2: 2.6 days

Q95 H1/2: 55.2 days

reliable model fit: yes

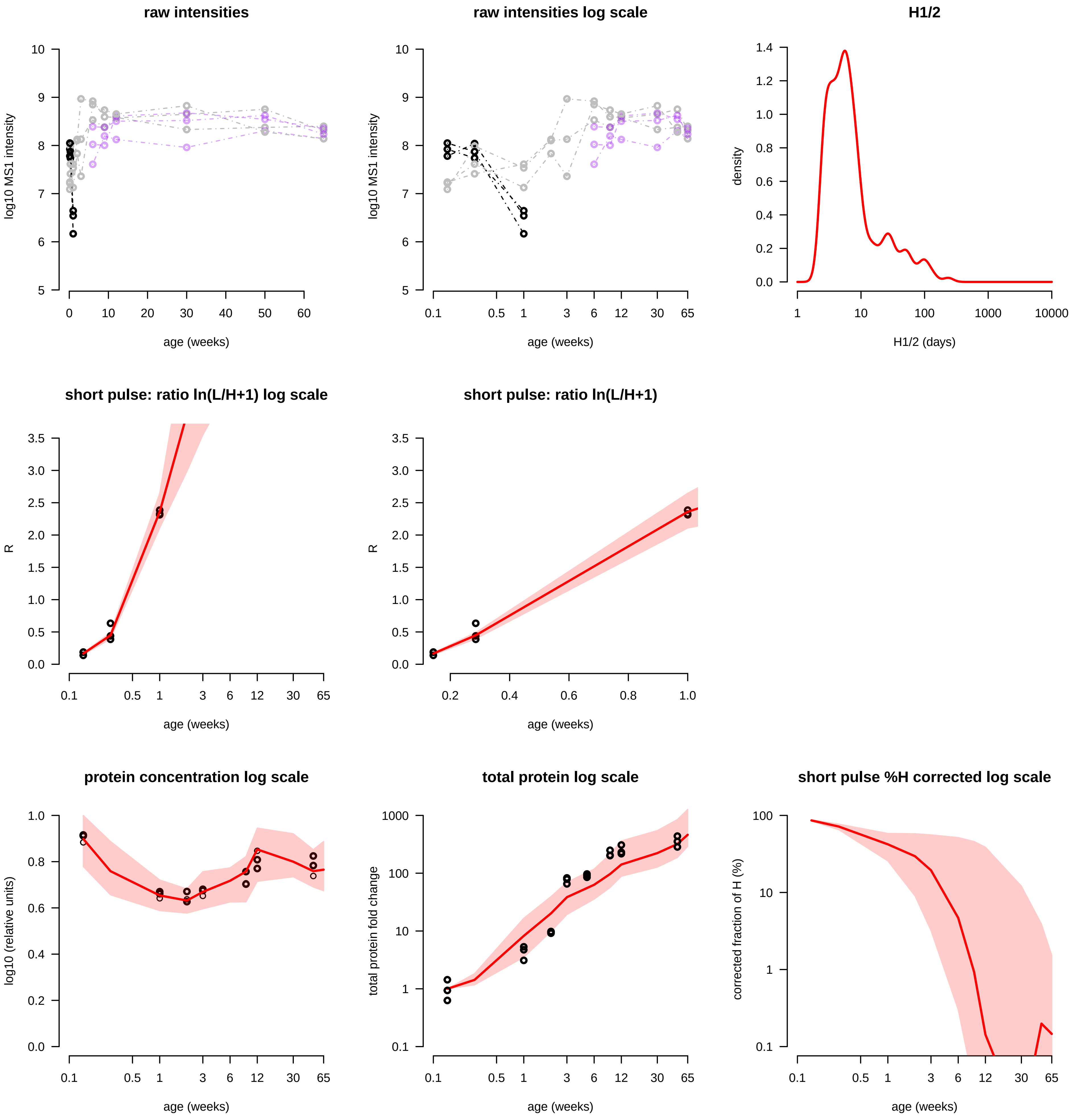

P47941

>sp|P47941|CRKL_MOUSE

Crk−like protein

GN=Crkl

Med H1/2: 14.5 days

Q5 H1/2: 5.1 days

Q95 H1/2: 108.3 days

reliable model fit: yes

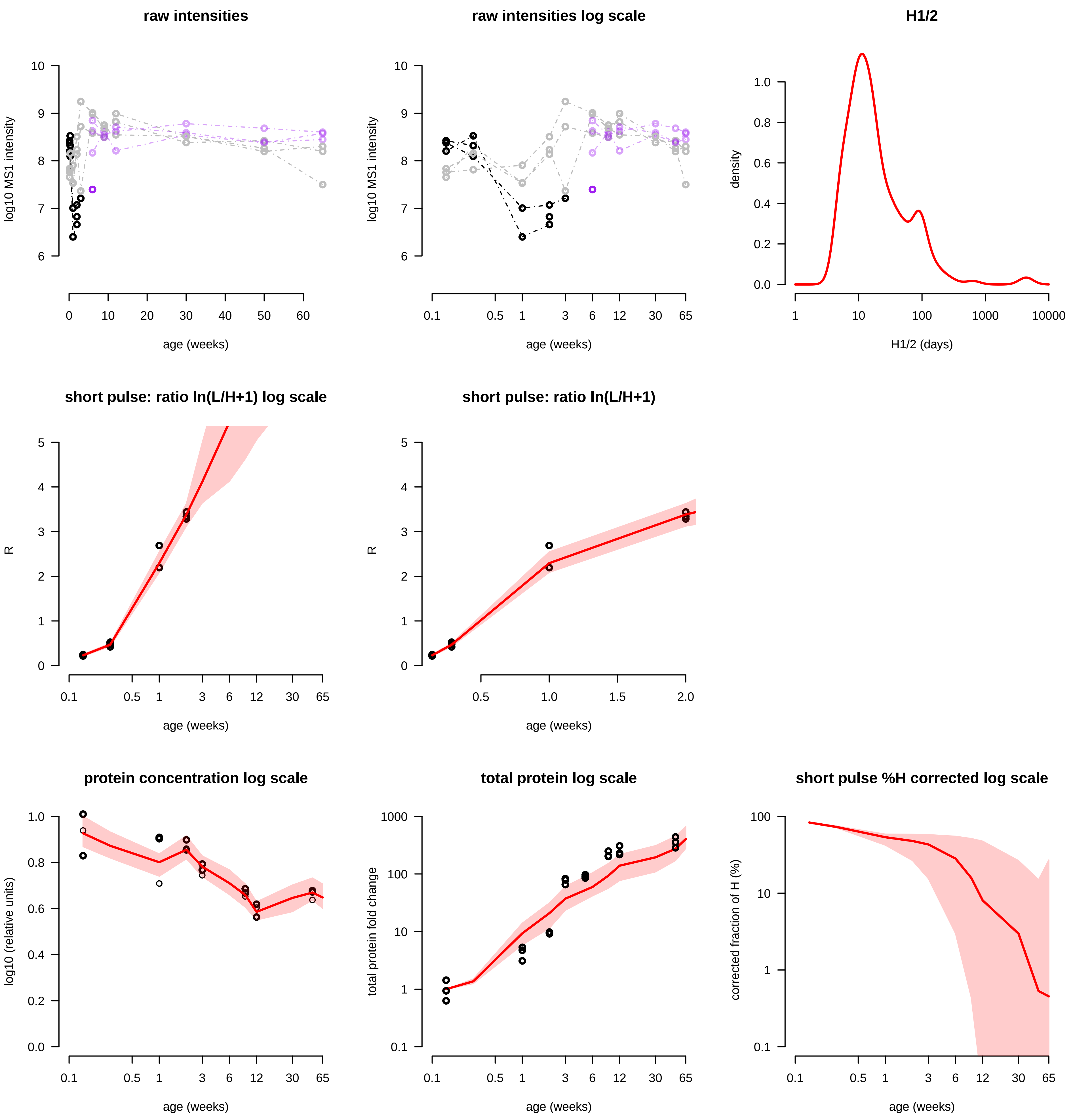

Q8CIG8

>sp|Q8CIG8|ANM5_MOUSE

Protein arginine N−methyltransferase 5

GN=Prmt5

Med H1/2: 12.7 days

Q5 H1/2: 5.9 days

Q95 H1/2: 57.2 days

reliable model fit: yes

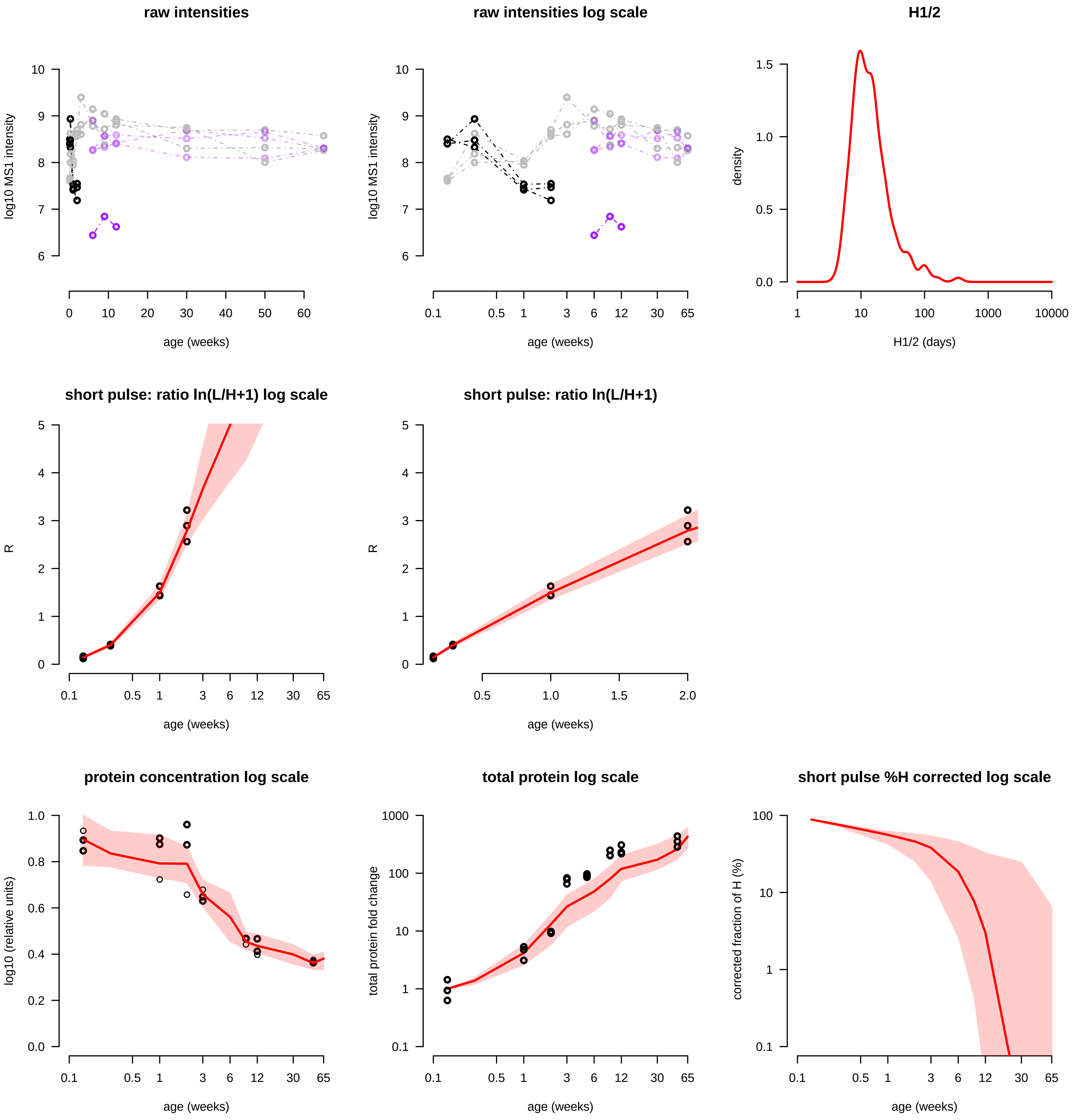

P70280

>sp|P70280|VAMP7_MOUSE

Vesicle−associated membrane protein 7

GN=Vamp7

Med H1/2: 58.5 days

Q5 H1/2: 18.6 days

Q95 H1/2: 549 days

reliable model fit: yes

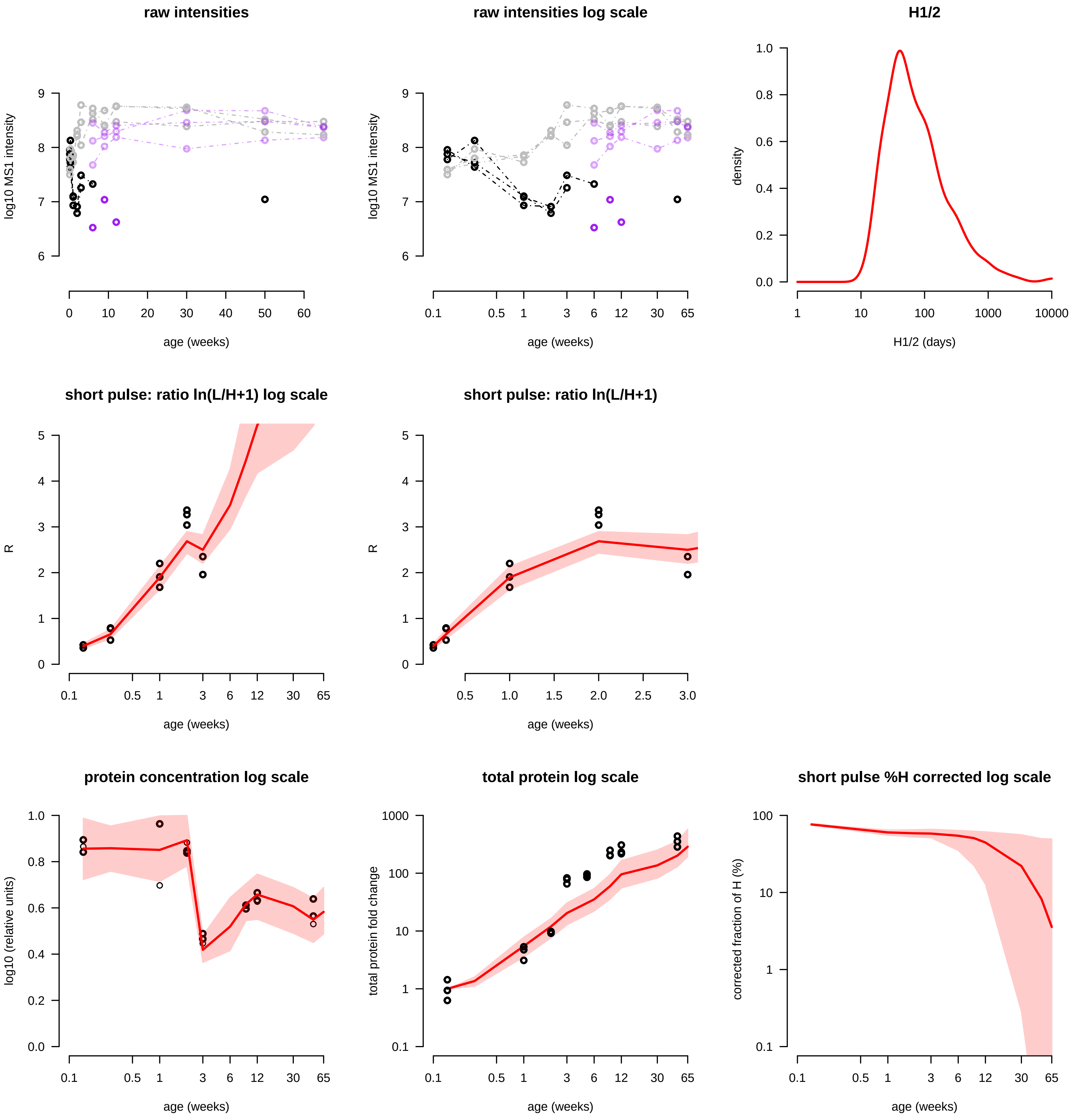

Q3UM45

>sp|Q3UM45|PP1R7_MOUSE

Protein phosphatase 1 regulatory subunit 7

GN=Ppp1r7

Med H1/2: 24.1 days

Q5 H1/2: 6.8 days

Q95 H1/2: 191 days

reliable model fit: yes

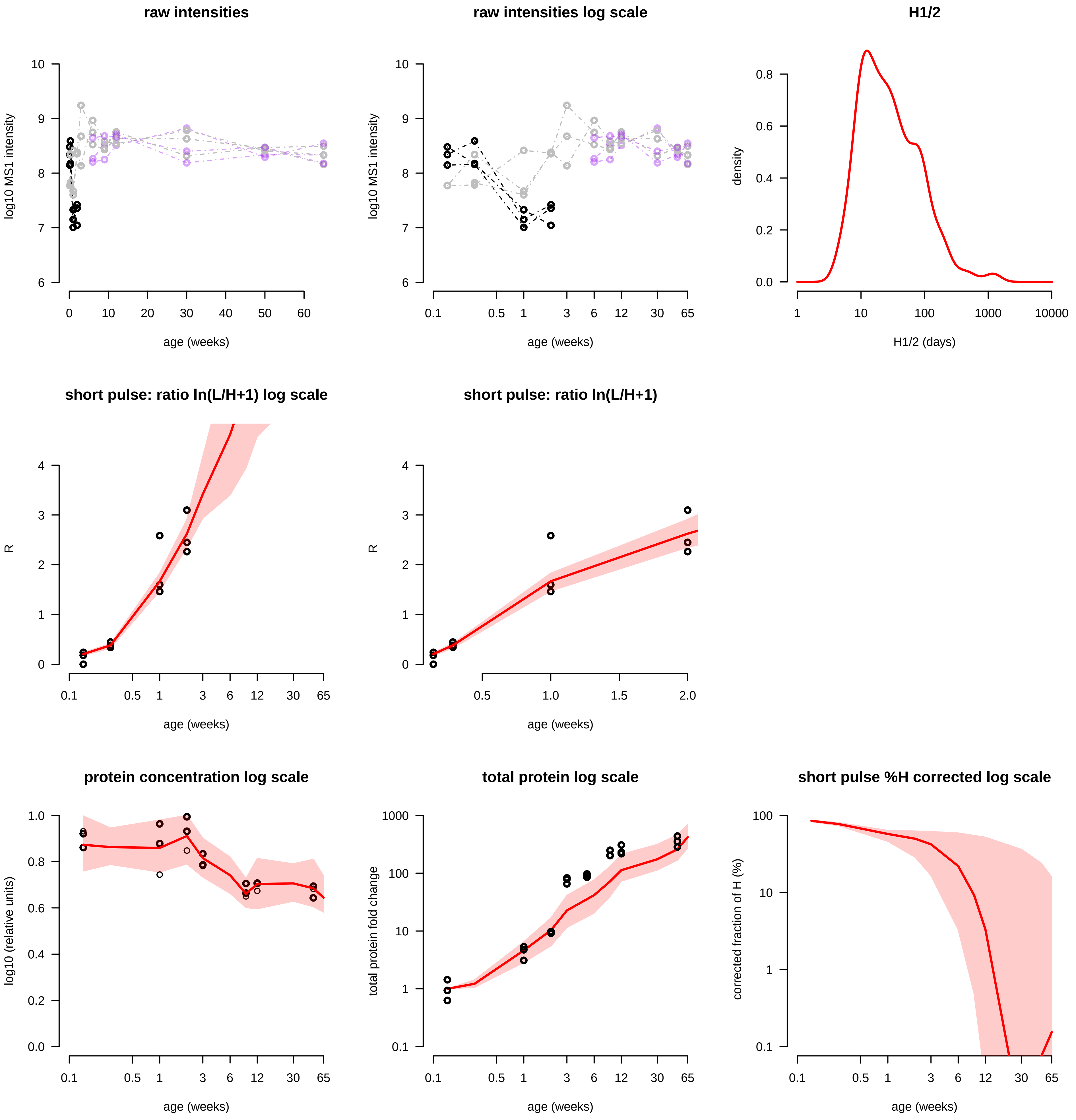

Q8R001

>sp|Q8R001|MARE2_MOUSE

Microtubule−associated protein RP/EB family member 2

GN=Mapre2

Med H1/2: 15.1 days

Q5 H1/2: 11.9 days

Q95 H1/2: 20.6 days

reliable model fit: yes

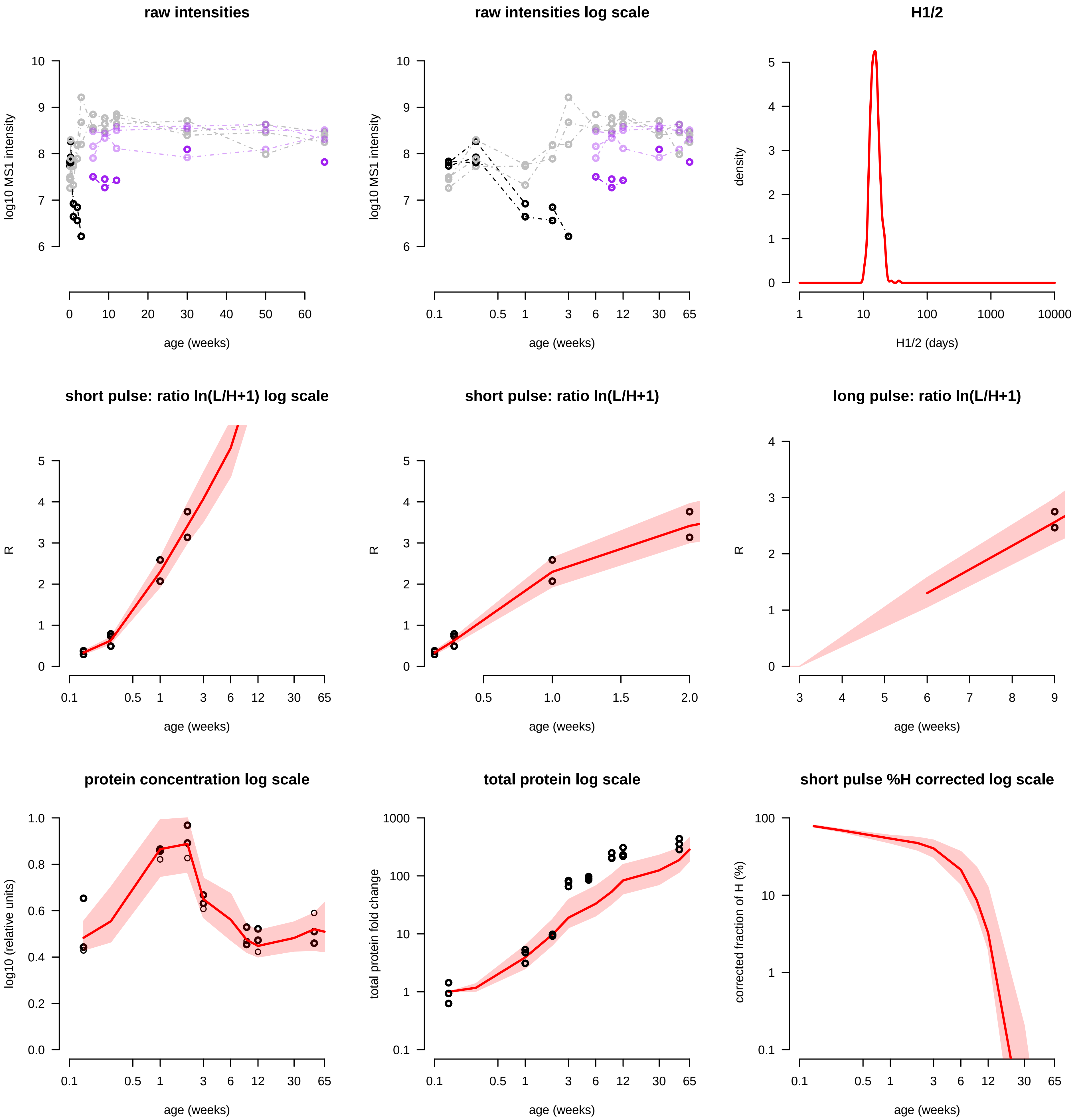

Q9DC11

>sp|Q9DC11|PXDC2_MOUSE

Plexin domain−containing protein 2

GN=Plxdc2

Med H1/2: 5.7 days

Q5 H1/2: 4.5 days

Q95 H1/2: 8.2 days

reliable model fit: yes

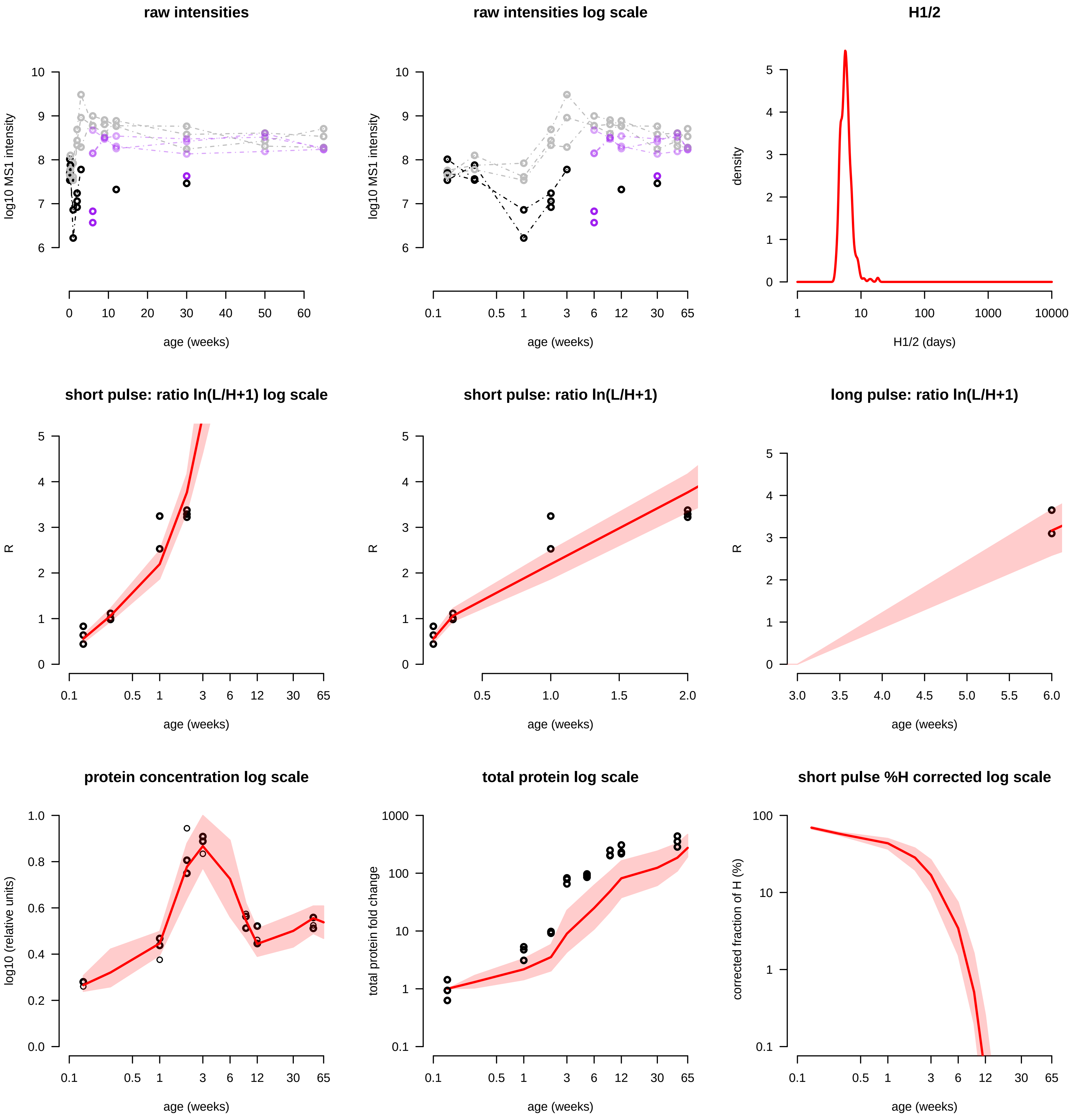

Q5M8N0

>sp|Q5M8N0|CNRP1_MOUSE

CB1 cannabinoid receptor−interacting protein 1

GN=Cnrip1

Med H1/2: 11 days

Q5 H1/2: 4.4 days

Q95 H1/2: 173.6 days

reliable model fit: yes

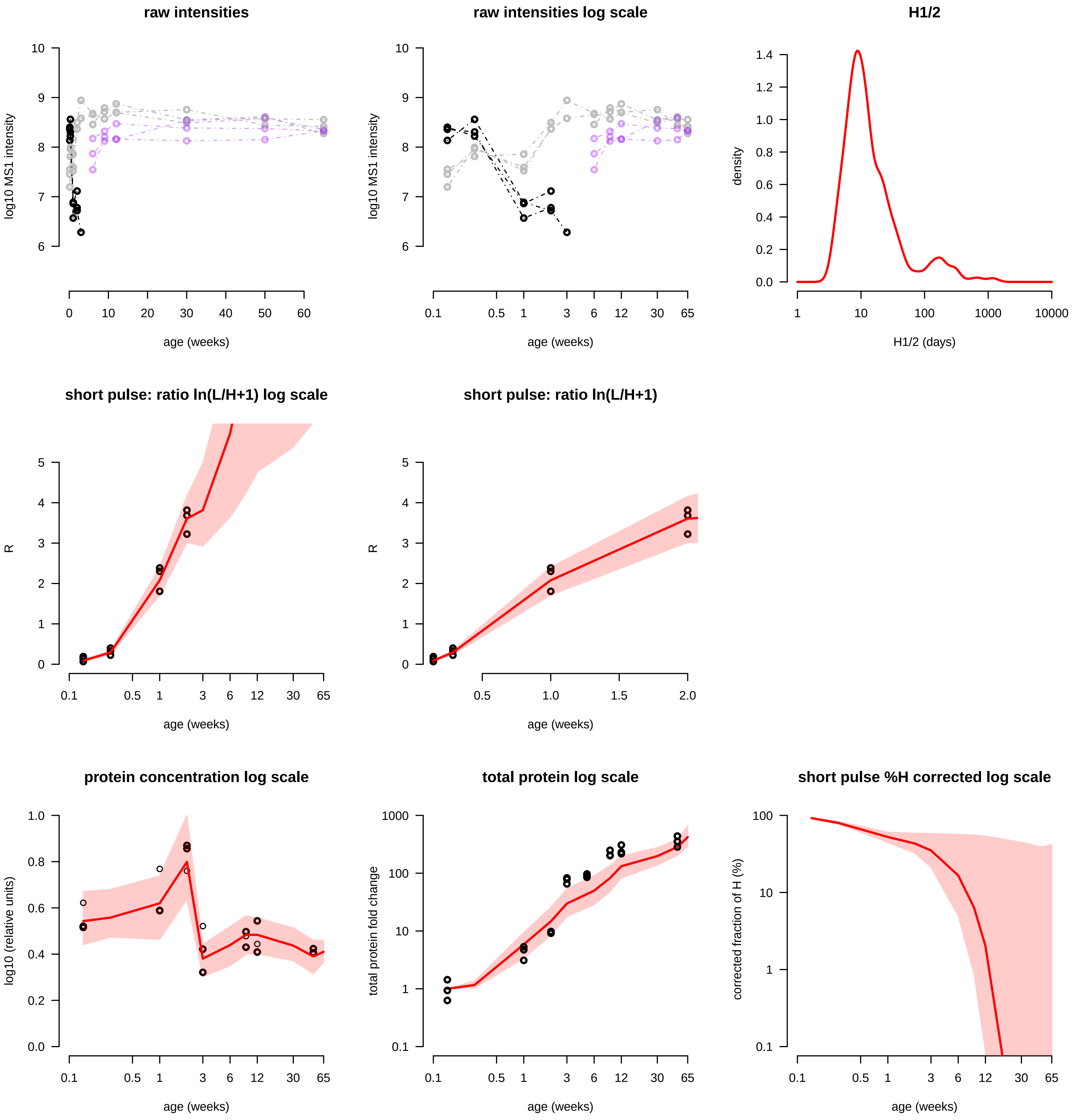

Q9D2V7

>sp|Q9D2V7|CORO7_MOUSE

Coronin−7

GN=Coro7

Med H1/2: 8.3 days

Q5 H1/2: 3.5 days

Q95 H1/2: 82.5 days

reliable model fit: yes

raw intensities

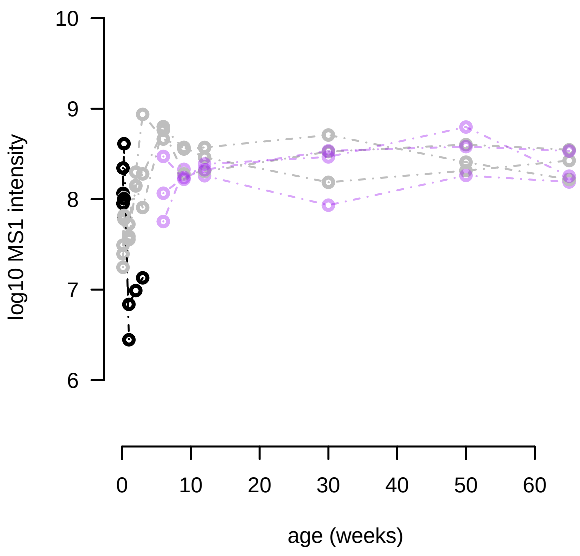

raw intensities log scale

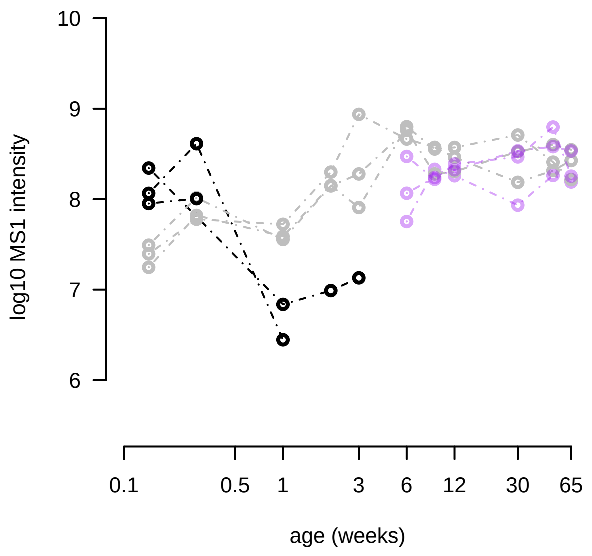

H1/2

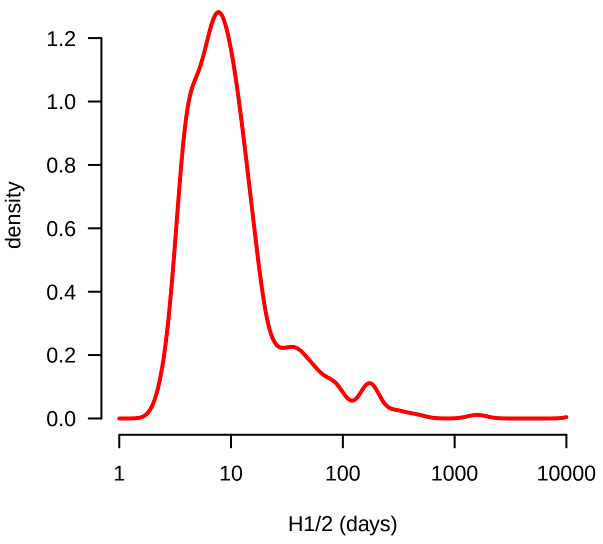

short pulse: ratio ln(L/H+1) log scale

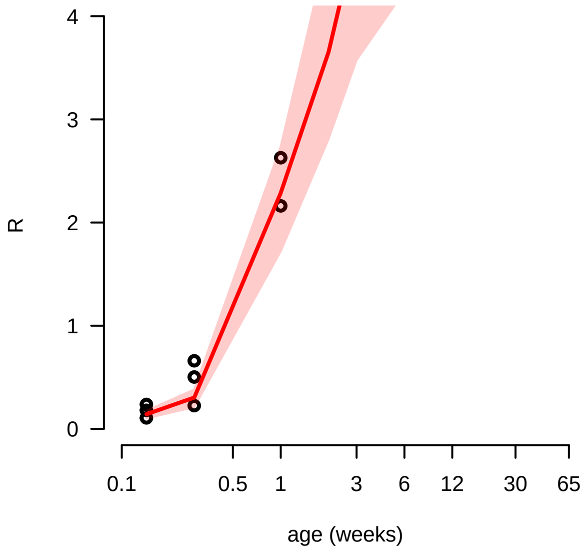

short pulse: ratio ln(L/H+1)

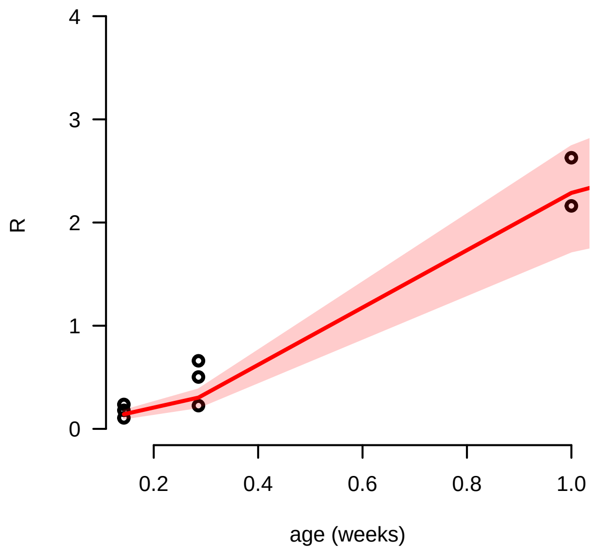

protein concentration log scale

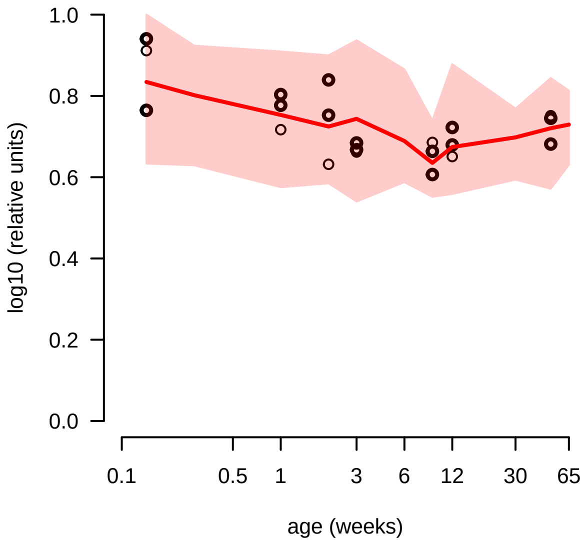

total protein log scale

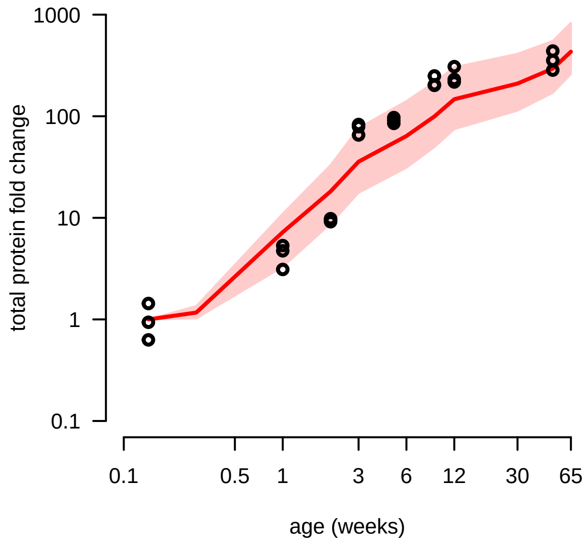

short pulse %H corrected log scale

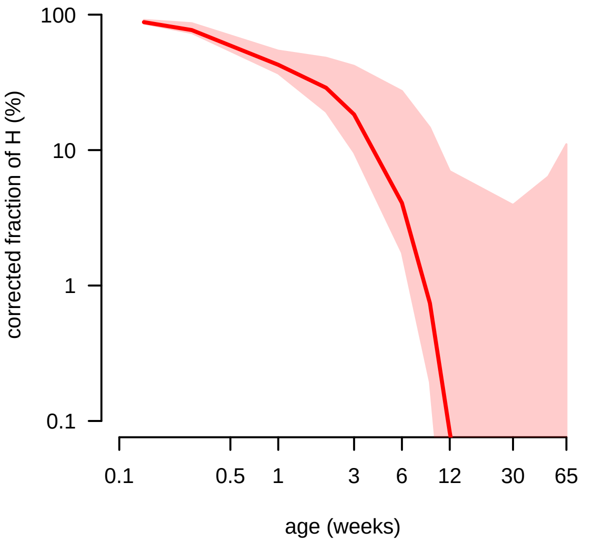

Q9R1T4−2;Q9R1T4−3;Q9R1T4

>sp|Q9R1T4−2|SEPT6_MOUSE

Isoform I of Septin−6

GN=Septin6

Med H1/2: 7.3 days

Q5 H1/2: 3.7 days

Q95 H1/2: 24.8 days

reliable model fit: yes

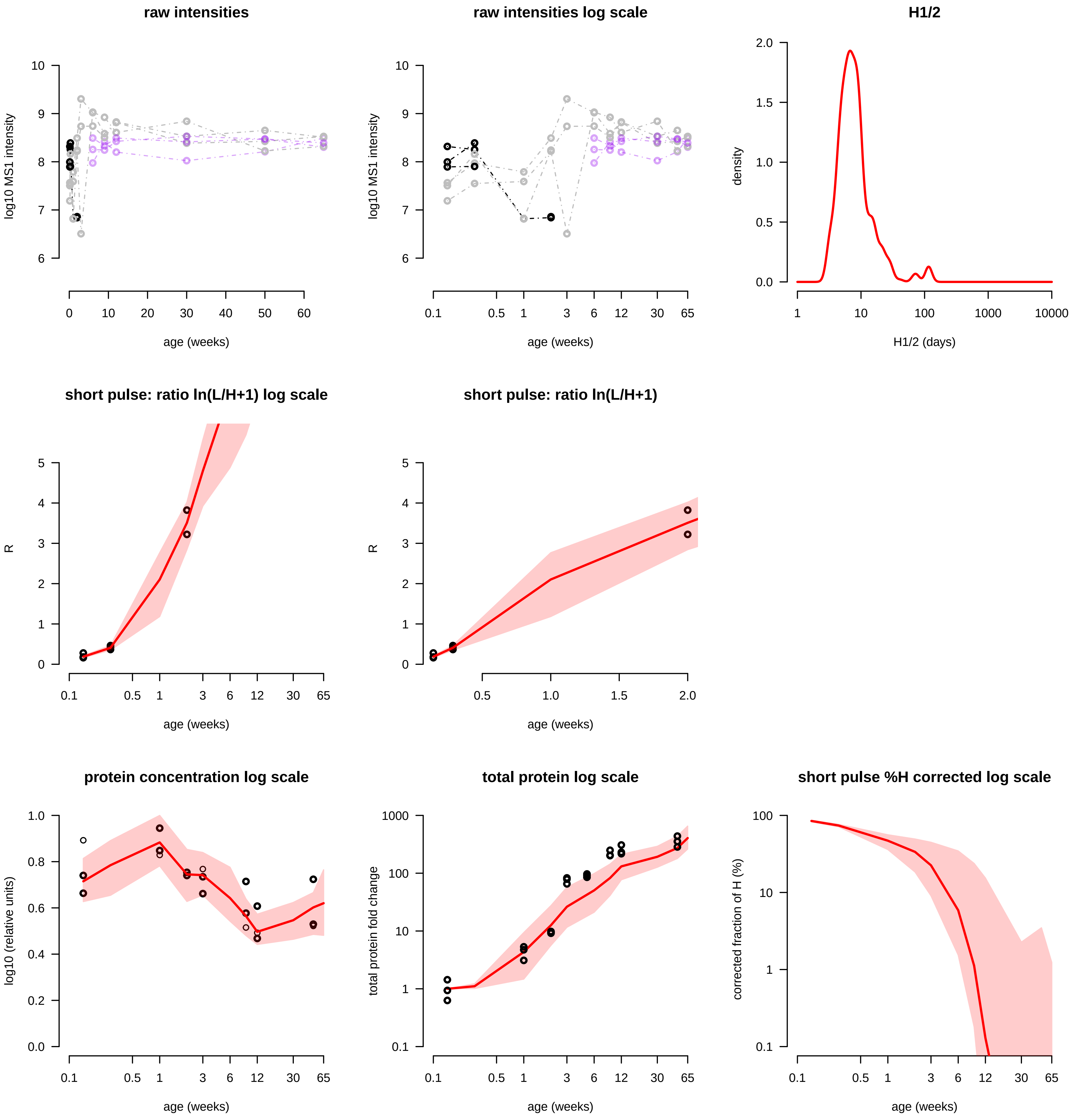

Q9R0M6

>sp|Q9R0M6|RAB9A_MOUSE

Ras−related protein Rab−9A

GN=Rab9a

Med H1/2: 4.8 days

Q5 H1/2: 4.1 days

Q95 H1/2: 6.3 days

reliable model fit: yes

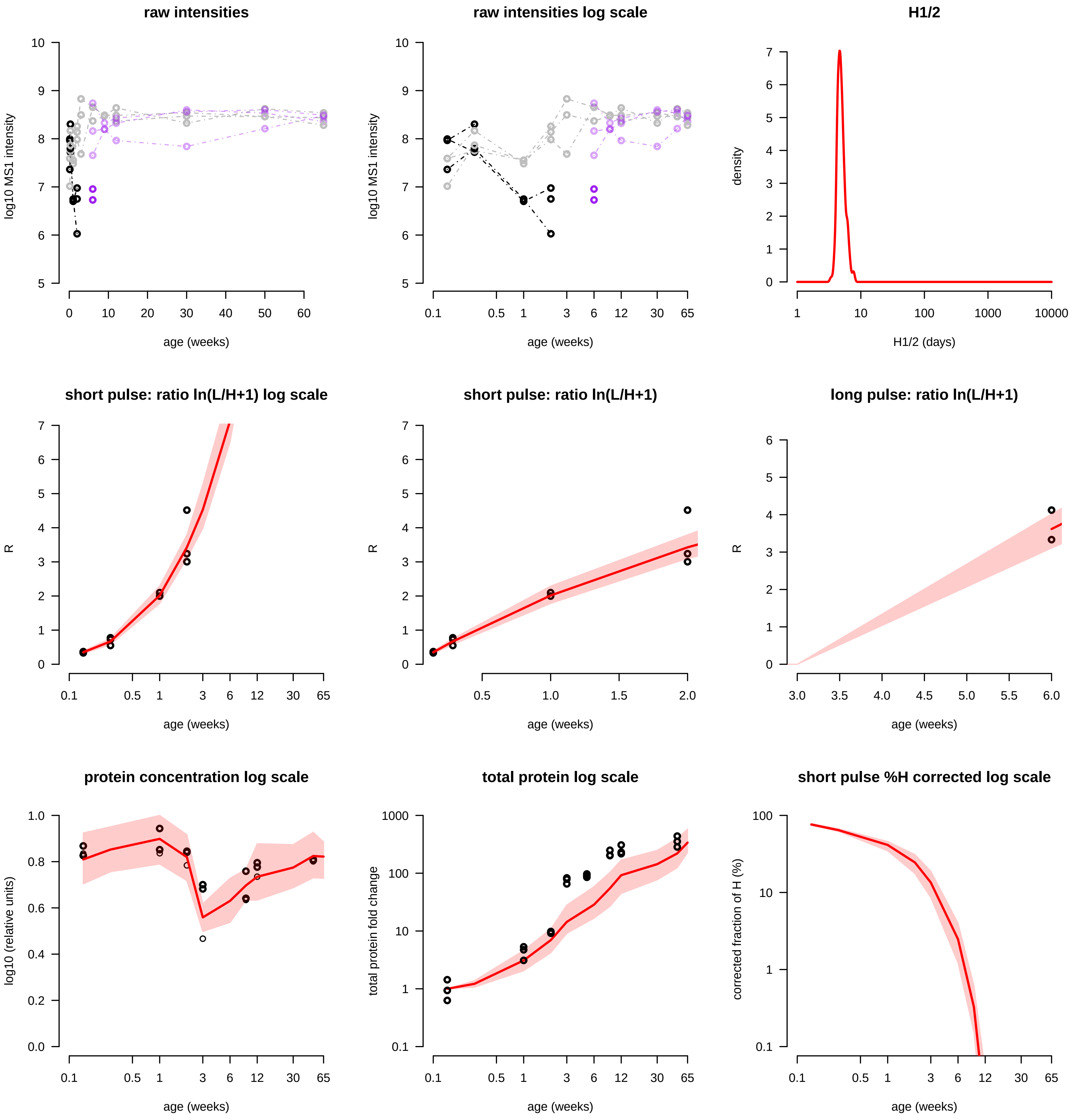

Q3UMY5;Q3UMY5−3

>sp|Q3UMY5|EMAL4_MOUSE

Echinoderm microtubule−associated protein−like 4

GN=Eml4

Med H1/2: 1823.4 days

Q5 H1/2: 424.3 days

Q95 H1/2: 21364.9 days

reliable model fit: no

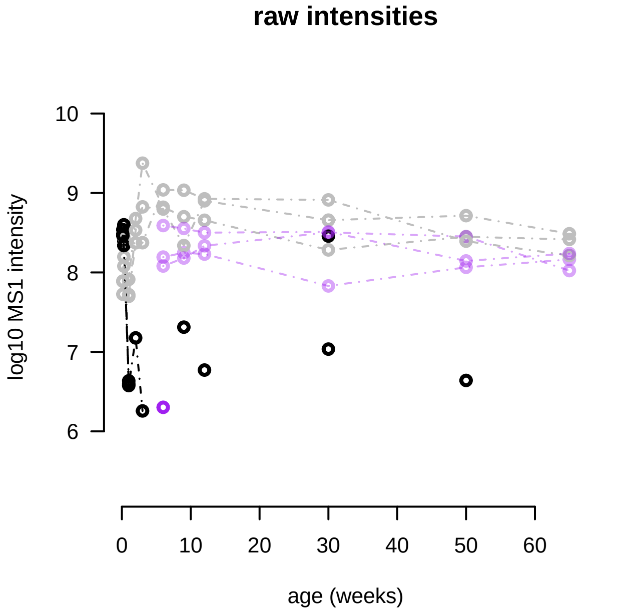

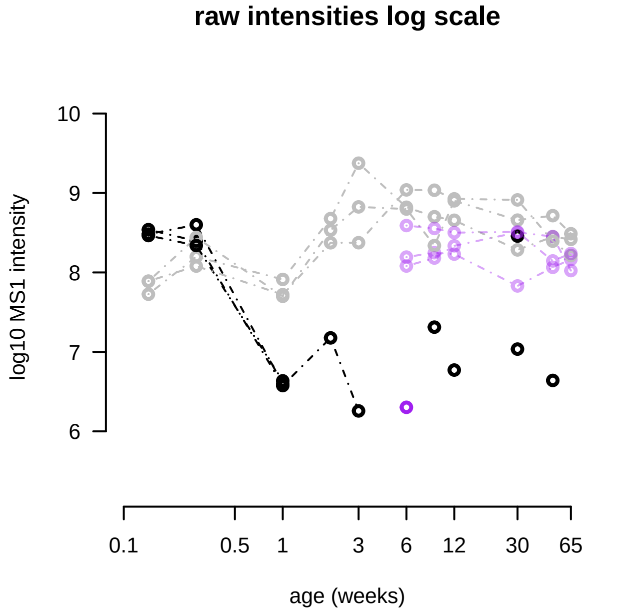

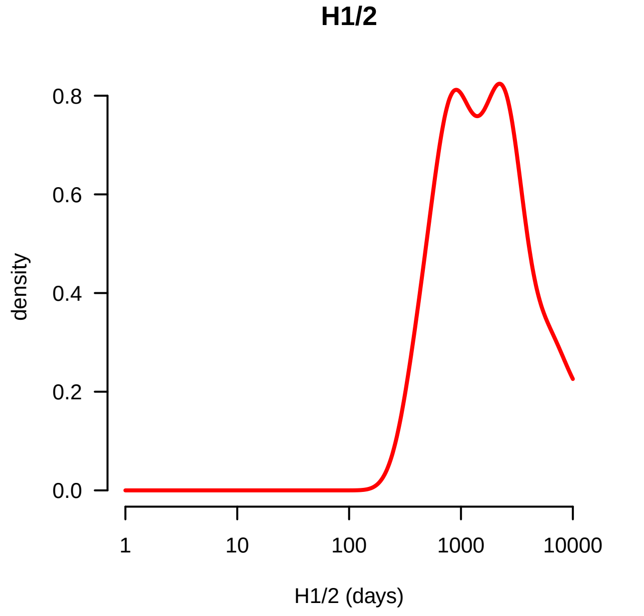

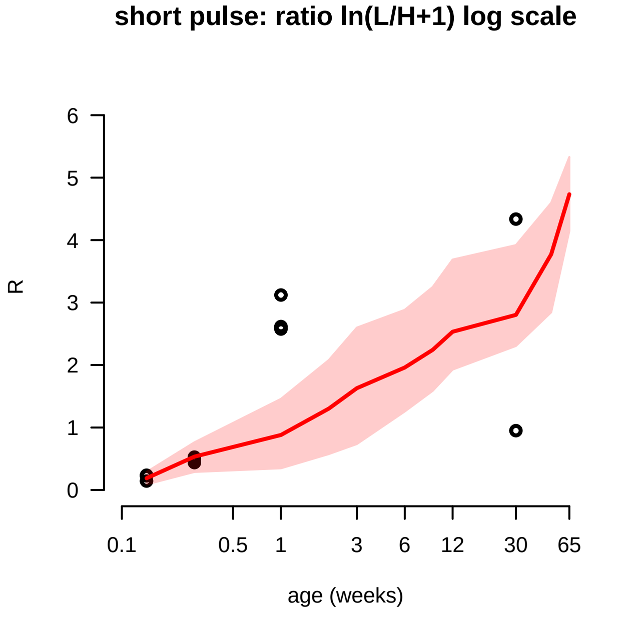

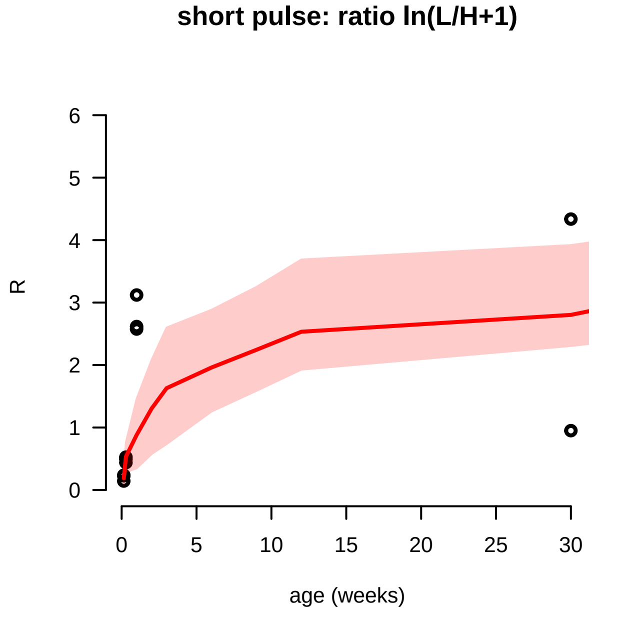

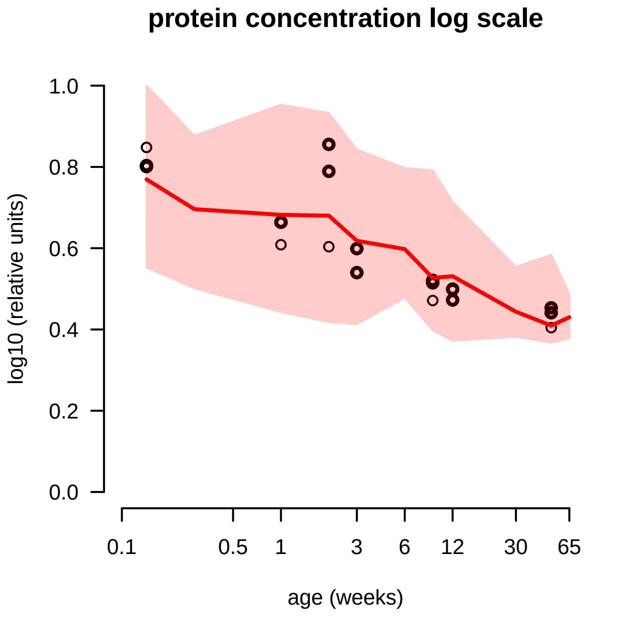

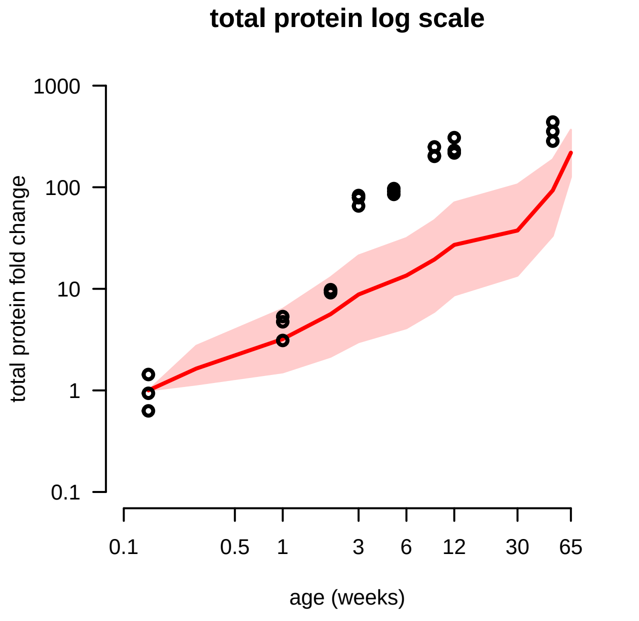

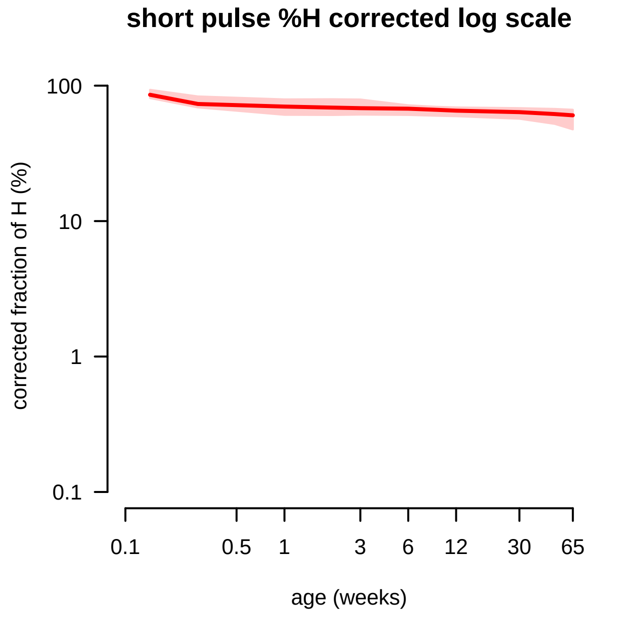

F8VPU2

>sp|F8VPU2|FARP1_MOUSE

FERM, ARHGEF and pleckstrin domain−containing protein 1

GN=Farp1

Med H1/2: 5.2 days

Q5 H1/2: 2.9 days

Q95 H1/2: 15.8 days

reliable model fit: yes

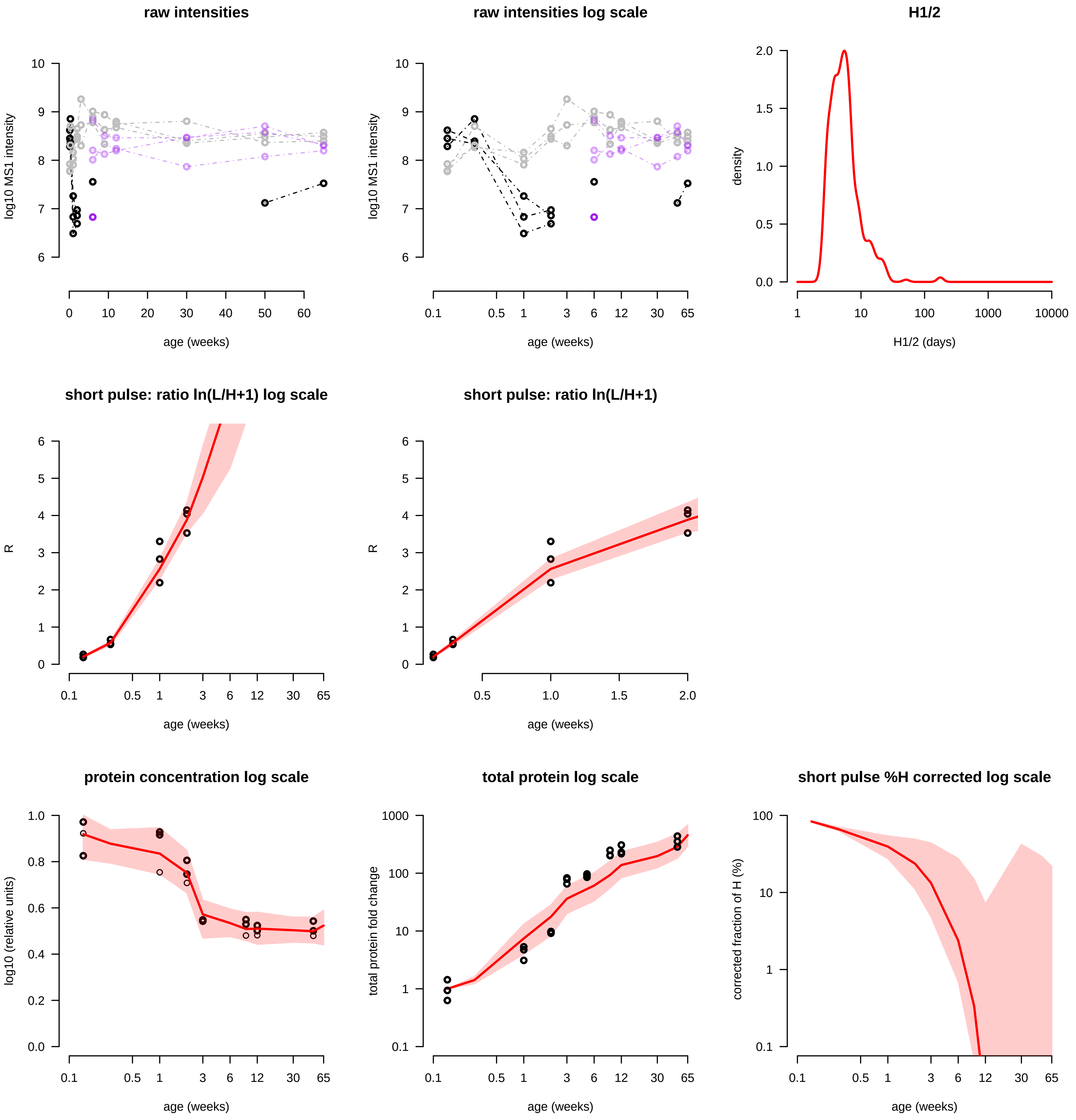

Q99LC8

>sp|Q99LC8|EI2BA_MOUSE

Translation initiation factor eIF−2B subunit alpha

GN=Eif2b1

Med H1/2: 6.2 days

Q5 H1/2: 2.8 days

Q95 H1/2: 23.1 days

reliable model fit: yes

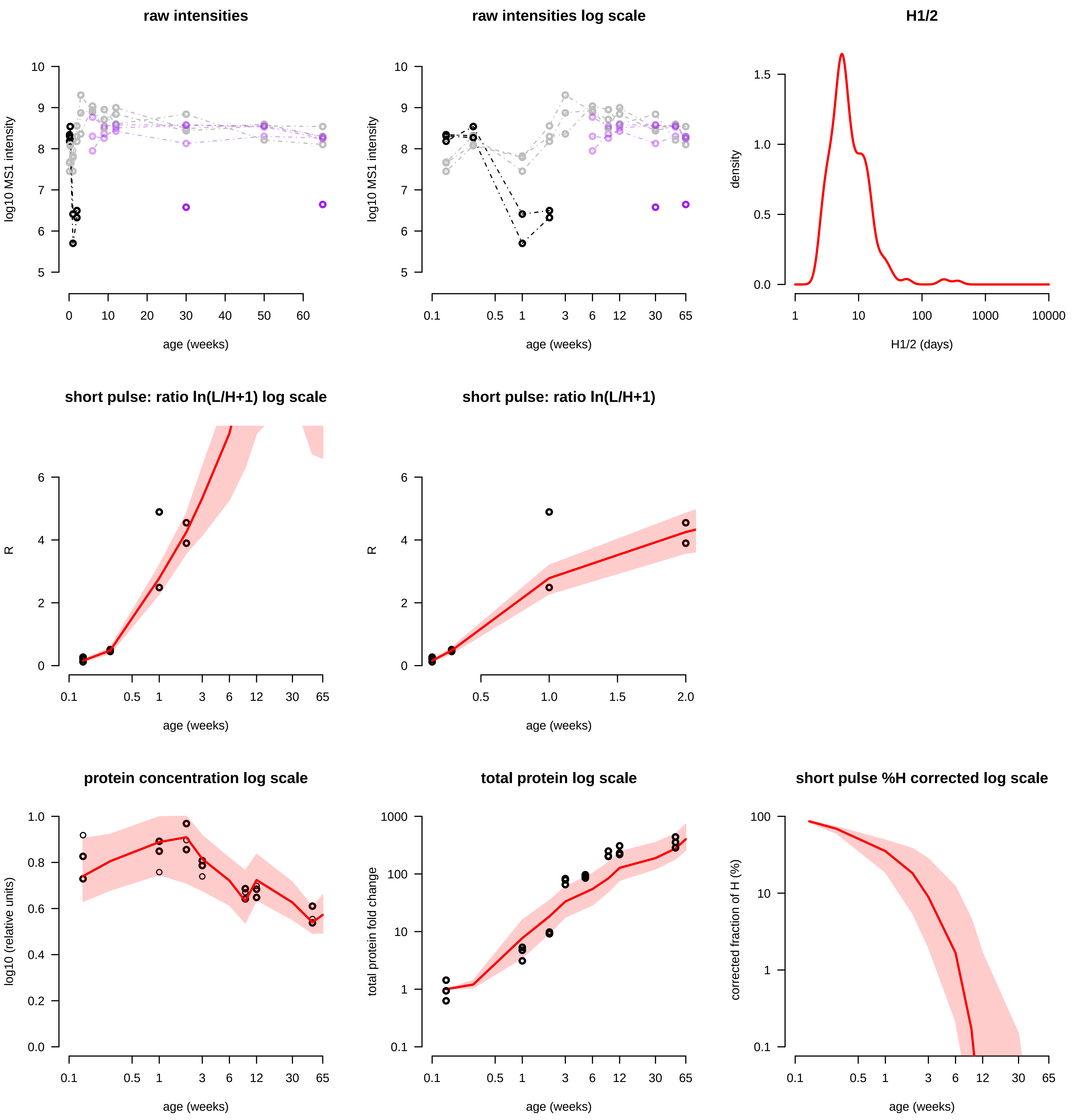

Q5XPI3;Q5XPI3−2

>sp|Q5XPI3|RN123_MOUSE

E3 ubiquitin−protein ligase RNF123

GN=Rnf123

Med H1/2: 23 days

Q5 H1/2: 12.4 days

Q95 H1/2: 192.9 days

reliable model fit: no

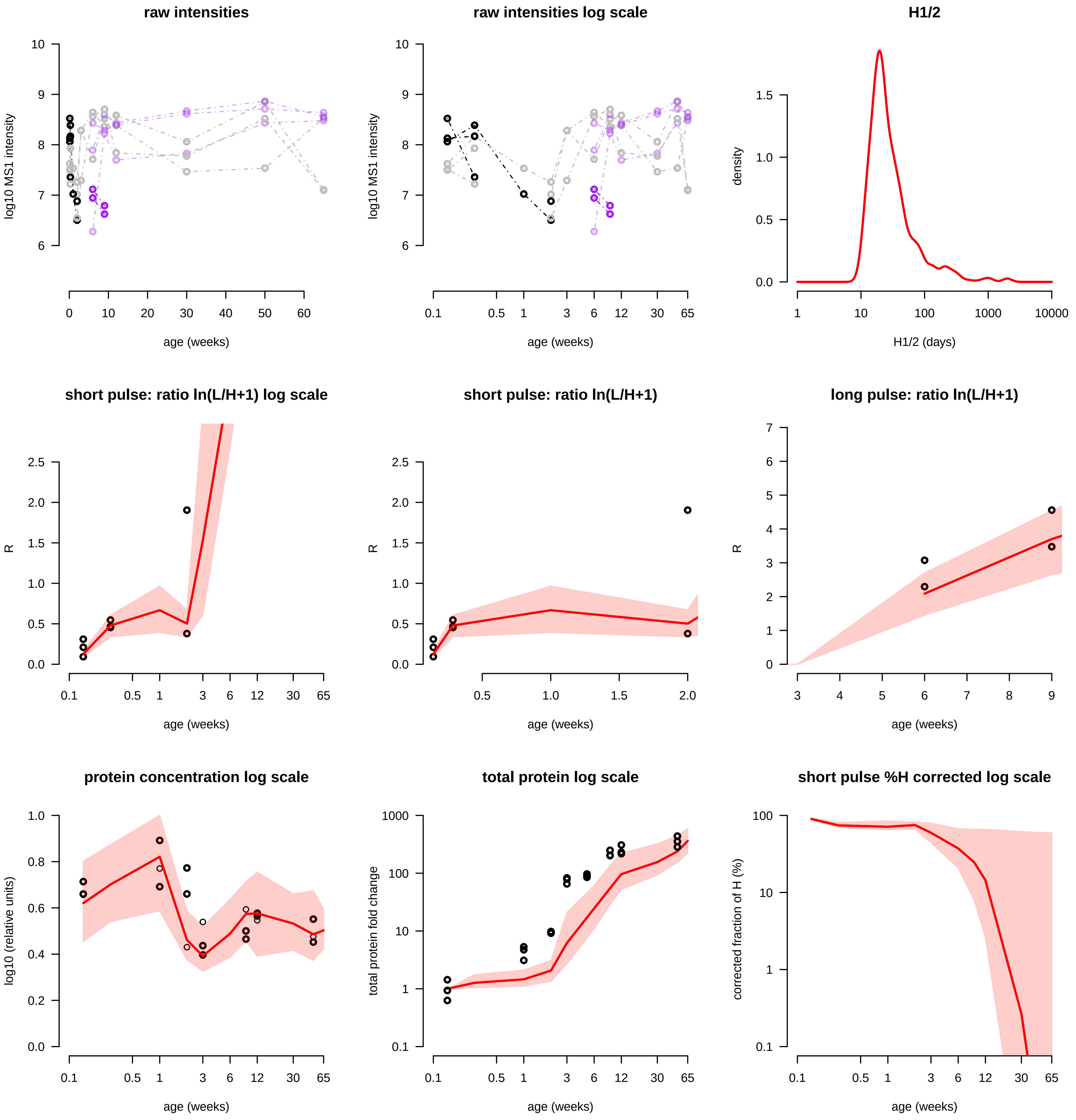

Q9R0Q9

>sp|Q9R0Q9|MPU1_MOUSE

Mannose−P−dolichol utilization defect 1 protein

GN=Mpdu1

Med H1/2: 9.9 days

Q5 H1/2: 7.7 days

Q95 H1/2: 13.2 days

reliable model fit: yes

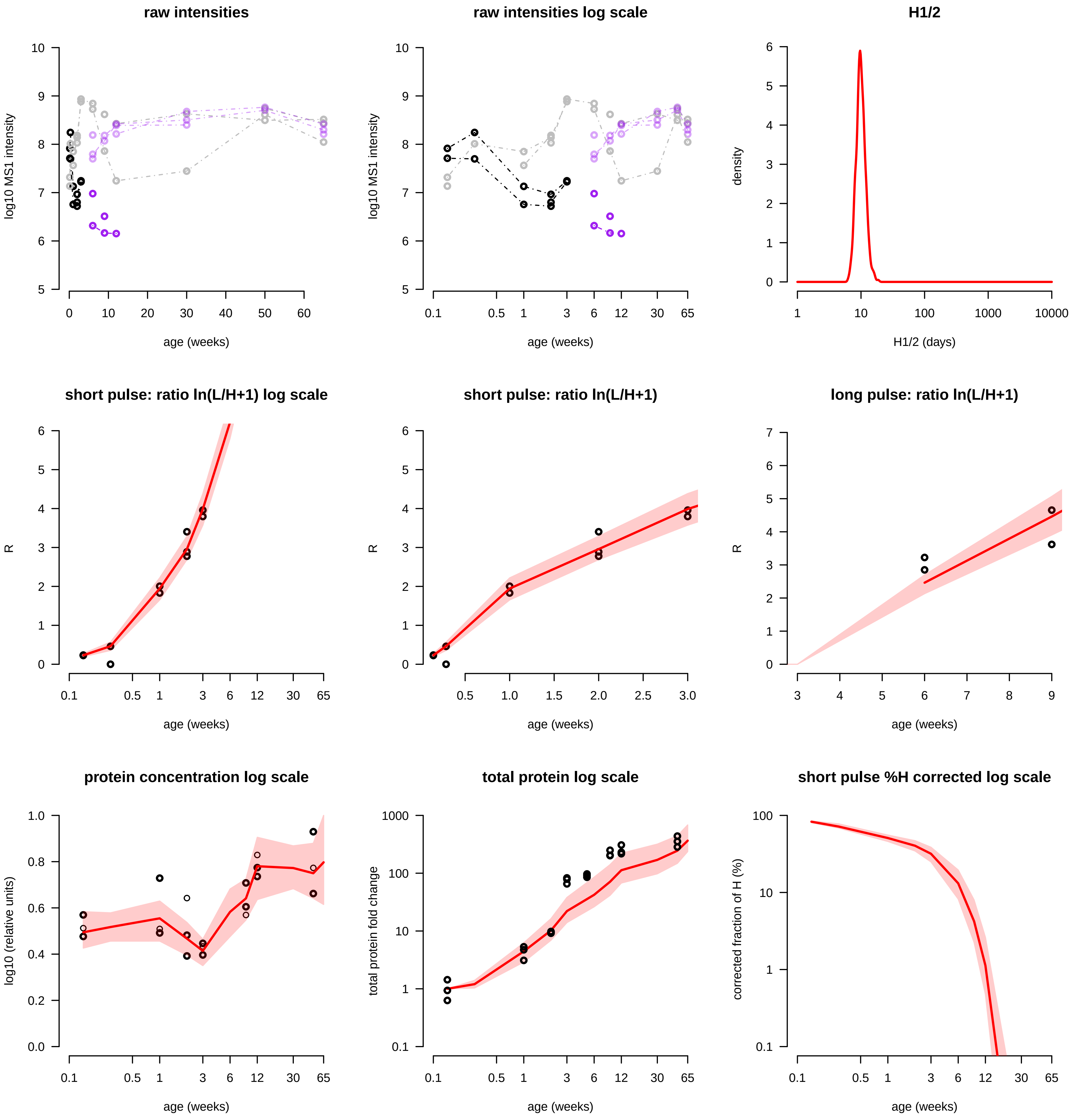

P41241

>sp|P41241|CSK_MOUSE

Tyrosine−protein kinase CSK

GN=Csk

Med H1/2: 4 days

Q5 H1/2: 2.5 days

Q95 H1/2: 21.5 days

reliable model fit: yes

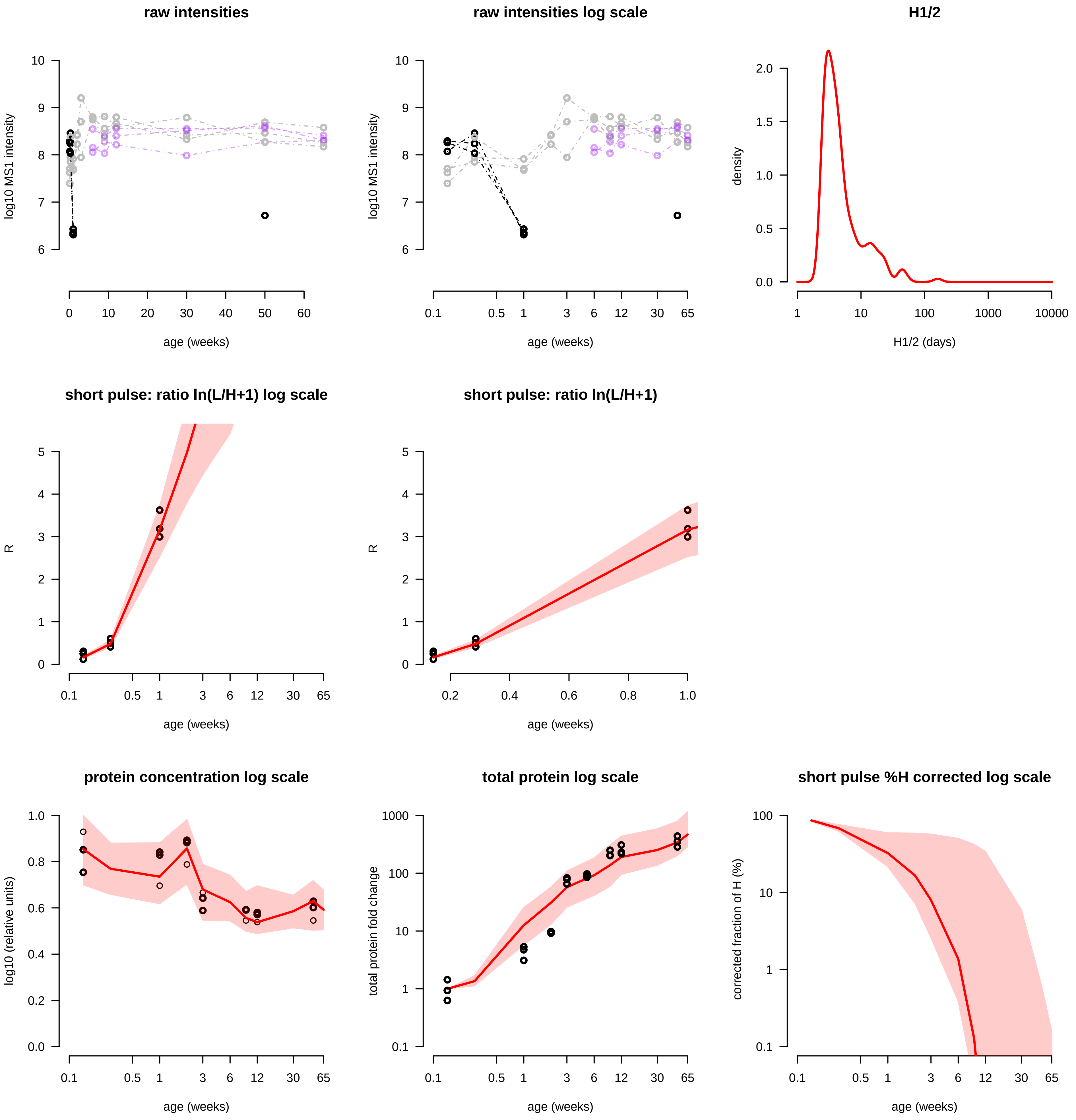

Q8CGZ0

>sp|Q8CGZ0|CHERP_MOUSE

Calcium homeostasis endoplasmic reticulum protein

GN=Cherp

Med H1/2: 4.7 days

Q5 H1/2: 2.6 days

Q95 H1/2: 54 days

reliable model fit: no

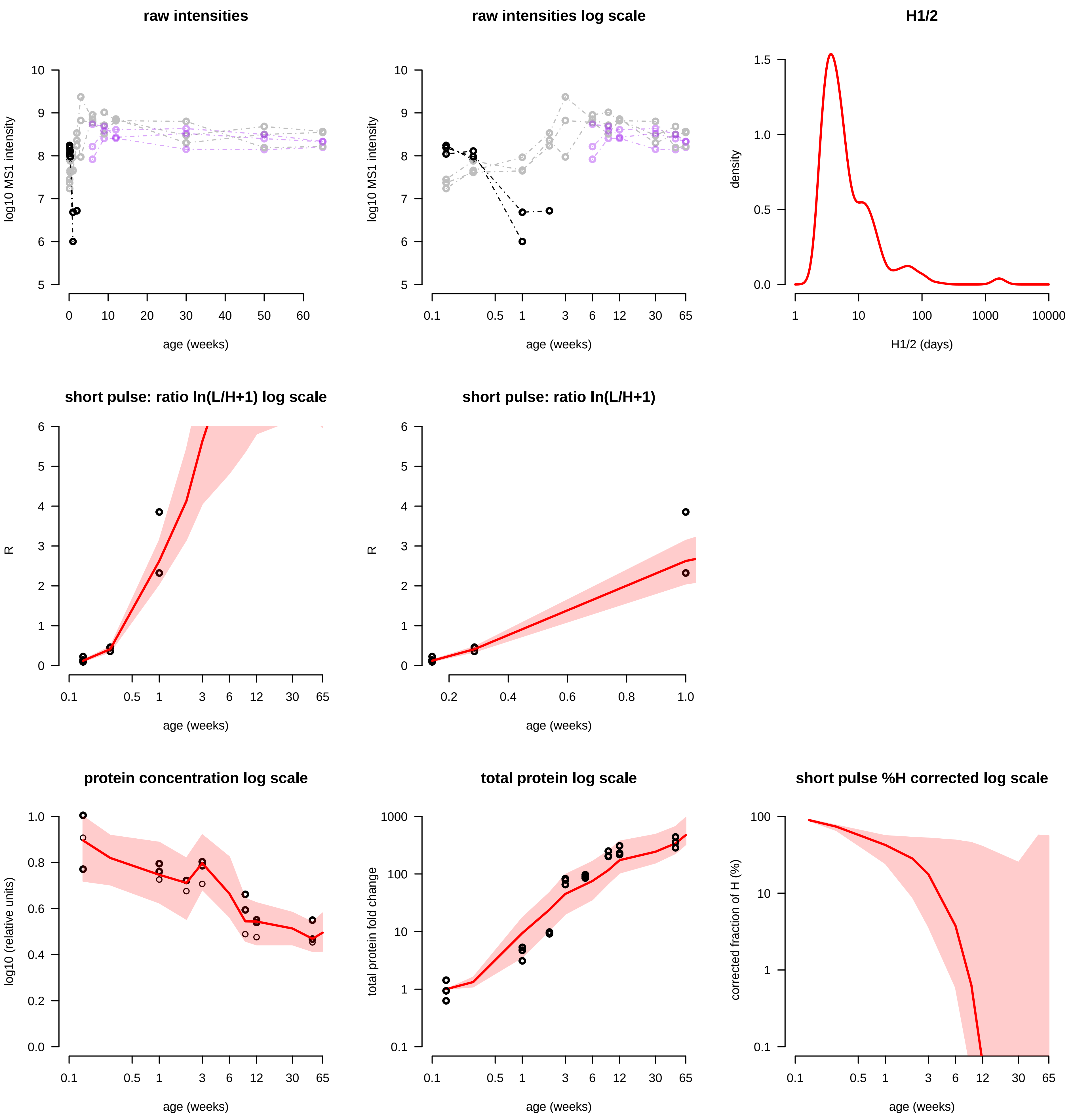

Q8CEC6

>sp|Q8CEC6|PPWD1_MOUSE

Peptidylprolyl isomerase domain and WD repeat−containing protein 1

GN=Ppwd1

Med H1/2: 12.5 days

Q5 H1/2: 5.8 days

Q95 H1/2: 83.1 days

reliable model fit: yes

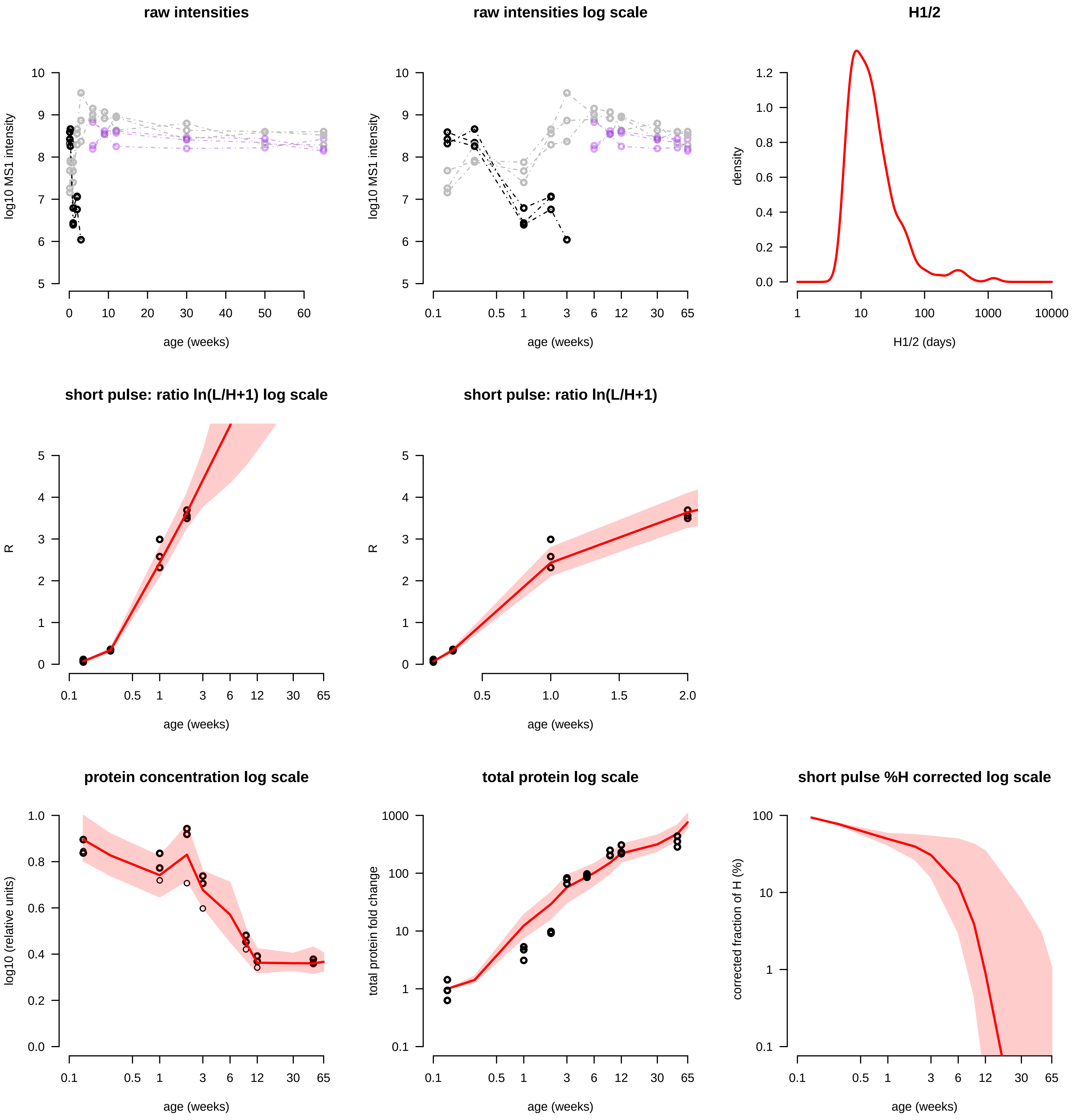

Q80XI4

>sp|Q80XI4|PI42B_MOUSE

Phosphatidylinositol 5−phosphate 4−kinase type−2 beta

GN=Pip4k2b

Med H1/2: 6.5 days

Q5 H1/2: 3 days

Q95 H1/2: 90.9 days

reliable model fit: no

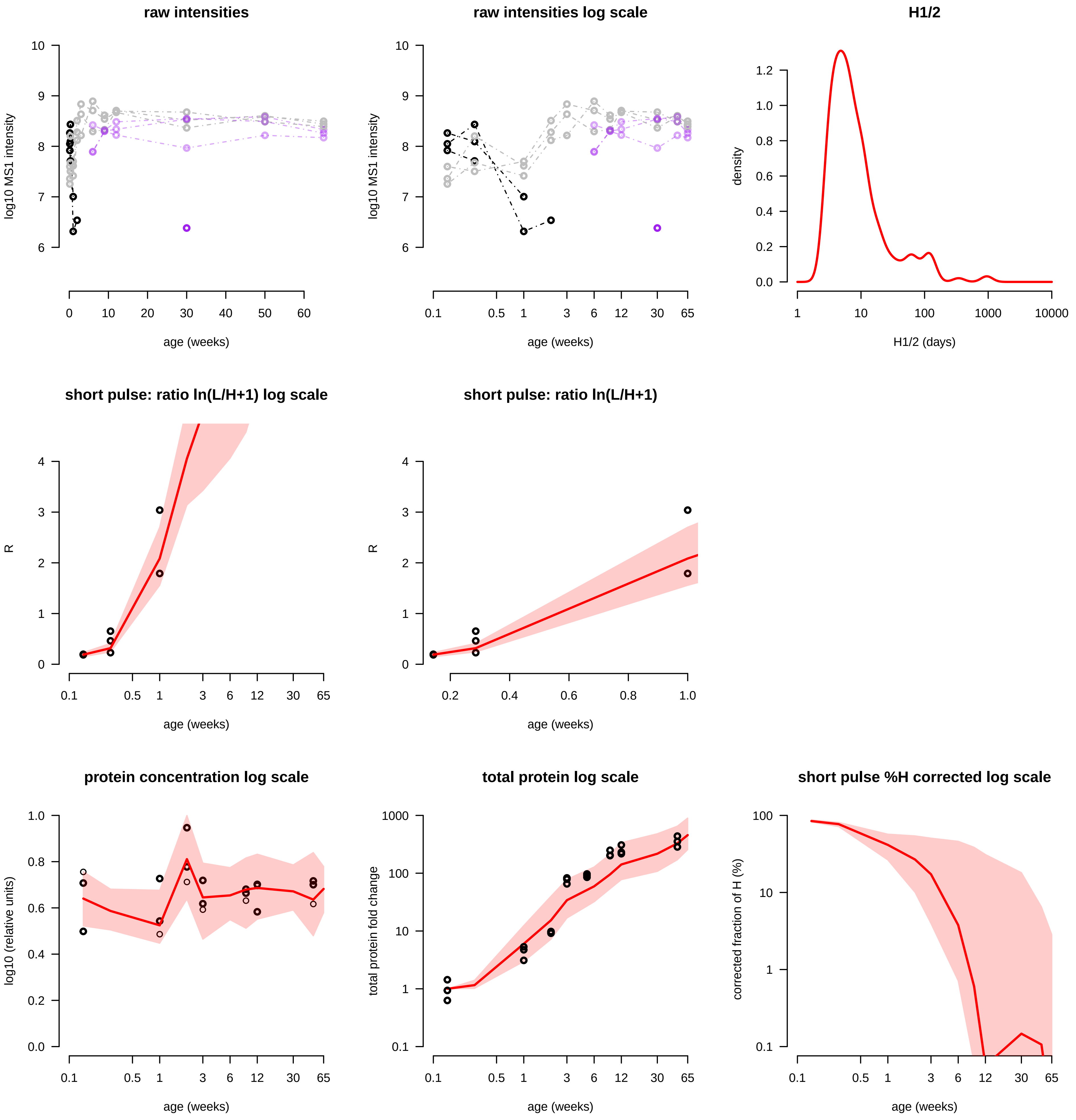

Q9EQQ9−3;Q9EQQ9

>sp|Q9EQQ9−3|OGA_MOUSE

Isoform 3 of Protein O−GlcNAcase

GN=Oga

Med H1/2: 22.3 days

Q5 H1/2: 7.5 days

Q95 H1/2: 272.3 days

reliable model fit: yes

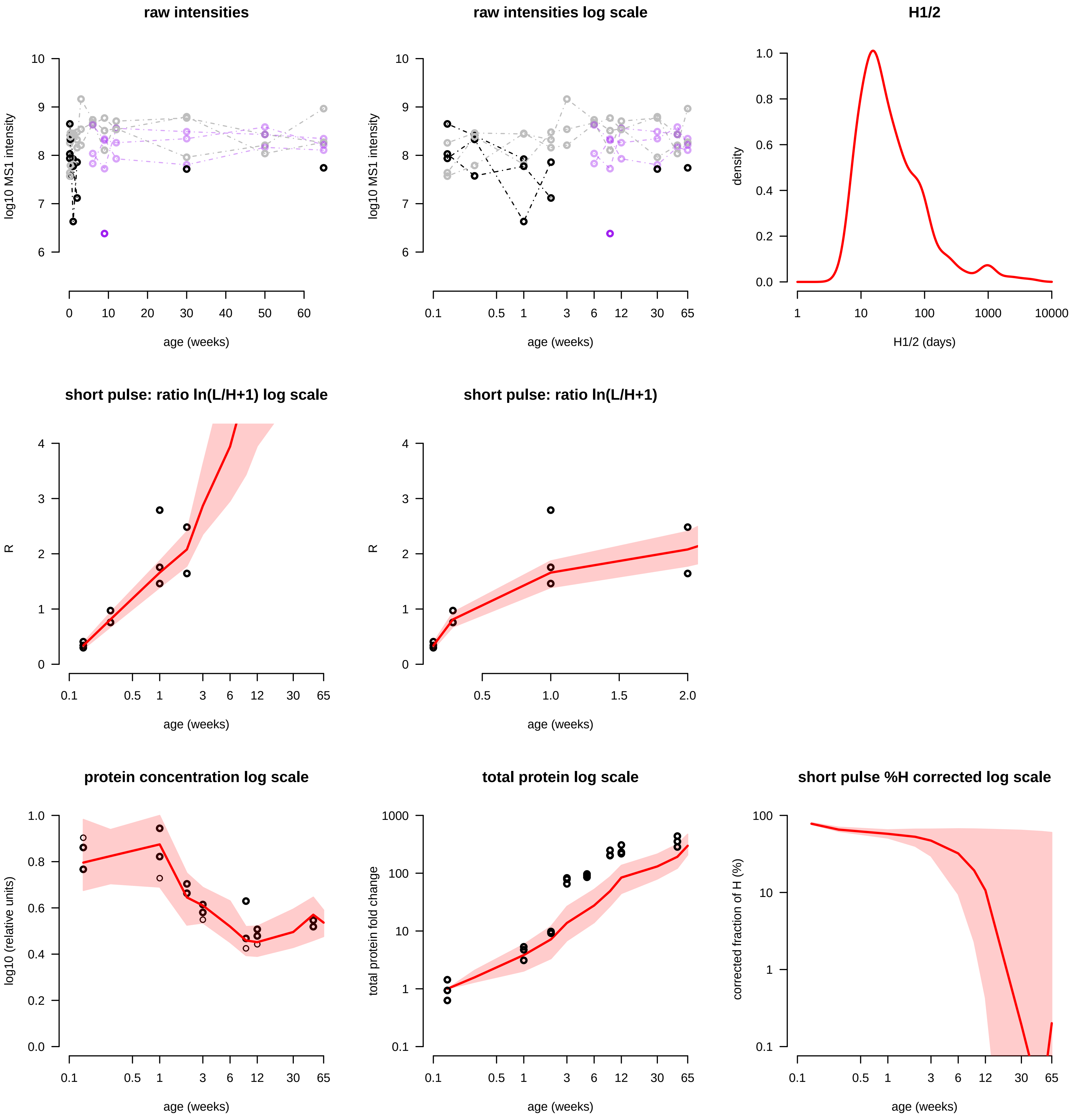

Q61733

>sp|Q61733|RT31_MOUSE

28S ribosomal protein S31, mitochondrial

GN=Mrps31

Med H1/2: 10.1 days

Q5 H1/2: 3.7 days

Q95 H1/2: 146.3 days

reliable model fit: no

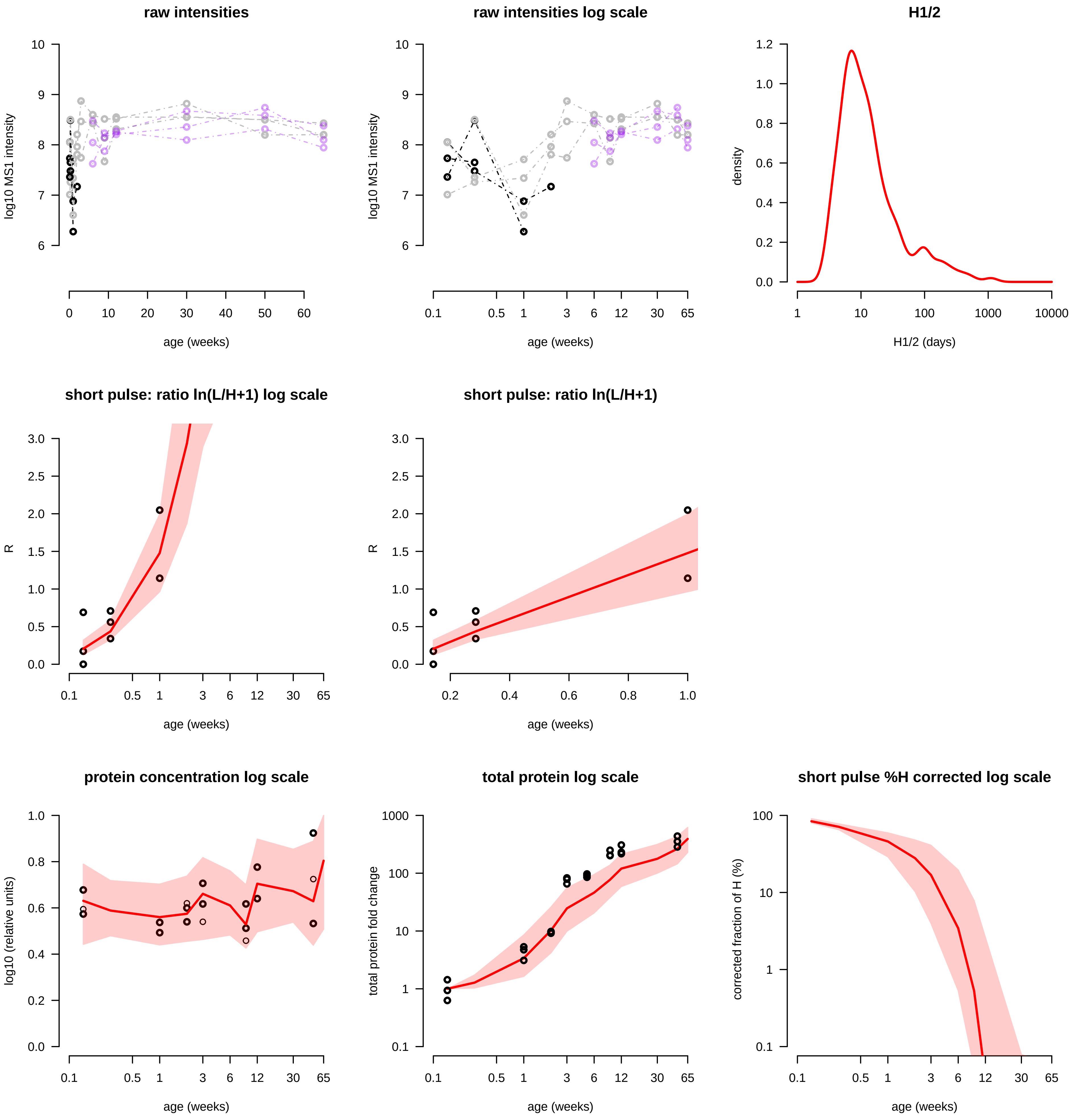

Q8K157

>sp|Q8K157|GALM_MOUSE

Galactose mutarotase

GN=Galm

Med H1/2: 20.6 days

Q5 H1/2: 5.7 days

Q95 H1/2: 201.3 days

reliable model fit: yes

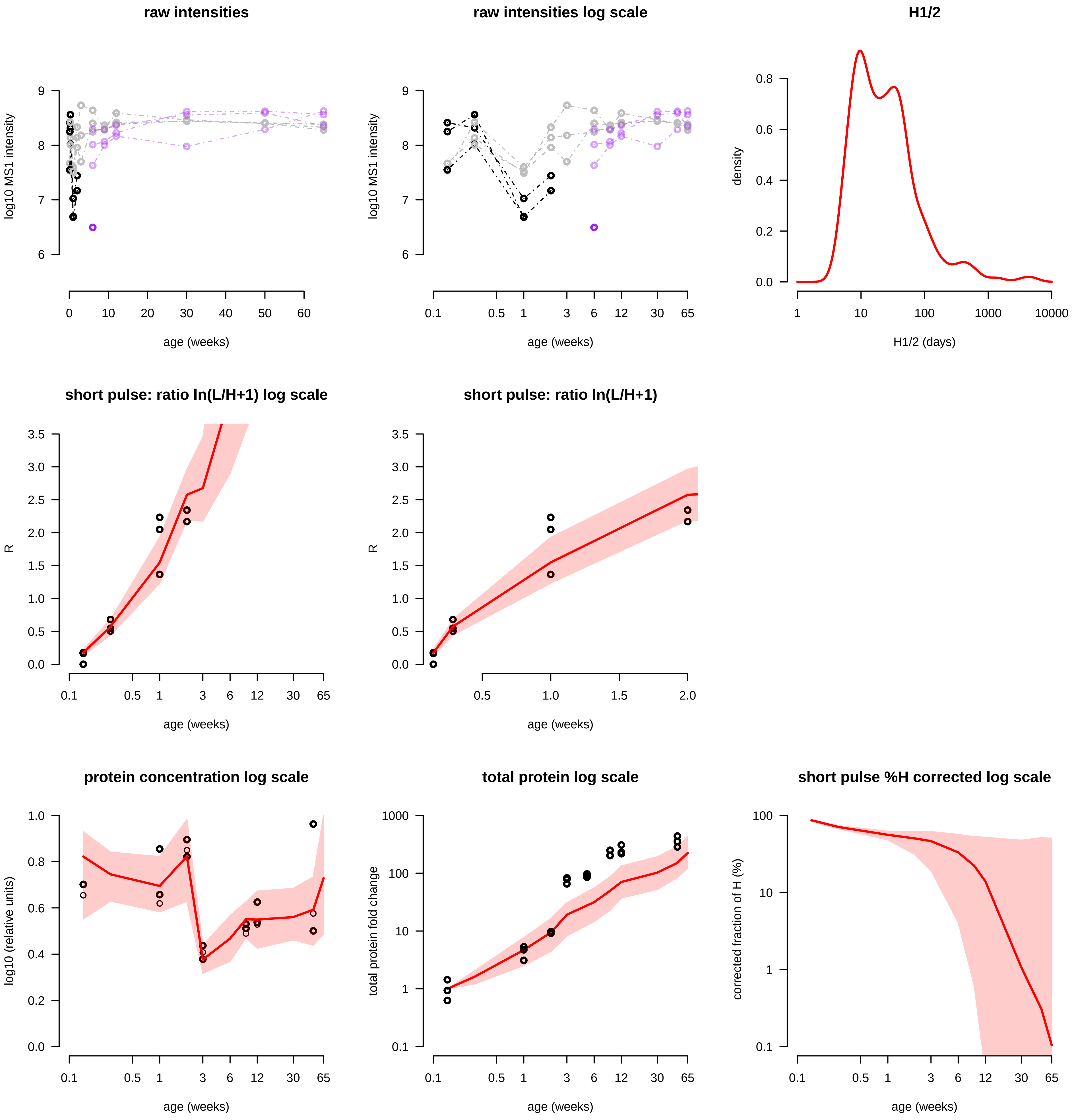

Q8BZQ7

>sp|Q8BZQ7|ANC2_MOUSE

Anaphase−promoting complex subunit 2

GN=Anapc2

Med H1/2: 5.8 days

Q5 H1/2: 2.9 days

Q95 H1/2: 25.6 days

reliable model fit: yes

raw intensities

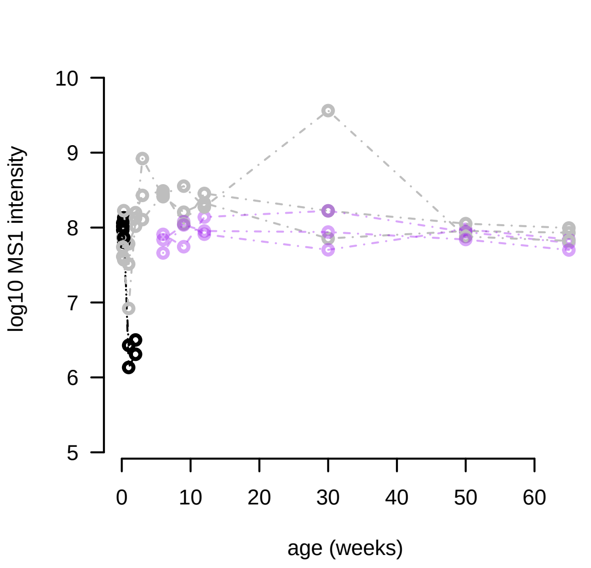

raw intensities log scale

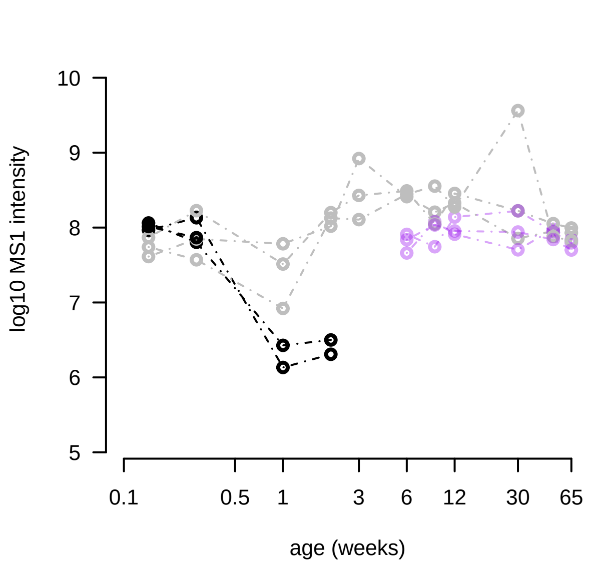

H1/2

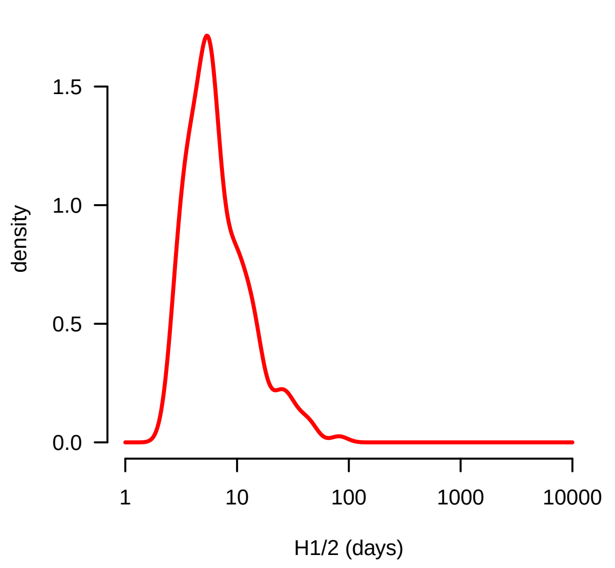

short pulse: ratio ln(L/H+1) log scale

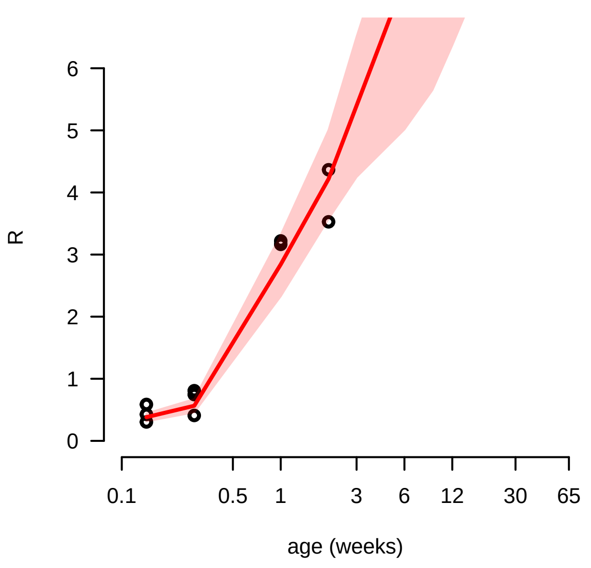

short pulse: ratio ln(L/H+1)

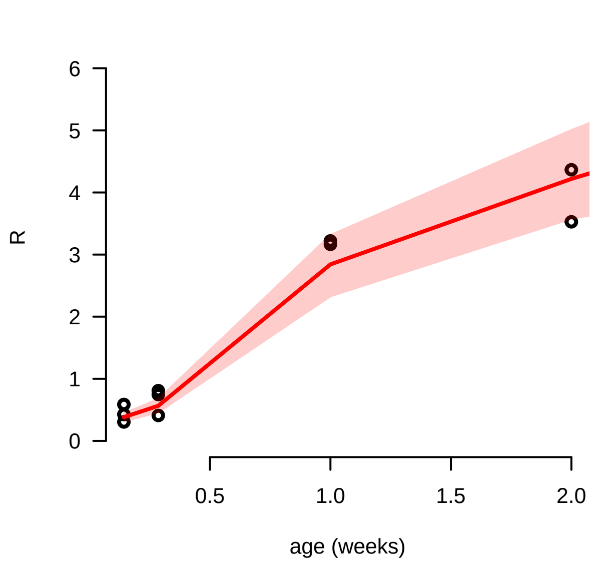

protein concentration log scale

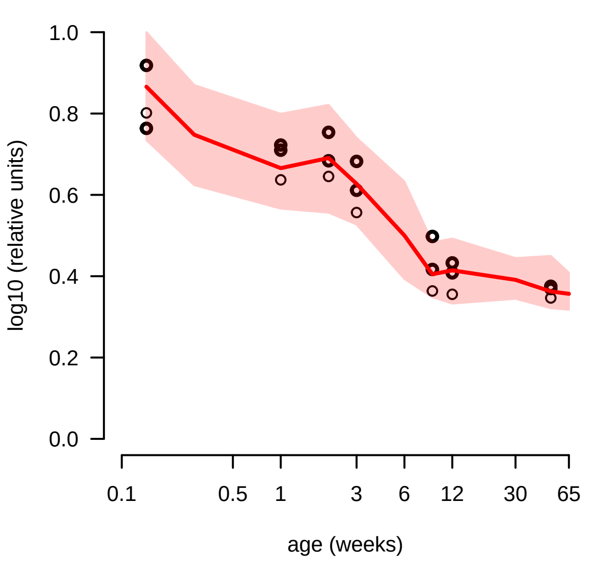

total protein log scale

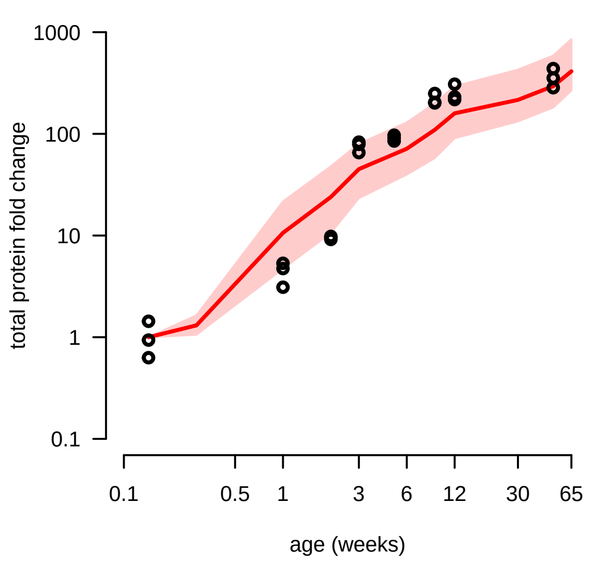

short pulse %H corrected log scale

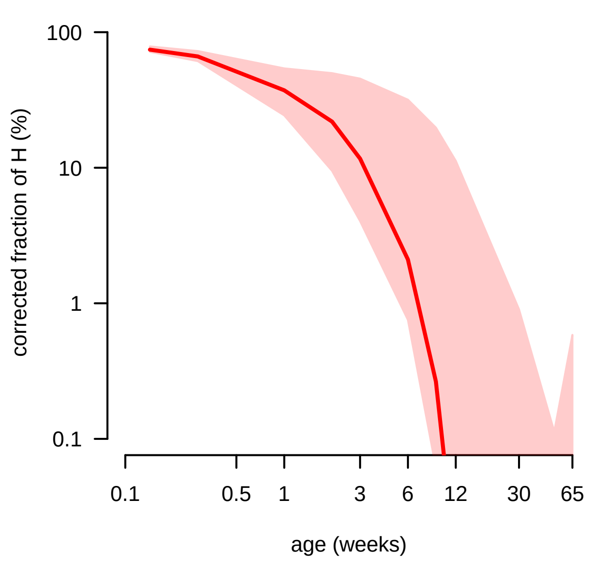

P52795

>sp|P52795|EFNB1_MOUSE

Ephrin−B1

GN=Efnb1

Med H1/2: 6.3 days

Q5 H1/2: 2.7 days

Q95 H1/2: 62.1 days

reliable model fit: yes

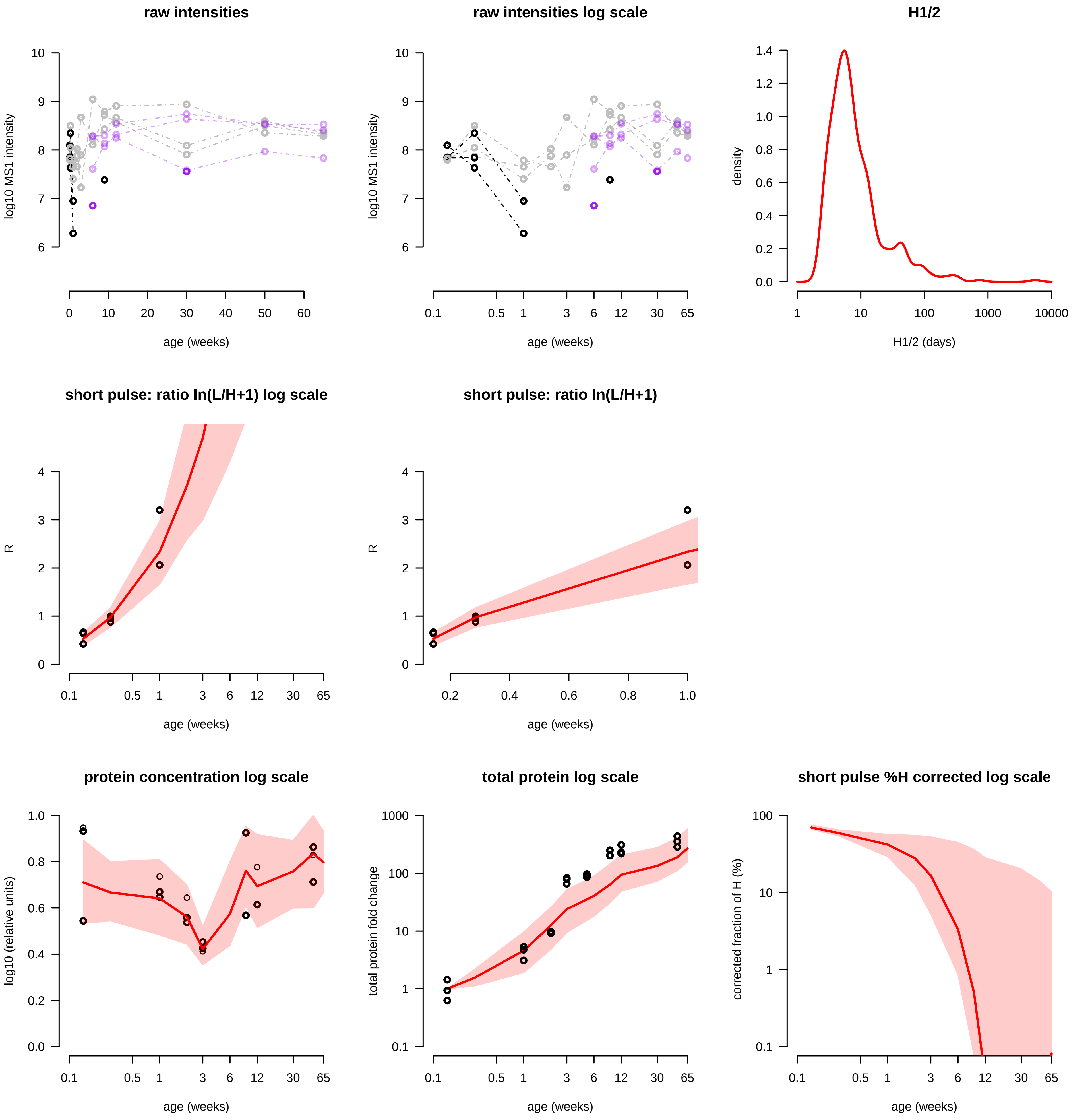

Q60770;Q60770−2

>sp|Q60770|STXB3_MOUSE

Syntaxin−binding protein 3

GN=Stxbp3

Med H1/2: 7.3 days

Q5 H1/2: 2.6 days

Q95 H1/2: 58.3 days

reliable model fit: no

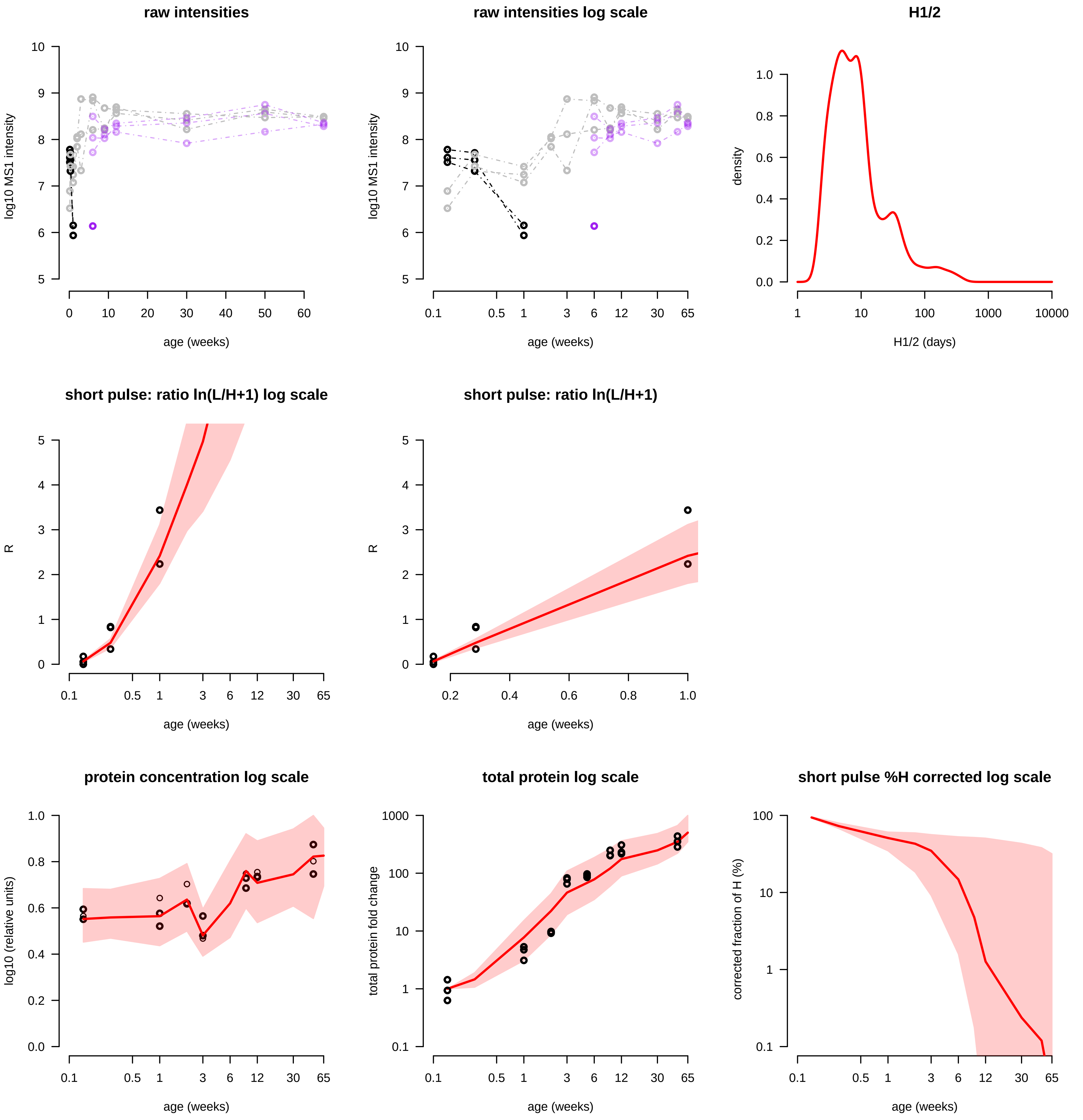

O55128

>sp|O55128|SAP18_MOUSE

Histone deacetylase complex subunit SAP18

GN=Sap18

Med H1/2: 9 days

Q5 H1/2: 3.5 days

Q95 H1/2: 217.3 days

reliable model fit: yes

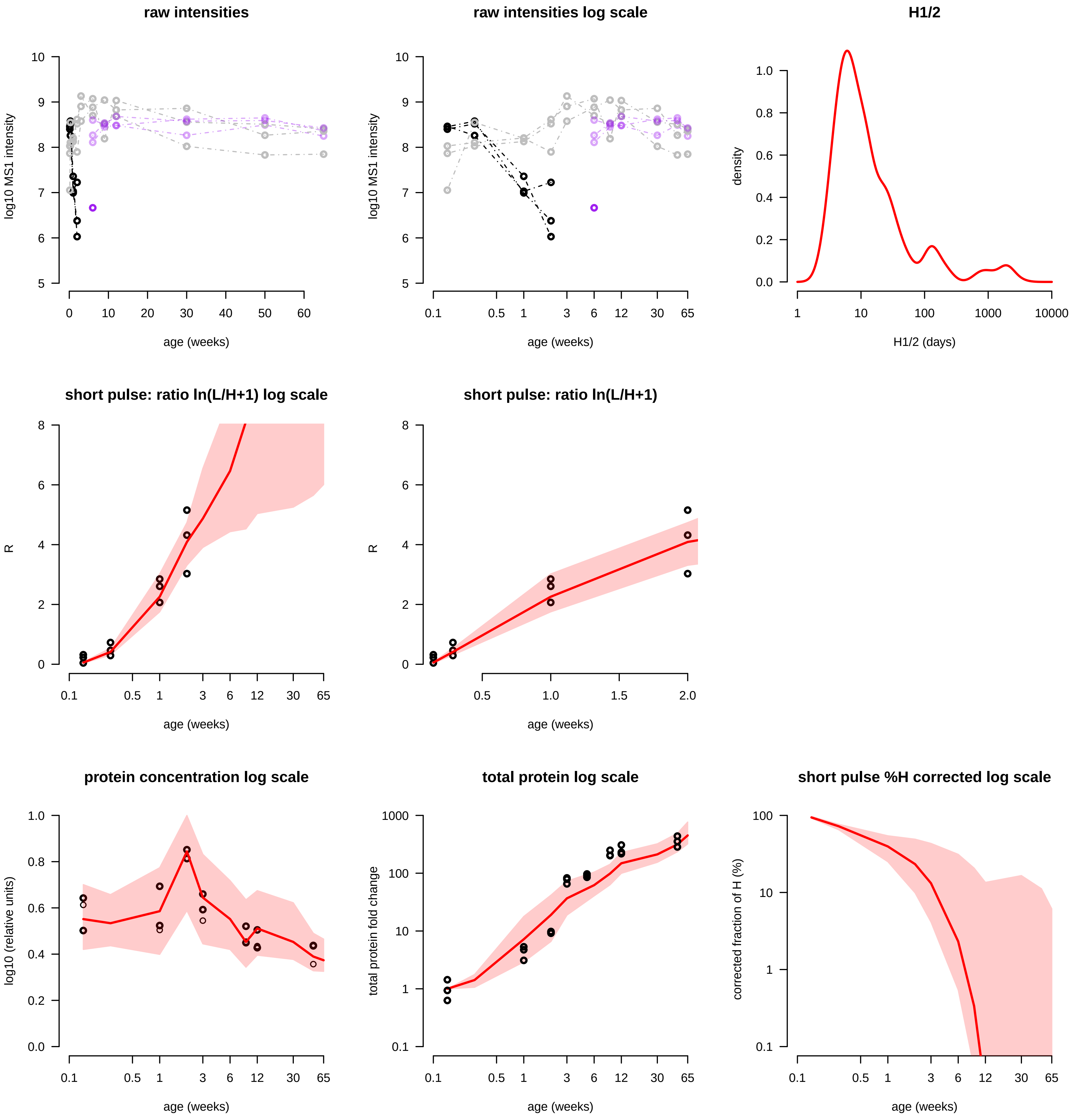

Q8BXQ2

>sp|Q8BXQ2|PIGT_MOUSE

GPI transamidase component PIG−T

GN=Pigt

Med H1/2: 5.2 days

Q5 H1/2: 2.8 days

Q95 H1/2: 47 days

reliable model fit: yes

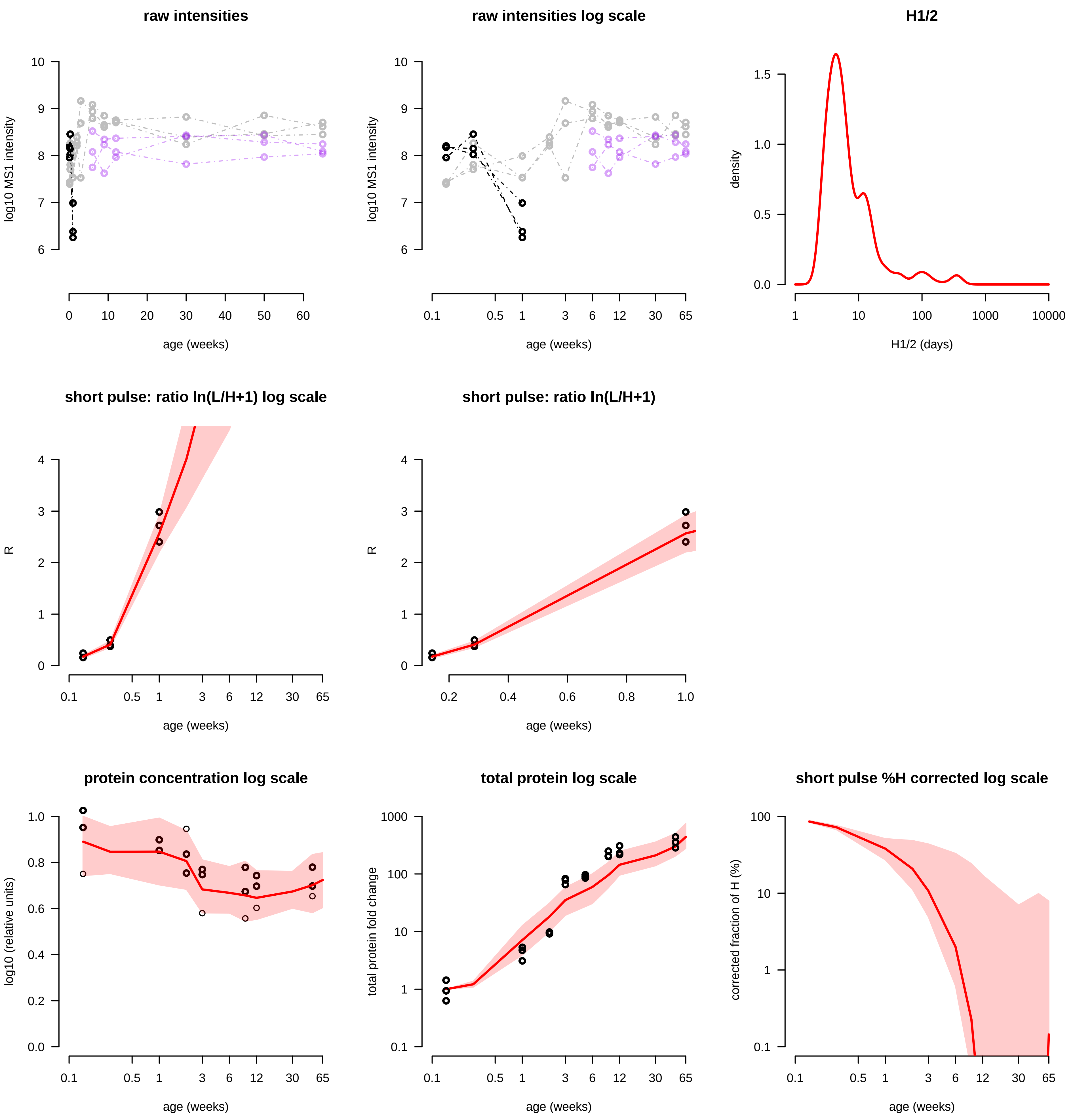

Q9DBE0

>sp|Q9DBE0|CSAD_MOUSE

Cysteine sulfinic acid decarboxylase

GN=Csad

Med H1/2: 10 days

Q5 H1/2: 3.3 days

Q95 H1/2: 109.7 days

reliable model fit: yes

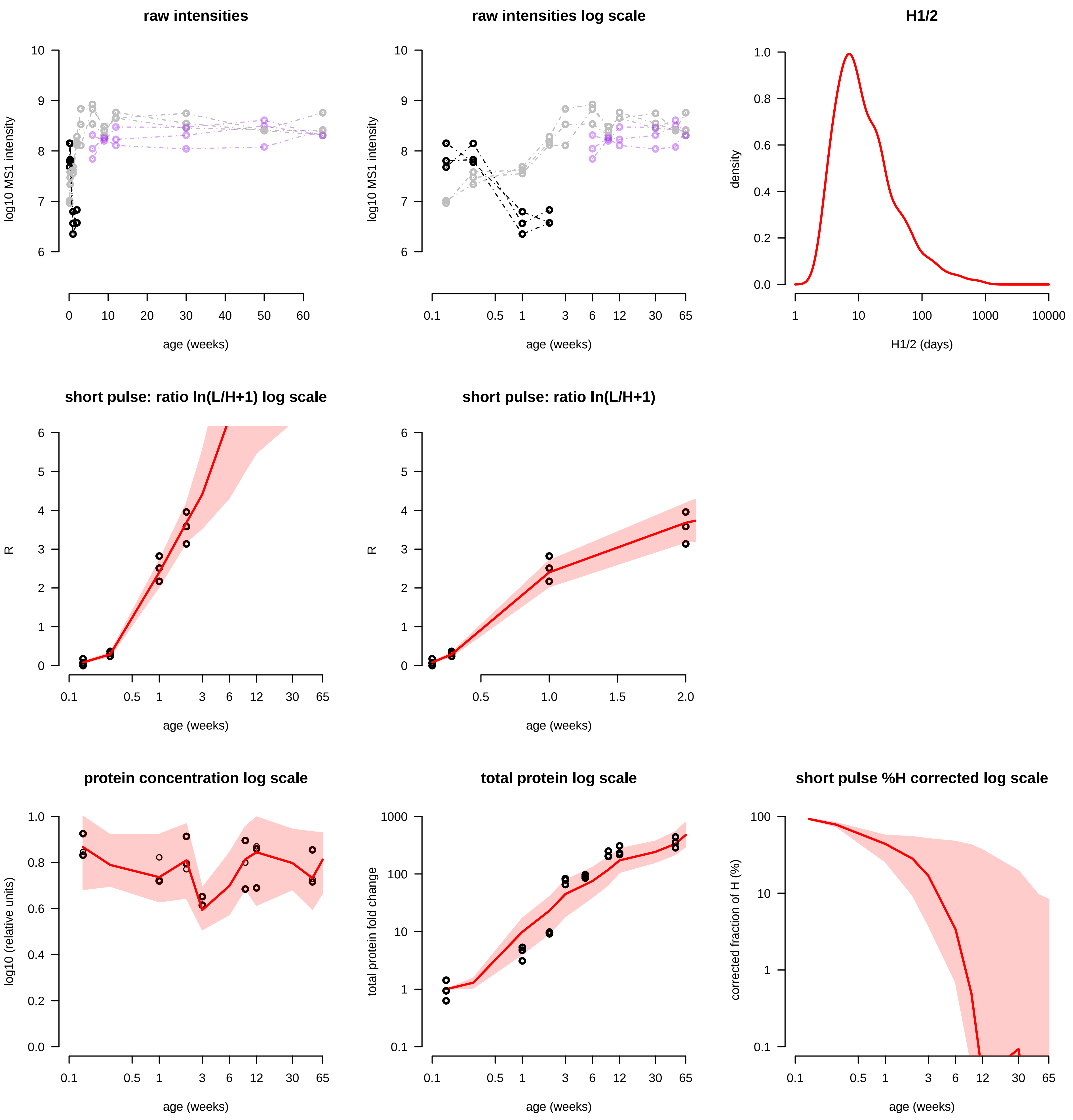

Q99PL6

>sp|Q99PL6|UBXN6_MOUSE

UBX domain−containing protein 6

GN=Ubxn6

Med H1/2: 5.3 days

Q5 H1/2: 2.7 days

Q95 H1/2: 47.5 days

reliable model fit: yes

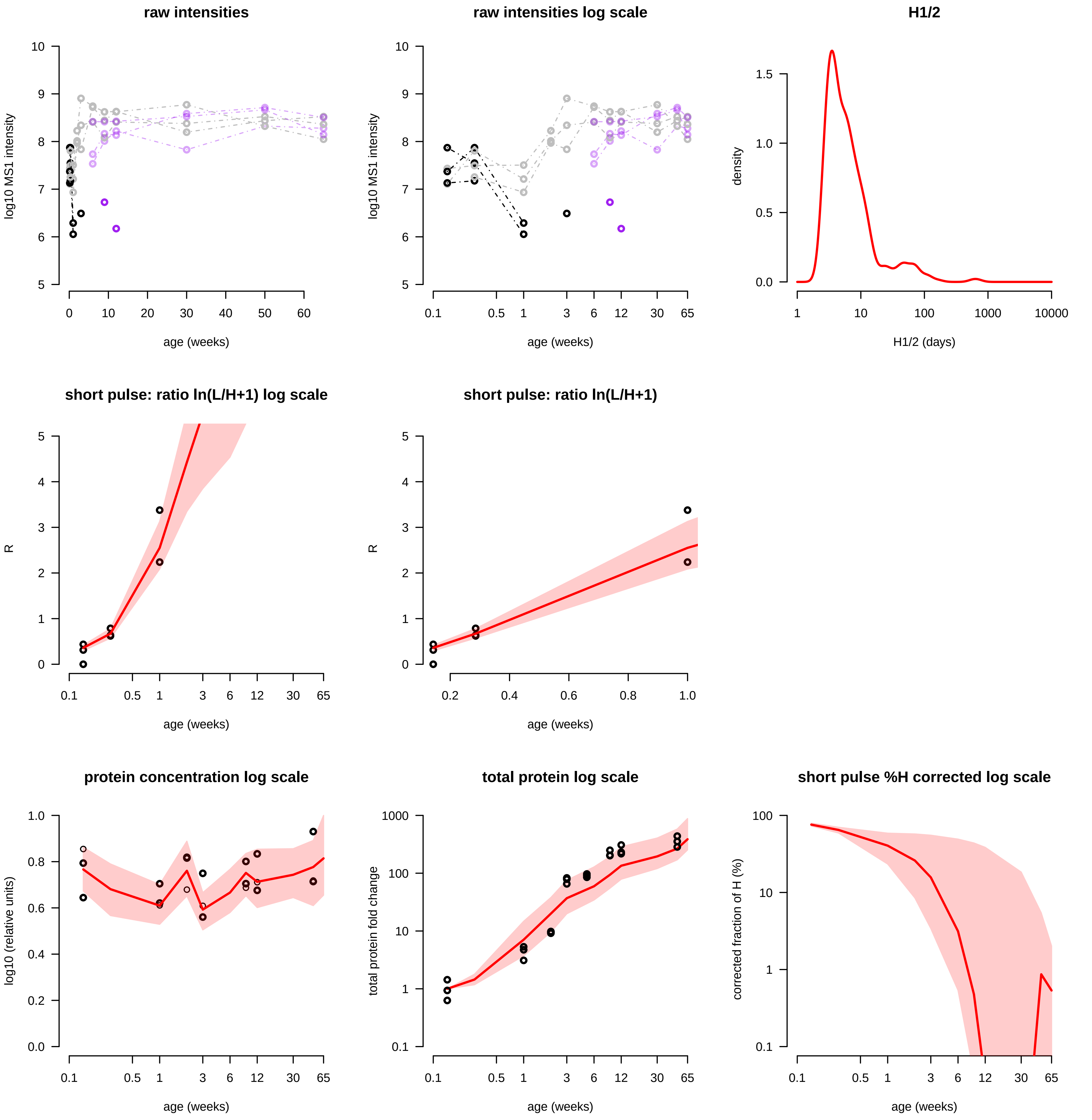

P12367

>sp|P12367|KAP2_MOUSE

cAMP−dependent protein kinase type II−alpha regulatory subunit

GN=Prkar2a

Med H1/2: 10.7 days

Q5 H1/2: 4 days

Q95 H1/2: 167.1 days

reliable model fit: yes

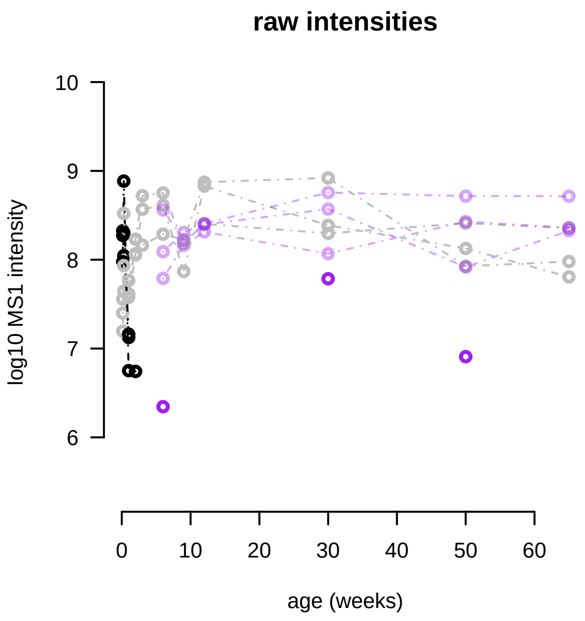

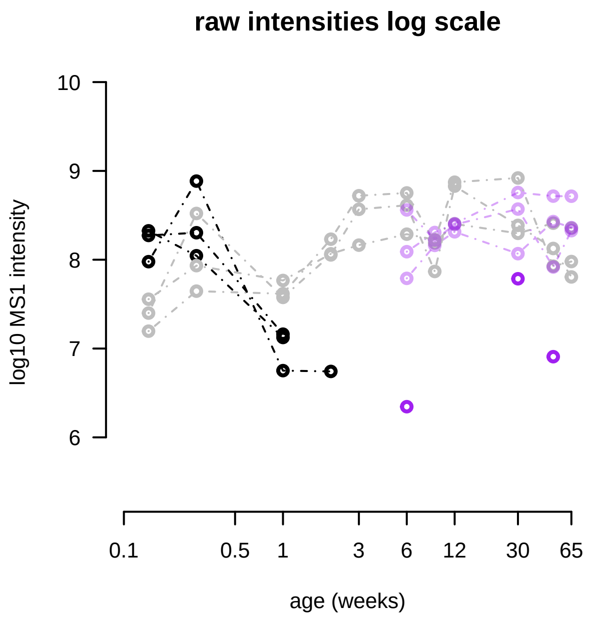

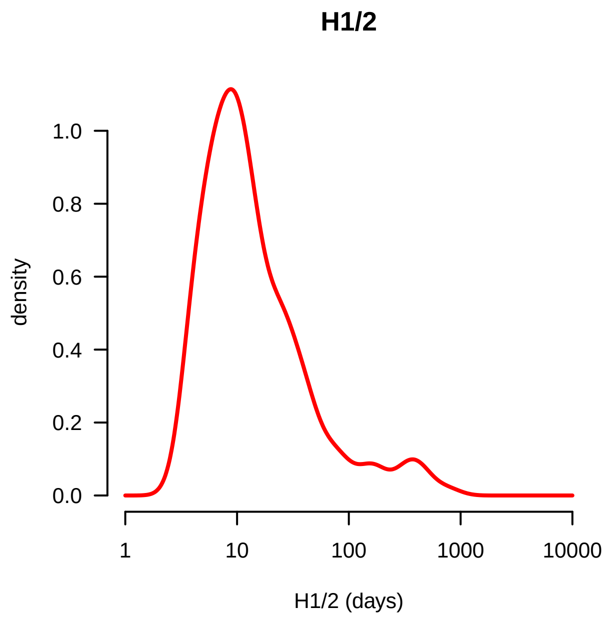

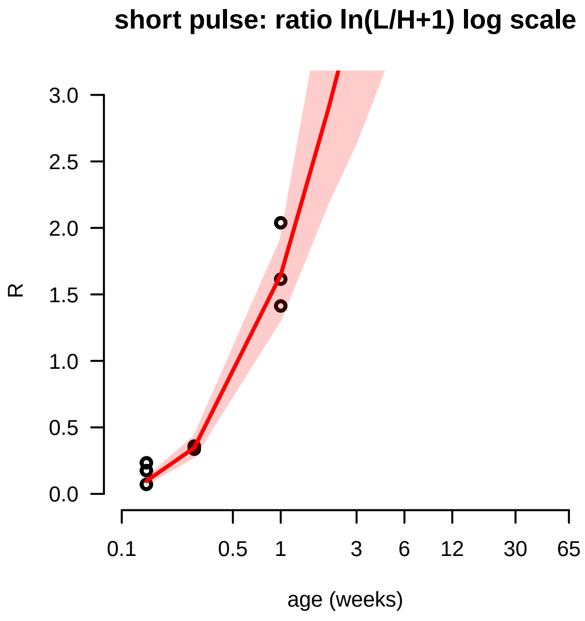

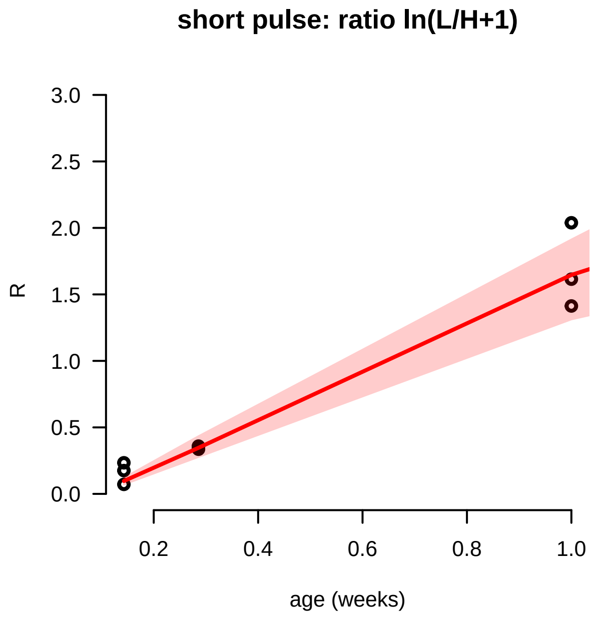

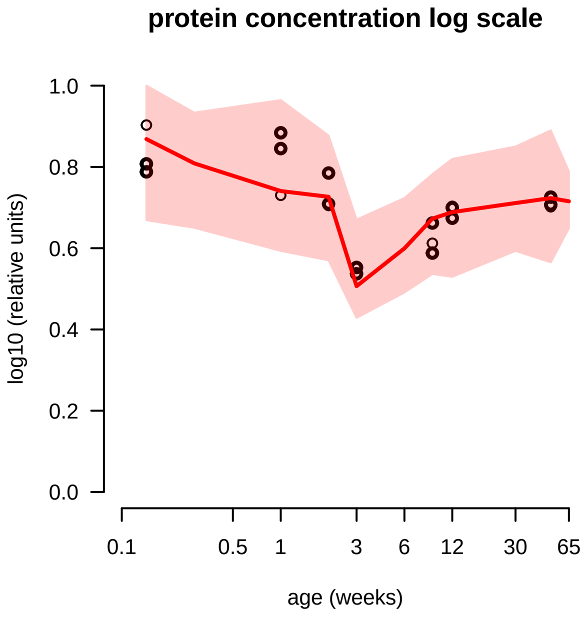

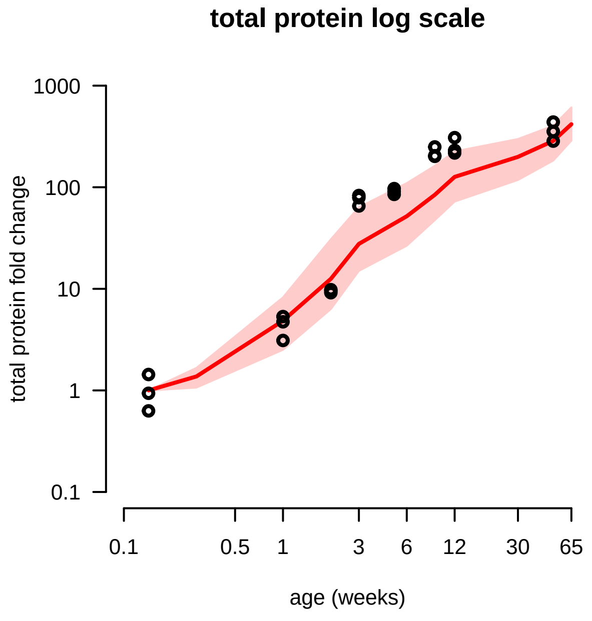

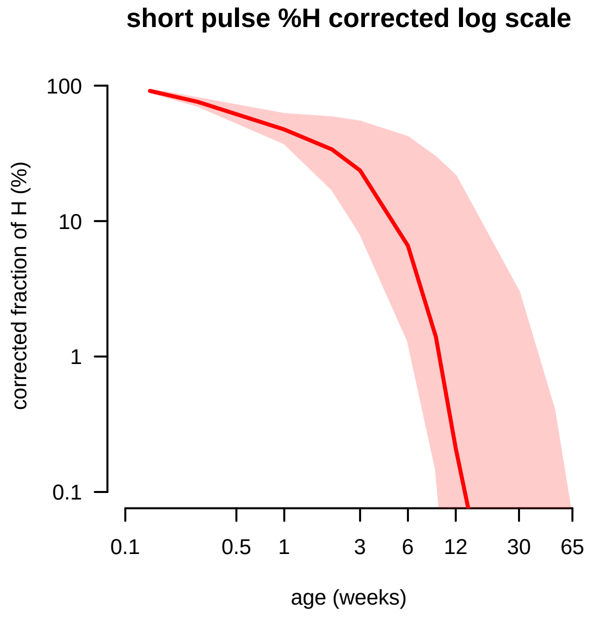

Q63810−2;Q63810

>sp|Q63810−2|CANB1_MOUSE

Isoform 2 of Calcineurin subunit B type 1

GN=Ppp3r1

Med H1/2: 274.6 days

Q5 H1/2: 95.5 days

Q95 H1/2: 2897.1 days

reliable model fit: no

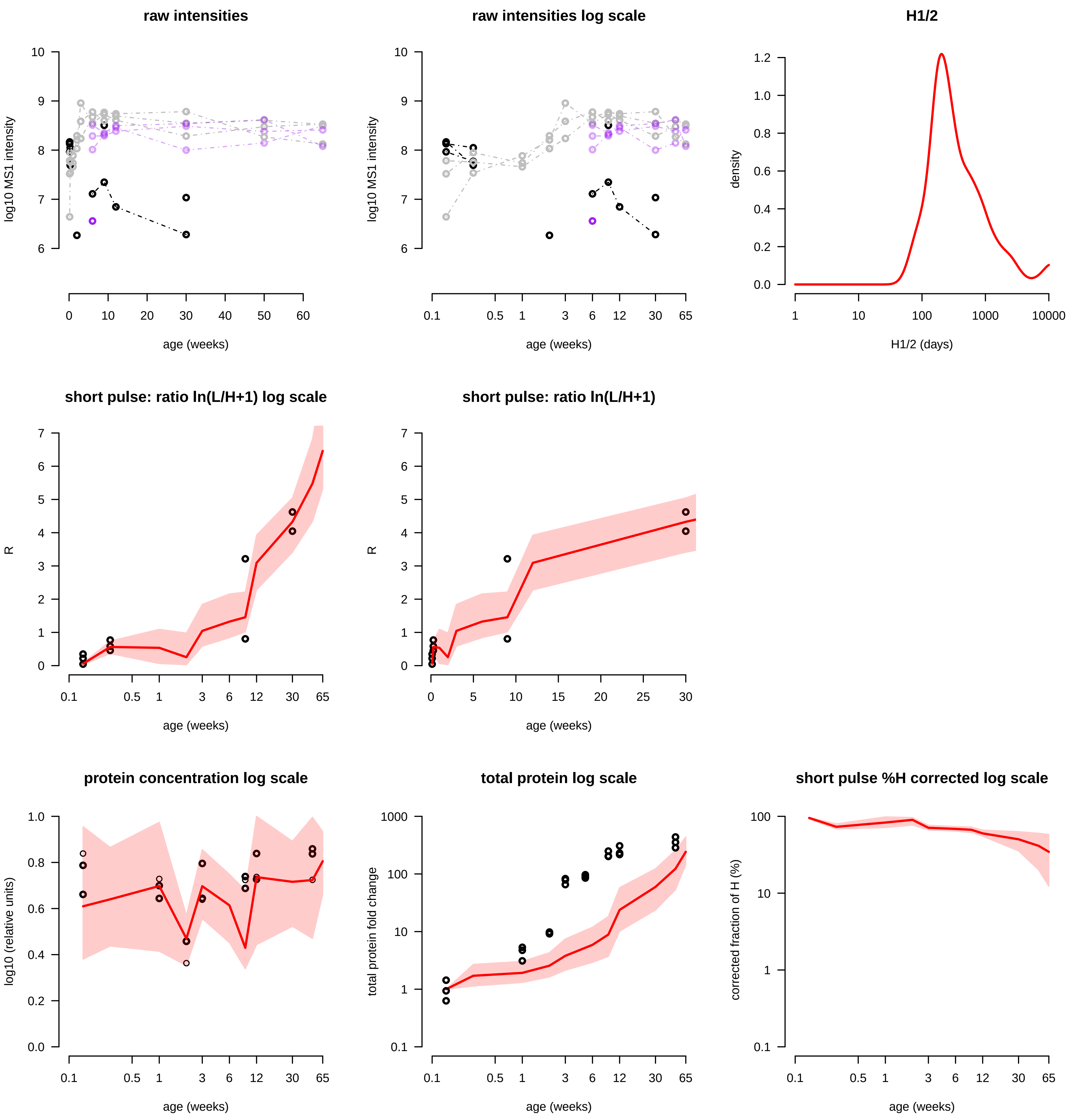

Q99M31;Q99M31−2

>sp|Q99M31|HSP7E_MOUSE

Heat shock 70 kDa protein 14

GN=Hspa14

Med H1/2: 7.8 days

Q5 H1/2: 3.1 days

Q95 H1/2: 51.4 days

reliable model fit: yes

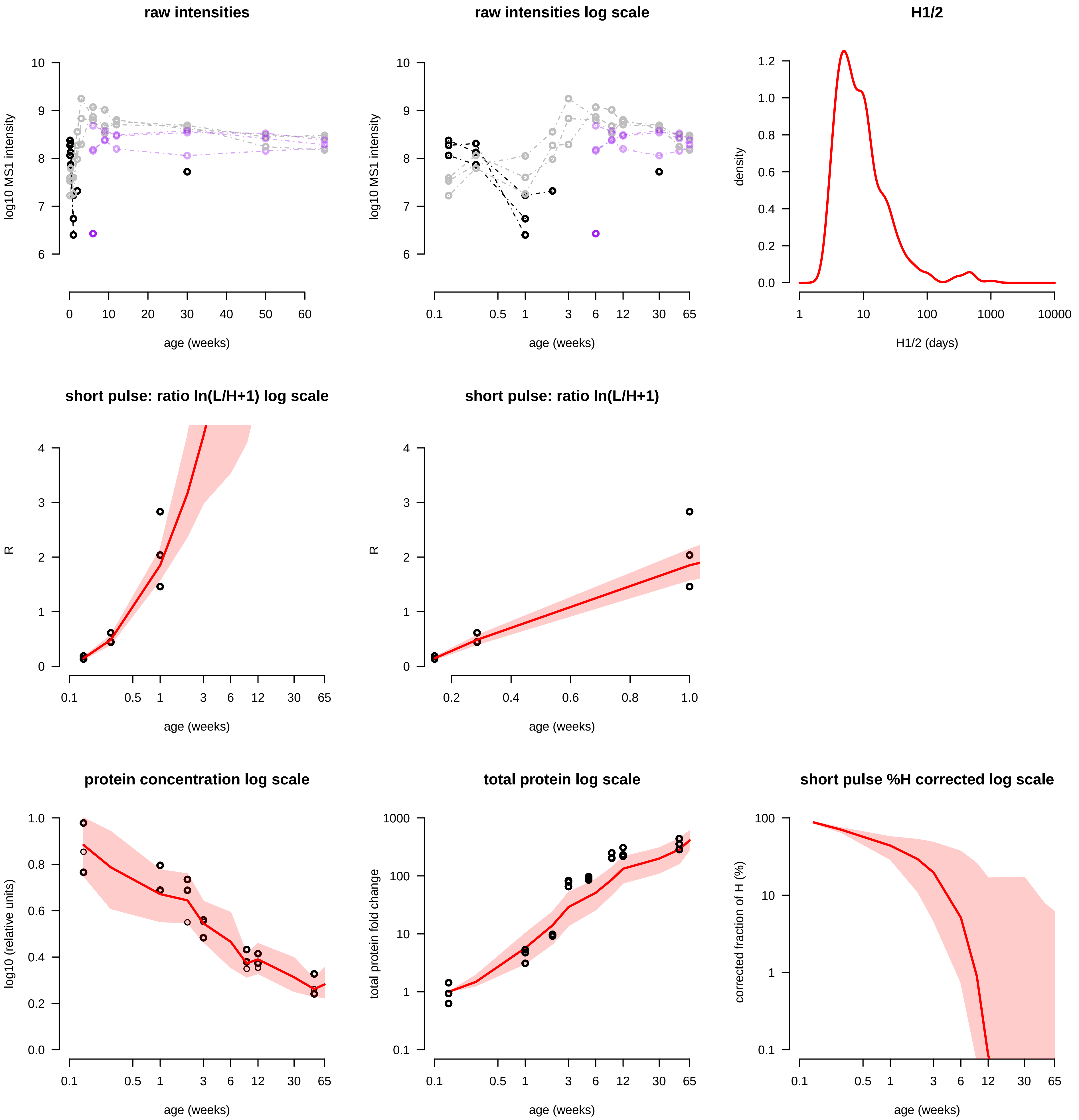

Q6A028

>sp|Q6A028|SWP70_MOUSE

Switch−associated protein 70

GN=Swap70

Med H1/2: 9.3 days

Q5 H1/2: 3.9 days

Q95 H1/2: 36.3 days

reliable model fit: yes

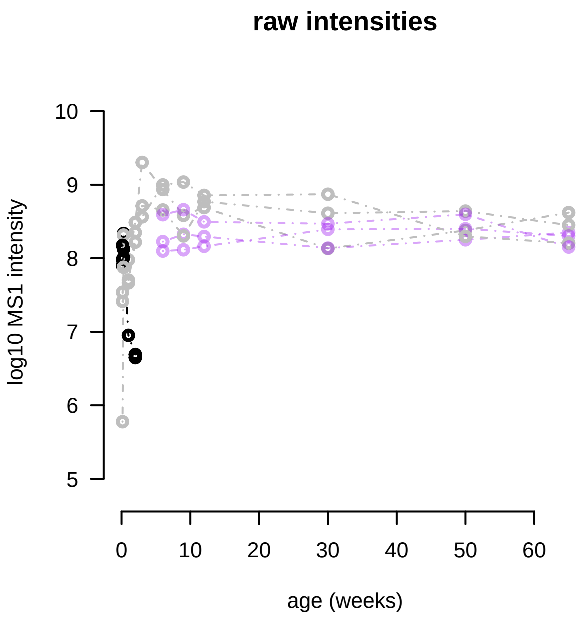

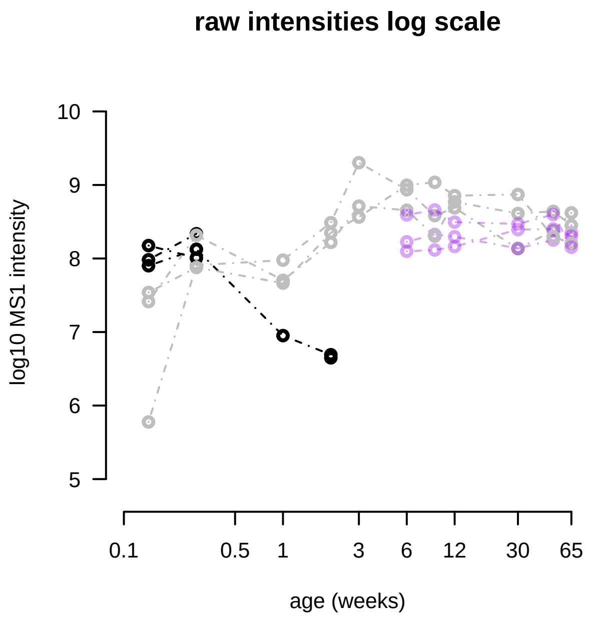

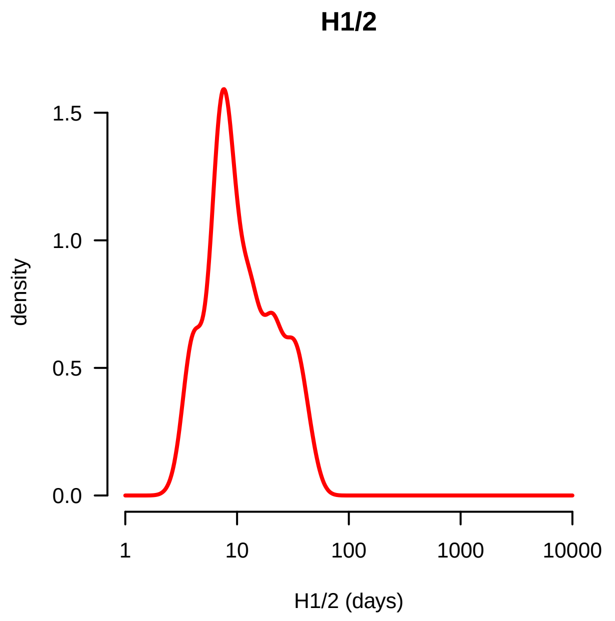

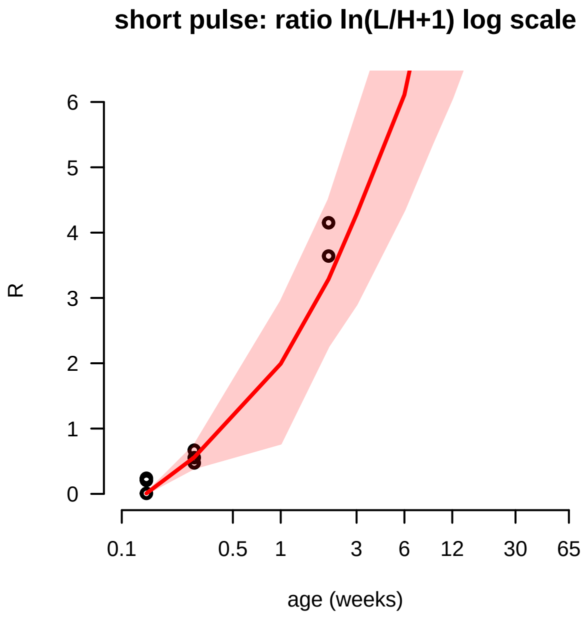

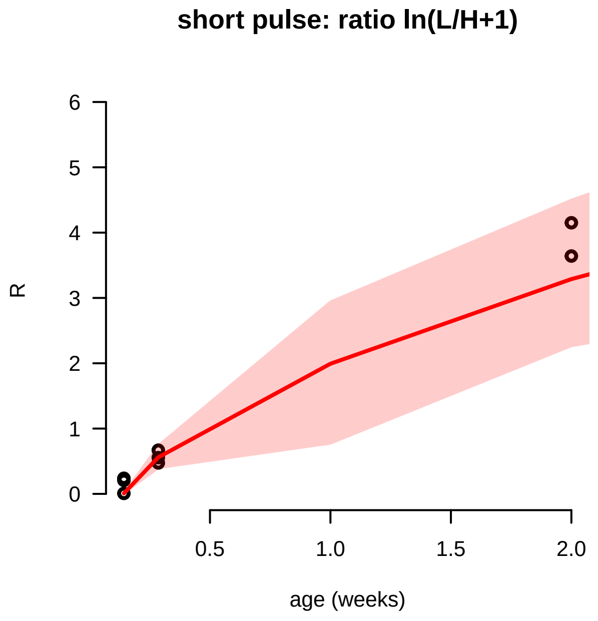

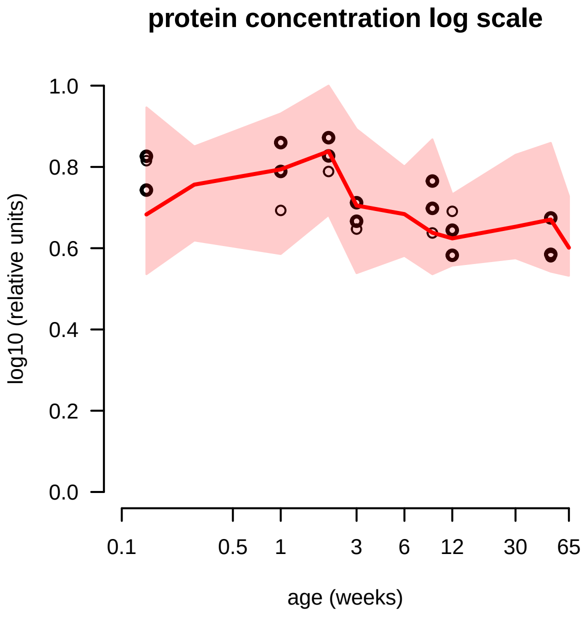

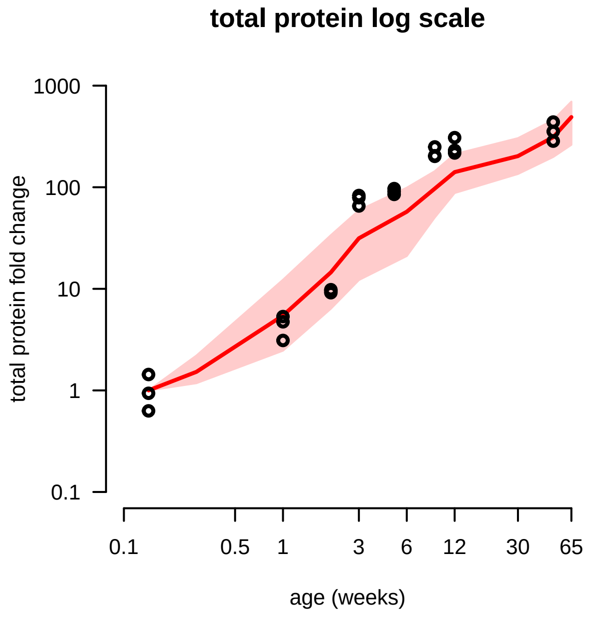

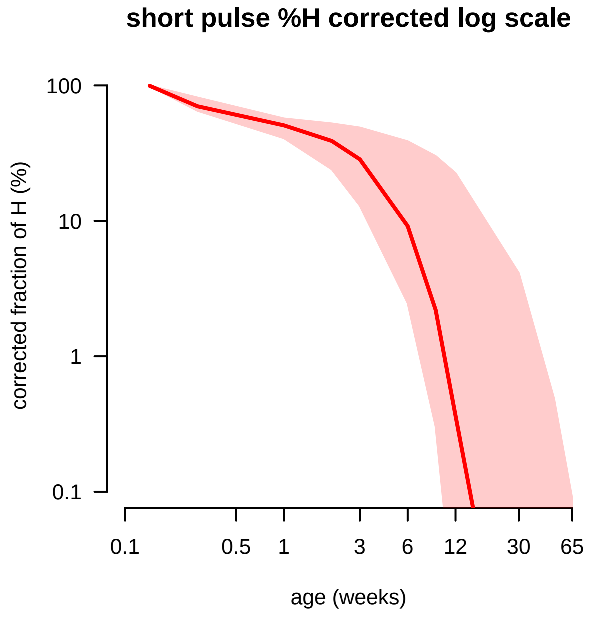

O54890

>sp|O54890|ITB3_MOUSE

Integrin beta−3

GN=Itgb3

Med H1/2: 1710.9 days

Q5 H1/2: 358.9 days

Q95 H1/2: 17250.7 days

reliable model fit: no

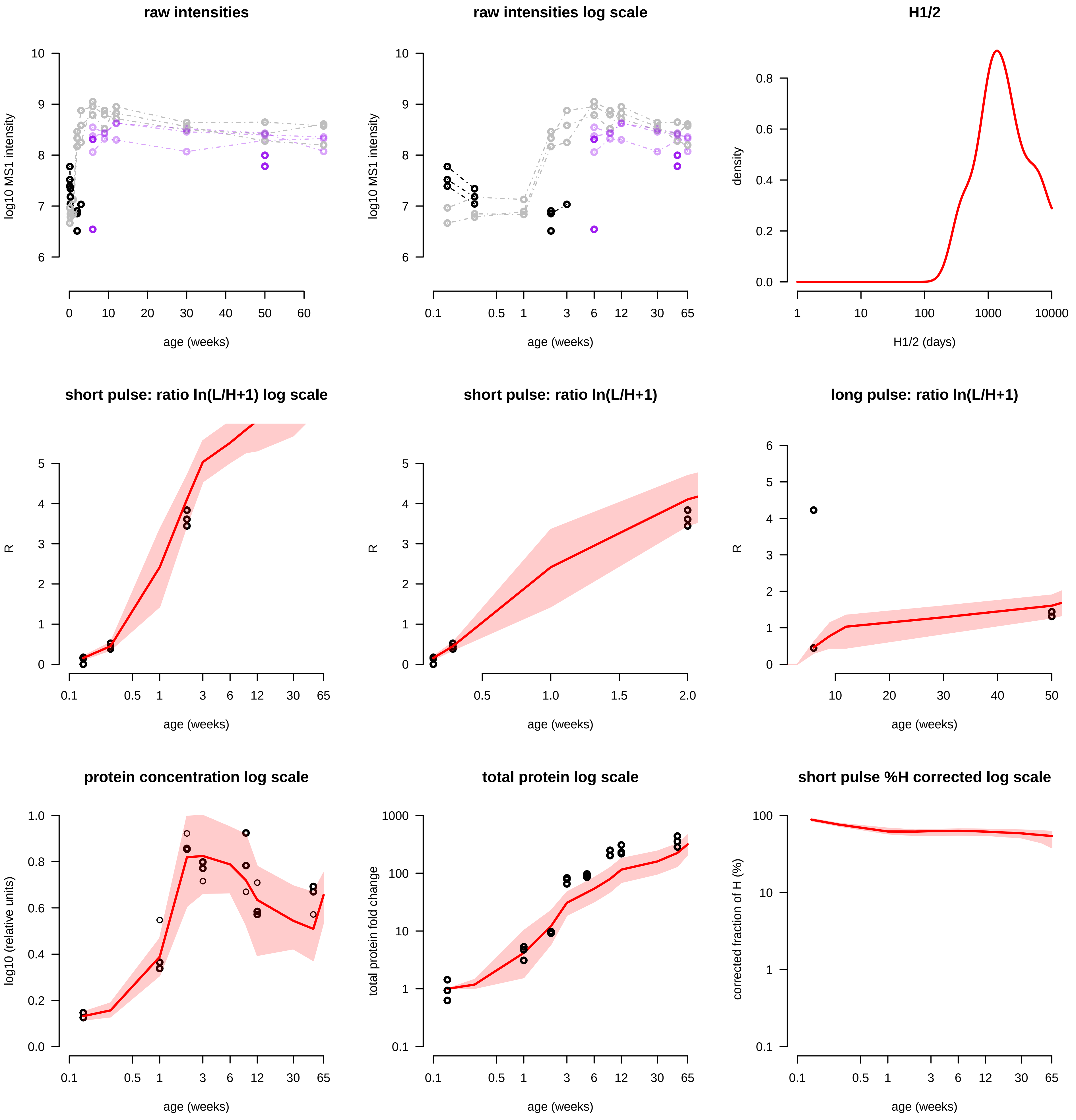

Q8VE70

>sp|Q8VE70|PDC10_MOUSE

Programmed cell death protein 10

GN=Pdcd10

Med H1/2: 5.7 days

Q5 H1/2: 2.5 days

Q95 H1/2: 28.7 days

reliable model fit: yes

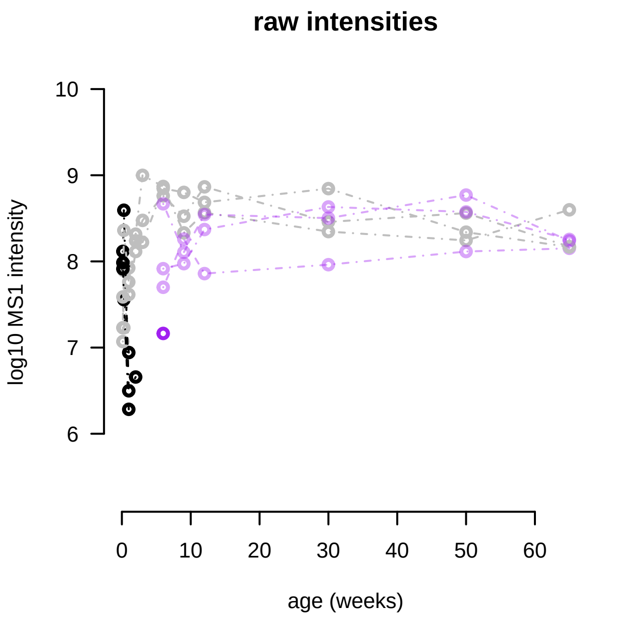

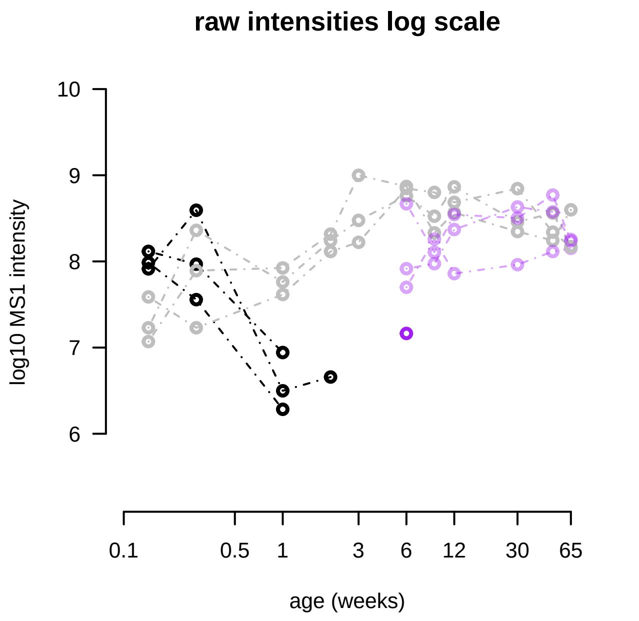

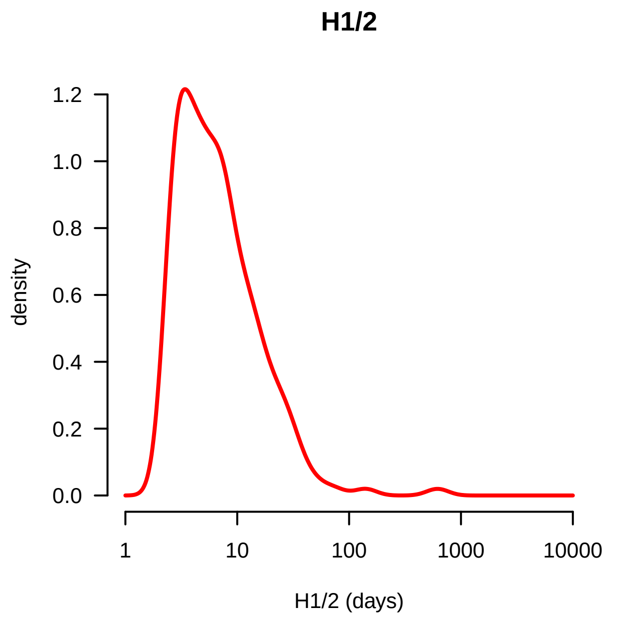

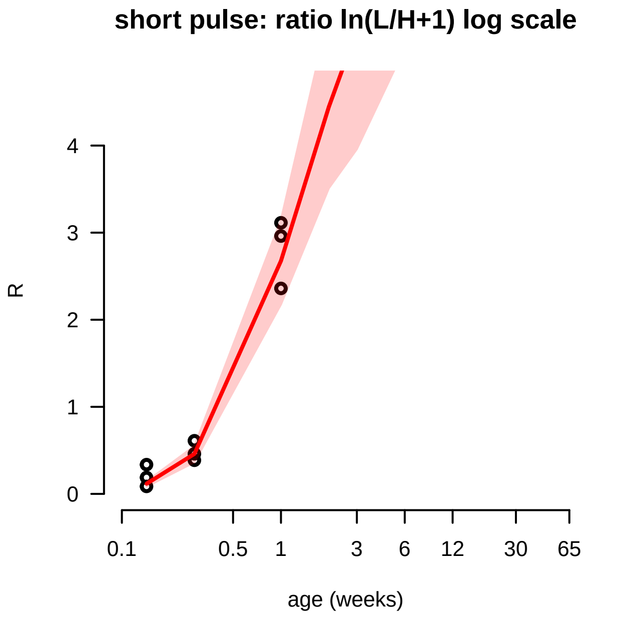

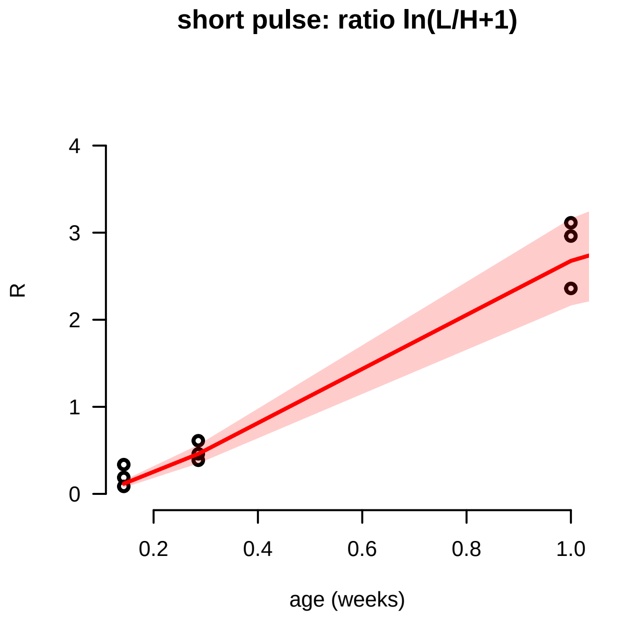

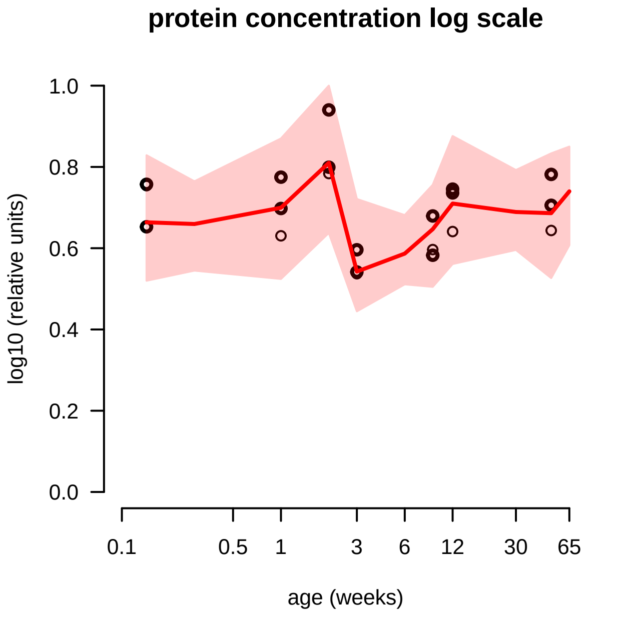

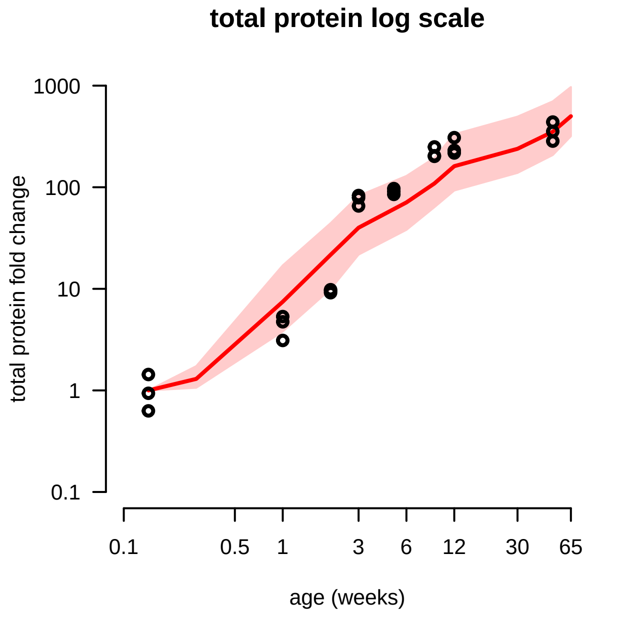

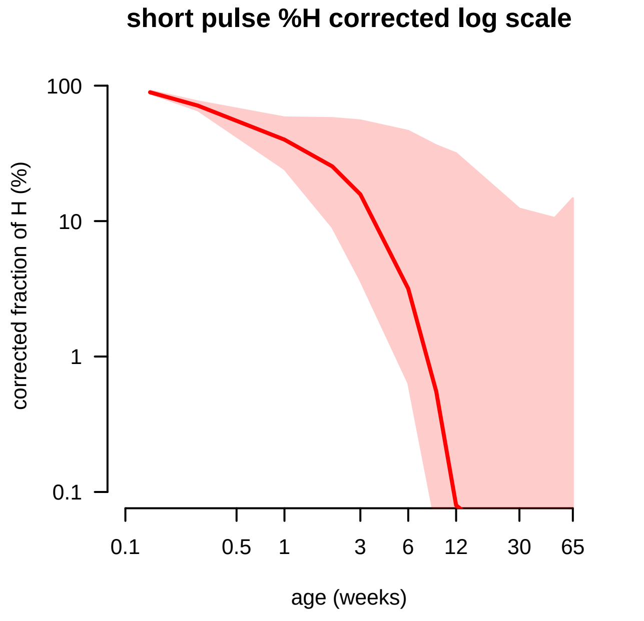

Q6R891

>sp|Q6R891|NEB2_MOUSE

Neurabin−2

GN=Ppp1r9b

Med H1/2: 4.2 days

Q5 H1/2: 2.6 days

Q95 H1/2: 19.6 days

reliable model fit: yes

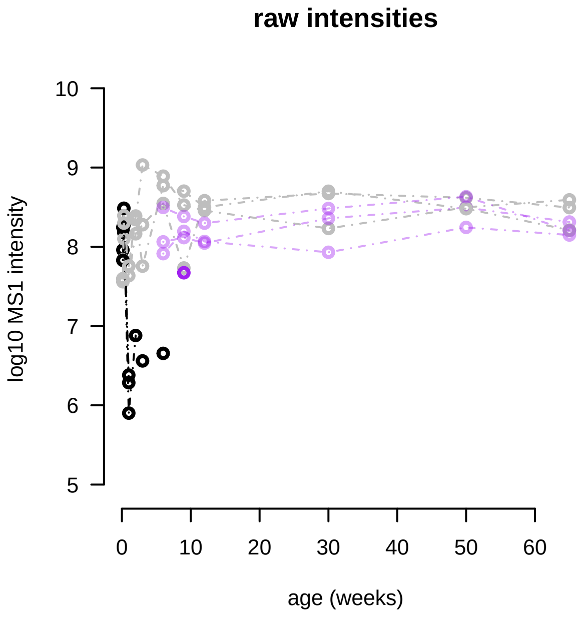

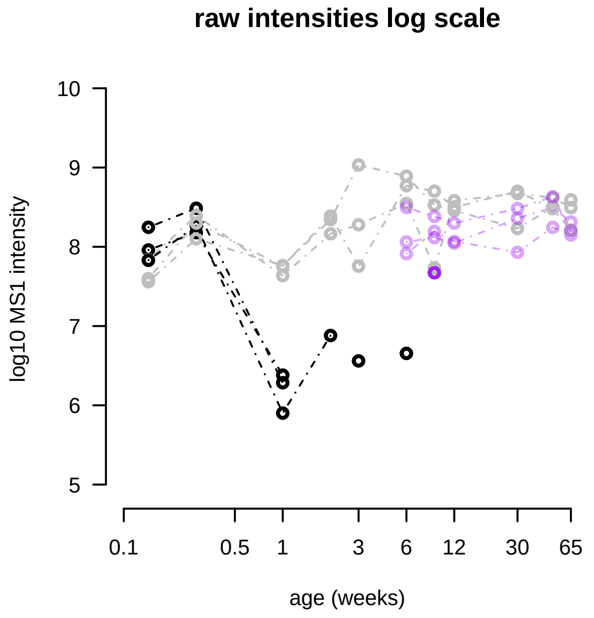

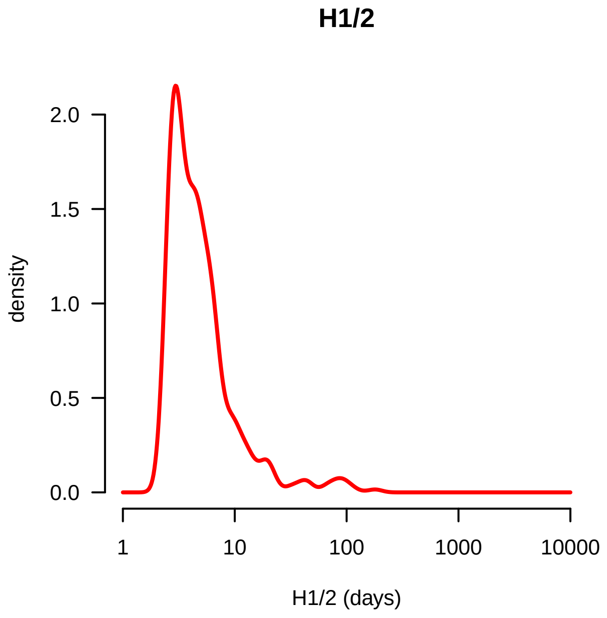

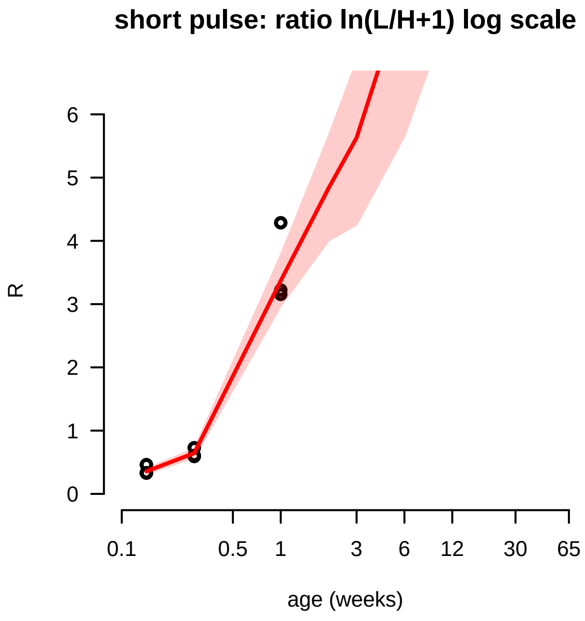

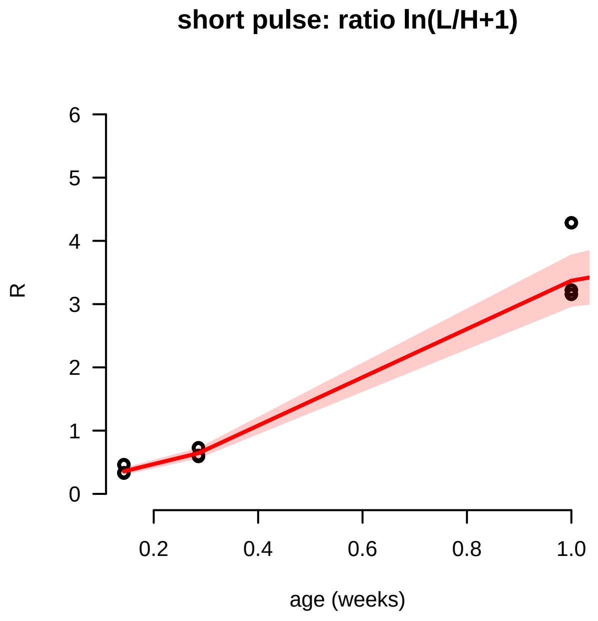

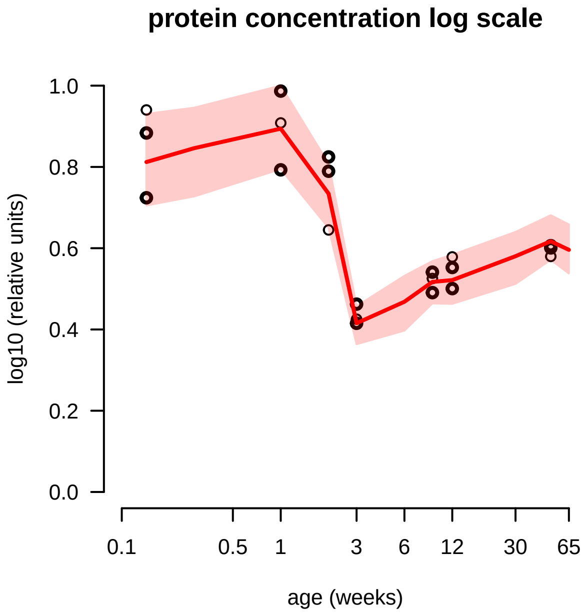

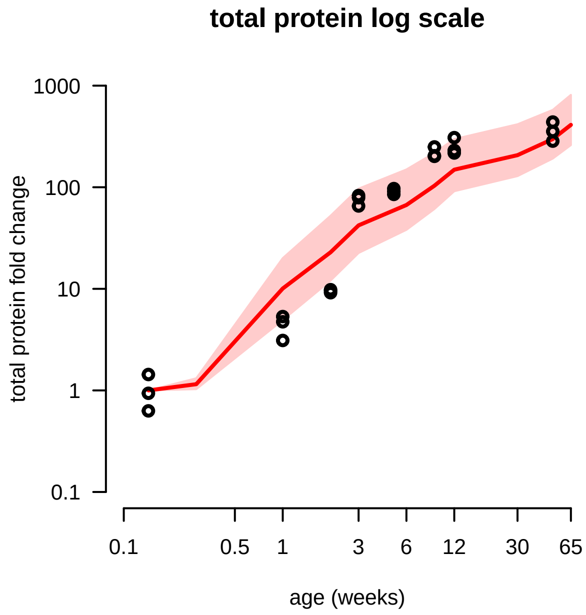

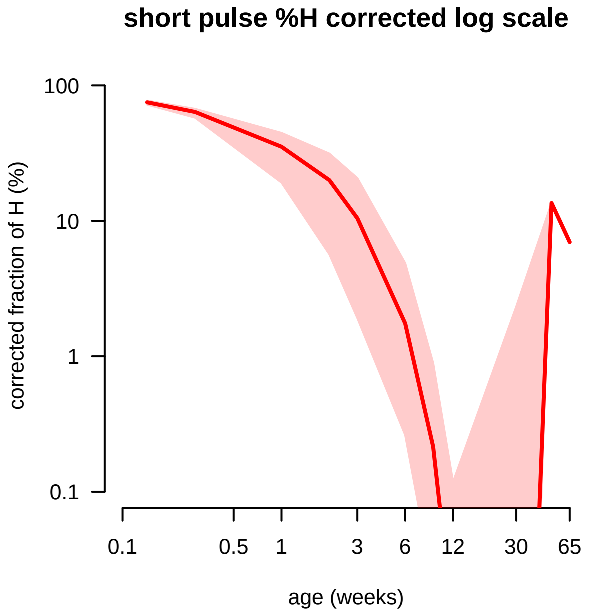

Q9Z0R6;Q9Z0R6−2

>sp|Q9Z0R6|ITSN2_MOUSE

Intersectin−2

GN=Itsn2

Med H1/2: 3.2 days

Q5 H1/2: 3.2 days

Q95 H1/2: 3.2 days

reliable model fit: no

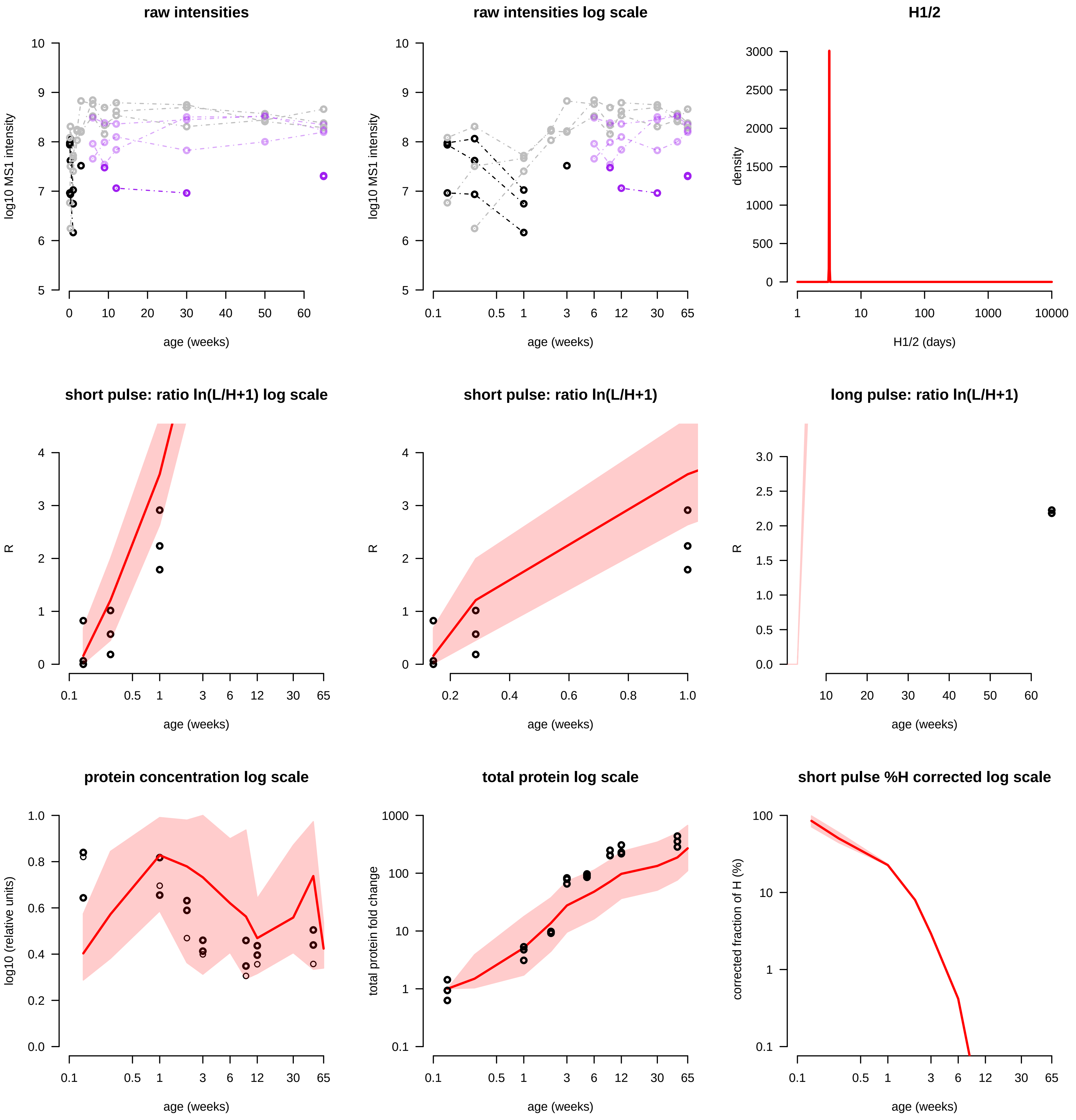

Q9R069

>sp|Q9R069|BCAM_MOUSE

Basal cell adhesion molecule

GN=Bcam

Med H1/2: 5.7 days

Q5 H1/2: 3.1 days

Q95 H1/2: 20.2 days

reliable model fit: yes

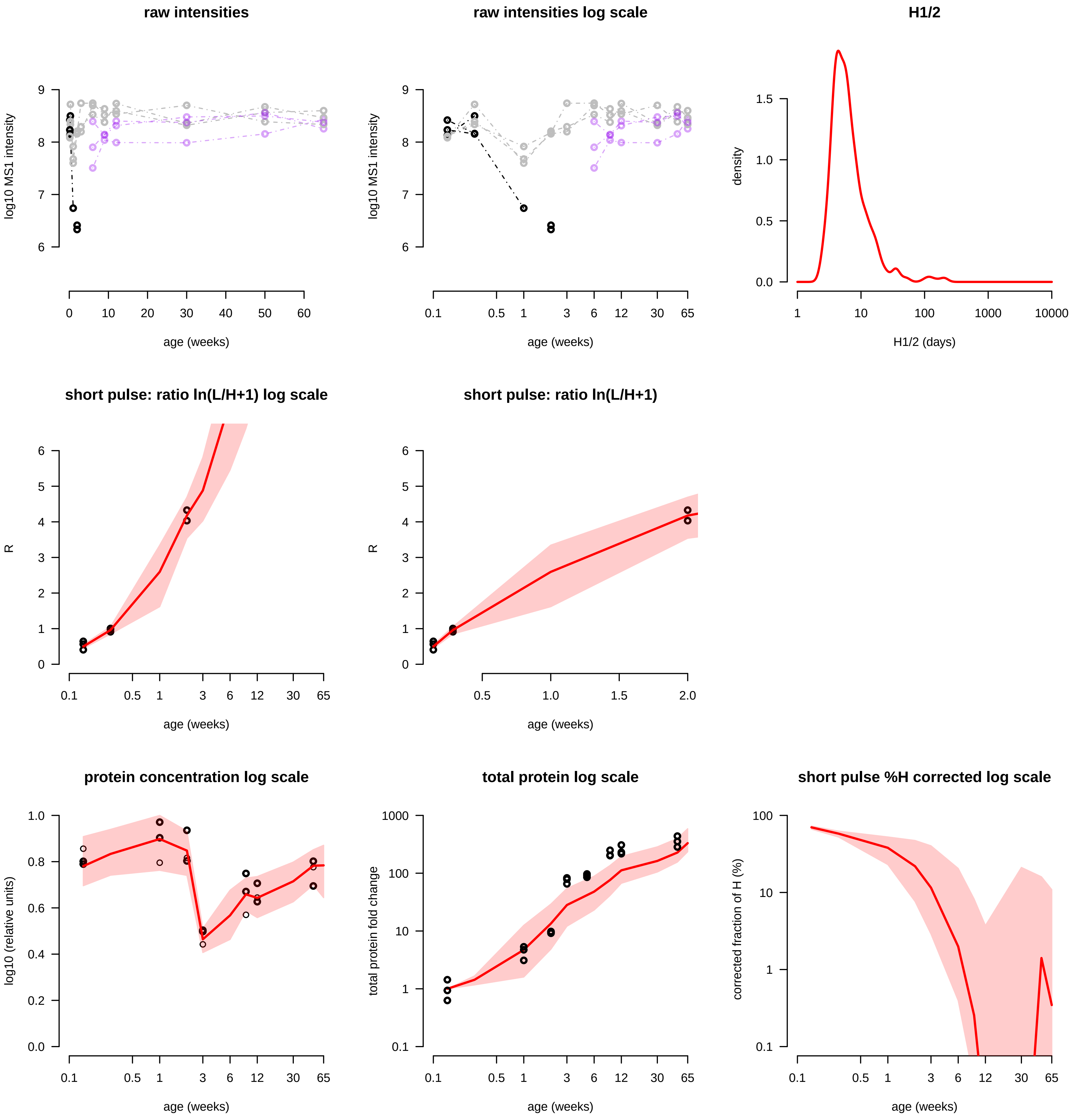

P42232

>sp|P42232|STA5B_MOUSE

Signal transducer and activator of transcription 5B

GN=Stat5b

Med H1/2: 4.4 days

Q5 H1/2: 3.8 days

Q95 H1/2: 5.6 days

reliable model fit: yes

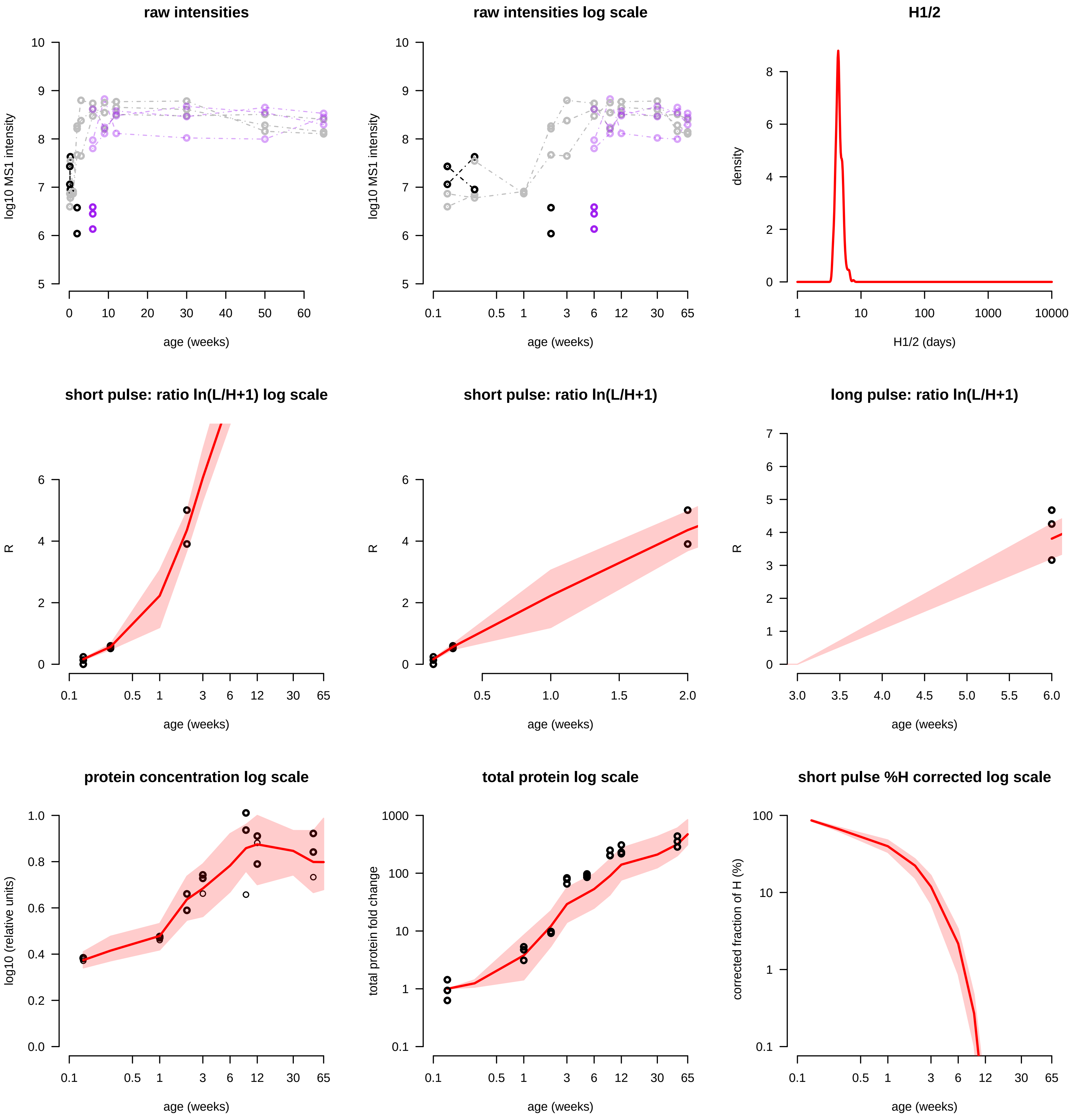

Q8VDK1−2

>sp|Q8VDK1−2|NIT1_MOUSE

Isoform 2 of Deaminated glutathione amidase

GN=Nit1

Med H1/2: 23 days

Q5 H1/2: 9.5 days

Q95 H1/2: 284.9 days

reliable model fit: yes

raw intensities

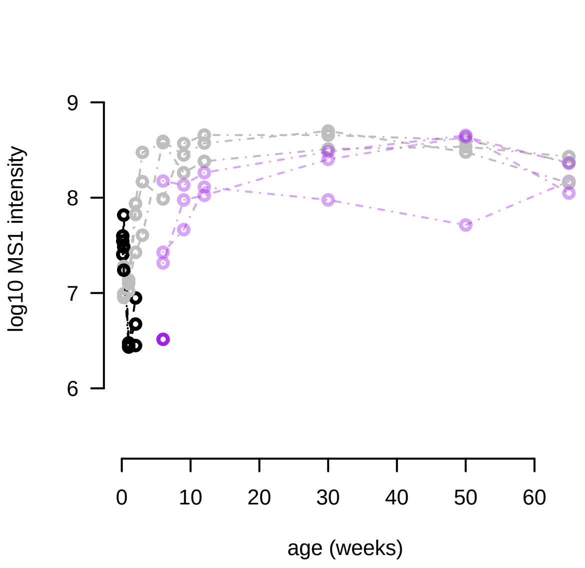

raw intensities log scale

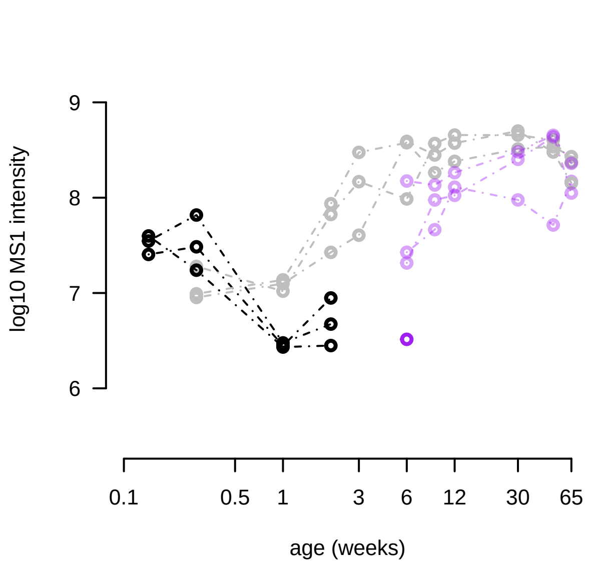

H1/2

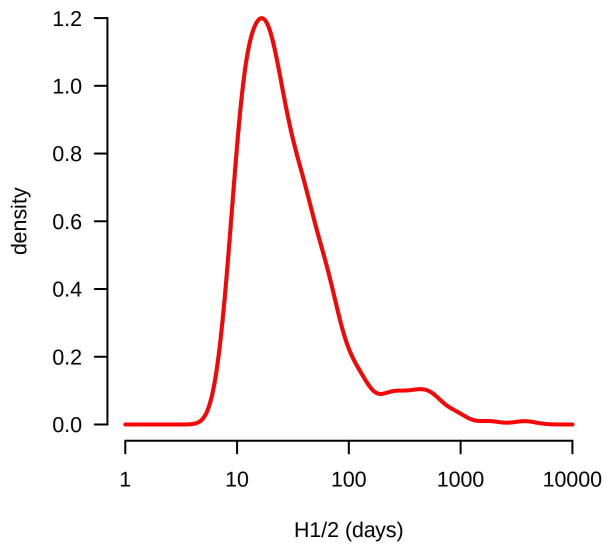

short pulse: ratio ln(L/H+1) log scale

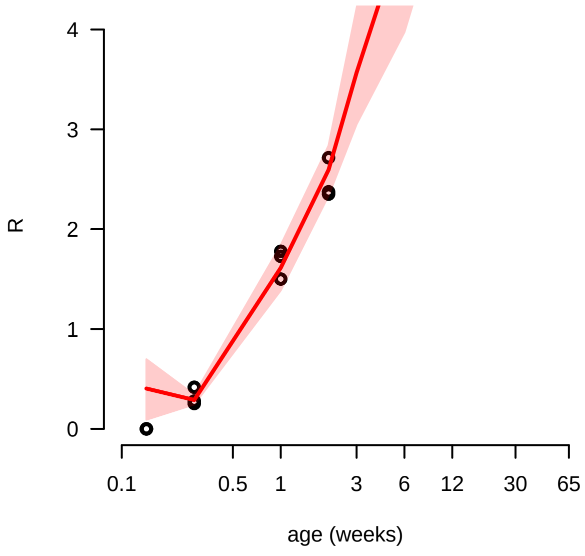

short pulse: ratio ln(L/H+1)

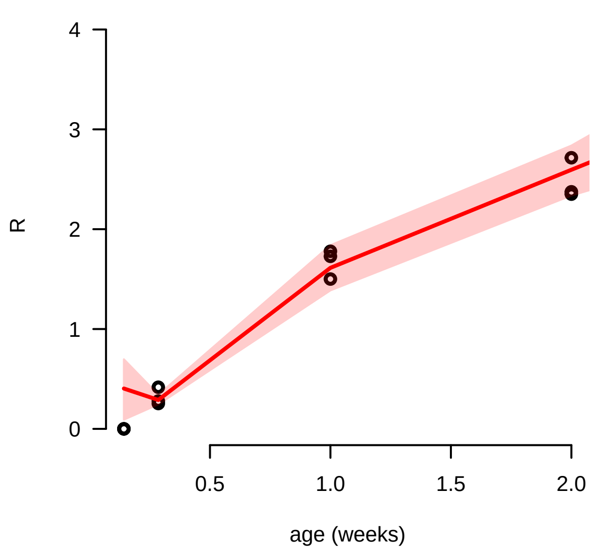

protein concentration log scale

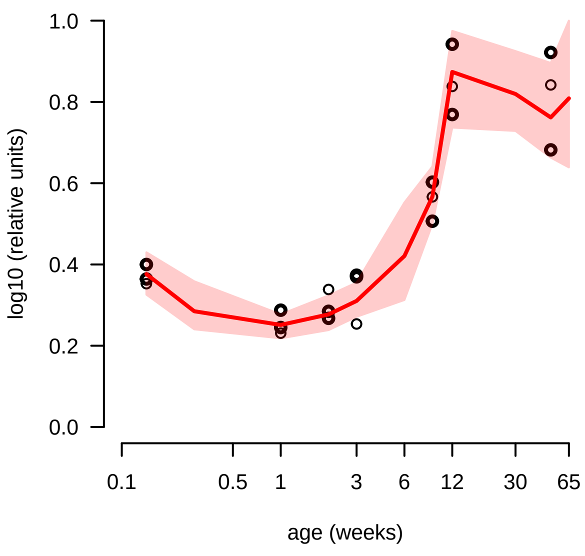

total protein log scale

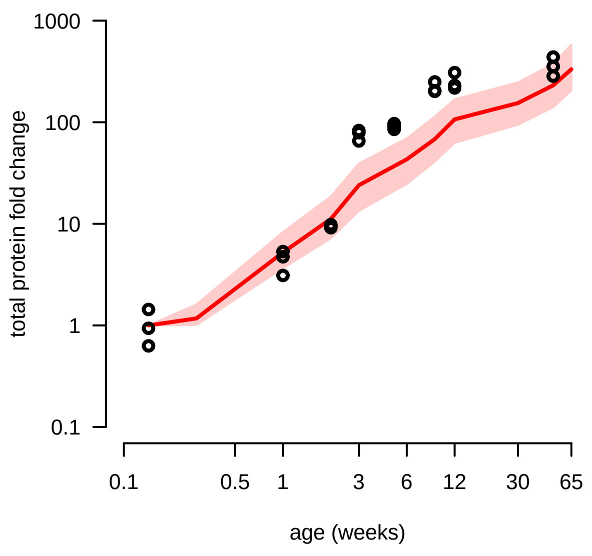

short pulse %H corrected log scale

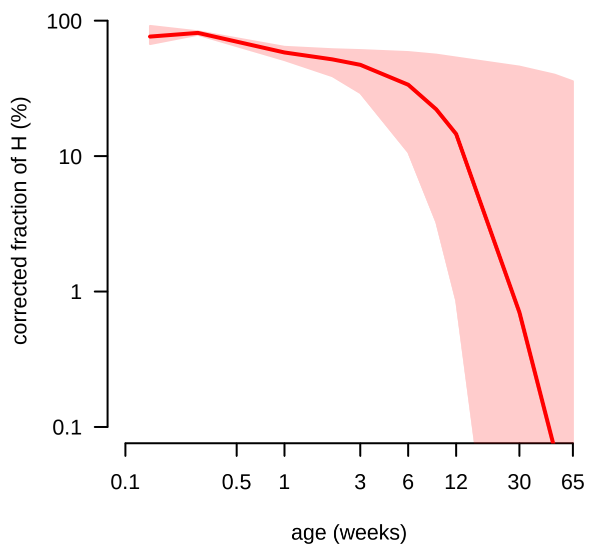

Q8K297

>sp|Q8K297|GT251_MOUSE

Procollagen galactosyltransferase 1

GN=Colgalt1

Med H1/2: 2029.4 days

Q5 H1/2: 533.2 days

Q95 H1/2: 48817.8 days

reliable model fit: no

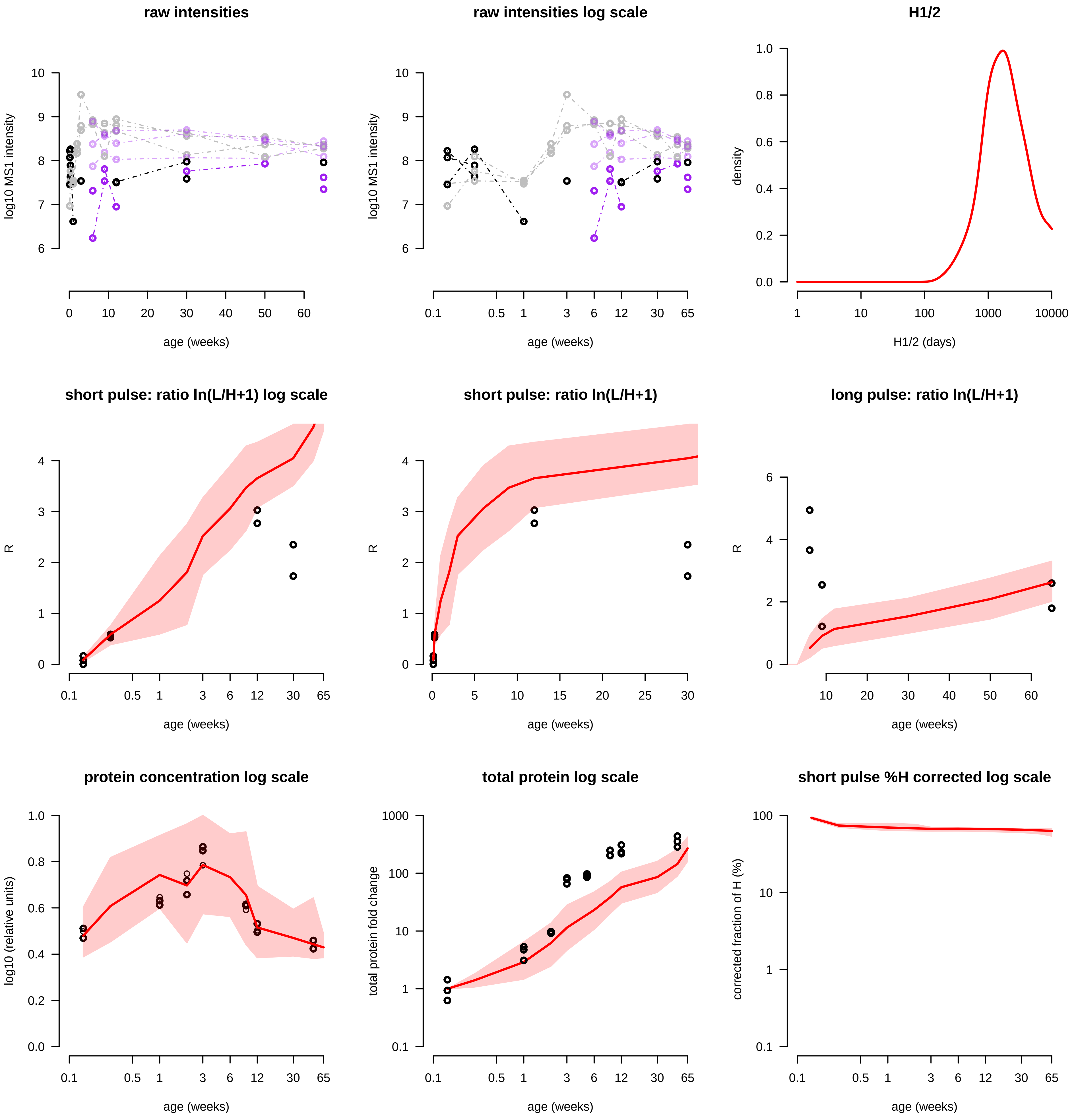

Q9JJK2

>sp|Q9JJK2|LANC2_MOUSE

LanC−like protein 2

GN=Lancl2

Med H1/2: 5.5 days

Q5 H1/2: 4.2 days

Q95 H1/2: 8.9 days

reliable model fit: no

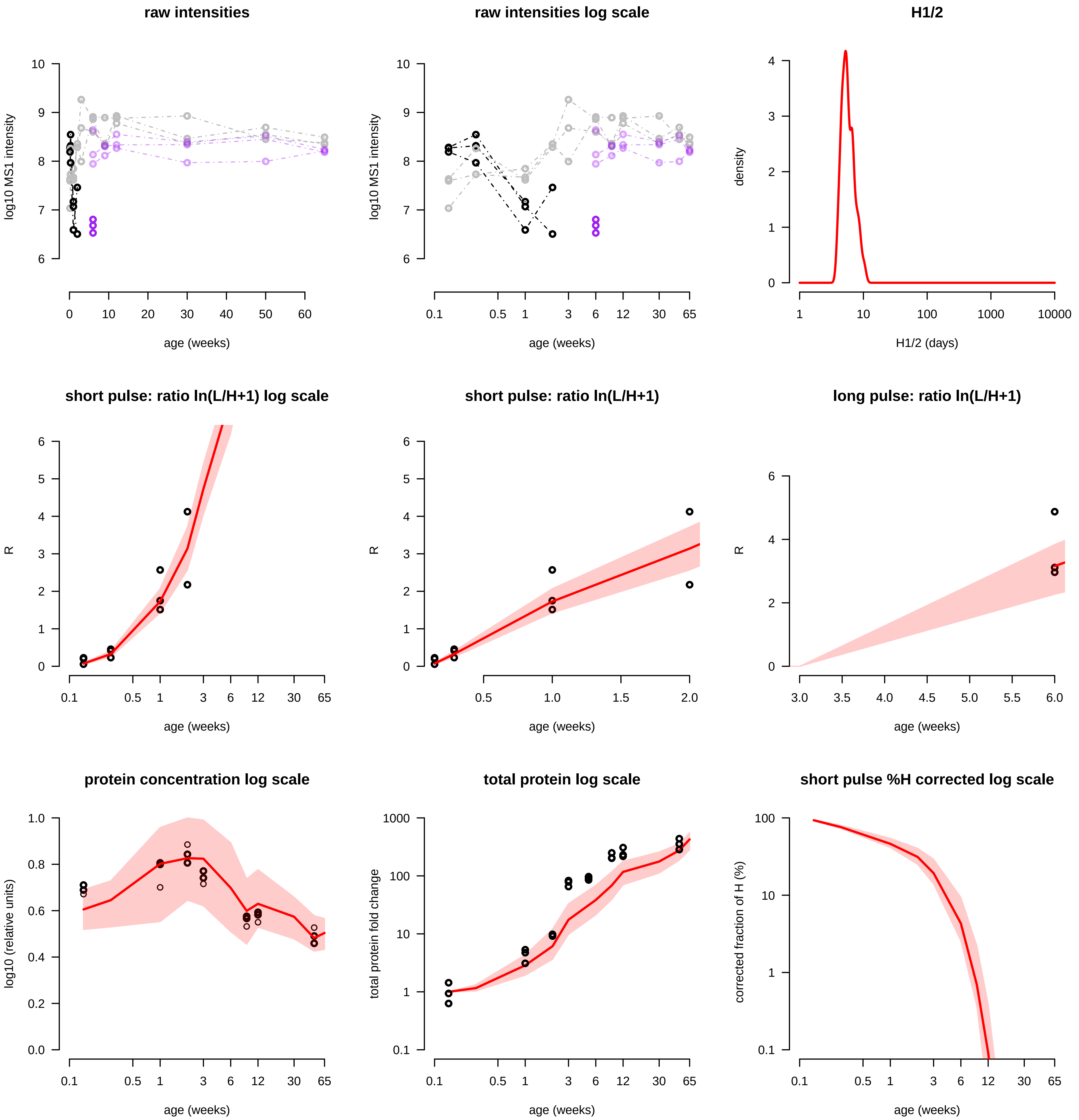

Q3USZ8

>sp|Q3USZ8|DIK2A_MOUSE

Divergent protein kinase domain 2A

GN=Dipk2a

Med H1/2: 9.7 days

Q5 H1/2: 3.7 days

Q95 H1/2: 86.5 days

reliable model fit: no

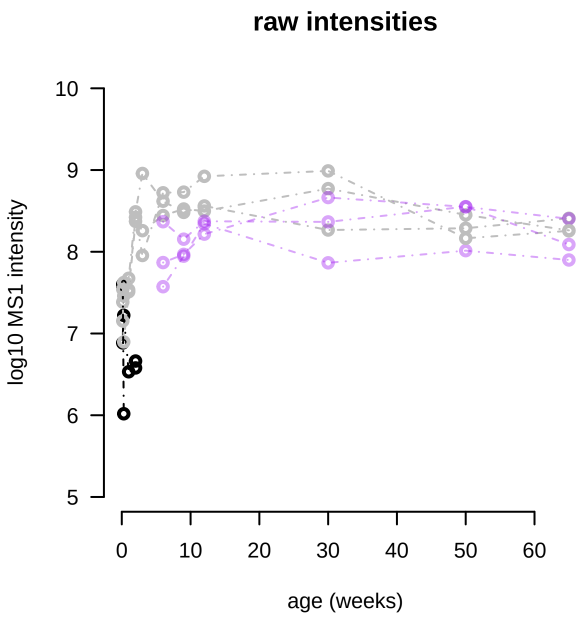

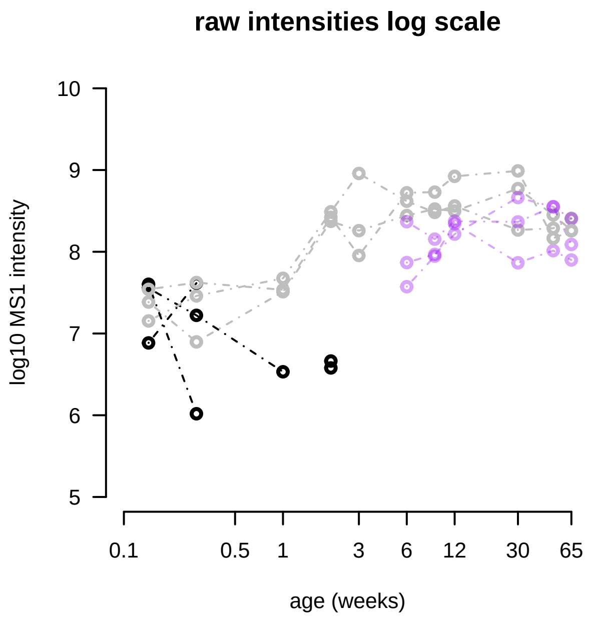

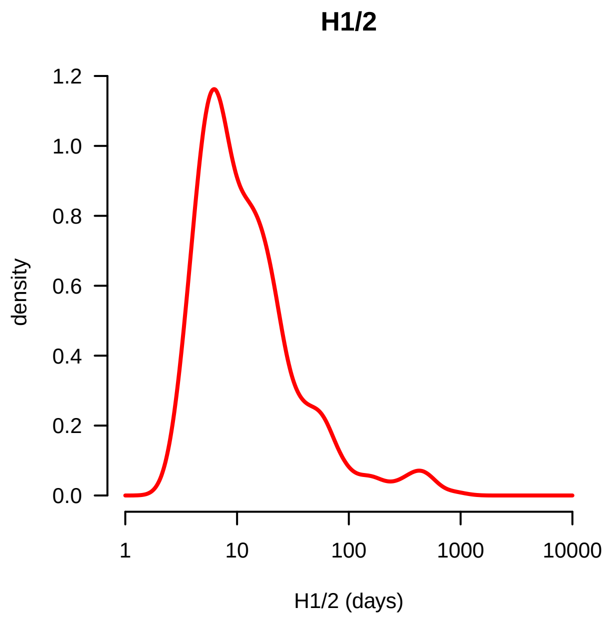

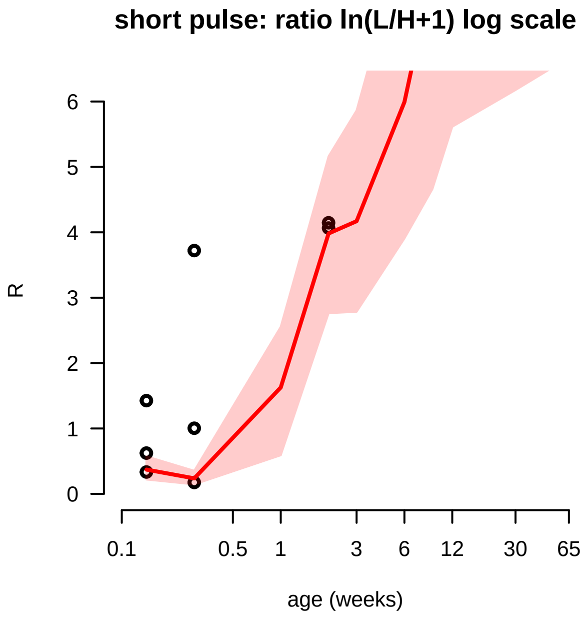

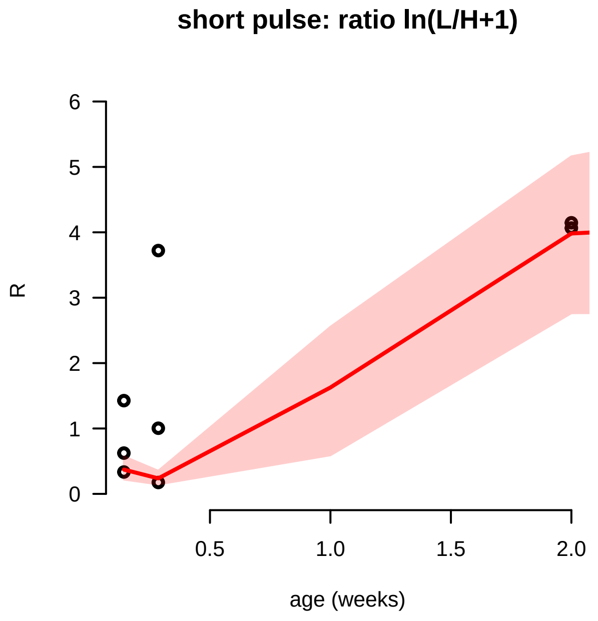

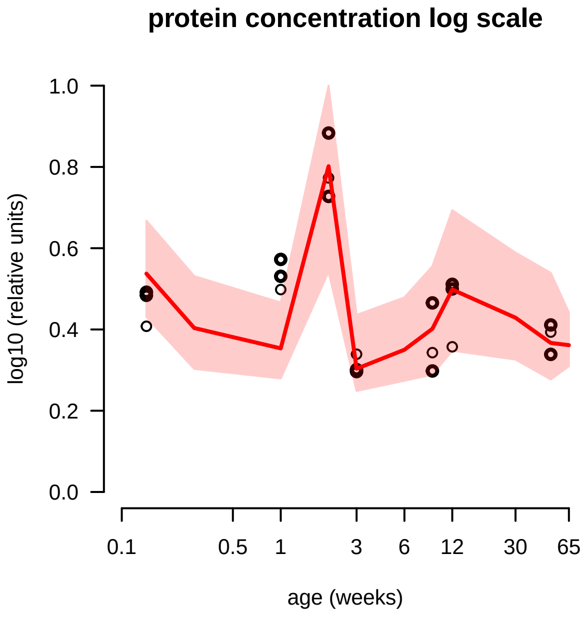

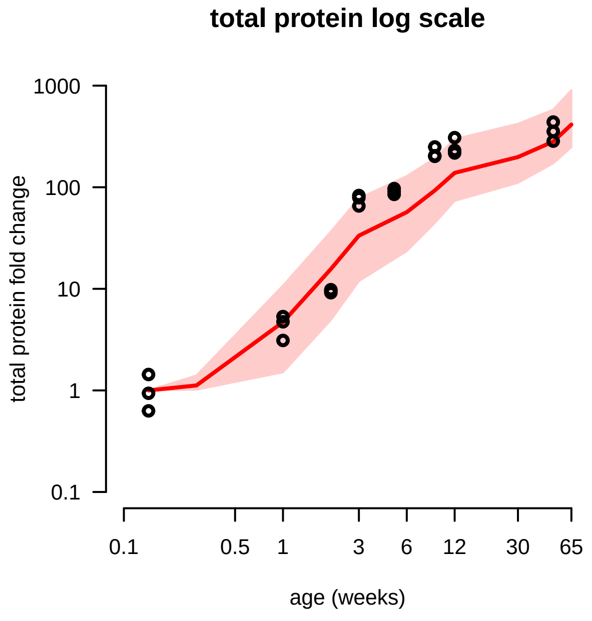

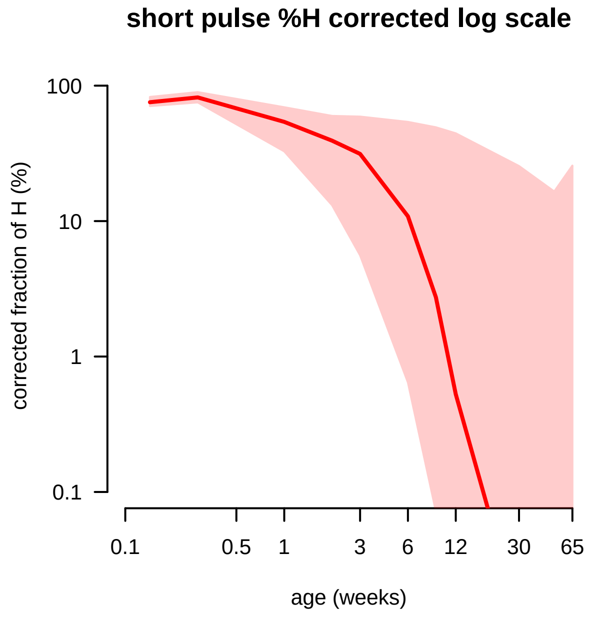

Q8K3J1

>sp|Q8K3J1|NDUS8_MOUSE

NADH dehydrogenase [ubiquinone] iron−sulfur protein 8, mitochondrial

GN=Ndufs8

Med H1/2: 10.3 days

Q5 H1/2: 4 days

Q95 H1/2: 75.5 days

reliable model fit: yes

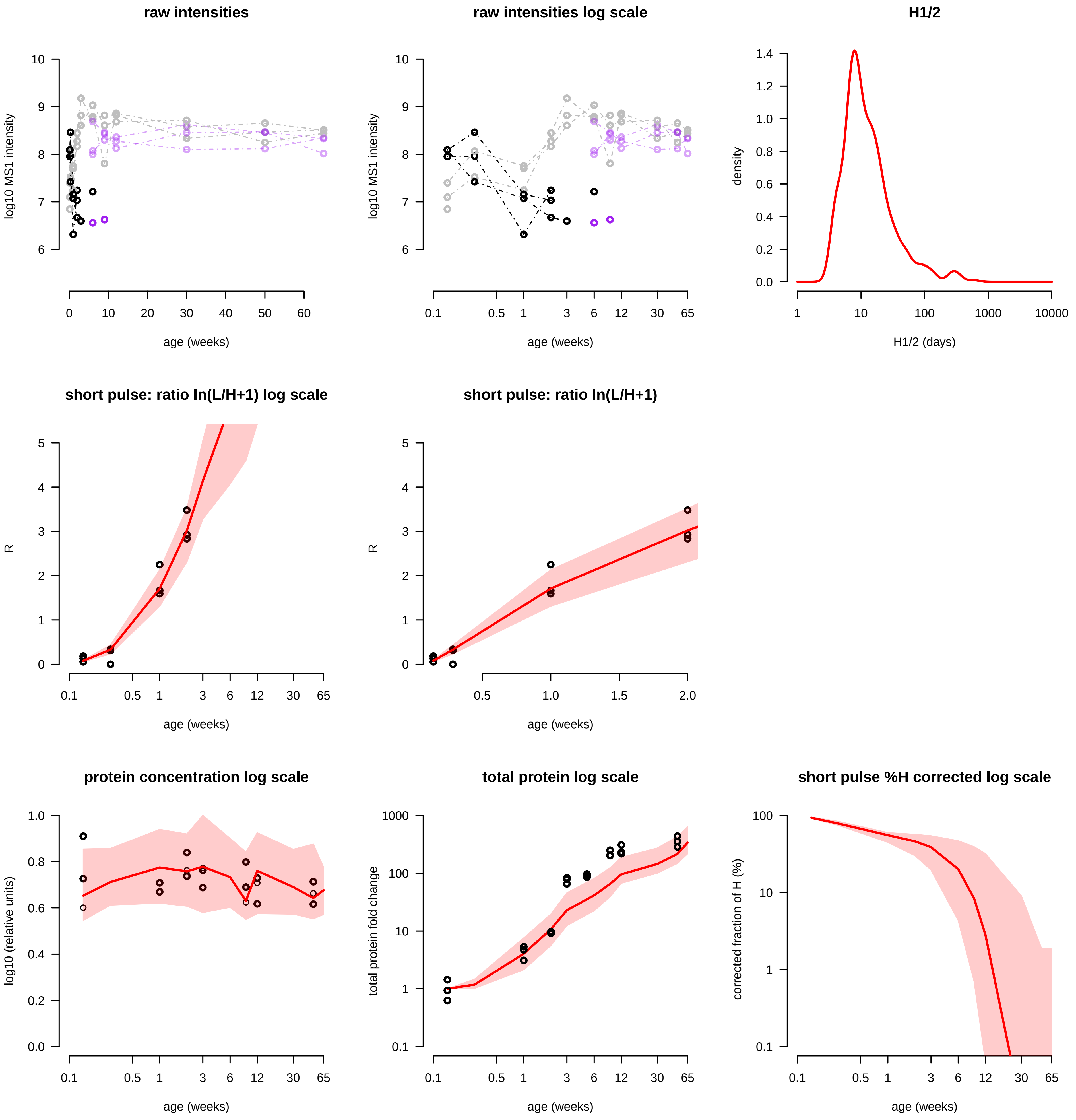

Q8R0A0

>sp|Q8R0A0|T2FB_MOUSE

General transcription factor IIF subunit 2

GN=Gtf2f2

Med H1/2: 4.7 days

Q5 H1/2: 3.6 days

Q95 H1/2: 6.3 days

reliable model fit: yes

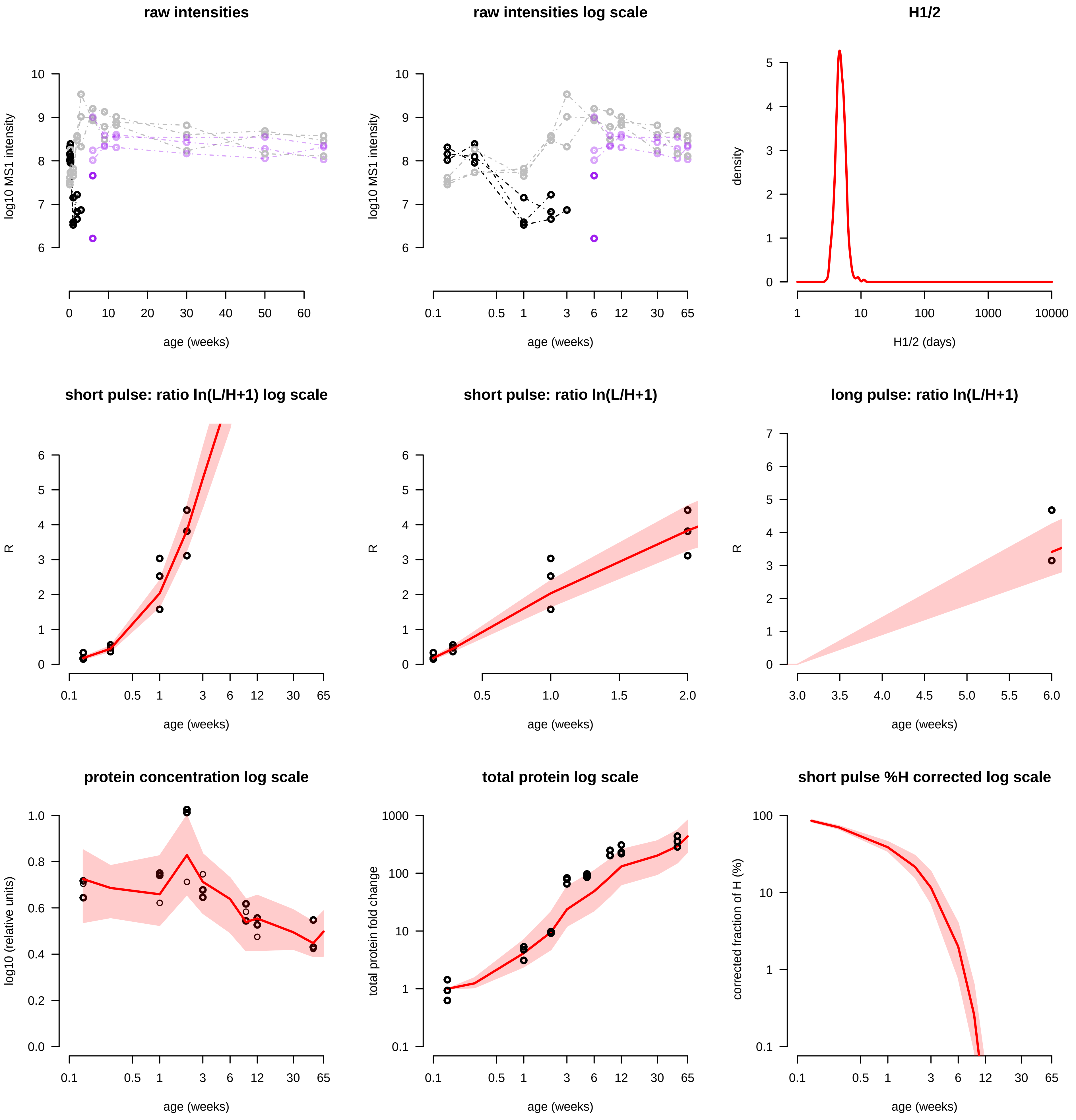

Q9CQ54

>sp|Q9CQ54|NDUC2_MOUSE

NADH dehydrogenase [ubiquinone] 1 subunit C2

GN=Ndufc2

Med H1/2: 5 days

Q5 H1/2: 3.5 days

Q95 H1/2: 7.7 days

reliable model fit: yes

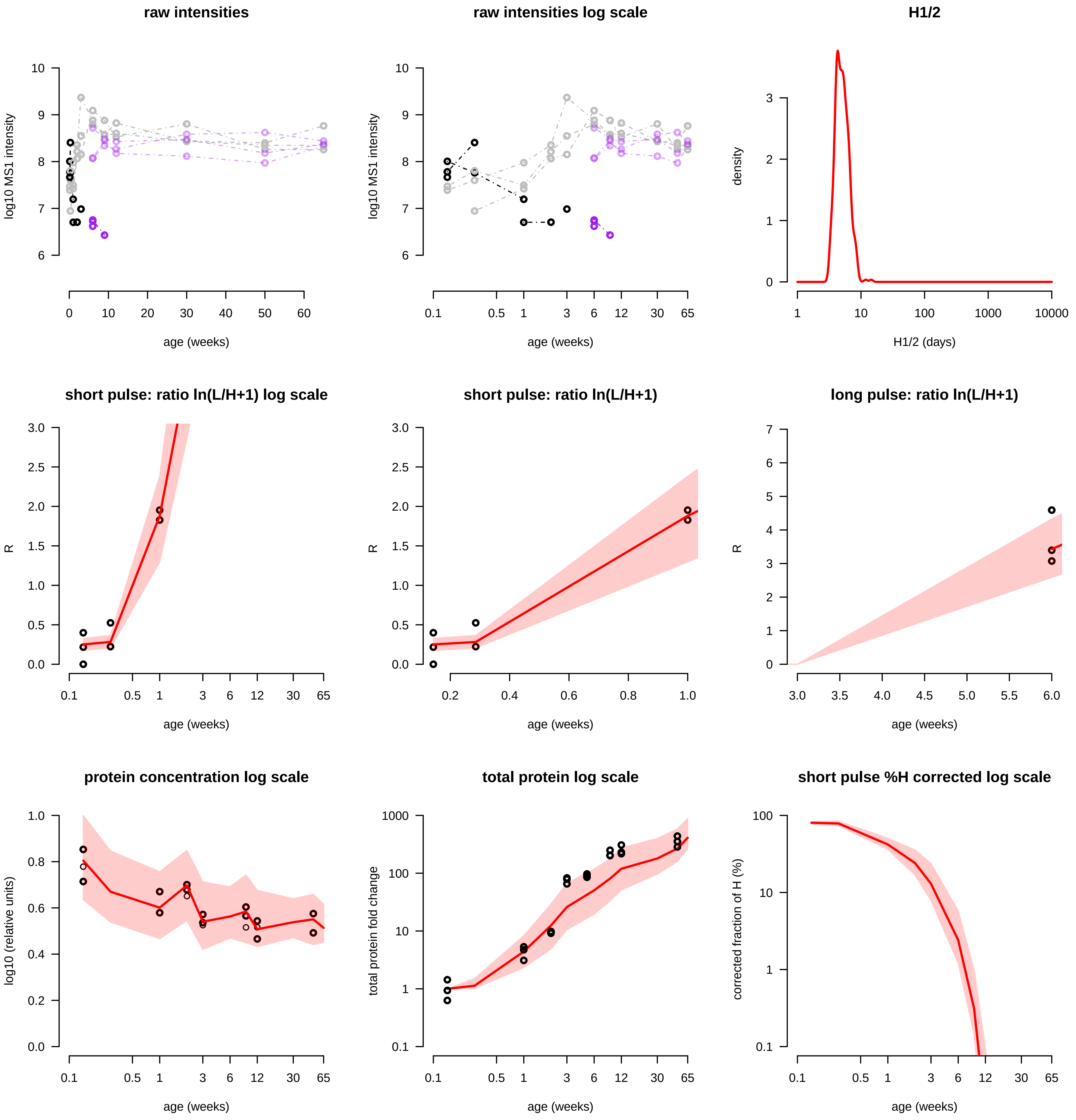

Q9ES97−2;Q9ES97;Q9ES97−5

>sp|Q9ES97−2|RTN3_MOUSE

Isoform 2 of Reticulon−3

GN=Rtn3

Med H1/2: 4.7 days

Q5 H1/2: 2.5 days

Q95 H1/2: 28.6 days

reliable model fit: yes

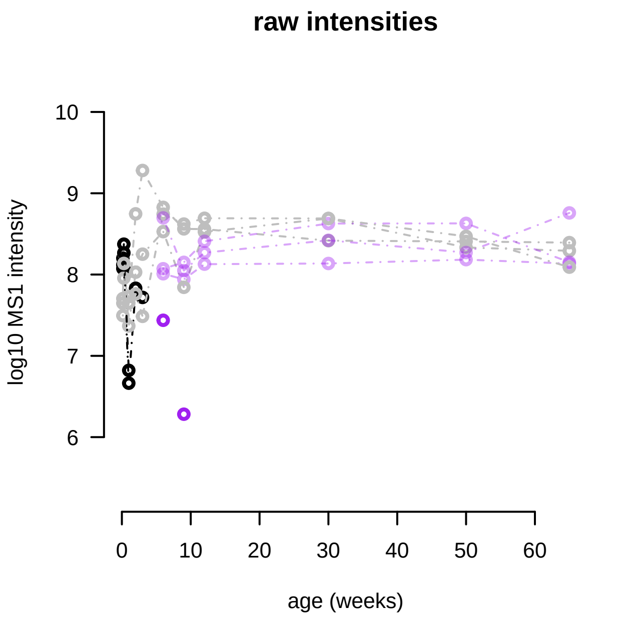

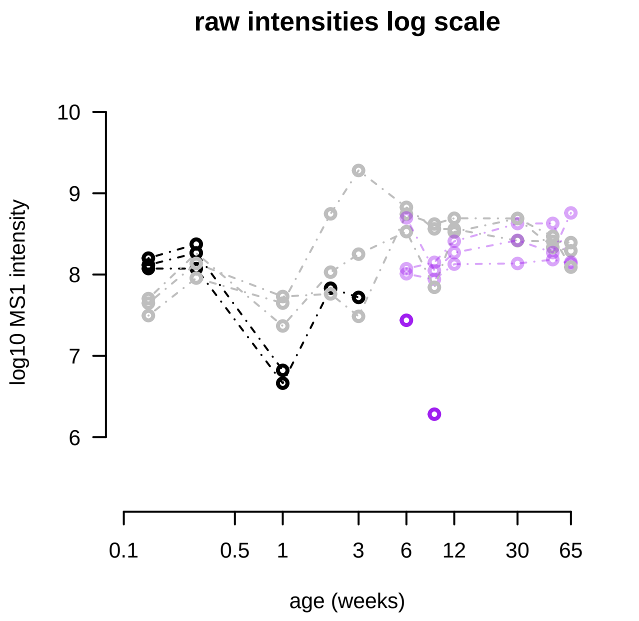

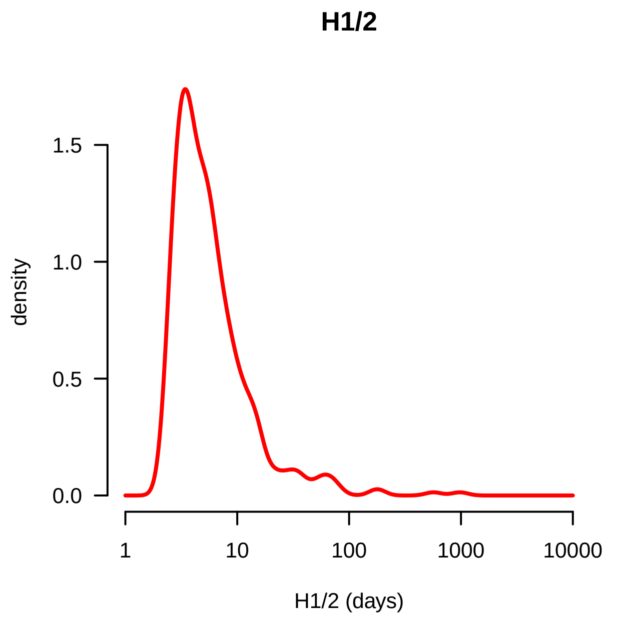

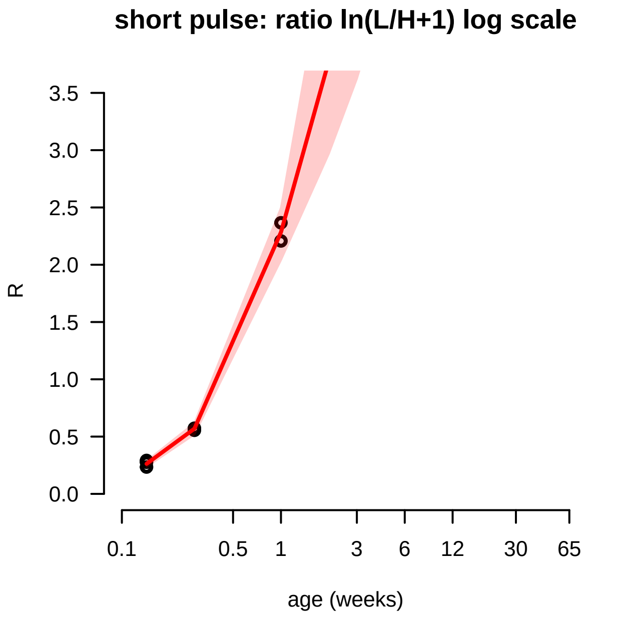

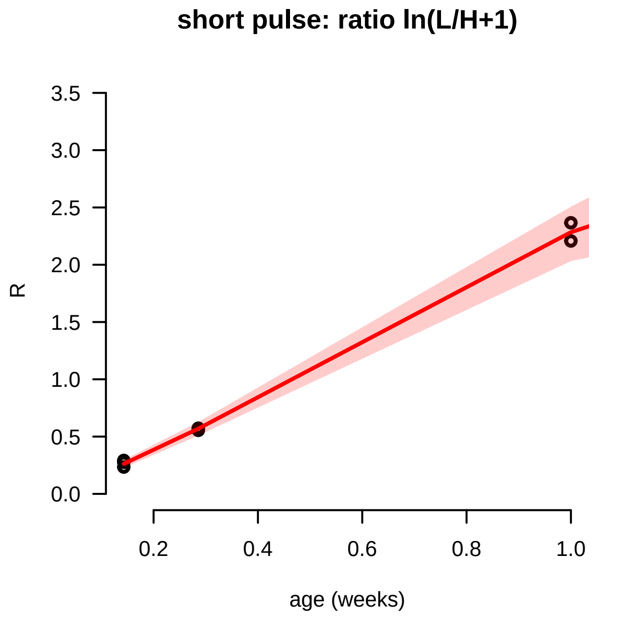

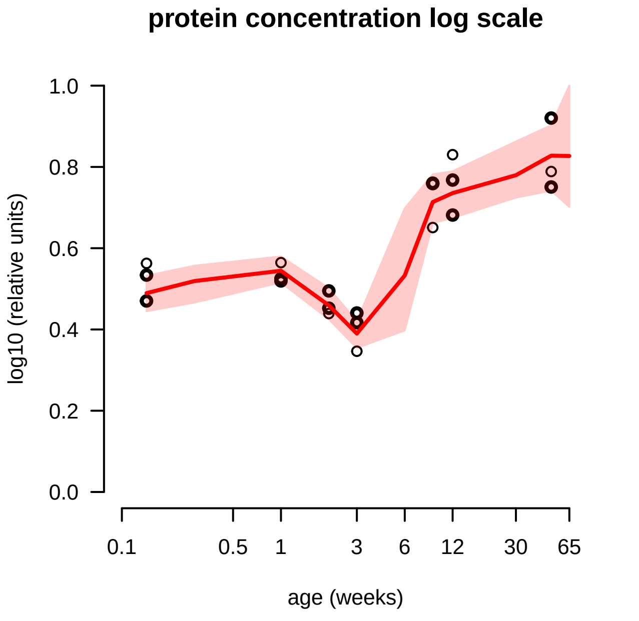

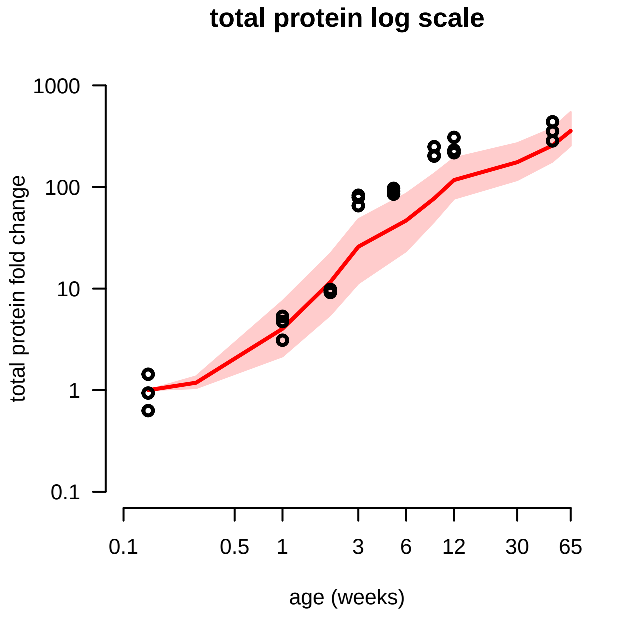

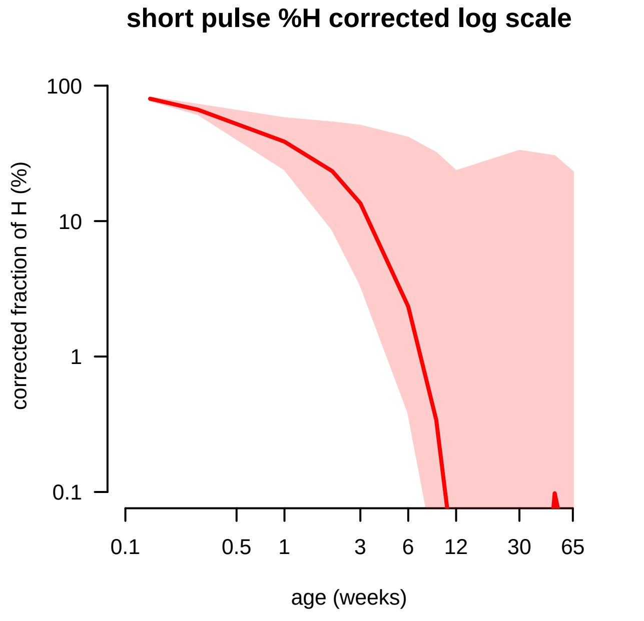

P62313

>sp|P62313|LSM6_MOUSE

U6 snRNA−associated Sm−like protein LSm6

GN=Lsm6

Med H1/2: 10.5 days

Q5 H1/2: 5.1 days

Q95 H1/2: 42.9 days

reliable model fit: yes

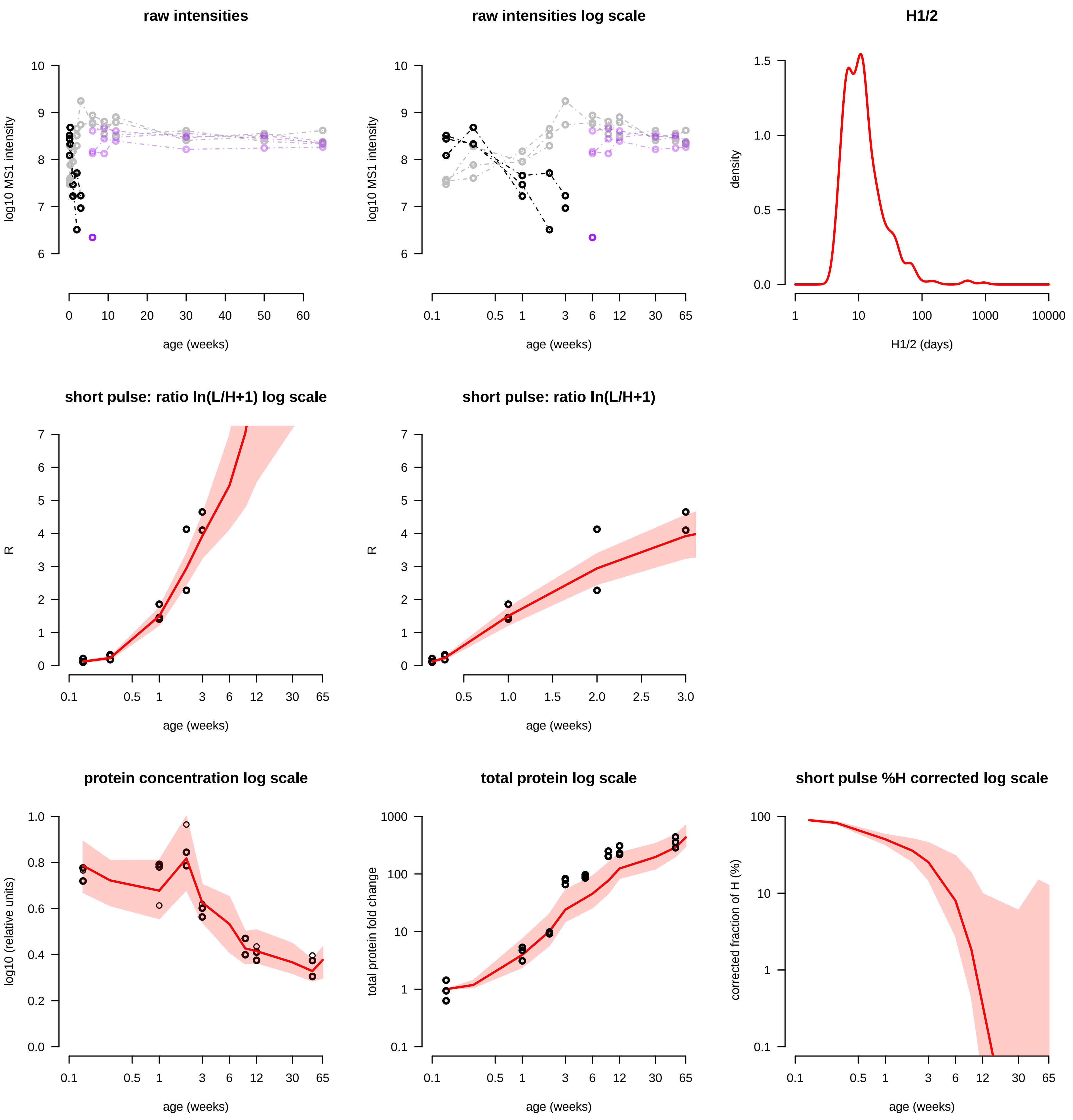

Q3UBX0

>sp|Q3UBX0|TM109_MOUSE

Transmembrane protein 109

GN=Tmem109

Med H1/2: 7.2 days

Q5 H1/2: 3 days

Q95 H1/2: 70.5 days

reliable model fit: yes

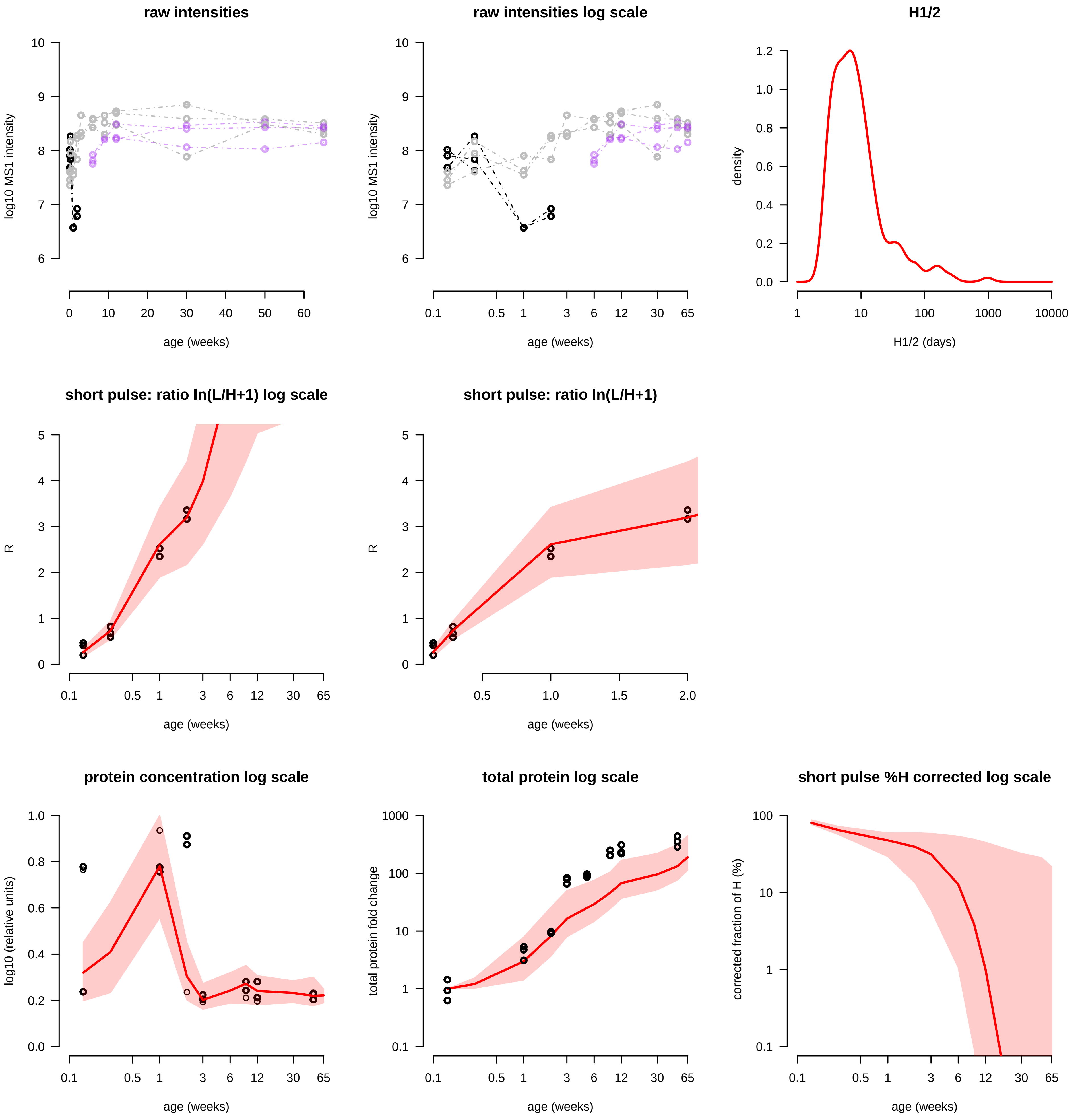

O55013

>sp|O55013|TPPC3_MOUSE

Trafficking protein particle complex subunit 3

GN=Trappc3

Med H1/2: 7 days

Q5 H1/2: 2.7 days

Q95 H1/2: 31.6 days

reliable model fit: yes

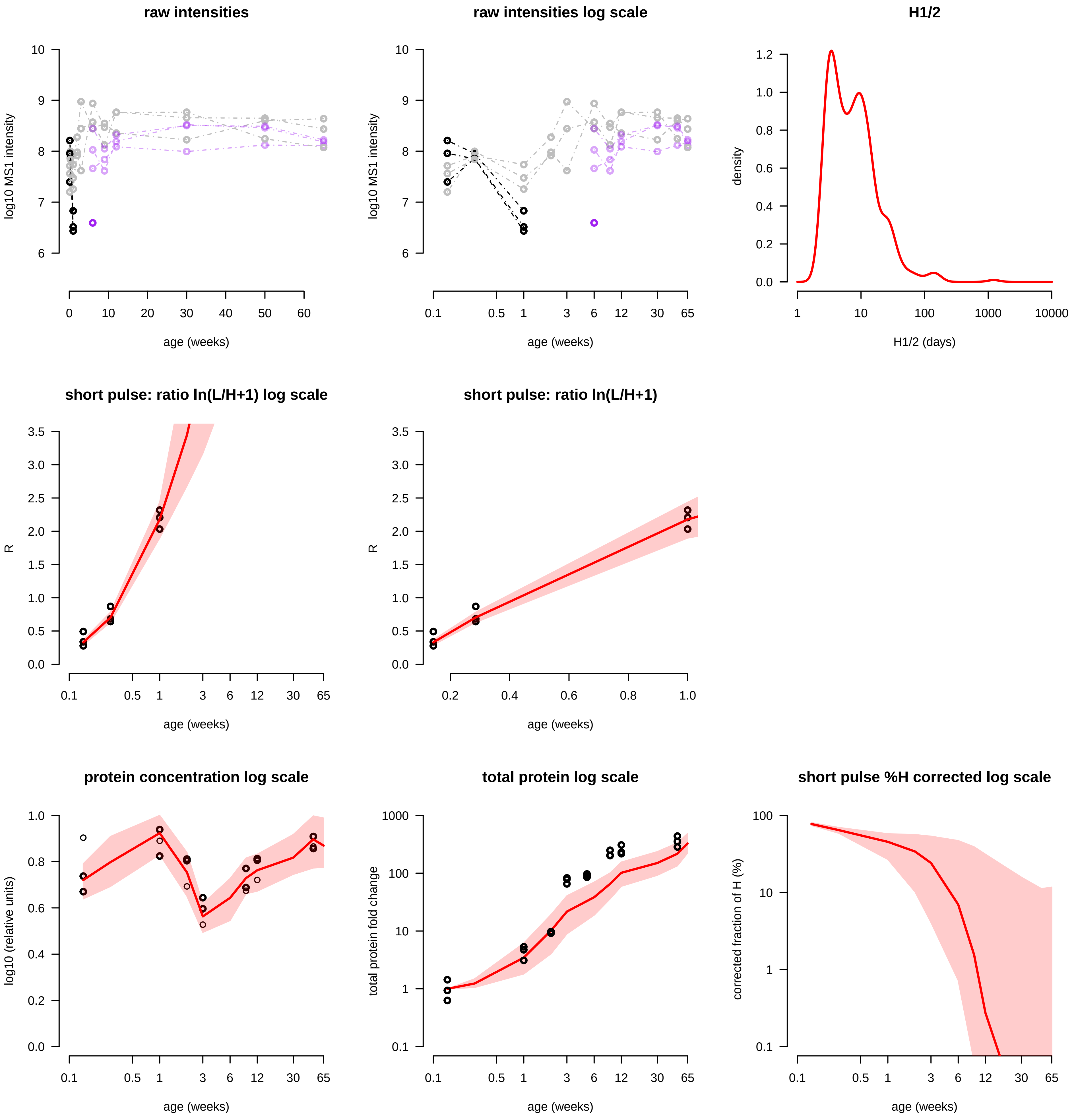

Q6PGC1

>sp|Q6PGC1|DHX29_MOUSE

ATP−dependent RNA helicase DHX29

GN=Dhx29

Med H1/2: 8.4 days

Q5 H1/2: 3.7 days

Q95 H1/2: 59.8 days

reliable model fit: yes

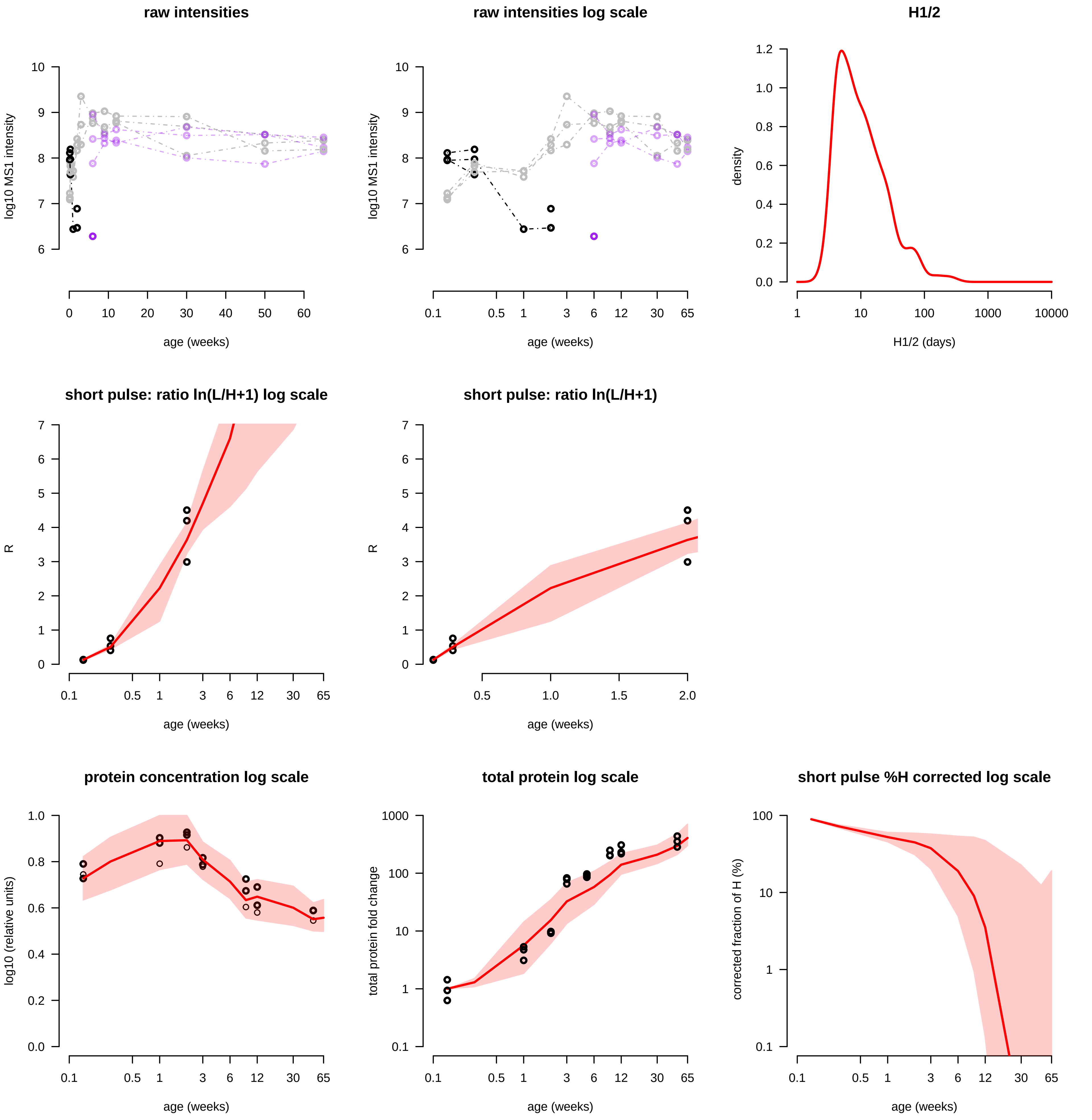

Q64726

>sp|Q64726|ZA2G_MOUSE

Zinc−alpha−2−glycoprotein

GN=Azgp1

Med H1/2: 40.4 days

Q5 H1/2: 9.9 days

Q95 H1/2: 563 days

reliable model fit: yes

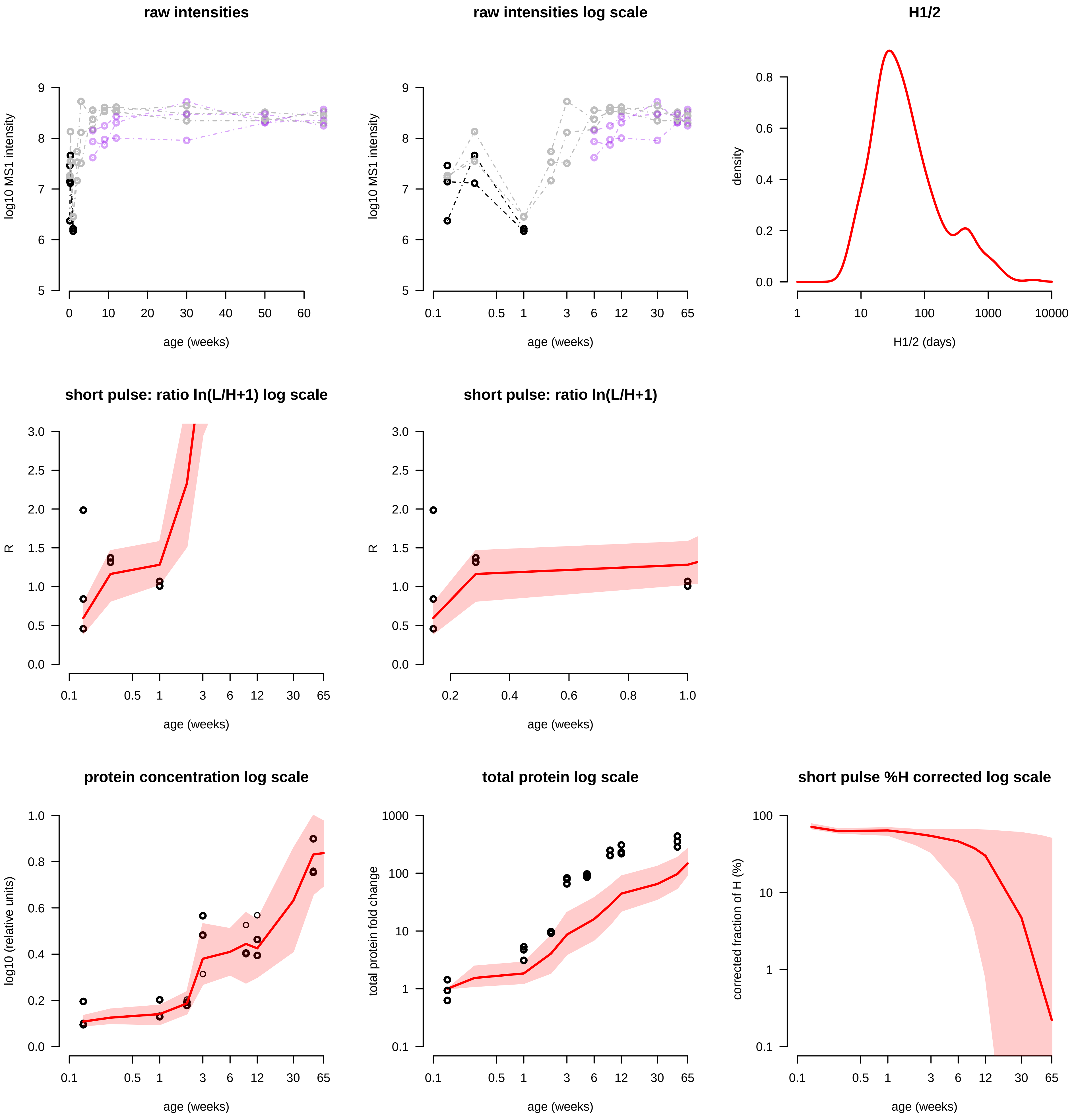

Q63850

>sp|Q63850|NUP62_MOUSE

Nuclear pore glycoprotein p62

GN=Nup62

Med H1/2: 7.9 days

Q5 H1/2: 6.7 days

Q95 H1/2: 11.1 days

reliable model fit: yes

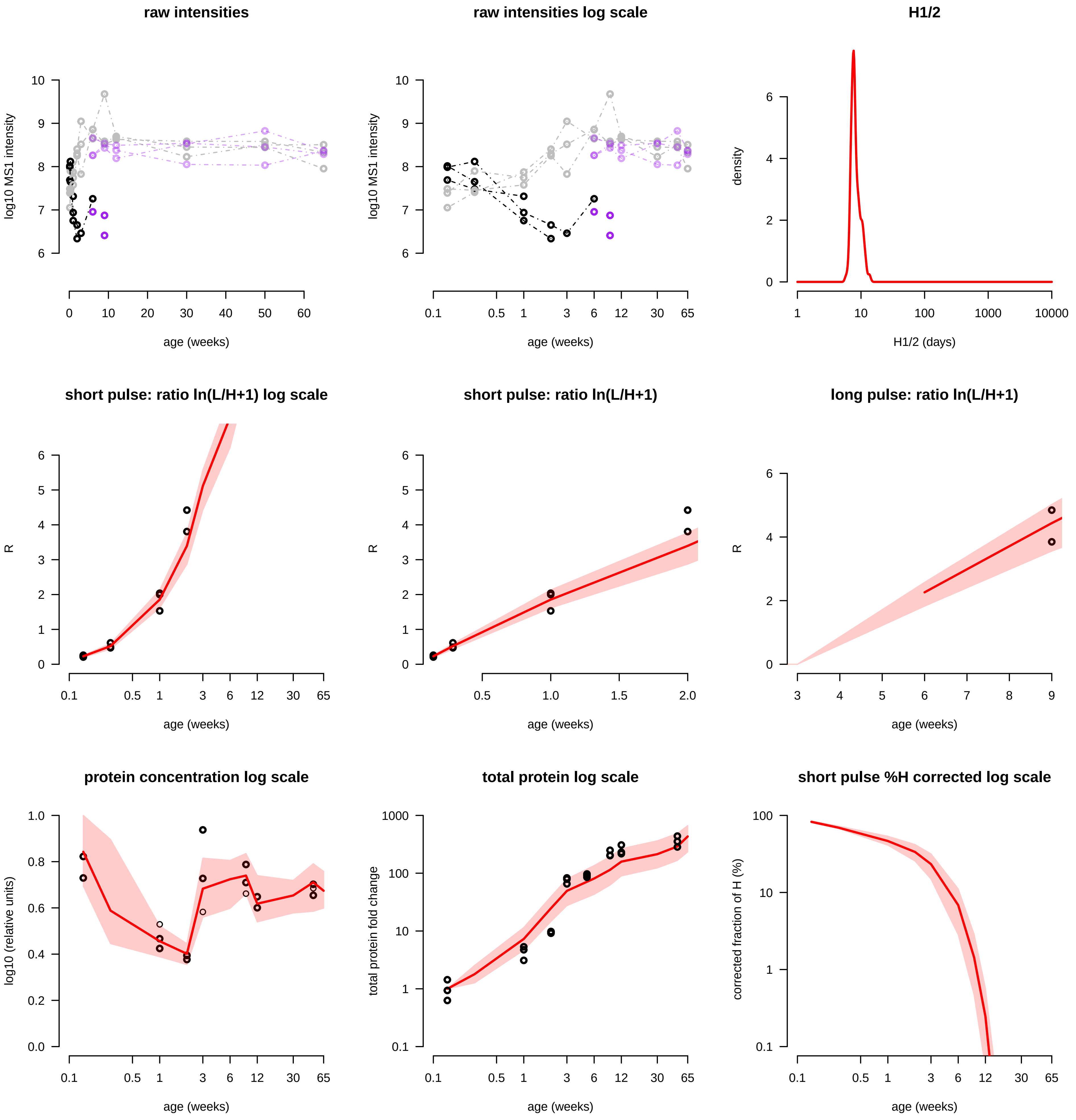

Q9DC50

>sp|Q9DC50|OCTC_MOUSE

Peroxisomal carnitine O−octanoyltransferase

GN=Crot

Med H1/2: 6.1 days

Q5 H1/2: 2.8 days

Q95 H1/2: 89.5 days

reliable model fit: yes

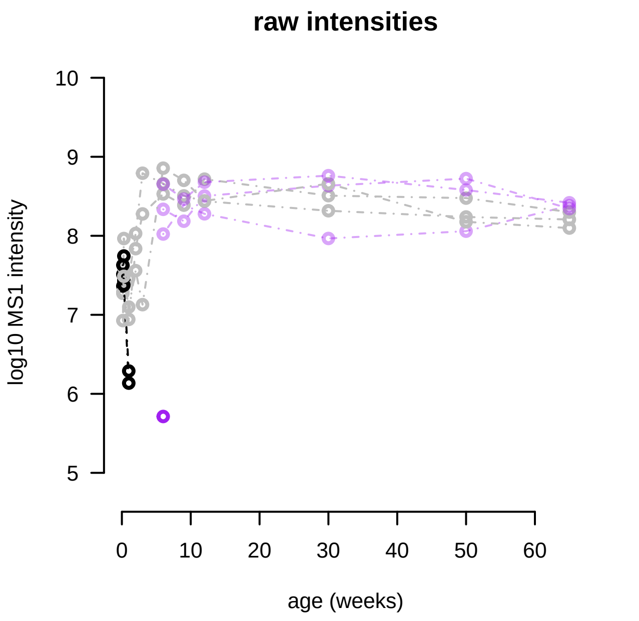

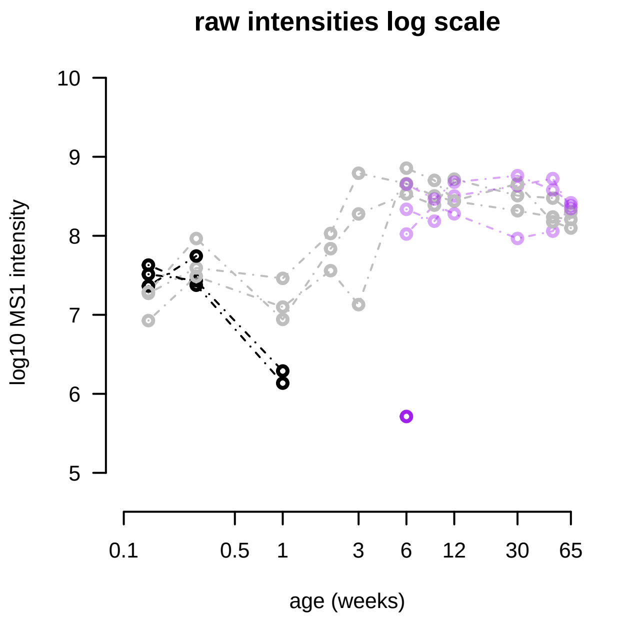

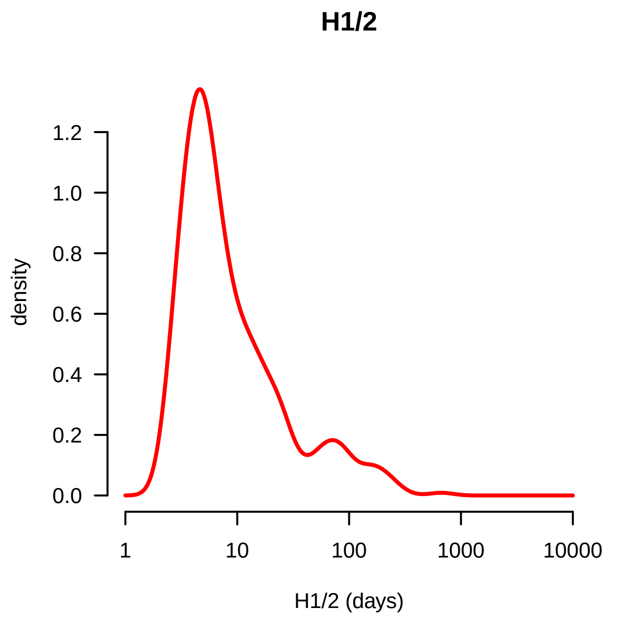

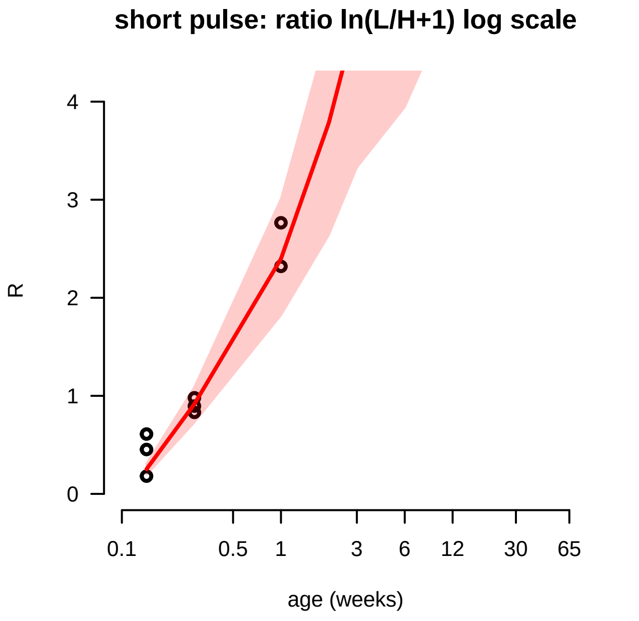

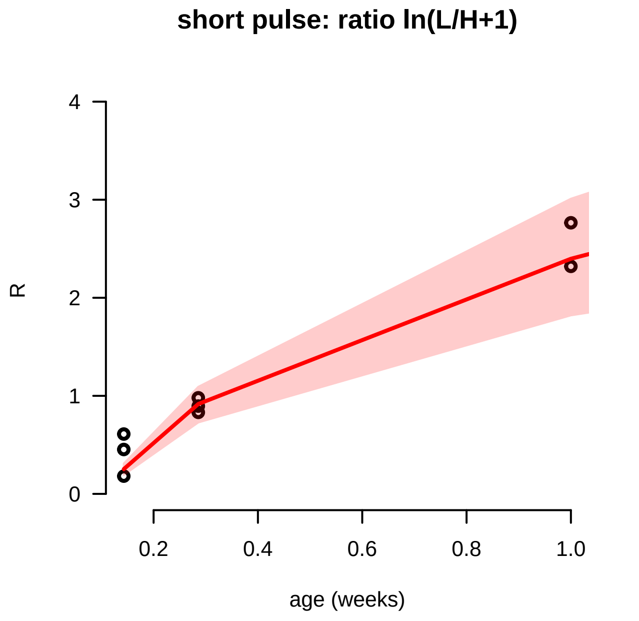

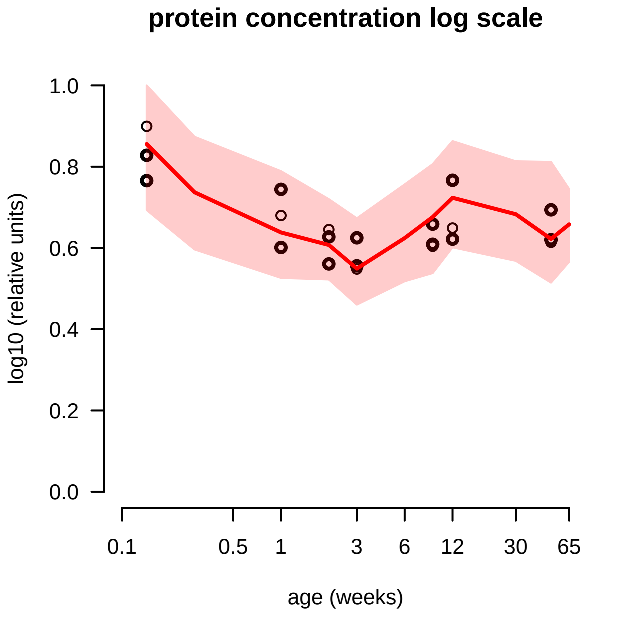

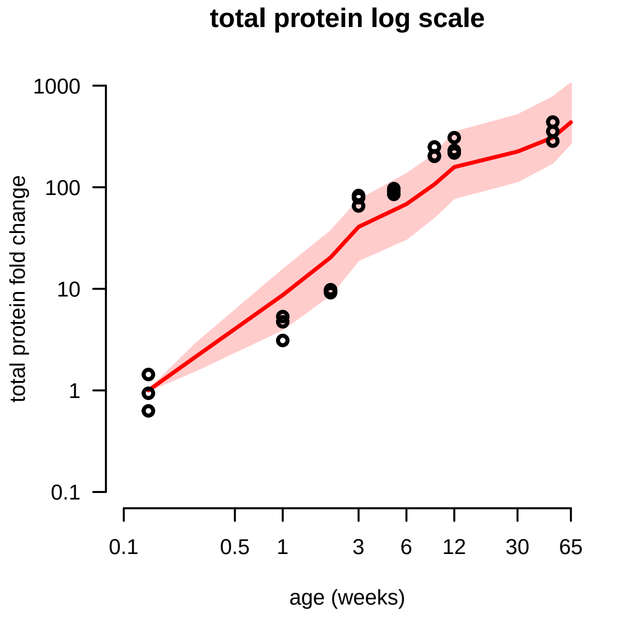

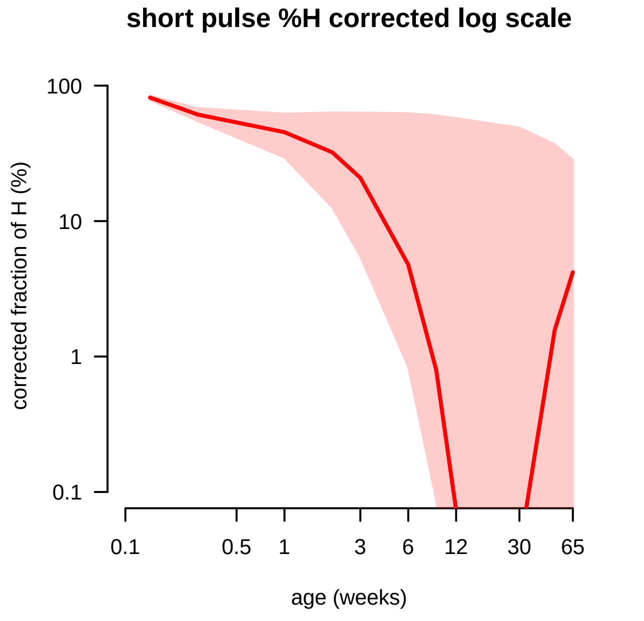

Q9CQ22

>sp|Q9CQ22|LTOR1_MOUSE

Ragulator complex protein LAMTOR1

GN=Lamtor1

Med H1/2: 19.6 days

Q5 H1/2: 7.3 days

Q95 H1/2: 199.2 days

reliable model fit: yes

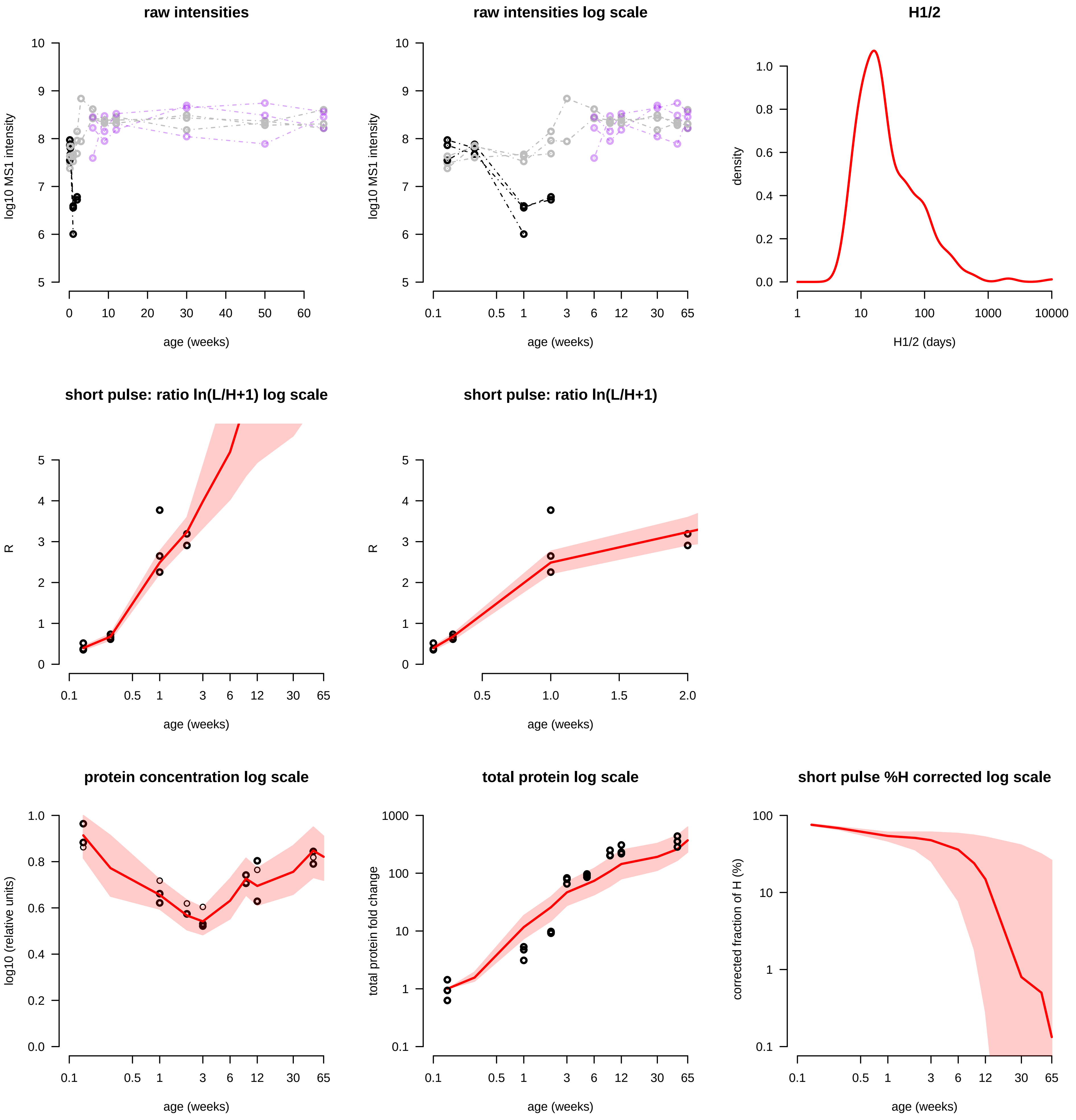

Q9D281

>sp|Q9D281|NXP20_MOUSE

Protein Noxp20

GN=Fam114a1

Med H1/2: 5.8 days

Q5 H1/2: 2.9 days

Q95 H1/2: 48.3 days

reliable model fit: yes

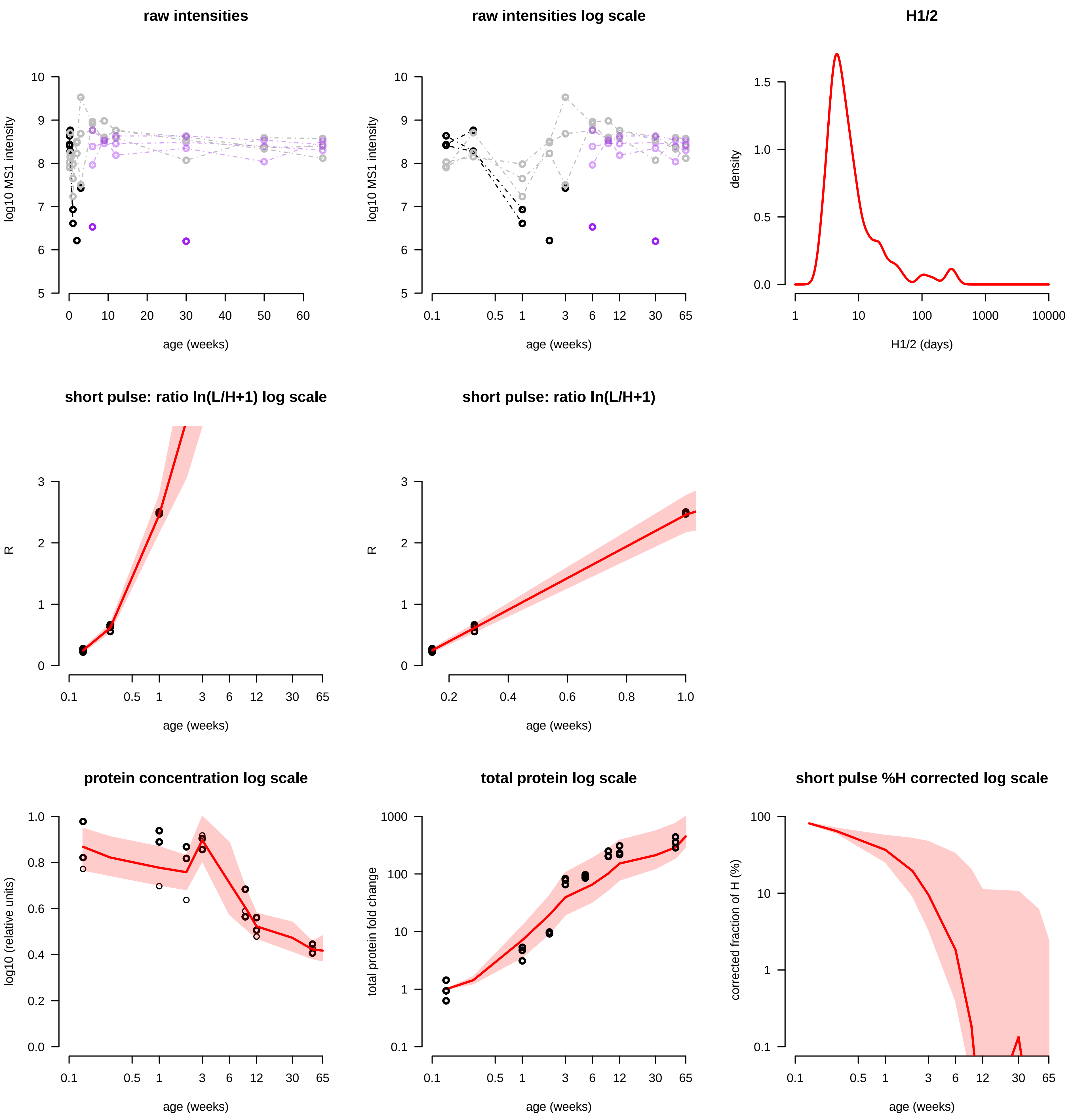

ₗM5;Q9R1M5−3;Q9R1M5−2;Q9R1M5−4;Q9R1M5−7;Q9R1I

>sp|Q9R1M5|NALP5_MOUSE

NACHT, LRR and PYD domains−containing protein 5

GN=Nlrp5

Med H1/2: 8.2 days

Q5 H1/2: 5.8 days

Q95 H1/2: 14.2 days

reliable model fit: yes

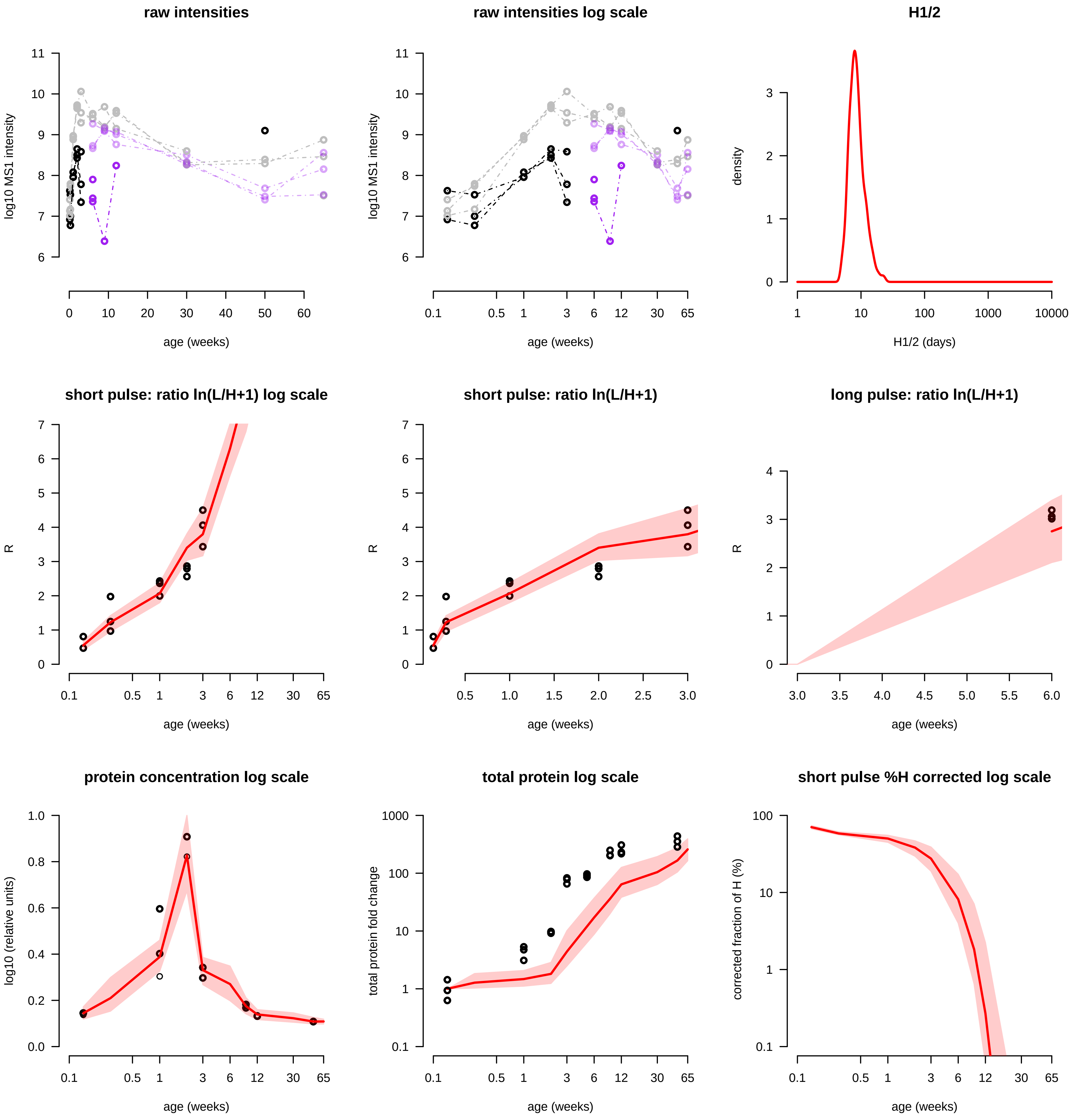

Q8K4L3

>sp|Q8K4L3|SVIL_MOUSE

Supervillin

GN=Svil

Med H1/2: 235.5 days

Q5 H1/2: 164.9 days

Q95 H1/2: 510.6 days

reliable model fit: yes

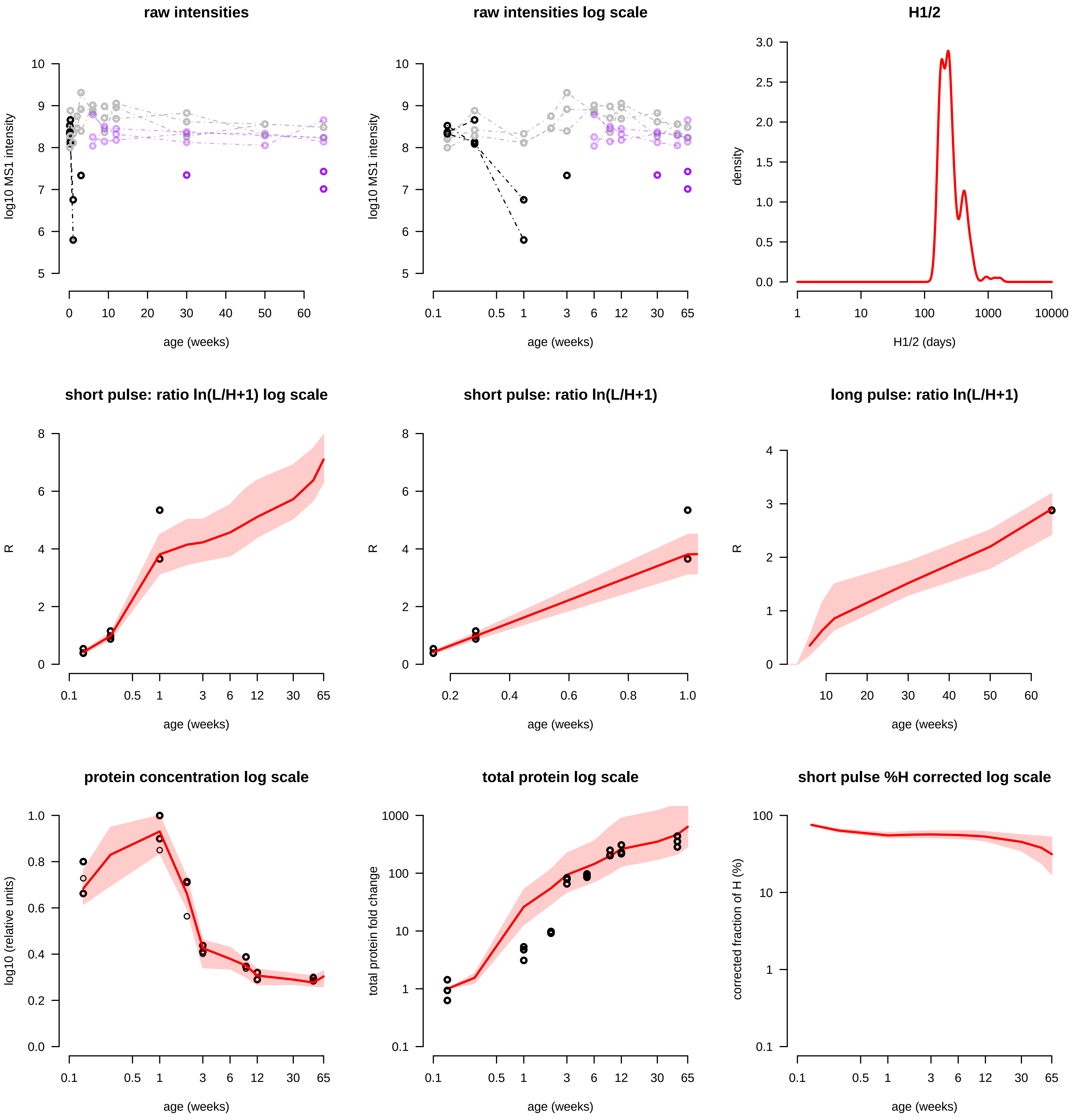

Q9D8X1

>sp|Q9D8X1|CUTC_MOUSE

Copper homeostasis protein cutC homolog

GN=Cutc

Med H1/2: 22.7 days

Q5 H1/2: 7.5 days

Q95 H1/2: 668.2 days

reliable model fit: yes

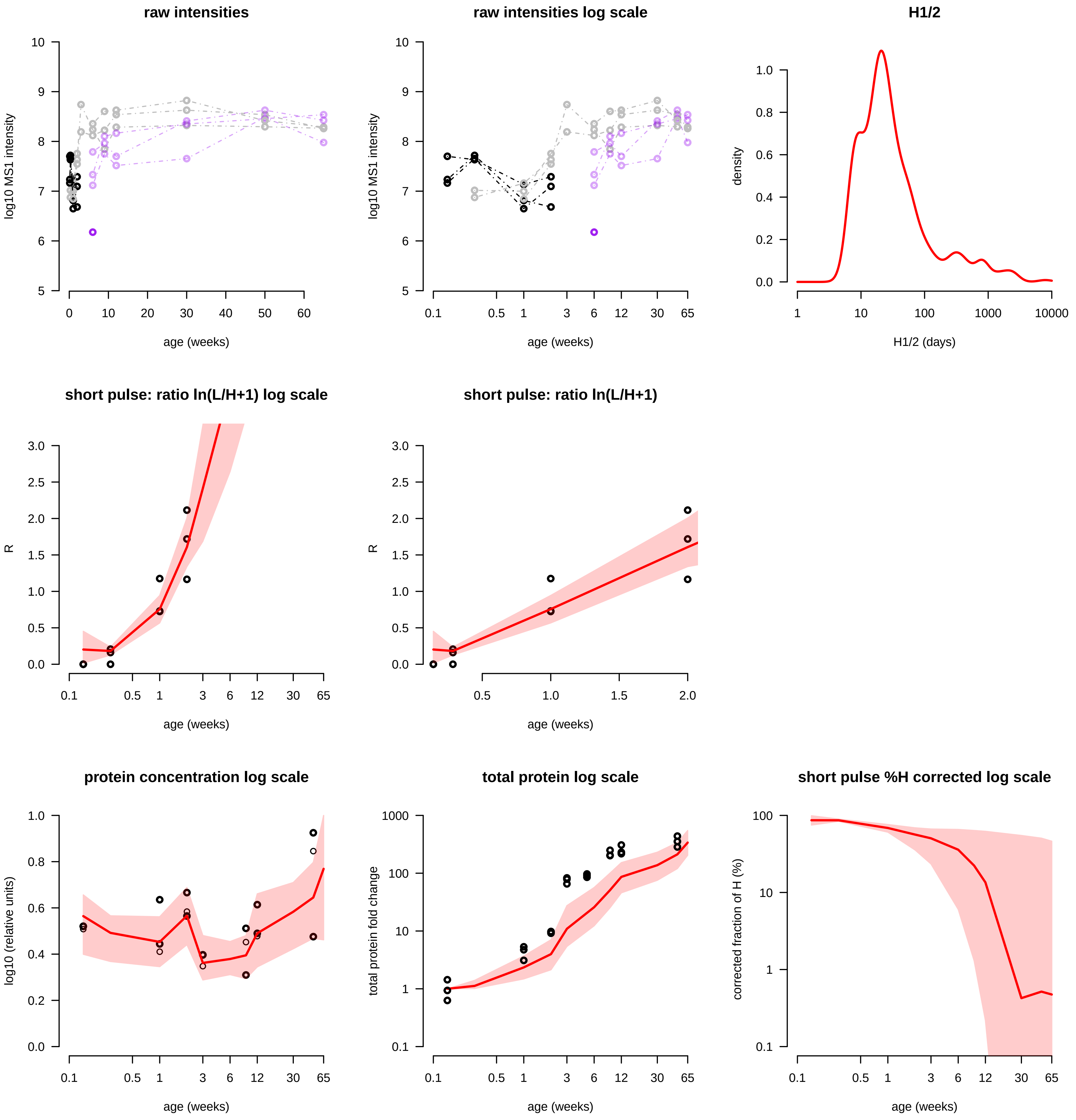

Q80TM9;Q80TM9−2;Q80TM9−3;Q80TM9−6

>sp|Q80TM9|NISCH_MOUSE

Nischarin

GN=Nisch

Med H1/2: 2411.8 days

Q5 H1/2: 936.8 days

Q95 H1/2: 23370.6 days

reliable model fit: yes

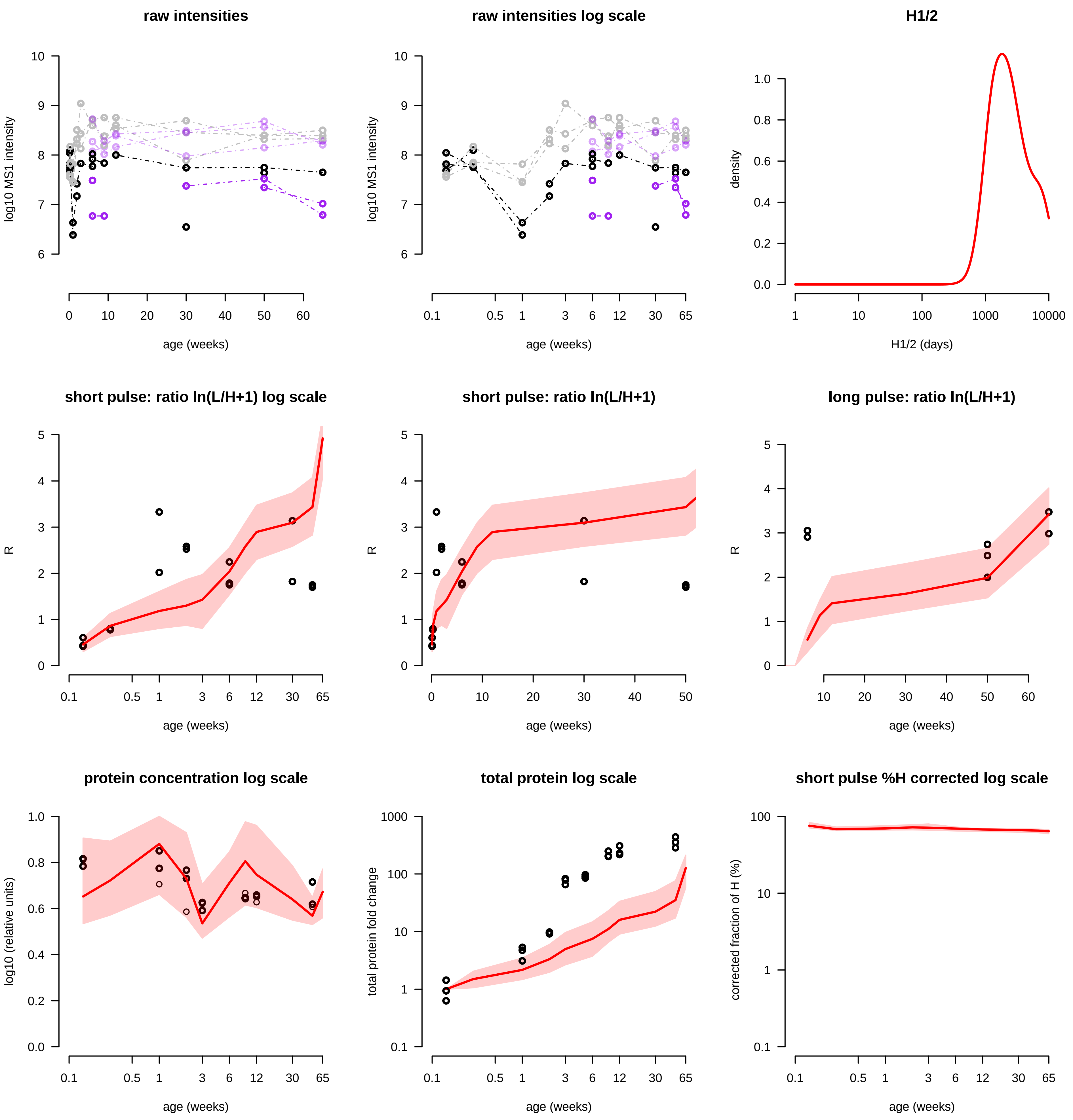

P53564−3

>sp|P53564−3|CUX1_MOUSE

Isoform 3 of Homeobox protein cut−like 1

GN=Cux1

Med H1/2: 20.8 days

Q5 H1/2: 7.7 days

Q95 H1/2: 158.1 days

reliable model fit: yes

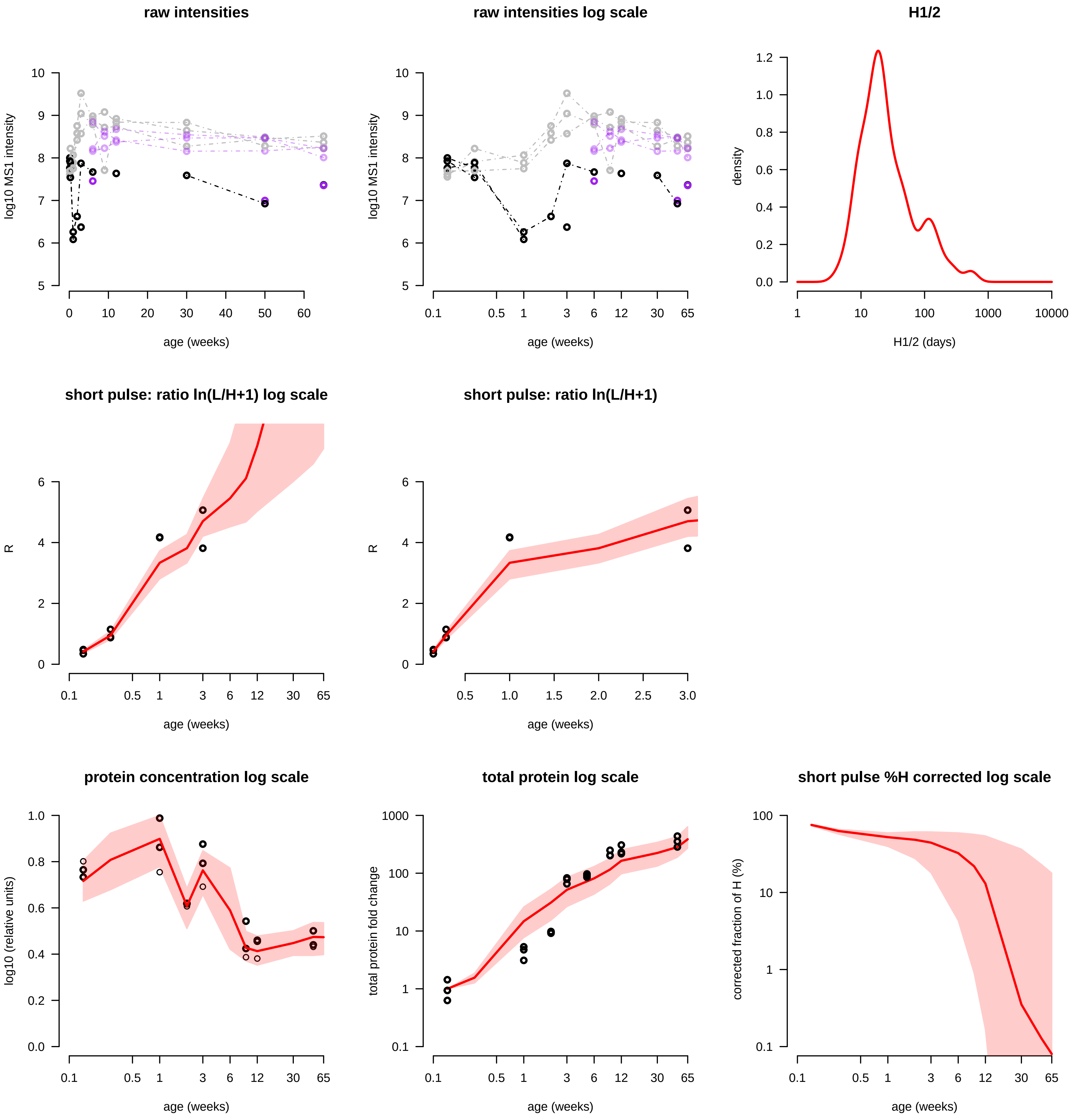

Q8CH25;Q8CH25−2

>sp|Q8CH25|SLTM_MOUSE

SAFB−like transcription modulator

GN=Sltm

Med H1/2: 5.2 days

Q5 H1/2: 3.3 days

Q95 H1/2: 22.5 days

reliable model fit: yes

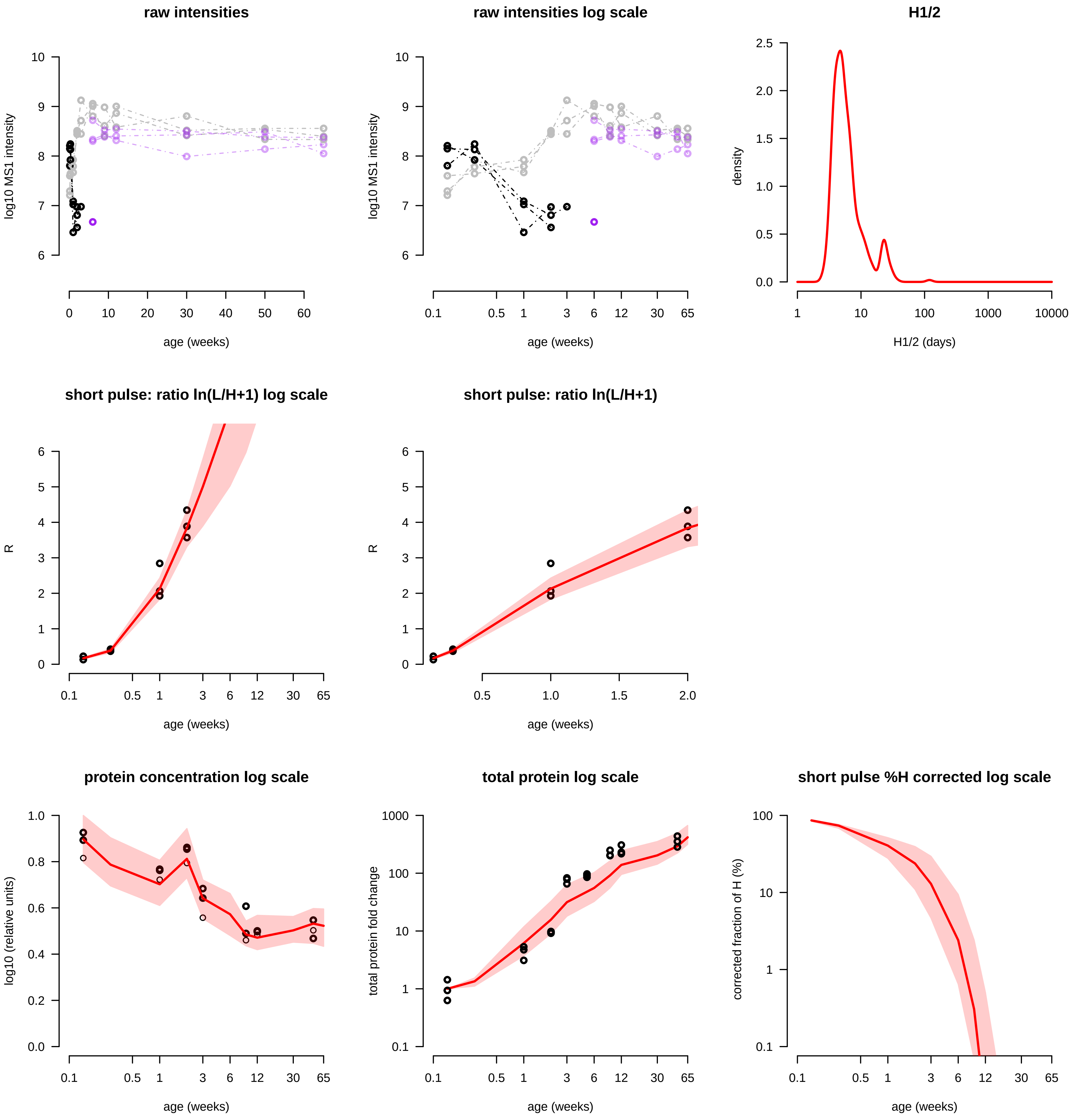

P48193;P48193−2

>sp|P48193|EPB41_MOUSE

Protein 4.1

GN=Epb41

Med H1/2: 5.2 days

Q5 H1/2: 3.9 days

Q95 H1/2: 8.3 days

reliable model fit: yes

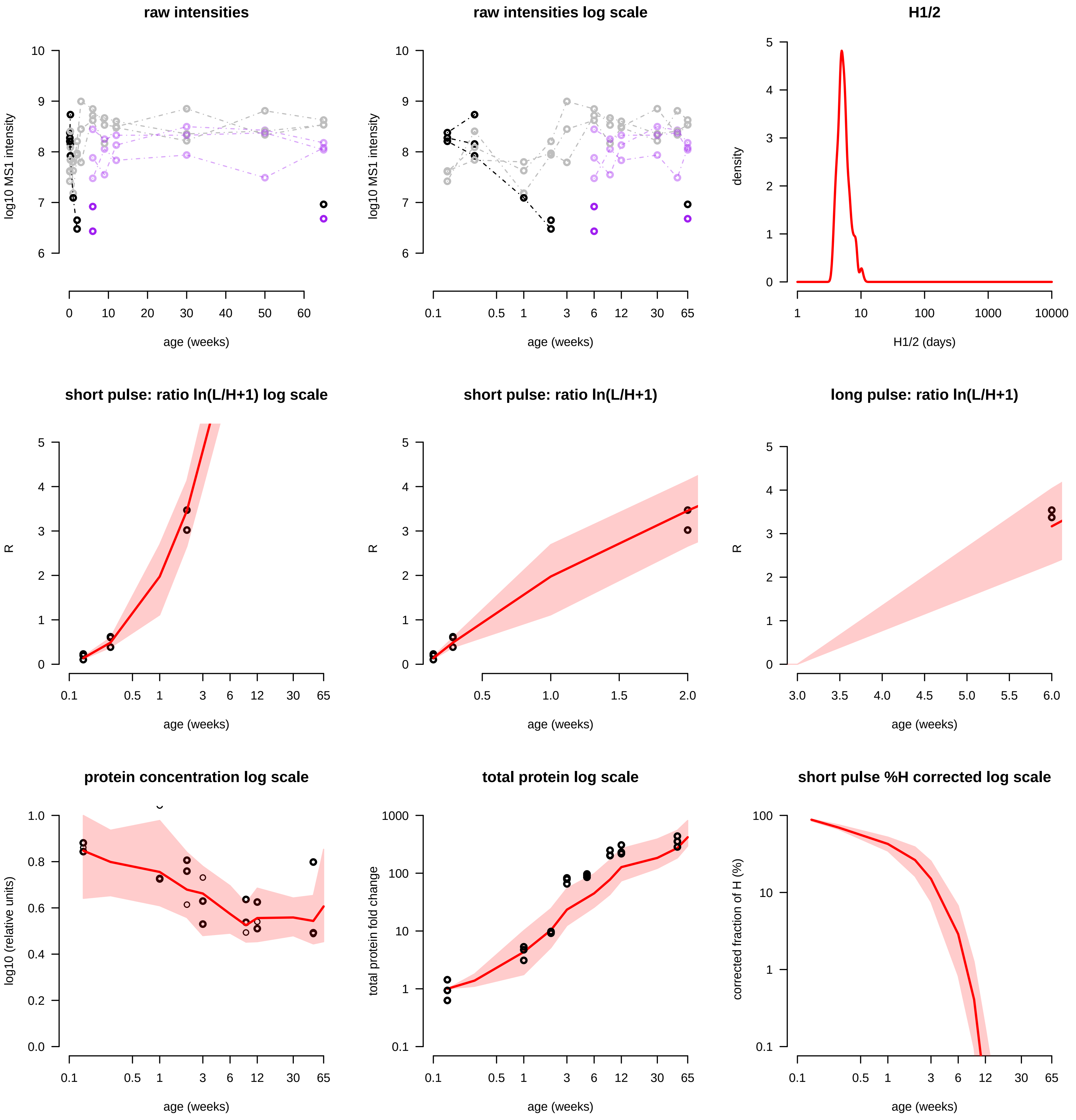

Q9D883

>sp|Q9D883|U2AF1_MOUSE

Splicing factor U2AF 35 kDa subunit

GN=U2af1

Med H1/2: 5 days

Q5 H1/2: 3.8 days

Q95 H1/2: 7.4 days

reliable model fit: yes

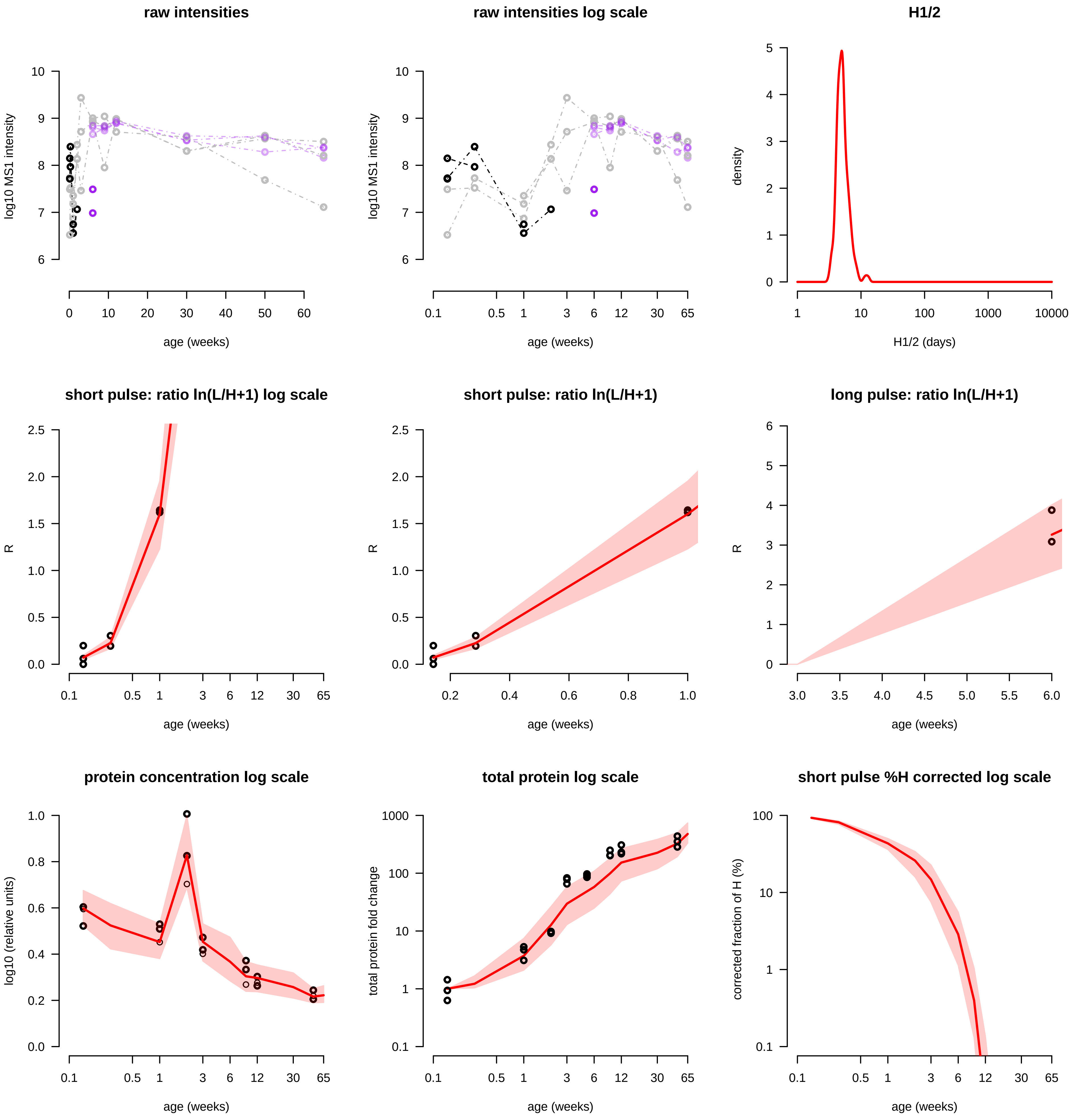

Q8VDD9

>sp|Q8VDD9|PHIP_MOUSE

PH−interacting protein

GN=Phip

Med H1/2: 5188.6 days

Q5 H1/2: 1085 days

Q95 H1/2: 127147.3 days

reliable model fit: yes

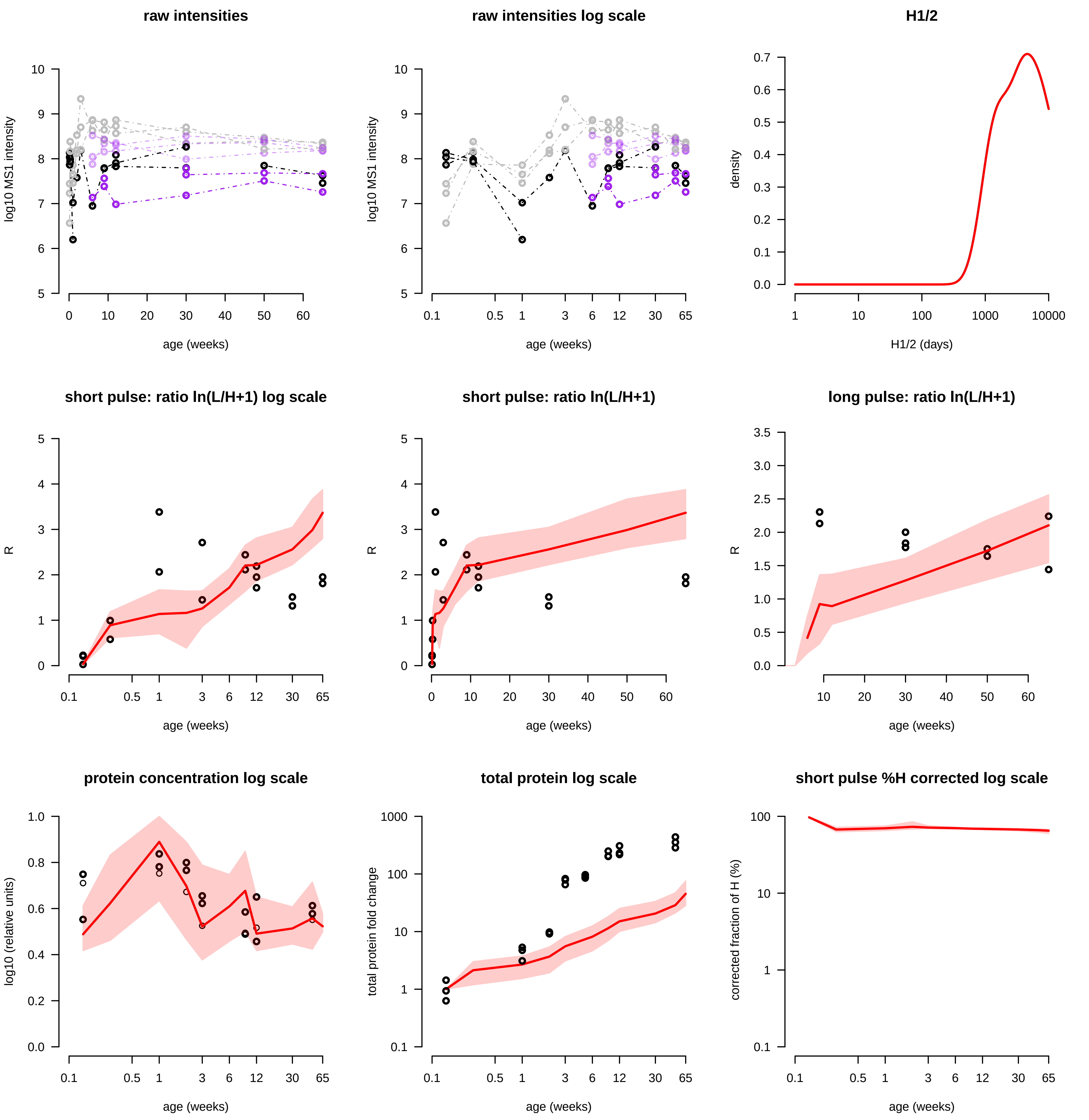

Q80U30−2;Q80U30

>sp|Q80U30−2|CL16A_MOUSE

Isoform 2 of Protein CLEC16A

GN=Clec16a

Med H1/2: 26.4 days

Q5 H1/2: 16.4 days

Q95 H1/2: 101.6 days

reliable model fit: no

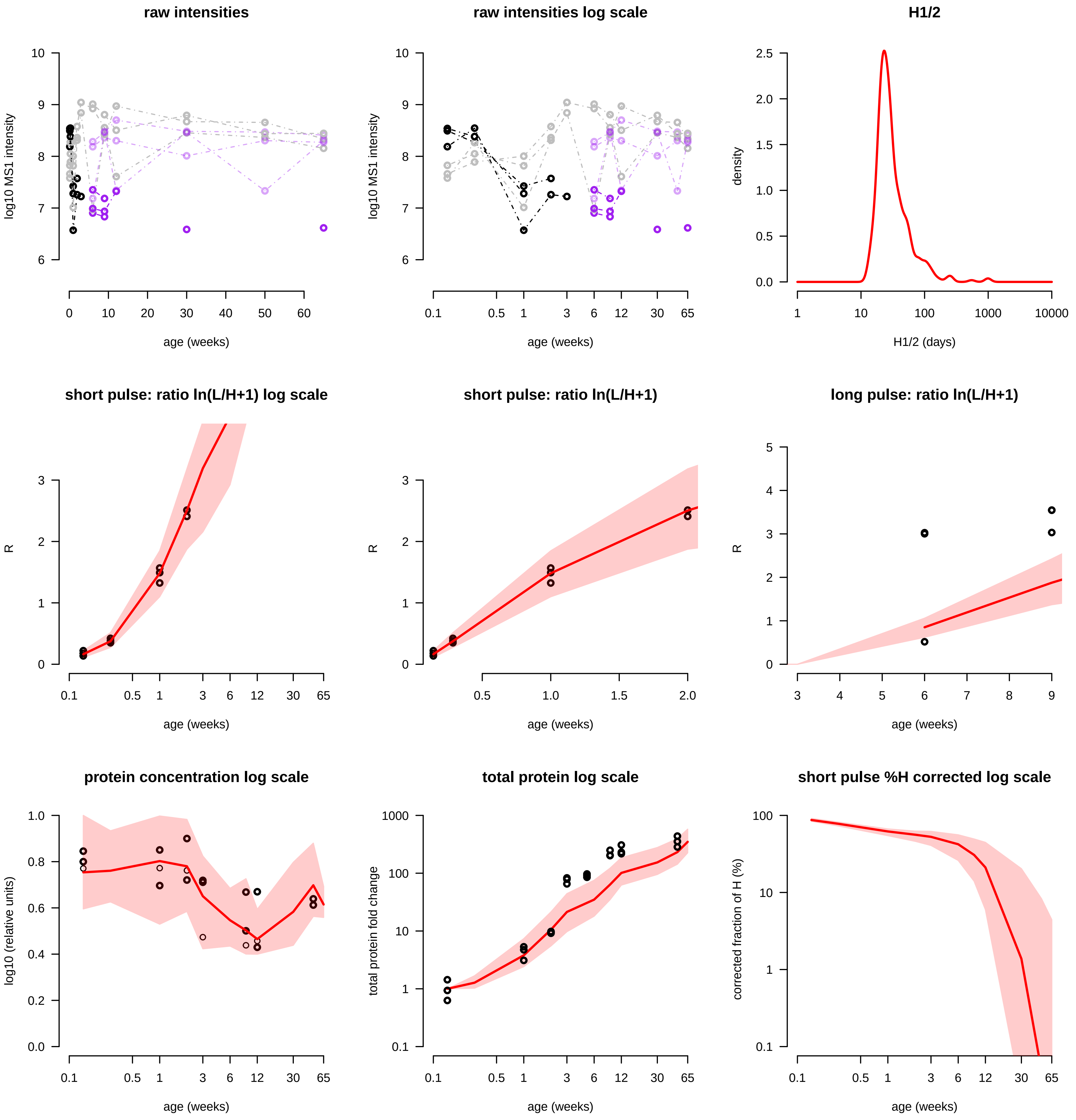

Q8C0L8

>sp|Q8C0L8|COG5_MOUSE

Conserved oligomeric Golgi complex subunit 5

GN=Cog5

Med H1/2: 2671.1 days

Q5 H1/2: 893.1 days

Q95 H1/2: 18161.2 days

reliable model fit: no

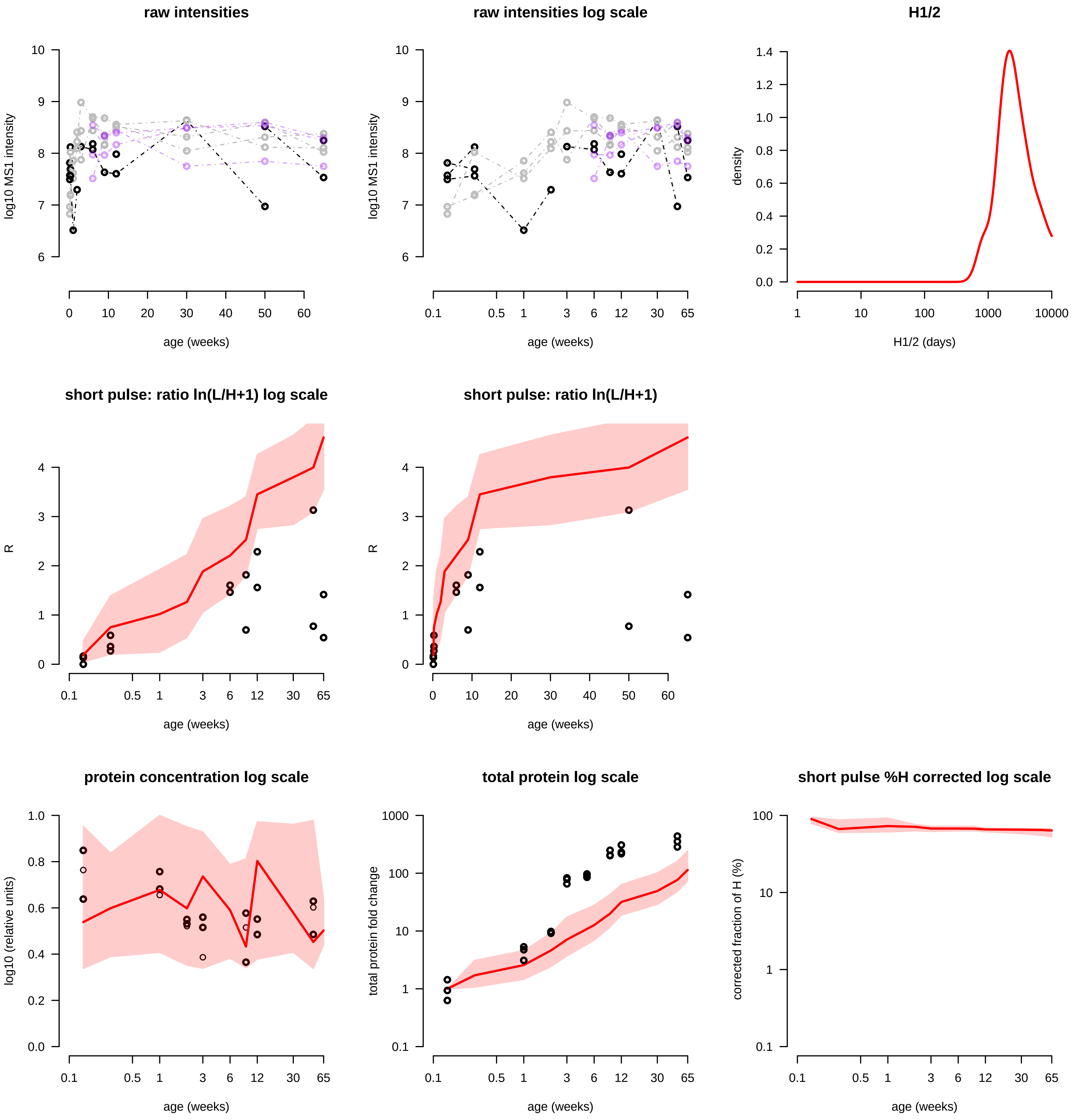

Q6PIP5

>sp|Q6PIP5|NUDC1_MOUSE

NudC domain−containing protein 1

GN=Nudcd1

Med H1/2: 15.8 days

Q5 H1/2: 5.7 days

Q95 H1/2: 180.5 days

reliable model fit: no

raw intensities

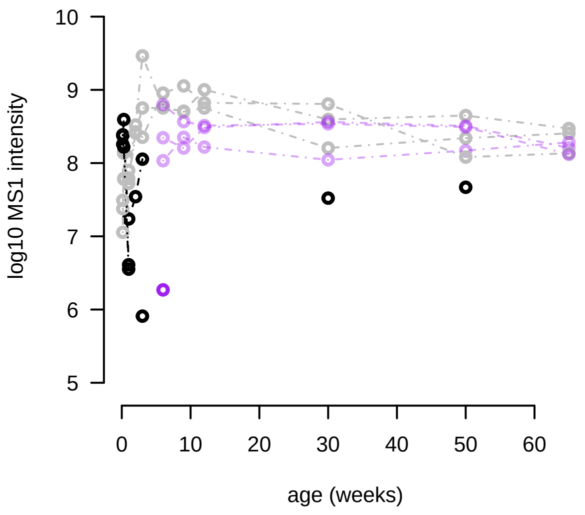

raw intensities log scale

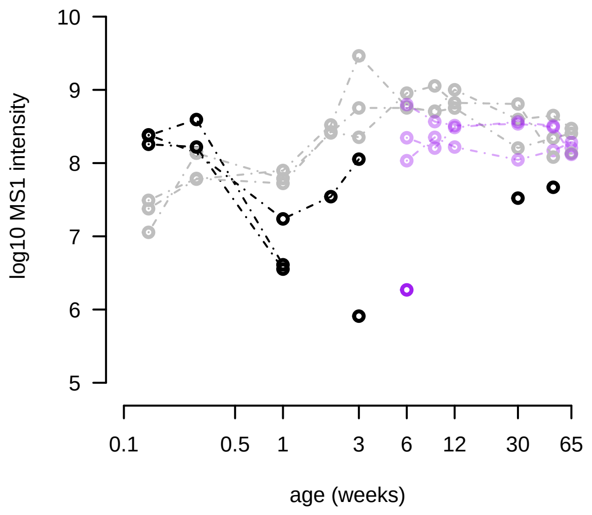

H1/2

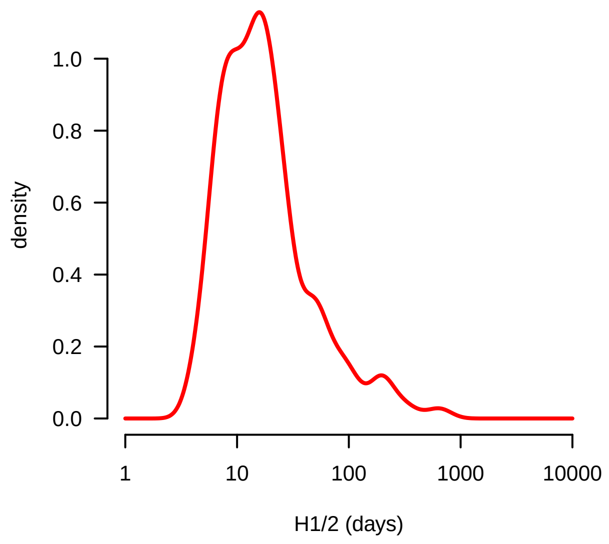

short pulse: ratio ln(L/H+1) log scale

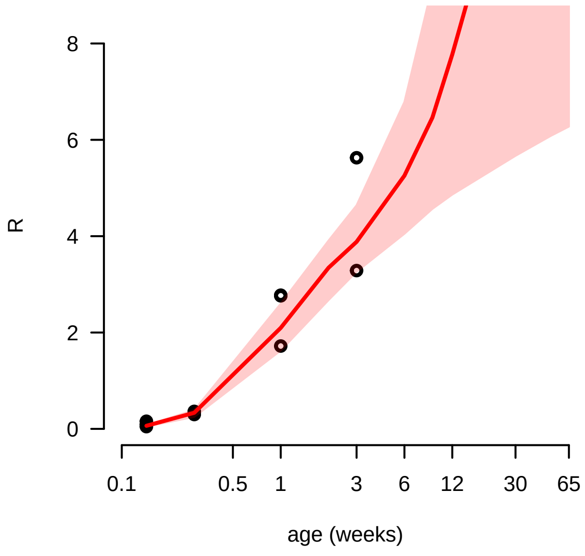

short pulse: ratio ln(L/H+1)

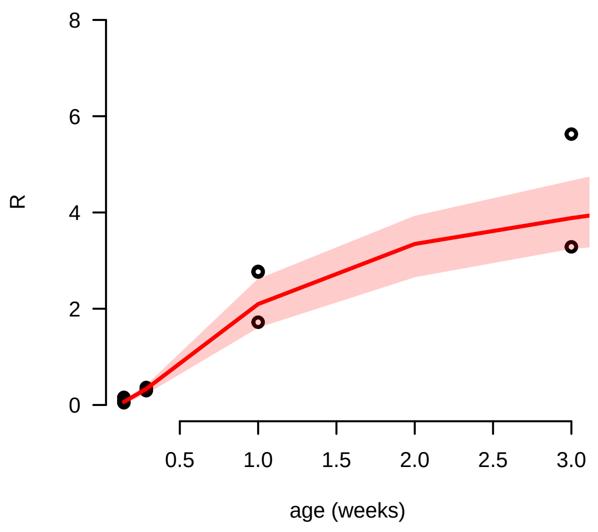

protein concentration log scale

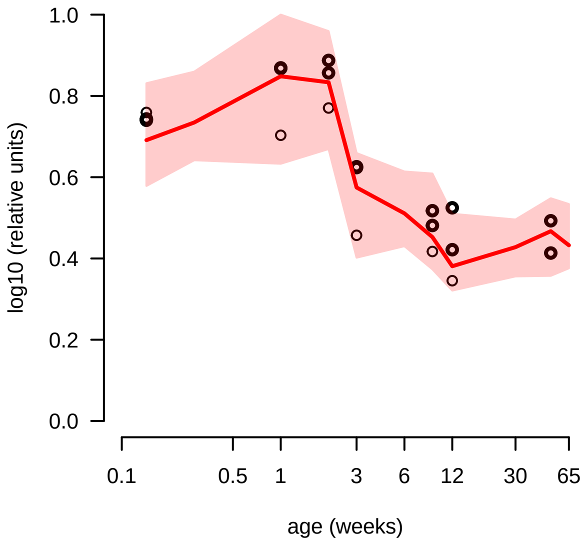

total protein log scale

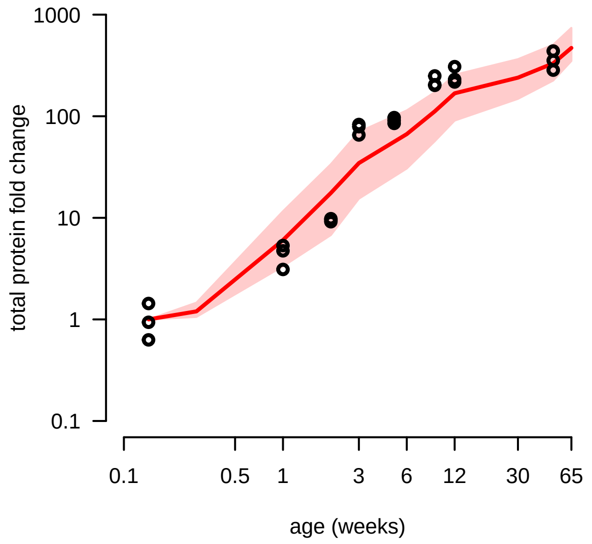

short pulse %H corrected log scale

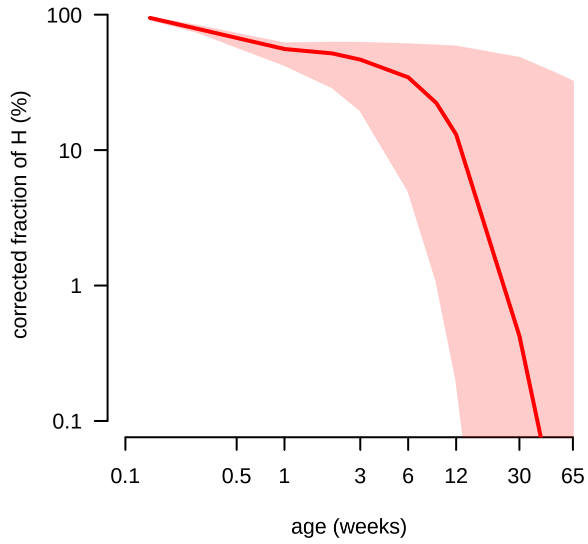

Q9CQE3

>sp|Q9CQE3|RT17_MOUSE

28S ribosomal protein S17, mitochondrial

GN=Mrps17

Med H1/2: 12.9 days

Q5 H1/2: 4.6 days

Q95 H1/2: 97.5 days

reliable model fit: yes

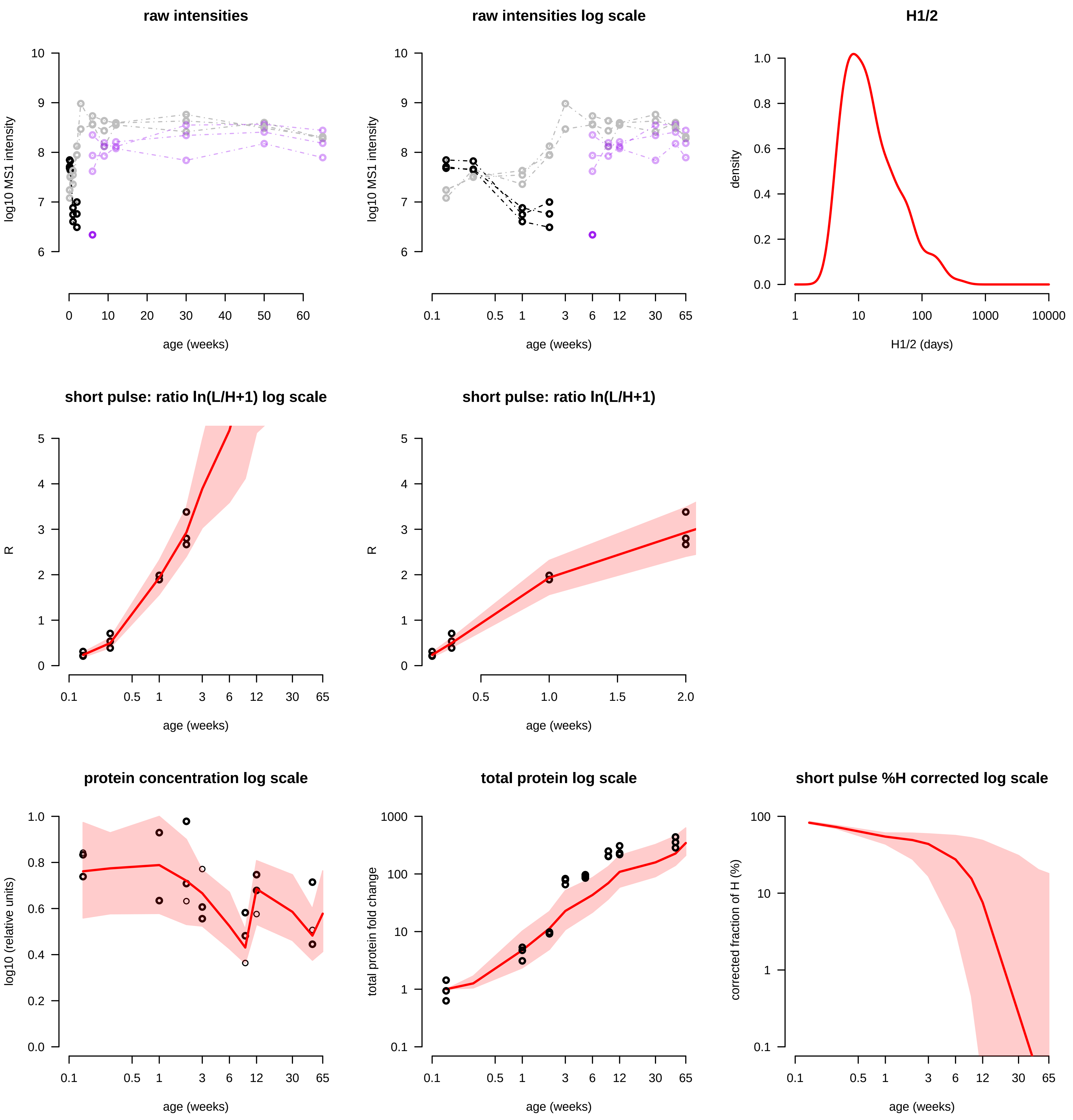

Q9JK81

>sp|Q9JK81|MYG1_MOUSE

MYG1 exonuclease

GN=Myg1

Med H1/2: 9.1 days

Q5 H1/2: 3.5 days

Q95 H1/2: 274.6 days

reliable model fit: no

raw intensities

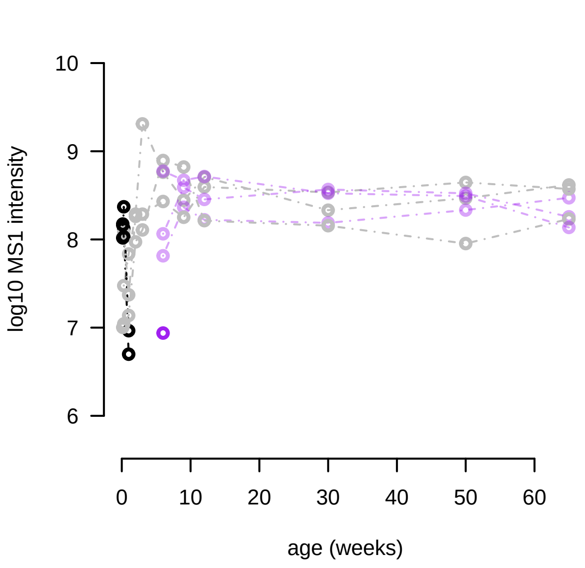

raw intensities log scale

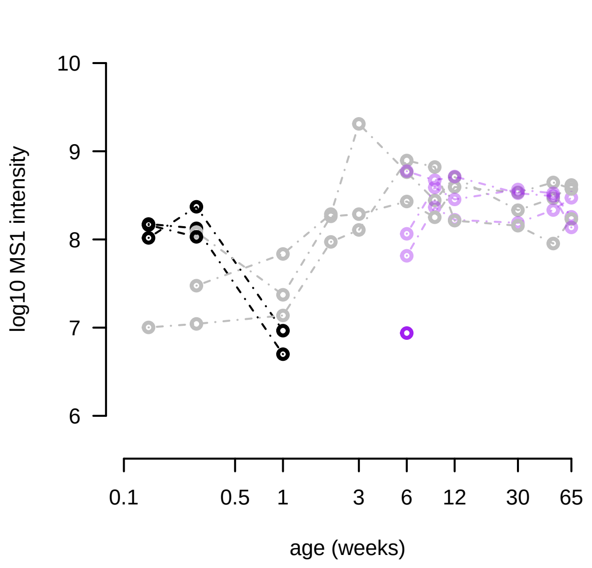

H1/2

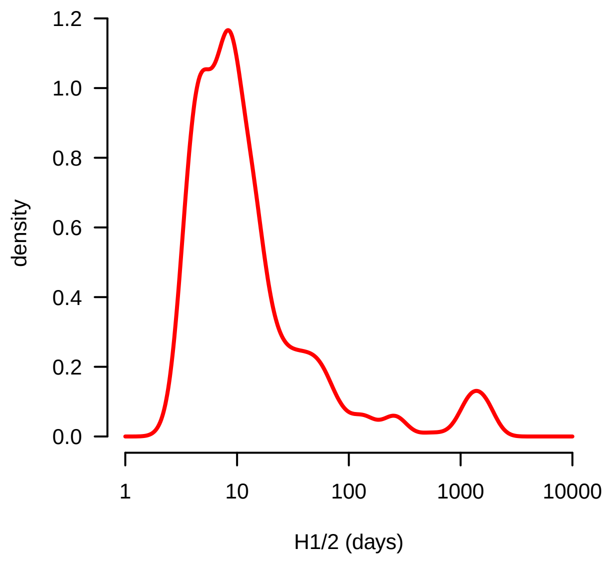

short pulse: ratio ln(L/H+1) log scale

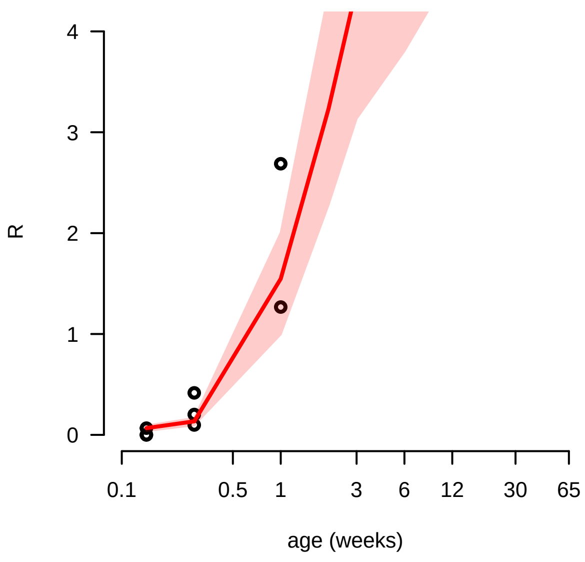

short pulse: ratio ln(L/H+1)

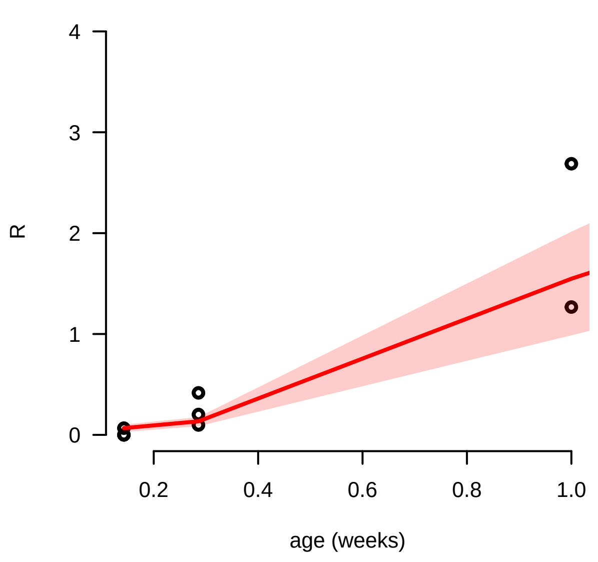

protein concentration log scale

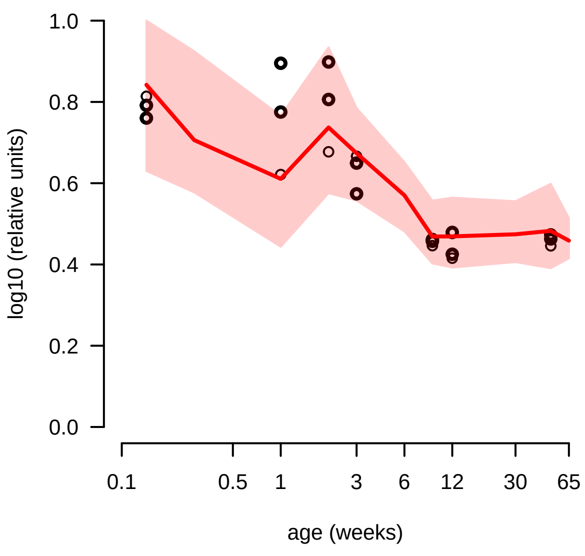

total protein log scale

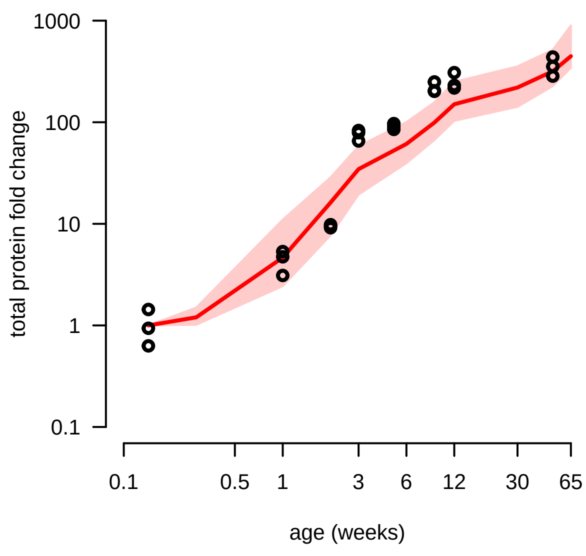

short pulse %H corrected log scale

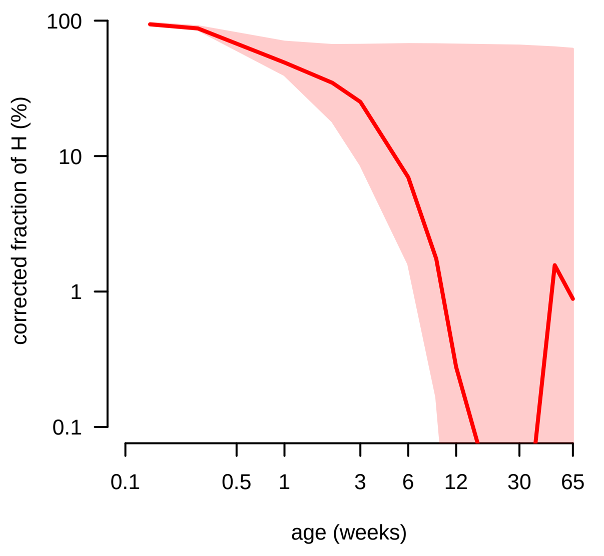

Q921S7

>sp|Q921S7|RM37_MOUSE

39S ribosomal protein L37, mitochondrial

GN=Mrpl37

Med H1/2: 13.4 days

Q5 H1/2: 4.9 days

Q95 H1/2: 127.5 days

reliable model fit: no

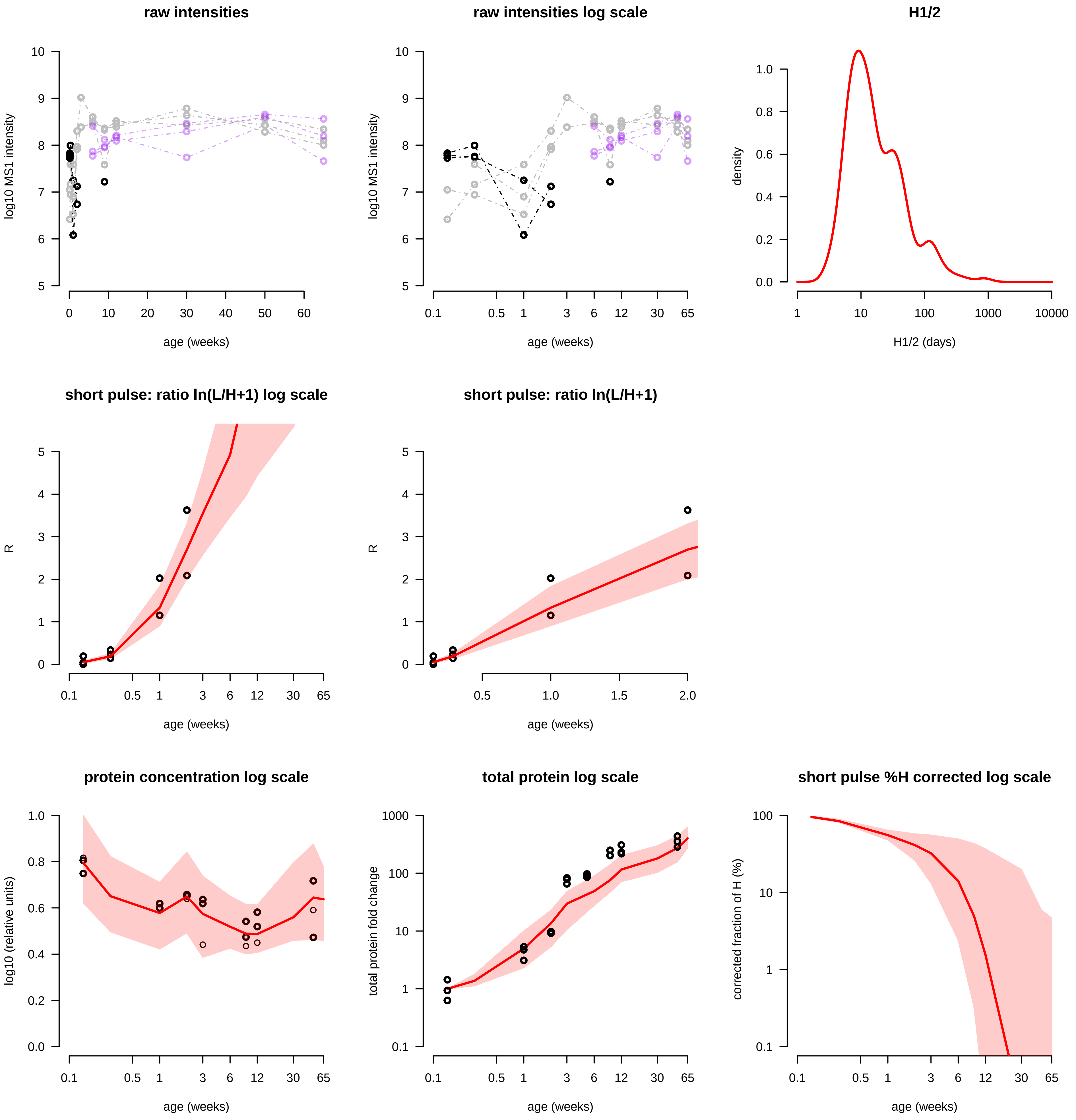

Q9ERS2

>sp|Q9ERS2|NDUAD_MOUSE

NADH dehydrogenase [ubiquinone] 1 alpha subcomplex subunit 13

GN=Ndufa13

Med H1/2: 7.4 days

Q5 H1/2: 5 days

Q95 H1/2: 12.9 days

reliable model fit: yes

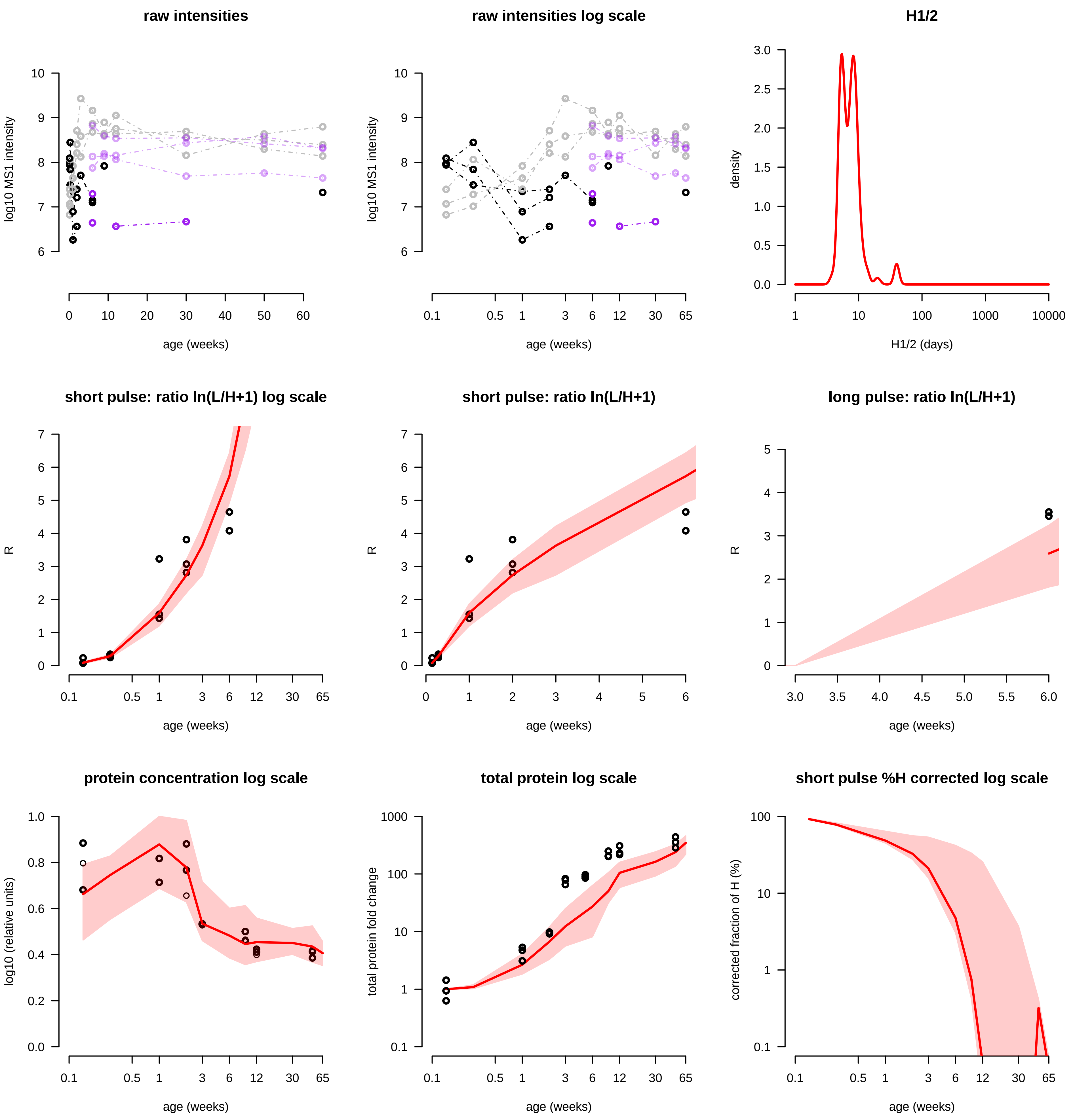

Q61550

>sp|Q61550|RAD21_MOUSE

Double−strand−break repair protein rad21 homolog

GN=Rad21

Med H1/2: 8.6 days

Q5 H1/2: 4.7 days

Q95 H1/2: 44 days

reliable model fit: yes

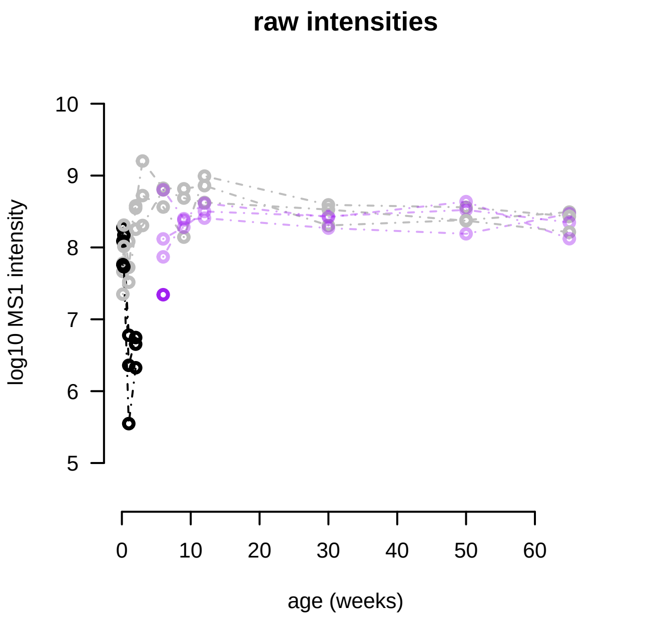

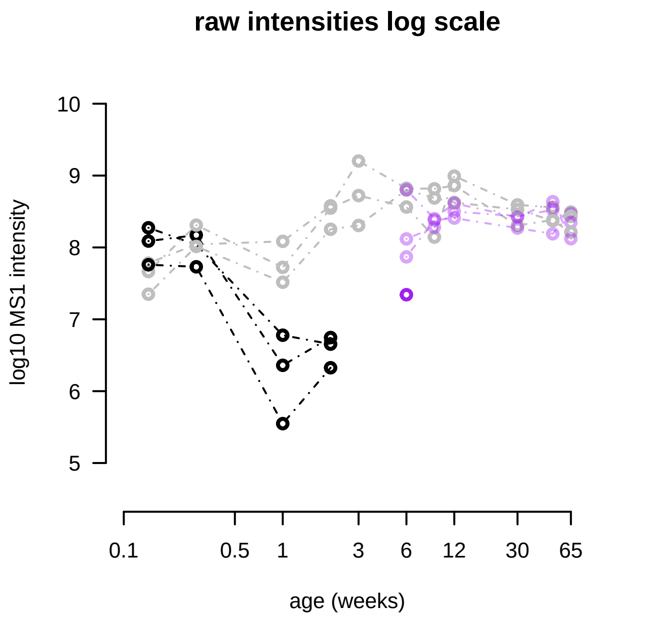

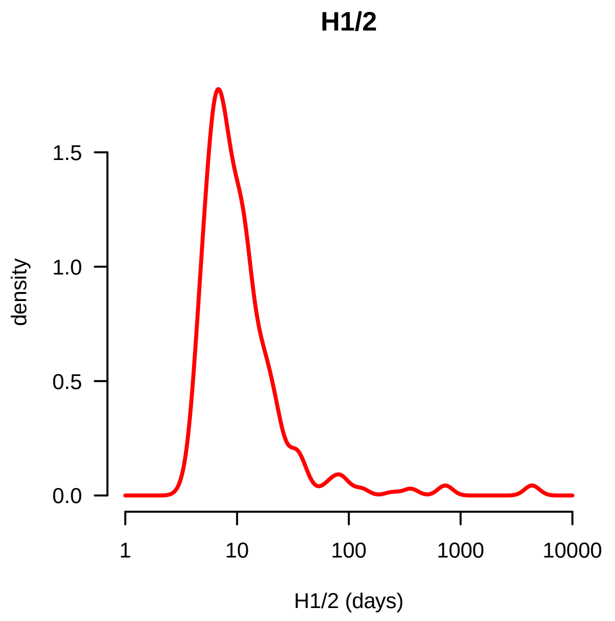

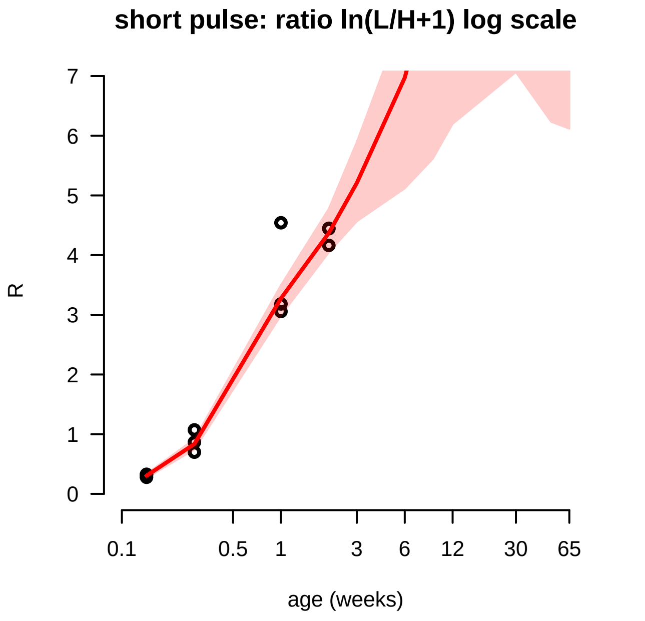

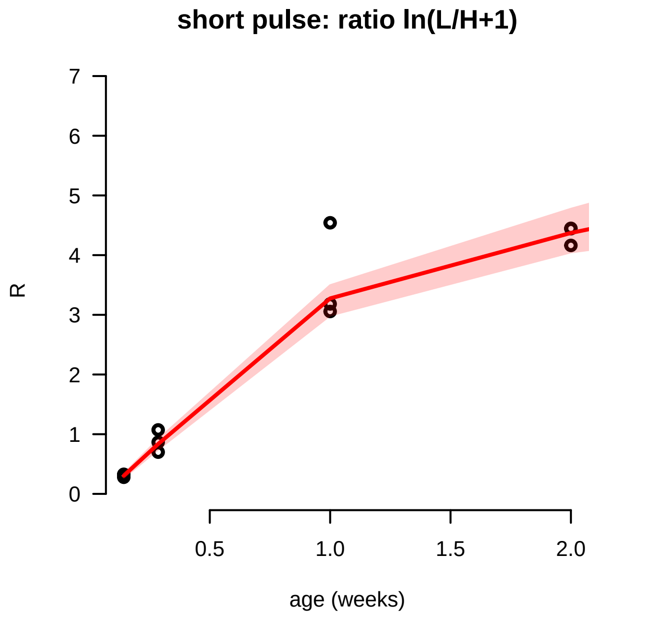

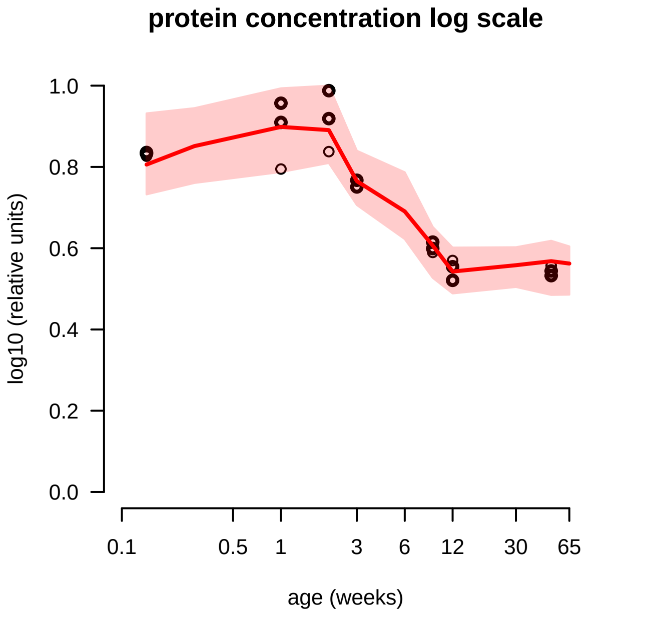

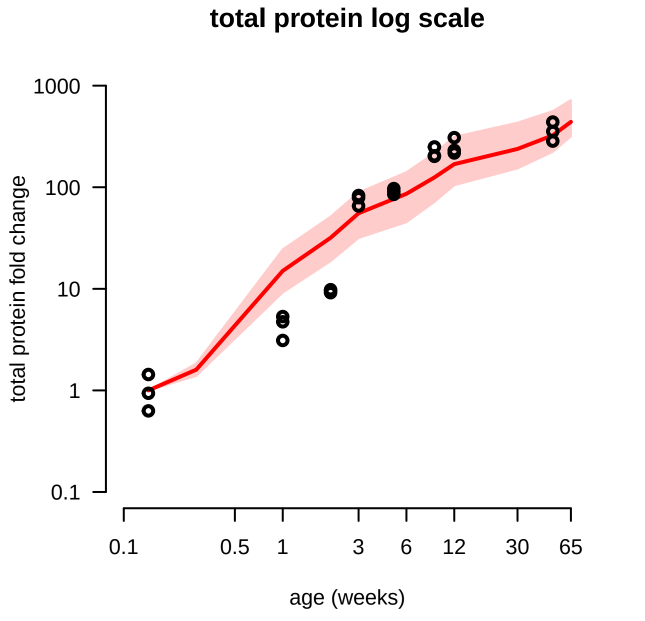

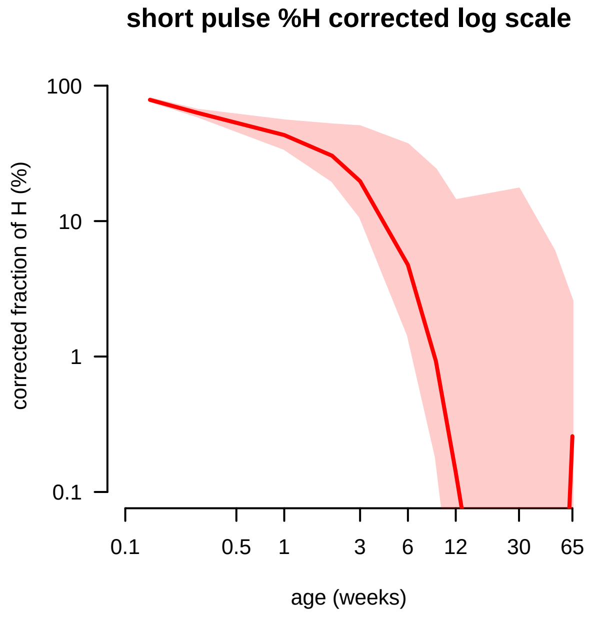

Q6NZN0−5;Q6NZN0−3;Q6NZN0−4;Q6NZN0−2;Q6NZN0

>sp|Q6NZN0−5|RBM26_MOUSE

Isoform 5 of RNA−binding protein 26

GN=Rbm26

Med H1/2: 4.8 days

Q5 H1/2: 2.9 days

Q95 H1/2: 10.7 days

reliable model fit: yes

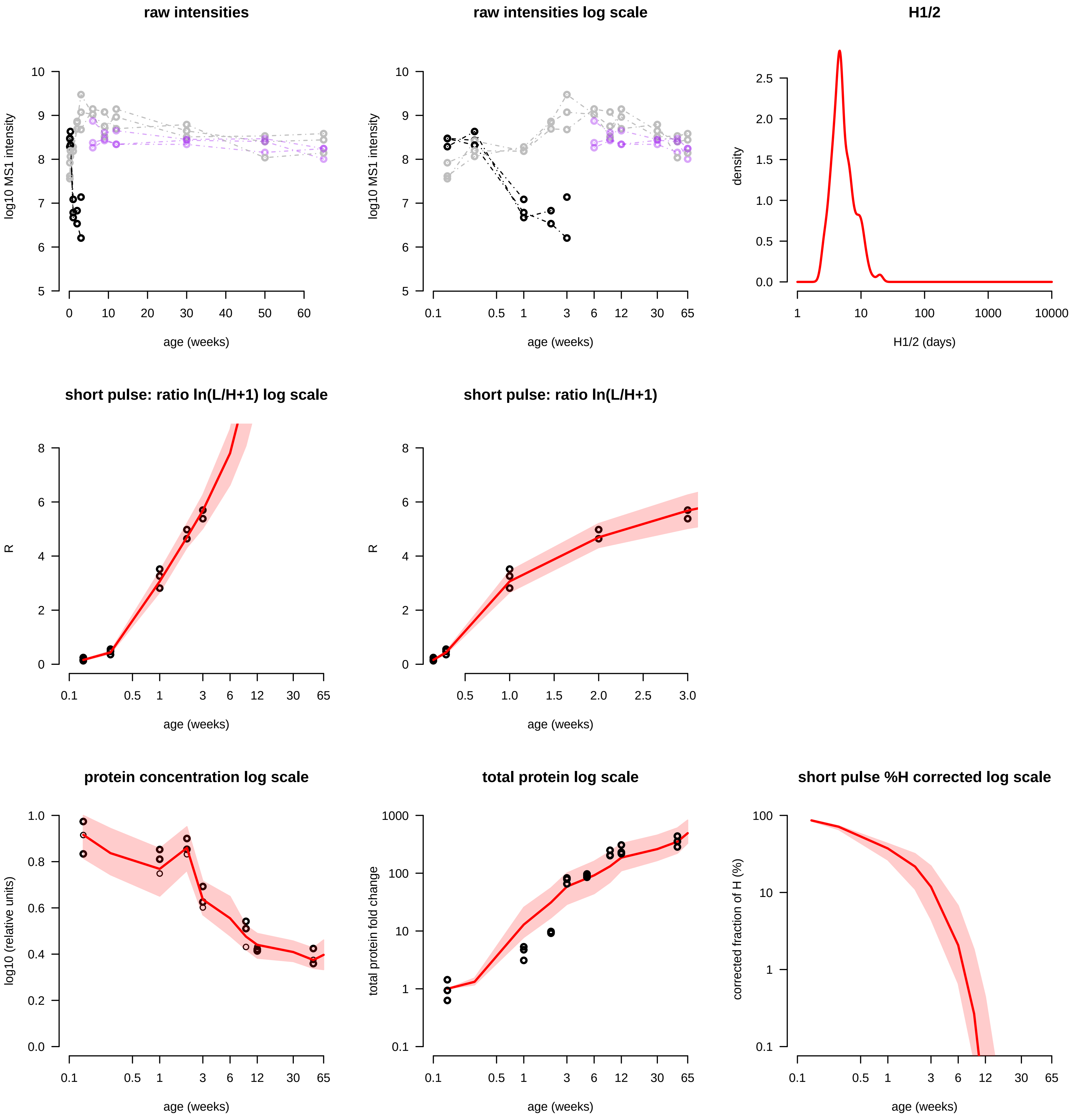

A2ATU0

>sp|A2ATU0|DHTK1_MOUSE

Probable 2−oxoglutarate dehydrogenase E1 component DHKTD1, mitochondria

GN=Dhtkd1

Med H1/2: 8.7 days

Q5 H1/2: 3.2 days

Q95 H1/2: 90.7 days

reliable model fit: yes

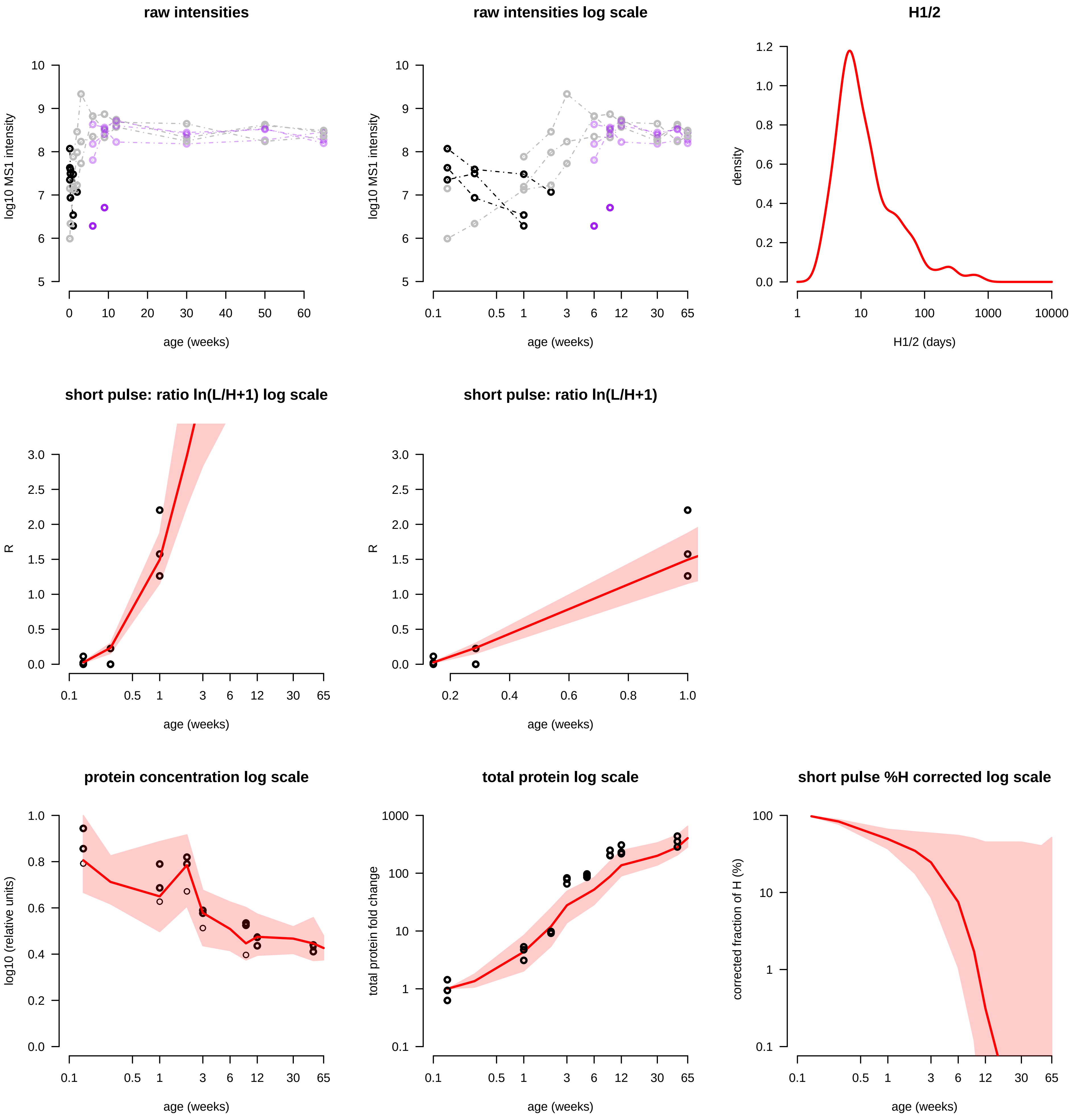

Q3UQA7

>sp|Q3UQA7|SELH_MOUSE

Selenoprotein H

GN=Selenoh

Med H1/2: 12.2 days

Q5 H1/2: 5.1 days

Q95 H1/2: 277.8 days

reliable model fit: yes

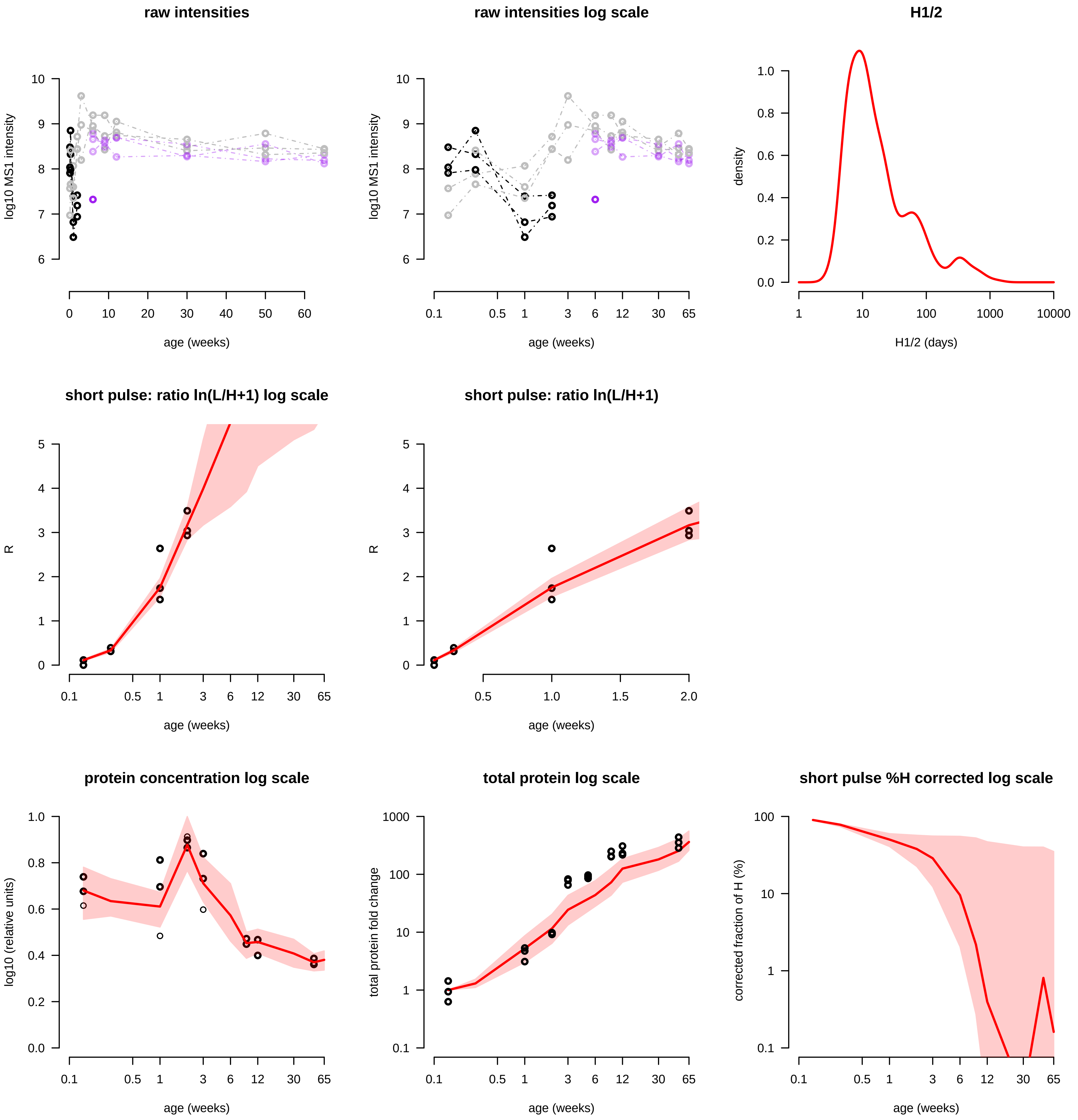

Q9R0Q6

>sp|Q9R0Q6|ARC1A_MOUSE

Actin−related protein 2/3 complex subunit 1A

GN=Arpc1a

Med H1/2: 7.4 days

Q5   H1/2: 6.1 days

Q95 H1/2: 9.8 days

reliable model fit: yes

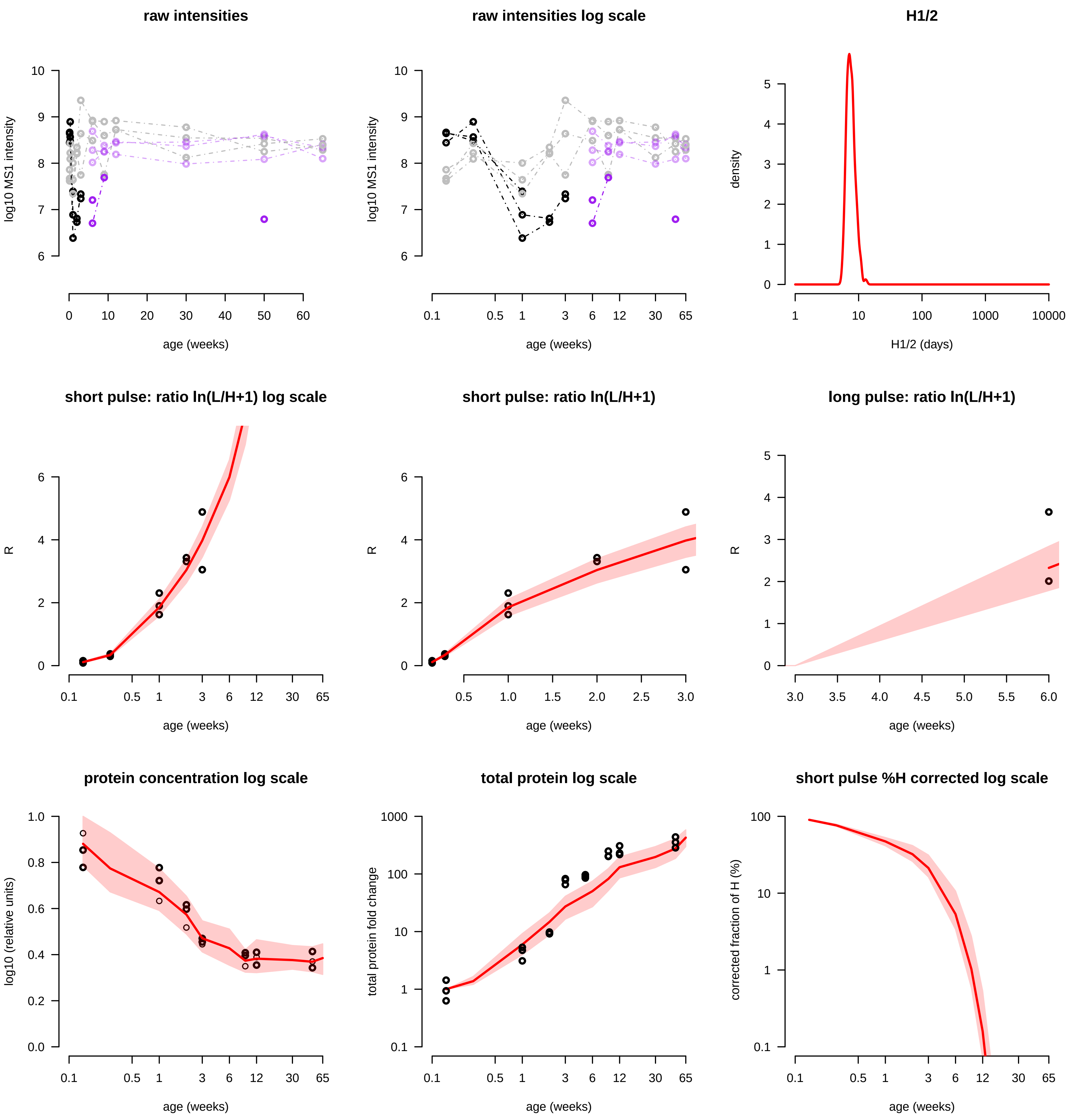

Q9CPX6

>sp|Q9CPX6|ATG3_MOUSE

Ubiquitin−like−conjugating enzyme ATG3

GN=Atg3

Med H1/2: 6 days

Q5 H1/2: 3 days

Q95 H1/2: 26.2 days

reliable model fit: yes

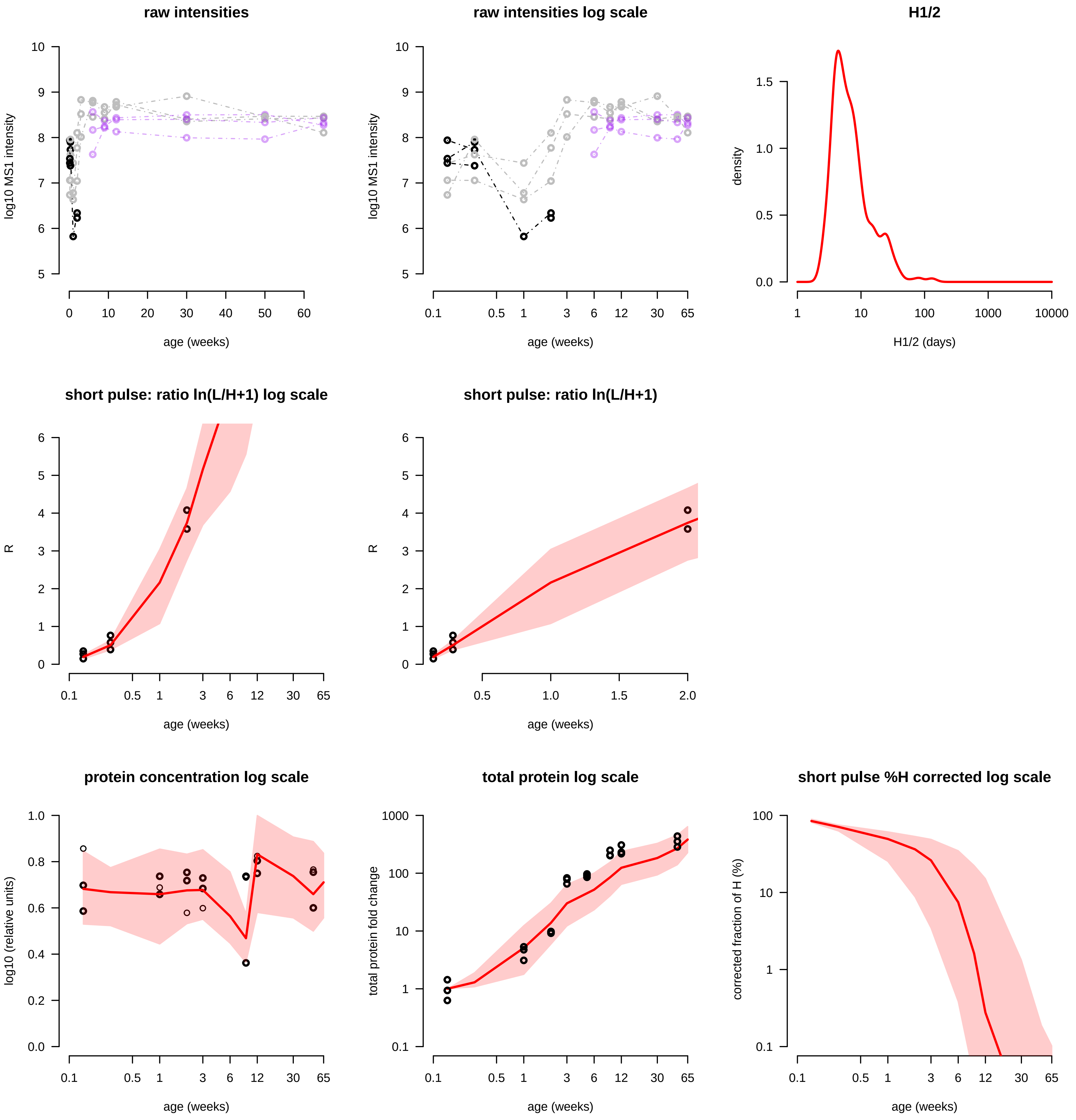

Q3TWW8

>sp|Q3TWW8|SRSF6_MOUSE

Serine/arginine−rich splicing factor 6

GN=Srsf6

Med H1/2: 6.5 days

Q5 H1/2: 5.4 days

Q95 H1/2: 8 days

reliable model fit: yes

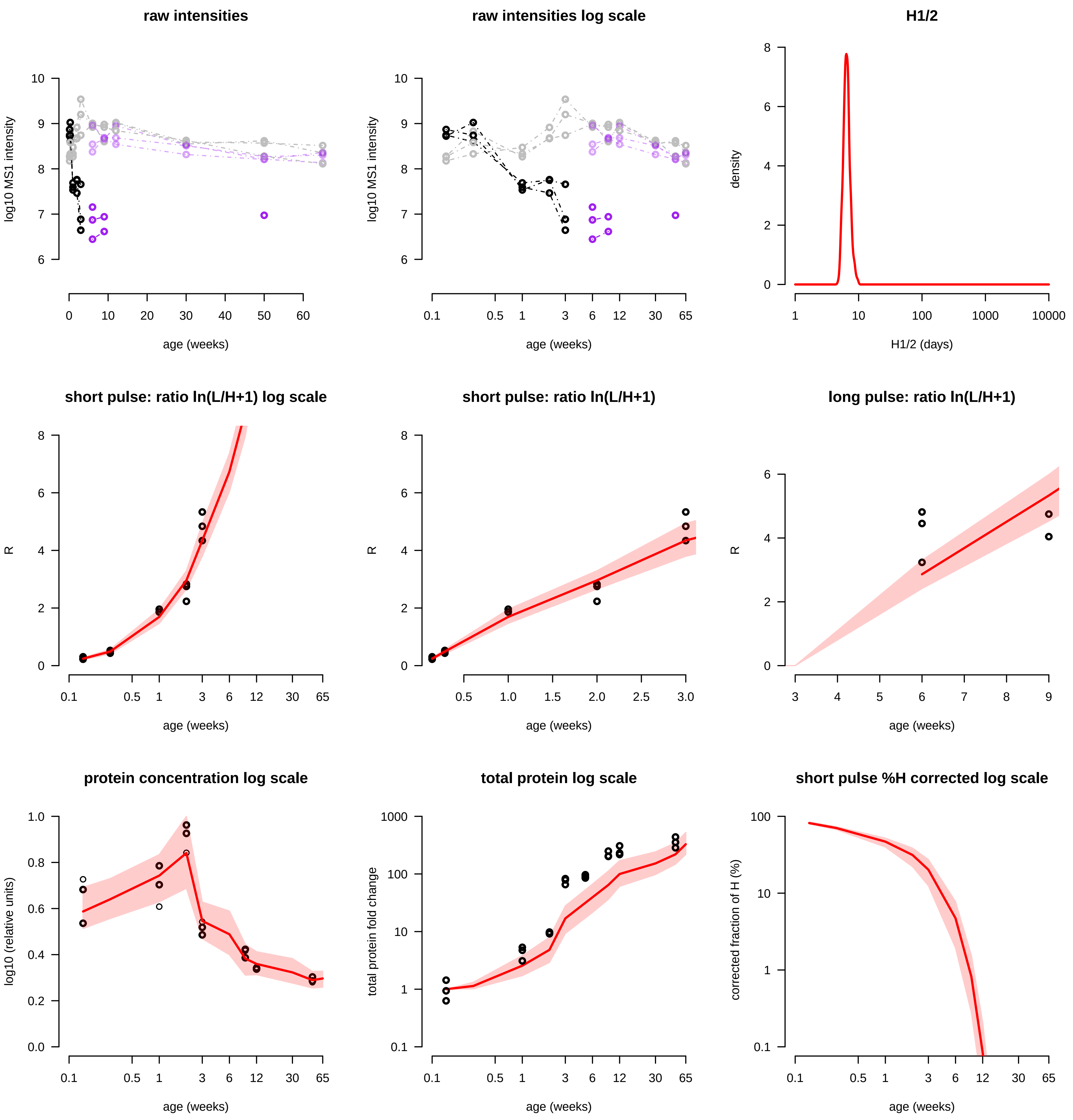

P01631

>sp|P01631|KV2A7_MOUSE

Ig kappa chain V−II region 26−10

Med H1/2: 21.7 days

Q5 H1/2: 15 days

Q95 H1/2: 37.3 days

reliable model fit: yes

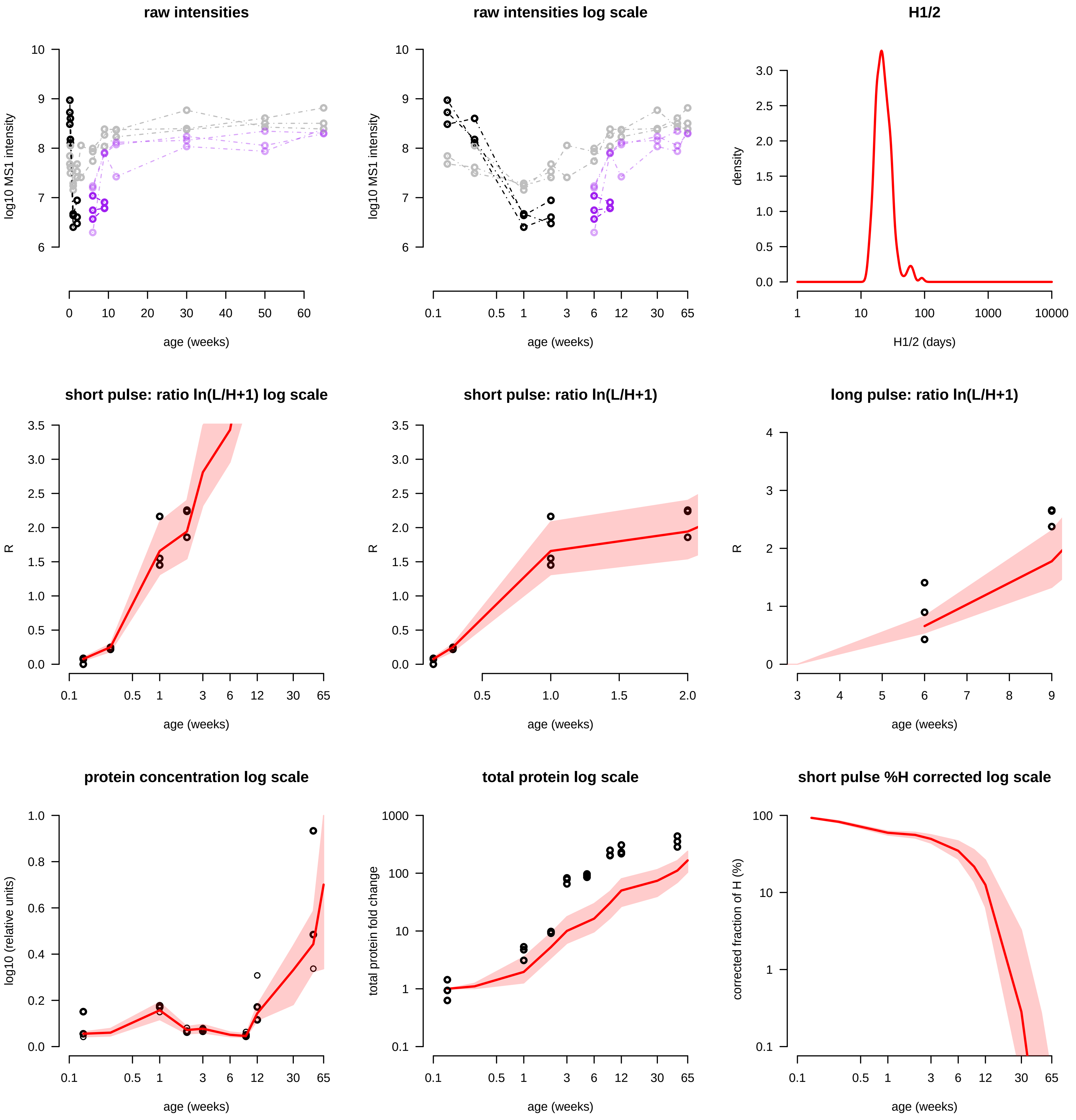

Q99JX7

>sp|Q99JX7|NXF1_MOUSE

Nuclear RNA export factor 1

GN=Nxf1

Med H1/2: 46 days

Q5 H1/2: 11.9 days

Q95 H1/2: 298.4 days

reliable model fit: no

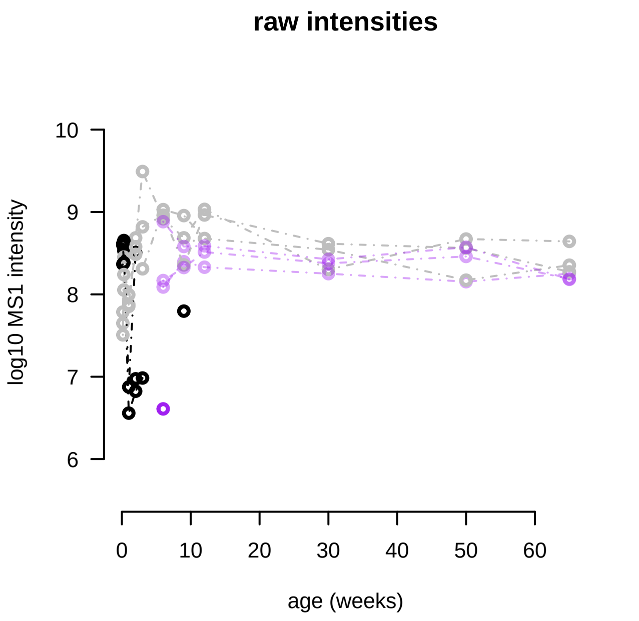

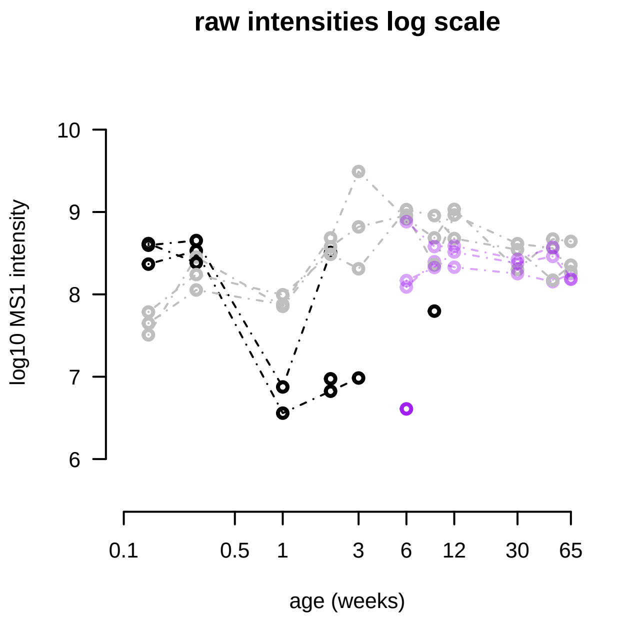

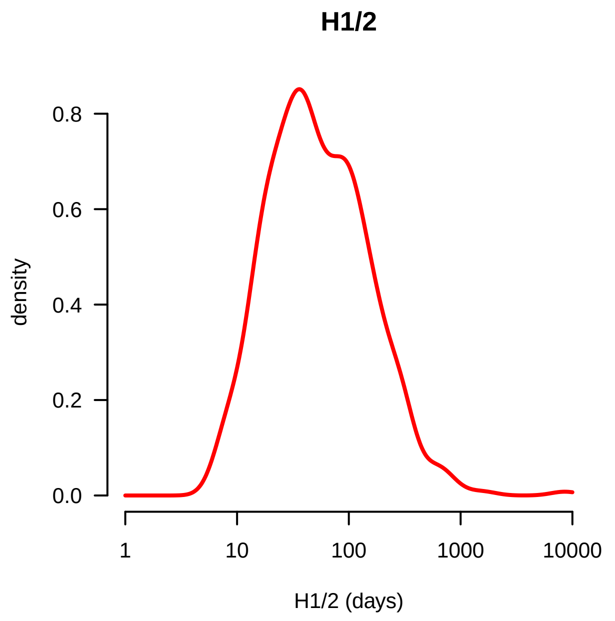

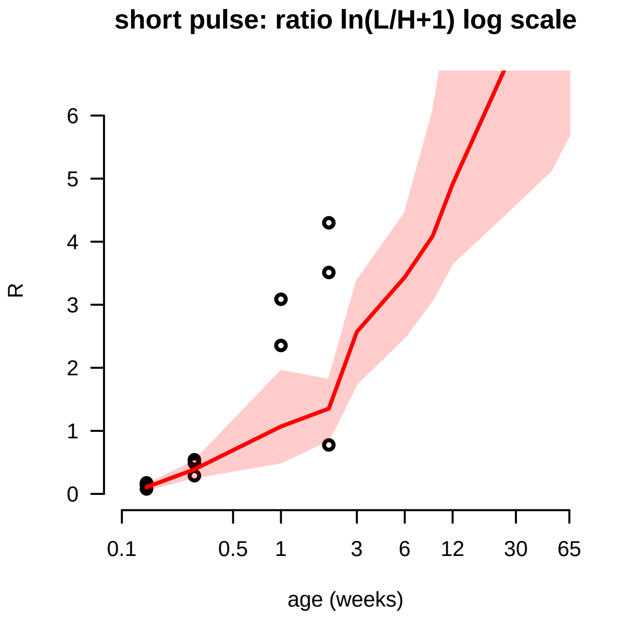

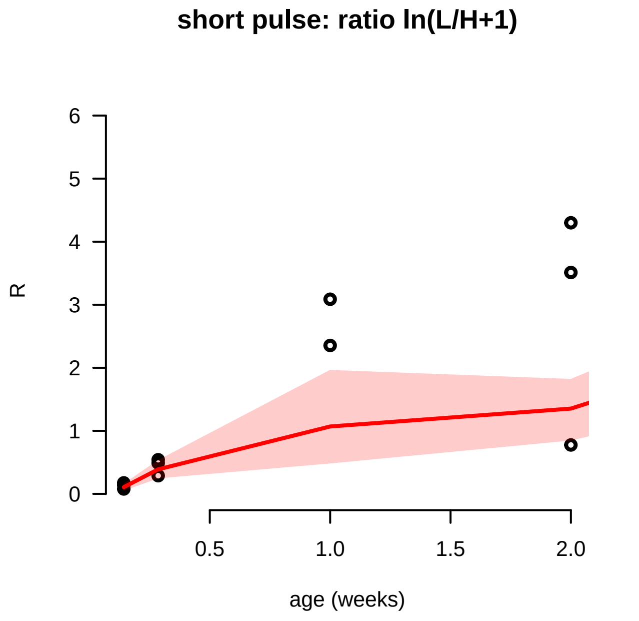

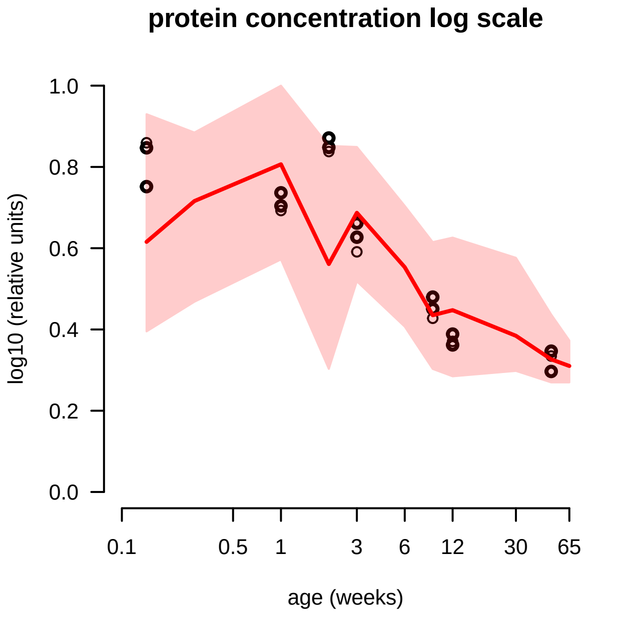

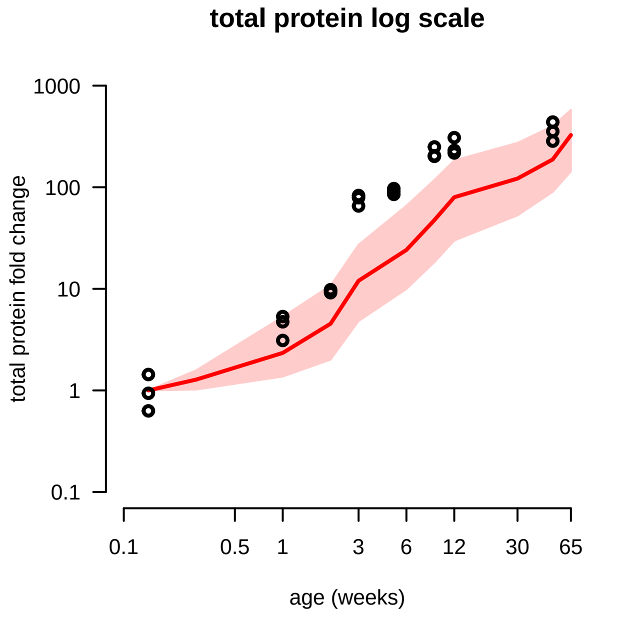

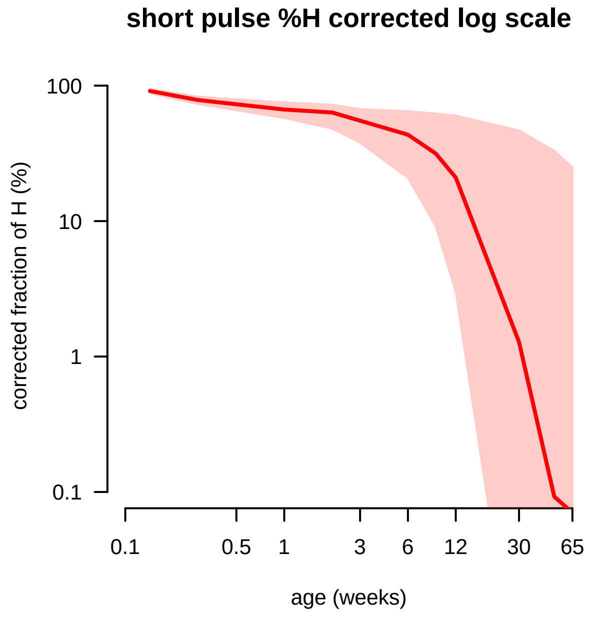

Q9EPJ9;Q9EPJ9−2

>sp|Q9EPJ9|ARFG1_MOUSE

ADP−ribosylation factor GTPase−activating protein 1

GN=Arfgap1

Med H1/2: 6.7 days

Q5 H1/2: 2.7 days

Q95 H1/2: 38.8 days

reliable model fit: no

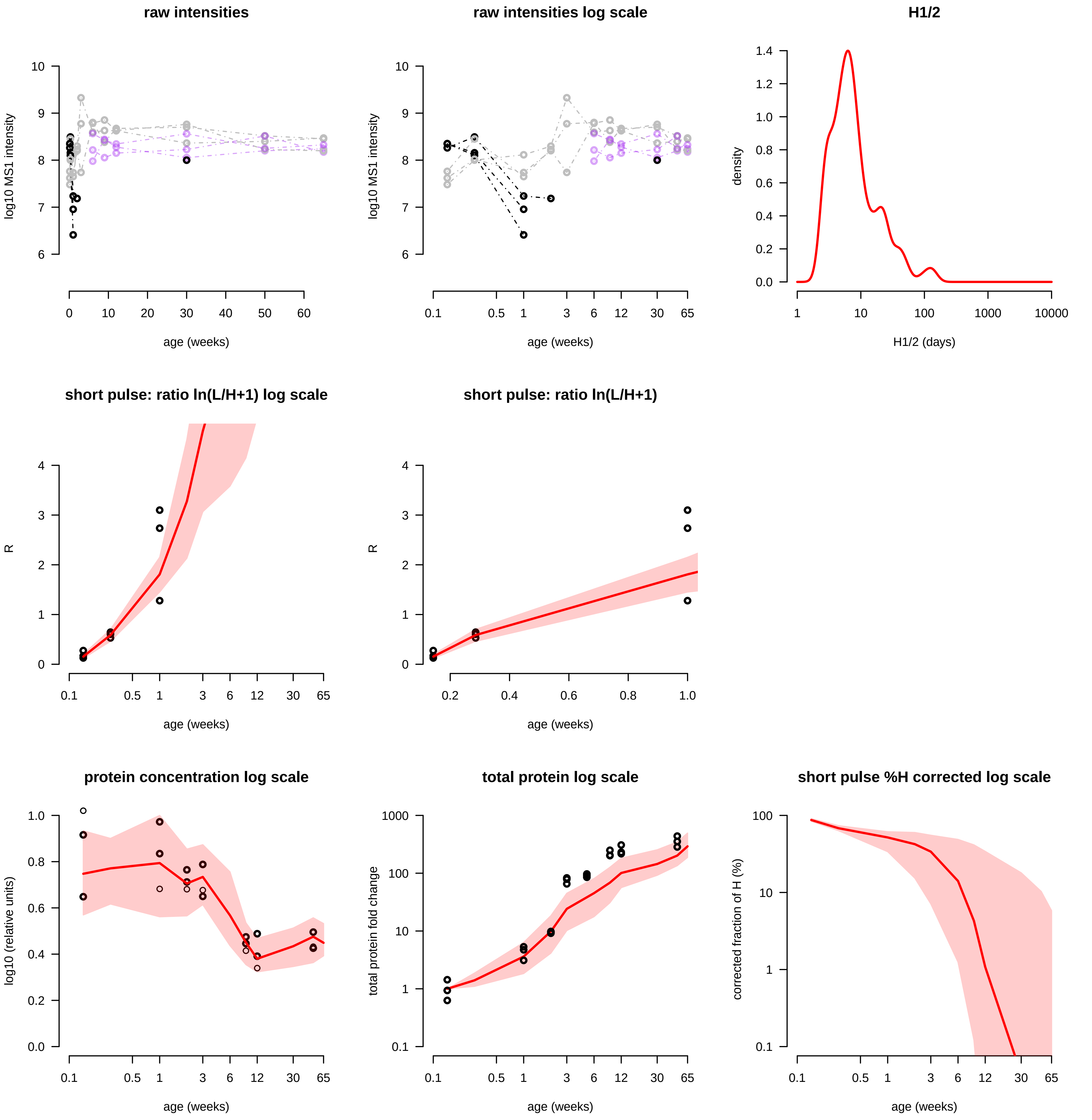

Q9CWE0

>sp|Q9CWE0|MFR1L_MOUSE

Mitochondrial fission regulator 1−like

GN=Mtfr1l

Med H1/2: 824.3 days

Q5 H1/2: 231.5 days

Q95 H1/2: 10802.7 days

reliable model fit: yes

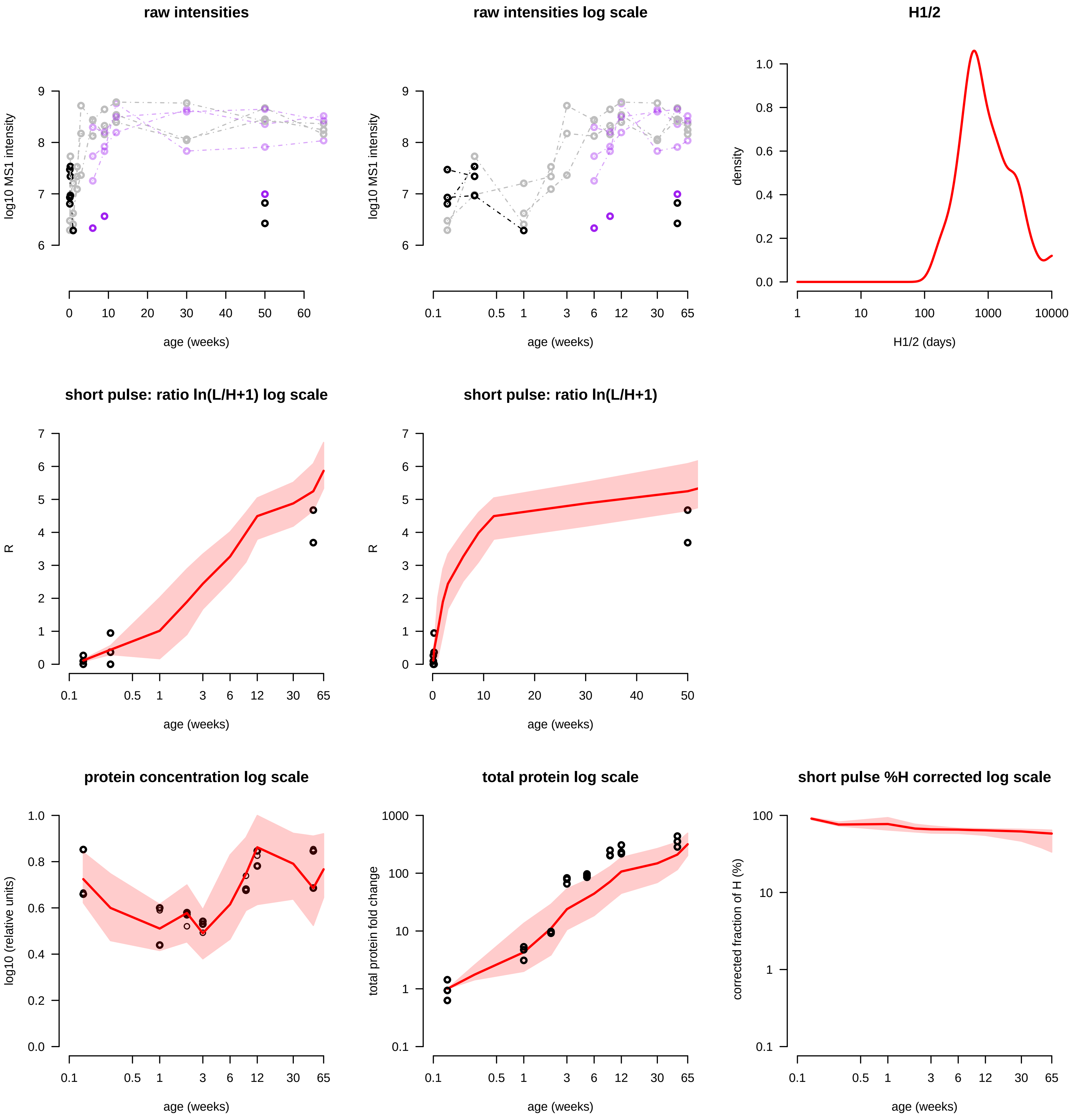

Q9D1M0

>sp|Q9D1M0|SEC13_MOUSE

Protein SEC13 homolog

GN=Sec13

Med H1/2: 6.6 days

Q5 H1/2: 2.9 days

Q95 H1/2: 47.2 days

reliable model fit: yes

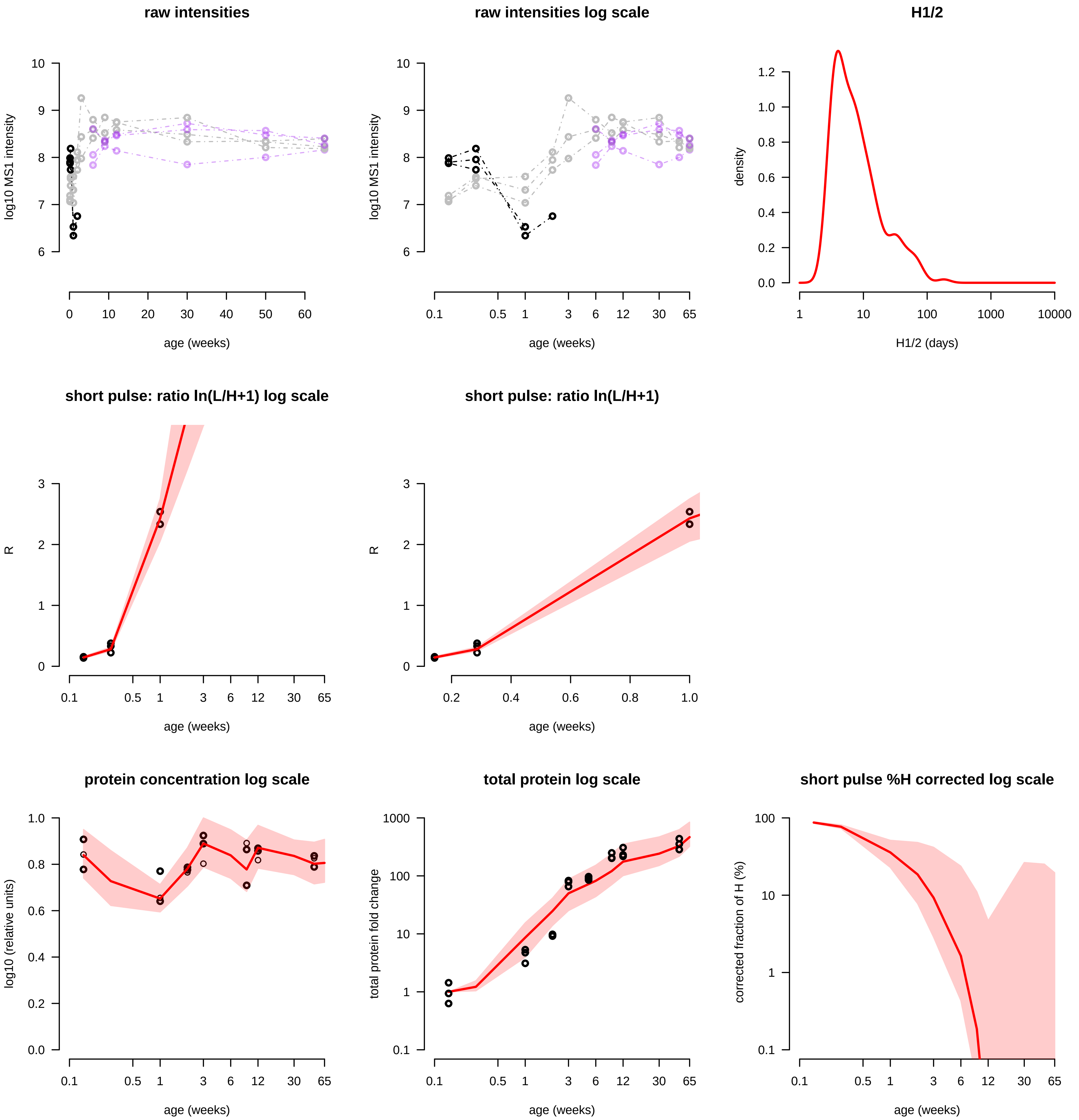

Q9CQJ4

>sp|Q9CQJ4|RING2_MOUSE

E3 ubiquitin−protein ligase RING2

GN=Rnf2

Med H1/2: 5.8 days

Q5 H1/2: 4.7 days

Q95 H1/2: 7.6 days

reliable model fit: yes

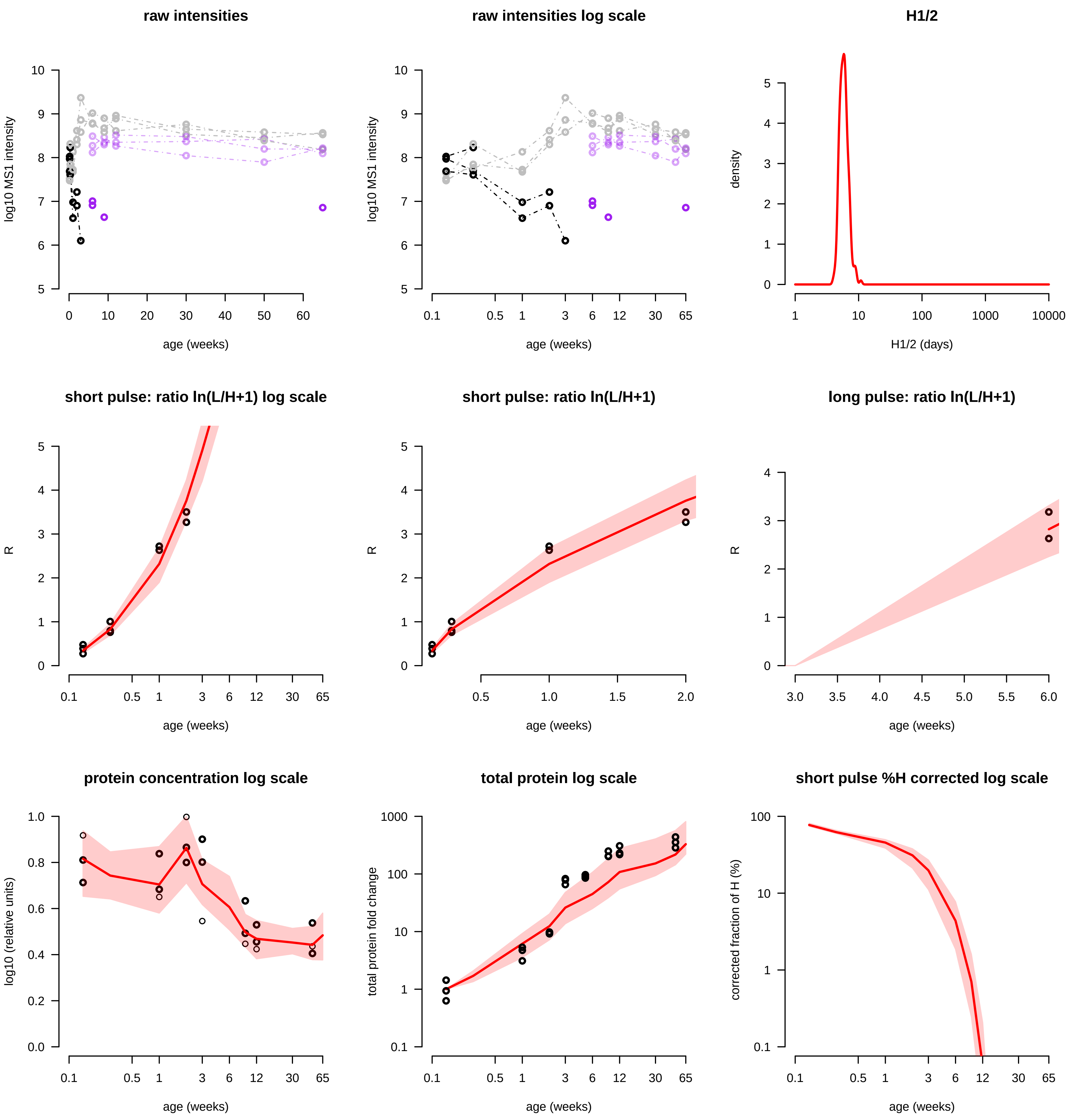

Q9DBC3

>sp|Q9DBC3|CMTR1_MOUSE

Cap−specific mRNA (nucleoside−2'−O−)−methyltransferase 1

GN=Cmtr1

Med H1/2: 34.4 days

Q5 H1/2: 10.9 days

Q95 H1/2: 944.8 days

reliable model fit: yes

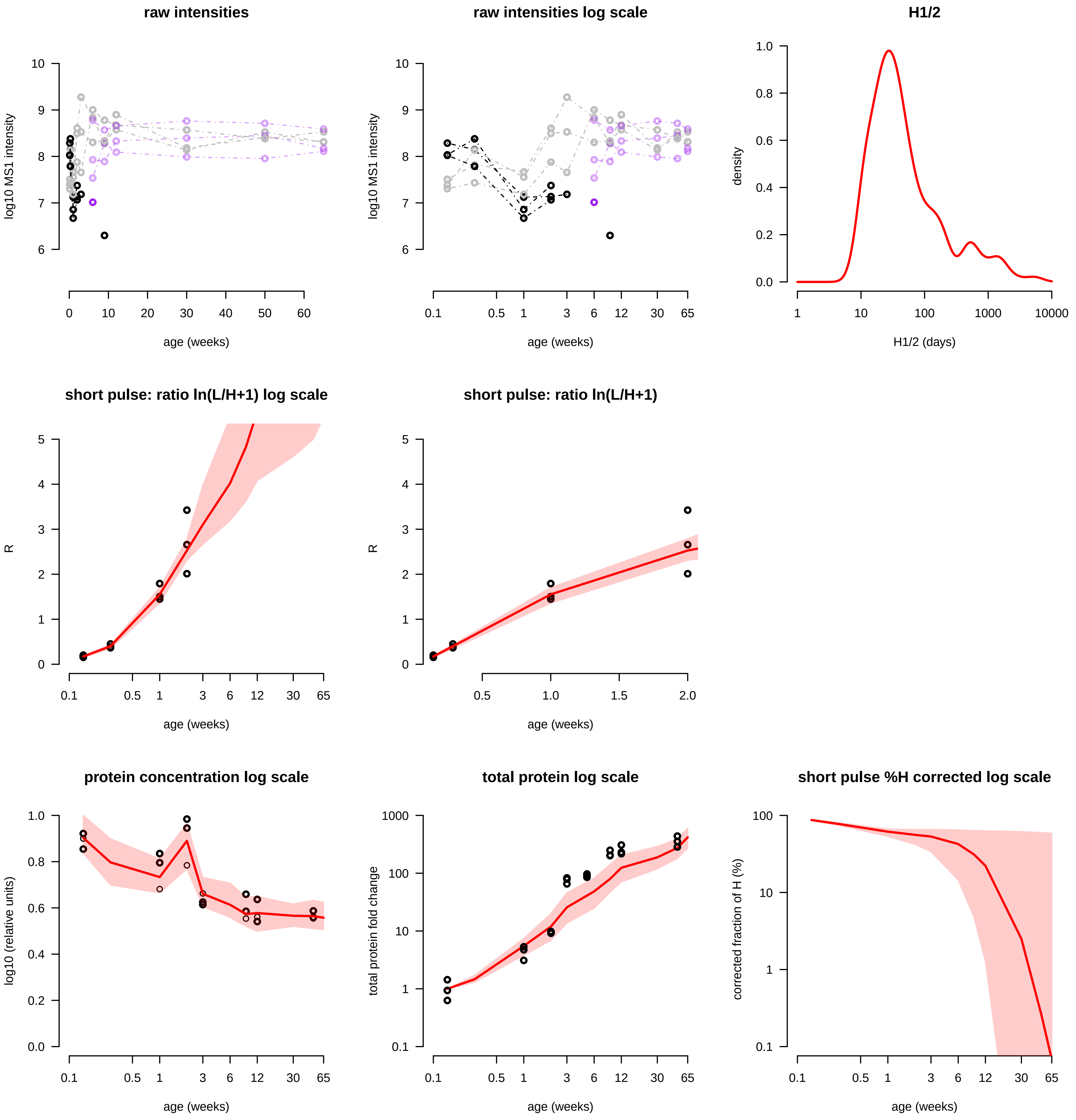

Q9Z0W3;Q9Z0W3−2

>sp|Q9Z0W3|NU160_MOUSE

Nuclear pore complex protein Nup160

GN=Nup160

Med H1/2: 12.3 days

Q5 H1/2: 4 days

Q95 H1/2: 44.4 days

reliable model fit: yes

raw intensities

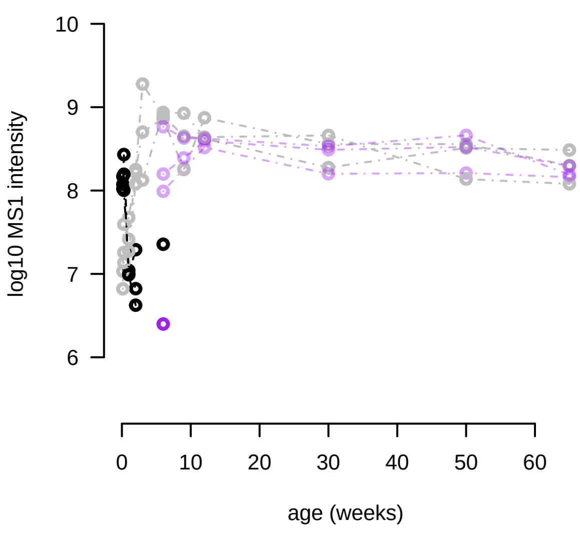

raw intensities log scale

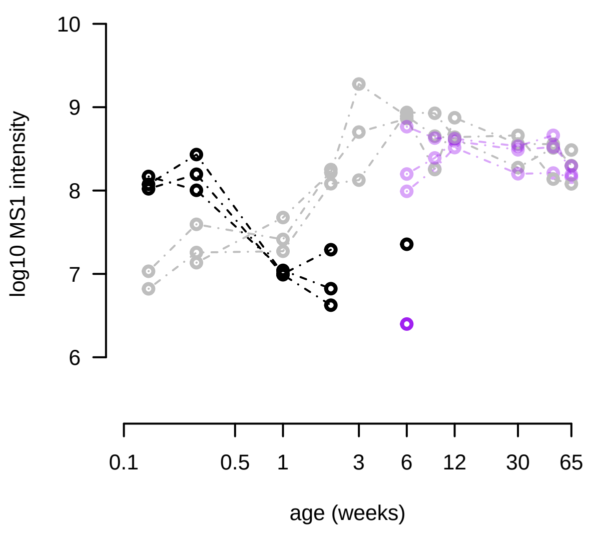

H1/2

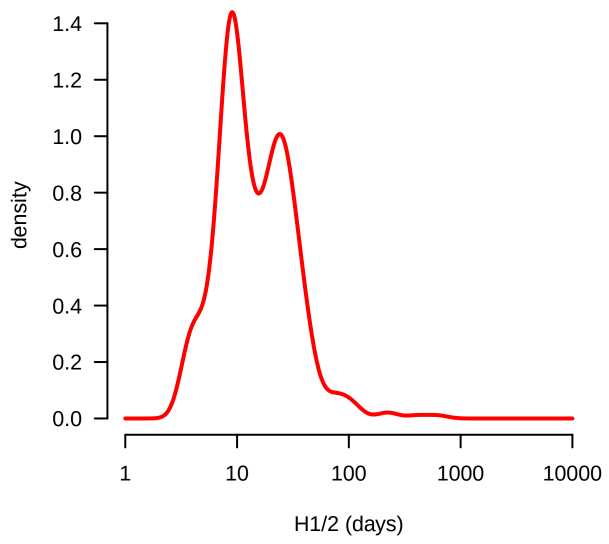

short pulse: ratio ln(L/H+1) log scale

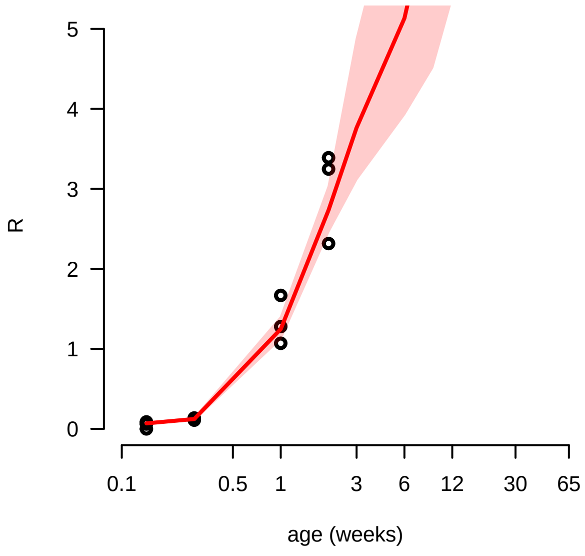

short pulse: ratio ln(L/H+1)

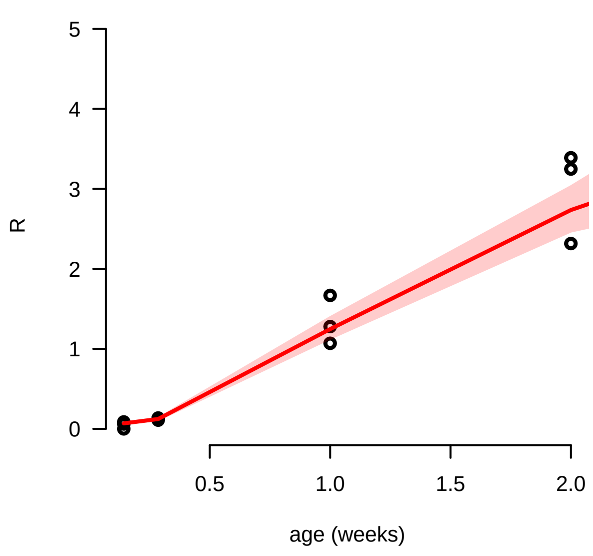

protein concentration log scale

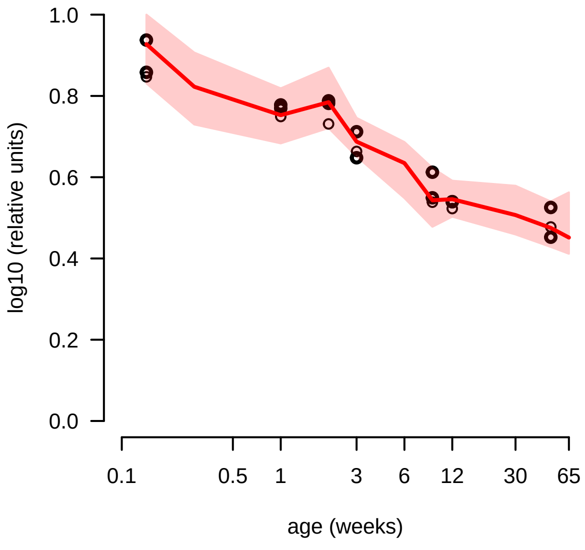

total protein log scale

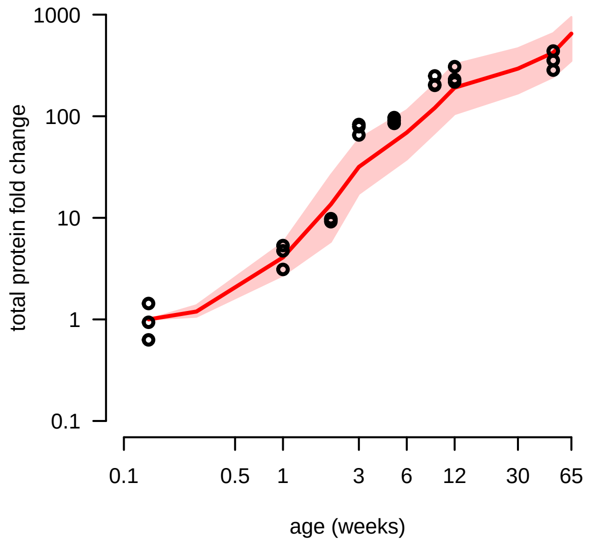

short pulse %H corrected log scale

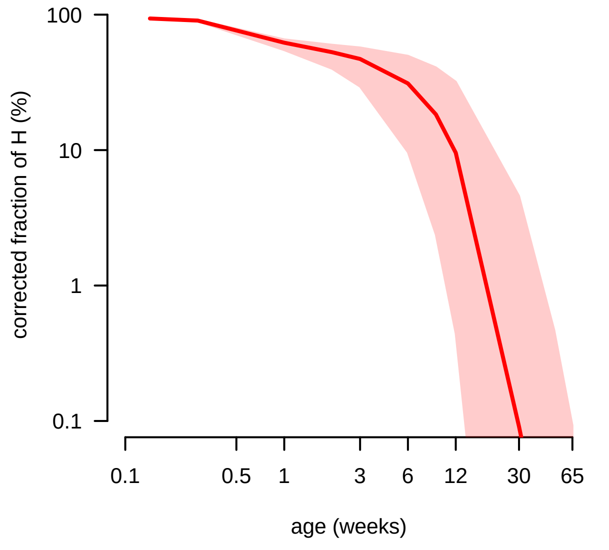

Q9D8S4

>sp|Q9D8S4|ORN_MOUSE

Oligoribonuclease, mitochondrial

GN=Rexo2

Med H1/2: 16.6 days

Q5 H1/2: 7.2 days

Q95 H1/2: 160.5 days

reliable model fit: yes

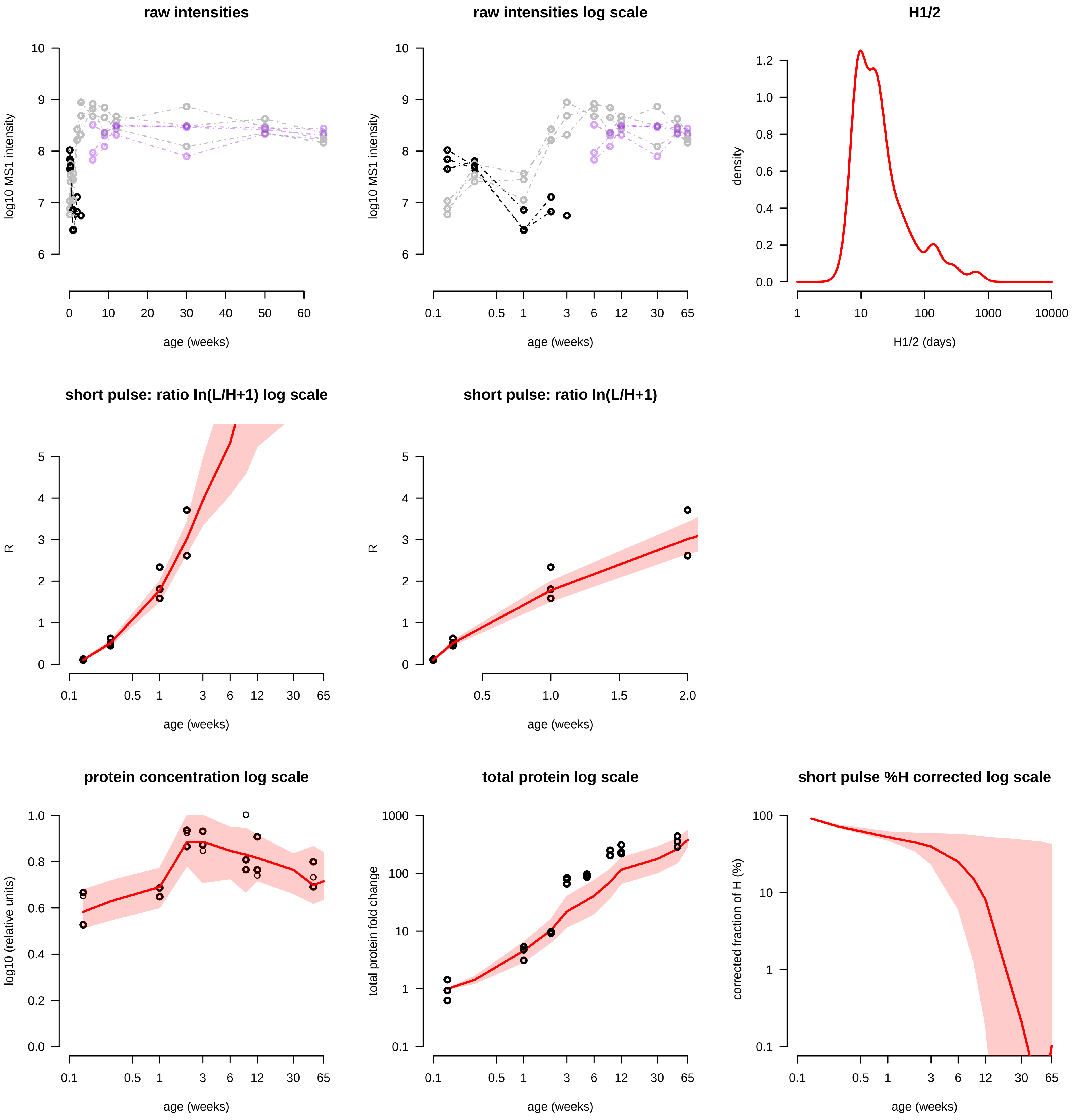

P47713

>sp|P47713|PA24A_MOUSE

Cytosolic phospholipase A2

GN=Pla2g4a

Med H1/2: 518.4 days

Q5 H1/2: 178 days

Q95 H1/2: 1842 days

reliable model fit: yes

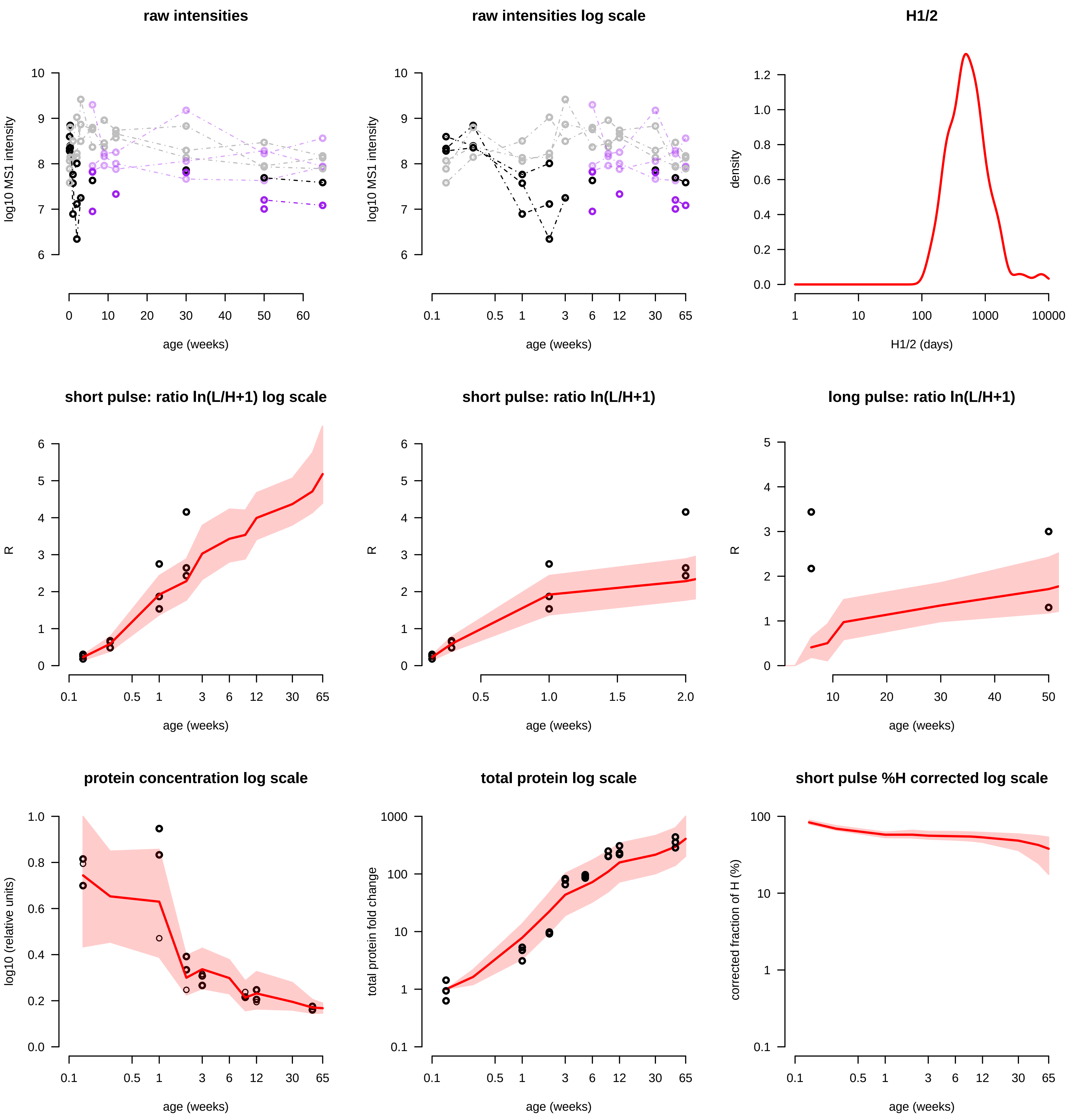

Q9CQF4

>sp|Q9CQF4|MRES1_MOUSE

Mitochondrial transcription rescue factor 1

GN=Mtres1

Med H1/2: 9.3 days

Q5 H1/2: 3.9 days

Q95 H1/2: 55.8 days

reliable model fit: yes

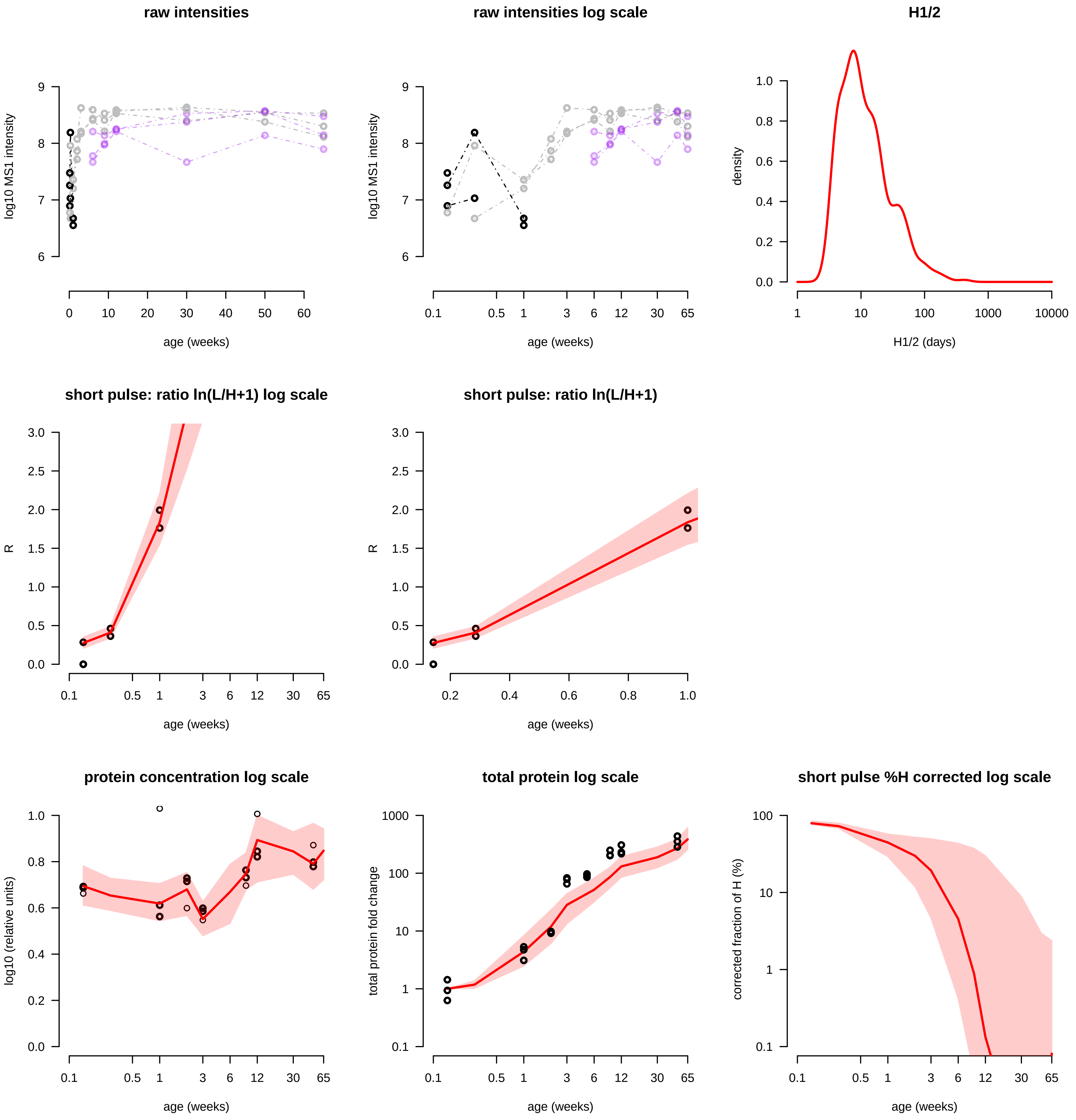

O35566

>sp|O35566|CD151_MOUSE

CD151 antigen

GN=Cd151

Med H1/2: 5.8 days

Q5 H1/2: 4.6 days

Q95 H1/2: 8 days

reliable model fit: yes

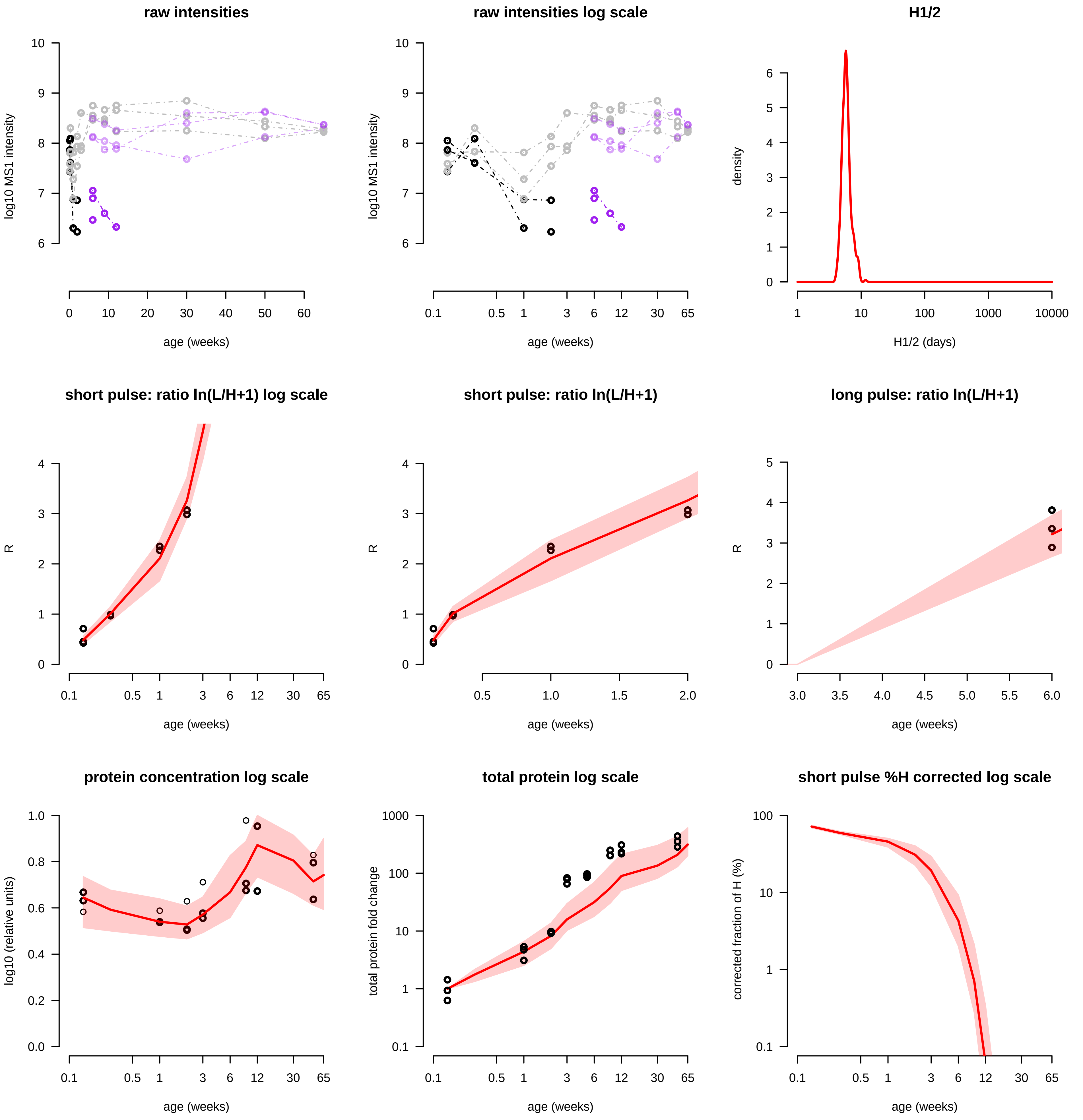

Q8BGZ4;Q8BGZ4−2

>sp|Q8BGZ4|CDC23_MOUSE

Cell division cycle protein 23 homolog

GN=Cdc23

Med H1/2: 14.5 days

Q5 H1/2: 6.4 days

Q95 H1/2: 47.3 days

reliable model fit: yes

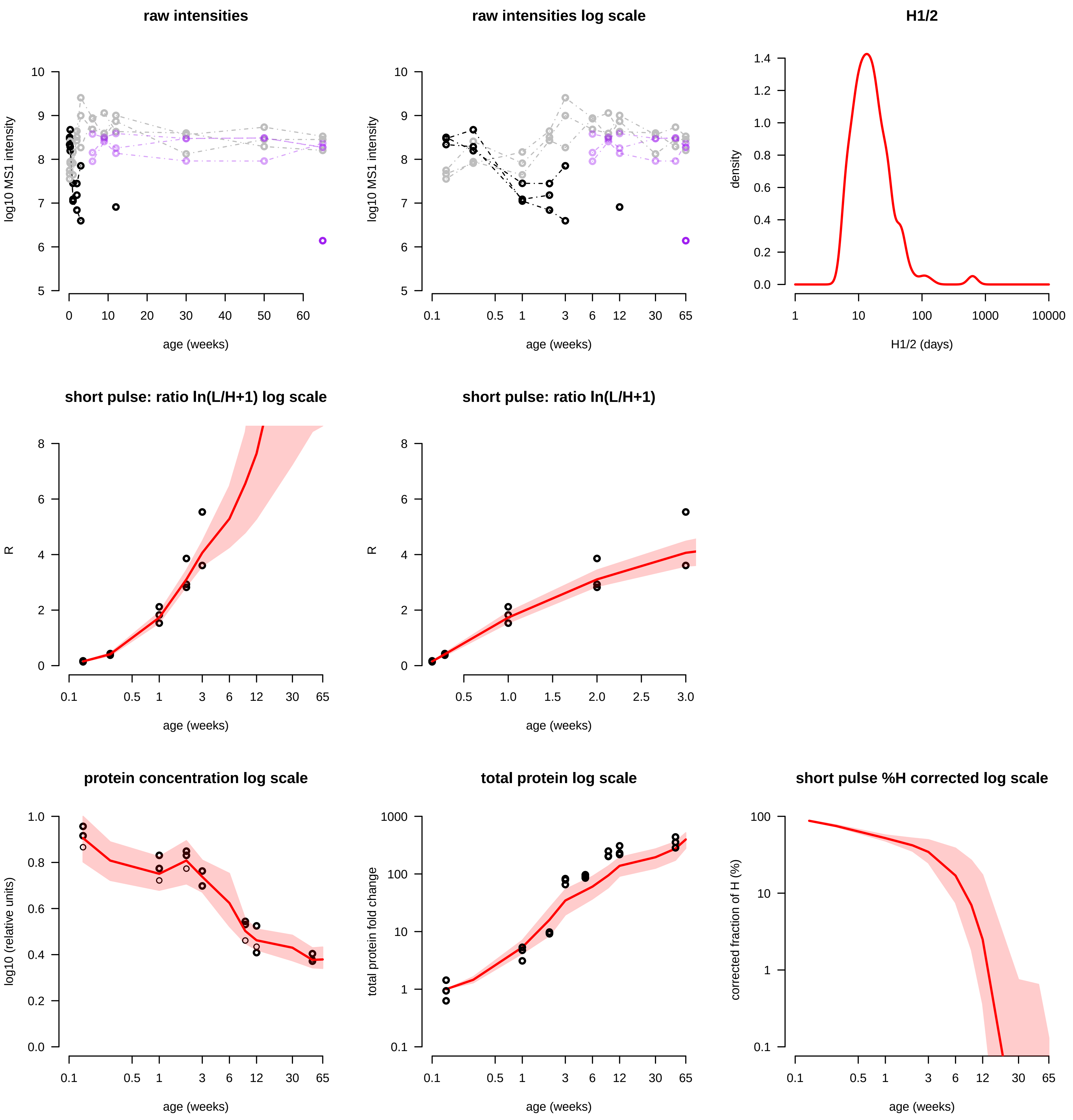

Q78XF5

>sp|Q78XF5|OSTC_MOUSE

Oligosaccharyltransferase complex subunit OSTC

GN=Ostc

Med H1/2: 5.2 days

Q5 H1/2: 4.1 days

Q95 H1/2: 7 days

reliable model fit: yes

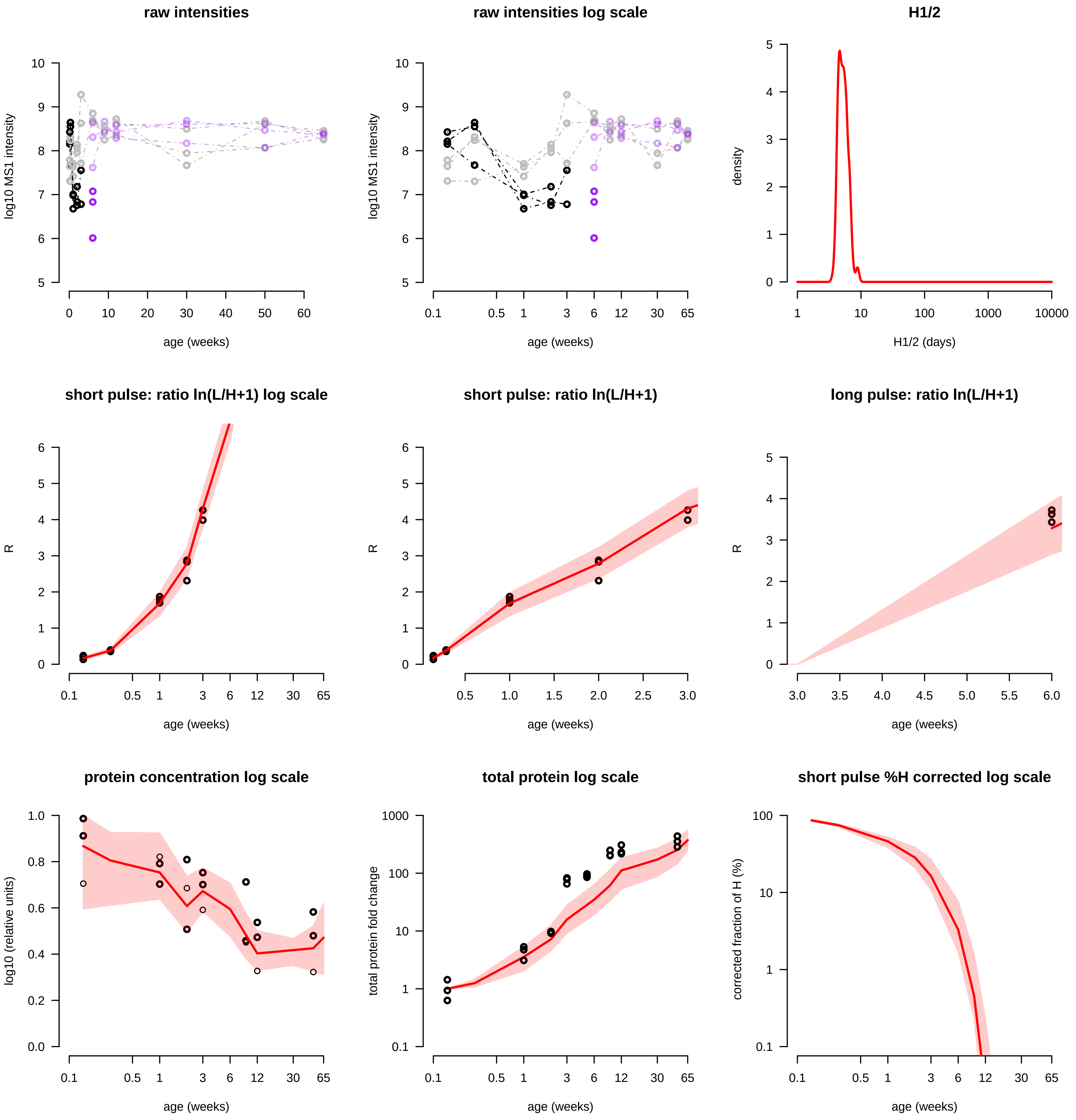

O88587−2;O88587

>sp|O88587−2|COMT_MOUSE

Isoform Soluble of Catechol O−methyltransferase

GN=Comt

Med H1/2: 25.2 days

Q5 H1/2: 15.1 days

Q95 H1/2: 110.3 days

reliable model fit: no

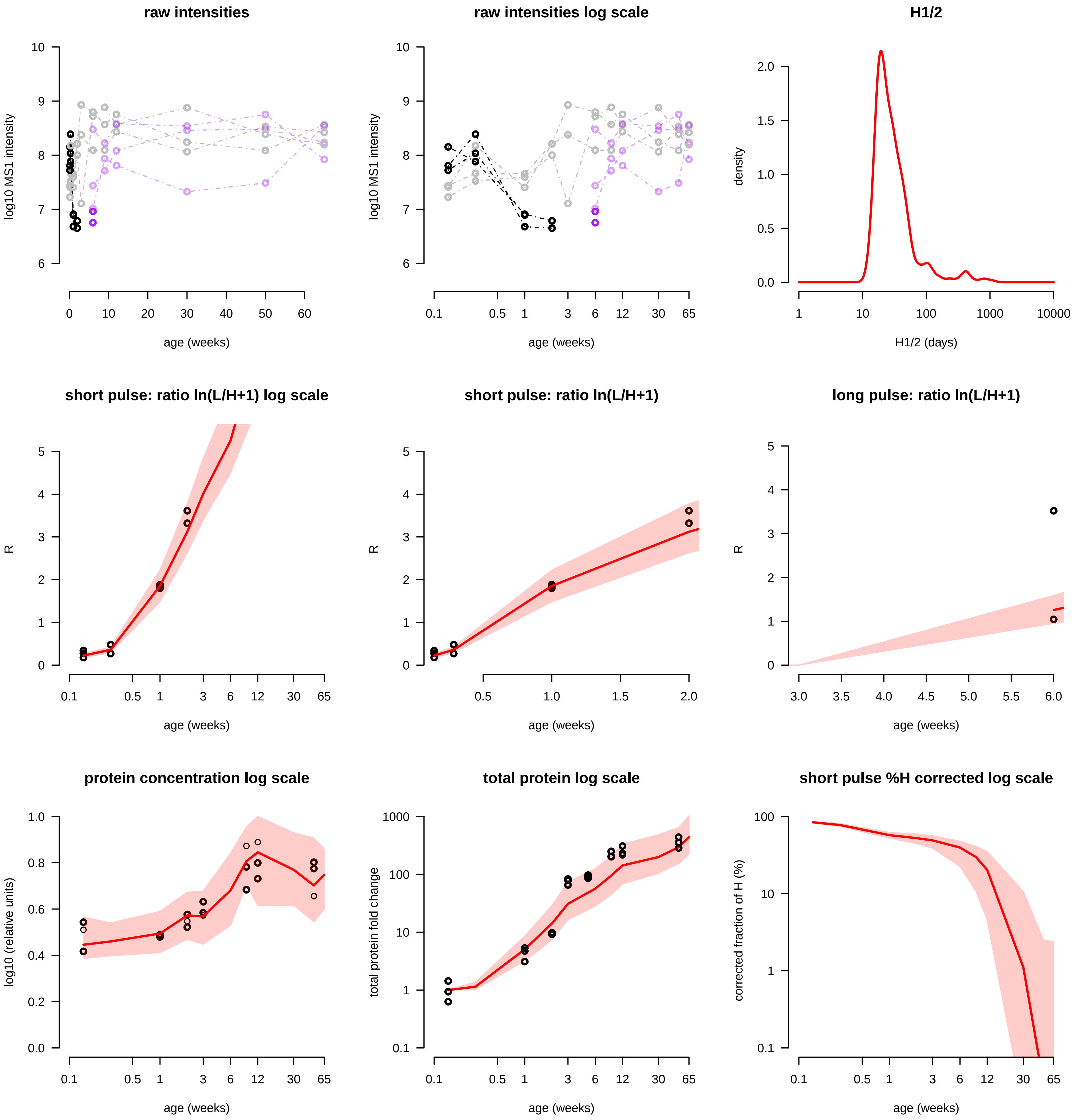

Q9CYR6

>sp|Q9CYR6|AGM1_MOUSE

Phosphoacetylglucosamine mutase

GN=Pgm3

Med H1/2: 4.5 days

Q5 H1/2: 2.9 days

Q95 H1/2: 10.6 days

reliable model fit: no

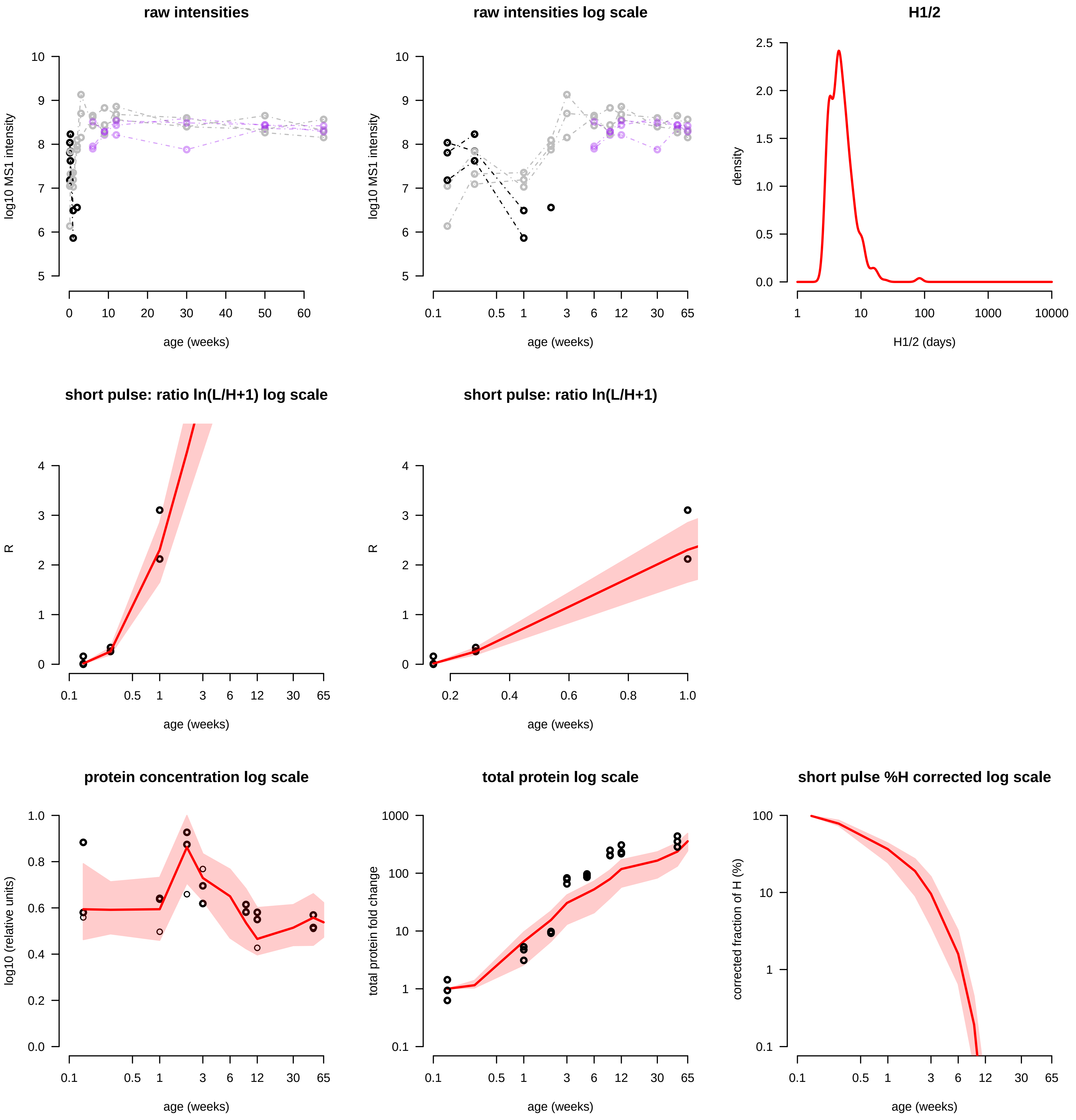

P25911

>sp|P25911|LYN_MOUSE

Tyrosine−protein kinase Lyn

GN=Lyn

Med H1/2: 2075.5 days

Q5 H1/2: 617 days

Q95 H1/2: 21487.8 days

reliable model fit: no

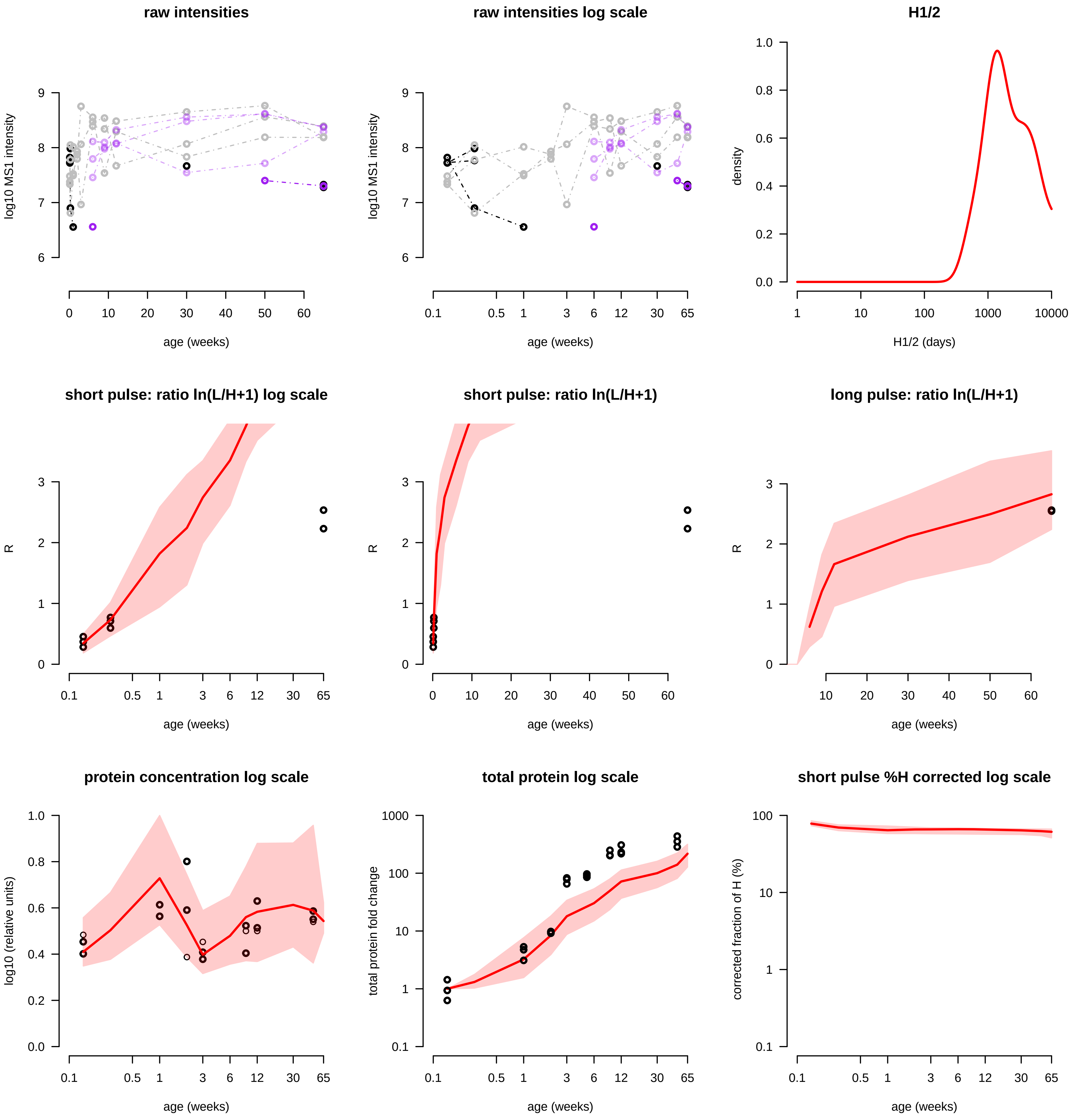

Q8VEB4

>sp|Q8VEB4|PAG15_MOUSE

Phospholipase A2 group XV

GN=Pla2g15

Med H1/2: 9.6 days

Q5 H1/2: 4 days

Q95 H1/2: 68.4 days

reliable model fit: yes

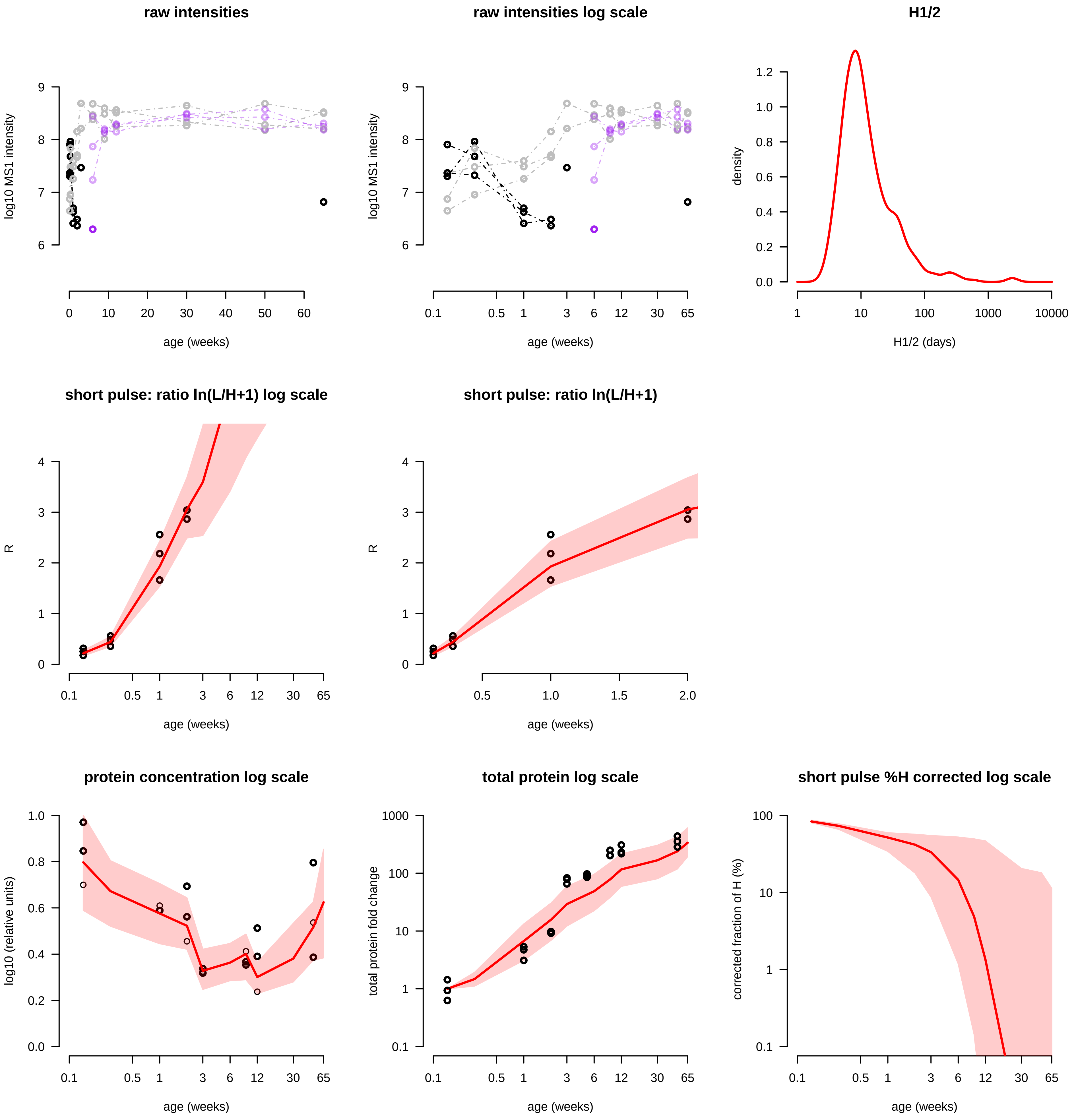

Q9DBB8

>sp|Q9DBB8|DHDH_MOUSE

Trans−1,2−dihydrobenzene−1,2−diol dehydrogenase

GN=Dhdh

Med H1/2: 1860.3 days

Q5 H1/2: 472.2 days

Q95 H1/2: 35162.9 days

reliable model fit: no

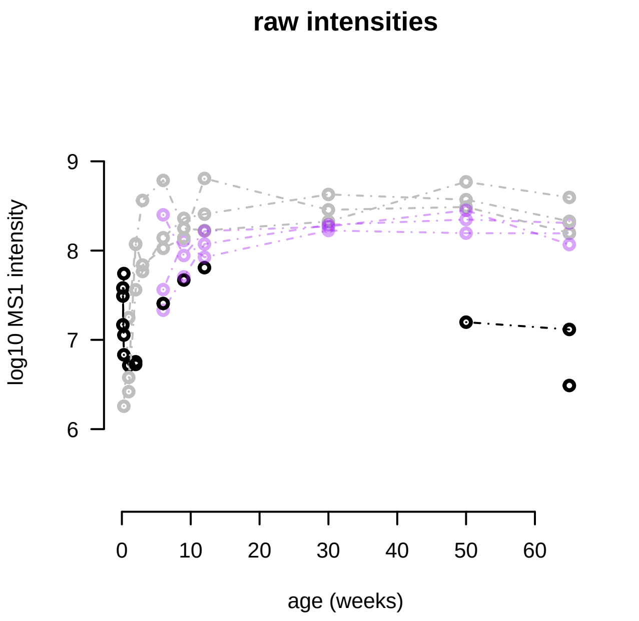

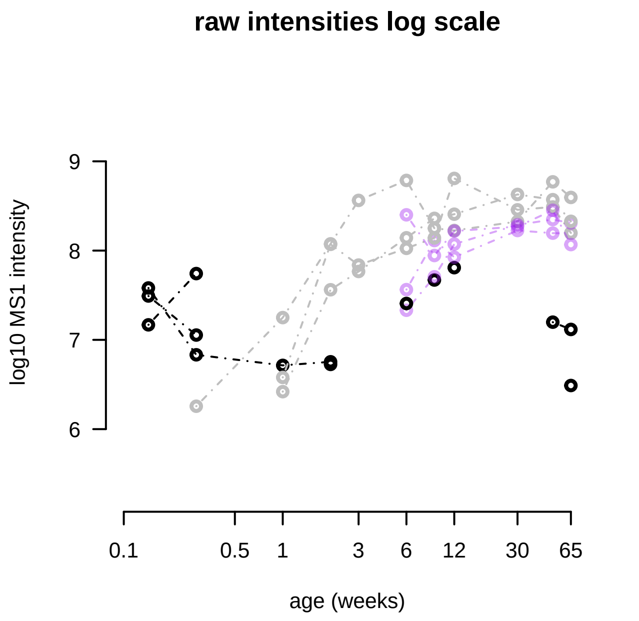

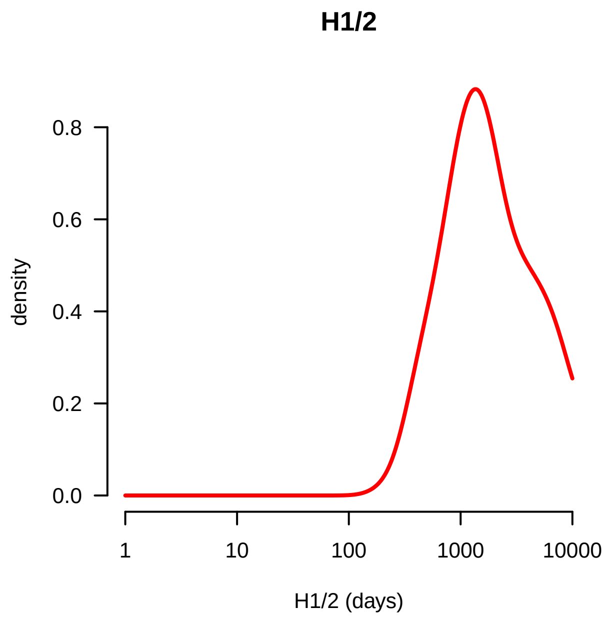

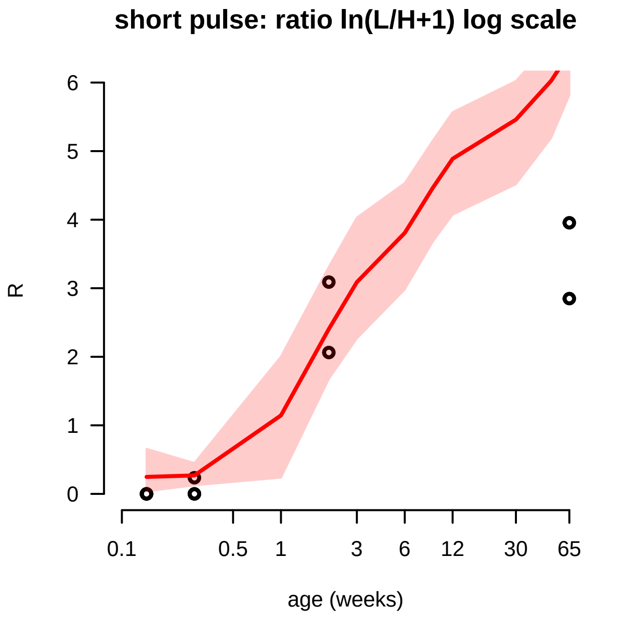

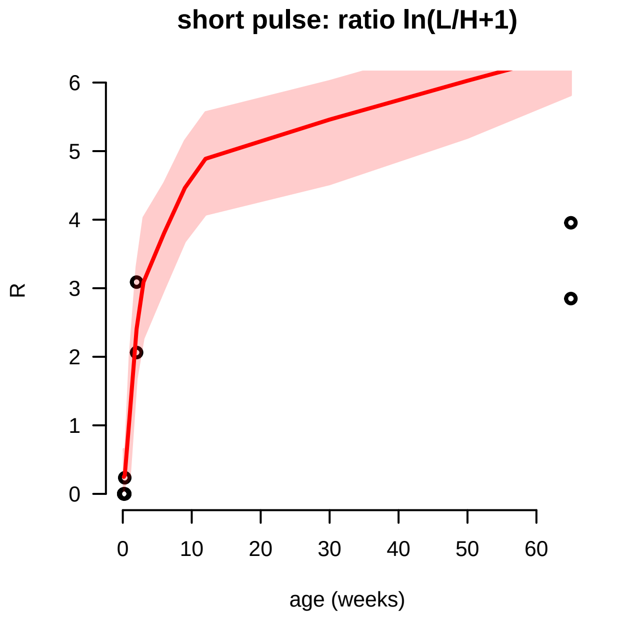

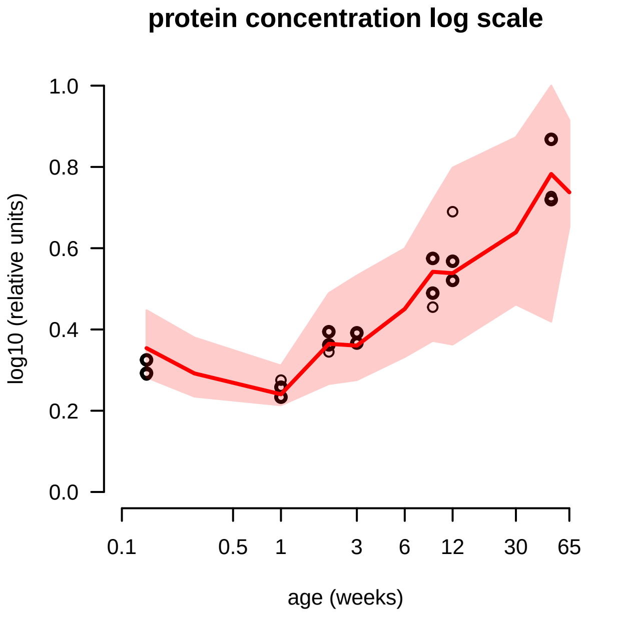

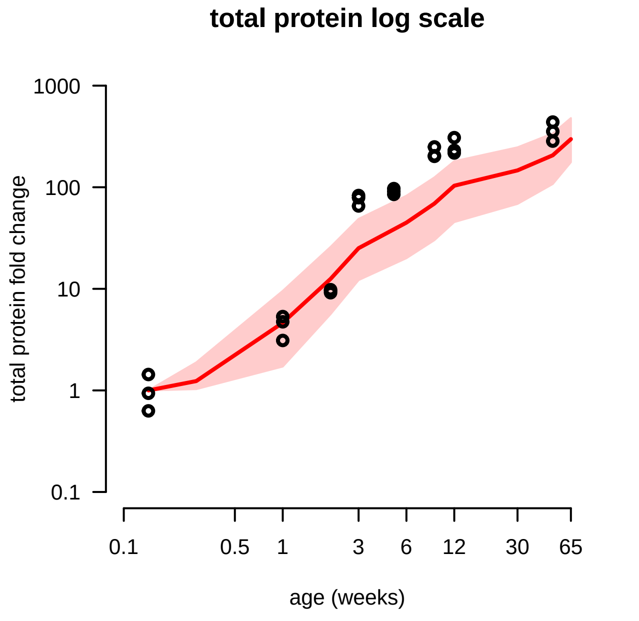

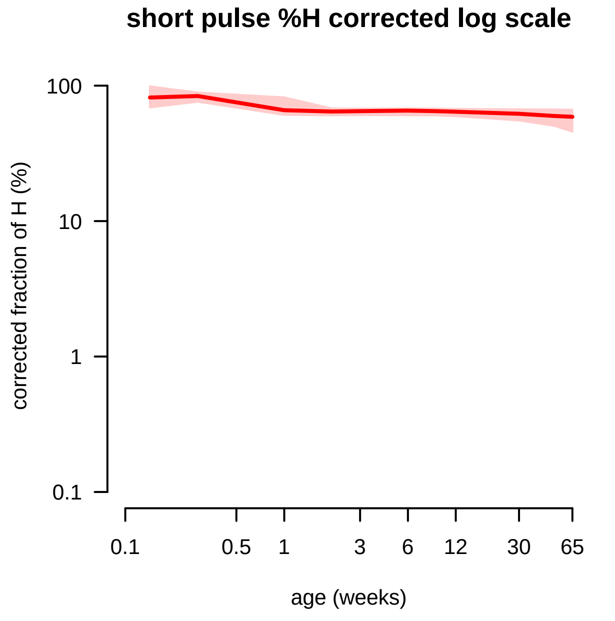

Q8BXN9;Q8BXN9−2;Q8BXN9−3

>sp|Q8BXN9|TM87A_MOUSE

Transmembrane protein 87A

GN=Tmem87a

Med H1/2: 9.3 days

Q5 H1/2: 3.7 days

Q95 H1/2: 81.8 days

reliable model fit: yes

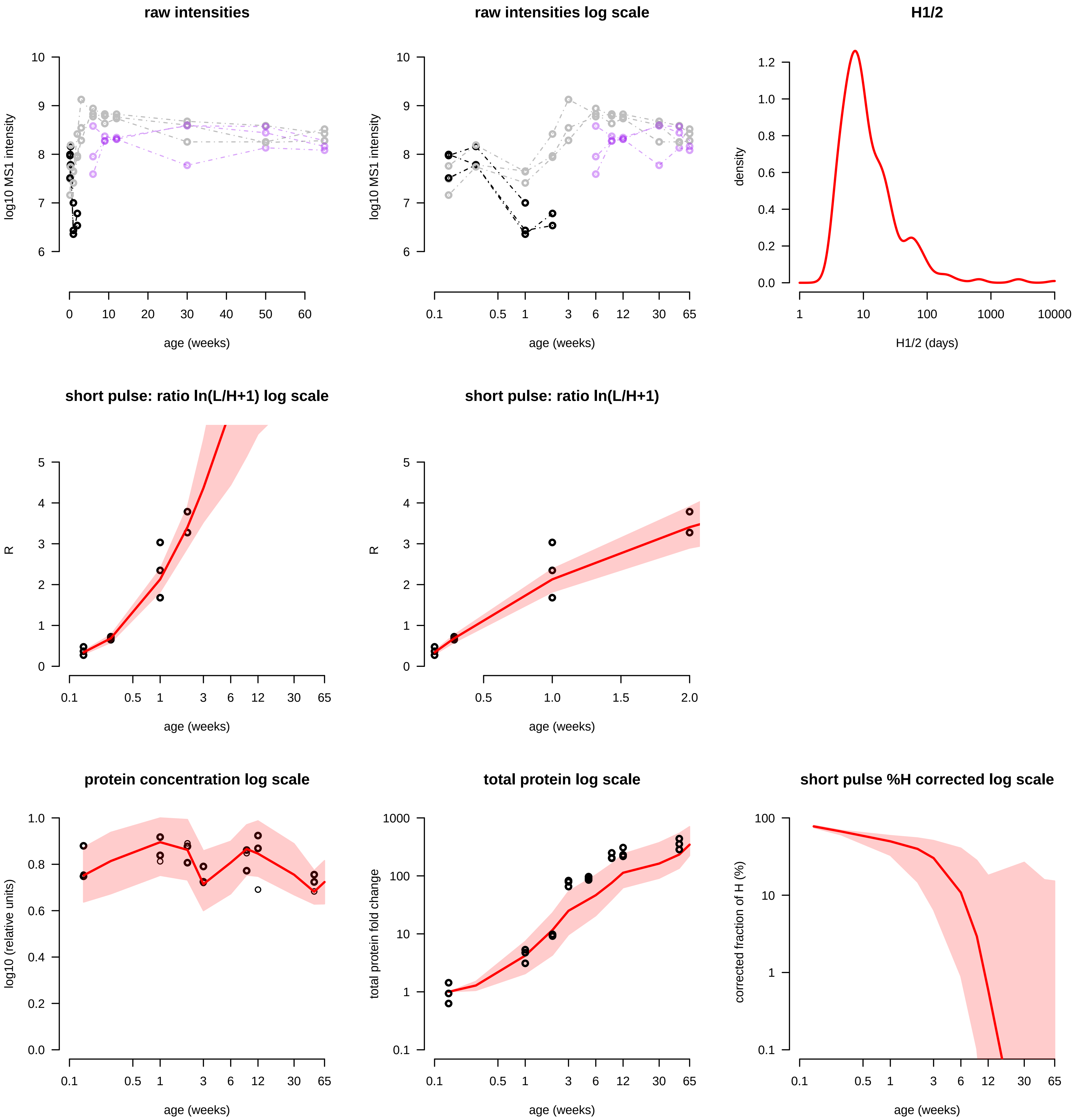

P23591

>sp|P23591|FCL_MOUSE

GDP−L−fucose synthase

GN=Gfus

Med H1/2: 10.1 days

Q5   H1/2: 4.7 days

Q95 H1/2: 184.2 days

reliable model fit: yes

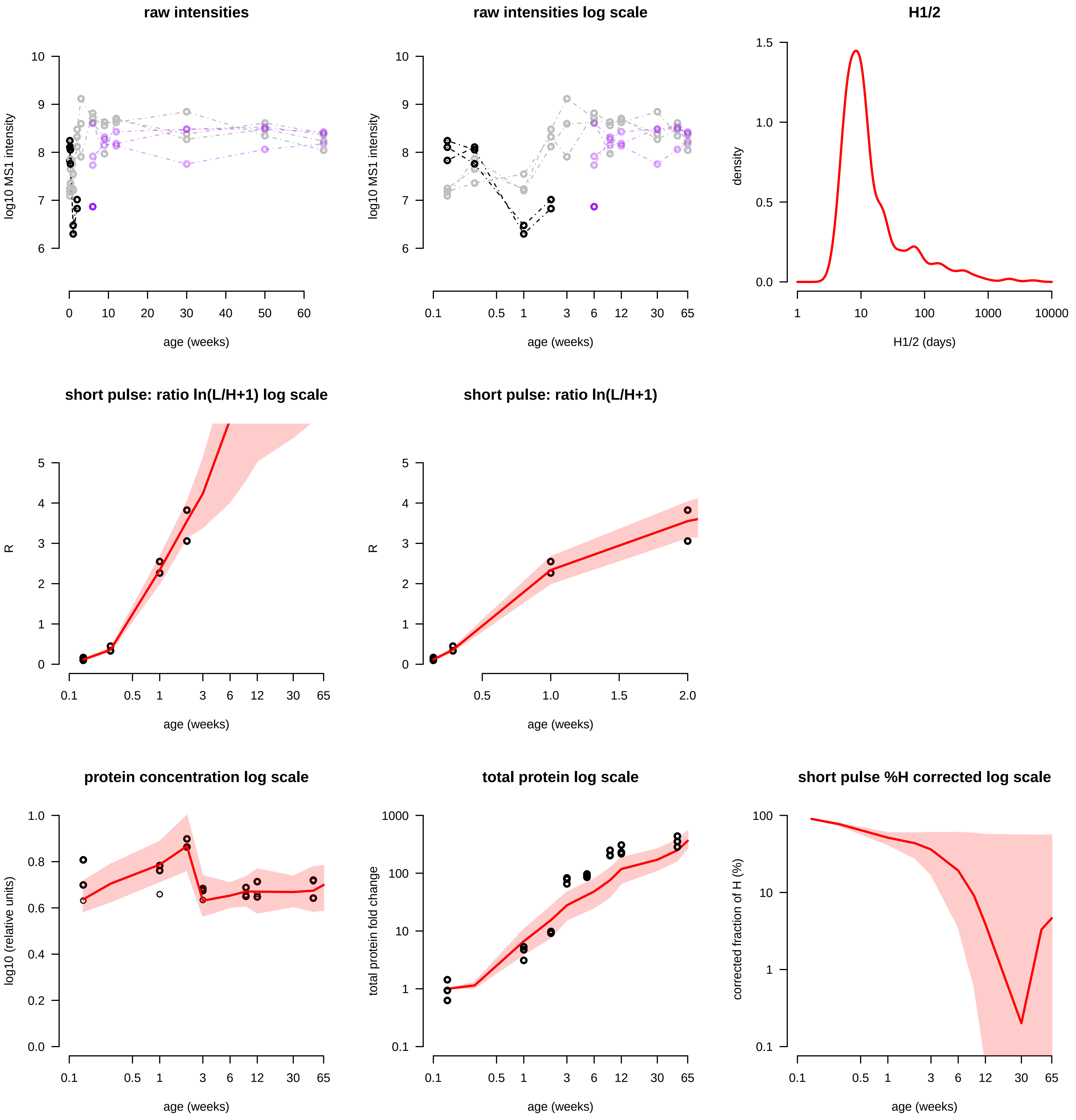

Q05CL8

>sp|Q05CL8|LARP7_MOUSE

La−related protein 7

GN=Larp7

Med H1/2: 4.7 days

Q5 H1/2: 2.6 days

Q95 H1/2: 15.2 days

reliable model fit: yes

raw intensities

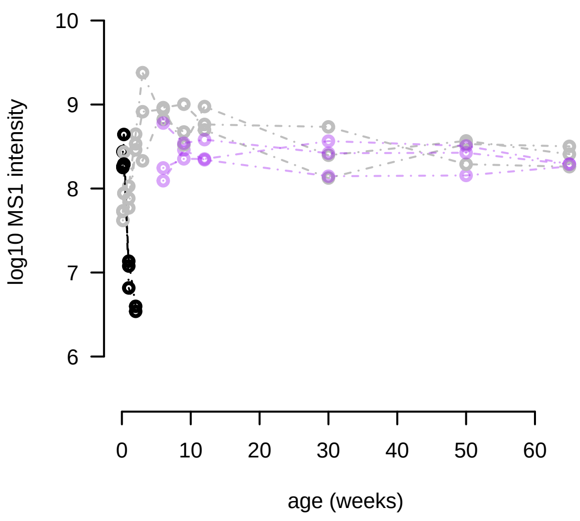

raw intensities log scale

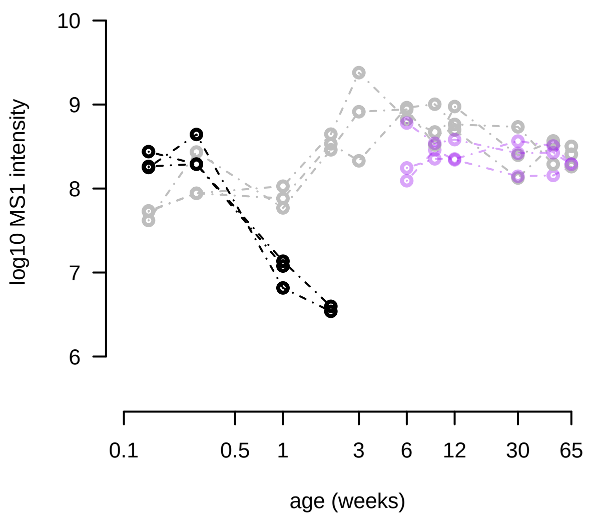

H1/2

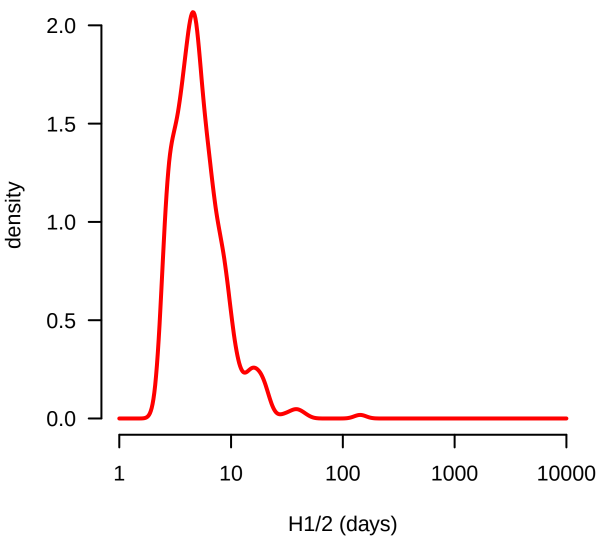

short pulse: ratio ln(L/H+1) log scale

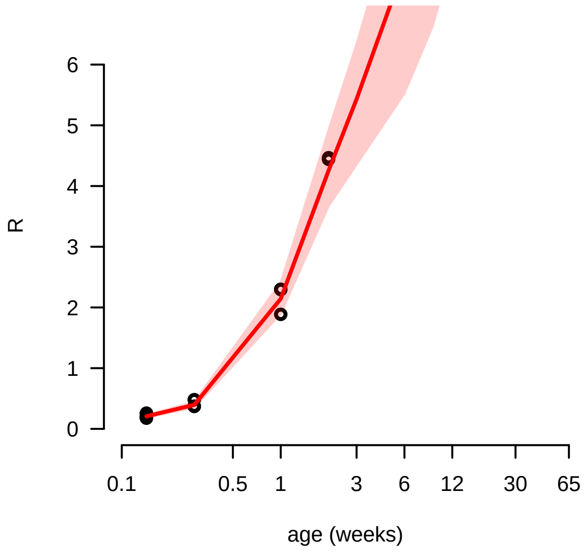

short pulse: ratio ln(L/H+1)

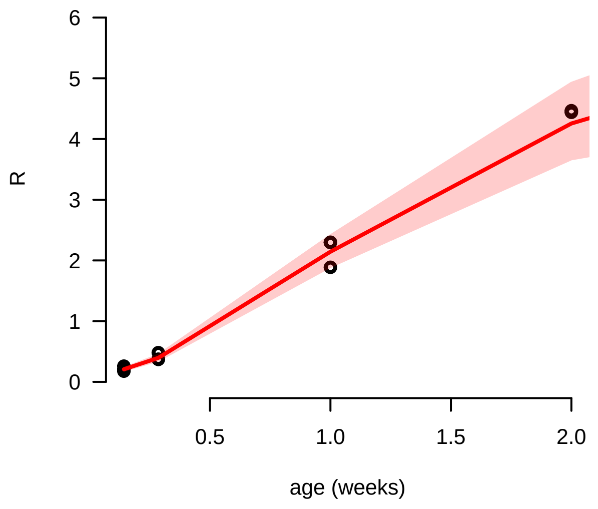

protein concentration log scale

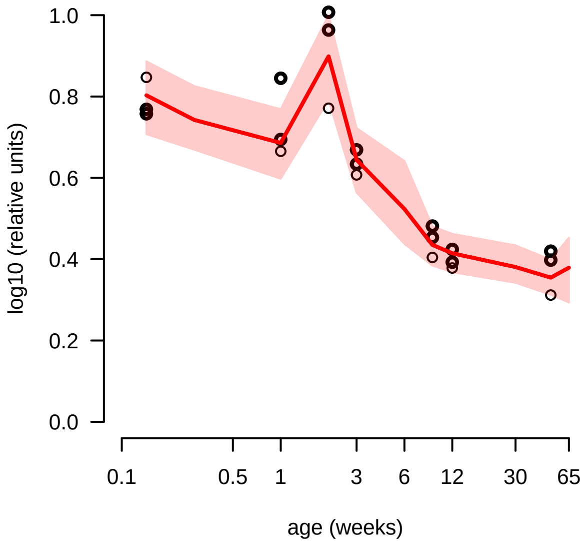

total protein log scale

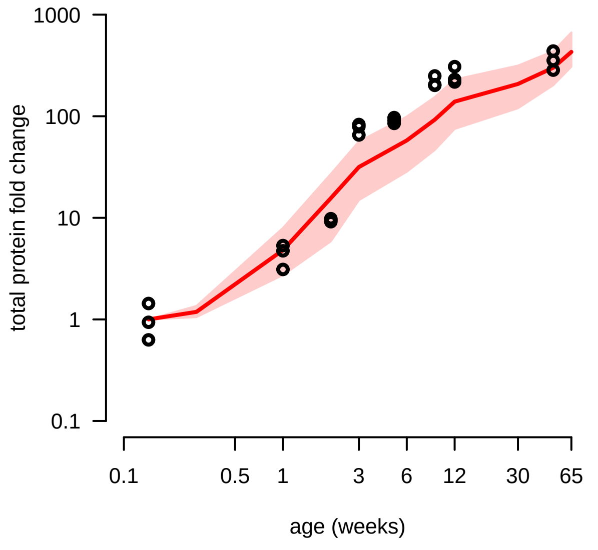

short pulse %H corrected log scale

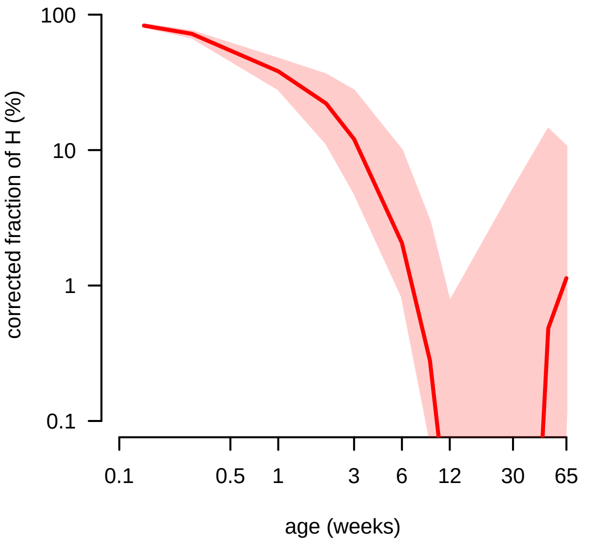

G5E897

>sp|G5E897|PLGT3_MOUSE

Protein O−glucosyltransferase 3

GN=Poglut3

Med H1/2: 9.4 days

Q5 H1/2: 3.6 days

Q95 H1/2: 40.3 days

reliable model fit: yes

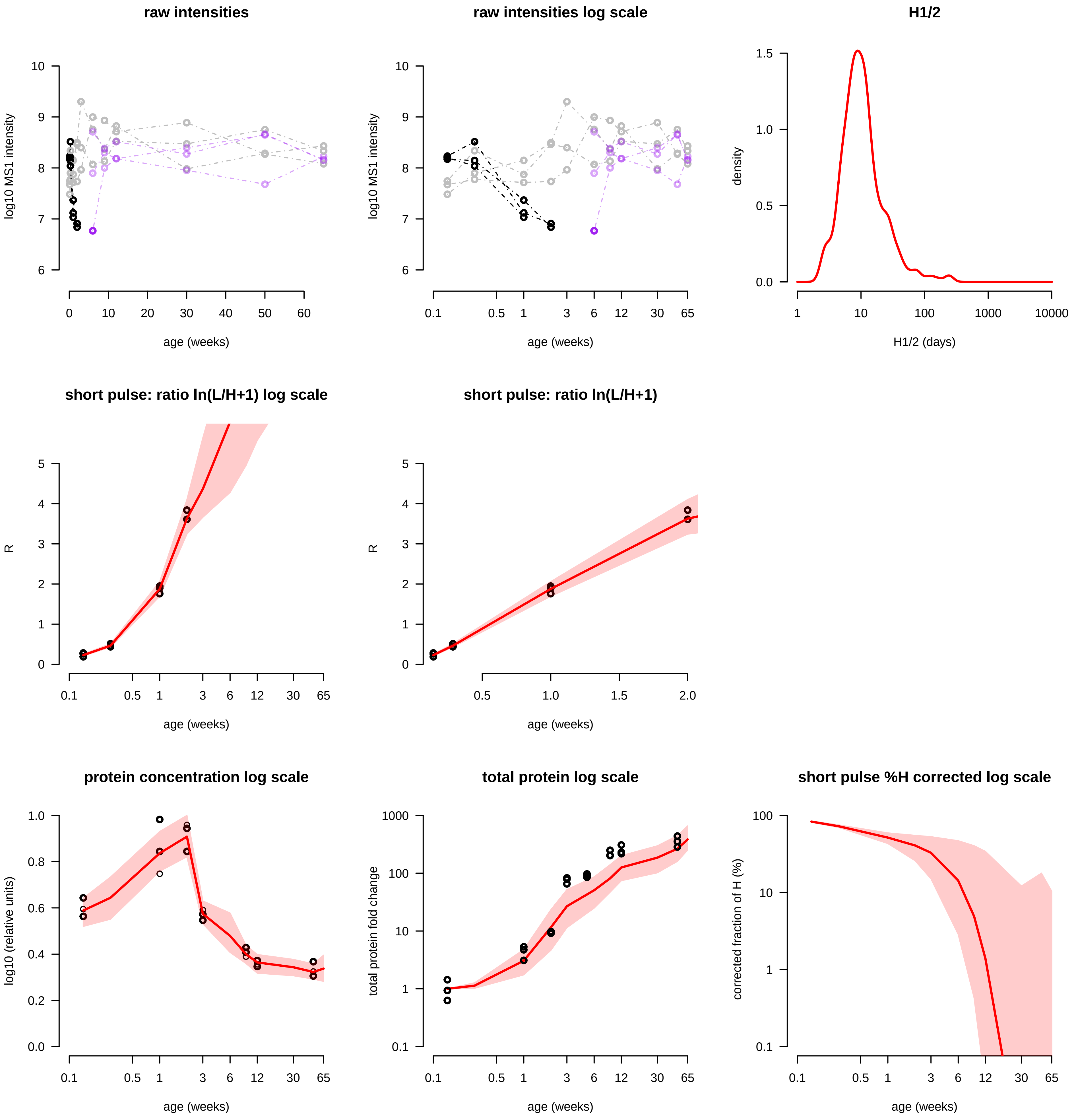

Q8BFS6;Q8BFS6−2;Q8BFS6−4

>sp|Q8BFS6|CPPED_MOUSE

Serine/threonine−protein phosphatase CPPED1

GN=Cpped1

Med H1/2: 14 days

Q5 H1/2: 5.8 days

Q95 H1/2: 124.5 days

reliable model fit: yes

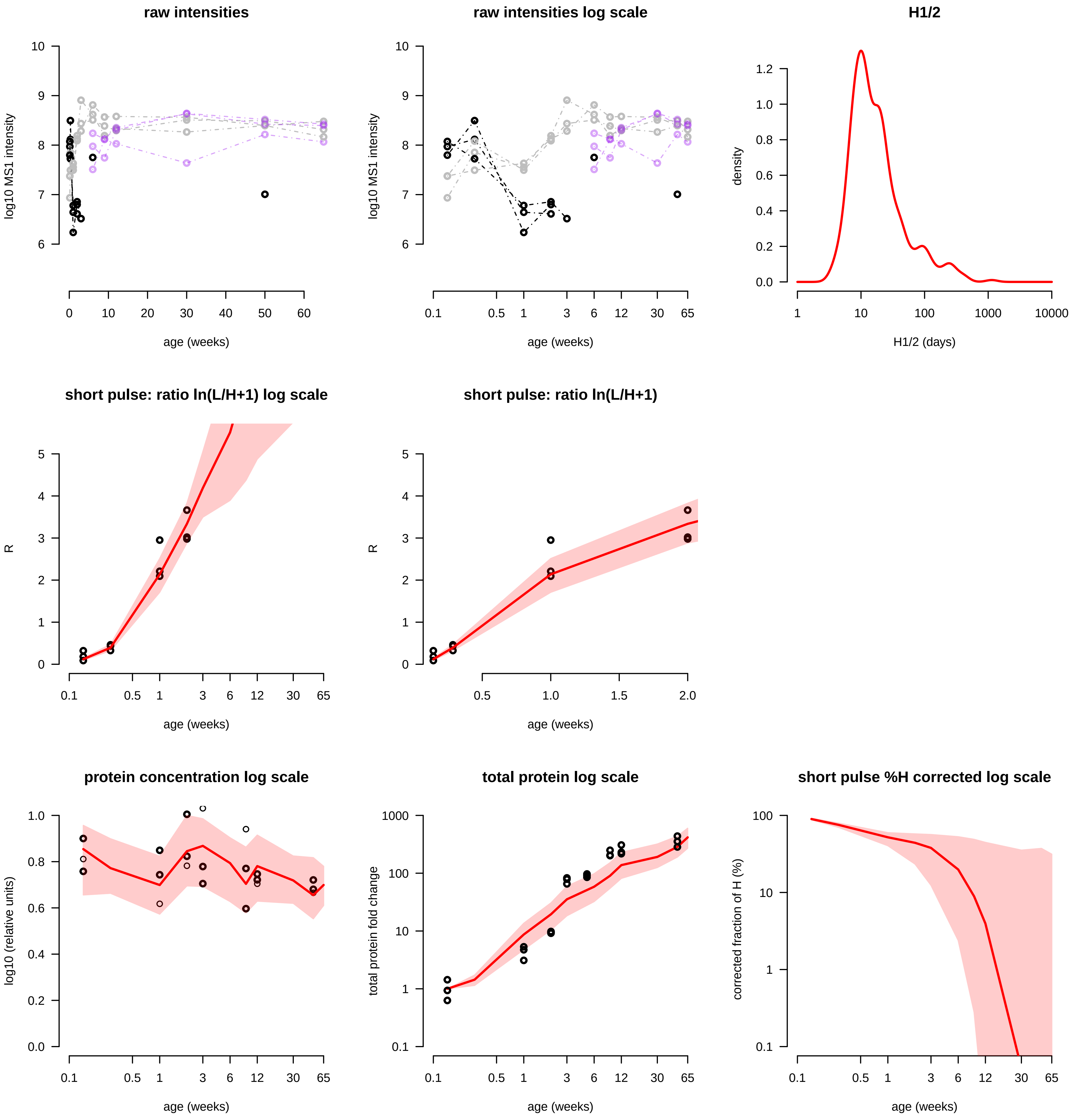

Q64008

>sp|Q64008|RAB34_MOUSE

Ras−related protein Rab−34

GN=Rab34

Med H1/2: 10.2 days

Q5 H1/2: 3.8 days

Q95 H1/2: 55.4 days

reliable model fit: yes

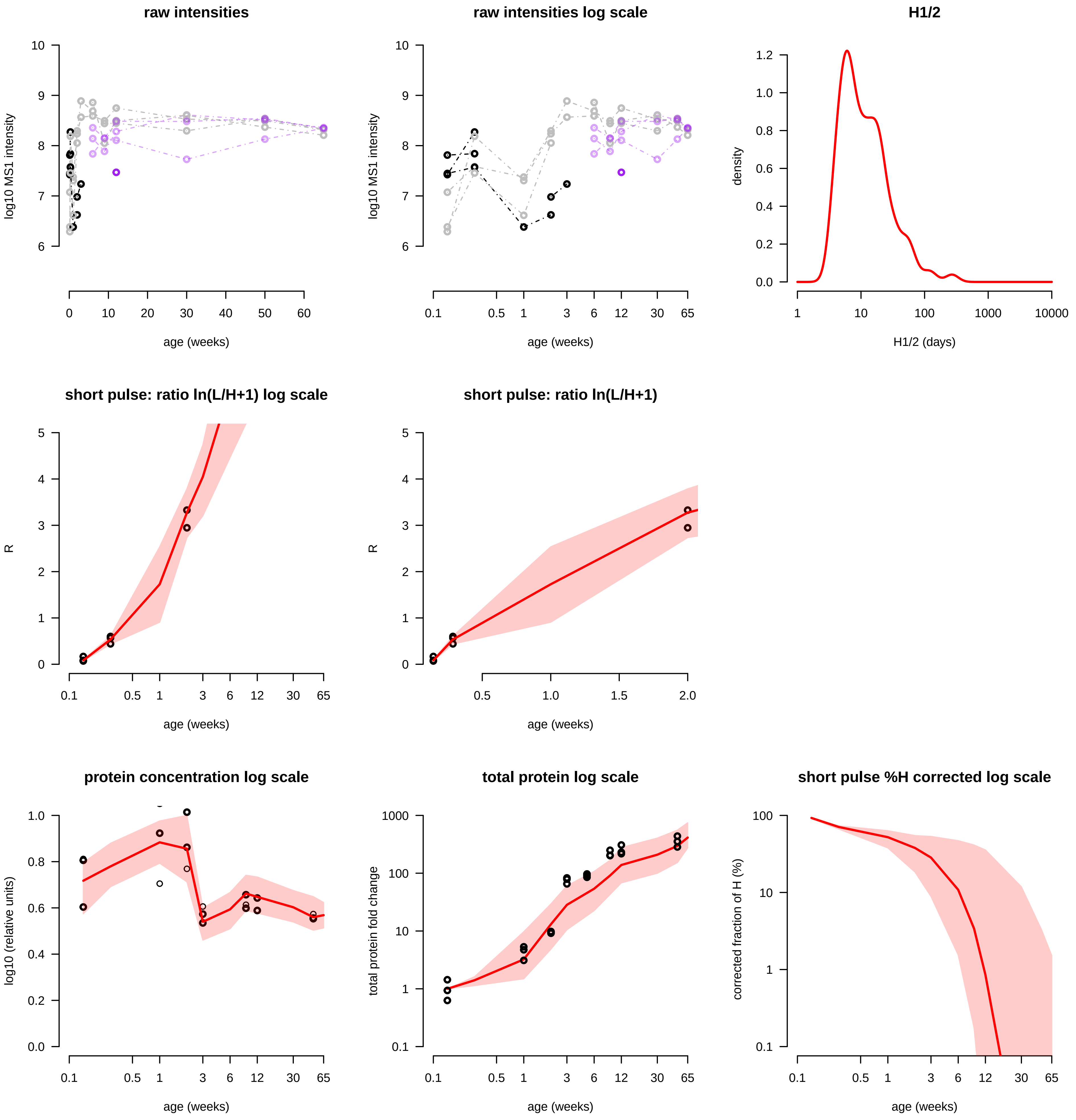

Q99J45

>sp|Q99J45|NRBP_MOUSE

Nuclear receptor−binding protein

GN=Nrbp1

Med H1/2: 3.8 days

Q5 H1/2: 3 days

Q95 H1/2: 4.9 days

reliable model fit: yes

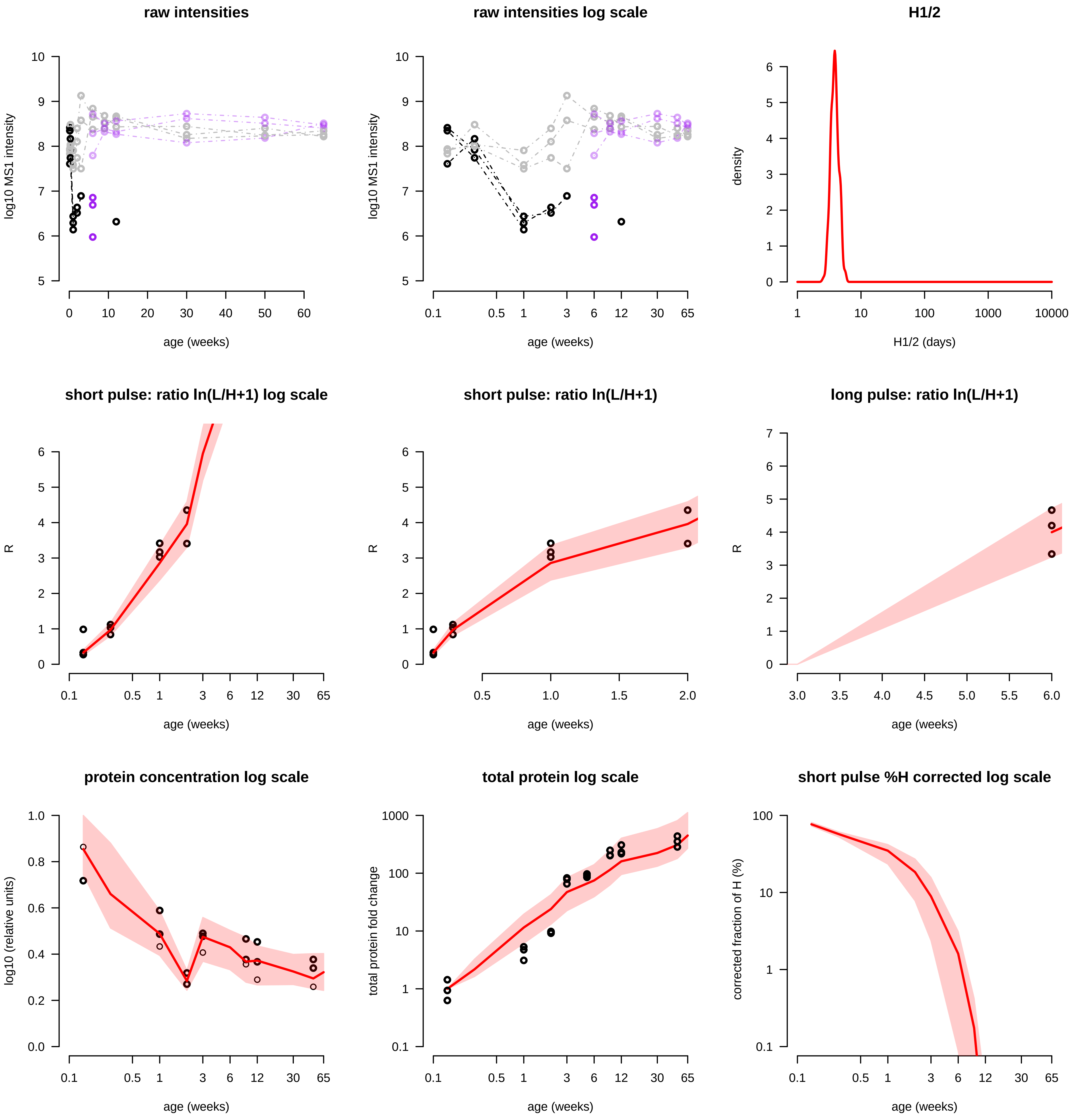

Q8K284;Q8K284−2

>sp|Q8K284|TF3C1_MOUSE

General transcription factor 3C polypeptide 1

GN=Gtf3c1

Med H1/2: 3.4 days

Q5 H1/2: 2.5 days

Q95 H1/2: 10.7 days

reliable model fit: yes

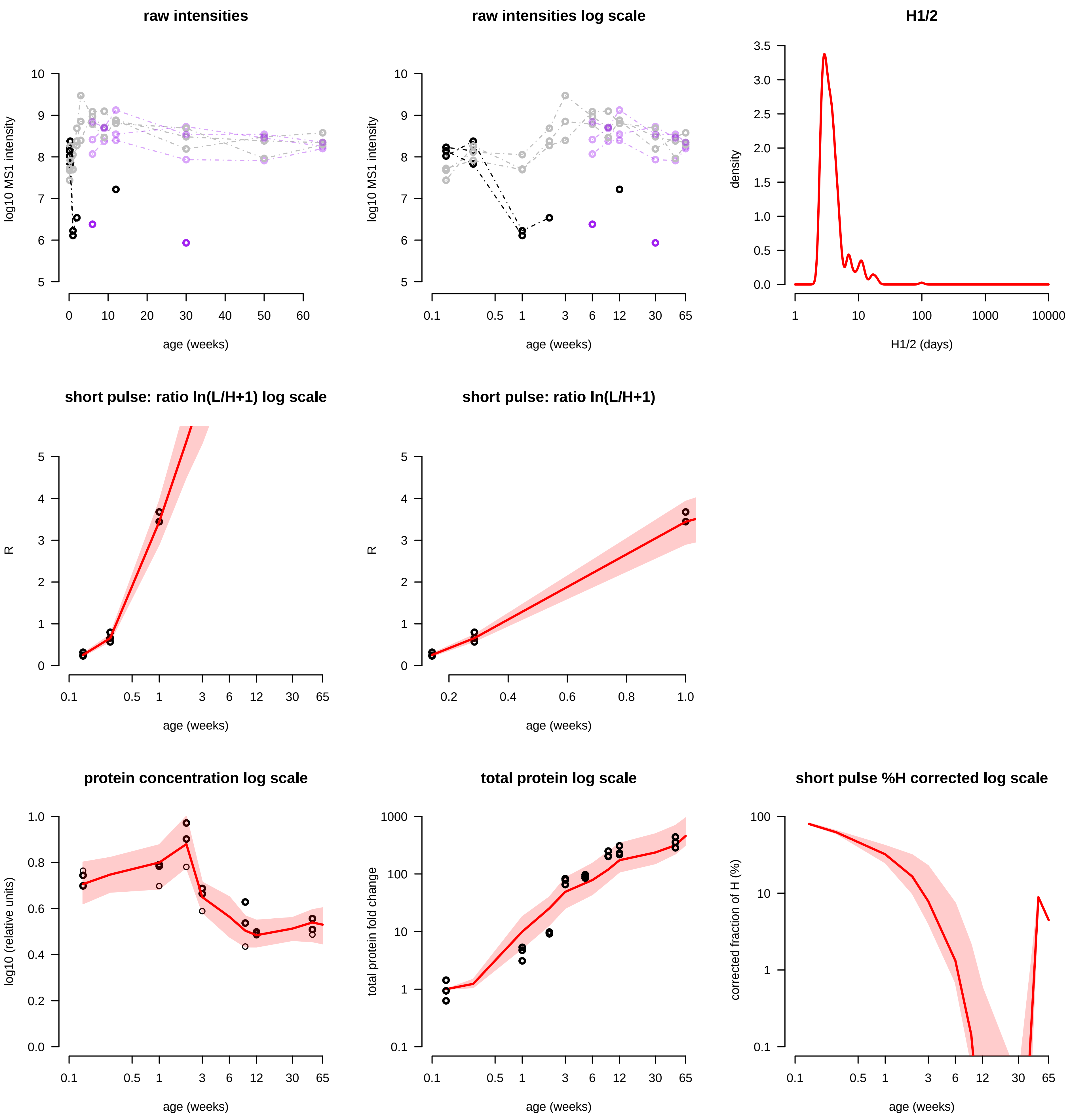

Q8BJZ4

>sp|Q8BJZ4|RT35_MOUSE

28S ribosomal protein S35, mitochondrial

GN=Mrps35

Med H1/2: 9.2 days

Q5 H1/2: 3.8 days

Q95 H1/2: 49.6 days

reliable model fit: yes

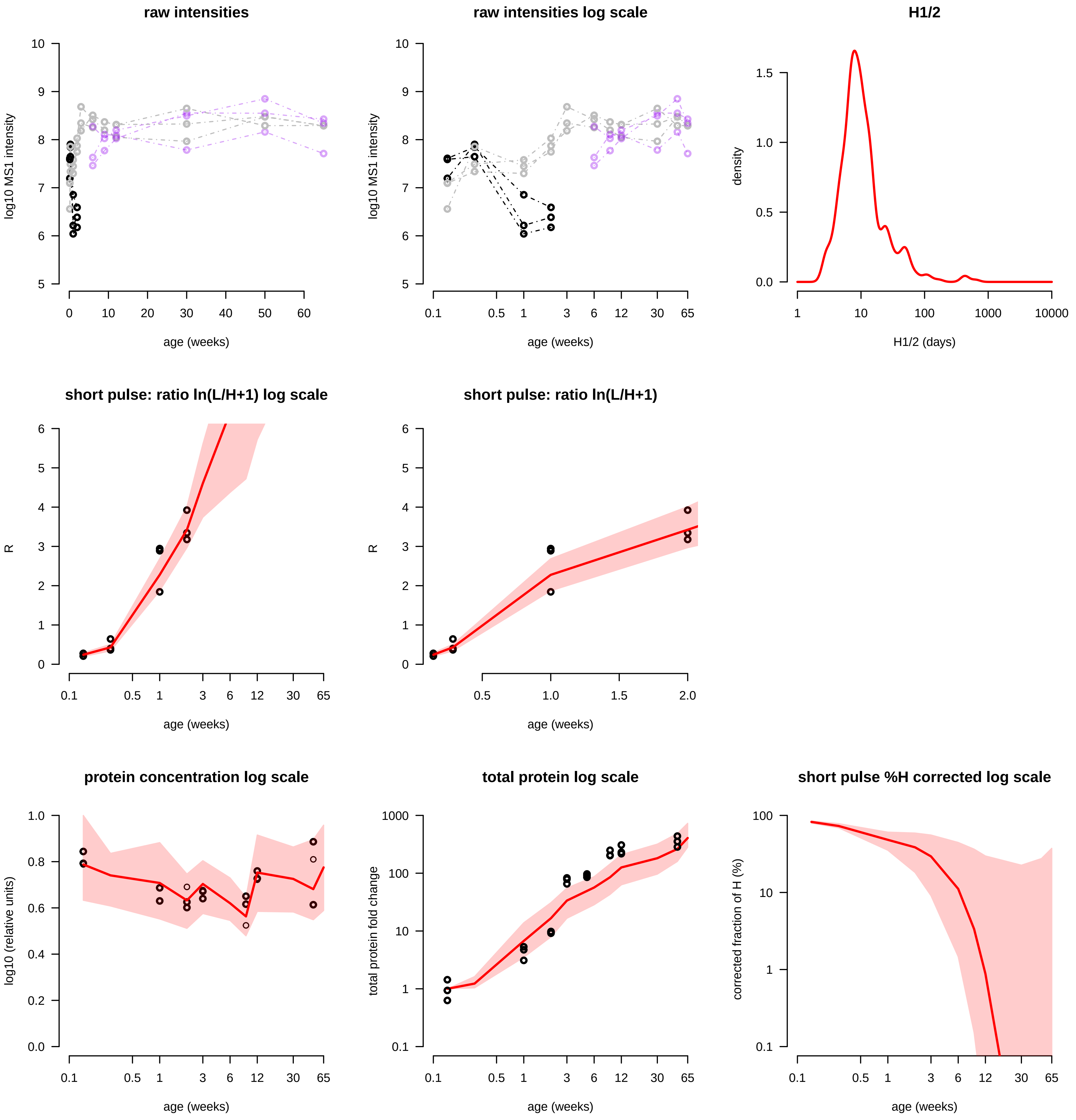

P19258

>sp|P19258|MPV17_MOUSE

Protein Mpv17

GN=Mpv17

Med H1/2: 15.8 days

Q5 H1/2: 9 days

Q95 H1/2: 42.5 days

reliable model fit: no

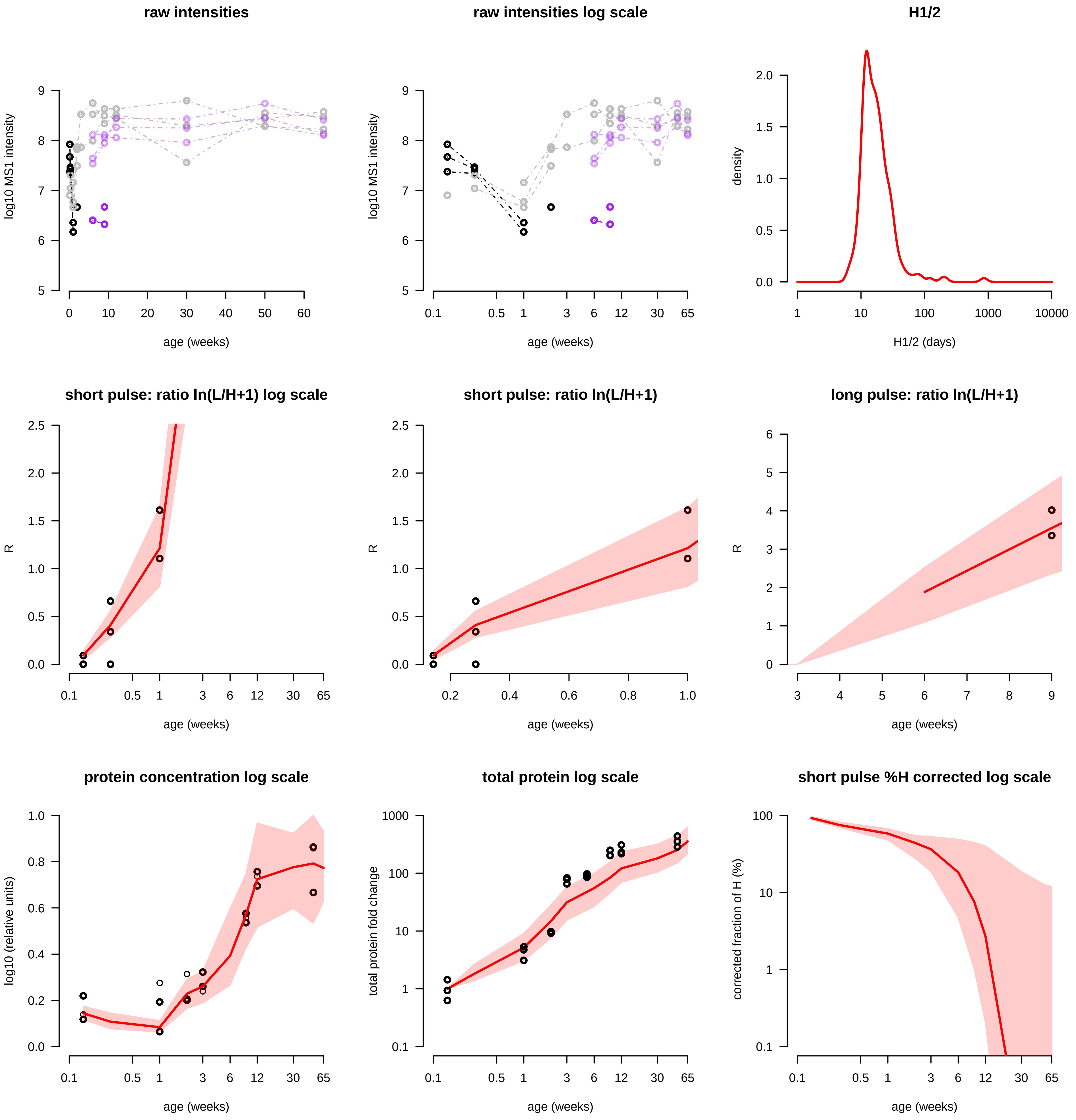

Q9DC70

>sp|Q9DC70|NDUS7_MOUSE

NADH dehydrogenase [ubiquinone] iron−sulfur protein 7, mitochondrial

GN=Ndufs7

Med H1/2: 8.1 days

Q5 H1/2: 6.5 days

Q95 H1/2: 11.3 days

reliable model fit: yes

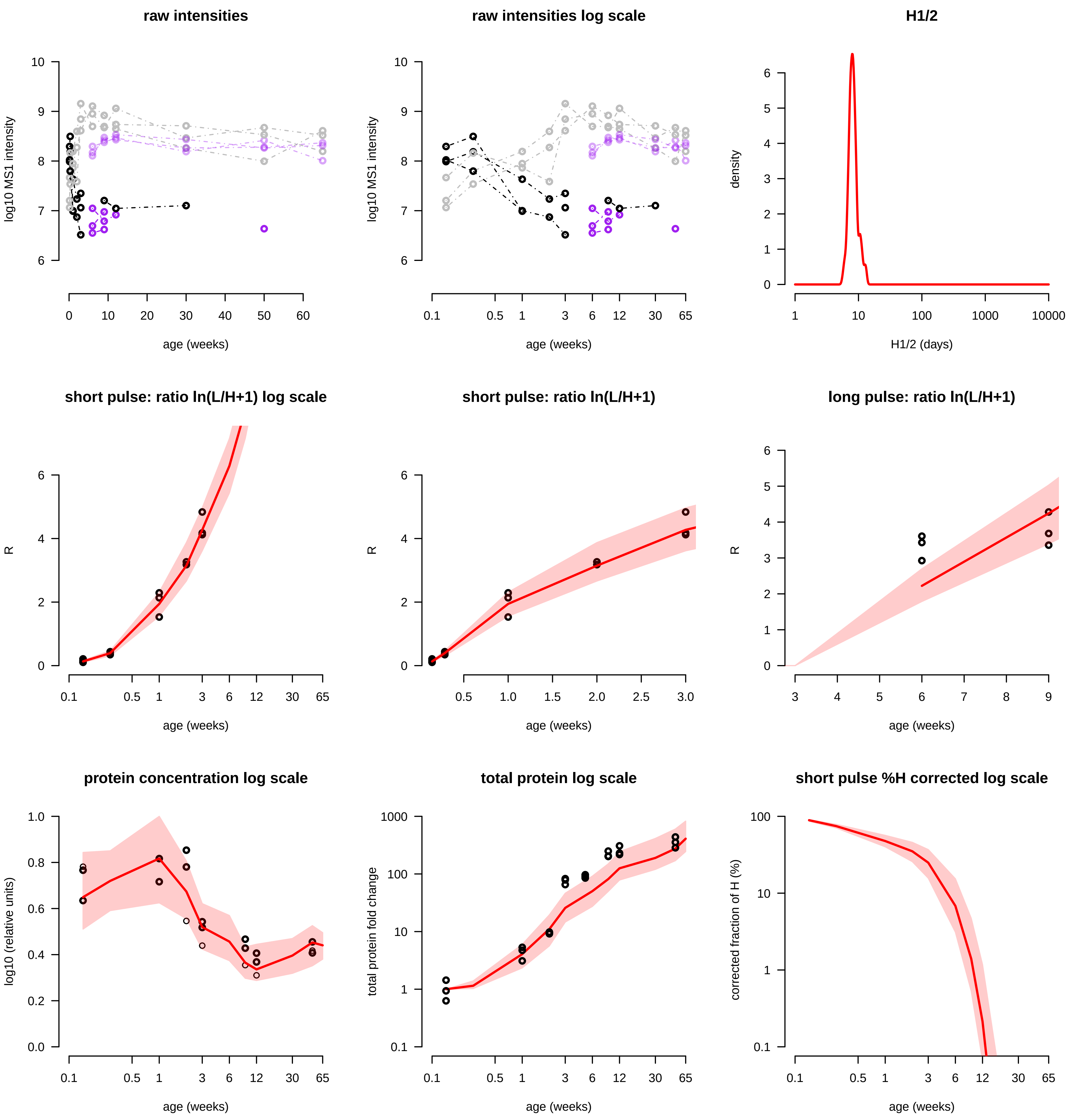

Q0VGB7

>sp|Q0VGB7|PP4R2_MOUSE

Serine/threonine−protein phosphatase 4 regulatory subunit 2

GN=Ppp4r2

Med H1/2: 937.6 days

Q5 H1/2: 231.7 days

Q95 H1/2: 20054 days

reliable model fit: no

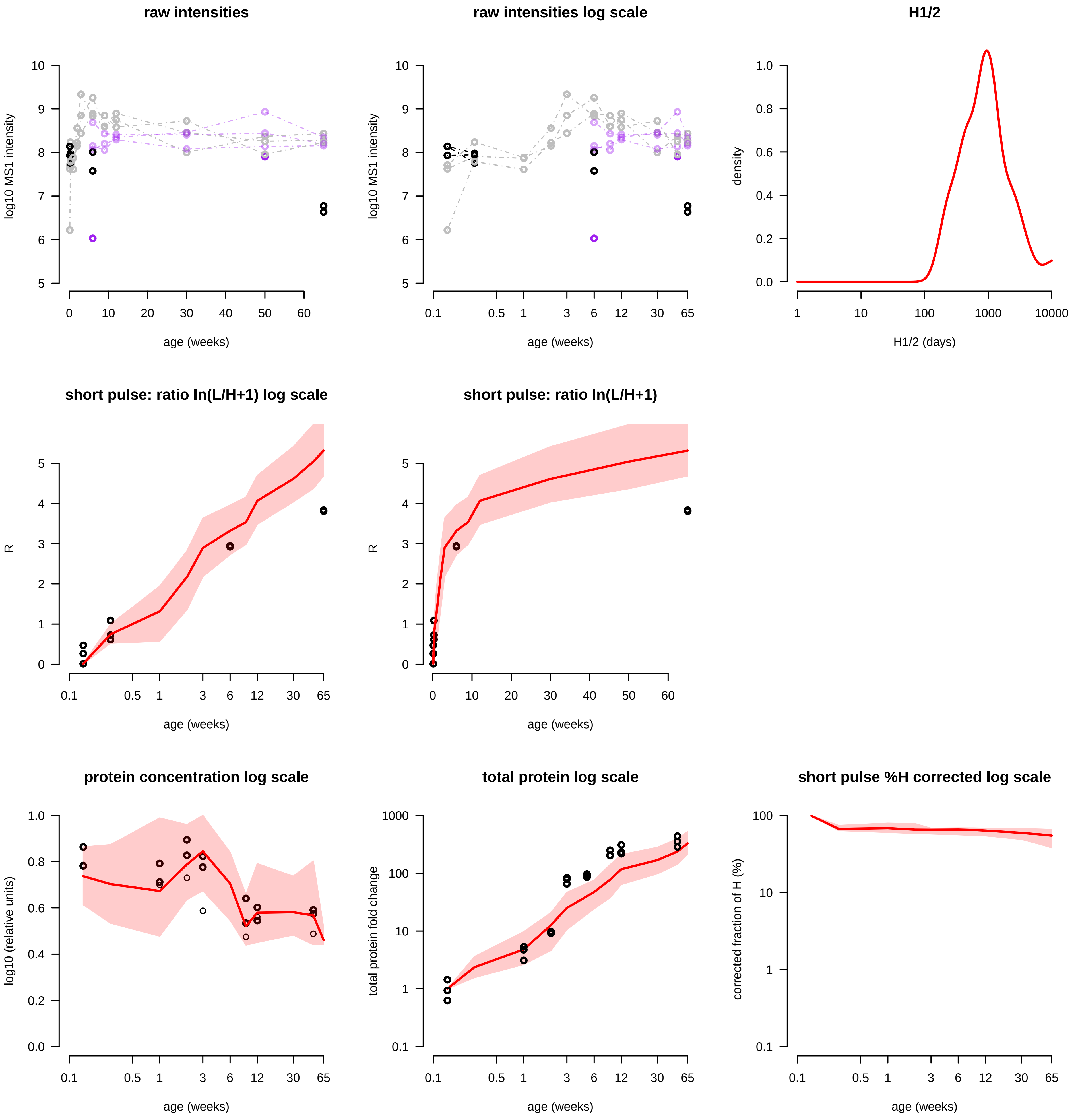

P97765;P97765−2

>sp|P97765|WBP2_MOUSE

WW domain−binding protein 2

GN=Wbp2

Med H1/2: 5.2 days

Q5 H1/2: 3.8 days

Q95 H1/2: 7.5 days

reliable model fit: yes

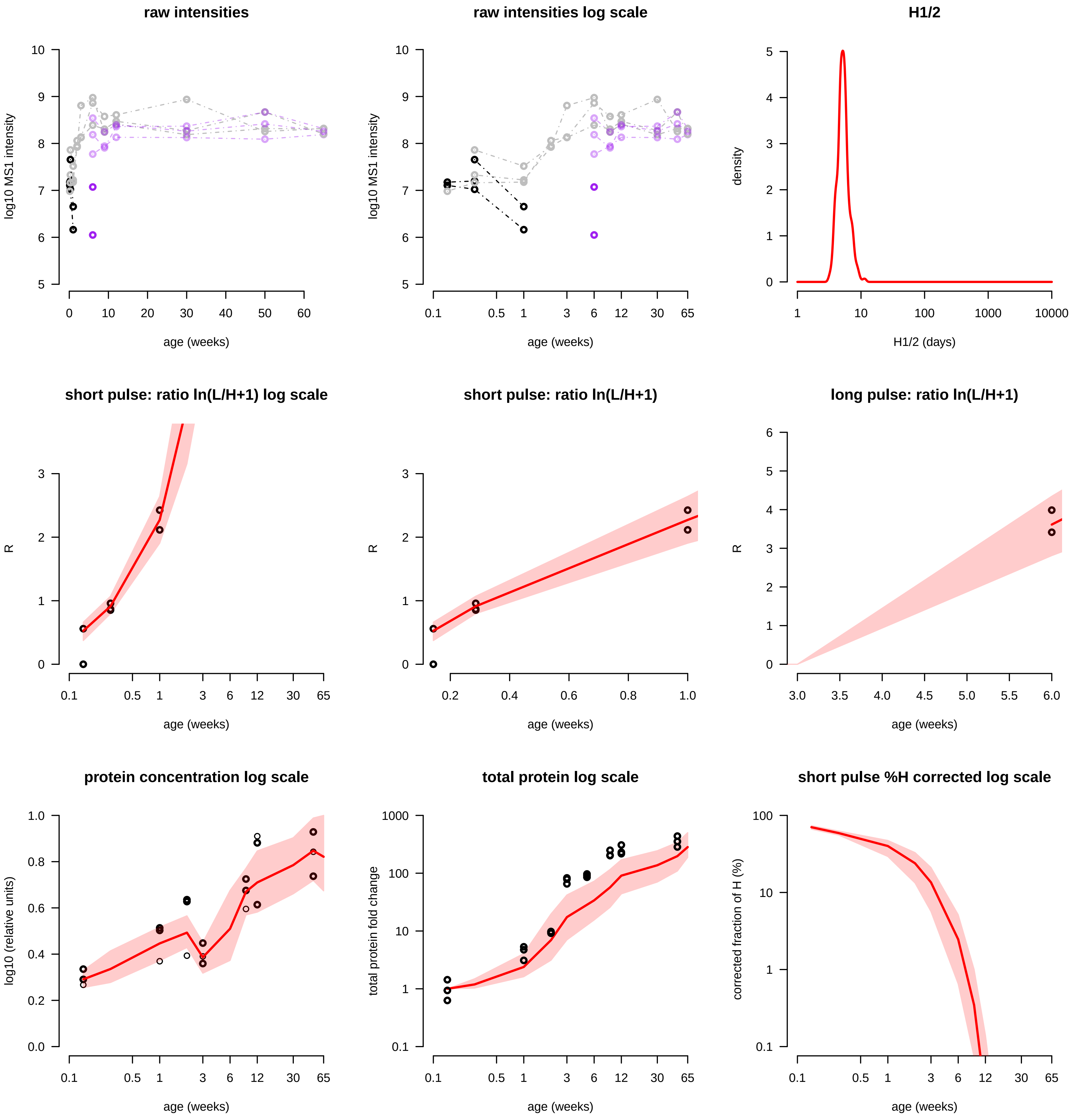

P47963

>sp|P47963|RL13_MOUSE

60S ribosomal protein L13

GN=Rpl13

Med H1/2: 6.8 days

Q5 H1/2: 5 days

Q95 H1/2: 10.9 days

reliable model fit: yes

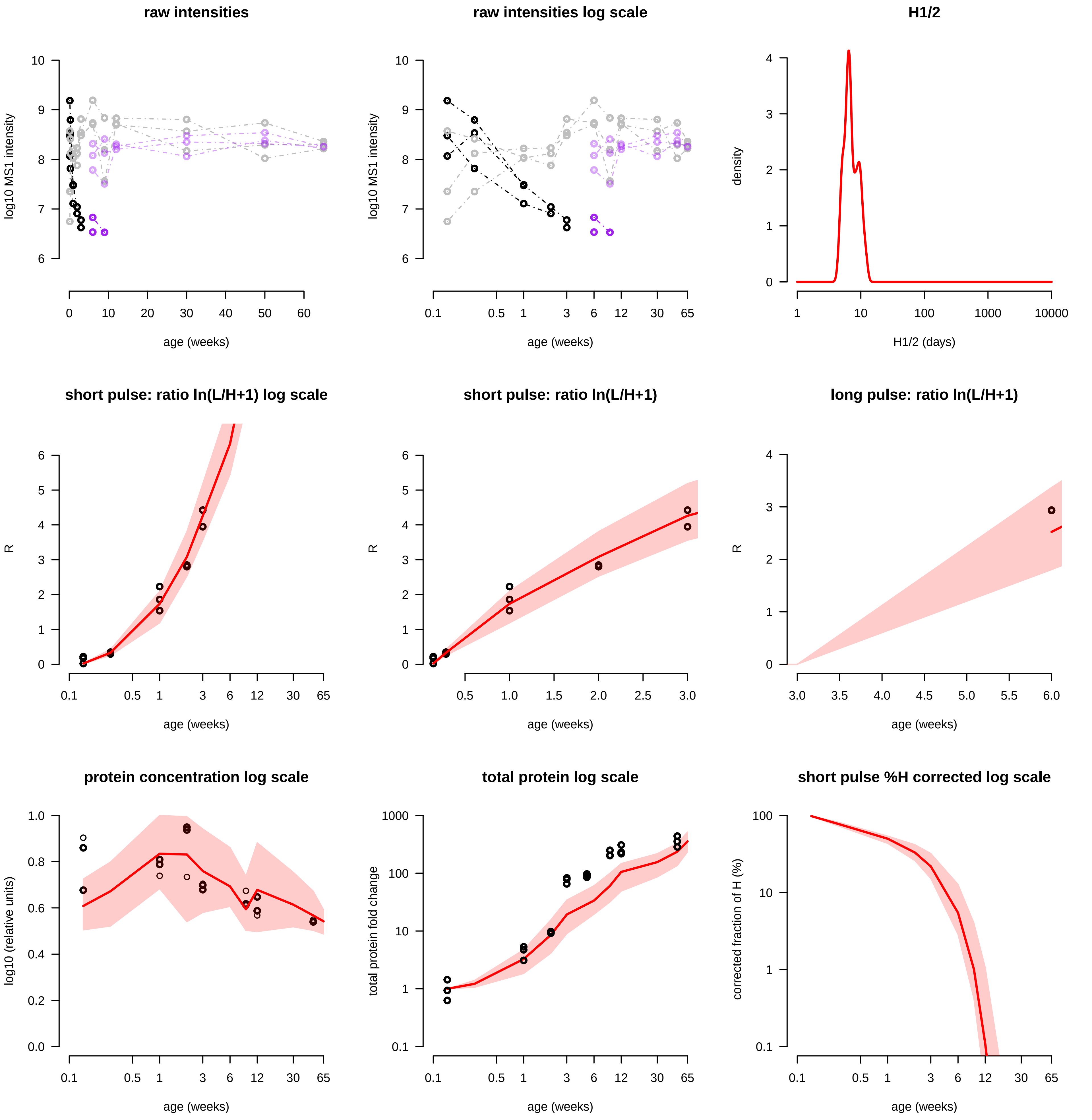

O35598

>sp|O35598|ADA10_MOUSE

Disintegrin and metalloproteinase domain−containing protein 10

GN=Adam10

Med H1/2: 5.3 days

Q5   H1/2: 2.8 days

Q95 H1/2: 31.5 days

reliable model fit: yes

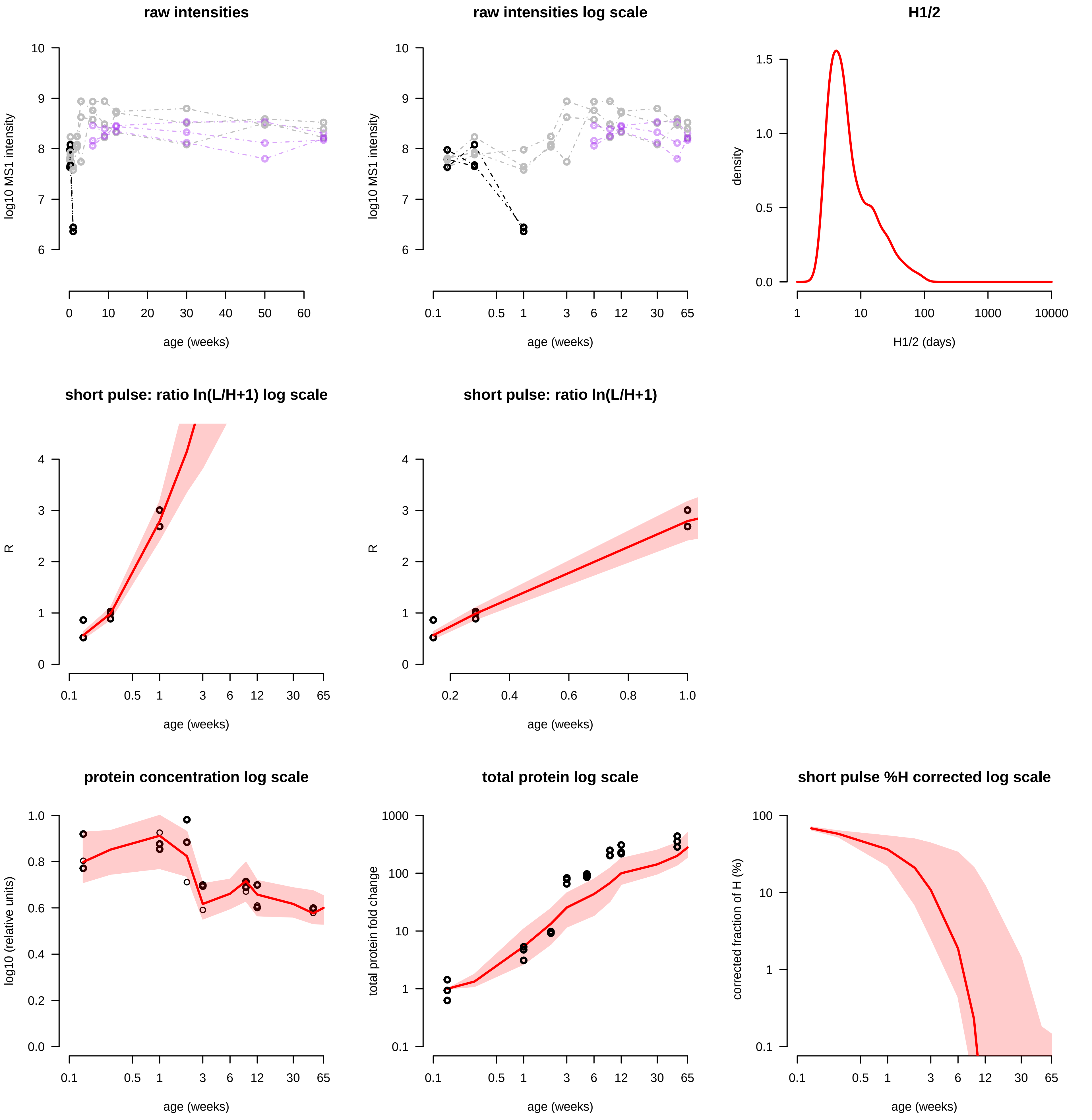

P49442

>sp|P49442|INPP_MOUSE

Inositol polyphosphate 1−phosphatase

GN=Inpp1

Med H1/2: 4.4 days

Q5 H1/2: 3.3 days

Q95 H1/2: 7 days

reliable model fit: yes

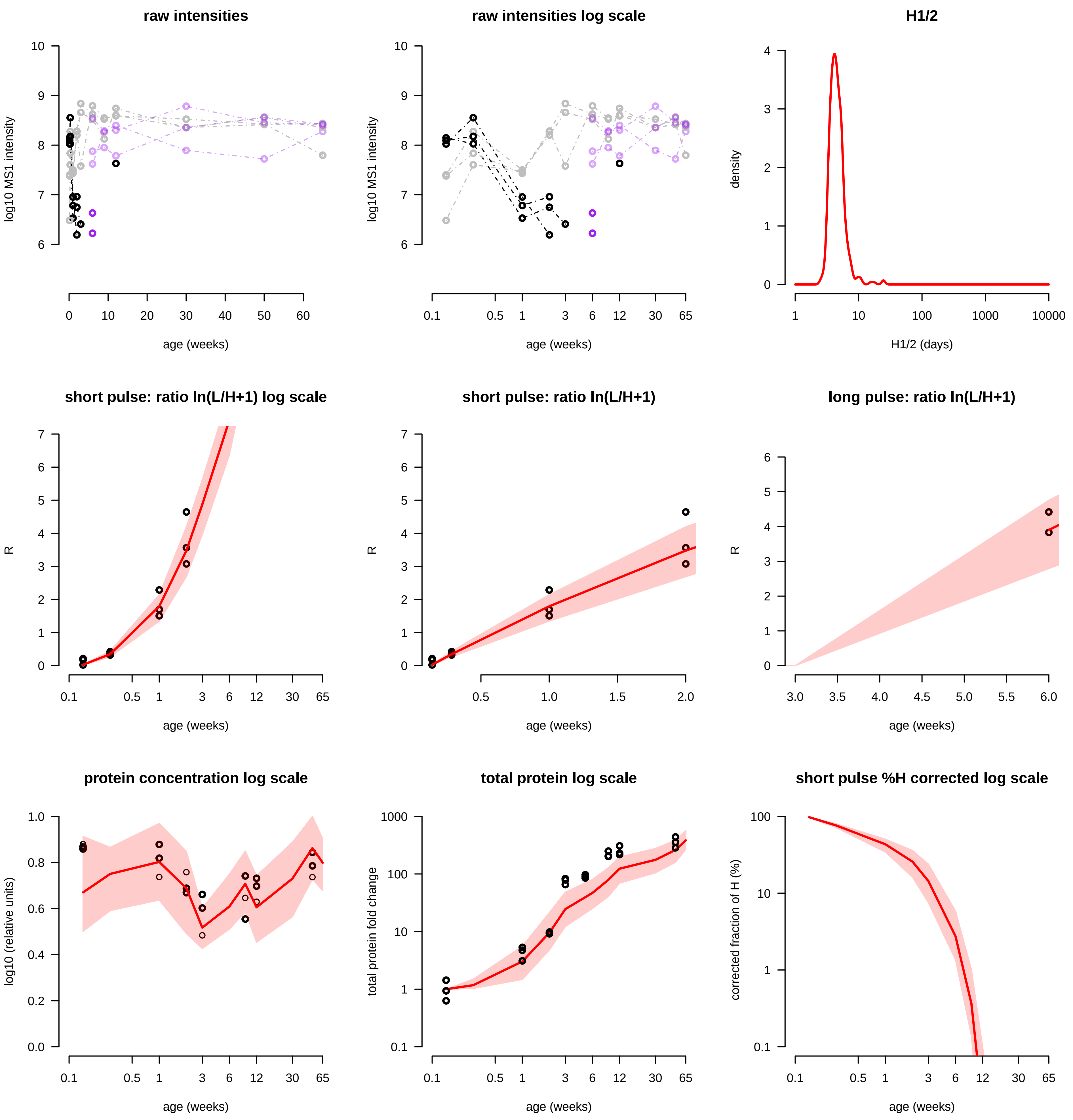

Q99N96

>sp|Q99N96|RM01_MOUSE

39S ribosomal protein L1, mitochondrial

GN=Mrpl1

Med H1/2: 6.8 days

Q5 H1/2: 3.5 days

Q95 H1/2: 21.5 days

reliable model fit: yes

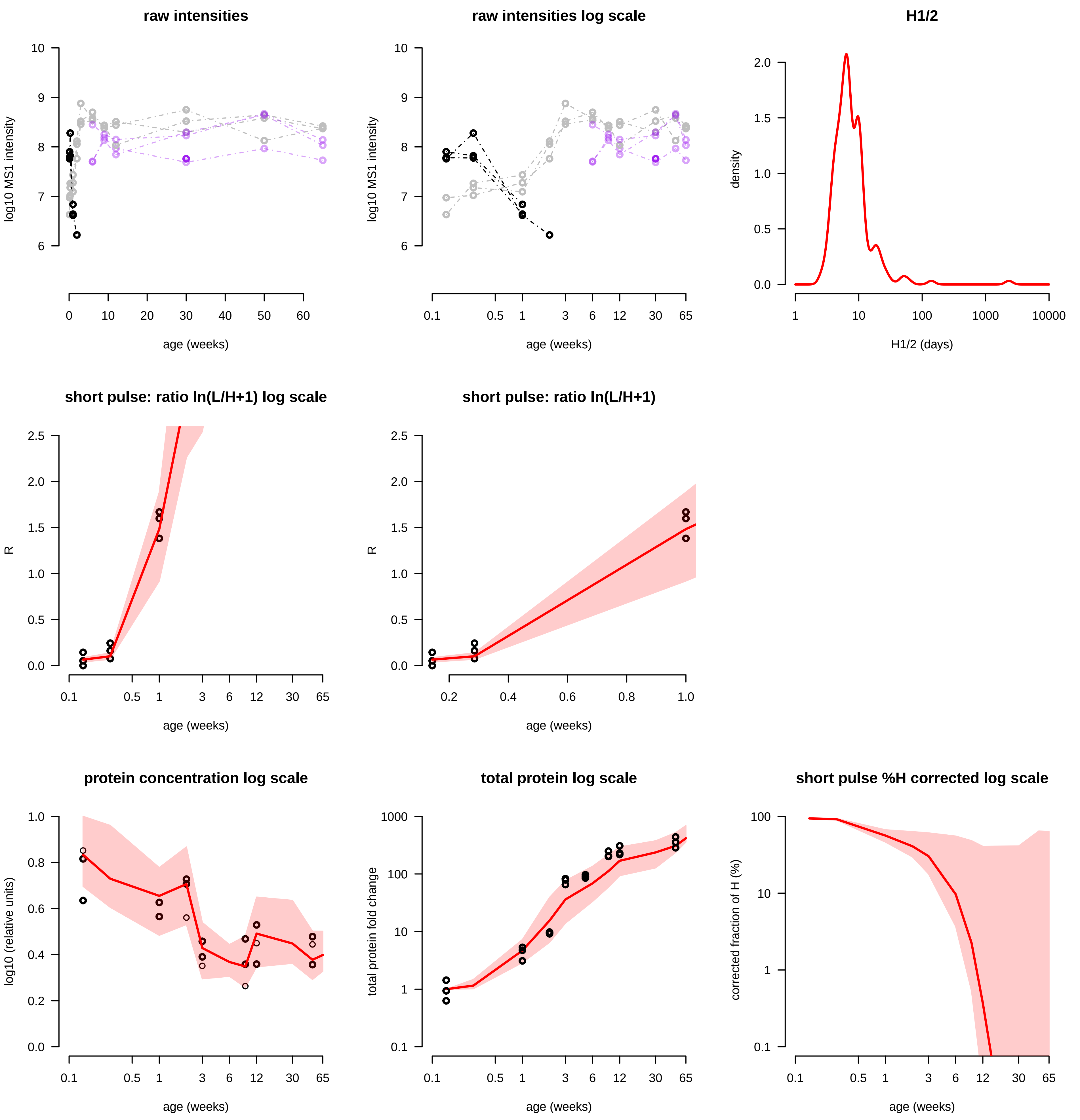

Q9Z247

>sp|Q9Z247|FKBP9_MOUSE

Peptidyl−prolyl cis−trans isomerase FKBP9

GN=Fkbp9

Med H1/2: 2.9 days

Q5 H1/2: 2.5 days

Q95 H1/2: 3.5 days

reliable model fit: yes

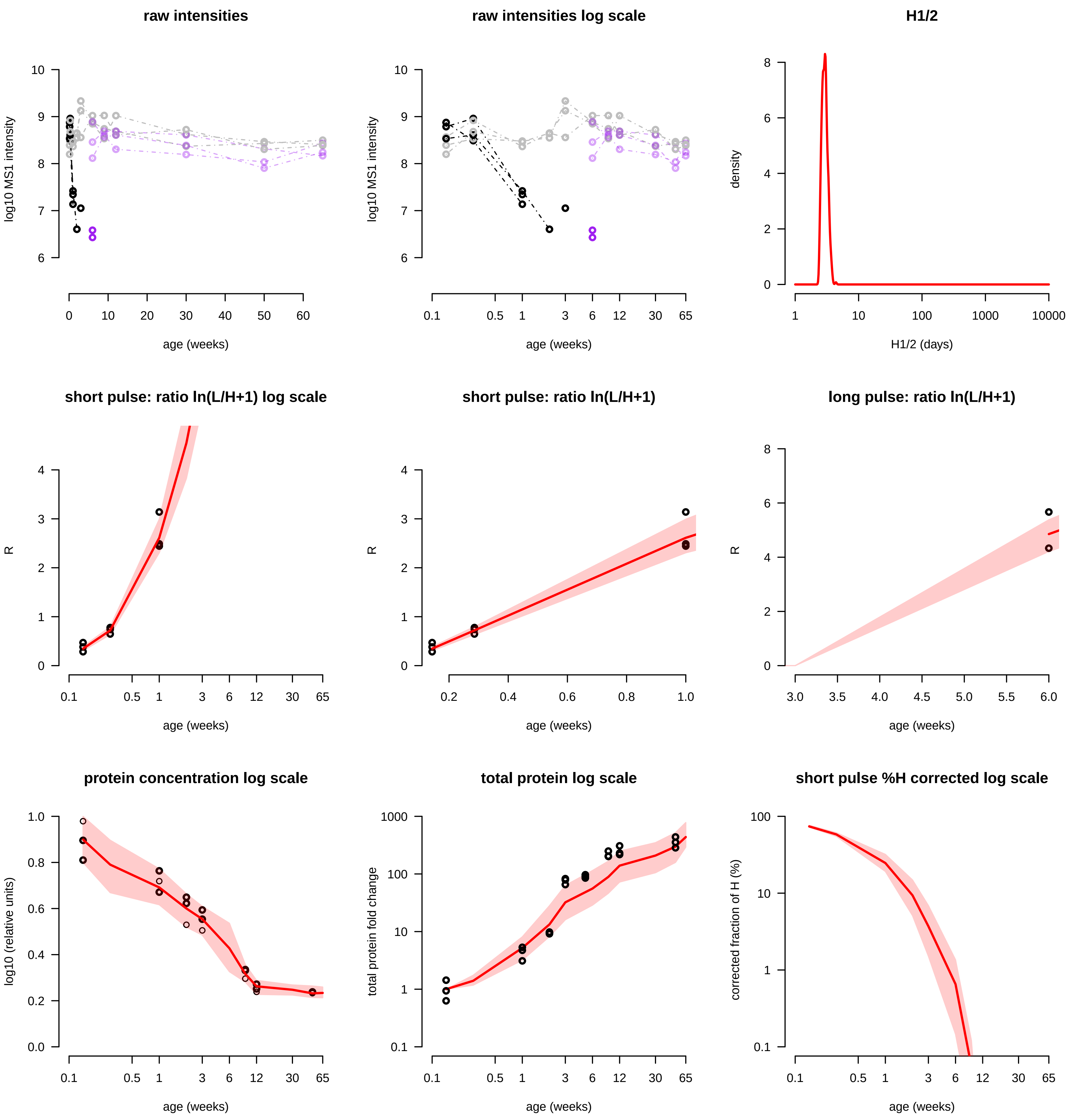

Q5XJY4

>sp|Q5XJY4|PARL_MOUSE

Presenilins−associated rhomboid−like protein, mitochondrial

GN=Parl

Med H1/2: 7.5 days

Q5 H1/2: 3.4 days

Q95 H1/2: 43.1 days

reliable model fit: yes

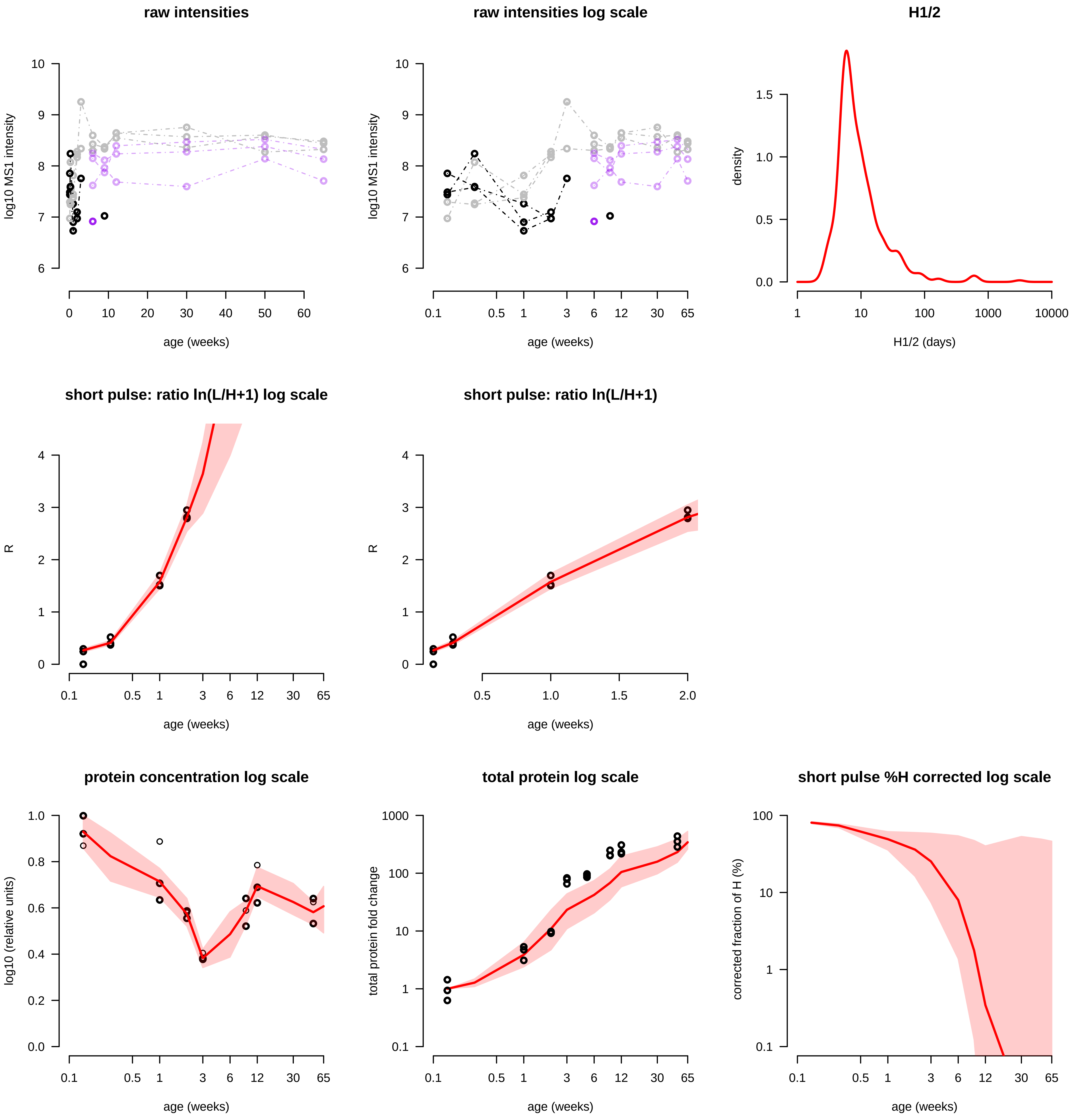

Q4KMM3−4;Q4KMM3−3

>sp|Q4KMM3−4|OXR1_MOUSE

Isoform 4 of Oxidation resistance protein 1

GN=Oxr1

Med H1/2: 484.5 days

Q5 H1/2: 145.6 days

Q95 H1/2: 4624.6 days

reliable model fit: no

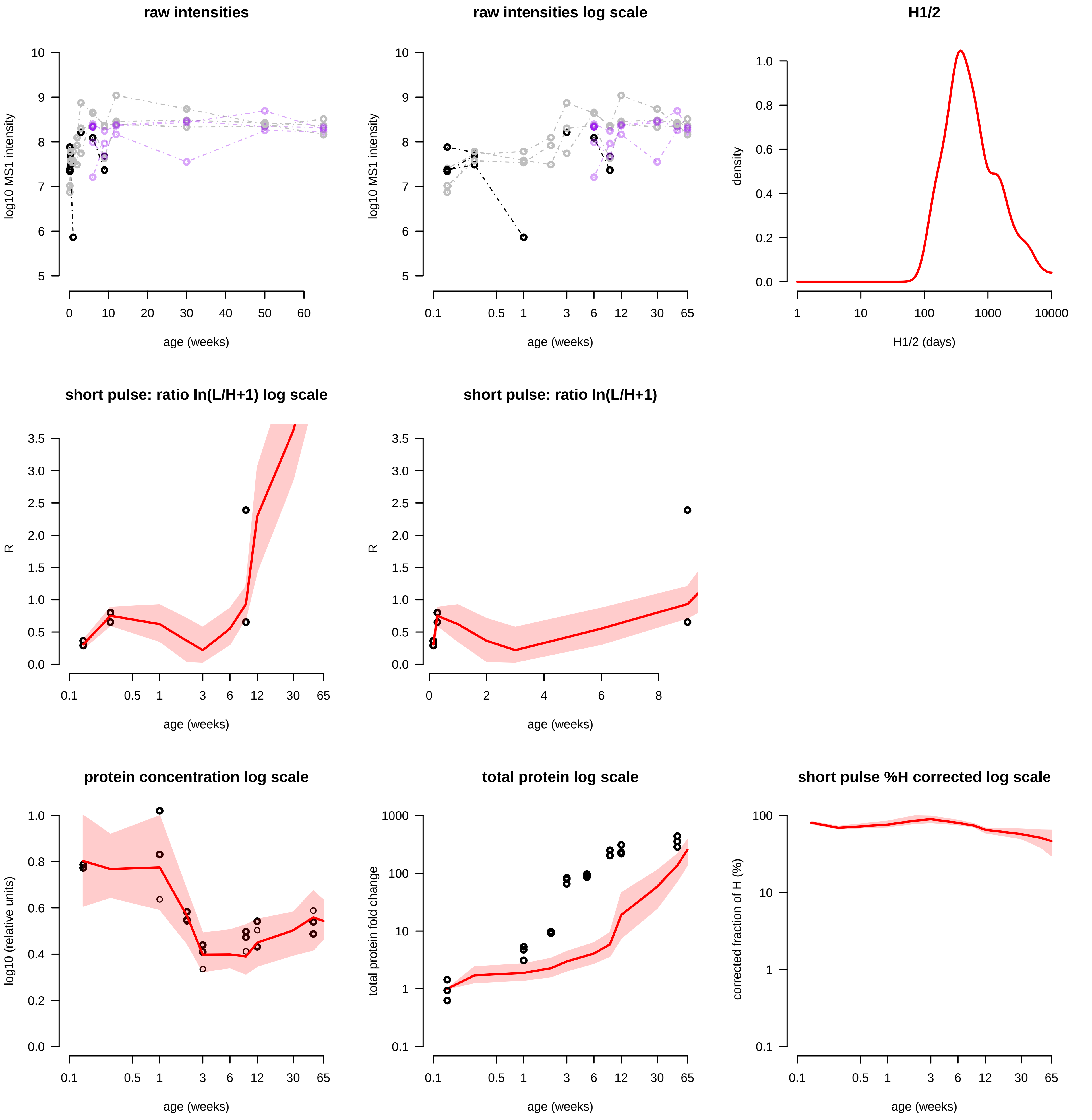

Q91WG4;Q91WG4−2

>sp|Q91WG4|ELP2_MOUSE

Elongator complex protein 2

GN=Elp2

Med H1/2: 913.4 days

Q5 H1/2: 239.3 days

Q95 H1/2: 23357.8 days

reliable model fit: yes

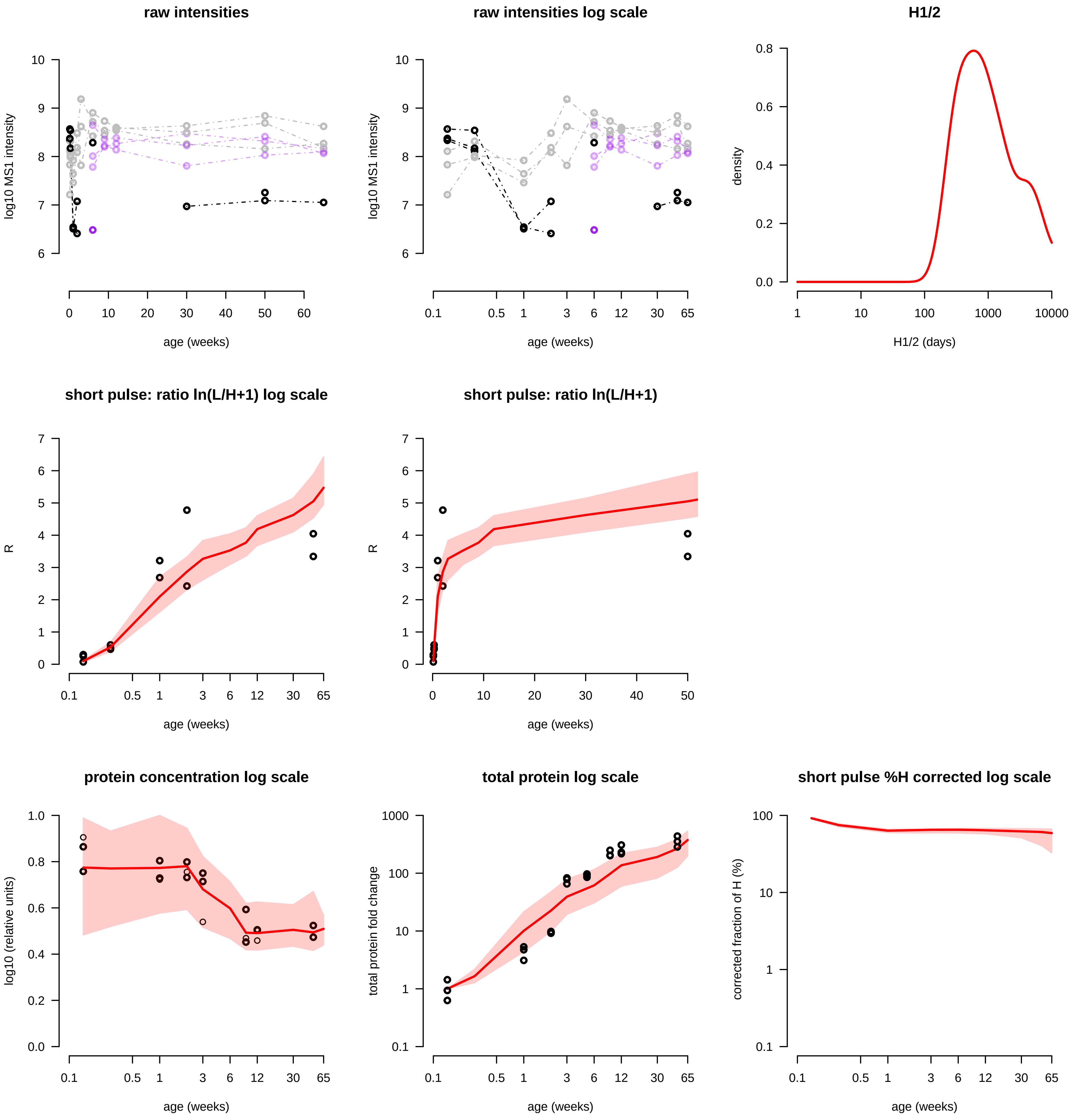

Q3V3R1

>sp|Q3V3R1|C1TM_MOUSE

Monofunctional C1−tetrahydrofolate synthase, mitochondrial

GN=Mthfd1l

Med H1/2: 3 days

Q5 H1/2: 2.5 days

Q95 H1/2: 4 days

reliable model fit: yes

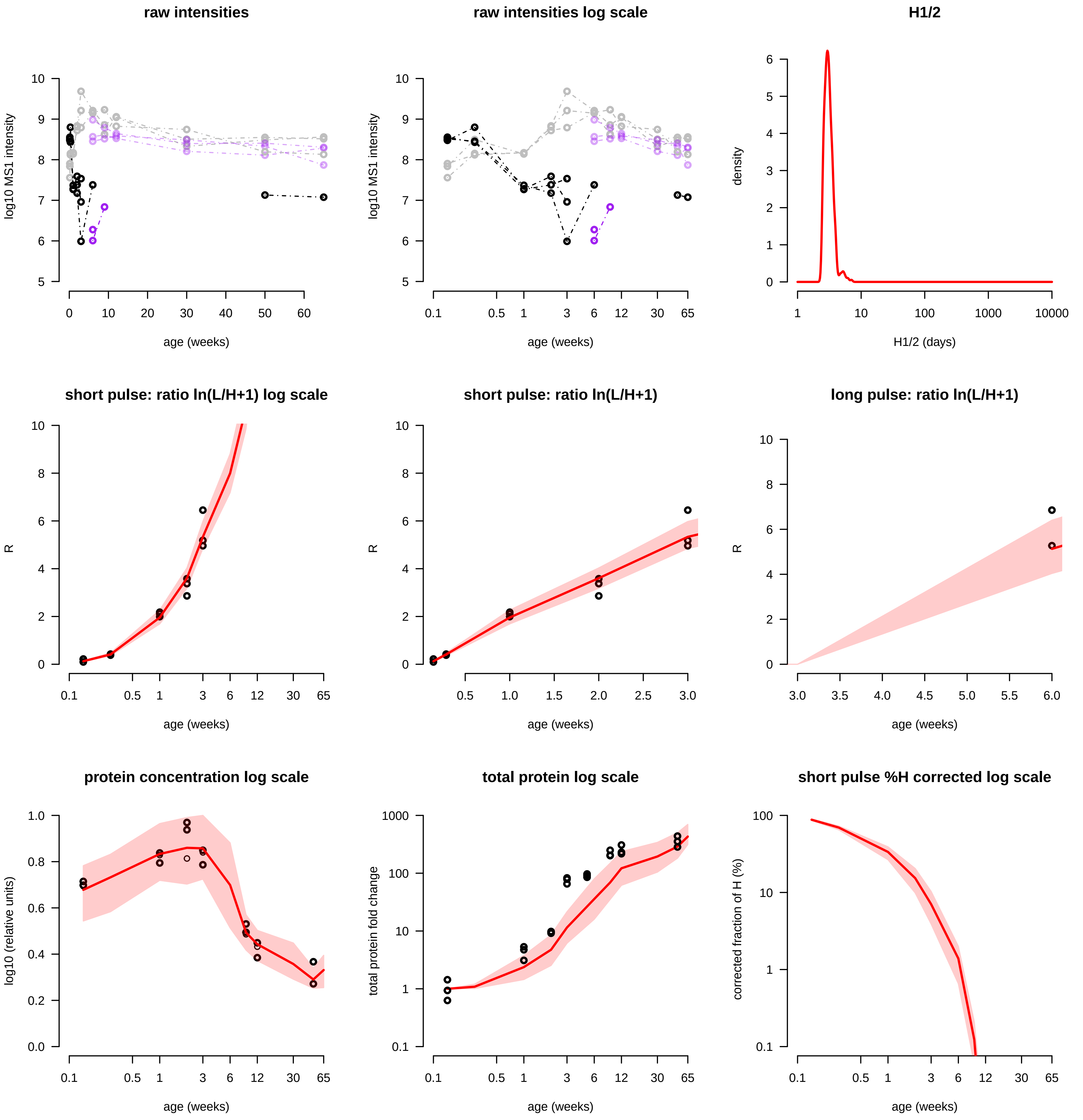

Q9R062

>sp|Q9R062|GLYG_MOUSE

Glycogenin−1

GN=Gyg1

Med H1/2: 5.6 days

Q5   H1/2: 4.4 days

Q95 H1/2: 7.6 days

reliable model fit: yes

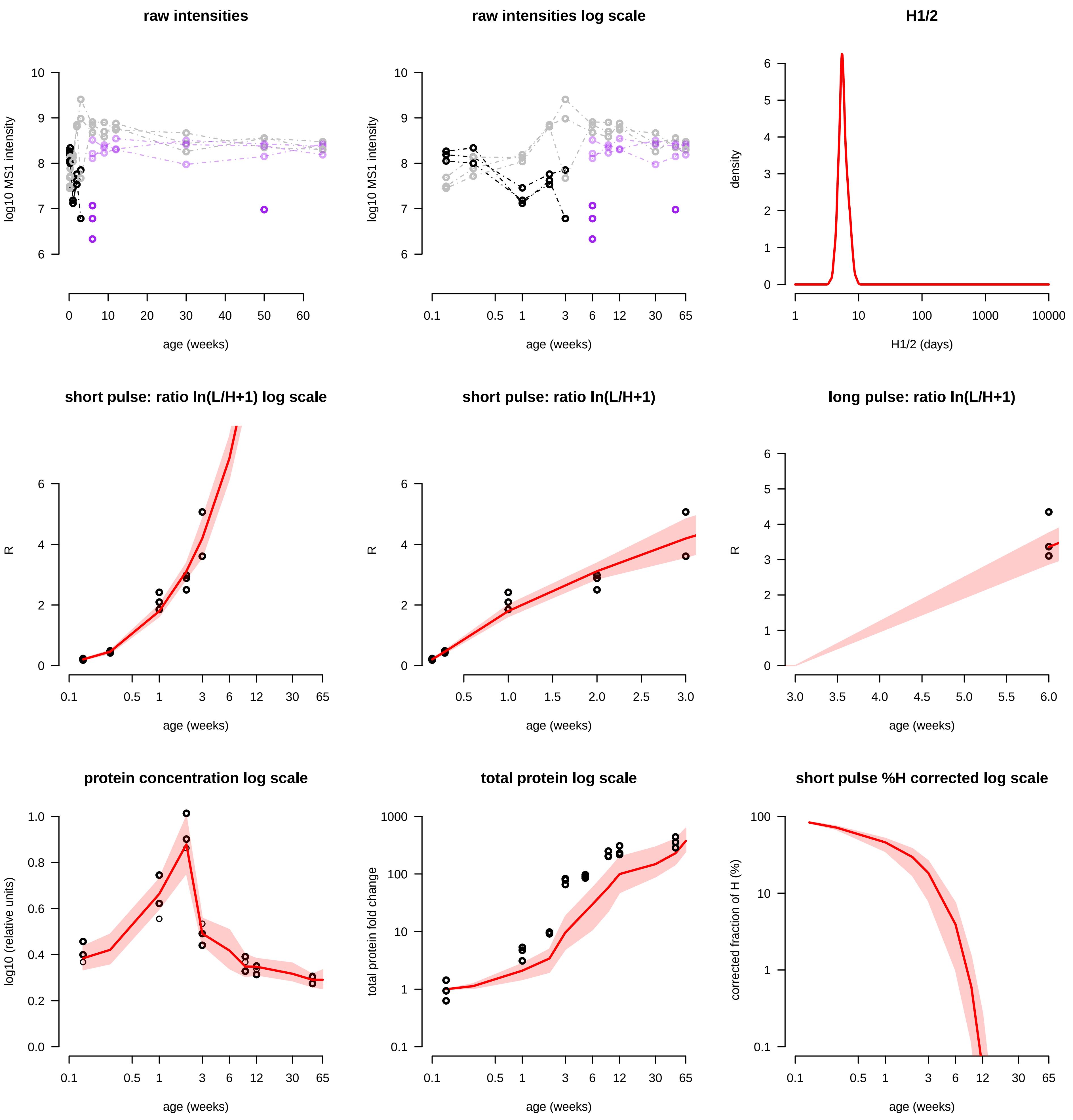

O88291

>sp|O88291|ZN326_MOUSE

DBIRD complex subunit ZNF326

GN=Znf326

Med H1/2: 13.8 days

Q5 H1/2: 5.2 days

Q95 H1/2: 133.9 days

reliable model fit: yes

raw intensities

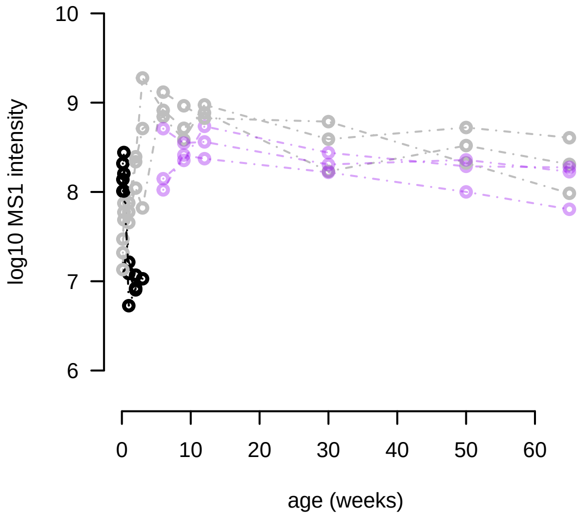

raw intensities log scale

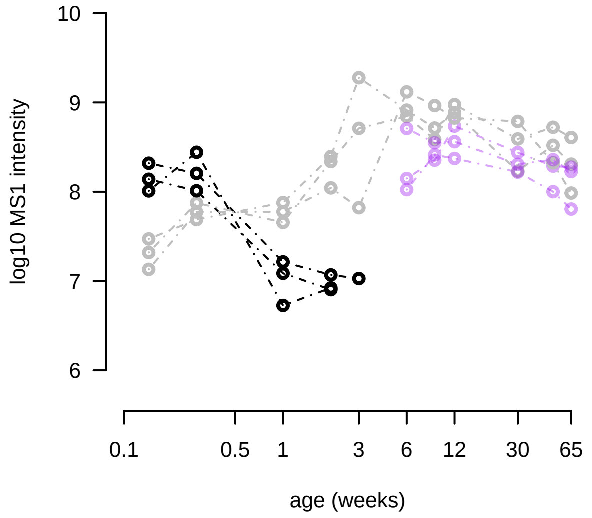

H1/2

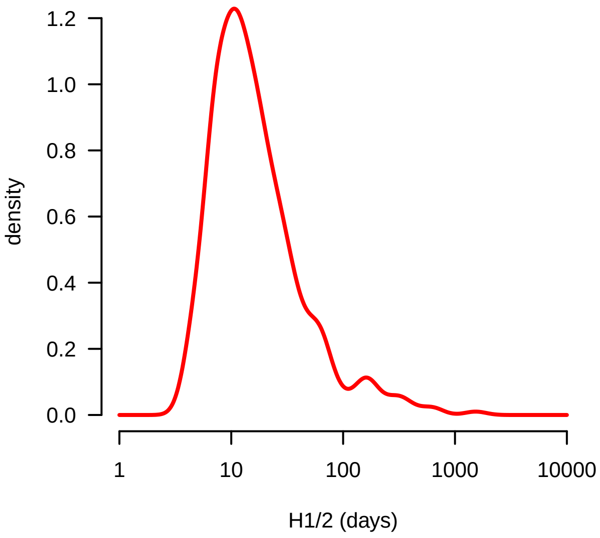

short pulse: ratio ln(L/H+1) log scale

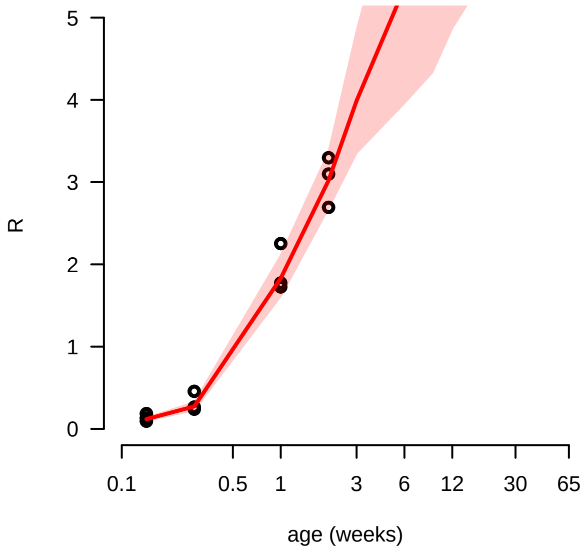

short pulse: ratio ln(L/H+1)

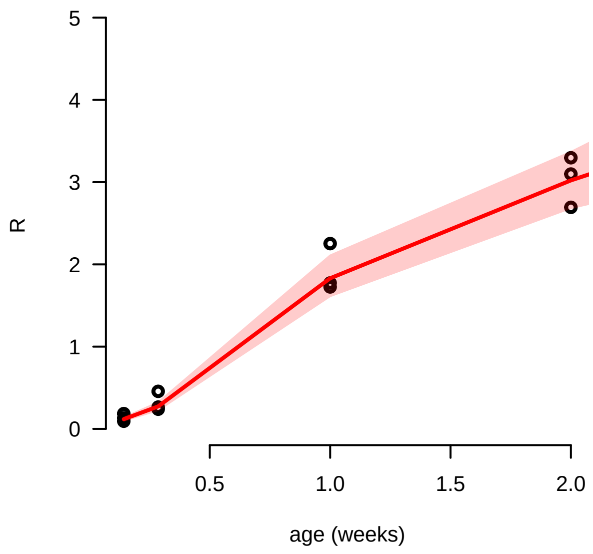

protein concentration log scale

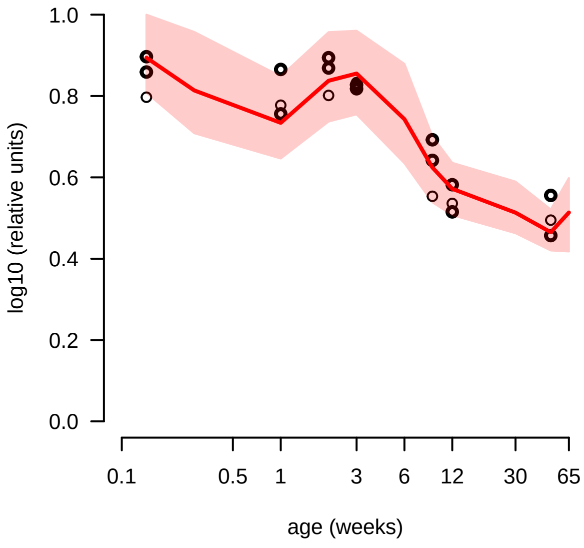

total protein log scale

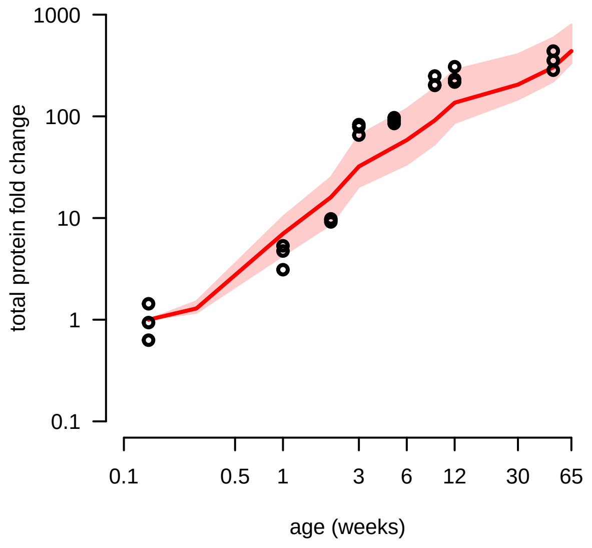

short pulse %H corrected log scale

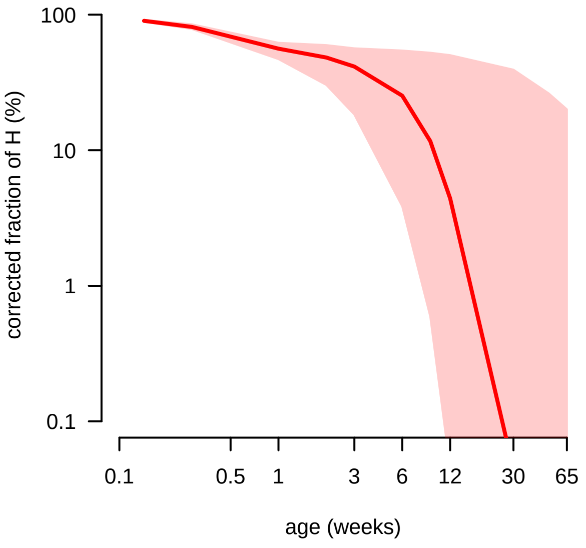

Q61136

>sp|Q61136|PRP4B_MOUSE

Serine/threonine−protein kinase PRP4 homolog

GN=Prpf4b

Med H1/2: 9.9 days

Q5 H1/2: 3.6 days

Q95 H1/2: 136.2 days

reliable model fit: yes

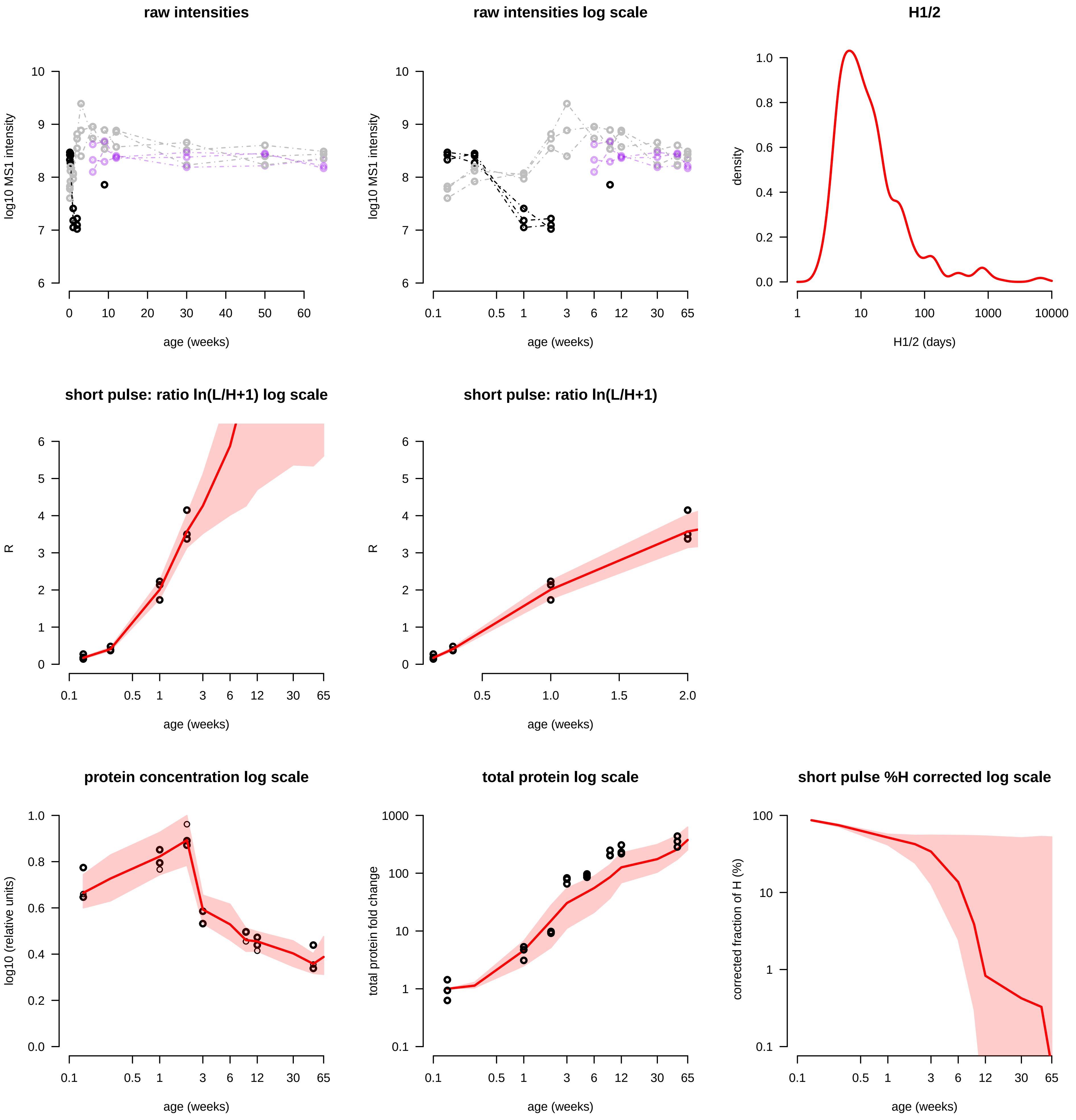

Q8BZF8

>sp|Q8BZF8|PGM5_MOUSE

Phosphoglucomutase−like protein 5

GN=Pgm5

Med H1/2: 7.7 days

Q5 H1/2: 3.5 days

Q95 H1/2: 94 days

reliable model fit: no

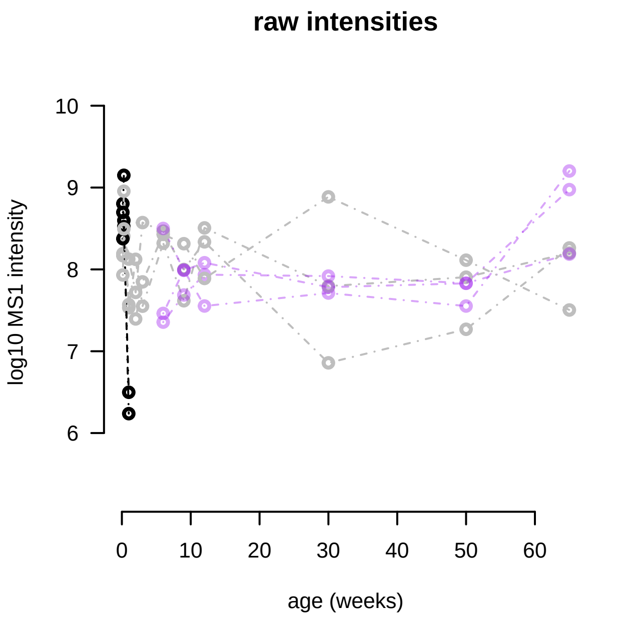

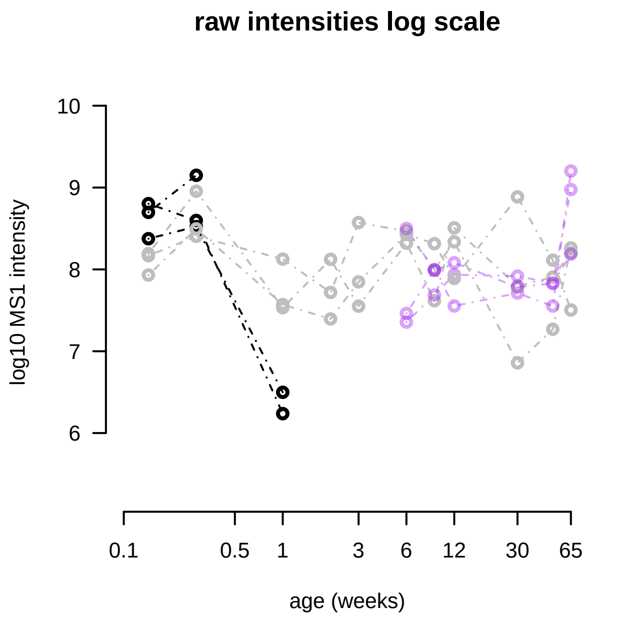

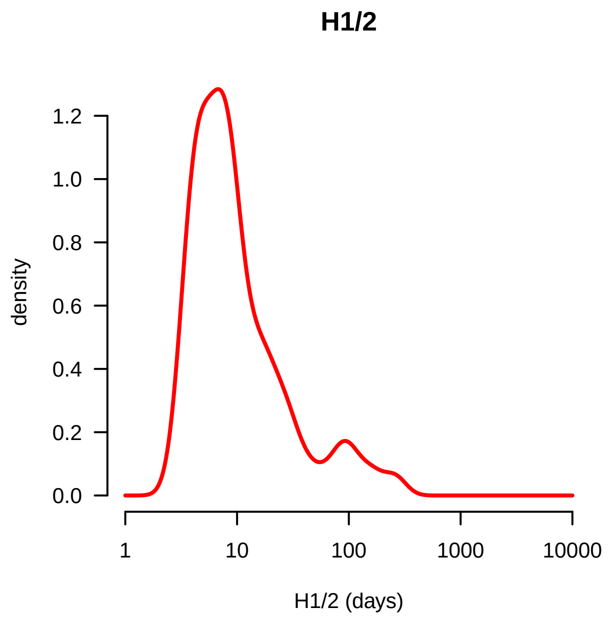

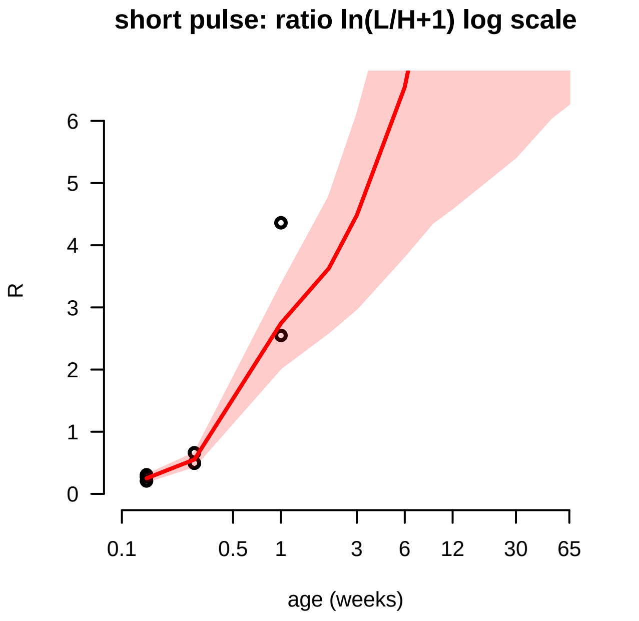

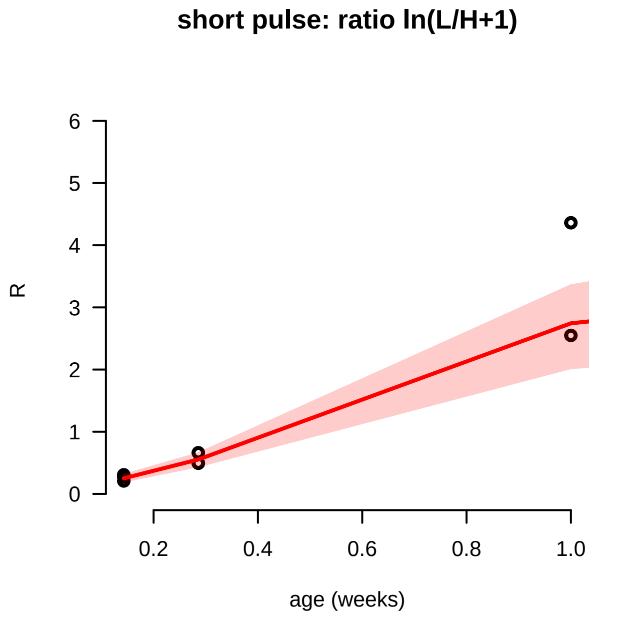

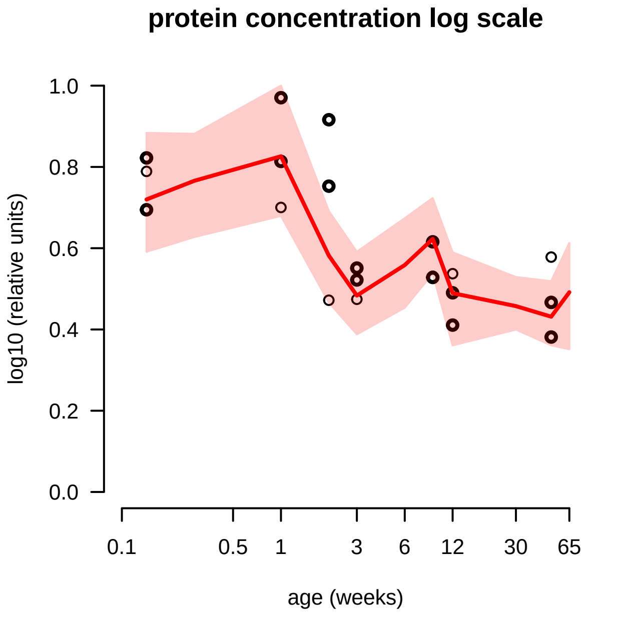

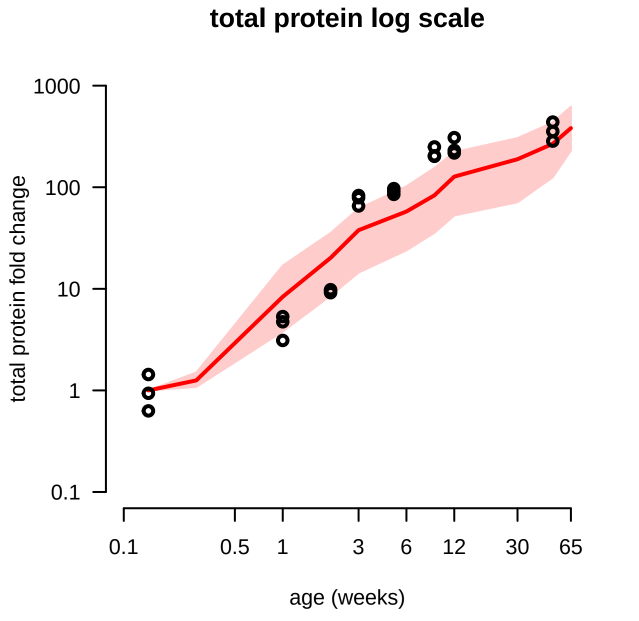

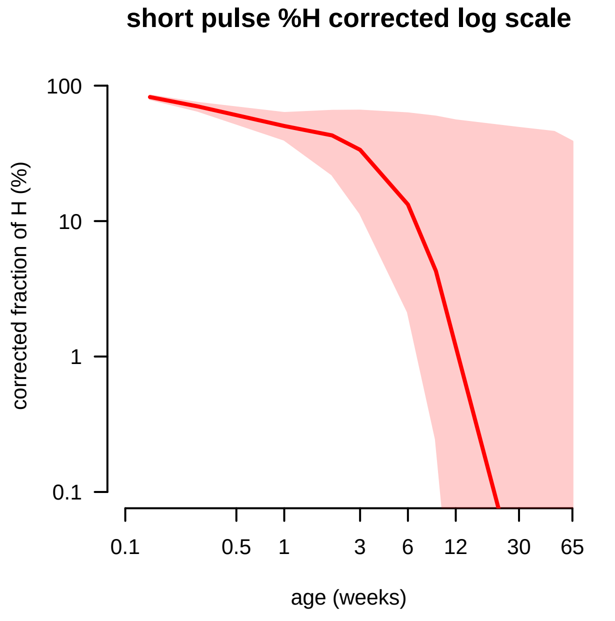

Q921W4;Q921W4−2

>sp|Q921W4|QORL1_MOUSE

Quinone oxidoreductase−like protein 1

GN=Cryzl1

Med H1/2: 3.7 days

Q5 H1/2: 2.9 days

Q95 H1/2: 5.7 days

reliable model fit: yes

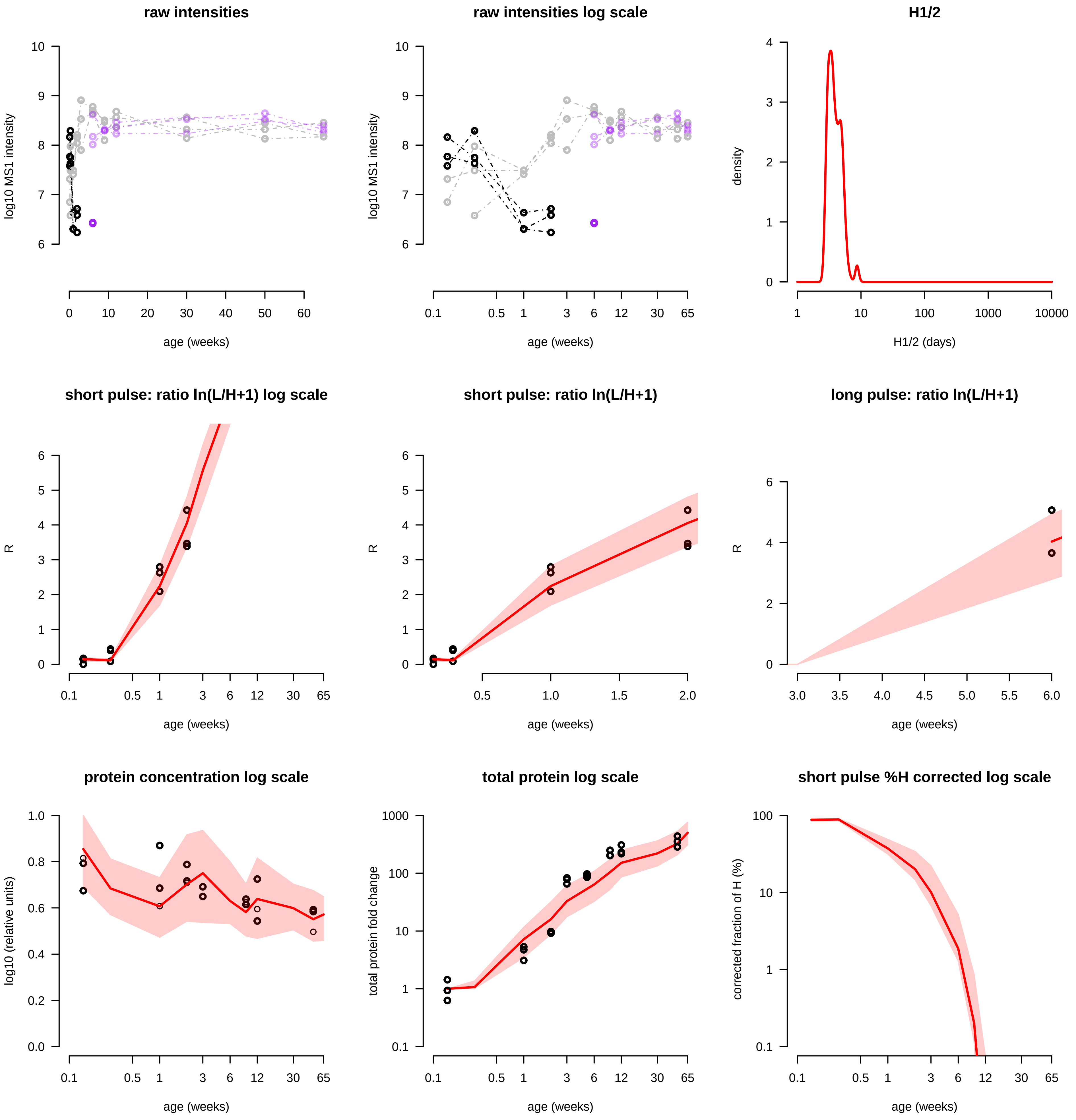

P57746

>sp|P57746|VATD_MOUSE

V−type proton ATPase subunit D

GN=Atp6v1d

Med H1/2: 6.2 days

Q5 H1/2: 2.7 days

Q95 H1/2: 44.9 days

reliable model fit: yes

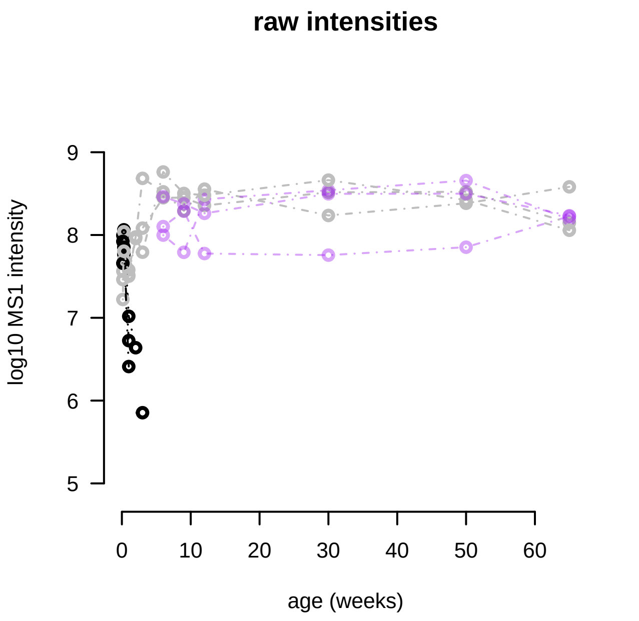

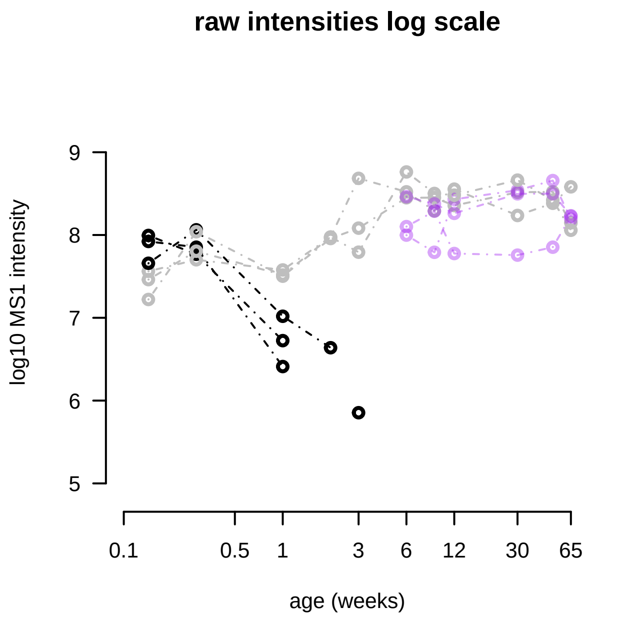

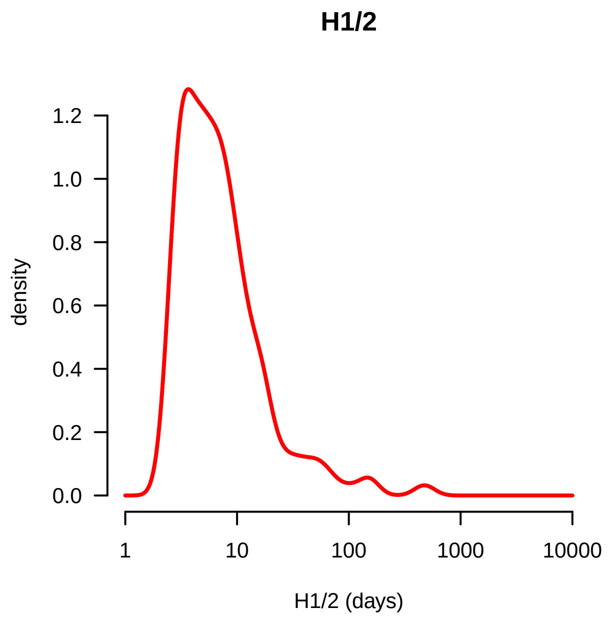

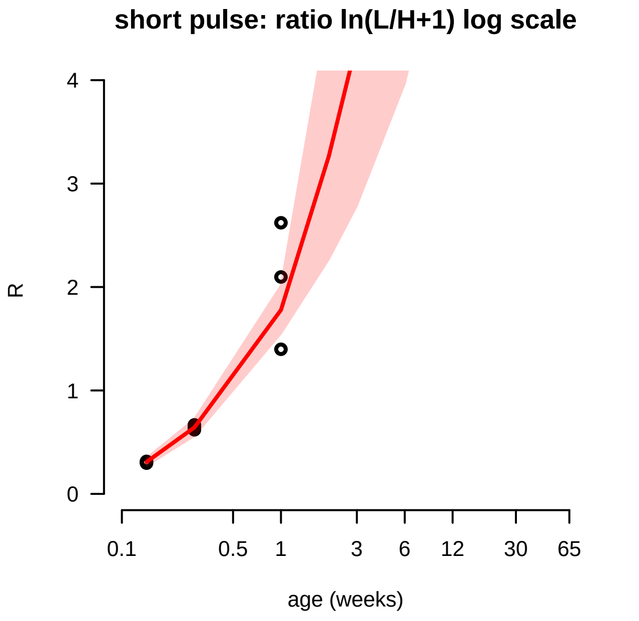

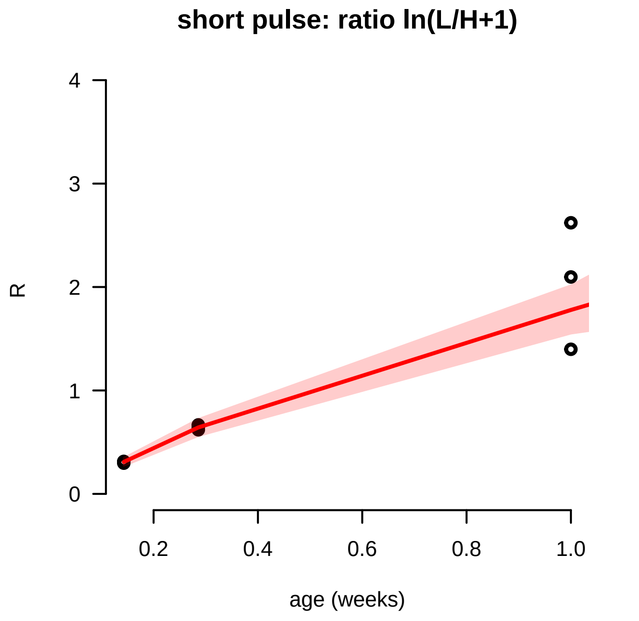

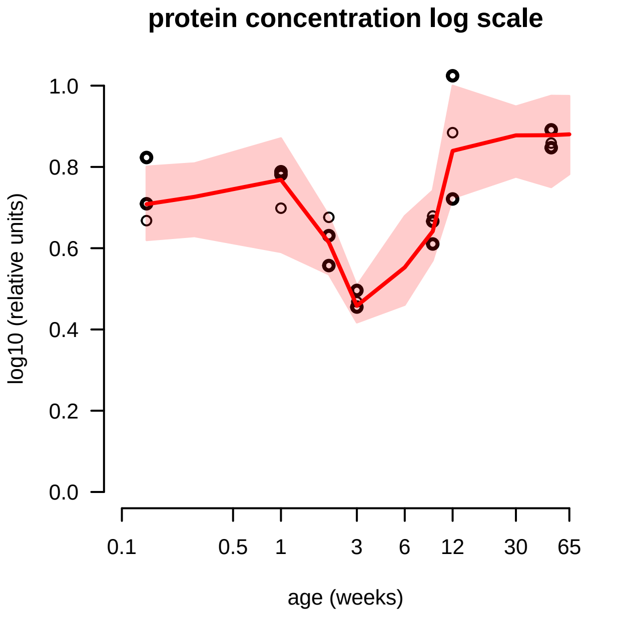

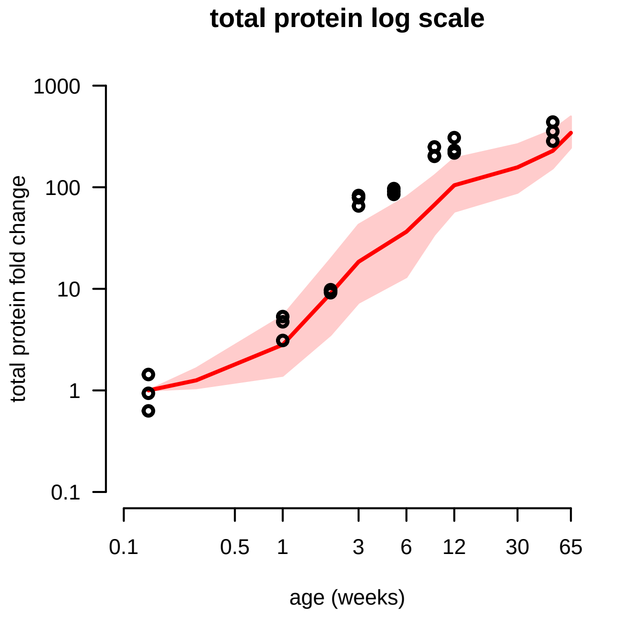

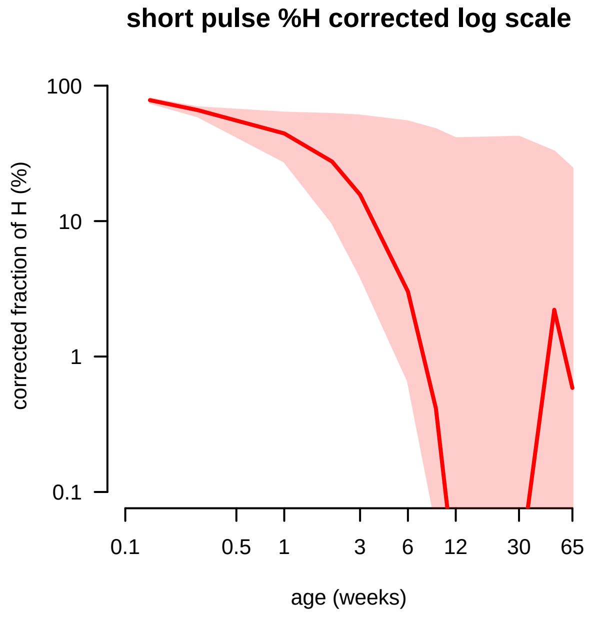

Q9CQF0

>sp|Q9CQF0|RM11_MOUSE

39S ribosomal protein L11, mitochondrial

GN=Mrpl11

Med H1/2: 3.9 days

Q5 H1/2: 2.6 days

Q95 H1/2: 8.8 days

reliable model fit: yes

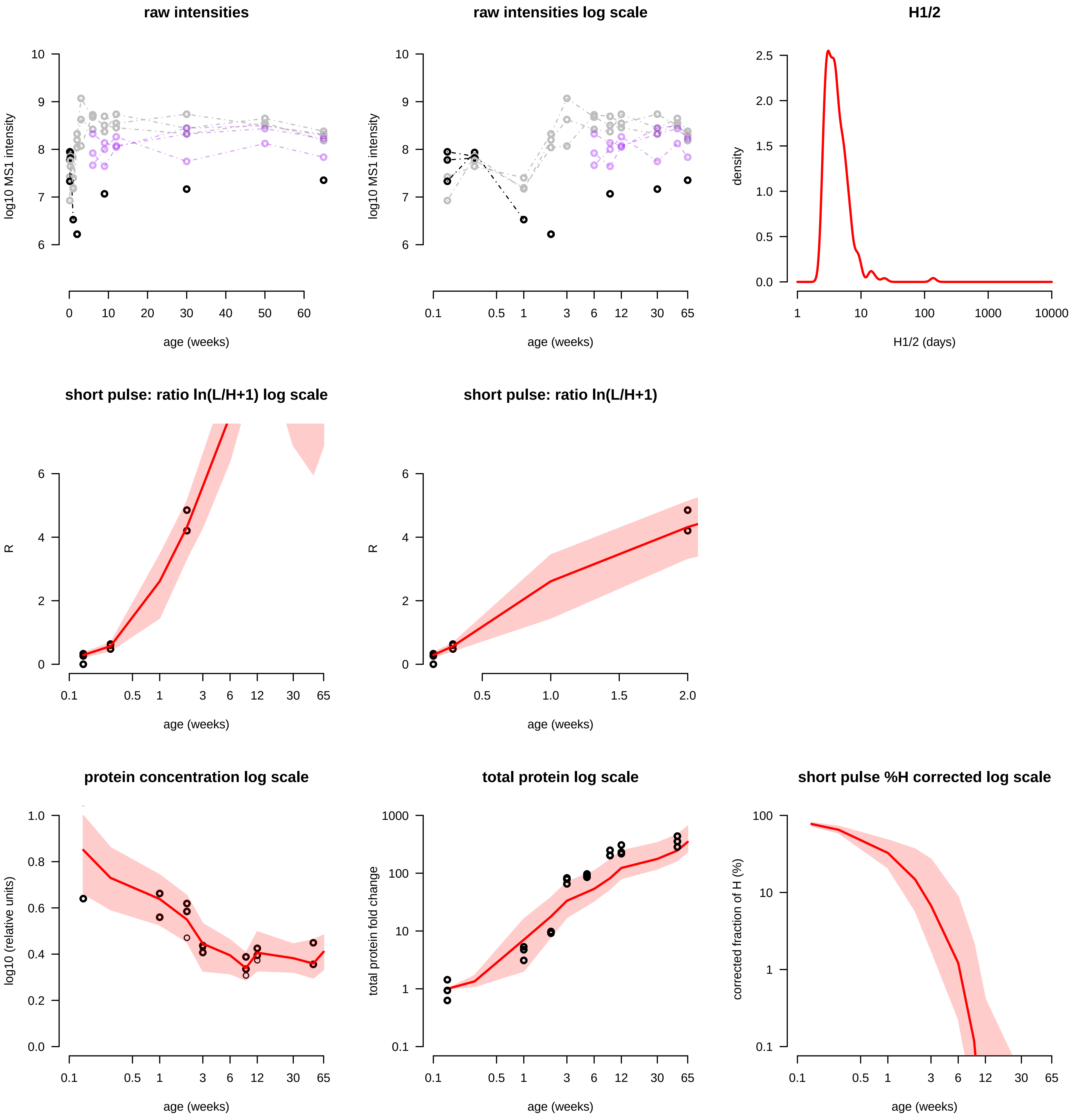

Q8VD65

>sp|Q8VD65|PI3R4_MOUSE

Phosphoinositide 3−kinase regulatory subunit 4

GN=Pik3r4

Med H1/2: 6.5 days

Q5 H1/2: 2.7 days

Q95 H1/2: 33.8 days

reliable model fit: yes

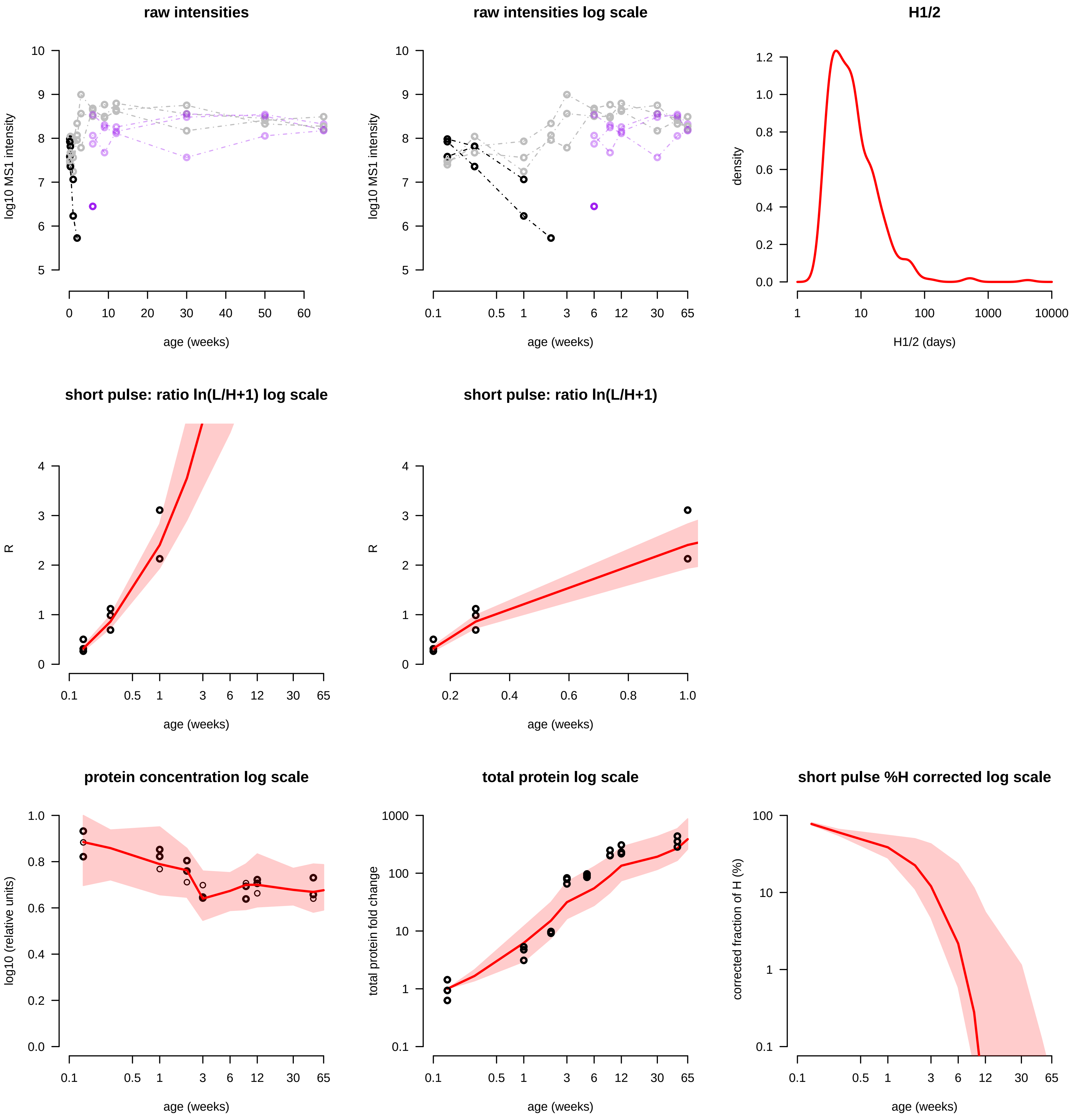

Q8BM55

>sp|Q8BM55|TM214_MOUSE

Transmembrane protein 214

GN=Tmem214

Med H1/2: 15.6 days

Q5 H1/2: 5.9 days

Q95 H1/2: 87.9 days

reliable model fit: yes

raw intensities

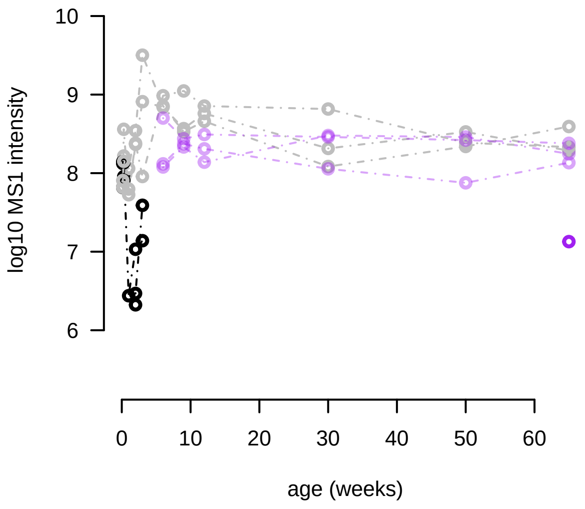

raw intensities log scale

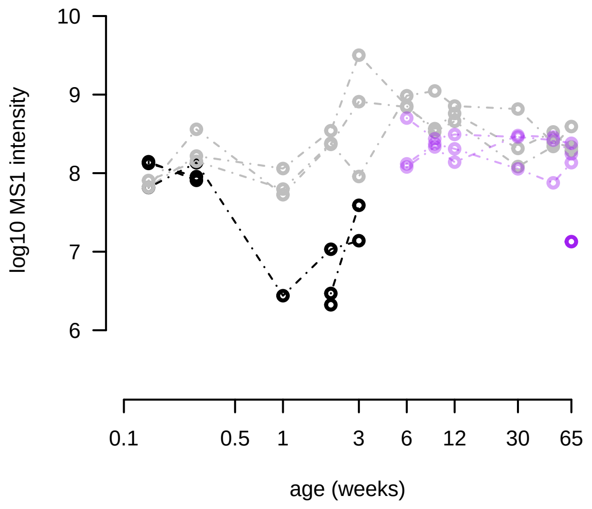

H1/2

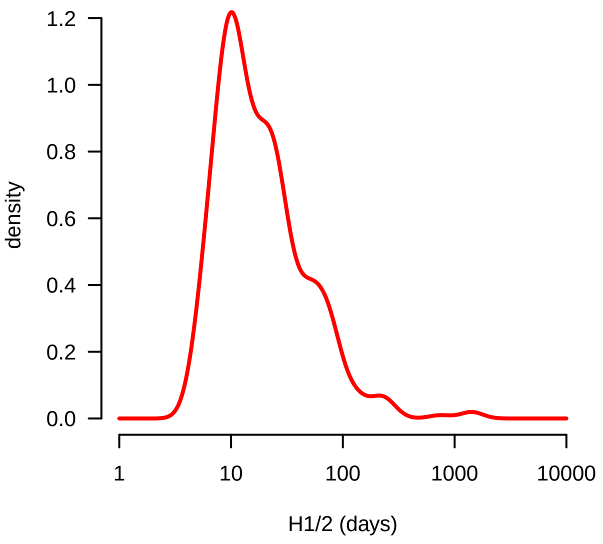

short pulse: ratio ln(L/H+1) log scale

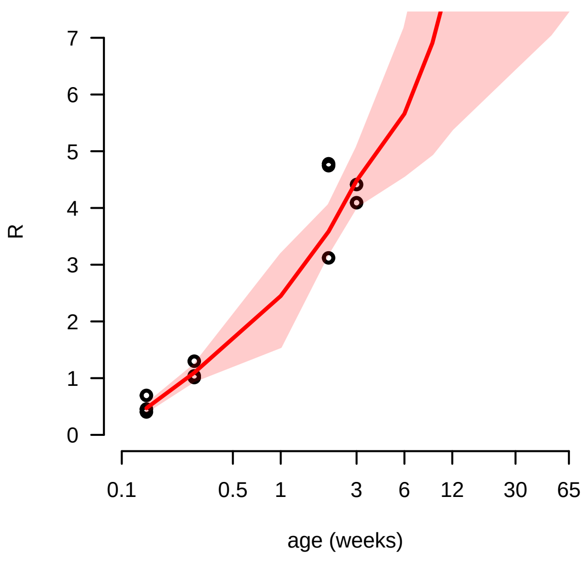

short pulse: ratio ln(L/H+1)

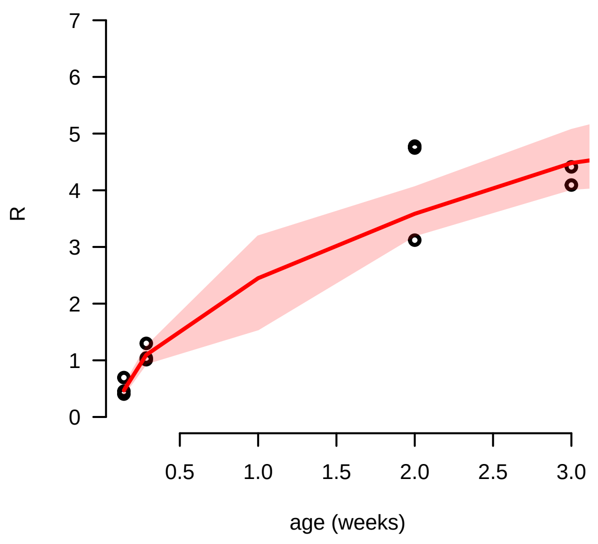

protein concentration log scale

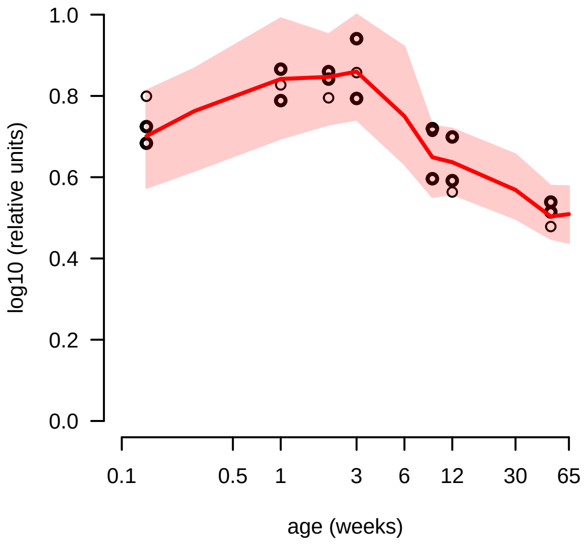

total protein log scale

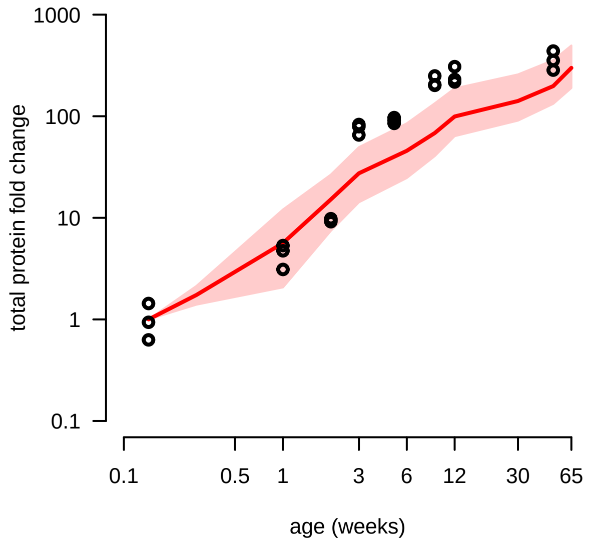

short pulse %H corrected log scale

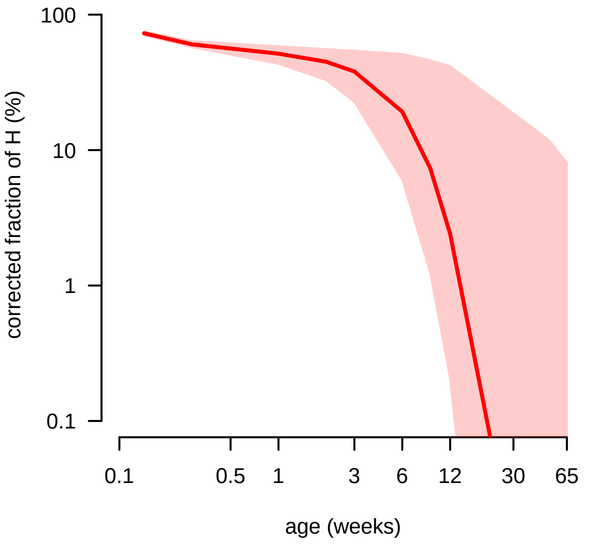

Q9QYJ3

>sp|Q9QYJ3|DNJB1_MOUSE

DnaJ homolog subfamily B member 1

GN=Dnajb1

Med H1/2: 3.9 days

Q5 H1/2: 3.2 days

Q95 H1/2: 4.8 days

reliable model fit: yes

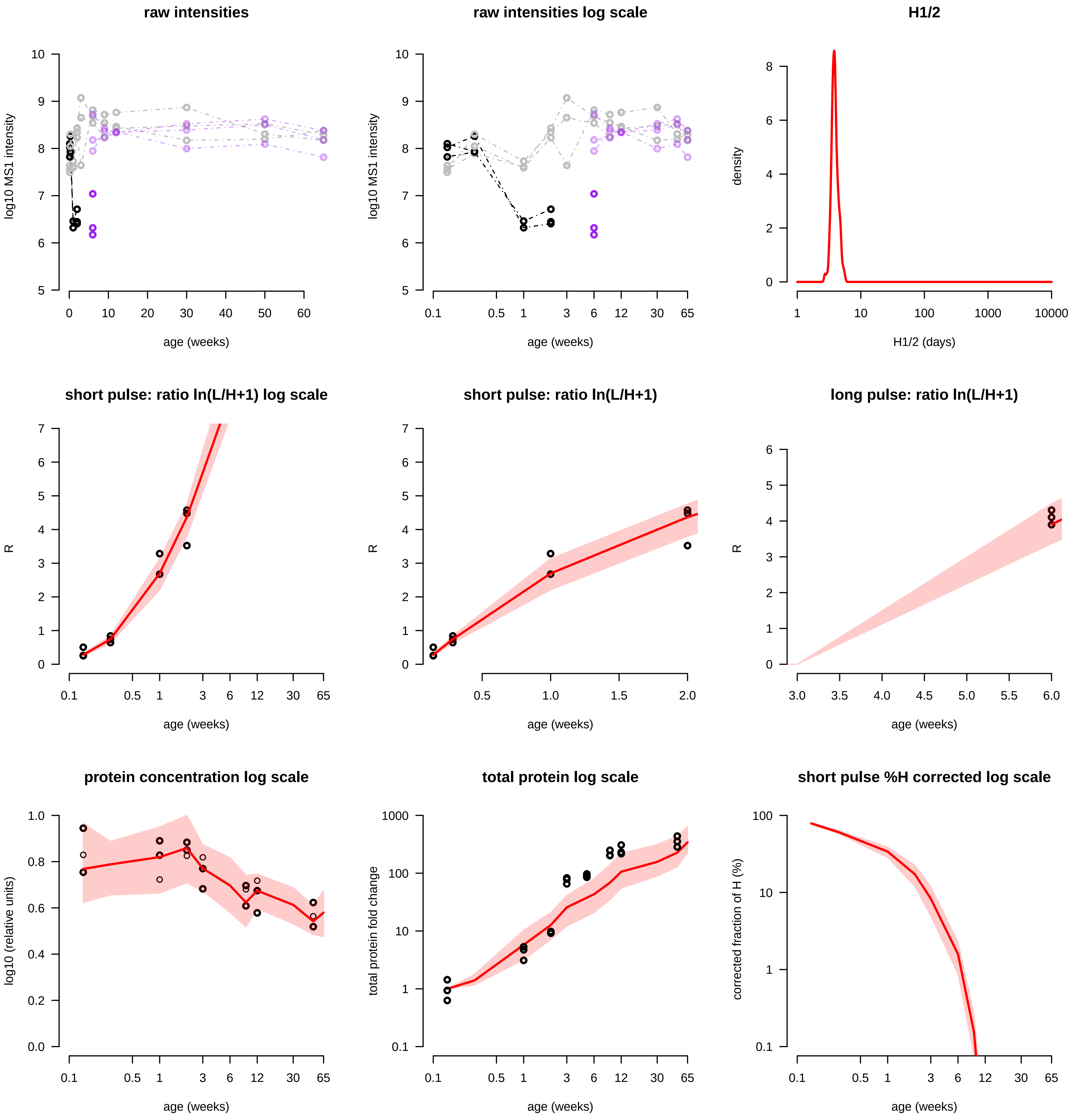

Q9Z1K5

>sp|Q9Z1K5|ARI1_MOUSE

E3 ubiquitin−protein ligase ARIH1

GN=Arih1

Med H1/2: 9.5 days

Q5   H1/2: 3.7 days

Q95 H1/2: 89.3 days

reliable model fit: yes

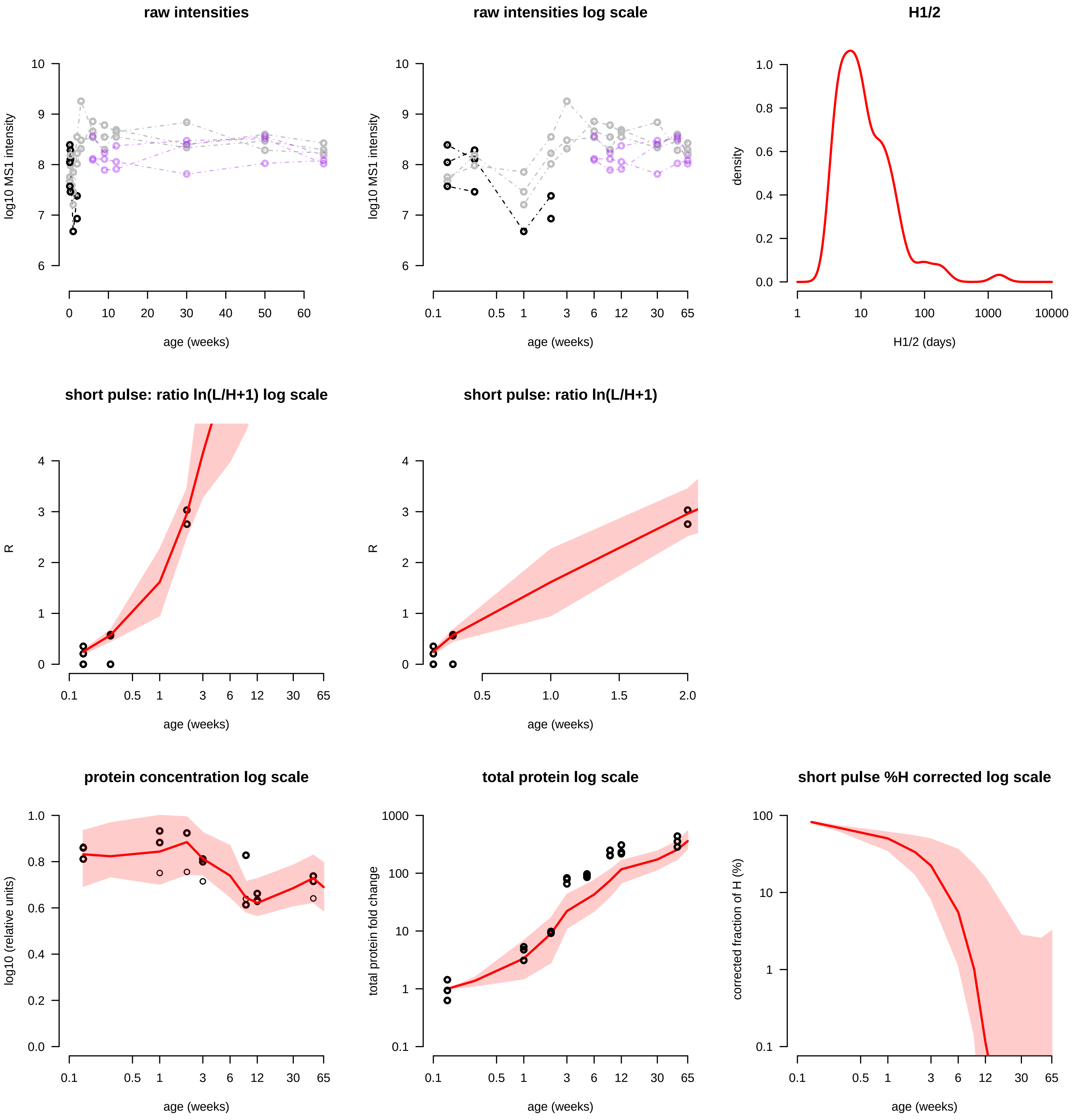

Q9JIH2

>sp|Q9JIH2|NUP50_MOUSE

Nuclear pore complex protein Nup50

GN=Nup50

Med H1/2: 4.9 days

Q5 H1/2: 3.8 days

Q95 H1/2: 6.4 days

reliable model fit: yes

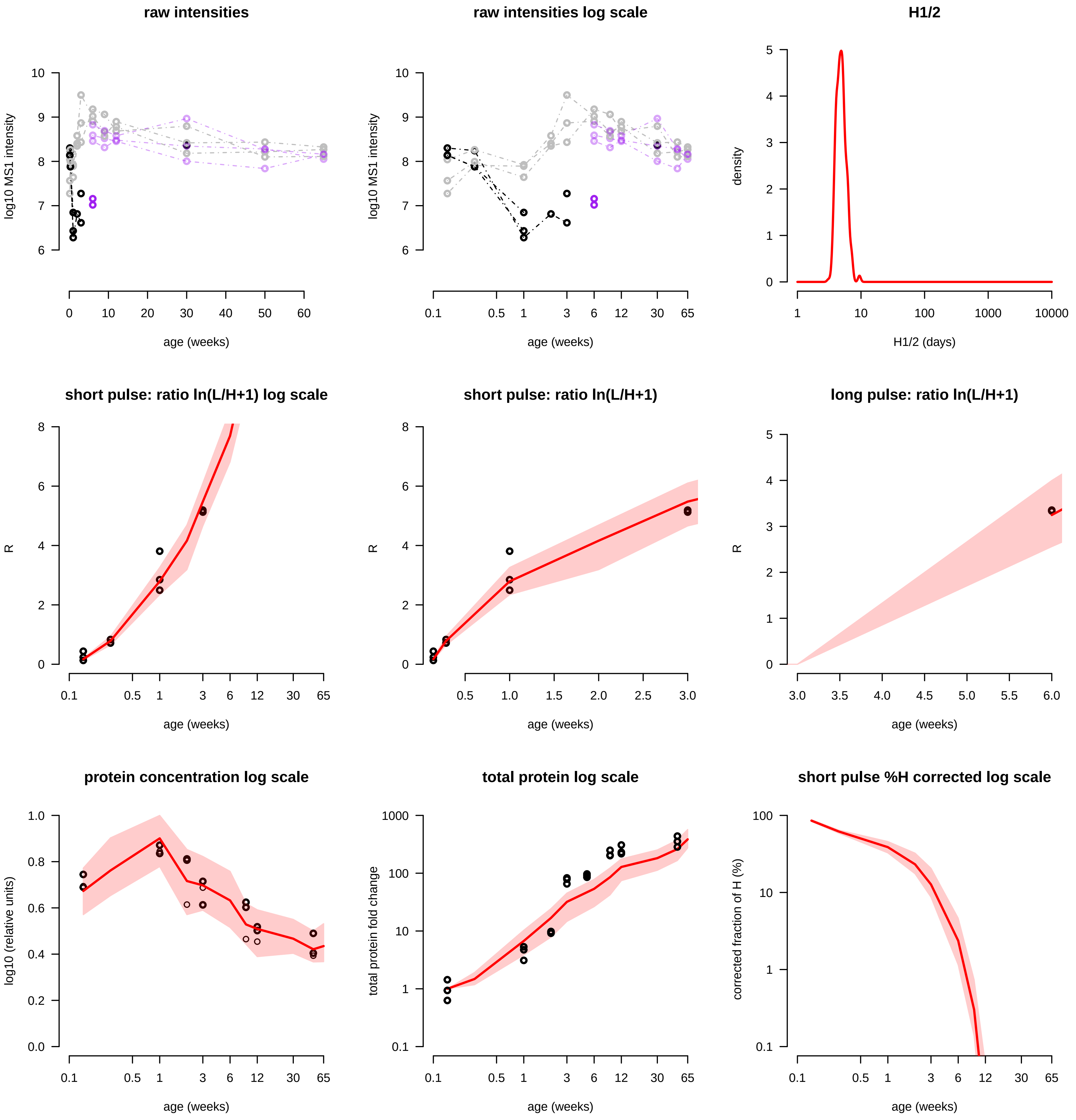

P62141

>sp|P62141|PP1B_MOUSE

Serine/threonine−protein phosphatase PP1−beta catalytic subunit

GN=Ppp1cb

Med H1/2: 4.8 days

Q5 H1/2: 2.7 days

Q95 H1/2: 22.1 days

reliable model fit: yes

raw intensities

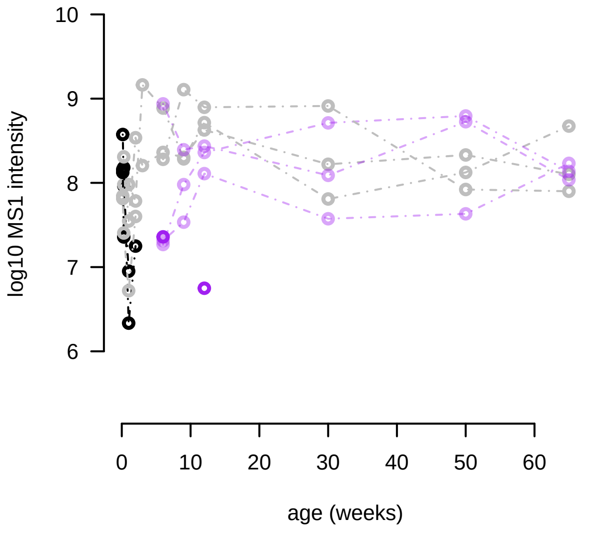

raw intensities log scale

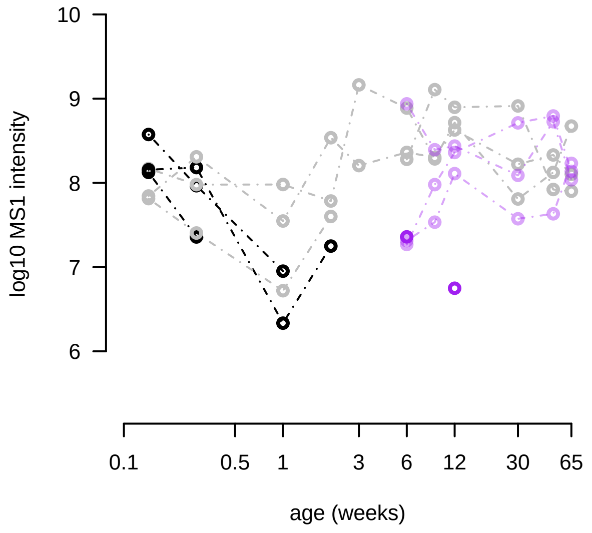

H1/2

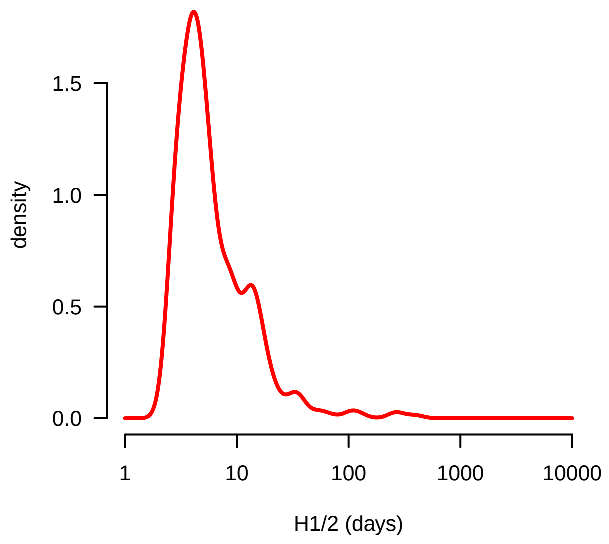

short pulse: ratio ln(L/H+1) log scale

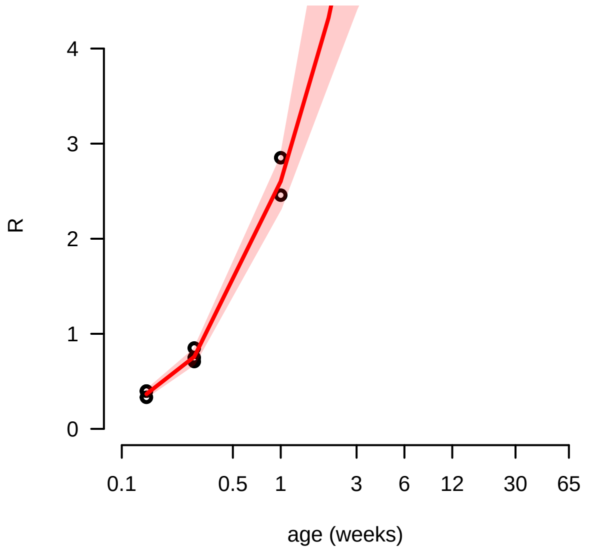

short pulse: ratio ln(L/H+1)

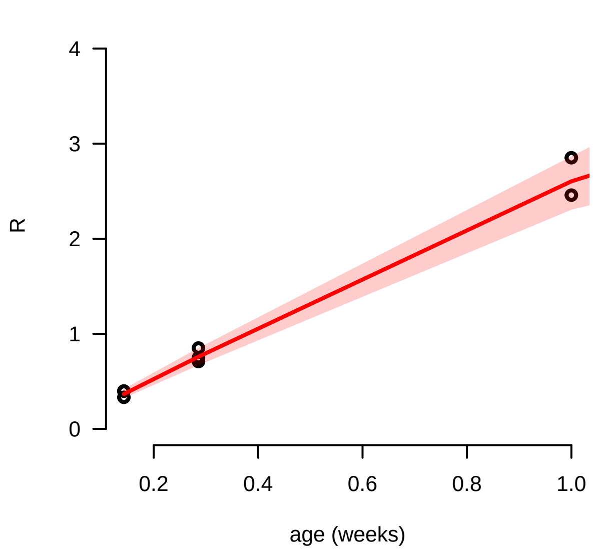

protein concentration log scale

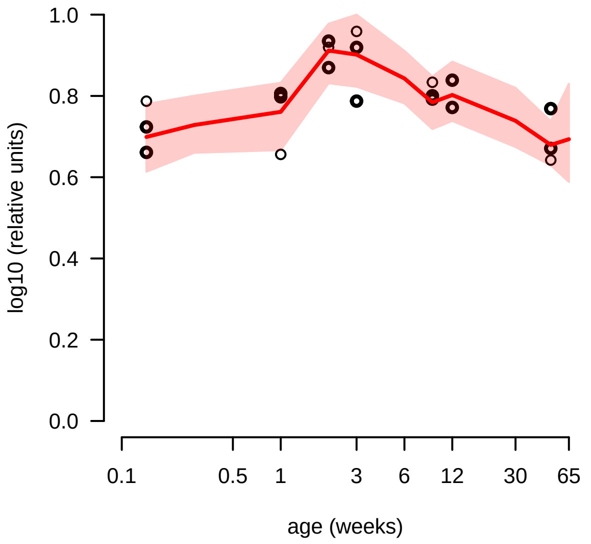

total protein log scale

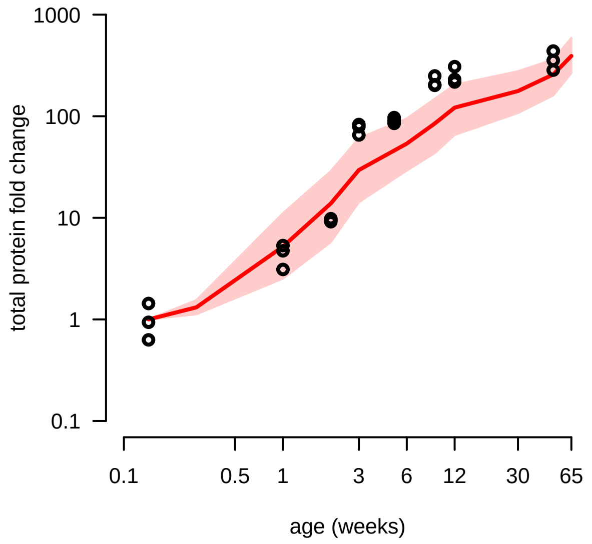

short pulse %H corrected log scale

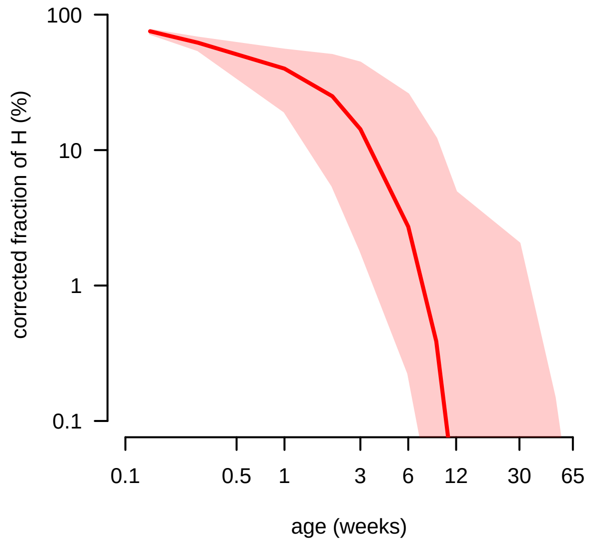

P49443

>sp|P49443|PPM1A_MOUSE

Protein phosphatase 1A

GN=Ppm1a

Med H1/2: 6 days

Q5 H1/2: 2.6 days

Q95 H1/2: 34.9 days

reliable model fit: yes

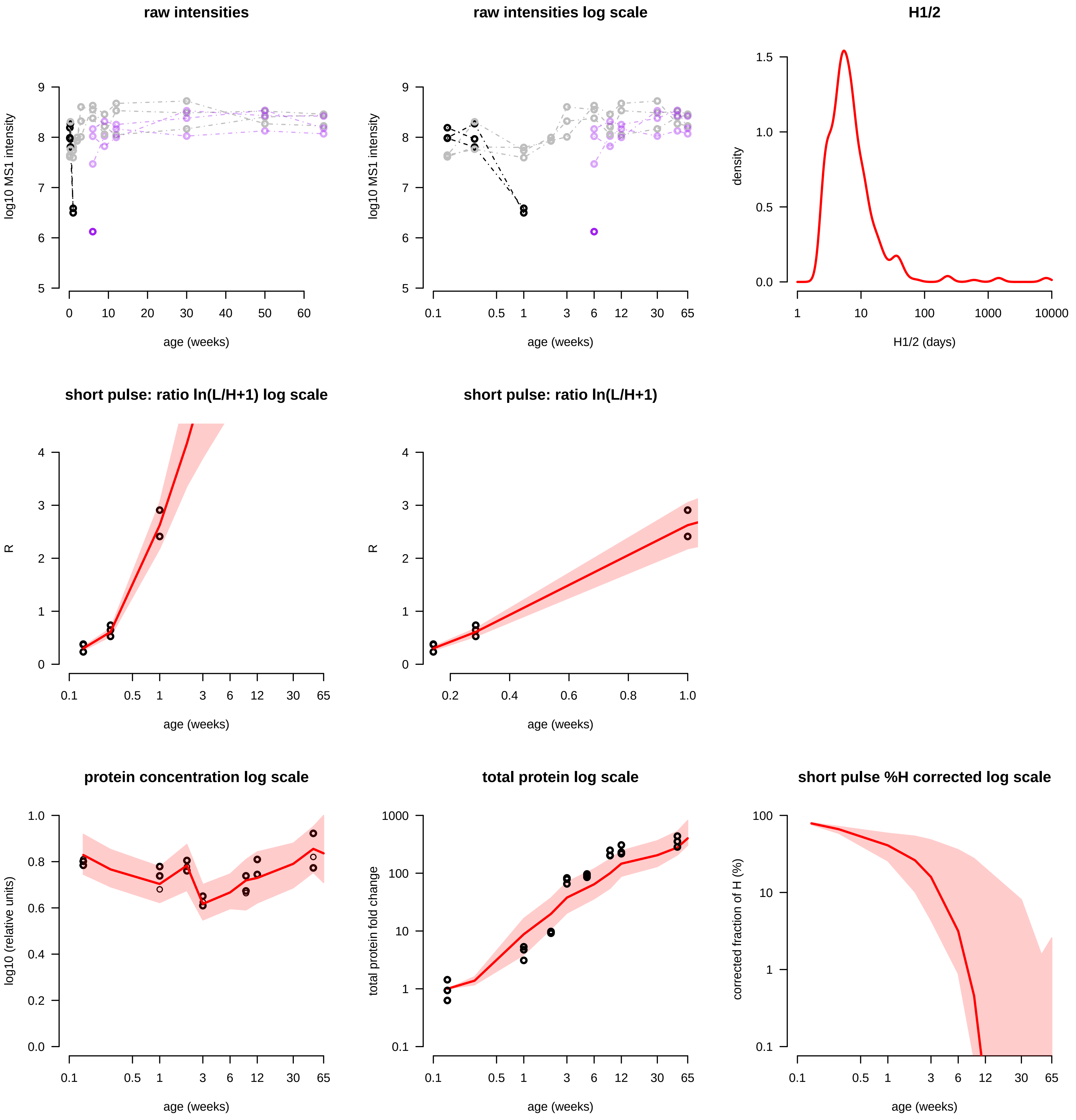

Q8BX10

>sp|Q8BX10|PGAM5_MOUSE

Serine/threonine−protein phosphatase PGAM5, mitochondrial

GN=Pgam5

Med H1/2: 4.5 days

Q5 H1/2: 3.8 days

Q95 H1/2: 5.7 days

reliable model fit: yes

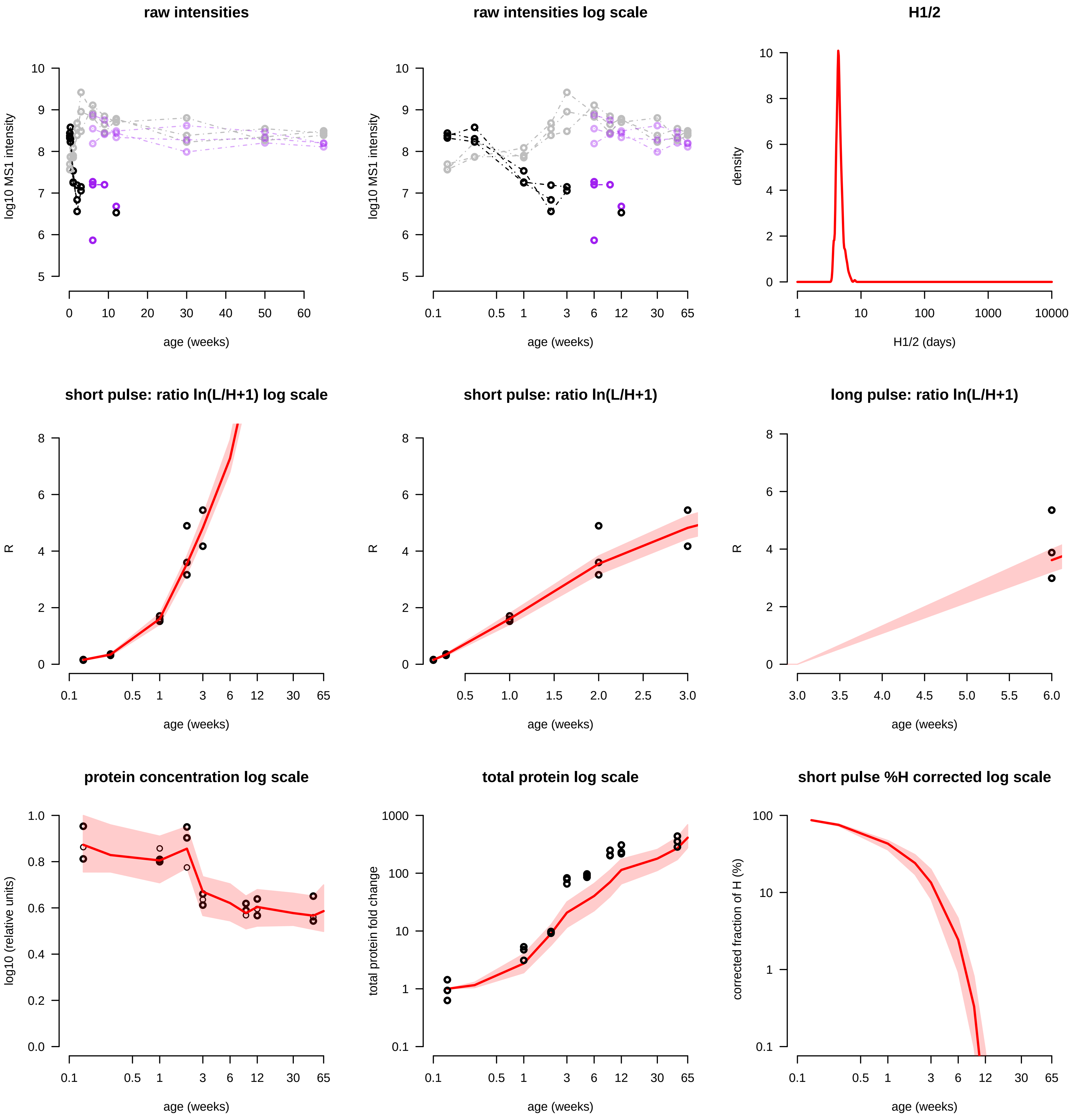

Q9CSU0;Q9CSU0−2;Q9CSU0−3

>sp|Q9CSU0|RPR1B_MOUSE

Regulation of nuclear pre−mRNA domain−containing protein 1B

GN=Rprd1b

Med H1/2: 4.2 days

Q5 H1/2: 2.9 days

Q95 H1/2: 7.7 days

reliable model fit: yes

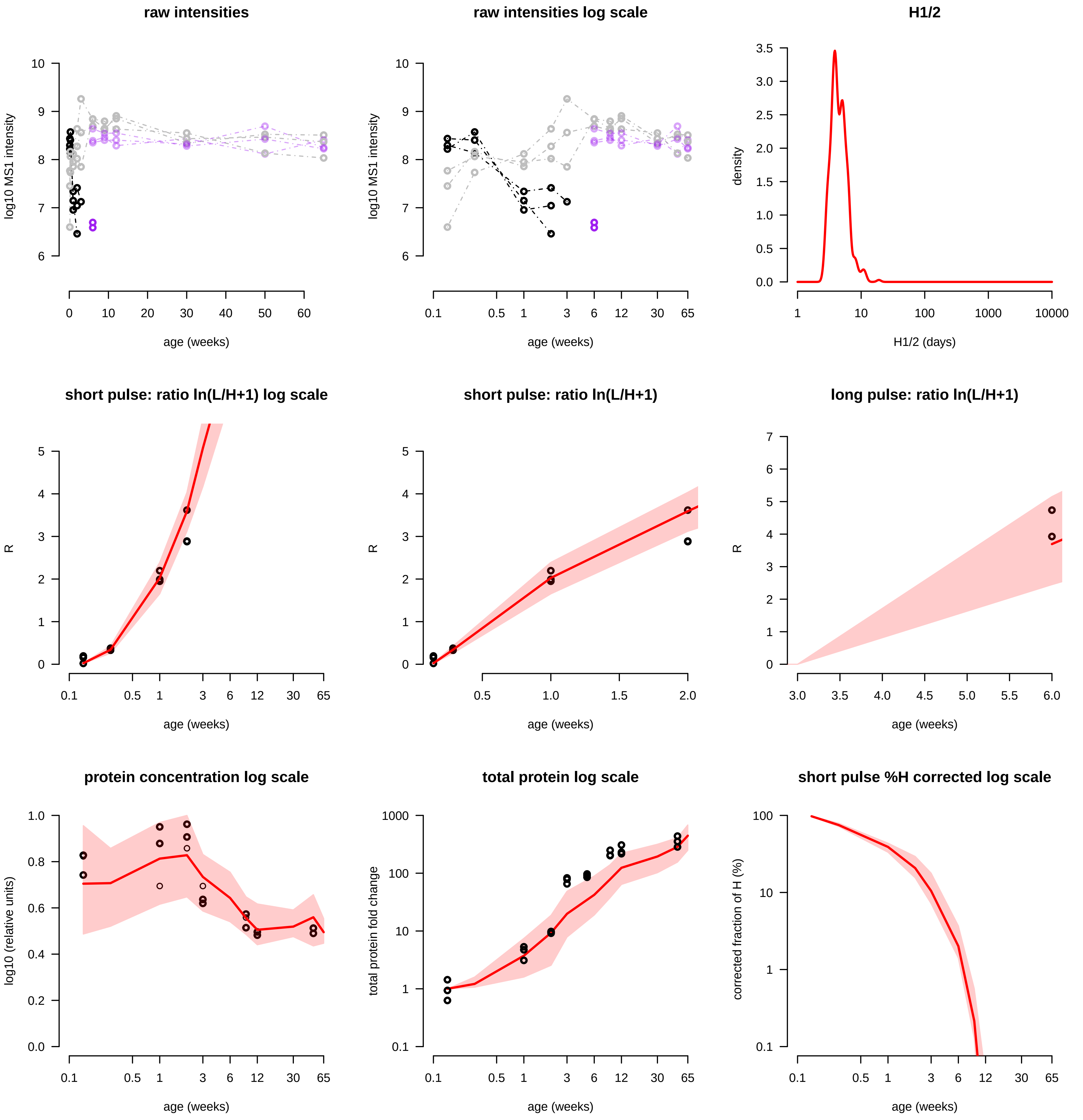

Q923D5

>sp|Q923D5|WBP11_MOUSE

WW domain−binding protein 11

GN=Wbp11

Med H1/2: 4.4 days

Q5 H1/2: 3.7 days

Q95 H1/2: 5.7 days

reliable model fit: yes

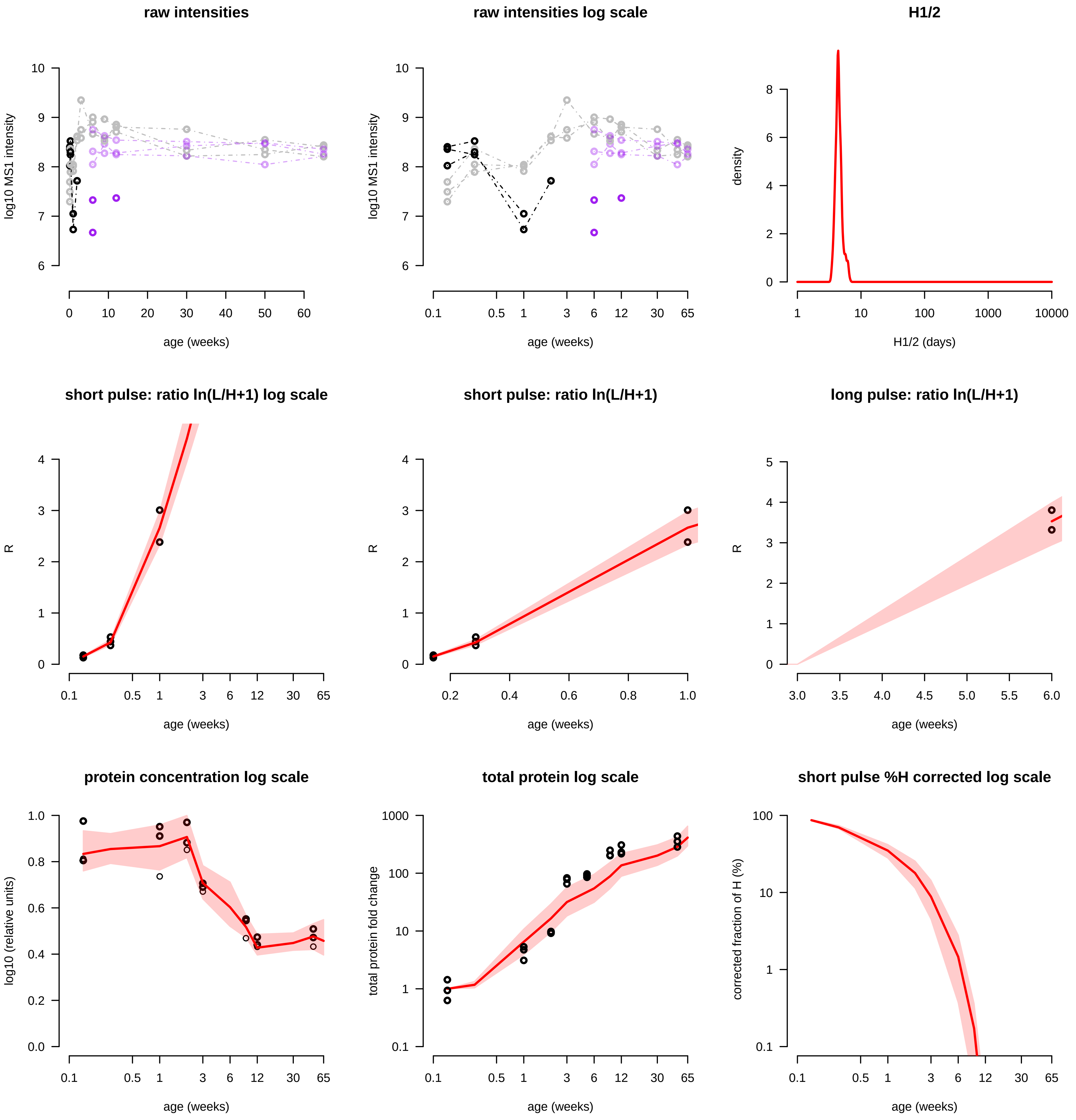

Q99JF5

>sp|Q99JF5|MVD1_MOUSE

Diphosphomevalonate decarboxylase

GN=Mvd

Med H1/2: 7.8 days

Q5 H1/2: 3.1 days

Q95 H1/2: 53 days

reliable model fit: yes

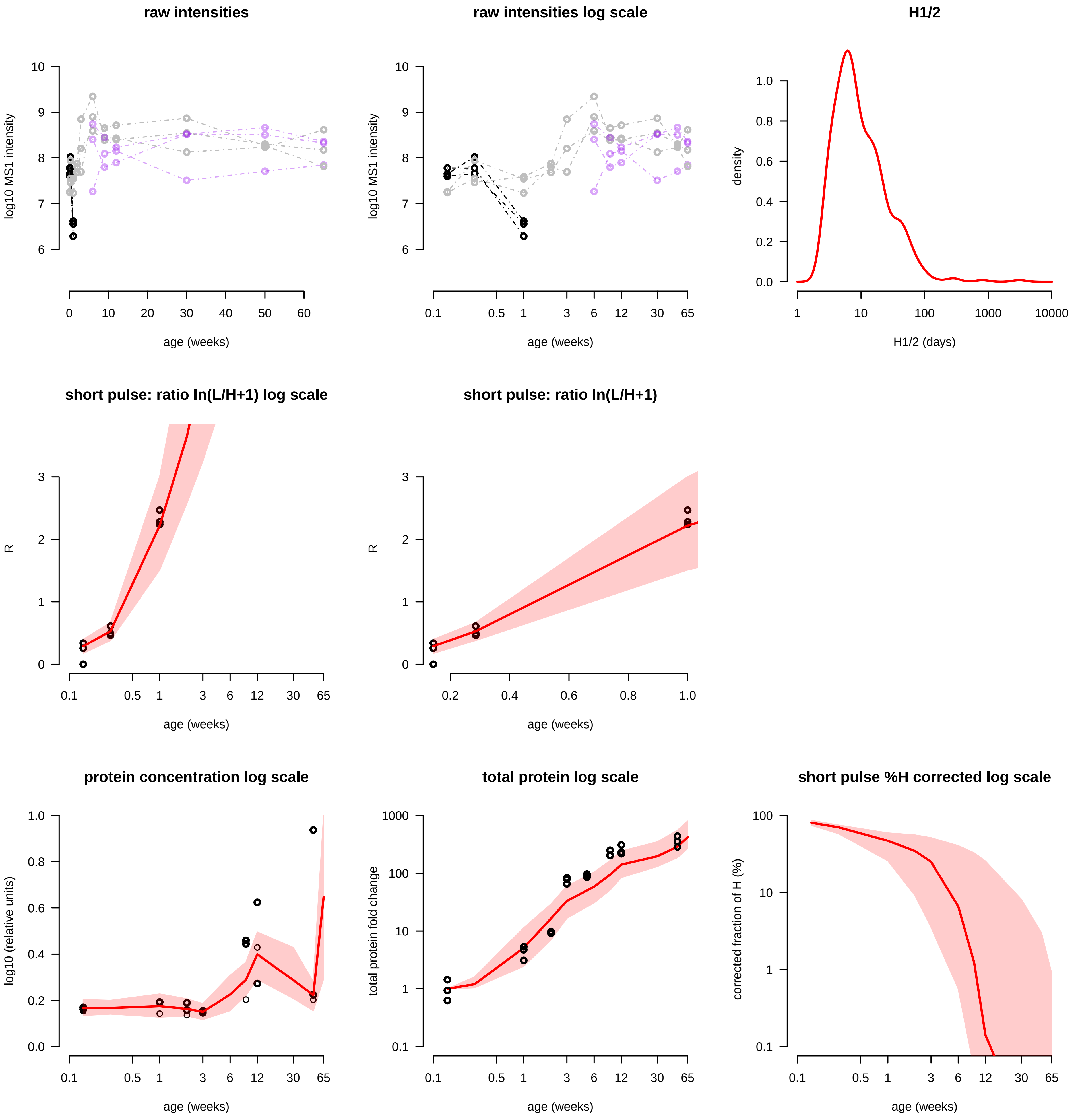

P70677

>sp|P70677|CASP3_MOUSE

Caspase−3

GN=Casp3

Med H1/2: 4.6 days

Q5 H1/2: 3.8 days

Q95 H1/2: 6.1 days

reliable model fit: yes

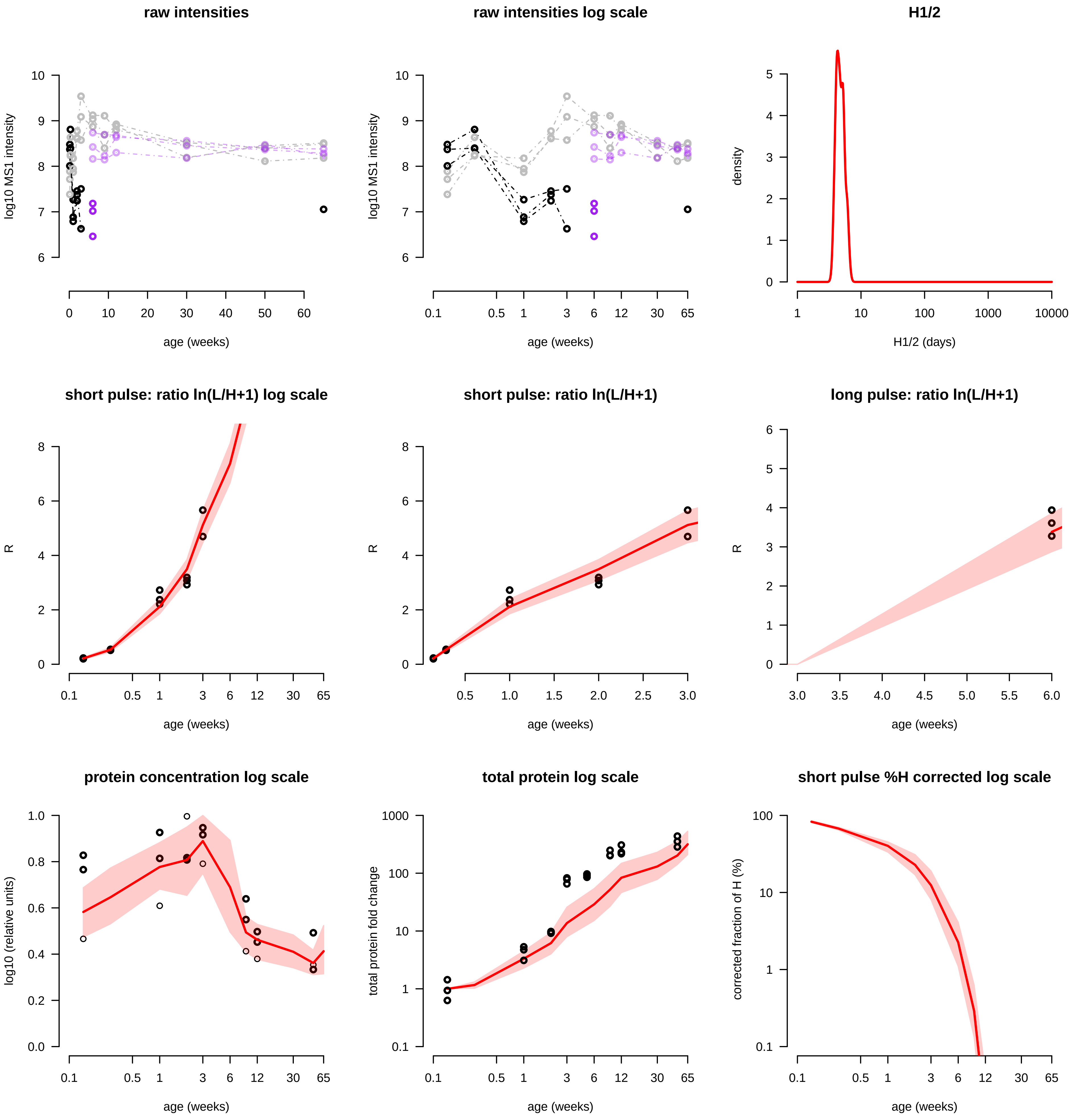

Q80YD1

>sp|Q80YD1|SUV3_MOUSE

ATP−dependent RNA helicase SUPV3L1, mitochondrial

GN=Supv3l1

Med H1/2: 13.3 days

Q5 H1/2: 4.6 days

Q95 H1/2: 140.5 days

reliable model fit: yes

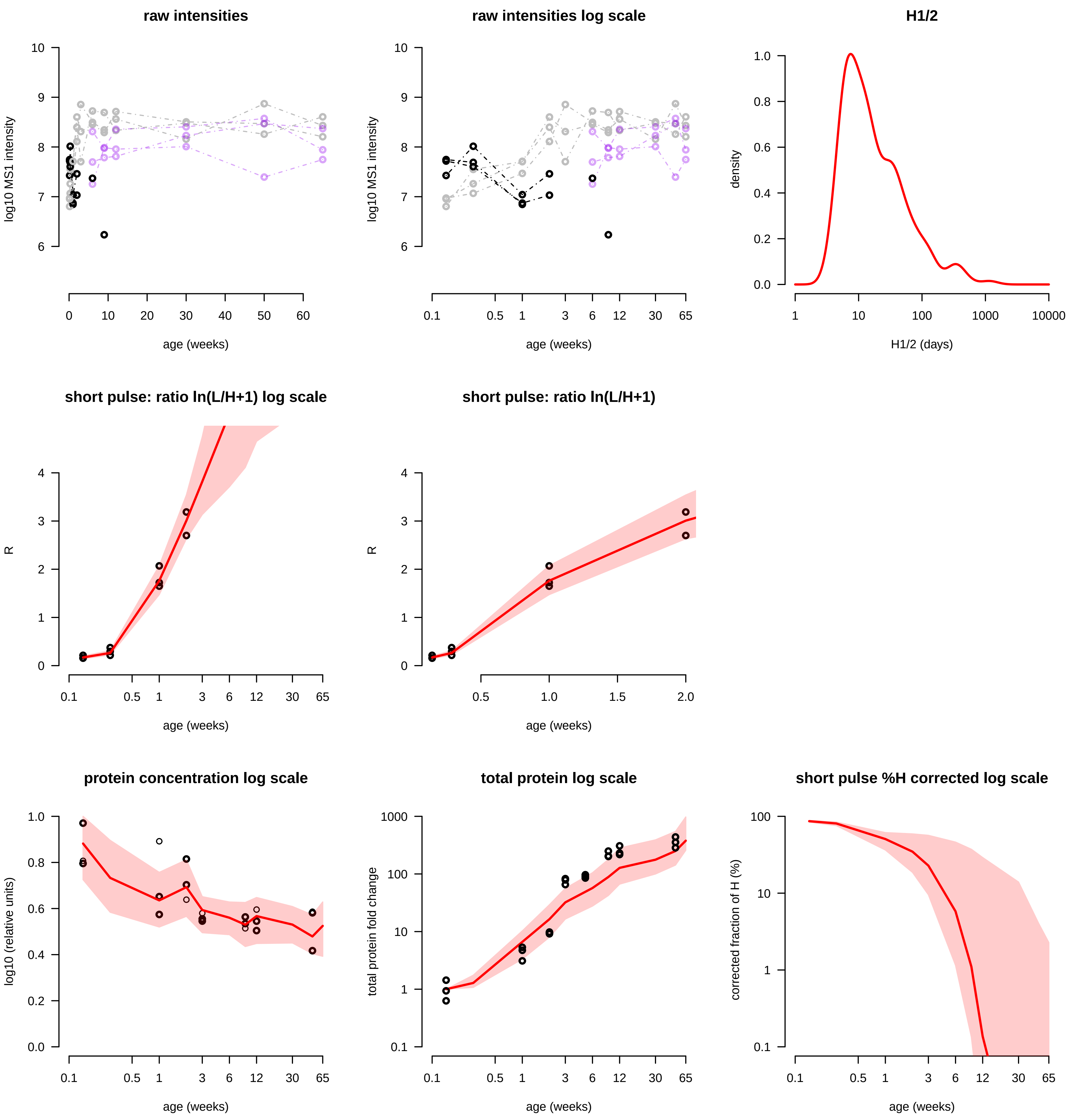

Q922Q9;Q922Q9−2

>sp|Q922Q9|CHID1_MOUSE

Chitinase domain−containing protein 1

GN=Chid1

Med H1/2: 49.6 days

Q5 H1/2: 13 days

Q95 H1/2: 487 days

reliable model fit: no

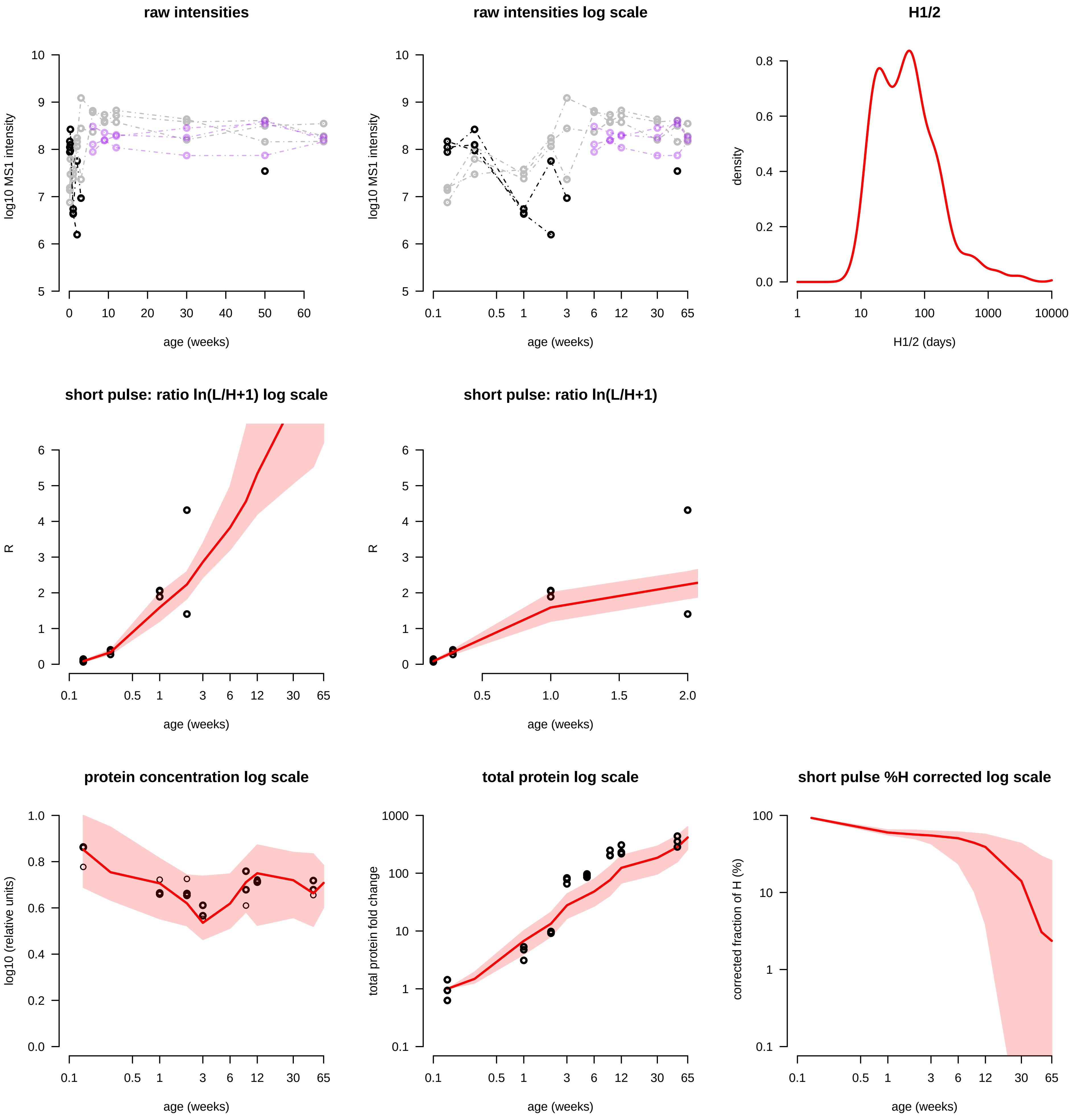

Q91YJ3

>sp|Q91YJ3|THYN1_MOUSE

Thymocyte nuclear protein 1

GN=Thyn1

Med H1/2: 10.8 days

Q5 H1/2: 3.6 days

Q95 H1/2: 39.2 days

reliable model fit: yes

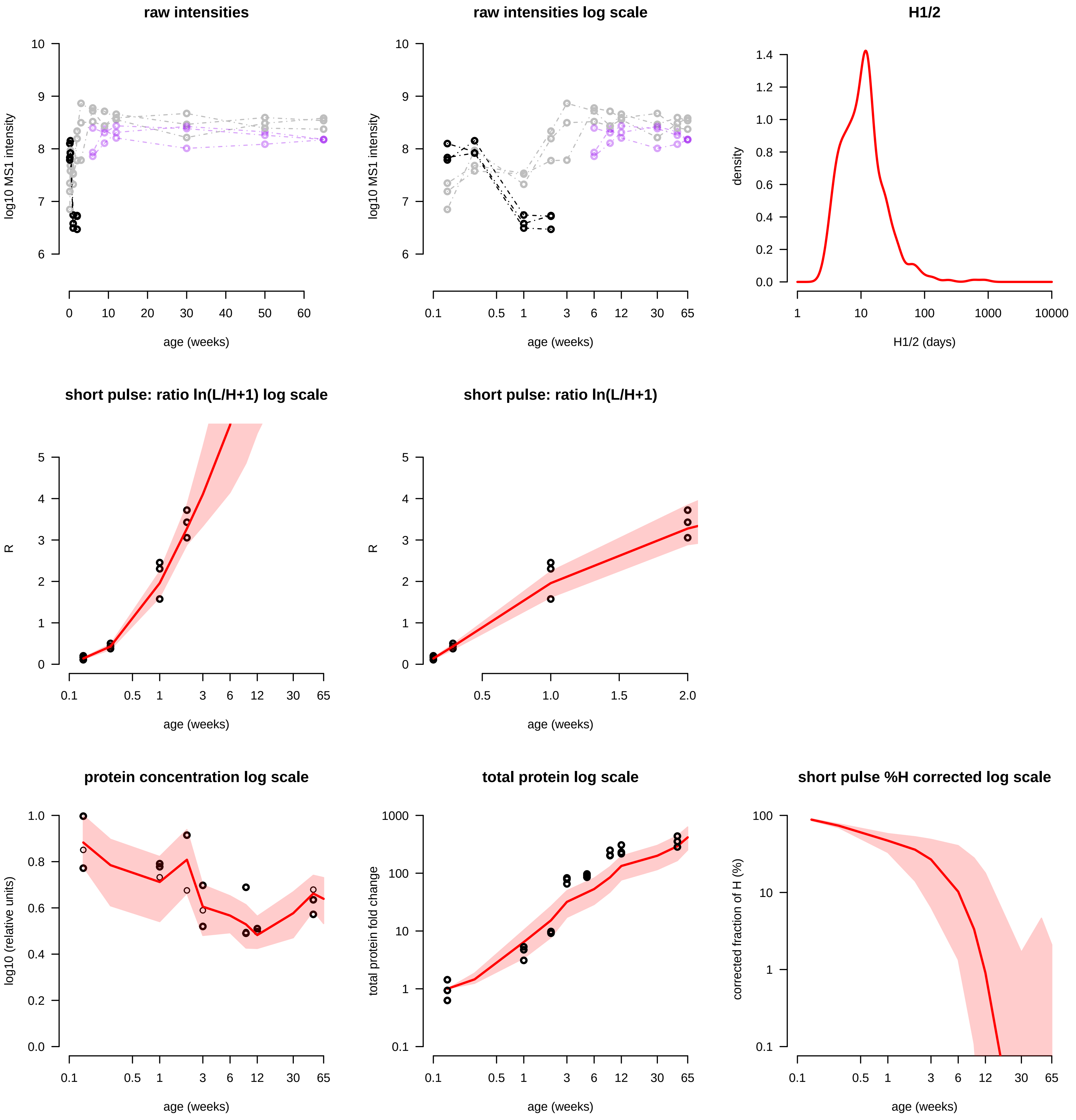

Q61466

>sp|Q61466|SMRD1_MOUSE

-related matrix−associated actin−dependent regulator of chromatin subfamily D

GN=Smarcd1

Med H1/2: 10.5 days

Q5 H1/2: 3.7 days

Q95 H1/2: 68.3 days

reliable model fit: yes

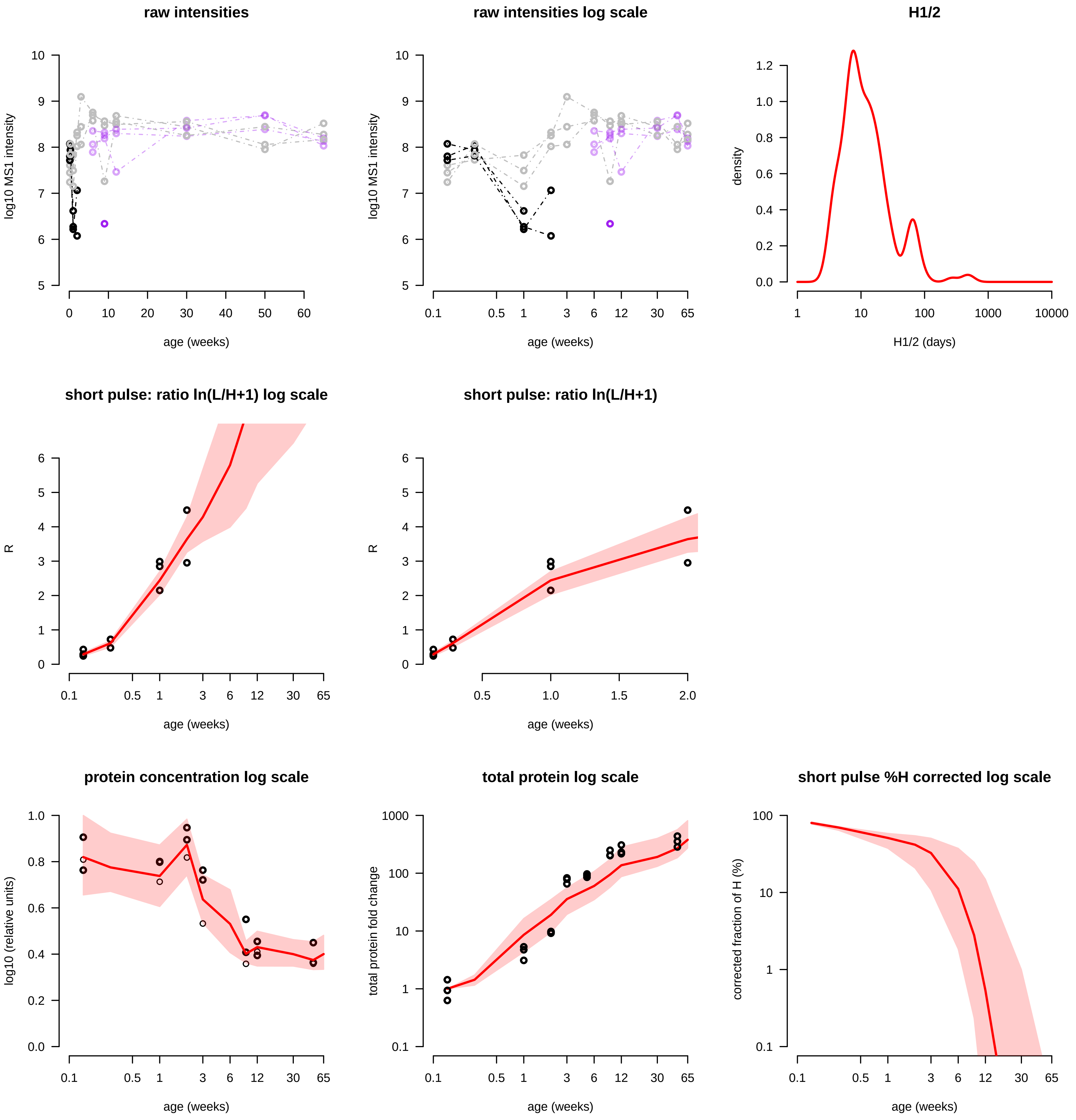

O35098;O35098−2

>sp|O35098|DPYL4_MOUSE

Dihydropyrimidinase−related protein 4

GN=Dpysl4

Med H1/2: 18.7 days

Q5 H1/2: 7.3 days

Q95 H1/2: 183.6 days

reliable model fit: no

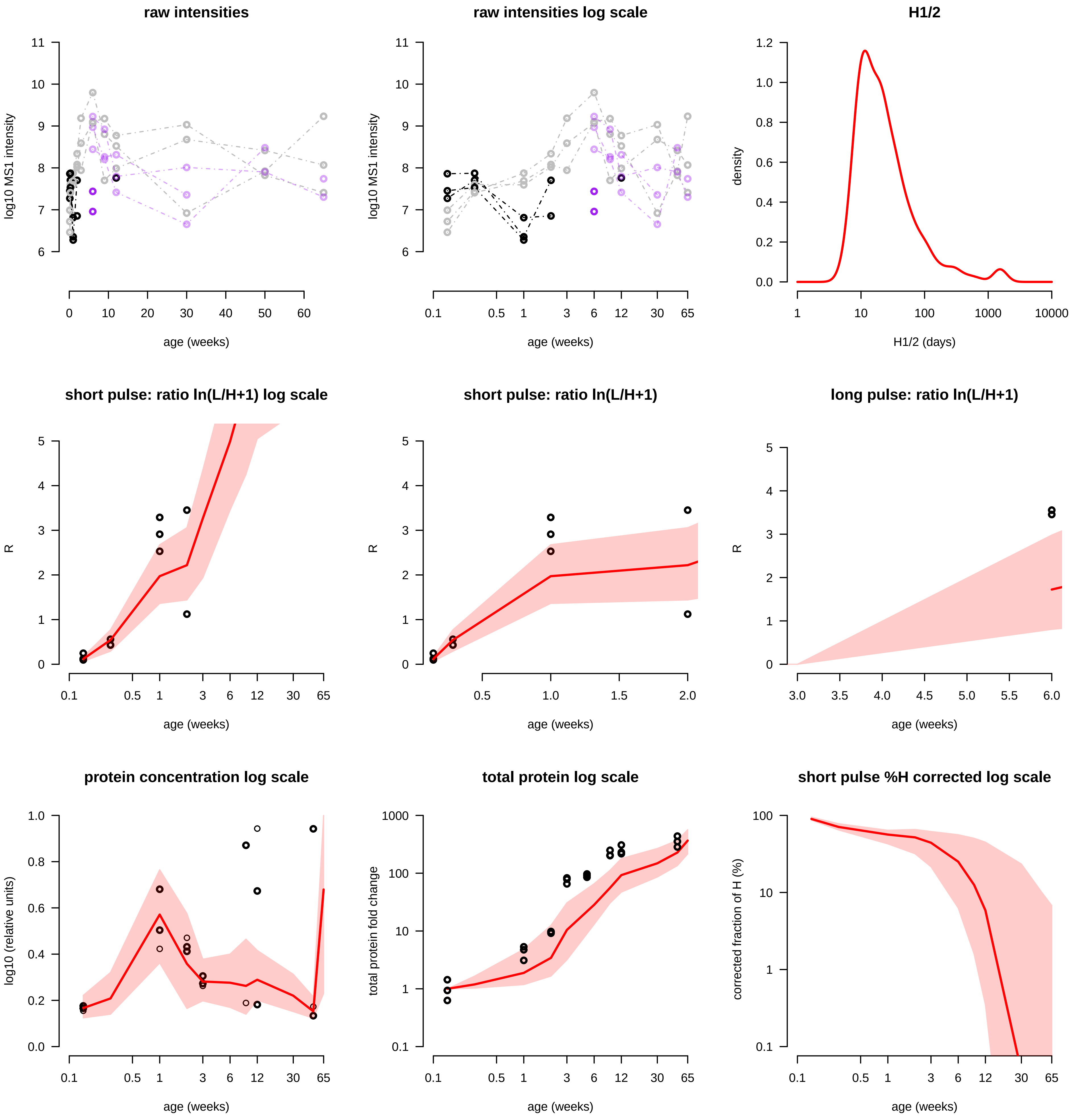

Q8BFR4

>sp|Q8BFR4|GNS_MOUSE

N−acetylglucosamine−6−sulfatase

GN=Gns

Med H1/2: 5.4 days

Q5 H1/2: 3 days

Q95 H1/2: 20.5 days

reliable model fit: yes

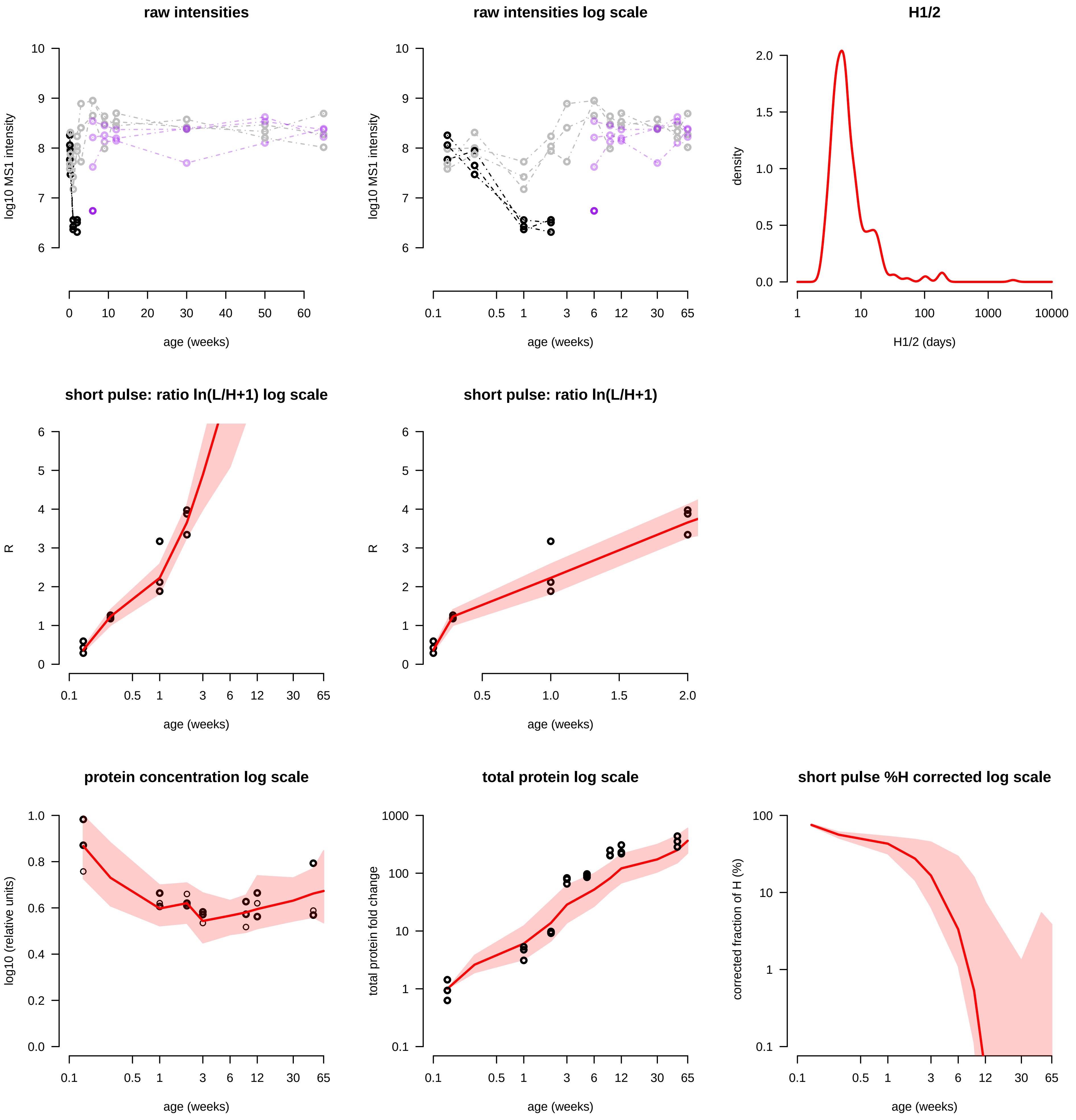

Q9QZH3

>sp|Q9QZH3|PPIE_MOUSE

Peptidyl−prolyl cis−trans isomerase E

GN=Ppie

Med H1/2: 15 days

Q5 H1/2: 6.6 days

Q95 H1/2: 98 days

reliable model fit: yes

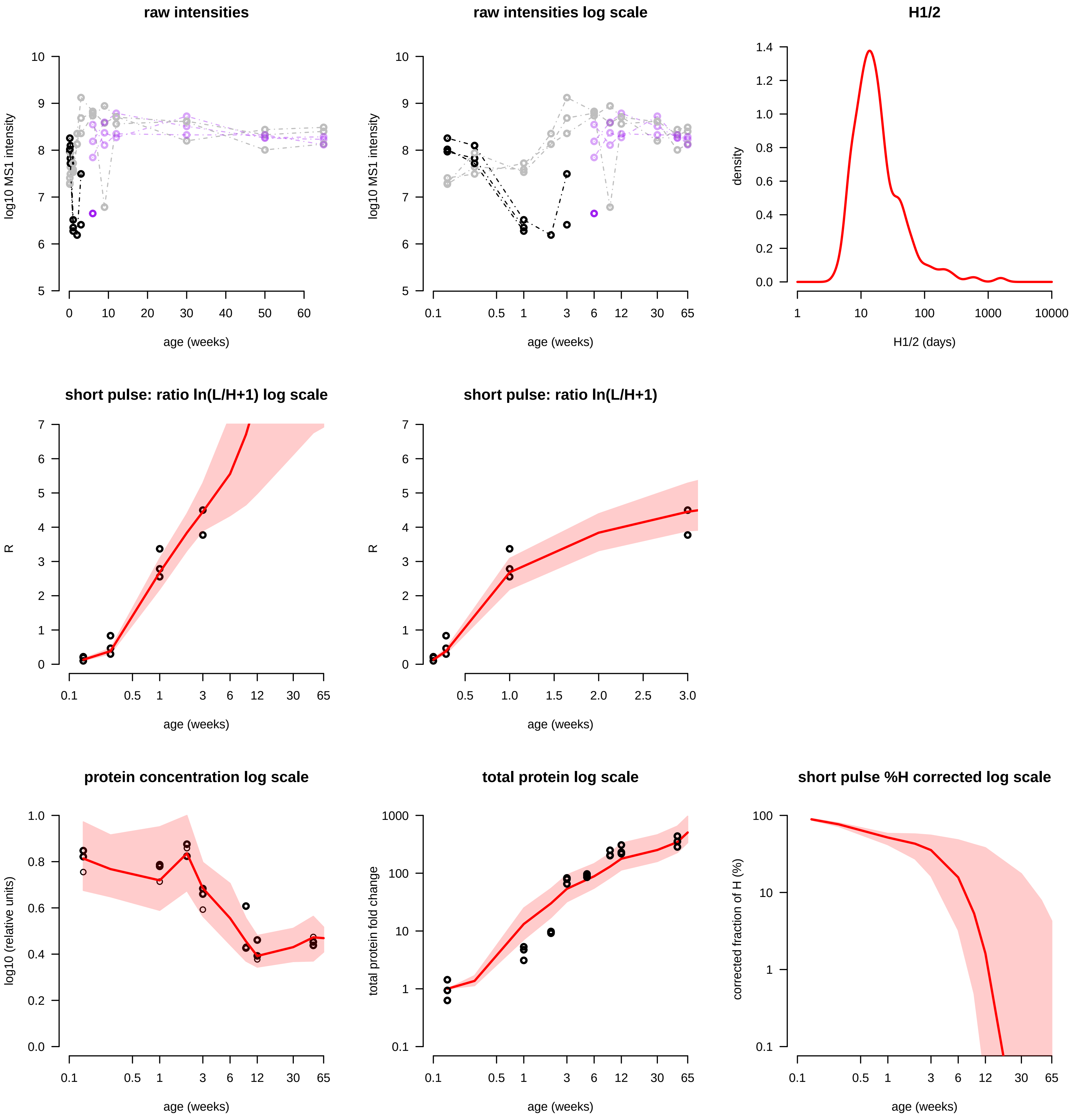

Q6NZF1

>sp|Q6NZF1|ZC11A_MOUSE

Zinc finger CCCH domain−containing protein 11A

GN=Zc3h11a

Med H1/2: 3.5 days

Q5 H1/2: 2.5 days

Q95 H1/2: 8.8 days

reliable model fit: yes

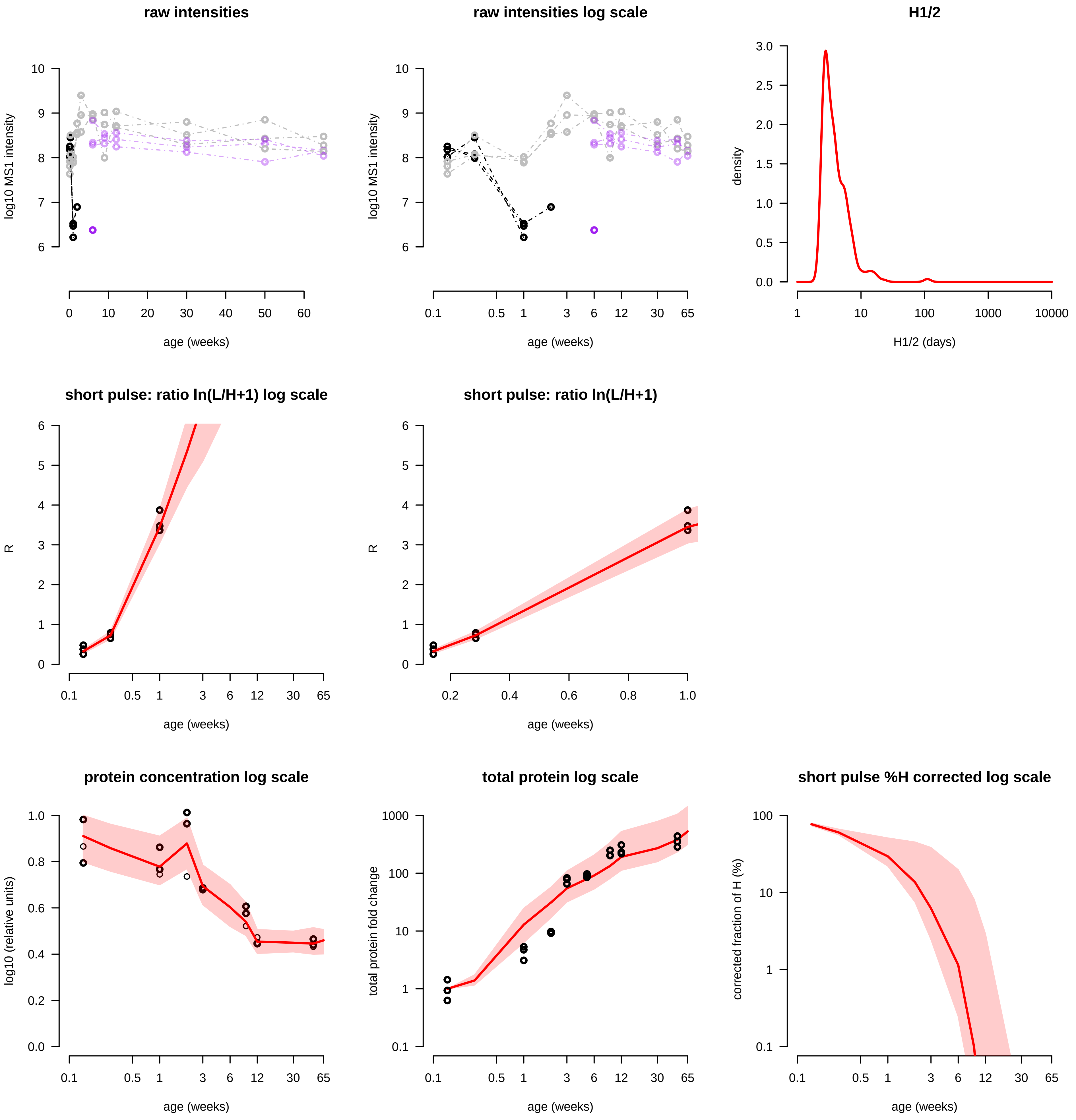

Q80XL6

>sp|Q80XL6|ACD11_MOUSE

Acyl−CoA dehydrogenase family member 11

GN=Acad11

Med H1/2: 1402.8 days

Q5 H1/2: 411.3 days

Q95 H1/2: 13388.2 days

reliable model fit: no

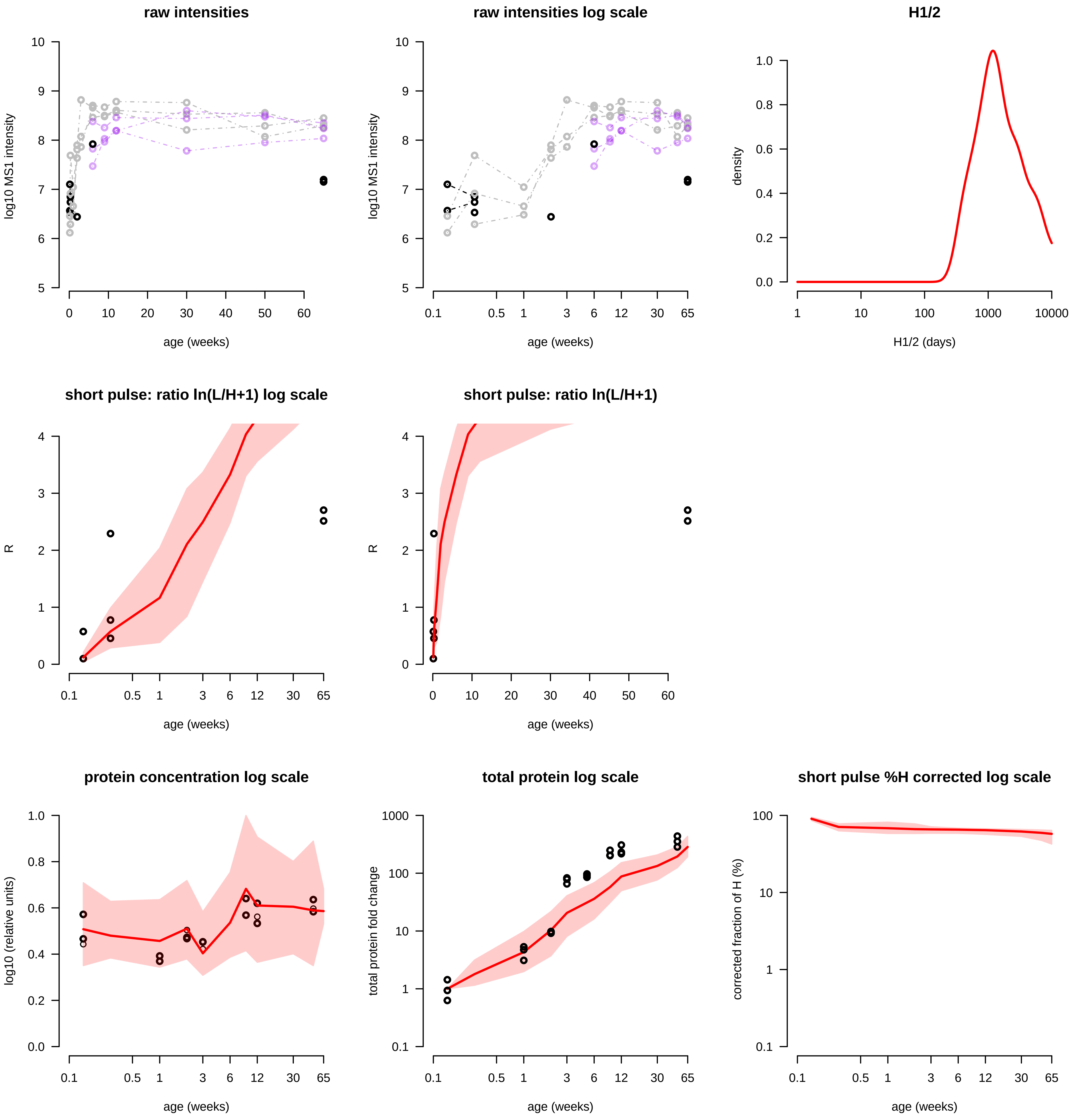

Q9Z2A5

>sp|Q9Z2A5|ATE1_MOUSE

Arginyl−tRNA−−protein transferase 1

GN=Ate1

Med H1/2: 497.6 days

Q5 H1/2: 162.1 days

Q95 H1/2: 8245.2 days

reliable model fit: yes

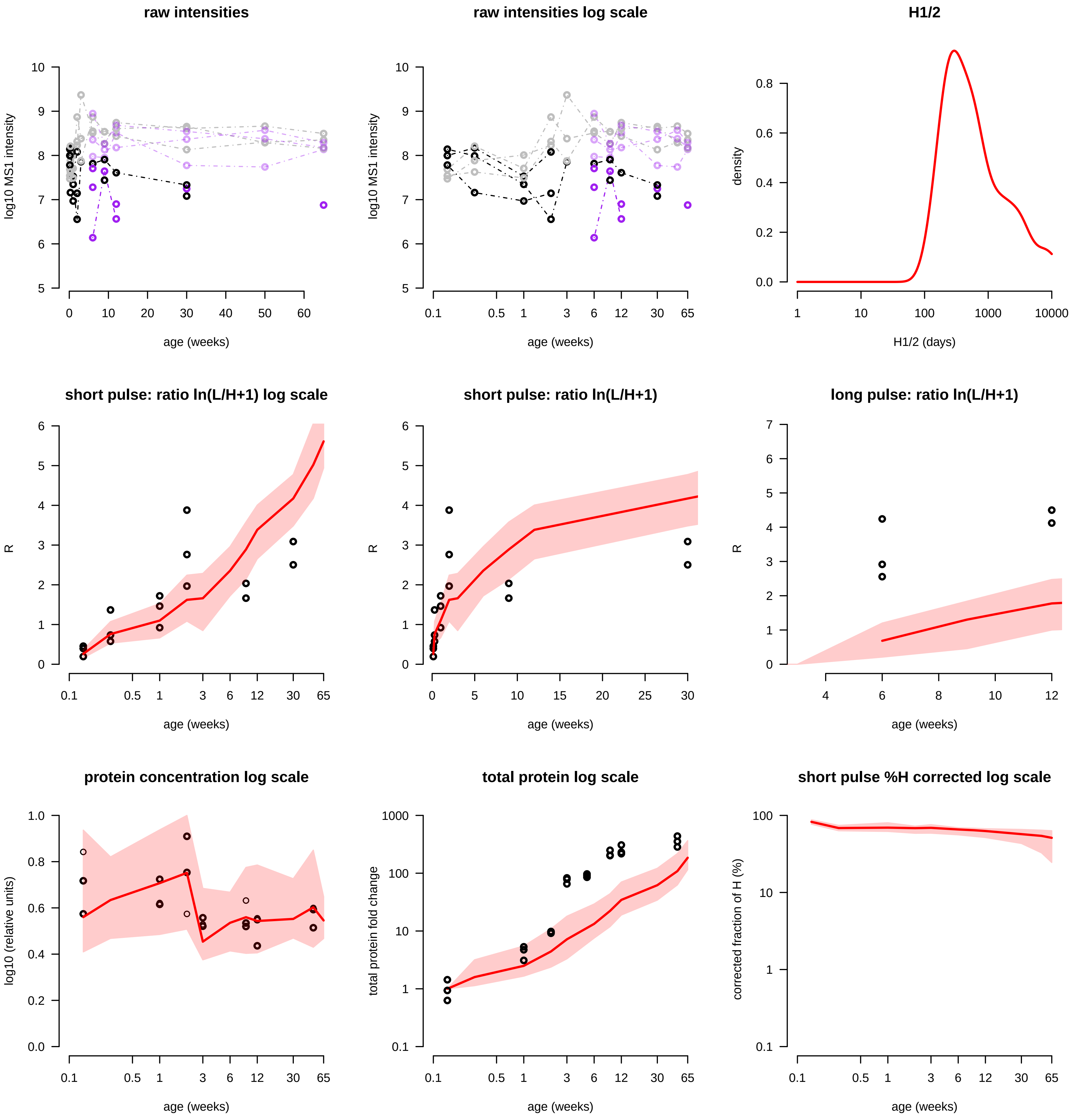

Q8VD04

>sp|Q8VD04|GRAP1_MOUSE

GRIP1−associated protein 1

GN=Gripap1

Med H1/2: 8.7 days

Q5 H1/2: 4.7 days

Q95 H1/2: 73.5 days

reliable model fit: yes

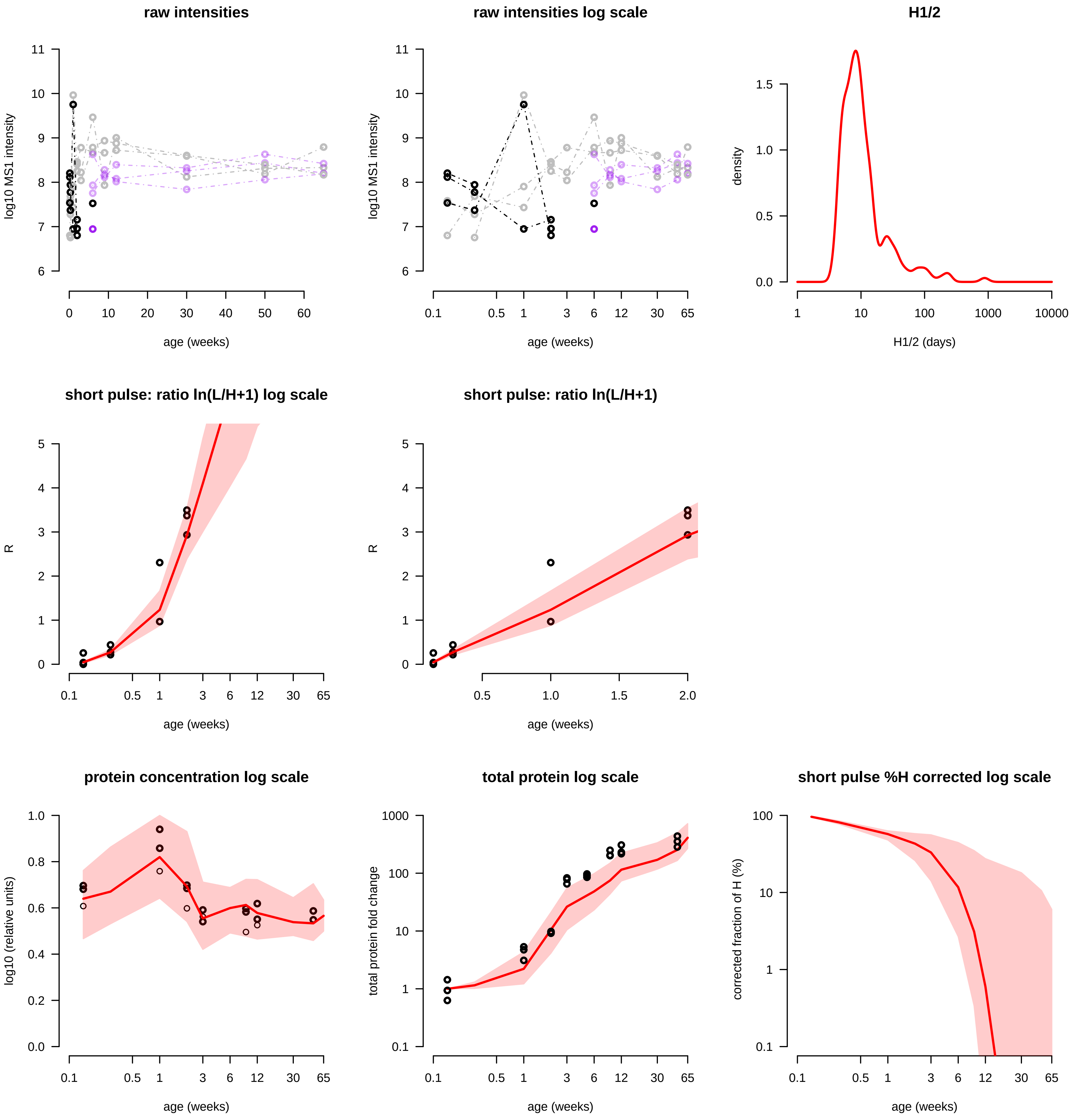

Q8K327

>sp|Q8K327|CHAP1_MOUSE

Chromosome alignment−maintaining phosphoprotein 1

GN=Champ1

Med H1/2: 4.1 days

Q5 H1/2: 2.6 days

Q95 H1/2: 7 days

reliable model fit: yes

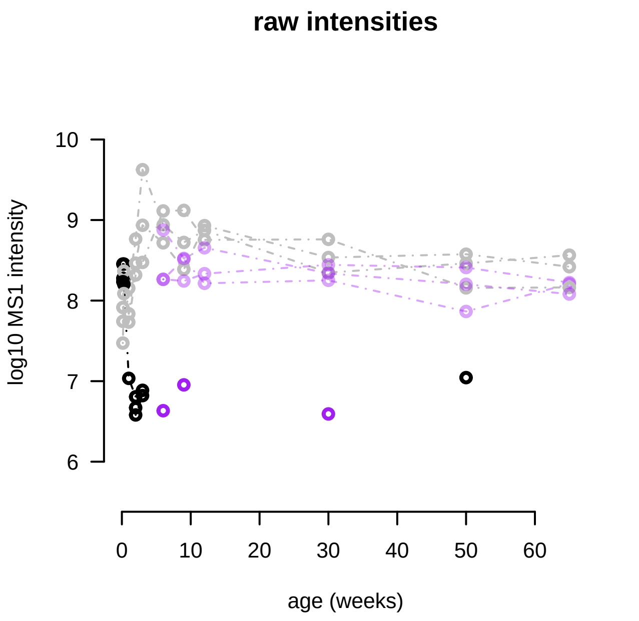

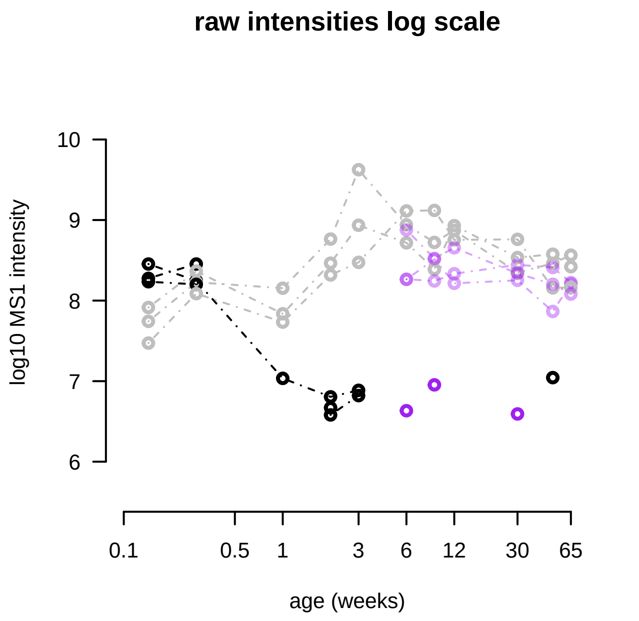

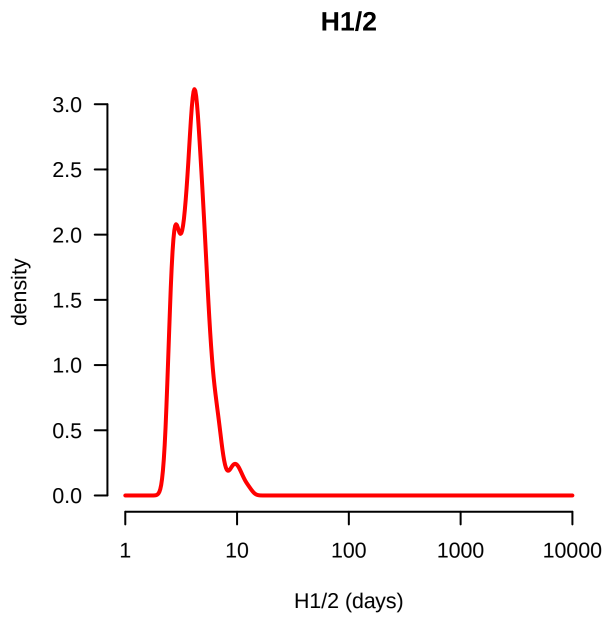

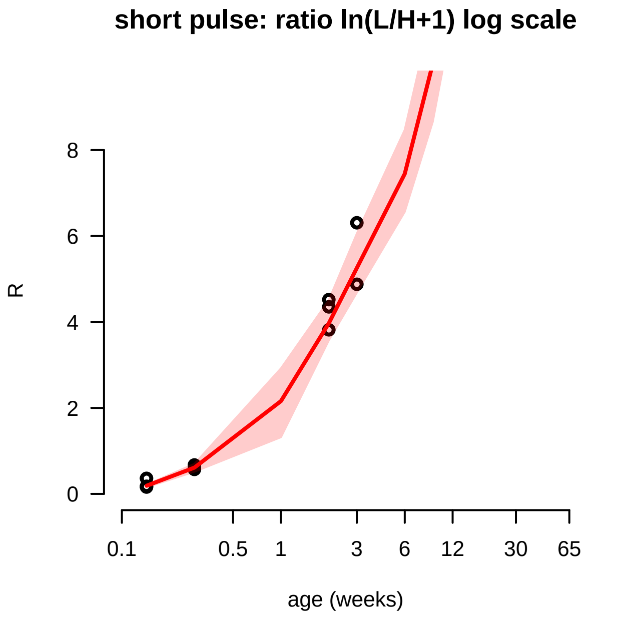

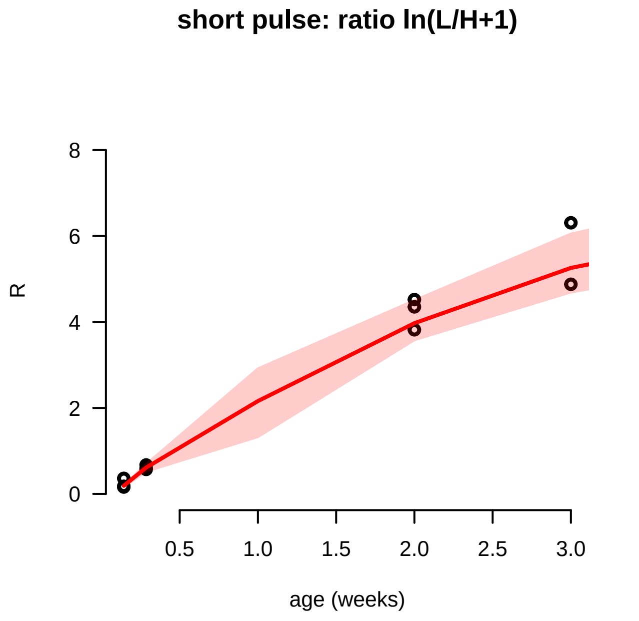

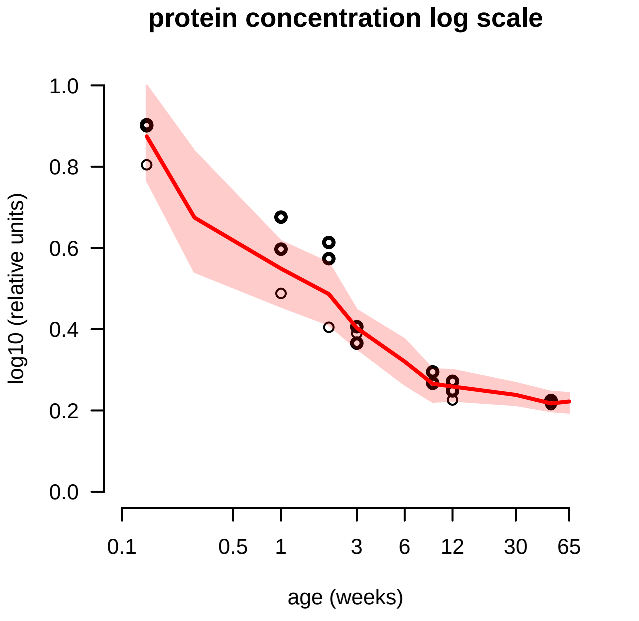

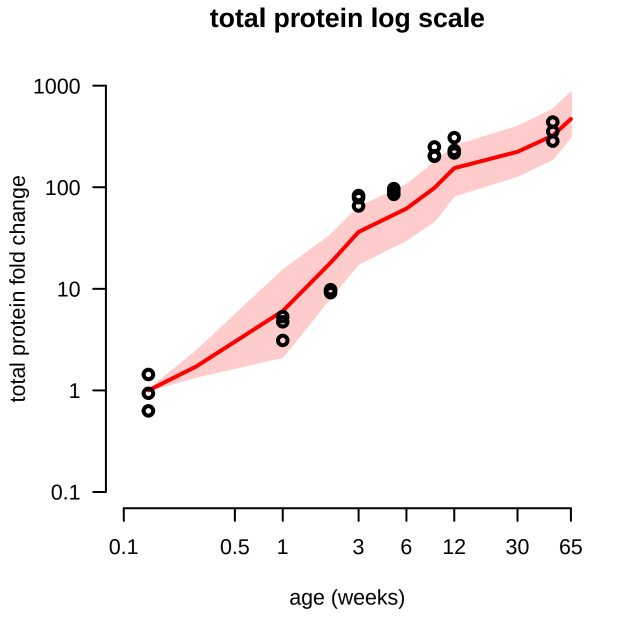

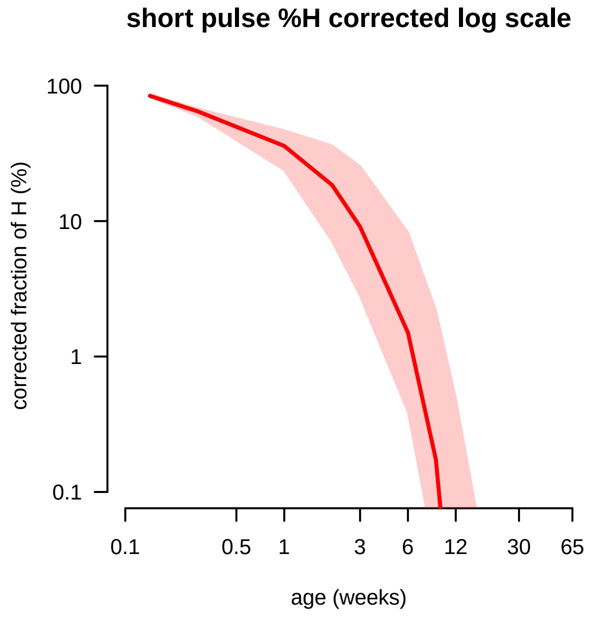

Q9EPU4

>sp|Q9EPU4|CPSF1_MOUSE

Cleavage and polyadenylation specificity factor subunit 1

GN=Cpsf1

Med H1/2: 4.8 days

Q5 H1/2: 2.8 days

Q95 H1/2: 30.2 days

reliable model fit: yes

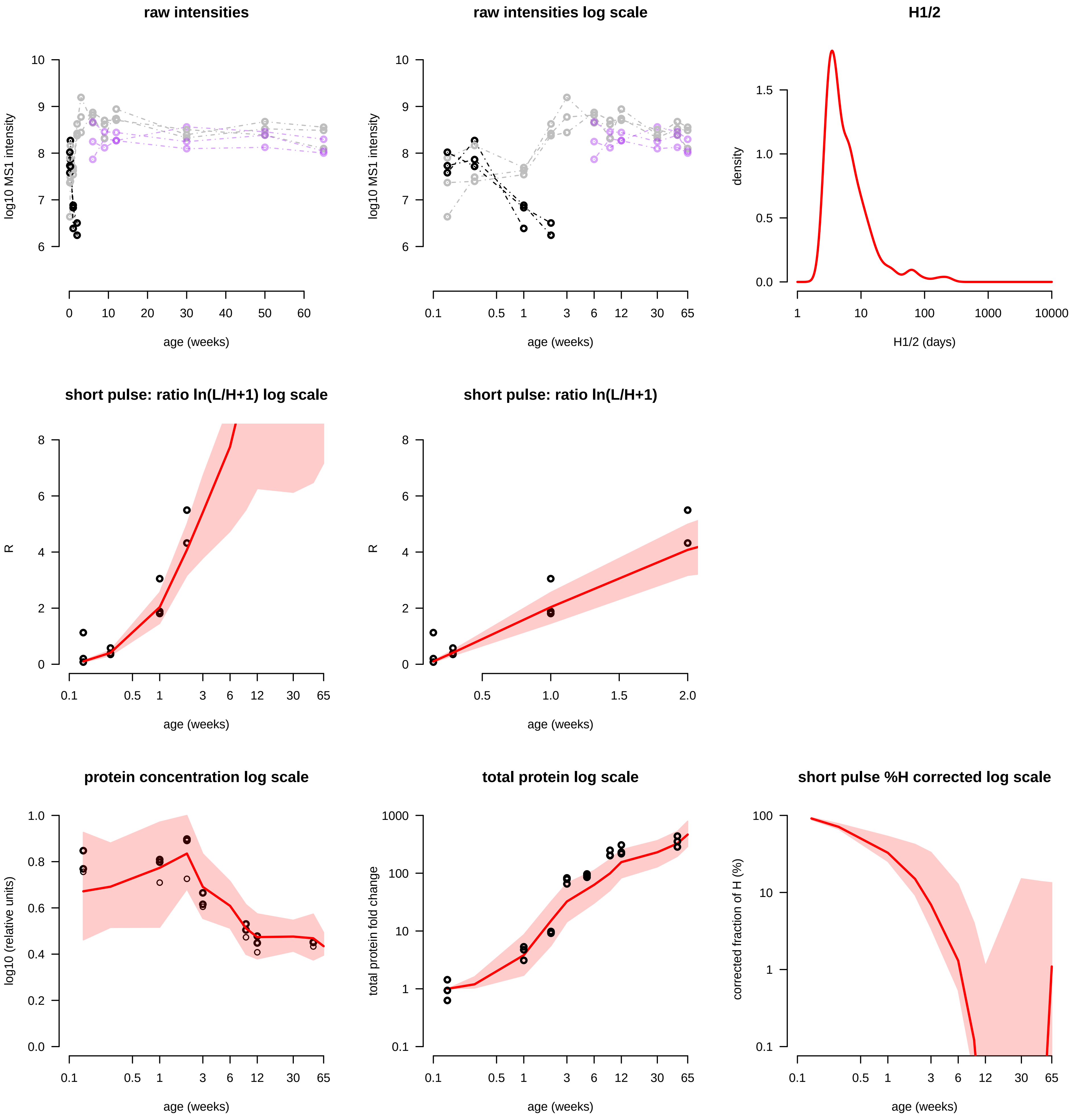

Q0GNC1;Q0GNC1−3

>sp|Q0GNC1|INF2_MOUSE

Inverted formin−2

GN=Inf2

Med H1/2: 2078.4 days

Q5 H1/2: 706.9 days

Q95 H1/2: 17796.4 days

reliable model fit: yes

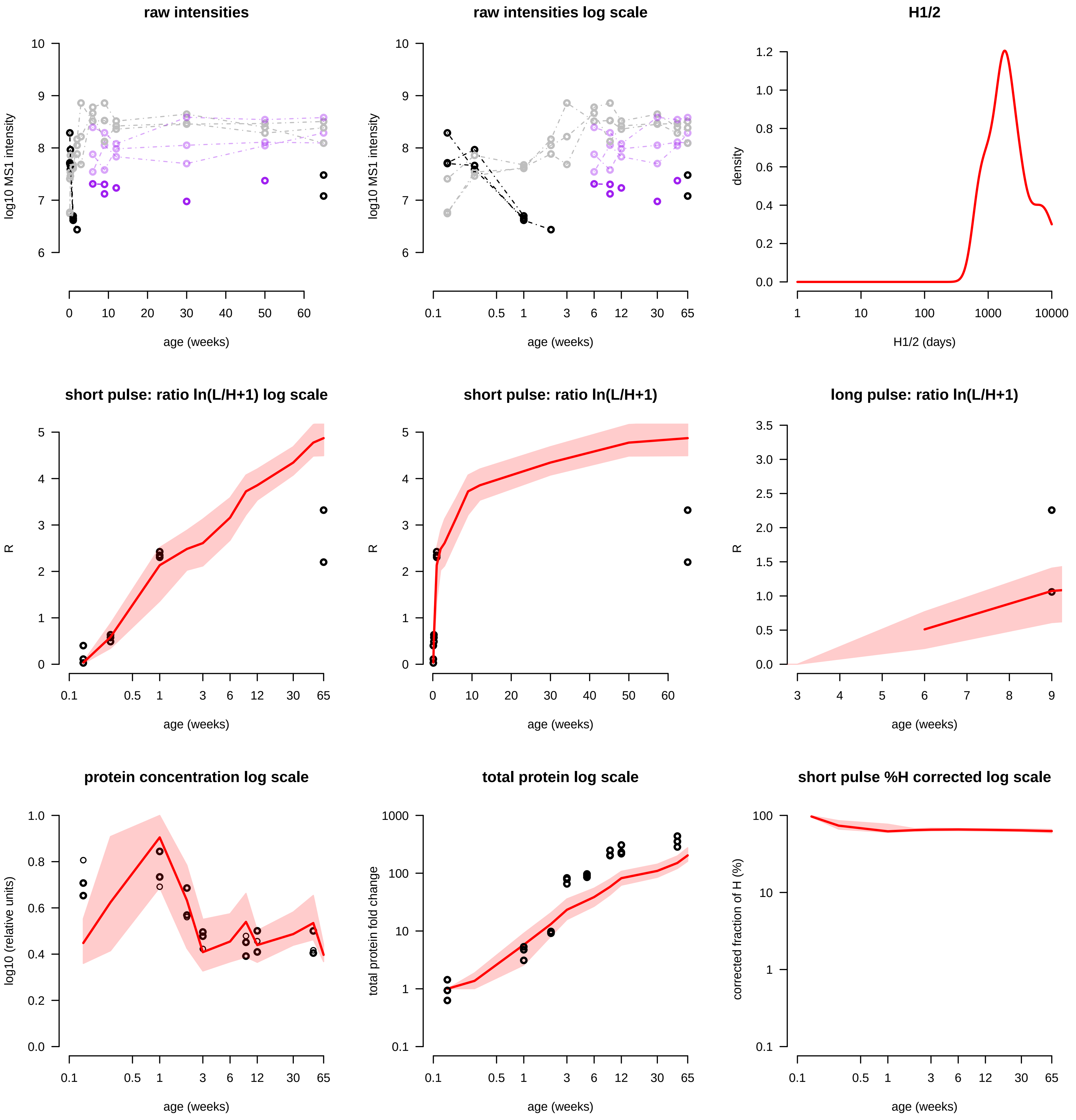

P54276

>sp|P54276|MSH6_MOUSE

DNA mismatch repair protein Msh6

GN=Msh6

Med H1/2: 131.1 days

Q5 H1/2: 57 days

Q95 H1/2: 1159.6 days

reliable model fit: yes

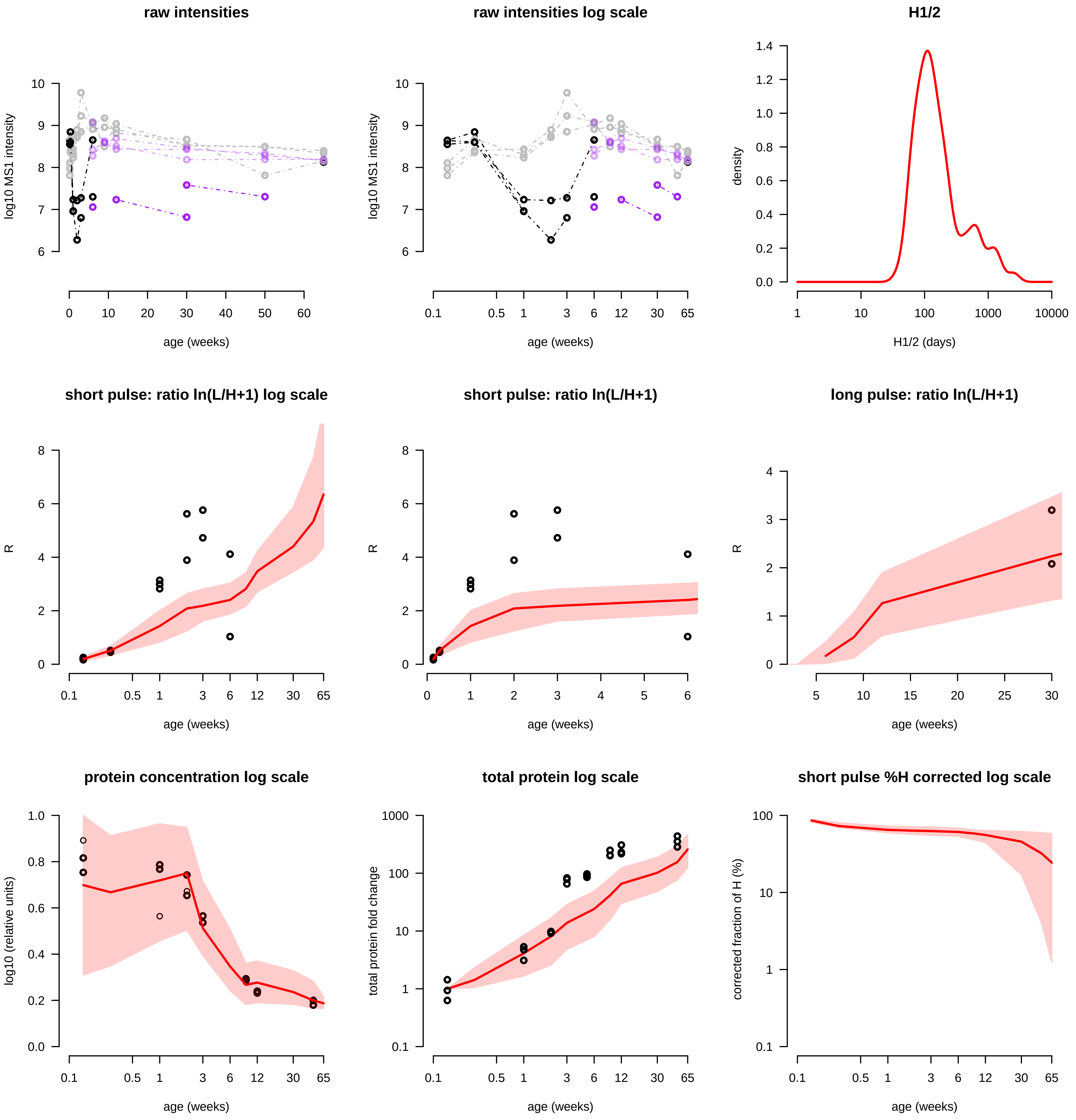

Q3UFY7;Q3UFY7−4;Q3UFY7−2

>sp|Q3UFY7|5NT3B_MOUSE

7−methylguanosine phosphate−specific 5'−nucleotidase

GN=Nt5c3b

Med H1/2: 21.2 days

Q5 H1/2: 7.7 days

Q95 H1/2: 172.4 days

reliable model fit: yes

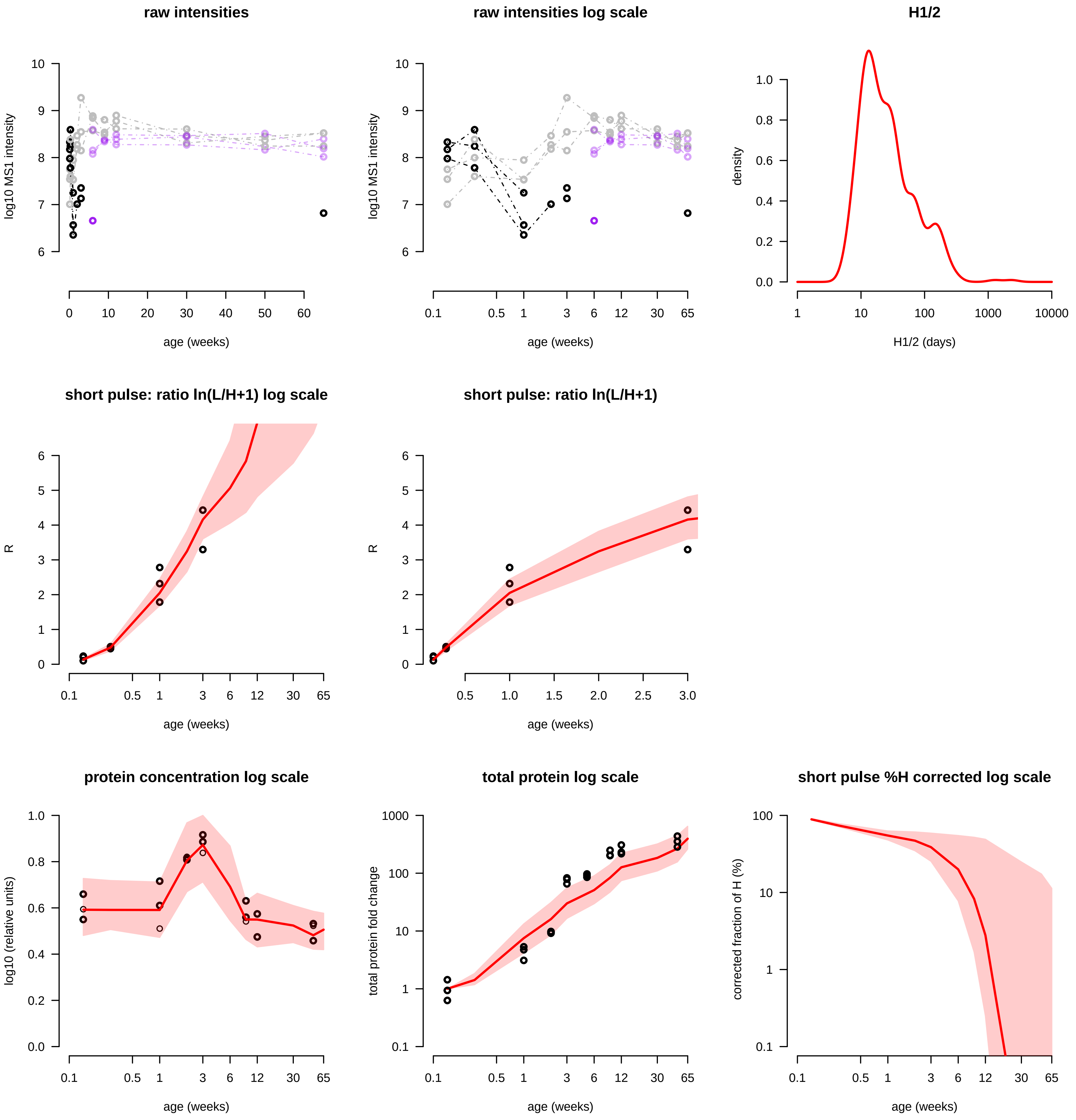

Q7TNP2

>sp|Q7TNP2|2AAB_MOUSE

erine/threonine−protein phosphatase 2A 65 kDa regulatory subunit A beta isofor

GN=Ppp2r1b

Med H1/2: 5.7 days

Q5 H1/2: 2.7 days

Q95 H1/2: 47.9 days

reliable model fit: yes

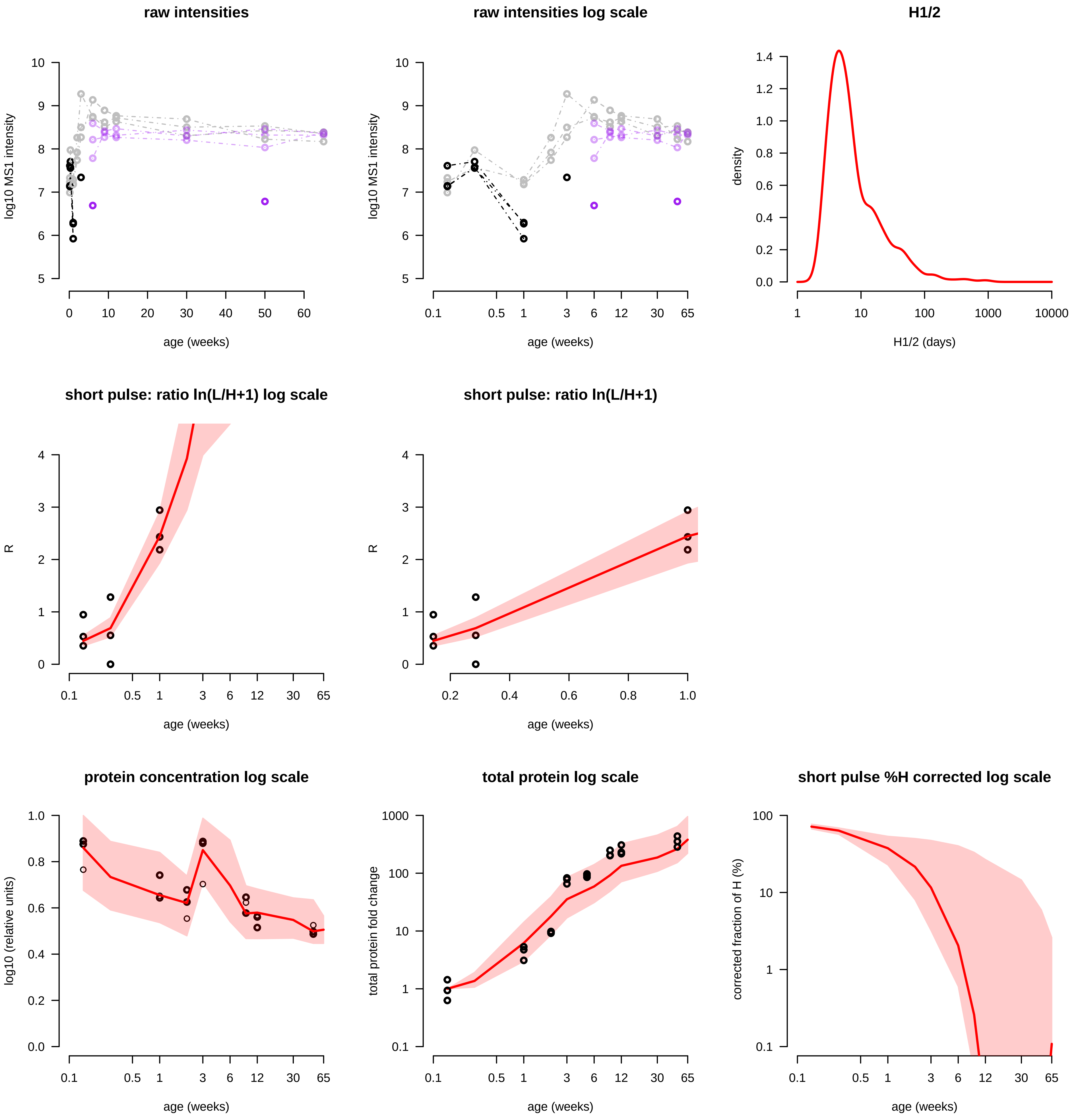

Q61133

>sp|Q61133|GSTT2_MOUSE

Glutathione S−transferase theta−2

GN=Gstt2

Med H1/2: 23.5 days

Q5 H1/2: 8.9 days

Q95 H1/2: 350.1 days

reliable model fit: yes

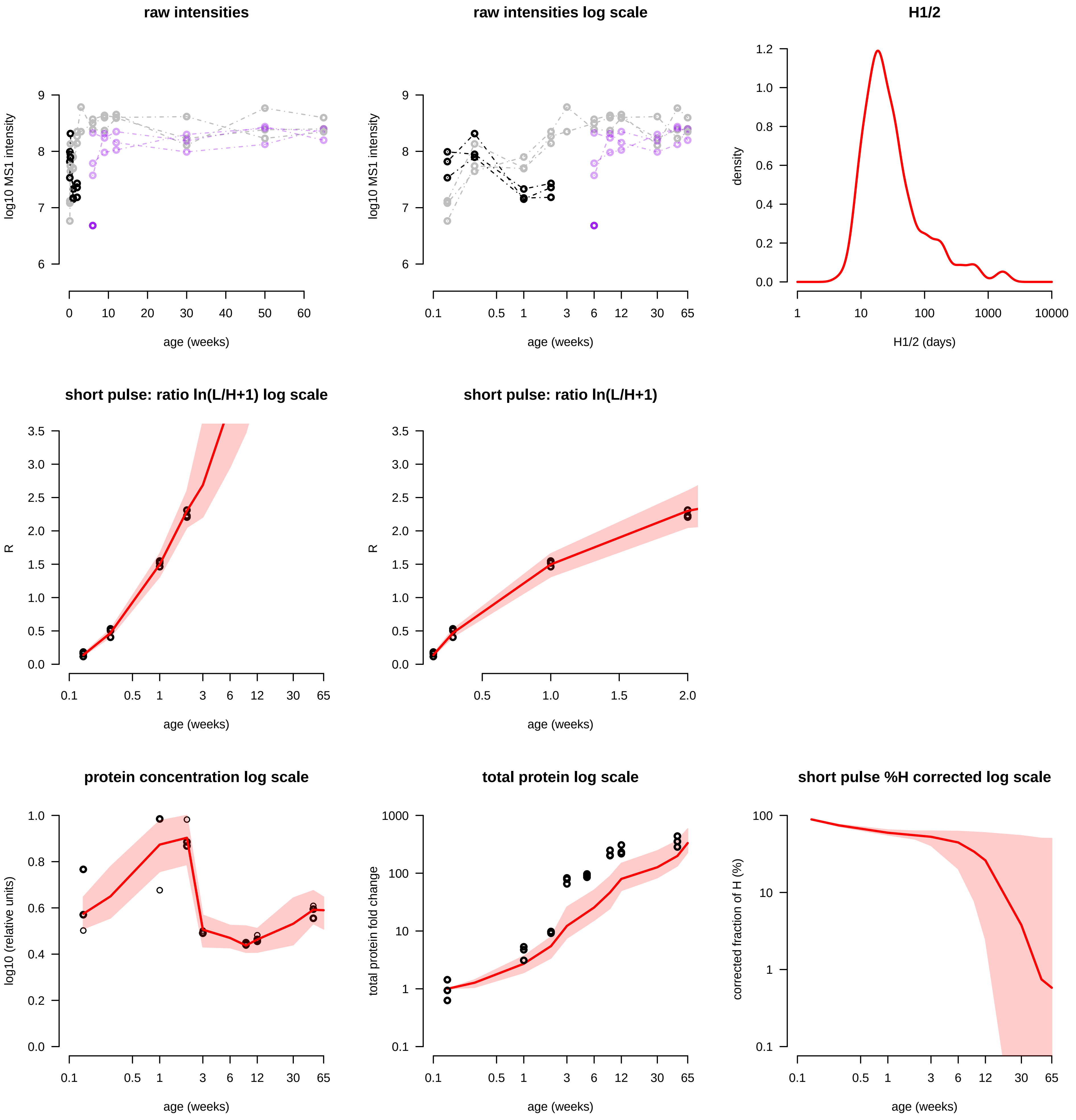

Q8CCP0;Q8CCP0−2;Q8CCP0−3

>sp|Q8CCP0|NEMF_MOUSE

Nuclear export mediator factor Nemf

GN=Nemf

Med H1/2: 5.1 days

Q5 H1/2: 2.7 days

Q95 H1/2: 16.6 days

reliable model fit: yes

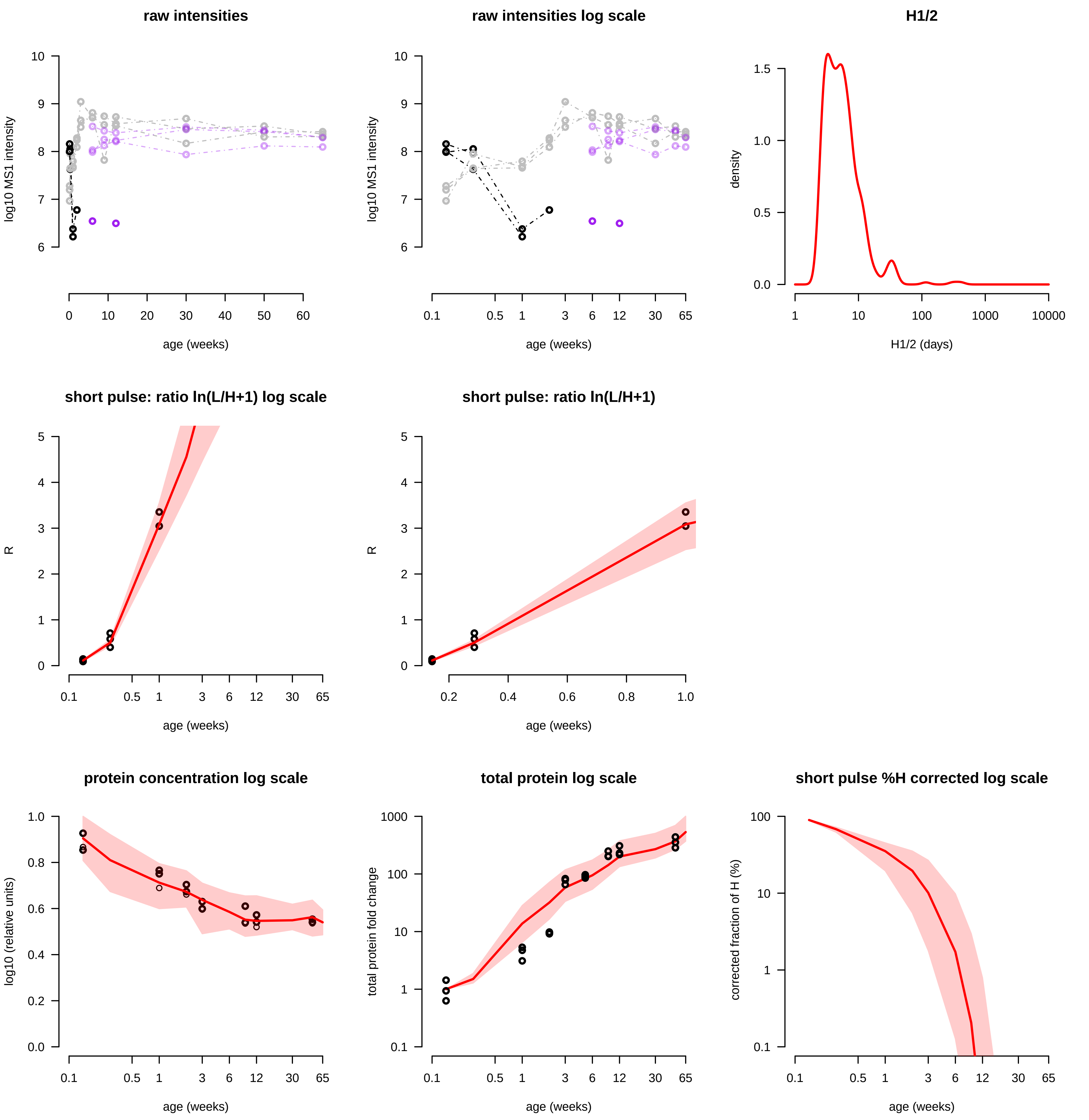

P82343;P82343−2

>sp|P82343|RENBP_MOUSE

N−acylglucosamine 2−epimerase

GN=Renbp

Med H1/2: 4.5 days

Q5 H1/2: 2.7 days

Q95 H1/2: 11 days

reliable model fit: yes

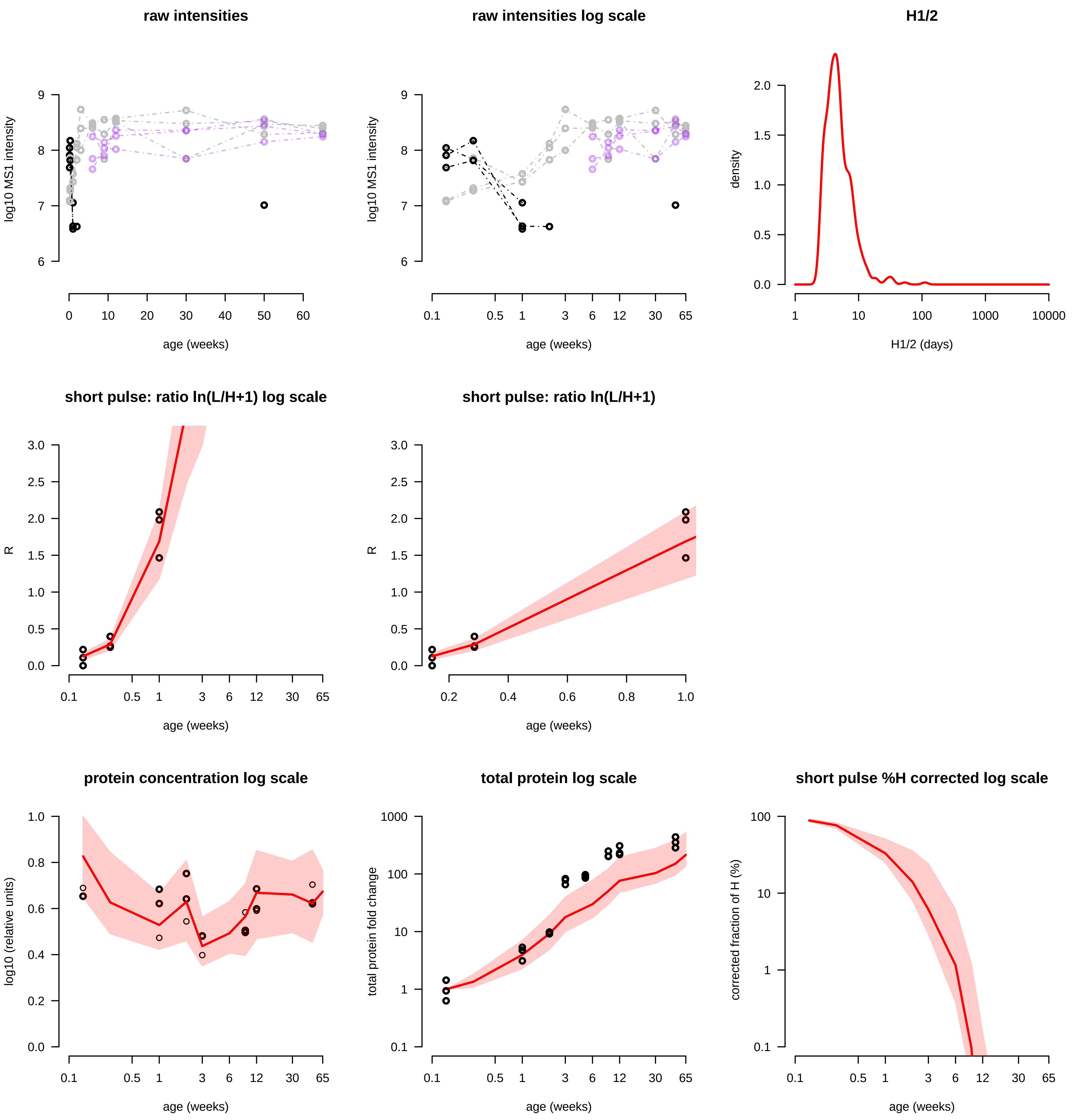

Q8R4X3

>sp|Q8R4X3|RBM12_MOUSE

RNA−binding protein 12

GN=Rbm12

Med H1/2: 19.7 days

Q5 H1/2: 6.2 days

Q95 H1/2: 149.2 days

reliable model fit: yes

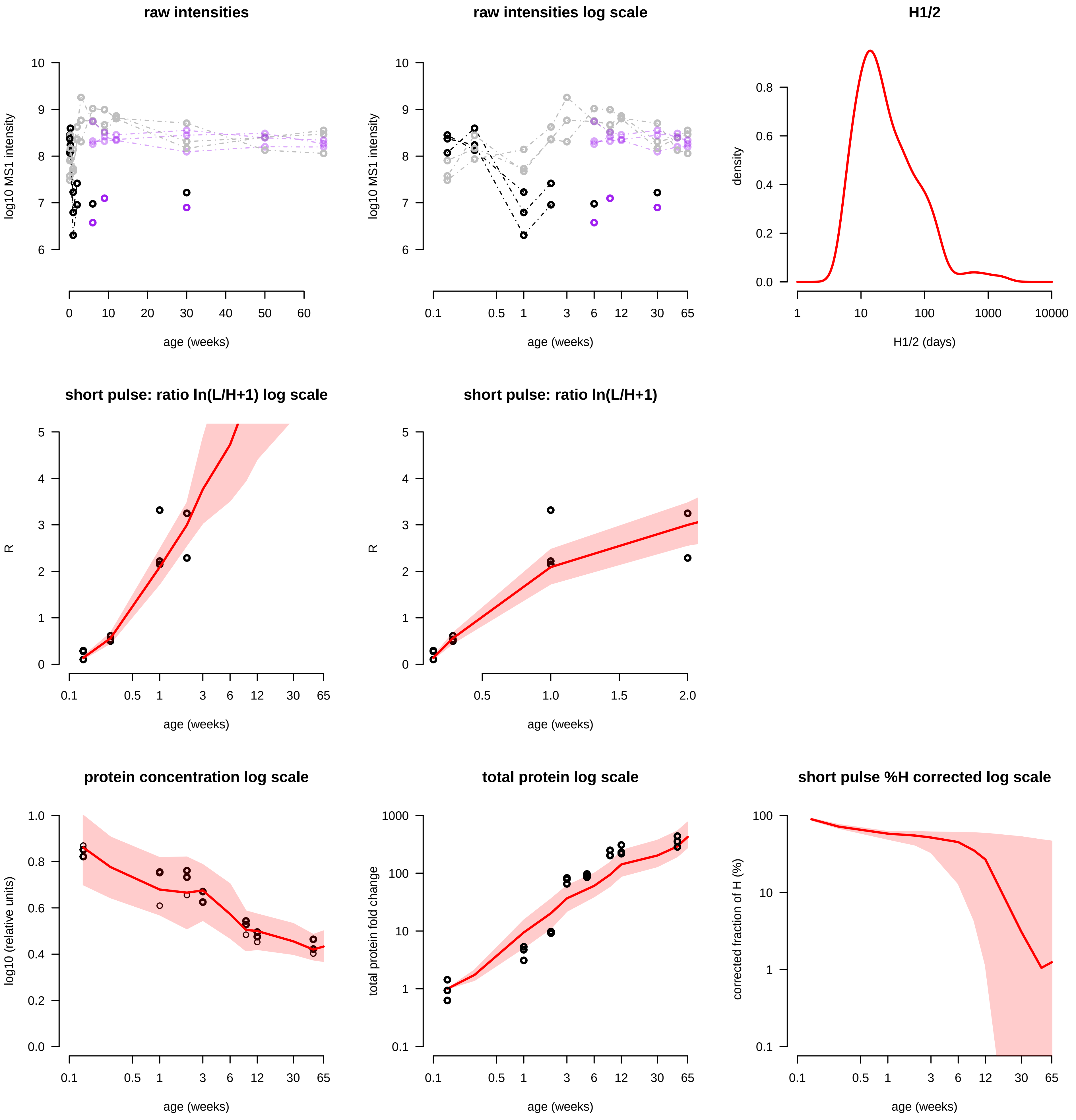

E9Q735

>sp|E9Q735|UBE4A_MOUSE

Ubiquitin conjugation factor E4 A

GN=Ube4a

Med H1/2: 2236.6 days

Q5 H1/2: 531.3 days

Q95 H1/2: 16588.4 days

reliable model fit: yes

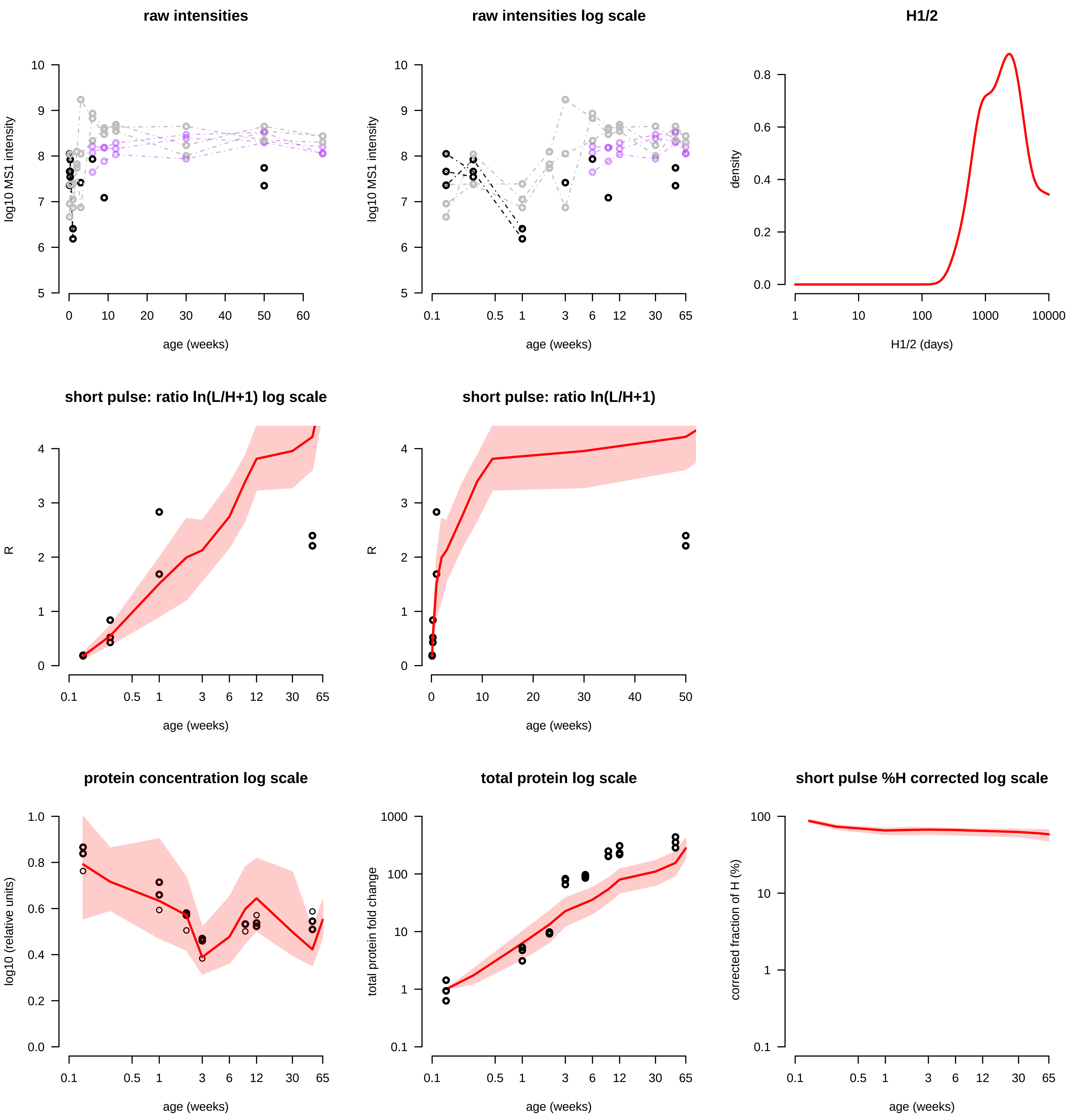

Q91ZR2

>sp|Q91ZR2|SNX18_MOUSE

Sorting nexin−18

GN=Snx18

Med H1/2: 5.7 days

Q5 H1/2: 4.7 days

Q95 H1/2: 7.6 days

reliable model fit: yes

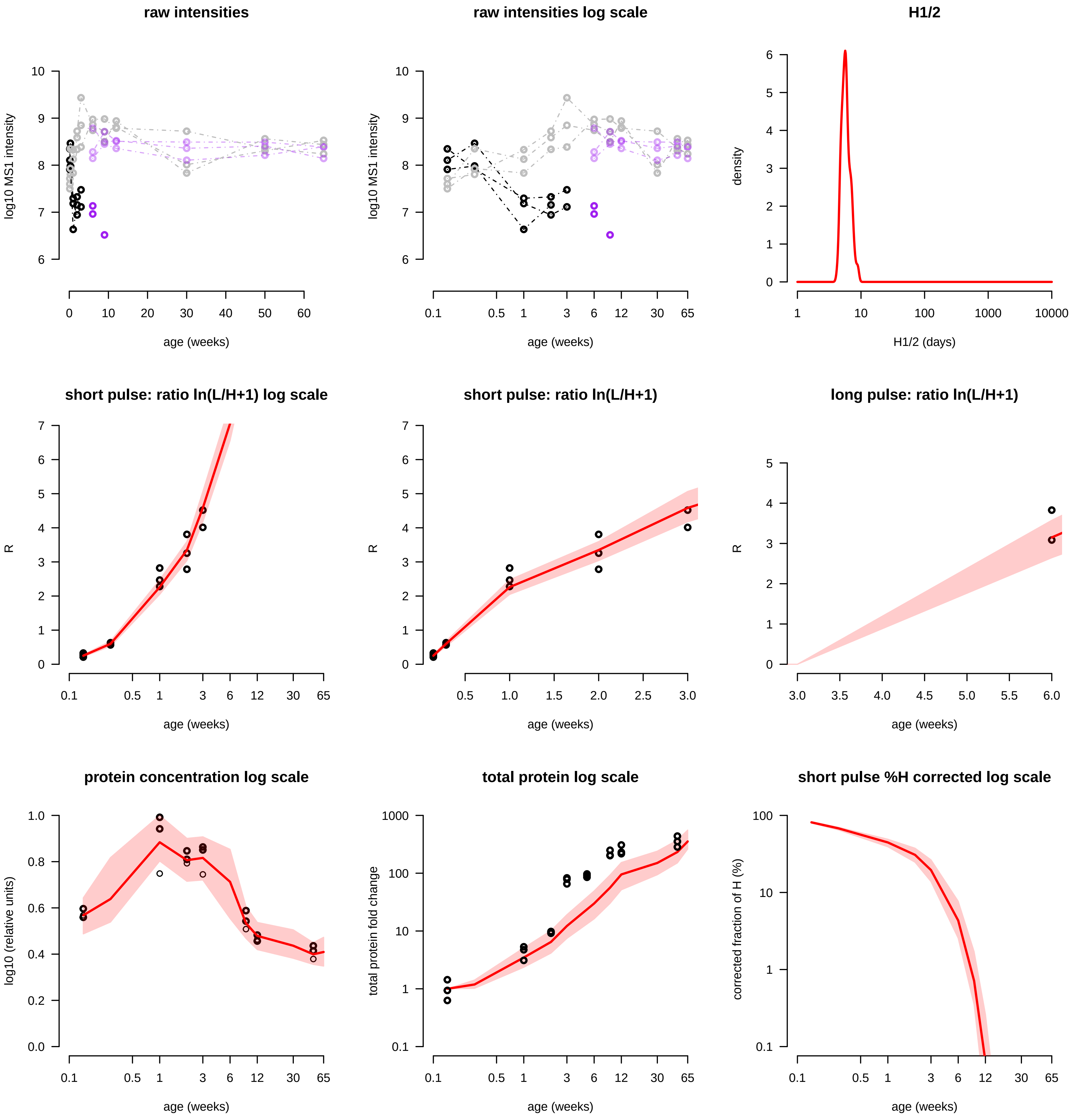

Q9QXK7

>sp|Q9QXK7|CPSF3_MOUSE

Cleavage and polyadenylation specificity factor subunit 3

GN=Cpsf3

Med H1/2: 36.1 days

Q5 H1/2: 10.1 days

Q95 H1/2: 522.1 days

reliable model fit: yes

raw intensities

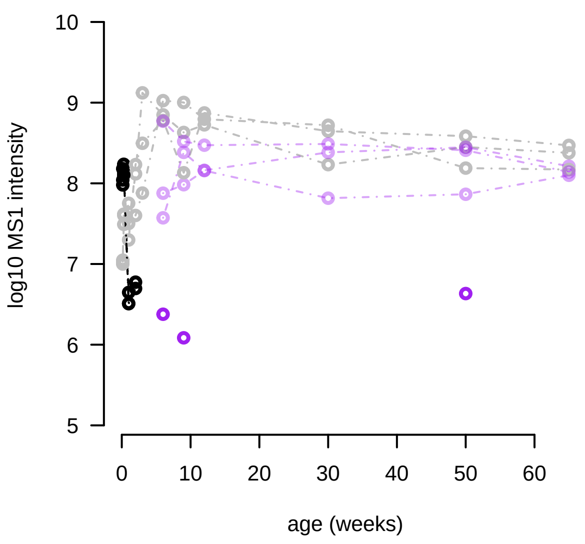

raw intensities log scale

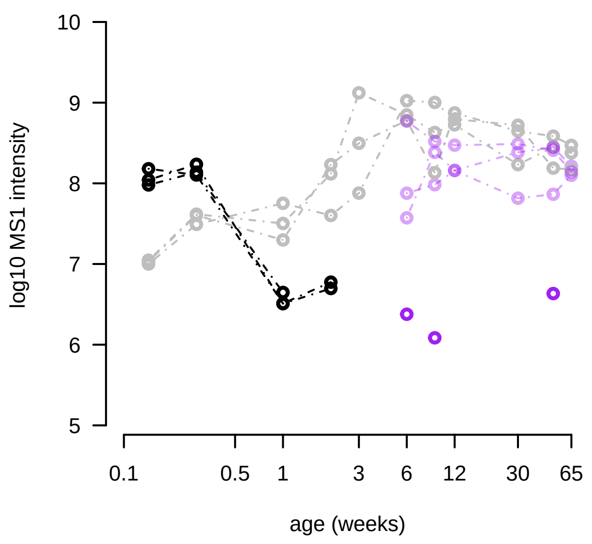

H1/2

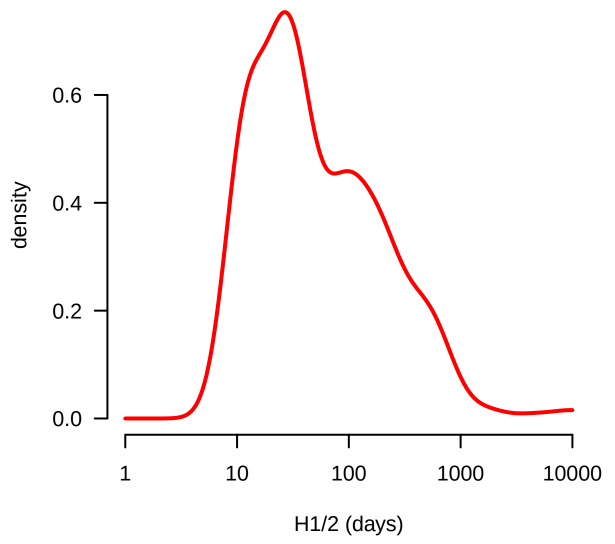

short pulse: ratio ln(L/H+1) log scale

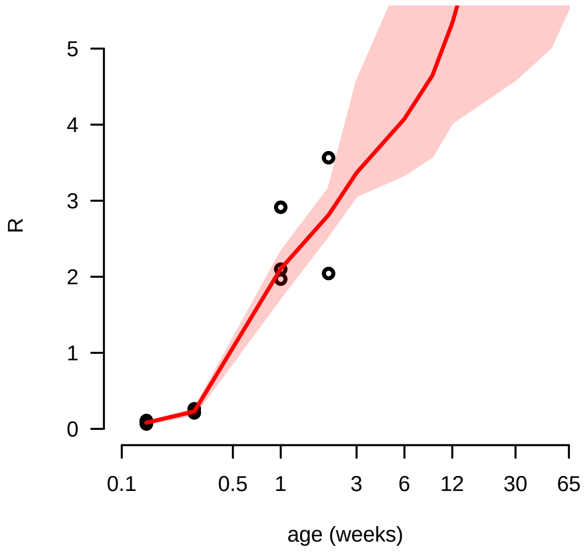

short pulse: ratio ln(L/H+1)

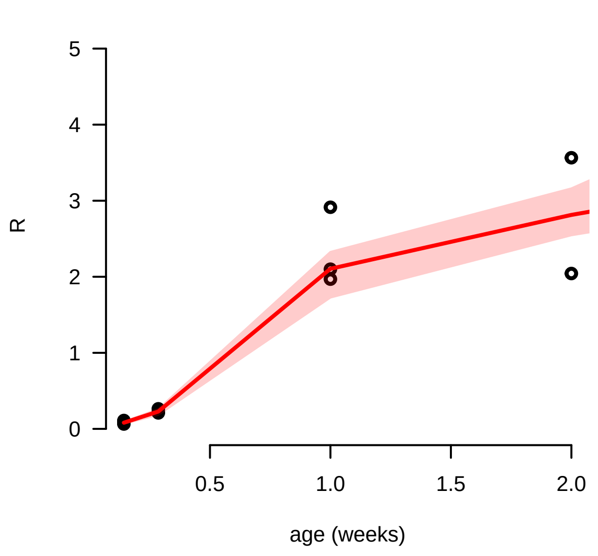

protein concentration log scale

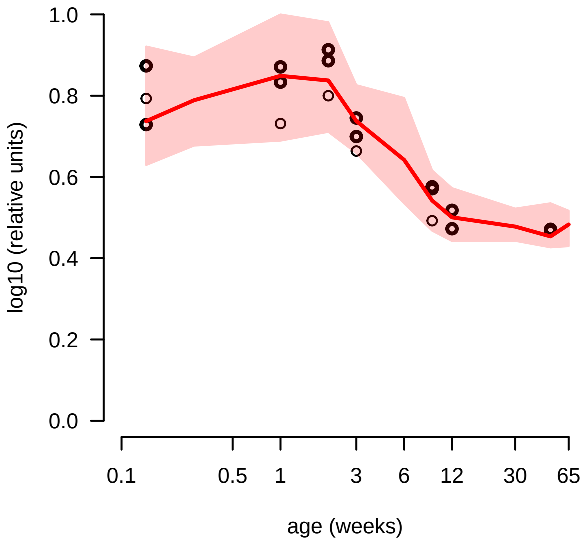

total protein log scale

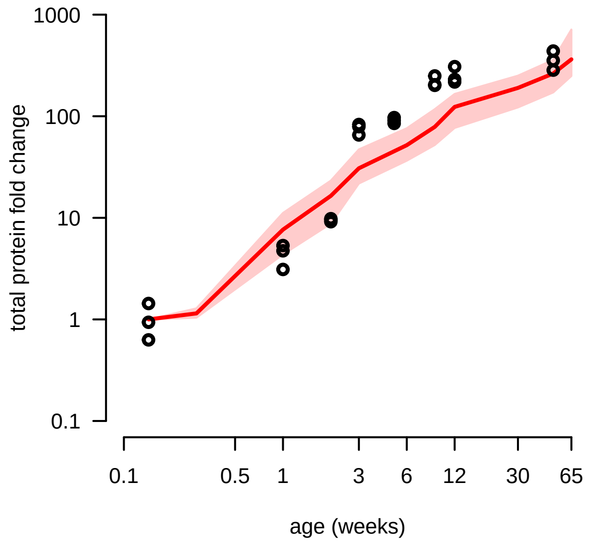

short pulse %H corrected log scale

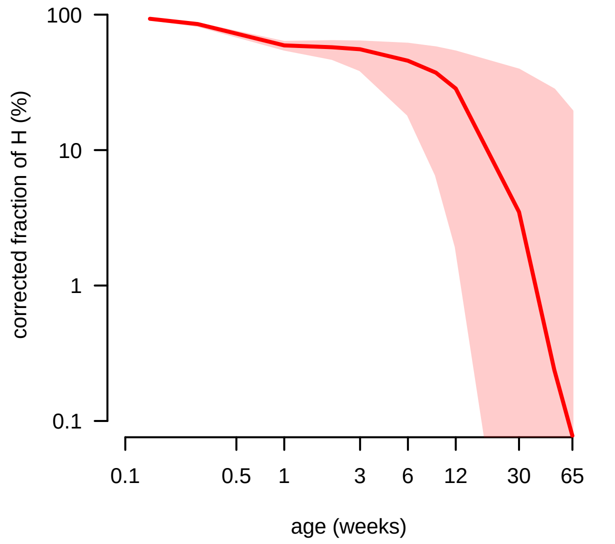

Q8R349

>sp|Q8R349|CDC16_MOUSE

Cell division cycle protein 16 homolog

GN=Cdc16

Med H1/2: 76.6 days

Q5 H1/2: 33.3 days

Q95 H1/2: 344.9 days

reliable model fit: no

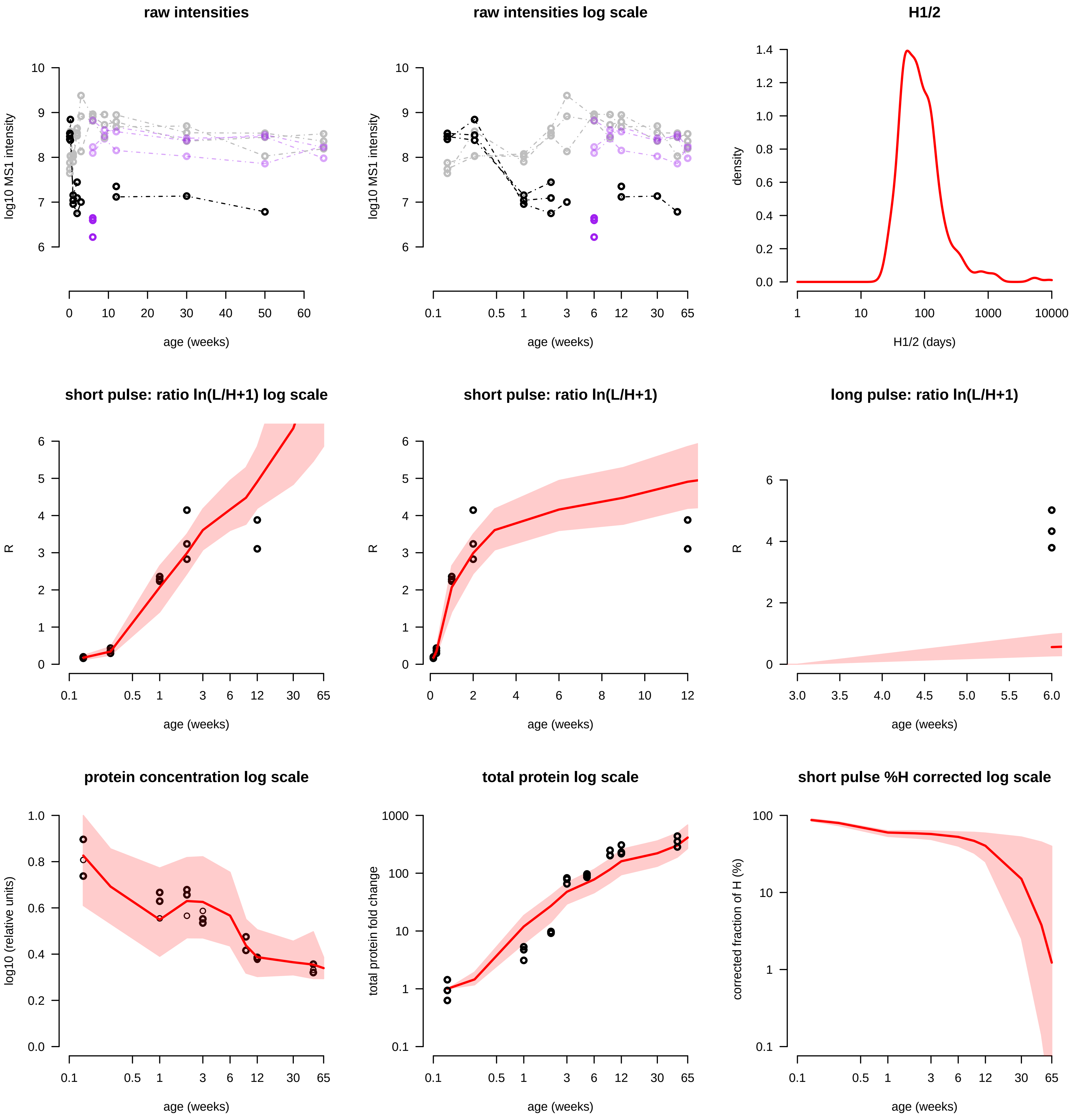

Q6WKZ8−2;Q6WKZ8−3

>sp|Q6WKZ8−2|UBR2_MOUSE

Isoform 2 of E3 ubiquitin−protein ligase UBR2

GN=Ubr2

Med H1/2: 8.7 days

Q5 H1/2: 7.2 days

Q95 H1/2: 11.8 days

reliable model fit: yes

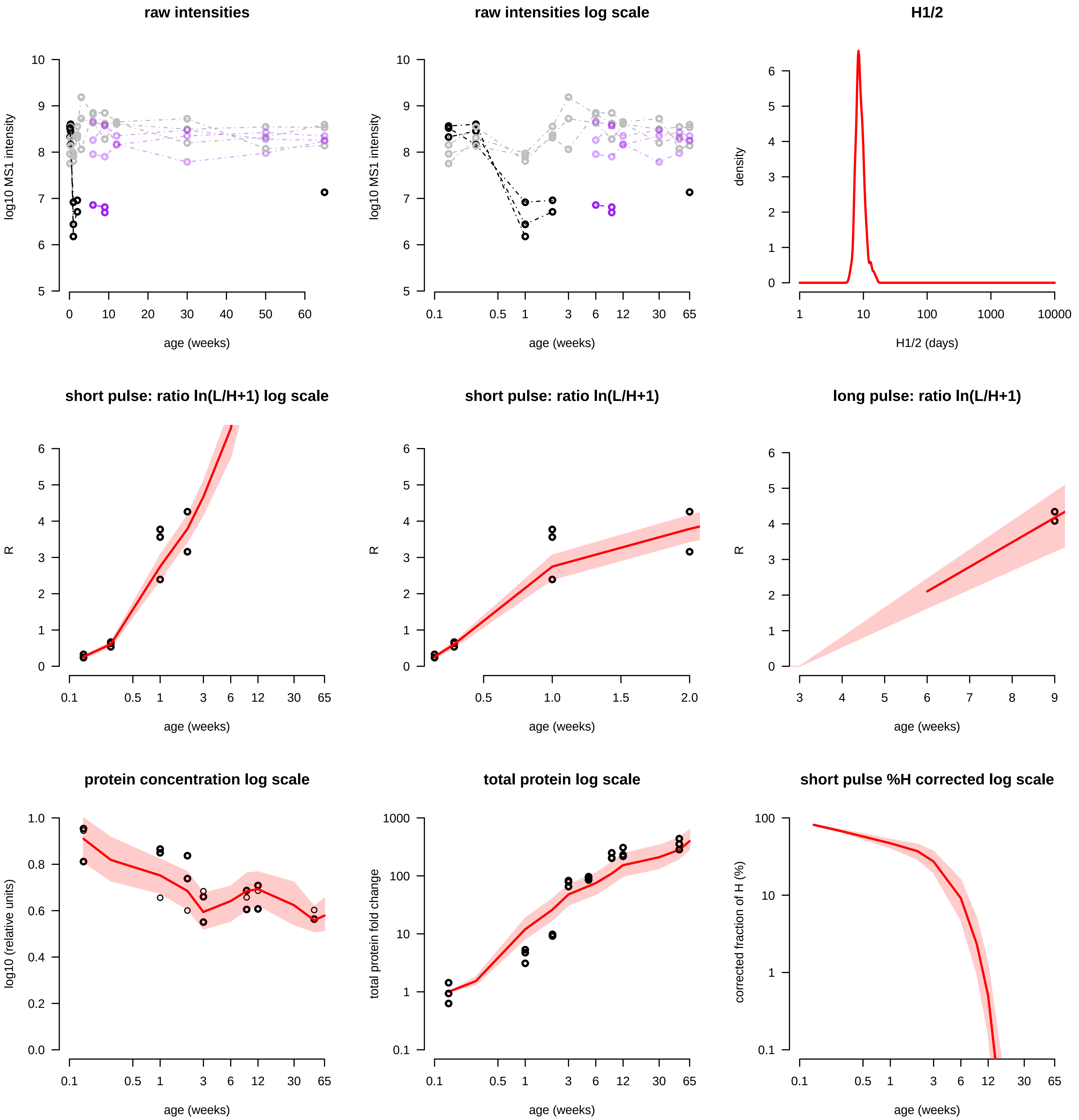

Q9Z172

>sp|Q9Z172|SUMO3_MOUSE

Small ubiquitin−related modifier 3

GN=Sumo3

Med H1/2: 21.1 days

Q5 H1/2: 10.2 days

Q95 H1/2: 154.1 days

reliable model fit: yes

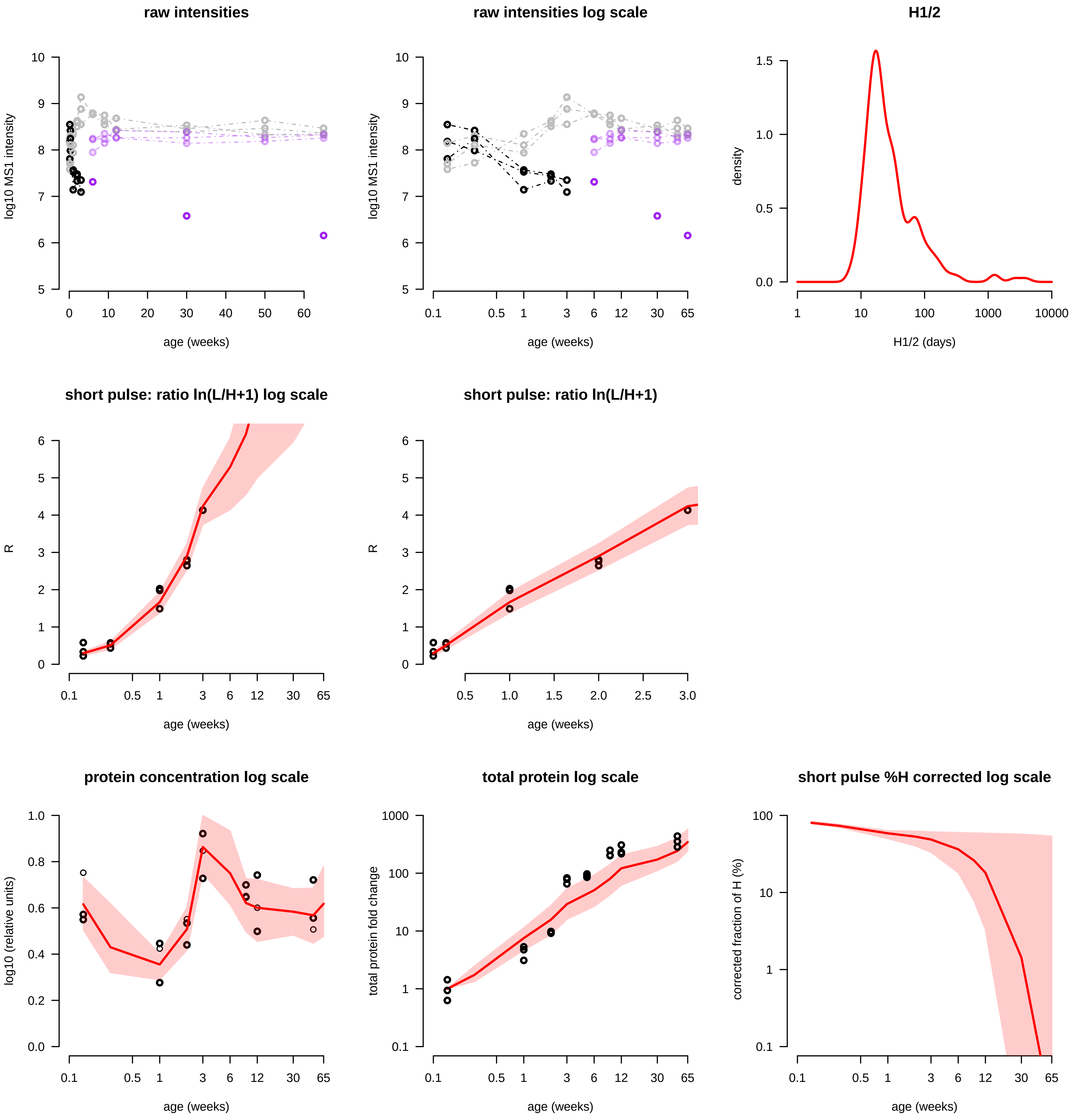

Q3V1T4;Q3V1T4−2

>sp|Q3V1T4|P3H1_MOUSE

Prolyl 3−hydroxylase 1

GN=P3h1

Med H1/2: 3.6 days

Q5 H1/2: 3 days

Q95 H1/2: 4.5 days

reliable model fit: yes

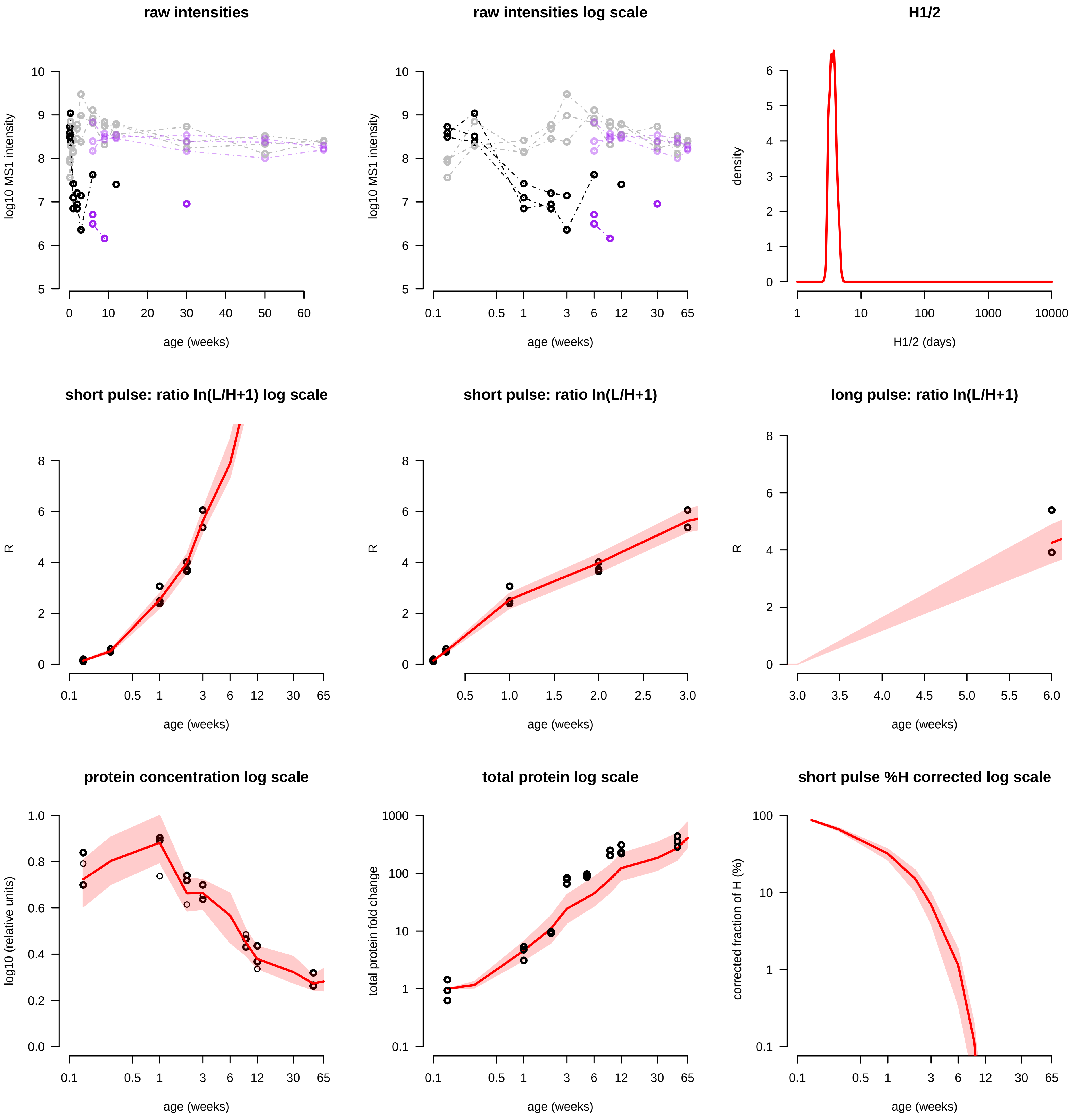

Q99KR7

>sp|Q99KR7|PPIF_MOUSE

Peptidyl−prolyl cis−trans isomerase F, mitochondrial

GN=Ppif

Med H1/2: 10.2 days

Q5   H1/2: 3.4 days

Q95 H1/2: 166.3 days

reliable model fit: yes

raw intensities

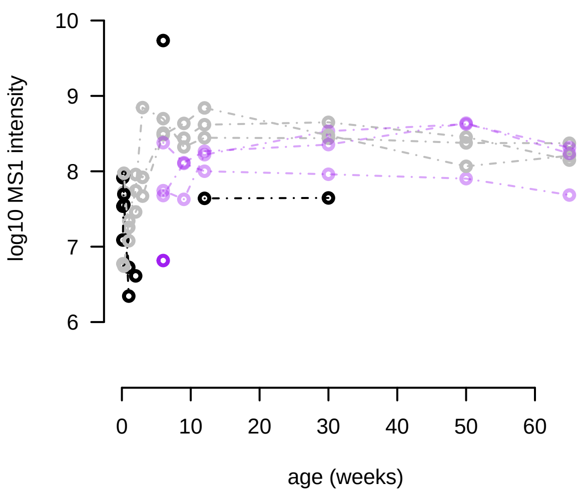

raw intensities log scale

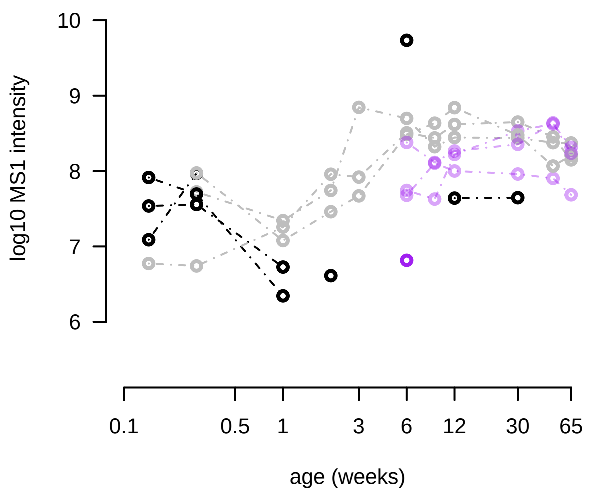

H1/2

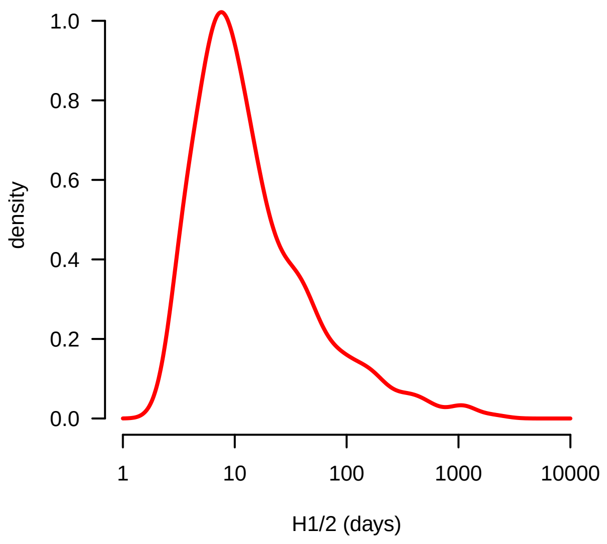

short pulse: ratio ln(L/H+1) log scale

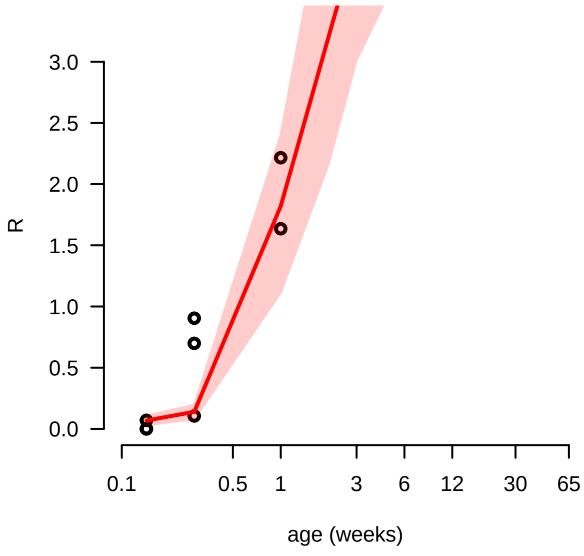

short pulse: ratio ln(L/H+1)

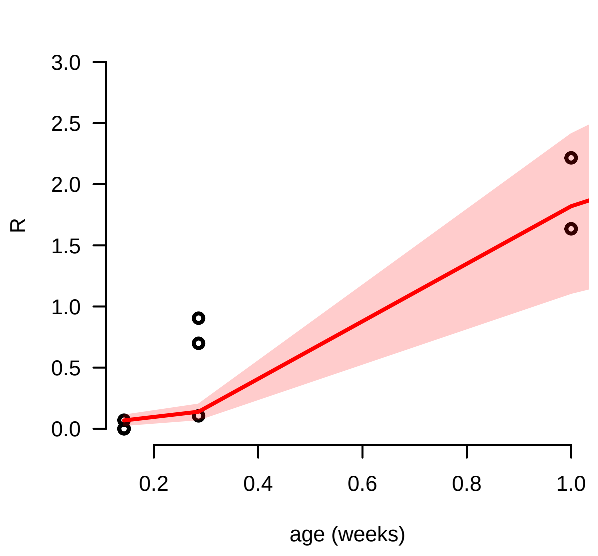

protein concentration log scale

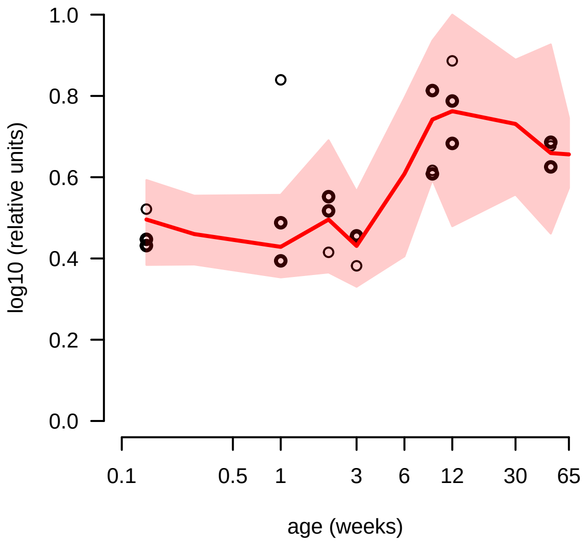

total protein log scale

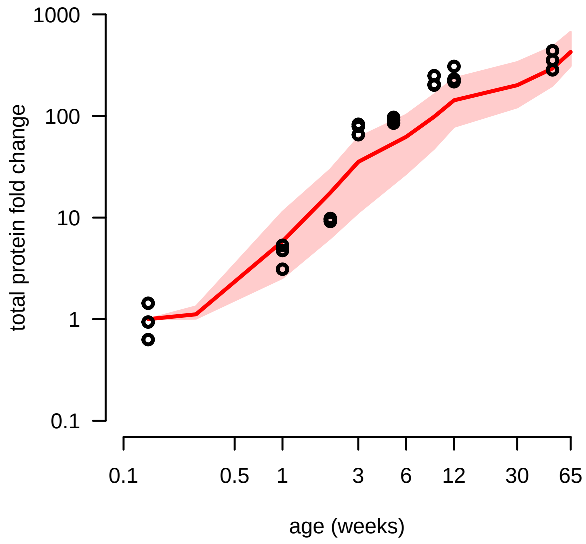

short pulse %H corrected log scale

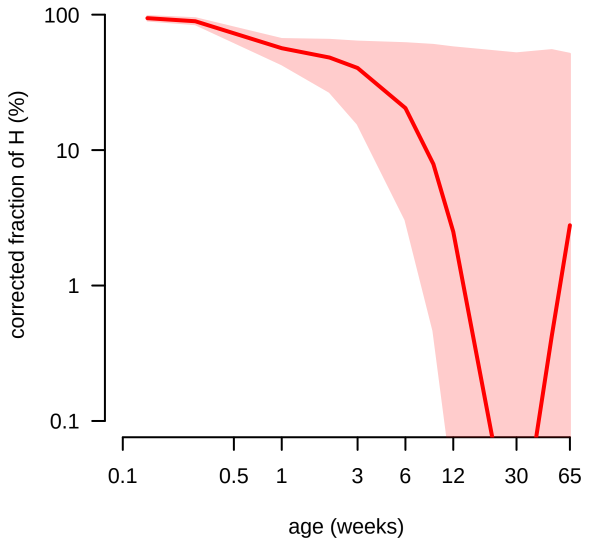

Q7TMY4;Q7TMY4−2

>sp|Q7TMY4|THOC7_MOUSE

THO complex subunit 7 homolog

GN=Thoc7

Med H1/2: 8.2 days

Q5 H1/2: 2.8 days

Q95 H1/2: 85.3 days

reliable model fit: yes

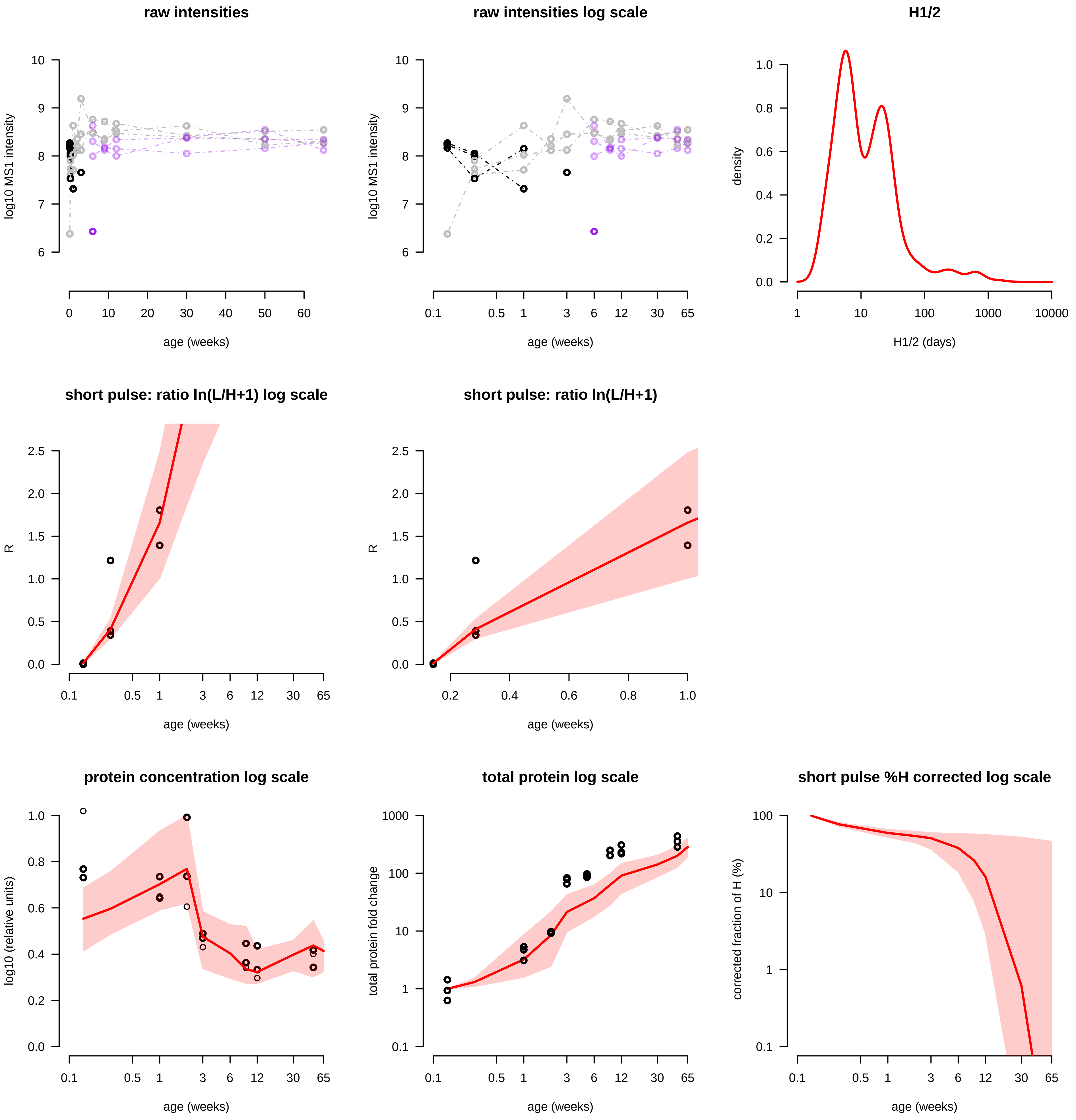

P13595;P13595−3;P13595−4

>sp|P13595|NCAM1_MOUSE

Neural cell adhesion molecule 1

GN=Ncam1

Med H1/2: 4.8 days

Q5 H1/2: 2.7 days

Q95 H1/2: 27.5 days

reliable model fit: yes

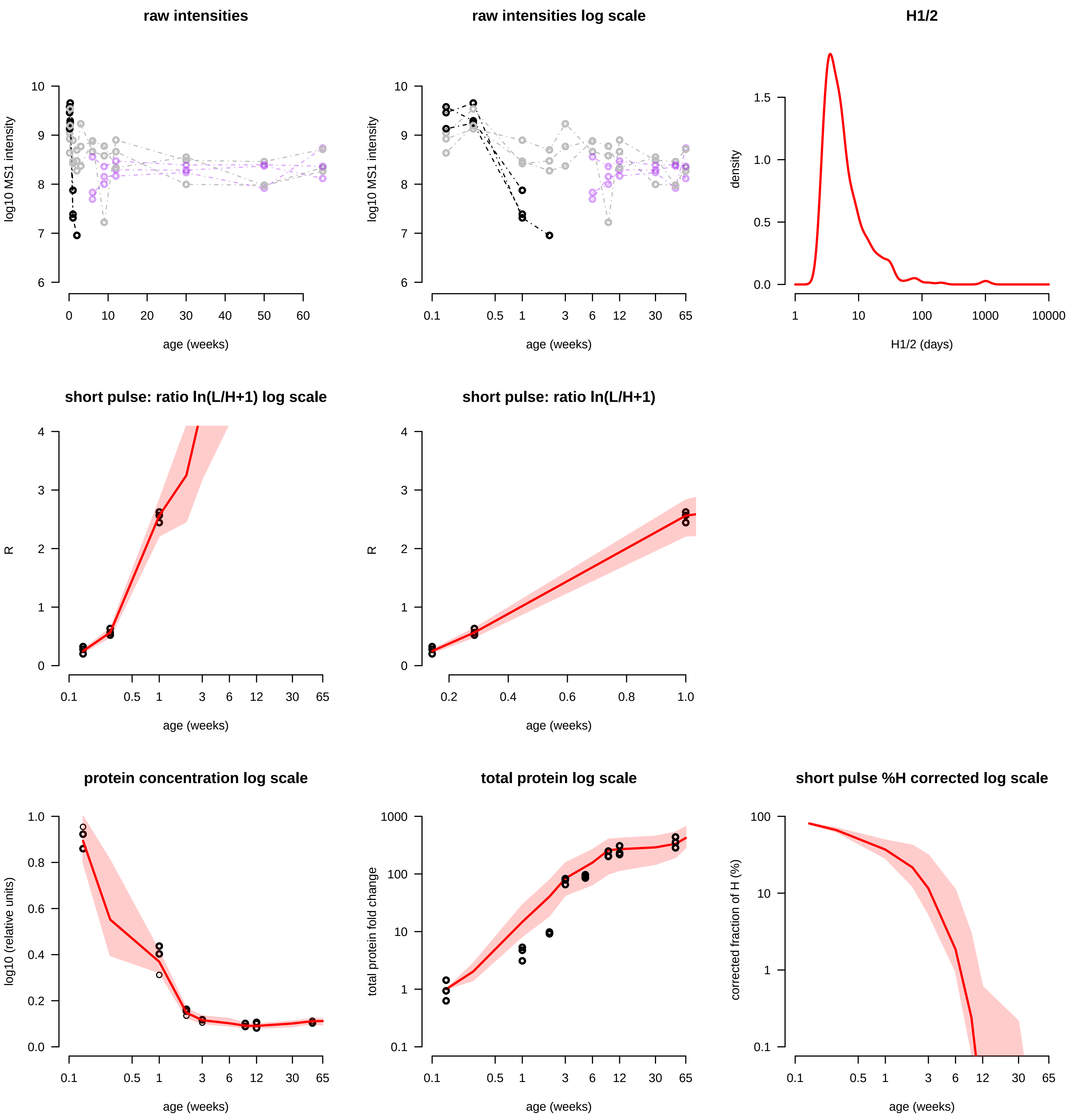

Q8BGR9

>sp|Q8BGR9|UBCP1_MOUSE

Ubiquitin−like domain−containing CTD phosphatase 1

GN=Ublcp1

Med H1/2: 4.5 days

Q5 H1/2: 3.7 days

Q95 H1/2: 8.3 days

reliable model fit: yes

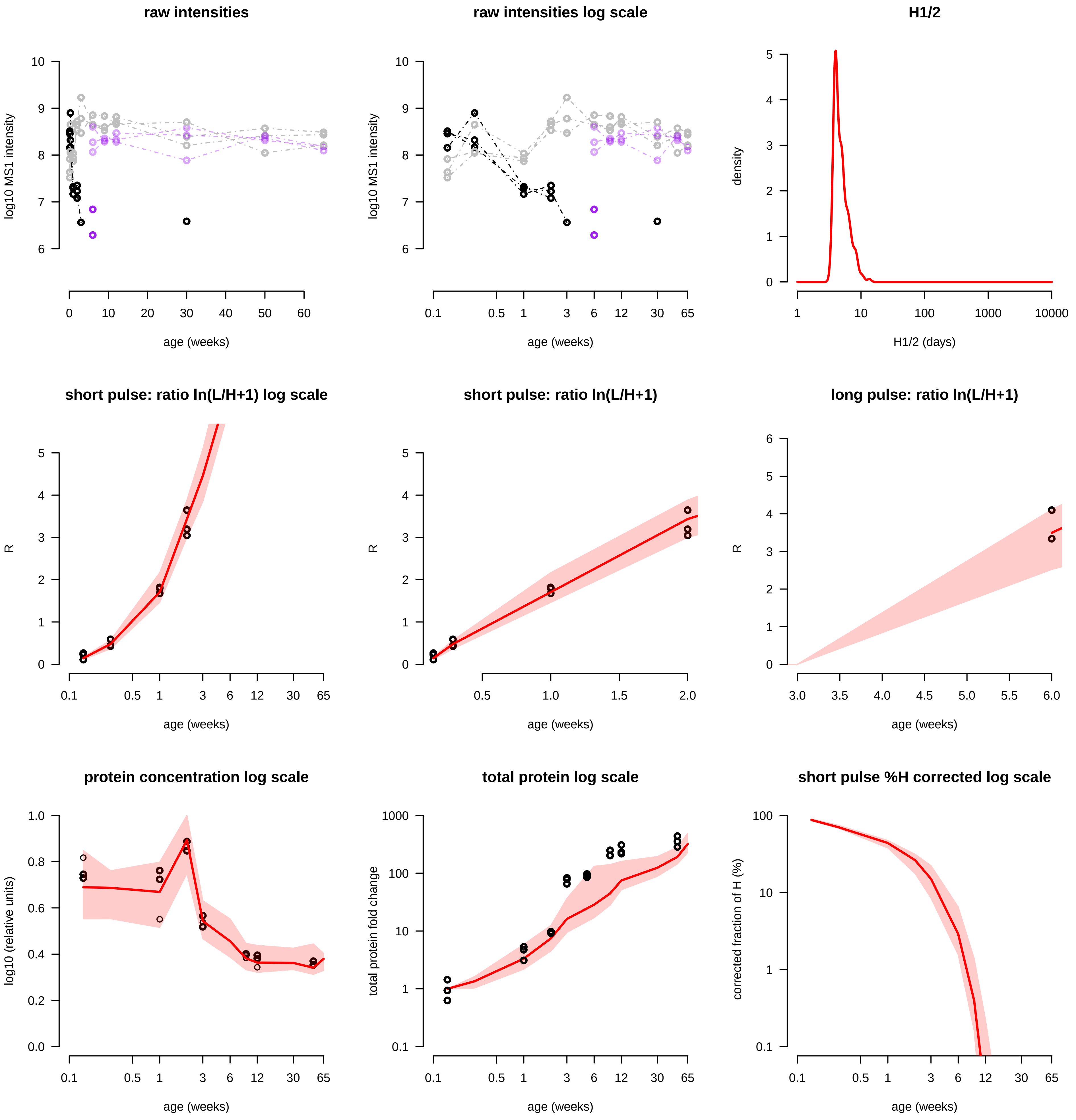

Q7TSQ8

>sp|Q7TSQ8|PDPR_MOUSE

Pyruvate dehydrogenase phosphatase regulatory subunit, mitochondrial

GN=Pdpr

Med H1/2: 8.1 days

Q5 H1/2: 2.8 days

Q95 H1/2: 78.7 days

reliable model fit: yes

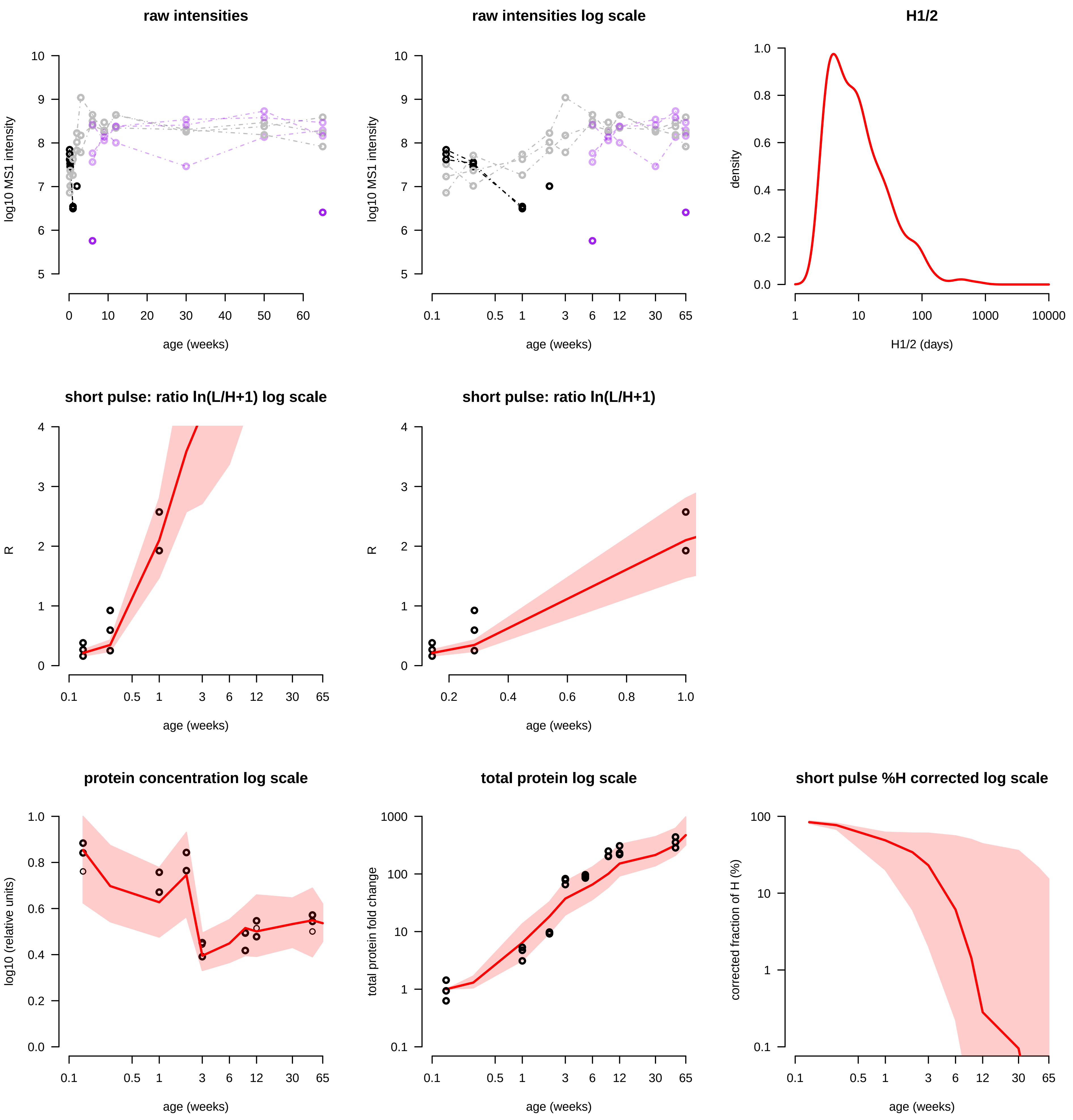

Q8CGA0

>sp|Q8CGA0|PPM1F_MOUSE

Protein phosphatase 1F

GN=Ppm1f

Med H1/2: 15.7 days

Q5 H1/2: 11.8 days

Q95 H1/2: 24.3 days

reliable model fit: yes

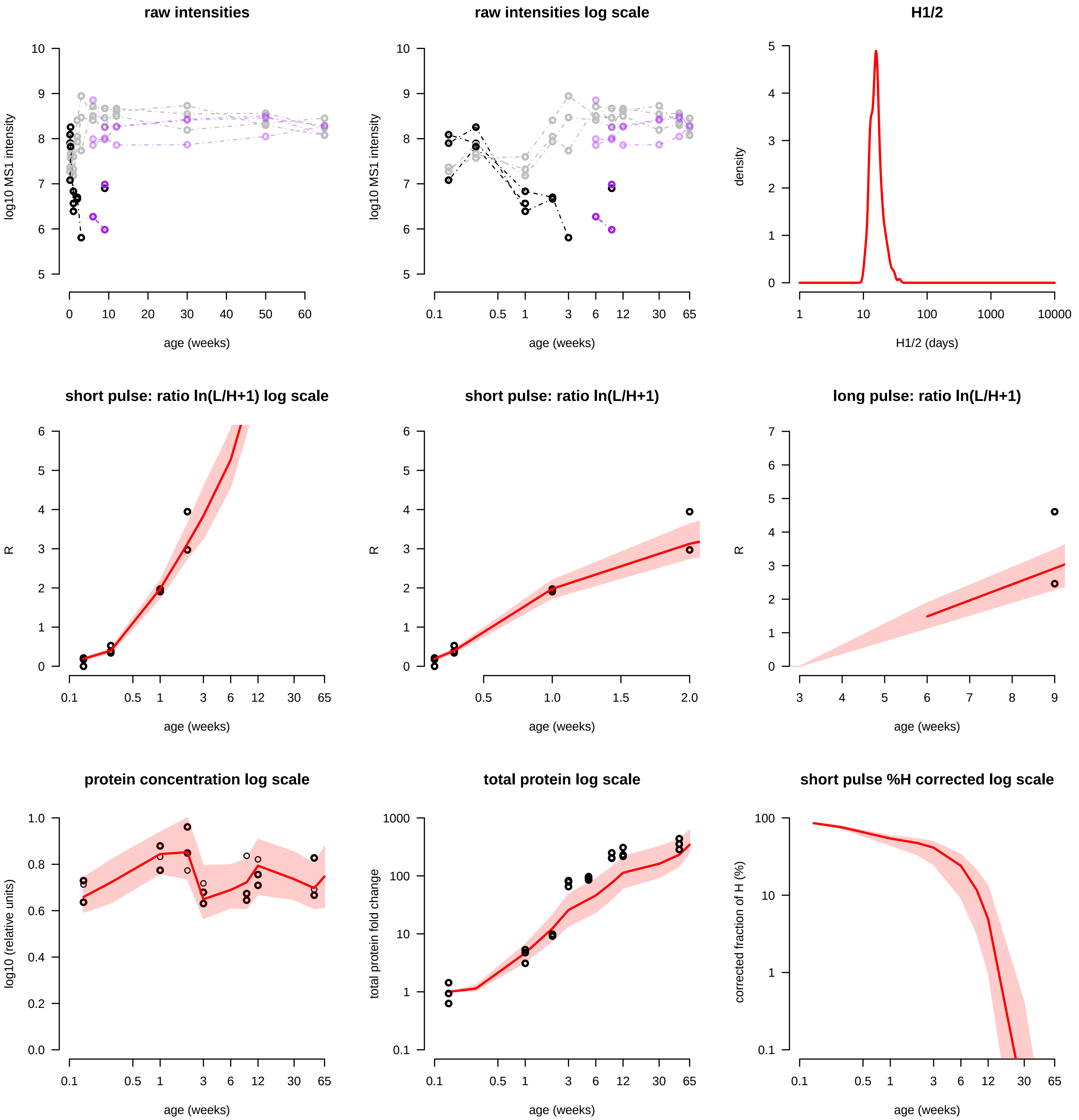

Q8BM72

>sp|Q8BM72|HSP13_MOUSE

Heat shock 70 kDa protein 13

GN=Hspa13

Med H1/2: 825.8 days

Q5 H1/2: 237.8 days

Q95 H1/2: 9949.1 days

reliable model fit: no

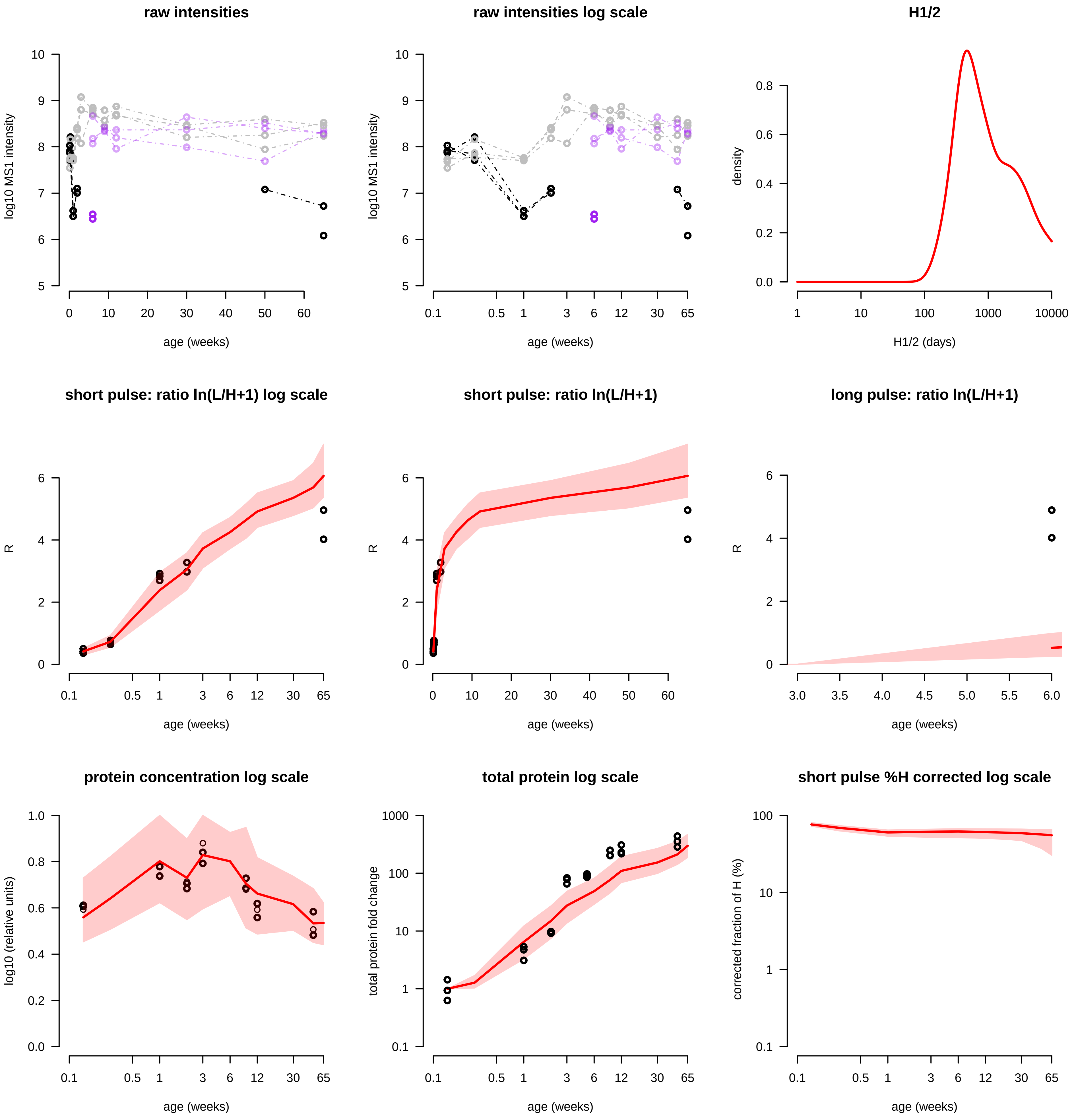

Q6NSR8

>sp|Q6NSR8|PEPL1_MOUSE

Probable aminopeptidase NPEPL1

GN=Npepl1

Med H1/2: 12.8 days

Q5 H1/2: 5.6 days

Q95 H1/2: 131.9 days

reliable model fit: yes

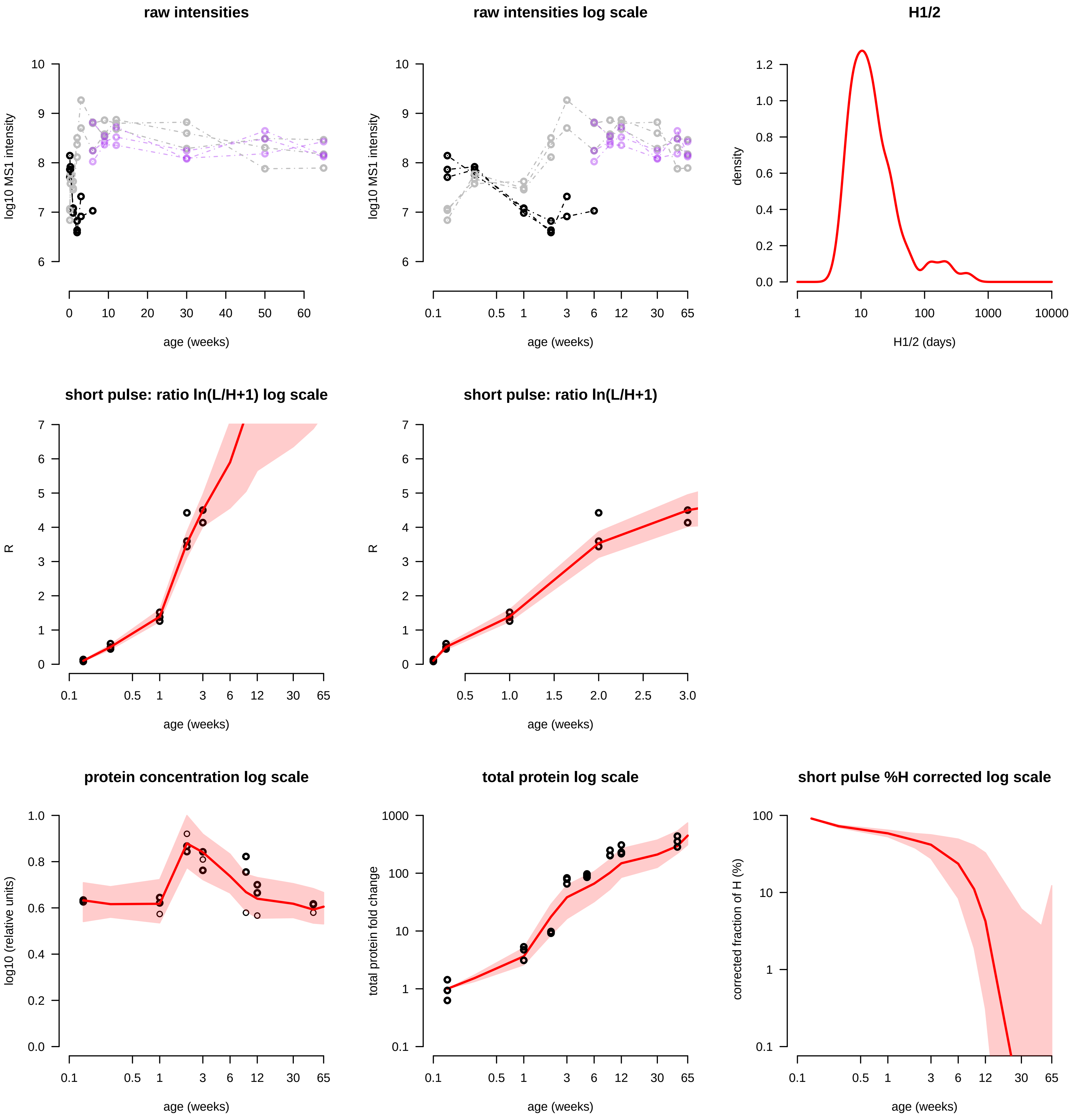

D3YYU8

>sp|D3YYU8|OBSL1_MOUSE

Obscurin−like protein 1

GN=Obsl1

Med H1/2: 8.6 days

Q5 H1/2: 3.9 days

Q95 H1/2: 31.7 days

reliable model fit: yes

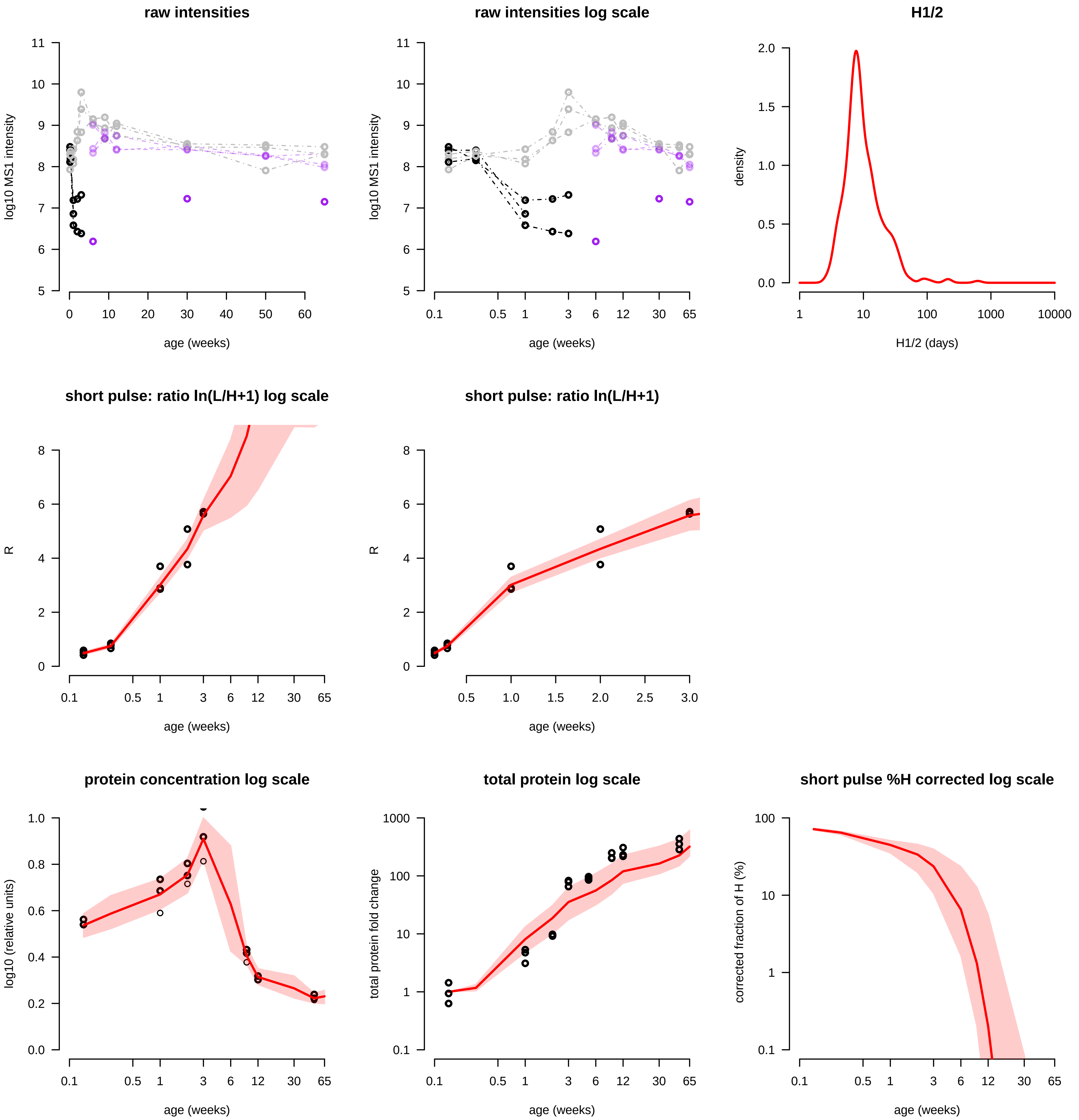

Q99K85

>sp|Q99K85|SERC_MOUSE

Phosphoserine aminotransferase

GN=Psat1

Med H1/2: 4 days

Q5 H1/2: 3.2 days

Q95 H1/2: 5.2 days

reliable model fit: yes

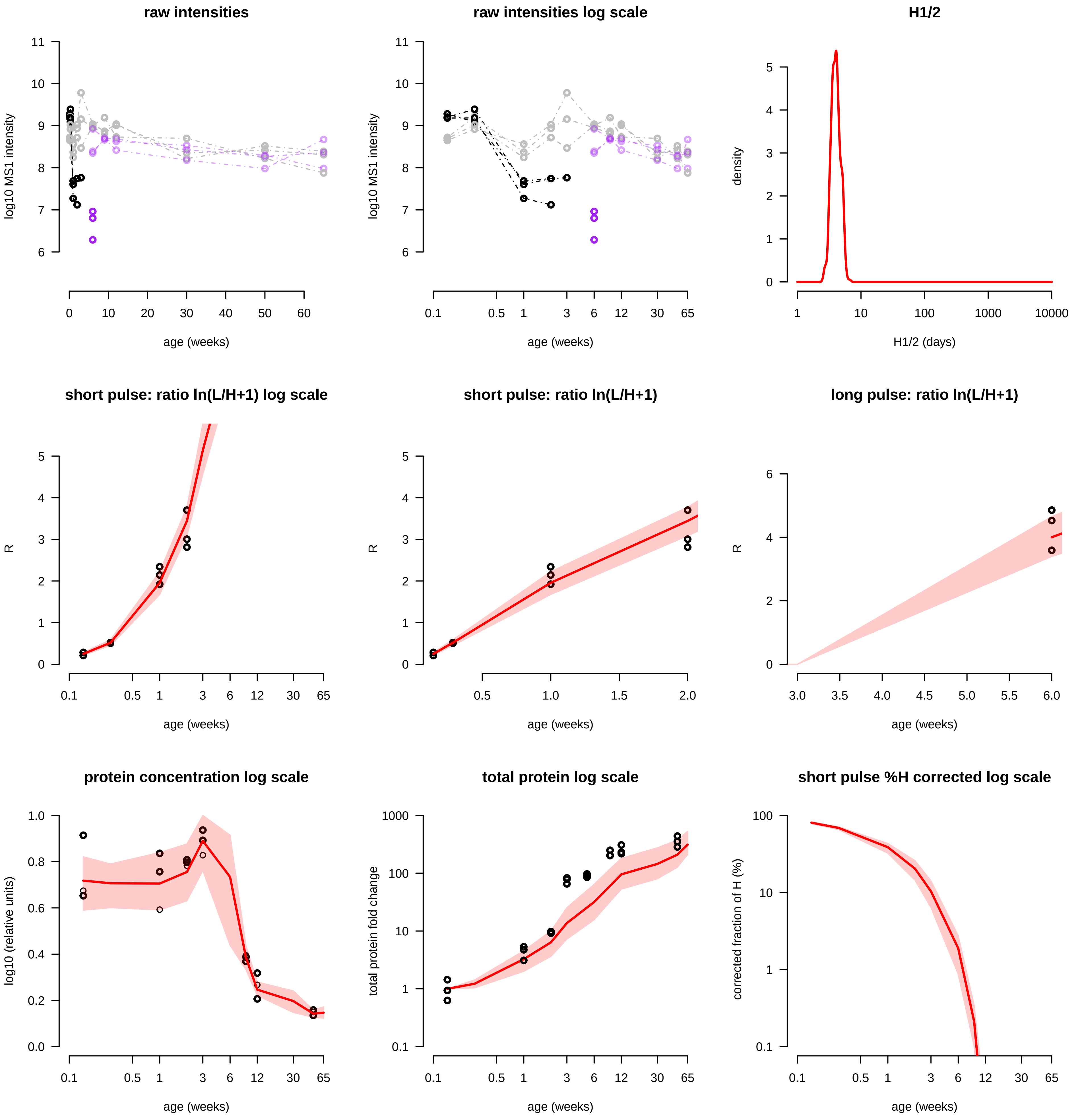

Q9R0I7

>sp|Q9R0I7|YLPM1_MOUSE

YLP motif−containing protein 1

GN=Ylpm1

Med H1/2: 4.3 days

Q5 H1/2: 2.7 days

Q95 H1/2: 19.8 days

reliable model fit: yes

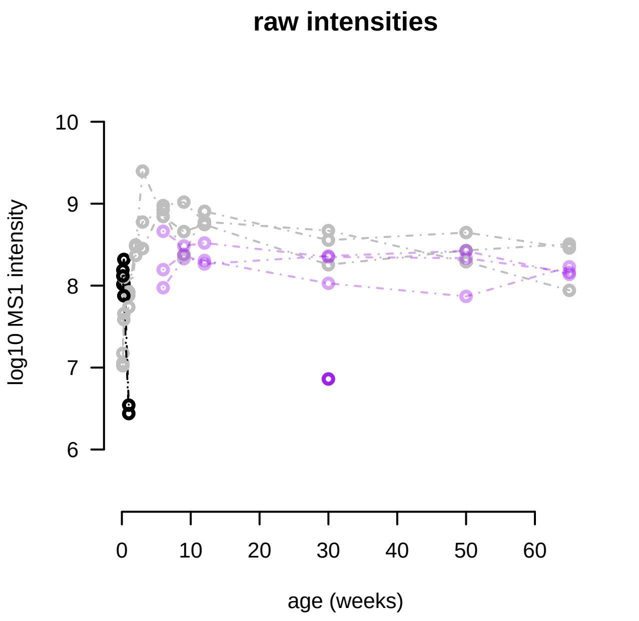

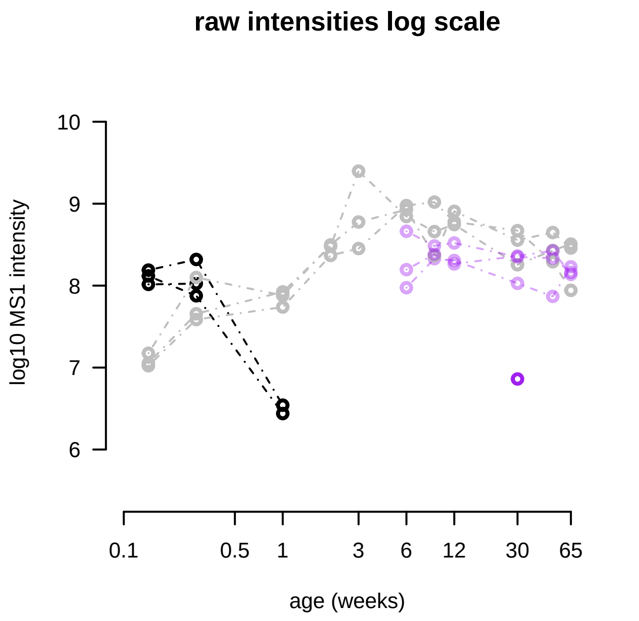

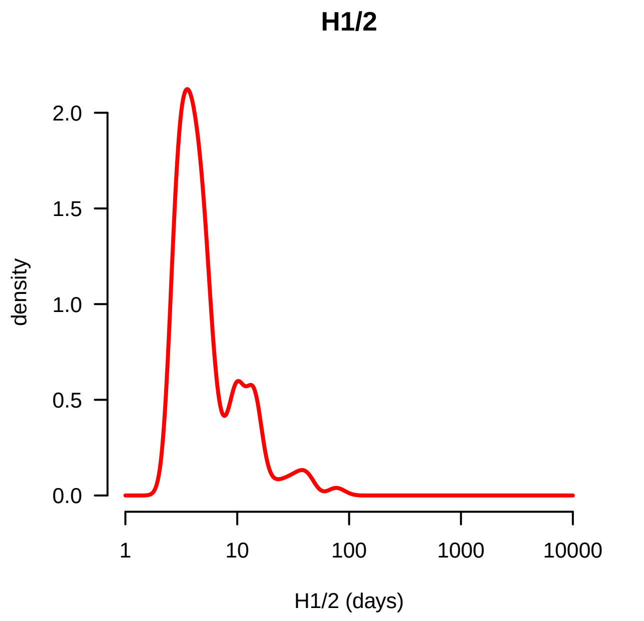

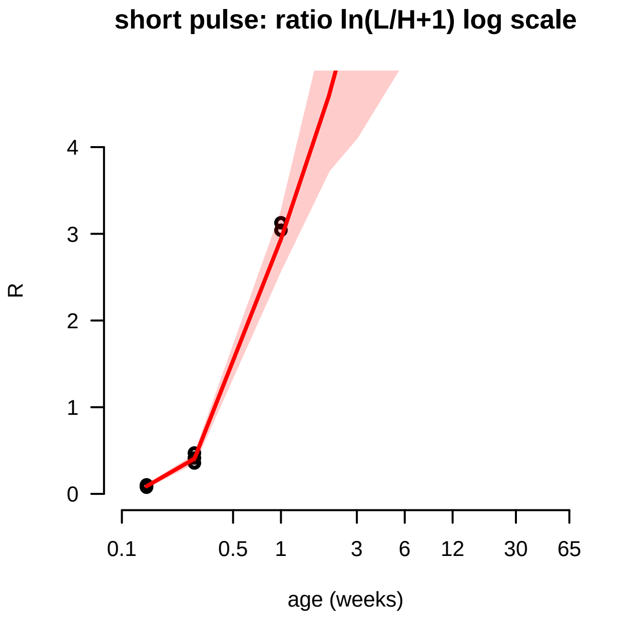

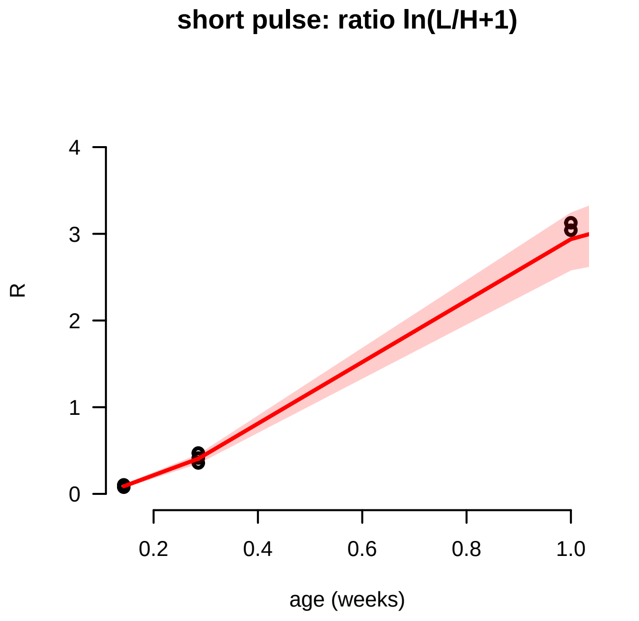

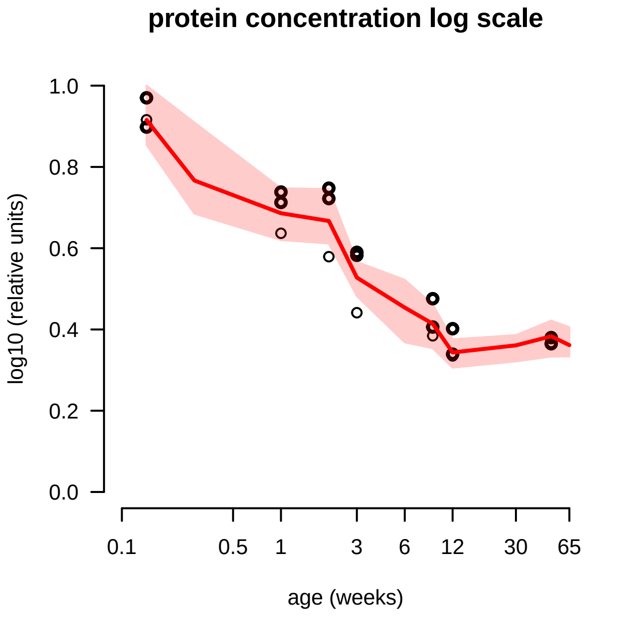

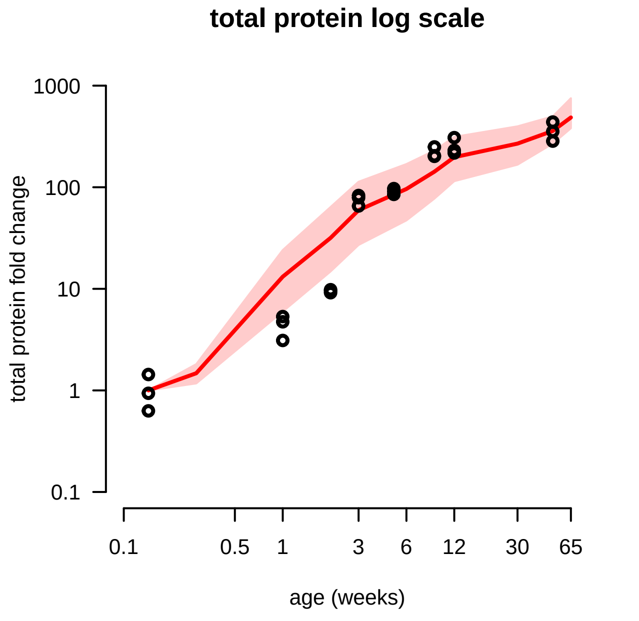

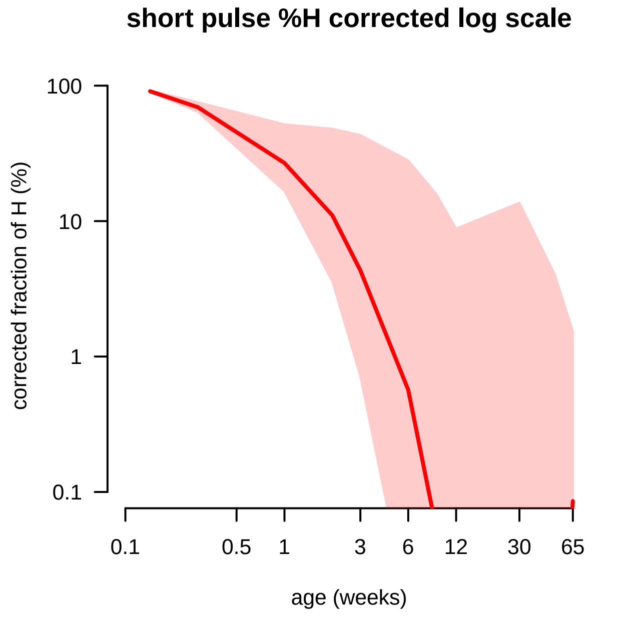

Q8BGT7

>sp|Q8BGT7|SPF30_MOUSE

Survival of motor neuron−related−splicing factor 30

GN=Smndc1

Med H1/2: 4.4 days

Q5 H1/2: 3.3 days

Q95 H1/2: 6.3 days

reliable model fit: yes

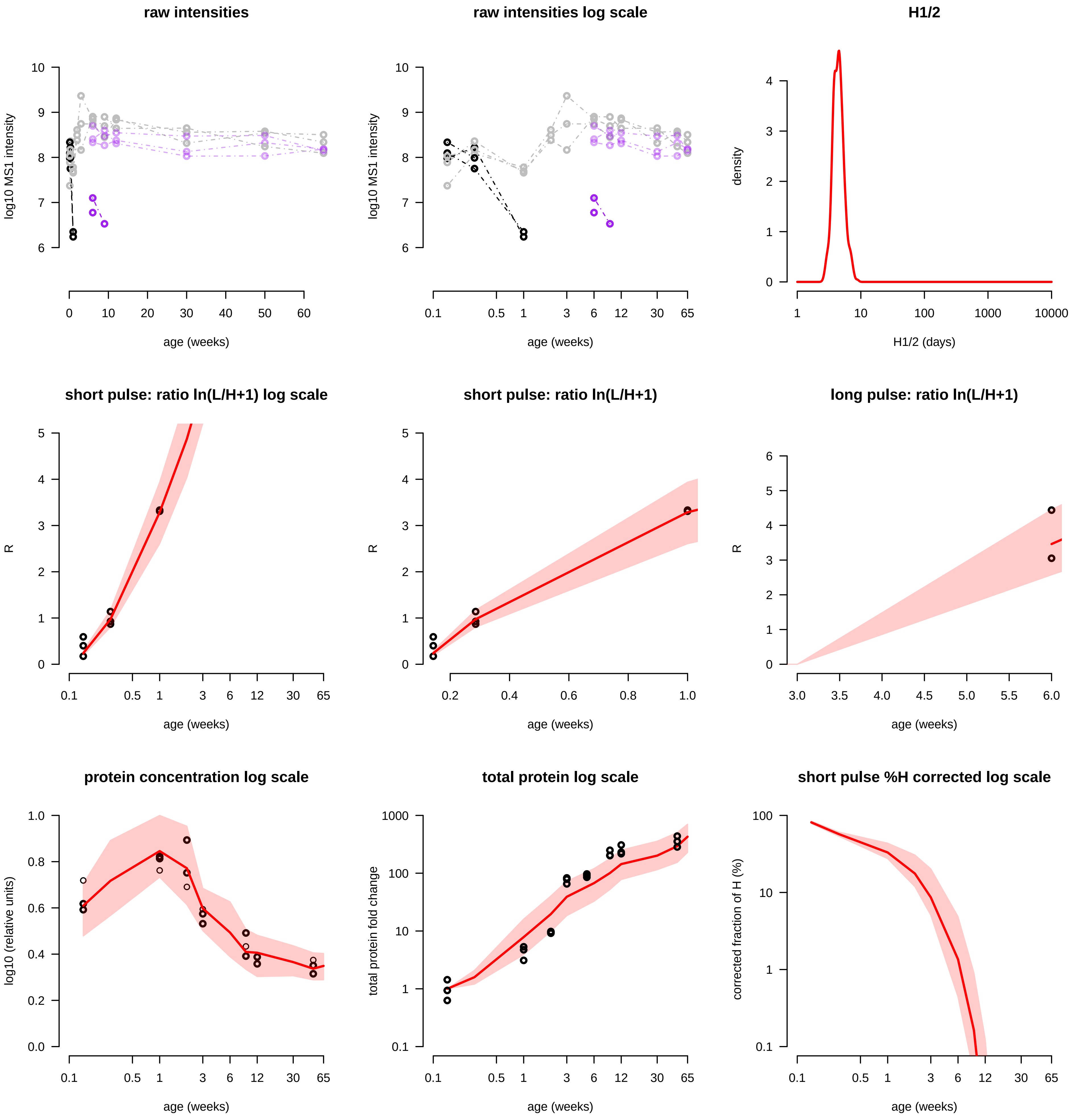

A6H630;A6H630−2;A6H630−4

>sp|A6H630|ARMT1_MOUSE

Damage−control phosphatase ARMT1

GN=Armt1

Med H1/2: 18.3 days

Q5 H1/2: 7.7 days

Q95 H1/2: 92.1 days

reliable model fit: yes

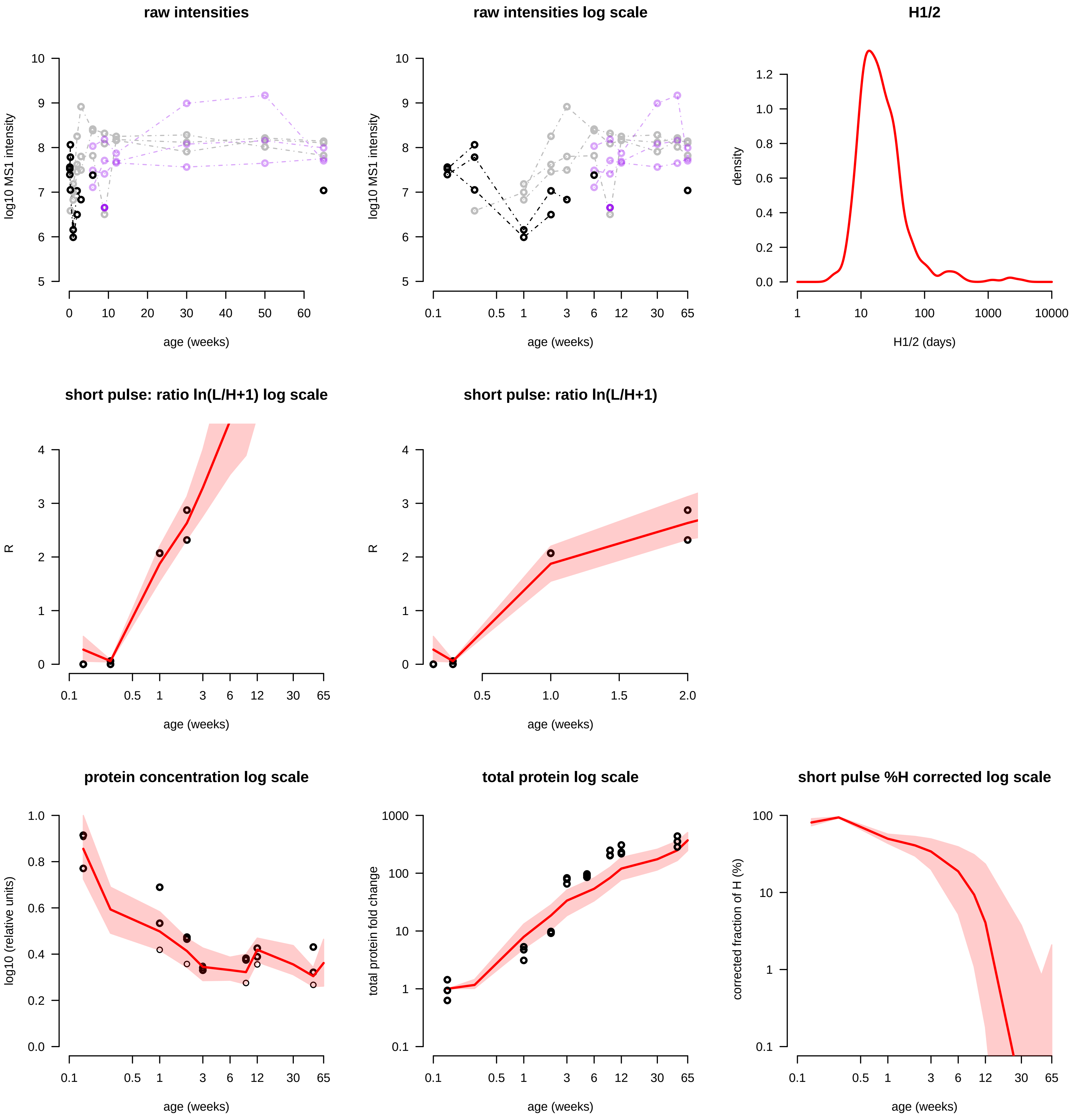

Q08460−5;Q08460−3;Q08460;Q08460−2;Q08460−4

>sp|Q08460−5|KCMA1_MOUSE

Isoform 5 of Calcium−activated potassium channel subunit alpha−1

GN=Kcnma1

Med H1/2: 3645.5 days

Q5 H1/2: 909.1 days

Q95 H1/2: 55103.6 days

reliable model fit: yes

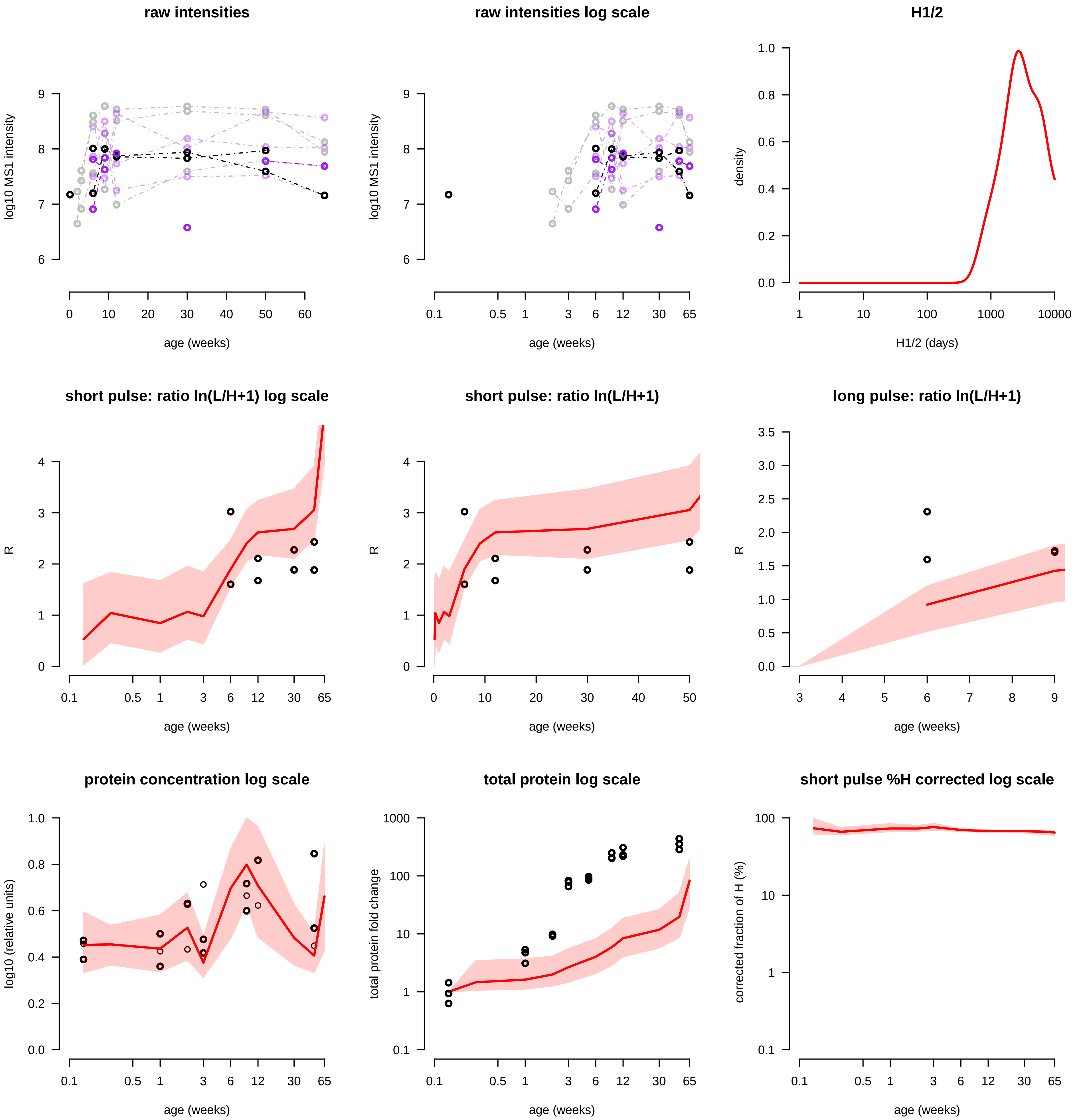

Q6P2B1;Q6P2B1−2

>sp|Q6P2B1|TNPO3_MOUSE

Transportin−3

GN=Tnpo3

Med H1/2: 3.5 days

Q5 H1/2: 2.7 days

Q95 H1/2: 5 days

reliable model fit: yes

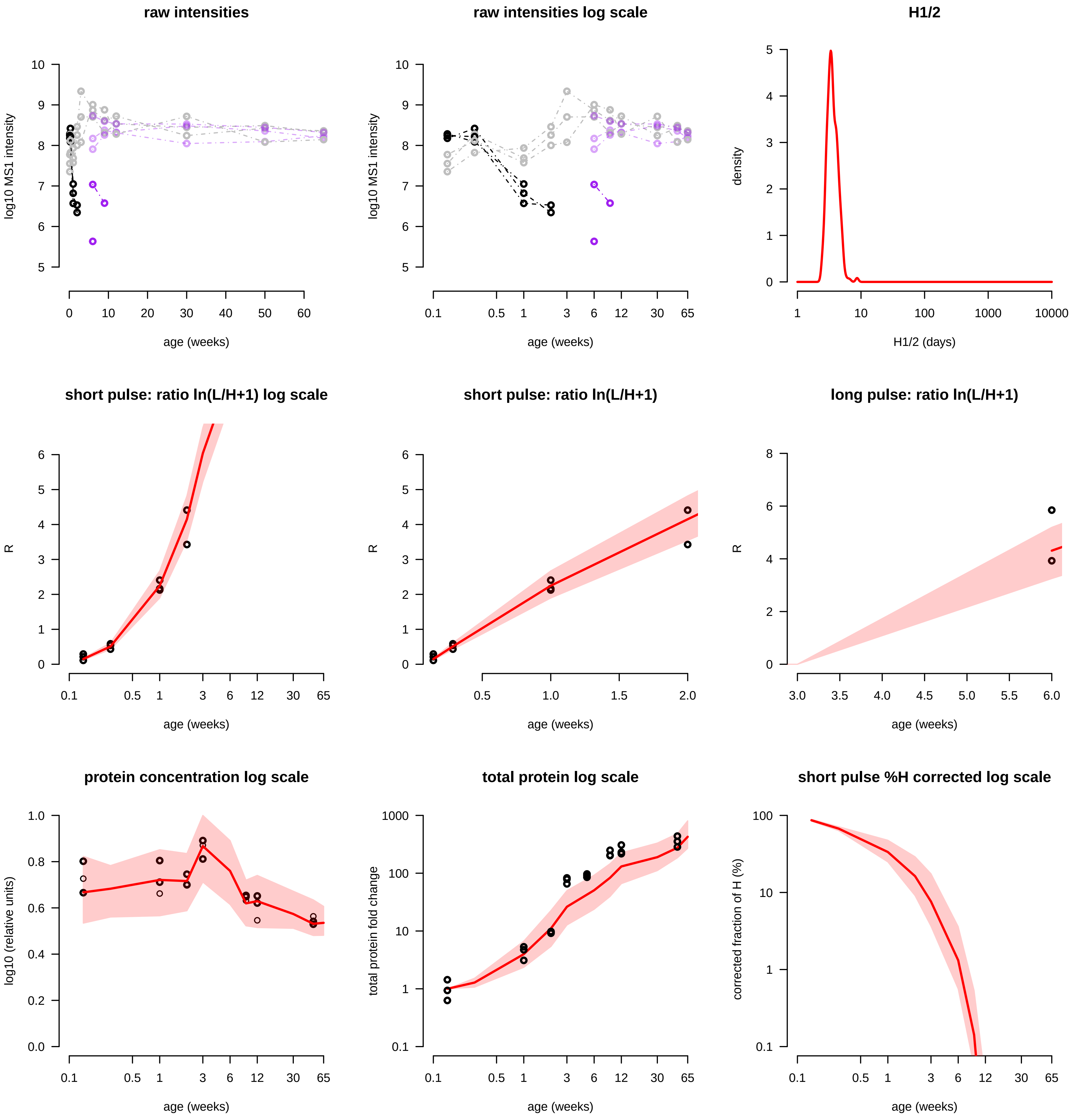

Q9WV91

>sp|Q9WV91|FPRP_MOUSE

Prostaglandin F2 receptor negative regulator

GN=Ptgfrn

Med H1/2: 6.4 days

Q5 H1/2: 2.8 days

Q95 H1/2: 42.5 days

reliable model fit: yes

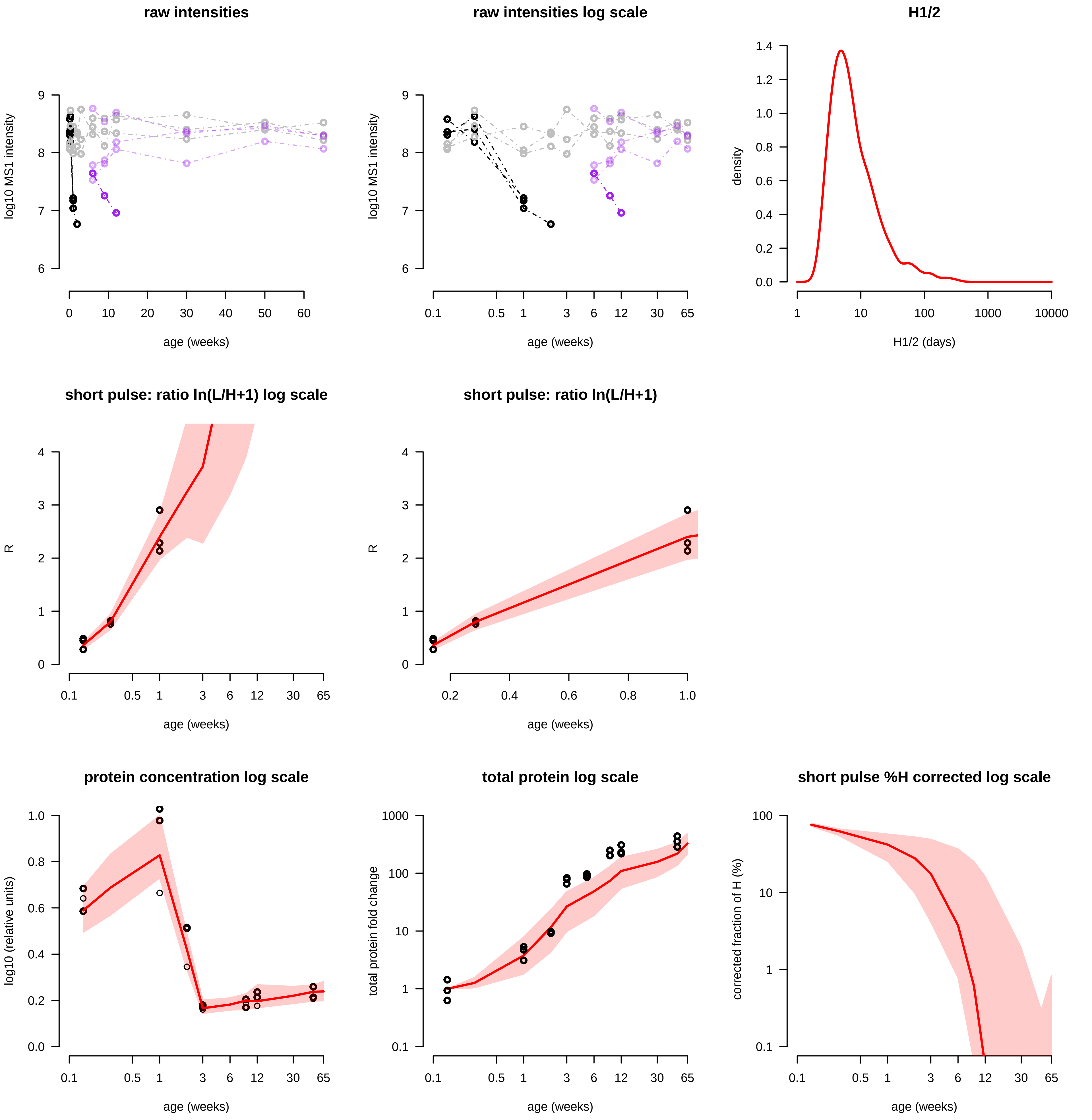

Q6P5G6

>sp|Q6P5G6|UBXN7_MOUSE

UBX domain−containing protein 7

GN=Ubxn7

Med H1/2: 10.9 days

Q5 H1/2: 5.6 days

Q95 H1/2: 37.2 days

reliable model fit: yes

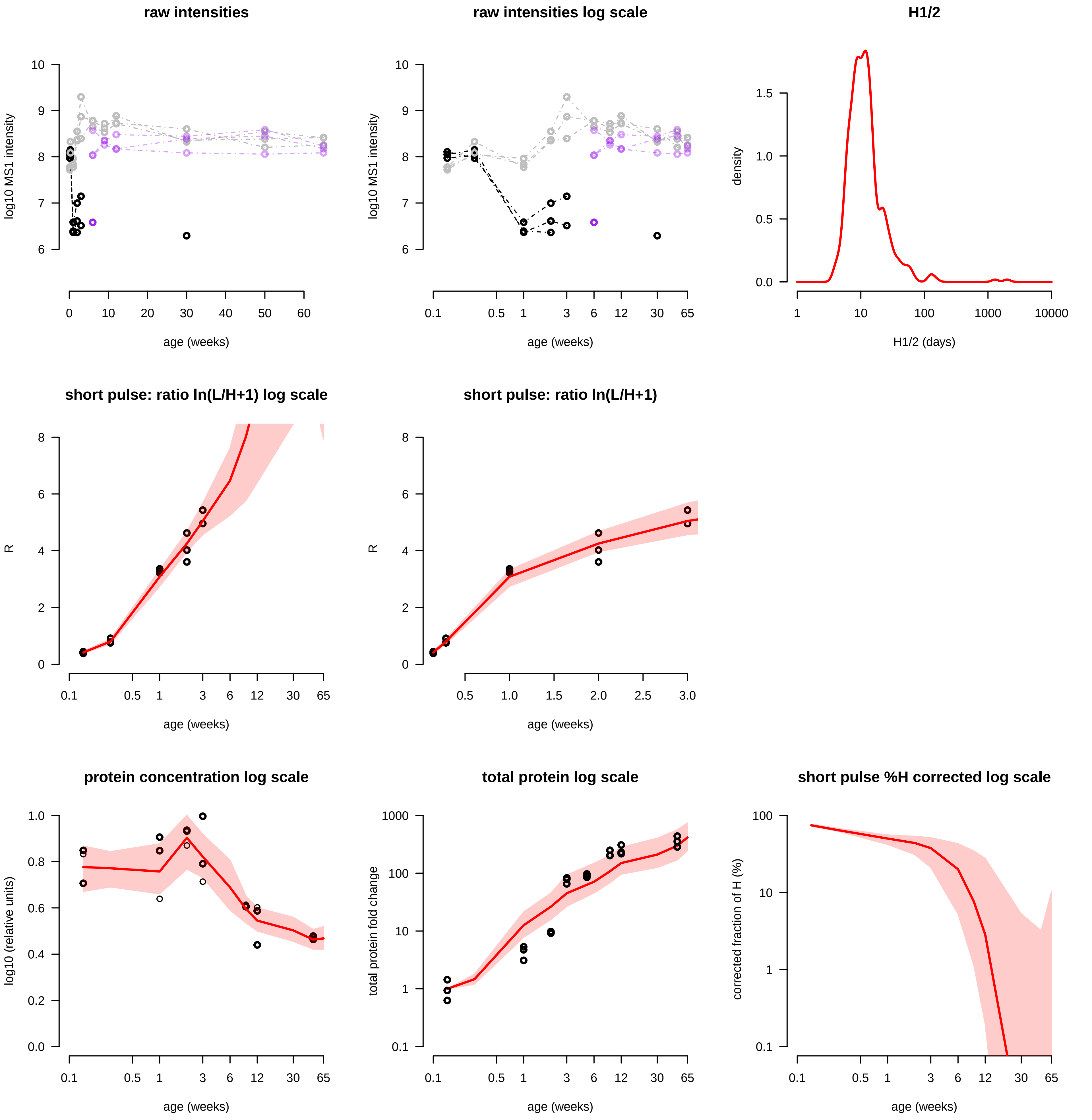

Q9CR67

>sp|Q9CR67|TMM33_MOUSE

Transmembrane protein 33

GN=Tmem33

Med H1/2: 6.8 days

Q5 H1/2: 5 days

Q95 H1/2: 10.6 days

reliable model fit: yes

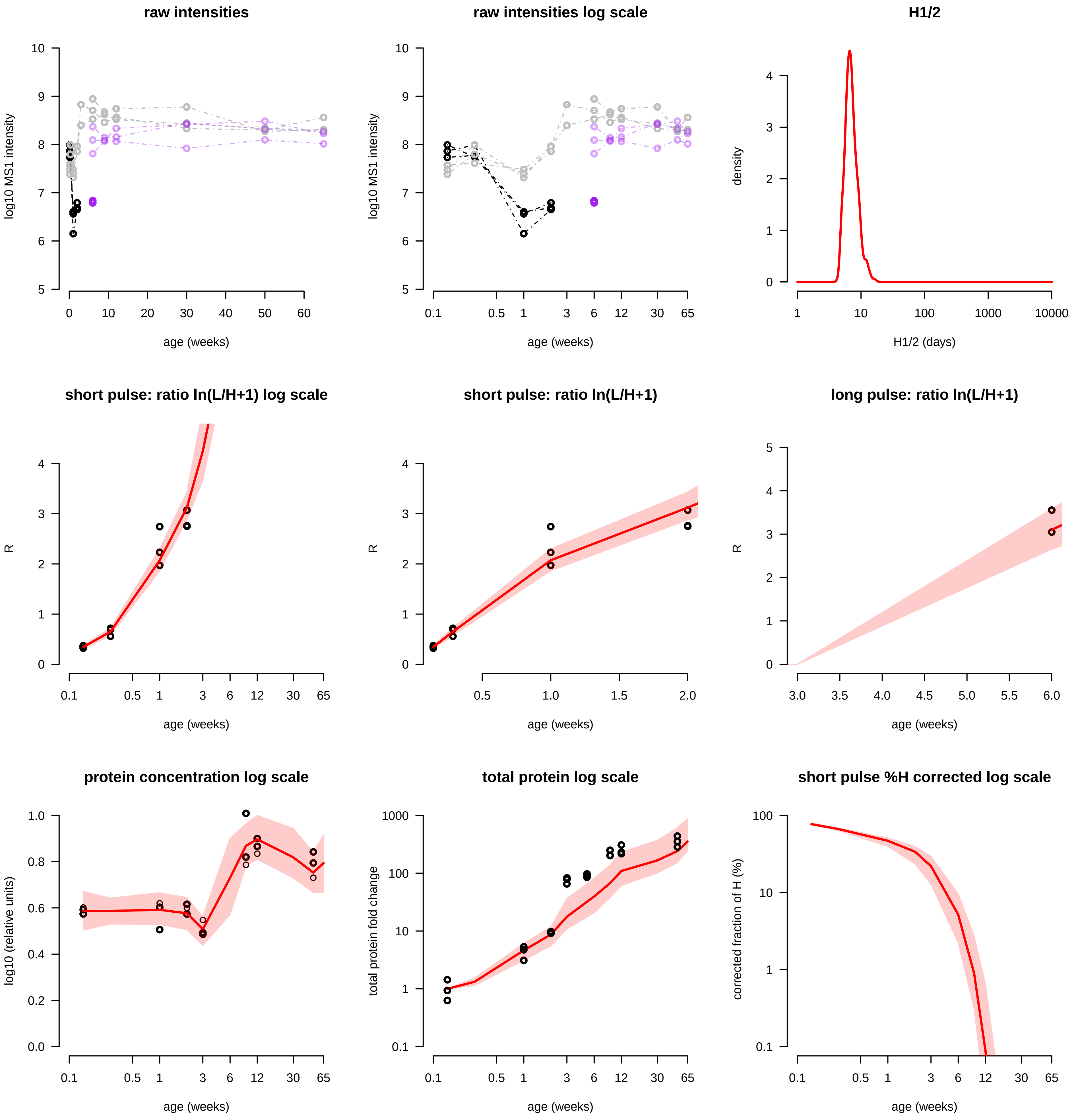

Q80W21

>sp|Q80W21|GSTM7_MOUSE

Glutathione S−transferase Mu 7

GN=Gstm7

Med H1/2: 7 days

Q5 H1/2: 3 days

Q95 H1/2: 27.3 days

reliable model fit: yes

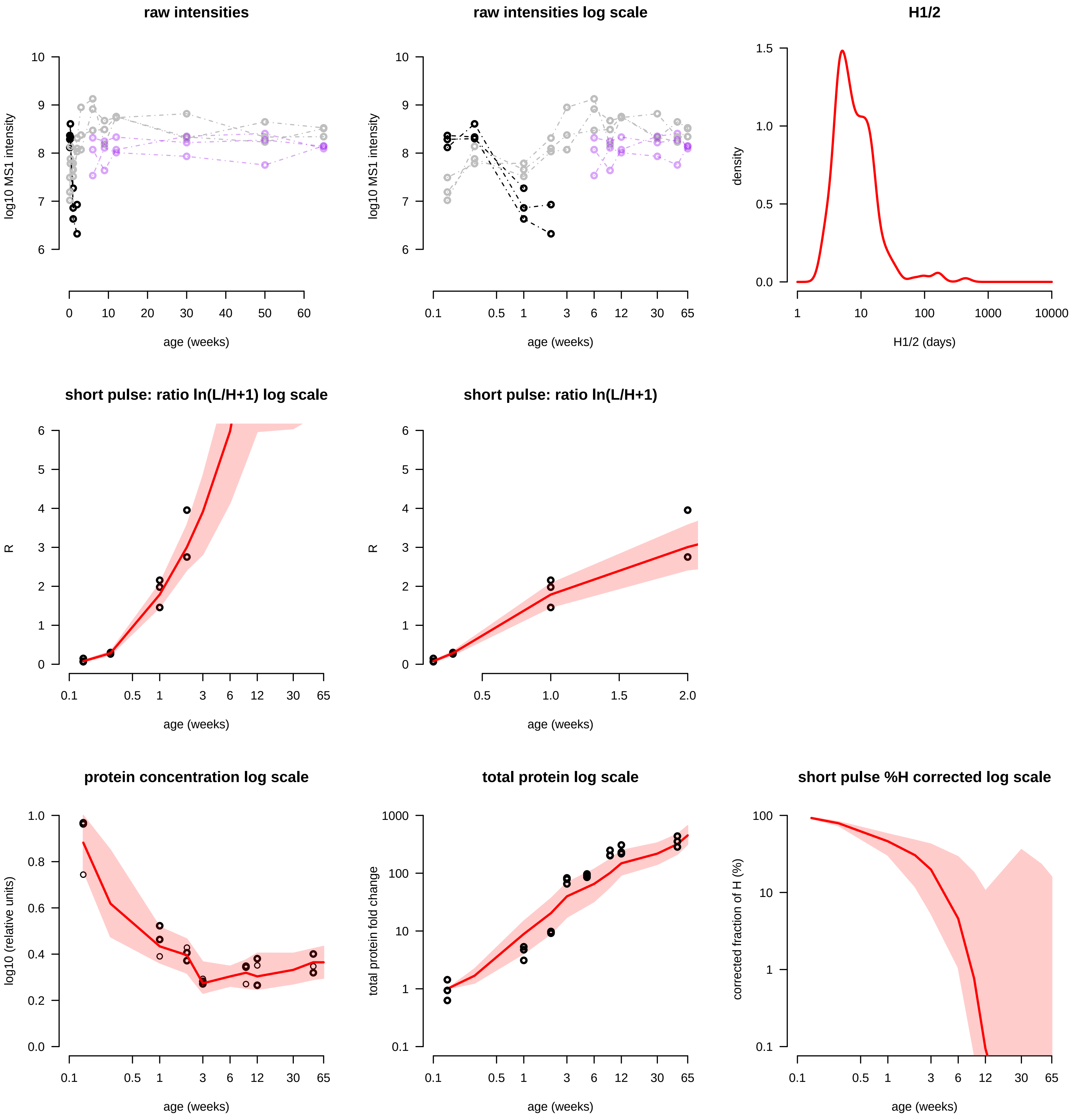

Q8QZY9

>sp|Q8QZY9|SF3B4_MOUSE

Splicing factor 3B subunit 4

GN=Sf3b4

Med H1/2: 3.9 days

Q5 H1/2: 2.8 days

Q95 H1/2: 5.8 days

reliable model fit: yes

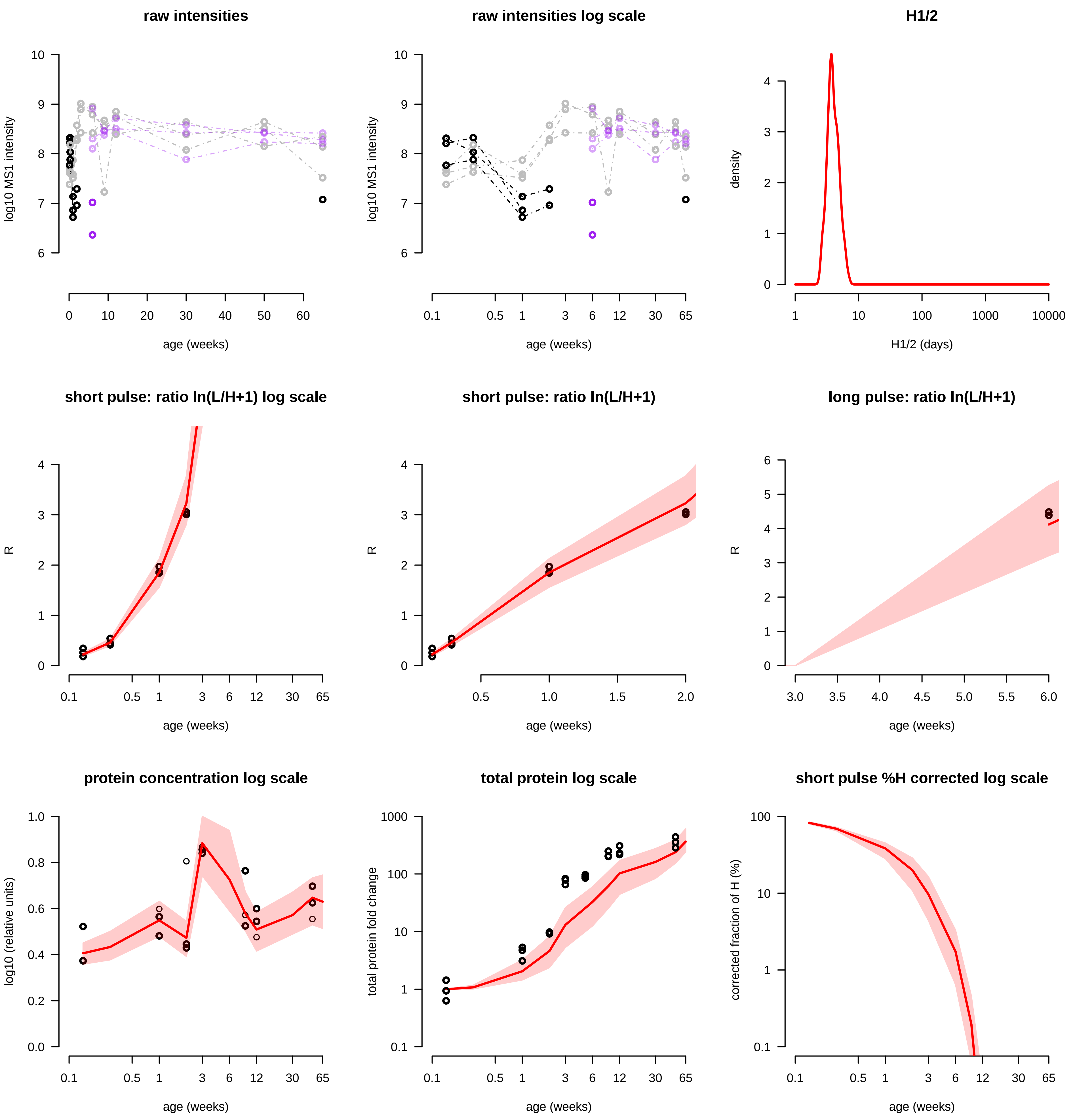

Q6B966

>sp|Q6B966|NAL14_MOUSE

NACHT, LRR and PYD domains−containing protein 14

GN=Nlrp14

Med H1/2: 10.6 days

Q5 H1/2: 8 days

Q95 H1/2: 13.7 days

reliable model fit: yes

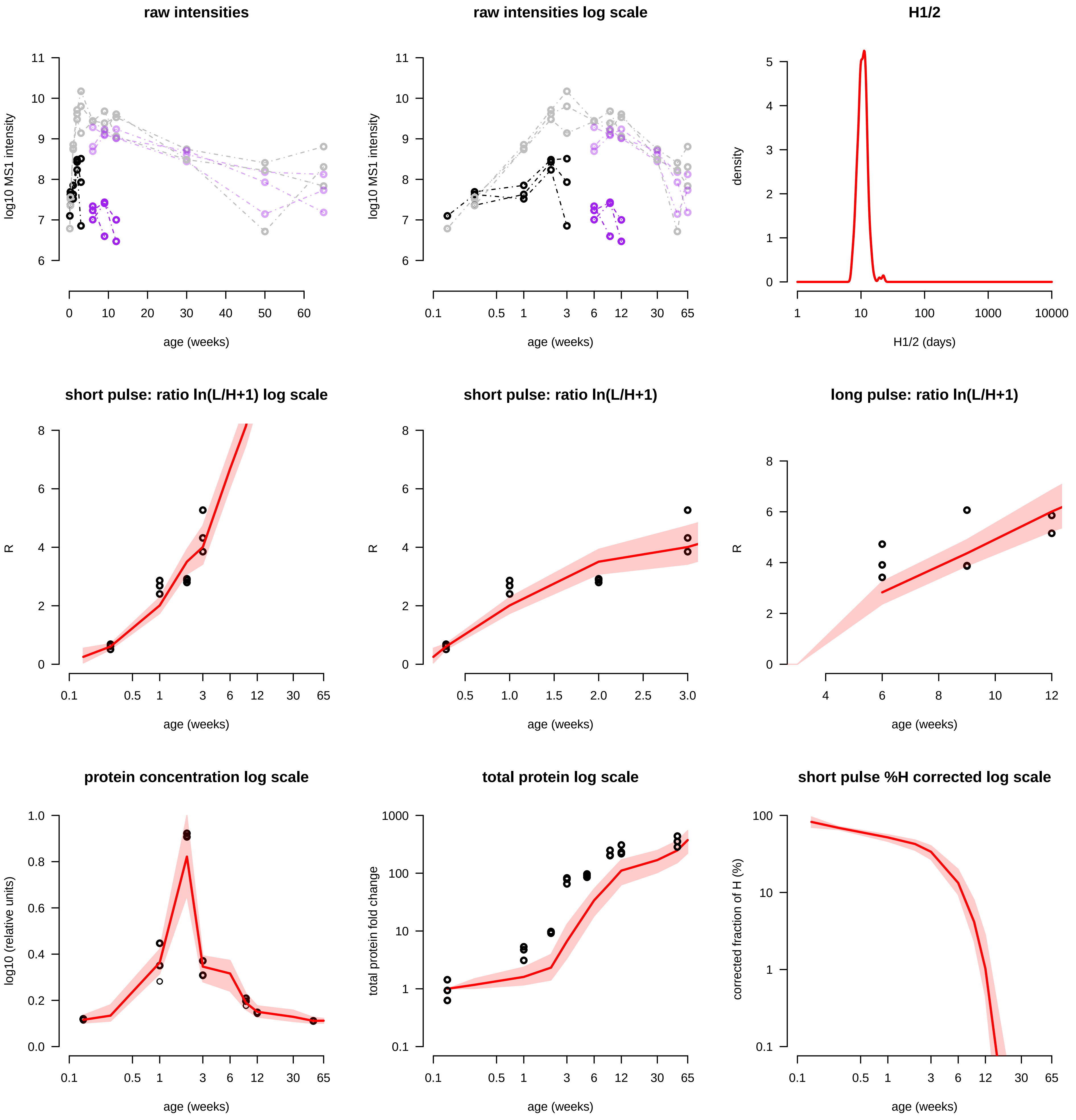

Q8BTY8;Q8BTY8−2;Q8BTY8−3

>sp|Q8BTY8|SCFD2_MOUSE

Sec1 family domain−containing protein 2

GN=Scfd2

Med H1/2: 4.4 days

Q5 H1/2: 2.6 days

Q95 H1/2: 34.2 days

reliable model fit: yes

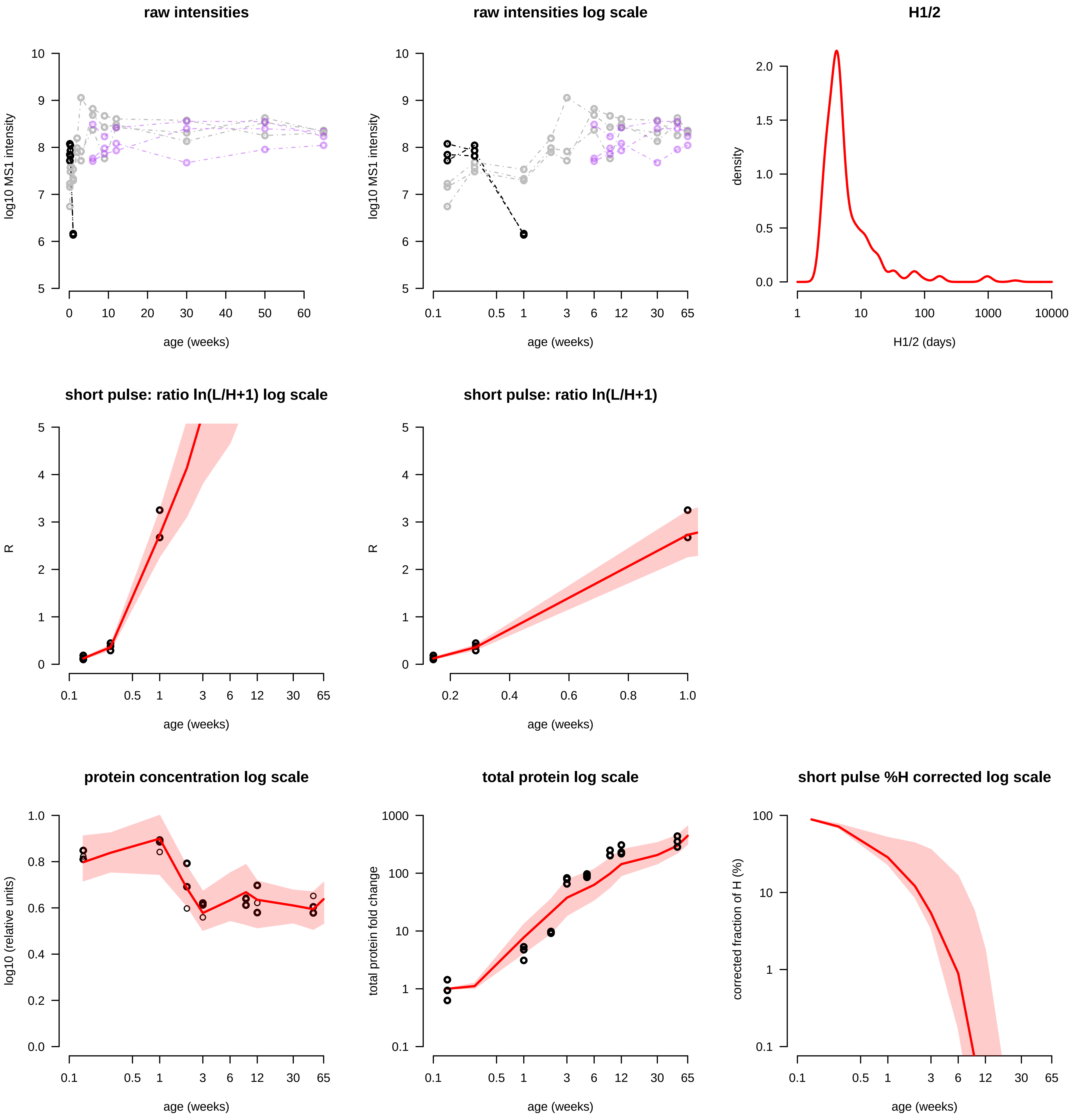

Q08481;Q08481−3;Q08481−4;Q08481−2

>sp|Q08481|PECA1_MOUSE

Platelet endothelial cell adhesion molecule

GN=Pecam1

Med H1/2: 648.4 days

Q5 H1/2: 174.8 days

Q95 H1/2: 7537.5 days

reliable model fit: no

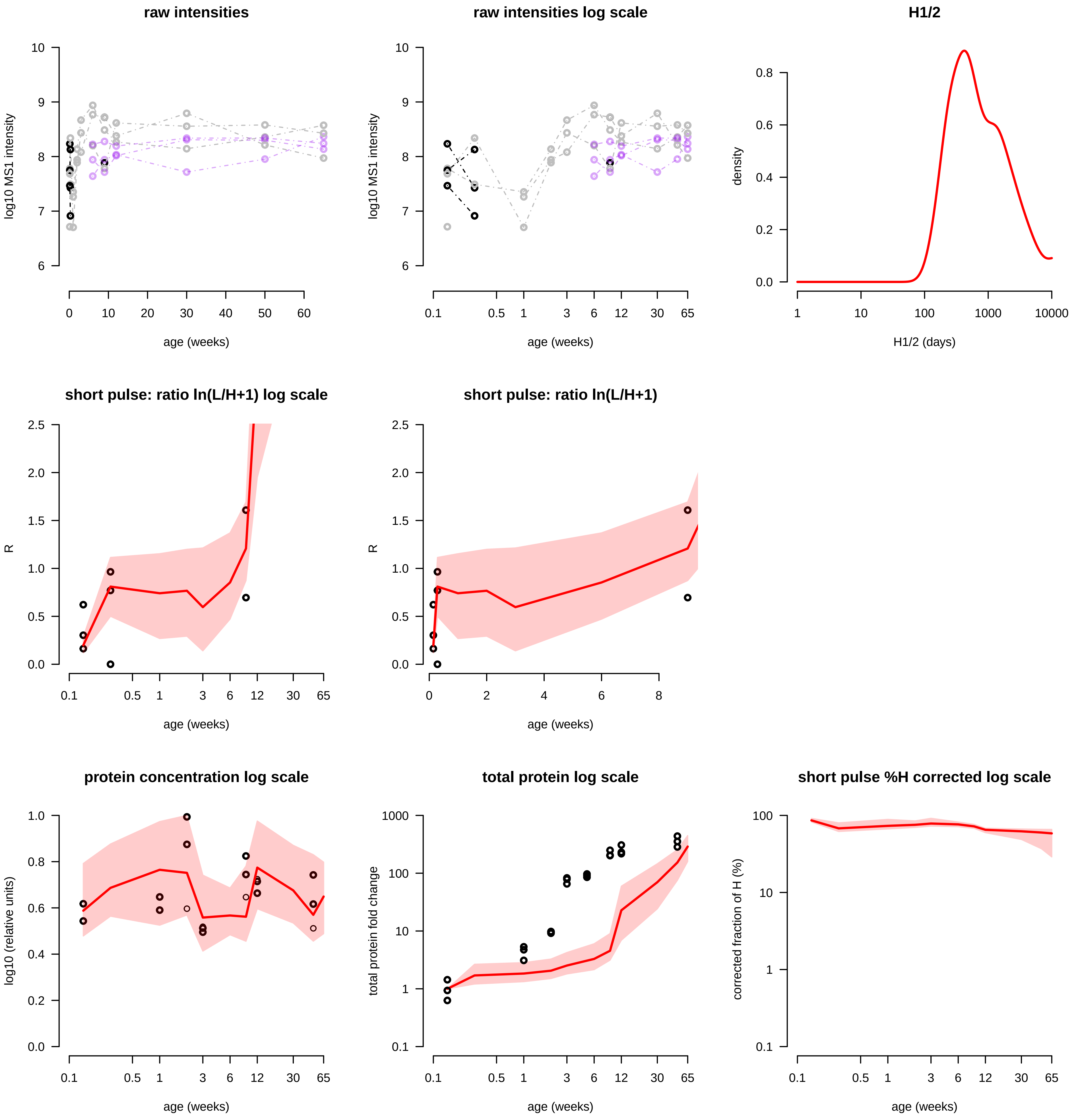

O09172

>sp|O09172|GSH0_MOUSE

Glutamate−−cysteine ligase regulatory subunit

GN=Gclm

Med H1/2: 9.1 days

Q5 H1/2: 6.7 days

Q95 H1/2: 12.8 days

reliable model fit: yes

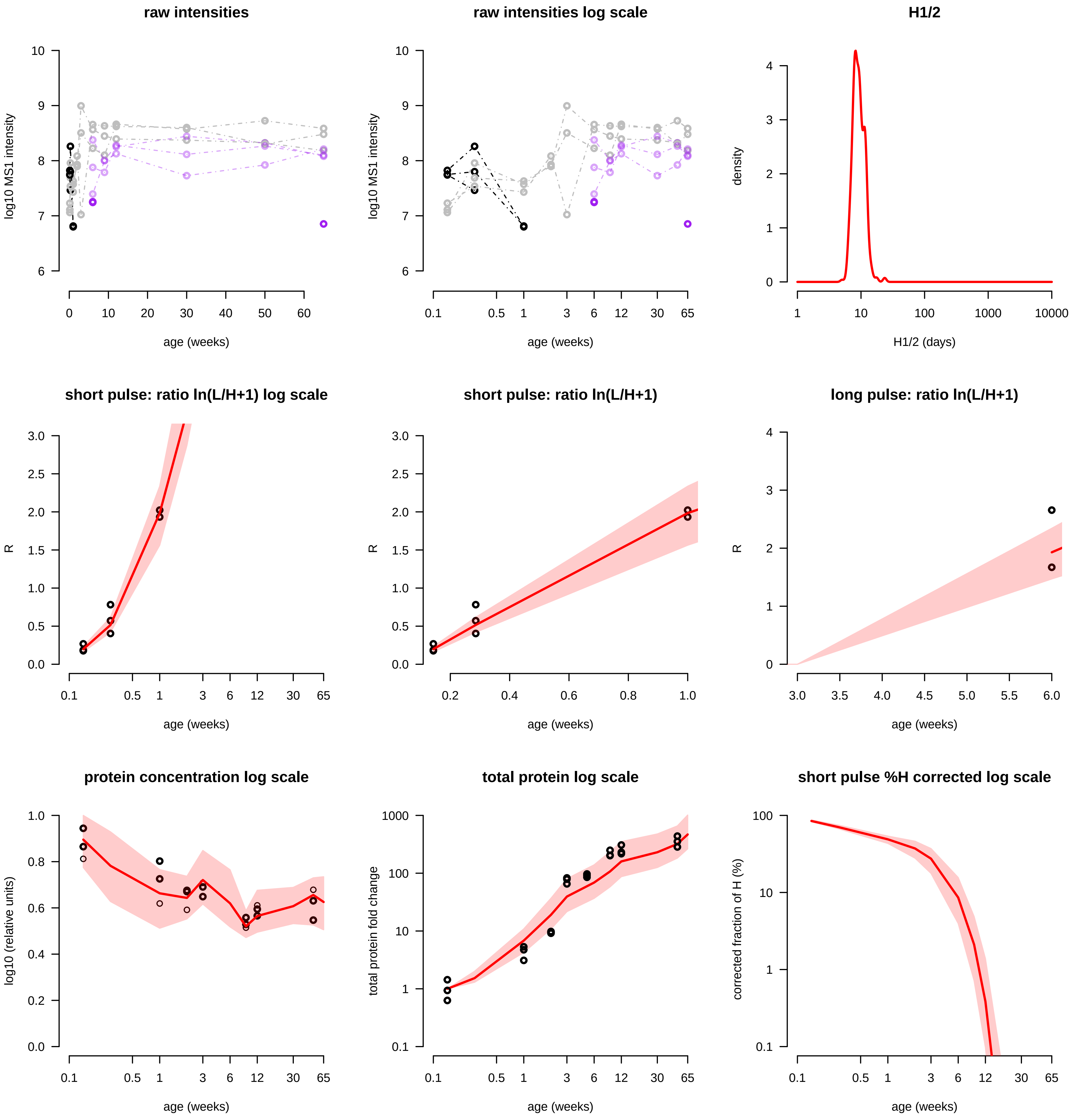

Q8BH24

>sp|Q8BH24|TM9S4_MOUSE

Transmembrane 9 superfamily member 4

GN=Tm9sf4

Med H1/2: 17.8 days

Q5 H1/2: 6.8 days

Q95 H1/2: 211.8 days

reliable model fit: yes

raw intensities

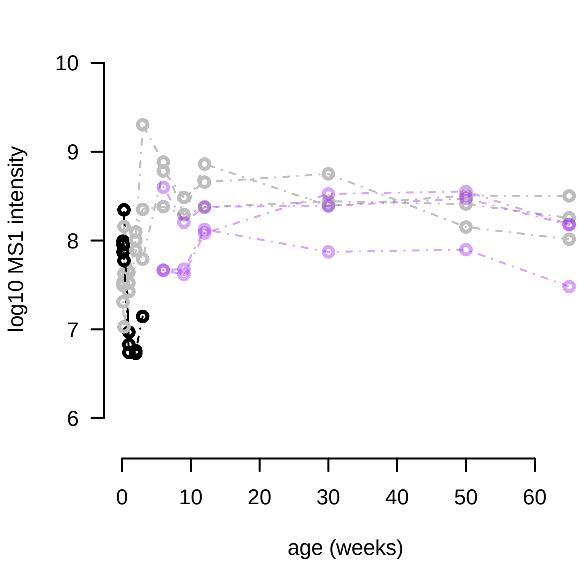

raw intensities log scale

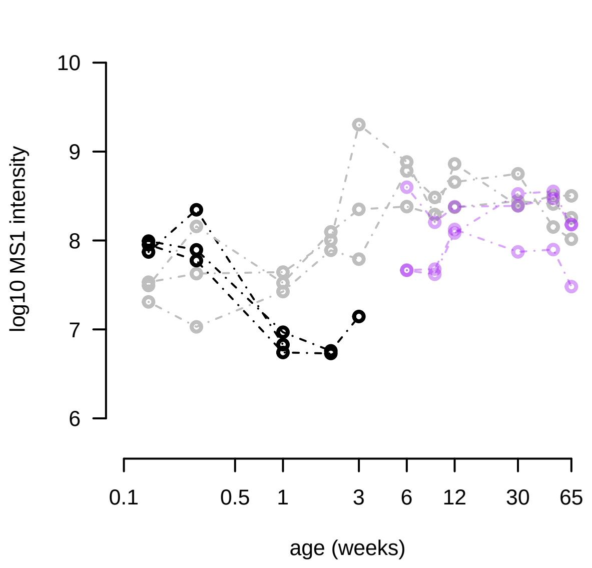

H1/2

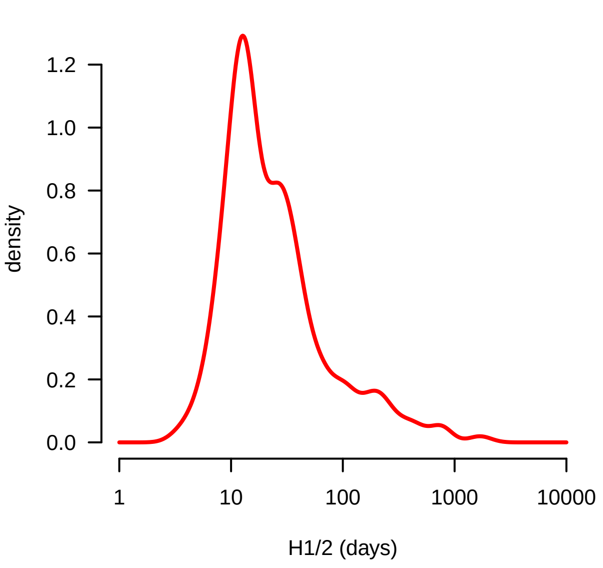

short pulse: ratio ln(L/H+1) log scale

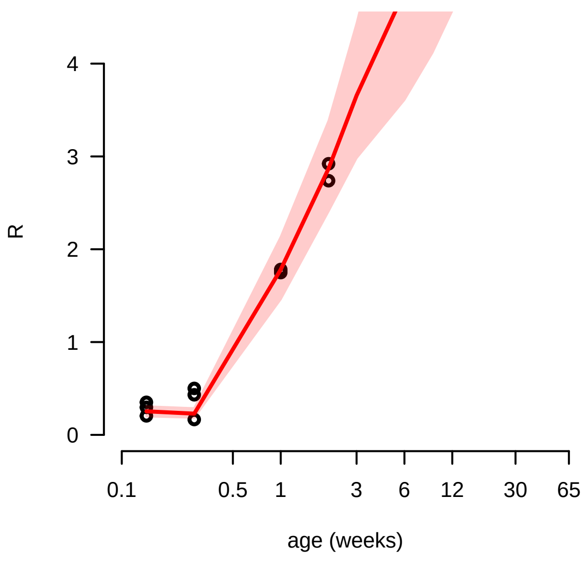

short pulse: ratio ln(L/H+1)

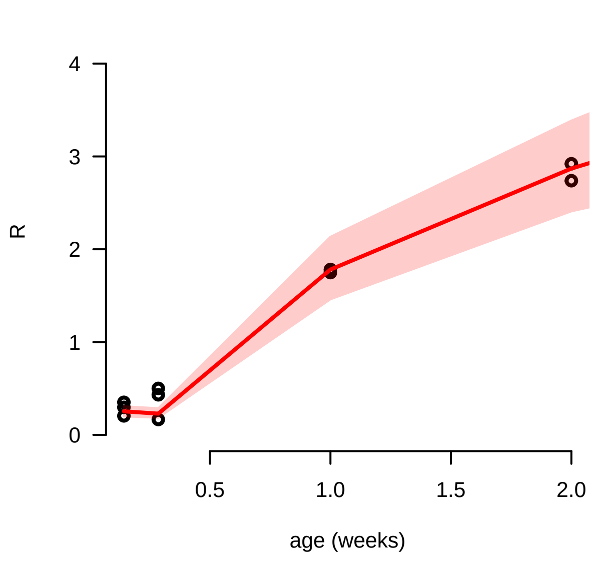

protein concentration log scale

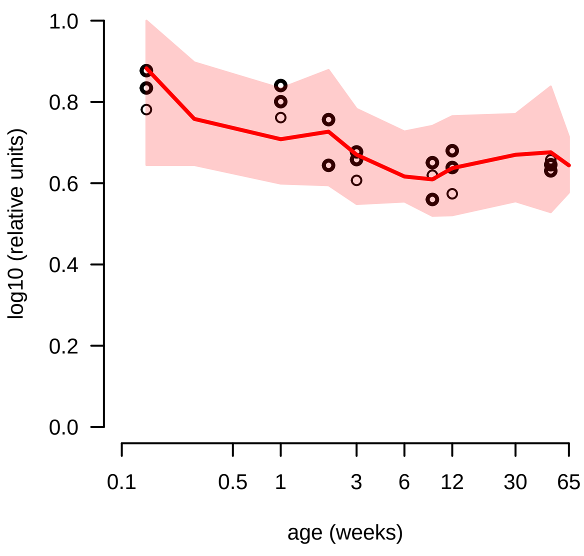

total protein log scale

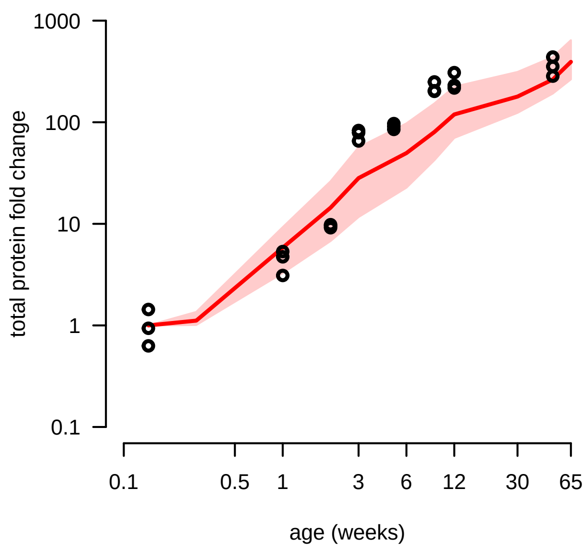

short pulse %H corrected log scale

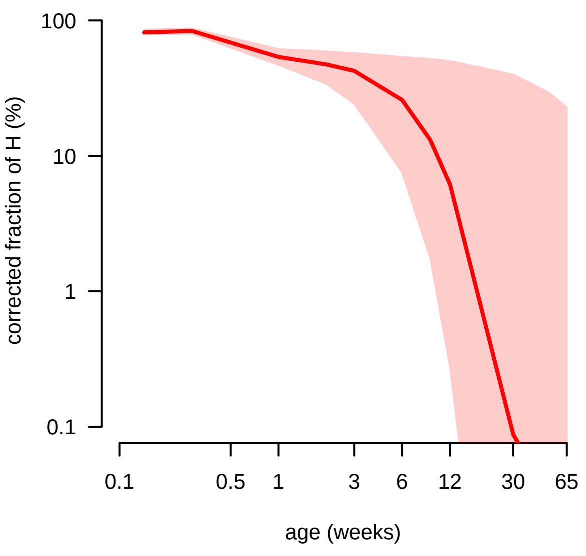

Q6PB93;Q6PB93−2

>sp|Q6PB93|GALT2_MOUSE

Polypeptide N−acetylgalactosaminyltransferase 2

GN=Galnt2

Med H1/2: 6.8 days

Q5 H1/2: 2.9 days

Q95 H1/2: 60.7 days

reliable model fit: yes

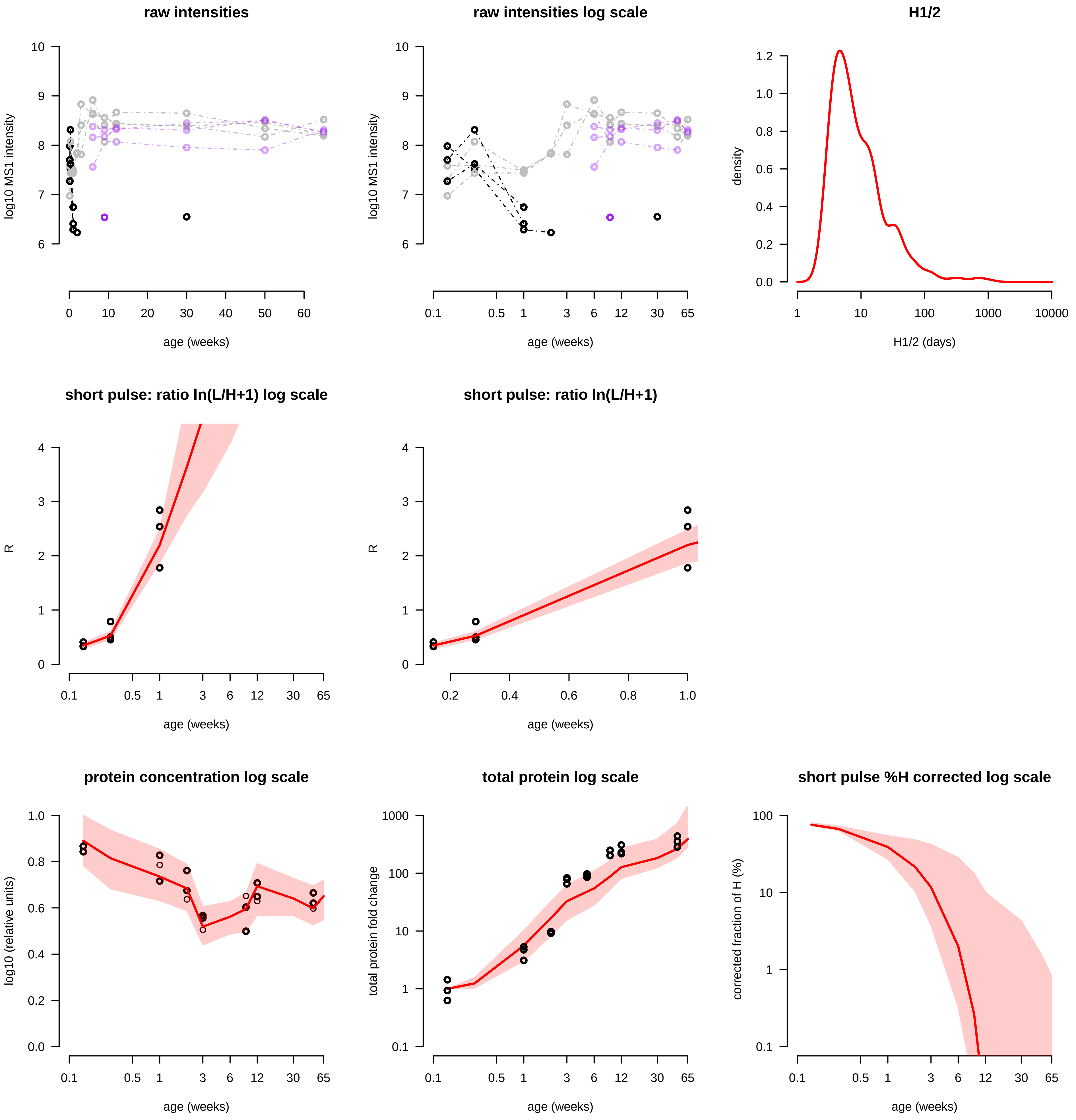

D3YXK2

>sp|D3YXK2|SAFB1_MOUSE

Scaffold attachment factor B1

GN=Safb

Med H1/2: 8.1 days

Q5 H1/2: 4.9 days

Q95 H1/2: 47.3 days

reliable model fit: yes

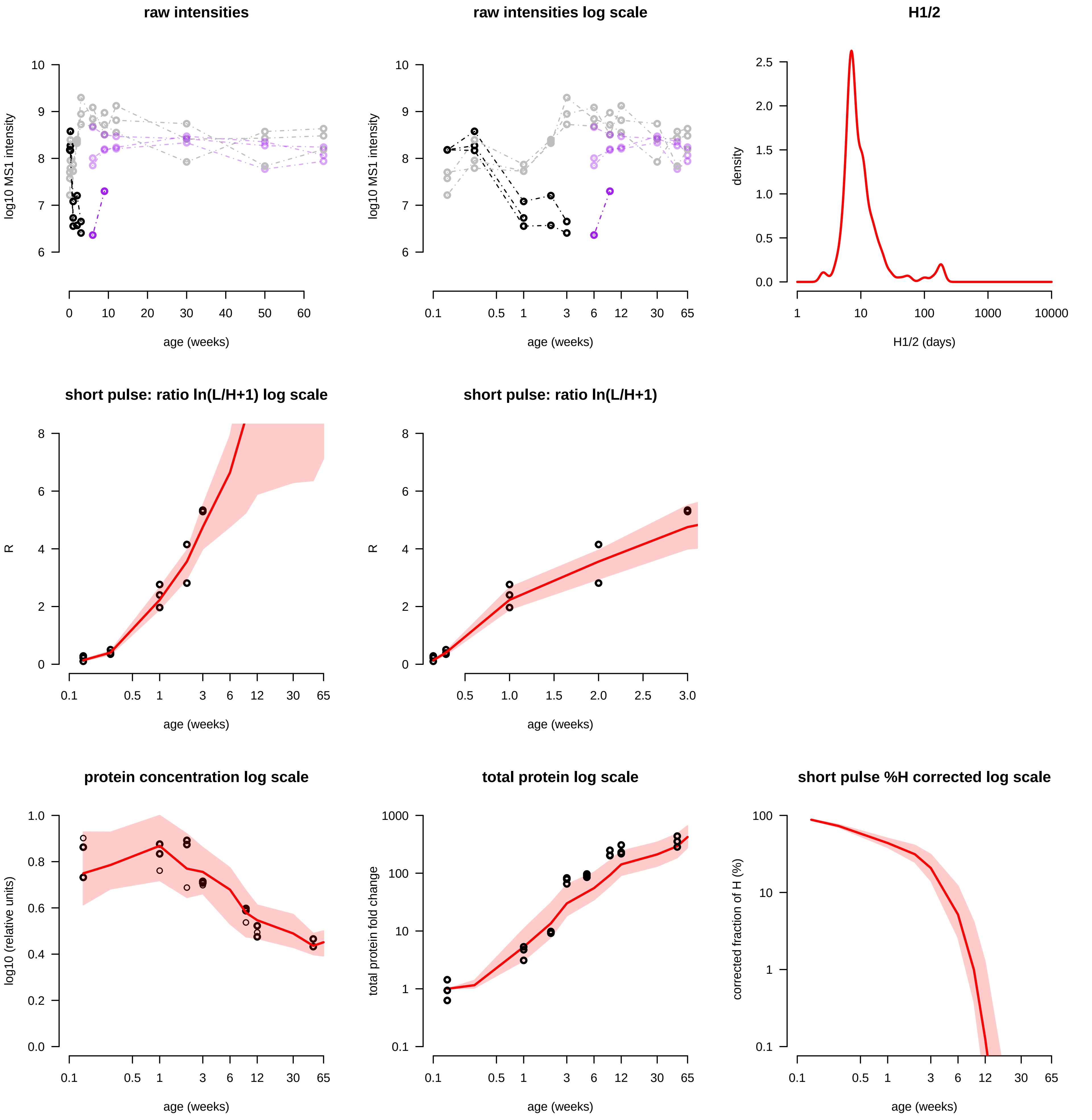

Q99LI7

>sp|Q99LI7|CSTF3_MOUSE

Cleavage stimulation factor subunit 3

GN=Cstf3

Med H1/2: 4.7 days

Q5 H1/2: 2.7 days

Q95 H1/2: 25.9 days

reliable model fit: yes

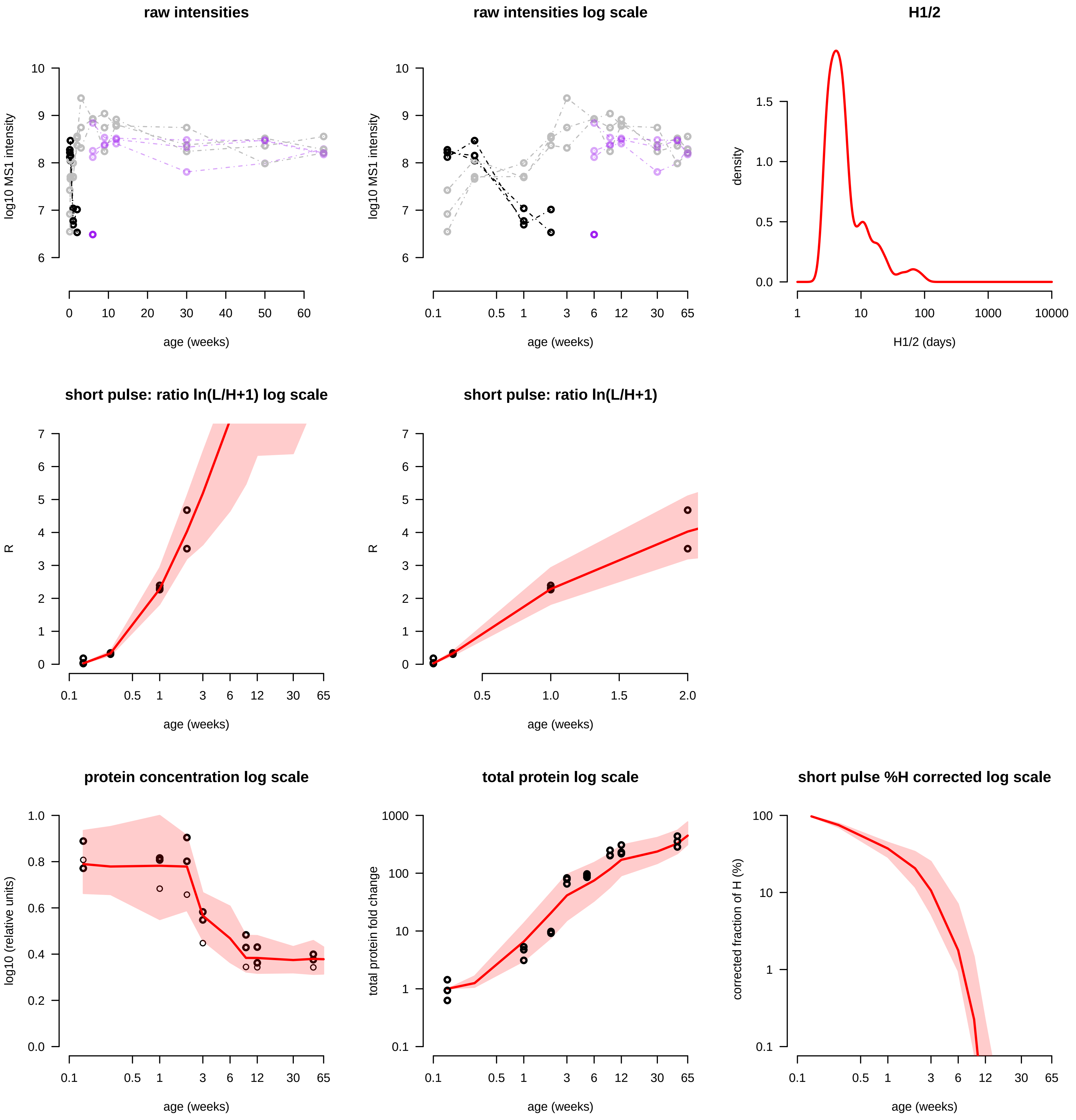

Q9EPB4

>sp|Q9EPB4|ASC_MOUSE

Apoptosis−associated speck−like protein containing a CARD

GN=Pycard

Med H1/2: 7 days

Q5 H1/2: 2.7 days

Q95 H1/2: 52.8 days

reliable model fit: yes

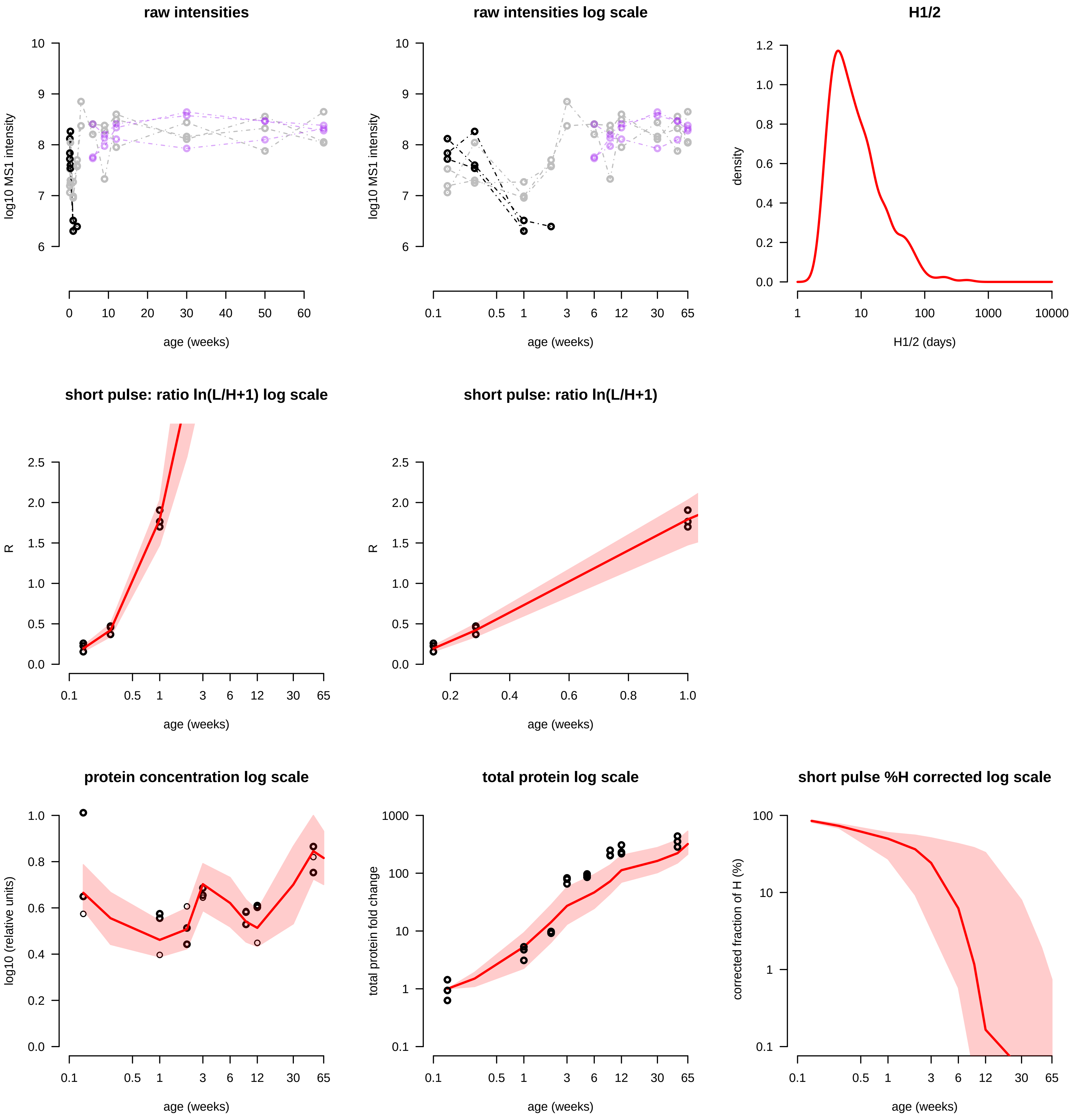

P25206

>sp|P25206|MCM3_MOUSE

DNA replication licensing factor MCM3

GN=Mcm3

Med H1/2: 3.1 days

Q5 H1/2: 2.6 days

Q95 H1/2: 3.8 days

reliable model fit: yes

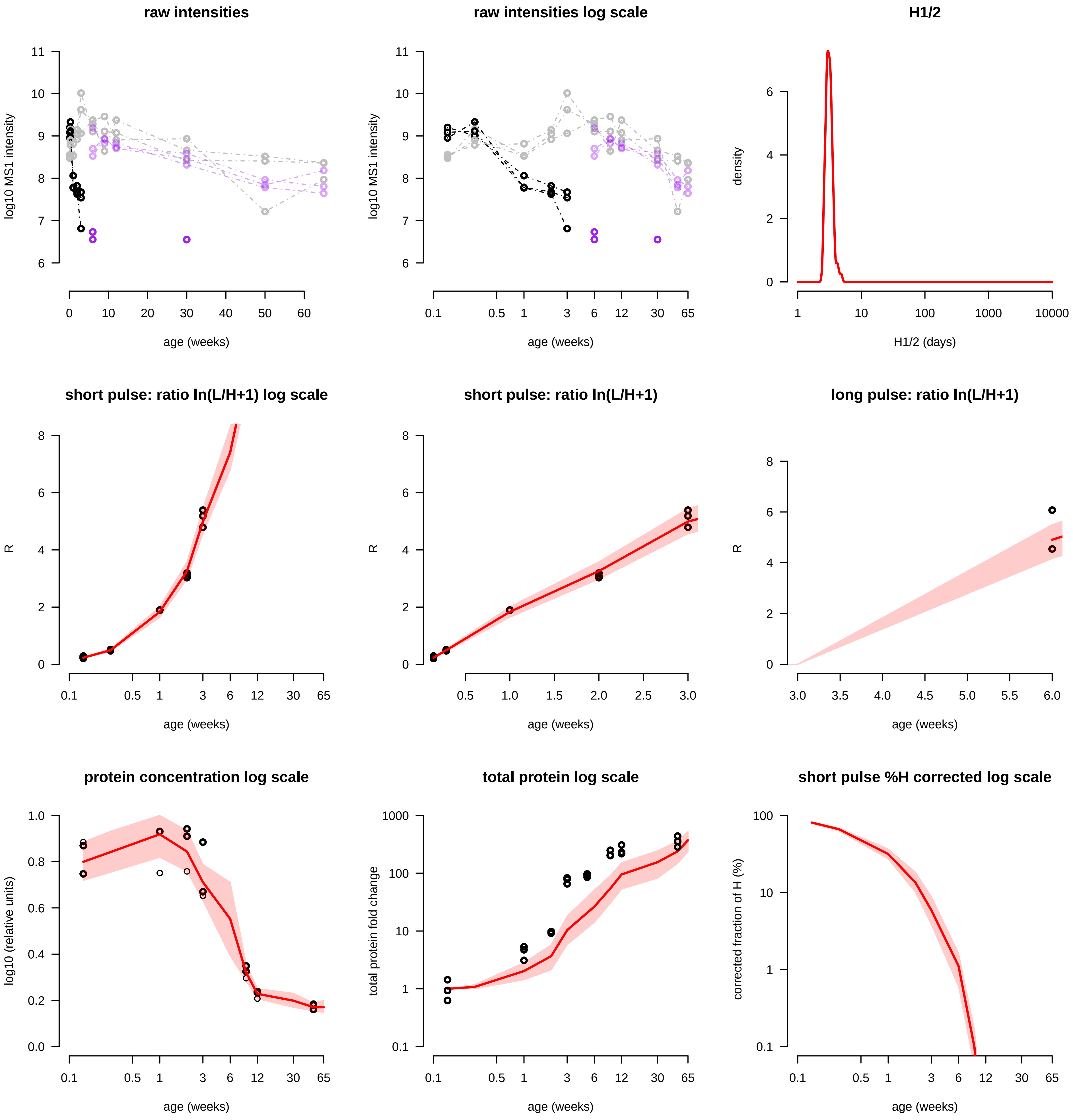

Q9R020;Q9R020−2

>sp|Q9R020|ZRAB2_MOUSE

Zinc finger Ran−binding domain−containing protein 2

GN=Zranb2

Med H1/2: 3.2 days

Q5 H1/2: 3.2 days

Q95 H1/2: 3.2 days

reliable model fit: no

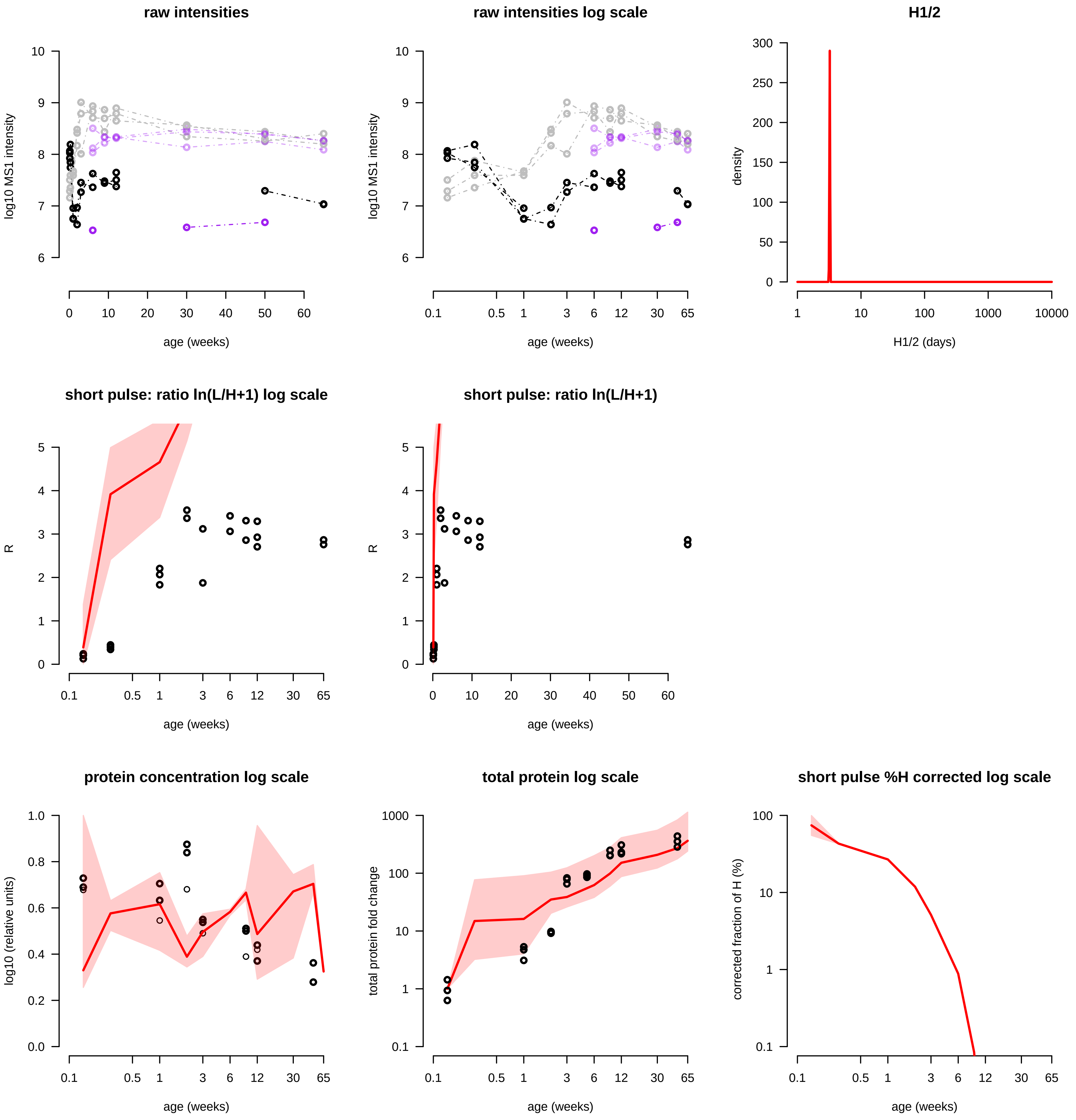

Q80U78−2

>sp|Q80U78−2|PUM1_MOUSE

Isoform 2 of Pumilio homolog 1

GN=Pum1

Med H1/2: 8.8 days

Q5 H1/2: 4.2 days

Q95 H1/2: 39.8 days

reliable model fit: yes

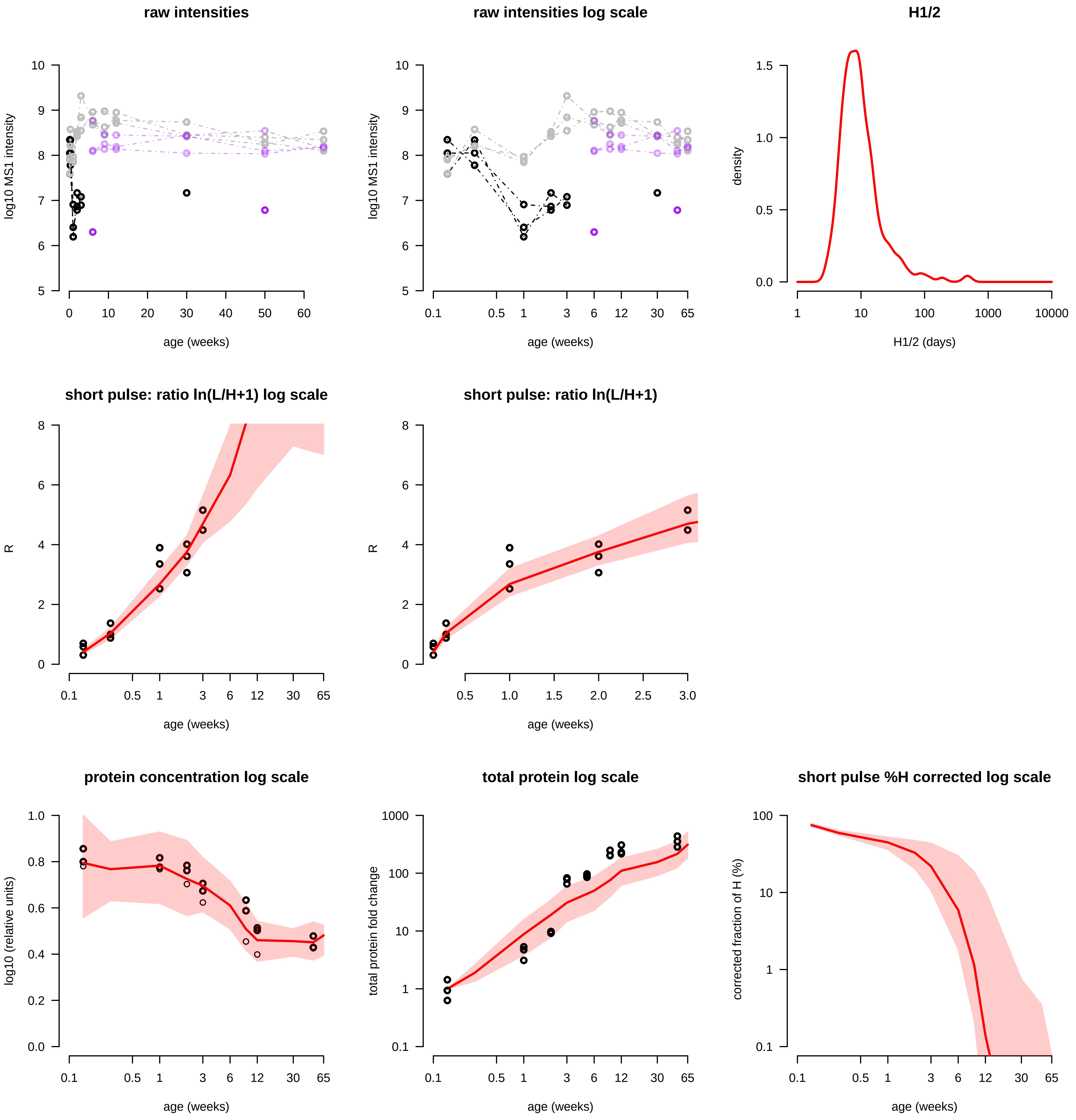

Q9D0I8

>sp|Q9D0I8|MRT4_MOUSE

mRNA turnover protein 4 homolog

GN=Mrto4

Med H1/2: 3.7 days

Q5 H1/2: 2.6 days

Q95 H1/2: 9.4 days

reliable model fit: yes

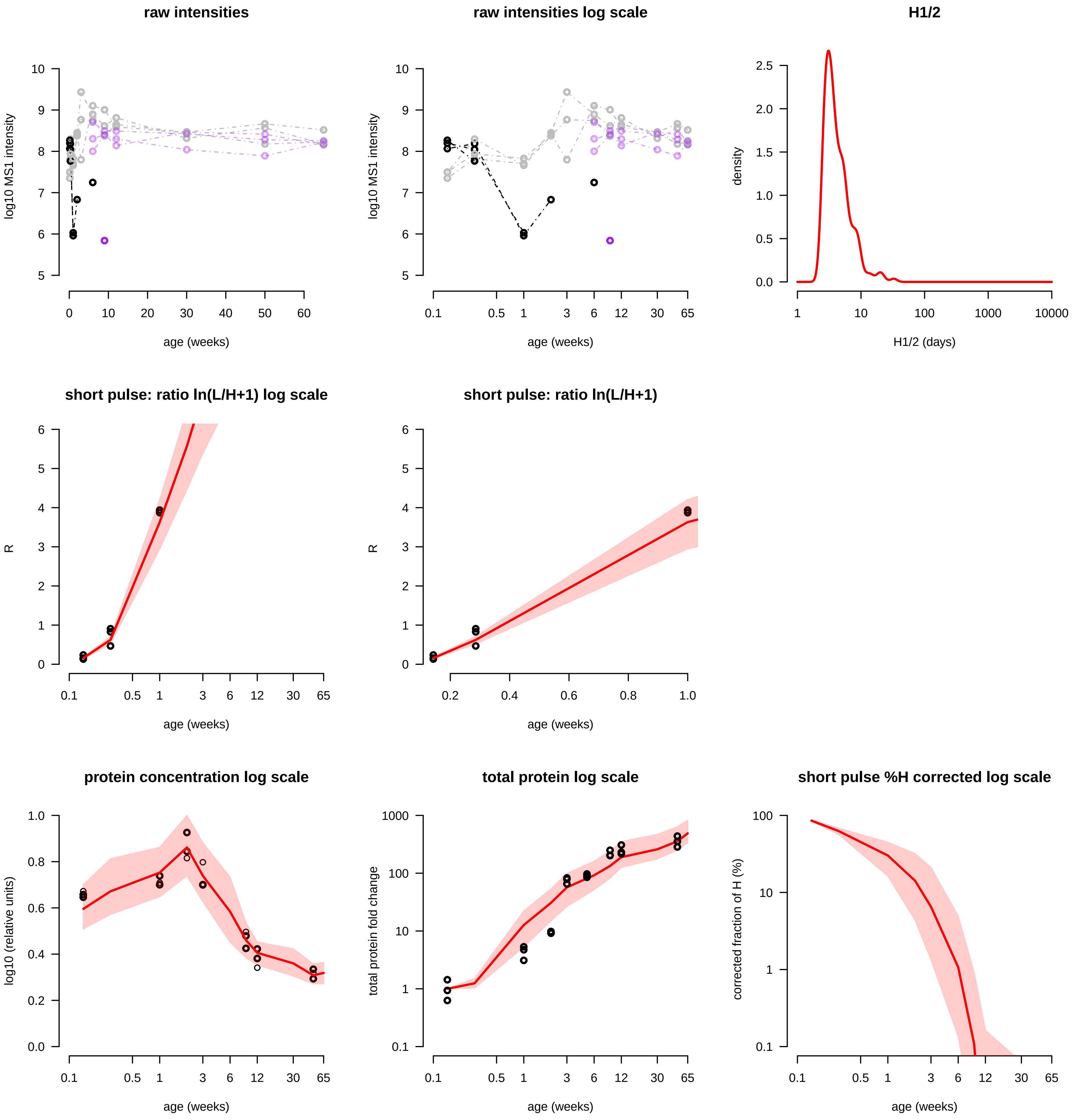

Q8CGC6

>sp|Q8CGC6|RBM28_MOUSE

RNA−binding protein 28

GN=Rbm28

Med H1/2: 12.1 days

Q5 H1/2: 4.7 days

Q95 H1/2: 62.2 days

reliable model fit: yes

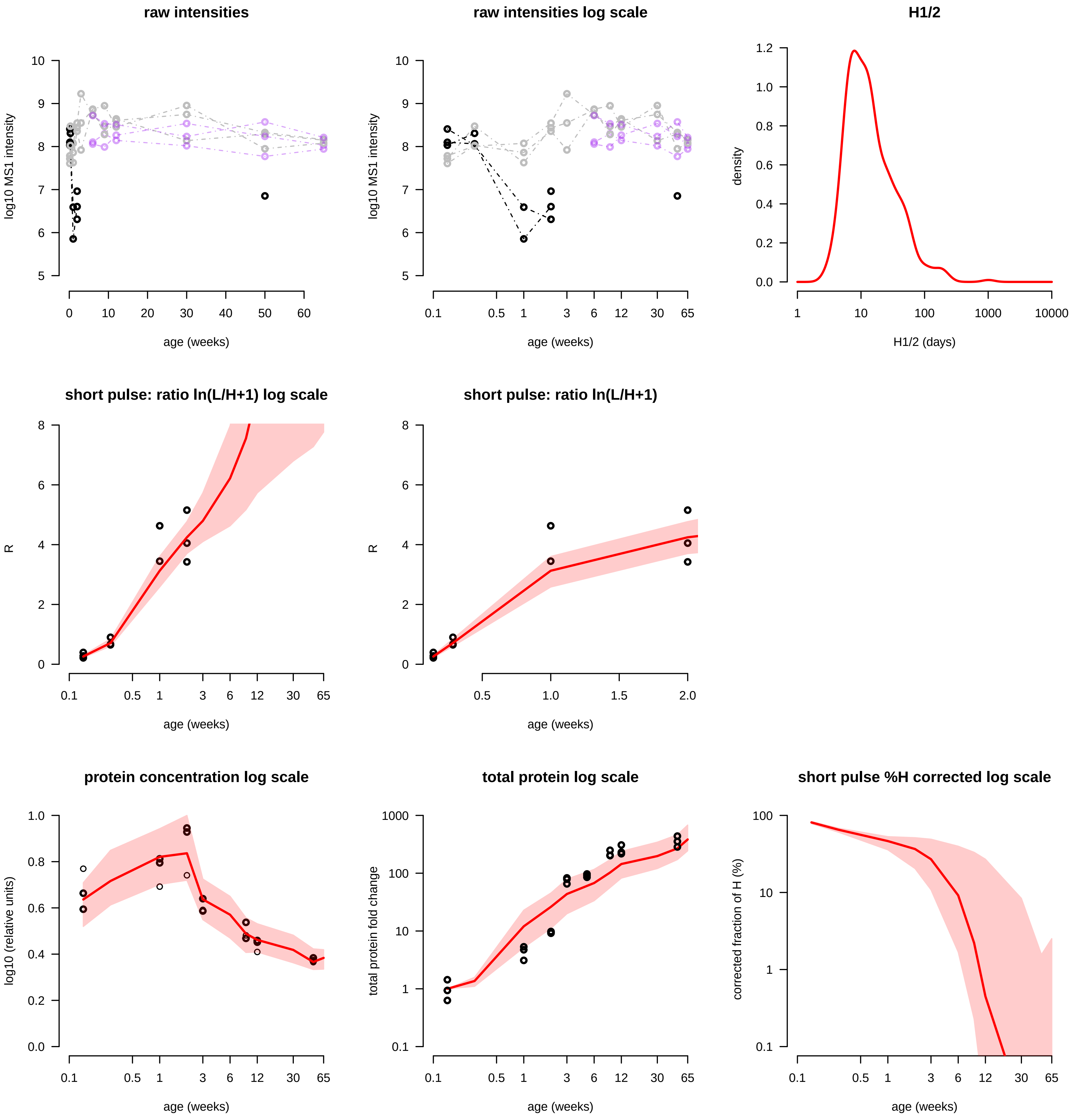

Q9JHQ5

>sp|Q9JHQ5|LZTL1_MOUSE

Leucine zipper transcription factor−like protein 1

GN=Lztfl1

Med H1/2: 191.6 days

Q5 H1/2: 130.5 days

Q95 H1/2: 345.6 days

reliable model fit: yes

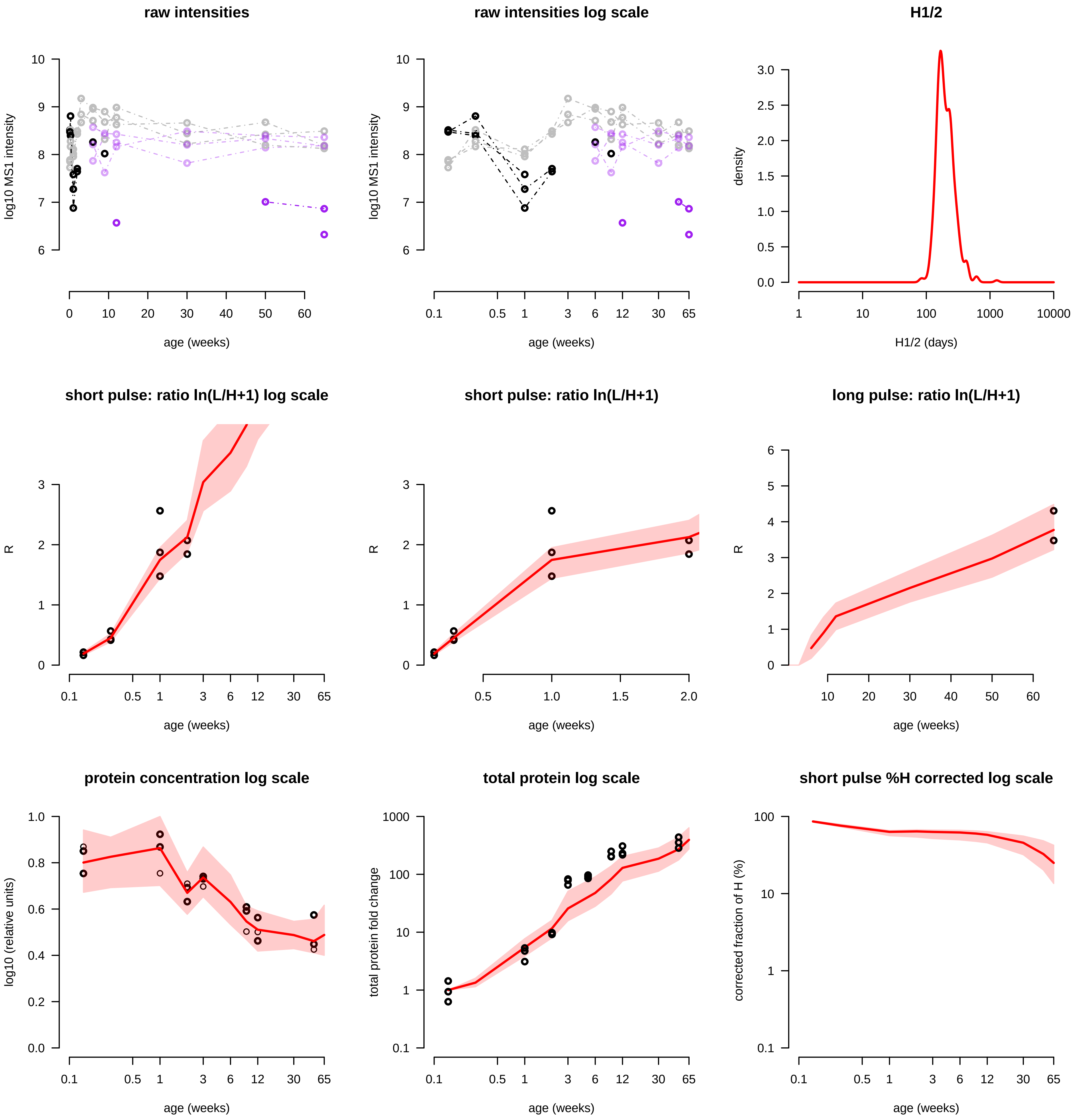

Q9WUK4

>sp|Q9WUK4|RFC2_MOUSE

Replication factor C subunit 2

GN=Rfc2

Med H1/2: 4.7 days

Q5 H1/2: 3.9 days

Q95 H1/2: 6 days

reliable model fit: yes

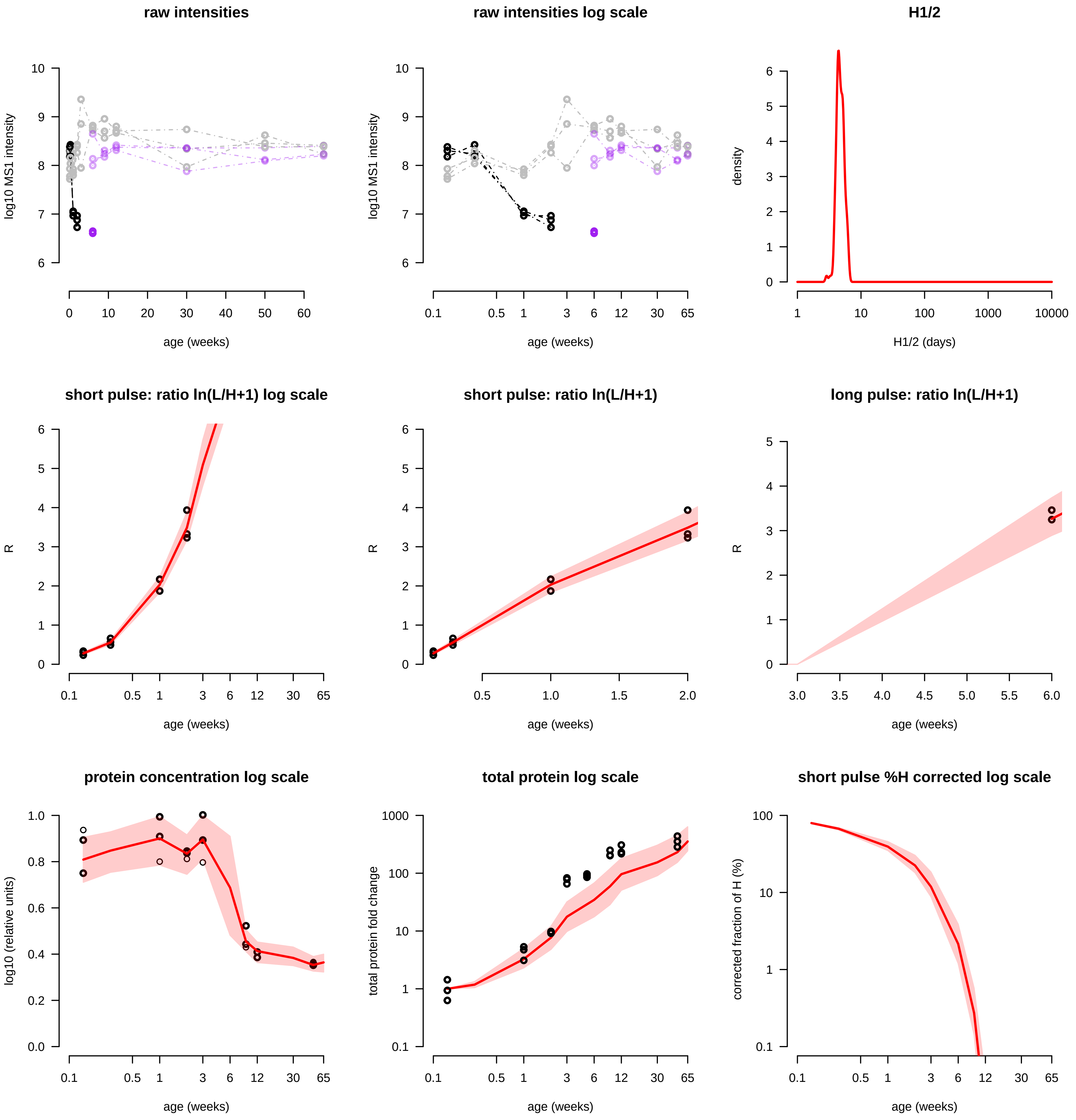

Q9QY06;Q9QY06−2;Q9QY06−3

>sp|Q9QY06|MYO9B_MOUSE

Unconventional myosin−IXb

GN=Myo9b

Med H1/2: 4.2 days

Q5 H1/2: 3.4 days

Q95 H1/2: 5.4 days

reliable model fit: yes

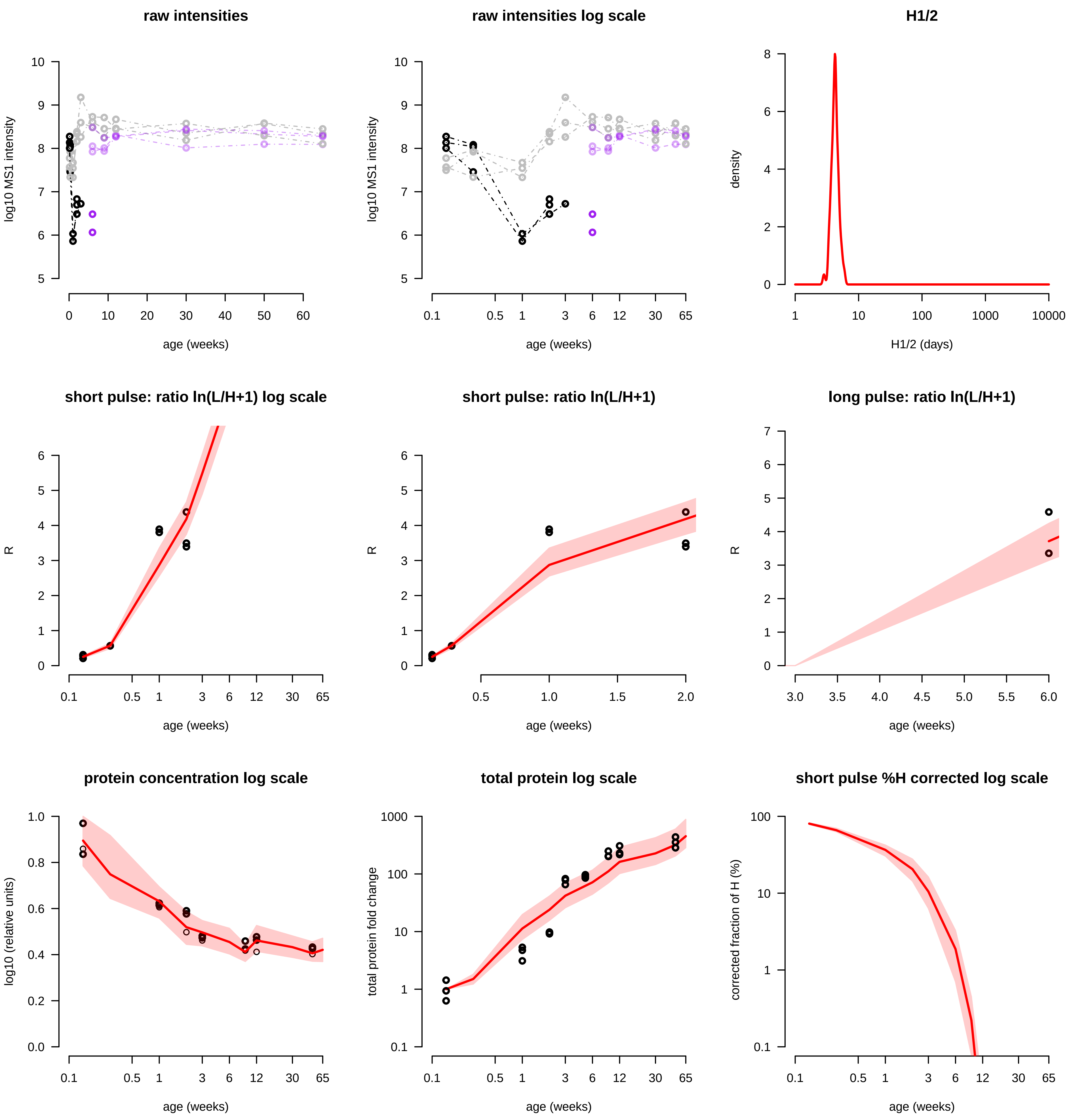

Q91WL8;Q91WL8−3;Q91WL8−4

>sp|Q91WL8|WWOX_MOUSE

WW domain−containing oxidoreductase

GN=Wwox

Med H1/2: 303.4 days

Q5 H1/2: 115.6 days

Q95 H1/2: 3652.3 days

reliable model fit: no

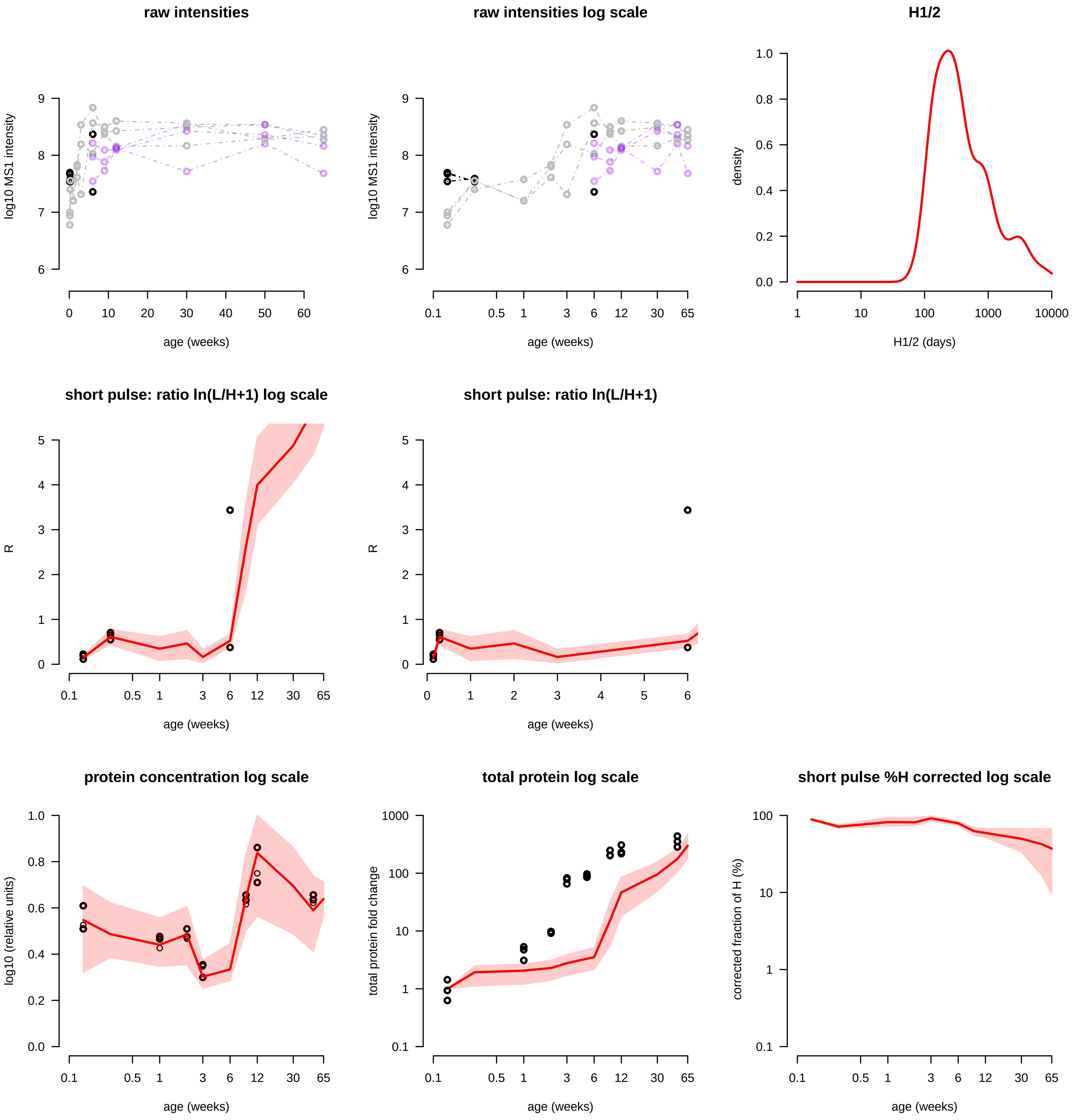

Q9JM51

>sp|Q9JM51|PTGES_MOUSE

Prostaglandin E synthase

GN=Ptges

Med H1/2: 13.1 days

Q5 H1/2: 8.3 days

Q95 H1/2: 35.1 days

reliable model fit: yes

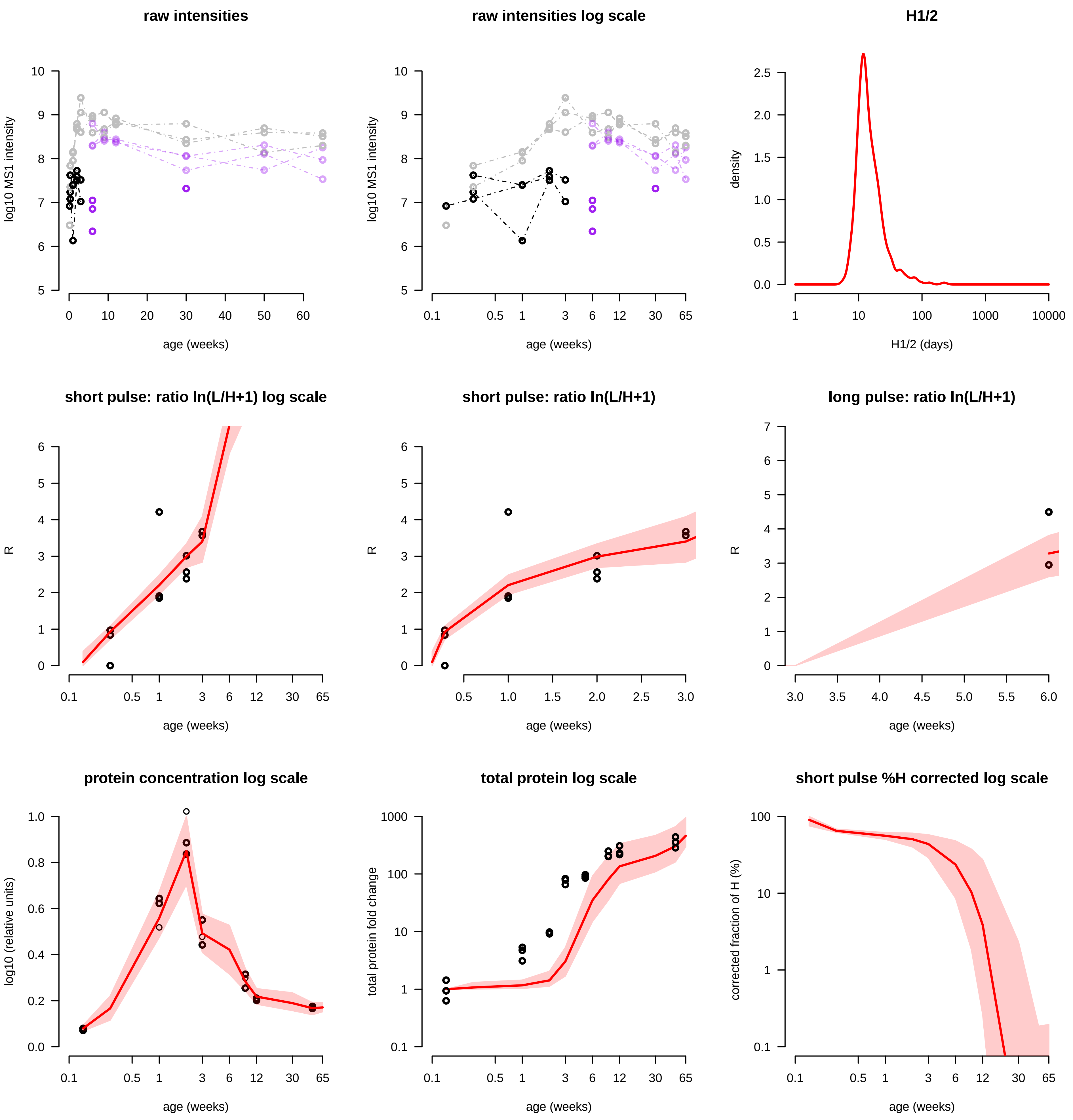

Q9CZR8

>sp|Q9CZR8|EFTS_MOUSE

Elongation factor Ts, mitochondrial

GN=Tsfm

Med H1/2: 7.5 days

Q5 H1/2: 3.3 days

Q95 H1/2: 64.1 days

reliable model fit: yes

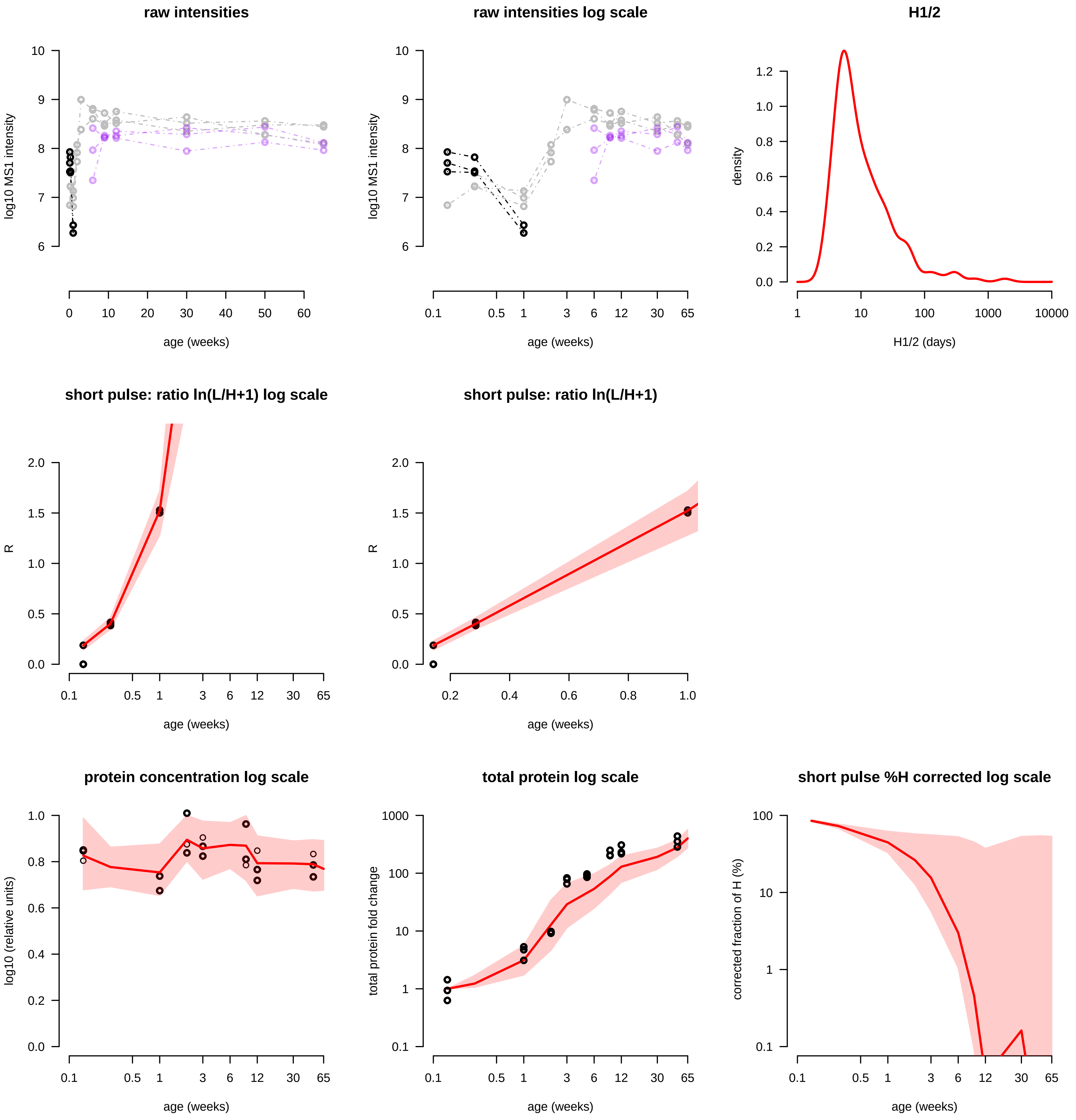

Q9D0B6;Q9D0B6−2

>sp|Q9D0B6|PBDC1_MOUSE

Protein PBDC1

GN=Pbdc1

Med H1/2: 3507.4 days

Q5 H1/2: 612.6 days

Q95 H1/2: 39844.6 days

reliable model fit: yes

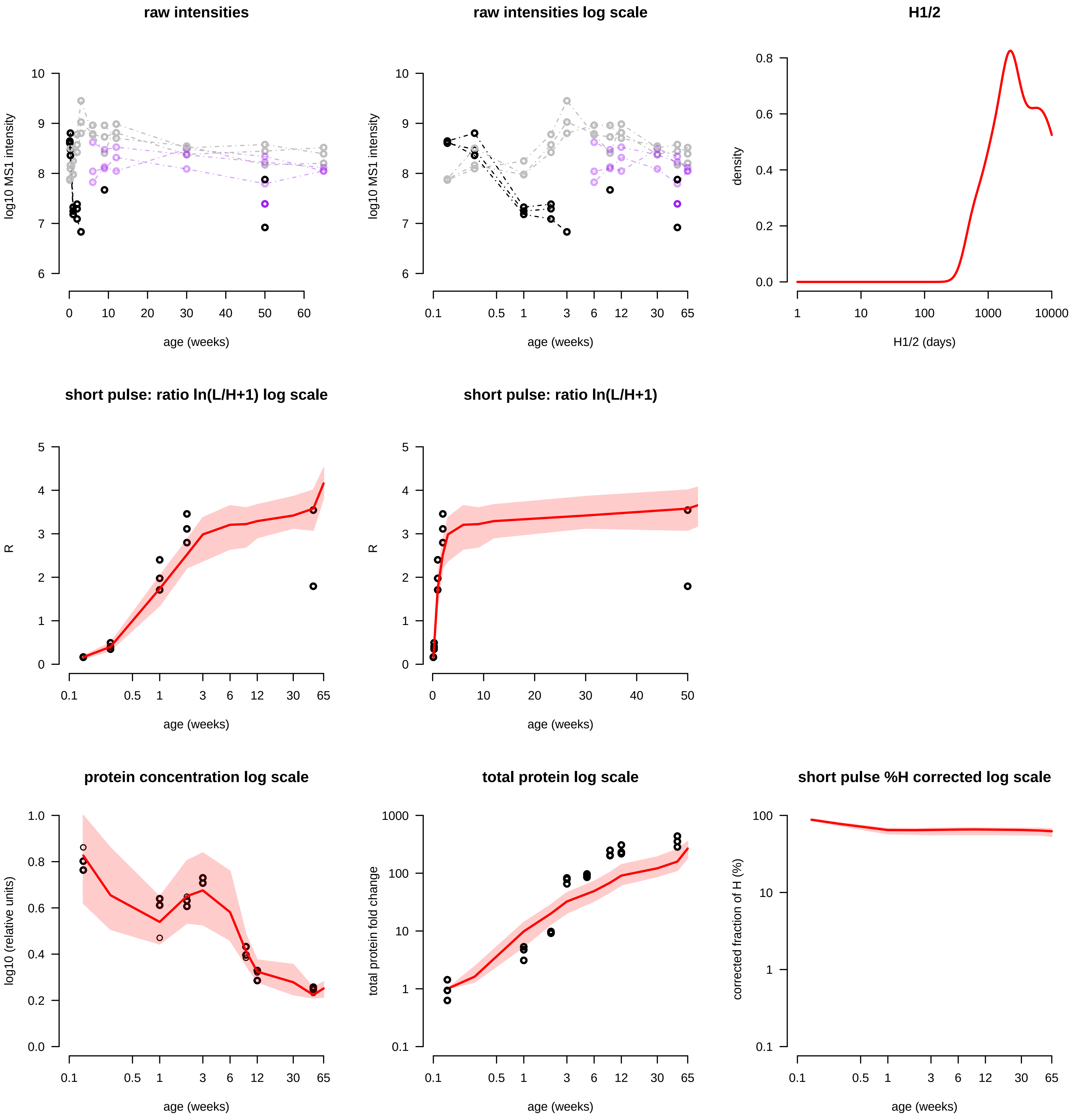

Q9CR00

>sp|Q9CR00|PSMD9_MOUSE

26S proteasome non−ATPase regulatory subunit 9

GN=Psmd9

Med H1/2: 5.4 days

Q5 H1/2: 2.8 days

Q95 H1/2: 47.8 days

reliable model fit: yes

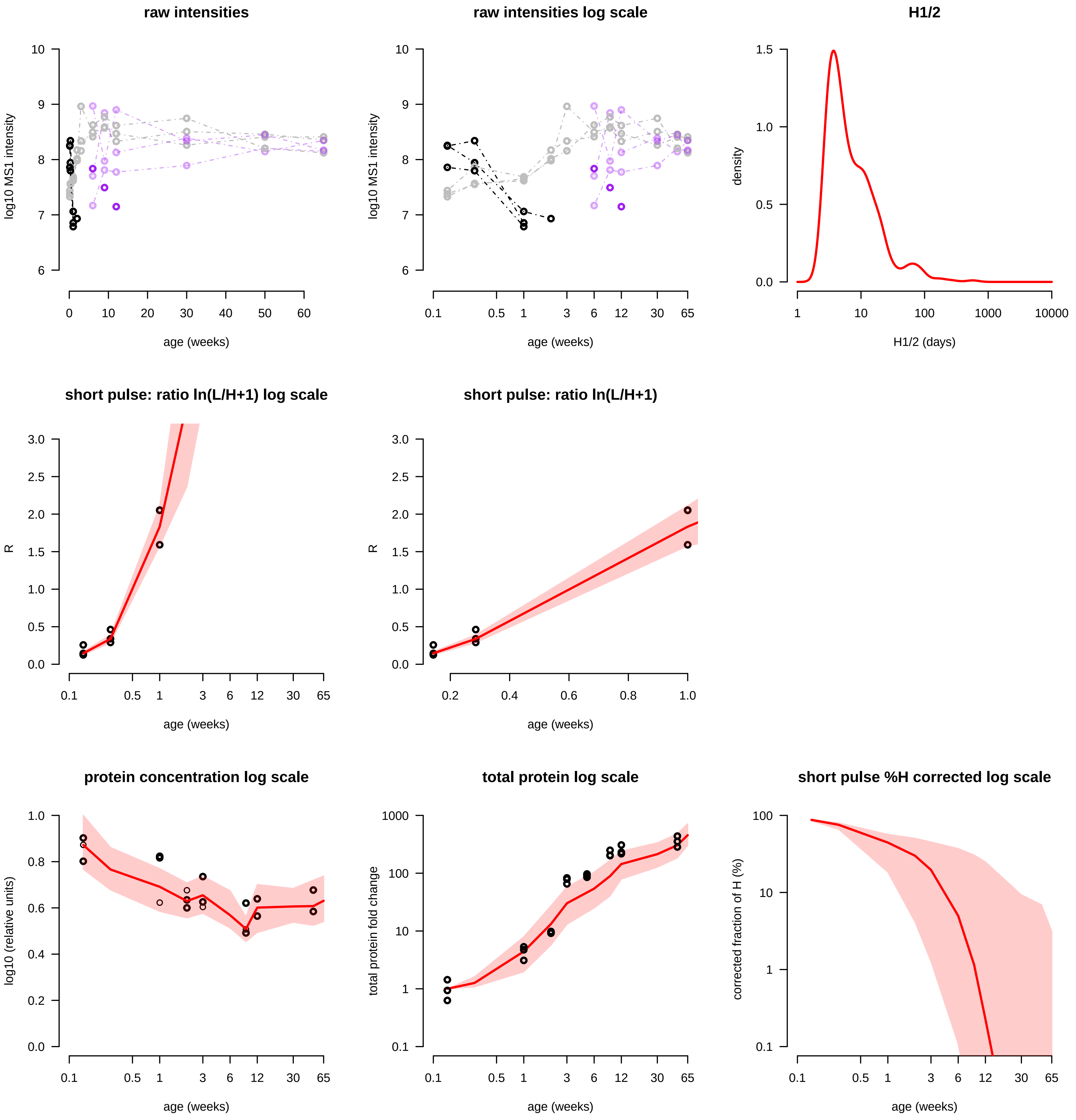

Q8CI95

>sp|Q8CI95|OSB11_MOUSE

Oxysterol−binding protein−related protein 11

GN=Osbpl11

Med H1/2: 6 days

Q5 H1/2: 3.1 days

Q95 H1/2: 33.7 days

reliable model fit: yes

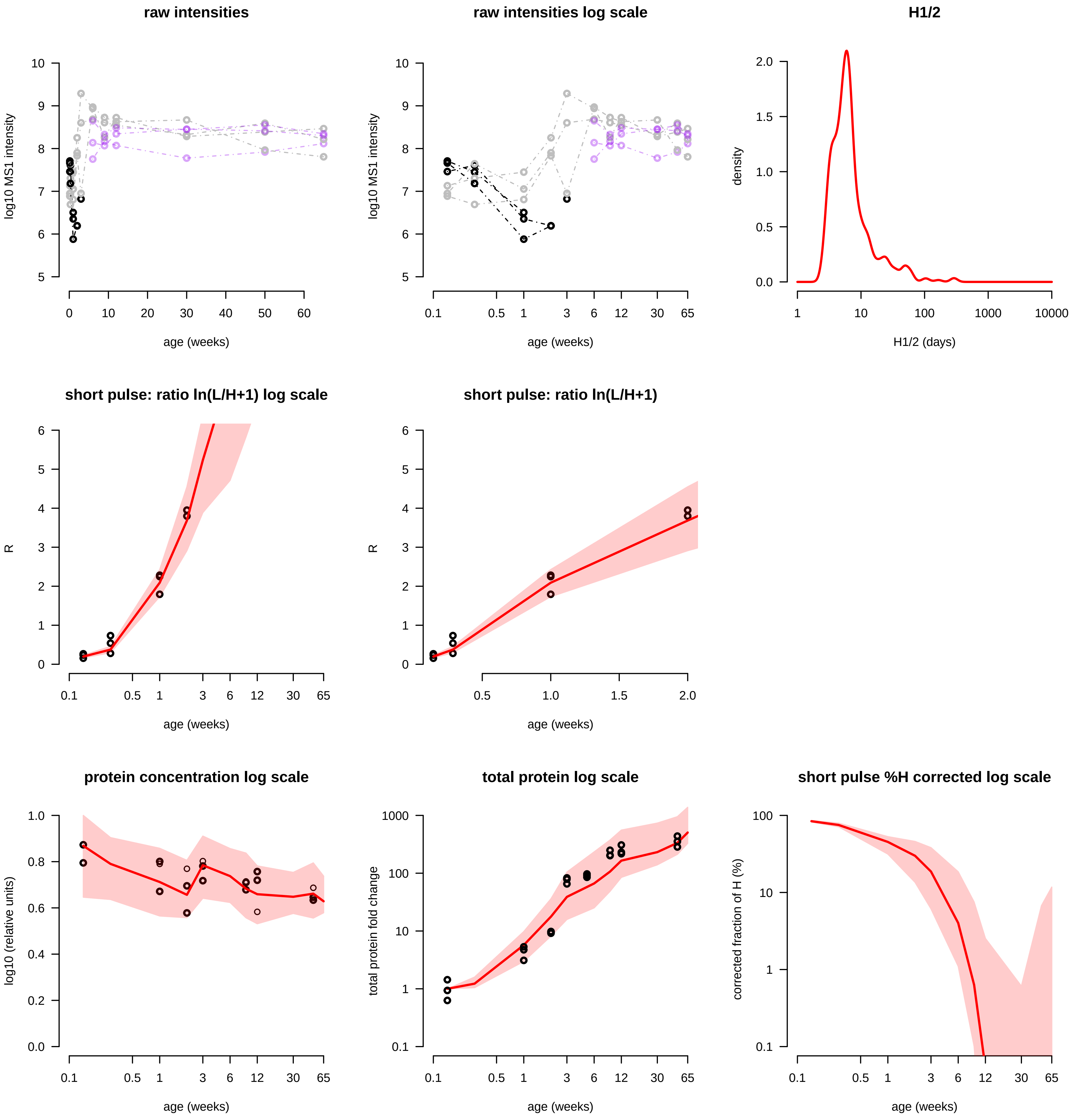

Q9EPQ7

>sp|Q9EPQ7|STAR5_MOUSE

StAR−related lipid transfer protein 5

GN=Stard5

Med H1/2: 156.6 days

Q5 H1/2: 42.7 days

Q95 H1/2: 3217.2 days

reliable model fit: yes

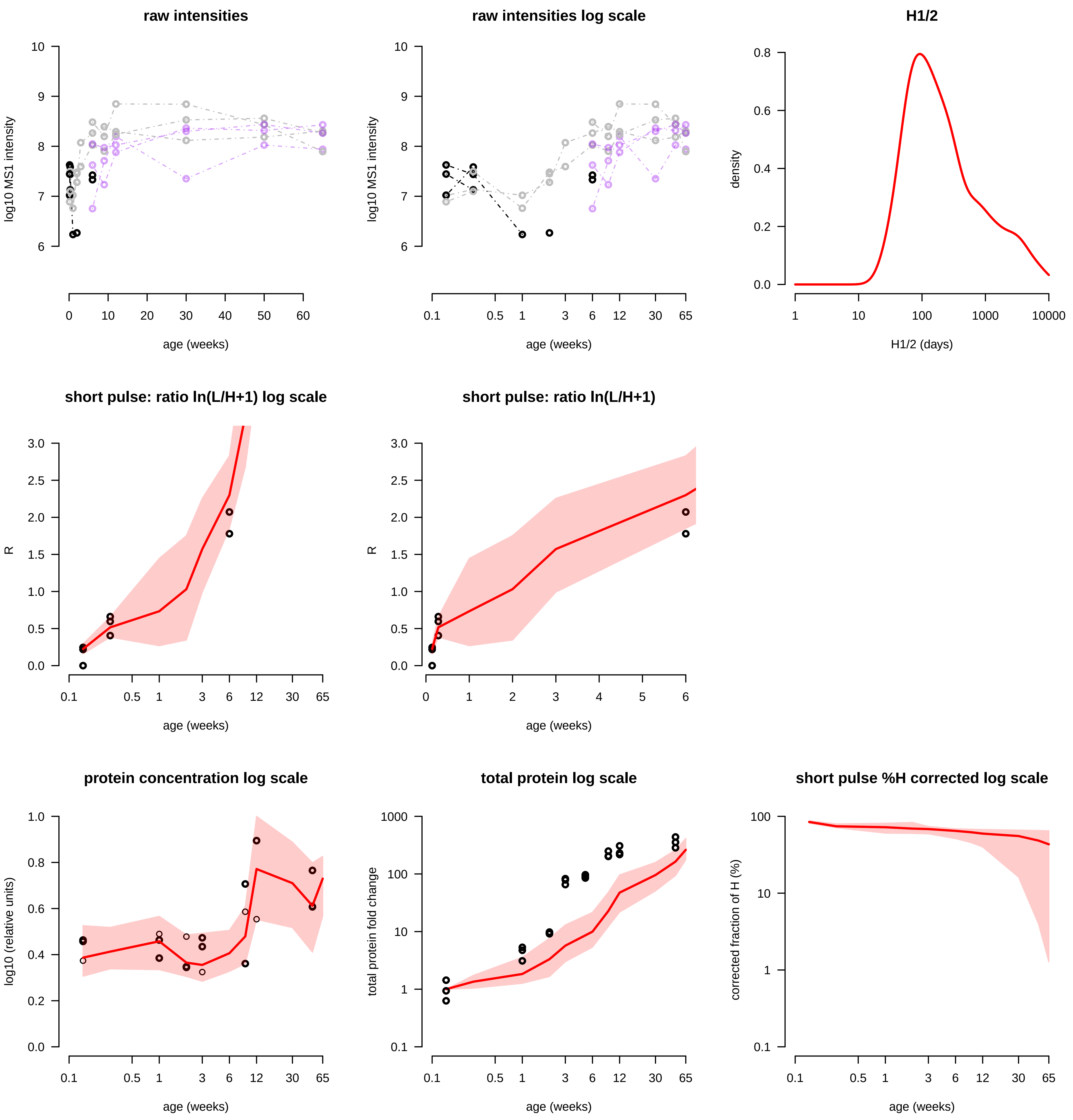

Q91WG5;Q91WG5−2

>sp|Q91WG5|AAKG2_MOUSE

5'−AMP−activated protein kinase subunit gamma−2

GN=Prkag2

Med H1/2: 7.7 days

Q5 H1/2: 3.1 days

Q95 H1/2: 91.8 days

reliable model fit: yes

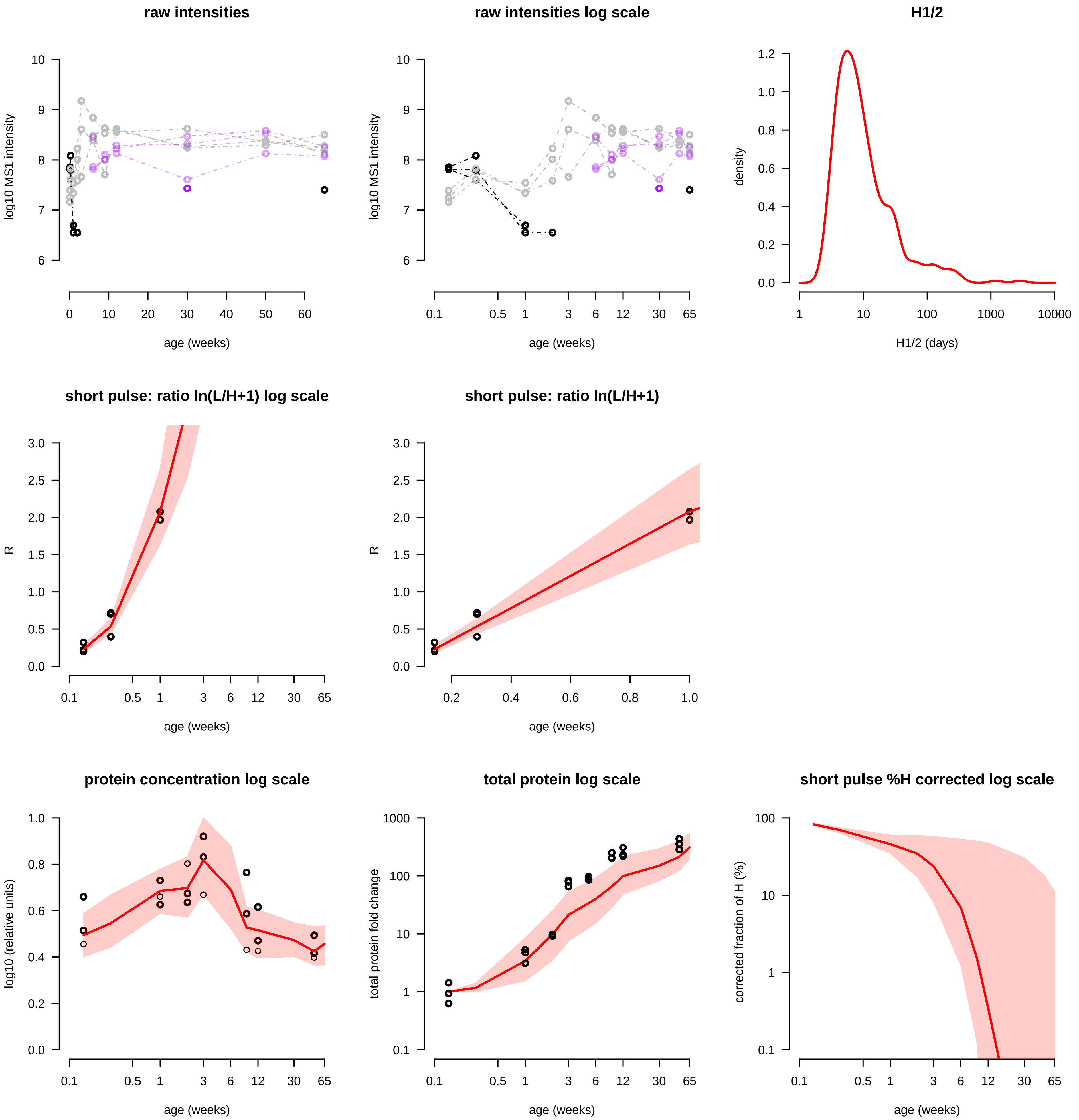

Q05512;Q05512−3;Q05512−4;Q05512−2

>sp|Q05512|MARK2_MOUSE

Serine/threonine−protein kinase MARK2

GN=Mark2

Med H1/2: 3.7 days

Q5 H1/2: 2.5 days

Q95 H1/2: 15.6 days

reliable model fit: yes

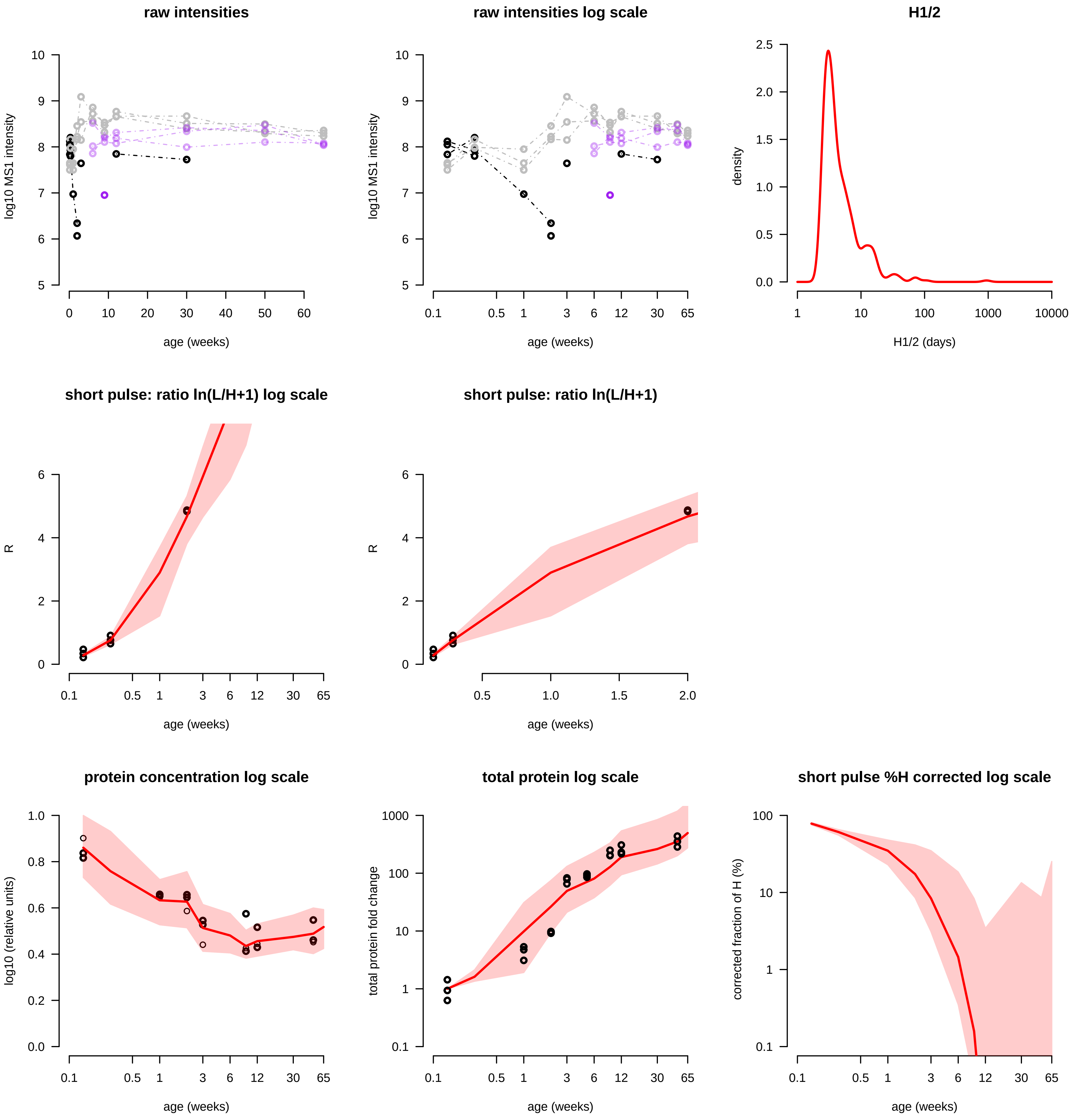

Q8VHR5;Q8VHR5−2

>sp|Q8VHR5|P66B_MOUSE

Transcriptional repressor p66−beta

GN=Gatad2b

Med H1/2: 3.3 days

Q5 H1/2: 2.5 days

Q95 H1/2: 12.8 days

reliable model fit: yes

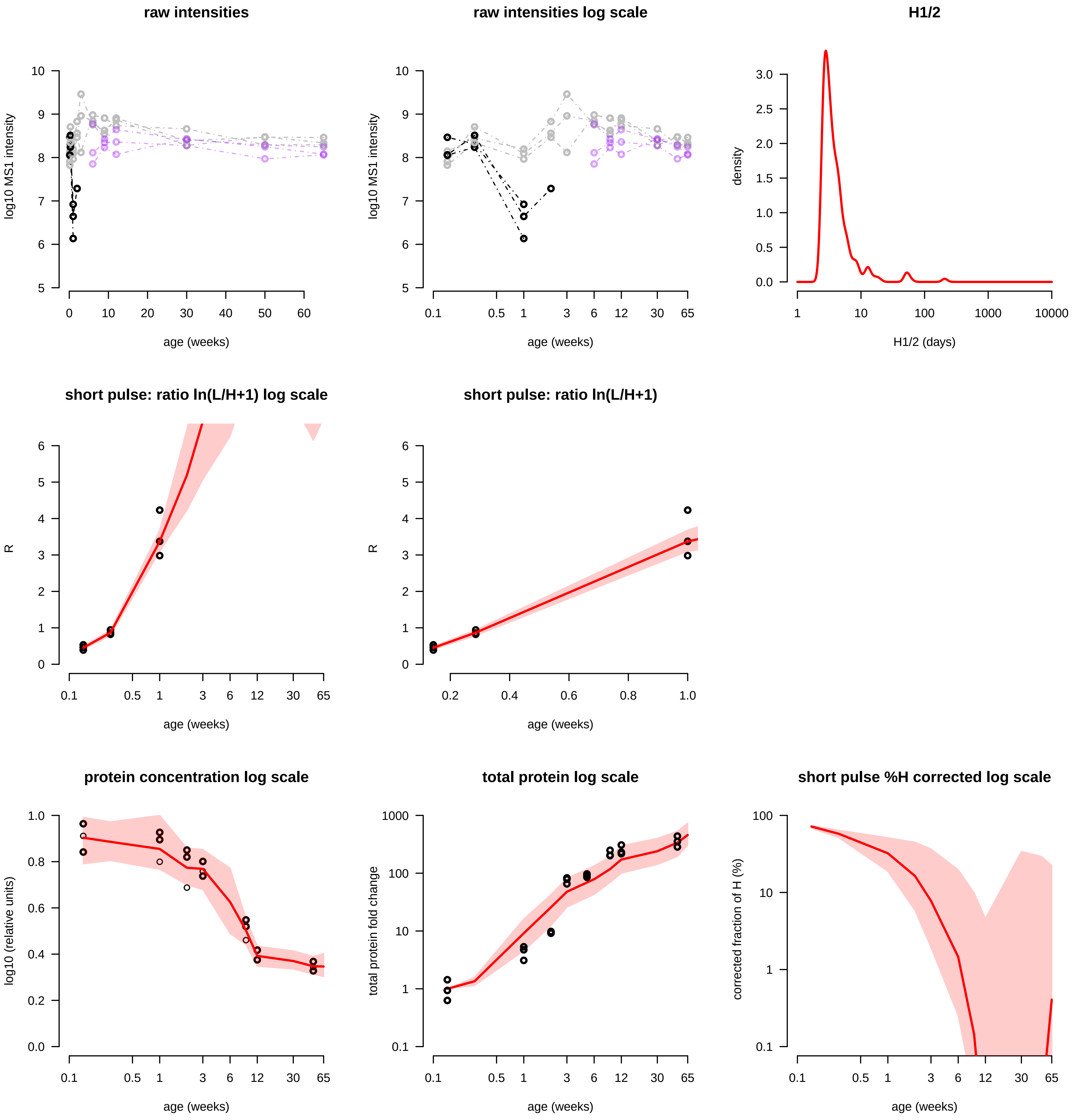

Q9D6J5

>sp|Q9D6J5|NDUB8_MOUSE

NADH dehydrogenase [ubiquinone] 1 beta subcomplex subunit 8, mitochondrial

GN=Ndufb8

Med H1/2: 17.1 days

Q5 H1/2: 4.9 days

Q95 H1/2: 191.3 days

reliable model fit: yes

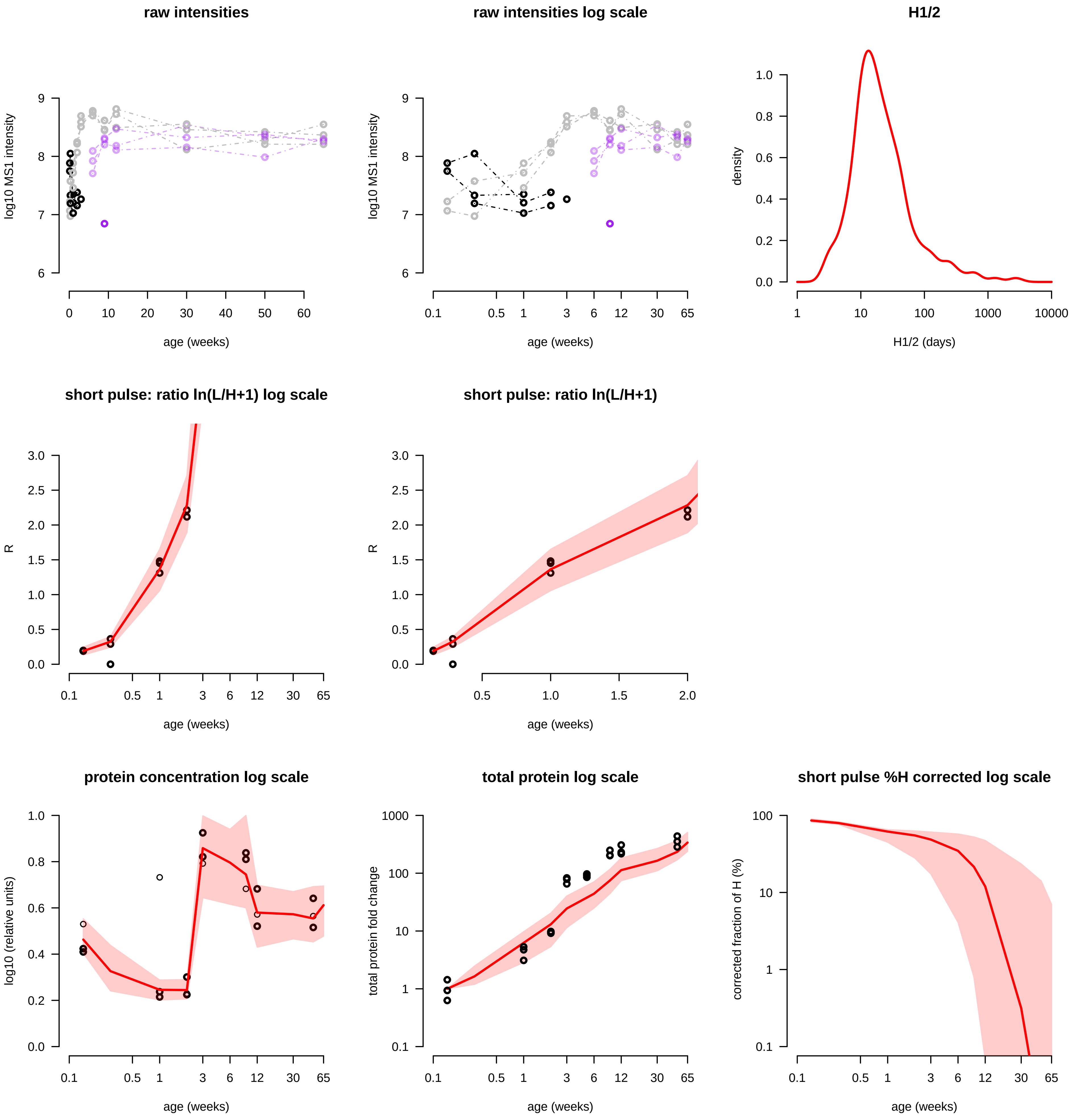

O08599;O08599−2

>sp|O08599|STXB1_MOUSE

Syntaxin−binding protein 1

GN=Stxbp1

Med H1/2: 19.7 days

Q5 H1/2: 13.9 days

Q95 H1/2: 28.9 days

reliable model fit: yes

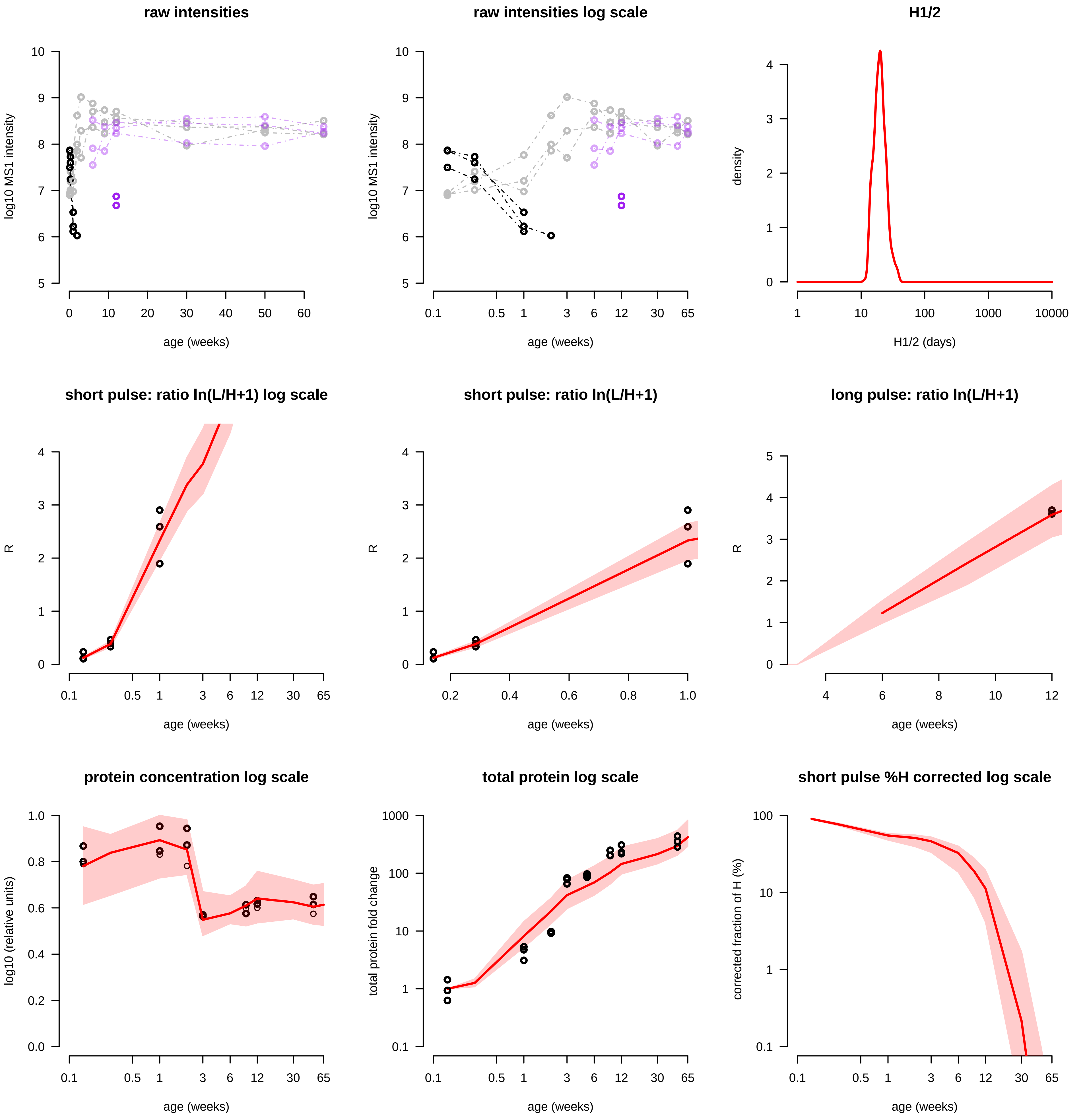

Q8JZM7

>sp|Q8JZM7|CDC73_MOUSE

Parafibromin

GN=Cdc73

Med H1/2: 5.2 days

Q5 H1/2: 2.7 days

Q95 H1/2: 17.4 days

reliable model fit: yes

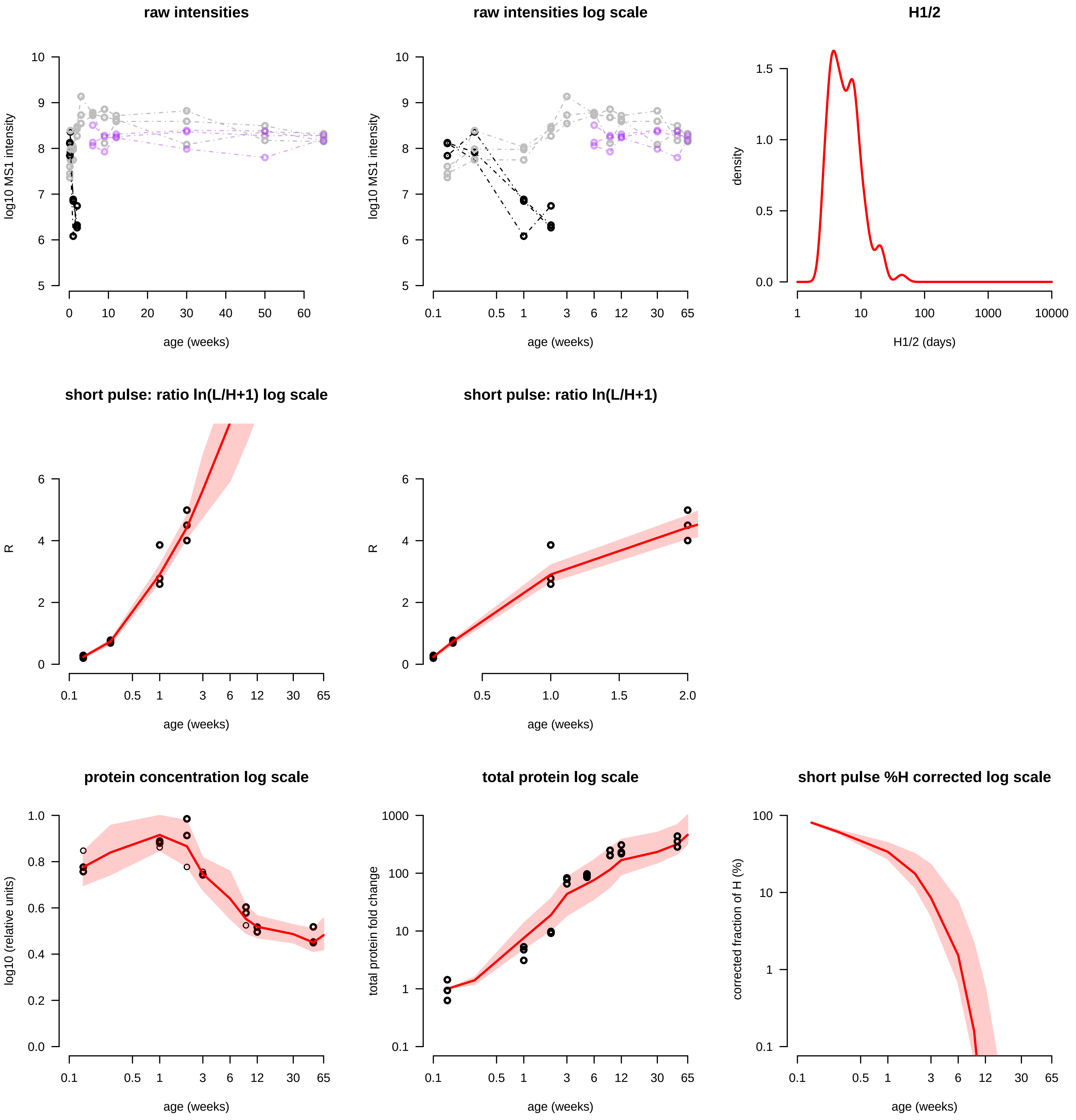

Q99LD9

>sp|Q99LD9|EI2BB_MOUSE

Translation initiation factor eIF−2B subunit beta

GN=Eif2b2

Med H1/2: 10.6 days

Q5 H1/2: 3.7 days

Q95 H1/2: 71.3 days

reliable model fit: yes

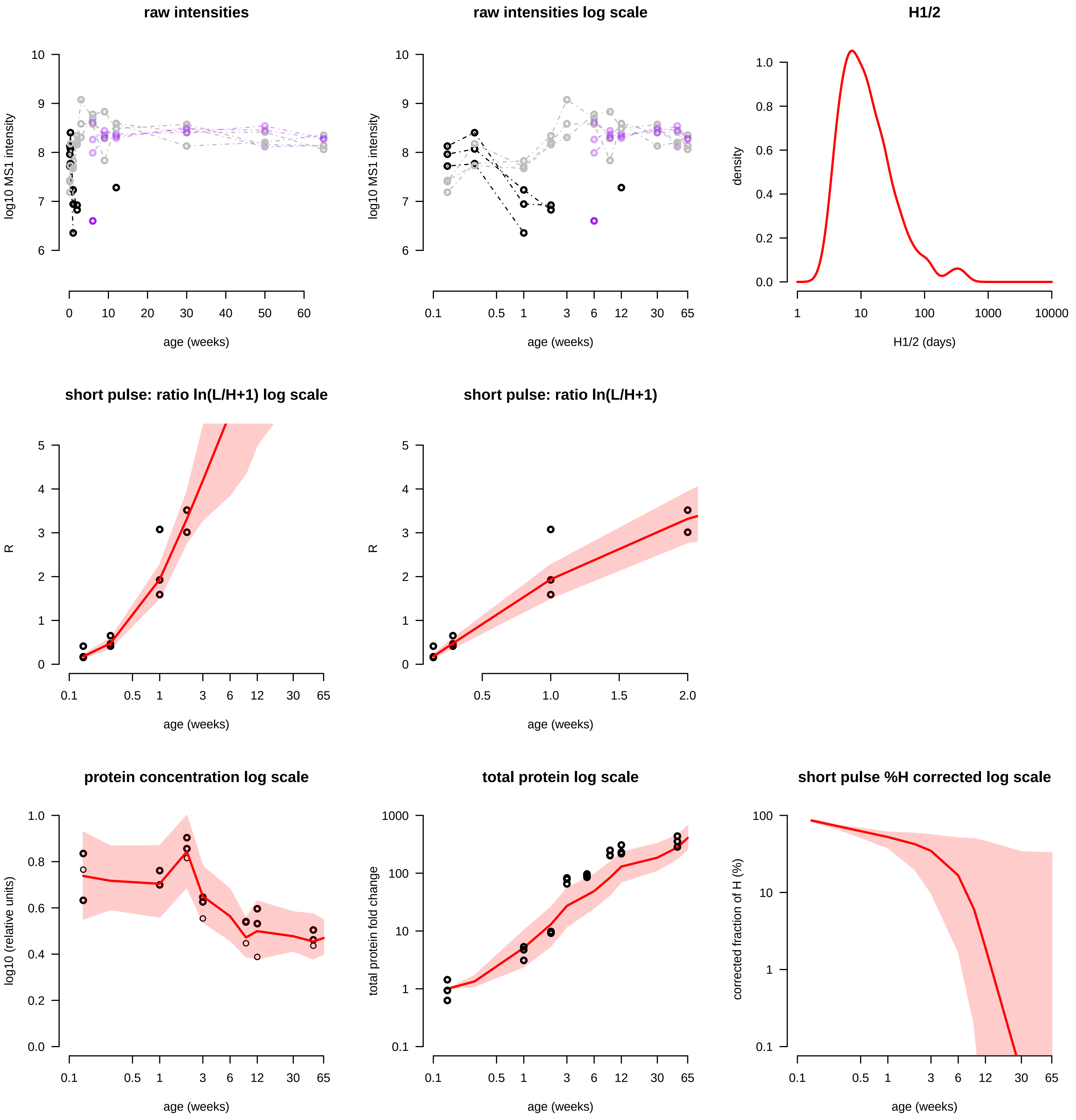

Q3THG9

>sp|Q3THG9|AASD1_MOUSE

Alanyl−tRNA editing protein Aarsd1

GN=Aarsd1

Med H1/2: 9.6 days

Q5 H1/2: 2.8 days

Q95 H1/2: 81.6 days

reliable model fit: yes

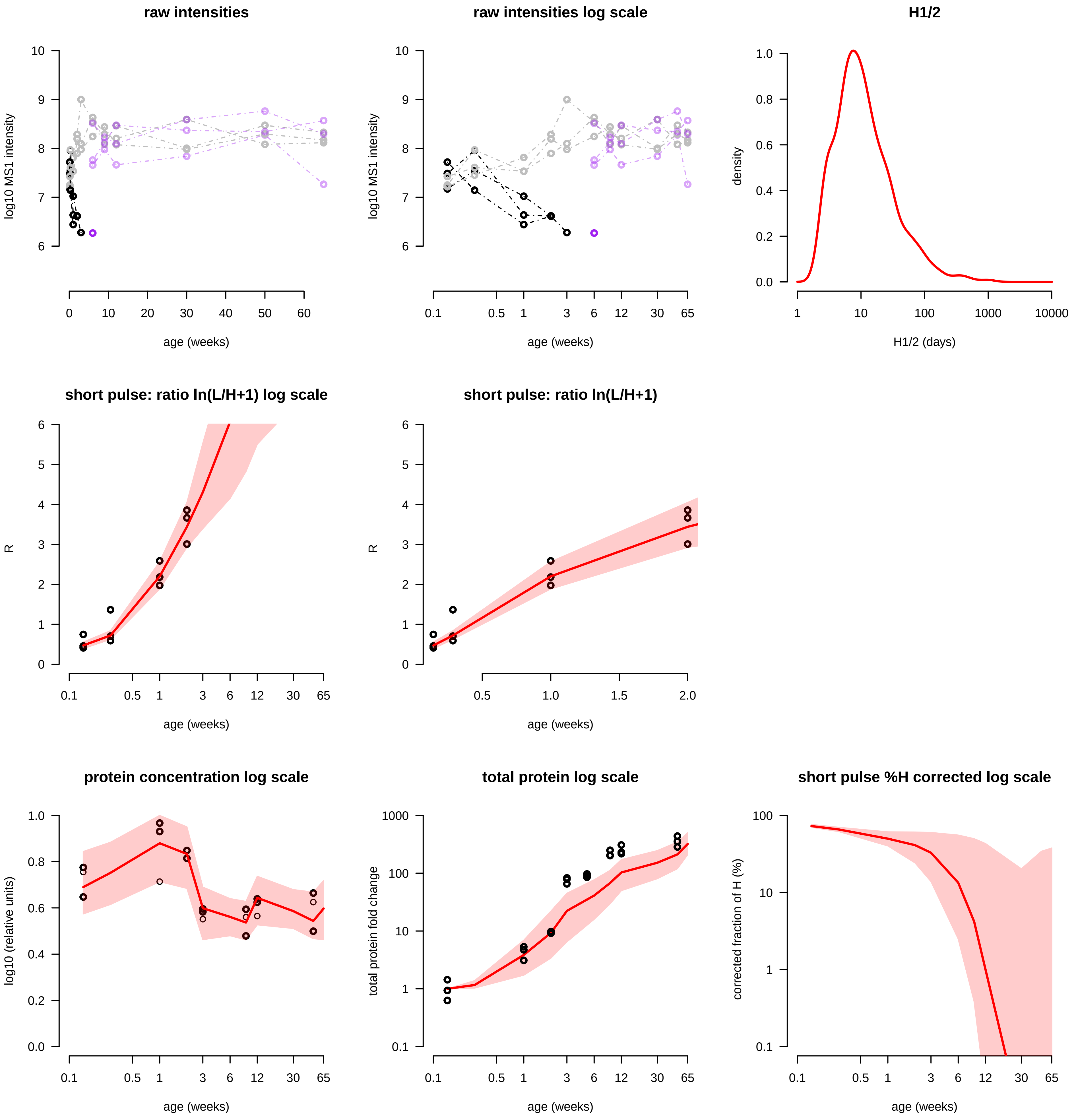

O09174

>sp|O09174|AMACR_MOUSE

Alpha−methylacyl−CoA racemase

GN=Amacr

Med H1/2: 5.8 days

Q5 H1/2: 2.8 days

Q95 H1/2: 26.1 days

reliable model fit: yes

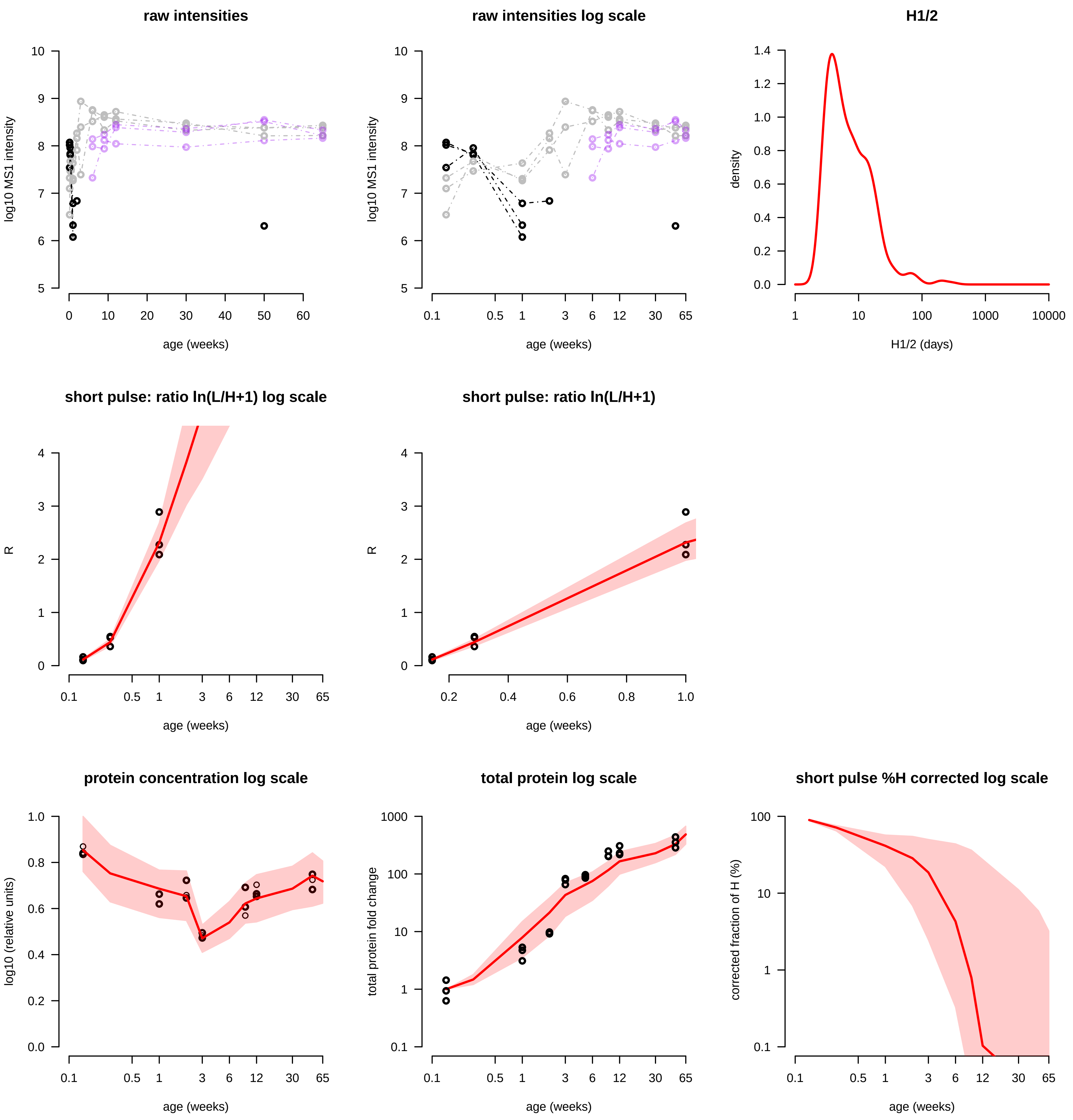

Q9QY81

>sp|Q9QY81|PO210_MOUSE

Nuclear pore membrane glycoprotein 210

GN=Nup210

Med H1/2: 10.6 days

Q5 H1/2: 4.1 days

Q95 H1/2: 48 days

reliable model fit: yes

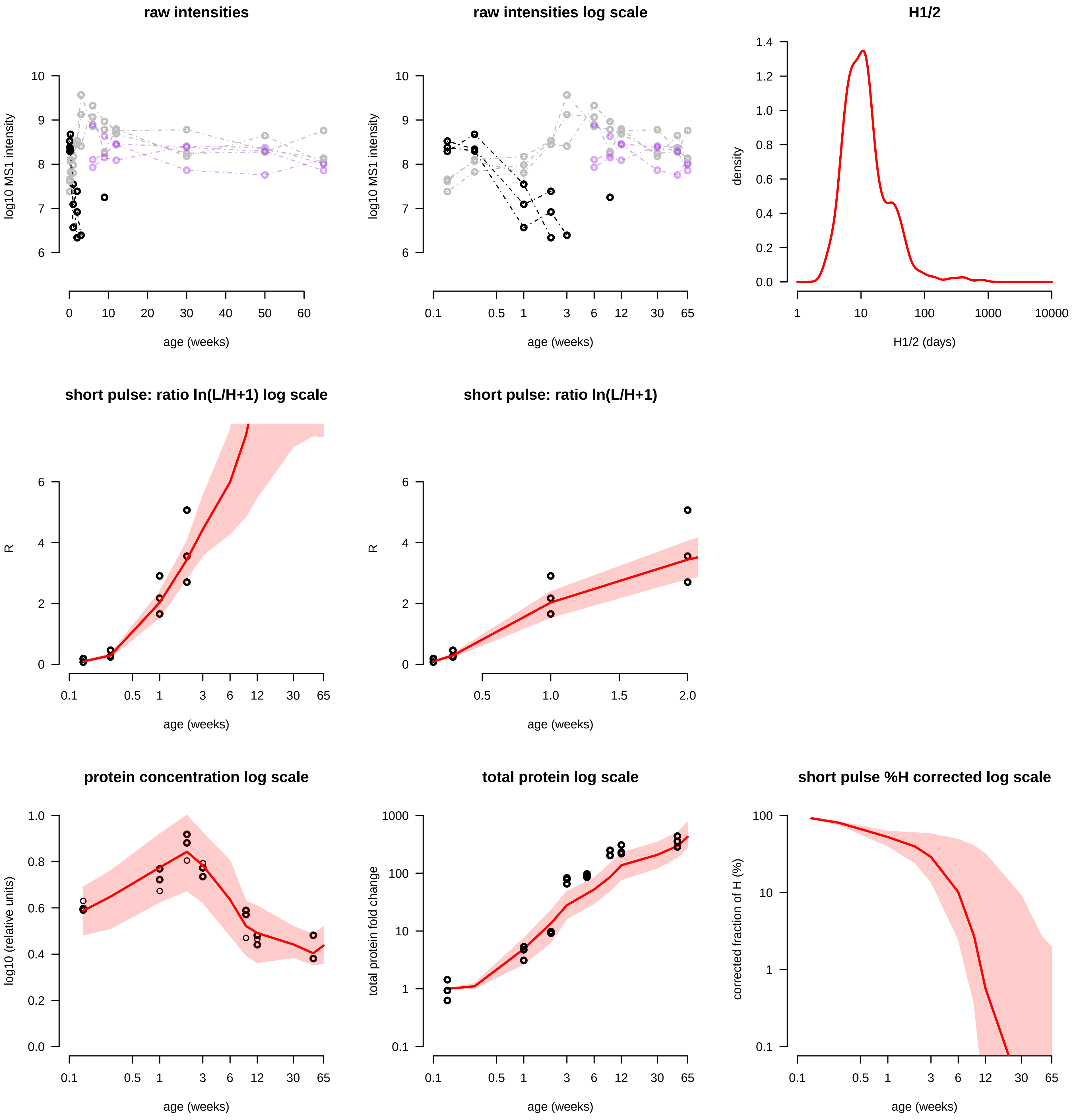

Q6ZWR6−4;Q6ZWR6

>sp|Q6ZWR6−4|SYNE1_MOUSE

Isoform 4 of Nesprin−1

GN=Syne1

Med H1/2: 41.6 days

Q5 H1/2: 20.3 days

Q95 H1/2: 222 days

reliable model fit: yes

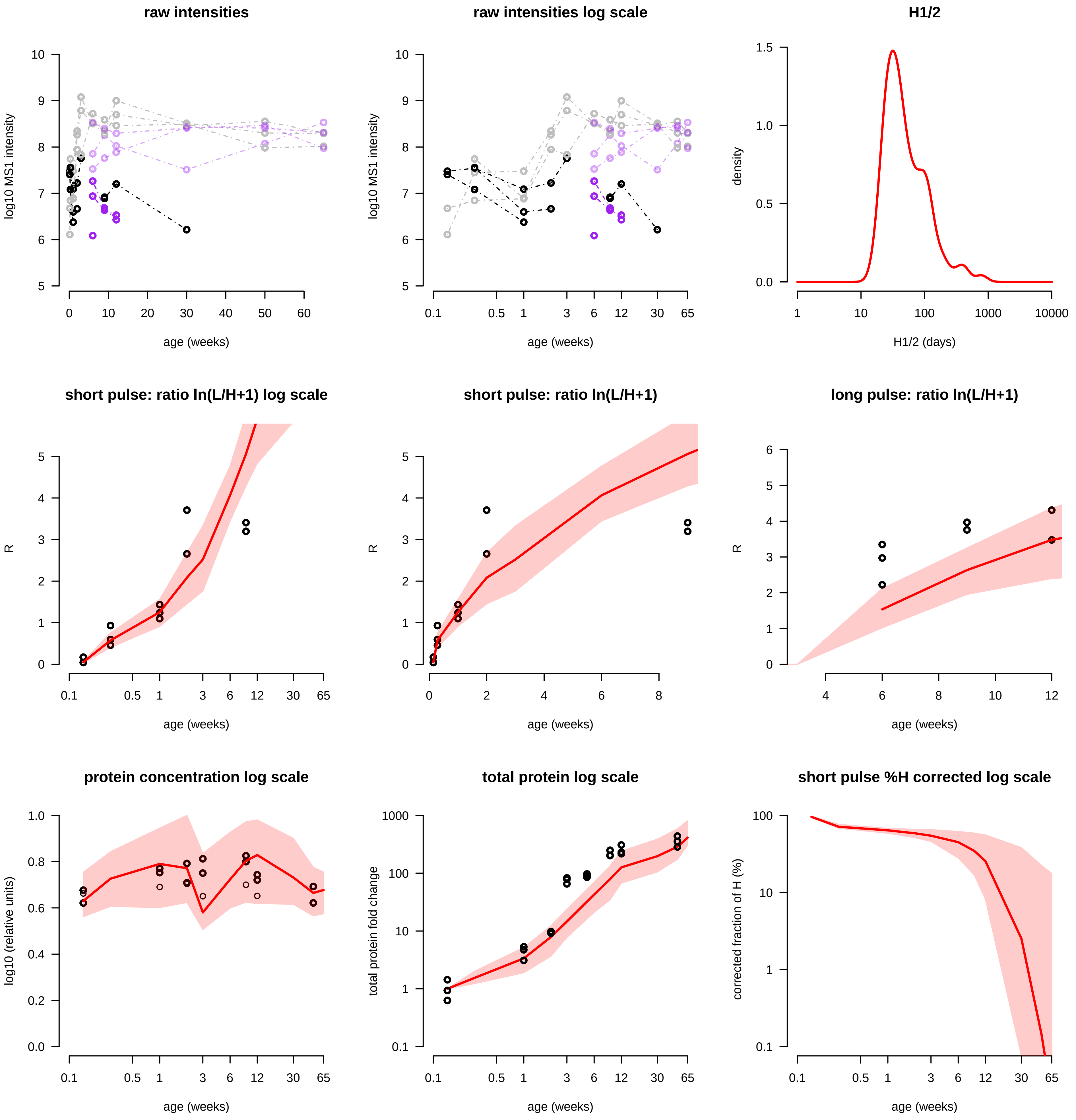

E9Q557

>sp|E9Q557|DESP_MOUSE

Desmoplakin

GN=Dsp

Med H1/2: 14.4 days

Q5 H1/2: 5.6 days

Q95 H1/2: 112.8 days

reliable model fit: yes

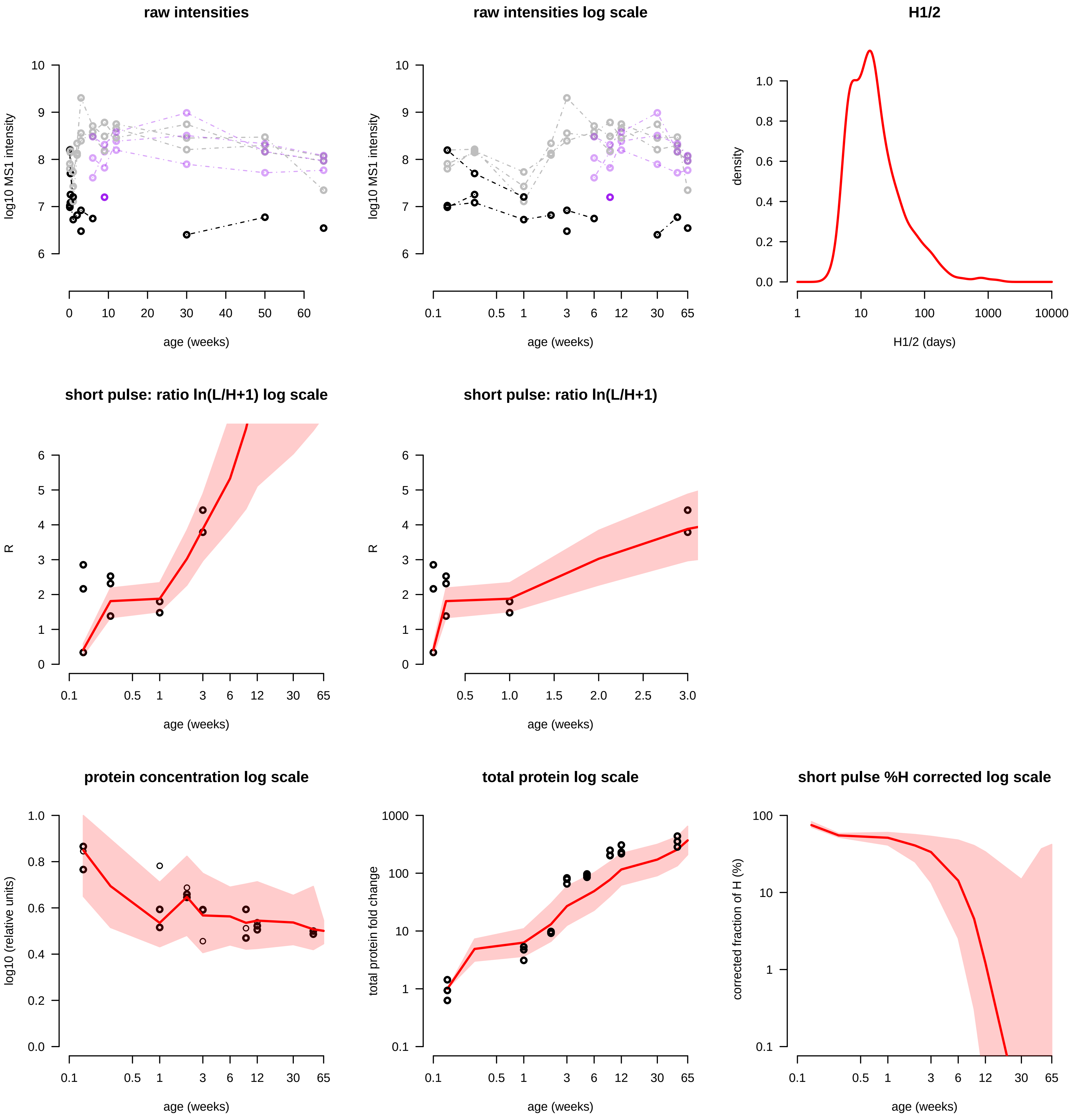

P97868;P97868−2

>sp|P97868|RBBP6_MOUSE

E3 ubiquitin−protein ligase RBBP6

GN=Rbbp6

Med H1/2: 8.8 days

Q5 H1/2: 4.6 days

Q95 H1/2: 52 days

reliable model fit: yes

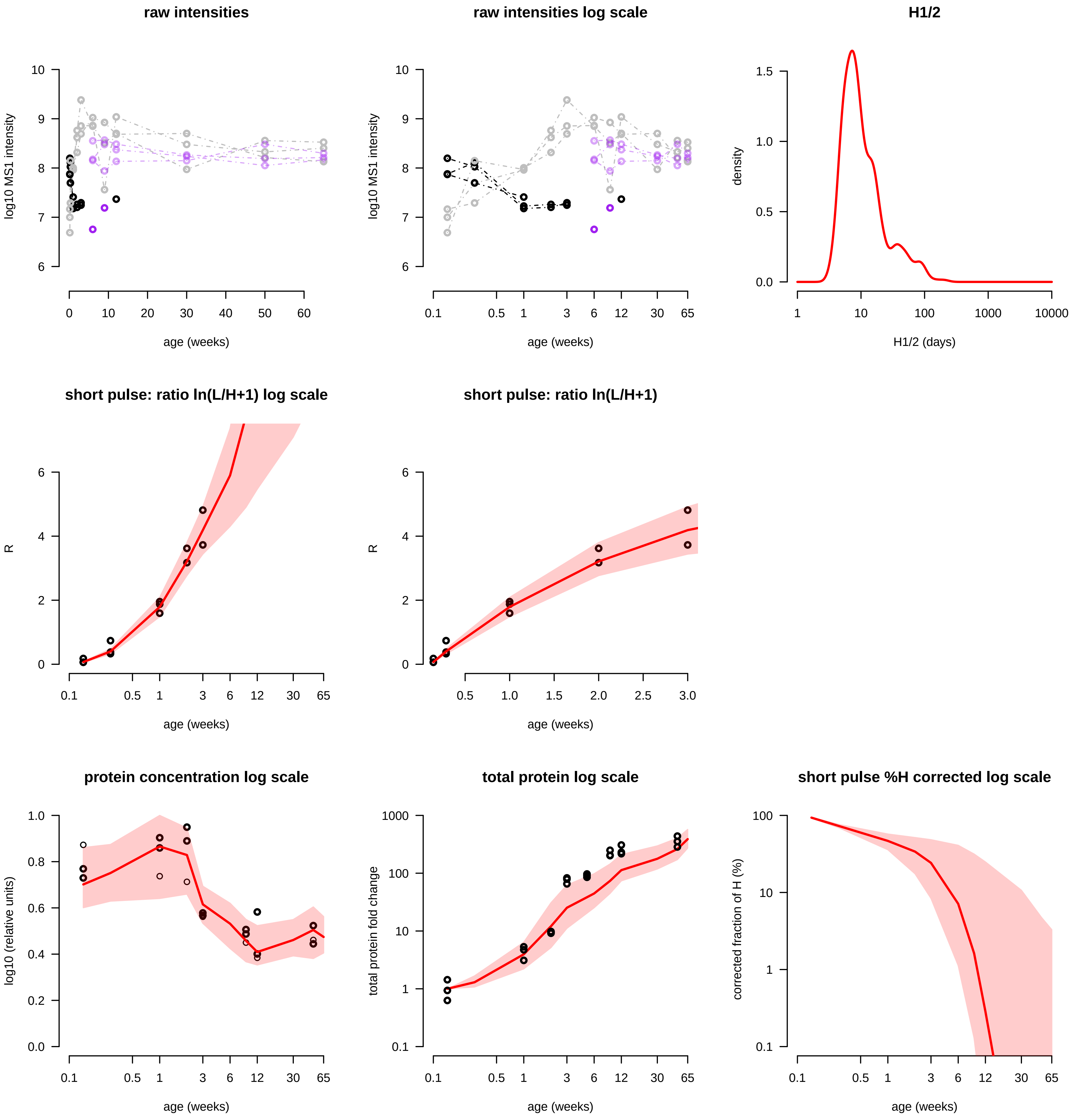

Q80Y81;Q80Y81−2

>sp|Q80Y81|RNZ2_MOUSE

Zinc phosphodiesterase ELAC protein 2

GN=Elac2

Med H1/2: 9.3 days

Q5 H1/2: 3.3 days

Q95 H1/2: 73.5 days

reliable model fit: yes

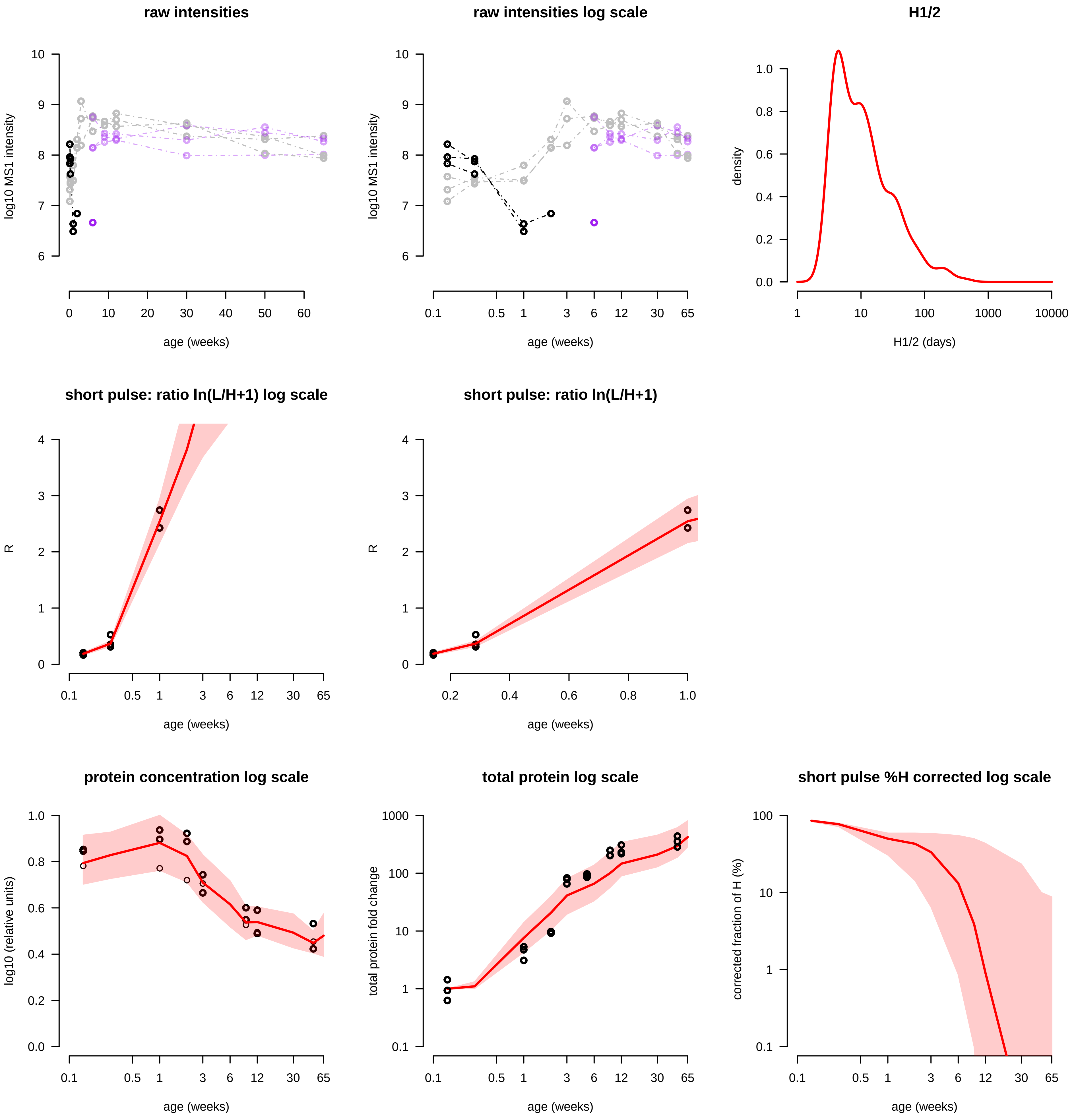

Q8VHK9

>sp|Q8VHK9|DHX36_MOUSE

ATP−dependent DNA/RNA helicase DHX36

GN=Dhx36

Med H1/2: 4283.3 days

Q5 H1/2: 938.5 days

Q95 H1/2: 37397.3 days

reliable model fit: yes

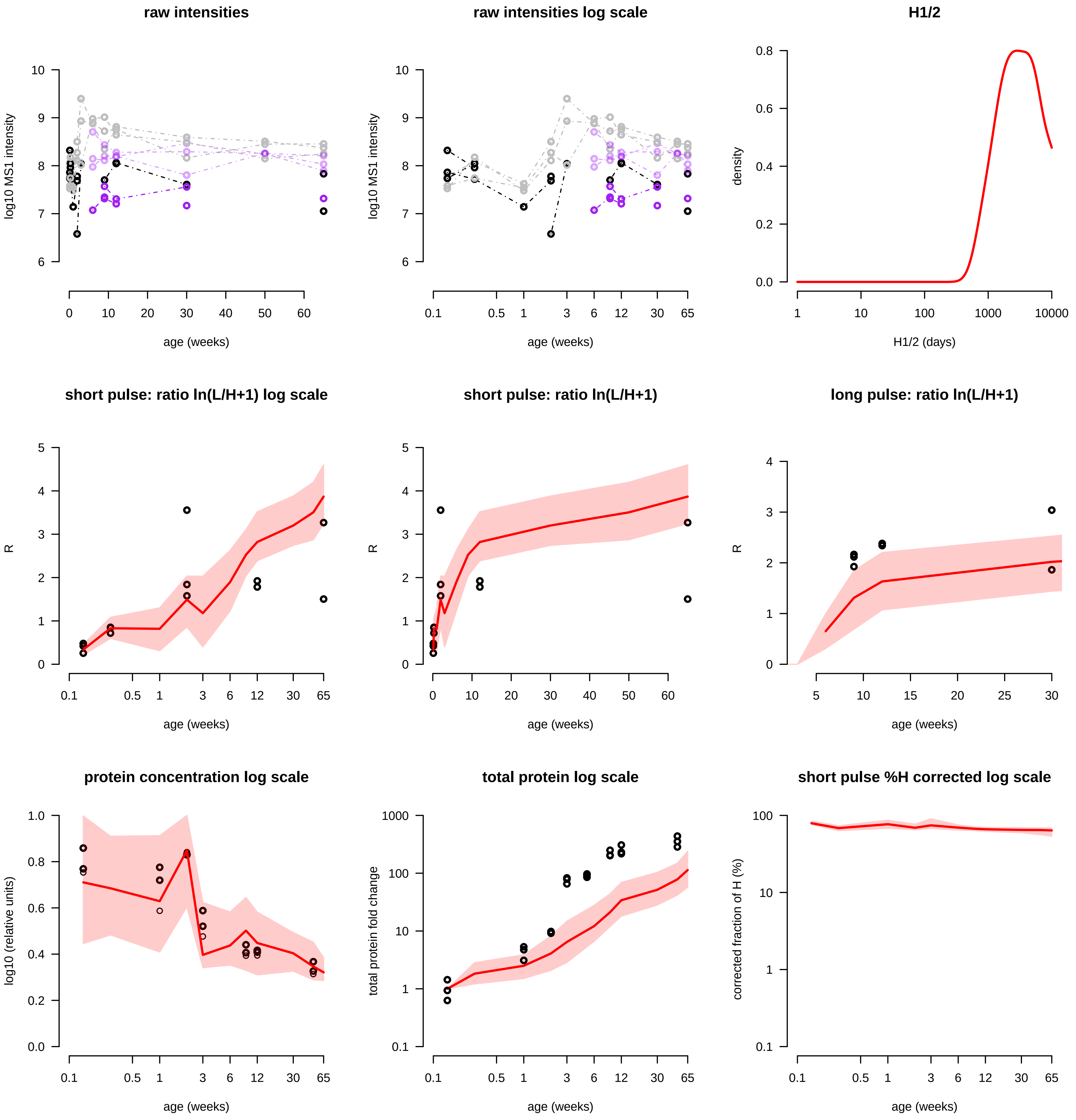

Q8BYA0

>sp|Q8BYA0|TBCD_MOUSE

Tubulin−specific chaperone D

GN=Tbcd

Med H1/2: 10.2 days

Q5 H1/2: 4.7 days

Q95 H1/2: 75.3 days

reliable model fit: yes

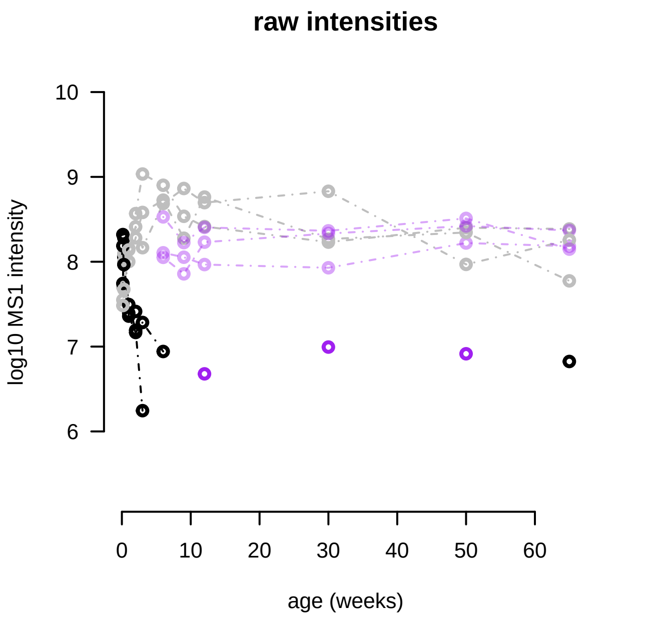

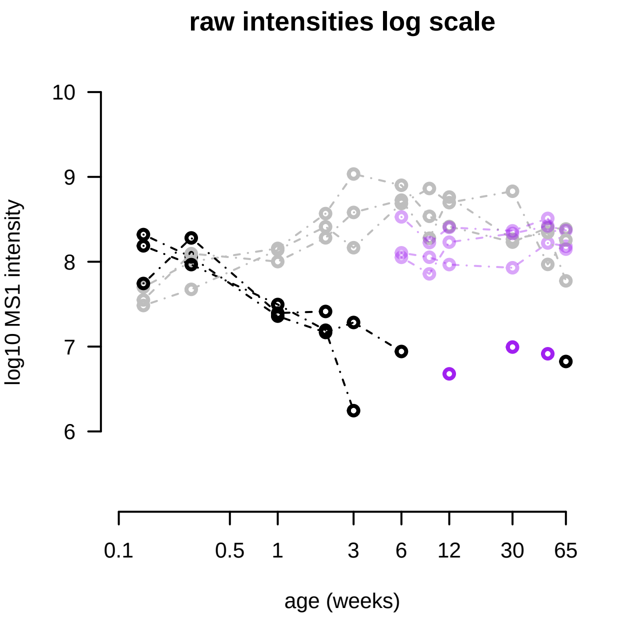

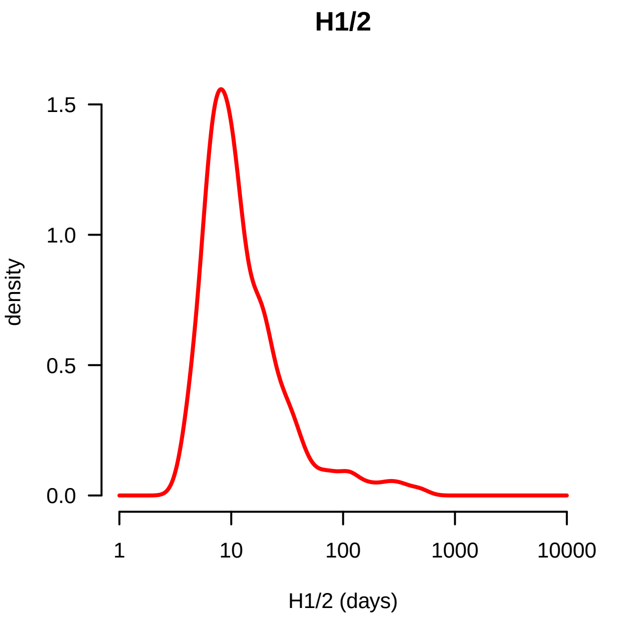

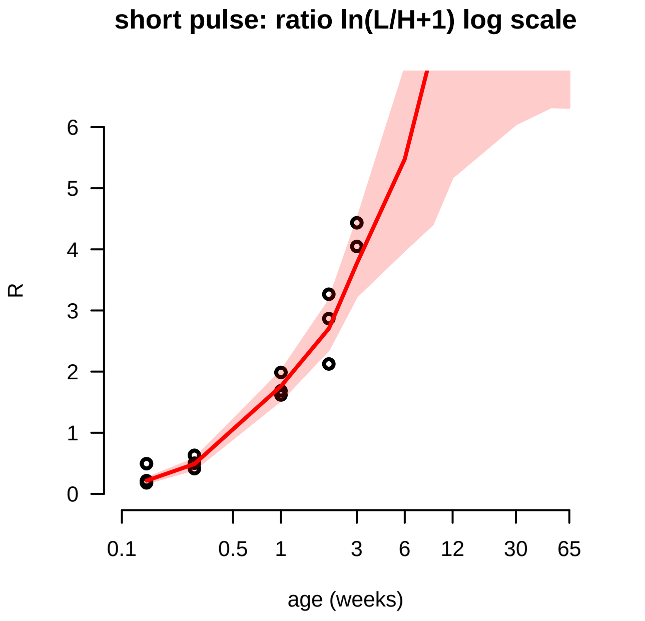

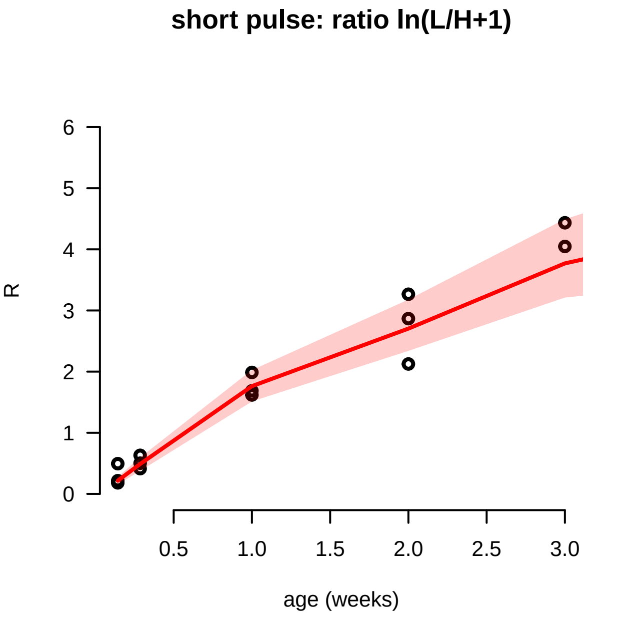

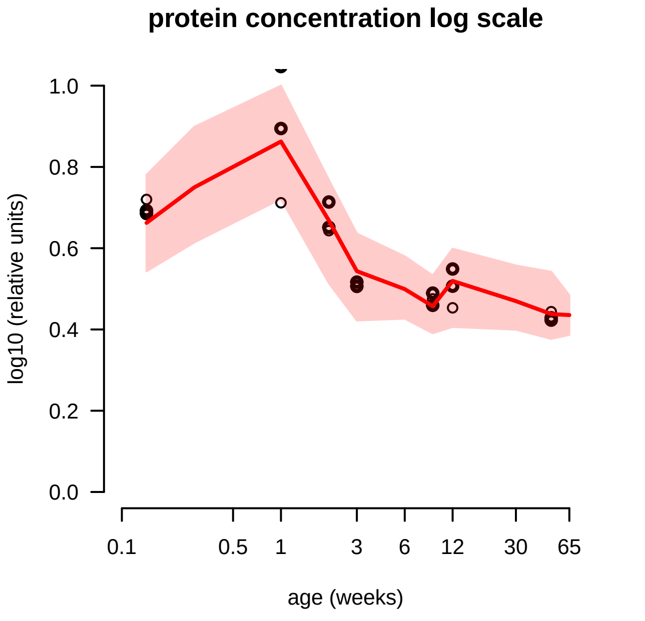

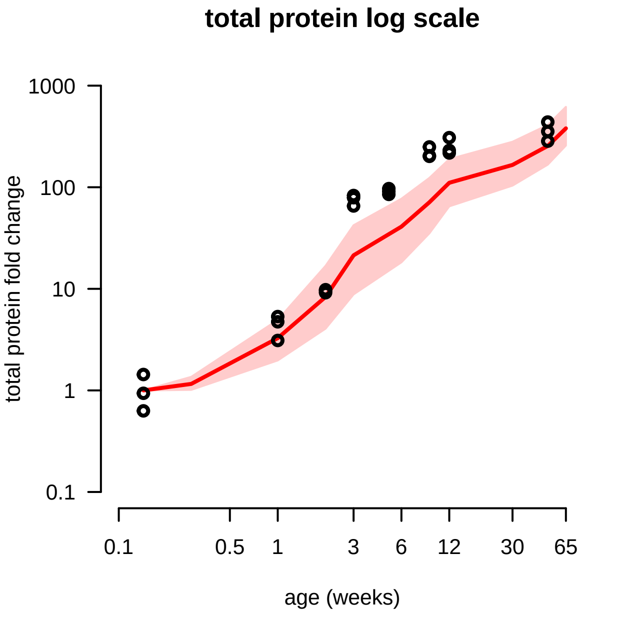

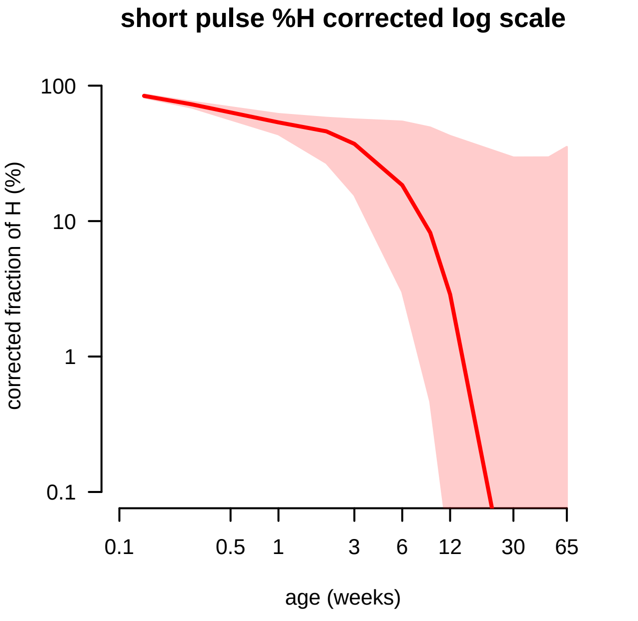

Q9Z0U1

>sp|Q9Z0U1|ZO2_MOUSE

Tight junction protein ZO−2

GN=Tjp2

Med H1/2: 345.8 days

Q5 H1/2: 126.6 days

Q95 H1/2: 2715.2 days

reliable model fit: yes

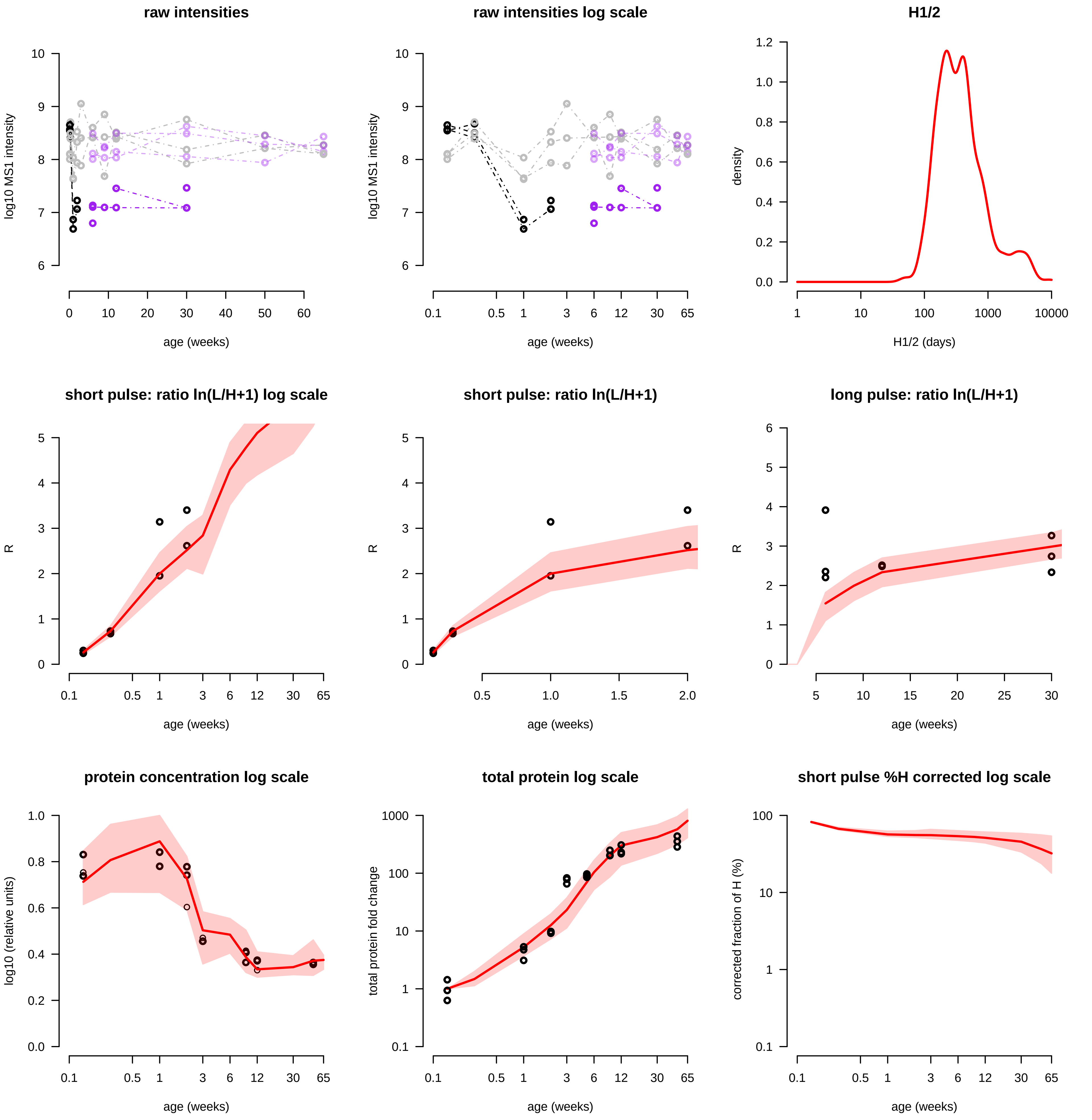

Q8BU31

>sp|Q8BU31|RAP2C_MOUSE

Ras−related protein Rap−2c

GN=Rap2c

Med H1/2: 5.1 days

Q5 H1/2: 4.2 days

Q95 H1/2: 6.6 days

reliable model fit: yes

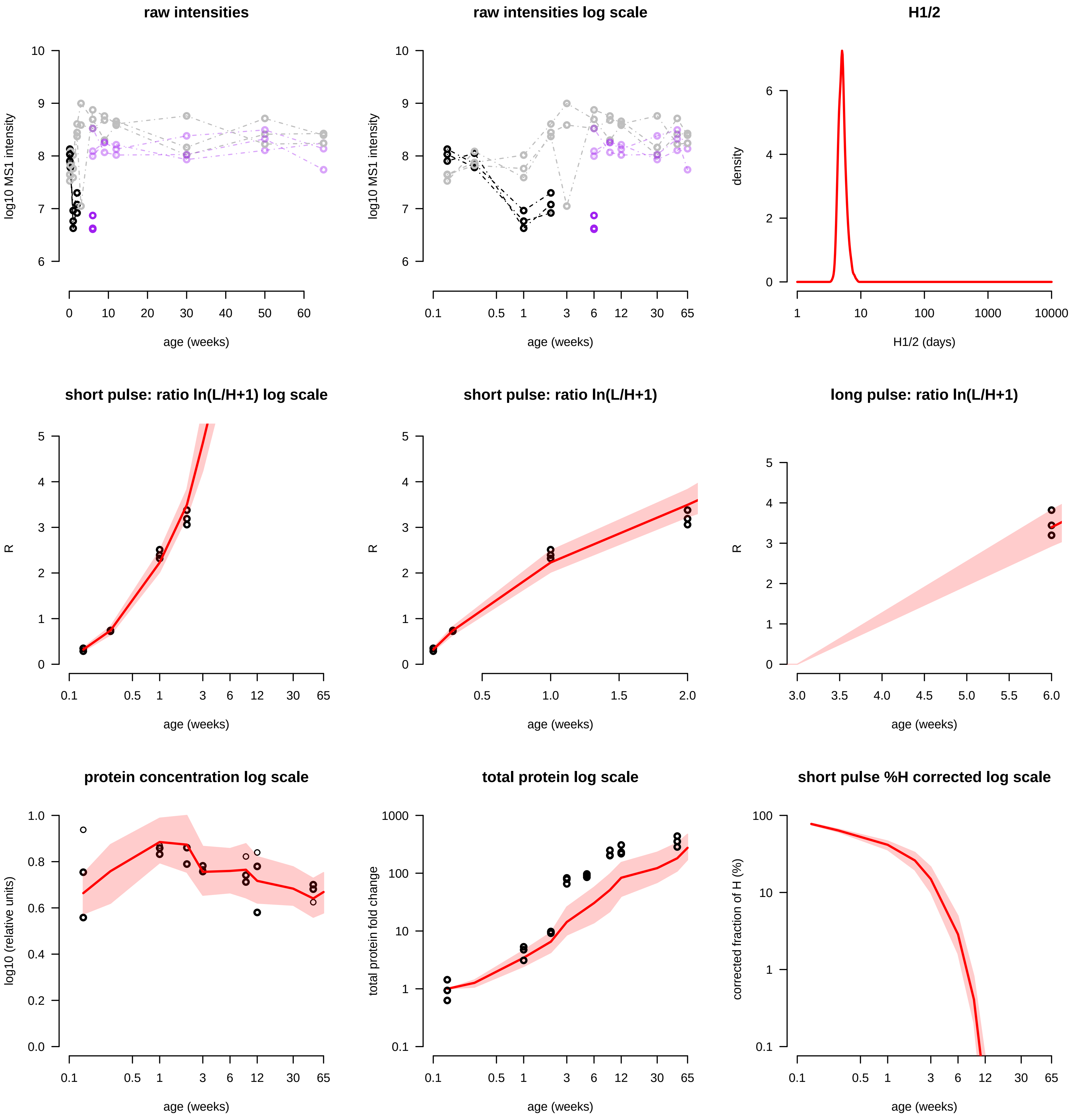

Q99N57;Q99N57−2

>sp|Q99N57|RAF1_MOUSE

RAF proto−oncogene serine/threonine−protein kinase

GN=Raf1

Med H1/2: 4.5 days

Q5 H1/2: 3.4 days

Q95 H1/2: 6.7 days

reliable model fit: yes

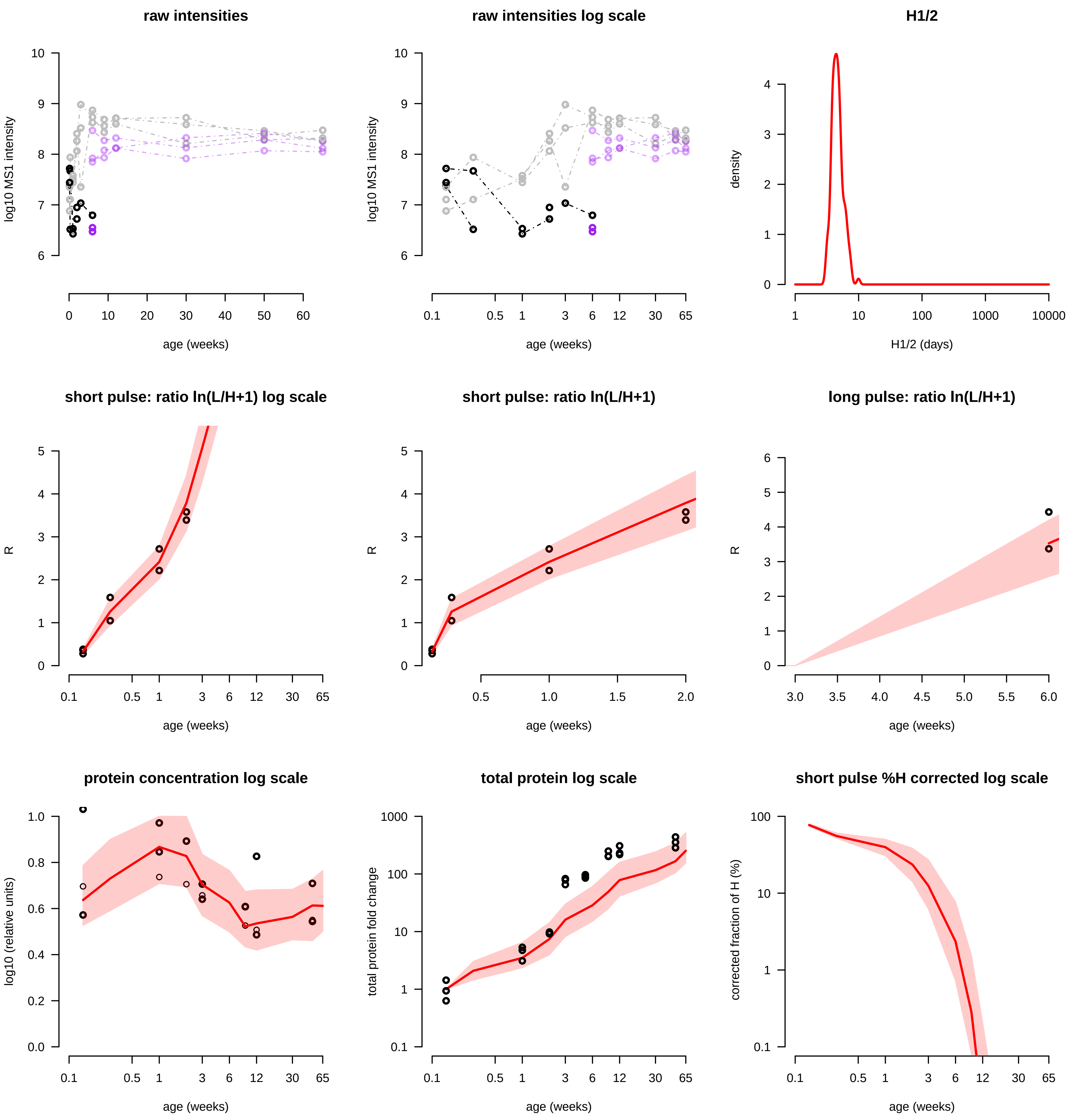

Q9D902

>sp|Q9D902|T2EB_MOUSE

General transcription factor IIE subunit 2

GN=Gtf2e2

Med H1/2: 9.5 days

Q5 H1/2: 4.1 days

Q95 H1/2: 61.9 days

reliable model fit: yes

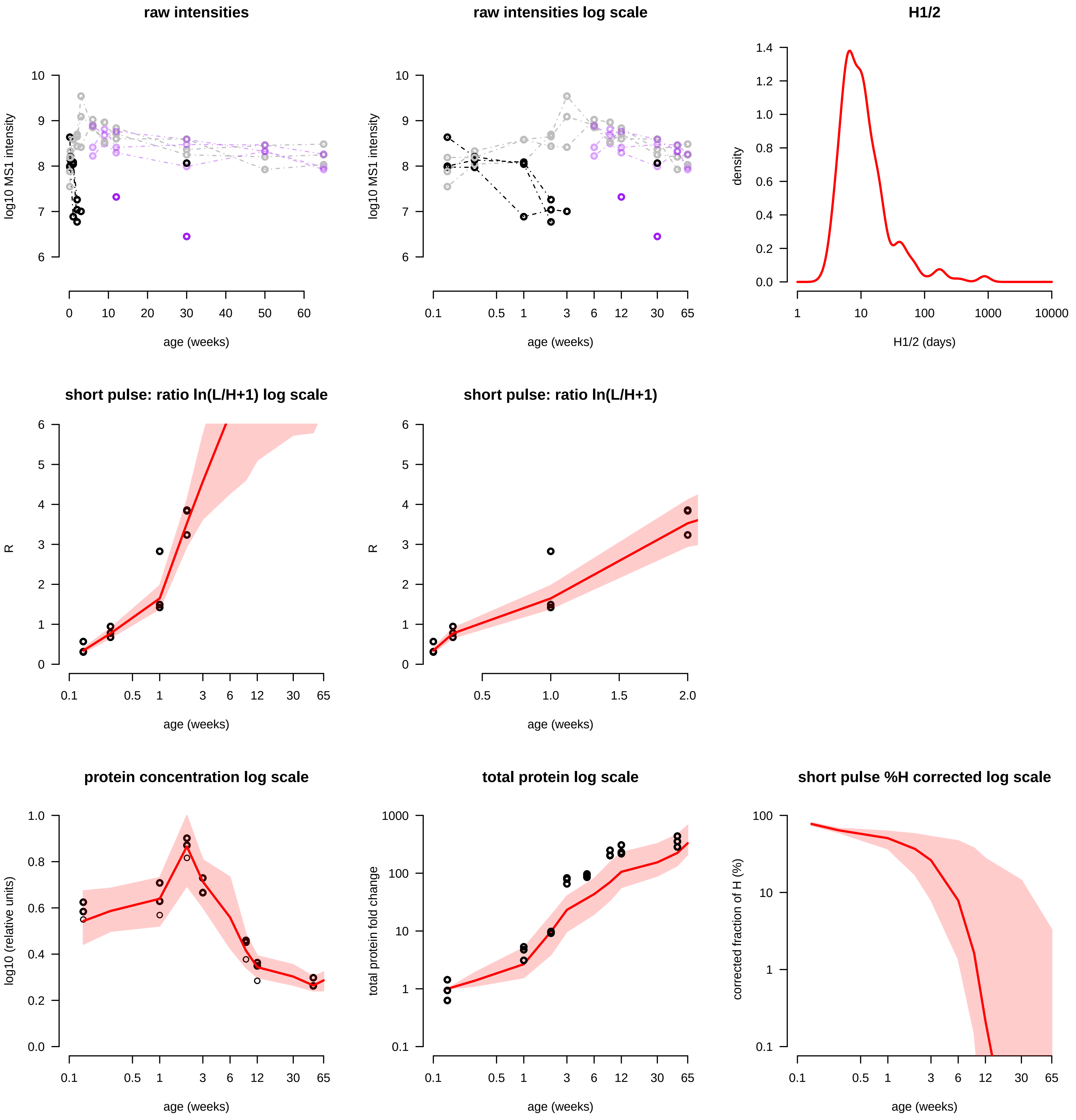

Q8CI11;Q8CI11−2

>sp|Q8CI11|GNL3_MOUSE

Guanine nucleotide−binding protein−like 3

GN=Gnl3

Med H1/2: 5.7 days

Q5 H1/2: 3.9 days

Q95 H1/2: 9.7 days

reliable model fit: yes

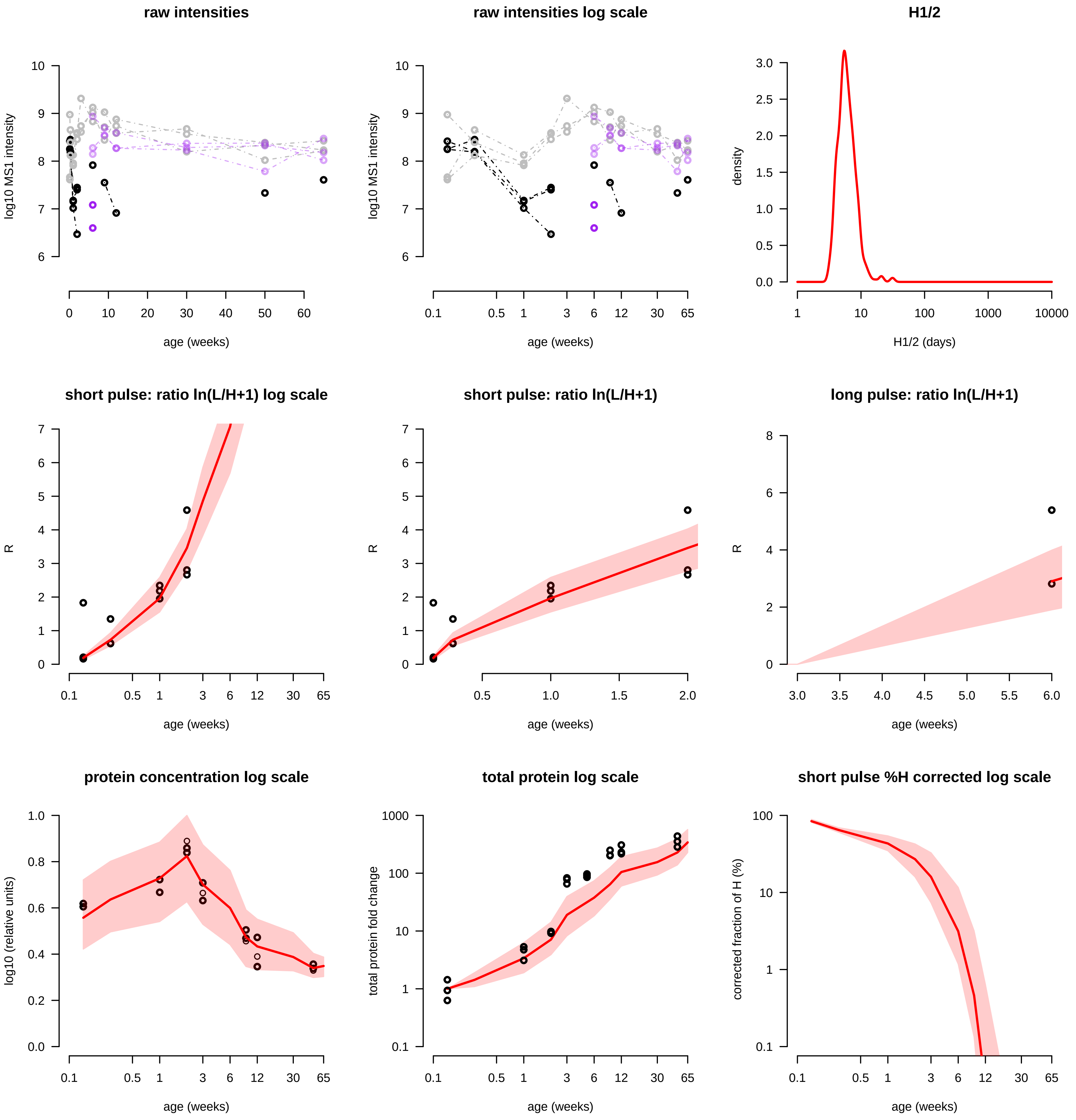

Q9JJA4

>sp|Q9JJA4|WDR12_MOUSE

Ribosome biogenesis protein WDR12

GN=Wdr12

Med H1/2: 3.9 days

Q5 H1/2: 2.8 days

Q95 H1/2: 5.3 days

reliable model fit: yes

raw intensities

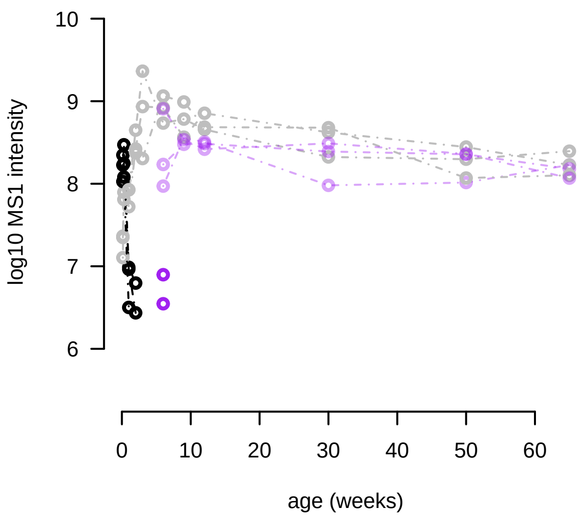

raw intensities log scale

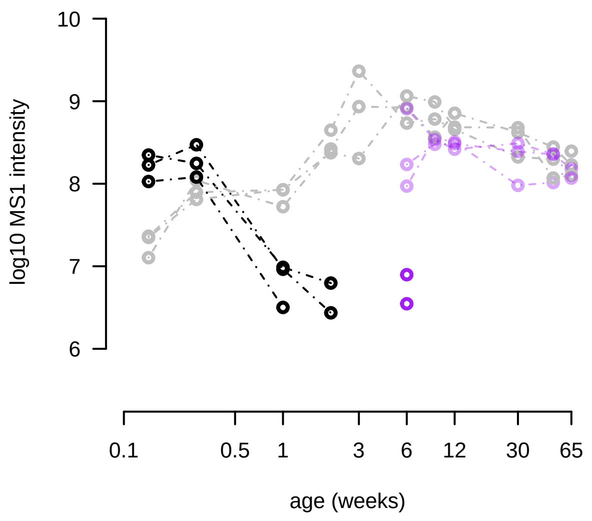

H1/2

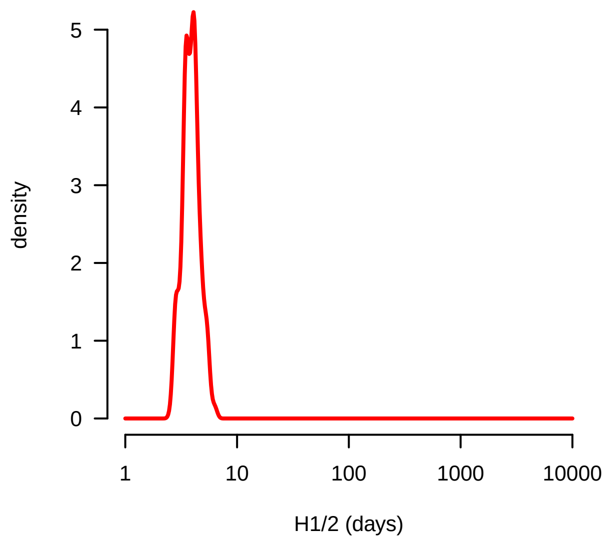

short pulse: ratio ln(L/H+1) log scale

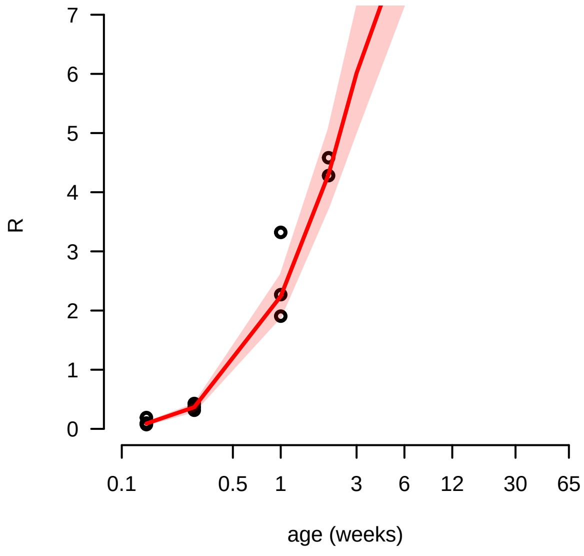

short pulse: ratio ln(L/H+1)

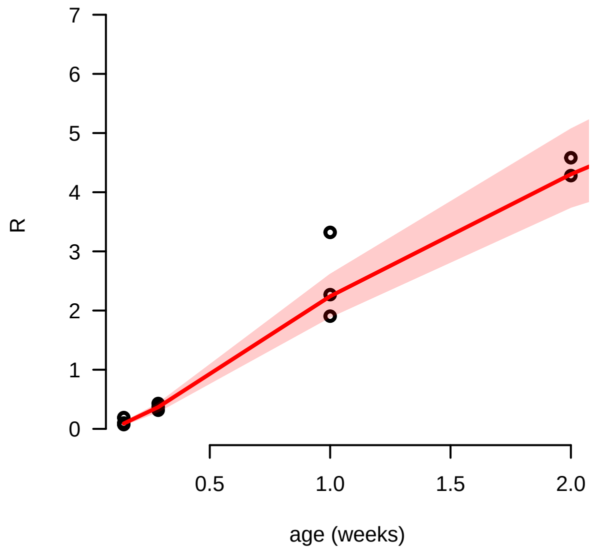

long pulse: ratio ln(L/H+1)

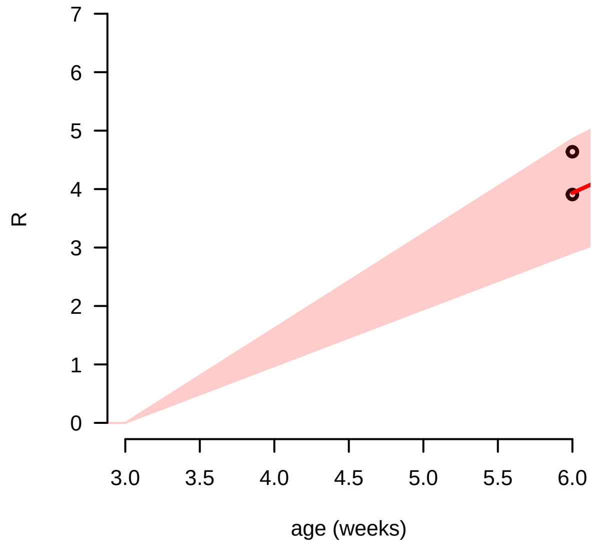

protein concentration log scale

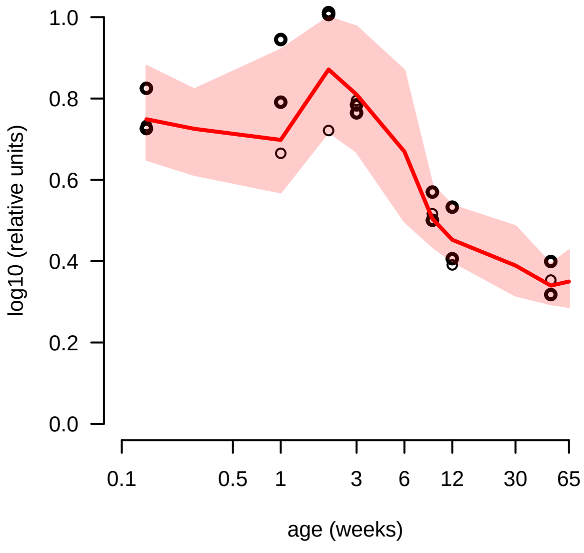

total protein log scale

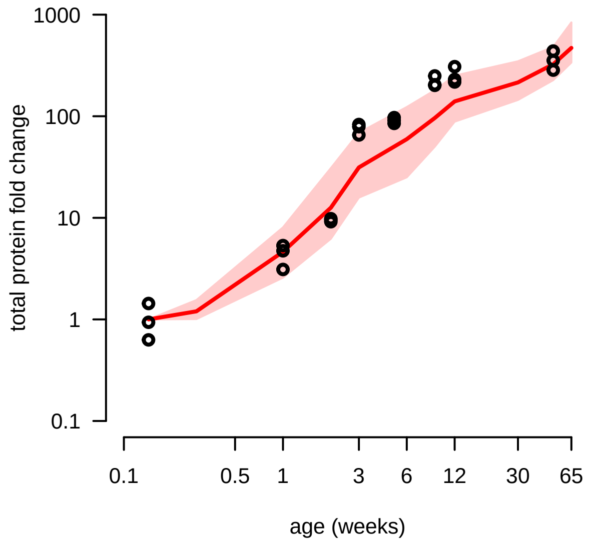

short pulse %H corrected log scale

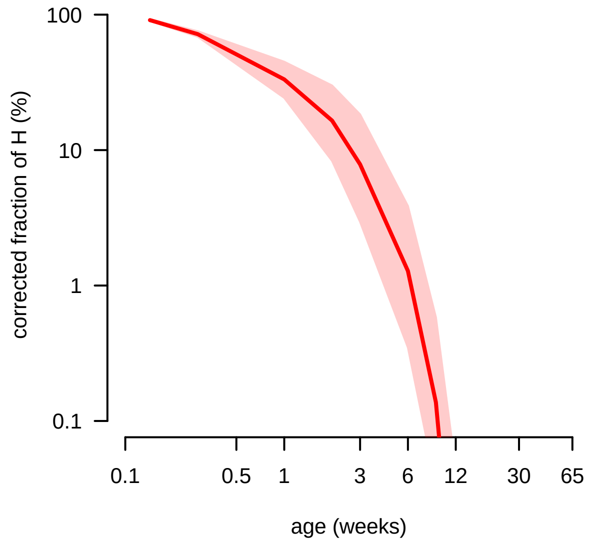

Q9CQN7

>sp|Q9CQN7|RM41_MOUSE

39S ribosomal protein L41, mitochondrial

GN=Mrpl41

Med H1/2: 7.1 days

Q5 H1/2: 2.7 days

Q95 H1/2: 50.2 days

reliable model fit: yes

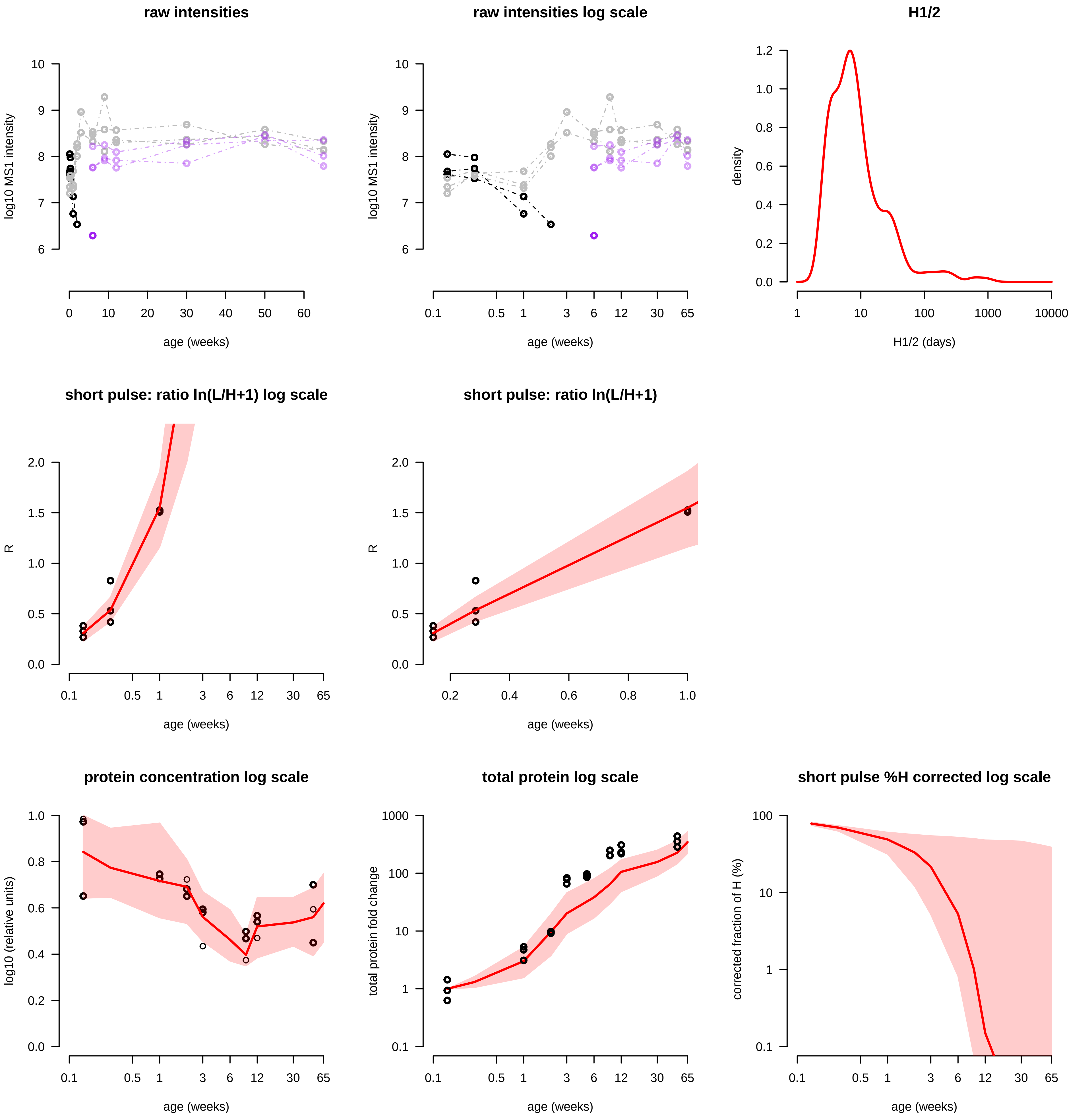

Q9CPU4

>sp|Q9CPU4|MGST3_MOUSE

Microsomal glutathione S−transferase 3

GN=Mgst3

Med H1/2: 7.6 days

Q5 H1/2: 6.2 days

Q95 H1/2: 10.2 days

reliable model fit: yes

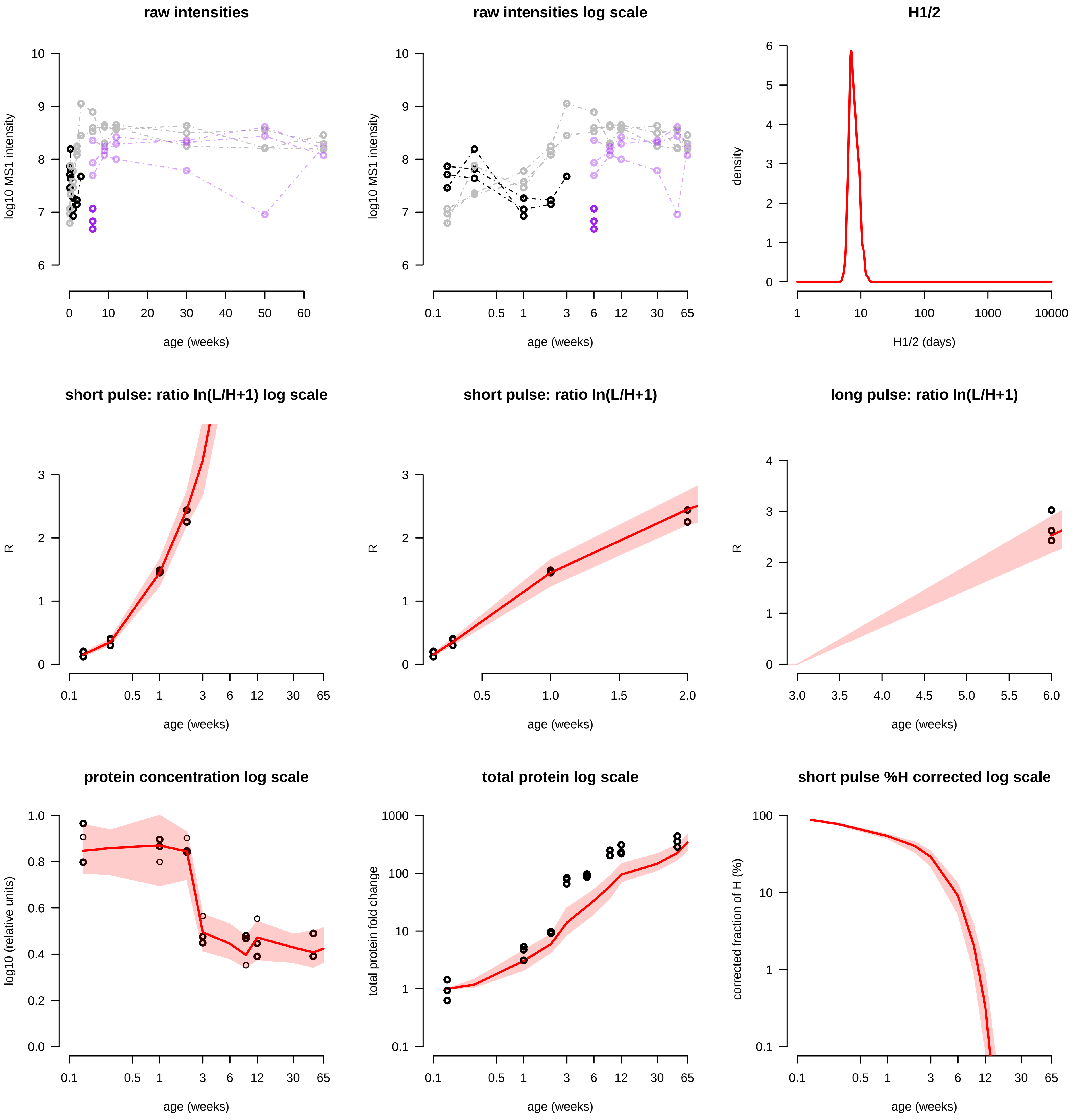

Q8CEC0−2;Q8CEC0;Q8CEC0−3

>sp|Q8CEC0−2|NUP88_MOUSE

Isoform 2 of Nuclear pore complex protein Nup88

GN=Nup88

Med H1/2: 16.8 days

Q5 H1/2: 6.5 days

Q95 H1/2: 134.7 days

reliable model fit: yes

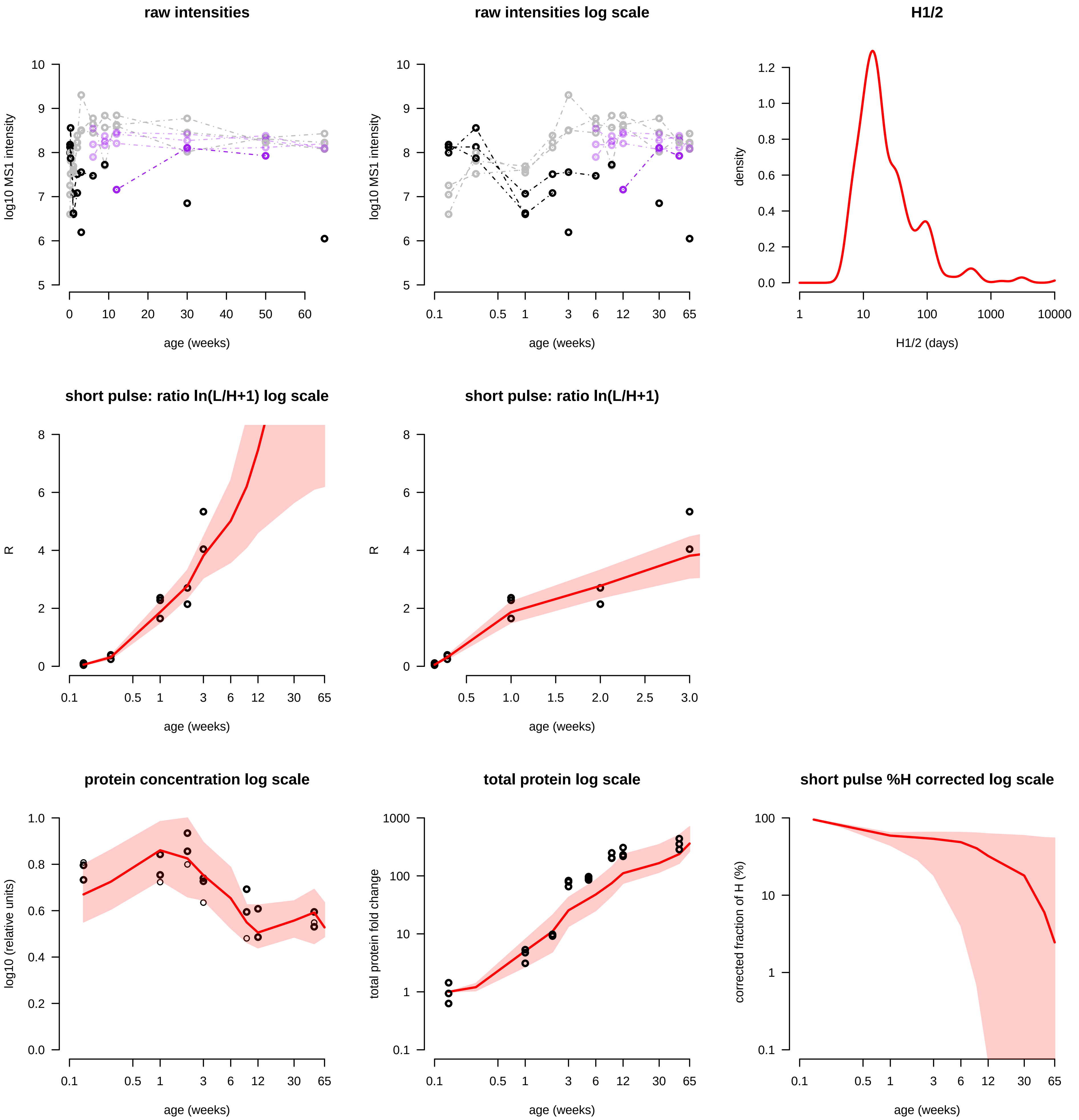

Q6KCD5;Q6KCD5−2

>sp|Q6KCD5|NIPBL_MOUSE

Nipped−B−like protein

GN=Nipbl

Med H1/2: 3710.8 days

Q5 H1/2: 715.7 days

Q95 H1/2: 36376.4 days

reliable model fit: no

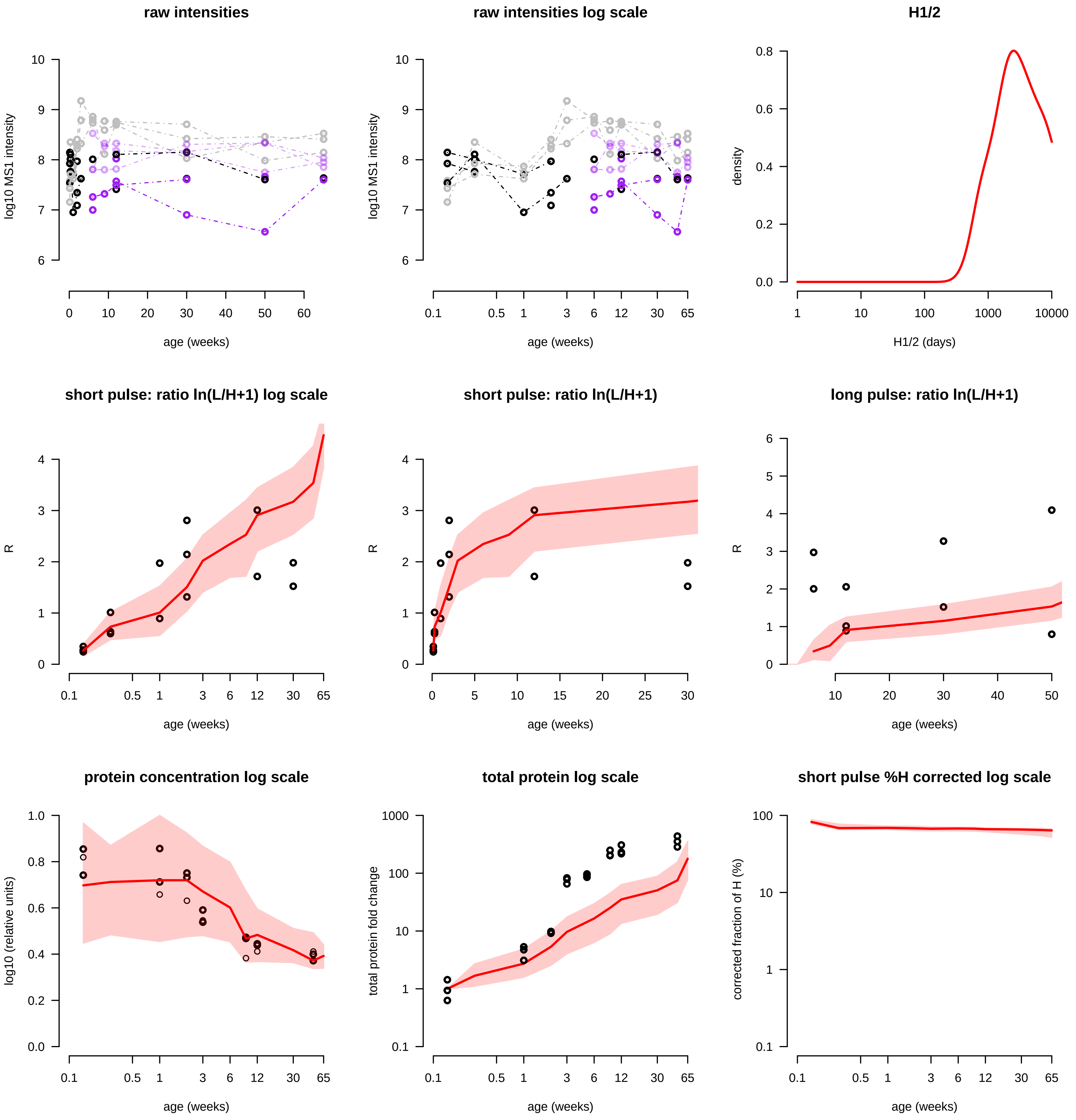

Q99KG3−3;Q99KG3

>sp|Q99KG3−3|RBM10_MOUSE

Isoform 3 of RNA−binding protein 10

GN=Rbm10

Med H1/2: 9.2 days

Q5 H1/2: 4.2 days

Q95 H1/2: 84 days

reliable model fit: yes

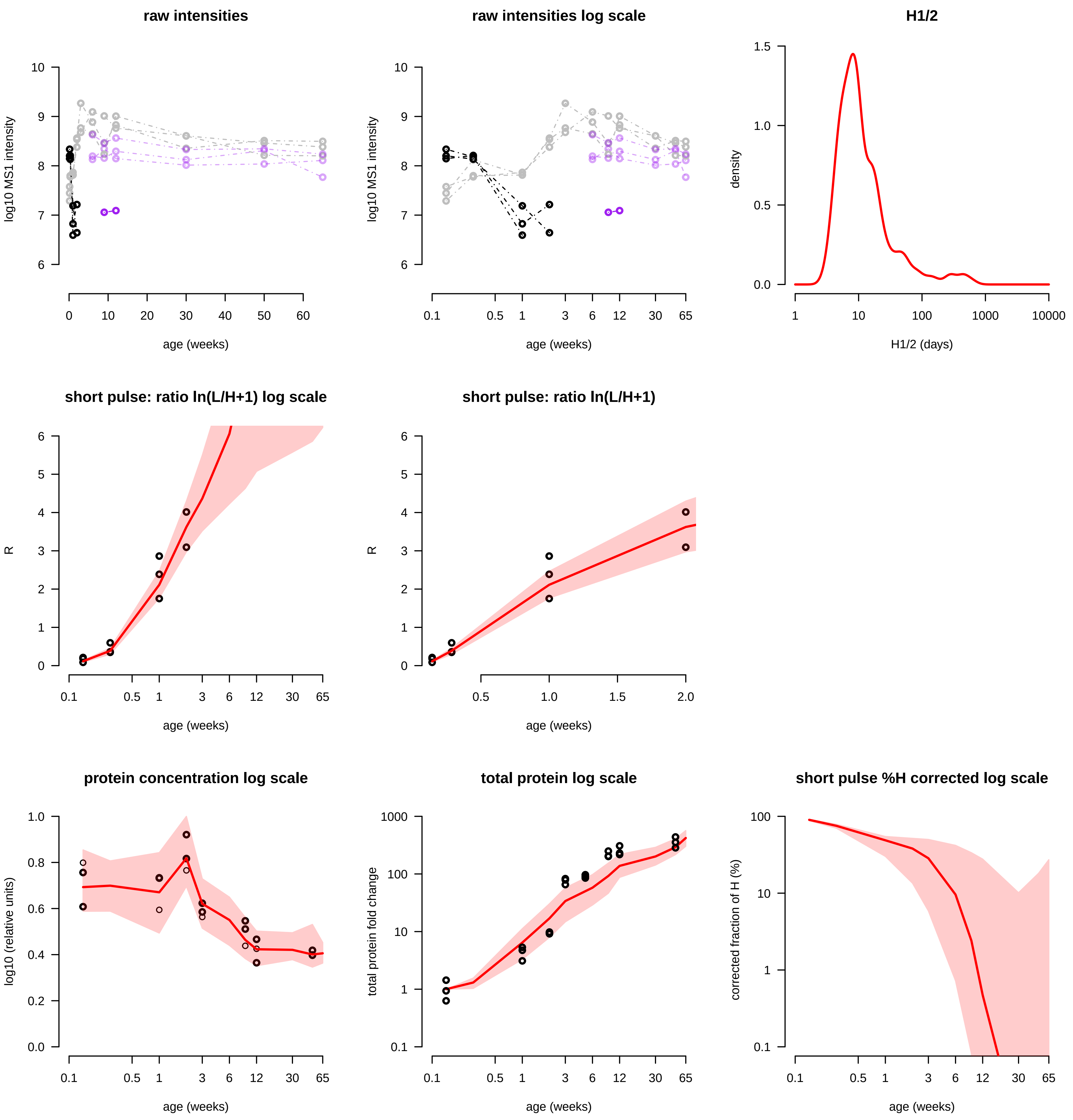

Q6NWW9

>sp|Q6NWW9|FND3B_MOUSE

Fibronectin type III domain−containing protein 3B

GN=Fndc3b

Med H1/2: 6.3 days

Q5 H1/2: 2.7 days

Q95 H1/2: 59.2 days

reliable model fit: yes

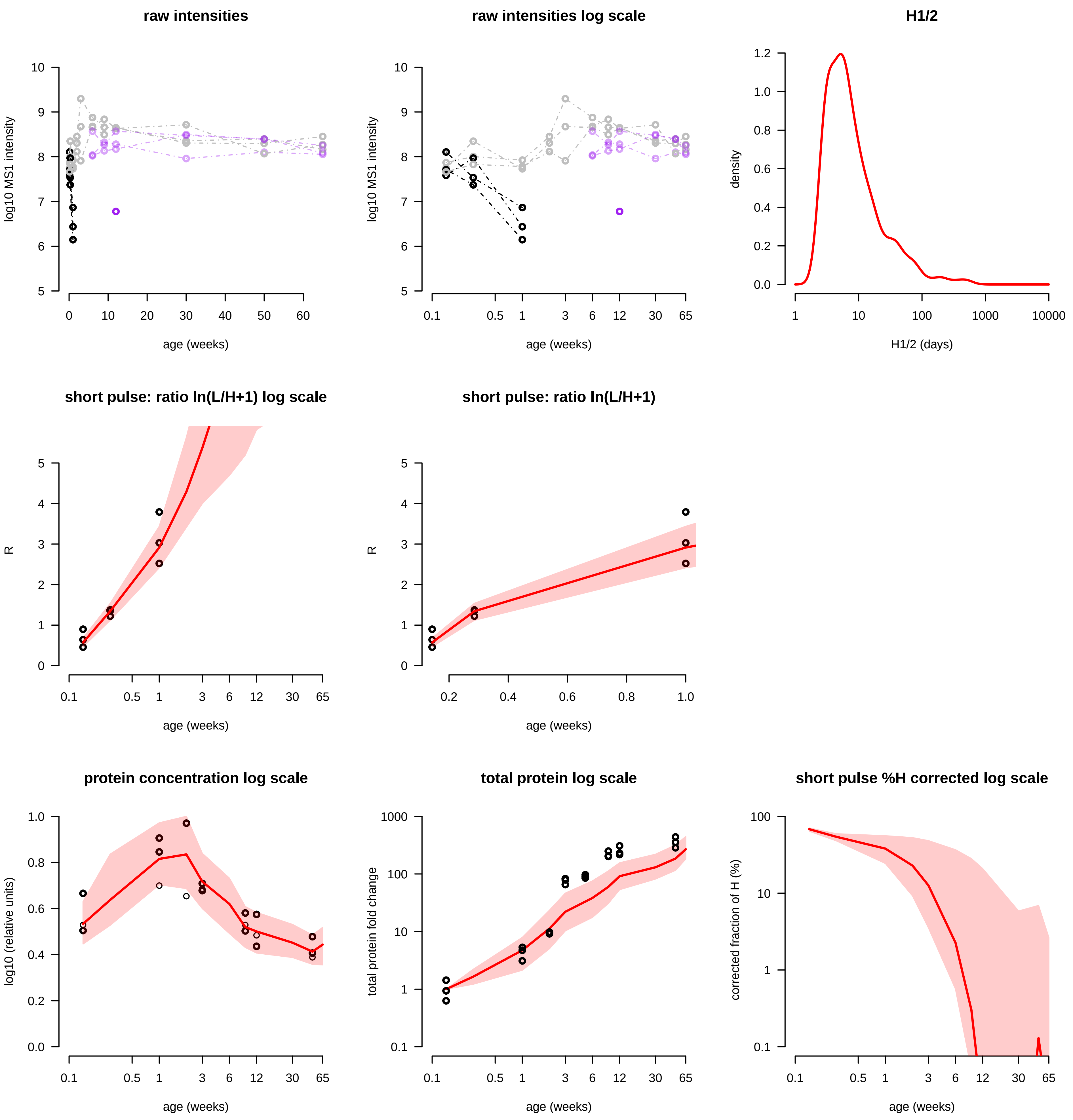

Q99K41

>sp|Q99K41|EMIL1_MOUSE

EMILIN−1

GN=Emilin1

Med H1/2: 11.4 days

Q5 H1/2: 3.9 days

Q95 H1/2: 155.5 days

reliable model fit: yes

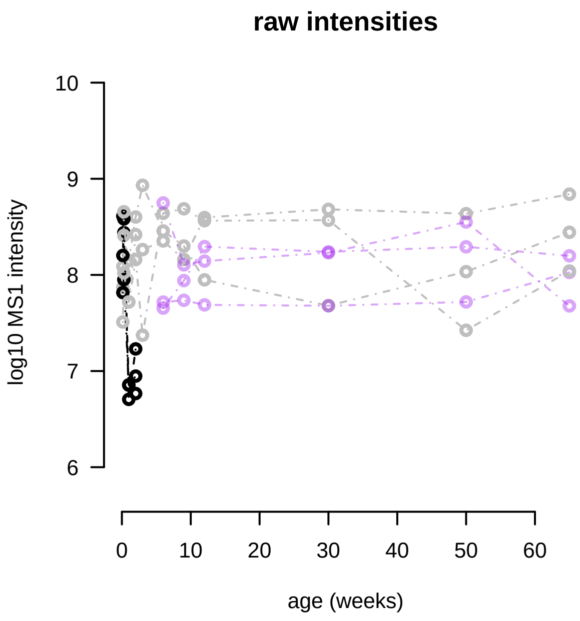

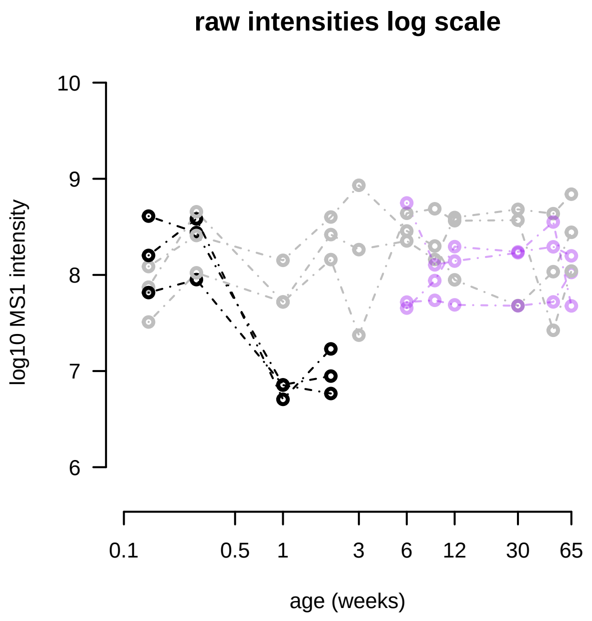

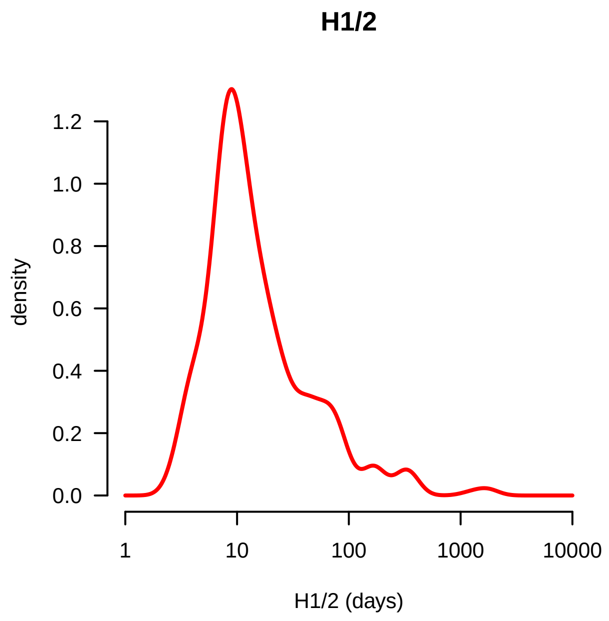

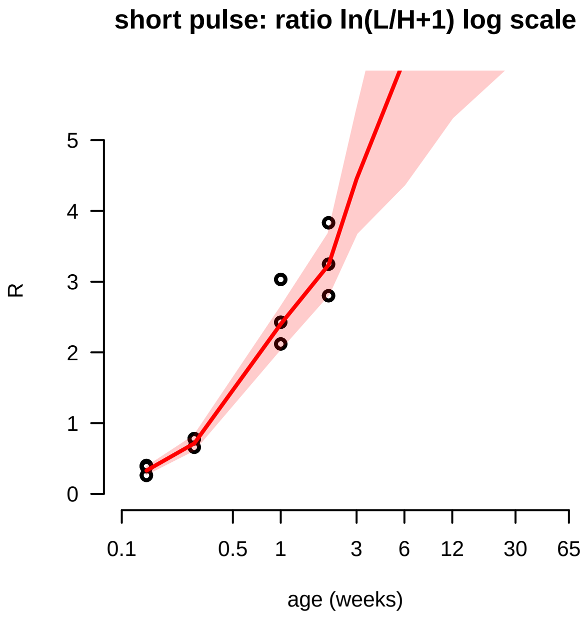

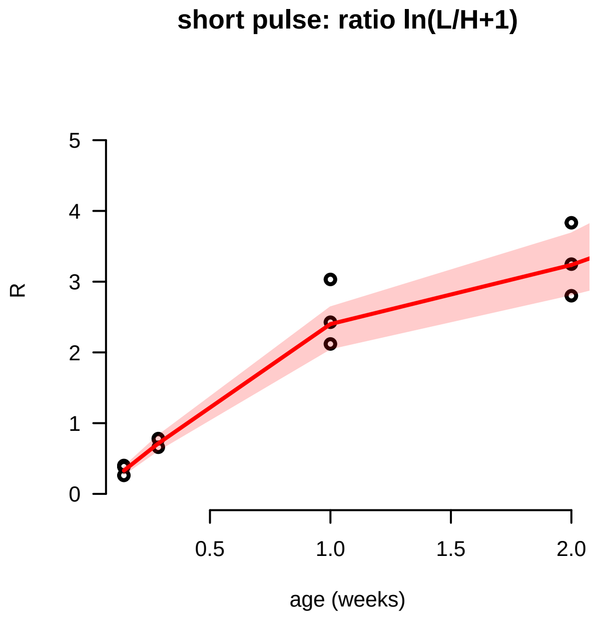

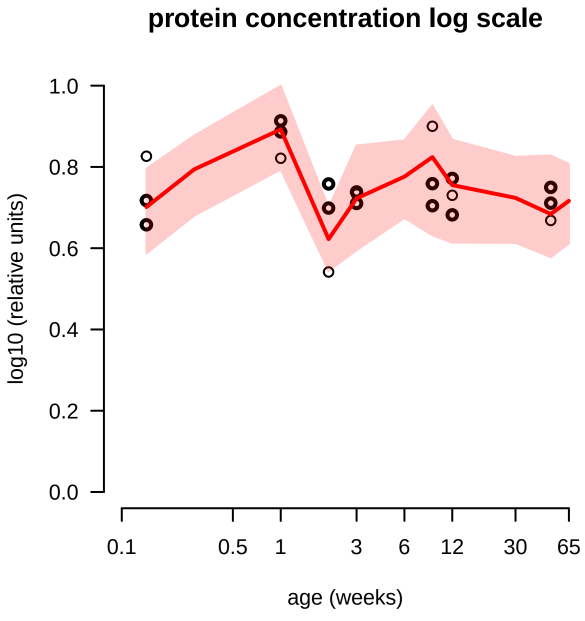

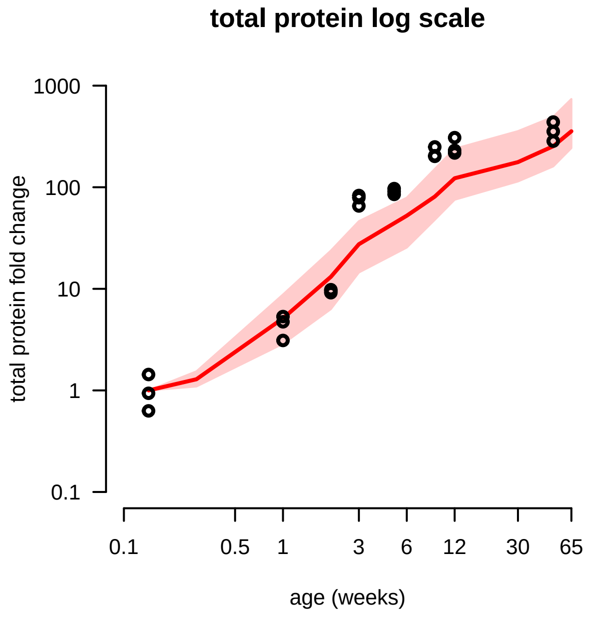

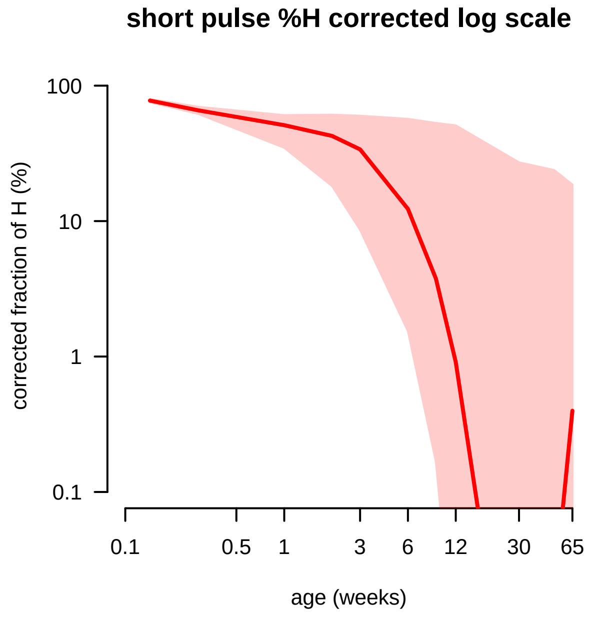

Q99LR1

>sp|Q99LR1|ABD12_MOUSE

Lysophosphatidylserine lipase ABHD12

GN=Abhd12

Med H1/2: 6.6 days

Q5 H1/2: 3 days

Q95 H1/2: 42.6 days

reliable model fit: yes

raw intensities

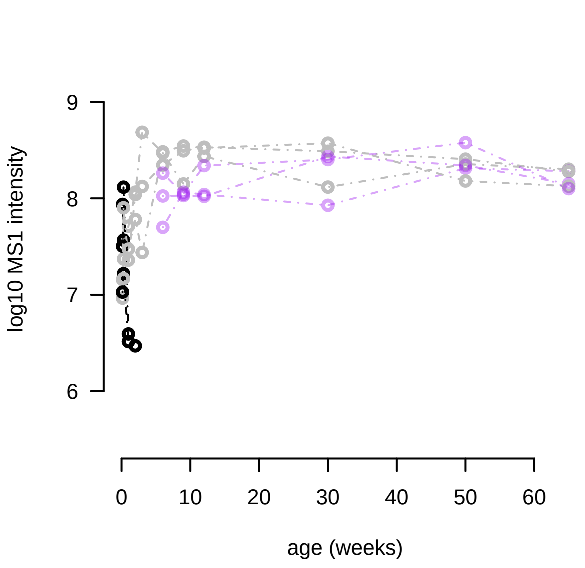

raw intensities log scale

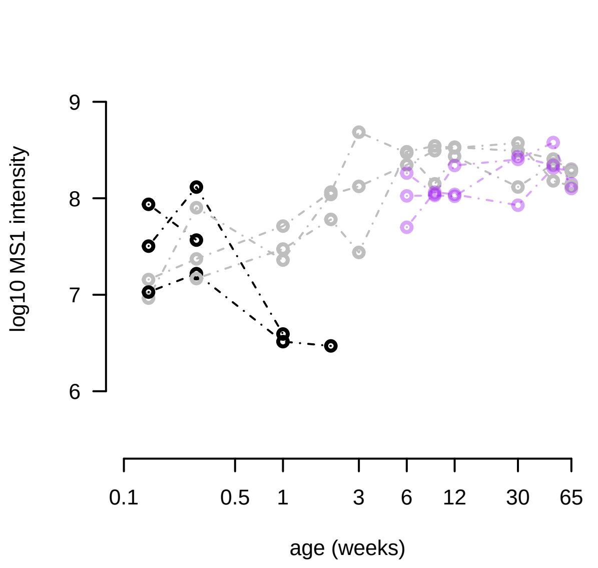

H1/2

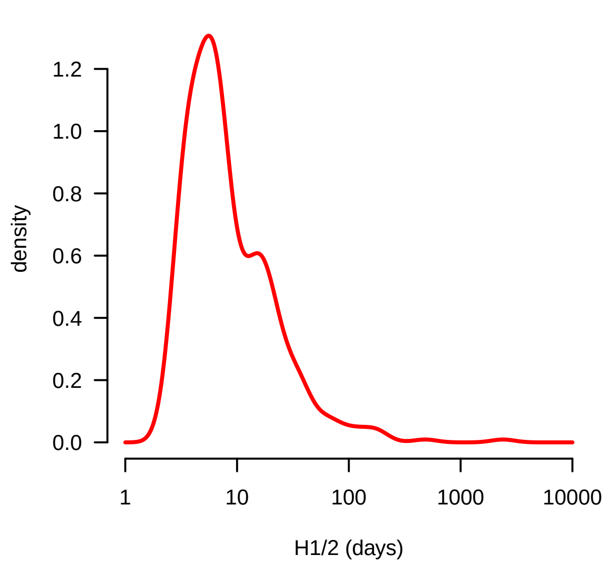

short pulse: ratio ln(L/H+1) log scale

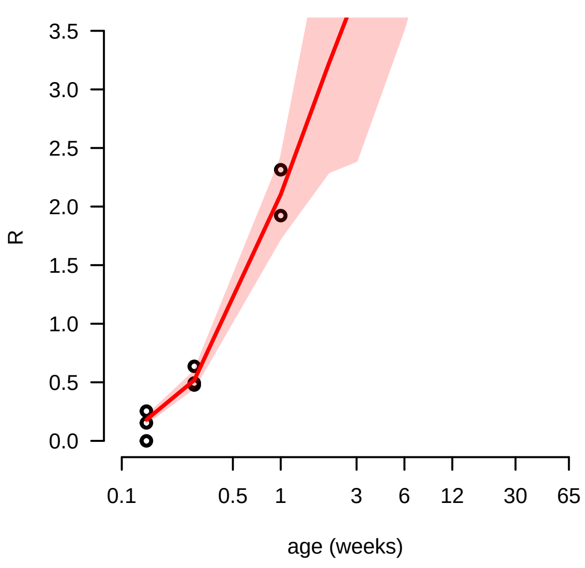

short pulse: ratio ln(L/H+1)

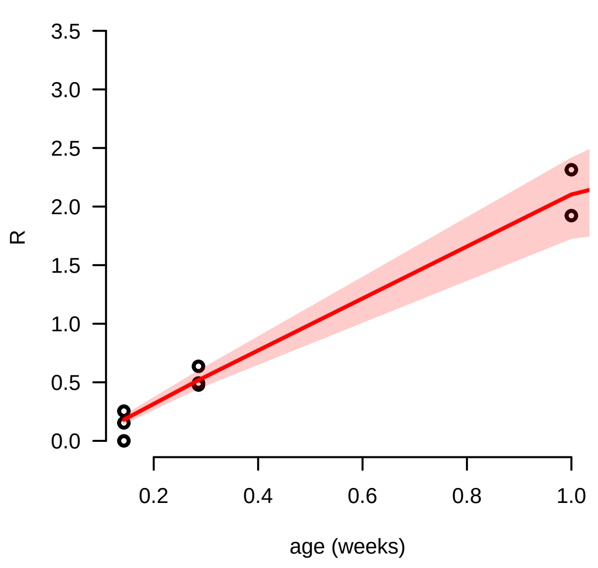

protein concentration log scale

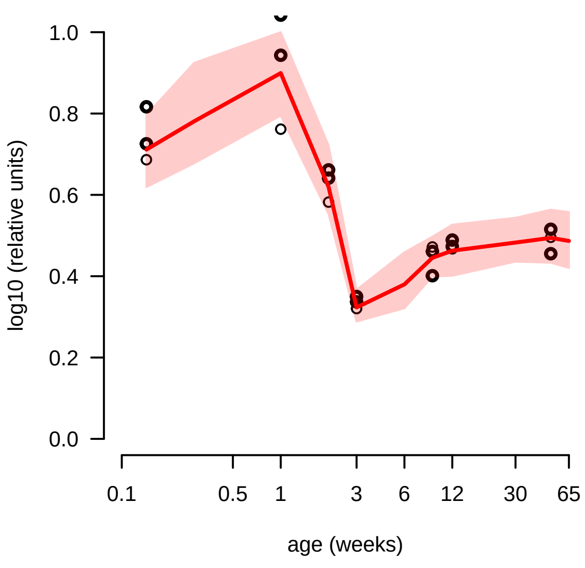

total protein log scale

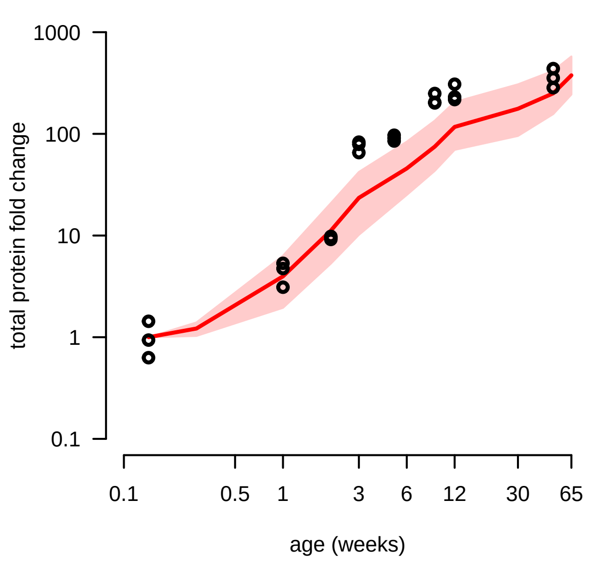

short pulse %H corrected log scale

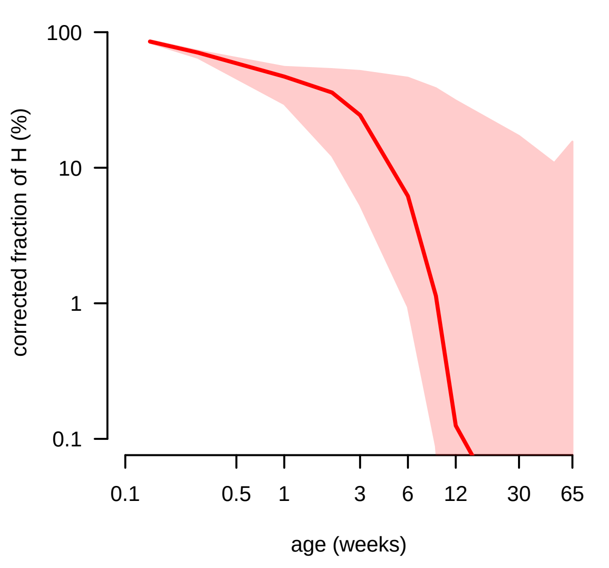

Q8CJG1

>sp|Q8CJG1|AGO1_MOUSE

Protein argonaute−1

GN=Ago1

Med H1/2: 11.6 days

Q5 H1/2: 4.6 days

Q95 H1/2: 89 days

reliable model fit: yes

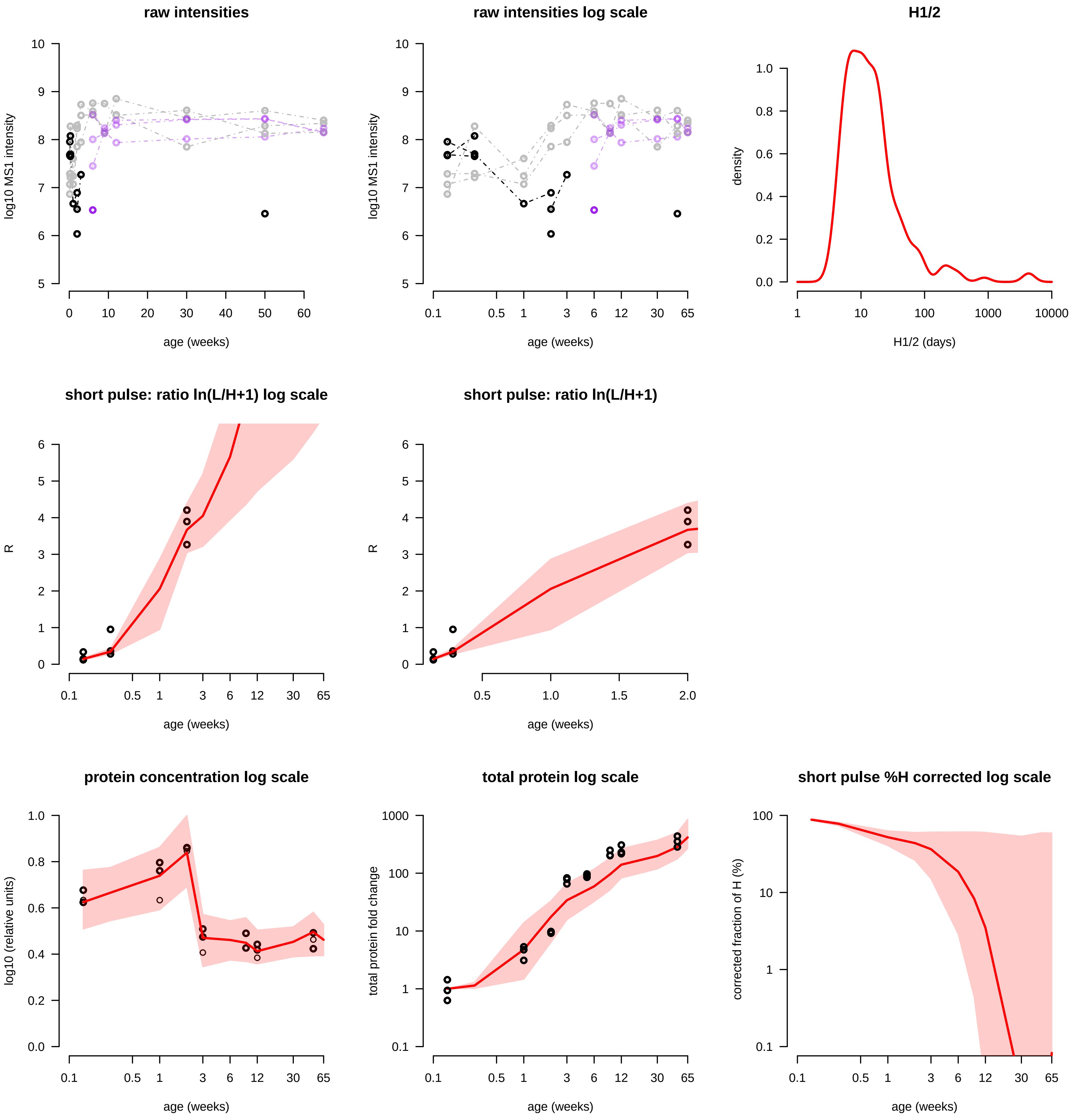

Q8BIK4−2;Q8BIK4

>sp|Q8BIK4−2|DOCK9_MOUSE

Isoform 2 of Dedicator of cytokinesis protein 9

GN=Dock9

Med H1/2: 6.2 days

Q5 H1/2: 3 days

Q95 H1/2: 29.4 days

reliable model fit: yes

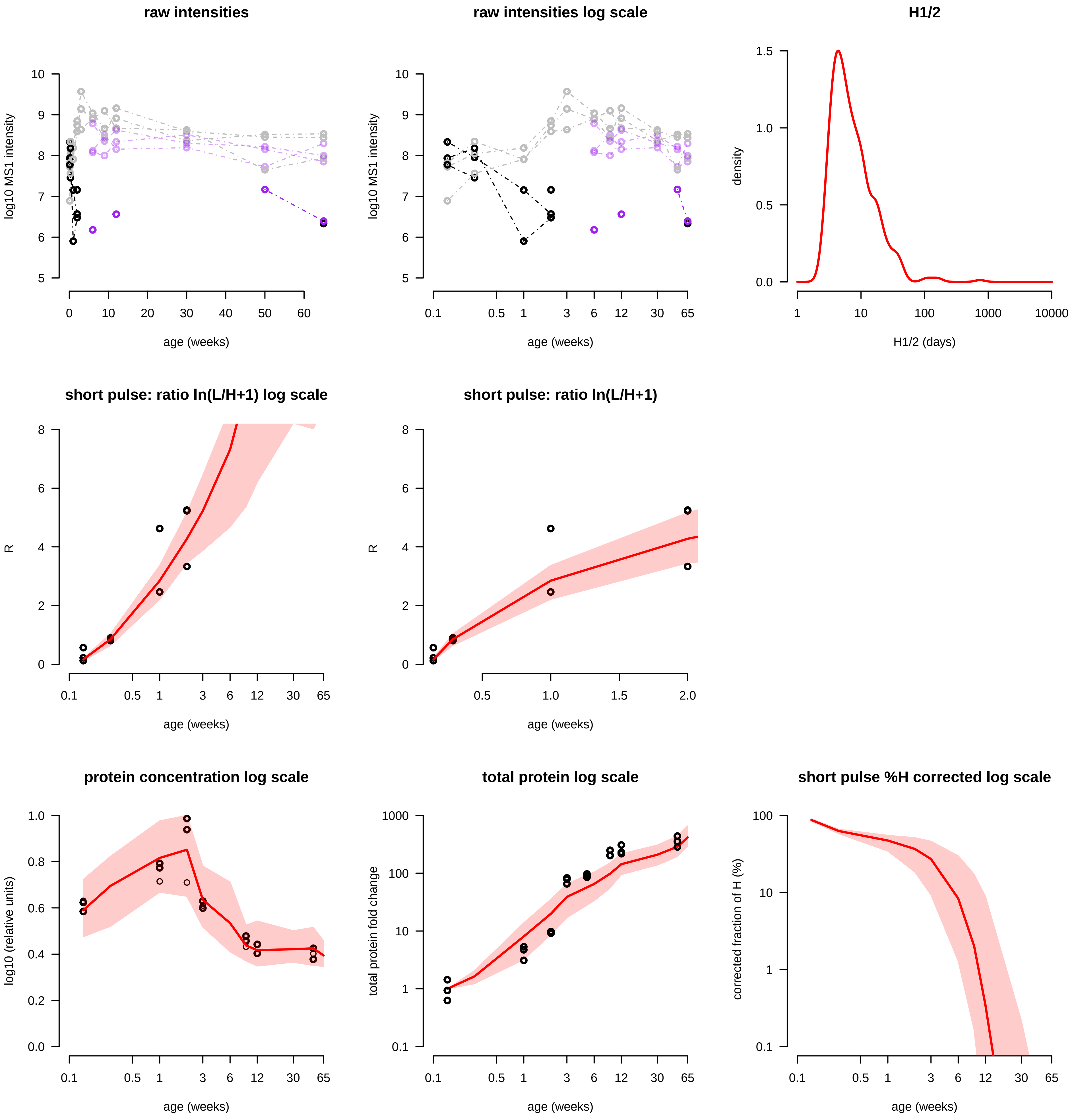

Q3TCJ1

>sp|Q3TCJ1|ABRX2_MOUSE

BRISC complex subunit Abraxas 2

GN=Abraxas2

Med H1/2: 17.1 days

Q5 H1/2: 6.6 days

Q95 H1/2: 197.7 days

reliable model fit: yes

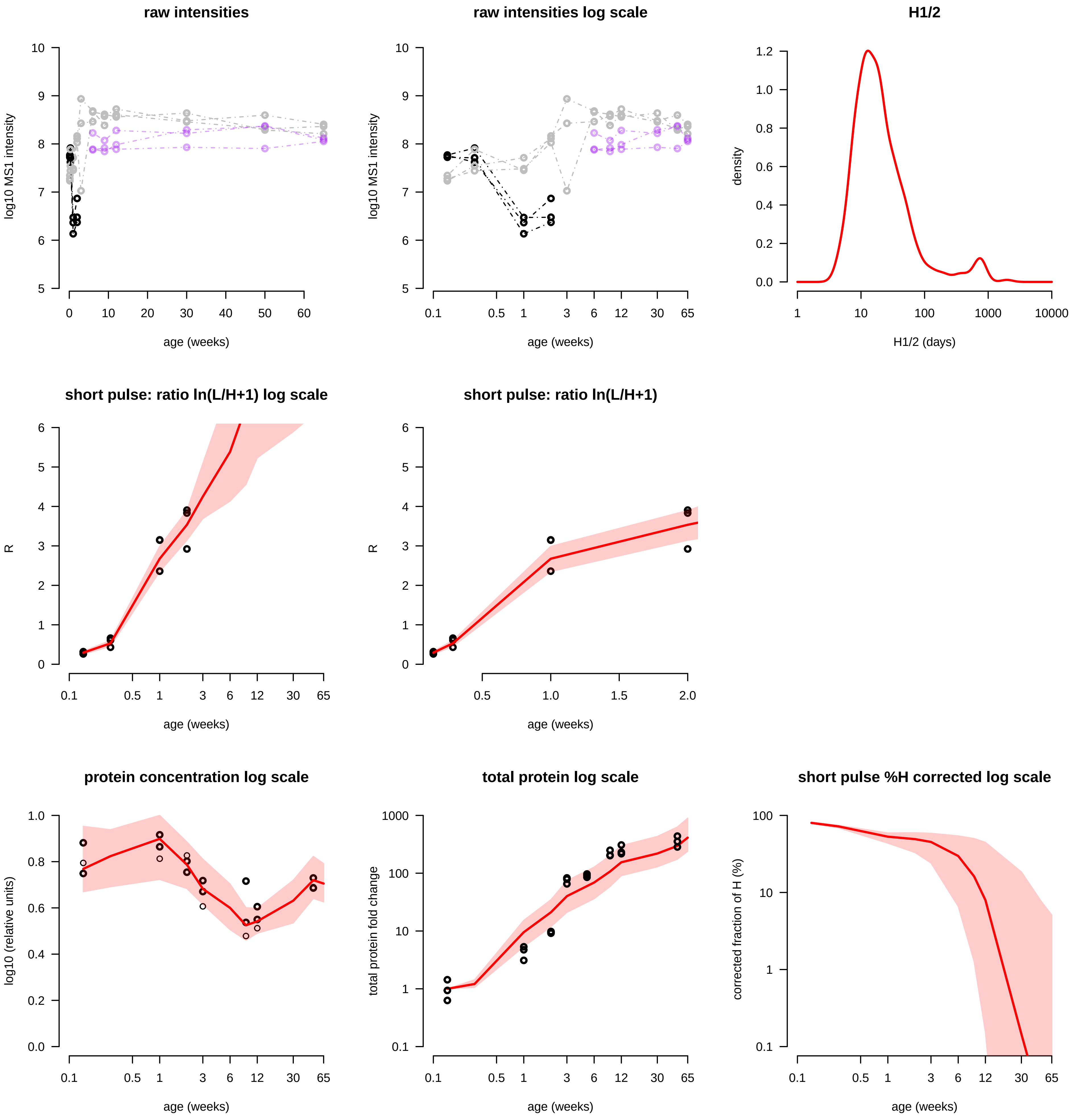

Q9D824;Q9D824−3;Q9D824−2

>sp|Q9D824|FIP1_MOUSE

Pre−mRNA 3'−end−processing factor FIP1

GN=Fip1l1

Med H1/2: 28.3 days

Q5 H1/2: 8.7 days

Q95 H1/2: 203.4 days

reliable model fit: yes

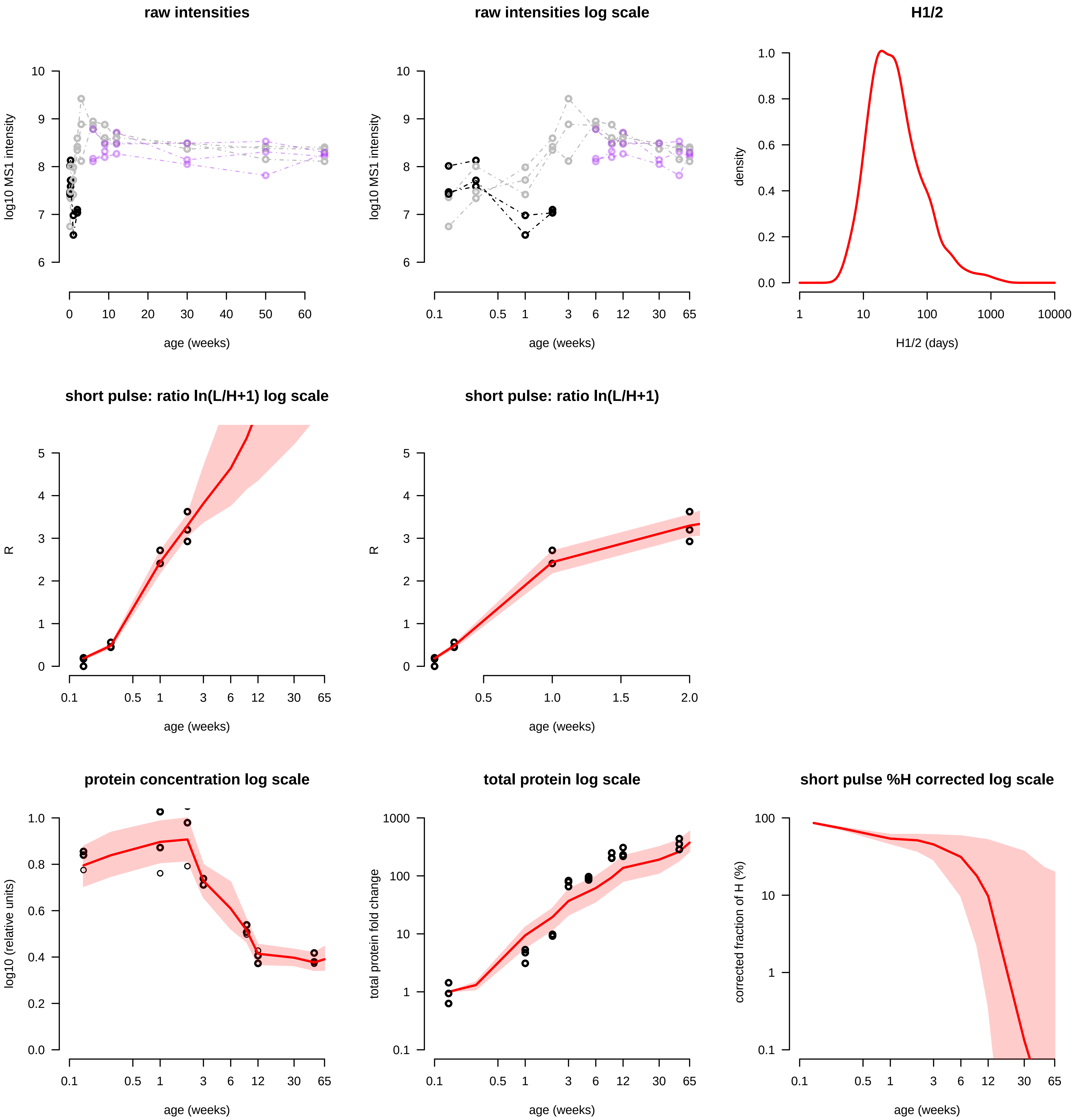

P16254

>sp|P16254|SRP14_MOUSE

Signal recognition particle 14 kDa protein

GN=Srp14

Med H1/2: 19.6 days

Q5 H1/2: 5.9 days

Q95 H1/2: 308.3 days

reliable model fit: yes

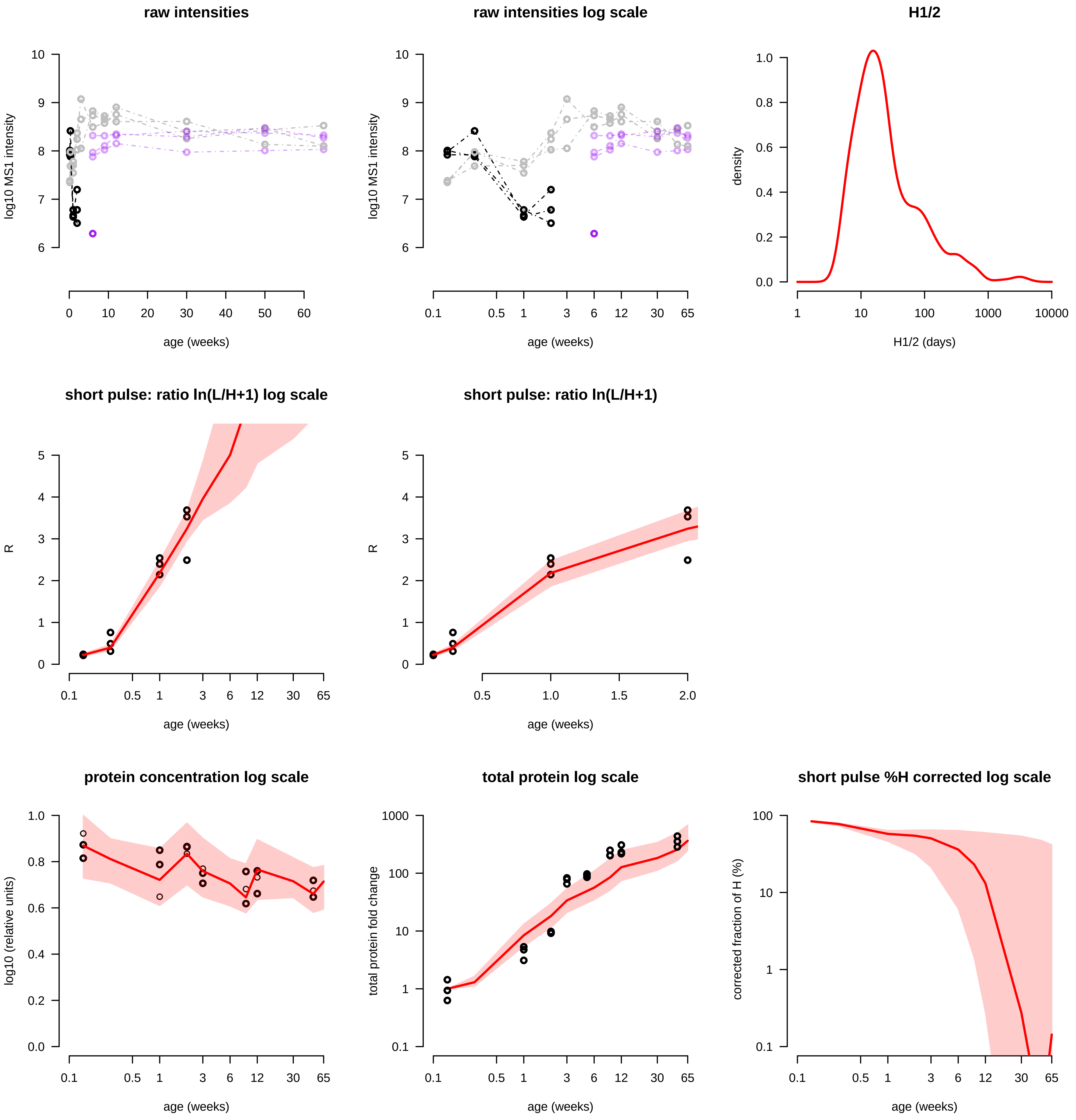

Q7TSC1

>sp|Q7TSC1|PRC2A_MOUSE

Protein PRRC2A

GN=Prrc2a

Med H1/2: 4.2 days

Q5 H1/2: 2.6 days

Q95 H1/2: 13.7 days

reliable model fit: yes

raw intensities

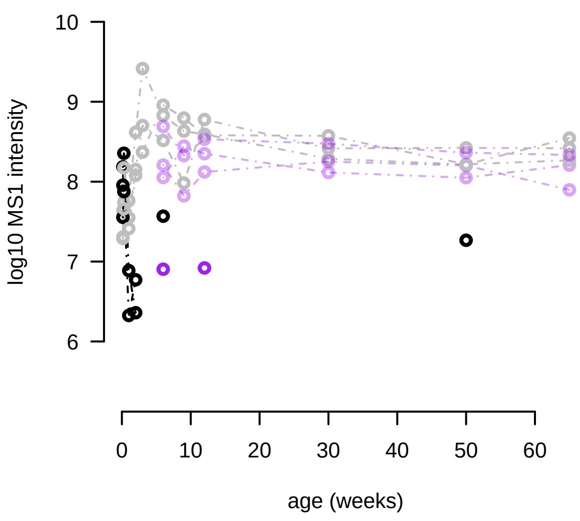

raw intensities log scale

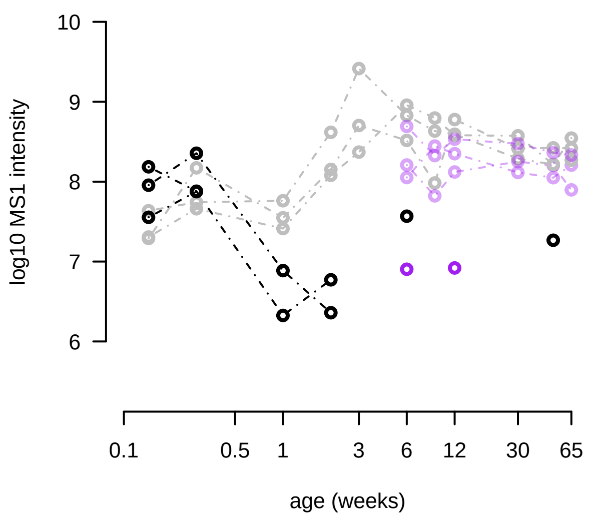

H1/2

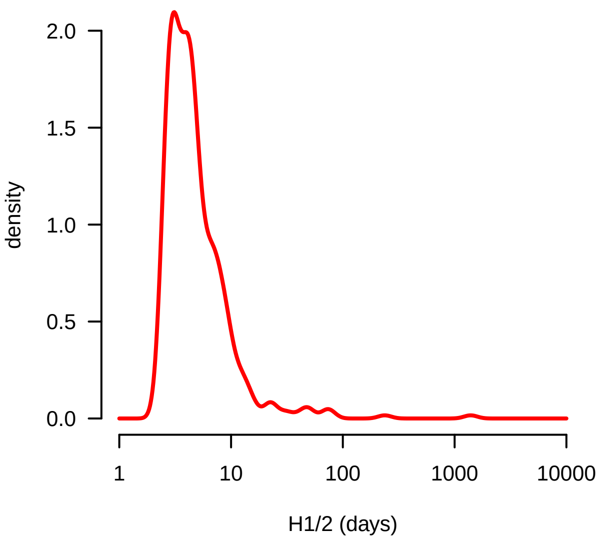

short pulse: ratio ln(L/H+1) log scale

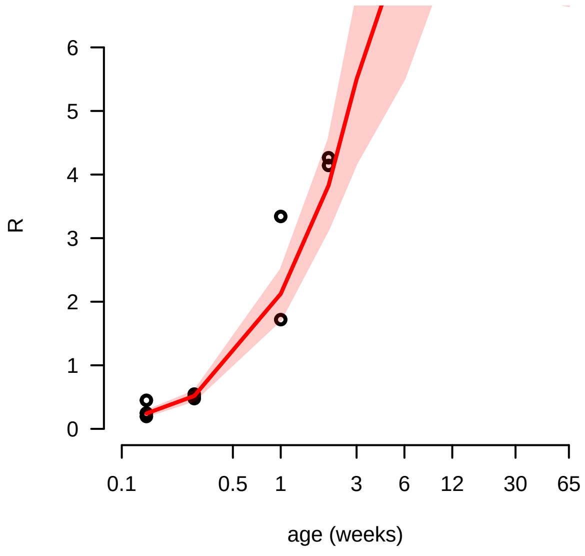

short pulse: ratio ln(L/H+1)

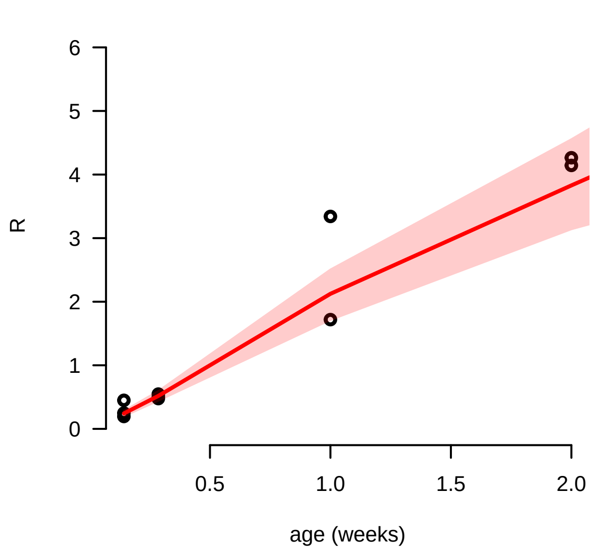

protein concentration log scale

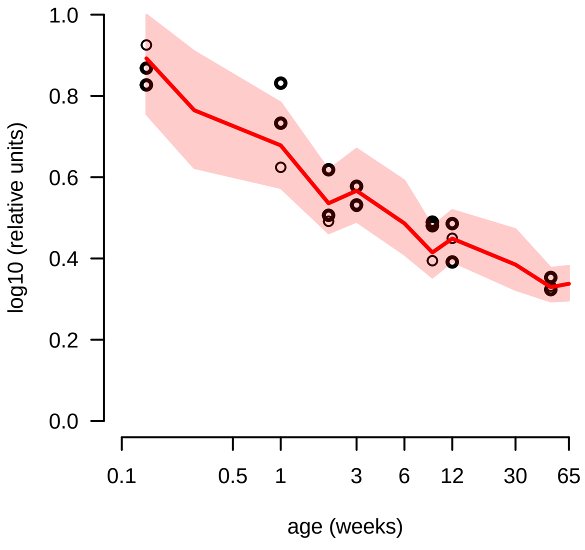

total protein log scale

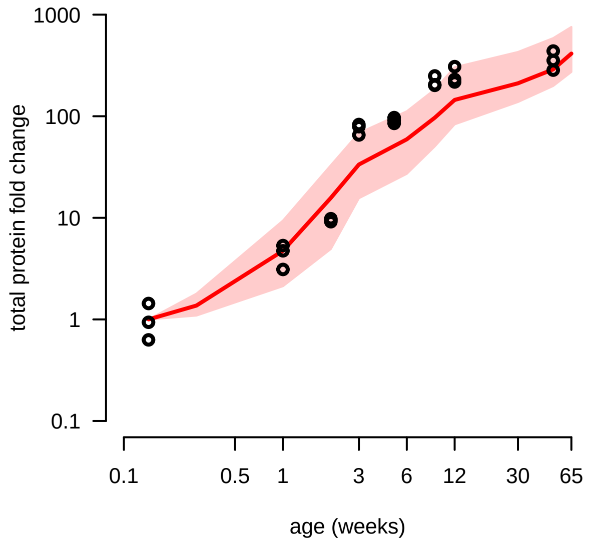

short pulse %H corrected log scale

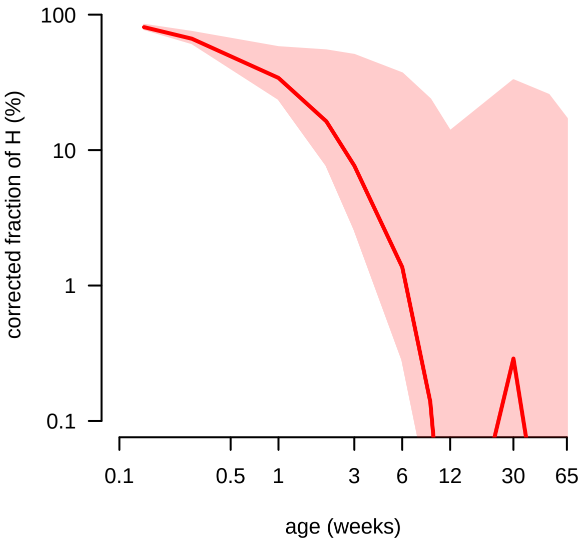

Q9DCE5

>sp|Q9DCE5|PK1IP_MOUSE

p21−activated protein kinase−interacting protein 1

GN=Pak1ip1

Med H1/2: 8.1 days

Q5 H1/2: 3.1 days

Q95 H1/2: 51.1 days

reliable model fit: yes

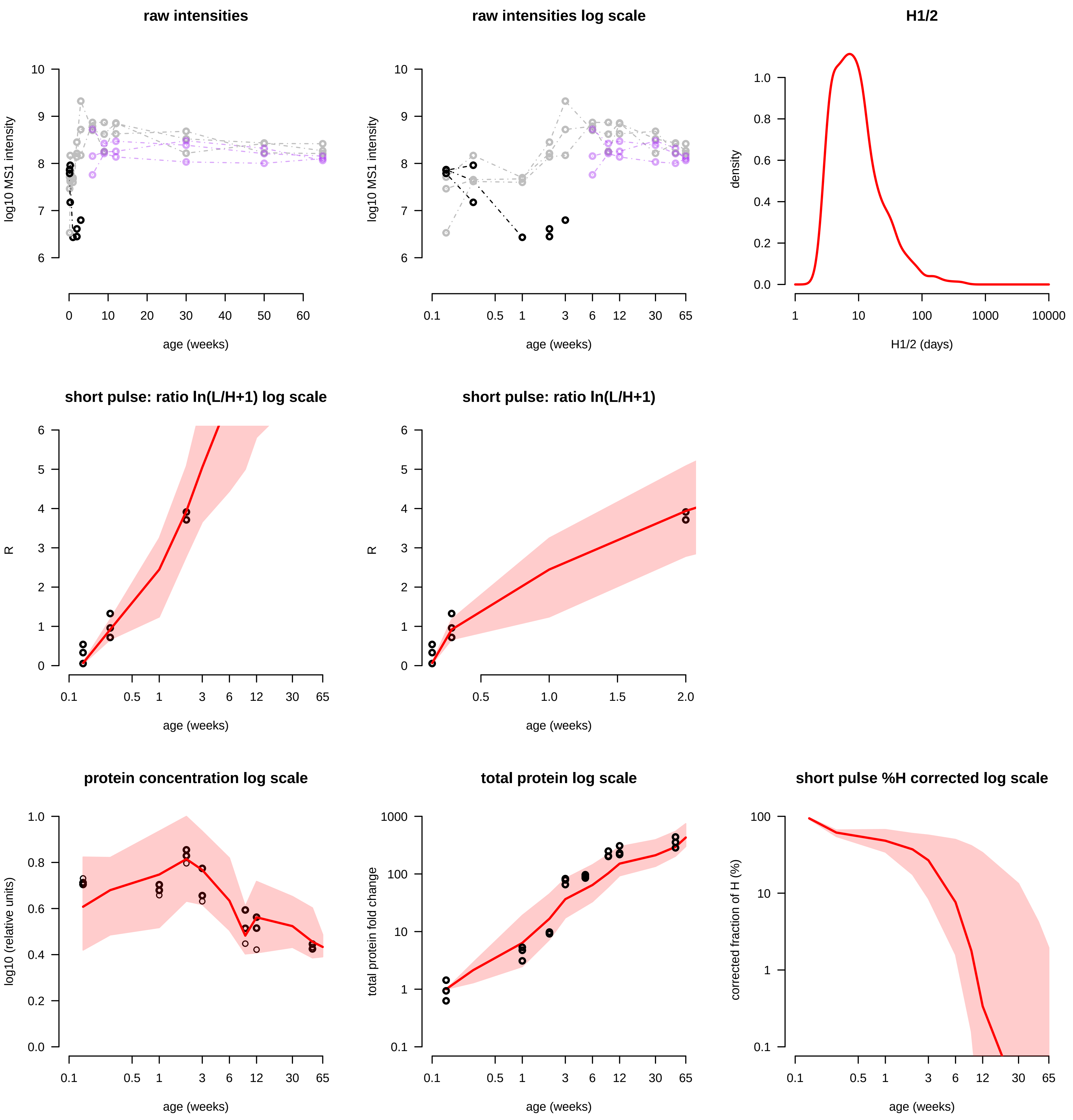

O54901

>sp|O54901|OX2G_MOUSE

OX−2 membrane glycoprotein

GN=Cd200

Med H1/2: 15.6 days

Q5 H1/2: 5.9 days

Q95 H1/2: 209.3 days

reliable model fit: yes

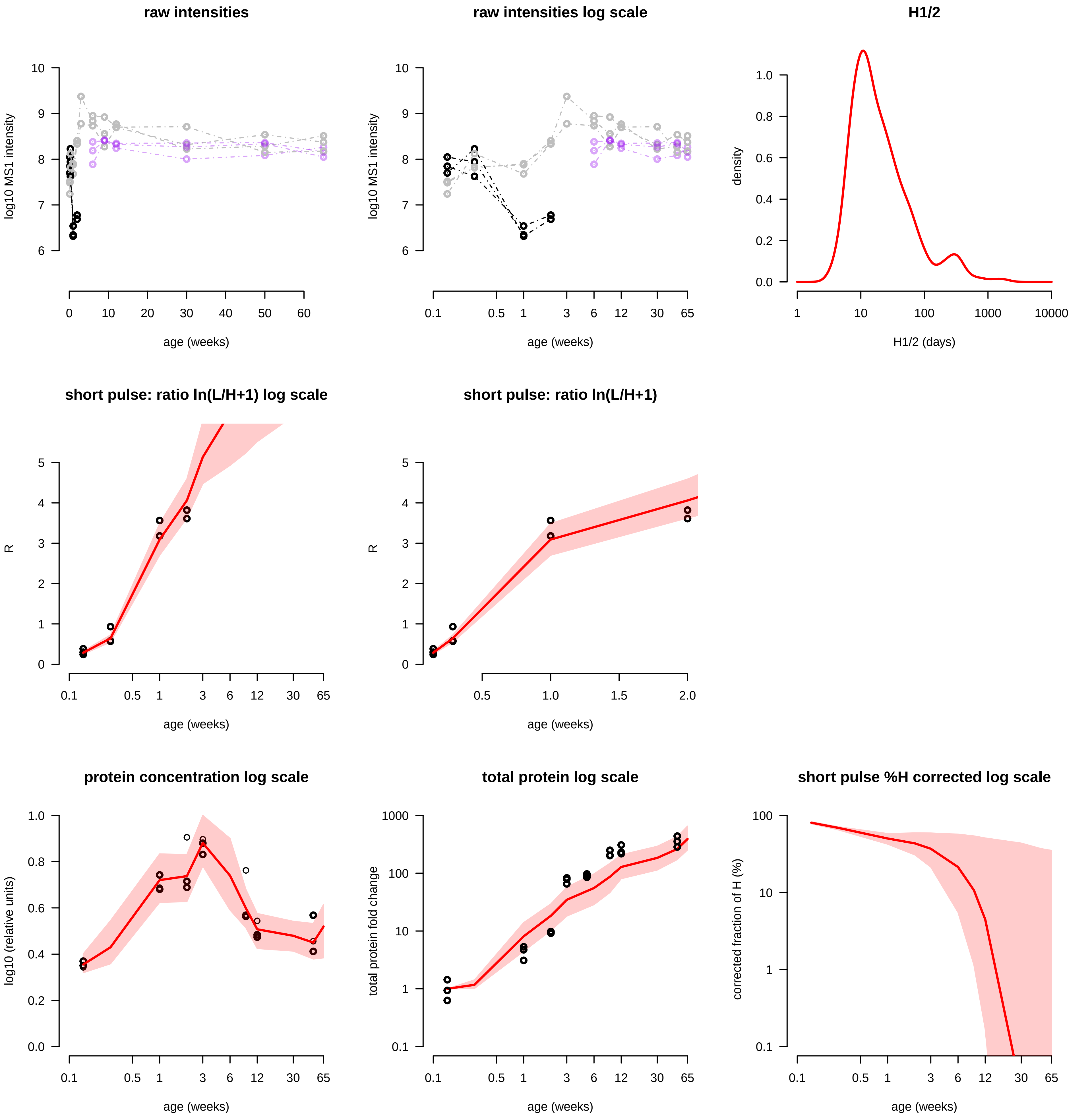

P50431

>sp|P50431|GLYC_MOUSE

Serine hydroxymethyltransferase, cytosolic

GN=Shmt1

Med H1/2: 13.3 days

Q5 H1/2: 5.7 days

Q95 H1/2: 85.2 days

reliable model fit: yes

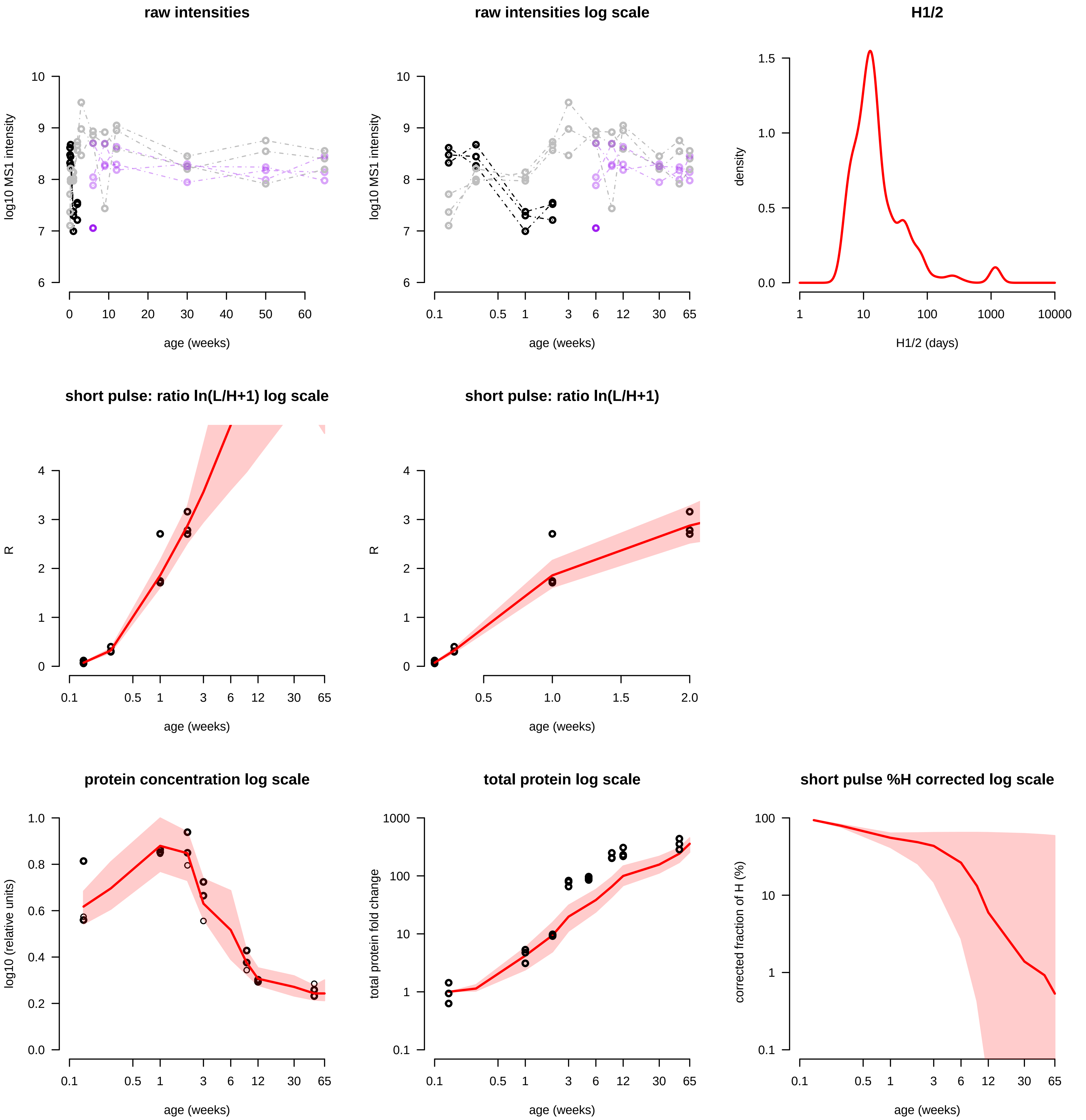

Q4VBE8

>sp|Q4VBE8|WDR18_MOUSE

WD repeat−containing protein 18

GN=Wdr18

Med H1/2: 14.4 days

Q5 H1/2: 5.6 days

Q95 H1/2: 107.4 days

reliable model fit: yes

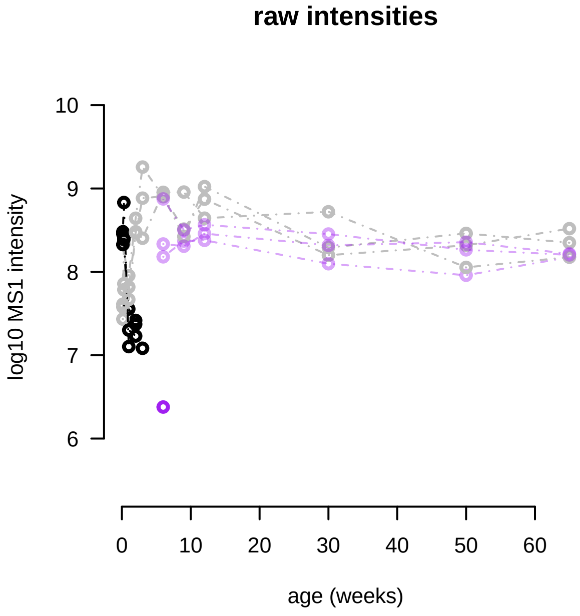

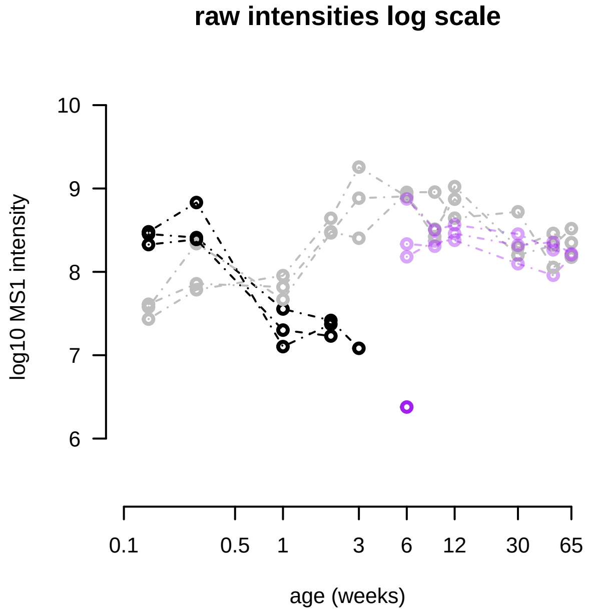

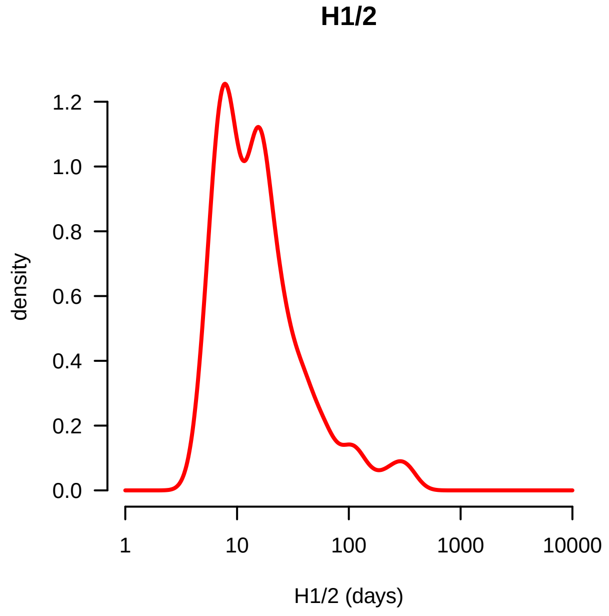

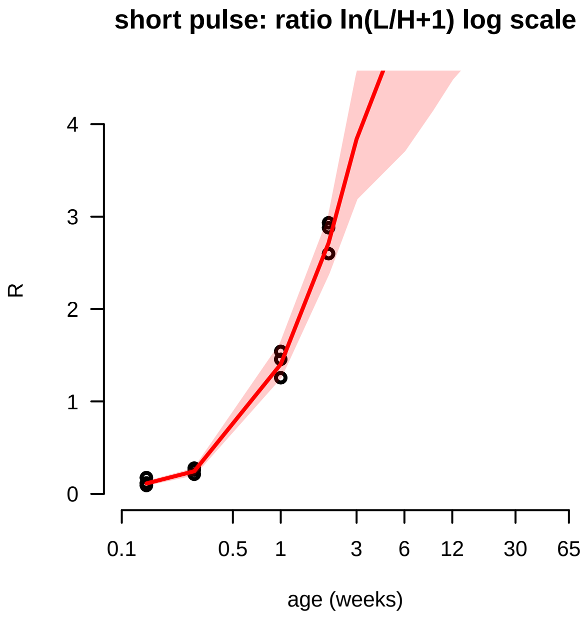

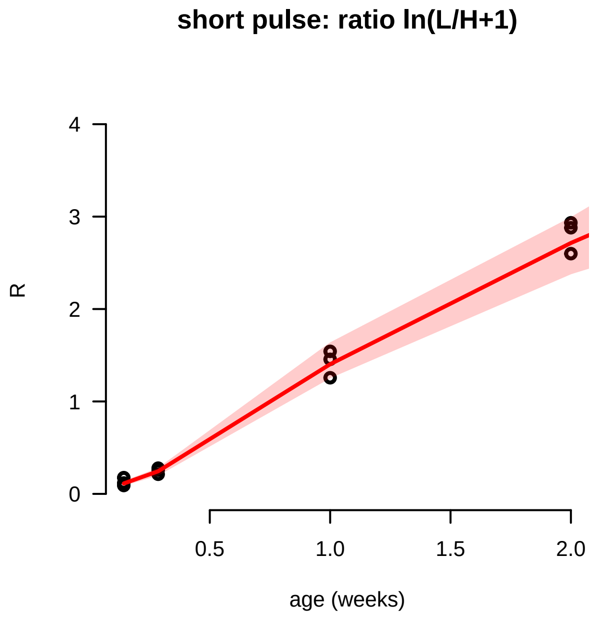

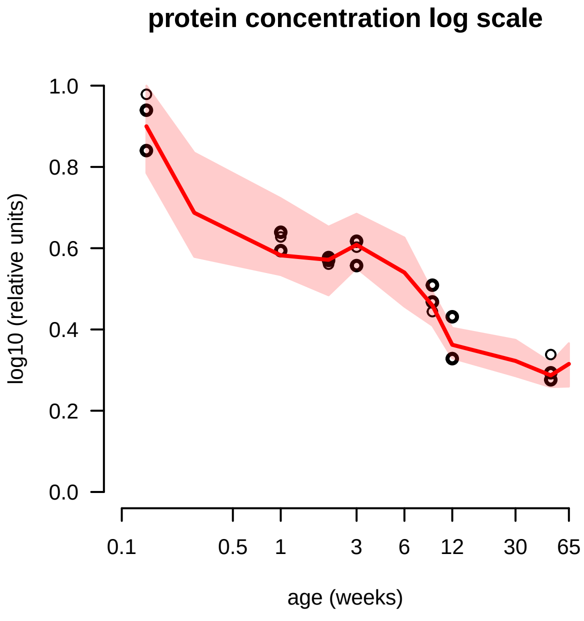

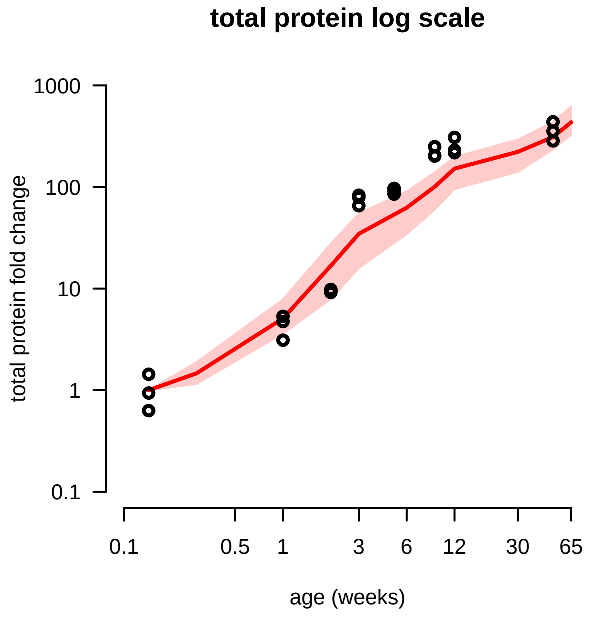

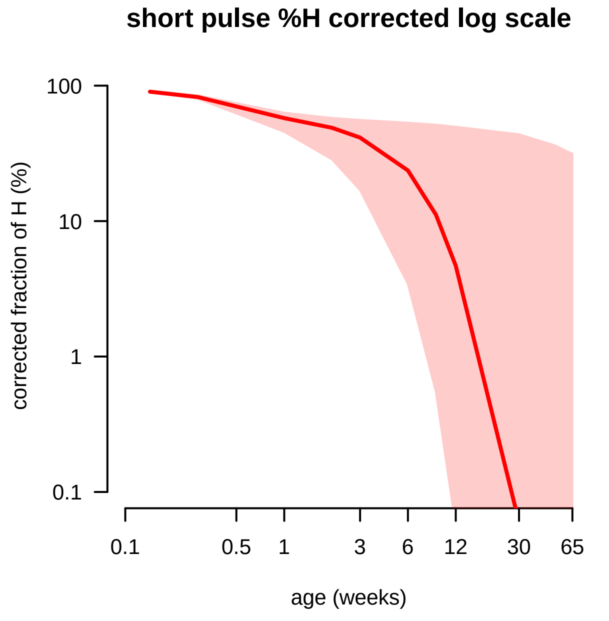

P23249

>sp|P23249|MOV10_MOUSE

Putative helicase MOV−10

GN=Mov10

Med H1/2: 5.2 days

Q5 H1/2: 2.6 days

Q95 H1/2: 11.8 days

reliable model fit: yes

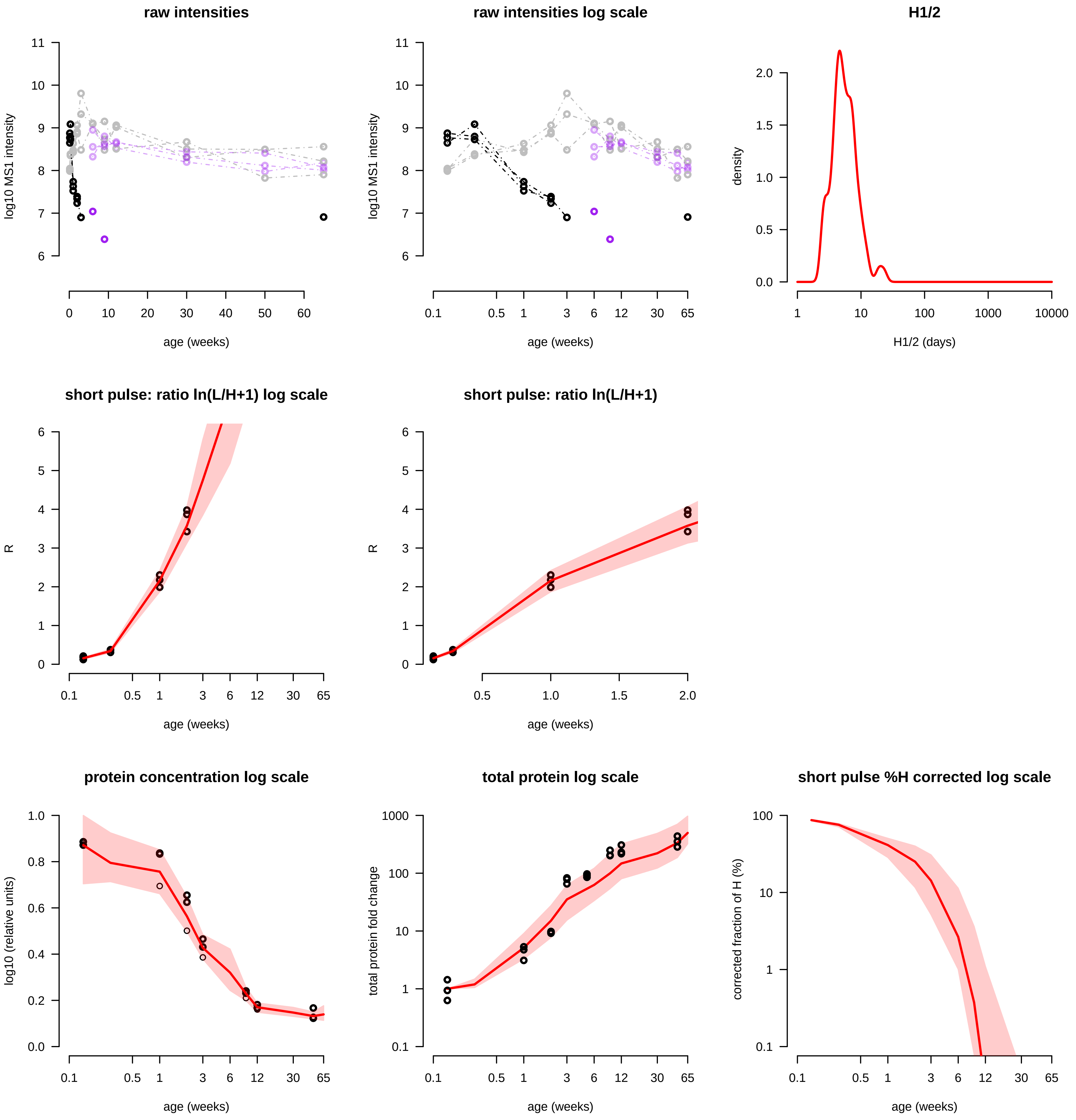

Q8C0L6

>sp|Q8C0L6|PAOX_MOUSE

Peroxisomal N(1)−acetyl−spermine/spermidine oxidase

GN=Paox

Med H1/2: 7.2 days

Q5 H1/2: 4.1 days

Q95 H1/2: 17.5 days

reliable model fit: yes

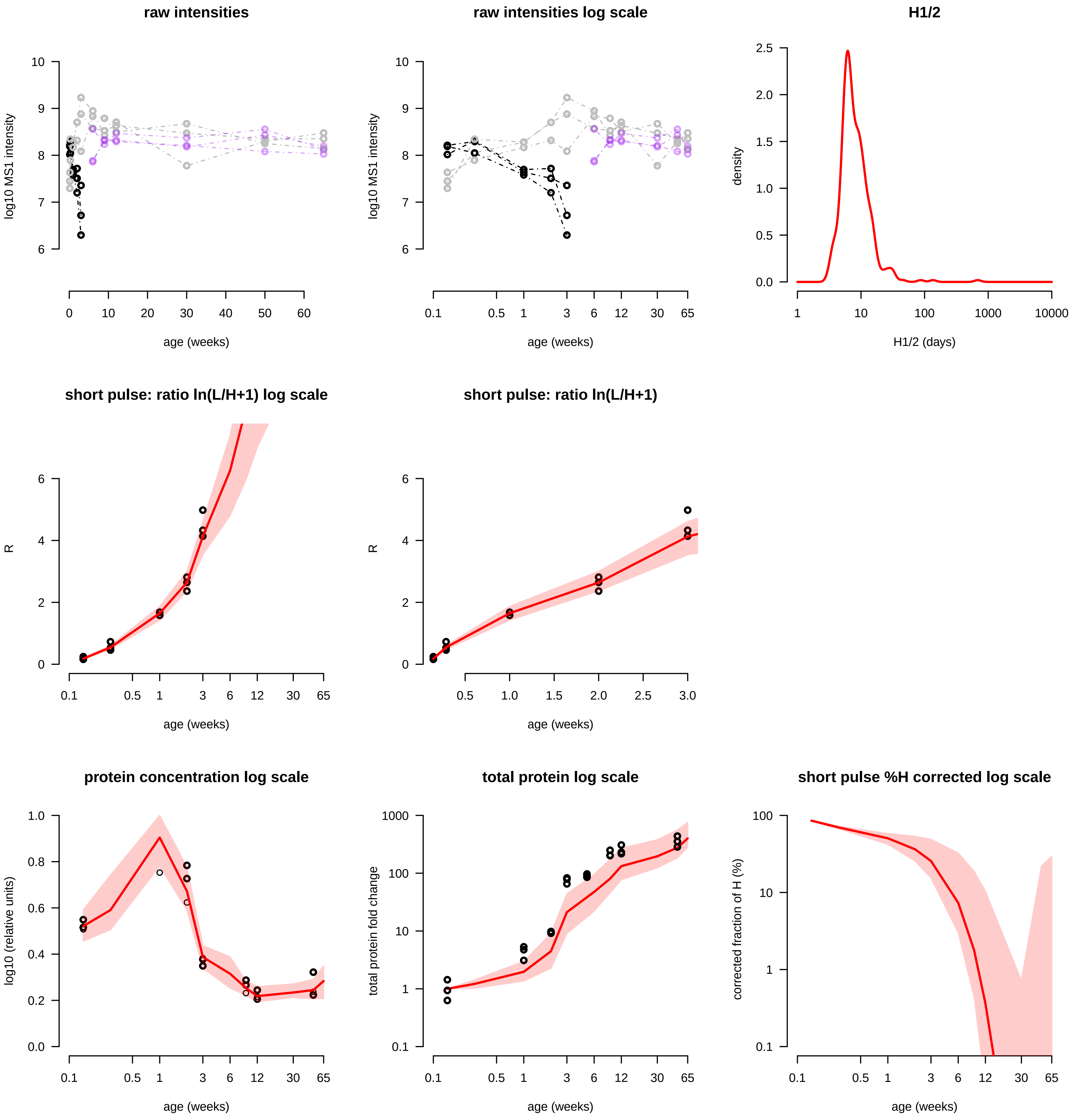

P97770−2;P97770;P97770−3

>sp|P97770−2|THUM3_MOUSE

Isoform 2 of THUMP domain−containing protein 3

GN=Thumpd3

Med H1/2: 9.3 days

Q5 H1/2: 6 days

Q95 H1/2: 17.4 days

reliable model fit: yes

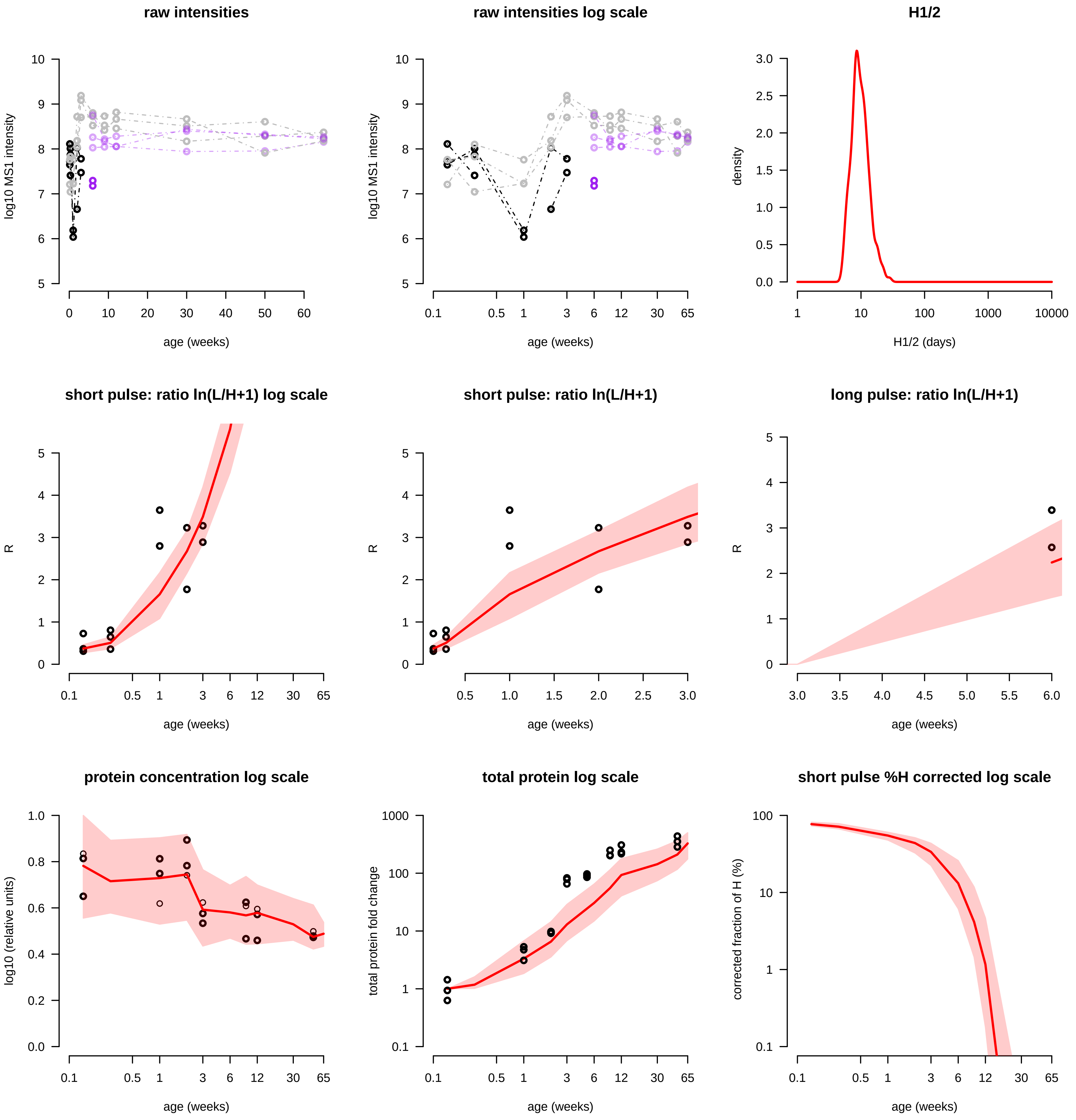

Q9DCA5

>sp|Q9DCA5|BRX1_MOUSE

Ribosome biogenesis protein BRX1 homolog

GN=Brix1

Med H1/2: 26.9 days

Q5 H1/2: 5.8 days

Q95 H1/2: 350.3 days

reliable model fit: yes

raw intensities

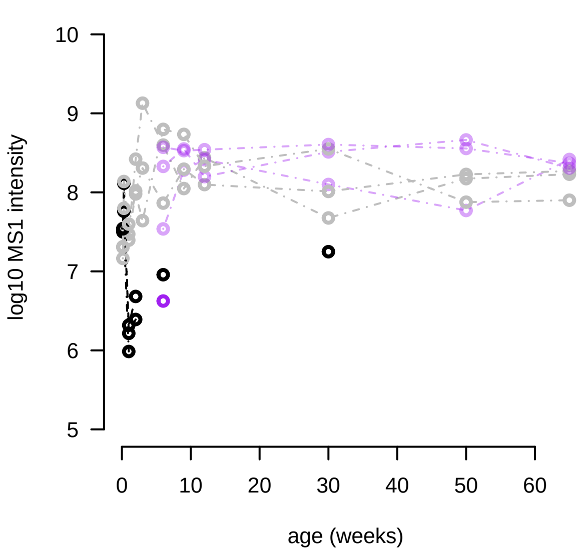

raw intensities log scale

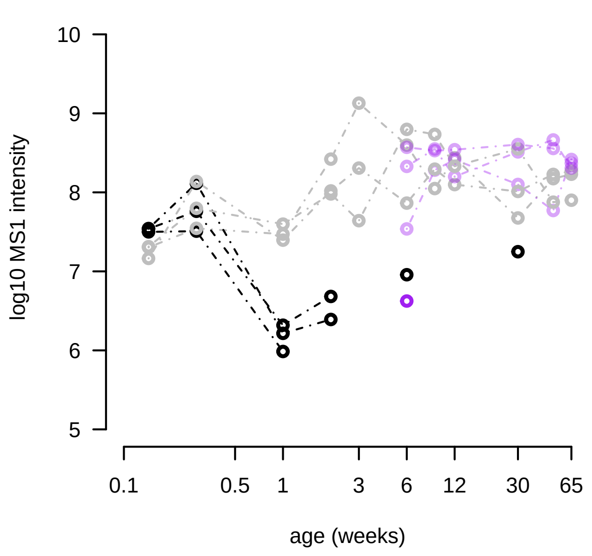

H1/2

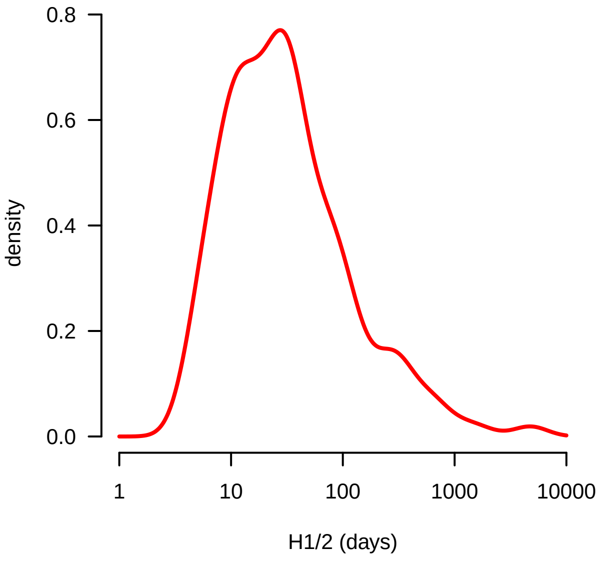

short pulse: ratio ln(L/H+1) log scale

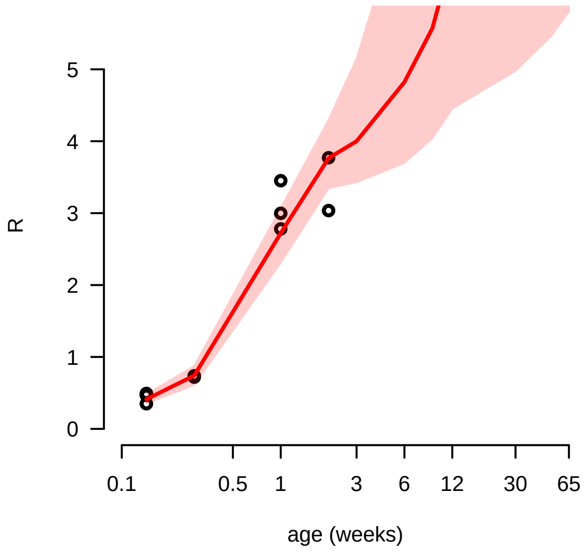

short pulse: ratio ln(L/H+1)

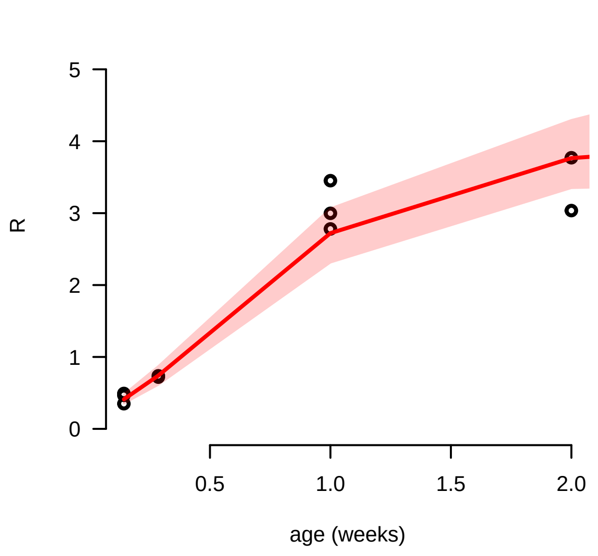

protein concentration log scale

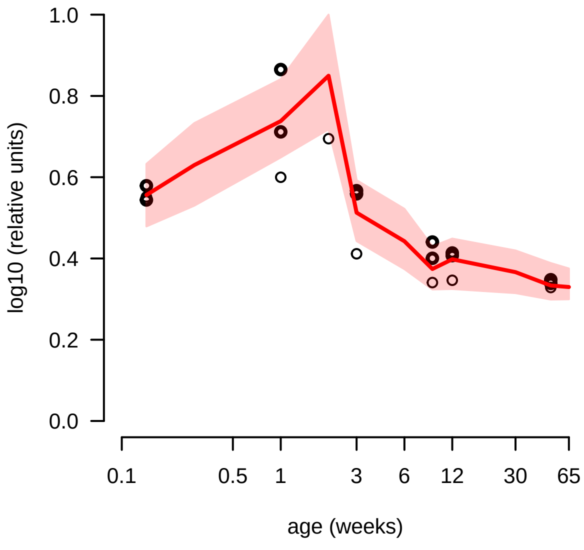

total protein log scale

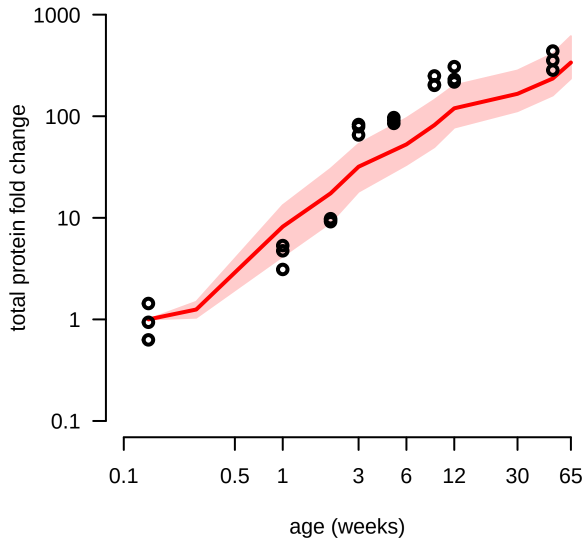

short pulse %H corrected log scale

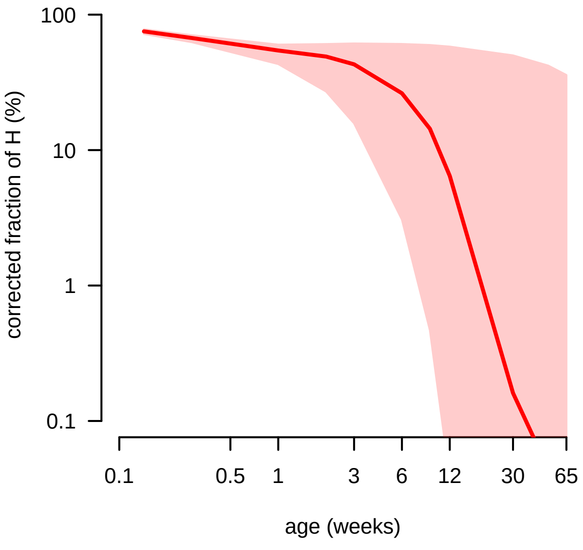

Q60675

>sp|Q60675|LAMA2_MOUSE

Laminin subunit alpha−2

GN=Lama2

Med H1/2: 39.9 days

Q5 H1/2: 13.8 days

Q95 H1/2: 457.9 days

reliable model fit: yes

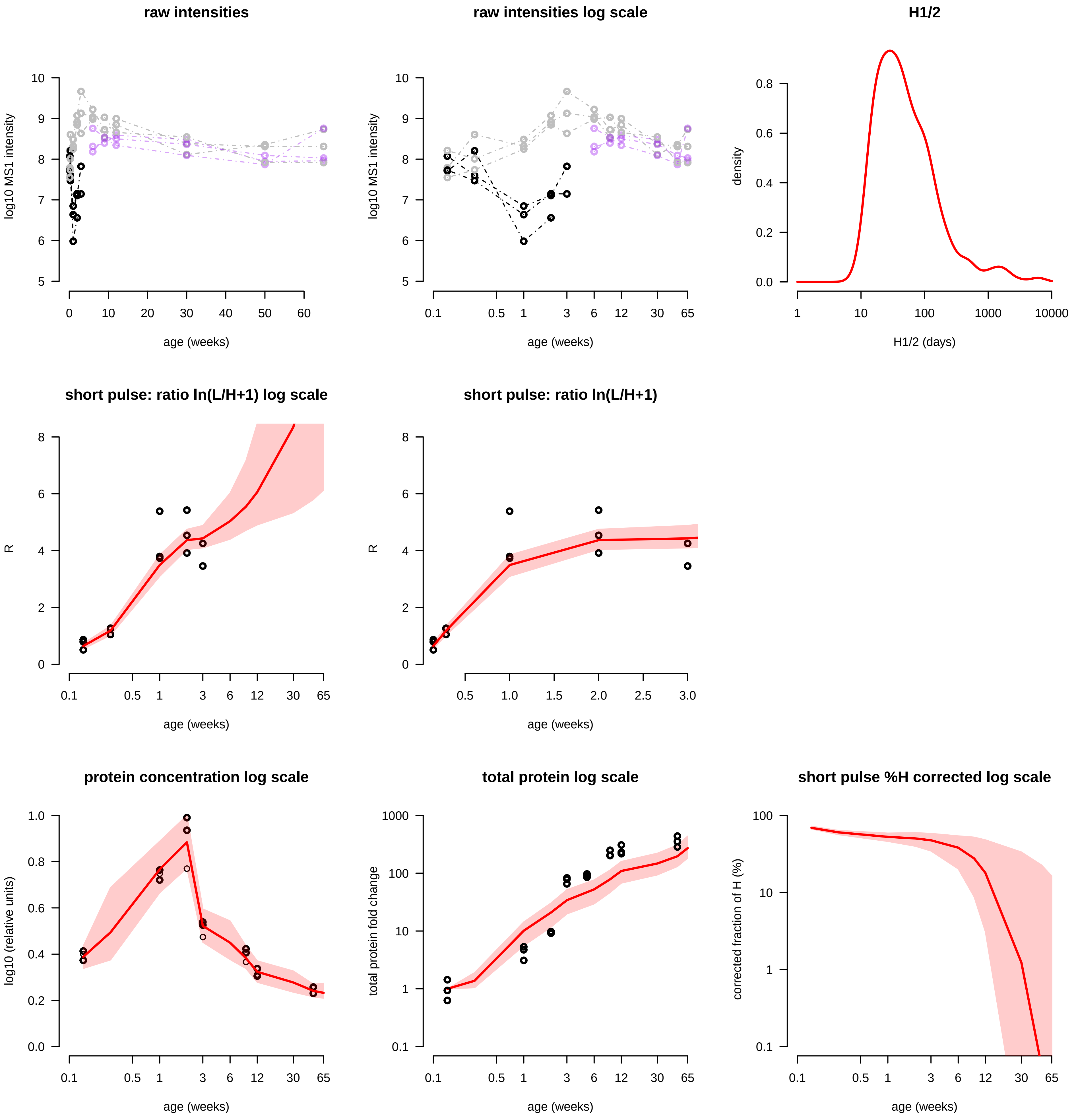

Q9WVG6−2;Q9WVG6

>sp|Q9WVG6−2|CARM1_MOUSE

Isoform 2 of Histone−arginine methyltransferase CARM1

GN=Carm1

Med H1/2: 8.6 days

Q5 H1/2: 3.1 days

Q95 H1/2: 64.6 days

reliable model fit: yes

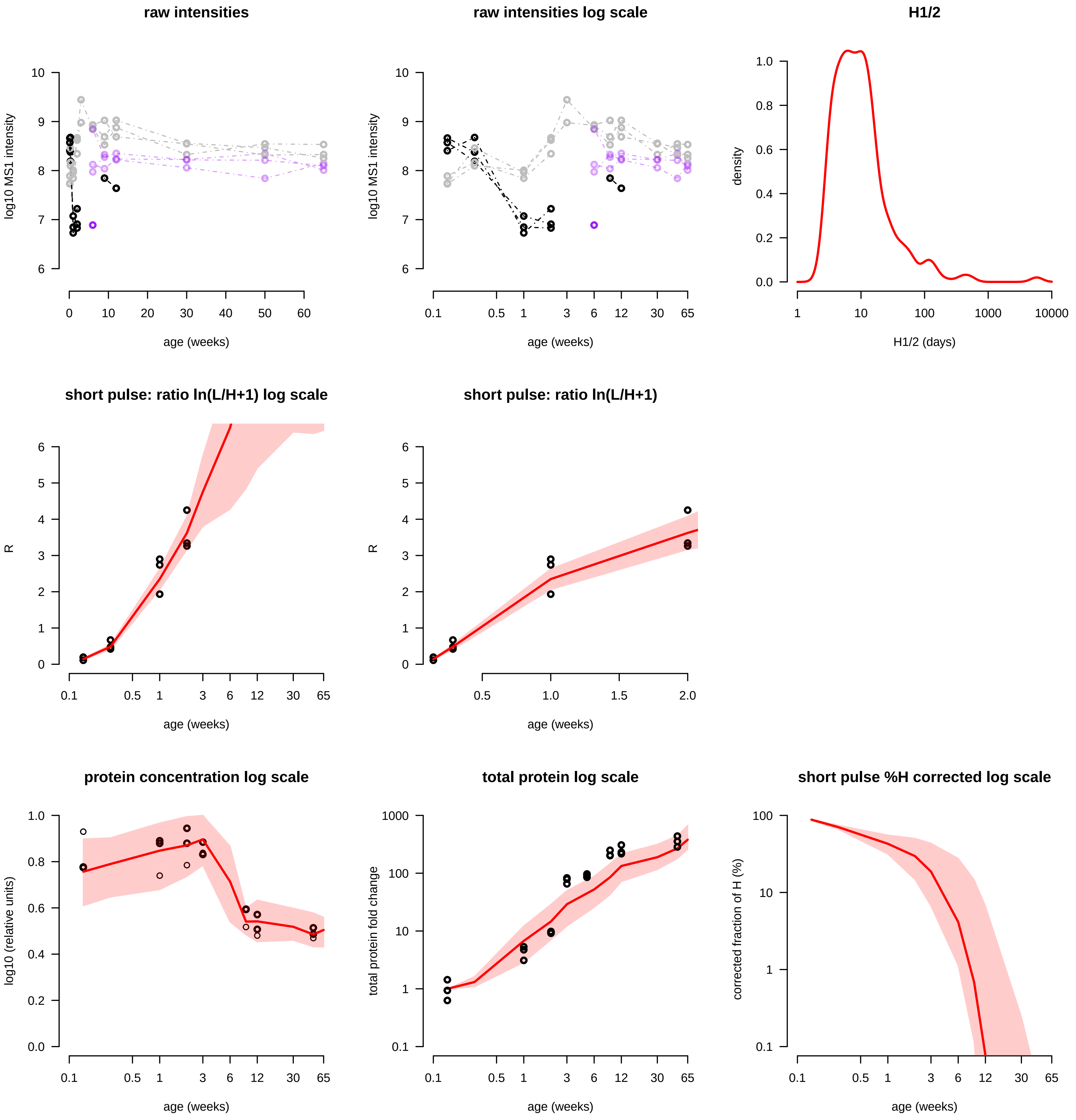

P18052;P18052−2

>sp|P18052|PTPRA_MOUSE

Receptor−type tyrosine−protein phosphatase alpha

GN=Ptpra

Med H1/2: 5.5 days

Q5 H1/2: 2.7 days

Q95 H1/2: 21.3 days

reliable model fit: yes

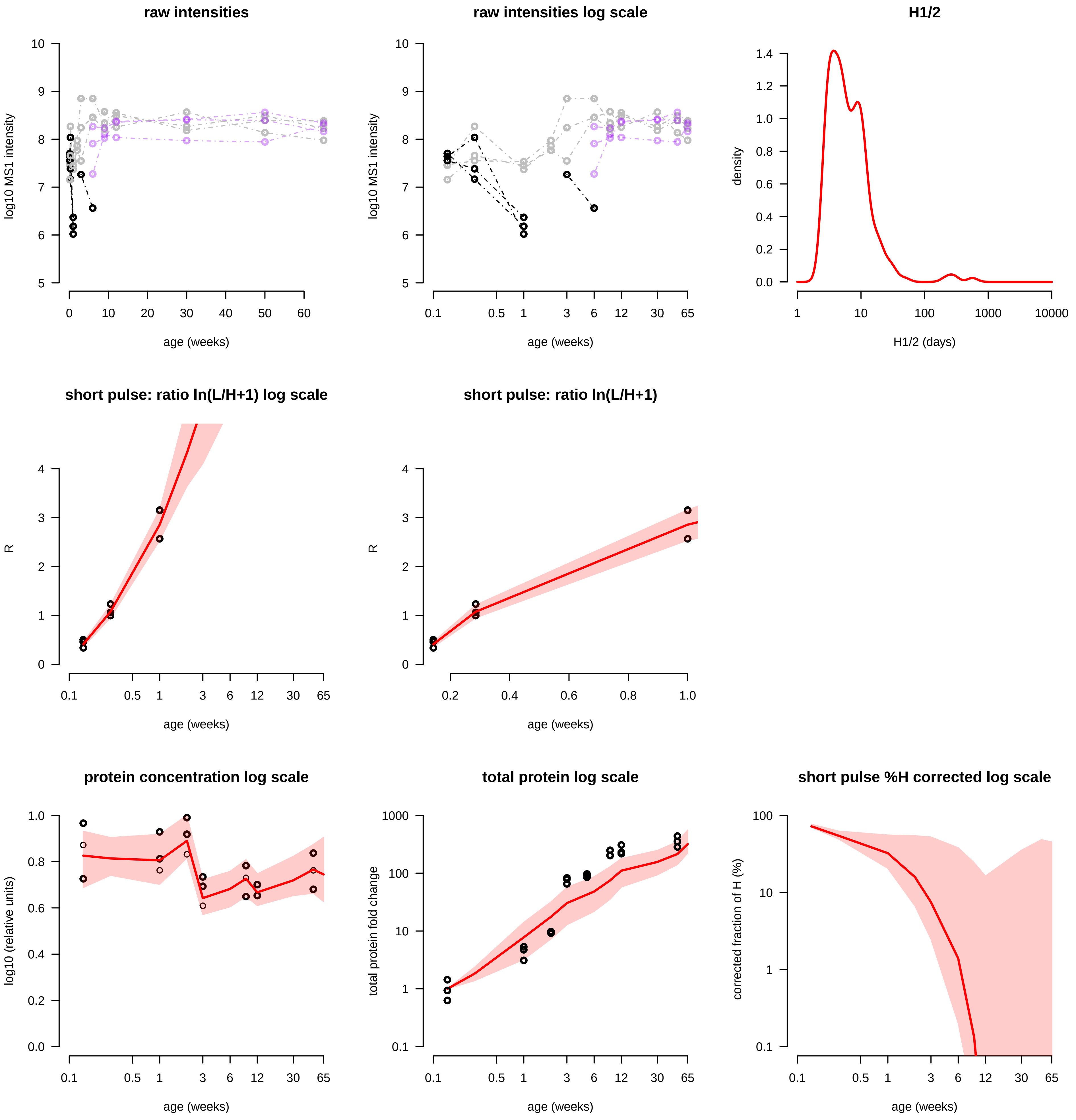

P97310

>sp|P97310|MCM2_MOUSE

DNA replication licensing factor MCM2

GN=Mcm2

Med H1/2: 4.2 days

Q5 H1/2: 3.3 days

Q95 H1/2: 5.3 days

reliable model fit: yes

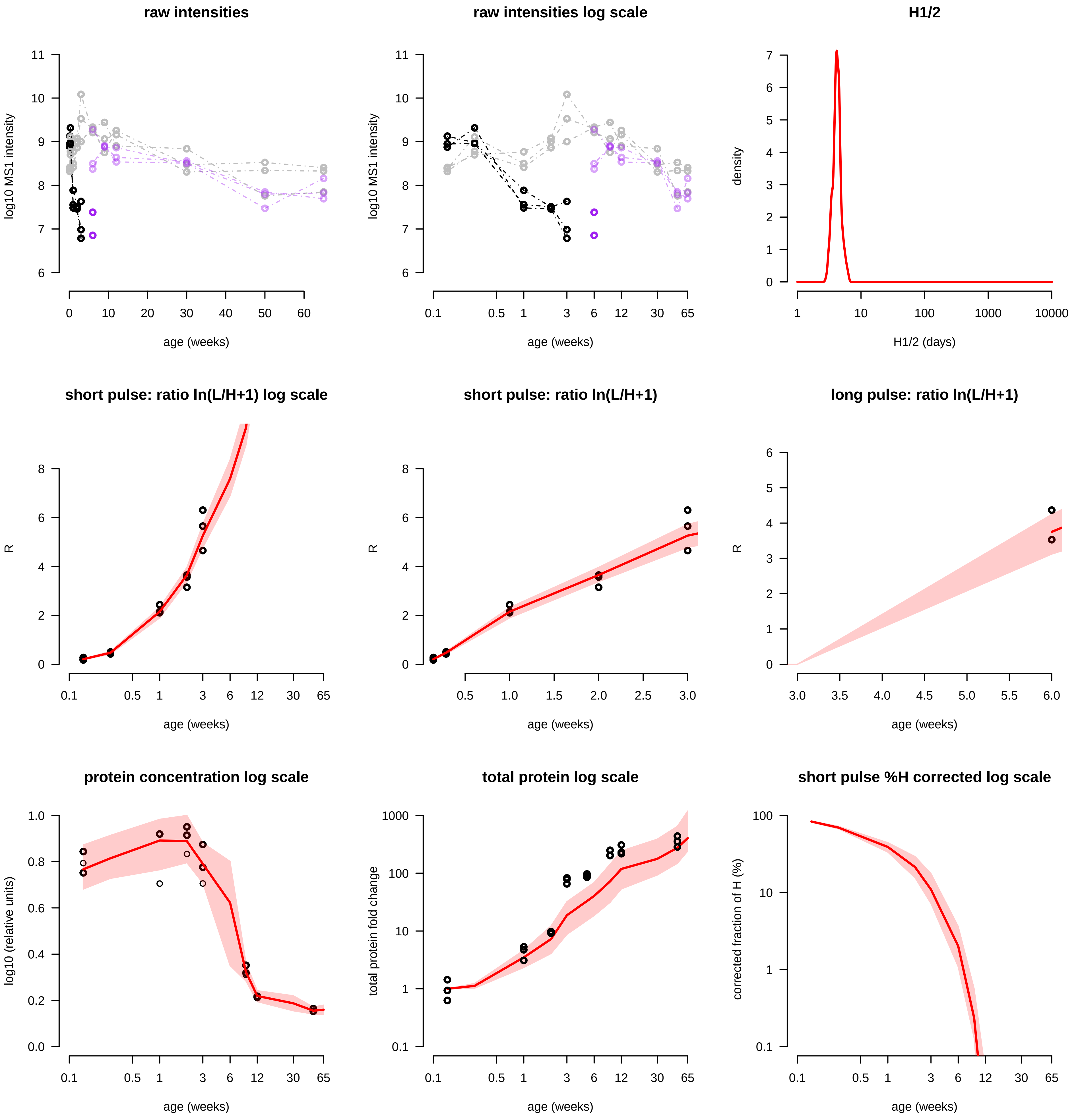

Q8C166

>sp|Q8C166|CPNE1_MOUSE

Copine−1

GN=Cpne1

Med H1/2: 24.8 days

Q5 H1/2: 7.2 days

Q95 H1/2: 249.1 days

reliable model fit: yes

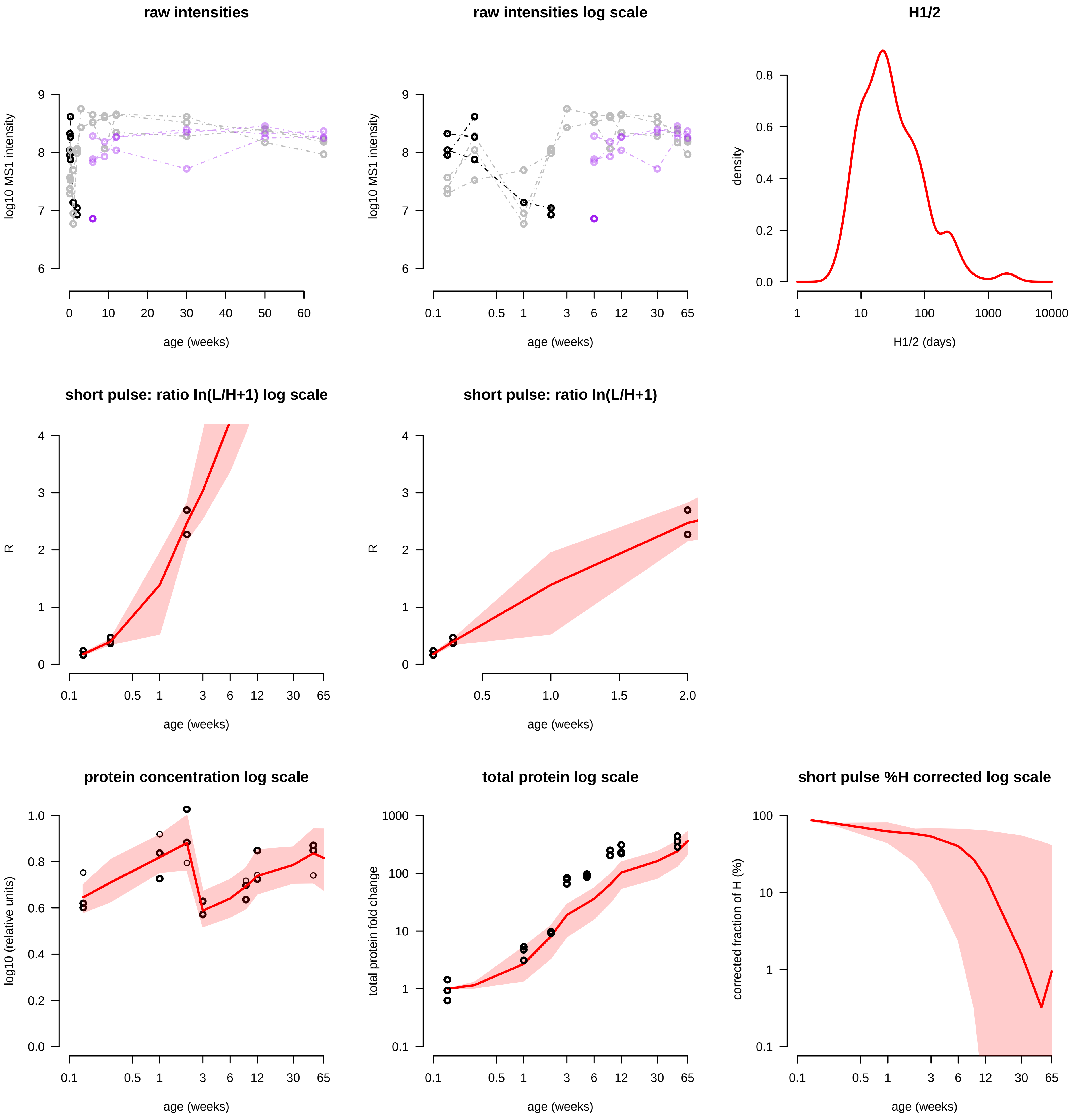

P53996

>sp|P53996|CNBP_MOUSE

Cellular nucleic acid−binding protein

GN=Cnbp

Med H1/2: 5.5 days

Q5 H1/2: 4.2 days

Q95 H1/2: 7.8 days

reliable model fit: yes

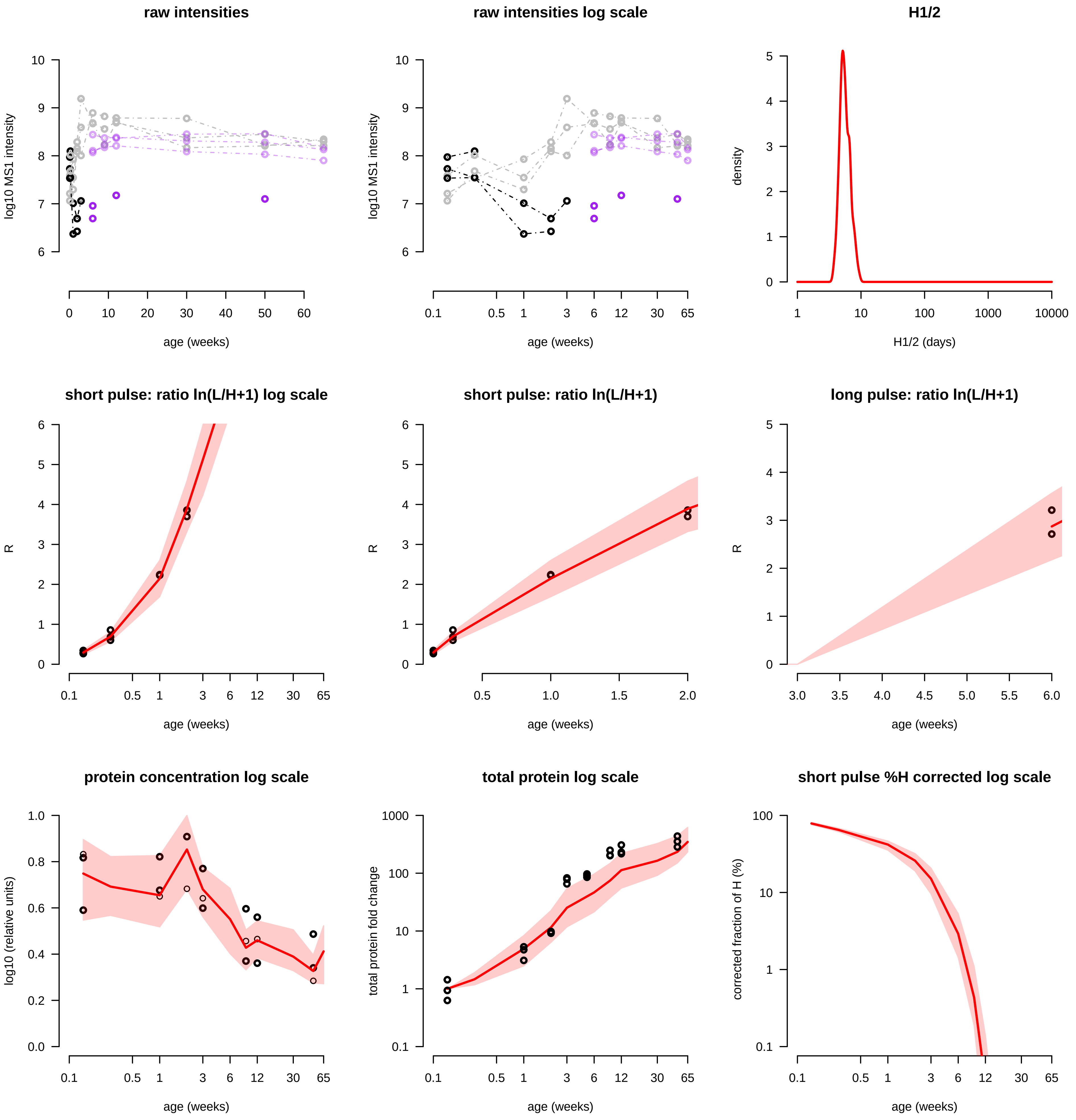

Q8VDG3

>sp|Q8VDG3|PARN_MOUSE

Poly(A)−specific ribonuclease PARN

GN=Parn

Med H1/2: 3.6 days

Q5 H1/2: 2.7 days

Q95 H1/2: 4.8 days

reliable model fit: yes

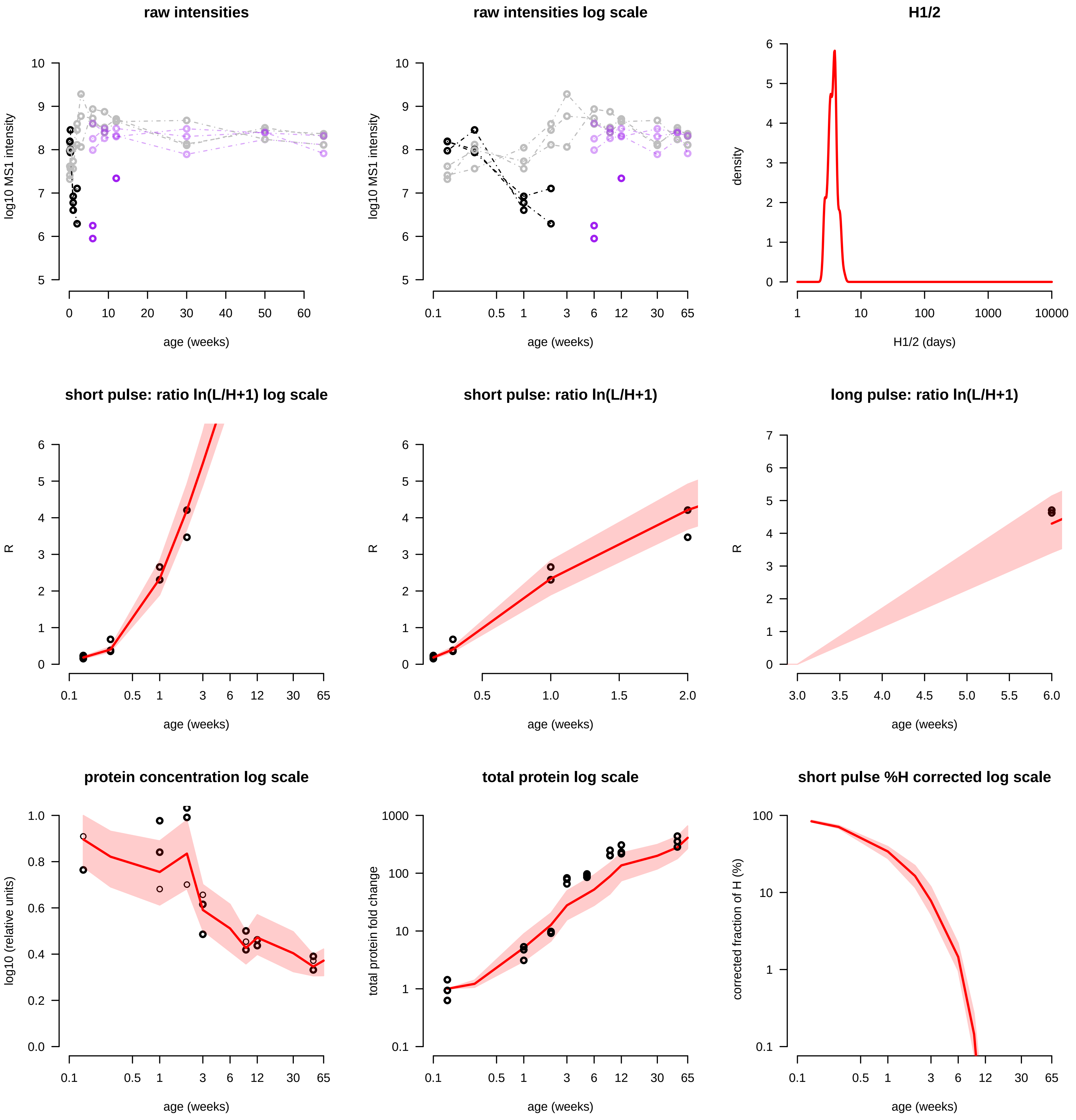

Q5DTM8;Q5DTM8−2

>sp|Q5DTM8|BRE1A_MOUSE

E3 ubiquitin−protein ligase BRE1A

GN=Rnf20

Med H1/2: 3.9 days

Q5 H1/2: 2.5 days

Q95 H1/2: 10.9 days

reliable model fit: yes

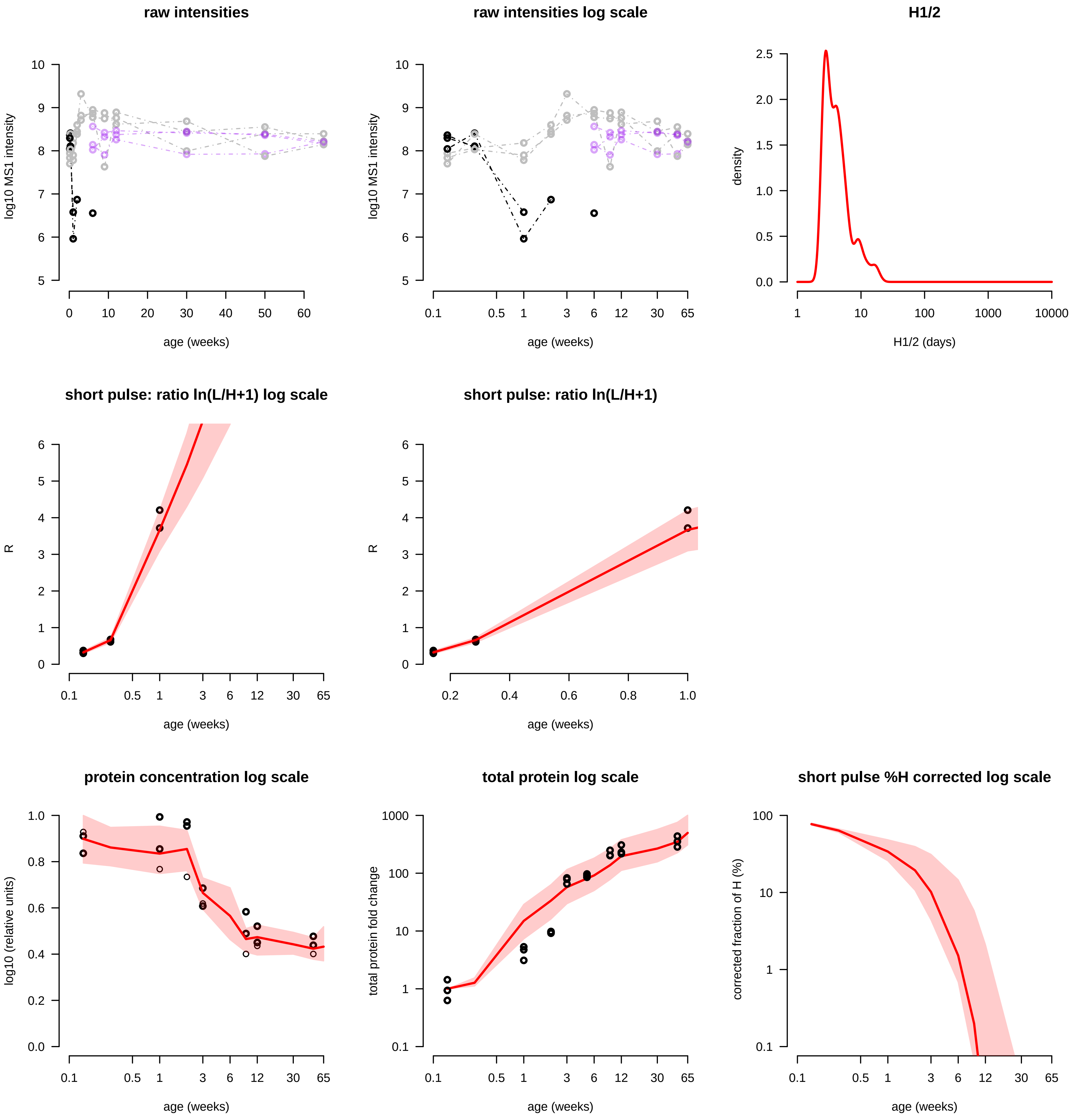

E9Q5C9;E9Q5C9−2

>sp|E9Q5C9|NOLC1_MOUSE

Nucleolar and coiled−body phosphoprotein 1

GN=Nolc1

Med H1/2: 3.7 days

Q5 H1/2: 2.9 days

Q95 H1/2: 5.5 days

reliable model fit: yes

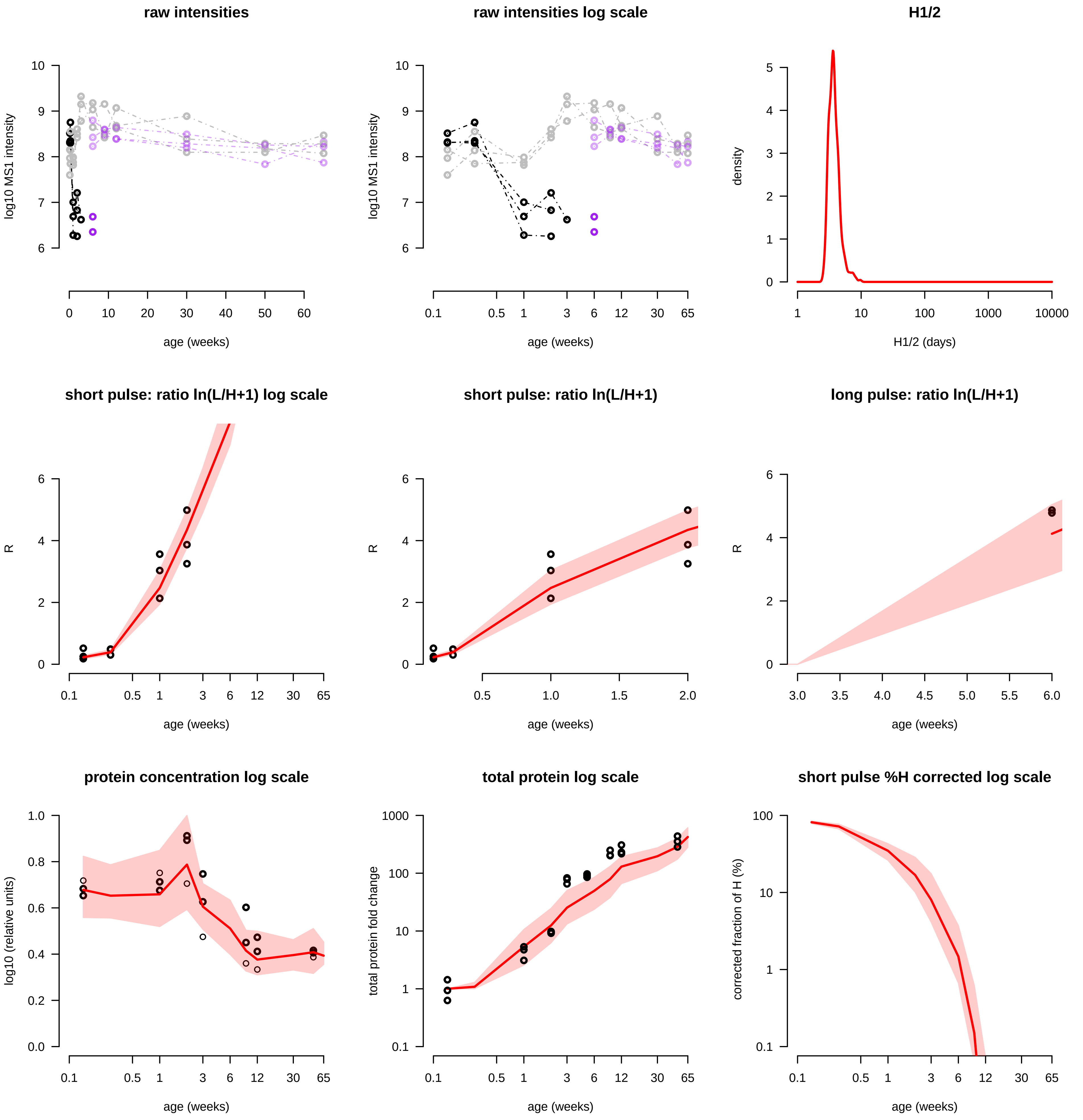

O88384

>sp|O88384|VTI1B_MOUSE

Vesicle transport through interaction with t−SNAREs homolog 1B

GN=Vti1b

Med H1/2: 6.9 days

Q5 H1/2: 3.2 days

Q95 H1/2: 34.6 days

reliable model fit: yes

raw intensities

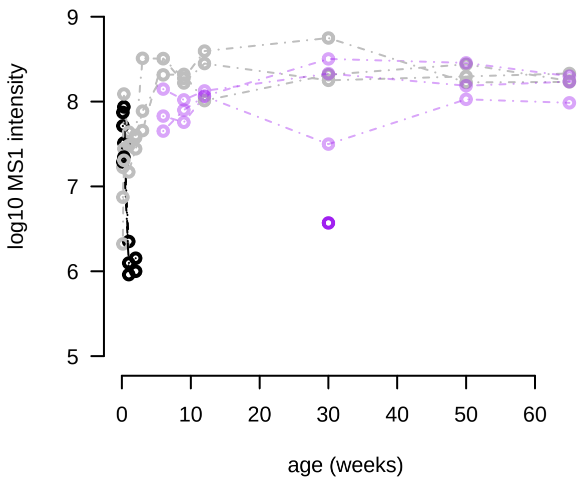

raw intensities log scale

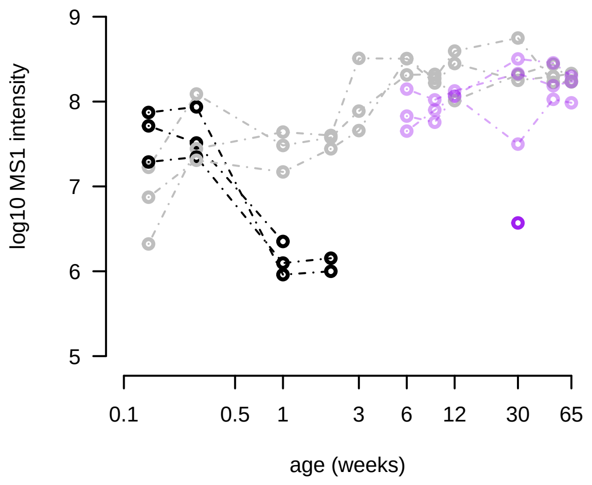

H1/2

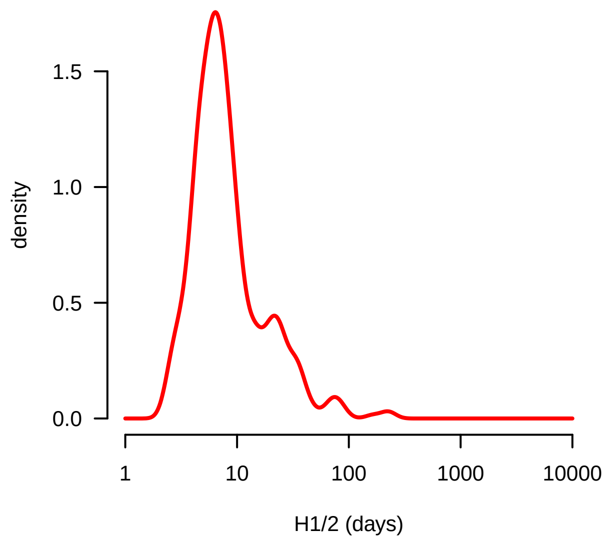

short pulse: ratio ln(L/H+1) log scale

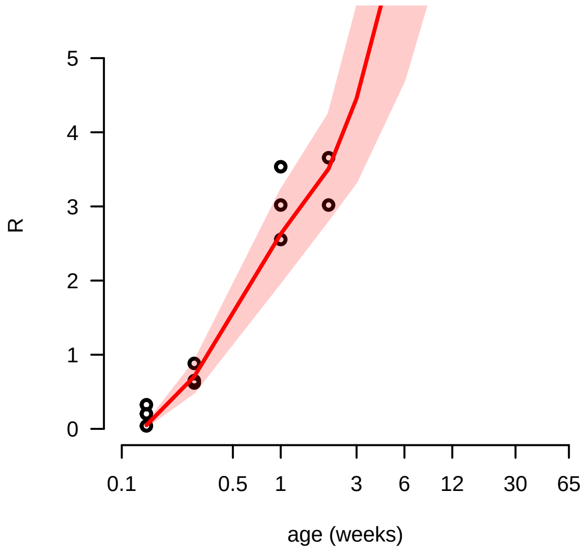

short pulse: ratio ln(L/H+1)

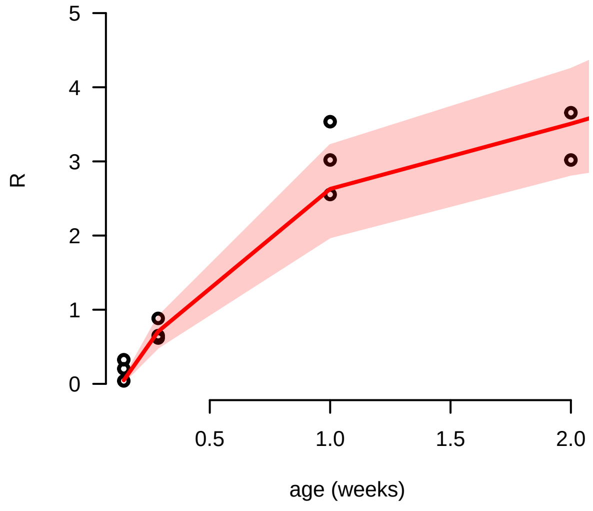

protein concentration log scale

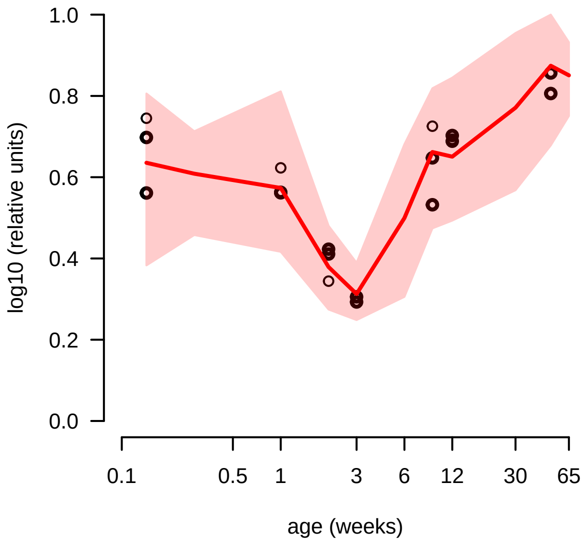

total protein log scale

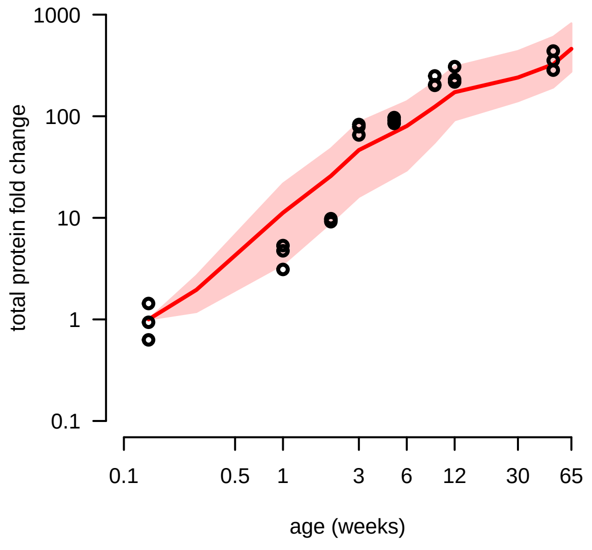

short pulse %H corrected log scale

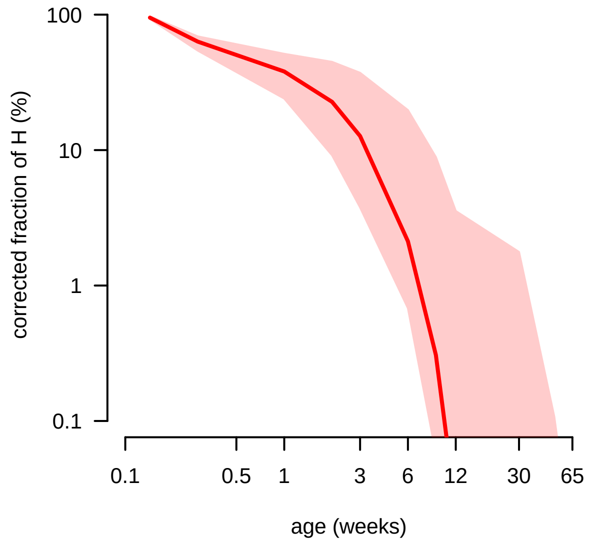

Q8BZW8

>sp|Q8BZW8|NHLC2_MOUSE

NHL repeat−containing protein 2

GN=Nhlrc2

Med H1/2: 7.9 days

Q5 H1/2: 2.7 days

Q95 H1/2: 43.1 days

reliable model fit: yes

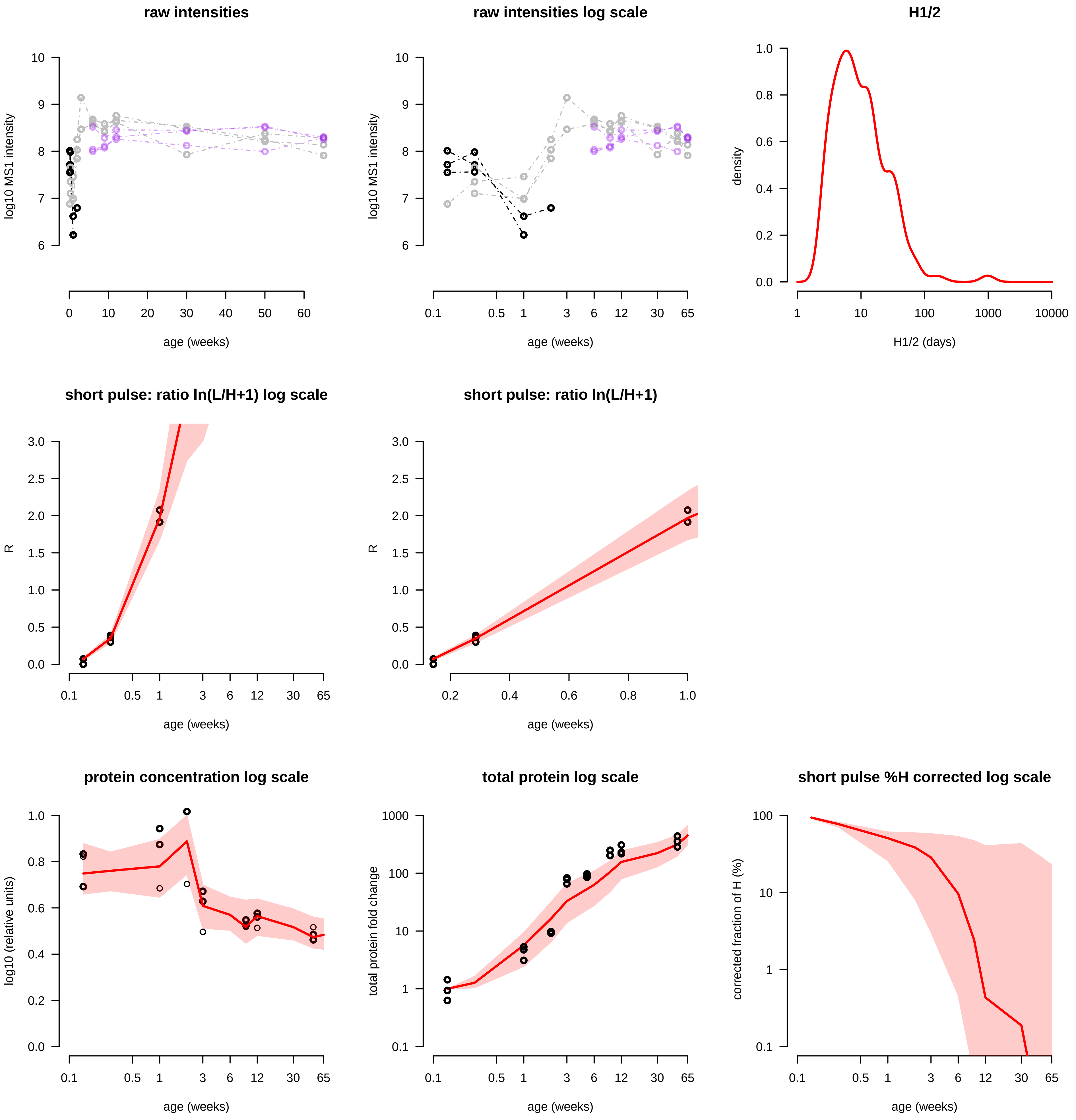

P35285

>sp|P35285|RB22A_MOUSE

Ras−related protein Rab−22A

GN=Rab22a

Med H1/2: 5.6 days

Q5 H1/2: 2.9 days

Q95 H1/2: 23.1 days

reliable model fit: yes

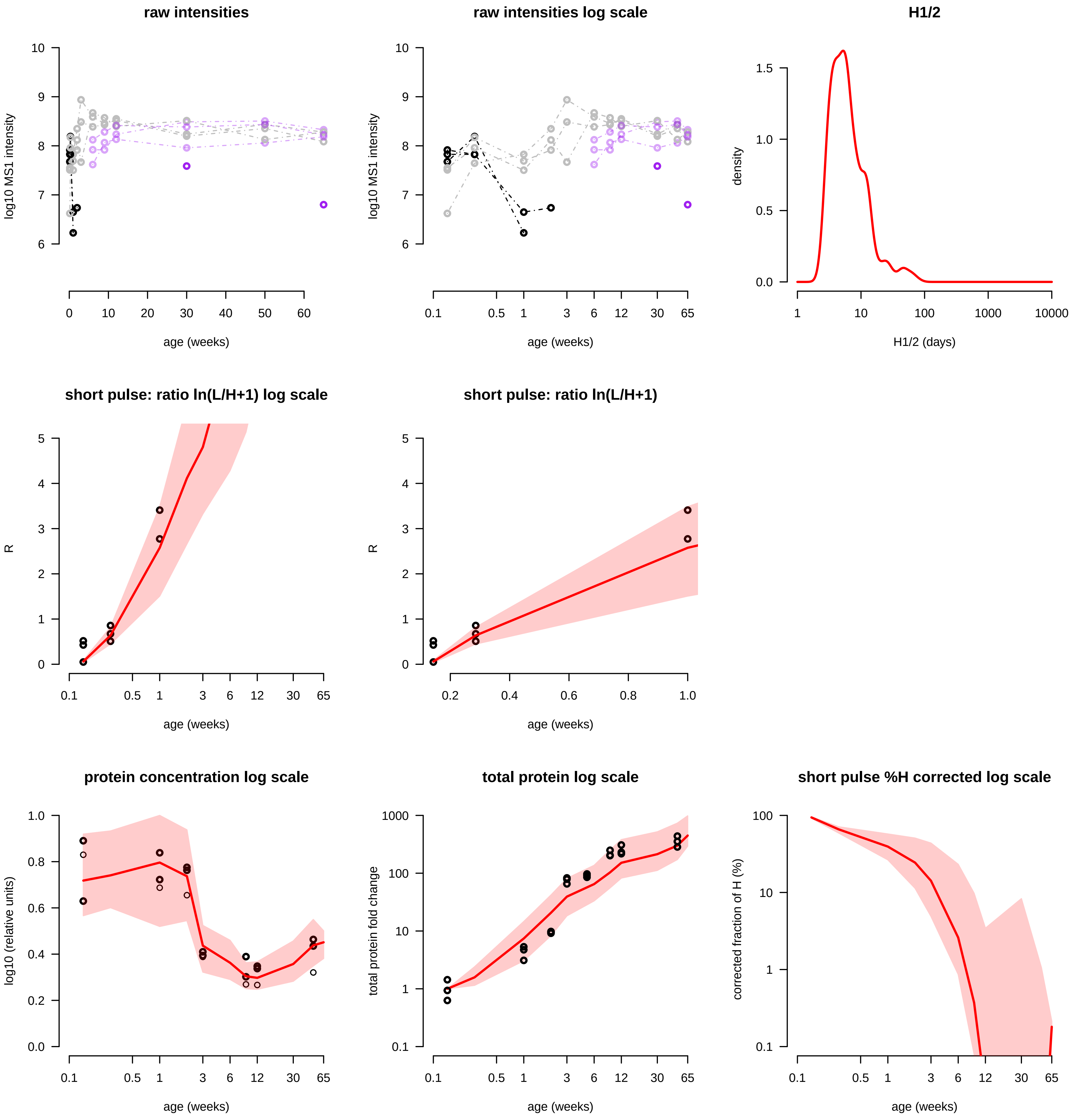

Q9D2C7

>sp|Q9D2C7|BI1_MOUSE

Bax inhibitor 1

GN=Tmbim6

Med H1/2: 12.1 days

Q5 H1/2: 4.6 days

Q95 H1/2: 147.9 days

reliable model fit: yes

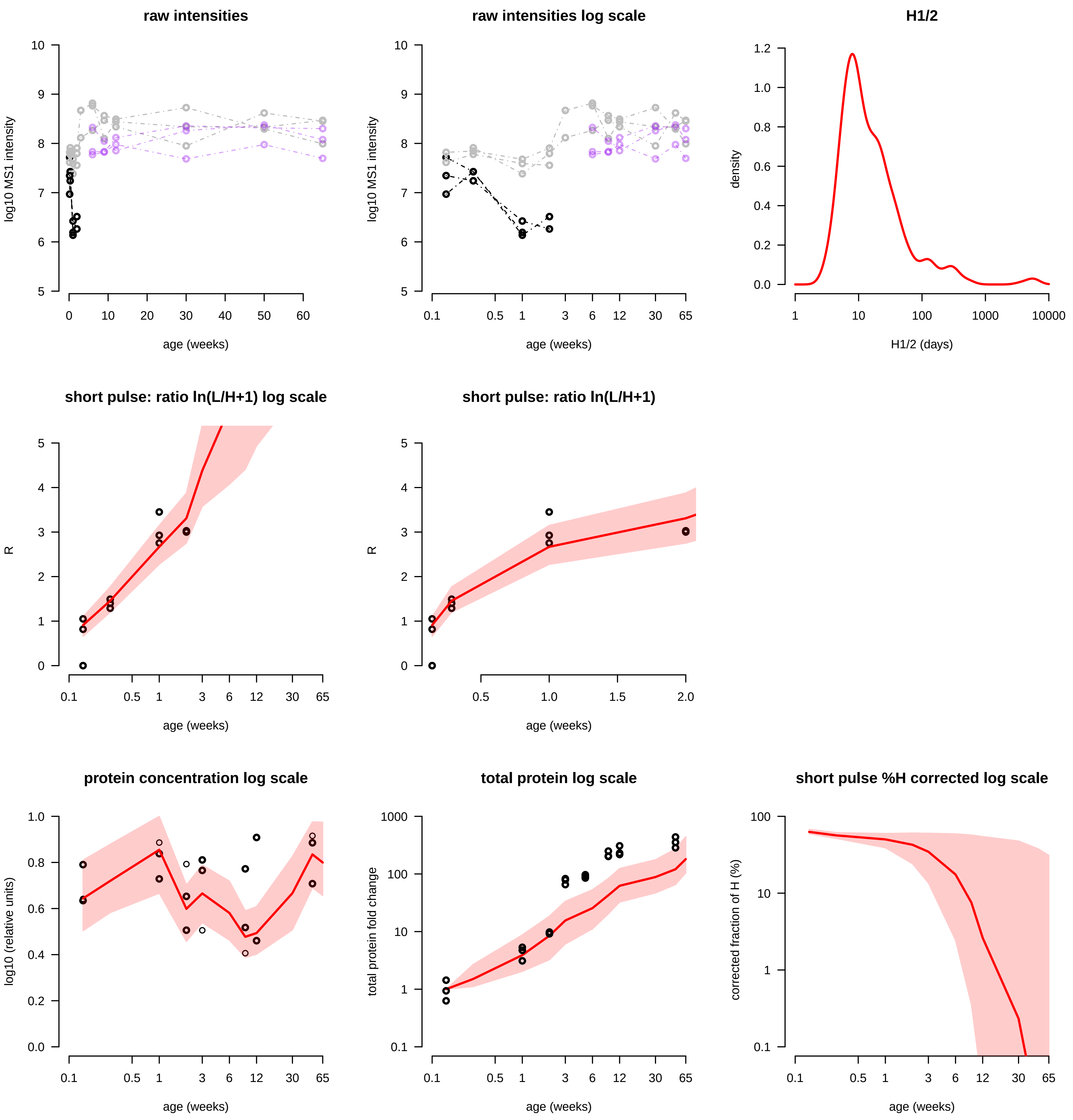

Q9DAU1

>sp|Q9DAU1|CNPY3_MOUSE

Protein canopy homolog 3

GN=Cnpy3

Med H1/2: 881 days

Q5 H1/2: 130.4 days

Q95 H1/2: 12470.4 days

reliable model fit: no

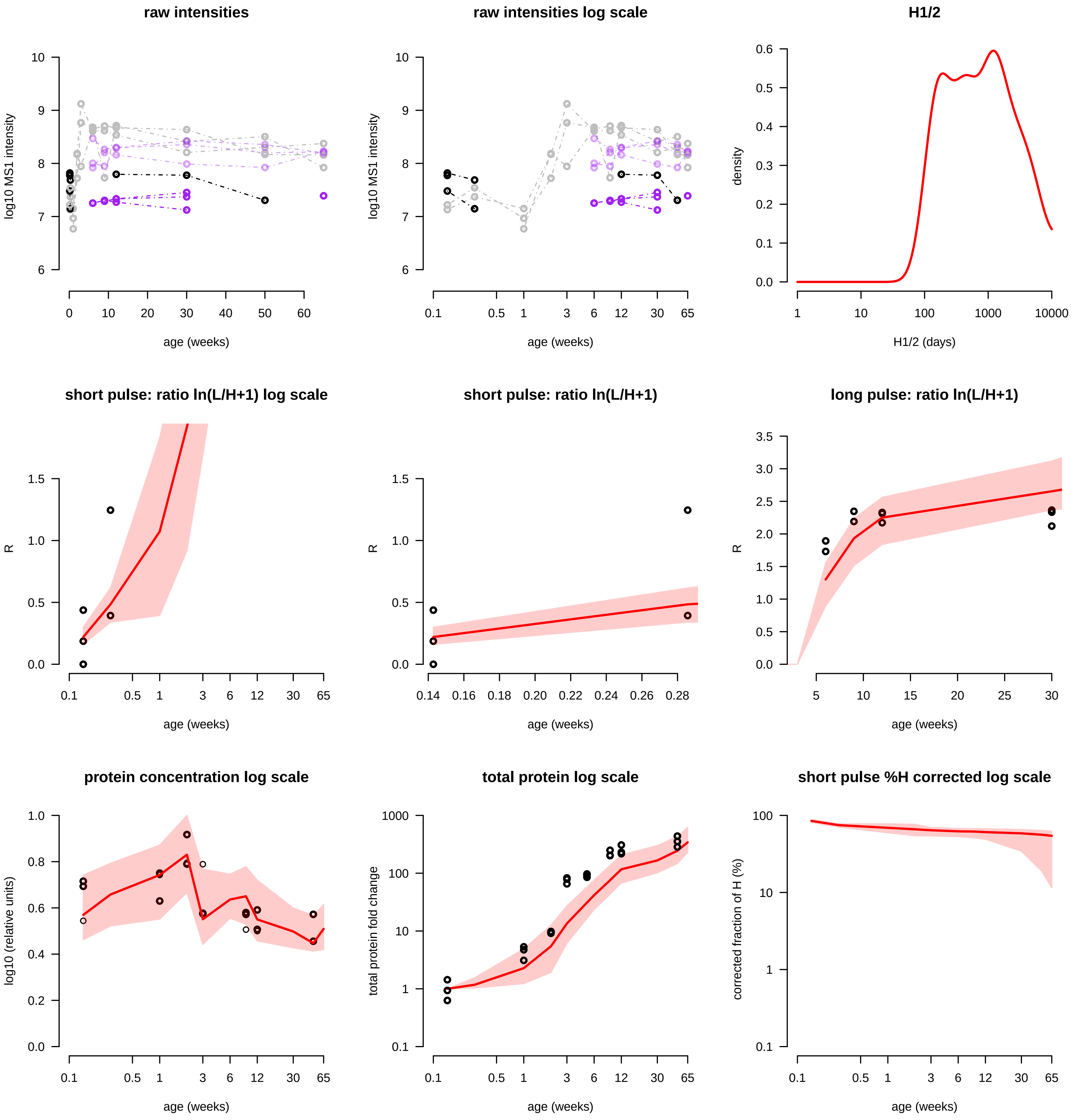

P49717

>sp|P49717|MCM4_MOUSE

DNA replication licensing factor MCM4

GN=Mcm4

Med H1/2: 3.5 days

Q5 H1/2: 2.7 days

Q95 H1/2: 4.8 days

reliable model fit: yes

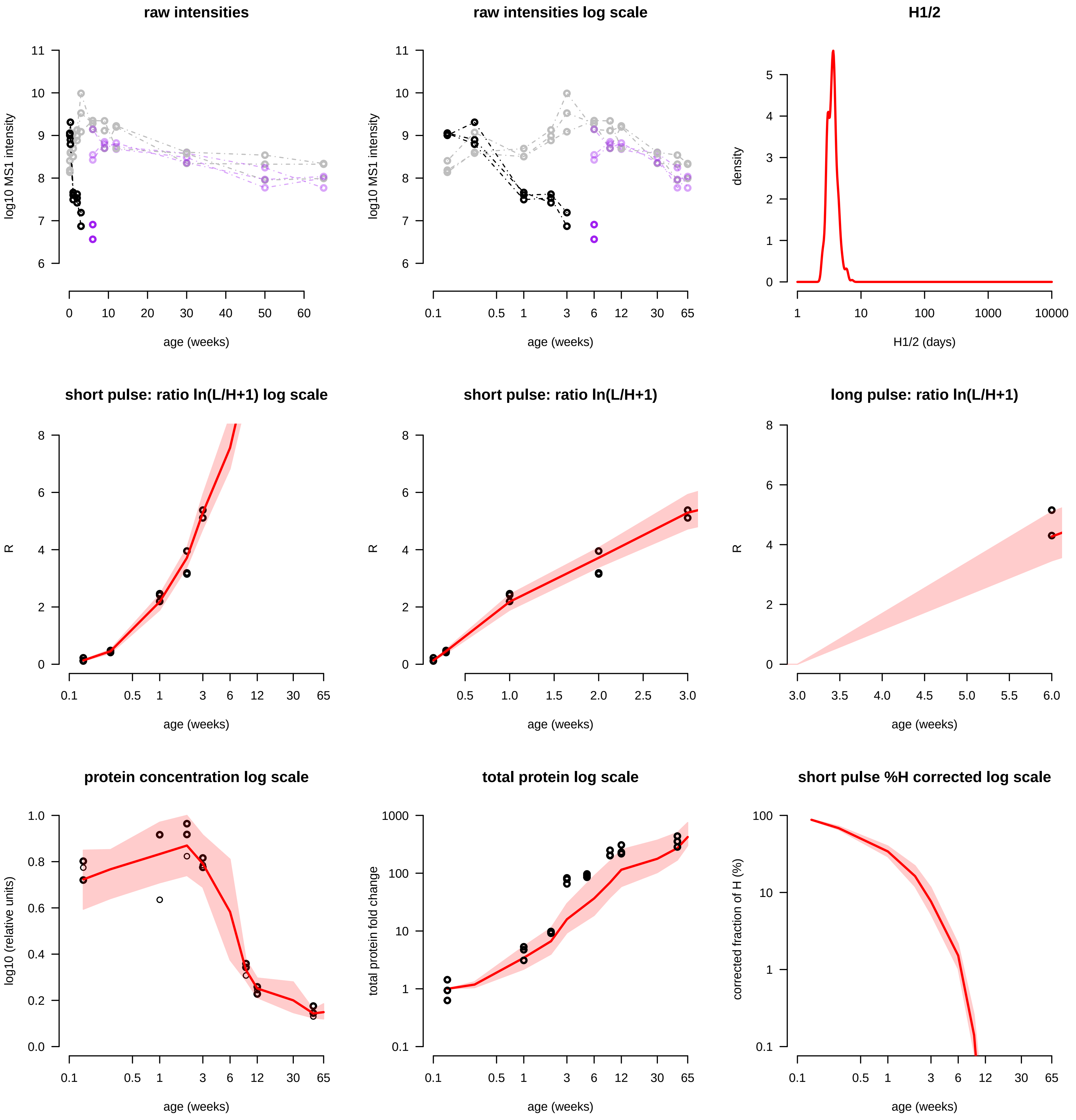

Q9DCT2

>sp|Q9DCT2|NDUS3_MOUSE

NADH dehydrogenase [ubiquinone] iron−sulfur protein 3, mitochondrial

GN=Ndufs3

Med H1/2: 4.7 days

Q5 H1/2: 3.6 days

Q95 H1/2: 6.8 days

reliable model fit: yes

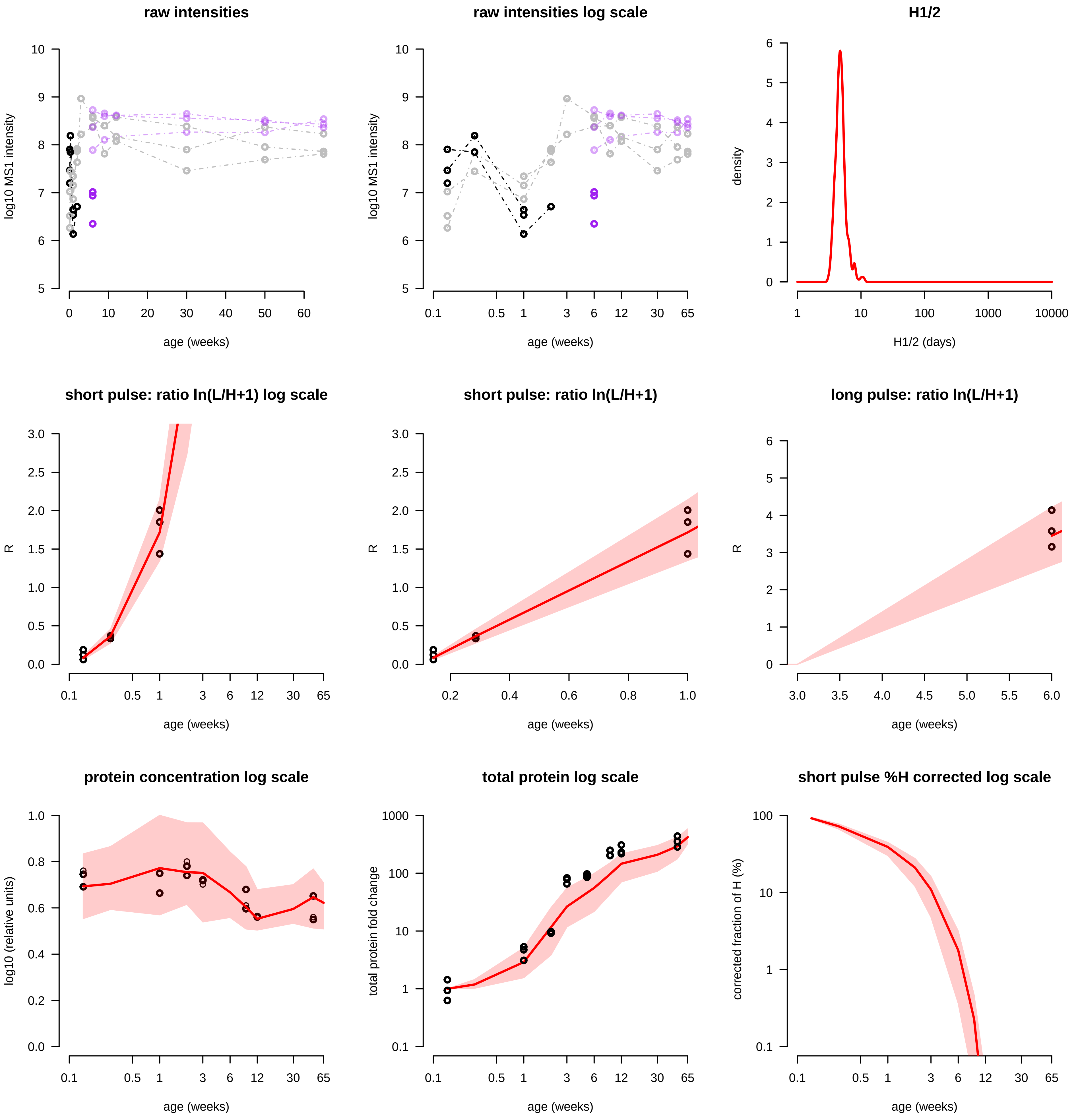

Q99JW2

>sp|Q99JW2|ACY1_MOUSE

Aminoacylase−1

GN=Acy1

Med H1/2: 7.9 days

Q5 H1/2: 5 days

Q95 H1/2: 27.1 days

reliable model fit: no

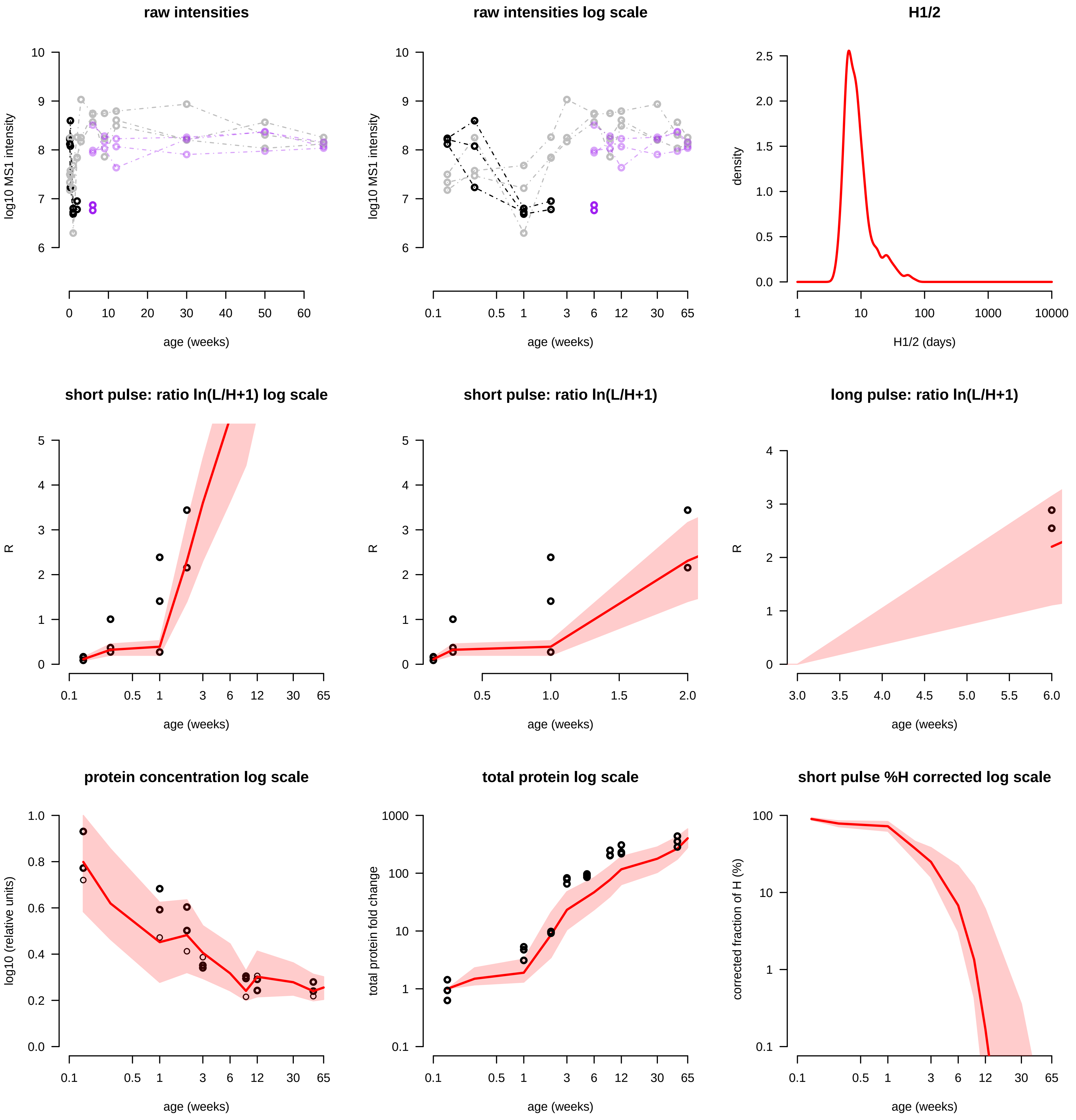

Q6PAM1;Q6PAM1−2

>sp|Q6PAM1|TXLNA_MOUSE

Alpha−taxilin

GN=Txlna

Med H1/2: 6.8 days

Q5 H1/2: 3.9 days

Q95 H1/2: 20.3 days

reliable model fit: yes

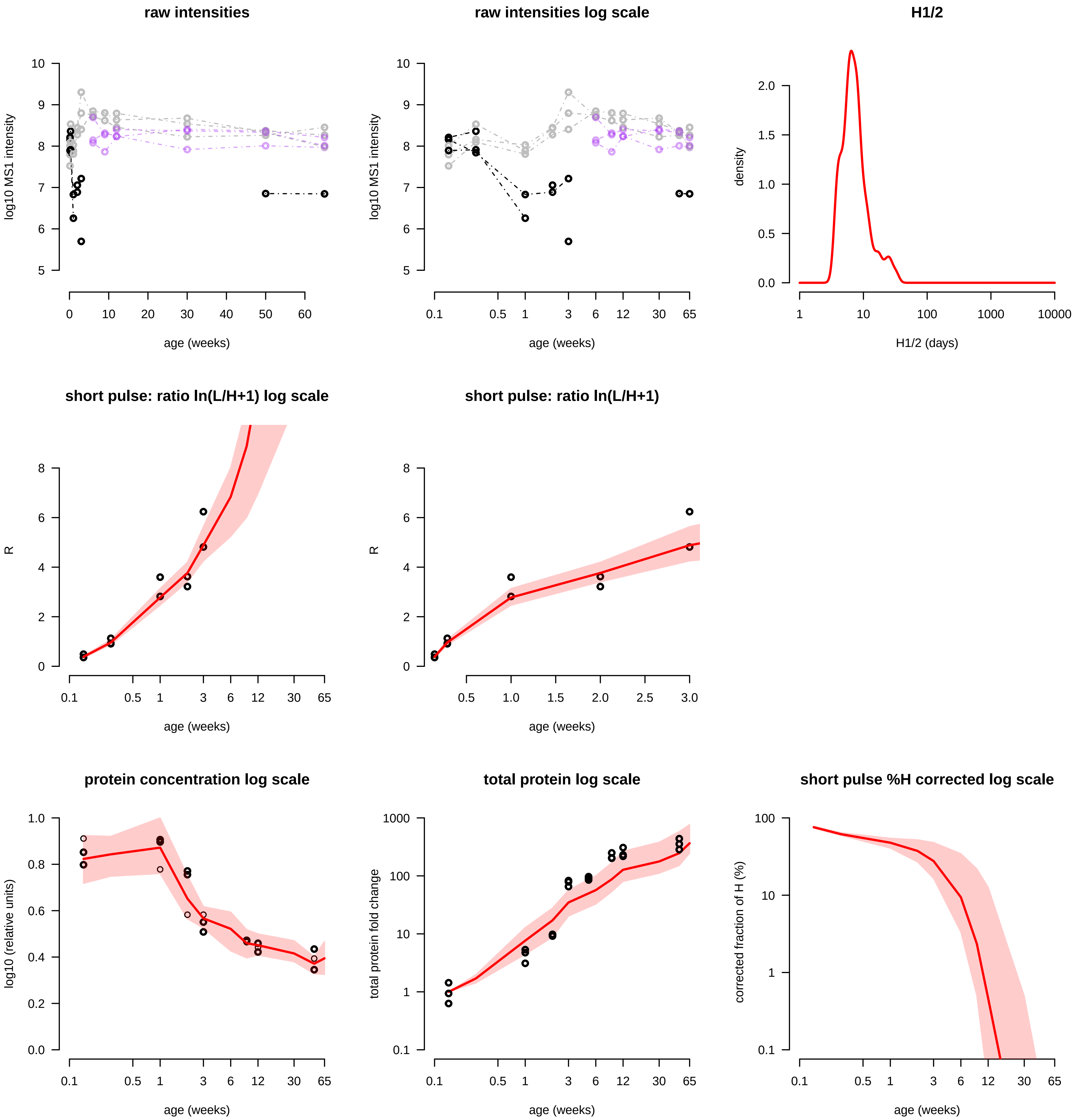

Q8C052

>sp|Q8C052|MAP1S_MOUSE

Microtubule−associated protein 1S

GN=Map1s

Med H1/2: 6.9 days

Q5 H1/2: 2.8 days

Q95 H1/2: 41.3 days

reliable model fit: yes

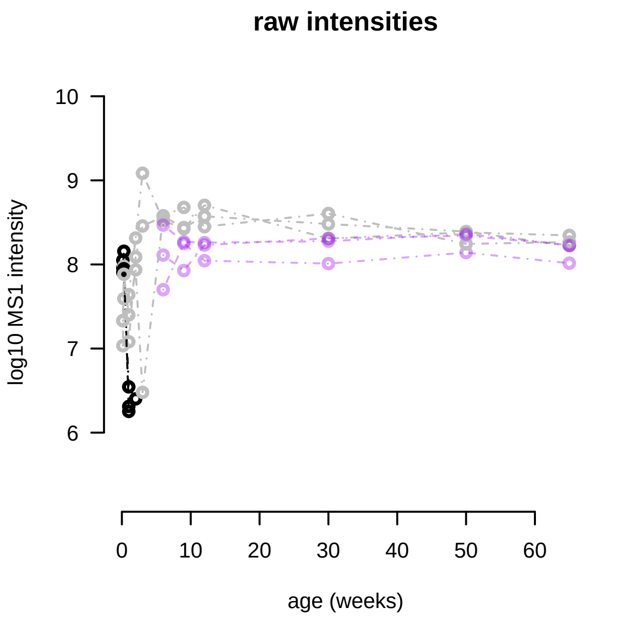

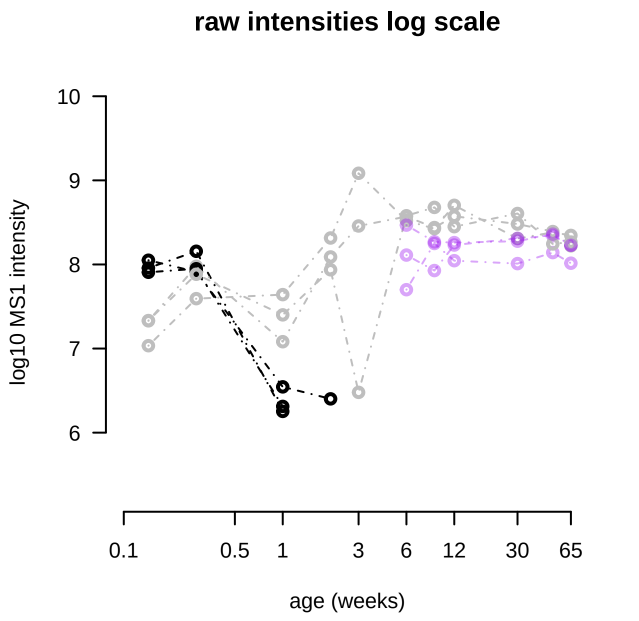

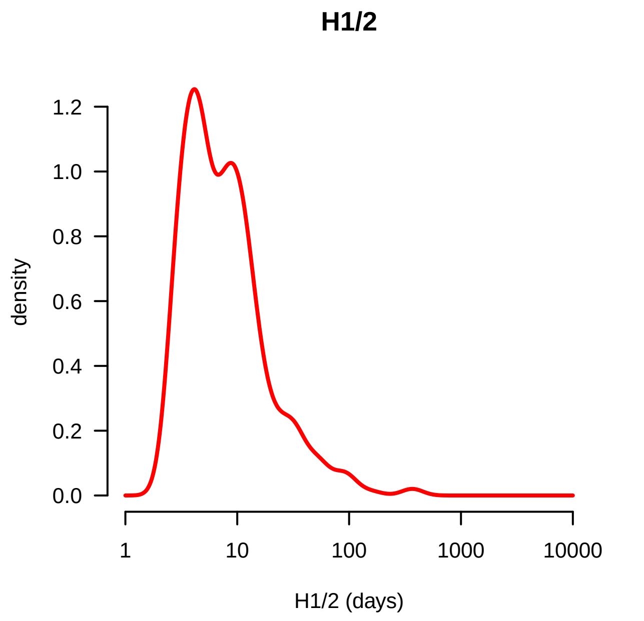

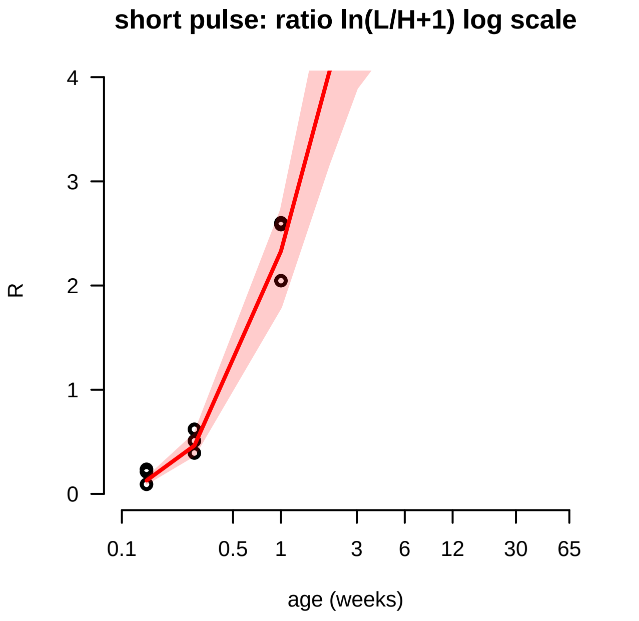

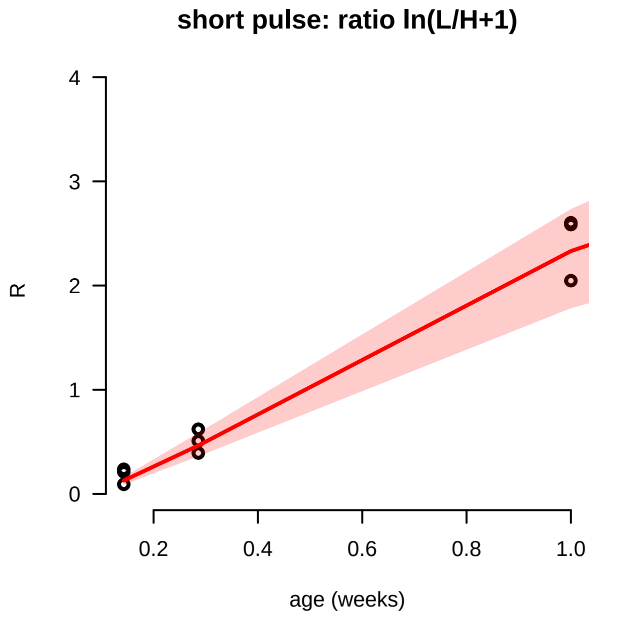

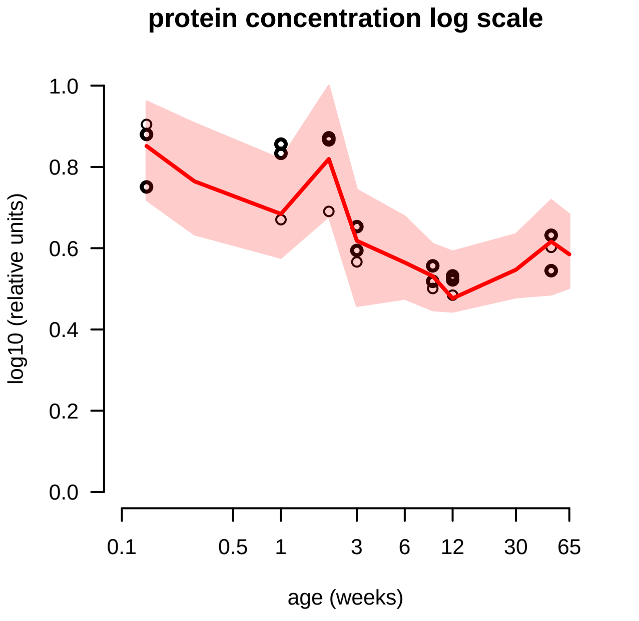

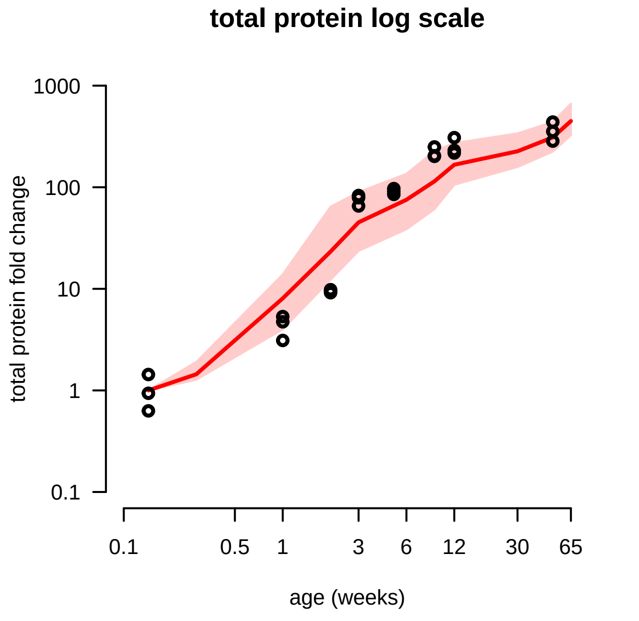

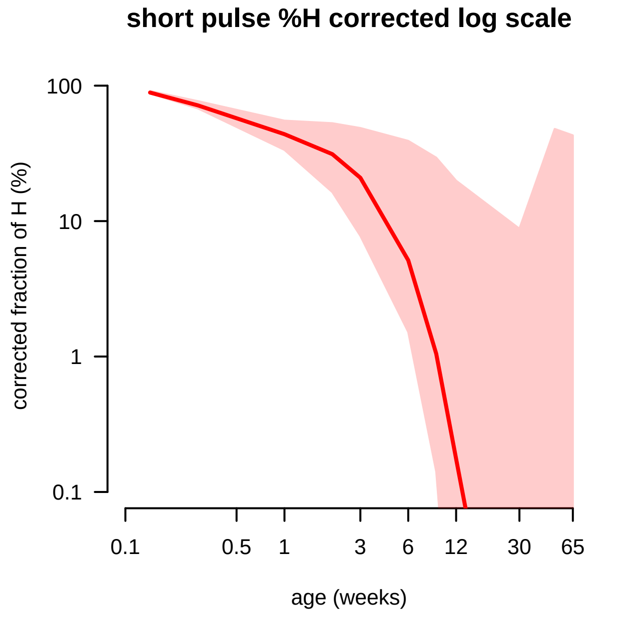

Q9D7N3

>sp|Q9D7N3|RT09_MOUSE

28S ribosomal protein S9, mitochondrial

GN=Mrps9

Med H1/2: 2988.4 days

Q5 H1/2: 937.1 days

Q95 H1/2: 31452 days

reliable model fit: yes

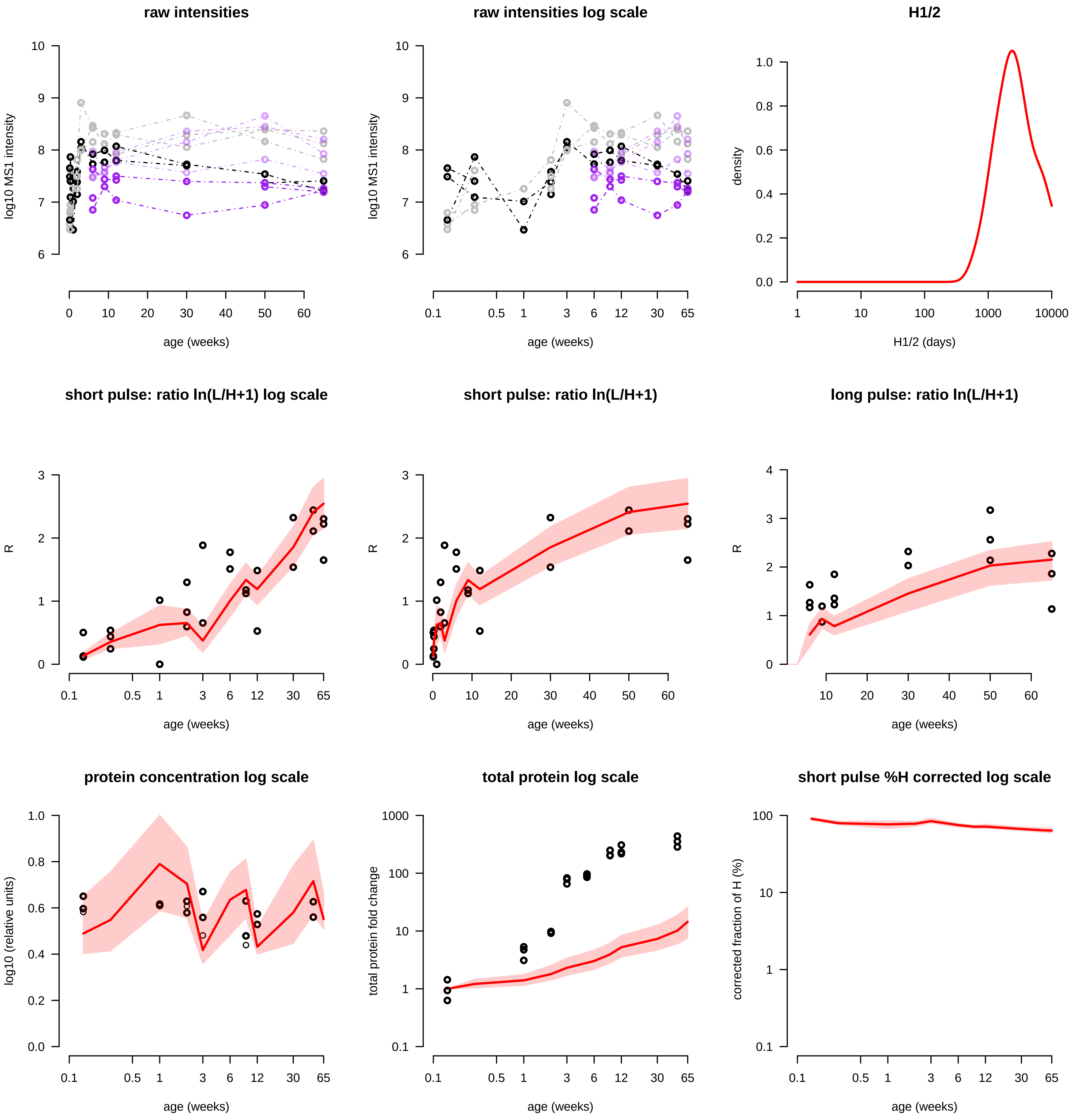

Q80SY5

>sp|Q80SY5|PR38B_MOUSE

Pre−mRNA−splicing factor 38B

GN=Prpf38b

Med H1/2: 10.6 days

Q5 H1/2: 3.4 days

Q95 H1/2: 79.7 days

reliable model fit: yes

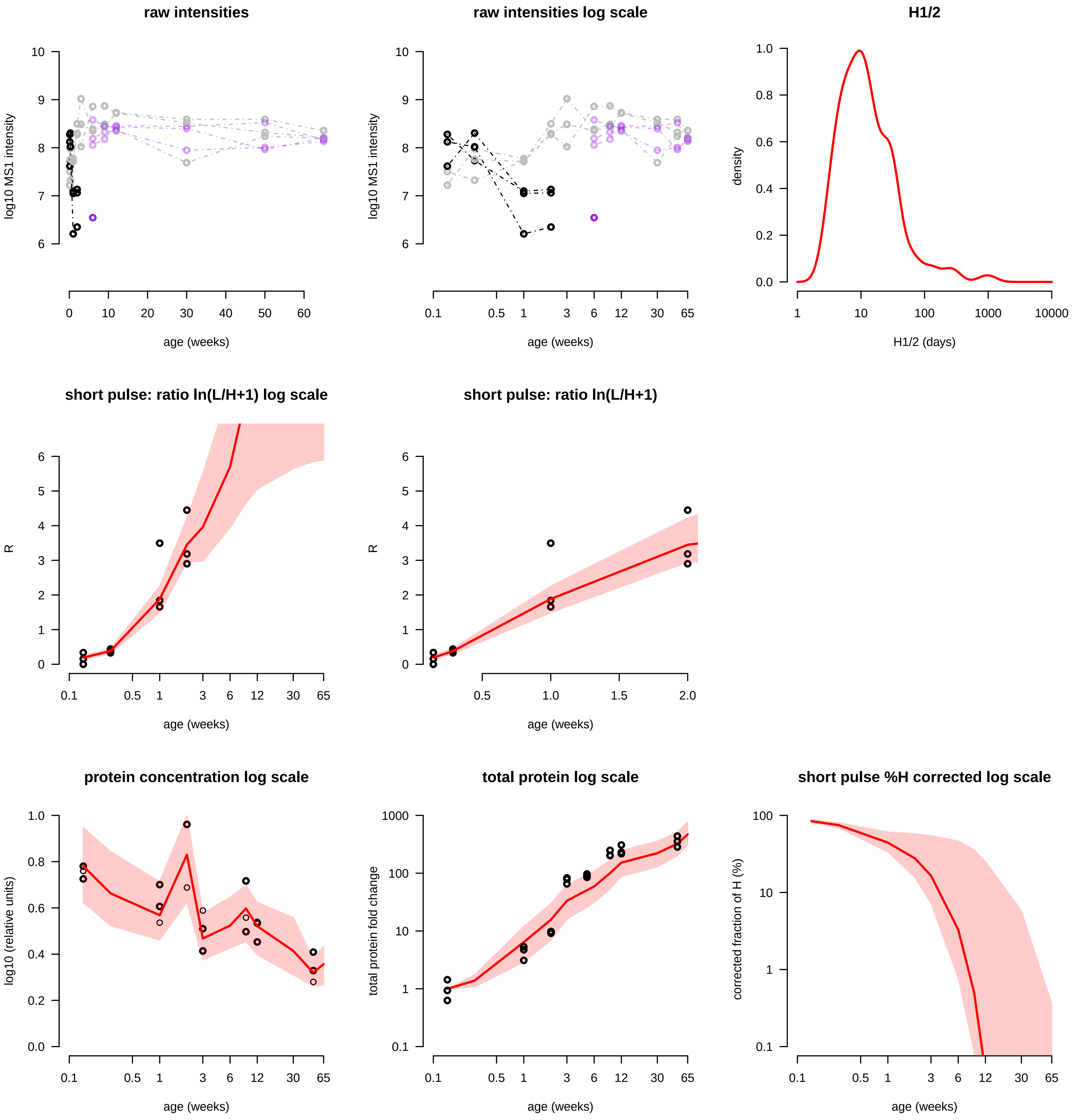

Q99N92

>sp|Q99N92|RM27_MOUSE

39S ribosomal protein L27, mitochondrial

GN=Mrpl27

Med H1/2: 14.2 days

Q5 H1/2: 4.9 days

Q95 H1/2: 207.6 days

reliable model fit: yes

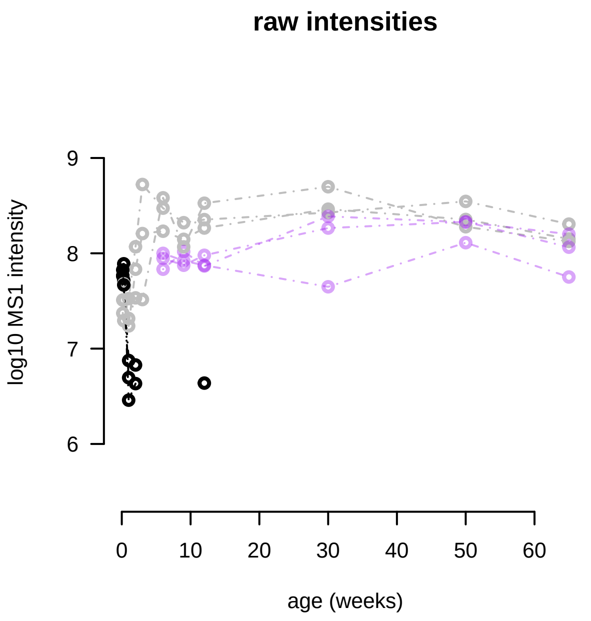

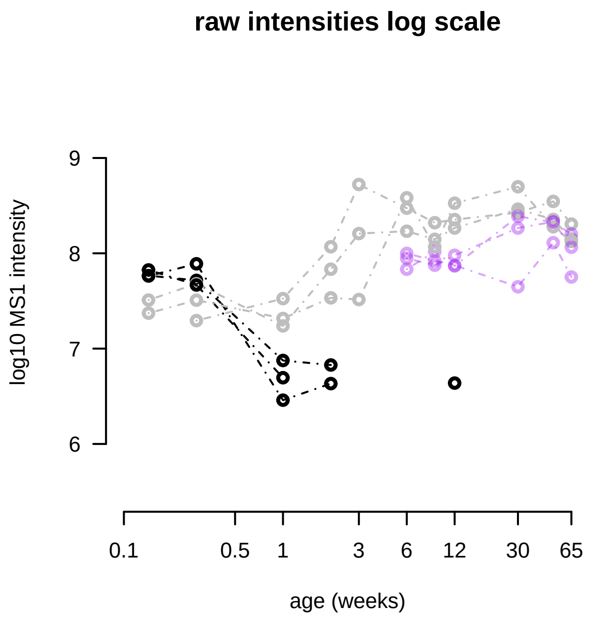

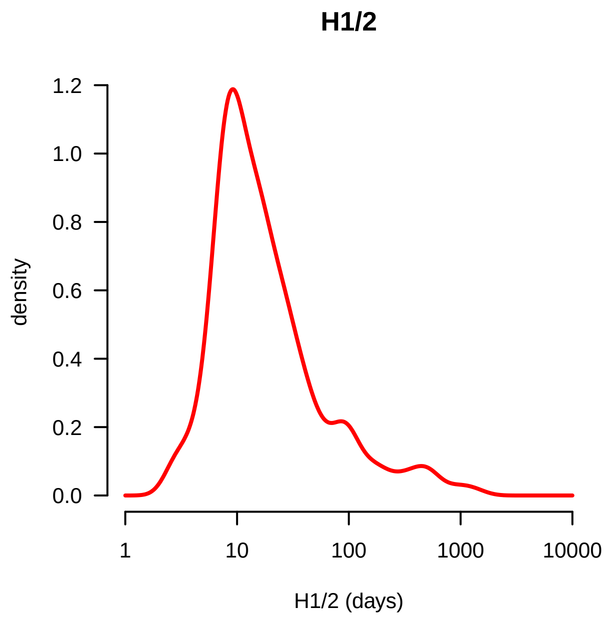

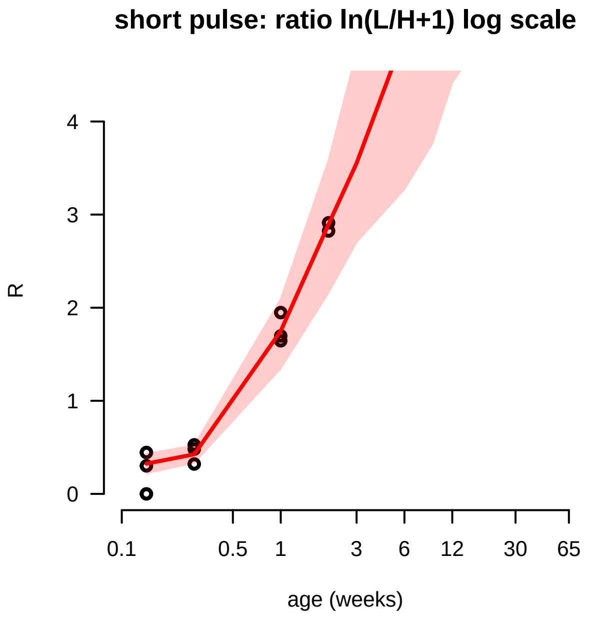

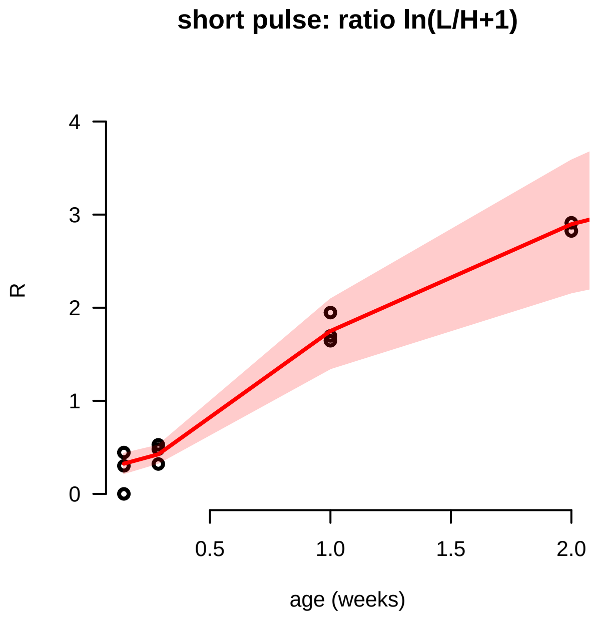

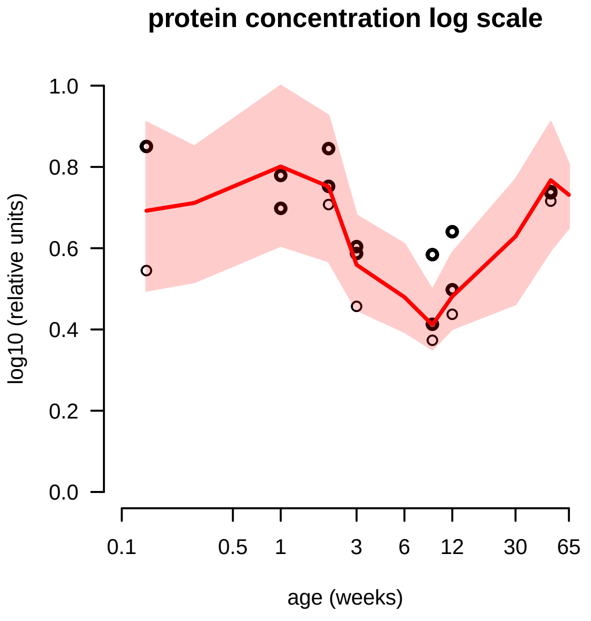

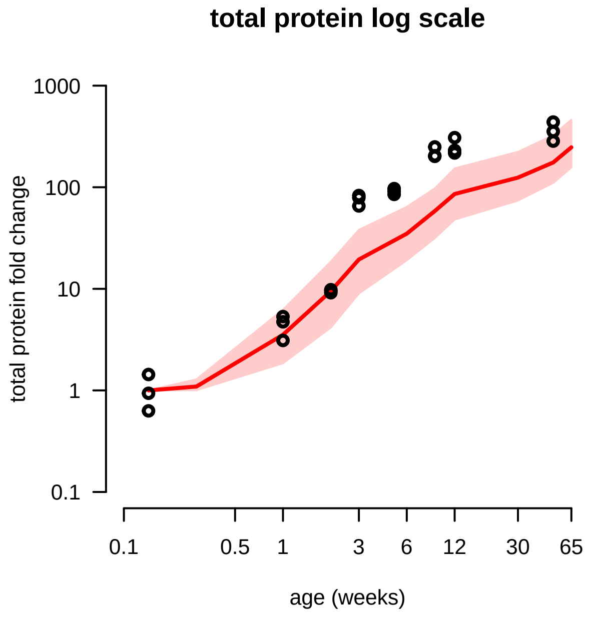

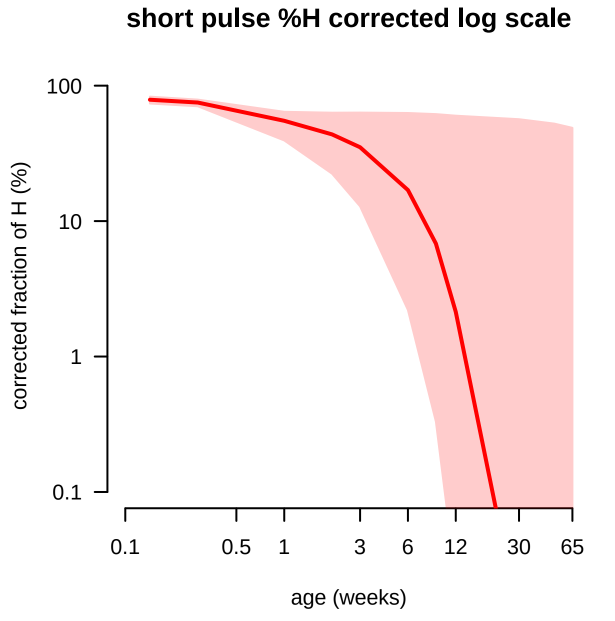

Q8BTZ4−2;Q8BTZ4

>sp|Q8BTZ4−2|APC5_MOUSE

Isoform 2 of Anaphase−promoting complex subunit 5

GN=Anapc5

Med H1/2: 320.8 days

Q5 H1/2: 140.7 days

Q95 H1/2: 2389.9 days

reliable model fit: yes

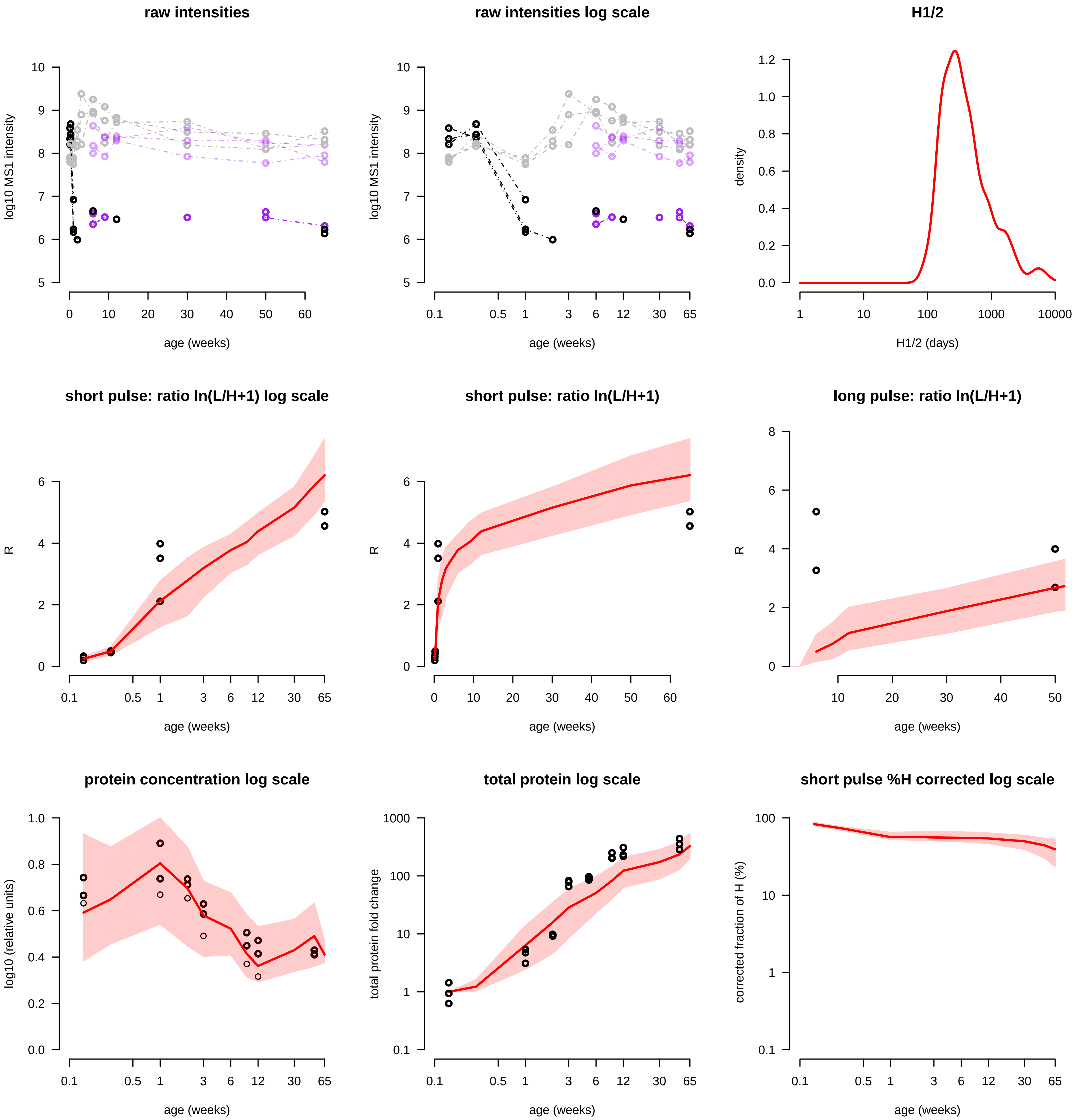

Q80U35

>sp|Q80U35|ARHGH_MOUSE

Rho guanine nucleotide exchange factor 17

GN=Arhgef17

Med H1/2: 329.8 days

Q5 H1/2: 135.5 days

Q95 H1/2: 1994.9 days

reliable model fit: no

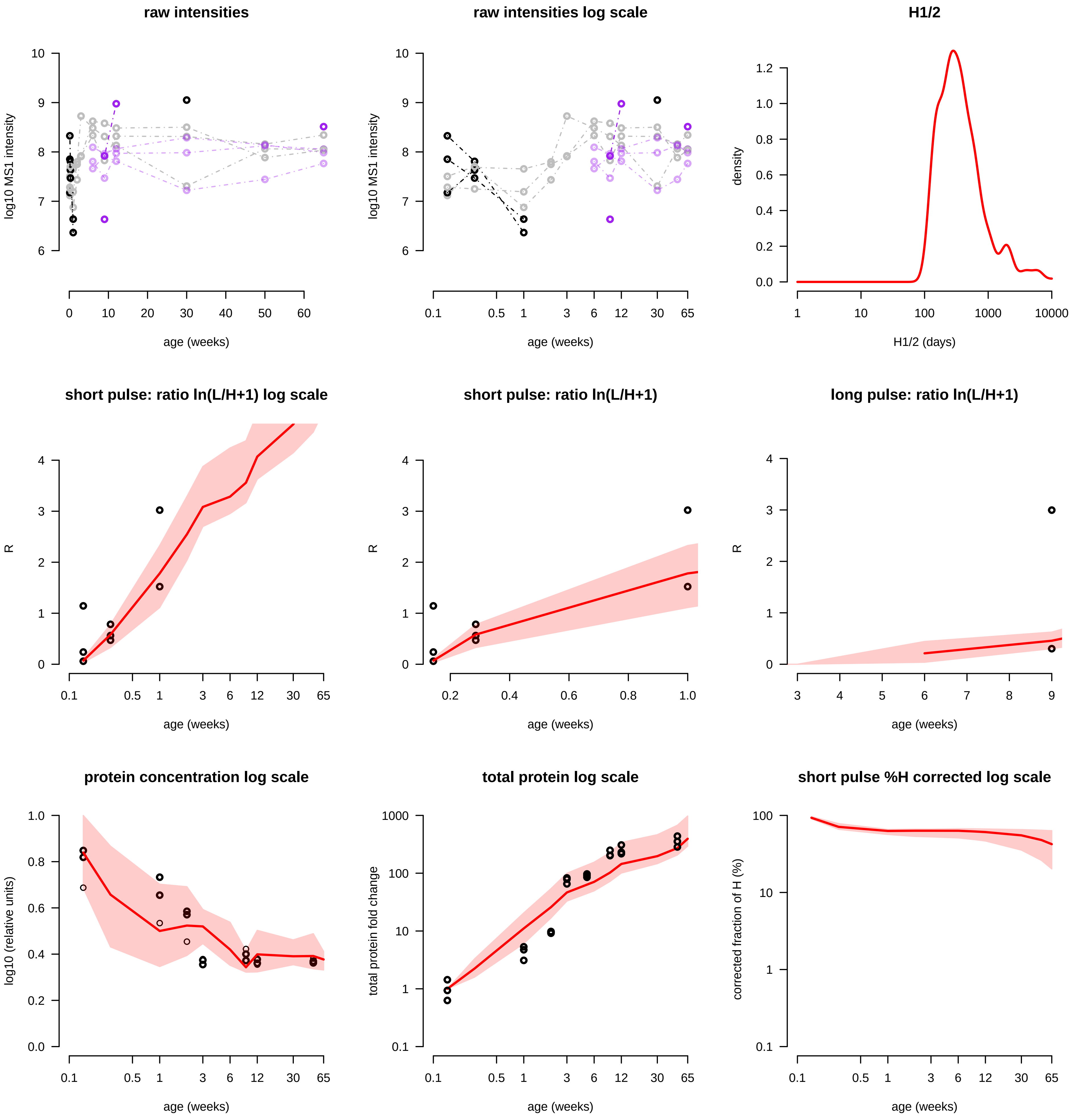

Q61881

>sp|Q61881|MCM7_MOUSE

DNA replication licensing factor MCM7

GN=Mcm7

Med H1/2: 3.1 days

Q5 H1/2: 2.6 days

Q95 H1/2: 4.1 days

reliable model fit: yes

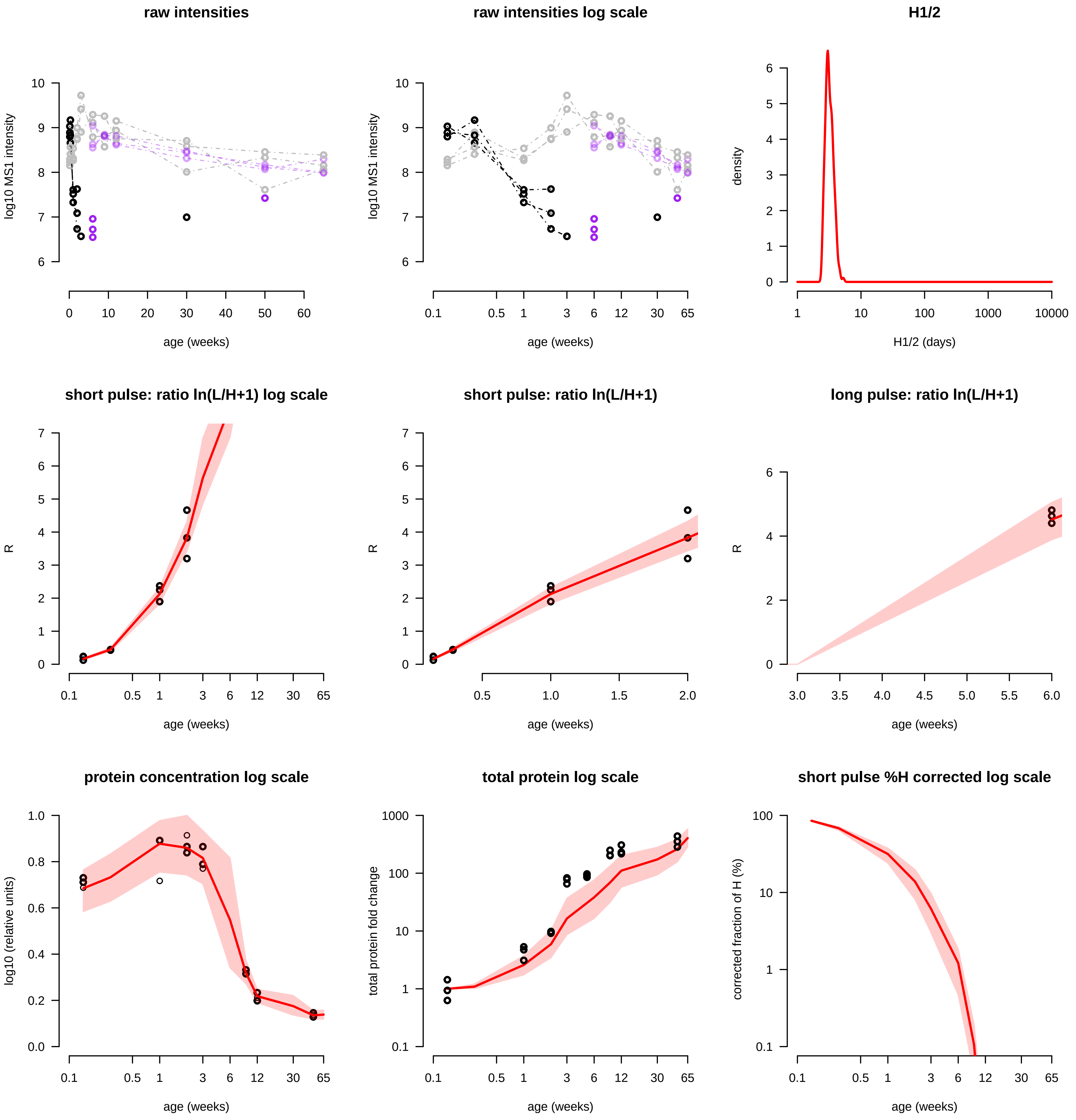

Q69ZS7;Q69ZS7−2

>sp|Q69ZS7|HBS1L_MOUSE

HBS1−like protein

GN=Hbs1l

Med H1/2: 5 days

Q5 H1/2: 2.5 days

Q95 H1/2: 31.7 days

reliable model fit: yes

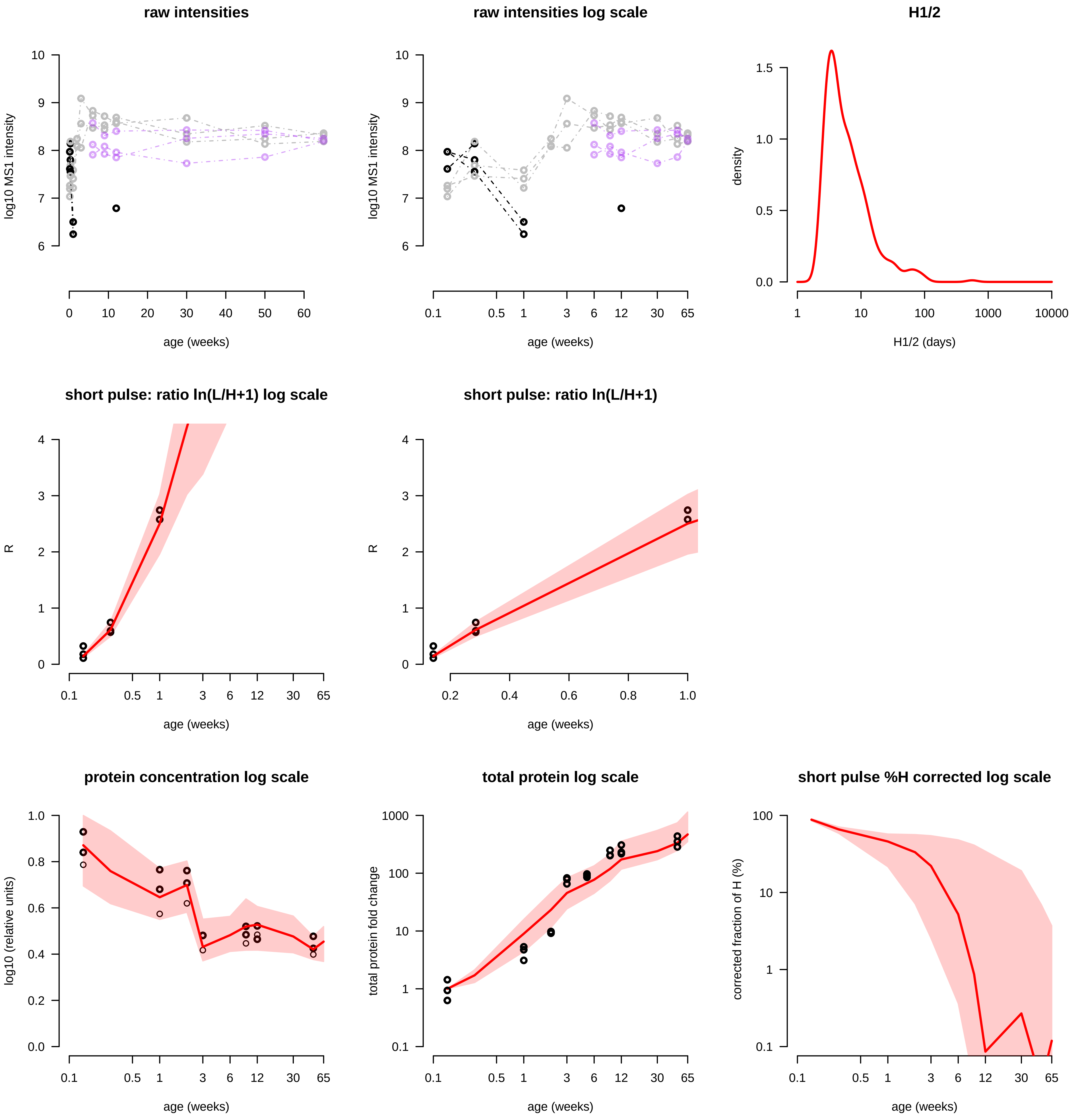

Q8VI75

>sp|Q8VI75|IPO4_MOUSE

Importin−4

GN=Ipo4

Med H1/2: 6.2 days

Q5 H1/2: 4.8 days

Q95 H1/2: 8.7 days

reliable model fit: yes

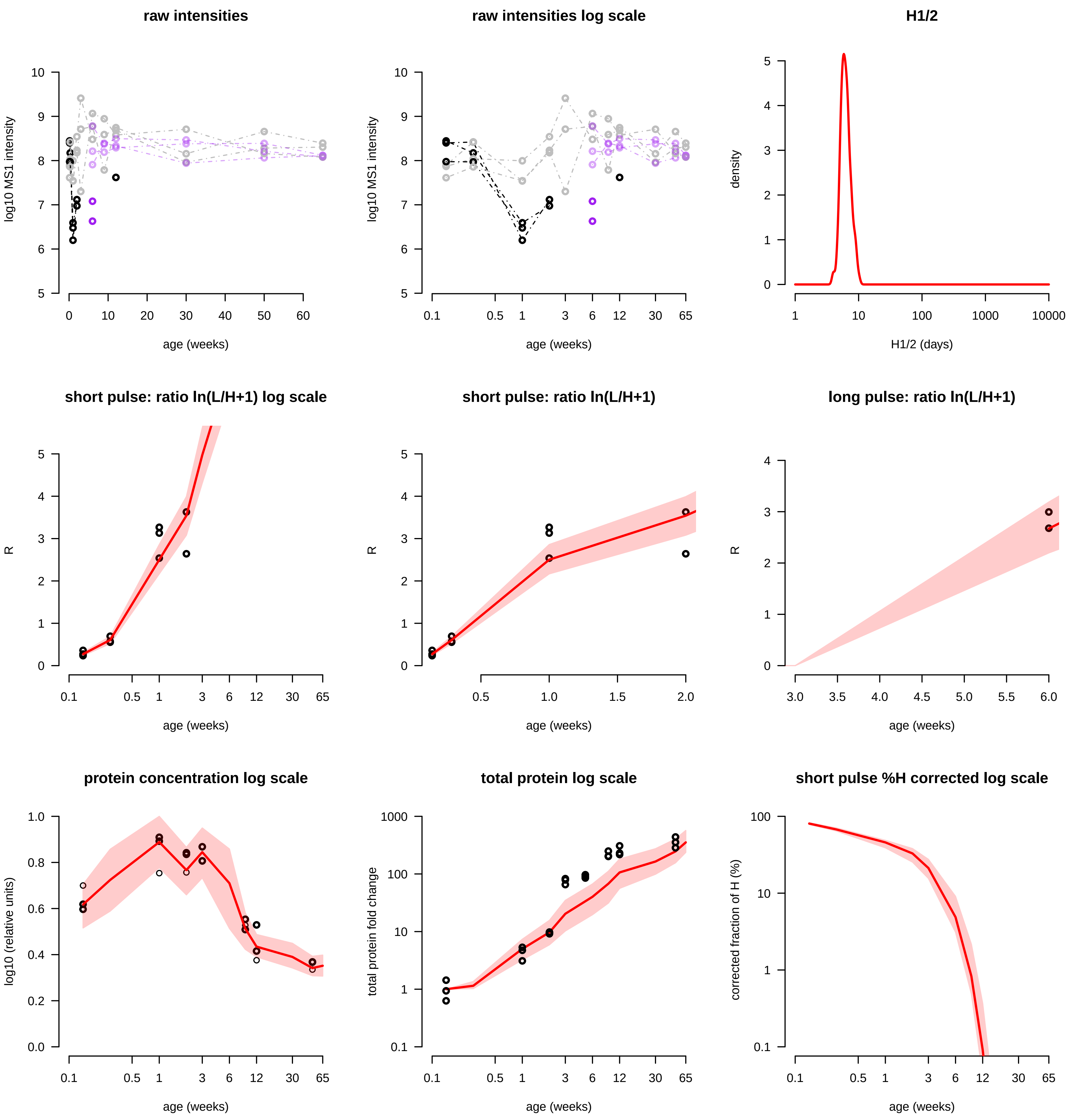

Q91X20

>sp|Q91X20|ASH2L_MOUSE

Set1/Ash2 histone methyltransferase complex subunit ASH2

GN=Ash2l

Med H1/2: 5.9 days

Q5 H1/2: 3.5 days

Q95 H1/2: 22.2 days

reliable model fit: yes

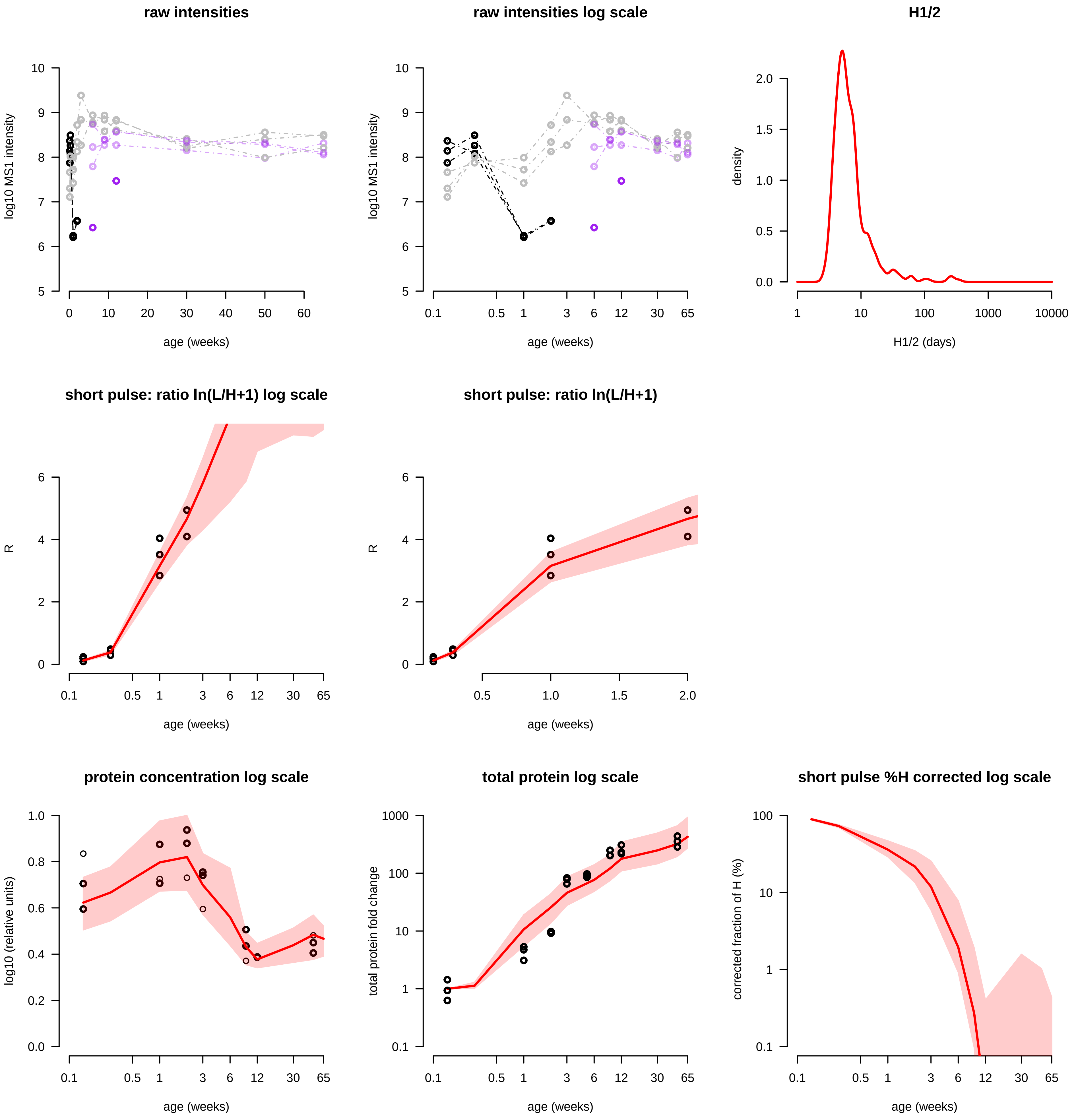

Q99M28;Q99M28−2

>sp|Q99M28|RNPS1_MOUSE

RNA−binding protein with serine−rich domain 1

GN=Rnps1

Med H1/2: 10.7 days

Q5 H1/2: 8.2 days

Q95 H1/2: 14.7 days

reliable model fit: yes

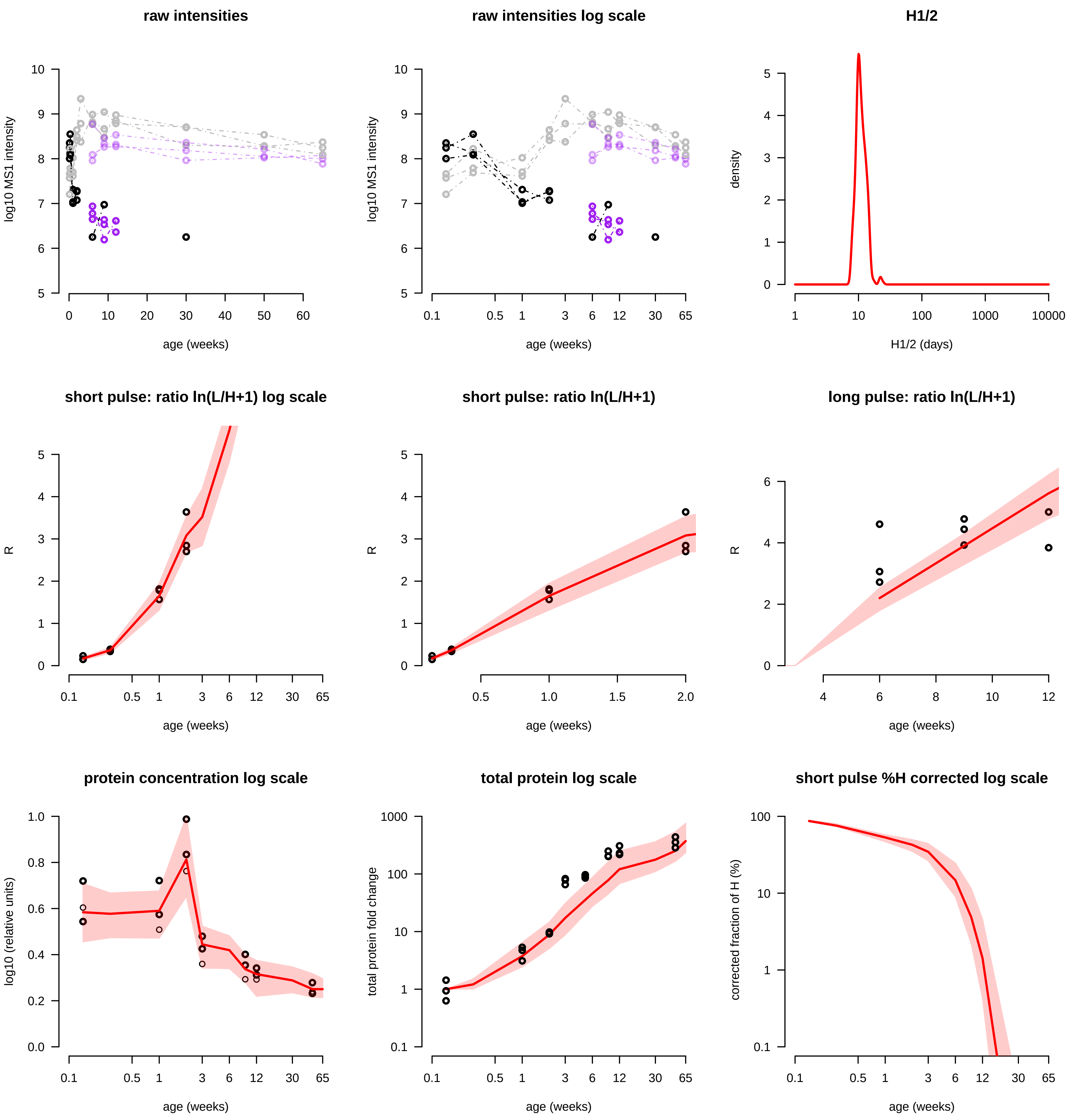

Q8VE22

>sp|Q8VE22|RT23_MOUSE

28S ribosomal protein S23, mitochondrial

GN=Mrps23

Med H1/2: 8.4 days

Q5 H1/2: 4.8 days

Q95 H1/2: 45.3 days

reliable model fit: yes

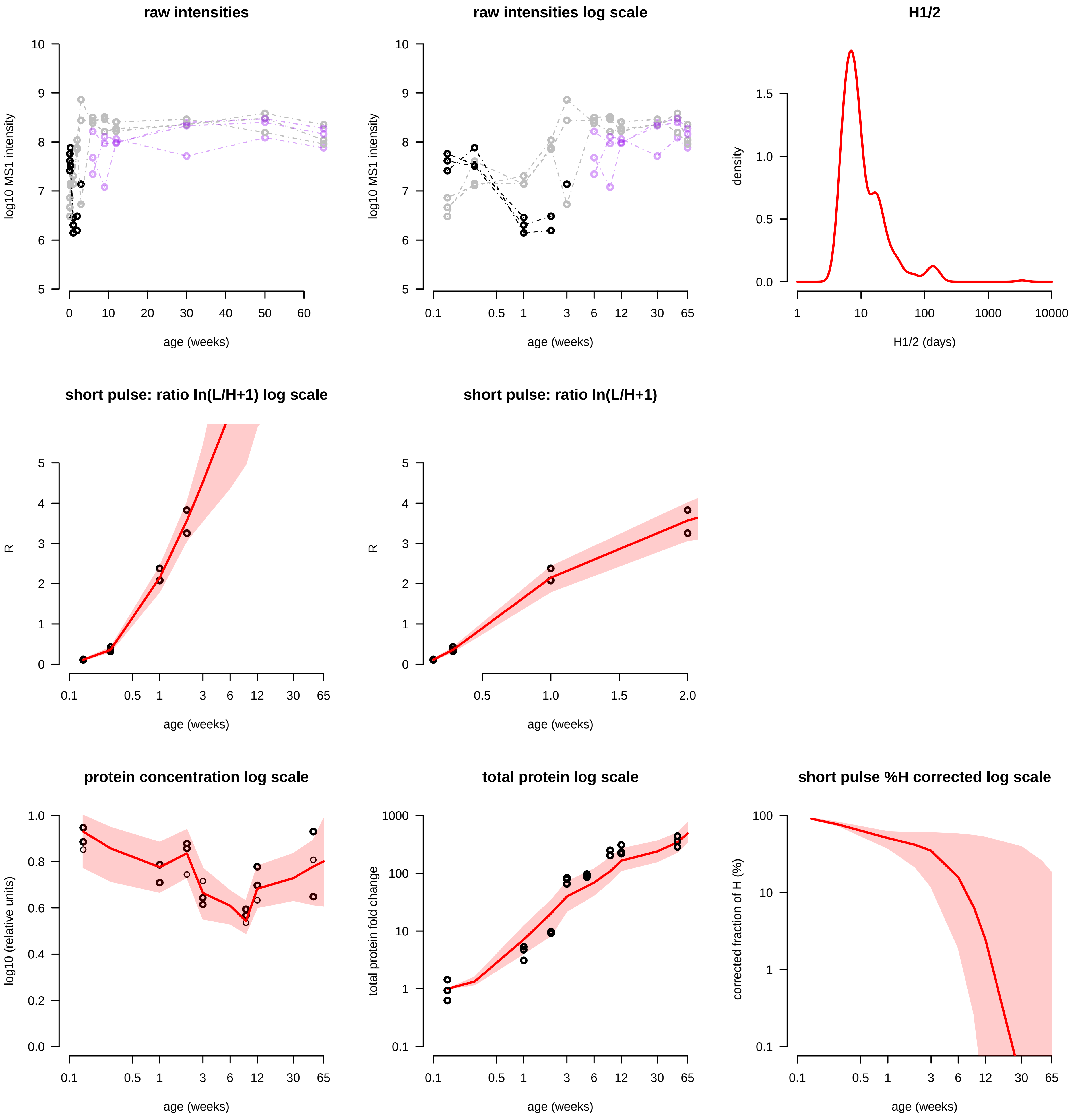

Q8K274

>sp|Q8K274|KT3K_MOUSE

Ketosamine−3−kinase

GN=Fn3krp

Med H1/2: 5 days

Q5 H1/2: 2.9 days

Q95 H1/2: 49.2 days

reliable model fit: yes

raw intensities

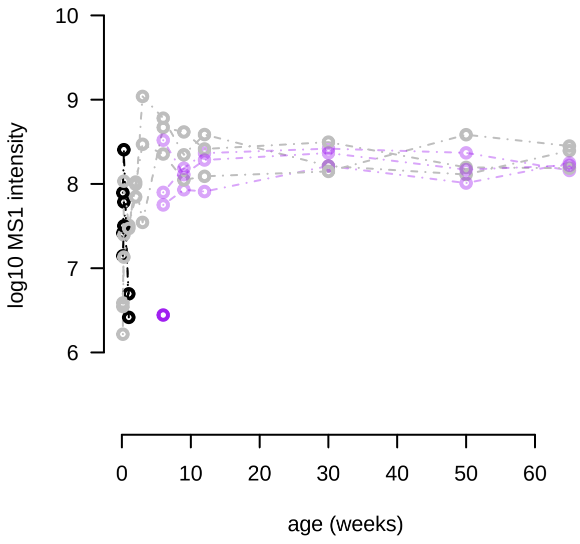

raw intensities log scale

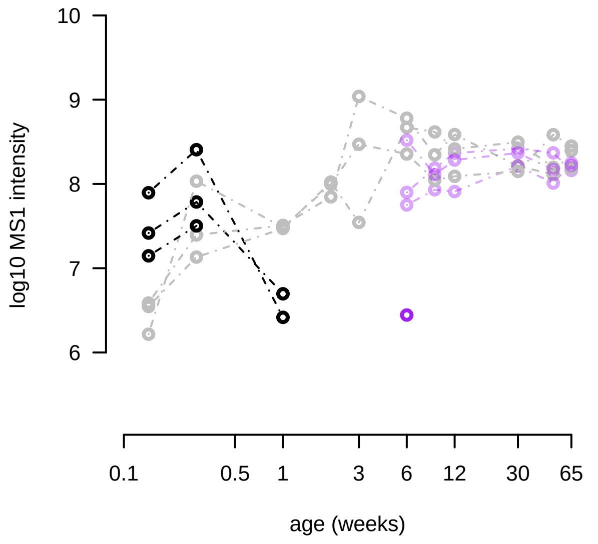

H1/2

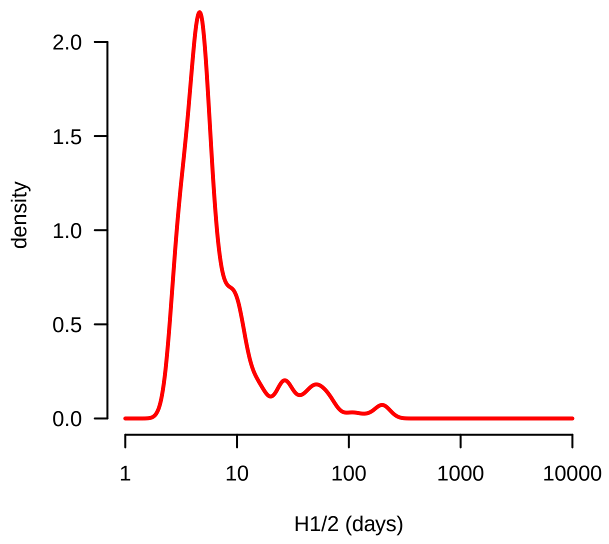

short pulse: ratio ln(L/H+1) log scale

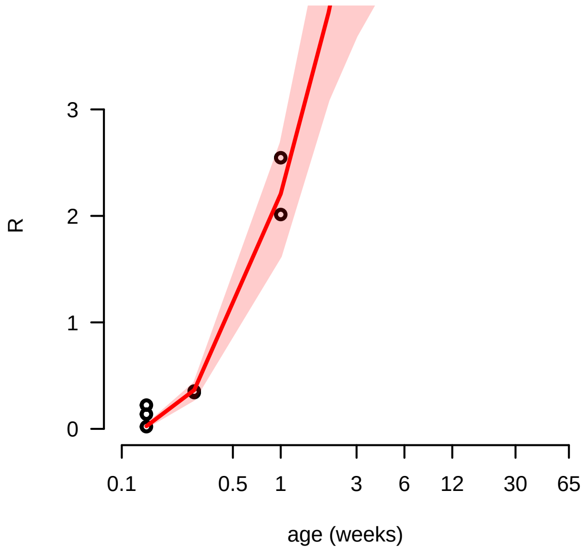

short pulse: ratio ln(L/H+1)

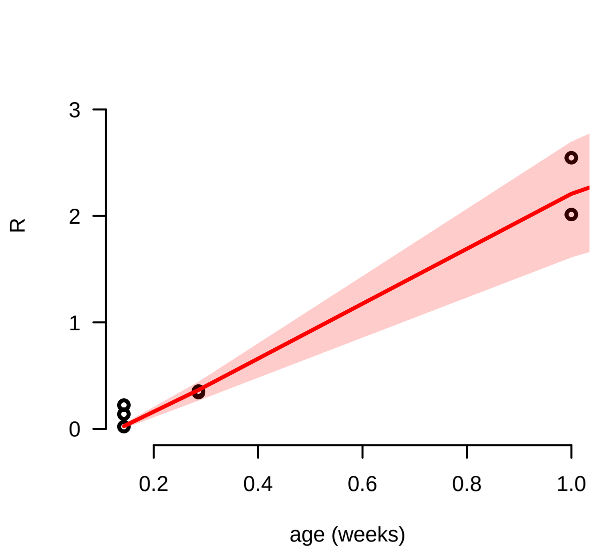

protein concentration log scale

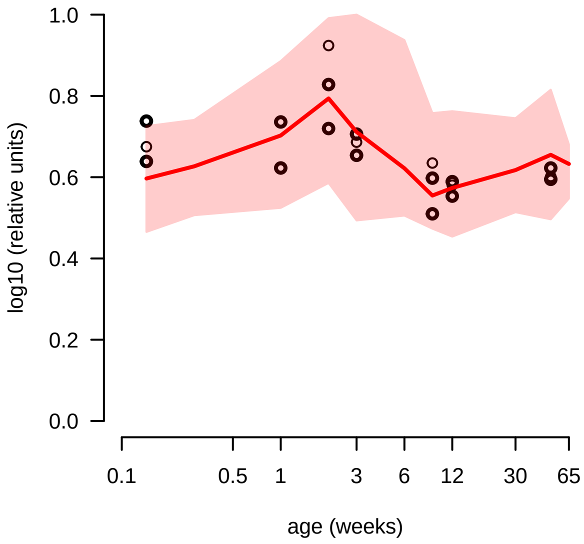

total protein log scale

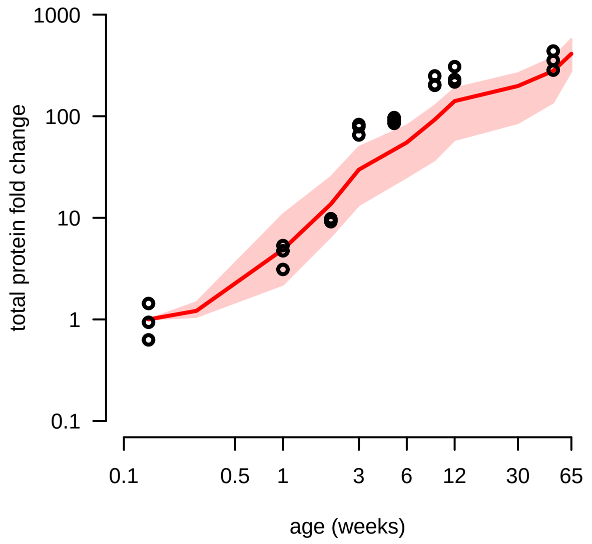

short pulse %H corrected log scale

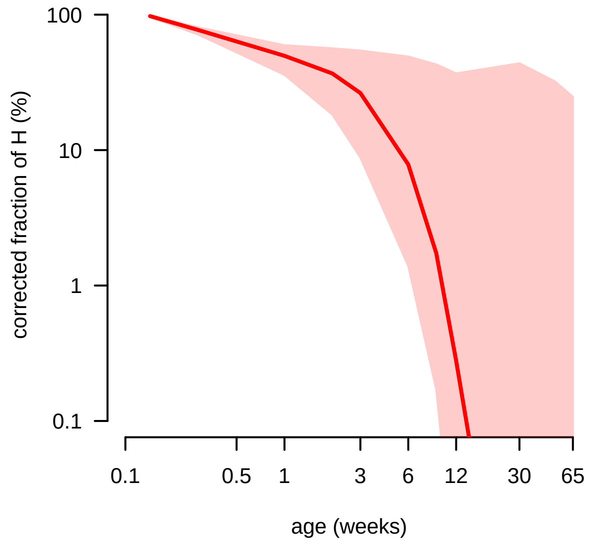

Q9CQZ6

>sp|Q9CQZ6|NDUB3_MOUSE

NADH dehydrogenase [ubiquinone] 1 beta subcomplex subunit 3

GN=Ndufb3

Med H1/2: 8.4 days

Q5 H1/2: 6 days

Q95 H1/2: 12.7 days

reliable model fit: yes

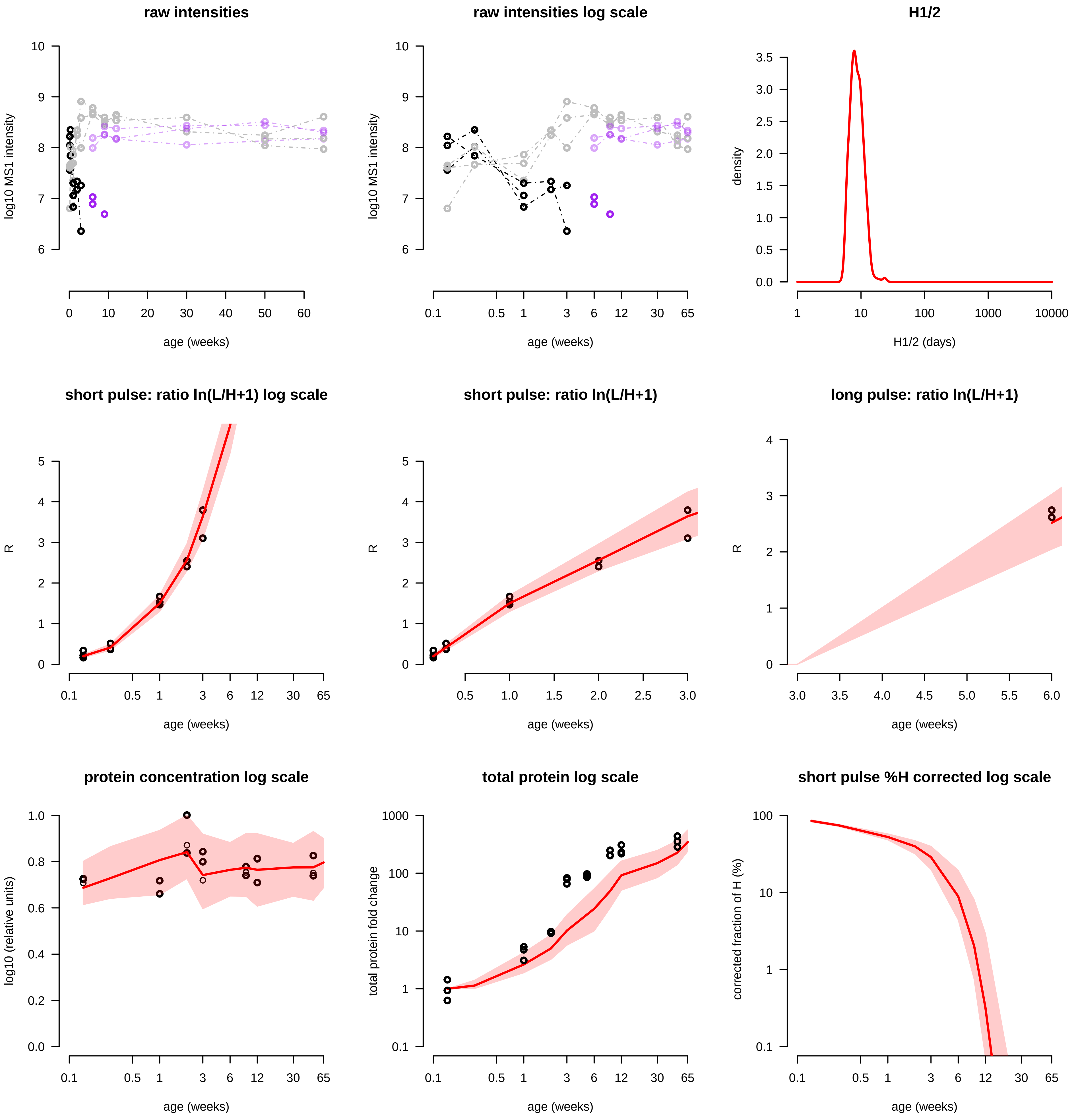

Q9JMD0−4;Q9JMD0−3;Q9JMD0−2;Q9JMD0

>sp|Q9JMD0−4|ZN207_MOUSE

Isoform 4 of BUB3−interacting and GLEBS motif−containing protein ZNF207

GN=Znf207

Med H1/2: 4.3 days

Q5 H1/2: 3.6 days

Q95 H1/2: 5.5 days

reliable model fit: yes

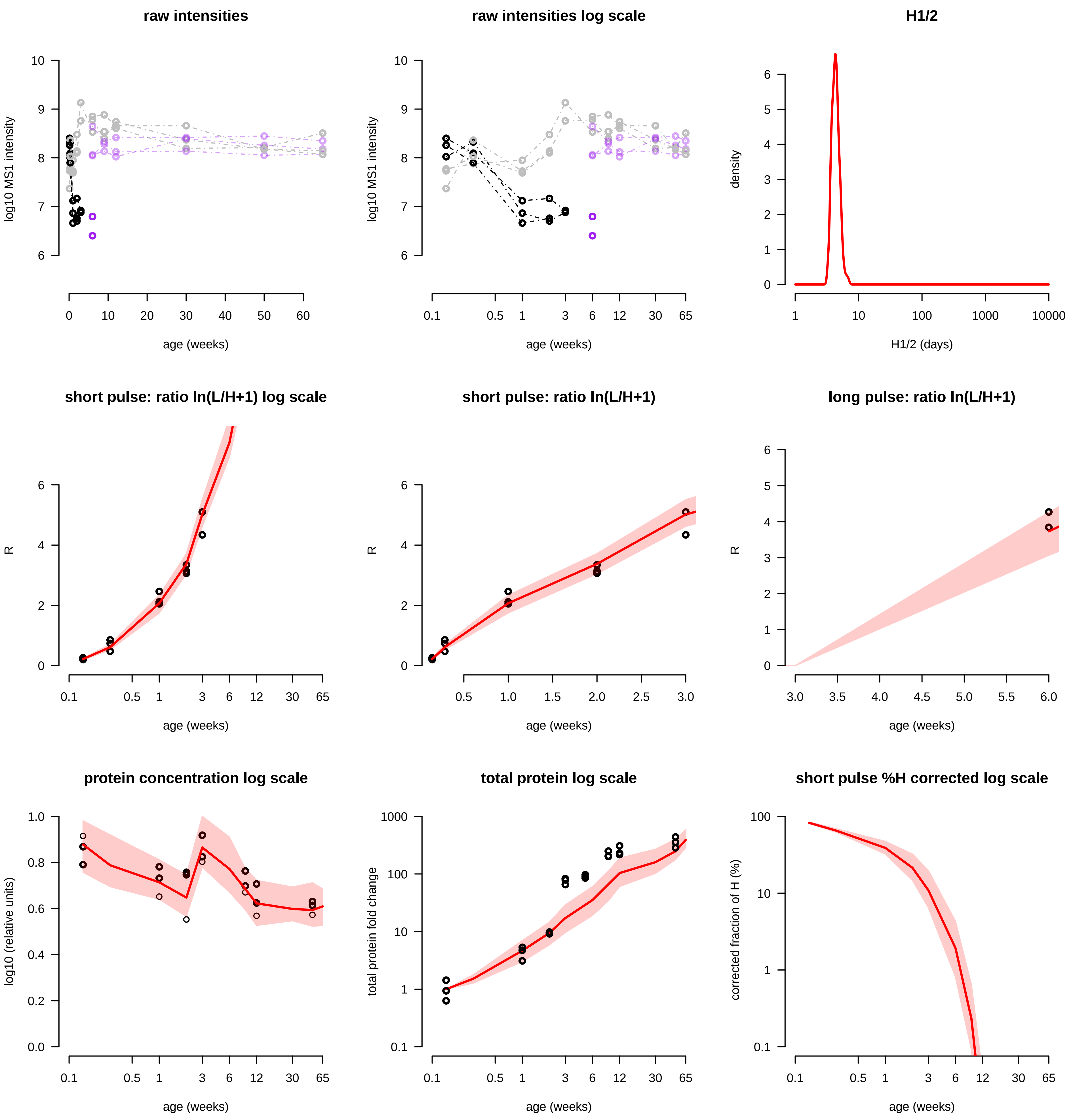

Q8VDF2

>sp|Q8VDF2|UHRF1_MOUSE

E3 ubiquitin−protein ligase UHRF1

GN=Uhrf1

Med H1/2: 6.4 days

Q5 H1/2: 4.4 days

Q95 H1/2: 9.2 days

reliable model fit: yes

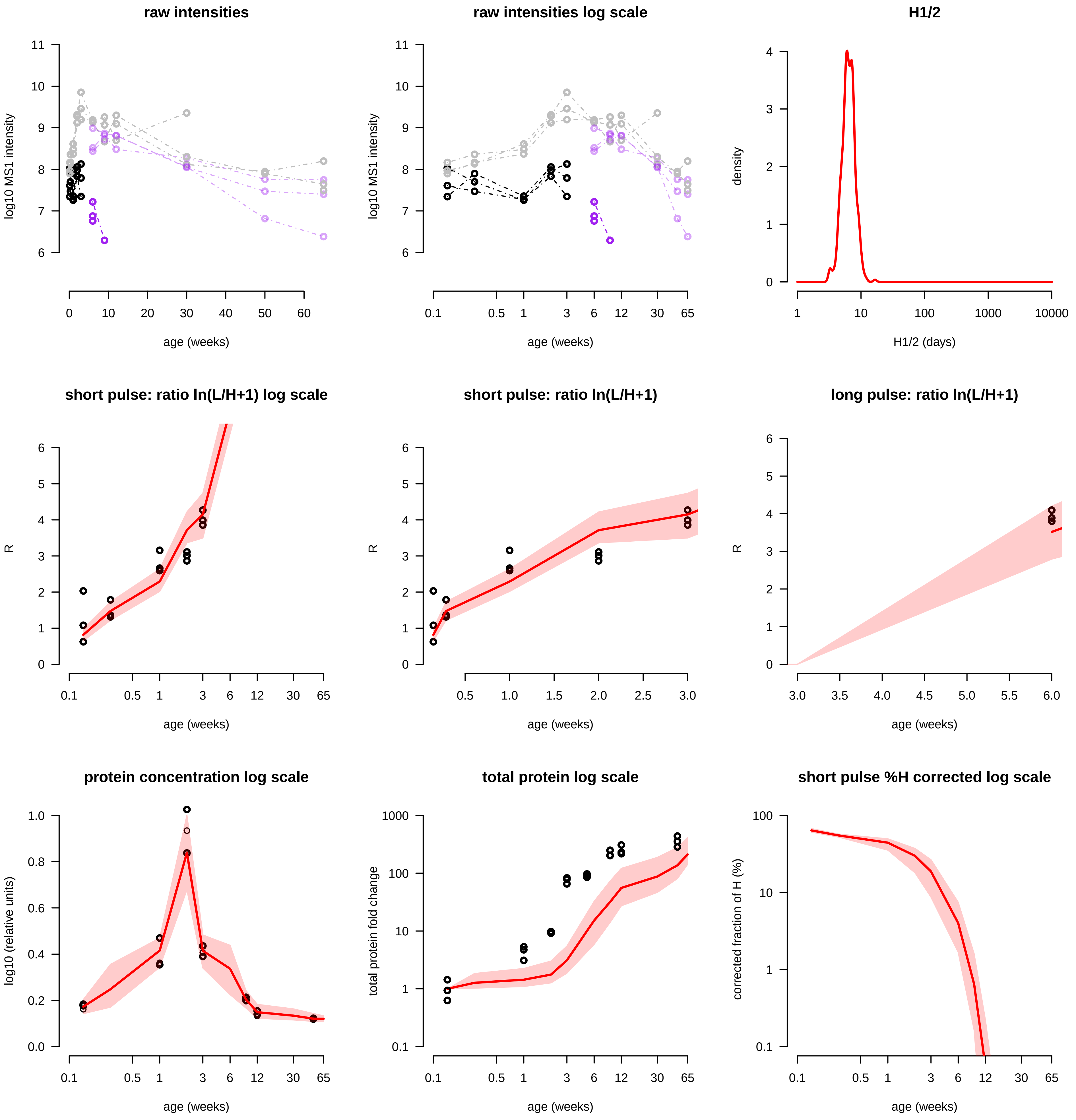

Q9DBS2

>sp|Q9DBS2|TPRGL_MOUSE

Tumor protein p63−regulated gene 1−like protein

GN=Tprg1l

Med H1/2: 5.9 days

Q5 H1/2: 4.4 days

Q95 H1/2: 9.5 days

reliable model fit: yes

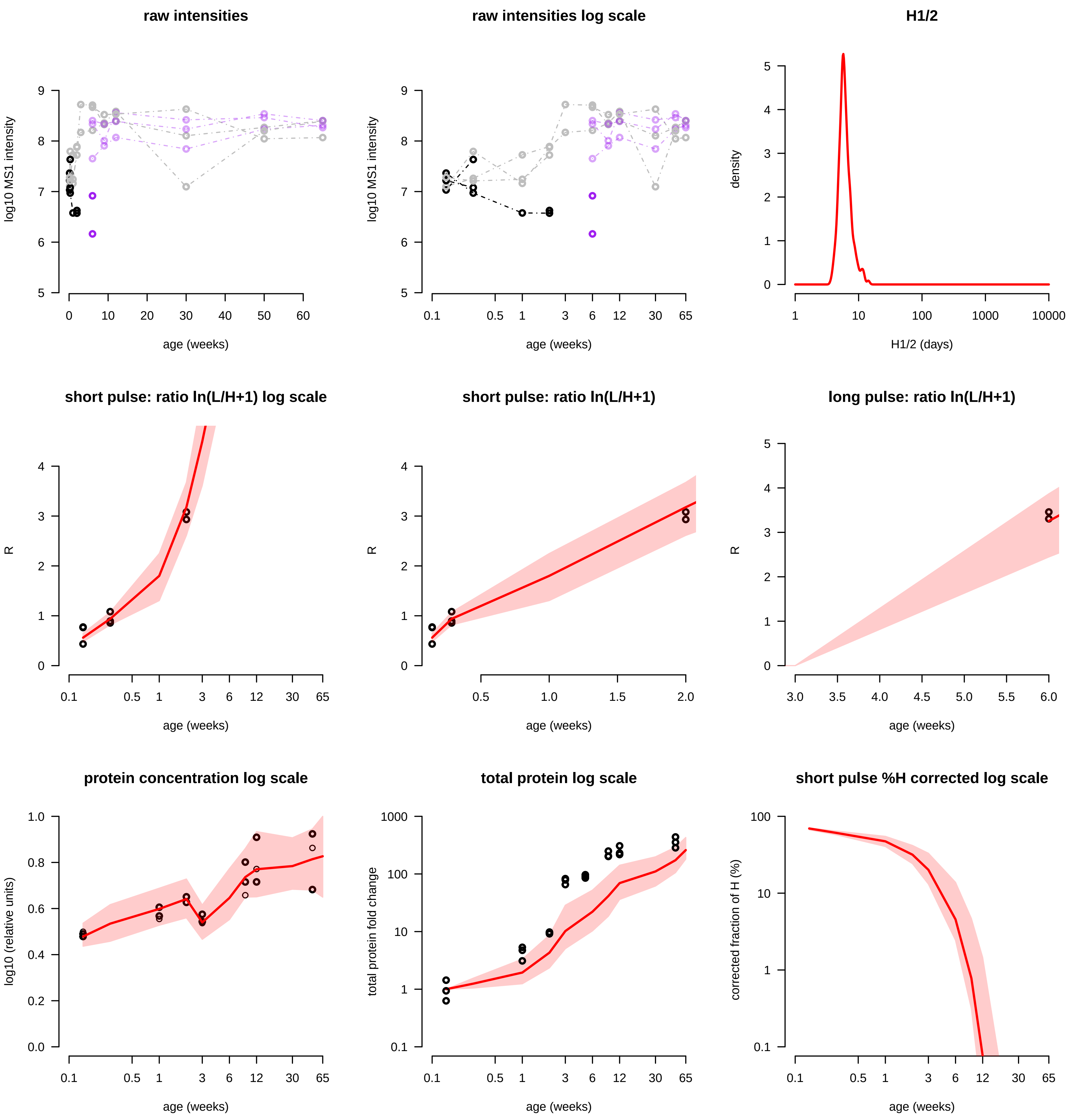

Q80X19−2;Q80X19

>sp|Q80X19−2|COEA1_MOUSE

Isoform 2 of Collagen alpha−1(XIV) chain

GN=Col14a1

Med H1/2: 6.8 days

Q5 H1/2: 3.8 days

Q95 H1/2: 12 days

reliable model fit: yes

raw intensities

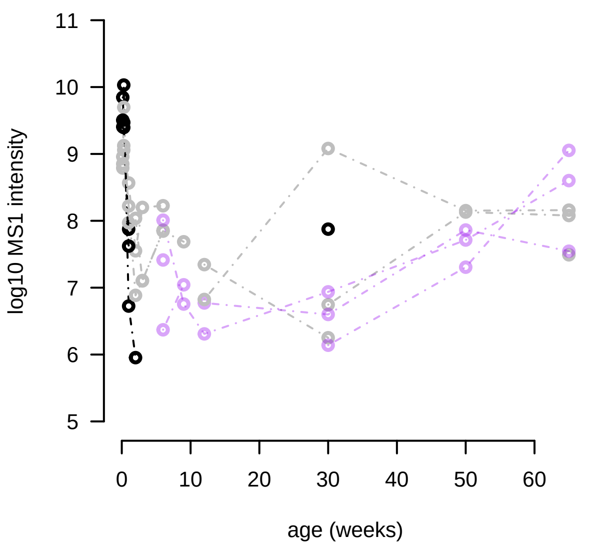

raw intensities log scale

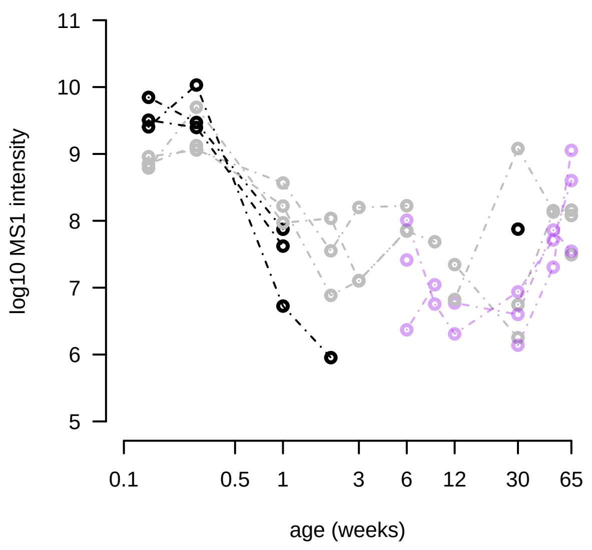

H1/2

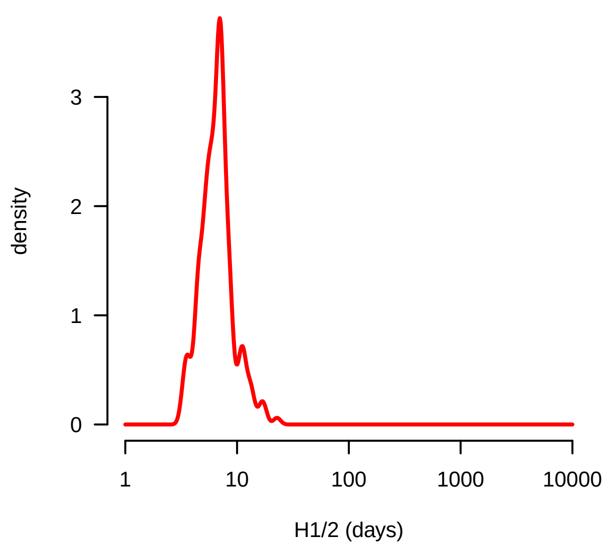

short pulse: ratio ln(L/H+1) log scale

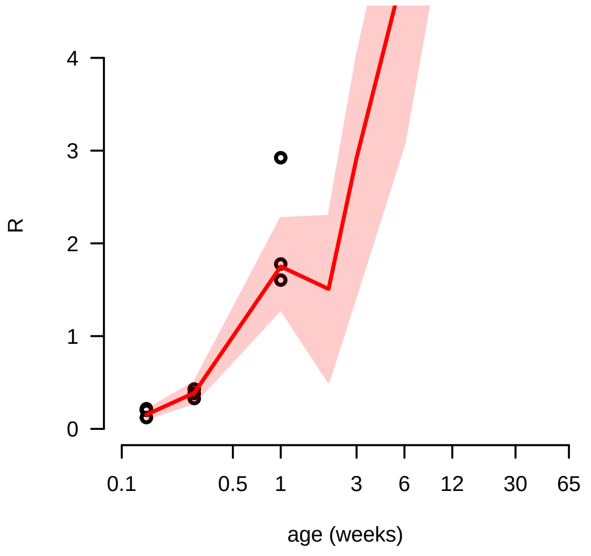

short pulse: ratio ln(L/H+1)

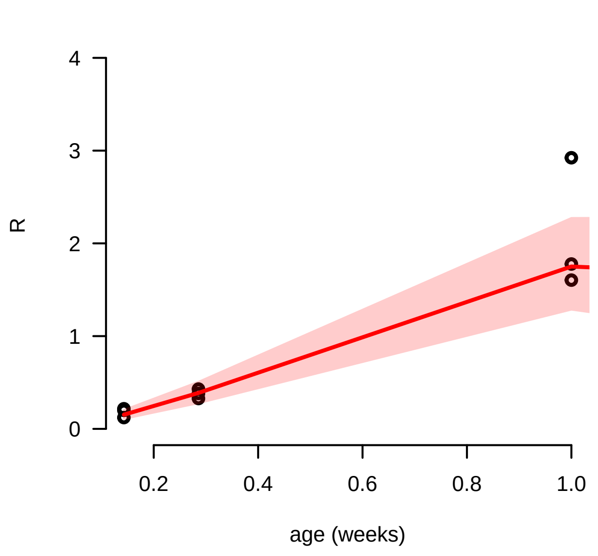

protein concentration log scale

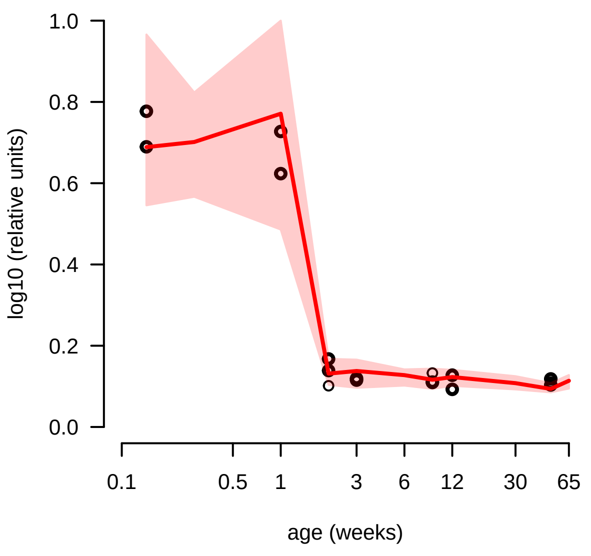

total protein log scale

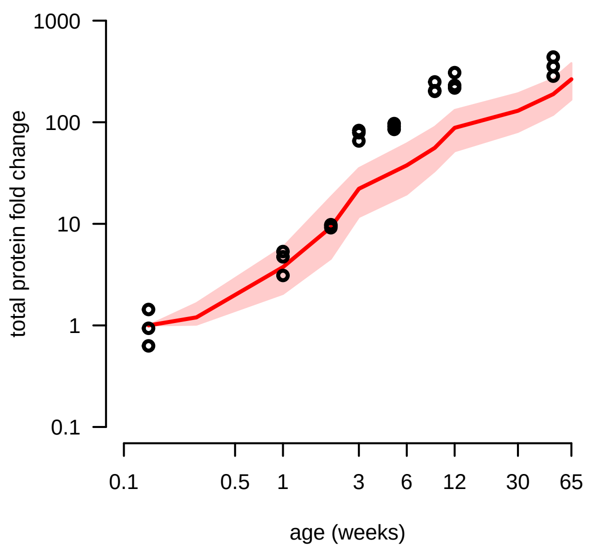

short pulse %H corrected log scale

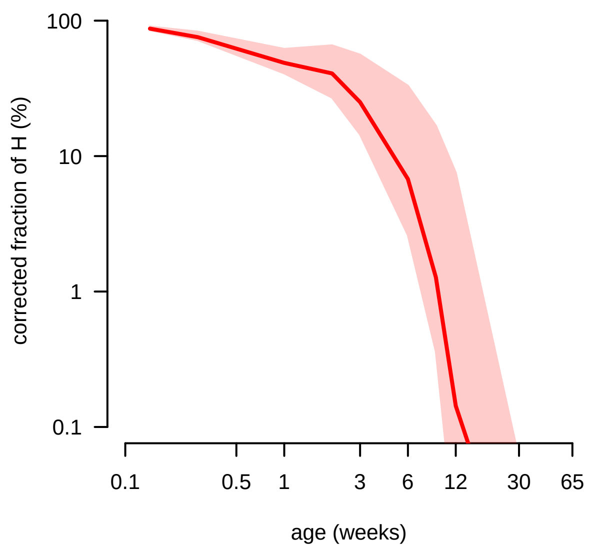

Q6GV12

>sp|Q6GV12|KDSR_MOUSE

3−ketodihydrosphingosine reductase

GN=Kdsr

Med H1/2: 11 days

Q5 H1/2: 3.9 days

Q95 H1/2: 93.5 days

reliable model fit: yes

raw intensities

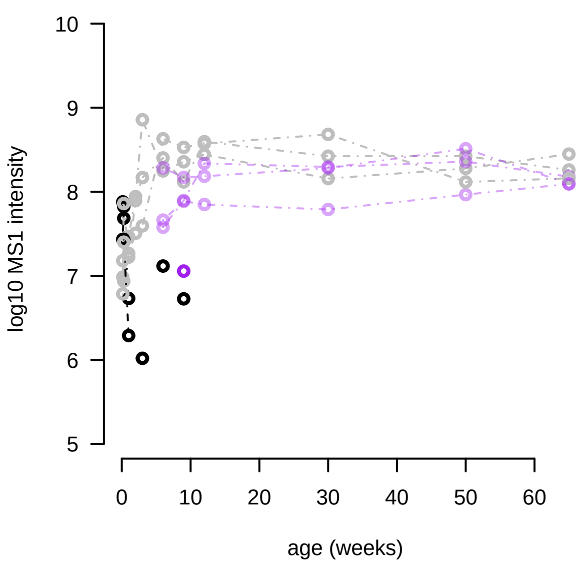

raw intensities log scale

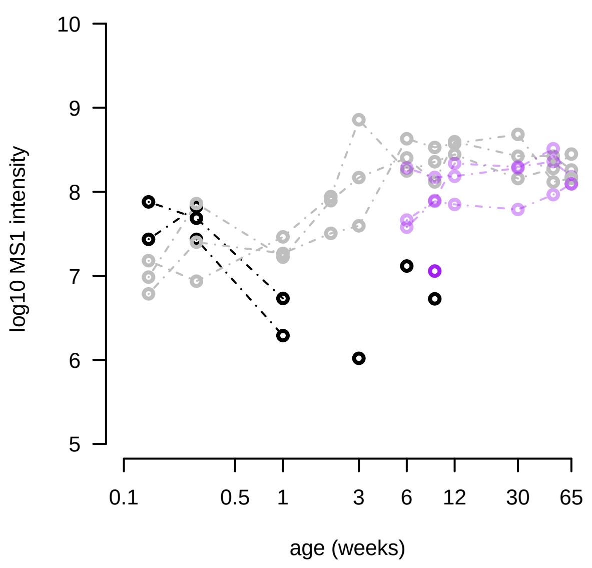

H1/2

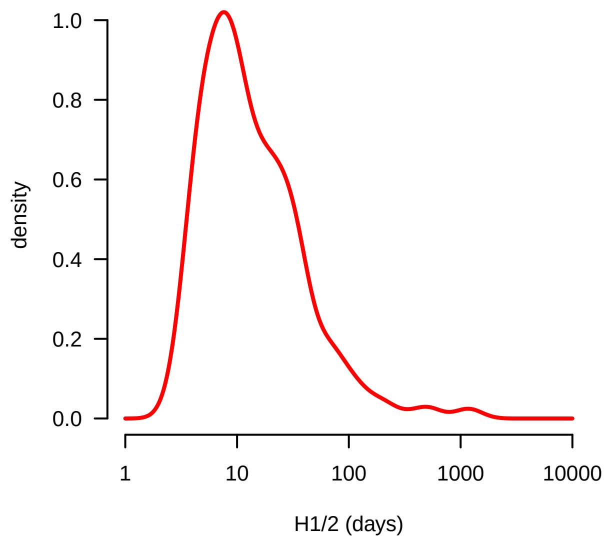

short pulse: ratio ln(L/H+1) log scale

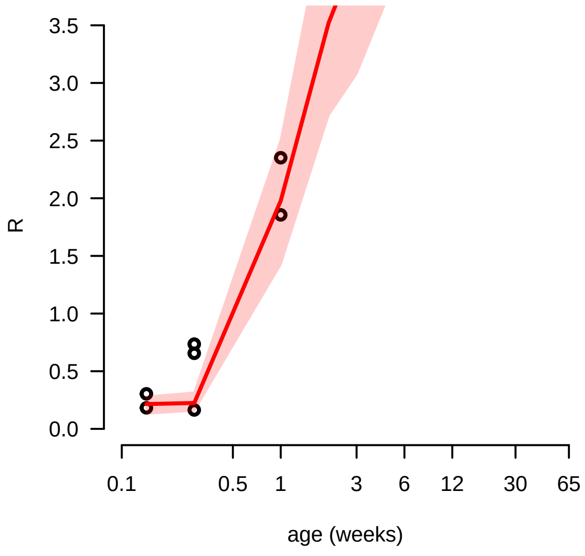

short pulse: ratio ln(L/H+1)

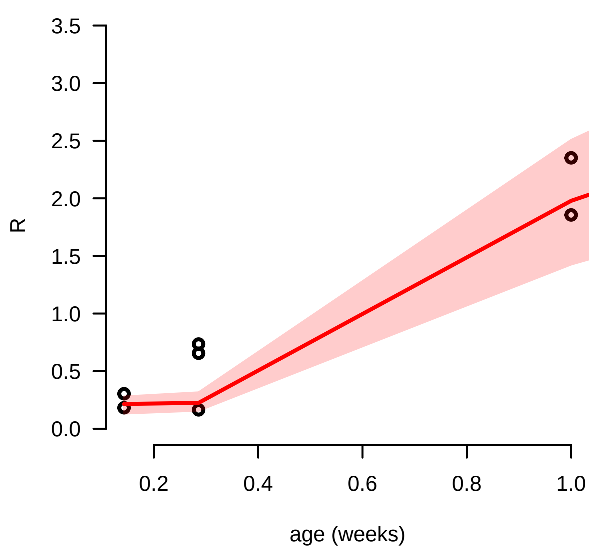

protein concentration log scale

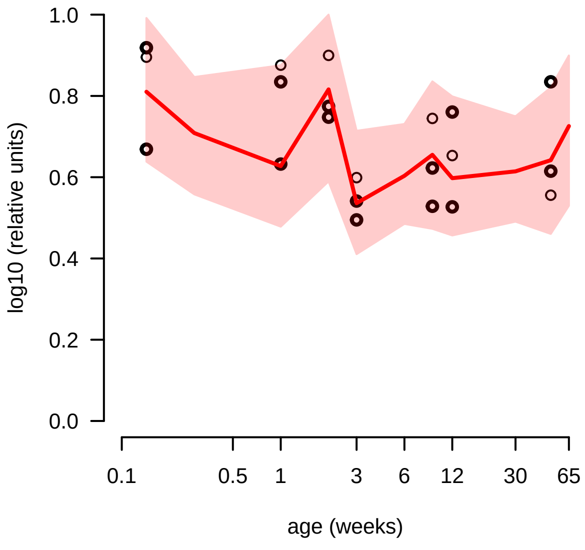

total protein log scale

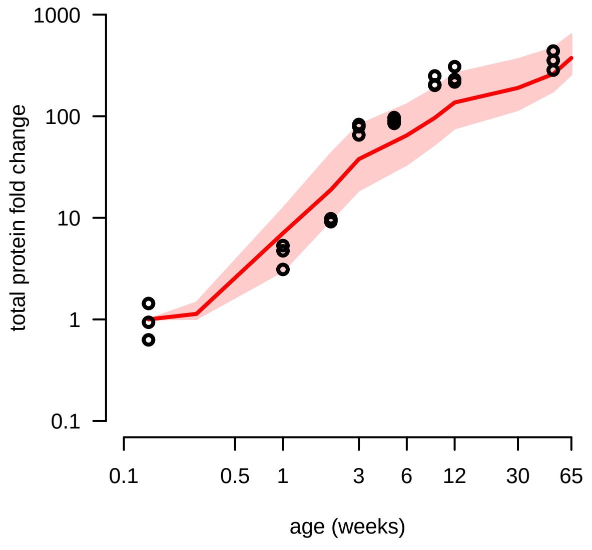

short pulse %H corrected log scale

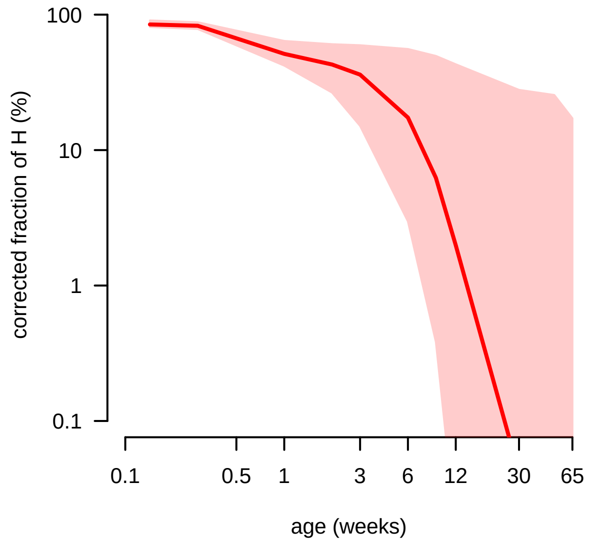

Q9CPR5;Q9CPR5−2

>sp|Q9CPR5|RM15_MOUSE

39S ribosomal protein L15, mitochondrial

GN=Mrpl15

Med H1/2: 18.3 days

Q5 H1/2: 6.2 days

Q95 H1/2: 159.1 days

reliable model fit: yes

raw intensities

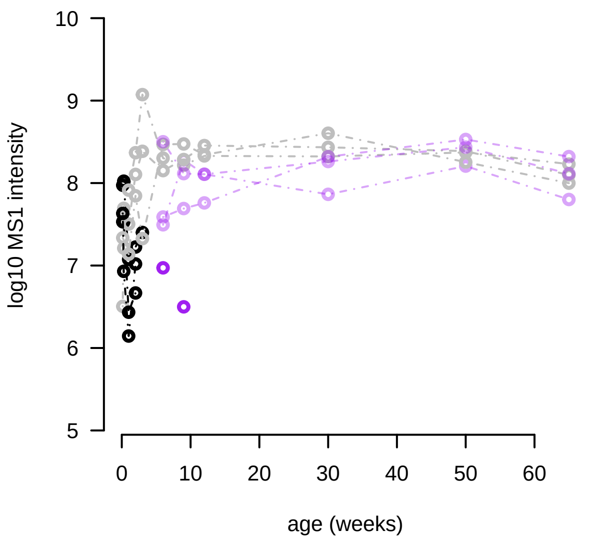

raw intensities log scale

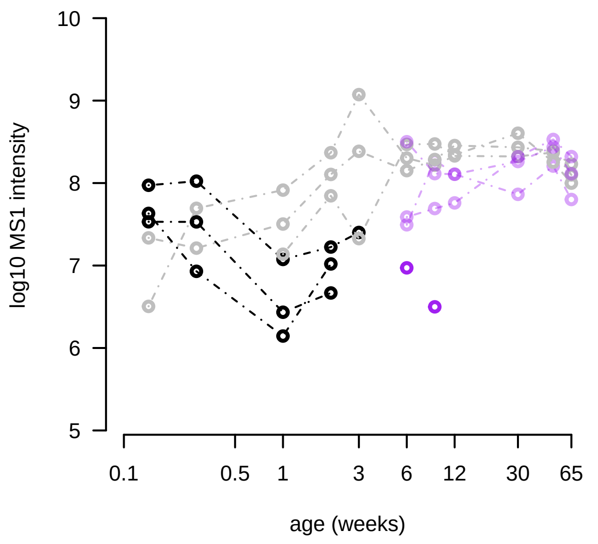

H1/2

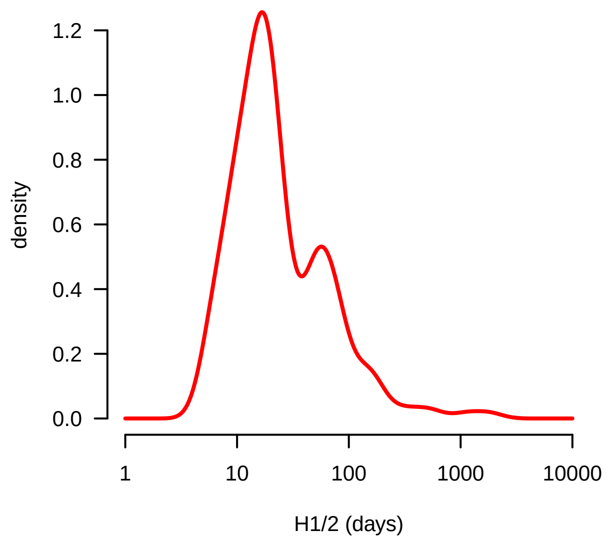

short pulse: ratio ln(L/H+1) log scale

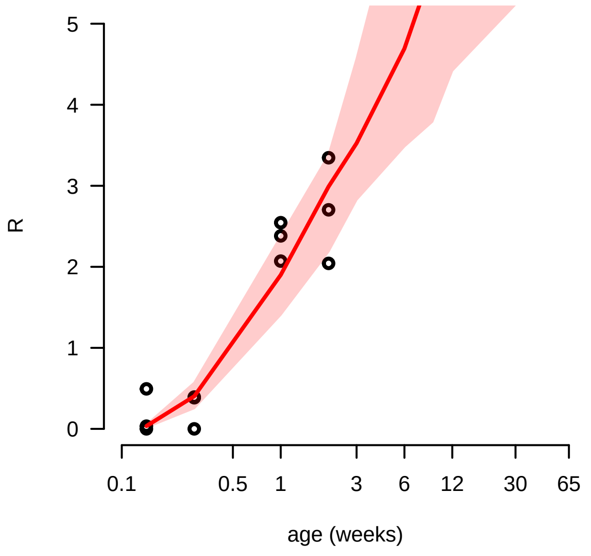

short pulse: ratio ln(L/H+1)

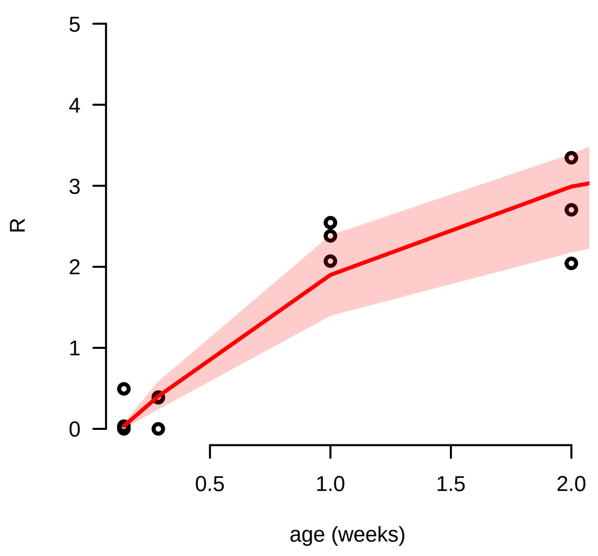

protein concentration log scale

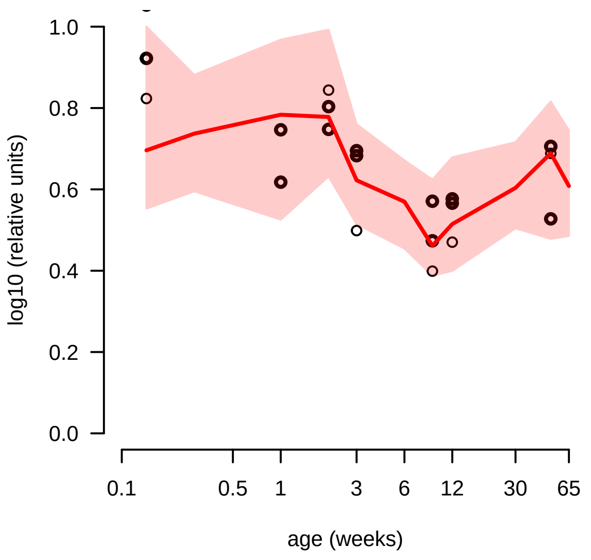

total protein log scale

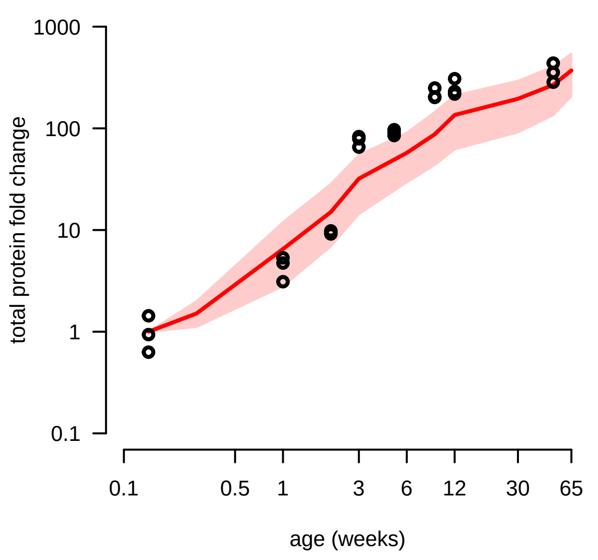

short pulse %H corrected log scale

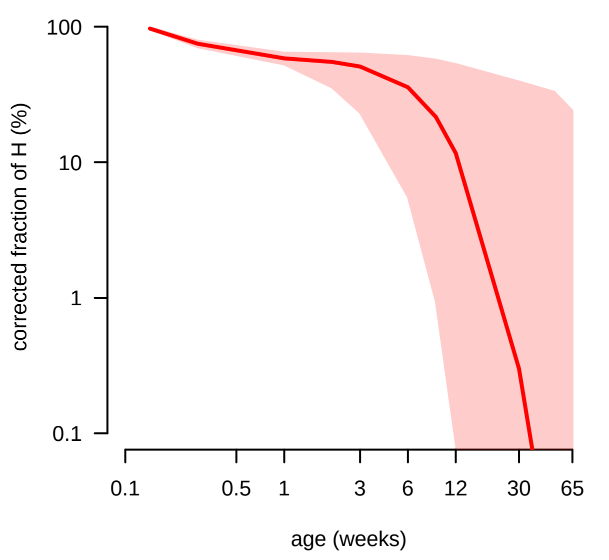

Q8C5W3−2;Q8C5W3

>sp|Q8C5W3−2|TBCEL_MOUSE

Isoform 2 of Tubulin−specific chaperone cofactor E−like protein

GN=Tbcel

Med H1/2: 7.4 days

Q5 H1/2: 2.6 days

Q95 H1/2: 61.1 days

reliable model fit: yes

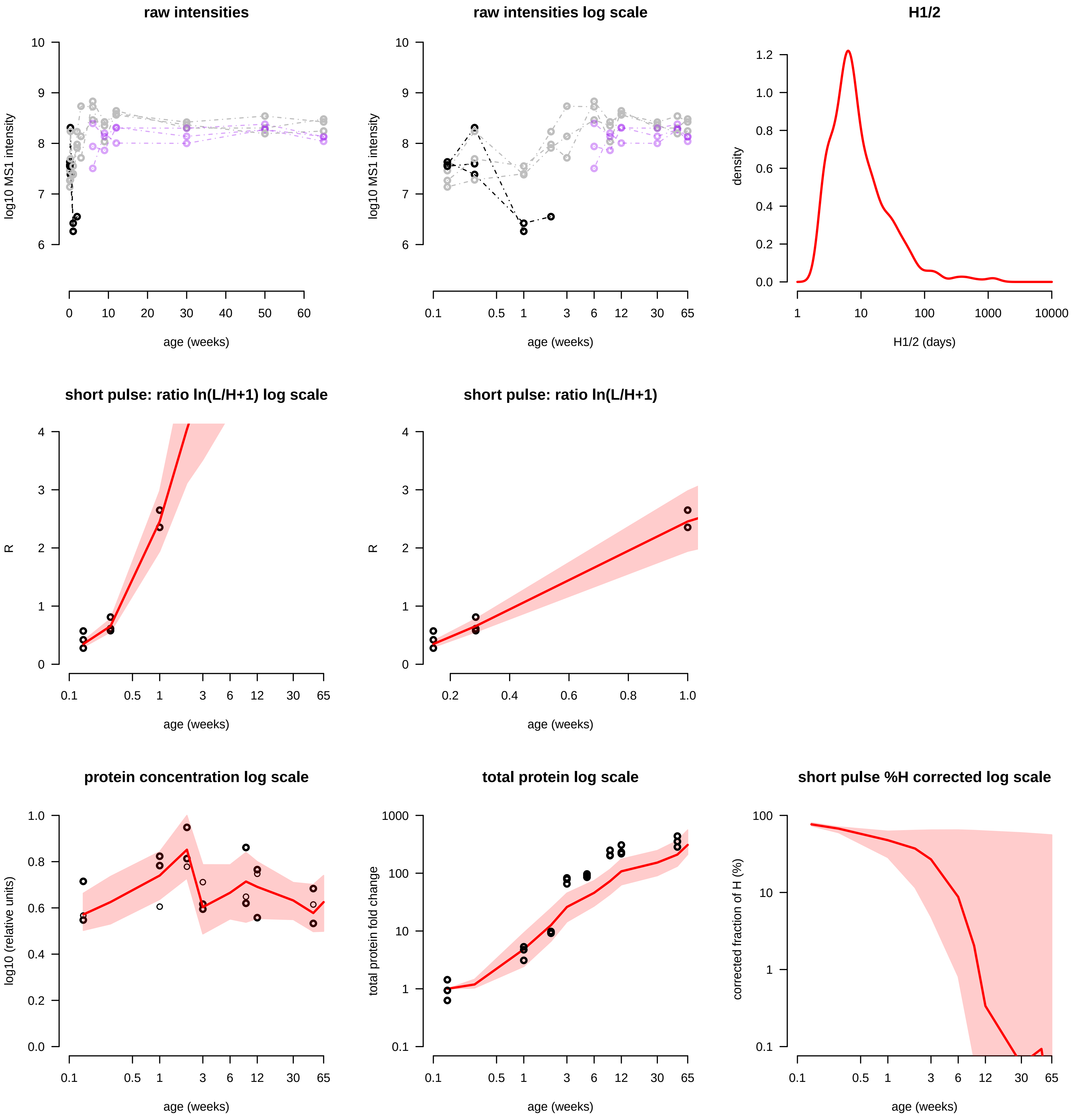

Q07456

>sp|Q07456|AMBP_MOUSE

Protein AMBP

GN=Ambp

Med H1/2: 4.2 days

Q5 H1/2: 2.5 days

Q95 H1/2: 25.2 days

reliable model fit: yes

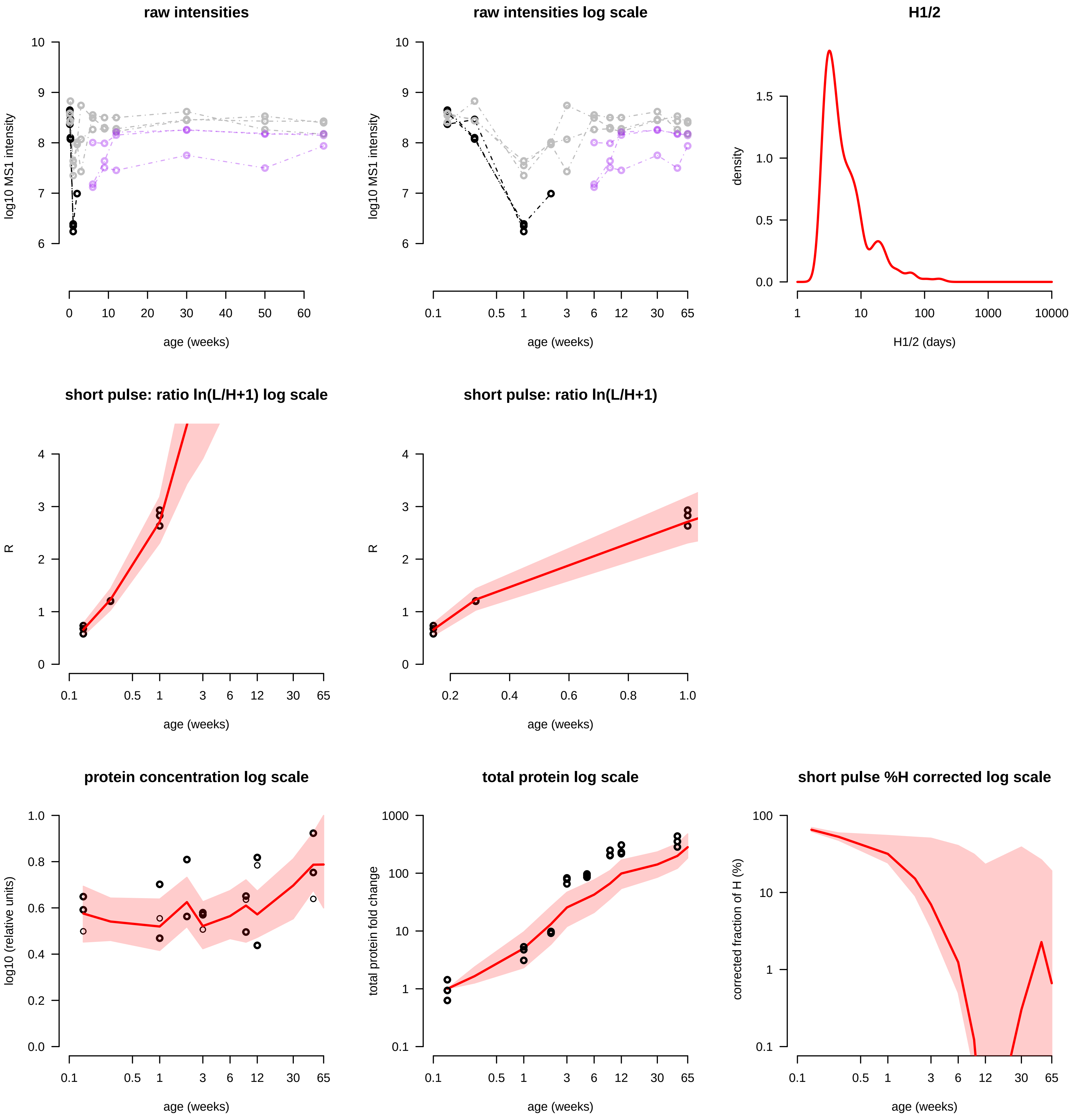

Q6PD26

>sp|Q6PD26|PIGS_MOUSE

GPI transamidase component PIG−S

GN=Pigs

Med H1/2: 5 days

Q5 H1/2: 2.8 days

Q95 H1/2: 20.9 days

reliable model fit: yes

raw intensities

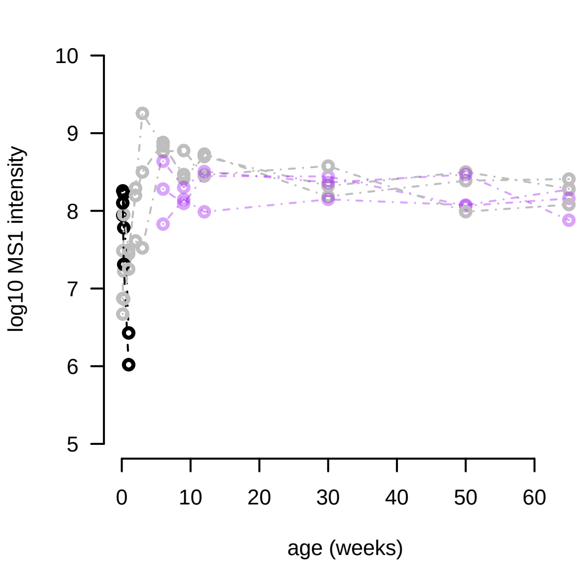

raw intensities log scale

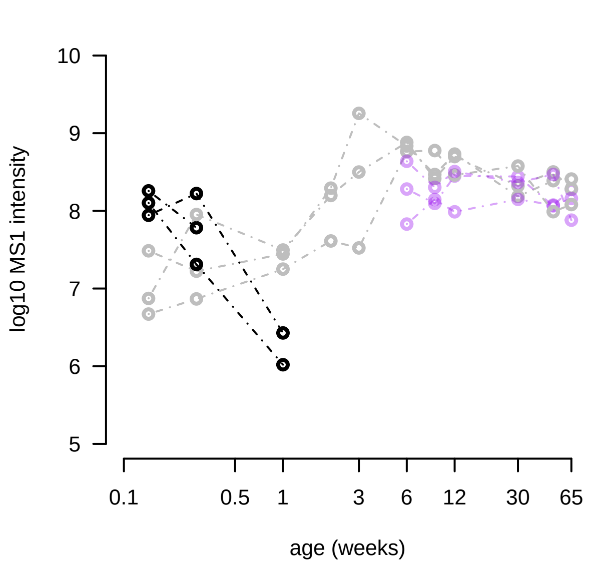

H1/2

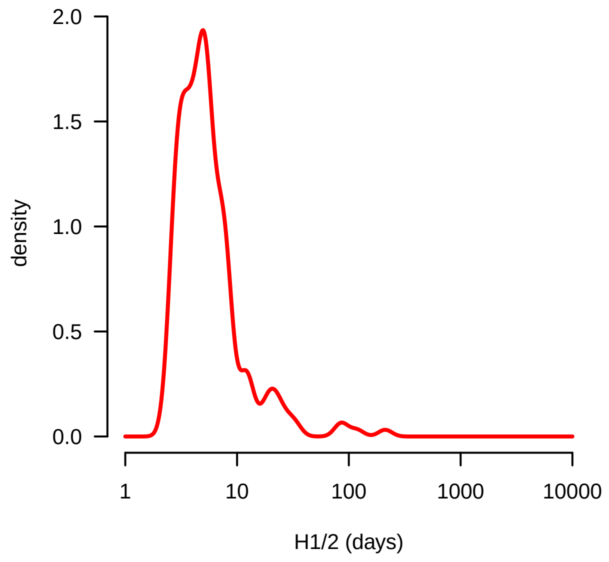

short pulse: ratio ln(L/H+1) log scale

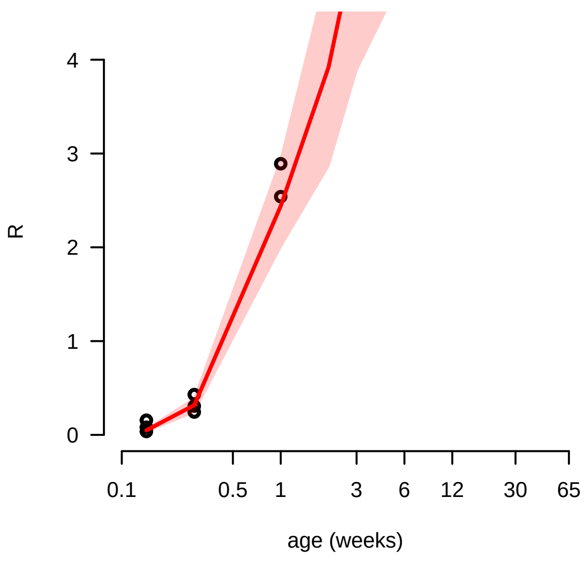

short pulse: ratio ln(L/H+1)

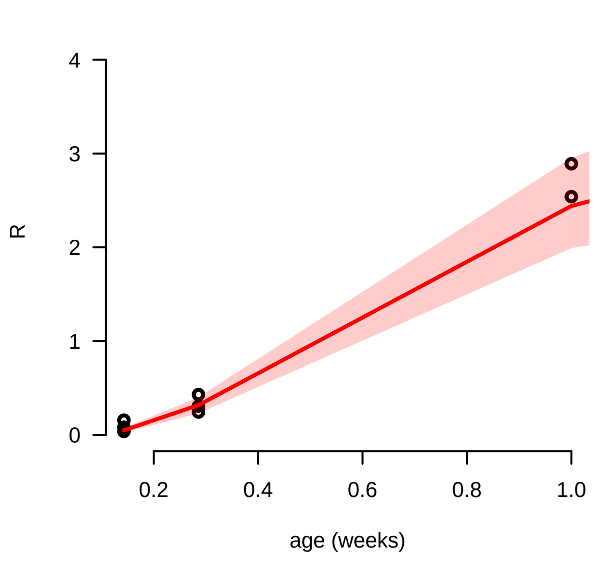

protein concentration log scale

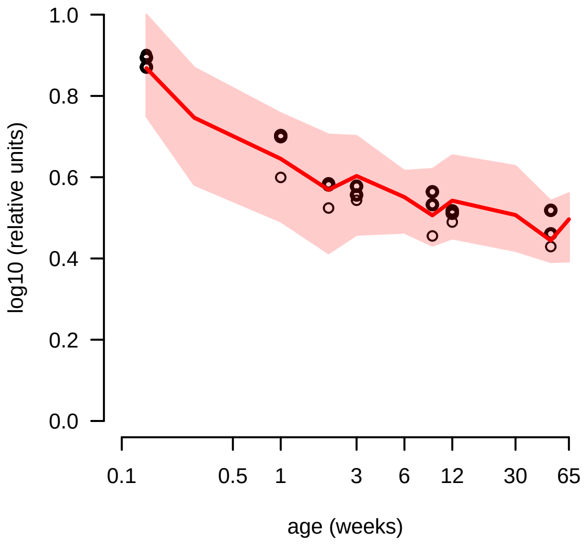

total protein log scale

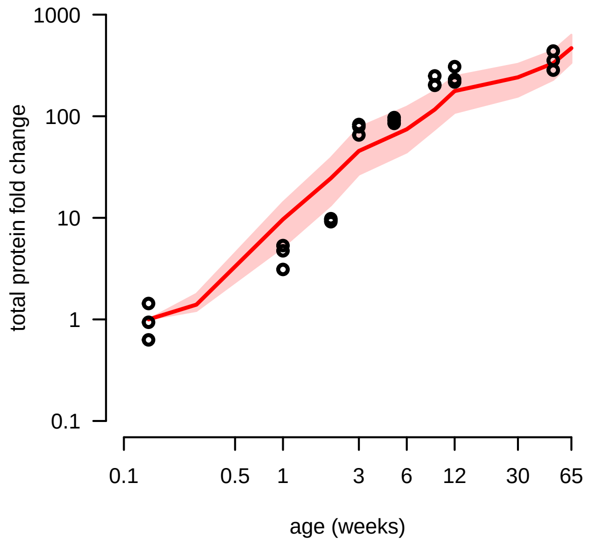

short pulse %H corrected log scale

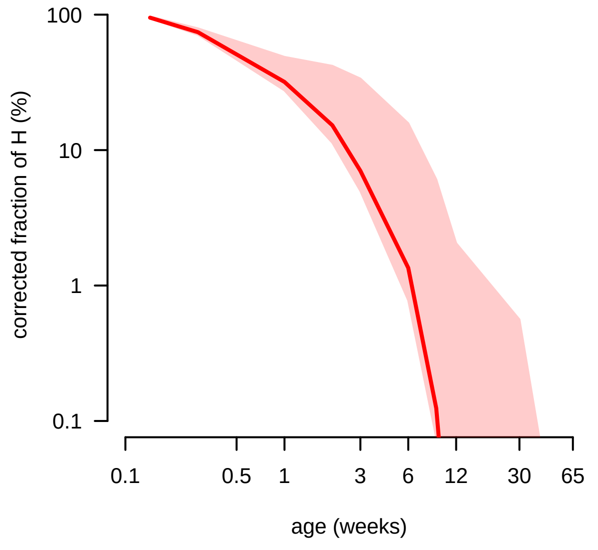

Q8BX17

>sp|Q8BX17|GEMI5_MOUSE

Gem−associated protein 5

GN=Gemin5

Med H1/2: 8546.5 days

Q5 H1/2: 1799.9 days

Q95 H1/2: 110831.9 days

reliable model fit: no

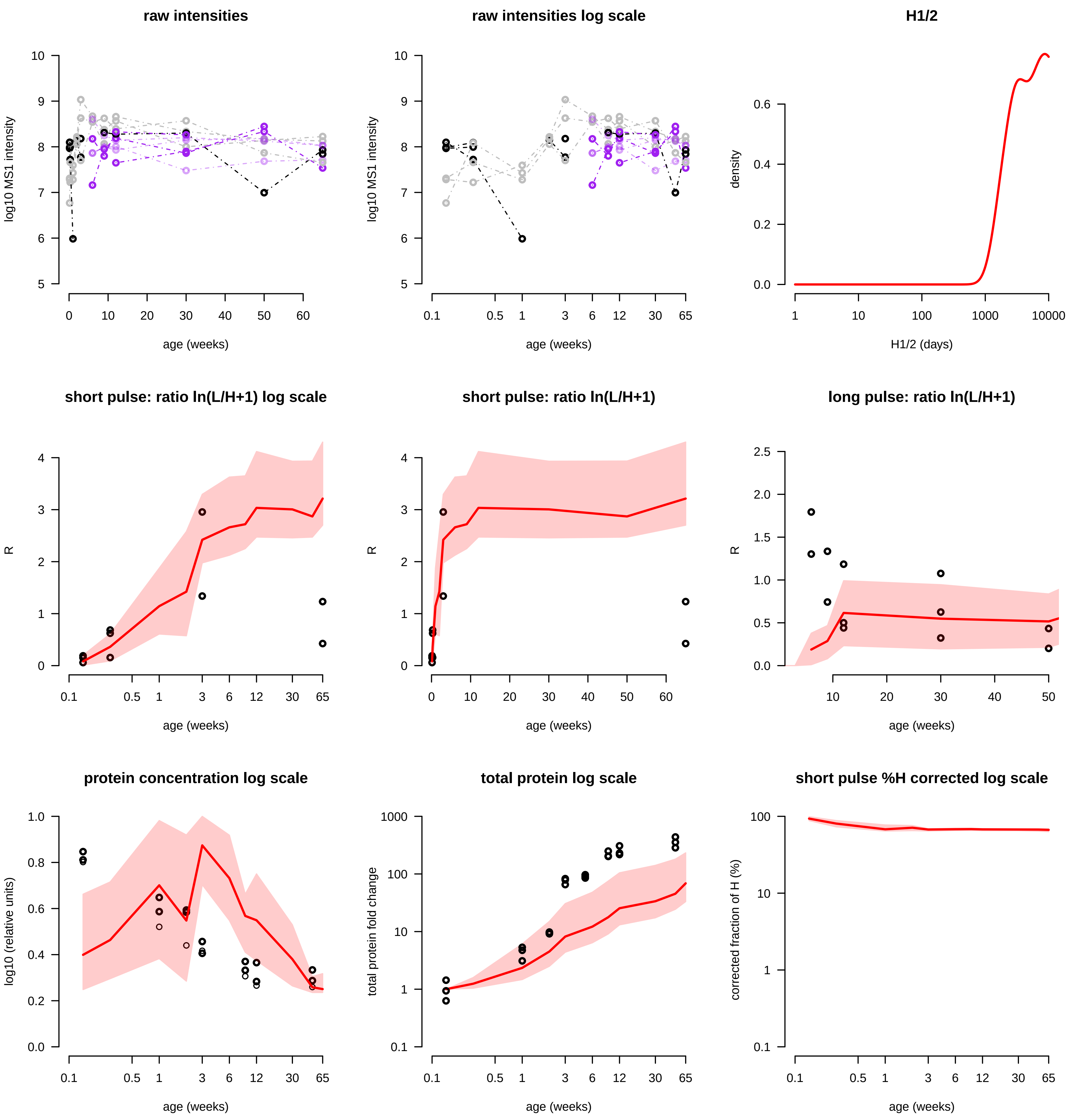

Q9JIX0

>sp|Q9JIX0|ENY2_MOUSE

Transcription and mRNA export factor ENY2

GN=Eny2

Med H1/2: 5.4 days

Q5 H1/2: 3.9 days

Q95 H1/2: 8 days

reliable model fit: yes

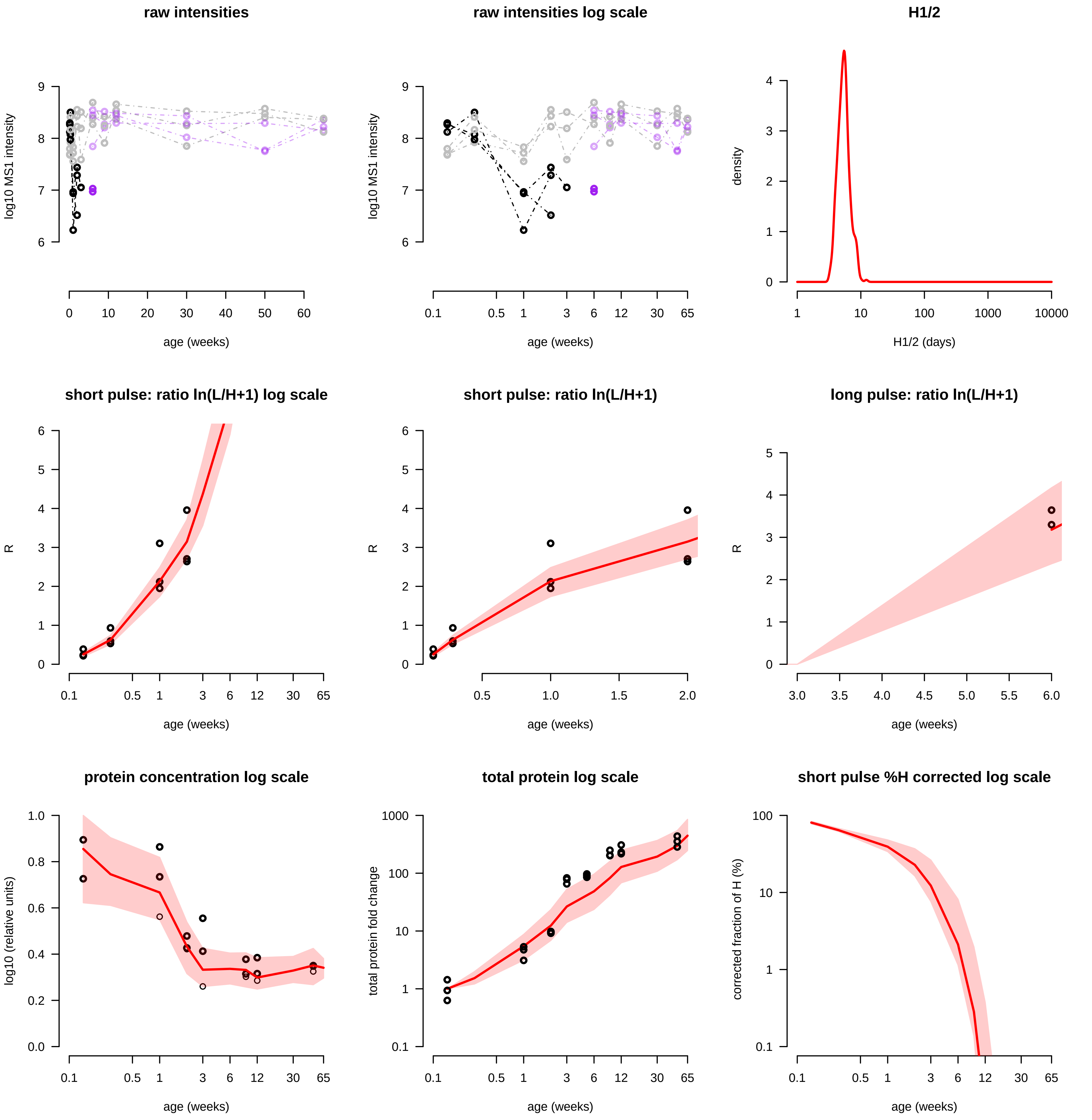

P58064

>sp|P58064|RT06_MOUSE

28S ribosomal protein S6, mitochondrial

GN=Mrps6

Med H1/2: 8.9 days

Q5 H1/2: 3.5 days

Q95 H1/2: 84 days

reliable model fit: yes

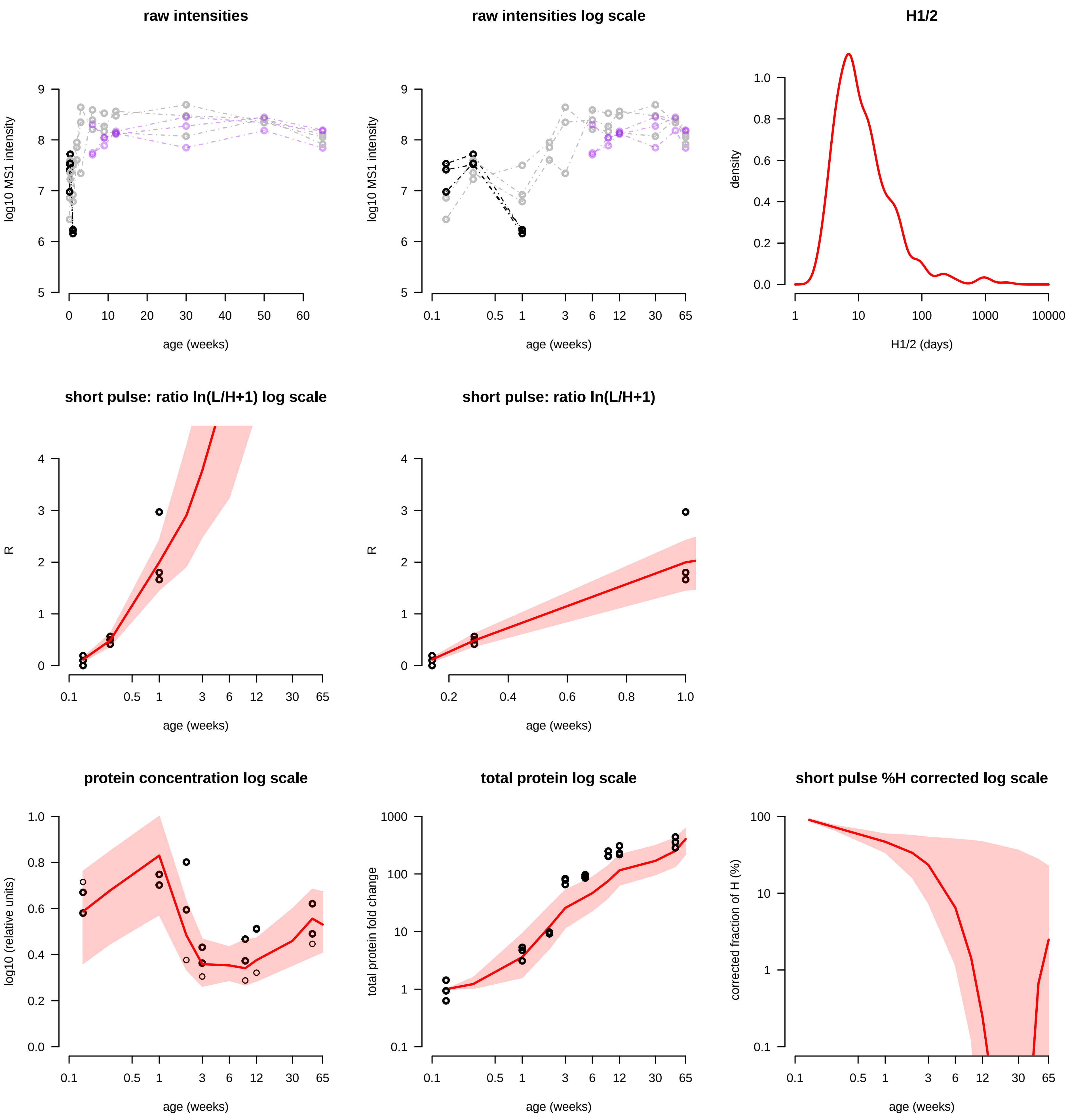

Q922H2

>sp|Q922H2|PDK3_MOUSE

[Pyruvate dehydrogenase (acetyl−transferring)] kinase isozyme 3, mitochondrial

GN=Pdk3

Med H1/2: 13.8 days

Q5 H1/2: 4.7 days

Q95 H1/2: 174.1 days

reliable model fit: yes

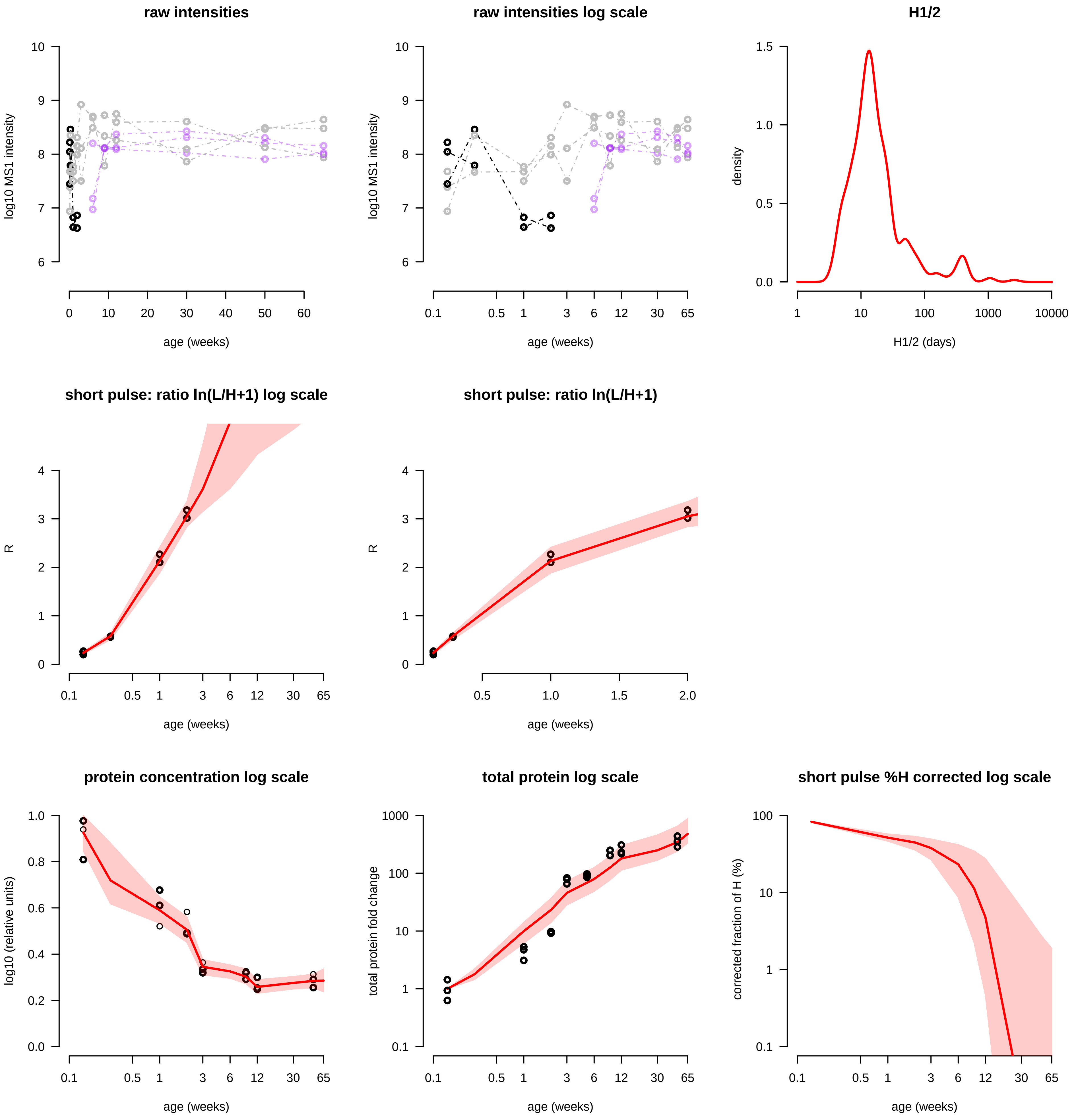

P43247

>sp|P43247|MSH2_MOUSE

DNA mismatch repair protein Msh2

GN=Msh2

Med H1/2: 8.4 days

Q5 H1/2: 4.8 days

Q95 H1/2: 25.9 days

reliable model fit: yes

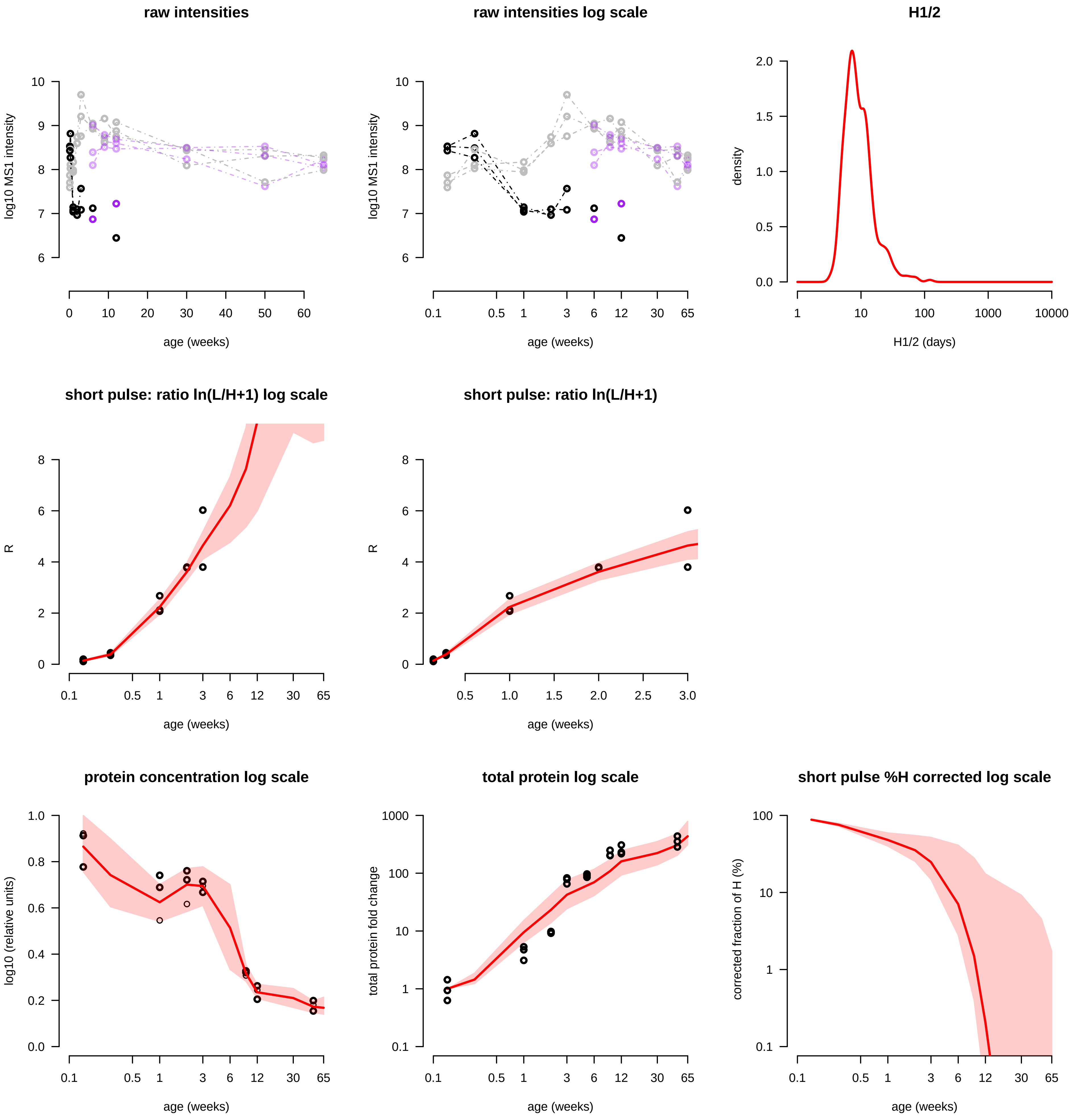

Q9CQ75

>sp|Q9CQ75|NDUA2_MOUSE

NADH dehydrogenase [ubiquinone] 1 alpha subcomplex subunit 2

GN=Ndufa2

Med H1/2: 4.5 days

Q5 H1/2: 3.2 days

Q95 H1/2: 6.7 days

reliable model fit: yes

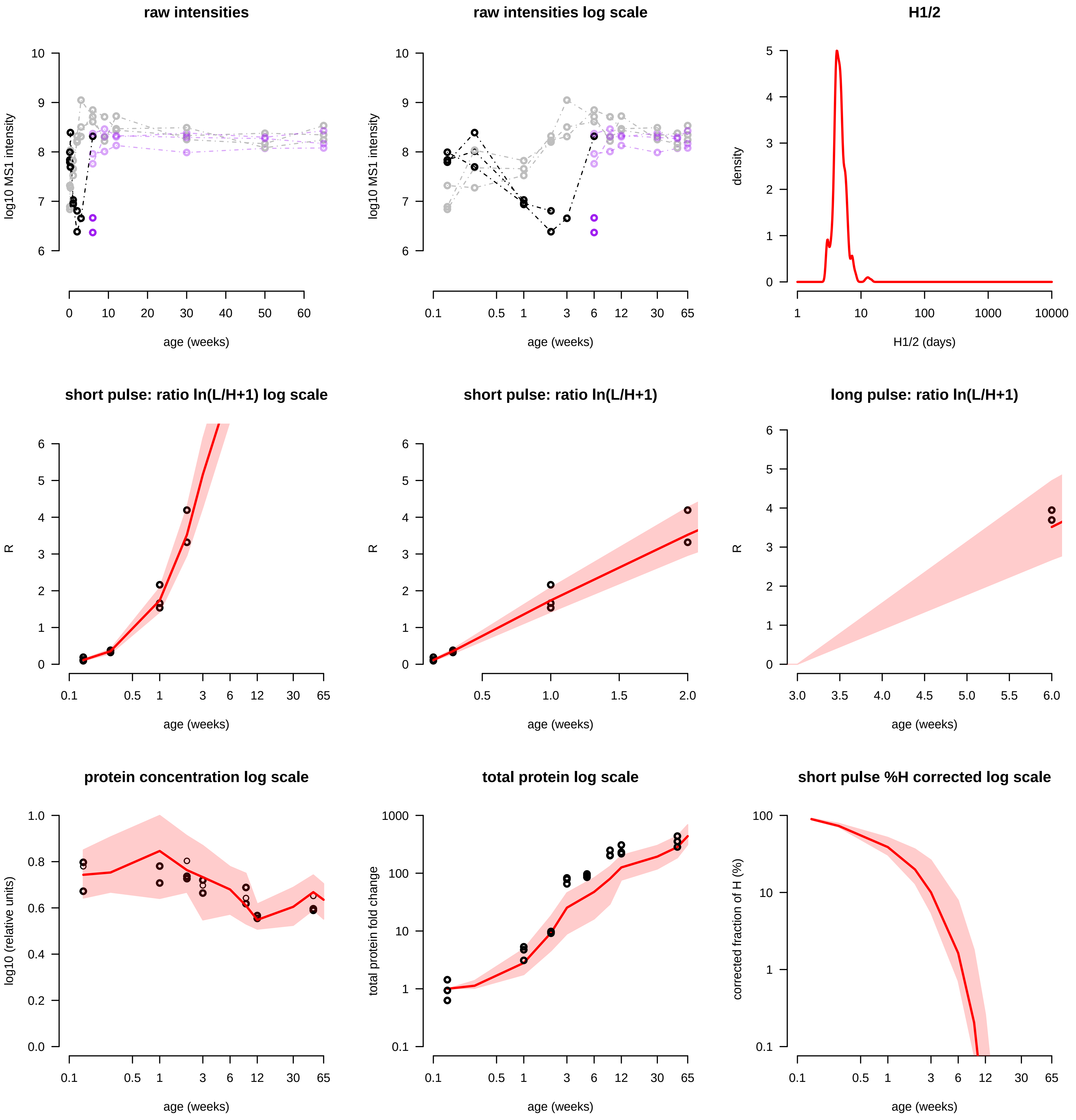

Q9JJV2;Q9JJV2−3

>sp|Q9JJV2|PROF2_MOUSE

Profilin−2

GN=Pfn2

Med H1/2: 9.9 days

Q5 H1/2: 4.2 days

Q95 H1/2: 37.2 days

reliable model fit: yes

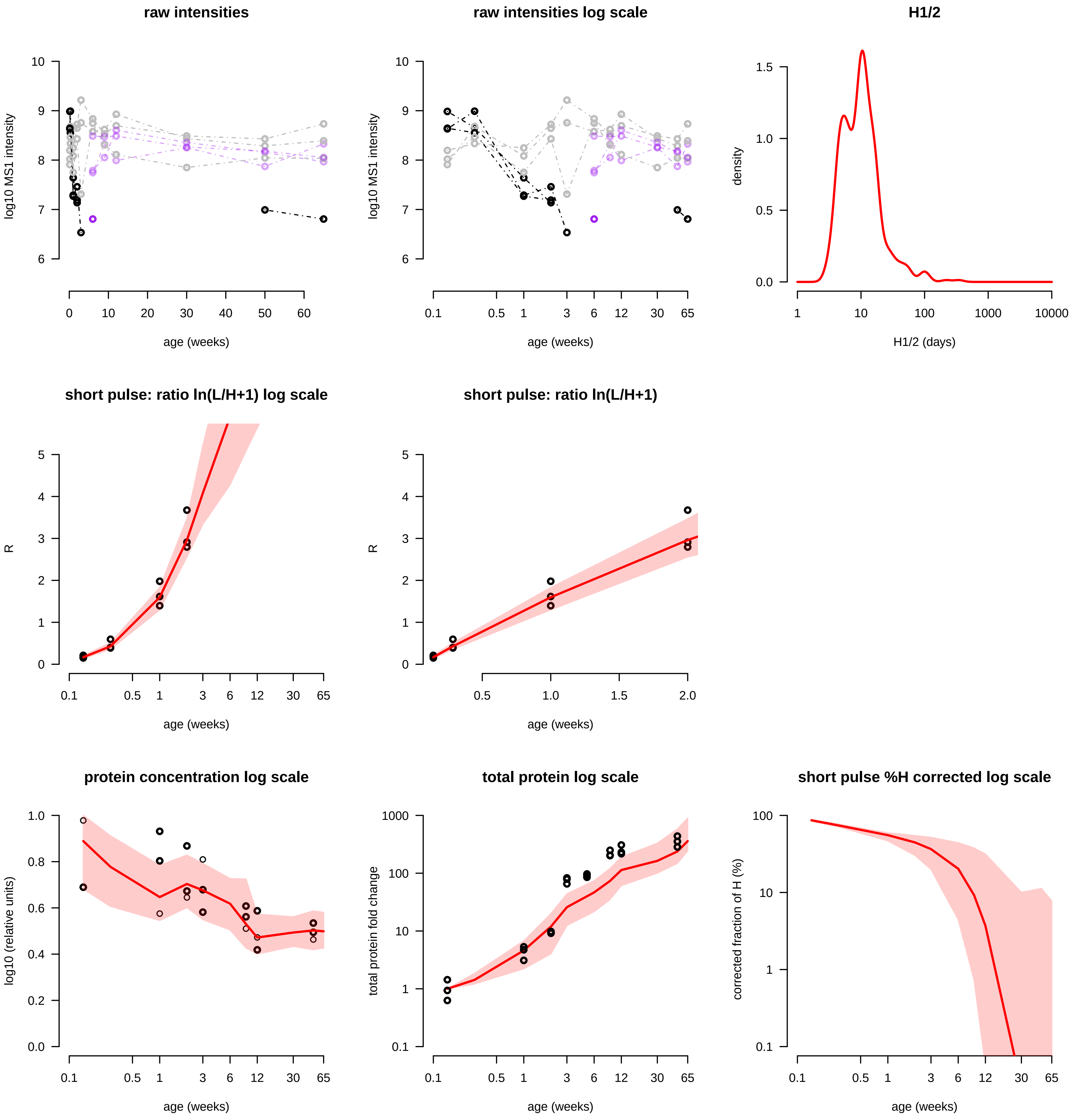

Q91XD6

>sp|Q91XD6|VPS36_MOUSE

Vacuolar protein−sorting−associated protein 36

GN=Vps36

Med H1/2: 4.9 days

Q5 H1/2: 2.7 days

Q95 H1/2: 124.7 days

reliable model fit: yes

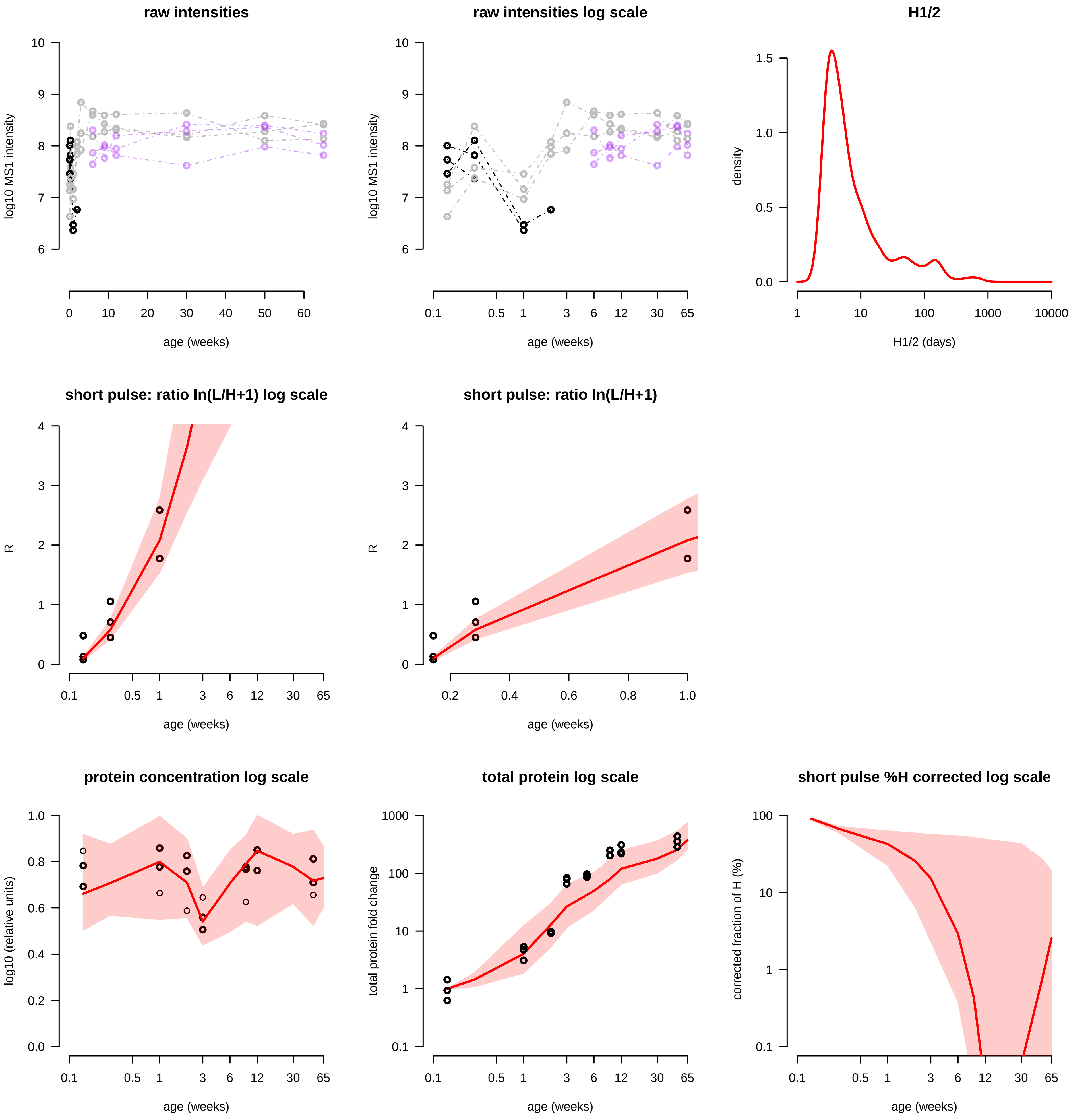

Q6PHQ8

>sp|Q6PHQ8|NAA35_MOUSE

N−alpha−acetyltransferase 35, NatC auxiliary subunit

GN=Naa35

Med H1/2: 6.2 days

Q5 H1/2: 2.9 days

Q95 H1/2: 26.9 days

reliable model fit: yes

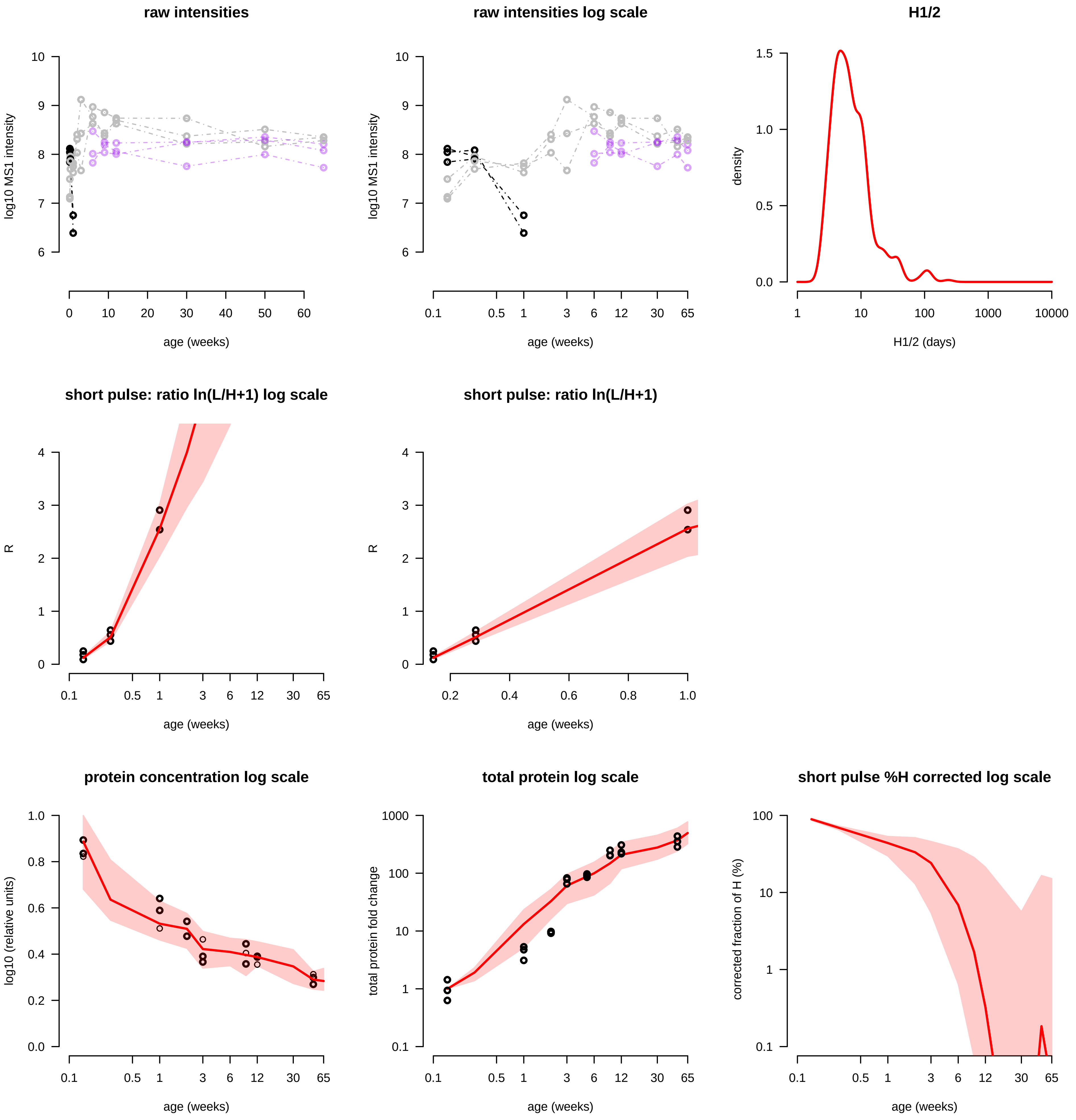

Q9JKF7

>sp|Q9JKF7|RM39_MOUSE

39S ribosomal protein L39, mitochondrial

GN=Mrpl39

Med H1/2: 14.7 days

Q5 H1/2: 9 days

Q95 H1/2: 35 days

reliable model fit: yes

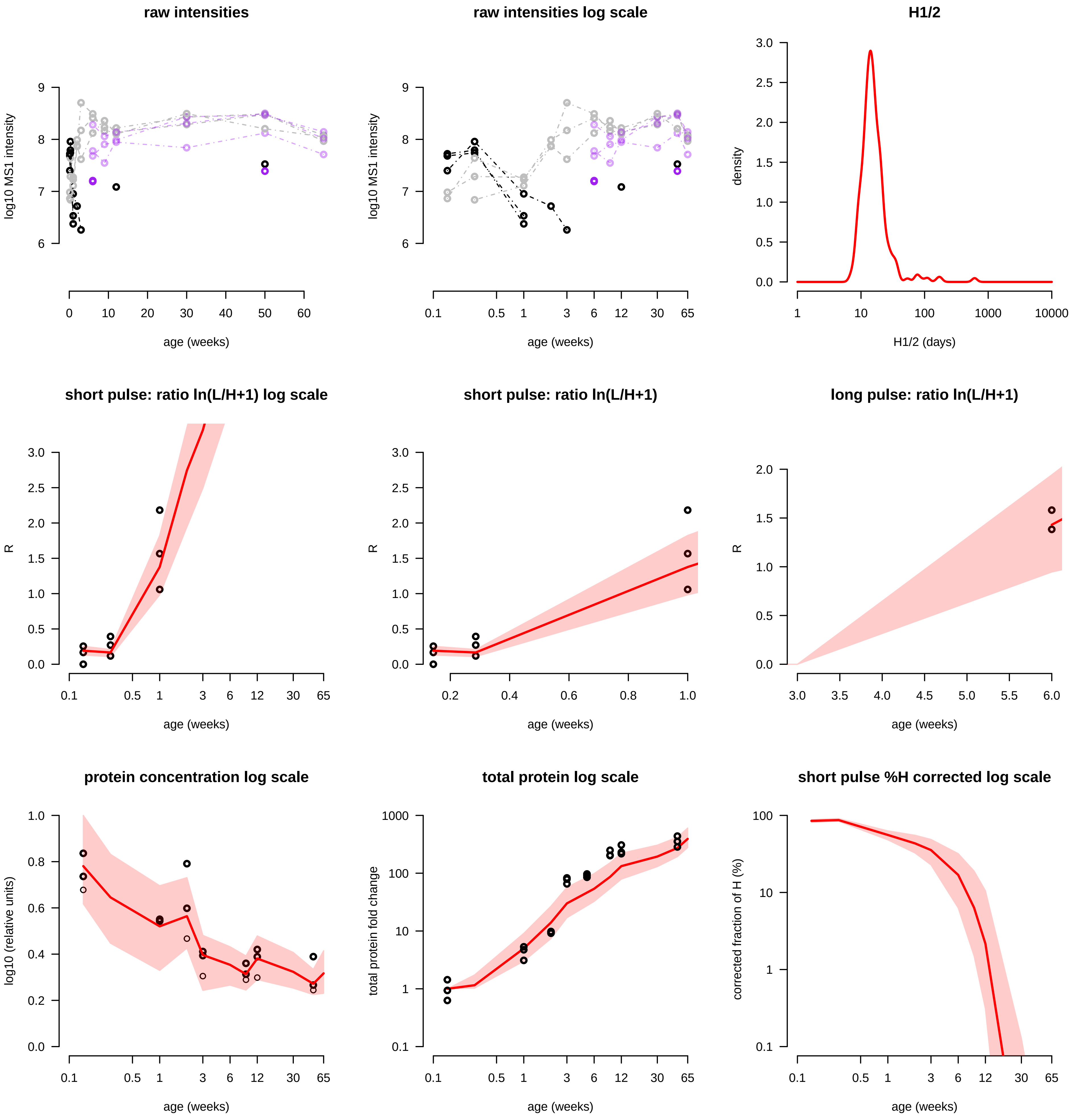

Q8CFE3

>sp|Q8CFE3|RCOR1_MOUSE

REST corepressor 1

GN=Rcor1

Med H1/2: 3.8 days

Q5 H1/2: 2.9 days

Q95 H1/2: 5.2 days

reliable model fit: yes

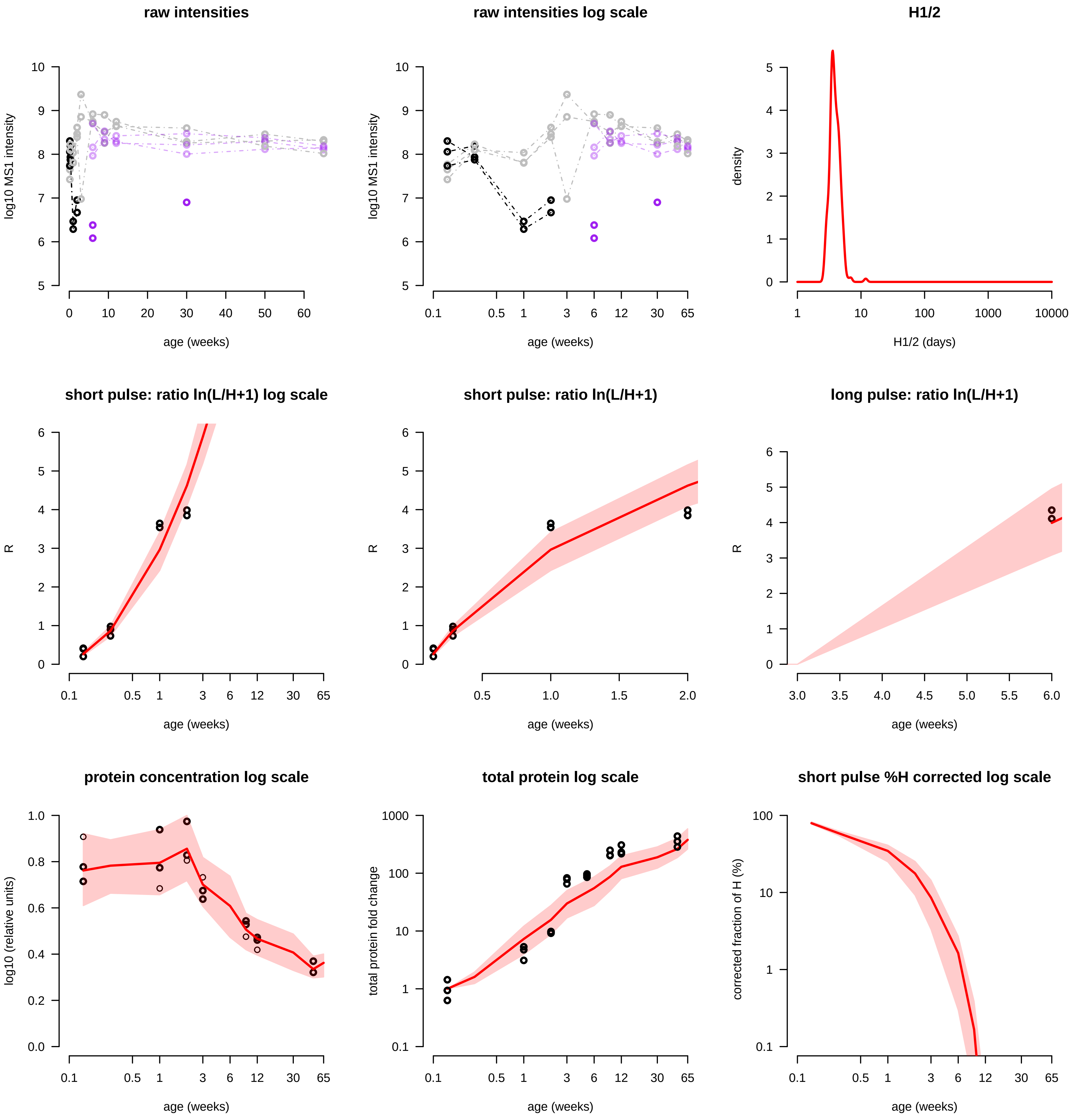

P51175

>sp|P51175|PPOX_MOUSE

Protoporphyrinogen oxidase

GN=Ppox

Med H1/2: 6.9 days

Q5 H1/2: 2.9 days

Q95 H1/2: 74.7 days

reliable model fit: yes

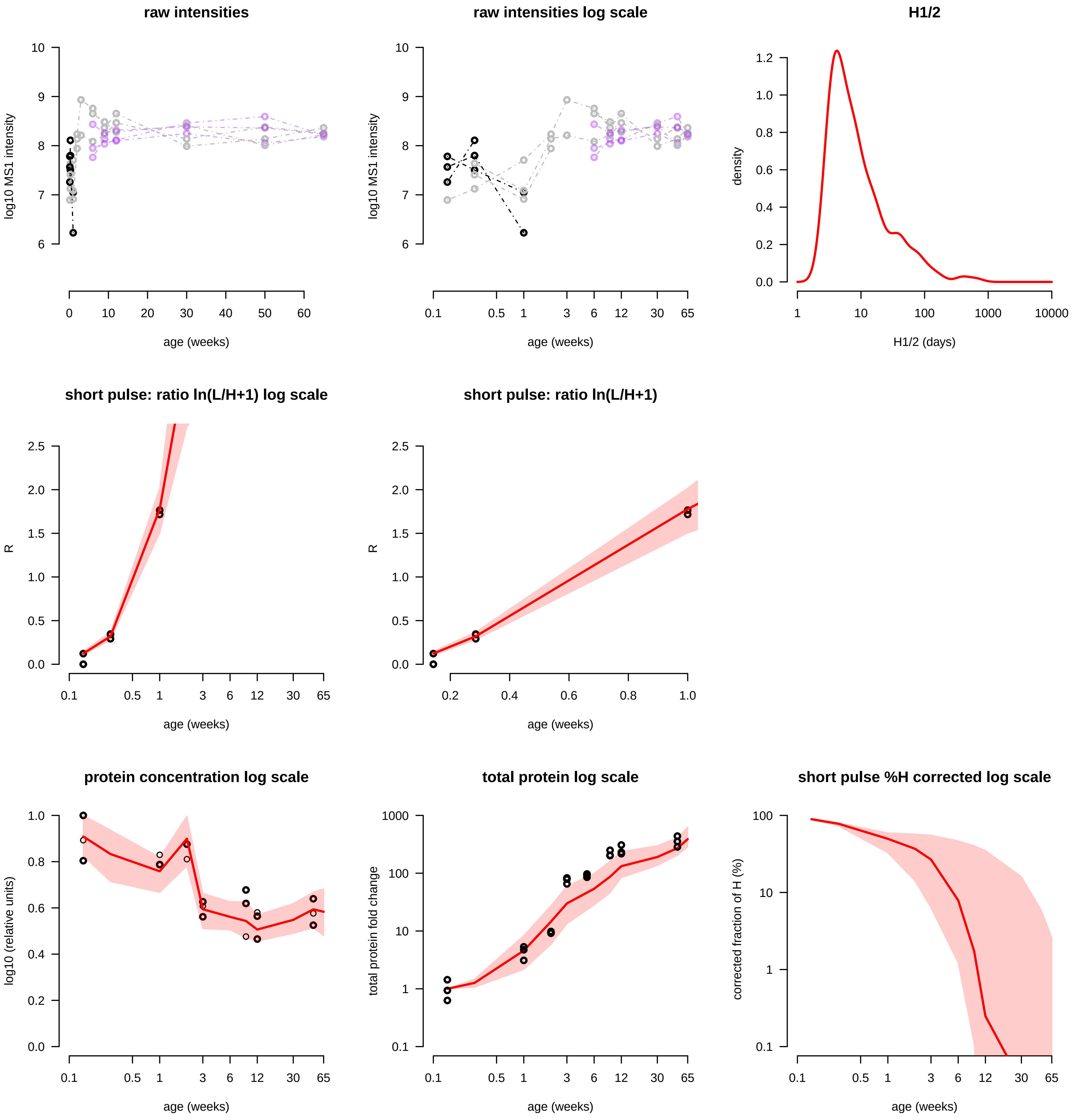

Q91VU0

>sp|Q91VU0|FAM3C_MOUSE

Protein FAM3C

GN=Fam3c

Med H1/2: 5.6 days

Q5 H1/2: 3.2 days

Q95 H1/2: 14.1 days

reliable model fit: yes

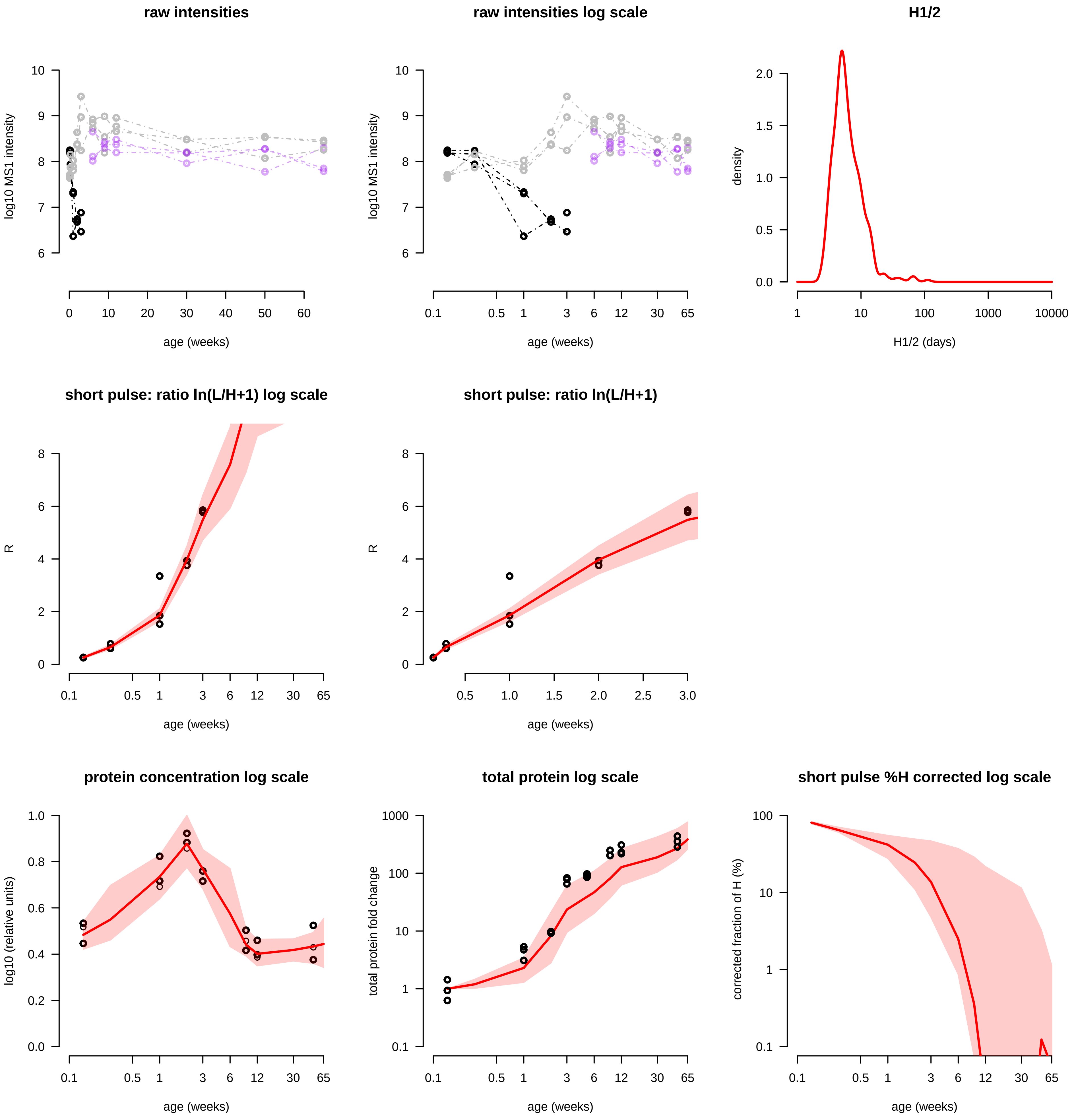

P97311

>sp|P97311|MCM6_MOUSE

DNA replication licensing factor MCM6

GN=Mcm6

Med H1/2: 3.7 days

Q5 H1/2: 2.9 days

Q95 H1/2: 4.5 days

reliable model fit: yes

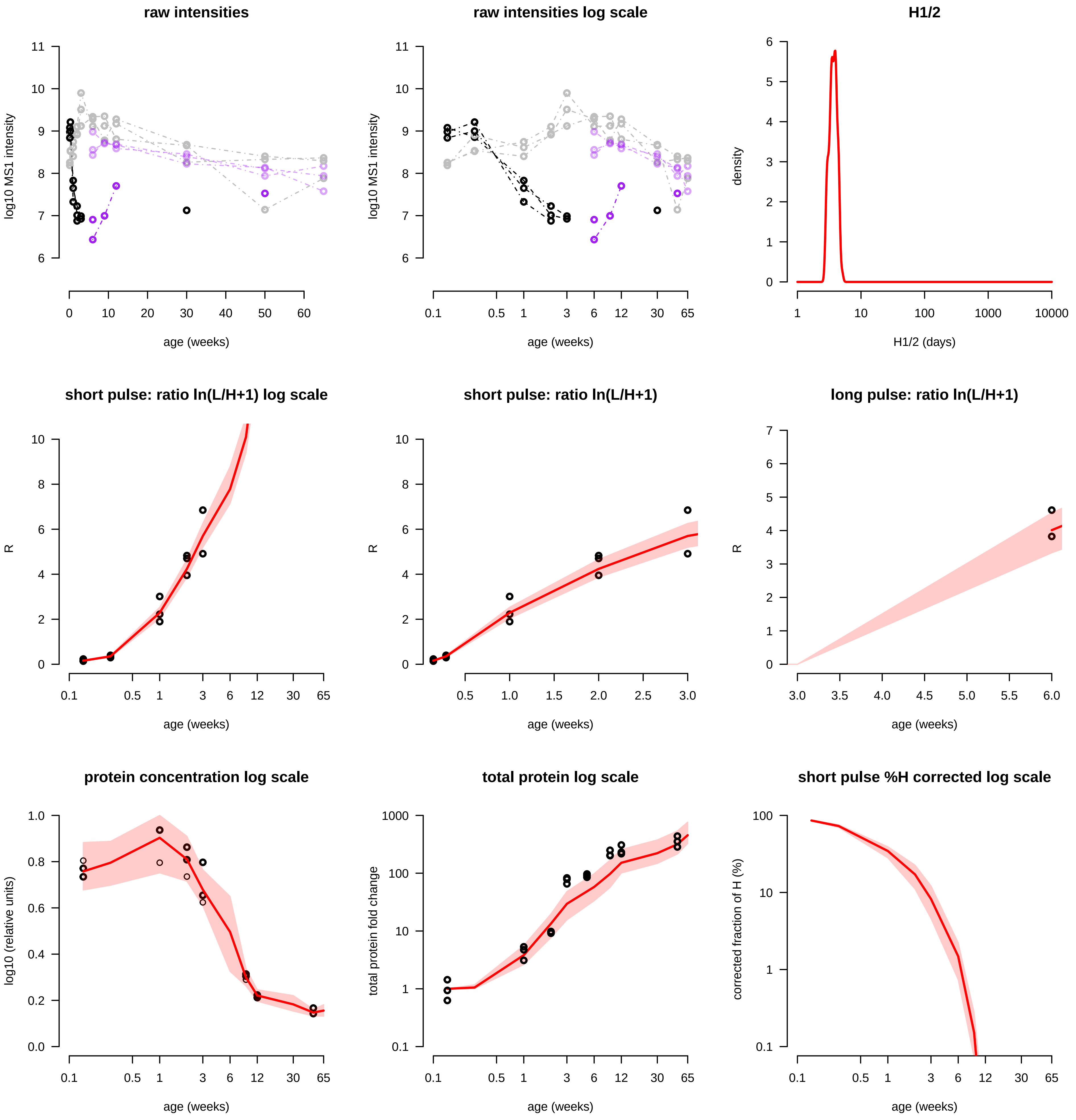

O54692

>sp|O54692|ZW10_MOUSE

Centromere/kinetochore protein zw10 homolog

GN=Zw10

Med H1/2: 7 days

Q5 H1/2: 3.2 days

Q95 H1/2: 26.9 days

reliable model fit: yes

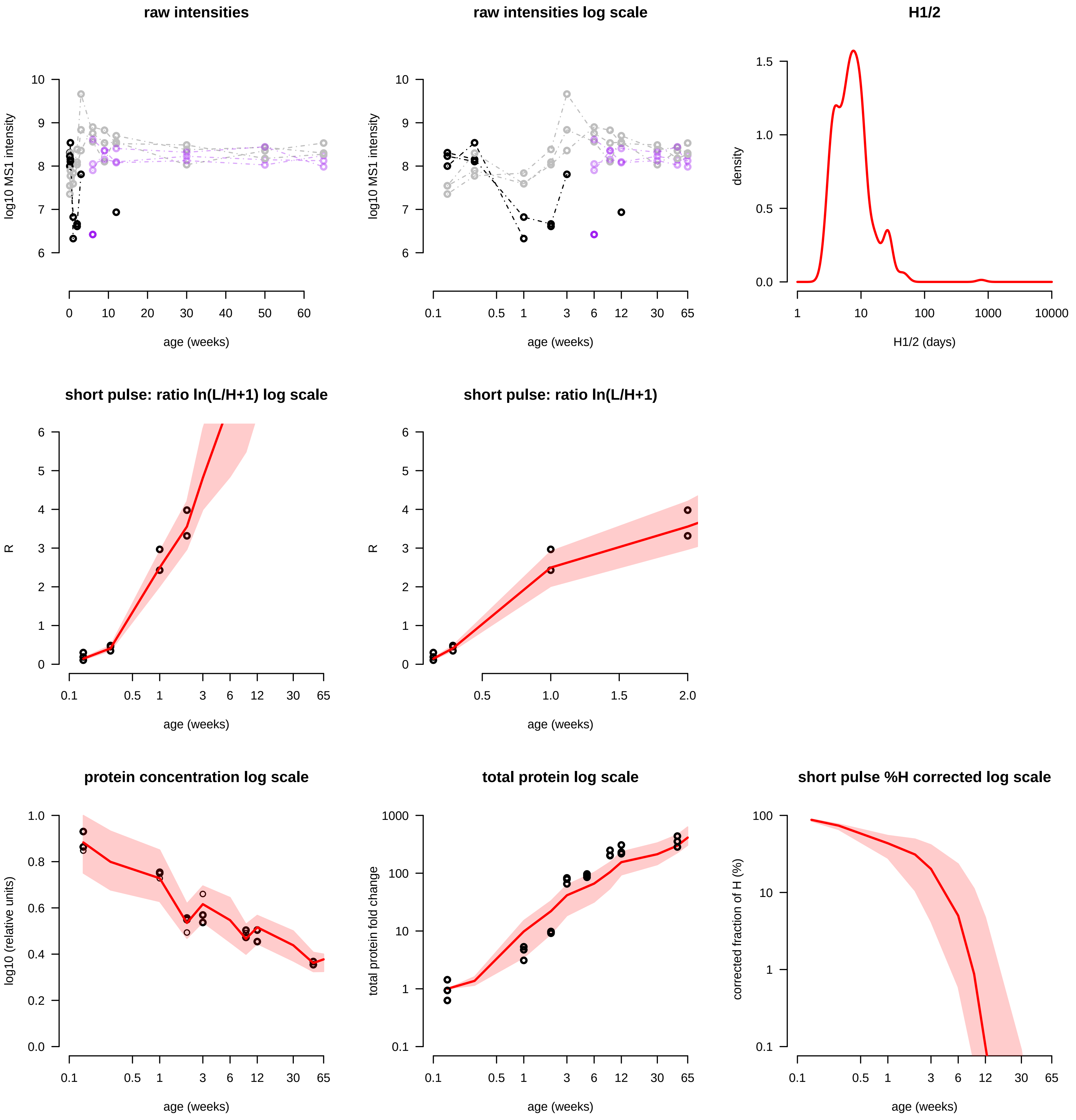

Q9D0M1

>sp|Q9D0M1|KPRA_MOUSE

Phosphoribosyl pyrophosphate synthase−associated protein 1

GN=Prpsap1

Med H1/2: 6.4 days

Q5 H1/2: 4.8 days

Q95 H1/2: 8.7 days

reliable model fit: yes

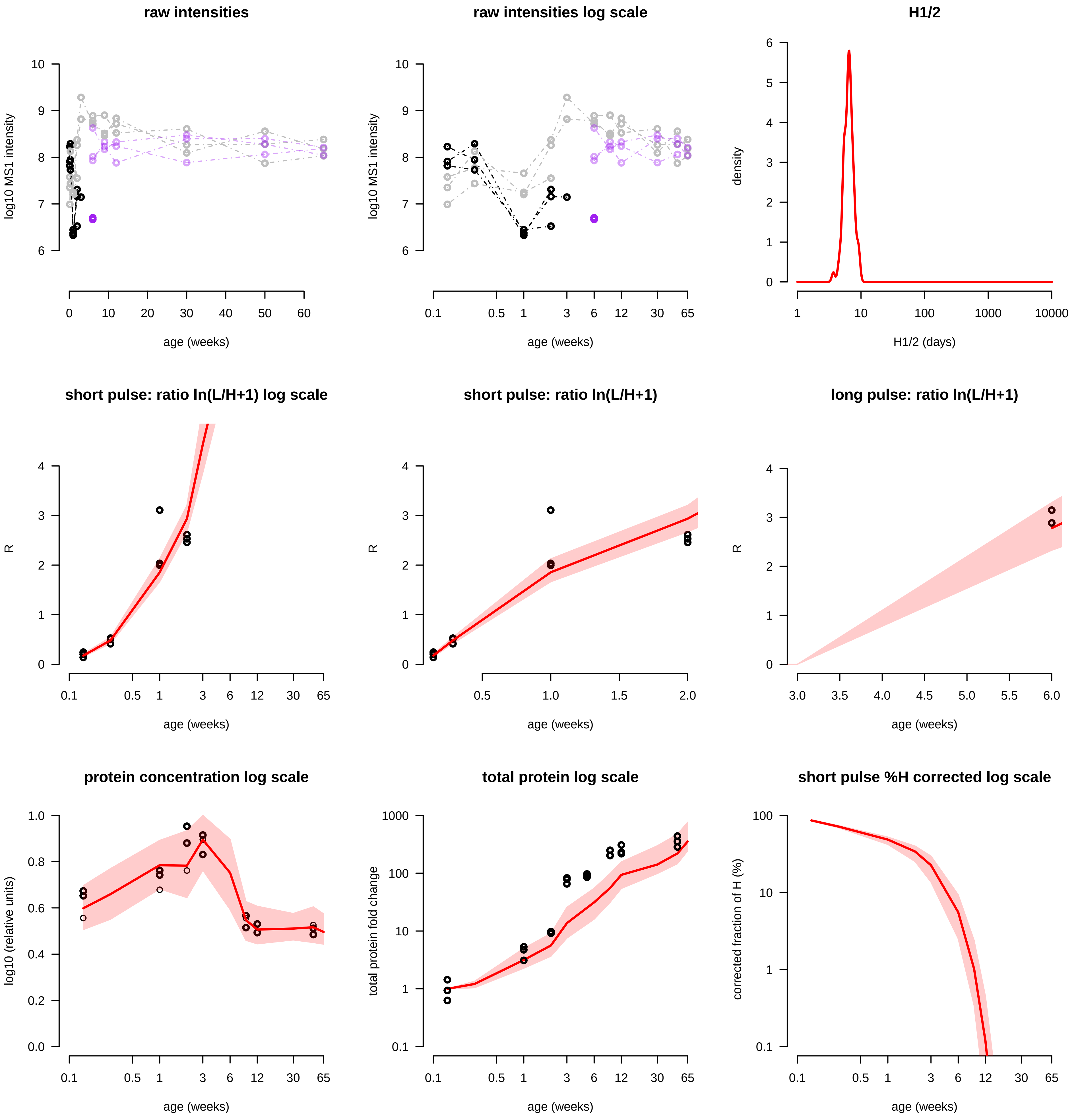

Q9EQI8

>sp|Q9EQI8|RM46_MOUSE

39S ribosomal protein L46, mitochondrial

GN=Mrpl46

Med H1/2: 12.3 days

Q5 H1/2: 3.3 days

Q95 H1/2: 100.4 days

reliable model fit: yes

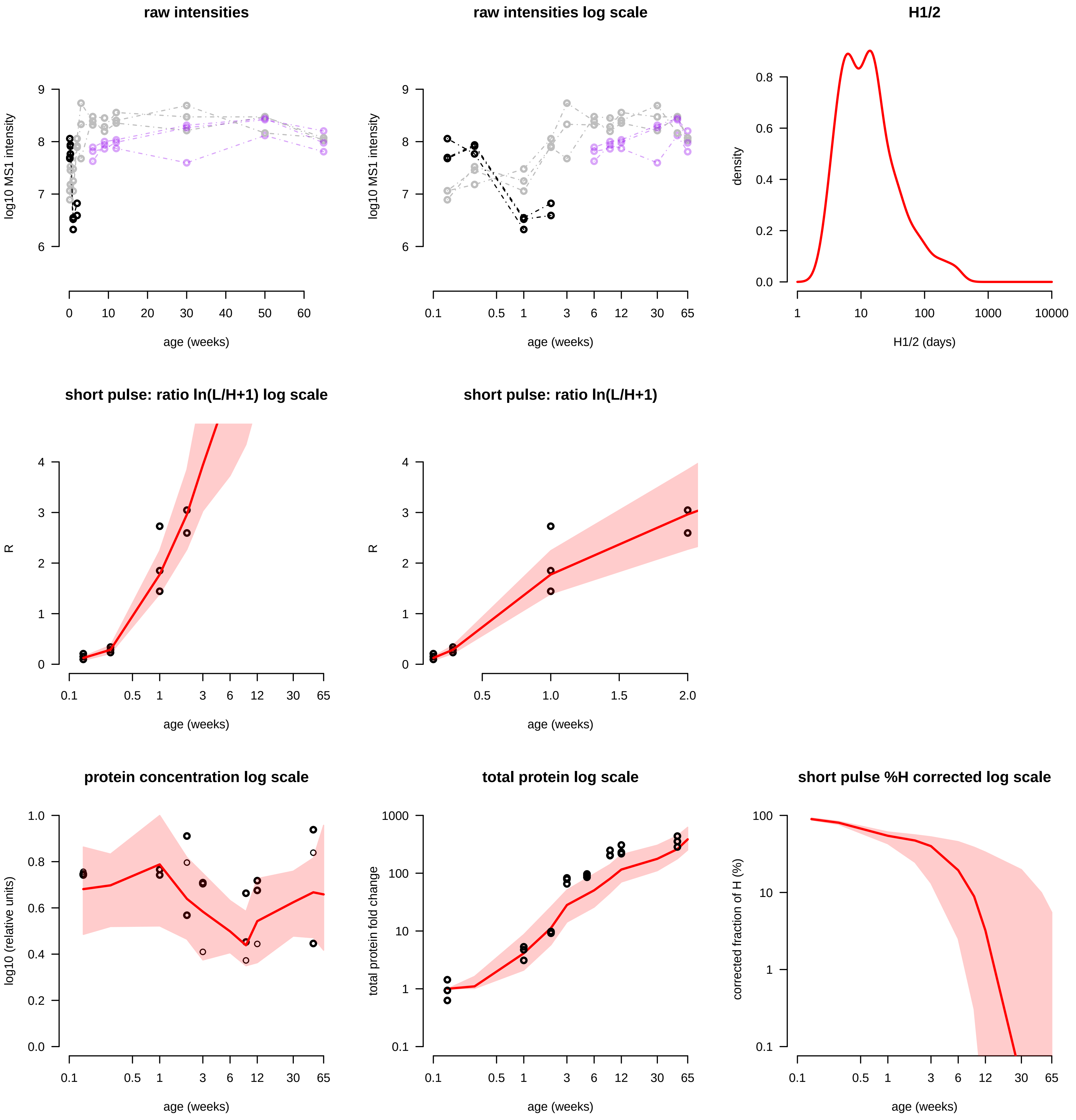

Q9WTU0

>sp|Q9WTU0|PHF2_MOUSE

Lysine−specific demethylase PHF2

GN=Phf2

Med H1/2: 11.1 days

Q5 H1/2: 4 days

Q95 H1/2: 109.2 days

reliable model fit: yes

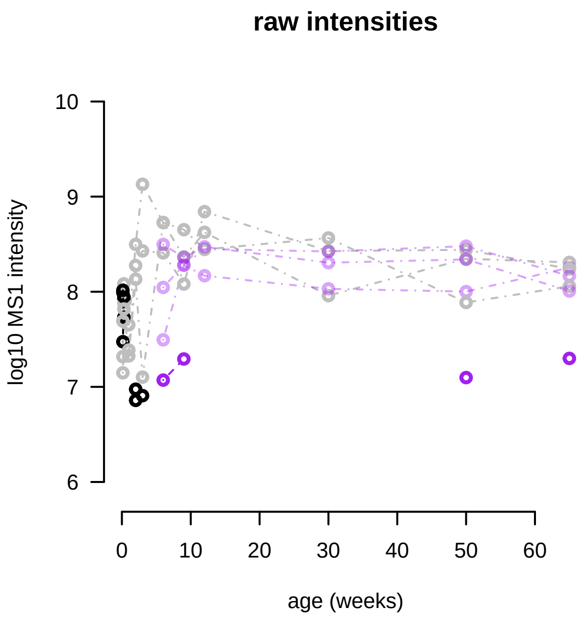

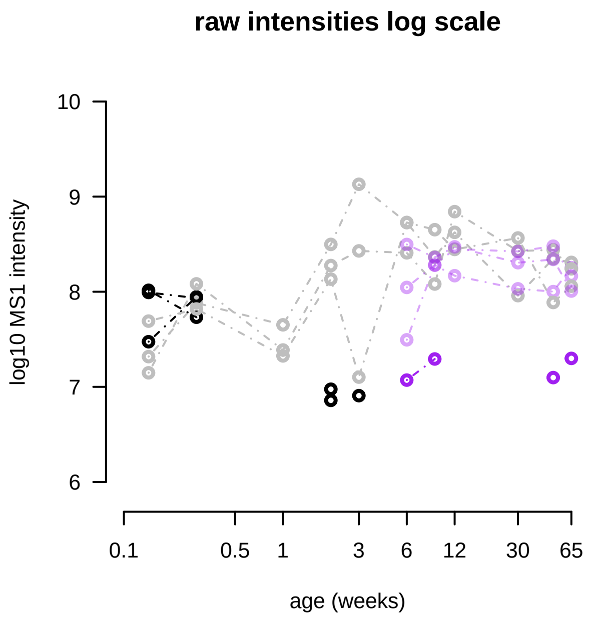

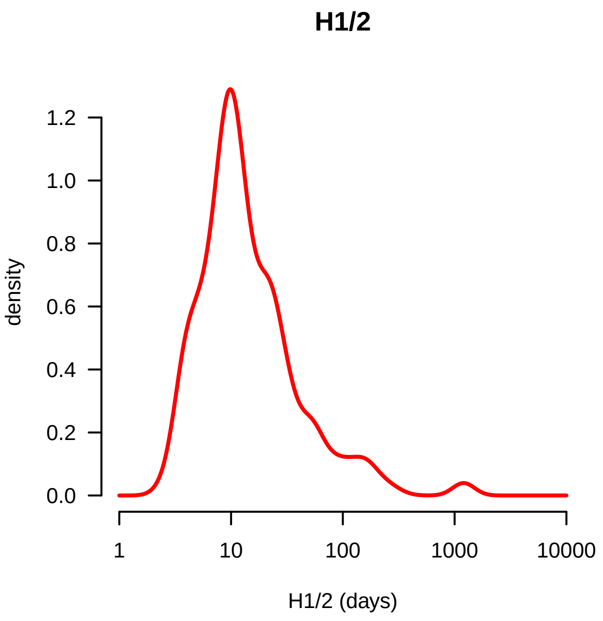

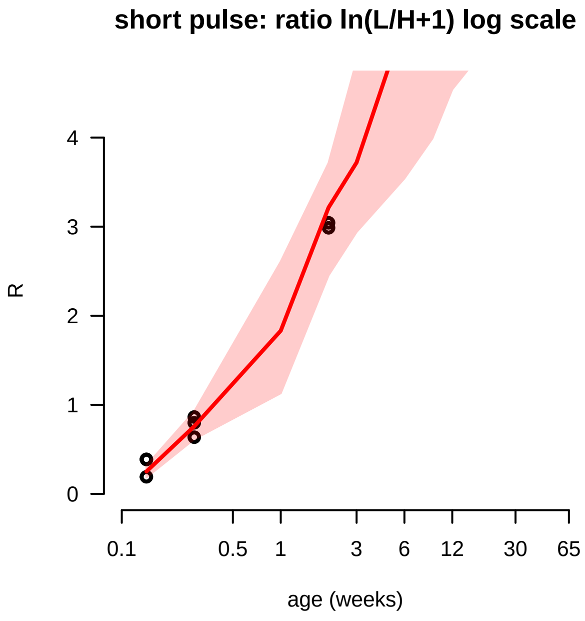

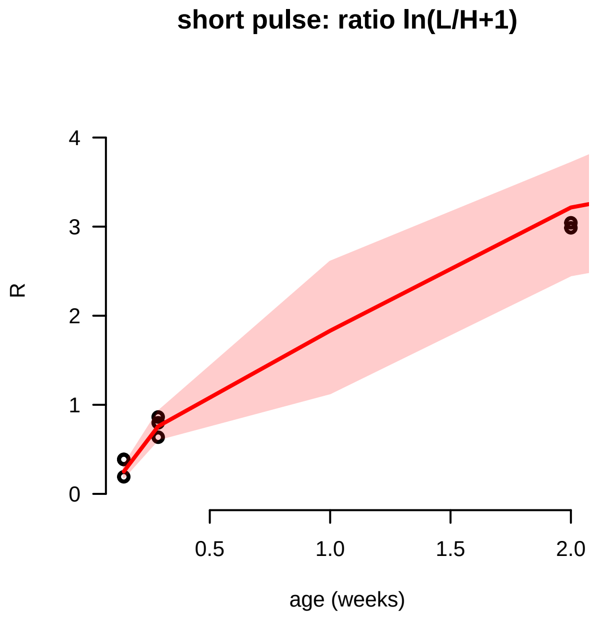

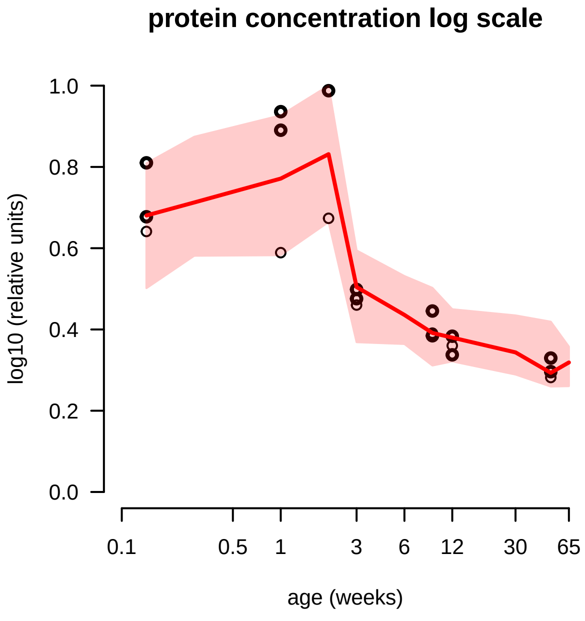

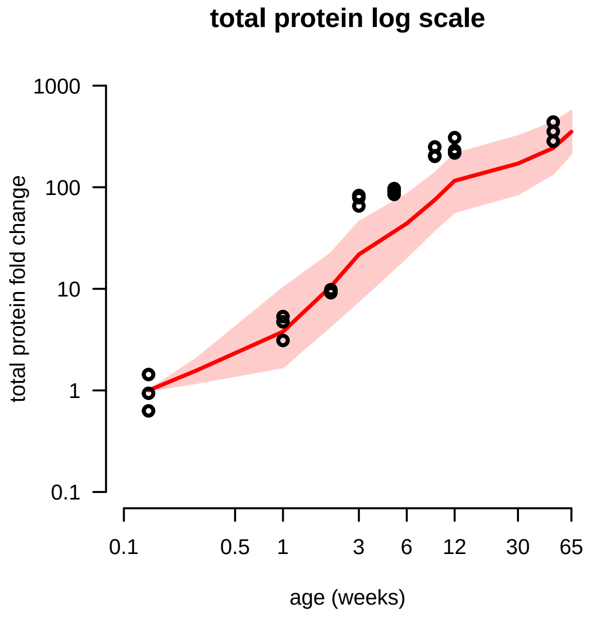

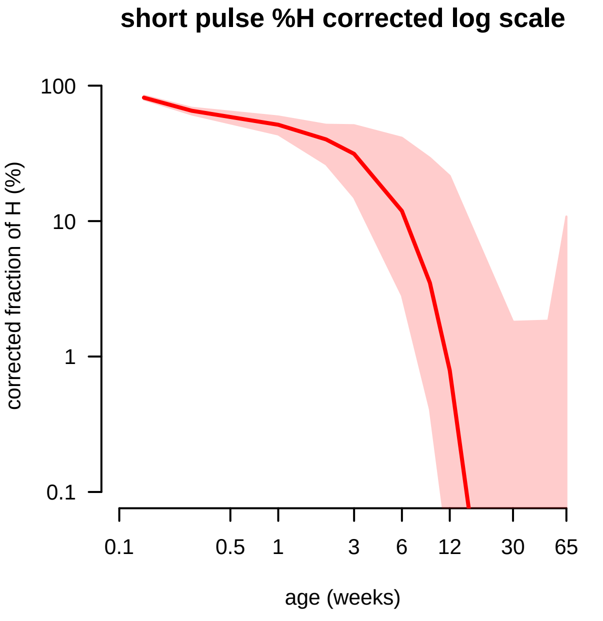

Q8CFQ3

>sp|Q8CFQ3|AQR_MOUSE

RNA helicase aquarius

GN=Aqr

Med H1/2: 4.3 days

Q5 H1/2: 2.6 days

Q95 H1/2: 9.1 days

reliable model fit: yes

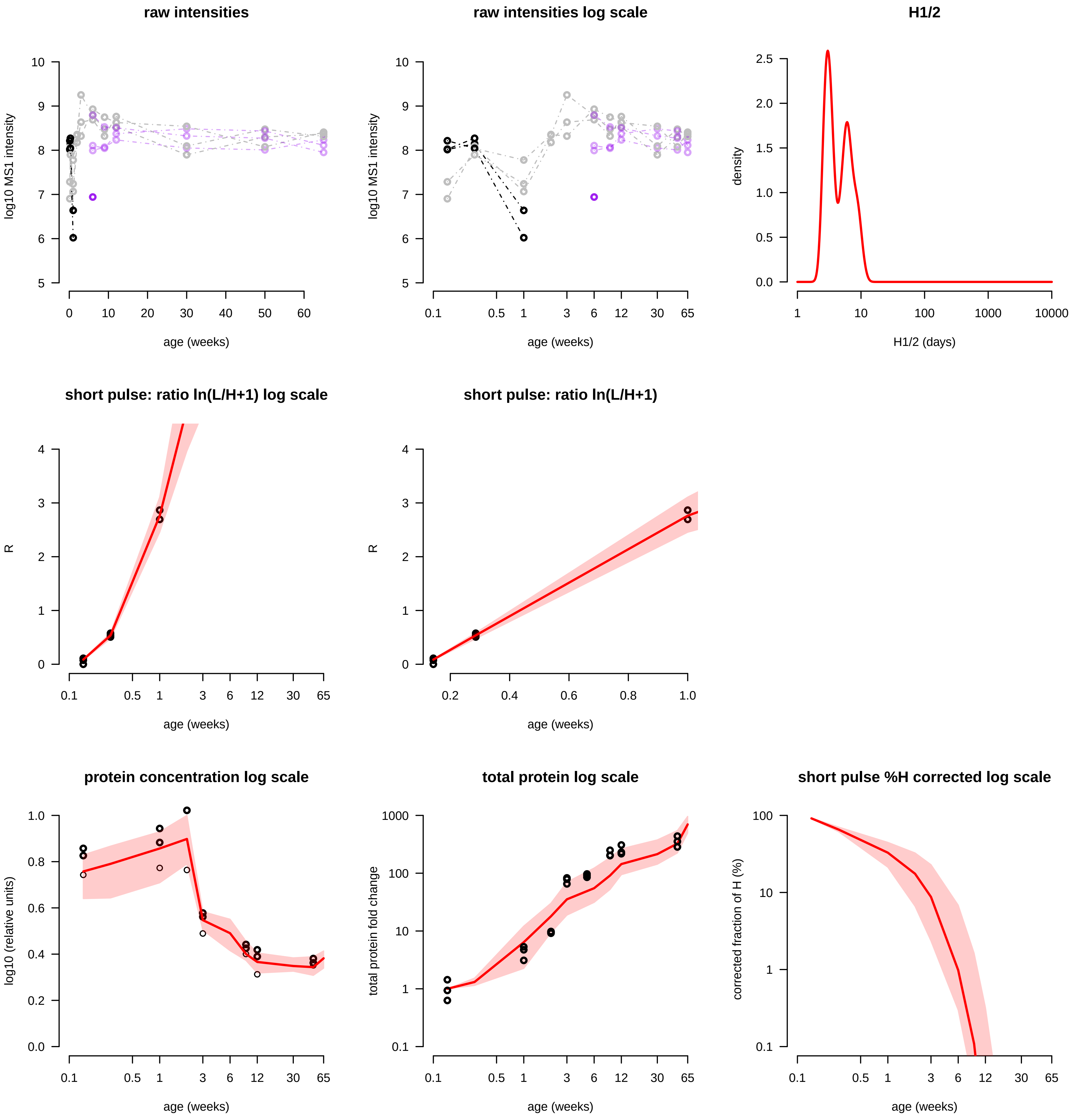

Q03958

>sp|Q03958|PFD6_MOUSE

Prefoldin subunit 6

GN=Pfdn6

Med H1/2: 5.4 days

Q5 H1/2: 2.6 days

Q95 H1/2: 22.3 days

reliable model fit: yes

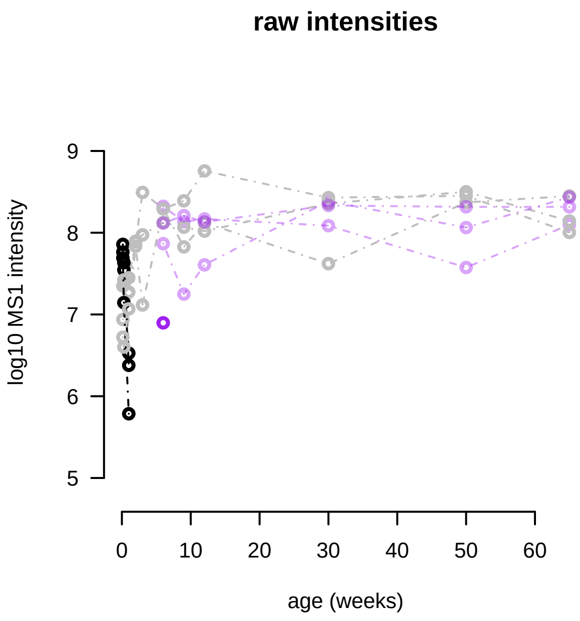

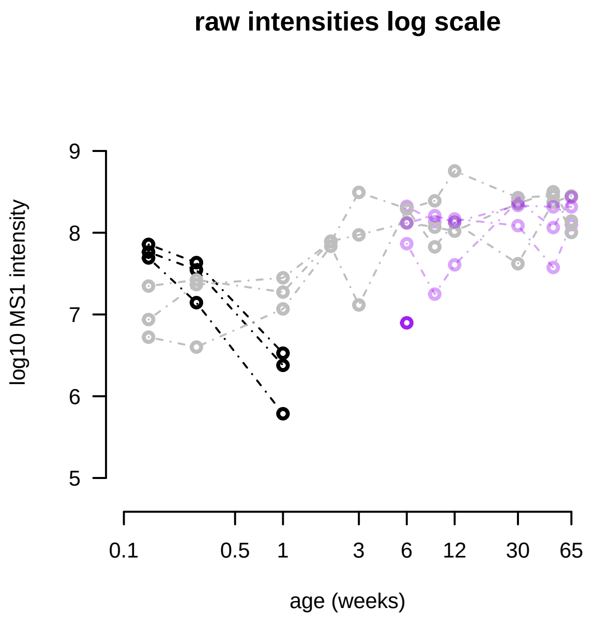

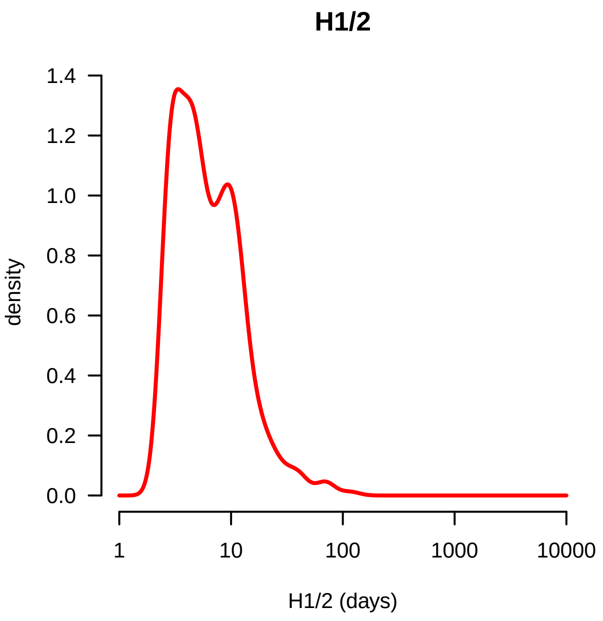

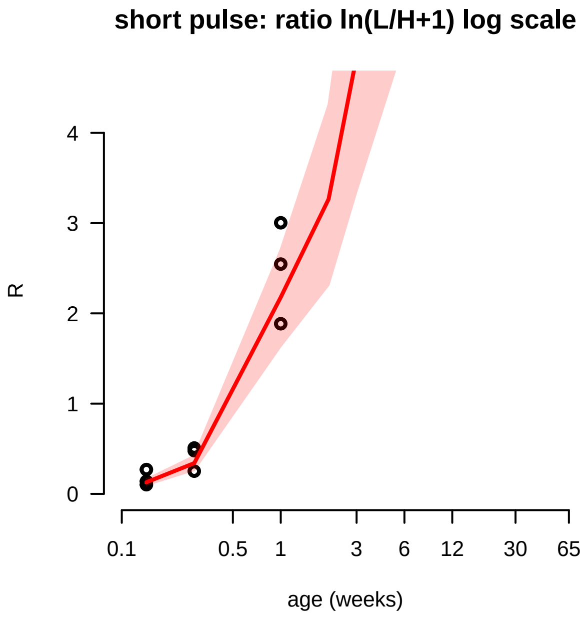

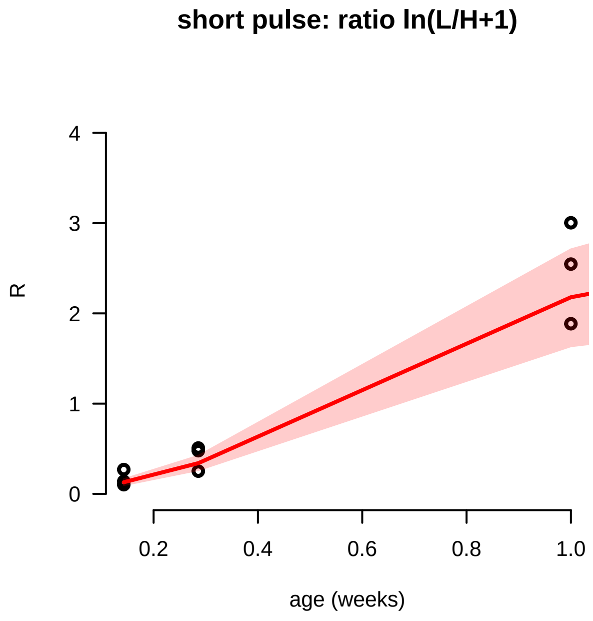

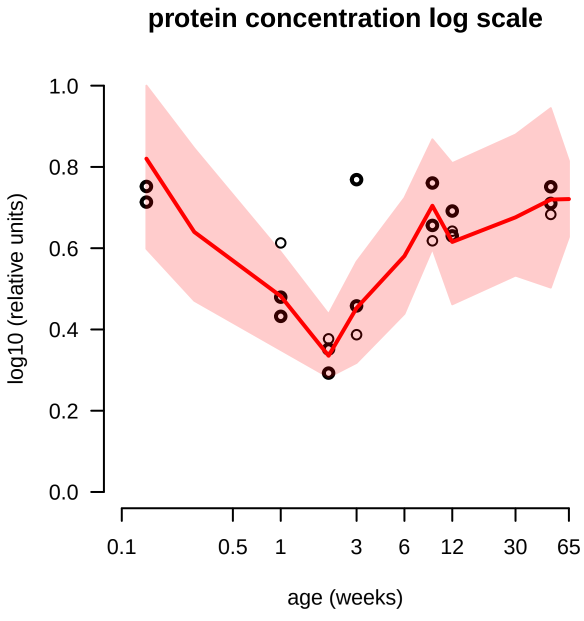

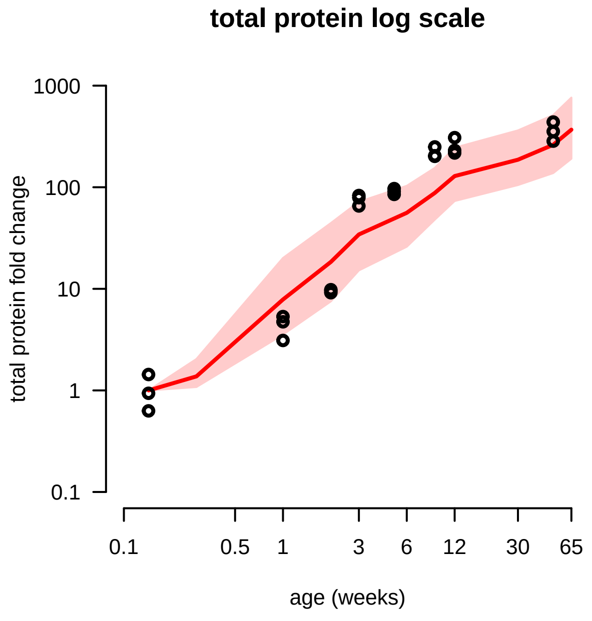

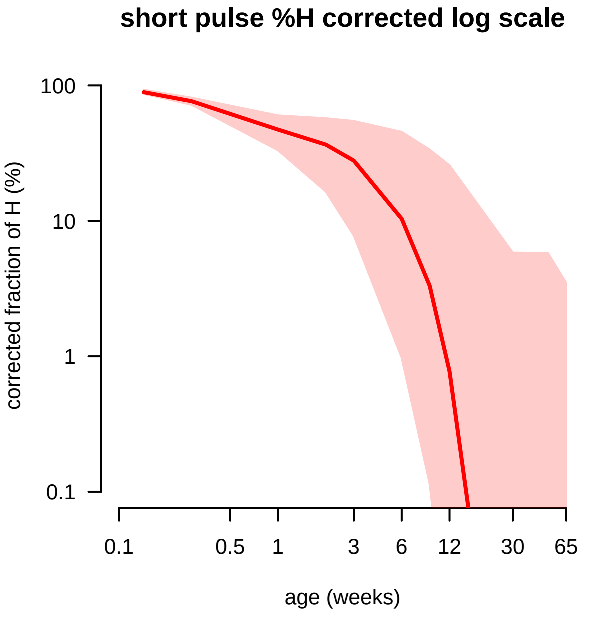

Q6PGB8;Q6PGB8−2

>sp|Q6PGB8|SMCA1_MOUSE

Probable global transcription activator SNF2L1

GN=Smarca1

Med H1/2: 2074.6 days

Q5 H1/2: 730.1 days

Q95 H1/2: 19213.5 days

reliable model fit: no

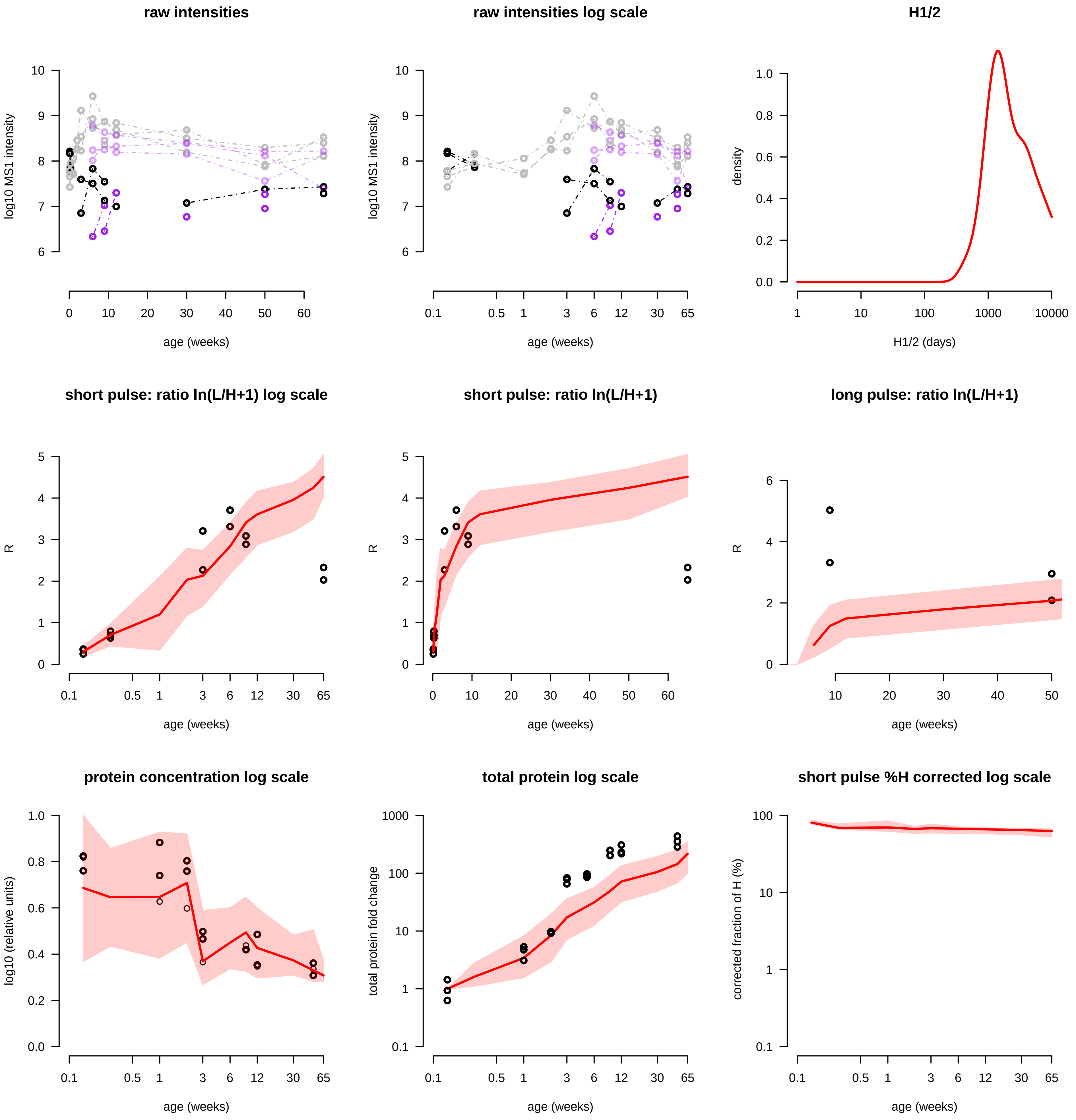

P63087−2;P63087

>sp|P63087−2|PP1G_MOUSE

soform 2 of Serine/threonine−protein phosphatase PP1−gamma catalytic subun

GN=Ppp1cc

Med H1/2: 4.5 days

Q5 H1/2: 3.7 days

Q95 H1/2: 5.5 days

reliable model fit: yes

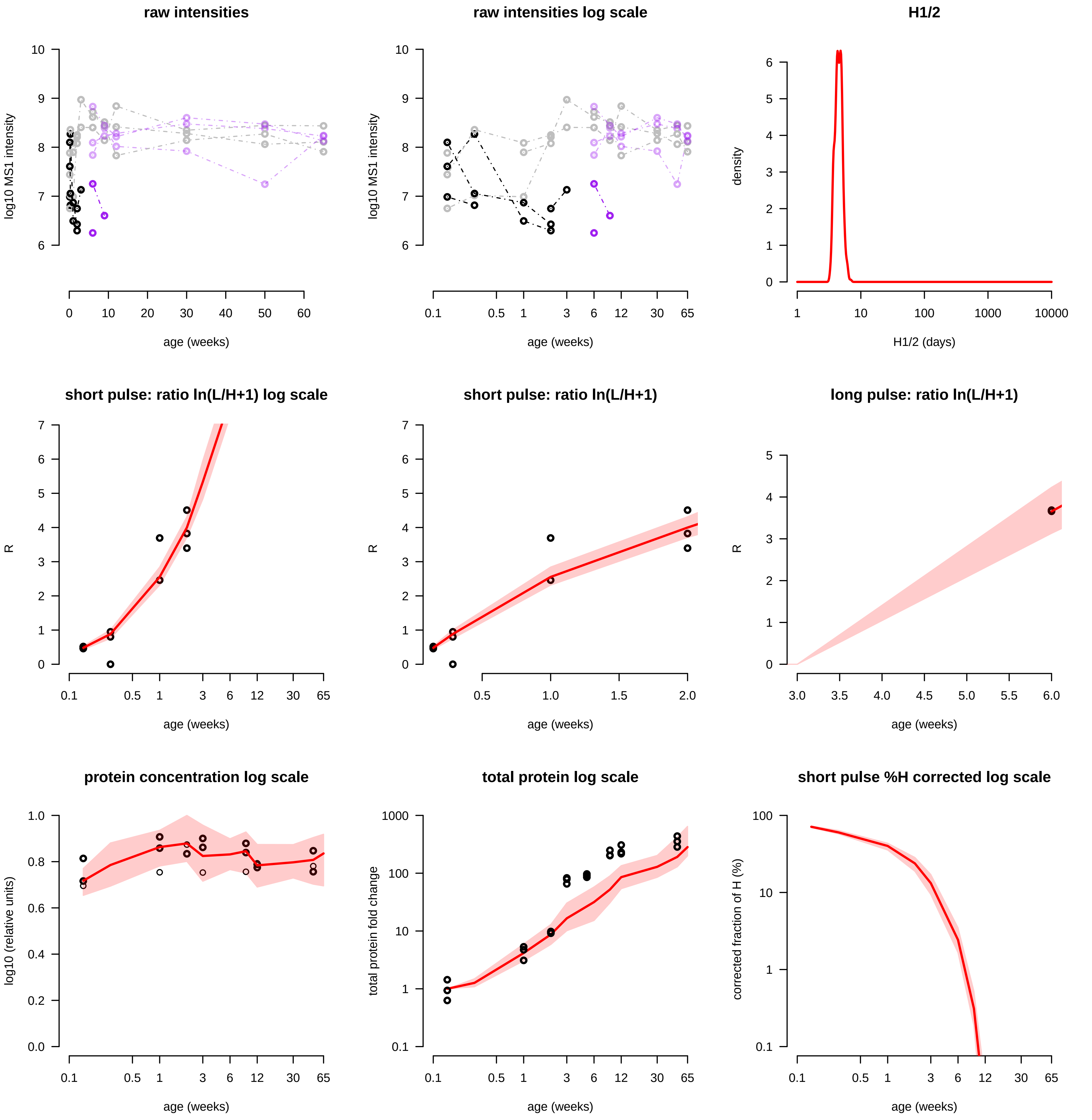

Q8K0C9

>sp|Q8K0C9|GMDS_MOUSE

GDP−mannose 4,6 dehydratase

GN=Gmds

Med H1/2: 14.5 days

Q5   H1/2: 5.1 days

Q95 H1/2: 76.1 days

reliable model fit: yes

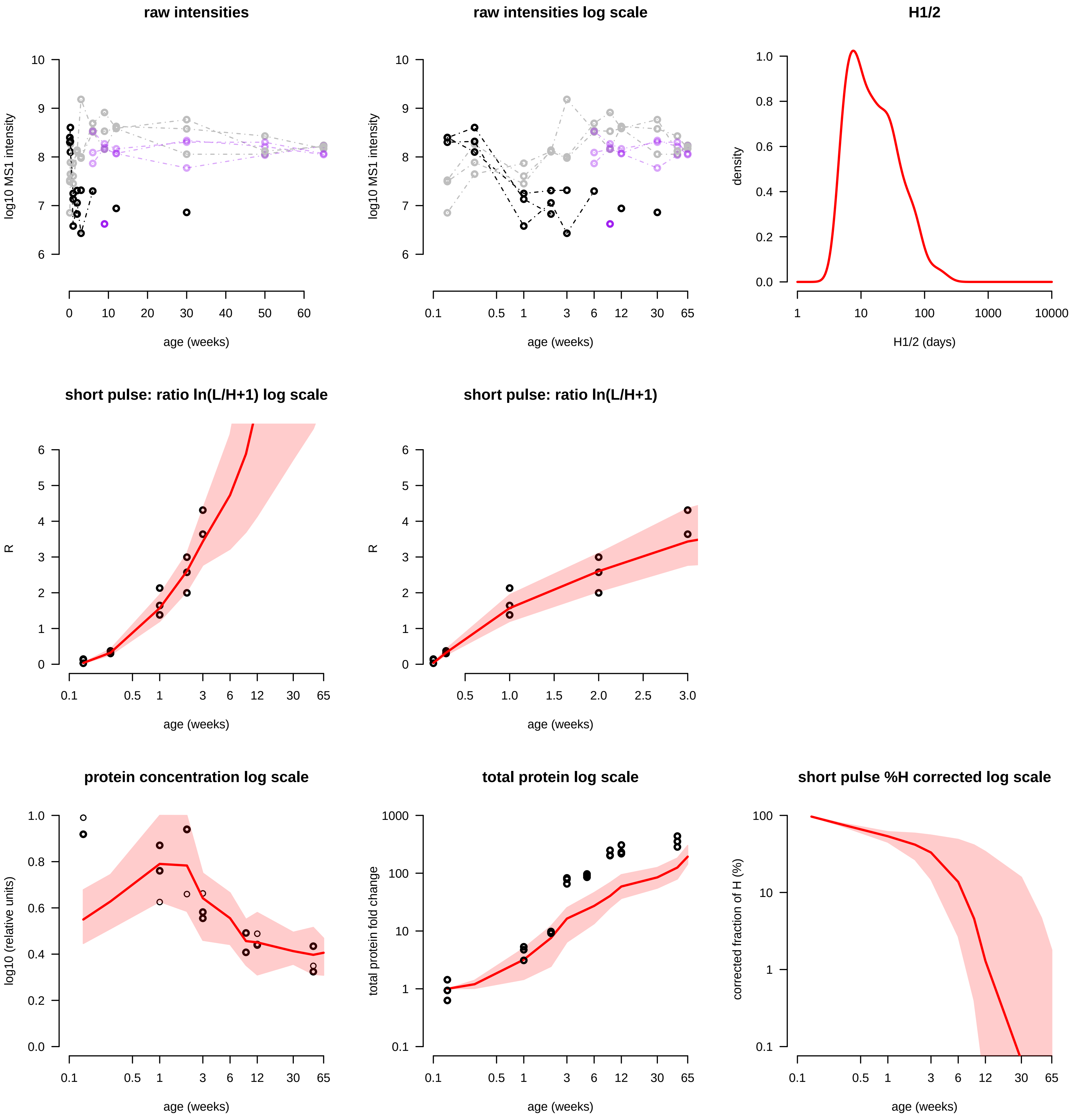

Q91X97

>sp|Q91X97|NCALD_MOUSE

Neurocalcin−delta

GN=Ncald

Med H1/2: 13.9 days

Q5 H1/2: 4.6 days

Q95 H1/2: 348 days

reliable model fit: yes

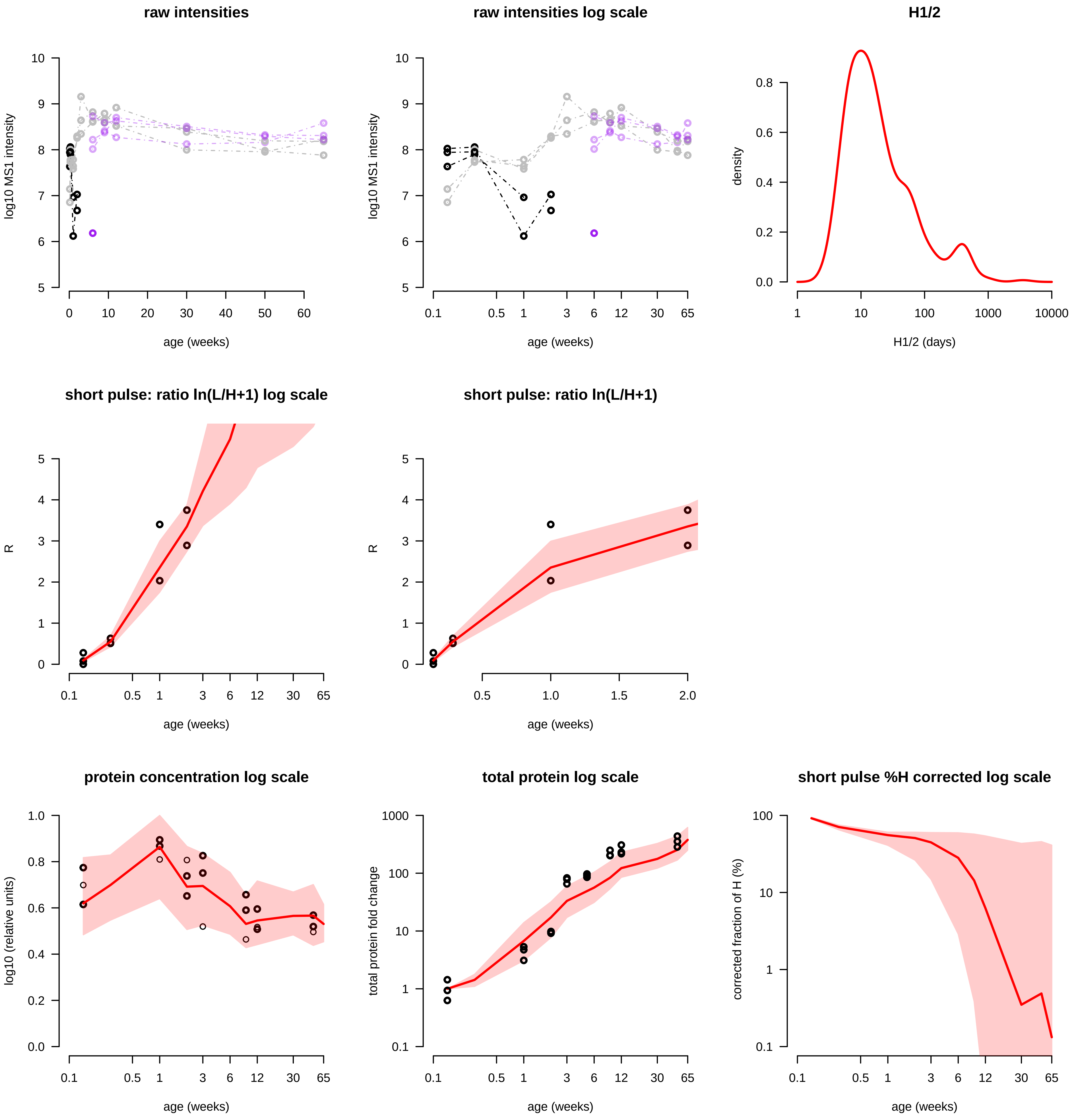

P61458

>sp|P61458|PHS_MOUSE

Pterin−4−alpha−carbinolamine dehydratase

GN=Pcbd1

Med H1/2: 5.5 days

Q5 H1/2: 4.2 days

Q95 H1/2: 8.6 days

reliable model fit: yes

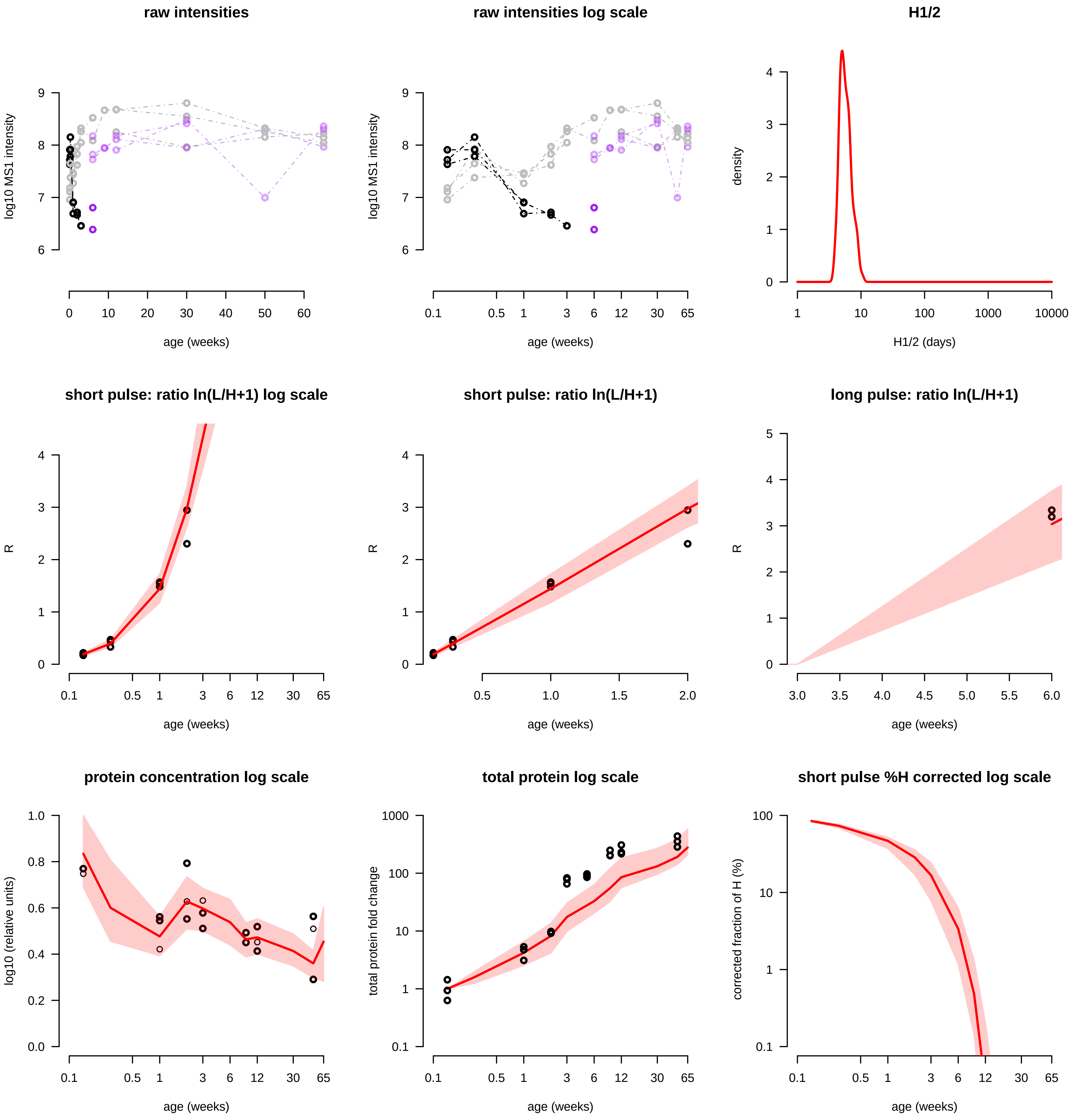

Q8C0D5

>sp|Q8C0D5|EFL1_MOUSE

Elongation factor−like GTPase 1

GN=Efl1

Med H1/2: 23.8 days

Q5 H1/2: 5.3 days

Q95 H1/2: 450.9 days

reliable model fit: no

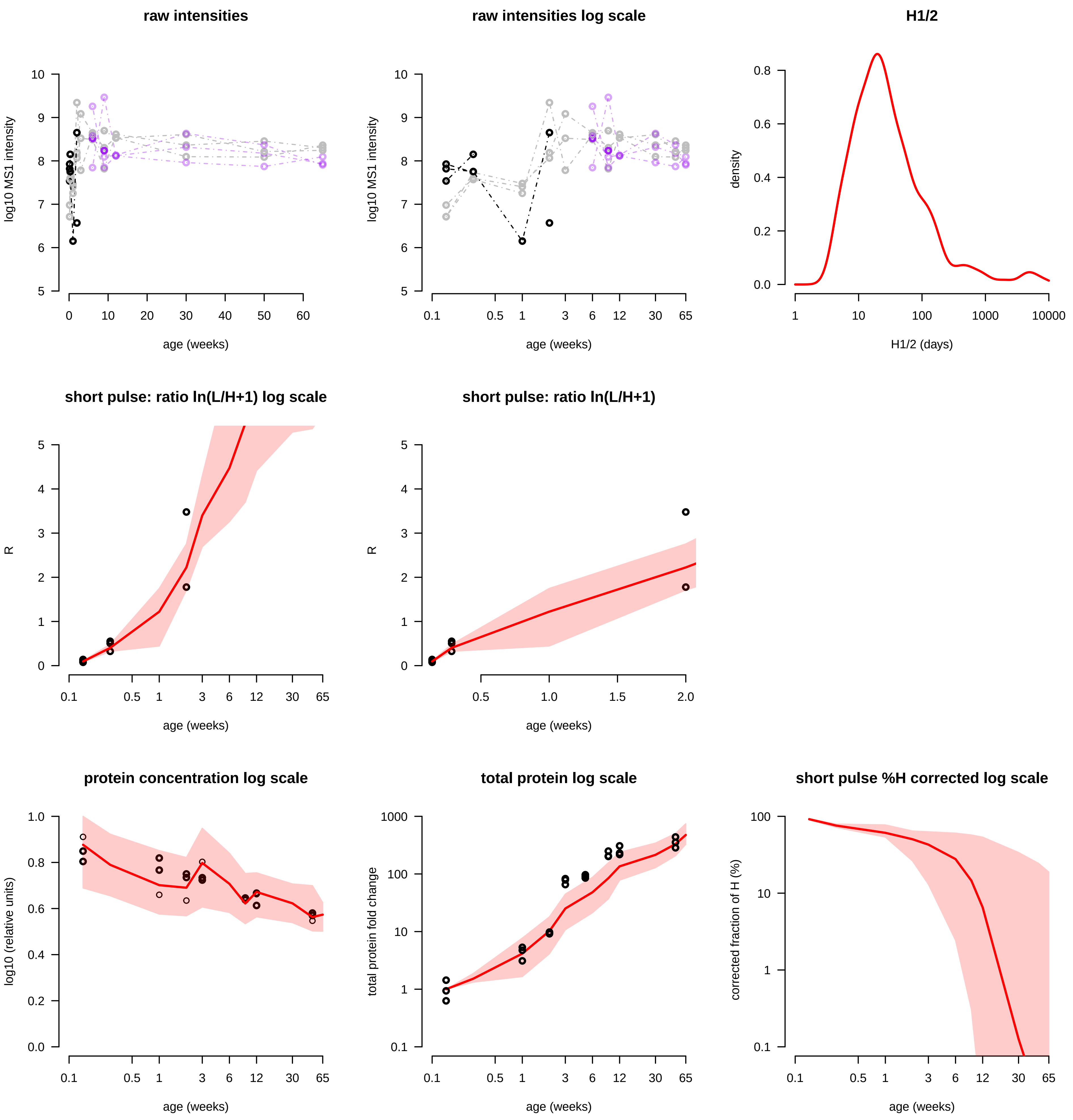

Q9D2U9;Q8CGP0

>sp|Q9D2U9|H2B3A_MOUSE

Histone H2B type 3−A

GN=Hist3h2ba

Med H1/2: 27.4 days

Q5 H1/2: 8.1 days

Q95 H1/2: 267 days

reliable model fit: yes

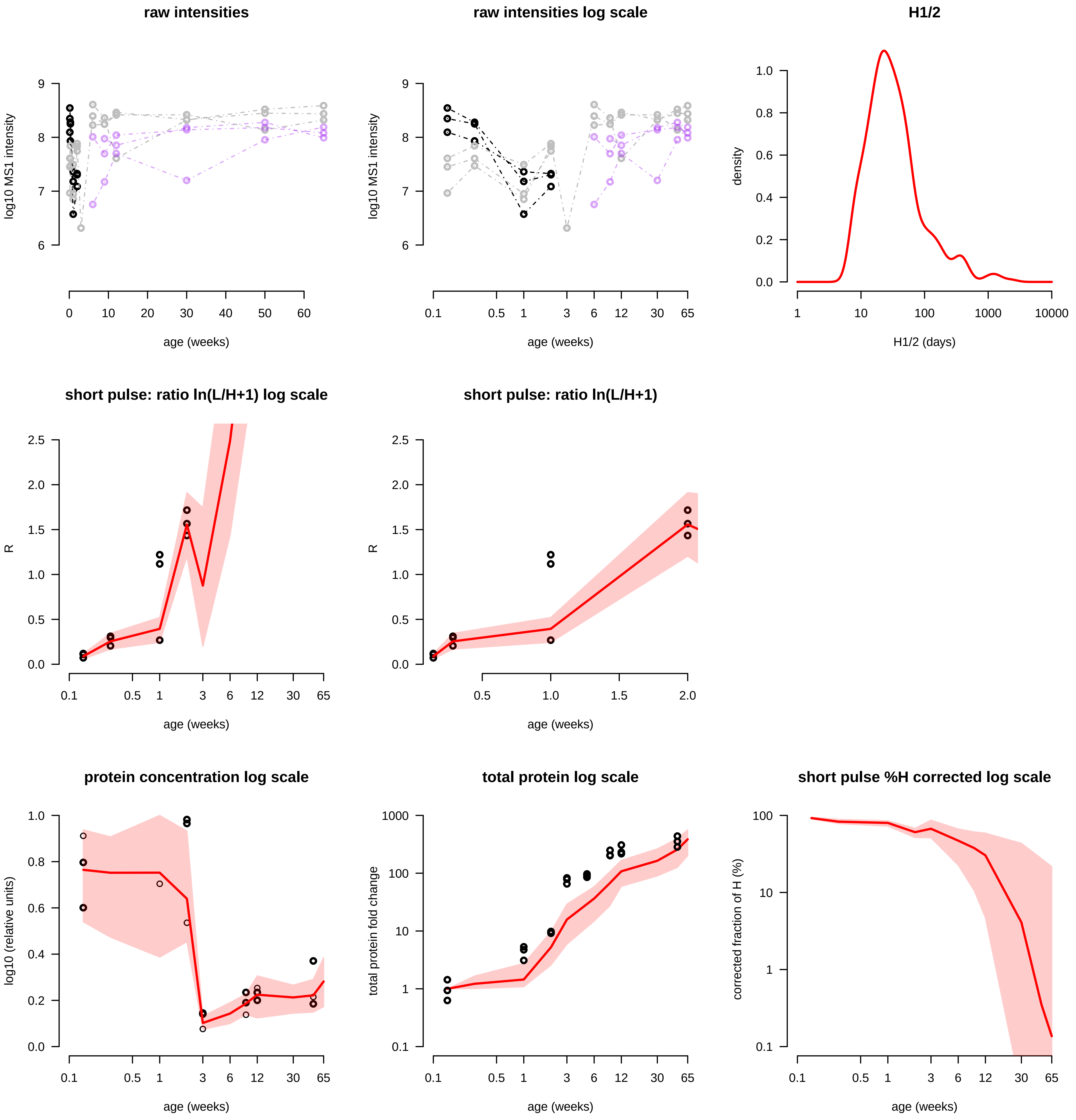

P97760

>sp|P97760|RPB3_MOUSE

DNA−directed RNA polymerase II subunit RPB3

GN=Polr2c

Med H1/2: 4 days

Q5 H1/2: 3.2 days

Q95 H1/2: 5.4 days

reliable model fit: yes

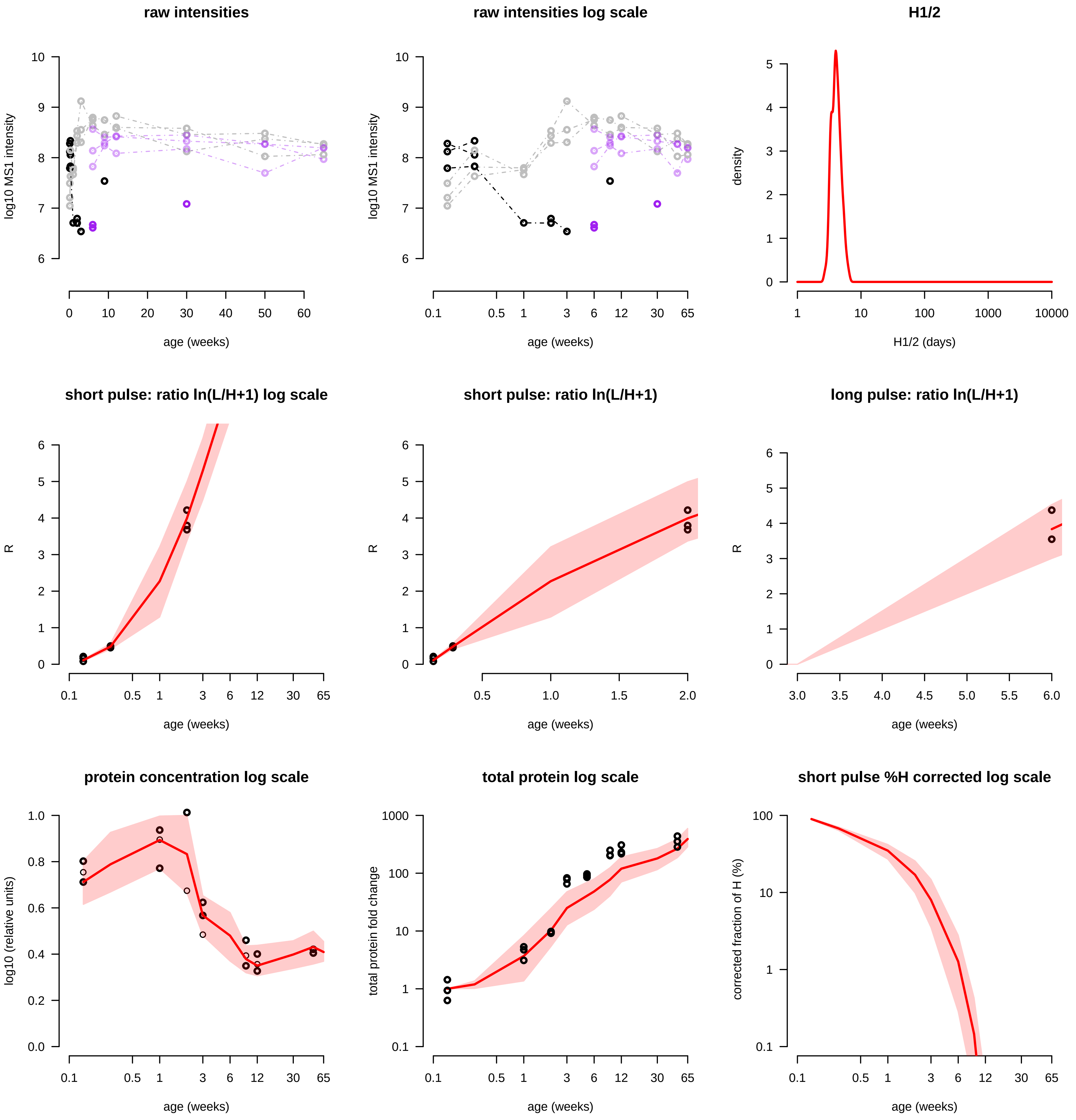

Q9CQP0

>sp|Q9CQP0|RM33_MOUSE

39S ribosomal protein L33, mitochondrial

GN=Mrpl33

Med H1/2: 9.4 days

Q5 H1/2: 3 days

Q95 H1/2: 71.5 days

reliable model fit: yes

raw intensities

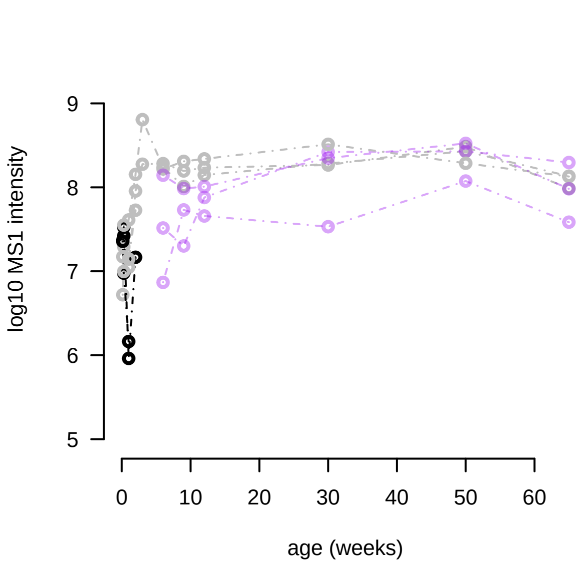

raw intensities log scale

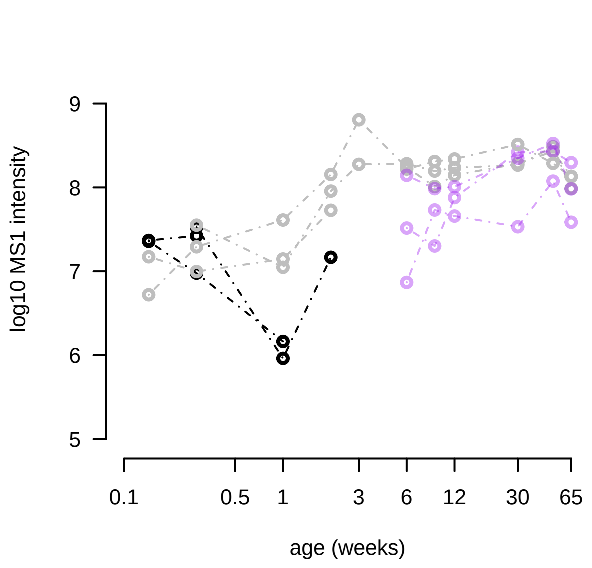

H1/2

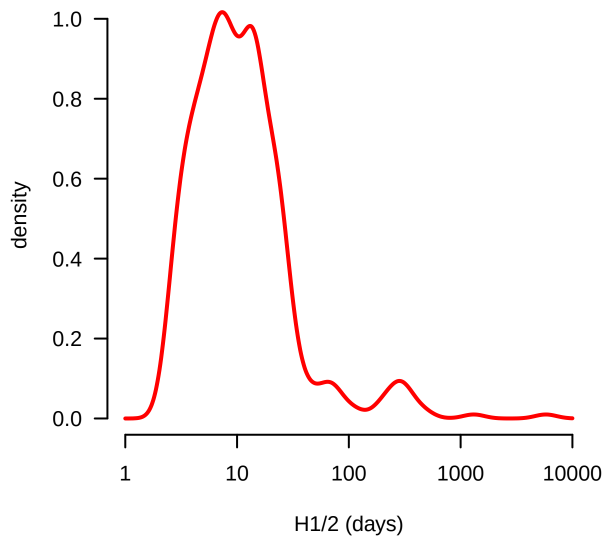

short pulse: ratio ln(L/H+1) log scale

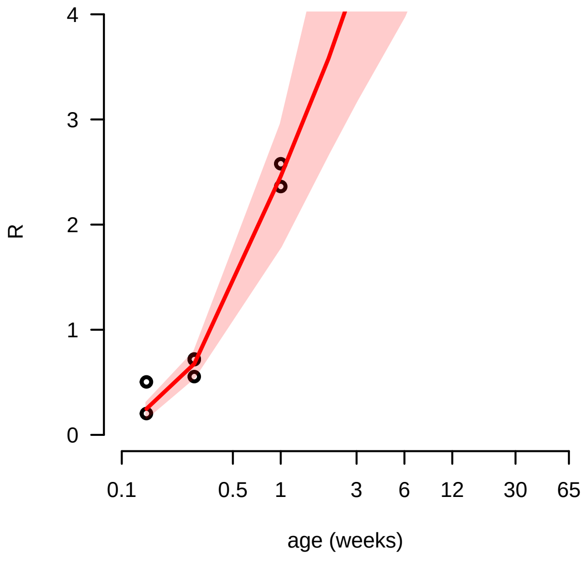

short pulse: ratio ln(L/H+1)

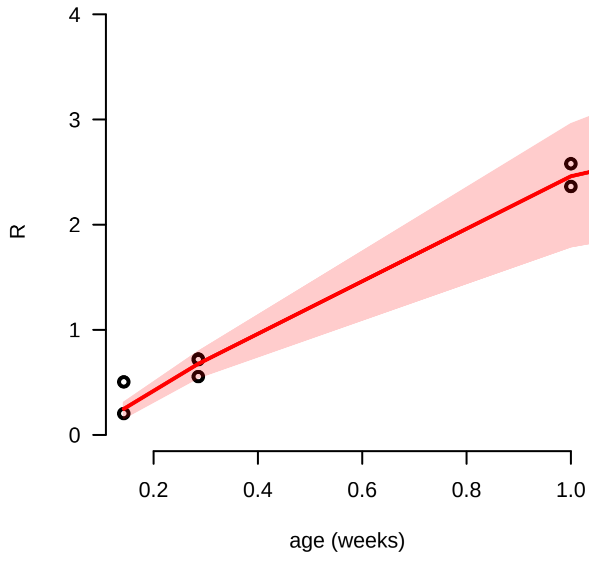

protein concentration log scale

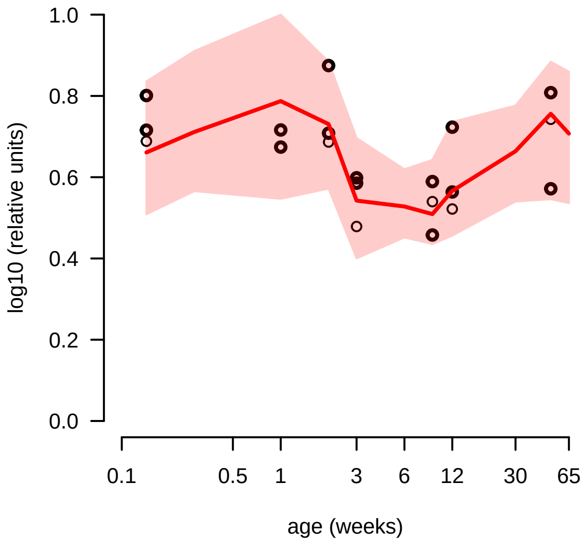

total protein log scale

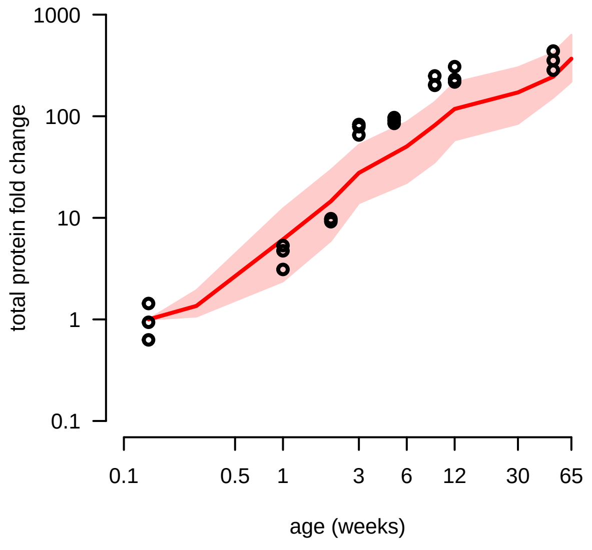

short pulse %H corrected log scale

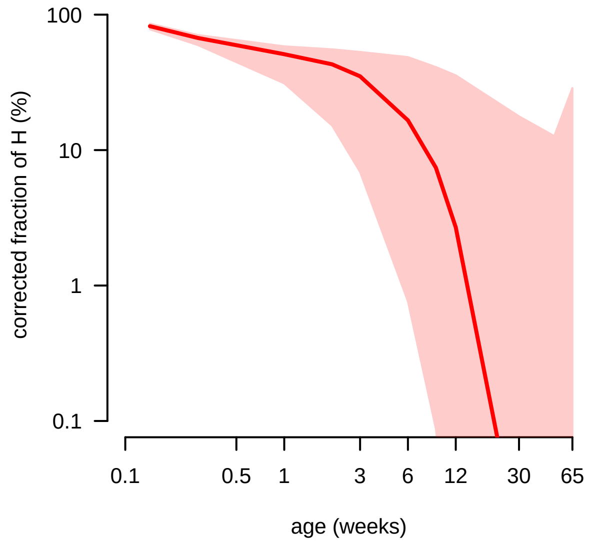

Q9D937

>sp|Q9D937|CK098_MOUSE

Uncharacterized protein C11orf98 homolog

Med H1/2: 6.9 days

Q5 H1/2: 2.8 days

Q95 H1/2: 36.9 days

reliable model fit: yes

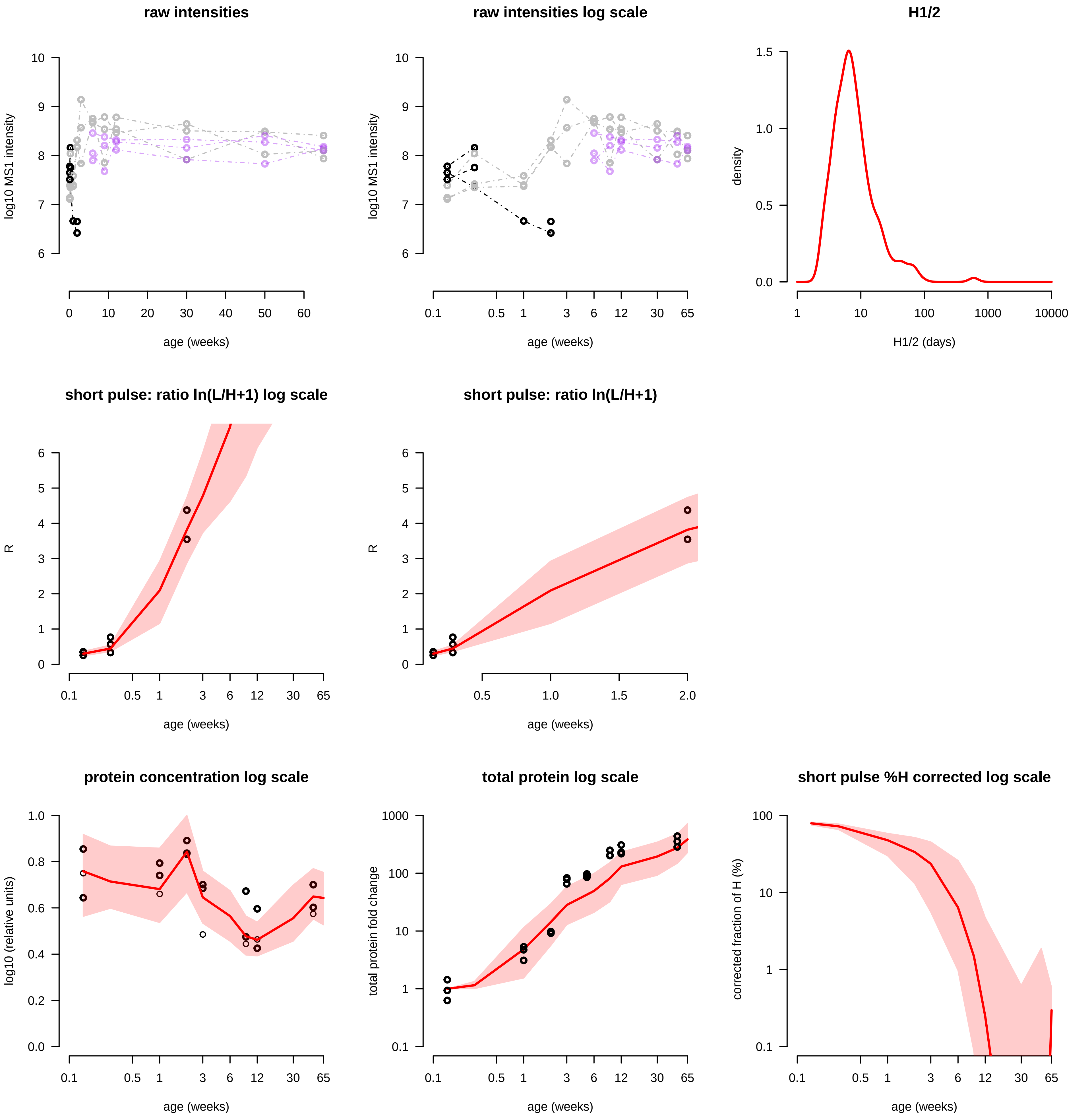

Q9D964

>sp|Q9D964|GATM_MOUSE

Glycine amidinotransferase, mitochondrial

GN=Gatm

Med H1/2: 4.2 days

Q5 H1/2: 2.5 days

Q95 H1/2: 7.6 days

reliable model fit: yes

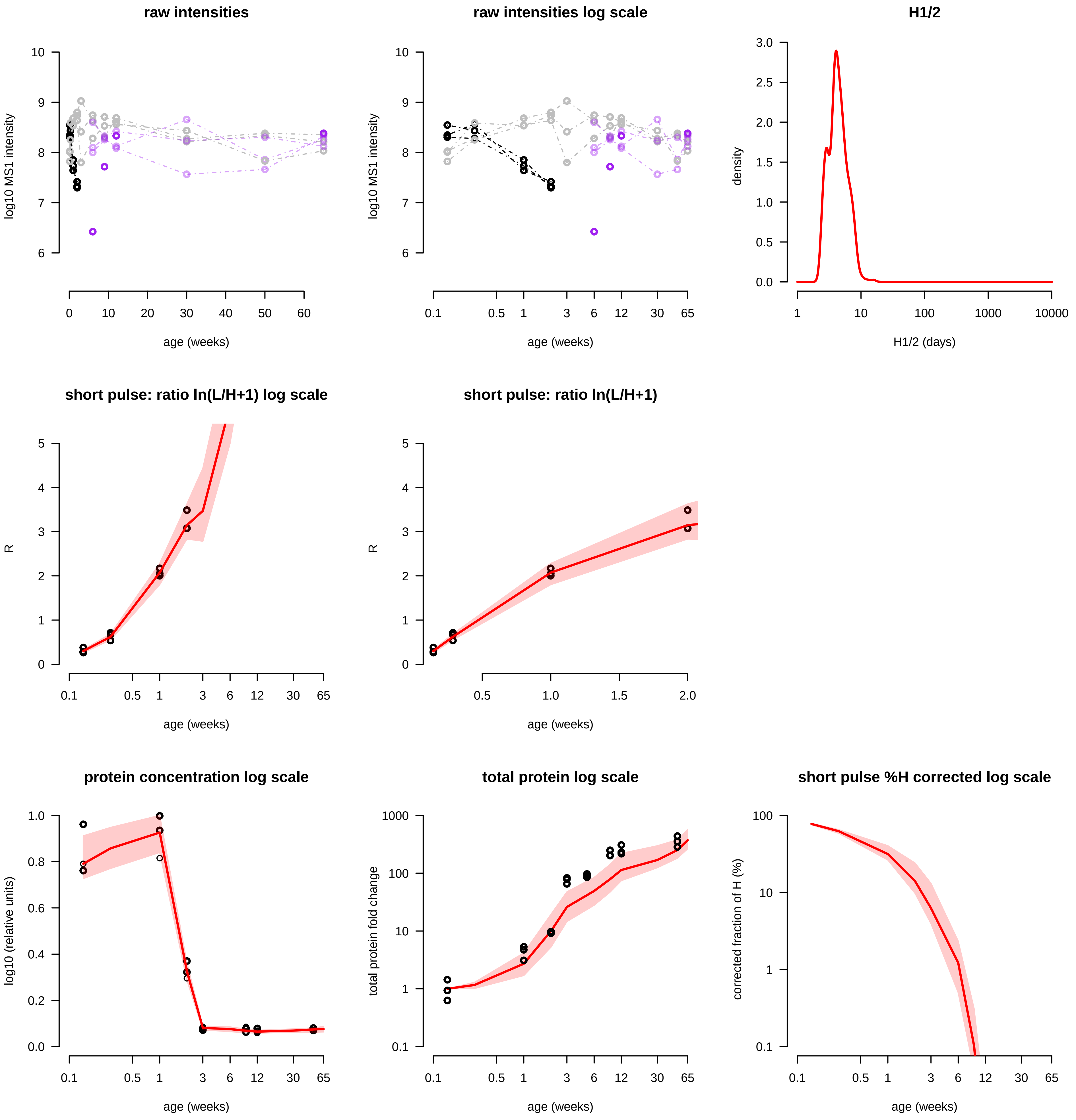

Q9ESJ0

>sp|Q9ESJ0|XPO4_MOUSE

Exportin−4

GN=Xpo4

Med H1/2: 11.5 days

Q5 H1/2: 4.3 days

Q95 H1/2: 49.9 days

reliable model fit: no

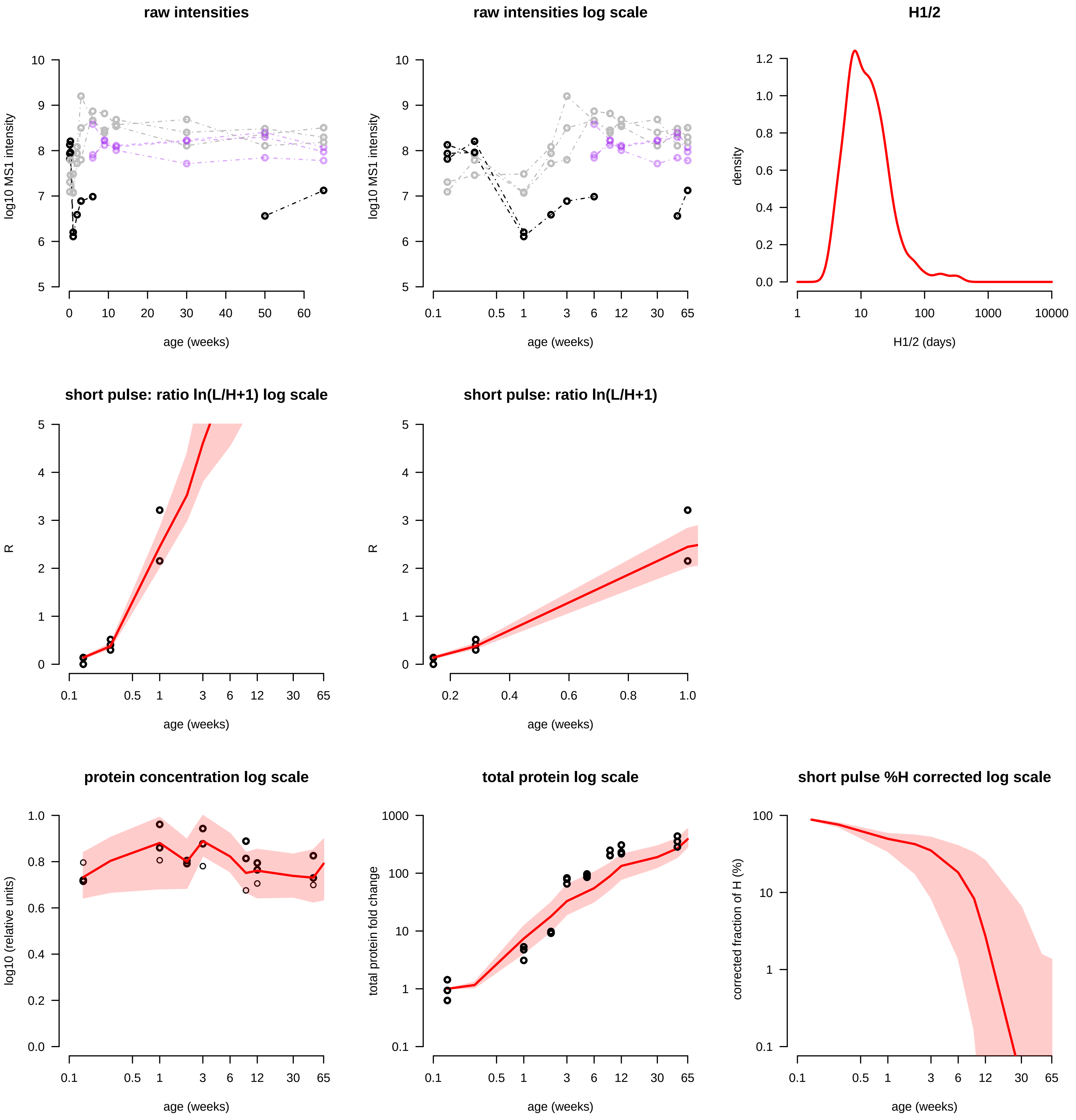

Q9CQK7

>sp|Q9CQK7|RWDD1_MOUSE

RWD domain−containing protein 1

GN=Rwdd1

Med H1/2: 5.6 days

Q5 H1/2: 2.8 days

Q95 H1/2: 68.7 days

reliable model fit: yes

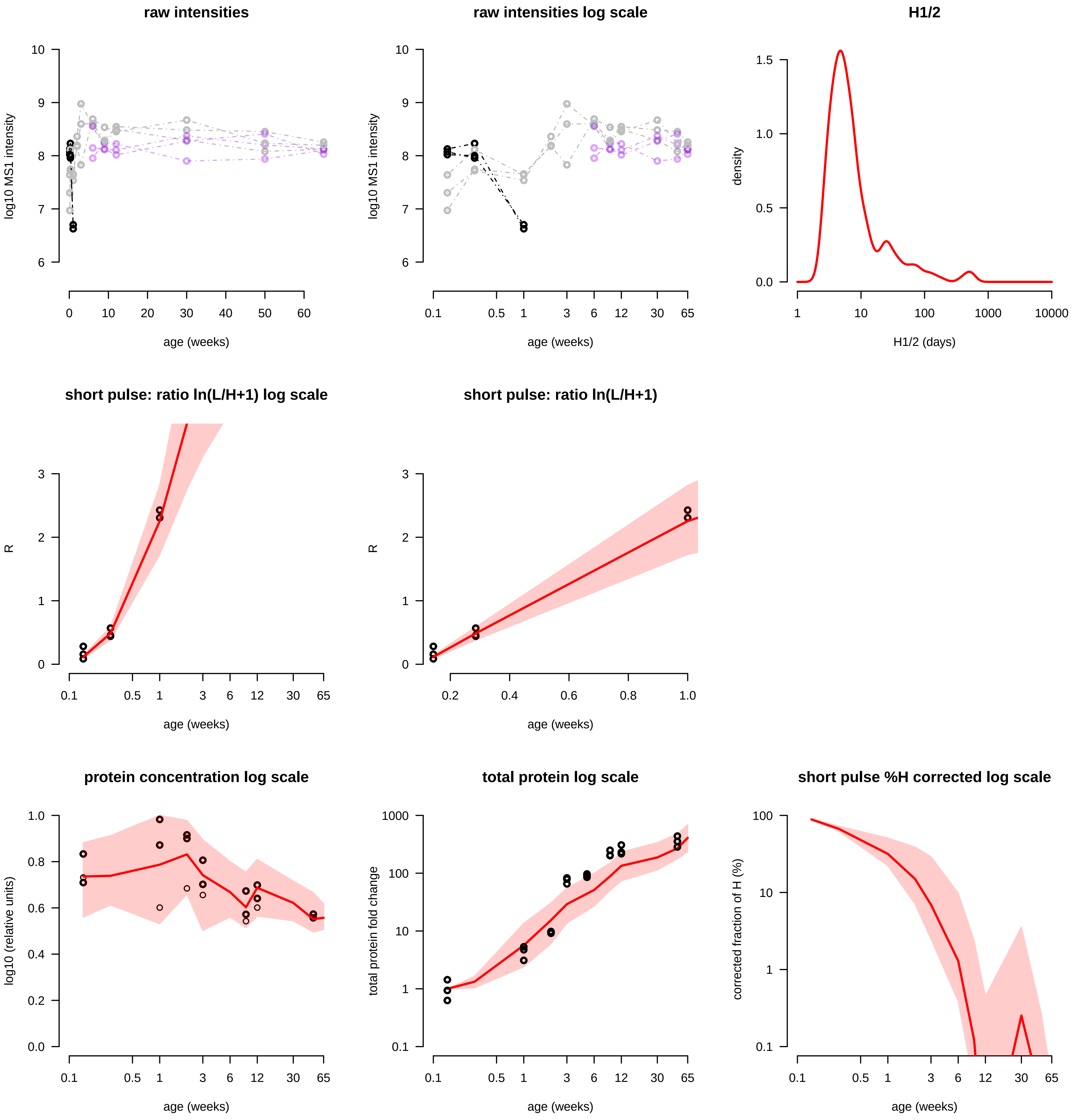

Q8BZH4

>sp|Q8BZH4|POGZ_MOUSE

Pogo transposable element with ZNF domain

GN=Pogz

Med H1/2: 3 days

Q5 H1/2: 2.5 days

Q95 H1/2: 6.1 days

reliable model fit: yes

raw intensities

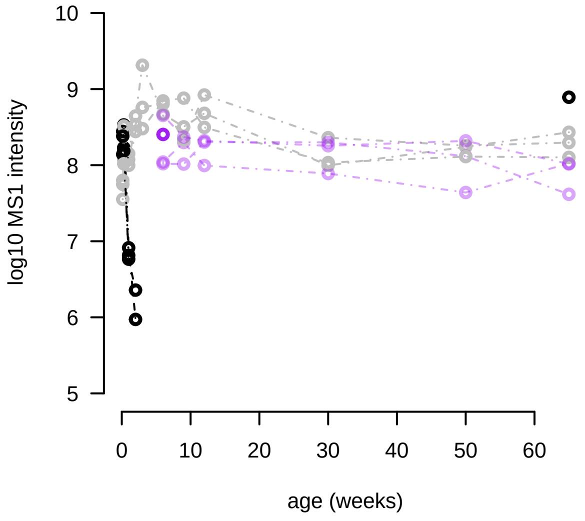

raw intensities log scale

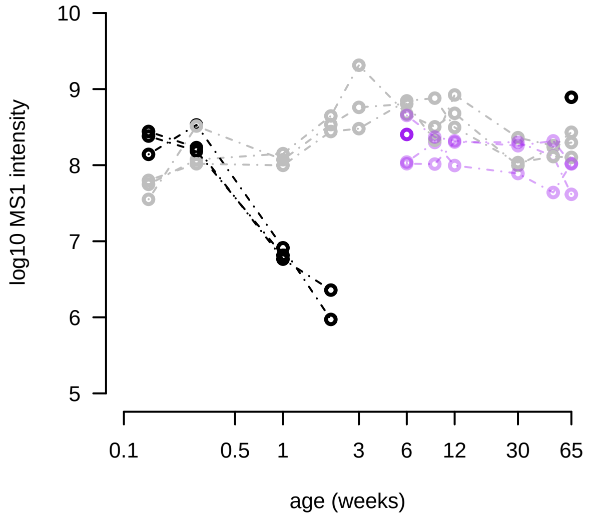

H1/2

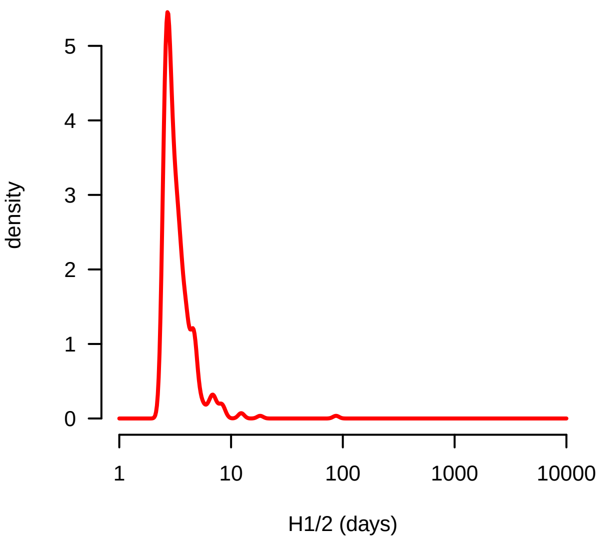

short pulse: ratio ln(L/H+1) log scale

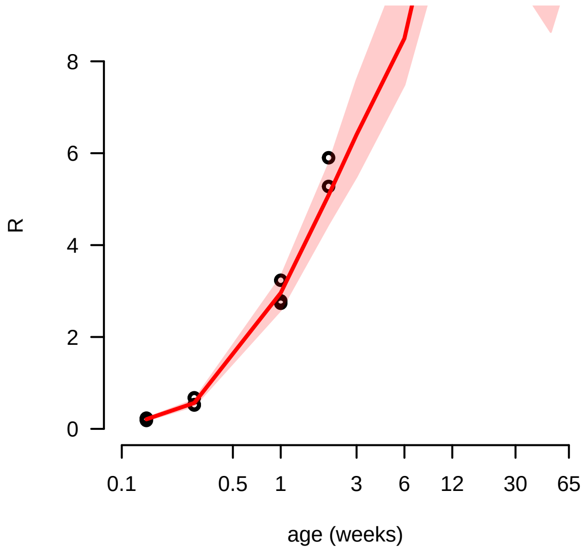

short pulse: ratio ln(L/H+1)

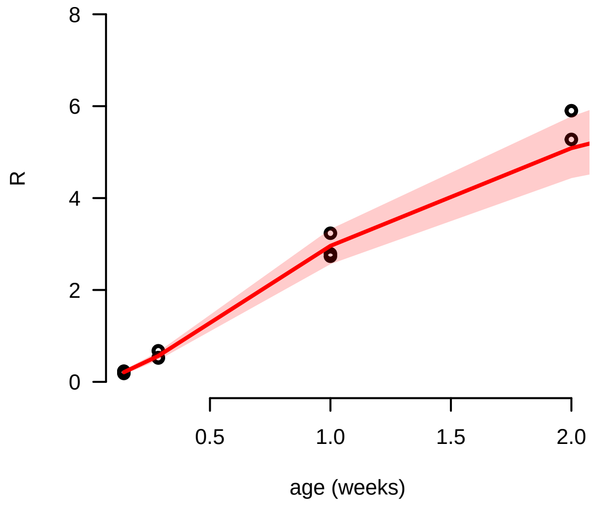

protein concentration log scale

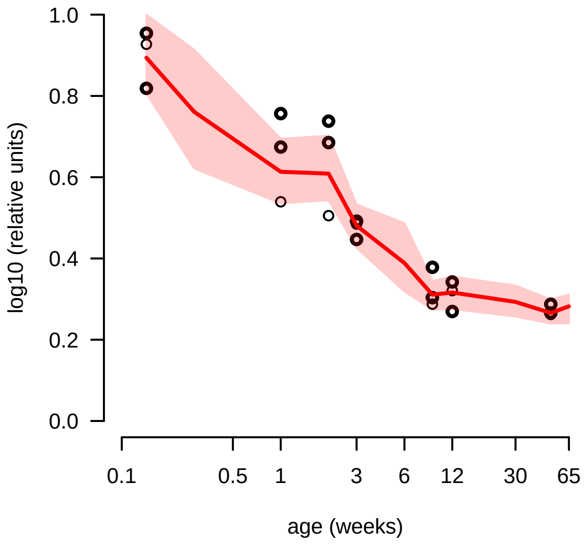

total protein log scale

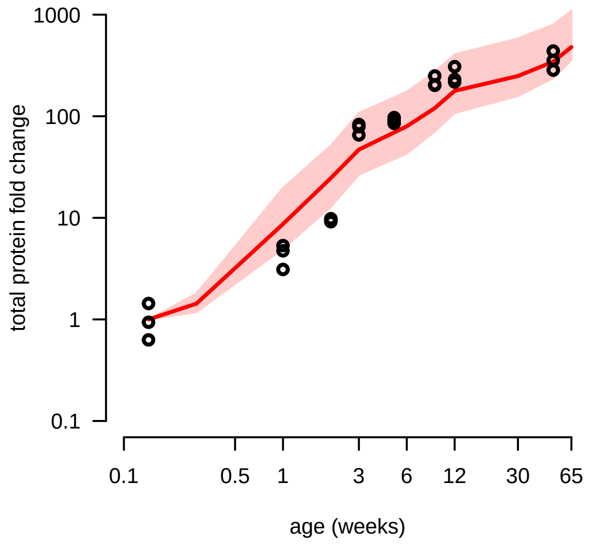

short pulse %H corrected log scale

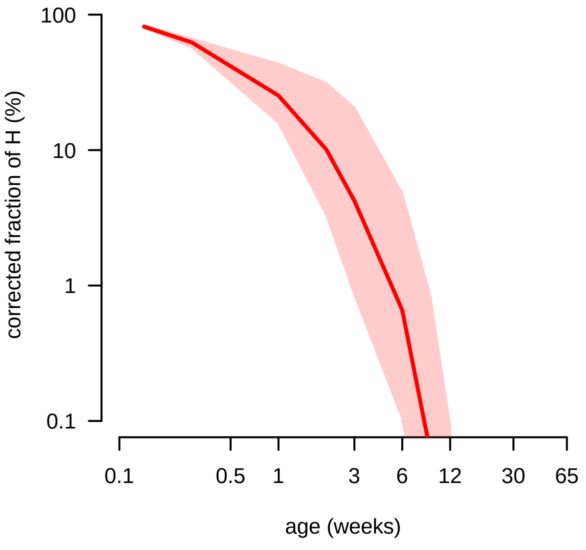

Q52KI8;Q52KI8−2

>sp|Q52KI8|SRRM1_MOUSE

Serine/arginine repetitive matrix protein 1

GN=Srrm1

Med H1/2: 9.3 days

Q5 H1/2: 6.1 days

Q95 H1/2: 25 days

reliable model fit: yes

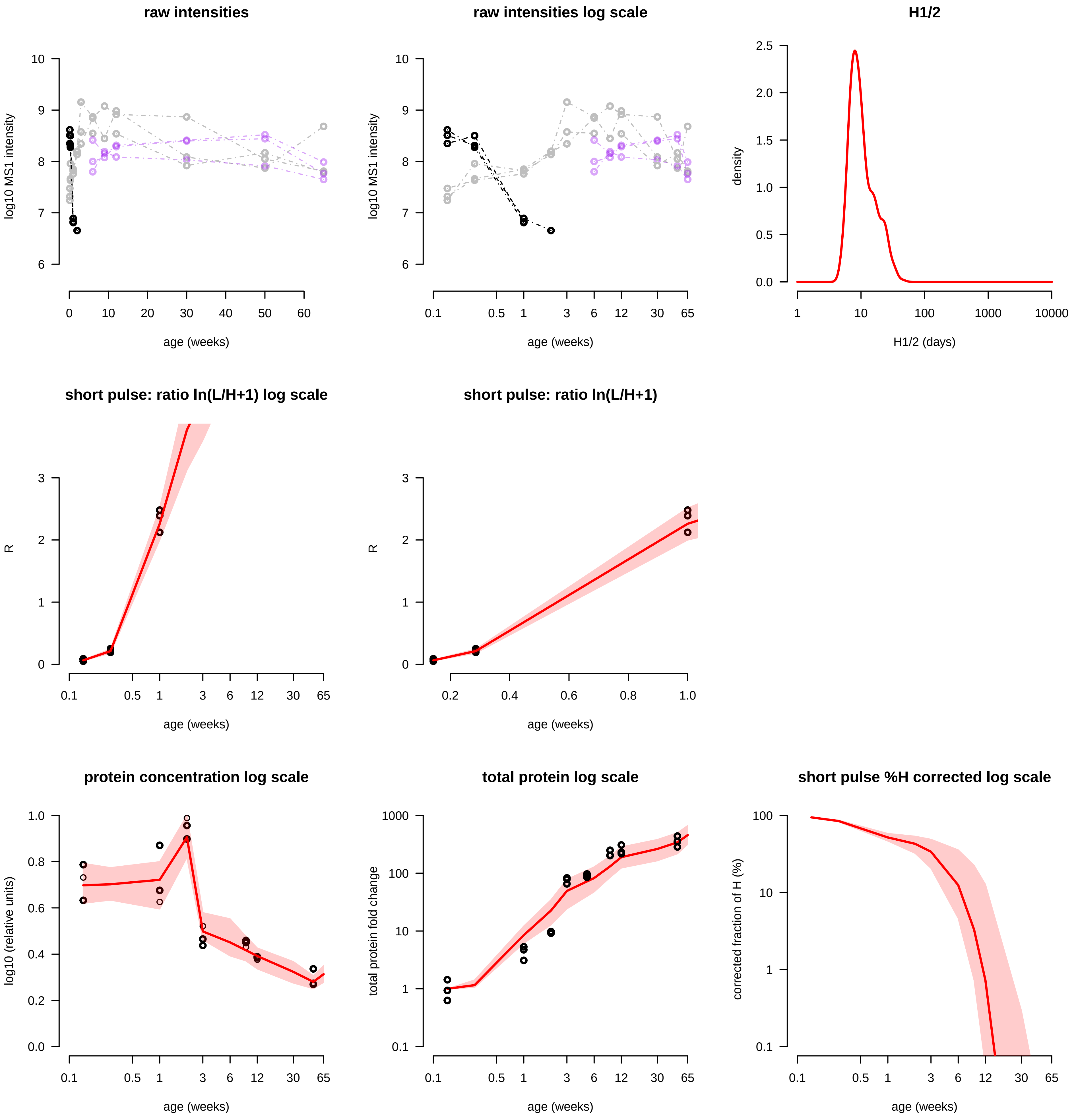

Q9ET22

>sp|Q9ET22|DPP2_MOUSE

Dipeptidyl peptidase 2

GN=Dpp7

Med H1/2: 4.4 days

Q5 H1/2: 2.5 days

Q95 H1/2: 12.1 days

reliable model fit: yes

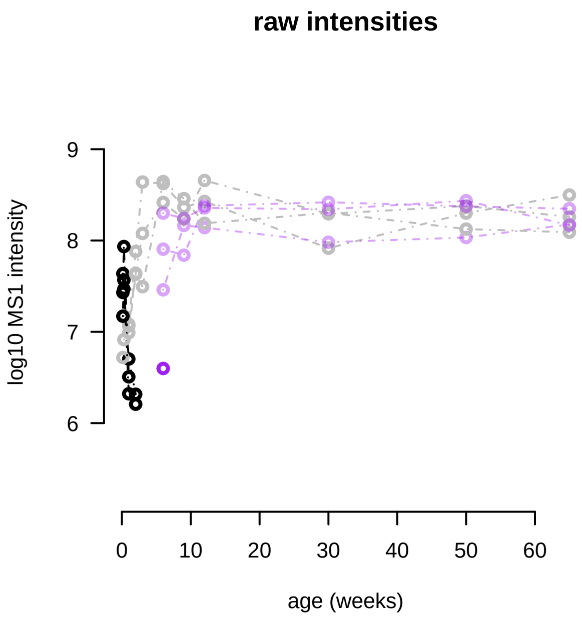

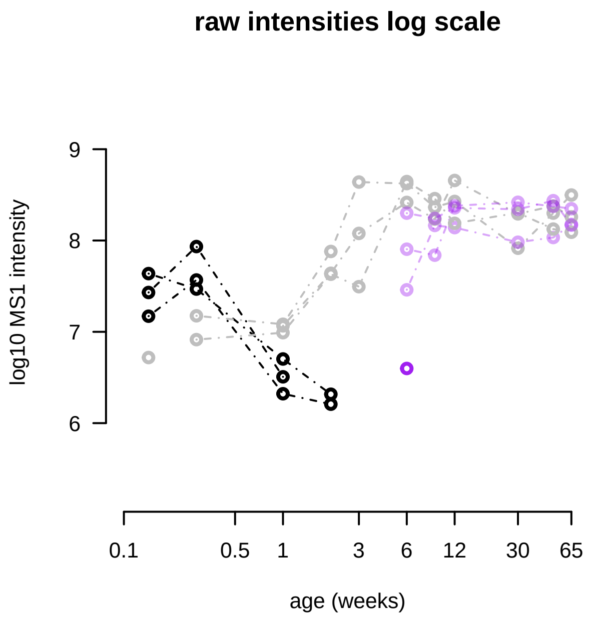

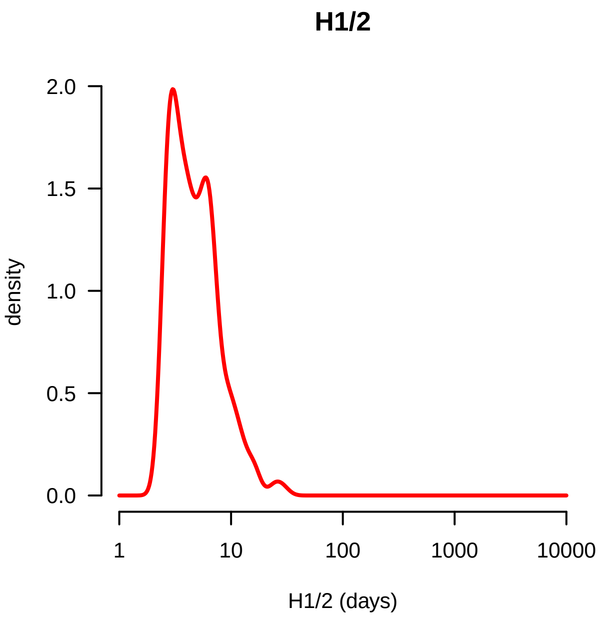

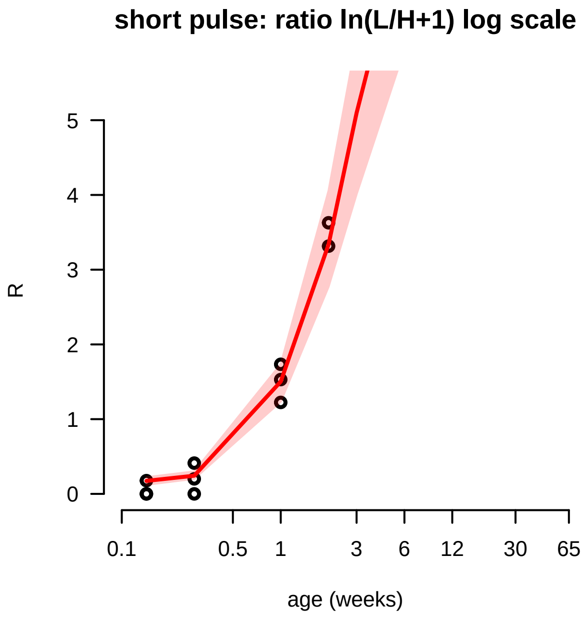

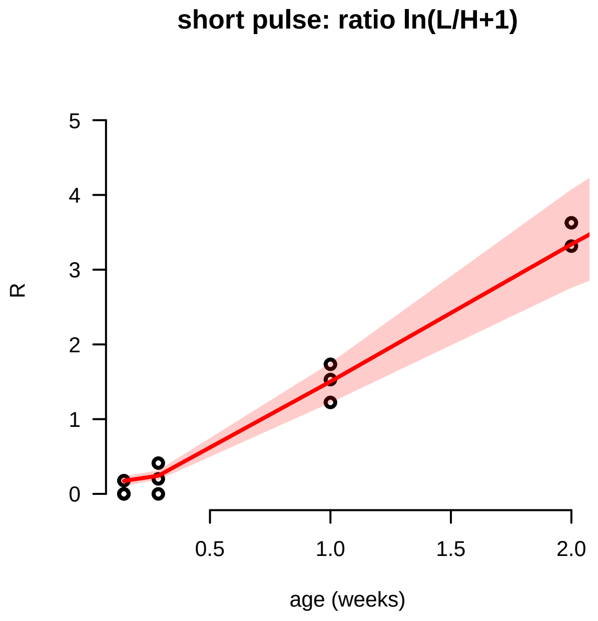

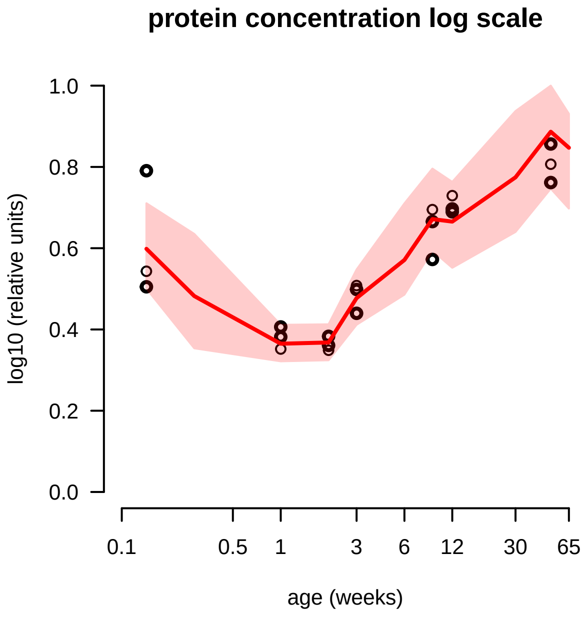

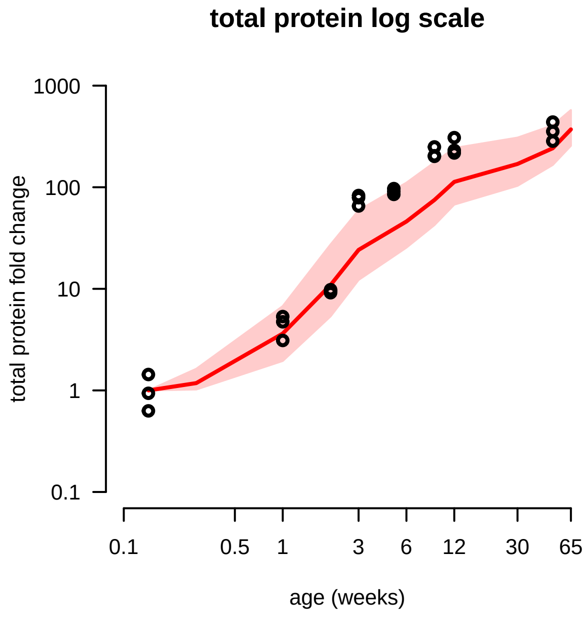

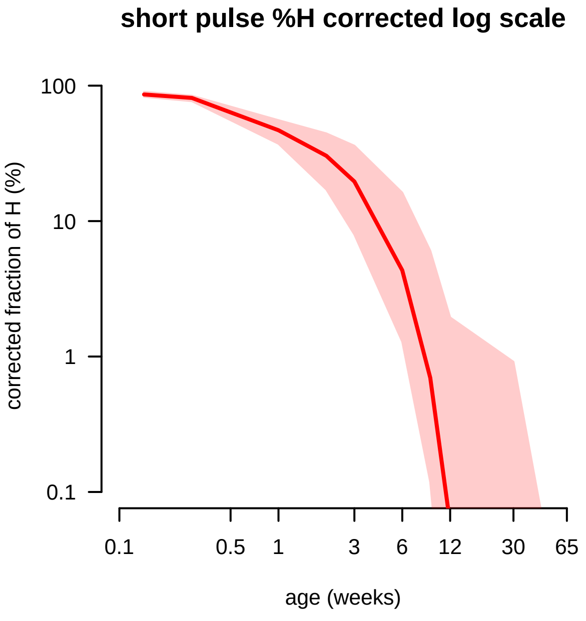

Q924T2

>sp|Q924T2|RT02_MOUSE

28S ribosomal protein S2, mitochondrial

GN=Mrps2

Med H1/2: 7.1 days

Q5 H1/2: 2.7 days

Q95 H1/2: 54.3 days

reliable model fit: yes

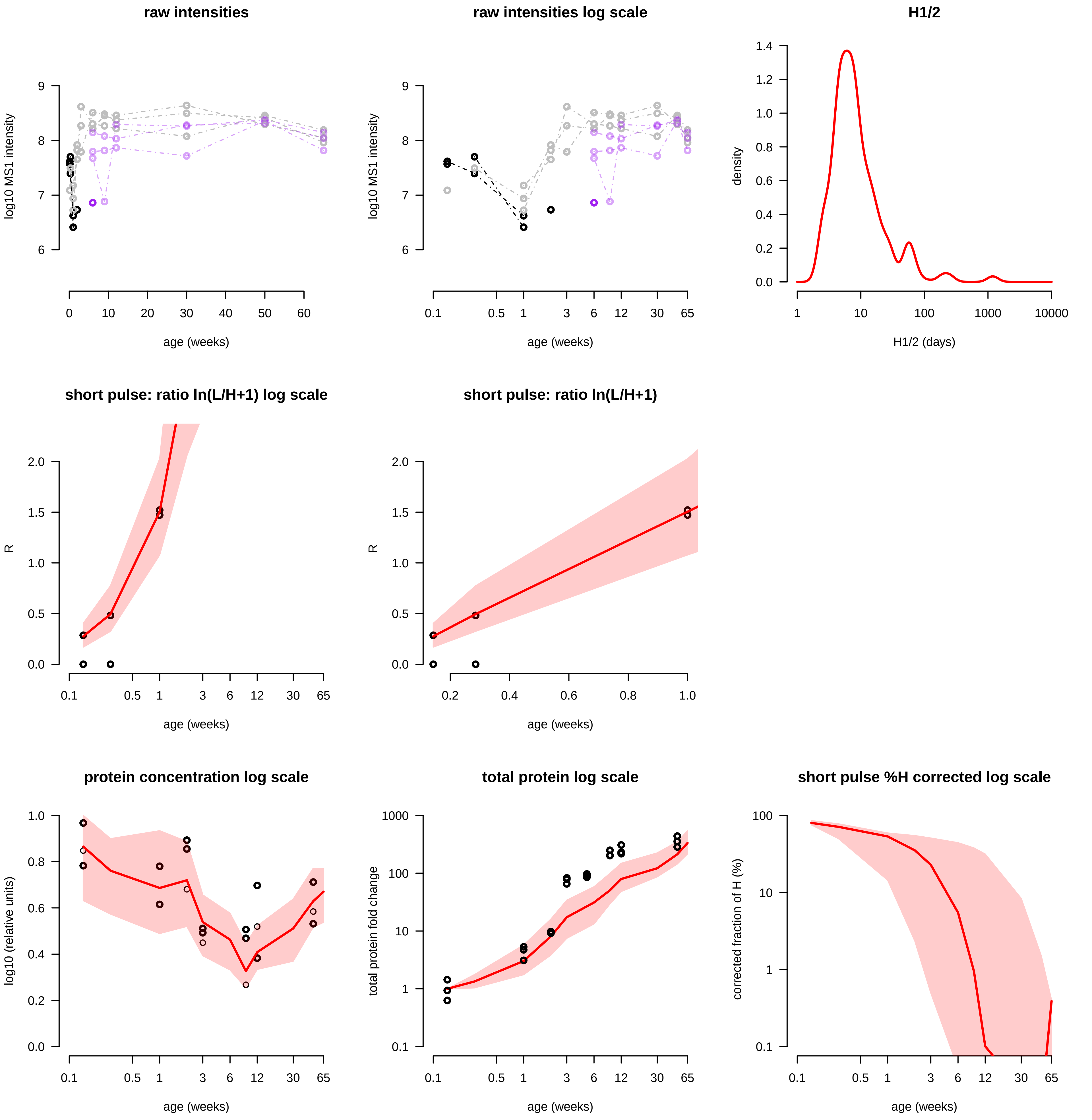

Q5RL79

>sp|Q5RL79|KTAP2_MOUSE

Keratinocyte−associated protein 2

GN=Krtcap2

Med H1/2: 5.8 days

Q5 H1/2: 4.3 days

Q95 H1/2: 8.1 days

reliable model fit: yes

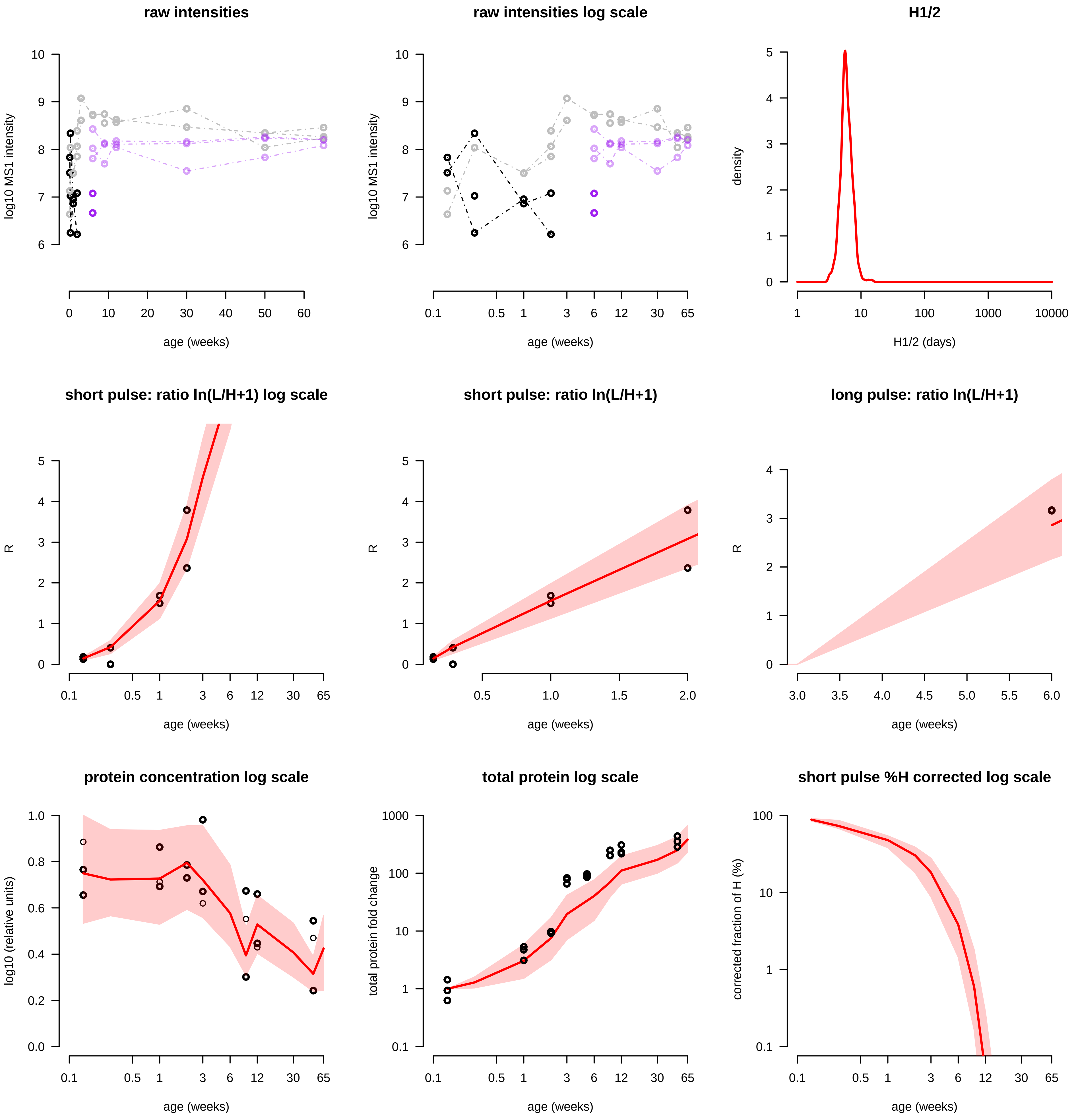

P62320

>sp|P62320|SMD3_MOUSE

Small nuclear ribonucleoprotein Sm D3

GN=Snrpd3

Med H1/2: 6.4 days

Q5 H1/2: 5 days

Q95 H1/2: 9 days

reliable model fit: yes

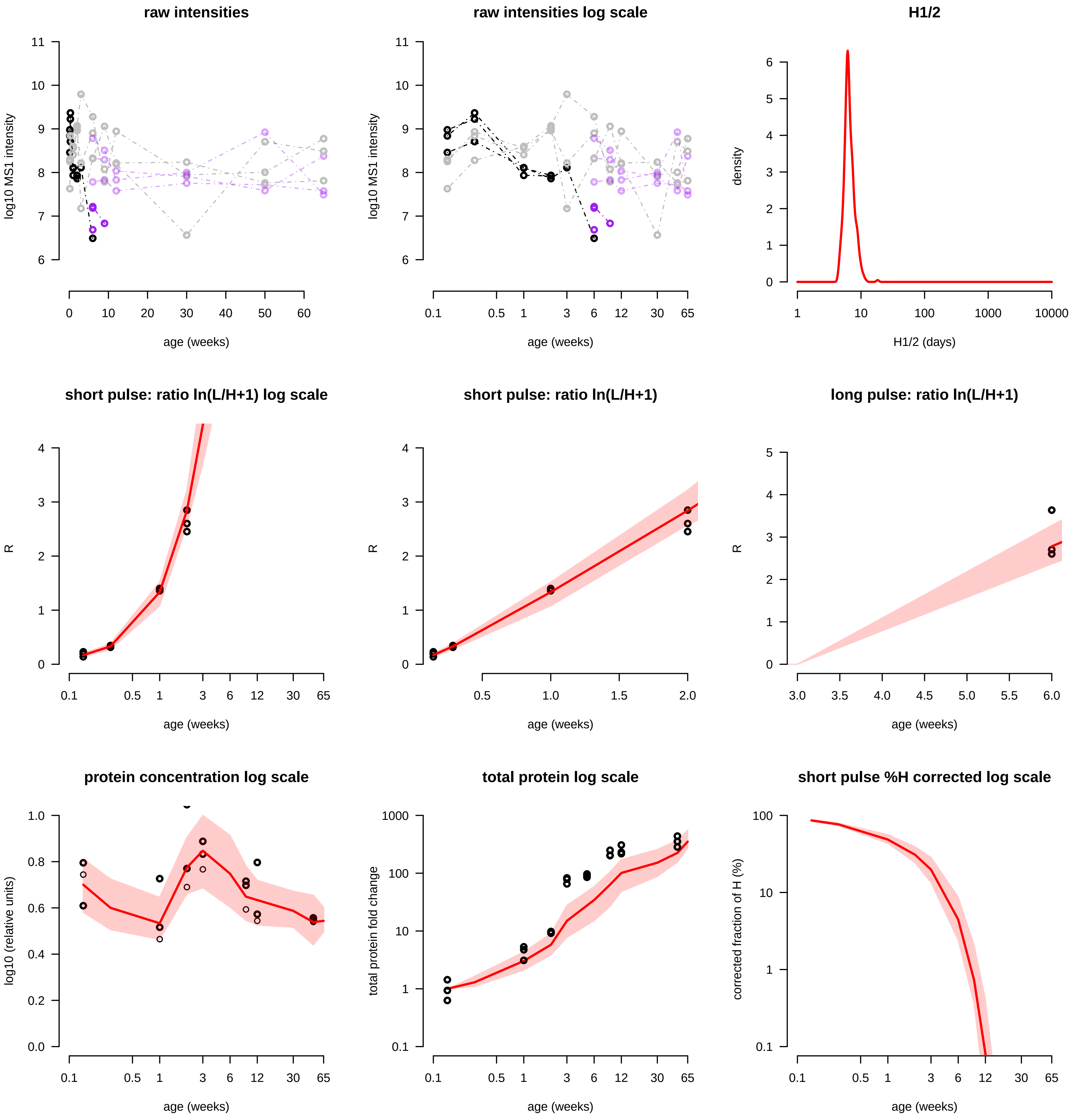

P35283

>sp|P35283|RAB12_MOUSE

Ras−related protein Rab−12

GN=Rab12

Med H1/2: 6.2 days

Q5 H1/2: 2.7 days

Q95 H1/2: 81 days

reliable model fit: yes

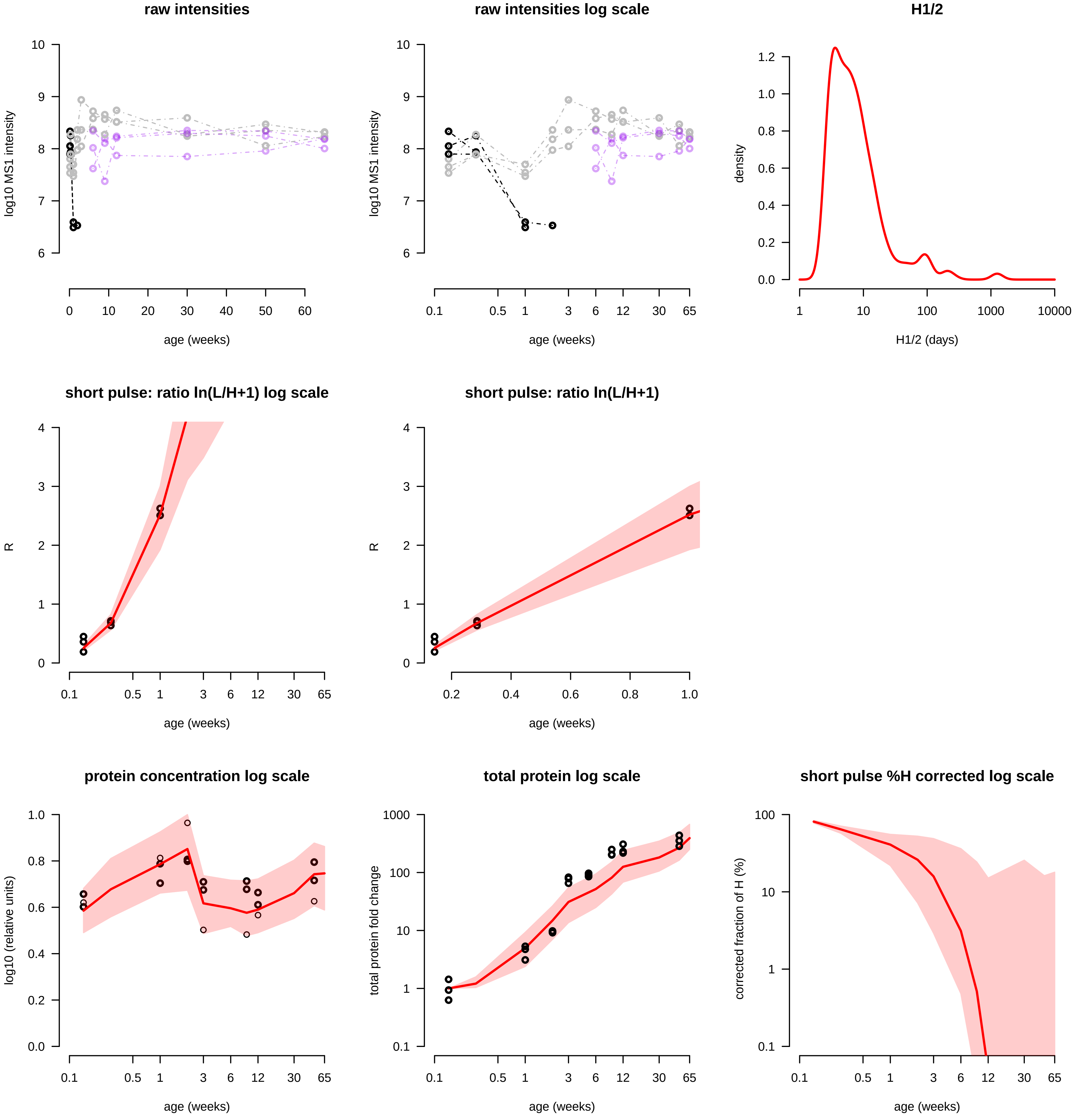

P54731

>sp|P54731|FAF1_MOUSE

FAS−associated factor 1

GN=Faf1

Med H1/2: 6.1 days

Q5 H1/2: 2.7 days

Q95 H1/2: 55 days

reliable model fit: yes

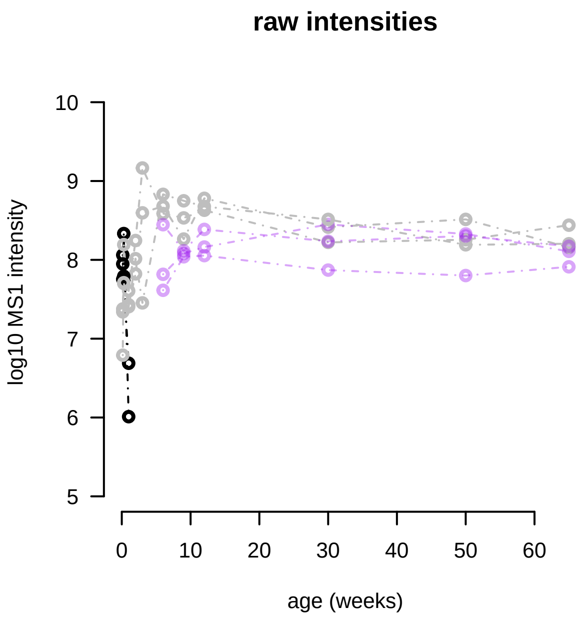

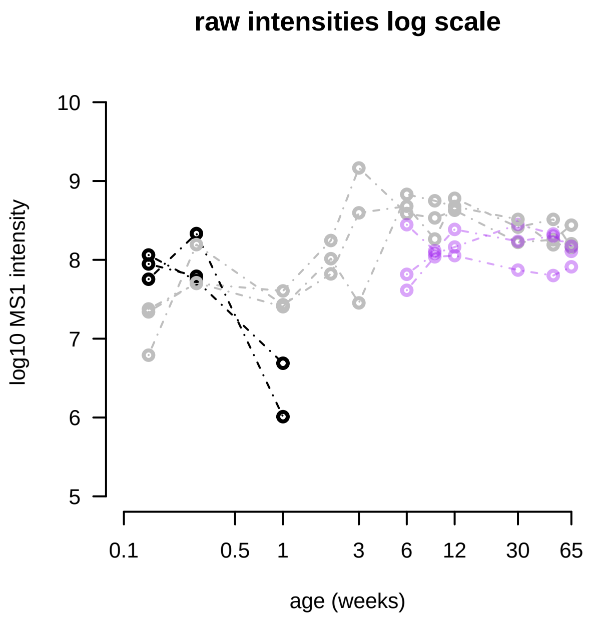

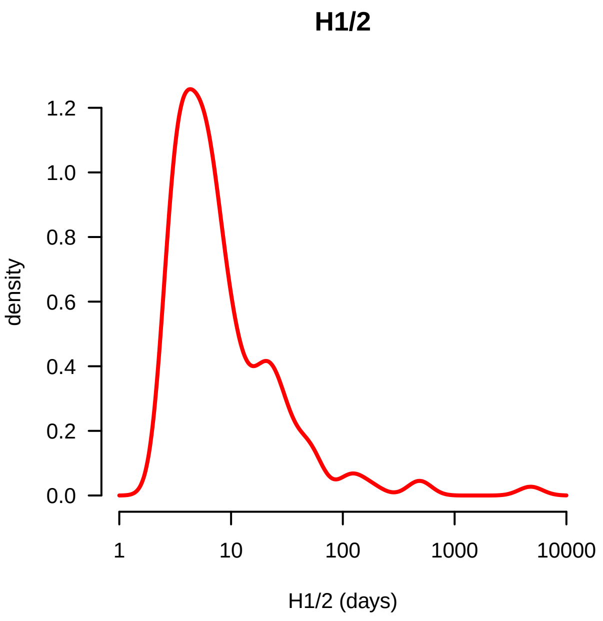

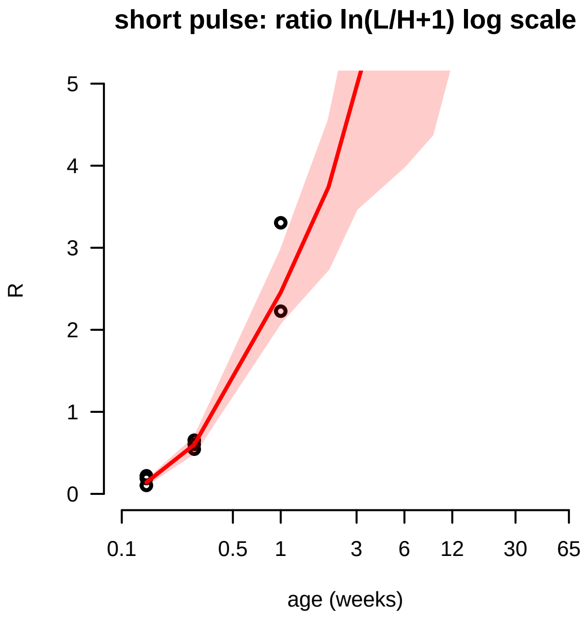

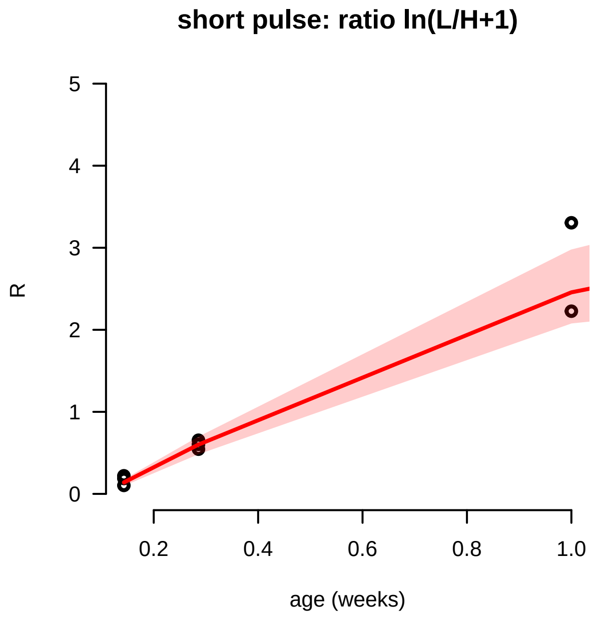

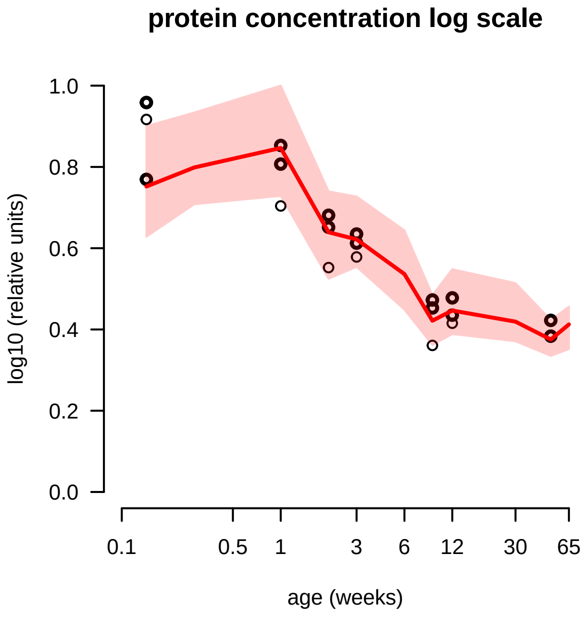

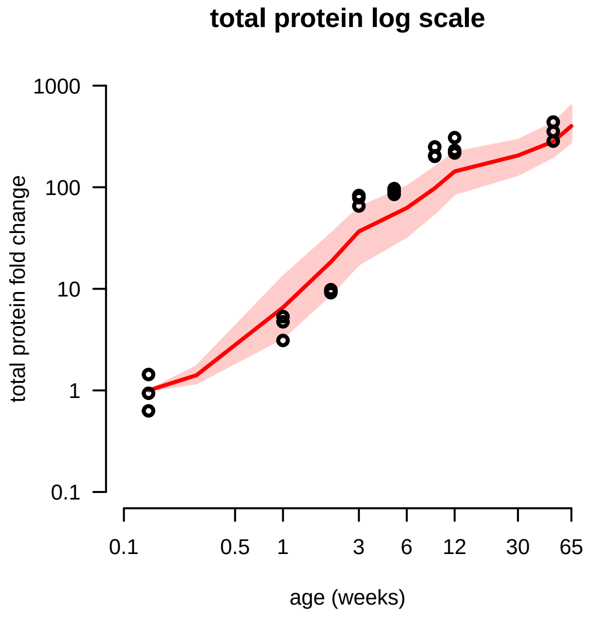

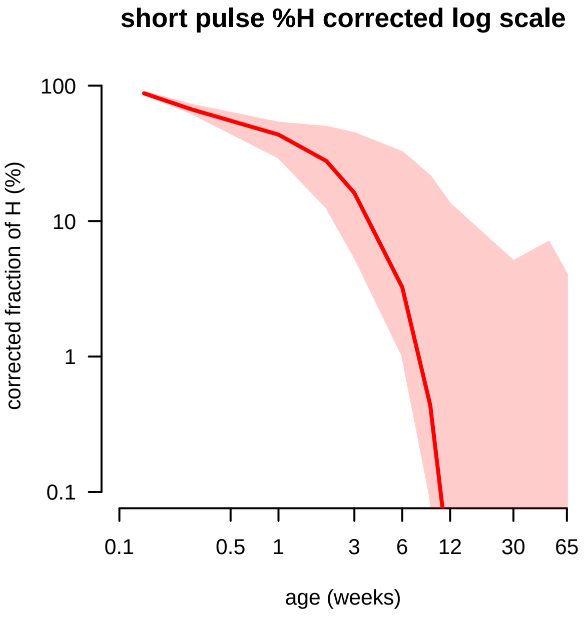

A6H5Y3

>sp|A6H5Y3|METH_MOUSE

Methionine synthase

GN=Mtr

Med H1/2: 3.2 days

Q5 H1/2: 2.5 days

Q95 H1/2: 5.6 days

reliable model fit: yes

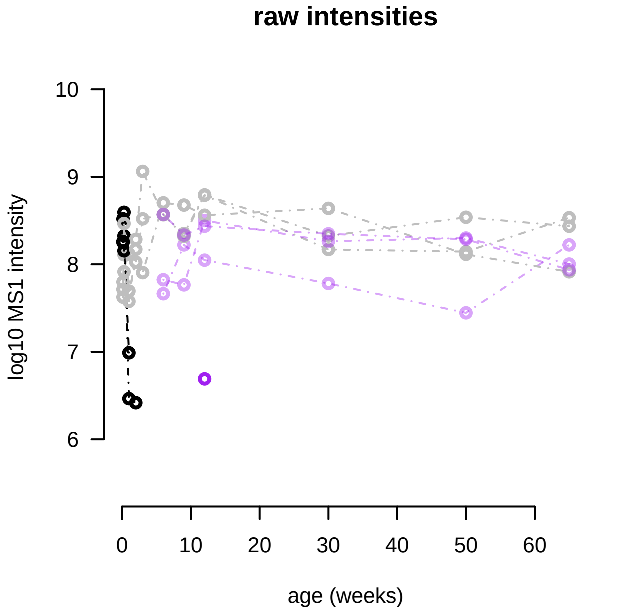

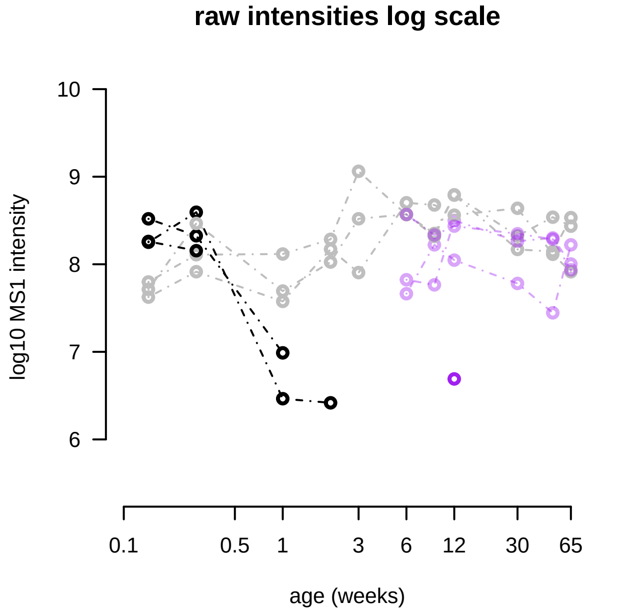

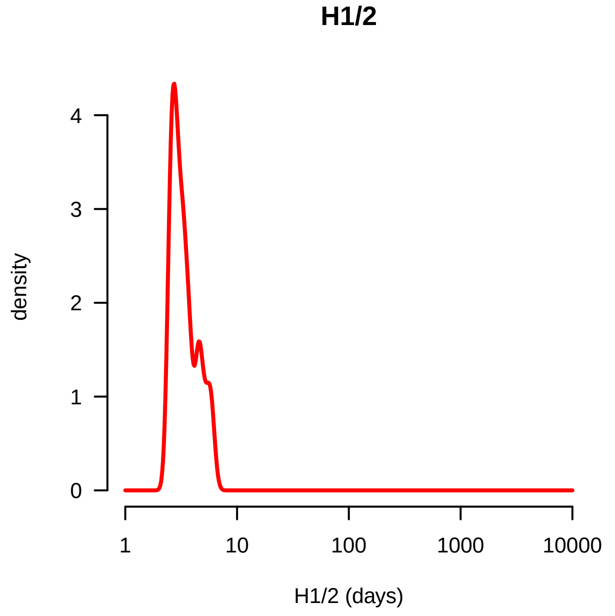

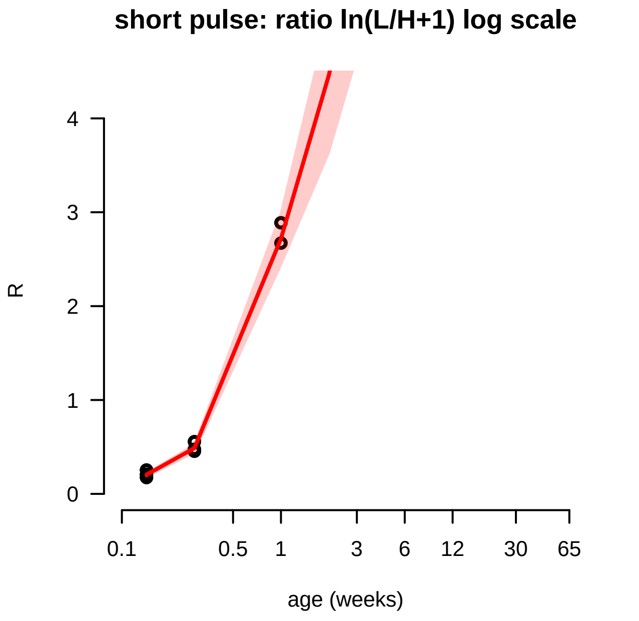

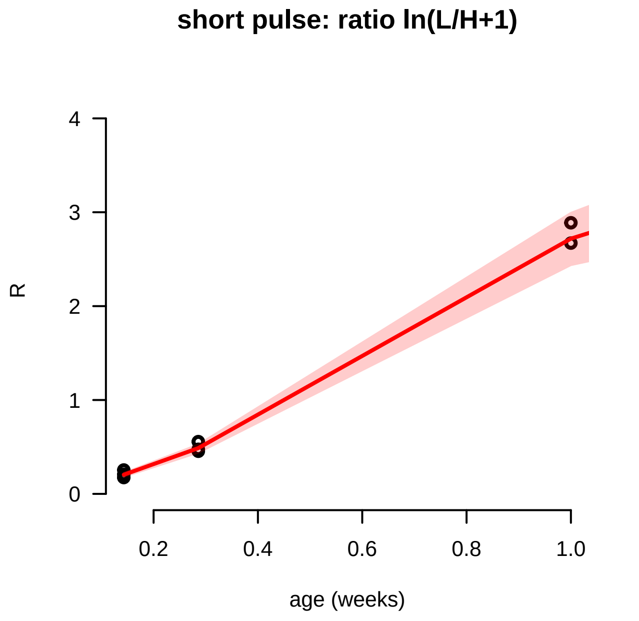

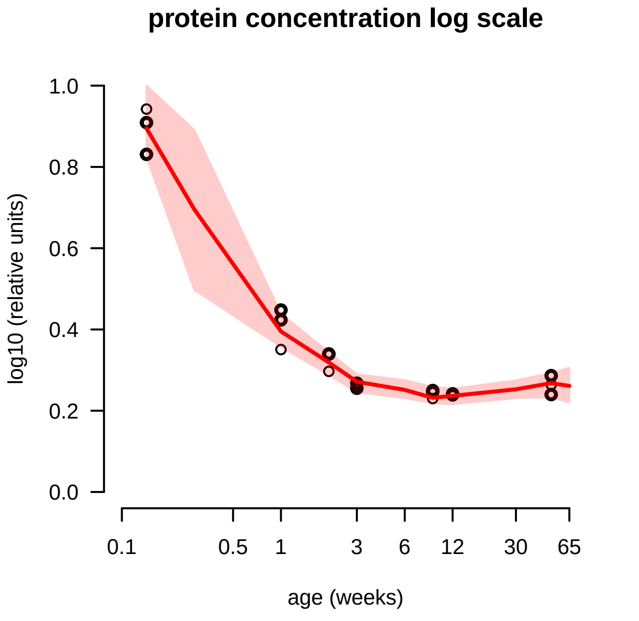

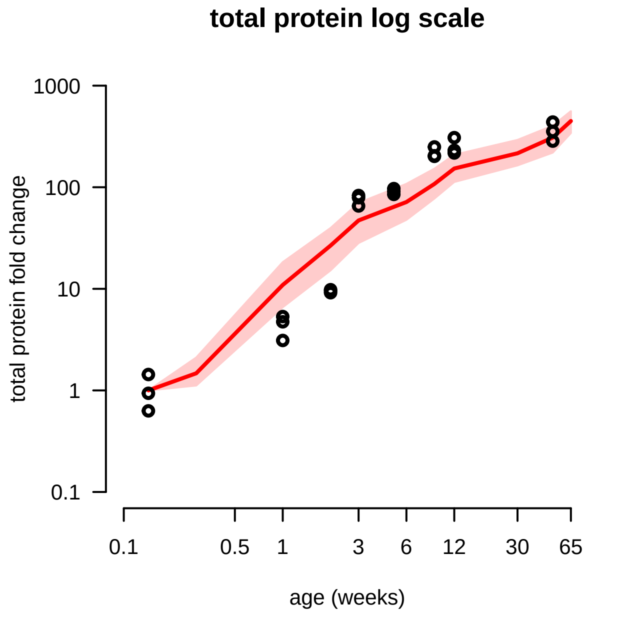

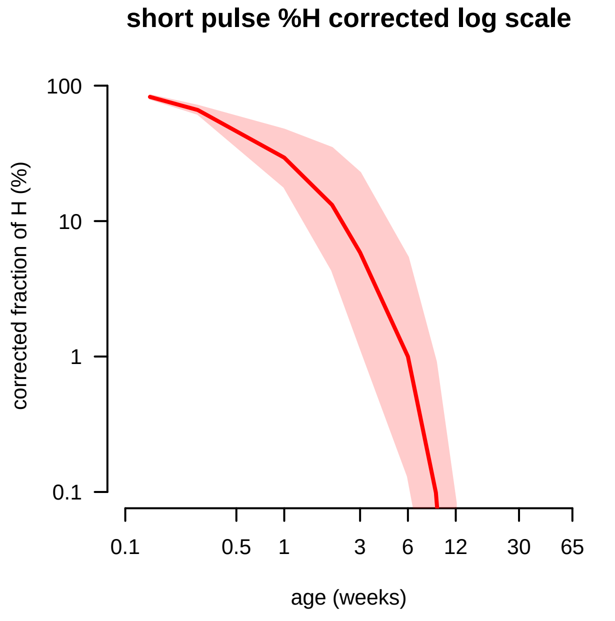

Q6W8Q3

>sp|Q6W8Q3|PC4L1_MOUSE

Purkinje cell protein 4−like protein 1

GN=Pcp4l1

Med H1/2: 5.6 days

Q5 H1/2: 2.9 days

Q95 H1/2: 38.8 days

reliable model fit: yes

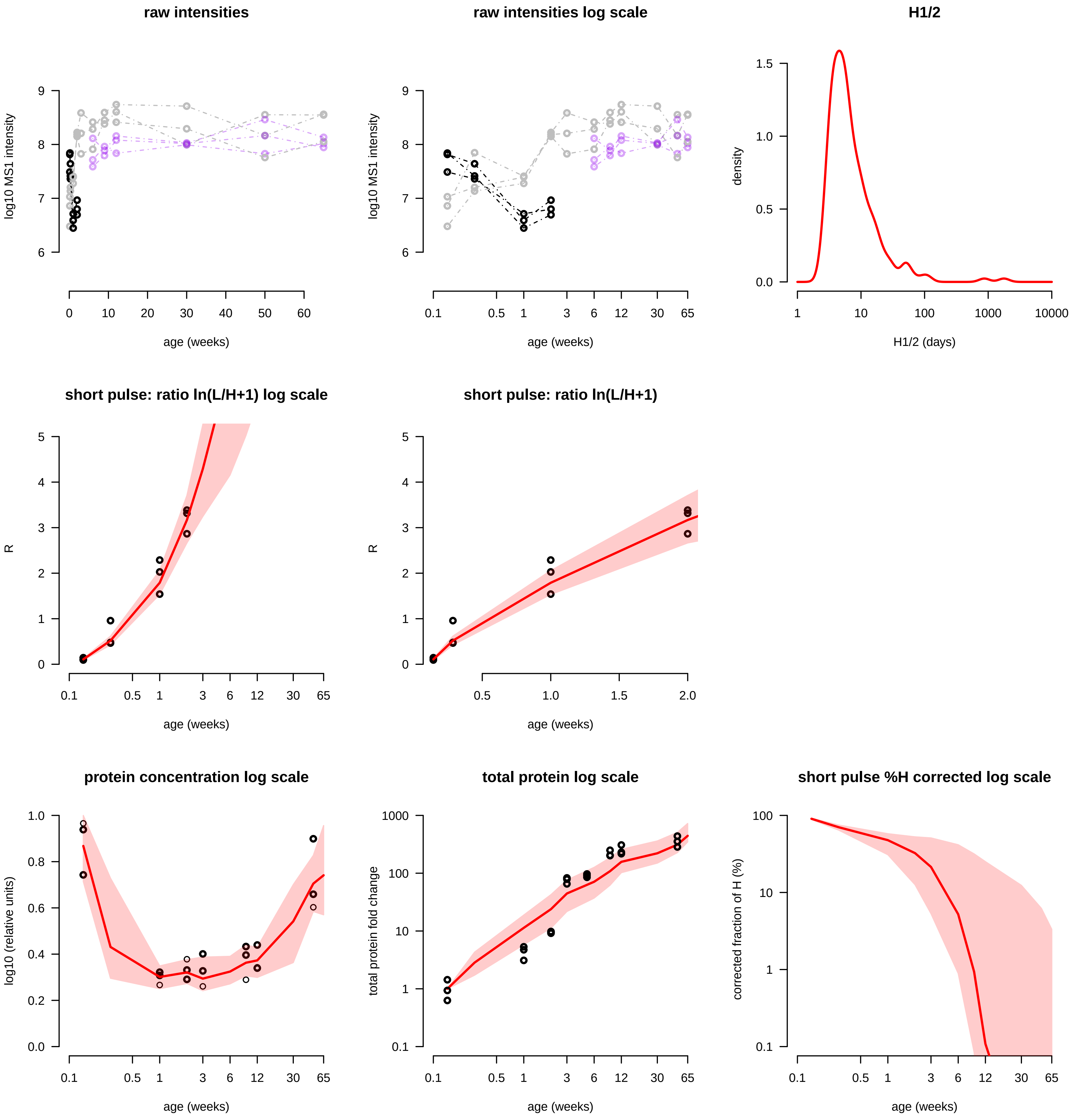

Q91WC0;Q91WC0−2

>sp|Q91WC0|SETD3_MOUSE

Actin−histidine N−methyltransferase

GN=Setd3

Med H1/2: 34.4 days

Q5 H1/2: 17.7 days

Q95 H1/2: 181.6 days

reliable model fit: no

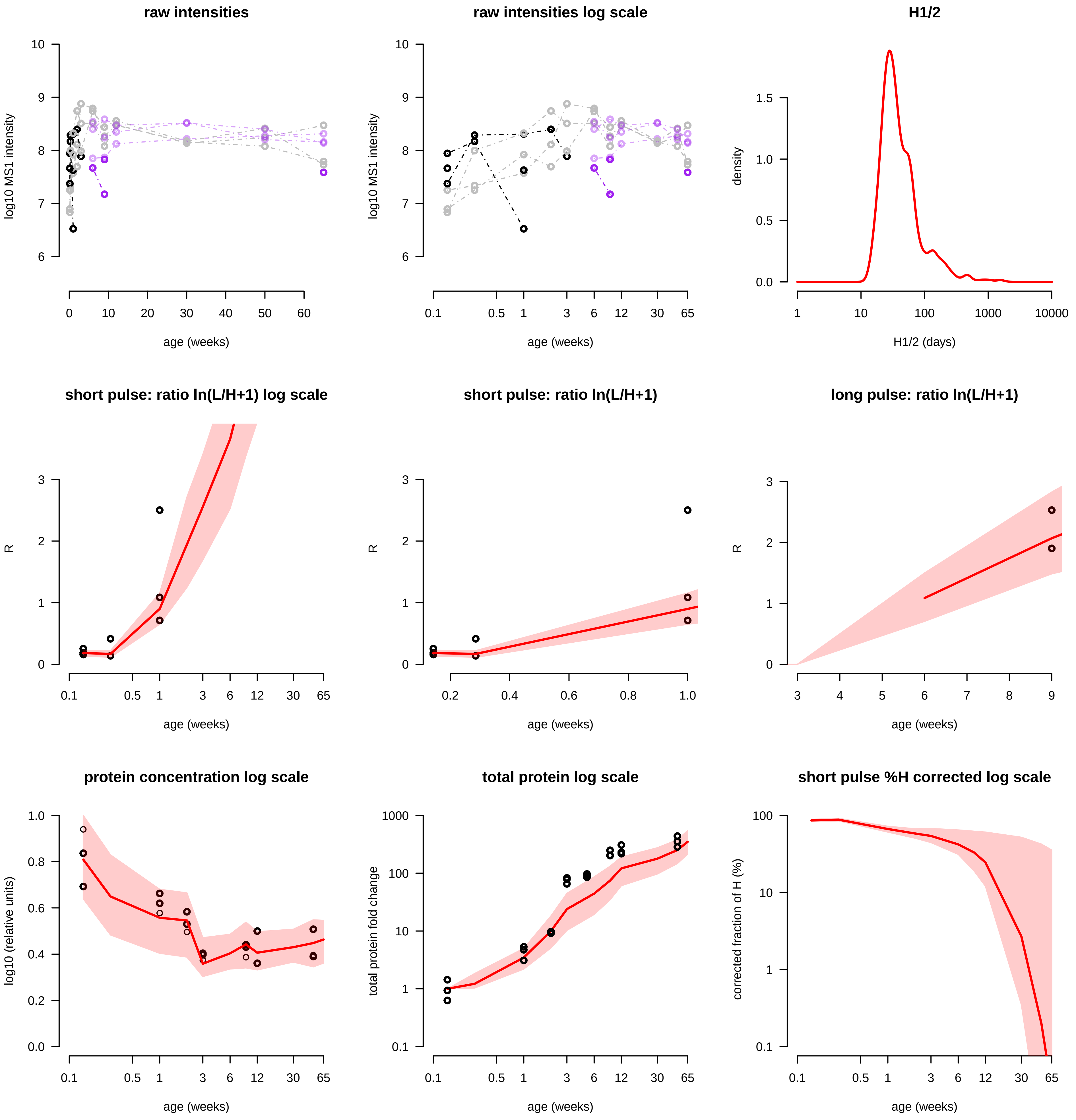

Q6Q477;Q6Q477−2

>sp|Q6Q477|AT2B4_MOUSE

Plasma membrane calcium−transporting ATPase 4

GN=Atp2b4

Med H1/2: 8 days

Q5 H1/2: 3.3 days

Q95 H1/2: 38.3 days

reliable model fit: yes

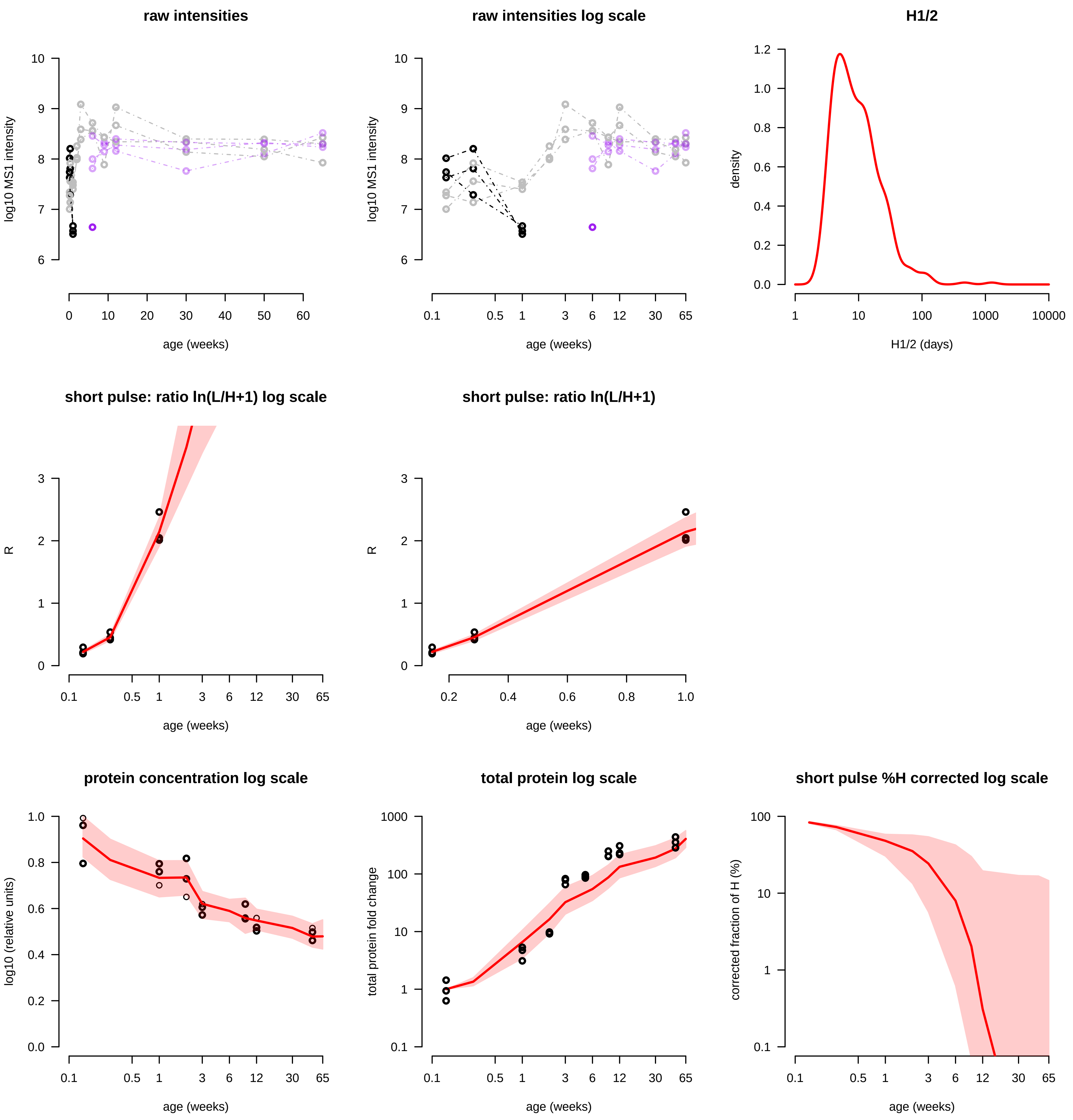

P34152;P34152−4;P34152−6;P34152−5

>sp|P34152|FAK1_MOUSE

Focal adhesion kinase 1

GN=Ptk2

Med H1/2: 3575.2 days

Q5 H1/2: 804.4 days

Q95 H1/2: 37629.4 days

reliable model fit: no

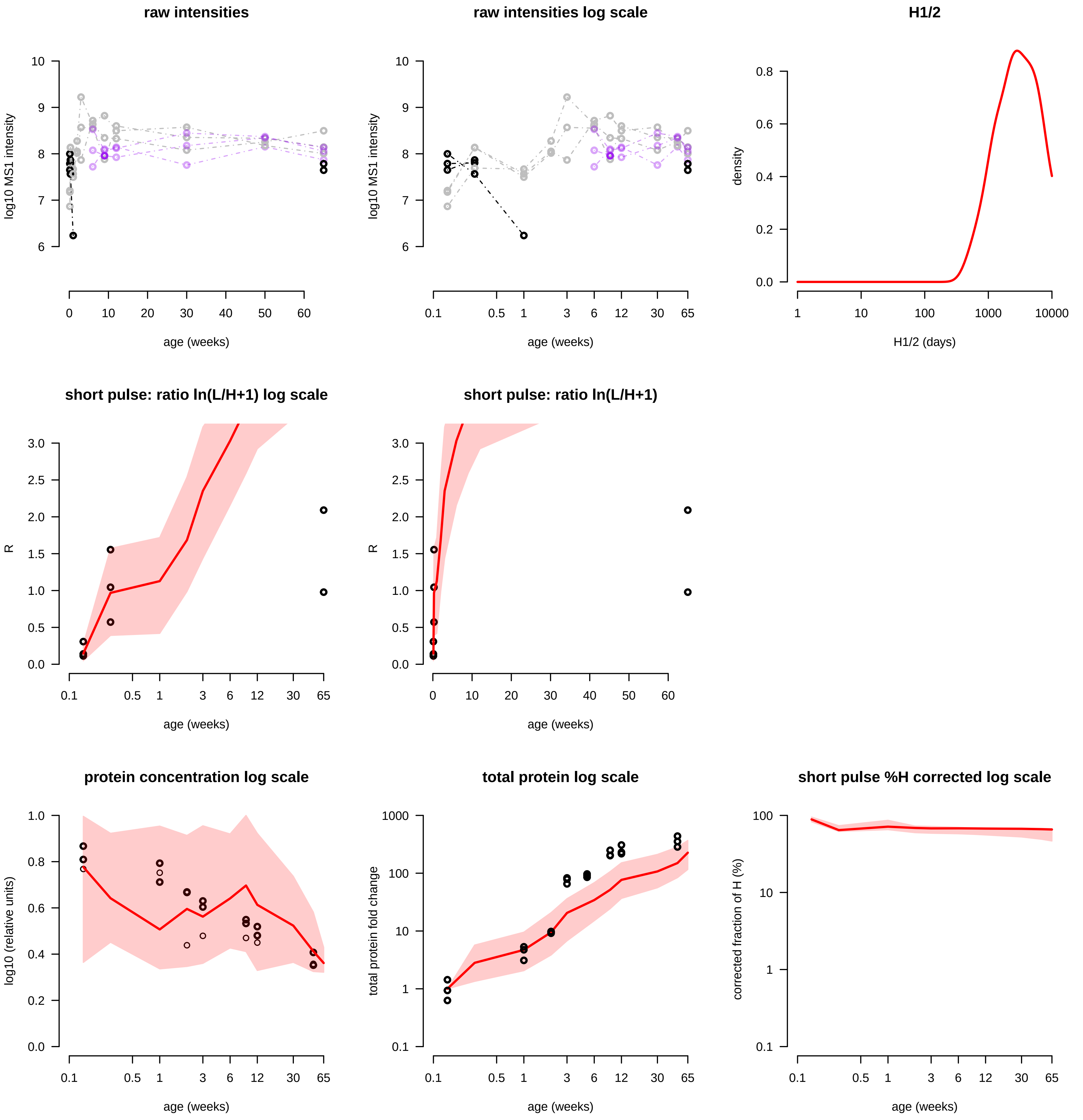

Q9Z108

>sp|Q9Z108|STAU1_MOUSE

Double−stranded RNA−binding protein Staufen homolog 1

GN=Stau1

Med H1/2: 5.6 days

Q5 H1/2: 3 days

Q95 H1/2: 13.7 days

reliable model fit: yes

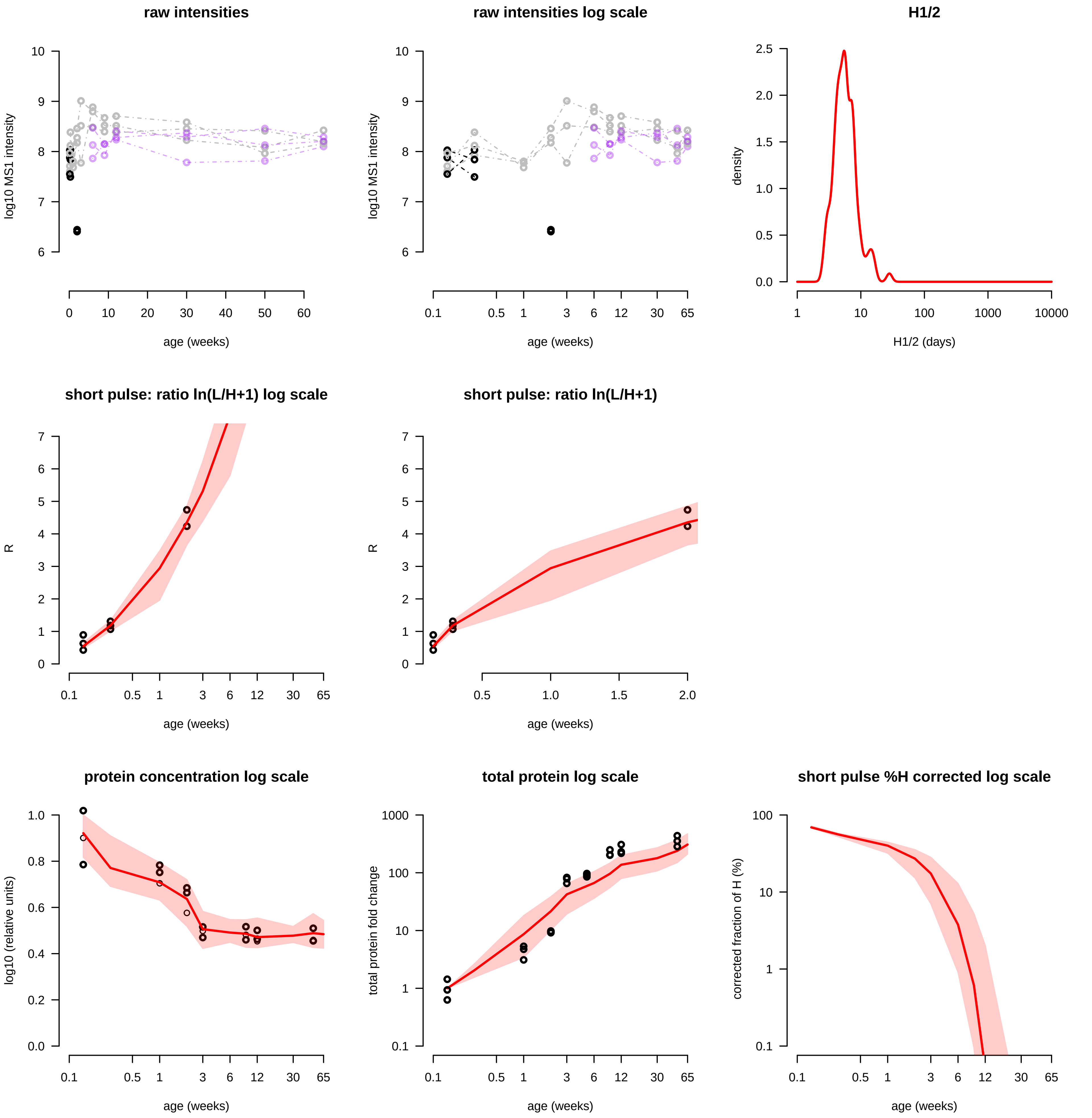

O70209

>sp|O70209|PDLI3_MOUSE

PDZ and LIM domain protein 3

GN=Pdlim3

Med H1/2: 10.4 days

Q5 H1/2: 4.2 days

Q95 H1/2: 100.9 days

reliable model fit: yes

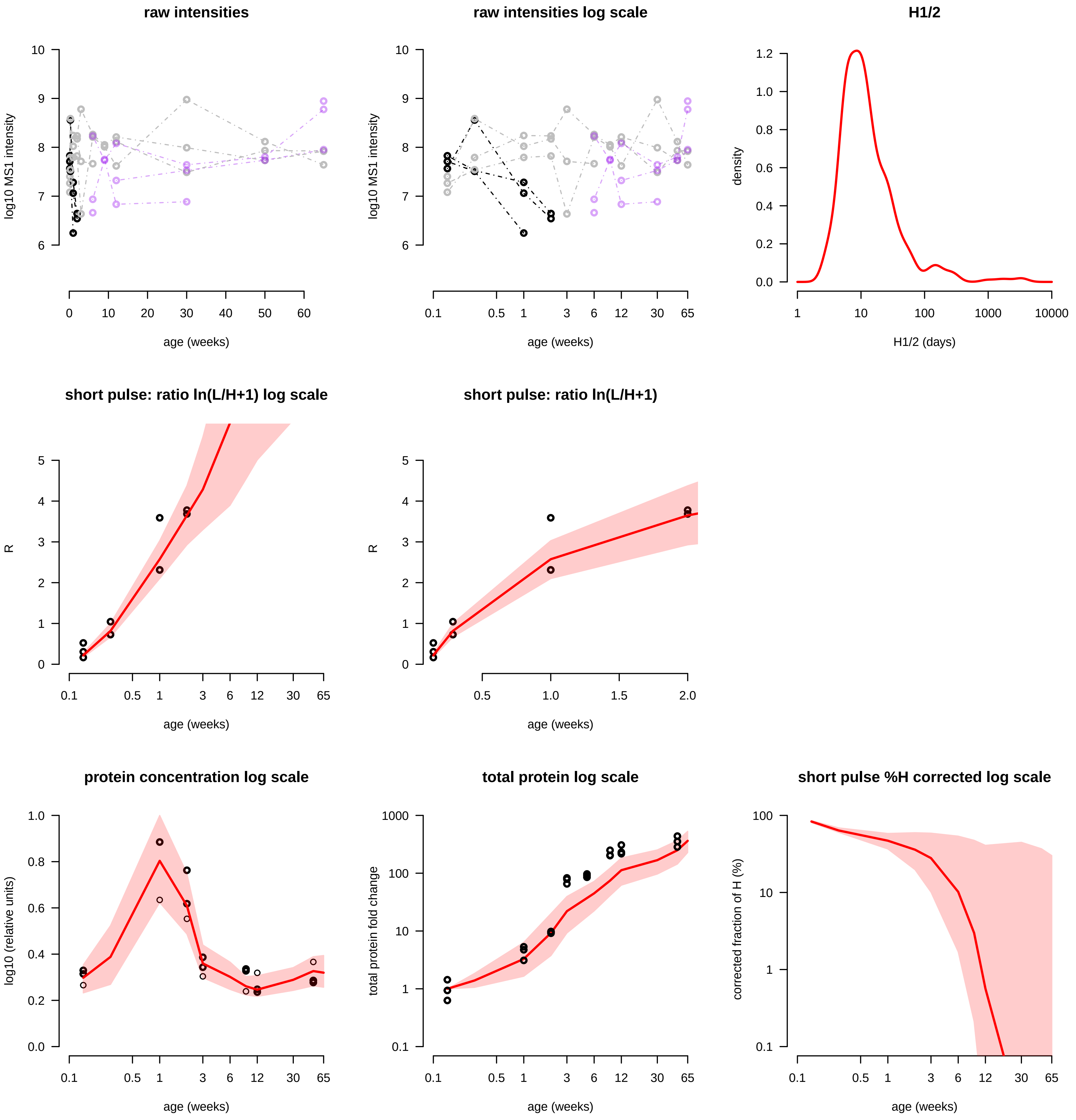

P49813

>sp|P49813|TMOD1_MOUSE

Tropomodulin−1

GN=Tmod1

Med H1/2: 14.4 days

Q5 H1/2: 8.8 days

Q95 H1/2: 30.9 days

reliable model fit: yes

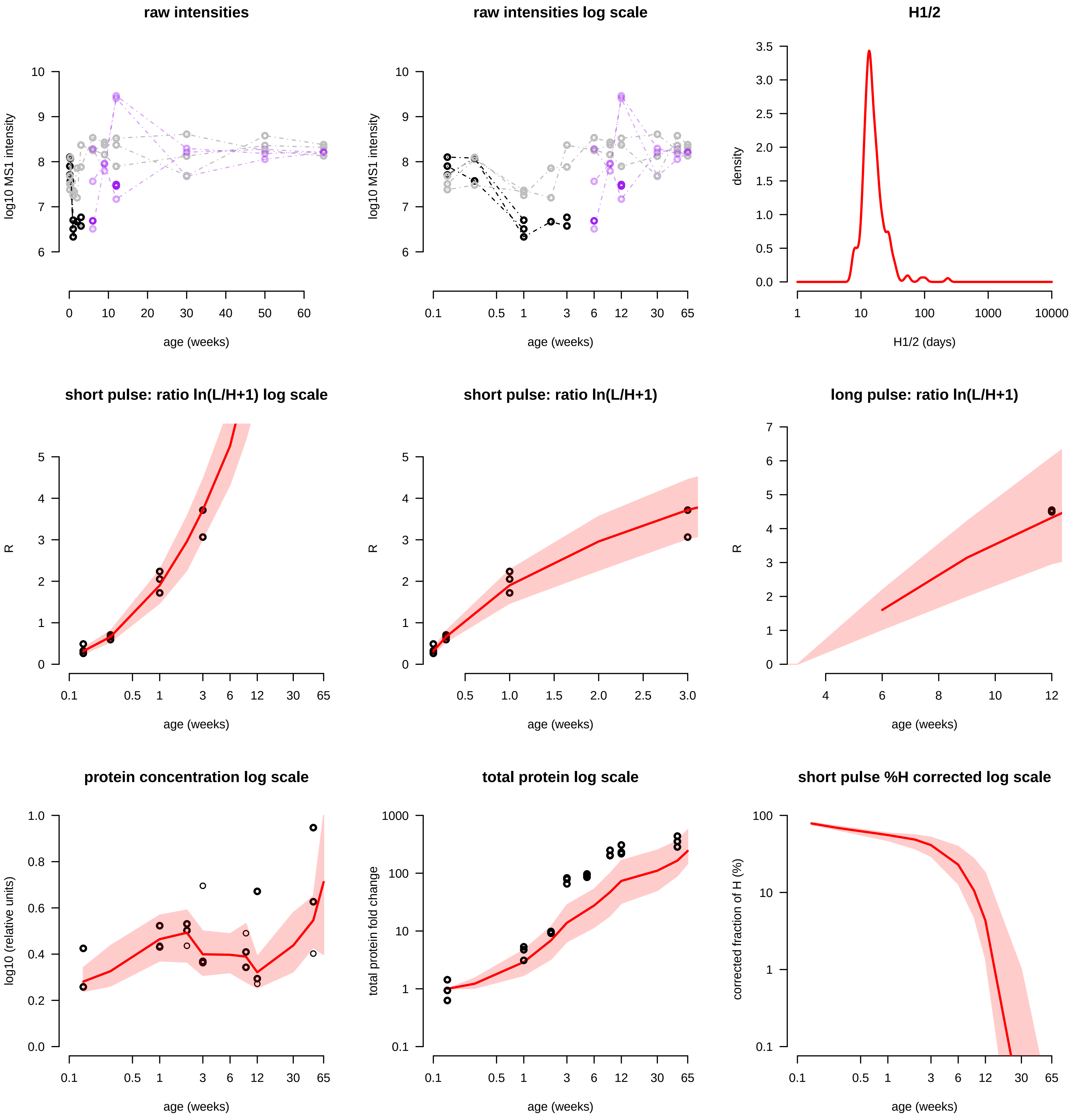

Q8C5H8

>sp|Q8C5H8|NAKD2_MOUSE

NAD kinase 2, mitochondrial

GN=Nadk2

Med H1/2: 12.6 days

Q5 H1/2: 6.3 days

Q95 H1/2: 165.6 days

reliable model fit: yes

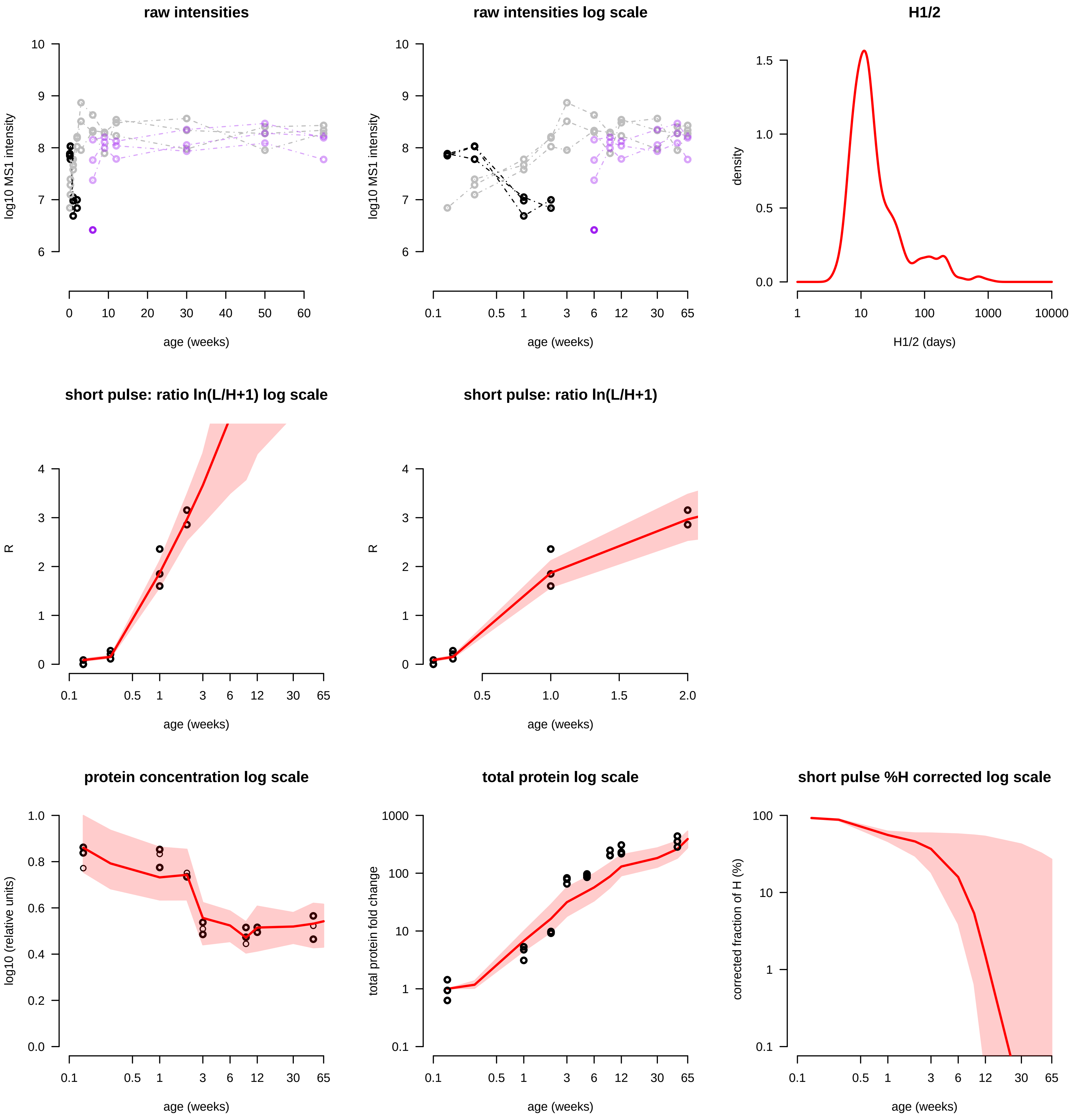

P00375

>sp|P00375|DYR_MOUSE

Dihydrofolate reductase

GN=Dhfr

Med H1/2: 4.6 days

Q5 H1/2: 3.6 days

Q95 H1/2: 6.2 days

reliable model fit: yes

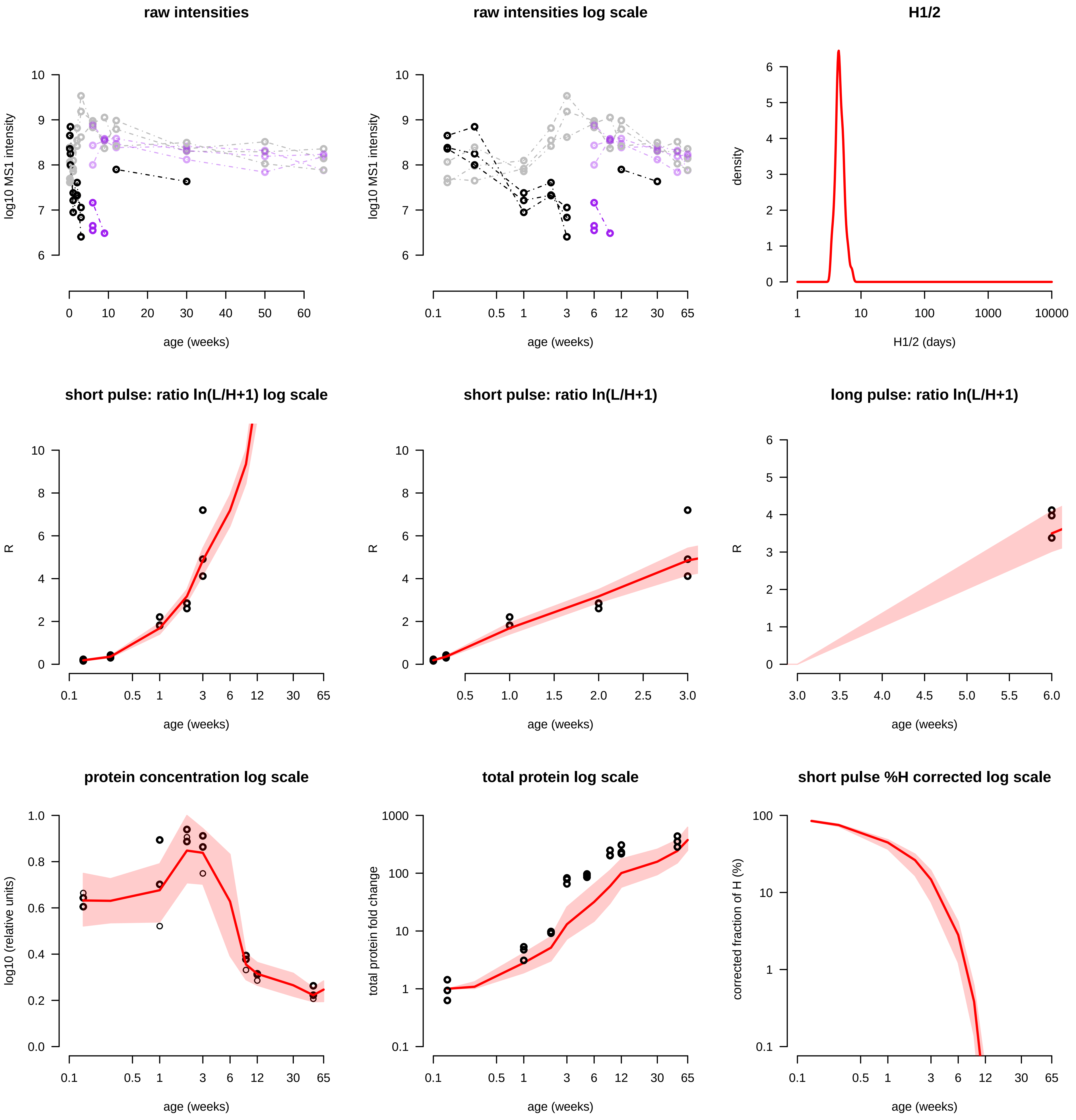

Q8BGB7;Q8BGB7−2

>sp|Q8BGB7|ENOPH_MOUSE

Enolase−phosphatase E1

GN=Enoph1

Med H1/2: 21.4 days

Q5 H1/2: 6.9 days

Q95 H1/2: 187.8 days

reliable model fit: yes

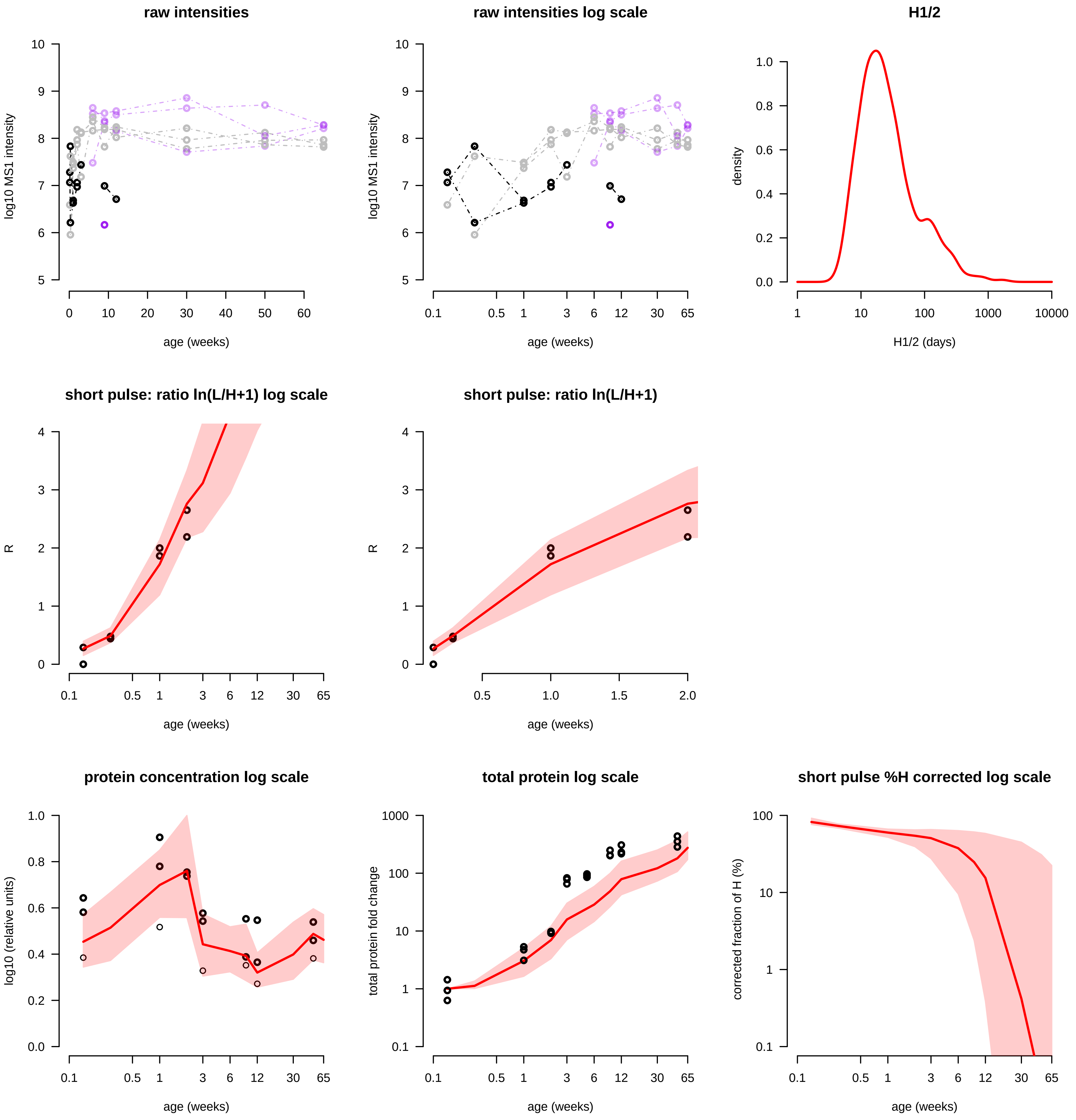

Q8BMQ2;Q8BMQ2−2

>sp|Q8BMQ2|TF3C4_MOUSE

General transcription factor 3C polypeptide 4

GN=Gtf3c4

Med H1/2: 4 days

Q5 H1/2: 2.5 days

Q95 H1/2: 13 days

reliable model fit: yes

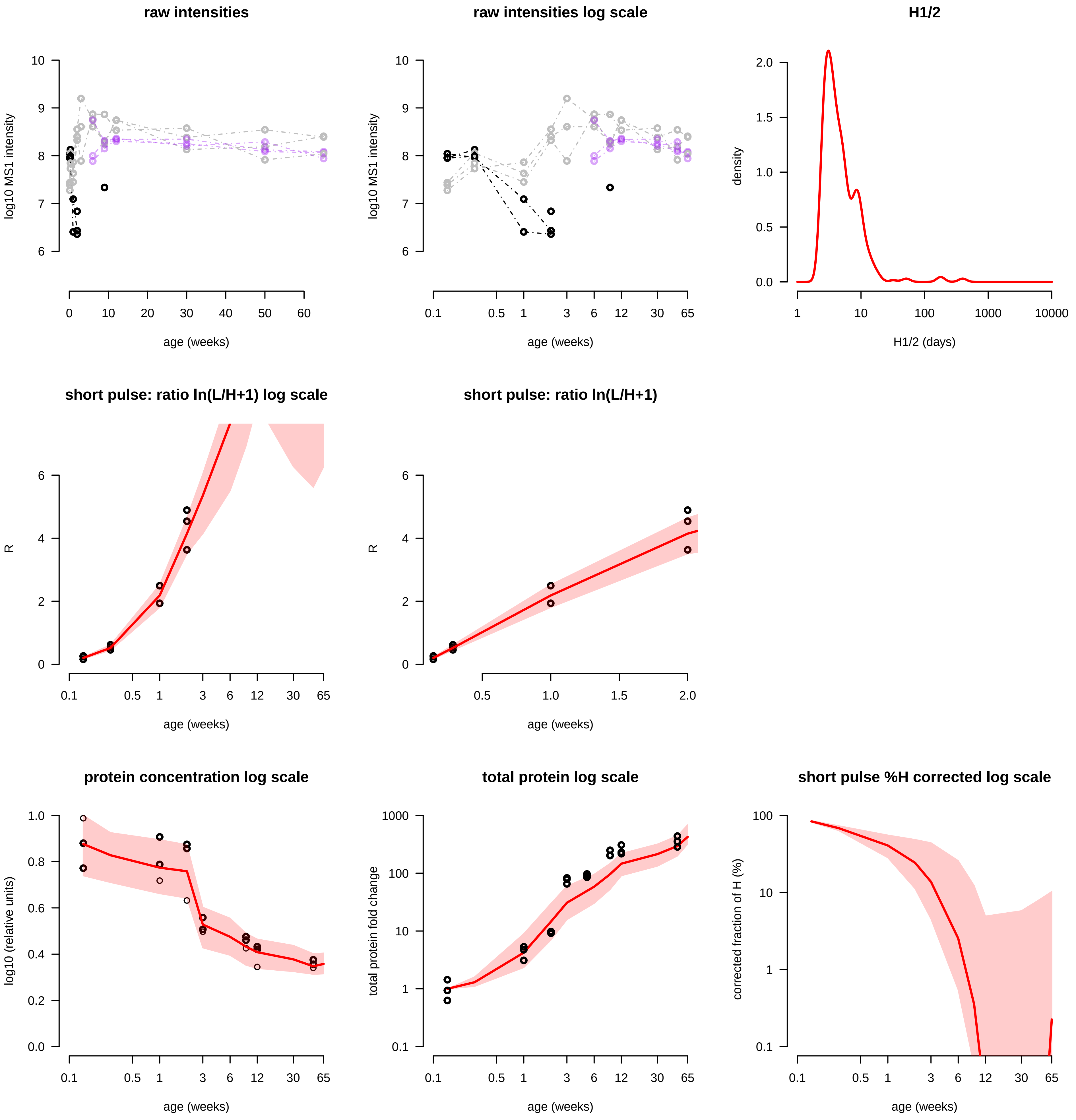

Q8BTV2−2;Q8BTV2;Q8BTV2−3

>sp|Q8BTV2−2|CPSF7_MOUSE

Isoform 2 of Cleavage and polyadenylation specificity factor subunit 7

GN=Cpsf7

Med H1/2: 4.8 days

Q5 H1/2: 4 days

Q95 H1/2: 6.5 days

reliable model fit: yes

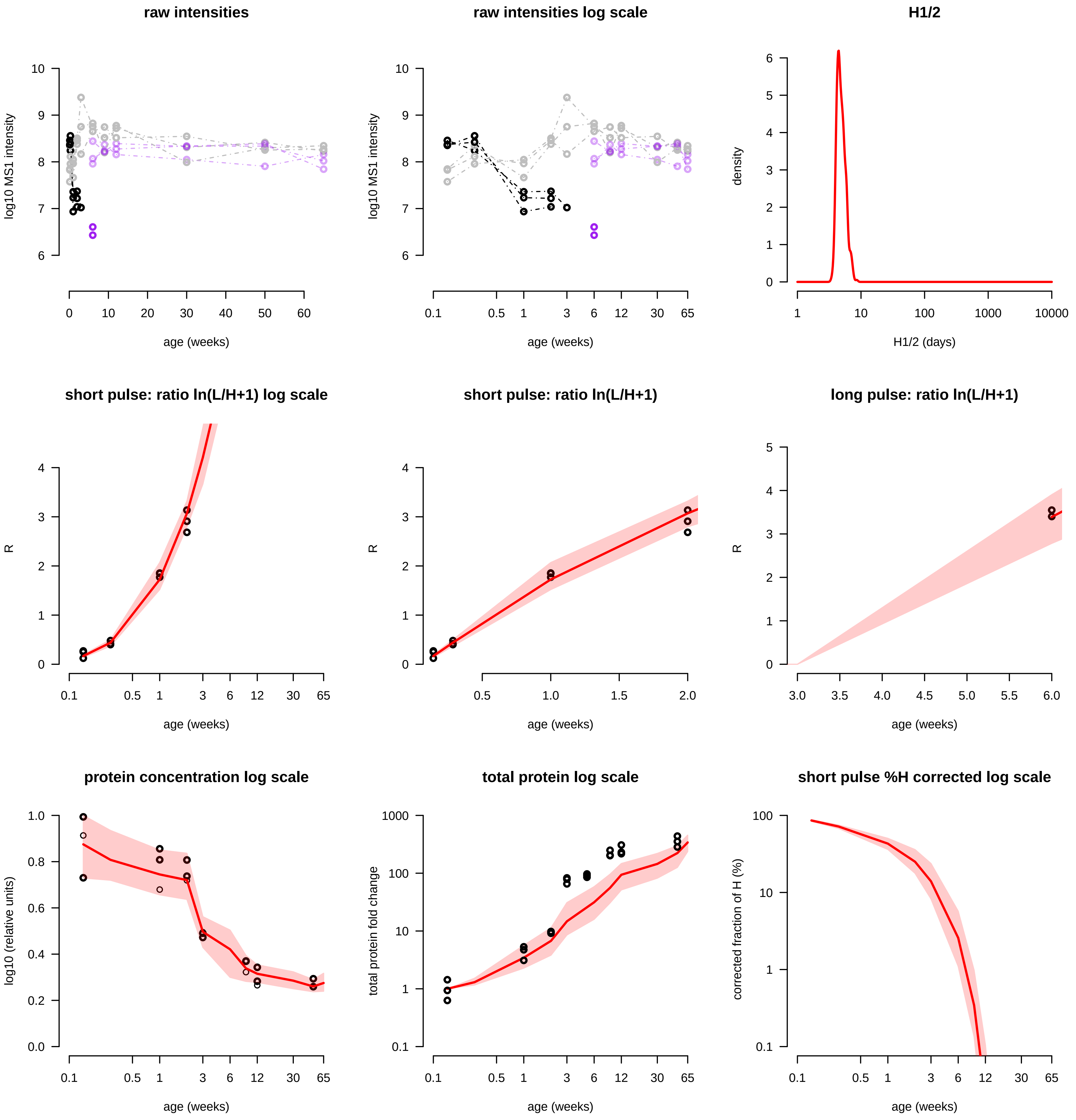

P70255−2;P70255;P70255−3;P70255−5;P70255−6

>sp|P70255−2|NFIC_MOUSE

Isoform 2 of Nuclear factor 1 C−type

GN=Nfic

Med H1/2: 2616 days

Q5 H1/2: 1010.4 days

Q95 H1/2: 17602.6 days

reliable model fit: no

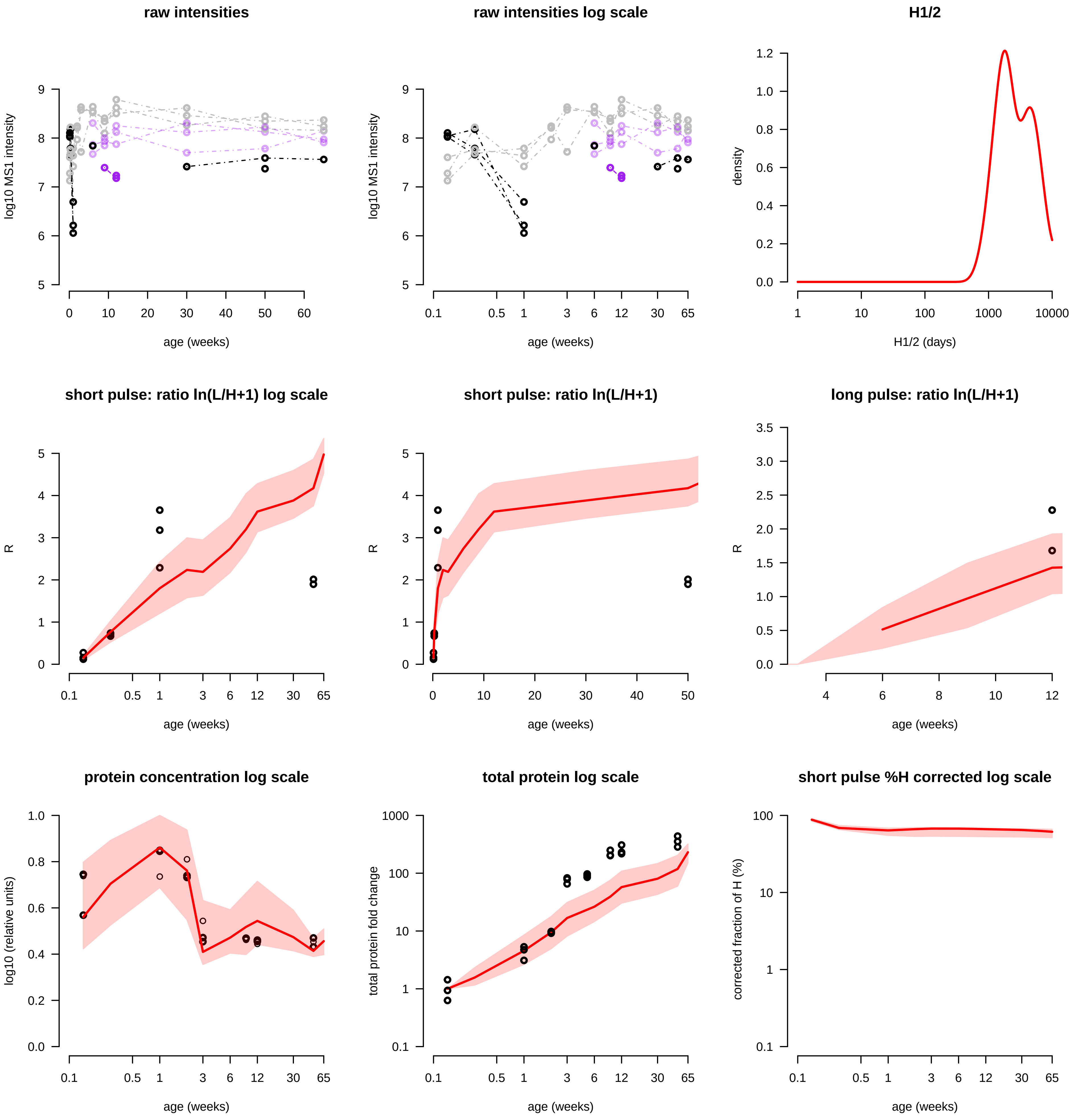

P49138

>sp|P49138|MAPK2_MOUSE

MAP kinase−activated protein kinase 2

GN=Mapkapk2

Med H1/2: 6.1 days

Q5 H1/2: 3 days

Q95 H1/2: 88.1 days

reliable model fit: yes

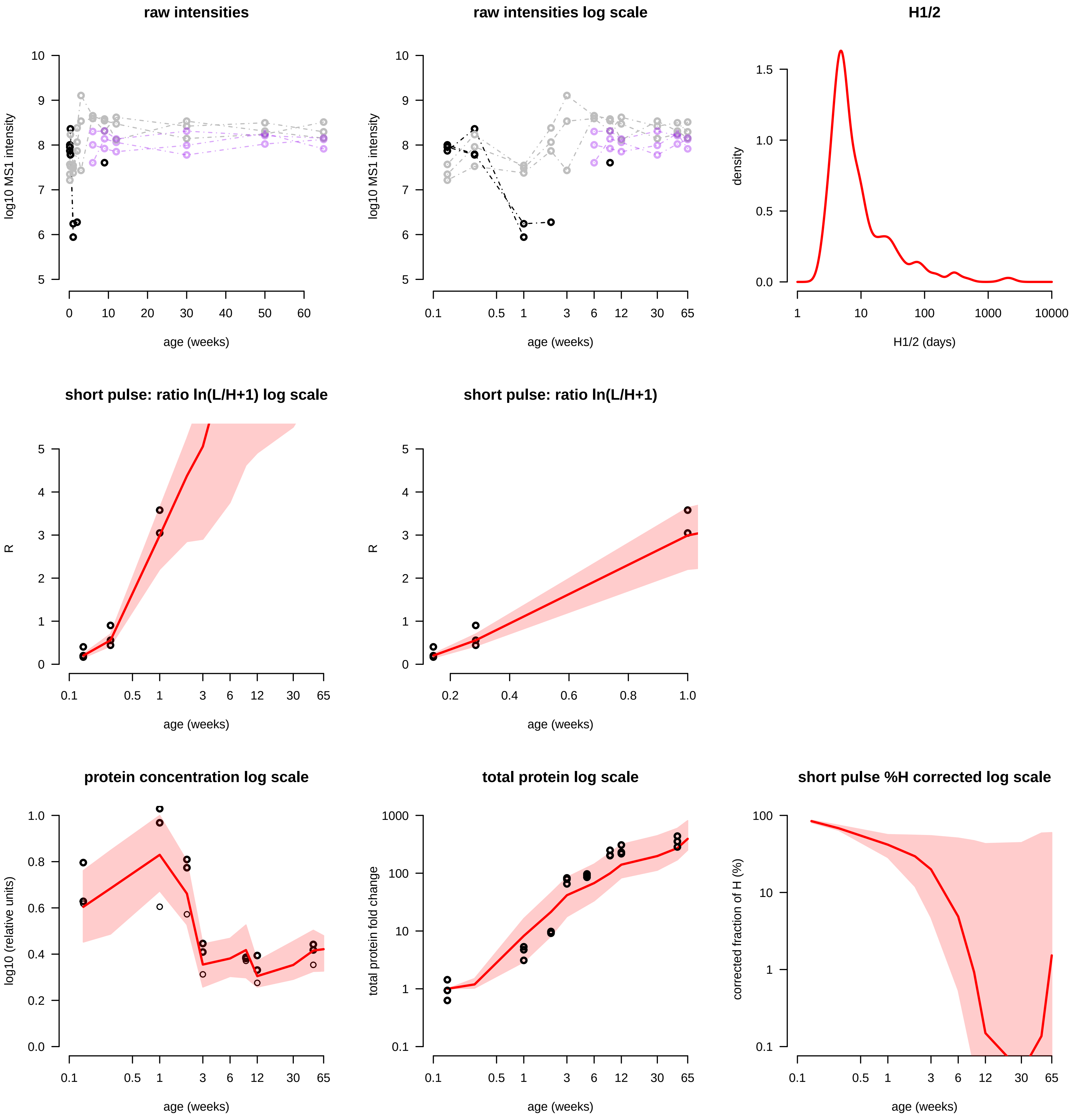

Q9CRY7

>sp|Q9CRY7|GDPD1_MOUSE

Lysophospholipase D GDPD1

GN=Gdpd1

Med H1/2: 4.5 days

Q5 H1/2: 3.7 days

Q95 H1/2: 5.4 days

reliable model fit: yes

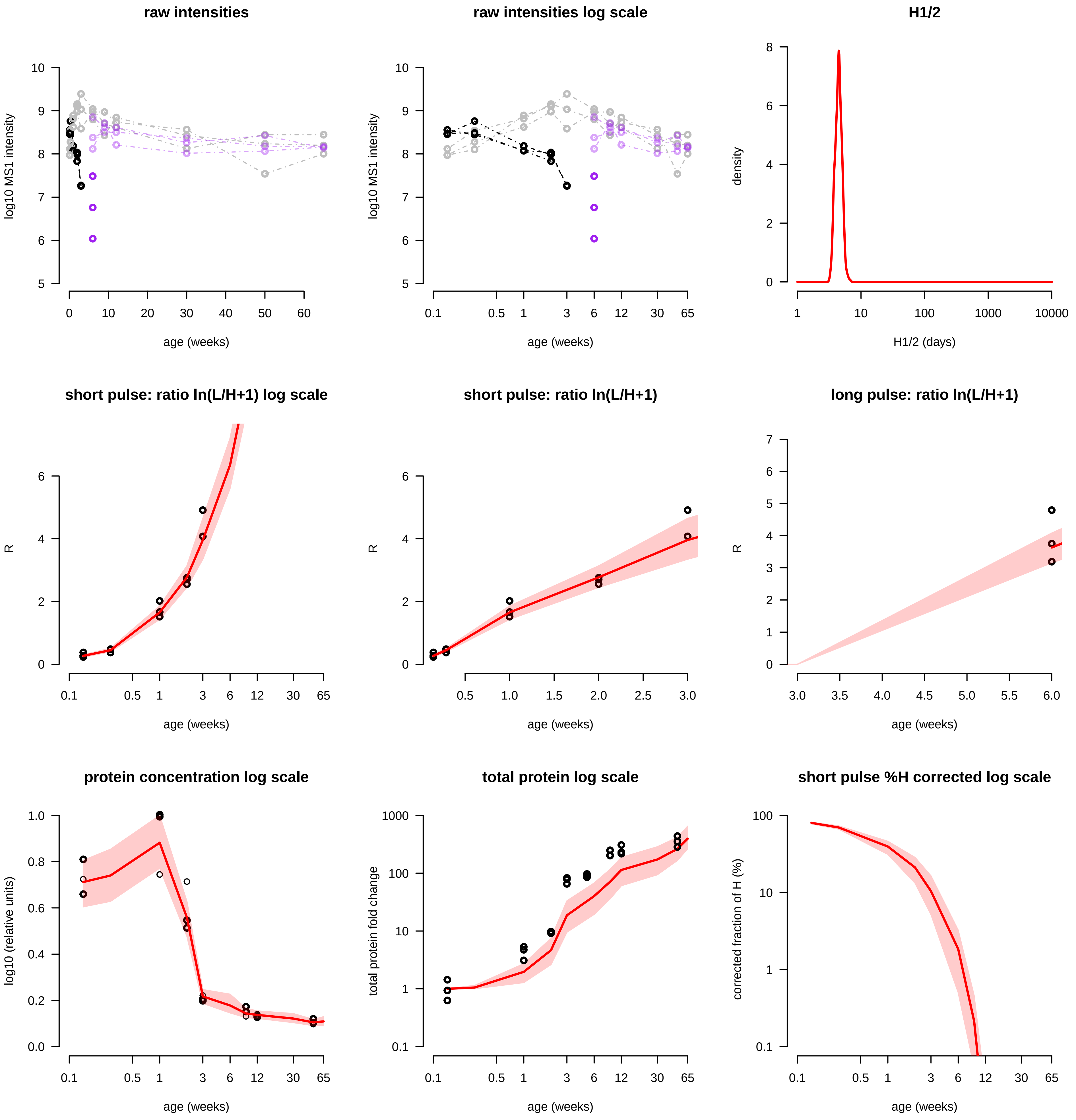

Q00422

>sp|Q00422|GABPA_MOUSE

GA−binding protein alpha chain

GN=Gabpa

Med H1/2: 3.5 days

Q5 H1/2: 2.8 days

Q95 H1/2: 4.5 days

reliable model fit: yes

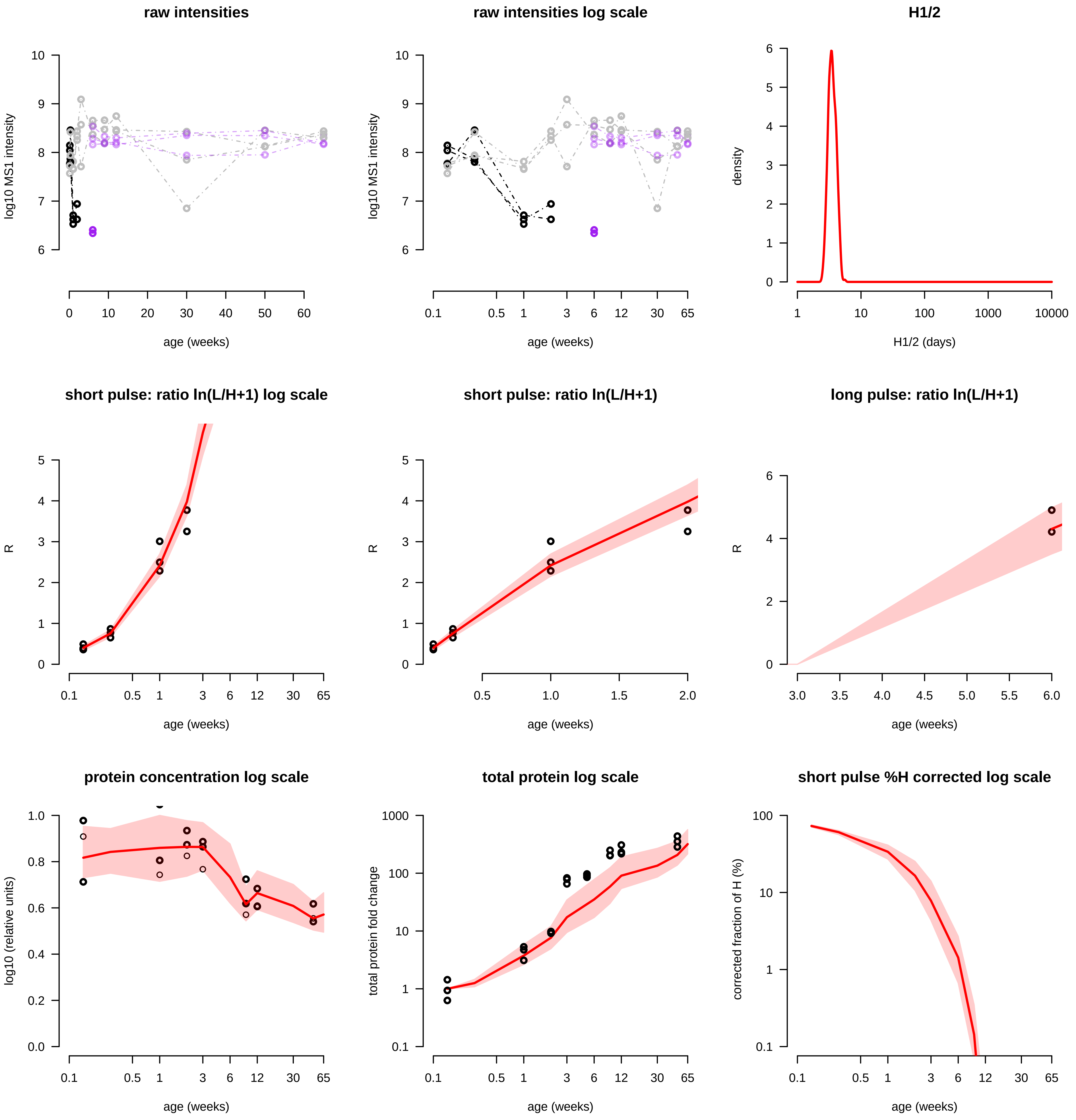

Q9CQ80;Q9CQ80−2

>sp|Q9CQ80|VPS25_MOUSE

Vacuolar protein−sorting−associated protein 25

GN=Vps25

Med H1/2: 3216.4 days

Q5 H1/2: 861 days

Q95 H1/2: 29913.3 days

reliable model fit: no

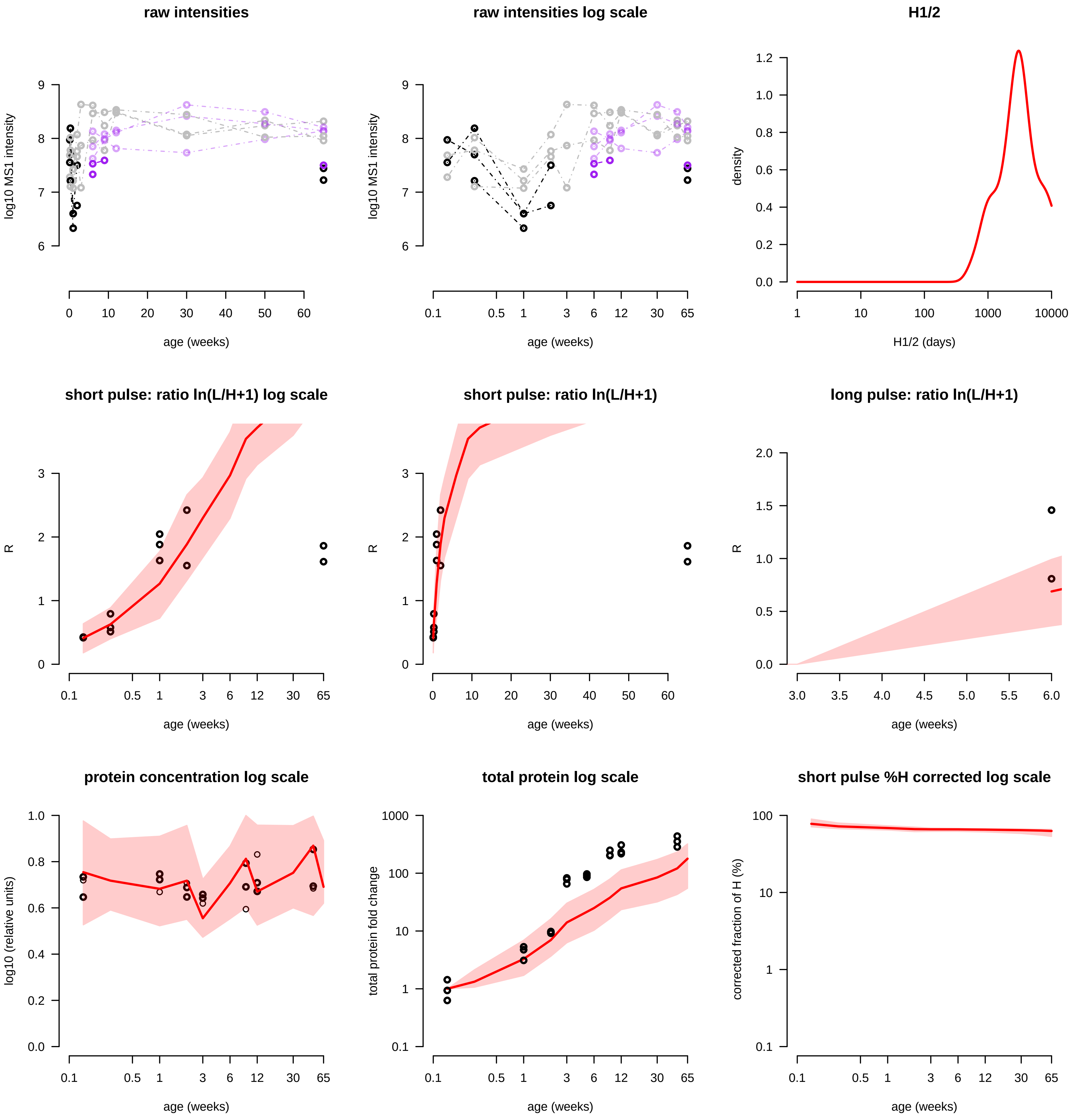

E9PYK3

>sp|E9PYK3|PARP4_MOUSE

Protein mono−ADP−ribosyltransferase PARP4

GN=Parp4

Med H1/2: 996.3 days

Q5 H1/2: 286.4 days

Q95 H1/2: 13704.9 days

reliable model fit: no

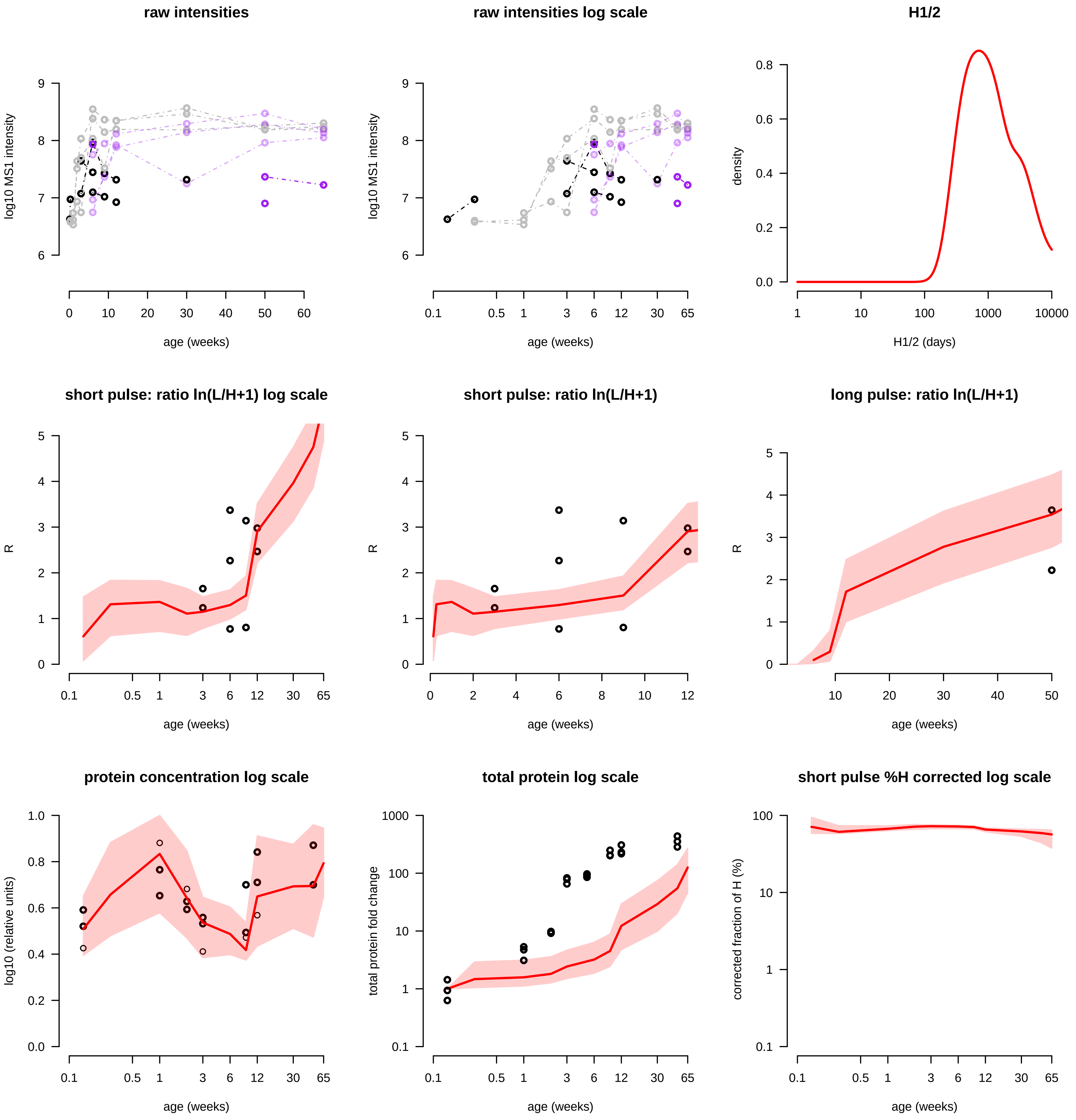

Q99N95

>sp|Q99N95|RM03_MOUSE

39S ribosomal protein L3, mitochondrial

GN=Mrpl3

Med H1/2: 7.5 days

Q5 H1/2: 3.8 days

Q95 H1/2: 32.7 days

reliable model fit: yes

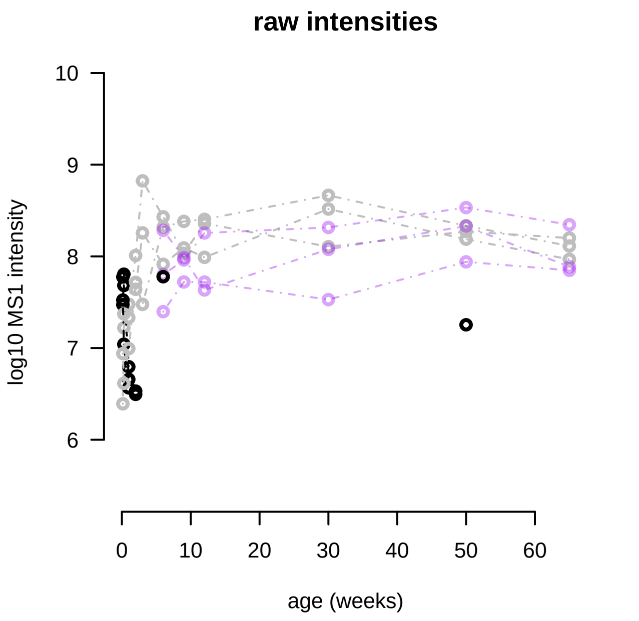

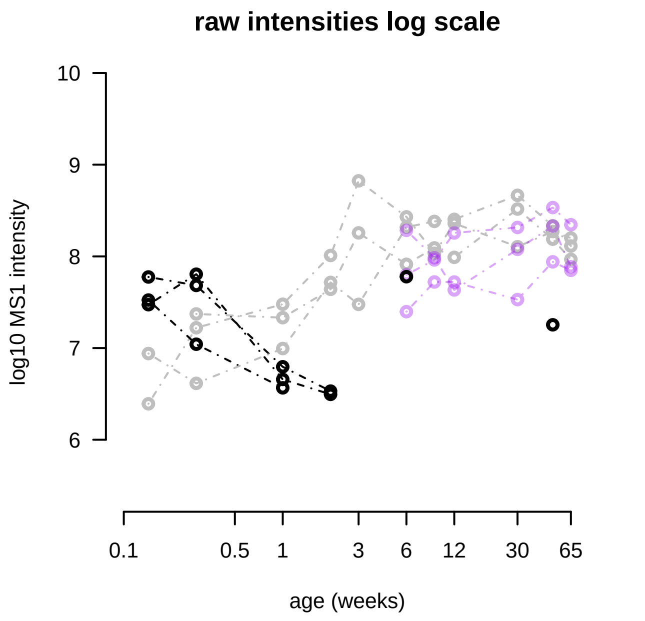

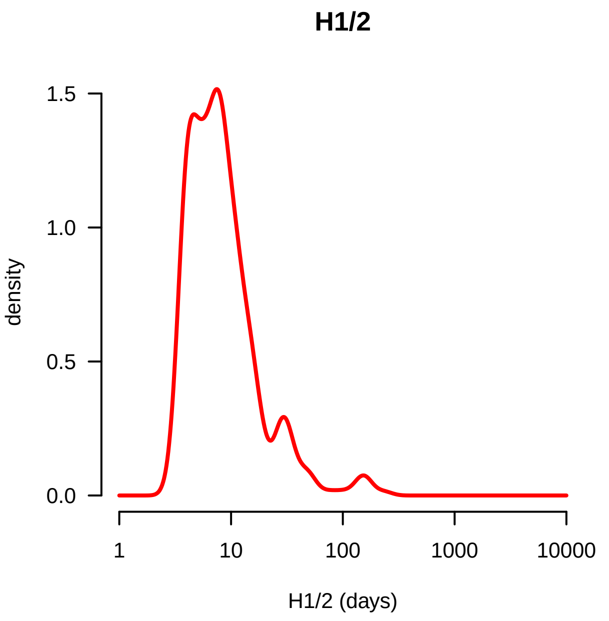

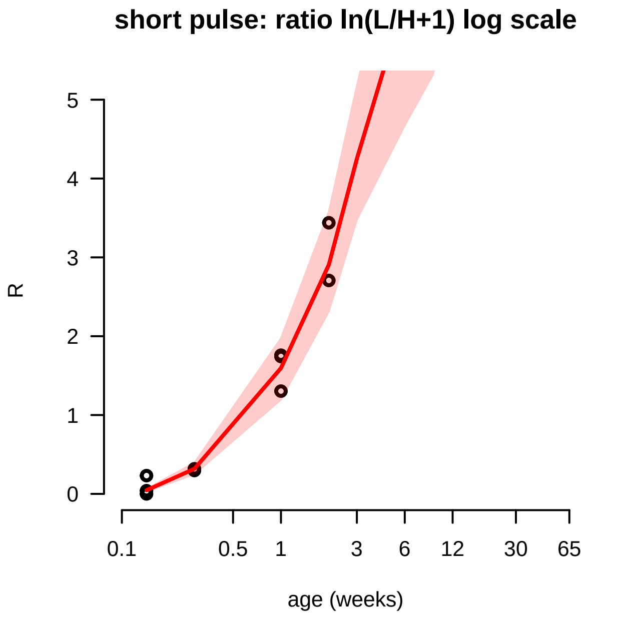

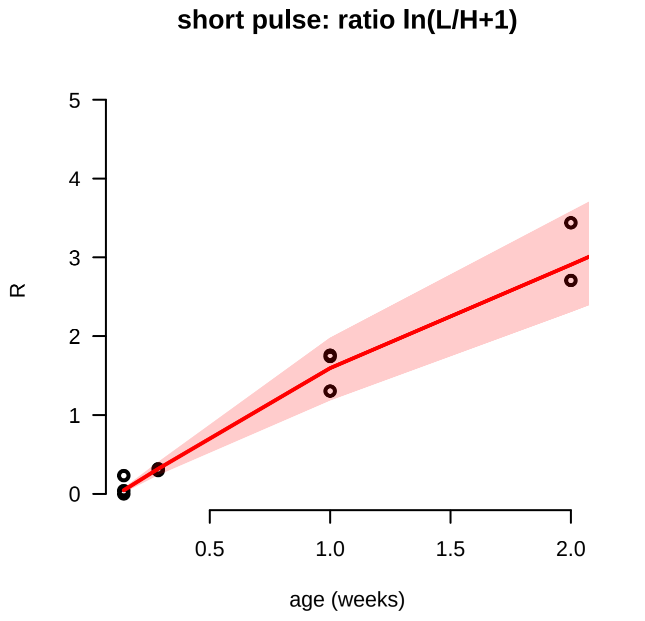

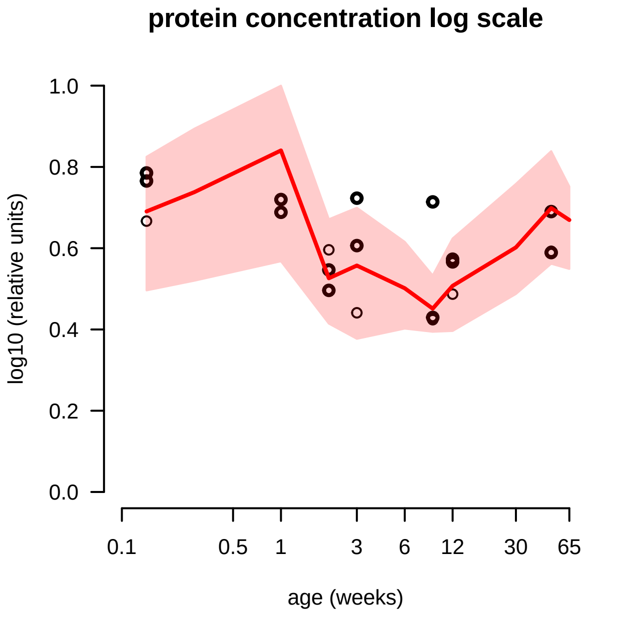

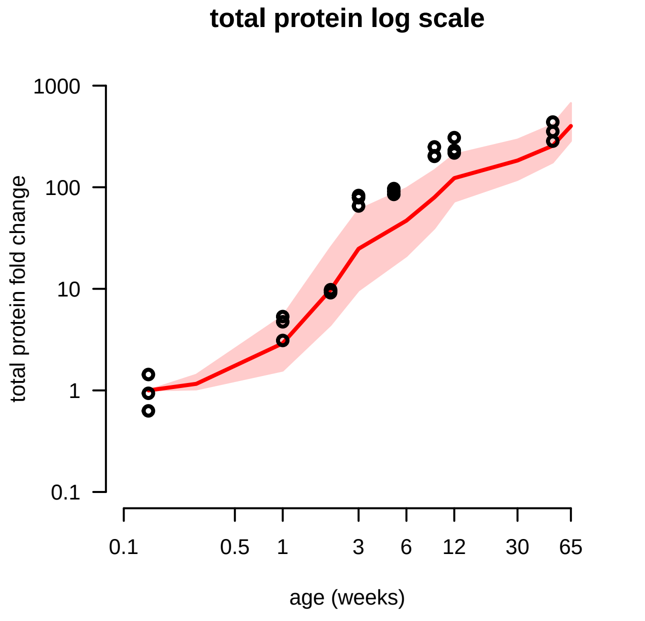

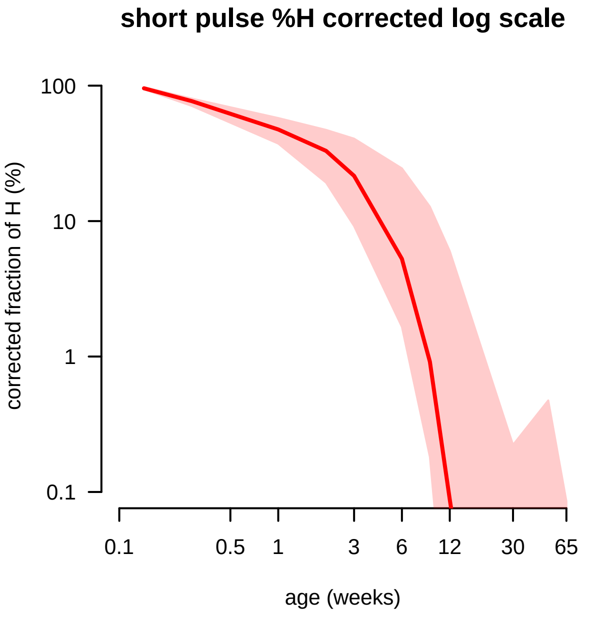

Q04690−2;Q04690;Q04690−4;Q04690−3

>sp|Q04690−2|NF1_MOUSE

Isoform I of Neurofibromin

GN=Nf1

Med H1/2: 1666.5 days

Q5 H1/2: 485.6 days

Q95 H1/2: 23024.4 days

reliable model fit: yes

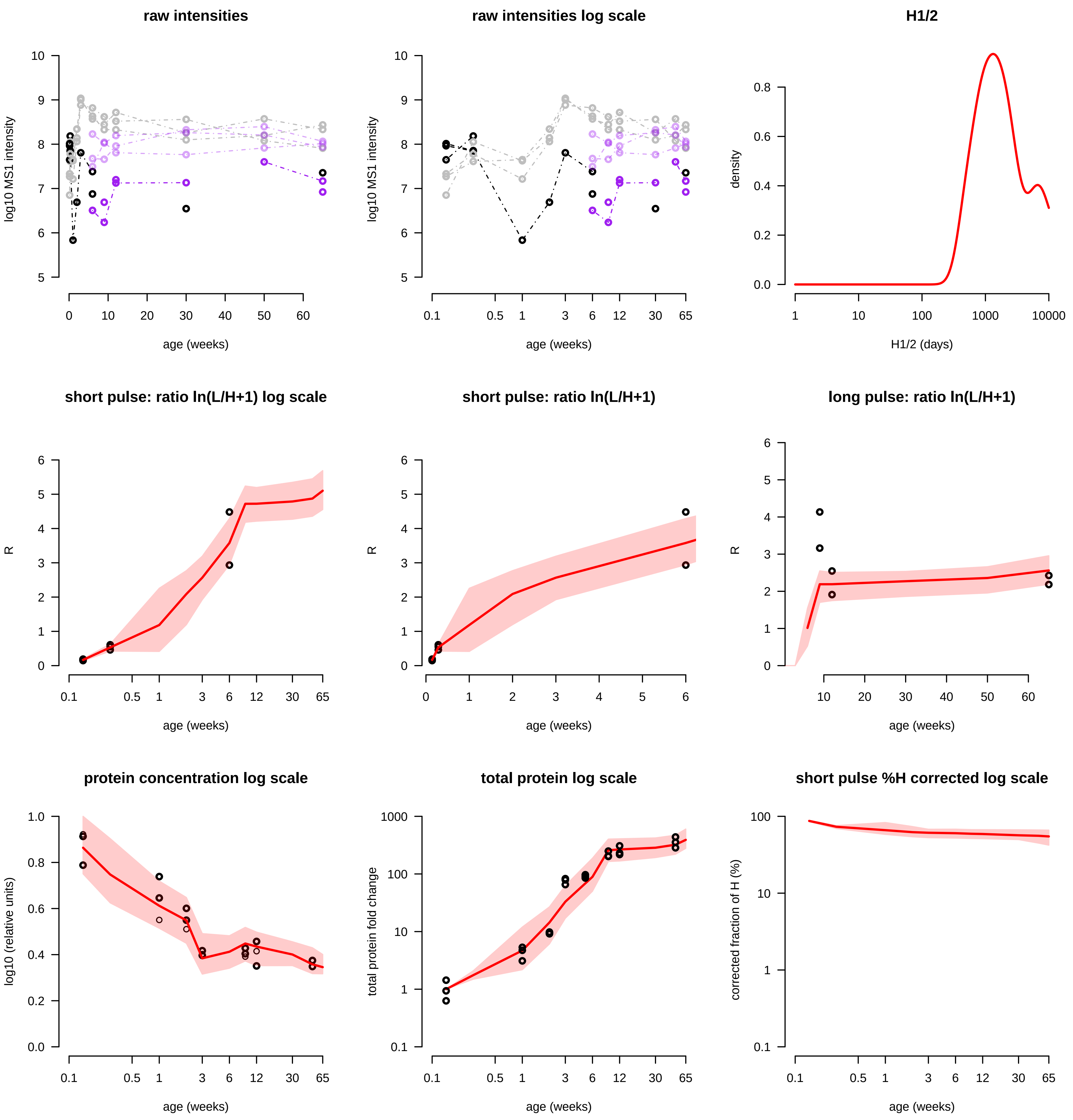

O35226;O35226−4

>sp|O35226|PSMD4_MOUSE

26S proteasome non−ATPase regulatory subunit 4

GN=Psmd4

Med H1/2: 6.6 days

Q5 H1/2: 2.7 days

Q95 H1/2: 52.8 days

reliable model fit: yes

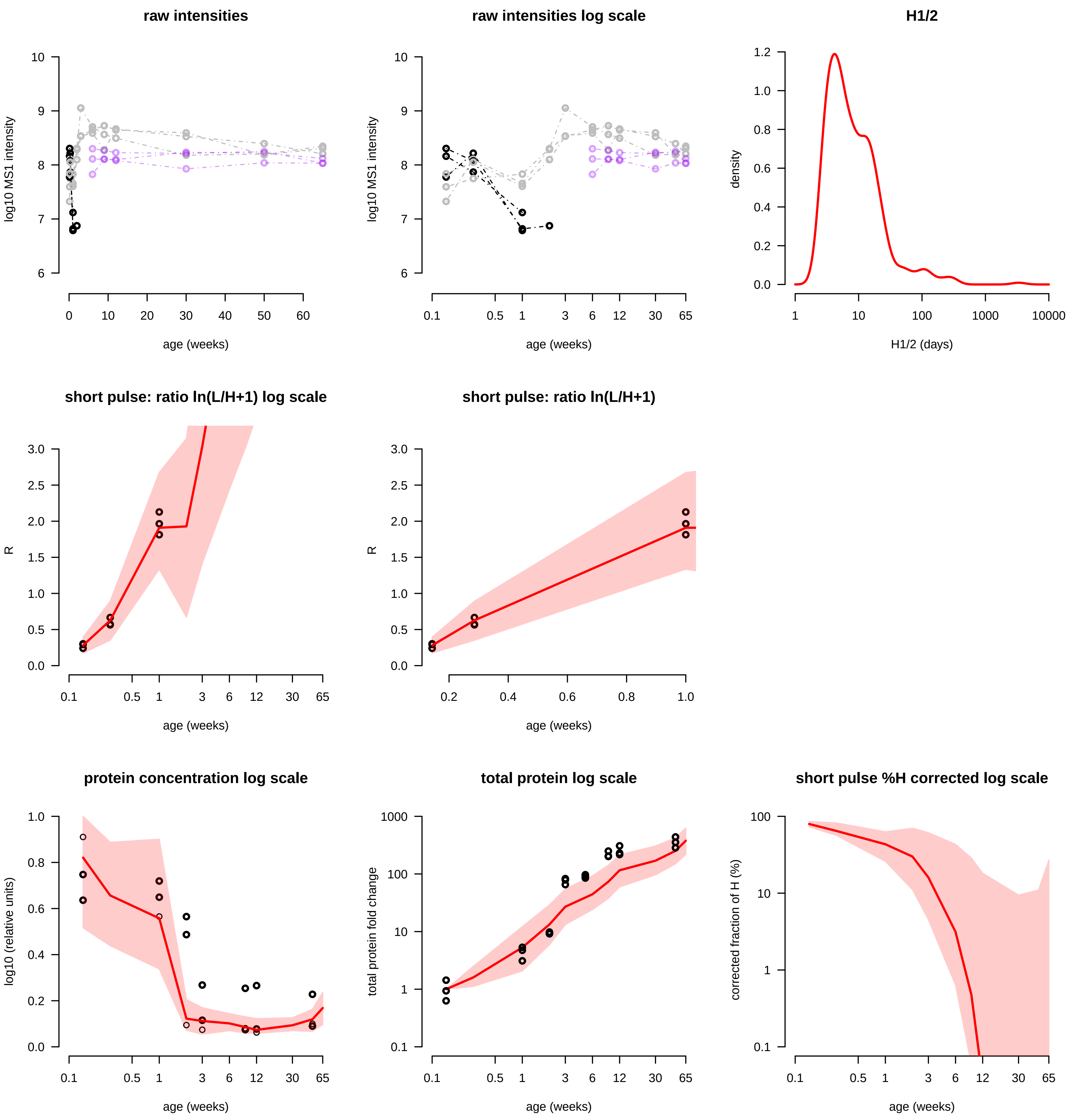

Q9QZ73

>sp|Q9QZ73|DCNL1_MOUSE

DCN1−like protein 1

GN=Dcun1d1

Med H1/2: 8.4 days

Q5 H1/2: 2.6 days

Q95 H1/2: 42 days

reliable model fit: yes

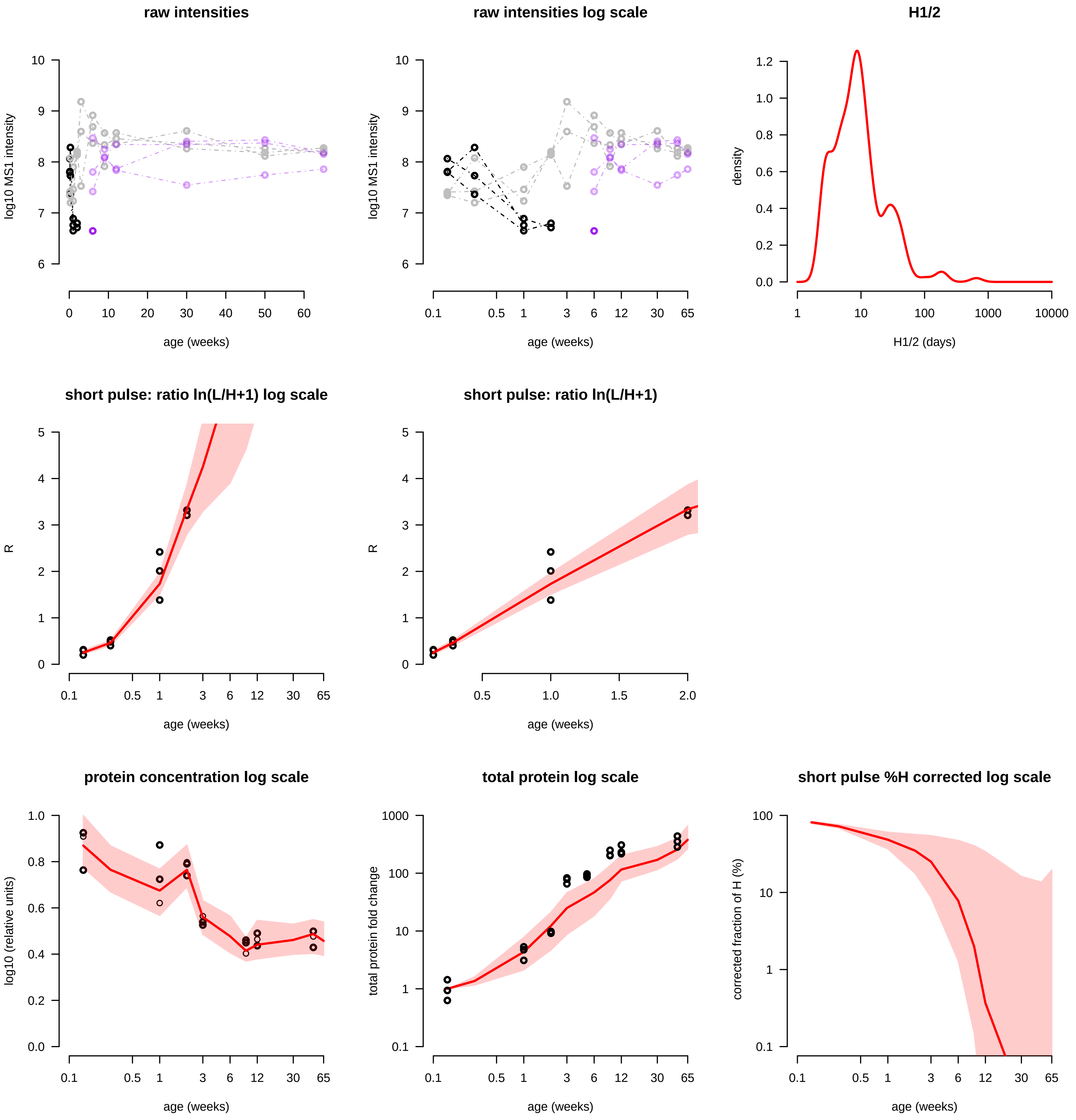

Q9D3P8

>sp|Q9D3P8|PLRKT_MOUSE

Plasminogen receptor (KT)

GN=Plgrkt

Med H1/2: 5.8 days

Q5 H1/2: 4.4 days

Q95 H1/2: 8.6 days

reliable model fit: yes

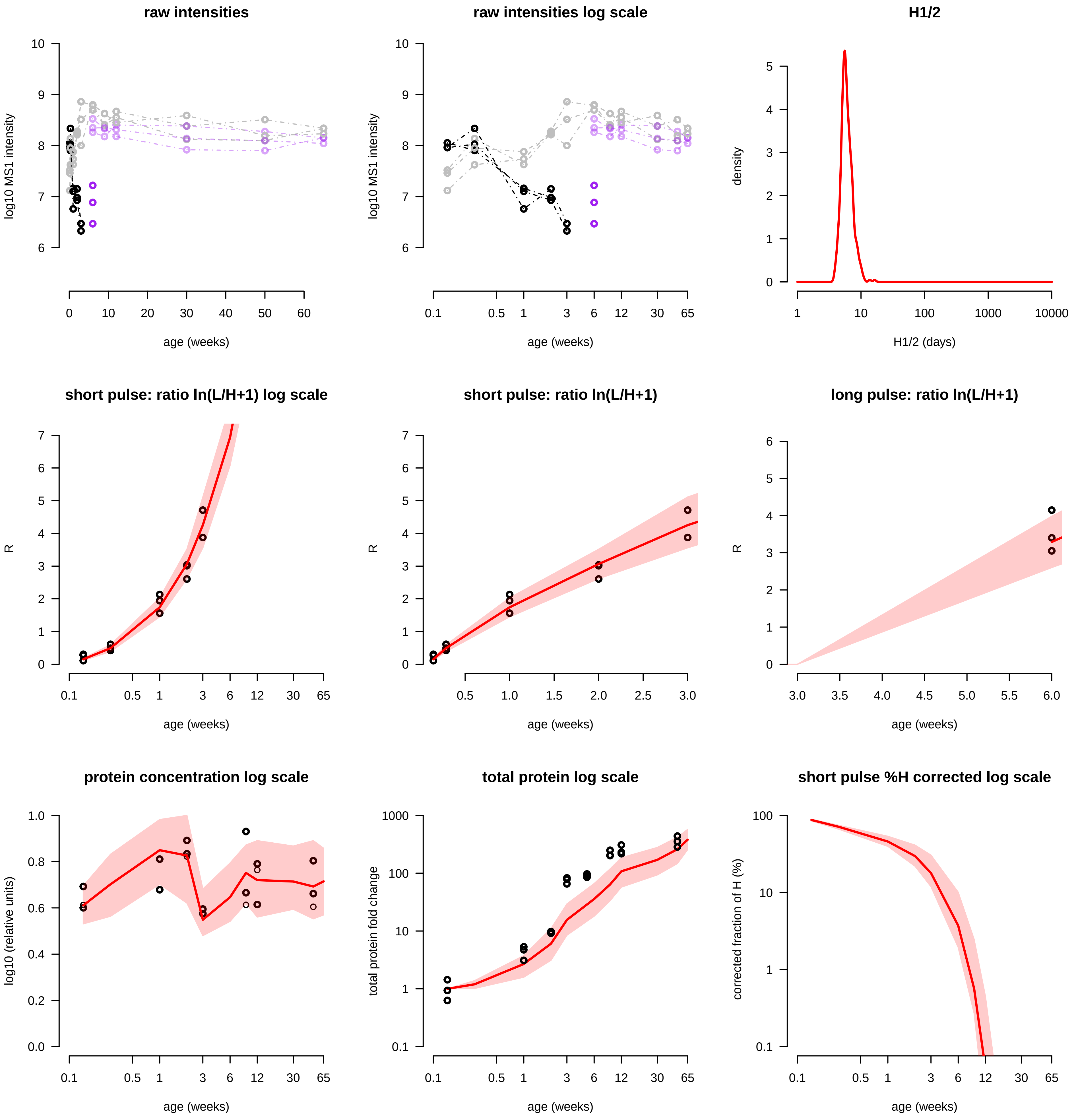

Q921M7

>sp|Q921M7|CYRIB_MOUSE

CYFIP−related Rac1 interactor B

GN=Cyrib

Med H1/2: 5.1 days

Q5 H1/2: 3.8 days

Q95 H1/2: 8 days

reliable model fit: yes

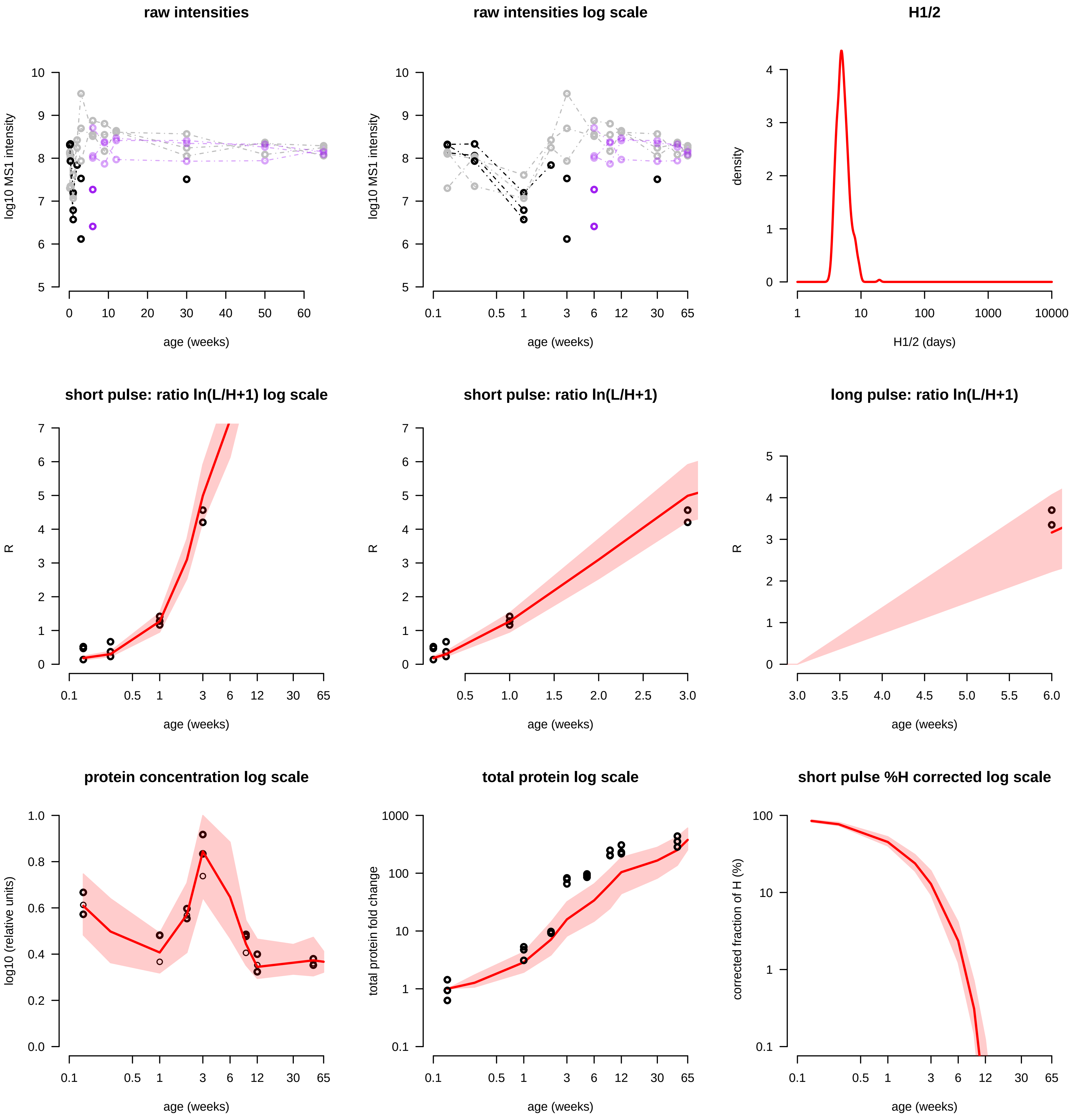

P97393

>sp|P97393|RHG05_MOUSE

Rho GTPase−activating protein 5

GN=Arhgap5

Med H1/2: 641.7 days

Q5 H1/2: 269.4 days

Q95 H1/2: 5611.2 days

reliable model fit: yes

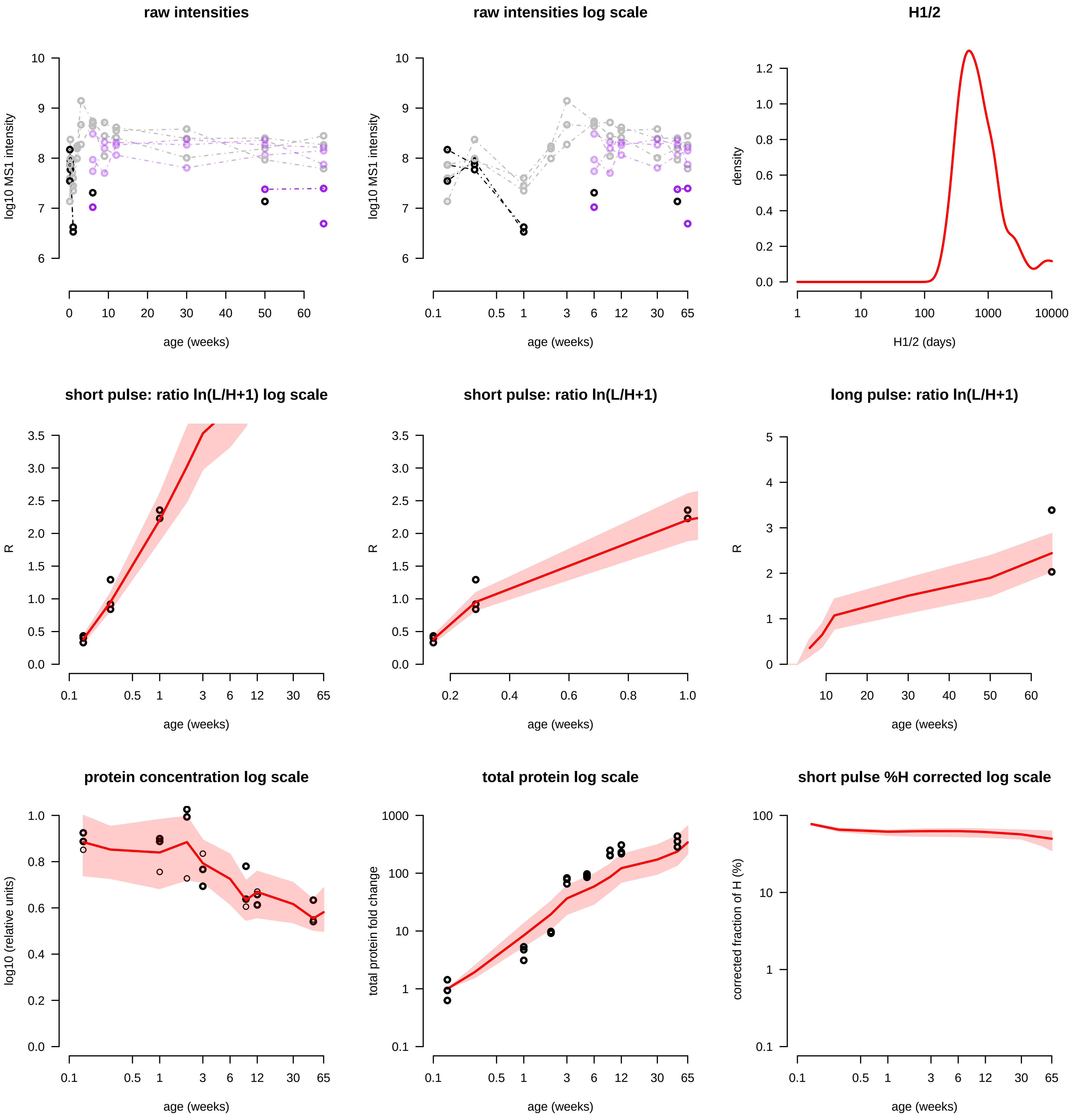

Q80TH2−1;Q80TH2;Q80TH2−2

>sp|Q80TH2−1|ERBIN_MOUSE

Isoform 1 of Erbin

GN=Erbin

Med H1/2: 1673.5 days

Q5 H1/2: 401.6 days

Q95 H1/2: 28382.7 days

reliable model fit: no

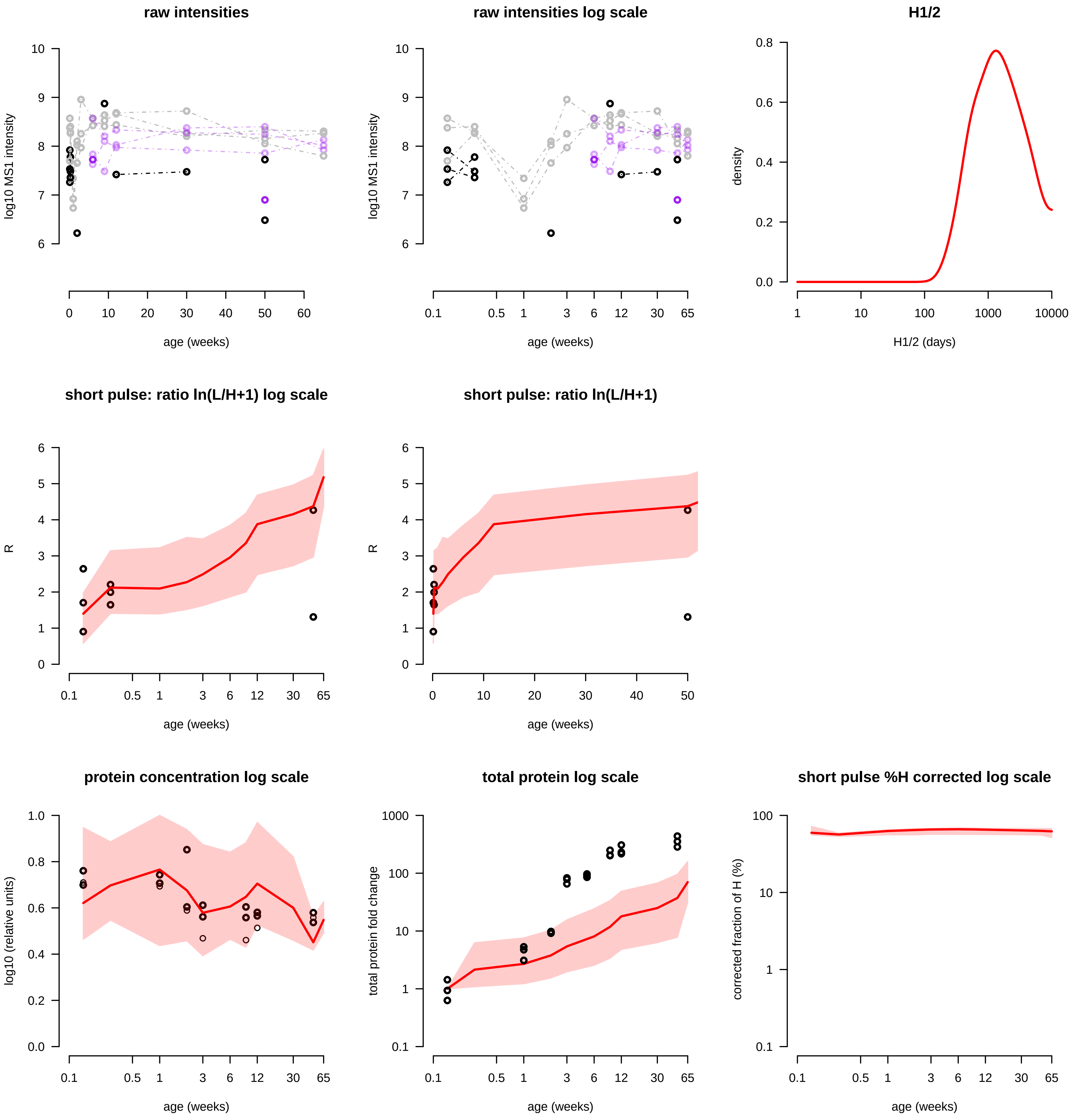

Q9D706

>sp|Q9D706|RPAP3_MOUSE

RNA polymerase II−associated protein 3

GN=Rpap3

Med H1/2: 3.1 days

Q5 H1/2: 2.5 days

Q95 H1/2: 8.8 days

reliable model fit: yes

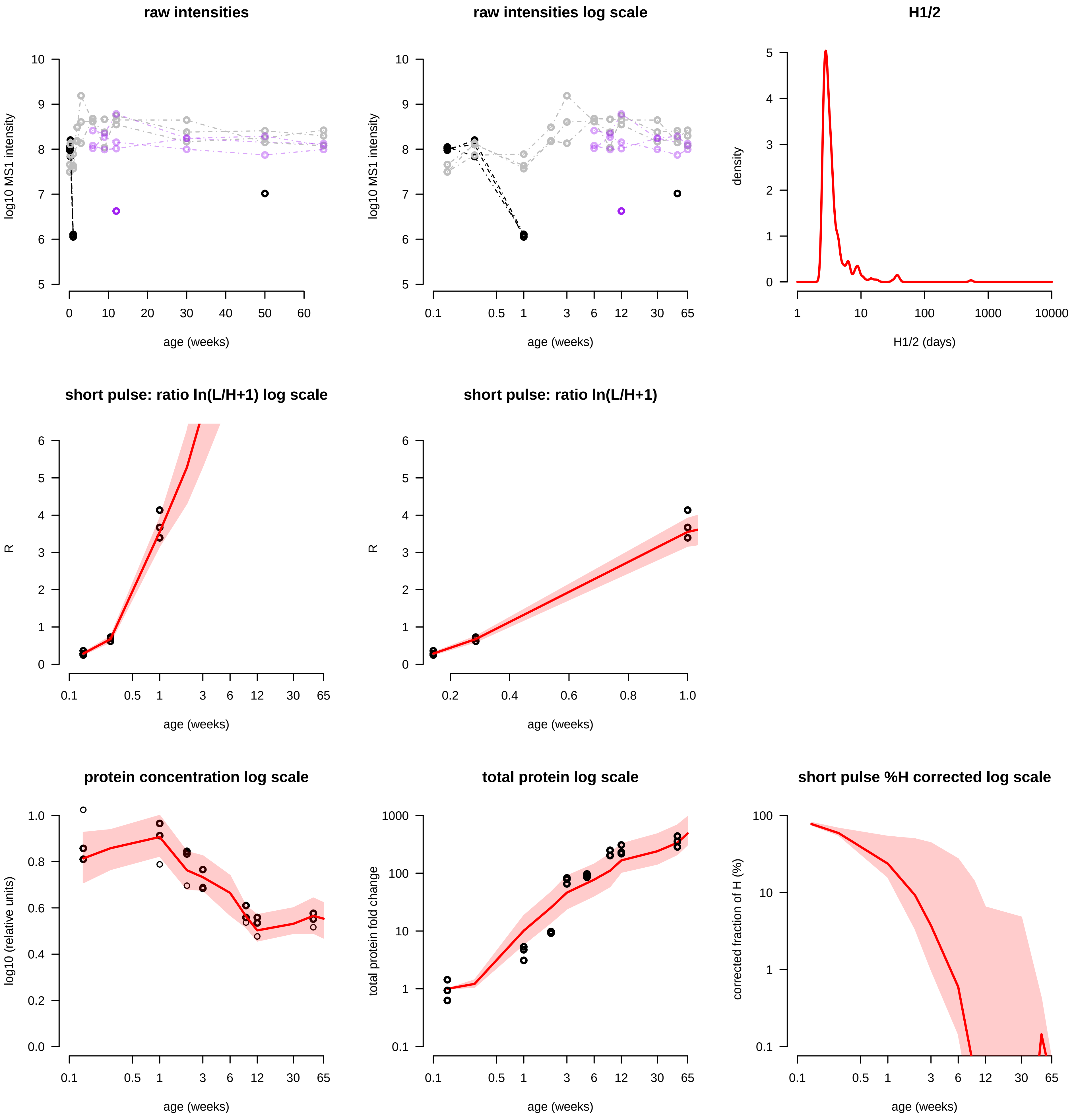

Q9D338

>sp|Q9D338|RM19_MOUSE

39S ribosomal protein L19, mitochondrial

GN=Mrpl19

Med H1/2: 13.3 days

Q5 H1/2: 4.1 days

Q95 H1/2: 66.9 days

reliable model fit: yes

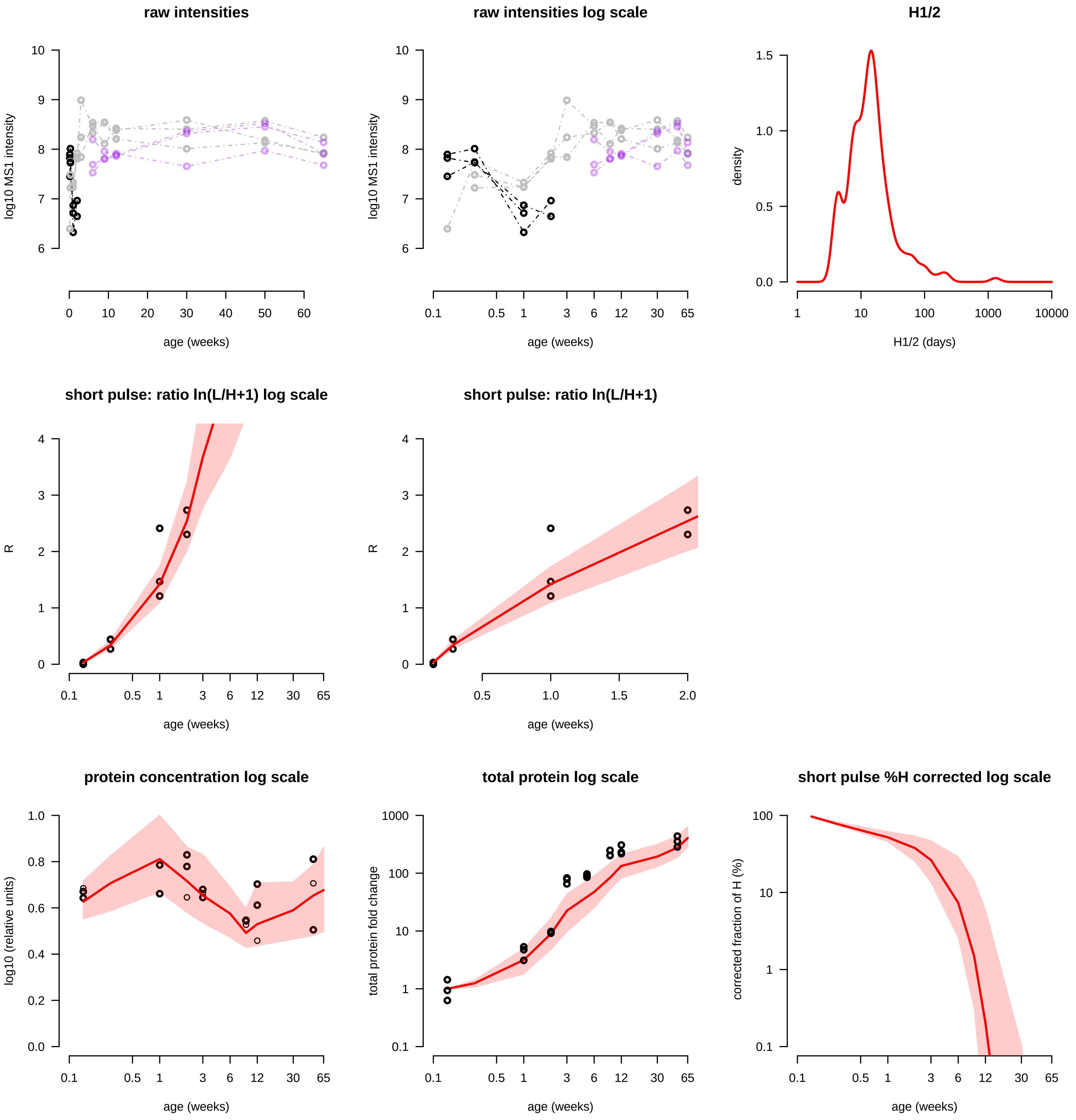

P50171;P50171−2

>sp|P50171|DHB8_MOUSE

(3R)−3−hydroxyacyl−CoA dehydrogenase

GN=Hsd17b8

Med H1/2: 19 days

Q5 H1/2: 7.4 days

Q95 H1/2: 97.4 days

reliable model fit: yes

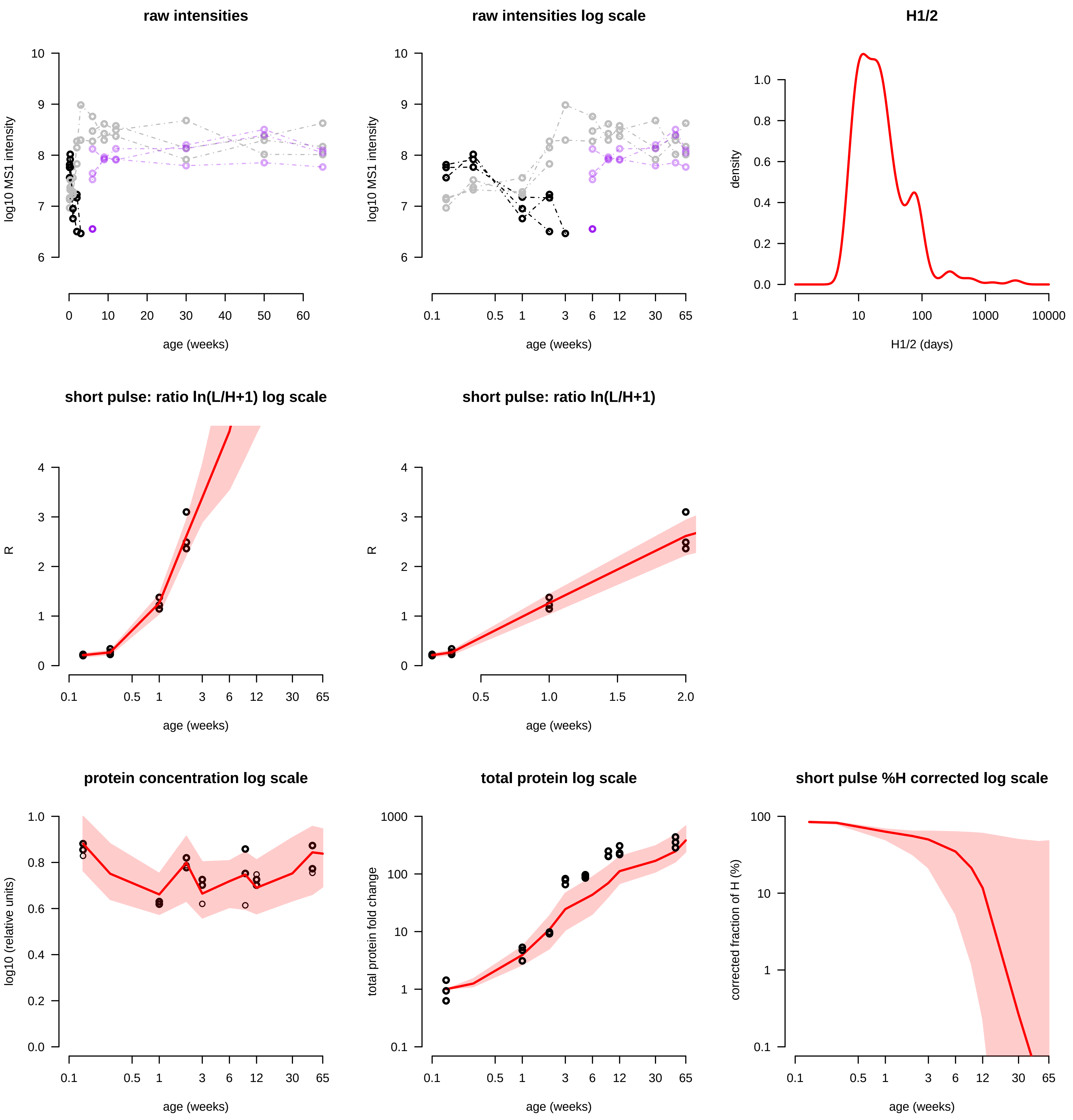

Q811U4

>sp|Q811U4|MFN1_MOUSE

Mitofusin−1

GN=Mfn1

Med H1/2: 41.5 days

Q5 H1/2: 9.5 days

Q95 H1/2: 541.5 days

reliable model fit: no

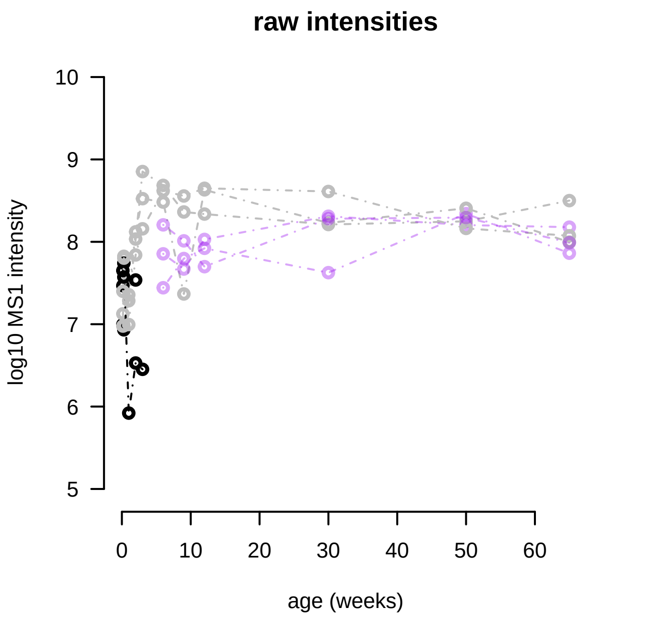

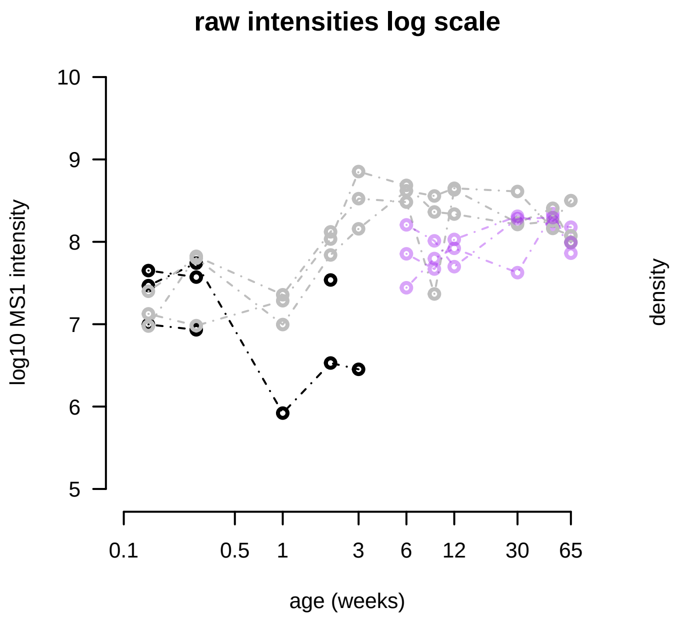

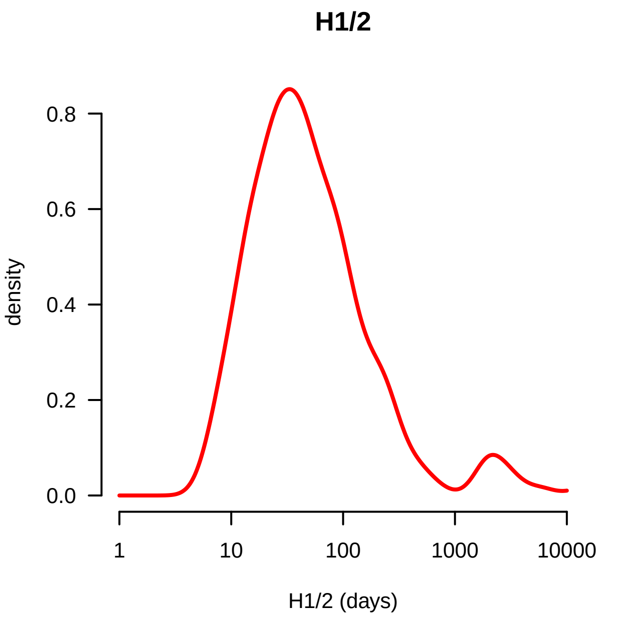

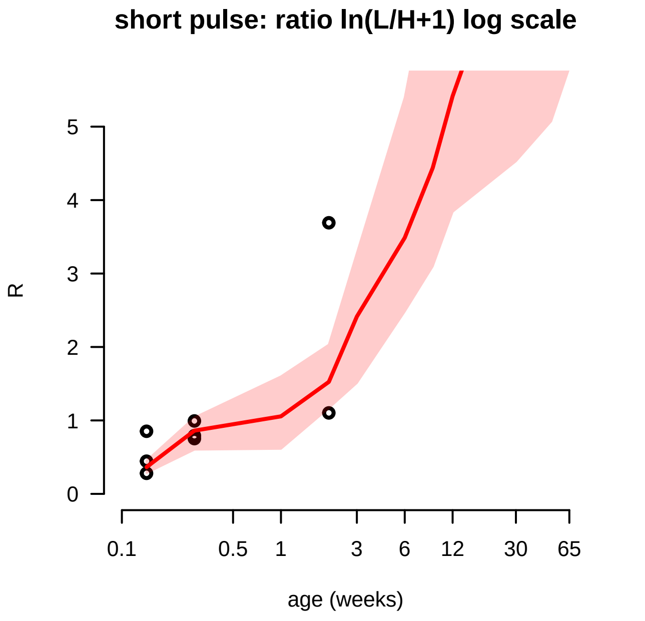

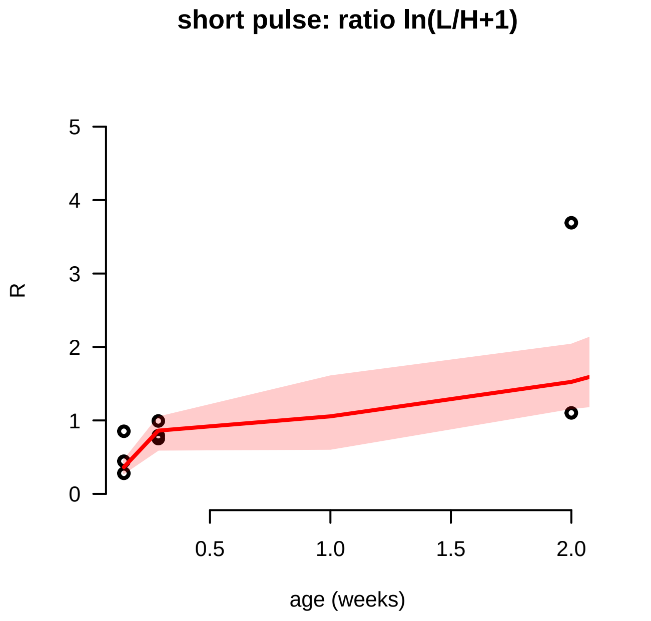

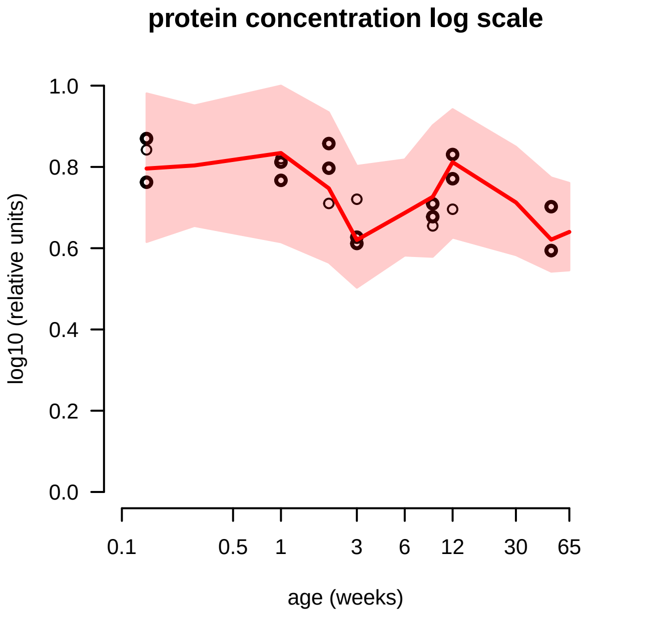

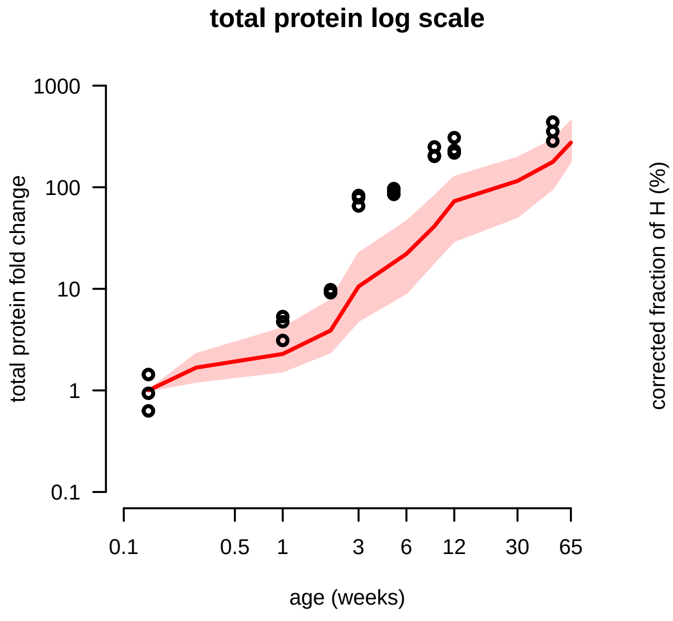

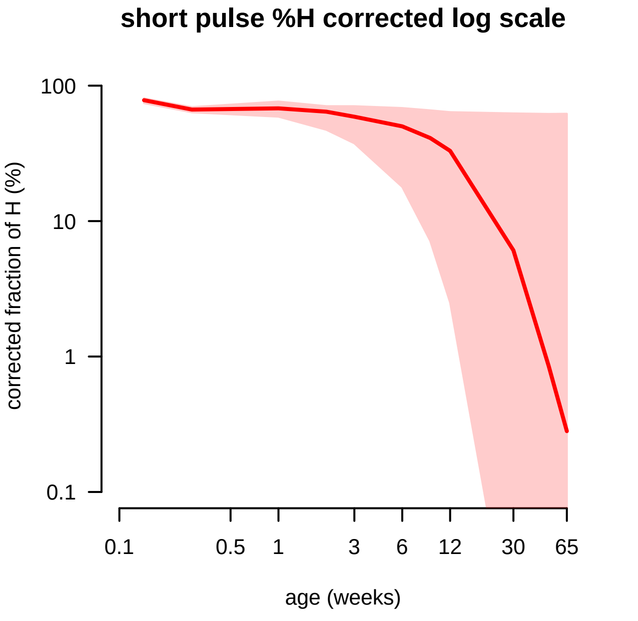

Q9D7S9

>sp|Q9D7S9|CHMP5_MOUSE

Charged multivesicular body protein 5

GN=Chmp5

Med H1/2: 25 days

Q5 H1/2: 7.5 days

Q95 H1/2: 285.1 days

reliable model fit: yes

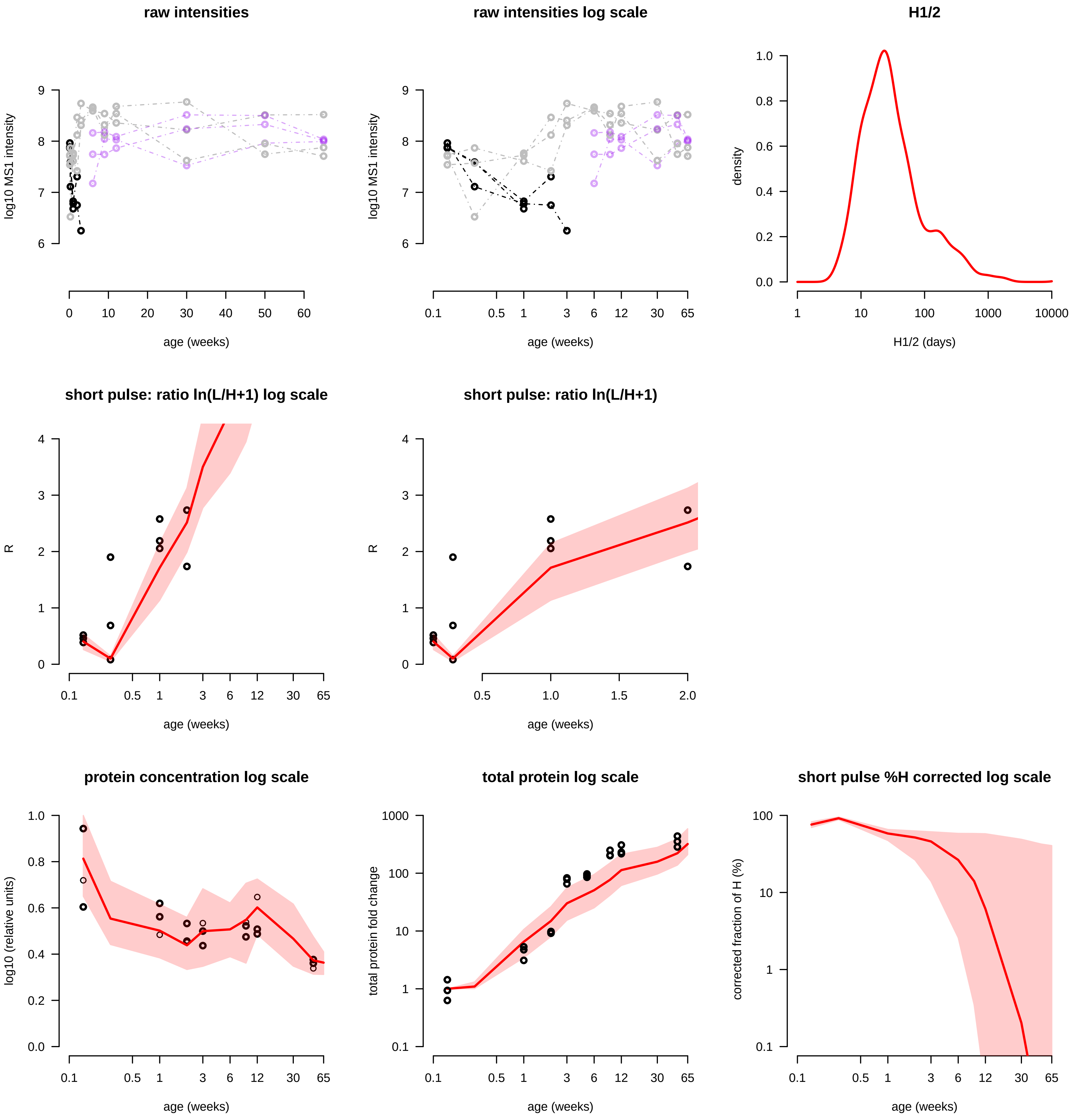

Q8BRH0;Q8BRH0−2

>sp|Q8BRH0|TMTC3_MOUSE

Protein O−mannosyl−transferase TMTC3

GN=Tmtc3

Med H1/2: 4.6 days

Q5 H1/2: 3.1 days

Q95 H1/2: 11.4 days

reliable model fit: yes

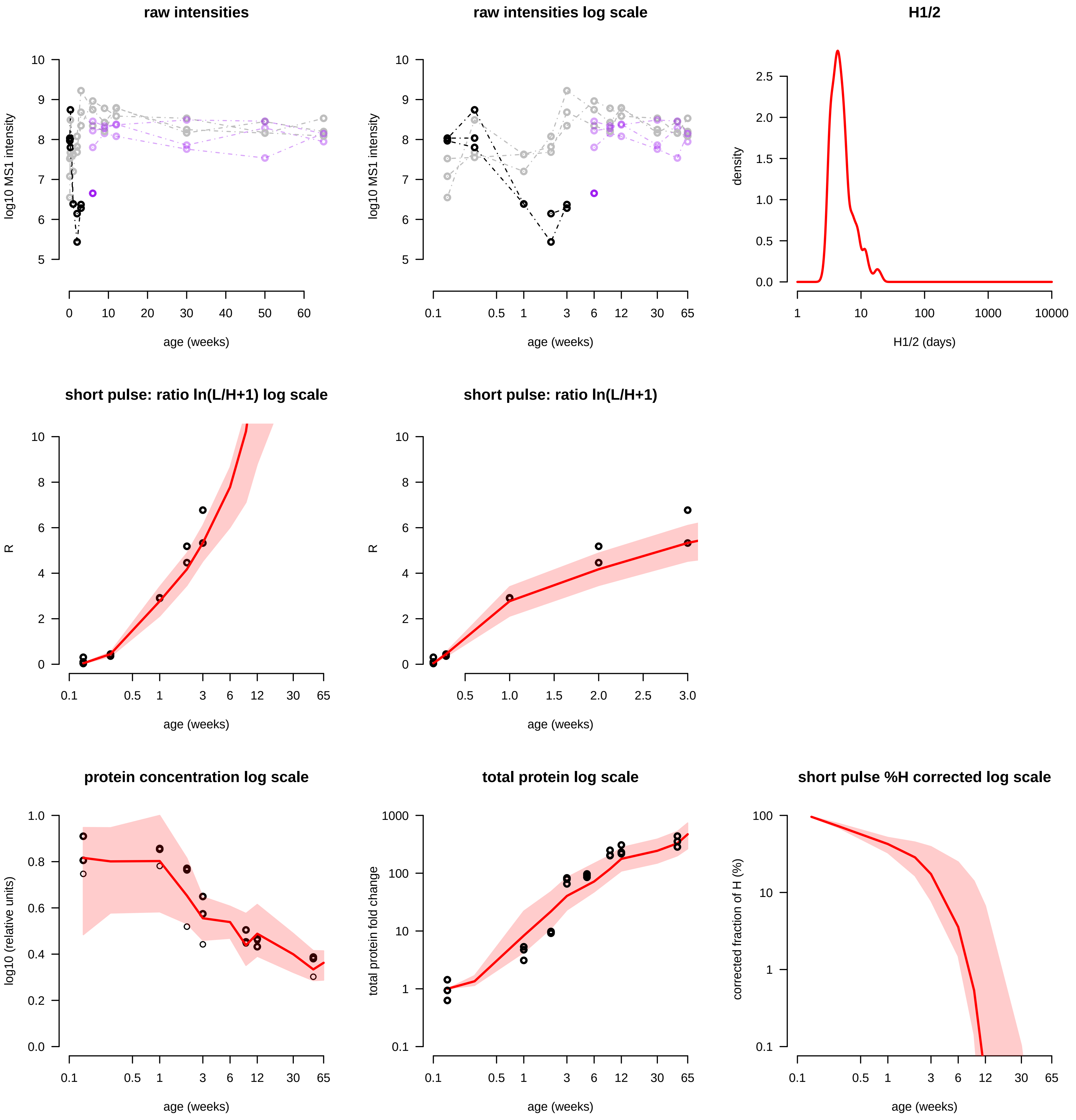

Q8BI72

>sp|Q8BI72|CARF_MOUSE

CDKN2A−interacting protein

GN=Cdkn2aip

Med H1/2: 6.4 days

Q5 H1/2: 3.1 days

Q95 H1/2: 1308.5 days

reliable model fit: yes

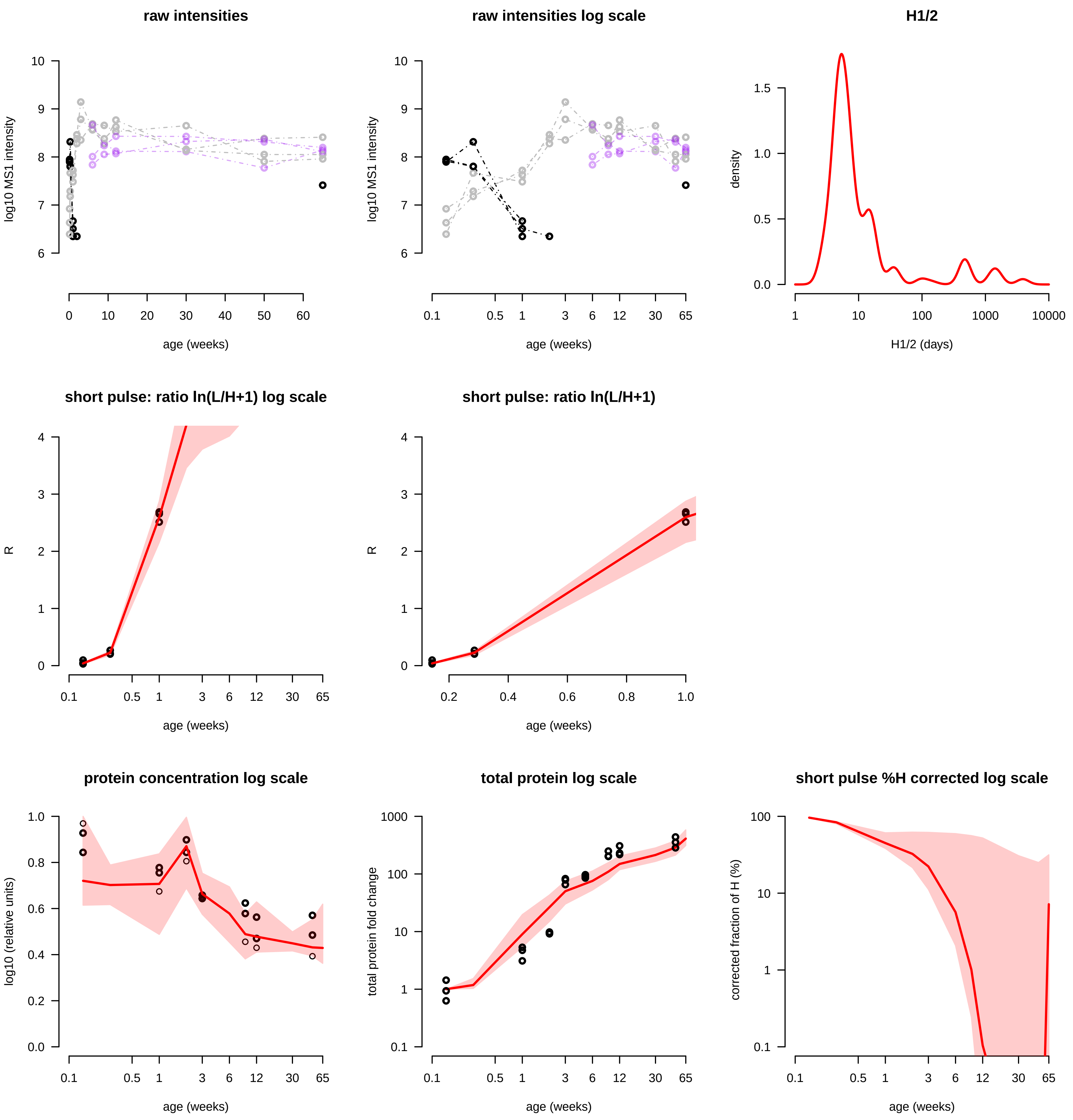

Q8K2A1;Q8K2A1−2

>sp|Q8K2A1|GULP1_MOUSE

PTB domain−containing engulfment adapter protein 1

GN=Gulp1

Med H1/2: 38.5 days

Q5 H1/2: 9.8 days

Q95 H1/2: 591.1 days

reliable model fit: no

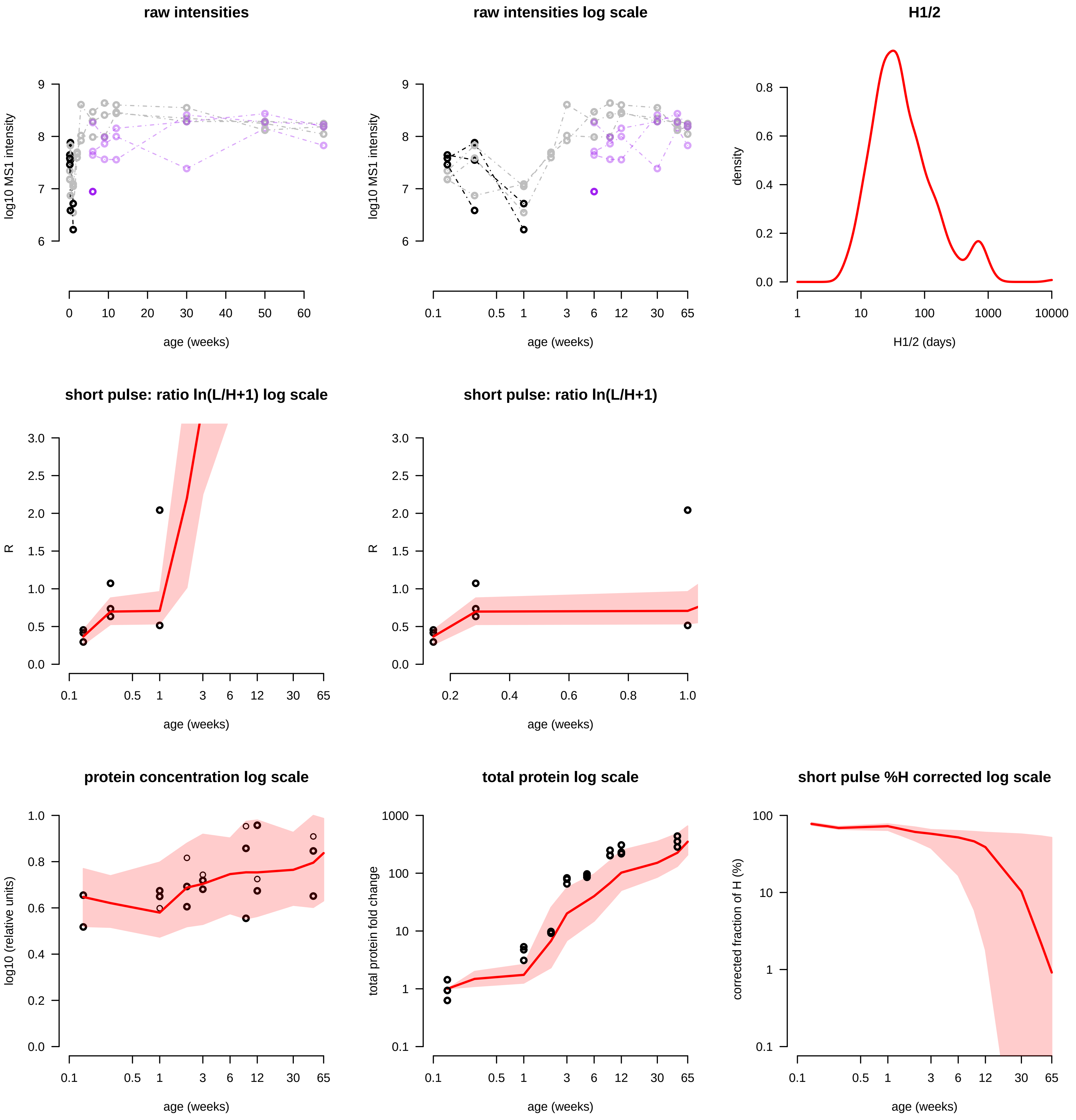

O08912

>sp|O08912|GALT1_MOUSE

Polypeptide N−acetylgalactosaminyltransferase 1

GN=Galnt1

Med H1/2: 5.4 days

Q5 H1/2: 2.7 days

Q95 H1/2: 37 days

reliable model fit: yes

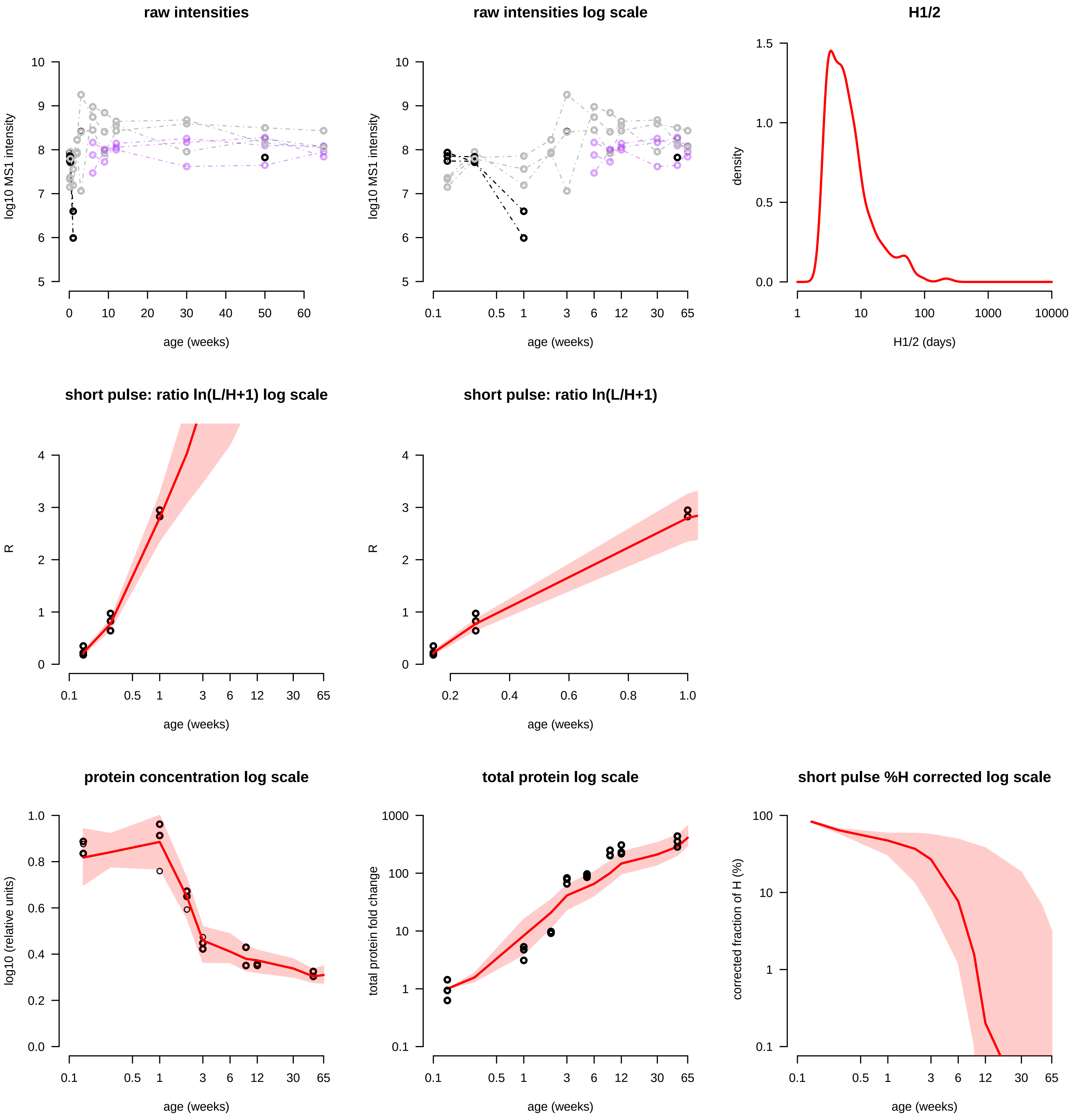

Q8BGA5

>sp|Q8BGA5|KRR1_MOUSE

KRR1 small subunit processome component homolog

GN=Krr1

Med H1/2: 22.8 days

Q5 H1/2: 8.1 days

Q95 H1/2: 353.1 days

reliable model fit: yes

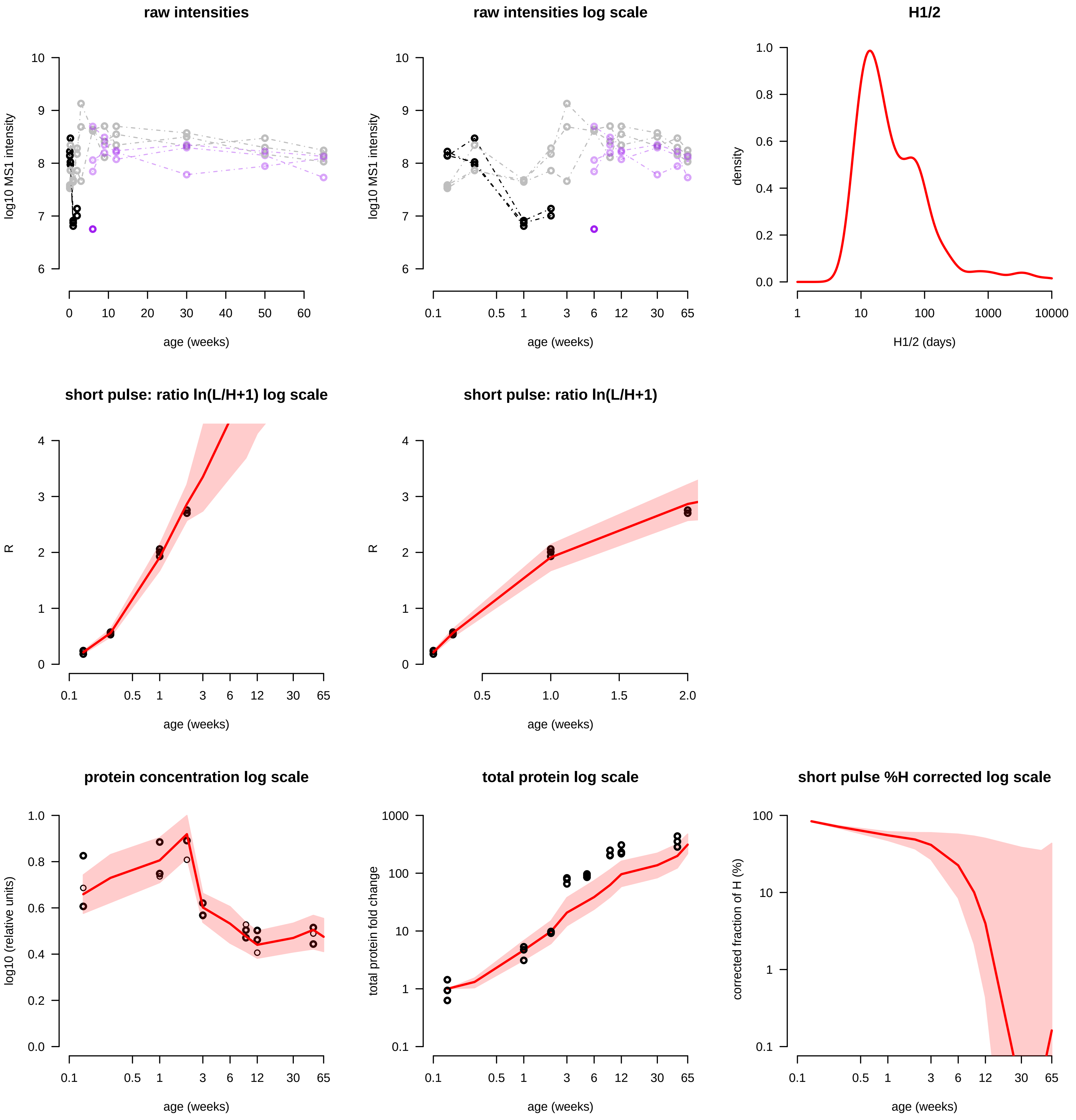

Q9Z210

>sp|Q9Z210|PX11B_MOUSE

Peroxisomal membrane protein 11B

GN=Pex11b

Med H1/2: 9.5 days

Q5 H1/2: 2.9 days

Q95 H1/2: 121.7 days

reliable model fit: yes

raw intensities

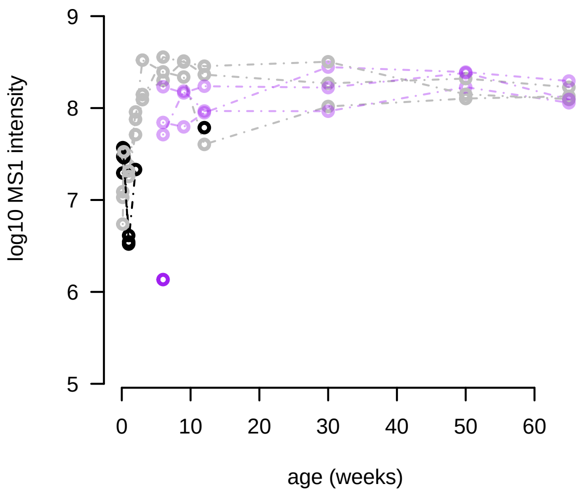

raw intensities log scale

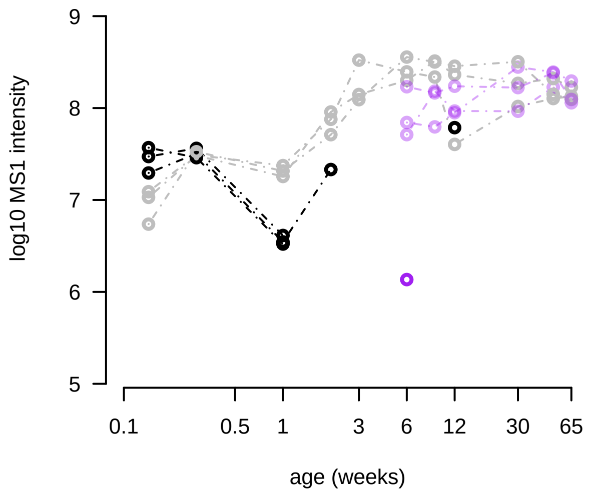

H1/2

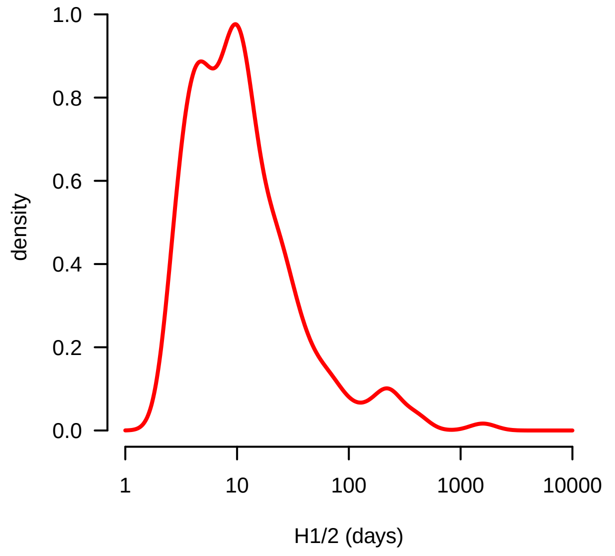

short pulse: ratio ln(L/H+1) log scale

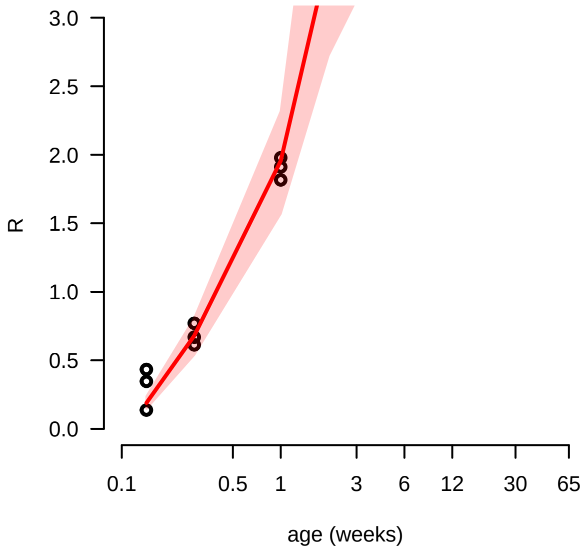

short pulse: ratio ln(L/H+1)

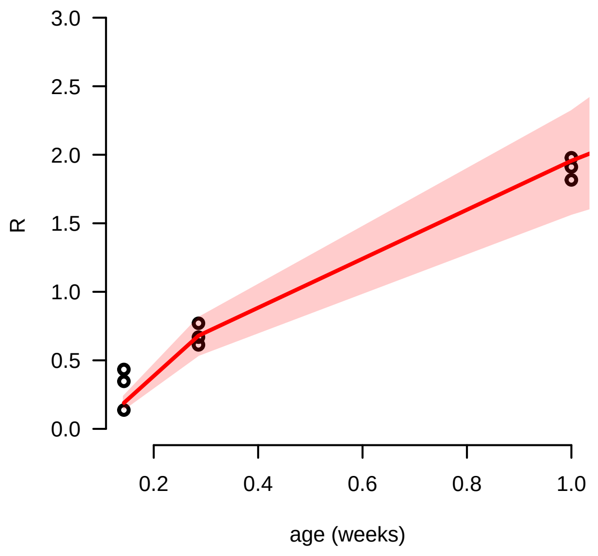

protein concentration log scale

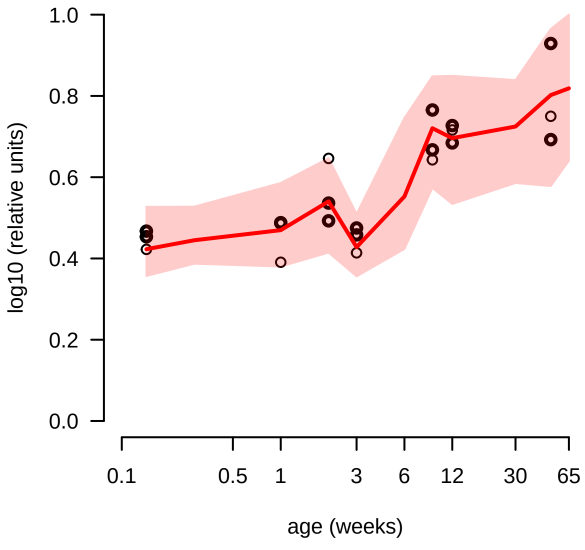

total protein log scale

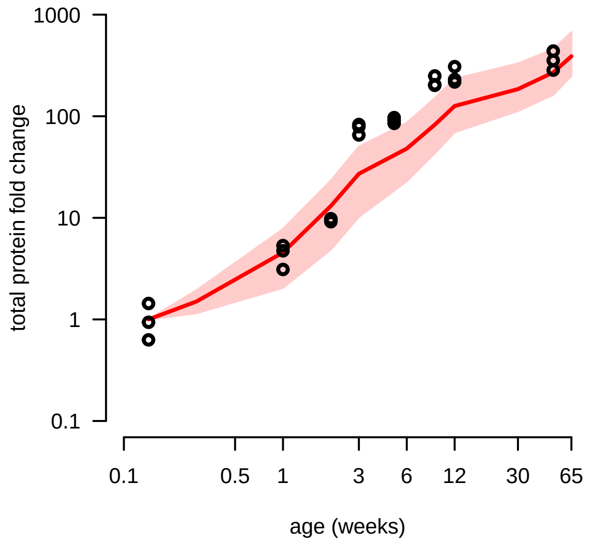

short pulse %H corrected log scale

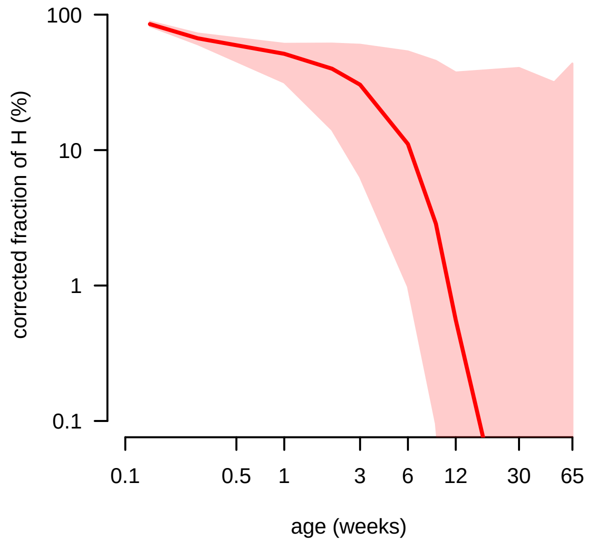

Q8R2Q8

>sp|Q8R2Q8|BST2_MOUSE

Bone marrow stromal antigen 2

GN=Bst2

Med H1/2: 26.1 days

Q5 H1/2: 8.9 days

Q95 H1/2: 269.1 days

reliable model fit: no

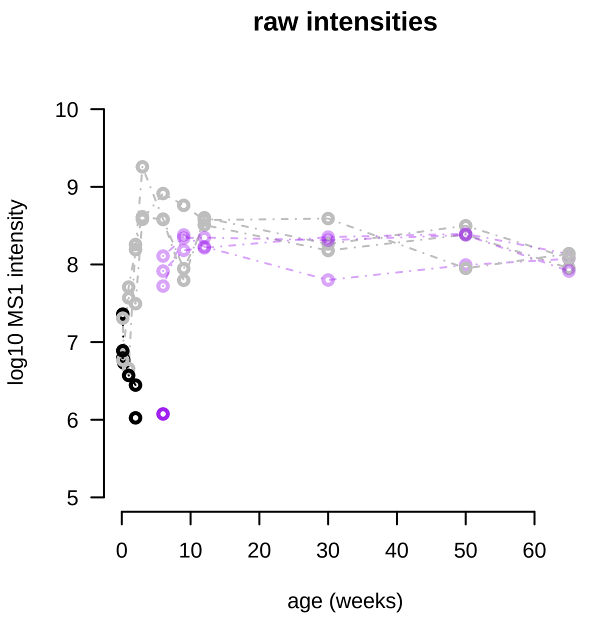

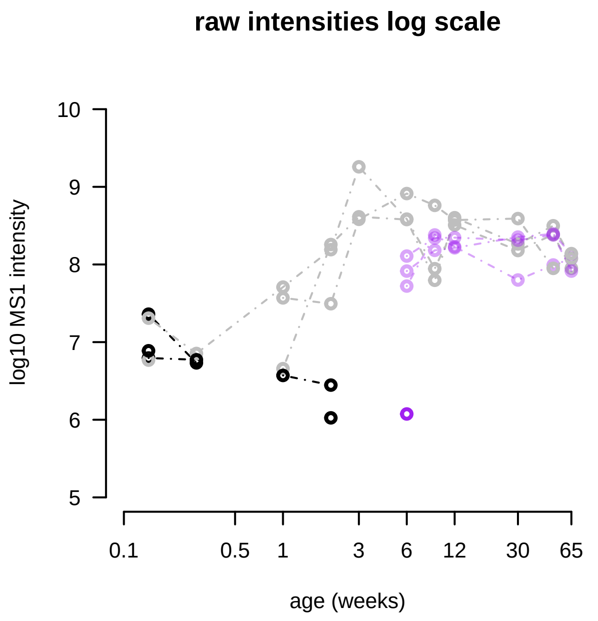

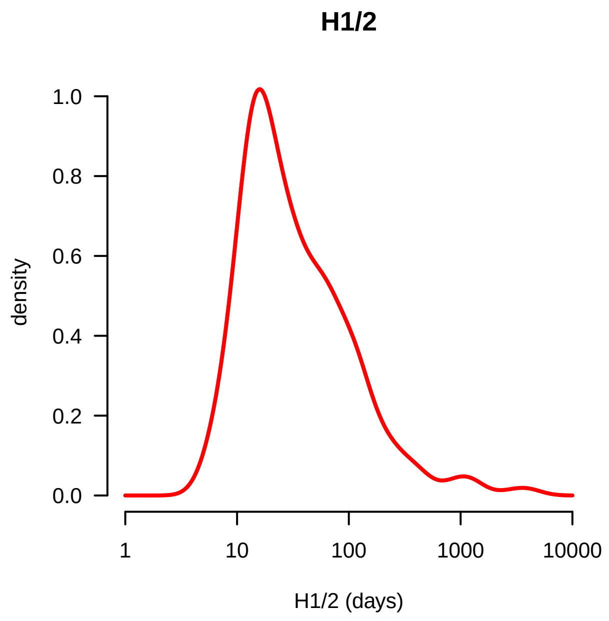

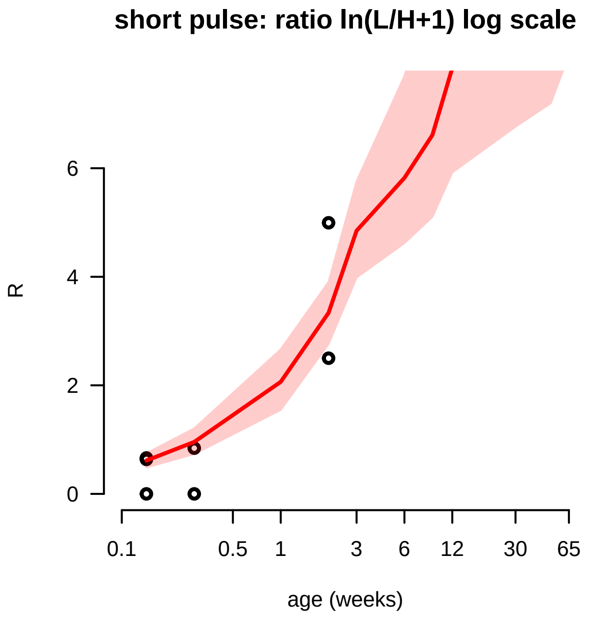

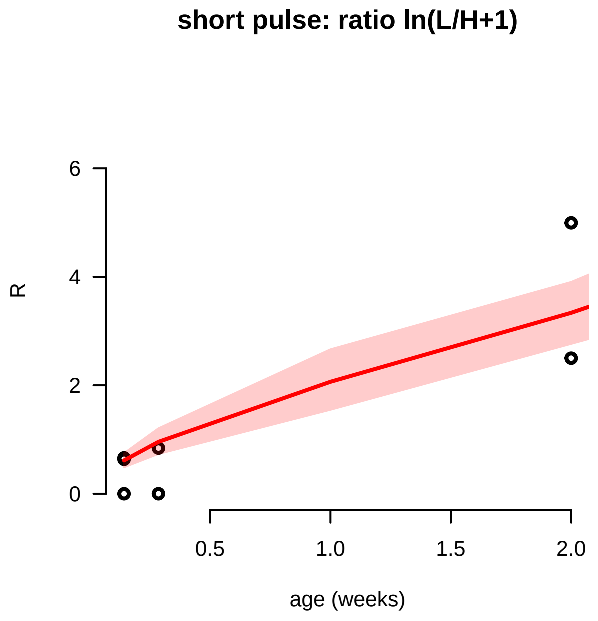

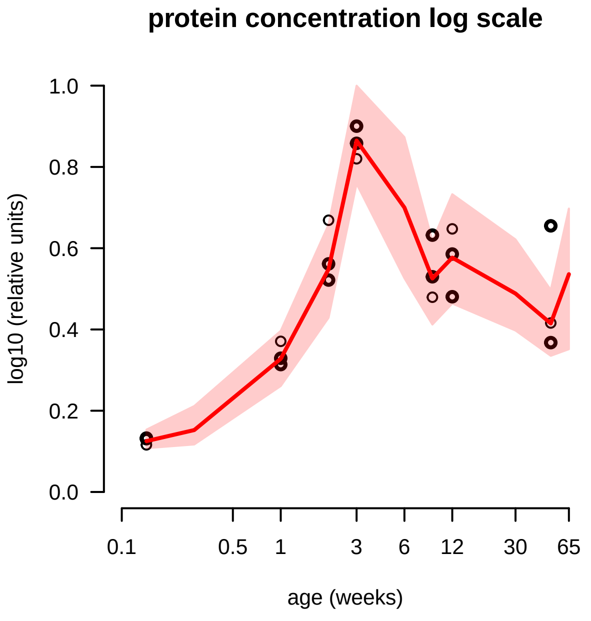

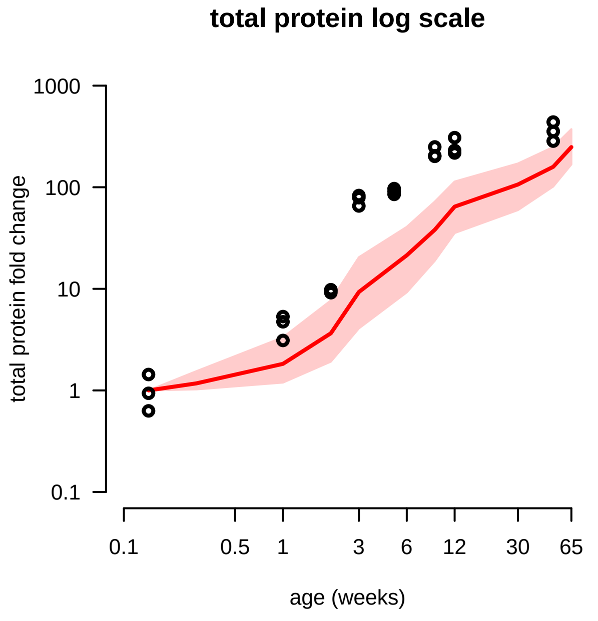

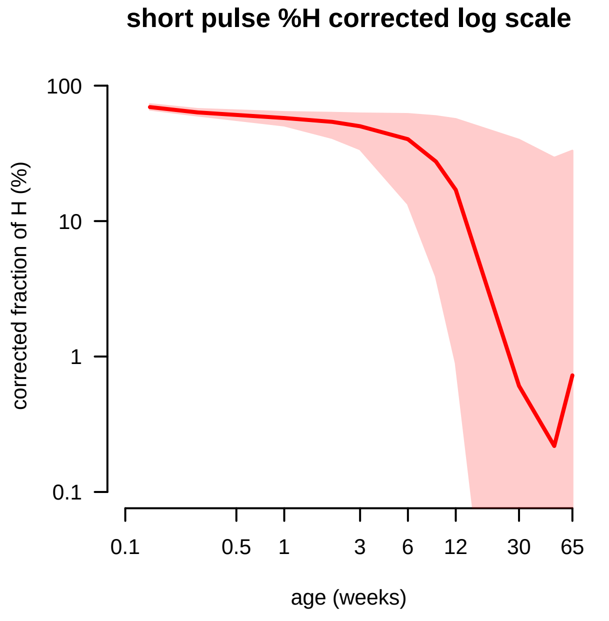

Q3UHK6−2

>sp|Q3UHK6−2|TEN4_MOUSE

Isoform 2 of Teneurin−4

GN=Tenm4

Med H1/2: 3885.1 days

Q5 H1/2: 1194.4 days

Q95 H1/2: 47477.6 days

reliable model fit: no

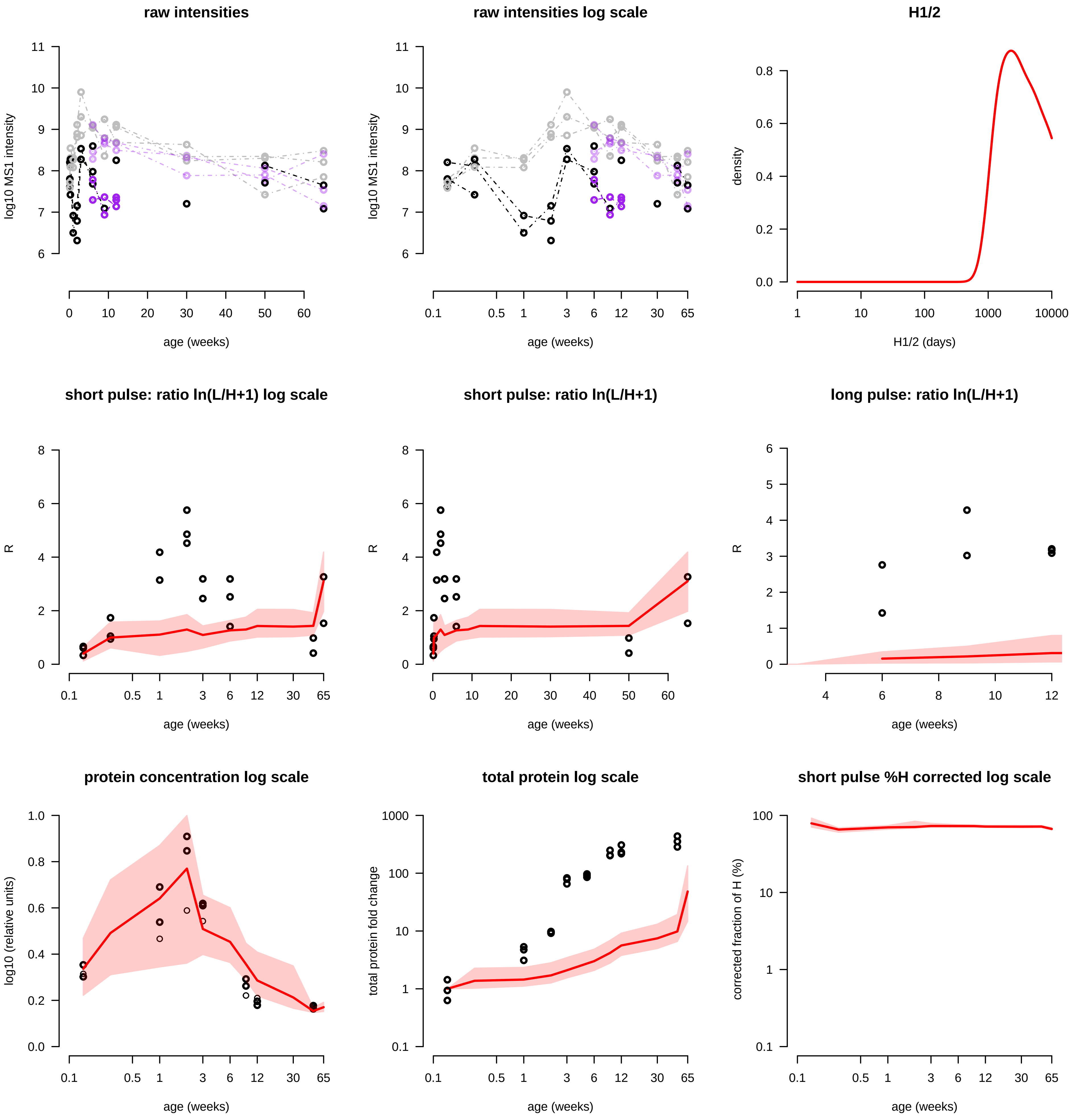

Q0P5W1;Q0P5W1−3;Q0P5W1−2

>sp|Q0P5W1|VPS8_MOUSE

Vacuolar protein sorting−associated protein 8 homolog

GN=Vps8

Med H1/2: 8.9 days

Q5 H1/2: 3 days

Q95 H1/2: 106.4 days

reliable model fit: yes

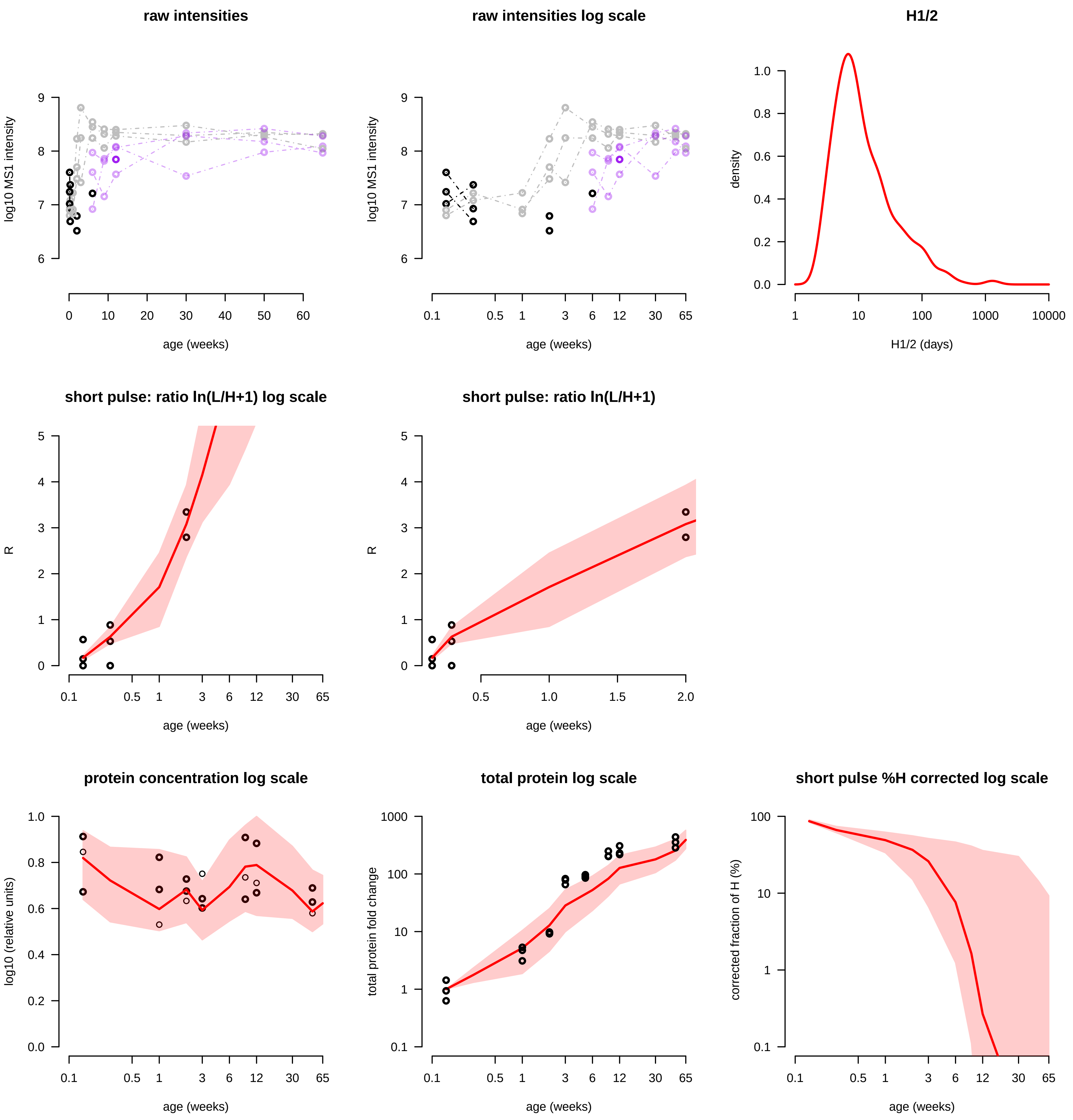

Q62018;Q62018−3

>sp|Q62018|CTR9_MOUSE

RNA polymerase−associated protein CTR9 homolog

GN=Ctr9

Med H1/2: 54.2 days

Q5 H1/2: 39.3 days

Q95 H1/2: 106.5 days

reliable model fit: yes

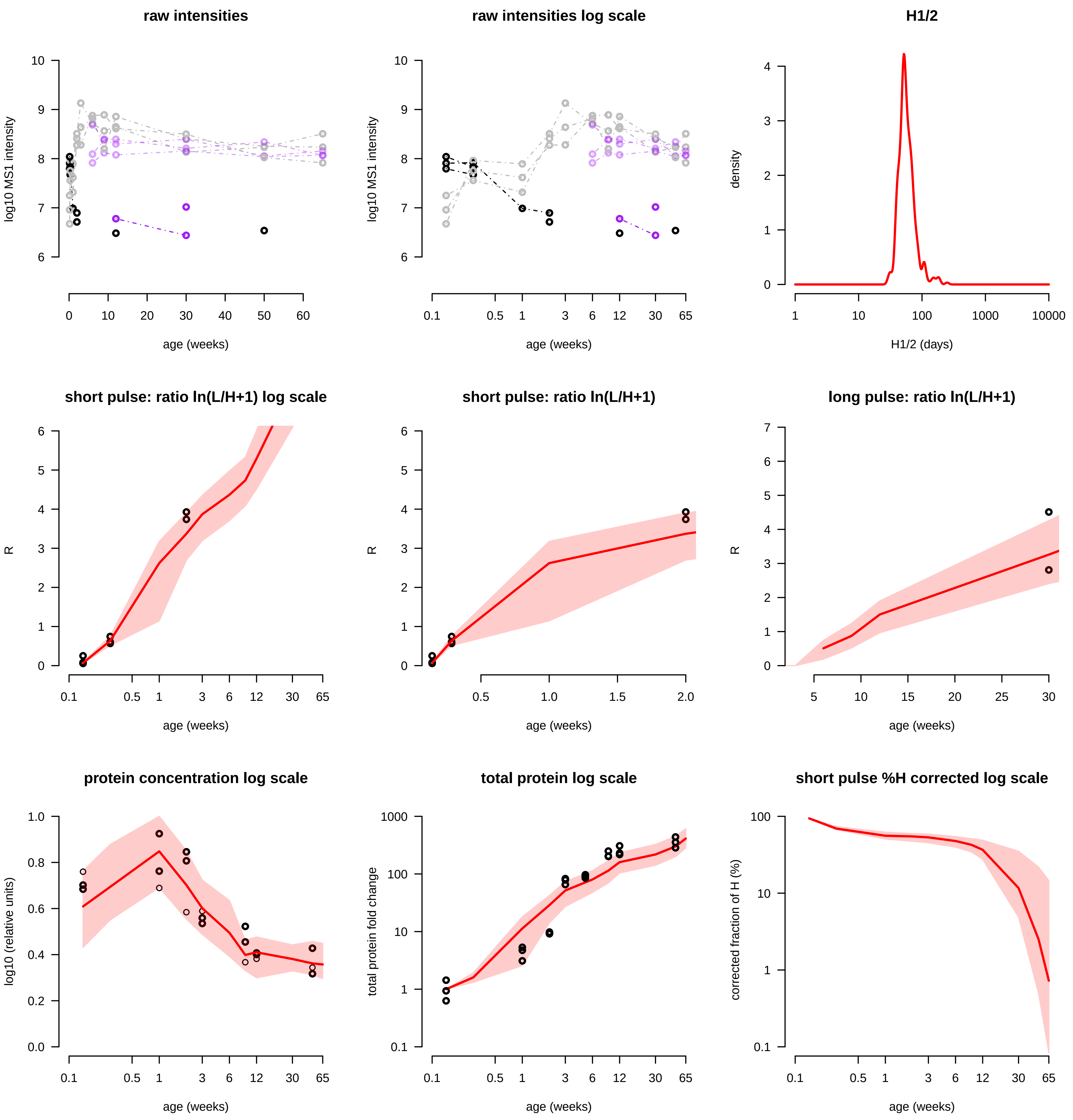

O55135

>sp|O55135|IF6_MOUSE

Eukaryotic translation initiation factor 6

GN=Eif6

Med H1/2: 4.3 days

Q5 H1/2: 3.6 days

Q95 H1/2: 5.8 days

reliable model fit: yes

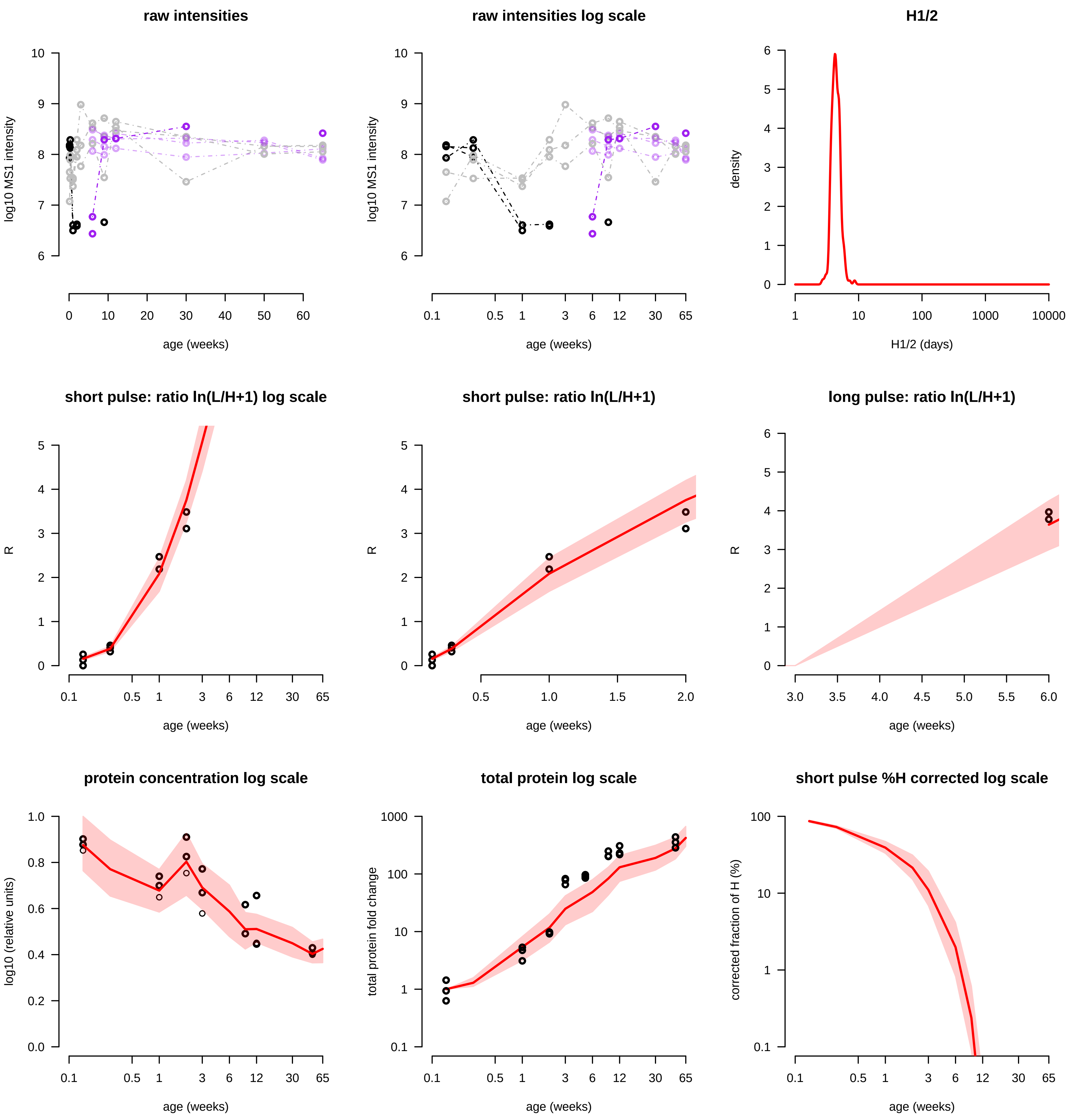

Q8BQ47

>sp|Q8BQ47|CNPY4_MOUSE

Protein canopy homolog 4

GN=Cnpy4

Med H1/2: 24.6 days

Q5 H1/2: 9.4 days

Q95 H1/2: 101.2 days

reliable model fit: yes

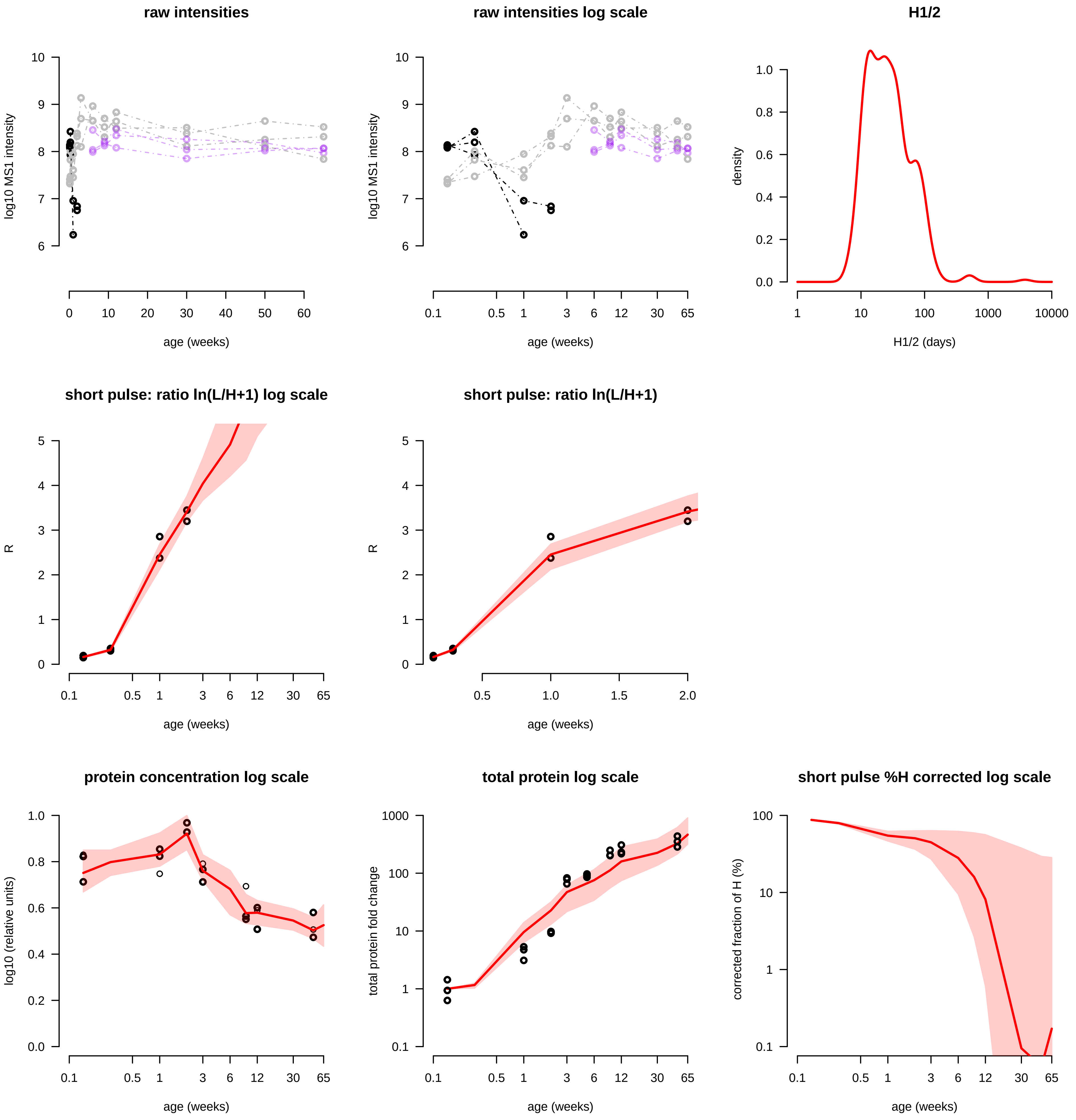

Q8K4G5;Q8K4G5−2;Q8K4G5−4;Q8K4G5−3

>sp|Q8K4G5|ABLM1_MOUSE

Actin−binding LIM protein 1

GN=Ablim1

Med H1/2: 4.8 days

Q5 H1/2: 2.9 days

Q95 H1/2: 22.2 days

reliable model fit: yes

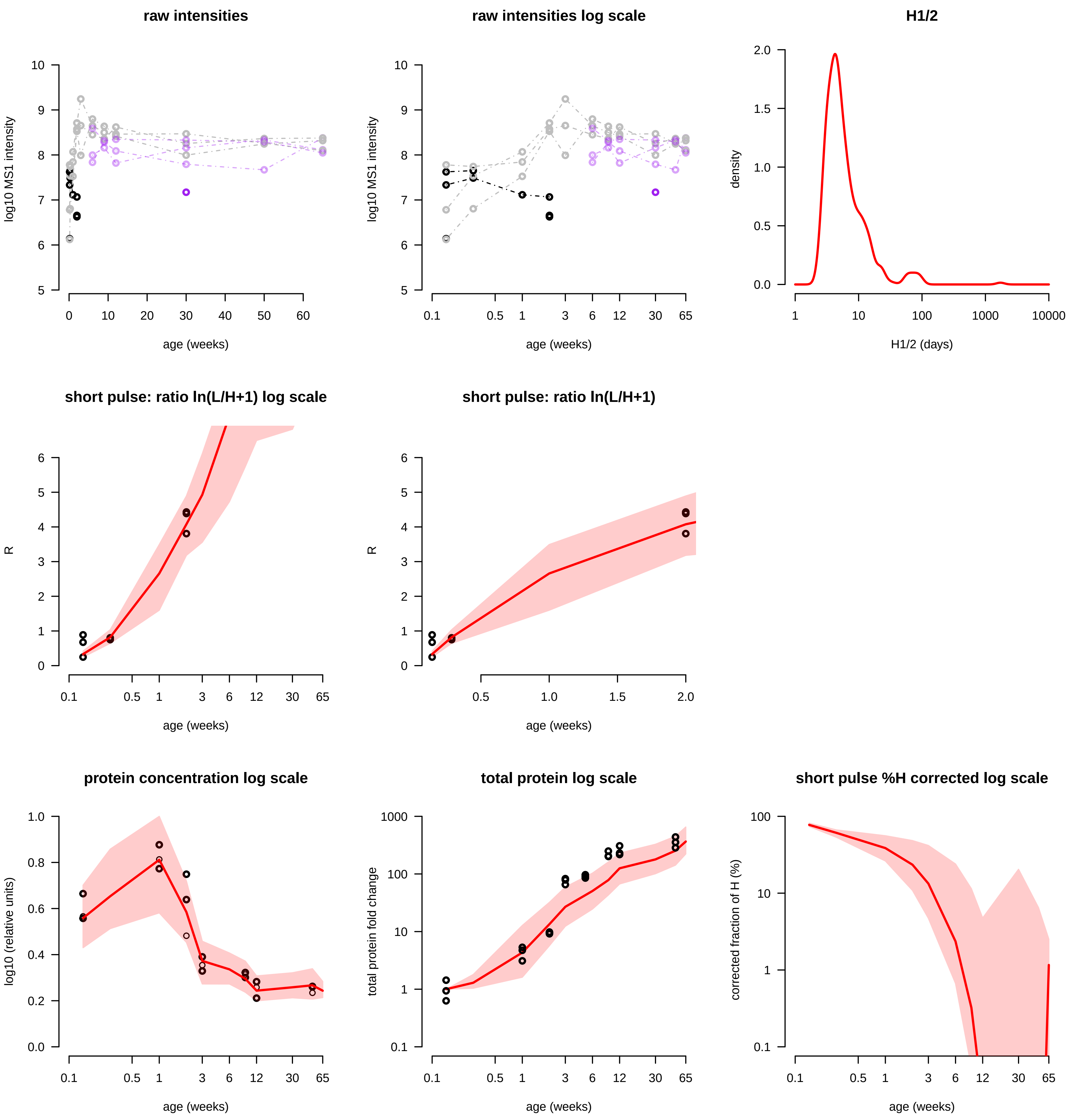

Q922H4

>sp|Q922H4|GMPPA_MOUSE

Mannose−1−phosphate guanyltransferase alpha

GN=Gmppa

Med H1/2: 8.2 days

Q5 H1/2: 3 days

Q95 H1/2: 93.7 days

reliable model fit: yes

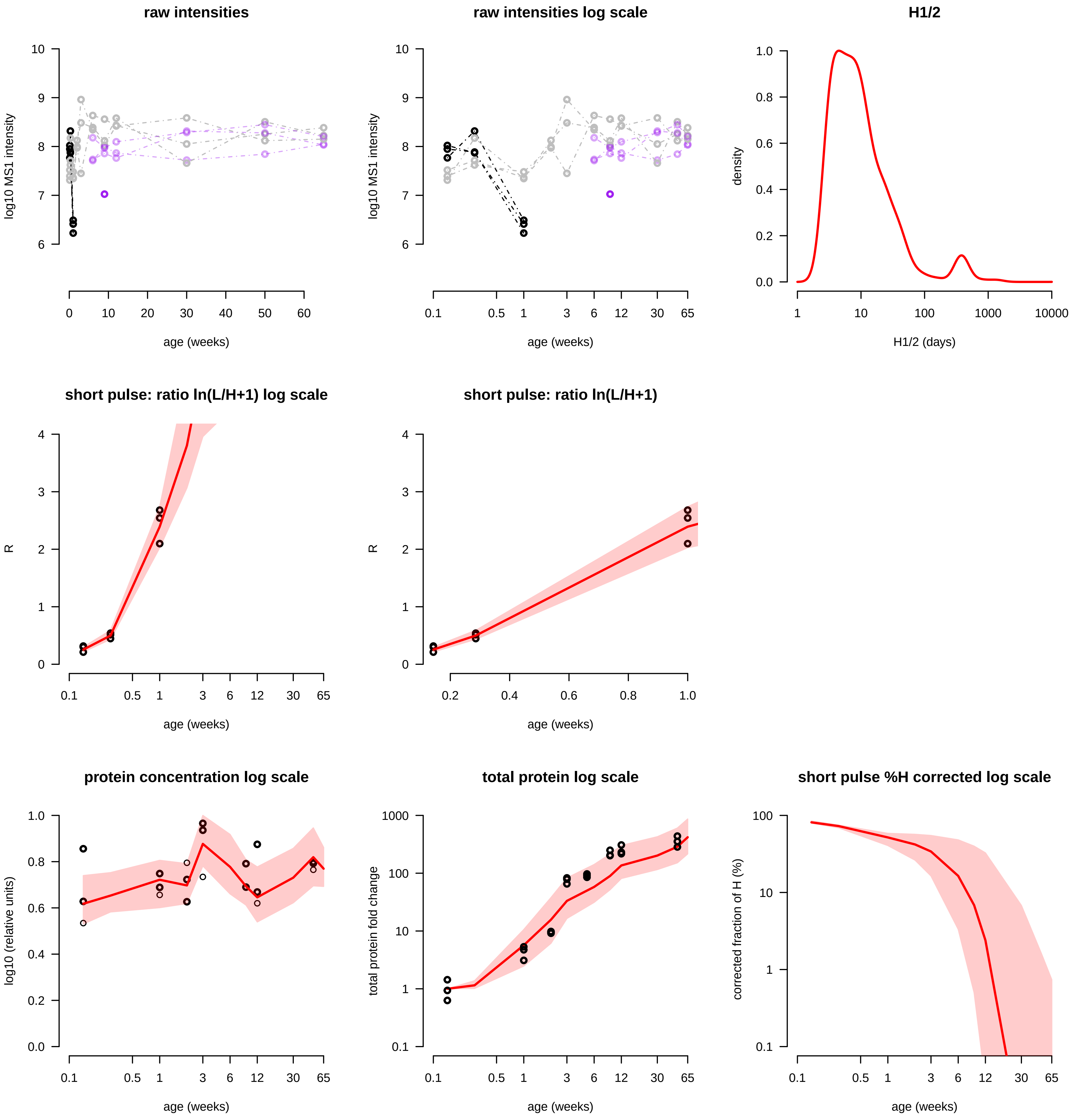

Q61103

>sp|Q61103|REQU_MOUSE

Zinc finger protein ubi−d4

GN=Dpf2

Med H1/2: 5.5 days

Q5 H1/2: 2.7 days

Q95 H1/2: 38.6 days

reliable model fit: yes

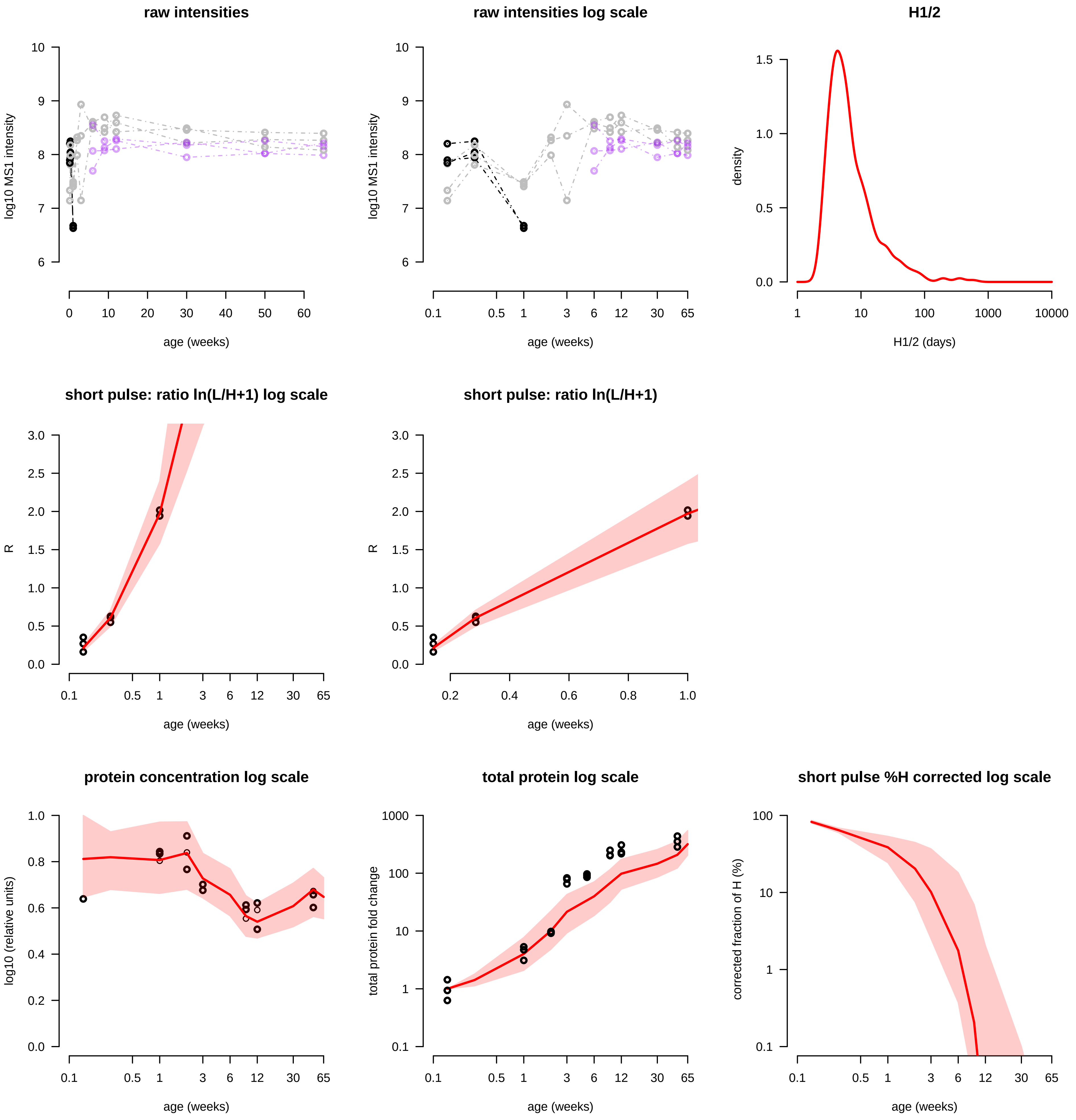

Q01147−1;Q01147

>sp|Q01147−1|CREB1_MOUSE

Isoform 2 of Cyclic AMP−responsive element−binding protein 1

GN=Creb1

Med H1/2: 7.4 days

Q5 H1/2: 3 days

Q95 H1/2: 30.9 days

reliable model fit: yes

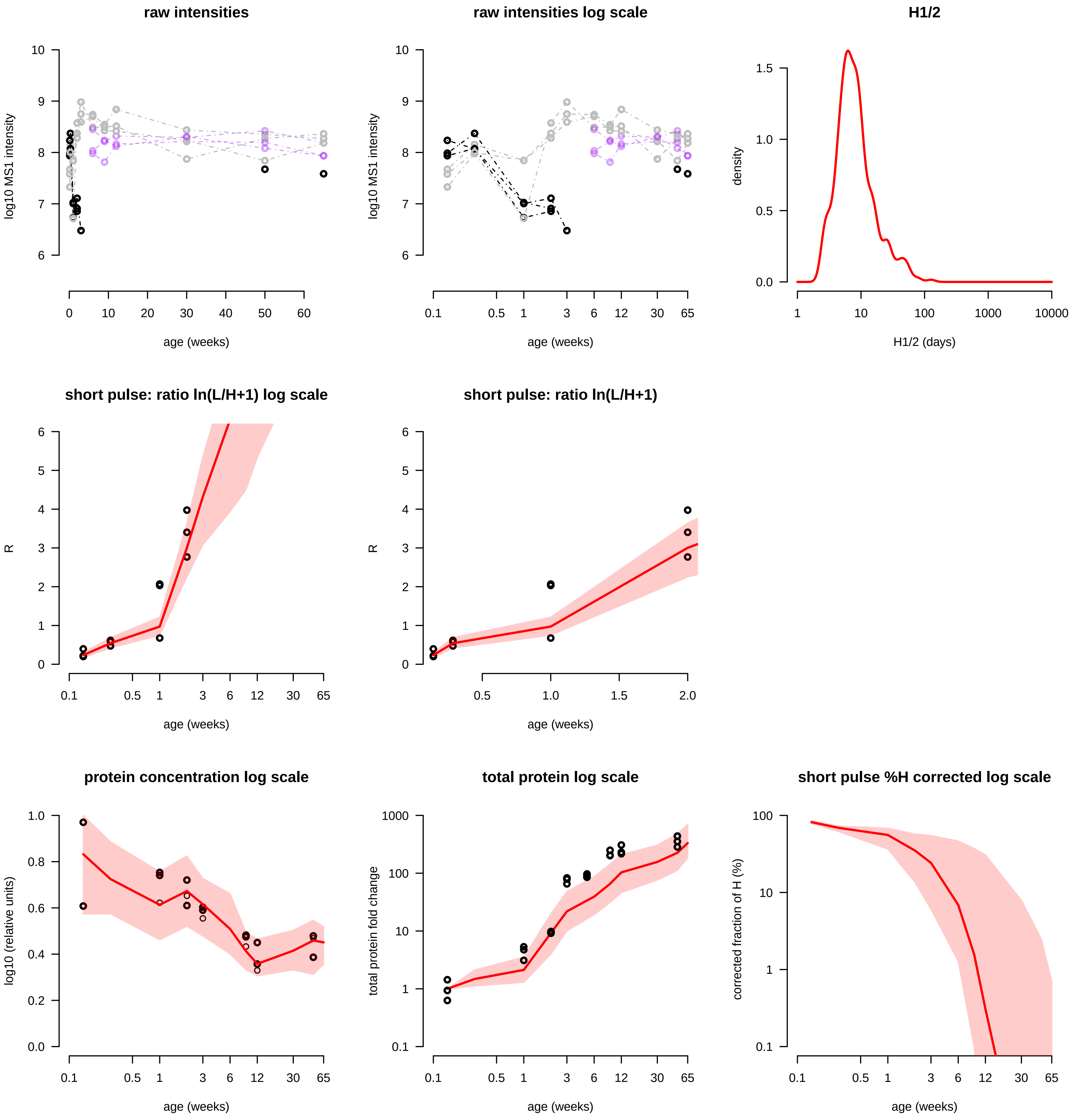

P59242

>sp|P59242|CING_MOUSE

Cingulin

GN=Cgn

Med H1/2: 5.4 days

Q5 H1/2: 4.6 days

Q95 H1/2: 6.6 days

reliable model fit: yes

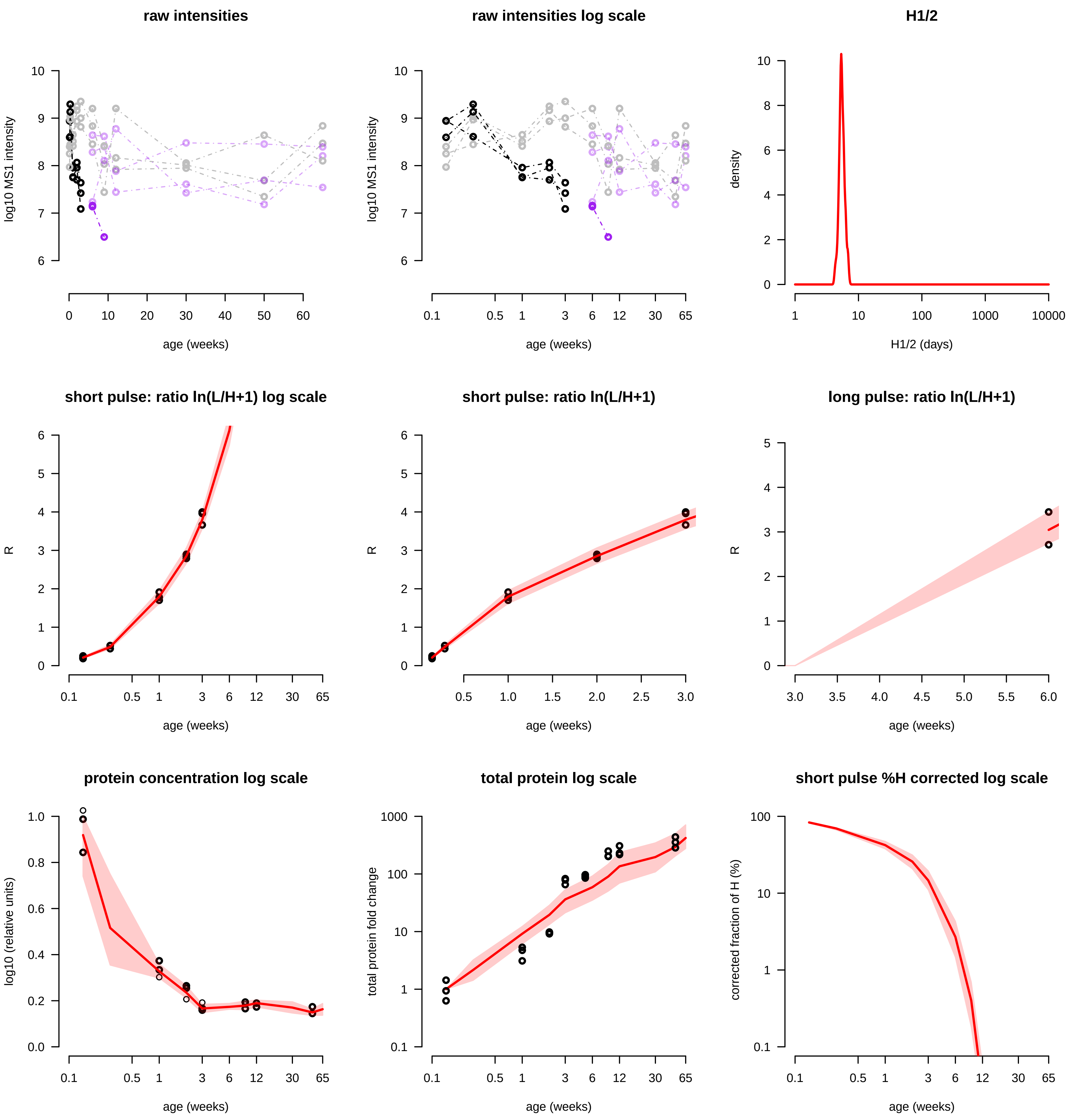

Q9D903

>sp|Q9D903|EBP2_MOUSE

Probable rRNA−processing protein EBP2

GN=Ebna1bp2

Med H1/2: 3.4 days

Q5 H1/2: 2.5 days

Q95 H1/2: 11.9 days

reliable model fit: yes

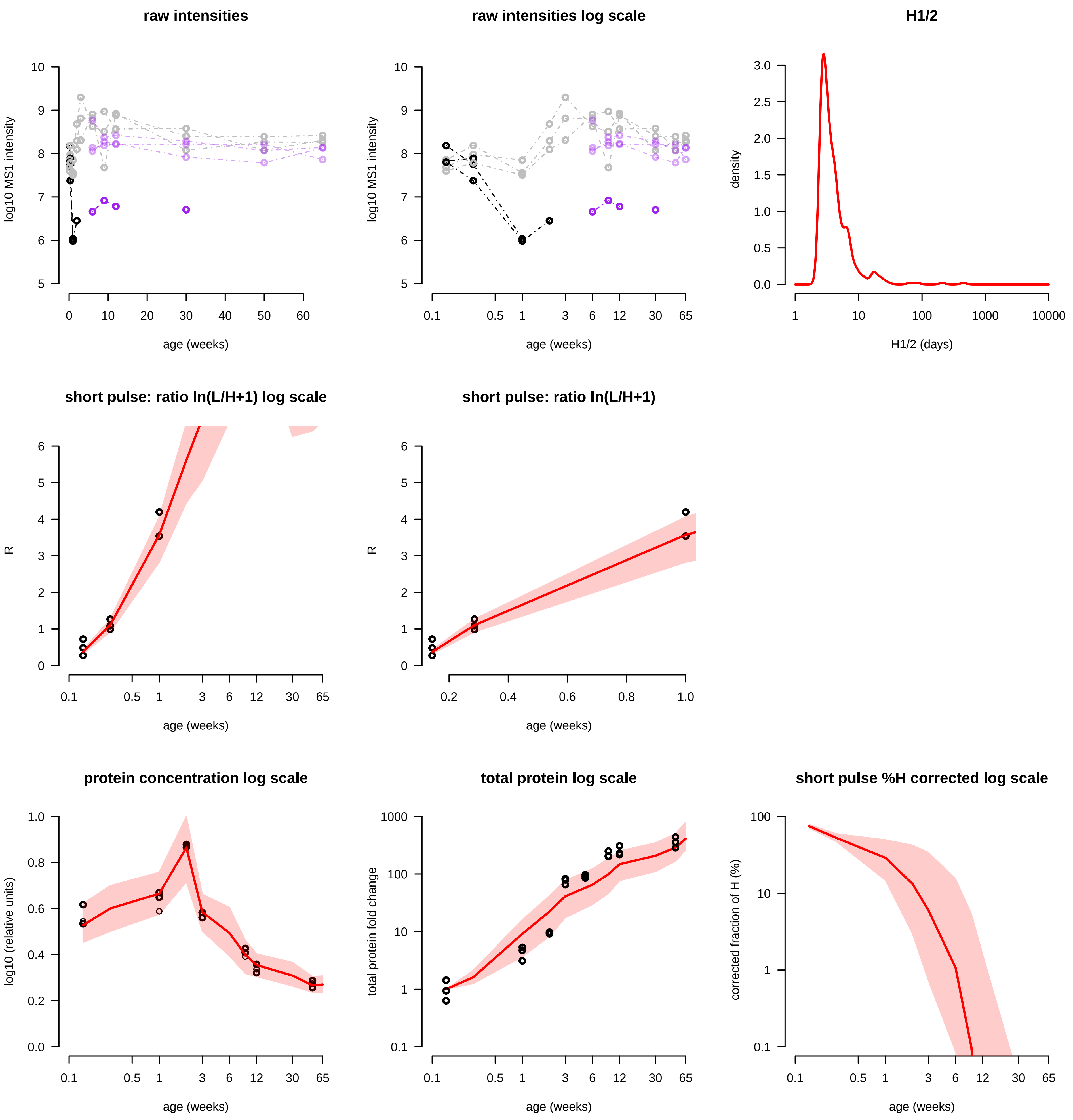

Q99LY9

>sp|Q99LY9|NDUS5_MOUSE

NADH dehydrogenase [ubiquinone] iron−sulfur protein 5

GN=Ndufs5

Med H1/2: 6.7 days

Q5 H1/2: 4.7 days

Q95 H1/2: 14.1 days

reliable model fit: yes

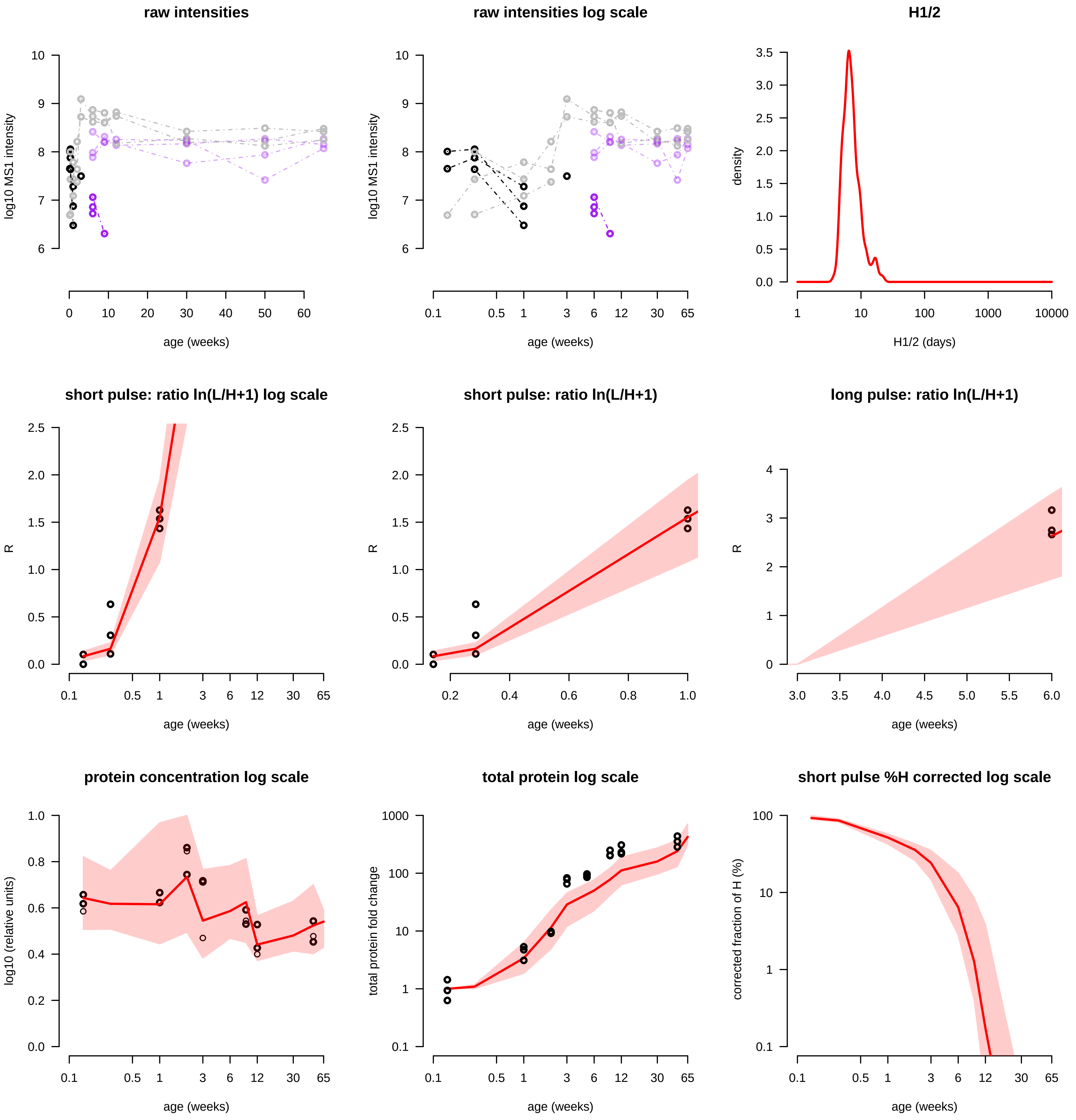

Q9D0R8

>sp|Q9D0R8|LSM12_MOUSE

Protein LSM12 homolog

GN=Lsm12

Med H1/2: 12.8 days

Q5 H1/2: 4.4 days

Q95 H1/2: 98.8 days

reliable model fit: yes

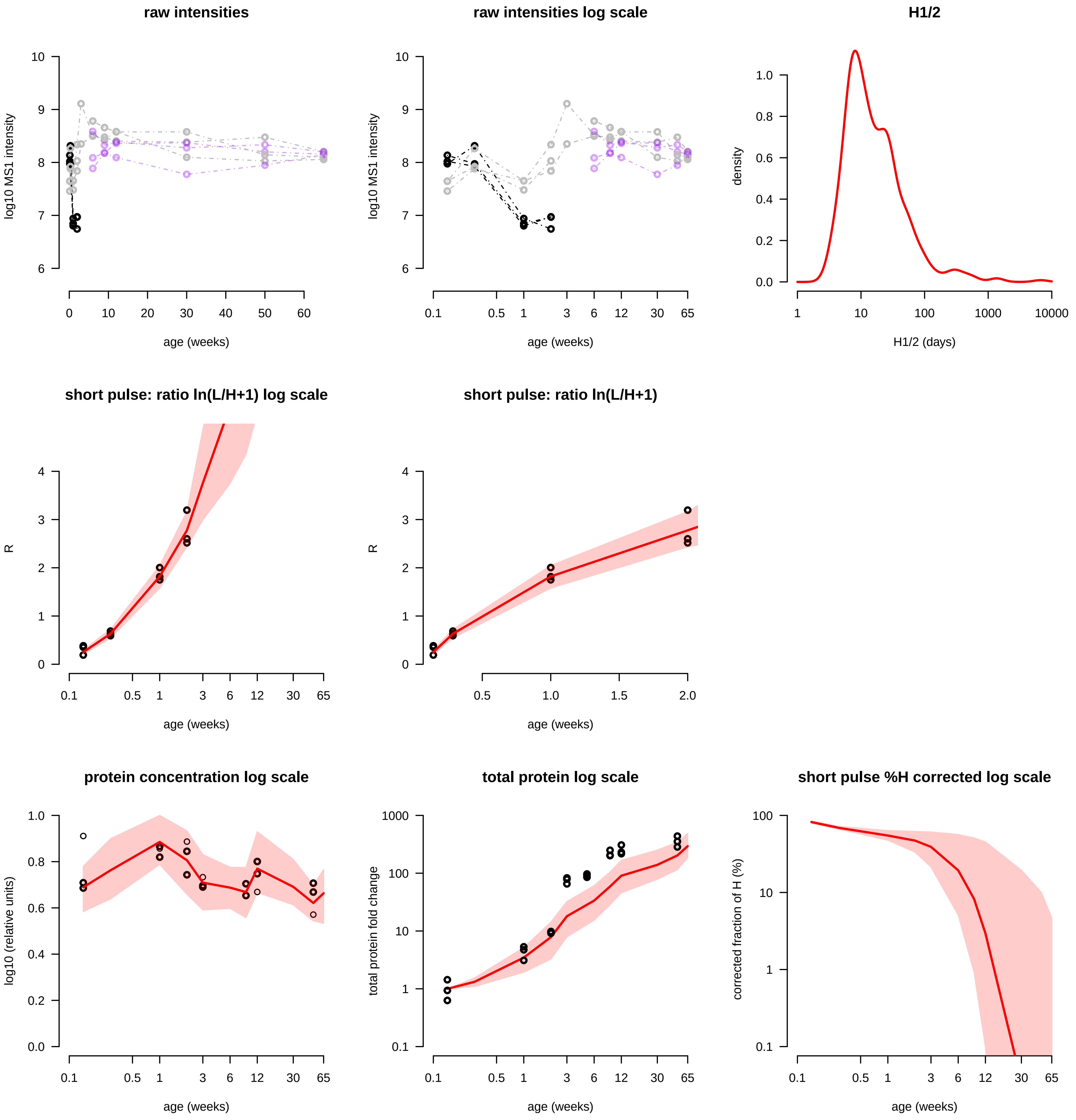

P39749

>sp|P39749|FEN1_MOUSE

Flap endonuclease 1

GN=Fen1

Med H1/2: 9.3 days

Q5 H1/2: 4.2 days

Q95 H1/2: 39.9 days

reliable model fit: yes

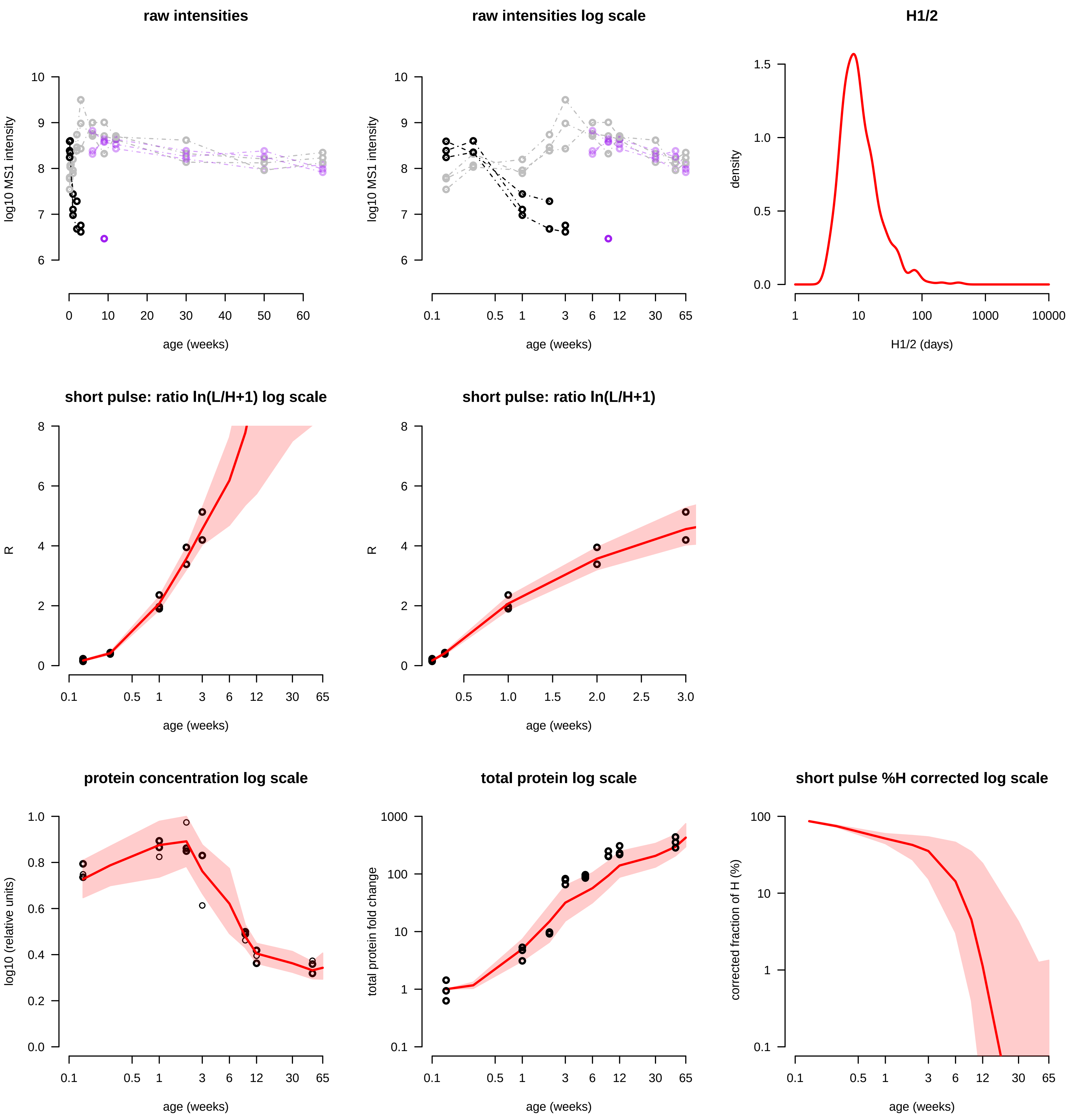

Q91YX5

>sp|Q91YX5|LGAT1_MOUSE

Acyl−CoA:lysophosphatidylglycerol acyltransferase 1

GN=Lpgat1

Med H1/2: 203.1 days

Q5 H1/2: 57.6 days

Q95 H1/2: 1682.8 days

reliable model fit: no

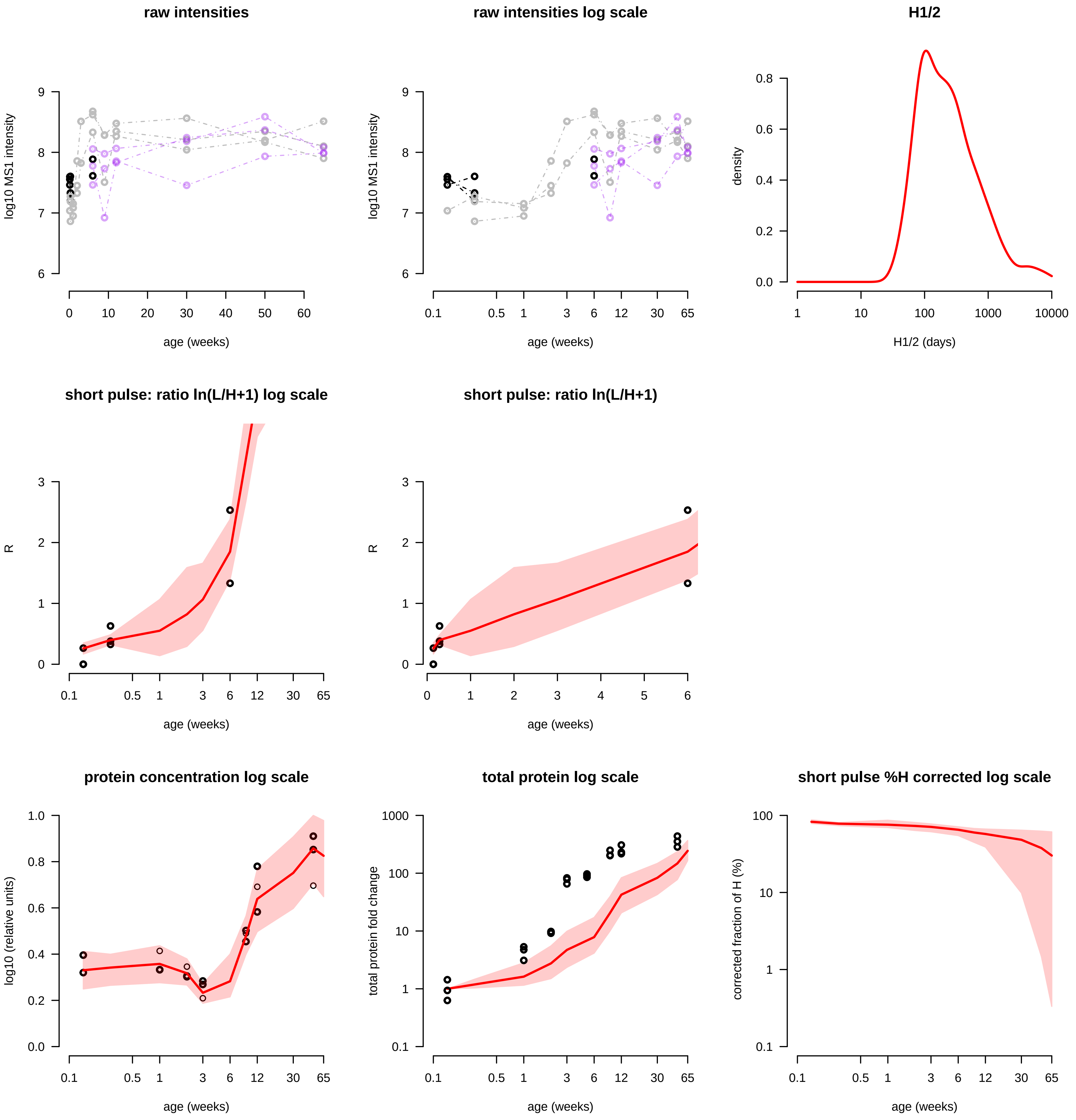

Q8K441

>sp|Q8K441|ABCA6_MOUSE

ATP−binding cassette sub−family A member 6

GN=Abca6

Med H1/2: 135.5 days

Q5 H1/2: 83 days

Q95 H1/2: 336.6 days

reliable model fit: yes

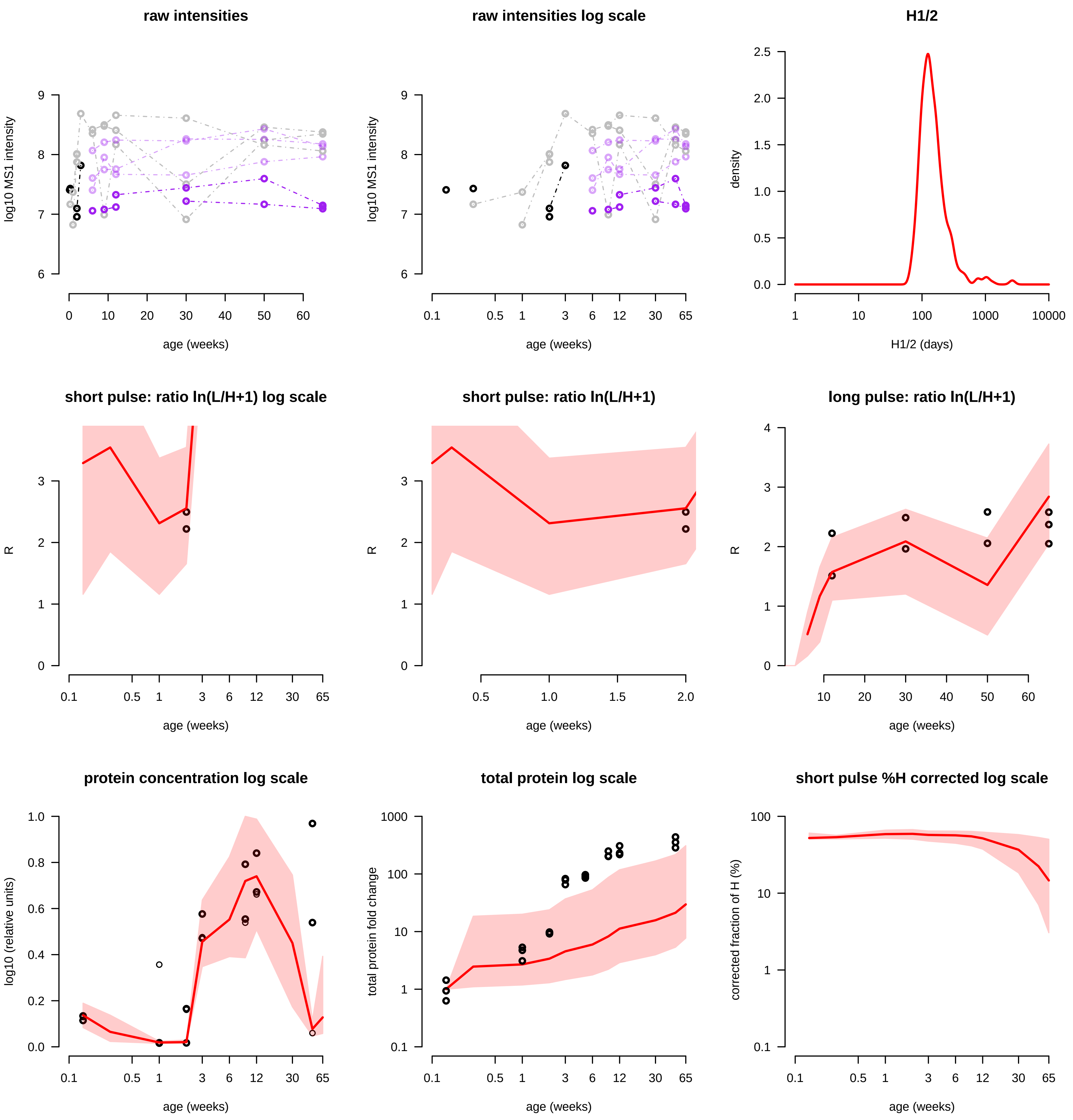

Q8R1U1

>sp|Q8R1U1|COG4_MOUSE

Conserved oligomeric Golgi complex subunit 4

GN=Cog4

Med H1/2: 12.3 days

Q5 H1/2: 7.9 days

Q95 H1/2: 27.5 days

reliable model fit: yes

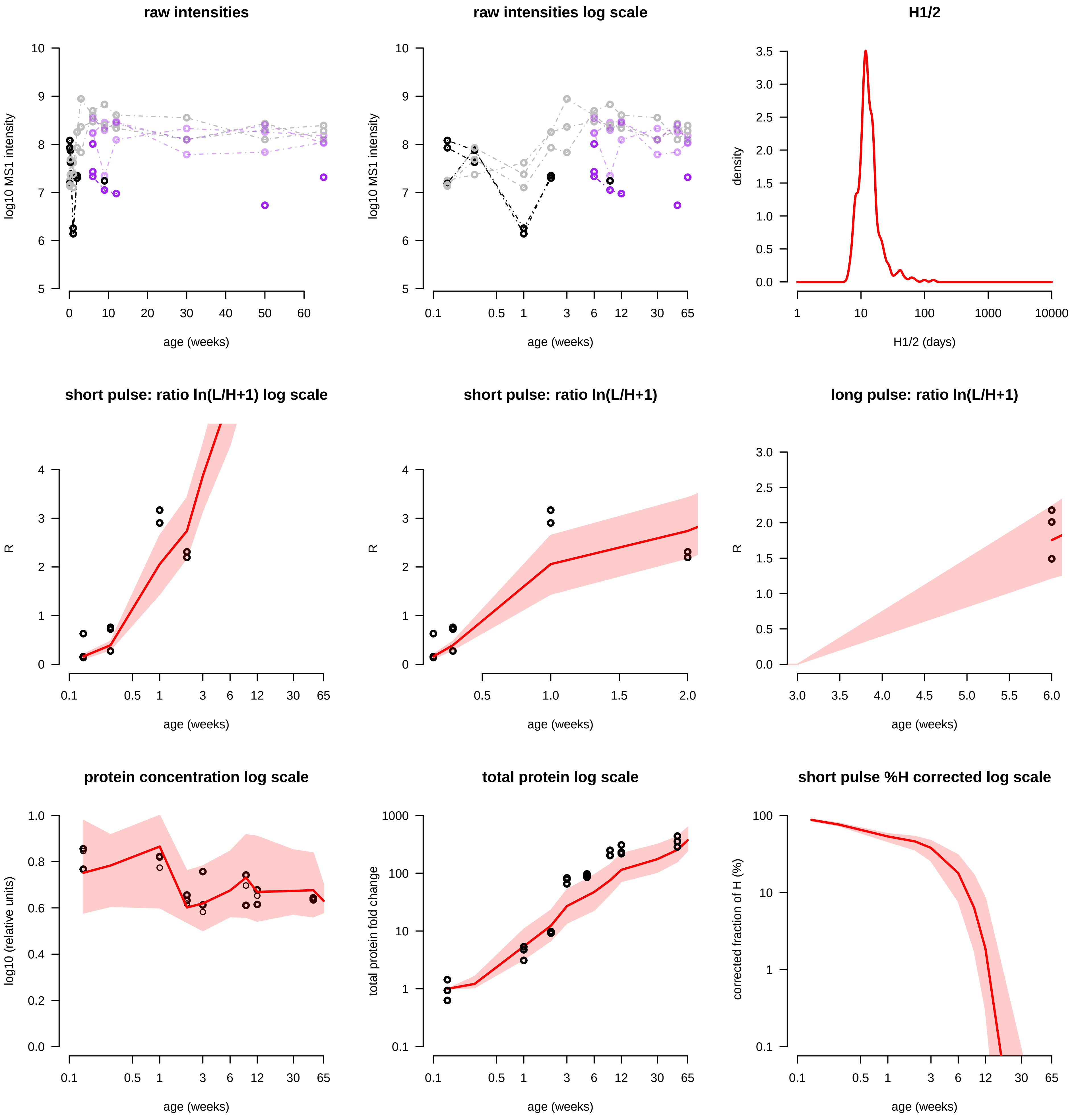

Q9CZJ2

>sp|Q9CZJ2|HS12B_MOUSE

Heat shock 70 kDa protein 12B

GN=Hspa12b

Med H1/2: 15.7 days

Q5 H1/2: 5.6 days

Q95 H1/2: 143.8 days

reliable model fit: yes

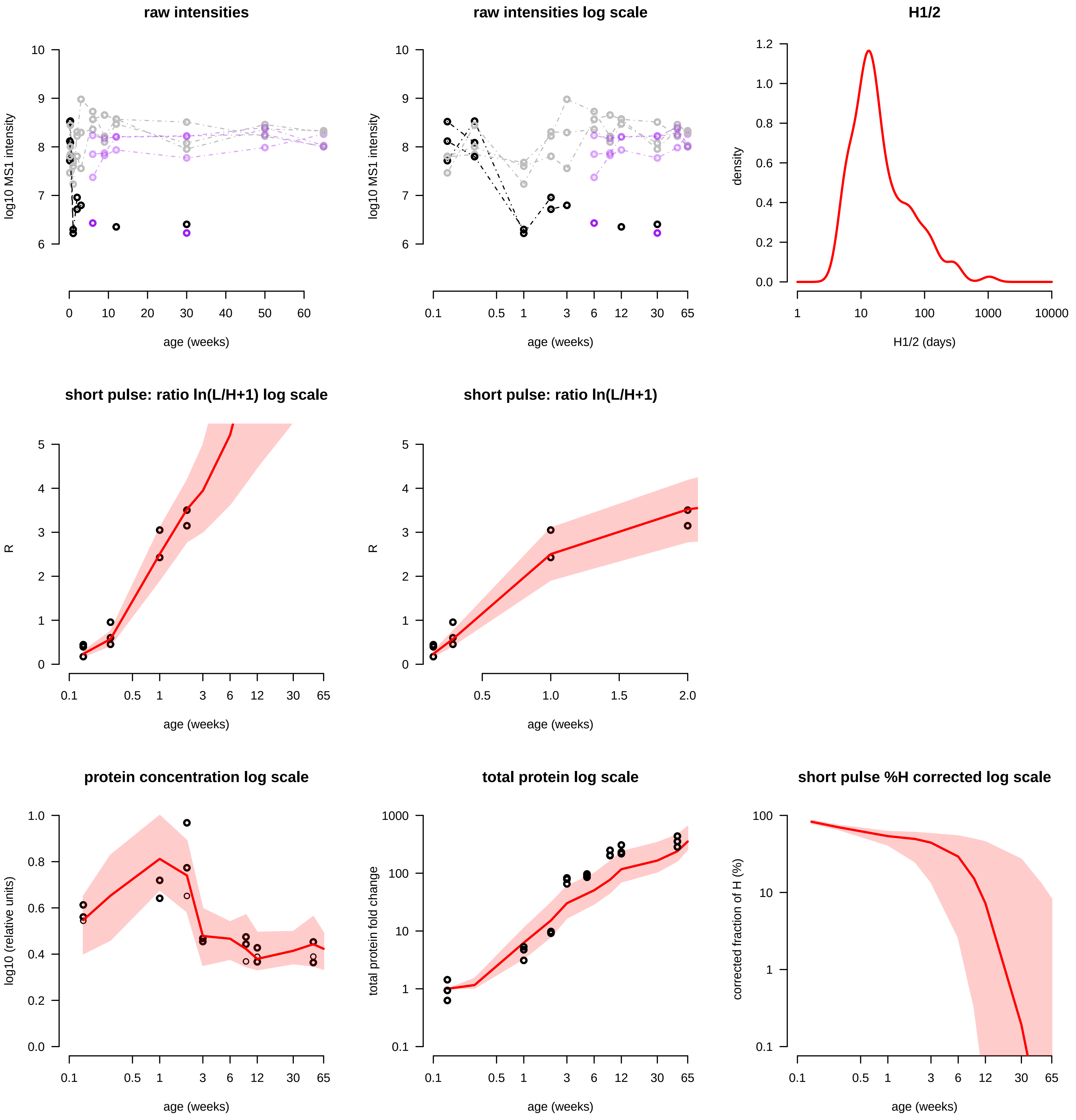

;C0HKE5;C0HKE4;C0HKE3;C0HKE2;C0HKE1;Q8CGP6;Q

>sp|C0HKE9|H2A1P_MOUSE

Histone H2A type 1−P

GN=Hist1h2ap

Med H1/2: 8.7 days

Q5 H1/2: 6 days

Q95 H1/2: 17.5 days

reliable model fit: yes

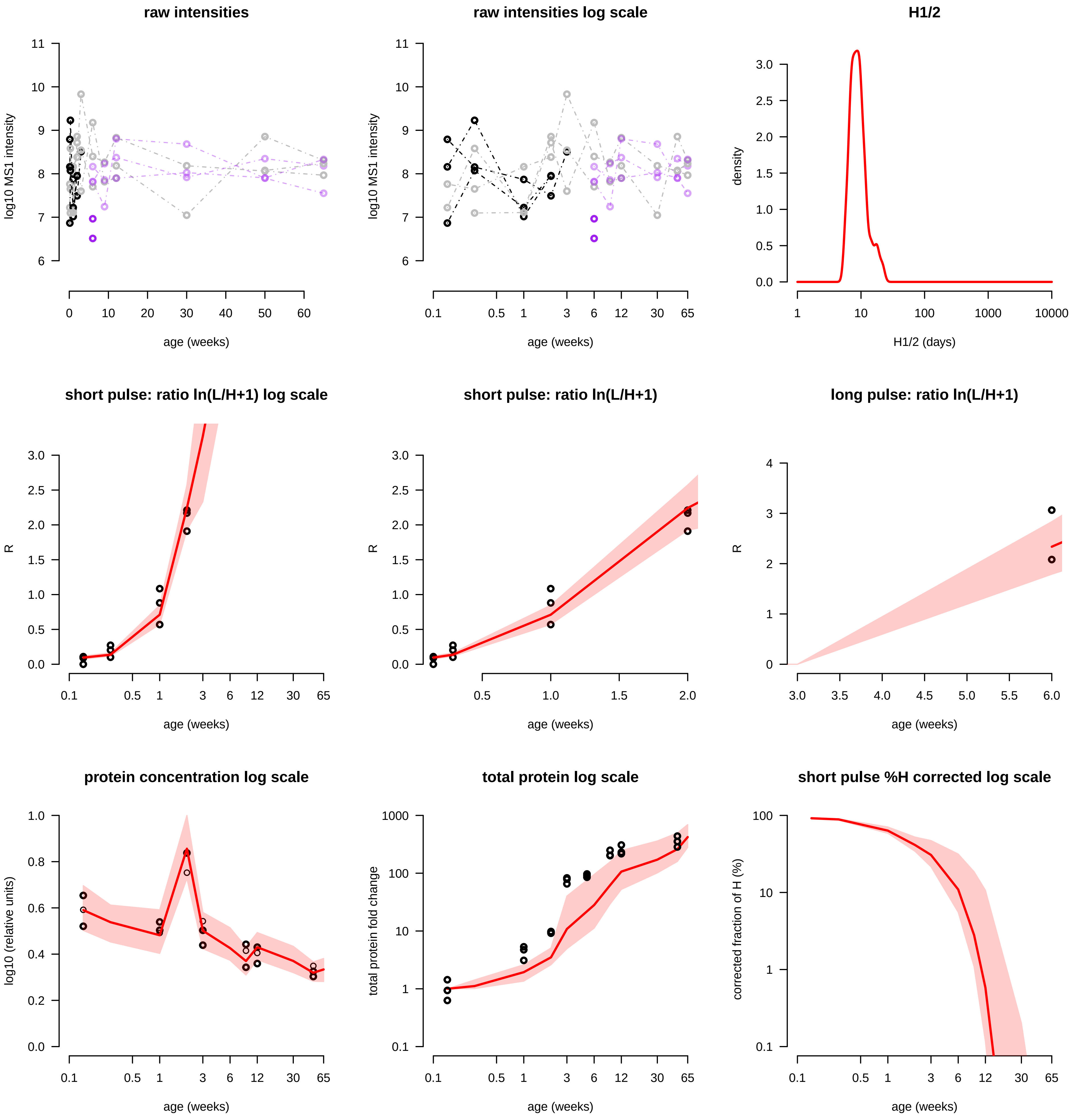

Q9CRA5

>sp|Q9CRA5|GOLP3_MOUSE

Golgi phosphoprotein 3

GN=Golph3

Med H1/2: 8.3 days

Q5 H1/2: 3.4 days

Q95 H1/2: 56.8 days

reliable model fit: yes

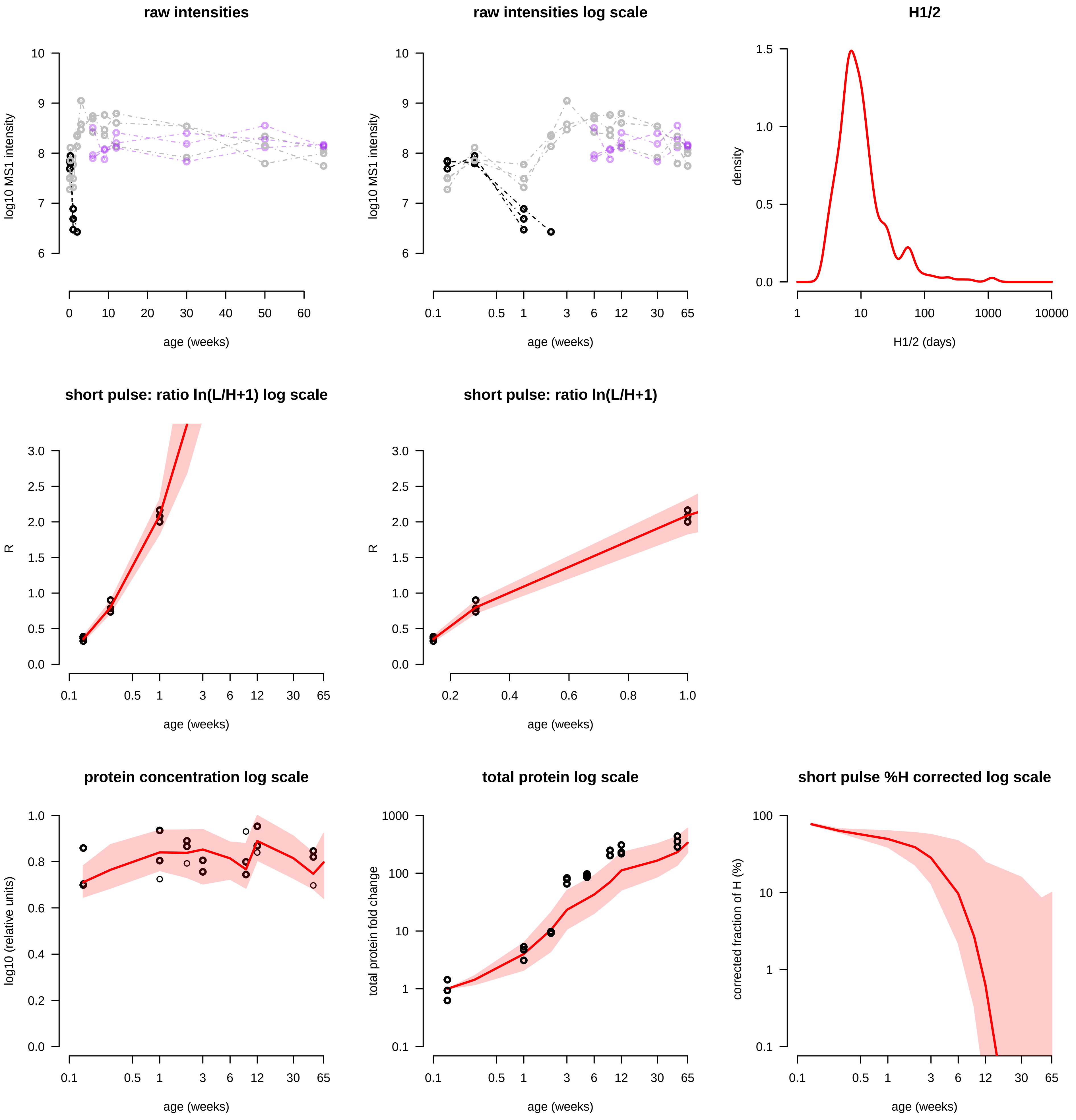

O09110;O09110−2

>sp|O09110|MP2K3_MOUSE

Dual specificity mitogen−activated protein kinase kinase 3

GN=Map2k3

Med H1/2: 12.9 days

Q5 H1/2: 4.5 days

Q95 H1/2: 135.9 days

reliable model fit: yes

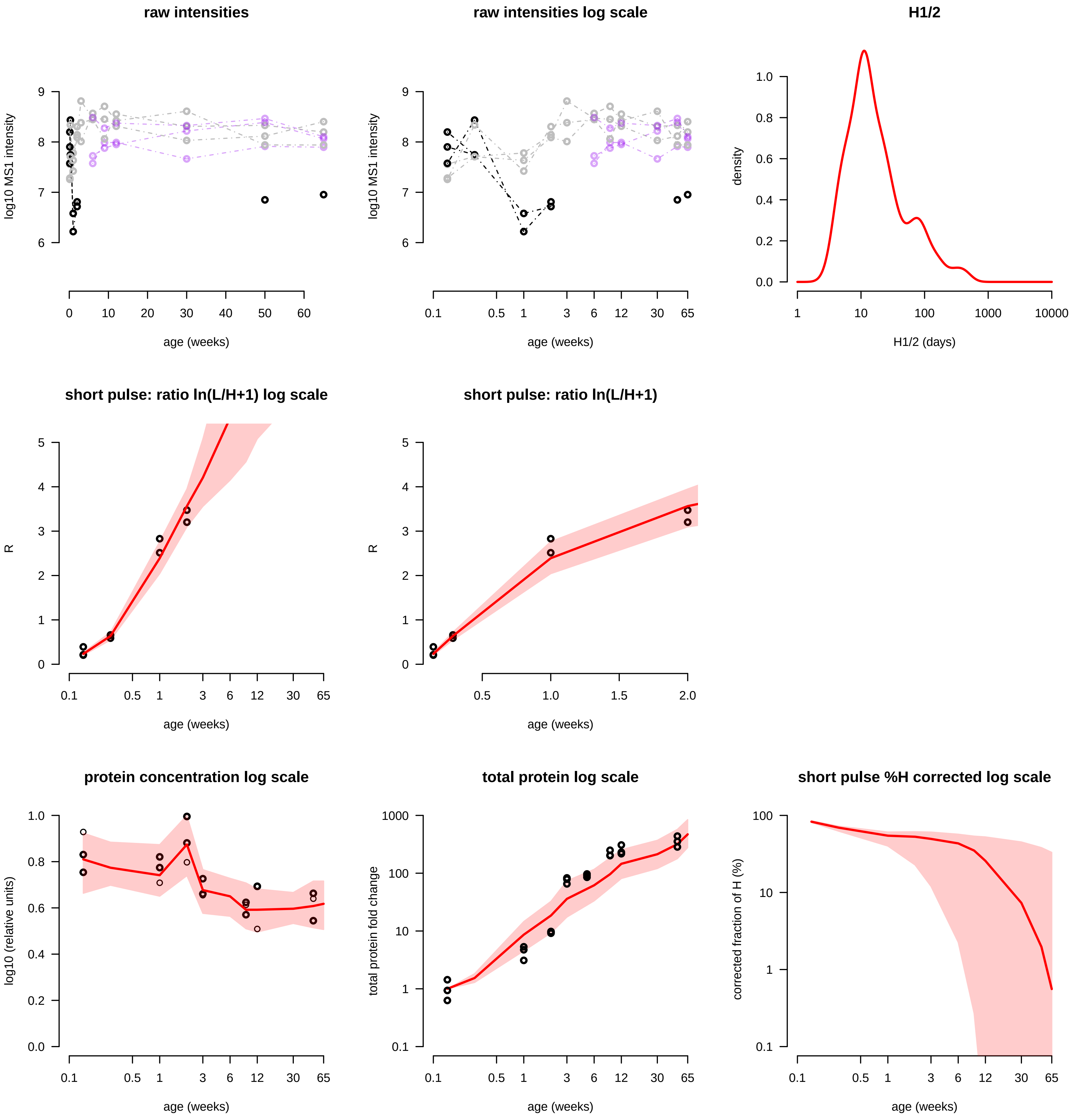

Q9ERL9

>sp|Q9ERL9|GCYA1_MOUSE

Guanylate cyclase soluble subunit alpha−1

GN=Gucy1a1

Med H1/2: 8 days

Q5 H1/2: 3.2 days

Q95 H1/2: 63.9 days

reliable model fit: yes

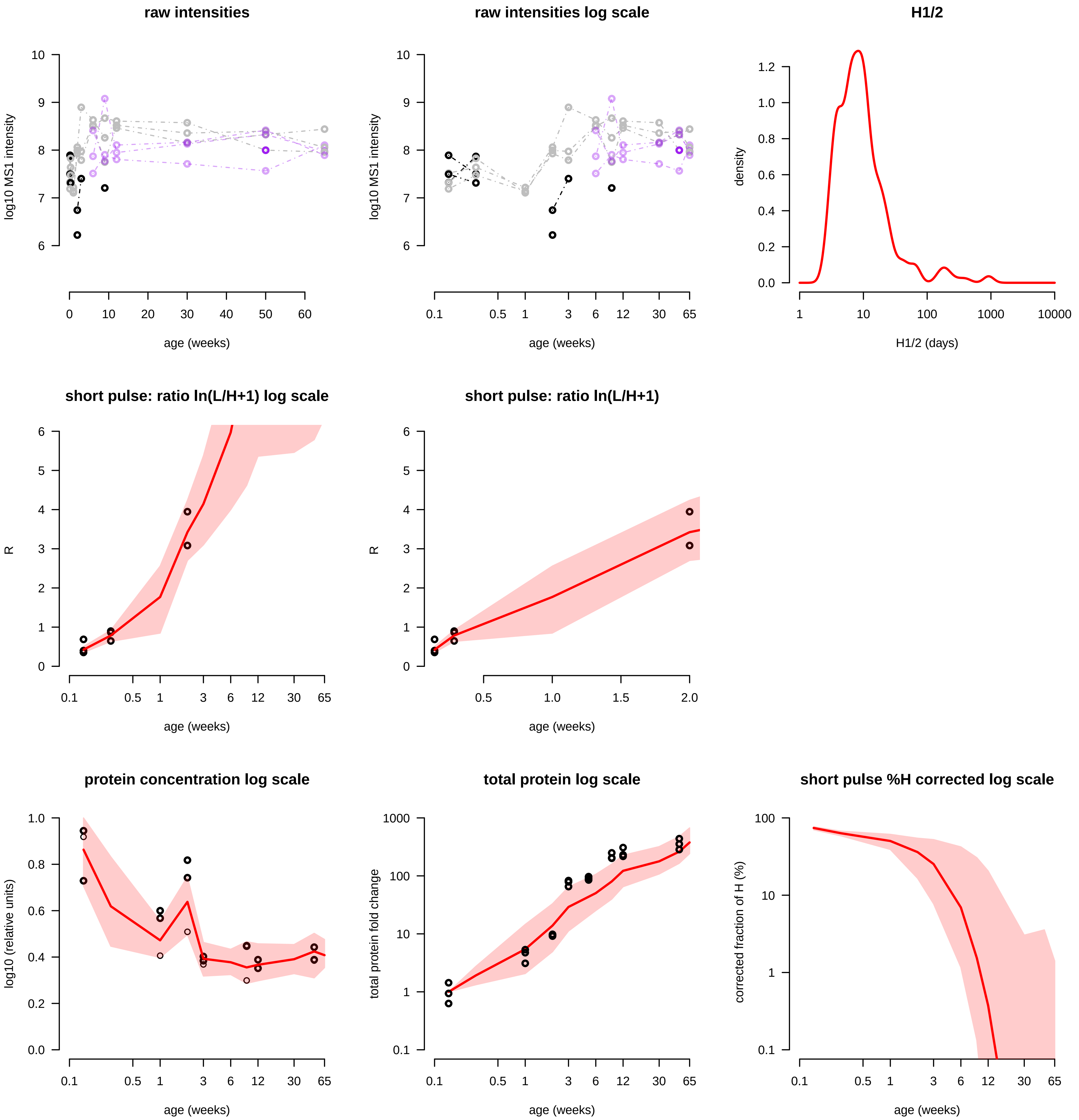

P52760

>sp|P52760|RIDA_MOUSE

2−iminobutanoate/2−iminopropanoate deaminase

GN=Rida

Med H1/2: 19.5 days

Q5 H1/2: 7.2 days

Q95 H1/2: 130.6 days

reliable model fit: yes

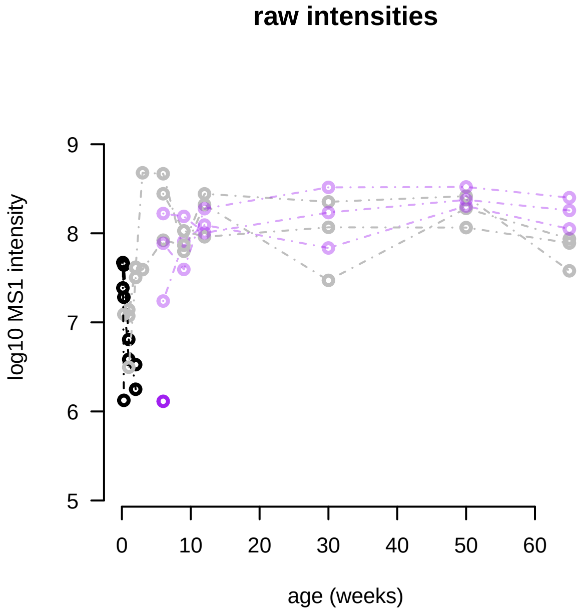

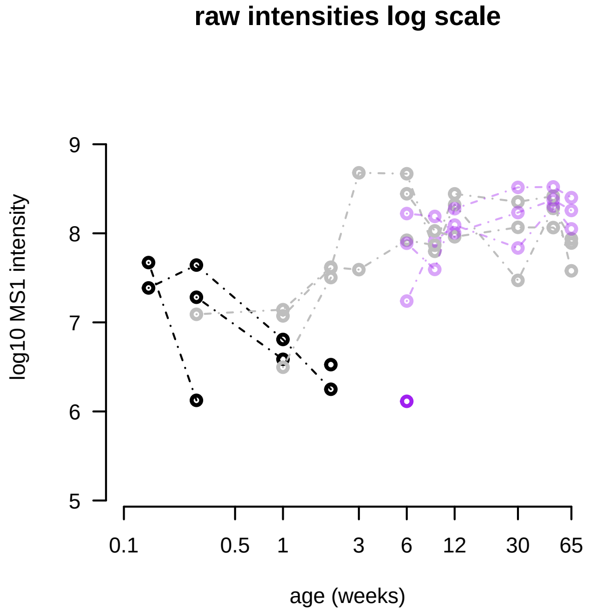

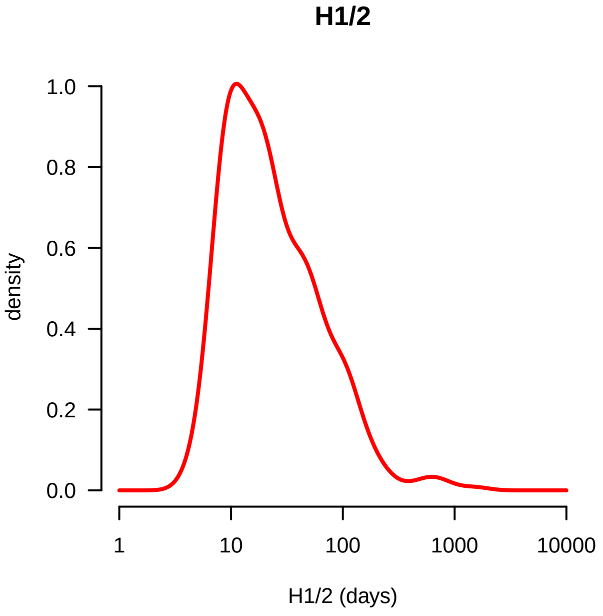

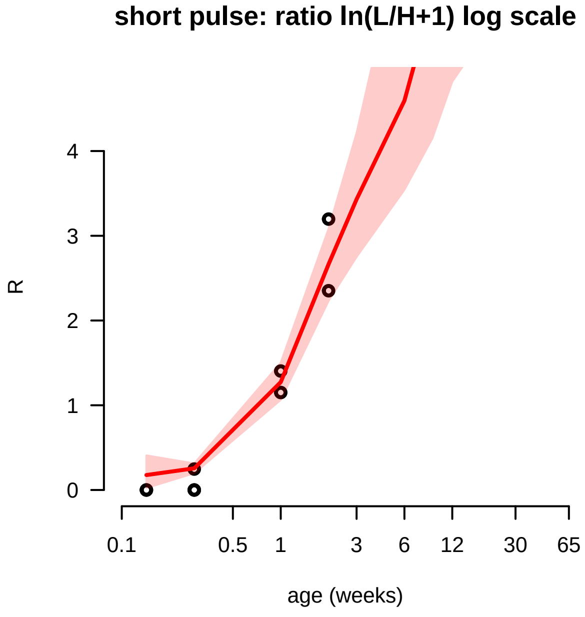

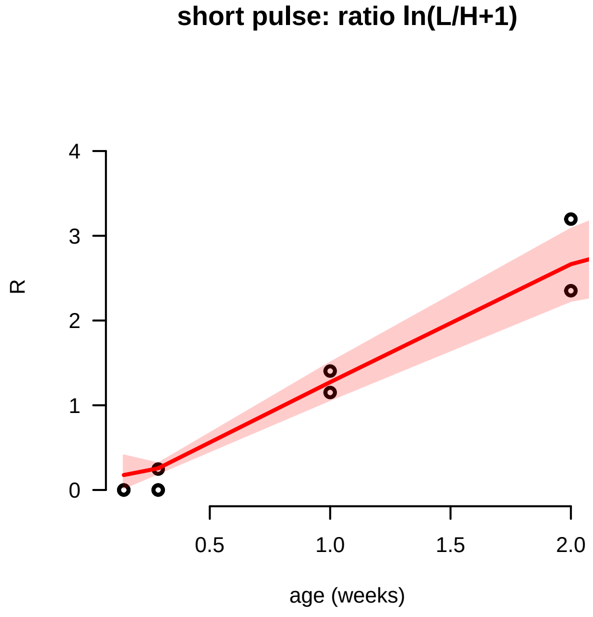

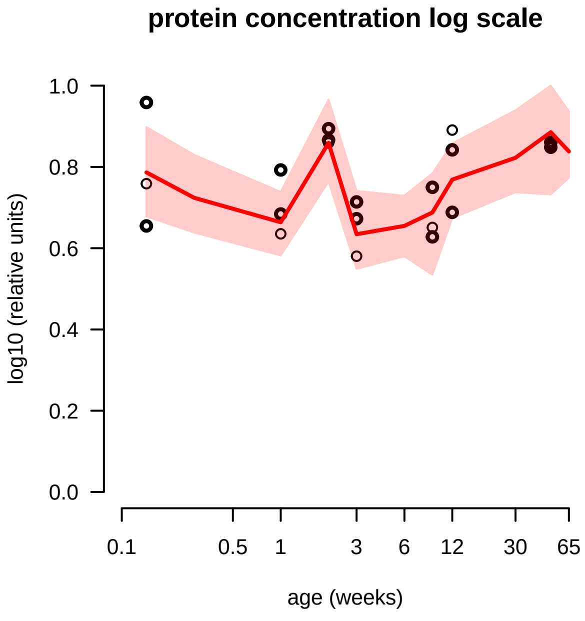

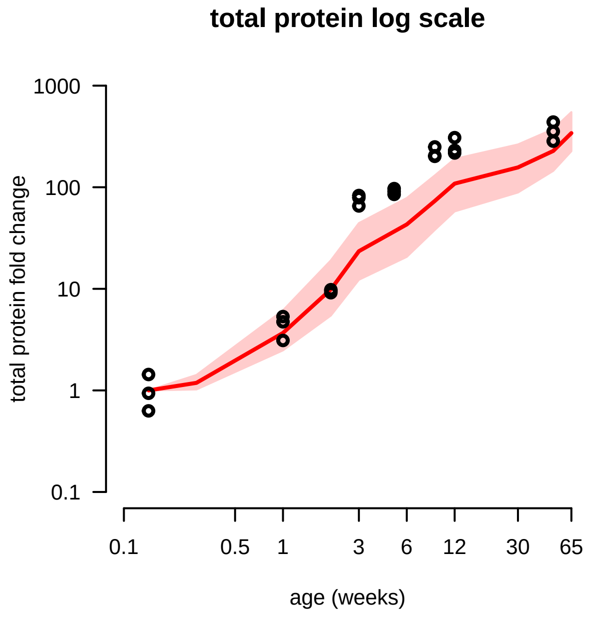

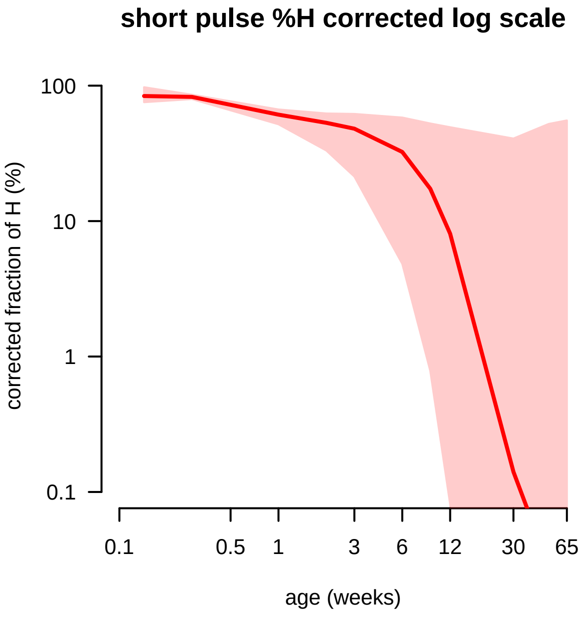

Q6P5B0

>sp|Q6P5B0|RRP12_MOUSE

RRP12−like protein

GN=Rrp12

Med H1/2: 7.2 days

Q5 H1/2: 2.8 days

Q95 H1/2: 97 days

reliable model fit: yes

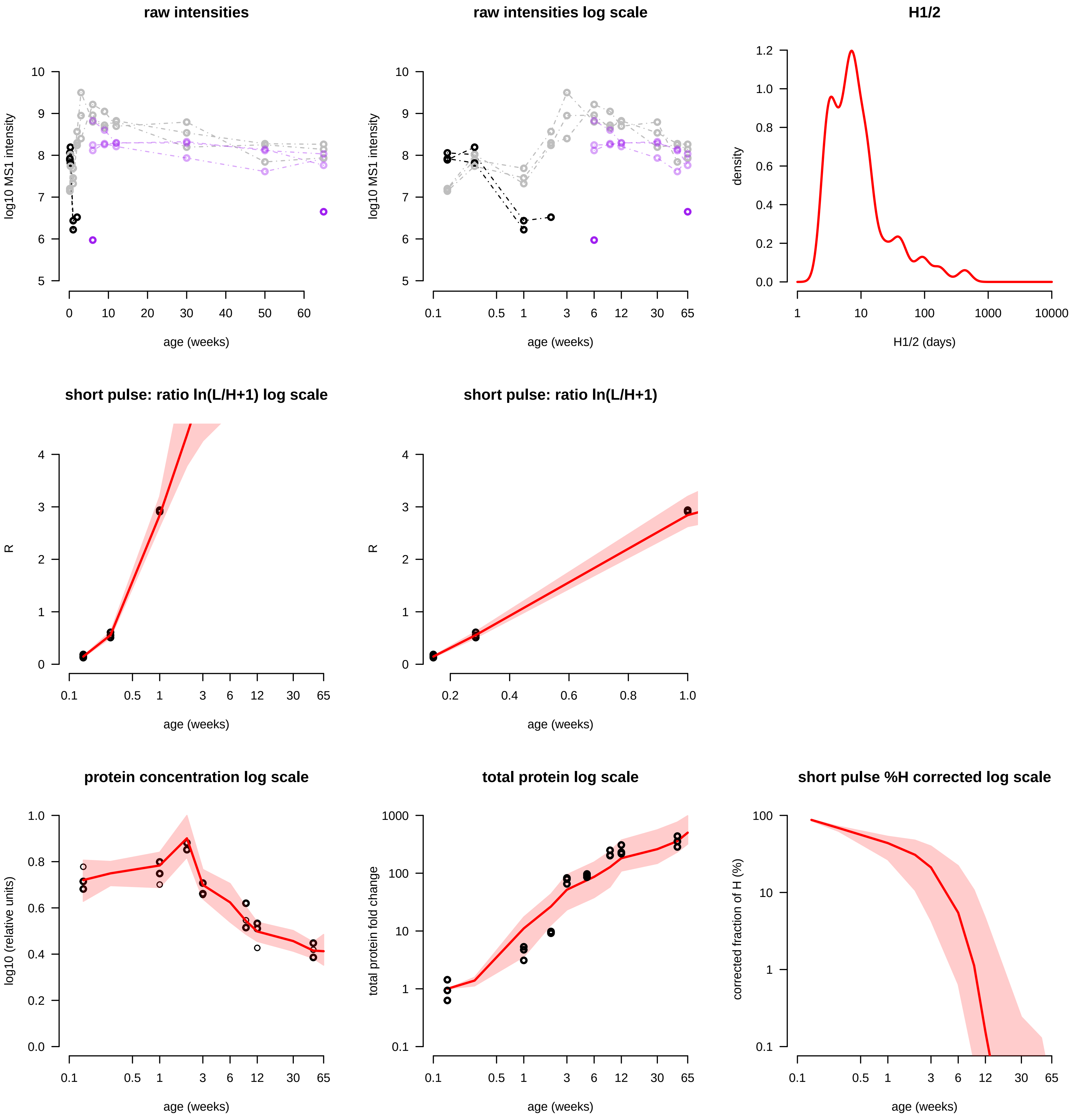

O54916;O54916−4

>sp|O54916|REPS1_MOUSE

RalBP1−associated Eps domain−containing protein 1

GN=Reps1

Med H1/2: 7.6 days

Q5 H1/2: 2.9 days

Q95 H1/2: 72.9 days

reliable model fit: yes

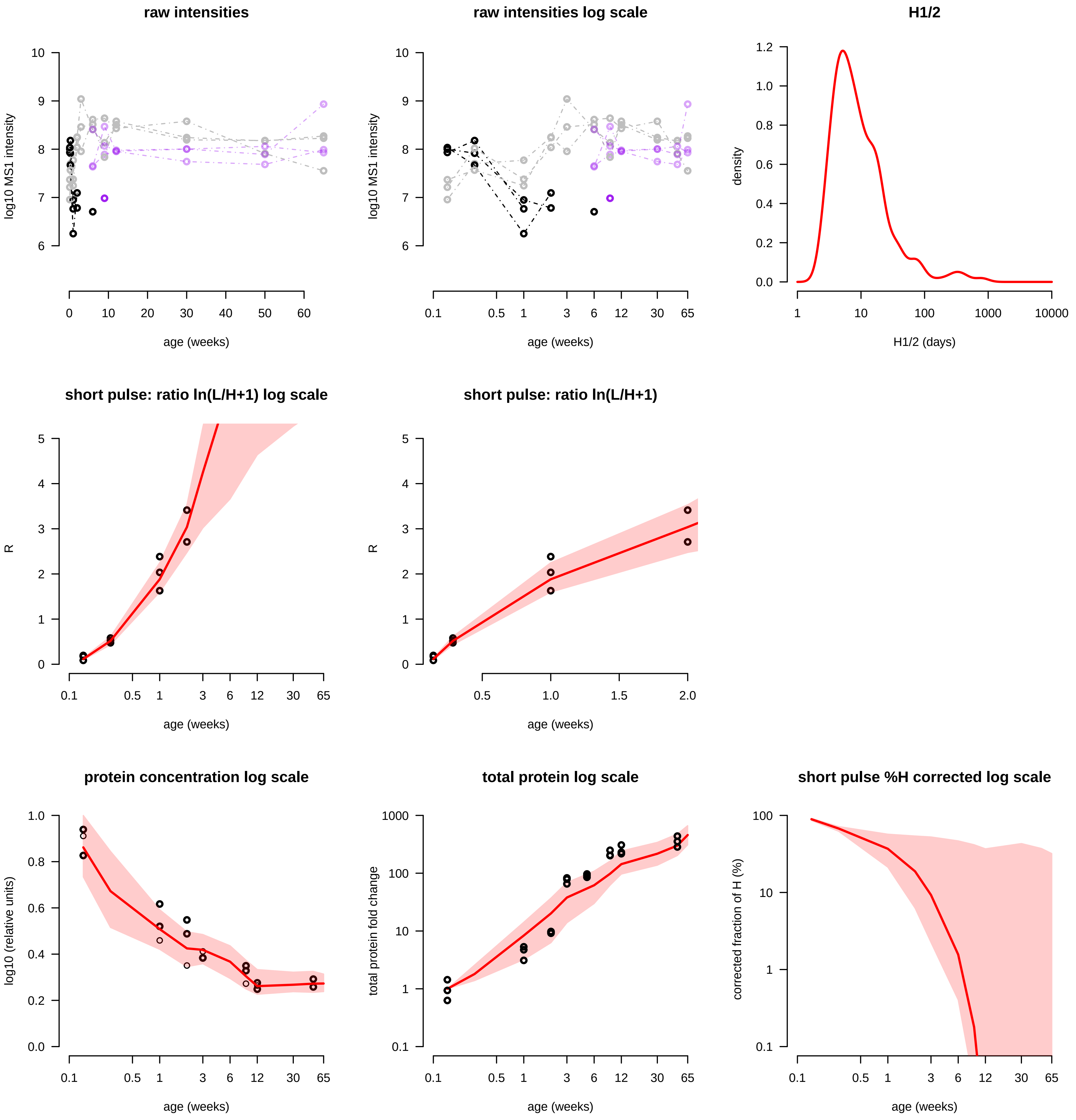

Q3U487

>sp|Q3U487|HECD3_MOUSE

E3 ubiquitin−protein ligase HECTD3

GN=Hectd3

Med H1/2: 2514 days

Q5 H1/2: 575.4 days

Q95 H1/2: 14001.8 days

reliable model fit: yes

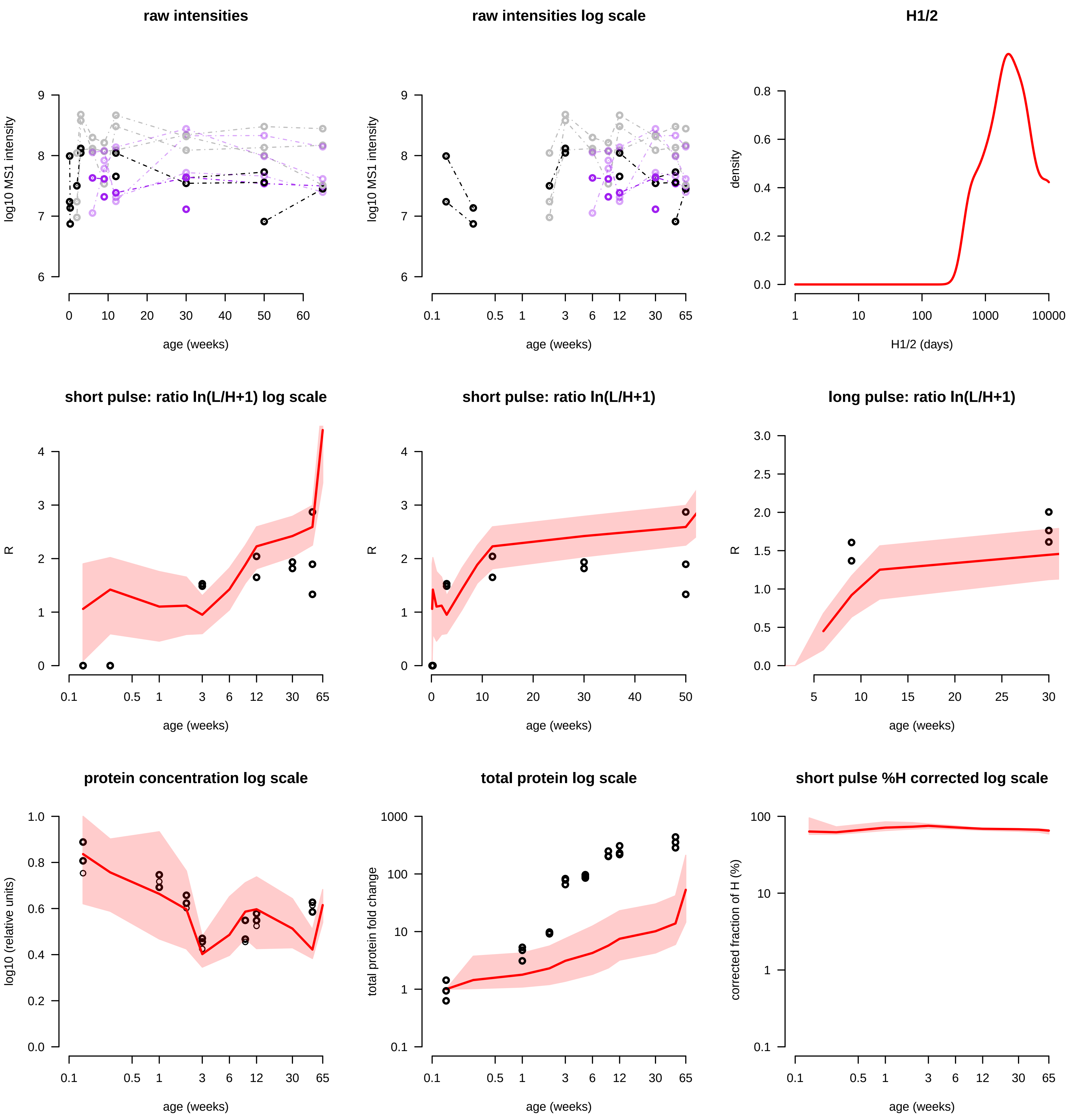

Q8BJ05

>sp|Q8BJ05|ZC3HE_MOUSE

Zinc finger CCCH domain−containing protein 14

GN=Zc3h14

Med H1/2: 4.1 days

Q5 H1/2: 2.5 days

Q95 H1/2: 16.1 days

reliable model fit: yes

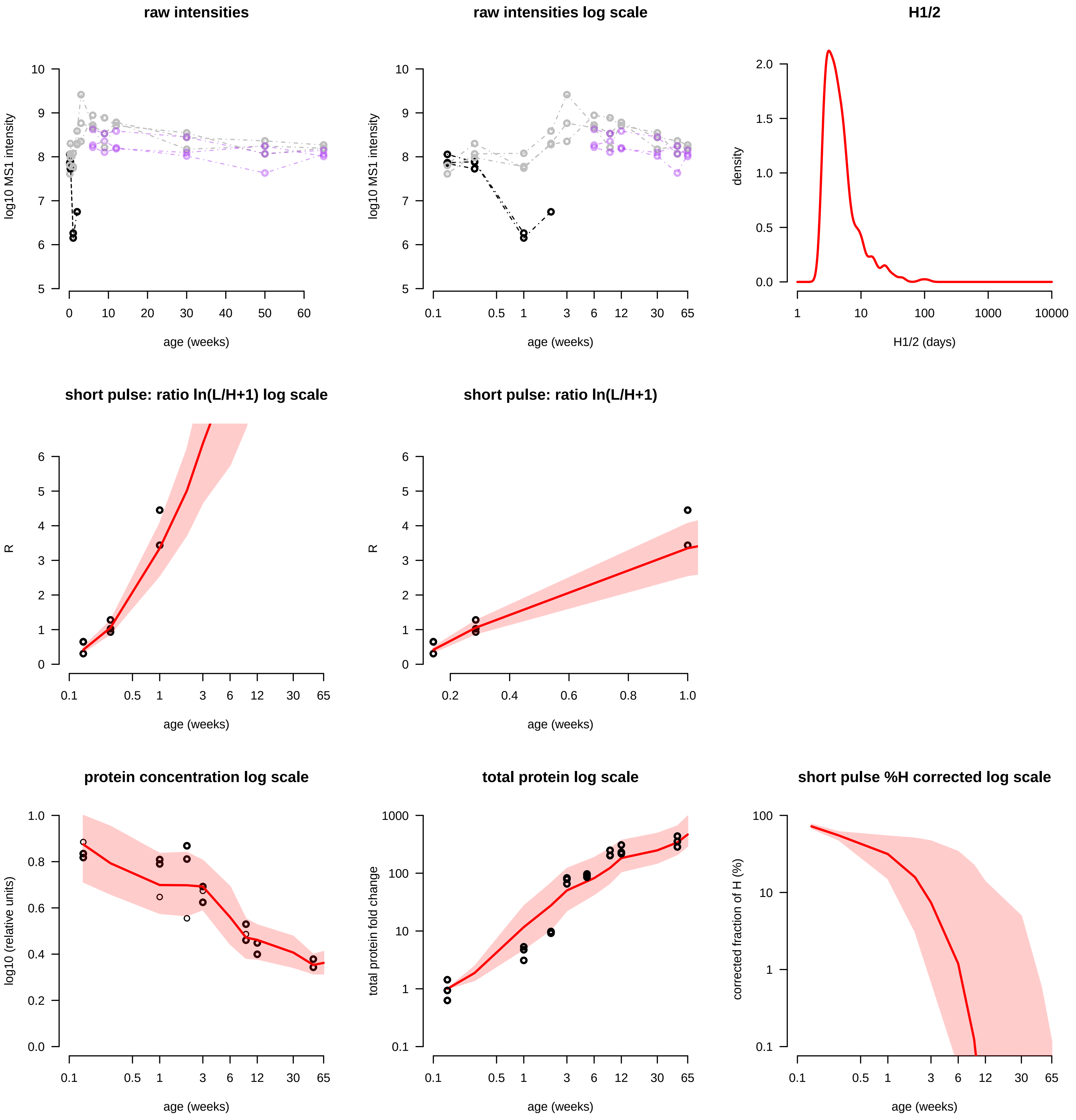

Q9D820;Q9D820−2

>sp|Q9D820|PRXD1_MOUSE

Prolyl−tRNA synthetase associated domain−containing protein 1

GN=Prorsd1

Med H1/2: 9.9 days

Q5 H1/2: 4.9 days

Q95 H1/2: 69.3 days

reliable model fit: yes

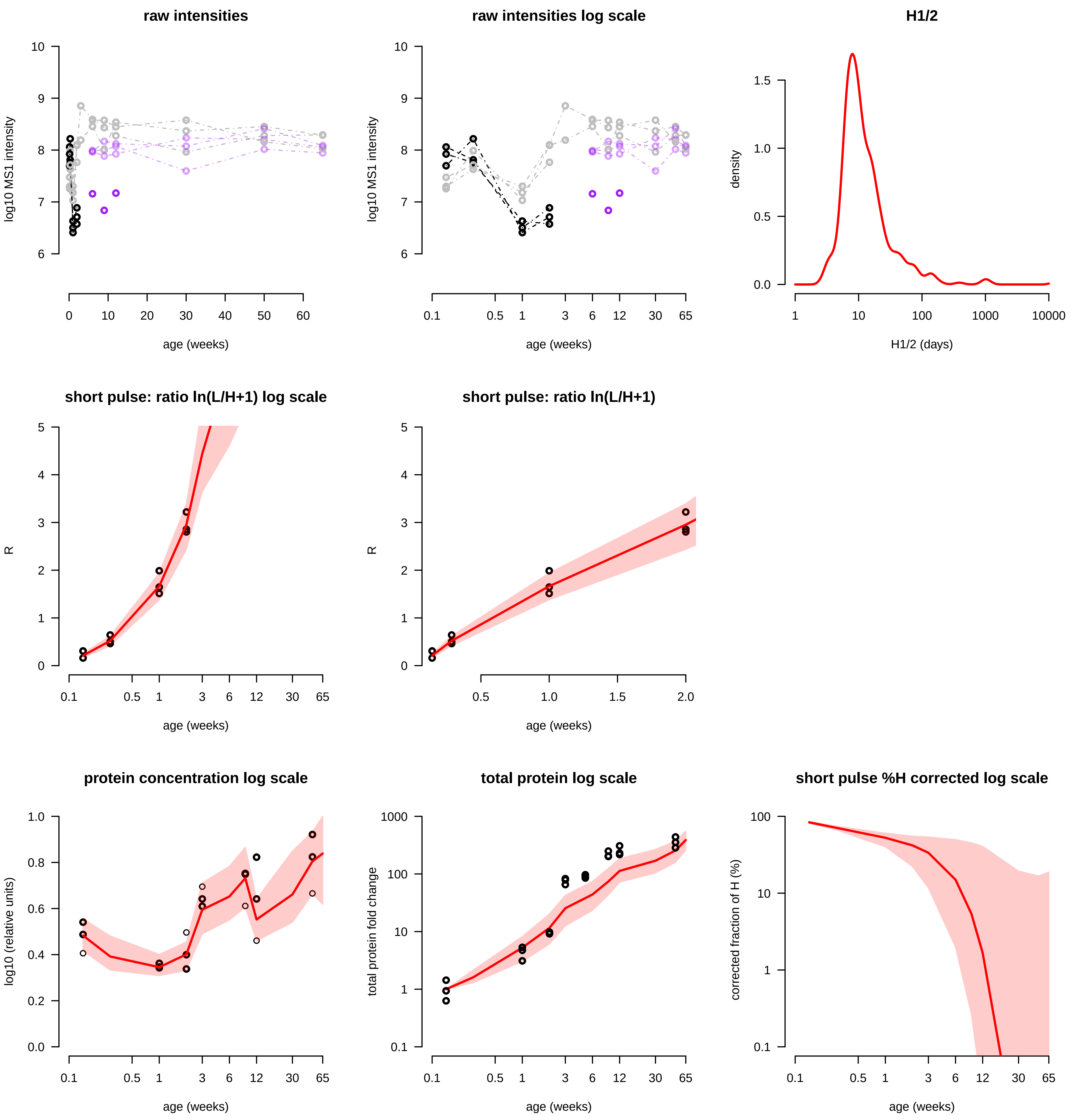

Q3U319

>sp|Q3U319|BRE1B_MOUSE

E3 ubiquitin−protein ligase BRE1B

GN=Rnf40

Med H1/2: 4.6 days

Q5 H1/2: 2.6 days

Q95 H1/2: 22.9 days

reliable model fit: yes

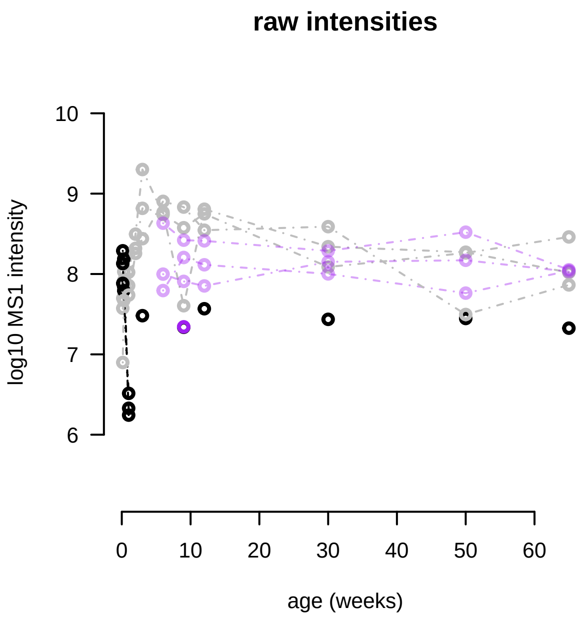

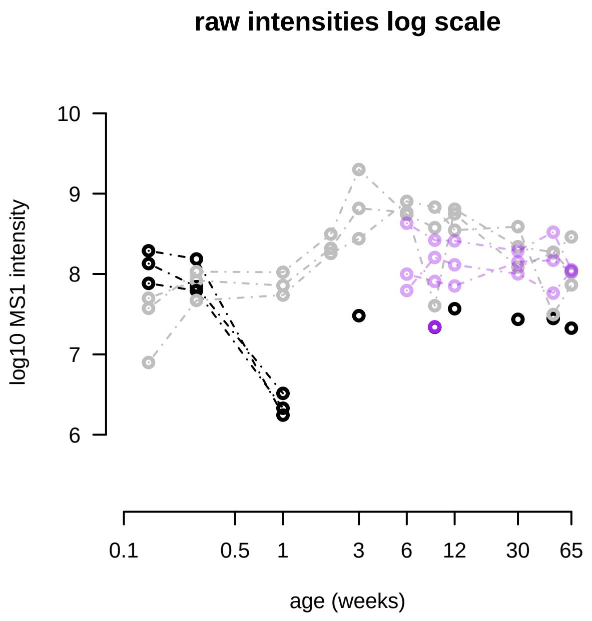

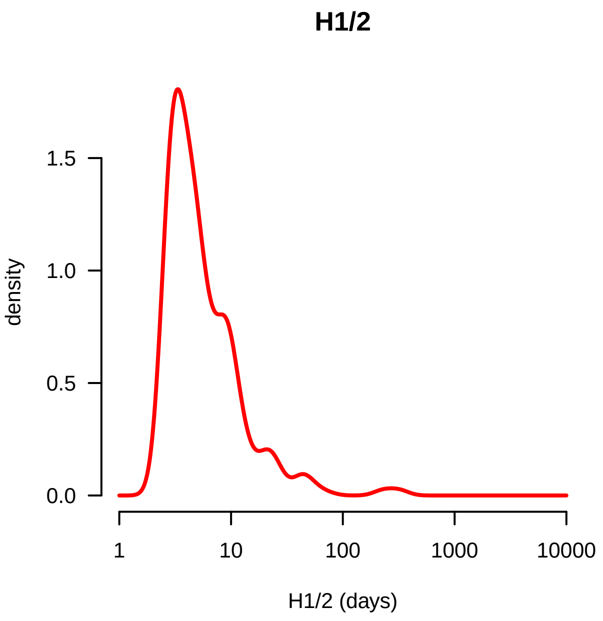

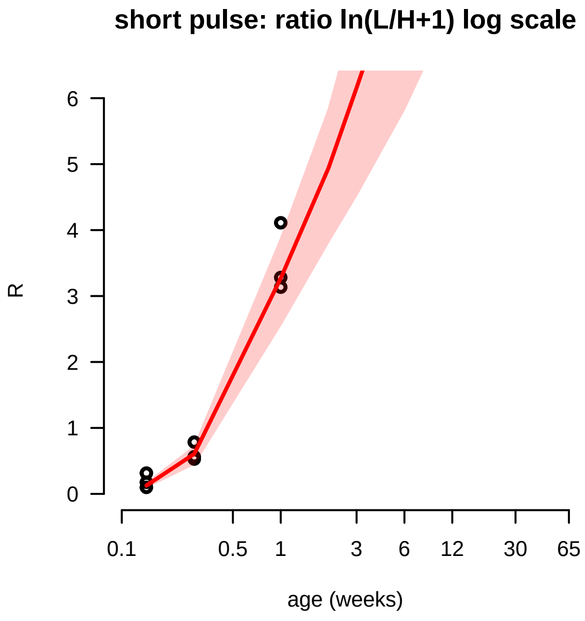

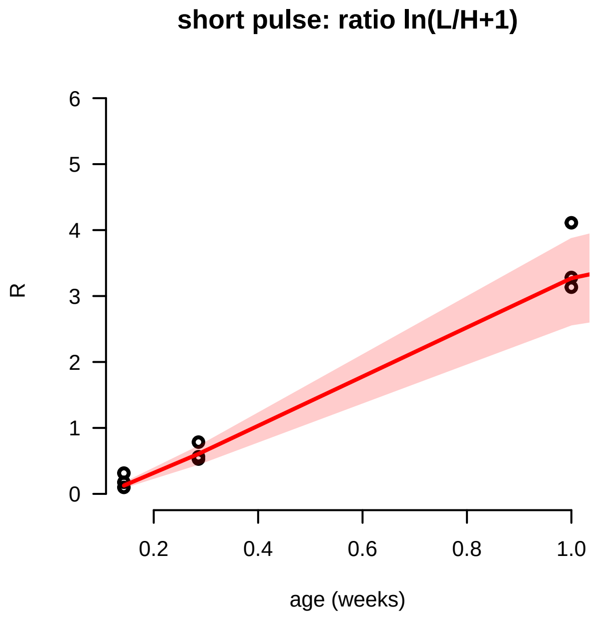

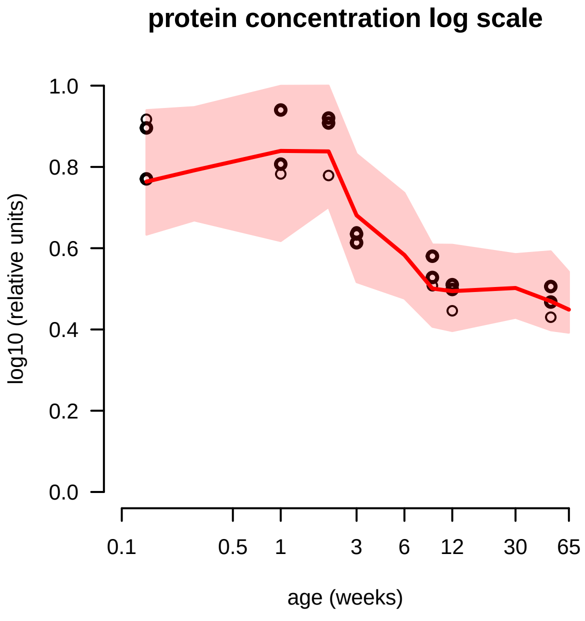

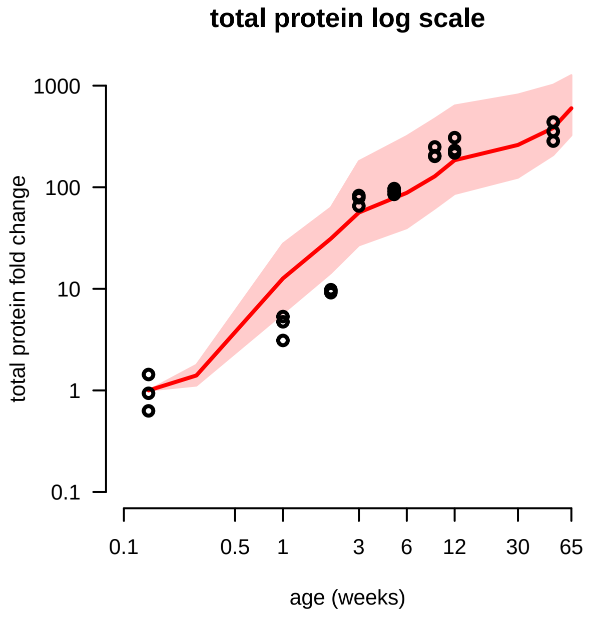

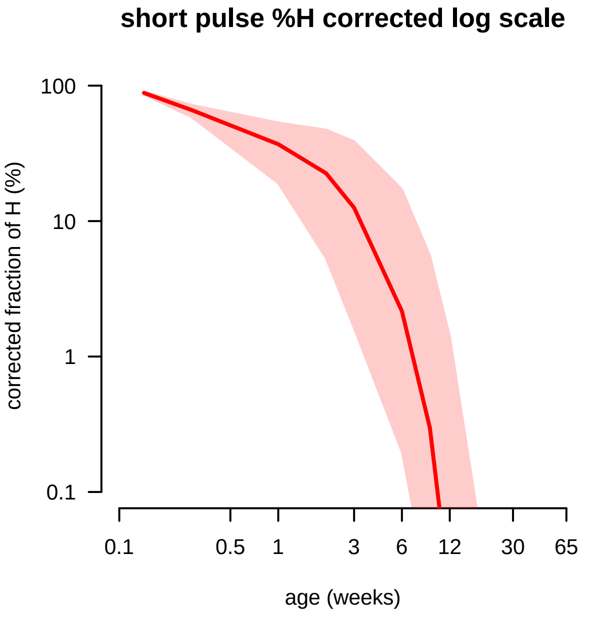

Q9ERU3

>sp|Q9ERU3|ZNF22_MOUSE

Zinc finger protein 22

GN=Znf22

Med H1/2: 5.9 days

Q5 H1/2: 2.7 days

Q95 H1/2: 50.4 days

reliable model fit: yes

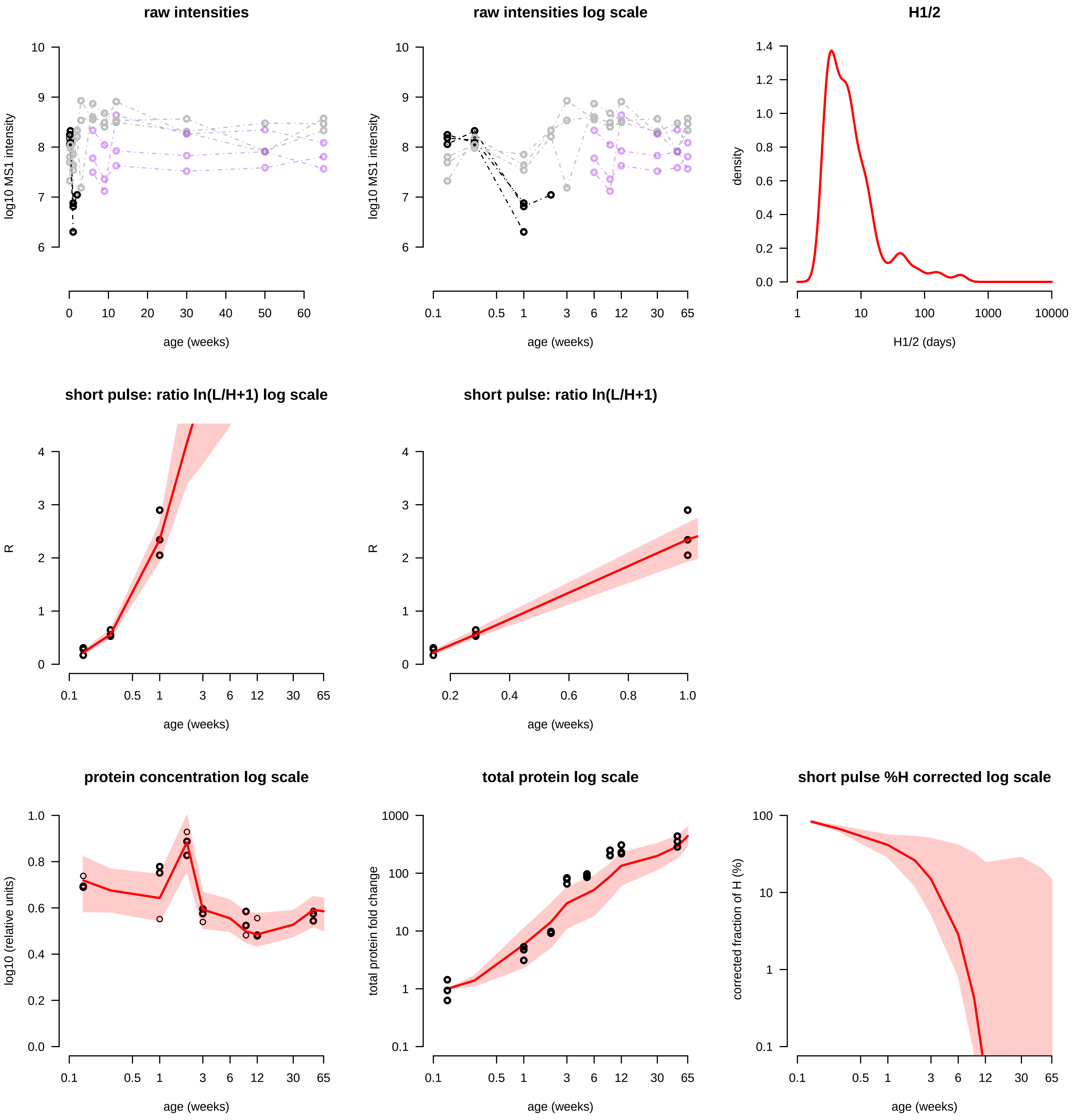

Q8BXA1

>sp|Q8BXA1|GOLI4_MOUSE

Golgi integral membrane protein 4

GN=Golim4

Med H1/2: 8 days

Q5 H1/2: 2.8 days

Q95 H1/2: 83.9 days

reliable model fit: yes

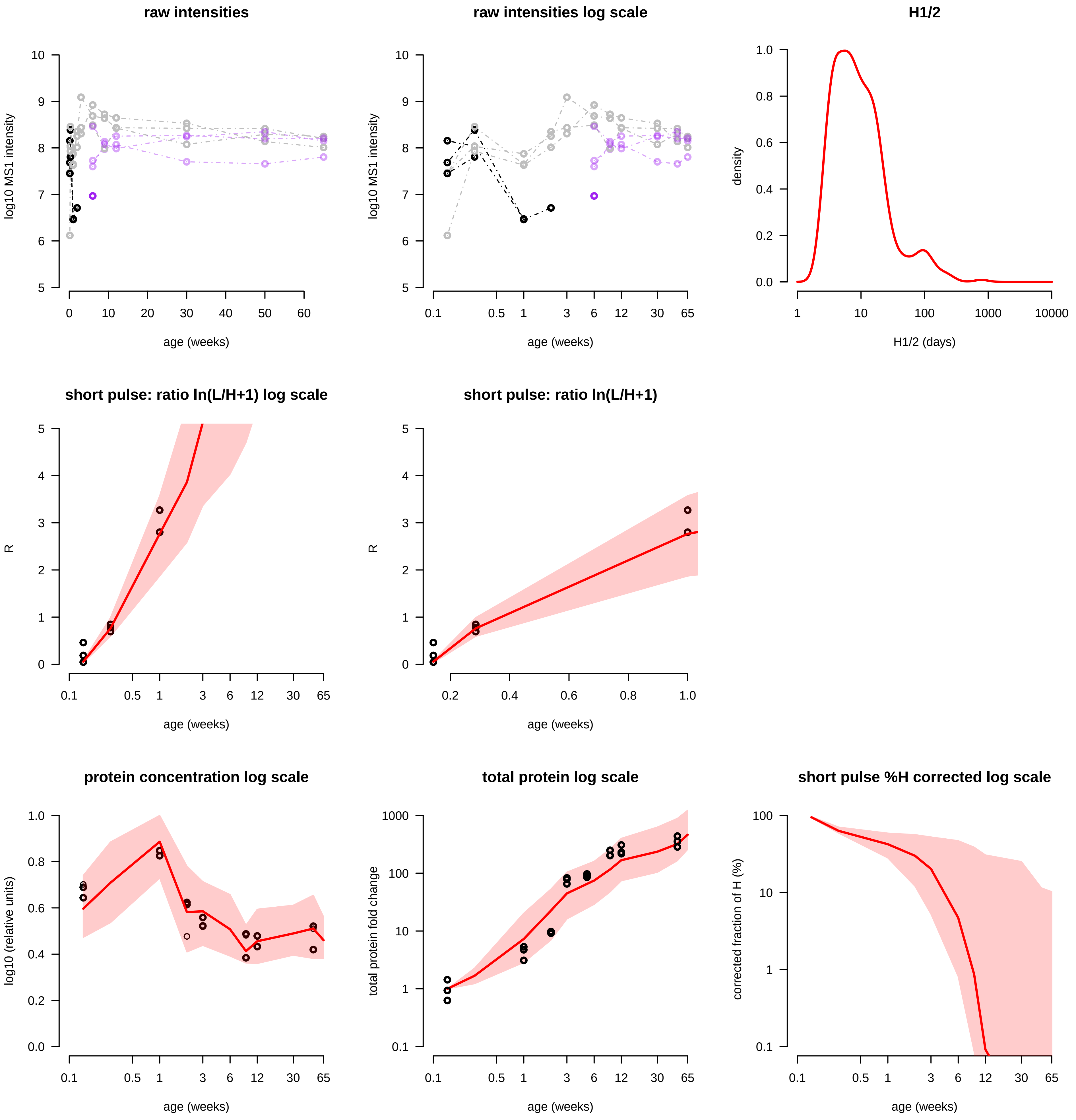

P97298

>sp|P97298|PEDF_MOUSE

Pigment epithelium−derived factor

GN=Serpinf1

Med H1/2: 6 days

Q5 H1/2: 3 days

Q95 H1/2: 22.1 days

reliable model fit: yes

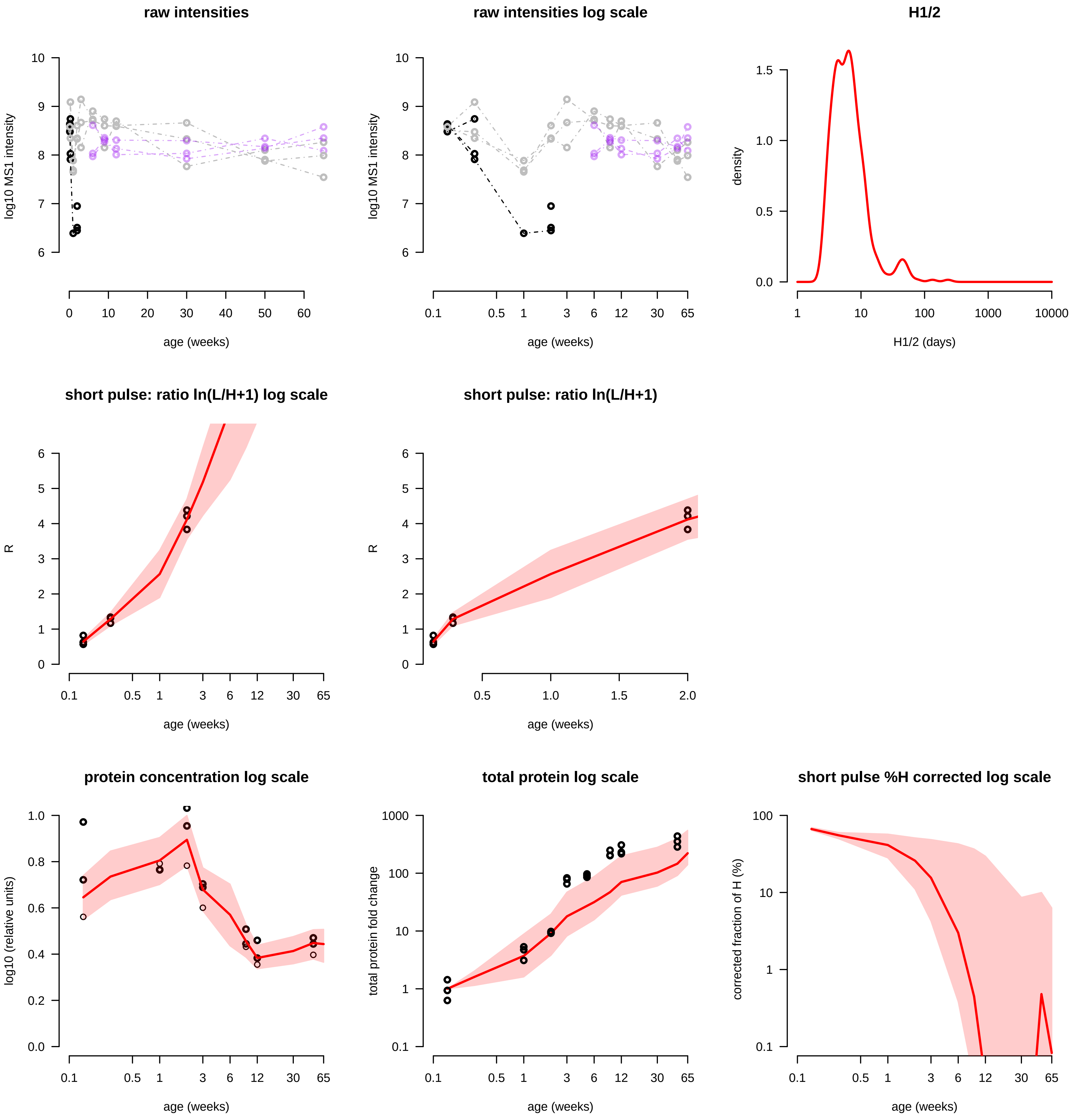

Q80YV3

>sp|Q80YV3|TRRAP_MOUSE

Transformation/transcription domain−associated protein

GN=Trrap

Med H1/2: 4.2 days

Q5 H1/2: 2.8 days

Q95 H1/2: 17.2 days

reliable model fit: yes

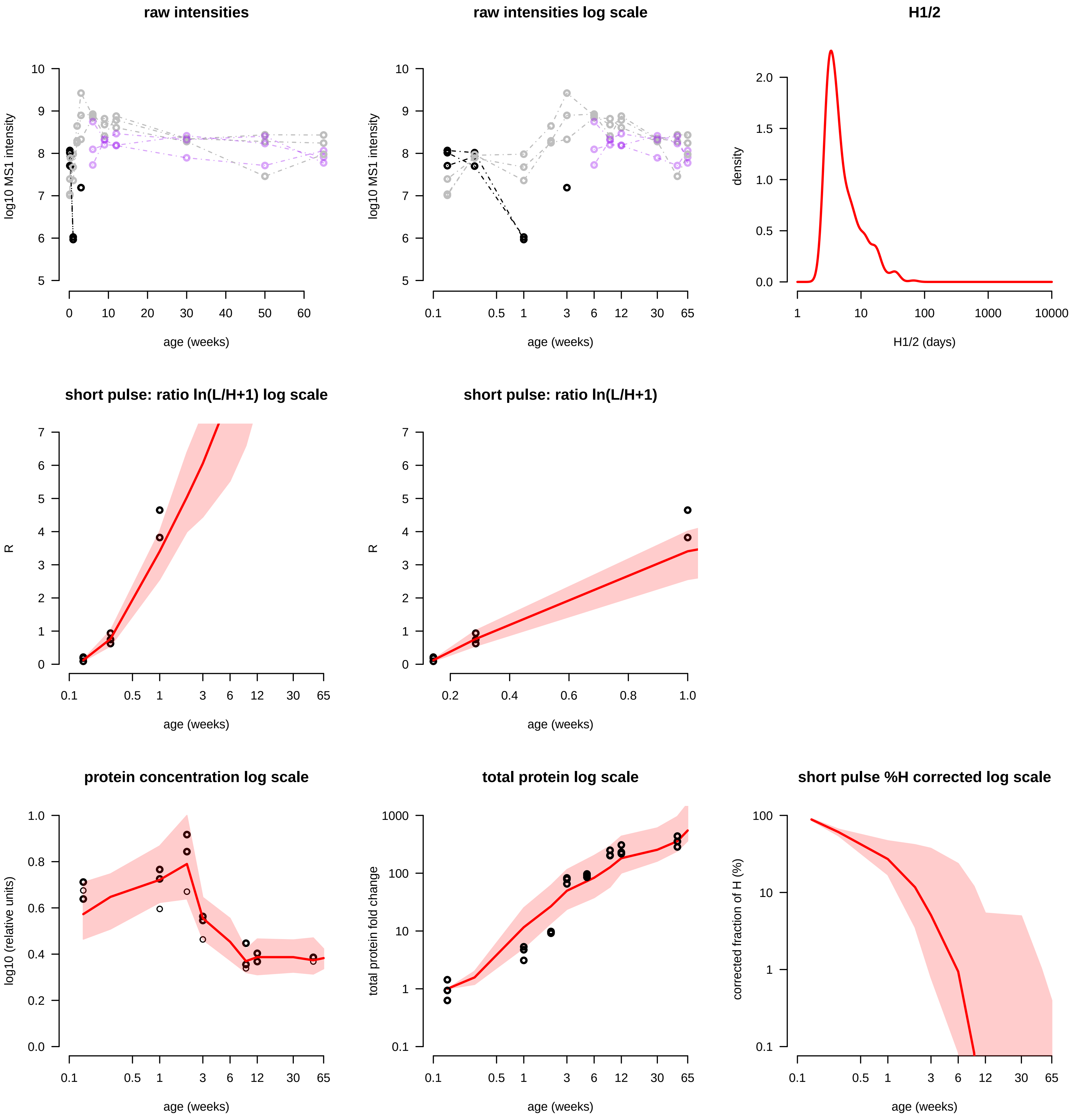

Q9QXA5

>sp|Q9QXA5|LSM4_MOUSE

U6 snRNA−associated Sm−like protein LSm4

GN=Lsm4

Med H1/2: 24 days

Q5 H1/2: 7.9 days

Q95 H1/2: 216.8 days

reliable model fit: yes

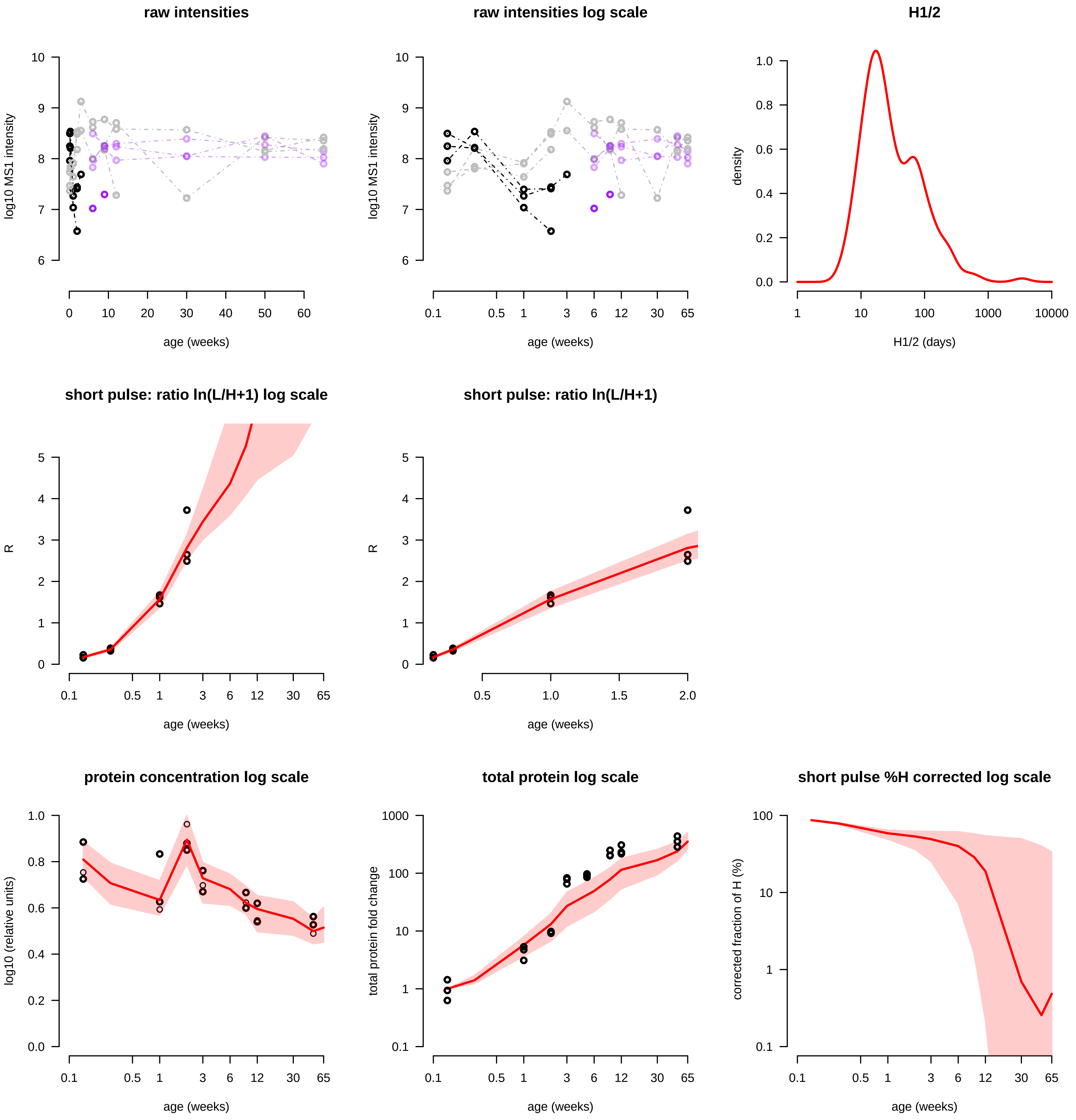

Q9WTS2

>sp|Q9WTS2|FUT8_MOUSE

Alpha−(1,6)−fucosyltransferase

GN=Fut8

Med H1/2: 7.6 days

Q5 H1/2: 2.9 days

Q95 H1/2: 30.3 days

reliable model fit: yes

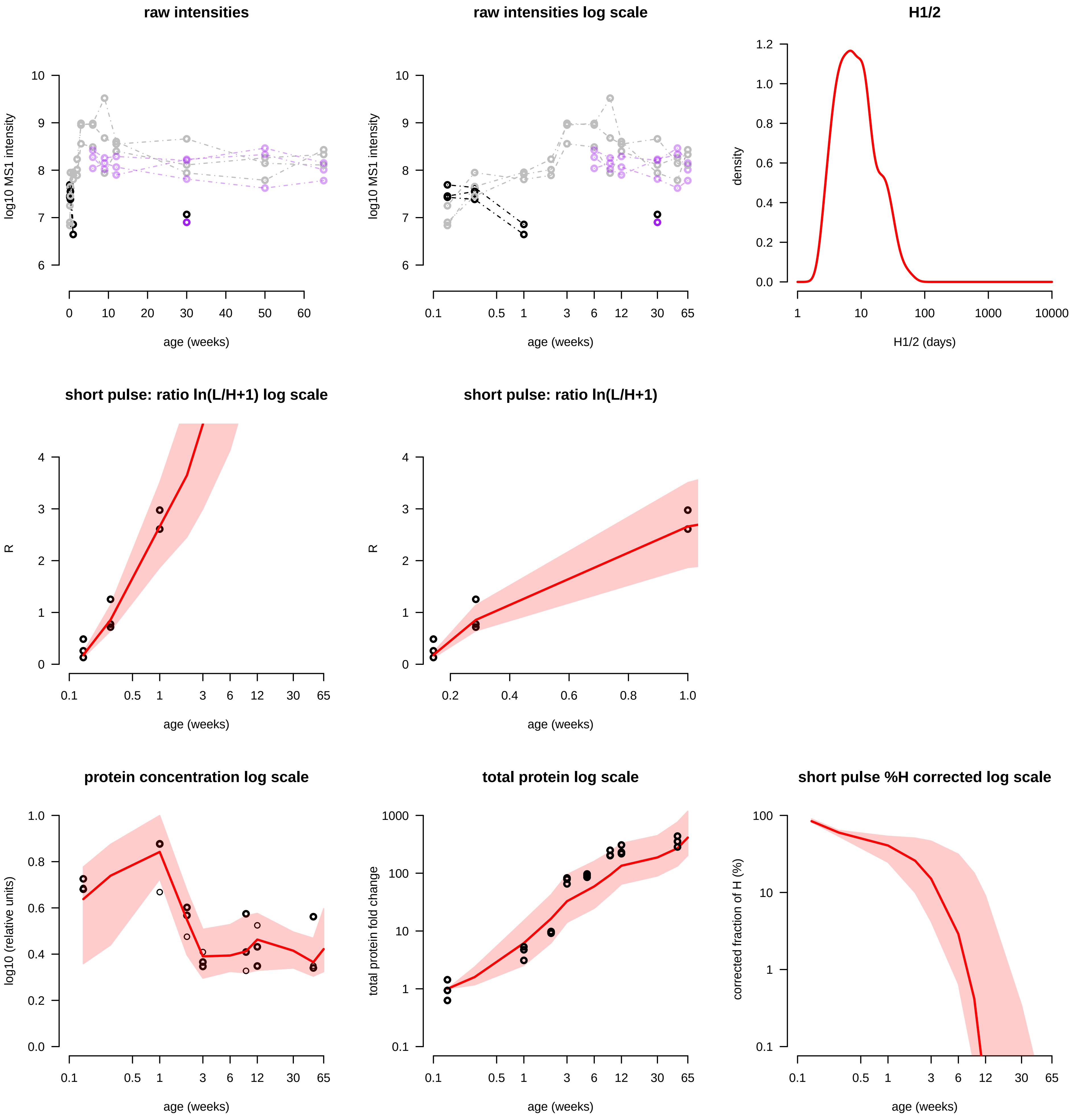

P57716

>sp|P57716|NICA_MOUSE

Nicastrin

GN=Ncstn

Med H1/2: 10.7 days

Q5 H1/2: 4.7 days

Q95 H1/2: 66 days

reliable model fit: yes

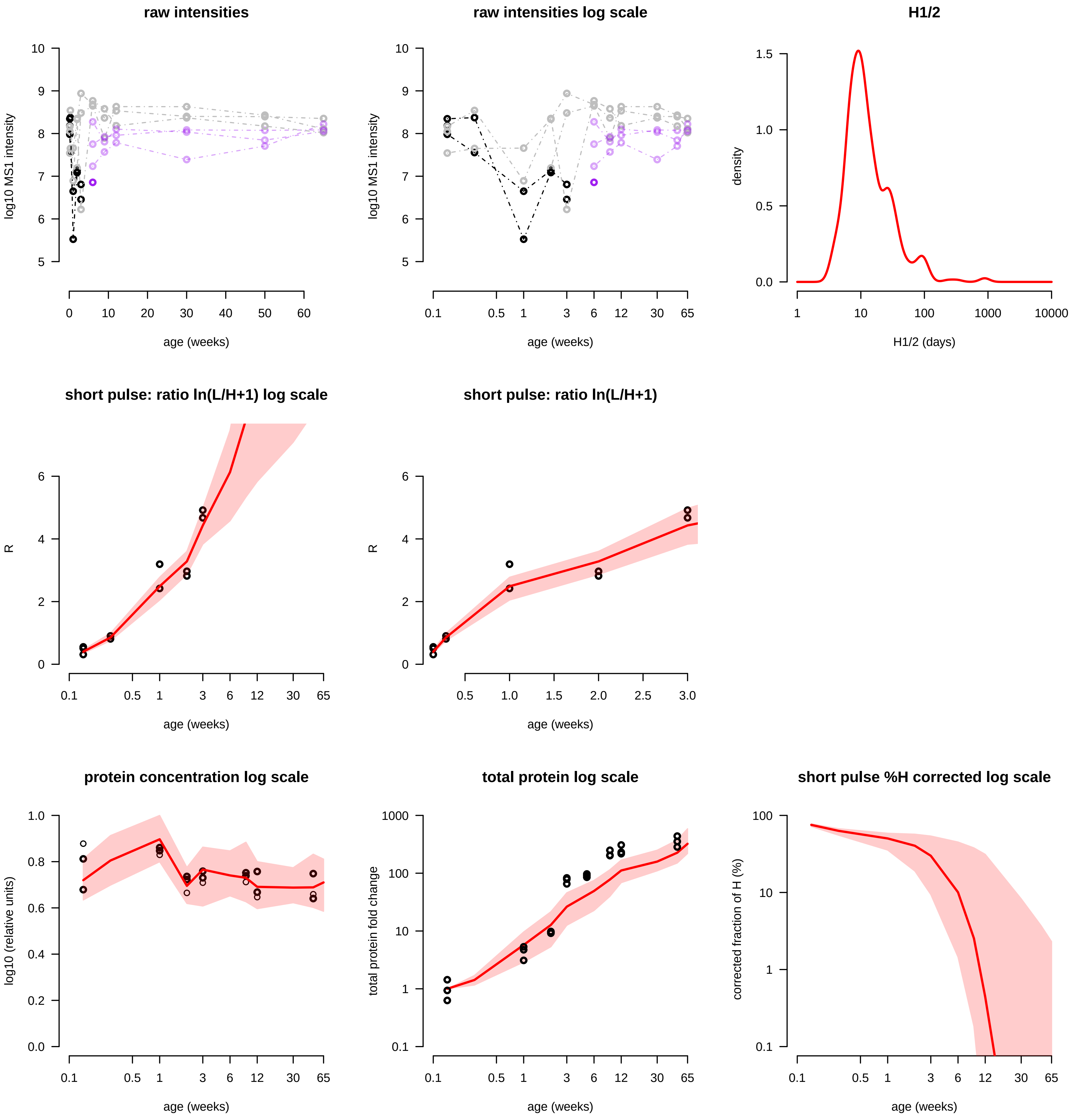

Q8BWU5

>sp|Q8BWU5|OSGEP_MOUSE

Probable tRNA N6−adenosine threonylcarbamoyltransferase

GN=Osgep

Med H1/2: 6.5 days

Q5 H1/2: 2.8 days

Q95 H1/2: 46.1 days

reliable model fit: yes

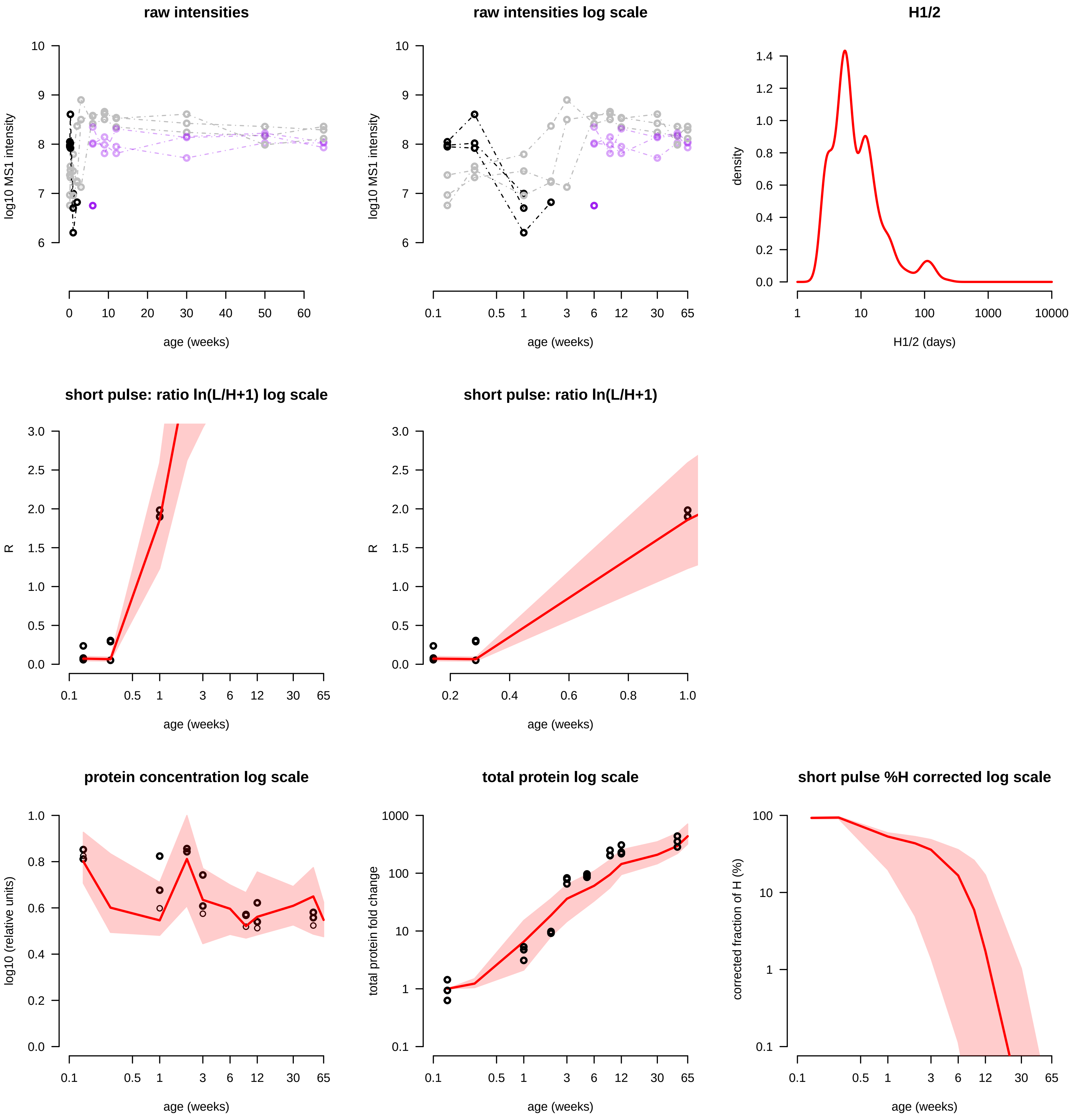

C0HKD9;C0HKD8

>sp|C0HKD9|MFA1B_MOUSE

Microfibrillar−associated protein 1B

GN=Mfap1b

Med H1/2: 12.6 days

Q5 H1/2: 5 days

Q95 H1/2: 98.7 days

reliable model fit: yes

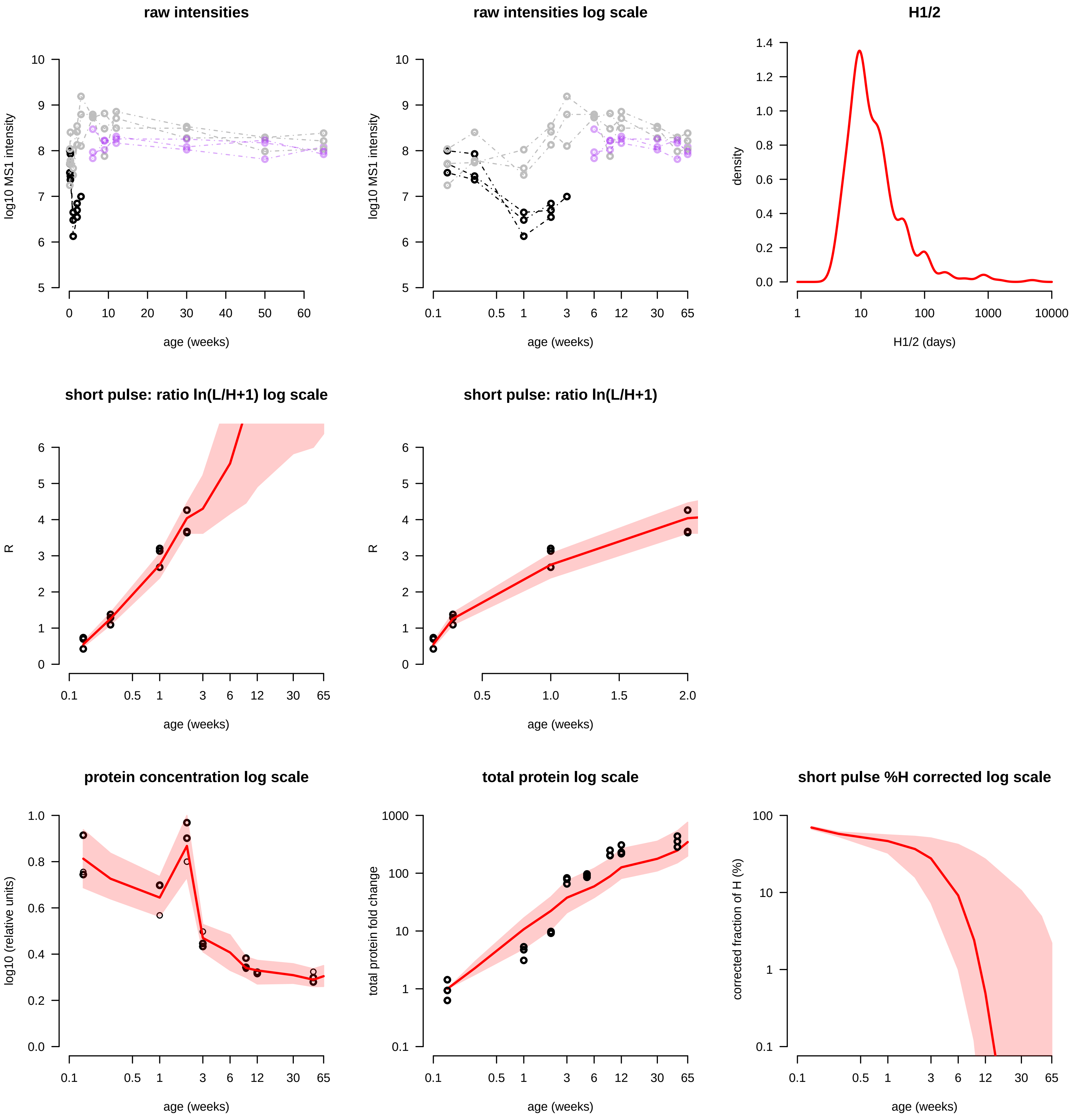

Q8K2T8

>sp|Q8K2T8|PAF1_MOUSE

RNA polymerase II−associated factor 1 homolog

GN=Paf1

Med H1/2: 4.8 days

Q5 H1/2: 2.5 days

Q95 H1/2: 22.3 days

reliable model fit: yes

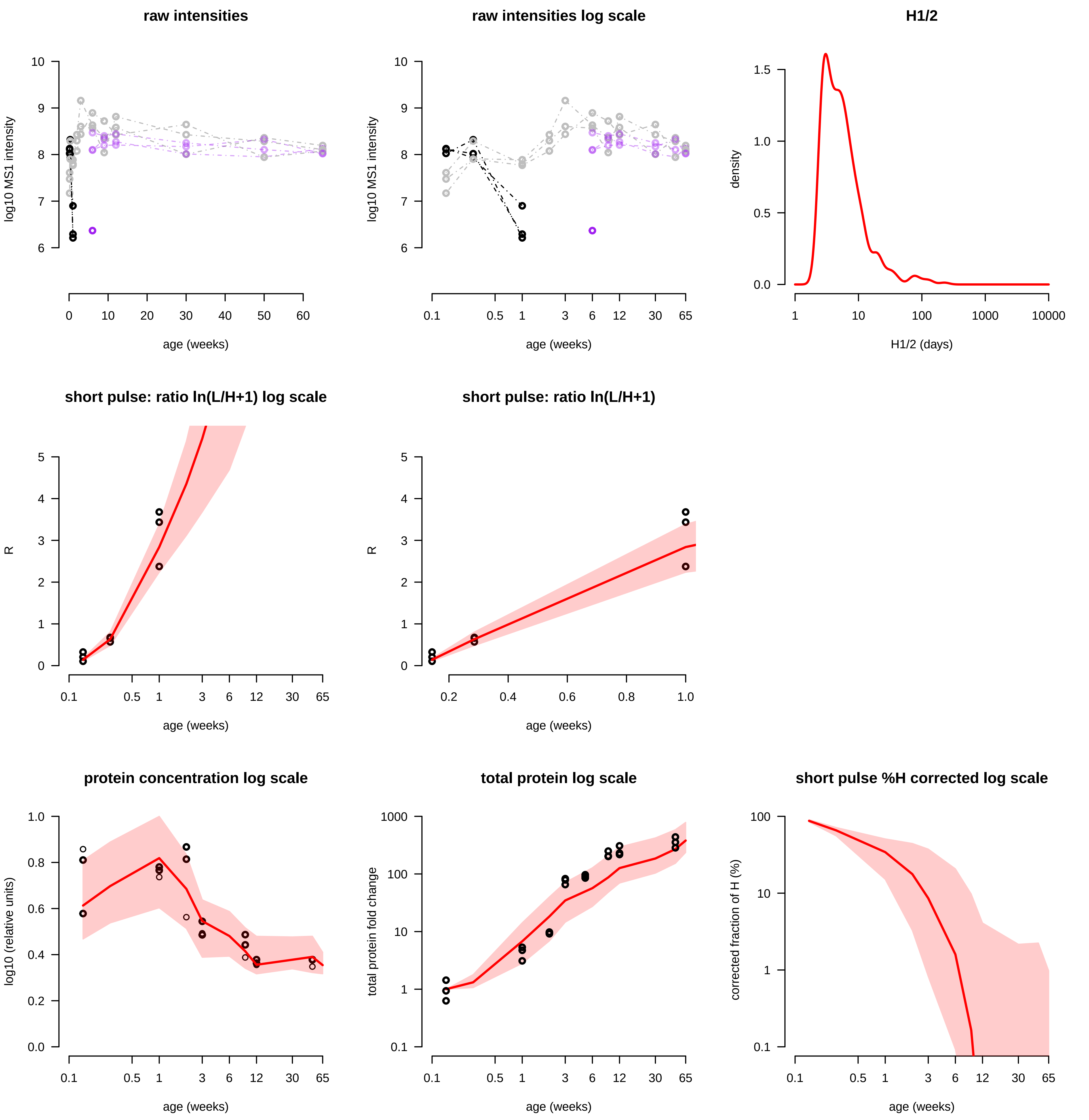

Q8R409

>sp|Q8R409|HEXI1_MOUSE

Protein HEXIM1

GN=Hexim1

Med H1/2: 13.2 days

Q5 H1/2: 4.2 days

Q95 H1/2: 143 days

reliable model fit: yes

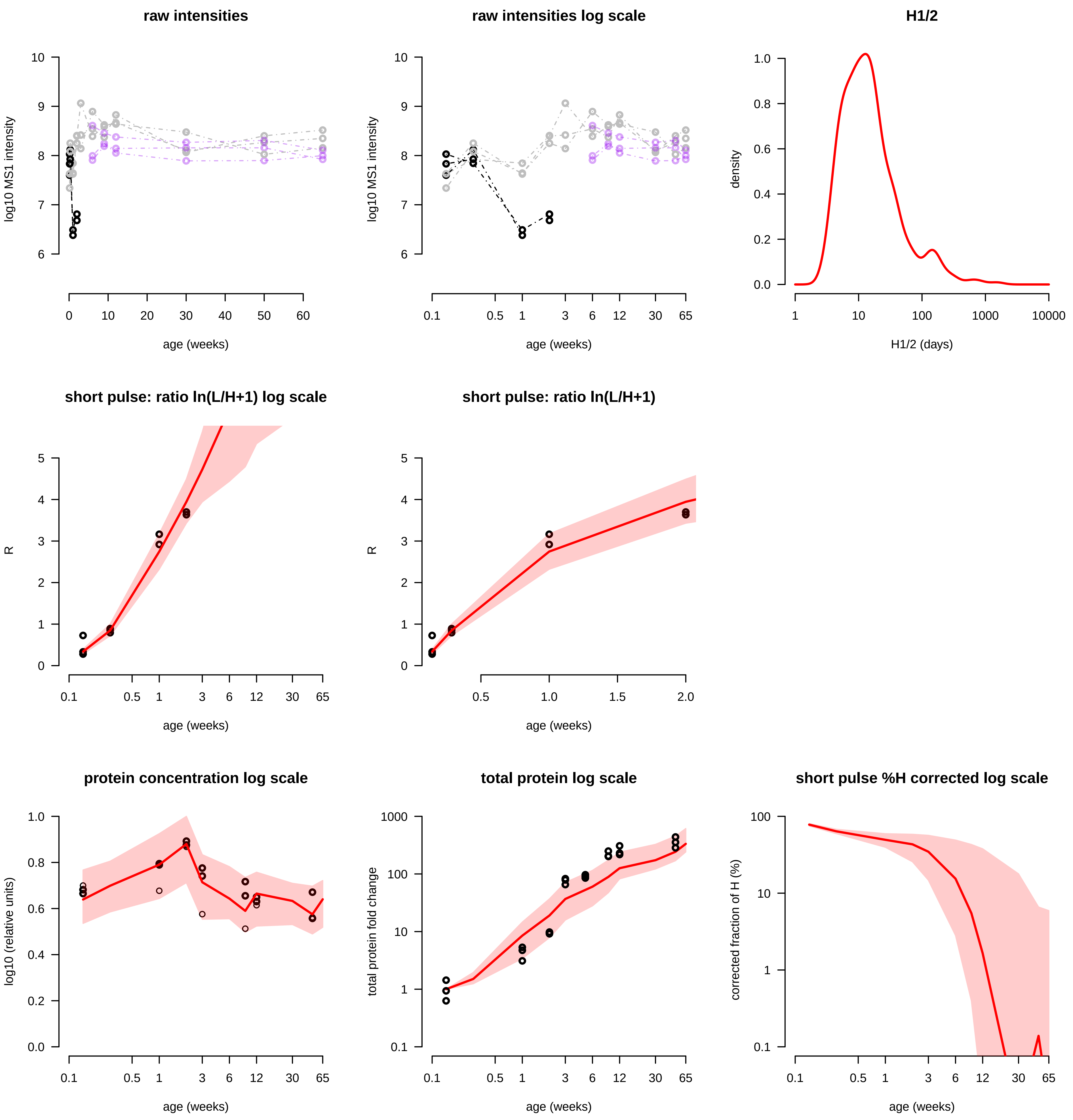

P36993;P36993−3;P36993−5

>sp|P36993|PPM1B_MOUSE

Protein phosphatase 1B

GN=Ppm1b

Med H1/2: 9.8 days

Q5 H1/2: 3.4 days

Q95 H1/2: 60.6 days

reliable model fit: yes

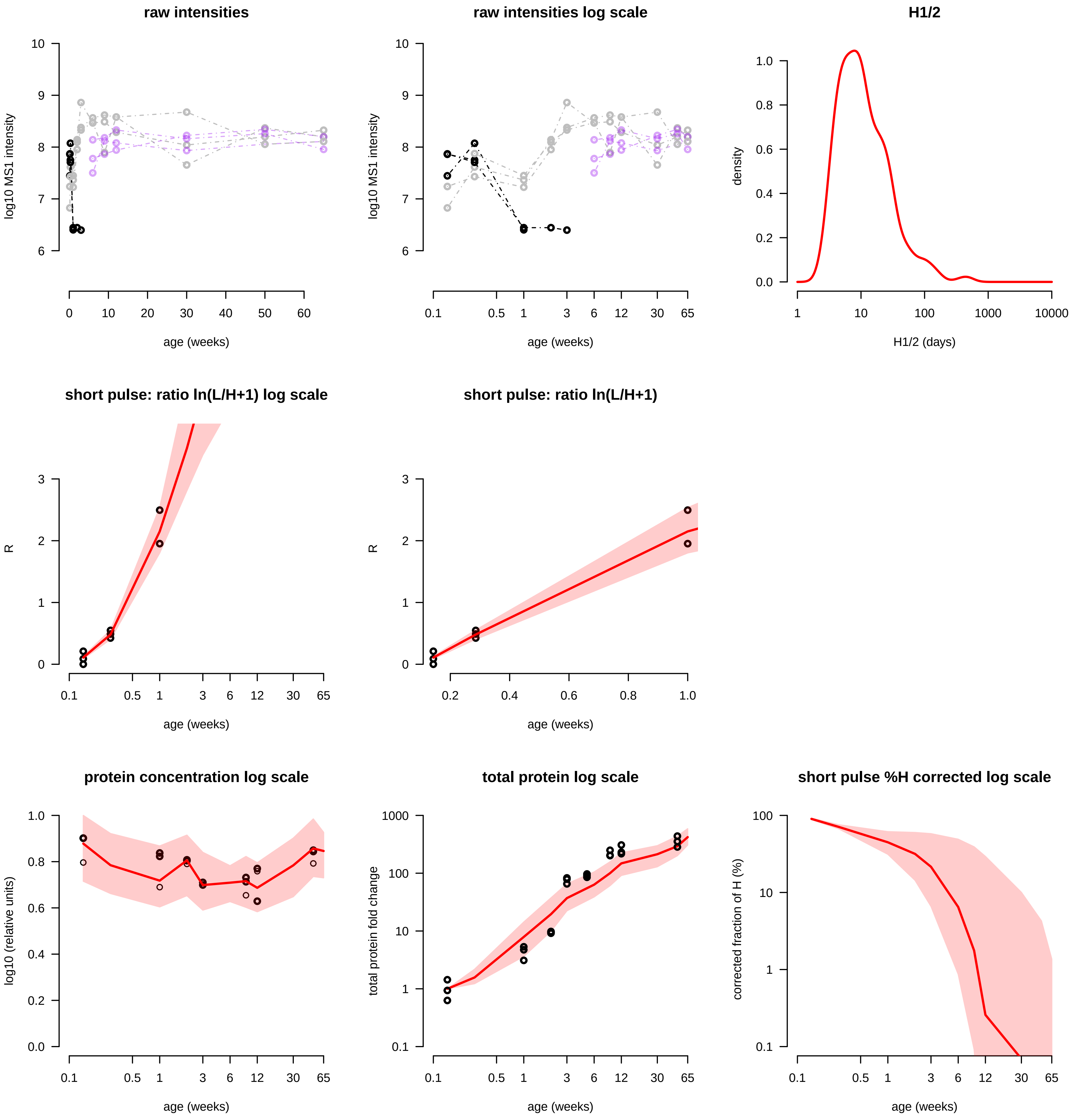

Q8VBT9;Q8VBT9−2;Q8VBT9−3

>sp|Q8VBT9|ASPC1_MOUSE

Tether containing UBX domain for GLUT4

GN=Aspscr1

Med H1/2: 7 days

Q5 H1/2: 2.8 days

Q95 H1/2: 50.5 days

reliable model fit: yes

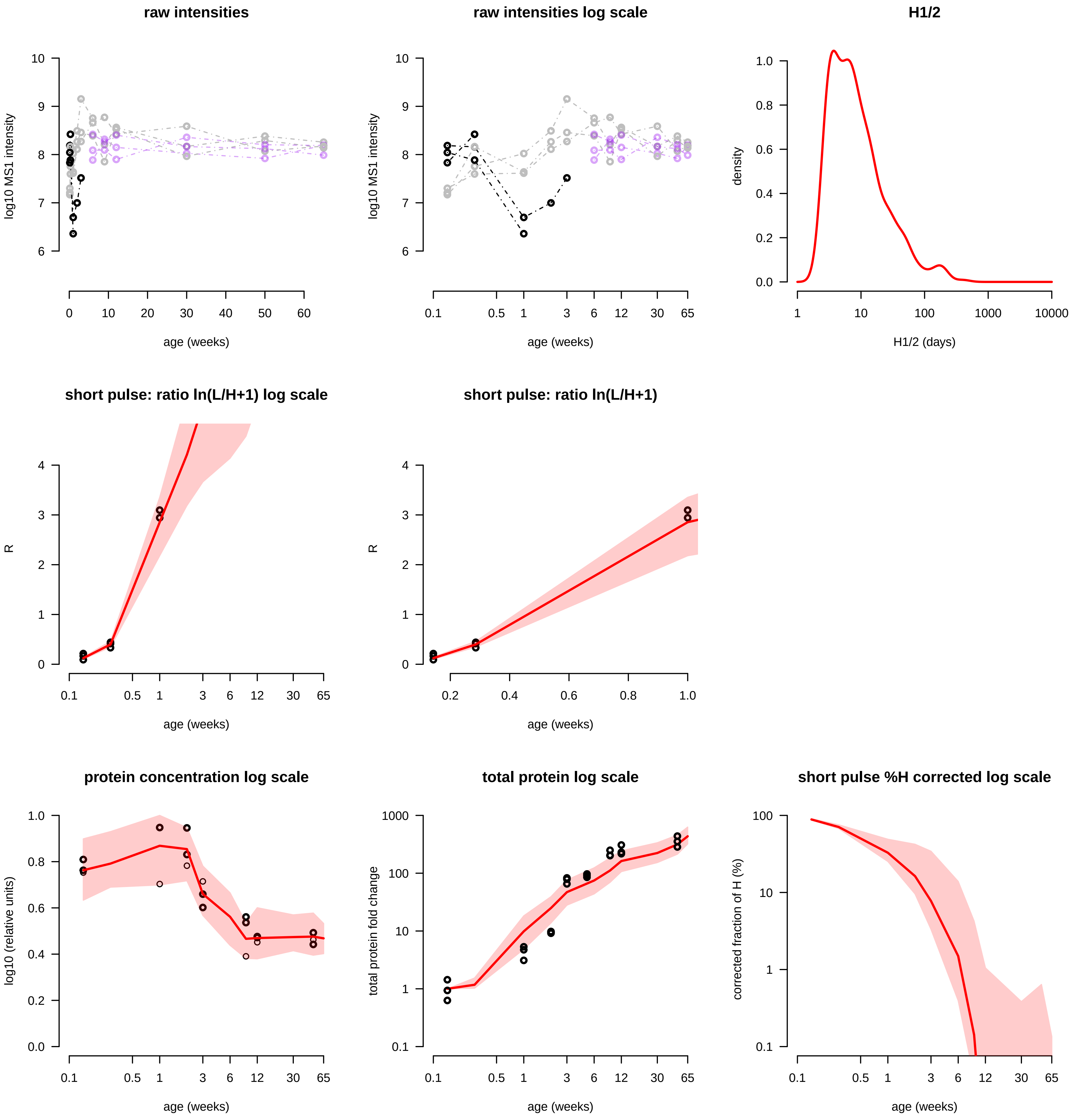

Q8C165

>sp|Q8C165|P20D1_MOUSE

N−fatty−acyl−amino acid synthase/hydrolase PM20D1

GN=Pm20d1

Med H1/2: 4.6 days

Q5 H1/2: 2.5 days

Q95 H1/2: 34.8 days

reliable model fit: yes

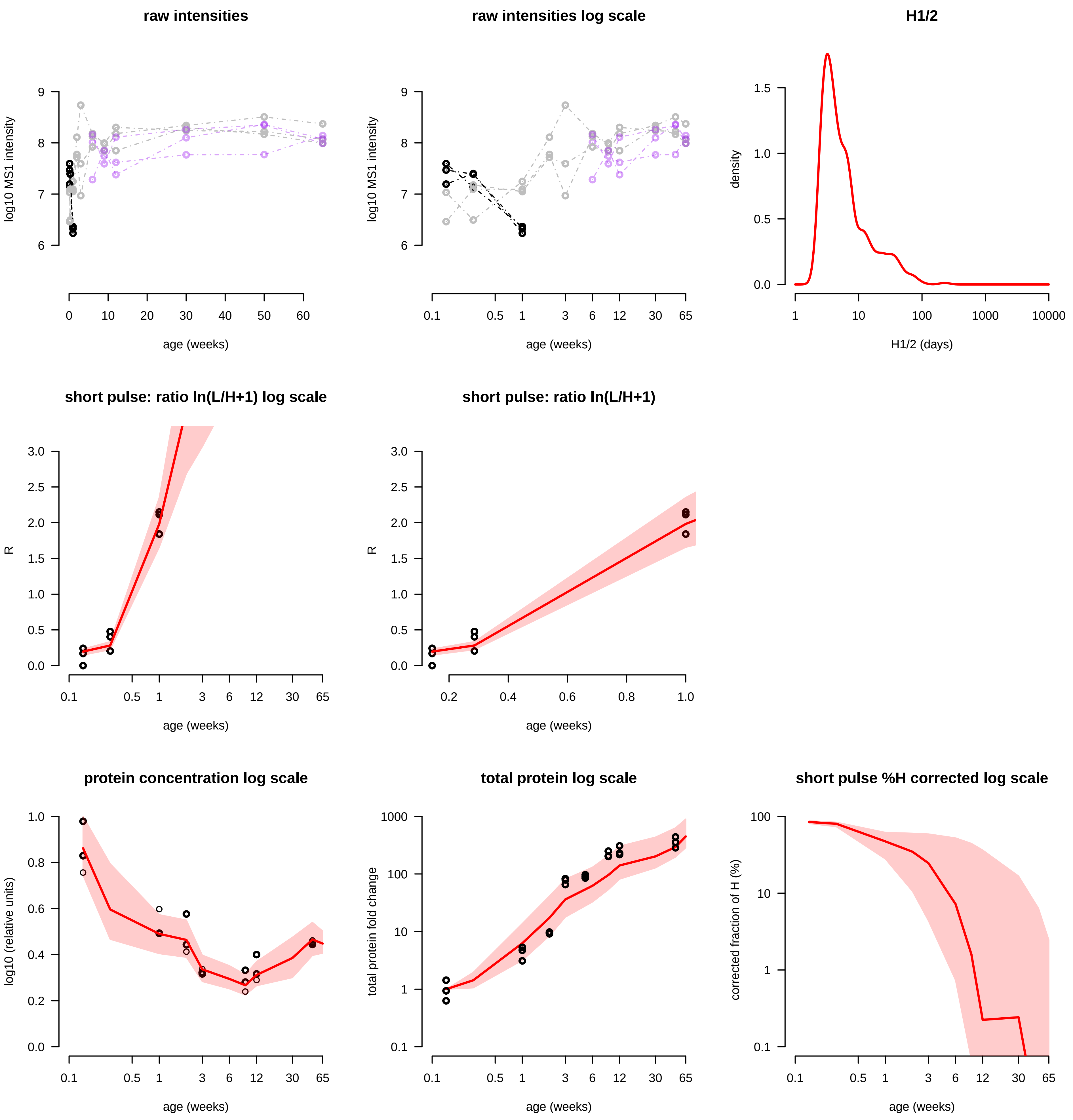

P55288

>sp|P55288|CAD11_MOUSE

Cadherin−11

GN=Cdh11

Med H1/2: 7.8 days

Q5 H1/2: 3.4 days

Q95 H1/2: 38 days

reliable model fit: yes

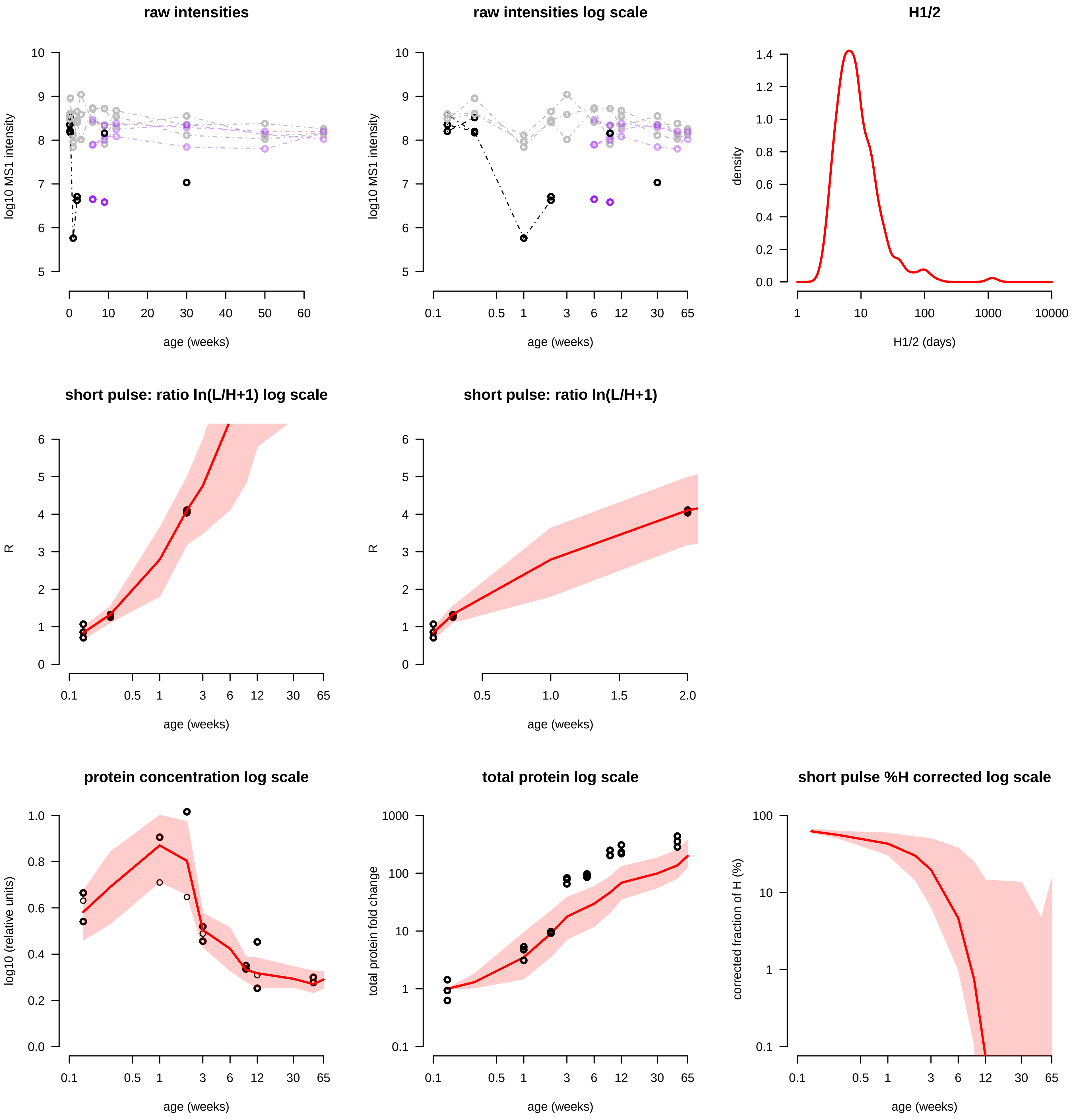

P03921

>sp|P03921|NU5M_MOUSE

NADH−ubiquinone oxidoreductase chain 5

GN=Mtnd5

Med H1/2: 11.6 days

Q5 H1/2: 3.9 days

Q95 H1/2: 134.4 days

reliable model fit: yes

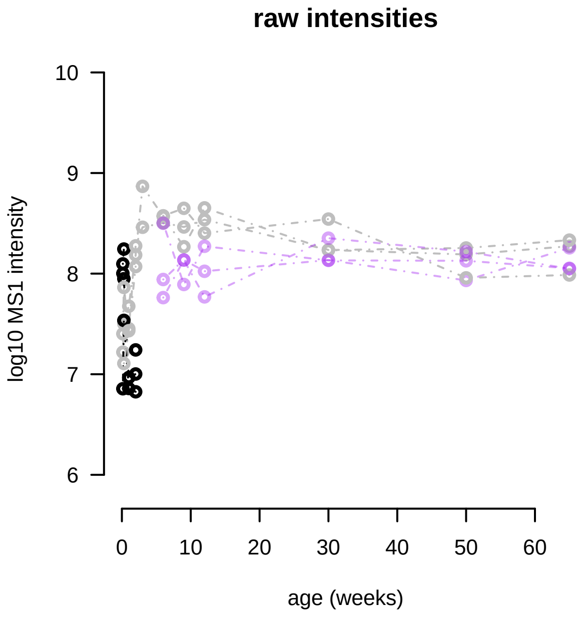

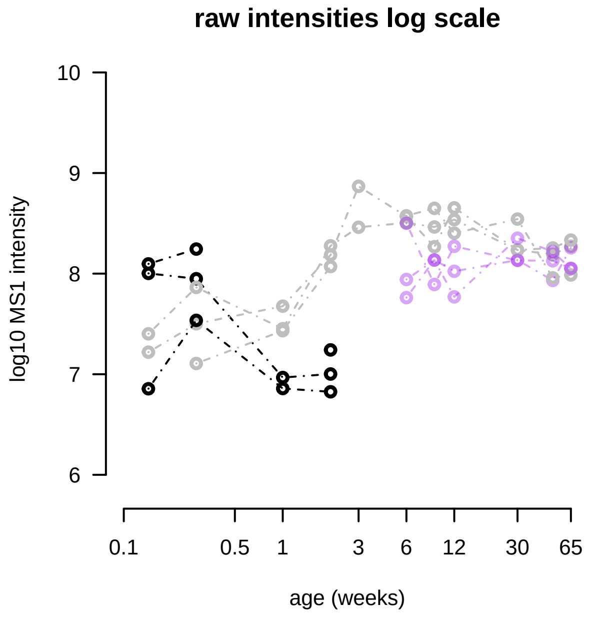

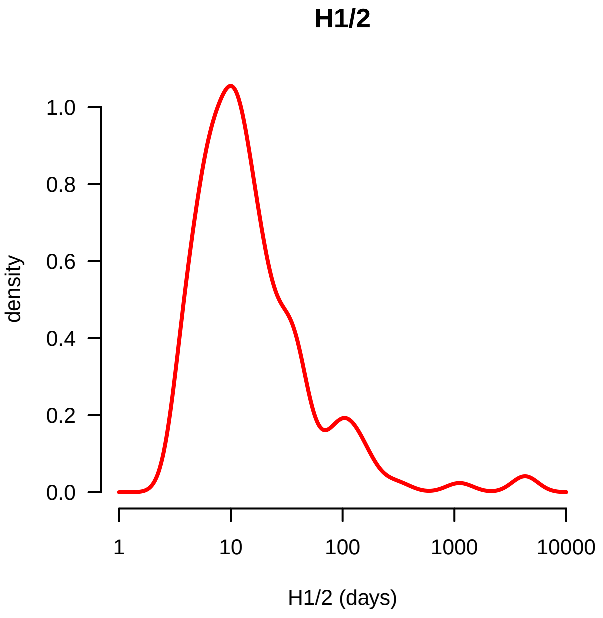

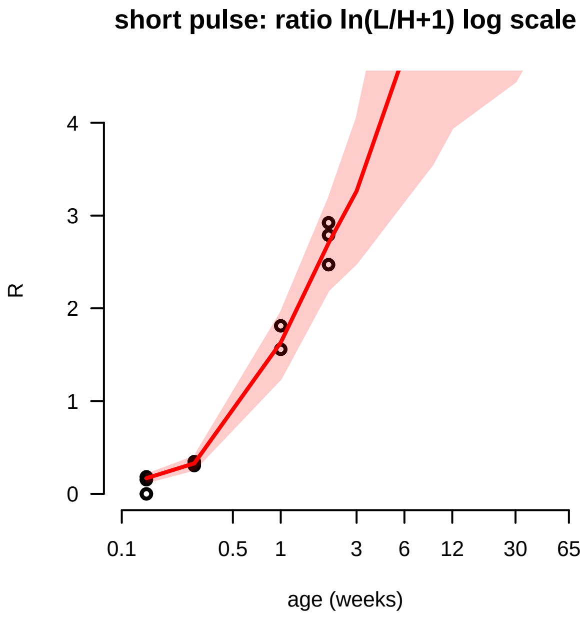

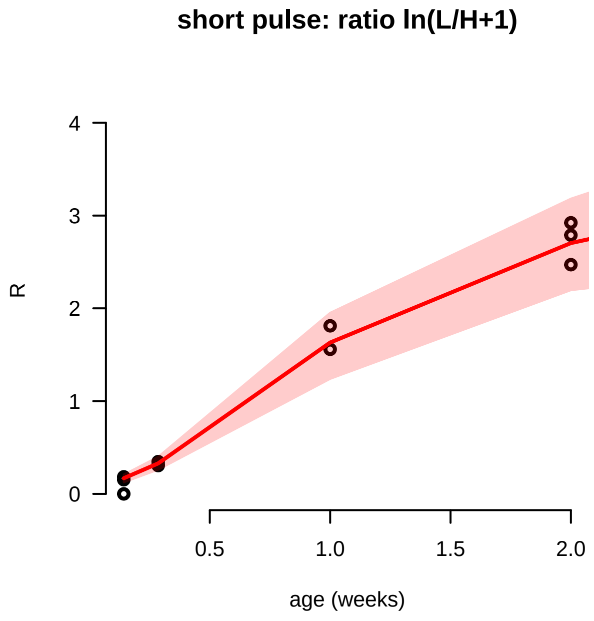

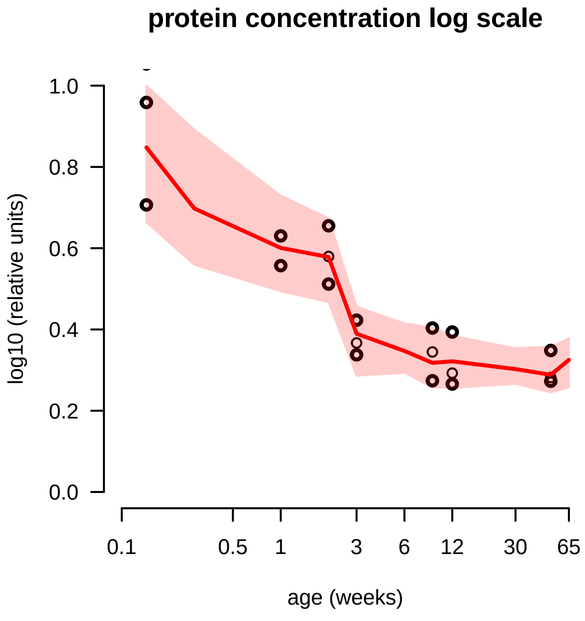

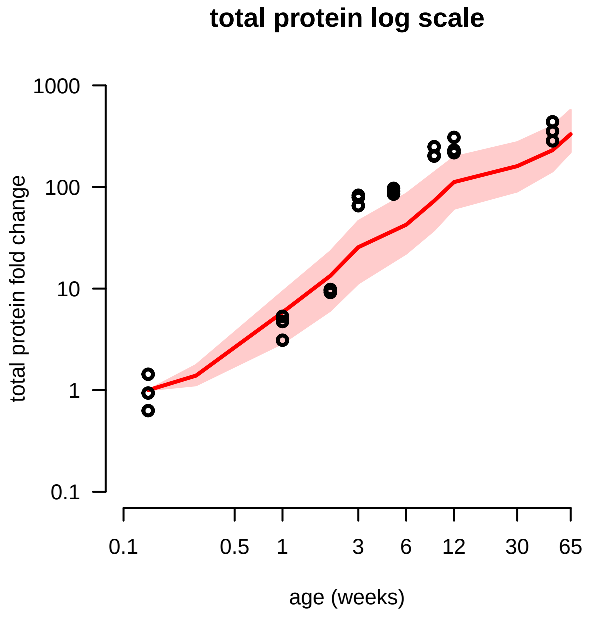

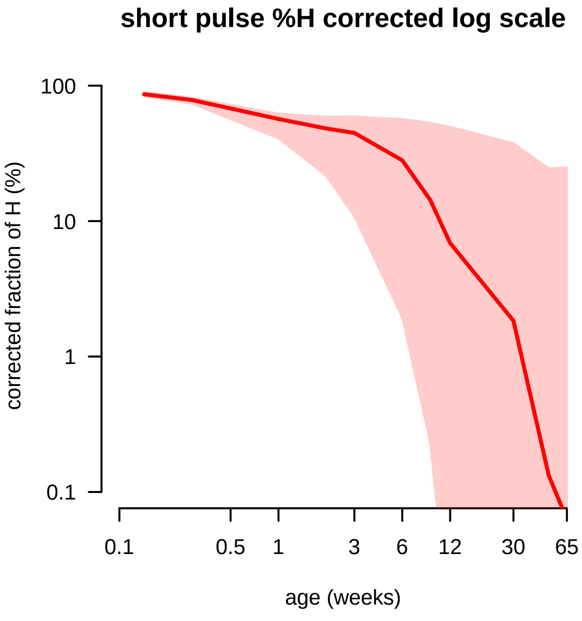

P02463

>sp|P02463|CO4A1_MOUSE

Collagen alpha−1(IV) chain

GN=Col4a1

Med H1/2: 2.8 days

Q5 H1/2: 2.8 days

Q95 H1/2: 2.8 days

reliable model fit: no

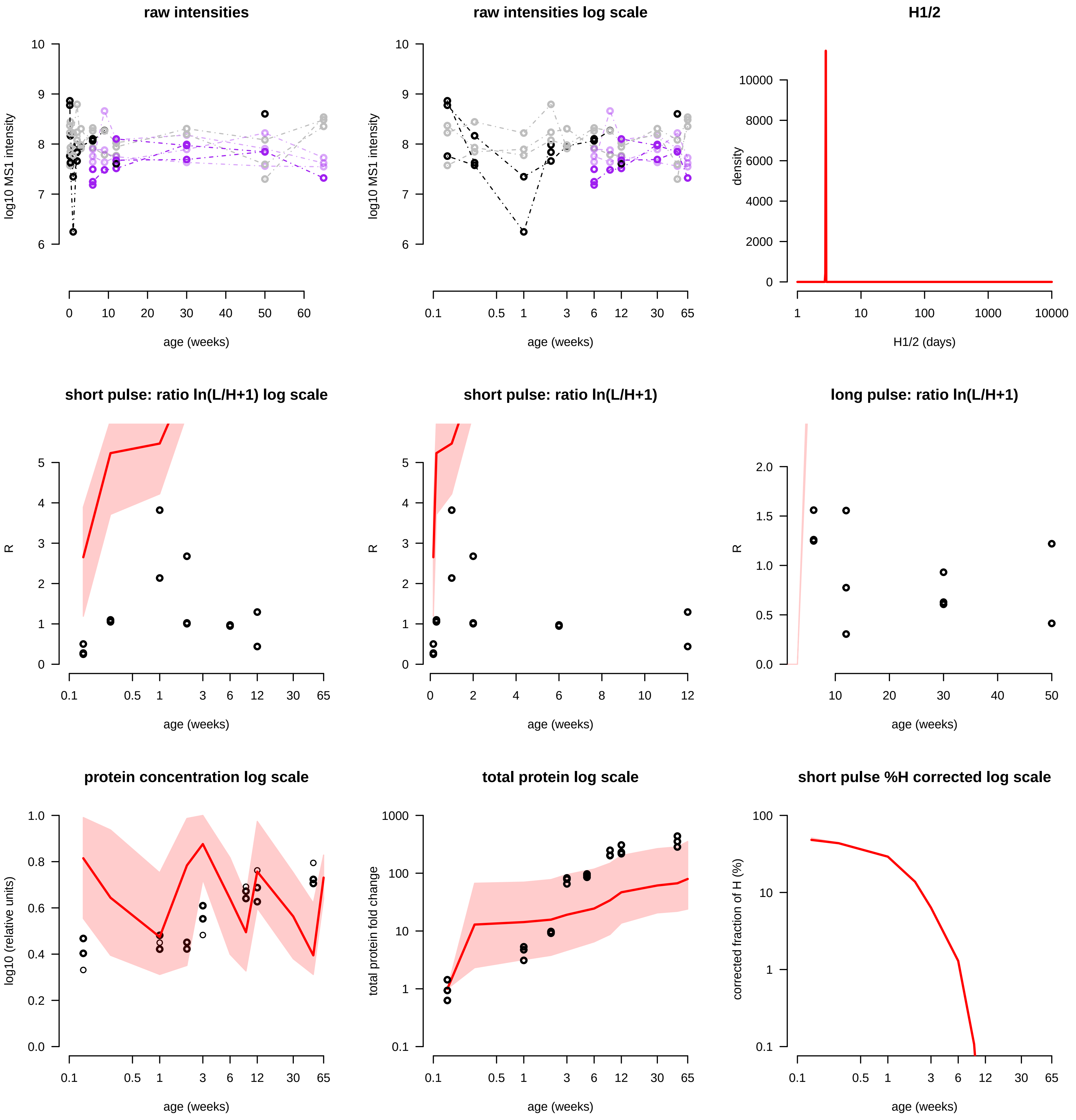

Q80TP3

>sp|Q80TP3|UBR5_MOUSE

E3 ubiquitin−protein ligase UBR5

GN=Ubr5

Med H1/2: 147.8 days

Q5 H1/2: 41.2 days

Q95 H1/2: 1421.9 days

reliable model fit: no

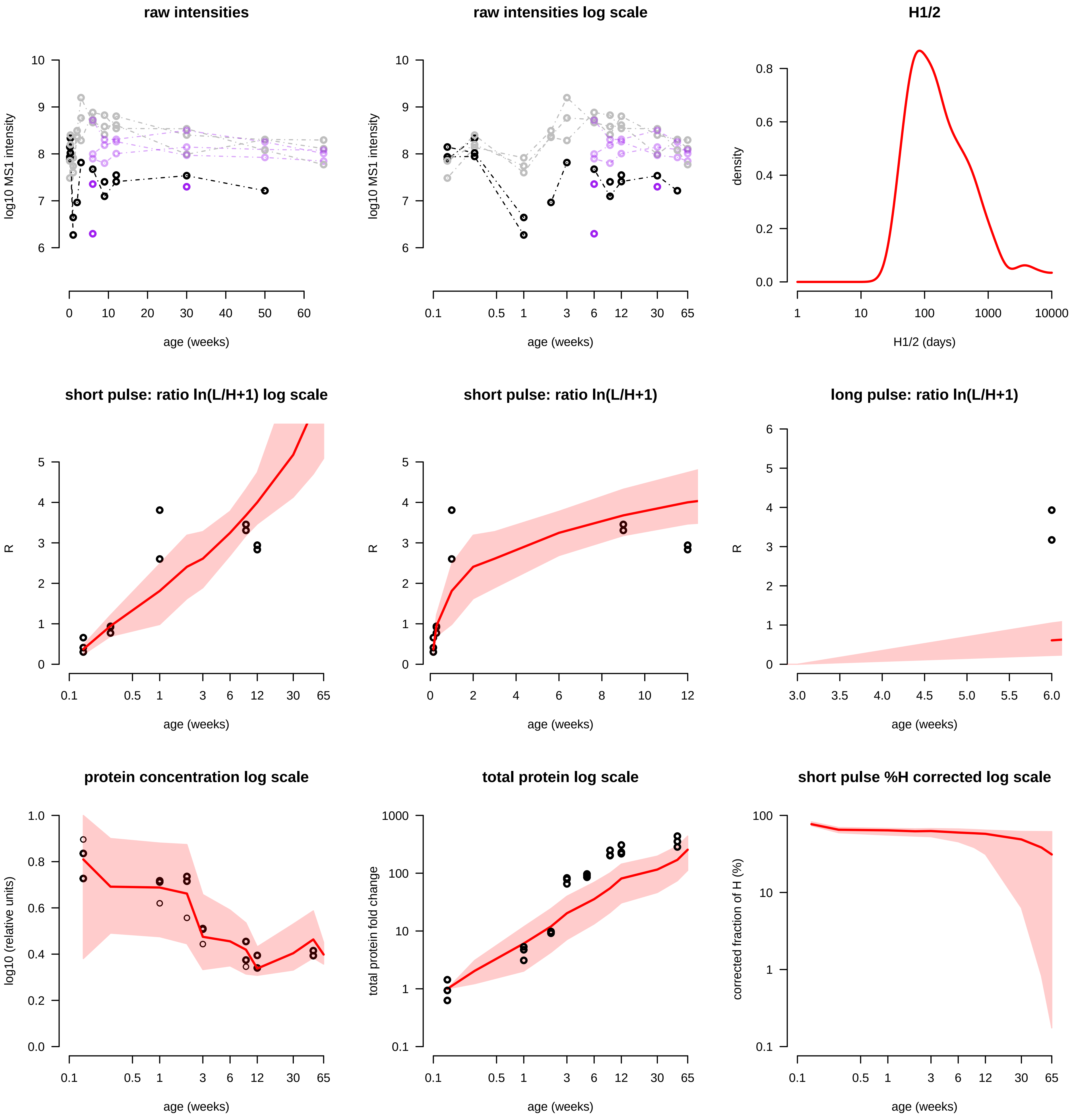

P97386;P97386−2

>sp|P97386|DNLI3_MOUSE

DNA ligase 3

GN=Lig3

Med H1/2: 43.6 days

Q5 H1/2: 17.7 days

Q95 H1/2: 311.3 days

reliable model fit: yes

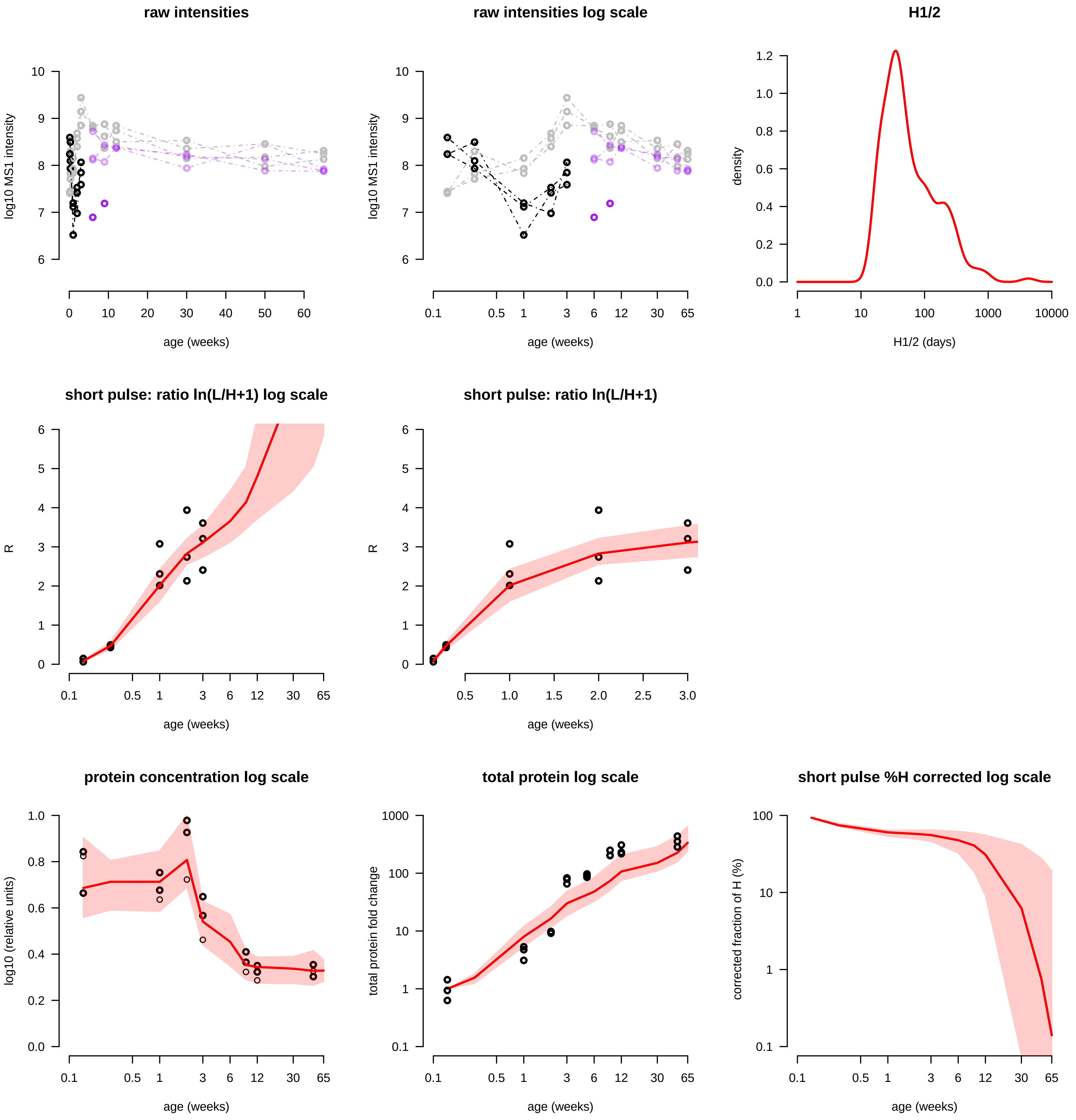

Q9DCT1

>sp|Q9DCT1|AKCL2_MOUSE

1,5−anhydro−D−fructose reductase

GN=Akr1e2

Med H1/2: 9.9 days

Q5 H1/2: 4 days

Q95 H1/2: 77.2 days

reliable model fit: yes

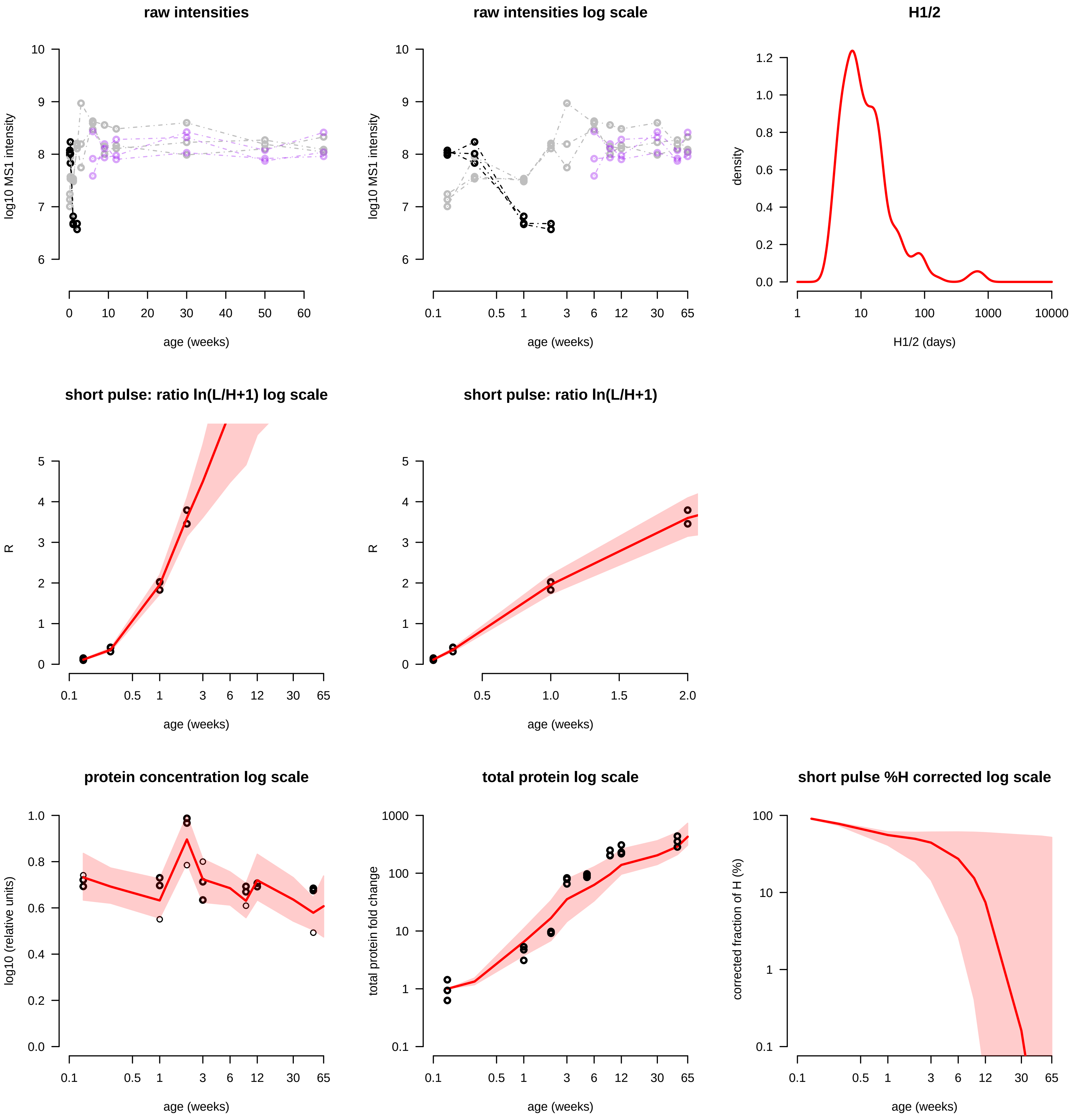

Q9CWM4

>sp|Q9CWM4|PFD1_MOUSE

Prefoldin subunit 1

GN=Pfdn1

Med H1/2: 3.9 days

Q5 H1/2: 2.5 days

Q95 H1/2: 11 days

reliable model fit: yes

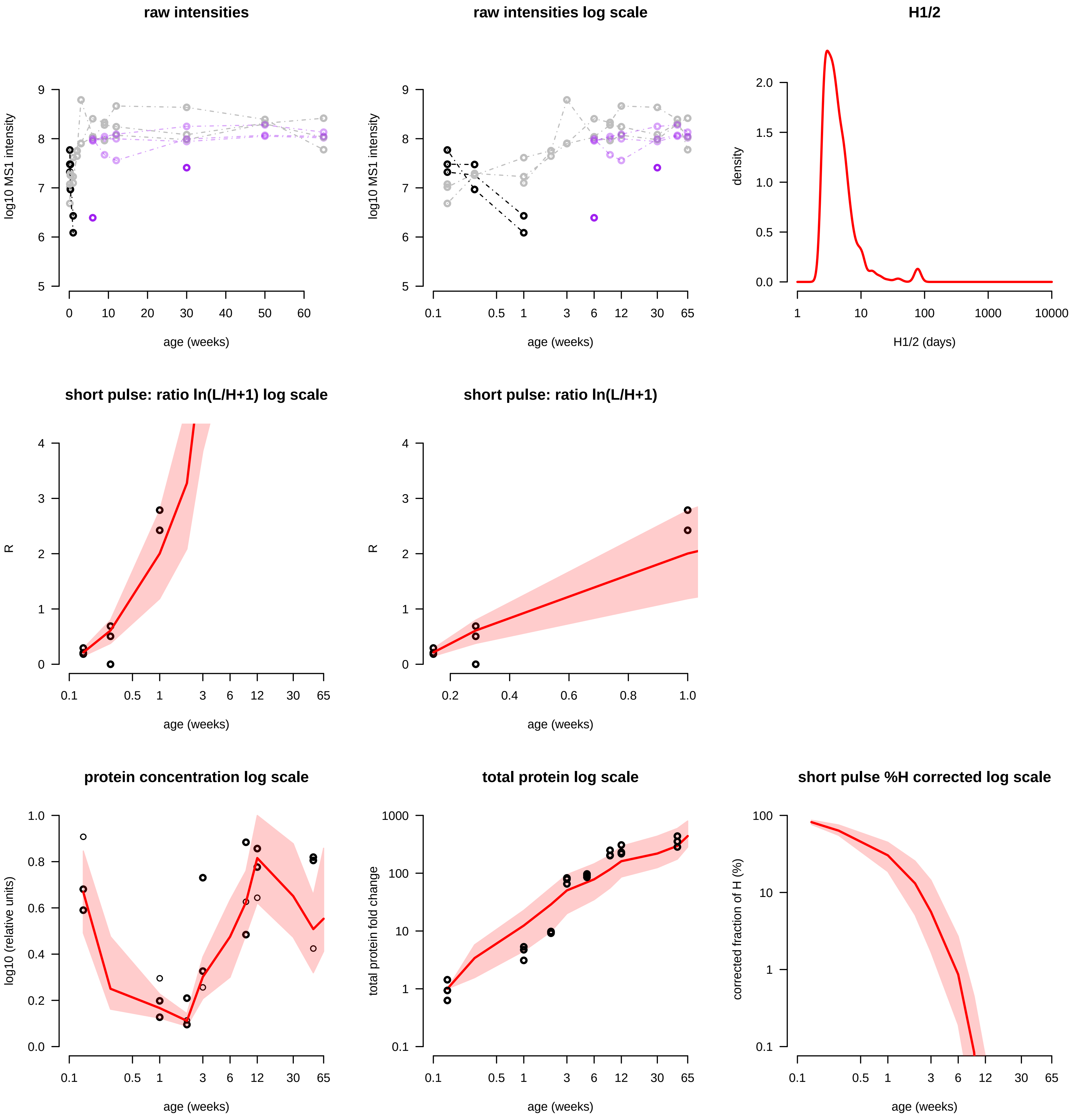

P49718

>sp|P49718|MCM5_MOUSE

DNA replication licensing factor MCM5

GN=Mcm5

Med H1/2: 3.5 days

Q5 H1/2: 2.9 days

Q95 H1/2: 4.3 days

reliable model fit: yes

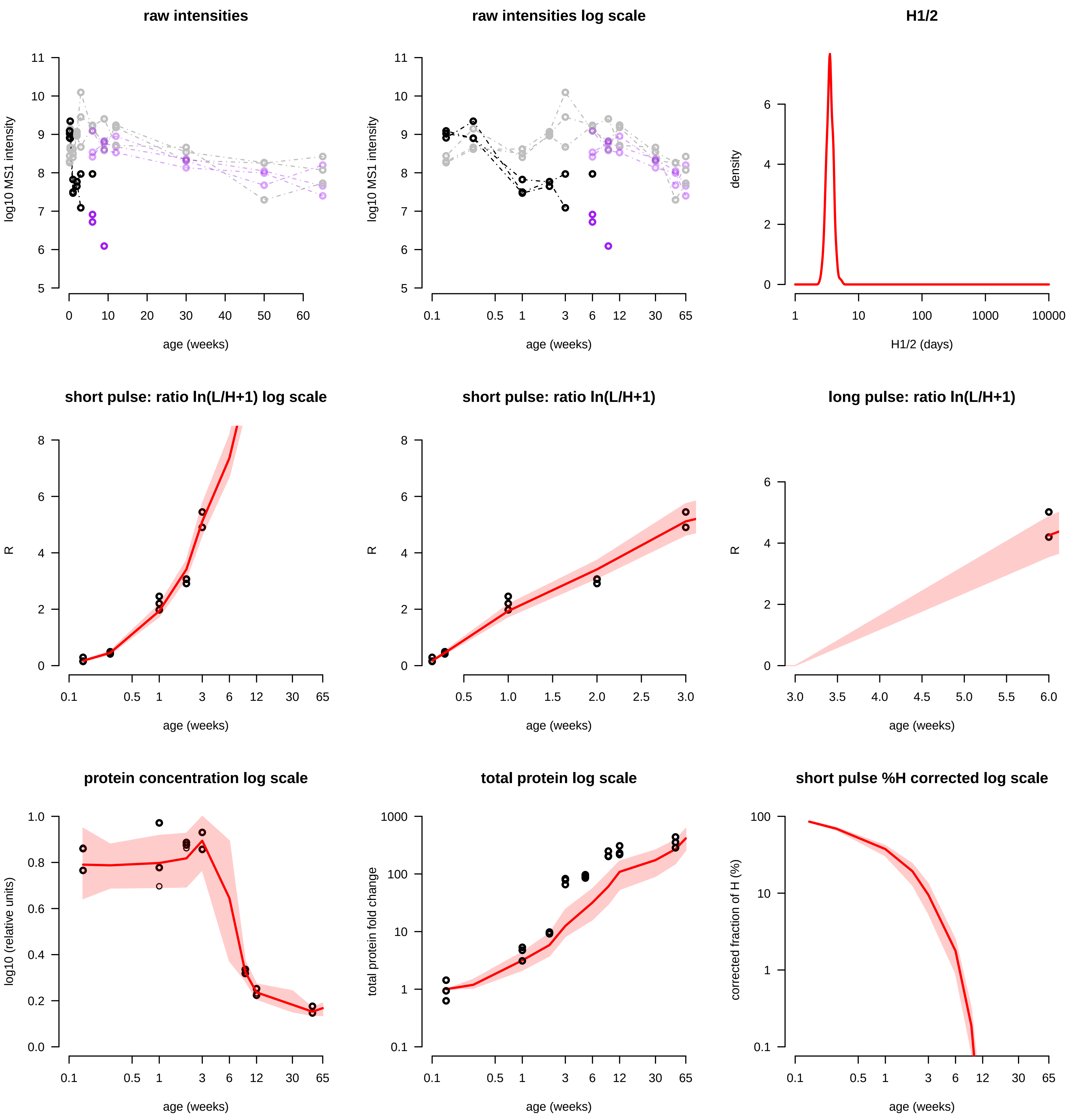

Q8C547

>sp|Q8C547|HTR5B_MOUSE

HEAT repeat−containing protein 5B

GN=Heatr5b

Med H1/2: 19.3 days

Q5 H1/2: 14.1 days

Q95 H1/2: 25.1 days

reliable model fit: yes

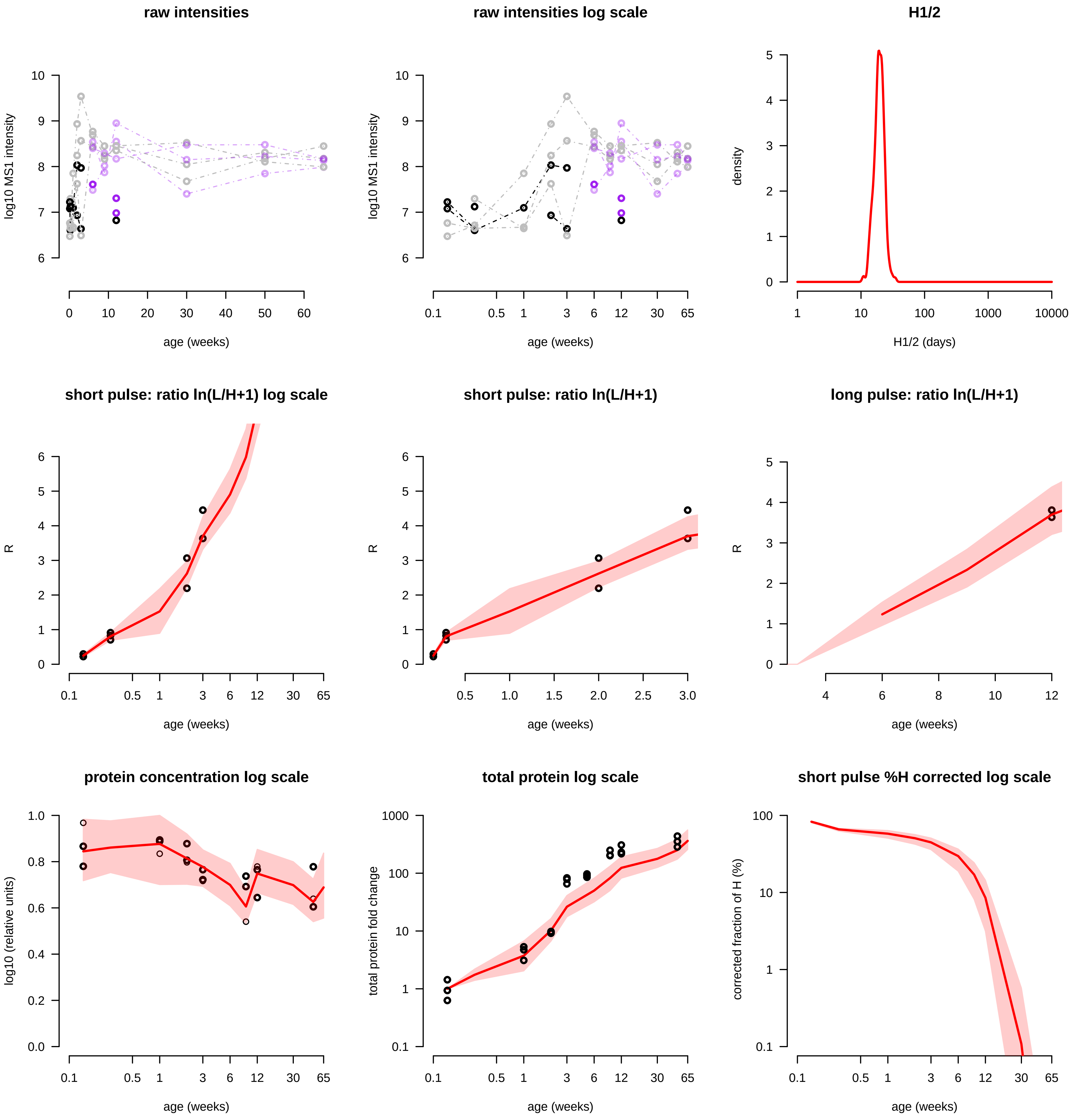

E9Q4N7

>sp|E9Q4N7|ARI1B_MOUSE

AT−rich interactive domain−containing protein 1B

GN=Arid1b

Med H1/2: 4.4 days

Q5 H1/2: 2.6 days

Q95 H1/2: 29.2 days

reliable model fit: yes

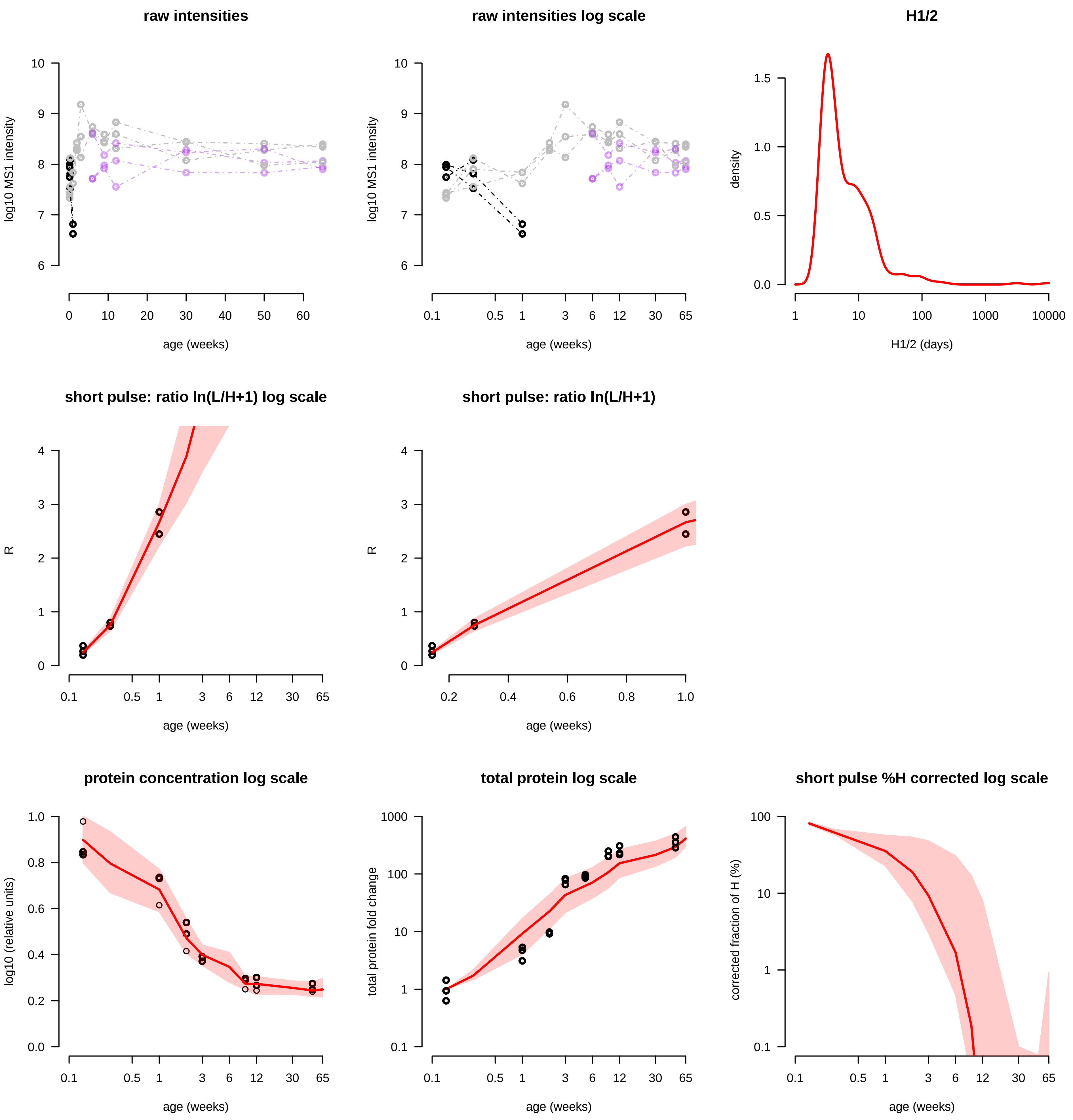

Q6ZQ73

>sp|Q6ZQ73|CAND2_MOUSE

Cullin−associated NEDD8−dissociated protein 2

GN=Cand2

Med H1/2: 7.8 days

Q5 H1/2: 3.3 days

Q95 H1/2: 65.1 days

reliable model fit: yes

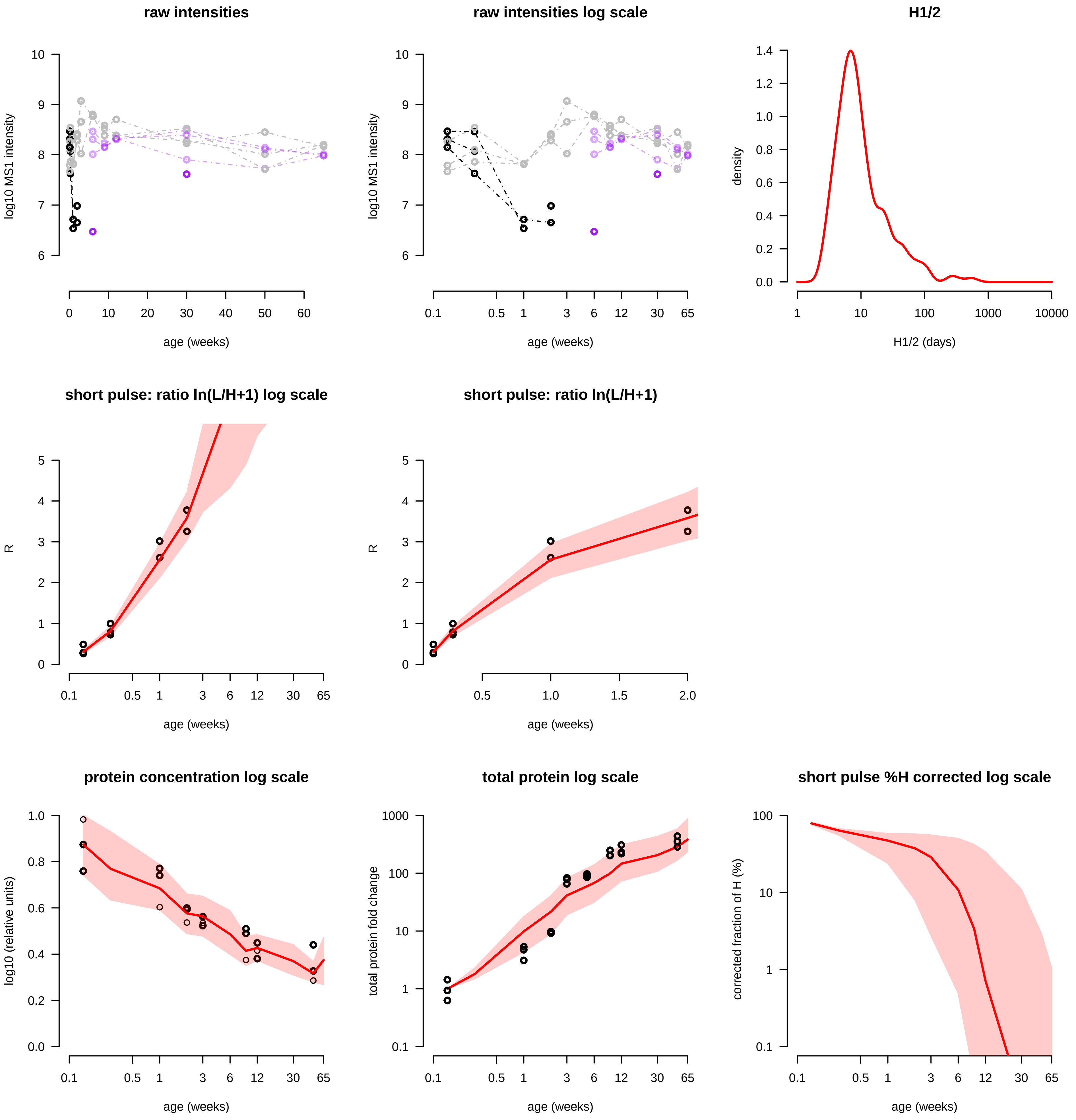

Q99PG2;Q99PG2−2

>sp|Q99PG2|OGFR_MOUSE

Opioid growth factor receptor

GN=Ogfr

Med H1/2: 7.5 days

Q5 H1/2: 3.7 days

Q95 H1/2: 41.9 days

reliable model fit: yes

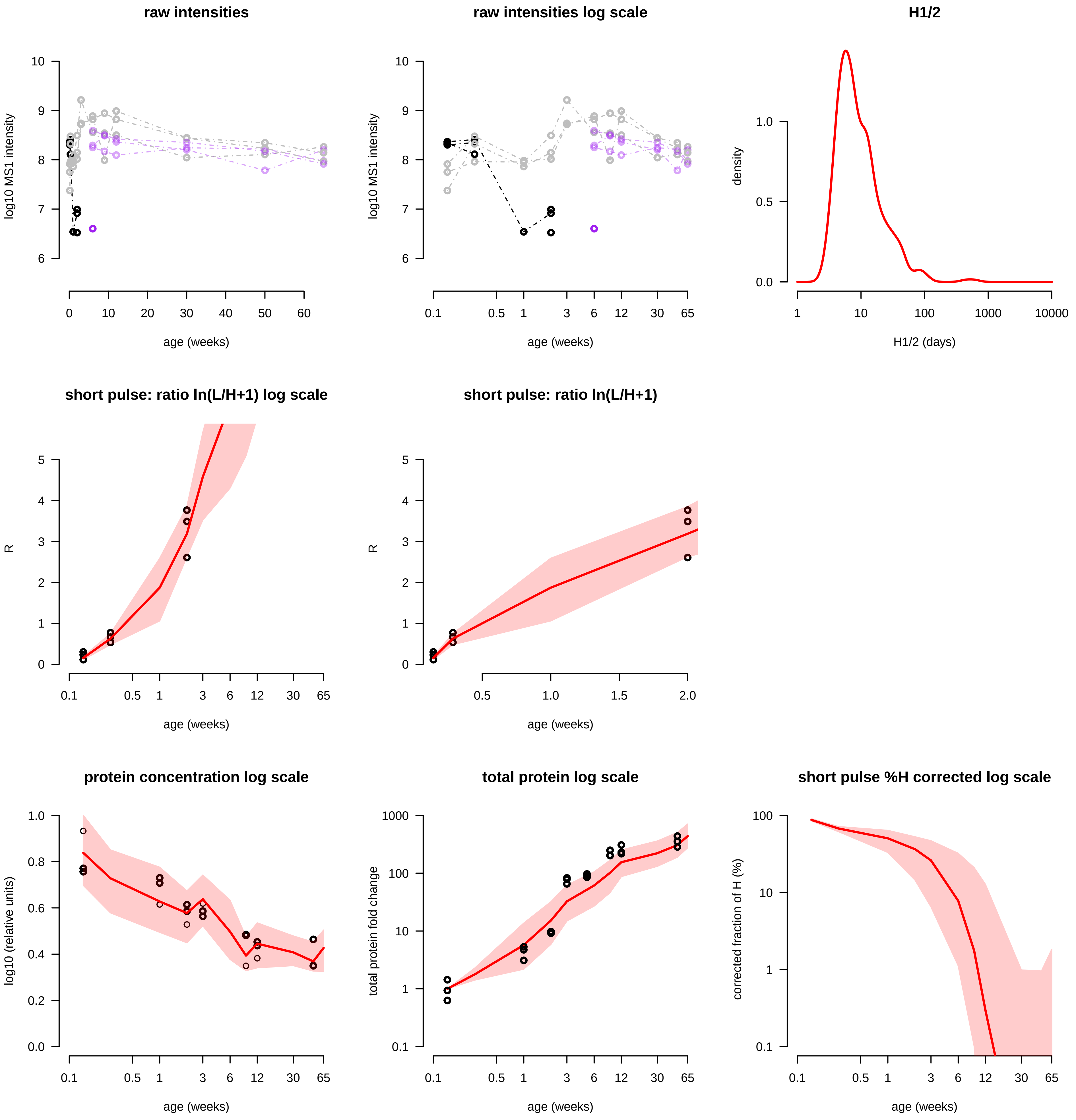

O70480

>sp|O70480|VAMP4_MOUSE

Vesicle−associated membrane protein 4

GN=Vamp4

Med H1/2: 15.1 days

Q5 H1/2: 9.7 days

Q95 H1/2: 31.4 days

reliable model fit: yes

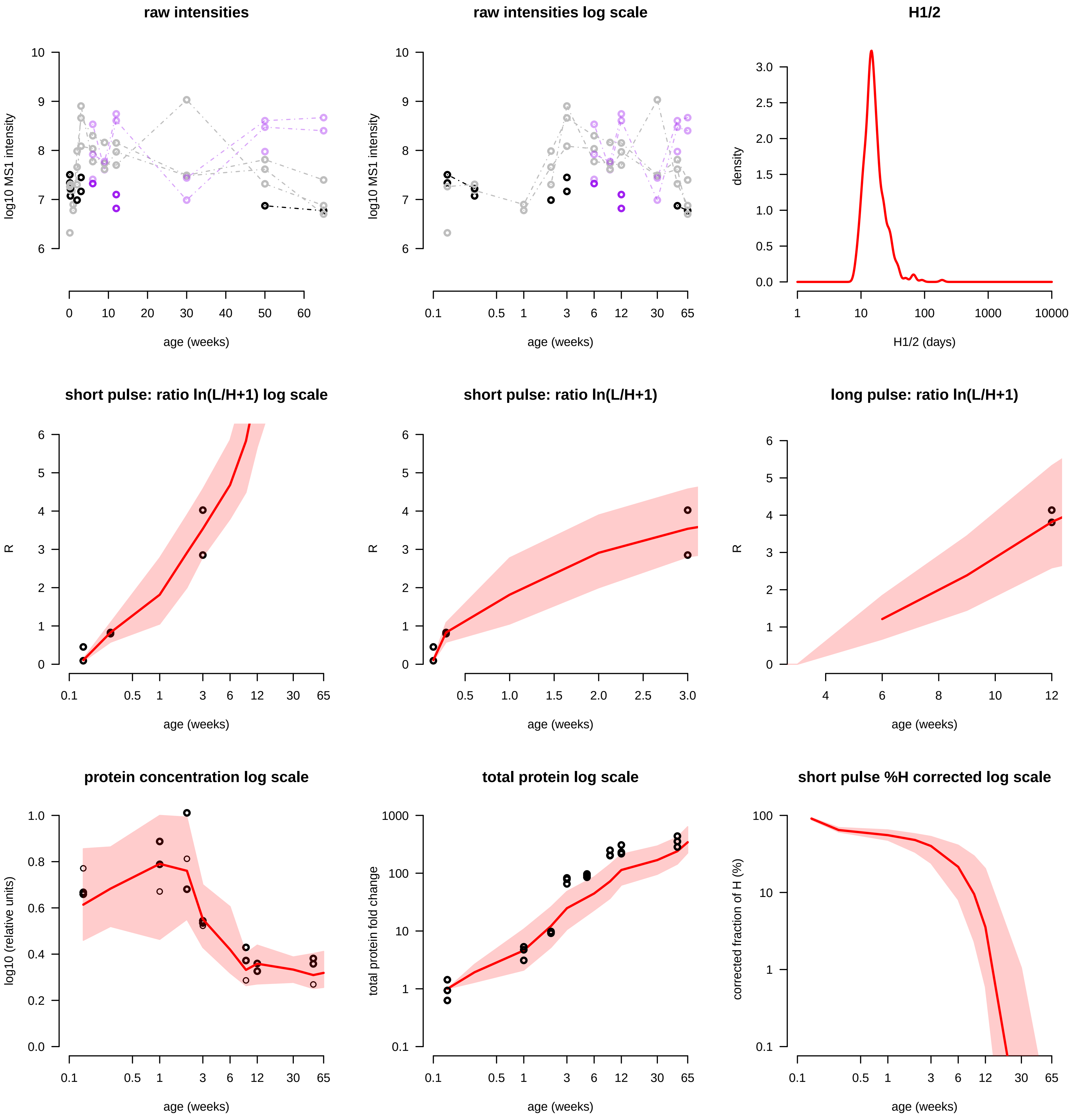

Q3UL36;Q3UL36−2

>sp|Q3UL36|ARGL1_MOUSE

Arginine and glutamate−rich protein 1

GN=Arglu1

Med H1/2: 5.4 days

Q5 H1/2: 4.1 days

Q95 H1/2: 8.3 days

reliable model fit: yes

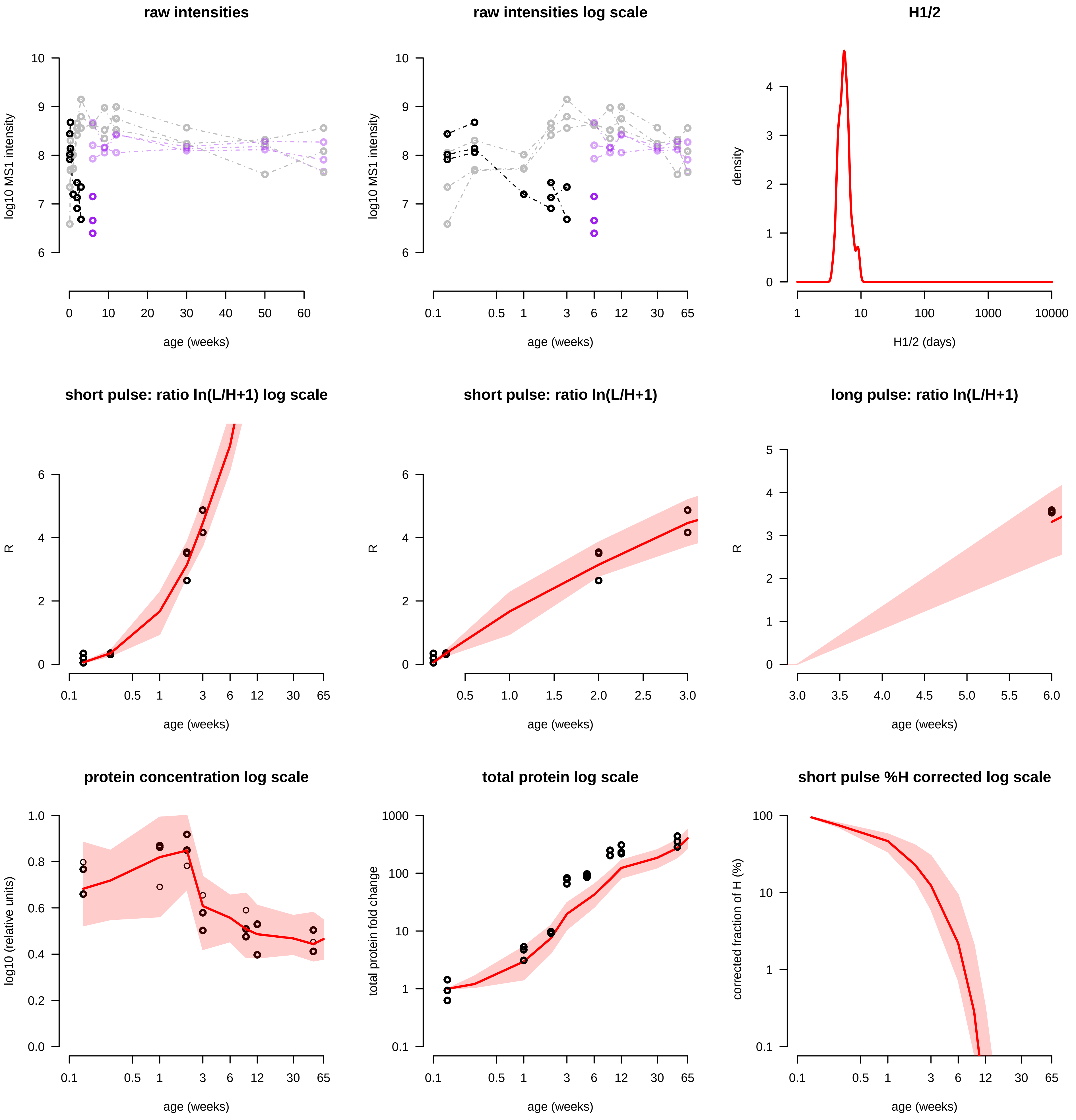

P97470

>sp|P97470|PP4C_MOUSE

Serine/threonine−protein phosphatase 4 catalytic subunit

GN=Ppp4c

Med H1/2: 21.5 days

Q5 H1/2: 7.2 days

Q95 H1/2: 160.2 days

reliable model fit: yes

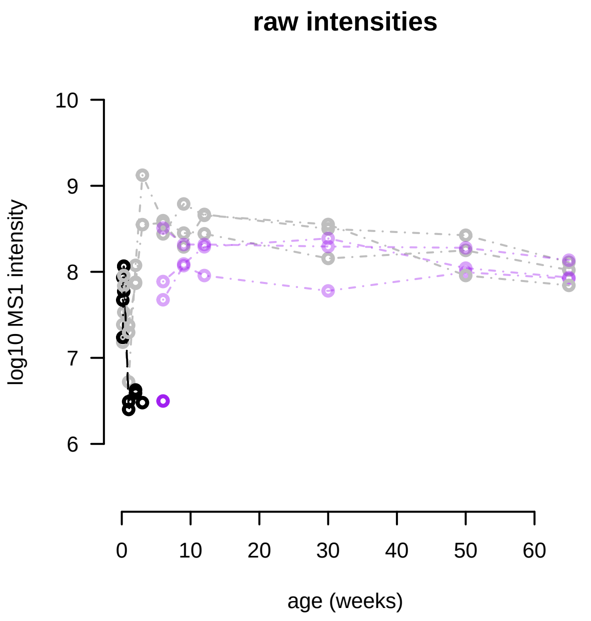

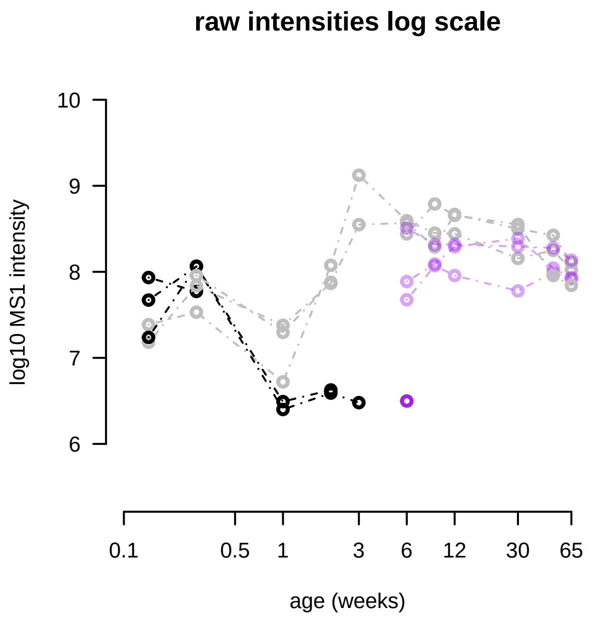

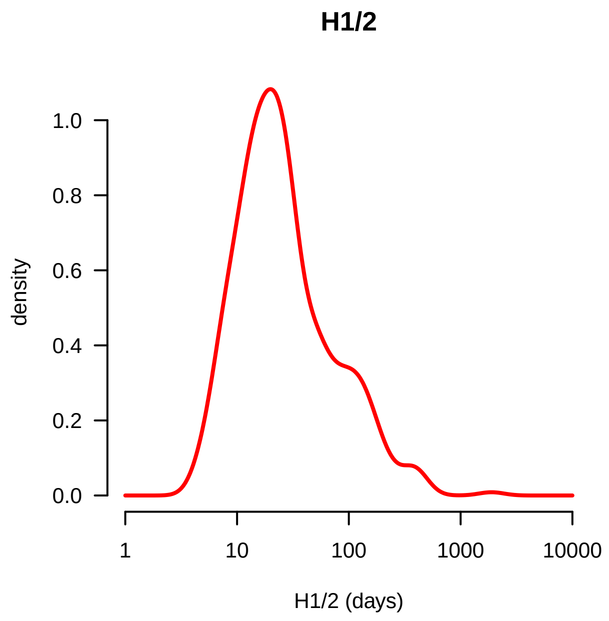

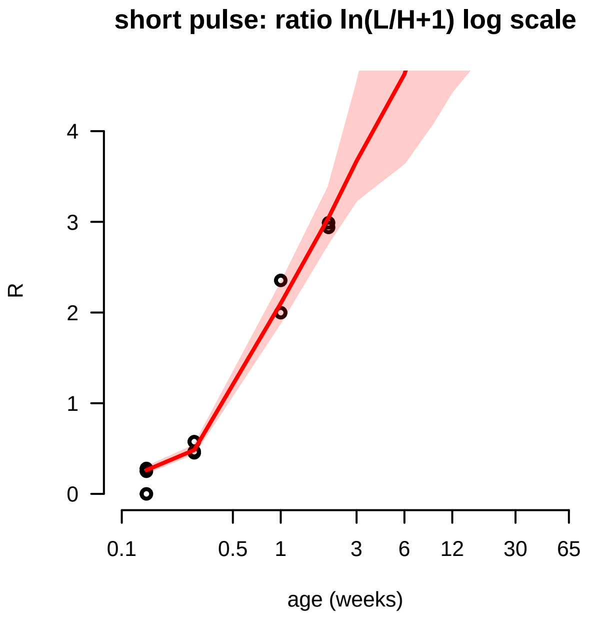

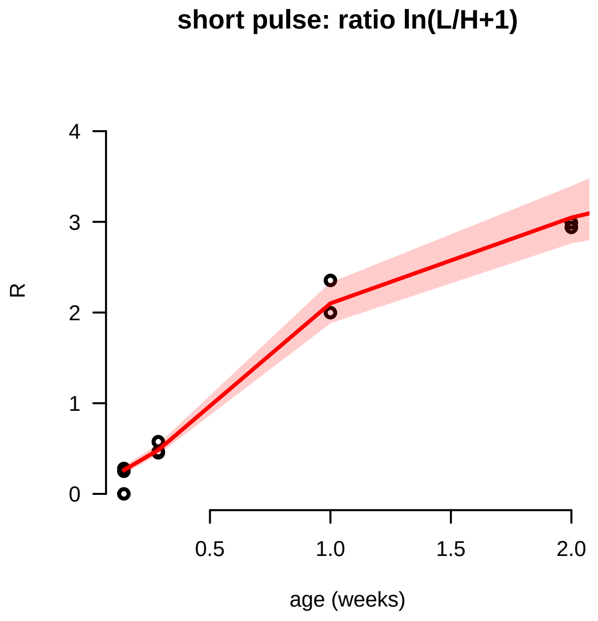

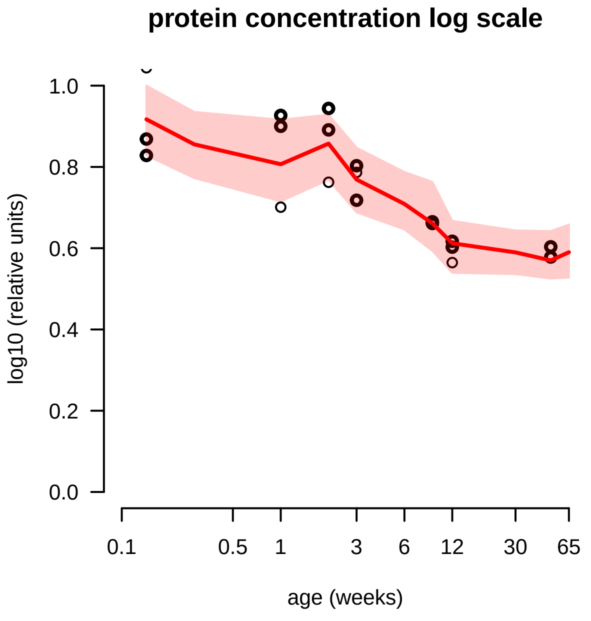

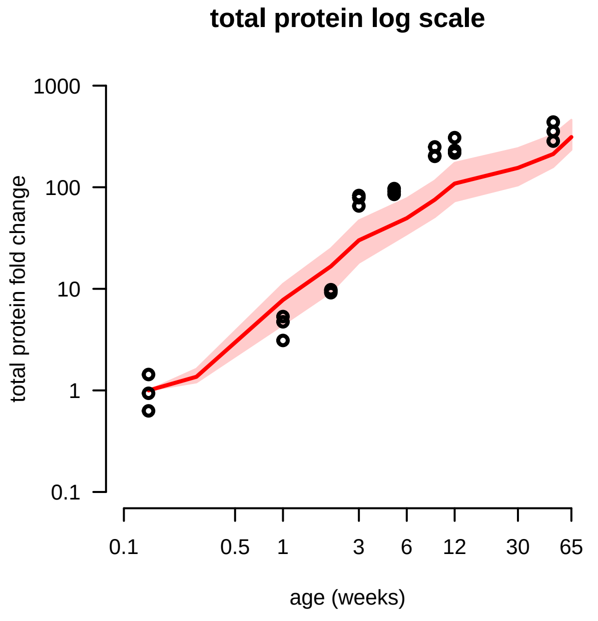

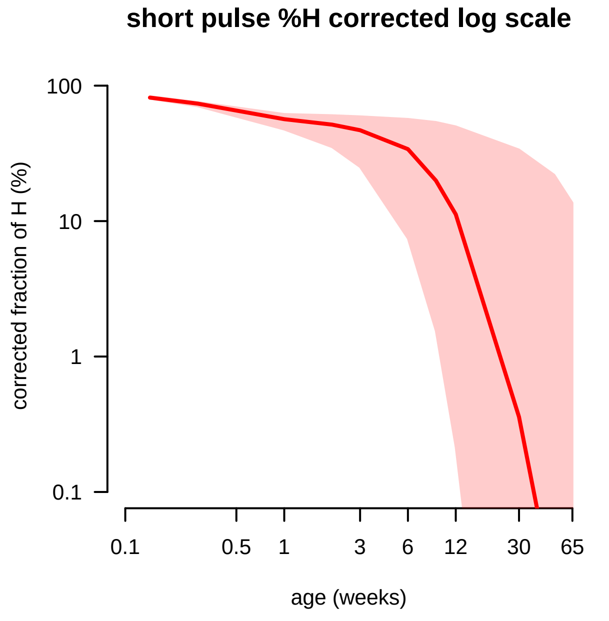

Q9Z321

>sp|Q9Z321|TOP3B_MOUSE

DNA topoisomerase 3−beta−1

GN=Top3b

Med H1/2: 1199.4 days

Q5 H1/2: 254.9 days

Q95 H1/2: 16002.8 days

reliable model fit: yes

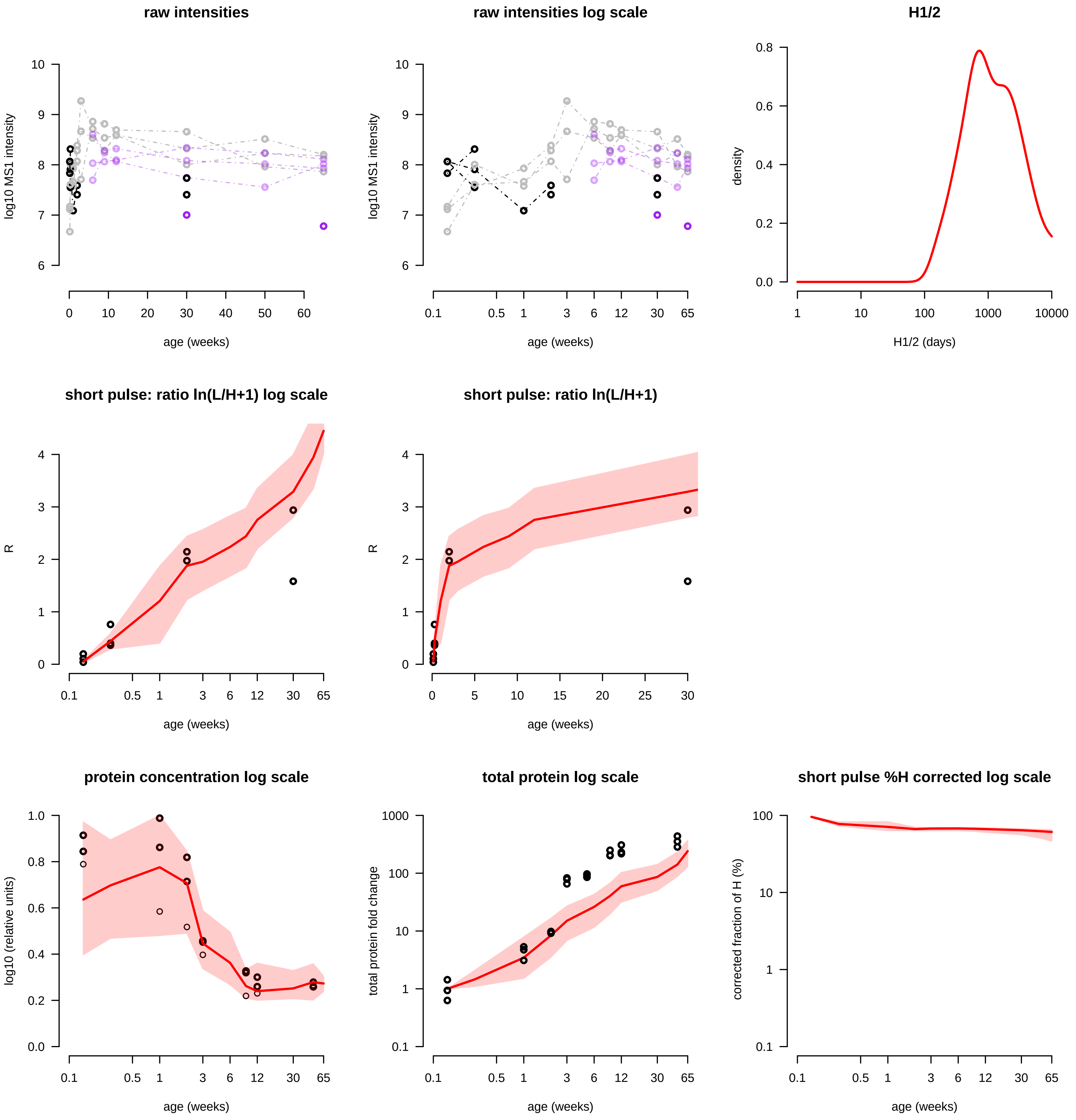

Q91WK1

>sp|Q91WK1|SPRY4_MOUSE

SPRY domain−containing protein 4

GN=Spryd4

Med H1/2: 4.1 days

Q5 H1/2: 3.1 days

Q95 H1/2: 5.4 days

reliable model fit: yes

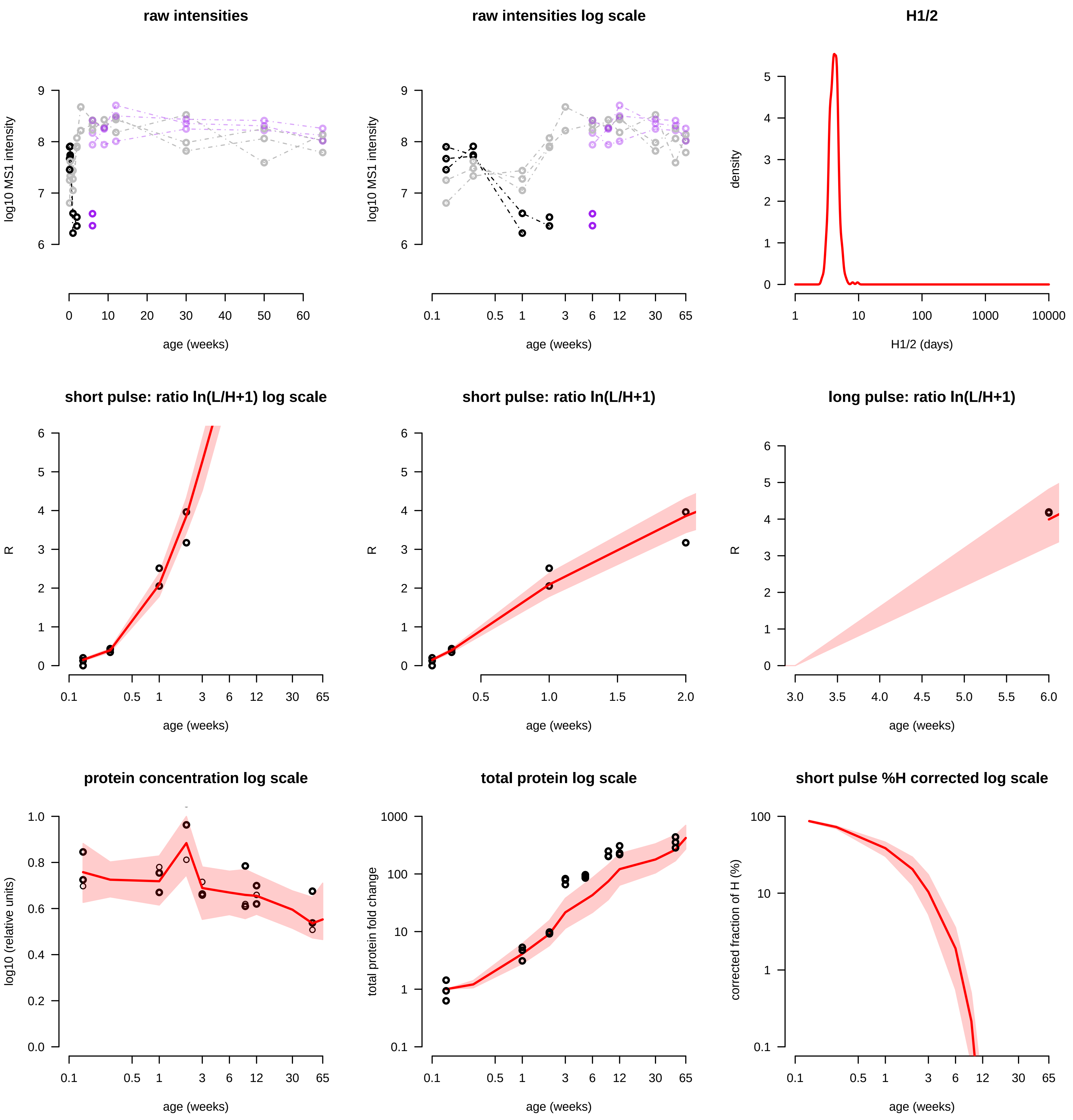

Q9D1E6

>sp|Q9D1E6|TBCB_MOUSE

Tubulin−folding cofactor B

GN=Tbcb

Med H1/2: 4.4 days

Q5 H1/2: 3.5 days

Q95 H1/2: 6 days

reliable model fit: yes

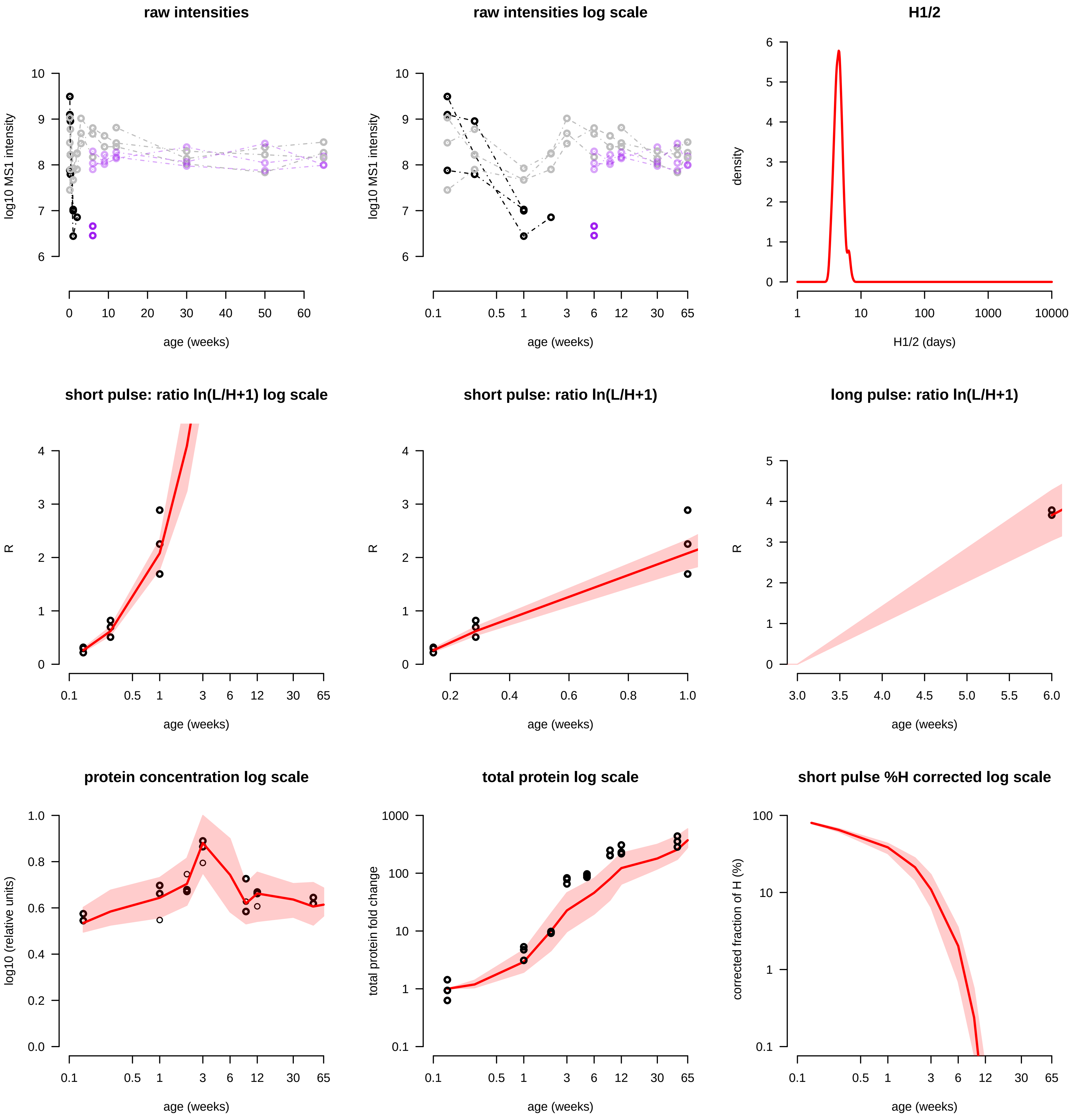

P41233

>sp|P41233|ABCA1_MOUSE

Phospholipid−transporting ATPase ABCA1

GN=Abca1

Med H1/2: 20.8 days

Q5 H1/2: 8.2 days

Q95 H1/2: 227.2 days

reliable model fit: yes

raw intensities

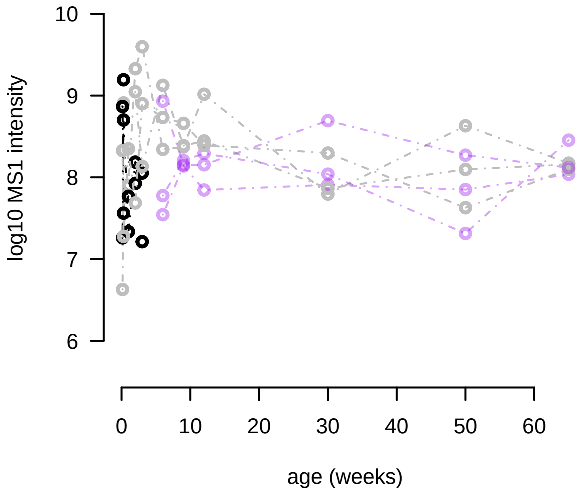

raw intensities log scale

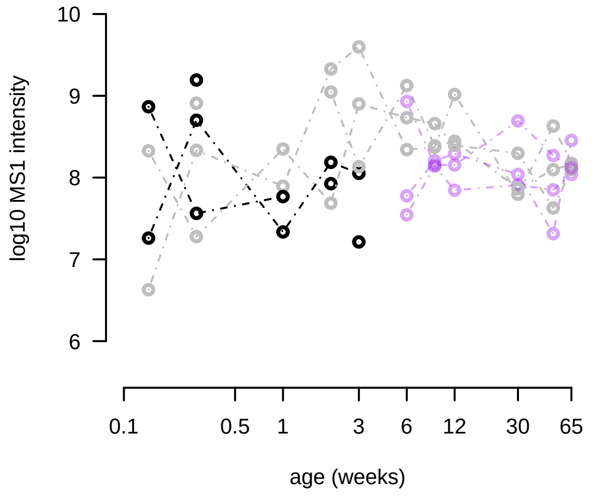

H1/2

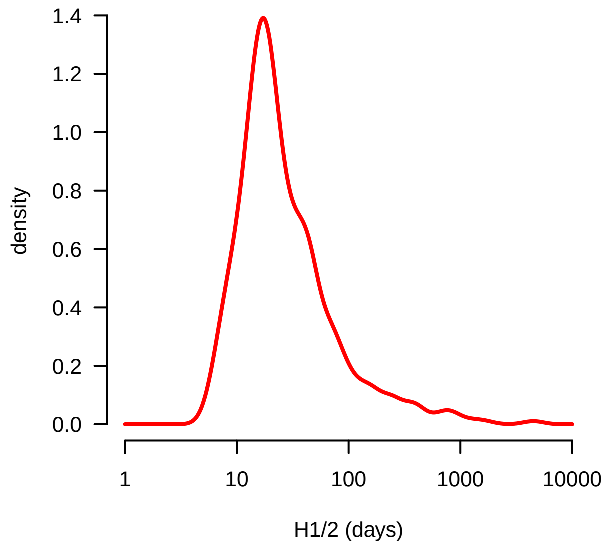

short pulse: ratio ln(L/H+1) log scale

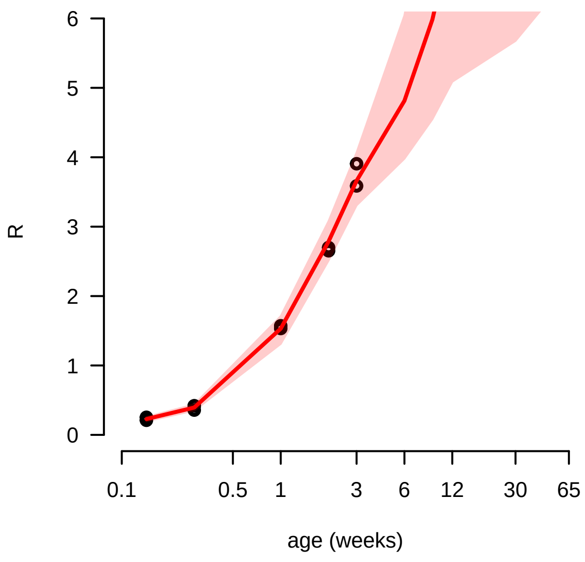

short pulse: ratio ln(L/H+1)

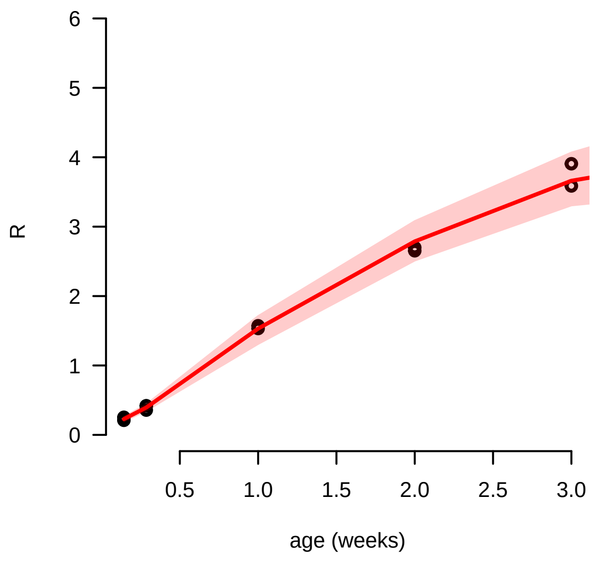

protein concentration log scale

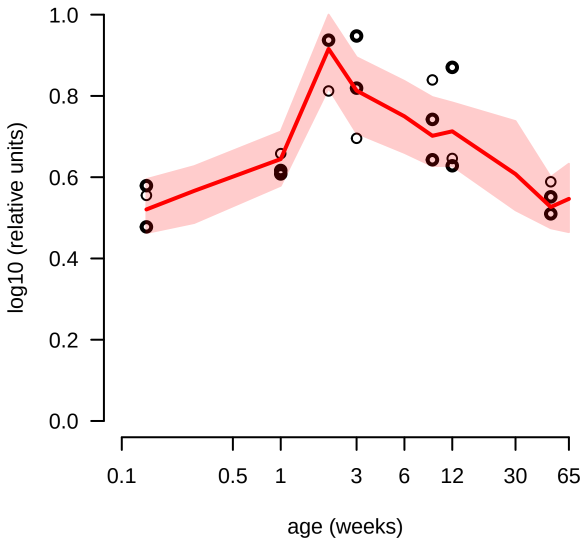

total protein log scale

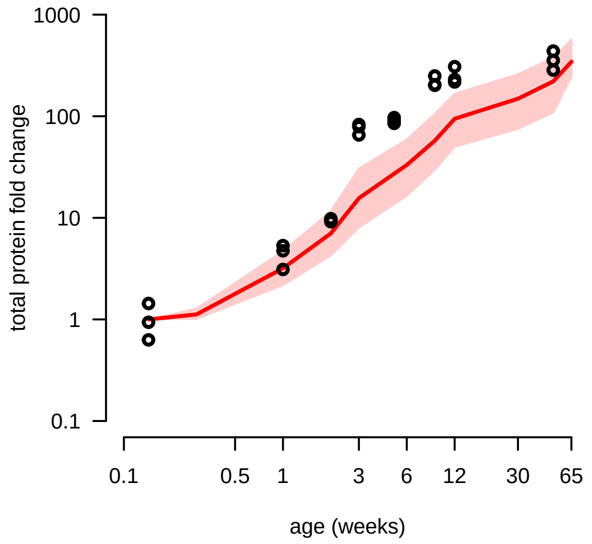

short pulse %H corrected log scale

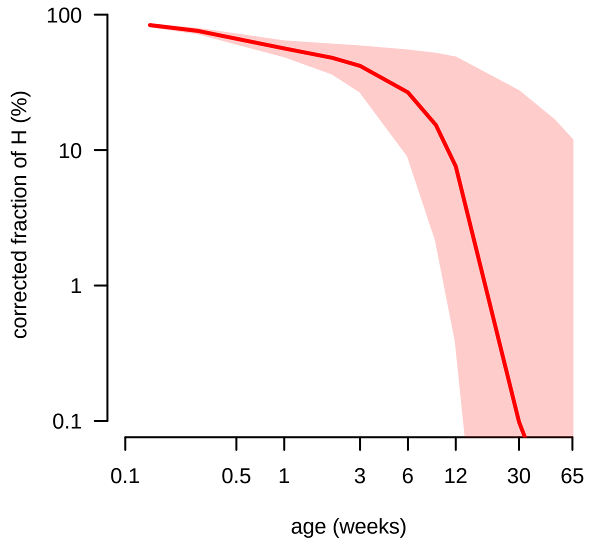

Q922K7

>sp|Q922K7|NOP2_MOUSE

Probable 28S rRNA (cytosine−C(5))−methyltransferase

GN=Nop2

Med H1/2: 4.6 days

Q5 H1/2: 2.7 days

Q95 H1/2: 32.4 days

reliable model fit: yes

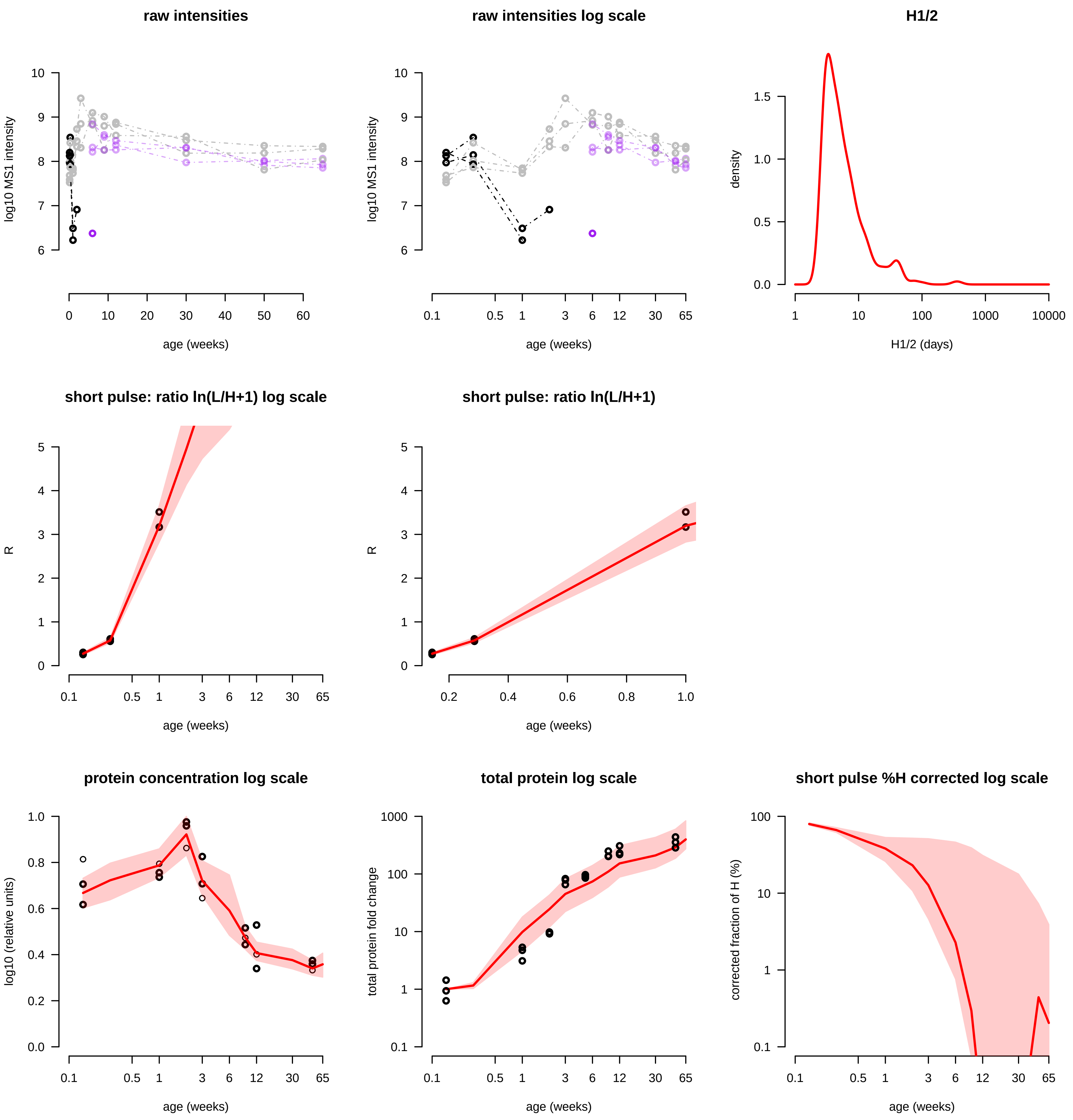

Q9CRC8

>sp|Q9CRC8|LRC40_MOUSE

Leucine−rich repeat−containing protein 40

GN=Lrrc40

Med H1/2: 114.1 days

Q5 H1/2: 44.3 days

Q95 H1/2: 580.8 days

reliable model fit: no

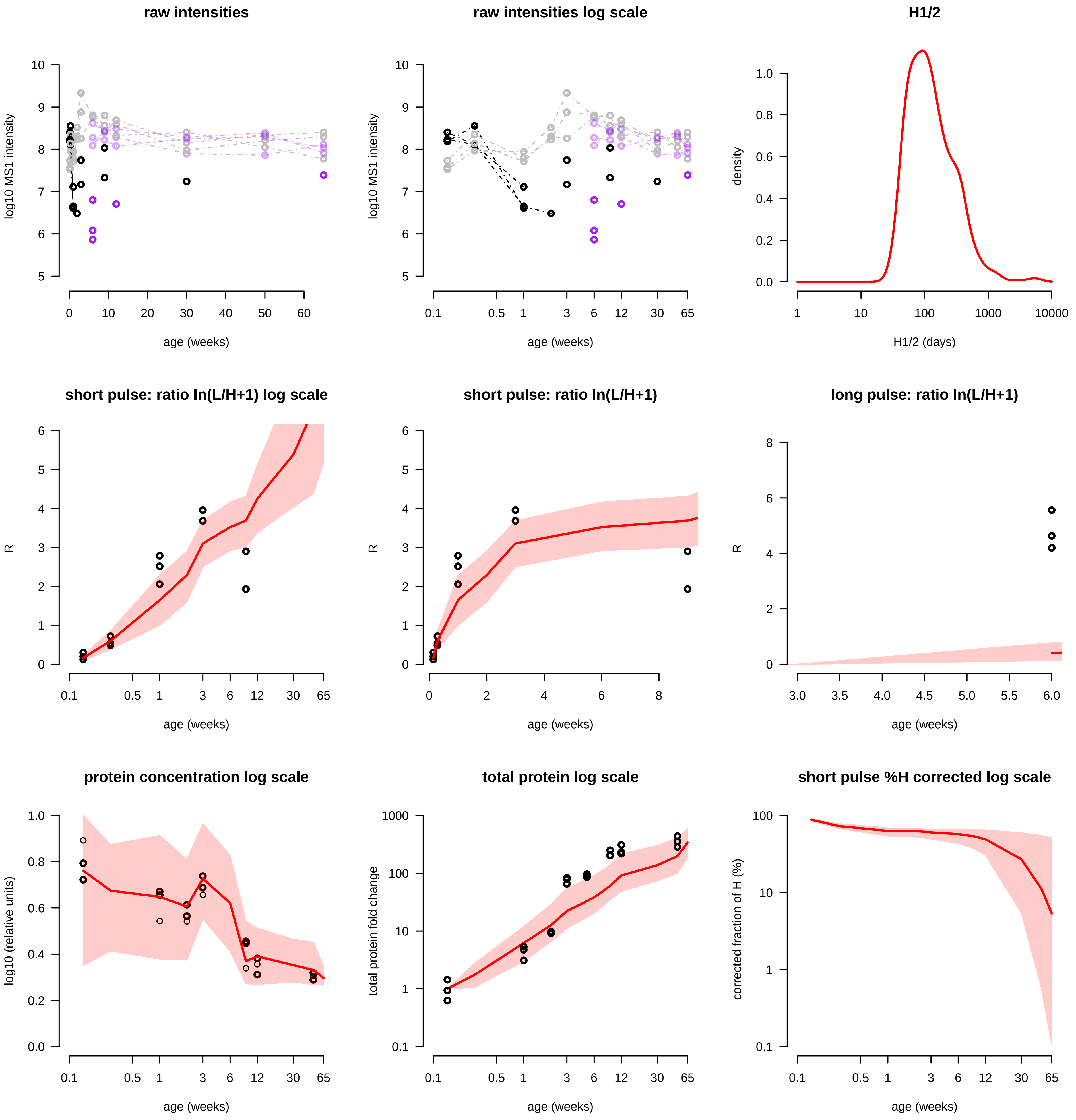

Q8BHG9

>sp|Q8BHG9|CGBP1_MOUSE

CGG triplet repeat−binding protein 1

GN=Cggbp1

Med H1/2: 4.8 days

Q5 H1/2: 2.6 days

Q95 H1/2: 21.1 days

reliable model fit: yes

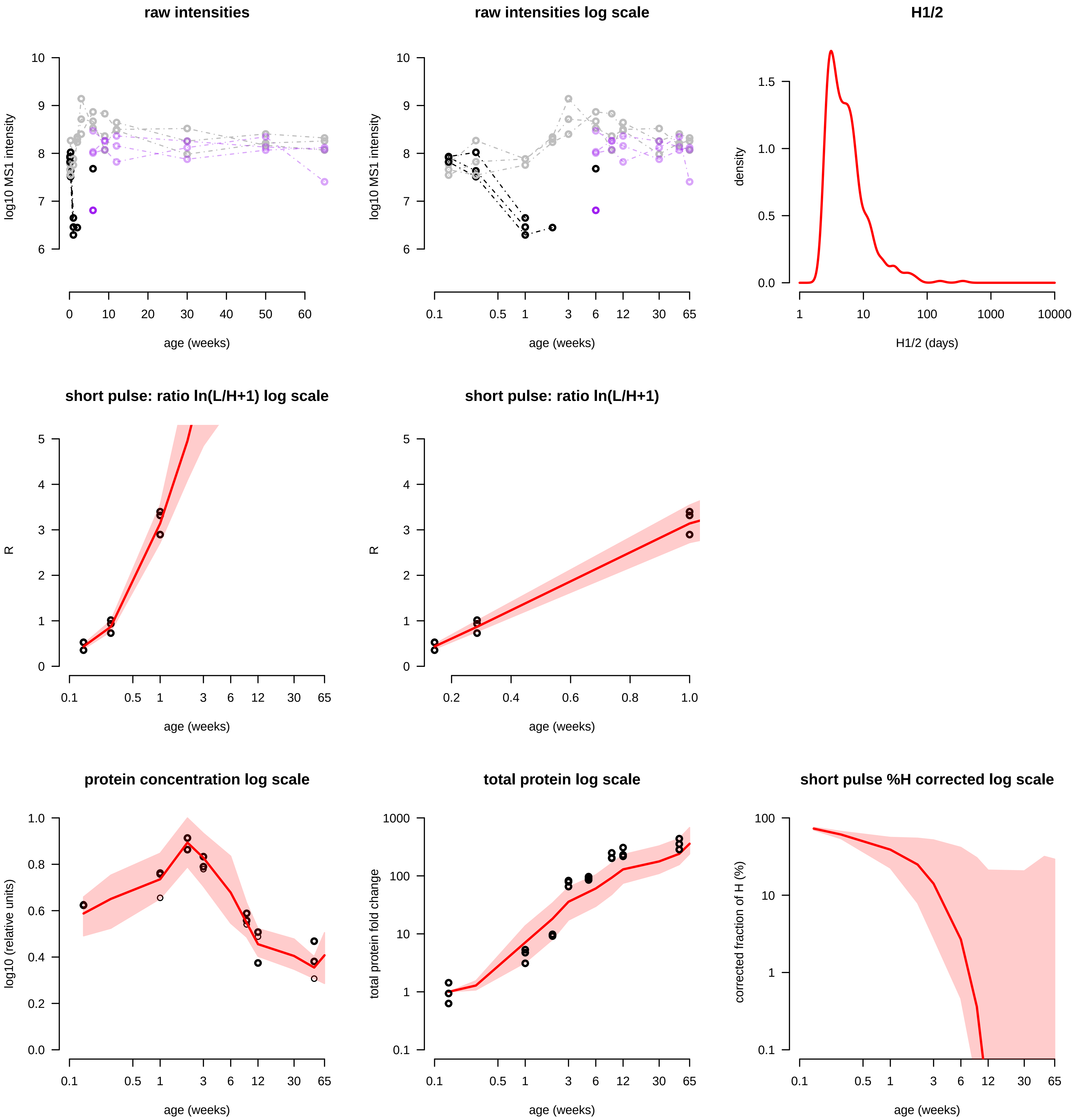

Q9D853

>sp|Q9D853|EFMT2_MOUSE

EEF1A lysine methyltransferase 2

GN=Eef1akmt2

Med H1/2: 4.7 days

Q5 H1/2: 2.5 days

Q95 H1/2: 25.9 days

reliable model fit: yes

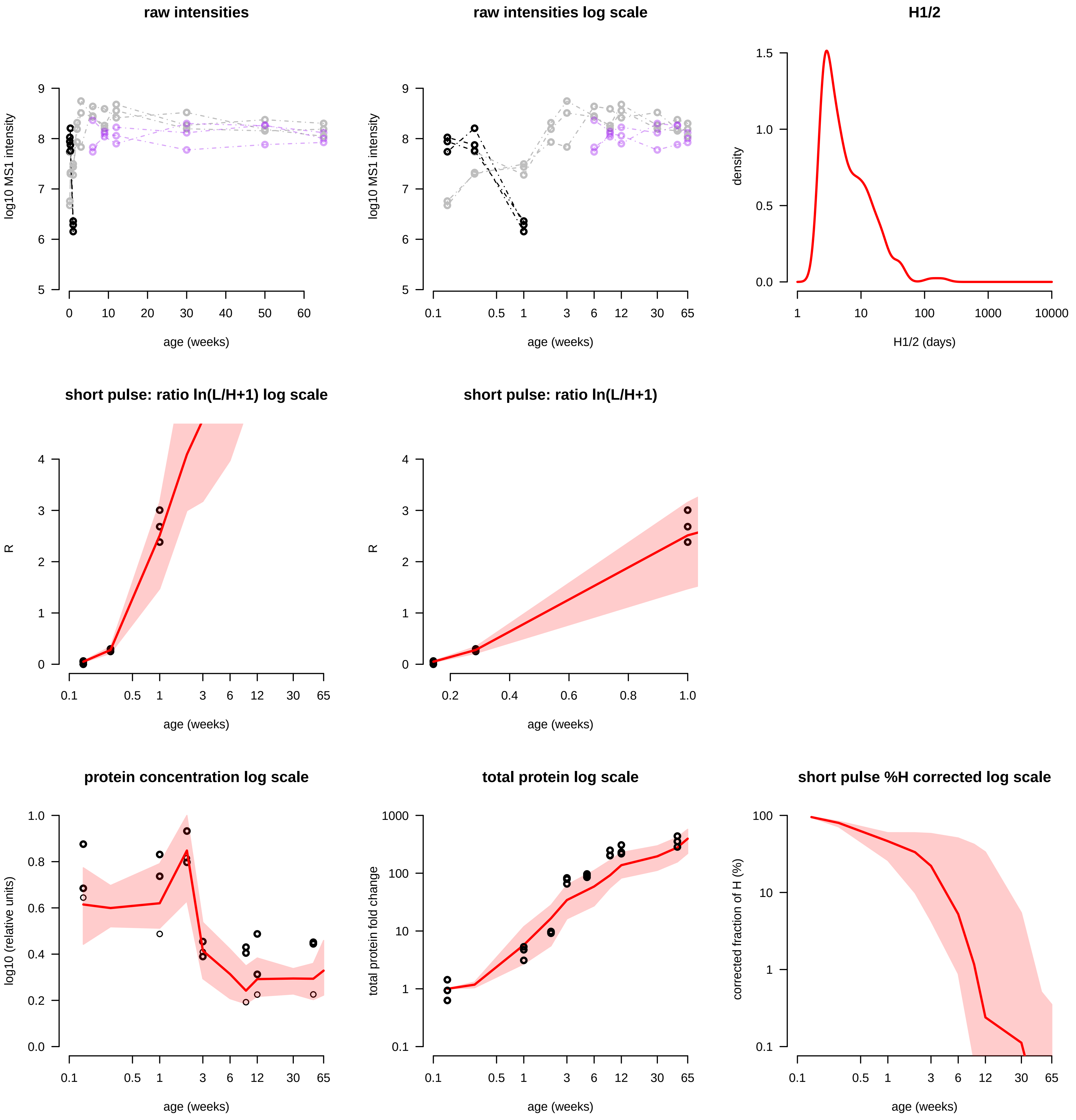

Q61823

>sp|Q61823|PDCD4_MOUSE

Programmed cell death protein 4

GN=Pdcd4

Med H1/2: 34.1 days

Q5 H1/2: 10.8 days

Q95 H1/2: 721.5 days

reliable model fit: yes

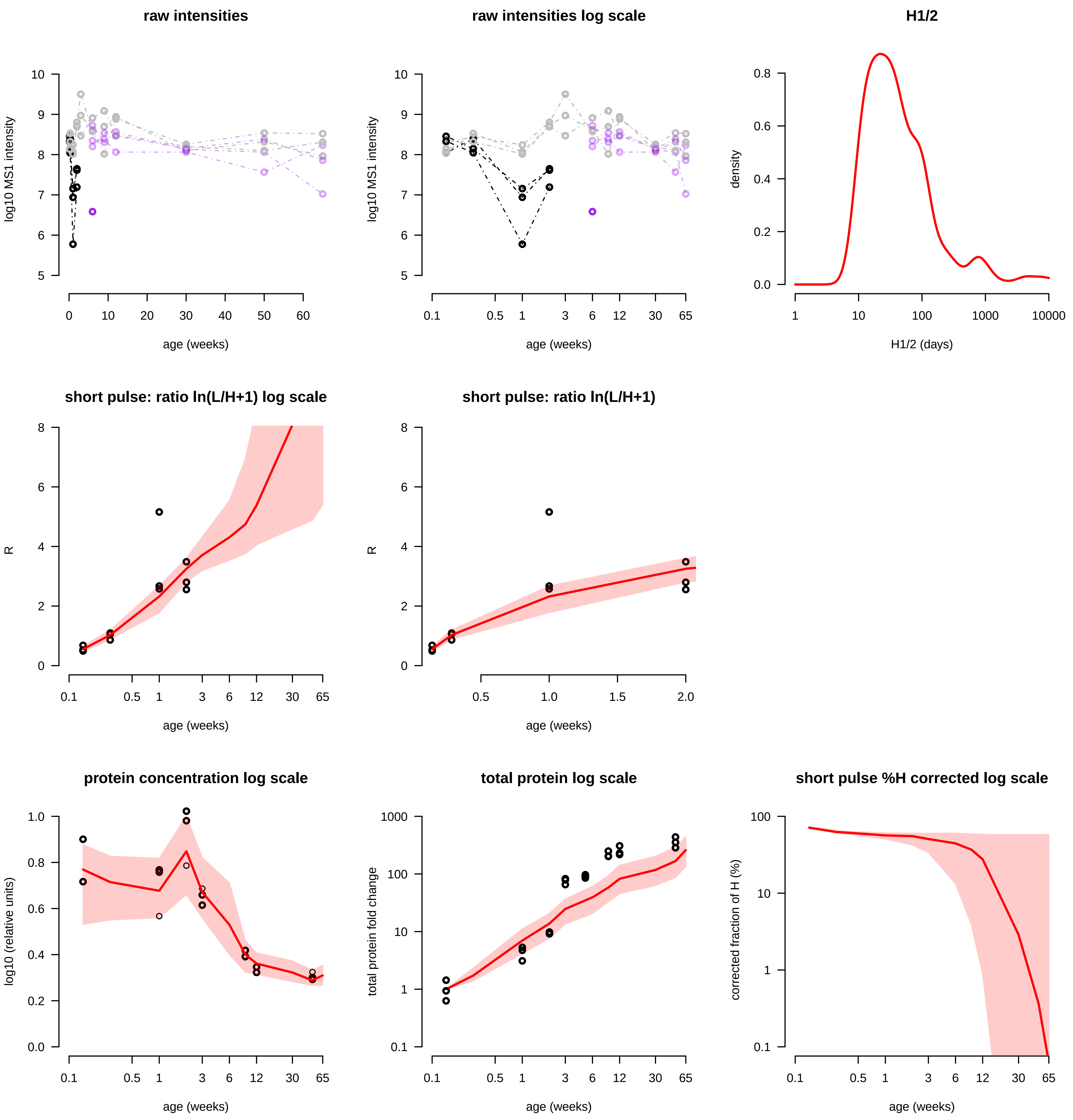

Q9CQJ8

>sp|Q9CQJ8|NDUB9_MOUSE

NADH dehydrogenase [ubiquinone] 1 beta subcomplex subunit 9

GN=Ndufb9

Med H1/2: 8.1 days

Q5 H1/2: 4.2 days

Q95 H1/2: 52.1 days

reliable model fit: yes

raw intensities

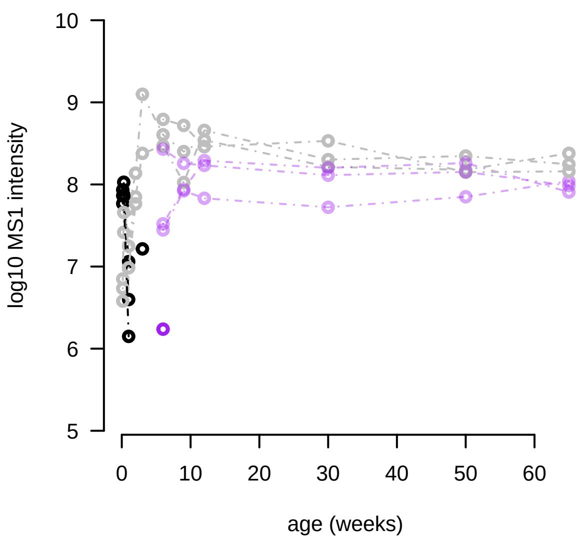

raw intensities log scale

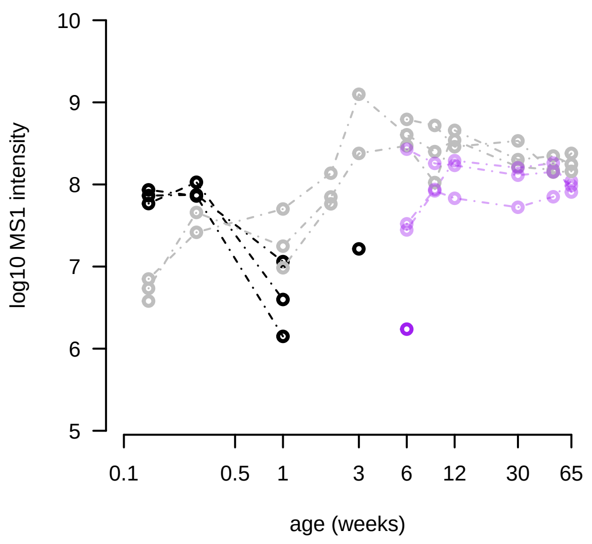

H1/2

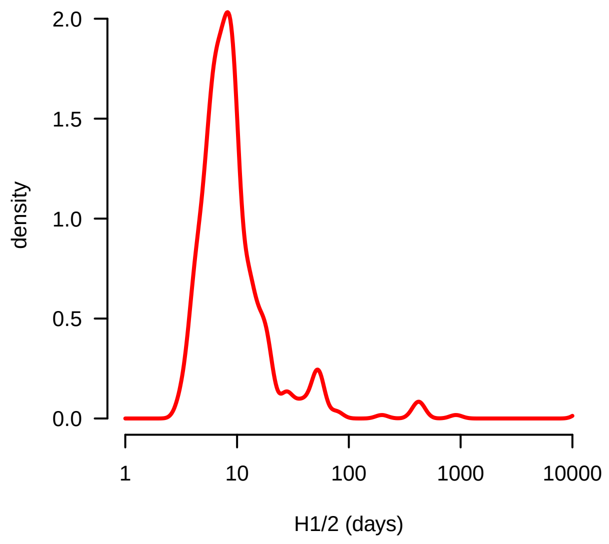

short pulse: ratio ln(L/H+1) log scale

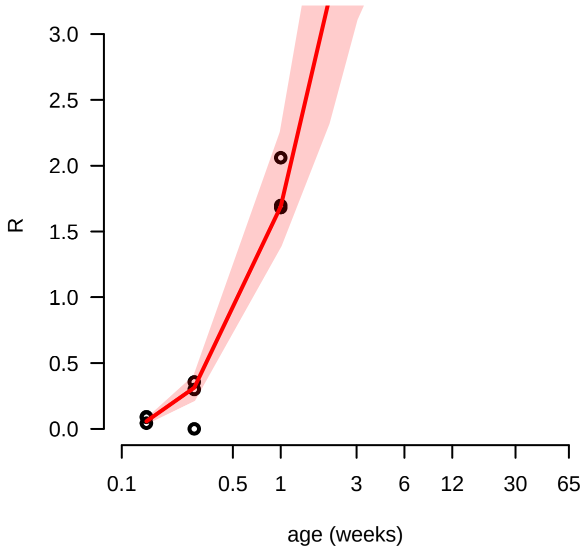

short pulse: ratio ln(L/H+1)

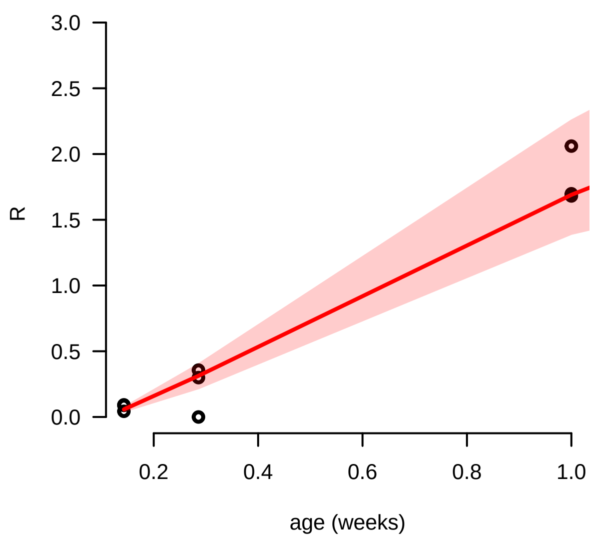

protein concentration log scale

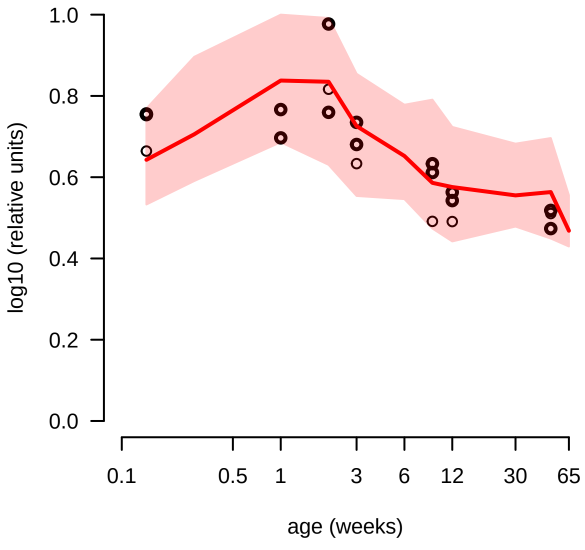

total protein log scale

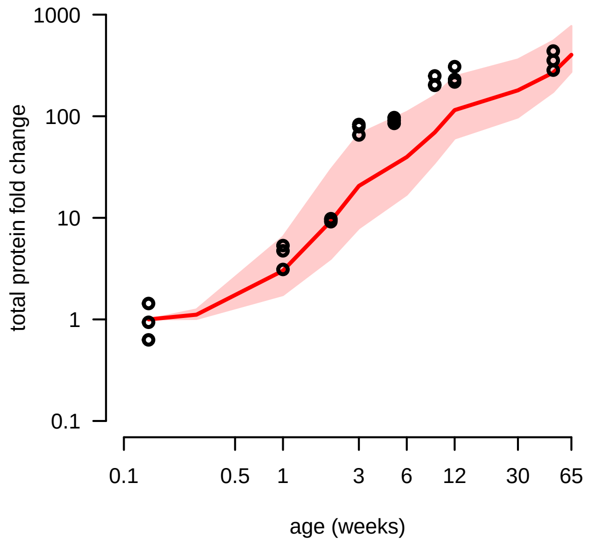

short pulse %H corrected log scale

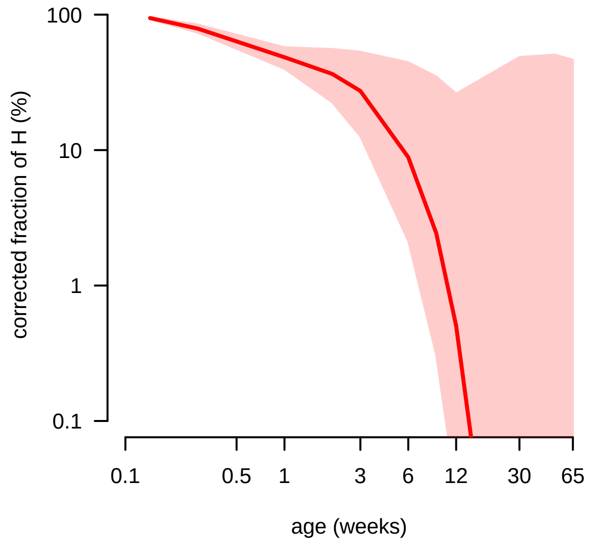

P32507

>sp|P32507|NECT2_MOUSE

Nectin−2

GN=Nectin2

Med H1/2: 2854.1 days

Q5 H1/2: 1286.8 days

Q95 H1/2: 31327.4 days

reliable model fit: no

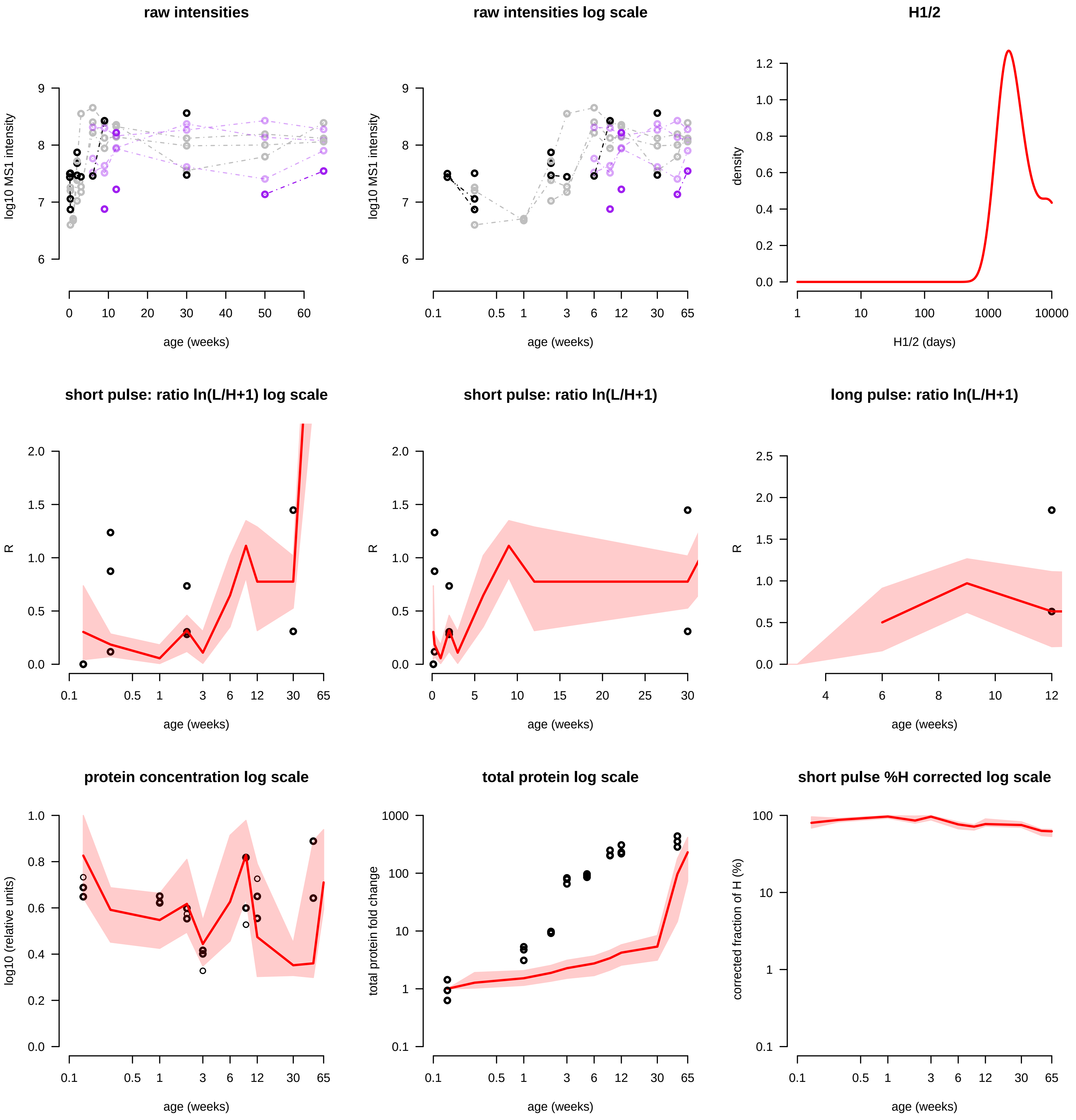

Q9D7X8

>sp|Q9D7X8|GGCT_MOUSE

Gamma−glutamylcyclotransferase

GN=Ggct

Med H1/2: 6.2 days

Q5 H1/2: 4.2 days

Q95 H1/2: 12.4 days

reliable model fit: yes

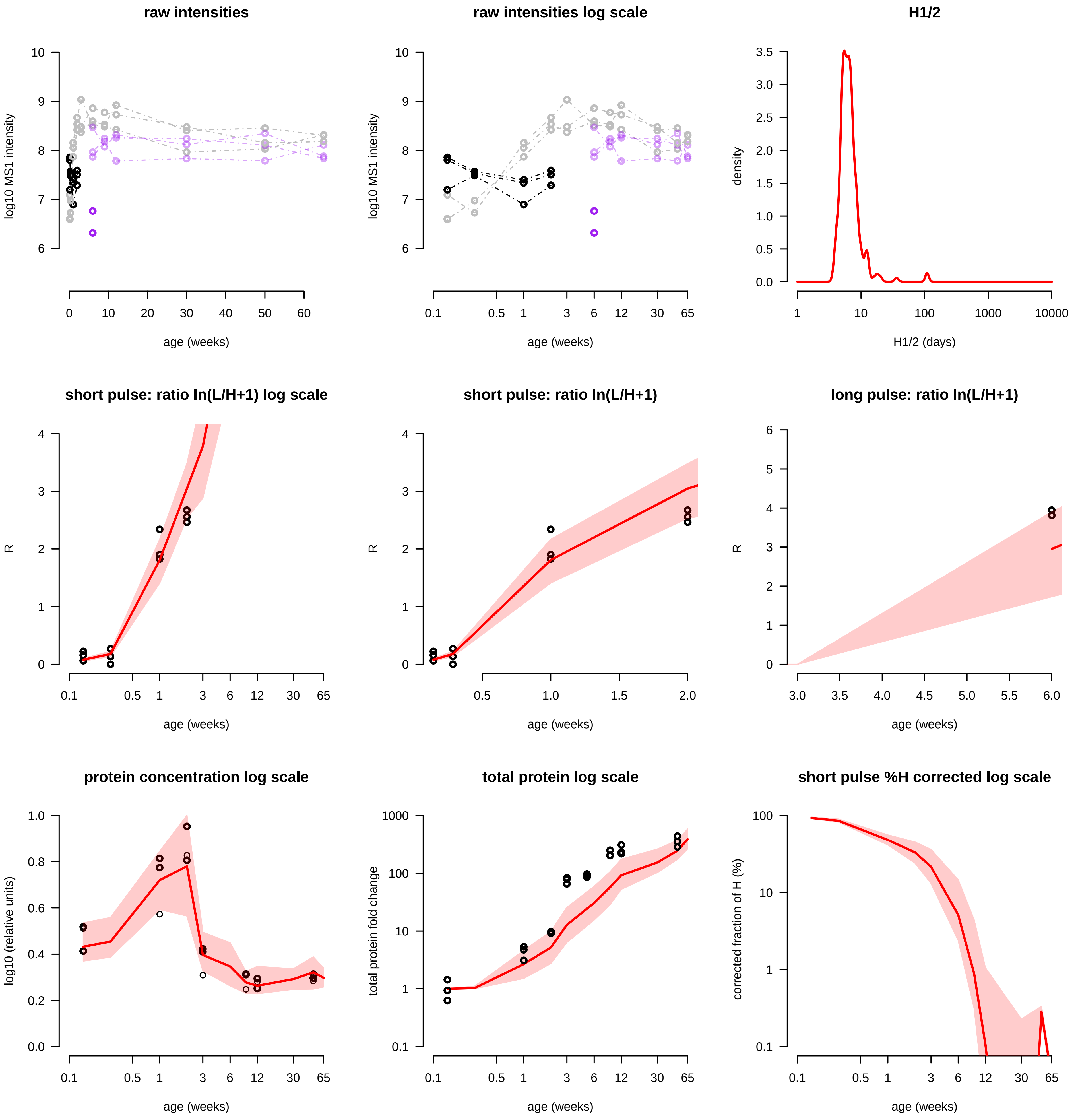

Q8CIV8

>sp|Q8CIV8|TBCE_MOUSE

Tubulin−specific chaperone E

GN=Tbce

Med H1/2: 3628.2 days

Q5 H1/2: 869.5 days

Q95 H1/2: 51978.2 days

reliable model fit: yes

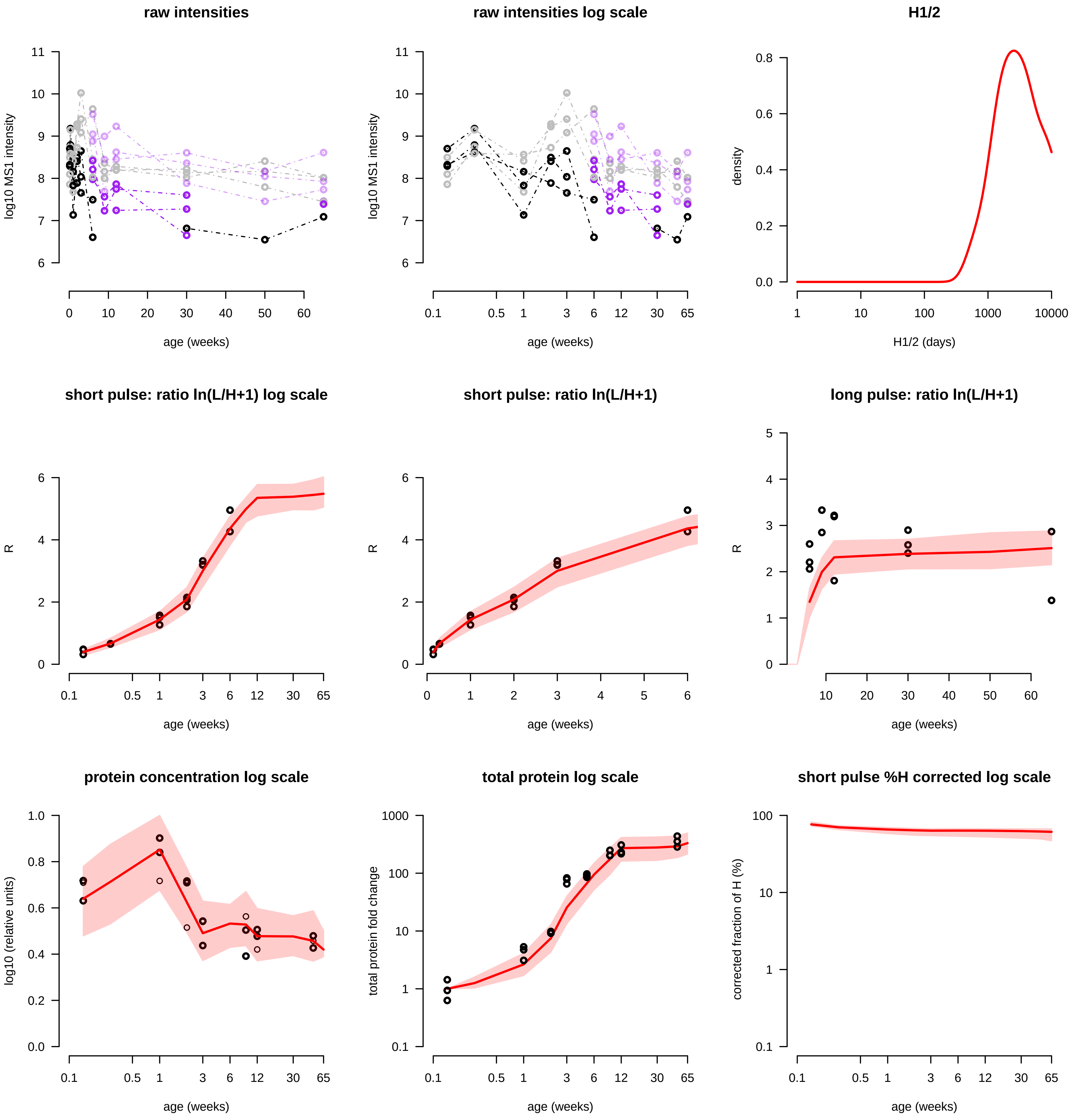

Q61391

>sp|Q61391|NEP_MOUSE

Neprilysin

GN=Mme

Med H1/2: 9.3 days

Q5 H1/2: 4.3 days

Q95 H1/2: 49.3 days

reliable model fit: yes

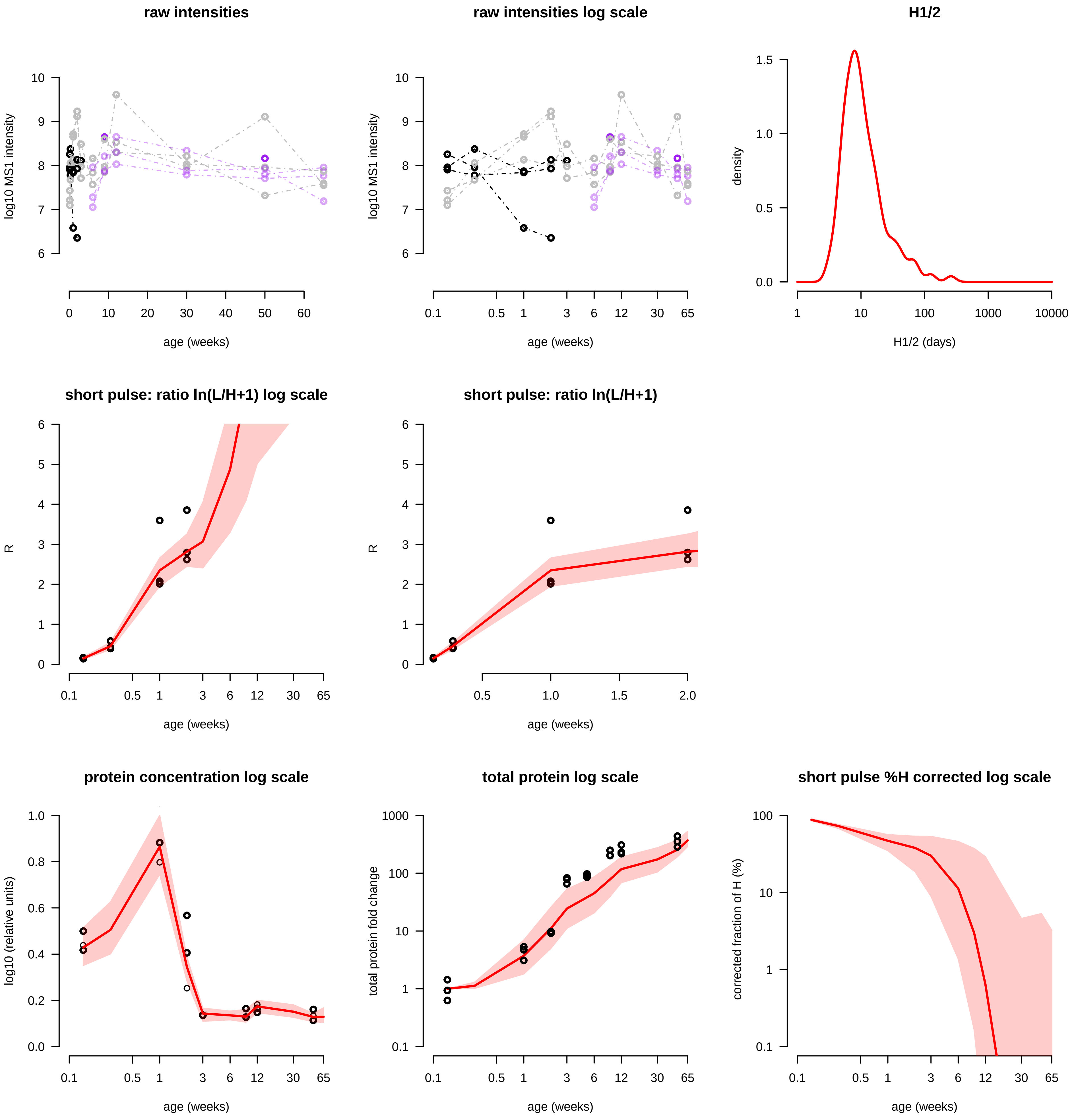

Q8VID5

>sp|Q8VID5|RECQ5_MOUSE

ATP−dependent DNA helicase Q5

GN=Recql5

Med H1/2: 8.9 days

Q5 H1/2: 6.3 days

Q95 H1/2: 15.1 days

reliable model fit: yes

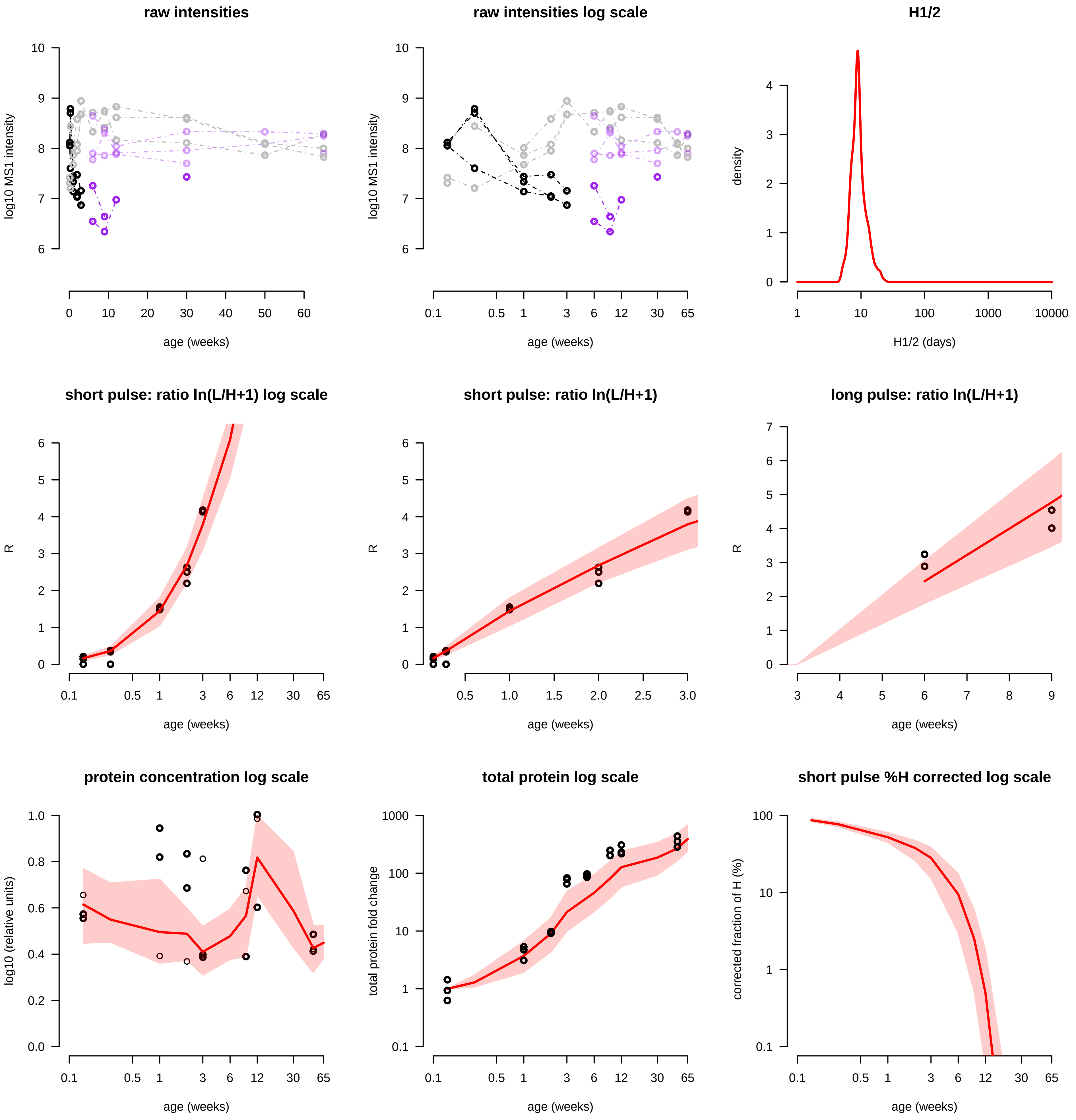

P83870

>sp|P83870|PHF5A_MOUSE

PHD finger−like domain−containing protein 5A

GN=Phf5a

Med H1/2: 21.9 days

Q5 H1/2: 7 days

Q95 H1/2: 141.5 days

reliable model fit: yes

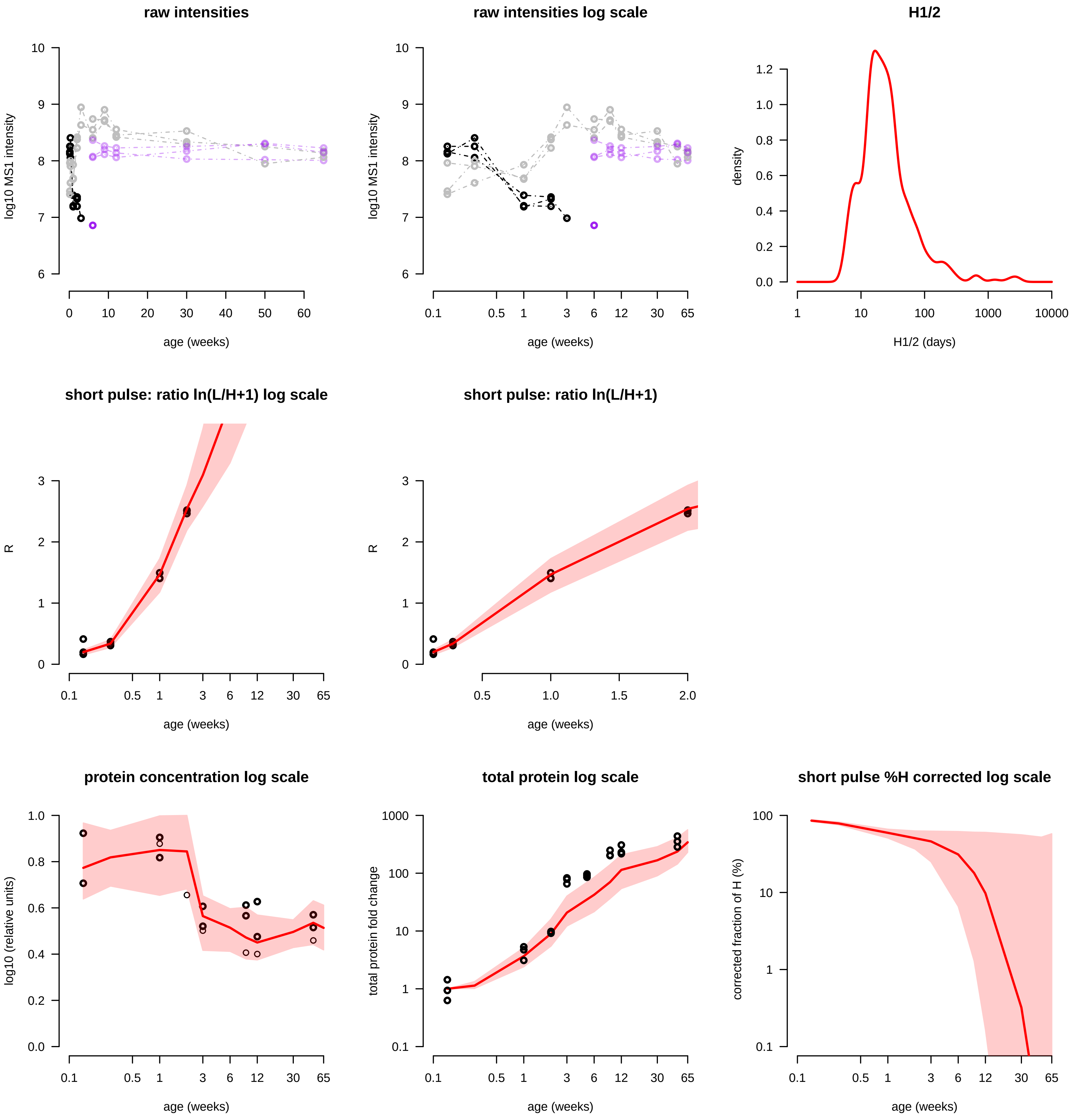

Q505D1

>sp|Q505D1|ANR28_MOUSE

Serine/threonine−protein phosphatase 6 regulatory ankyrin repeat subunit A

GN=Ankrd28

Med H1/2: 2177.1 days

Q5 H1/2: 580.1 days

Q95 H1/2: 37055.6 days

reliable model fit: no

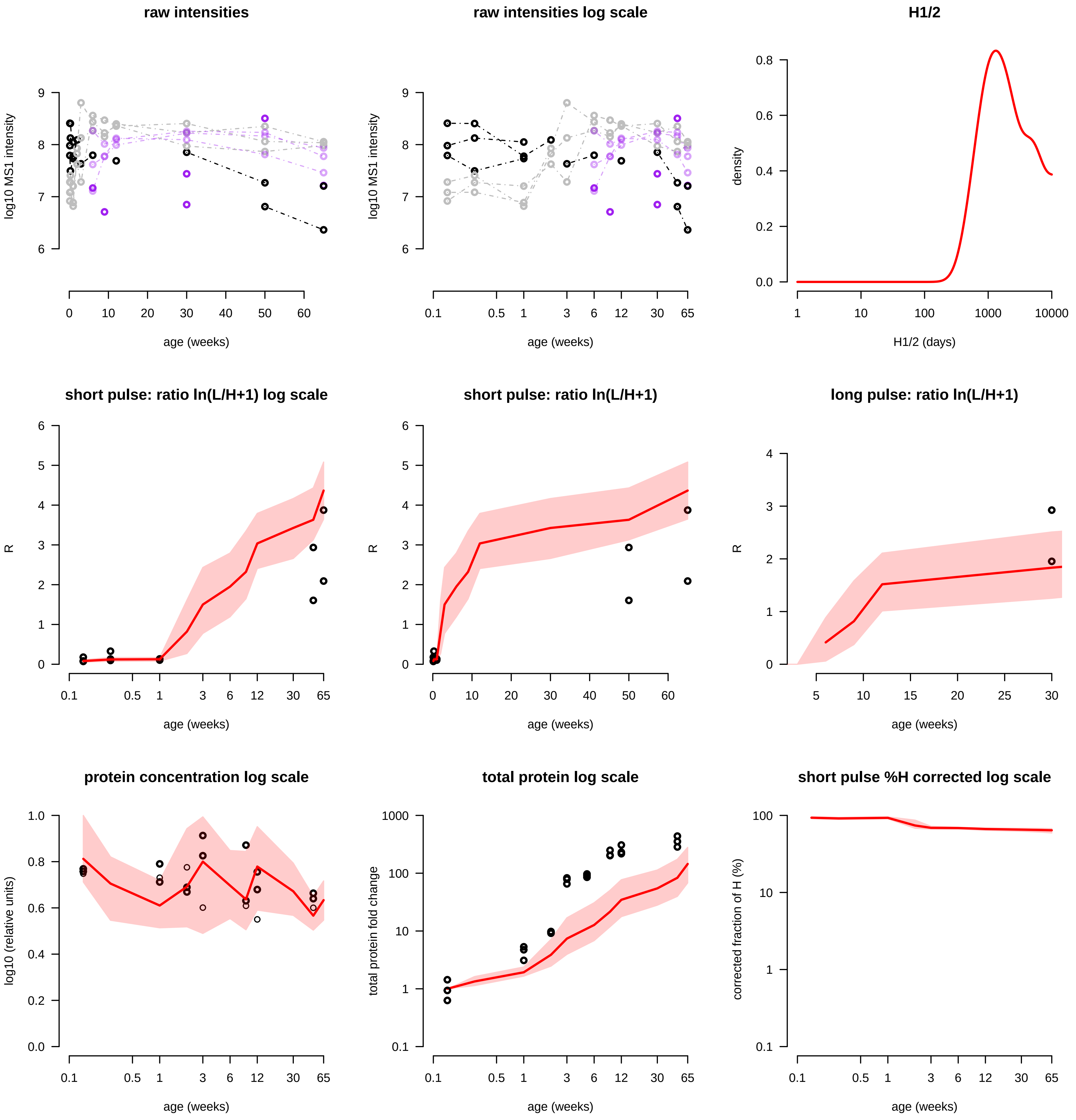

Q8CAS9−2

>sp|Q8CAS9−2|PARP9_MOUSE

Isoform 2 of Protein mono−ADP−ribosyltransferase PARP9

GN=Parp9

Med H1/2: 2537.7 days

Q5 H1/2: 656.3 days

Q95 H1/2: 46985.5 days

reliable model fit: no

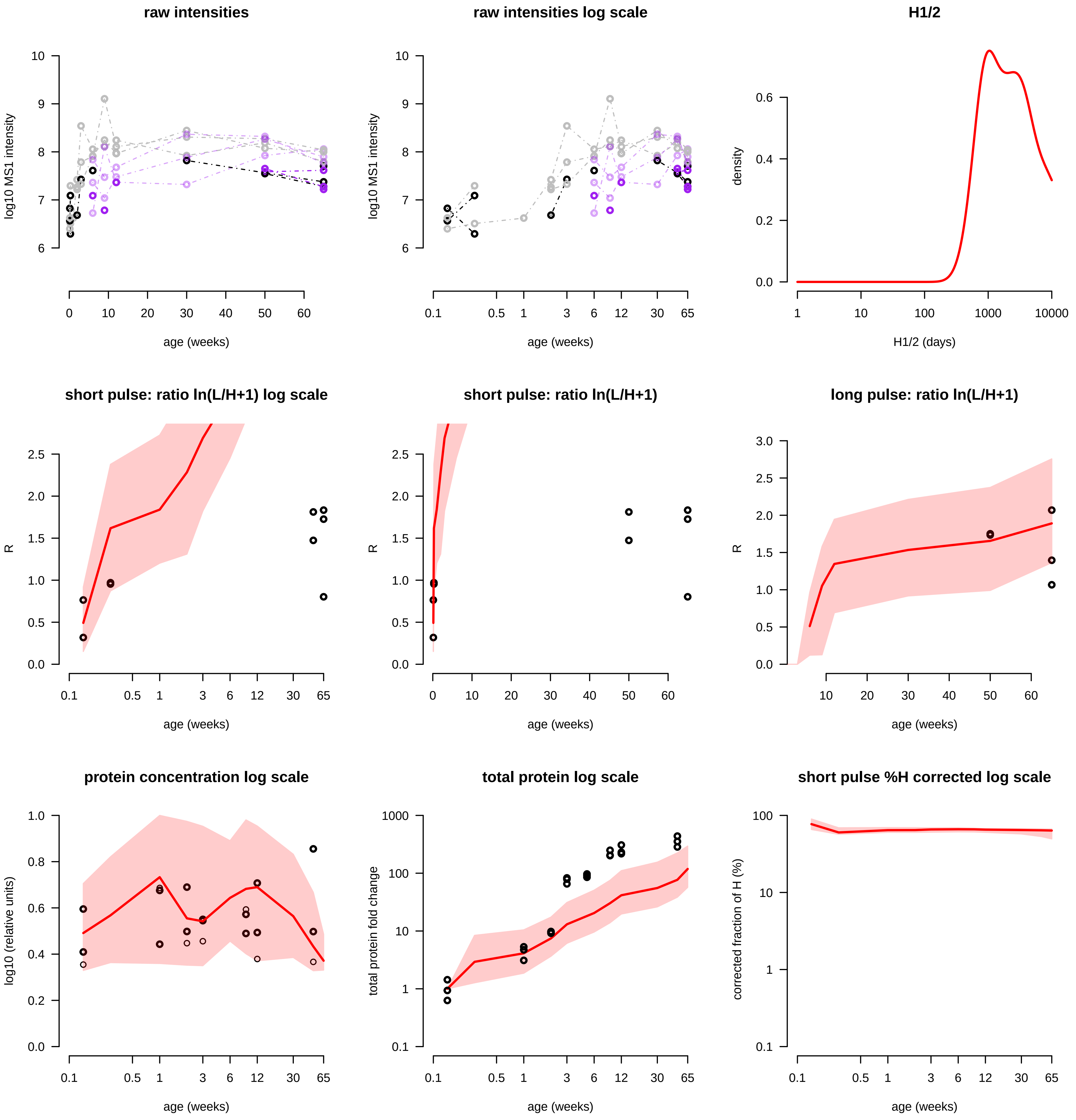

Q9D6K9;Q9D6K9−2

>sp|Q9D6K9|CERS5_MOUSE

Ceramide synthase 5

GN=Cers5

Med H1/2: 16.9 days

Q5 H1/2: 5.7 days

Q95 H1/2: 160 days

reliable model fit: yes

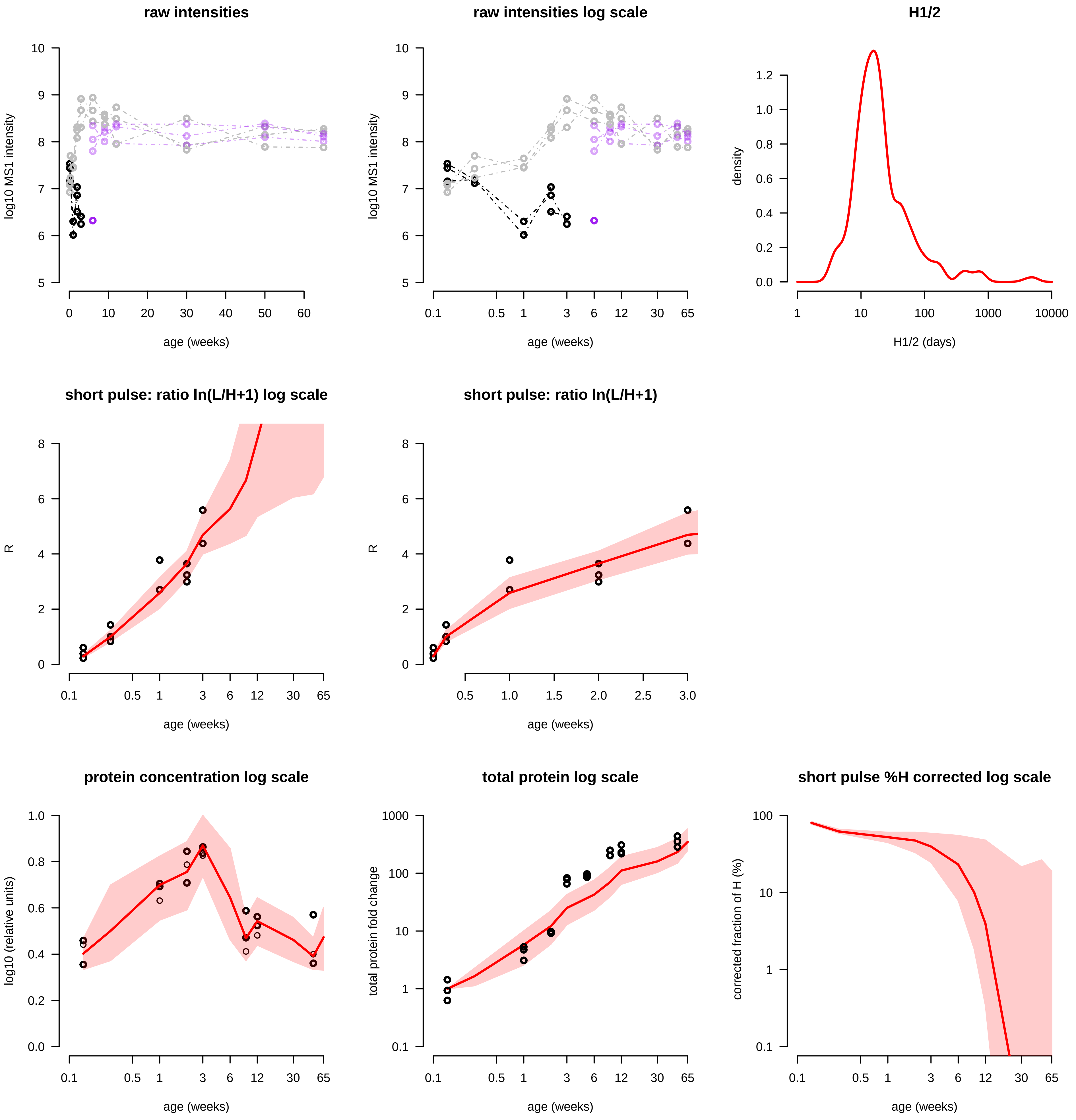

Q91VE6;Q91VE6−2

>sp|Q91VE6|MK67I_MOUSE

MKI67 FHA domain−interacting nucleolar phosphoprotein

GN=Nifk

Med H1/2: 16.5 days

Q5 H1/2: 7.5 days

Q95 H1/2: 86.2 days

reliable model fit: yes

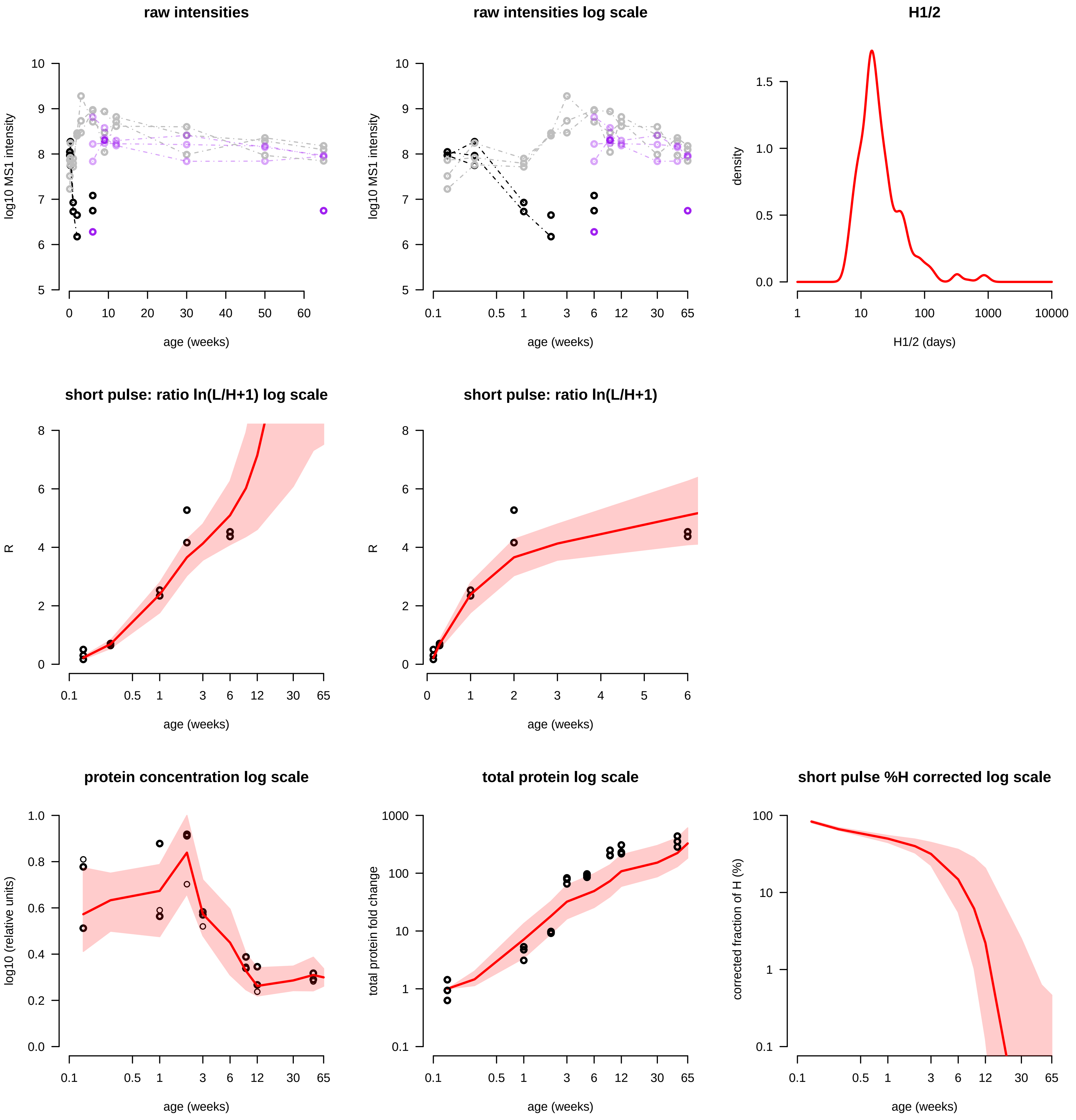

Q8CG48

>sp|Q8CG48|SMC2_MOUSE

Structural maintenance of chromosomes protein 2

GN=Smc2

Med H1/2: 65.1 days

Q5 H1/2: 22 days

Q95 H1/2: 621.4 days

reliable model fit: yes

raw intensities

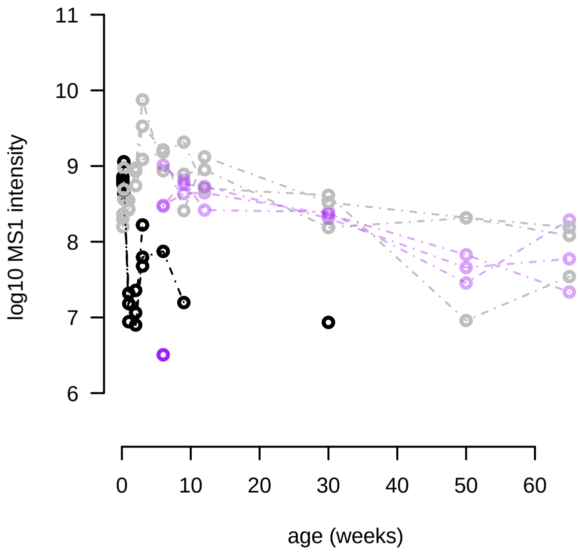

raw intensities log scale

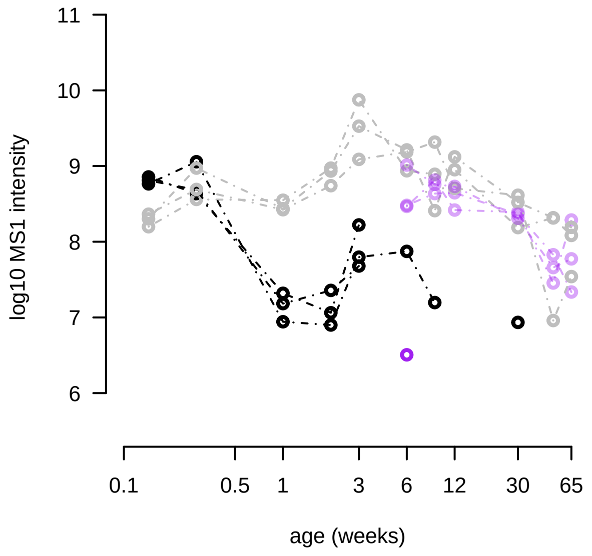

H1/2

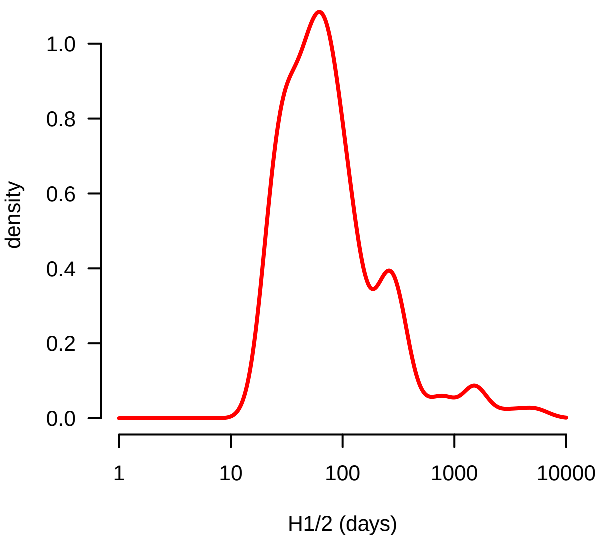

short pulse: ratio ln(L/H+1) log scale

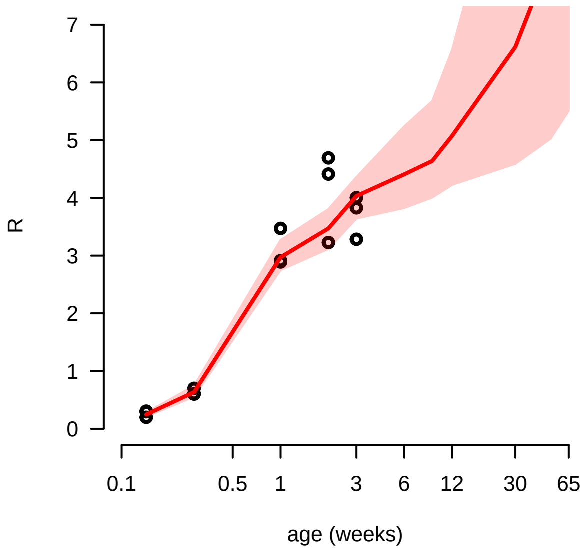

short pulse: ratio ln(L/H+1)

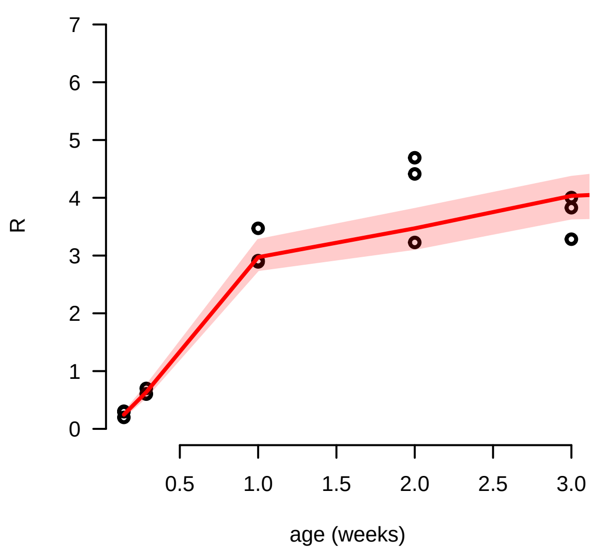

protein concentration log scale

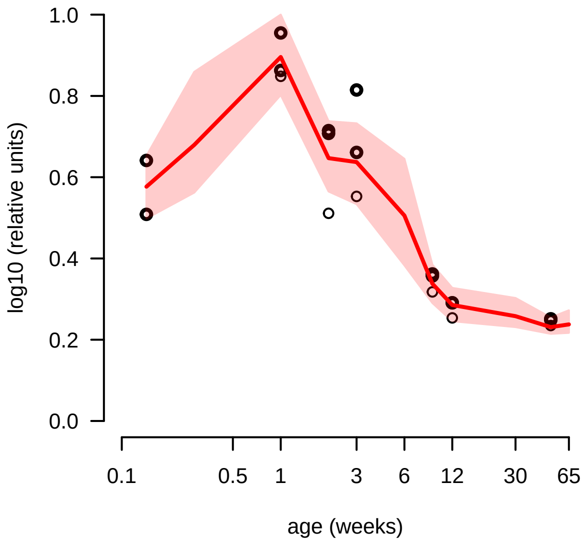

total protein log scale

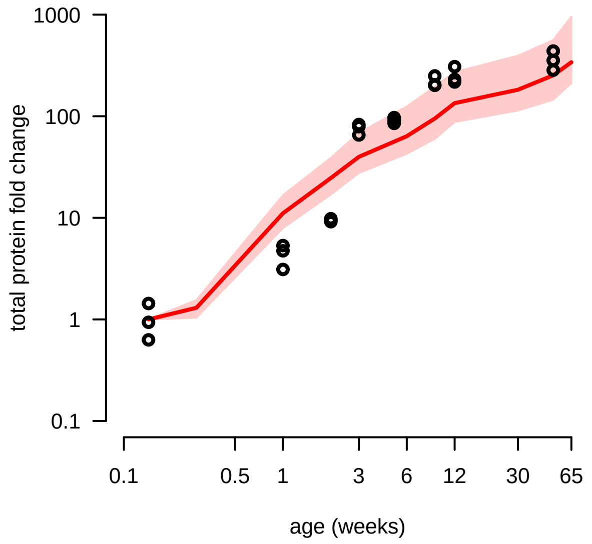

short pulse %H corrected log scale

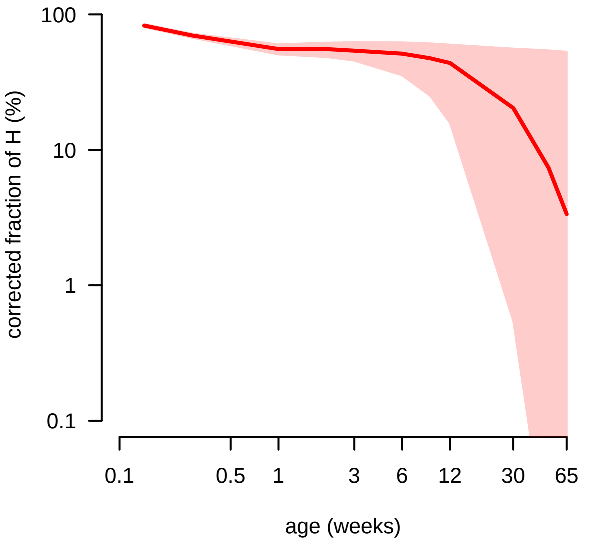

P55194

>sp|P55194|3BP1_MOUSE

SH3 domain−binding protein 1

GN=Sh3bp1

Med H1/2: 9 days

Q5 H1/2: 3.9 days

Q95 H1/2: 65.9 days

reliable model fit: yes

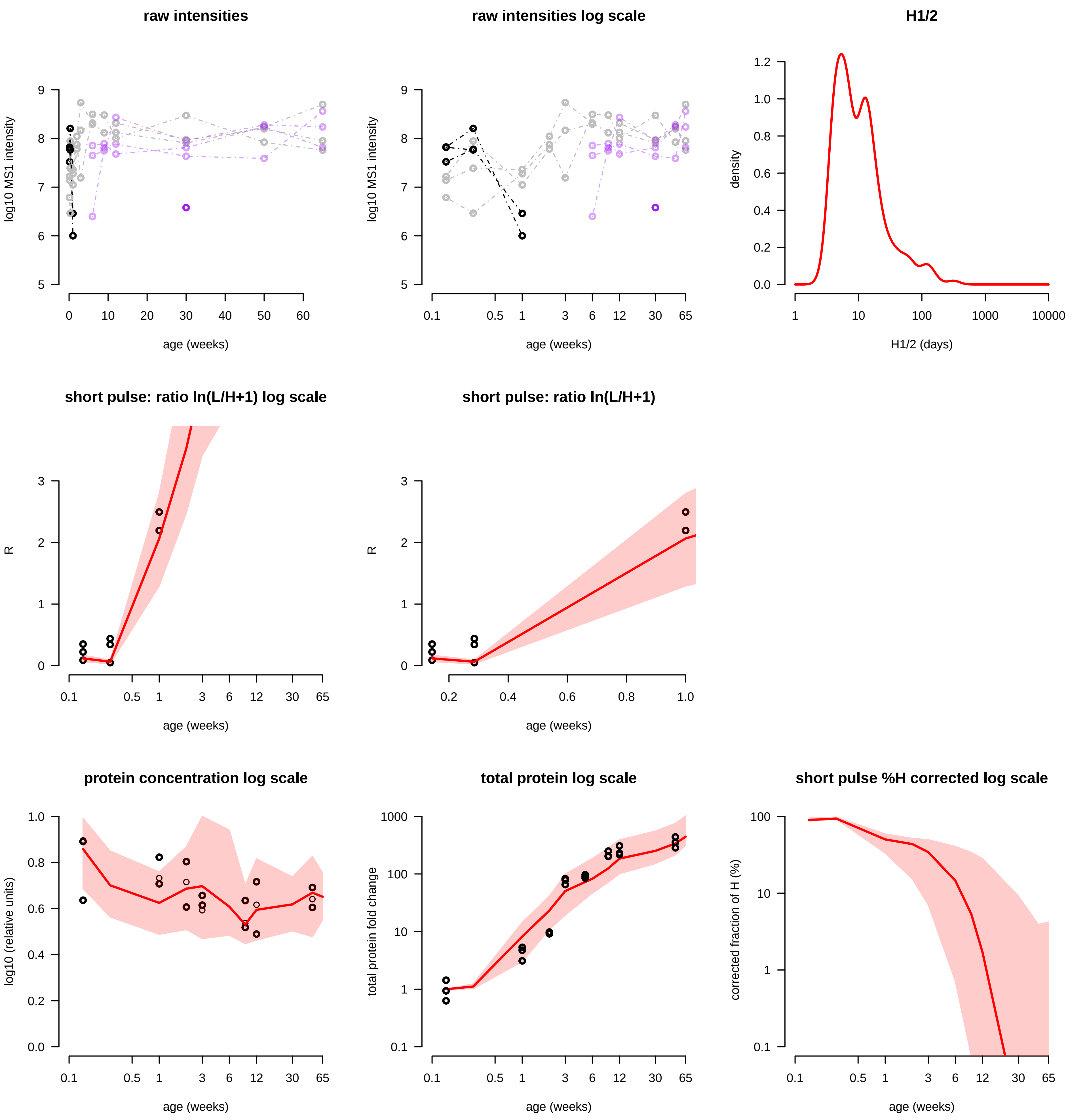

Q9ES46

>sp|Q9ES46|PARVB_MOUSE

Beta−parvin

GN=Parvb

Med H1/2: 3.6 days

Q5 H1/2: 2.5 days

Q95 H1/2: 15.5 days

reliable model fit: yes

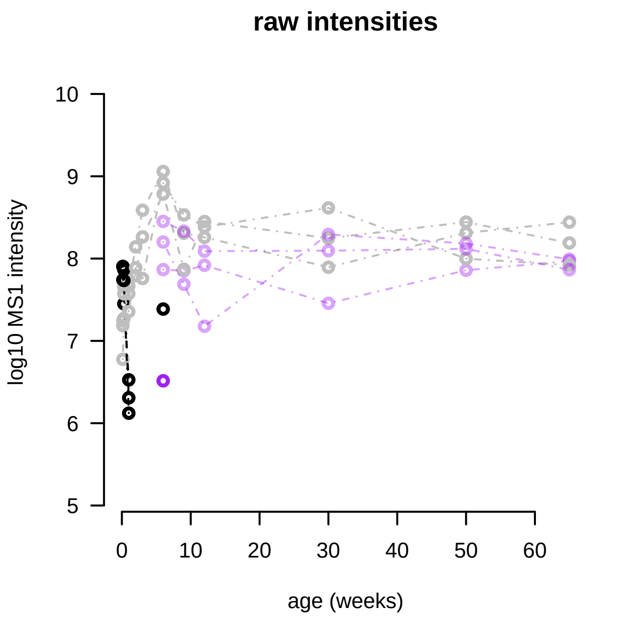

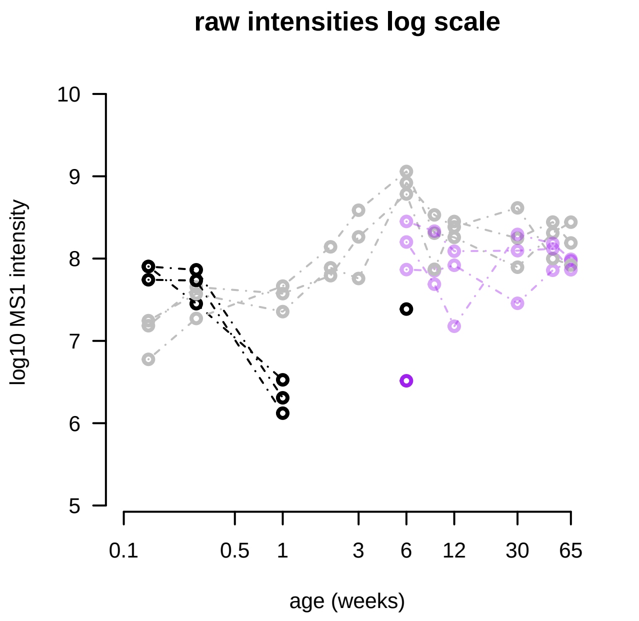

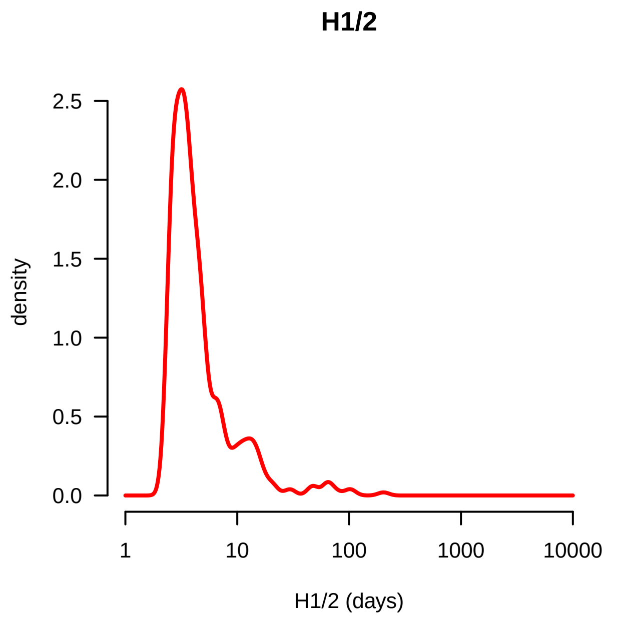

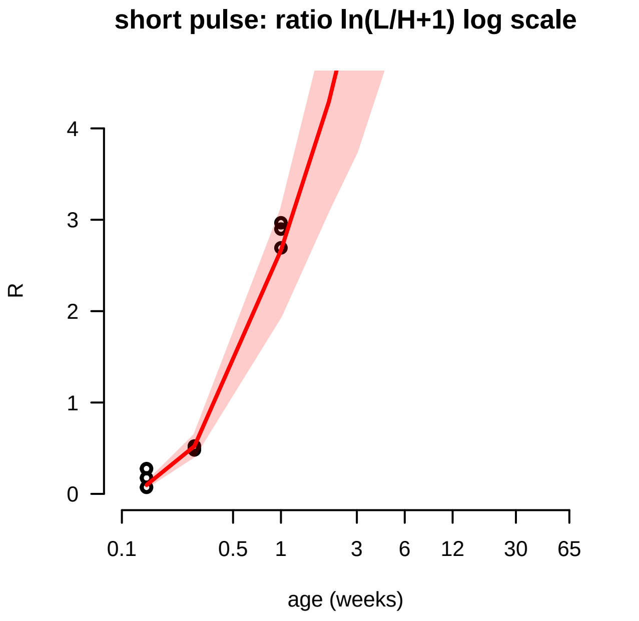

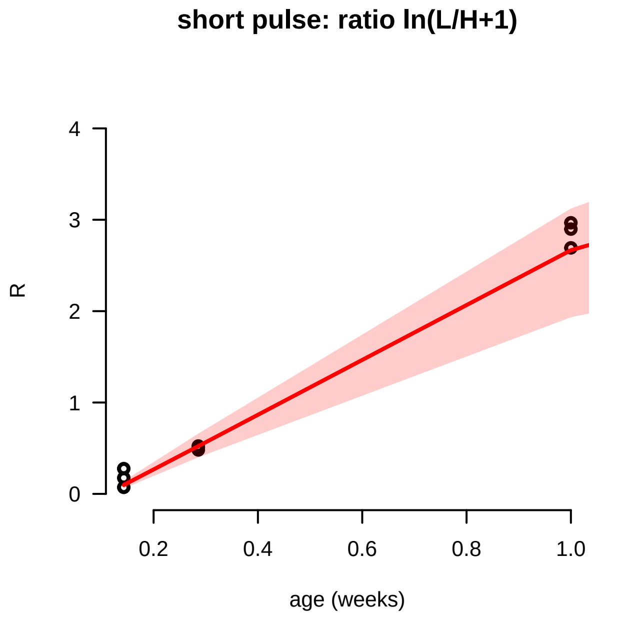

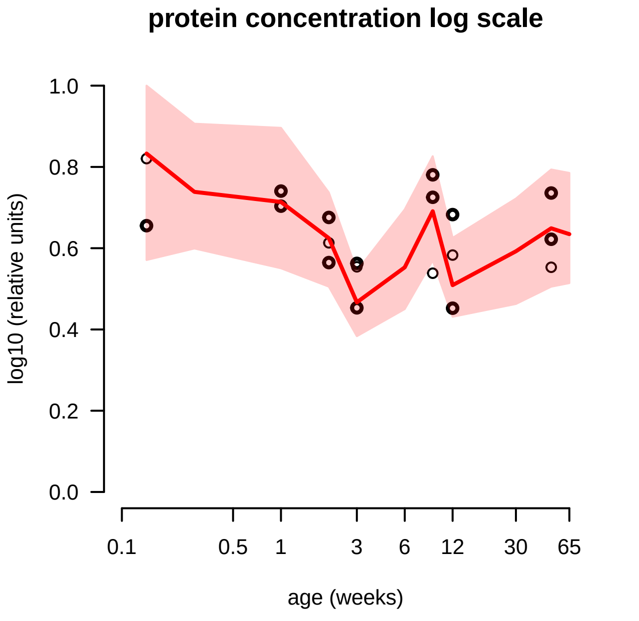

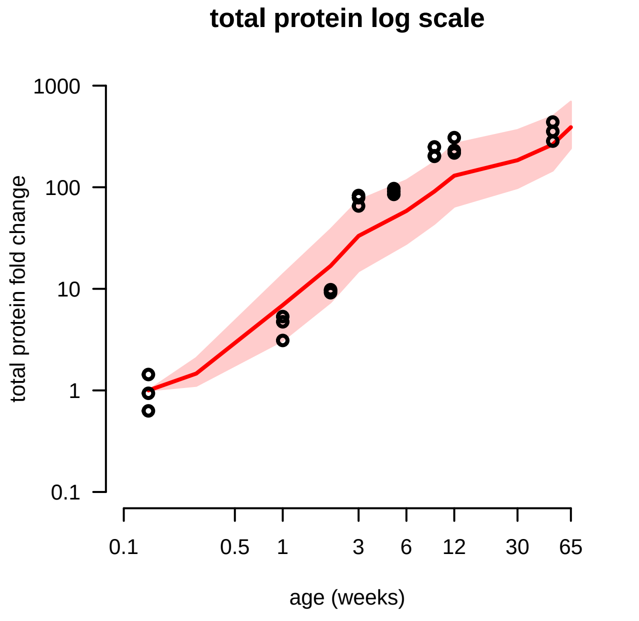

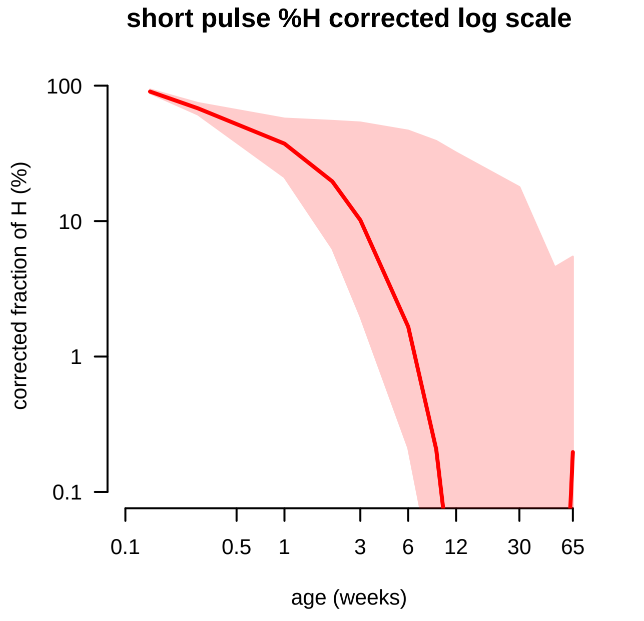

Q60823

>sp|Q60823|AKT2_MOUSE

RAC−beta serine/threonine−protein kinase

GN=Akt2

Med H1/2: 7.1 days

Q5 H1/2: 3.8 days

Q95 H1/2: 25.8 days

reliable model fit: yes

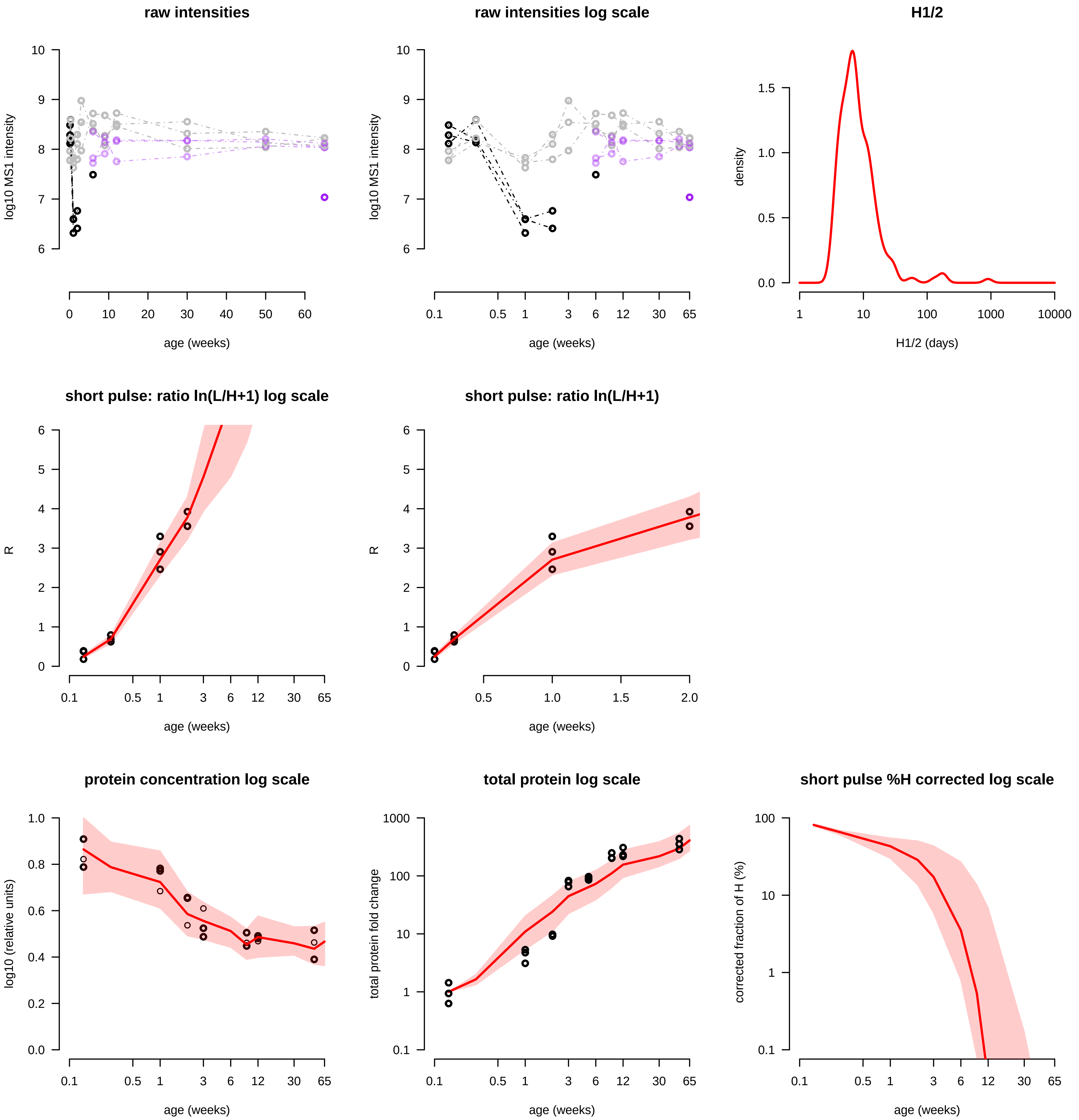

Q9Z2B9

>sp|Q9Z2B9|KS6A4_MOUSE

Ribosomal protein S6 kinase alpha−4

GN=Rps6ka4

Med H1/2: 8 days

Q5 H1/2: 3.4 days

Q95 H1/2: 63.2 days

reliable model fit: yes

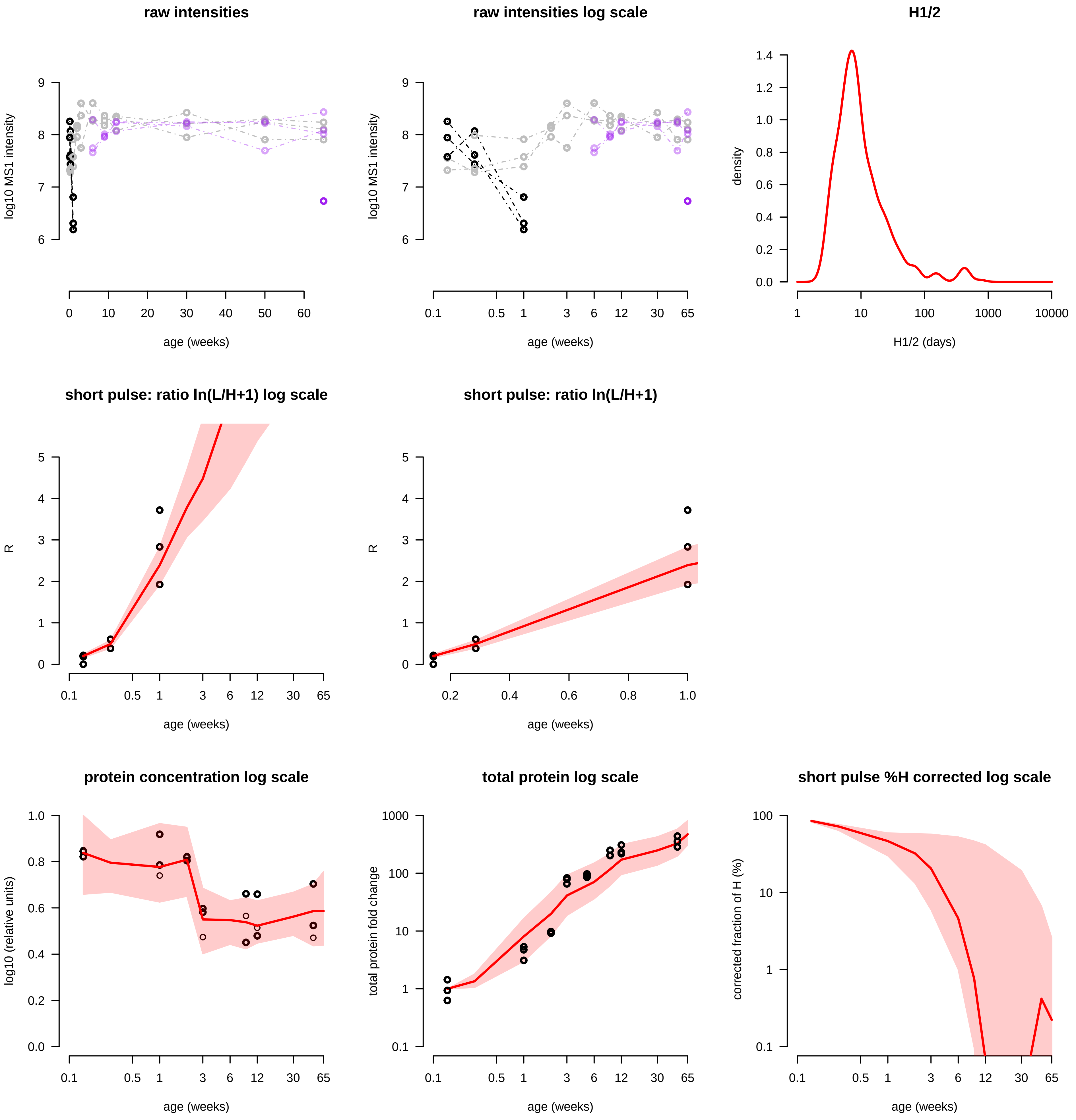

Q8K212

>sp|Q8K212|PACS1_MOUSE

Phosphofurin acidic cluster sorting protein 1

GN=Pacs1

Med H1/2: 45.7 days

Q5 H1/2: 11.8 days

Q95 H1/2: 428.3 days

reliable model fit: yes

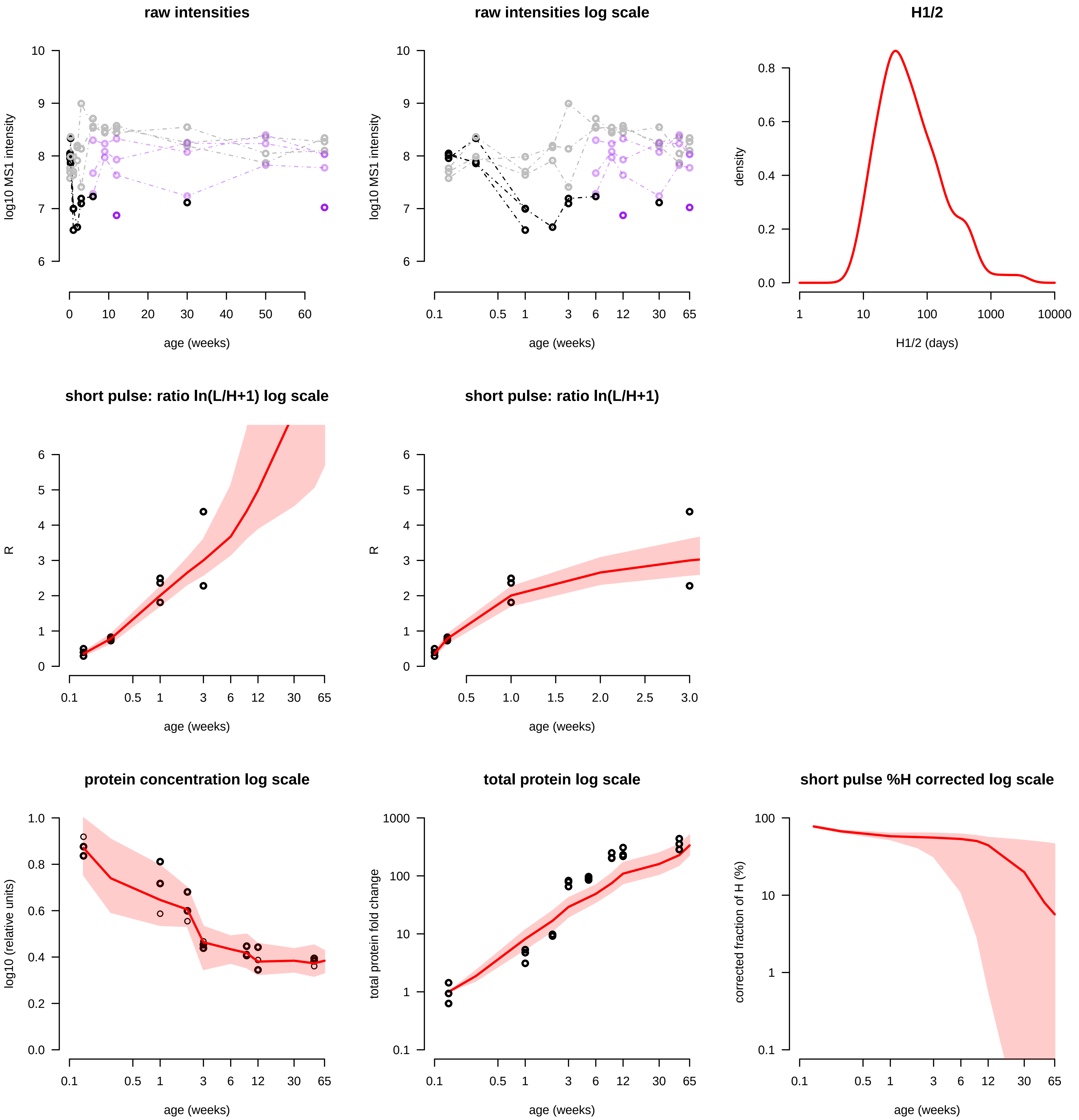

O88286−3

>sp|O88286−3|WIZ_MOUSE

Isoform 2 of Protein Wiz

GN=Wiz

Med H1/2: 8.7 days

Q5 H1/2: 7.1 days

Q95 H1/2: 11.9 days

reliable model fit: yes

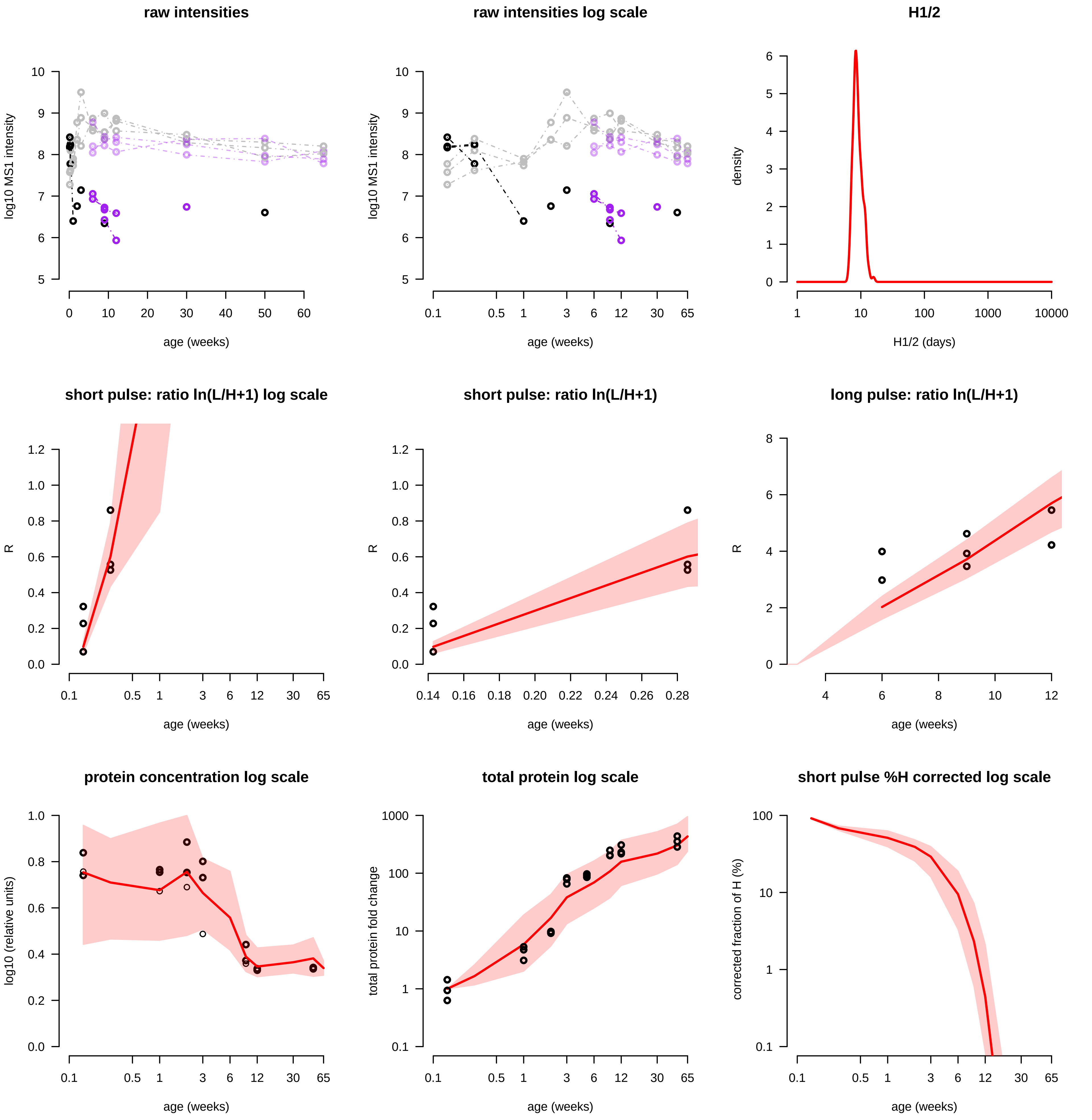

A6X8Z5

>sp|A6X8Z5|RHG31_MOUSE

Rho GTPase−activating protein 31

GN=Arhgap31

Med H1/2: 7.1 days

Q5 H1/2: 4.5 days

Q95 H1/2: 11.5 days

reliable model fit: yes

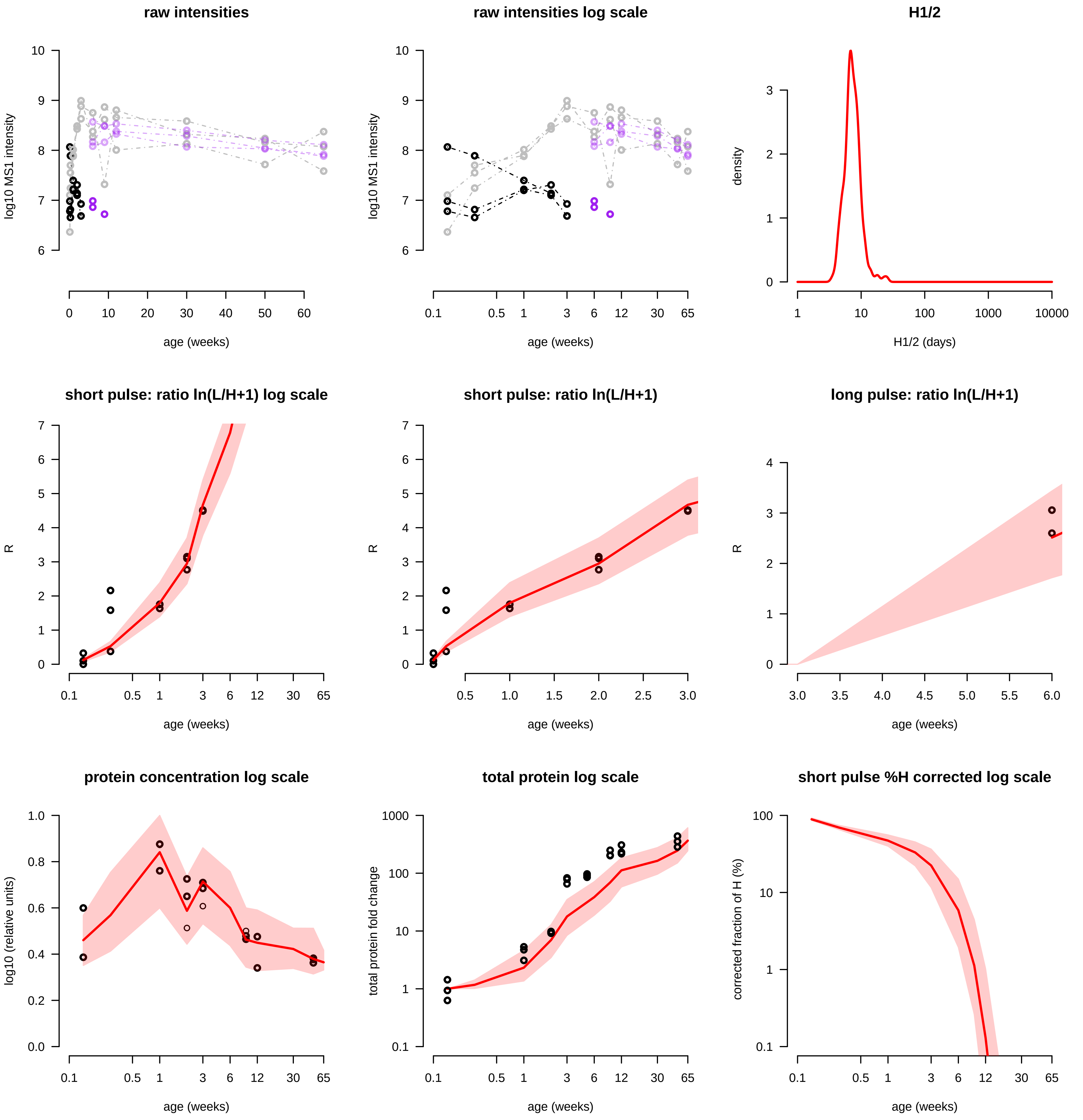

Q6PDH0;Q6PDH0−2

>sp|Q6PDH0|PHLB1_MOUSE

Pleckstrin homology−like domain family B member 1

GN=Phldb1

Med H1/2: 17.3 days

Q5 H1/2: 4.2 days

Q95 H1/2: 262.8 days

reliable model fit: yes

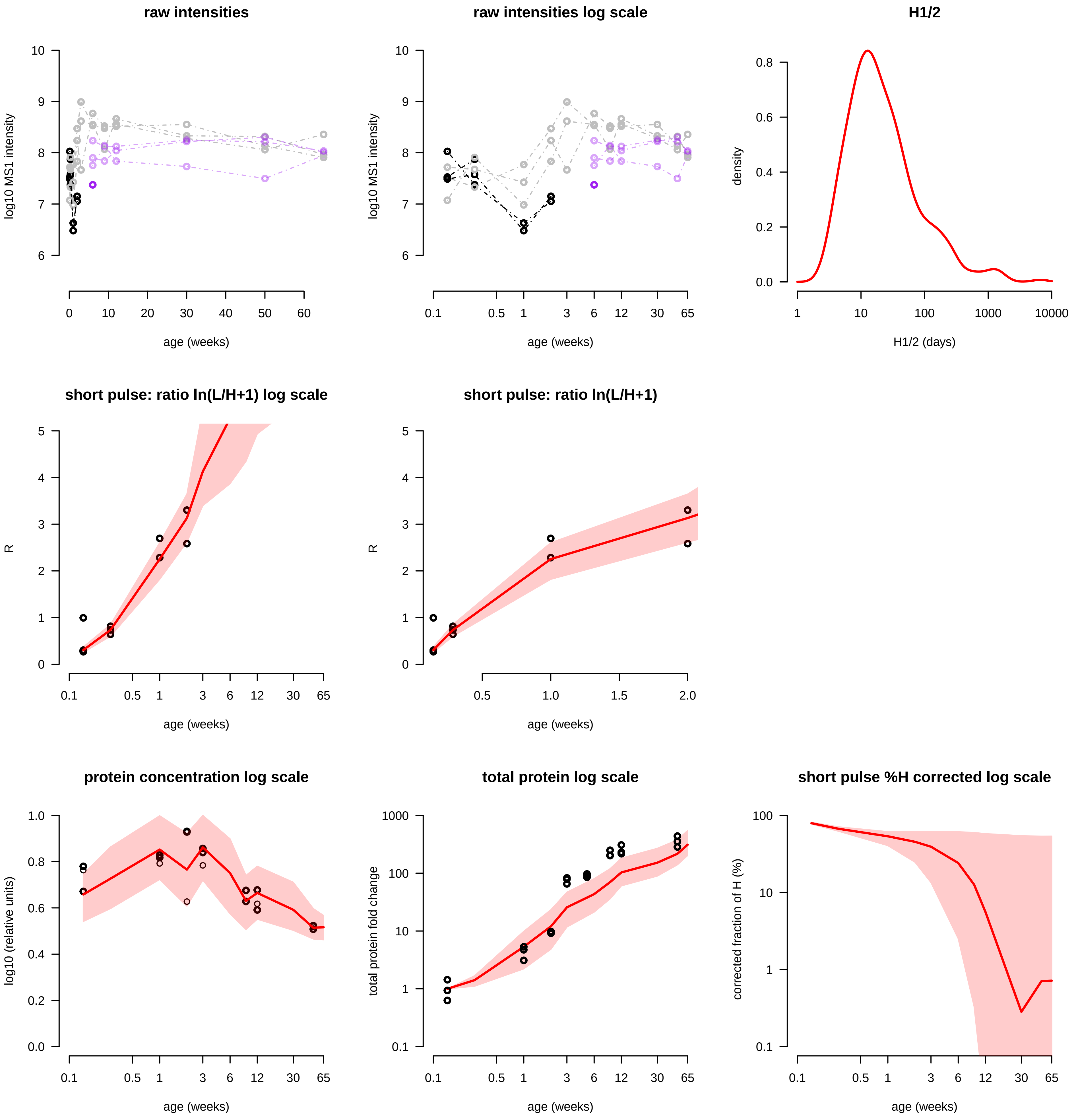

Q8VCL2

>sp|Q8VCL2|SCO2_MOUSE

Protein SCO2 homolog, mitochondrial

GN=Sco2

Med H1/2: 21.4 days

Q5 H1/2: 5.9 days

Q95 H1/2: 314.8 days

reliable model fit: yes

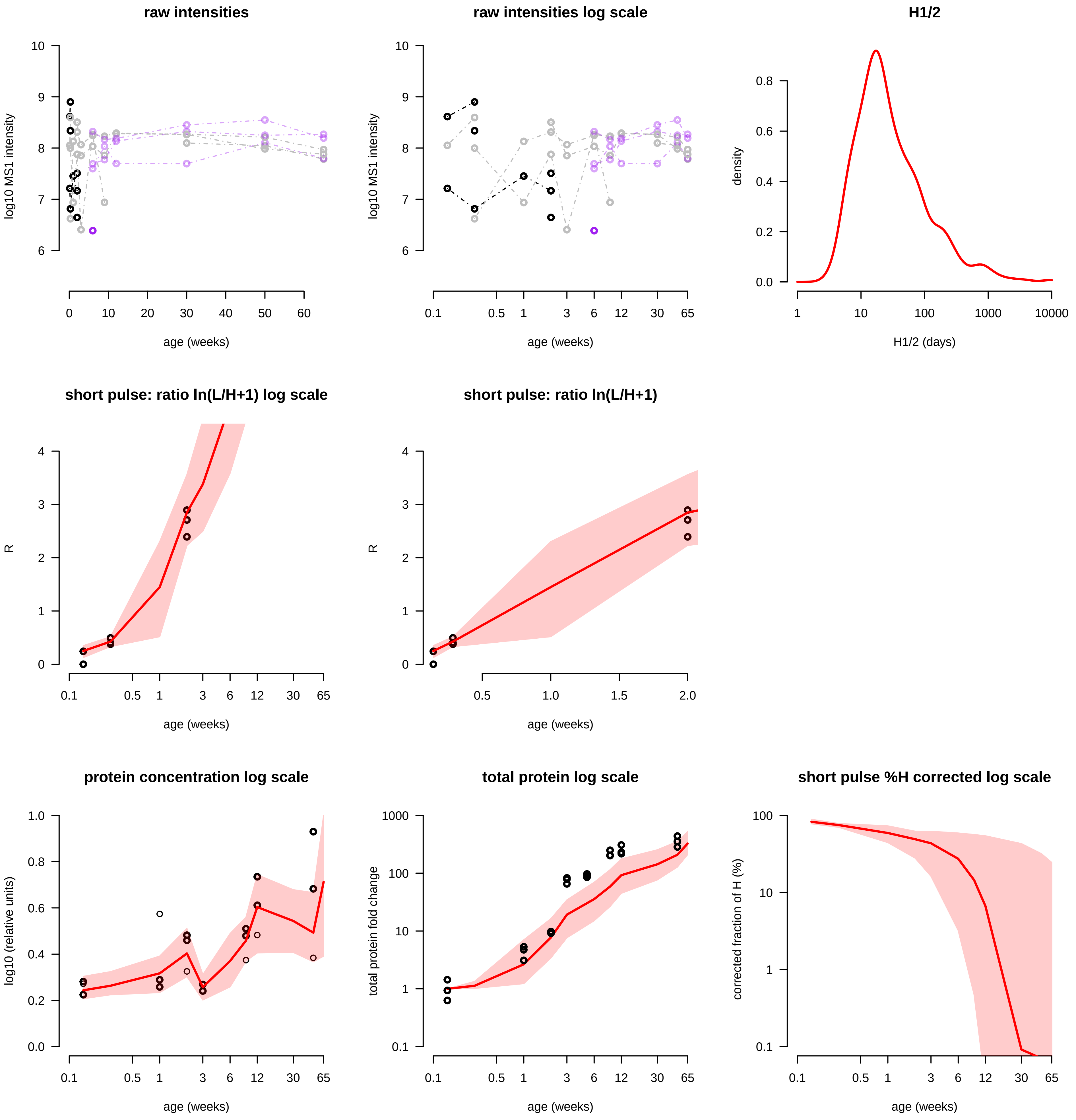

P70699

>sp|P70699|LYAG_MOUSE

Lysosomal alpha−glucosidase

GN=Gaa

Med H1/2: 17.4 days

Q5 H1/2: 6.4 days

Q95 H1/2: 107.1 days

reliable model fit: yes

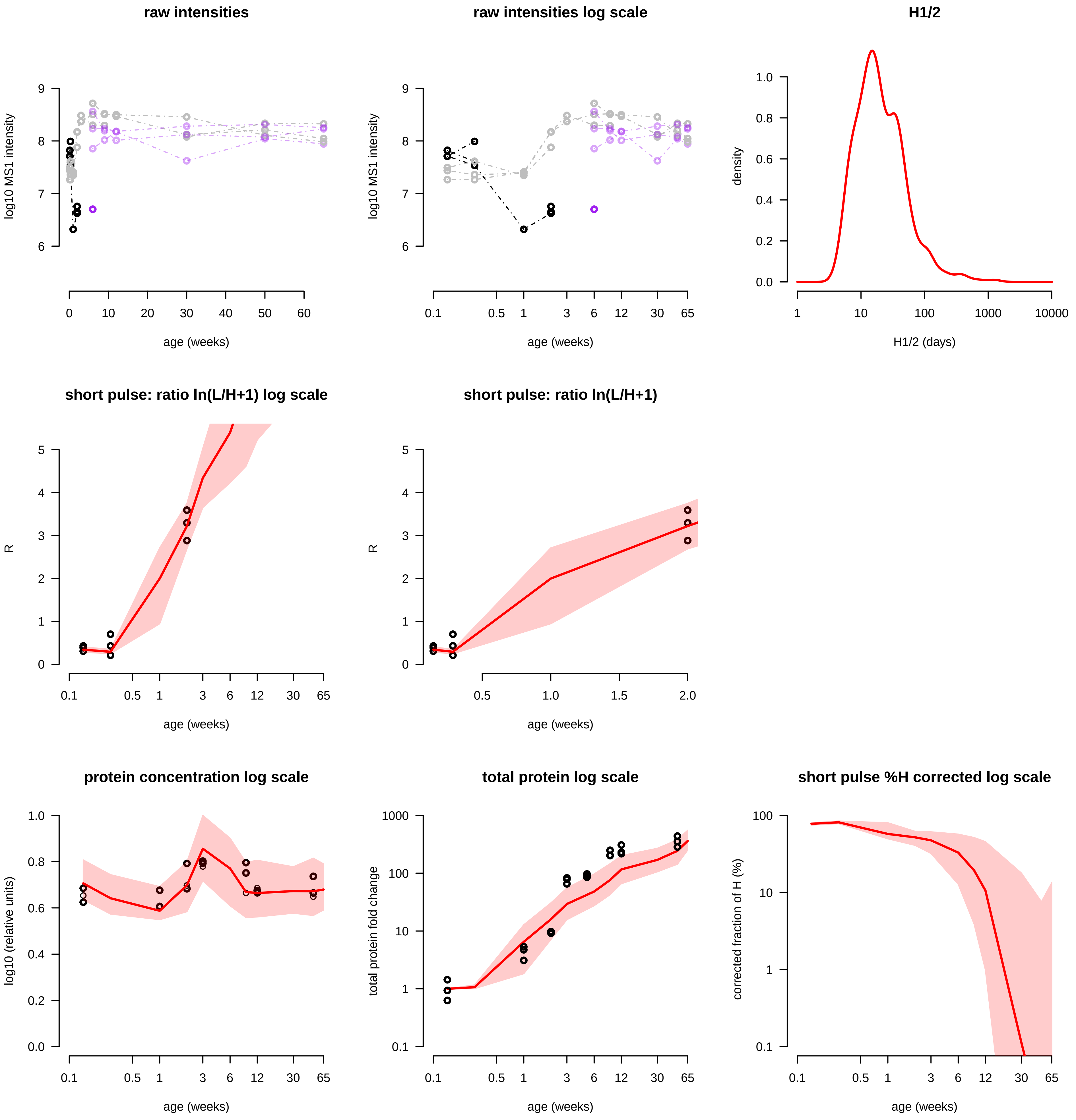

Q9ERA6

>sp|Q9ERA6|TFP11_MOUSE

Tuftelin−interacting protein 11

GN=Tfip11

Med H1/2: 4.9 days

Q5 H1/2: 3.9 days

Q95 H1/2: 5.8 days

reliable model fit: yes

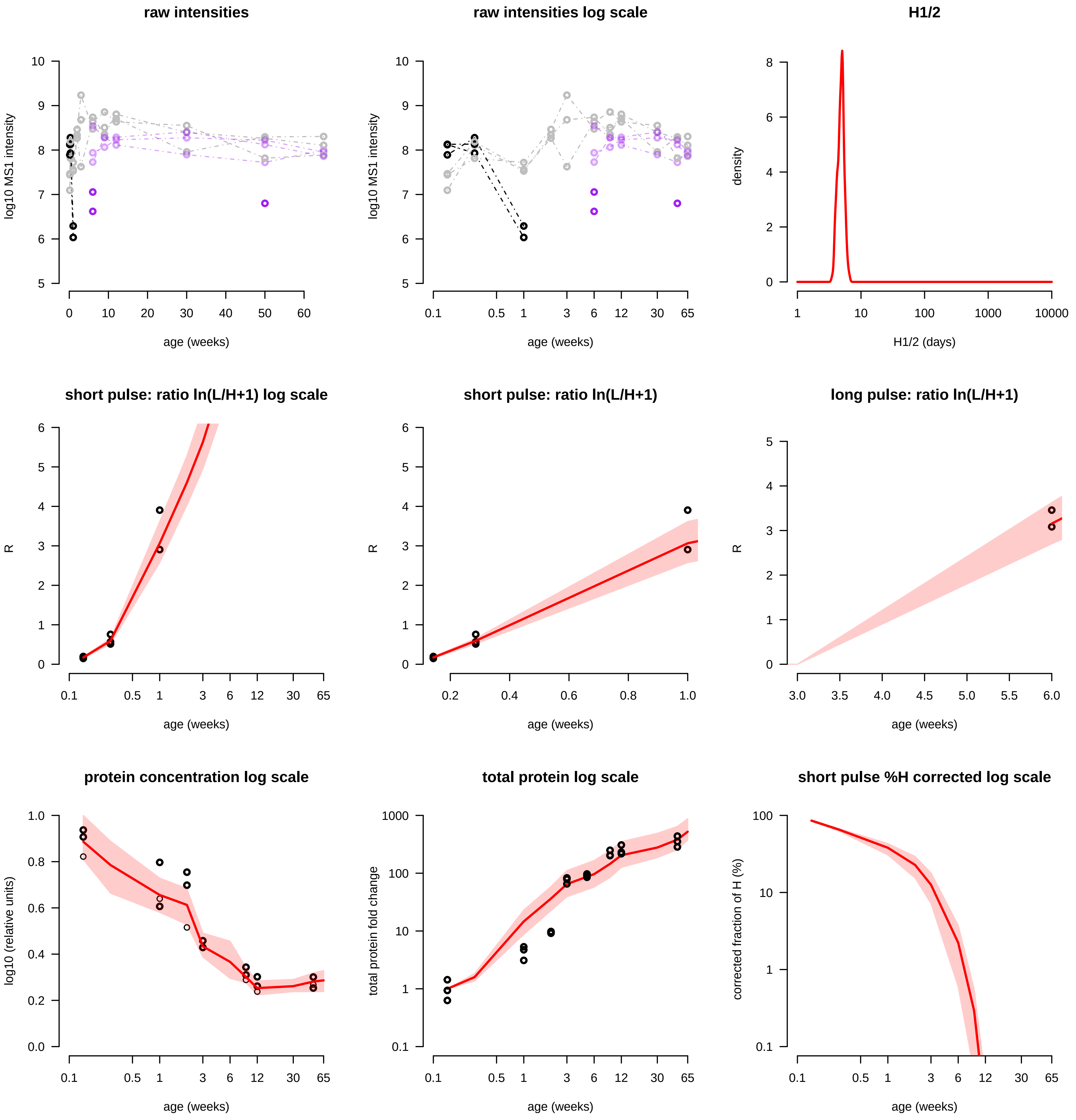

Q9D8B4

>sp|Q9D8B4|NDUAB_MOUSE

NADH dehydrogenase [ubiquinone] 1 alpha subcomplex subunit 11

GN=Ndufa11

Med H1/2: 8.4 days

Q5 H1/2: 6.5 days

Q95 H1/2: 12 days

reliable model fit: yes

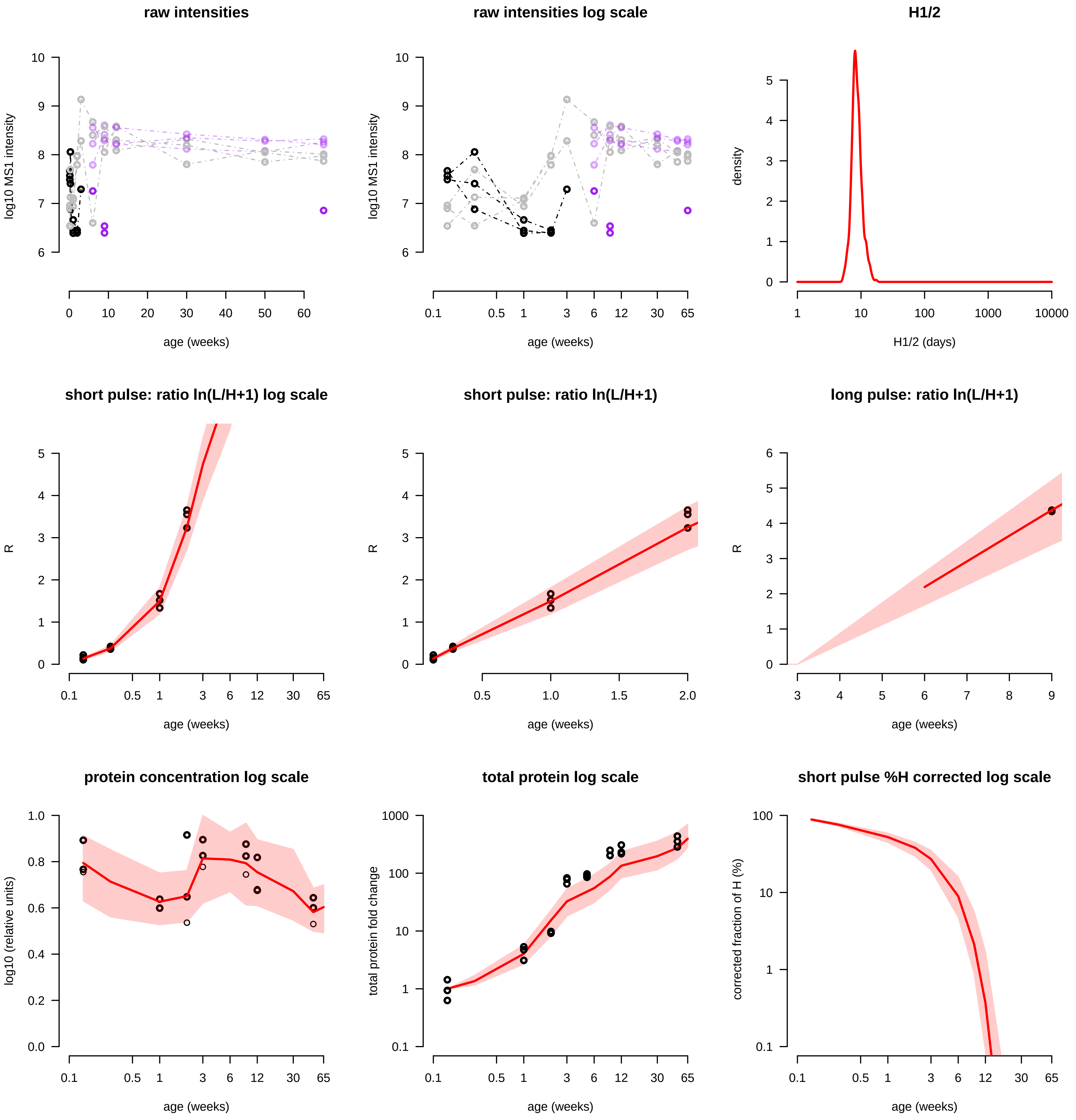

Q99L27

>sp|Q99L27|GMPR2_MOUSE

GMP reductase 2

GN=Gmpr2

Med H1/2: 85.5 days

Q5 H1/2: 24.9 days

Q95 H1/2: 807.7 days

reliable model fit: no

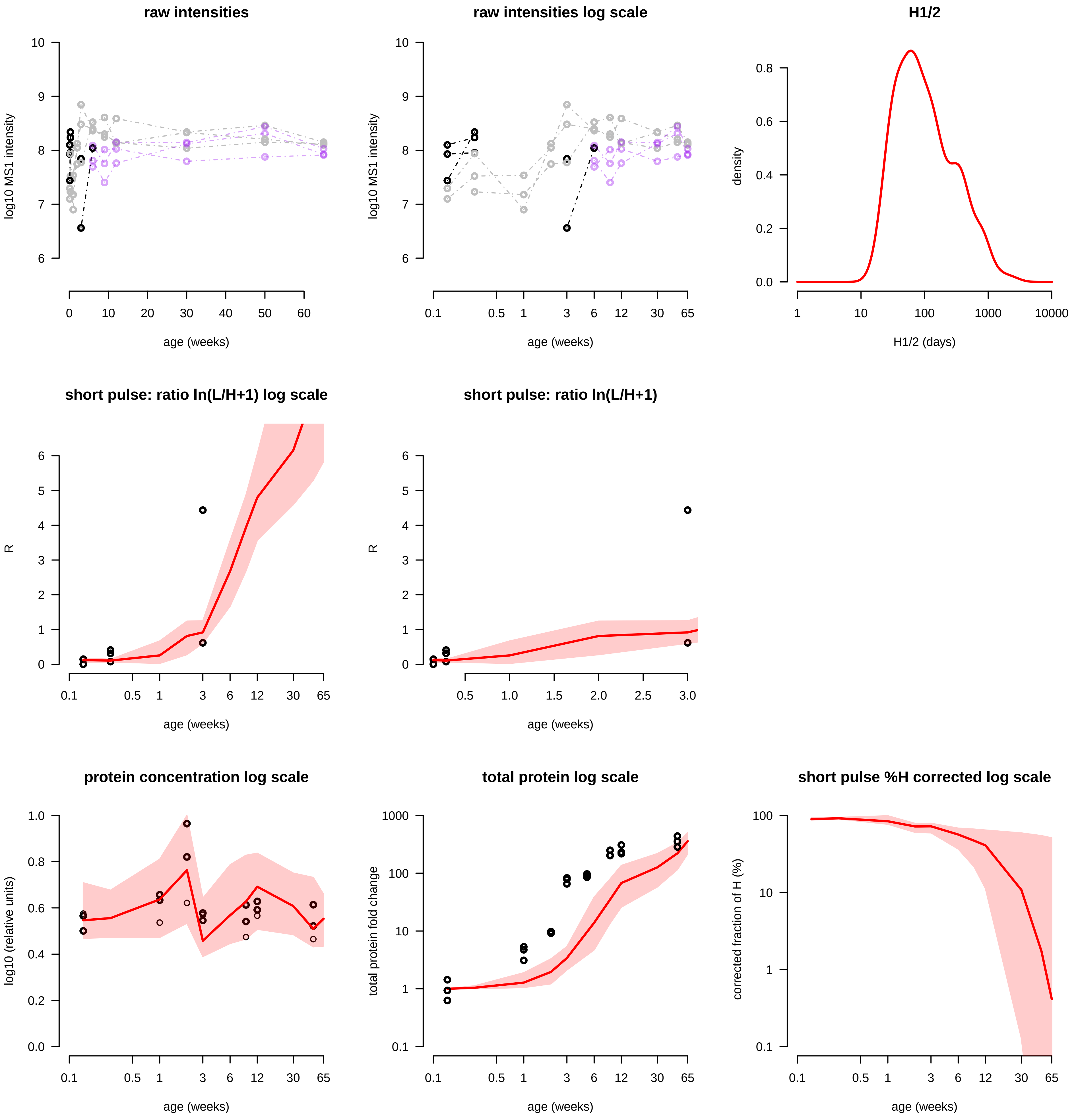

Q9CR61

>sp|Q9CR61|NDUB7_MOUSE

NADH dehydrogenase [ubiquinone] 1 beta subcomplex subunit 7

GN=Ndufb7

Med H1/2: 8.7 days

Q5 H1/2: 3.8 days

Q95 H1/2: 81.6 days

reliable model fit: yes

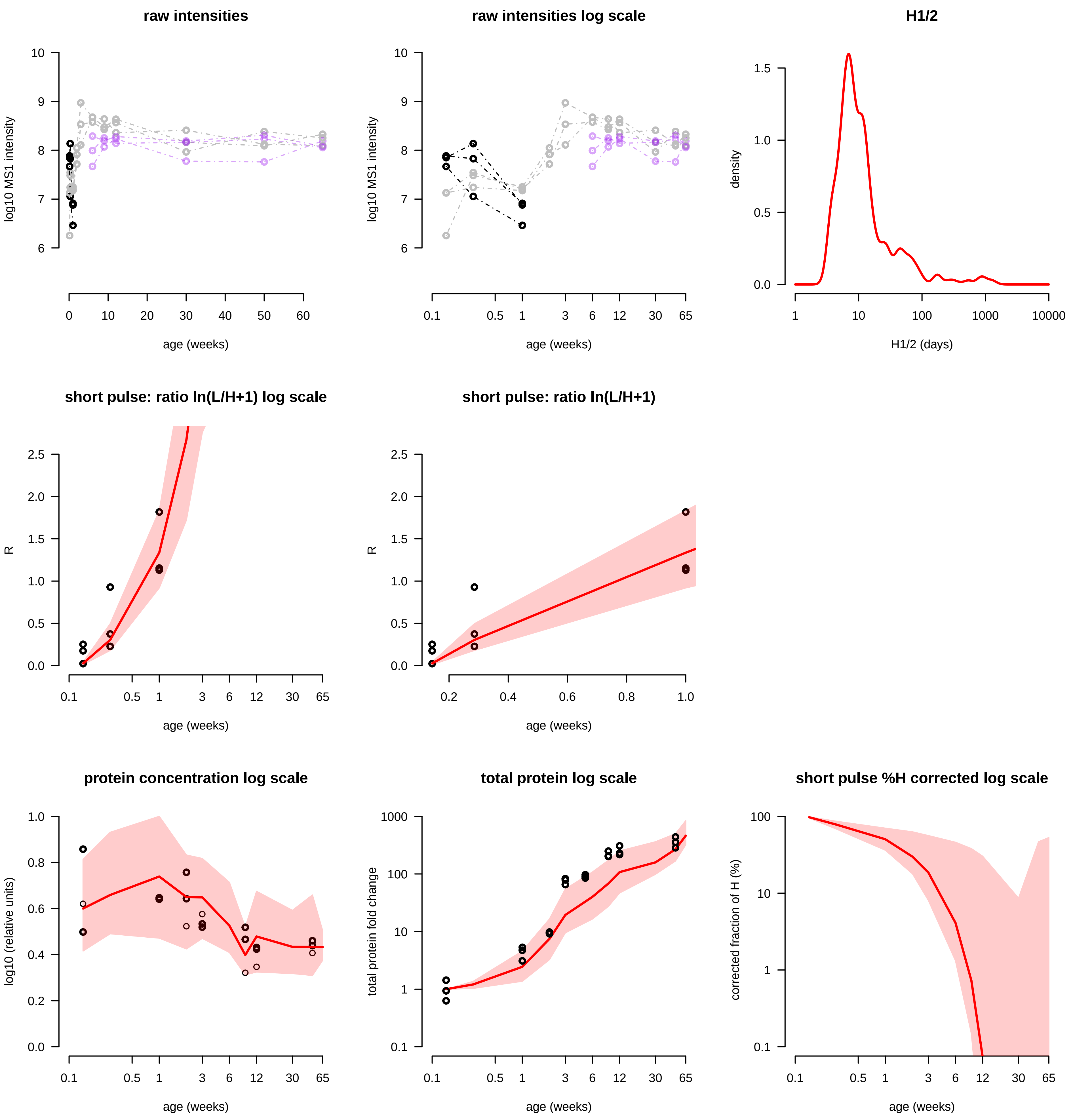

Q8K268

>sp|Q8K268|ABCF3_MOUSE

ATP−binding cassette sub−family F member 3

GN=Abcf3

Med H1/2: 27.5 days

Q5 H1/2: 5 days

Q95 H1/2: 345.3 days

reliable model fit: no

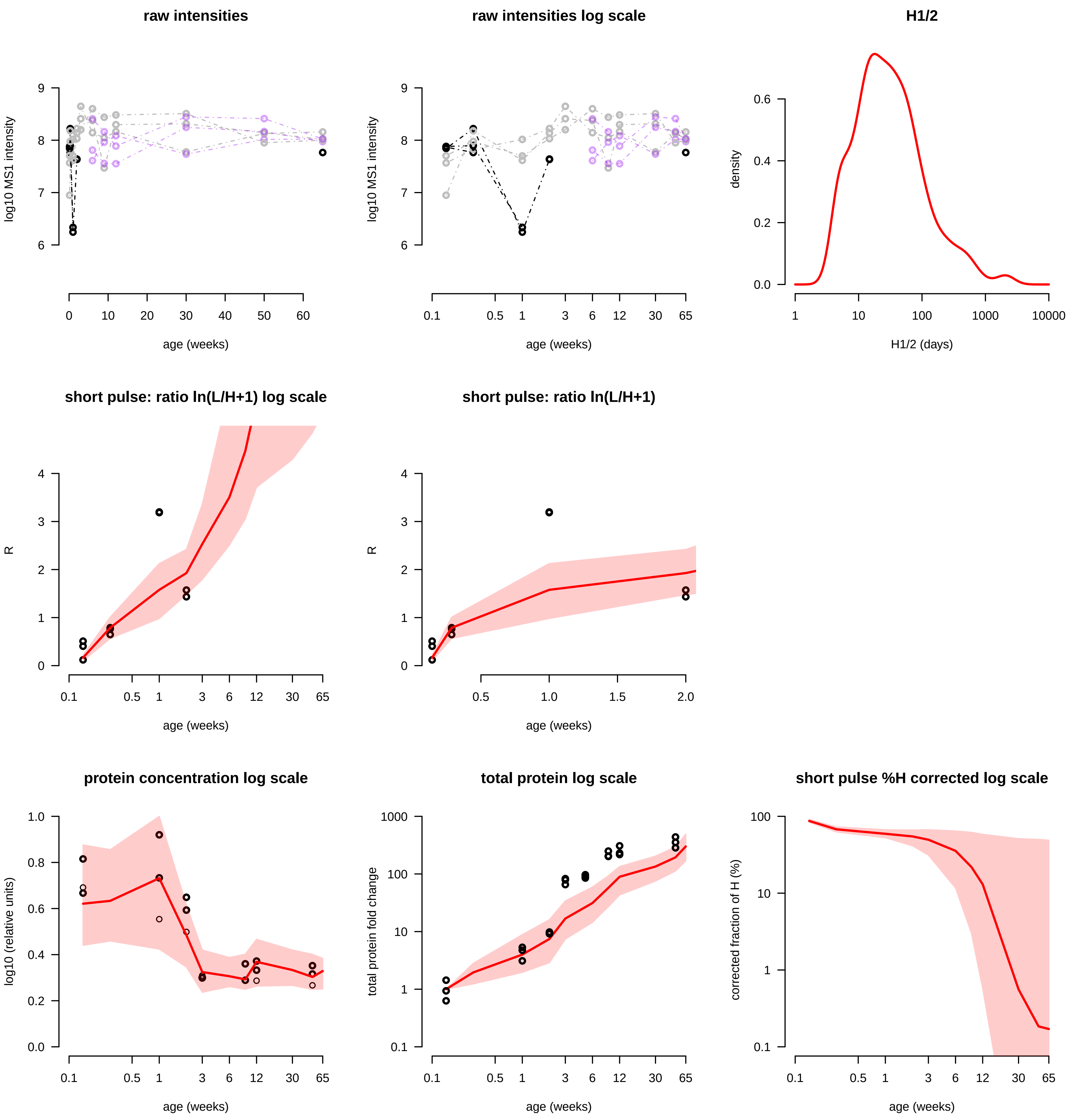

Q8R1X6;Q8R1X6−2

>sp|Q8R1X6|SPART_MOUSE

Spartin

GN=Spart

Med H1/2: 4.7 days

Q5 H1/2: 2.8 days

Q95 H1/2: 43.5 days

reliable model fit: yes

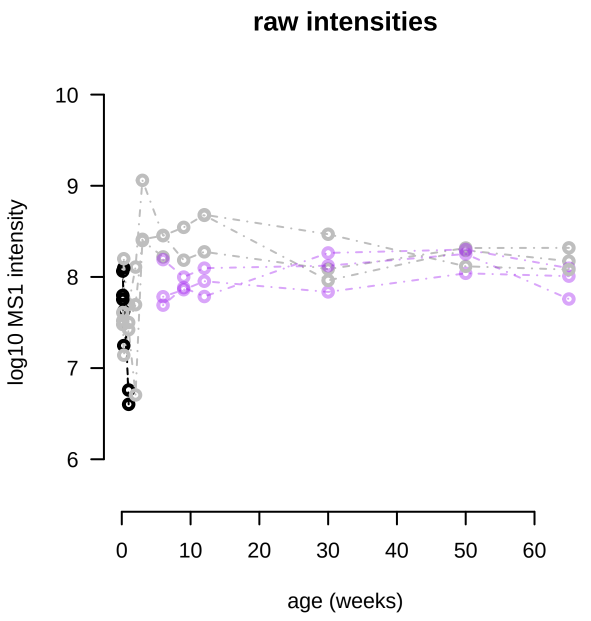

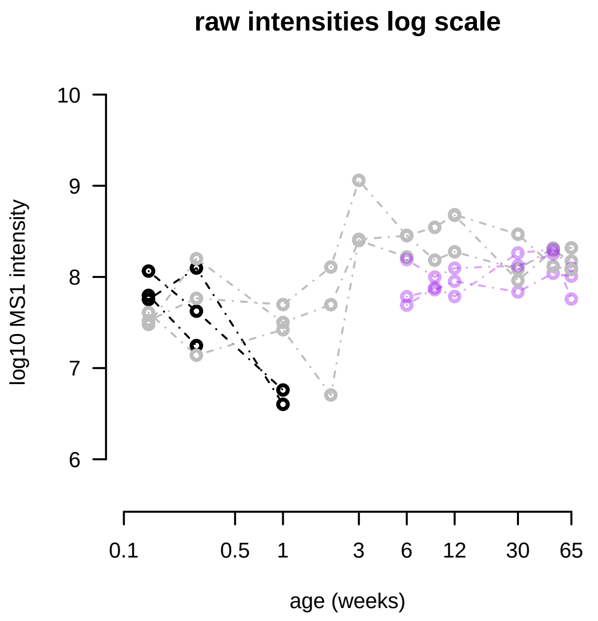

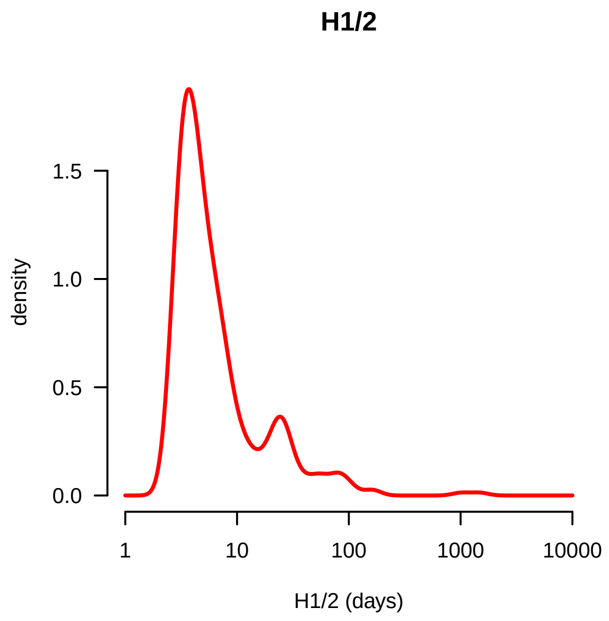

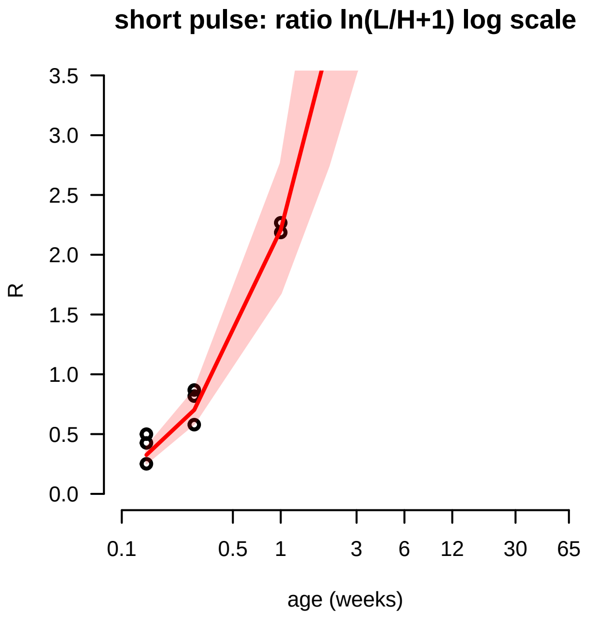

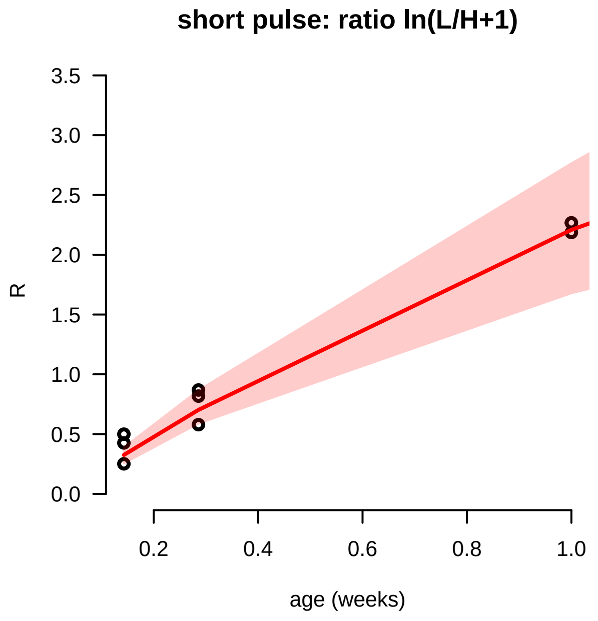

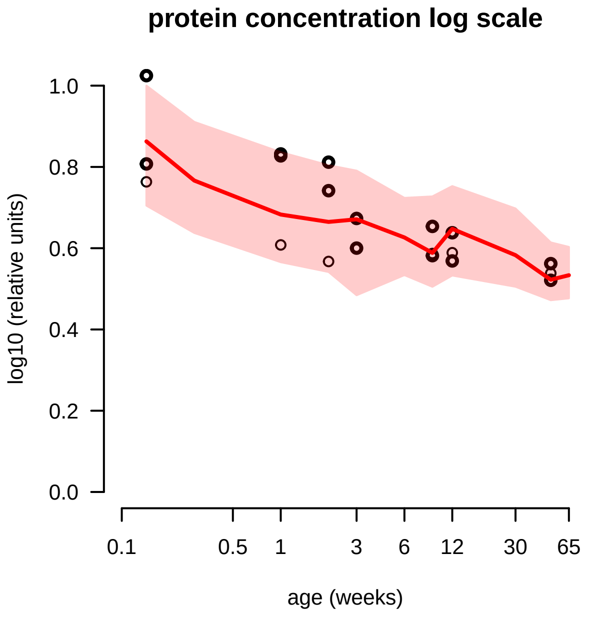

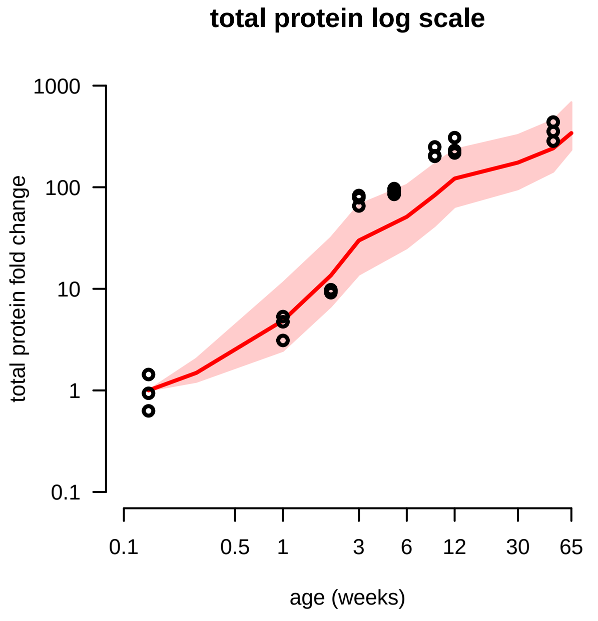

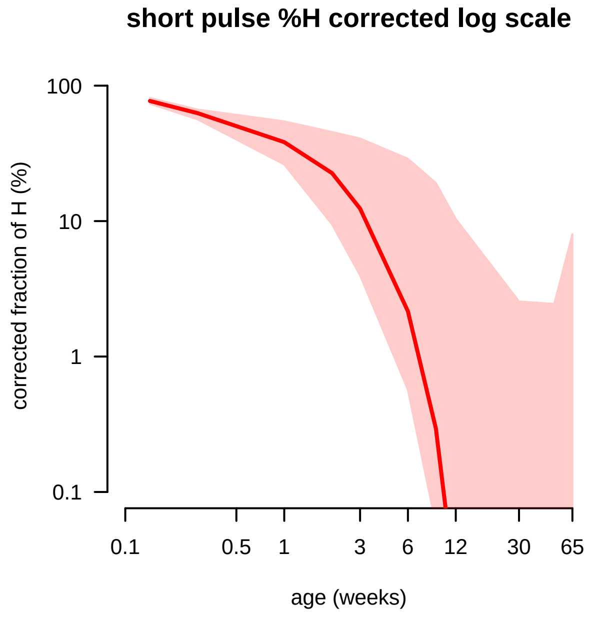

A2A690;A2A690−2

>sp|A2A690|TANC2_MOUSE

Protein TANC2

GN=Tanc2

Med H1/2: 19.9 days

Q5 H1/2: 8.9 days

Q95 H1/2: 133.8 days

reliable model fit: yes

raw intensities

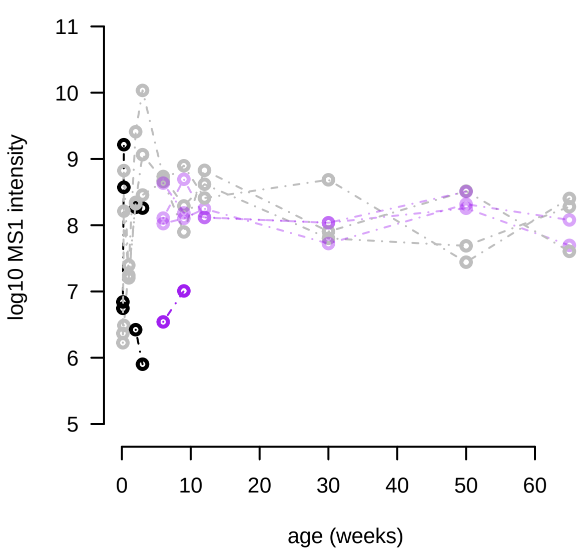

raw intensities log scale

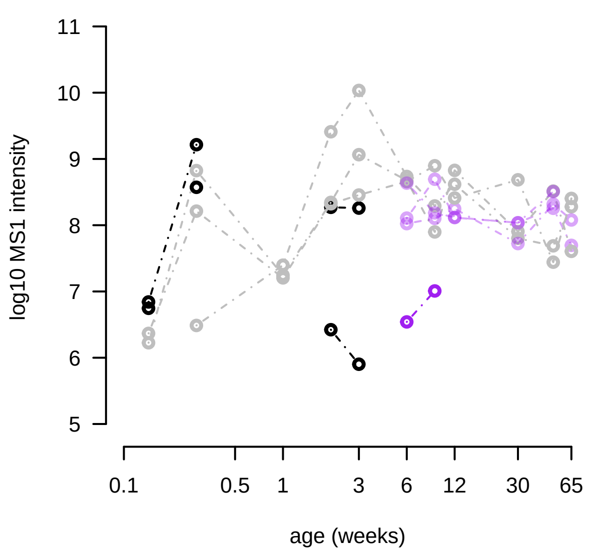

H1/2

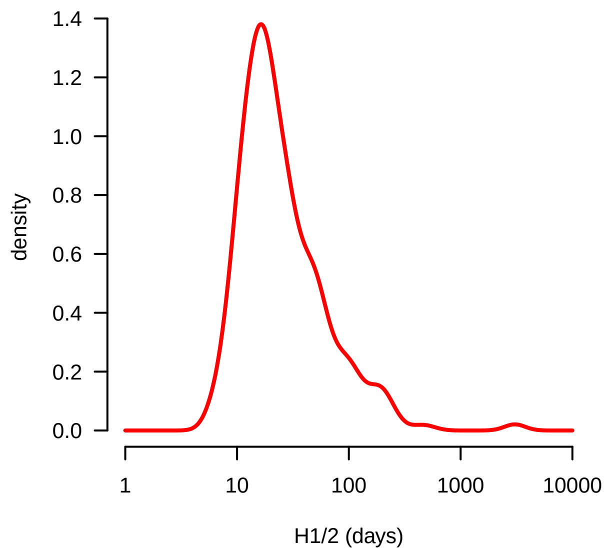

short pulse: ratio ln(L/H+1) log scale

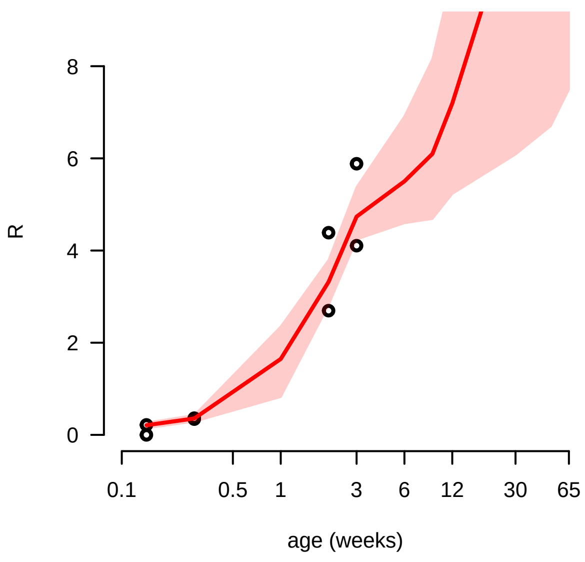

short pulse: ratio ln(L/H+1)

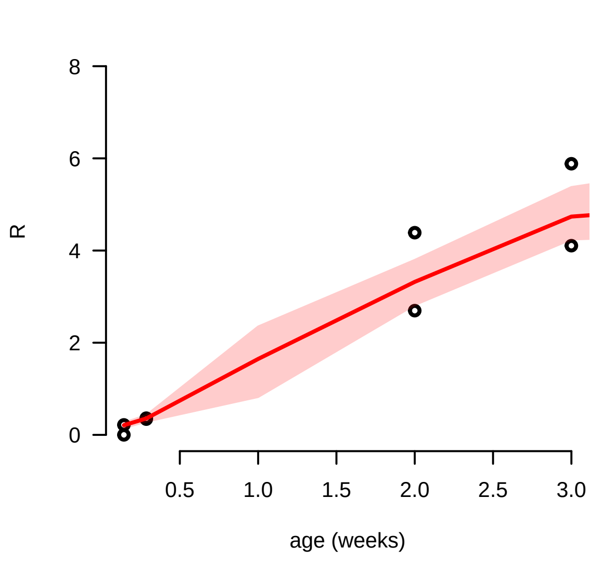

protein concentration log scale

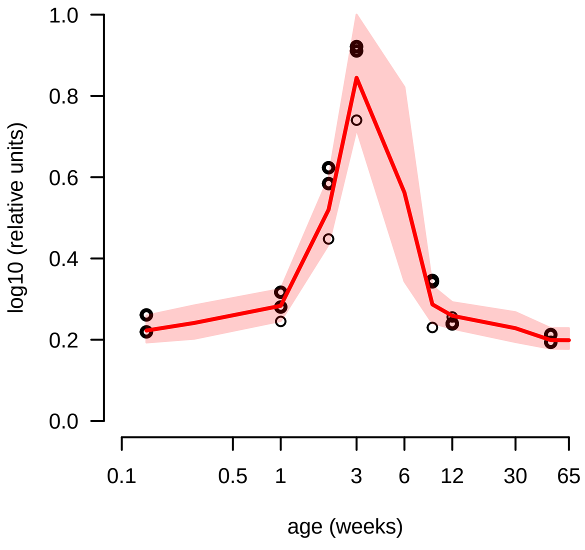

total protein log scale

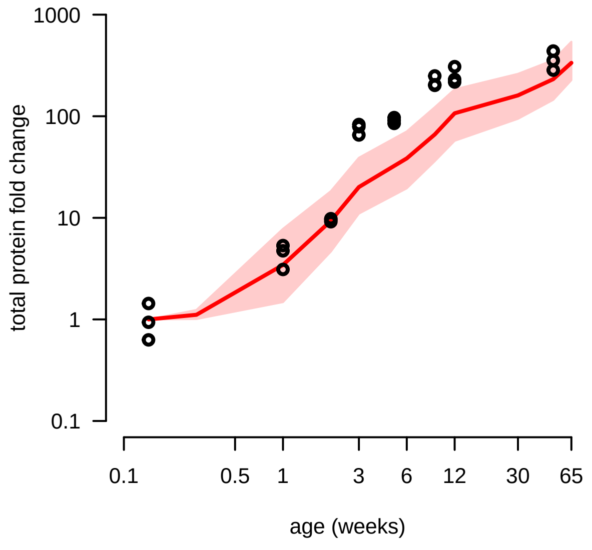

short pulse %H corrected log scale

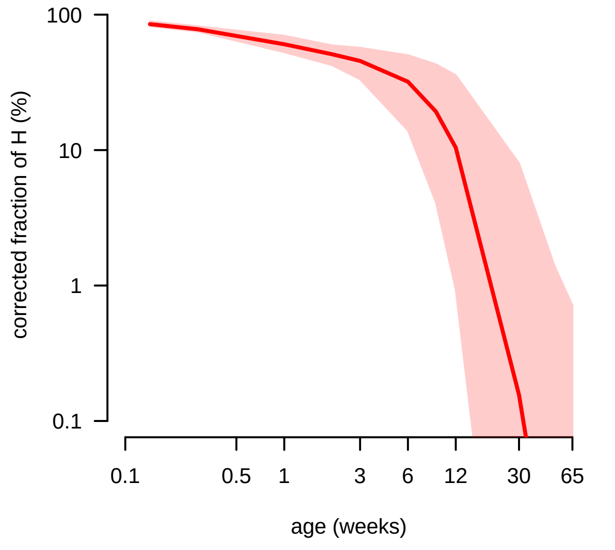

O89110

>sp|O89110|CASP8_MOUSE

Caspase−8

GN=Casp8

Med H1/2: 13.1 days

Q5 H1/2: 5 days

Q95 H1/2: 93.4 days

reliable model fit: no

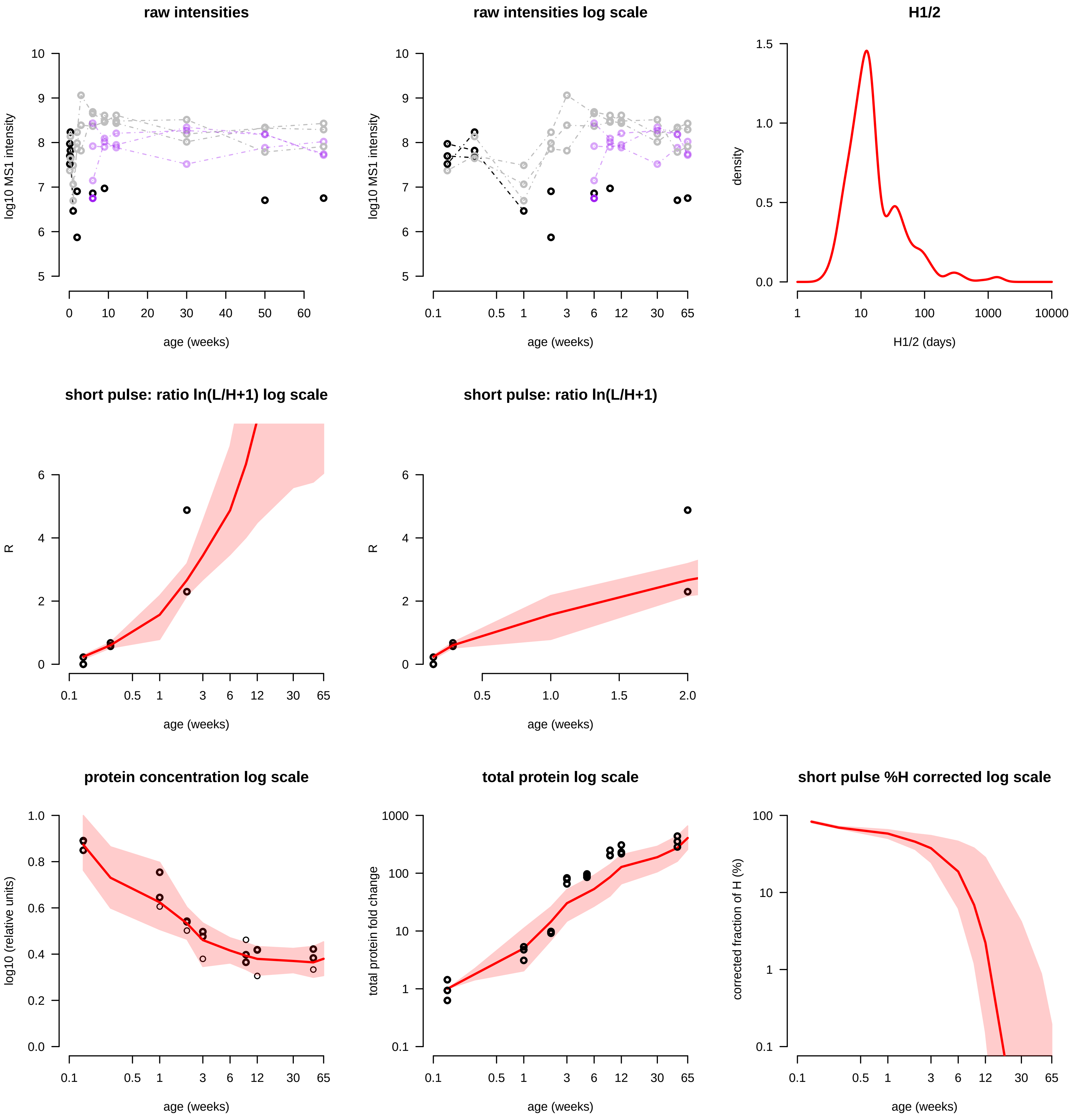

Q9Z0H8−2

>sp|Q9Z0H8−2|CLIP2_MOUSE

Isoform 2 of CAP−Gly domain−containing linker protein 2

GN=Clip2

Med H1/2: 4.7 days

Q5 H1/2: 2.6 days

Q95 H1/2: 24 days

reliable model fit: yes

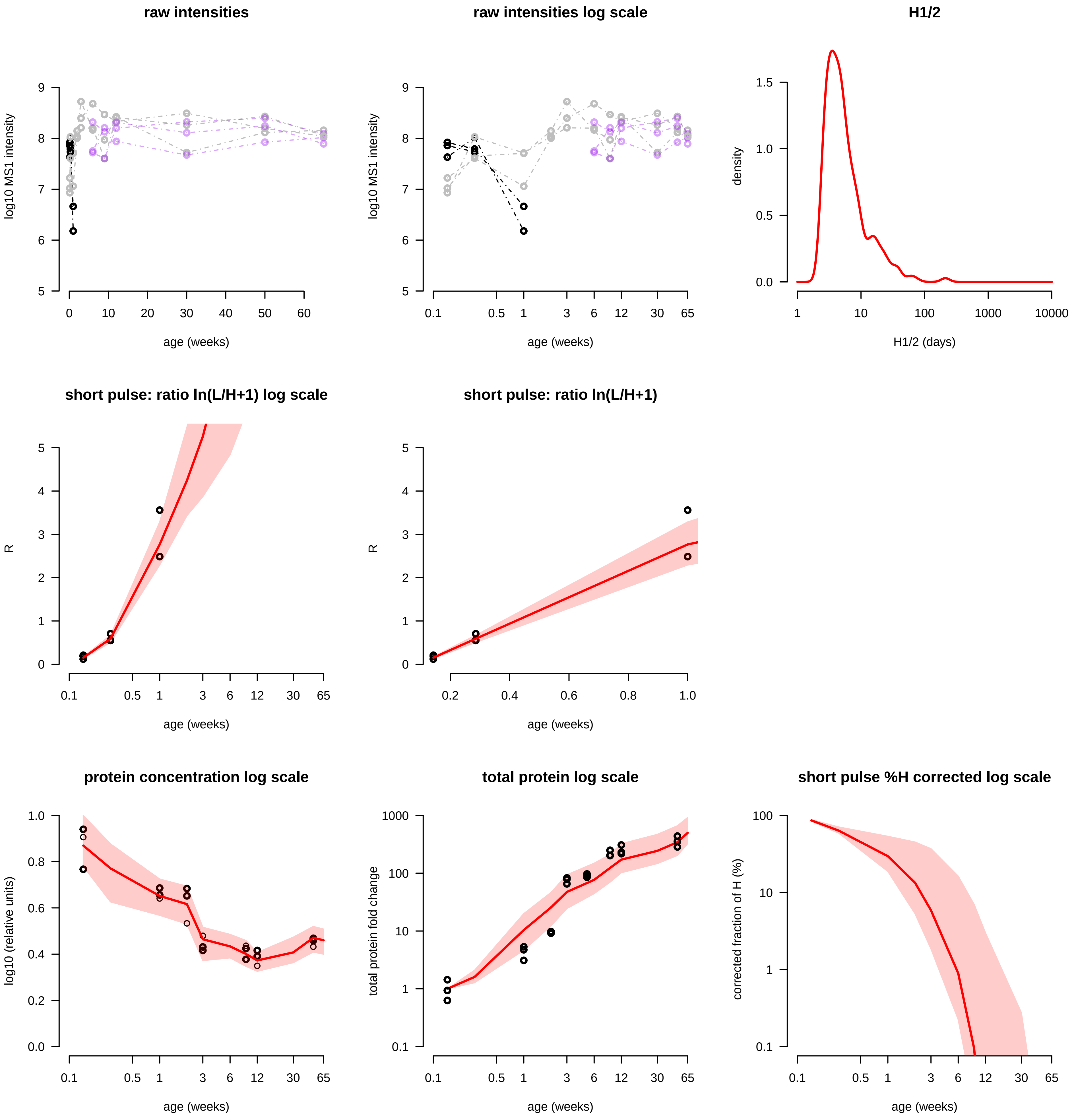

Q80U87

>sp|Q80U87|UBP8_MOUSE

Ubiquitin carboxyl−terminal hydrolase 8

GN=Usp8

Med H1/2: 4.6 days

Q5 H1/2: 2.6 days

Q95 H1/2: 19.7 days

reliable model fit: yes

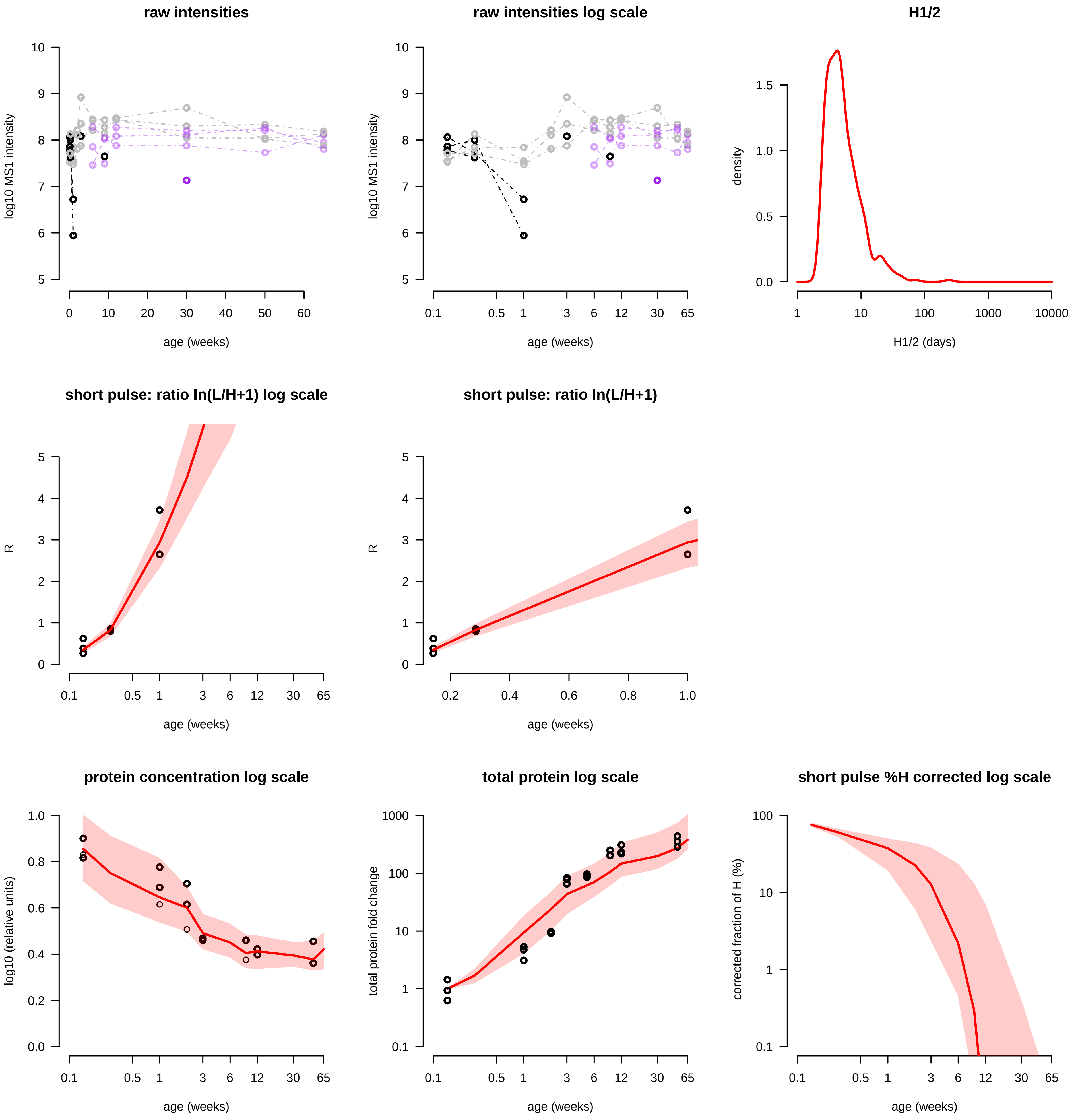

Q9CQE7;Q9CQE7−2

>sp|Q9CQE7|ERGI3_MOUSE

Endoplasmic reticulum−Golgi intermediate compartment protein 3

GN=Ergic3

Med H1/2: 8.1 days

Q5 H1/2: 3.1 days

Q95 H1/2: 119.5 days

reliable model fit: yes

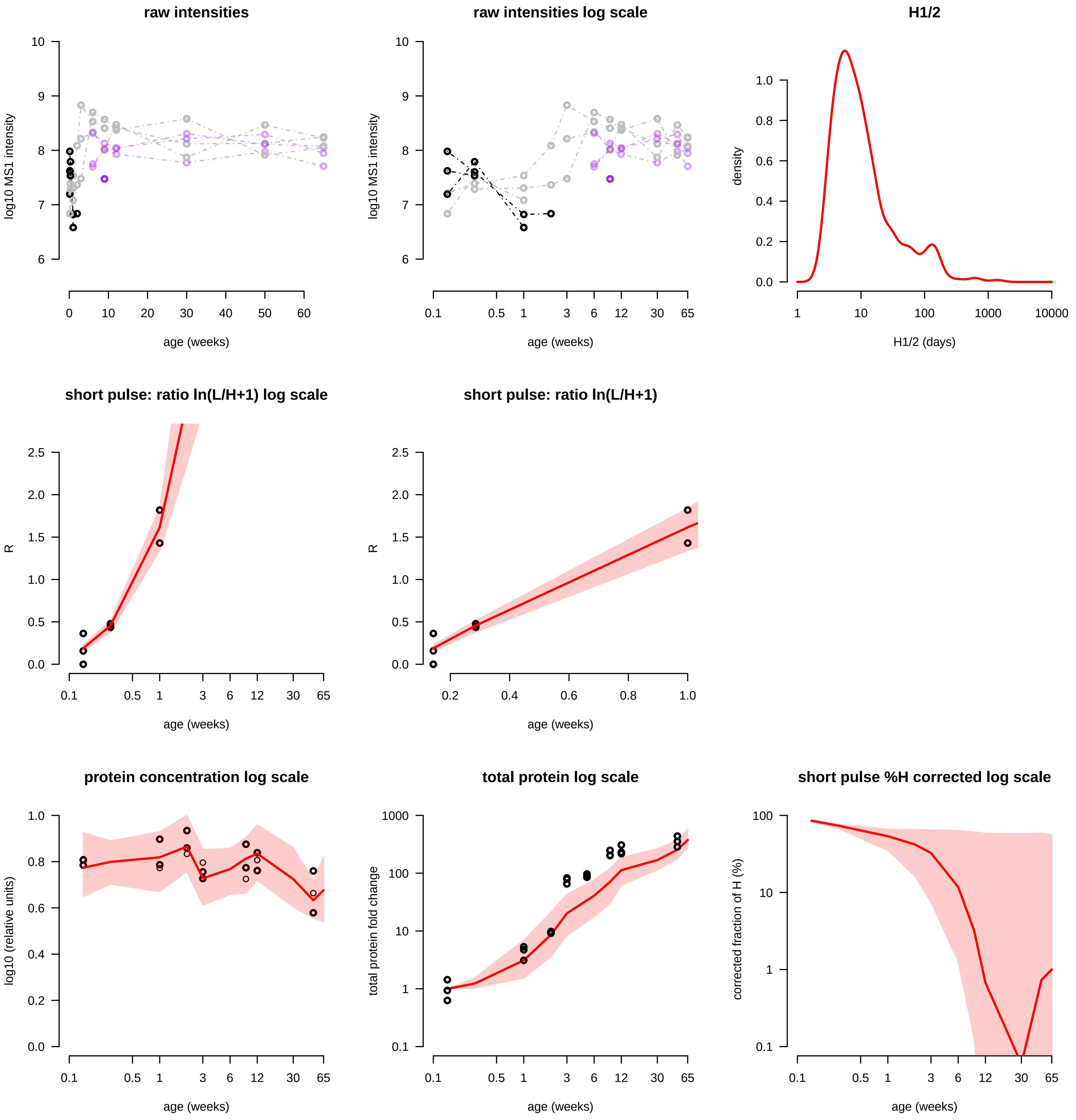

Q9DC48

>sp|Q9DC48|PRP17_MOUSE

Pre−mRNA−processing factor 17

GN=Cdc40

Med H1/2: 82.2 days

Q5 H1/2: 24.2 days

Q95 H1/2: 1010.8 days

reliable model fit: no

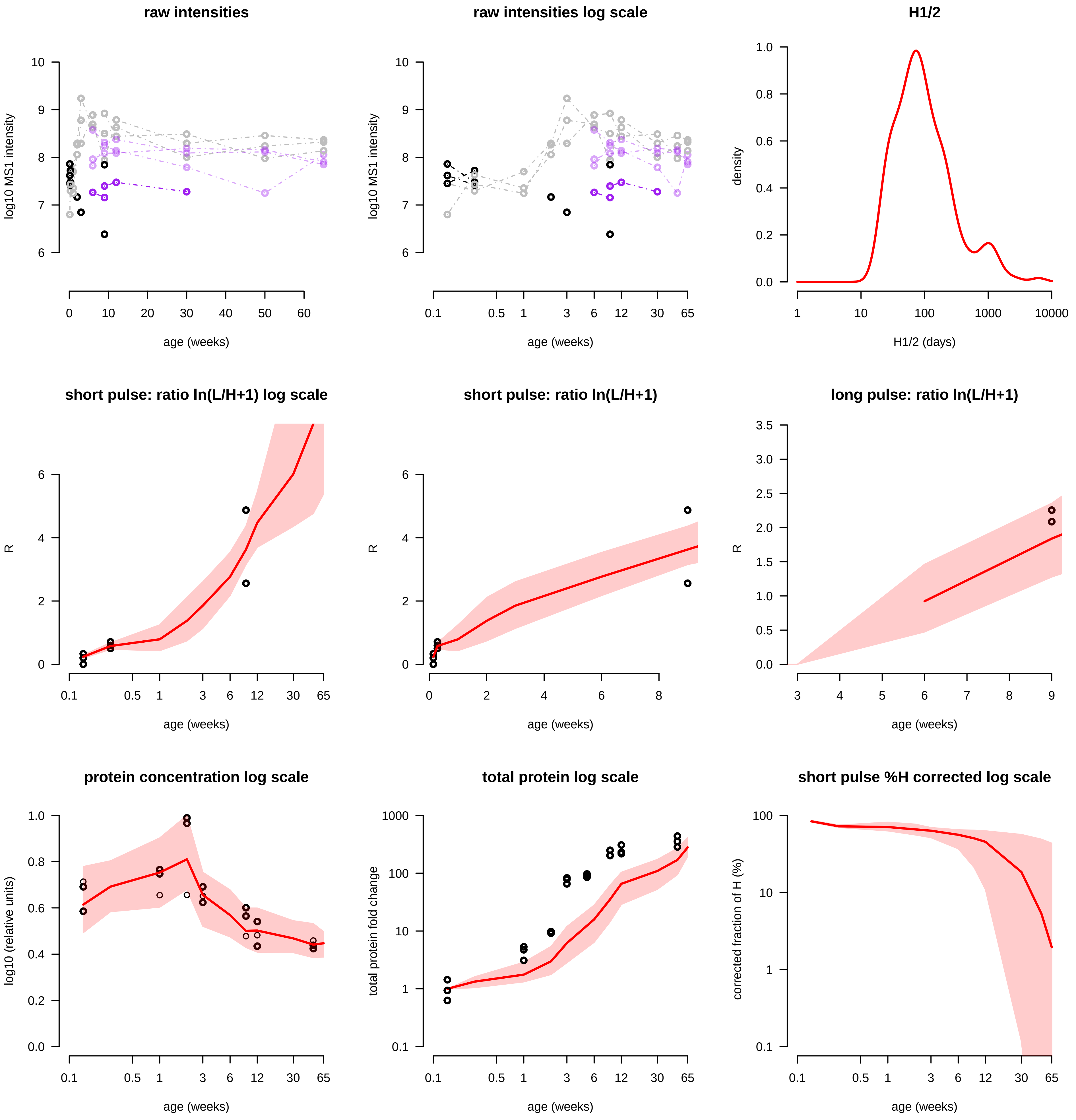

P40240

>sp|P40240|CD9_MOUSE

CD9 antigen

GN=Cd9

Med H1/2: 15.8 days

Q5 H1/2: 4.3 days

Q95 H1/2: 136.7 days

reliable model fit: yes

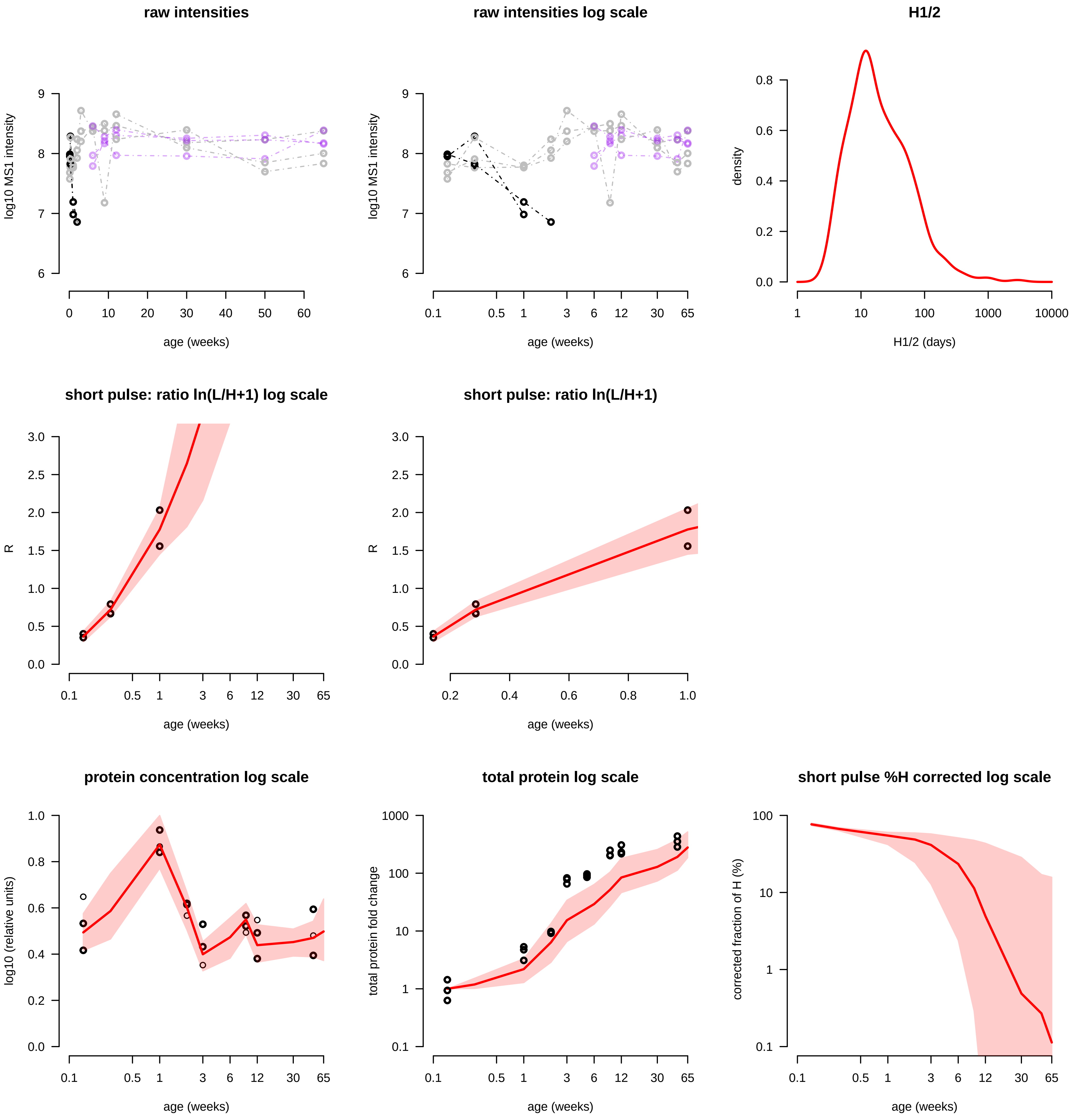

O55060

>sp|O55060|TPMT_MOUSE

Thiopurine S−methyltransferase

GN=Tpmt

Med H1/2: 39.4 days

Q5 H1/2: 19 days

Q95 H1/2: 150.2 days

reliable model fit: yes

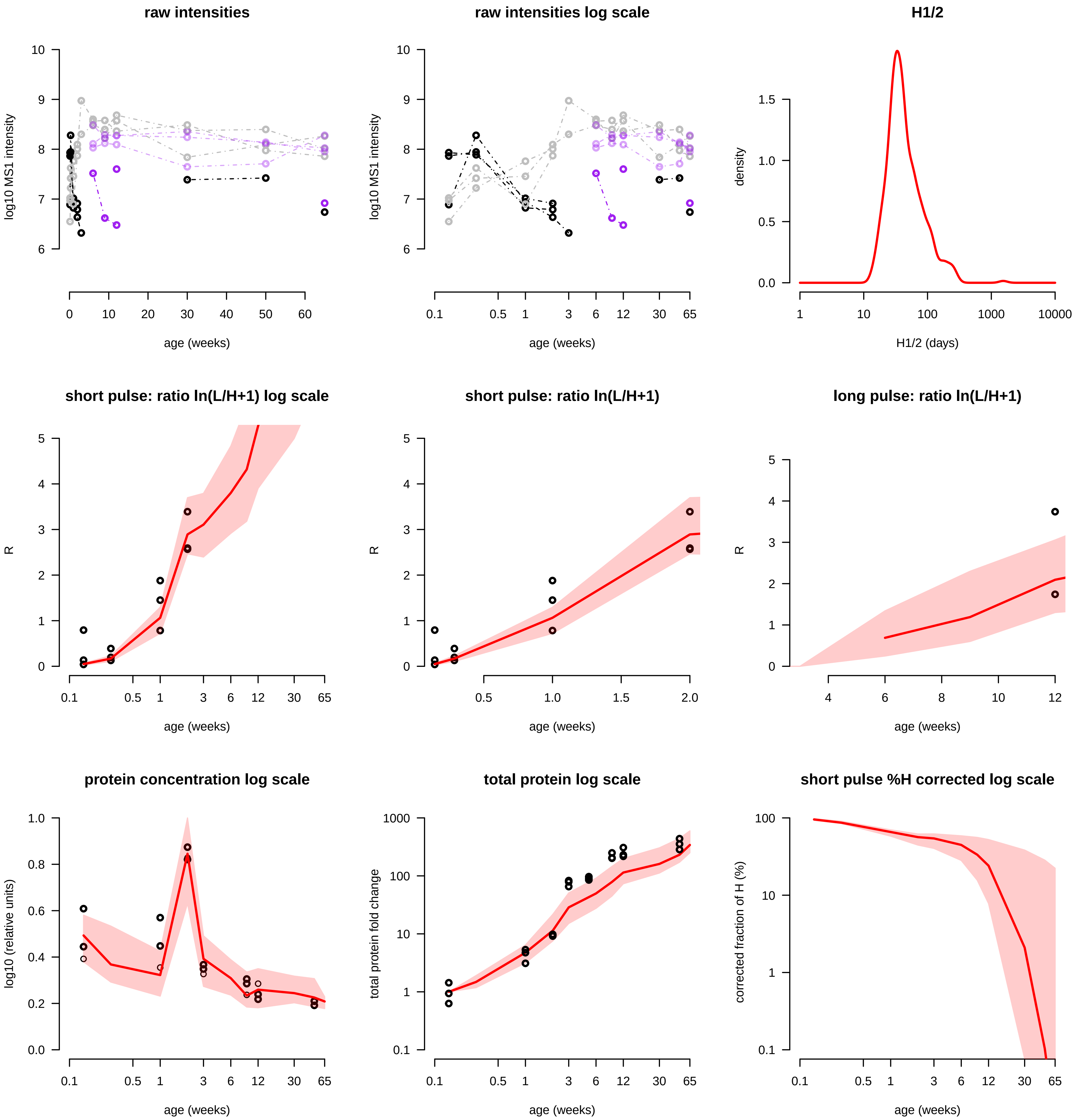

Q9CYW4

>sp|Q9CYW4|HDHD3_MOUSE

Haloacid dehalogenase−like hydrolase domain−containing protein 3

GN=Hdhd3

Med H1/2: 7.6 days

Q5 H1/2: 3.2 days

Q95 H1/2: 72.8 days

reliable model fit: yes

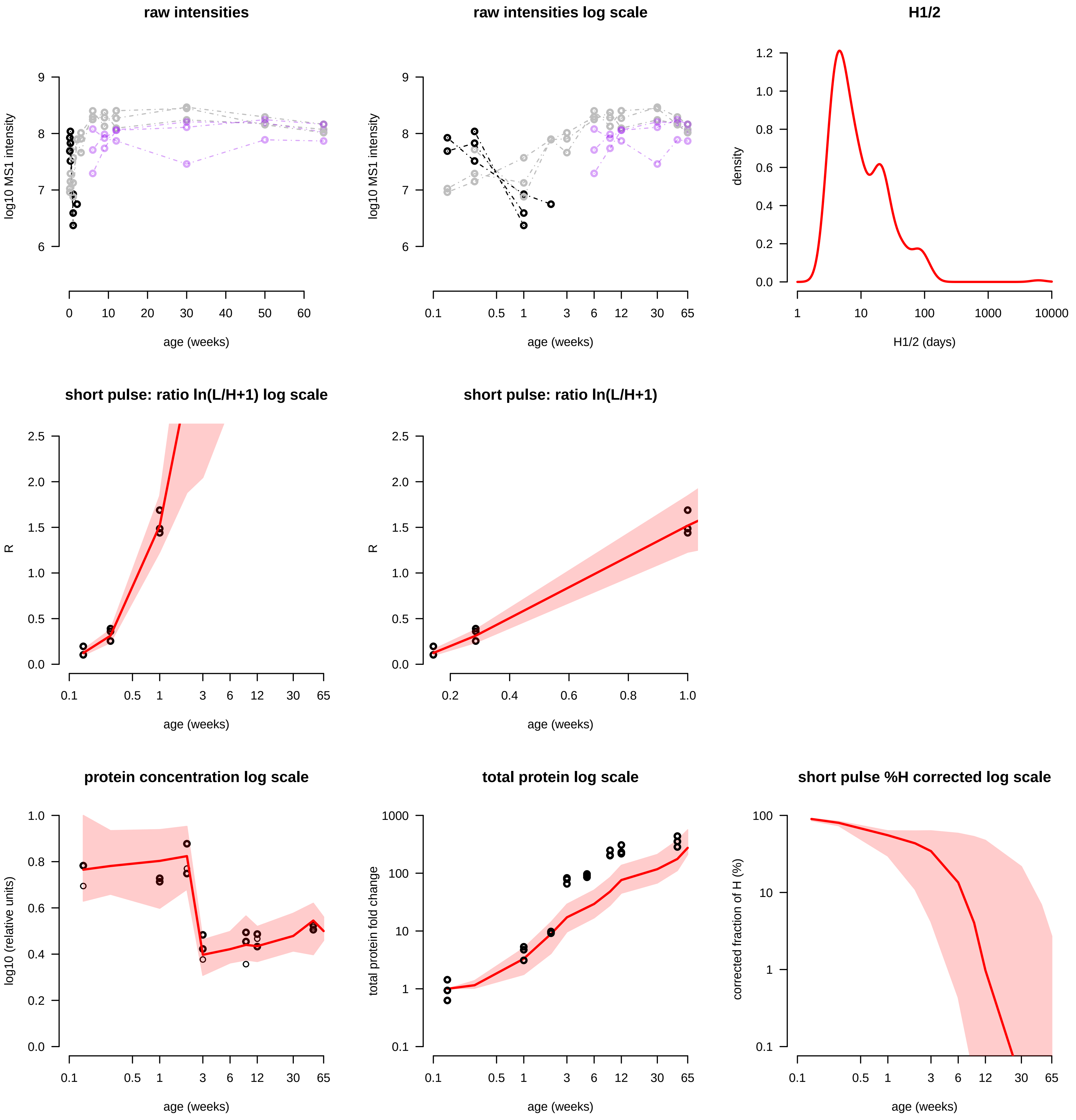

Q9D1H7;Q9D1H7−2

>sp|Q9D1H7|GET4_MOUSE

Golgi to ER traffic protein 4 homolog

GN=Get4

Med H1/2: 4.4 days

Q5 H1/2: 3.4 days

Q95 H1/2: 6 days

reliable model fit: yes

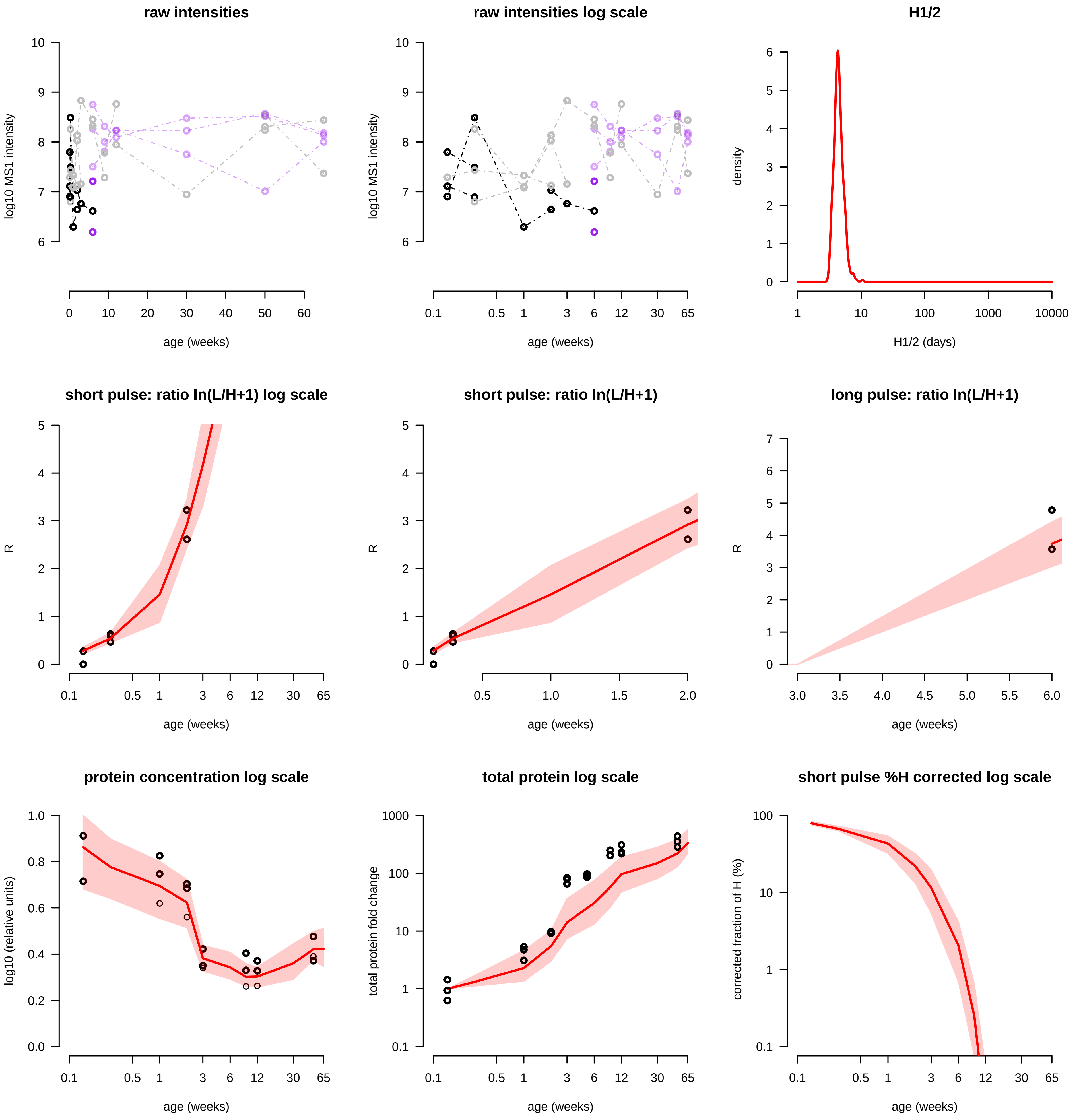

Q8R3C0

>sp|Q8R3C0|MCMBP_MOUSE

Mini−chromosome maintenance complex−binding protein

GN=Mcmbp

Med H1/2: 5.7 days

Q5 H1/2: 2.7 days

Q95 H1/2: 46 days

reliable model fit: yes

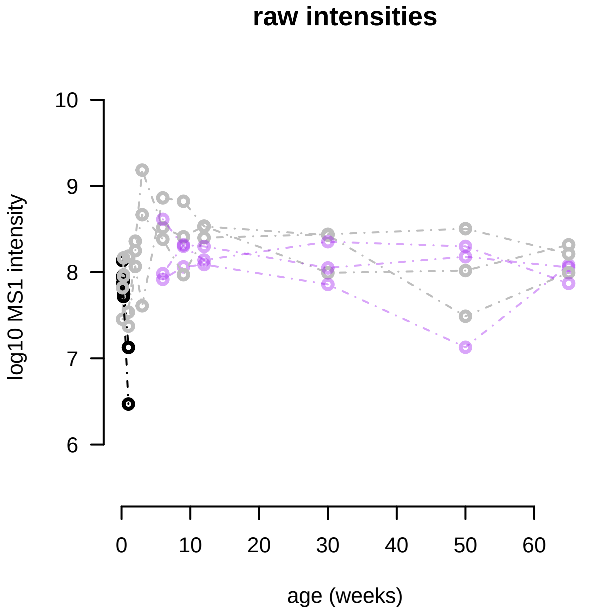

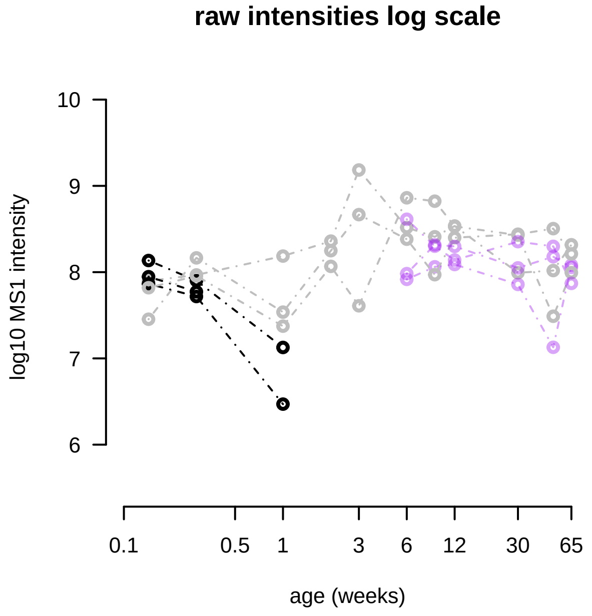

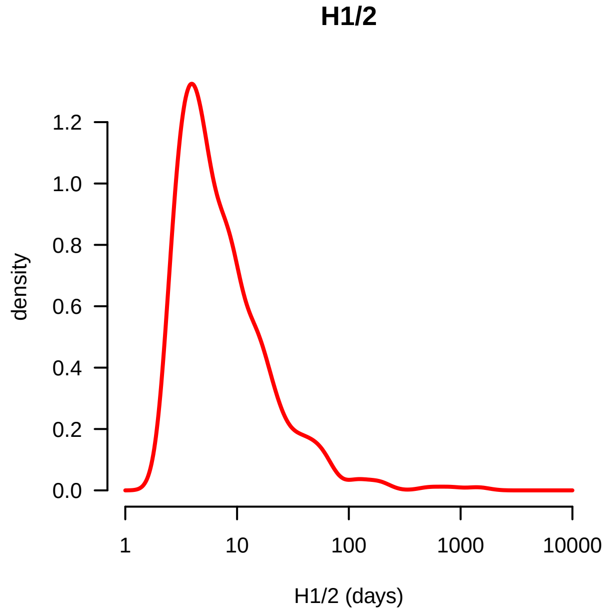

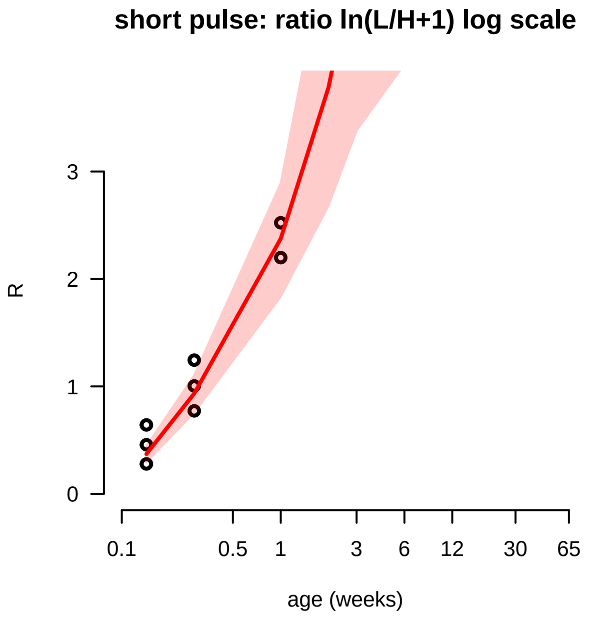

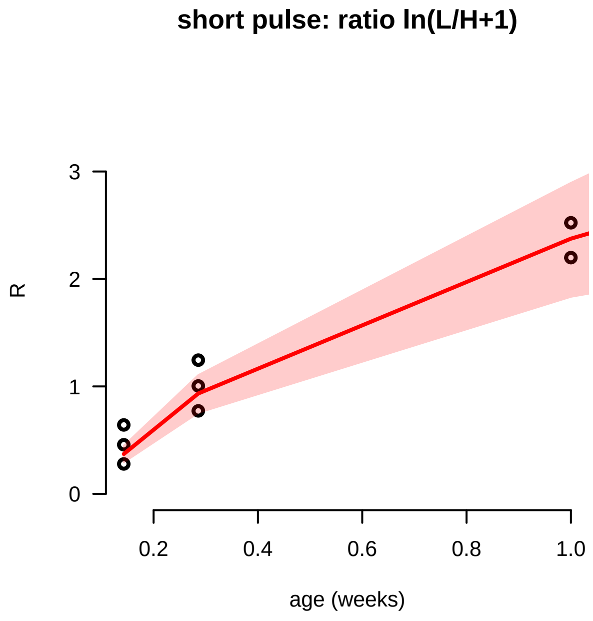

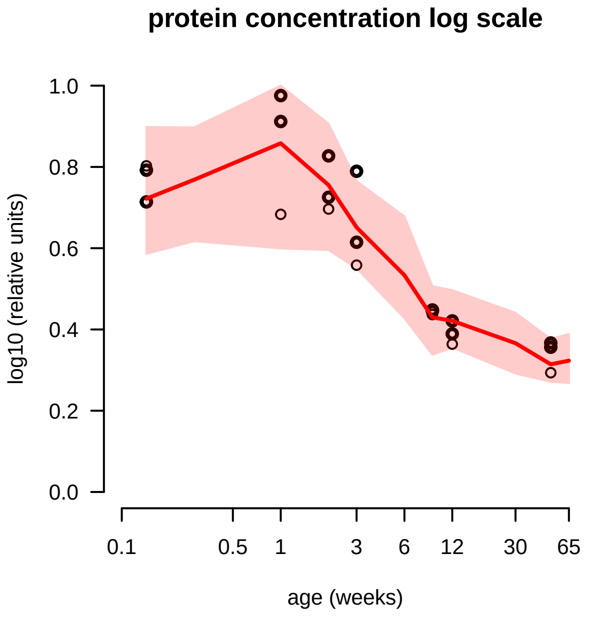

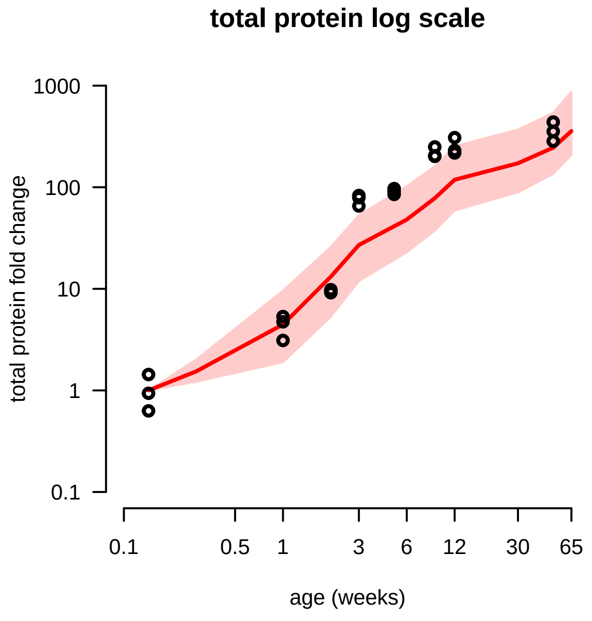

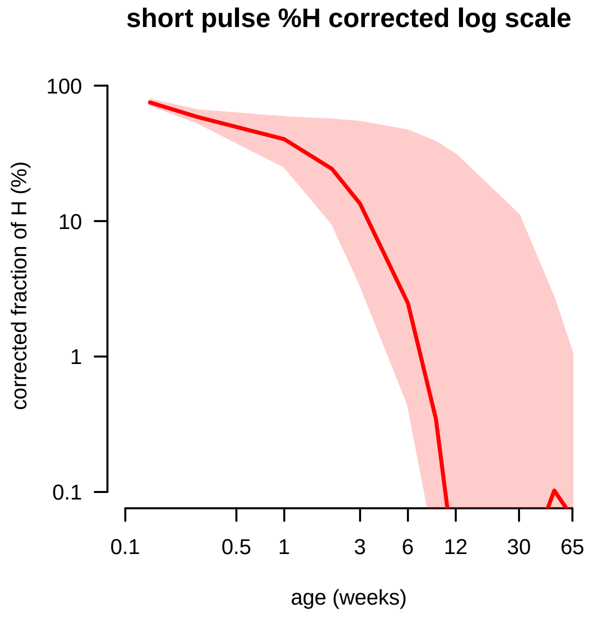

Q9WUK2−2;Q9WUK2

>sp|Q9WUK2−2|IF4H_MOUSE

Isoform Short of Eukaryotic translation initiation factor 4H

GN=Eif4h

Med H1/2: 12.6 days

Q5 H1/2: 3.7 days

Q95 H1/2: 149.2 days

reliable model fit: yes

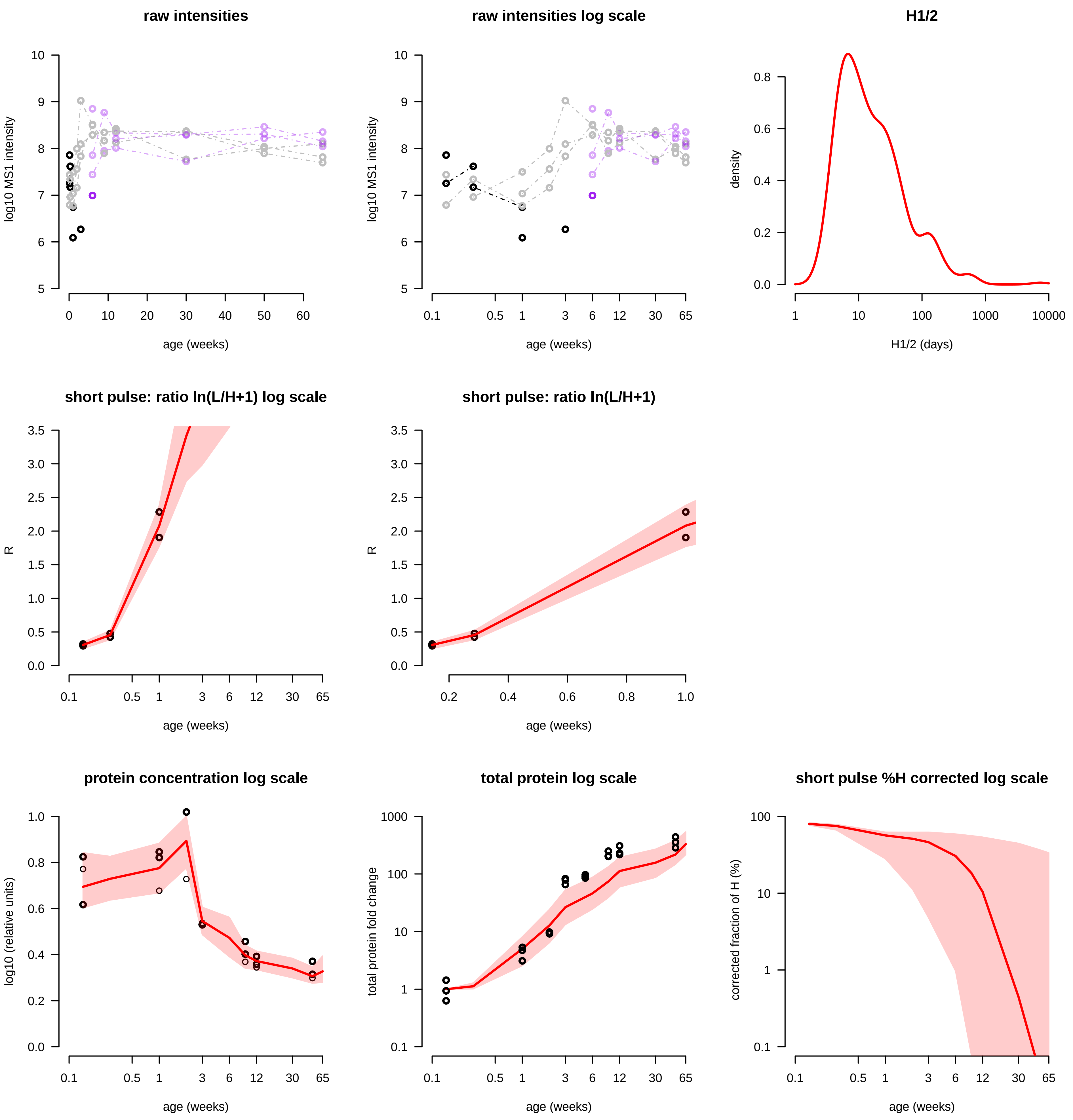

Q9JLB0−2;Q9JLB0

>sp|Q9JLB0−2|PALS2_MOUSE

Isoform Alpha of Protein PALS2

GN=Pals2

Med H1/2: 6.7 days

Q5 H1/2: 2.7 days

Q95 H1/2: 89.7 days

reliable model fit: yes

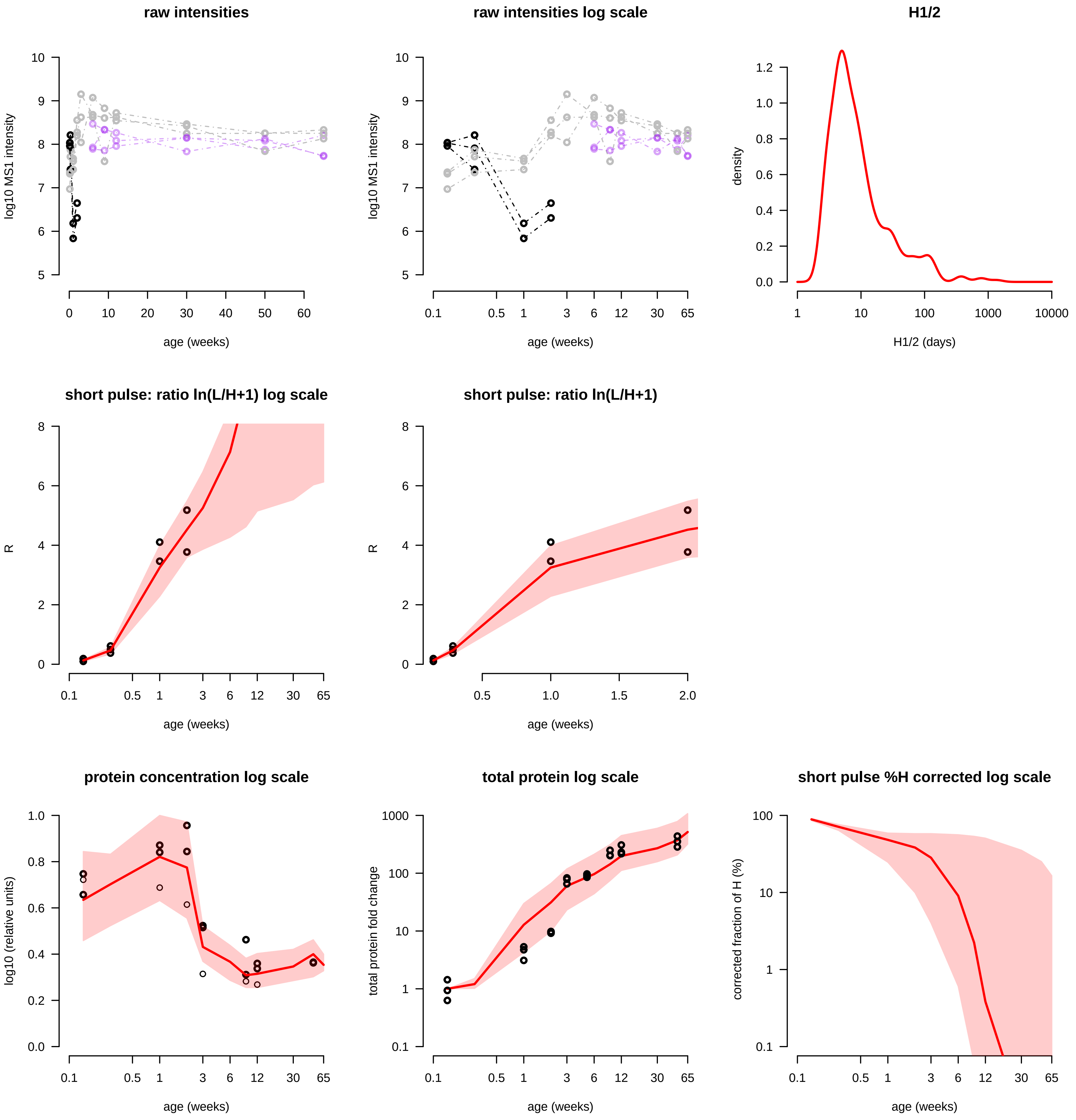

P70236

>sp|P70236|MP2K6_MOUSE

Dual specificity mitogen−activated protein kinase kinase 6

GN=Map2k6

Med H1/2: 10.8 days

Q5 H1/2: 3.7 days

Q95 H1/2: 71.1 days

reliable model fit: yes

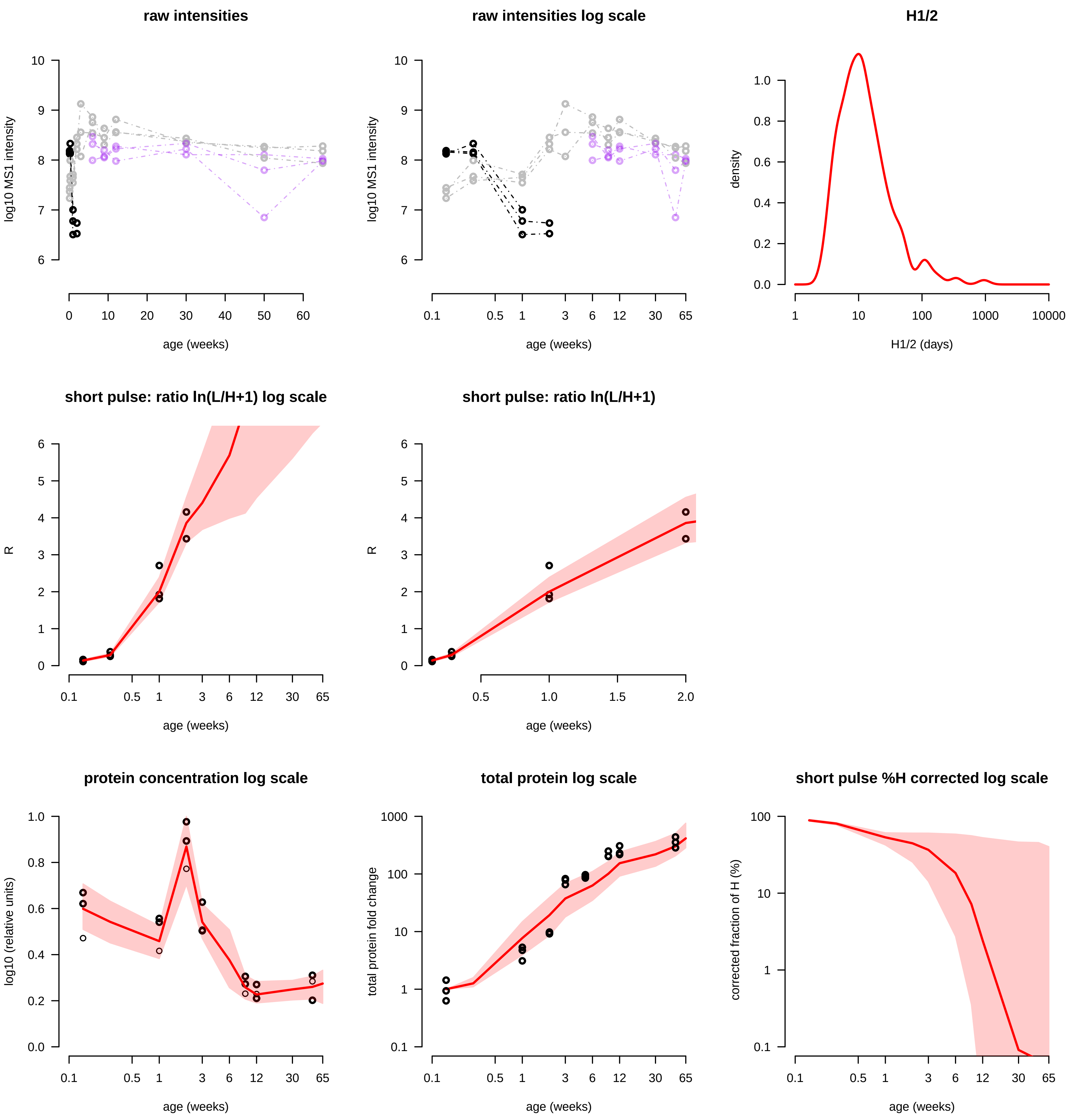

Q8K2V6;Q8K2V6−2

>sp|Q8K2V6|IPO11_MOUSE

Importin−11

GN=Ipo11

Med H1/2: 3.1 days

Q5 H1/2: 2.5 days

Q95 H1/2: 5.5 days

reliable model fit: yes

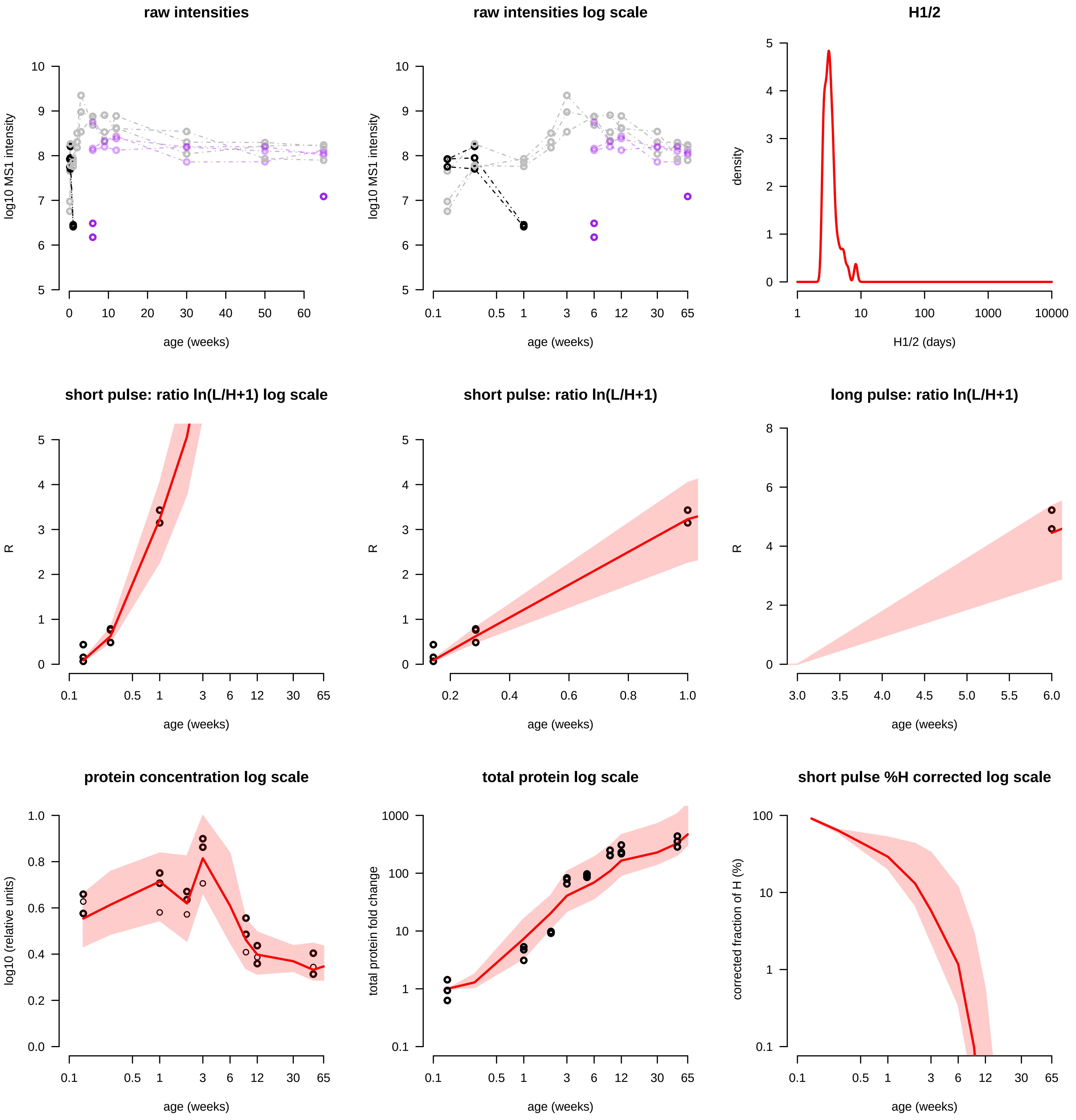

Q61474;Q61474−2

>sp|Q61474|MSI1H_MOUSE

RNA−binding protein Musashi homolog 1

GN=Msi1

Med H1/2: 480.9 days

Q5 H1/2: 140 days

Q95 H1/2: 5341 days

reliable model fit: no

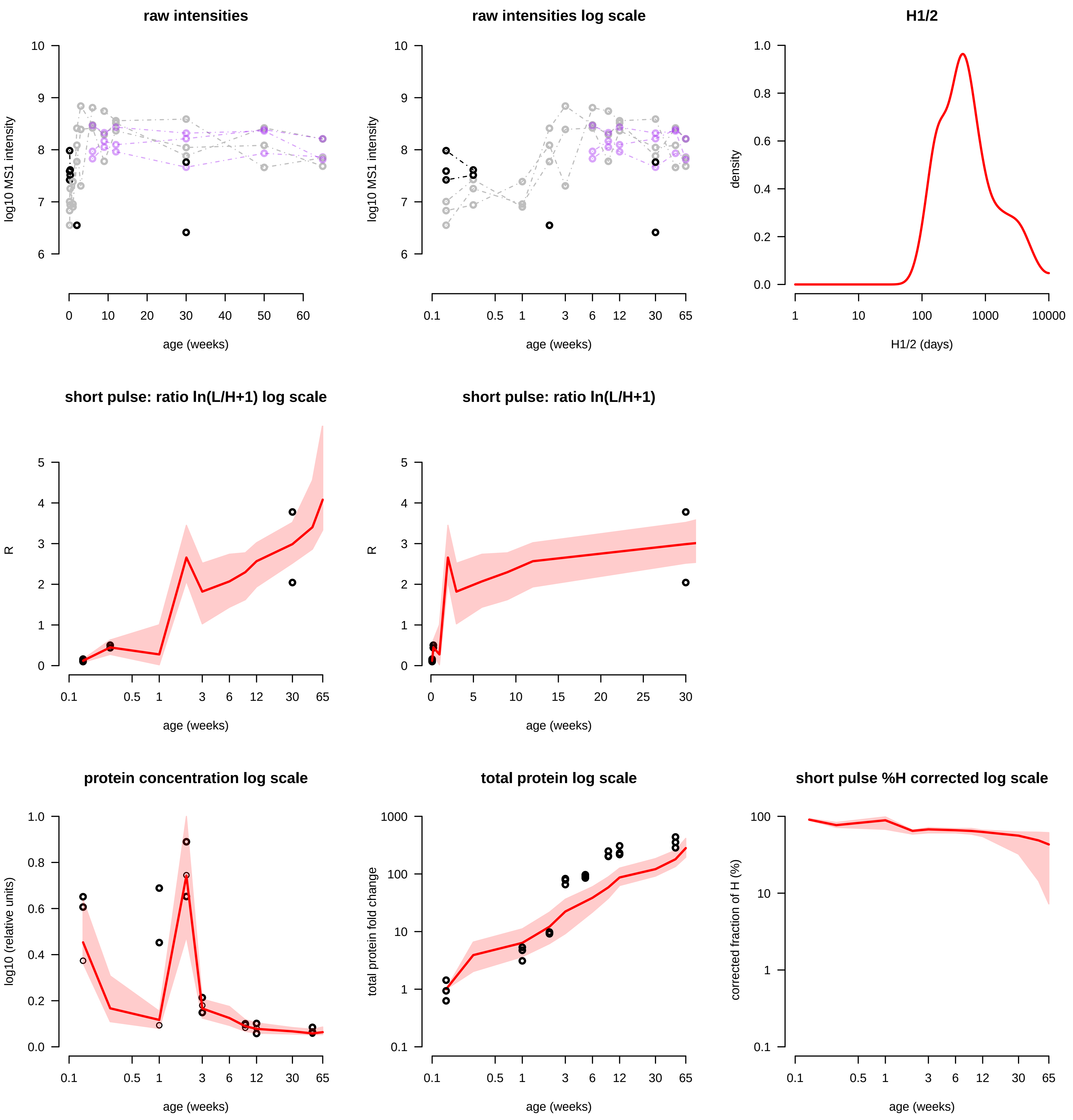

Q8CCM6

>sp|Q8CCM6|TIM21_MOUSE

Mitochondrial import inner membrane translocase subunit Tim21

GN=Timm21

Med H1/2: 8.4 days

Q5 H1/2: 3.4 days

Q95 H1/2: 59.8 days

reliable model fit: yes

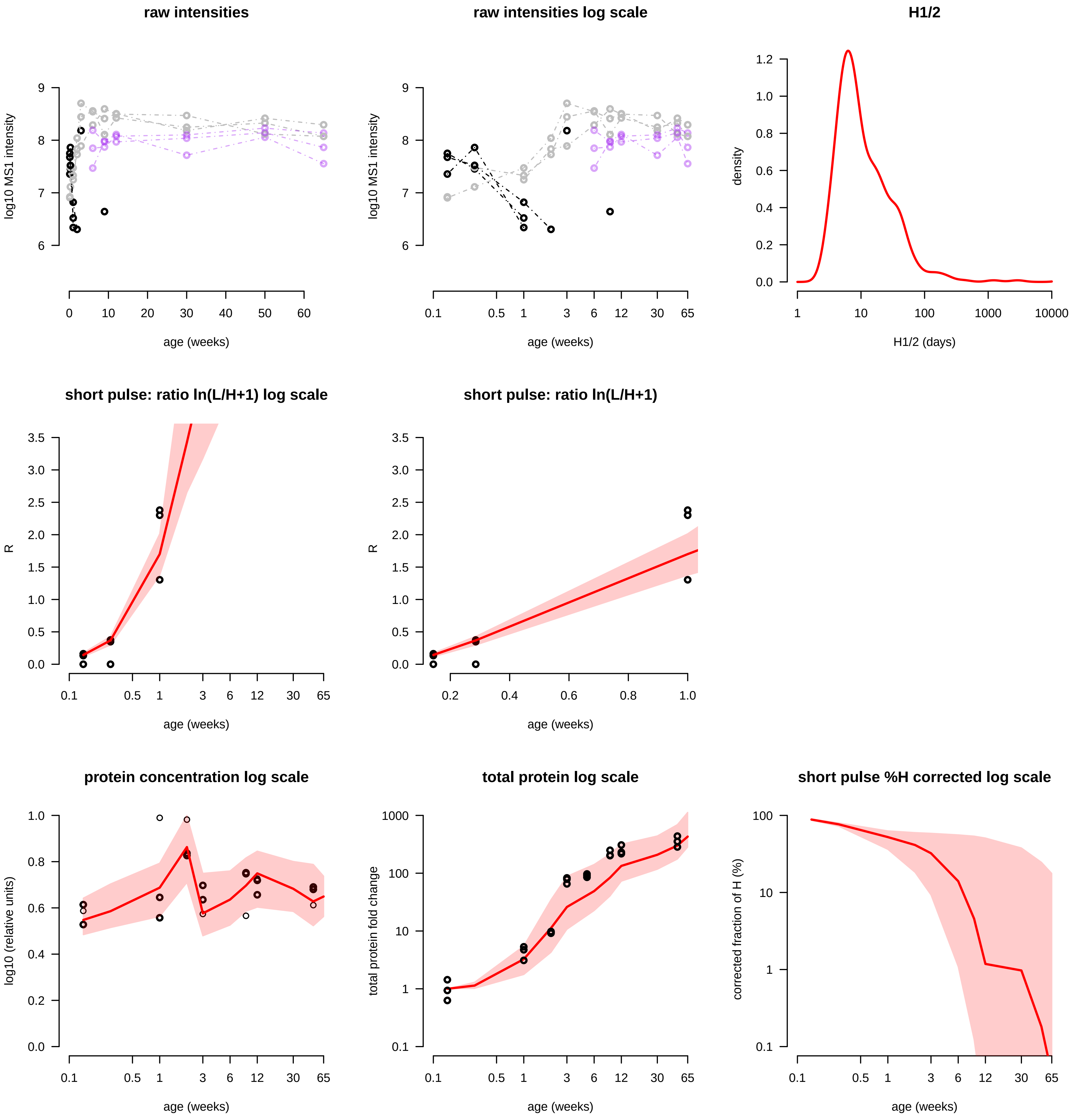

C0HK80;C0HK79

>sp|C0HK80|ARXS2_MOUSE

Adipocyte−related X−chromosome expressed sequence 2

GN=Arxes2

Med H1/2: 5.1 days

Q5 H1/2: 4.2 days

Q95 H1/2: 6.5 days

reliable model fit: yes

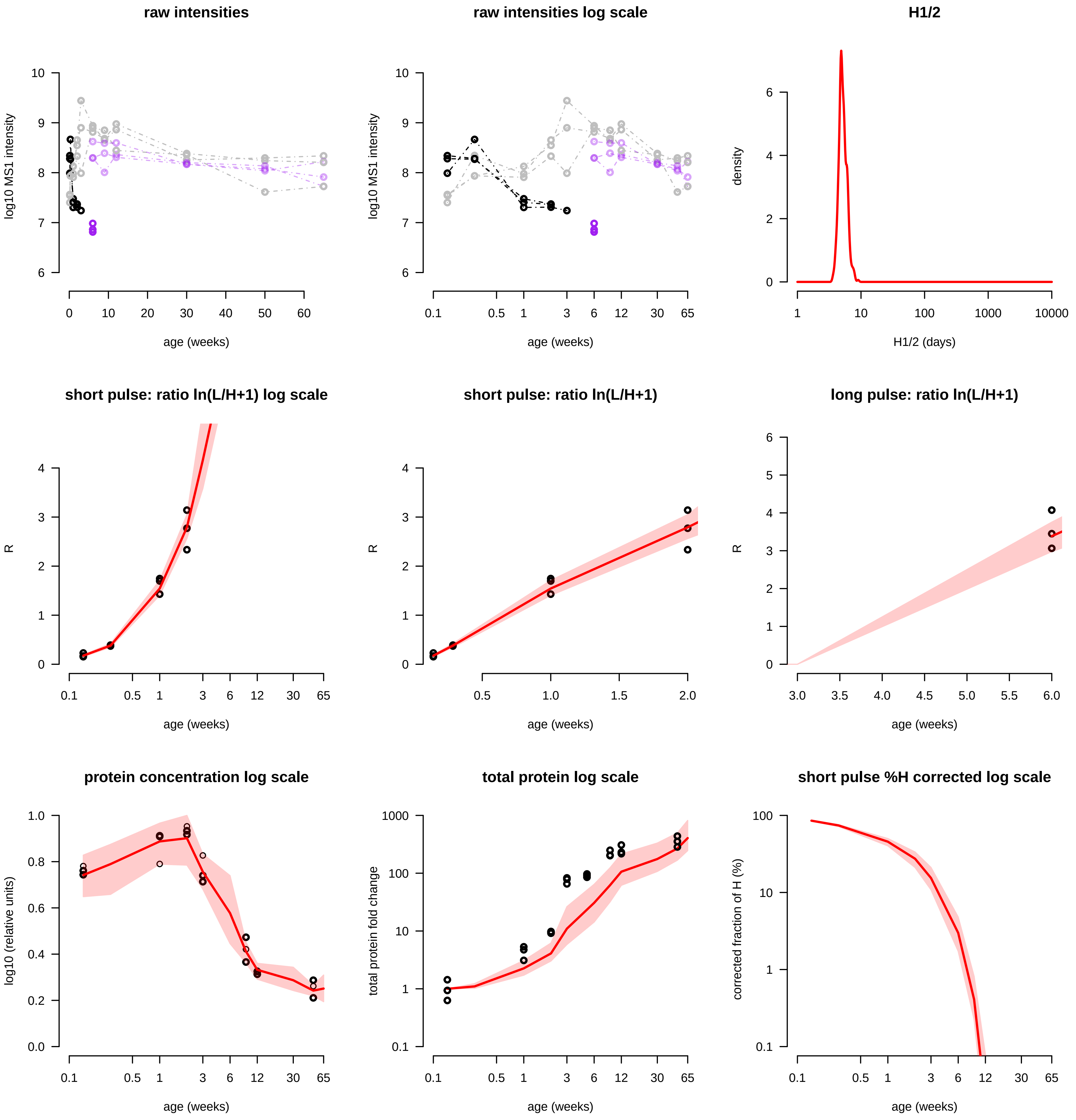

Q61301−2;Q61301−3;Q61301

>sp|Q61301−2|CTNA2_MOUSE

Isoform 2 of Catenin alpha−2

GN=Ctnna2

Med H1/2: 8.9 days

Q5 H1/2: 3.1 days

Q95 H1/2: 64 days

reliable model fit: yes

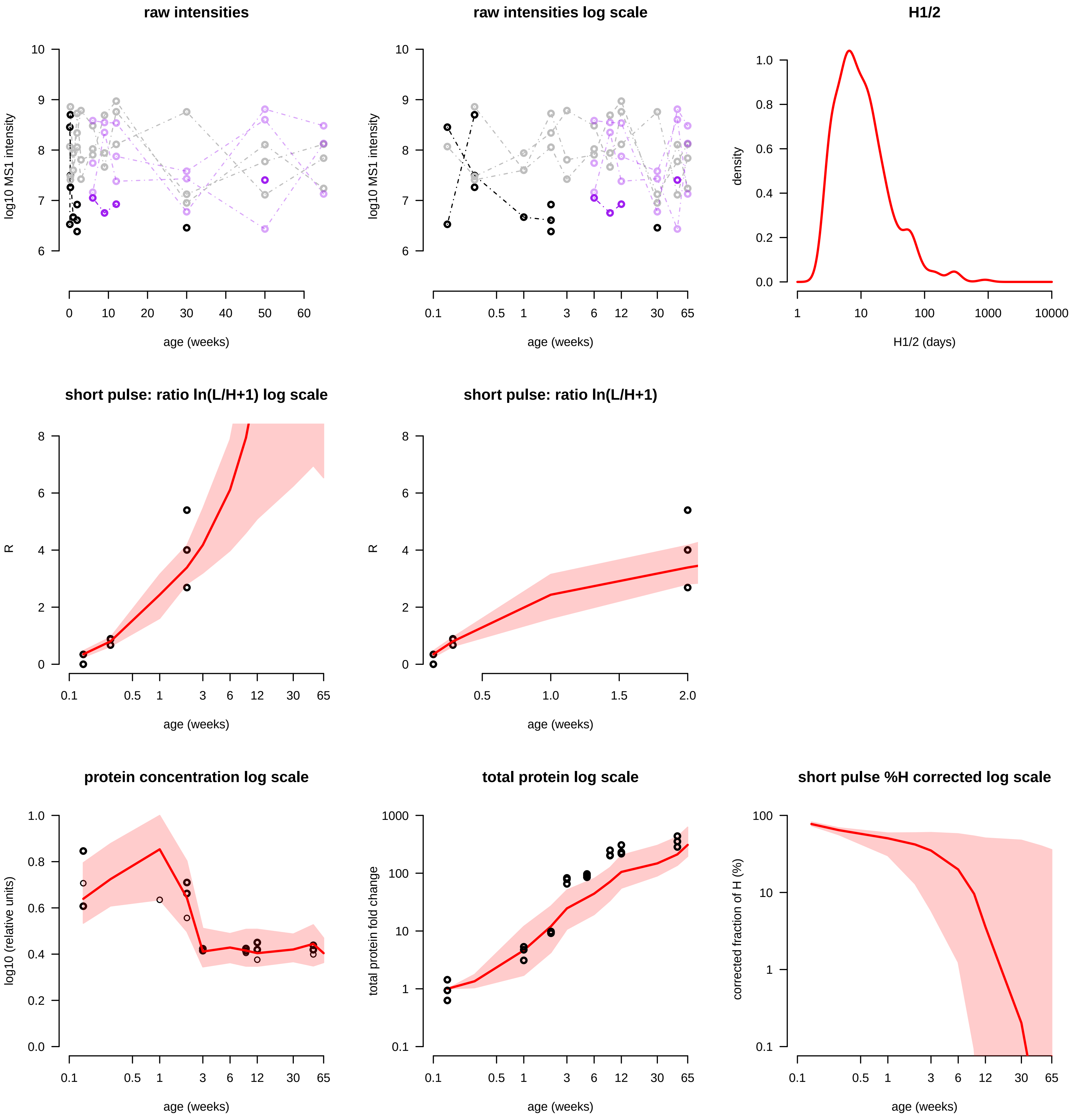

Q8BP27−2;Q8BP27;Q8BP27−3

>sp|Q8BP27−2|SFR1_MOUSE

Isoform 2 of Swi5−dependent recombination DNA repair protein 1 homolog

GN=Sfr1

Med H1/2: 38 days

Q5 H1/2: 9.1 days

Q95 H1/2: 607.1 days

reliable model fit: yes

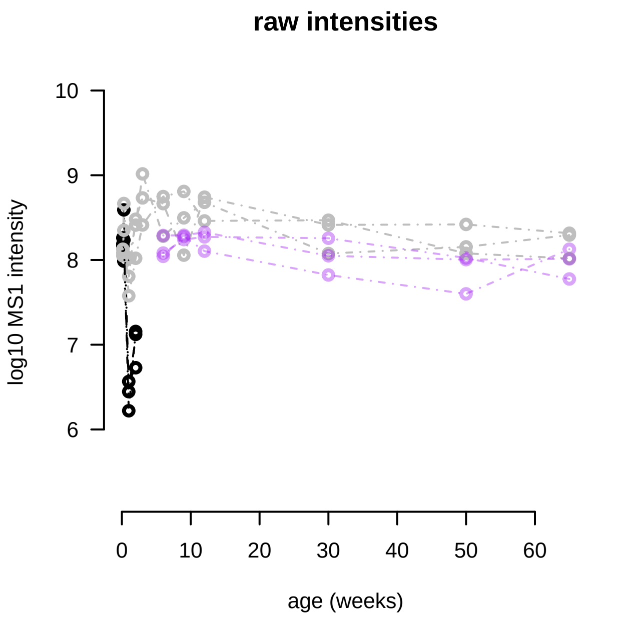

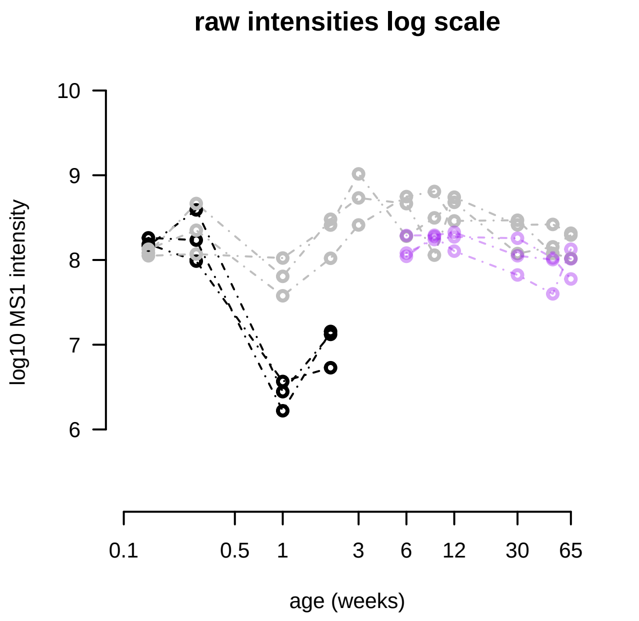

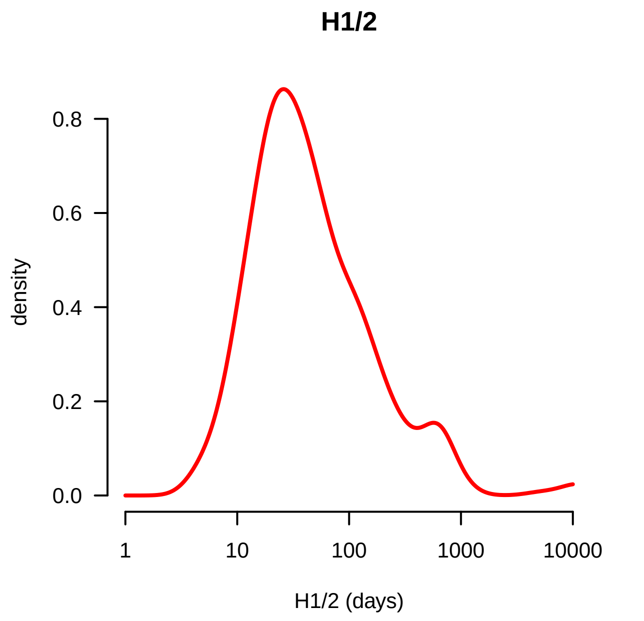

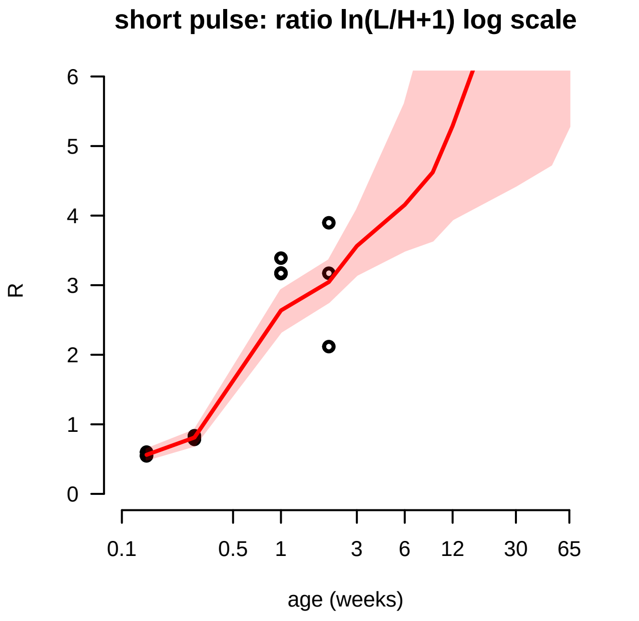

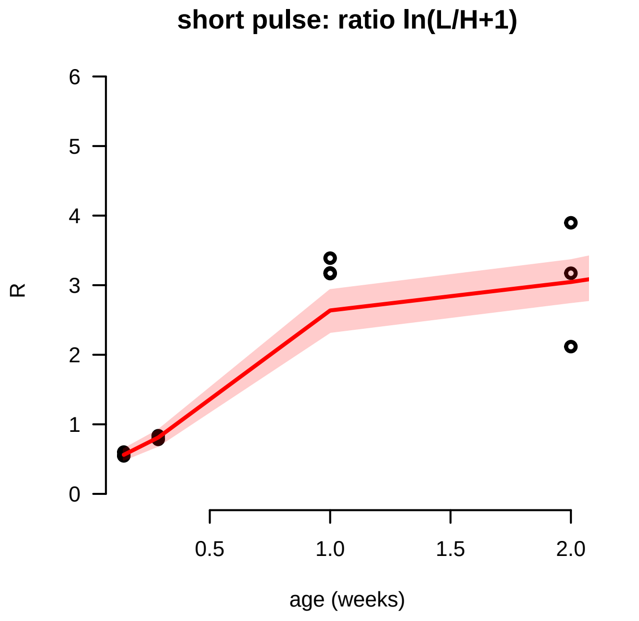

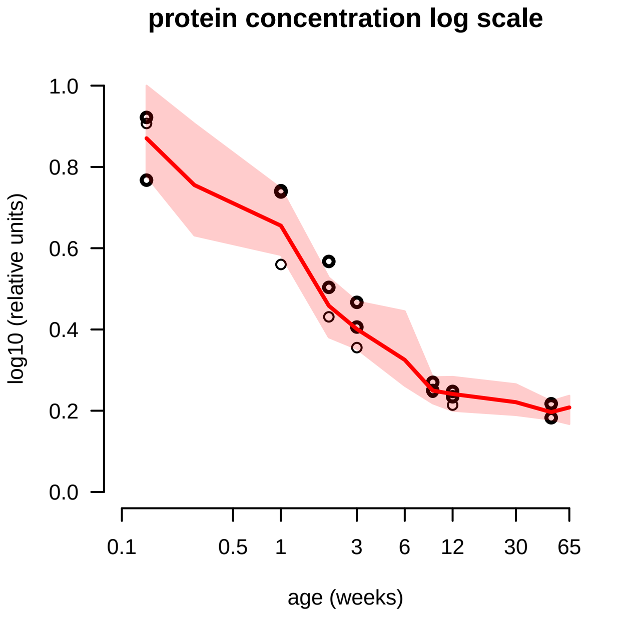

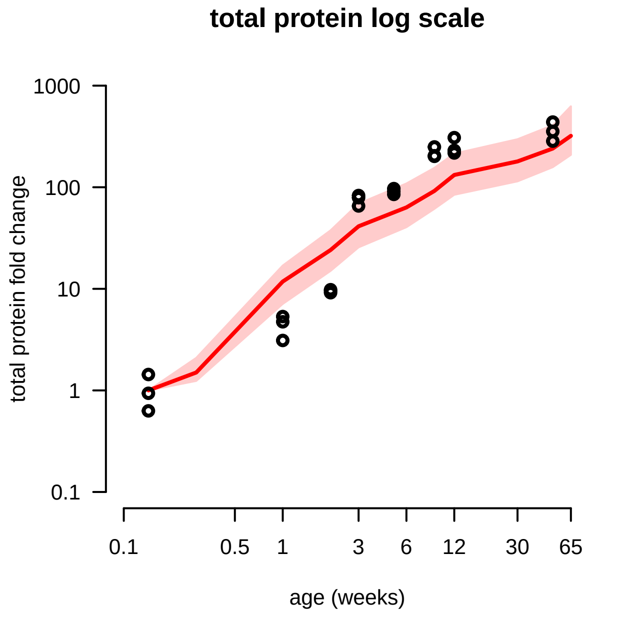

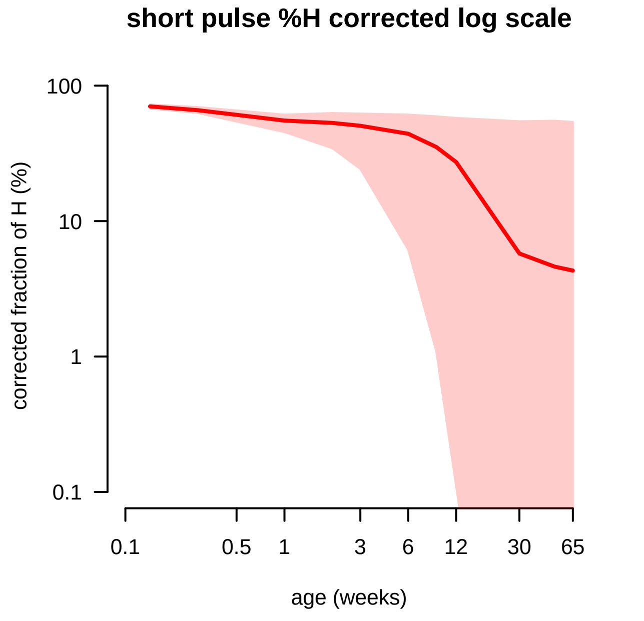

P30285

>sp|P30285|CDK4_MOUSE

Cyclin−dependent kinase 4

GN=Cdk4

Med H1/2: 5.6 days

Q5 H1/2: 2.6 days

Q95 H1/2: 34.4 days

reliable model fit: yes

raw intensities

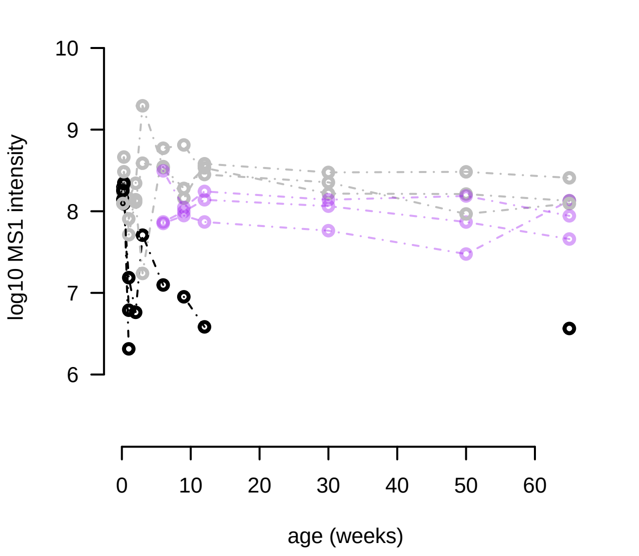

raw intensities log scale

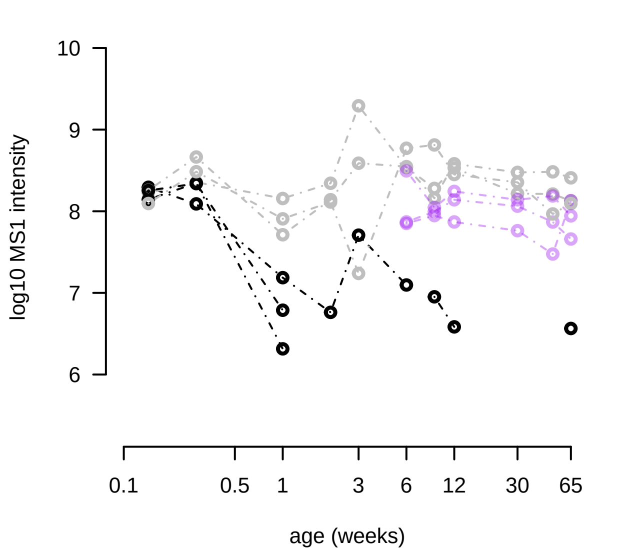

H1/2

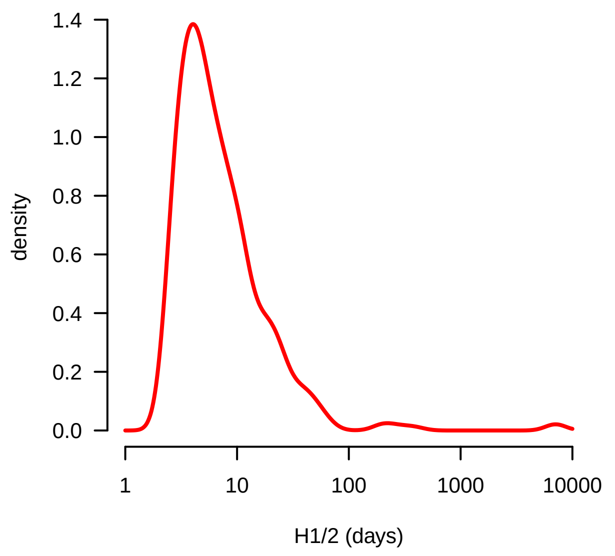

short pulse: ratio ln(L/H+1) log scale

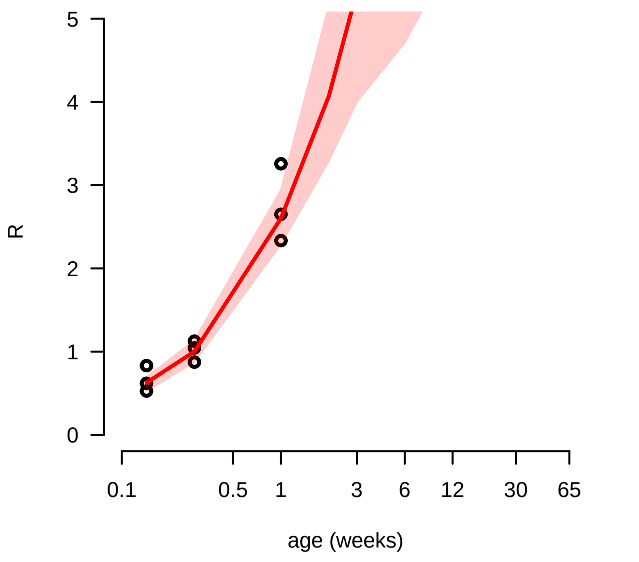

short pulse: ratio ln(L/H+1)

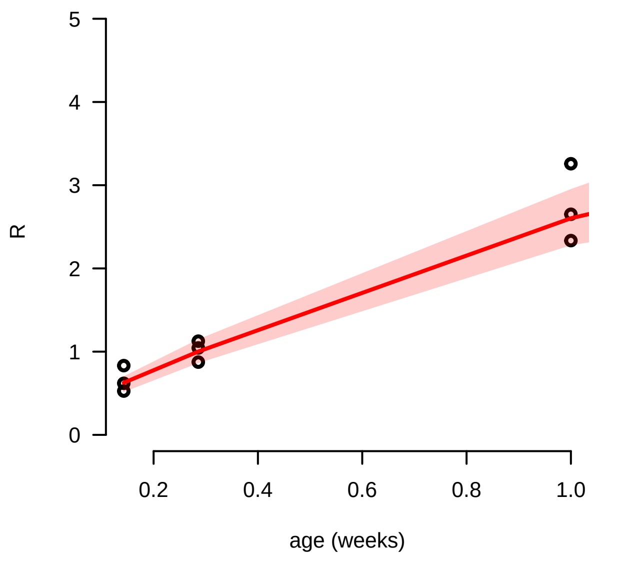

protein concentration log scale

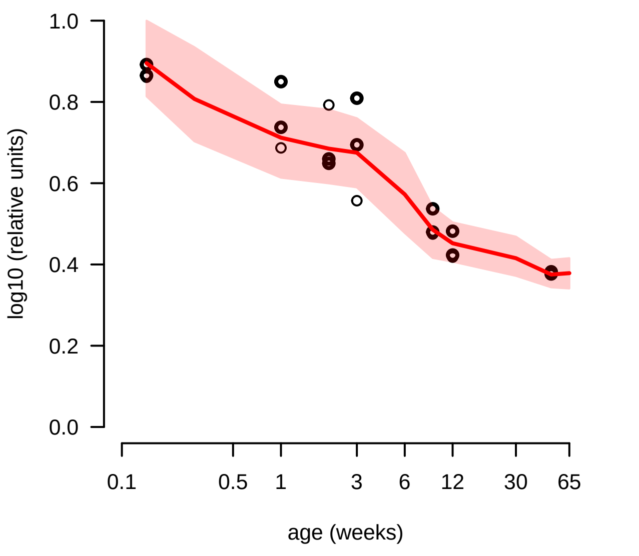

total protein log scale

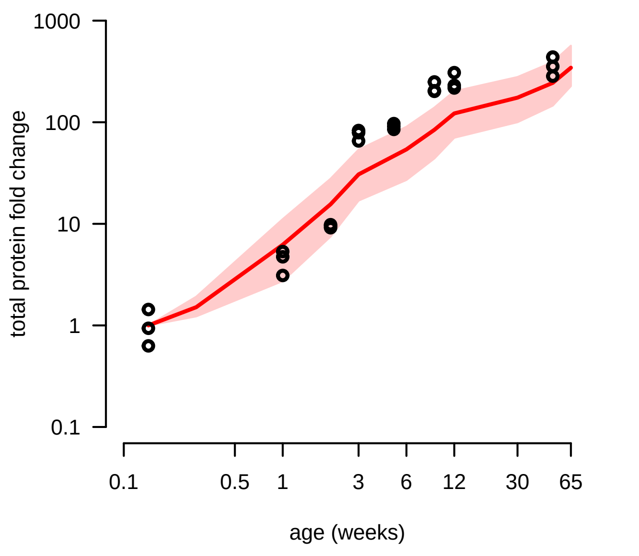

short pulse %H corrected log scale

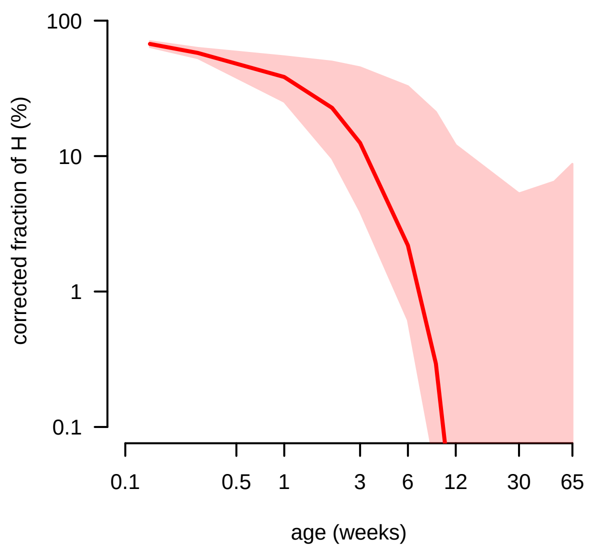

Q6ZPZ3;Q6ZPZ3−2

>sp|Q6ZPZ3|ZC3H4_MOUSE

Zinc finger CCCH domain−containing protein 4

GN=Zc3h4

Med H1/2: 6.1 days

Q5 H1/2: 3.1 days

Q95 H1/2: 72.1 days

reliable model fit: yes

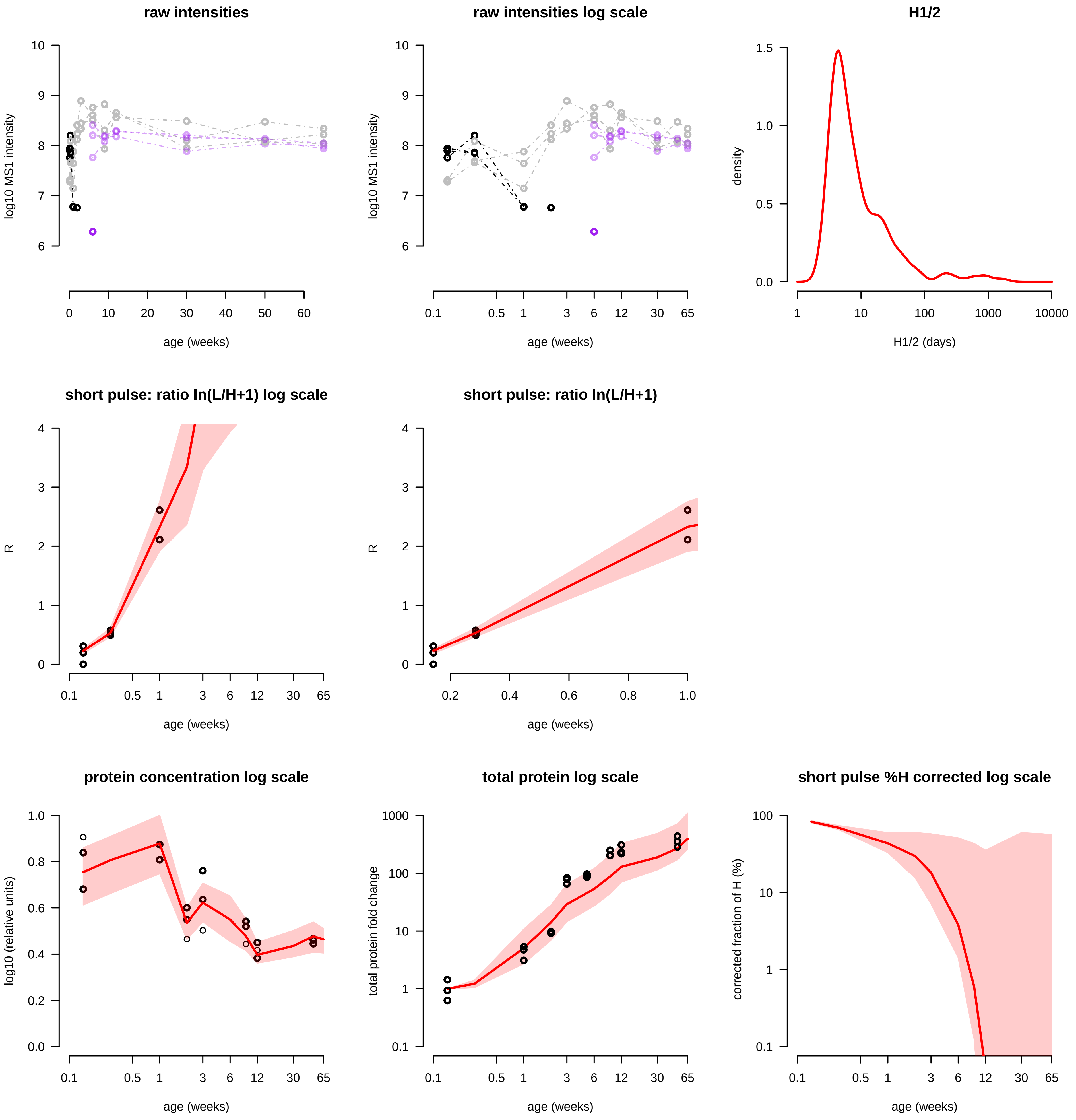

Q3UKC1

>sp|Q3UKC1|TAXB1_MOUSE

Tax1−binding protein 1 homolog

GN=Tax1bp1

Med H1/2: 5 days

Q5 H1/2: 2.5 days

Q95 H1/2: 31.9 days

reliable model fit: yes

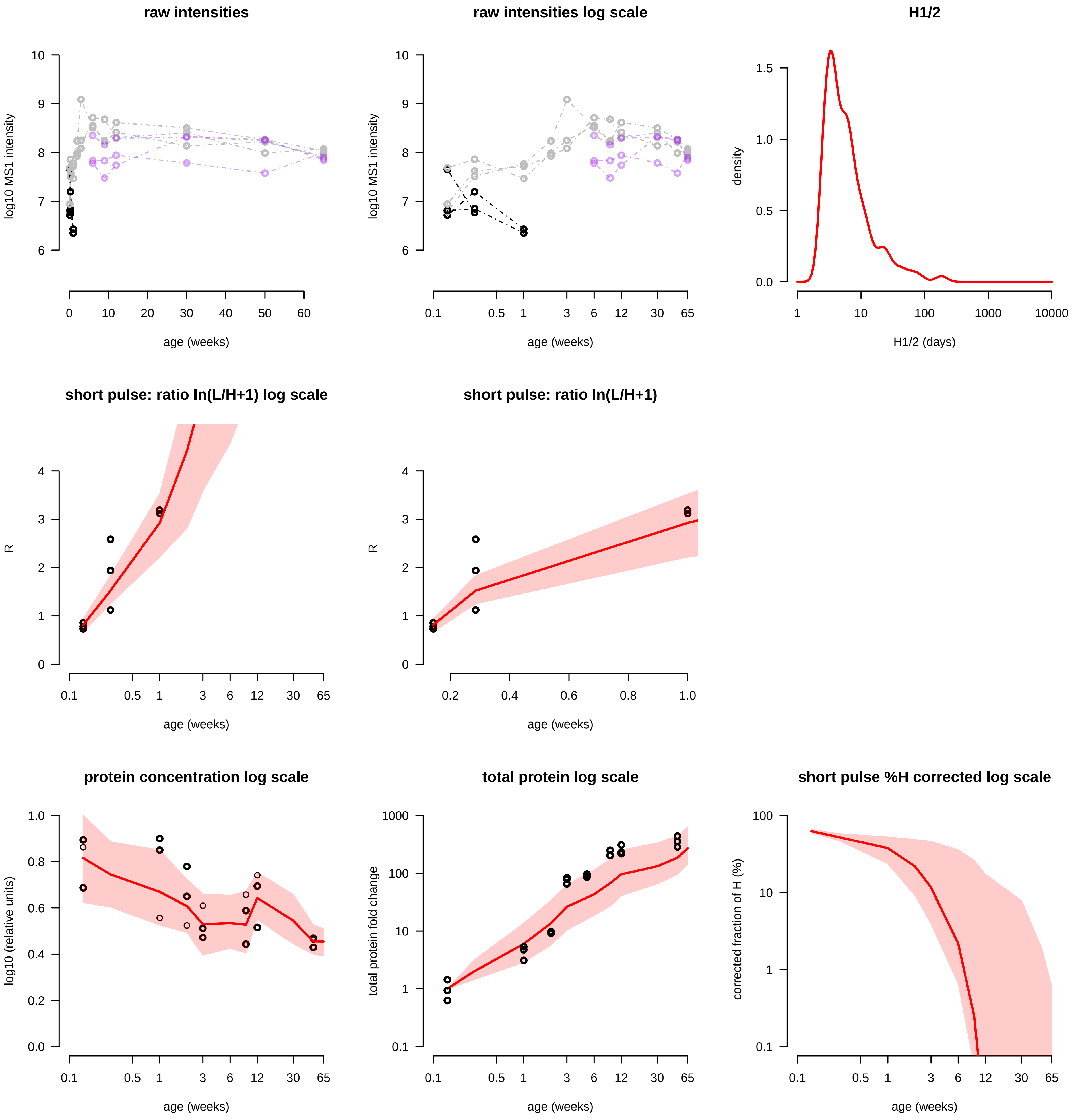

Q99MJ9

>sp|Q99MJ9|DDX50_MOUSE

ATP−dependent RNA helicase DDX50

GN=Ddx50

Med H1/2: 3.4 days

Q5 H1/2: 2.6 days

Q95 H1/2: 5.6 days

reliable model fit: no

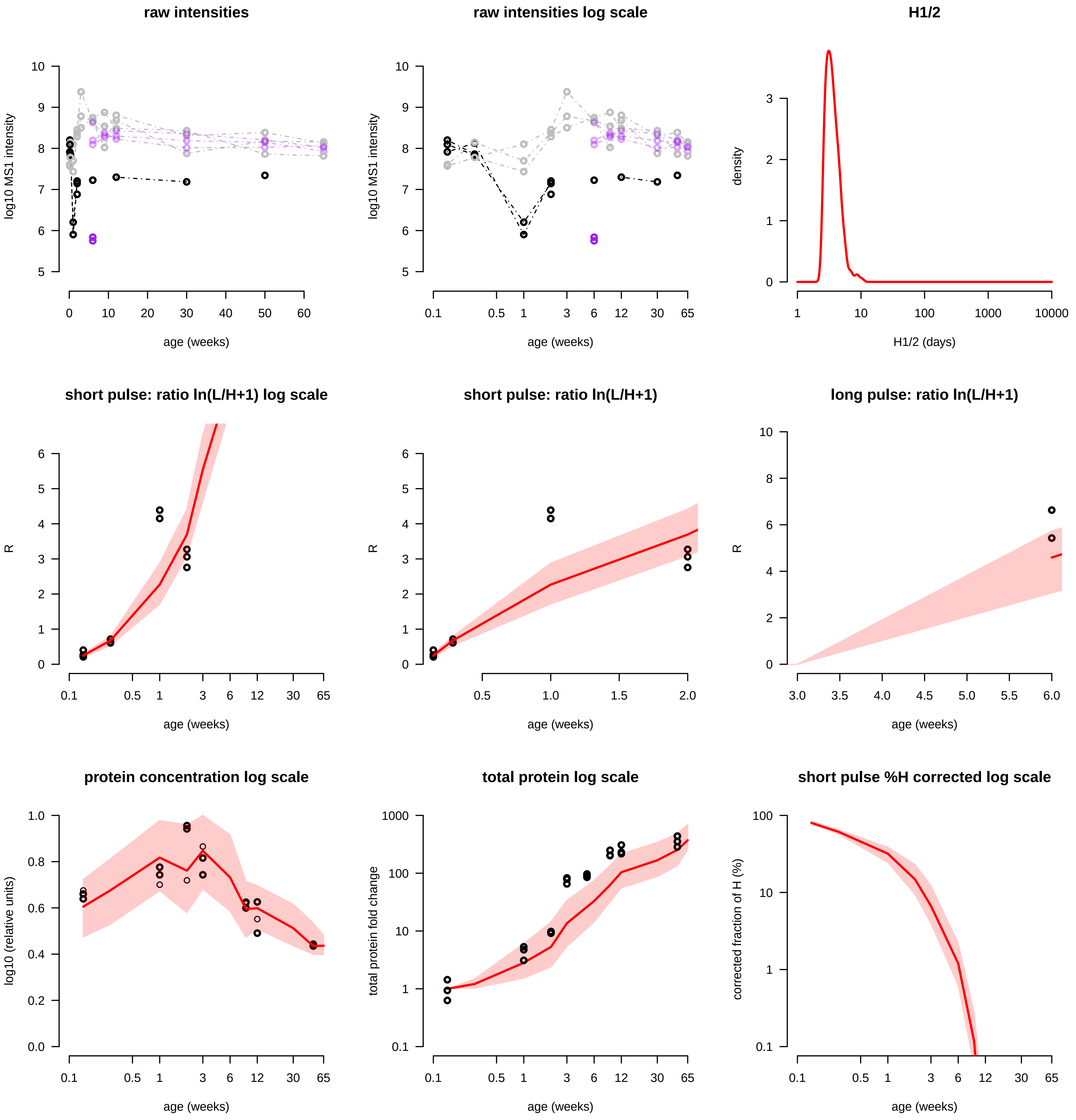

Q9WUN2

>sp|Q9WUN2|TBK1_MOUSE

Serine/threonine−protein kinase TBK1

GN=Tbk1

Med H1/2: 5.3 days

Q5 H1/2: 2.6 days

Q95 H1/2: 74.2 days

reliable model fit: yes

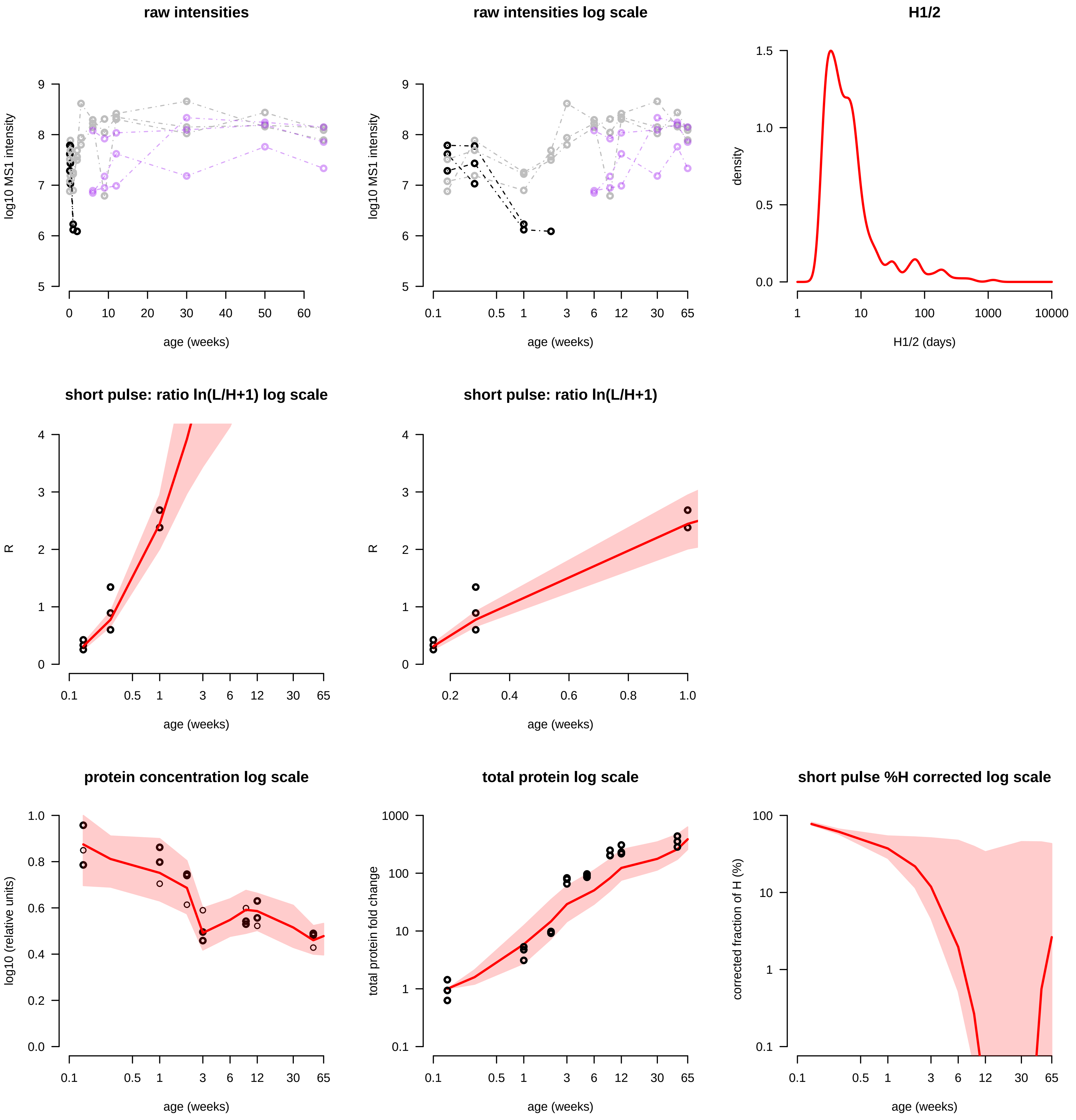

Q6ZPY7;Q6ZPY7−2

>sp|Q6ZPY7|KDM3B_MOUSE

Lysine−specific demethylase 3B

GN=Kdm3b

Med H1/2: 20.7 days

Q5 H1/2: 5.2 days

Q95 H1/2: 166.7 days

reliable model fit: yes

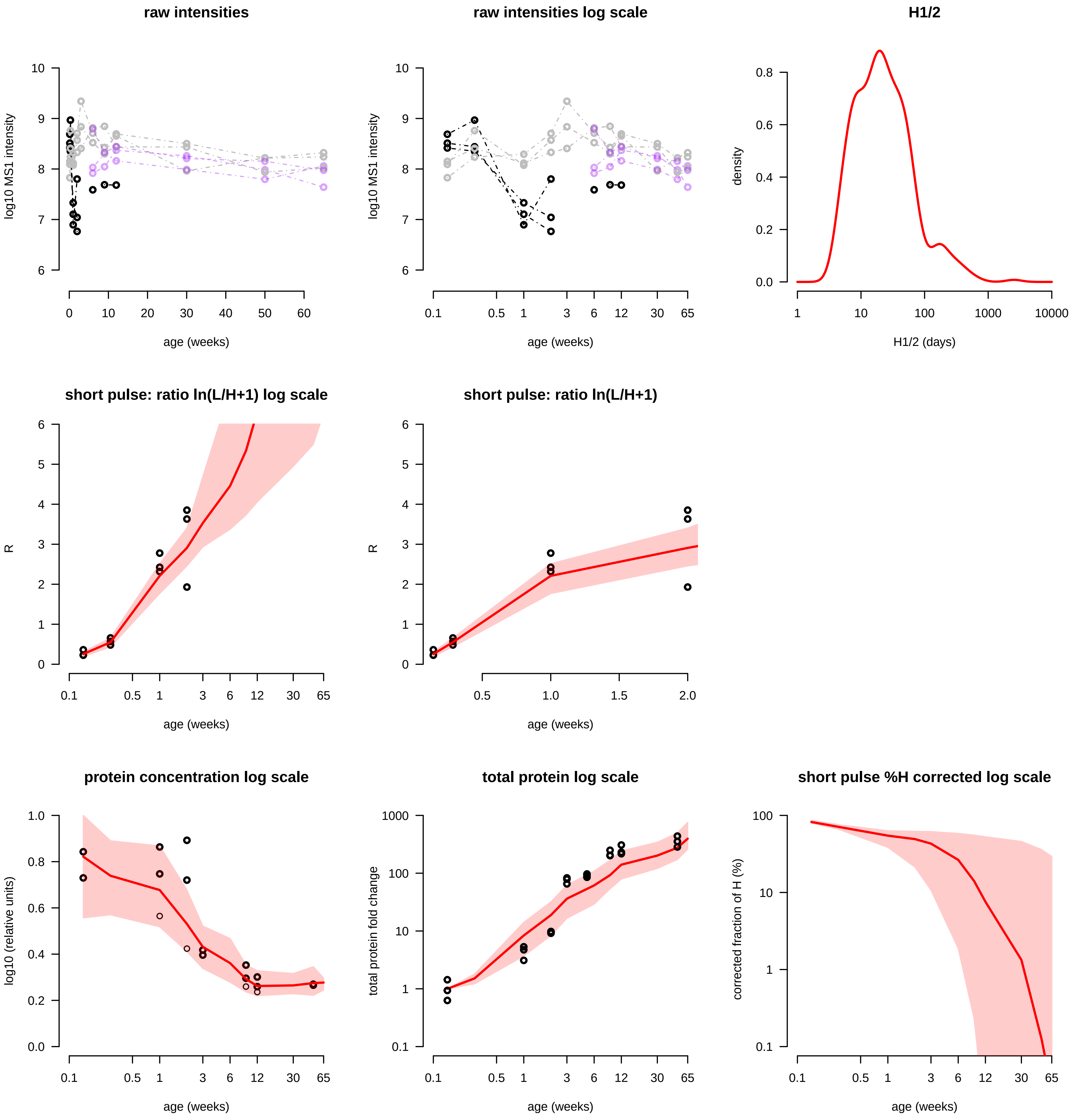

Q8CBE3

>sp|Q8CBE3|WDR37_MOUSE

WD repeat−containing protein 37

GN=Wdr37

Med H1/2: 9.5 days

Q5 H1/2: 3 days

Q95 H1/2: 108.9 days

reliable model fit: yes

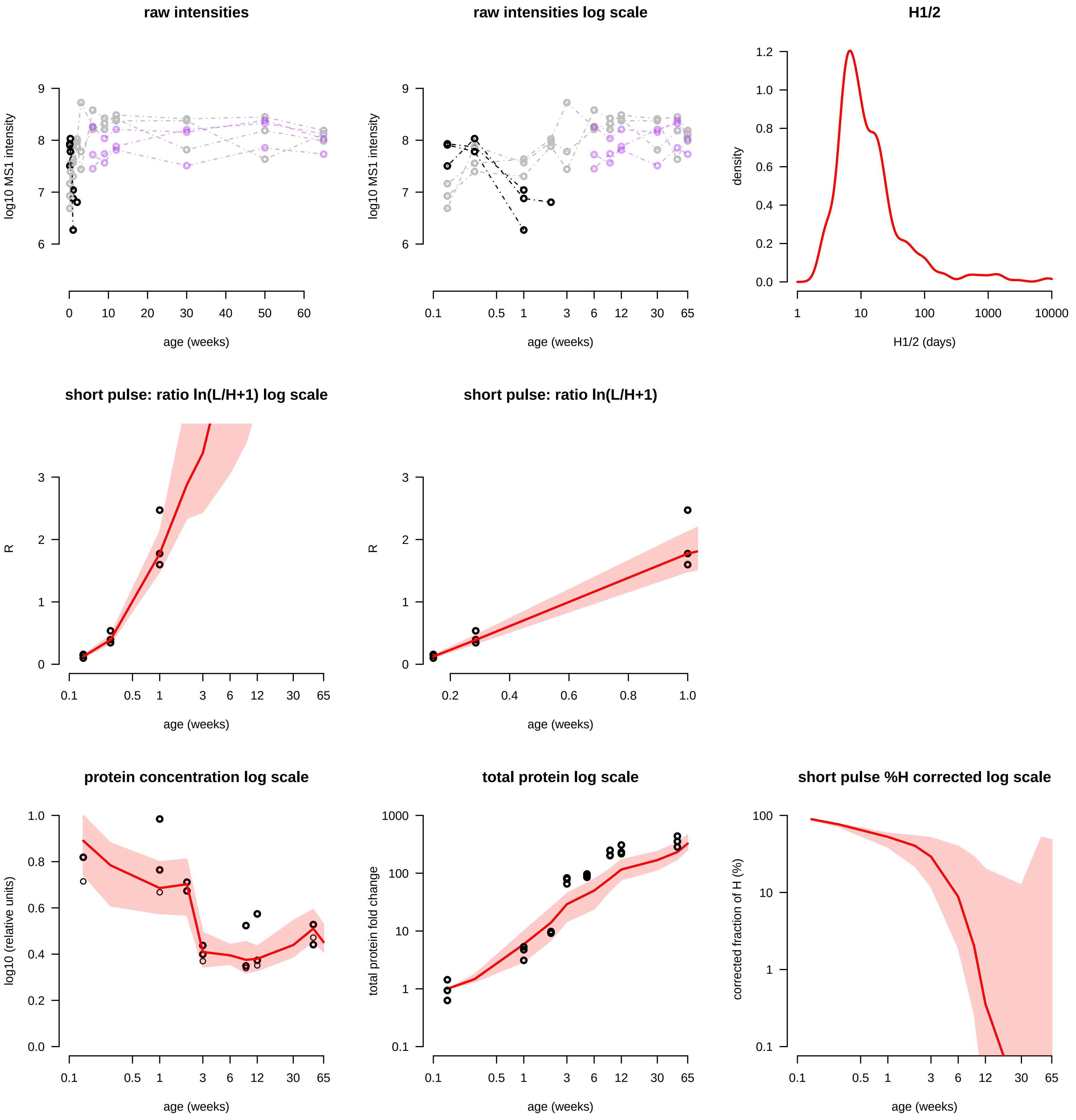

Q91VZ6

>sp|Q91VZ6|SMAP1_MOUSE

Stromal membrane−associated protein 1

GN=Smap1

Med H1/2: 20.8 days

Q5 H1/2: 5.2 days

Q95 H1/2: 181.5 days

reliable model fit: yes

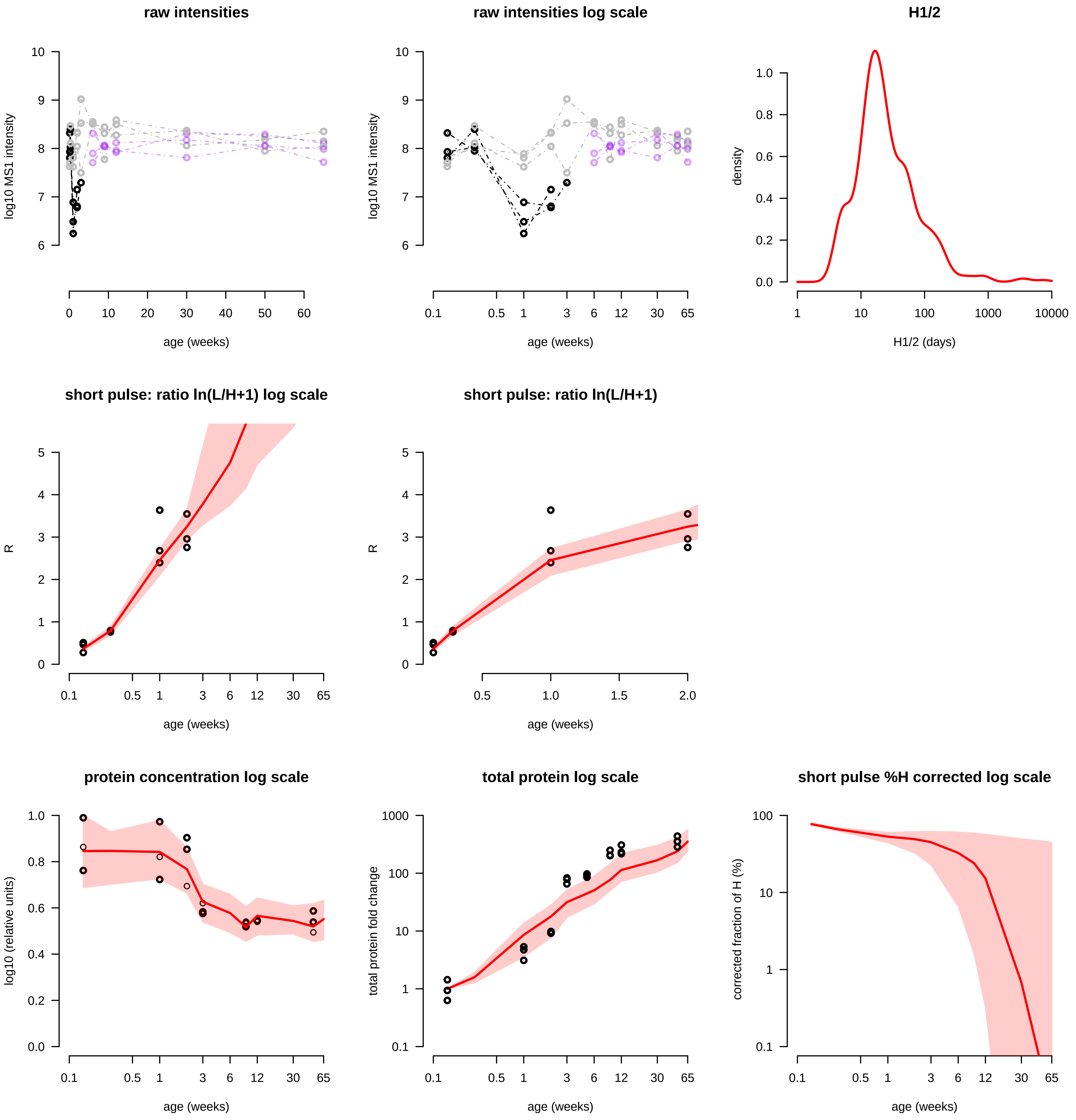

Q8BVU5

>sp|Q8BVU5|NUDT9_MOUSE

ADP−ribose pyrophosphatase, mitochondrial

GN=Nudt9

Med H1/2: 10.2 days

Q5 H1/2: 3.6 days

Q95 H1/2: 45.4 days

reliable model fit: yes

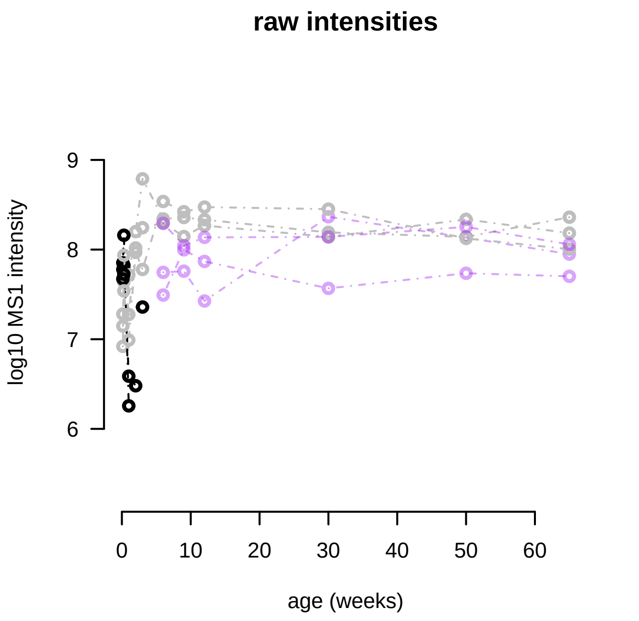

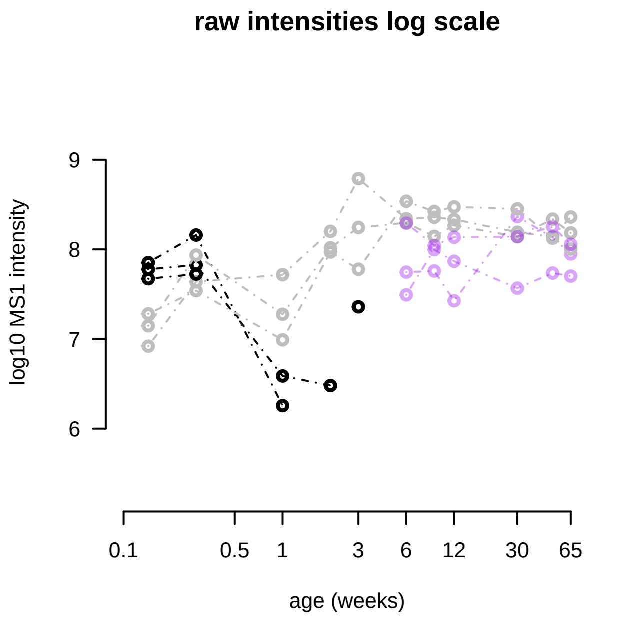

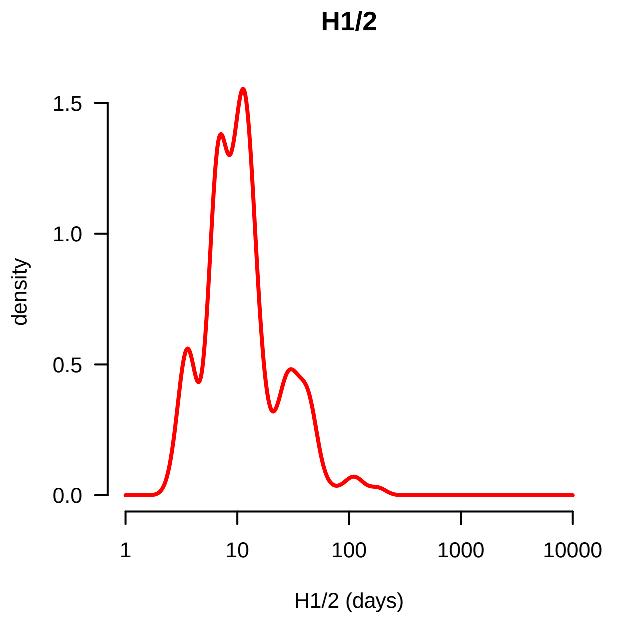

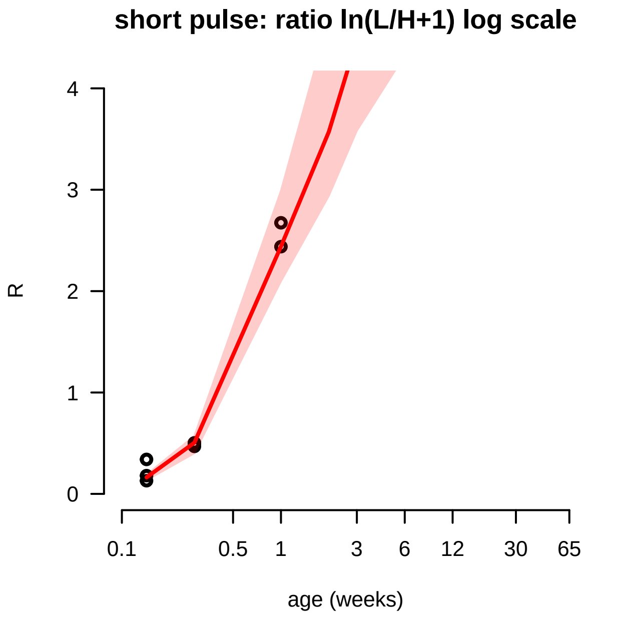

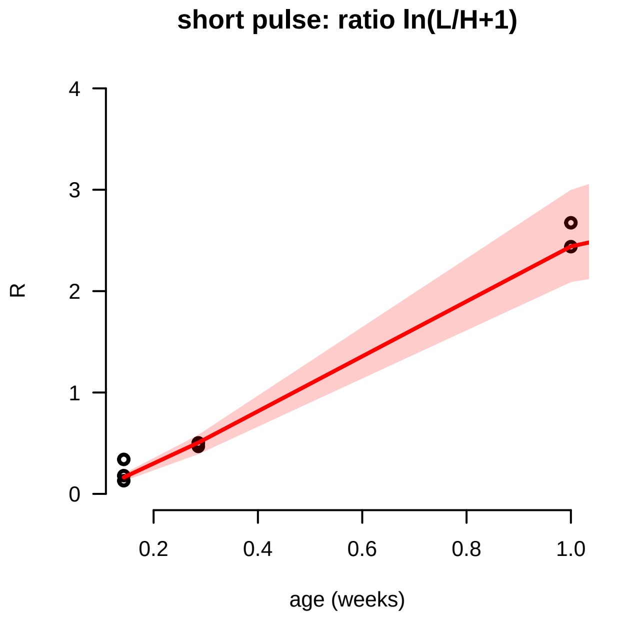

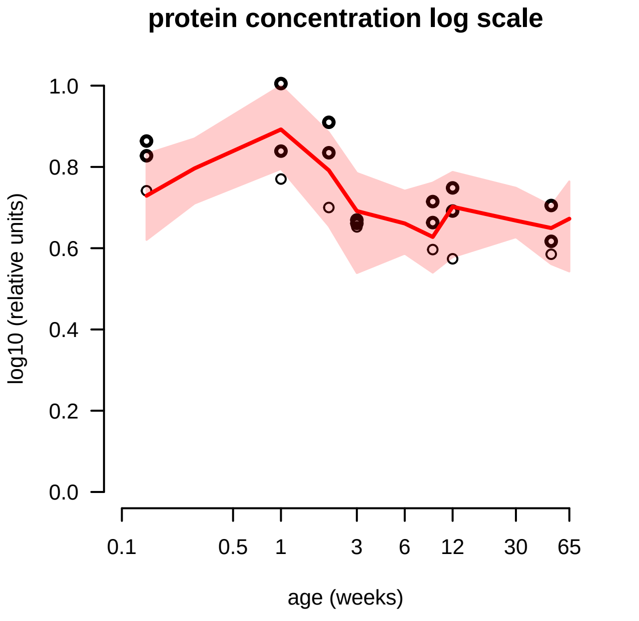

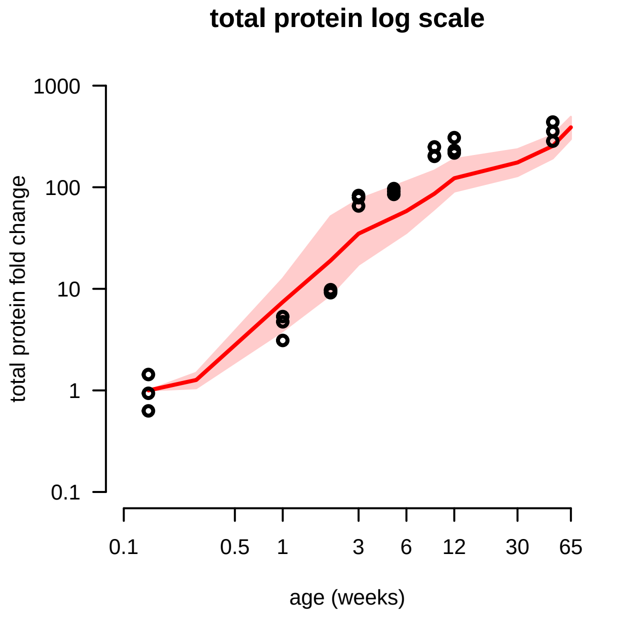

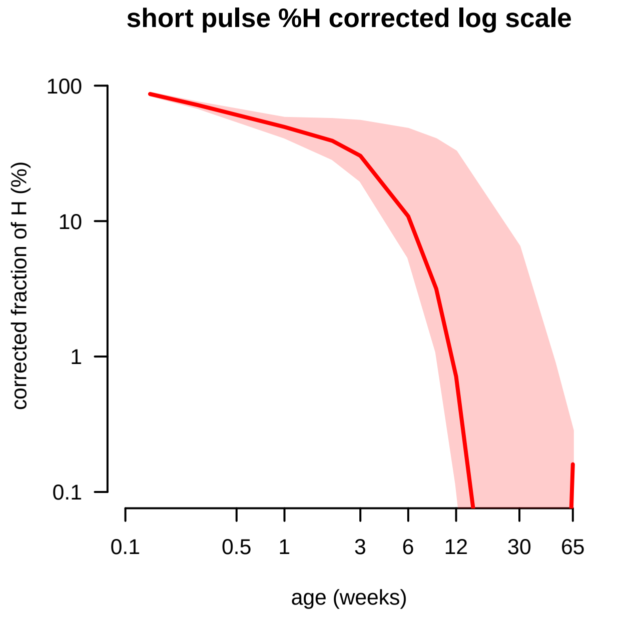

Q6ZPR5

>sp|Q6ZPR5|NSMA3_MOUSE

Sphingomyelin phosphodiesterase 4

GN=Smpd4

Med H1/2: 1626.5 days

Q5 H1/2: 542.1 days

Q95 H1/2: 24505.1 days

reliable model fit: no

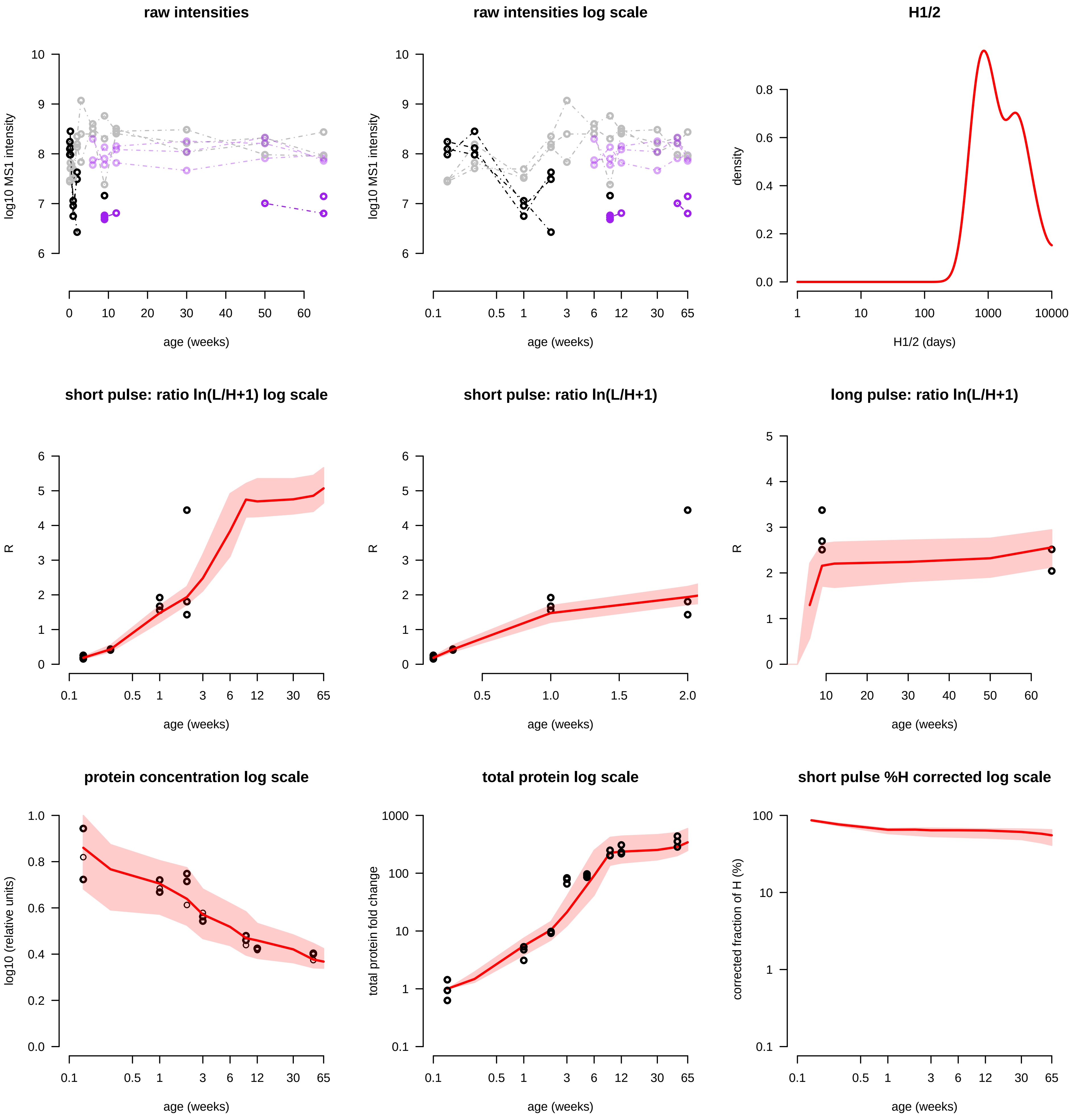

Q4PZA2−3;Q4PZA2−4;Q4PZA2

>sp|Q4PZA2−3|ECE1_MOUSE

Isoform C of Endothelin−converting enzyme 1

GN=Ece1

Med H1/2: 5.6 days

Q5 H1/2: 2.6 days

Q95 H1/2: 34.1 days

reliable model fit: yes

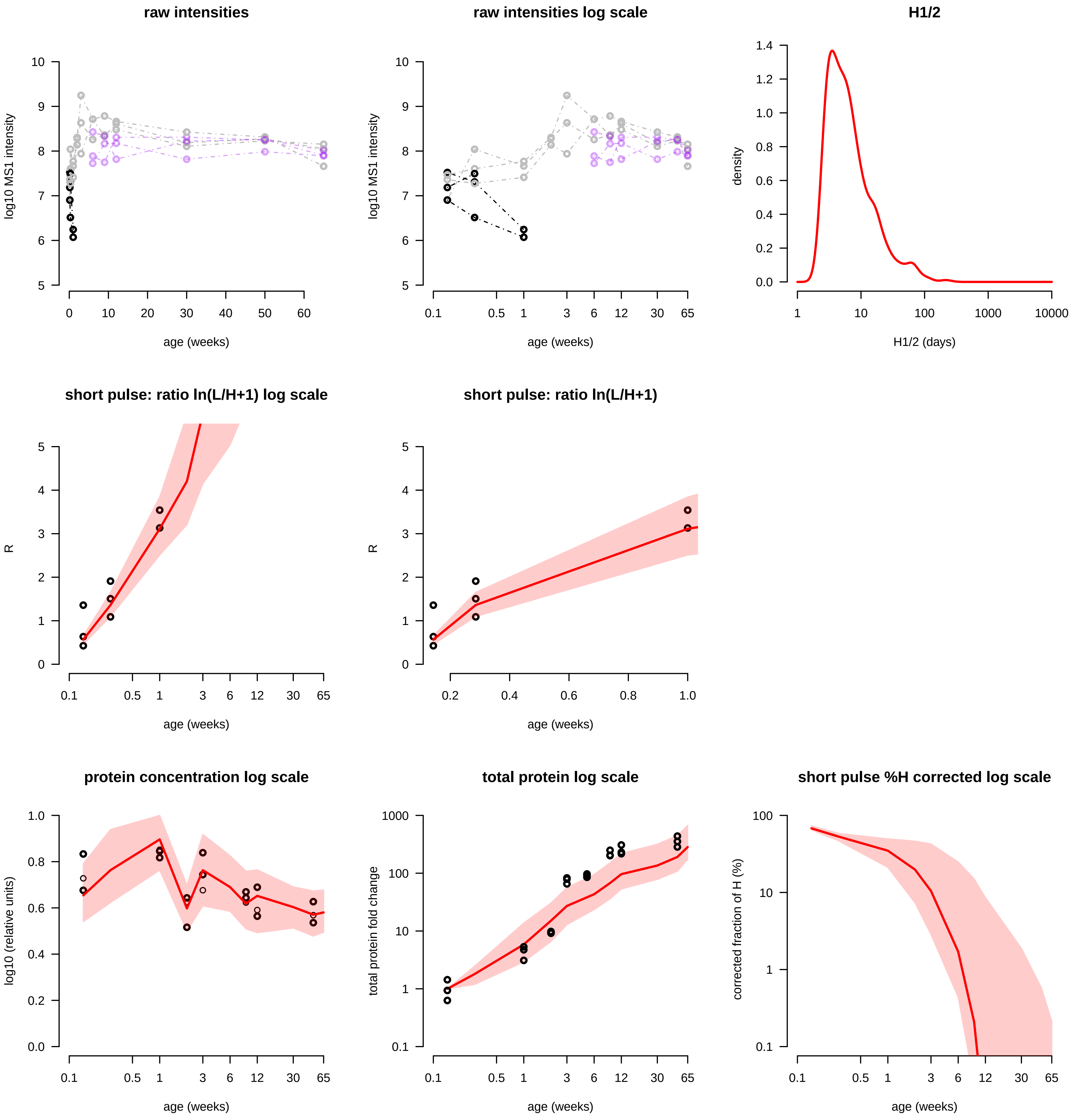

Q8CHY6

>sp|Q8CHY6|P66A_MOUSE

Transcriptional repressor p66 alpha

GN=Gatad2a

Med H1/2: 7.6 days

Q5 H1/2: 3 days

Q95 H1/2: 26.1 days

reliable model fit: yes

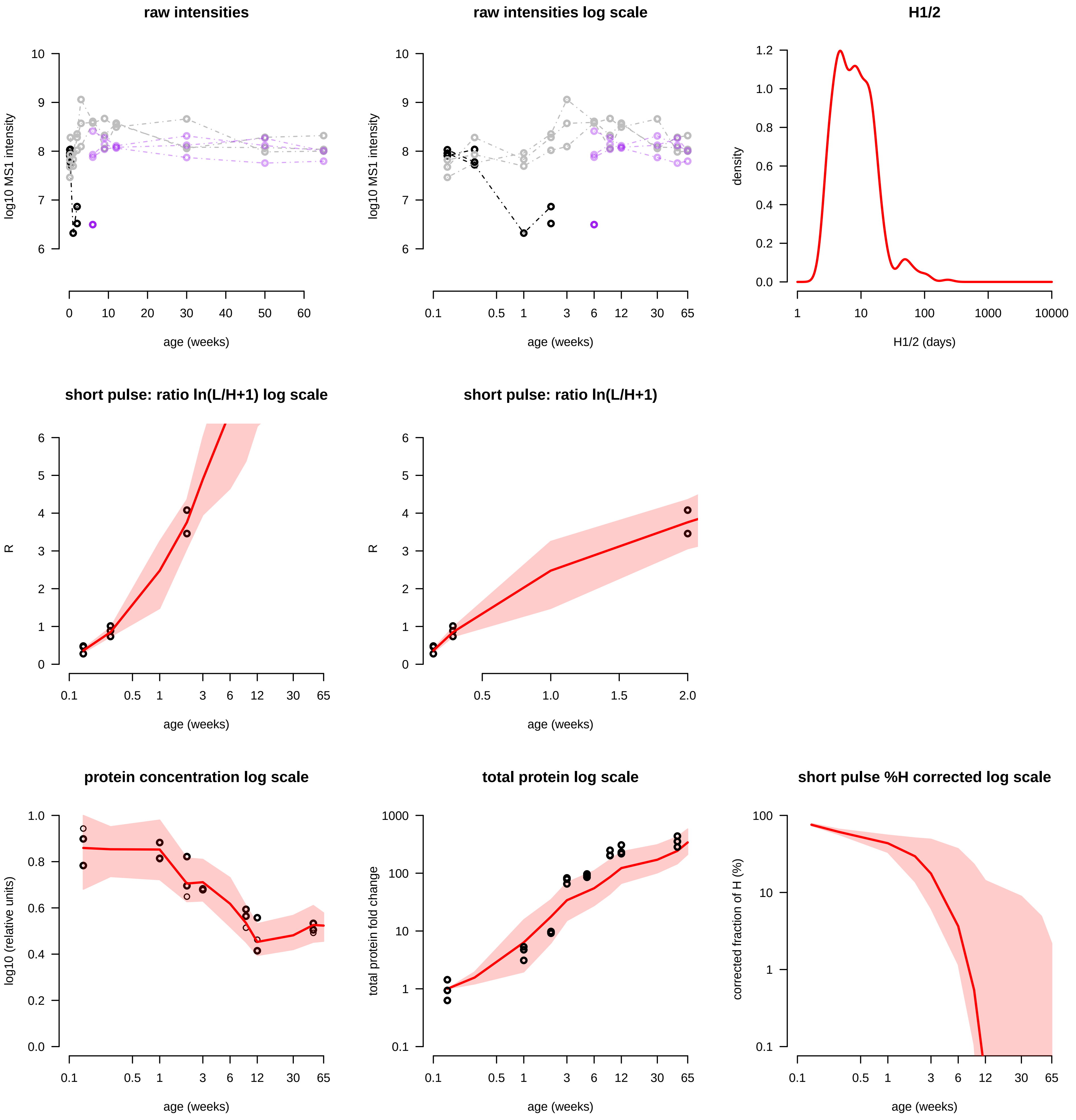

Q9JHH9

>sp|Q9JHH9|COPZ2_MOUSE

Coatomer subunit zeta−2

GN=Copz2

Med H1/2: 9.8 days

Q5 H1/2: 3.4 days

Q95 H1/2: 63.4 days

reliable model fit: no

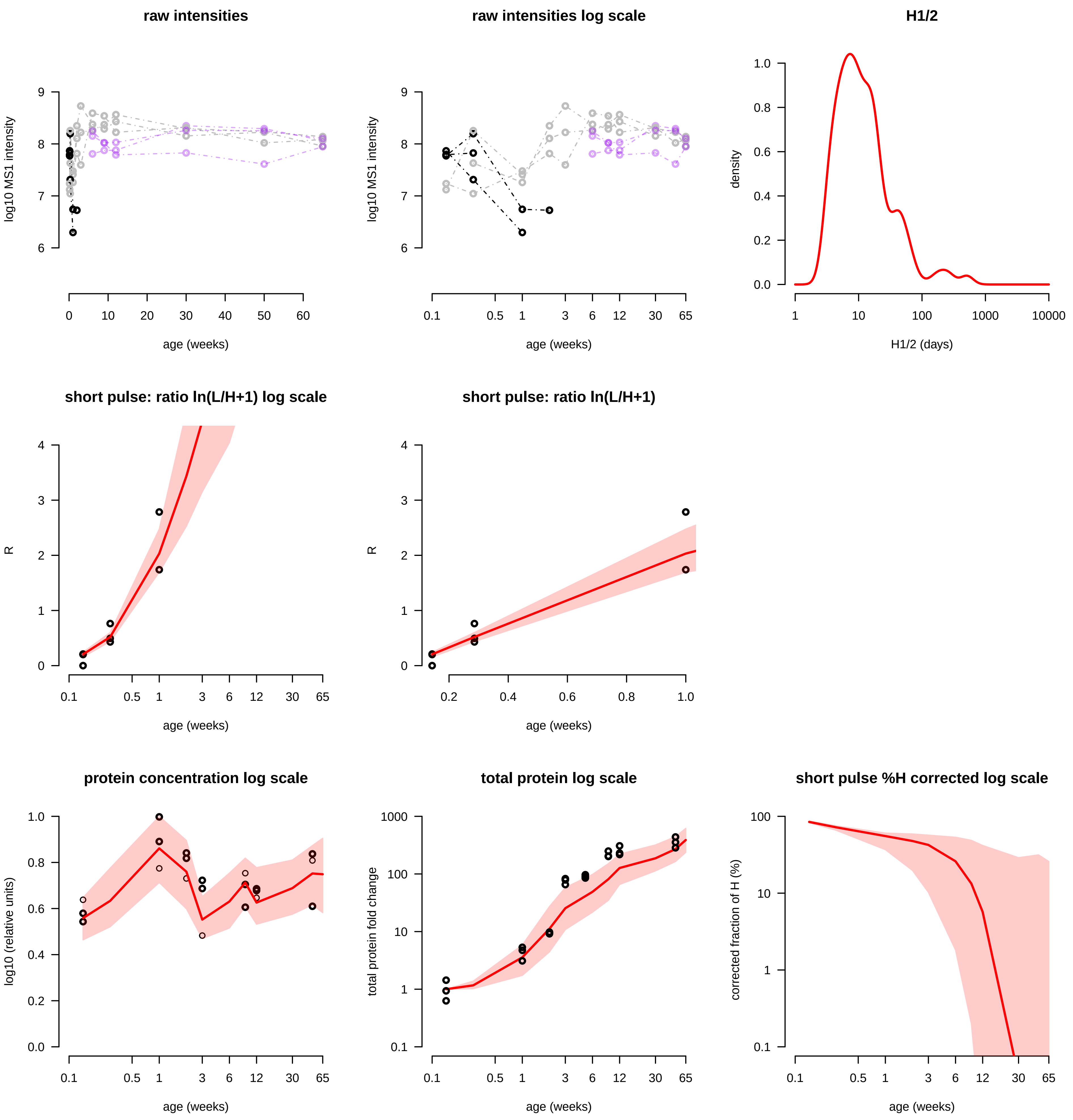

Q9JLC8−3;Q9JLC8;Q9JLC8−2

>sp|Q9JLC8−3|SACS_MOUSE

Isoform 3 of Sacsin

GN=Sacs

Med H1/2: 4858.6 days

Q5 H1/2: 1409.7 days

Q95 H1/2: 59799.7 days

reliable model fit: no

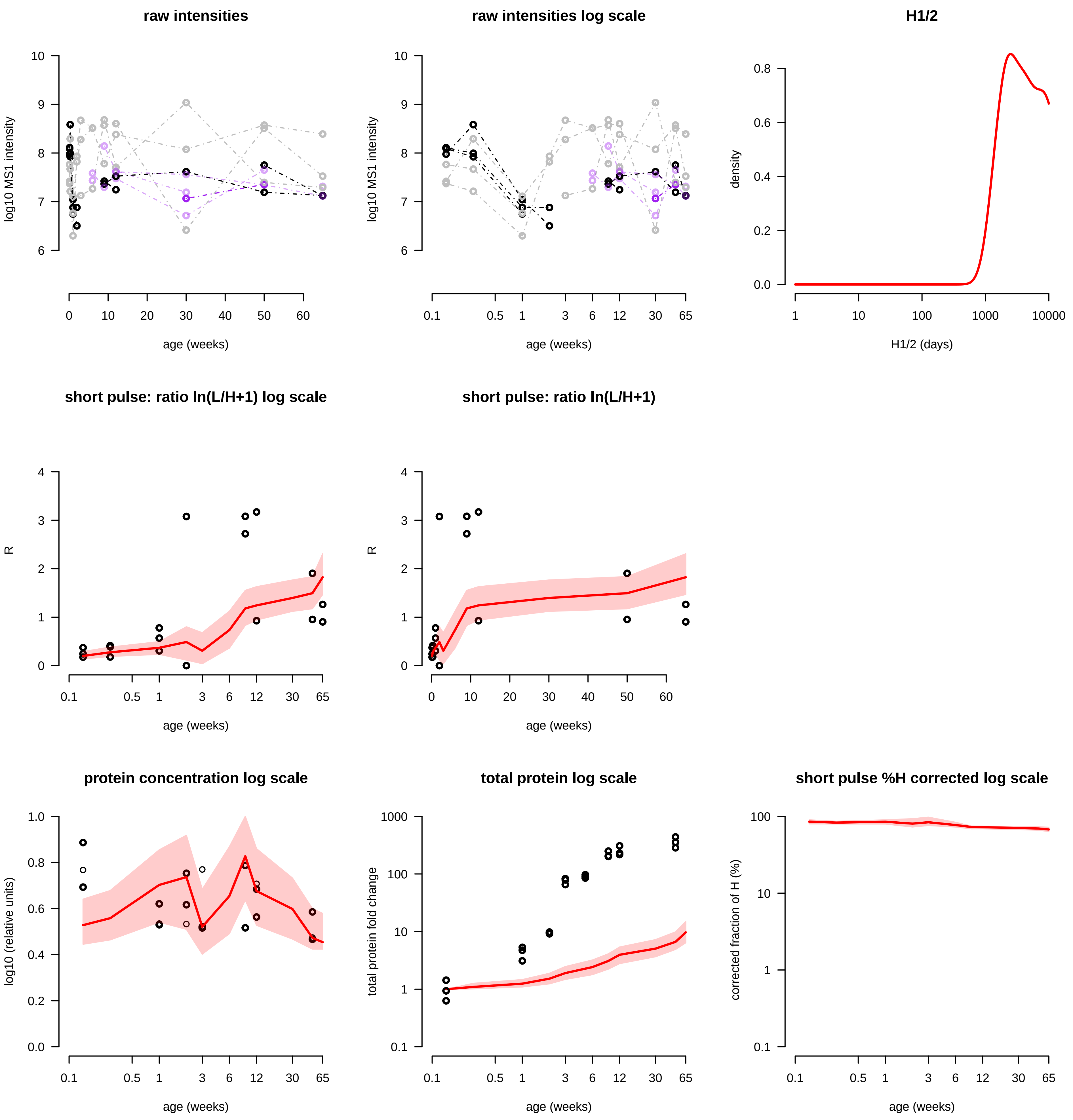

Q9DCA2

>sp|Q9DCA2|RT11_MOUSE

28S ribosomal protein S11, mitochondrial

GN=Mrps11

Med H1/2: 8.2 days

Q5 H1/2: 2.8 days

Q95 H1/2: 143.2 days

reliable model fit: yes

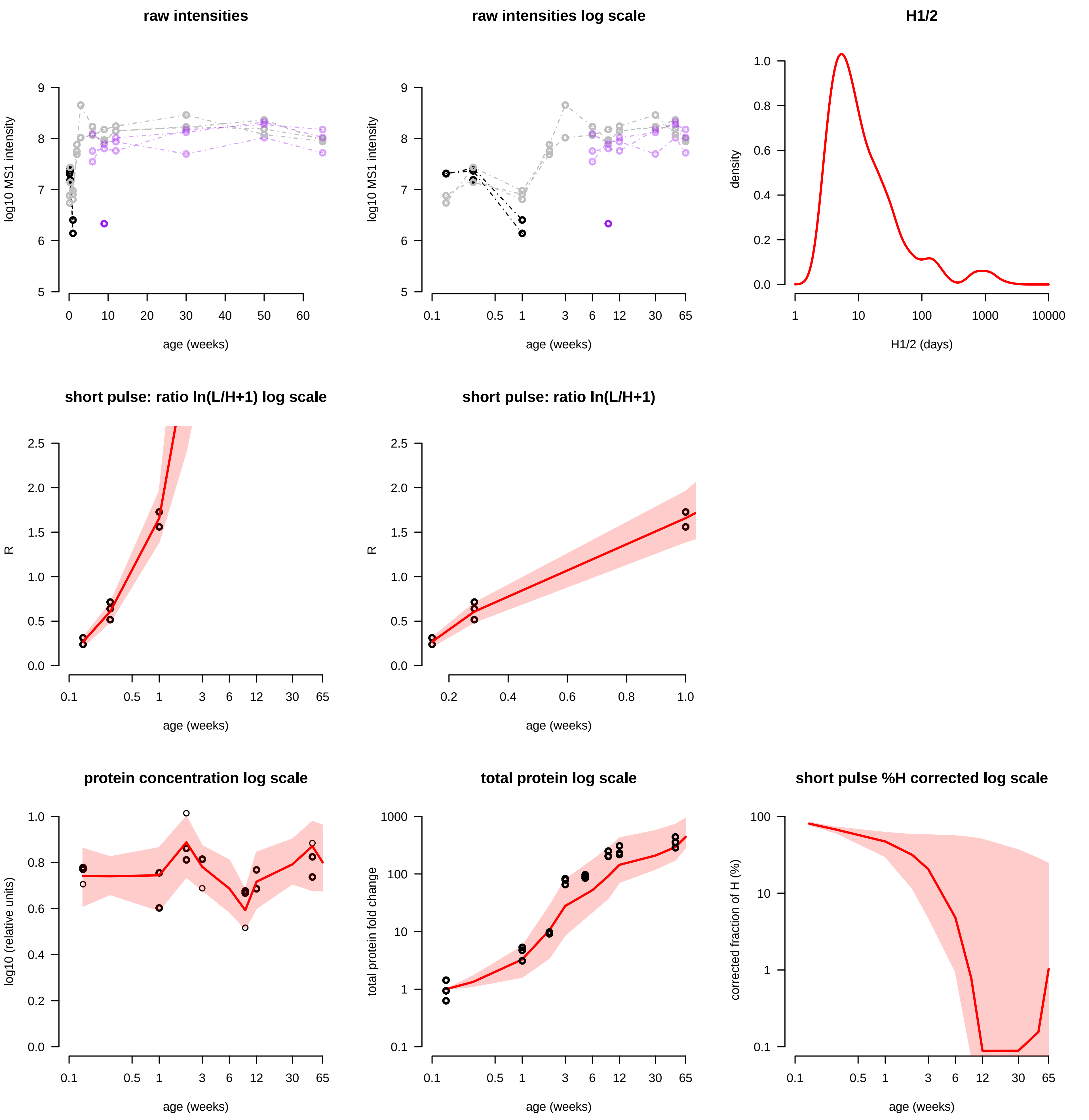

Q99N93

>sp|Q99N93|RM16_MOUSE

39S ribosomal protein L16, mitochondrial

GN=Mrpl16

Med H1/2: 7.5 days

Q5 H1/2: 3.2 days

Q95 H1/2: 67 days

reliable model fit: no

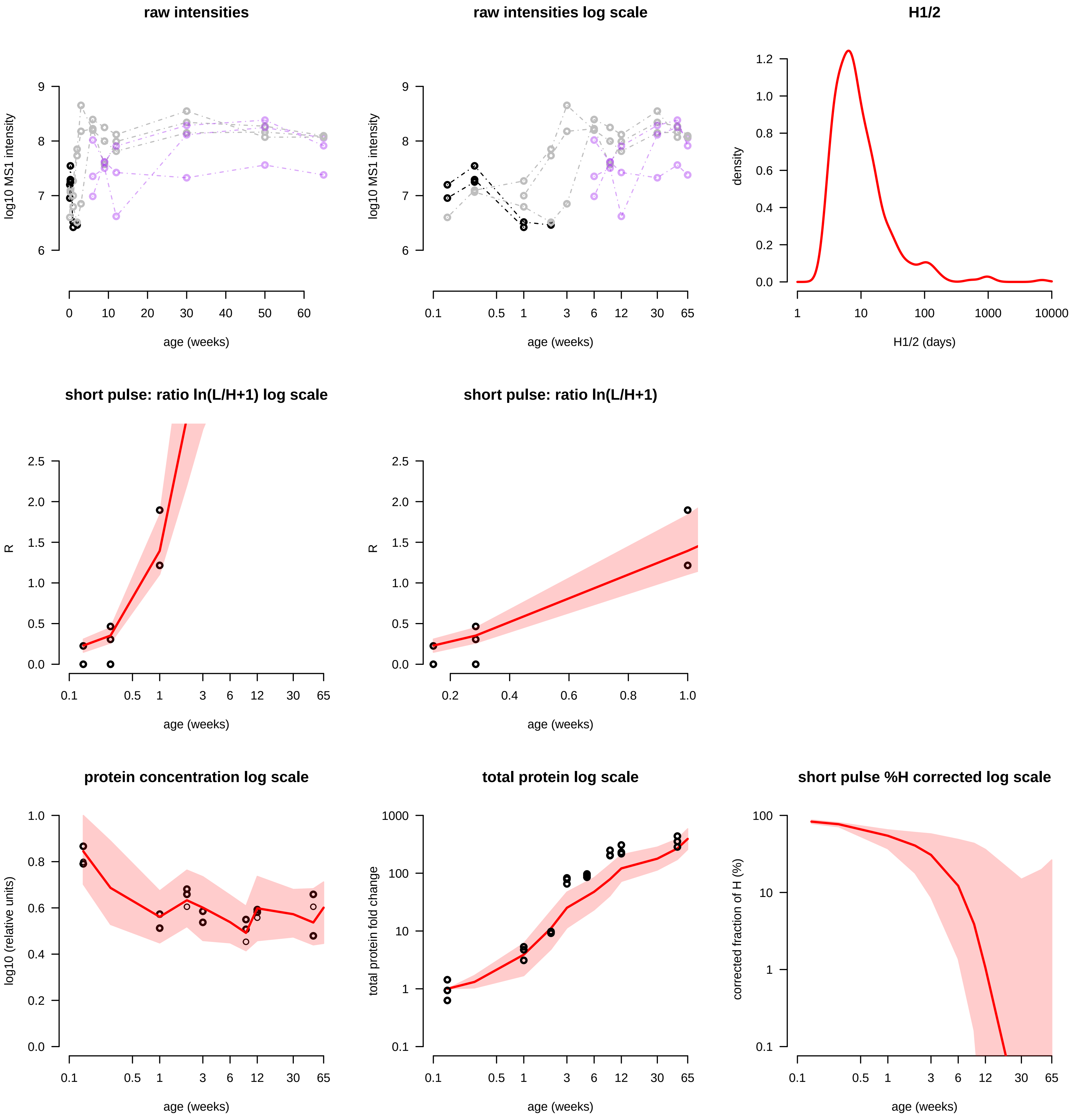

Q8K2T1;Q8K2T1−2;Q8K2T1−3

>sp|Q8K2T1|NMRL1_MOUSE

NmrA−like family domain−containing protein 1

GN=Nmral1

Med H1/2: 8.1 days

Q5 H1/2: 4.4 days

Q95 H1/2: 181.6 days

reliable model fit: yes

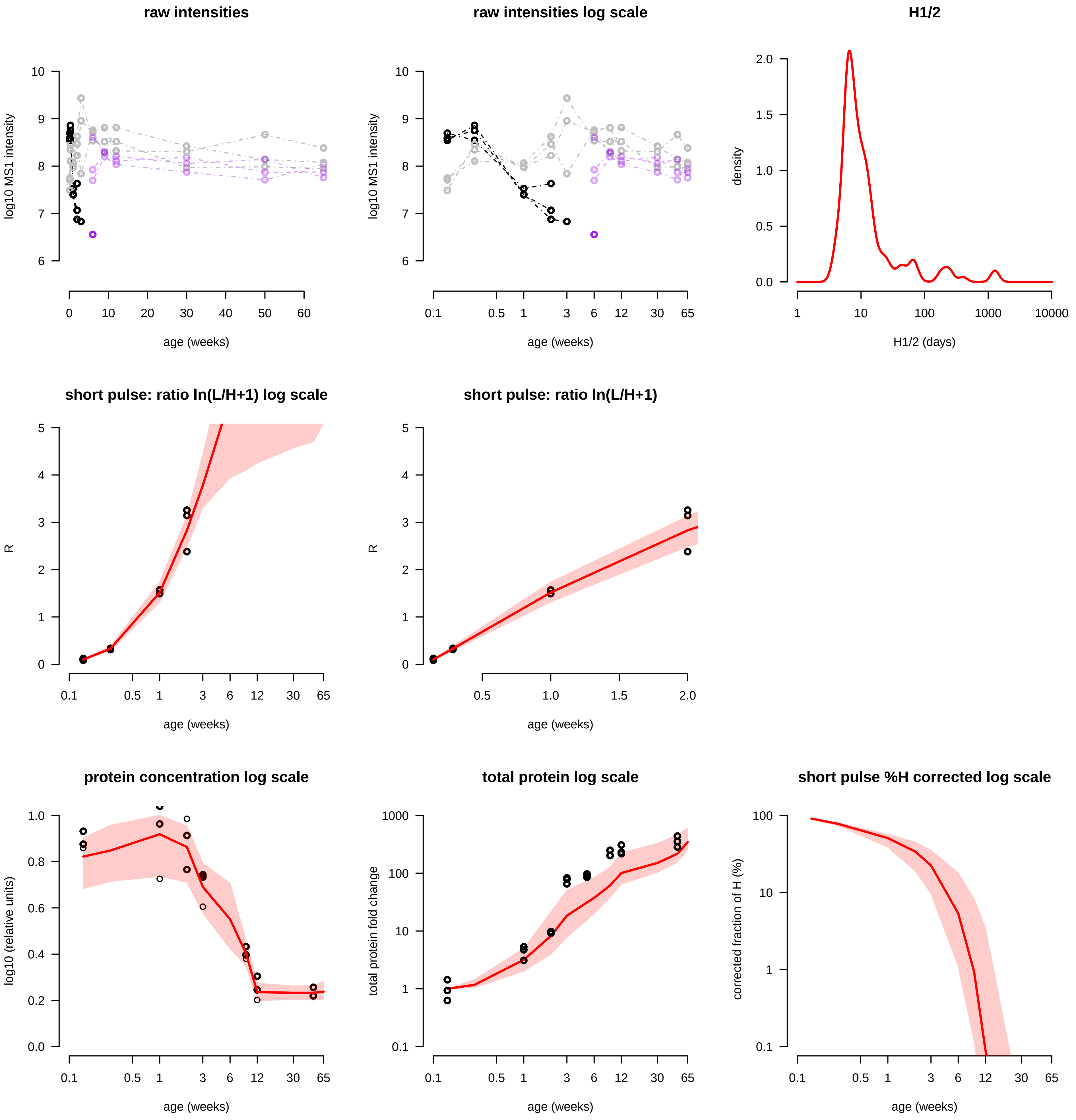

A2AQ19

>sp|A2AQ19|RTF1_MOUSE

RNA polymerase−associated protein RTF1 homolog

GN=Rtf1

Med H1/2: 5.4 days

Q5 H1/2: 2.7 days

Q95 H1/2: 26 days

reliable model fit: yes

raw intensities

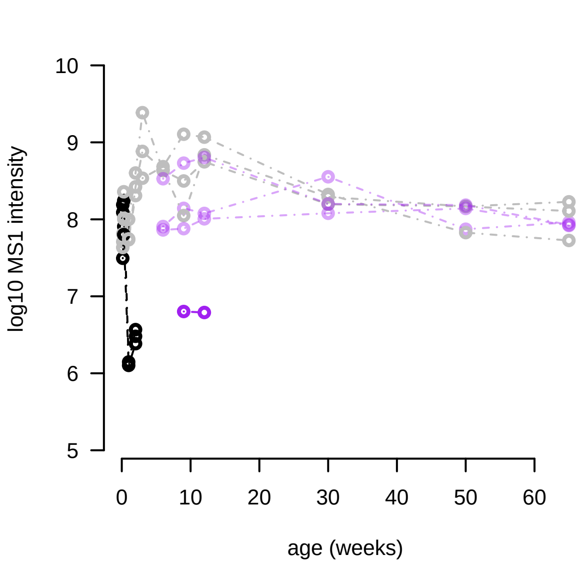

raw intensities log scale

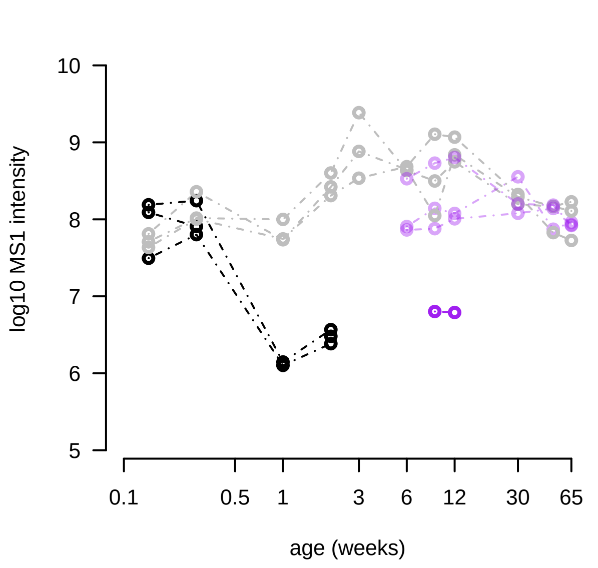

H1/2

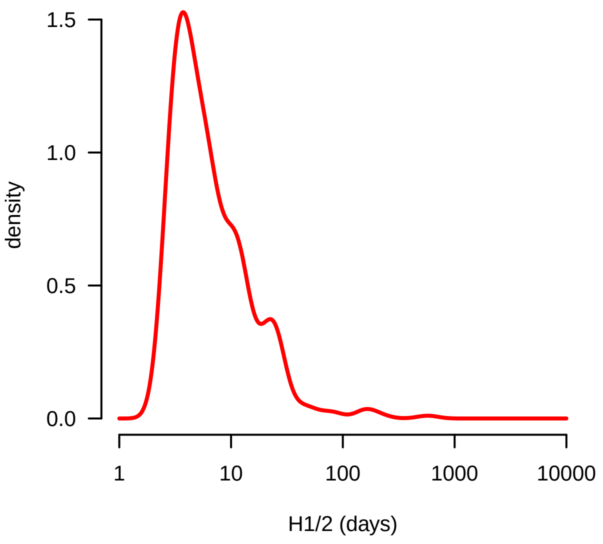

short pulse: ratio ln(L/H+1) log scale

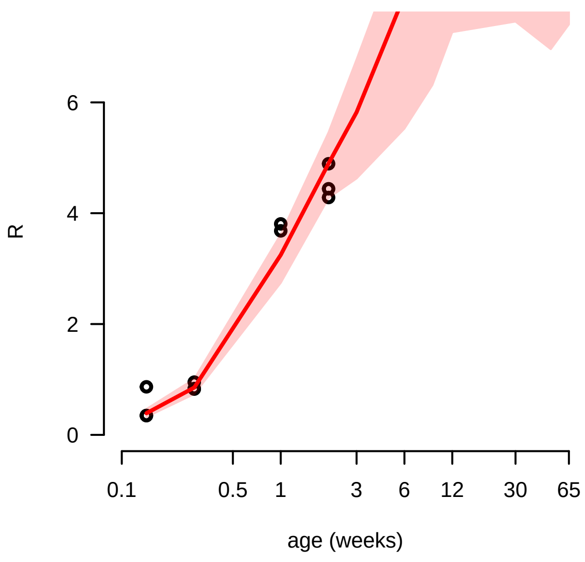

short pulse: ratio ln(L/H+1)

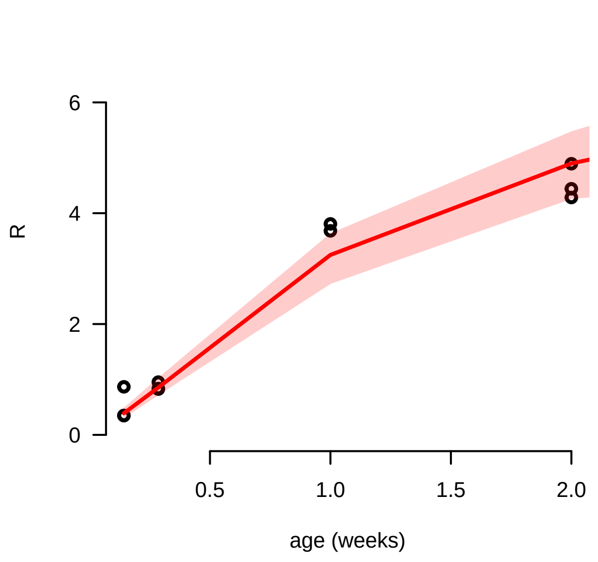

protein concentration log scale

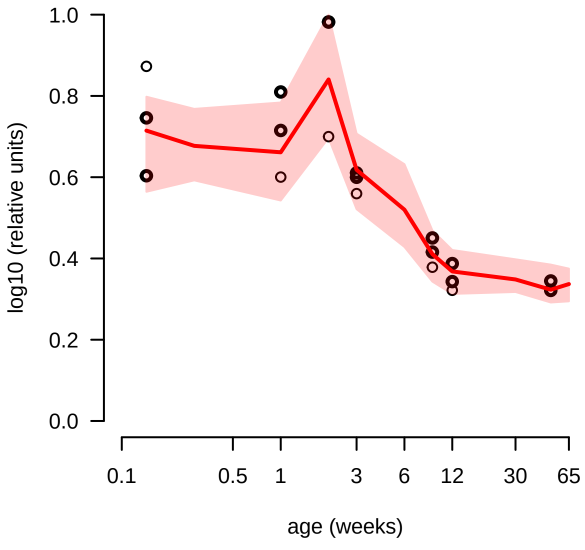

total protein log scale

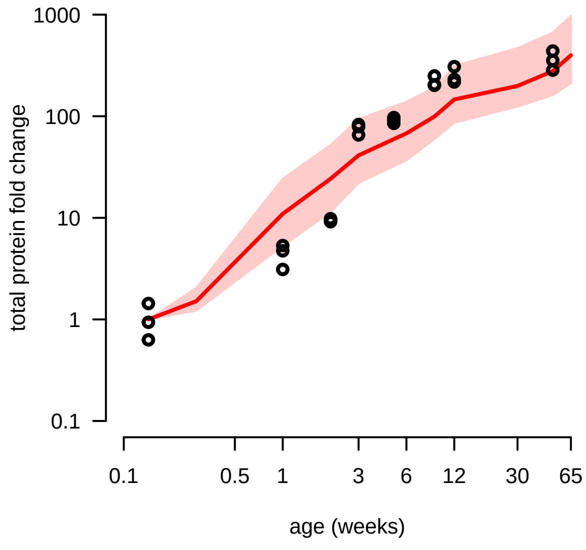

short pulse %H corrected log scale

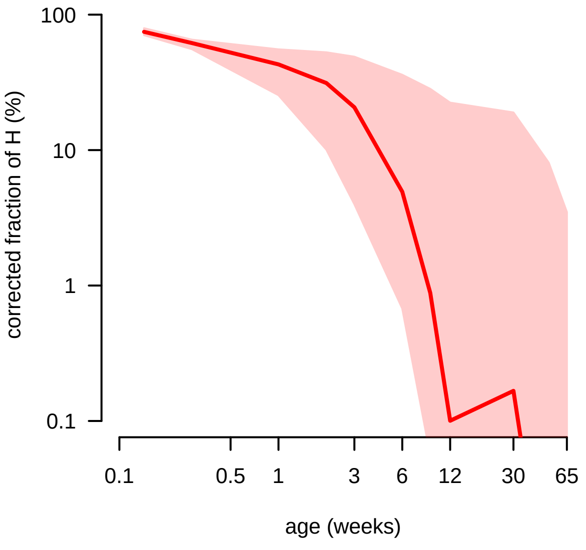

Q5SWD9

>sp|Q5SWD9|TSR1_MOUSE

Pre−rRNA−processing protein TSR1 homolog

GN=Tsr1

Med H1/2: 13.6 days

Q5 H1/2: 4.6 days

Q95 H1/2: 101.6 days

reliable model fit: yes

raw intensities

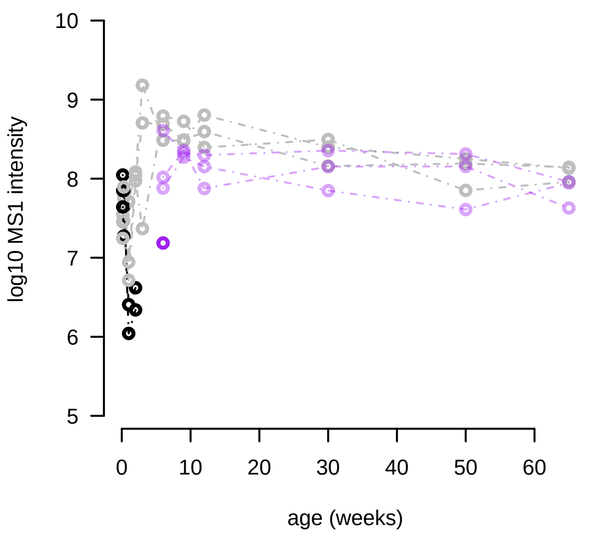

raw intensities log scale

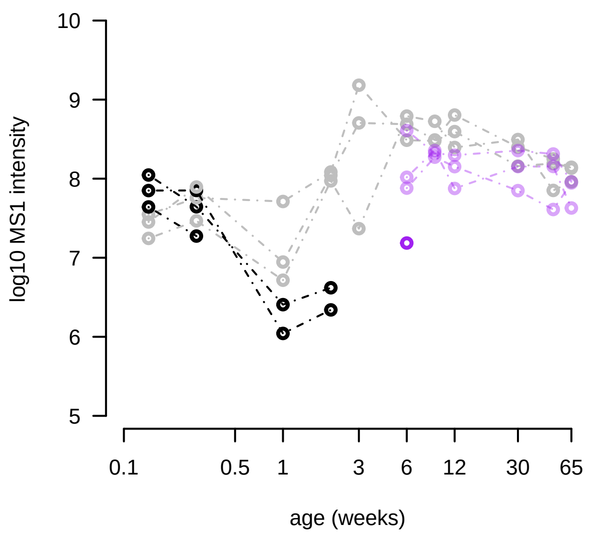

H1/2

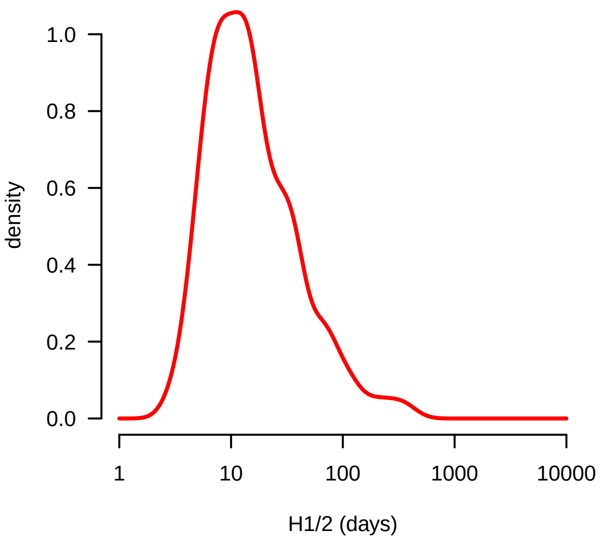

short pulse: ratio ln(L/H+1) log scale

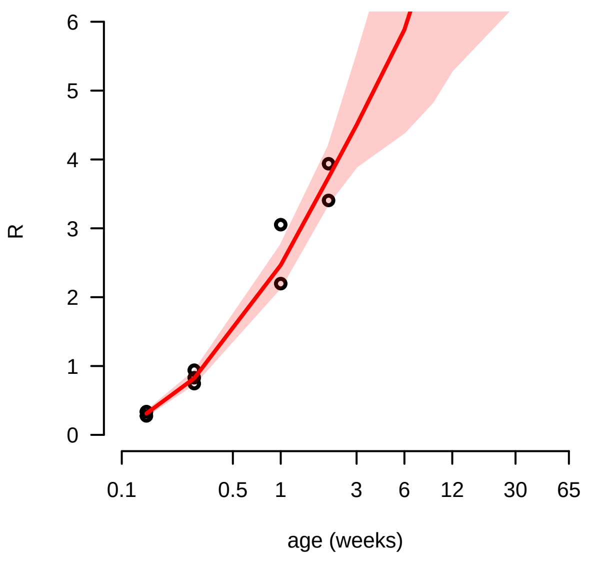

short pulse: ratio ln(L/H+1)

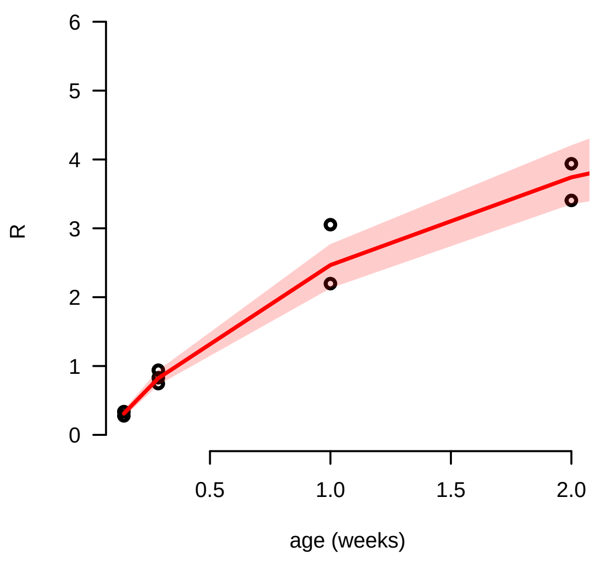

protein concentration log scale

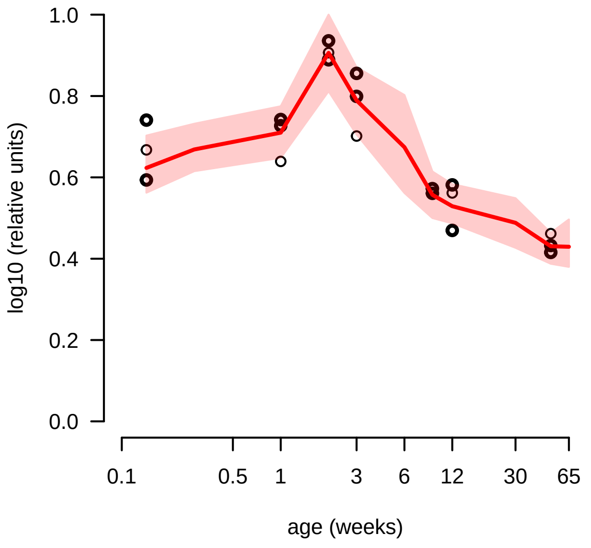

total protein log scale

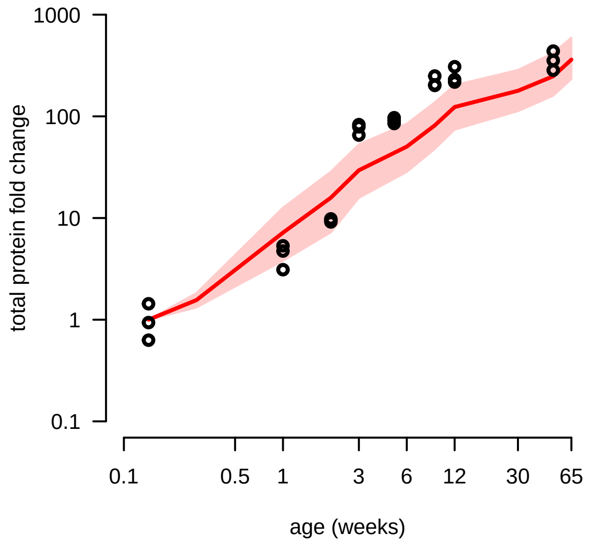

short pulse %H corrected log scale

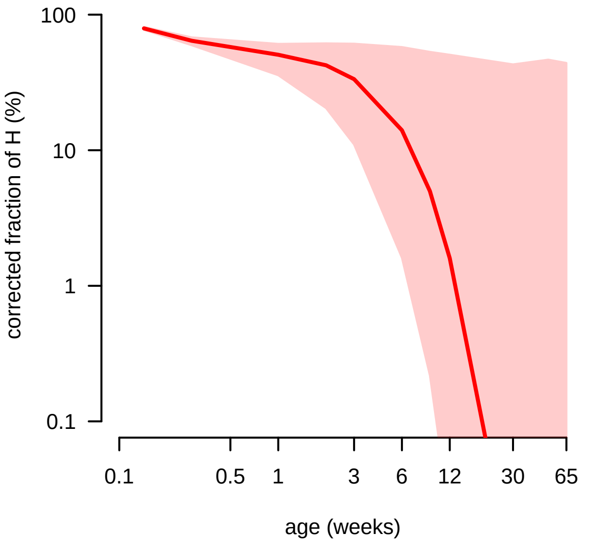

Q80W00;Q80W00−2

>sp|Q80W00|PP1RA_MOUSE

Serine/threonine−protein phosphatase 1 regulatory subunit 10

GN=Ppp1r10

Med H1/2: 4.5 days

Q5 H1/2: 2.6 days

Q95 H1/2: 9 days

reliable model fit: yes

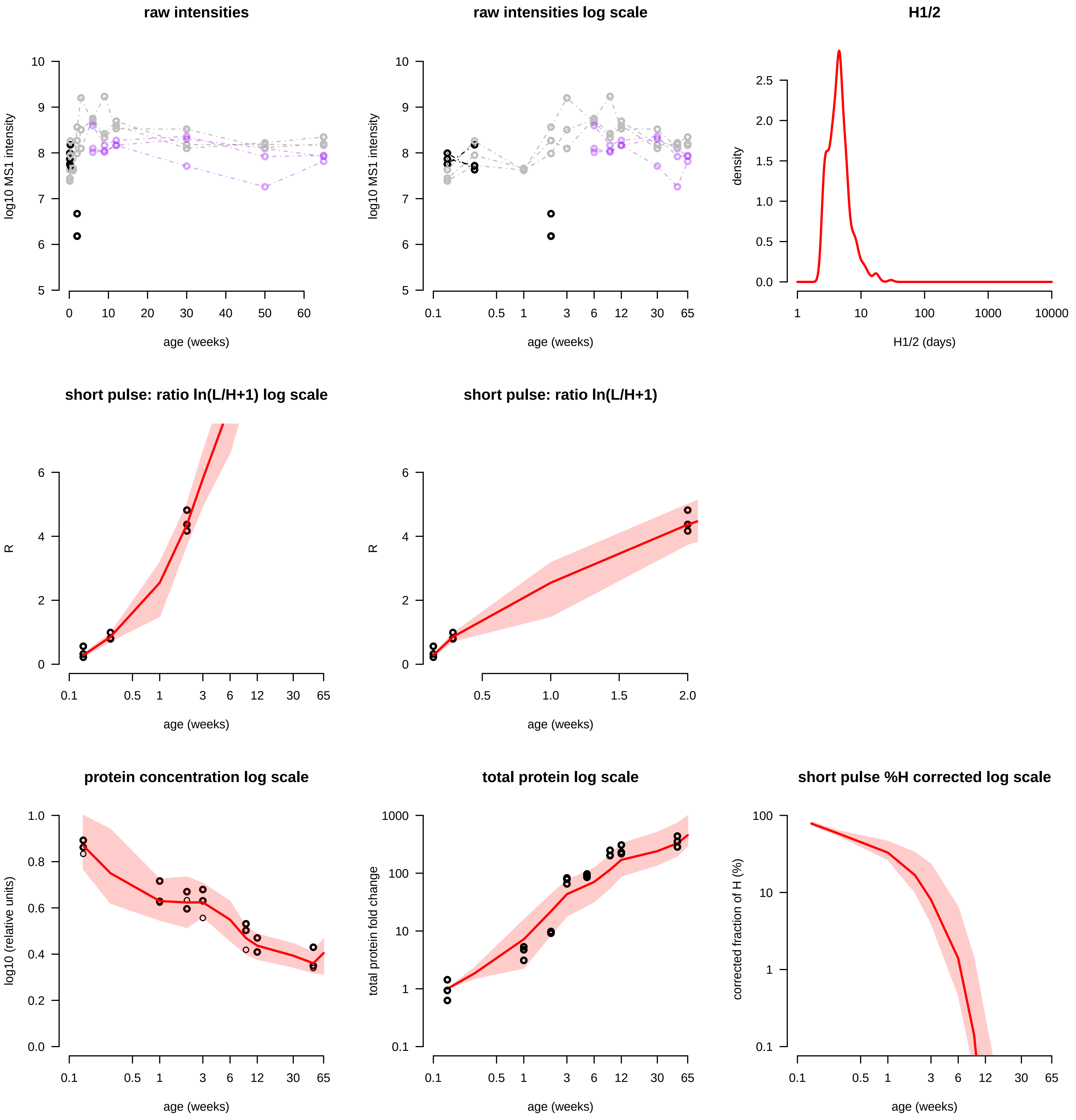

Q9CRB6

>sp|Q9CRB6|TPPP3_MOUSE

Tubulin polymerization−promoting protein family member 3

GN=Tppp3

Med H1/2: 8.5 days

Q5 H1/2: 6.1 days

Q95 H1/2: 15.7 days

reliable model fit: yes

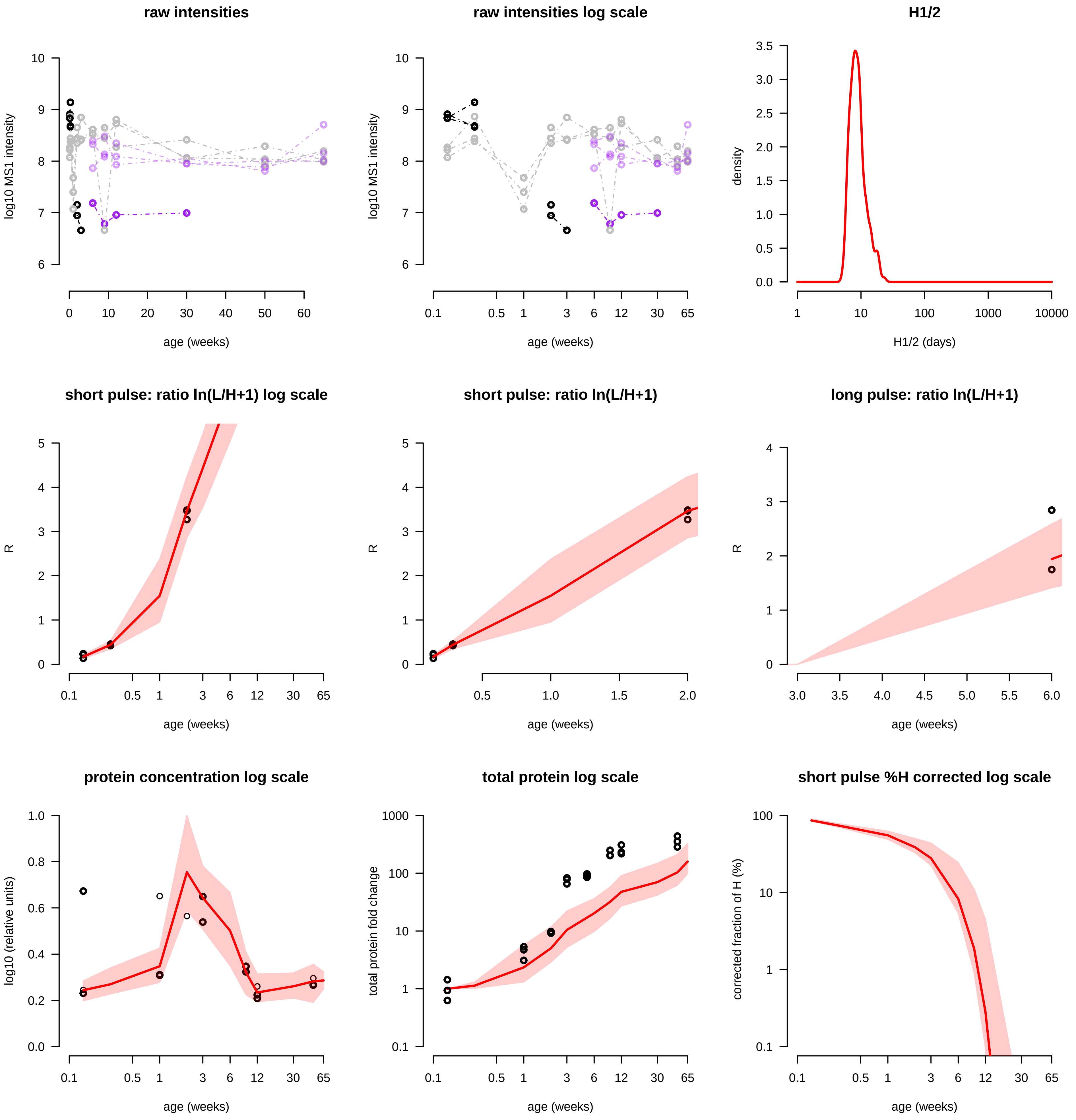

O89026

>sp|O89026|ROBO1_MOUSE

Roundabout homolog 1

GN=Robo1

Med H1/2: 11.1 days

Q5 H1/2: 7.8 days

Q95 H1/2: 17.2 days

reliable model fit: yes

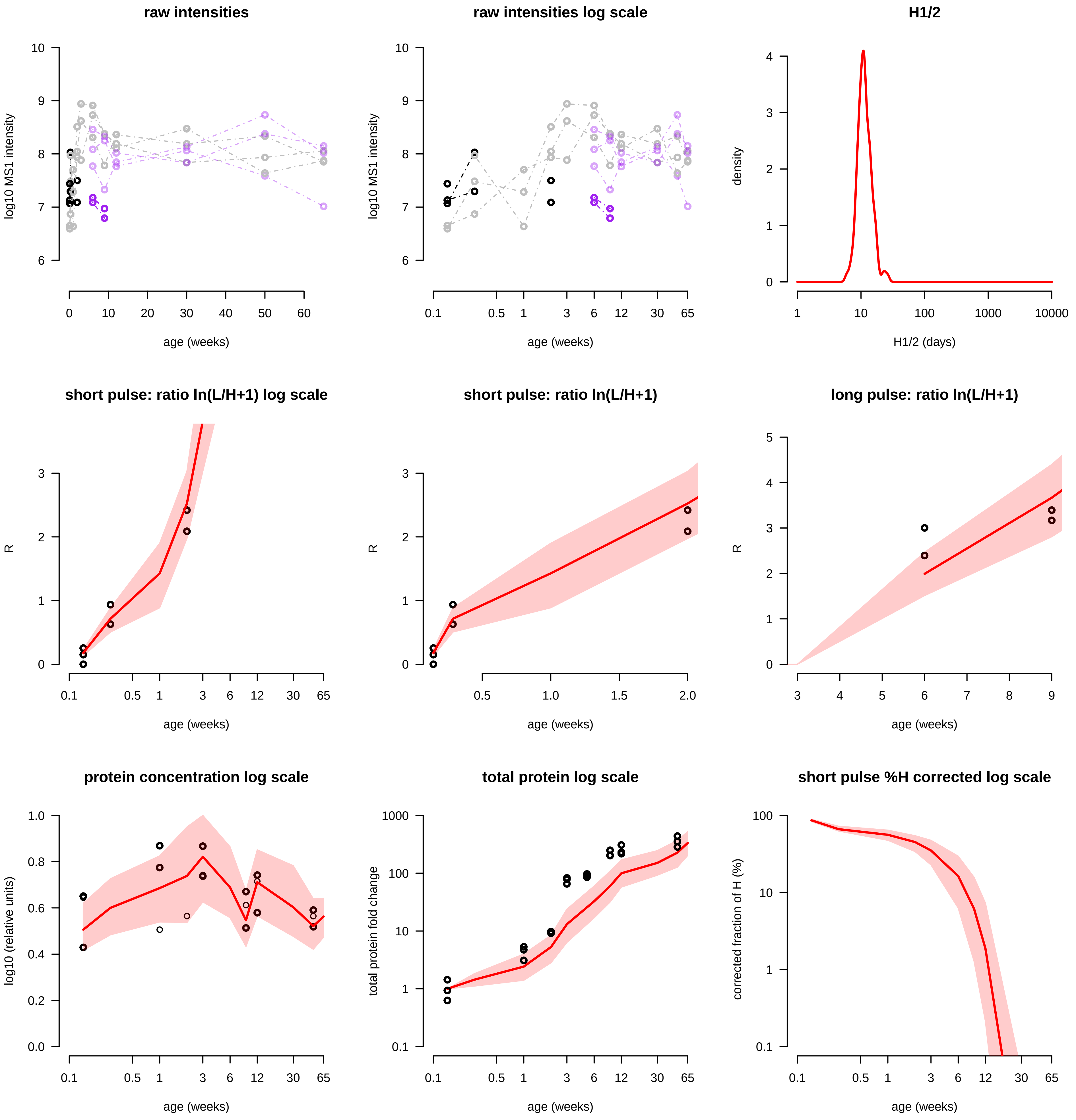

Q8C7V3

>sp|Q8C7V3|UTP15_MOUSE

U3 small nucleolar RNA−associated protein 15 homolog

GN=Utp15

Med H1/2: 2665.9 days

Q5 H1/2: 938 days

Q95 H1/2: 28154.9 days

reliable model fit: yes

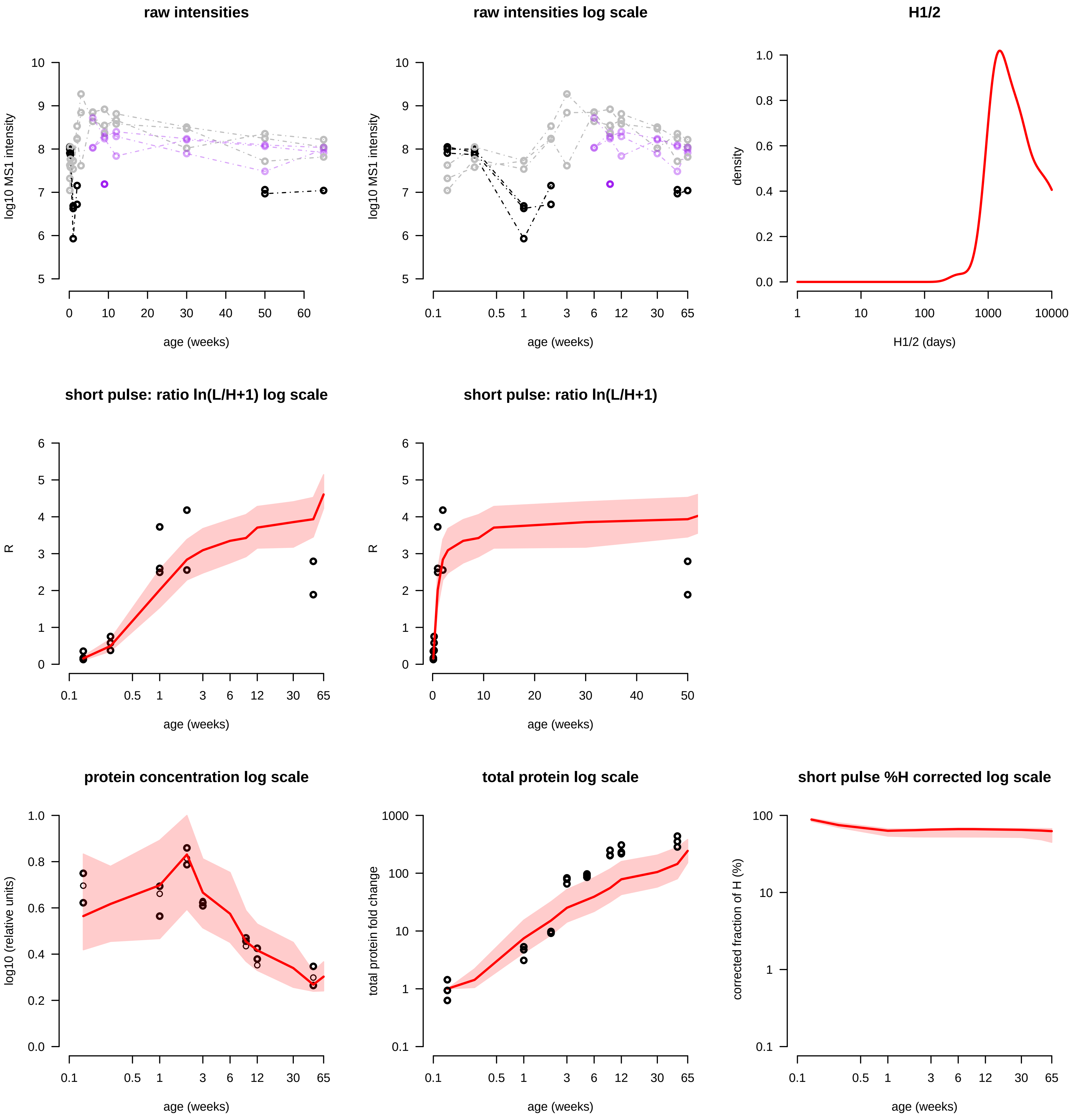

Q8K2Y7

>sp|Q8K2Y7|RM47_MOUSE

39S ribosomal protein L47, mitochondrial

GN=Mrpl47

Med H1/2: 11.2 days

Q5 H1/2: 4.1 days

Q95 H1/2: 84.9 days

reliable model fit: yes

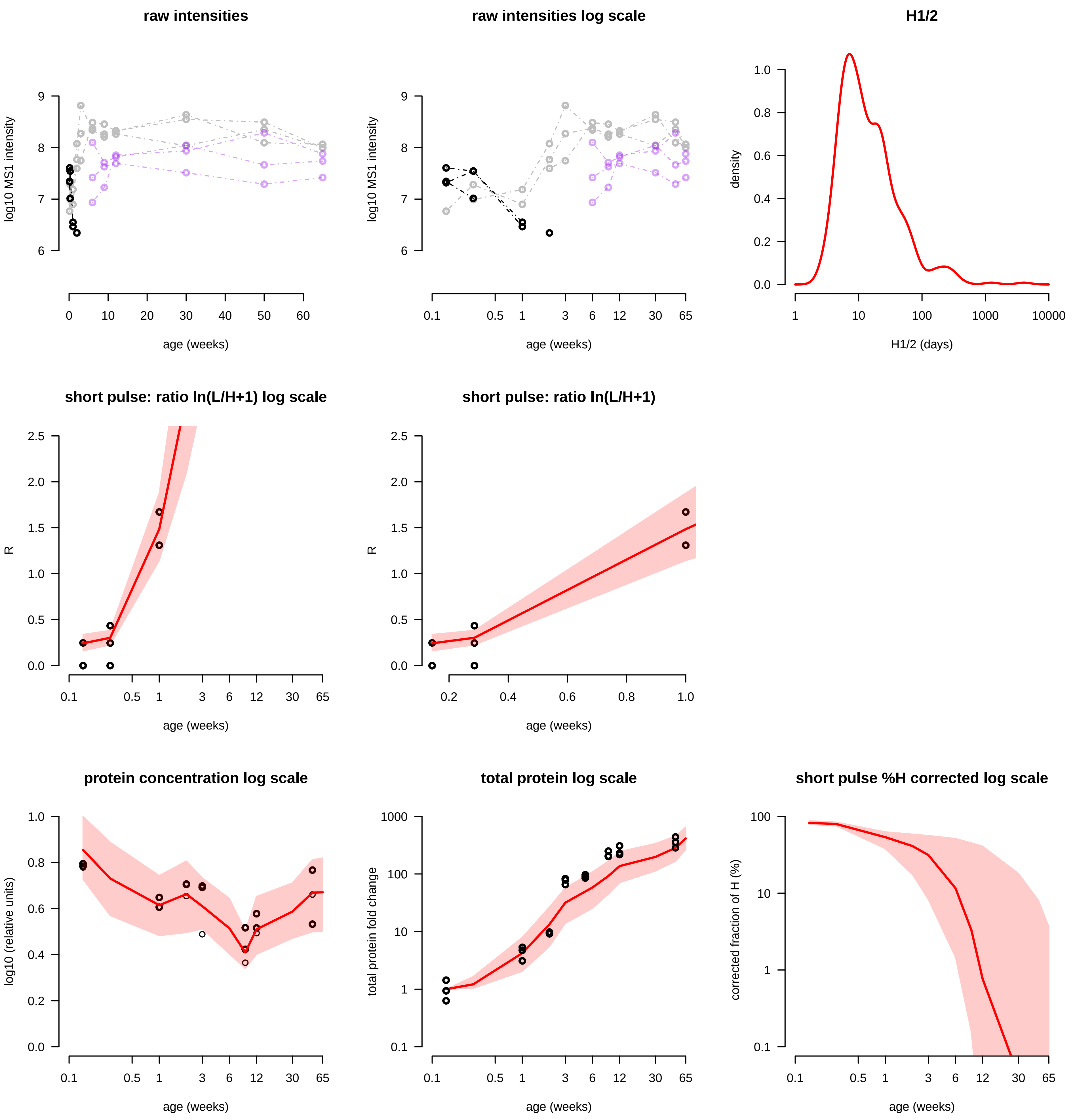

Q8C6K9;Q8C6K9−2

>sp|Q8C6K9|CO6A6_MOUSE

Collagen alpha−6(VI) chain

GN=Col6a6

Med H1/2: 6.2 days

Q5 H1/2: 3.2 days

Q95 H1/2: 13.9 days

reliable model fit: yes

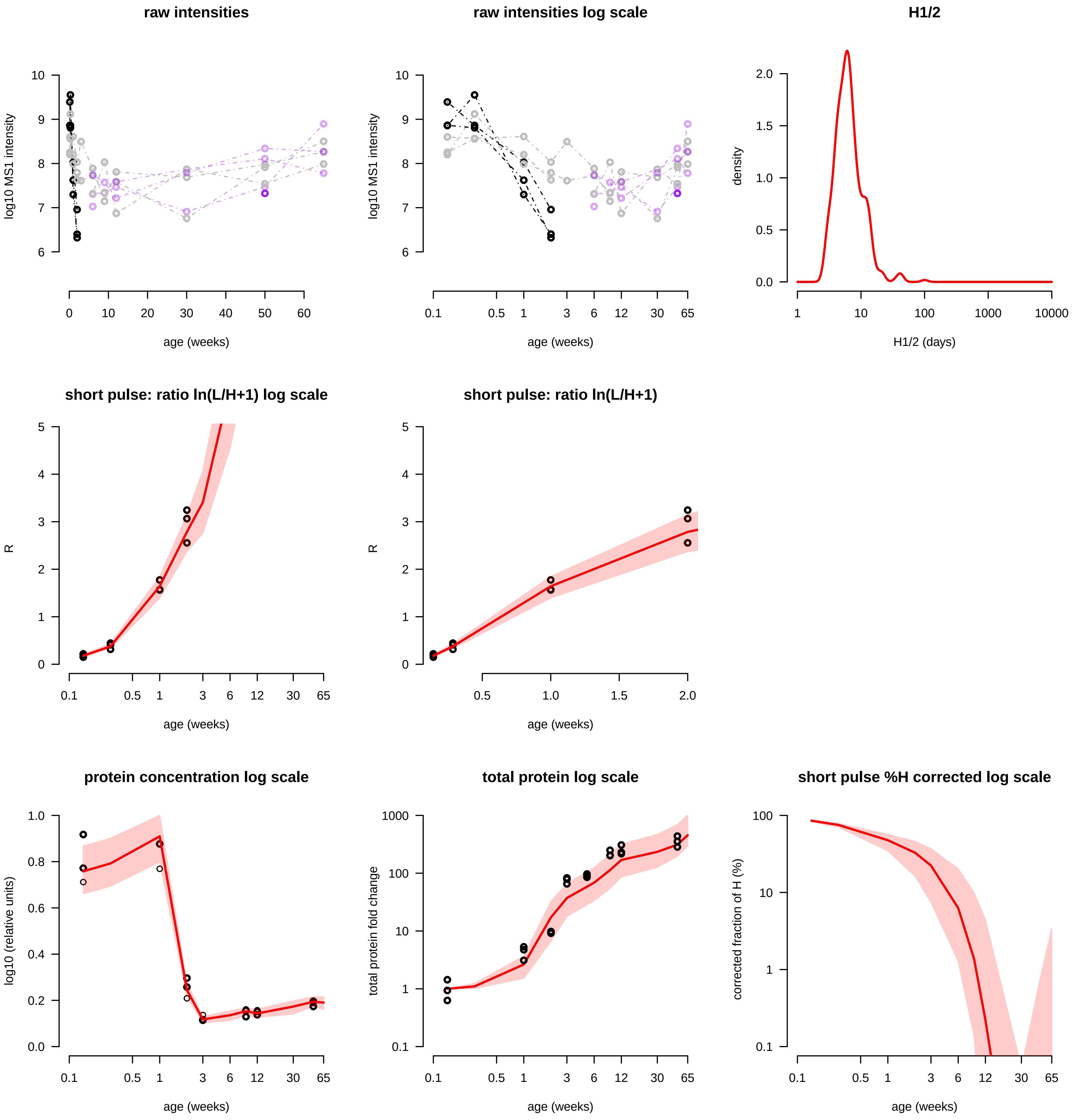

Q8R2U0−2;Q8R2U0

>sp|Q8R2U0−2|SEH1_MOUSE

Isoform 2 of Nucleoporin SEH1

GN=Seh1l

Med H1/2: 7.8 days

Q5 H1/2: 4.4 days

Q95 H1/2: 81.3 days

reliable model fit: yes

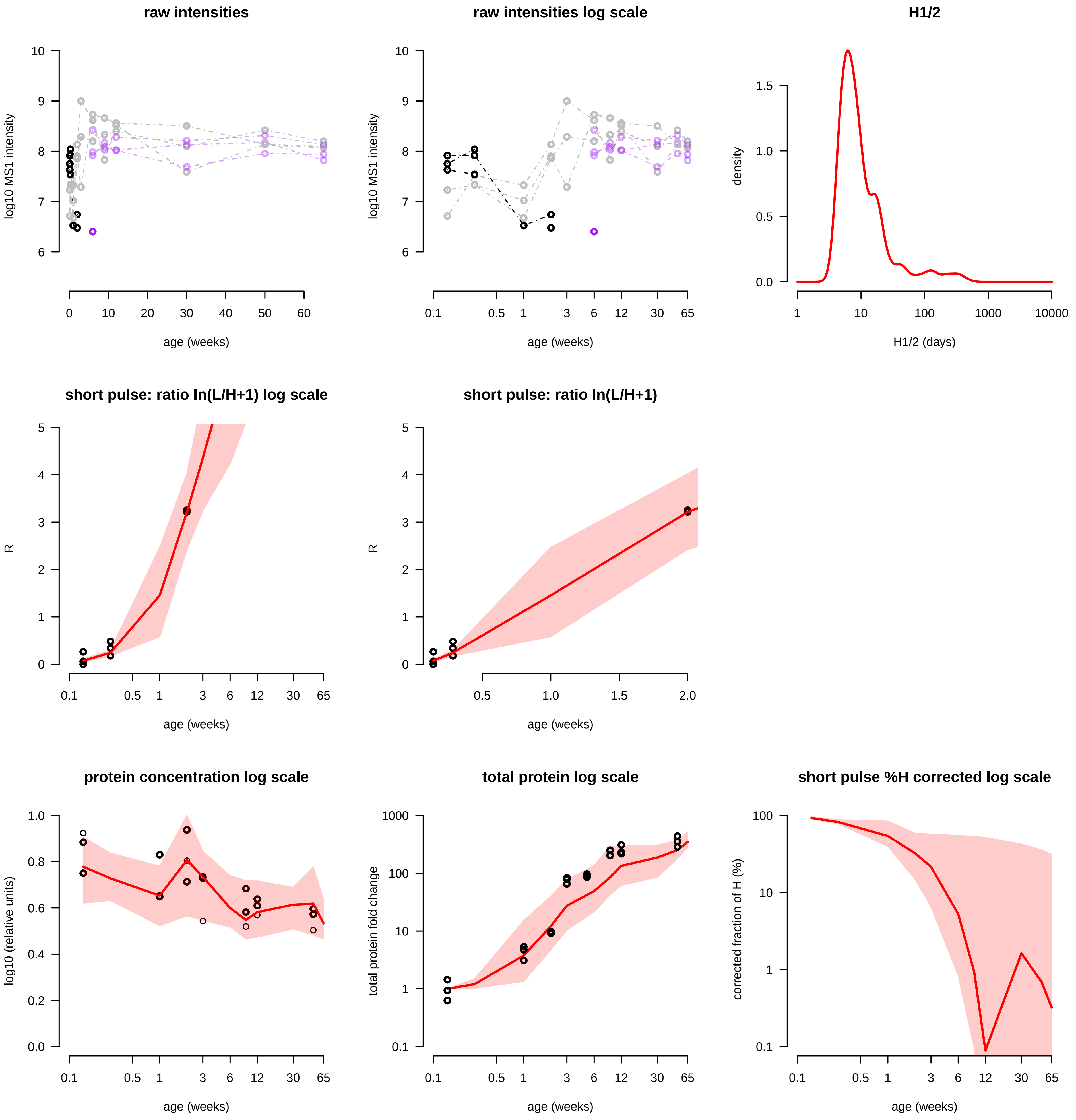

Q9Z0H3

>sp|Q9Z0H3|SNF5_MOUSE

-related matrix−associated actin−dependent regulator of chromatin subfamily B

GN=Smarcb1

Med H1/2: 4.6 days

Q5 H1/2: 3.5 days

Q95 H1/2: 7.1 days

reliable model fit: yes

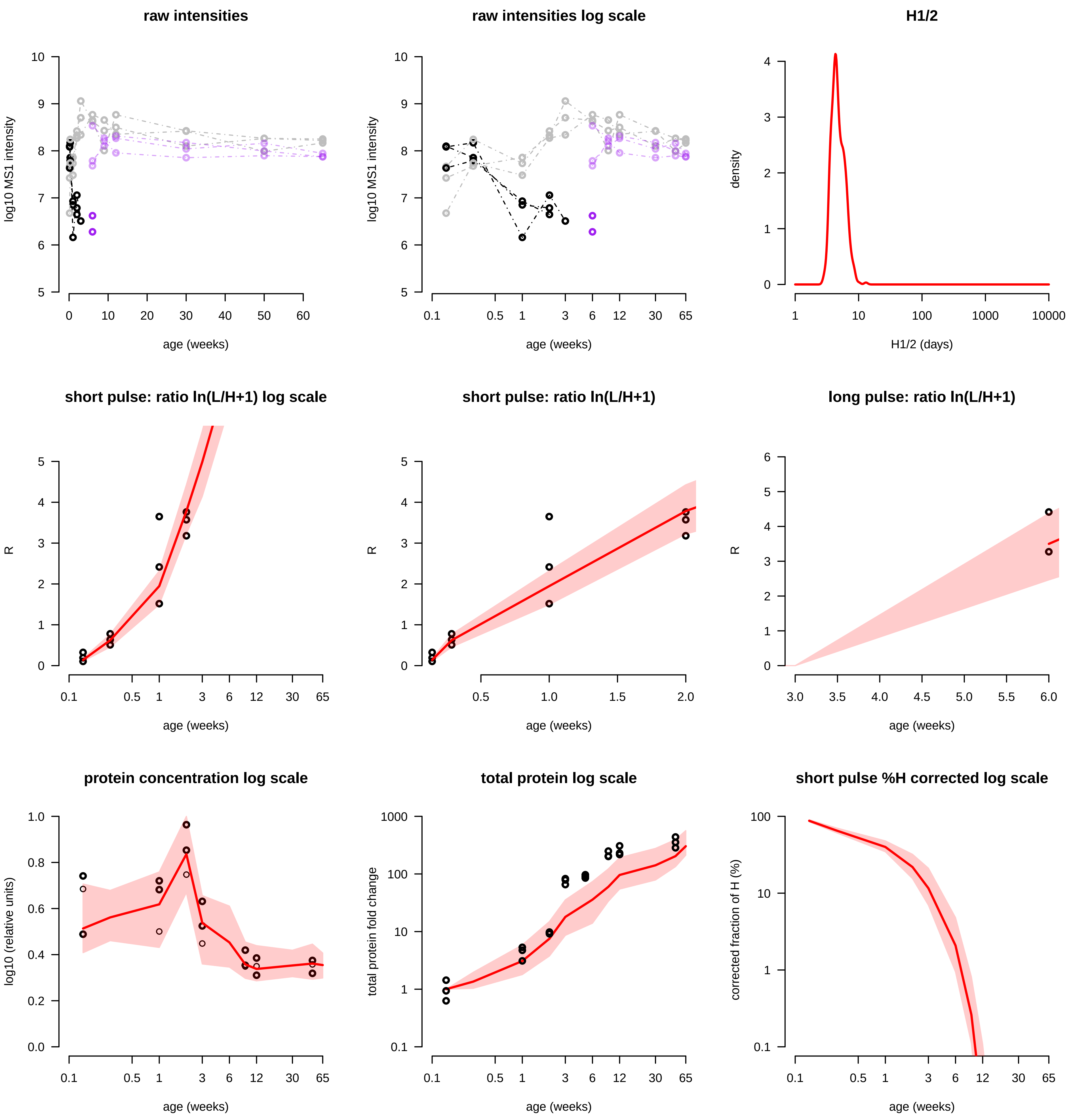

Q8K2F0;Q8K2F0−2

>sp|Q8K2F0|BRD3_MOUSE

Bromodomain−containing protein 3

GN=Brd3

Med H1/2: 6.7 days

Q5 H1/2: 4.7 days

Q95 H1/2: 12.3 days

reliable model fit: no

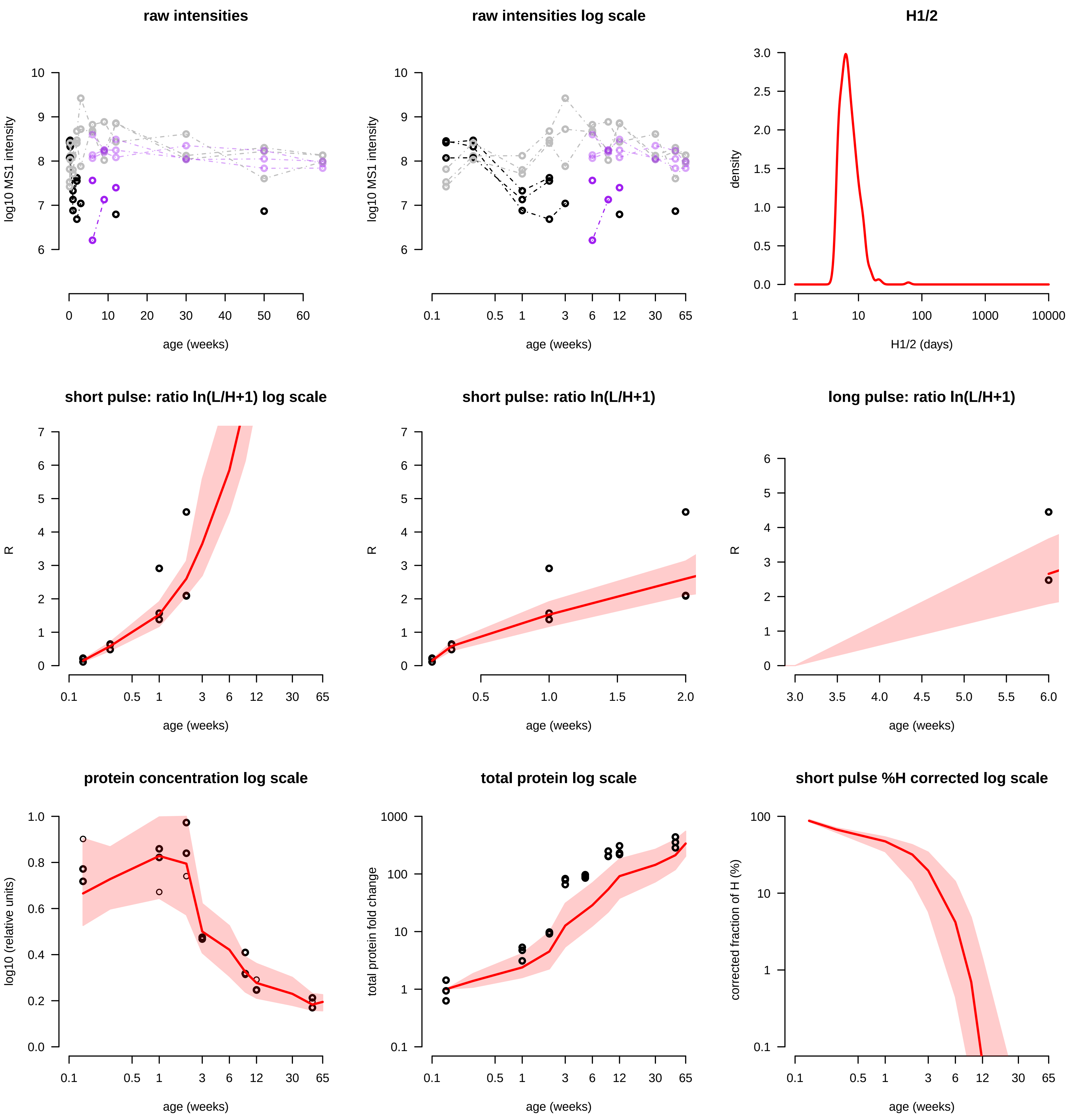

Q99NH0;Q99NH0−3;Q99NH0−2

>sp|Q99NH0|ANR17_MOUSE

Ankyrin repeat domain−containing protein 17

GN=Ankrd17

Med H1/2: 3.5 days

Q5 H1/2: 2.5 days

Q95 H1/2: 10.8 days

reliable model fit: yes

raw intensities

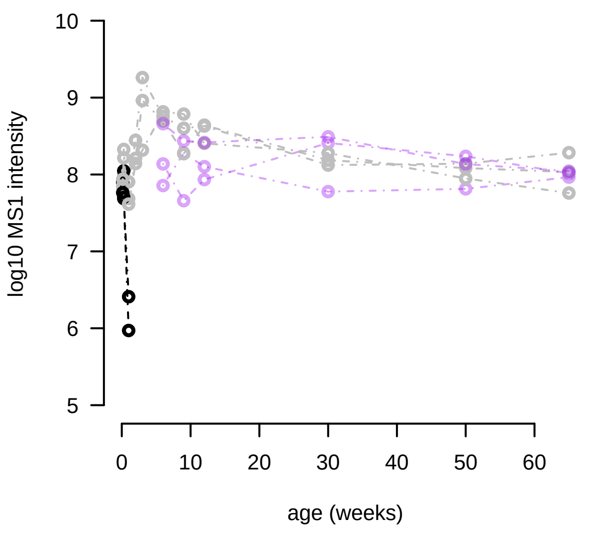

raw intensities log scale

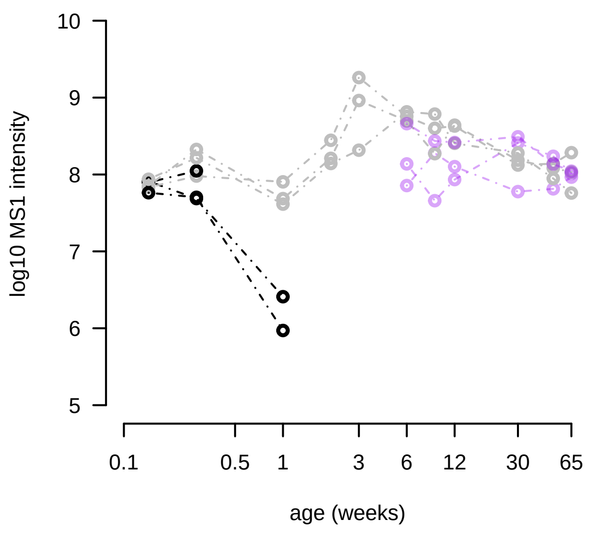

H1/2

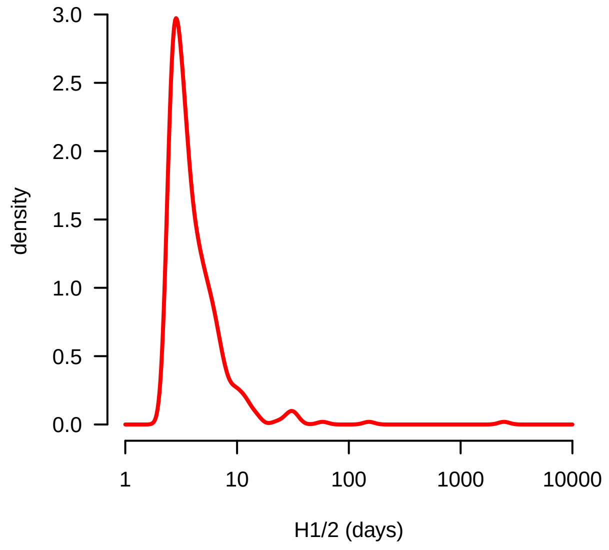

short pulse: ratio ln(L/H+1) log scale

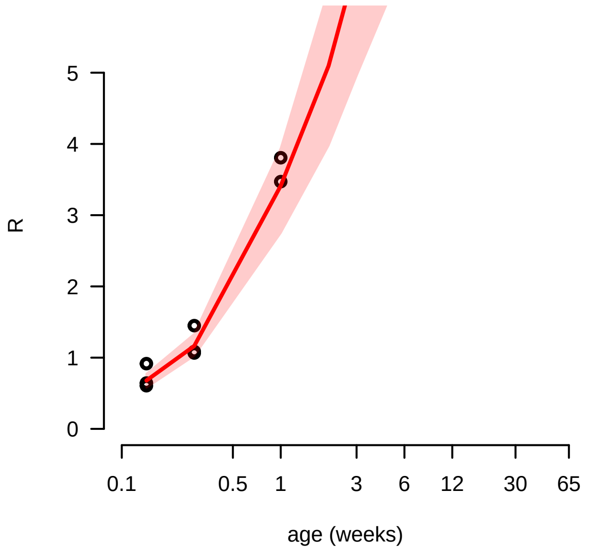

short pulse: ratio ln(L/H+1)

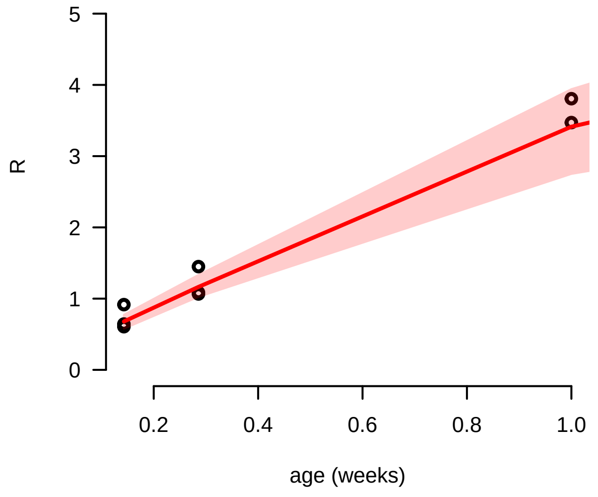

protein concentration log scale

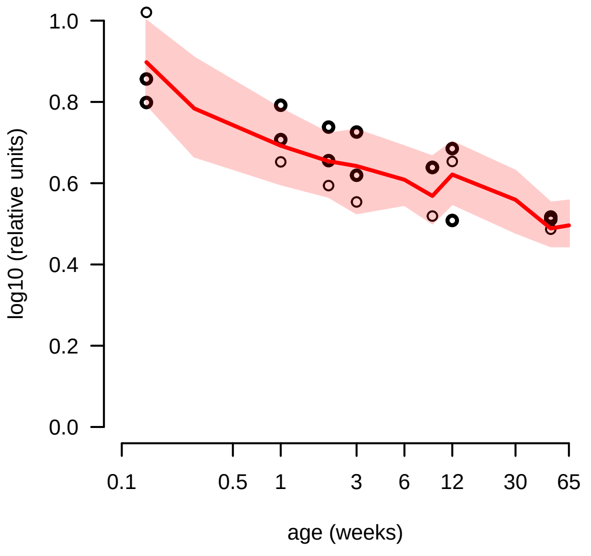

total protein log scale

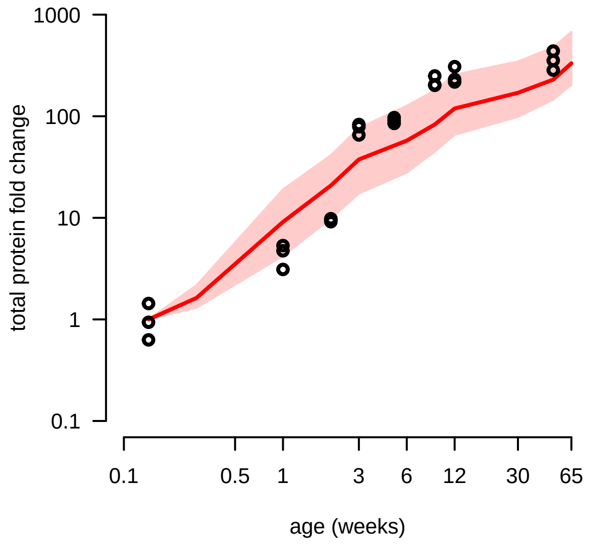

short pulse %H corrected log scale

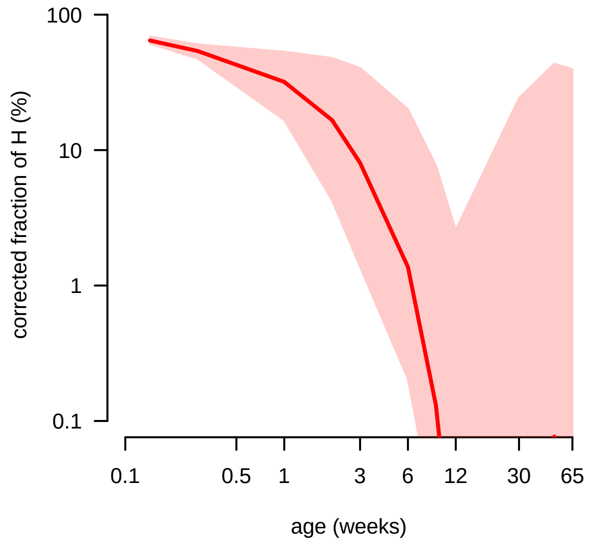

Q7TT18

>sp|Q7TT18|MCAF1_MOUSE

Activating transcription factor 7−interacting protein 1

GN=Atf7ip

Med H1/2: 3559.3 days

Q5 H1/2: 696.8 days

Q95 H1/2: 40345 days

reliable model fit: no

raw intensities

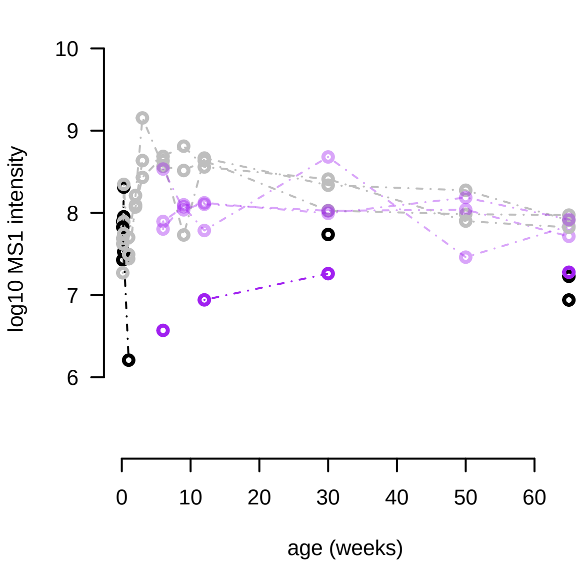

raw intensities log scale

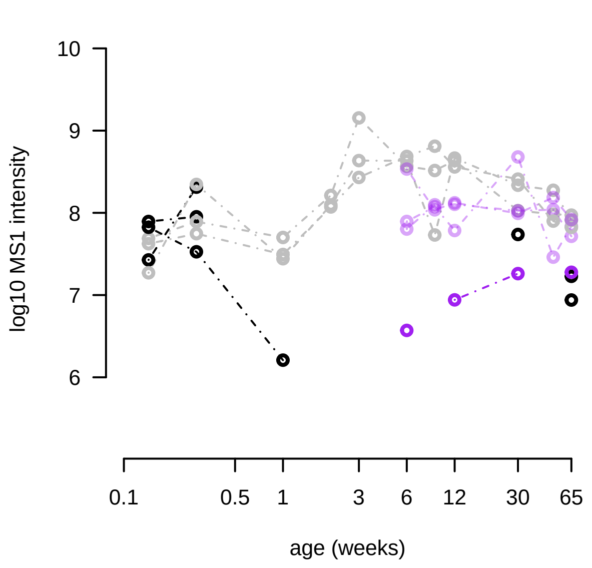

H1/2

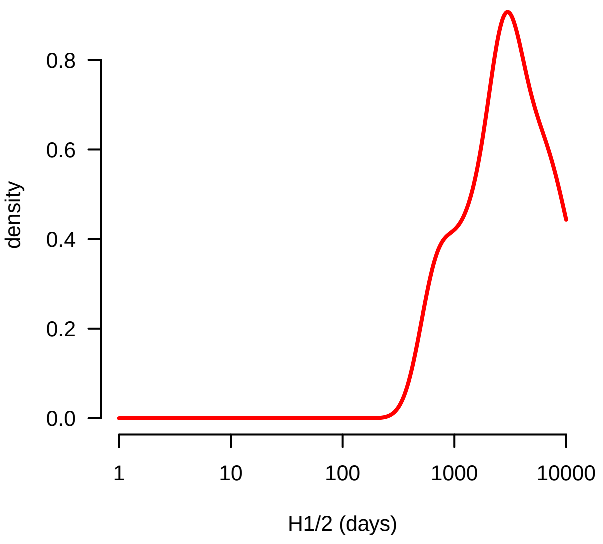

short pulse: ratio ln(L/H+1) log scale

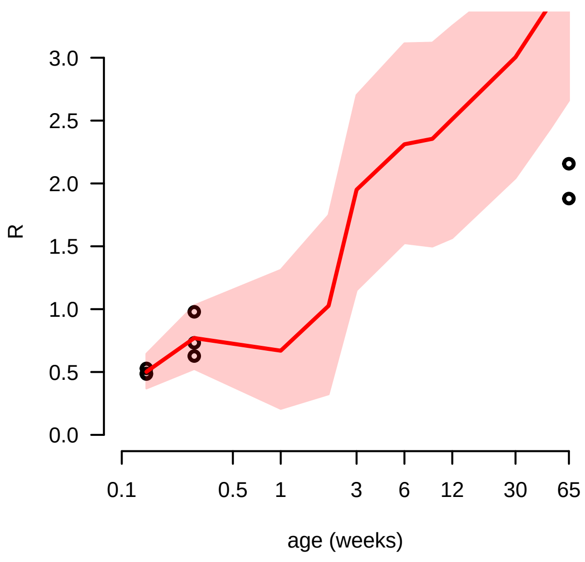

short pulse: ratio ln(L/H+1)

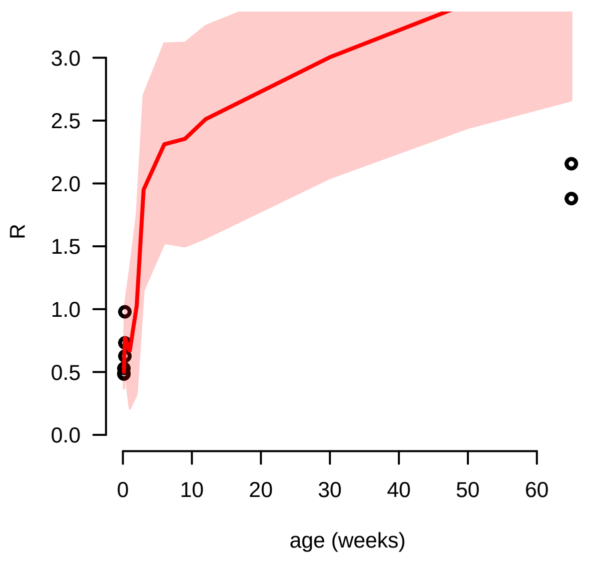

protein concentration log scale

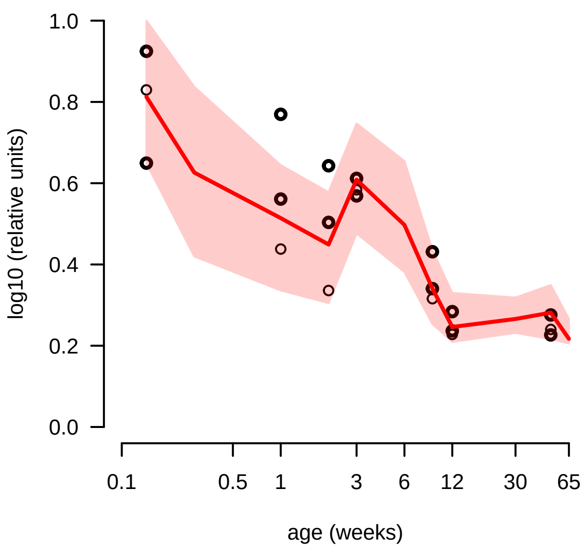

total protein log scale

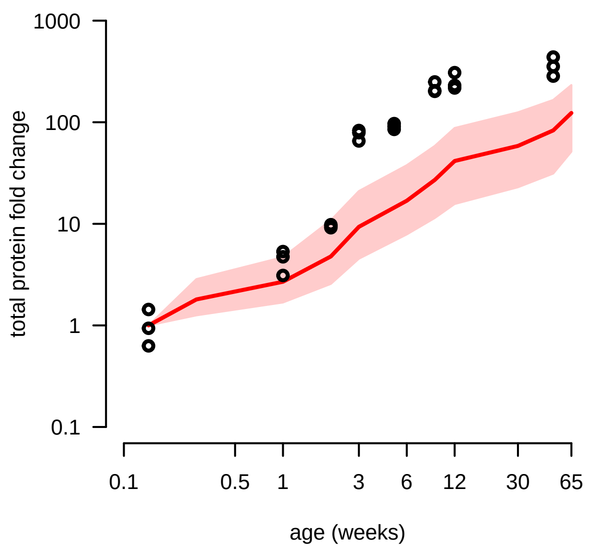

short pulse %H corrected log scale

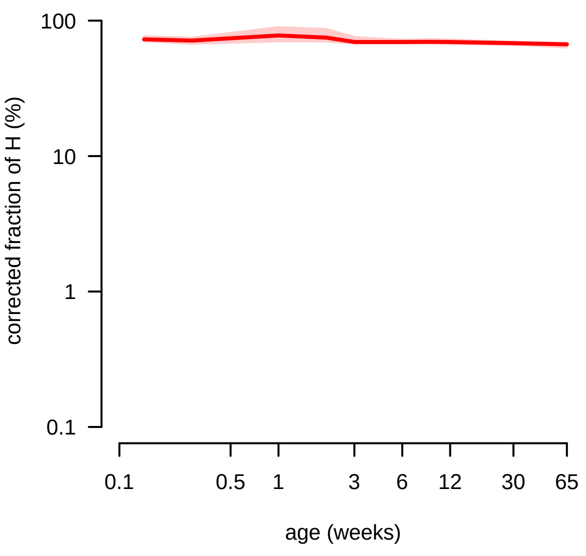

Q8R3C6;Q8R3C6−2

>sp|Q8R3C6|RBM19_MOUSE

Probable RNA−binding protein 19

GN=Rbm19

Med H1/2: 2849.1 days

Q5 H1/2: 698.1 days

Q95 H1/2: 43678.5 days

reliable model fit: yes

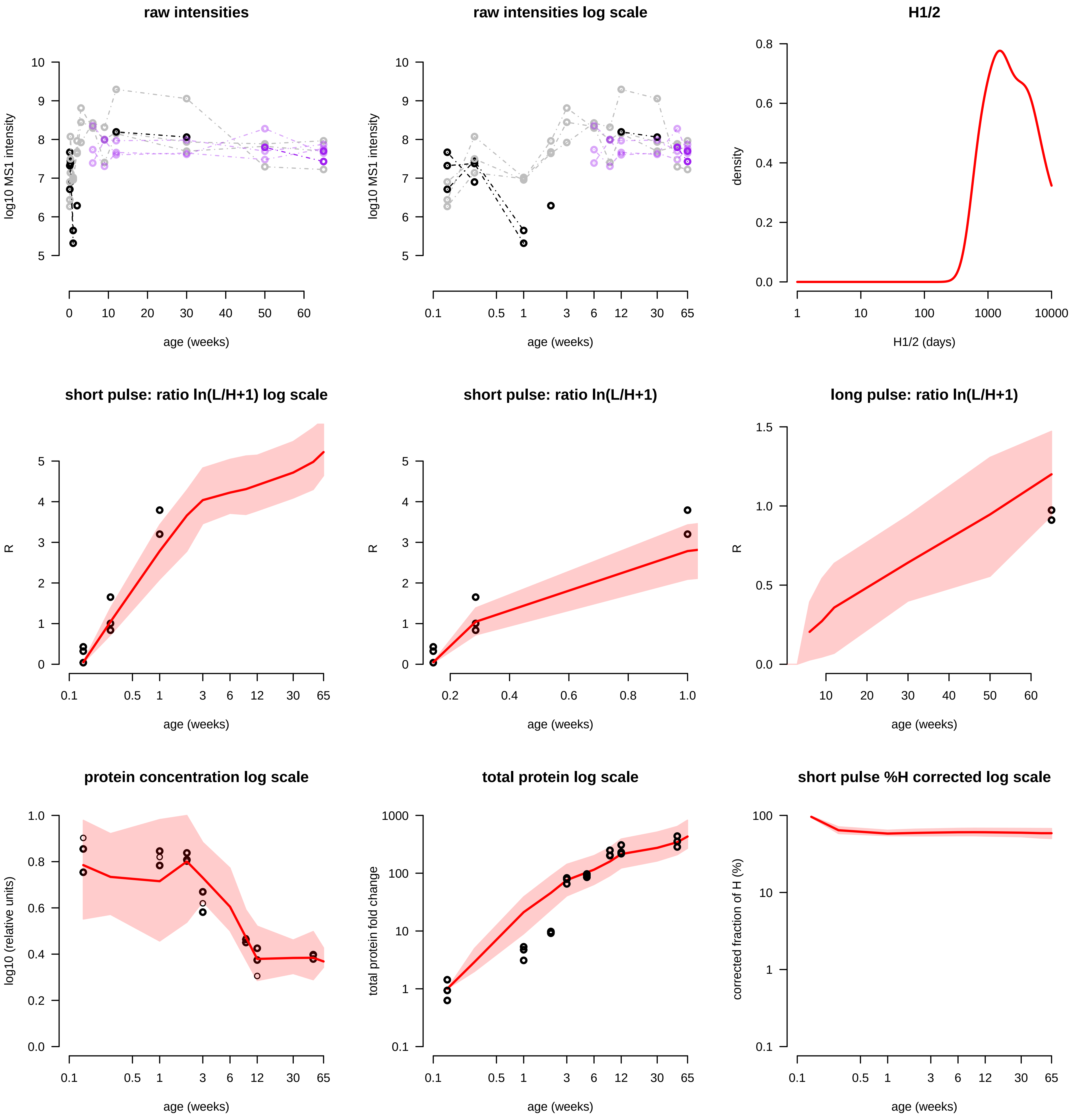

Q8CHP5−2

>sp|Q8CHP5−2|PYM1_MOUSE

Isoform 2 of Partner of Y14 and mago

GN=Pym1

Med H1/2: 18.6 days

Q5 H1/2: 6 days

Q95 H1/2: 357.6 days

reliable model fit: no

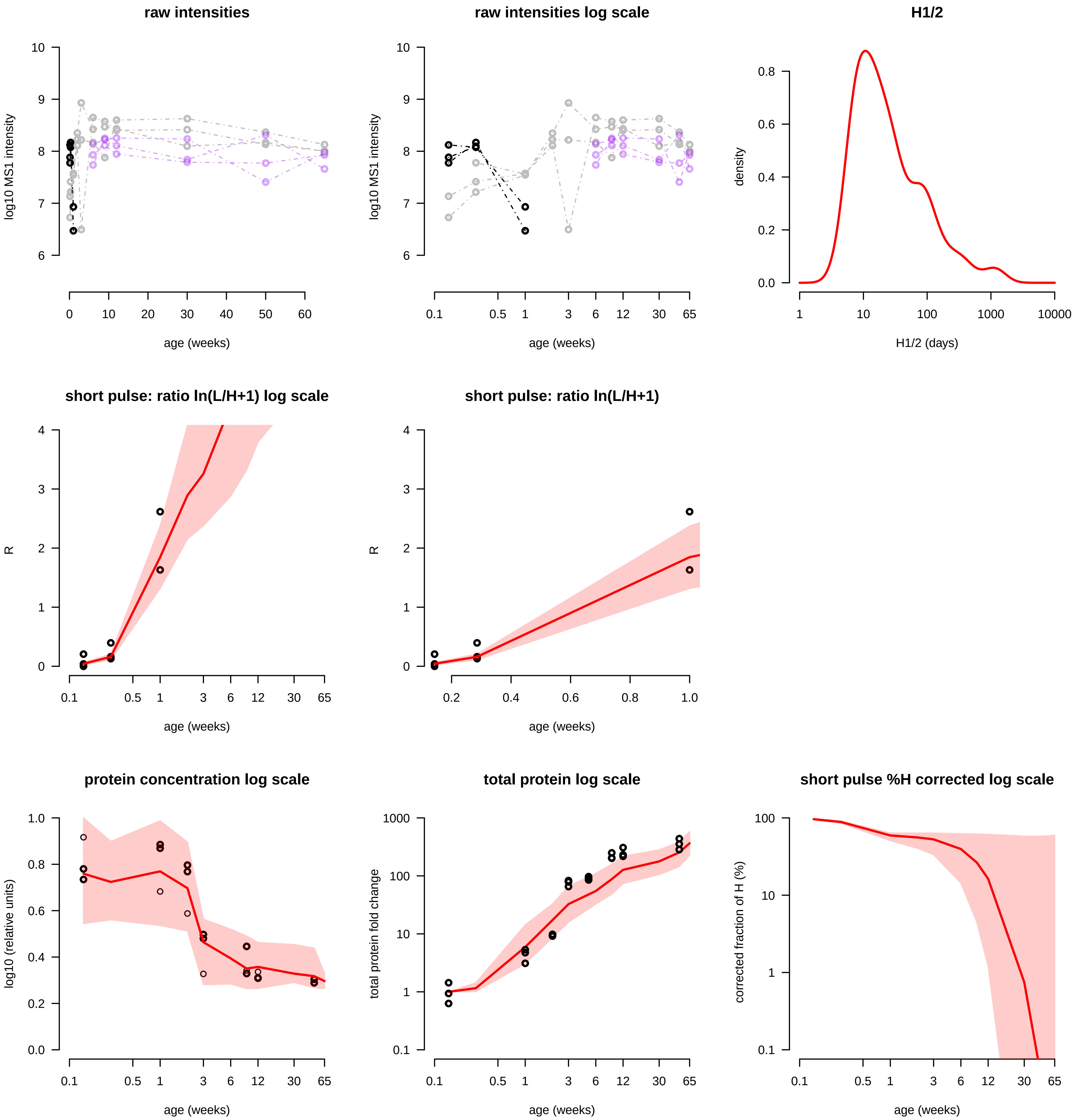

Q8BZR9

>sp|Q8BZR9|NCBP3_MOUSE

Nuclear cap−binding protein subunit 3

GN=Ncbp3

Med H1/2: 5.9 days

Q5 H1/2: 2.7 days

Q95 H1/2: 42.6 days

reliable model fit: yes

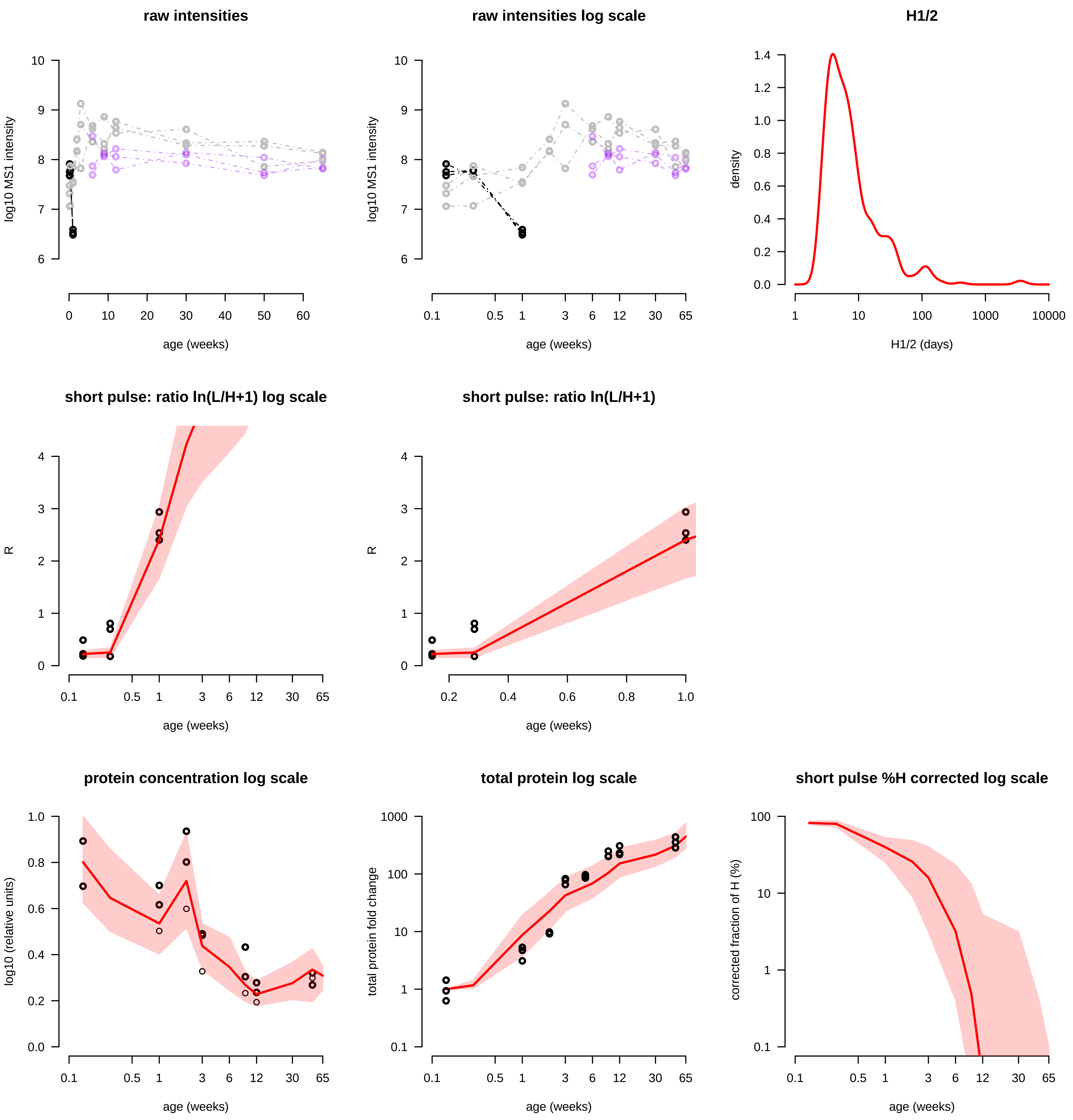

Q8CG47

>sp|Q8CG47|SMC4_MOUSE

Structural maintenance of chromosomes protein 4

GN=Smc4

Med H1/2: 4.2 days

Q5 H1/2: 2.7 days

Q95 H1/2: 10 days

reliable model fit: yes

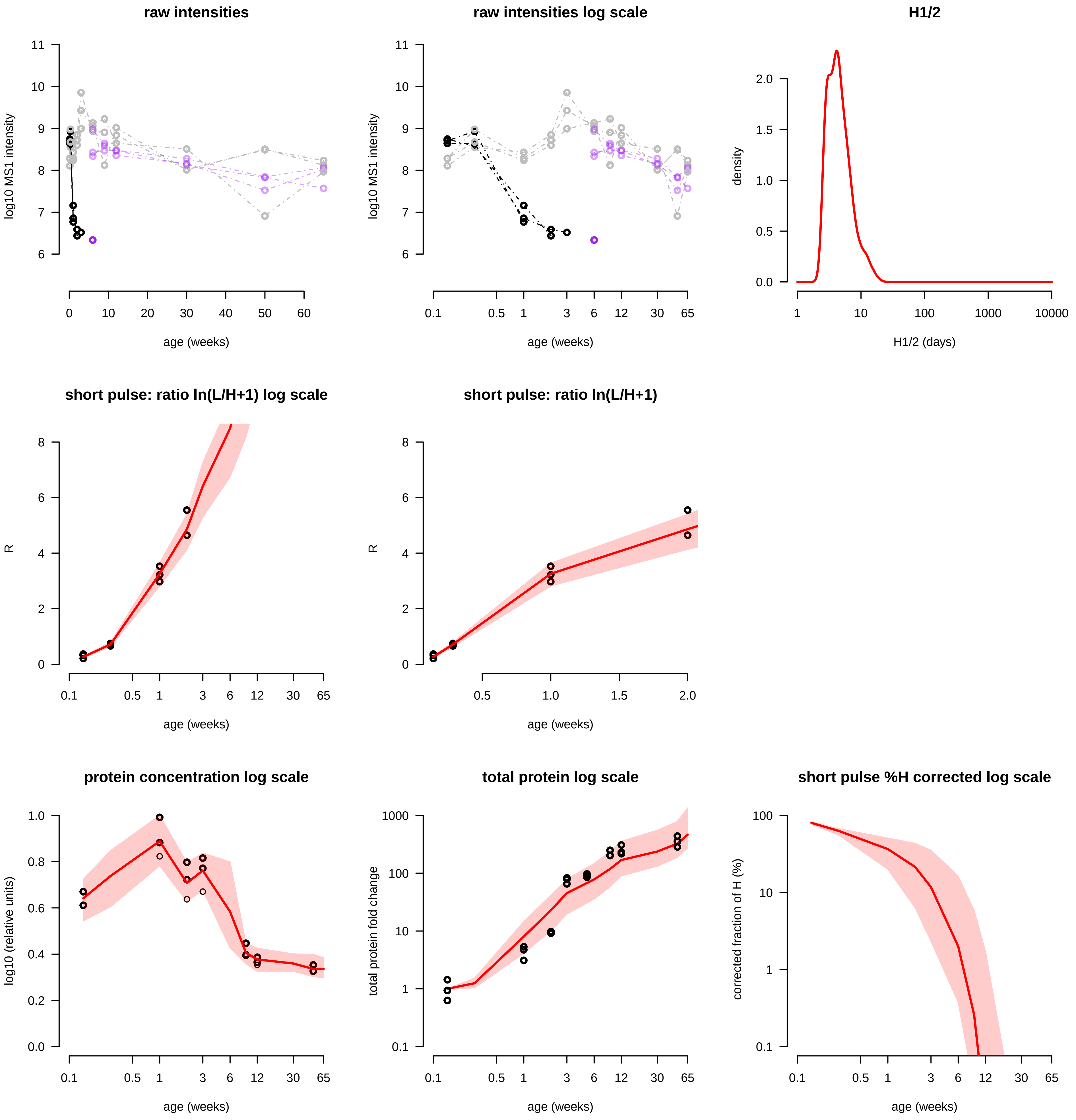

Q60575

>sp|Q60575|KIF1B_MOUSE

Kinesin−like protein KIF1B

GN=Kif1b

Med H1/2: 3737.5 days

Q5 H1/2: 782.1 days

Q95 H1/2: 53070.9 days

reliable model fit: yes

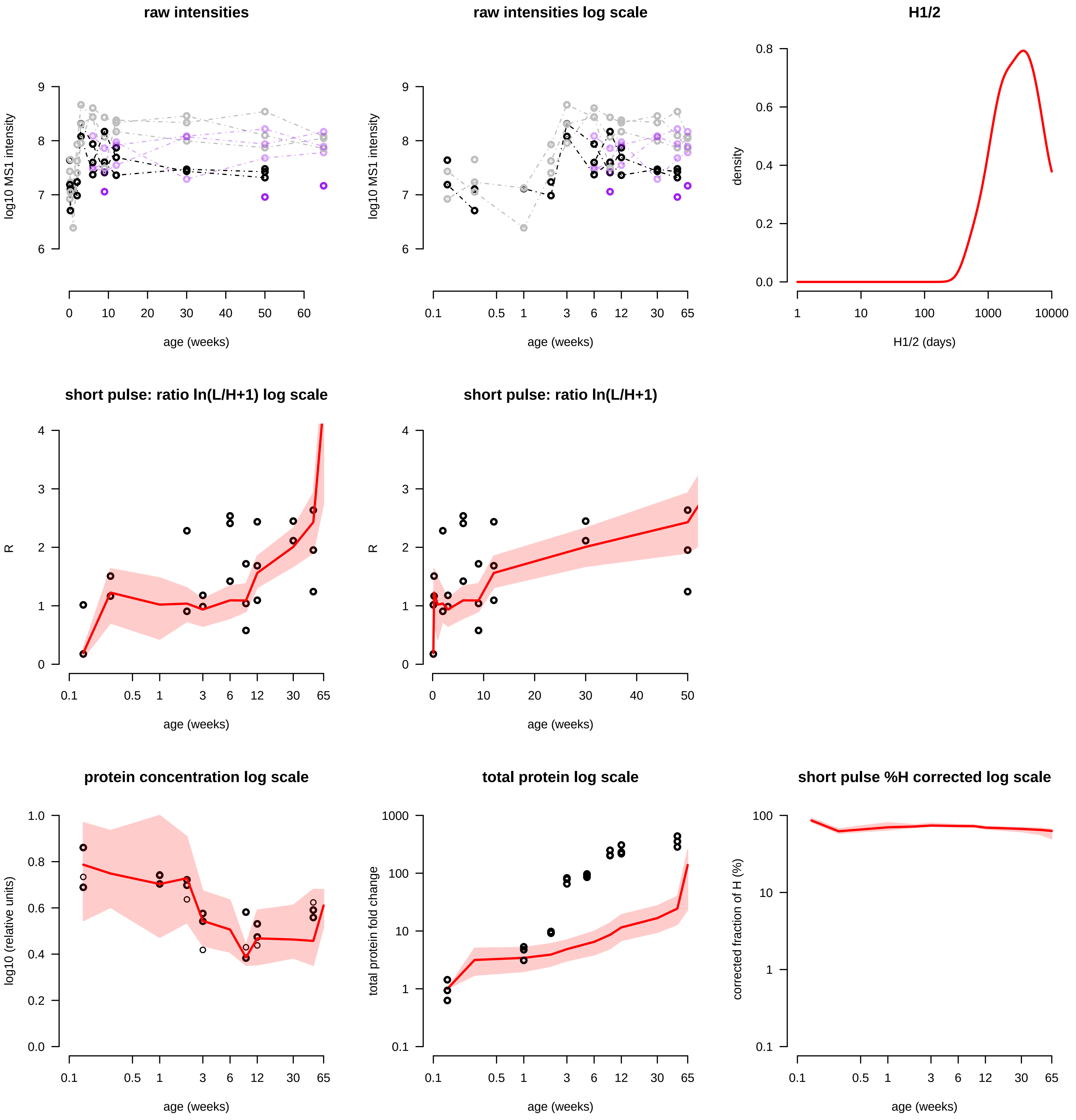

Q6ZQH8

>sp|Q6ZQH8|NU188_MOUSE

Nucleoporin NUP188

GN=Nup188

Med H1/2: 8.2 days

Q5 H1/2: 4.9 days

Q95 H1/2: 42.5 days

reliable model fit: yes

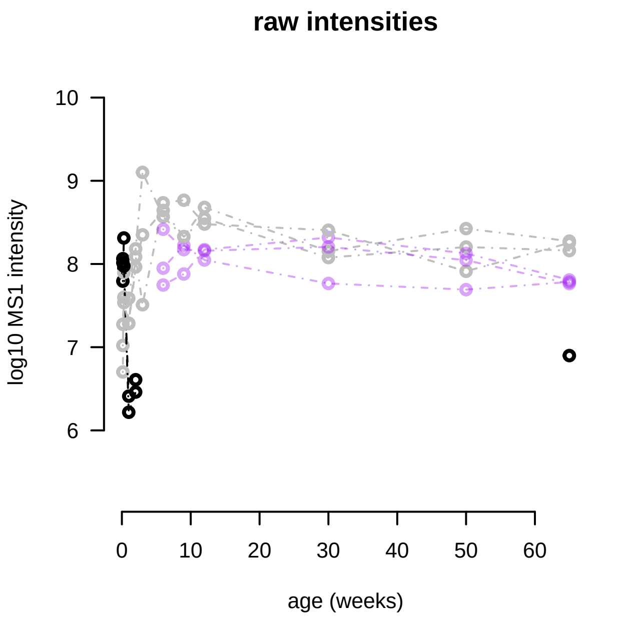

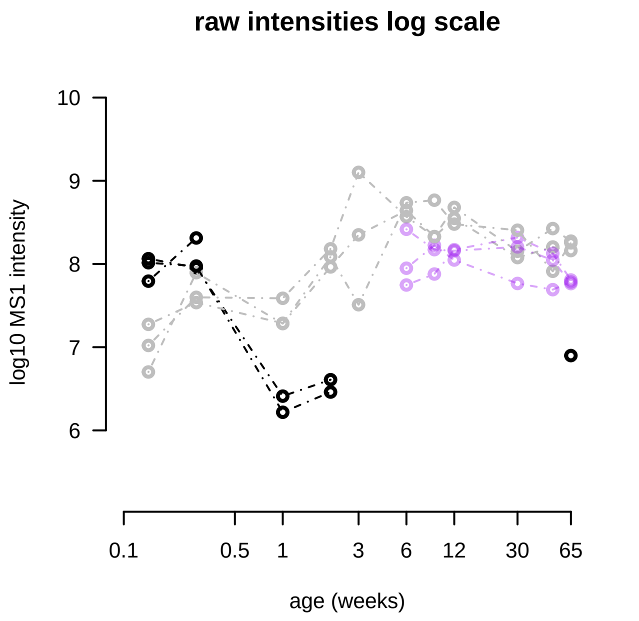

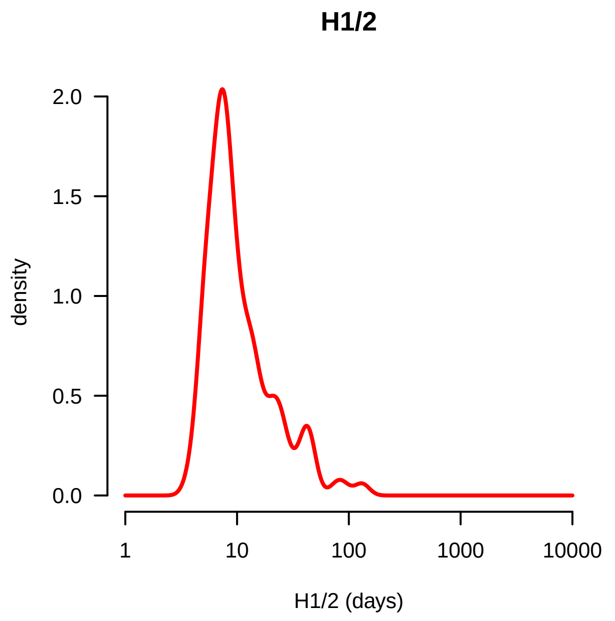

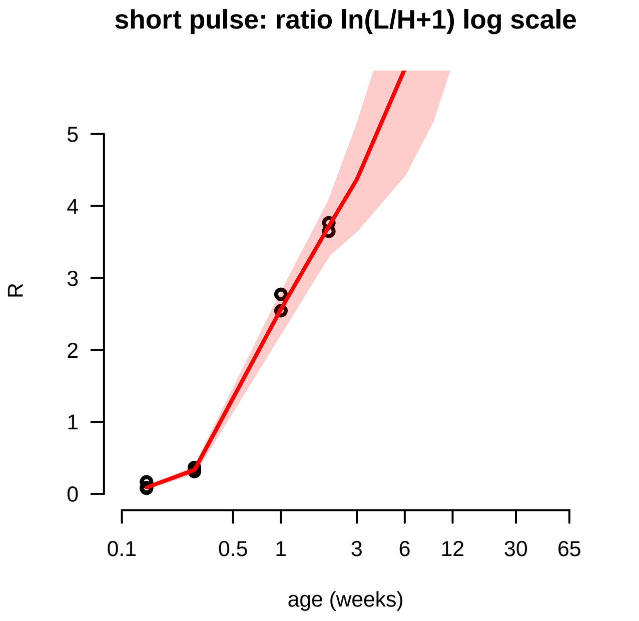

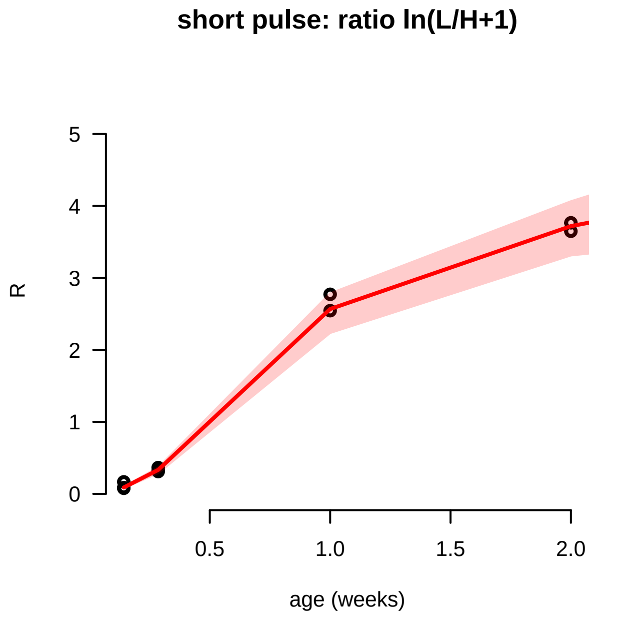

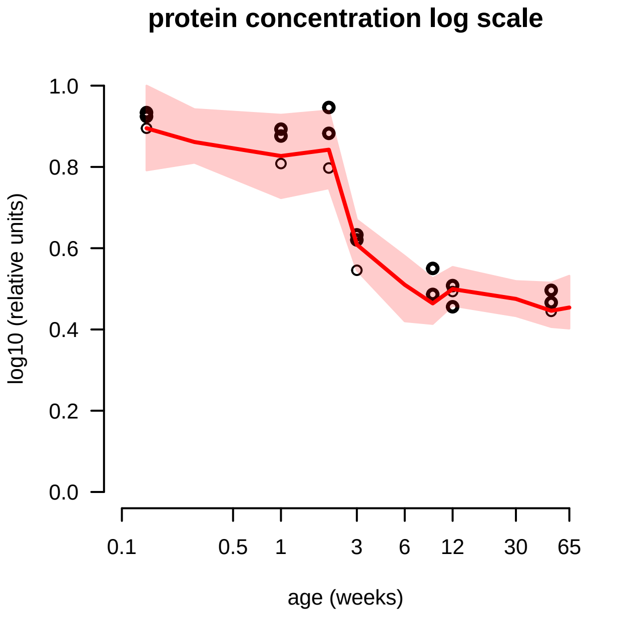

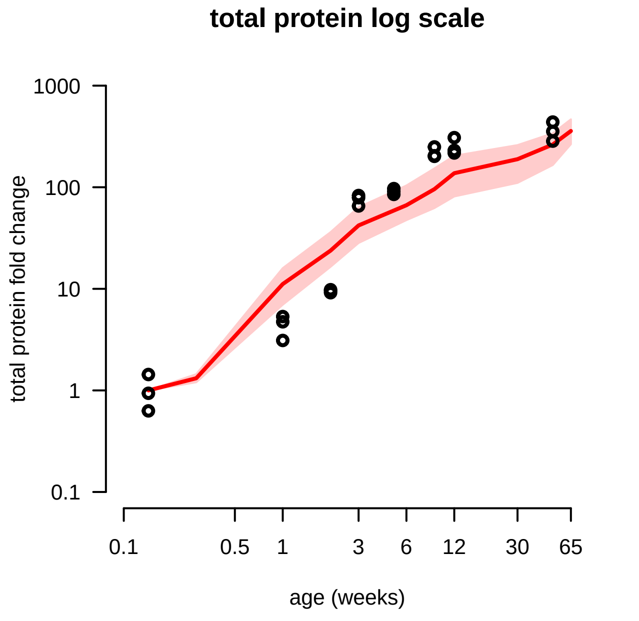

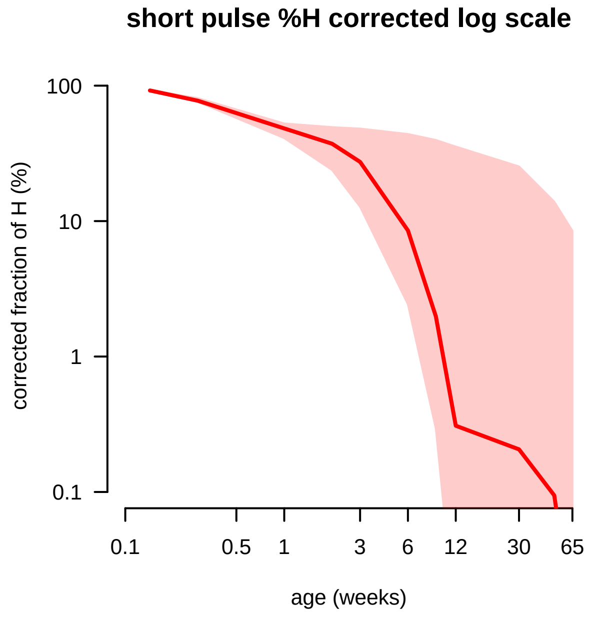

Q8K4P0

>sp|Q8K4P0|WDR33_MOUSE

pre−mRNA 3' end processing protein WDR33

GN=Wdr33

Med H1/2: 5.7 days

Q5 H1/2: 2.8 days

Q95 H1/2: 47.7 days

reliable model fit: yes

raw intensities

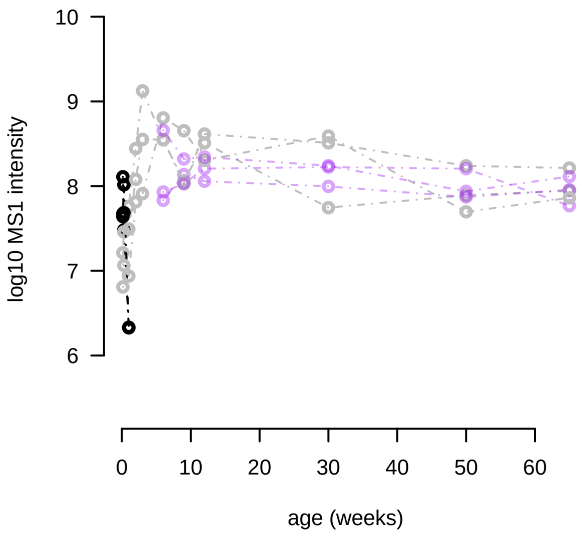

raw intensities log scale

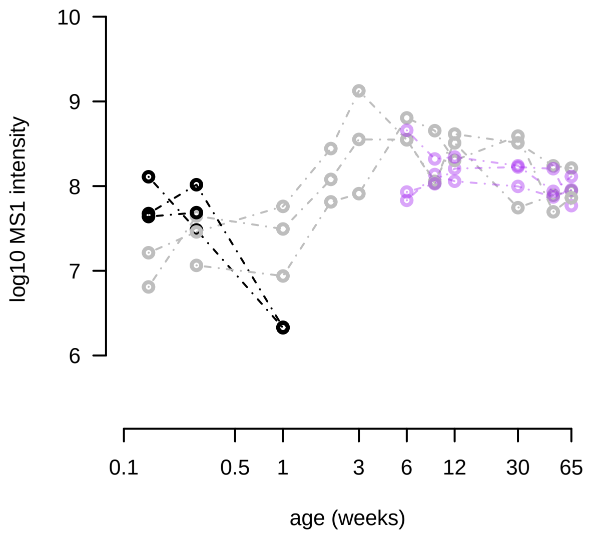

H1/2

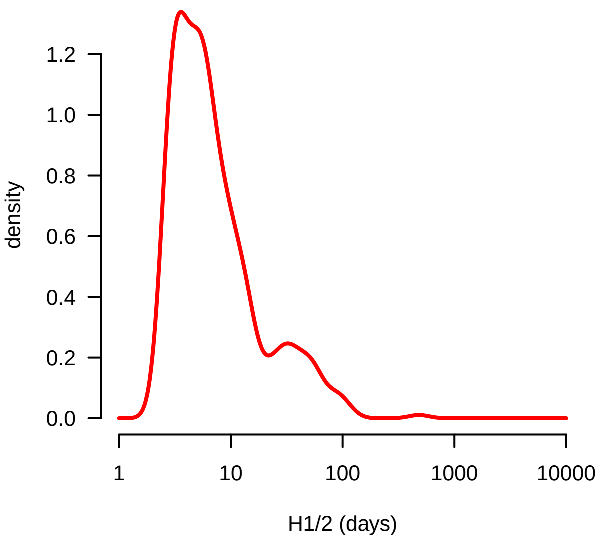

short pulse: ratio ln(L/H+1) log scale

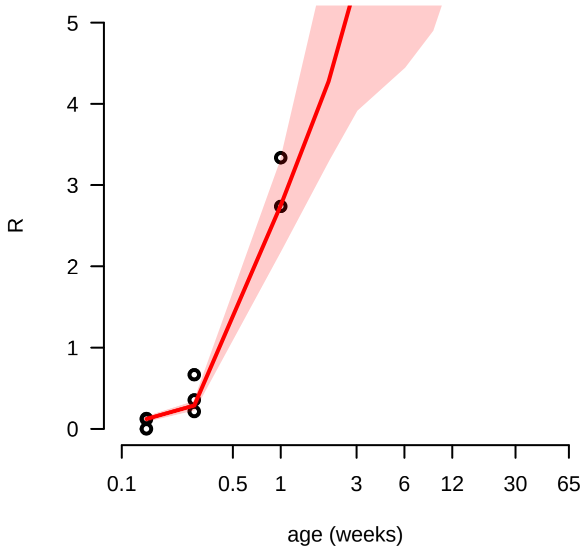

short pulse: ratio ln(L/H+1)

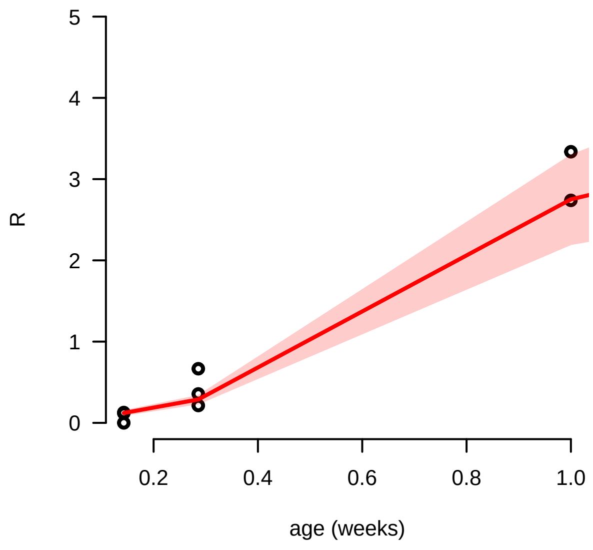

protein concentration log scale

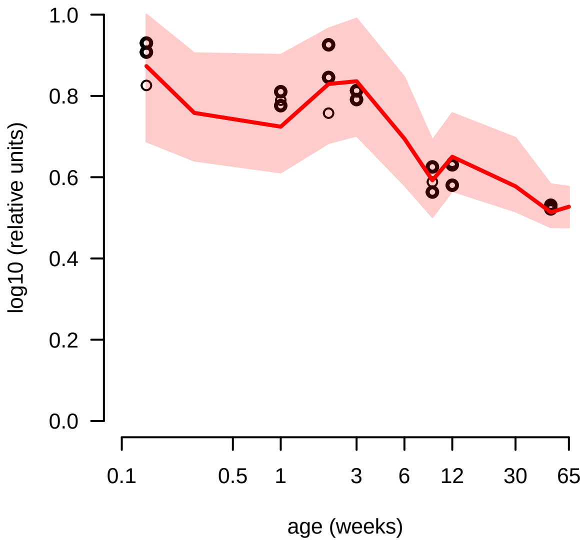

total protein log scale

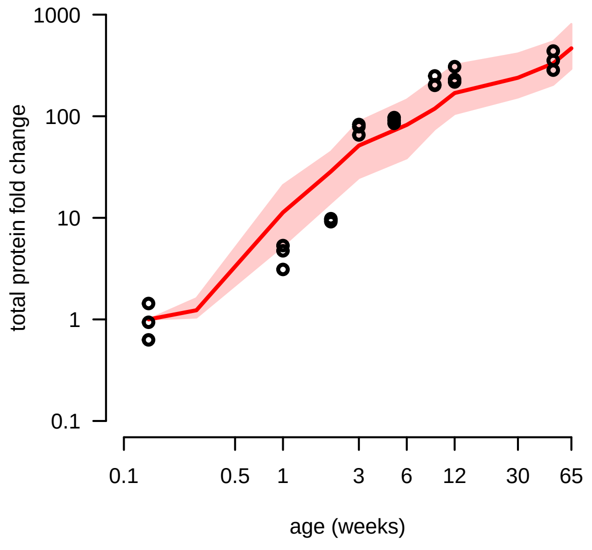

short pulse %H corrected log scale

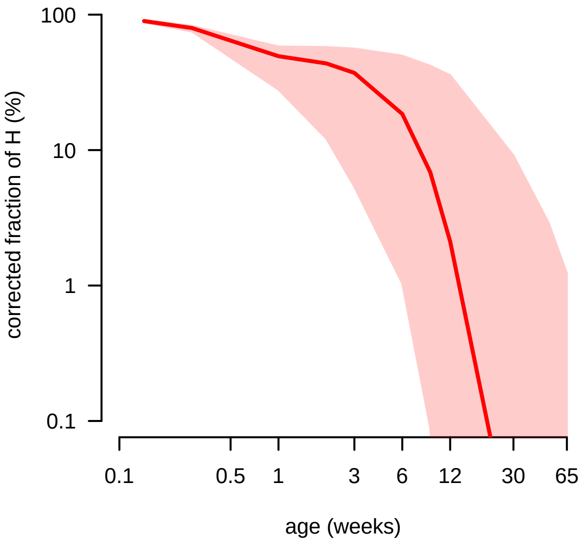

Q91W96

>sp|Q91W96|APC4_MOUSE

Anaphase−promoting complex subunit 4

GN=Anapc4

Med H1/2: 4.9 days

Q5 H1/2: 3.8 days

Q95 H1/2: 7 days

reliable model fit: yes

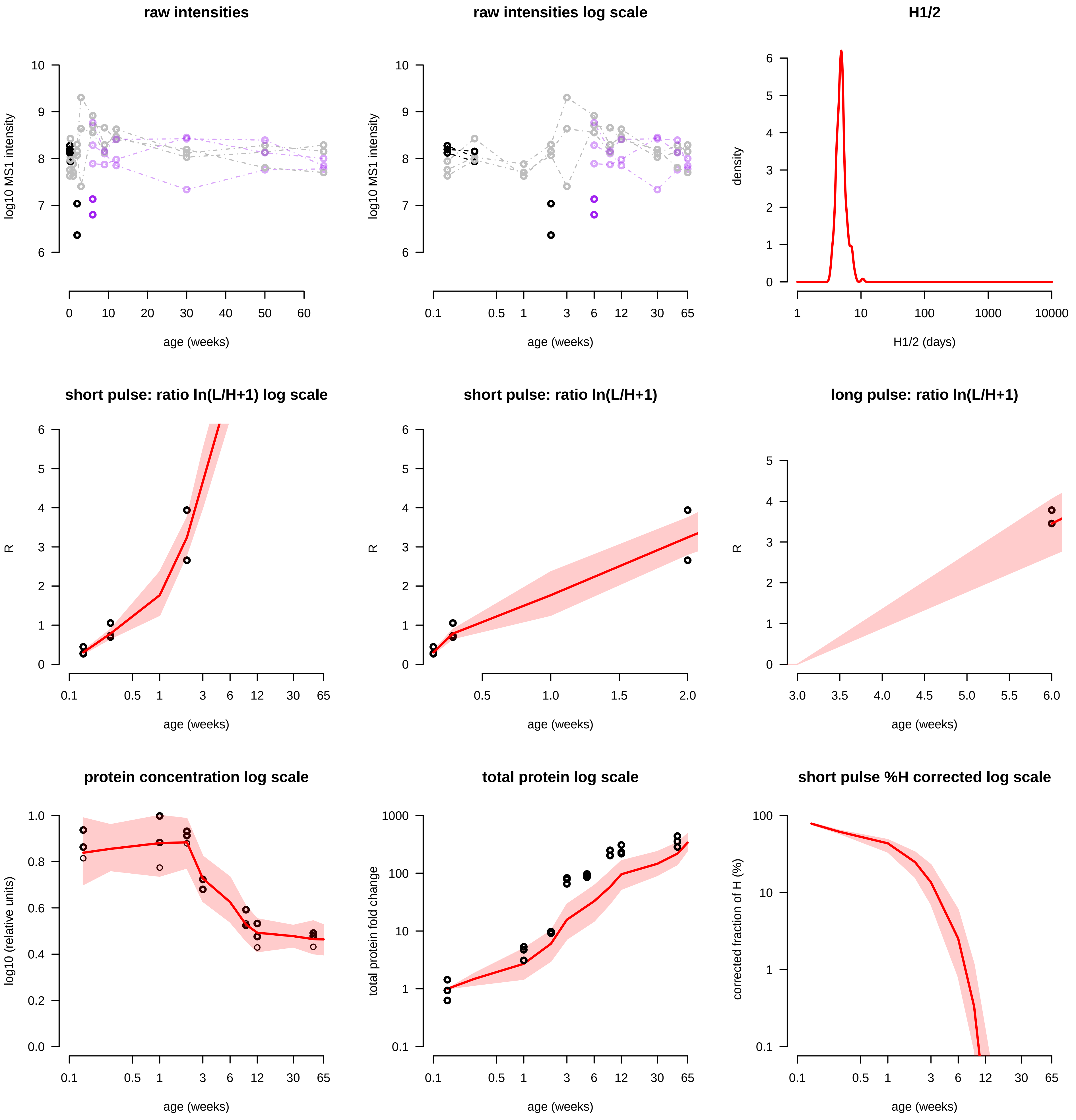

Q9CQT1

>sp|Q9CQT1|MTNA_MOUSE

Methylthioribose−1−phosphate isomerase

GN=Mri1

Med H1/2: 26 days

Q5 H1/2: 10.4 days

Q95 H1/2: 245.6 days

reliable model fit: yes

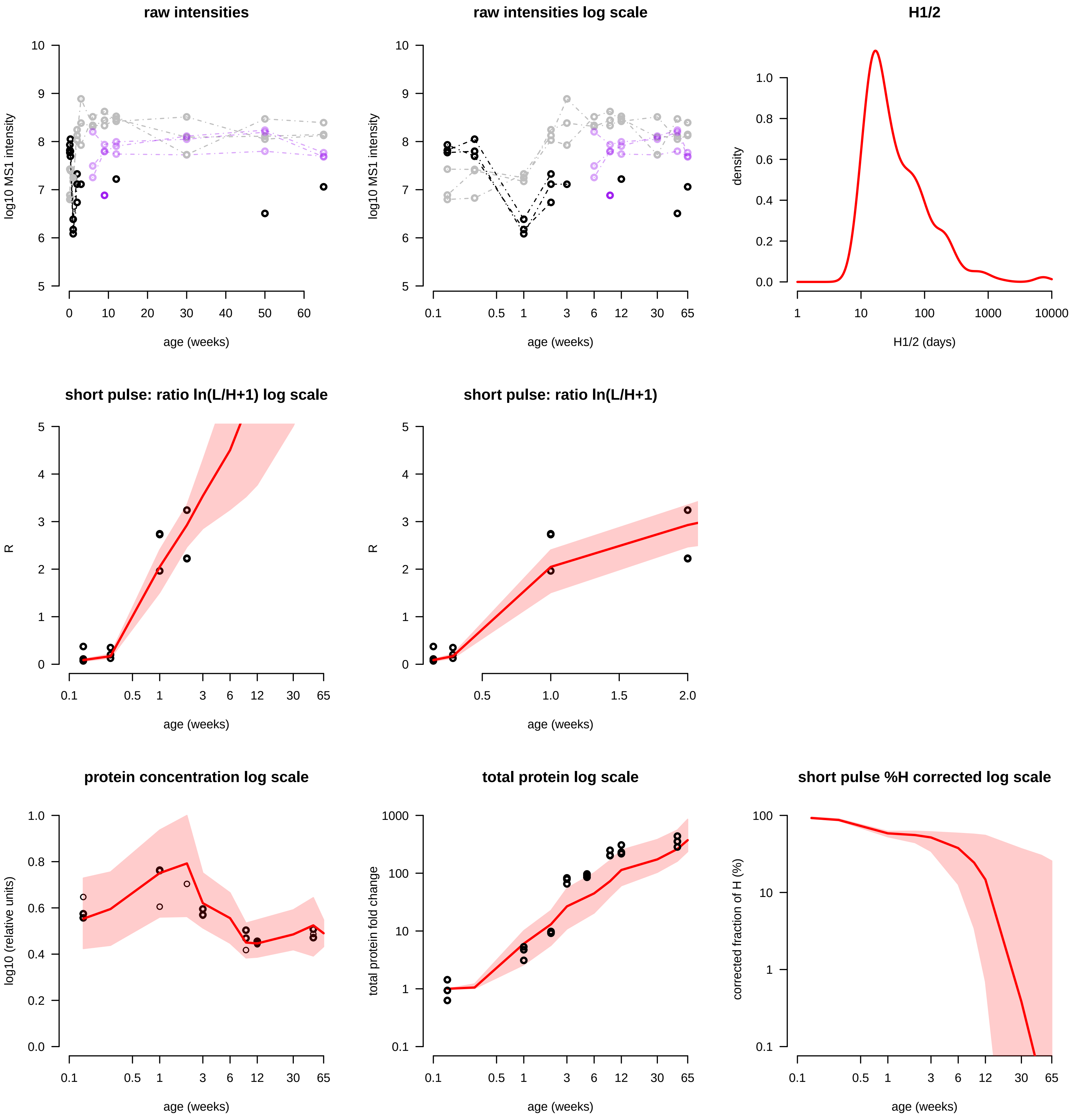

Q8VDW0

>sp|Q8VDW0|DX39A_MOUSE

ATP−dependent RNA helicase DDX39A

GN=Ddx39a

Med H1/2: 7.3 days

Q5 H1/2: 3.4 days

Q95 H1/2: 180.2 days

reliable model fit: yes

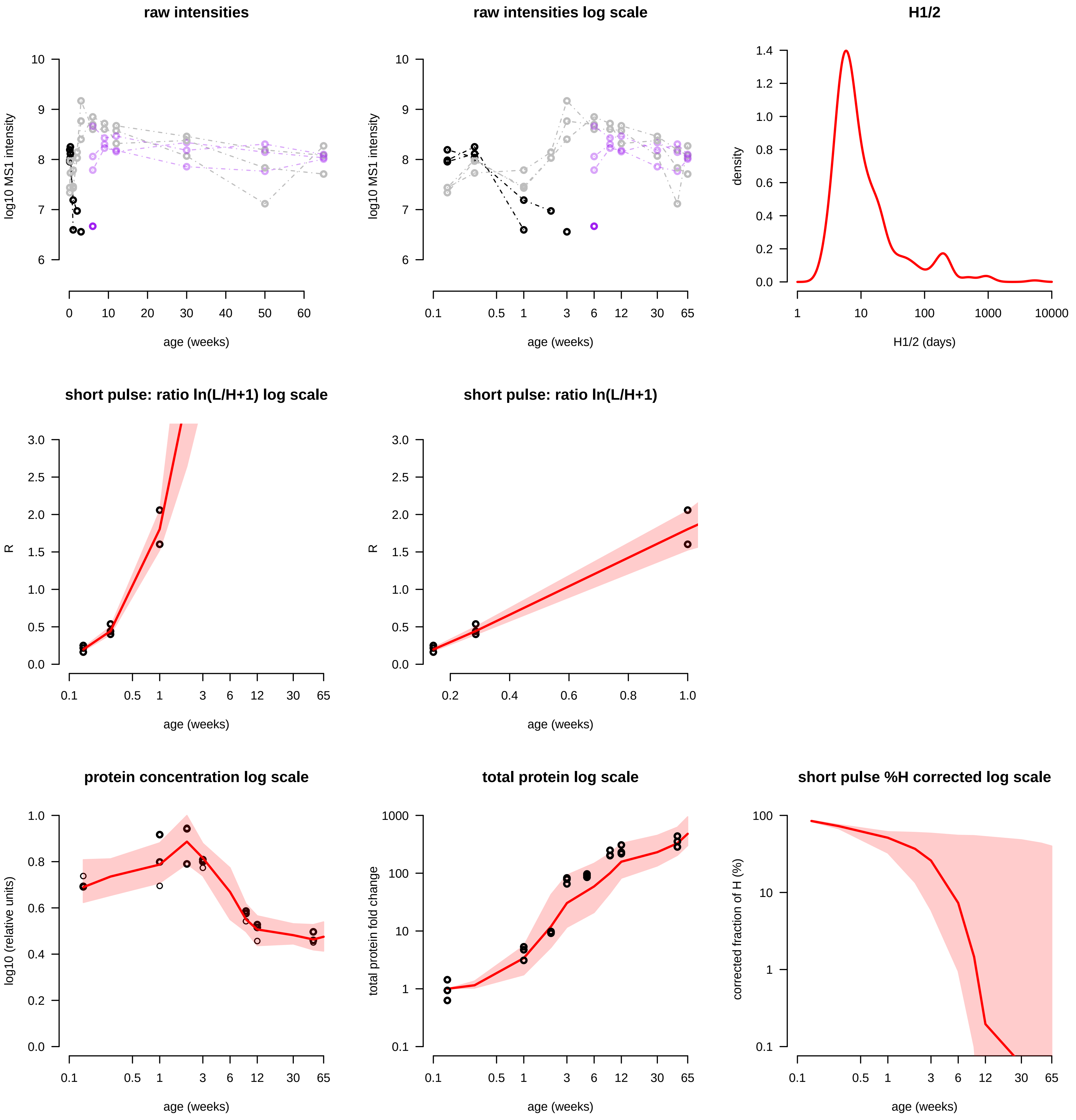

Q8CDA1;Q8CDA1−2

>sp|Q8CDA1|SAC2_MOUSE

Phosphatidylinositide phosphatase SAC2

GN=Inpp5f

Med H1/2: 11.8 days

Q5 H1/2: 3.7 days

Q95 H1/2: 68.5 days

reliable model fit: yes

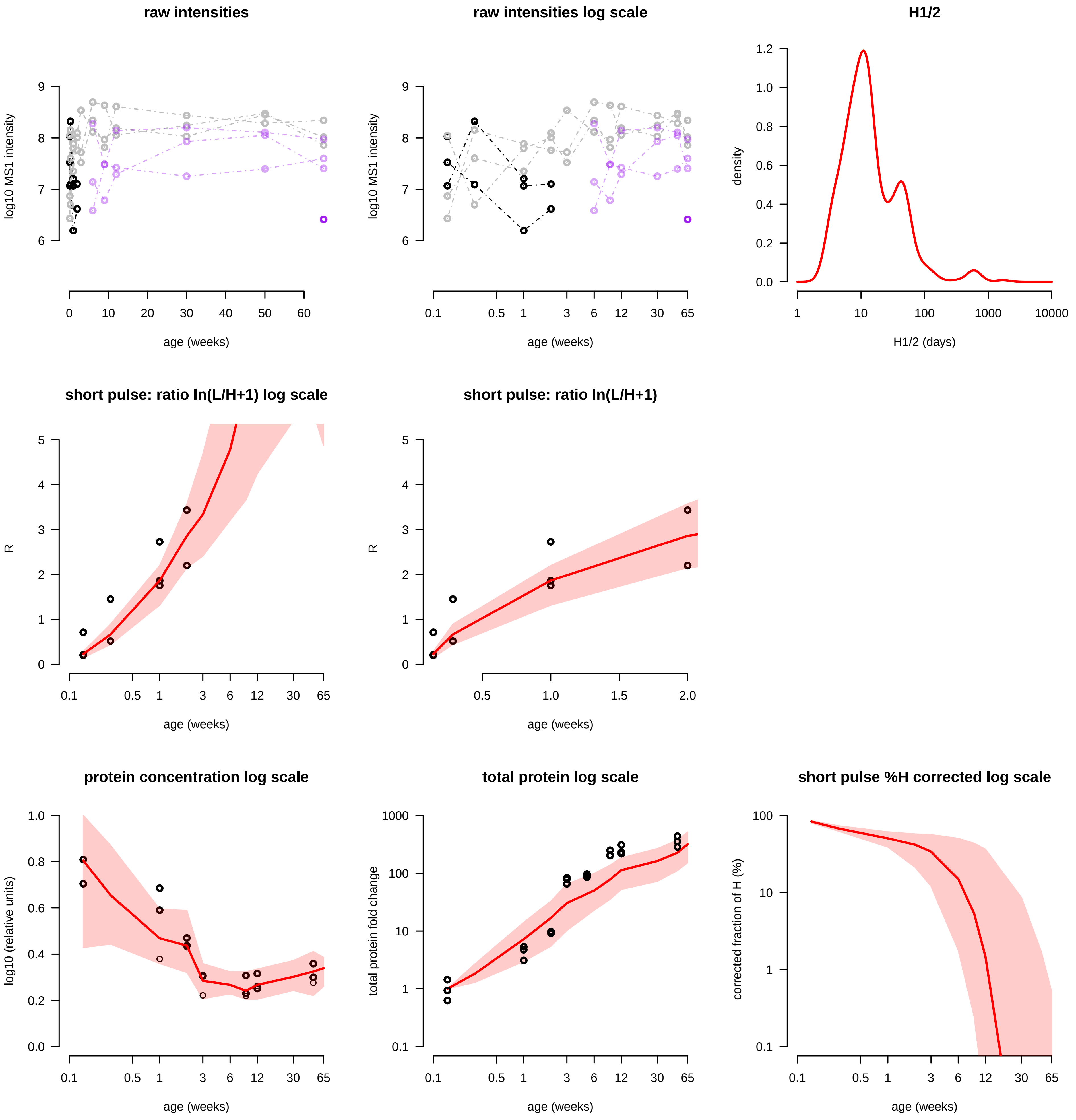

Q8R3E3−2;Q8R3E3

>sp|Q8R3E3−2|WIPI1_MOUSE

Isoform 2 of WD repeat domain phosphoinositide−interacting protein 1

GN=Wipi1

Med H1/2: 31.4 days

Q5 H1/2: 9.2 days

Q95 H1/2: 306.7 days

reliable model fit: yes

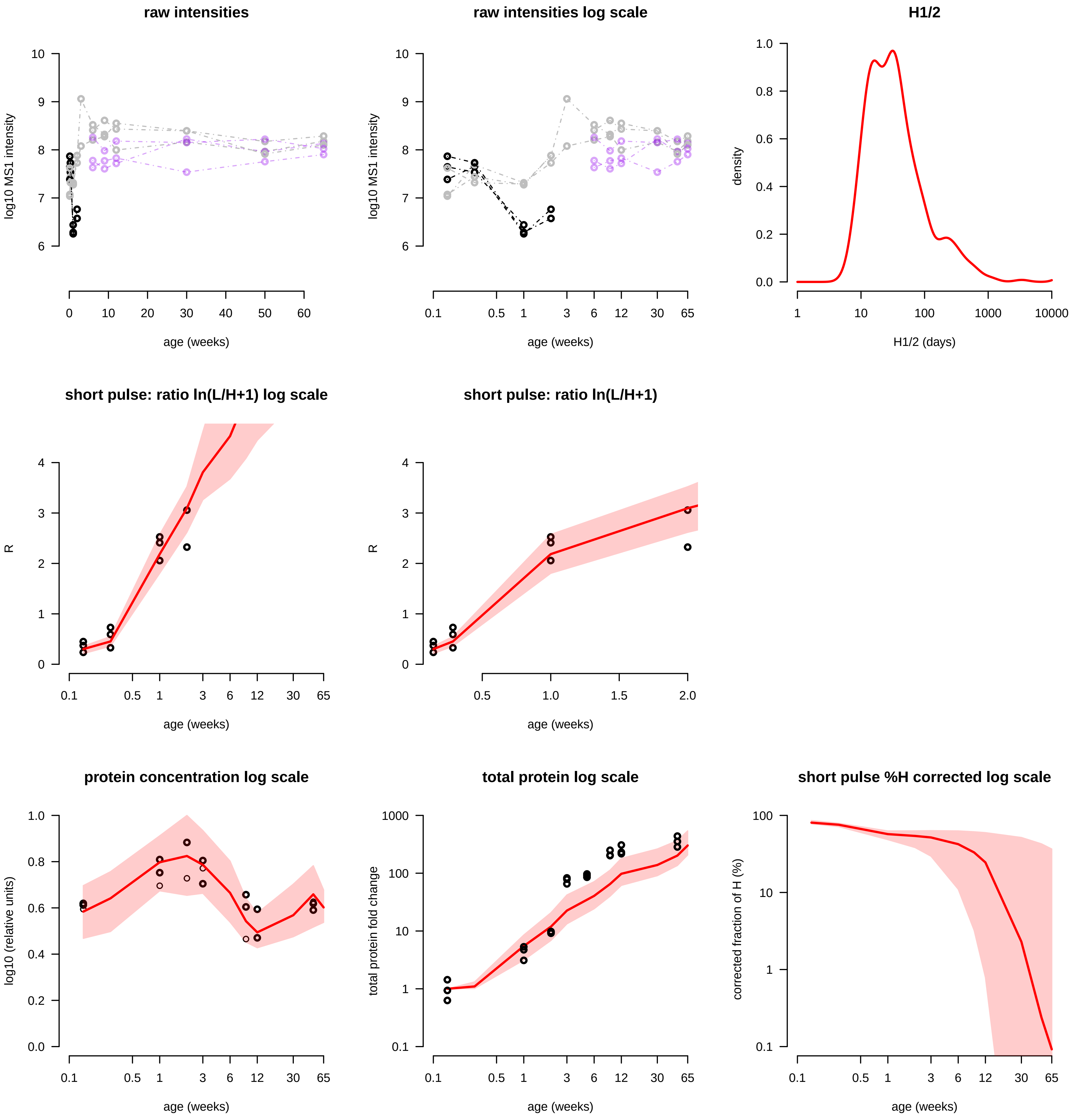

Q8K2F8

>sp|Q8K2F8|LS14A_MOUSE

Protein LSM14 homolog A

GN=Lsm14a

Med H1/2: 6.8 days

Q5 H1/2: 2.8 days

Q95 H1/2: 65.7 days

reliable model fit: yes

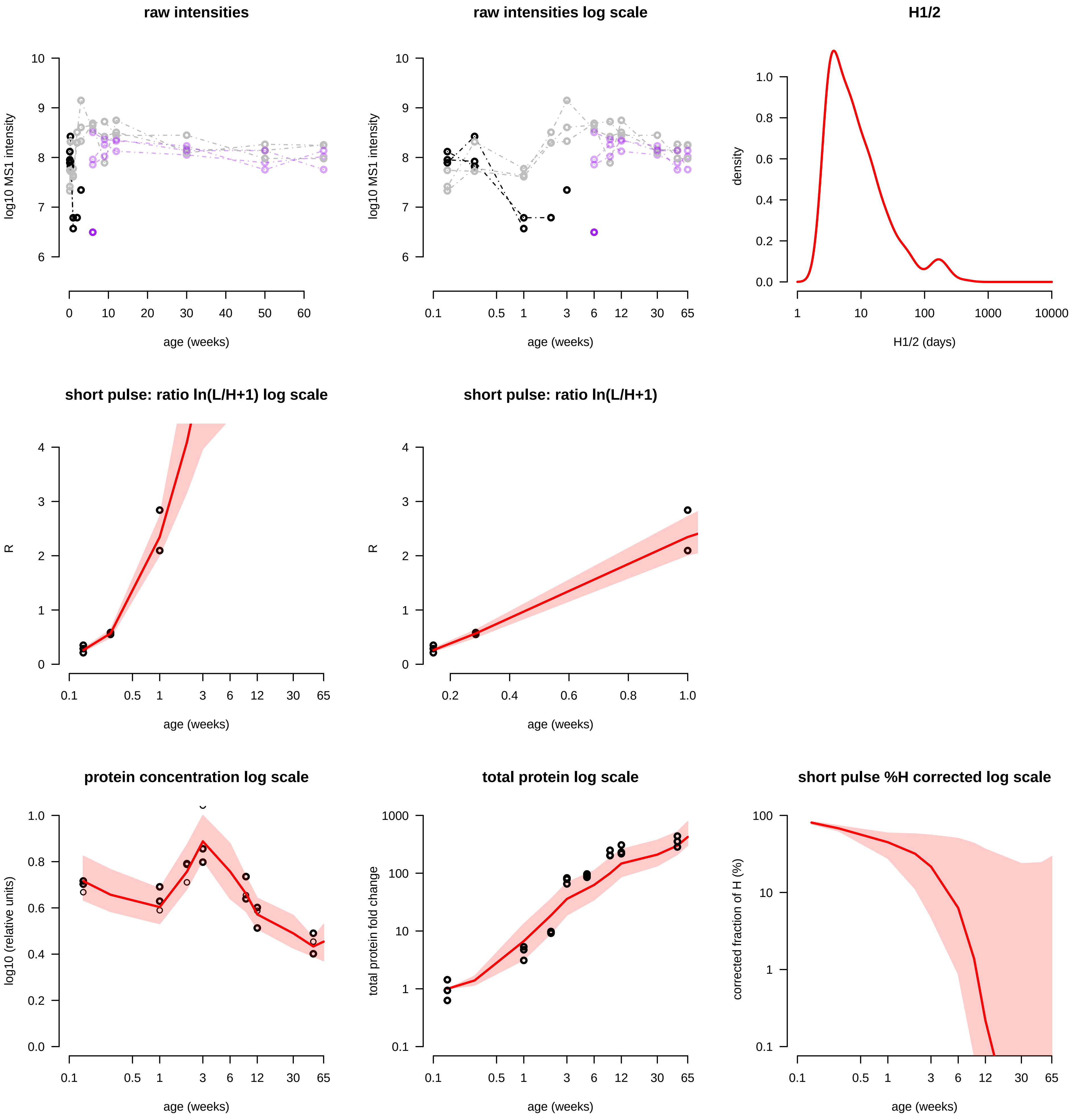

Q9ERD7

>sp|Q9ERD7|TBB3_MOUSE

Tubulin beta−3 chain

GN=Tubb3

Med H1/2: 18.3 days

Q5 H1/2: 4.7 days

Q95 H1/2: 122.3 days

reliable model fit: yes

raw intensities

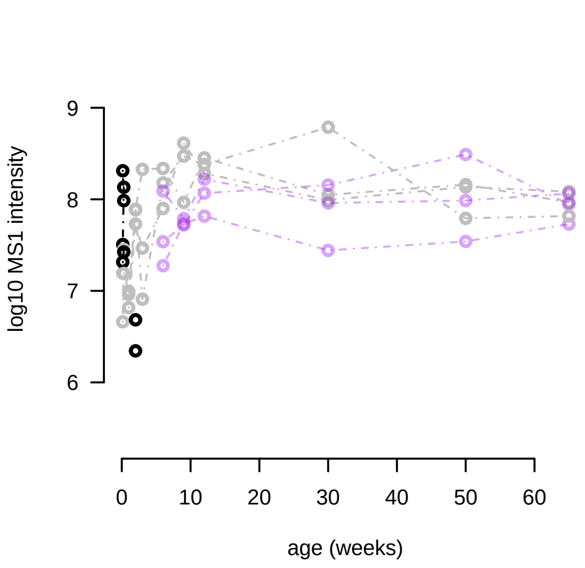

raw intensities log scale

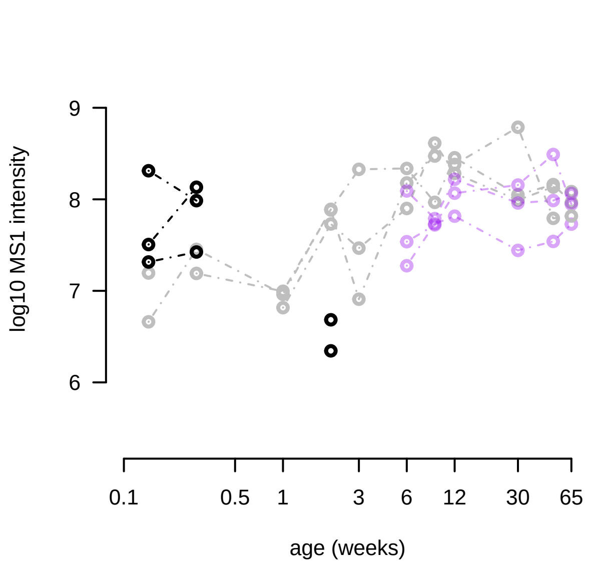

H1/2

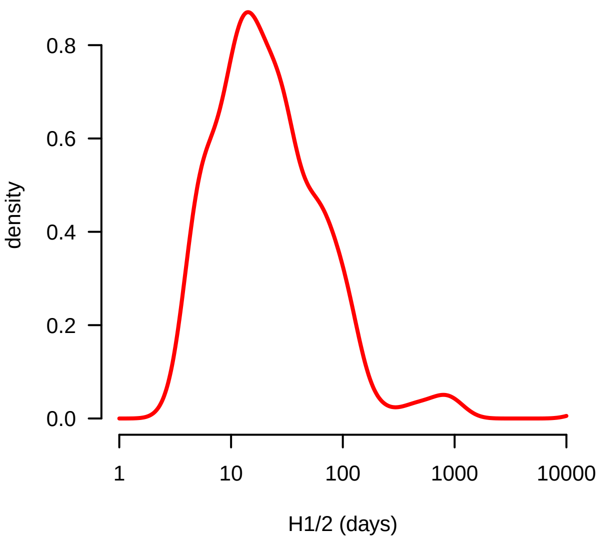

short pulse: ratio ln(L/H+1) log scale

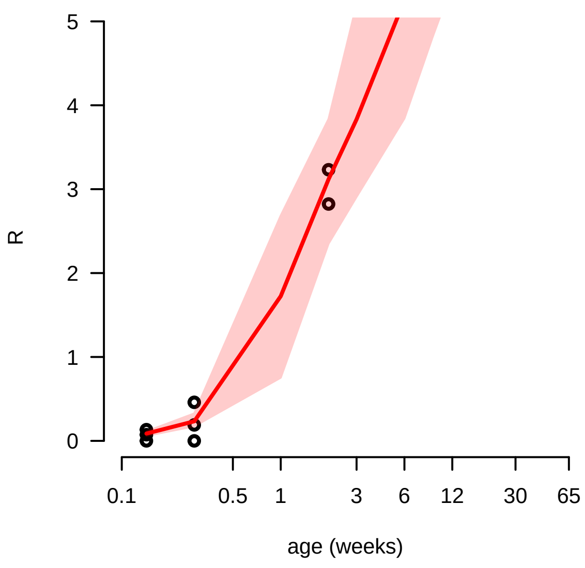

short pulse: ratio ln(L/H+1)

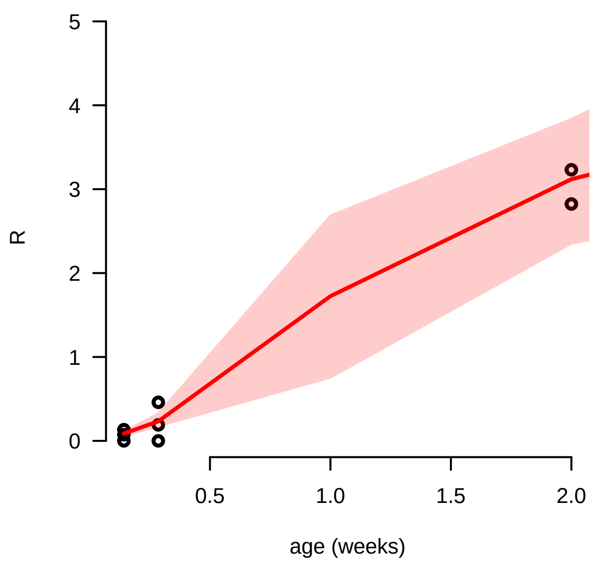

protein concentration log scale

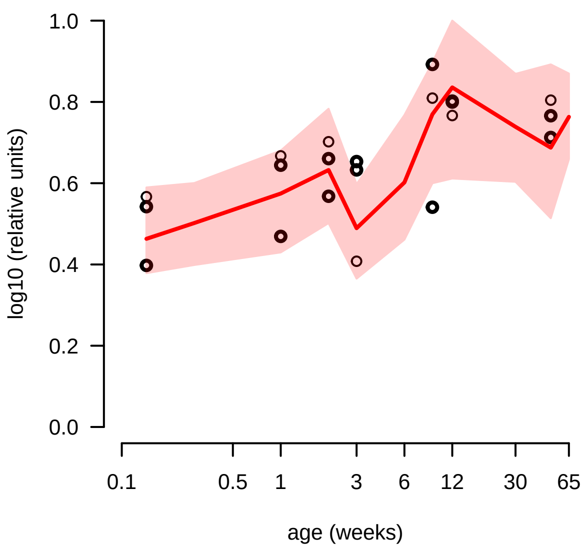

total protein log scale

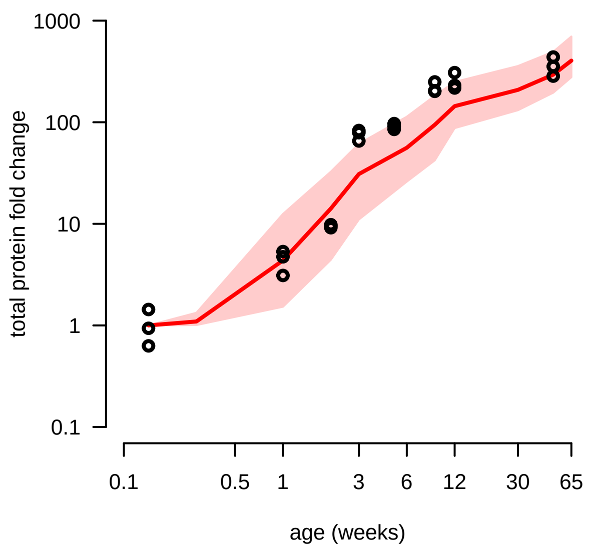

short pulse %H corrected log scale

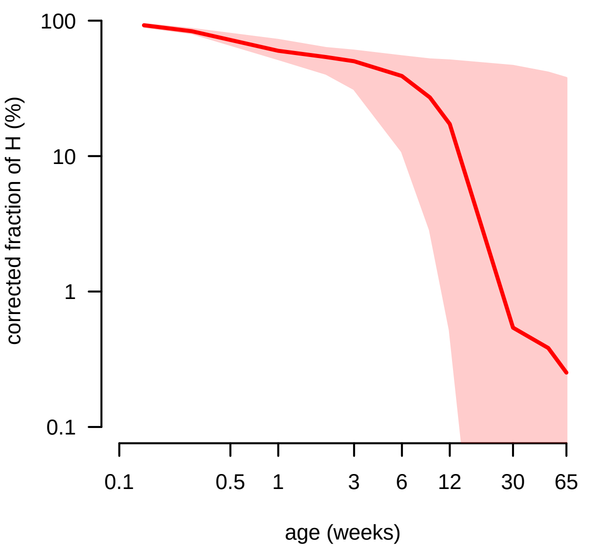

Q99LU0

>sp|Q99LU0|CH1B1_MOUSE

Charged multivesicular body protein 1b−1

GN=Chmp1b1

Med H1/2: 6 days

Q5 H1/2: 2.6 days

Q95 H1/2: 51.3 days

reliable model fit: yes

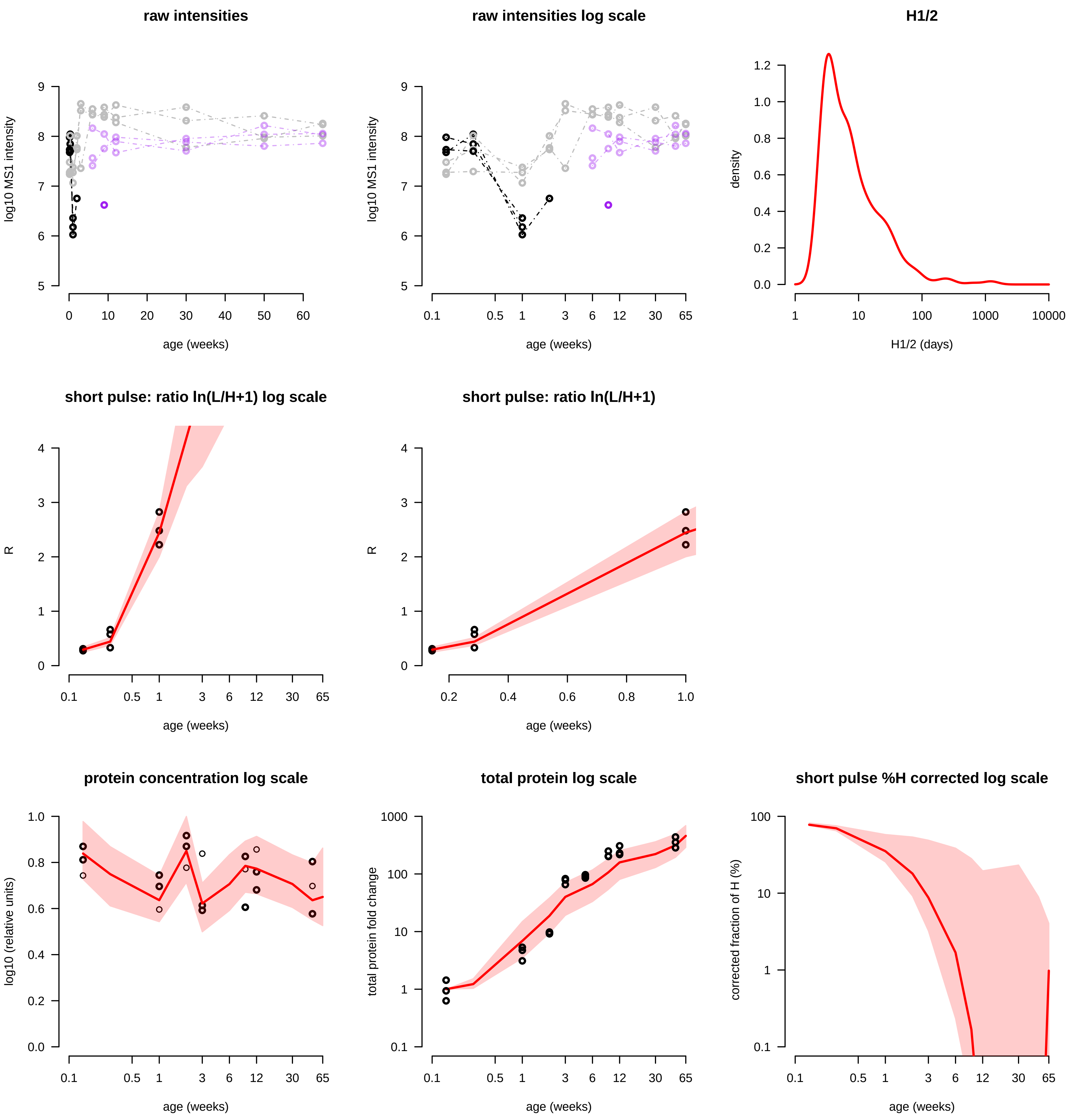

Q8BVA5;Q8BVA5−2

>sp|Q8BVA5|LDAH_MOUSE

Lipid droplet−associated hydrolase

GN=Ldah

Med H1/2: 8.3 days

Q5 H1/2: 3 days

Q95 H1/2: 49.2 days

reliable model fit: no

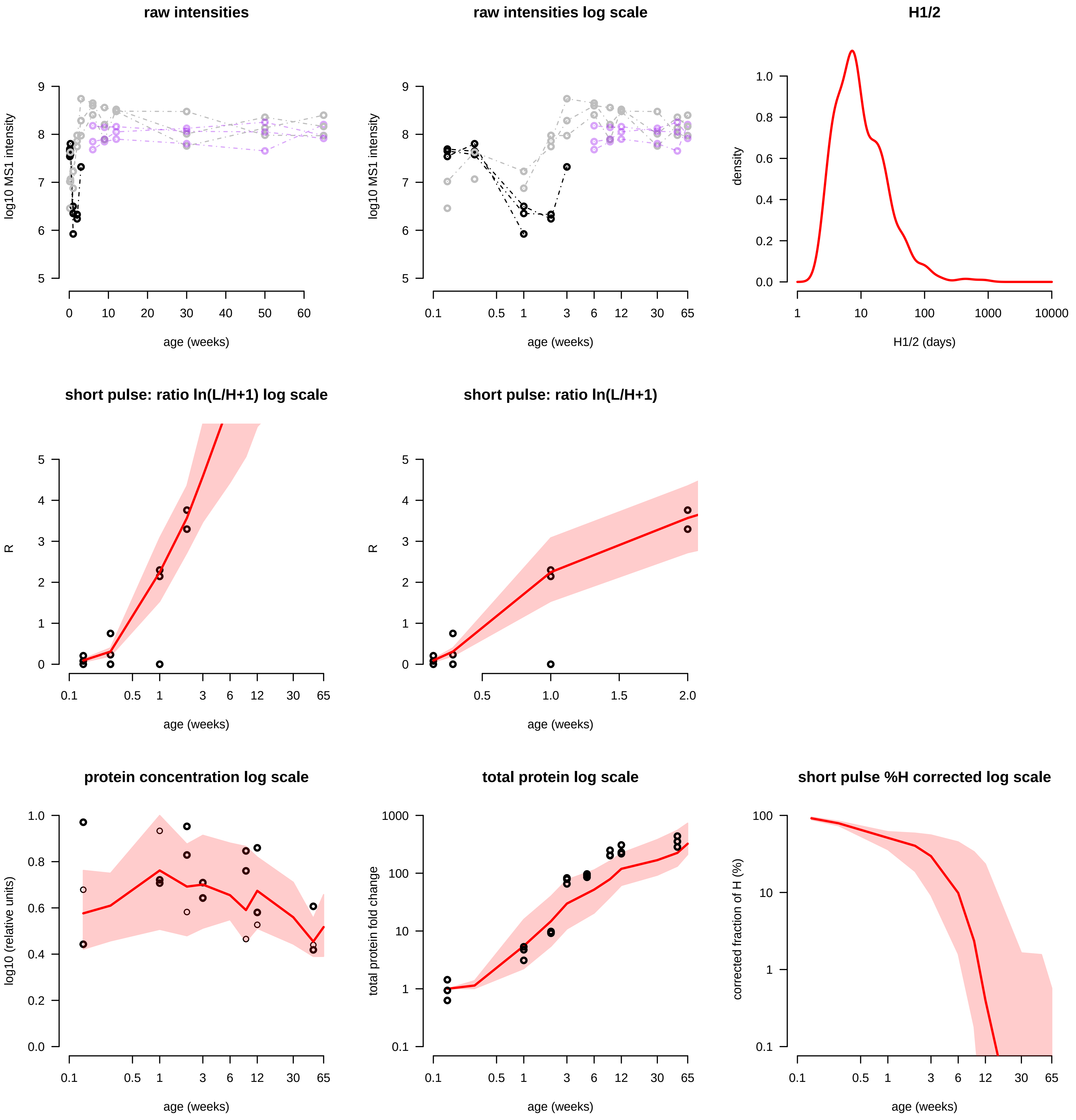

Q811L6

>sp|Q811L6|MAST4_MOUSE

Microtubule−associated serine/threonine−protein kinase 4

GN=Mast4

Med H1/2: 25.1 days

Q5 H1/2: 14.6 days

Q95 H1/2: 154.6 days

reliable model fit: yes

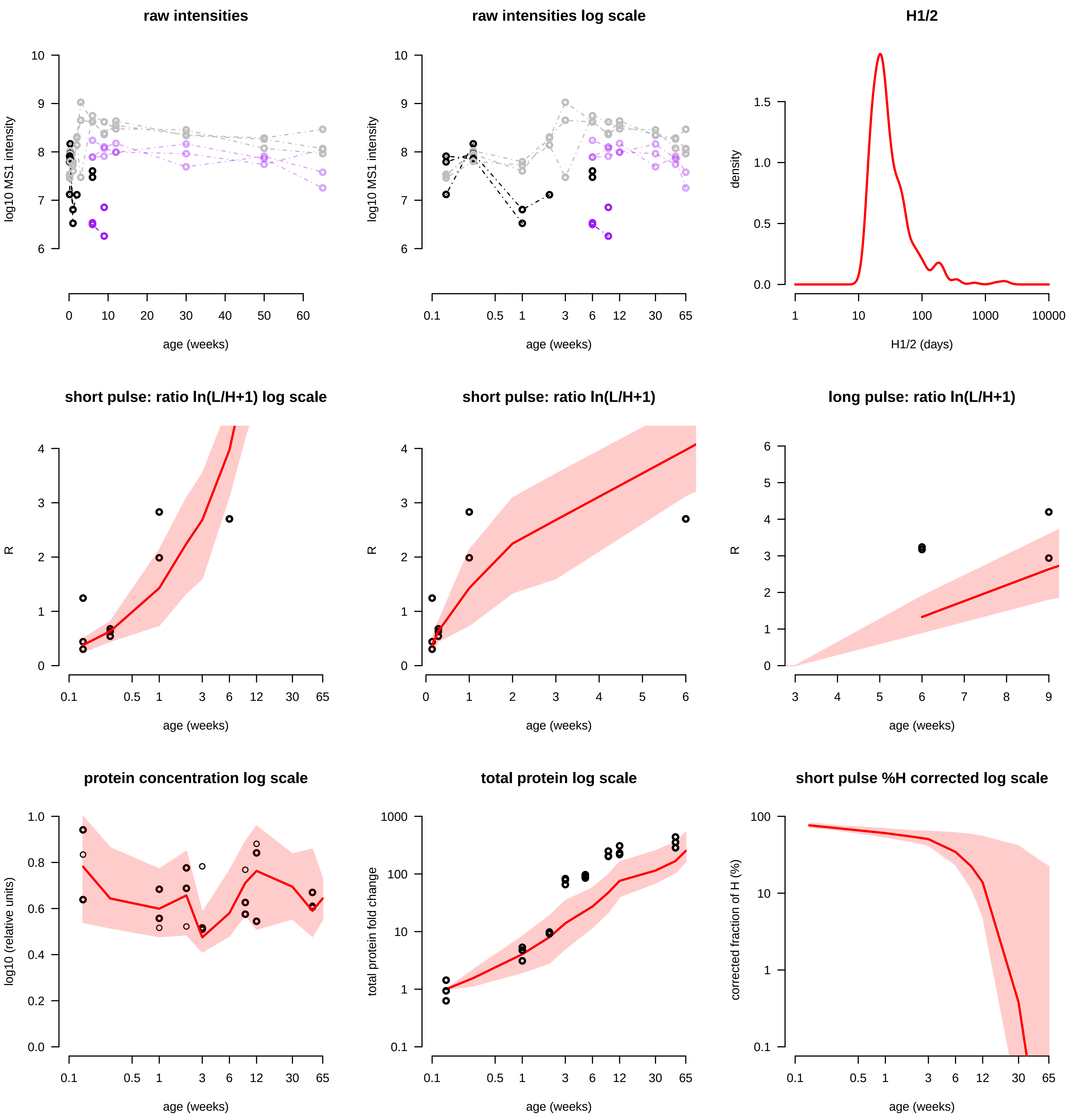

Q9CWW6

>sp|Q9CWW6|PIN4_MOUSE

Peptidyl−prolyl cis−trans isomerase NIMA−interacting 4

GN=Pin4

Med H1/2: 24.5 days

Q5 H1/2: 8.5 days

Q95 H1/2: 488.1 days

reliable model fit: yes

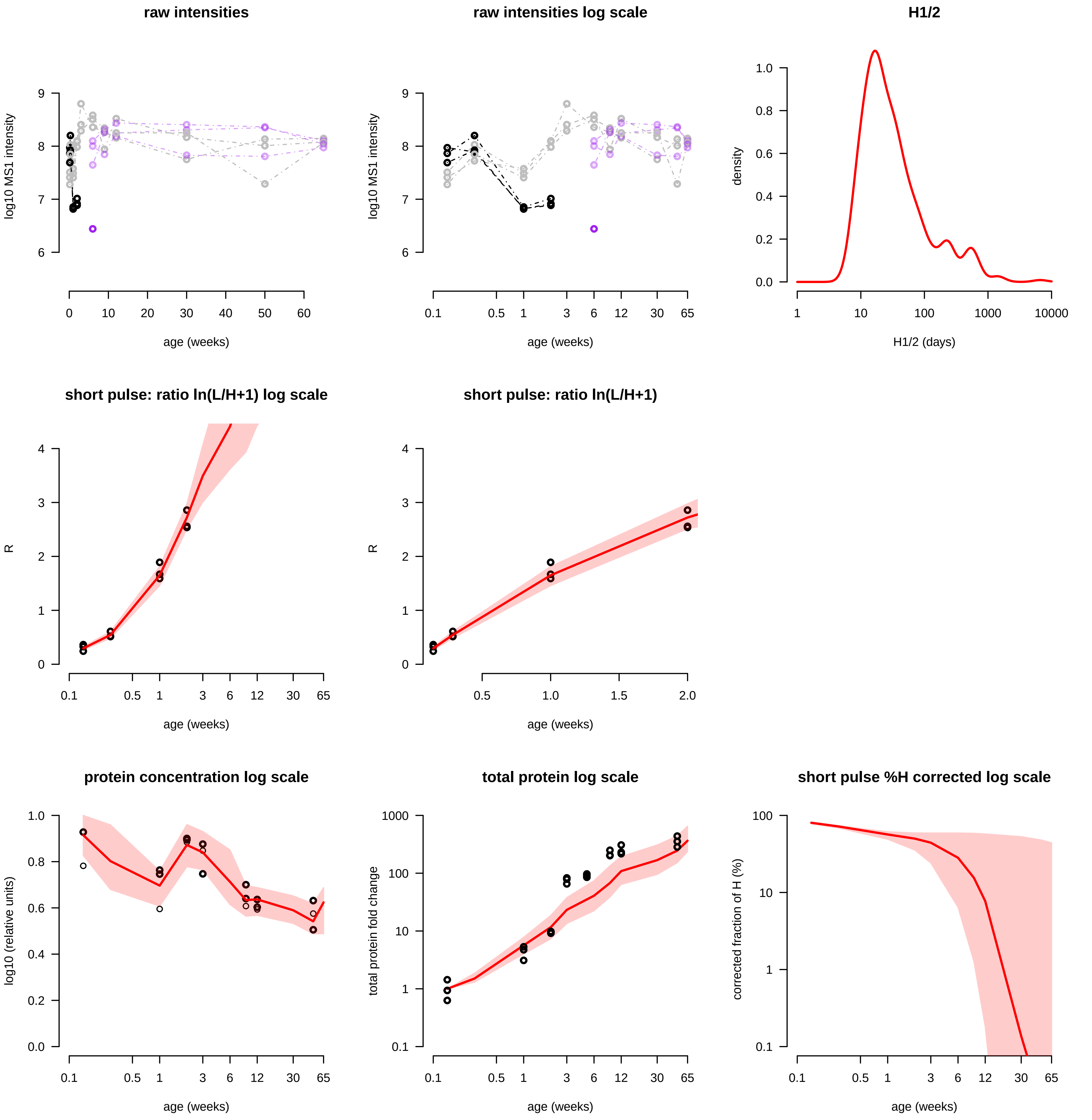

Q3TYS2

>sp|Q3TYS2|CYBC1_MOUSE

Cytochrome b−245 chaperone 1

GN=Cybc1

Med H1/2: 7.2 days

Q5 H1/2: 3.1 days

Q95 H1/2: 37.6 days

reliable model fit: yes

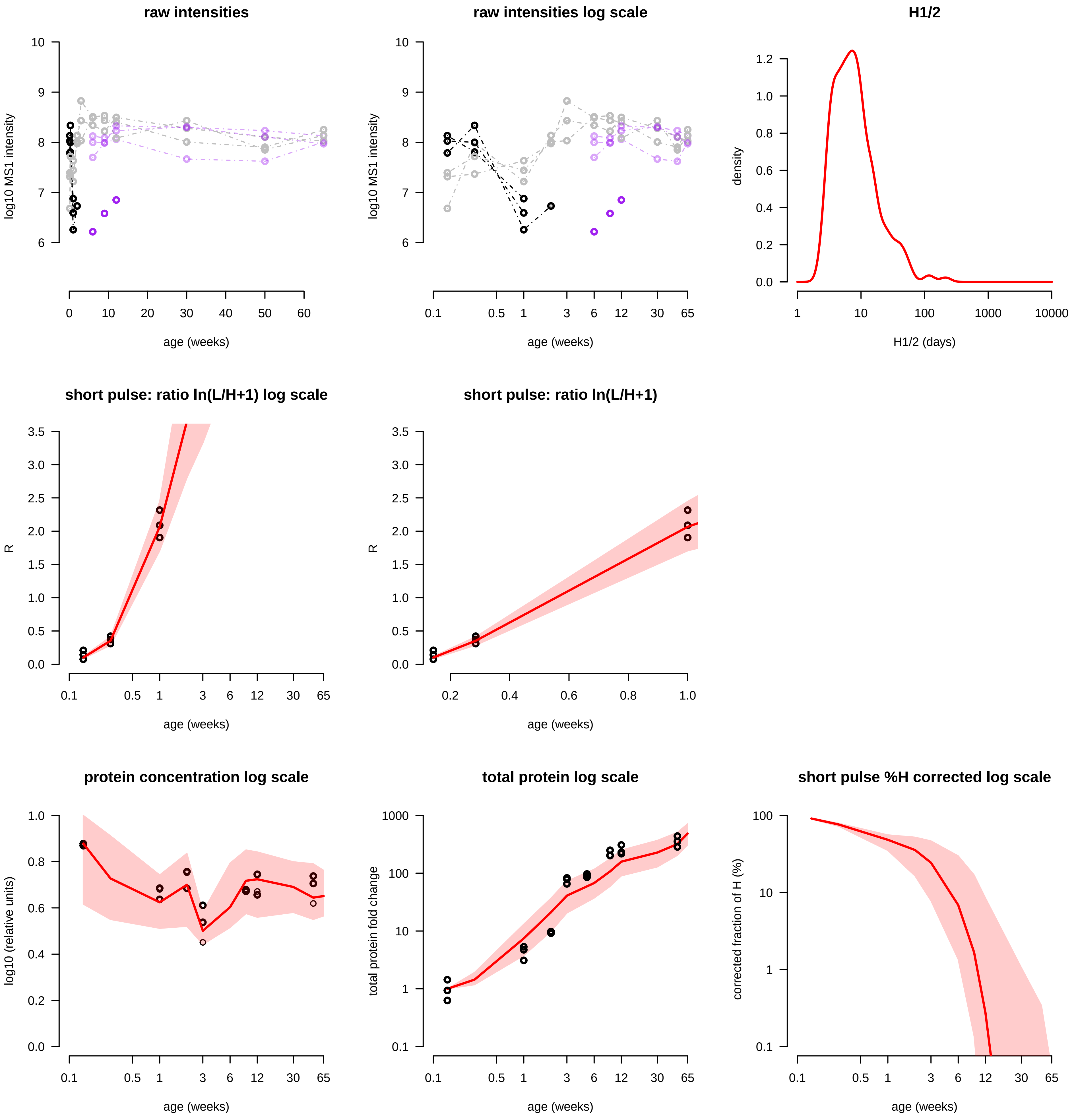

Q8CI33

>sp|Q8CI33|C19L1_MOUSE

CWF19−like protein 1

GN=Cwf19l1

Med H1/2: 16 days

Q5 H1/2: 4.4 days

Q95 H1/2: 100 days

reliable model fit: no

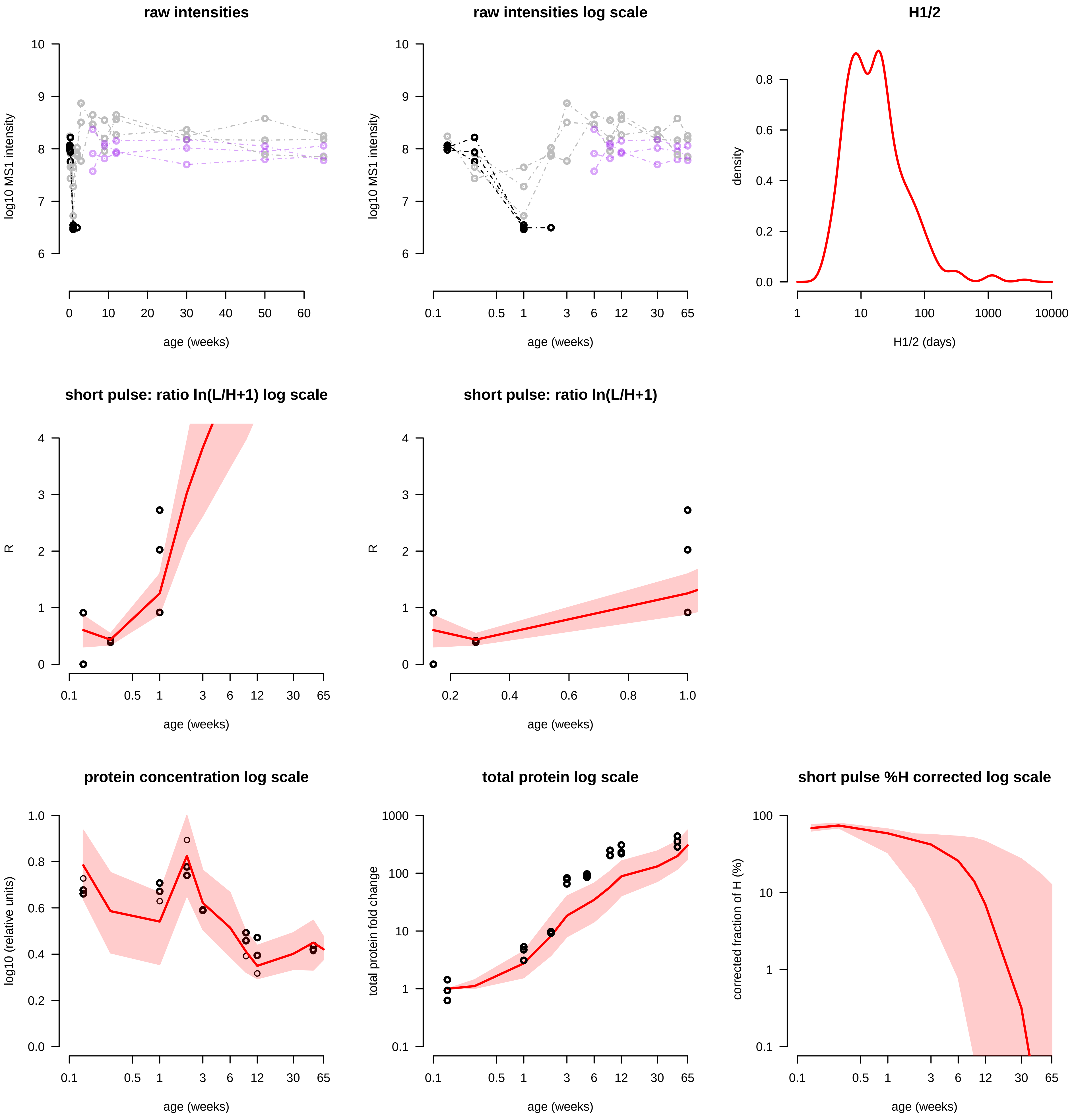

Q6PIU9

>sp|Q6PIU9|YJ005_MOUSE

Uncharacterized protein FLJ45252 homolog

Med H1/2: 72 days

Q5 H1/2: 22.7 days

Q95 H1/2: 1068.1 days

reliable model fit: no

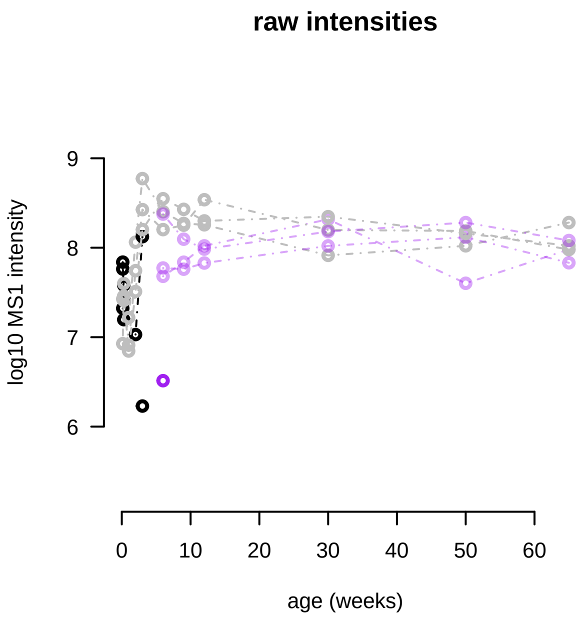

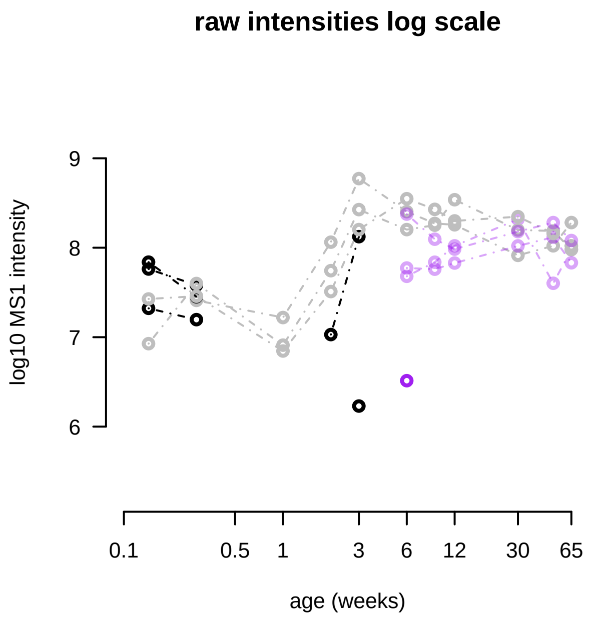

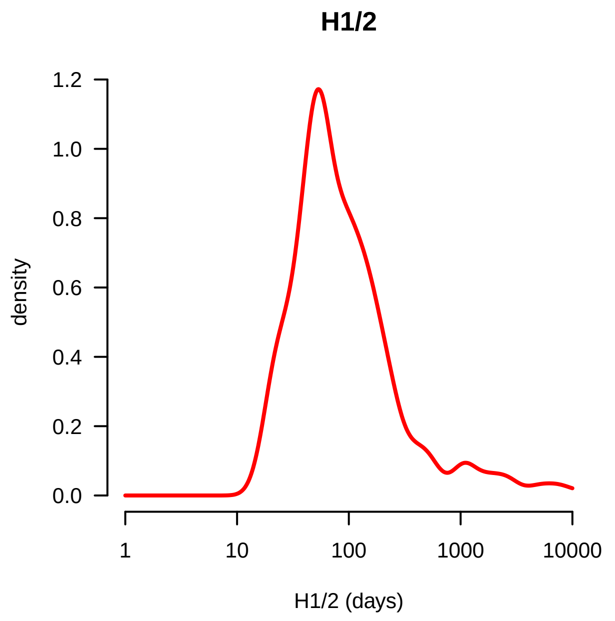

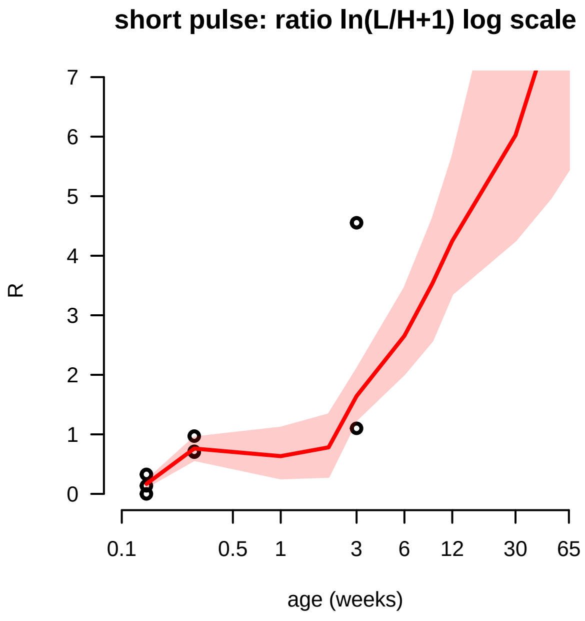

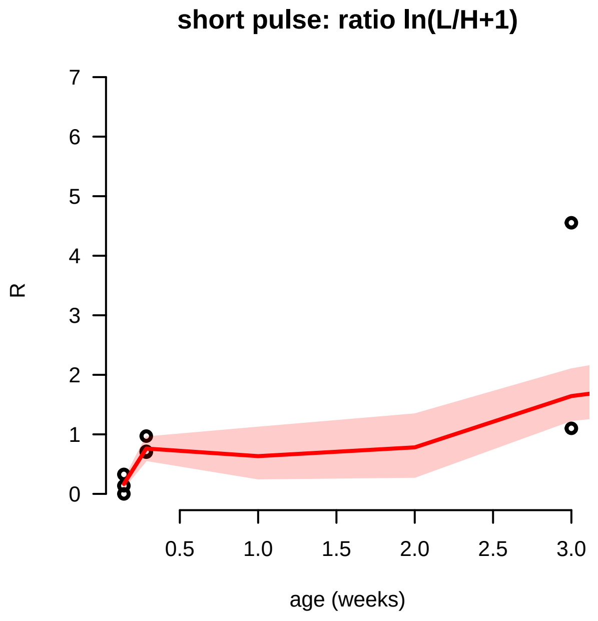

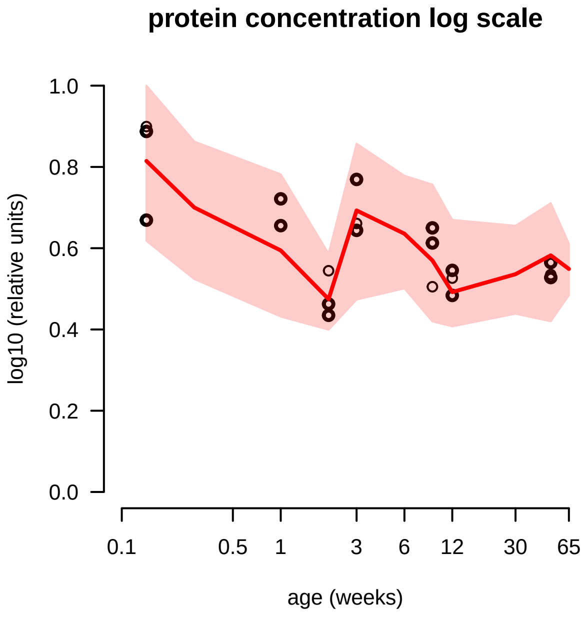

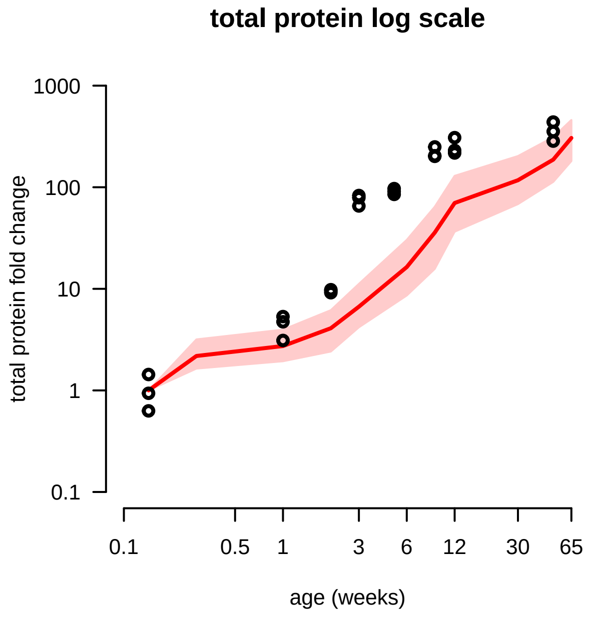

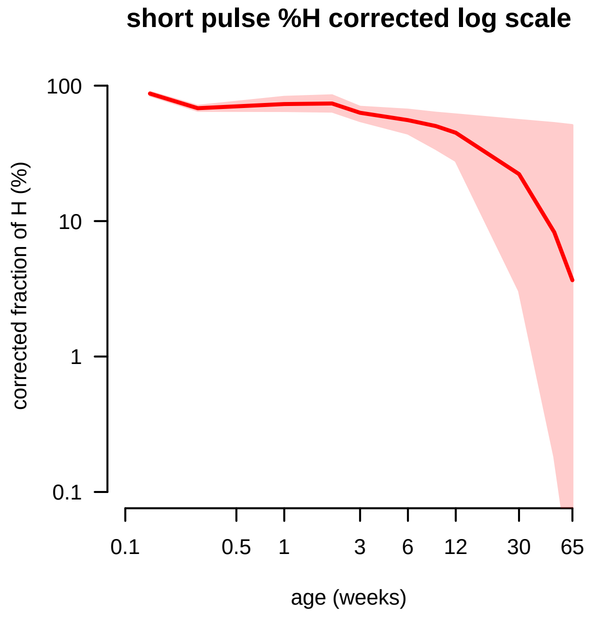

P58404−2;P58404

>sp|P58404−2|STRN4_MOUSE

Isoform 2 of Striatin−4

GN=Strn4

Med H1/2: 5.4 days

Q5 H1/2: 2.9 days

Q95 H1/2: 31.8 days

reliable model fit: yes

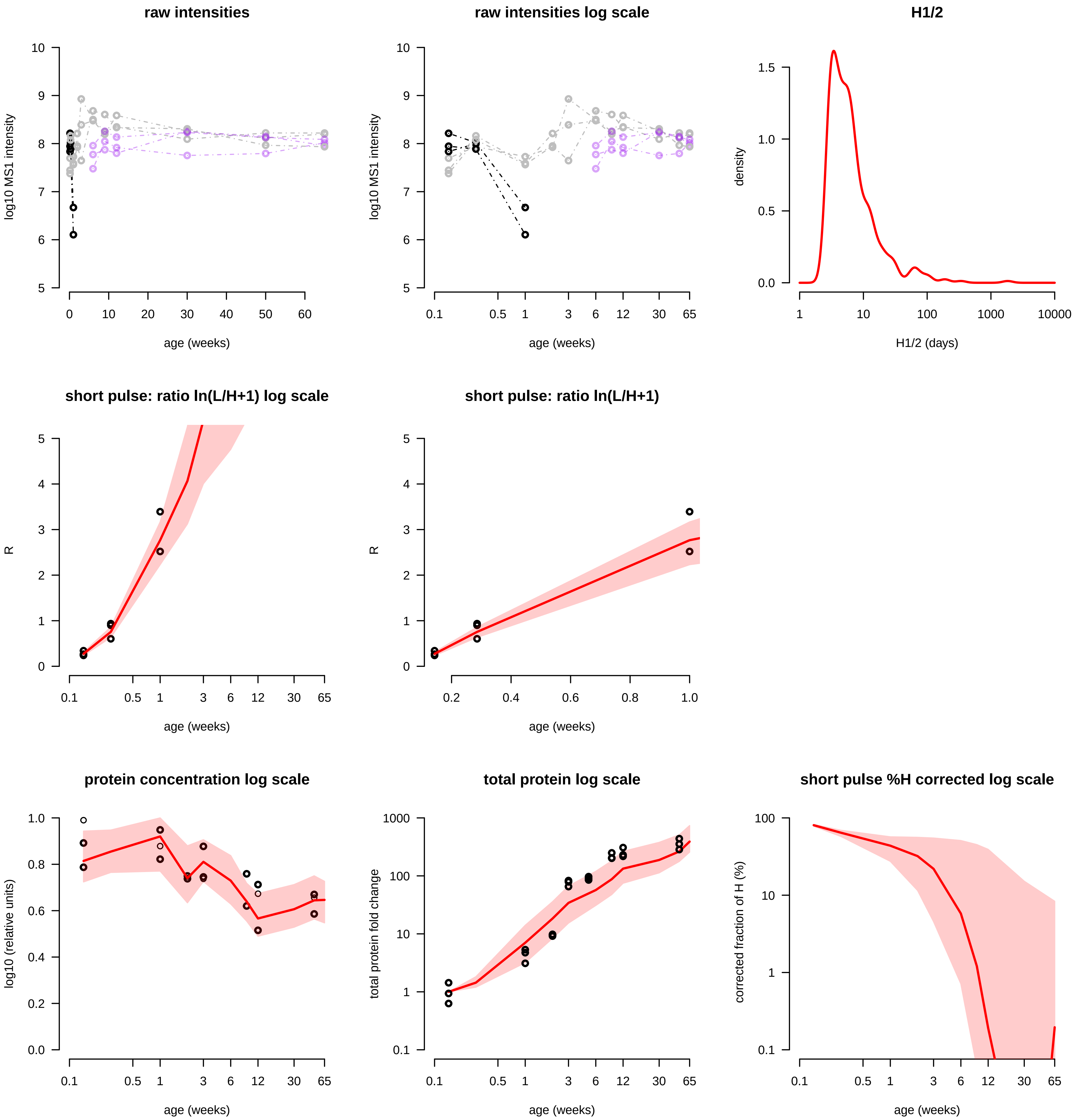

Q0VGY8

>sp|Q0VGY8|TANC1_MOUSE

Protein TANC1

GN=Tanc1

Med H1/2: 248.6 days

Q5 H1/2: 69.5 days

Q95 H1/2: 3059.2 days

reliable model fit: no

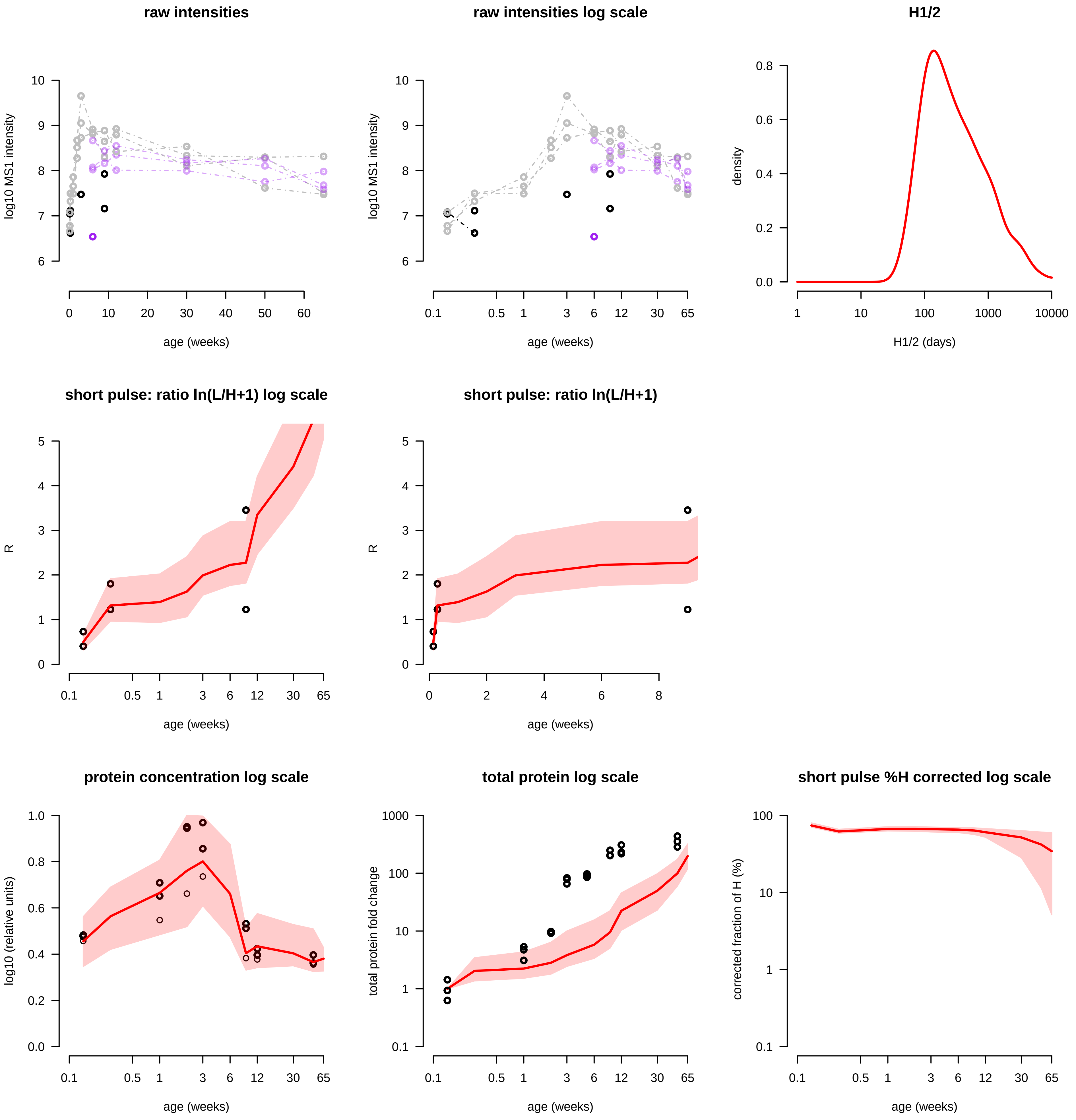

Q4VAE3

>sp|Q4VAE3|TMM65_MOUSE

Transmembrane protein 65

GN=Tmem65

Med H1/2: 10.3 days

Q5 H1/2: 4.9 days

Q95 H1/2: 31.1 days

reliable model fit: yes

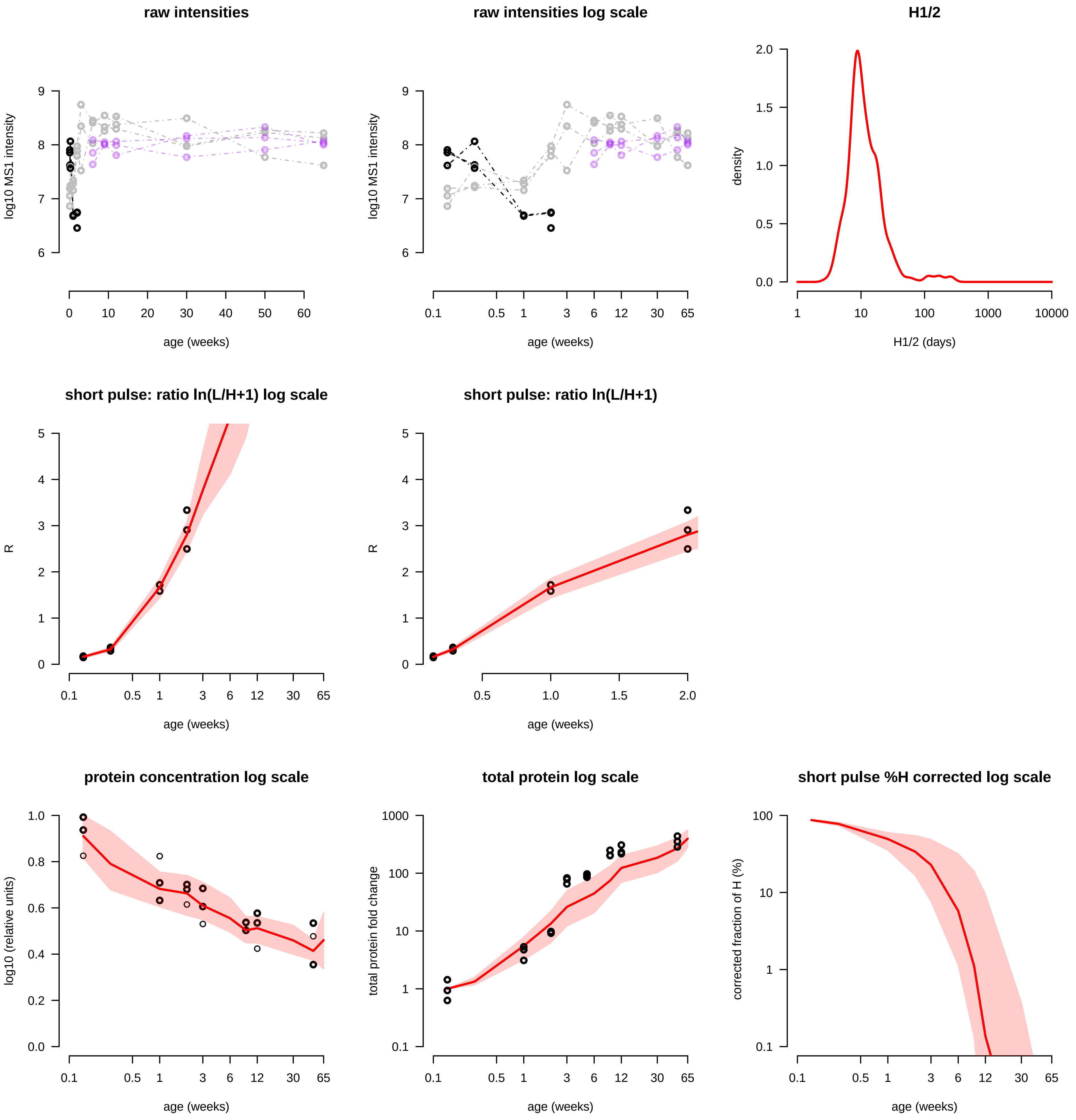

Q8VBV7

>sp|Q8VBV7|CSN8_MOUSE

COP9 signalosome complex subunit 8

GN=Cops8

Med H1/2: 8.6 days

Q5 H1/2: 6.8 days

Q95 H1/2: 13.7 days

reliable model fit: yes

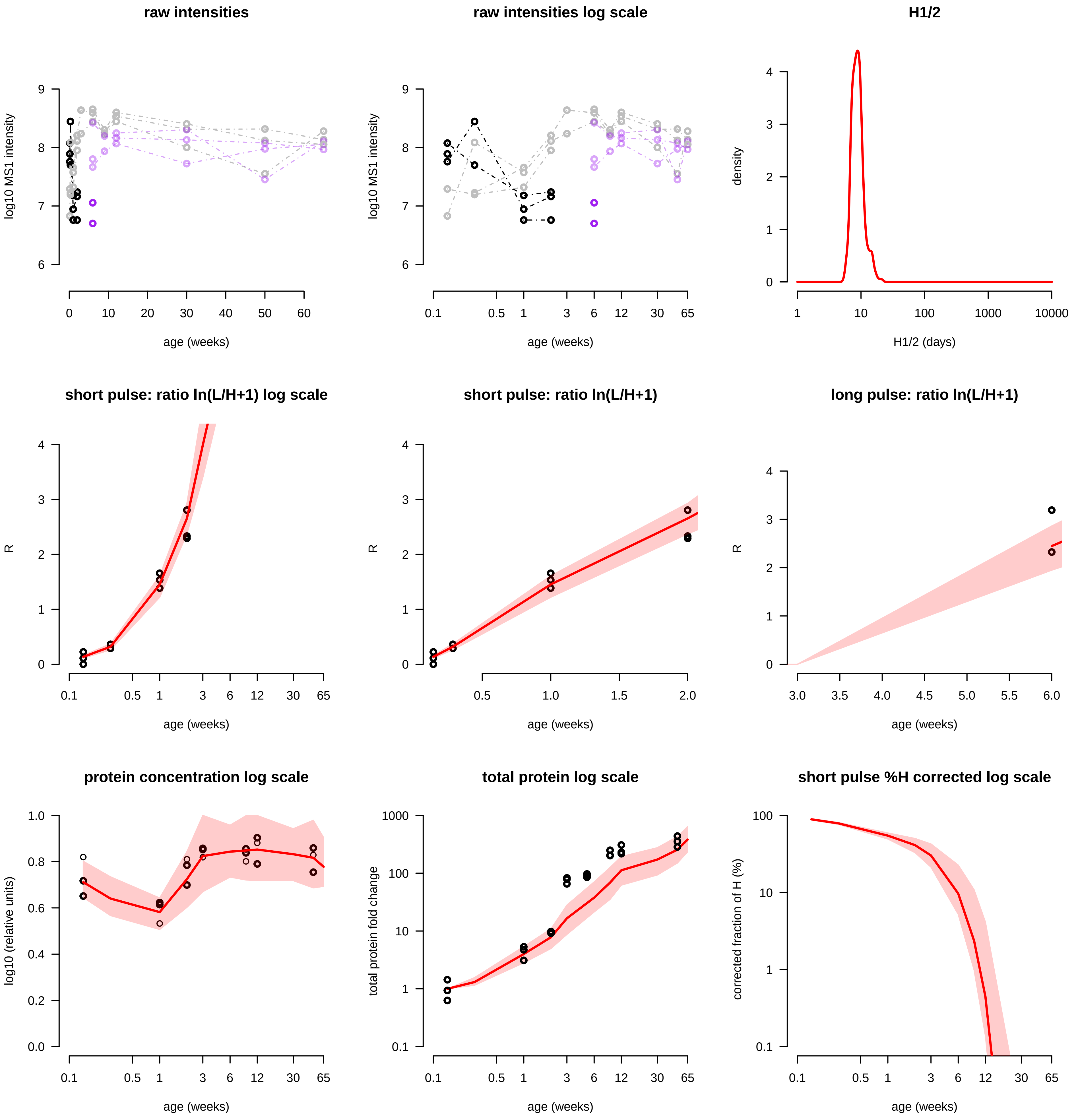

P70261

>sp|P70261|PALD_MOUSE

Paladin

GN=Pald1

Med H1/2: 7.1 days

Q5 H1/2: 3 days

Q95 H1/2: 43 days

reliable model fit: yes

raw intensities

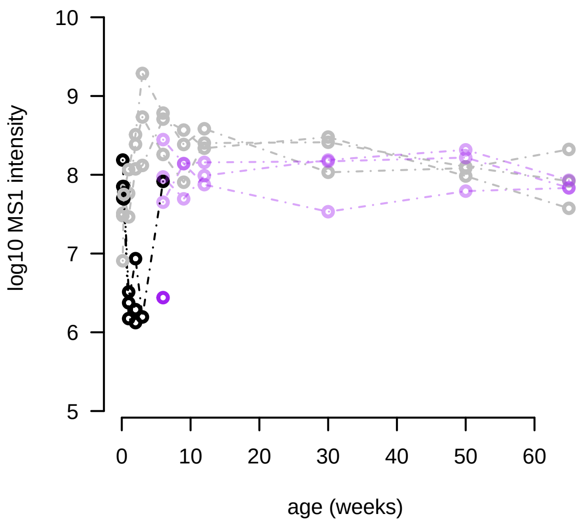

raw intensities log scale

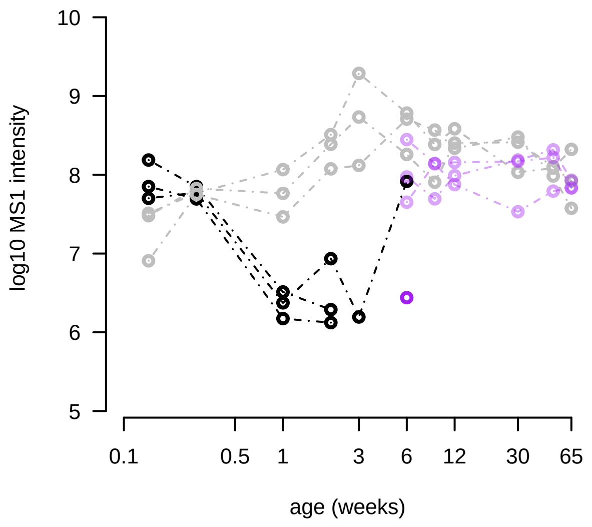

H1/2

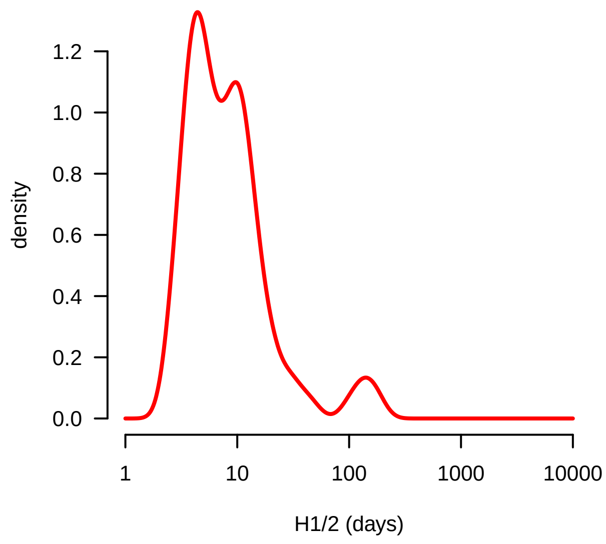

short pulse: ratio ln(L/H+1) log scale

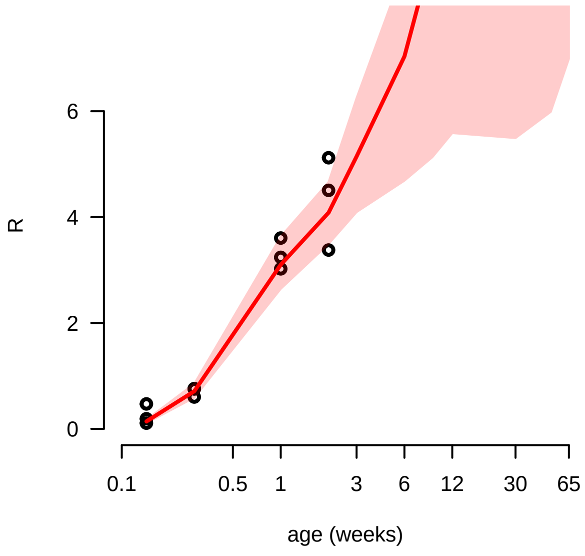

short pulse: ratio ln(L/H+1)

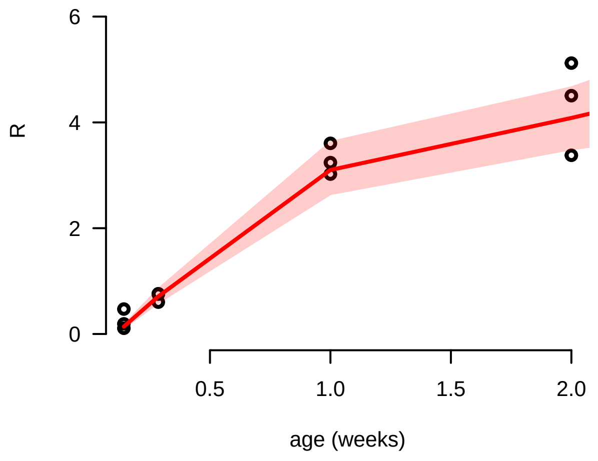

protein concentration log scale

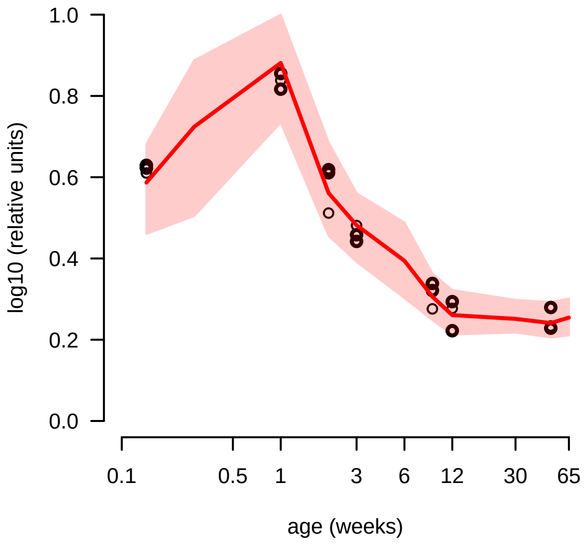

total protein log scale

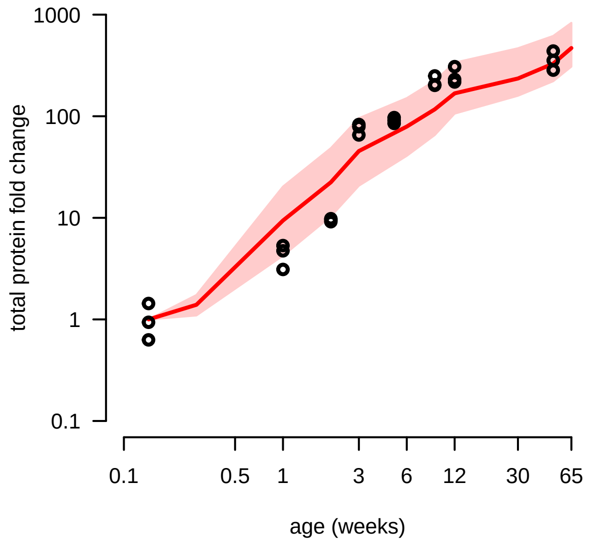

short pulse %H corrected log scale

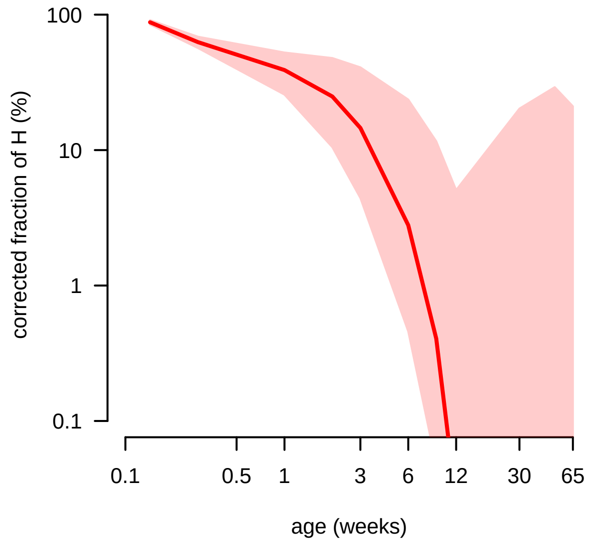

Q3UI43

>sp|Q3UI43|BABA1_MOUSE

BRISC and BRCA1−A complex member 1

GN=Babam1

Med H1/2: 12.8 days

Q5 H1/2: 4.7 days

Q95 H1/2: 77 days

reliable model fit: yes

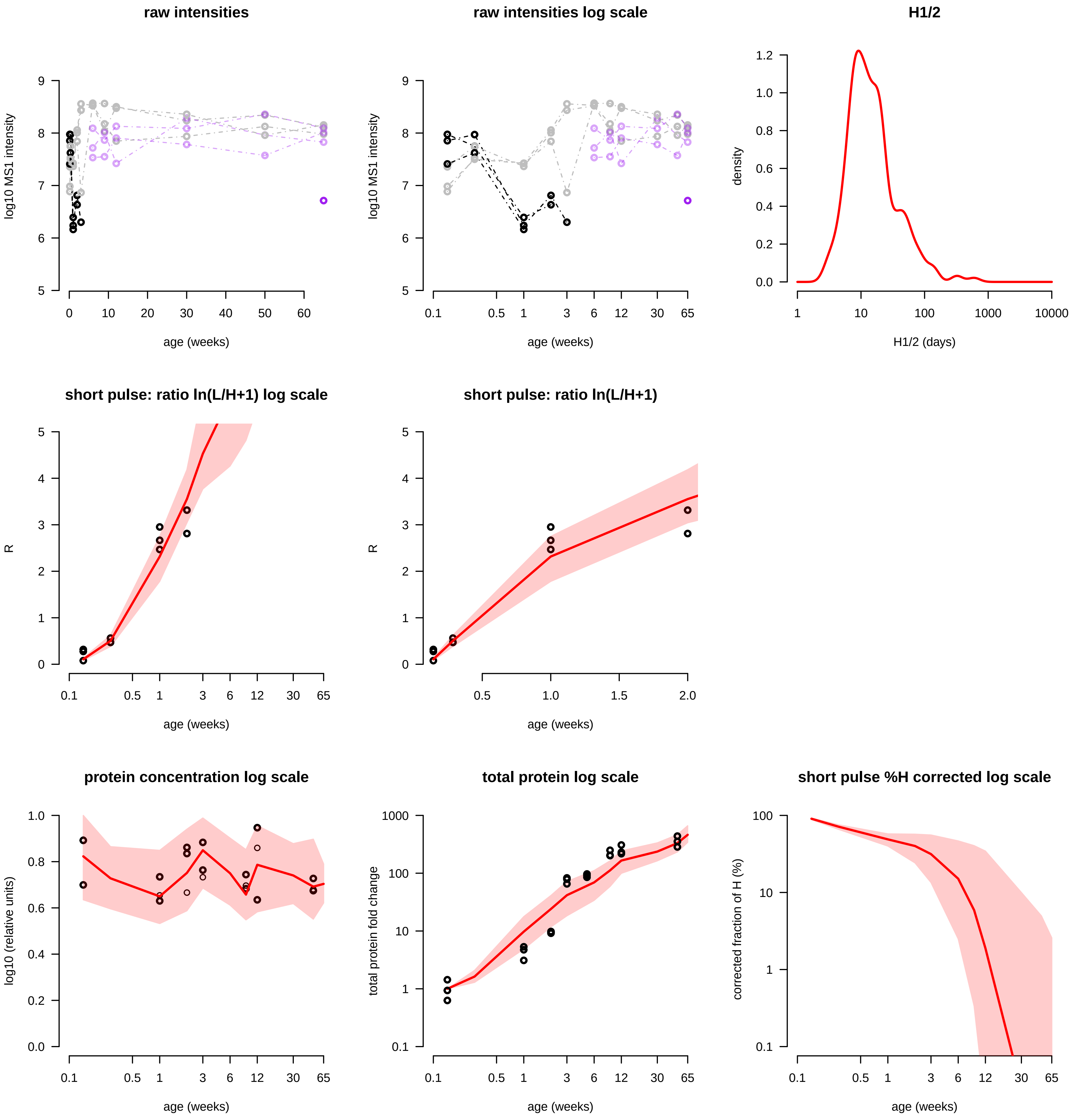

O35134

>sp|O35134|RPA1_MOUSE

DNA−directed RNA polymerase I subunit RPA1

GN=Polr1a

Med H1/2: 9.8 days

Q5 H1/2: 6.8 days

Q95 H1/2: 17.8 days

reliable model fit: no

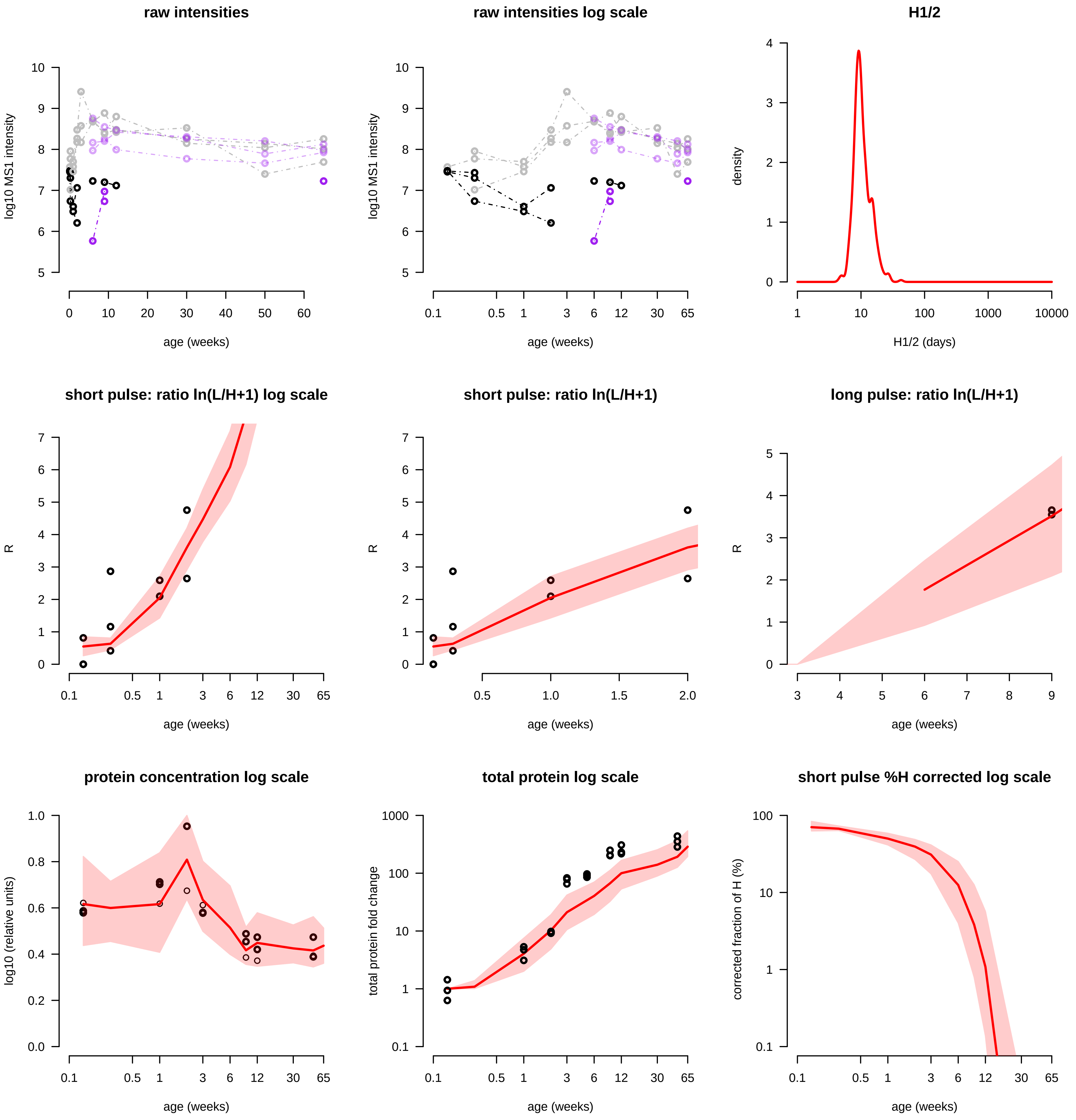

Q6Y5D8;Q6Y5D8−3;Q6Y5D8−2;Q6Y5D8−4

>sp|Q6Y5D8|RHG10_MOUSE

Rho GTPase−activating protein 10

GN=Arhgap10

Med H1/2: 21.4 days

Q5 H1/2: 6.5 days

Q95 H1/2: 470.7 days

reliable model fit: yes

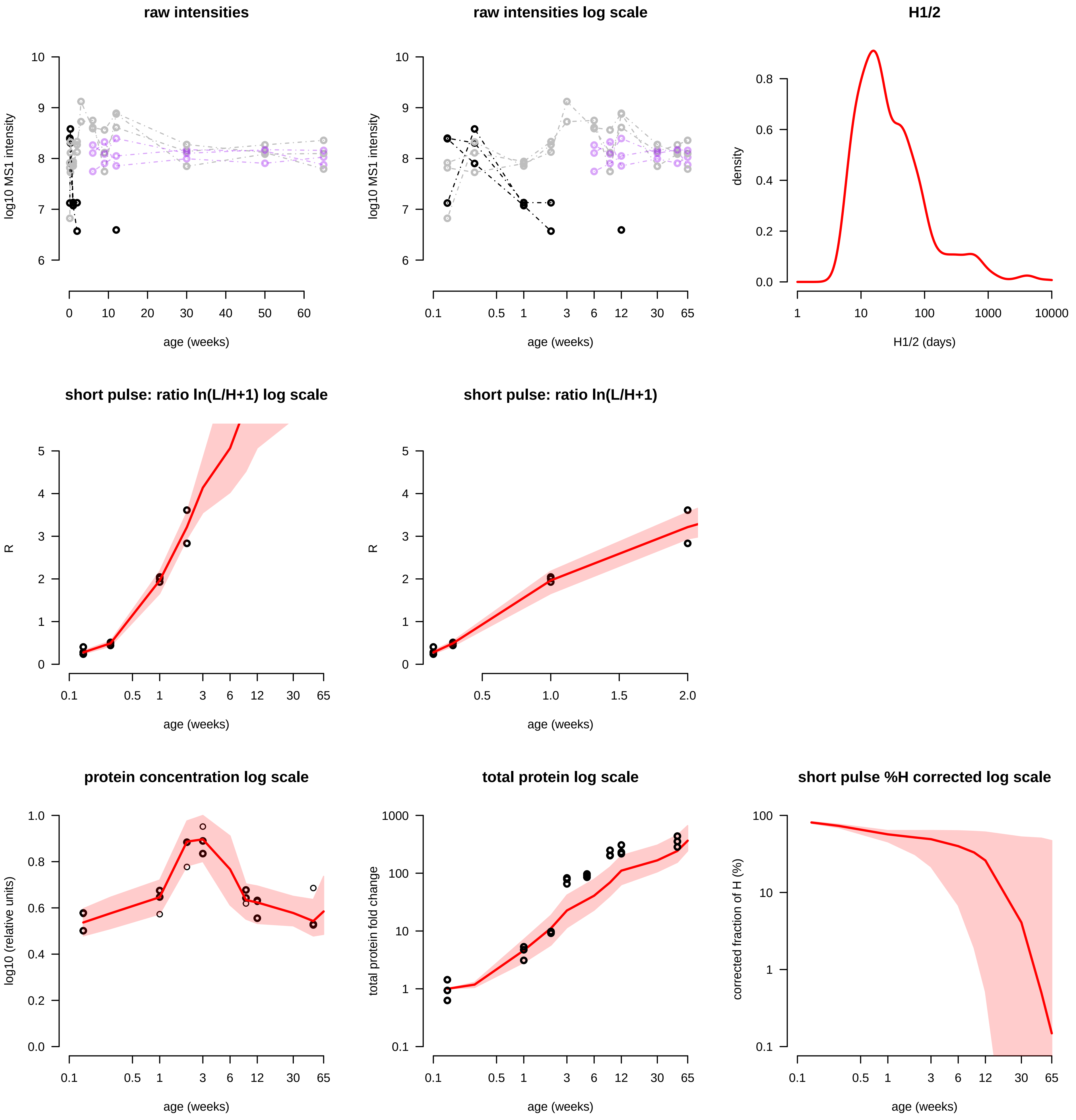

Q91Y86

>sp|Q91Y86|MK08_MOUSE

Mitogen−activated protein kinase 8

GN=Mapk8

Med H1/2: 4.7 days

Q5 H1/2: 2.7 days

Q95 H1/2: 40.5 days

reliable model fit: yes

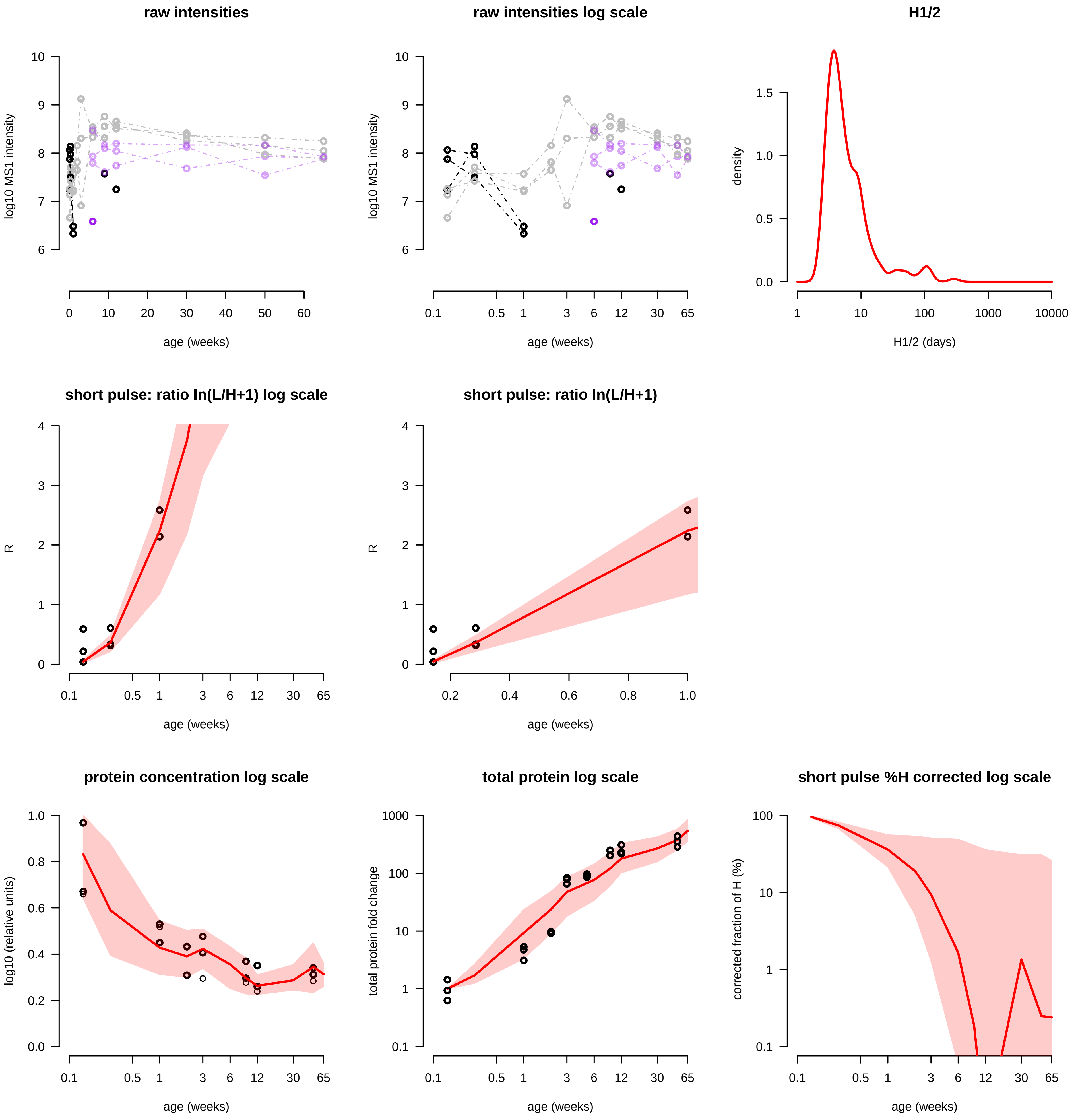

Q0VBL3−2;Q0VBL3

>sp|Q0VBL3−2|RBM15_MOUSE

Isoform 2 of RNA−binding protein 15

GN=Rbm15

Med H1/2: 20 days

Q5 H1/2: 7.4 days

Q95 H1/2: 197.6 days

reliable model fit: no

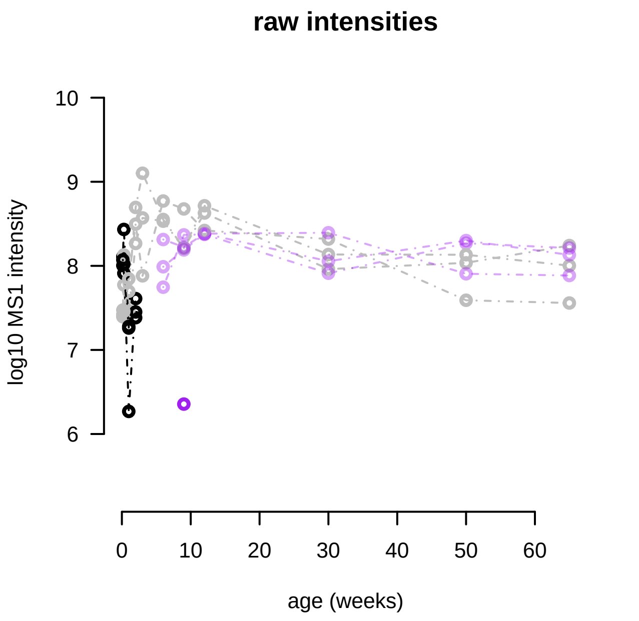

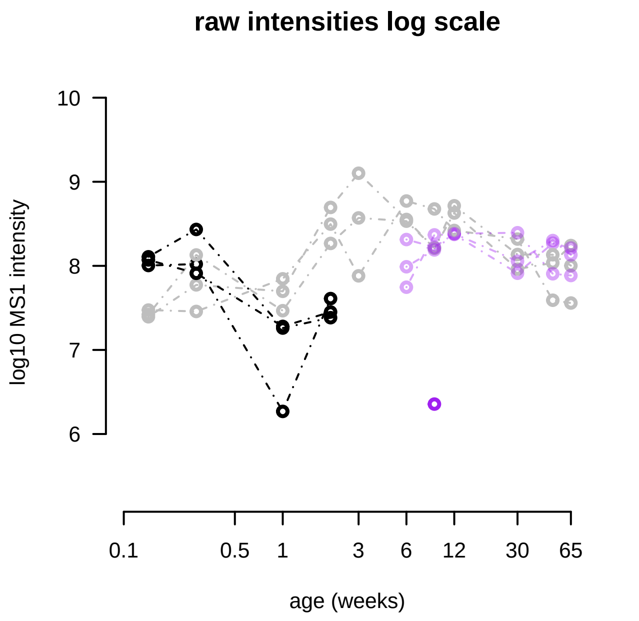

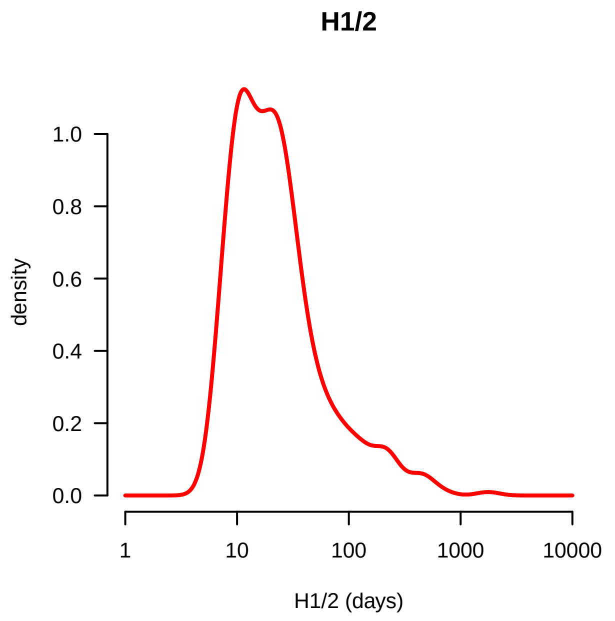

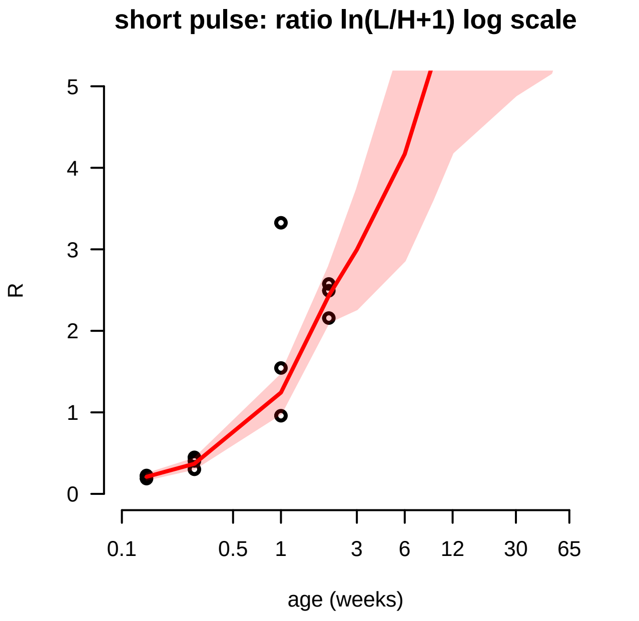

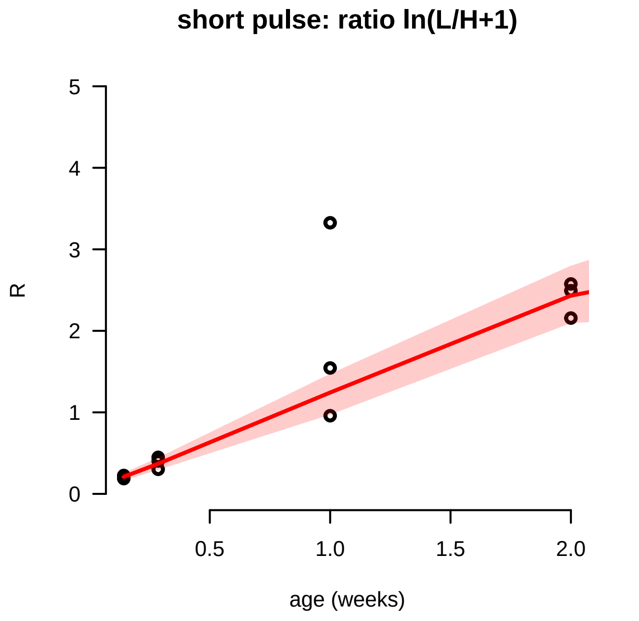

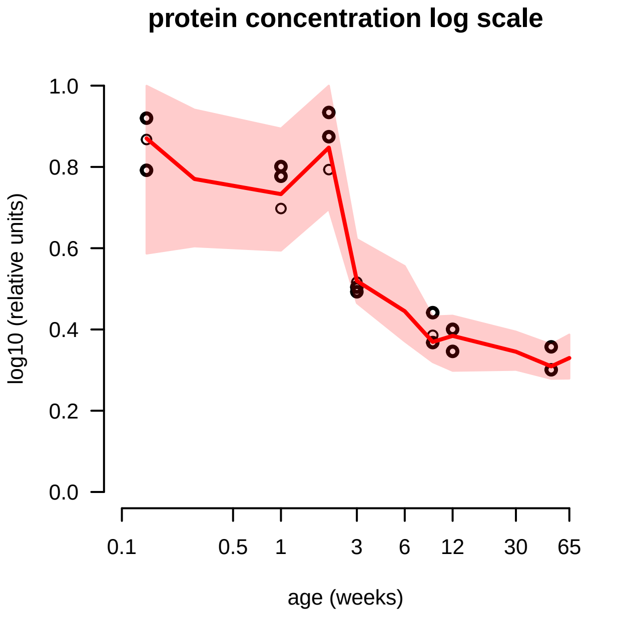

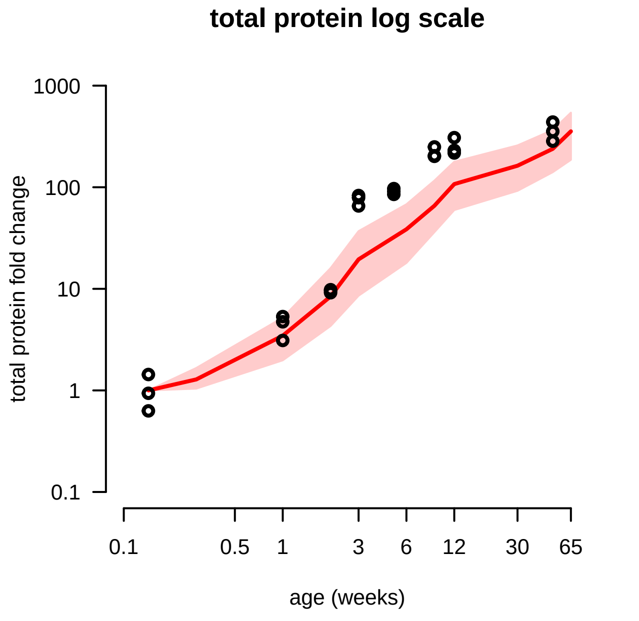

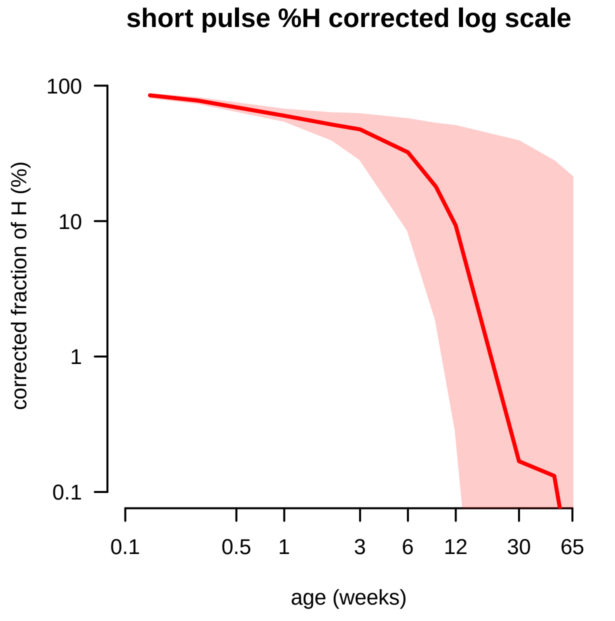

Q689Z5;Q689Z5−2

>sp|Q689Z5|SBNO1_MOUSE

Protein strawberry notch homolog 1

GN=Sbno1

Med H1/2: 4419.1 days

Q5 H1/2: 1008.4 days

Q95 H1/2: 24869.1 days

reliable model fit: no

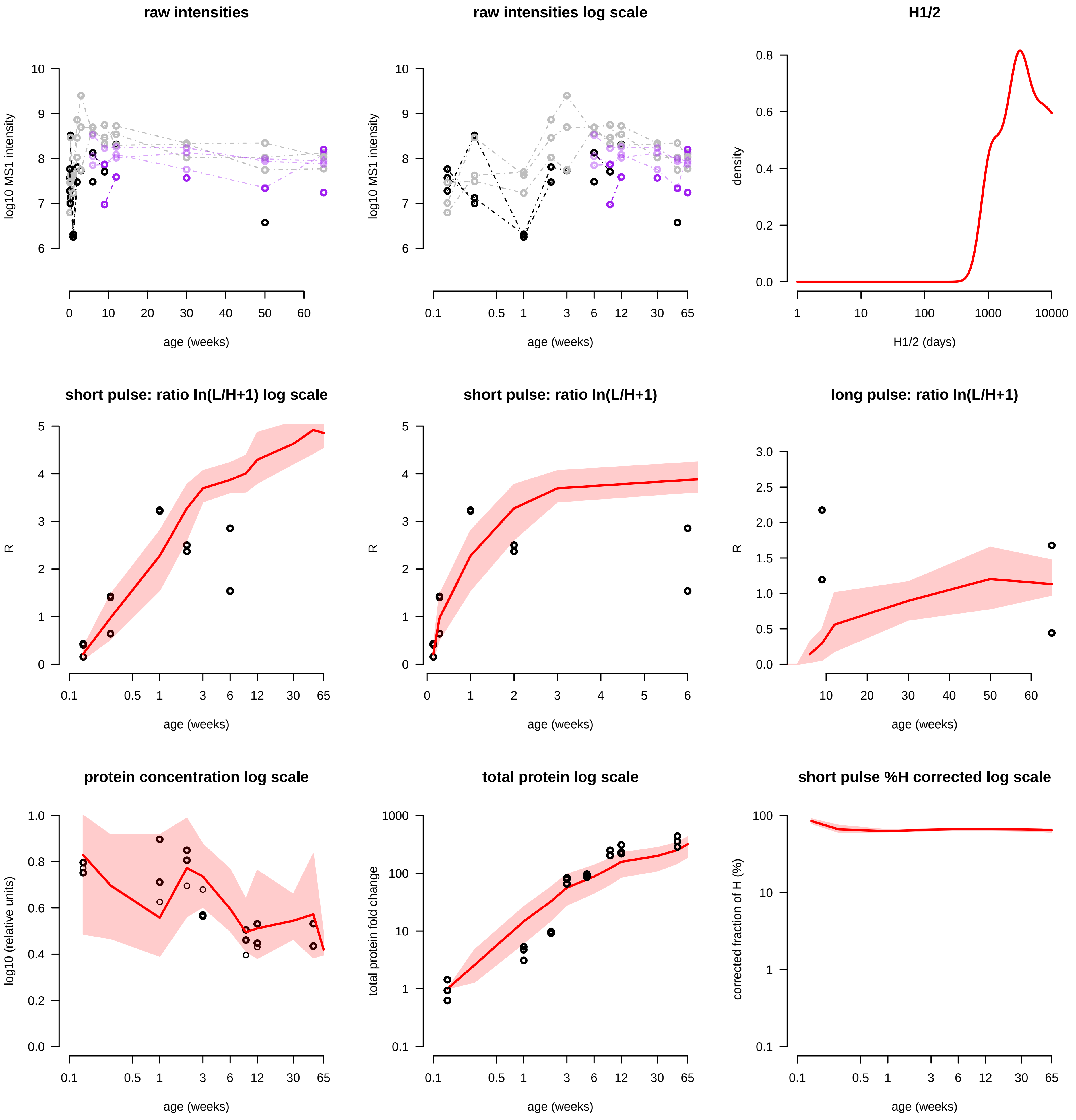

Q9Z2X2

>sp|Q9Z2X2|PSD10_MOUSE

26S proteasome non−ATPase regulatory subunit 10

GN=Psmd10

Med H1/2: 6.9 days

Q5 H1/2: 3.6 days

Q95 H1/2: 27.7 days

reliable model fit: yes

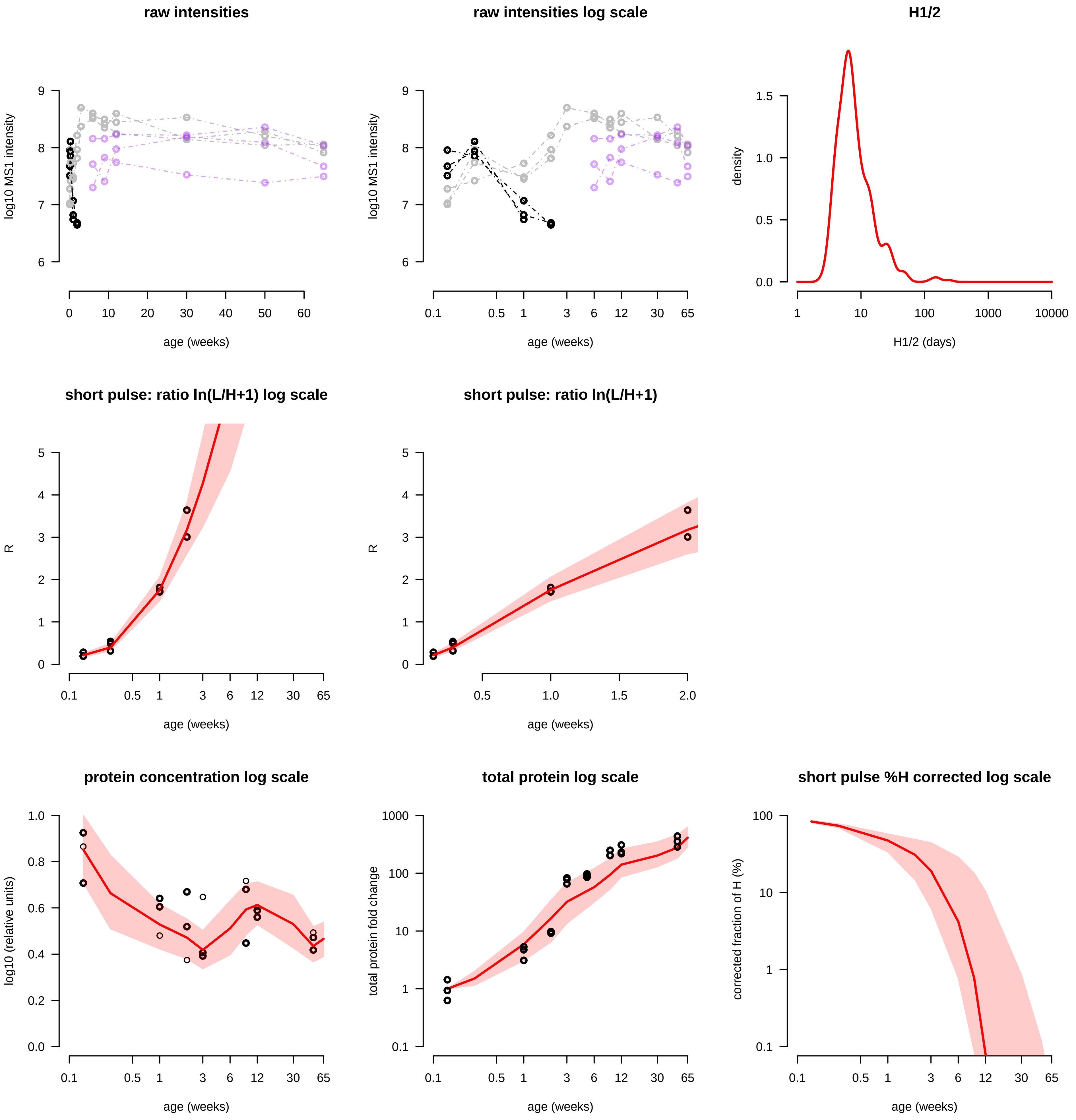

P52875

>sp|P52875|TM165_MOUSE

Transmembrane protein 165

GN=Tmem165

Med H1/2: 7 days

Q5 H1/2: 2.8 days

Q95 H1/2: 63.7 days

reliable model fit: yes

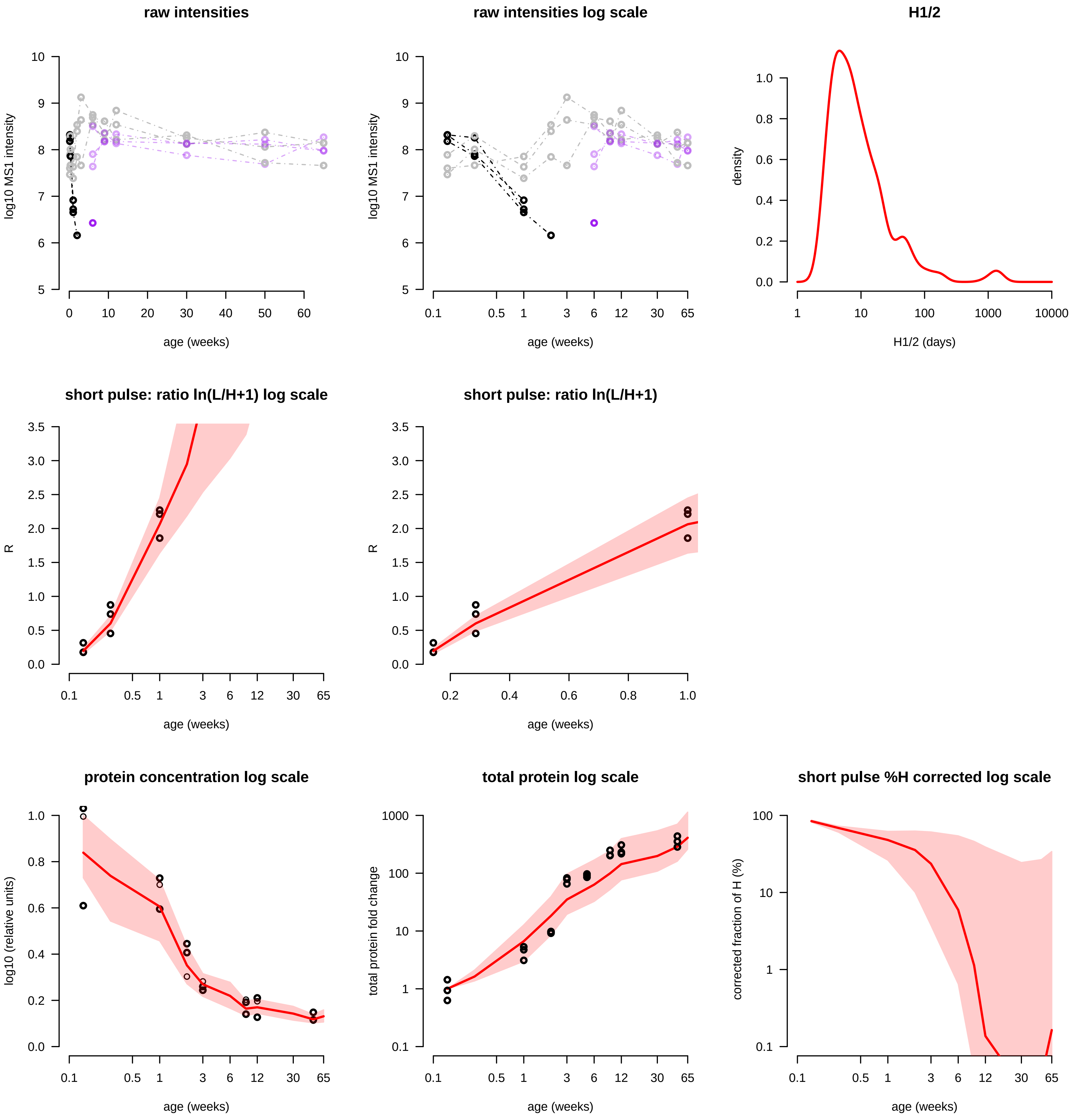

Q6P5H2−2;Q6P5H2

>sp|Q6P5H2−2|NEST_MOUSE

Isoform 2 of Nestin

GN=Nes

Med H1/2: 28.7 days

Q5 H1/2: 7.9 days

Q95 H1/2: 453.2 days

reliable model fit: yes

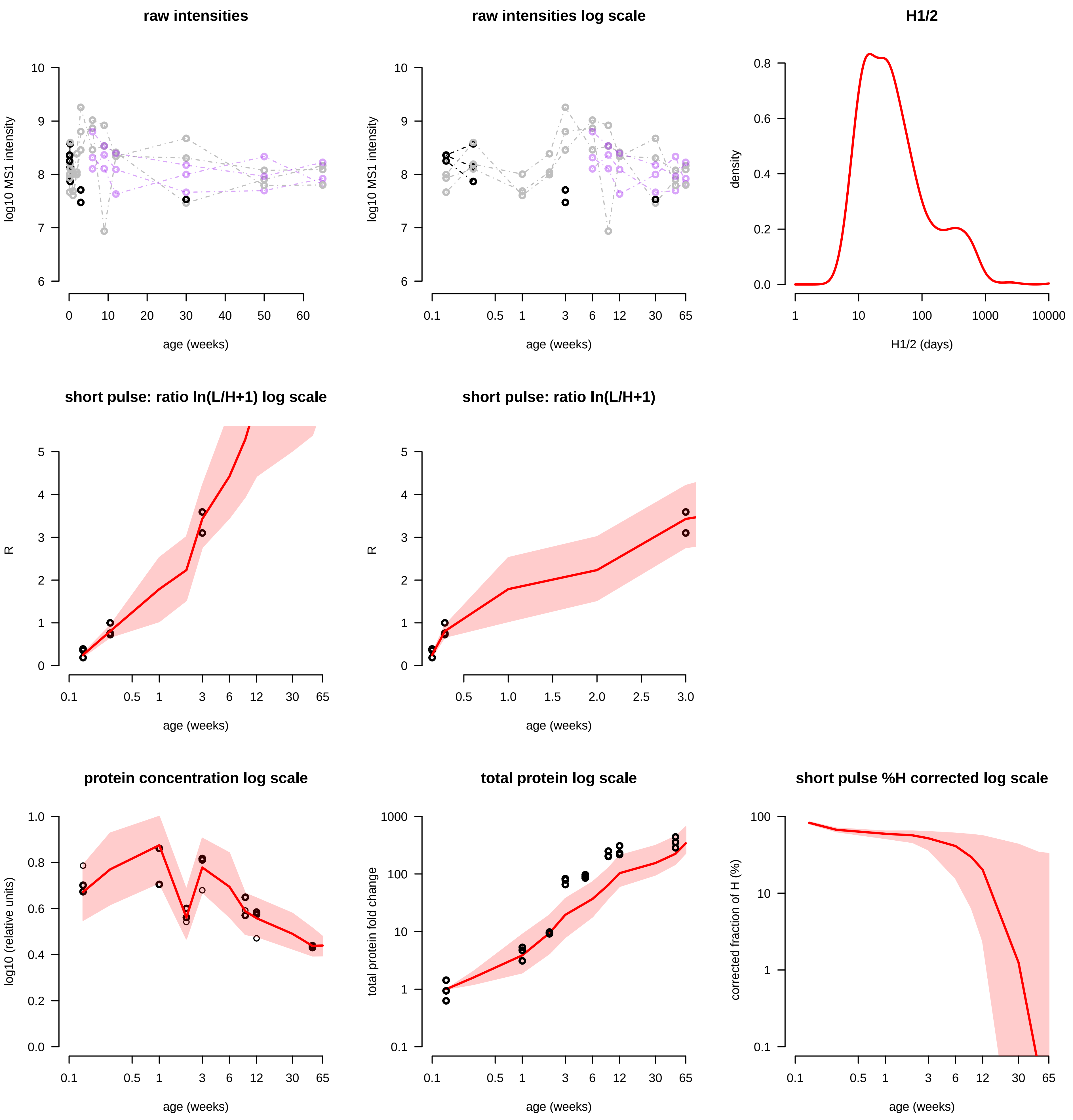

Q6QI06;Q6QI06−2

>sp|Q6QI06|RICTR_MOUSE

Rapamycin−insensitive companion of mTOR

GN=Rictor

Med H1/2: 33.7 days

Q5 H1/2: 8.1 days

Q95 H1/2: 731.7 days

reliable model fit: yes

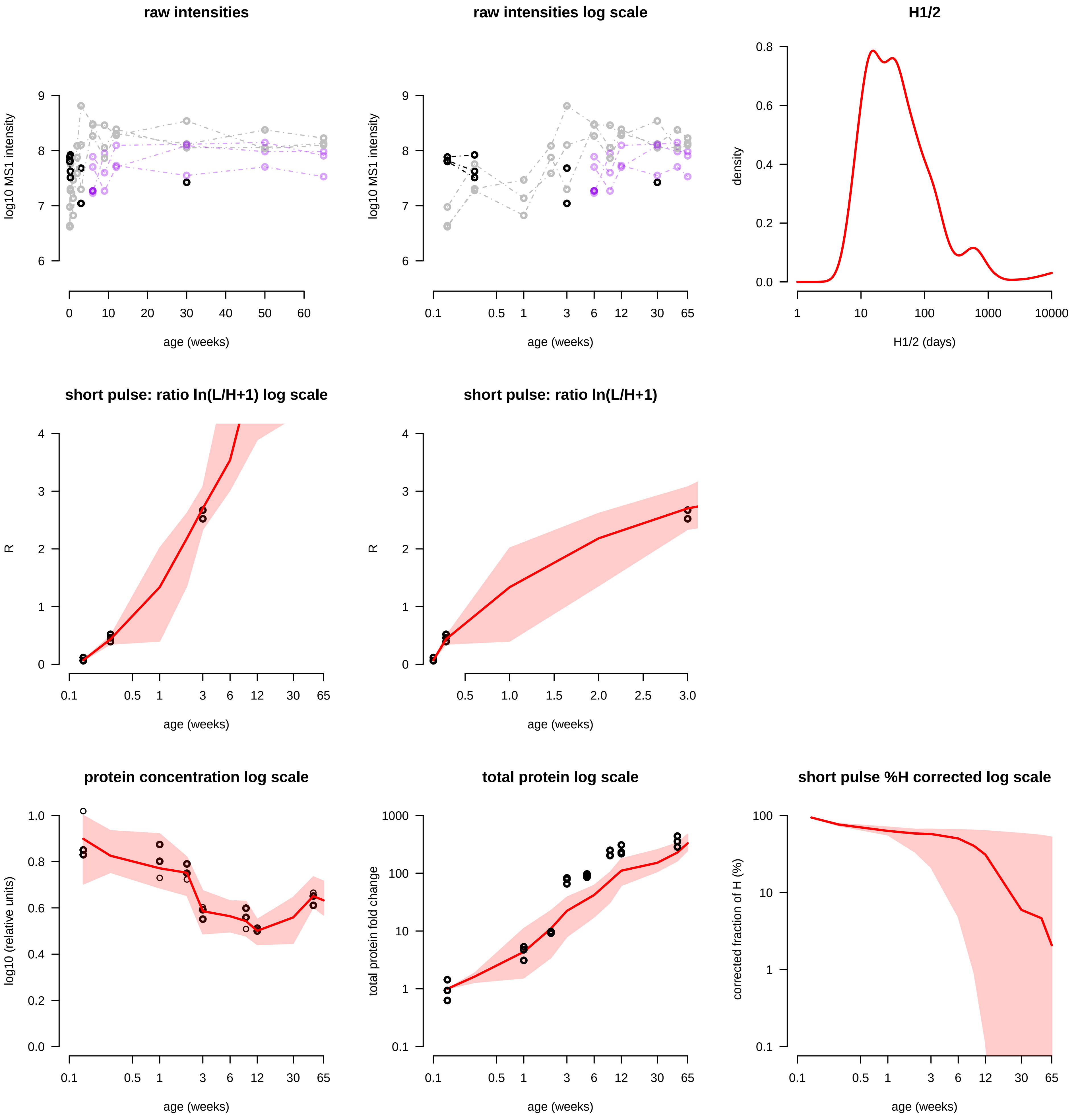

O70439

>sp|O70439|STX7_MOUSE

Syntaxin−7

GN=Stx7

Med H1/2: 7.6 days

Q5 H1/2: 2.8 days

Q95 H1/2: 155.3 days

reliable model fit: yes

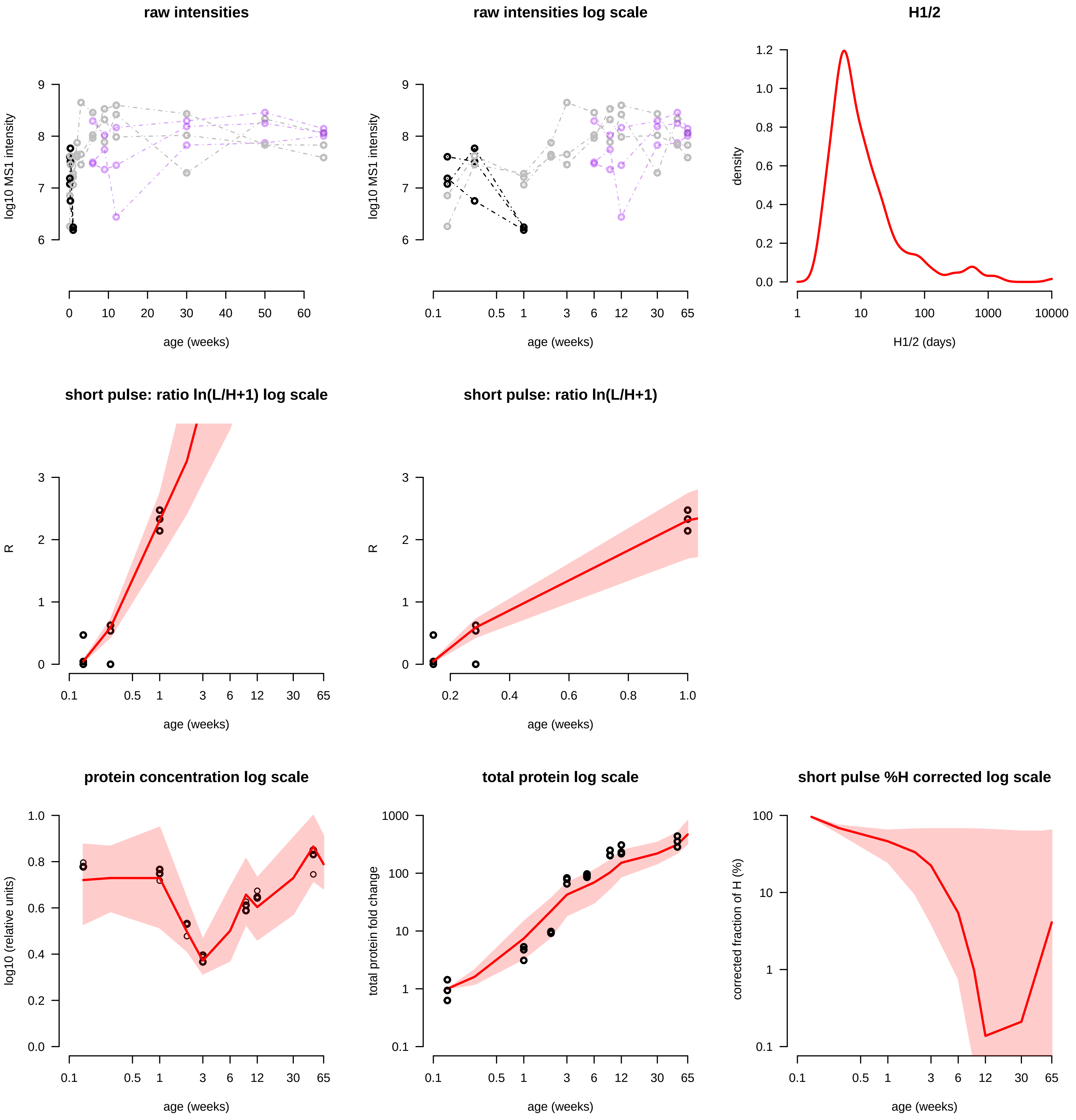

P08607

>sp|P08607|C4BPA_MOUSE

C4b−binding protein

GN=C4bpa

Med H1/2: 3189.6 days

Q5 H1/2: 935.9 days

Q95 H1/2: 35084.9 days

reliable model fit: yes

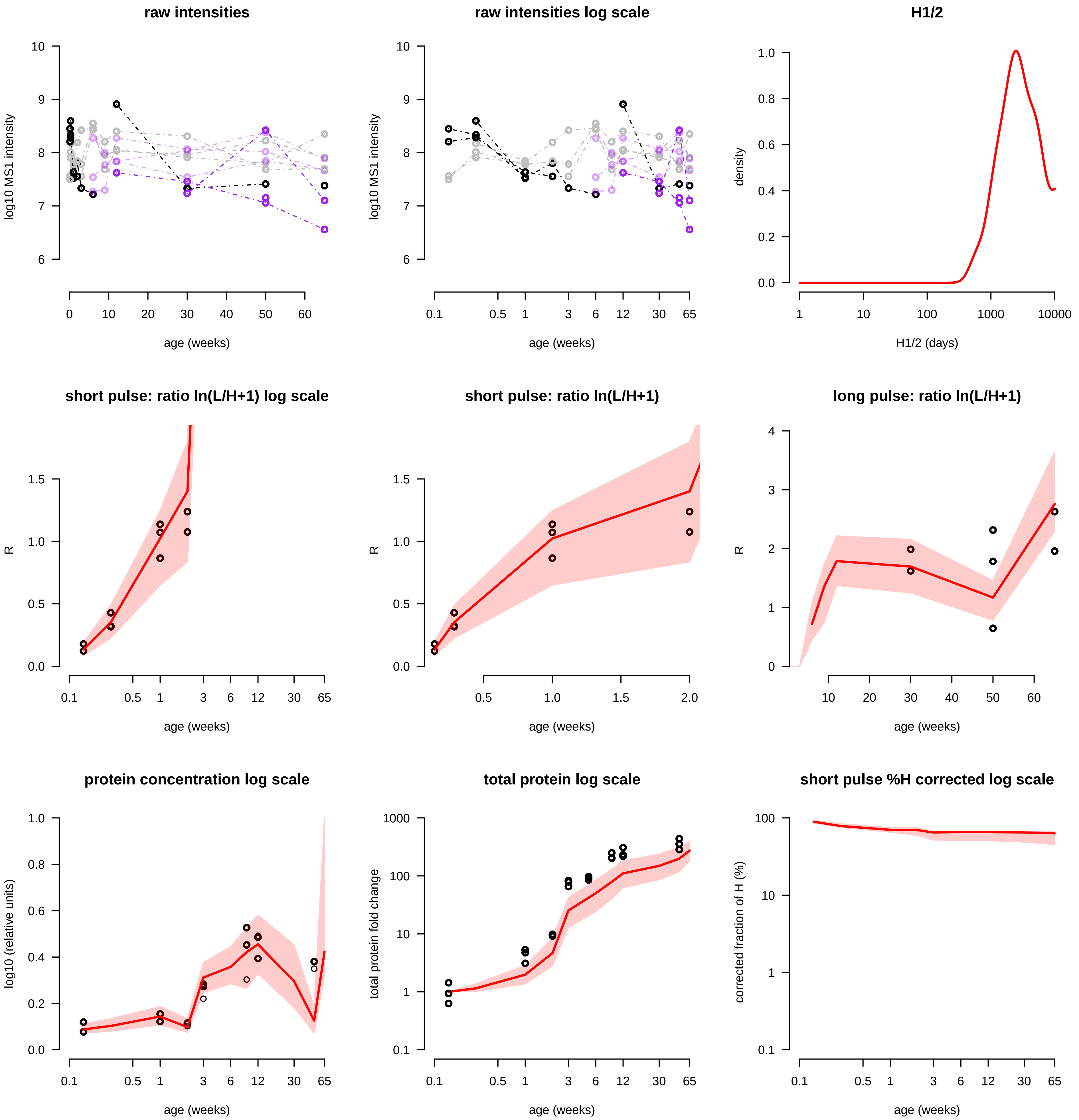

Q9DCM0

>sp|Q9DCM0|ETHE1_MOUSE

Persulfide dioxygenase ETHE1, mitochondrial

GN=Ethe1

Med H1/2: 7.8 days

Q5 H1/2: 6.1 days

Q95 H1/2: 12.3 days

reliable model fit: yes

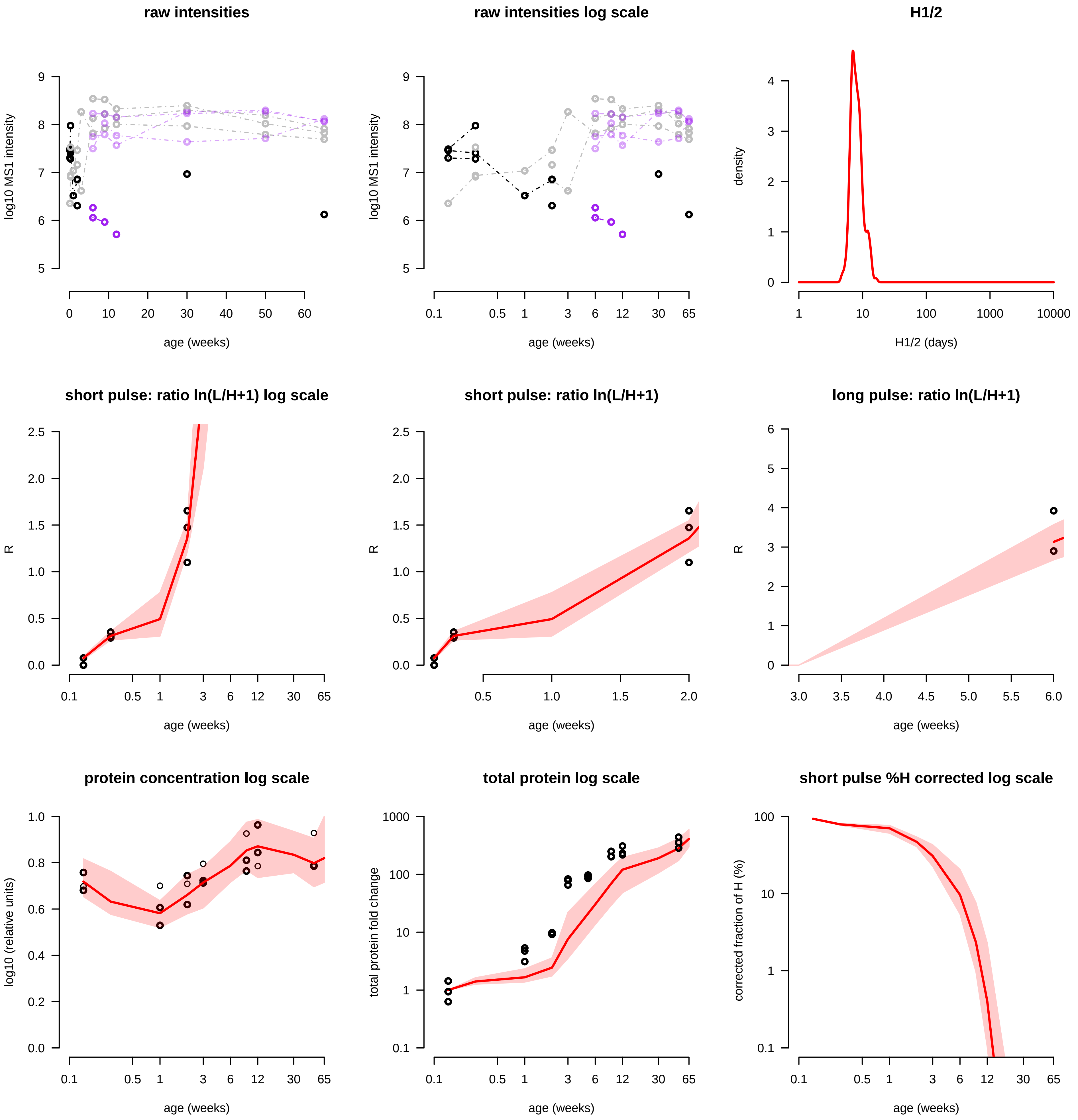

Q9CPX7

>sp|Q9CPX7|RT16_MOUSE

28S ribosomal protein S16, mitochondrial

GN=Mrps16

Med H1/2: 6.2 days

Q5 H1/2: 2.7 days

Q95 H1/2: 52.7 days

reliable model fit: no

raw intensities

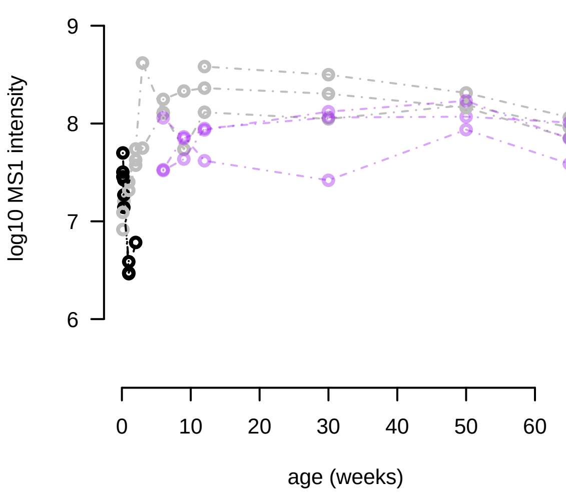

raw intensities log scale

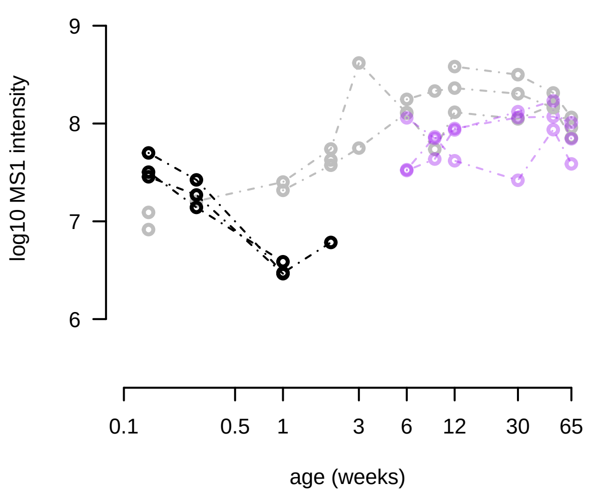

H1/2

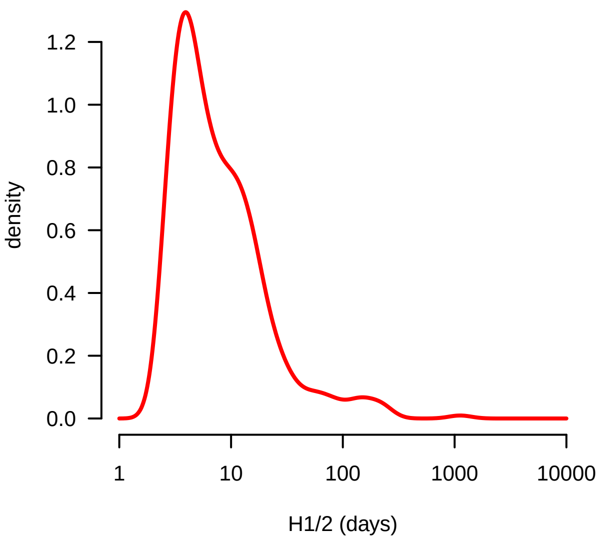

short pulse: ratio ln(L/H+1) log scale

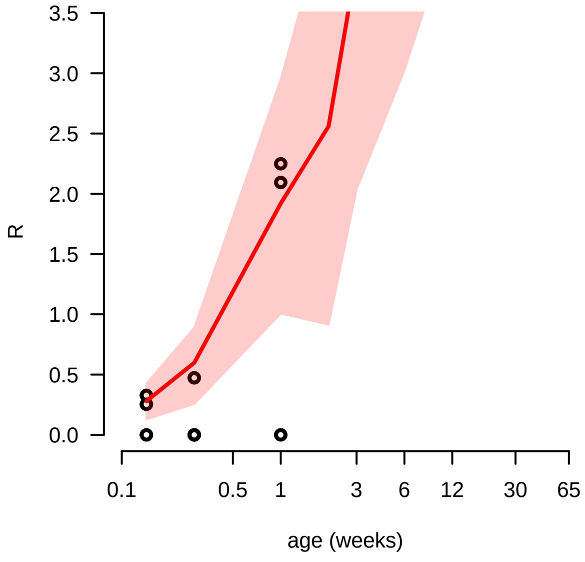

short pulse: ratio ln(L/H+1)

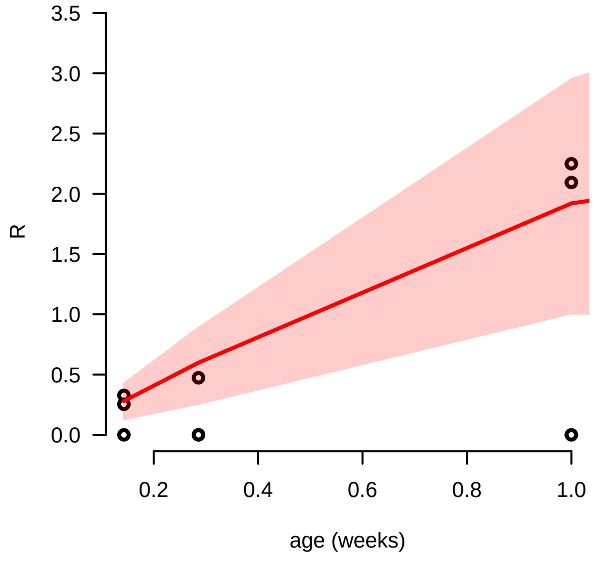

protein concentration log scale

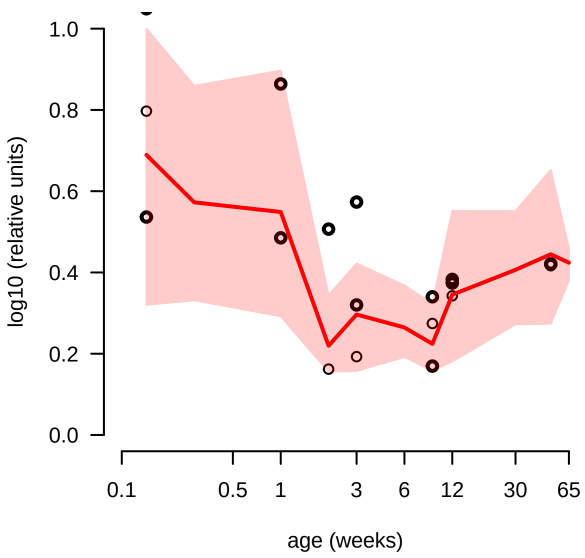

total protein log scale

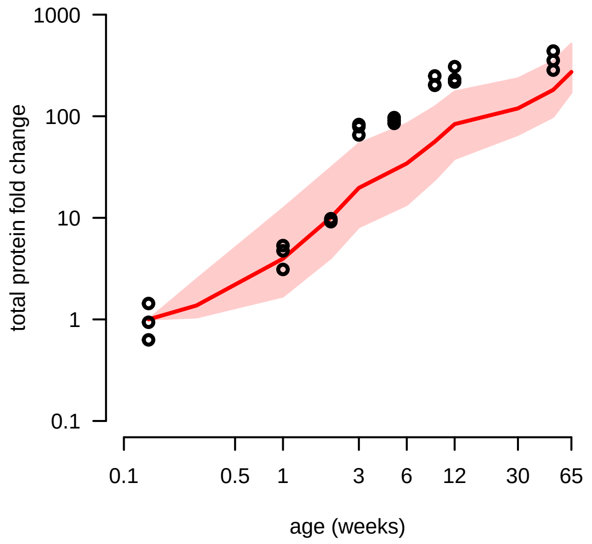

short pulse %H corrected log scale

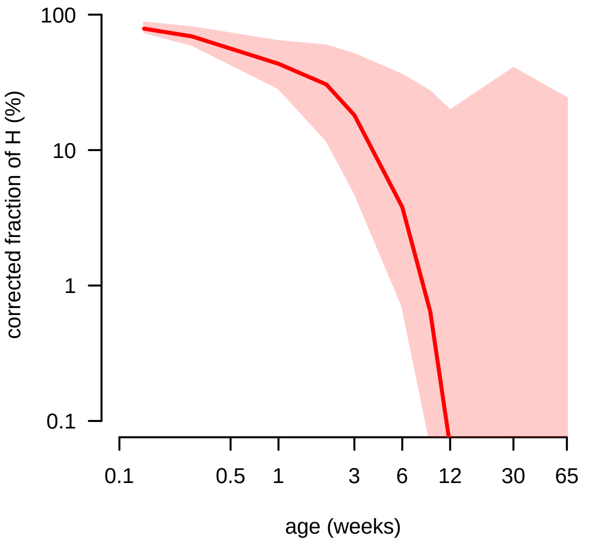

O35075

>sp|O35075|VP26C_MOUSE

Vacuolar protein sorting−associated protein 26C

GN=Vps26c

Med H1/2: 11.3 days

Q5 H1/2: 4.3 days

Q95 H1/2: 68.1 days

reliable model fit: yes

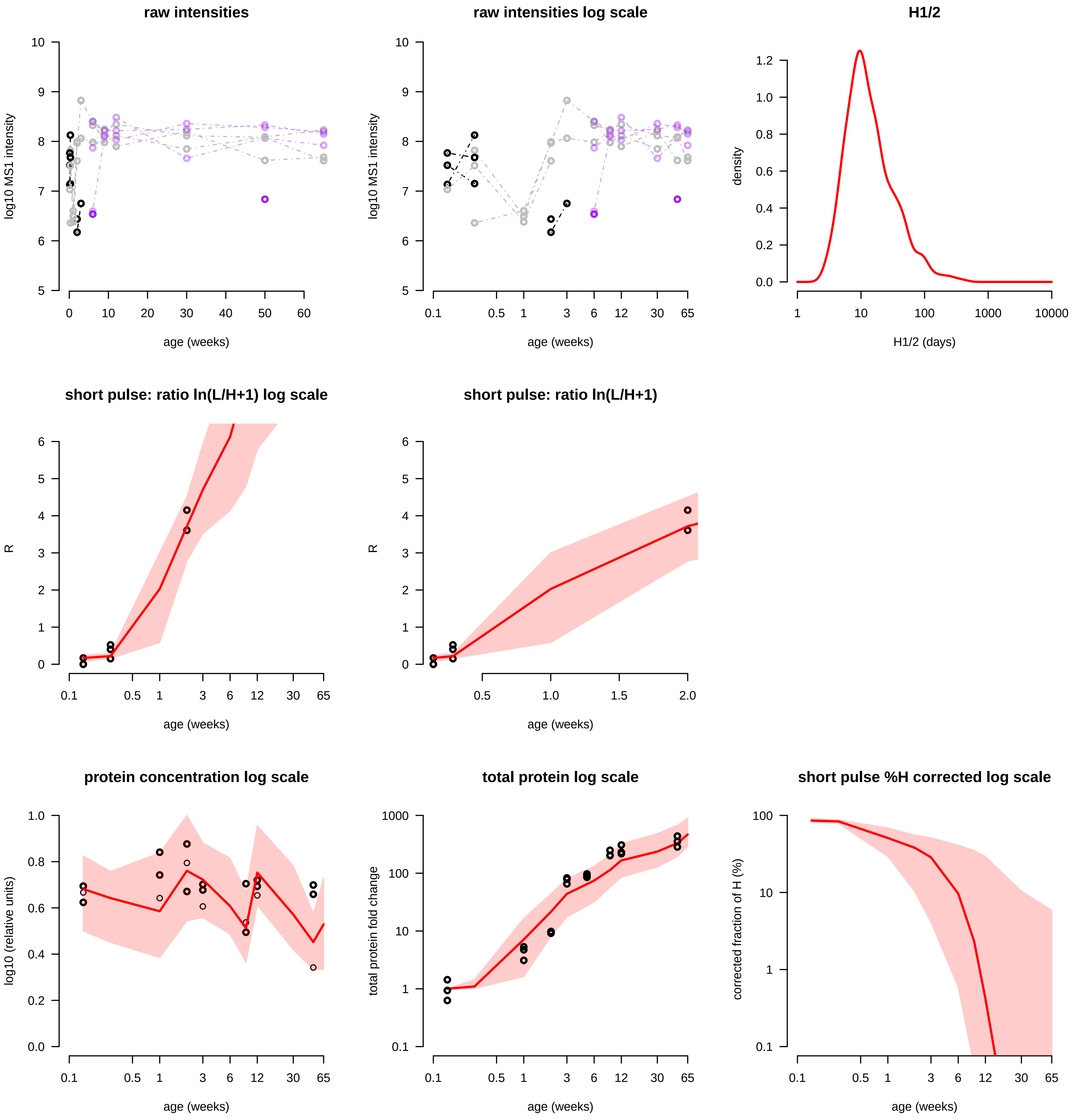

Q05909

>sp|Q05909|PTPRG_MOUSE

Receptor−type tyrosine−protein phosphatase gamma

GN=Ptprg

Med H1/2: 6.9 days

Q5 H1/2: 3.8 days

Q95 H1/2: 24.7 days

reliable model fit: yes

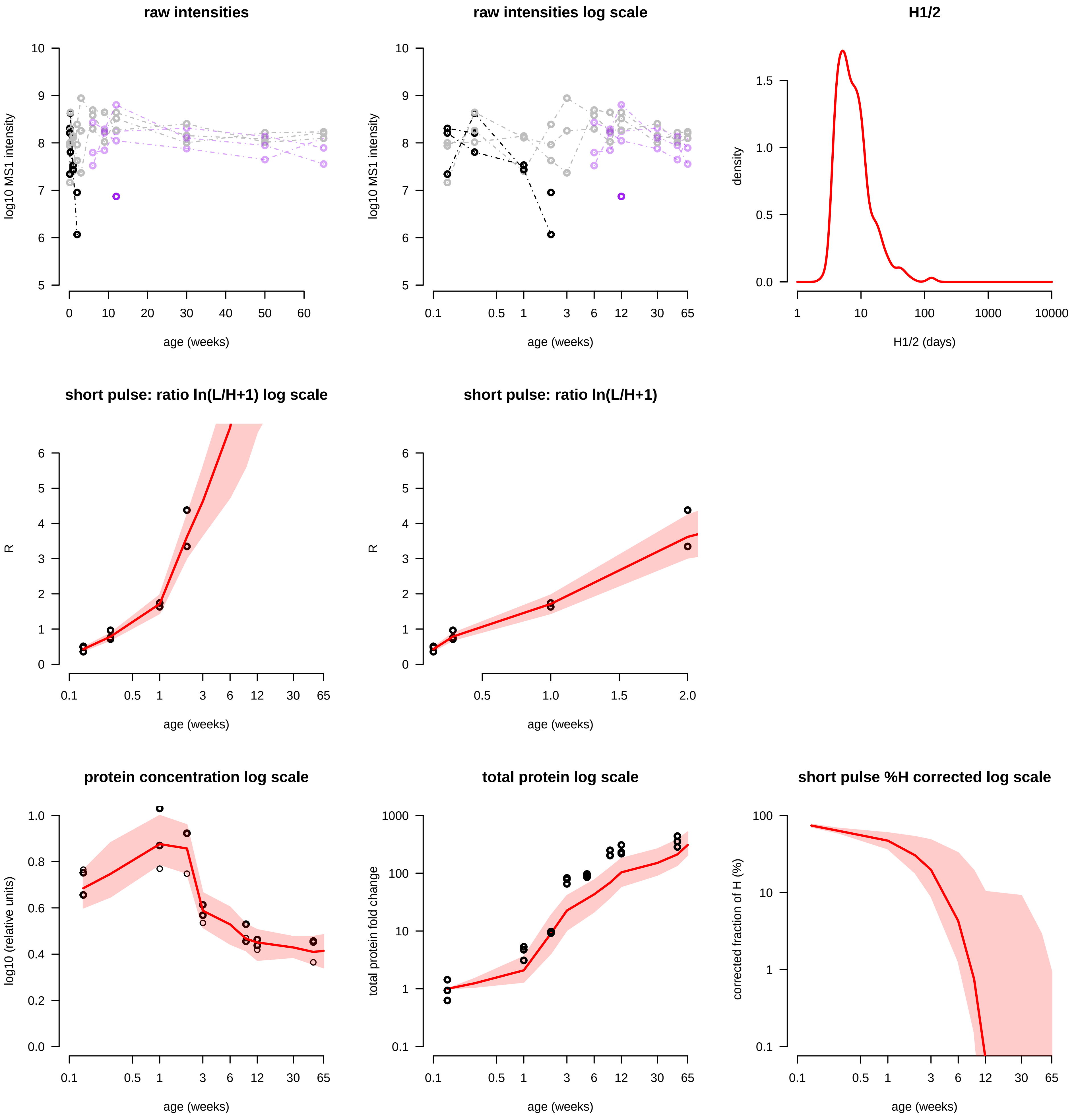

Q3URQ0

>sp|Q3URQ0|TEX10_MOUSE

Testis−expressed protein 10

GN=Tex10

Med H1/2: 5.7 days

Q5   H1/2: 4.1 days

Q95 H1/2: 9 days

reliable model fit: yes

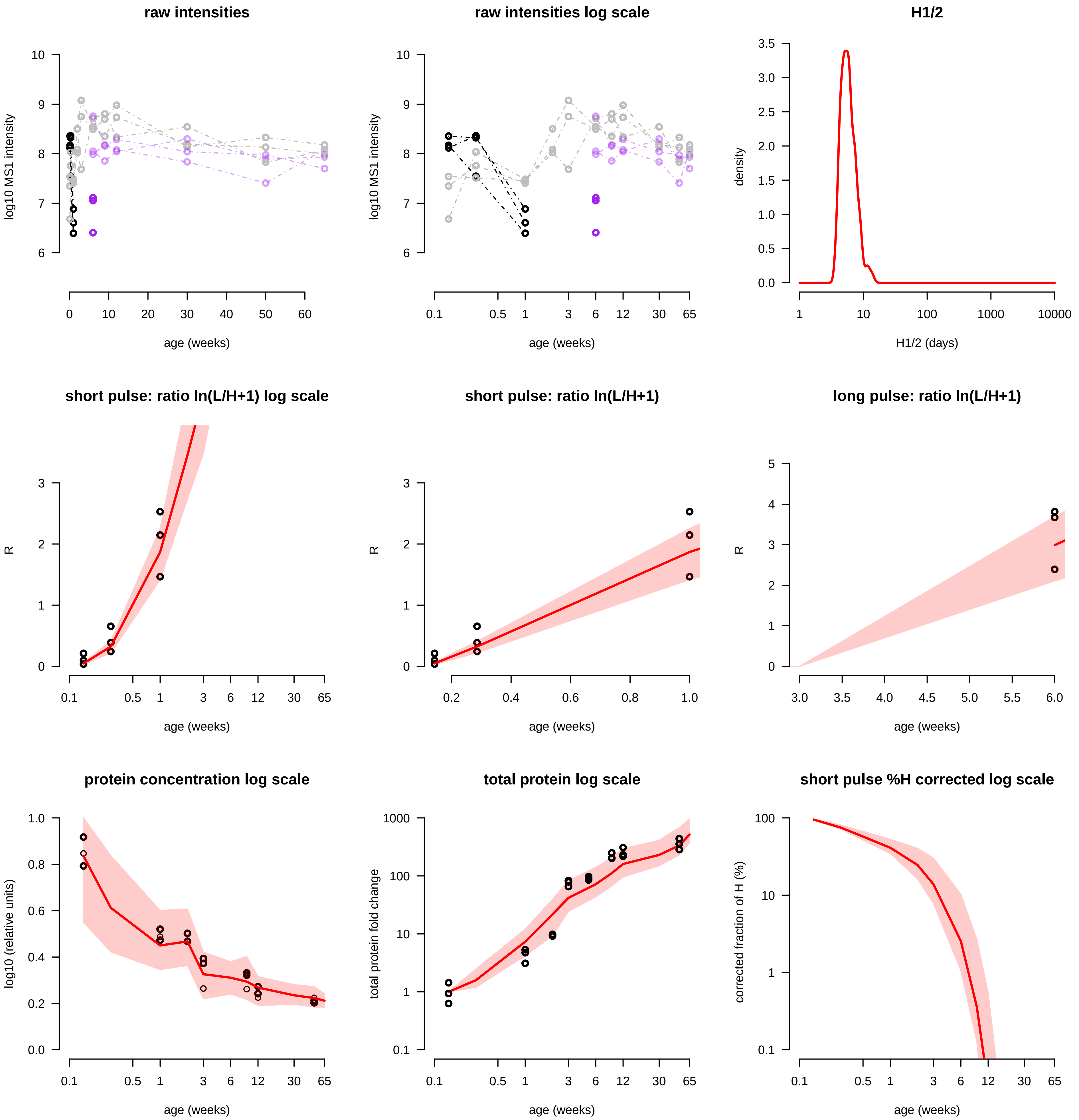

Q9CYA0

>sp|Q9CYA0|CREL2_MOUSE

Protein disulfide isomerase Creld2

GN=Creld2

Med H1/2: 2500.4 days

Q5 H1/2: 541 days

Q95 H1/2: 52921.8 days

reliable model fit: no

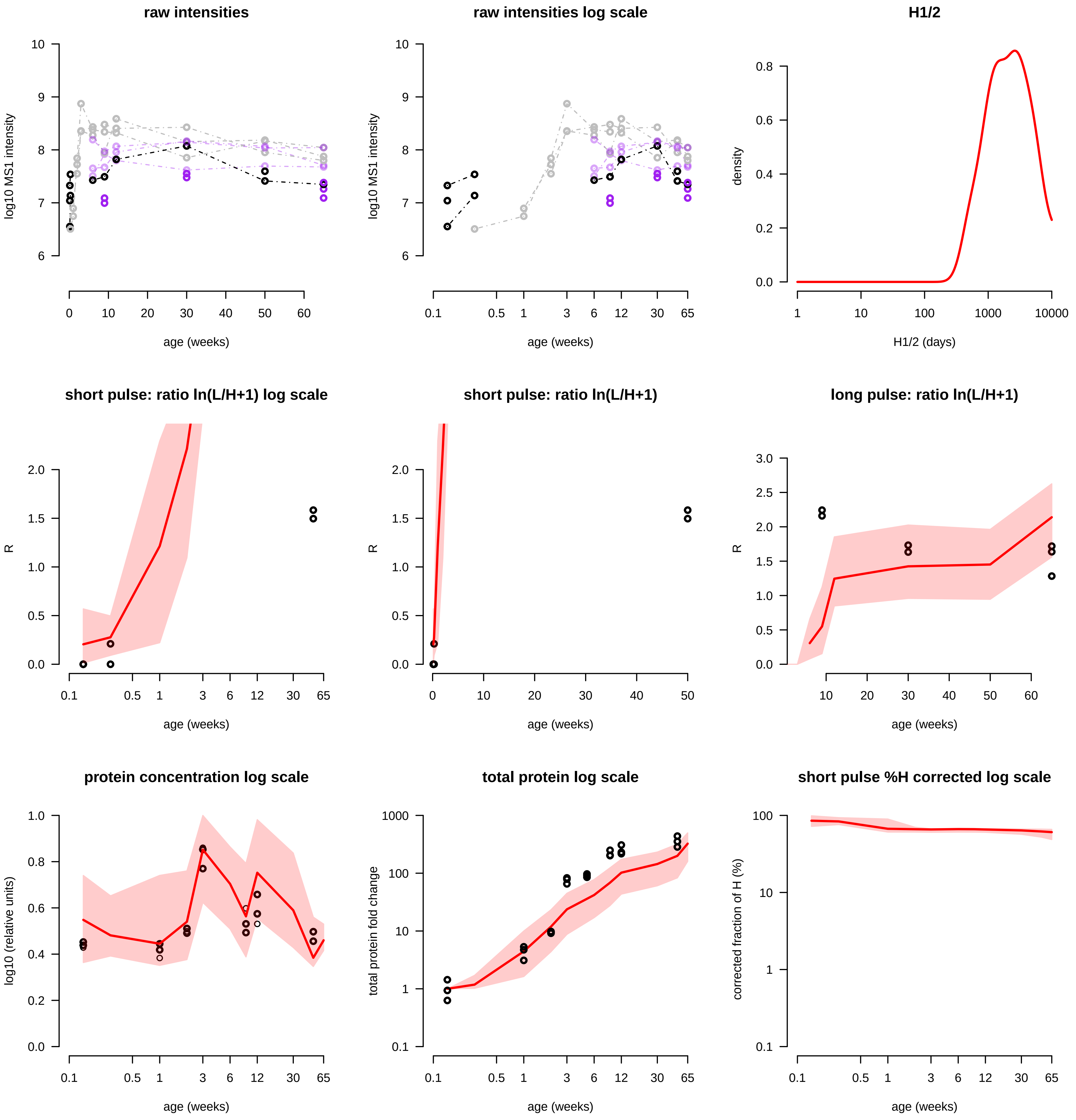

Q6WVG3

>sp|Q6WVG3|KCD12_MOUSE

BTB/POZ domain−containing protein KCTD12

GN=Kctd12

Med H1/2: 6.2 days

Q5 H1/2: 5.2 days

Q95 H1/2: 7.4 days

reliable model fit: yes

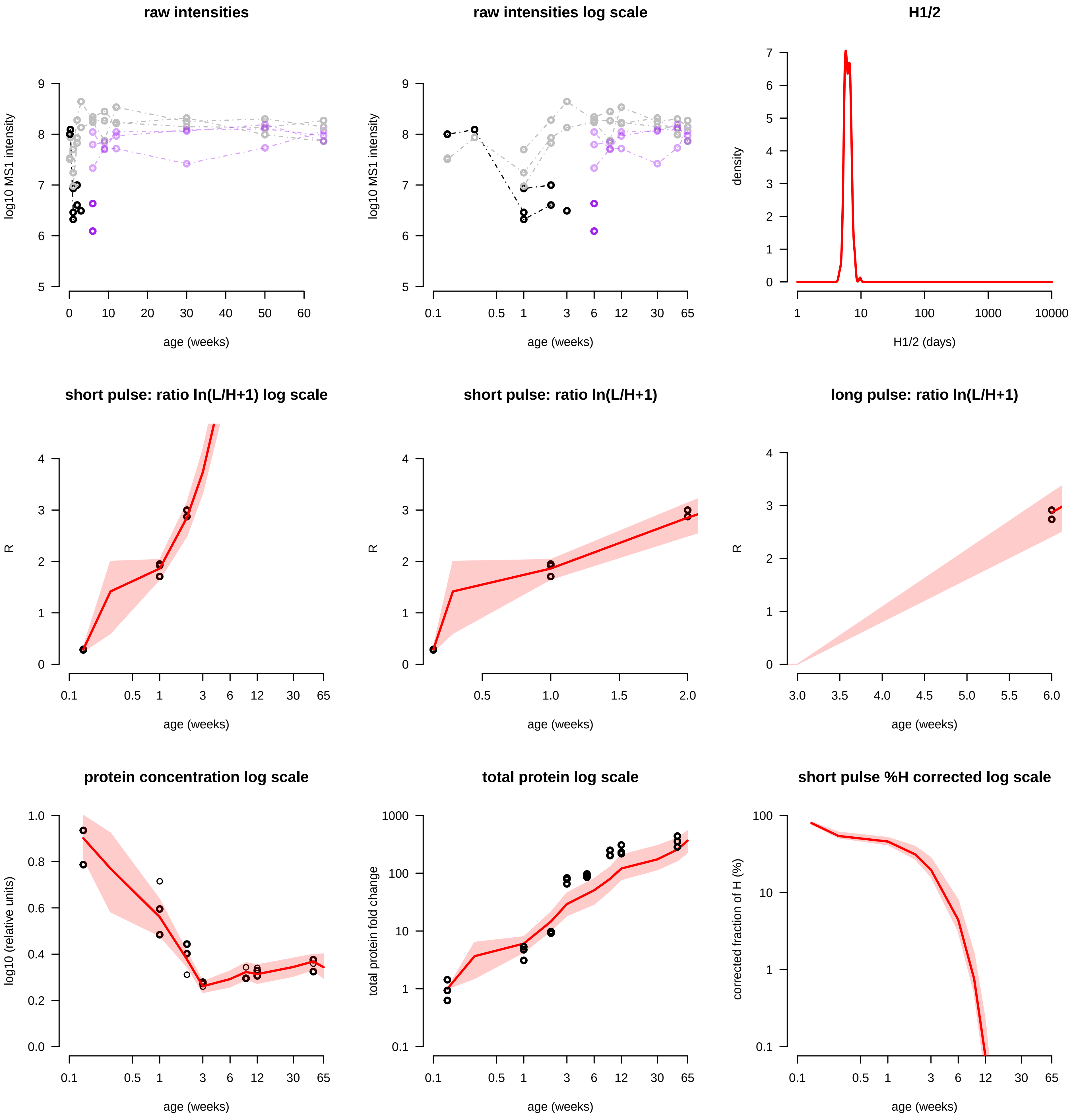

O09117−2;O09117

>sp|O09117−2|SYPL1_MOUSE

Isoform 2 of Synaptophysin−like protein 1

GN=Sypl1

Med H1/2: 10.4 days

Q5 H1/2: 4.6 days

Q95 H1/2: 99.7 days

reliable model fit: yes

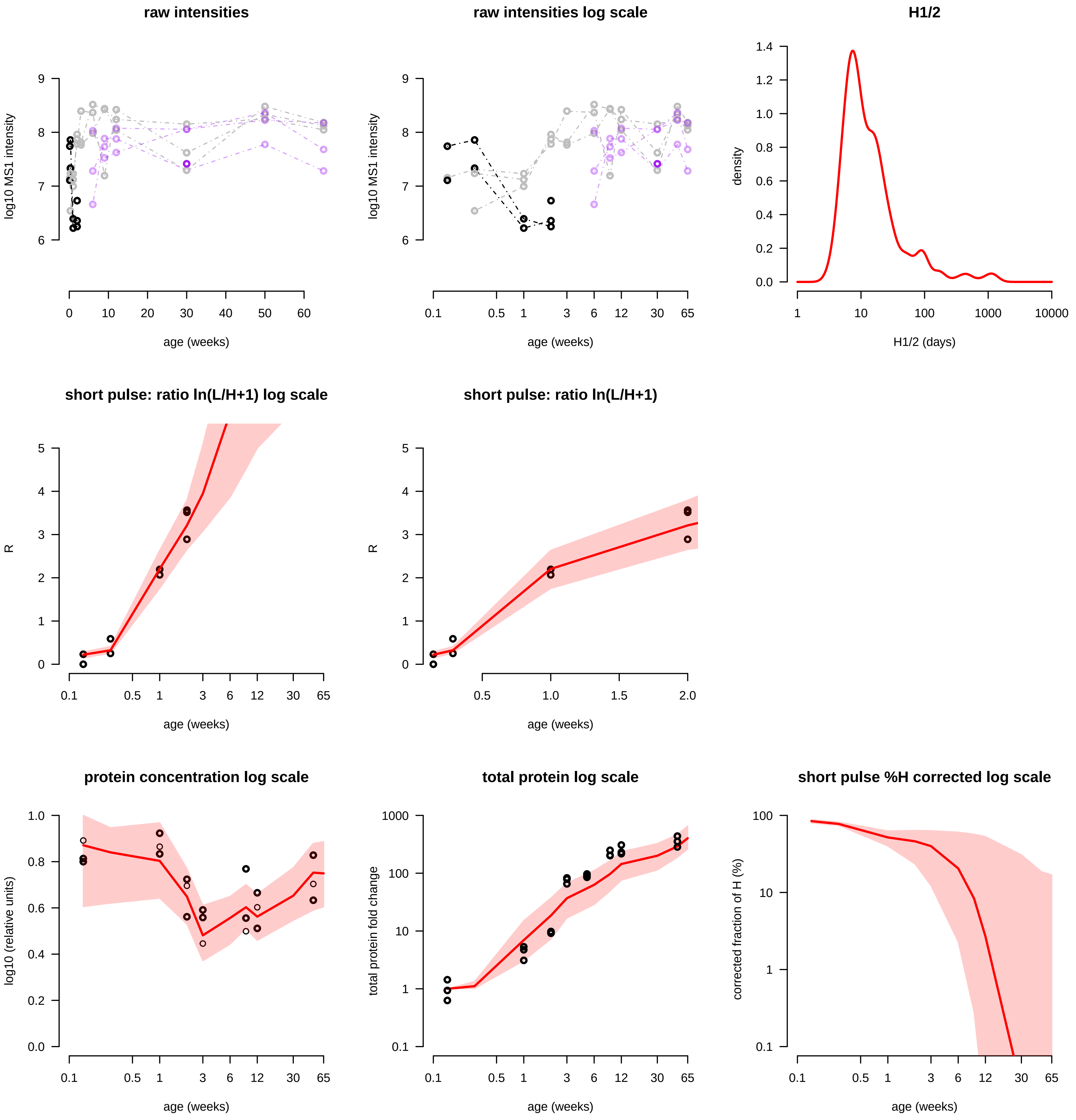

Q9NYQ2

>sp|Q9NYQ2|HAOX2_MOUSE

Hydroxyacid oxidase 2

GN=Hao2

Med H1/2: 16.5 days

Q5 H1/2: 3.7 days

Q95 H1/2: 143.5 days

reliable model fit: yes

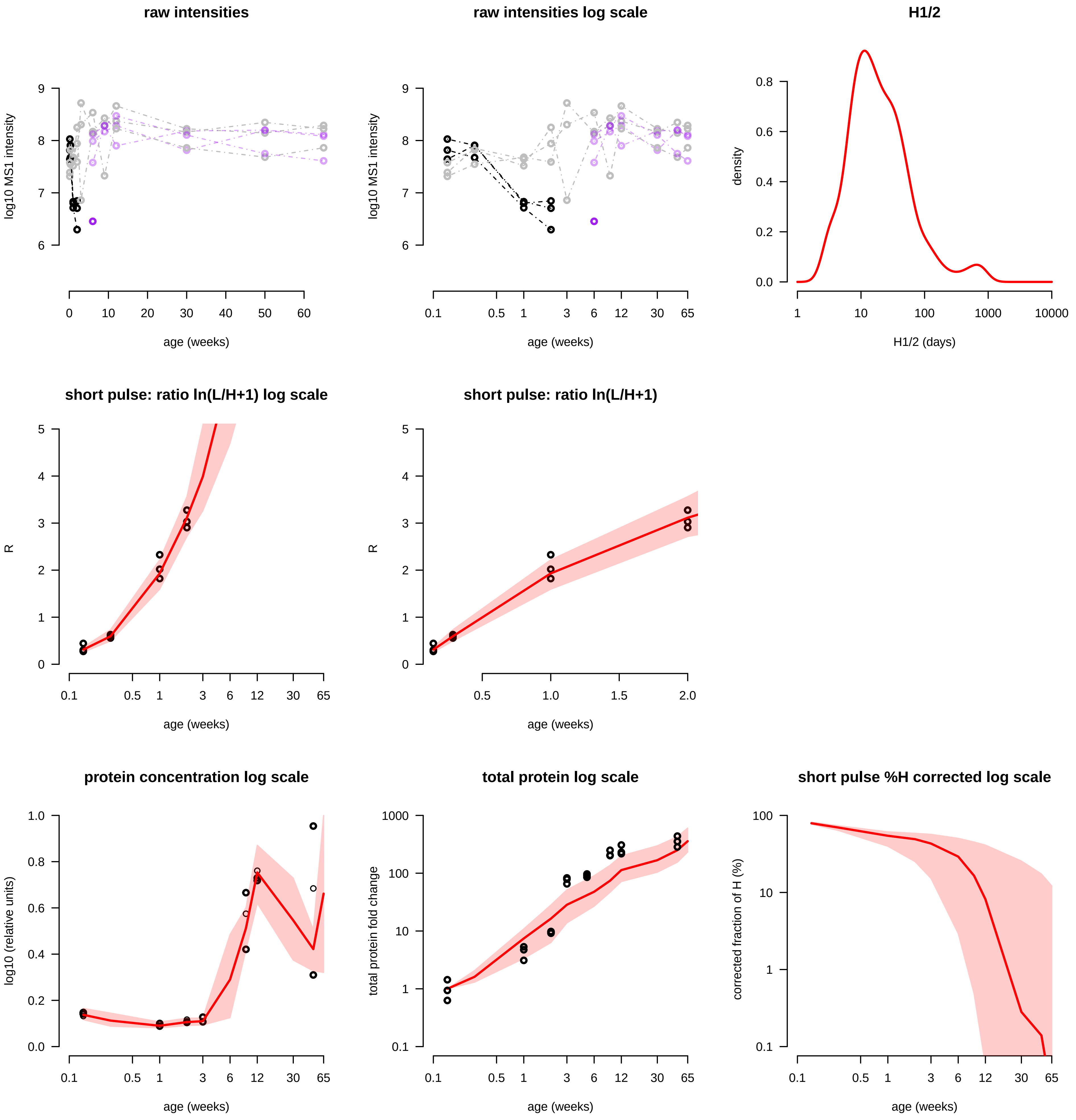

Q9DBE9

>sp|Q9DBE9|SPB1_MOUSE

pre−rRNA 2'−O−ribose RNA methyltransferase FTSJ3

GN=Ftsj3

Med H1/2: 3.3 days

Q5 H1/2: 2.5 days

Q95 H1/2: 8.7 days

reliable model fit: yes

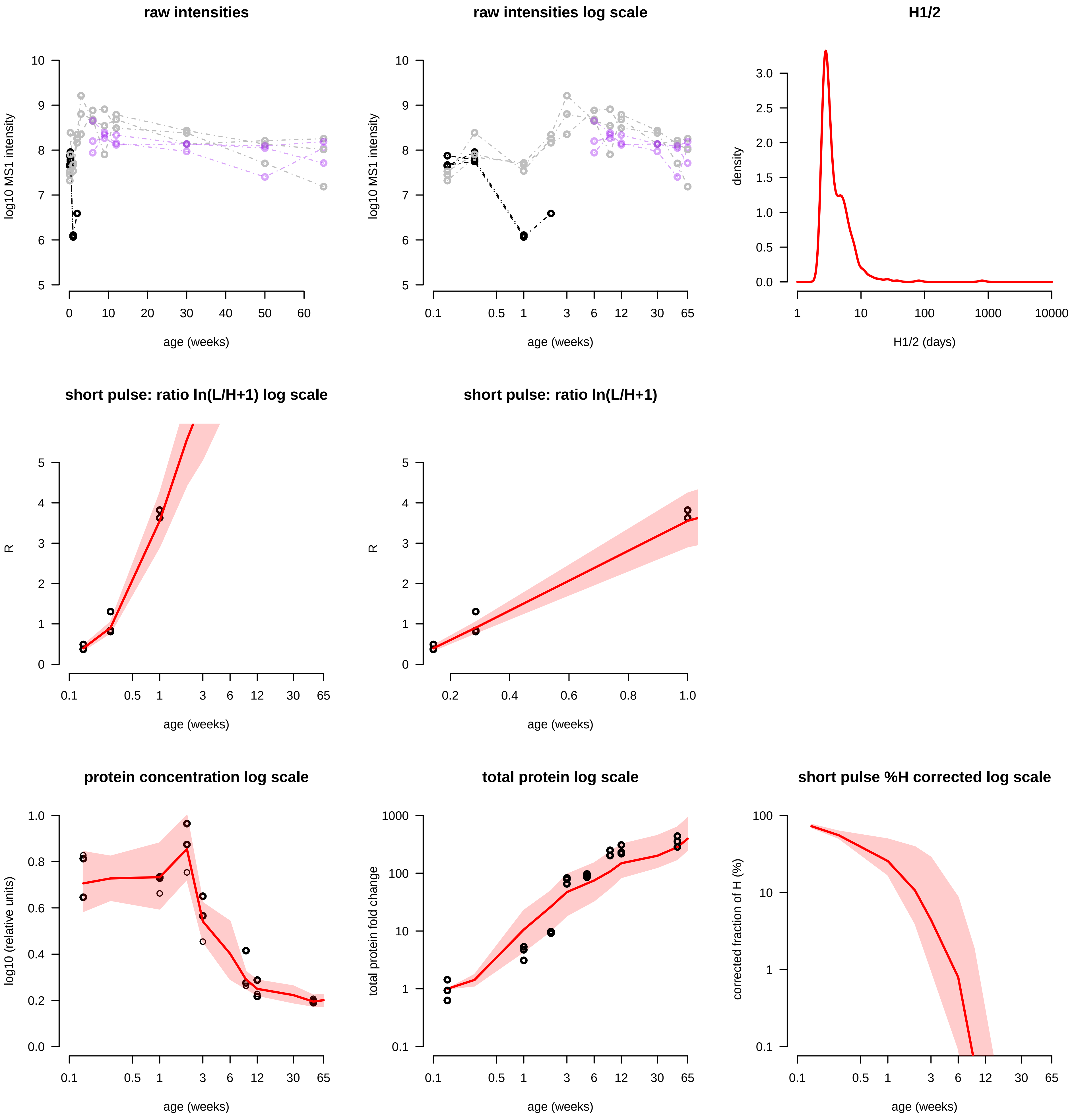

Q3UA37

>sp|Q3UA37|QRIC1_MOUSE

Transcriptional regulator QRICH1

GN=Qrich1

Med H1/2: 3.7 days

Q5 H1/2: 3.1 days

Q95 H1/2: 4.7 days

reliable model fit: yes

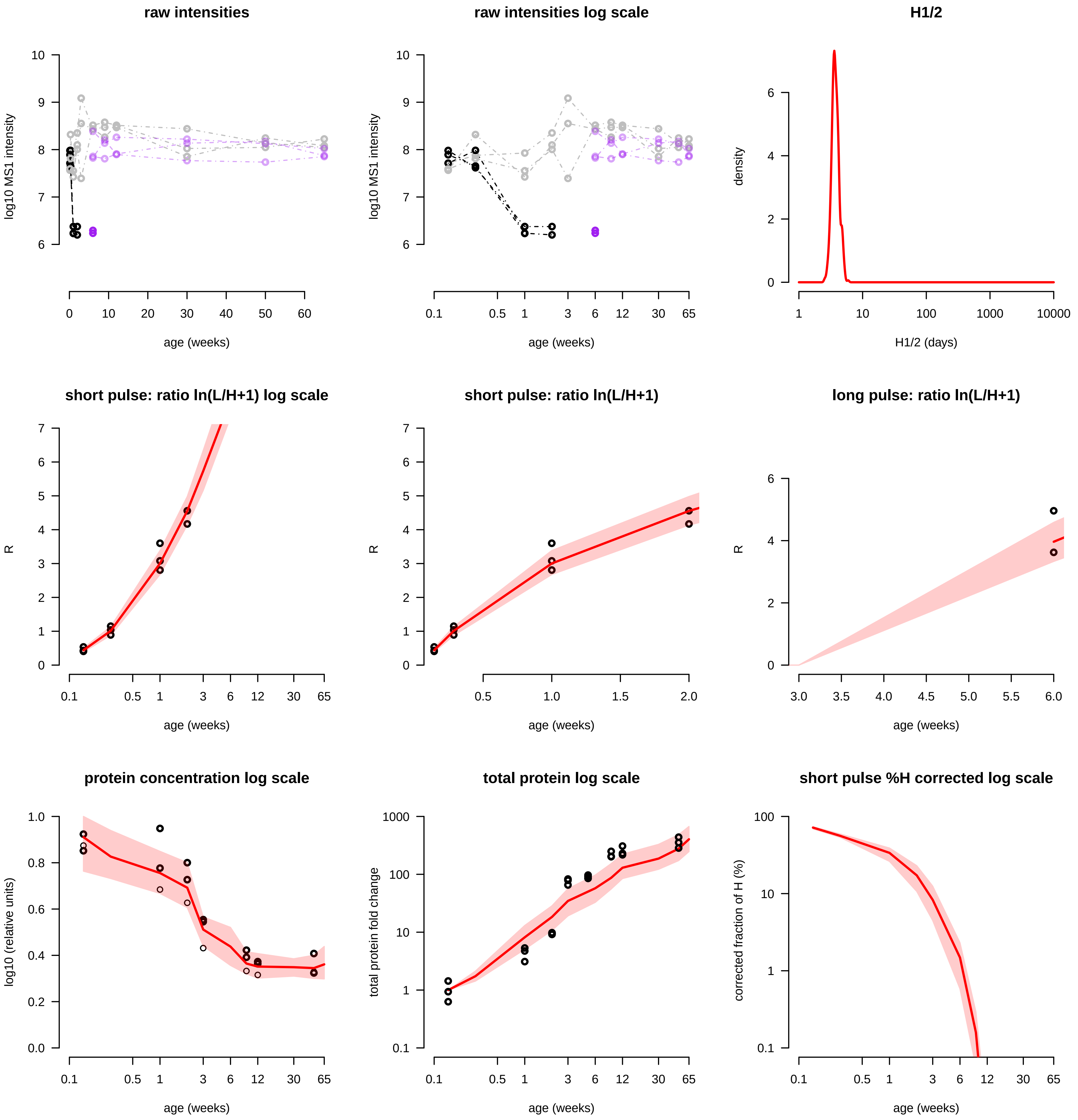

Q9CXG3

>sp|Q9CXG3|PPIL4_MOUSE

Peptidyl−prolyl cis−trans isomerase−like 4

GN=Ppil4

Med H1/2: 39.4 days

Q5 H1/2: 10.9 days

Q95 H1/2: 318 days

reliable model fit: no

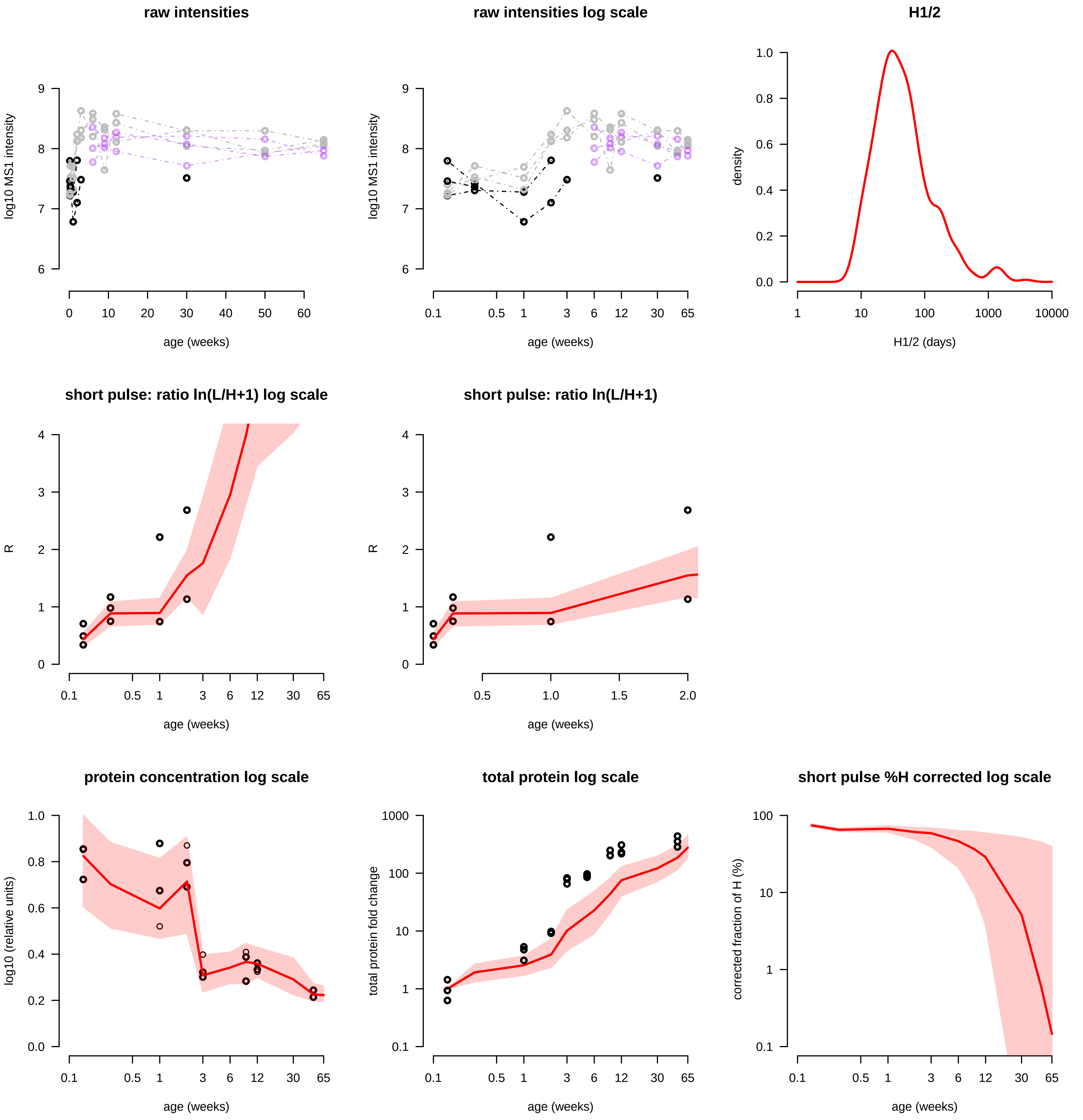

Q6PGH2

>sp|Q6PGH2|JUPI2_MOUSE

Jupiter microtubule associated homolog 2

GN=Jpt2

Med H1/2: 13.5 days

Q5 H1/2: 6.5 days

Q95 H1/2: 50.1 days

reliable model fit: yes

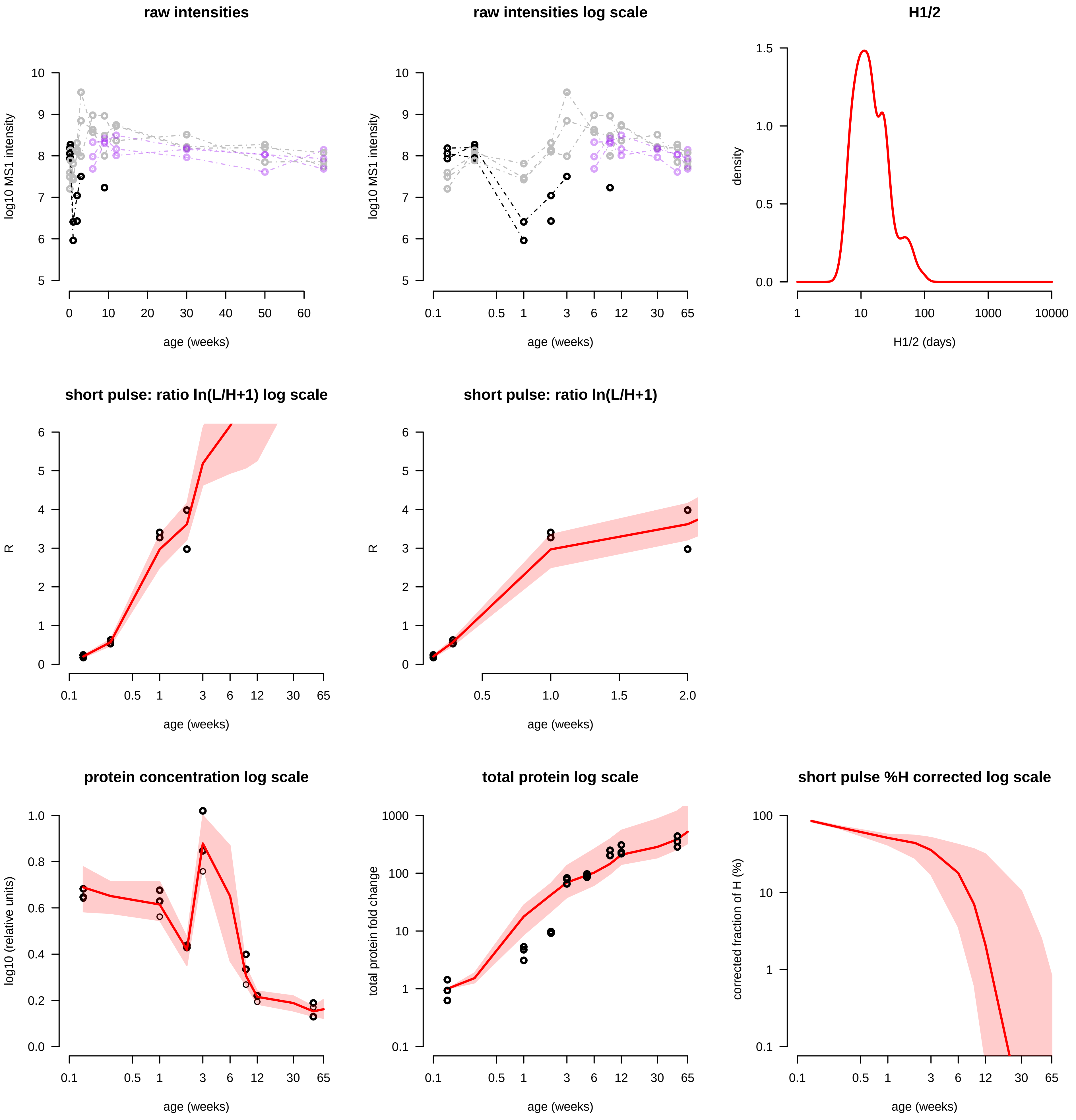

Q9EQ32;Q9EQ32−2

>sp|Q9EQ32|BCAP_MOUSE

Phosphoinositide 3−kinase adapter protein 1

GN=Pik3ap1

Med H1/2: 9.1 days

Q5 H1/2: 3.6 days

Q95 H1/2: 59.6 days

reliable model fit: yes

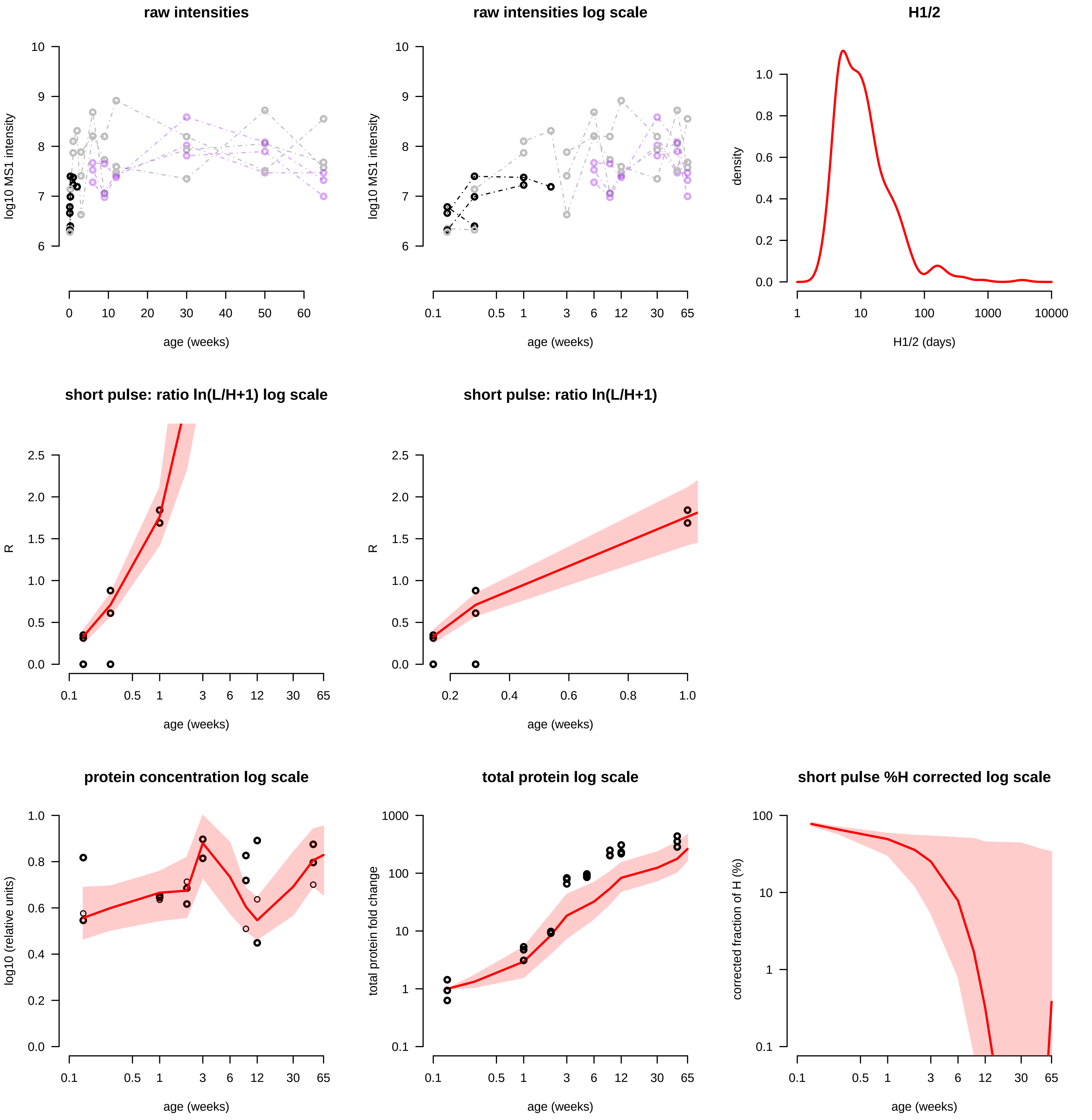

A2APV2;A2APV2−2

>sp|A2APV2|FMNL2_MOUSE

Formin−like protein 2

GN=Fmnl2

Med H1/2: 4.4 days

Q5 H1/2: 2.5 days

Q95 H1/2: 17.6 days

reliable model fit: yes

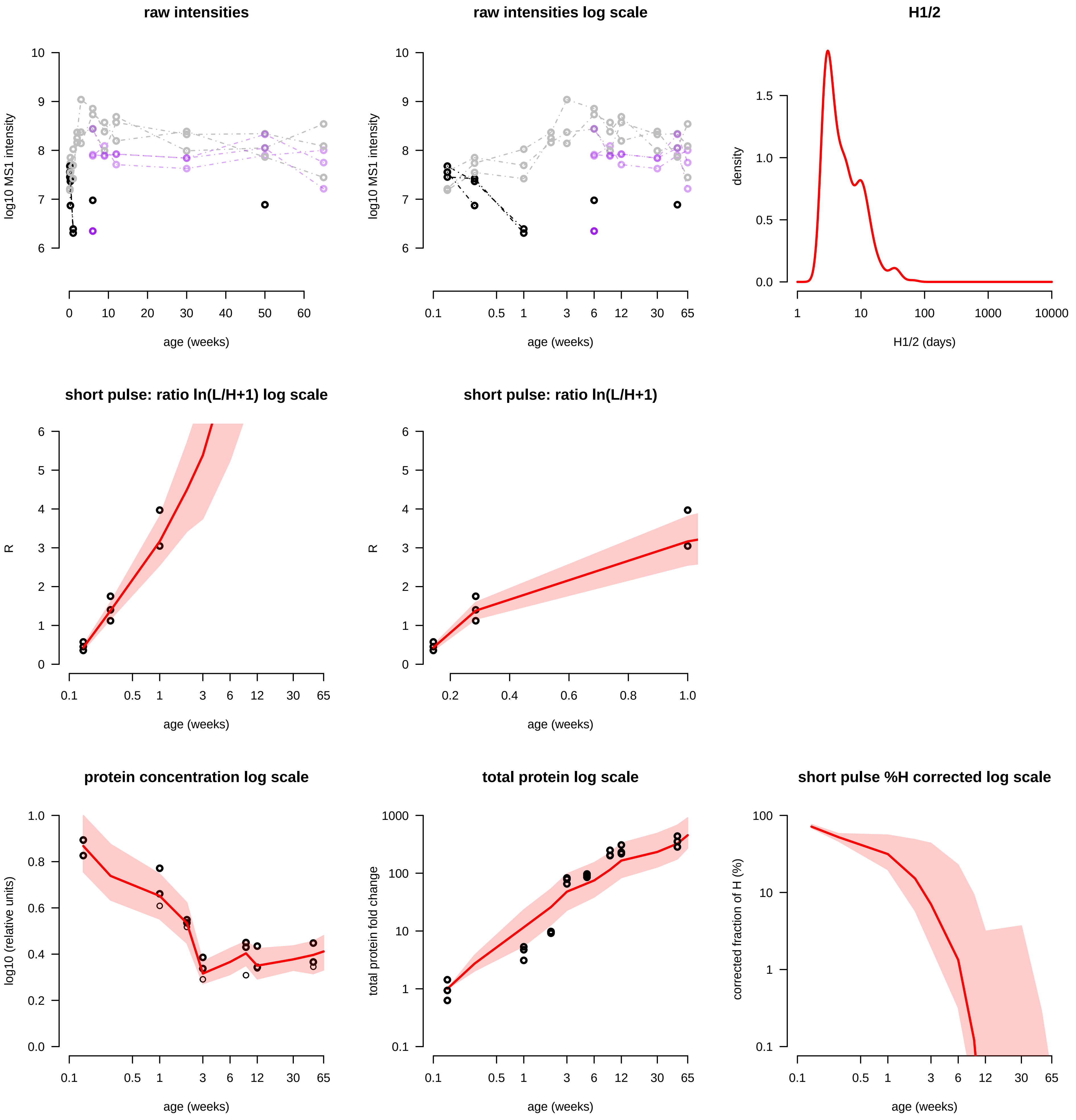

B1AZP2

>sp|B1AZP2|DLGP4_MOUSE

Disks large−associated protein 4

GN=Dlgap4

Med H1/2: 5.8 days

Q5 H1/2: 2.7 days

Q95 H1/2: 17 days

reliable model fit: yes

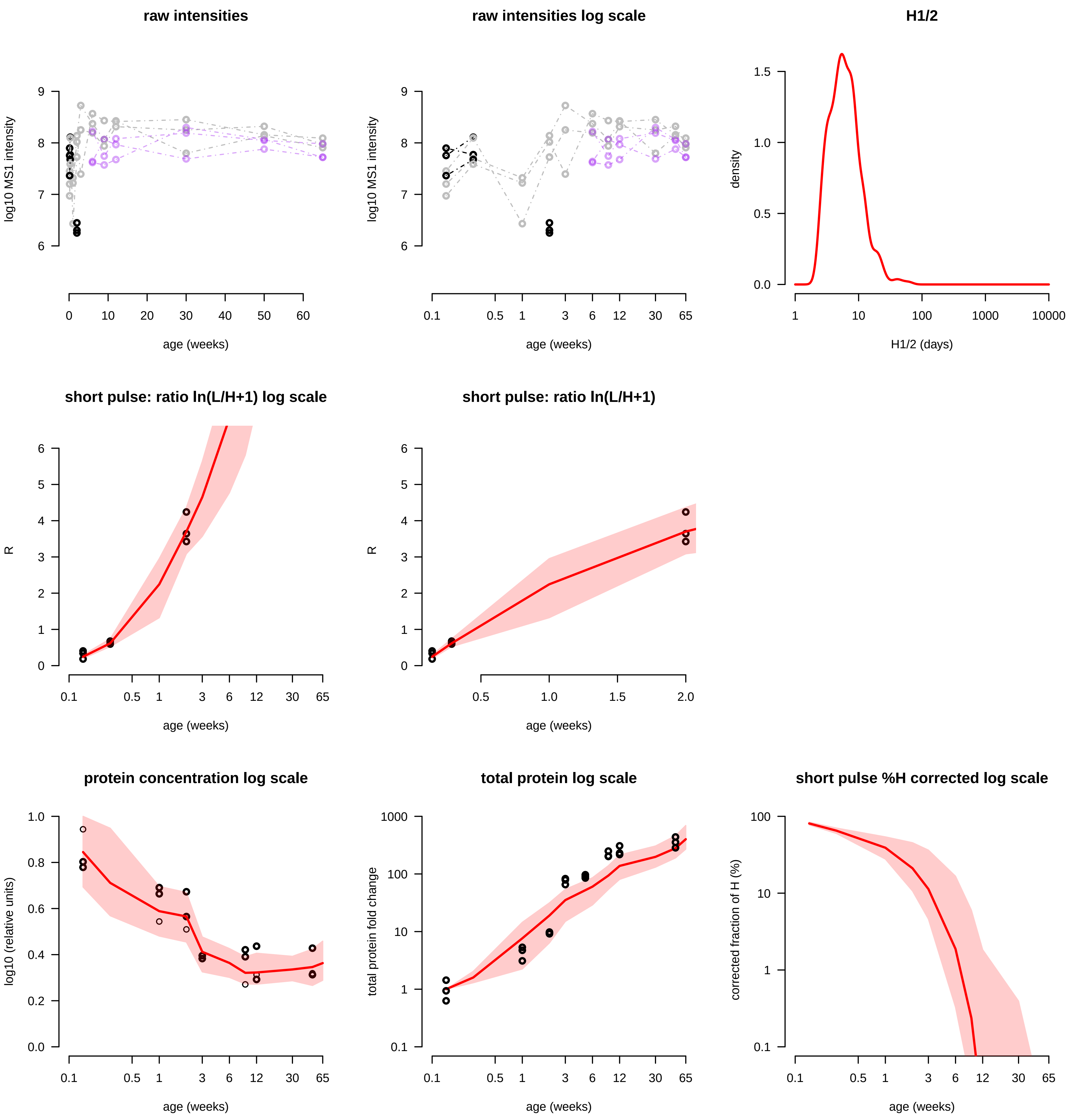

Q9JJL8

>sp|Q9JJL8|SYSM_MOUSE

Serine−−tRNA ligase, mitochondrial

GN=Sars2

Med H1/2: 7.8 days

Q5 H1/2: 3.2 days

Q95 H1/2: 83.5 days

reliable model fit: no

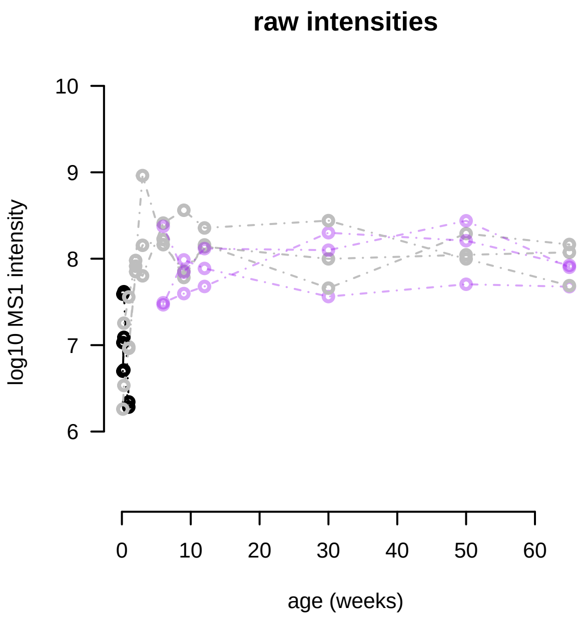

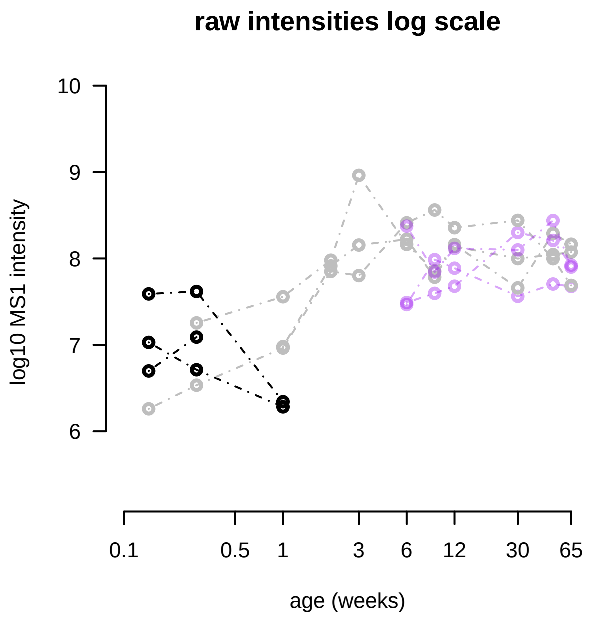

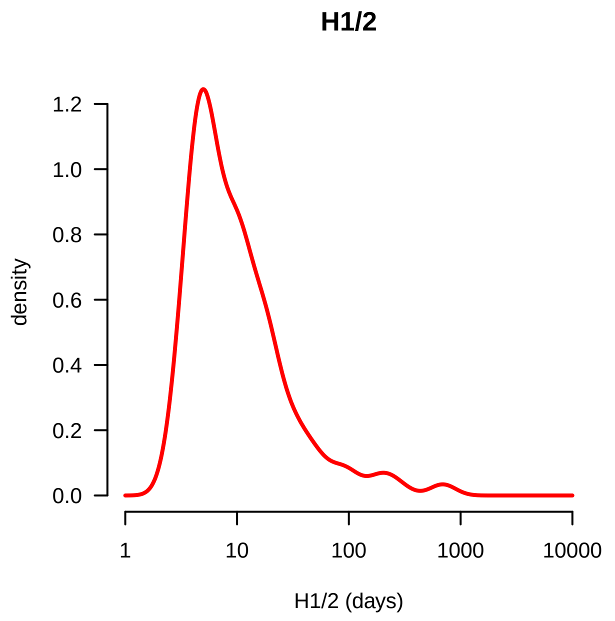

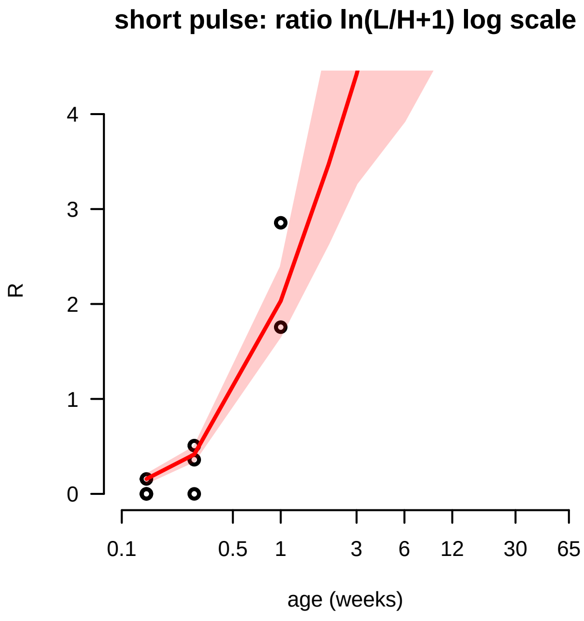

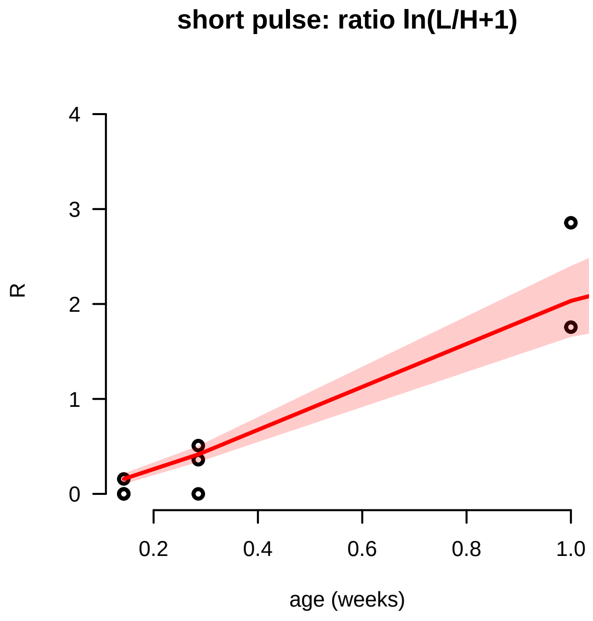

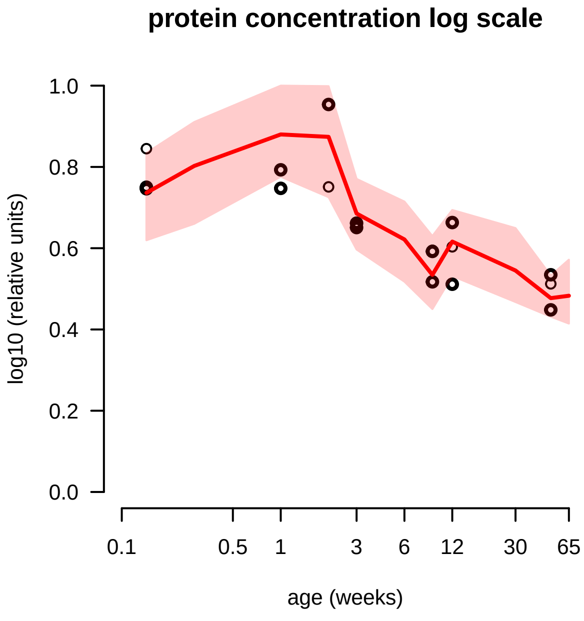

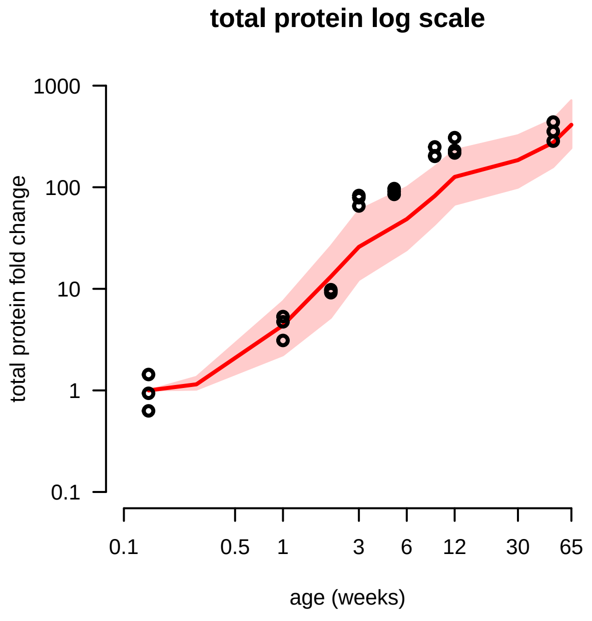

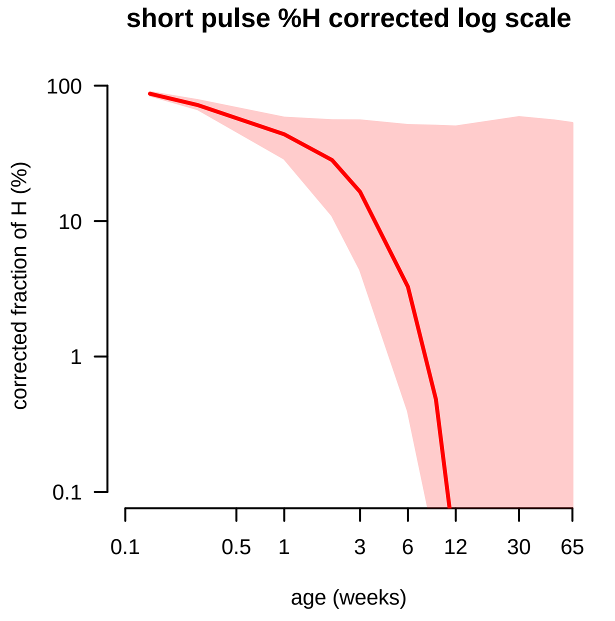

Q99J09

>sp|Q99J09|MEP50_MOUSE

Methylosome protein 50

GN=Wdr77

Med H1/2: 5.7 days

Q5 H1/2: 4.7 days

Q95 H1/2: 7.5 days

reliable model fit: yes

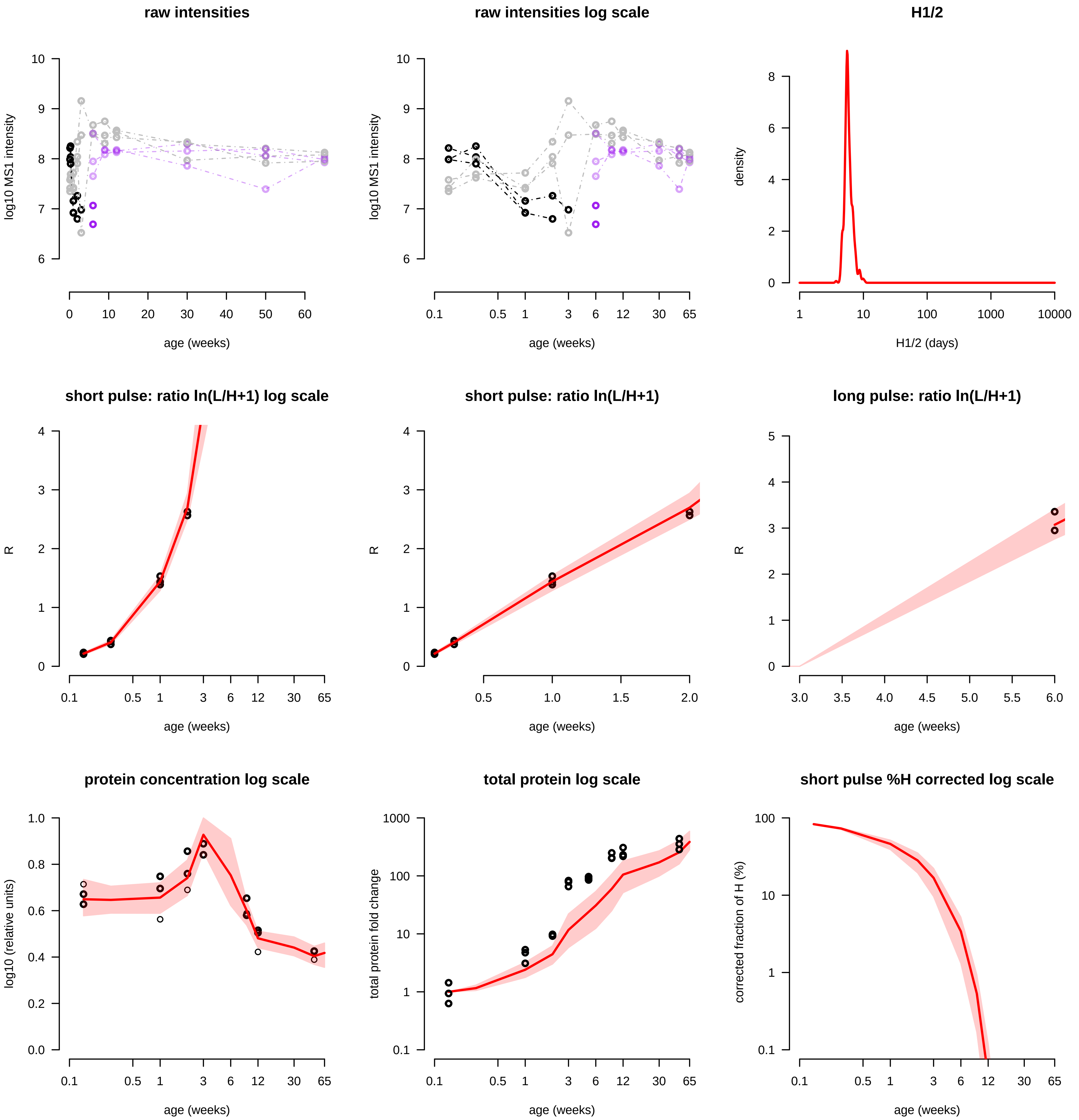

P15089

>sp|P15089|CBPA3_MOUSE

Mast cell carboxypeptidase A

GN=Cpa3

Med H1/2: 15.2 days

Q5 H1/2: 7.6 days

Q95 H1/2: 69.9 days

reliable model fit: yes

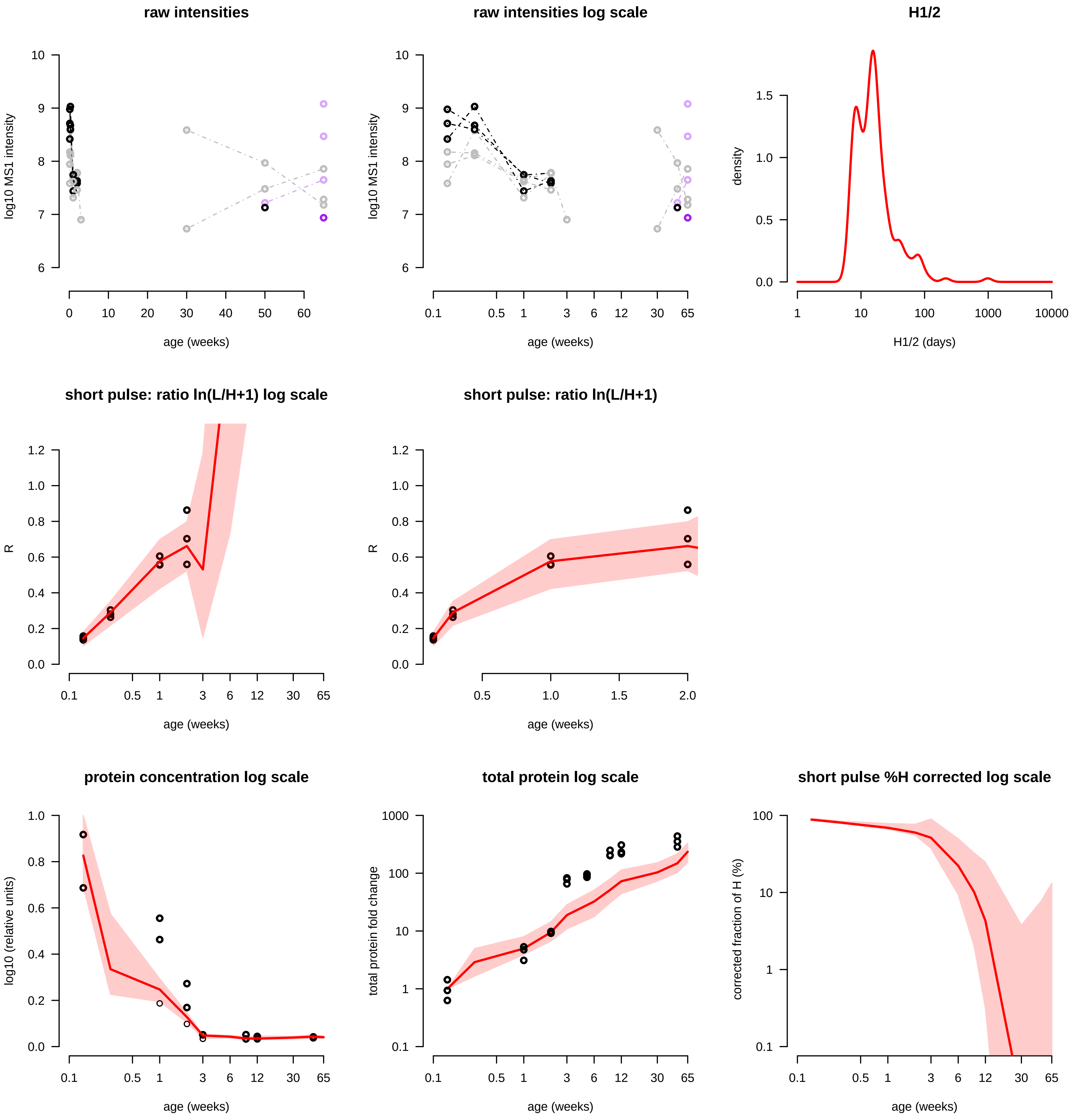

Q6Y7W8

>sp|Q6Y7W8|GGYF2_MOUSE

GRB10−interacting GYF protein 2

GN=Gigyf2

Med H1/2: 16.4 days

Q5 H1/2: 12.7 days

Q95 H1/2: 22.4 days

reliable model fit: yes

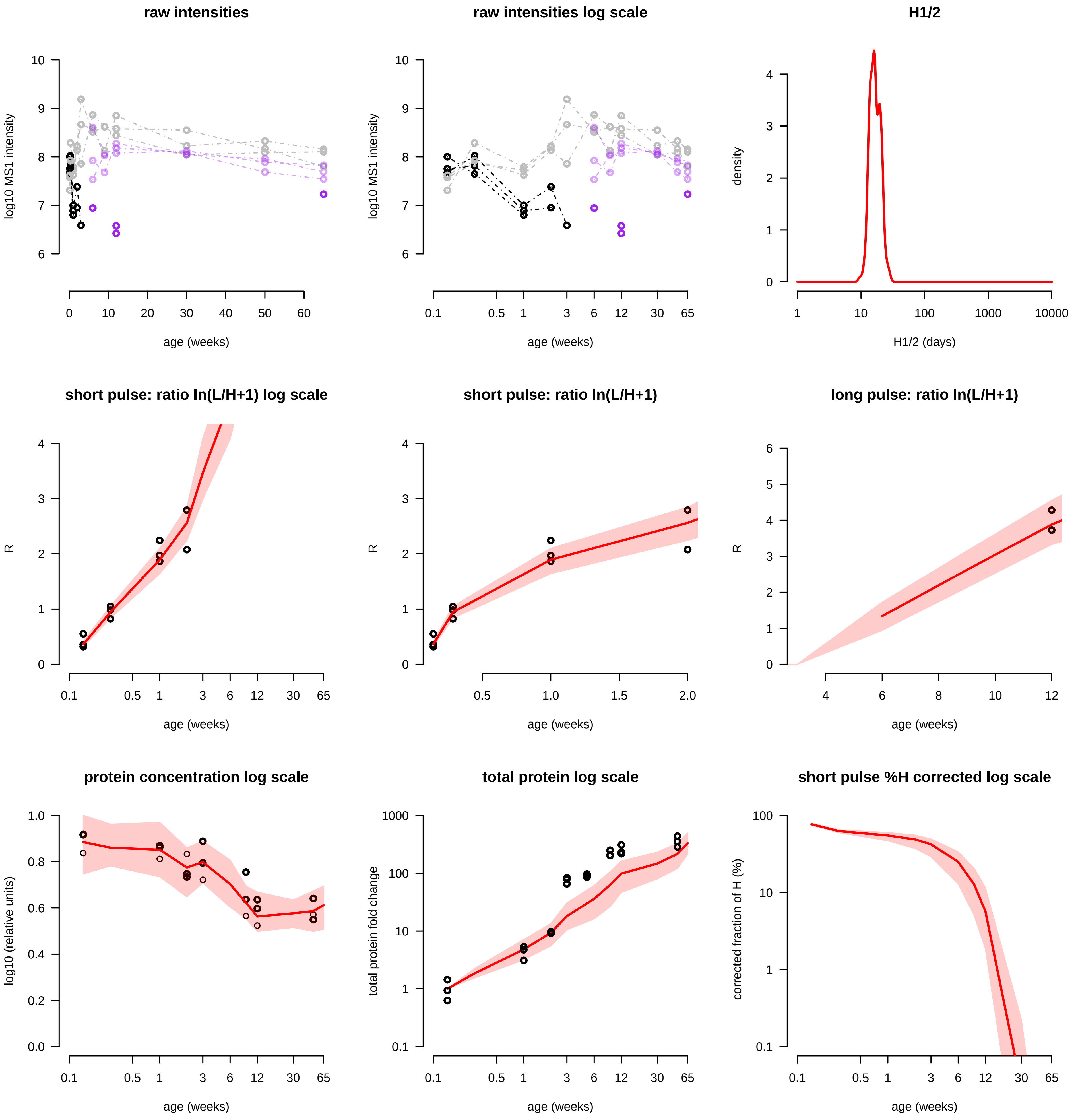

Q9Z1E4

>sp|Q9Z1E4|GYS1_MOUSE

Glycogen [starch] synthase, muscle

GN=Gys1

Med H1/2: 9.8 days

Q5 H1/2: 3.6 days

Q95 H1/2: 54.8 days

reliable model fit: yes

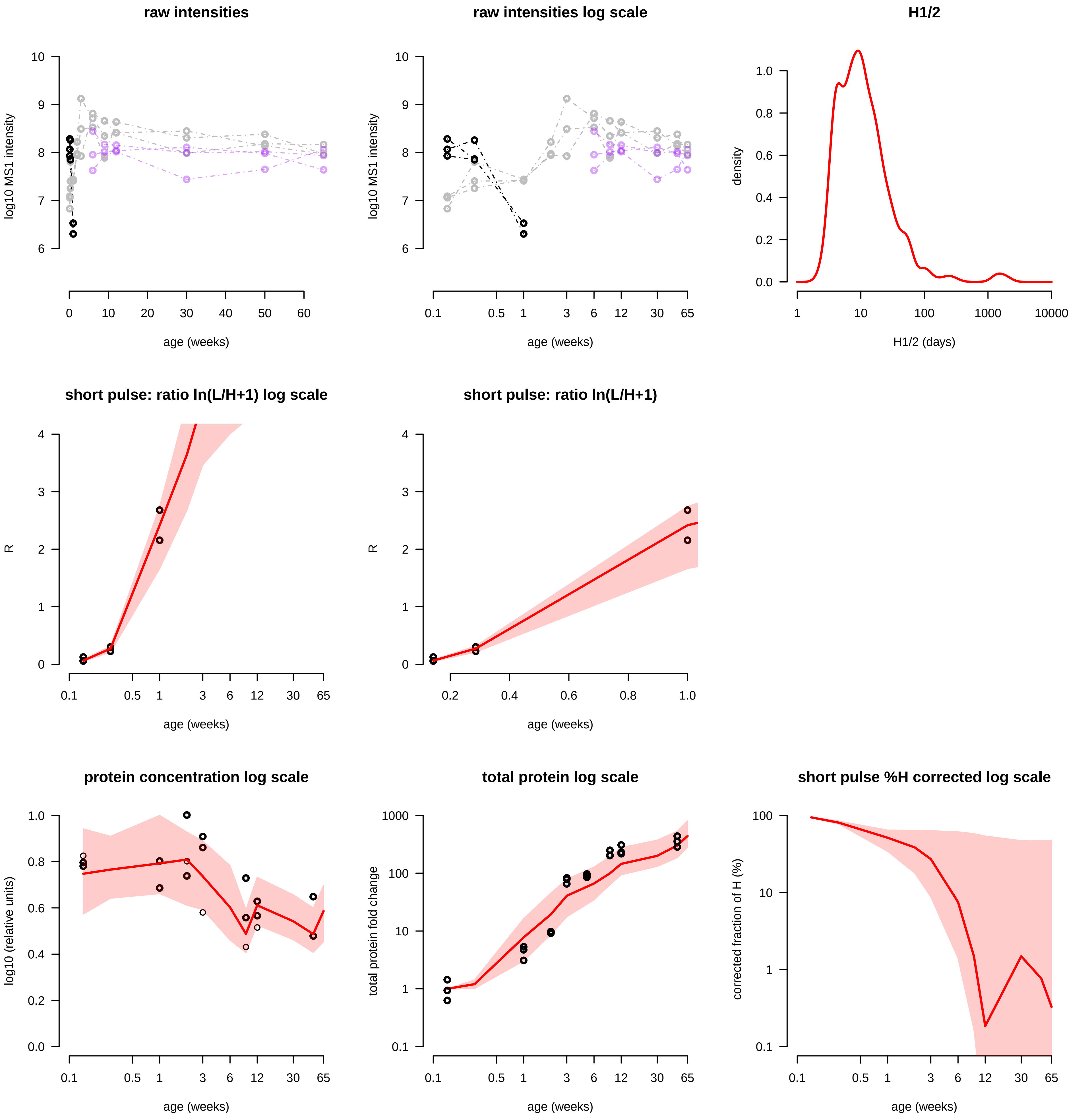

Q8C4Y3−3

>sp|Q8C4Y3−3|NELFB_MOUSE

Isoform 2 of Negative elongation factor B

GN=Nelfb

Med H1/2: 6.5 days

Q5 H1/2: 2.8 days

Q95 H1/2: 27.5 days

reliable model fit: yes

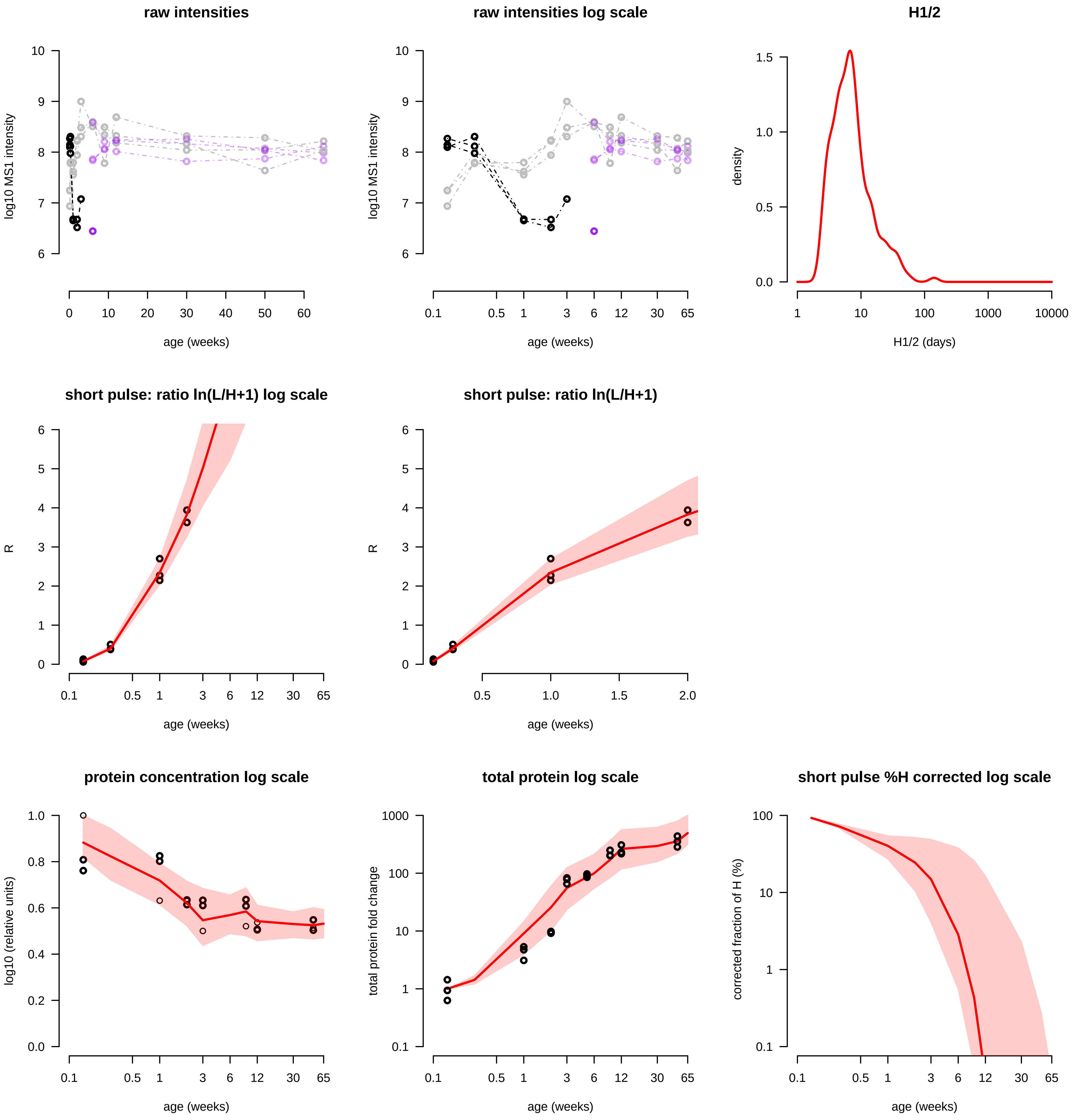

O54974

>sp|O54974|LEG7_MOUSE

Galectin−7

GN=Lgals7

Med H1/2: 9.7 days

Q5 H1/2: 4.3 days

Q95 H1/2: 92.2 days

reliable model fit: yes

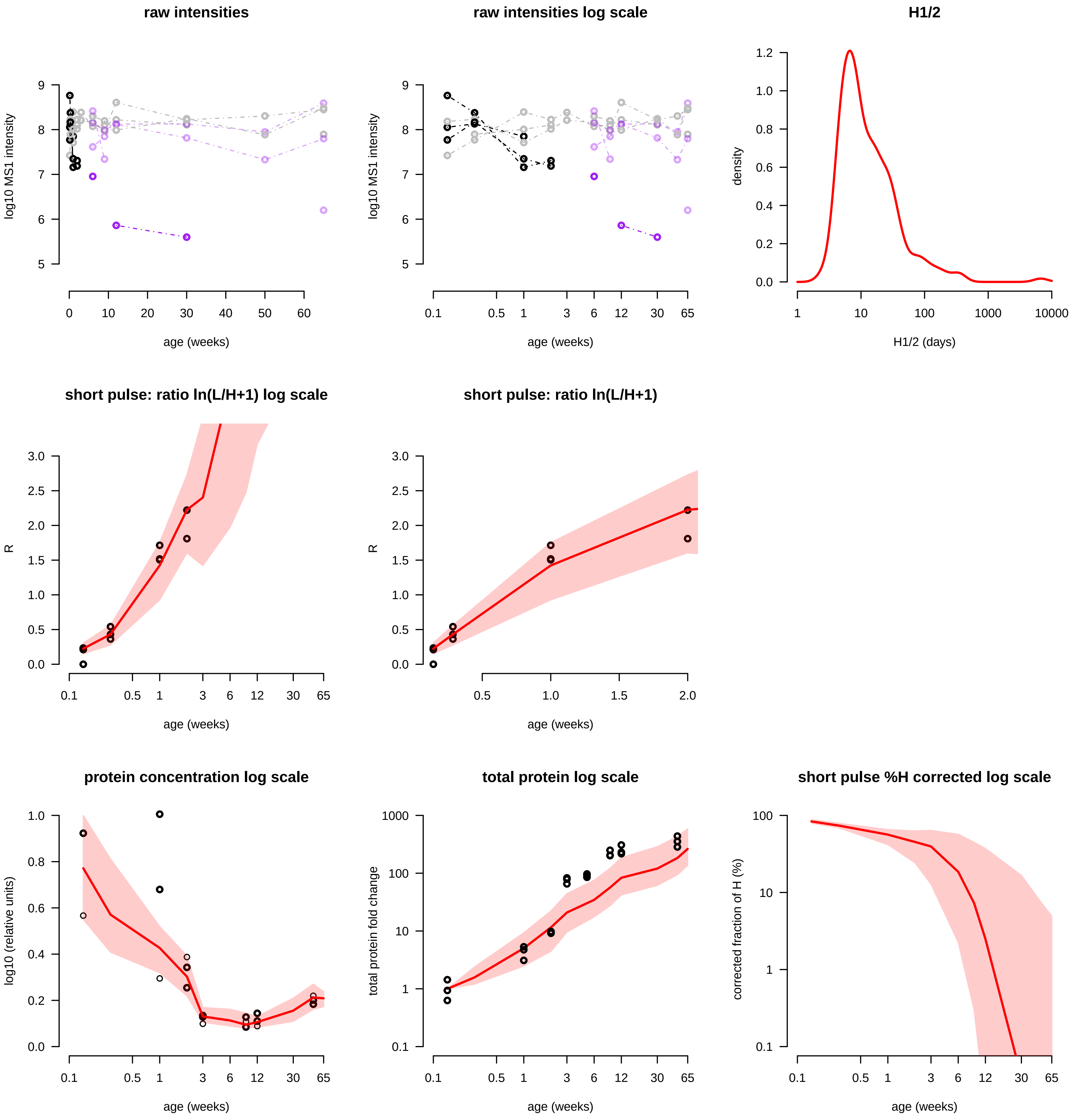

Q9JK23

>sp|Q9JK23|PSMG1_MOUSE

Proteasome assembly chaperone 1

GN=Psmg1

Med H1/2: 4.7 days

Q5 H1/2: 4 days

Q95 H1/2: 5.6 days

reliable model fit: yes

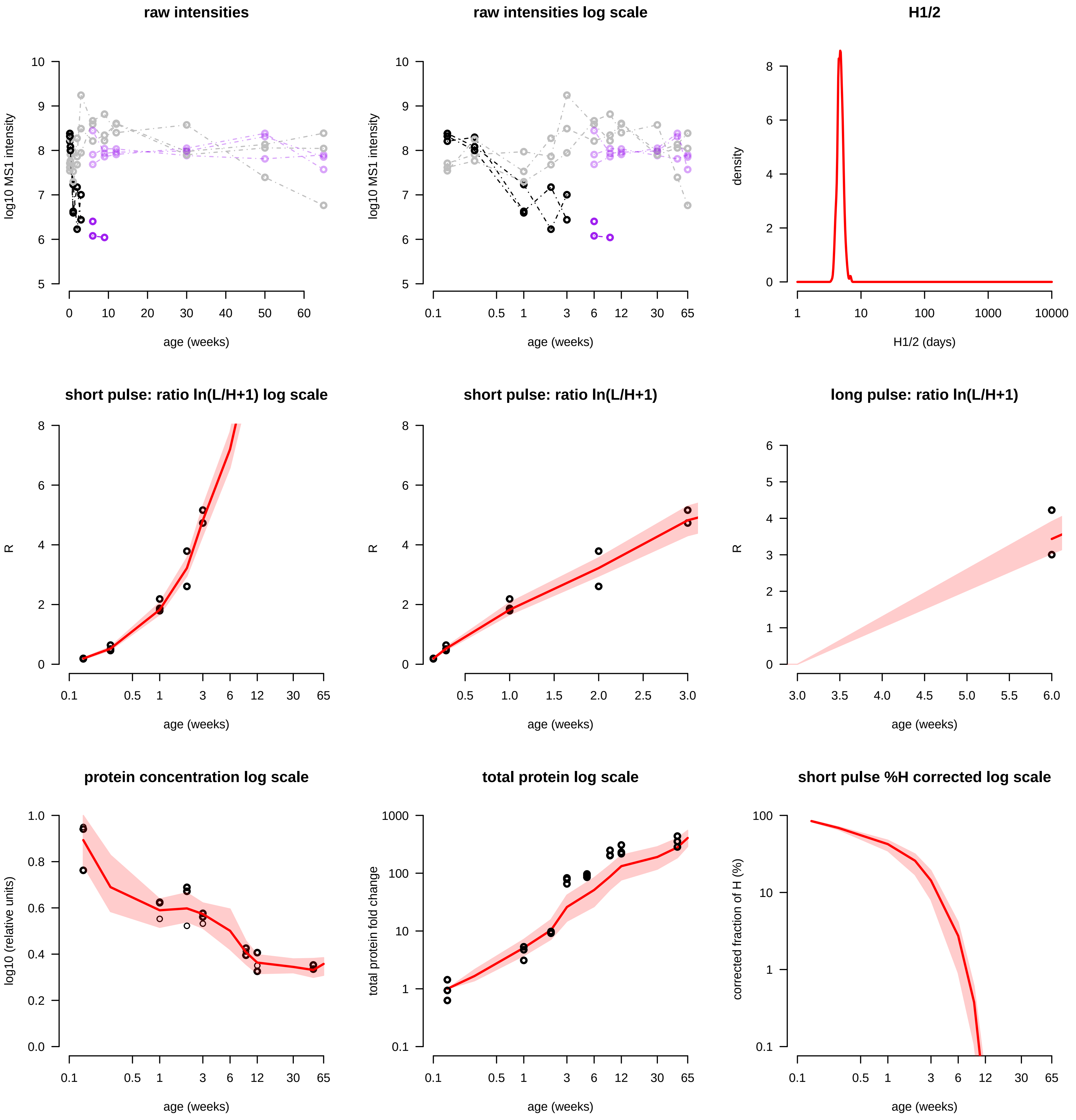

D3Z7P3−2

>sp|D3Z7P3−2|GLSK_MOUSE

Isoform 2 of Glutaminase kidney isoform, mitochondrial

GN=Gls

Med H1/2: 7.5 days

Q5 H1/2: 5.8 days

Q95 H1/2: 13.3 days

reliable model fit: yes

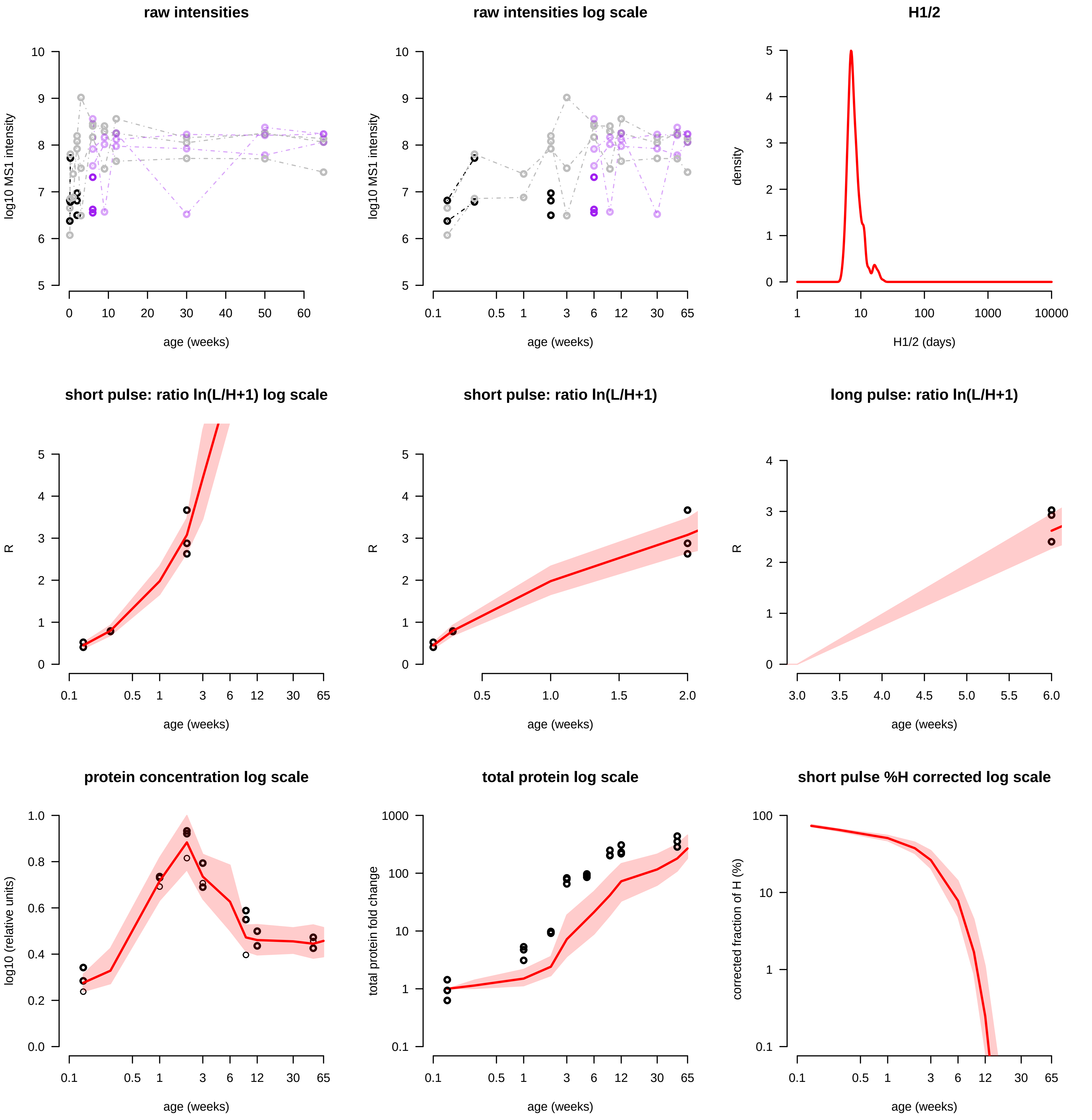

Q2KN98

>sp|Q2KN98|CYTSA_MOUSE

Cytospin−A

GN=Specc1l

Med H1/2: 3787.8 days

Q5 H1/2: 1039 days

Q95 H1/2: 63592.9 days

reliable model fit: no

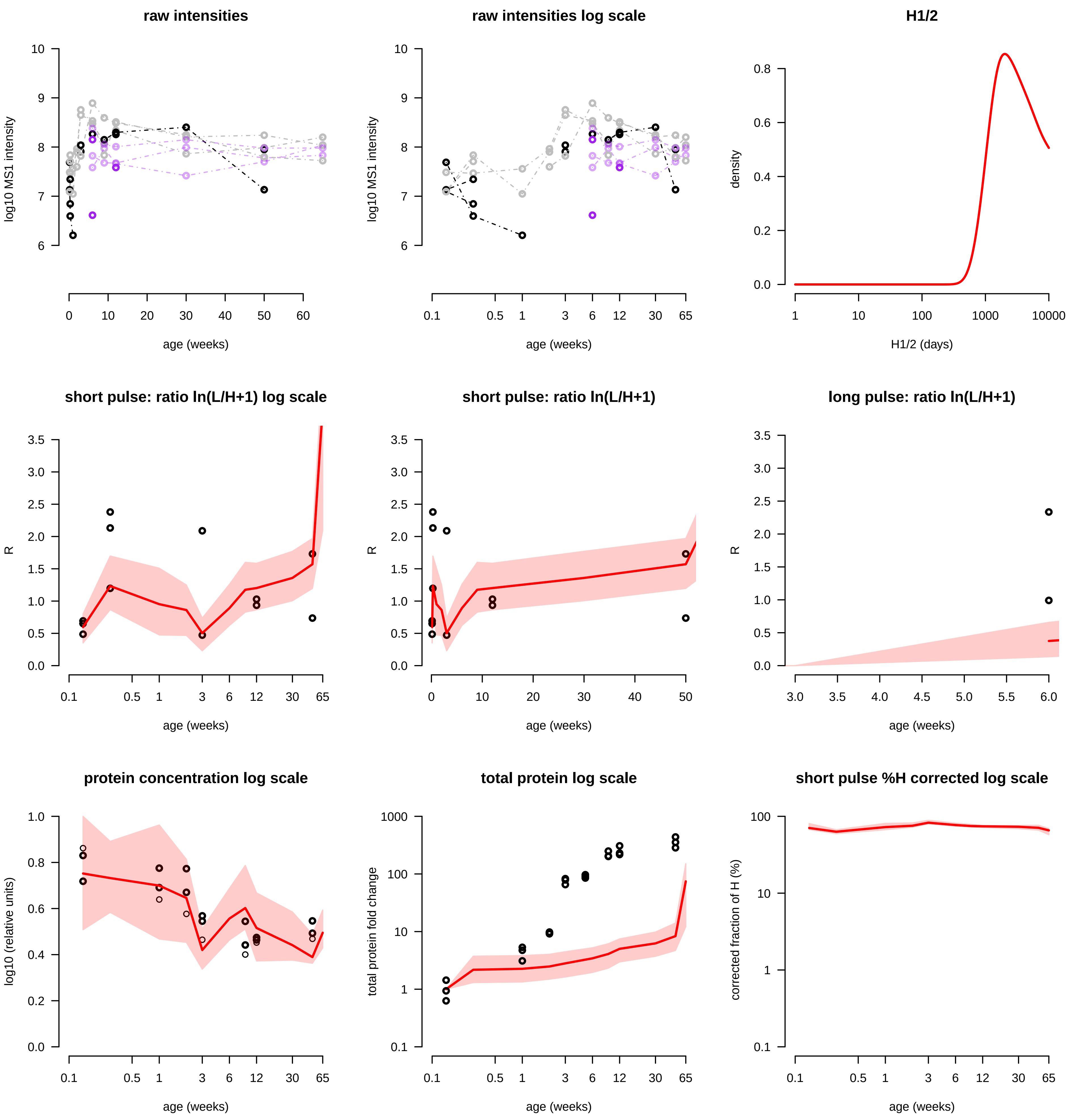

P70268;P70268−2

>sp|P70268|PKN1_MOUSE

Serine/threonine−protein kinase N1

GN=Pkn1

Med H1/2: 5.5 days

Q5 H1/2: 2.7 days

Q95 H1/2: 60.1 days

reliable model fit: yes

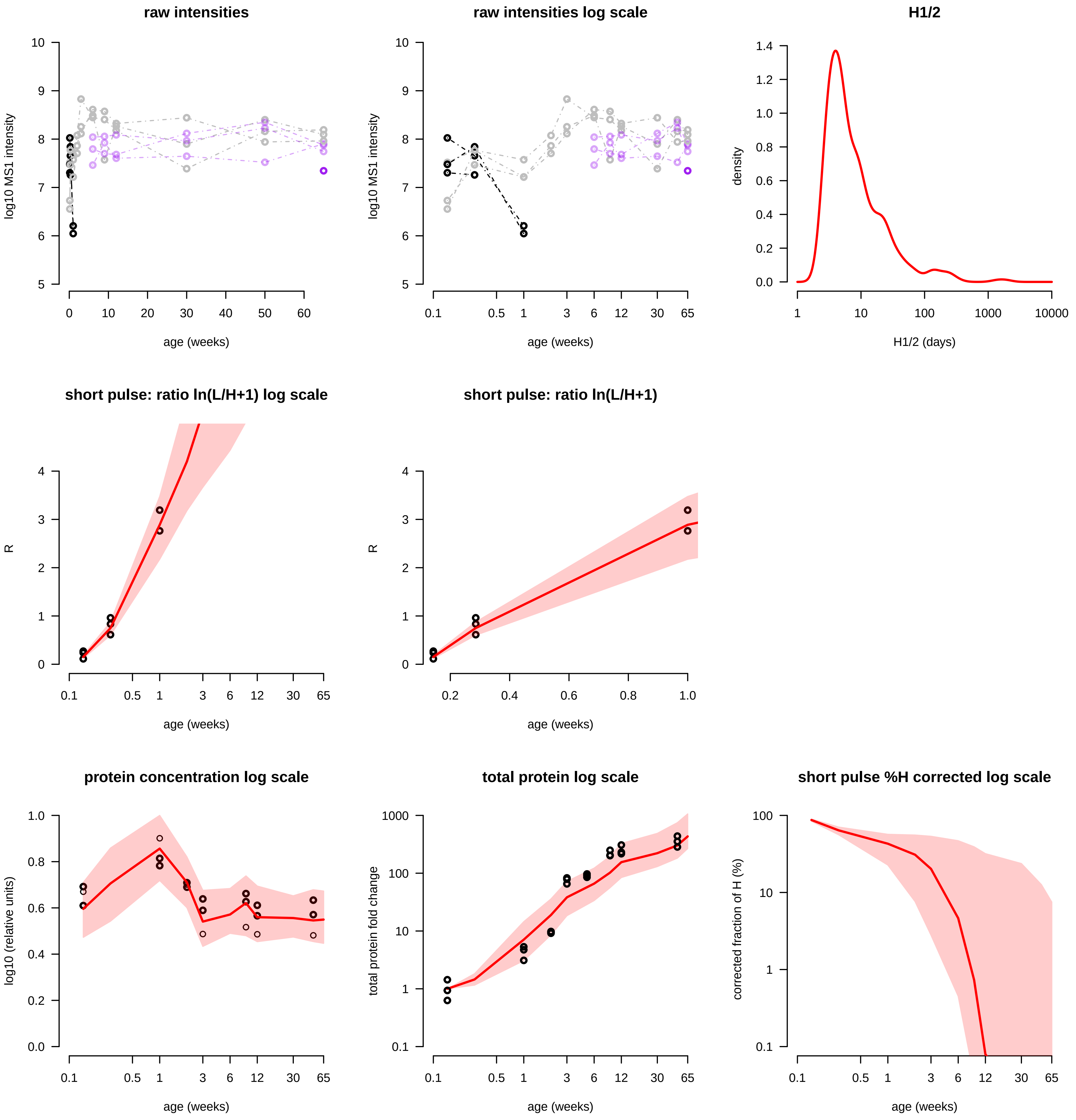

Q6NS57;Q6NS57−2

>sp|Q6NS57|MABP1_MOUSE

Mitogen−activated protein kinase−binding protein 1

GN=Mapkbp1

Med H1/2: 34.1 days

Q5 H1/2: 8.8 days

Q95 H1/2: 312.3 days

reliable model fit: yes

raw intensities

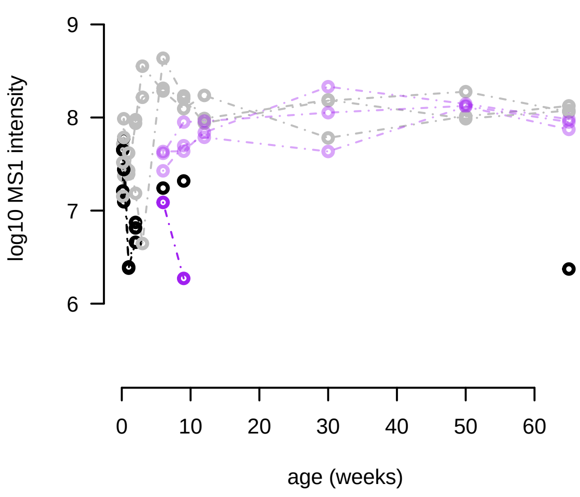

raw intensities log scale

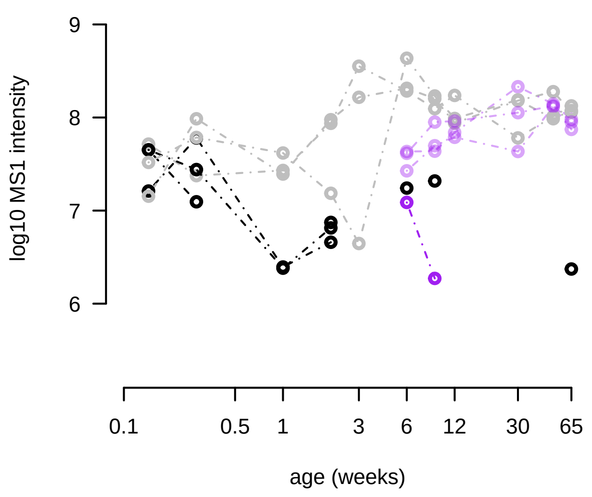

H1/2

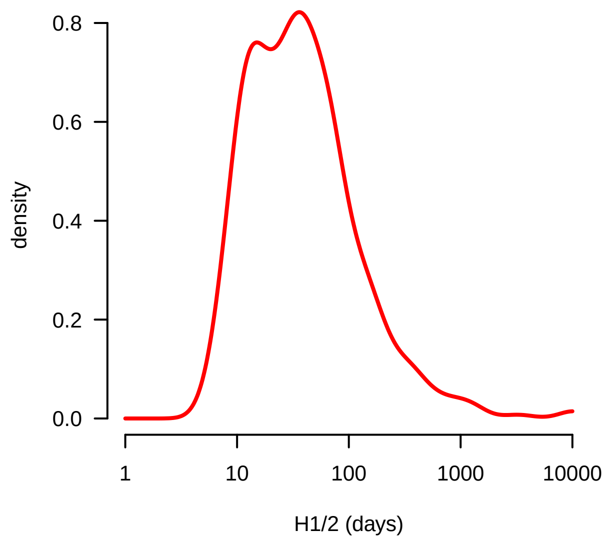

short pulse: ratio ln(L/H+1) log scale

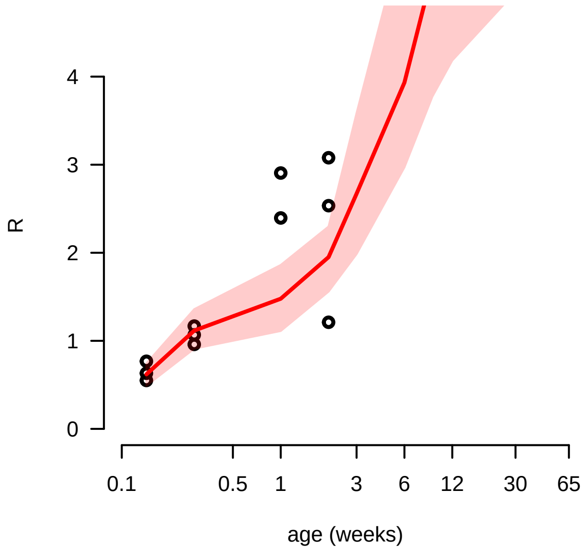

short pulse: ratio ln(L/H+1)

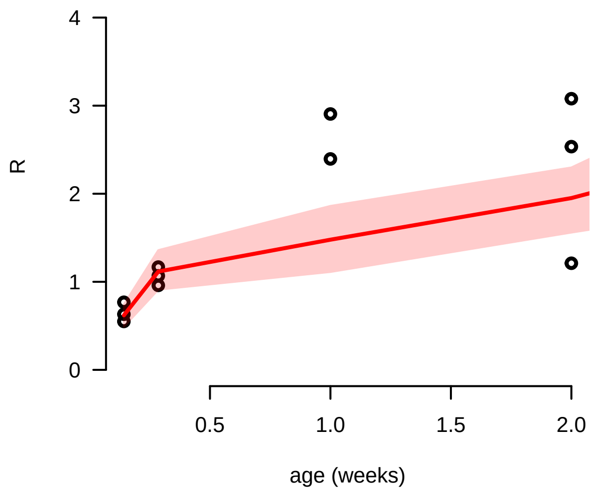

protein concentration log scale

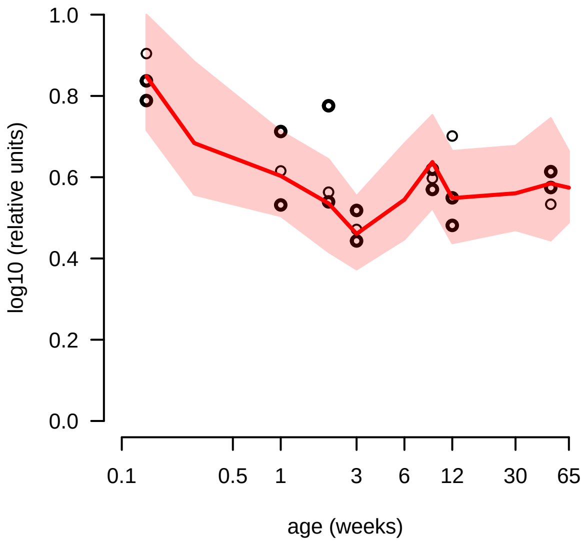

total protein log scale

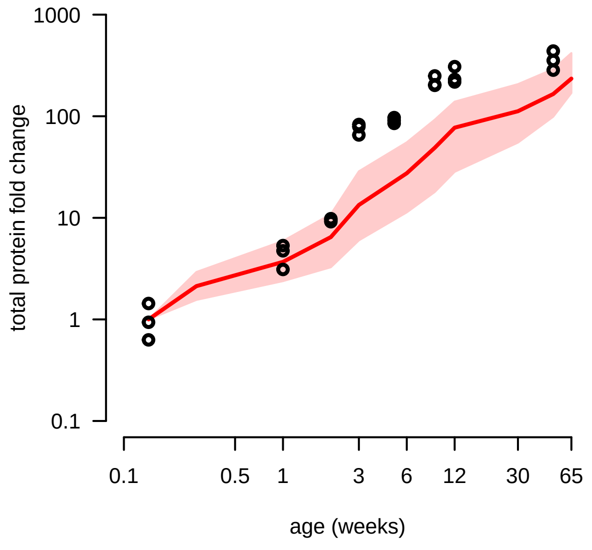

short pulse %H corrected log scale

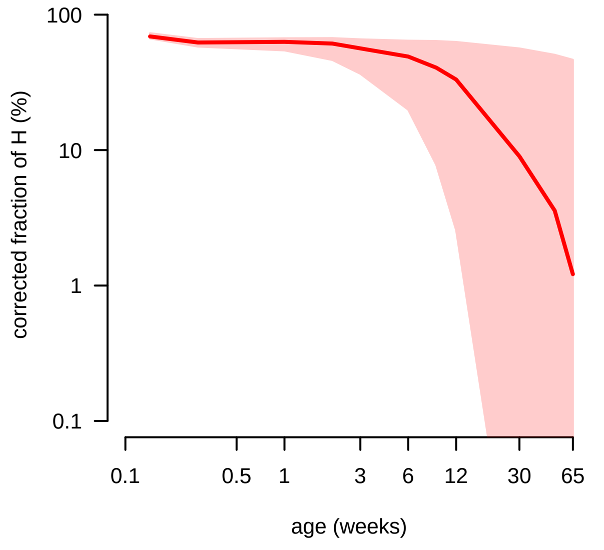

Q9D1M7

>sp|Q9D1M7|FKB11_MOUSE

Peptidyl−prolyl cis−trans isomerase FKBP11

GN=Fkbp11

Med H1/2: 16.3 days

Q5 H1/2: 5.4 days

Q95 H1/2: 326.6 days

reliable model fit: yes

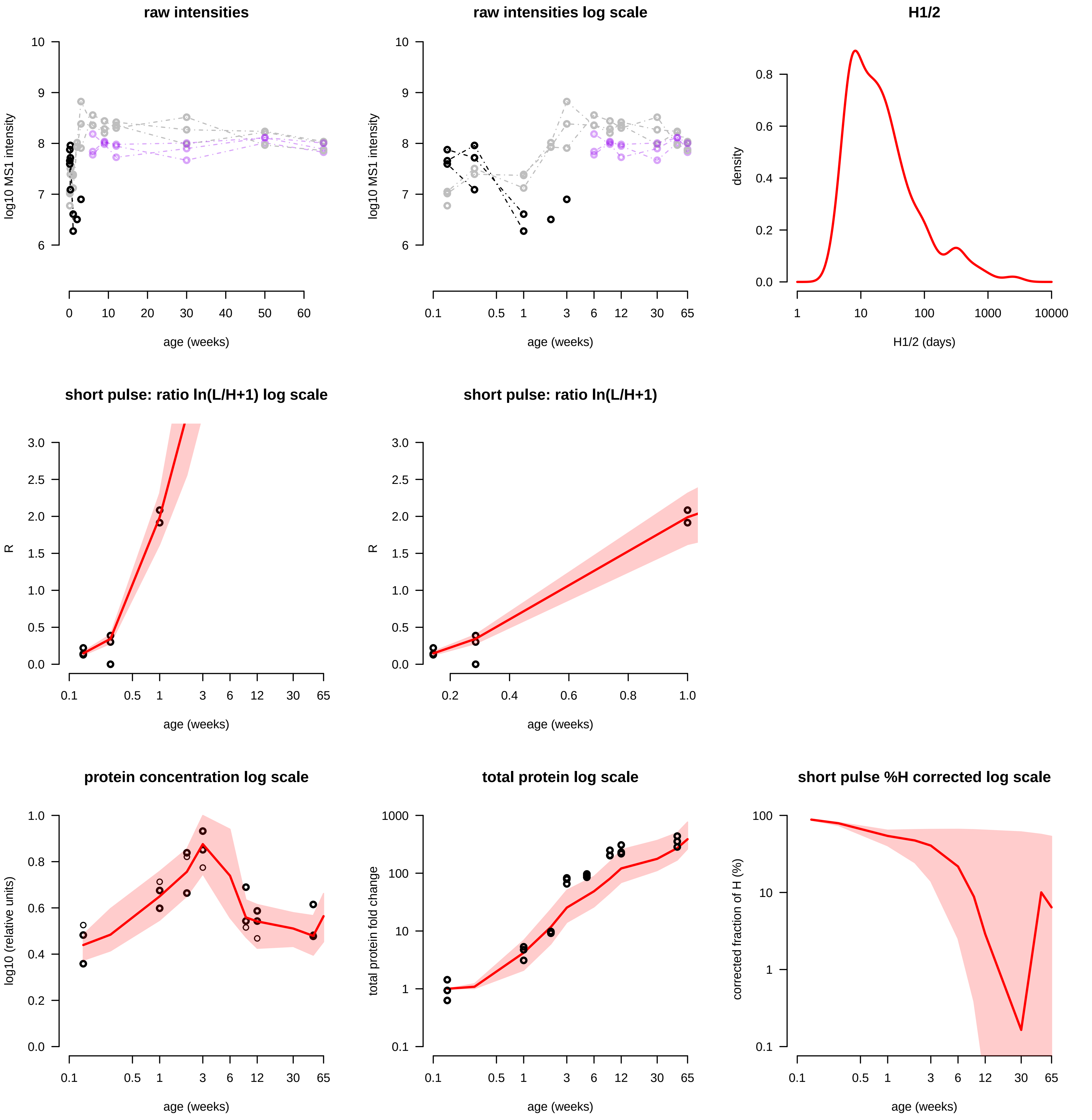

Q9DBD5

>sp|Q9DBD5|PELP1_MOUSE

Proline−, glutamic acid− and leucine−rich protein 1

GN=Pelp1

Med H1/2: 9.3 days

Q5 H1/2: 6.3 days

Q95 H1/2: 14.5 days

reliable model fit: yes

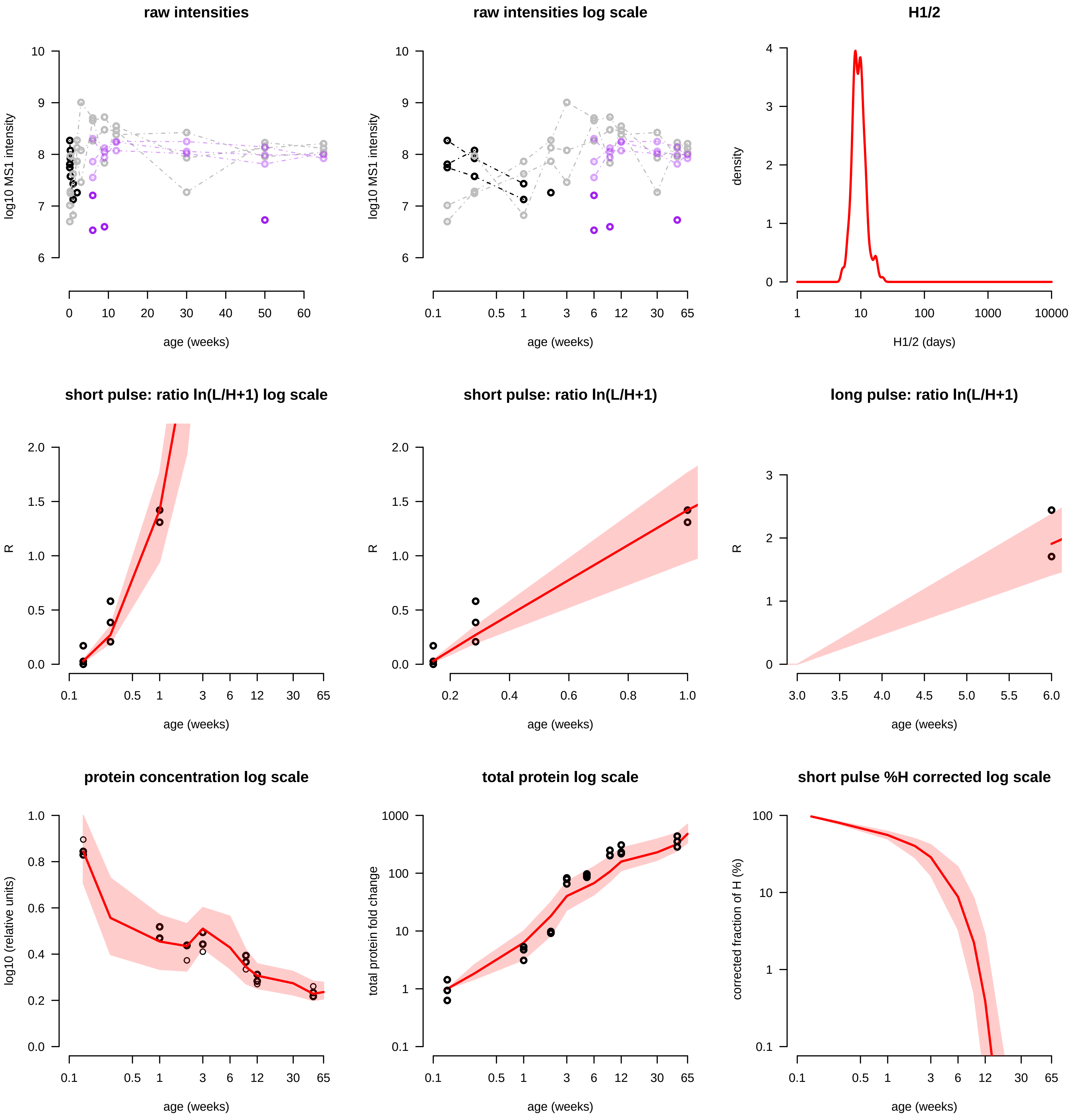

O35206

>sp|O35206|COFA1_MOUSE

Collagen alpha−1(XV) chain

GN=Col15a1

Med H1/2: 7.9 days

Q5 H1/2: 2.9 days

Q95 H1/2: 130.7 days

reliable model fit: yes

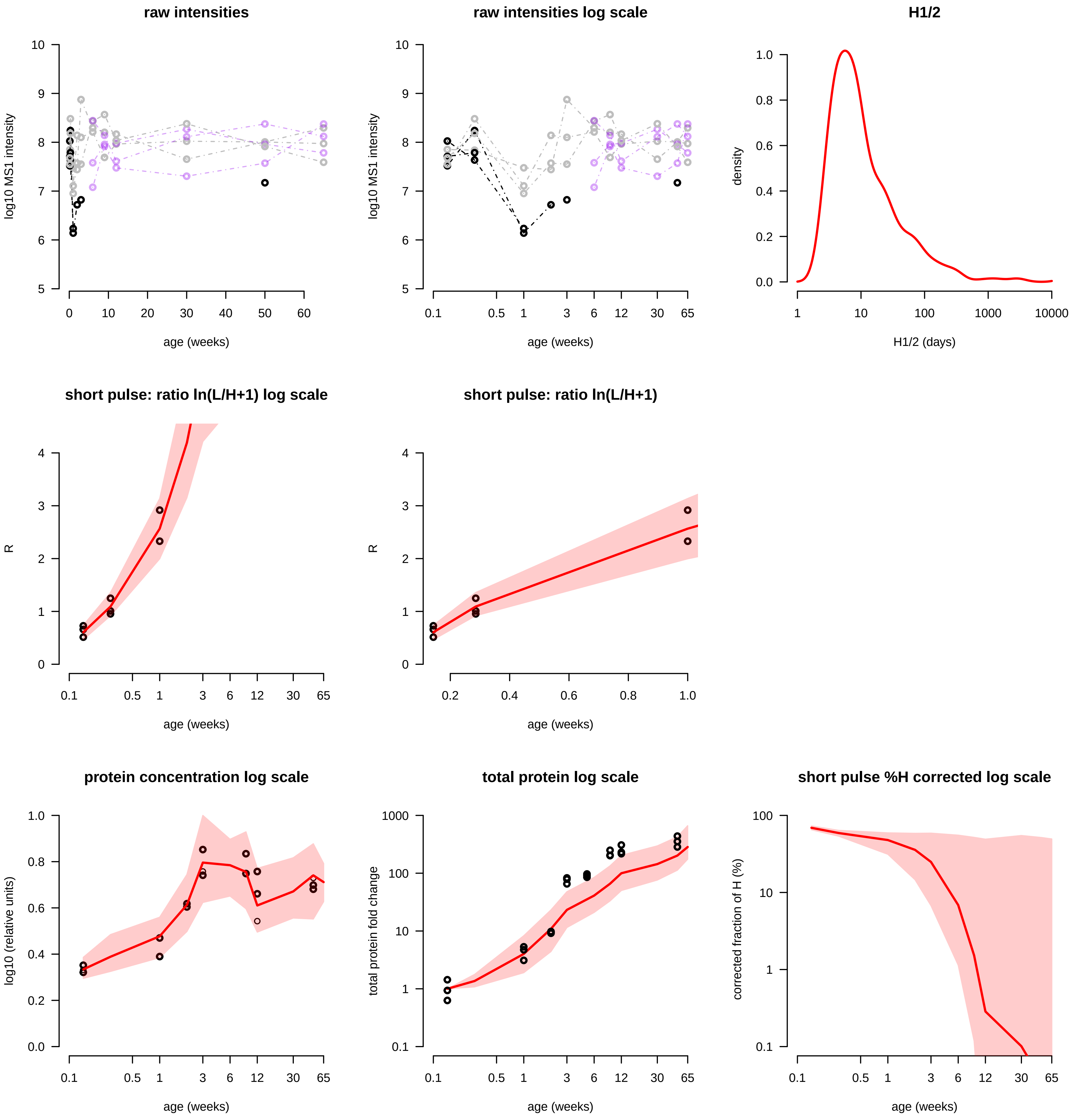

Q8CB77

>sp|Q8CB77|ELOA1_MOUSE

Elongin−A

GN=Eloa

Med H1/2: 4.5 days

Q5   H1/2: 2.6 days

Q95 H1/2: 15.3 days

reliable model fit: yes

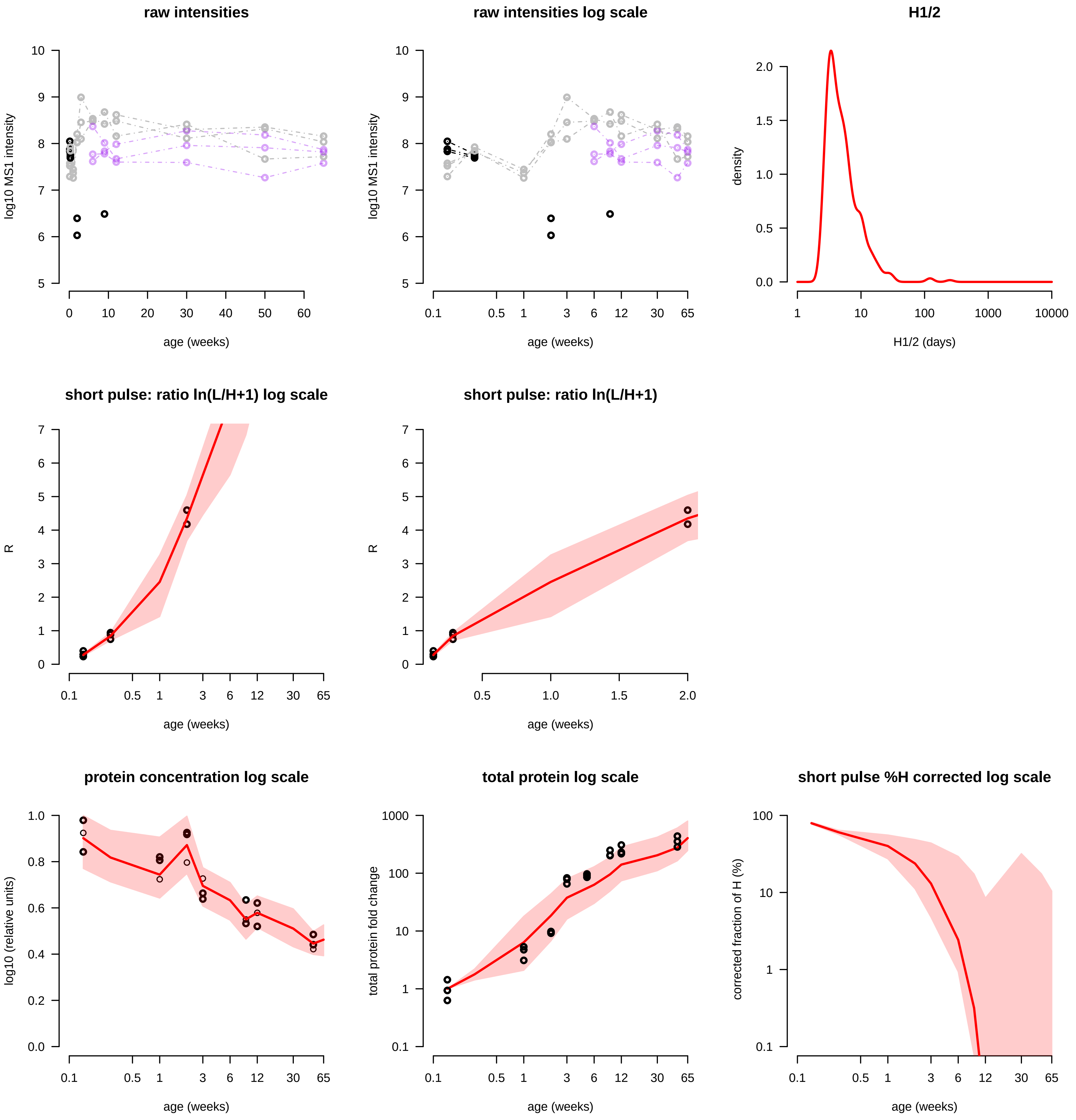

P60762−2;P60762

>sp|P60762−2|MO4L1_MOUSE

Isoform 2 of Mortality factor 4−like protein 1

GN=Morf4l1

Med H1/2: 5.3 days

Q5   H1/2: 2.8 days

Q95 H1/2: 32 days

reliable model fit: yes

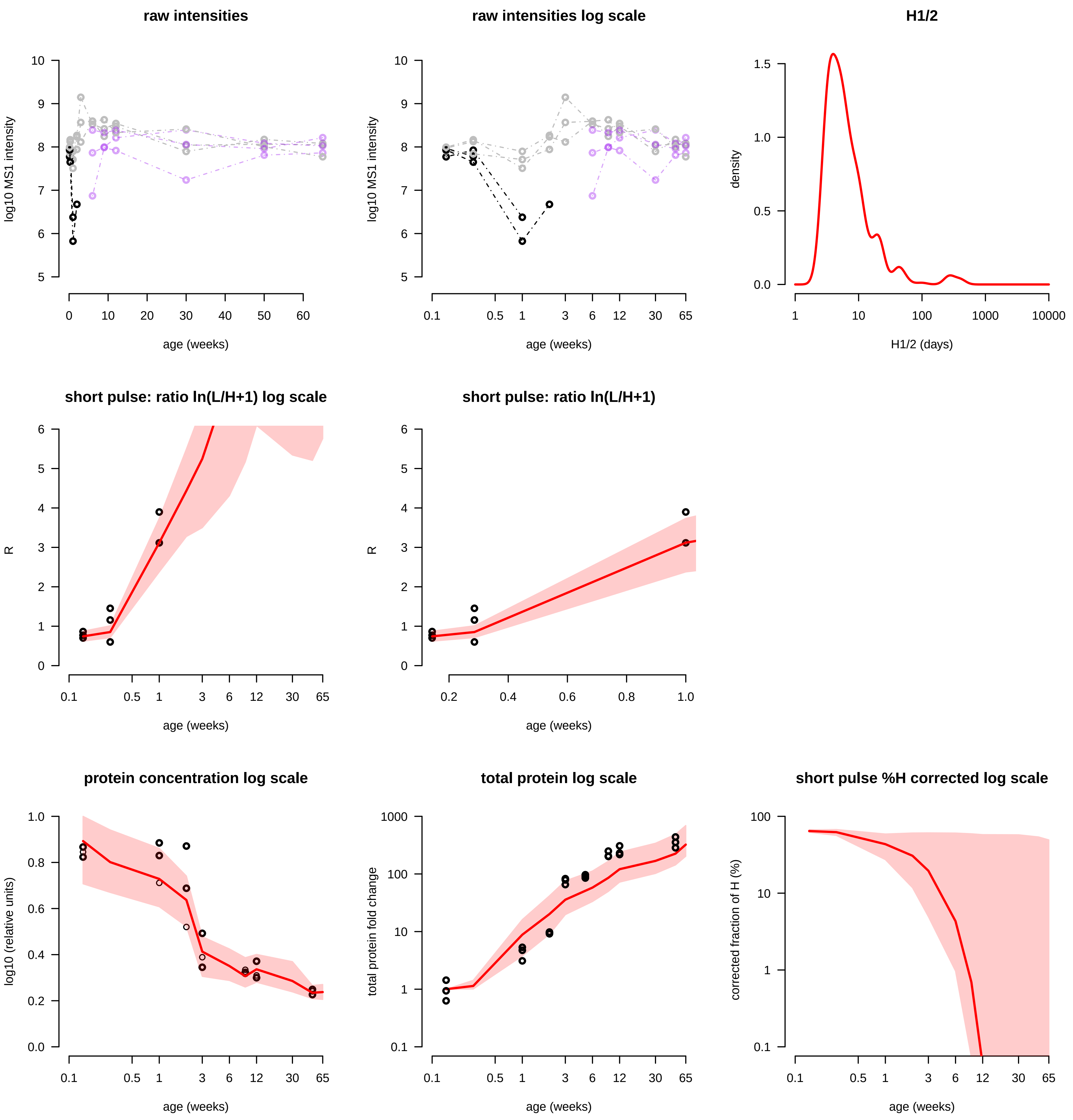

Q91W39

>sp|Q91W39|NCOA5_MOUSE

Nuclear receptor coactivator 5

GN=Ncoa5

Med H1/2: 5.6 days

Q5 H1/2: 3 days

Q95 H1/2: 66.5 days

reliable model fit: yes

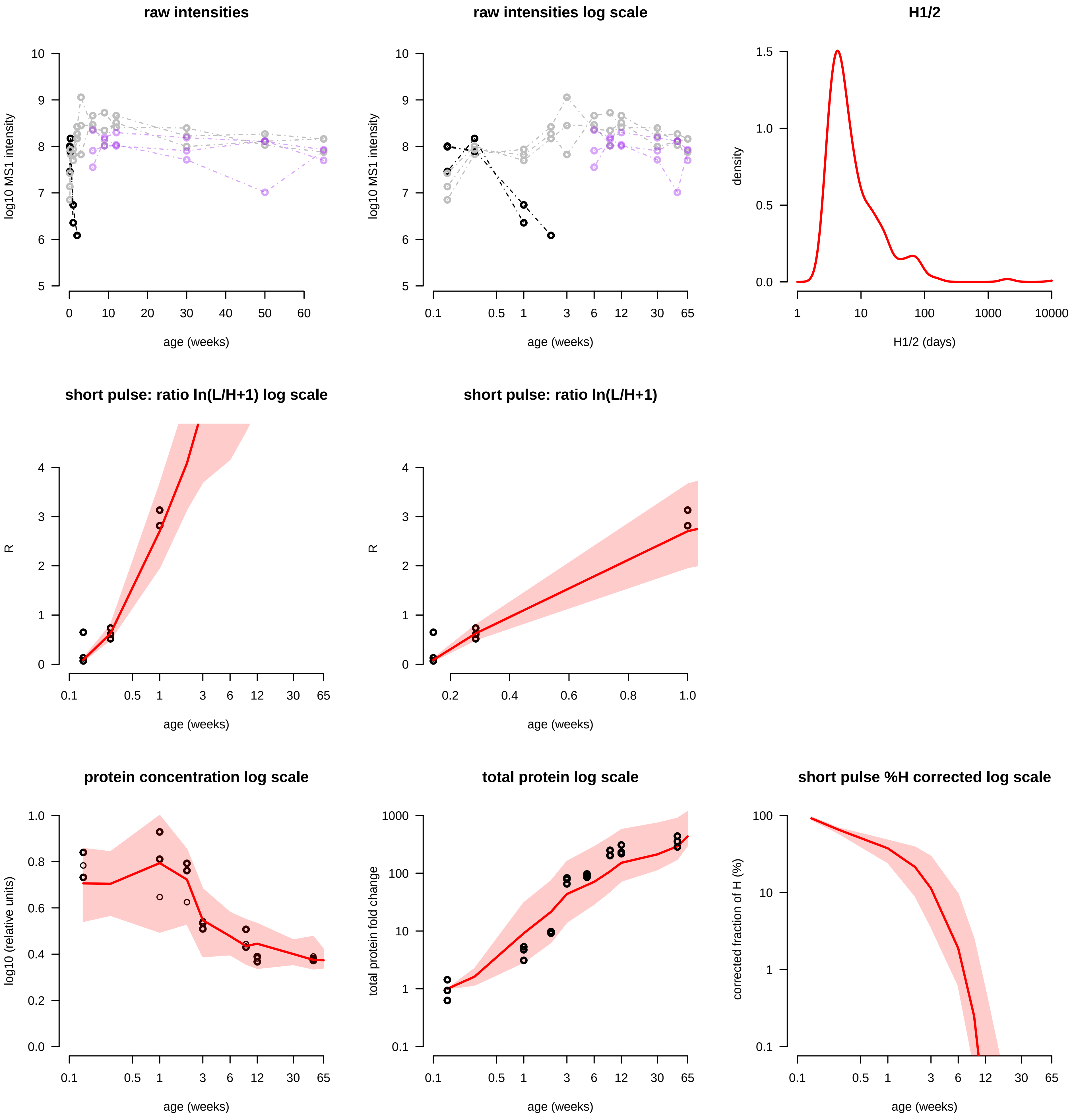

Q9CPW7

>sp|Q9CPW7|ZMAT2_MOUSE

Zinc finger matrin−type protein 2

GN=Zmat2

Med H1/2: 3.7 days

Q5 H1/2: 2.8 days

Q95 H1/2: 5.2 days

reliable model fit: yes

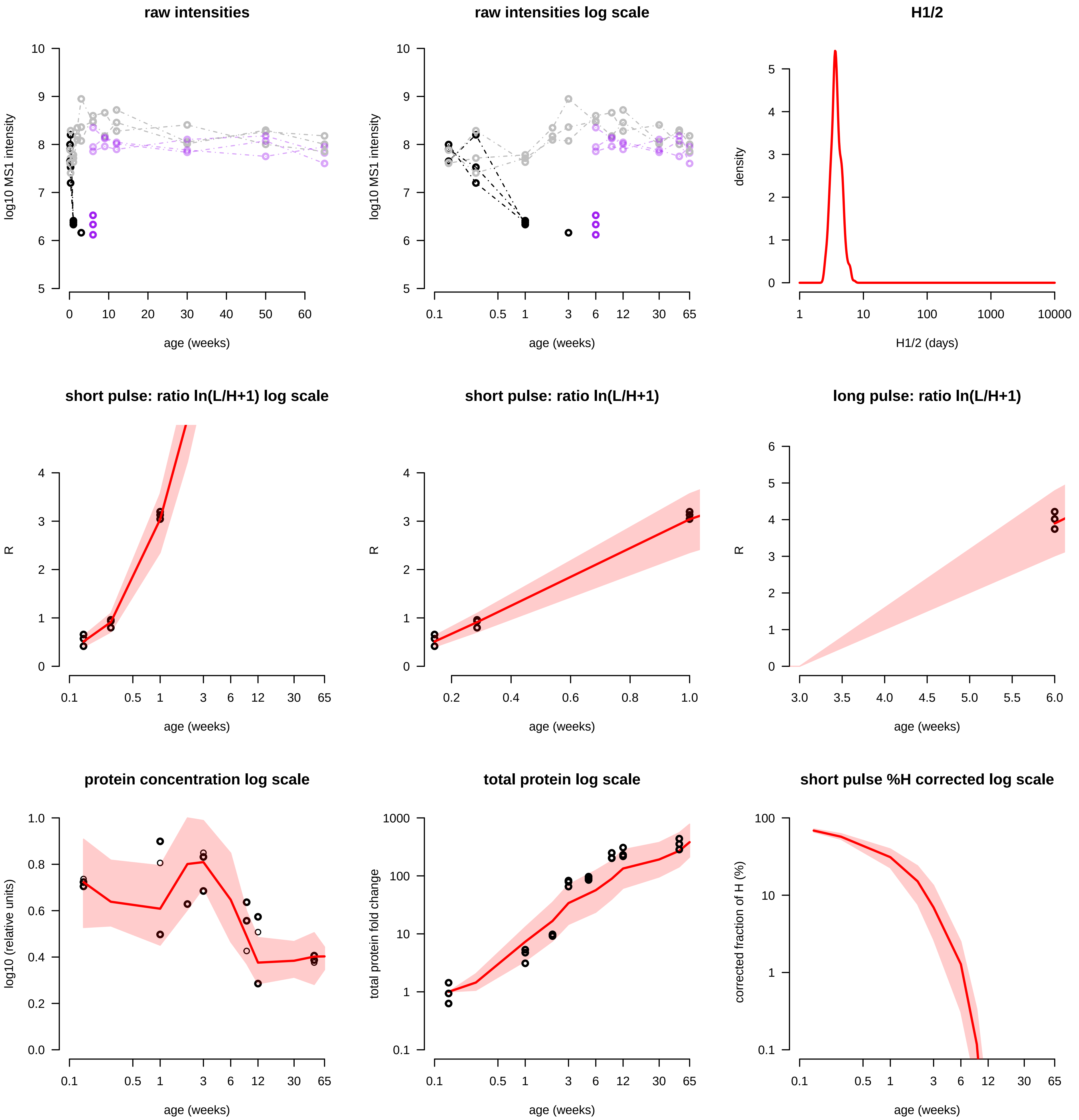

E9PZM4

>sp|E9PZM4|CHD2_MOUSE

Chromodomain−helicase−DNA−binding protein 2

GN=Chd2

Med H1/2: 5.8 days

Q5 H1/2: 2.7 days

Q95 H1/2: 39.3 days

reliable model fit: no

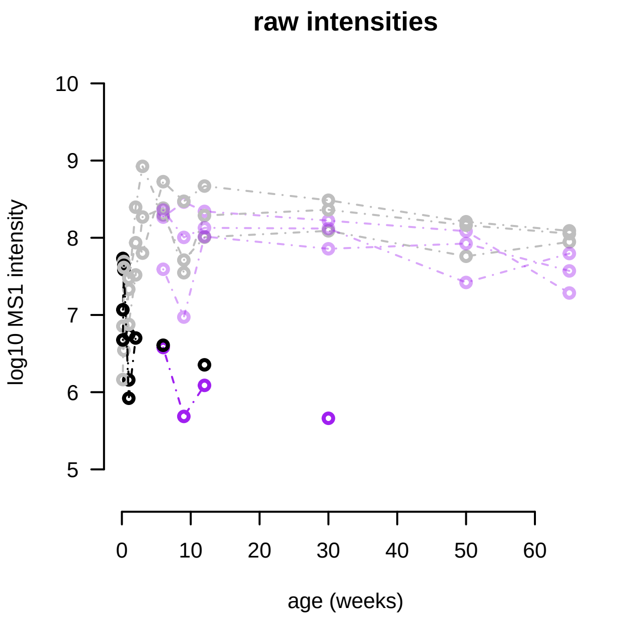

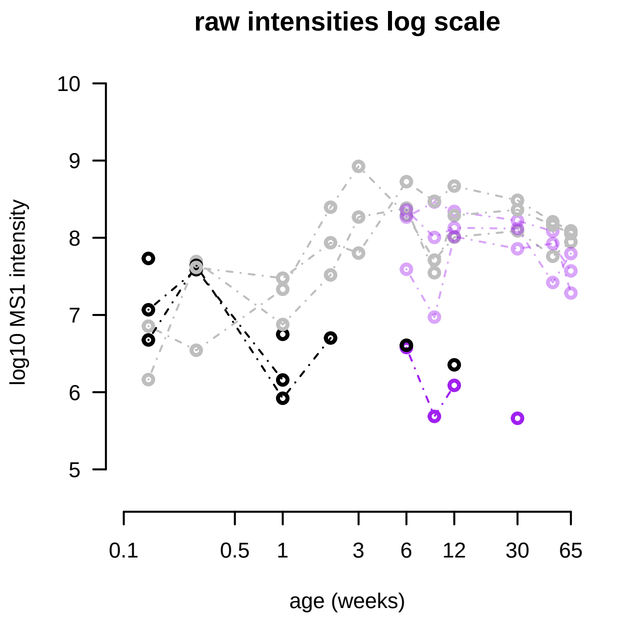

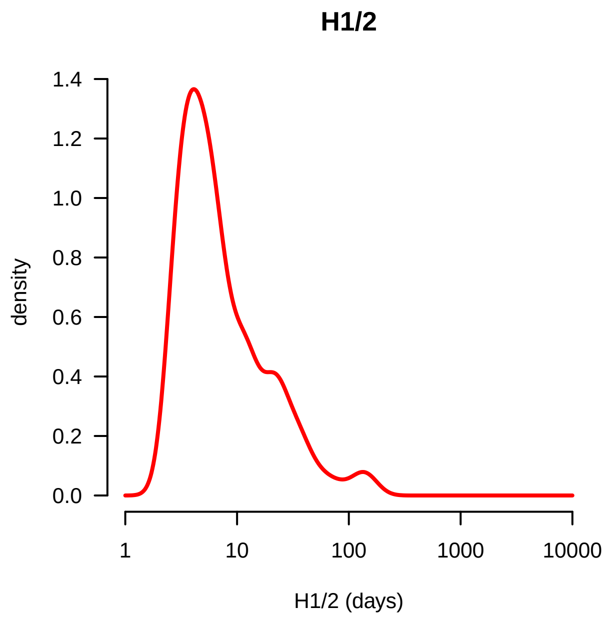

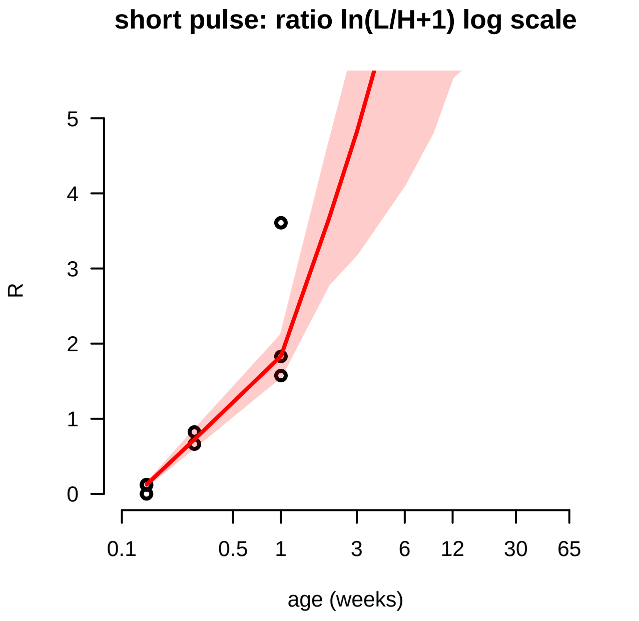

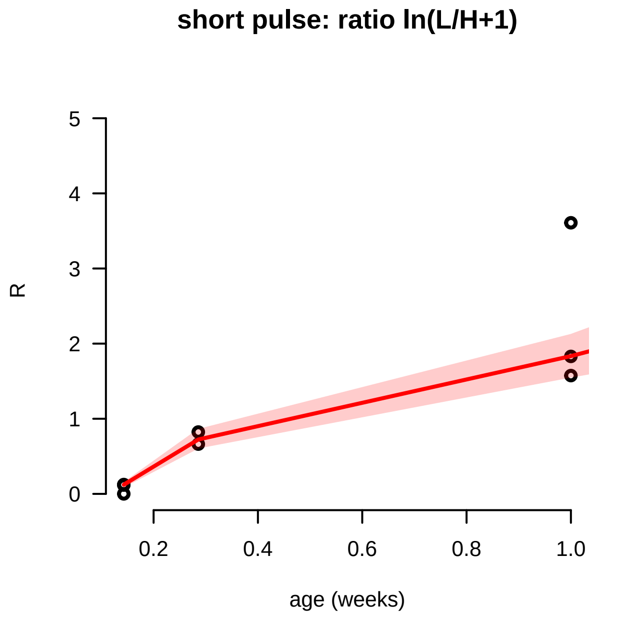

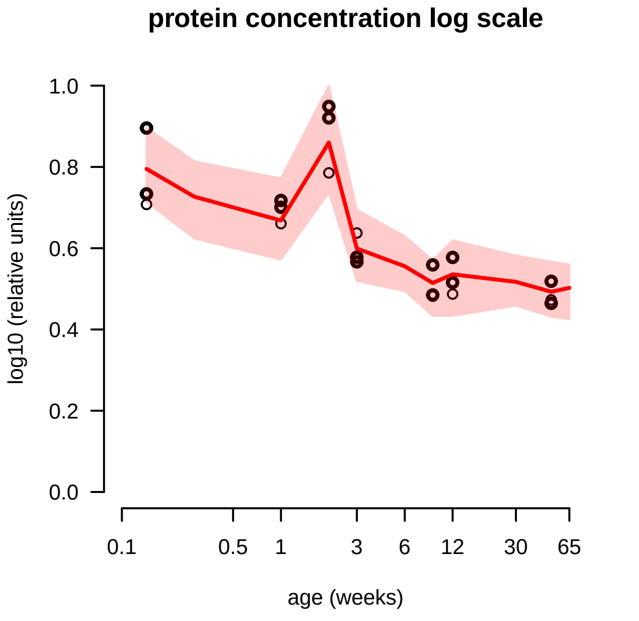

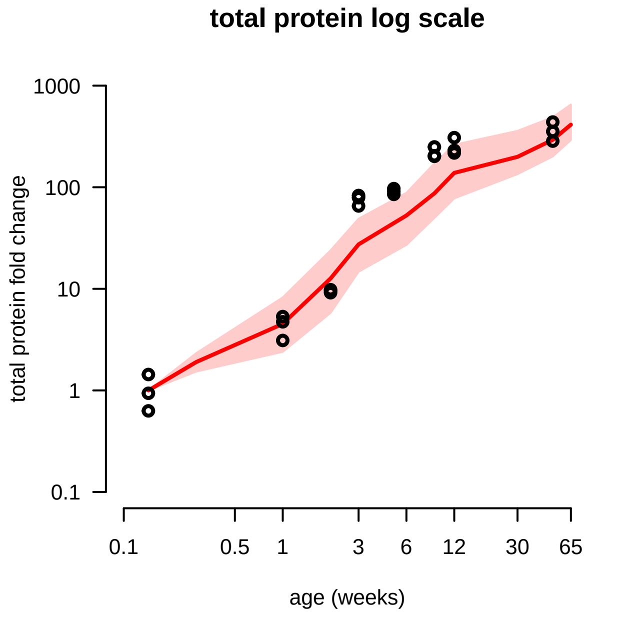

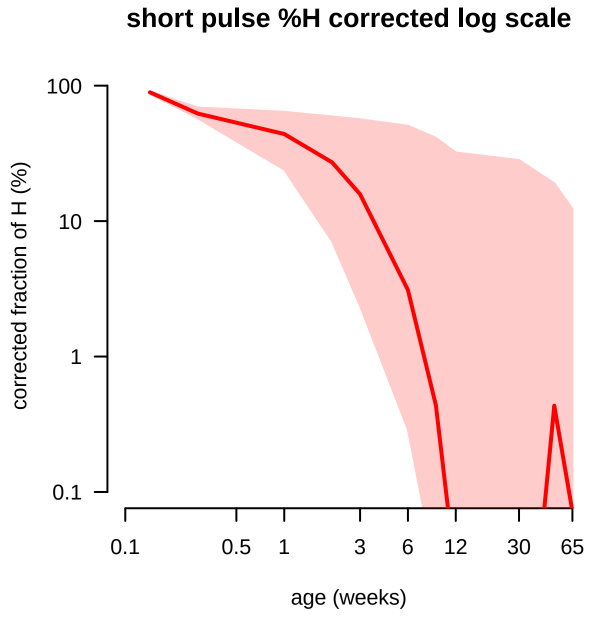

P58501

>sp|P58501|PAXB1_MOUSE

PAX3− and PAX7−binding protein 1

GN=Paxbp1

Med H1/2: 11 days

Q5 H1/2: 3.7 days

Q95 H1/2: 96.8 days

reliable model fit: no

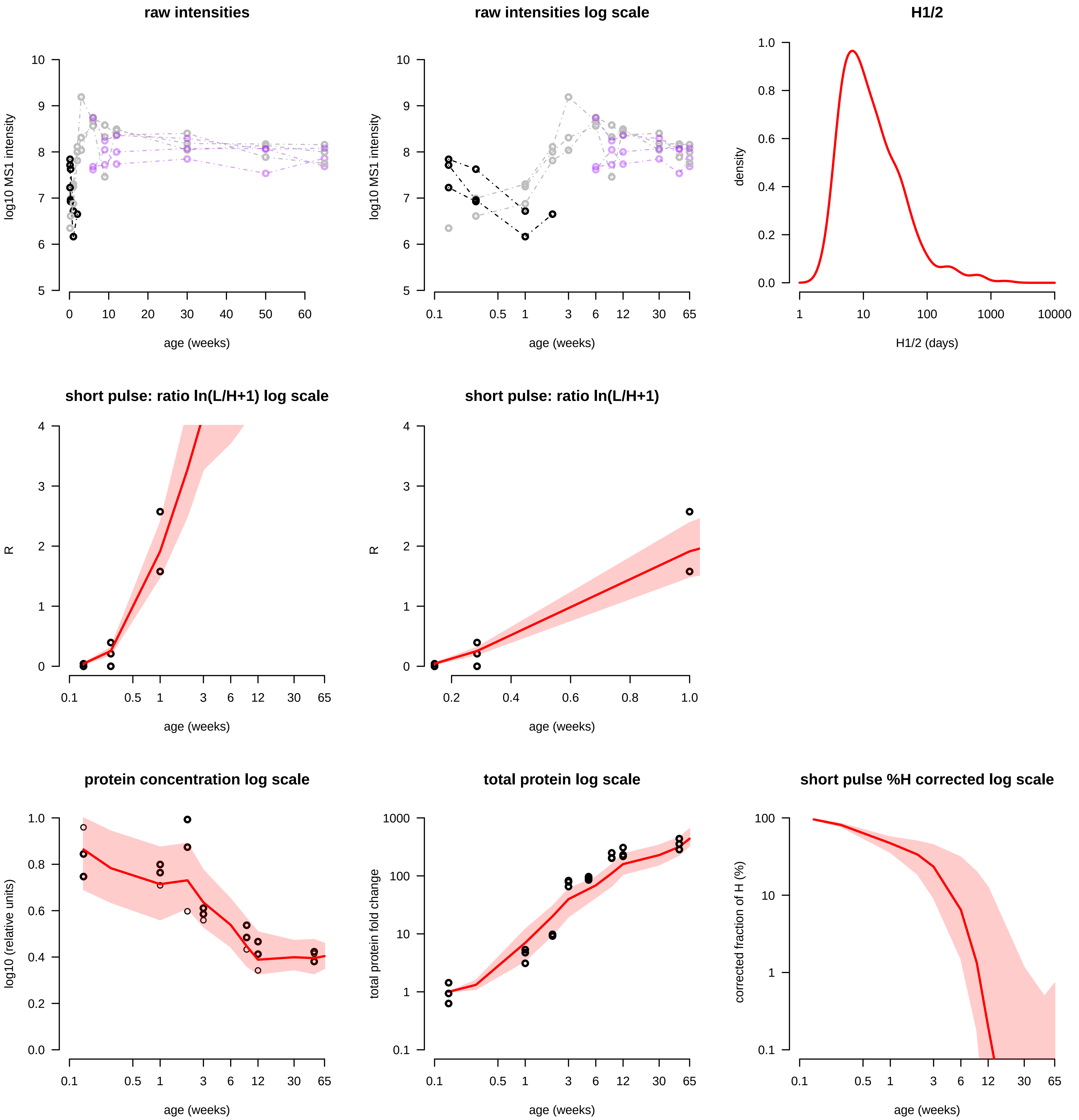

P59481

>sp|P59481|LMA2L_MOUSE

VIP36−like protein

GN=Lman2l

Med H1/2: 13.4 days

Q5 H1/2: 5.9 days

Q95 H1/2: 68.2 days

reliable model fit: yes

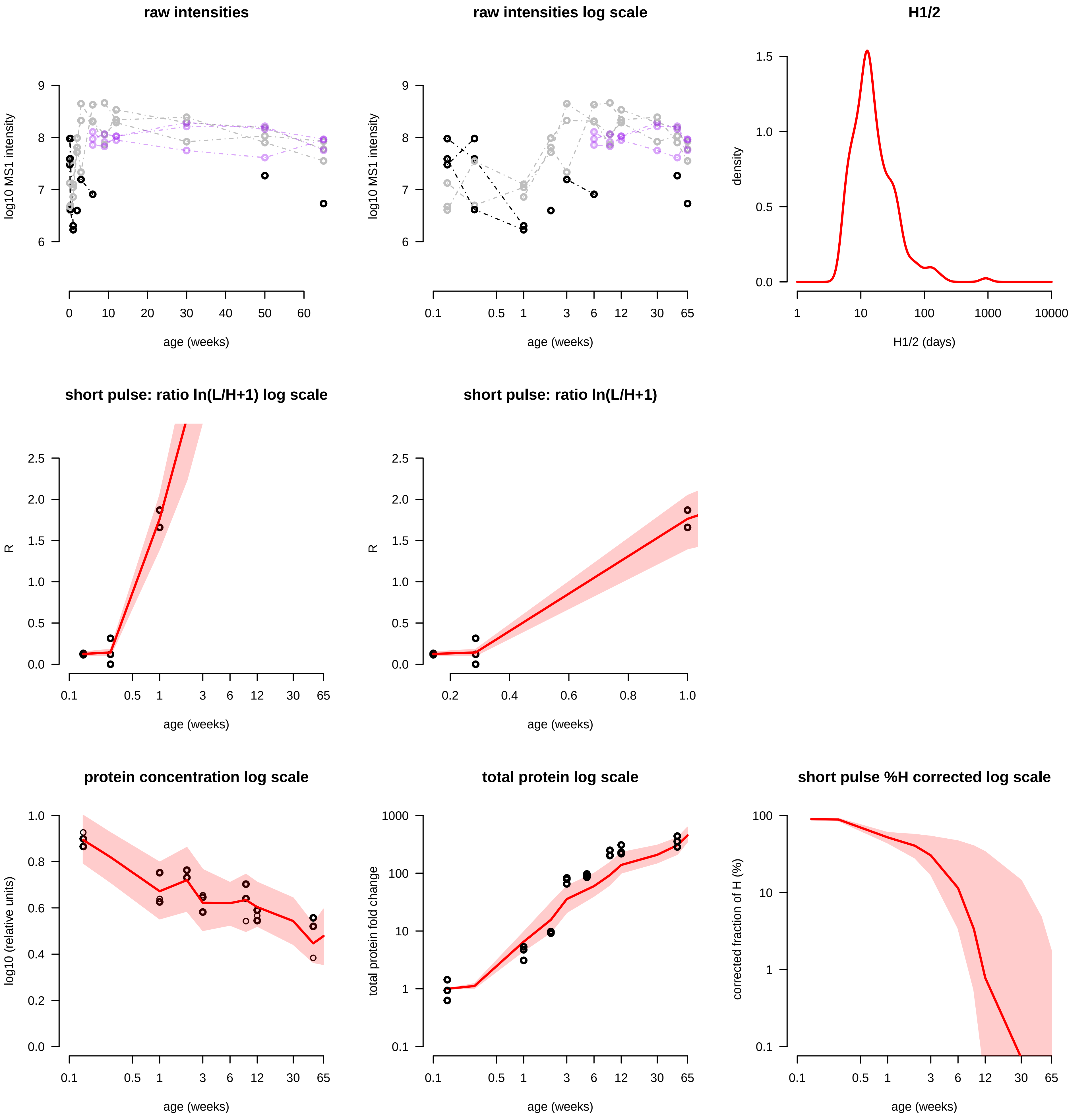

Q8BHT6

>sp|Q8BHT6|B3GLT_MOUSE

Beta−1,3−glucosyltransferase

GN=B3glct

Med H1/2: 5.6 days

Q5 H1/2: 3.6 days

Q95 H1/2: 22.7 days

reliable model fit: yes

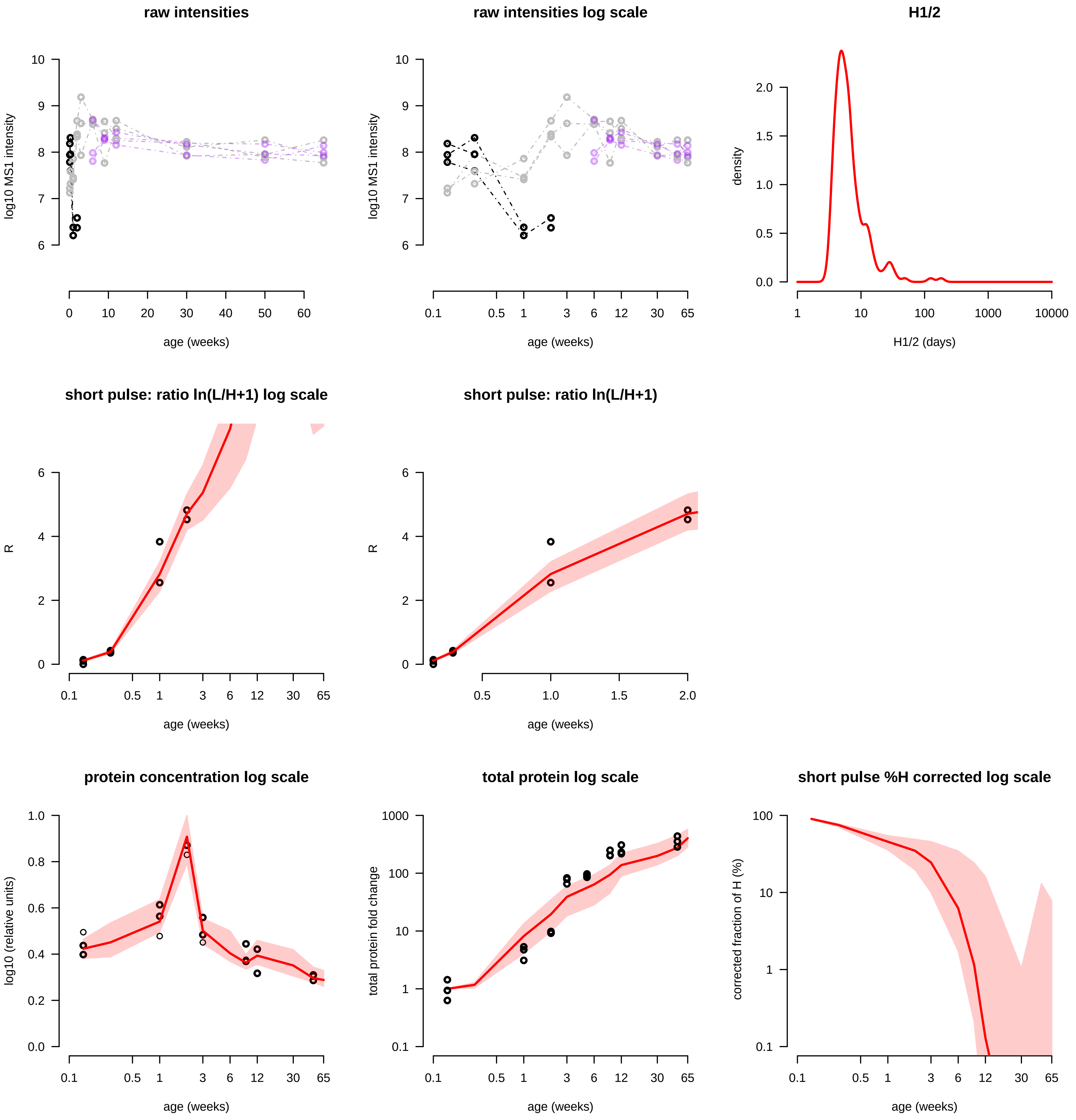

Q7TMQ7

>sp|Q7TMQ7|WDR91_MOUSE

WD repeat−containing protein 91

GN=Wdr91

Med H1/2: 22 days

Q5 H1/2: 6.5 days

Q95 H1/2: 183.4 days

reliable model fit: yes

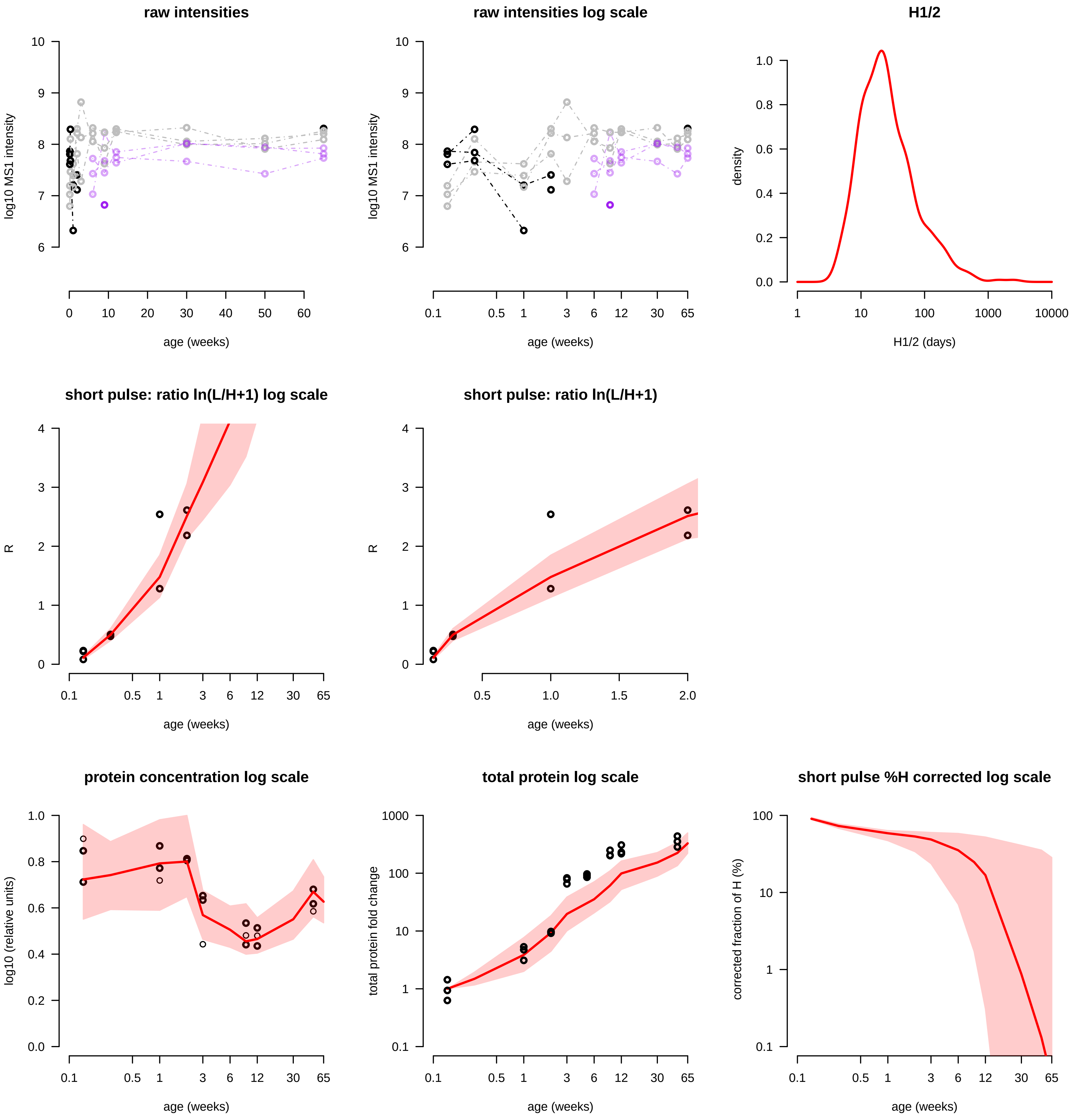

Q8VE62

>sp|Q8VE62|PAIP1_MOUSE

Polyadenylate−binding protein−interacting protein 1

GN=Paip1

Med H1/2: 6.9 days

Q5 H1/2: 2.7 days

Q95 H1/2: 35.6 days

reliable model fit: yes

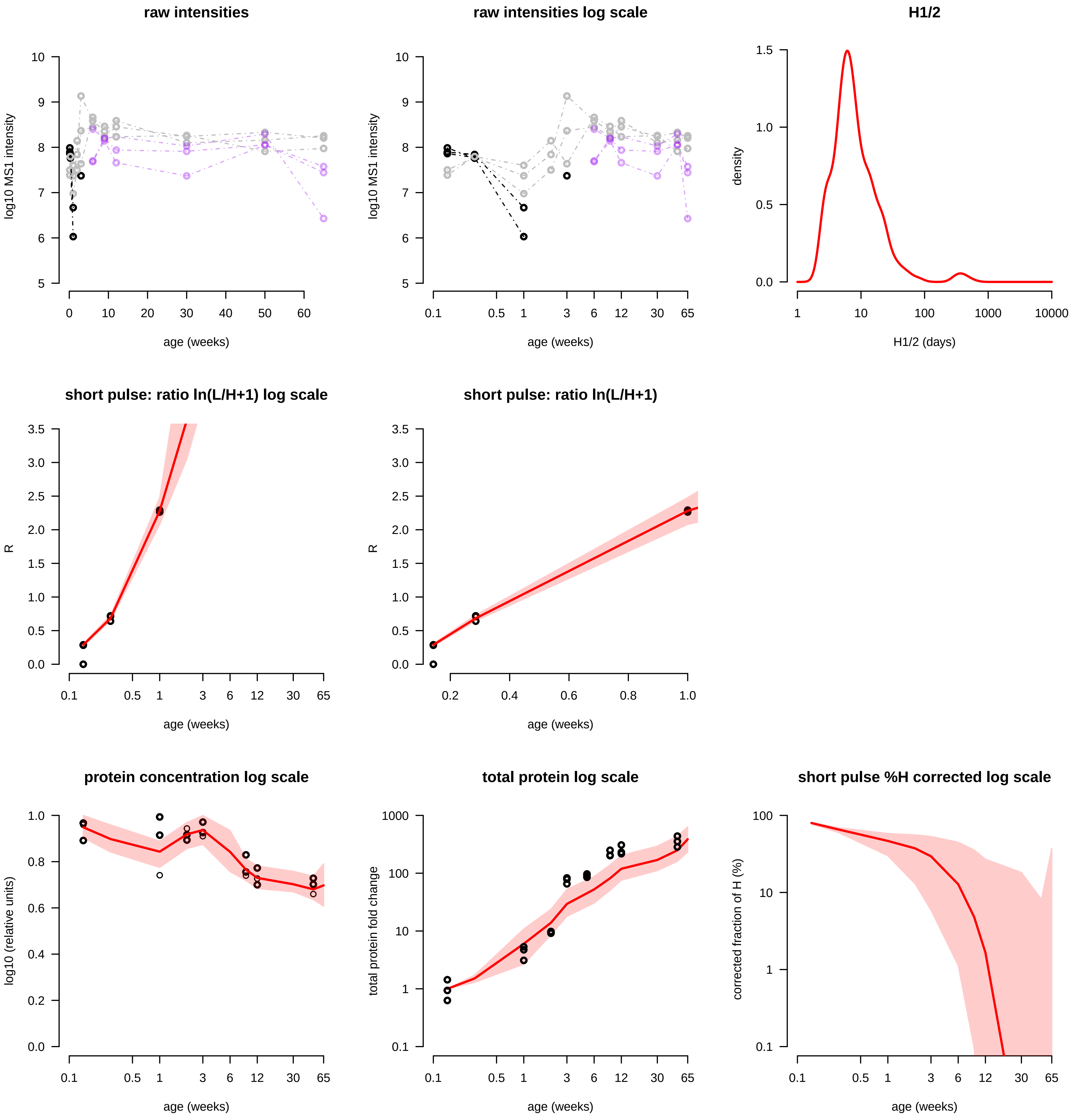

Q9R0G7

>sp|Q9R0G7|ZEB2_MOUSE

Zinc finger E−box−binding homeobox 2

GN=Zeb2

Med H1/2: 2117 days

Q5 H1/2: 725 days

Q95 H1/2: 13678.4 days

reliable model fit: no

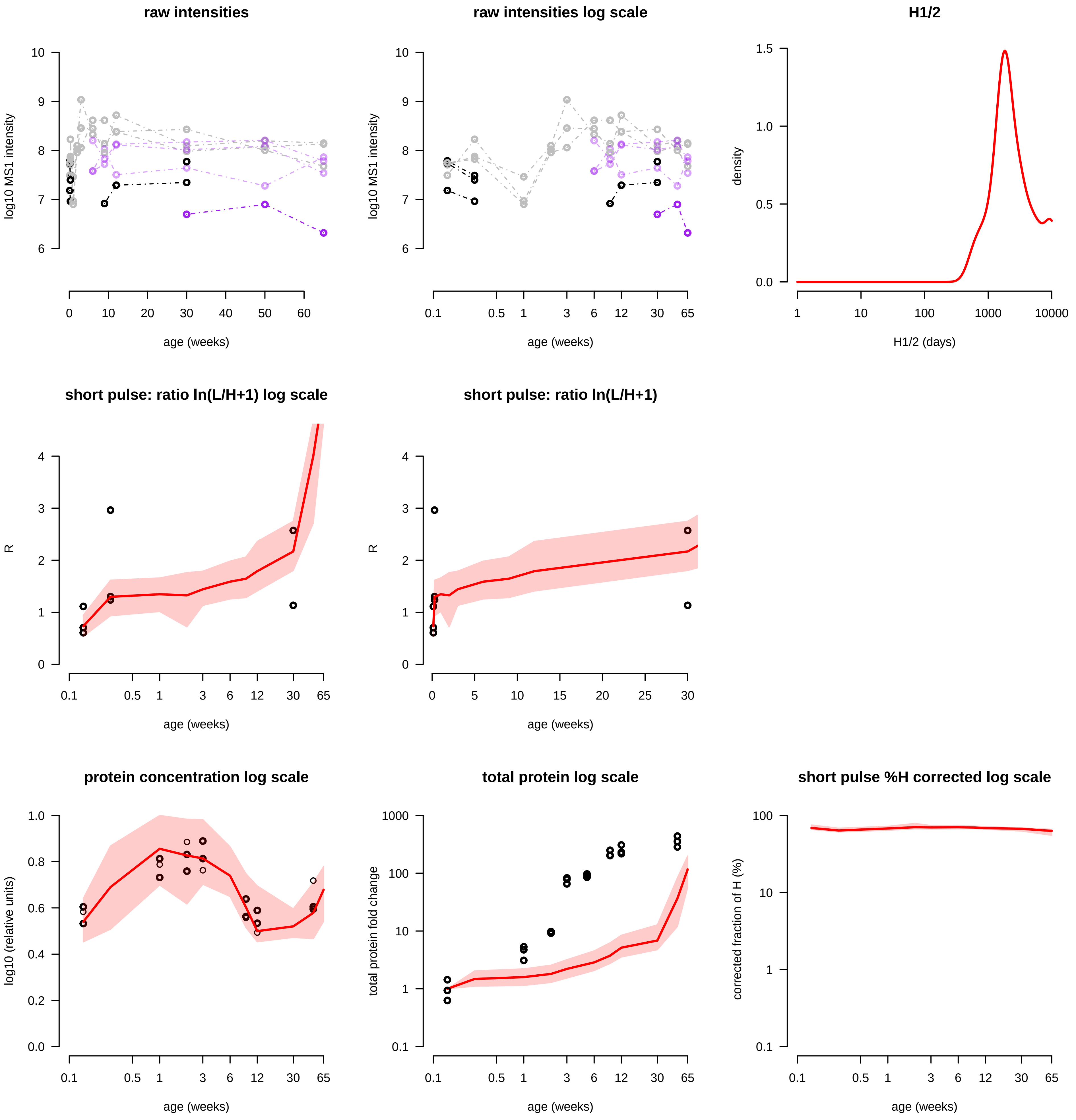

P97863;P97863−3;P97863−2

>sp|P97863|NFIB_MOUSE

Nuclear factor 1 B−type

GN=Nfib

Med H1/2: 14 days

Q5 H1/2: 4.1 days

Q95 H1/2: 368.9 days

reliable model fit: yes

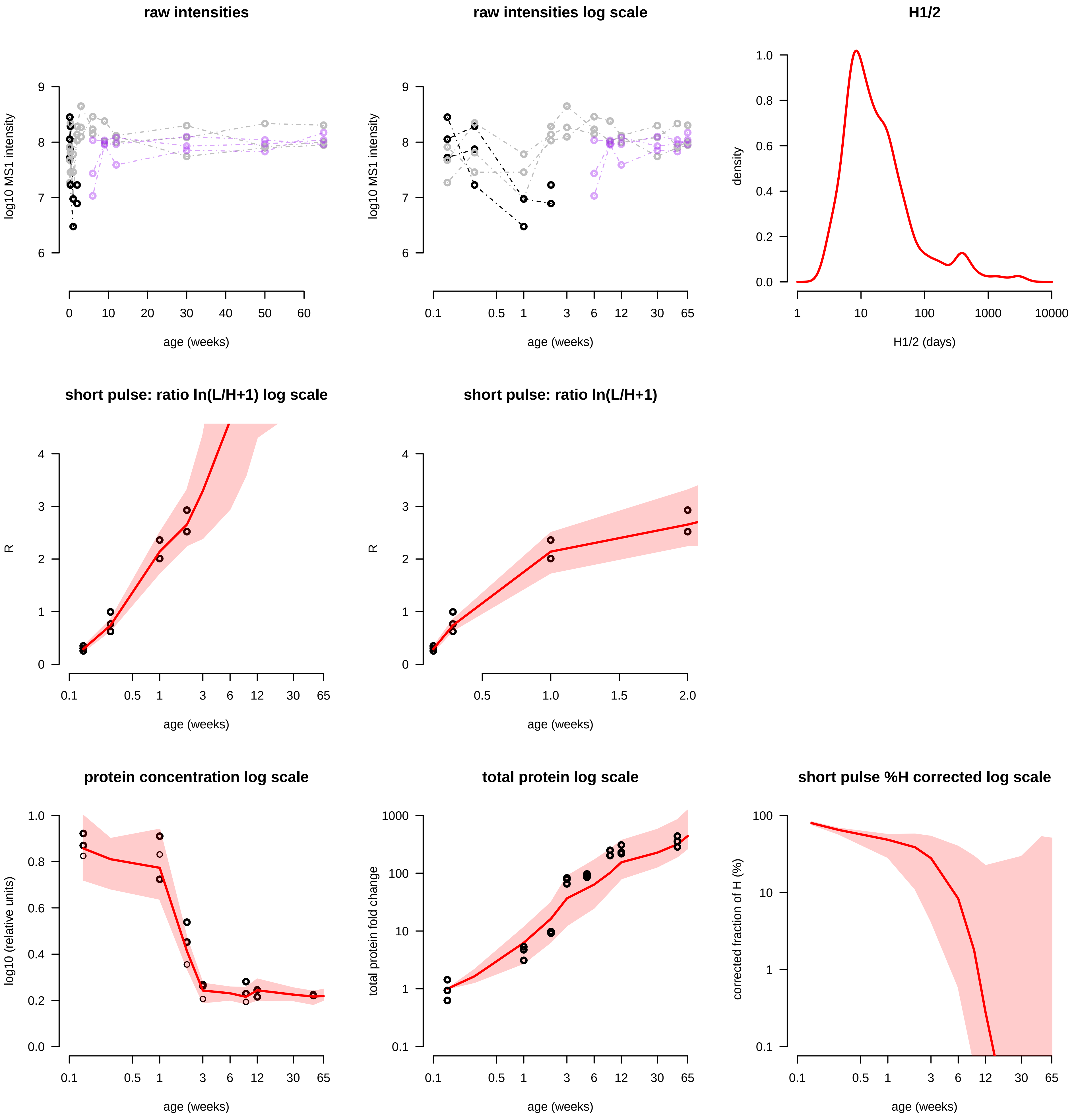

Q8CFE2

>sp|Q8CFE2|HPF1_MOUSE

Histone PARylation factor 1

GN=Hpf1

Med H1/2: 6 days

Q5 H1/2: 3 days

Q95 H1/2: 28.6 days

reliable model fit: yes

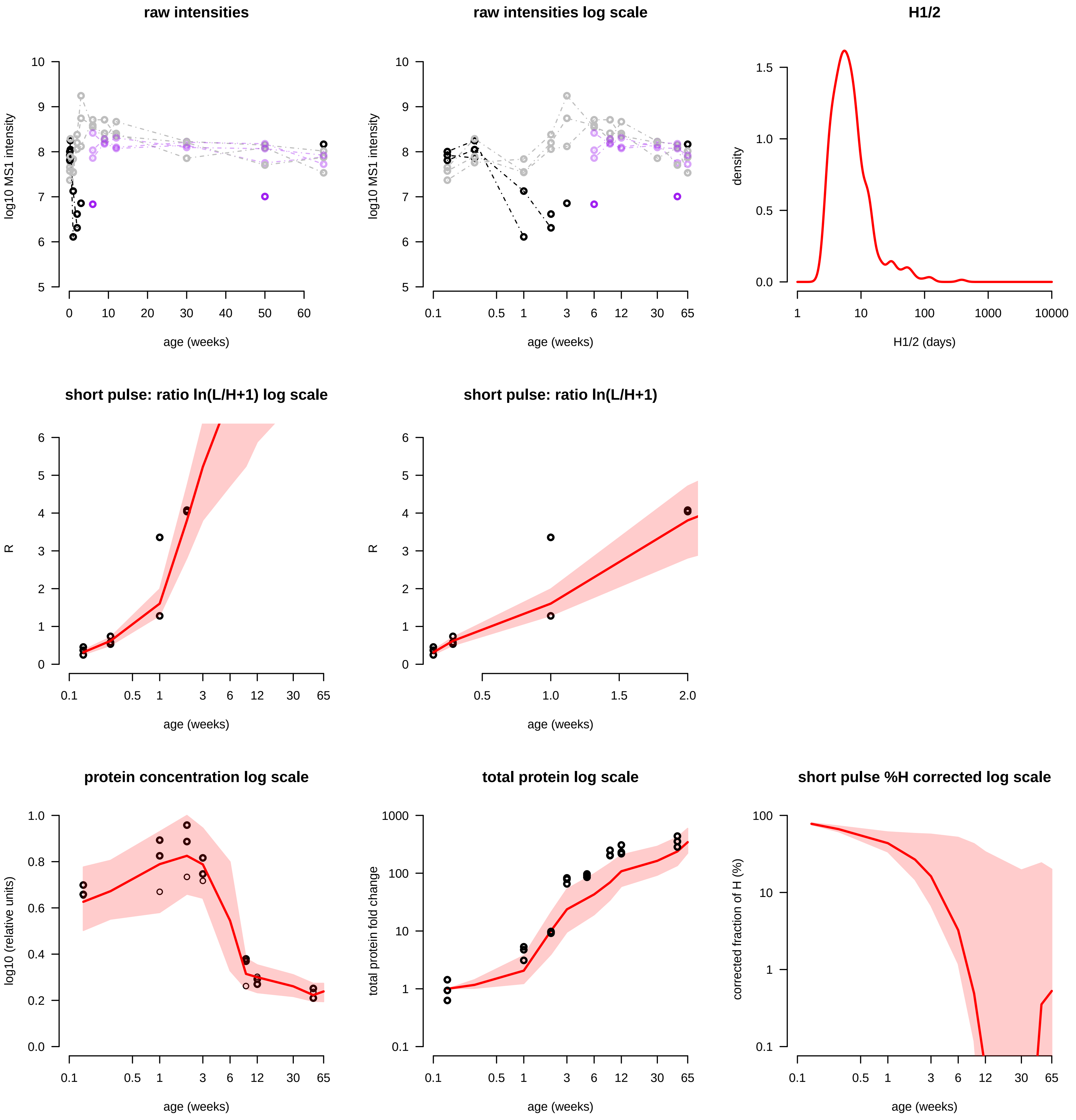

Q4LDD4−2;Q4LDD4;Q4LDD4−3

>sp|Q4LDD4−2|ARAP1_MOUSE

2 of Arf−GAP with Rho−GAP domain, ANK repeat and PH domain−containing p

GN=Arap1

Med H1/2: 4.5 days

Q5 H1/2: 2.7 days

Q95 H1/2: 40.6 days

reliable model fit: yes

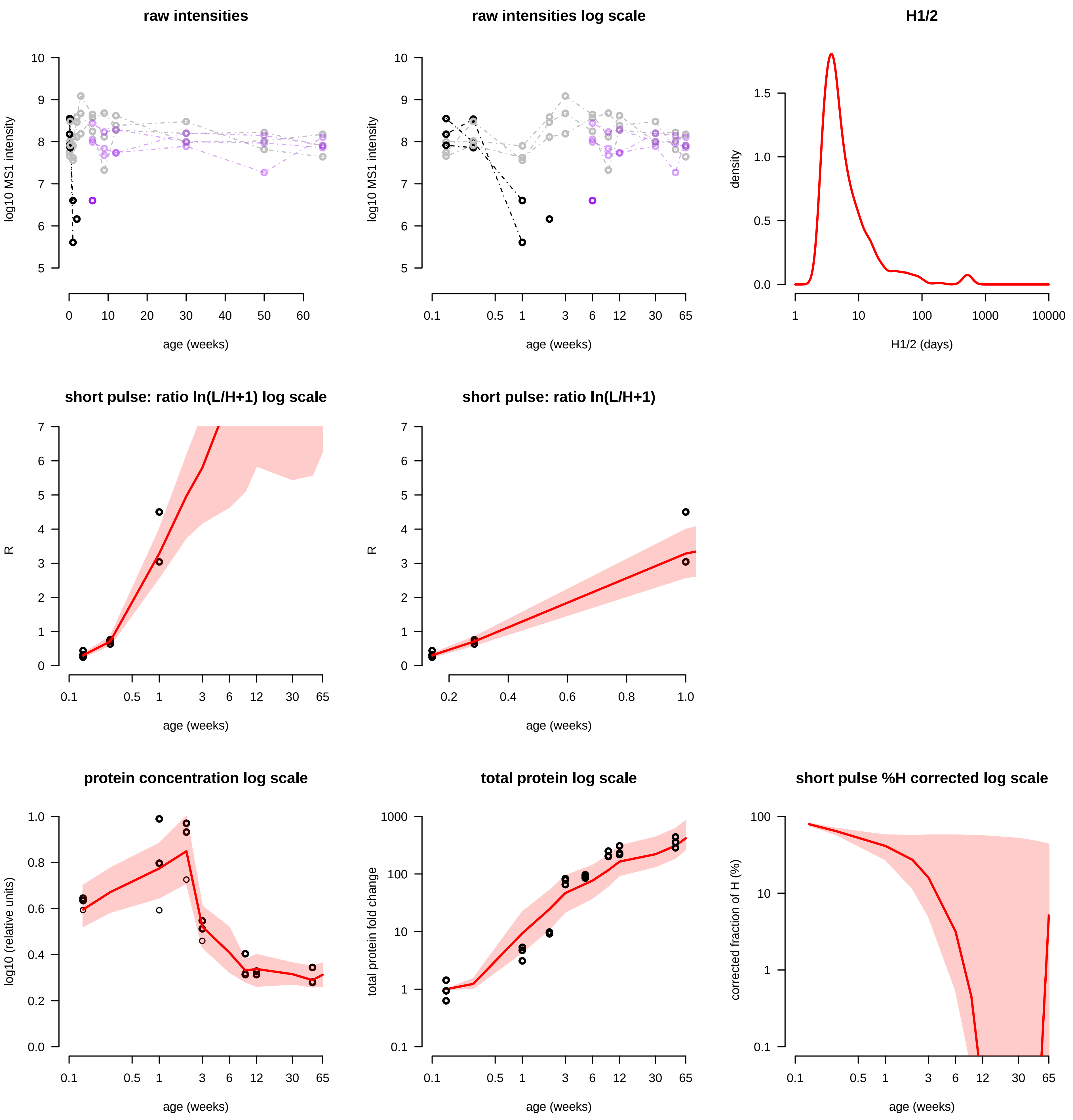

Q8R3L2;Q8R3L2−2;Q8R3L2−5;Q8R3L2−4;Q8R3L2−3

>sp|Q8R3L2|TCF25_MOUSE

Transcription factor 25

GN=Tcf25

Med H1/2: 13.2 days

Q5 H1/2: 4.9 days

Q95 H1/2: 192 days

reliable model fit: yes

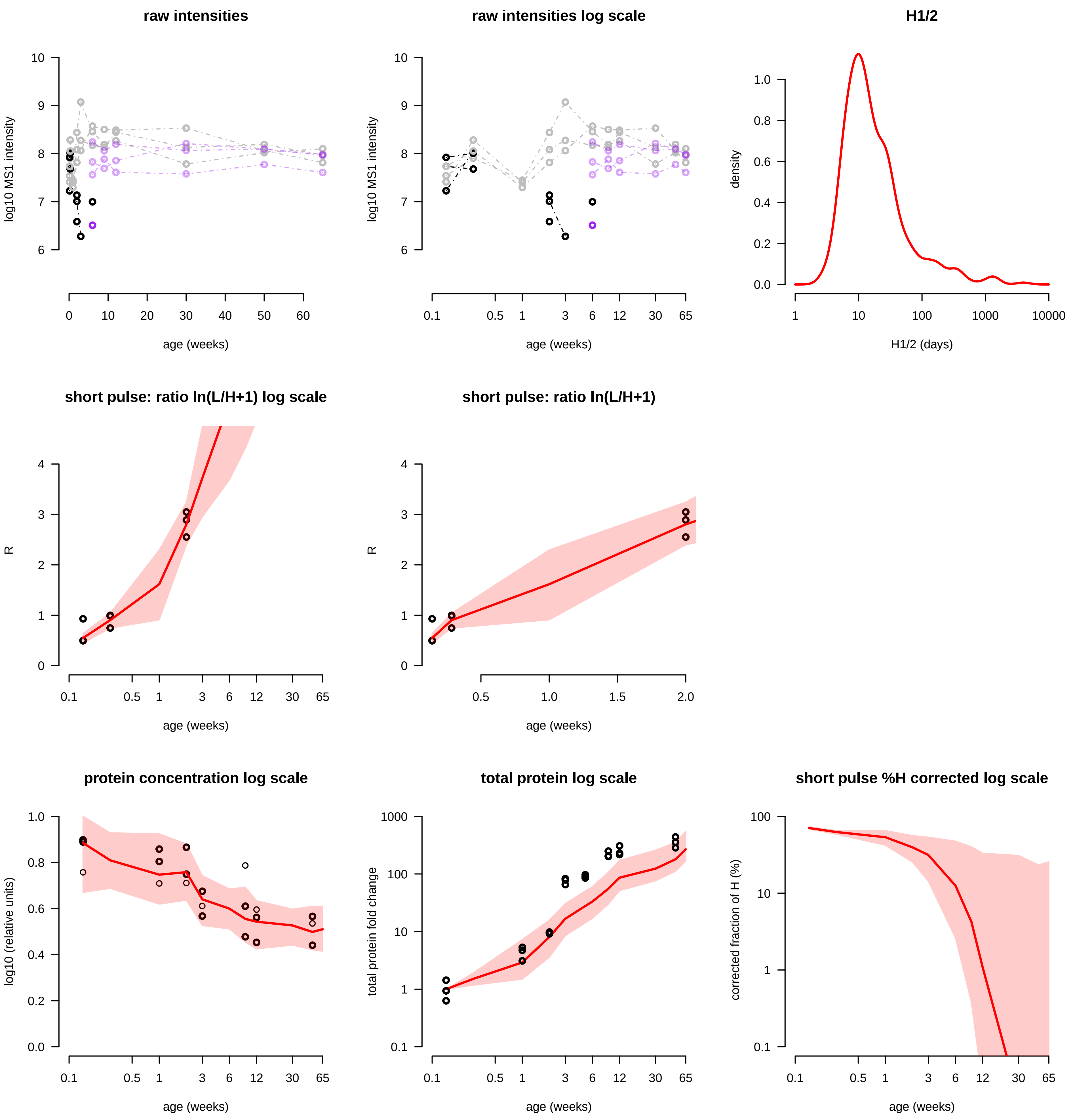

Q5XJE5

>sp|Q5XJE5|LEO1_MOUSE

RNA polymerase−associated protein LEO1

GN=Leo1

Med H1/2: 6 days

Q5 H1/2: 2.8 days

Q95 H1/2: 47.3 days

reliable model fit: yes

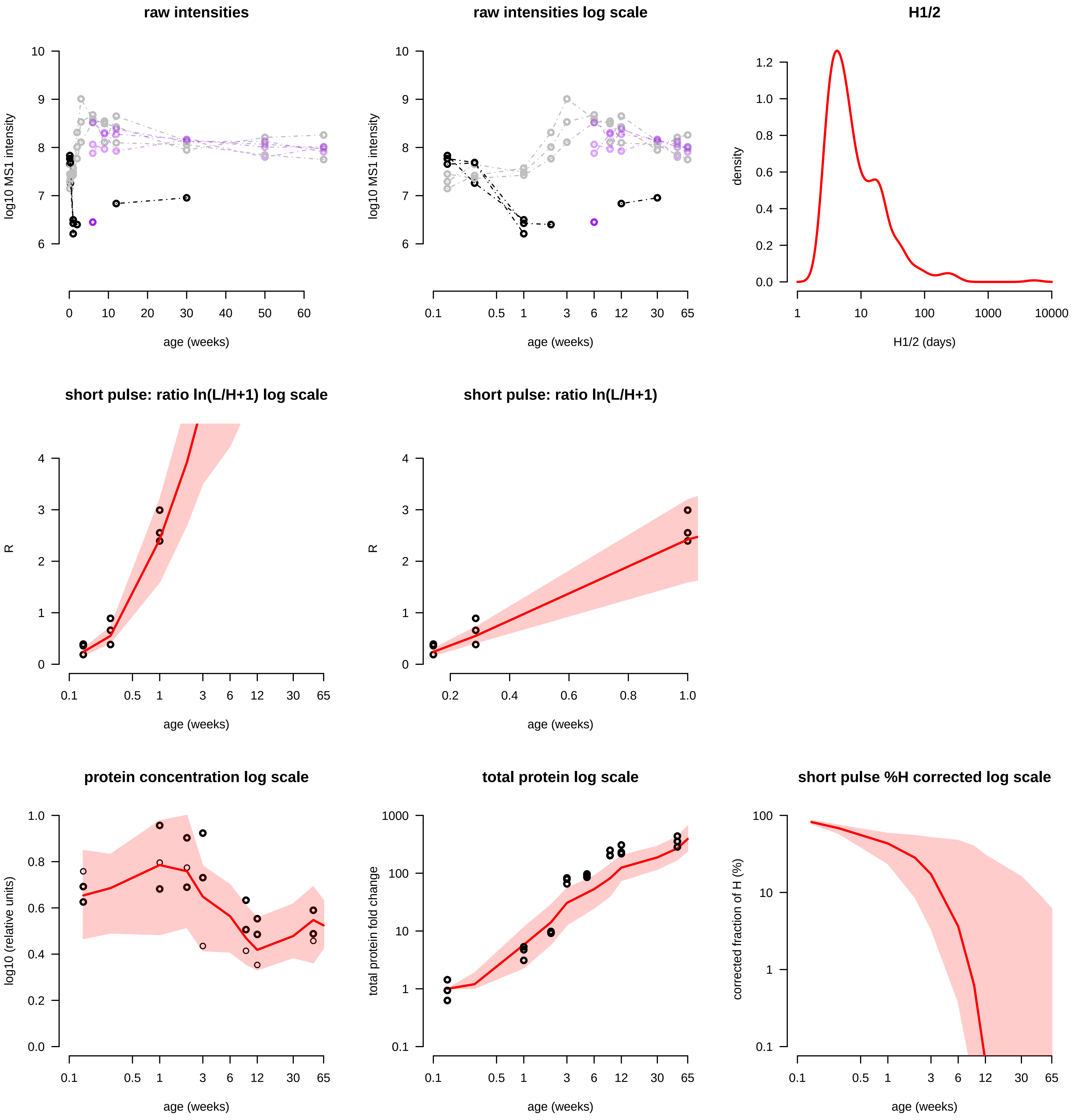

Q9D7H3

>sp|Q9D7H3|RTCA_MOUSE

RNA 3'−terminal phosphate cyclase

GN=RtcA

Med H1/2: 12.4 days

Q5 H1/2: 5.1 days

Q95 H1/2: 72.7 days

reliable model fit: yes

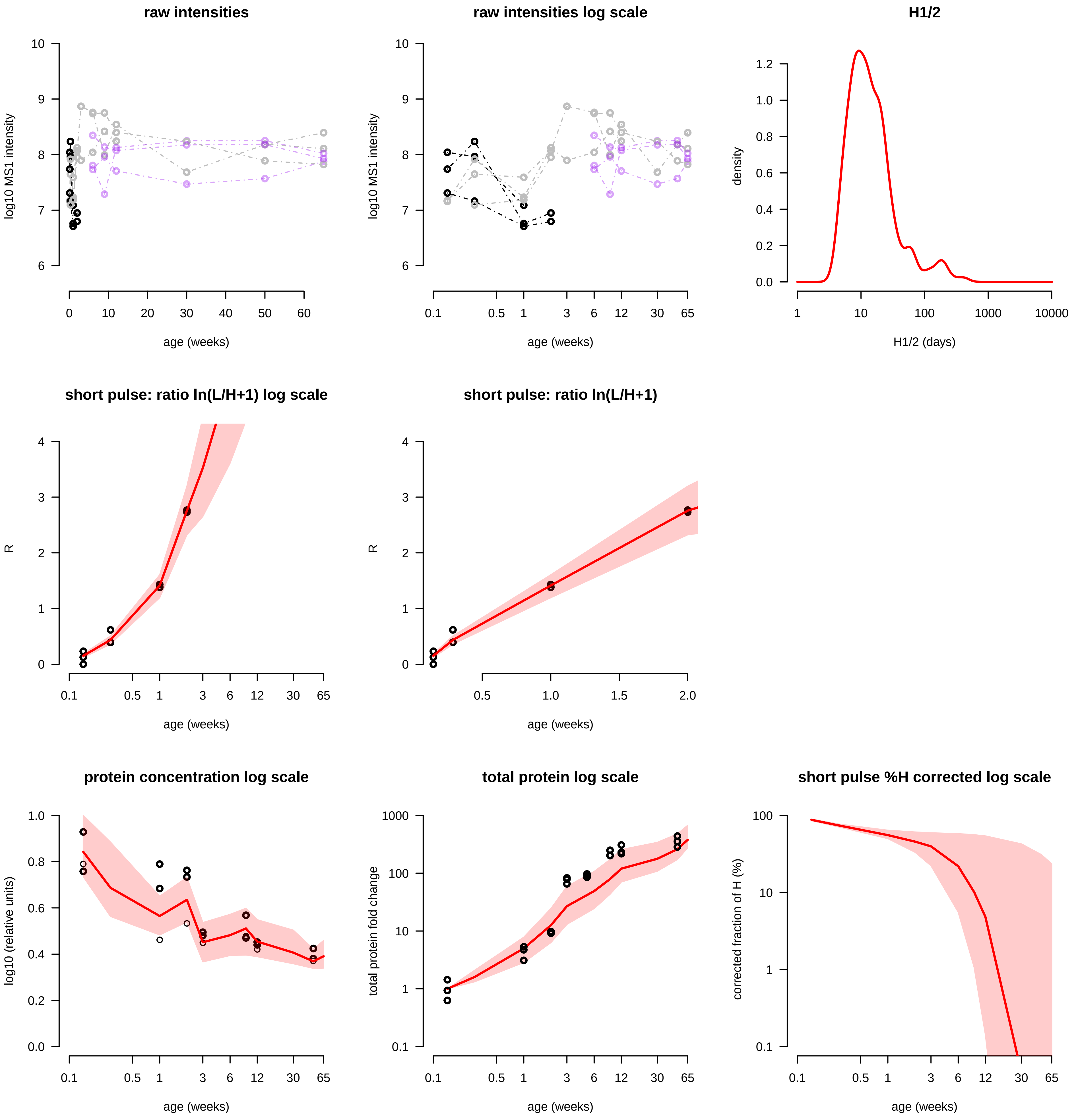

Q3UVL4;Q3UVL4−2

>sp|Q3UVL4|VPS51_MOUSE

Vacuolar protein sorting−associated protein 51 homolog

GN=Vps51

Med H1/2: 2279.7 days

Q5 H1/2: 749.8 days

Q95 H1/2: 14621.6 days

reliable model fit: yes

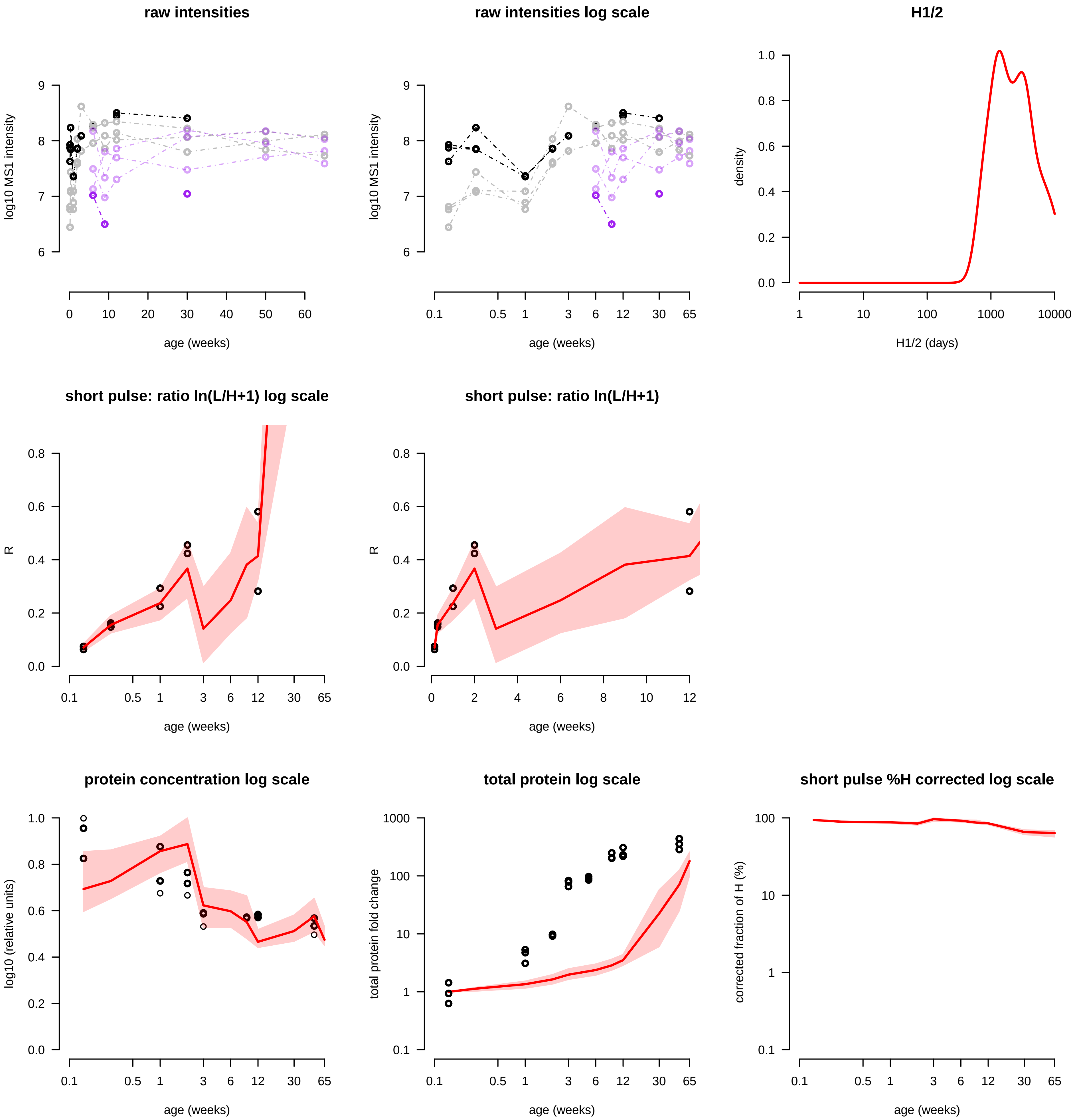

Q9CWN7

>sp|Q9CWN7|CNO11_MOUSE

CCR4−NOT transcription complex subunit 11

GN=Cnot11

Med H1/2: 8.3 days

Q5 H1/2: 3.5 days

Q95 H1/2: 71.2 days

reliable model fit: yes

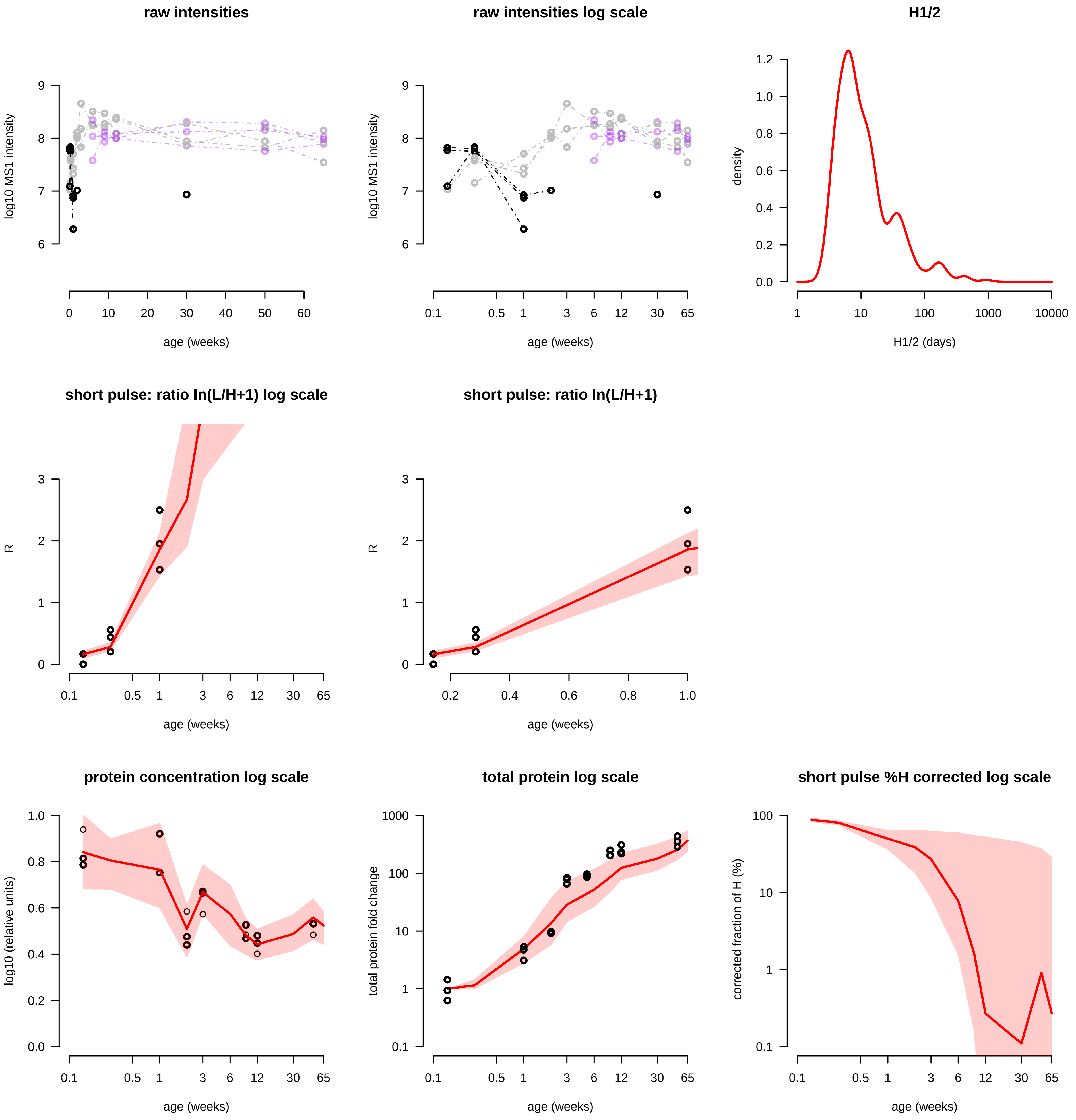

Q4FK66

>sp|Q4FK66|PR38A_MOUSE

Pre−mRNA−splicing factor 38A

GN=Prpf38a

Med H1/2: 7 days

Q5 H1/2: 3.7 days

Q95 H1/2: 28.7 days

reliable model fit: yes

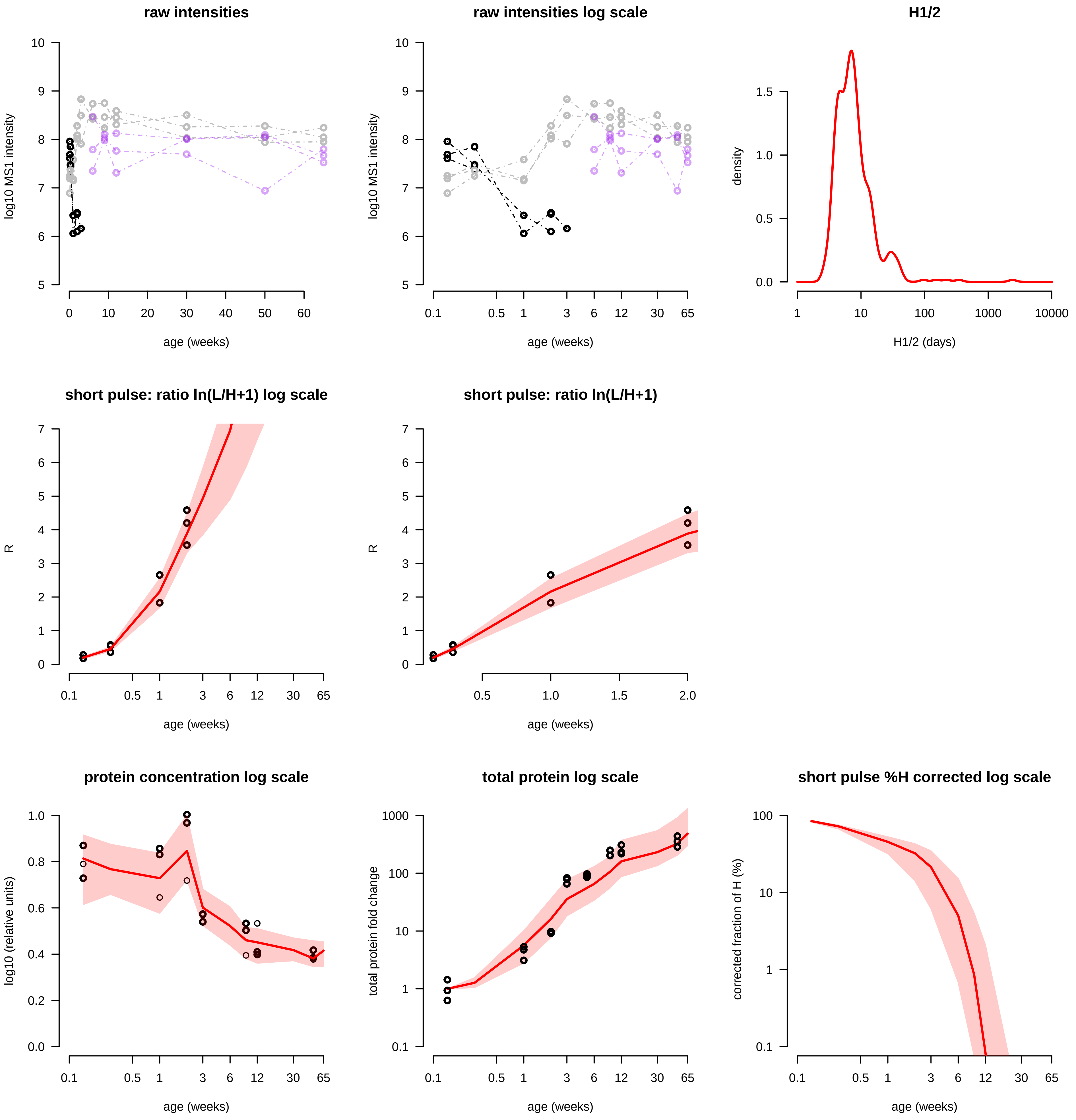

Q6P5E6

>sp|Q6P5E6|GGA2_MOUSE

ADP−ribosylation factor−binding protein GGA2

GN=Gga2

Med H1/2: 6.8 days

Q5 H1/2: 2.8 days

Q95 H1/2: 39.6 days

reliable model fit: yes

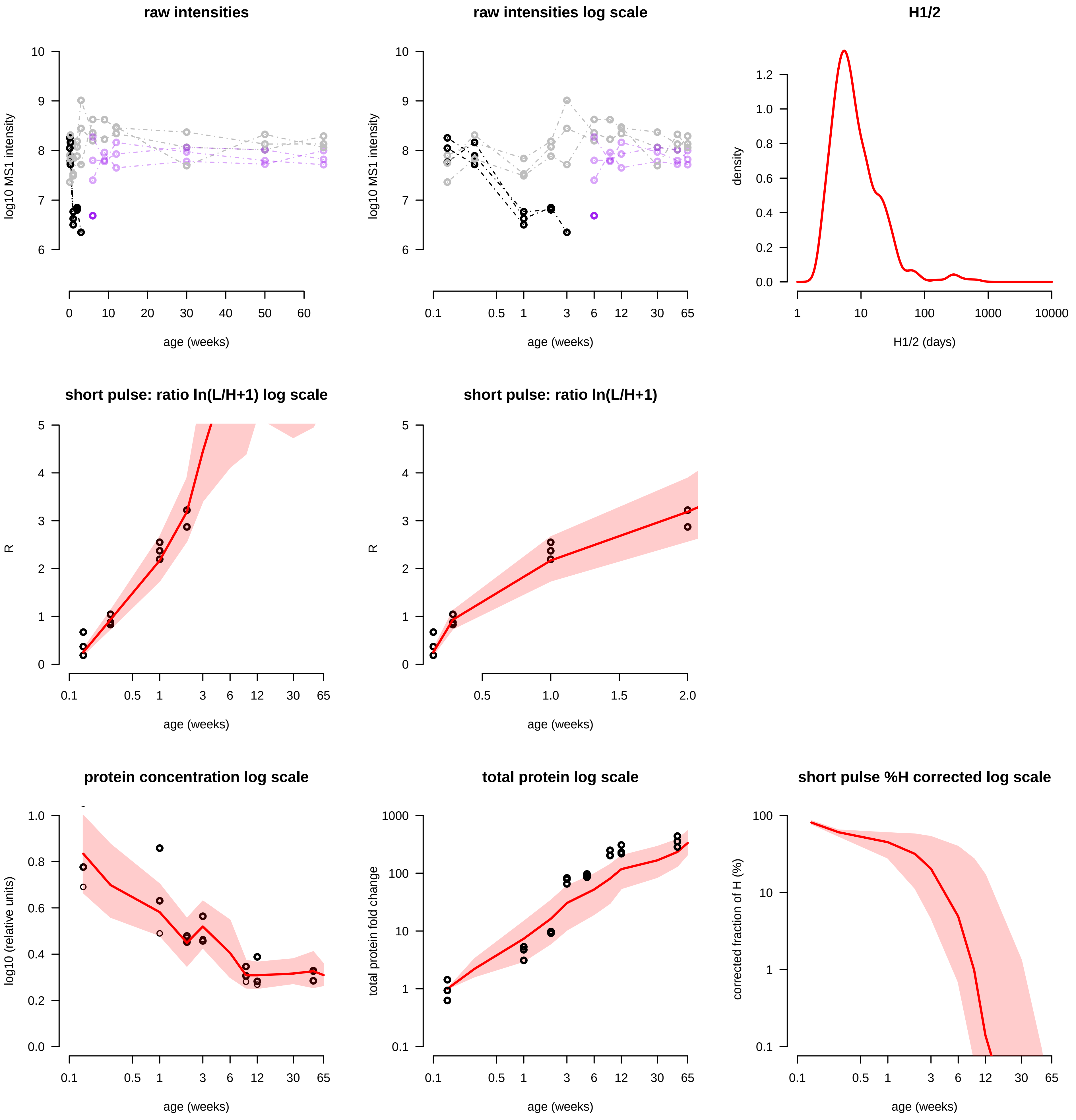

Q9WVB3

>sp|Q9WVB3|TLE6_MOUSE

Transducin−like enhancer protein 6

GN=Tle6

Med H1/2: 11.2 days

Q5 H1/2: 7.7 days

Q95 H1/2: 17 days

reliable model fit: yes

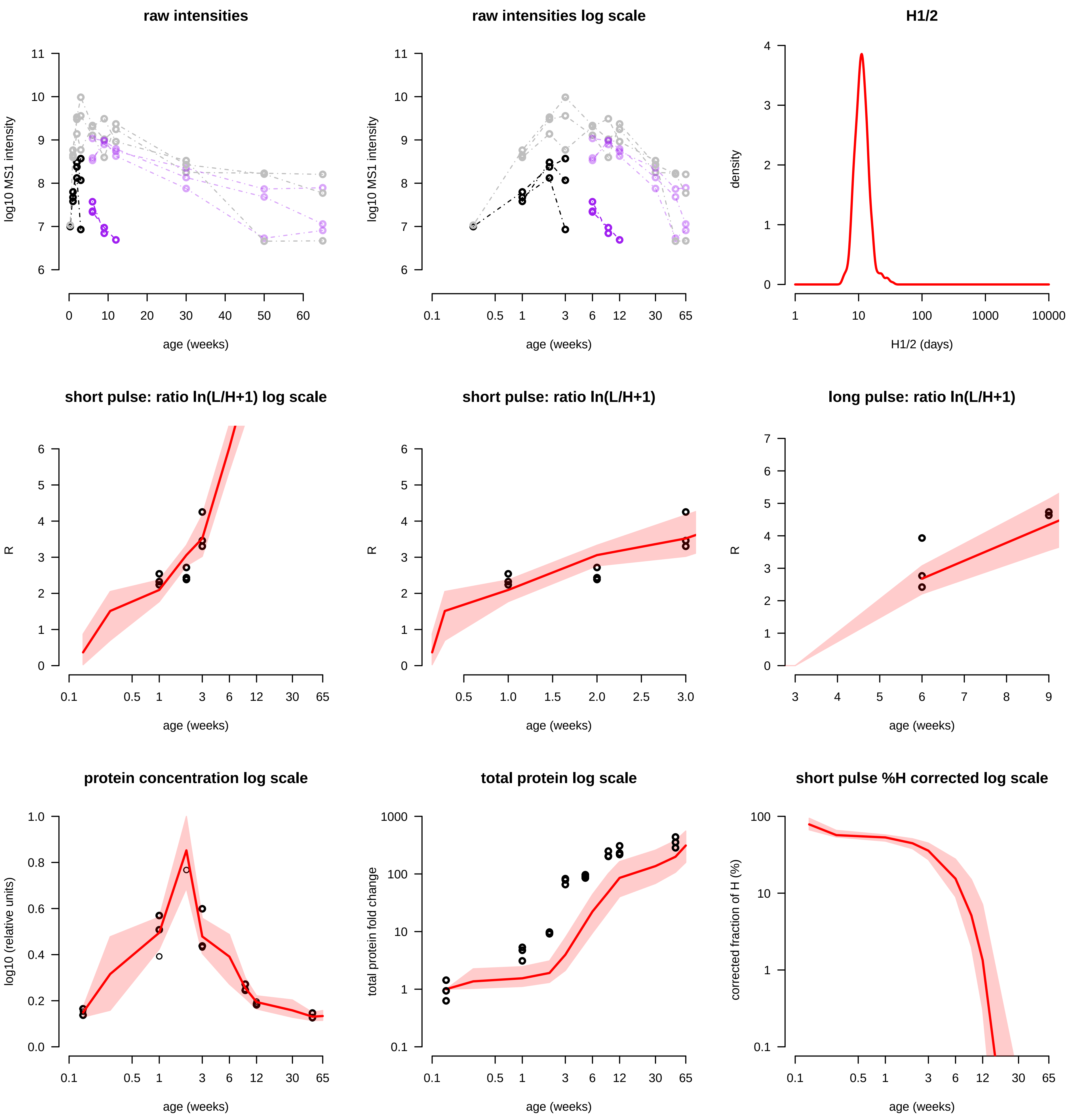

Q9DCR2

>sp|Q9DCR2|AP3S1_MOUSE

AP−3 complex subunit sigma−1

GN=Ap3s1

Med H1/2: 12.6 days

Q5 H1/2: 3.2 days

Q95 H1/2: 177.8 days

reliable model fit: yes

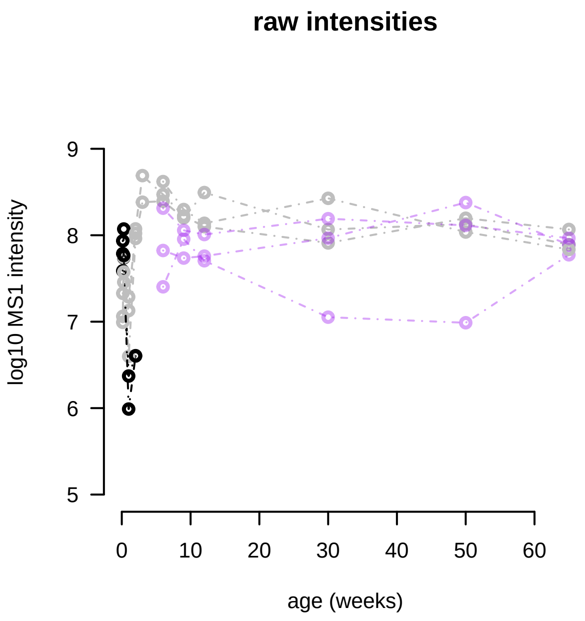

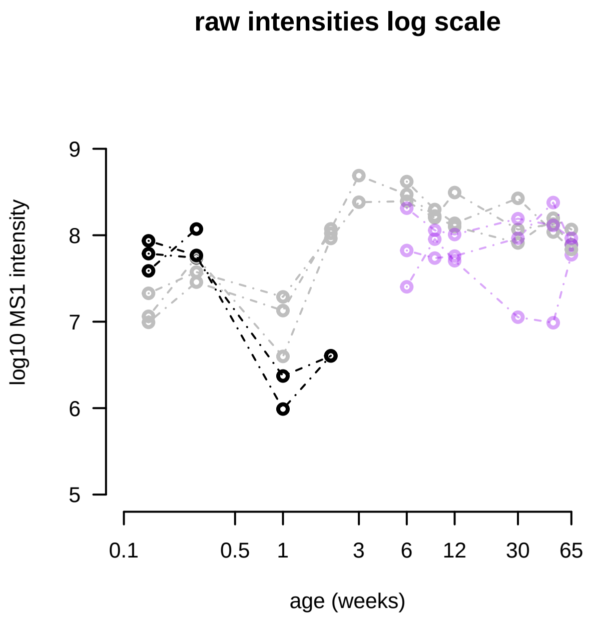

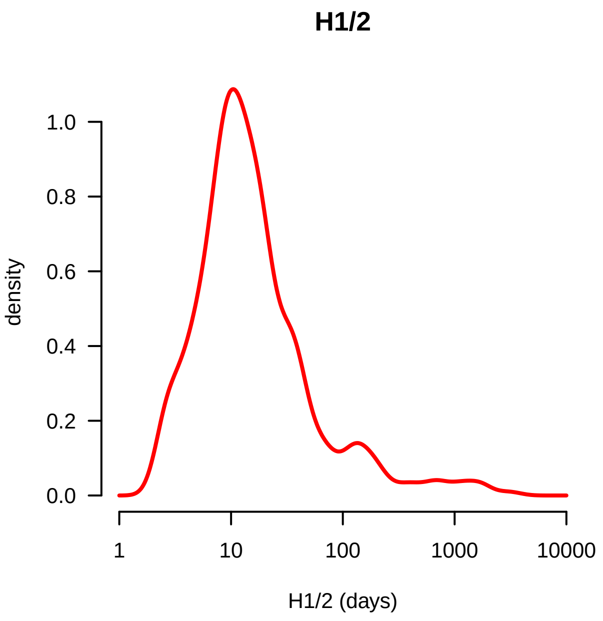

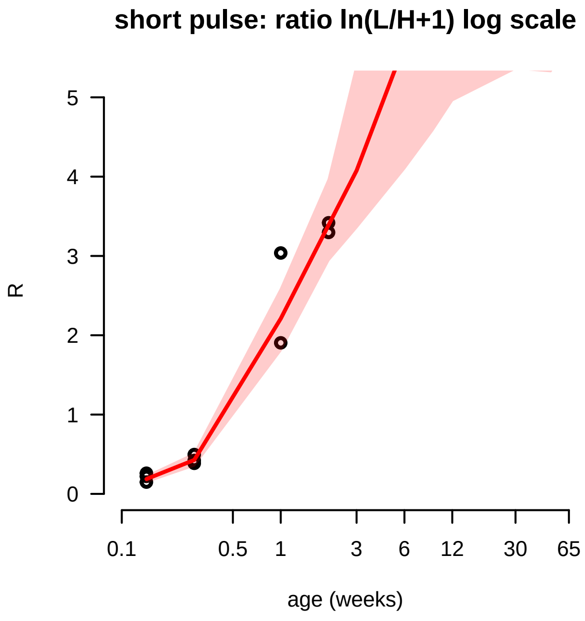

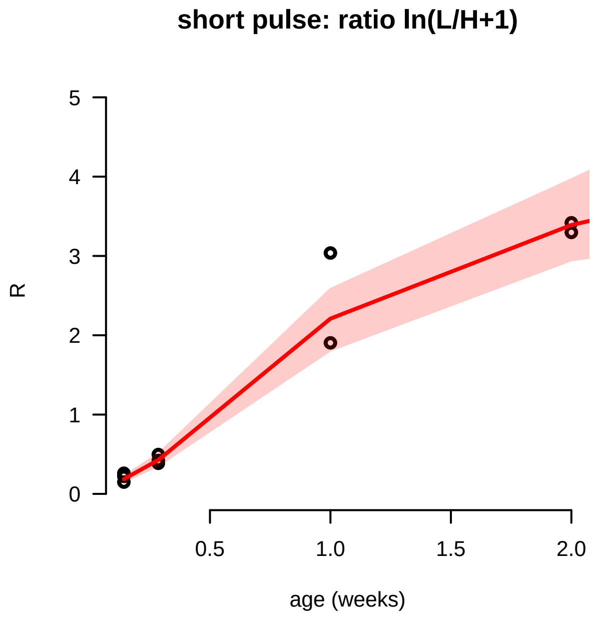

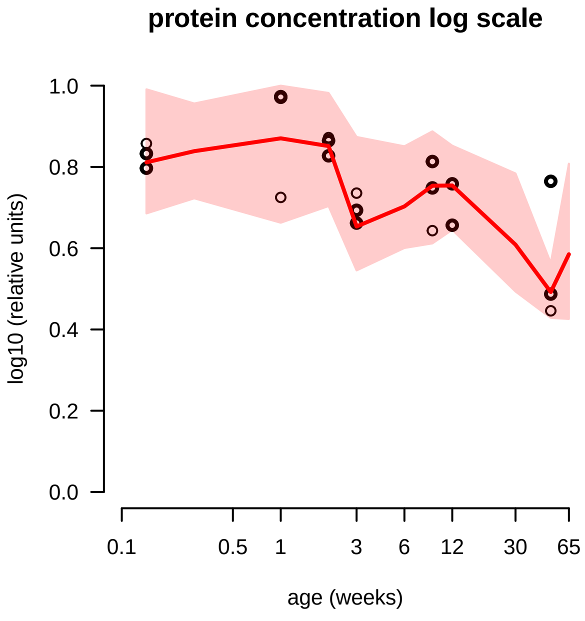

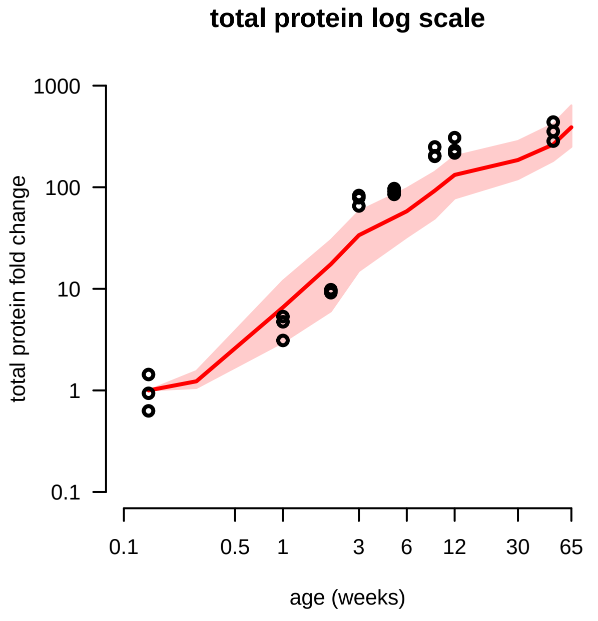

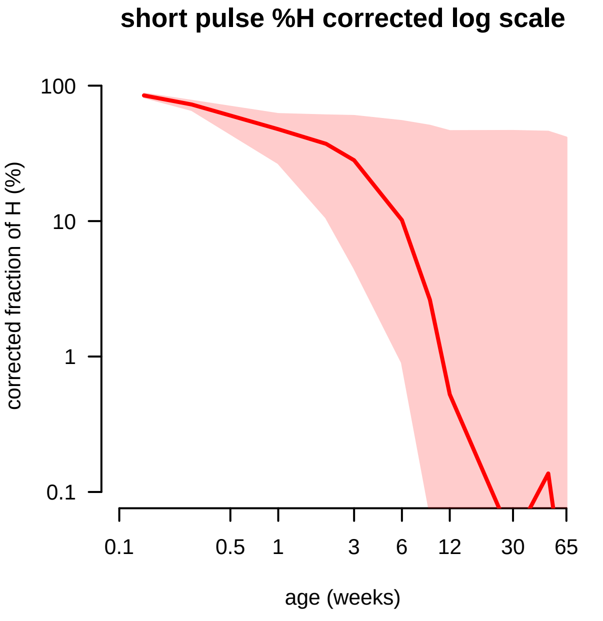

Q8QZV7

>sp|Q8QZV7|INT13_MOUSE

Integrator complex subunit 13

GN=IntS13

Med H1/2: 5.8 days

Q5 H1/2: 2.8 days

Q95 H1/2: 47.4 days

reliable model fit: yes

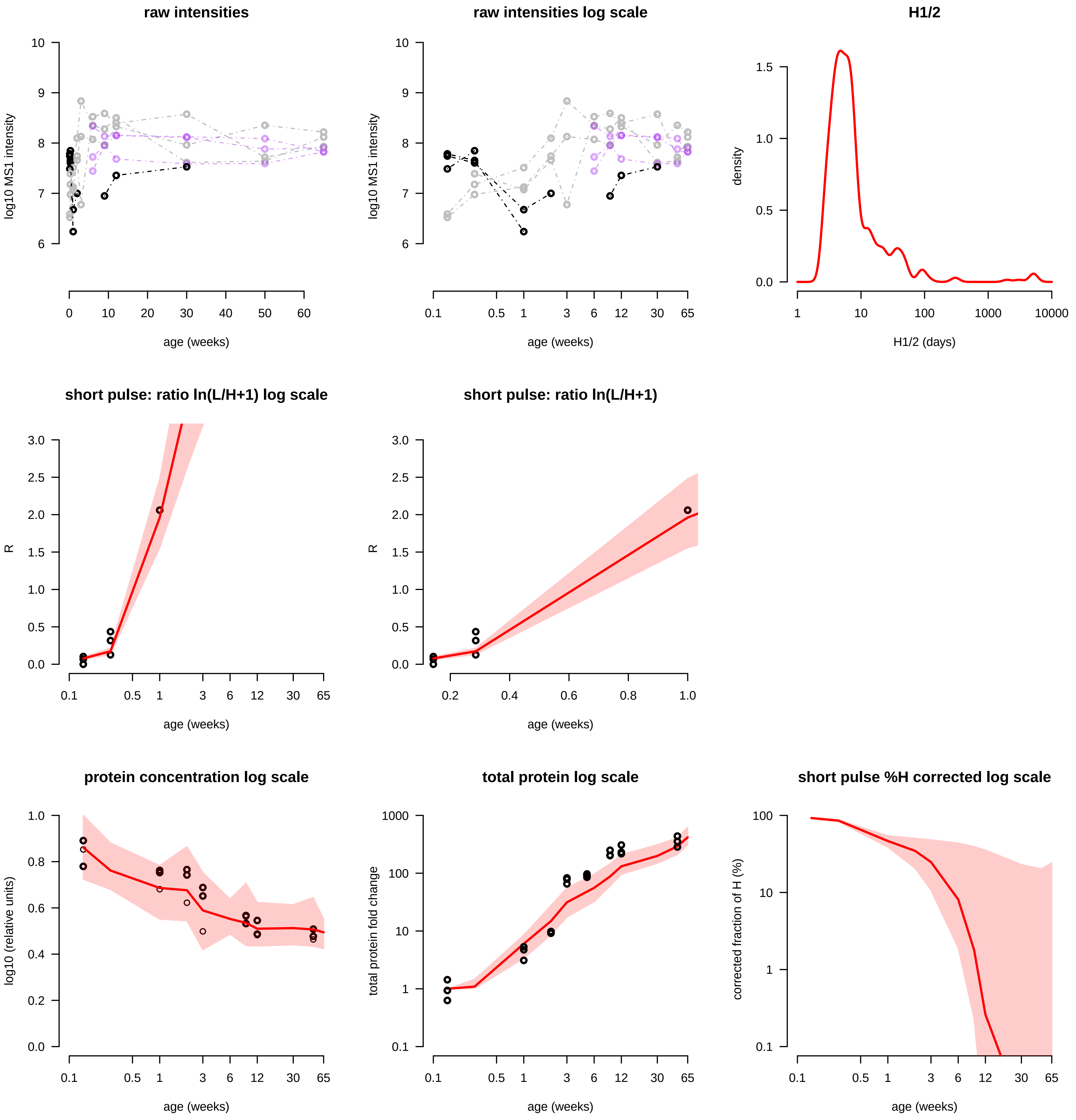

Q8VCY6

>sp|Q8VCY6|UTP6_MOUSE

U3 small nucleolar RNA−associated protein 6 homolog

GN=Utp6

Med H1/2: 2989.4 days

Q5 H1/2: 698.6 days

Q95 H1/2: 30397.6 days

reliable model fit: no

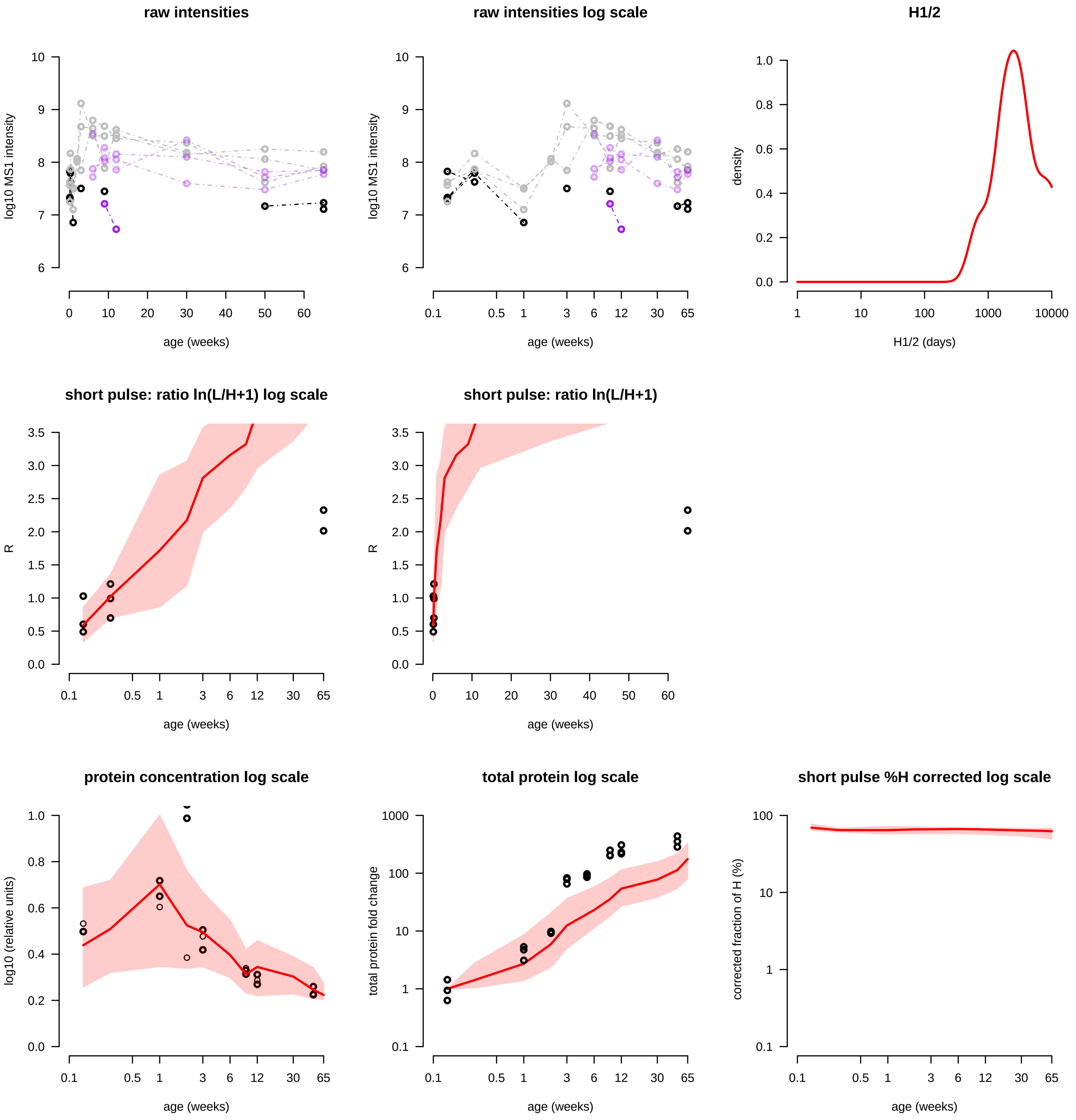

Q62193

>sp|Q62193|RFA2_MOUSE

Replication protein A 32 kDa subunit

GN=Rpa2

Med H1/2: 22.4 days

Q5 H1/2: 7.1 days

Q95 H1/2: 260.4 days

reliable model fit: yes

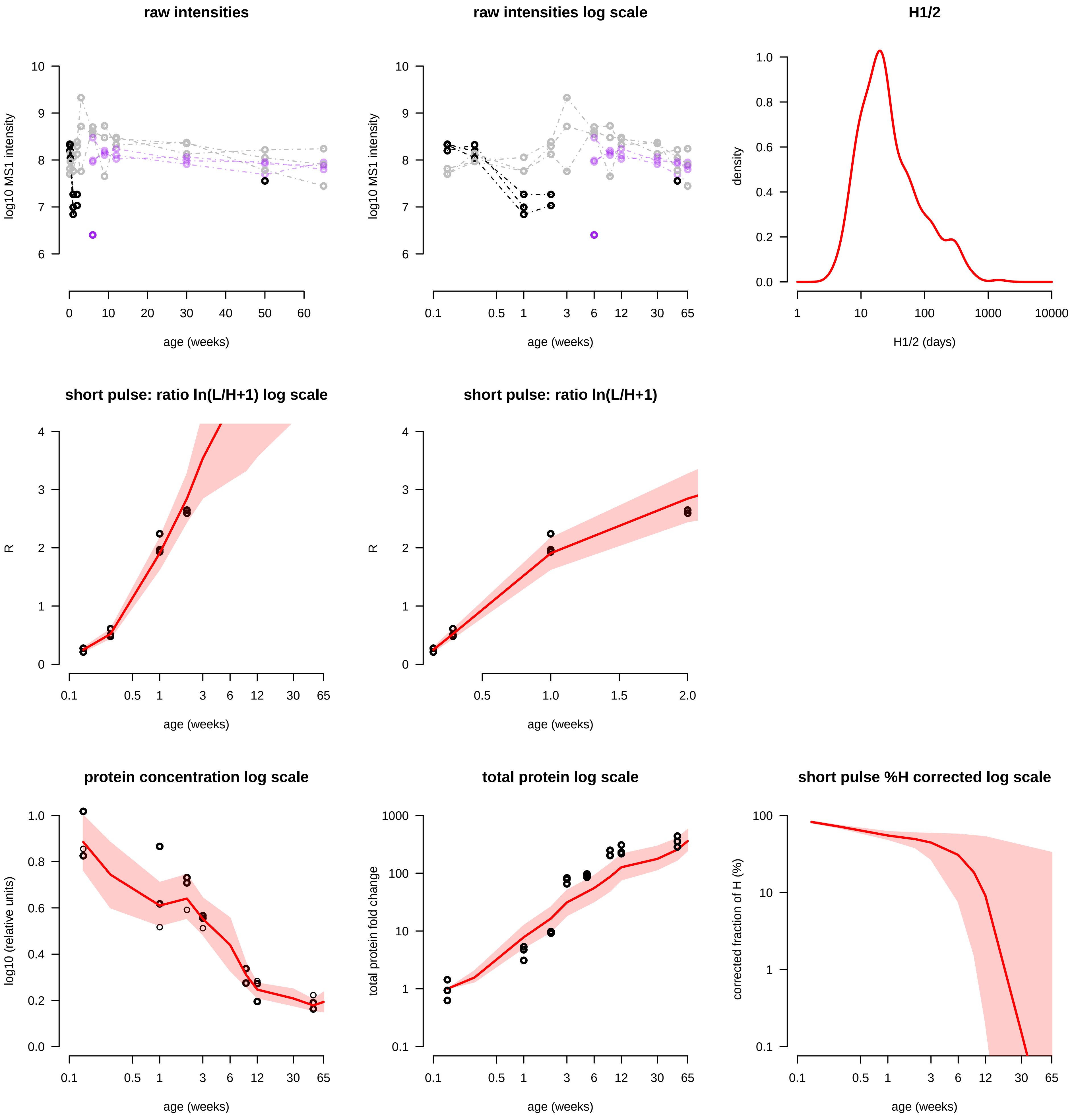

A2A8L5

>sp|A2A8L5|PTPRF_MOUSE

Receptor−type tyrosine−protein phosphatase F

GN=Ptprf

Med H1/2: 3.6 days

Q5 H1/2: 2.5 days

Q95 H1/2: 19.5 days

reliable model fit: yes

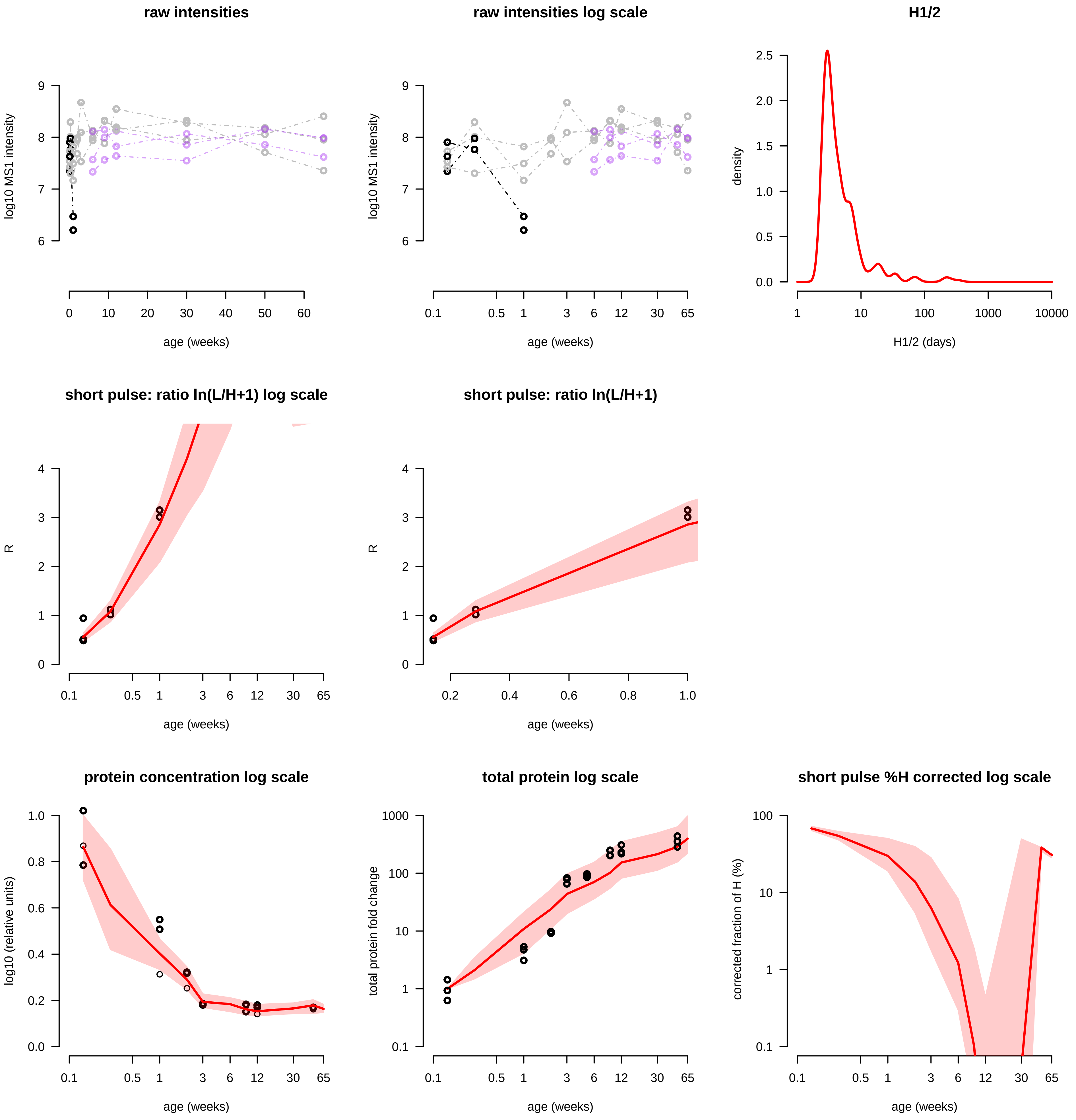

Q8C5N3−2;Q8C5N3

>sp|Q8C5N3−2|CWC22_MOUSE

Isoform 2 of Pre−mRNA−splicing factor CWC22 homolog

GN=Cwc22

Med H1/2: 5.9 days

Q5 H1/2: 4.6 days

Q95 H1/2: 8.6 days

reliable model fit: no

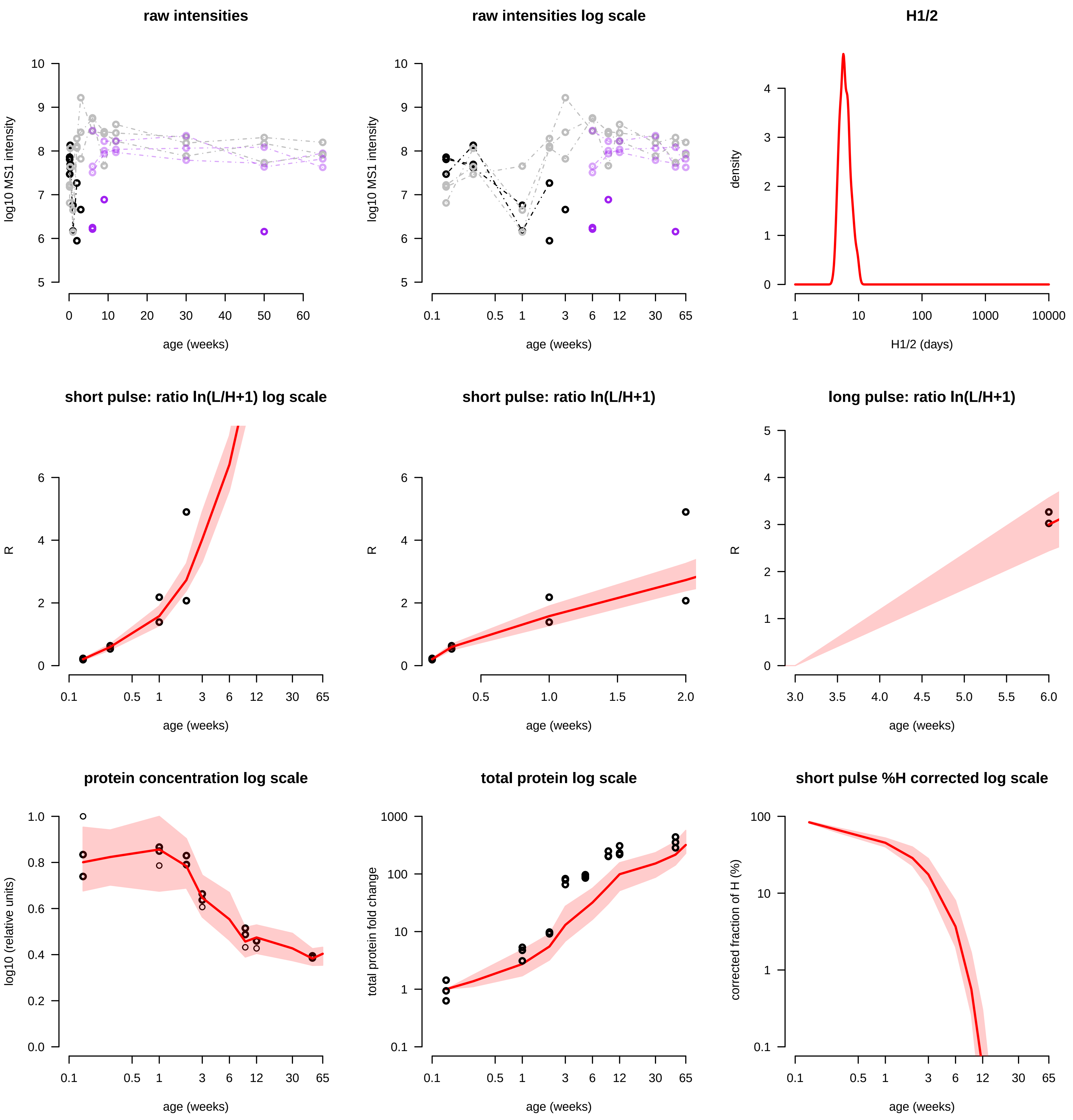

Q9R0M5;Q9R0M5−2

>sp|Q9R0M5|TPK1_MOUSE

Thiamin pyrophosphokinase 1

GN=Tpk1

Med H1/2: 7.4 days

Q5 H1/2: 2.9 days

Q95 H1/2: 99.8 days

reliable model fit: yes

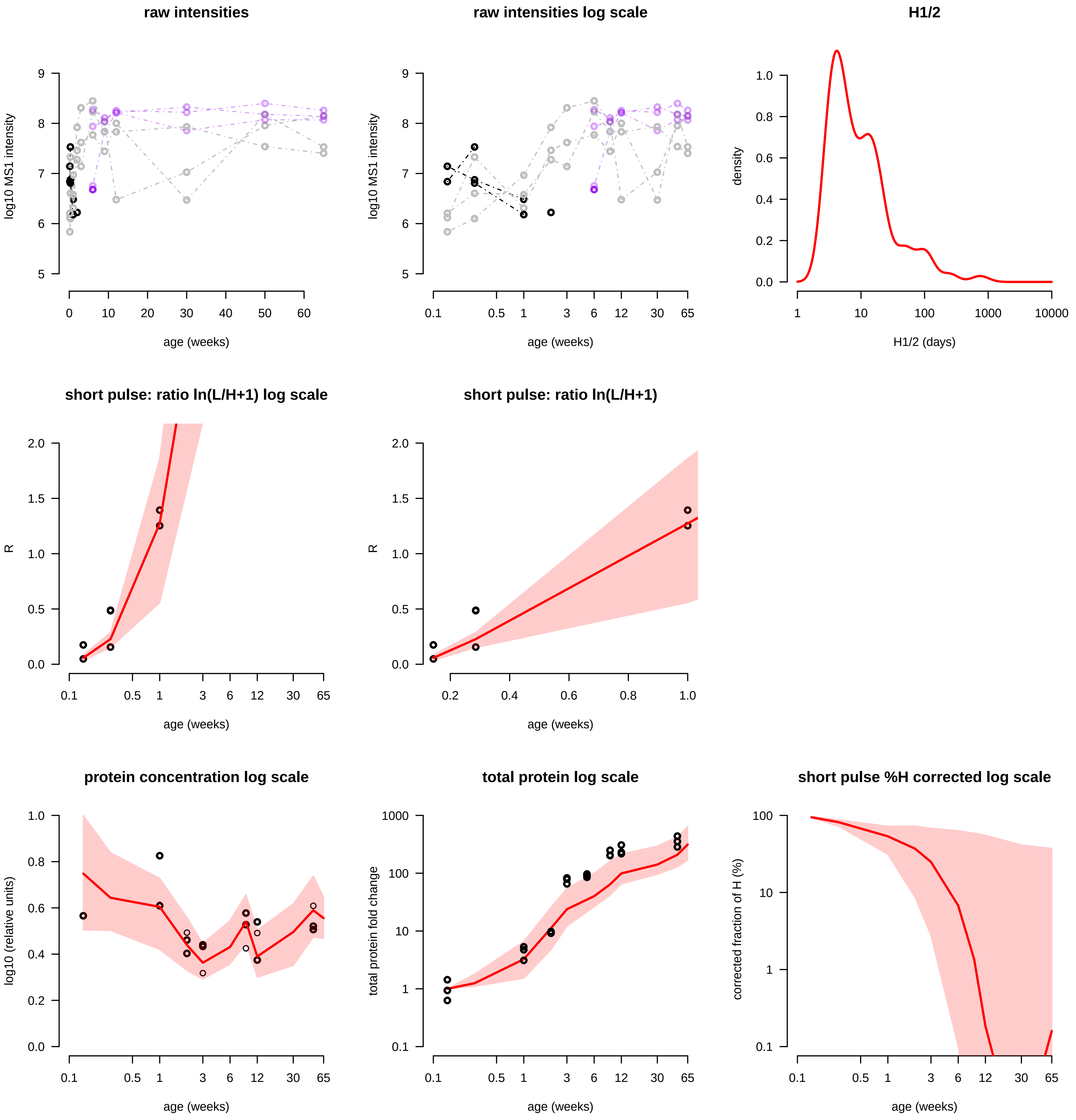

Q8CJ19;Q8CJ19−4

>sp|Q8CJ19|MICA3_MOUSE

[F−actin]−monooxygenase MICAL3

GN=Mical3

Med H1/2: 12.4 days

Q5 H1/2: 4.4 days

Q95 H1/2: 132.1 days

reliable model fit: yes

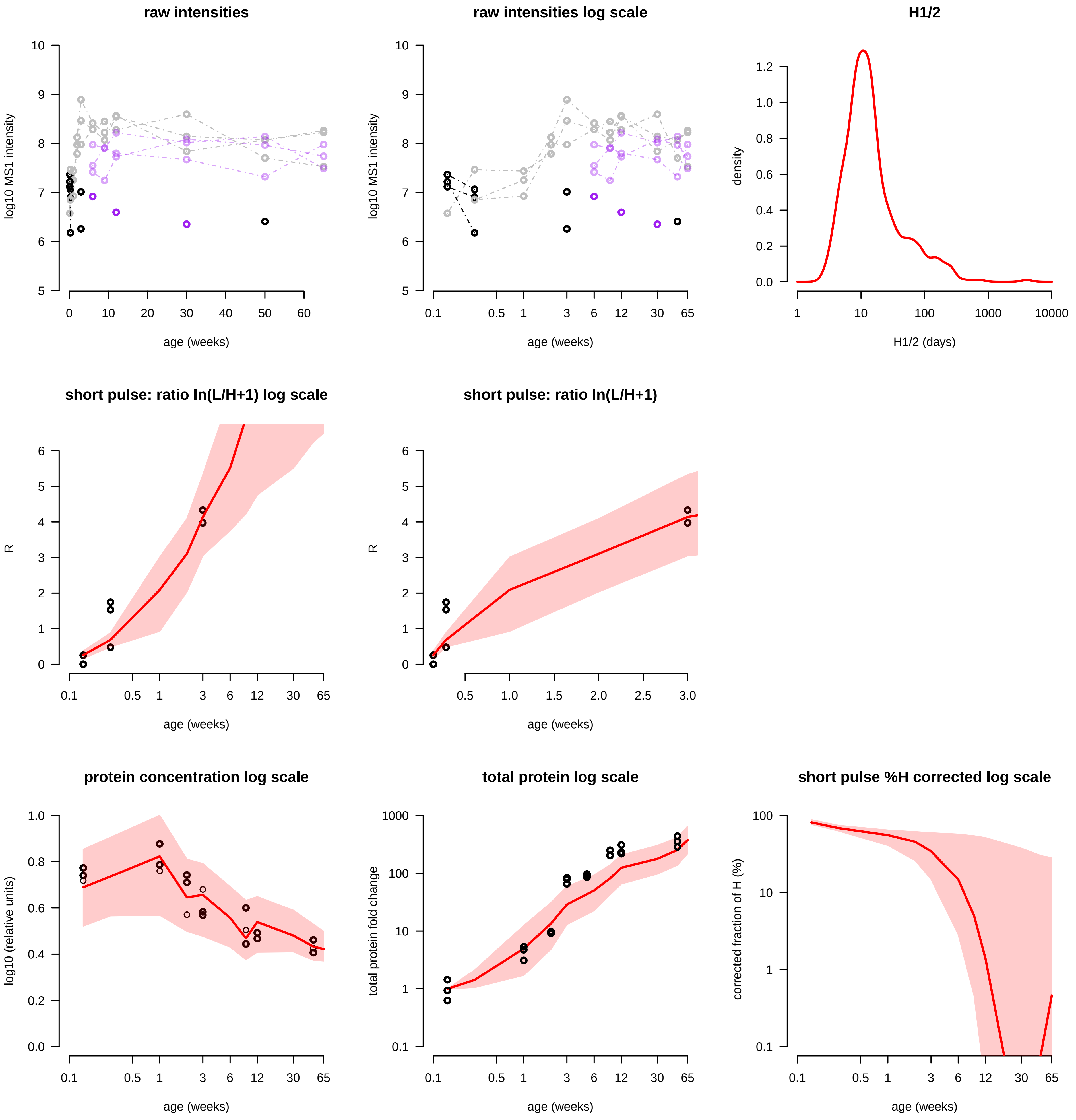

Q8R1F5

>sp|Q8R1F5|HYI_MOUSE

Putative hydroxypyruvate isomerase

GN=Hyi

Med H1/2: 11.1 days

Q5 H1/2: 3.9 days

Q95 H1/2: 168.2 days

reliable model fit: yes

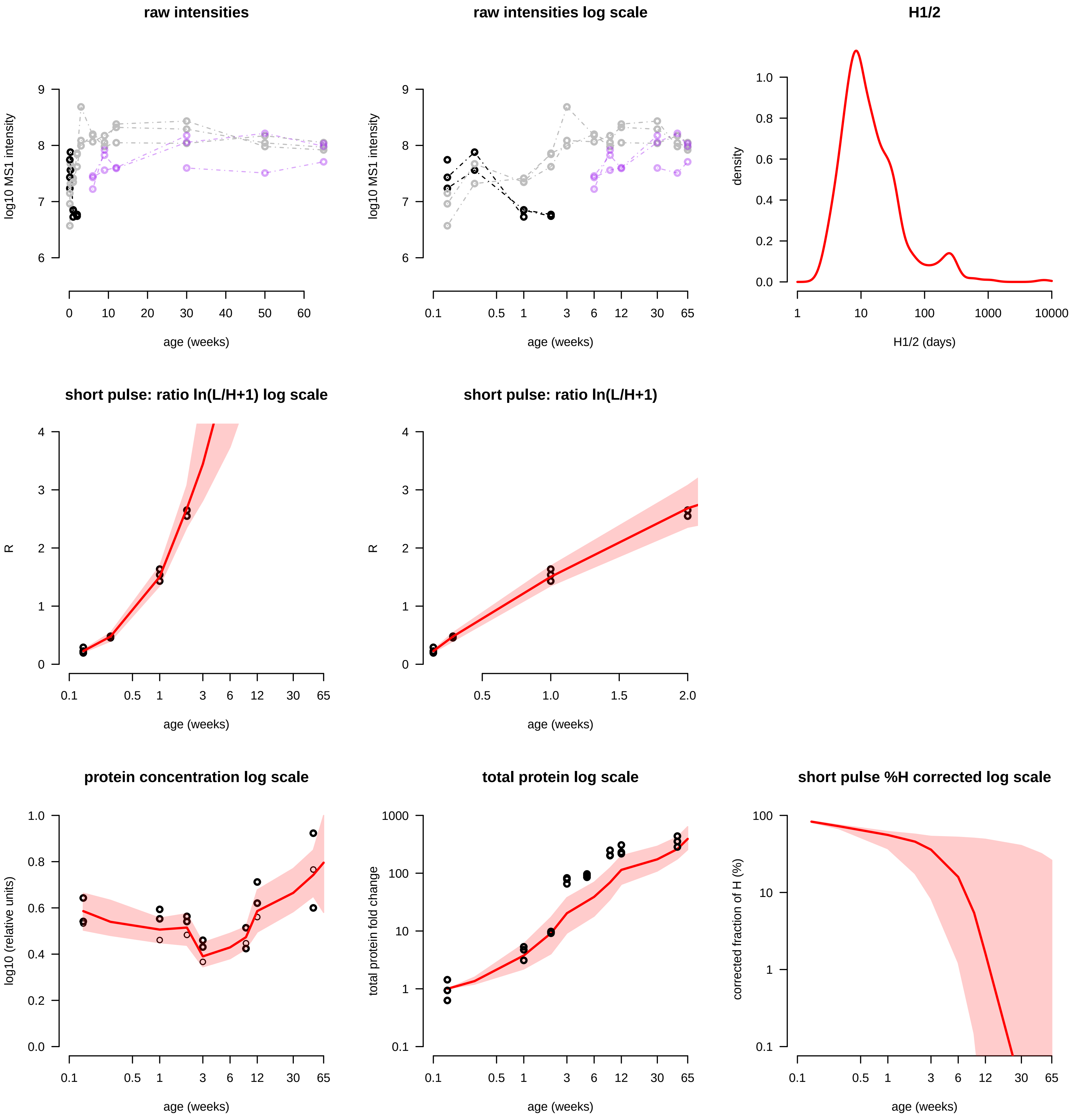

P50285

>sp|P50285|FMO1_MOUSE

Dimethylaniline monooxygenase [N−oxide−forming] 1

GN=Fmo1

Med H1/2: 7.4 days

Q5 H1/2: 2.6 days

Q95 H1/2: 76.1 days

reliable model fit: yes

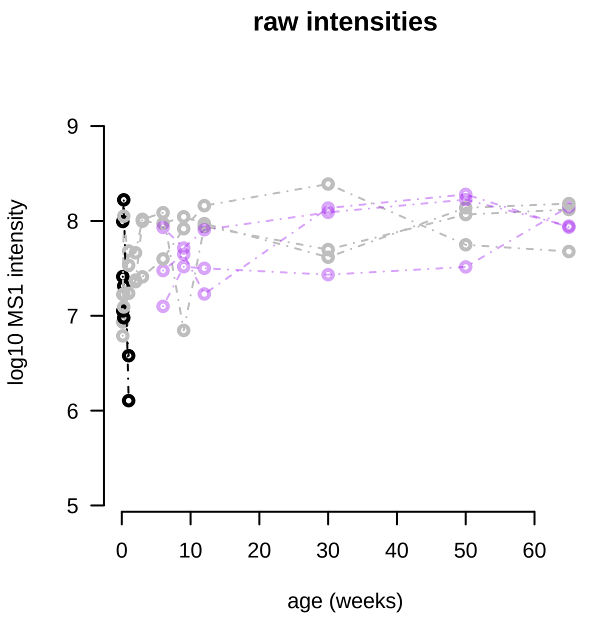

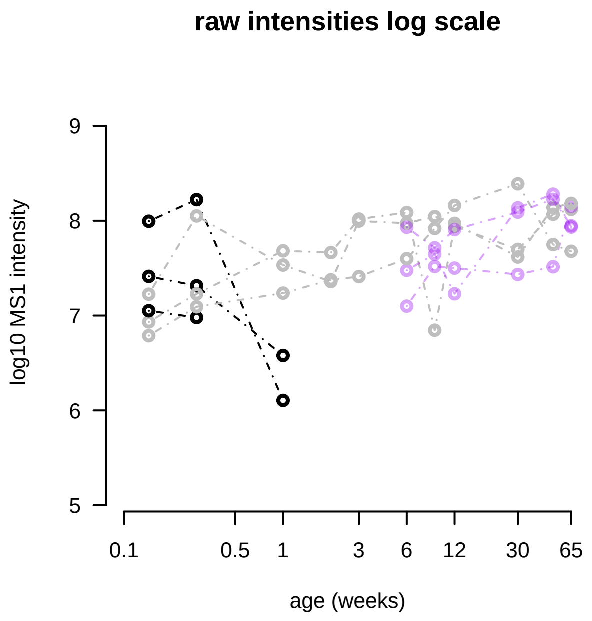

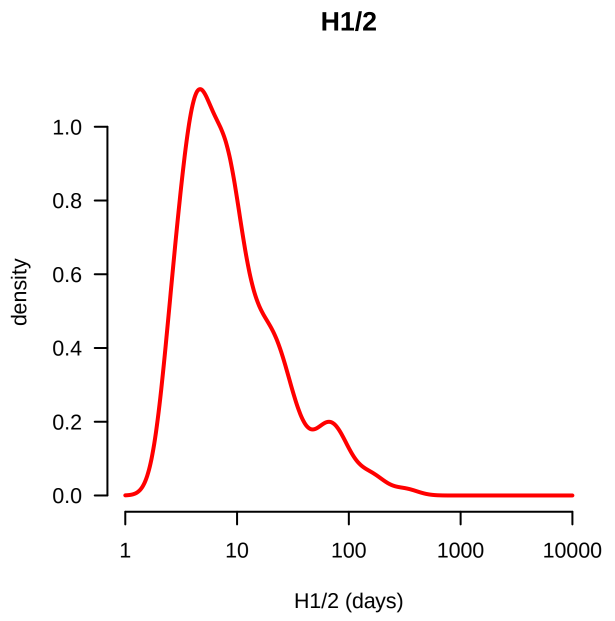

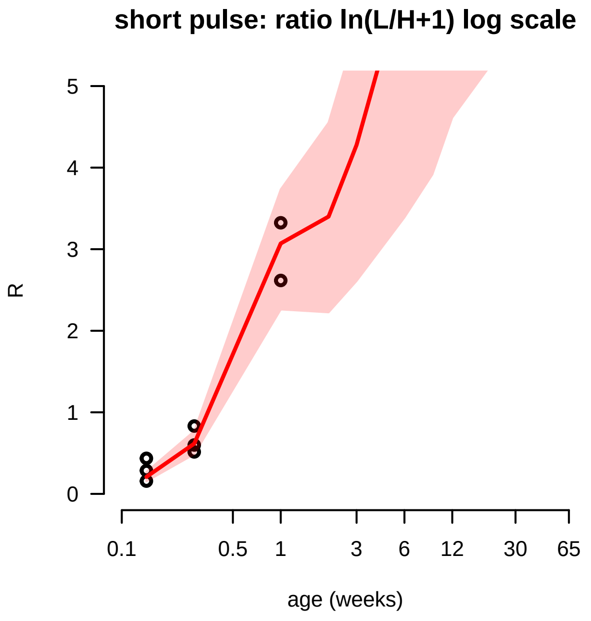

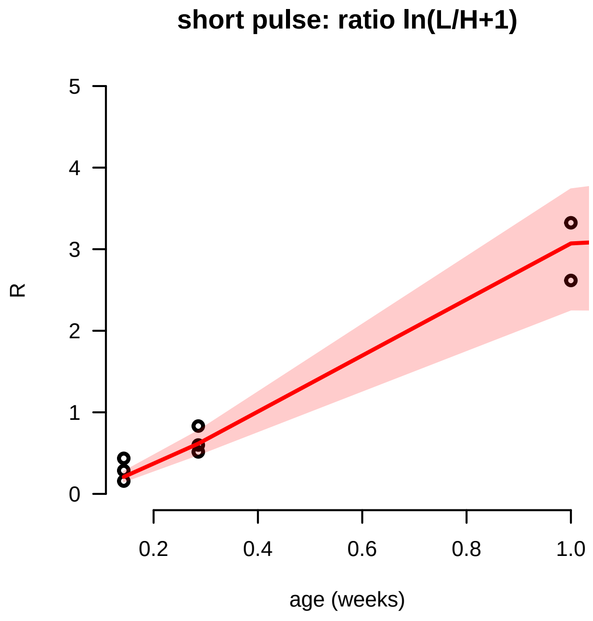

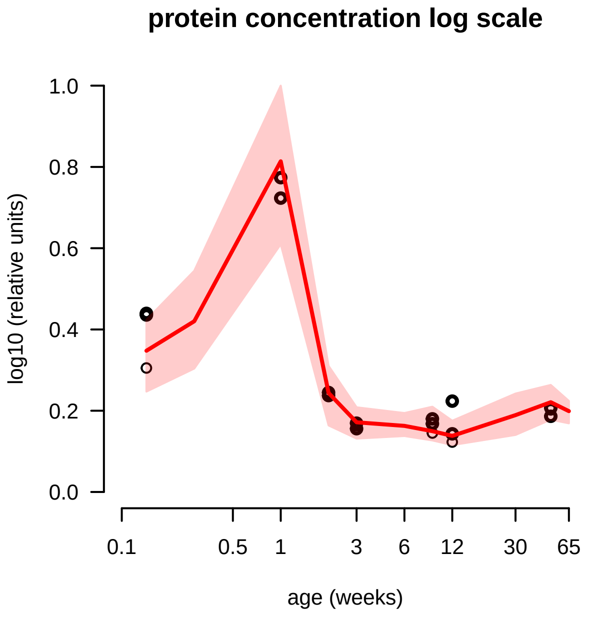

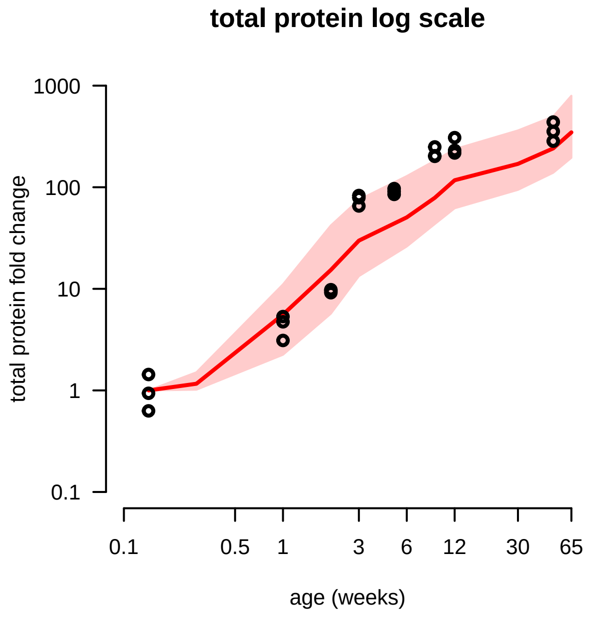

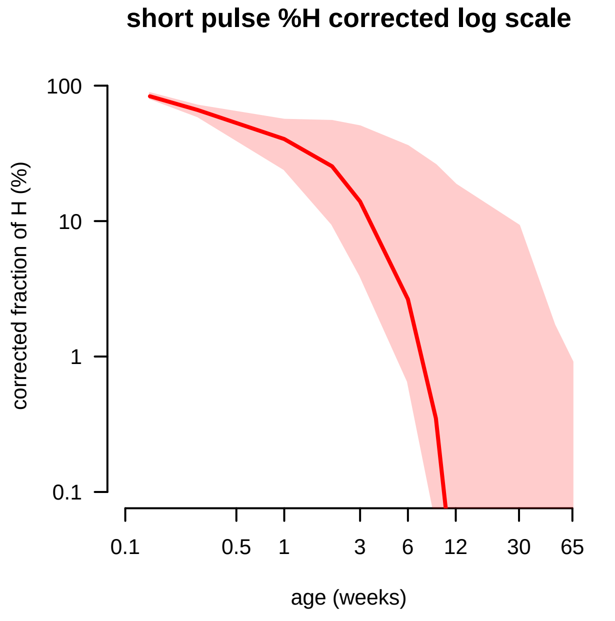

Q6P8M1;Q6P8M1−2

>sp|Q6P8M1|TATD1_MOUSE

Putative deoxyribonuclease TATDN1

GN=Tatdn1

Med H1/2: 8.6 days

Q5 H1/2: 3.6 days

Q95 H1/2: 74.8 days

reliable model fit: yes

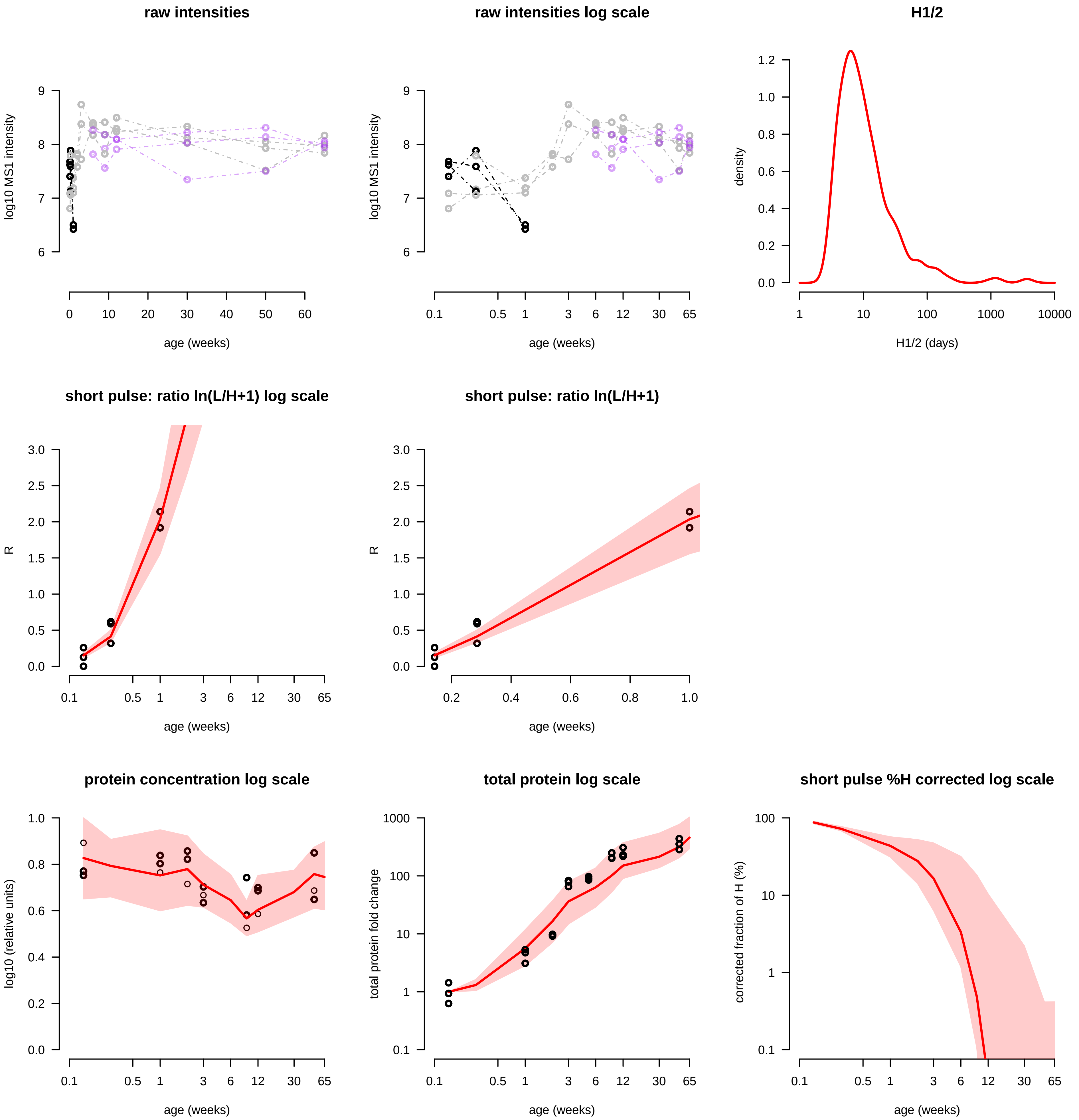

Q8C3P7;Q8C3P7−2

>sp|Q8C3P7|MTA70_MOUSE

N6−adenosine−methyltransferase subunit METTL3

GN=Mettl3

Med H1/2: 1873.7 days

Q5 H1/2: 723.5 days

Q95 H1/2: 14681.9 days

reliable model fit: no

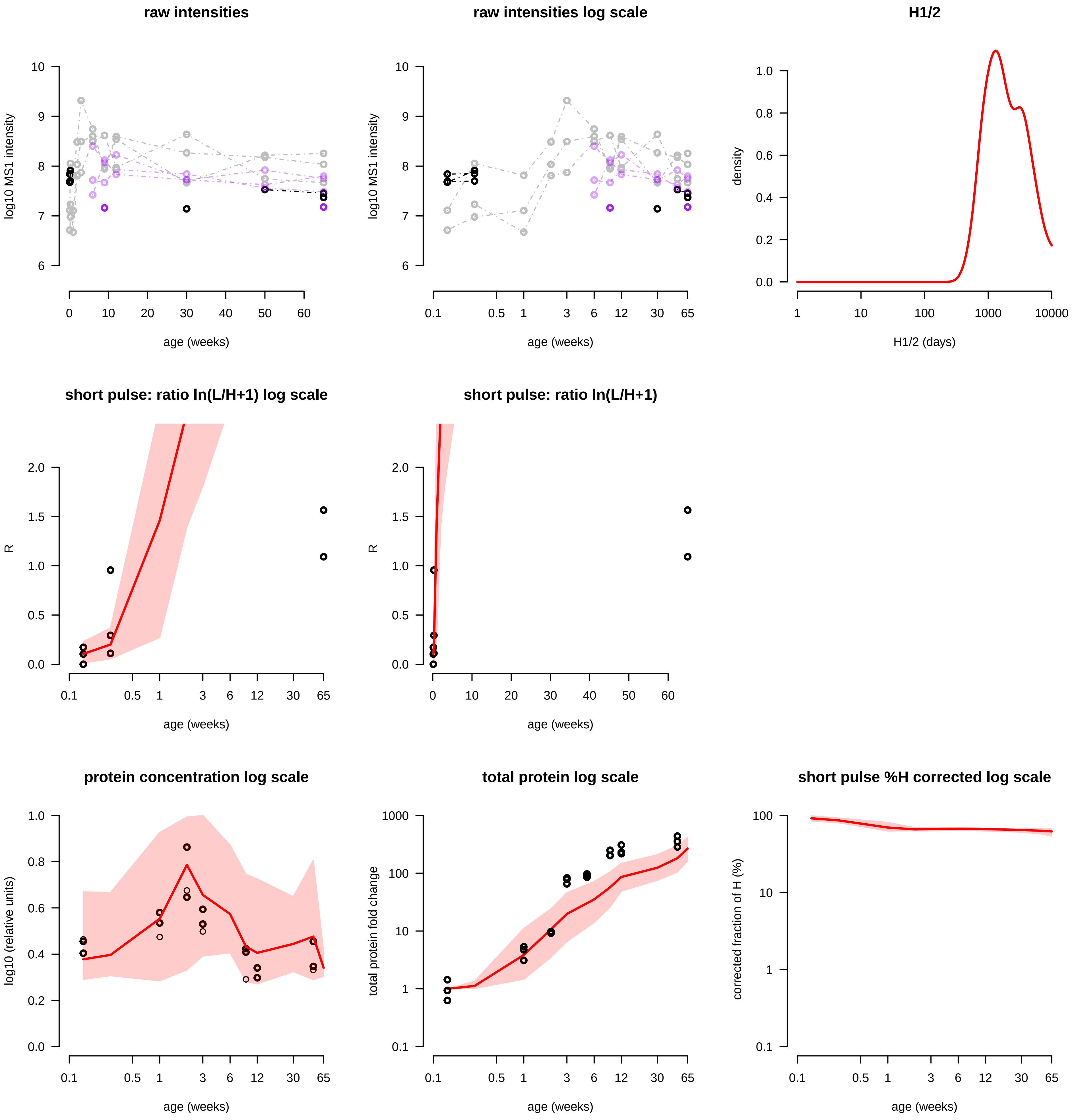

O35099

>sp|O35099|M3K5_MOUSE

Mitogen−activated protein kinase kinase kinase 5

GN=Map3k5

Med H1/2: 7.6 days

Q5 H1/2: 3.2 days

Q95 H1/2: 42.7 days

reliable model fit: yes

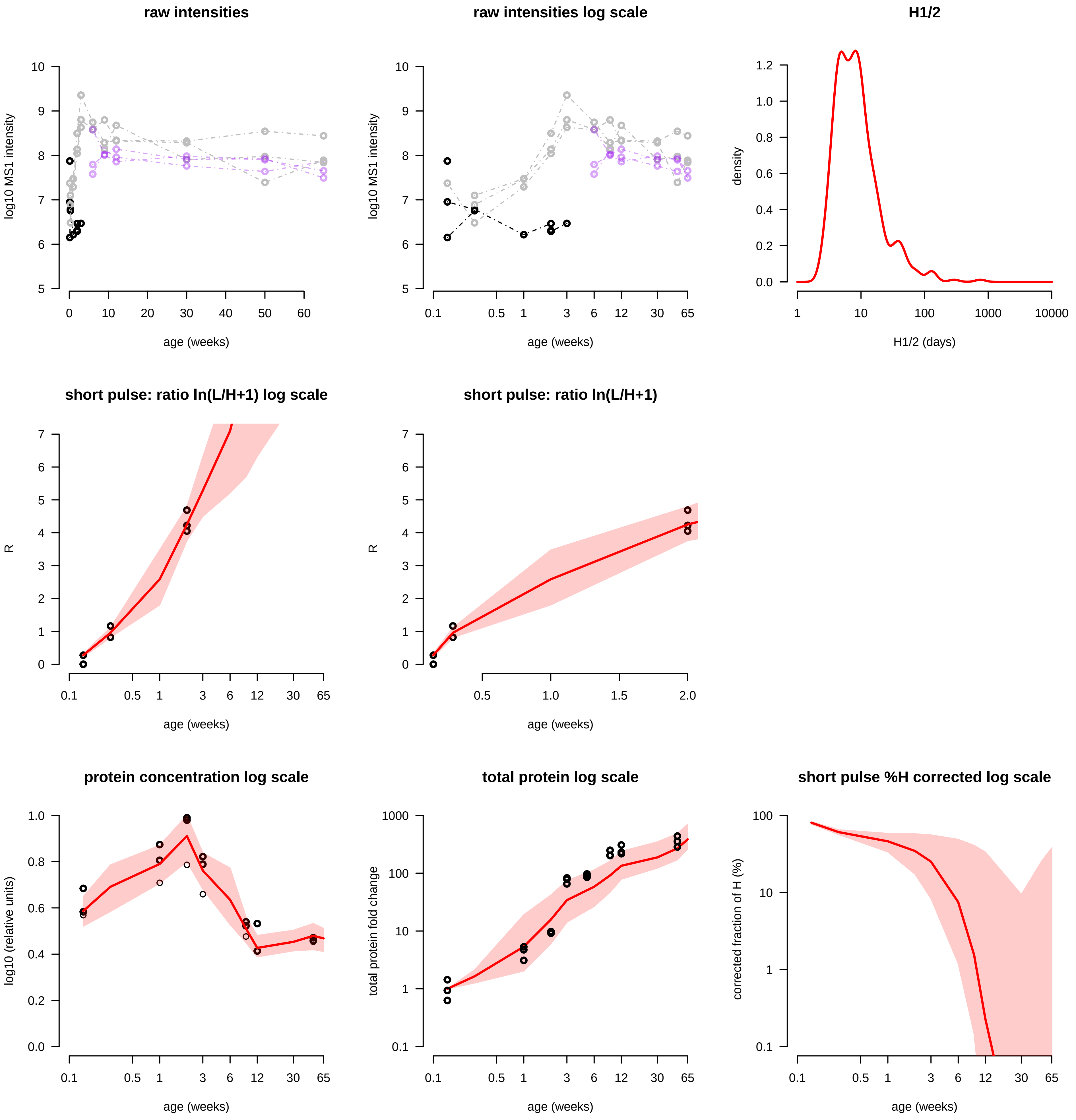

Q8R395

>sp|Q8R395|COMD5_MOUSE

COMM domain−containing protein 5

GN=Commd5

Med H1/2: 35 days

Q5 H1/2: 12.4 days

Q95 H1/2: 381.5 days

reliable model fit: yes

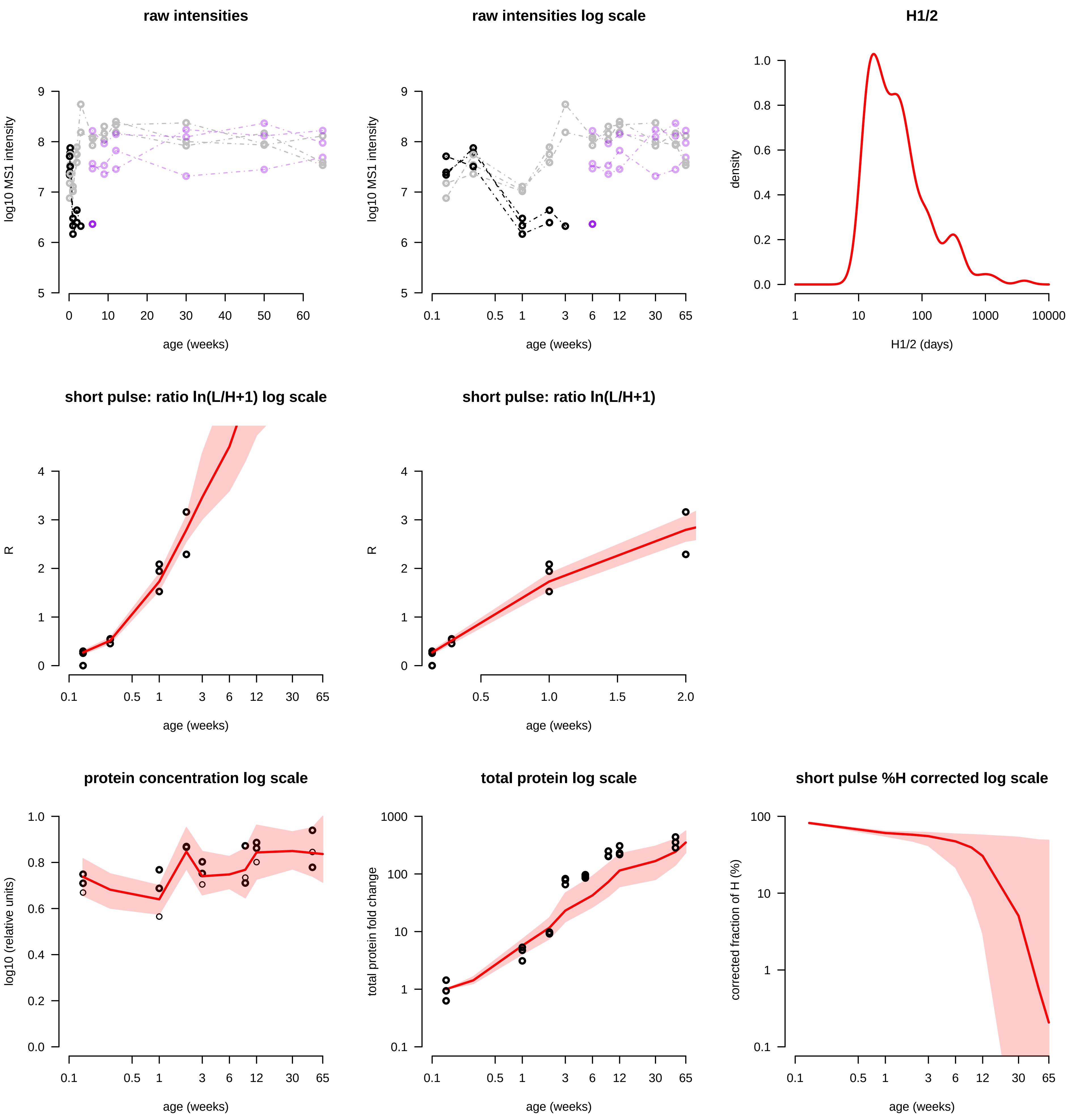

Q8K224

>sp|Q8K224|NAT10_MOUSE

RNA cytidine acetyltransferase

GN=Nat10

Med H1/2: 44 days

Q5 H1/2: 12.2 days

Q95 H1/2: 545.2 days

reliable model fit: no

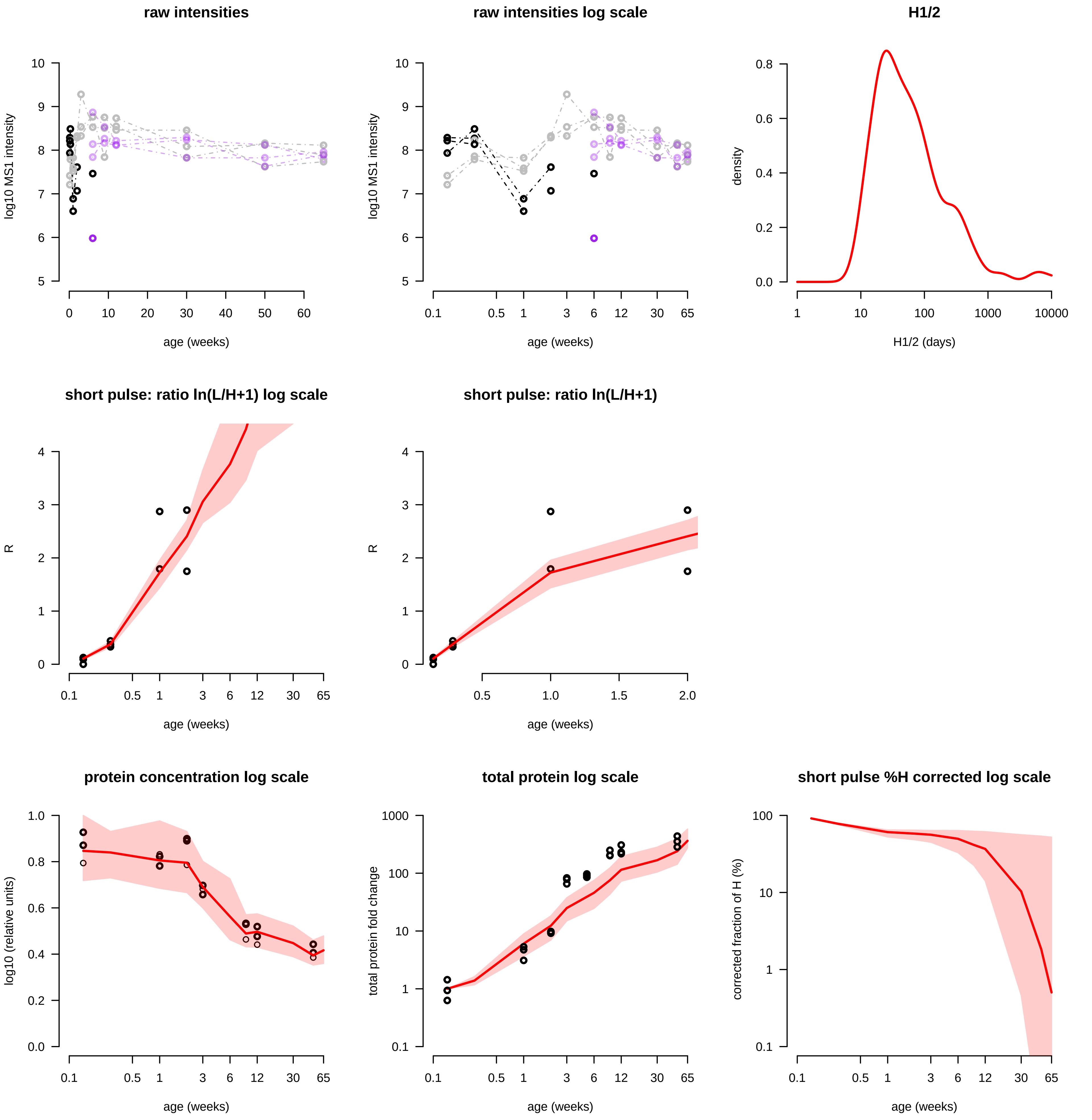

A2AR02

>sp|A2AR02|PPIG_MOUSE

Peptidyl−prolyl cis−trans isomerase G

GN=Ppig

Med H1/2: 8.3 days

Q5 H1/2: 4 days

Q95 H1/2: 90.1 days

reliable model fit: yes

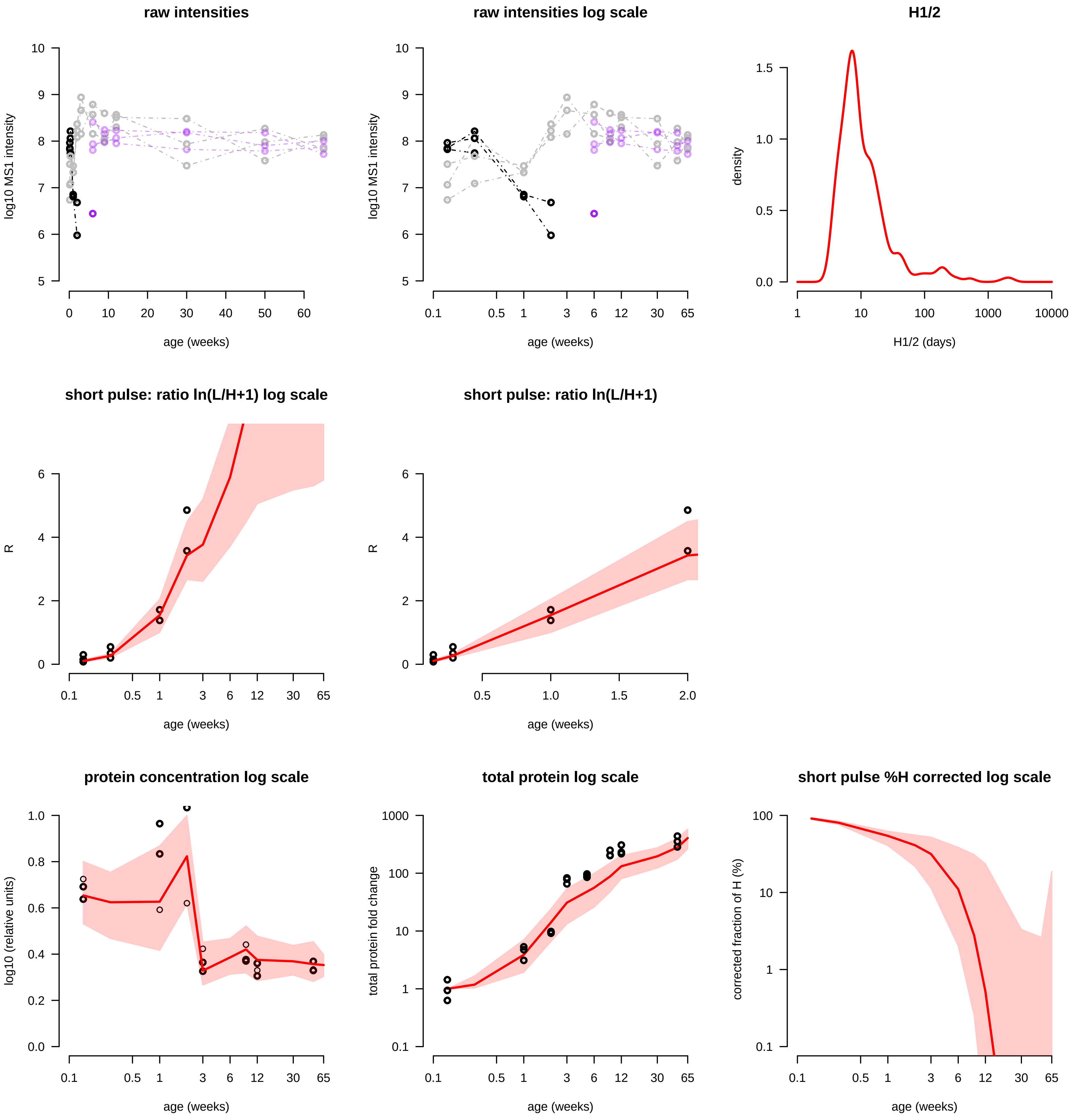

Q91XV3

>sp|Q91XV3|BASP1_MOUSE

Brain acid soluble protein 1

GN=Basp1

Med H1/2: 3.5 days

Q5 H1/2: 2.5 days

Q95 H1/2: 10.5 days

reliable model fit: no

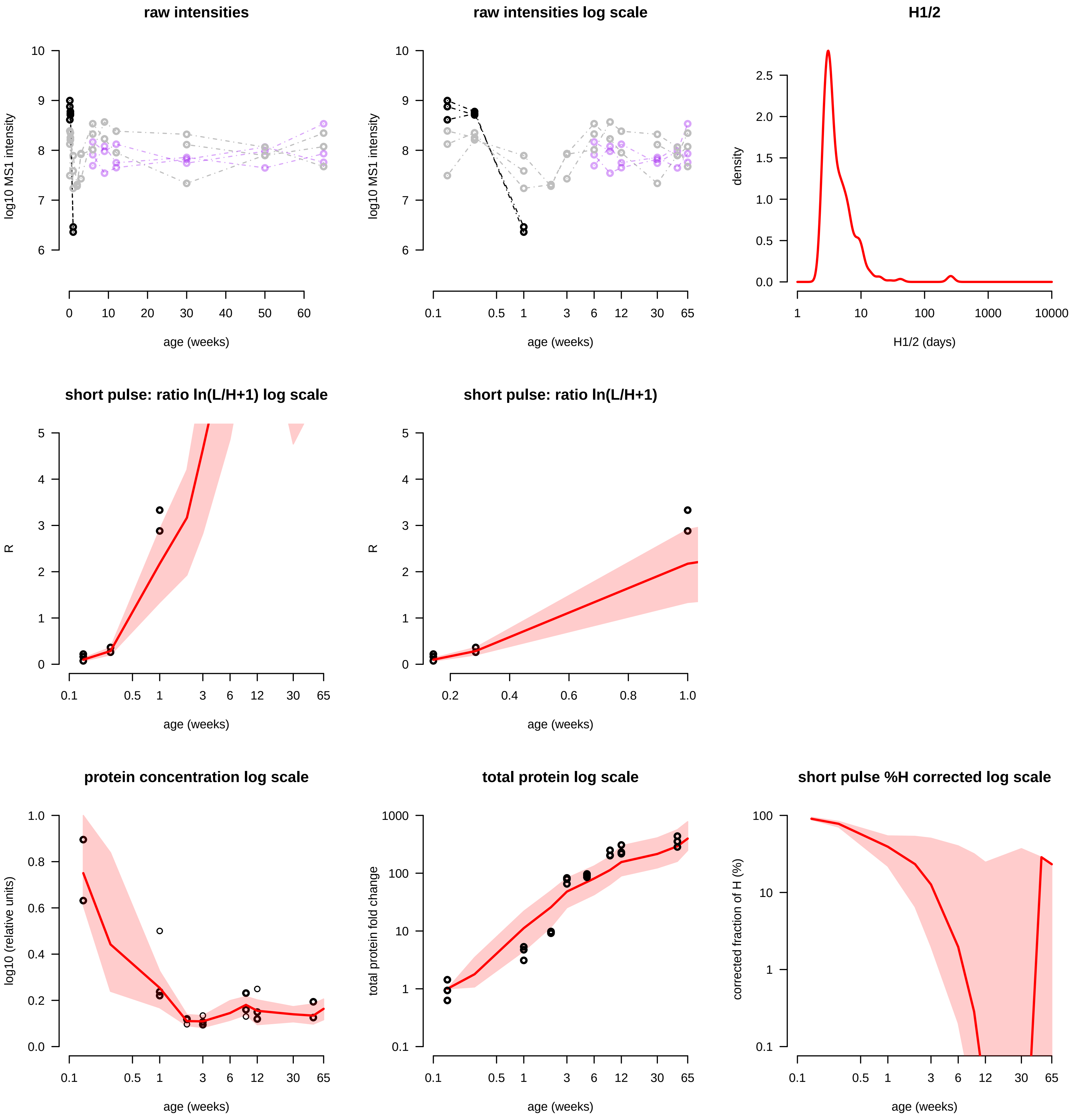

Q922S4−2;Q922S4−3;Q922S4

>sp|Q922S4−2|PDE2A_MOUSE

Isoform PDE2A1 of cGMP−dependent 3',5'−cyclic phosphodiesterase

GN=Pde2a

Med H1/2: 109.6 days

Q5 H1/2: 38 days

Q95 H1/2: 1078.1 days

reliable model fit: no

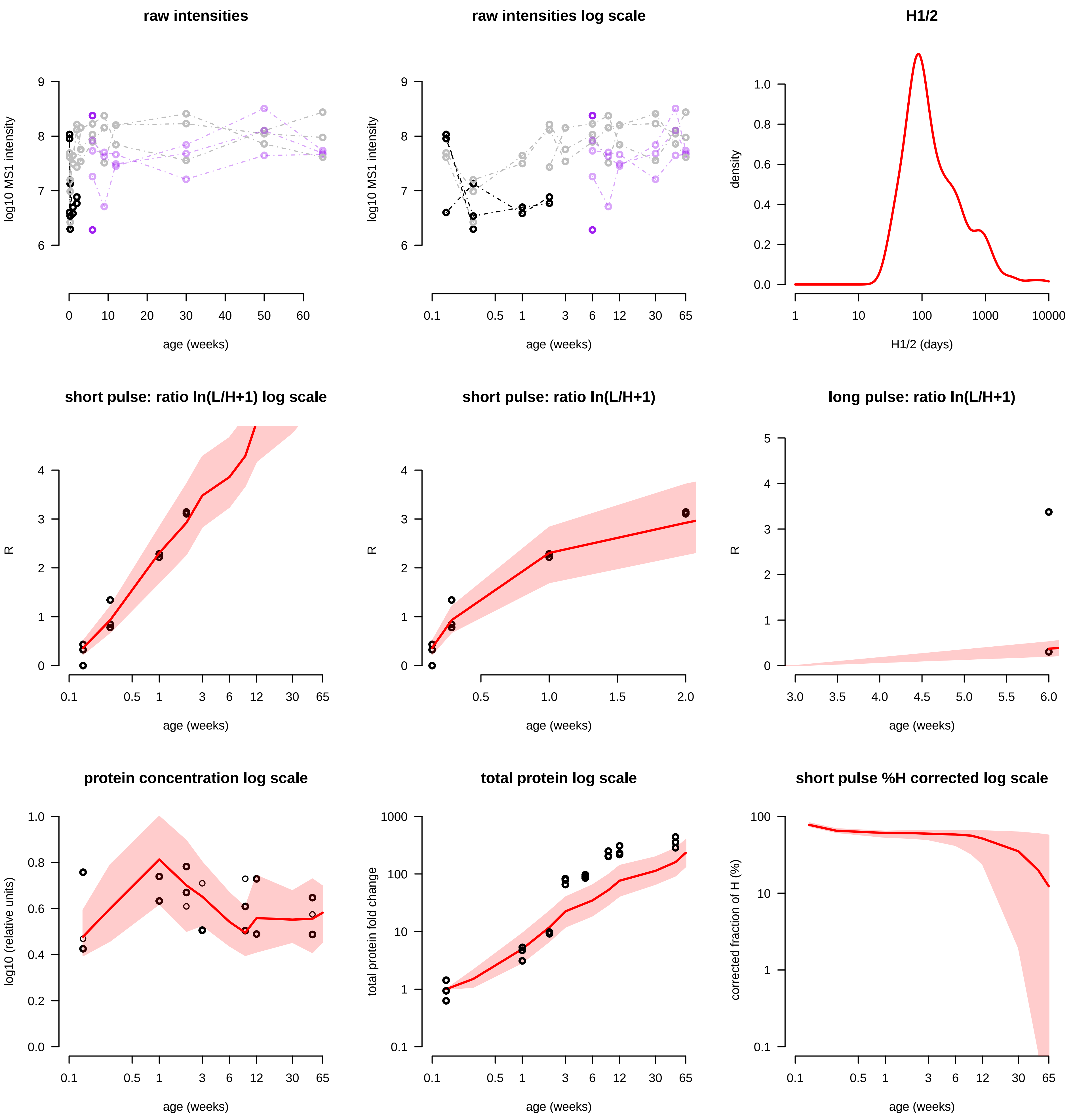

Q9Z2F7

>sp|Q9Z2F7|BNI3L_MOUSE

BCL2/adenovirus E1B 19 kDa protein−interacting protein 3−like

GN=Bnip3l

Med H1/2: 4.4 days

Q5 H1/2: 2.6 days

Q95 H1/2: 12.2 days

reliable model fit: yes

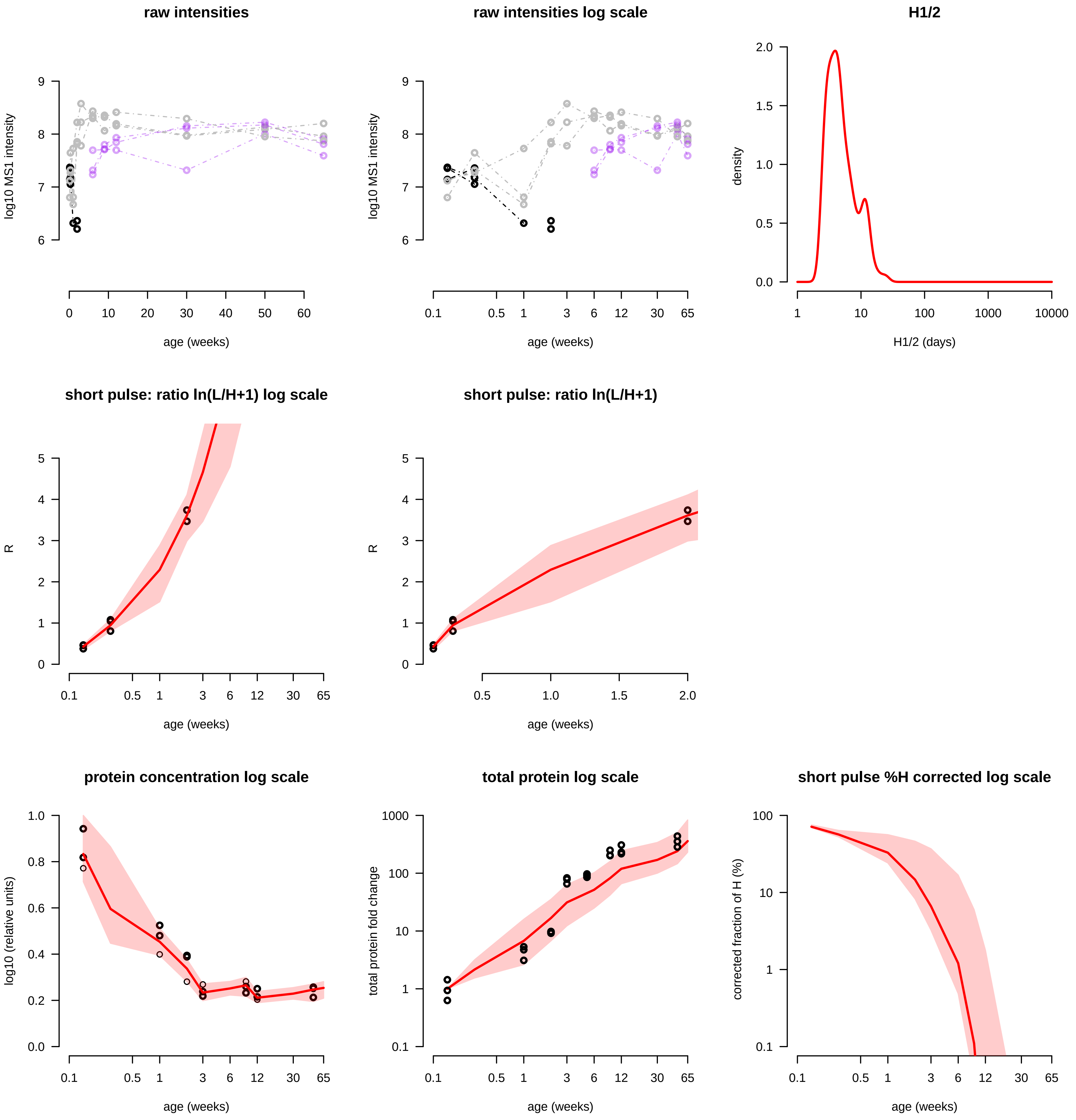

Q6PGH1;Q6PGH1−2

>sp|Q6PGH1|BUD31_MOUSE

Protein BUD31 homolog

GN=Bud31

Med H1/2: 253.9 days

Q5 H1/2: 49.4 days

Q95 H1/2: 2665 days

reliable model fit: no

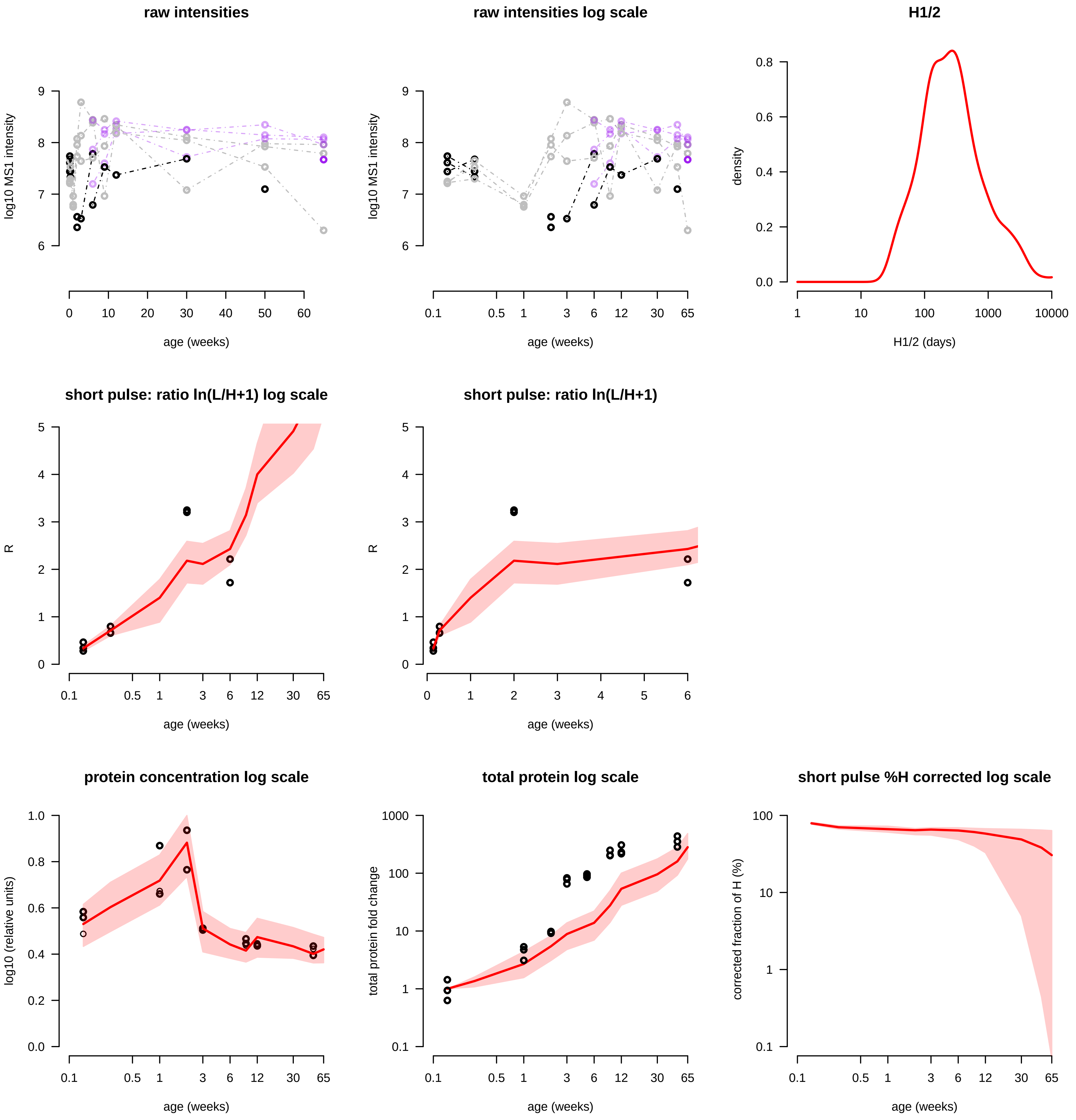

P62876

>sp|P62876|RPAB5_MOUSE

DNA−directed RNA polymerases I, II, and III subunit RPABC5

GN=Polr2l

Med H1/2: 5.1 days

Q5 H1/2: 4.2 days

Q95 H1/2: 6.8 days

reliable model fit: yes

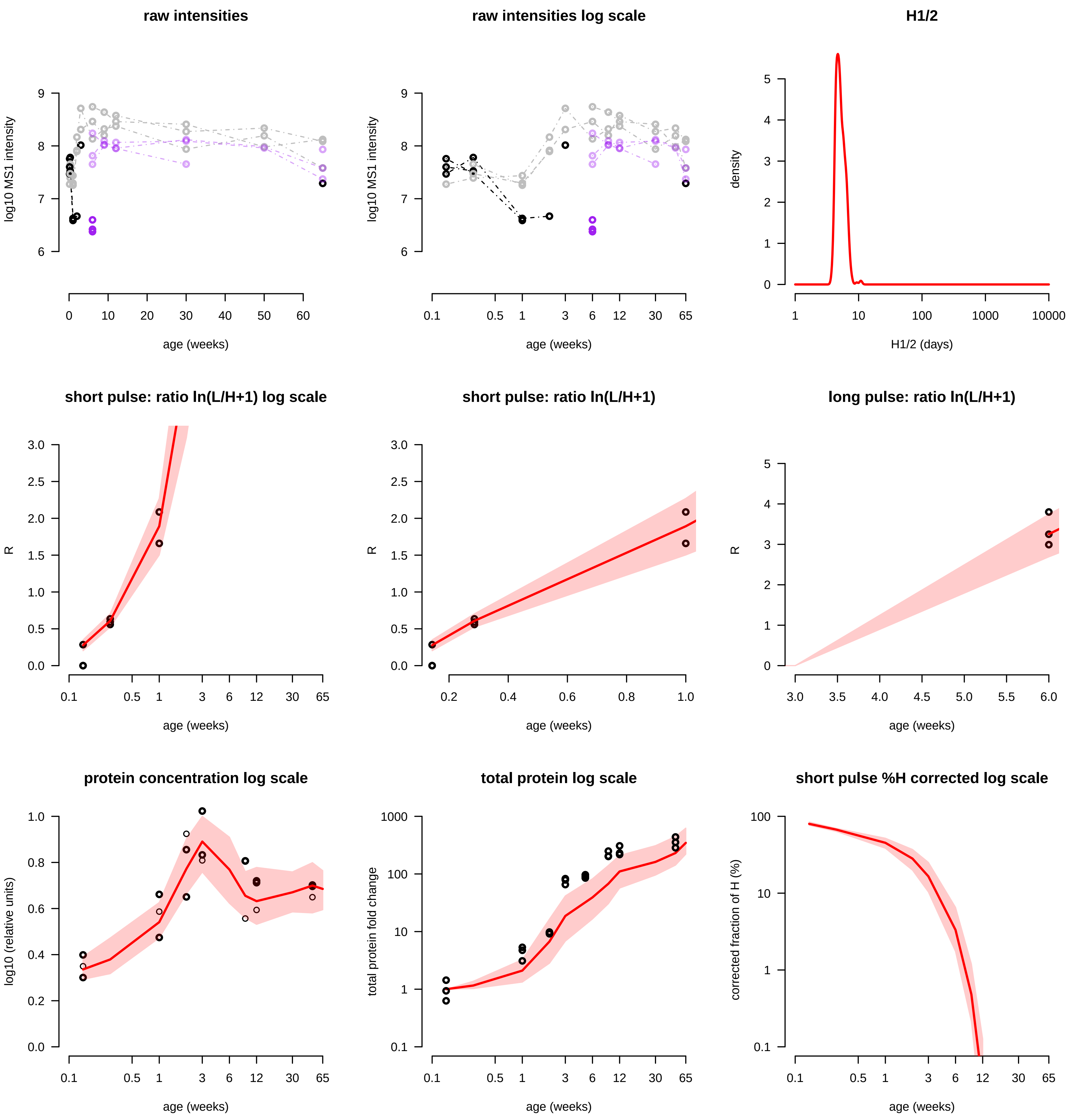

Q9EQG9;Q9EQG9−2;Q9EQG9−3

>sp|Q9EQG9|CERT_MOUSE

Ceramide transfer protein

GN=Cert1

Med H1/2: 5.4 days

Q5 H1/2: 2.8 days

Q95 H1/2: 34.7 days

reliable model fit: yes

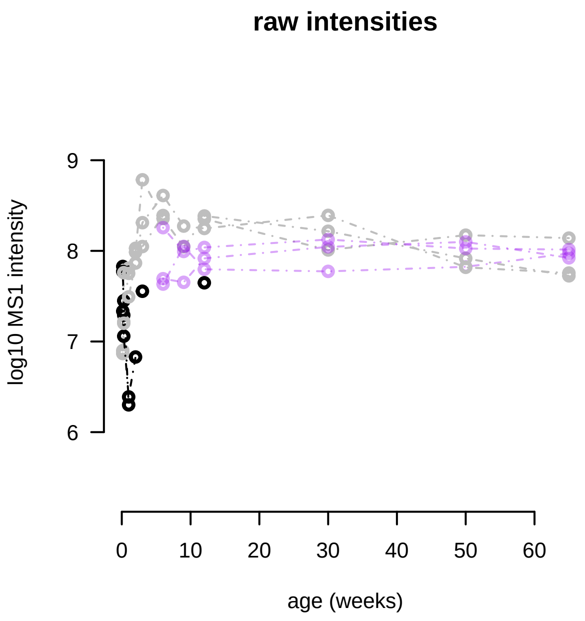

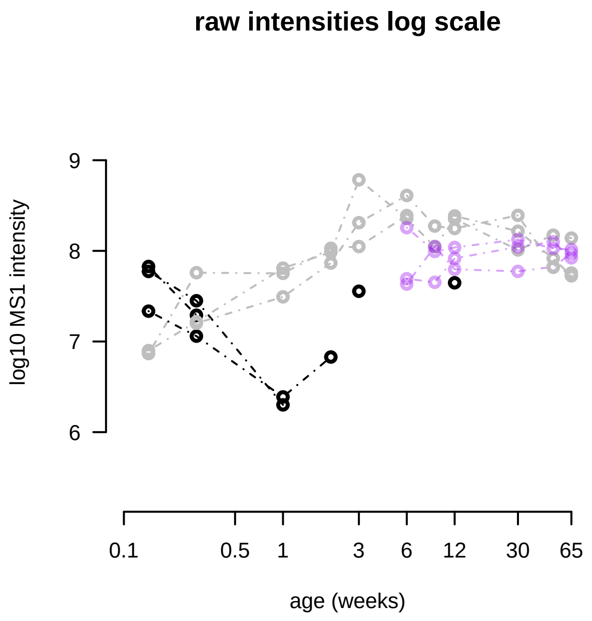

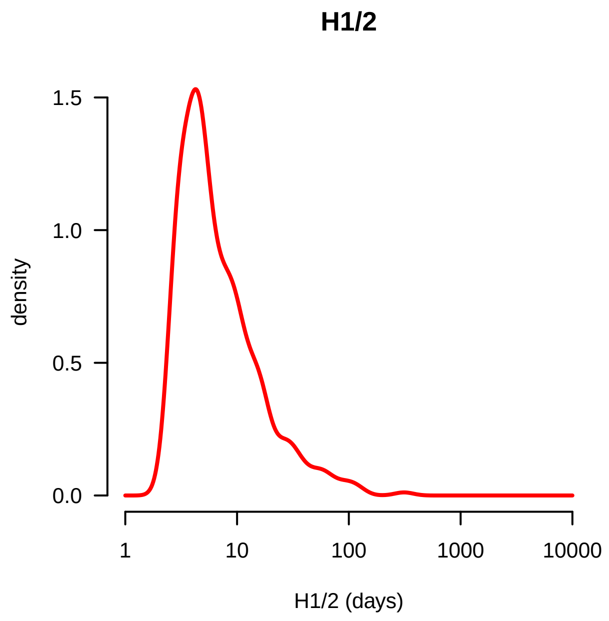

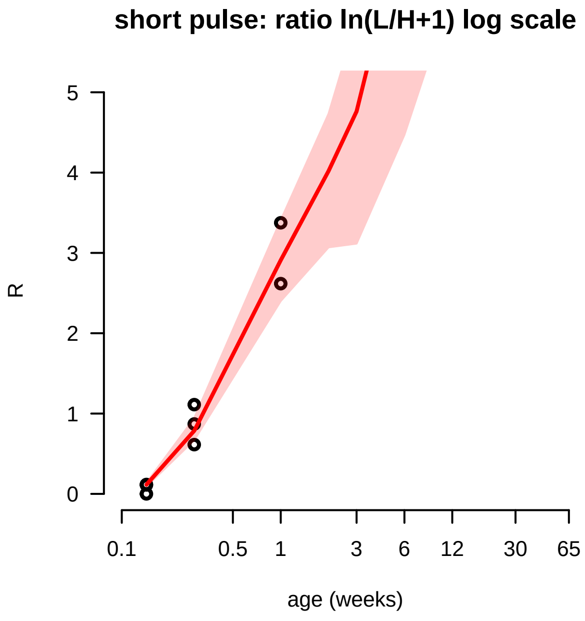

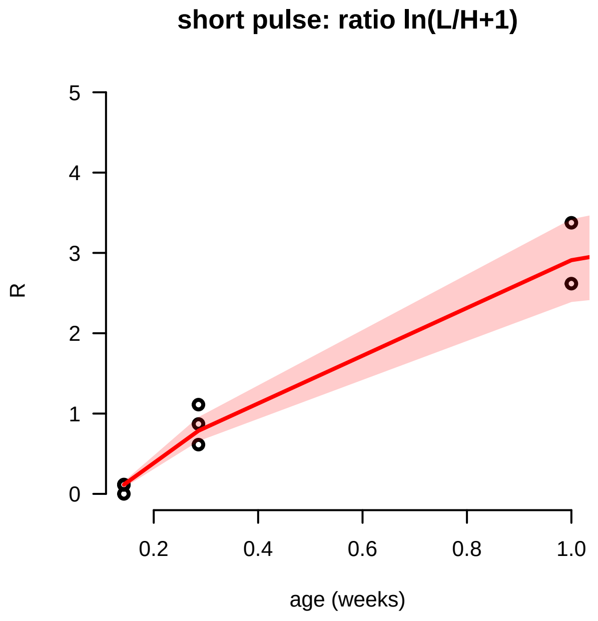

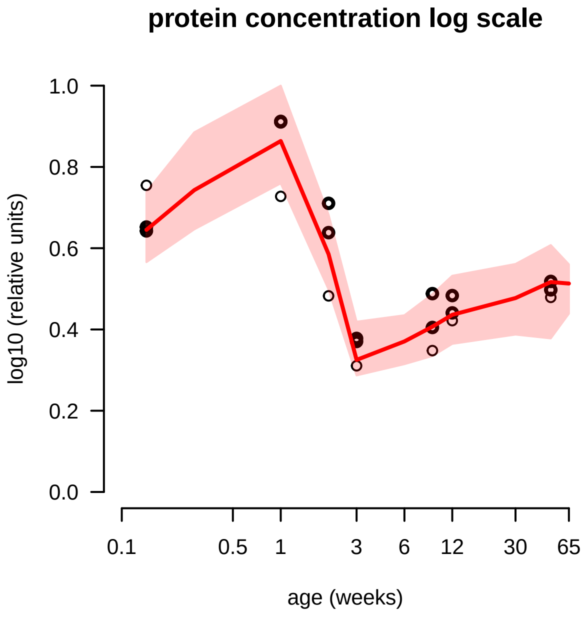

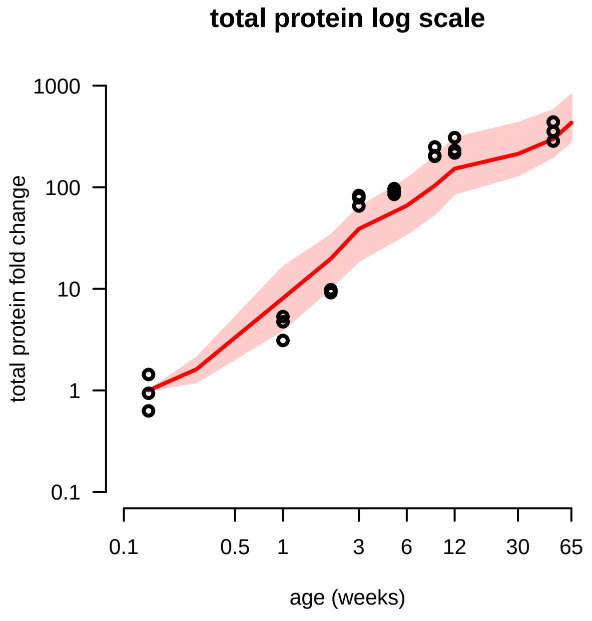

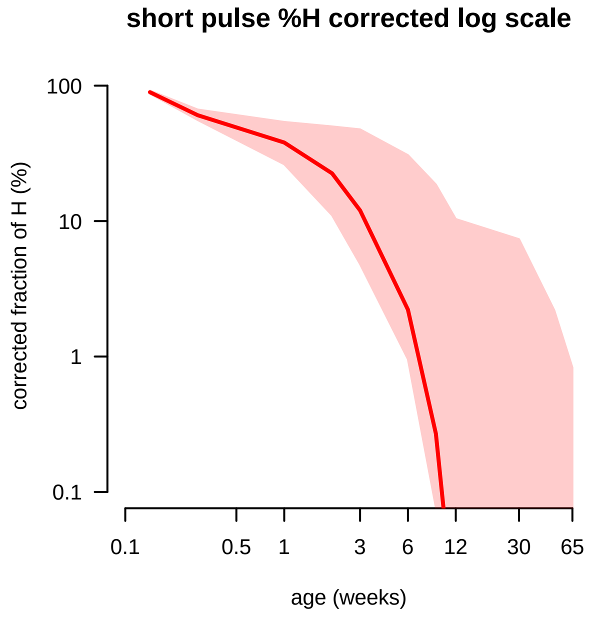

Q9CQ26

>sp|Q9CQ26|STABP_MOUSE

STAM−binding protein

GN=Stambp

Med H1/2: 8.5 days

Q5 H1/2: 4.3 days

Q95 H1/2: 61.1 days

reliable model fit: yes

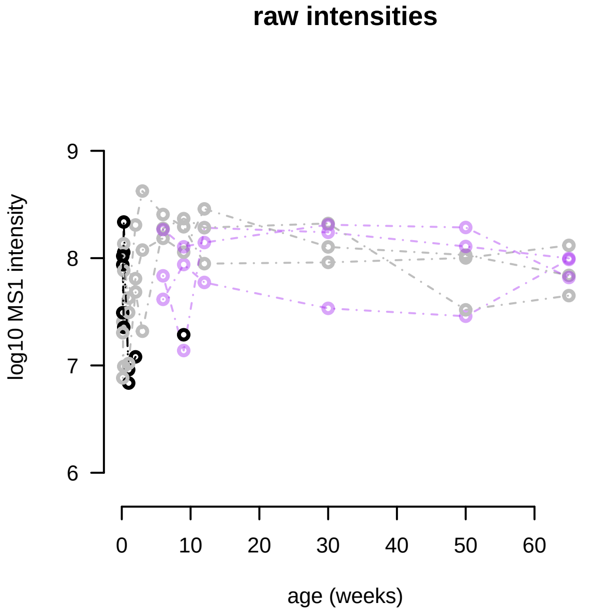

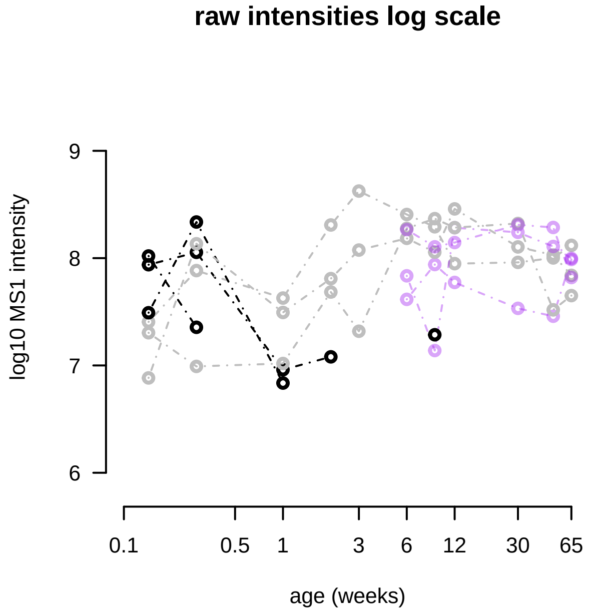

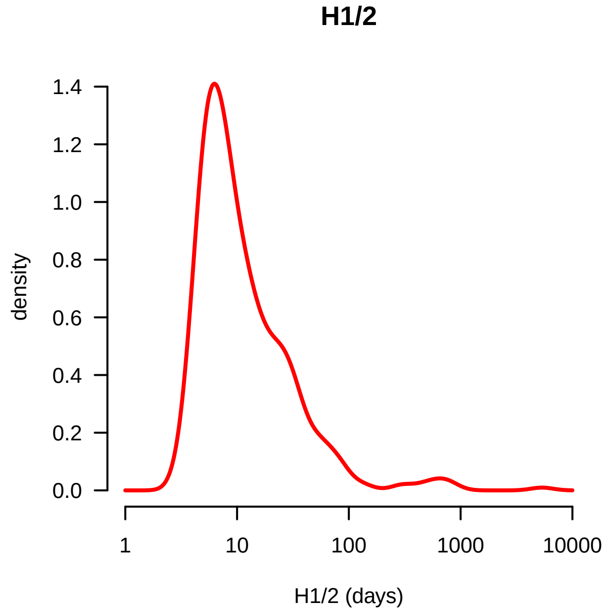

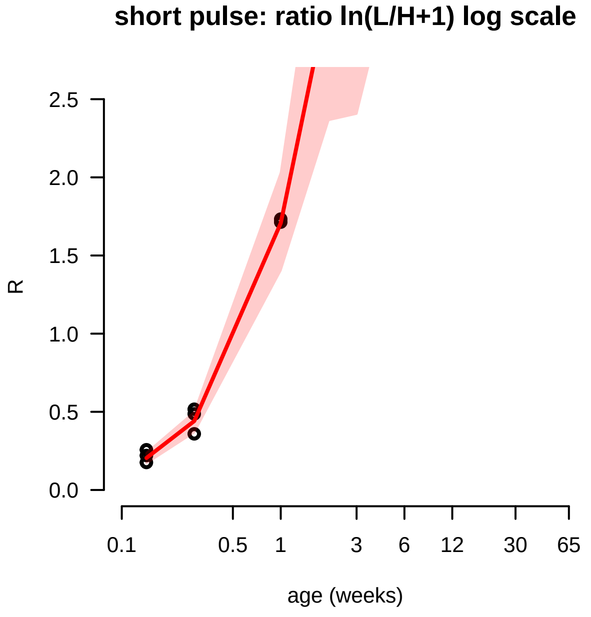

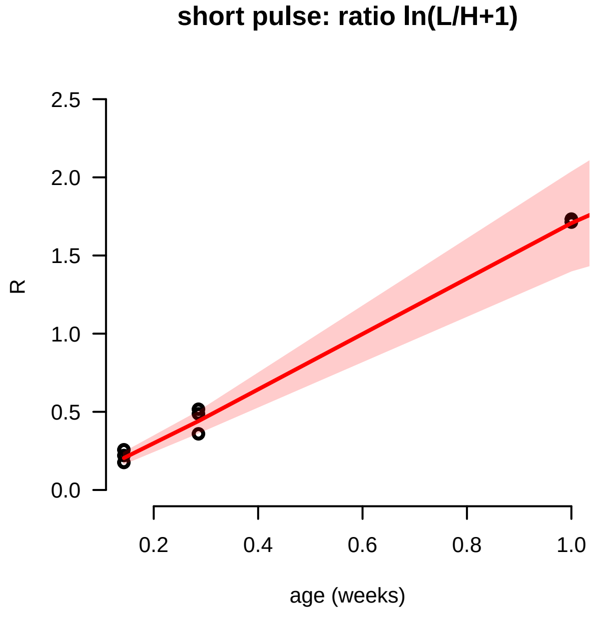

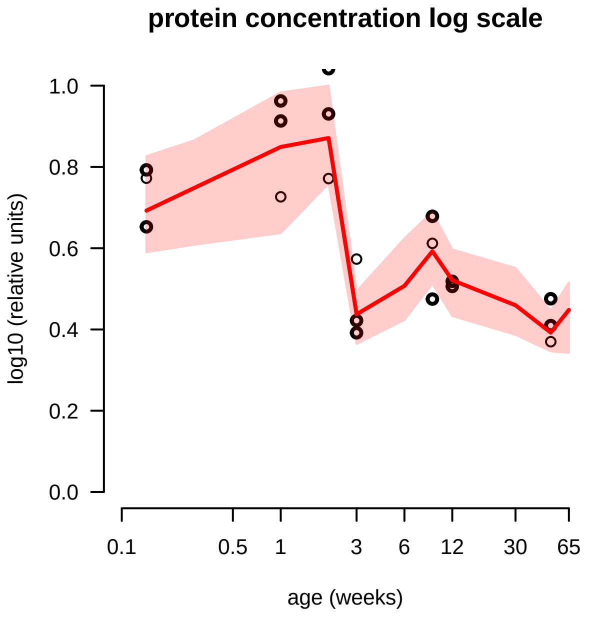

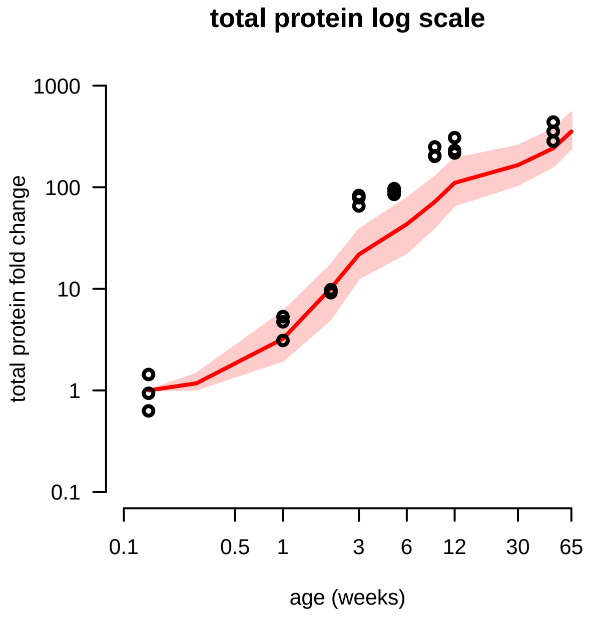

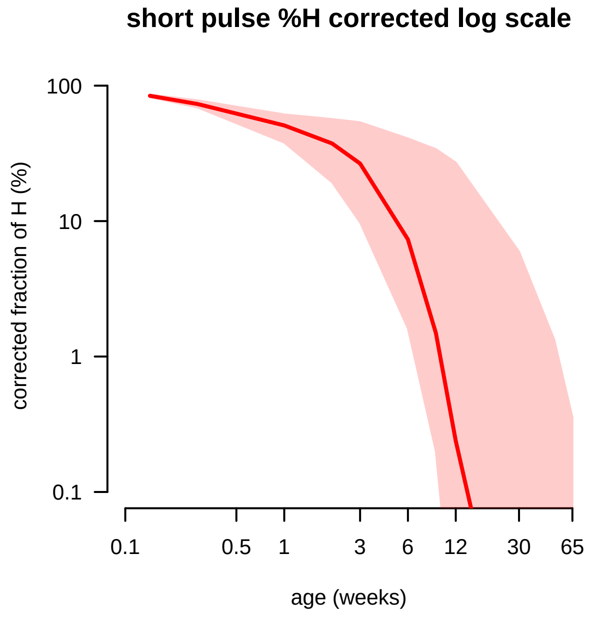

P54103

>sp|P54103|DNJC2_MOUSE

DnaJ homolog subfamily C member 2

GN=Dnajc2

Med H1/2: 7.1 days

Q5 H1/2: 2.6 days

Q95 H1/2: 80.1 days

reliable model fit: yes

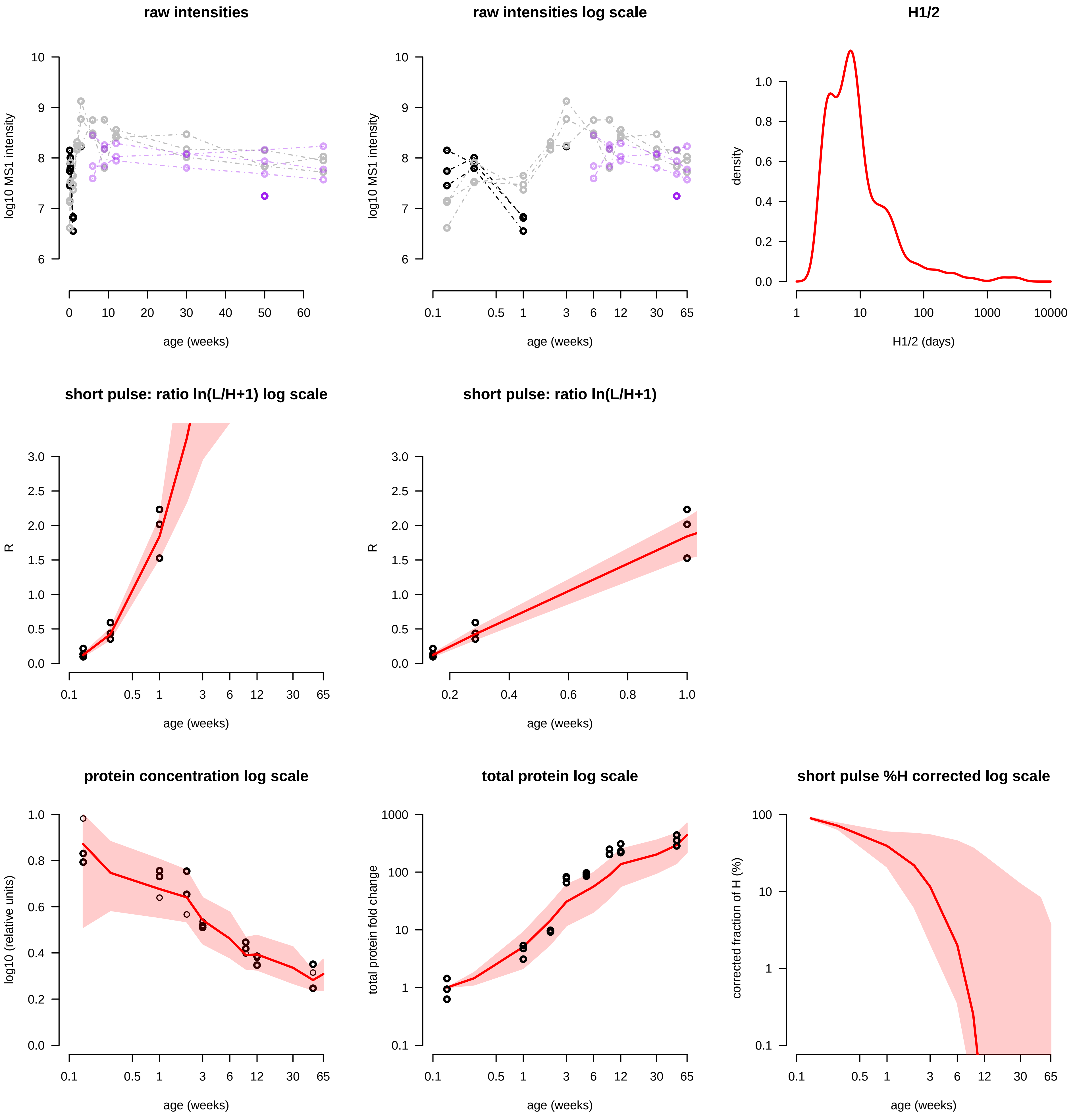

P60840−2;P60840;P60840−3

>sp|P60840−2|ENSA_MOUSE

Isoform 2 of Alpha−endosulfine

GN=Ensa

Med H1/2: 4.9 days

Q5 H1/2: 2.6 days

Q95 H1/2: 43.2 days

reliable model fit: yes

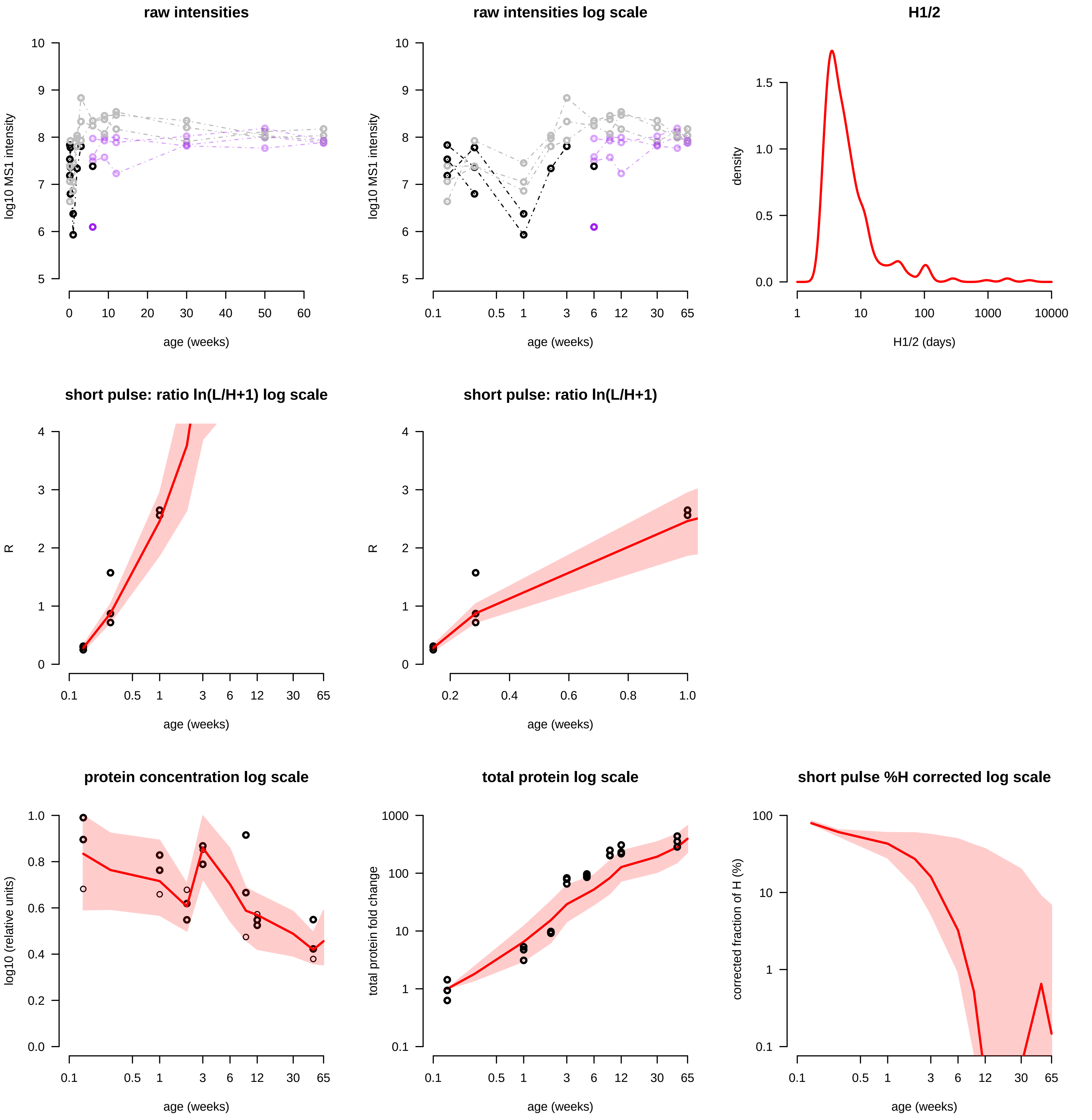

379−8;P15379−7;P15379−2;P15379−3;P15379−6;P15379−

>sp|P15379|CD44_MOUSE

CD44 antigen

GN=Cd44

Med H1/2: 4.6 days

Q5 H1/2: 2.7 days

Q95 H1/2: 30.1 days

reliable model fit: yes

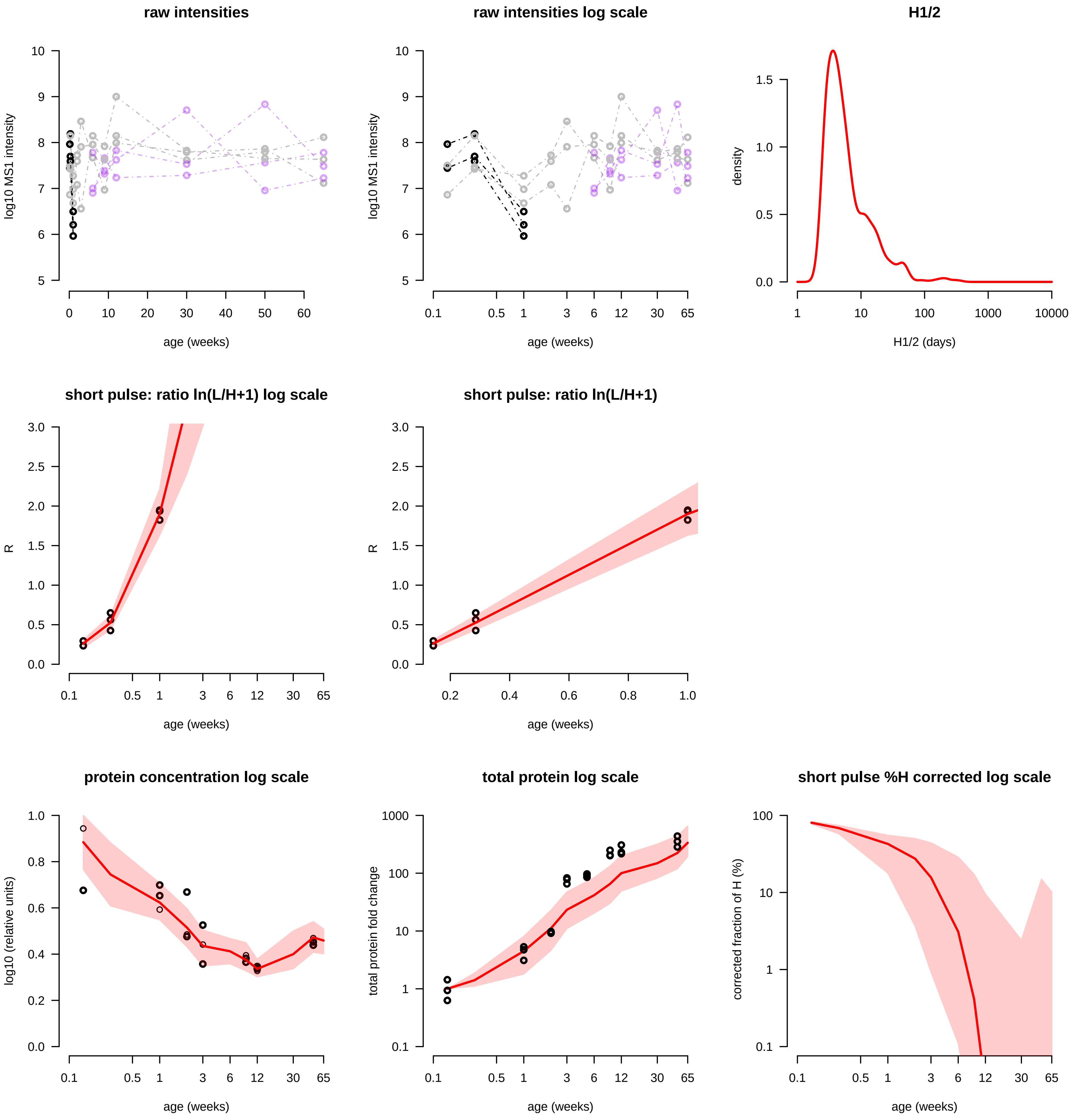

Q9QXG2

>sp|Q9QXG2|RAE1_MOUSE

Rab proteins geranylgeranyltransferase component A 1

GN=Chm

Med H1/2: 18.9 days

Q5 H1/2: 11.3 days

Q95 H1/2: 33.8 days

reliable model fit: yes

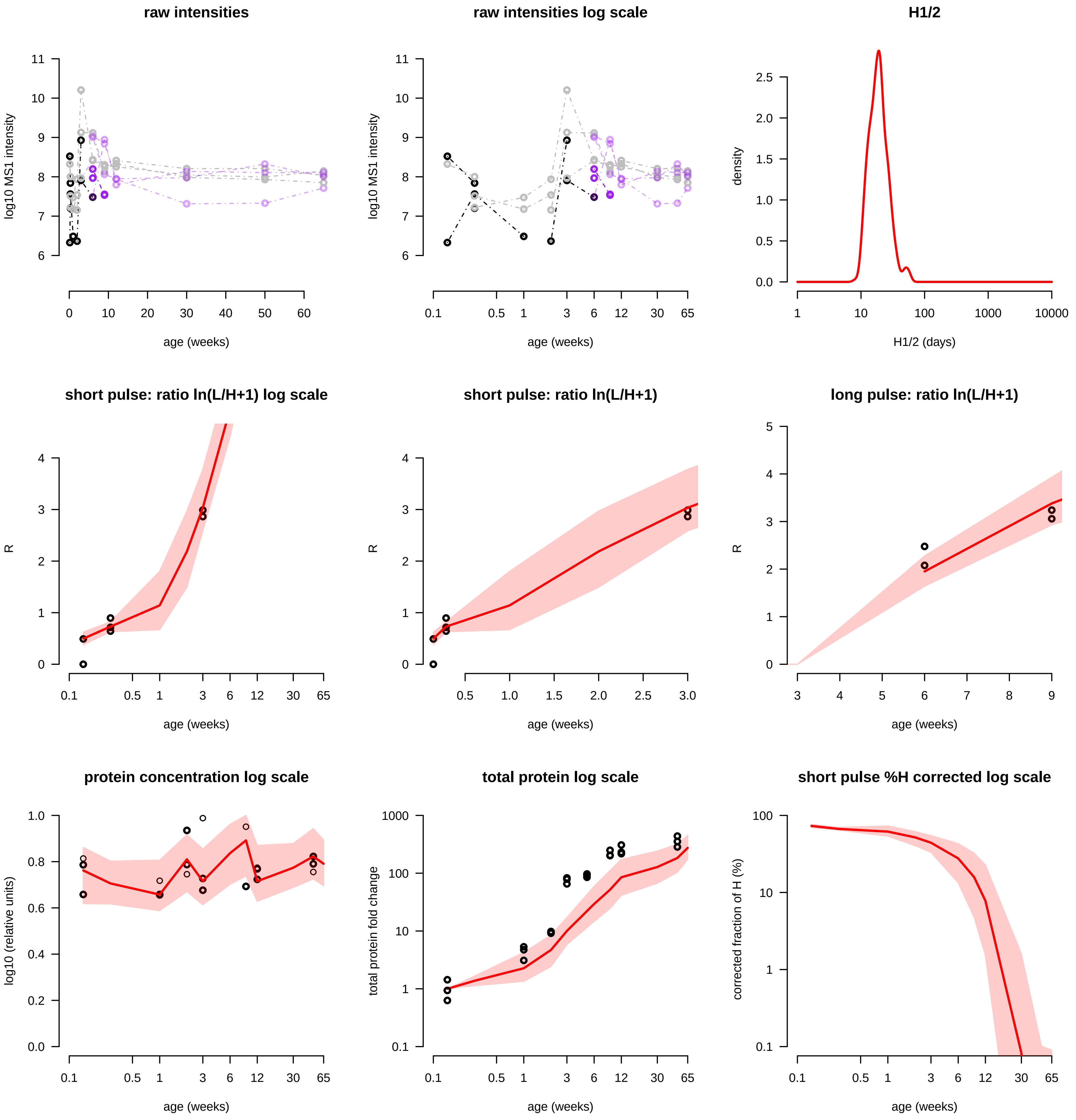

O55137

>sp|O55137|ACOT1_MOUSE

Acyl−coenzyme A thioesterase 1

GN=Acot1

Med H1/2: 9.6 days

Q5 H1/2: 4.6 days

Q95 H1/2: 32.8 days

reliable model fit: yes

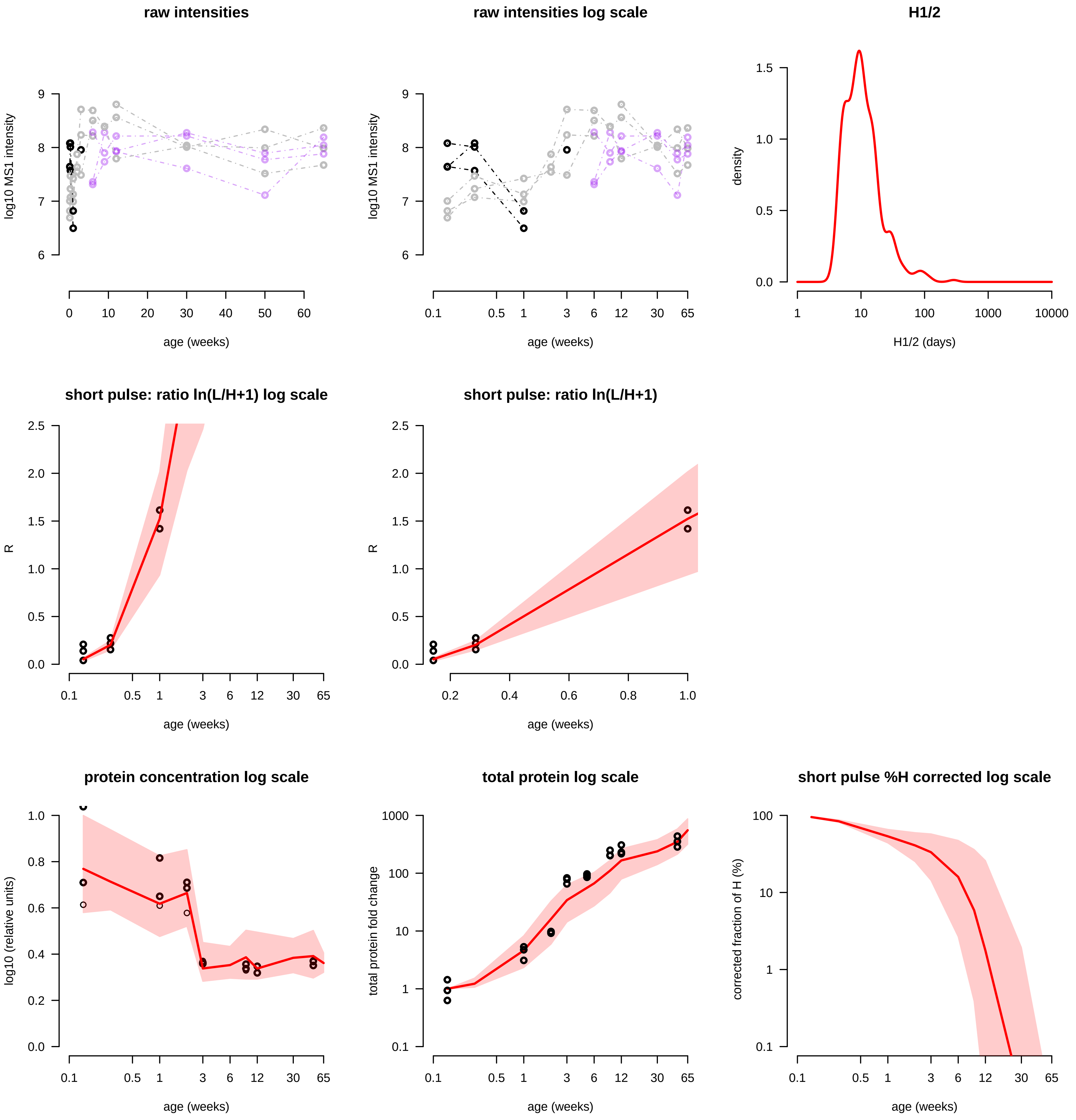

P52623

>sp|P52623|UCK1_MOUSE

Uridine−cytidine kinase 1

GN=Uck1

Med H1/2: 7.8 days

Q5 H1/2: 3.3 days

Q95 H1/2: 57.3 days

reliable model fit: yes

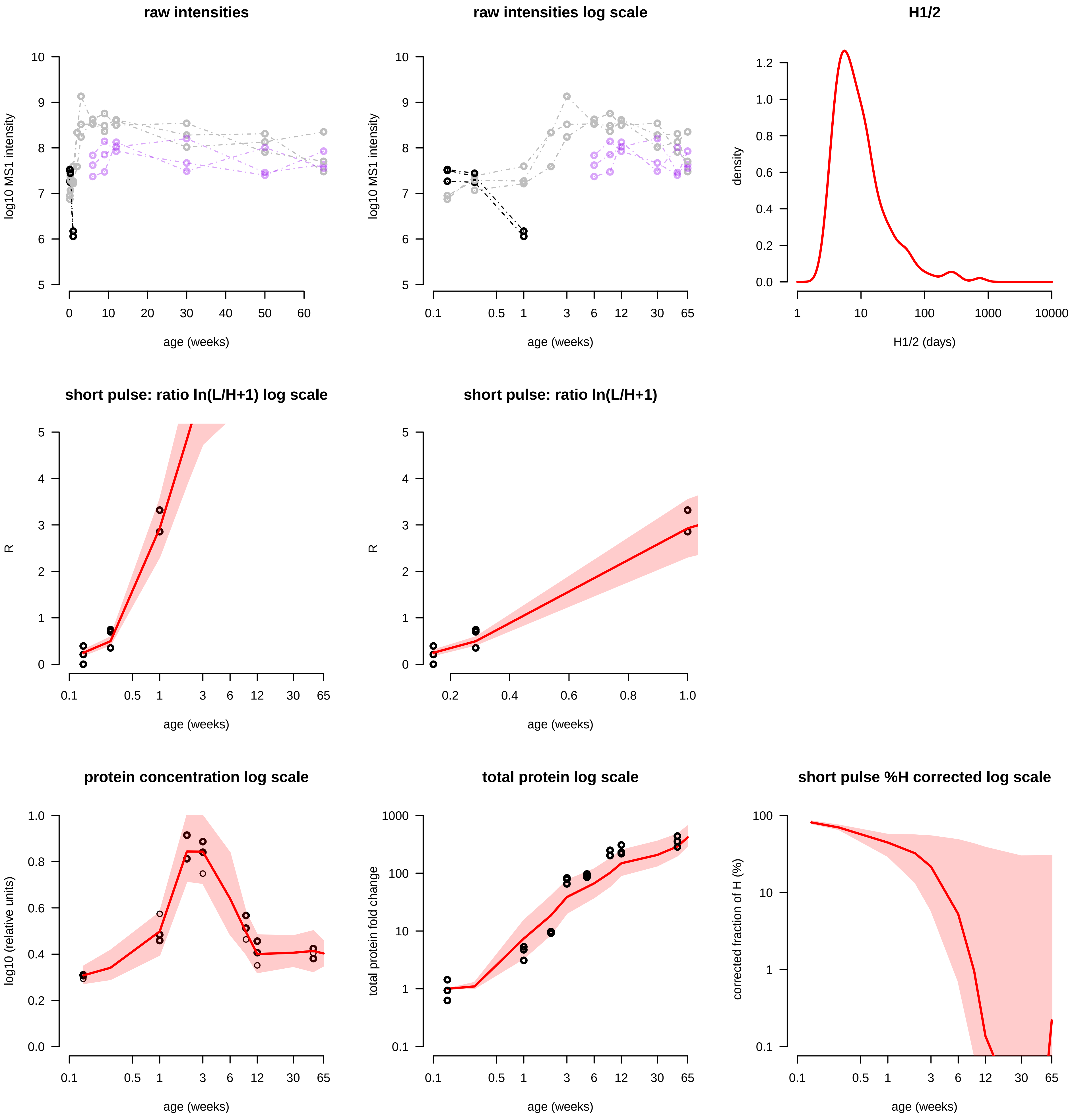

Q8BTJ4−2;Q8BTJ4

>sp|Q8BTJ4−2|ENPP4_MOUSE

Isoform 2 of Bis(5'−adenosyl)−triphosphatase enpp4

GN=Enpp4

Med H1/2: 10.6 days

Q5 H1/2: 3.2 days

Q95 H1/2: 83.9 days

reliable model fit: yes

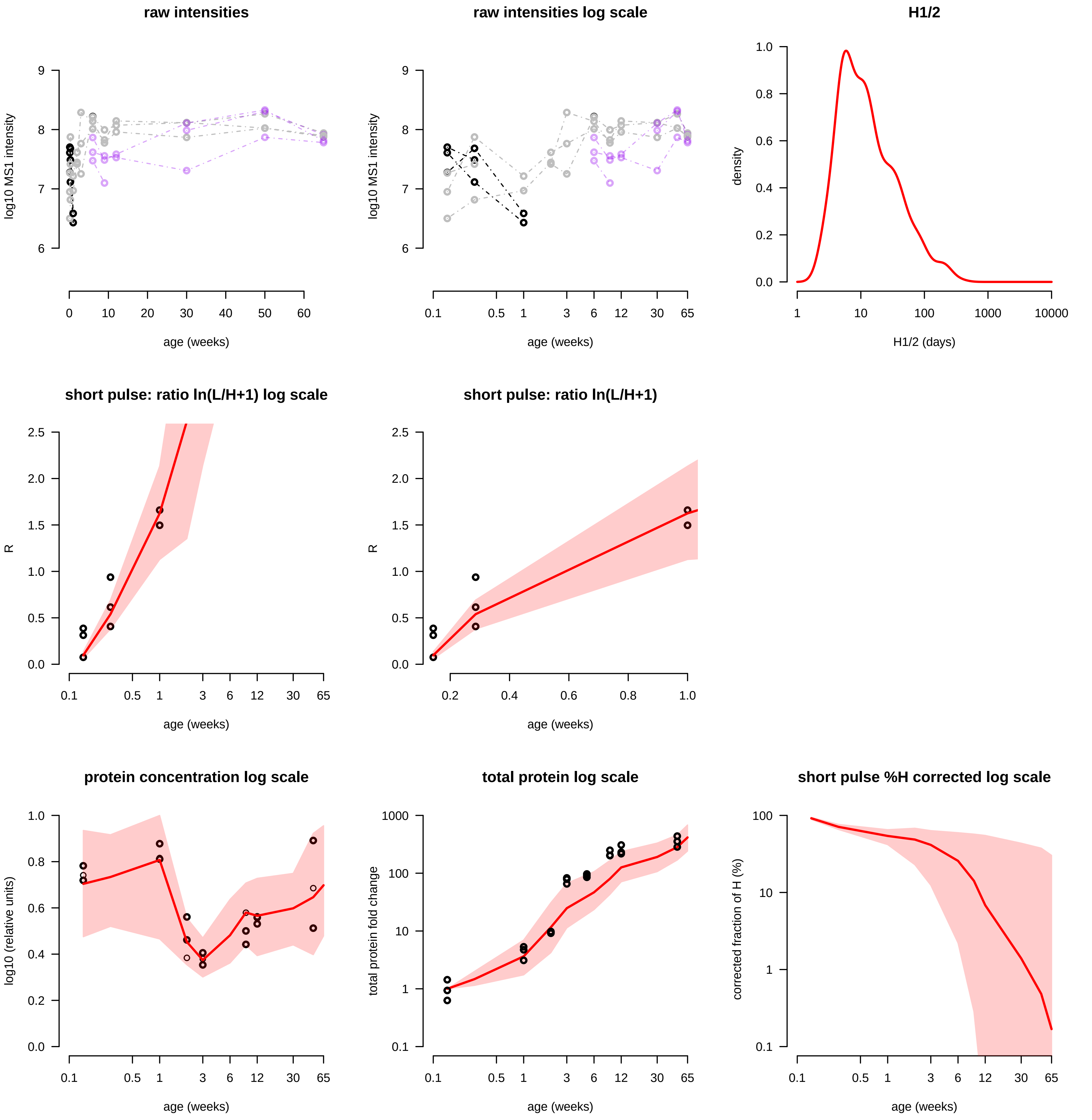

Q08024;Q08024−4;Q08024−3

>sp|Q08024|PEBB_MOUSE

Core−binding factor subunit beta

GN=Cbfb

Med H1/2: 8 days

Q5 H1/2: 3.2 days

Q95 H1/2: 61.5 days

reliable model fit: no

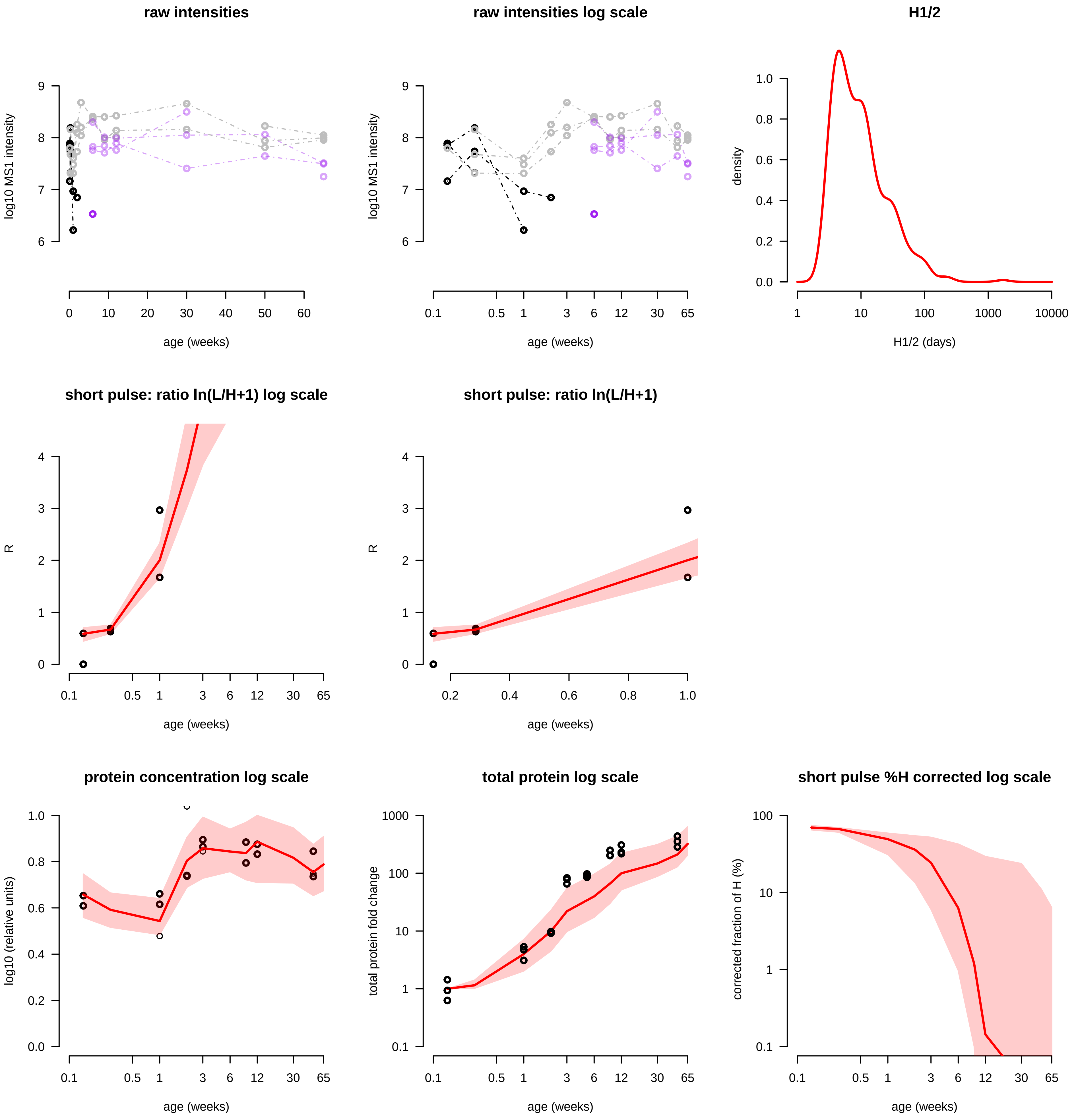

Q9JKY0

>sp|Q9JKY0|CNOT9_MOUSE

CCR4−NOT transcription complex subunit 9

GN=Cnot9

Med H1/2: 6.9 days

Q5 H1/2: 3.3 days

Q95 H1/2: 56.6 days

reliable model fit: yes

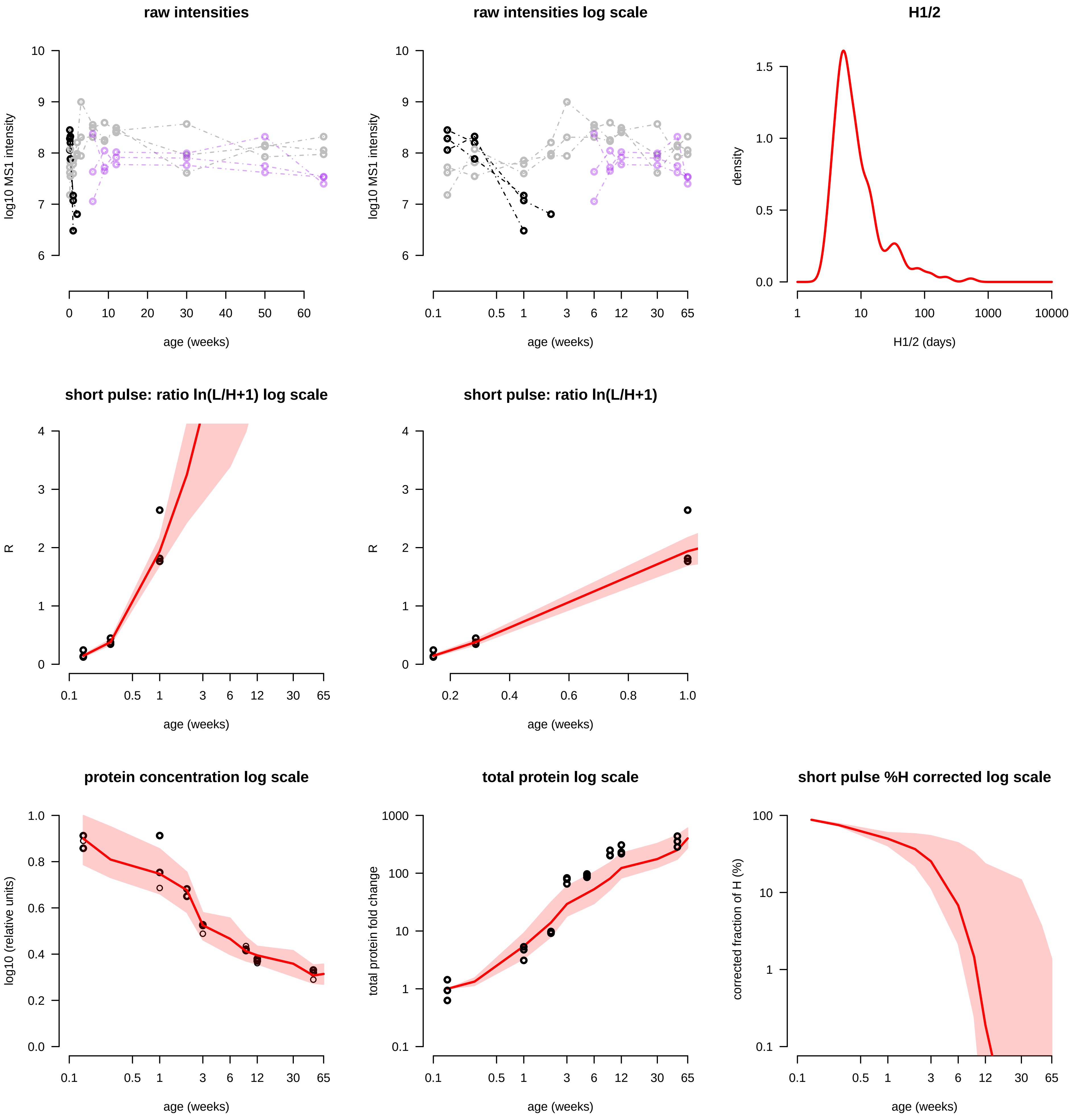

Q148V7;Q148V7−2;Q148V7−3

>sp|Q148V7|RELCH_MOUSE

RAB11−binding protein RELCH

GN=Relch

Med H1/2: 4.3 days

Q5 H1/2: 2.6 days

Q95 H1/2: 22.5 days

reliable model fit: yes

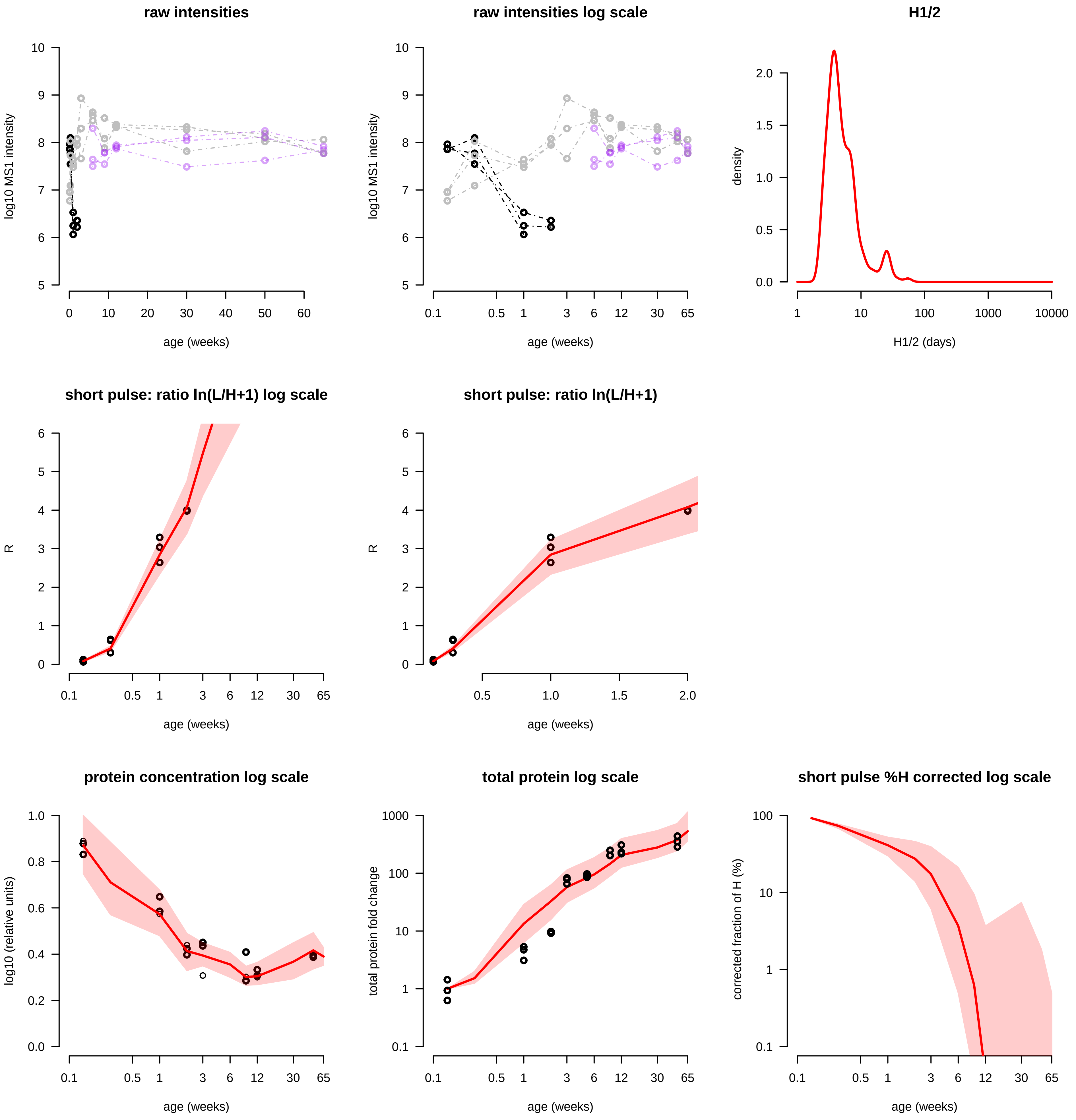

Q8R0F6;Q8R0F6−2

>sp|Q8R0F6|ILKAP_MOUSE

Integrin−linked kinase−associated serine/threonine phosphatase 2C

GN=Ilkap

Med H1/2: 4.1 days

Q5 H1/2: 2.6 days

Q95 H1/2: 13.1 days

reliable model fit: yes

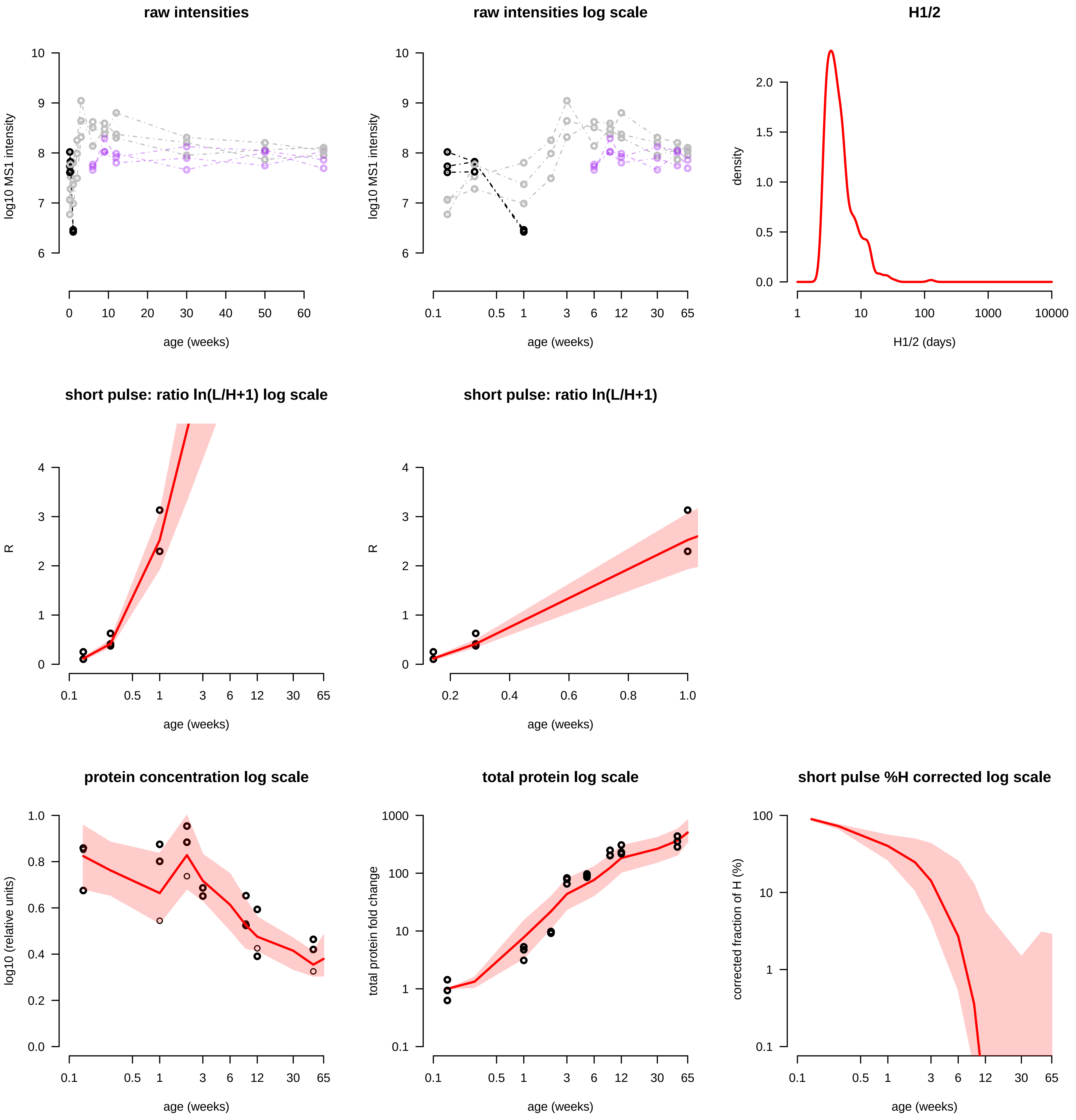

Q60996−3;Q60996

>sp|Q60996−3|2A5G_MOUSE

3 of Serine/threonine−protein phosphatase 2A 56 kDa regulatory subunit gamma

GN=Ppp2r5c

Med H1/2: 6.3 days

Q5 H1/2: 3 days

Q95 H1/2: 28.3 days

reliable model fit: yes

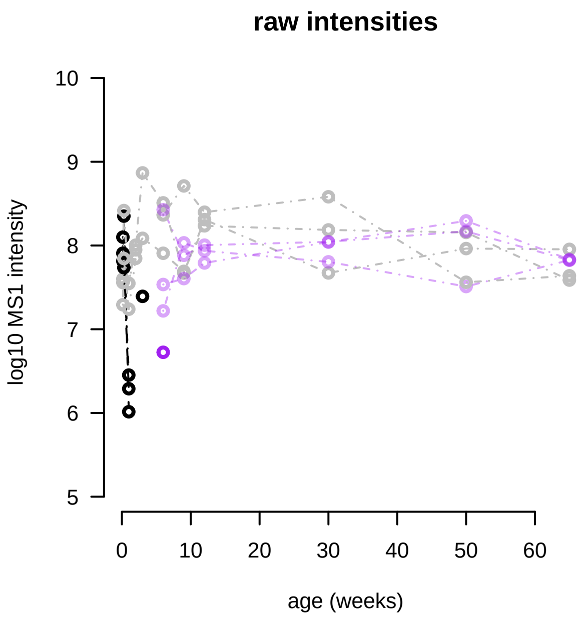

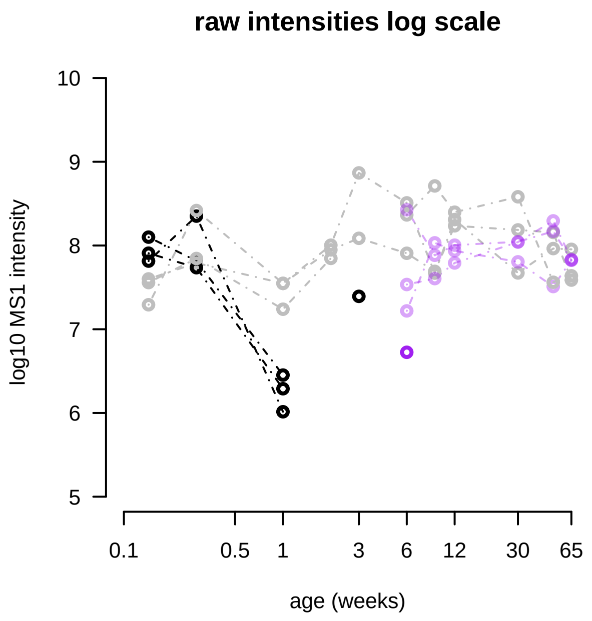

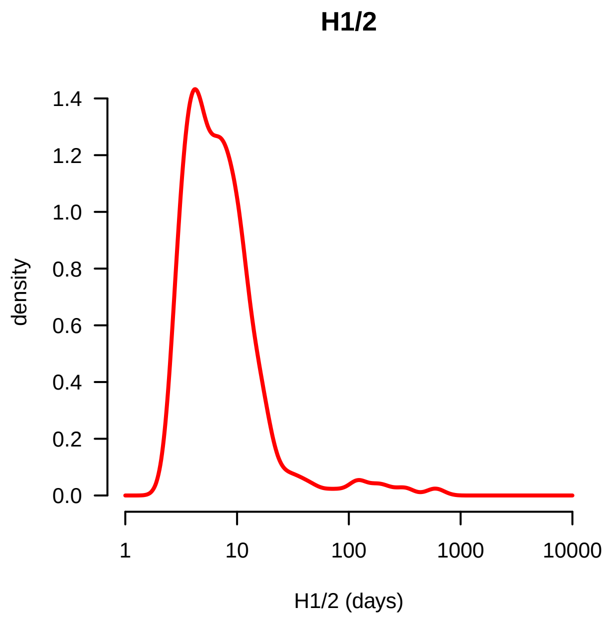

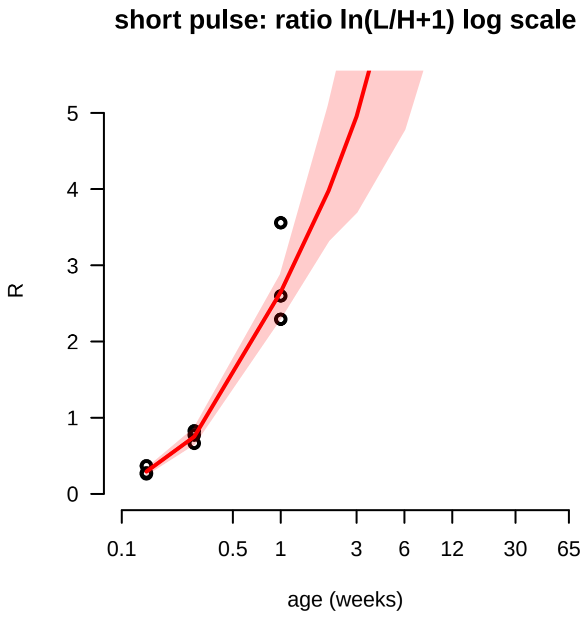

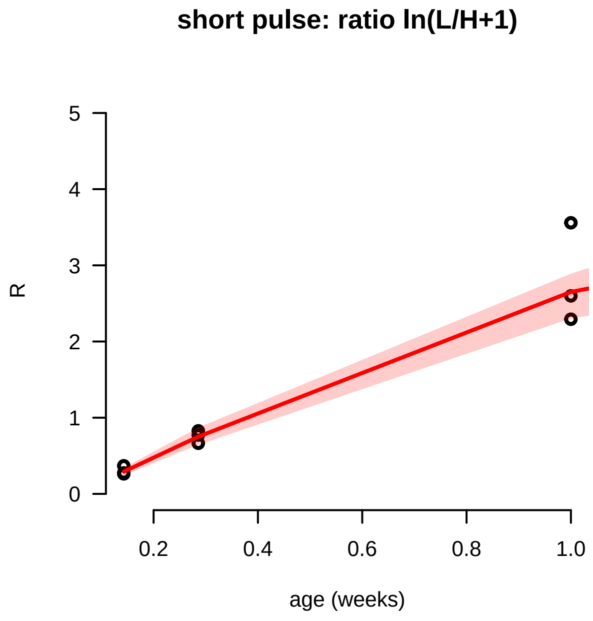

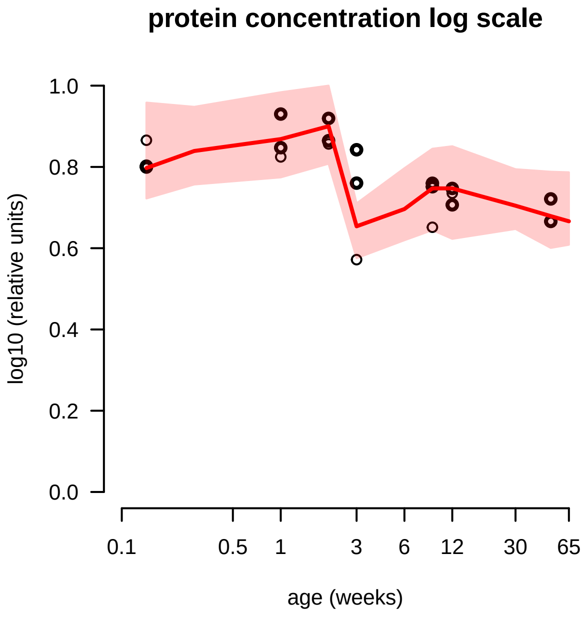

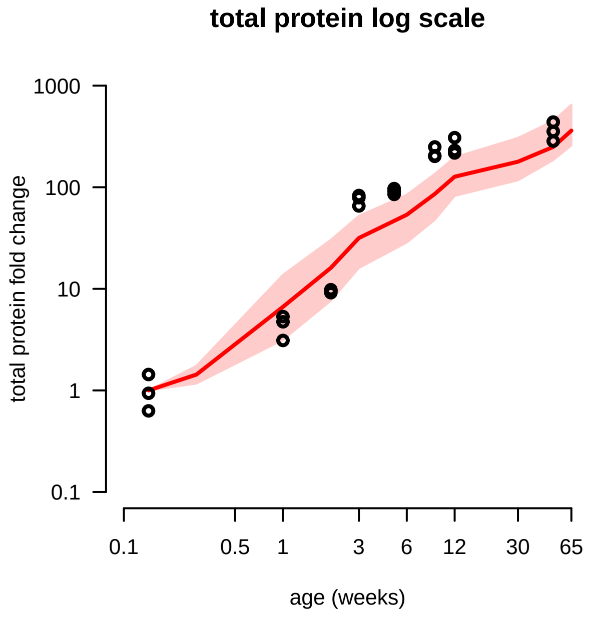

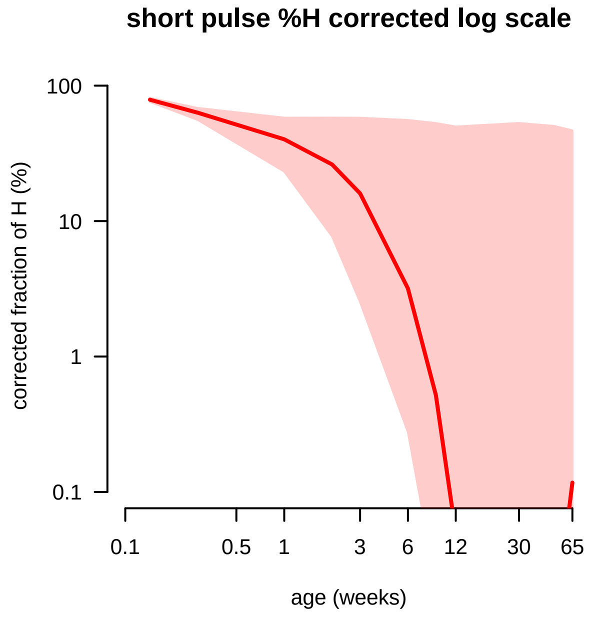

Q8VI63

>sp|Q8VI63|MOB2_MOUSE

MOB kinase activator 2

GN=Mob2

Med H1/2: 6.2 days

Q5 H1/2: 2.8 days

Q95 H1/2: 75.5 days

reliable model fit: yes

raw intensities

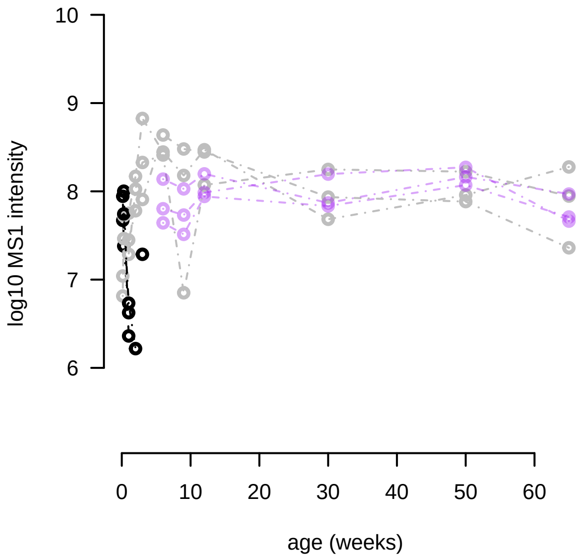

raw intensities log scale

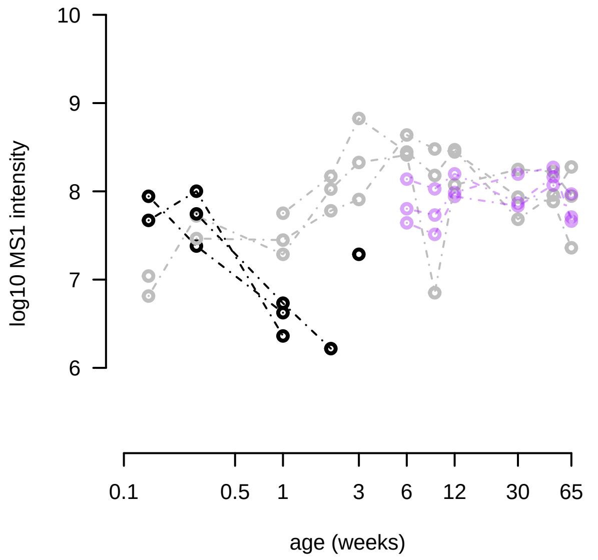

H1/2

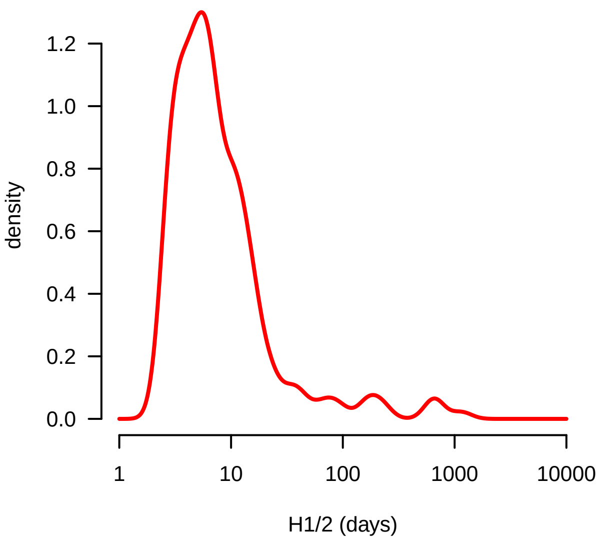

short pulse: ratio ln(L/H+1) log scale

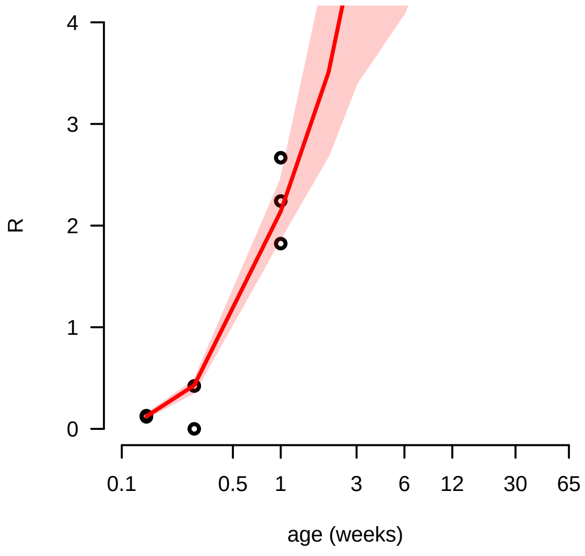

short pulse: ratio ln(L/H+1)

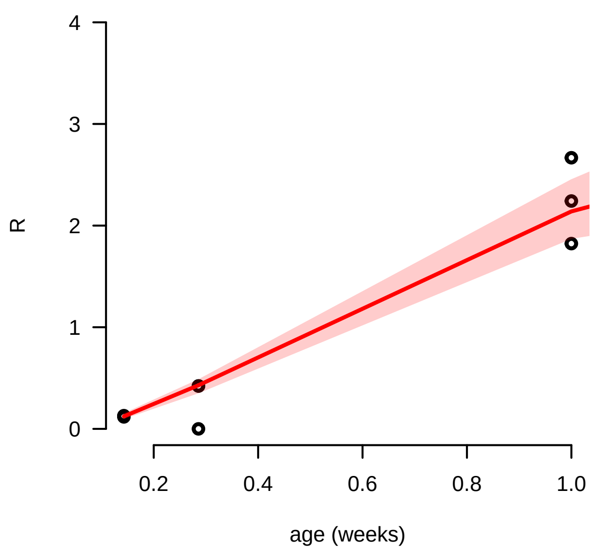

protein concentration log scale

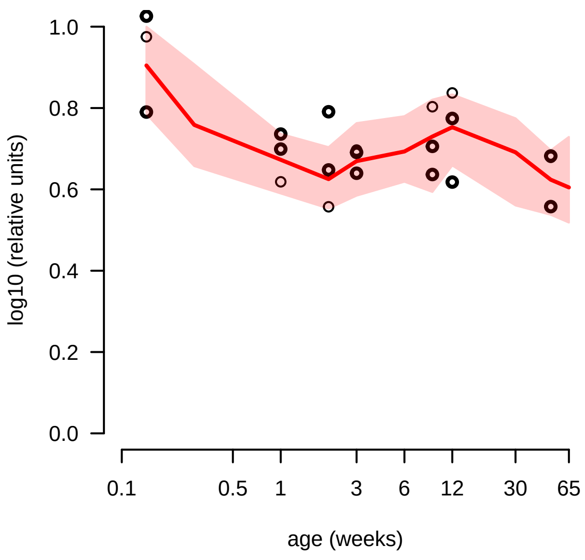

total protein log scale

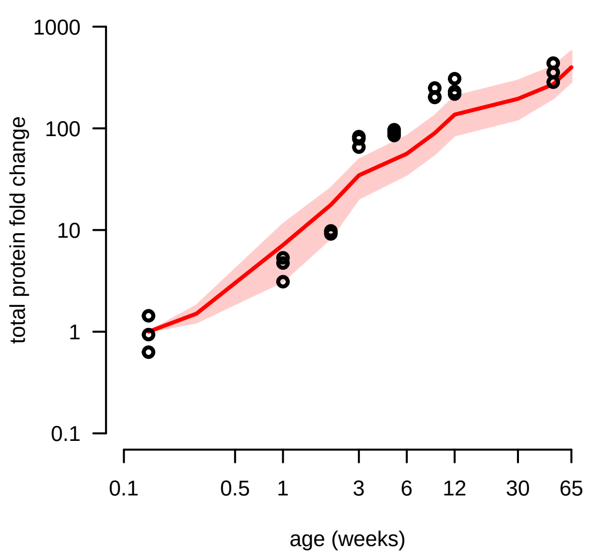

short pulse %H corrected log scale

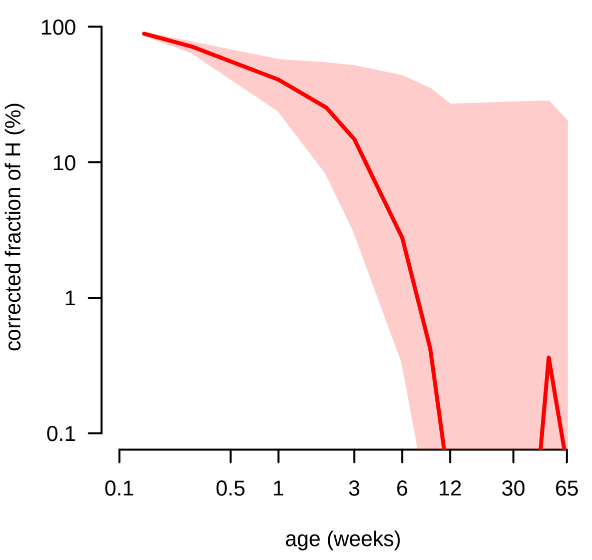

Q9D8T2

>sp|Q9D8T2|GSDMD_MOUSE

Gasdermin−D

GN=Gsdmd

Med H1/2: 5 days

Q5 H1/2: 2.6 days

Q95 H1/2: 73.9 days

reliable model fit: yes

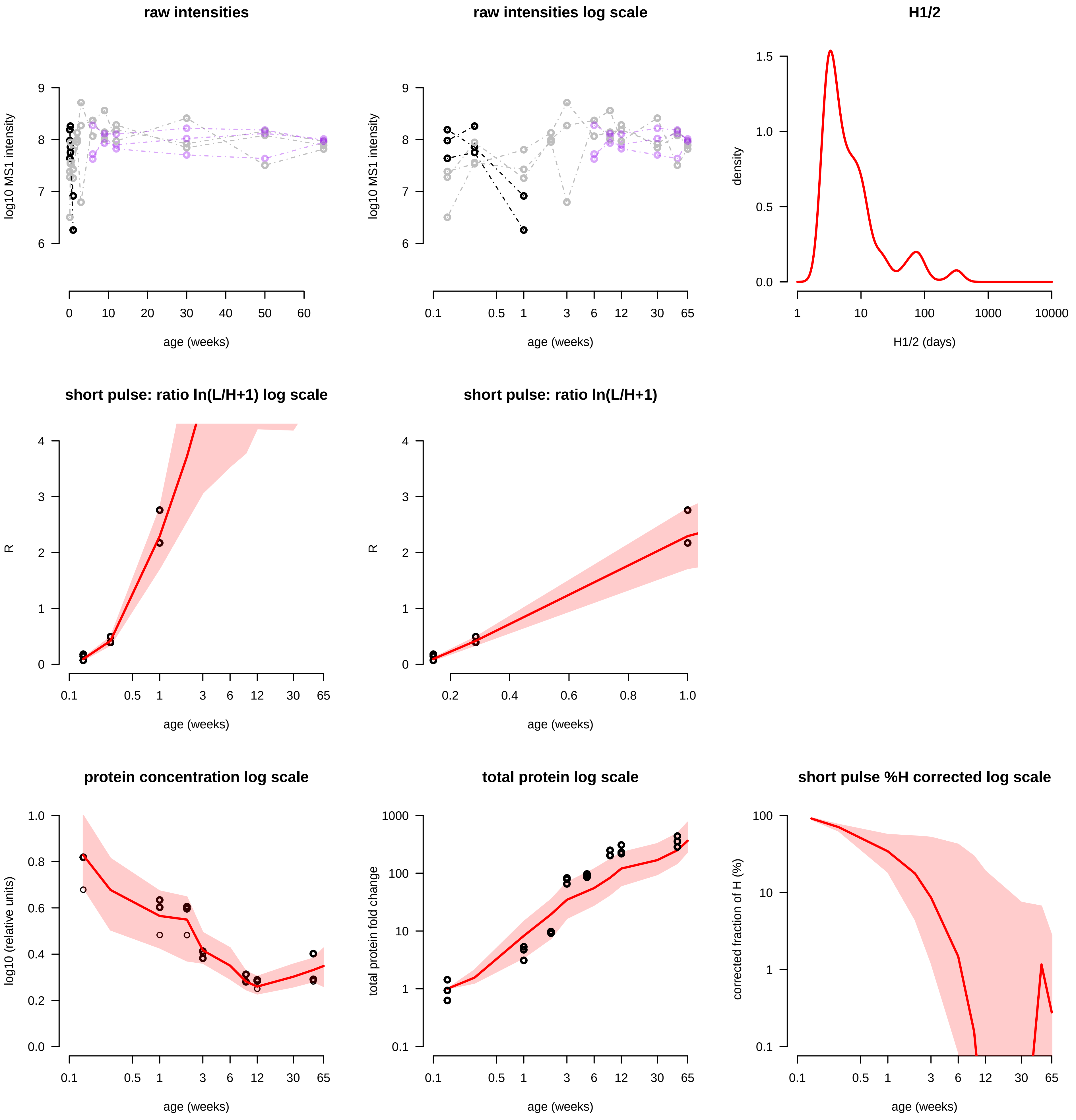

Q01279

>sp|Q01279|EGFR_MOUSE

Epidermal growth factor receptor

GN=Egfr

Med H1/2: 15.3 days

Q5 H1/2: 5.7 days

Q95 H1/2: 247.8 days

reliable model fit: yes

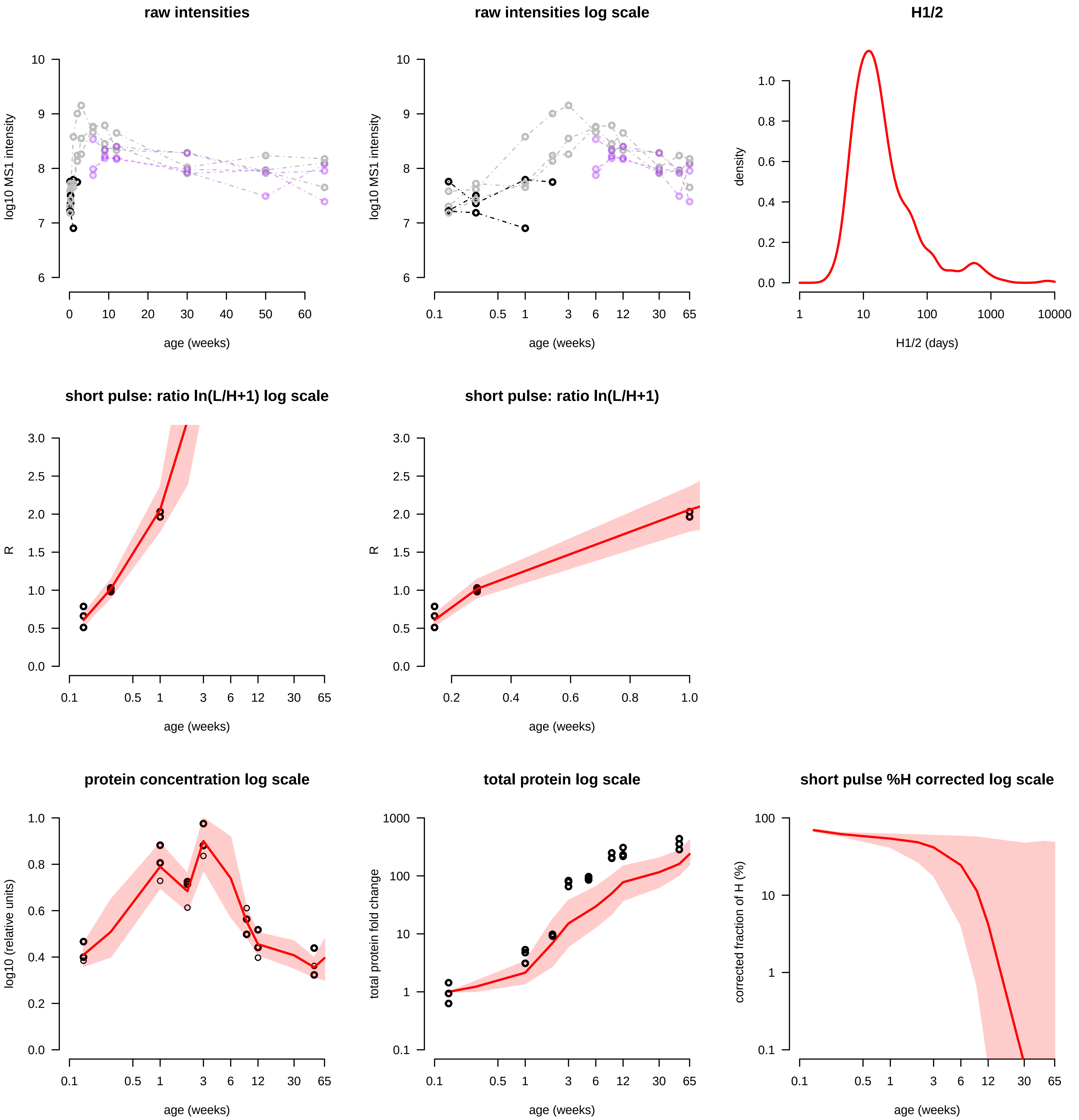

Q6PD19;Q6PD19−2;Q6PD19−3

>sp|Q6PD19|ARMD3_MOUSE

Armadillo−like helical domain−containing protein 3

GN=Armh3

Med H1/2: 26.7 days

Q5 H1/2: 10.1 days

Q95 H1/2: 257.5 days

reliable model fit: yes

raw intensities

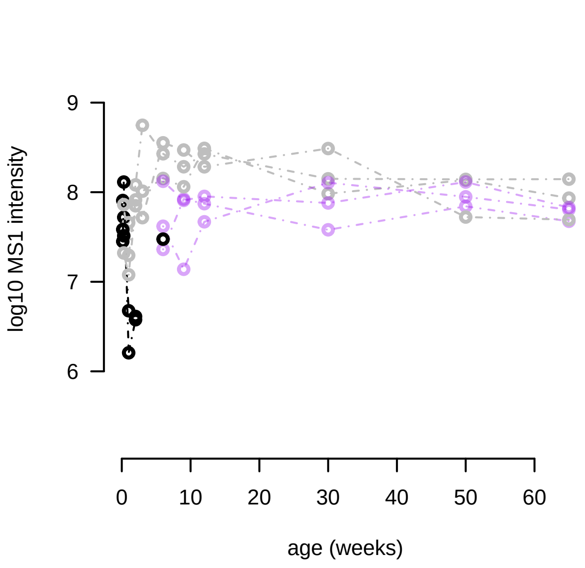

raw intensities log scale

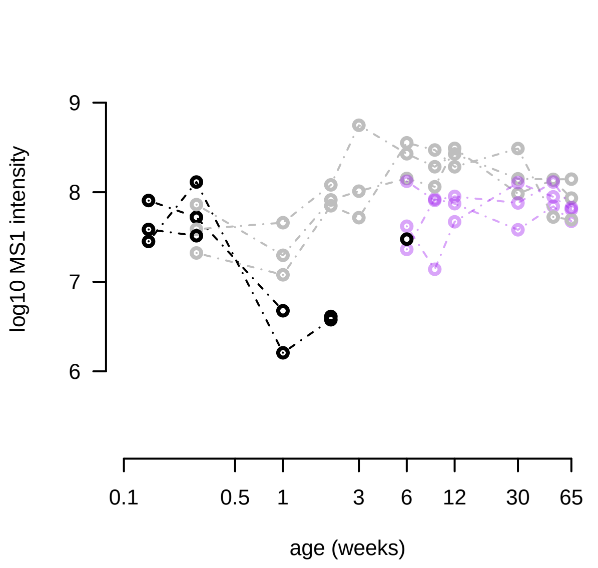

H1/2

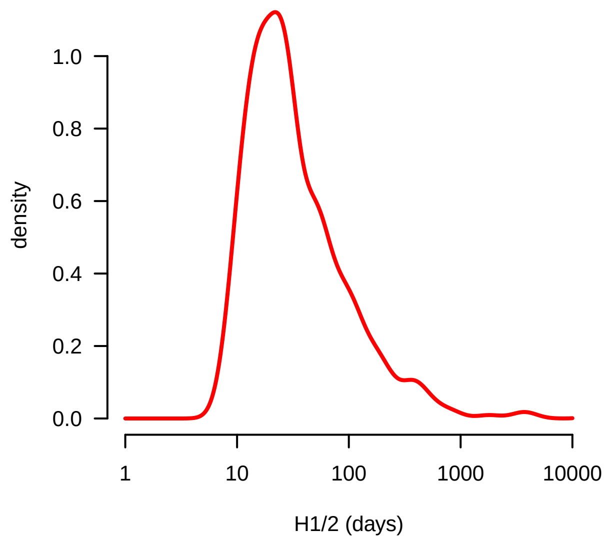

short pulse: ratio ln(L/H+1) log scale

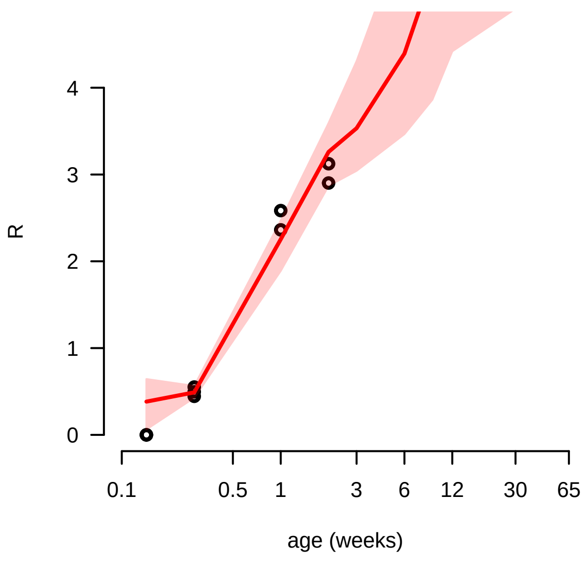

short pulse: ratio ln(L/H+1)

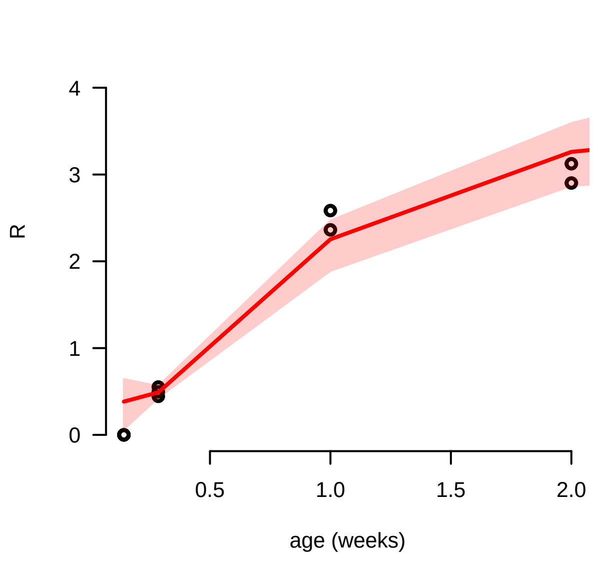

protein concentration log scale

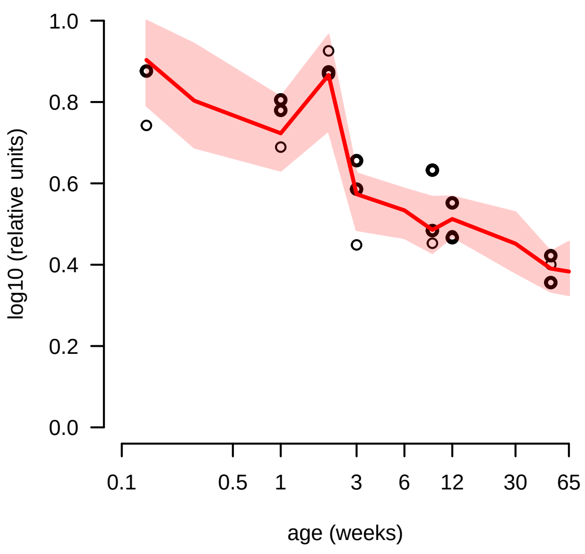

total protein log scale

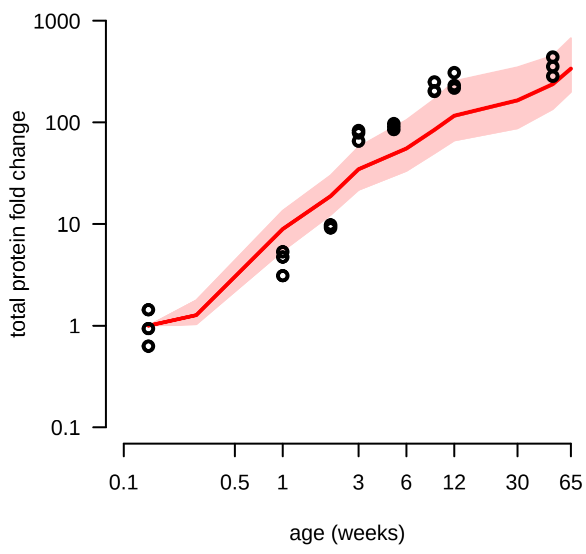

short pulse %H corrected log scale

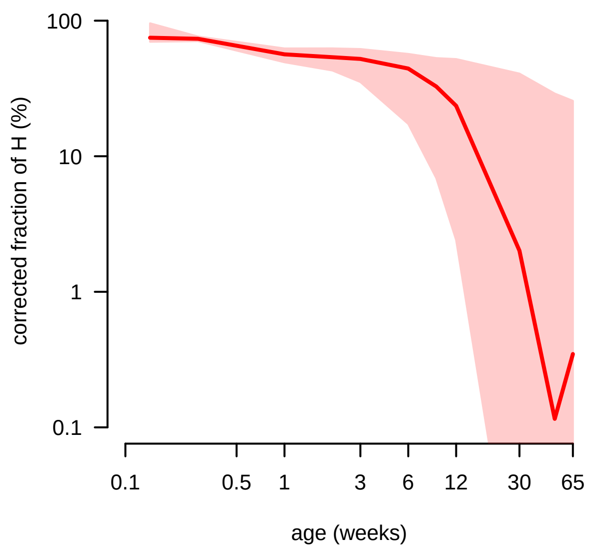

Q8BT14−3

>sp|Q8BT14−3|CNOT4_MOUSE

Isoform 3 of CCR4−NOT transcription complex subunit 4

GN=Cnot4

Med H1/2: 16.7 days

Q5 H1/2: 10.9 days

Q95 H1/2: 39 days

reliable model fit: yes

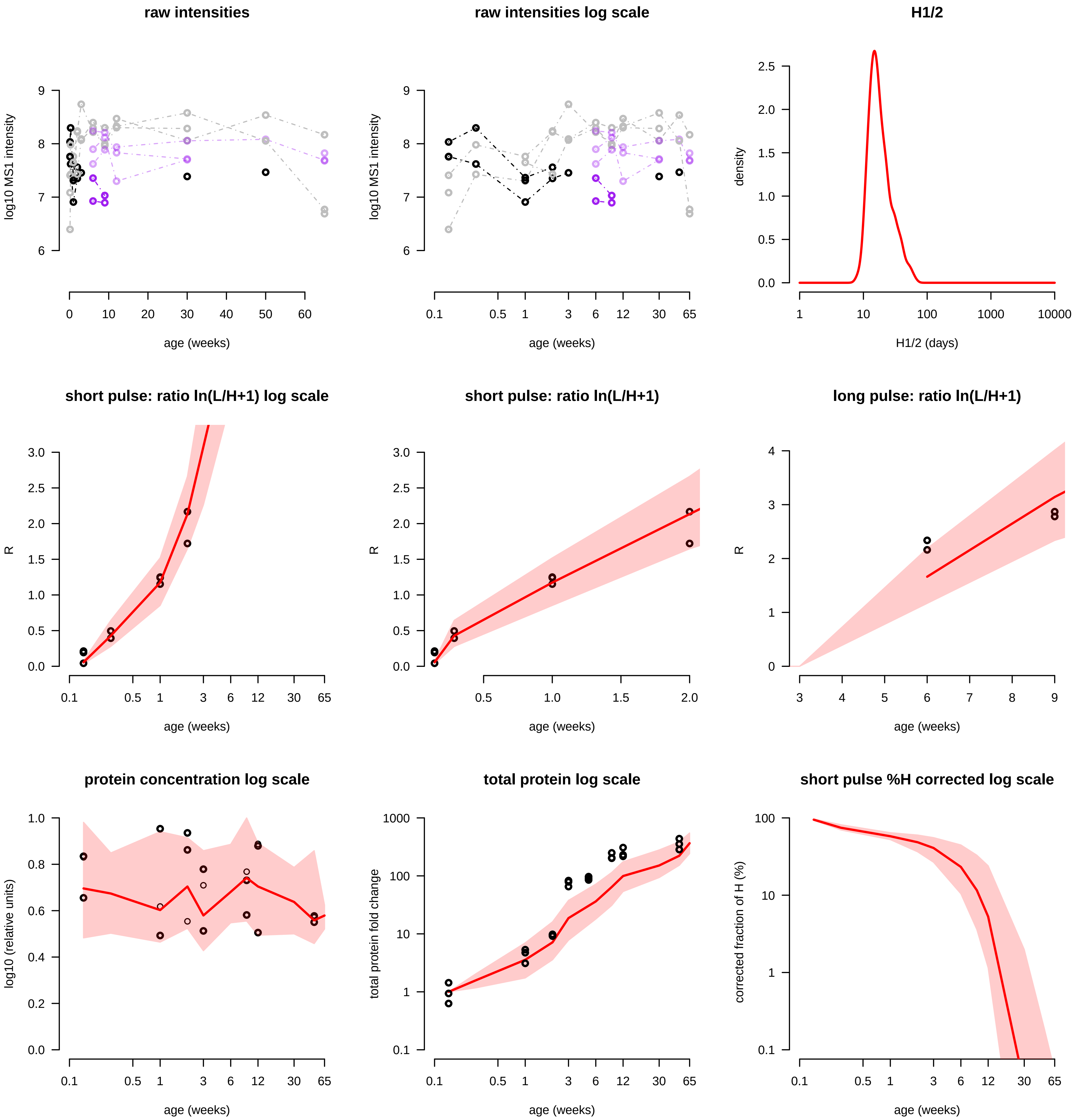

Q61189

>sp|Q61189|ICLN_MOUSE

Methylosome subunit pICln

GN=Clns1a

Med H1/2: 6.6 days

Q5 H1/2: 2.7 days

Q95 H1/2: 63.5 days

reliable model fit: yes

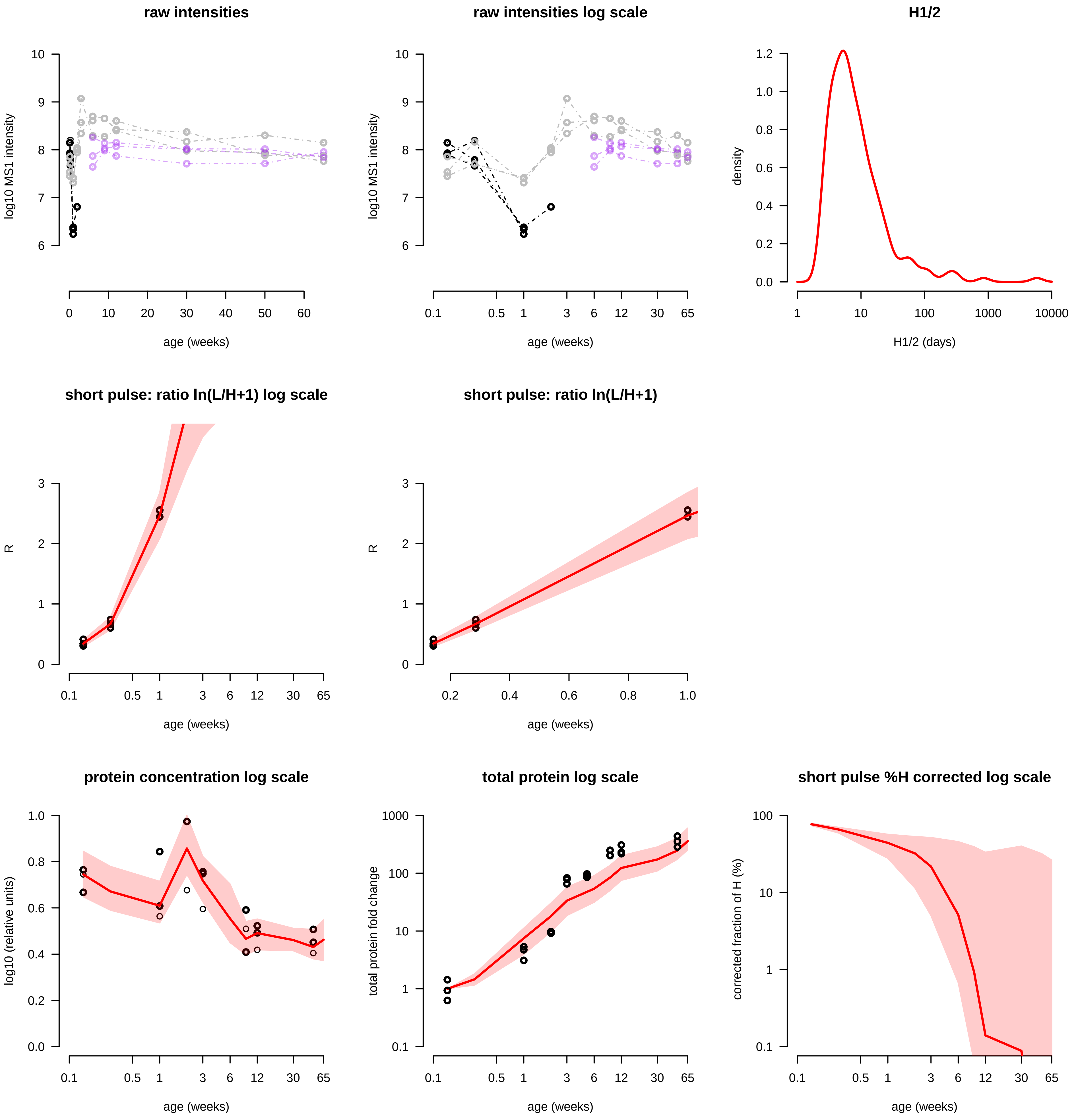

Q62245

>sp|Q62245|SOS1_MOUSE

Son of sevenless homolog 1

GN=Sos1

Med H1/2: 7.3 days

Q5 H1/2: 2.8 days

Q95 H1/2: 116.8 days

reliable model fit: yes

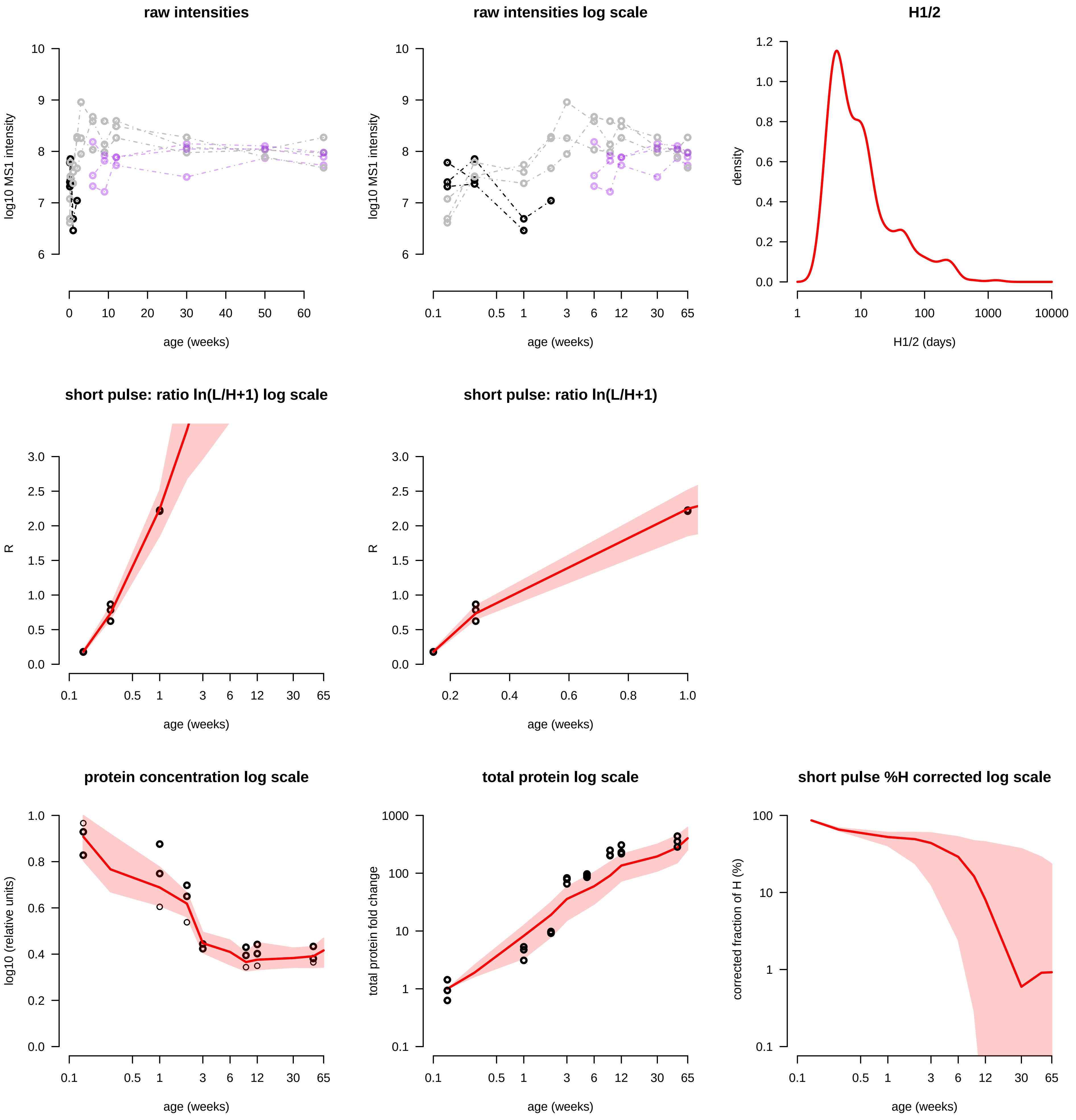

Q925J9−4;Q925J9;Q925J9−3;Q925J9−2

>sp|Q925J9−4|MED1_MOUSE

Isoform 4 of Mediator of RNA polymerase II transcription subunit 1

GN=Med1

Med H1/2: 5.6 days

Q5 H1/2: 2.8 days

Q95 H1/2: 52.4 days

reliable model fit: yes

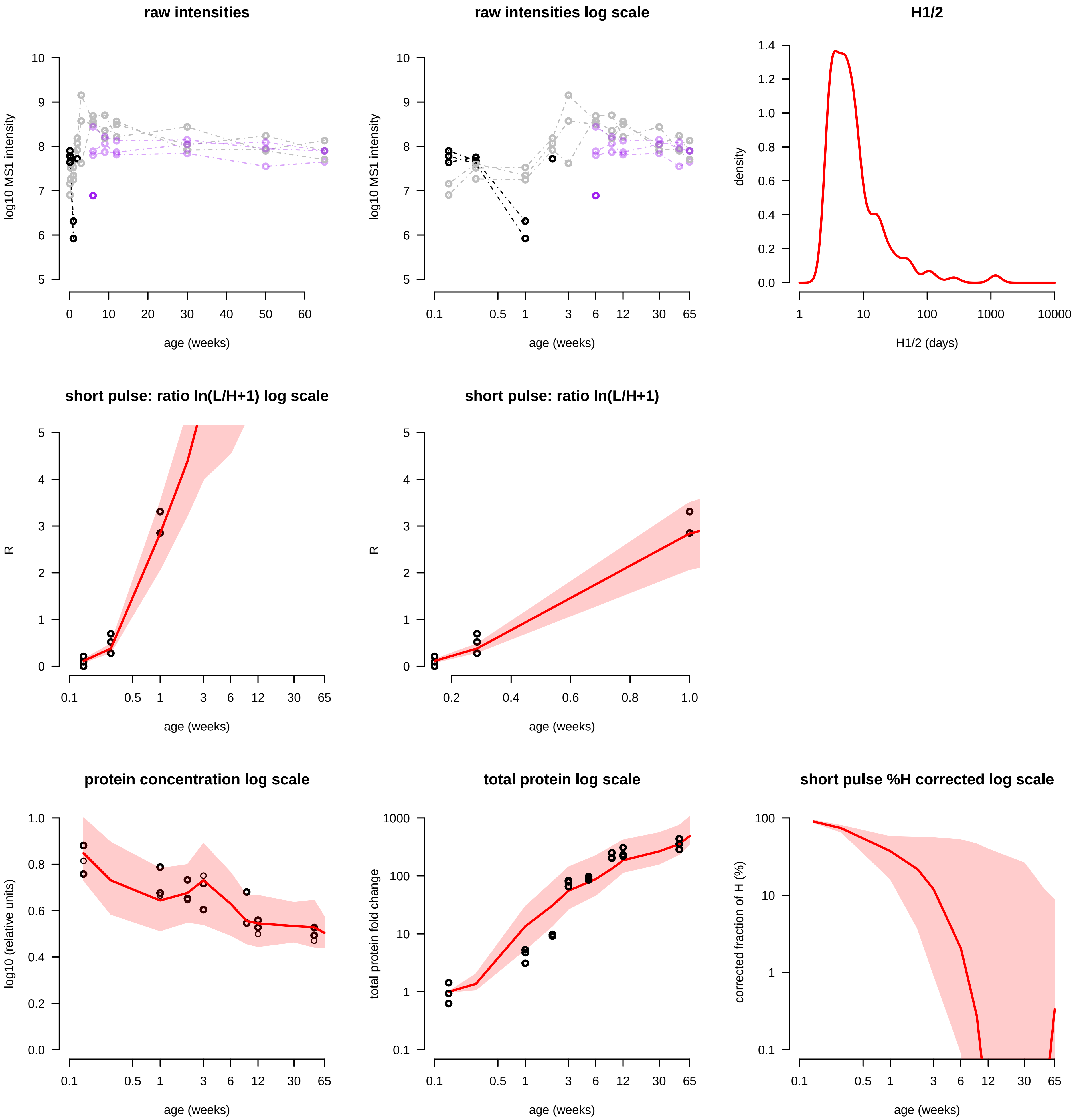

Q91YK2

>sp|Q91YK2|RRP1B_MOUSE

Ribosomal RNA processing protein 1 homolog B

GN=Rrp1b

Med H1/2: 8.1 days

Q5 H1/2: 3.3 days

Q95 H1/2: 68.7 days

reliable model fit: yes

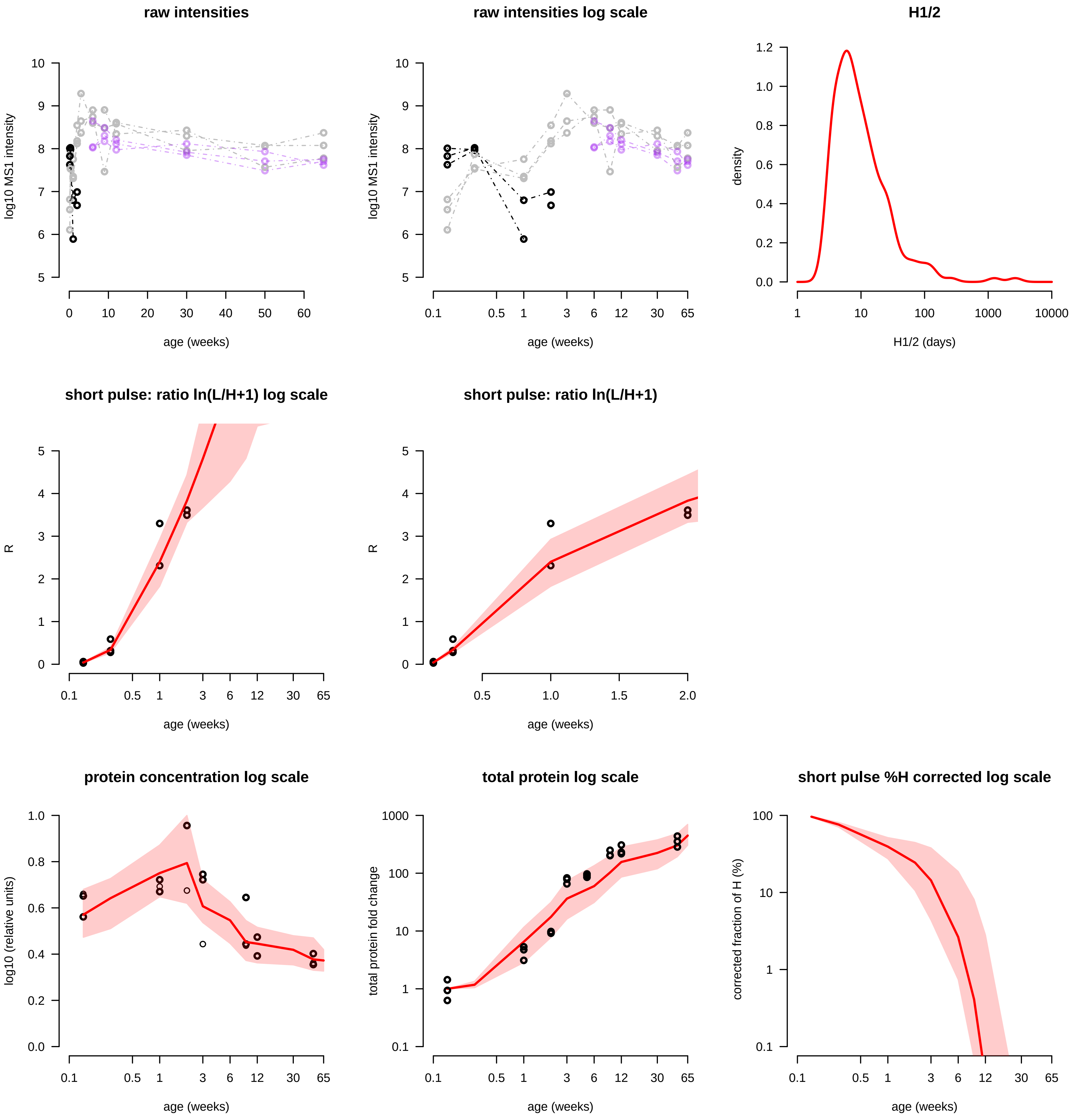

Q8BHL5−2;Q8BHL5;Q8BHL5−3

>sp|Q8BHL5−2|ELMO2_MOUSE

Isoform 2 of Engulfment and cell motility protein 2

GN=Elmo2

Med H1/2: 4.5 days

Q5 H1/2: 2.6 days

Q95 H1/2: 27.8 days

reliable model fit: yes

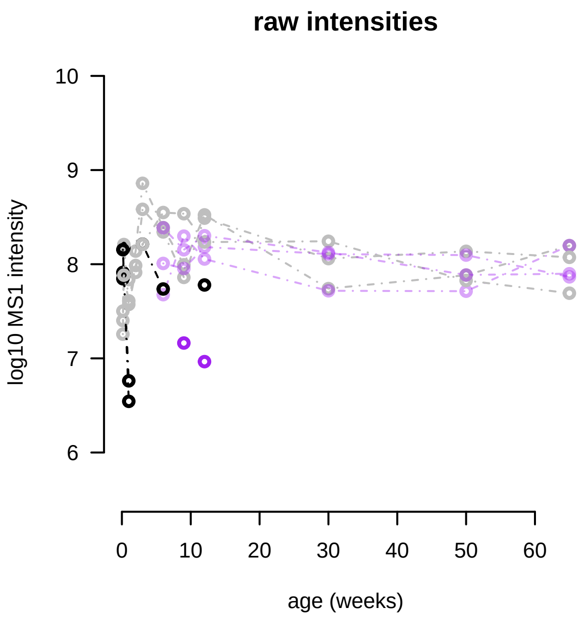

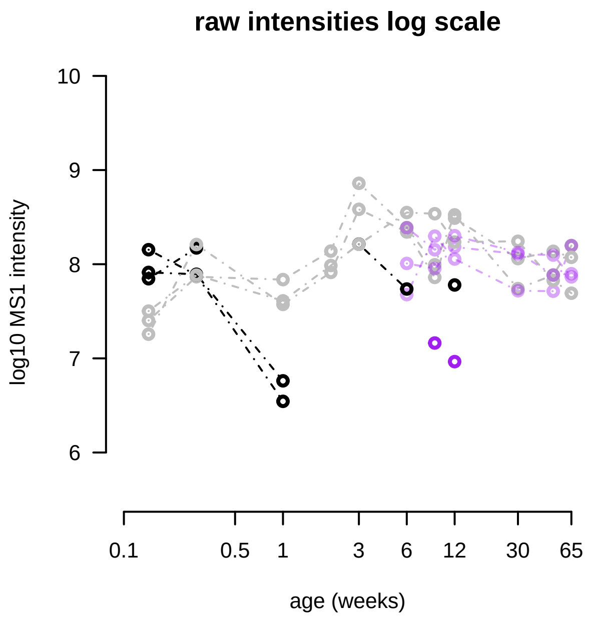

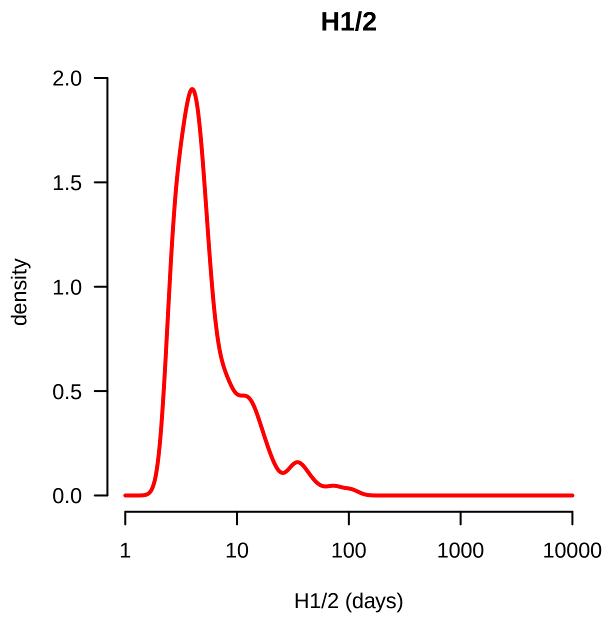

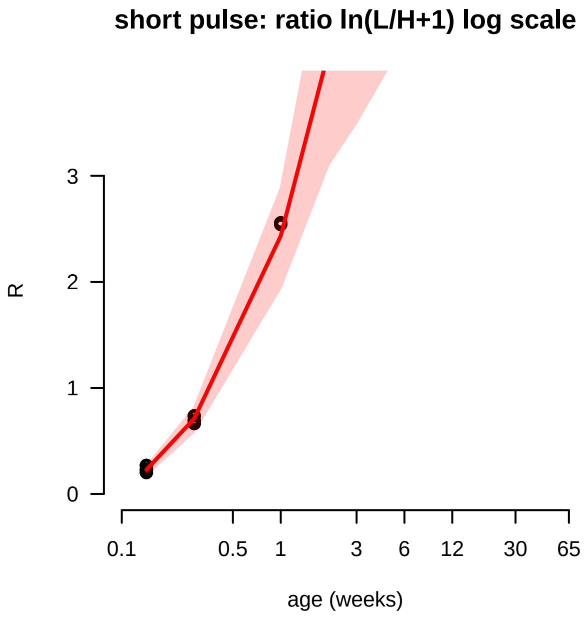

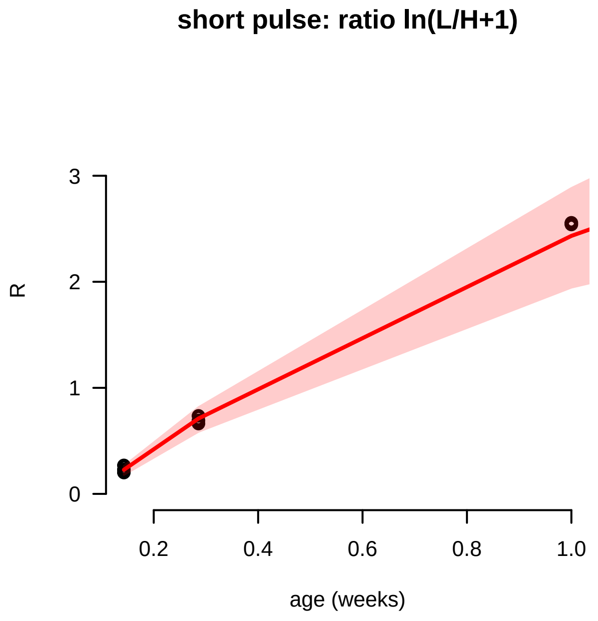

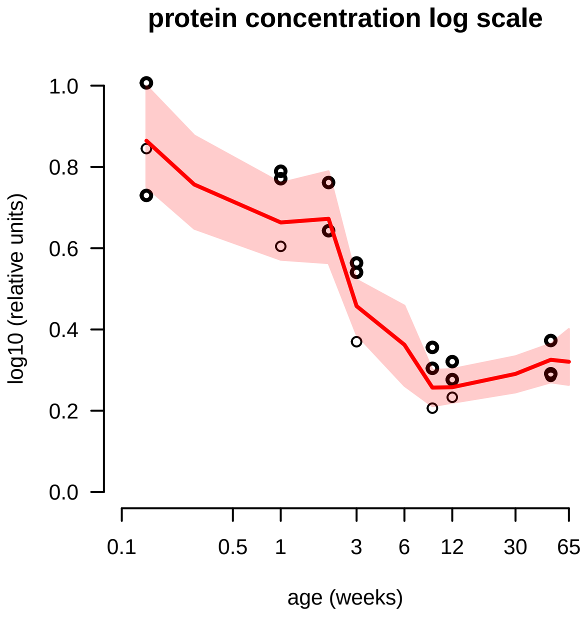

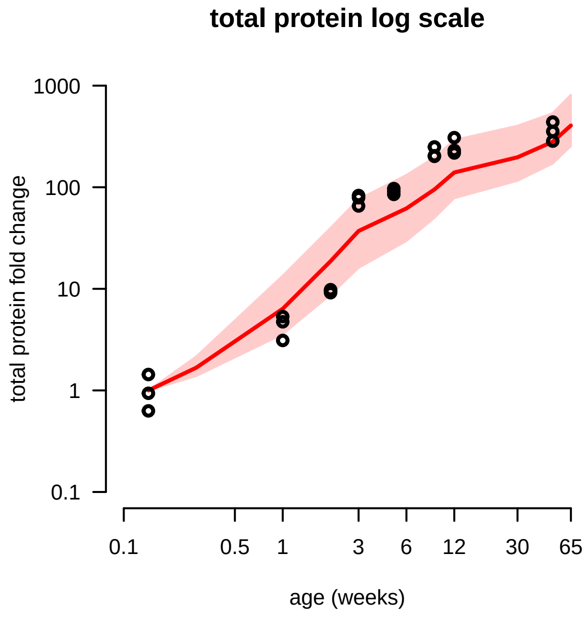

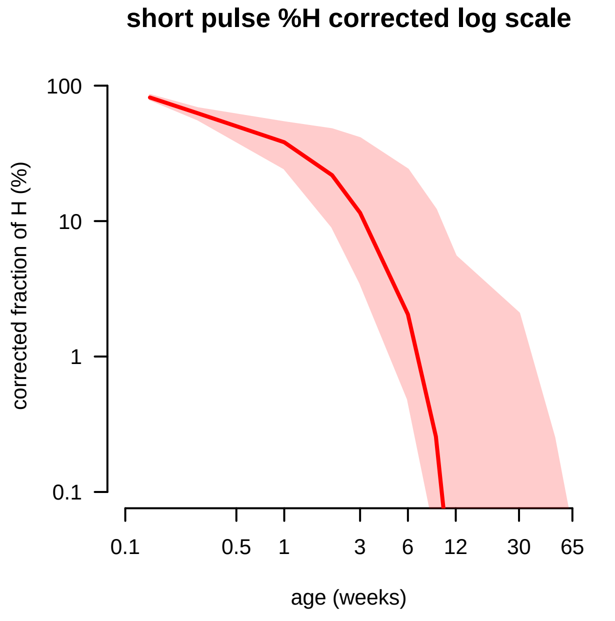

Q8BTU1;Q8BTU1−2

>sp|Q8BTU1|CFA20_MOUSE

Cilia− and flagella−associated protein 20

GN=Cfap20

Med H1/2: 9.9 days

Q5 H1/2: 4.2 days

Q95 H1/2: 99.1 days

reliable model fit: yes

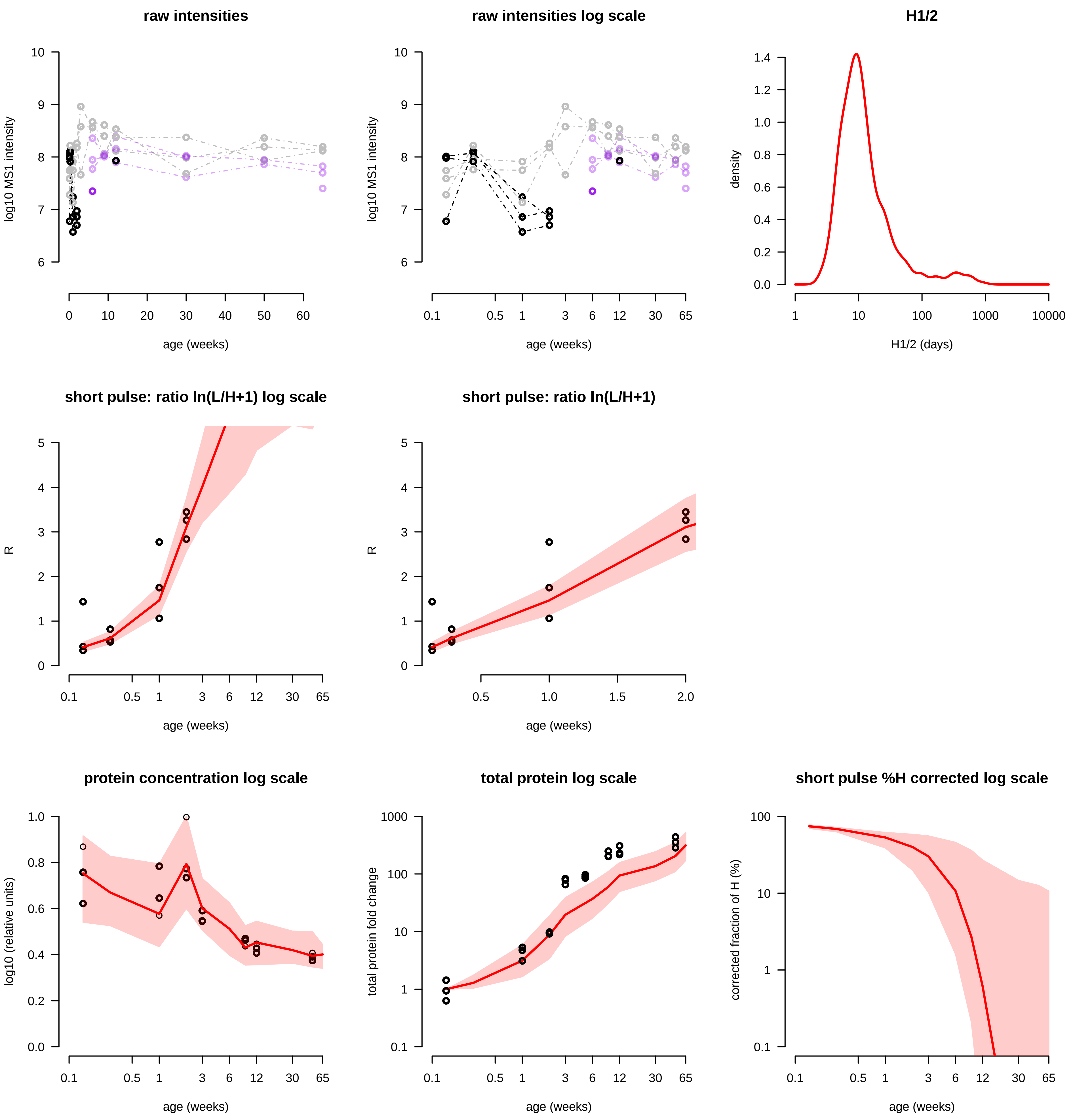

Q9CR39

>sp|Q9CR39|WIPI3_MOUSE

WD repeat domain phosphoinositide−interacting protein 3

GN=Wdr45b

Med H1/2: 3092.8 days

Q5   H1/2: 1017.3 days

Q95 H1/2: 28308.4 days

reliable model fit: yes

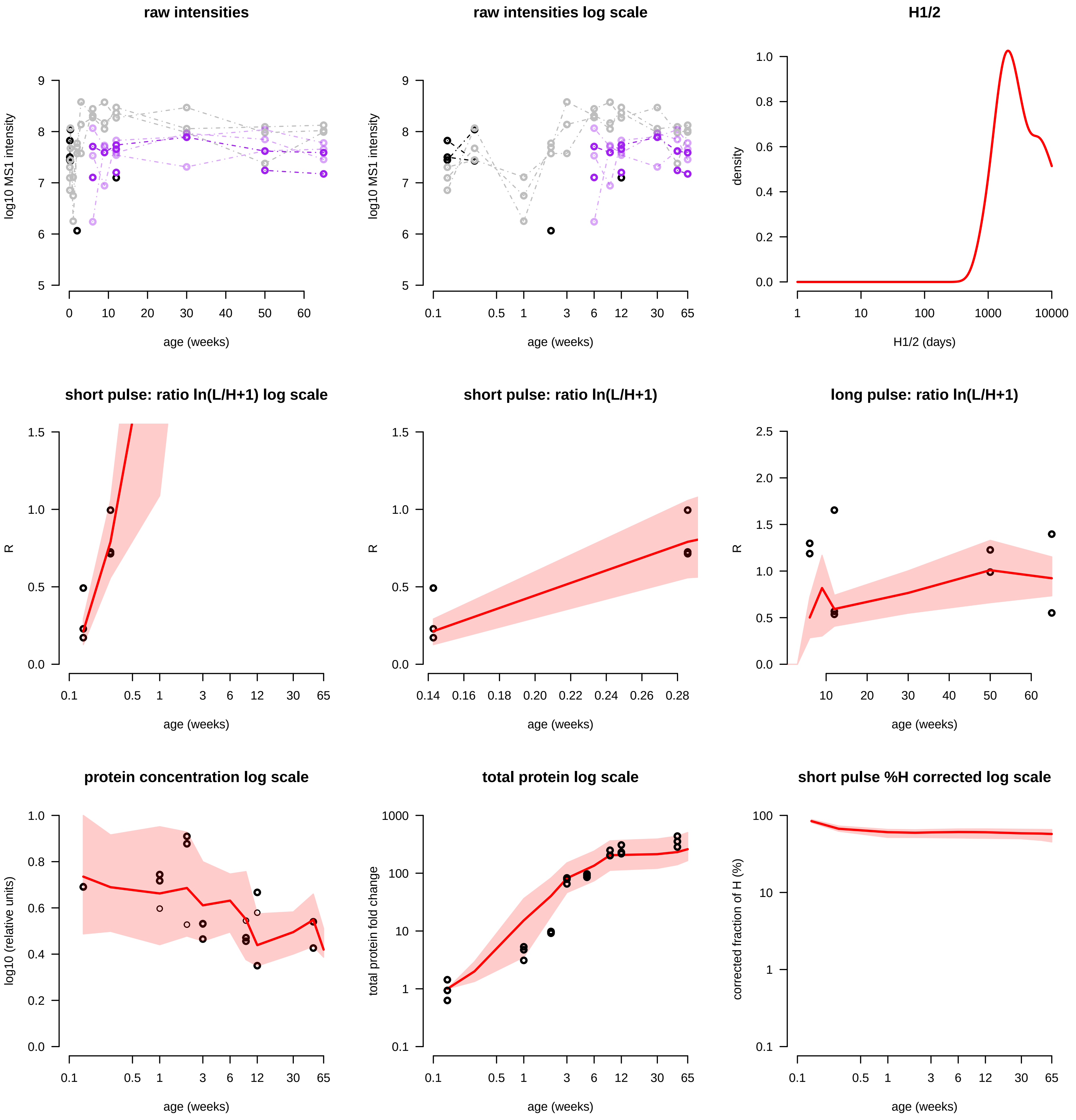

Q8VED9

>sp|Q8VED9|LEGL_MOUSE

Galectin−related protein

GN=Lgalsl

Med H1/2: 3724.5 days

Q5 H1/2: 627.3 days

Q95 H1/2: 16045.1 days

reliable model fit: no

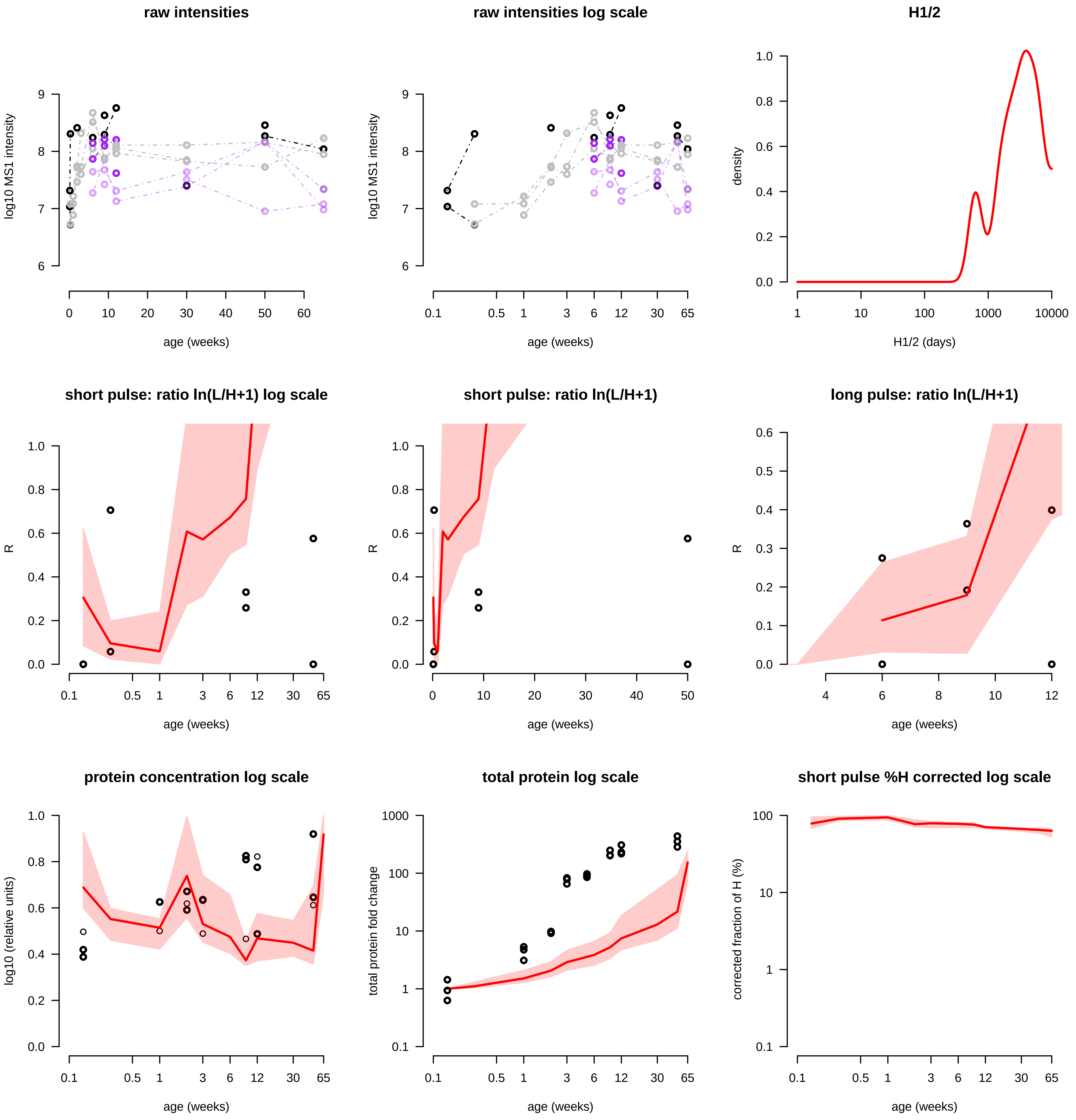

Q80TQ5;Q80TQ5−2;Q80TQ5−3

>sp|Q80TQ5|PKHM2_MOUSE

Pleckstrin homology domain−containing family M member 2

GN=Plekhm2

Med H1/2: 8.4 days

Q5 H1/2: 5.8 days

Q95 H1/2: 17.8 days

reliable model fit: yes

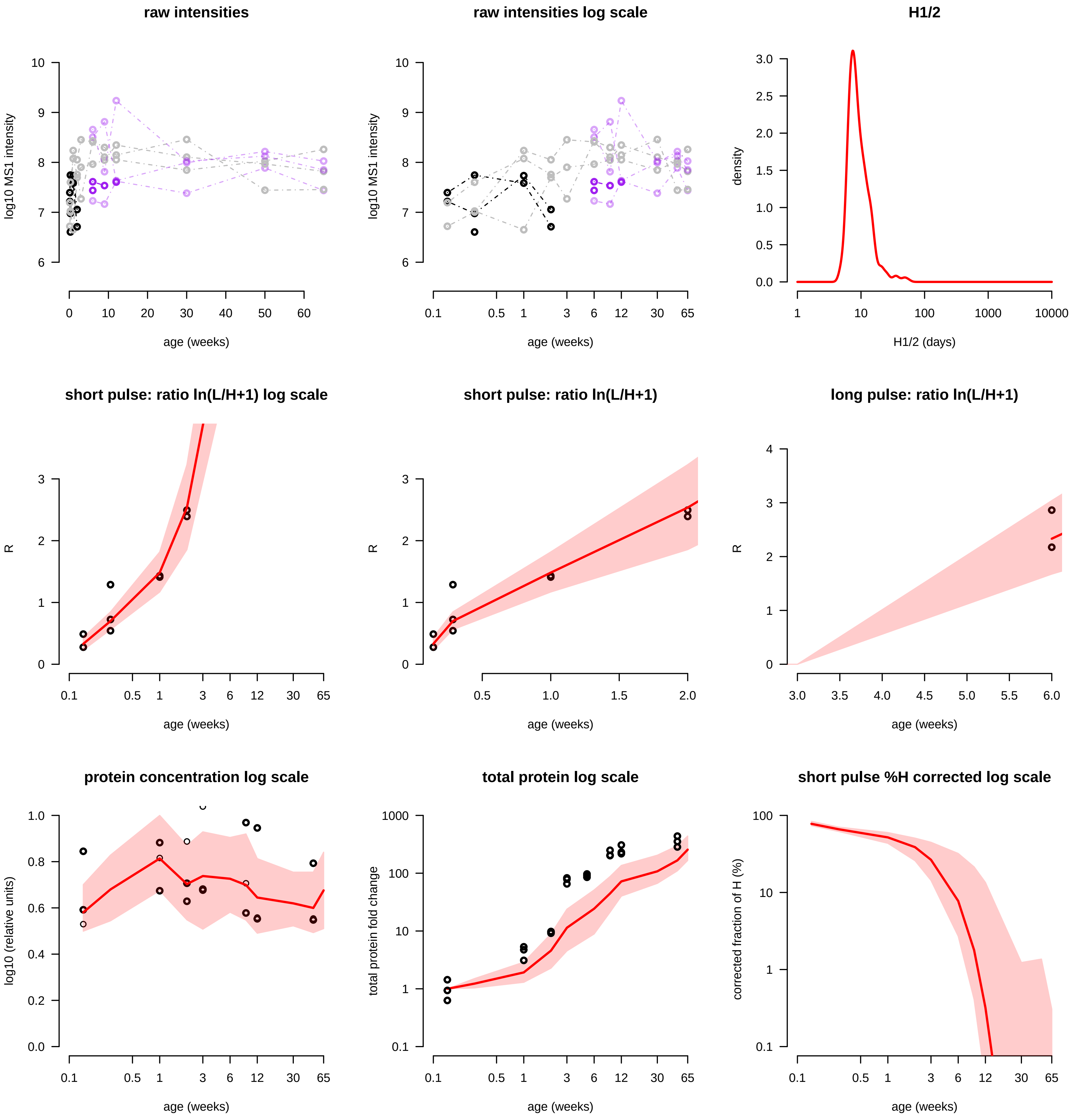

Q91YN5−3;Q91YN5;Q91YN5−2

>sp|Q91YN5−3|UAP1_MOUSE

Isoform 3 of UDP−N−acetylhexosamine pyrophosphorylase

GN=Uap1

Med H1/2: 6.1 days

Q5 H1/2: 2.8 days

Q95 H1/2: 47.7 days

reliable model fit: yes

raw intensities

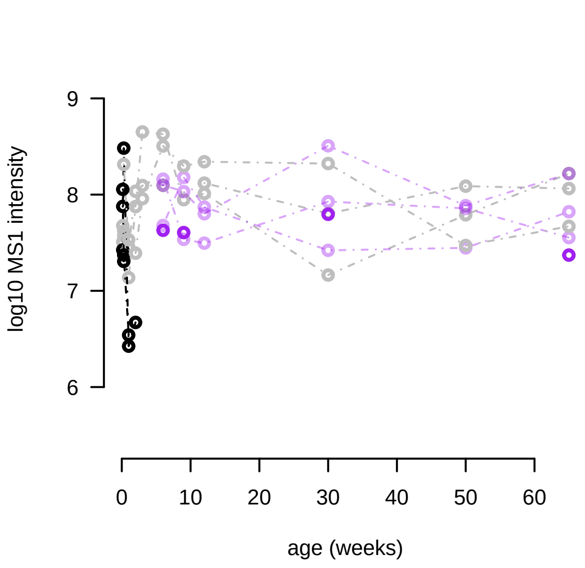

raw intensities log scale

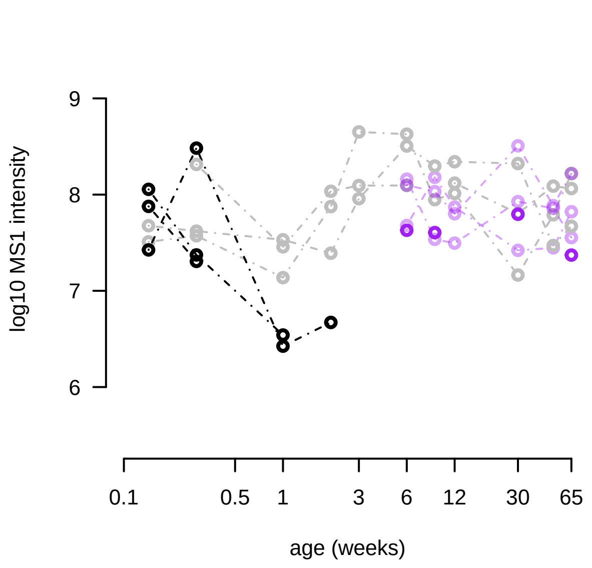

H1/2

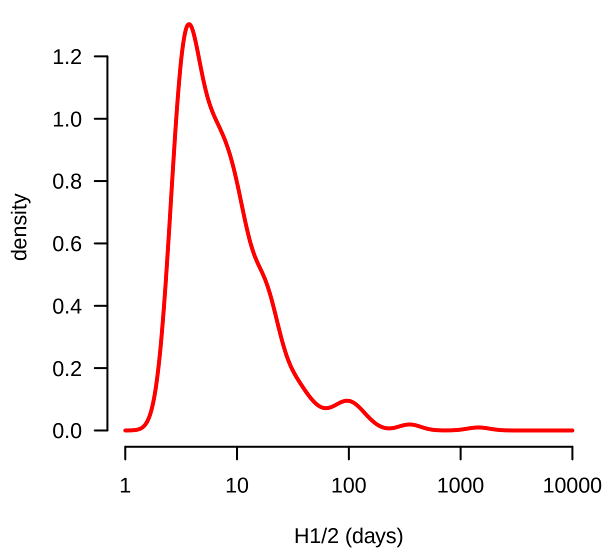

short pulse: ratio ln(L/H+1) log scale

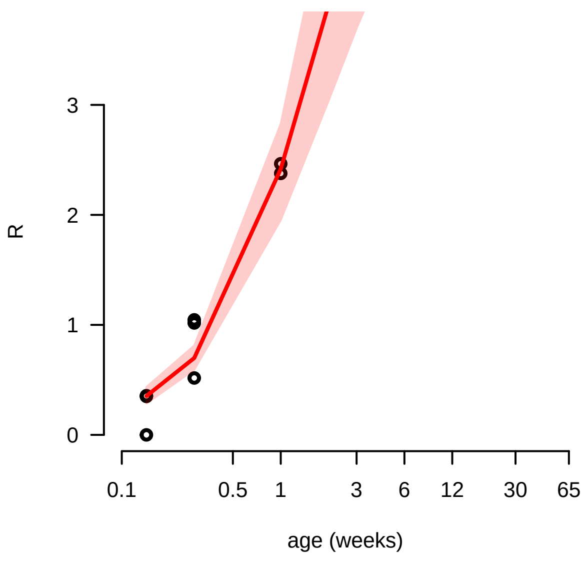

short pulse: ratio ln(L/H+1)

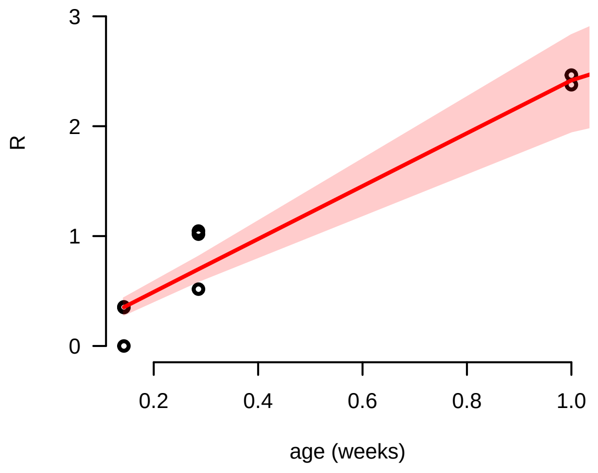

protein concentration log scale

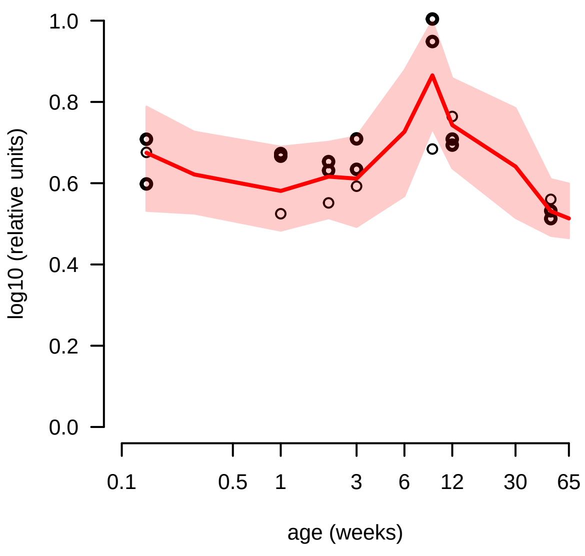

total protein log scale

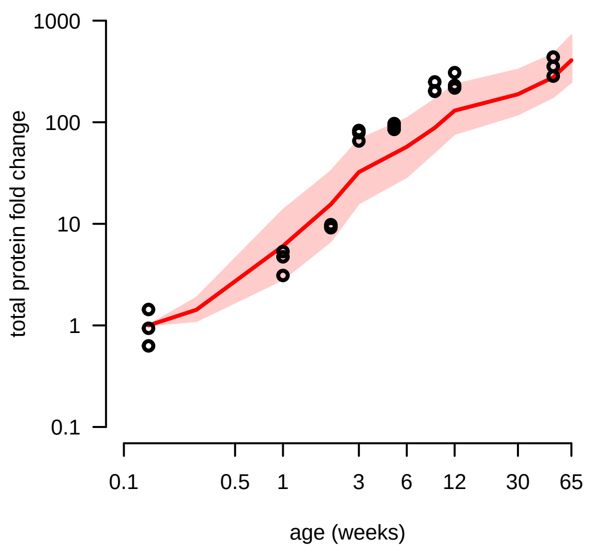

short pulse %H corrected log scale

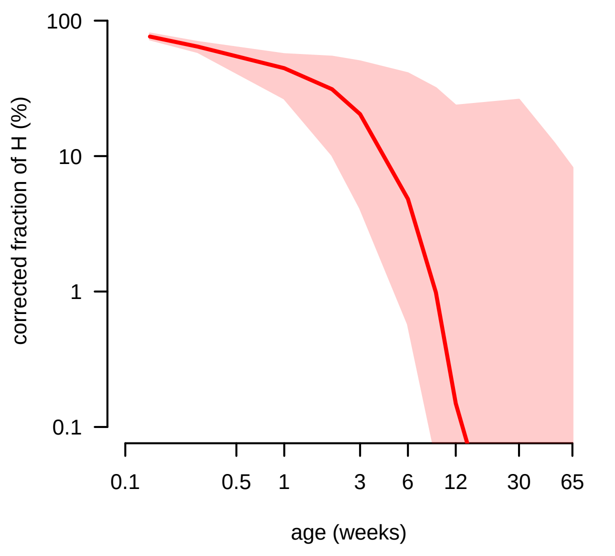

Q5U4D9

>sp|Q5U4D9|THOC6_MOUSE

THO complex subunit 6 homolog

GN=Thoc6

Med H1/2: 8.2 days

Q5 H1/2: 2.9 days

Q95 H1/2: 92 days

reliable model fit: yes

raw intensities

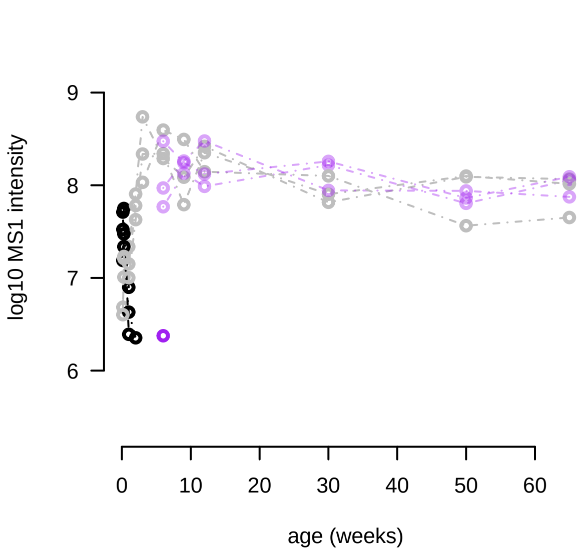

raw intensities log scale

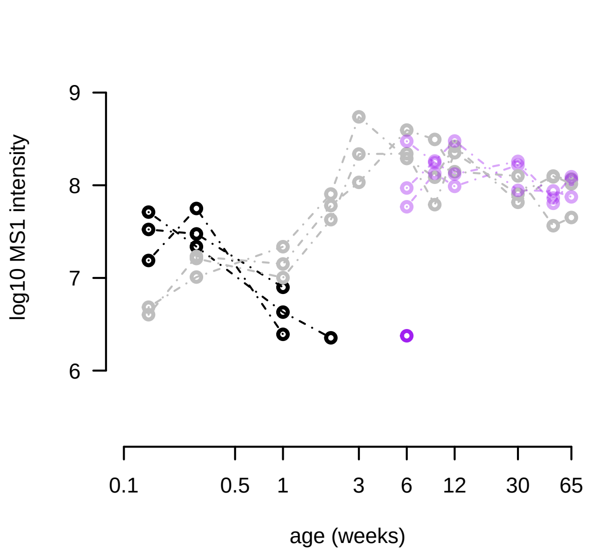

H1/2

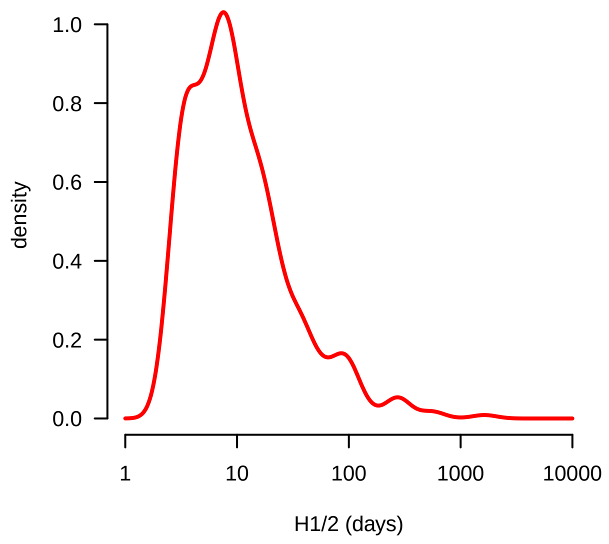

short pulse: ratio ln(L/H+1) log scale

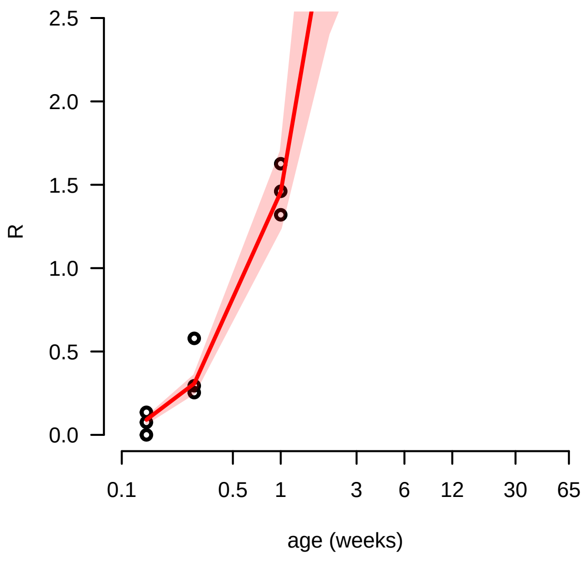

short pulse: ratio ln(L/H+1)

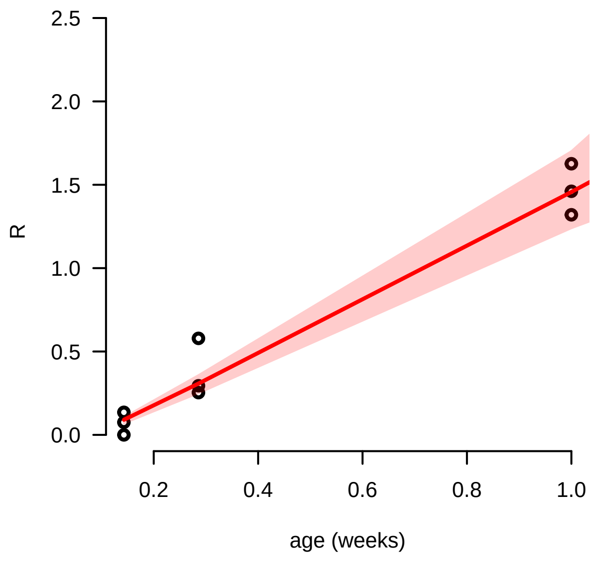

protein concentration log scale

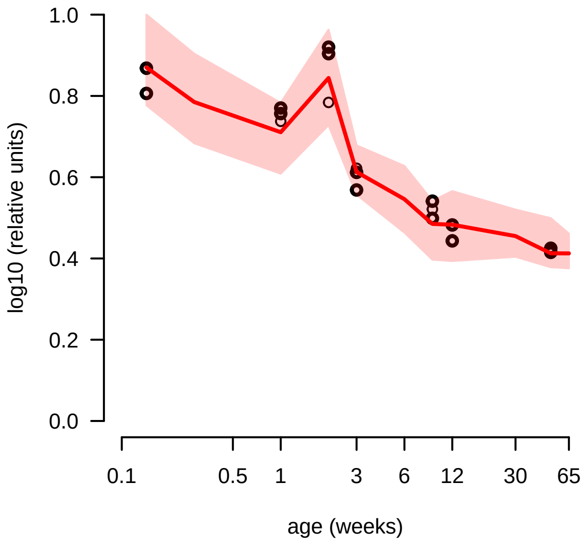

total protein log scale

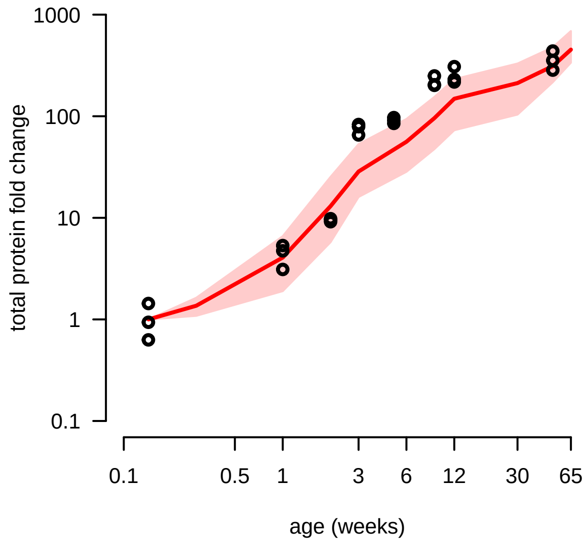

short pulse %H corrected log scale

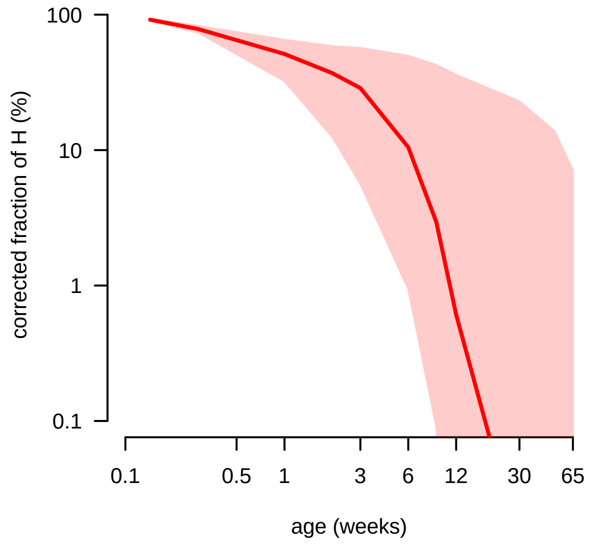

P21550

>sp|P21550|ENOB_MOUSE

Beta−enolase

GN=Eno3

Med H1/2: 4.8 days

Q5 H1/2: 2.8 days

Q95 H1/2: 20.2 days

reliable model fit: yes

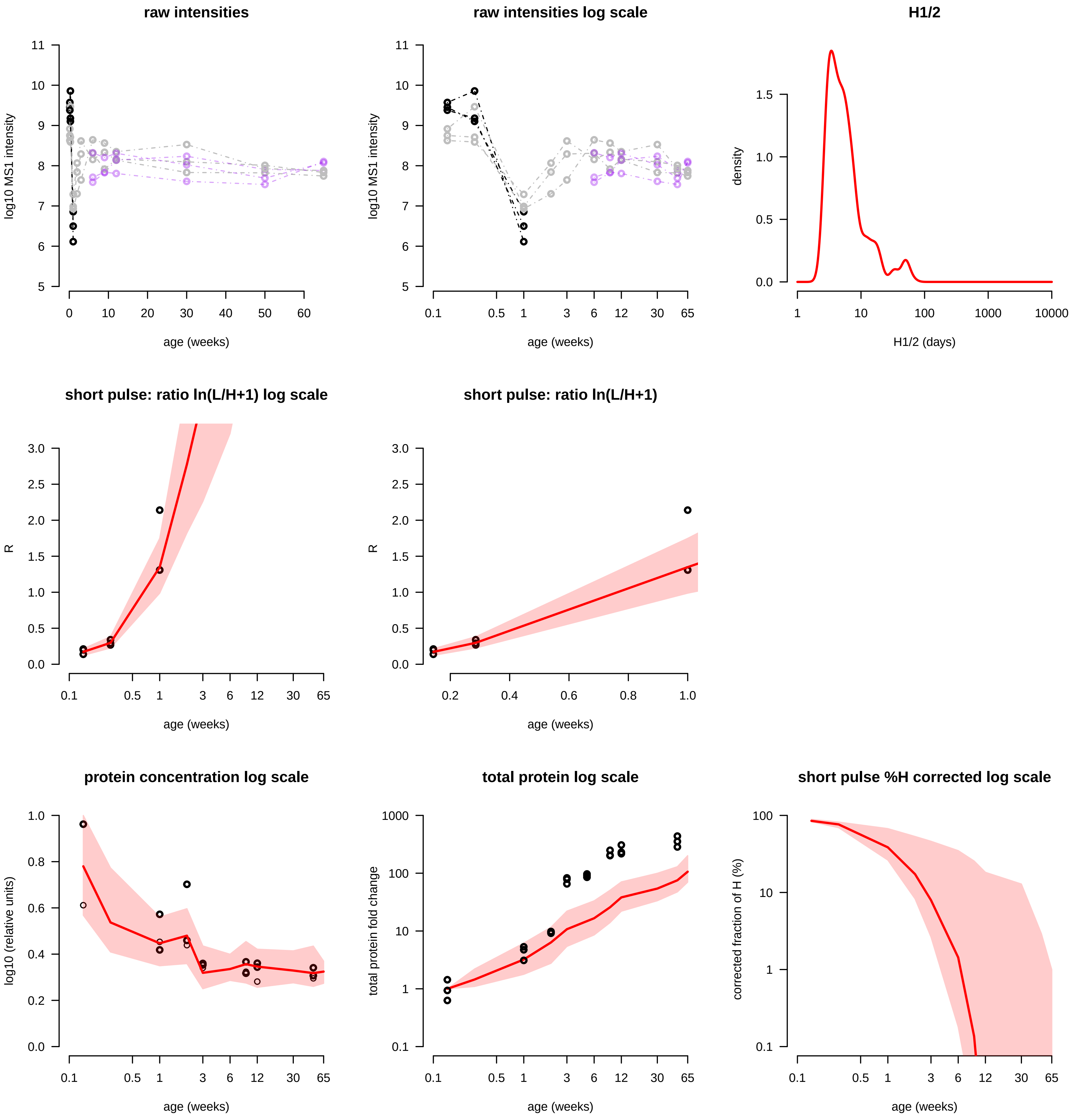

Q9DBS9

>sp|Q9DBS9|OSBL3_MOUSE

Oxysterol−binding protein−related protein 3

GN=Osbpl3

Med H1/2: 20.2 days

Q5 H1/2: 12.7 days

Q95 H1/2: 39.1 days

reliable model fit: yes

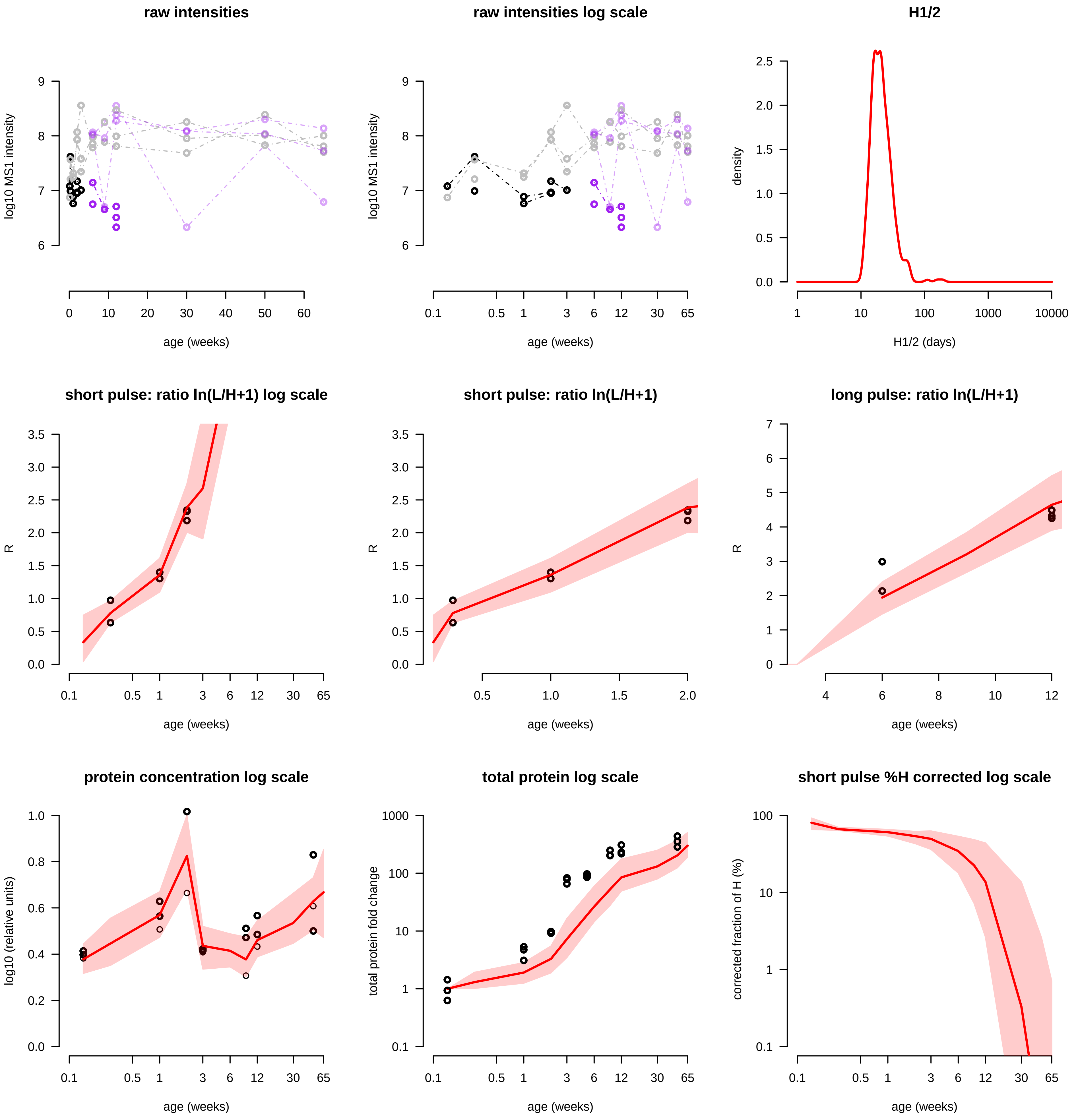

Q9D1K7

>sp|Q9D1K7|CT027_MOUSE

UPF0687 protein C20orf27 homolog

Med H1/2: 4.3 days

Q5 H1/2: 2.6 days

Q95 H1/2: 65 days

reliable model fit: yes

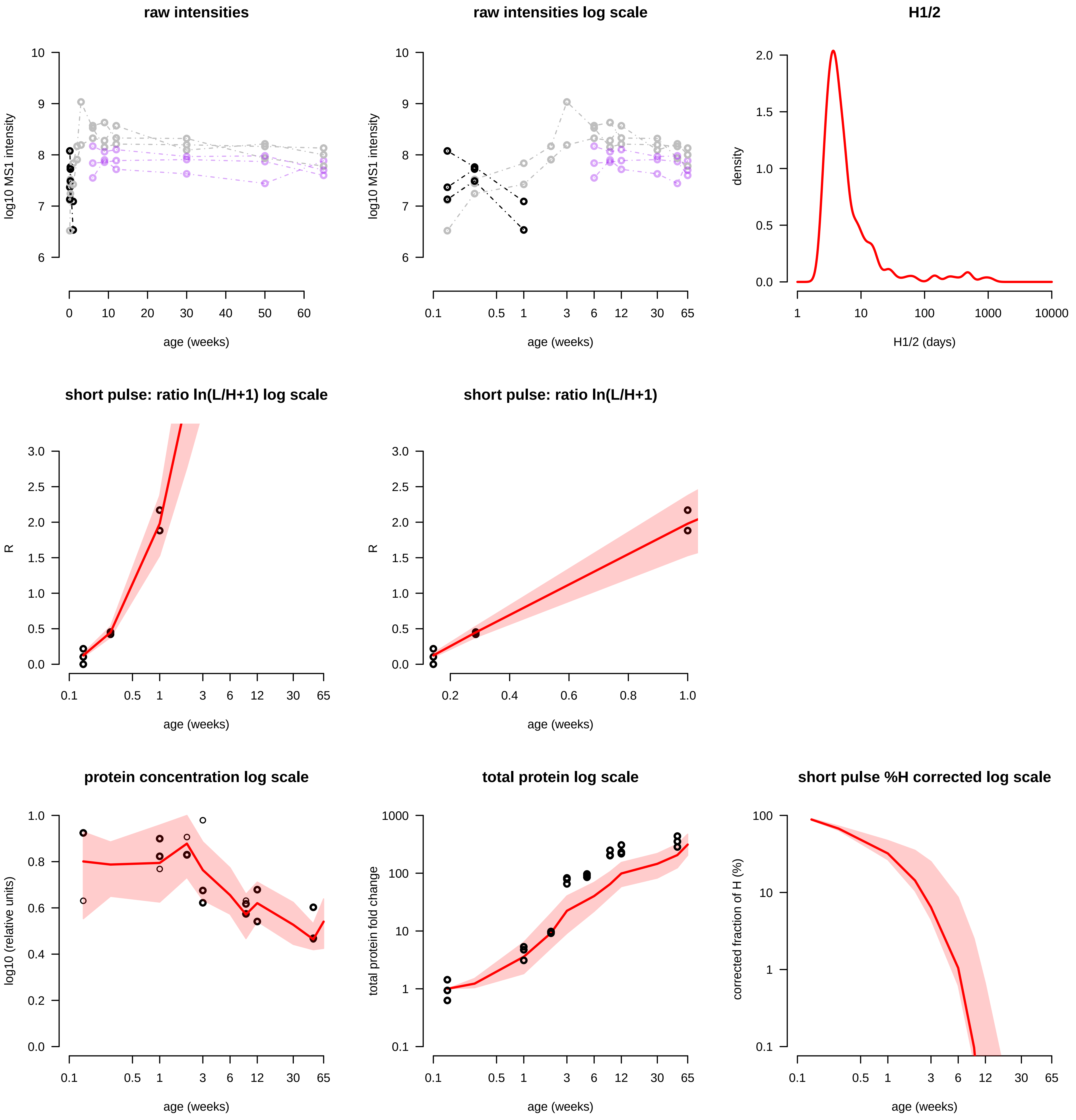

P70202

>sp|P70202|LXN_MOUSE

Latexin

GN=Lxn

Med H1/2: 11.1 days

Q5 H1/2: 5.3 days

Q95 H1/2: 68.9 days

reliable model fit: yes

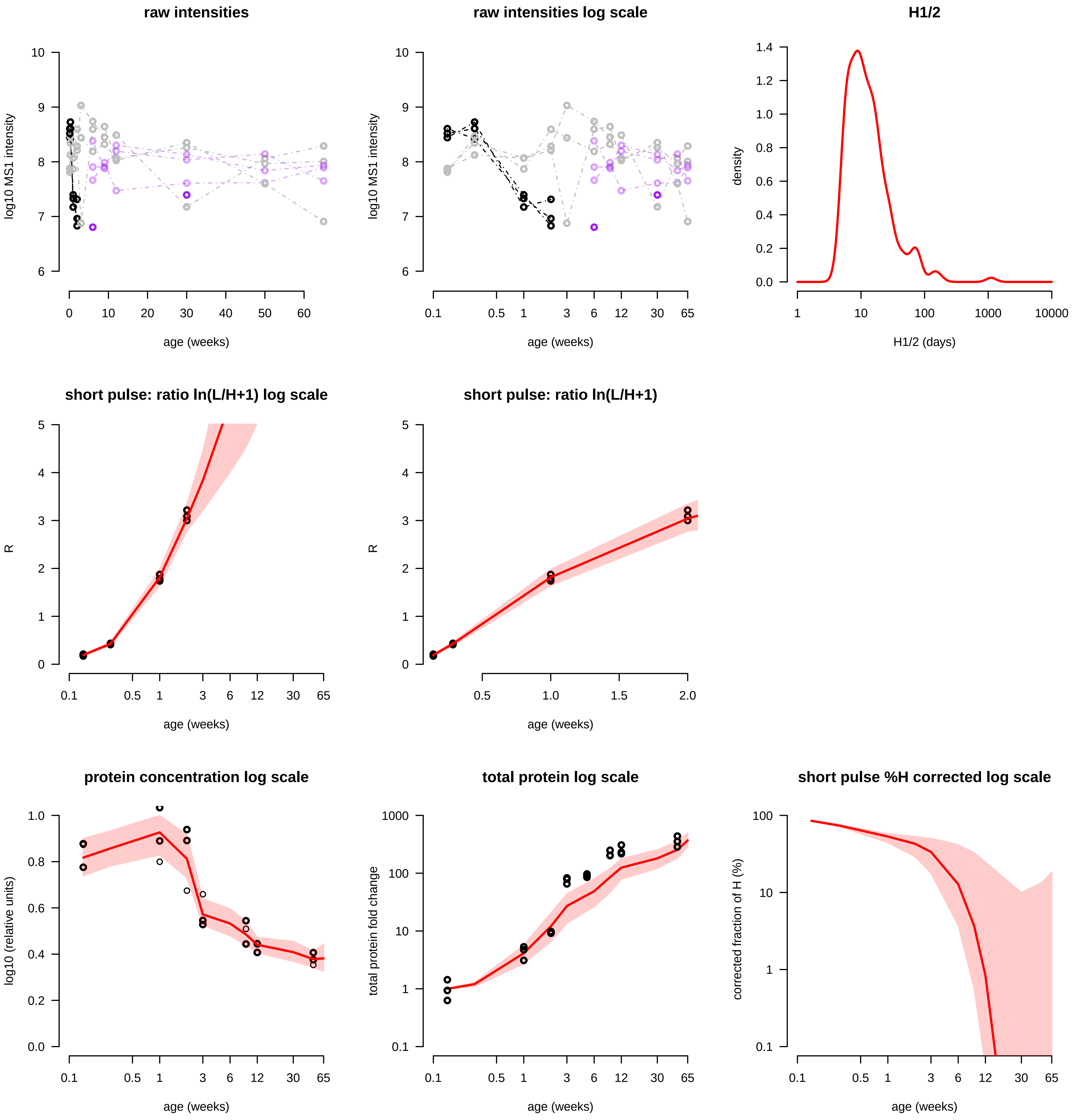

Q8R3F5

>sp|Q8R3F5|FABD_MOUSE

Malonyl−CoA−acyl carrier protein transacylase, mitochondrial

GN=Mcat

Med H1/2: 7.6 days

Q5 H1/2: 3.2 days

Q95 H1/2: 37.4 days

reliable model fit: yes

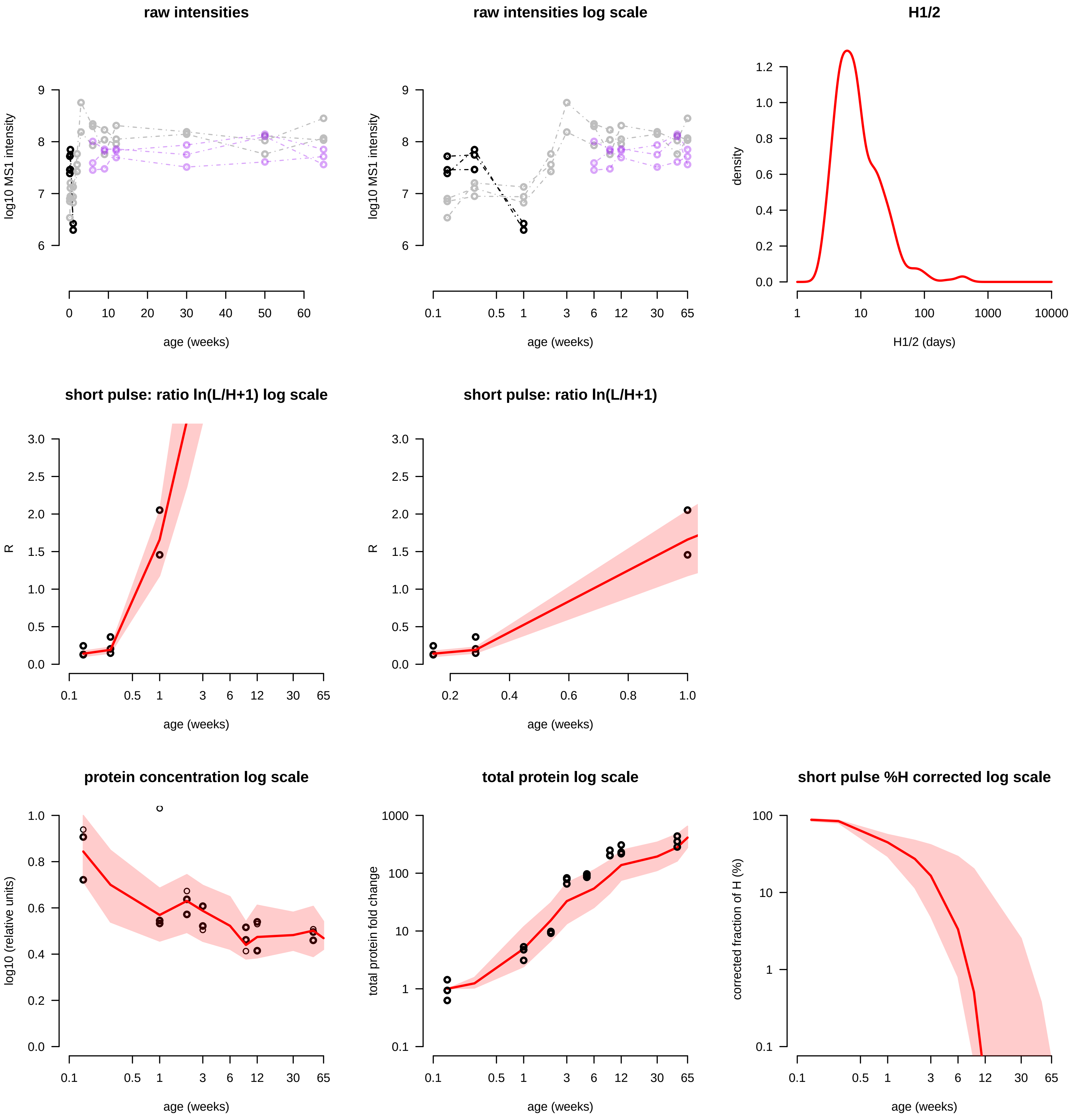

P97376

>sp|P97376|FRG1_MOUSE

Protein FRG1

GN=Frg1

Med H1/2: 5.3 days

Q5 H1/2: 2.6 days

Q95 H1/2: 40 days

reliable model fit: yes

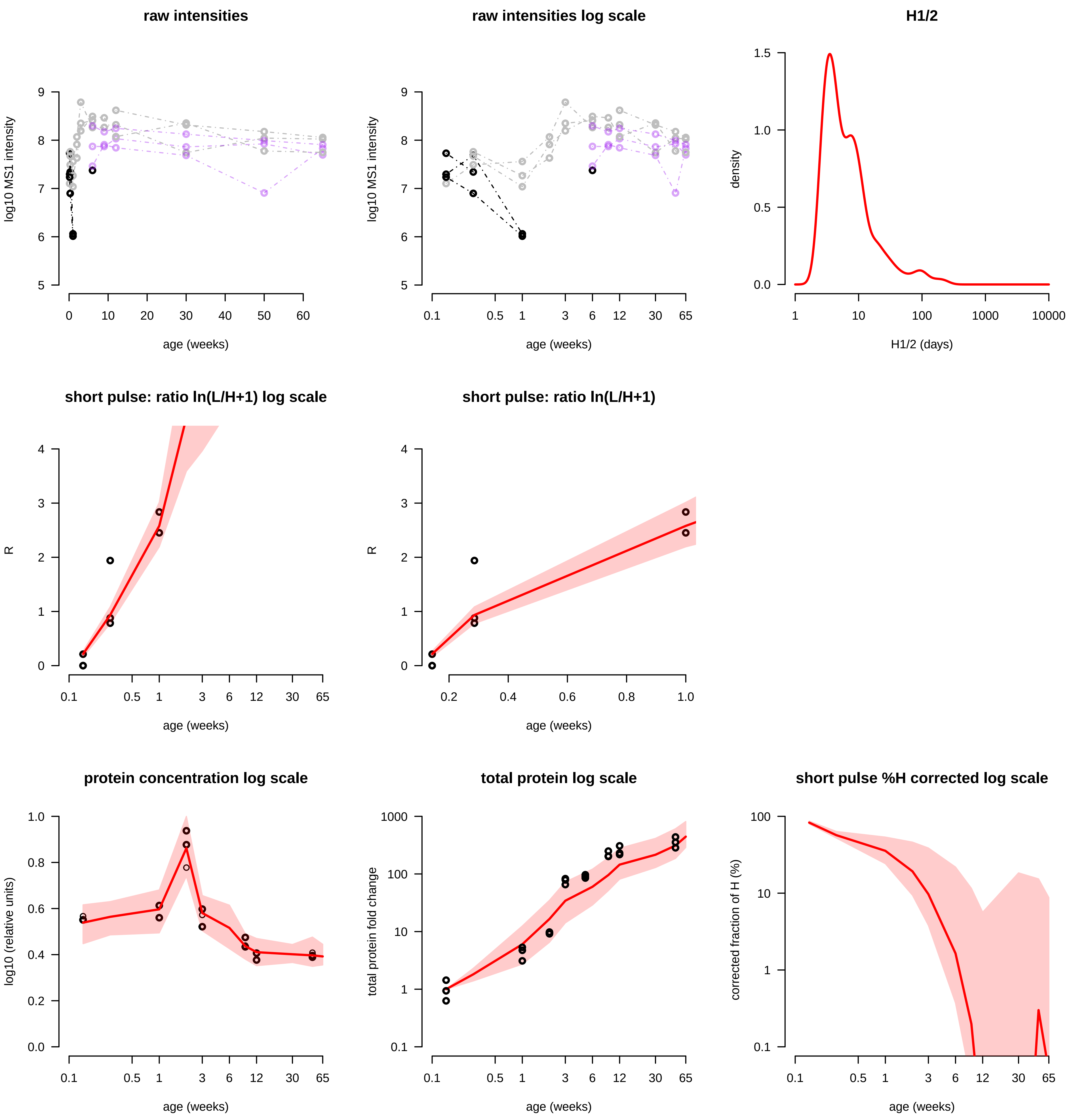

P26618;P26618−2

>sp|P26618|PGFRA_MOUSE

Platelet−derived growth factor receptor alpha

GN=Pdgfra

Med H1/2: 2540.1 days

Q5 H1/2: 629.8 days

Q95 H1/2: 79091.5 days

reliable model fit: yes

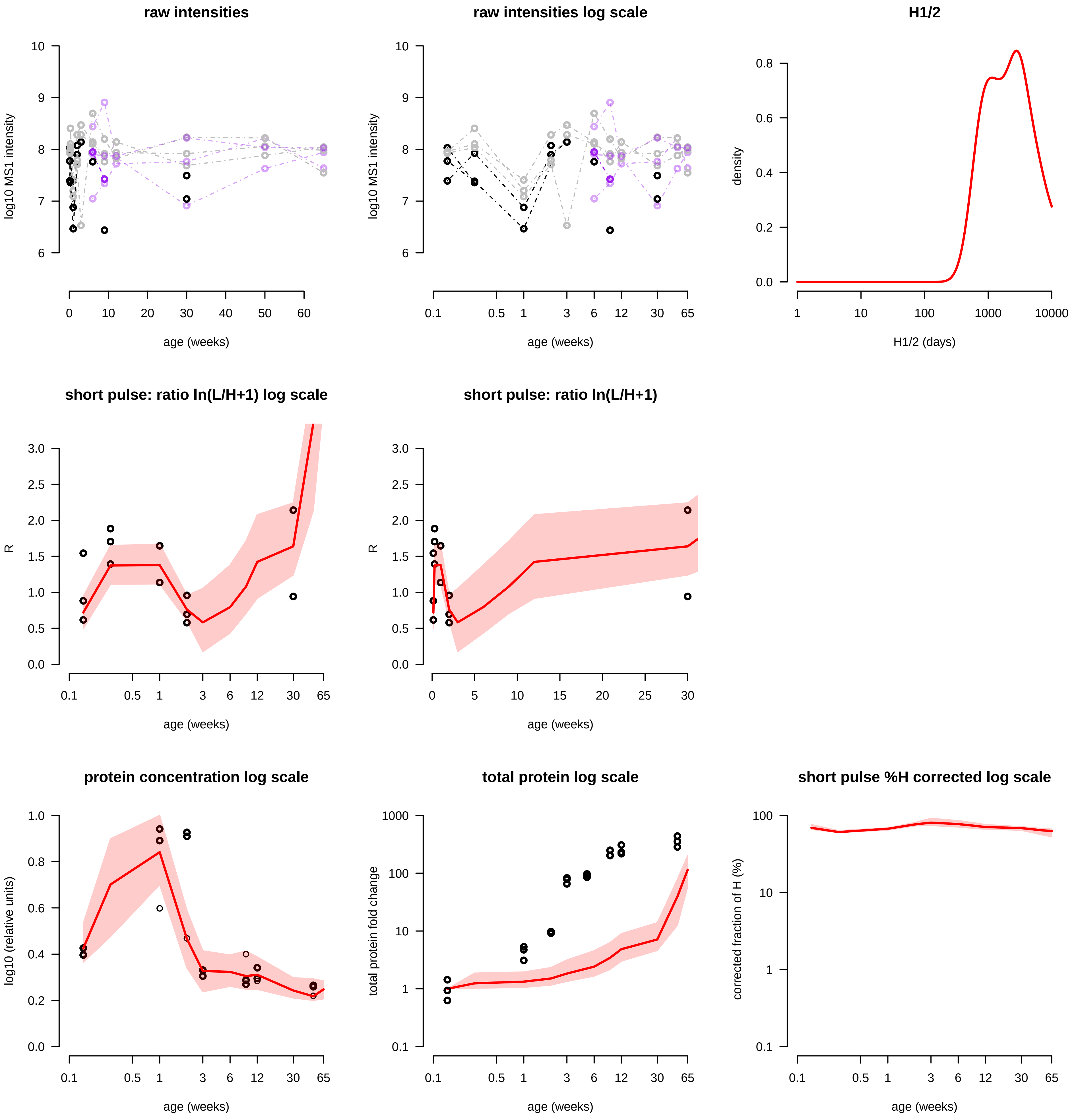

Q8BMS9

>sp|Q8BMS9|RASF2_MOUSE

Ras association domain−containing protein 2

GN=Rassf2

Med H1/2: 6.6 days

Q5 H1/2: 2.9 days

Q95 H1/2: 45.8 days

reliable model fit: yes

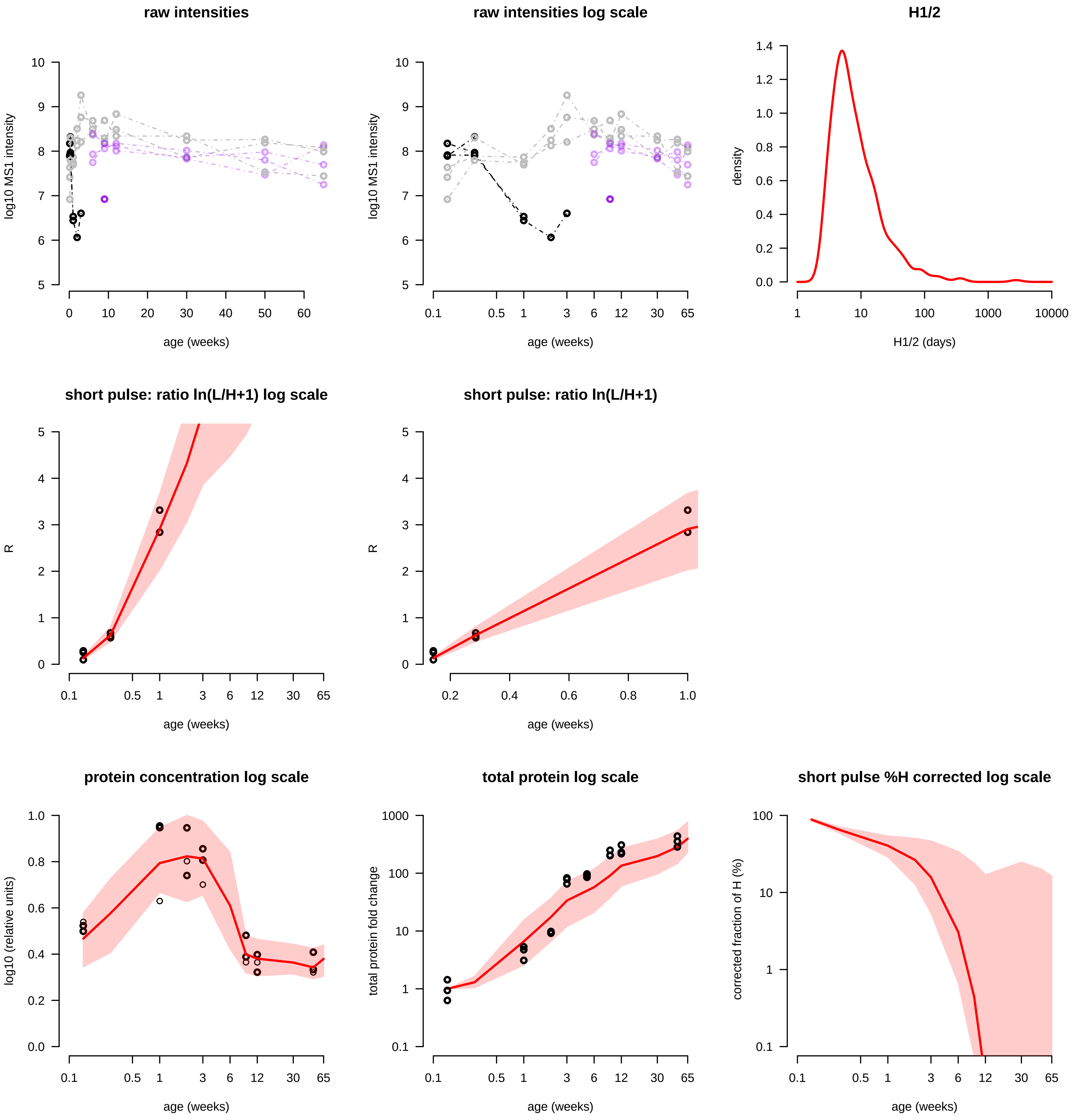

Q7TQK1;Q7TQK1−2

>sp|Q7TQK1|INT7_MOUSE

Integrator complex subunit 7

GN=Ints7

Med H1/2: 28.5 days

Q5 H1/2: 12.6 days

Q95 H1/2: 198.6 days

reliable model fit: yes

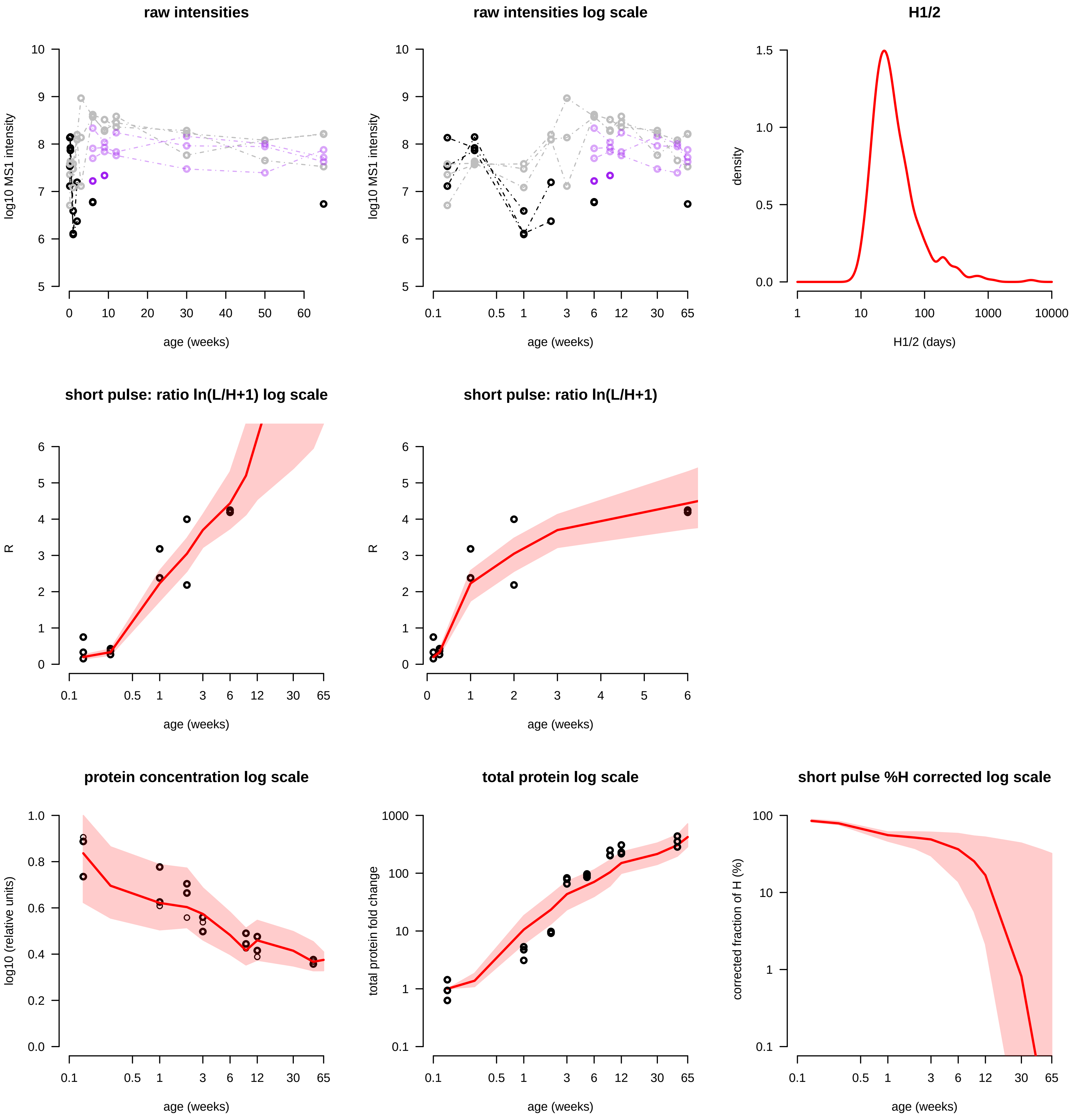

Q8C1D8

>sp|Q8C1D8|IWS1_MOUSE

Protein IWS1 homolog

GN=Iws1

Med H1/2: 7.1 days

Q5 H1/2: 2.9 days

Q95 H1/2: 68.5 days

reliable model fit: yes

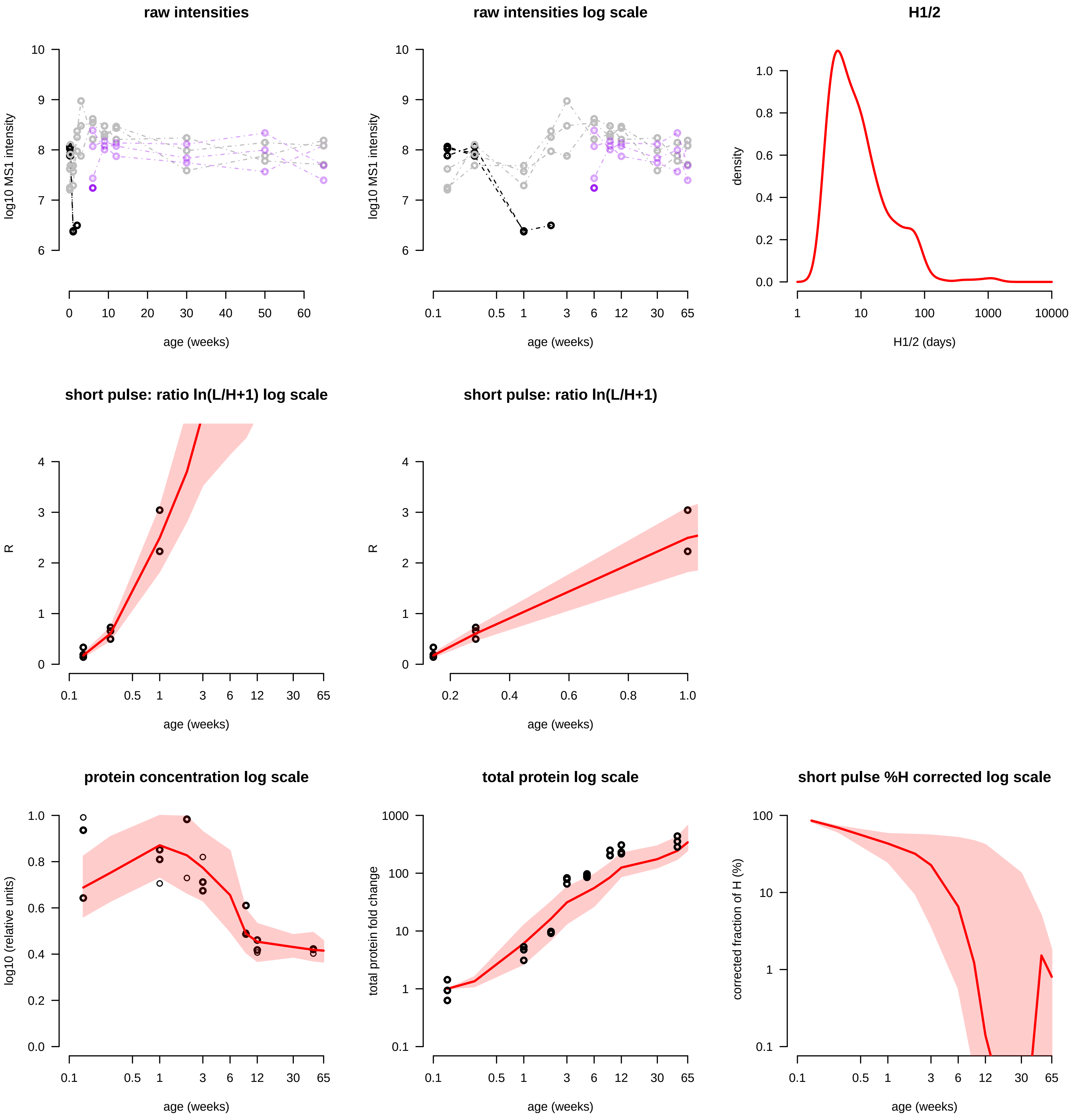

Q5DTT3

>sp|Q5DTT3|TASO2_MOUSE

Protein TASOR 2

GN=Tasor2

Med H1/2: 4844.7 days

Q5 H1/2: 1112.5 days

Q95 H1/2: 46417.6 days

reliable model fit: no

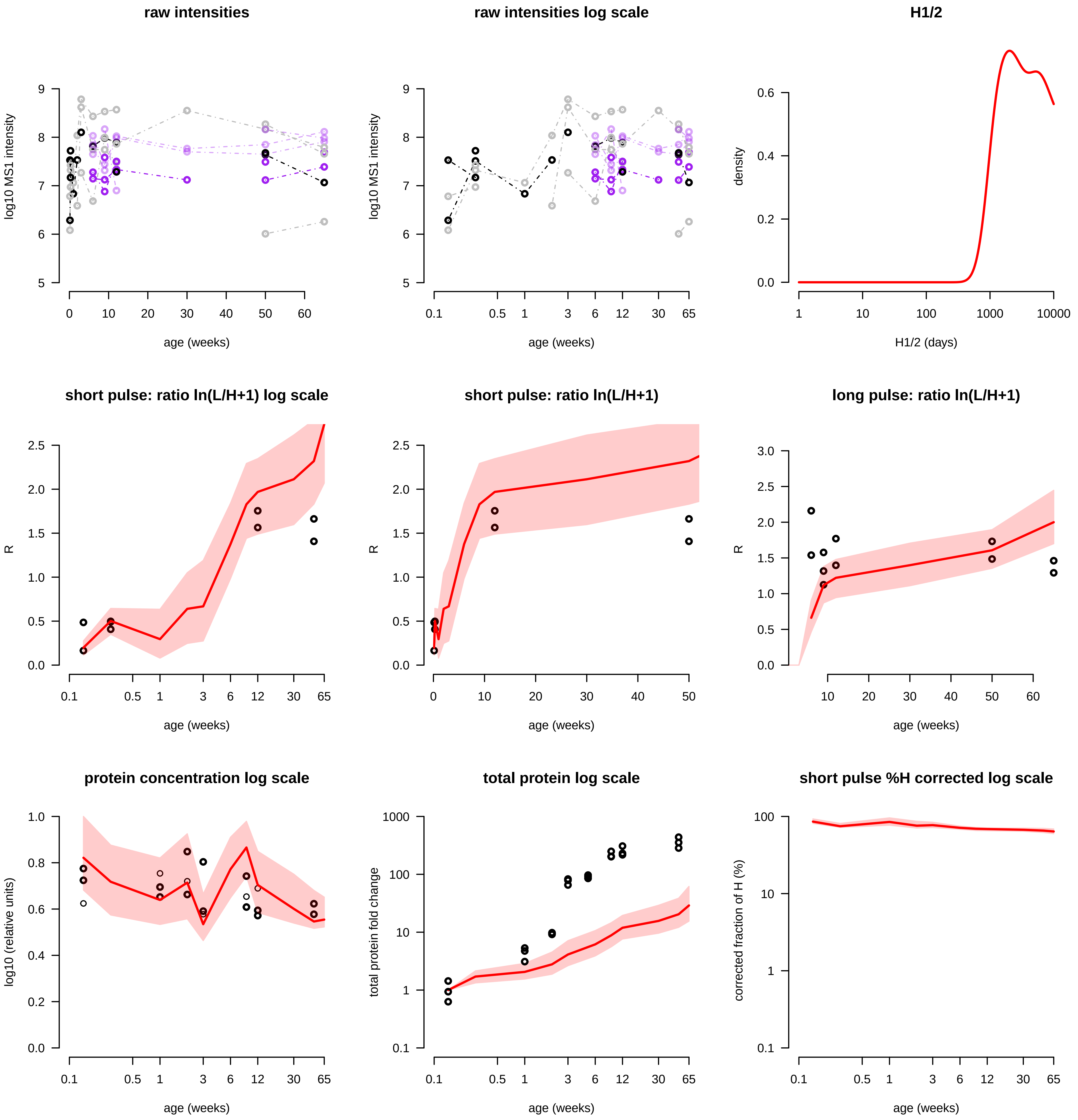

Q78PG9

>sp|Q78PG9|CCD25_MOUSE

Coiled−coil domain−containing protein 25

GN=Ccdc25

Med H1/2: 9.6 days

Q5 H1/2: 3.4 days

Q95 H1/2: 106.1 days

reliable model fit: yes

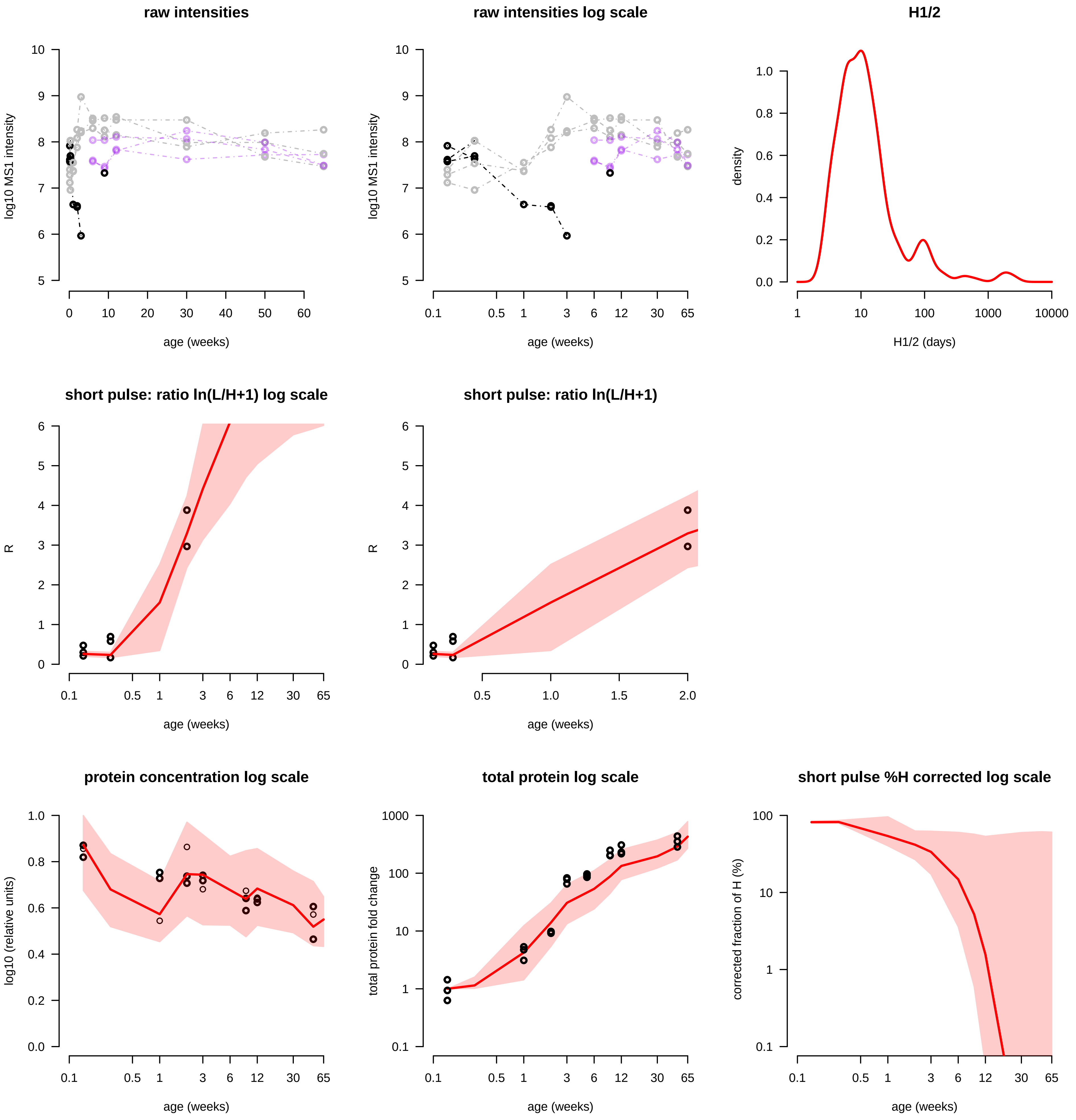

Q921G8

>sp|Q921G8|GCP2_MOUSE

Gamma−tubulin complex component 2

GN=Tubgcp2

Med H1/2: 43.5 days

Q5 H1/2: 12.1 days

Q95 H1/2: 465.8 days

reliable model fit: no

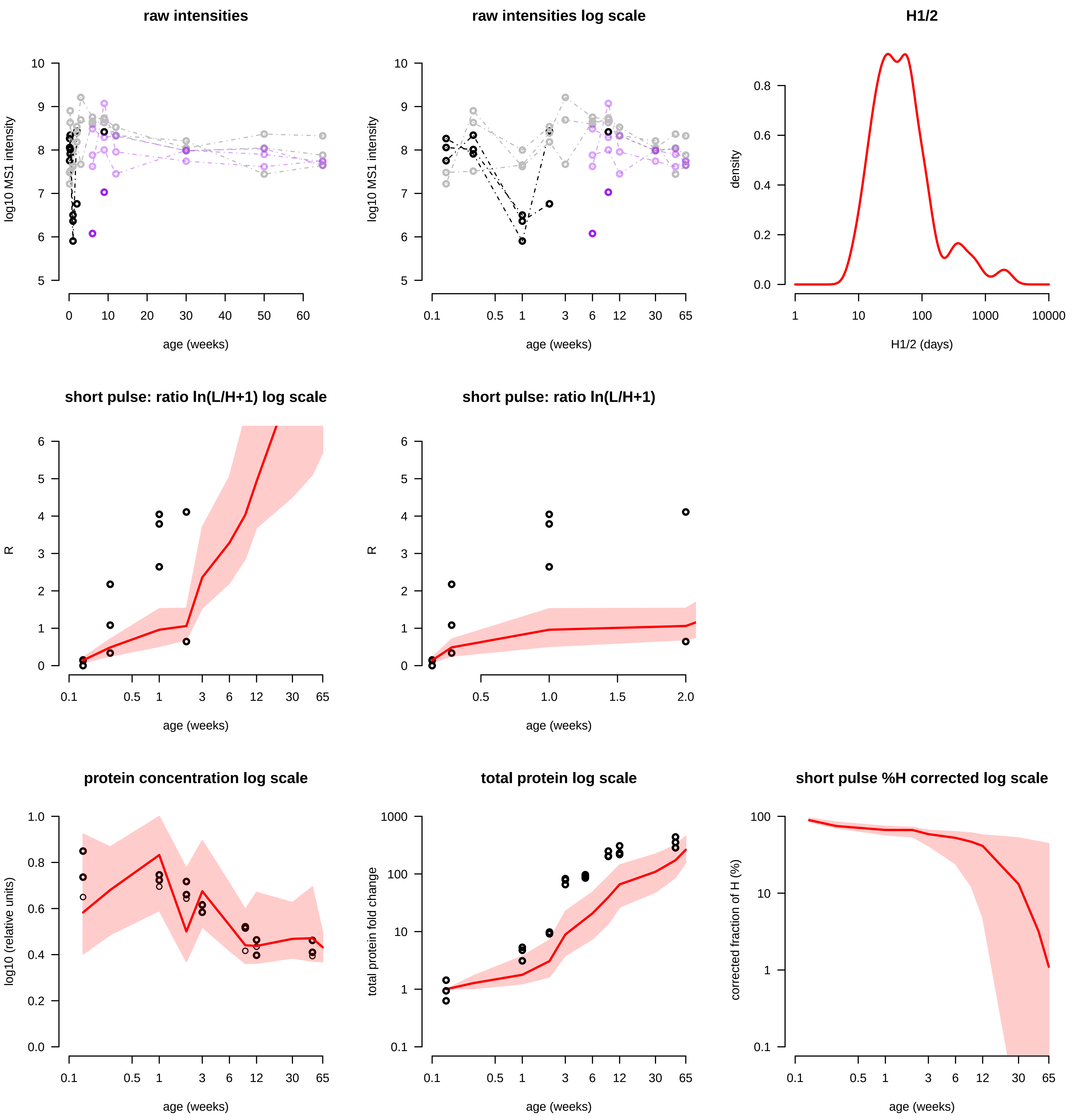

Q9R226

>sp|Q9R226|KHDR3_MOUSE

KH domain−containing, RNA−binding, signal transduction−associated protein 3

GN=Khdrbs3

Med H1/2: 5.2 days

Q5 H1/2: 4.1 days

Q95 H1/2: 7.5 days

reliable model fit: yes

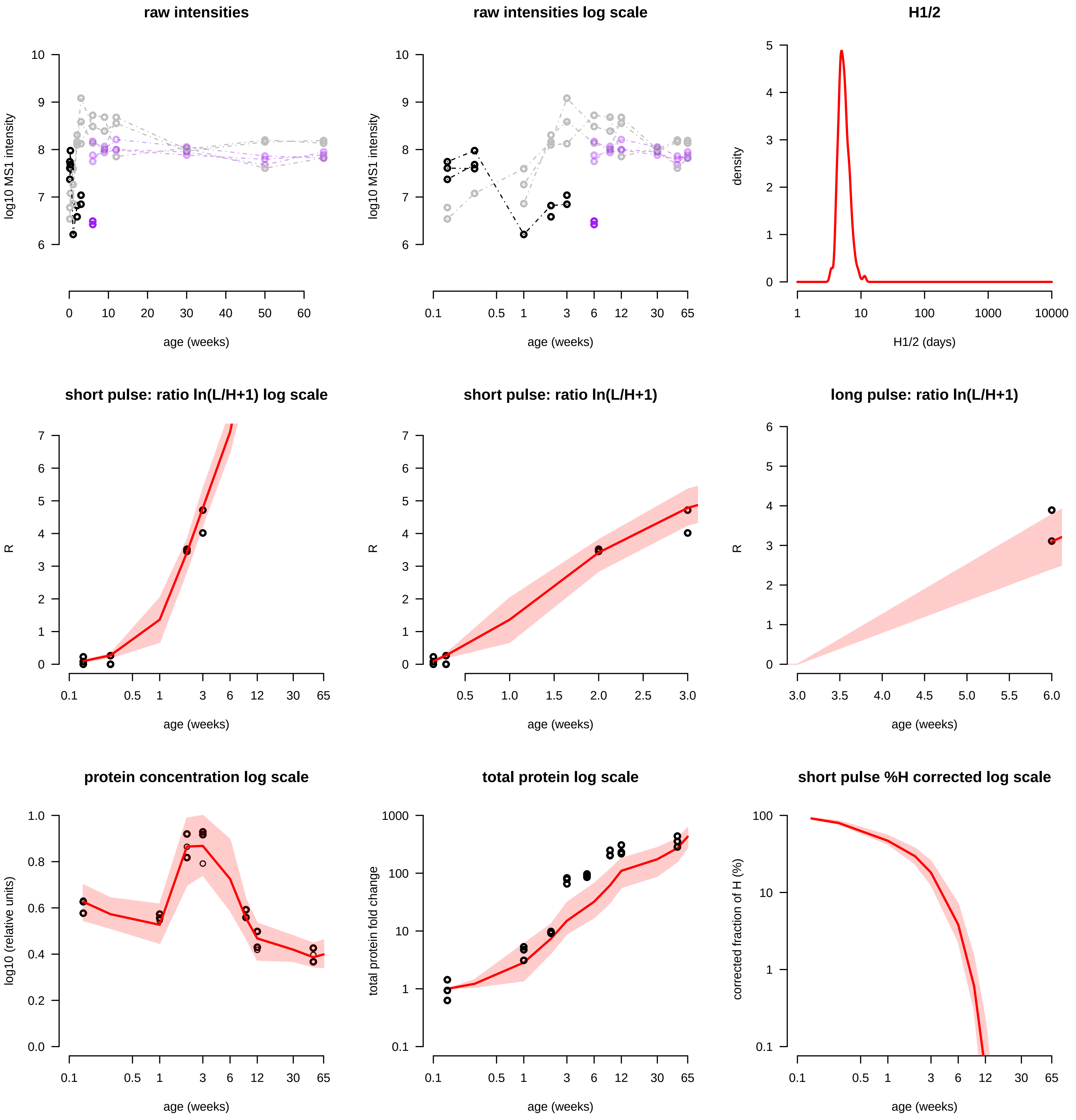

O35239

>sp|O35239|PTN9_MOUSE

Tyrosine−protein phosphatase non−receptor type 9

GN=Ptpn9

Med H1/2: 311.4 days

Q5 H1/2: 137.1 days

Q95 H1/2: 3206.3 days

reliable model fit: no

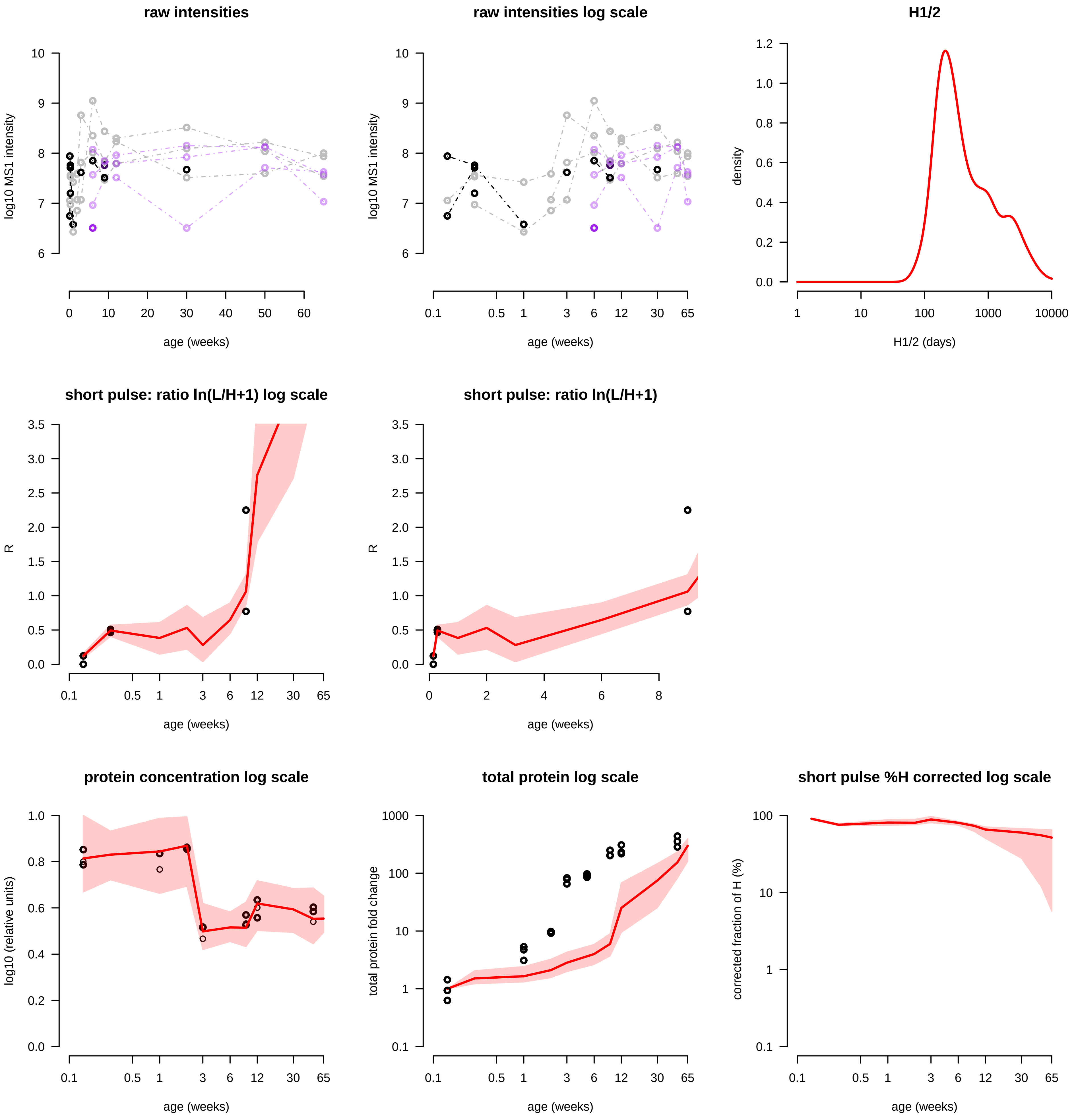

P47968

>sp|P47968|RPIA_MOUSE

Ribose−5−phosphate isomerase

GN=Rpia

Med H1/2: 6.9 days

Q5 H1/2: 3 days

Q95 H1/2: 51 days

reliable model fit: no

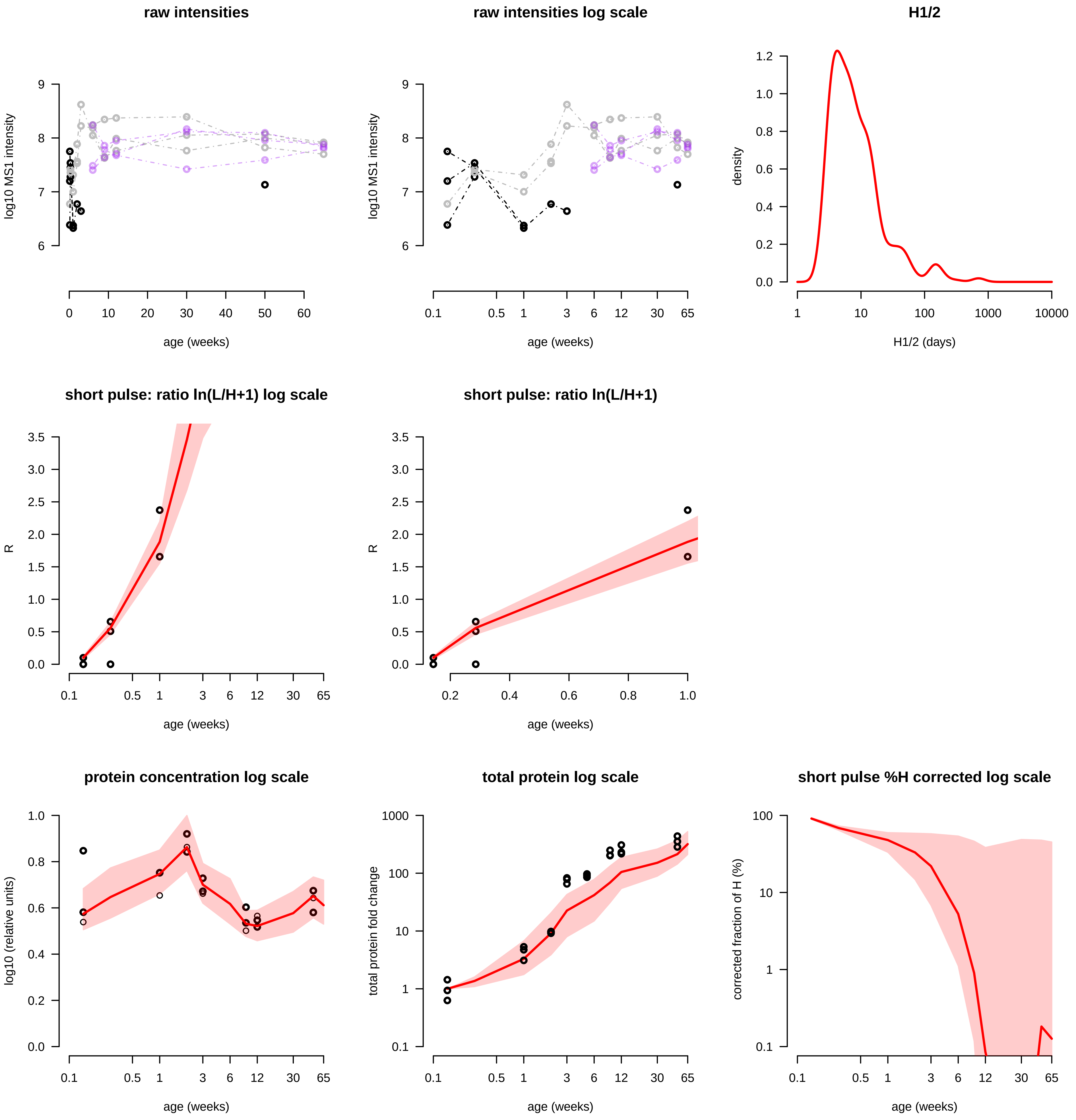

Q8VCX5;Q8VCX5−3;Q8VCX5−2

>sp|Q8VCX5|MICU1_MOUSE

Calcium uptake protein 1, mitochondrial

GN=Micu1

Med H1/2: 9 days

Q5 H1/2: 2.7 days

Q95 H1/2: 91.9 days

reliable model fit: yes

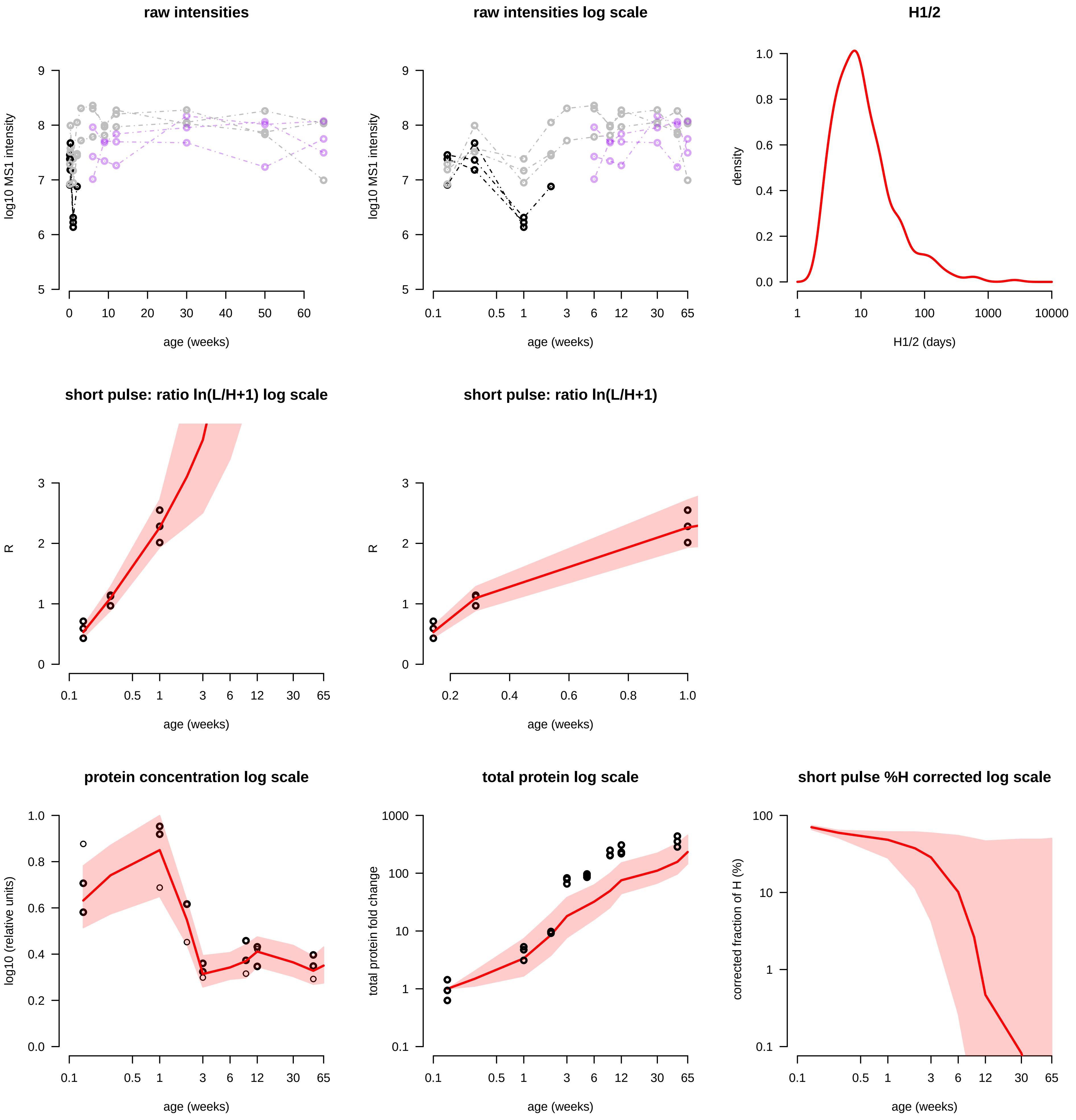

Q9CWI3

>sp|Q9CWI3|BCCIP_MOUSE

BRCA2 and CDKN1A−interacting protein

GN=Bccip

Med H1/2: 26.6 days

Q5 H1/2: 7.3 days

Q95 H1/2: 447.9 days

reliable model fit: yes

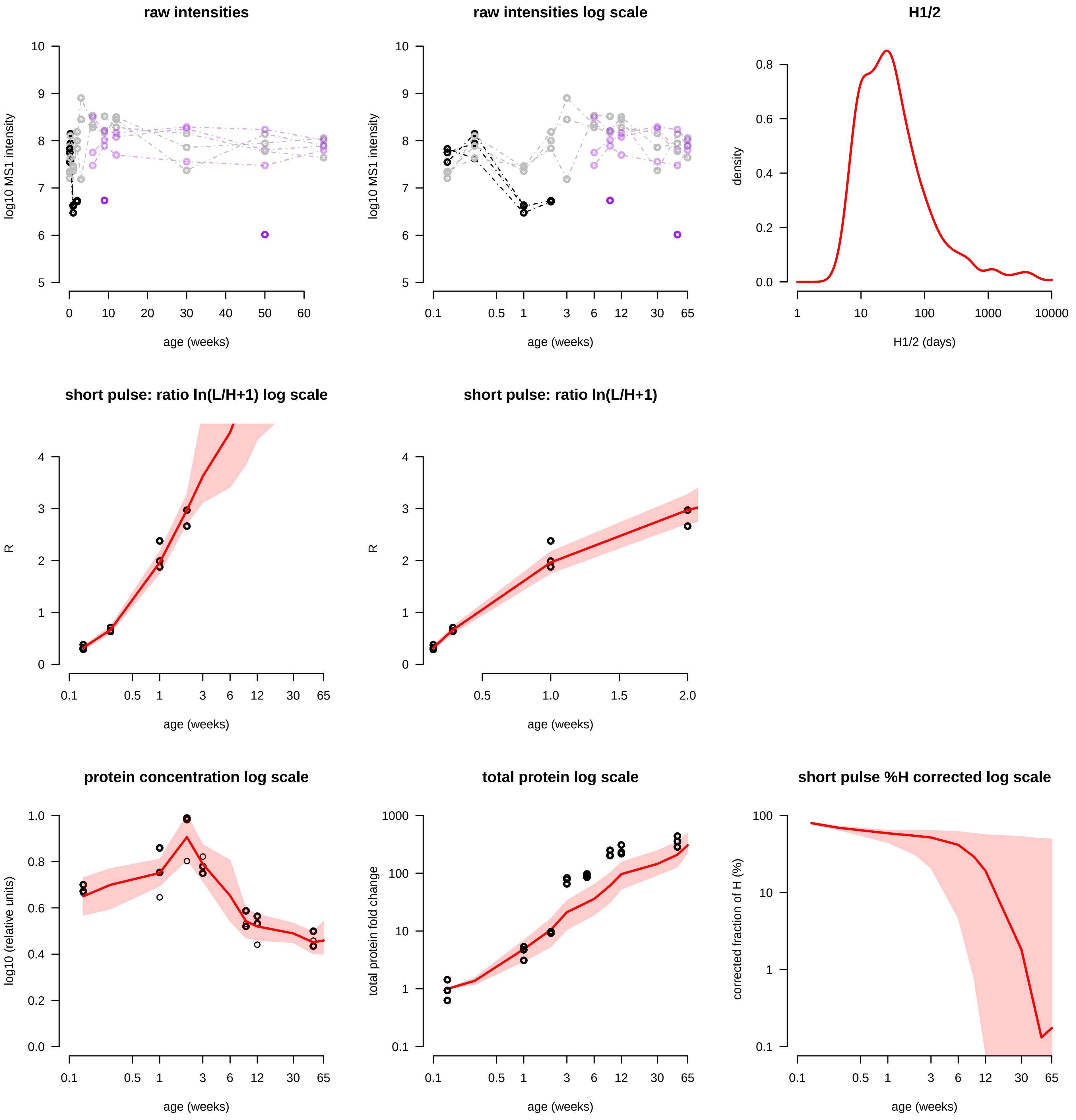

Q61216;Q61216−2

>sp|Q61216|MRE11_MOUSE

Double−strand break repair protein MRE11

GN=Mre11

Med H1/2: 6.7 days

Q5 H1/2: 2.7 days

Q95 H1/2: 125.9 days

reliable model fit: no

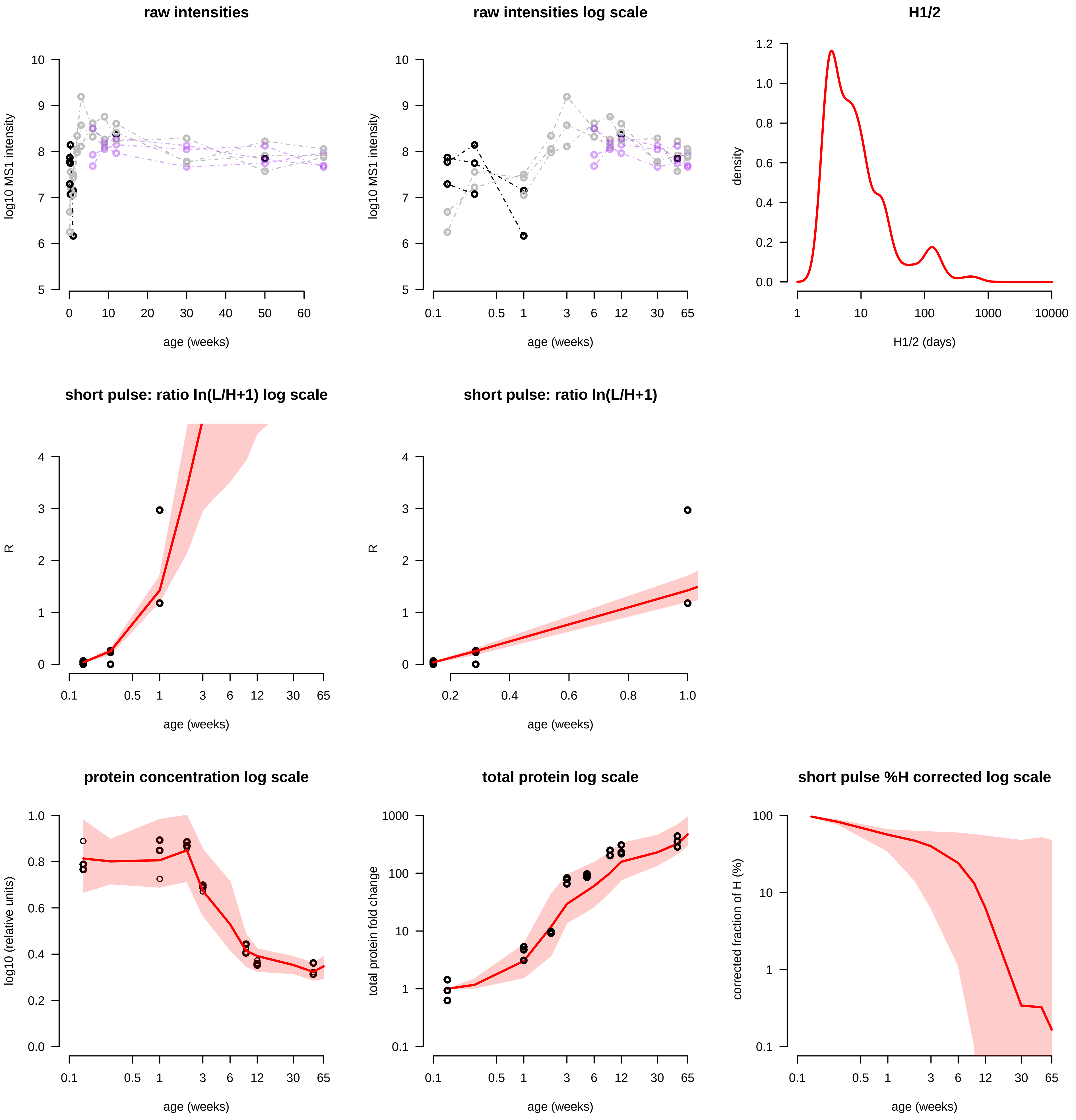

Q9D0A3

>sp|Q9D0A3|ARPIN_MOUSE

Arpin

GN=Arpin

Med H1/2: 8.7 days

Q5 H1/2: 3.1 days

Q95 H1/2: 57.8 days

reliable model fit: yes

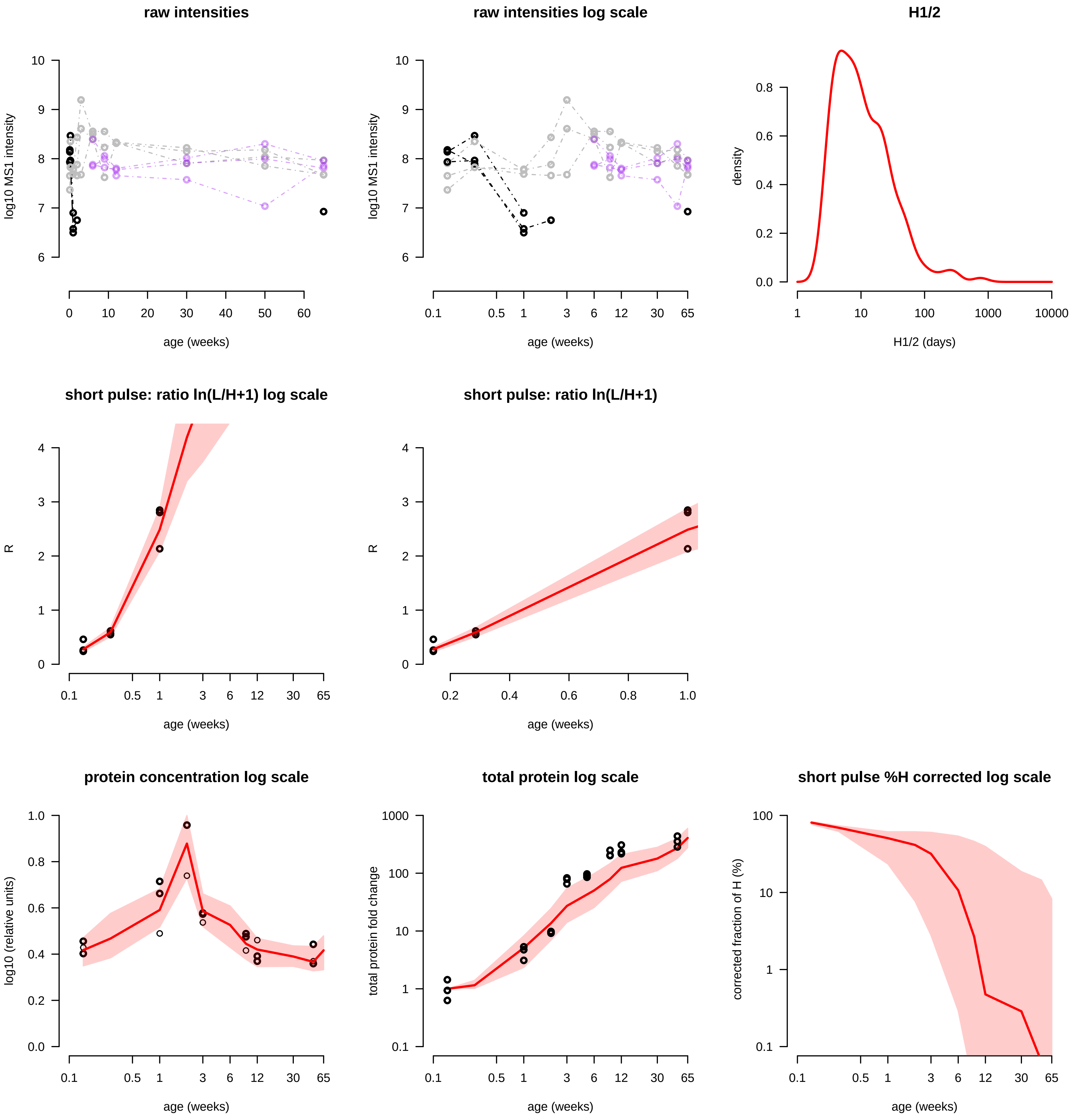

P97478

>sp|P97478|COQ7_MOUSE

5−demethoxyubiquinone hydroxylase, mitochondrial

GN=Coq7

Med H1/2: 7.9 days

Q5 H1/2: 3 days

Q95 H1/2: 62.1 days

reliable model fit: no

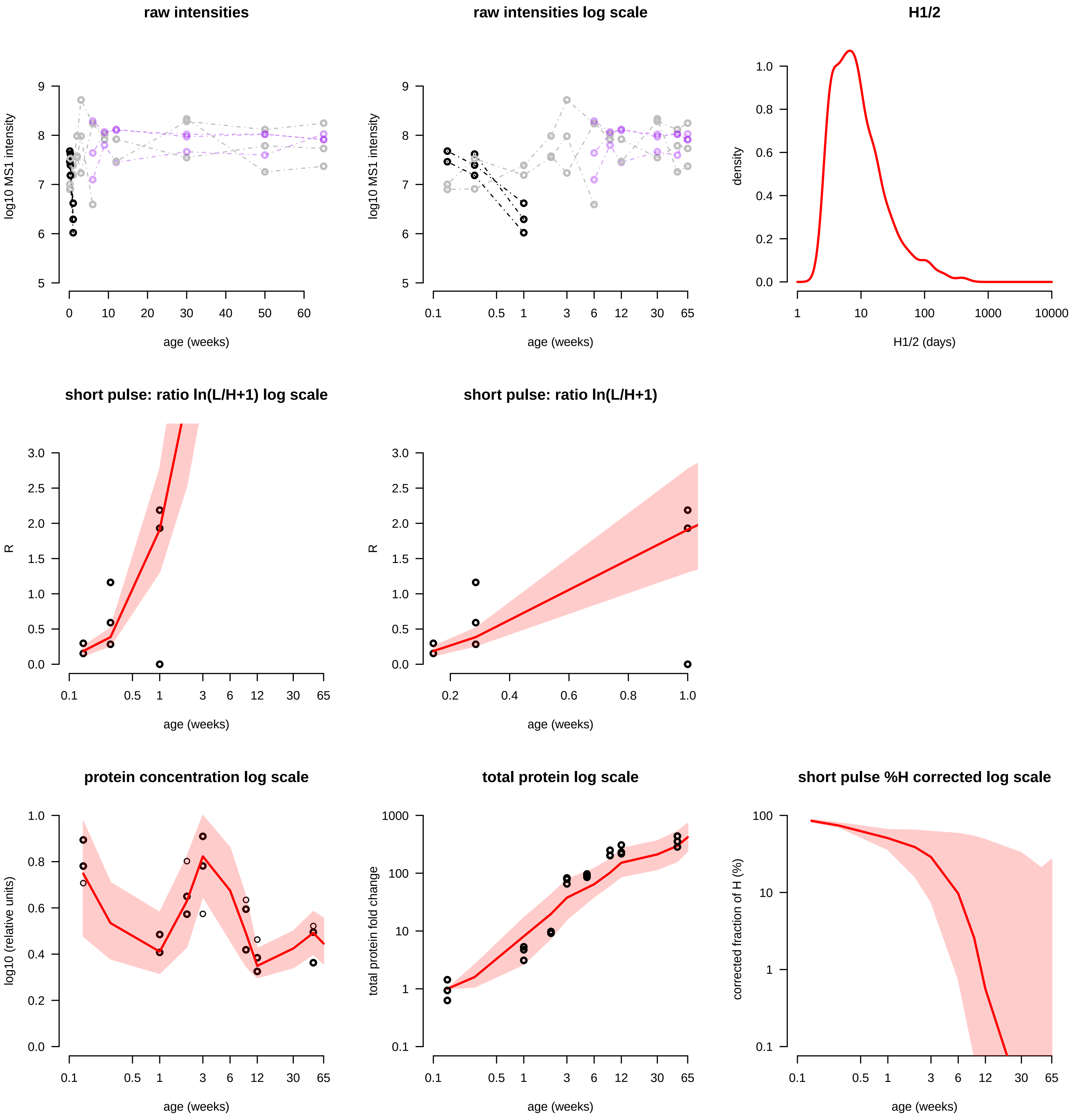

O55236

>sp|O55236|MCE1_MOUSE

mRNA−capping enzyme

GN=Rngtt

Med H1/2: 5.9 days

Q5 H1/2: 3.1 days

Q95 H1/2: 27.8 days

reliable model fit: yes

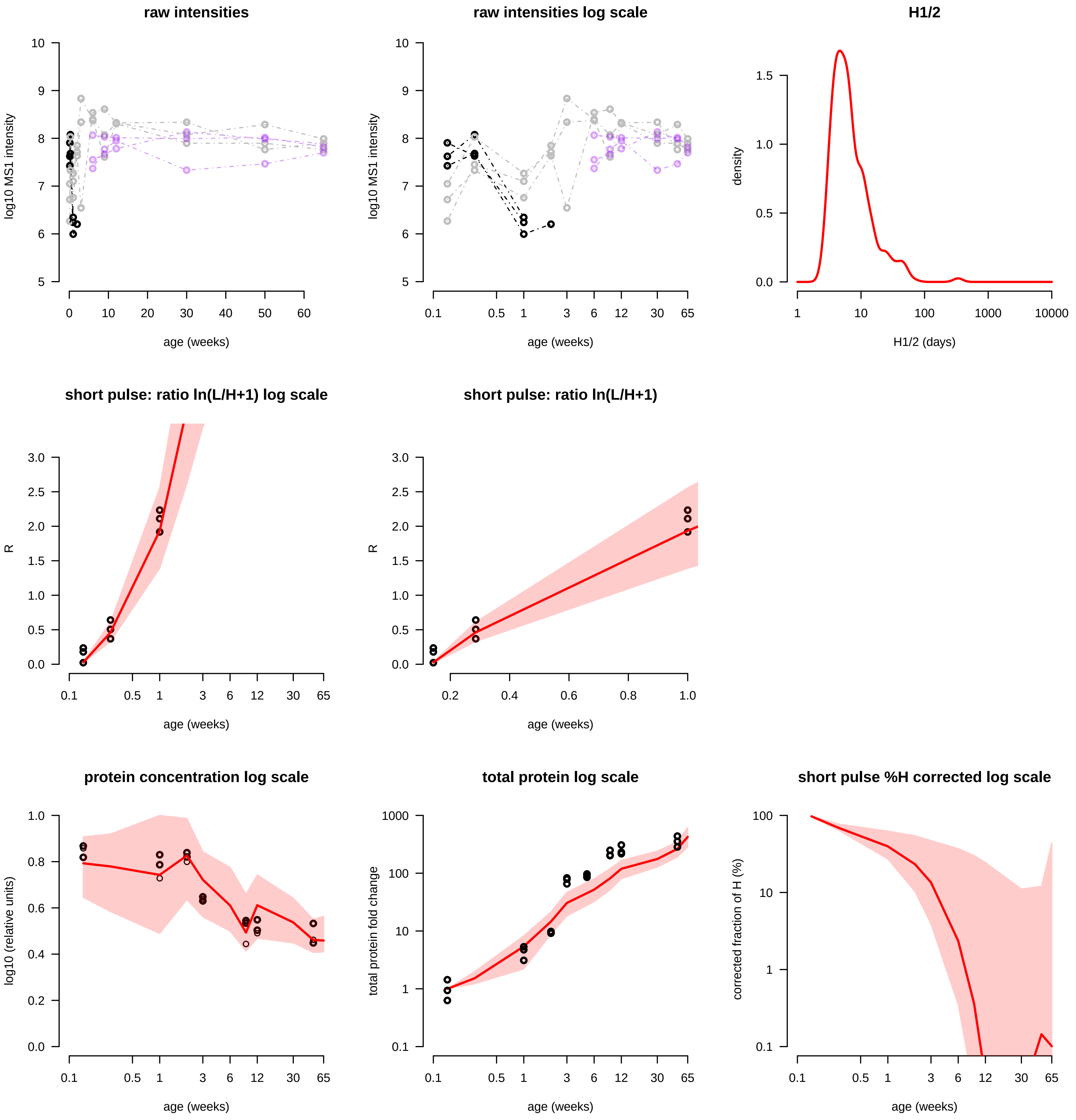

Q8R1S0

>sp|Q8R1S0|COQ6_MOUSE

Ubiquinone biosynthesis monooxygenase COQ6, mitochondrial

GN=Coq6

Med H1/2: 13.9 days

Q5 H1/2: 4.8 days

Q95 H1/2: 165.4 days

reliable model fit: yes

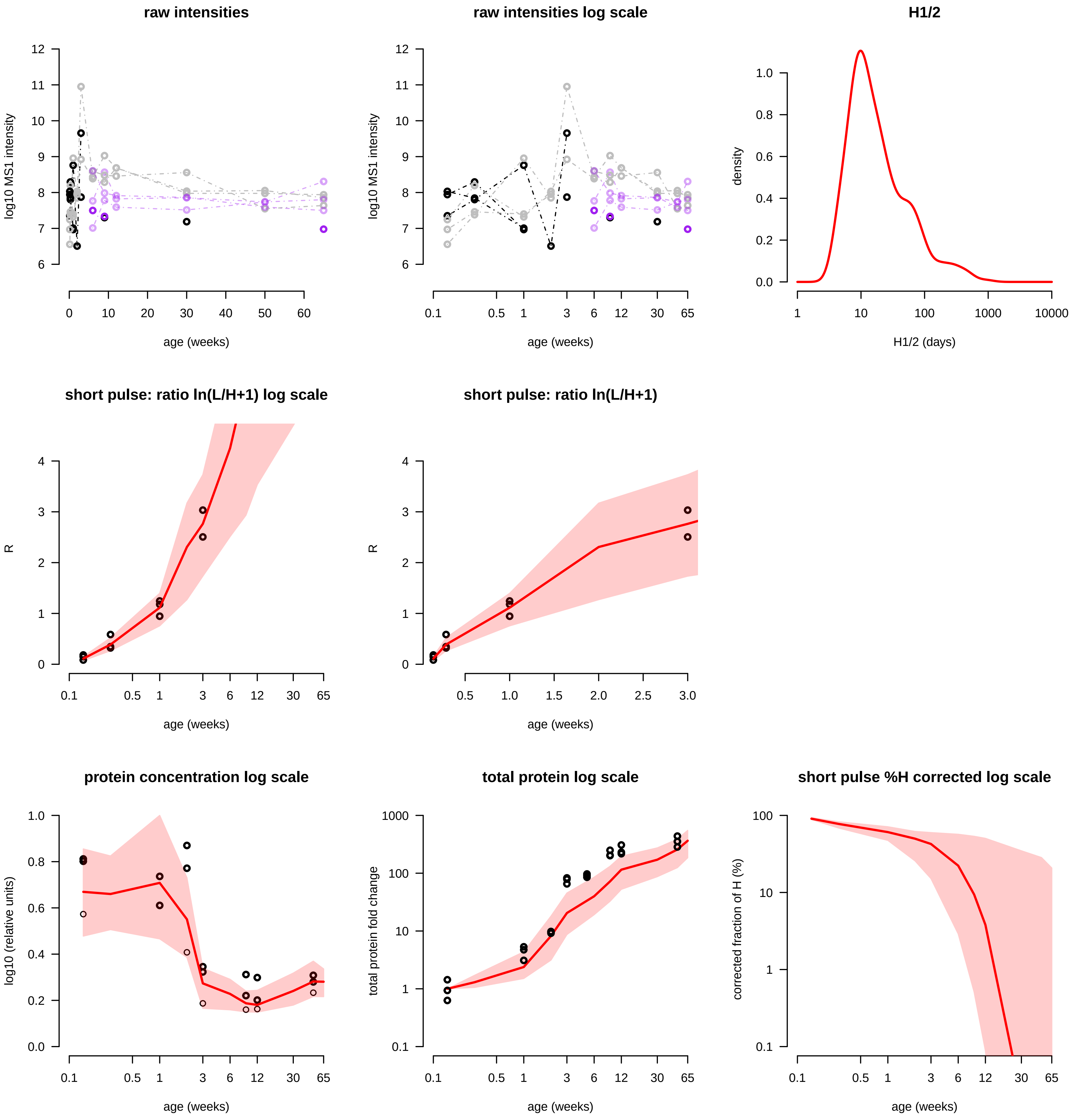

Q64512

>sp|Q64512|PTN13_MOUSE

Tyrosine−protein phosphatase non−receptor type 13

GN=Ptpn13

Med H1/2: 1168.1 days

Q5 H1/2: 349.8 days

Q95 H1/2: 14635.5 days

reliable model fit: no

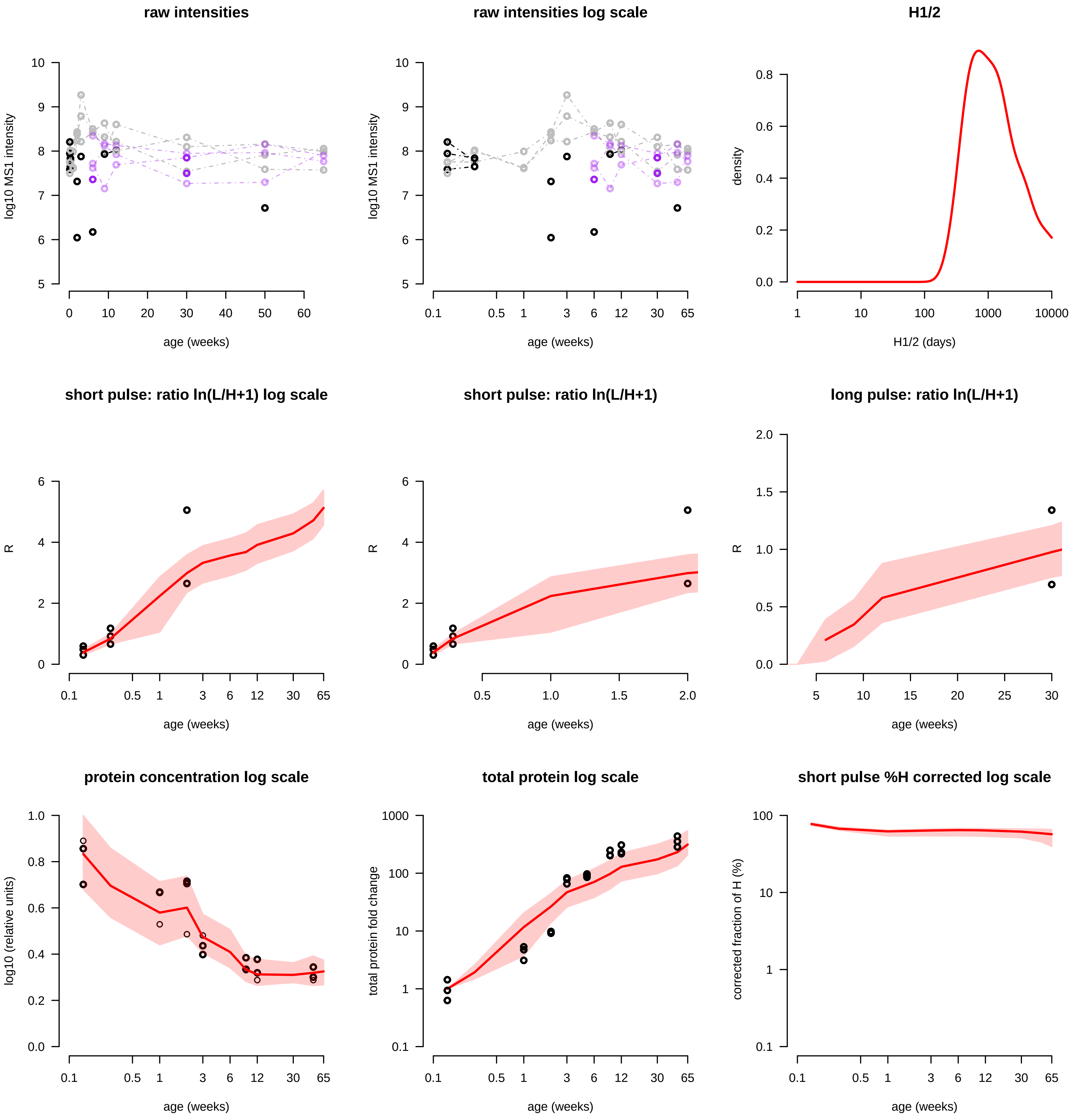

Q3UH66−7

>sp|Q3UH66−7|WNK2_MOUSE

Isoform 7 of Serine/threonine−protein kinase WNK2

GN=Wnk2

Med H1/2: 8.3 days

Q5 H1/2: 2.8 days

Q95 H1/2: 96 days

reliable model fit: yes

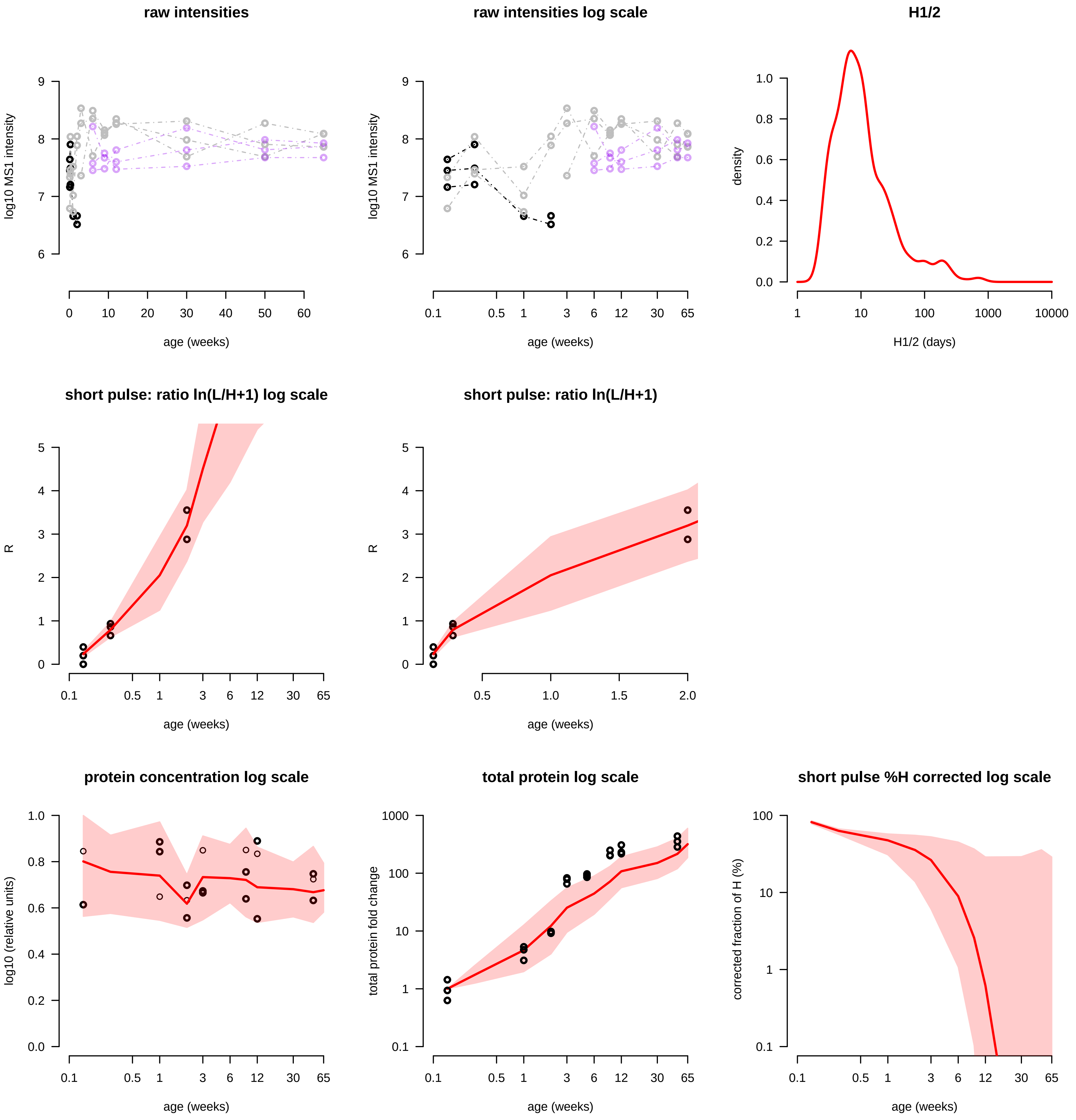

Q99LB7

>sp|Q99LB7|SARDH_MOUSE

Sarcosine dehydrogenase, mitochondrial

GN=Sardh

Med H1/2: 17.7 days

Q5 H1/2: 5.7 days

Q95 H1/2: 131.8 days

reliable model fit: yes

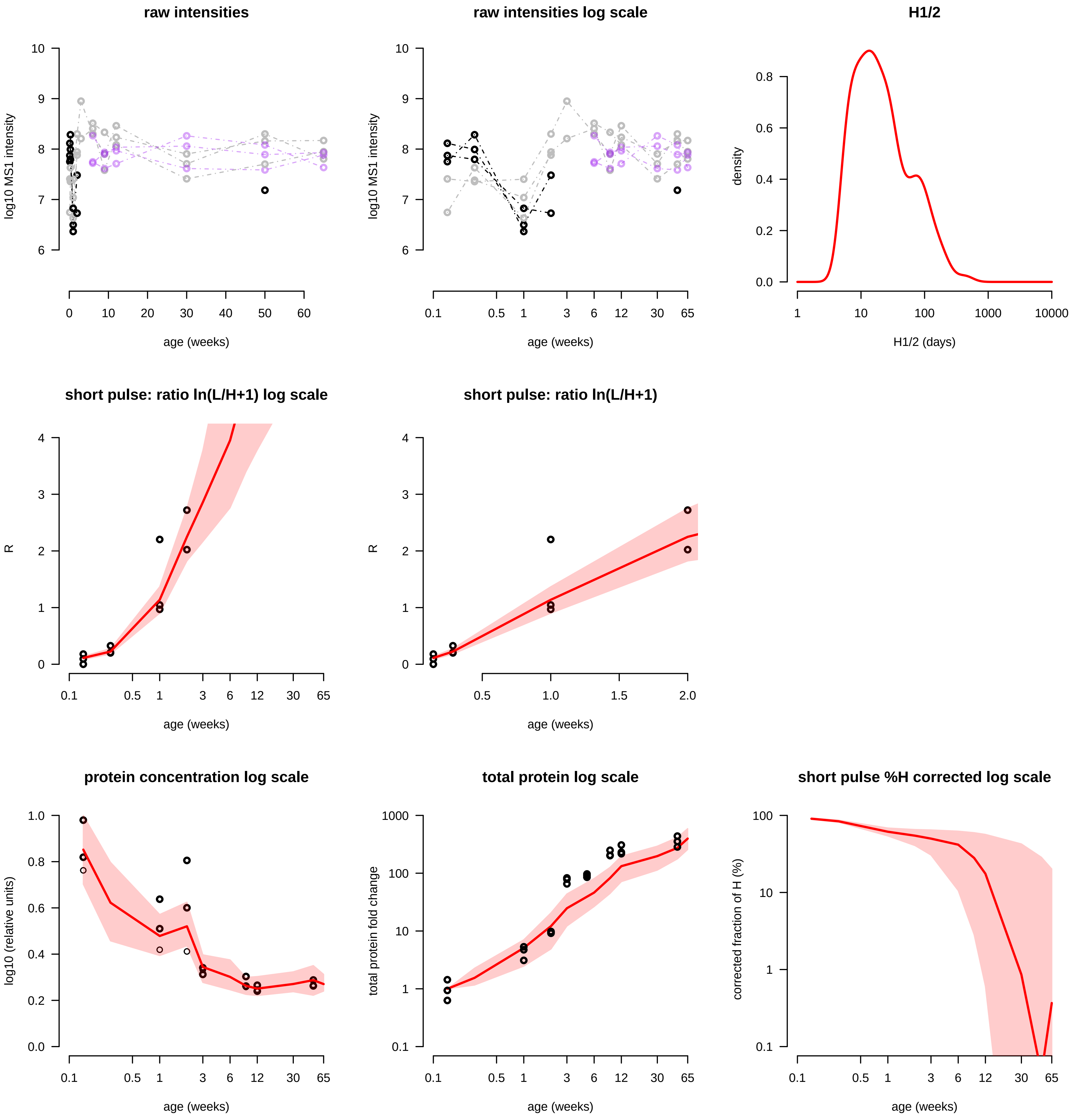

Q8K2I1

>sp|Q8K2I1|FNTB_MOUSE

Protein farnesyltransferase subunit beta

GN=Fntb

Med H1/2: 7.3 days

Q5 H1/2: 2.6 days

Q95 H1/2: 89.7 days

reliable model fit: yes

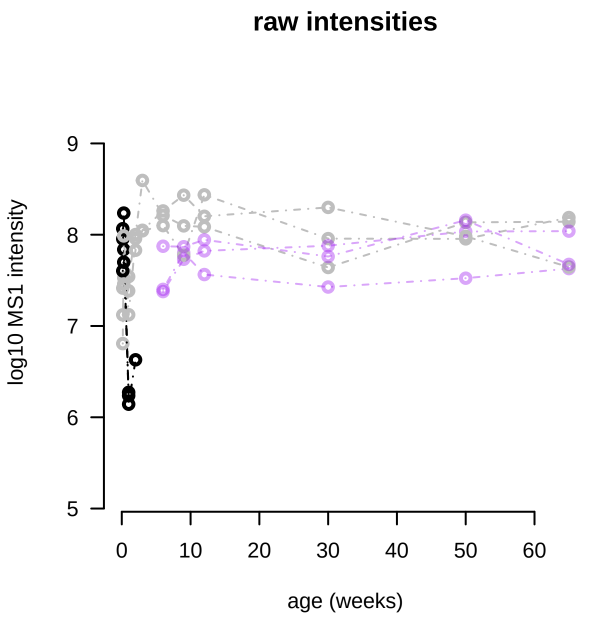

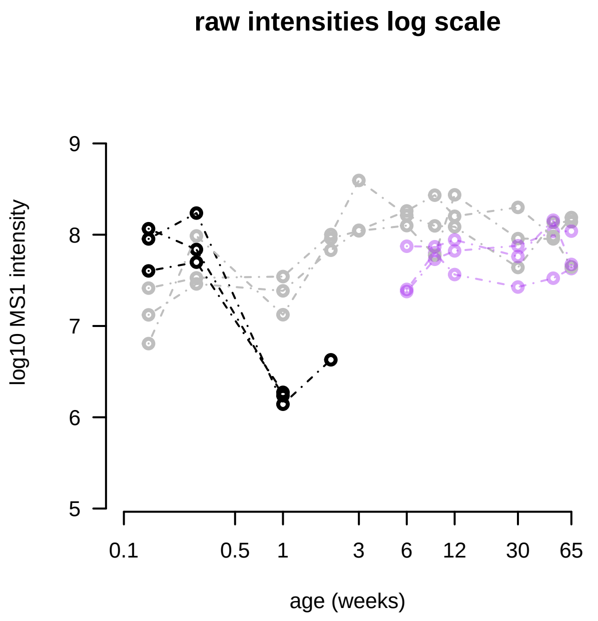

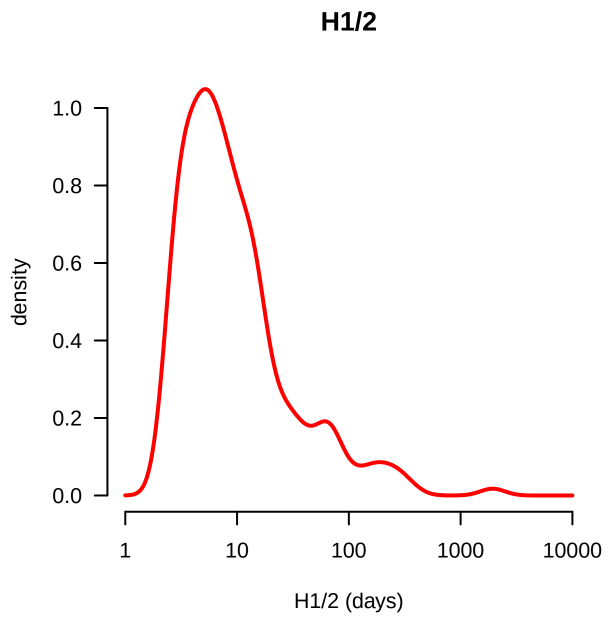

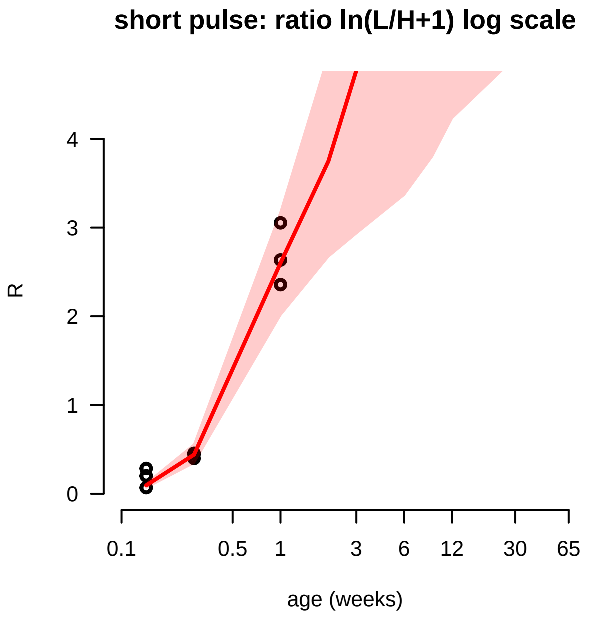

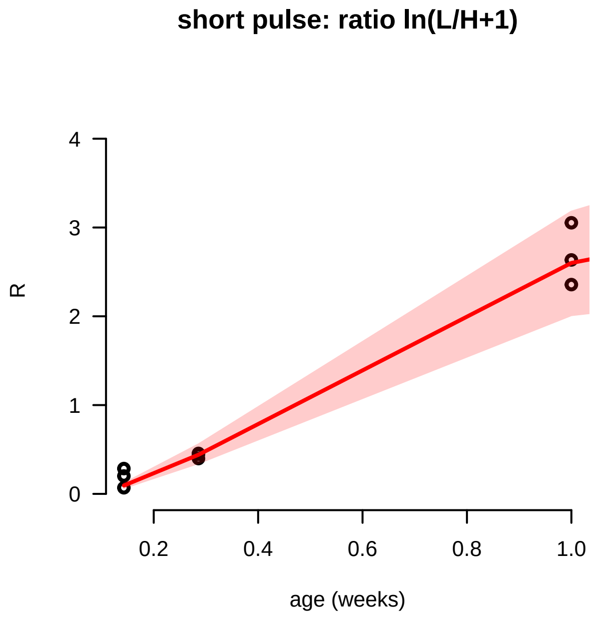

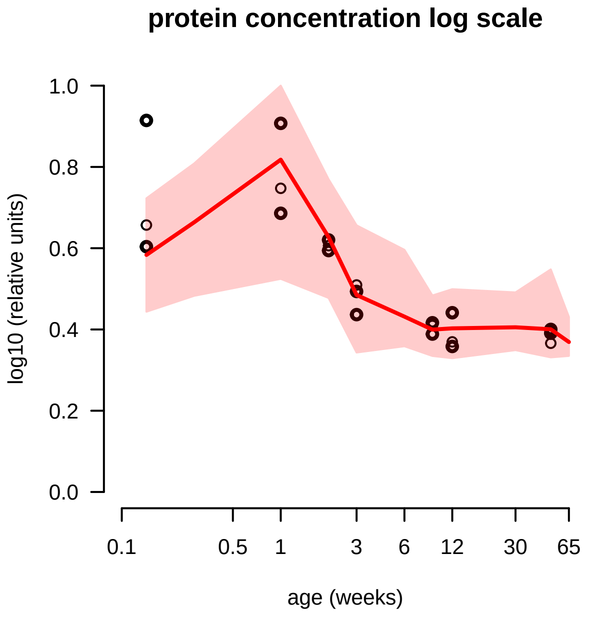

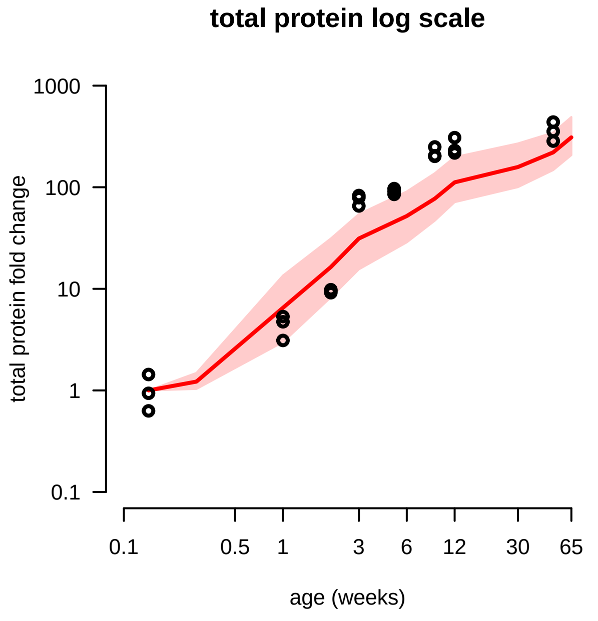

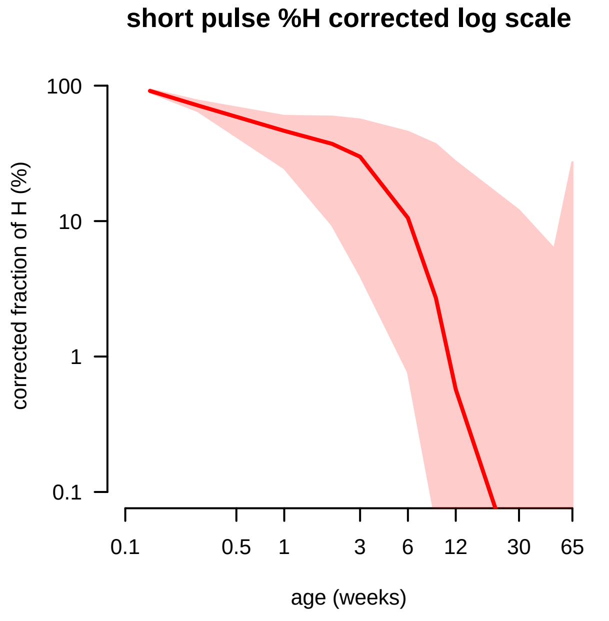

Q8CFI0−3;Q8CFI0

>sp|Q8CFI0−3|NED4L_MOUSE

Isoform 3 of E3 ubiquitin−protein ligase NEDD4−like

GN=Nedd4l

Med H1/2: 16.5 days

Q5 H1/2: 6.1 days

Q95 H1/2: 135.6 days

reliable model fit: yes

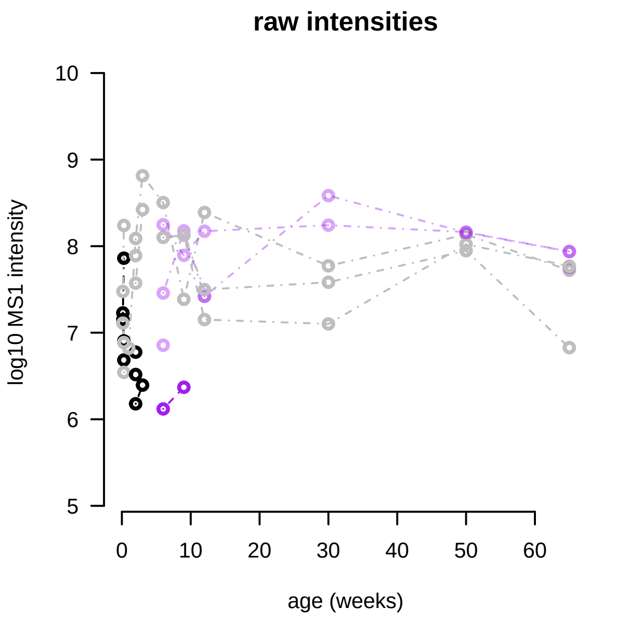

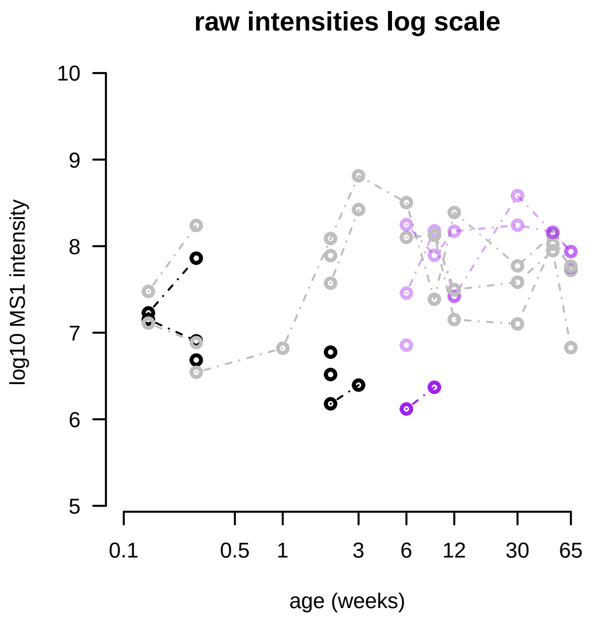

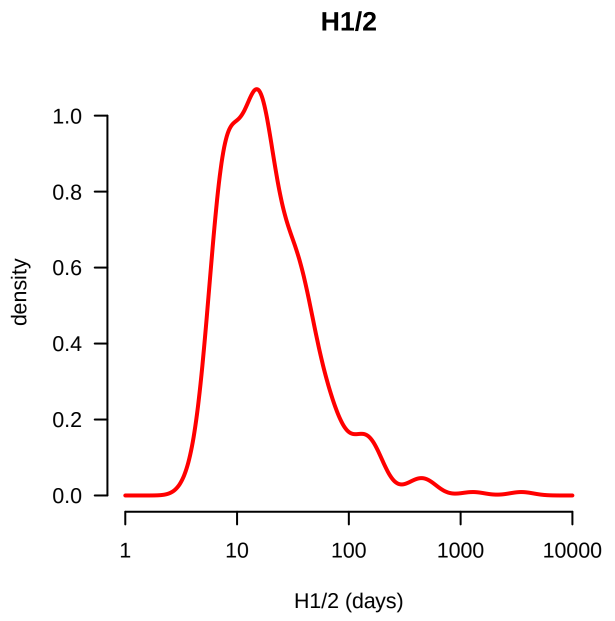

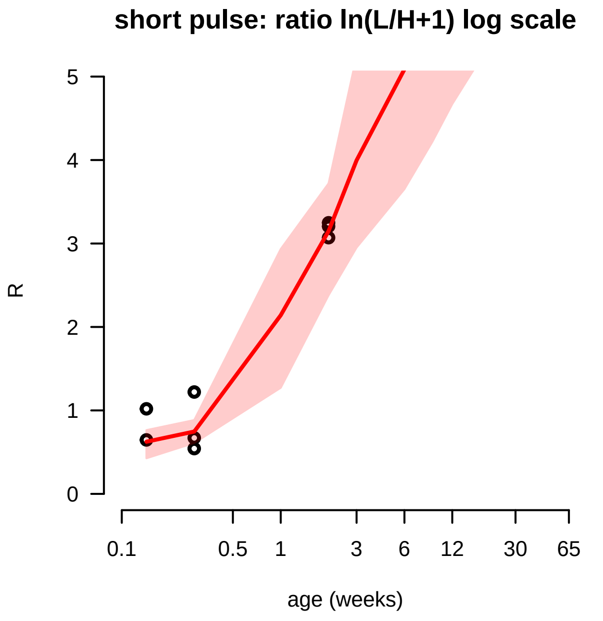

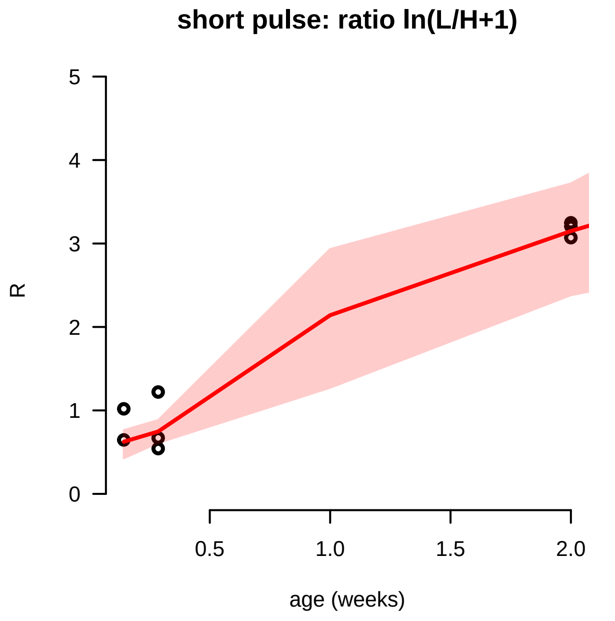

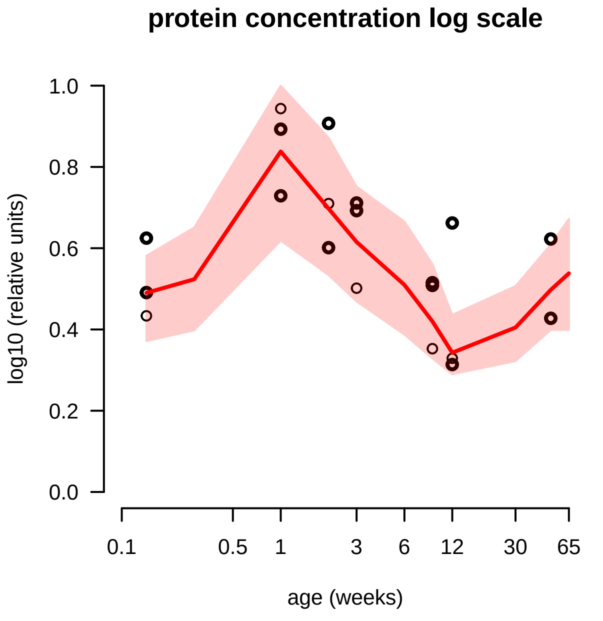

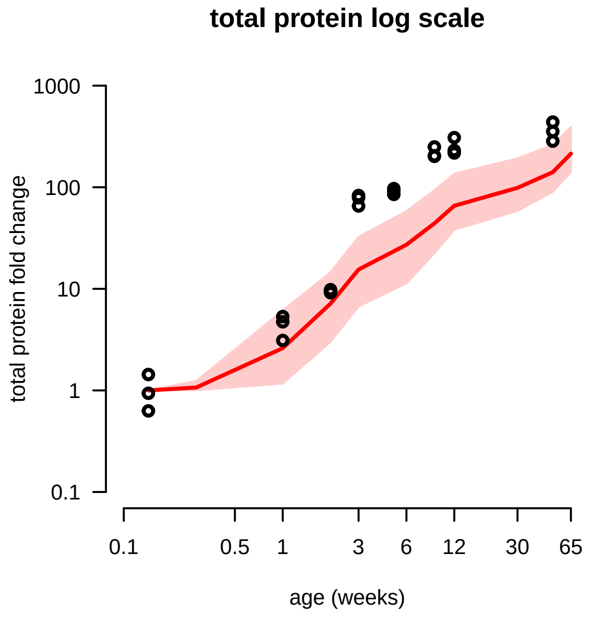

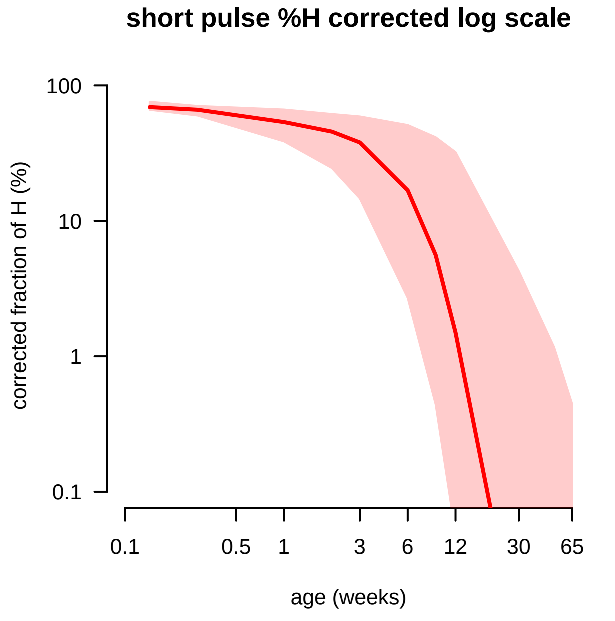

Q9D8M4

>sp|Q9D8M4|RL7L_MOUSE

60S ribosomal protein L7−like 1

GN=Rpl7l1

Med H1/2: 5.8 days

Q5 H1/2: 2.7 days

Q95 H1/2: 51 days

reliable model fit: yes

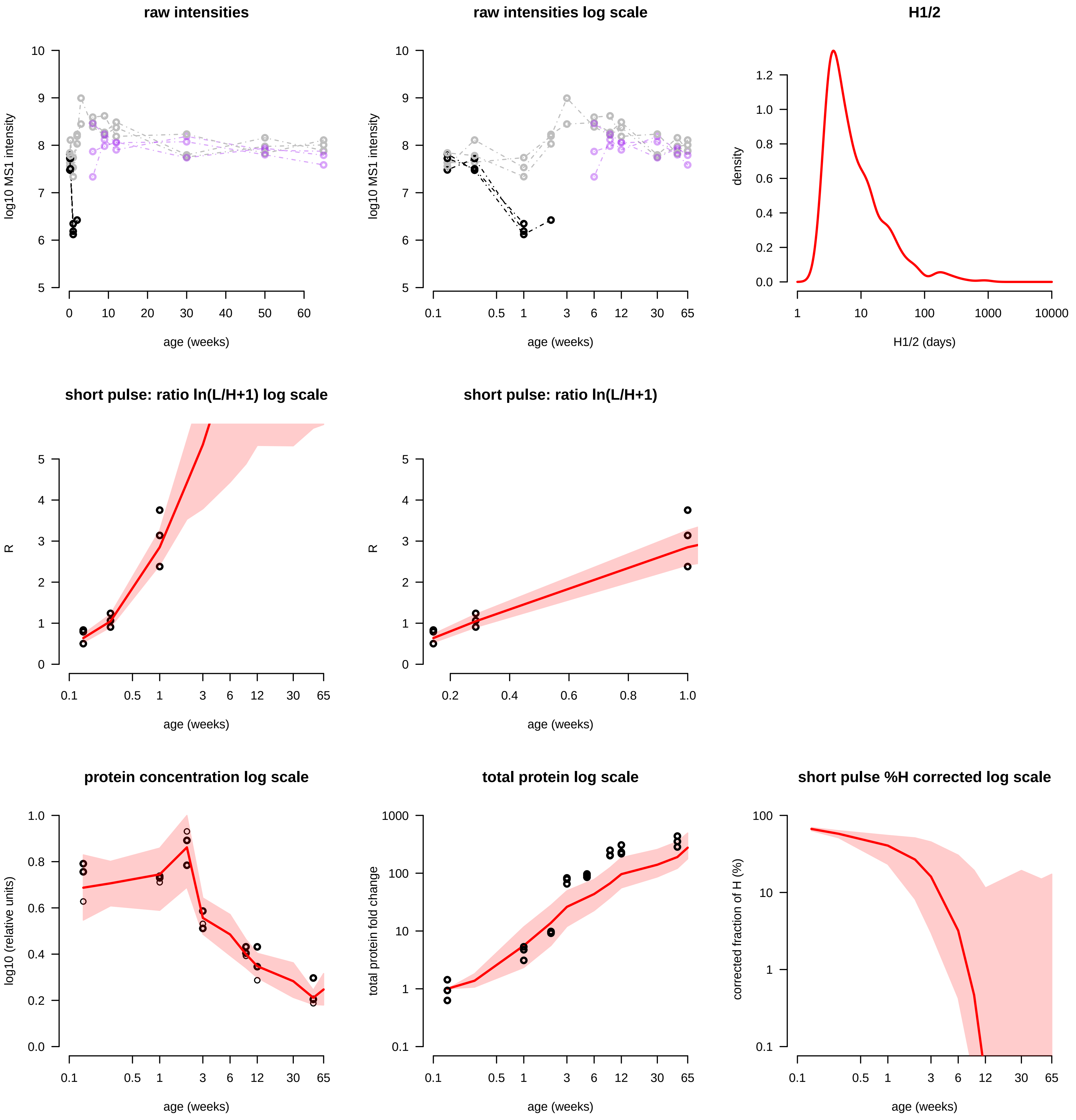

Q9CYI0

>sp|Q9CYI0|NJMU_MOUSE

Protein Njmu−R1

Med H1/2: 7.1 days

Q5 H1/2: 2.9 days

Q95 H1/2: 55.2 days

reliable model fit: yes

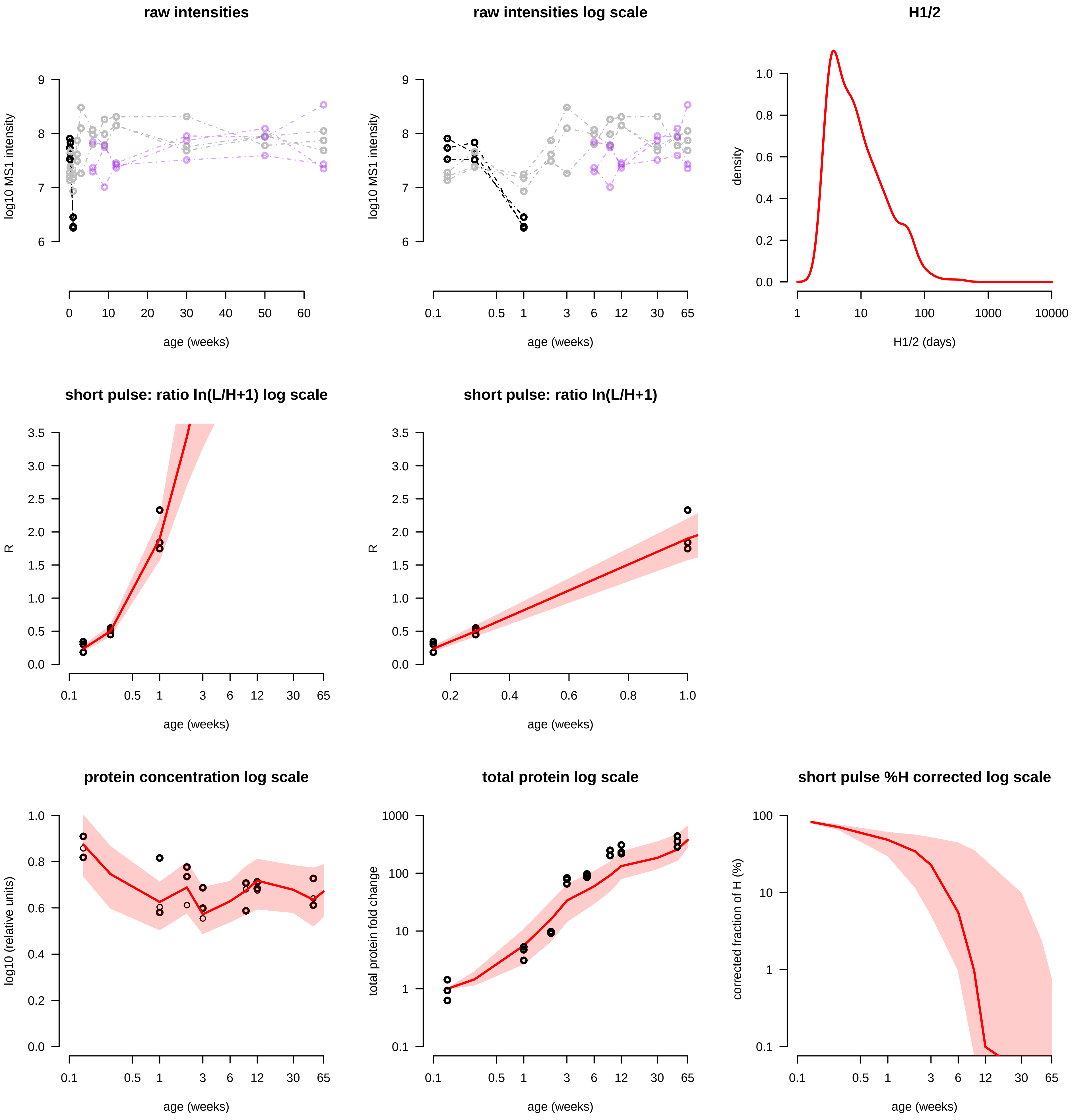

Q8K2Z2

>sp|Q8K2Z2|PRP39_MOUSE

Pre−mRNA−processing factor 39

GN=Prpf39

Med H1/2: 9.8 days

Q5 H1/2: 5 days

Q95 H1/2: 84.2 days

reliable model fit: yes

raw intensities

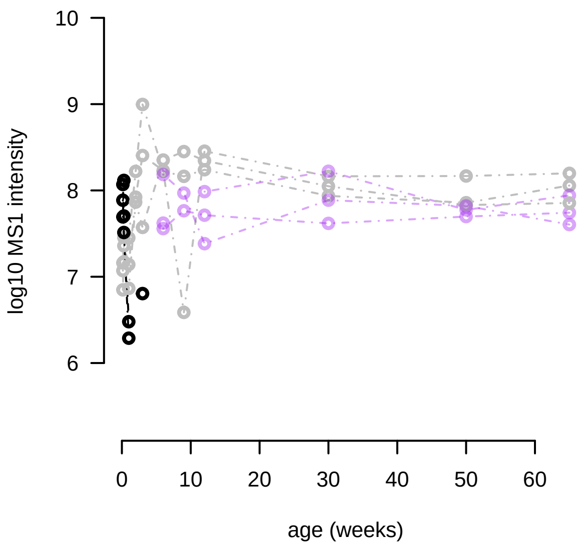

raw intensities log scale

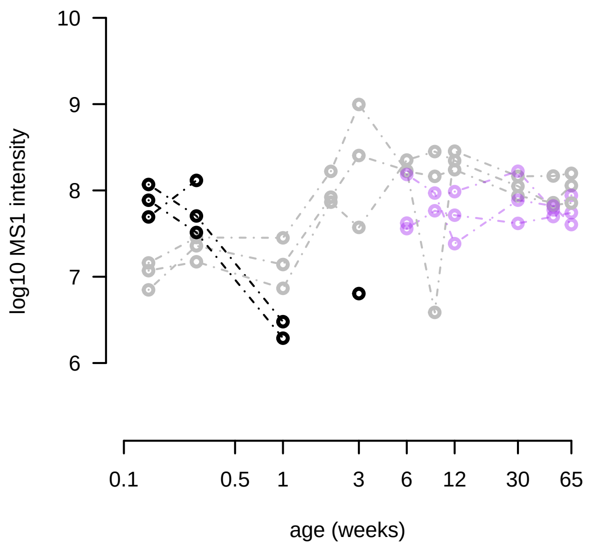

H1/2

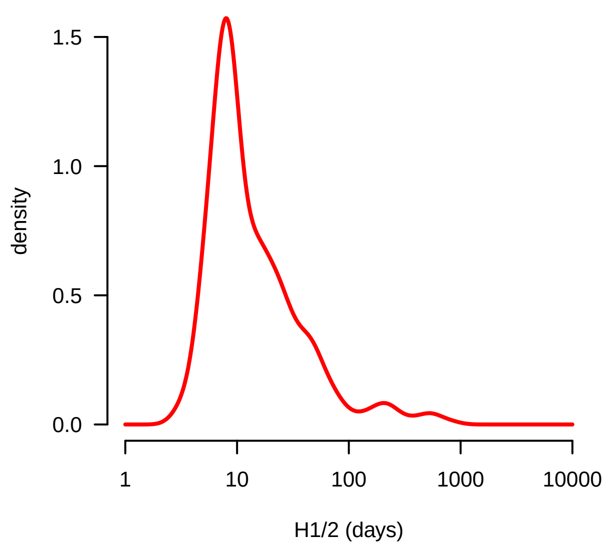

short pulse: ratio ln(L/H+1) log scale

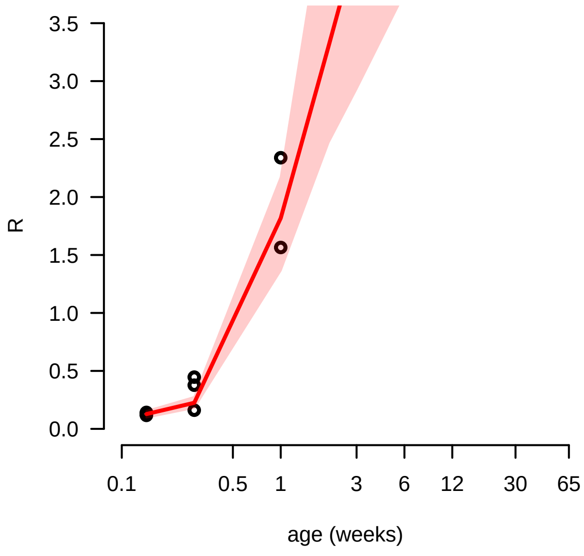

short pulse: ratio ln(L/H+1)

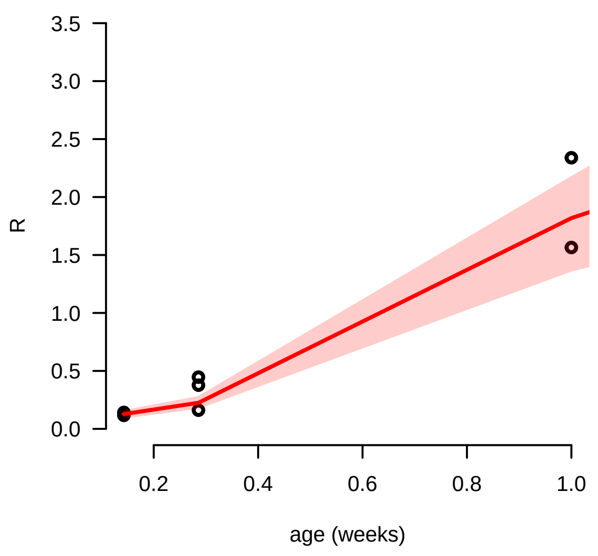

protein concentration log scale

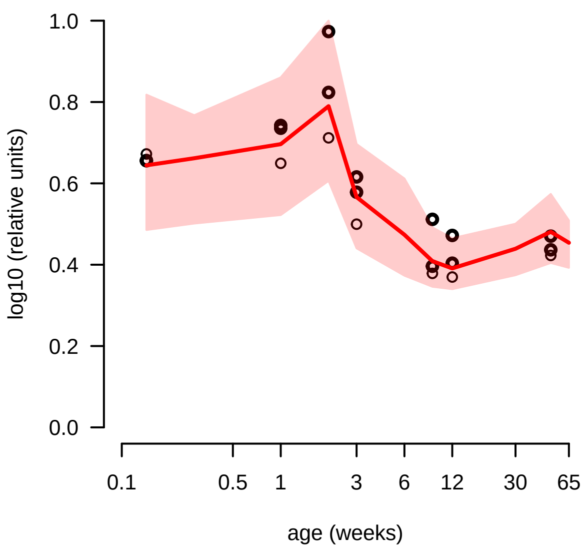

total protein log scale

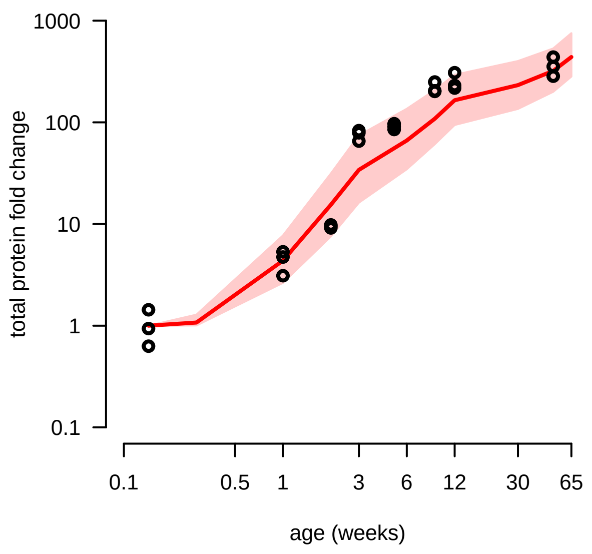

short pulse %H corrected log scale

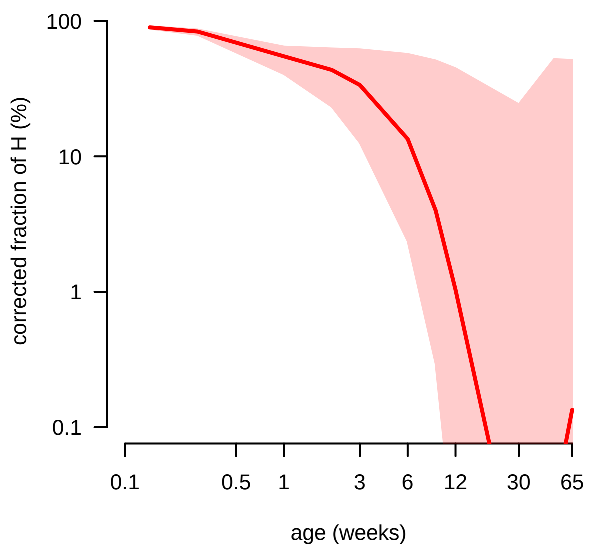

Q8CH02

>sp|Q8CH02|SUGP1_MOUSE

SURP and G−patch domain−containing protein 1

GN=Sugp1

Med H1/2: 24.3 days

Q5 H1/2: 15.5 days

Q95 H1/2: 60.4 days

reliable model fit: no

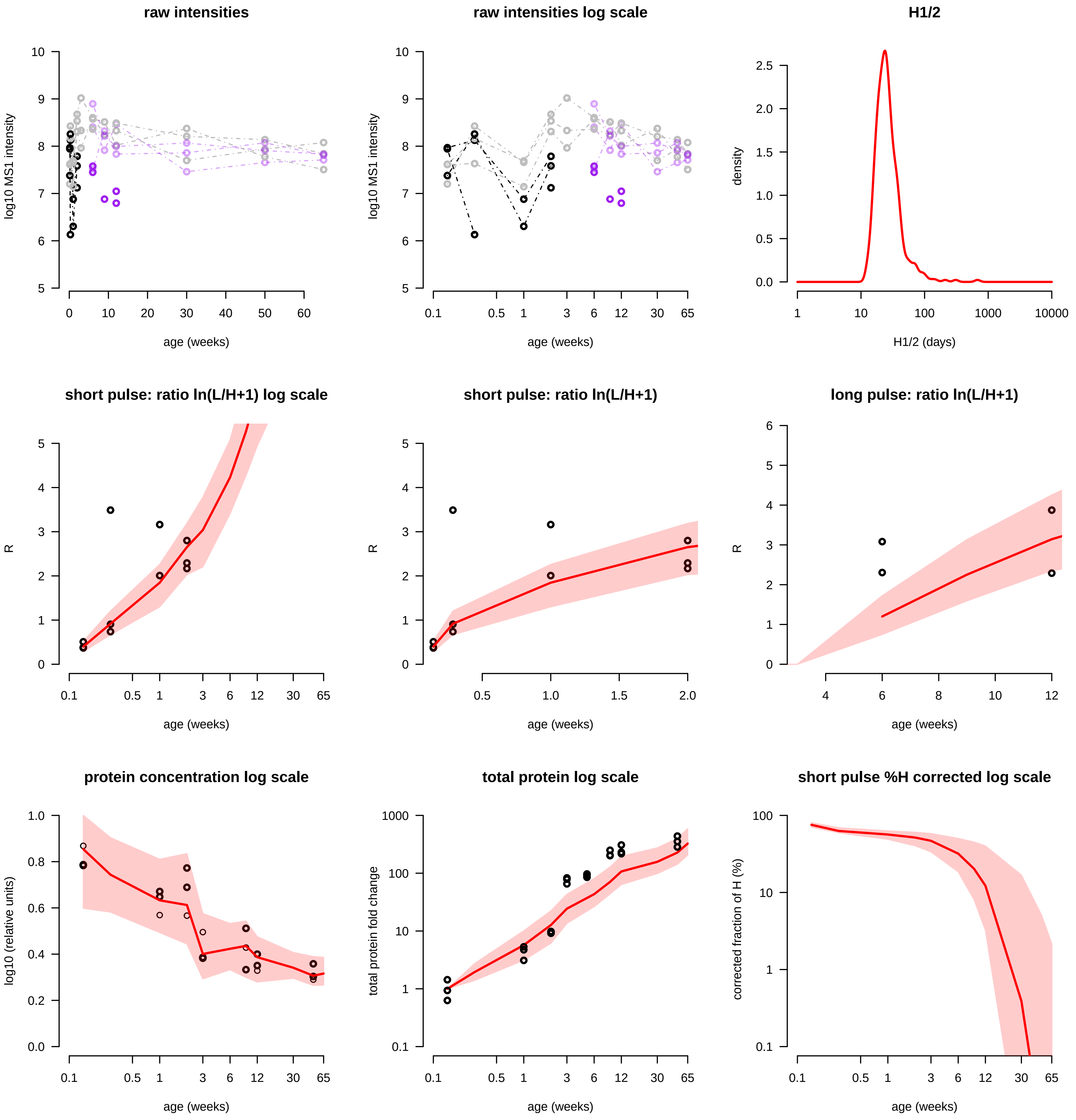

Q91Z31;Q91Z31−2

>sp|Q91Z31|PTBP2_MOUSE

Polypyrimidine tract−binding protein 2

GN=Ptbp2

Med H1/2: 5.7 days

Q5 H1/2: 2.9 days

Q95 H1/2: 14.6 days

reliable model fit: yes

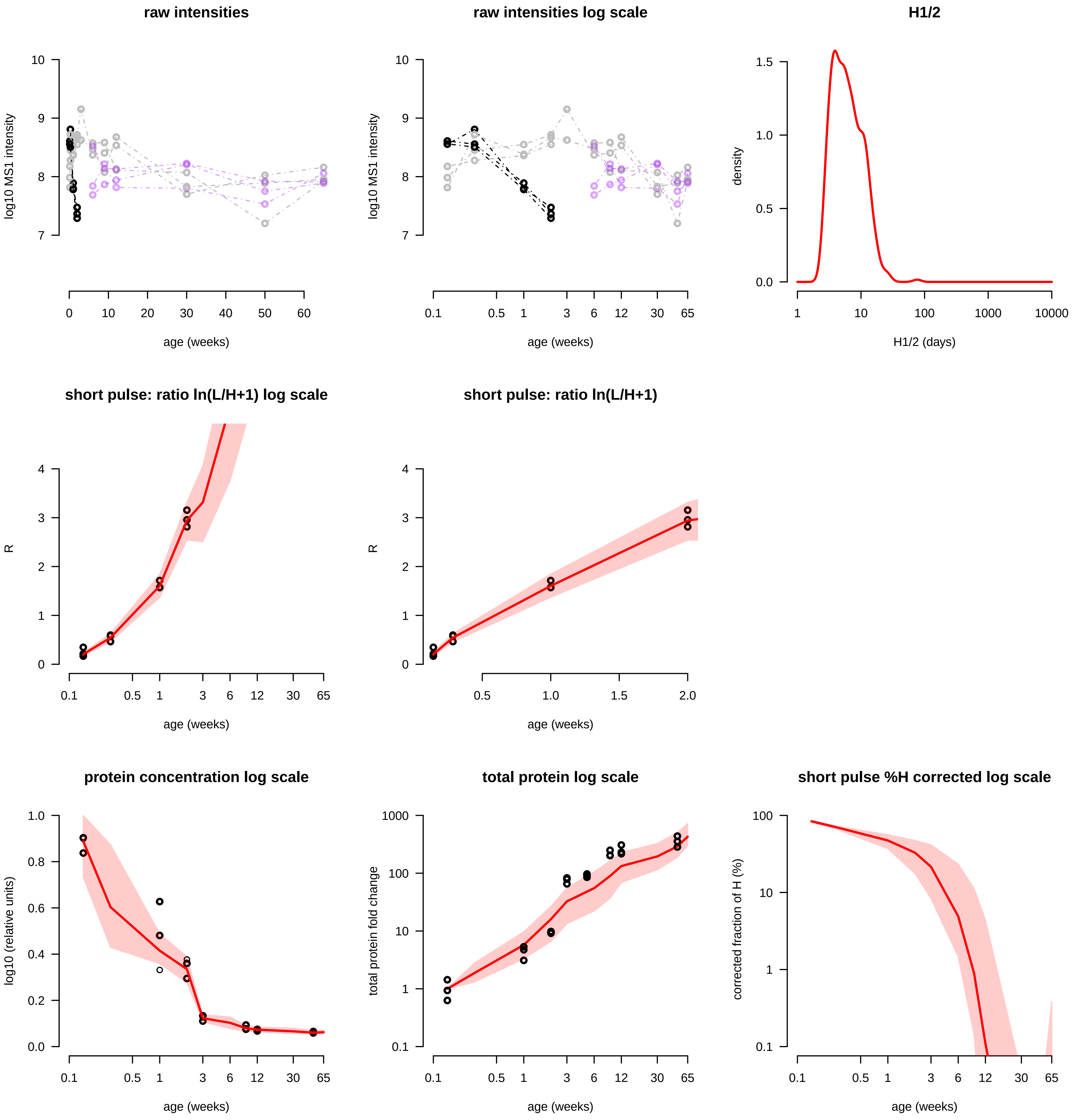

Q9Z2Q5

>sp|Q9Z2Q5|RM40_MOUSE

39S ribosomal protein L40, mitochondrial

GN=Mrpl40

Med H1/2: 8.7 days

Q5 H1/2: 3.3 days

Q95 H1/2: 57.1 days

reliable model fit: yes

raw intensities

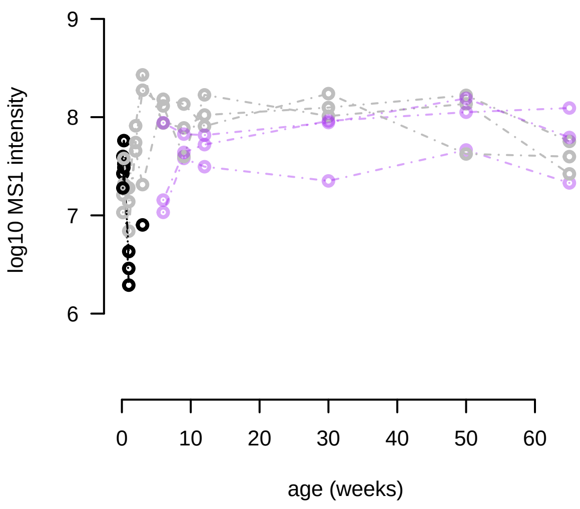

raw intensities log scale

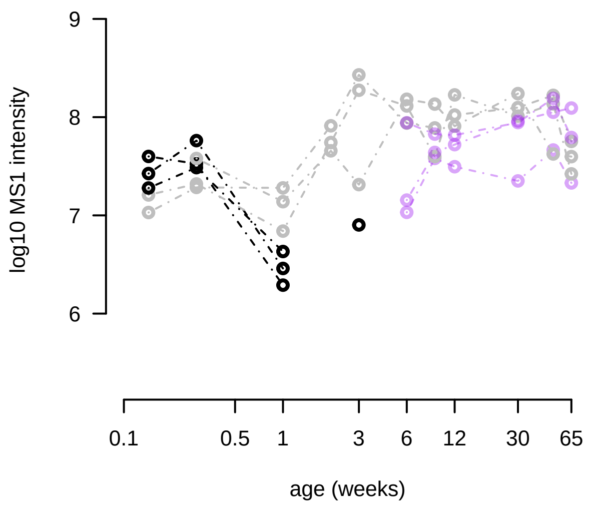

H1/2

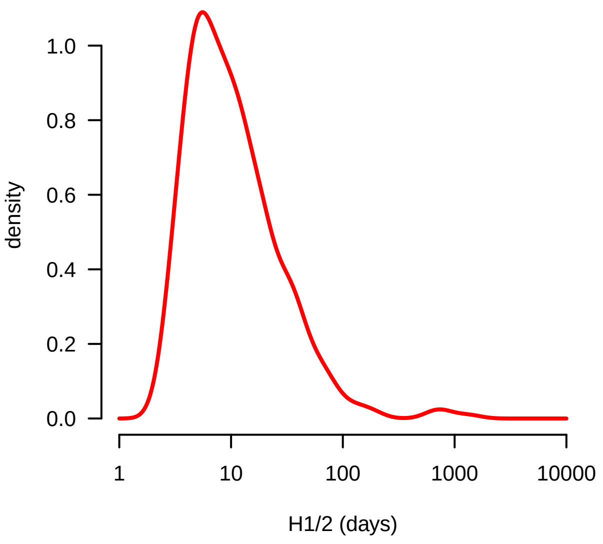

short pulse: ratio ln(L/H+1) log scale

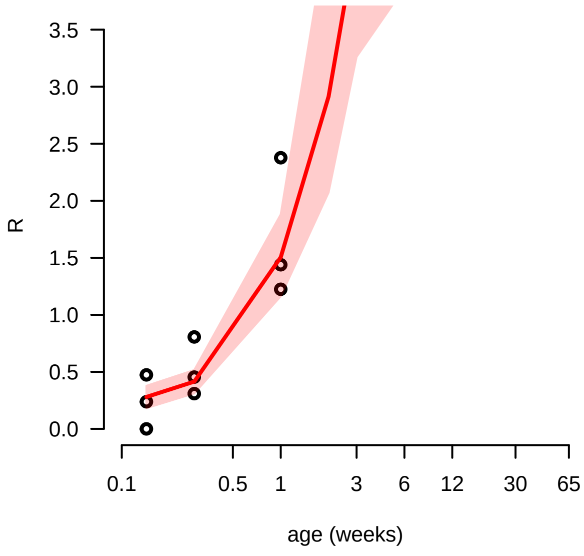

short pulse: ratio ln(L/H+1)

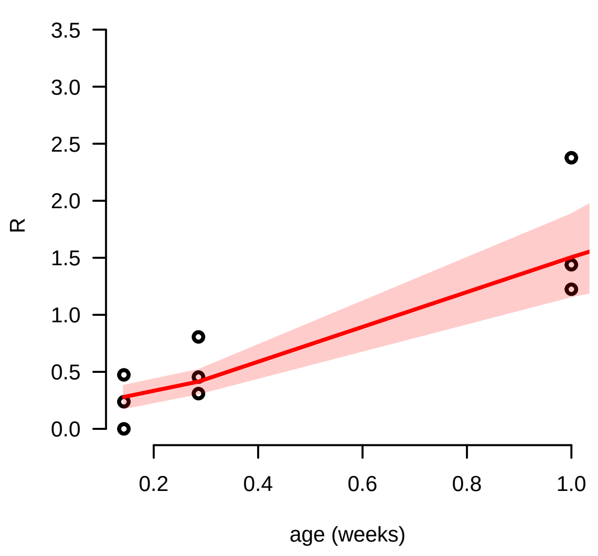

protein concentration log scale

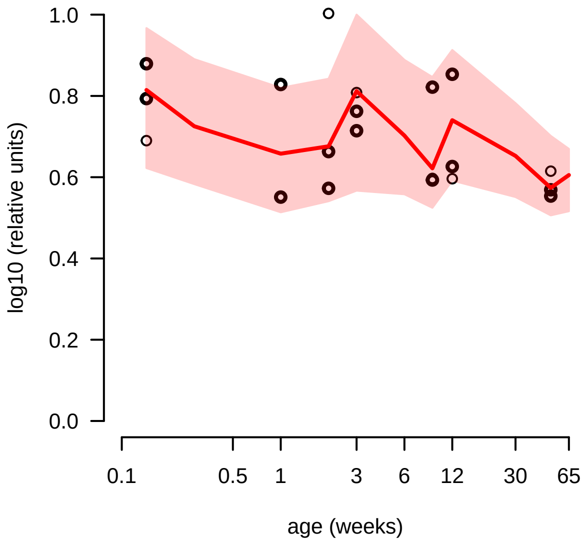

total protein log scale

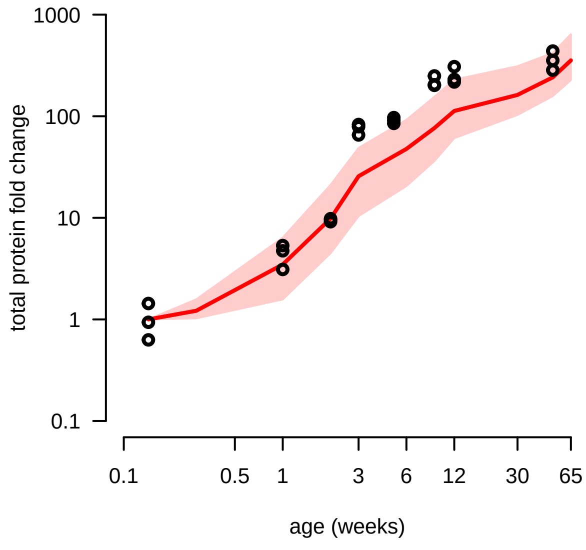

short pulse %H corrected log scale

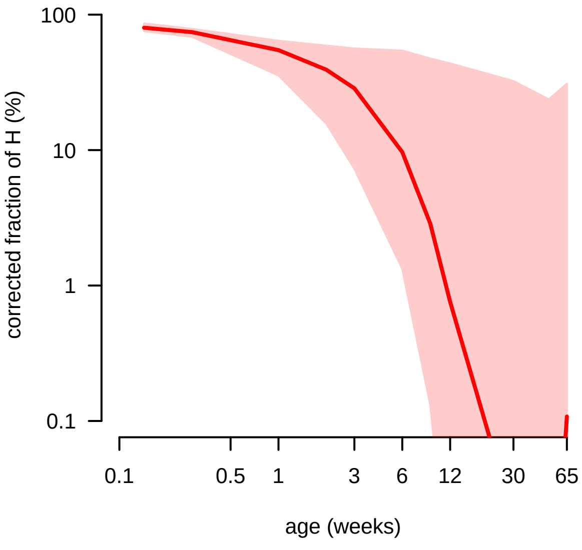

Q9D0M0

>sp|Q9D0M0|EXOS7_MOUSE

Exosome complex exonuclease RRP42

GN=Exosc7

Med H1/2: 16.3 days

Q5 H1/2: 7 days

Q95 H1/2: 123.4 days

reliable model fit: yes

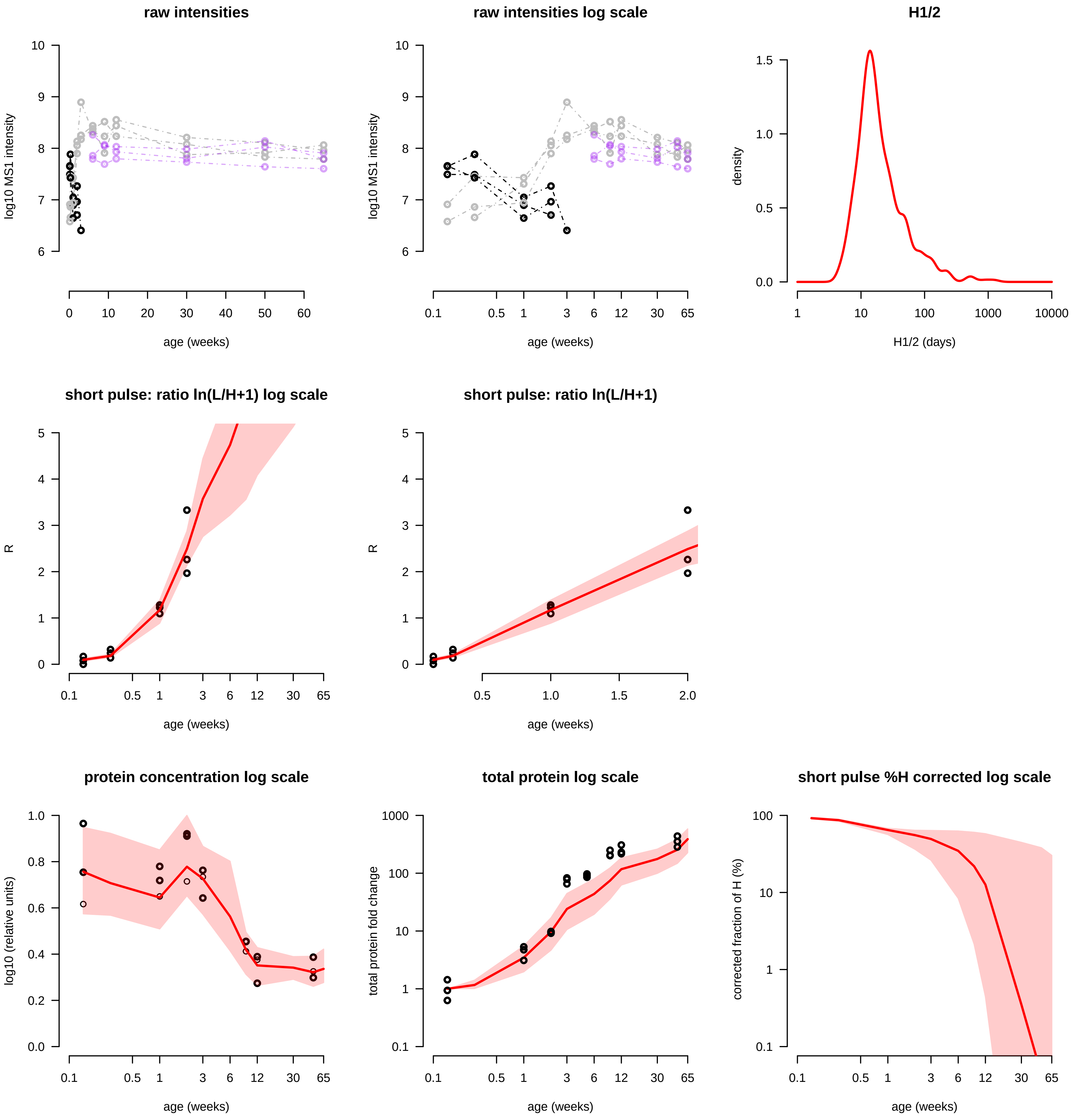

Q6PR54;Q6PR54−2;Q6PR54−3

>sp|Q6PR54|RIF1_MOUSE

Telomere−associated protein RIF1

GN=Rif1

Med H1/2: 8.2 days

Q5 H1/2: 4.2 days

Q95 H1/2: 49 days

reliable model fit: no

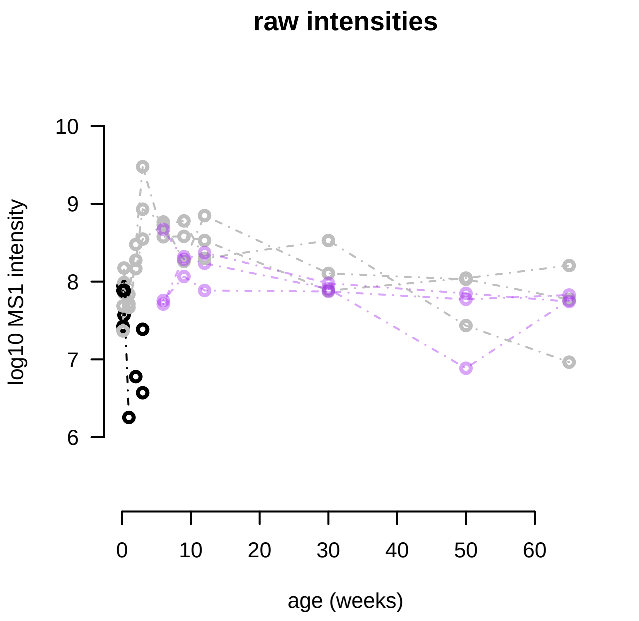

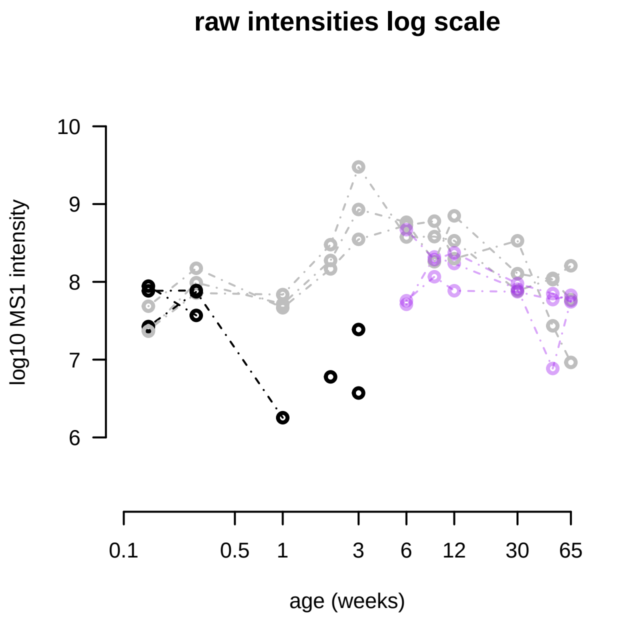

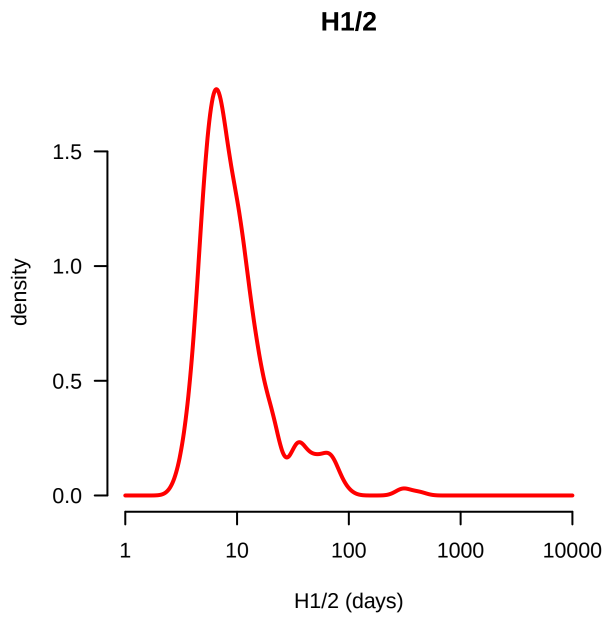

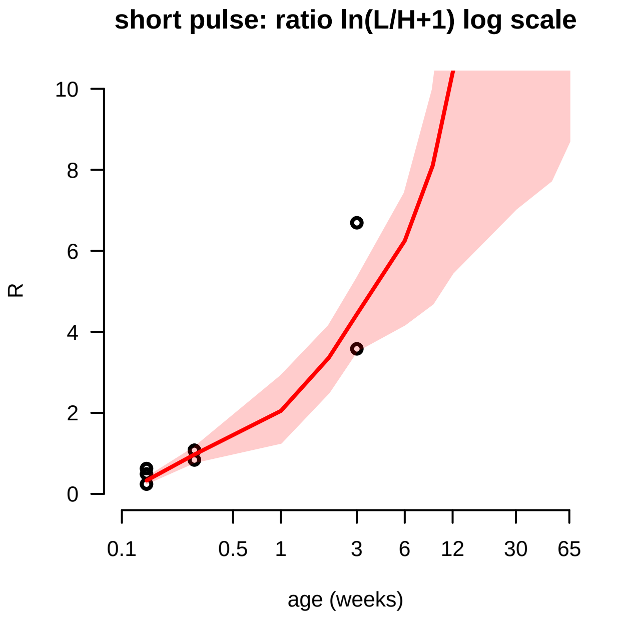

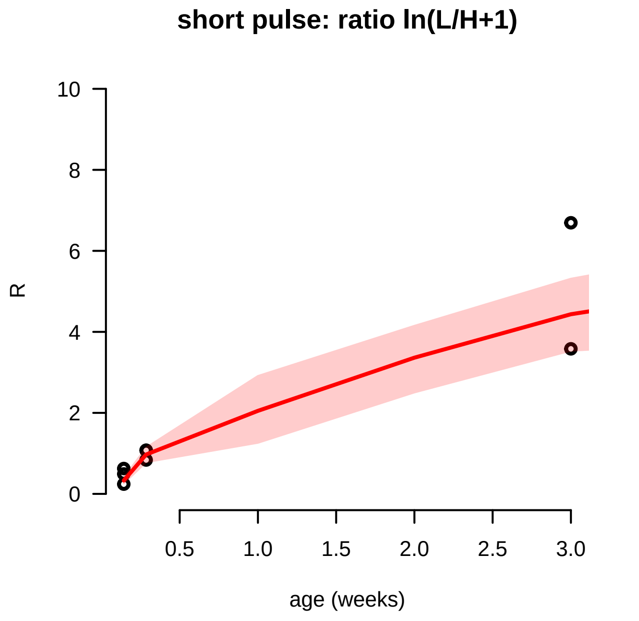

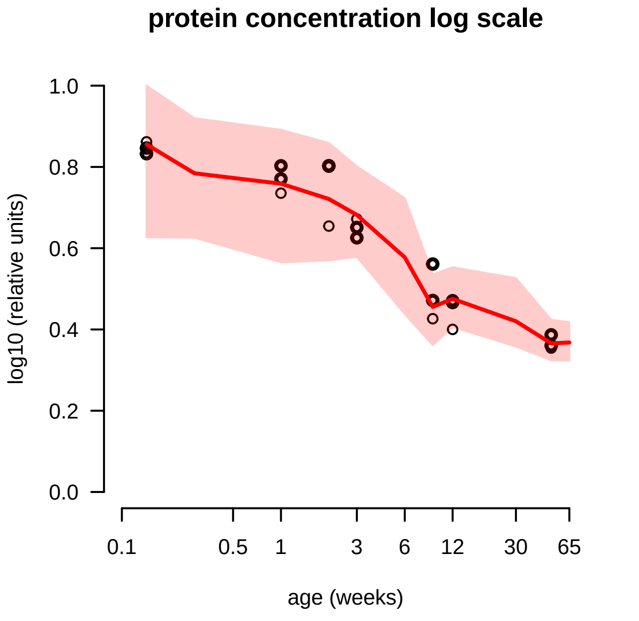

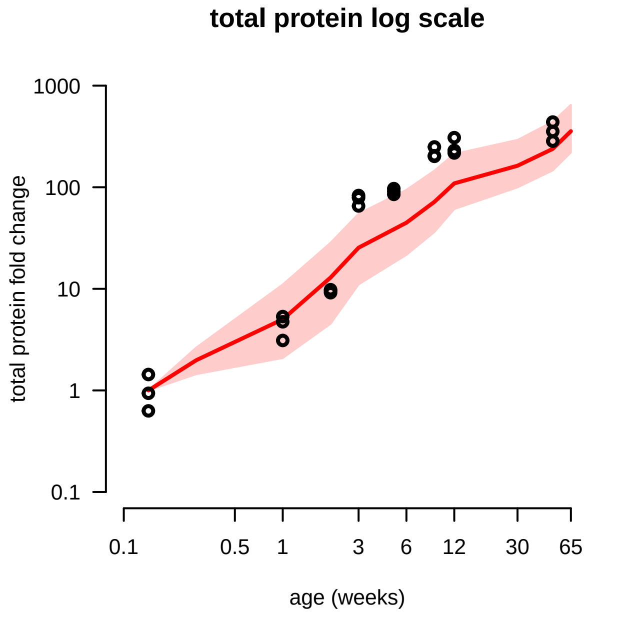

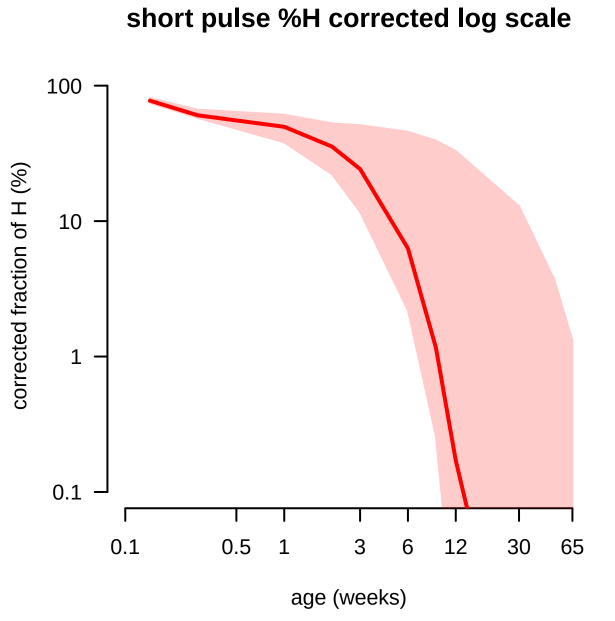

Q8K2Z4;Q8K2Z4−2

>sp|Q8K2Z4|CND1_MOUSE

Condensin complex subunit 1

GN=Ncapd2

Med H1/2: 4.5 days

Q5 H1/2: 2.6 days

Q95 H1/2: 20.5 days

reliable model fit: yes

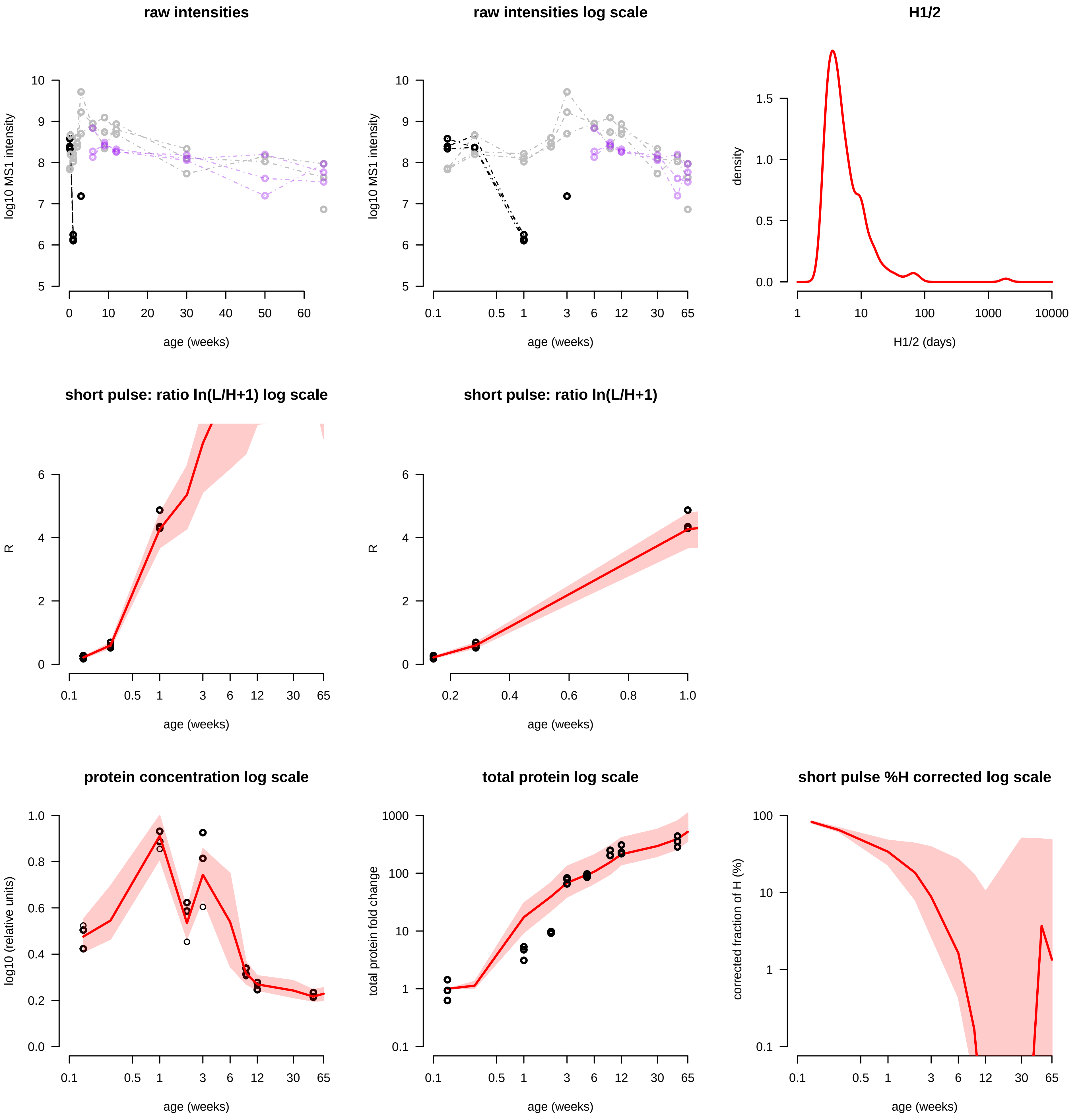

Q91Z96;Q91Z96−2

>sp|Q91Z96|BMP2K_MOUSE

BMP−2−inducible protein kinase

GN=Bmp2k

Med H1/2: 9 days

Q5 H1/2: 3.8 days

Q95 H1/2: 51.7 days

reliable model fit: yes

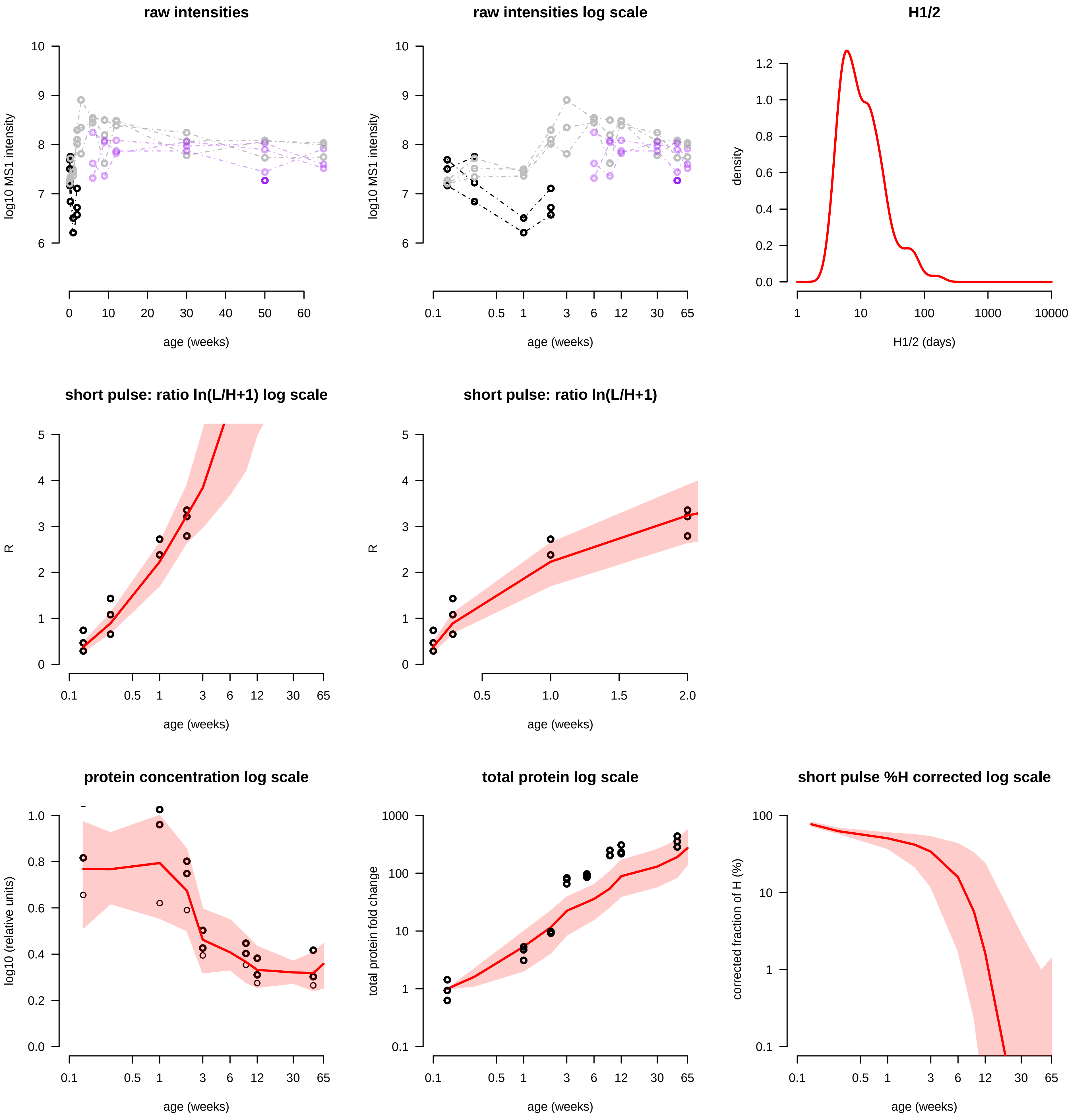

Q99MS7;Q99MS7−3;Q99MS7−2;Q99MS7−4

>sp|Q99MS7|EH1L1_MOUSE

EH domain−binding protein 1−like protein 1

GN=Ehbp1l1

Med H1/2: 8.6 days

Q5 H1/2: 3.2 days

Q95 H1/2: 92.8 days

reliable model fit: yes

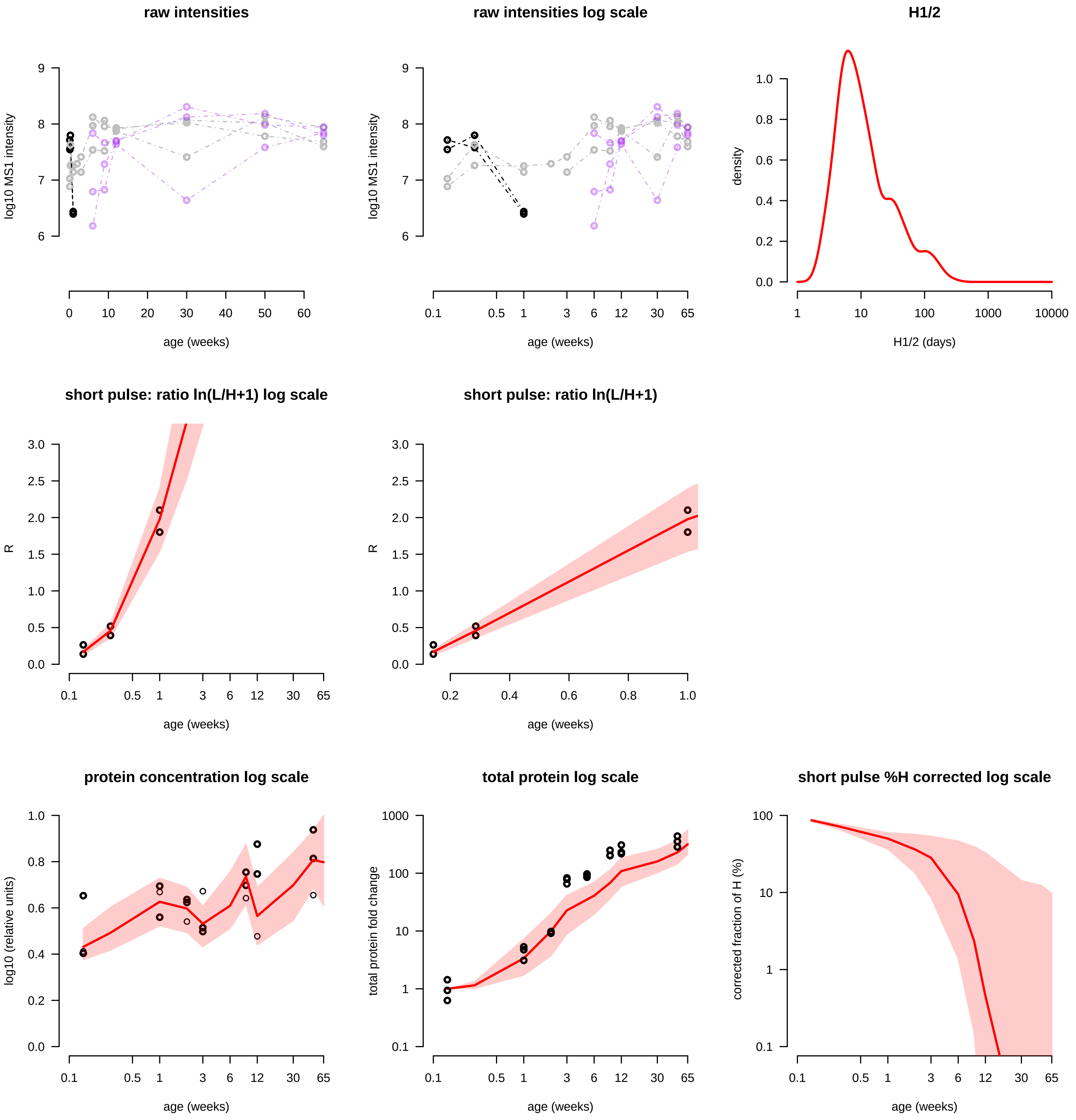

Q78JE5

>sp|Q78JE5|FBX22_MOUSE

F−box only protein 22

GN=Fbxo22

Med H1/2: 8.1 days

Q5 H1/2: 2.8 days

Q95 H1/2: 112.6 days

reliable model fit: yes

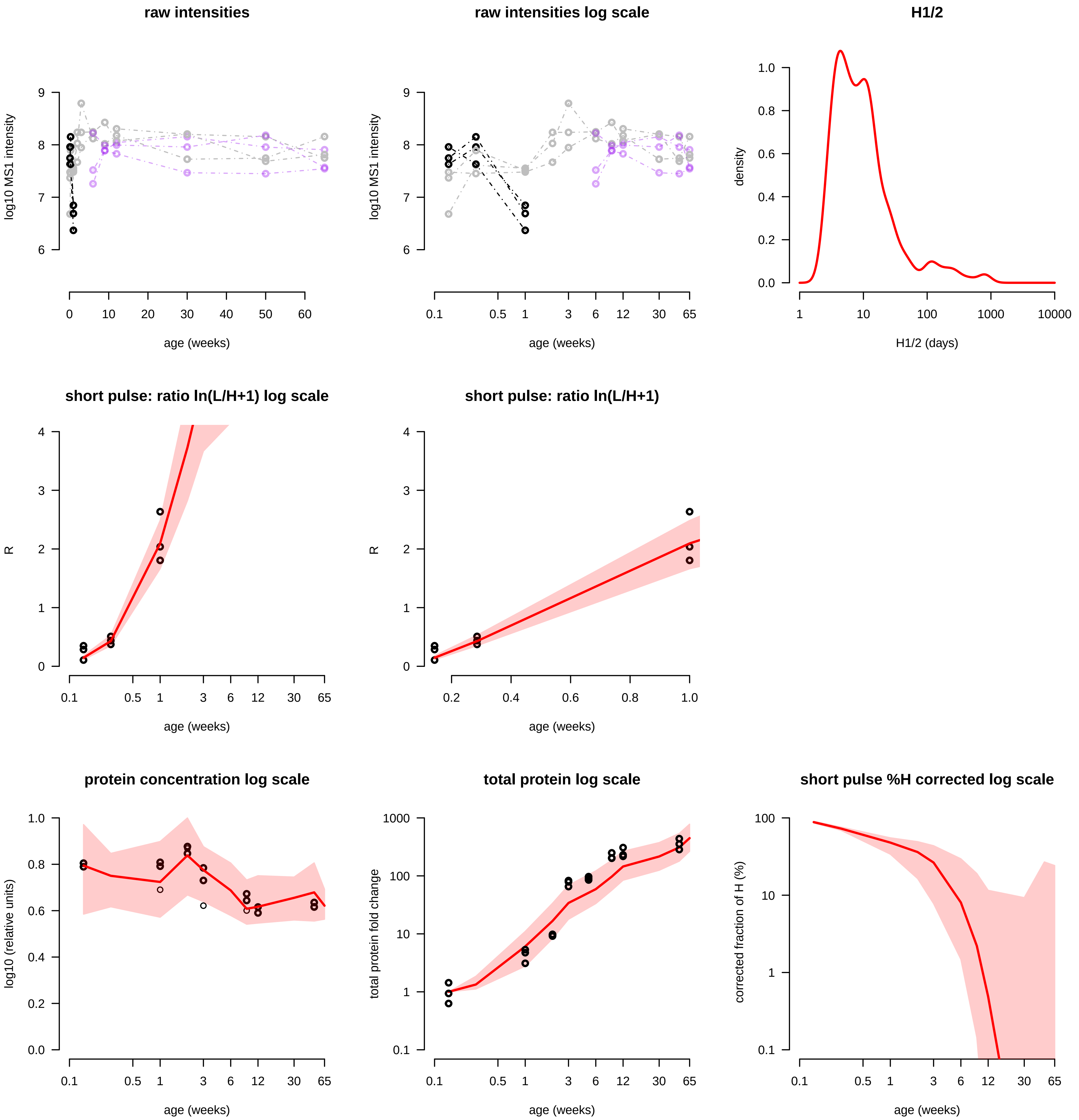

O88508

>sp|O88508|DNM3A_MOUSE

DNA (cytosine−5)−methyltransferase 3A

GN=Dnmt3a

Med H1/2: 70.6 days

Q5 H1/2: 29.9 days

Q95 H1/2: 272.5 days

reliable model fit: yes

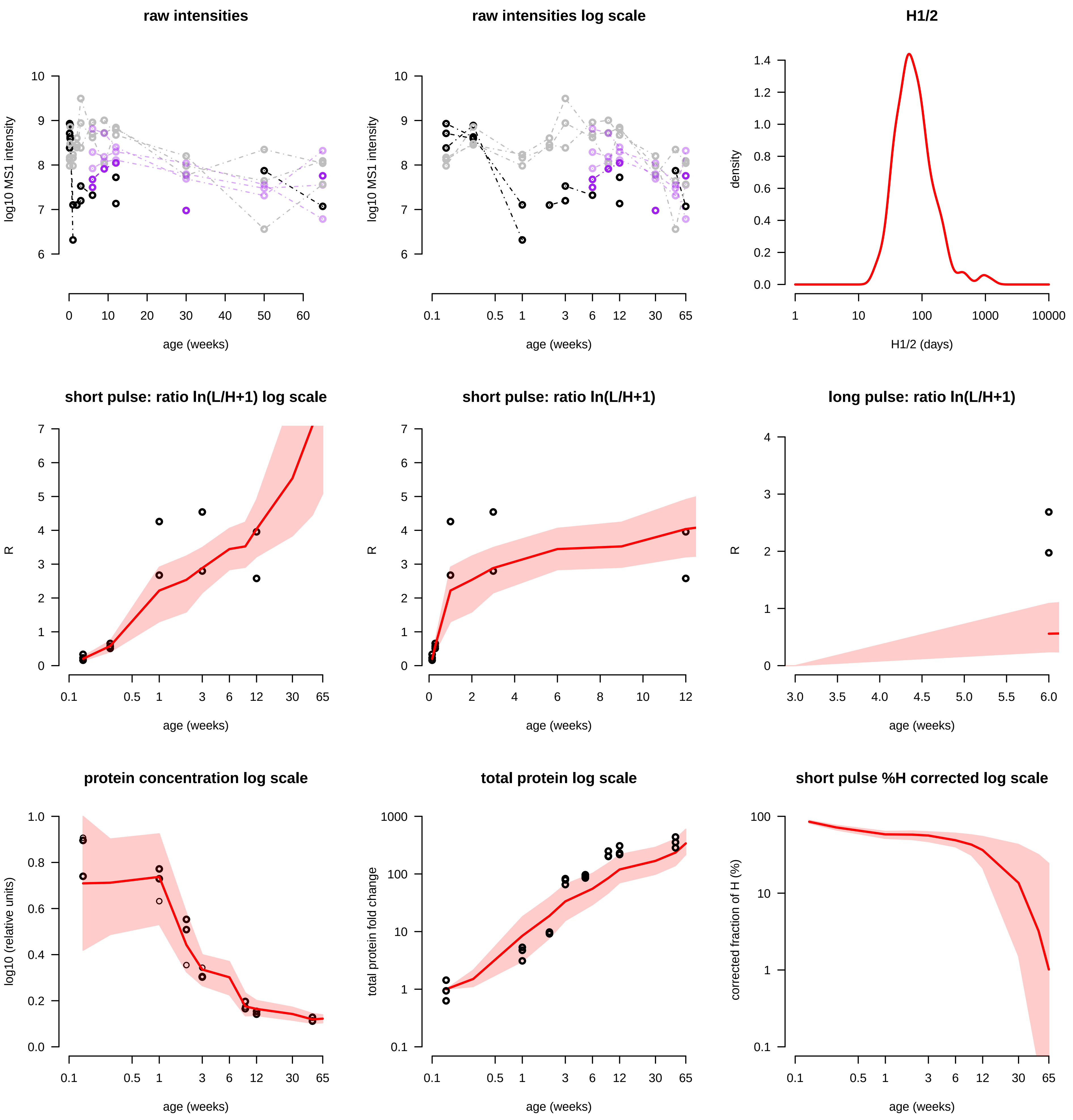

Q8VD62

>sp|Q8VD62|CK068_MOUSE

UPF0696 protein C11orf68 homolog

GN=Bles03

Med H1/2: 18.5 days

Q5 H1/2: 6.5 days

Q95 H1/2: 136.8 days

reliable model fit: yes

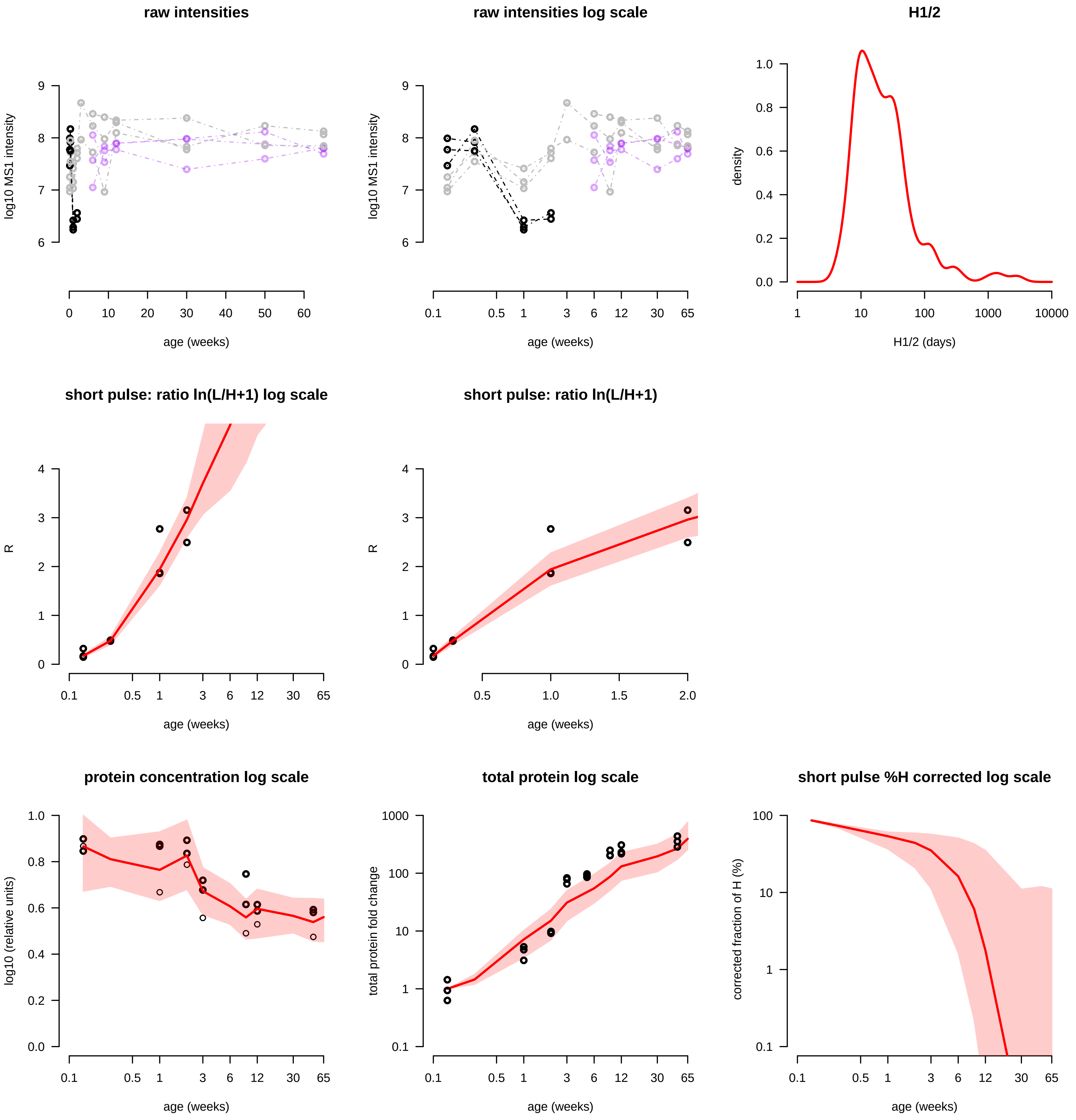

Q9D2R8

>sp|Q9D2R8|RT33_MOUSE

28S ribosomal protein S33, mitochondrial

GN=Mrps33

Med H1/2: 12.7 days

Q5 H1/2: 4.8 days

Q95 H1/2: 132.3 days

reliable model fit: yes

raw intensities

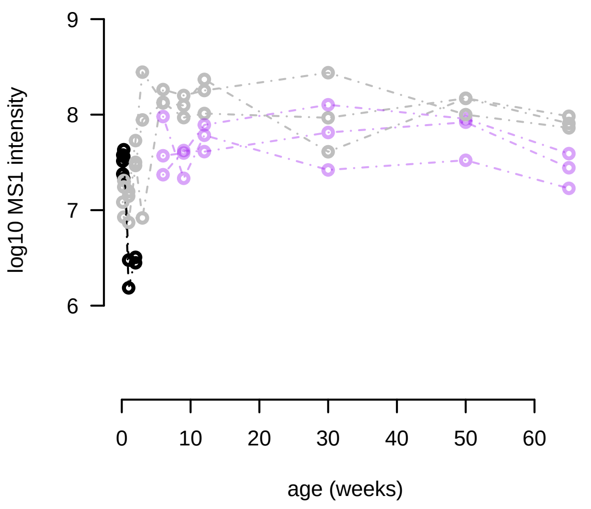

raw intensities log scale

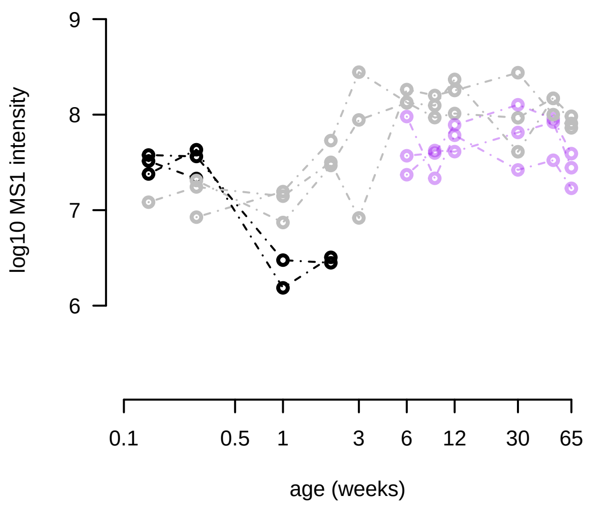

H1/2

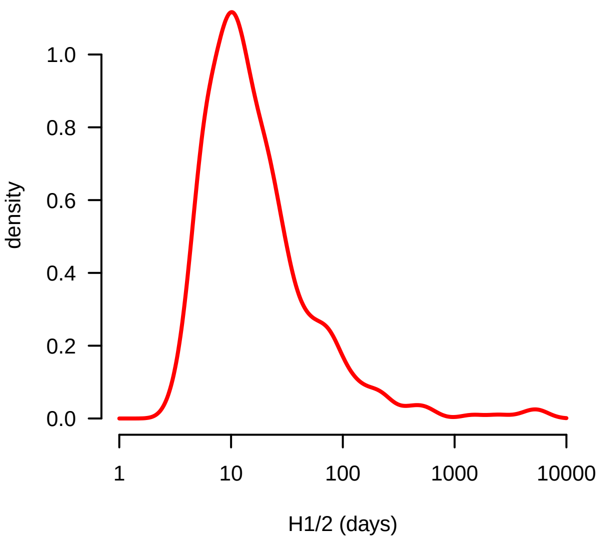

short pulse: ratio ln(L/H+1) log scale

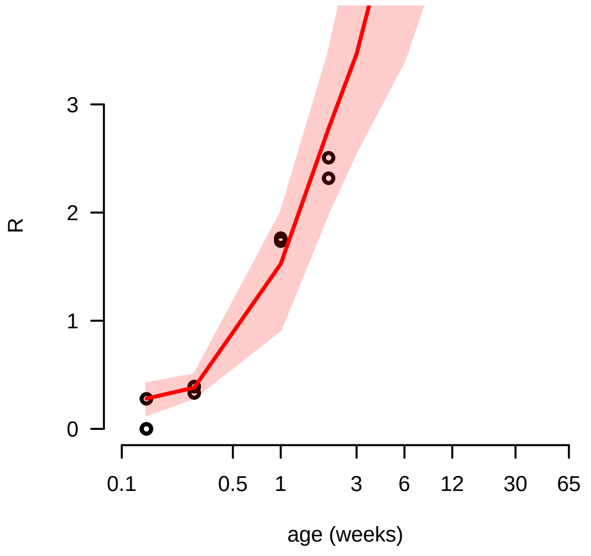

short pulse: ratio ln(L/H+1)

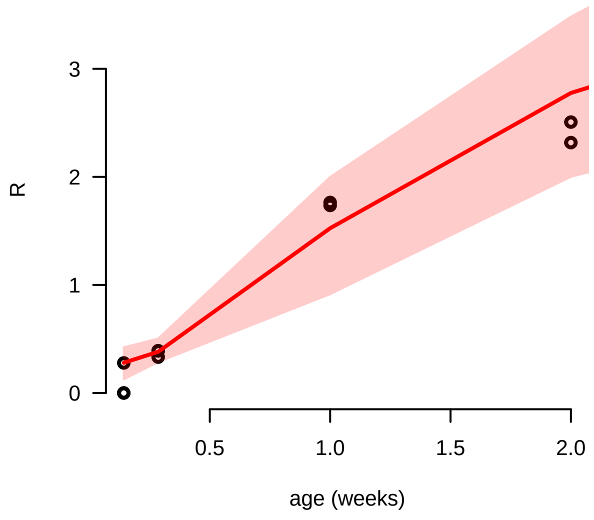

protein concentration log scale

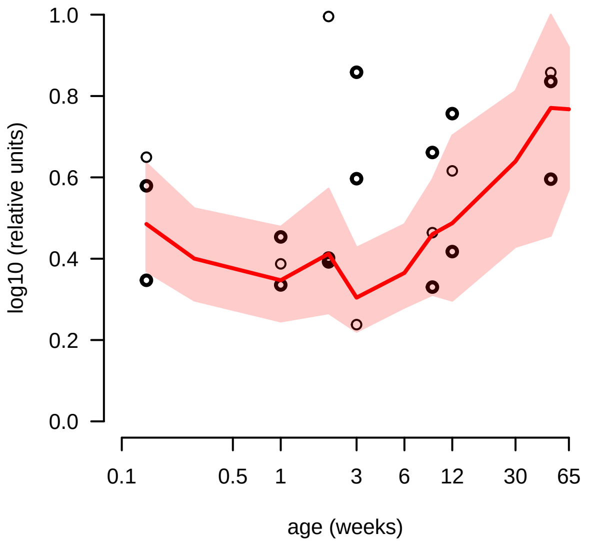

total protein log scale

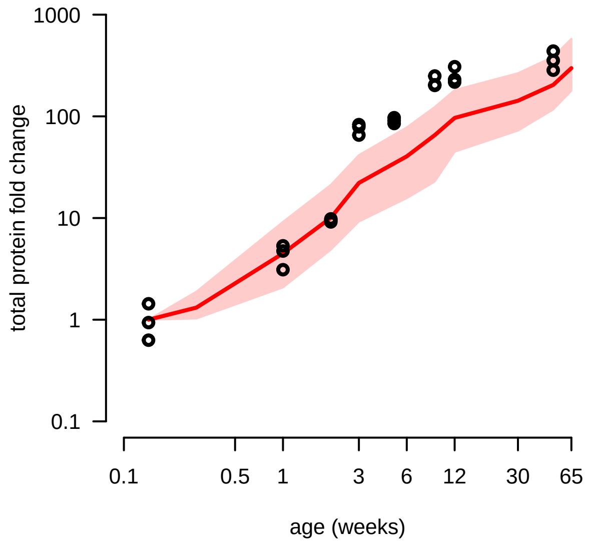

short pulse %H corrected log scale

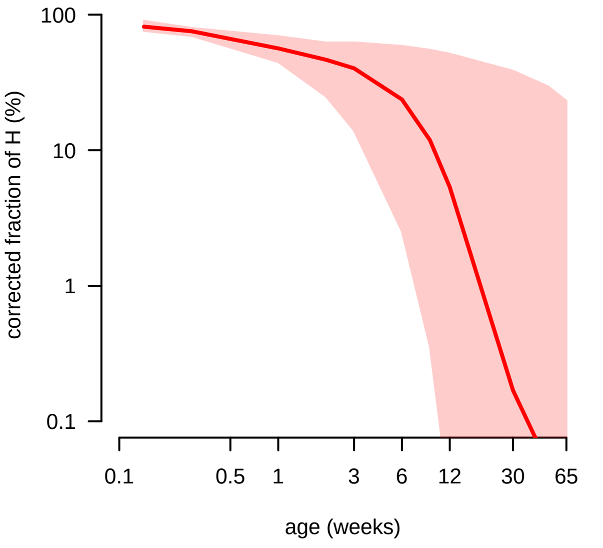

P40201

>sp|P40201|CHD1_MOUSE

Chromodomain−helicase−DNA−binding protein 1

GN=Chd1

Med H1/2: 2028.4 days

Q5 H1/2: 614.2 days

Q95 H1/2: 20368.8 days

reliable model fit: no

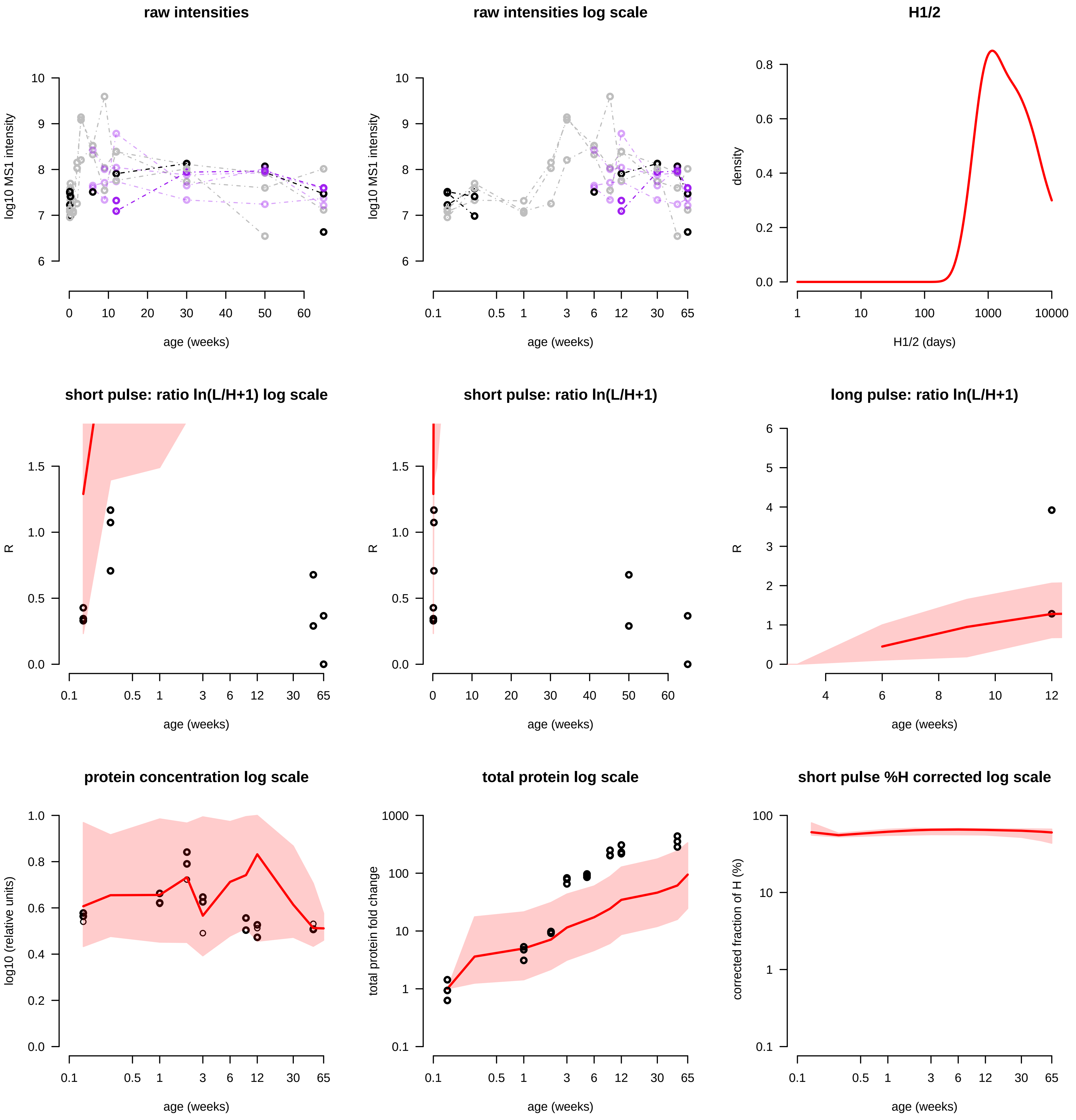

Q9CWX9

>sp|Q9CWX9|DDX47_MOUSE

Probable ATP−dependent RNA helicase DDX47

GN=Ddx47

Med H1/2: 7.4 days

Q5 H1/2: 2.7 days

Q95 H1/2: 67.9 days

reliable model fit: yes

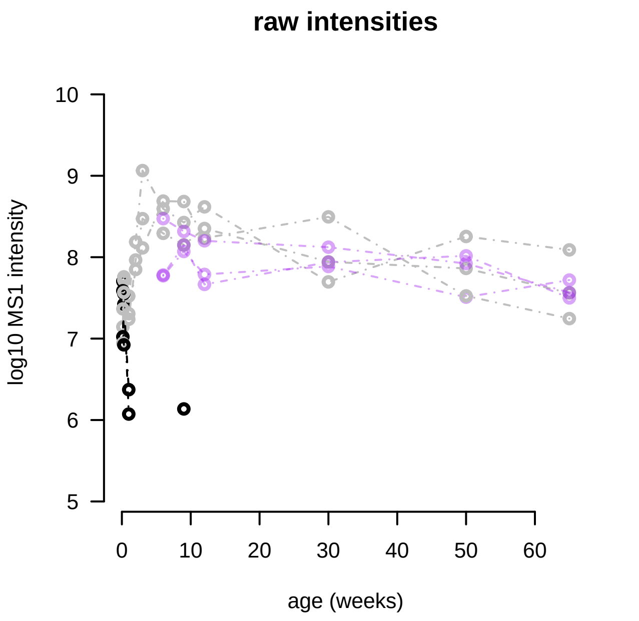

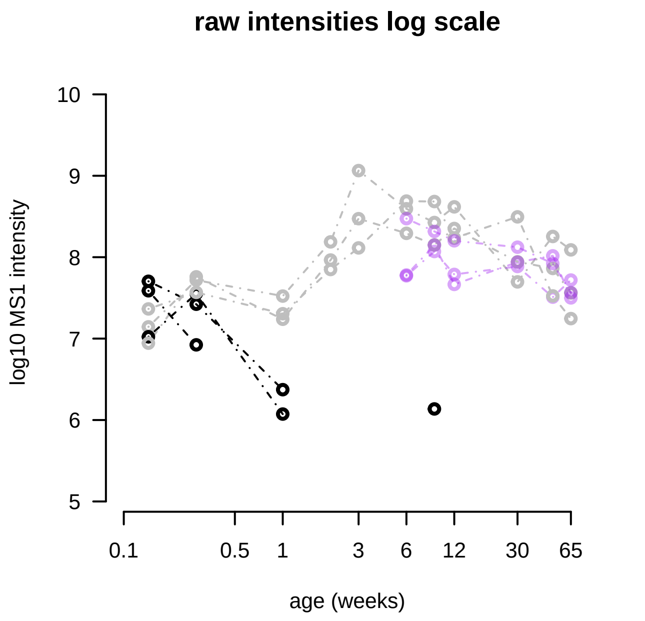

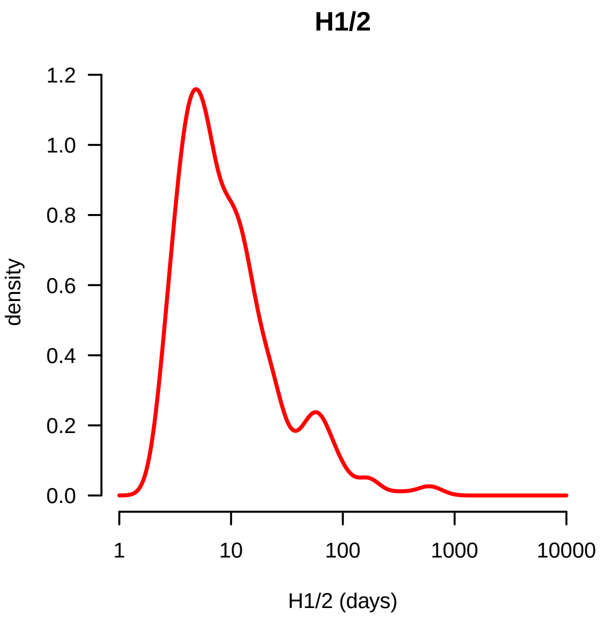

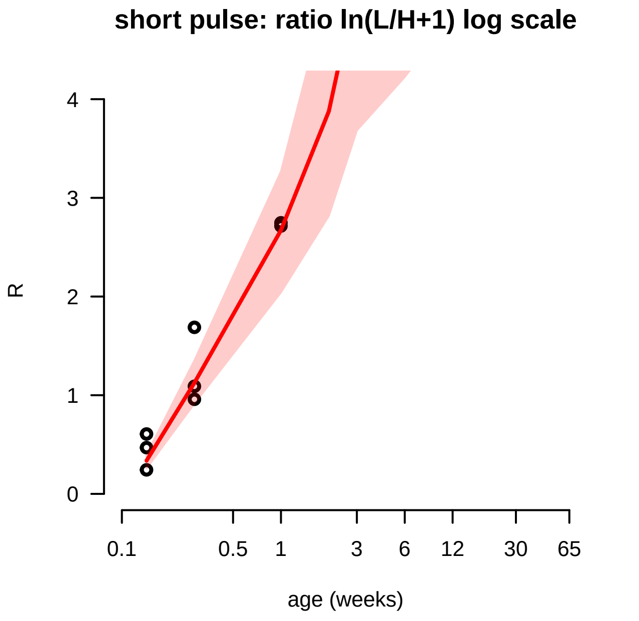

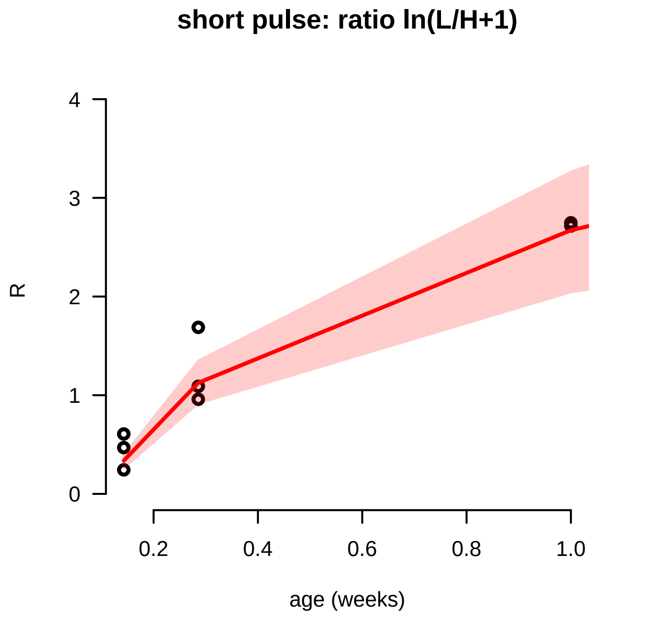

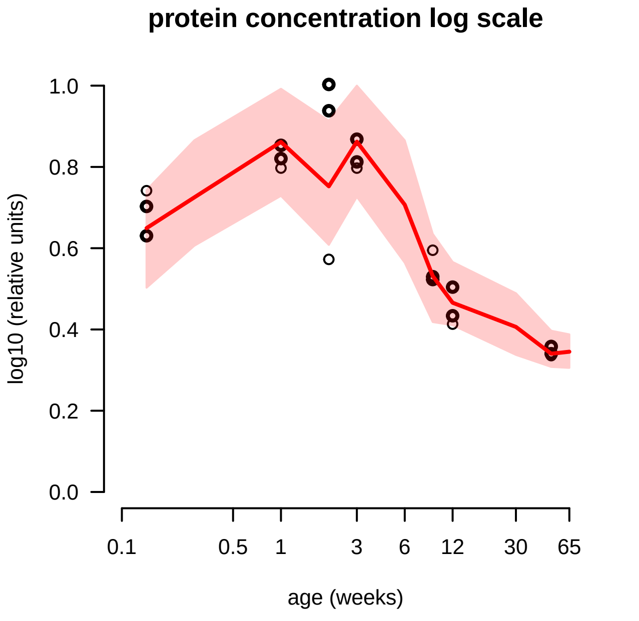

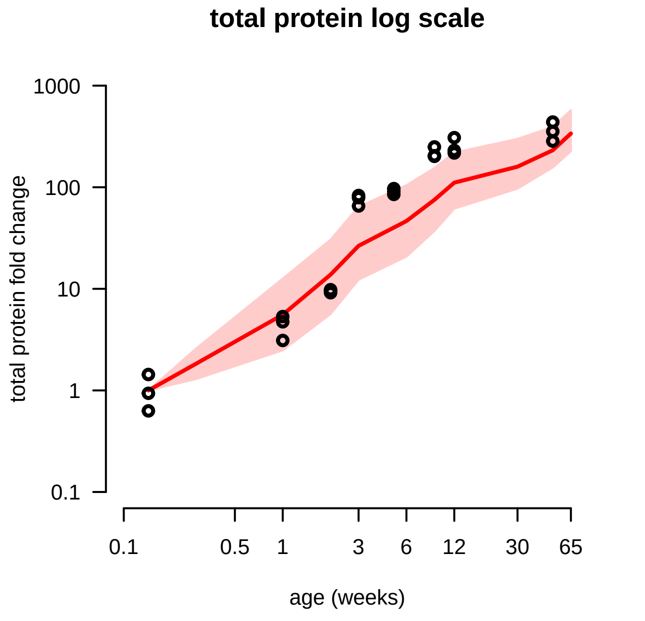

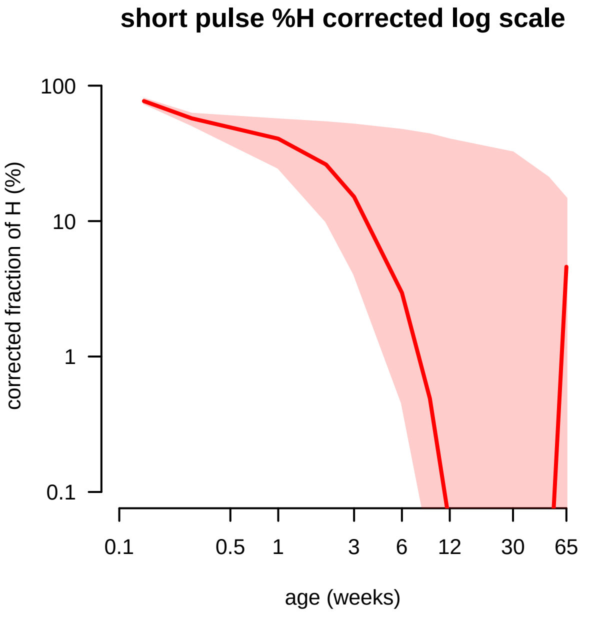

Q3TYX3

>sp|Q3TYX3|SMYD5_MOUSE

SET and MYND domain−containing protein 5

GN=Smyd5

Med H1/2: 10.6 days

Q5 H1/2: 3.1 days

Q95 H1/2: 100.4 days

reliable model fit: yes

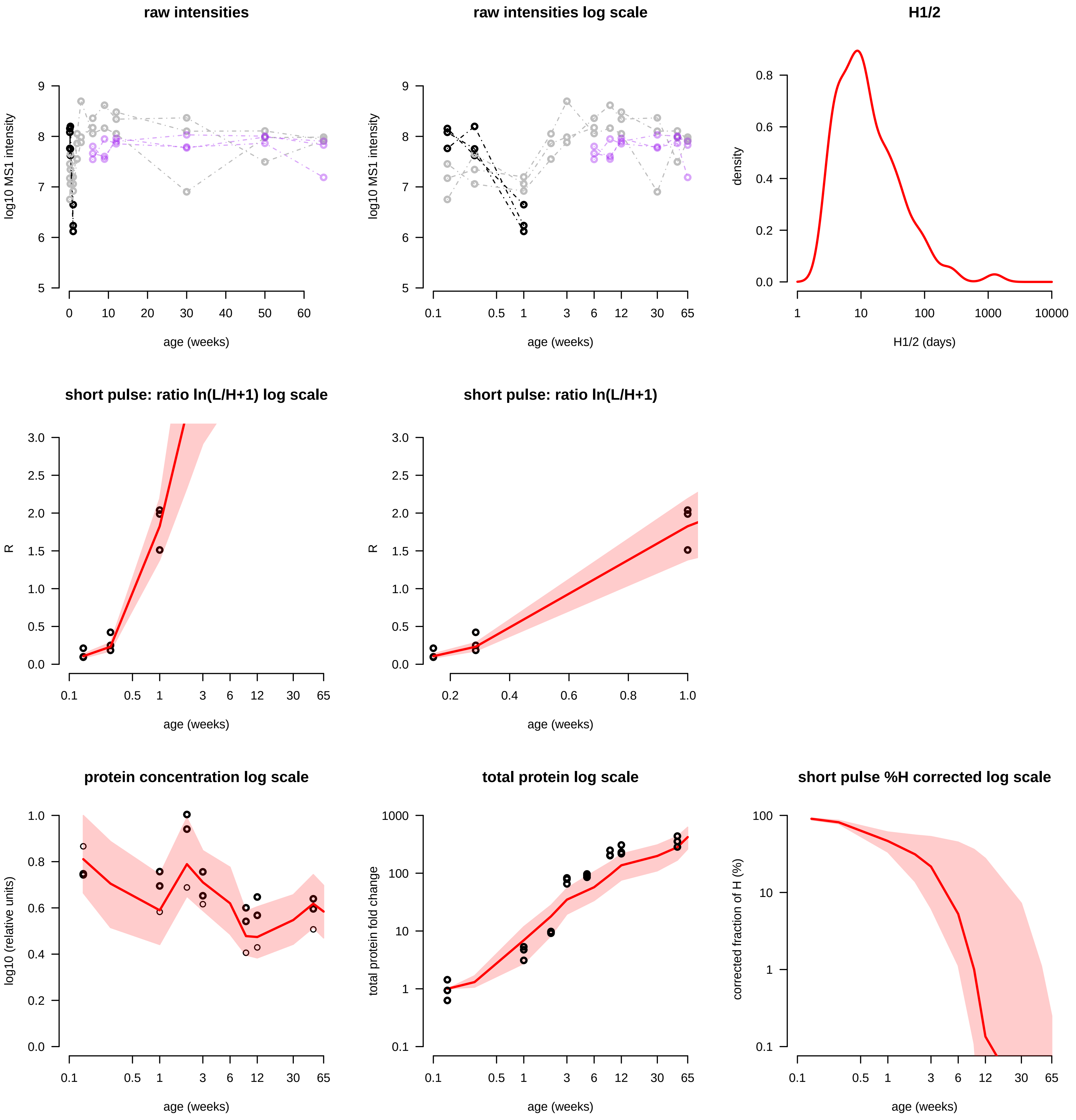

Q8R3Y8−2;Q8R3Y8

>sp|Q8R3Y8−2|I2BP1_MOUSE

Isoform 2 of Interferon regulatory factor 2−binding protein 1

GN=Irf2bp1

Med H1/2: 4.5 days

Q5 H1/2: 2.5 days

Q95 H1/2: 18.7 days

reliable model fit: yes

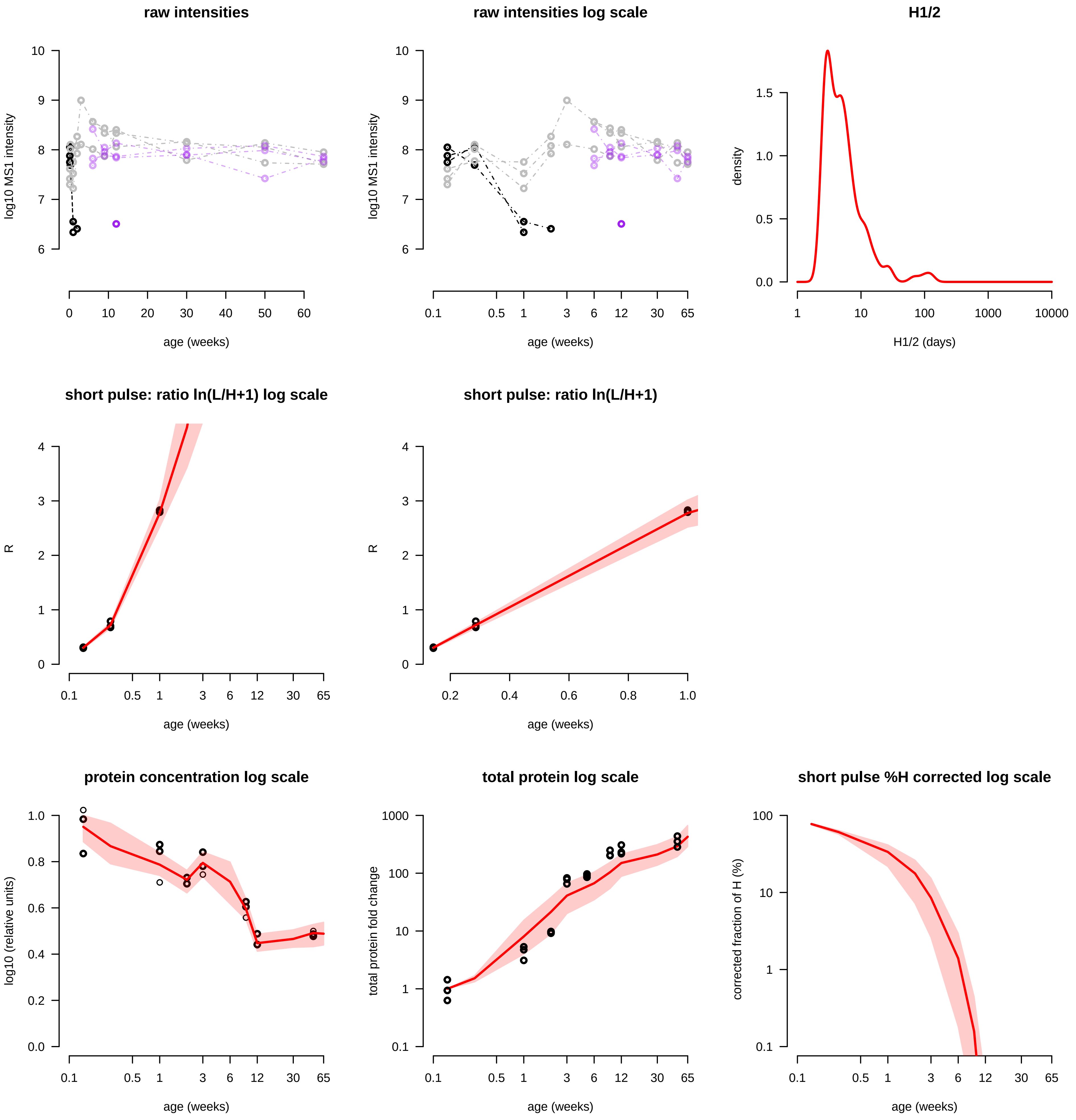

P09470;P09470−2

>sp|P09470|ACE_MOUSE

Angiotensin−converting enzyme

GN=Ace

Med H1/2: 6.6 days

Q5 H1/2: 2.9 days

Q95 H1/2: 49.8 days

reliable model fit: no

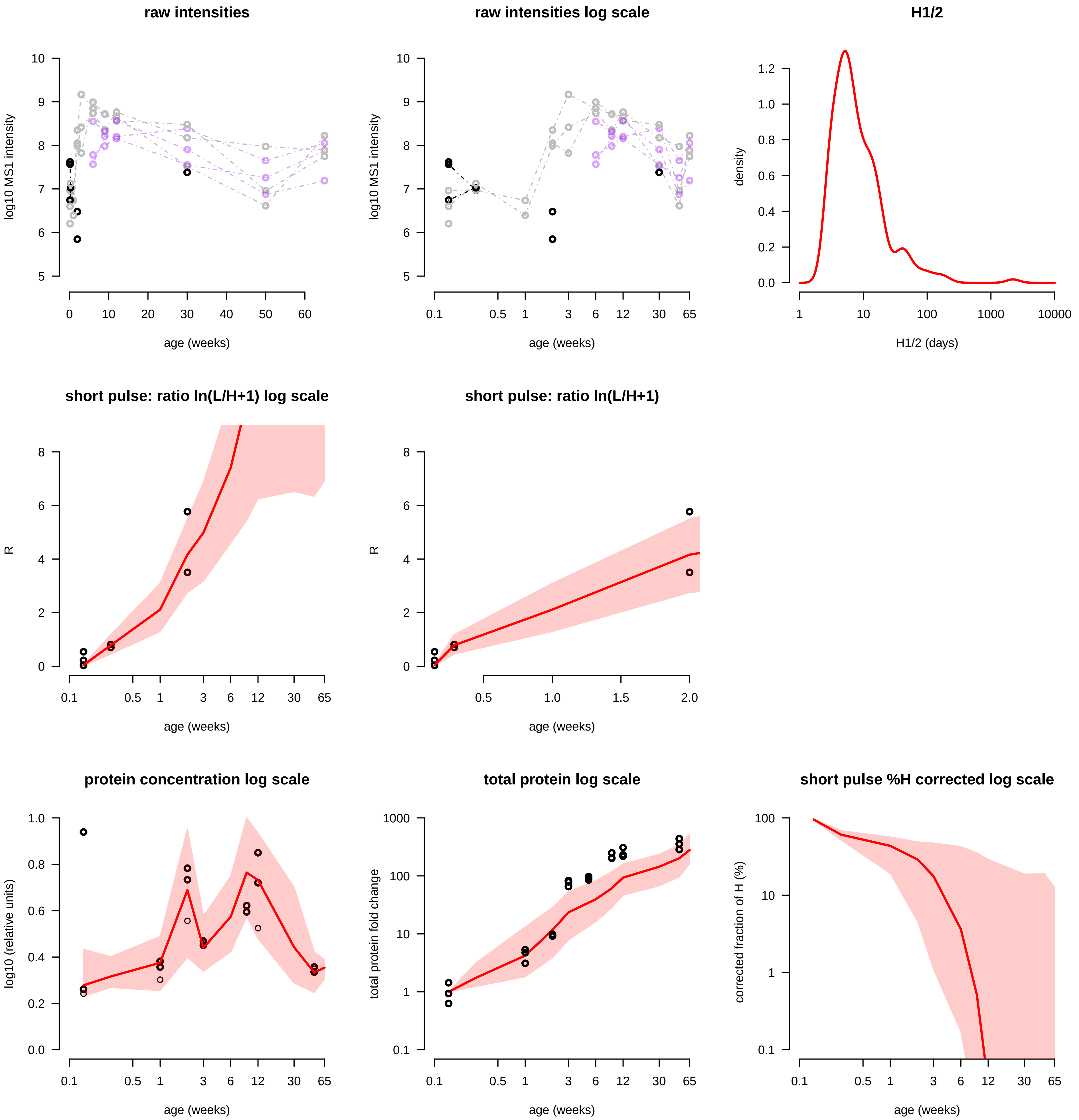

P68368

>sp|P68368|TBA4A_MOUSE

Tubulin alpha−4A chain

GN=Tuba4a

Med H1/2: 10.7 days

Q5 H1/2: 3.7 days

Q95 H1/2: 92.6 days

reliable model fit: yes

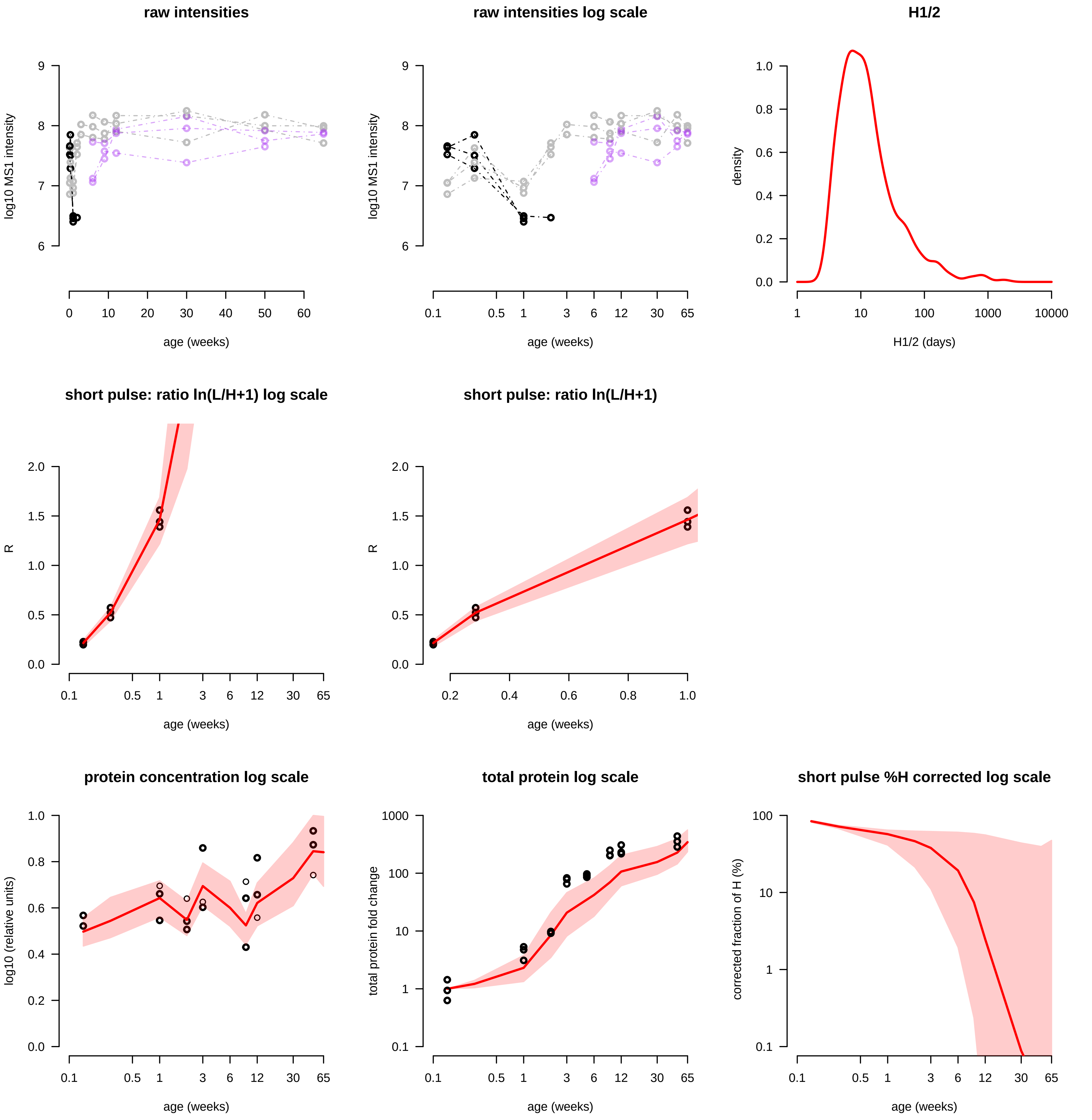

Q8VBX6−2;Q8VBX6;Q8VBX6−4

>sp|Q8VBX6−2|MPDZ_MOUSE

Isoform 2 of Multiple PDZ domain protein

GN=Mpdz

Med H1/2: 18.6 days

Q5 H1/2: 4.2 days

Q95 H1/2: 252 days

reliable model fit: yes

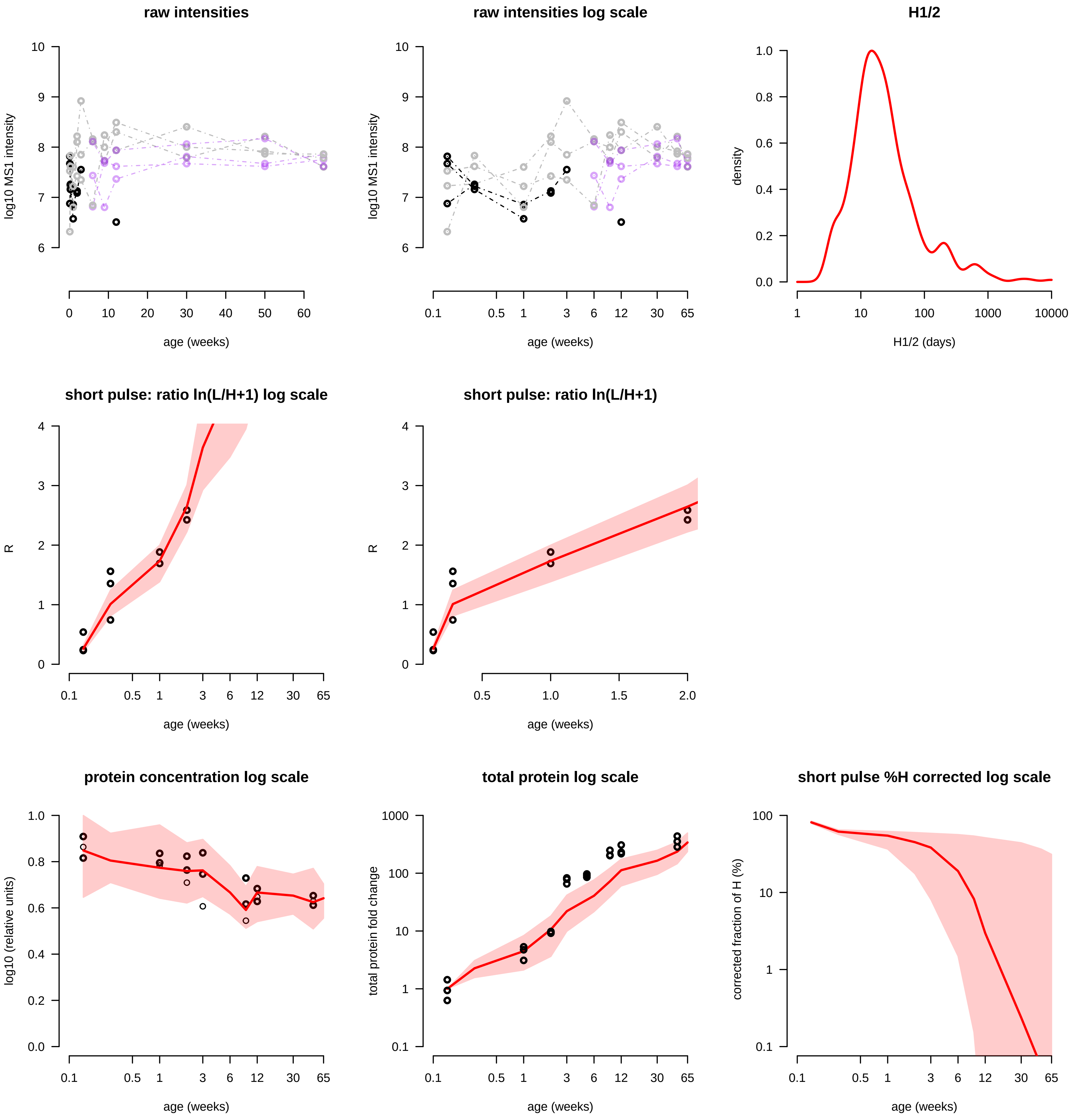

Q8BMS4

>sp|Q8BMS4|COQ3_MOUSE

Ubiquinone biosynthesis O−methyltransferase, mitochondrial

GN=Coq3

Med H1/2: 6.1 days

Q5 H1/2: 2.7 days

Q95 H1/2: 44.2 days

reliable model fit: yes

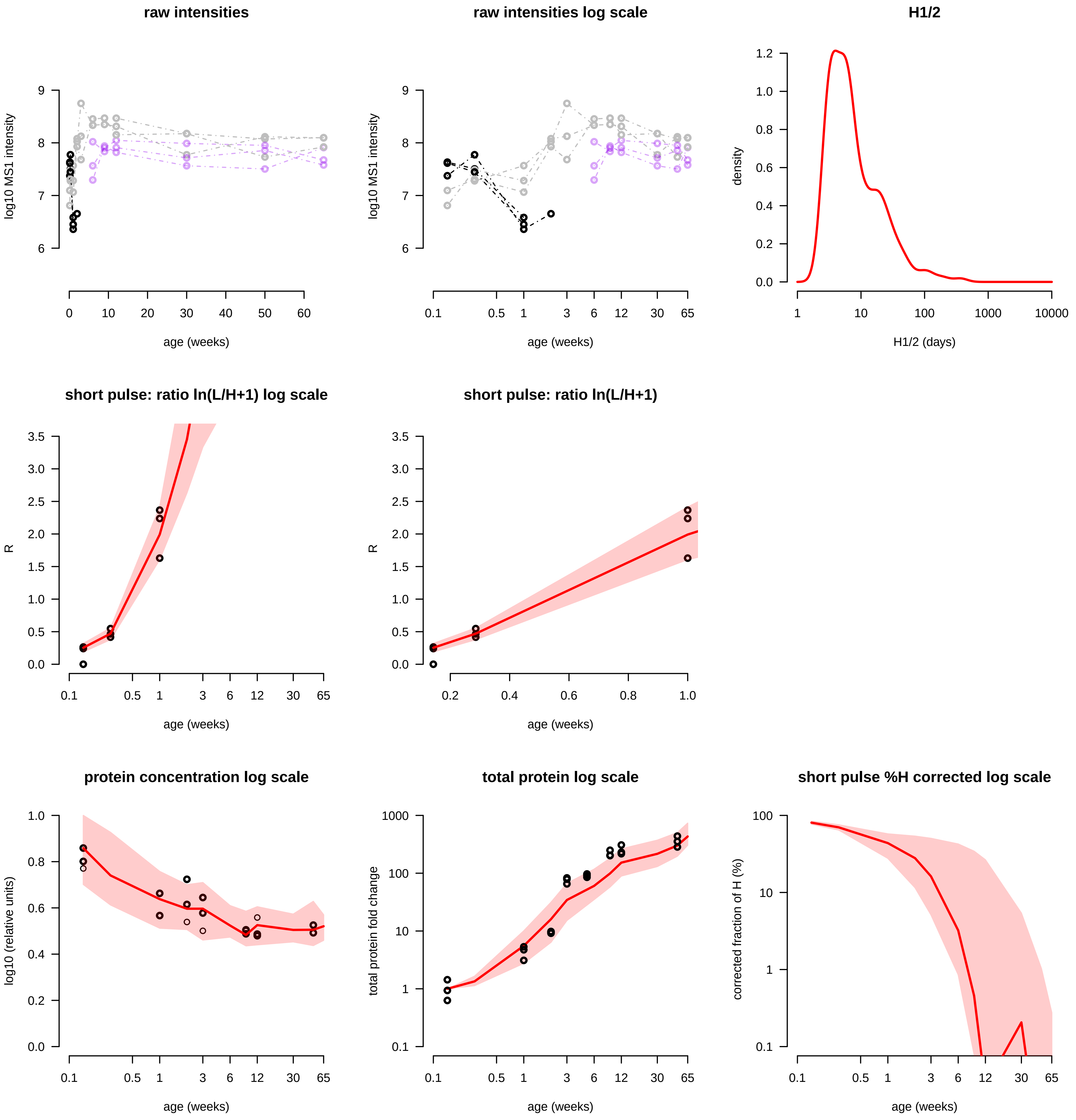

O88796

>sp|O88796|RPP30_MOUSE

Ribonuclease P protein subunit p30

GN=Rpp30

Med H1/2: 9.9 days

Q5 H1/2: 3.3 days

Q95 H1/2: 108.3 days

reliable model fit: yes

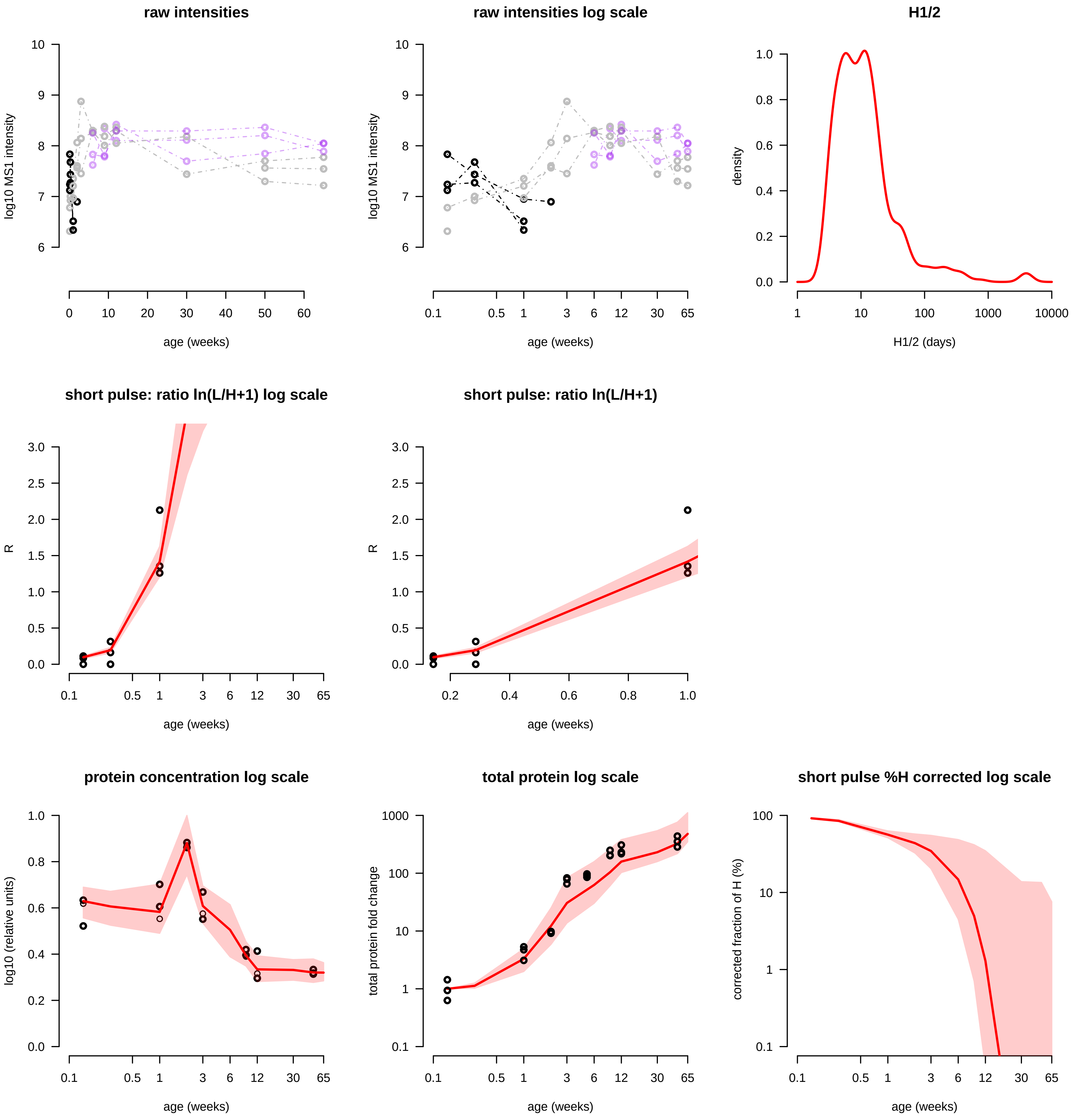

Q571I9

>sp|Q571I9|A16A1_MOUSE

Aldehyde dehydrogenase family 16 member A1

GN=Aldh16a1

Med H1/2: 15.1 days

Q5 H1/2: 3.7 days

Q95 H1/2: 103.7 days

reliable model fit: no

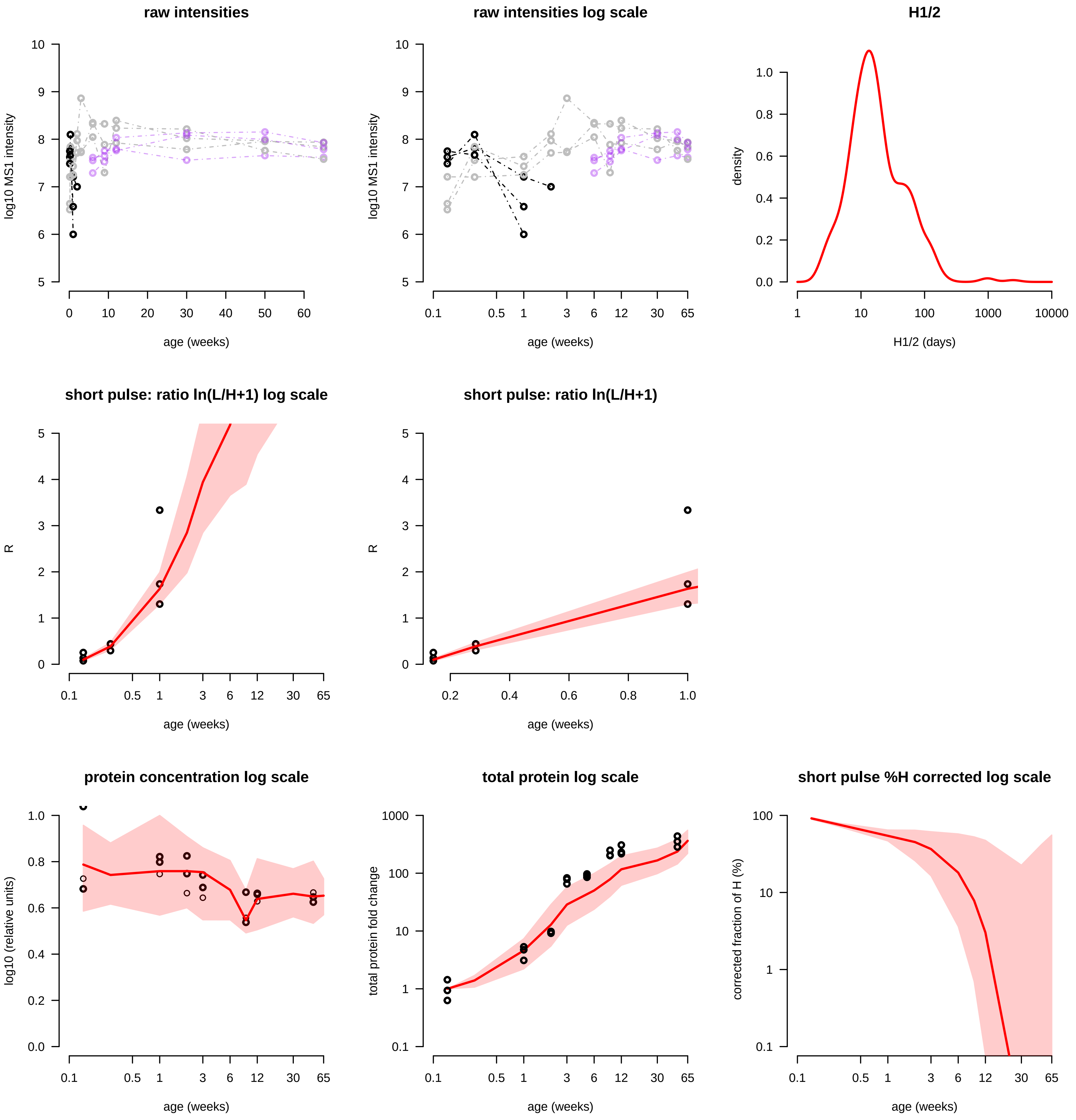

Q61398

>sp|Q61398|PCOC1_MOUSE

Procollagen C−endopeptidase enhancer 1

GN=Pcolce

Med H1/2: 5.4 days

Q5 H1/2: 2.9 days

Q95 H1/2: 22.7 days

reliable model fit: yes

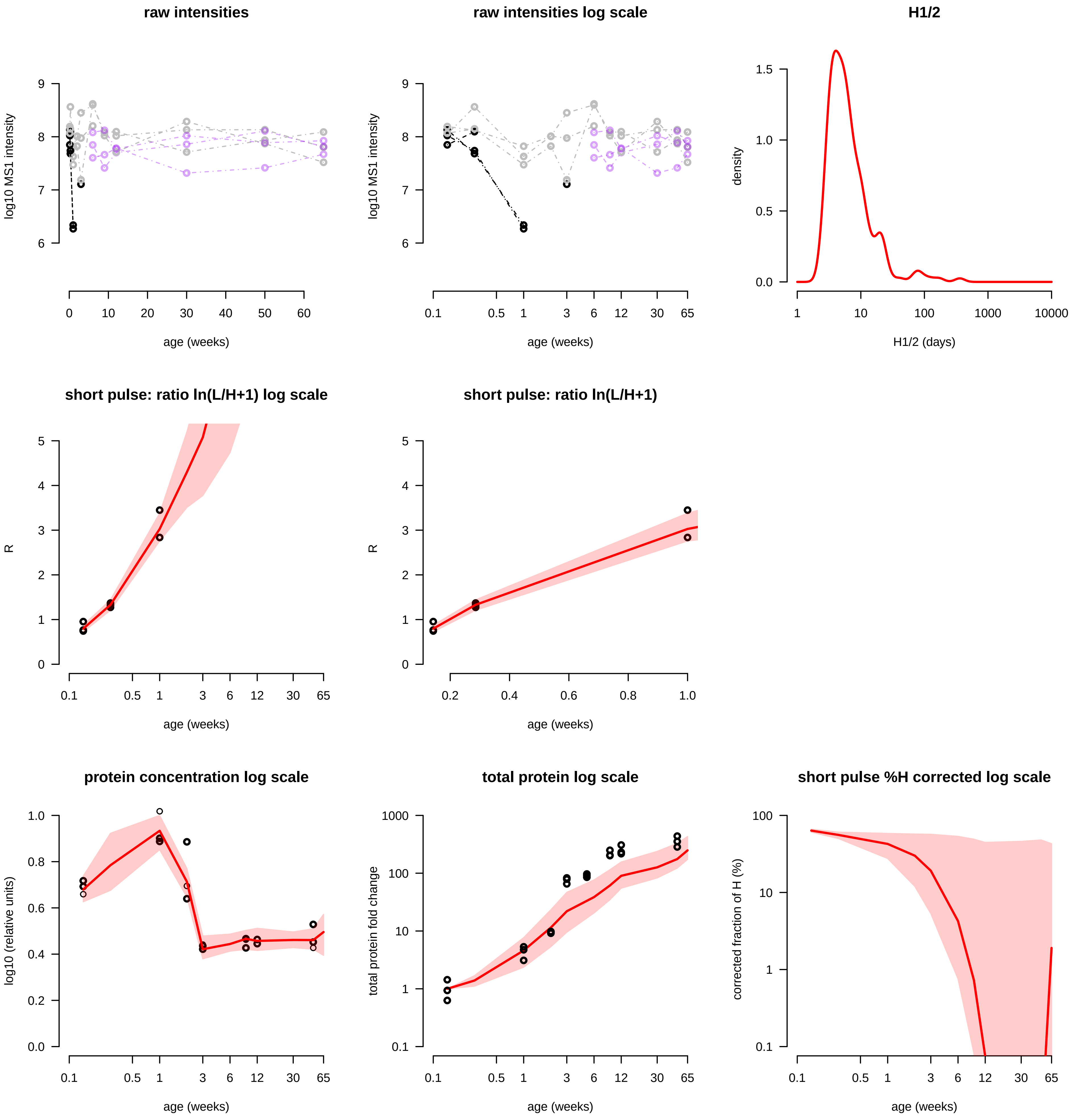

Q922L6

>sp|Q922L6|NELFD_MOUSE

Negative elongation factor D

GN=Nelfcd

Med H1/2: 4.2 days

Q5 H1/2: 2.6 days

Q95 H1/2: 9.8 days

reliable model fit: yes

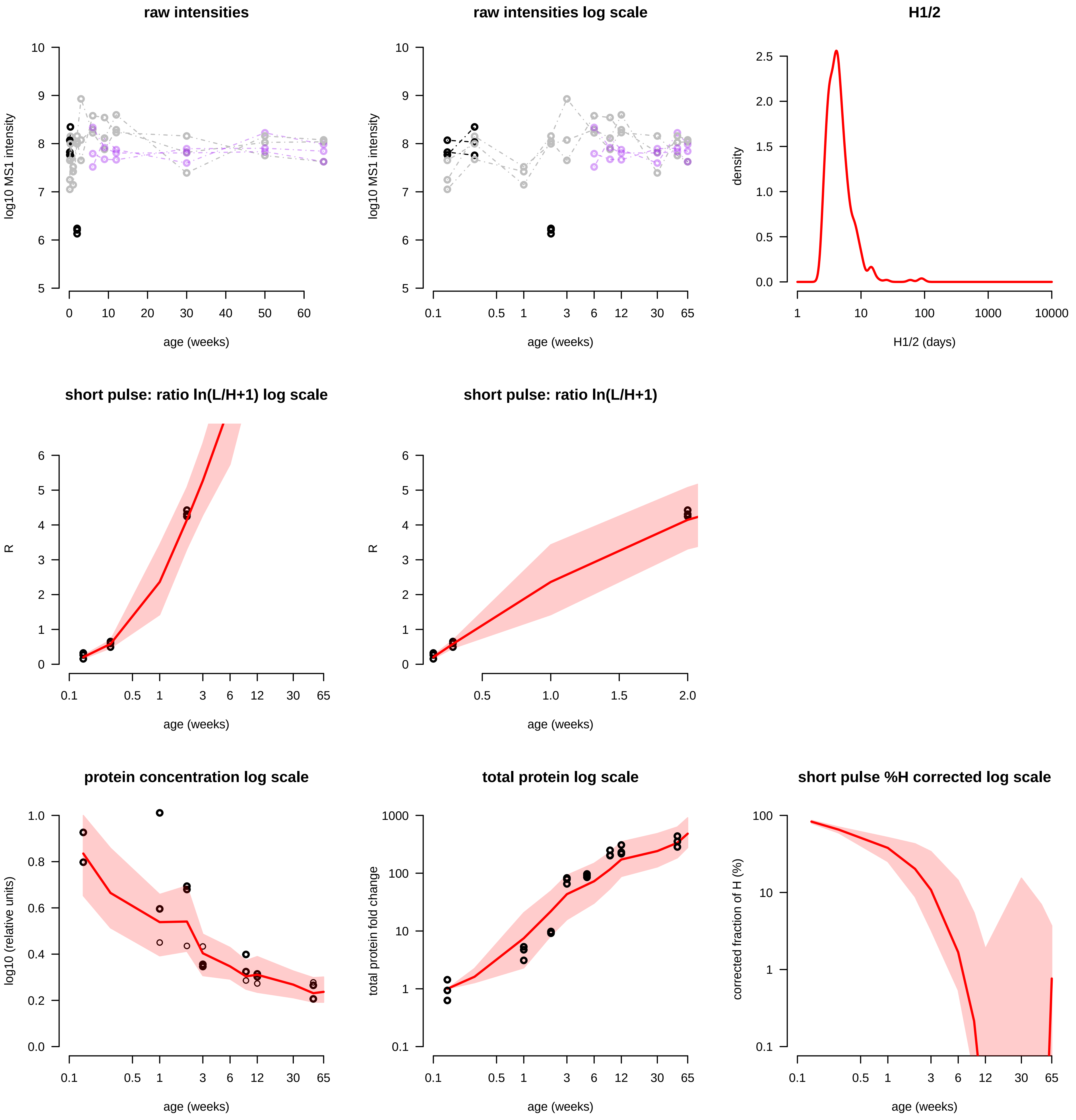

Q6A0A2;Q6A0A2−2;Q6A0A2−3

>sp|Q6A0A2|LAR4B_MOUSE

La−related protein 4B

GN=Larp4b

Med H1/2: 5.7 days

Q5 H1/2: 2.7 days

Q95 H1/2: 42.7 days

reliable model fit: yes

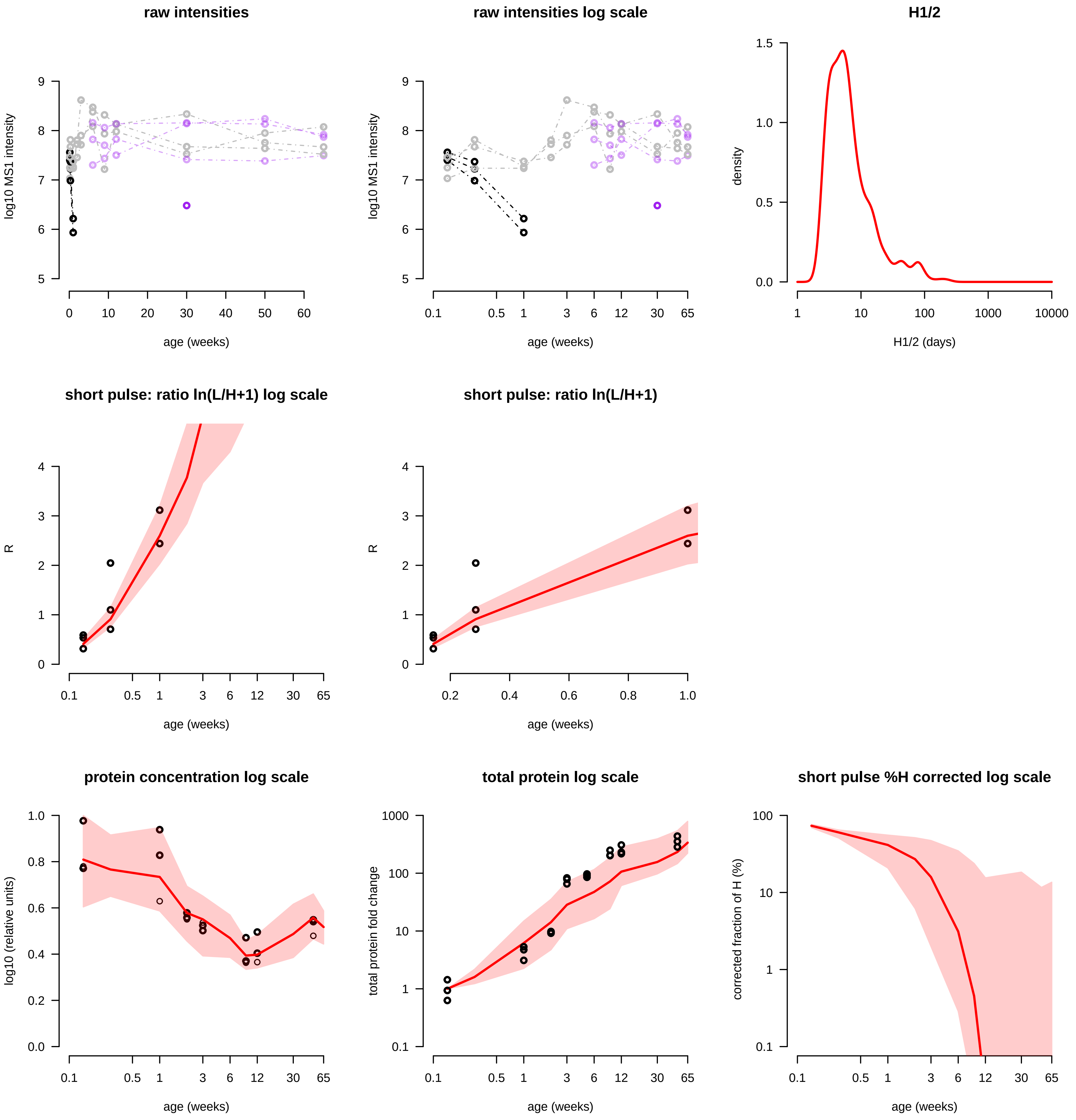

Q505B7−2;Q505B7

>sp|Q505B7−2|ARCH_MOUSE

Isoform 2 of Protein archease

GN=Zbtb8os

Med H1/2: 5.9 days

Q5 H1/2: 3.2 days

Q95 H1/2: 20.6 days

reliable model fit: yes

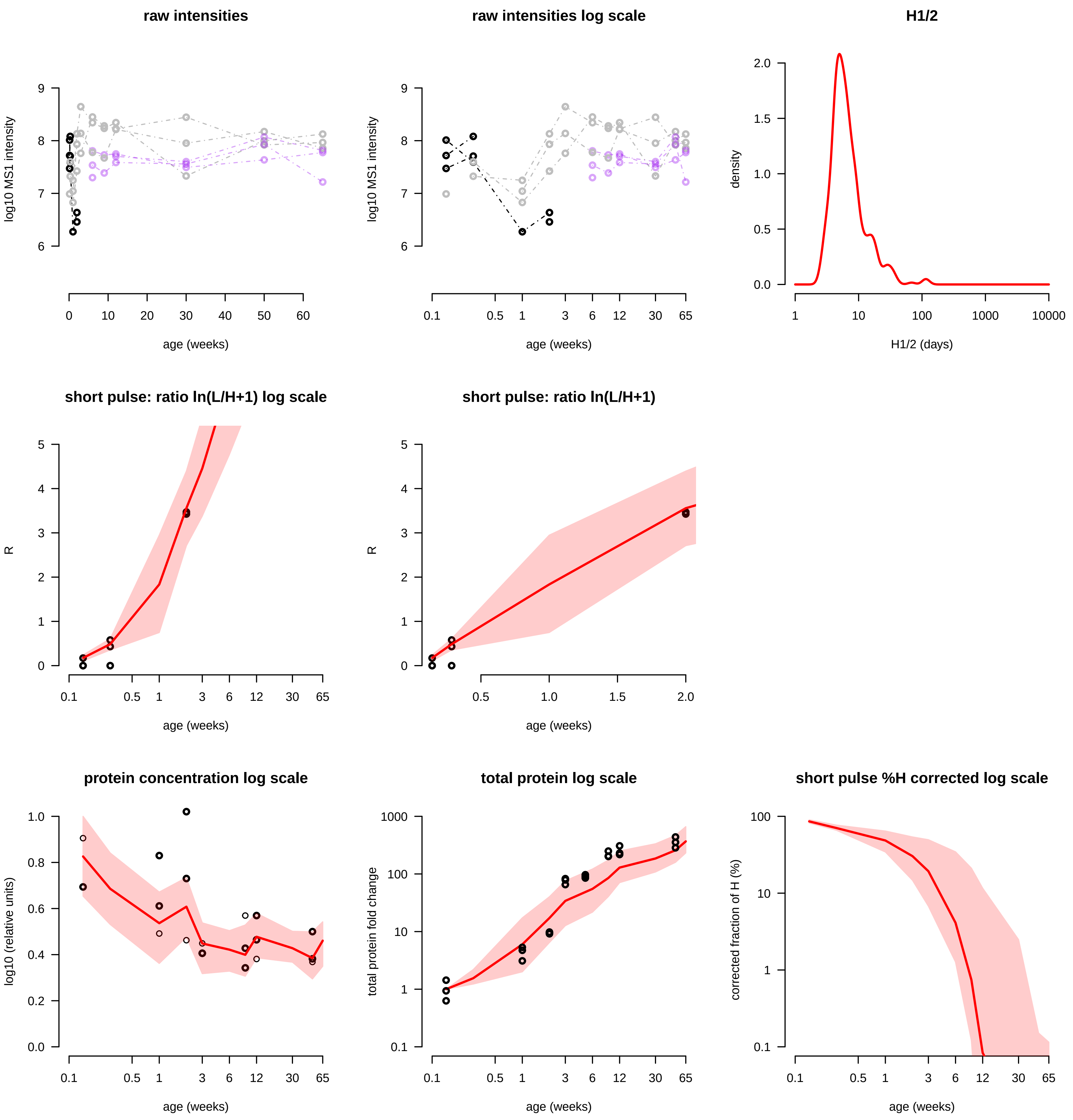

O70551

>sp|O70551|SRPK1_MOUSE

SRSF protein kinase 1

GN=Srpk1

Med H1/2: 3.5 days

Q5 H1/2: 2.5 days

Q95 H1/2: 8 days

reliable model fit: yes

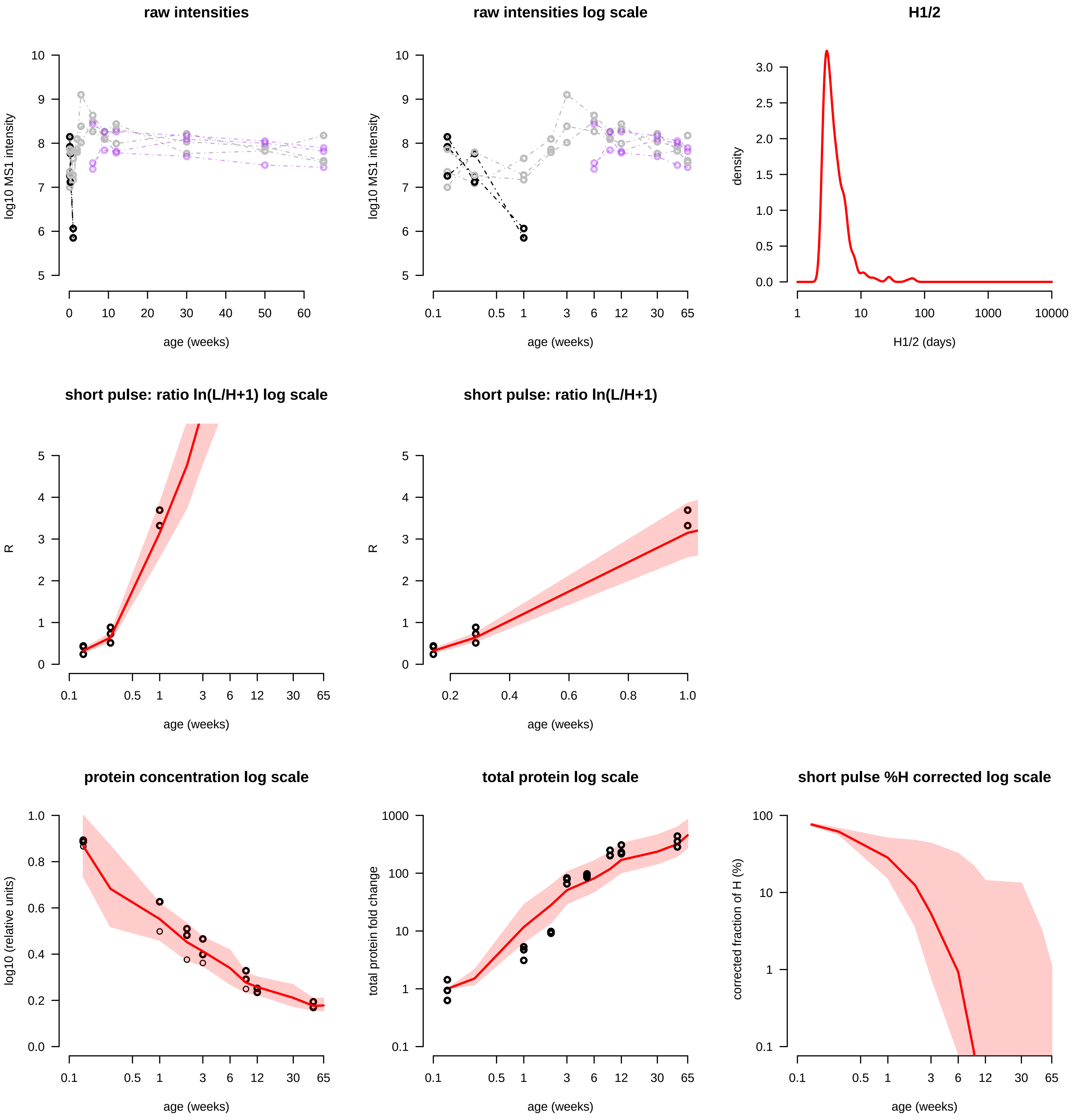

B2RX14

>sp|B2RX14|TUT4_MOUSE

Terminal uridylyltransferase 4

GN=Tut4

Med H1/2: 551.1 days

Q5 H1/2: 201.4 days

Q95 H1/2: 5662.2 days

reliable model fit: no

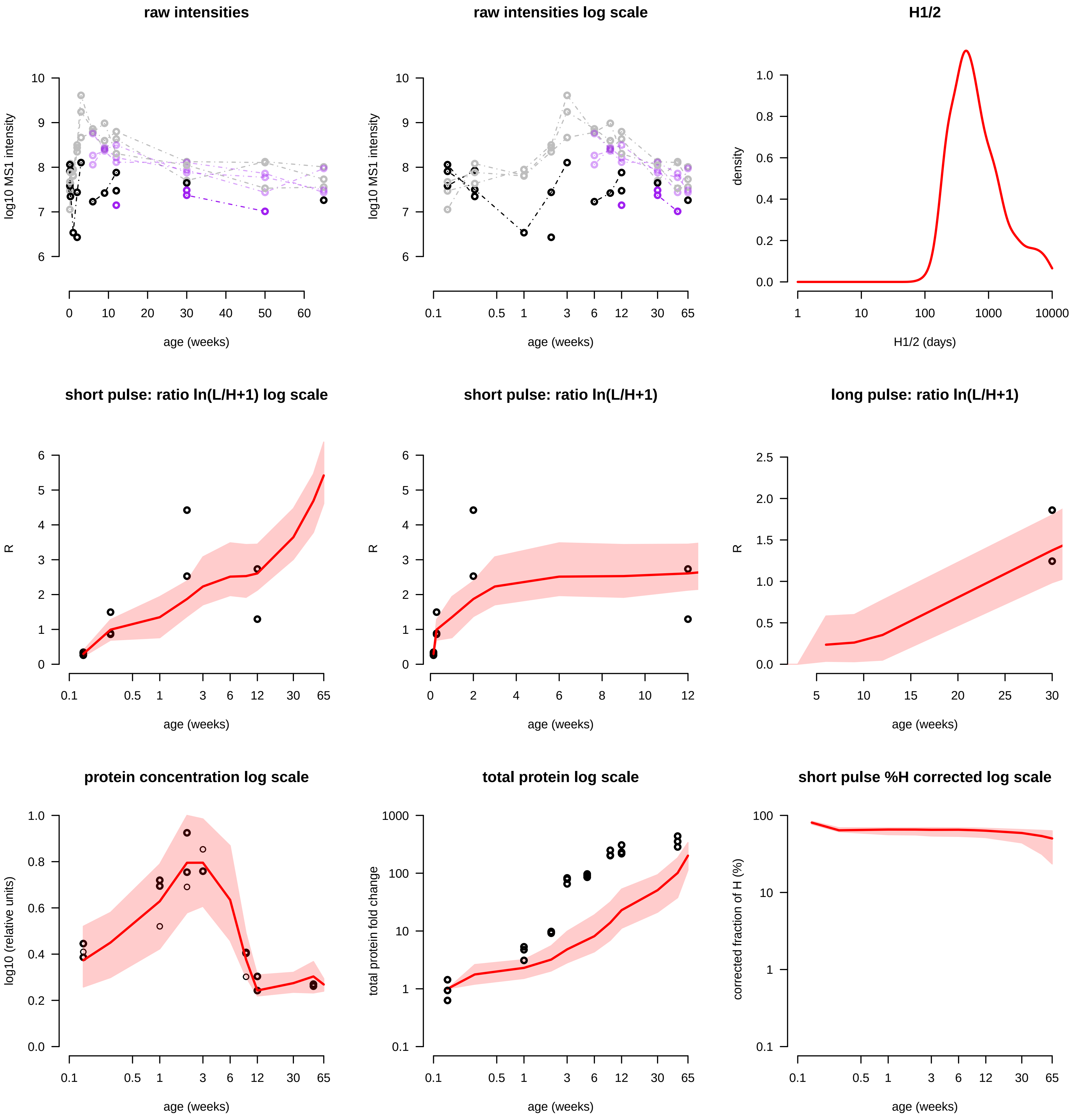

P12710

>sp|P12710|FABPL_MOUSE

Fatty acid−binding protein, liver

GN=Fabp1

Med H1/2: 95.9 days

Q5 H1/2: 37.6 days

Q95 H1/2: 487.6 days

reliable model fit: no

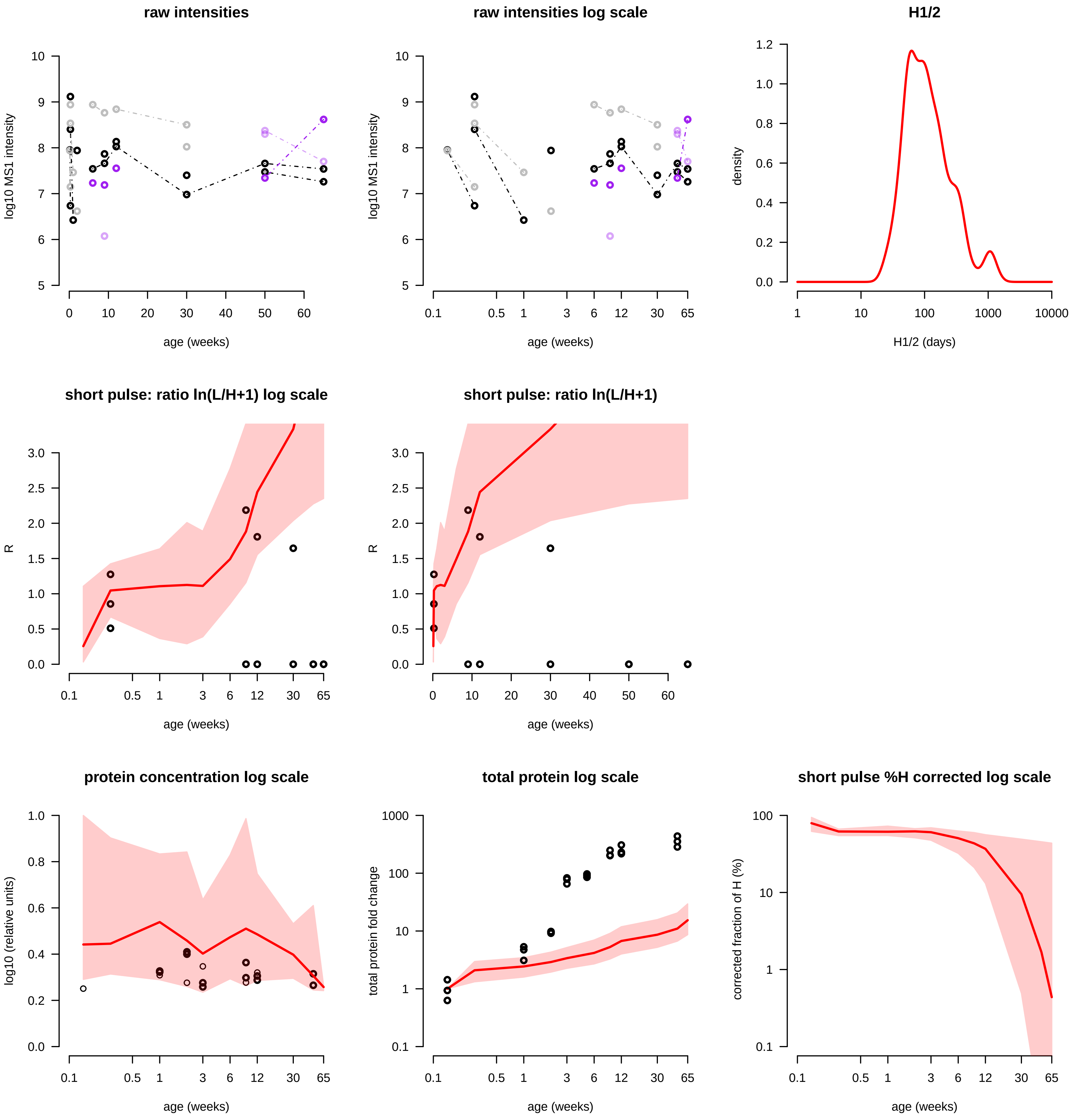

Q8K273

>sp|Q8K273|EMC5_MOUSE

ER membrane protein complex subunit 5

GN=Mmgt1

Med H1/2: 6.5 days

Q5 H1/2: 2.7 days

Q95 H1/2: 33.8 days

reliable model fit: yes

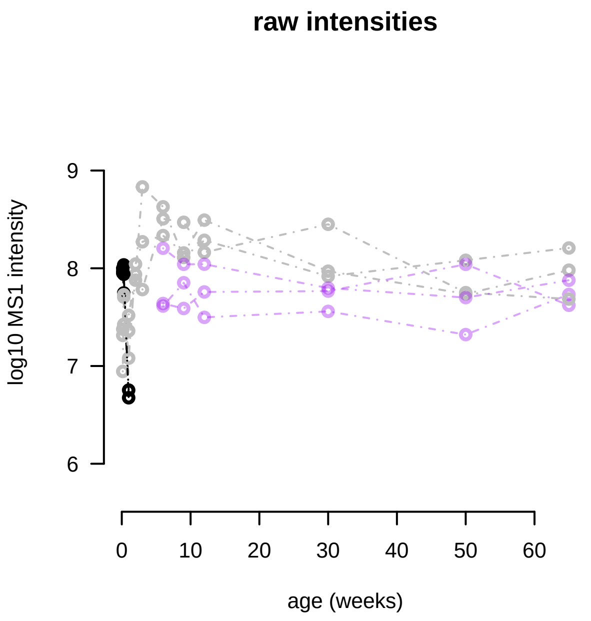

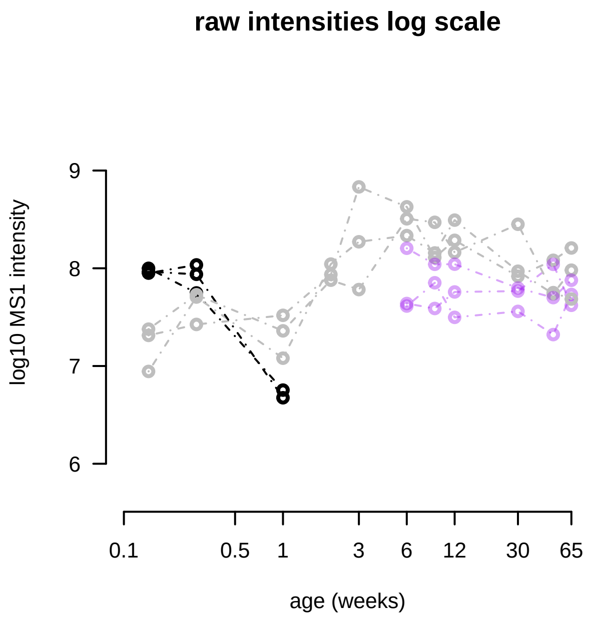

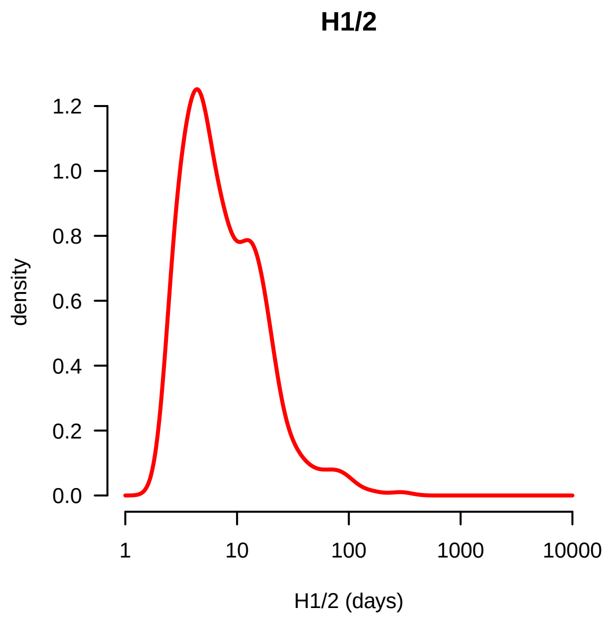

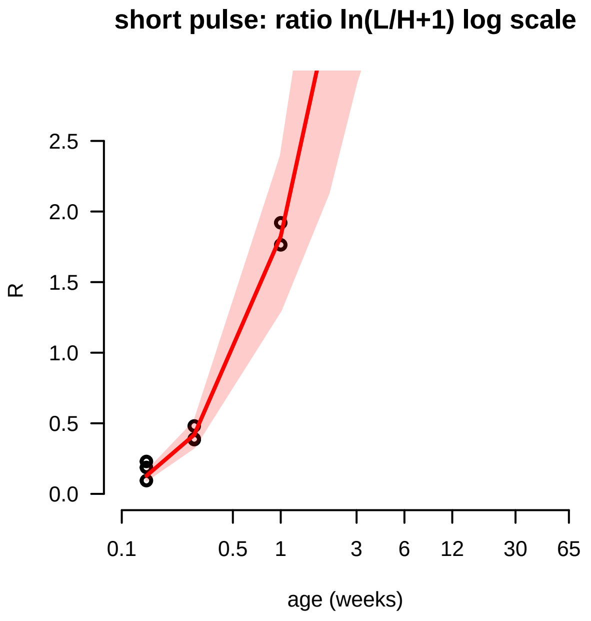

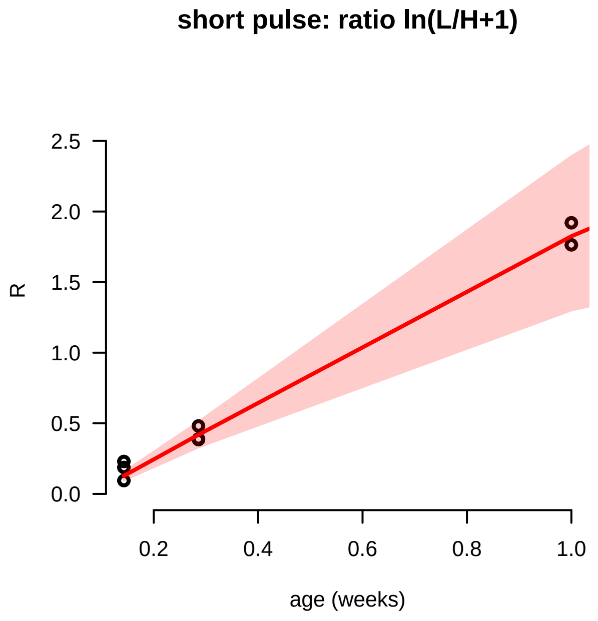

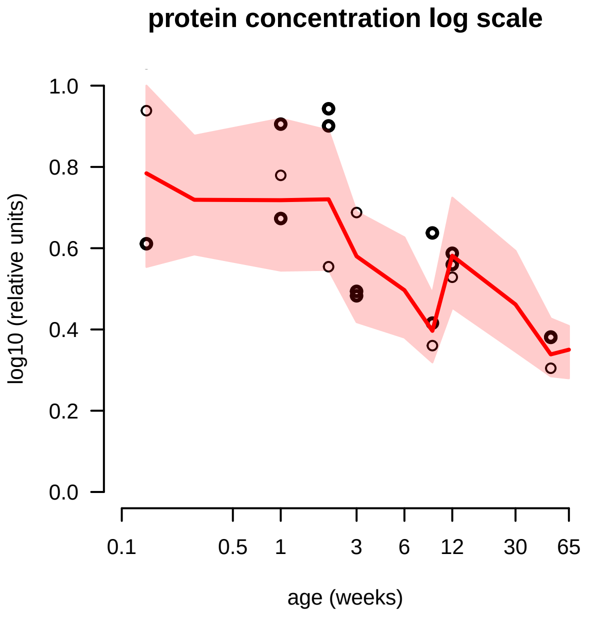

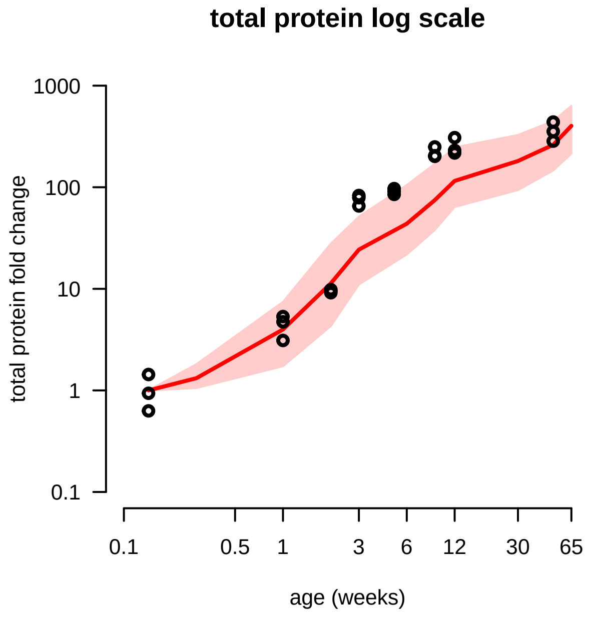

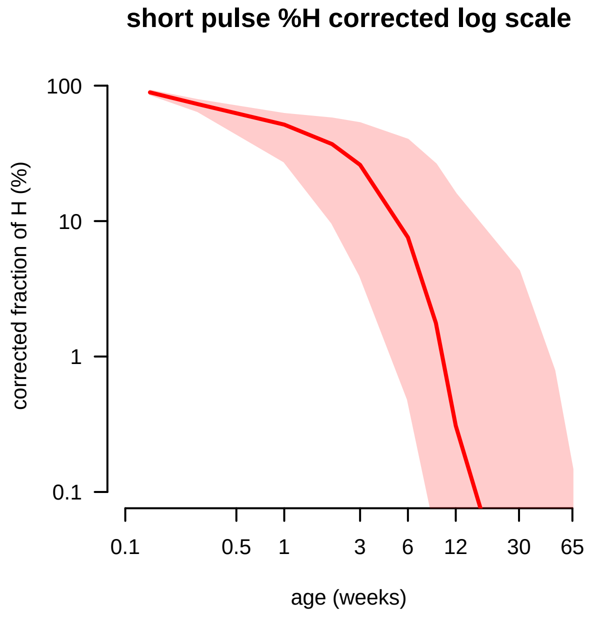

Q8BX57;Q8BX57−2;Q8BX57−3

>sp|Q8BX57|PXK_MOUSE

PX domain−containing protein kinase−like protein

GN=Pxk

Med H1/2: 2021.4 days

Q5 H1/2: 430.2 days

Q95 H1/2: 22533.2 days

reliable model fit: no

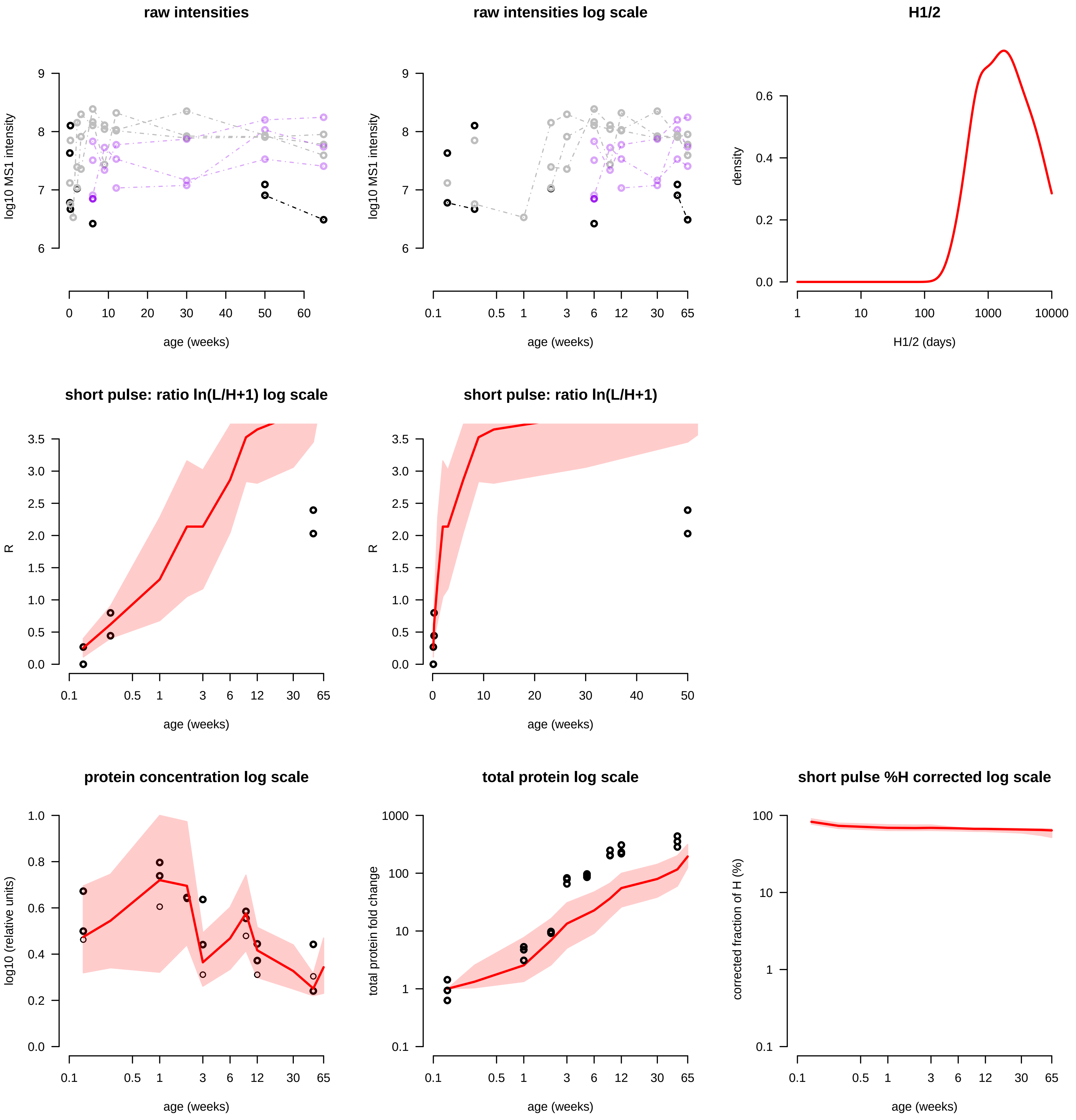

Q91YP3

>sp|Q91YP3|DEOC_MOUSE

Deoxyribose−phosphate aldolase

GN=Dera

Med H1/2: 17.8 days

Q5 H1/2: 5.7 days

Q95 H1/2: 102.5 days

reliable model fit: yes

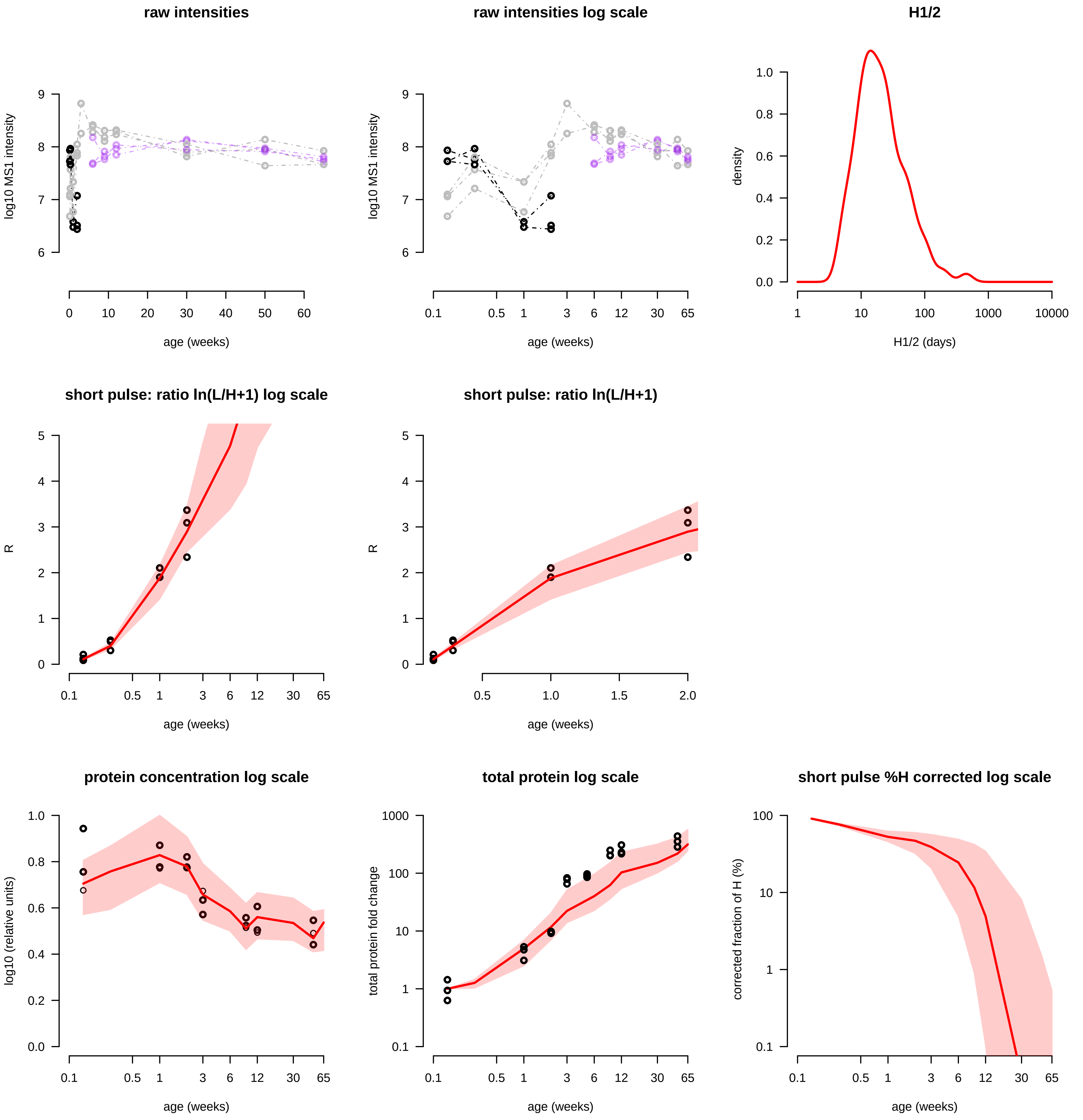

Q8VDS8−3;Q8VDS8;Q8VDS8−2

>sp|Q8VDS8−3|STX18_MOUSE

Isoform 3 of Syntaxin−18

GN=Stx18

Med H1/2: 6.2 days

Q5 H1/2: 3.4 days

Q95 H1/2: 34.3 days

reliable model fit: yes

raw intensities

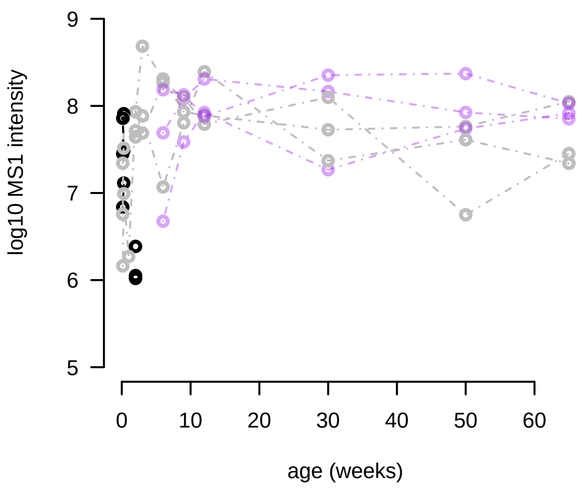

raw intensities log scale

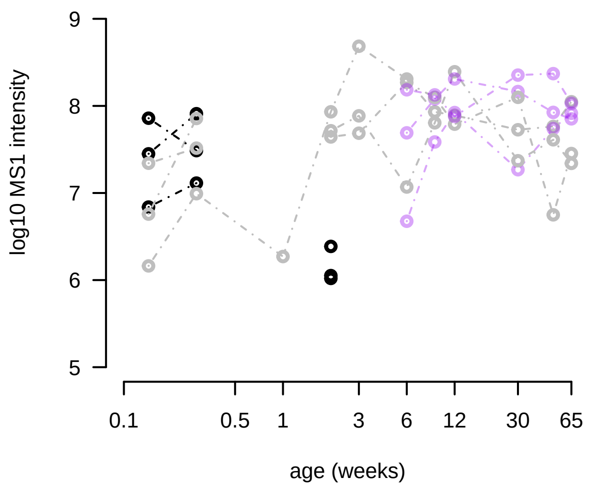

H1/2

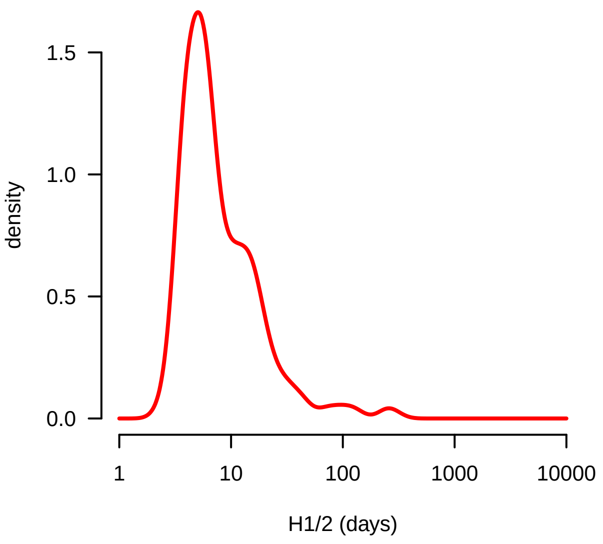

short pulse: ratio ln(L/H+1) log scale

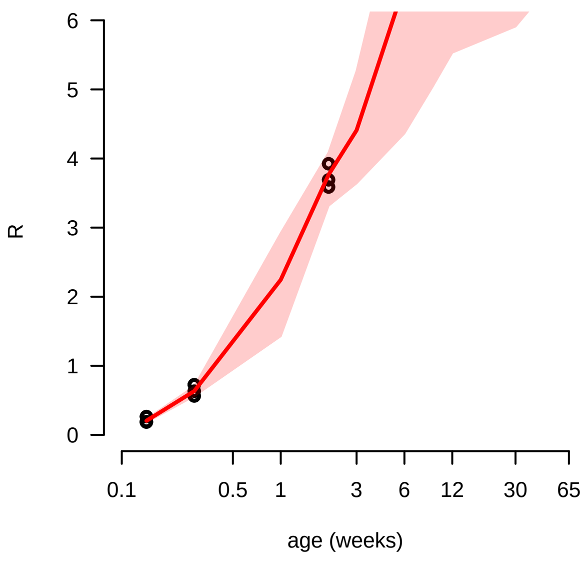

short pulse: ratio ln(L/H+1)

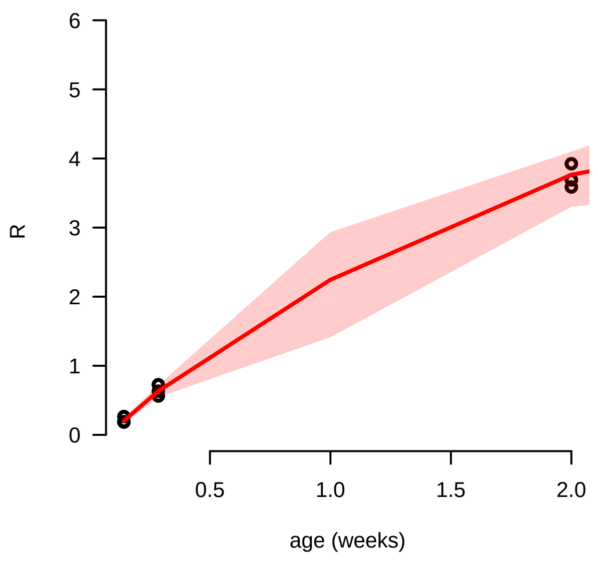

protein concentration log scale

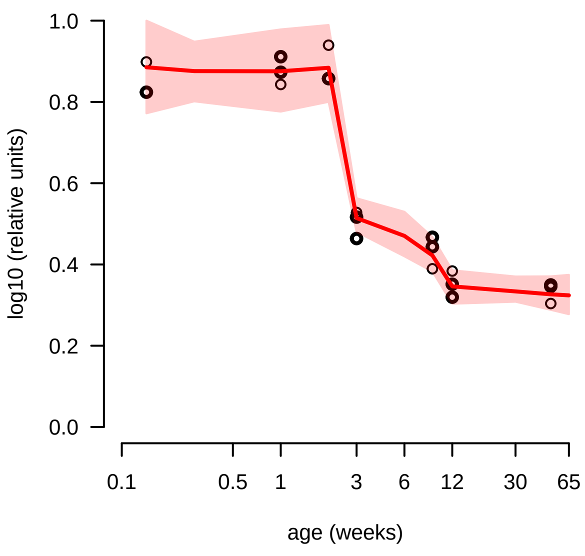

total protein log scale

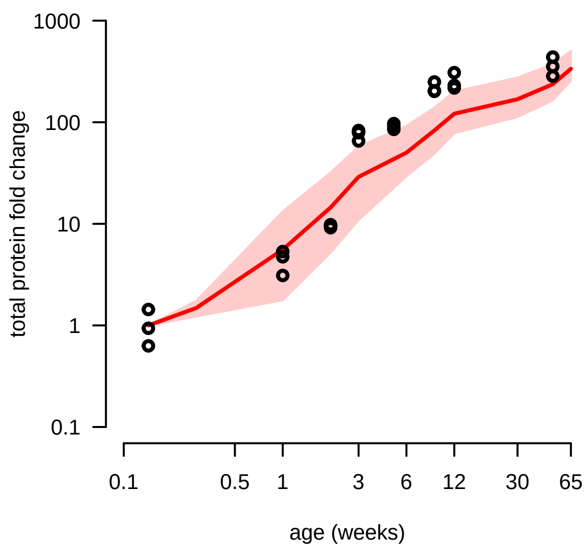

short pulse %H corrected log scale

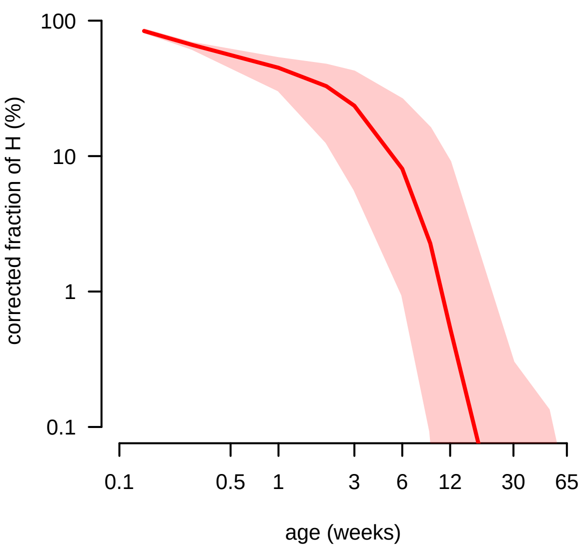

P07310

>sp|P07310|KCRM_MOUSE

Creatine kinase M−type

GN=Ckm

Med H1/2: 6.1 days

Q5 H1/2: 2.8 days

Q95 H1/2: 28.1 days

reliable model fit: no

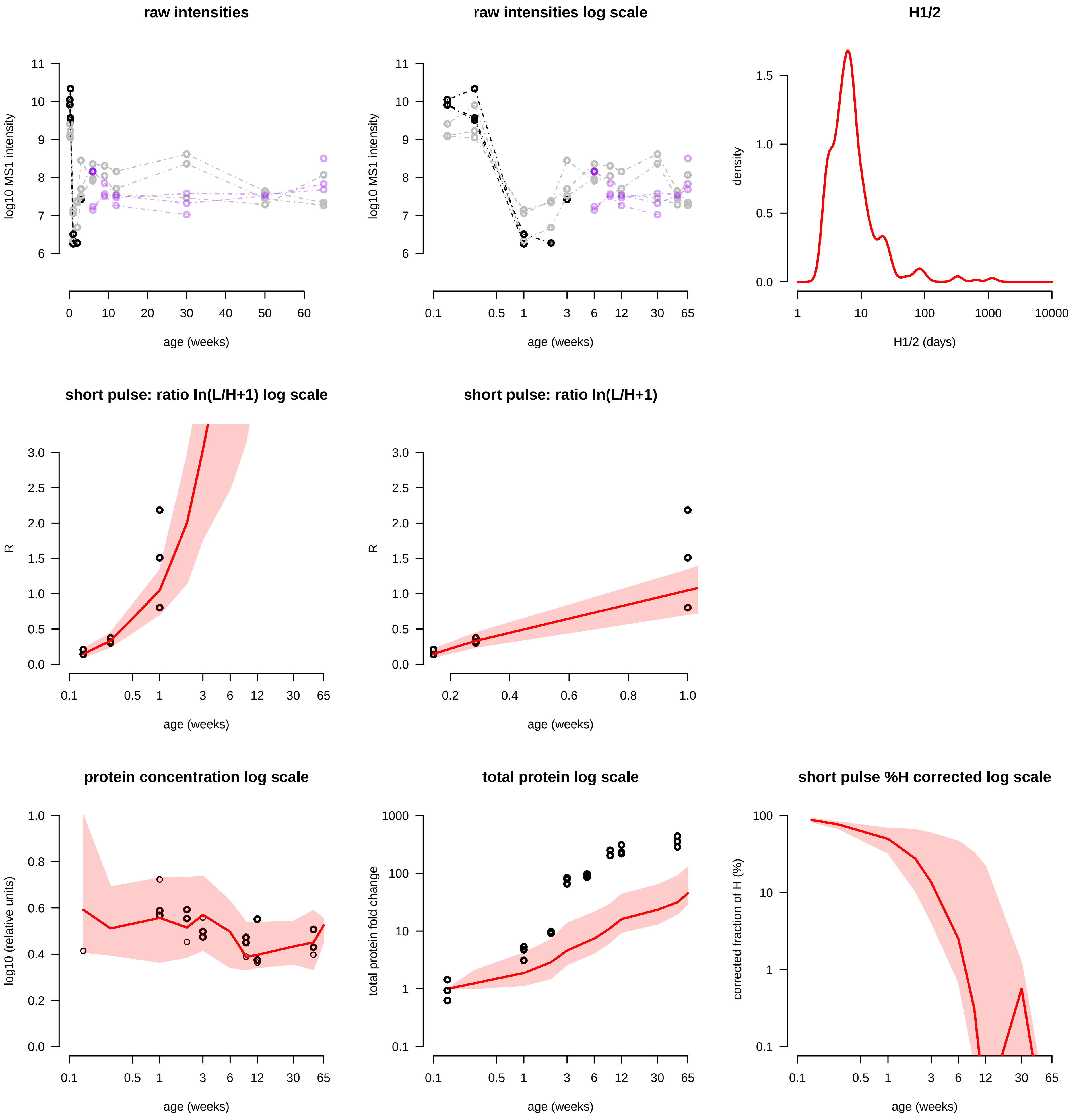

Q8BTW3

>sp|Q8BTW3|EXOS6_MOUSE

Exosome complex component MTR3

GN=Exosc6

Med H1/2: 35 days

Q5 H1/2: 11.2 days

Q95 H1/2: 243.6 days

reliable model fit: yes

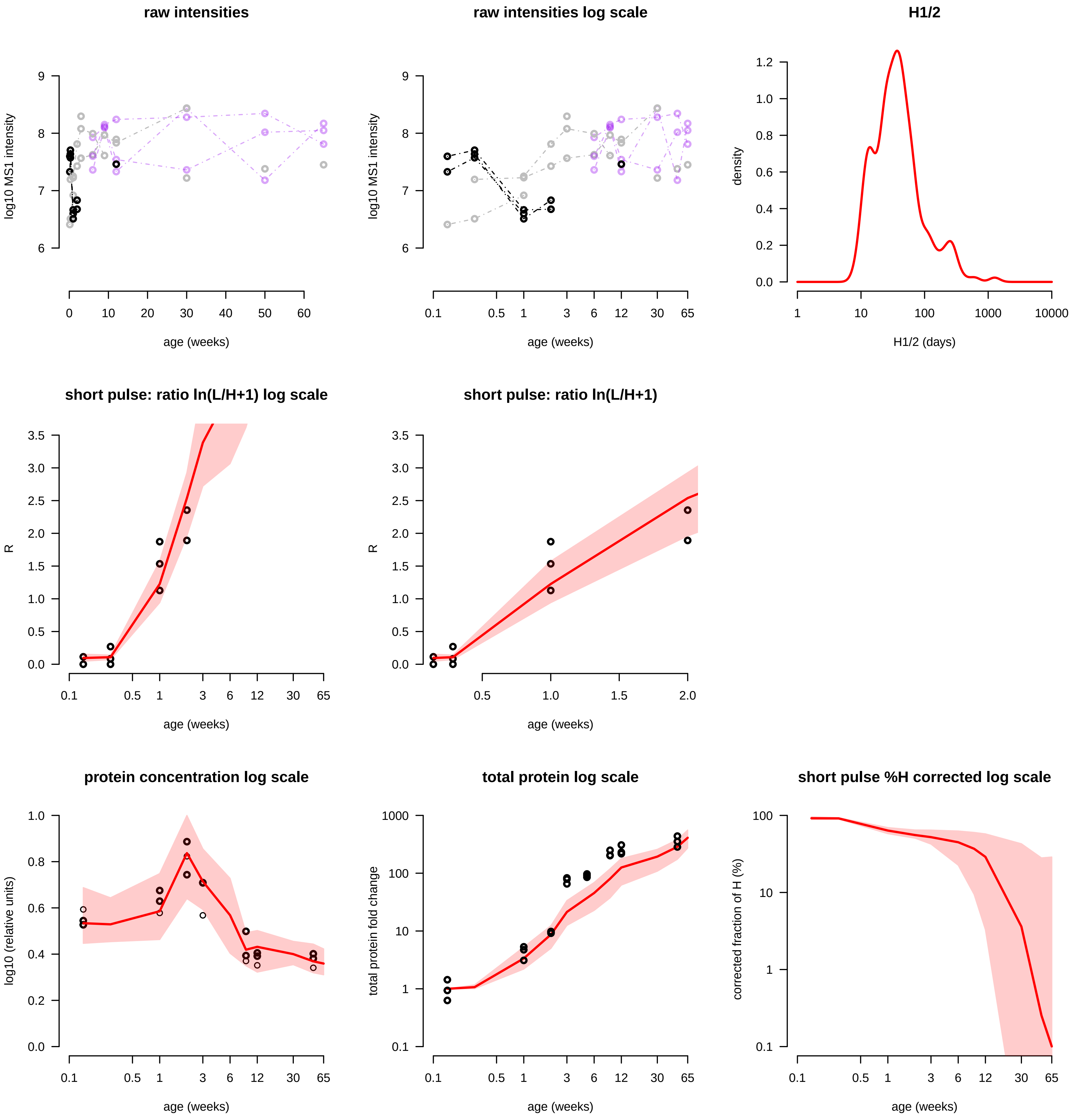

P45481

>sp|P45481|CBP_MOUSE

Histone lysine acetyltransferase CREBBP

GN=Crebbp

Med H1/2: 2022.4 days

Q5 H1/2: 532.1 days

Q95 H1/2: 27838.9 days

reliable model fit: yes

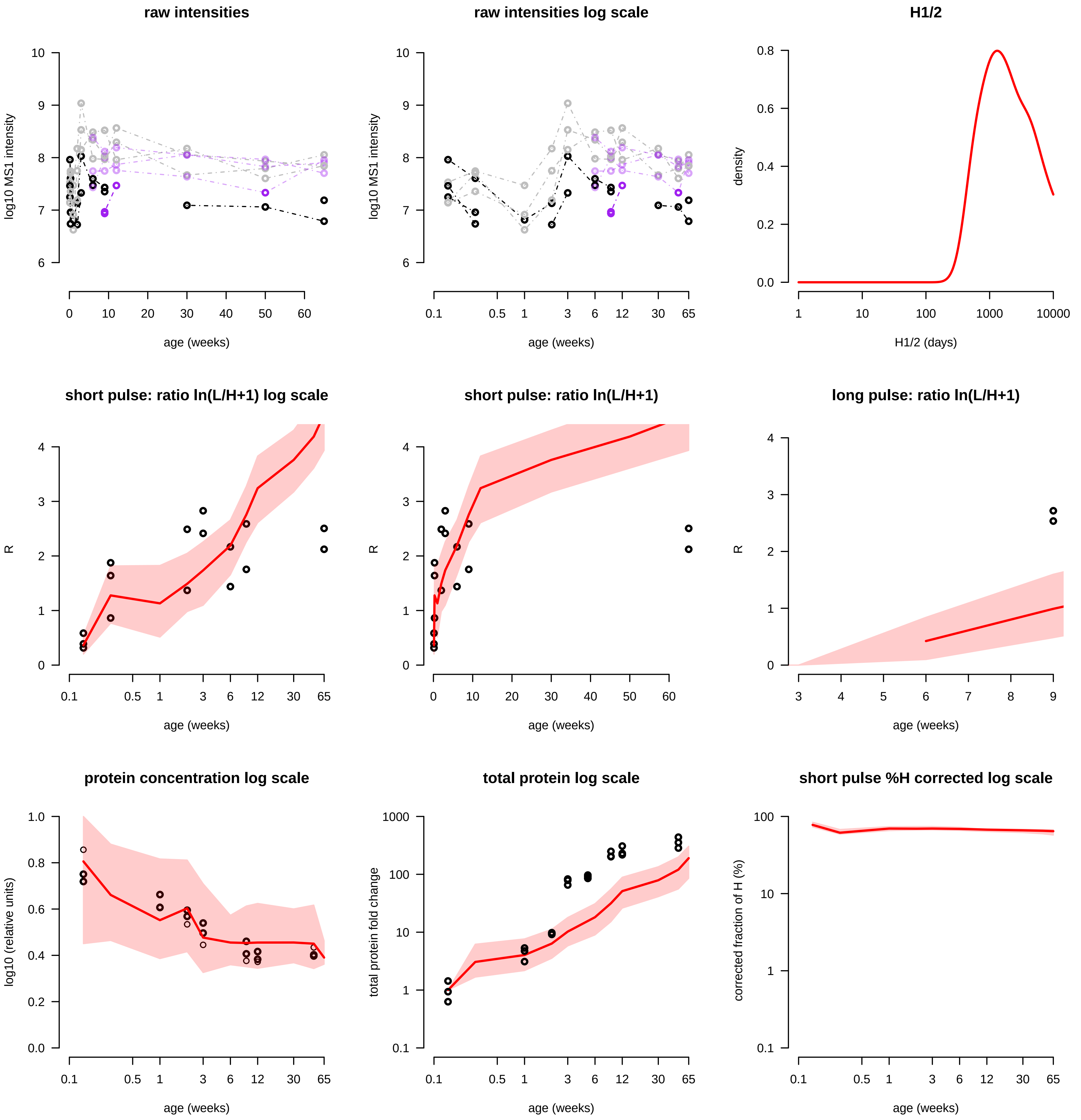

Q00899

>sp|Q00899|TYY1_MOUSE

Transcriptional repressor protein YY1

GN=Yy1

Med H1/2: 18.2 days

Q5 H1/2: 7.8 days

Q95 H1/2: 197.7 days

reliable model fit: yes

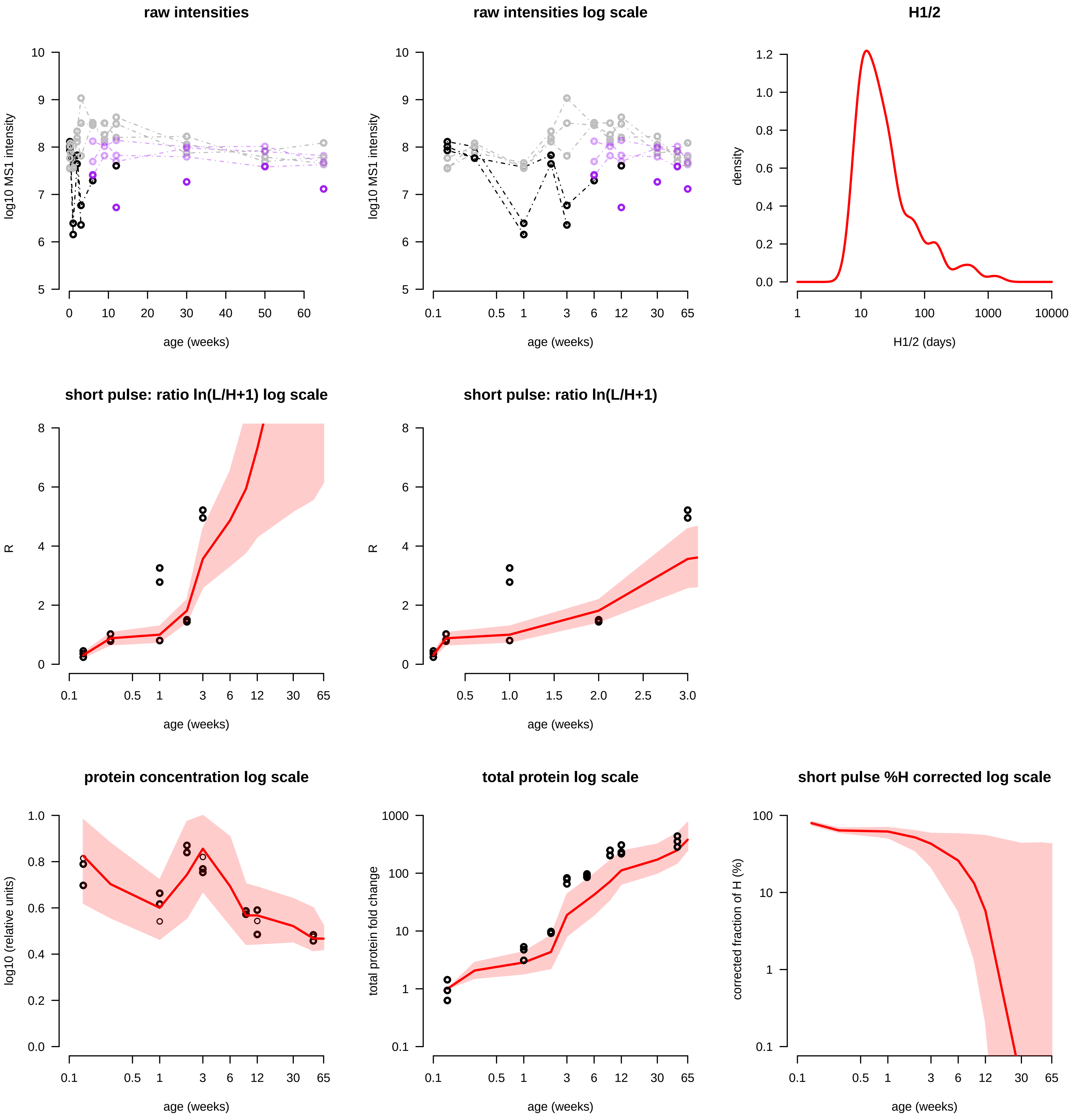

Q9DCD2

>sp|Q9DCD2|SYF1_MOUSE

Pre−mRNA−splicing factor SYF1

GN=Xab2

Med H1/2: 5.5 days

Q5 H1/2: 2.7 days

Q95 H1/2: 55.3 days

reliable model fit: yes

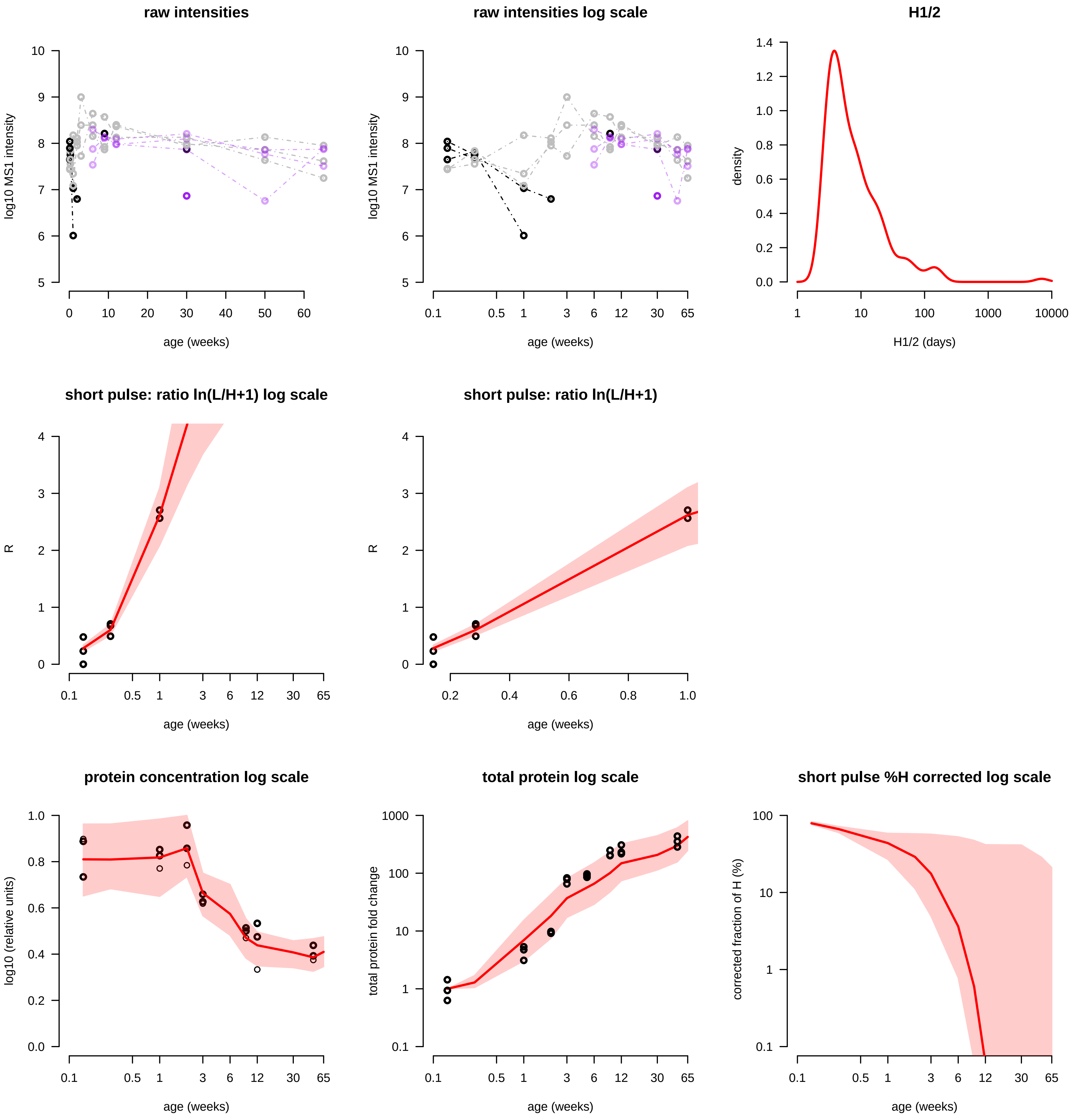

Q9CXL3

>sp|Q9CXL3|CG050_MOUSE

Uncharacterized protein C7orf50 homolog

Med H1/2: 32 days

Q5 H1/2: 21.9 days

Q95 H1/2: 58.3 days

reliable model fit: yes

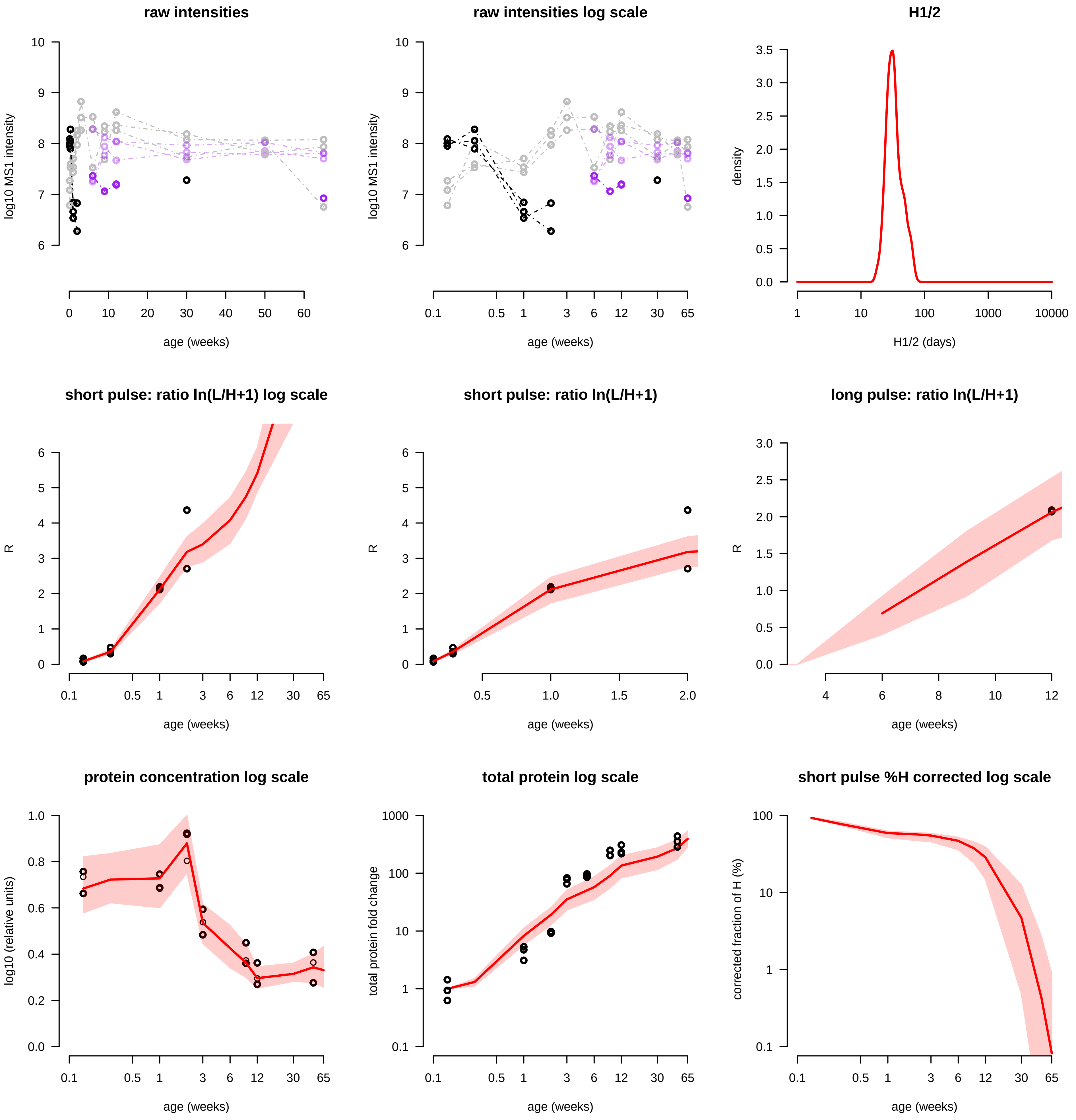

Q99MV7;Q99MV7−2;Q99MV7−3

>sp|Q99MV7|RNF17_MOUSE

RING finger protein 17

GN=Rnf17

Med H1/2: 11.5 days

Q5 H1/2: 8.5 days

Q95 H1/2: 20 days

reliable model fit: yes

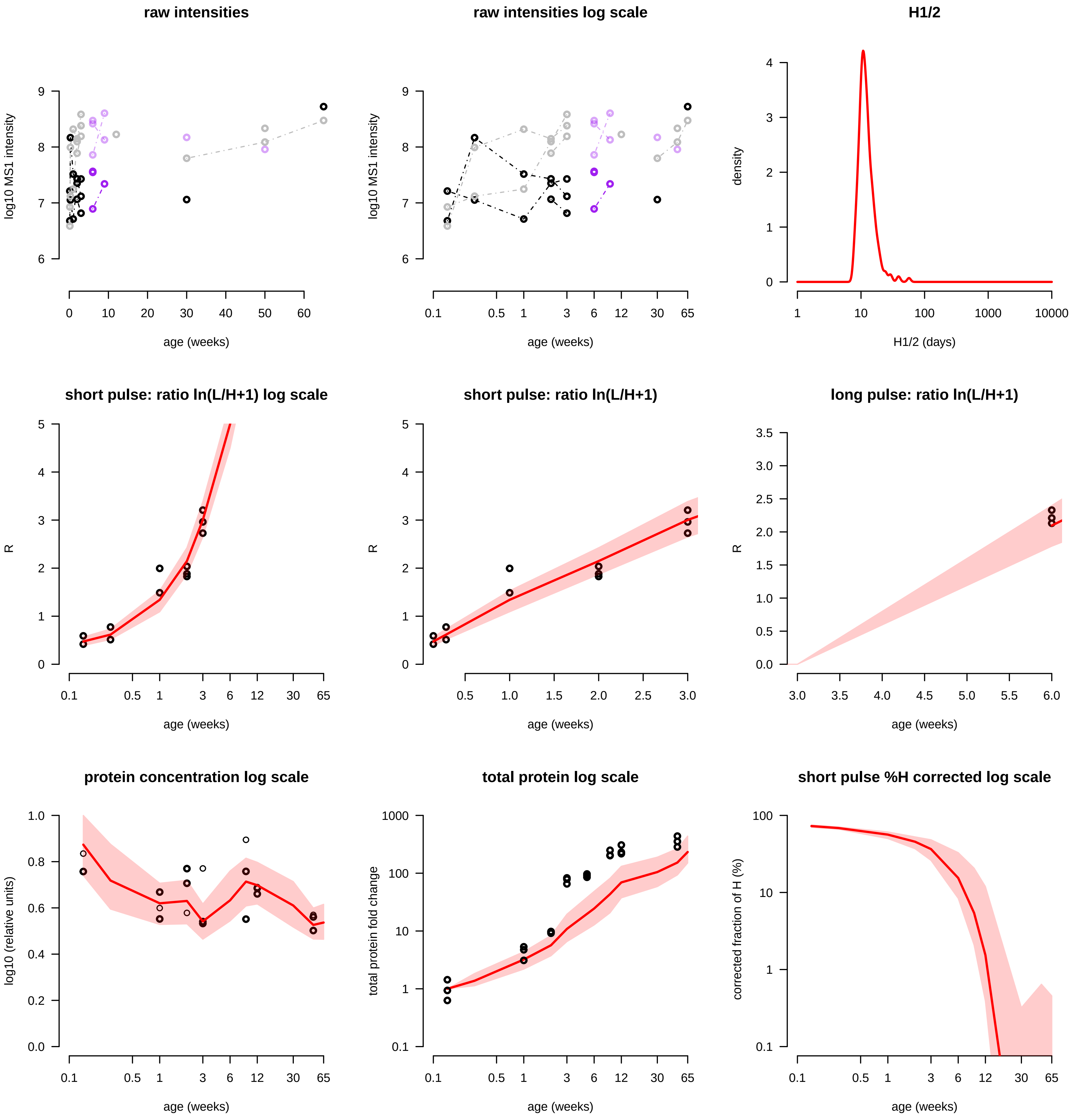

Q9CYD3

>sp|Q9CYD3|CRTAP_MOUSE

Cartilage−associated protein

GN=Crtap

Med H1/2: 5.6 days

Q5 H1/2: 2.8 days

Q95 H1/2: 32 days

reliable model fit: yes

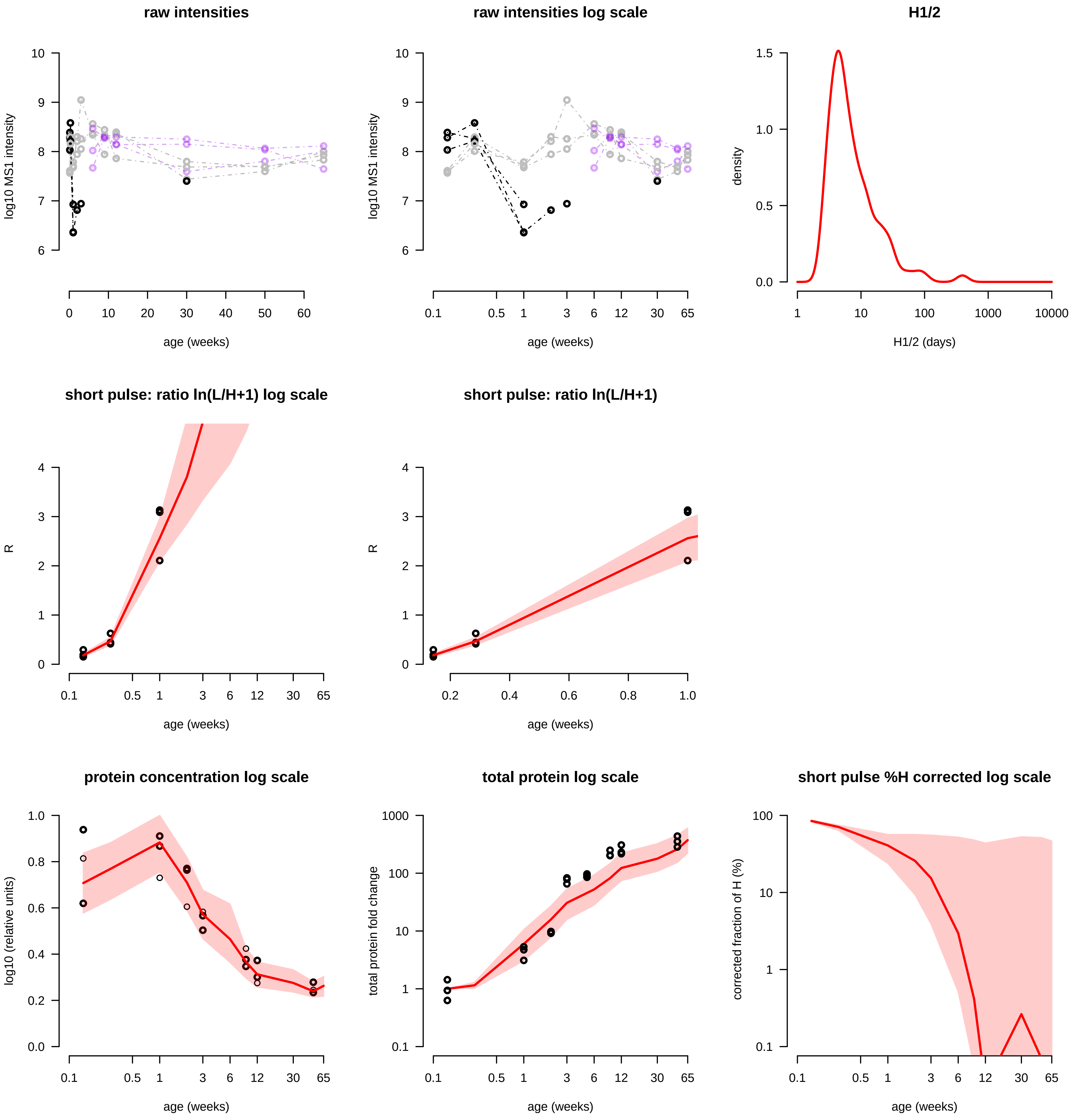

Q80V86

>sp|Q80V86|INT8_MOUSE

Integrator complex subunit 8

GN=Ints8

Med H1/2: 408.8 days

Q5 H1/2: 111.3 days

Q95 H1/2: 4601.7 days

reliable model fit: yes

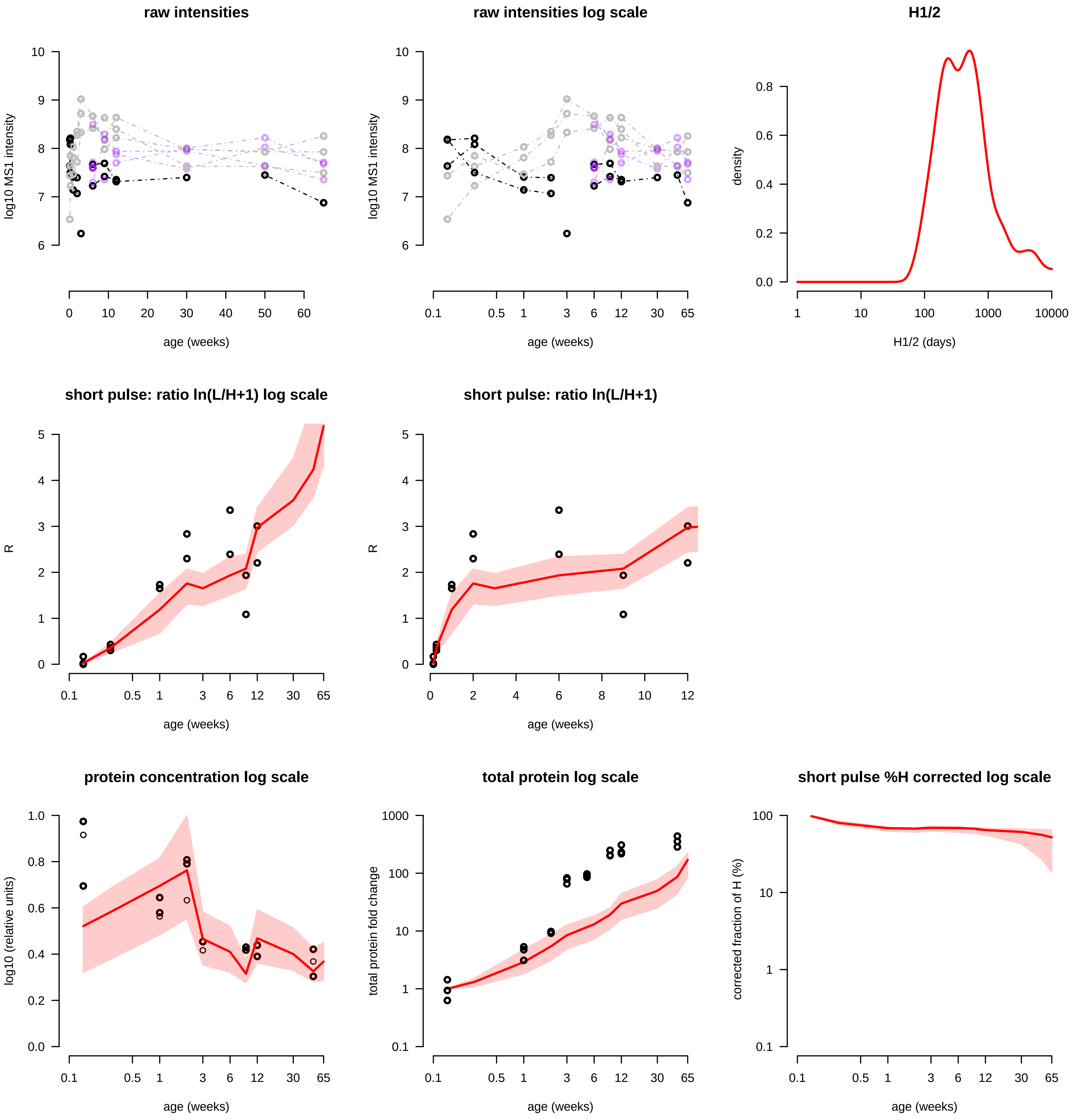

Q6IR34;Q6IR34−3;Q6IR34−5;Q6IR34−2

>sp|Q6IR34|GPSM1_MOUSE

G−protein−signaling modulator 1

GN=Gpsm1

Med H1/2: 14.4 days

Q5 H1/2: 4.6 days

Q95 H1/2: 109.9 days

reliable model fit: yes

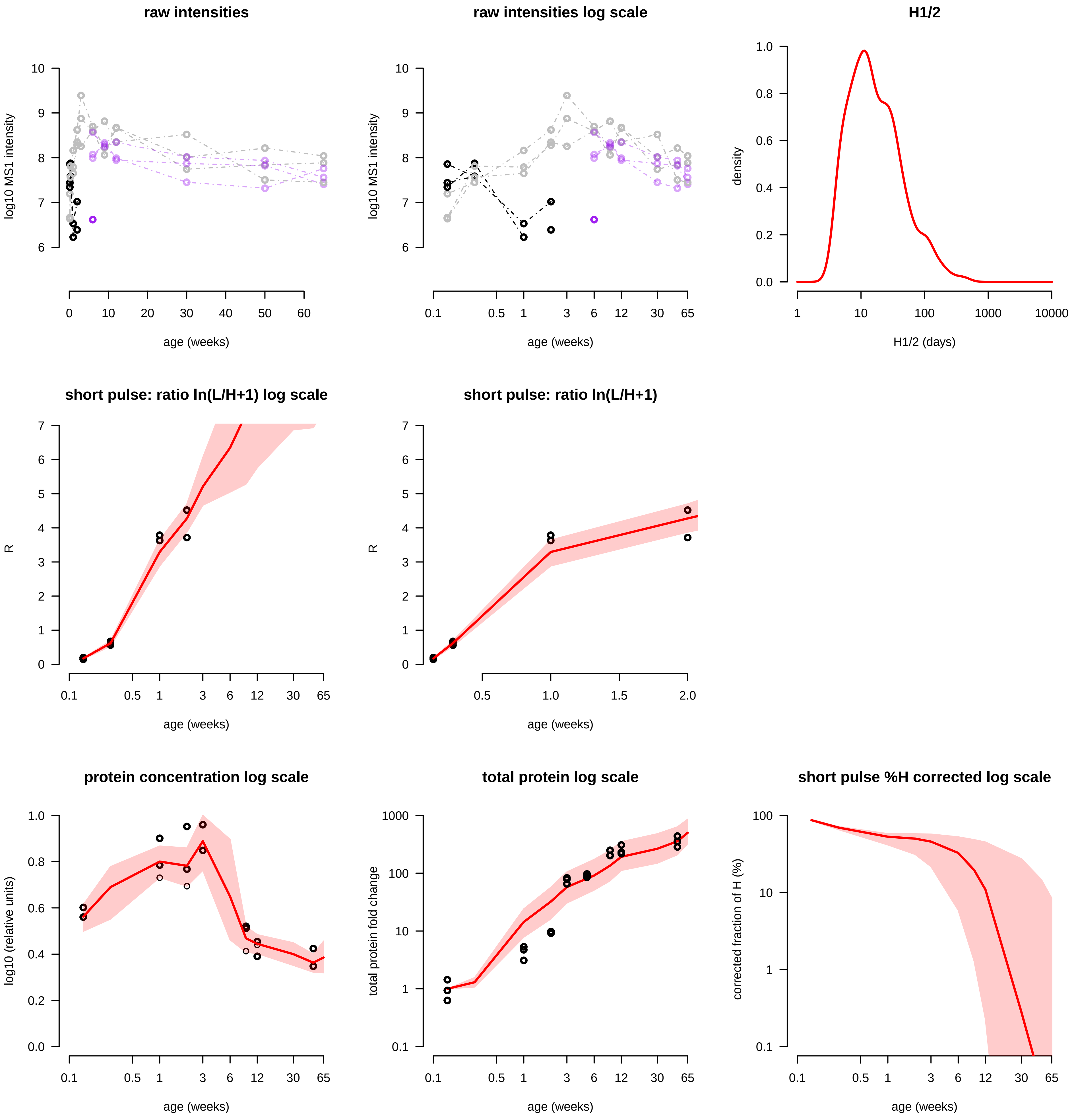

Q8R323

>sp|Q8R323|RFC3_MOUSE

Replication factor C subunit 3

GN=Rfc3

Med H1/2: 3.1 days

Q5 H1/2: 2.6 days

Q95 H1/2: 3.9 days

reliable model fit: yes

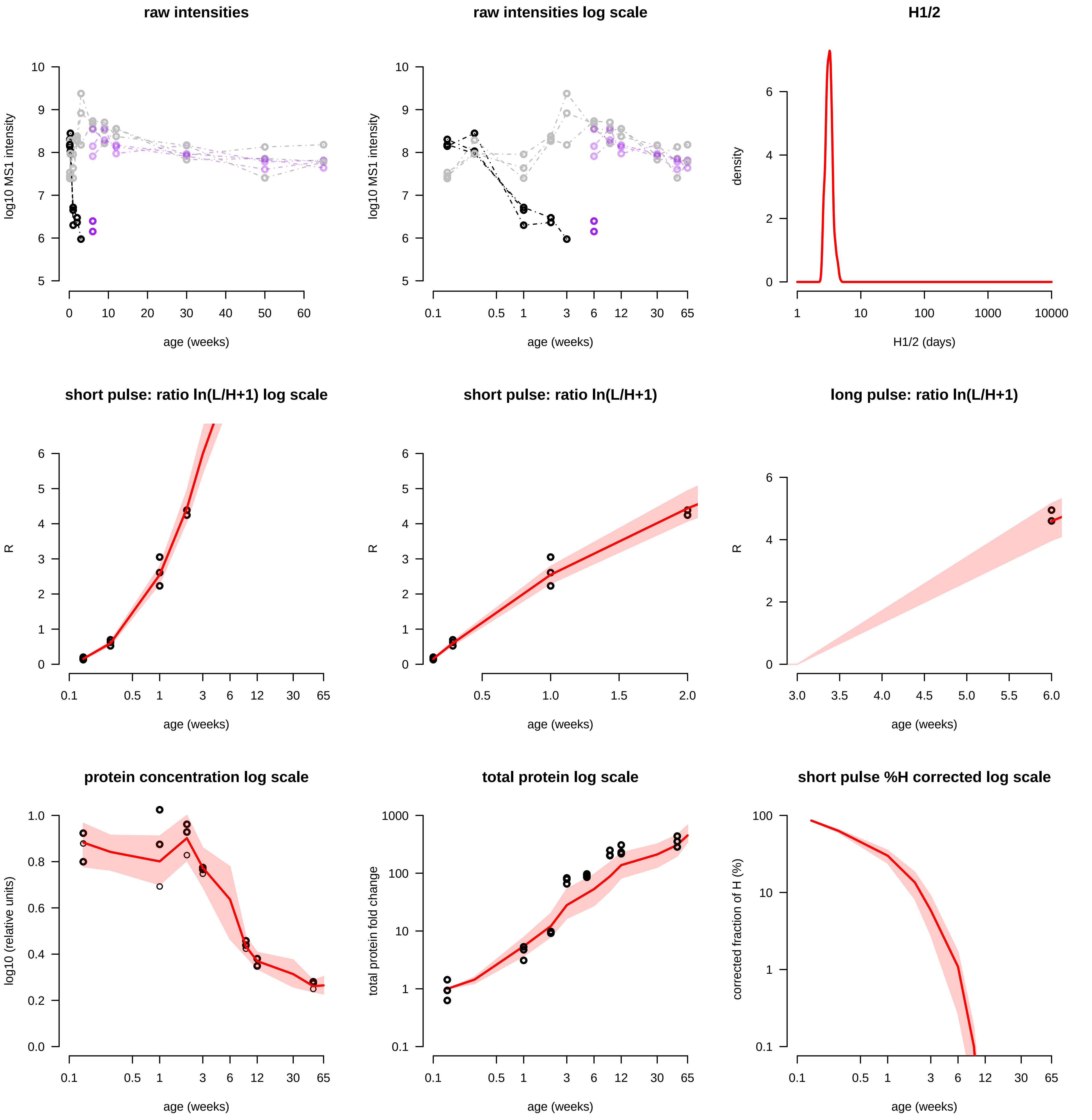

Q8C5P5−2;Q8C5P5;Q8C5P5−3

>sp|Q8C5P5−2|NT5D1_MOUSE

Isoform 2 of 5'−nucleotidase domain−containing protein 1

GN=Nt5dc1

Med H1/2: 10 days

Q5 H1/2: 3.8 days

Q95 H1/2: 94.9 days

reliable model fit: yes

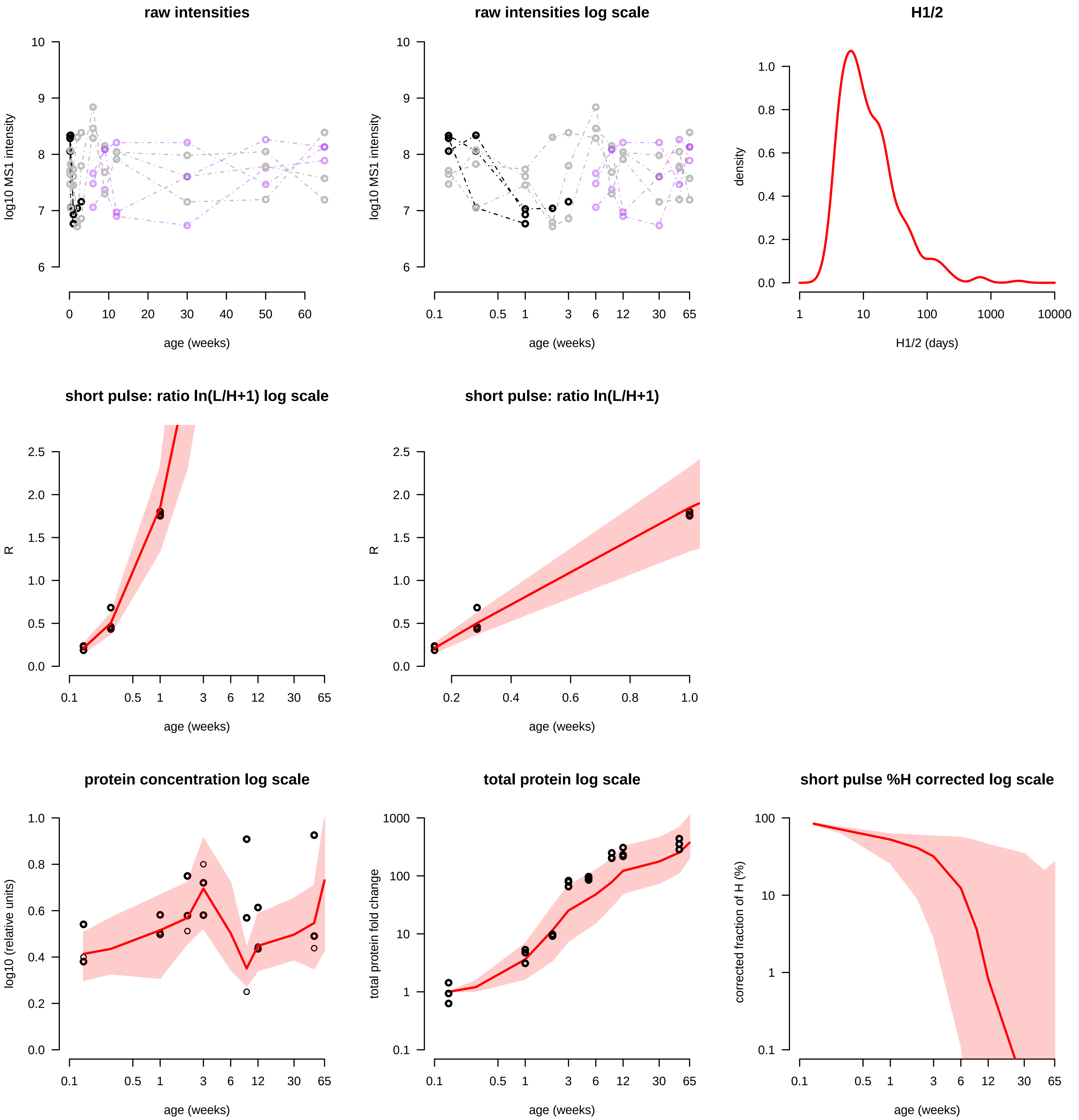

Q8JZV7

>sp|Q8JZV7|NAGA_MOUSE

N−acetylglucosamine−6−phosphate deacetylase

GN=Amdhd2

Med H1/2: 8.8 days

Q5 H1/2: 3.8 days

Q95 H1/2: 139.9 days

reliable model fit: yes

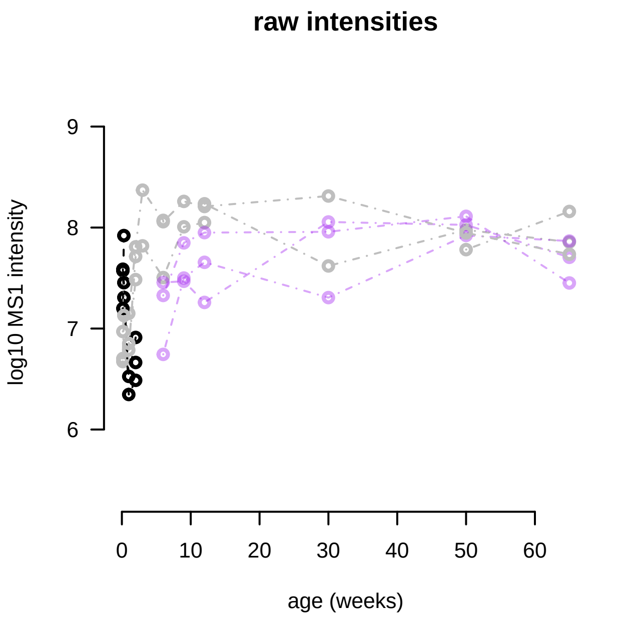

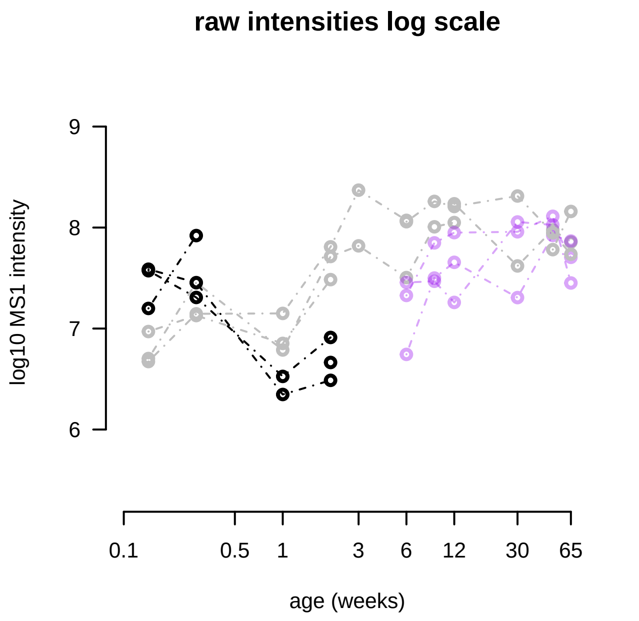

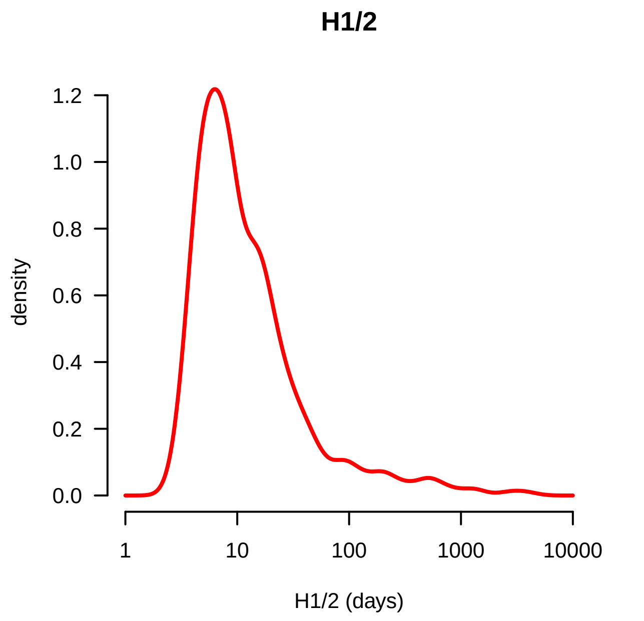

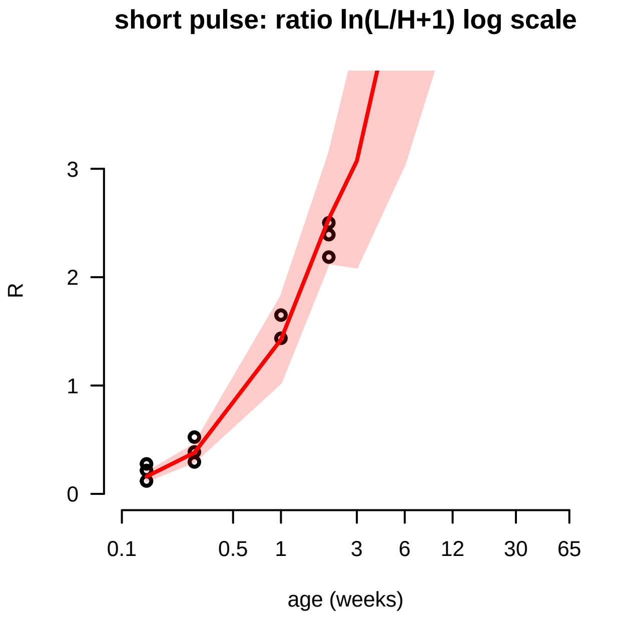

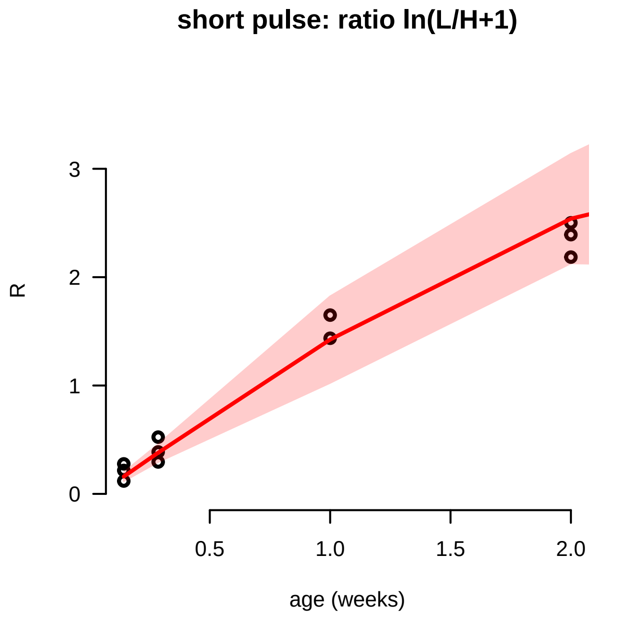

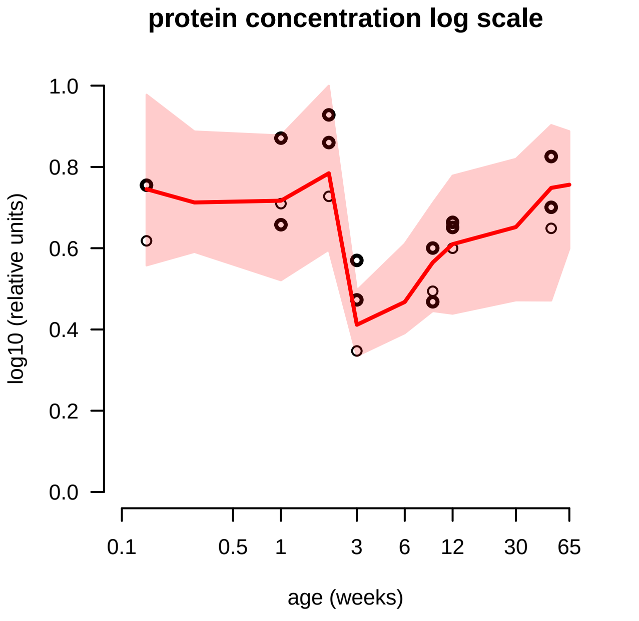

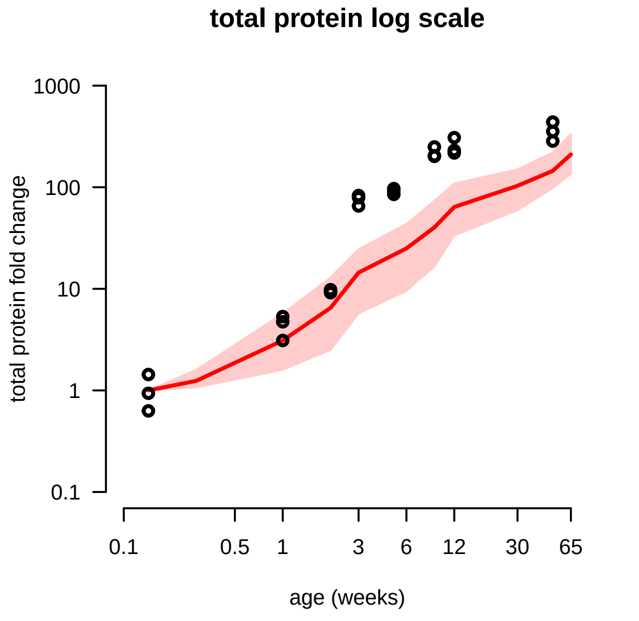

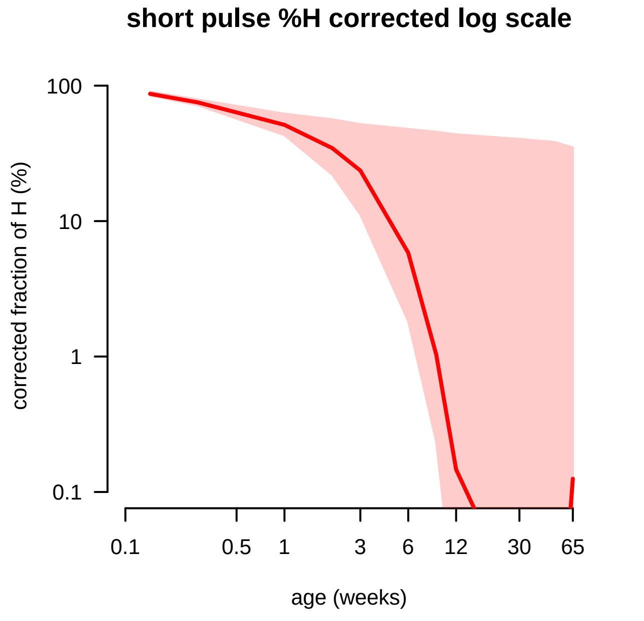

Q9R1Z7

>sp|Q9R1Z7|PTPS_MOUSE

6−pyruvoyl tetrahydrobiopterin synthase

GN=Pts

Med H1/2: 8.9 days

Q5 H1/2: 3 days

Q95 H1/2: 50.8 days

reliable model fit: yes

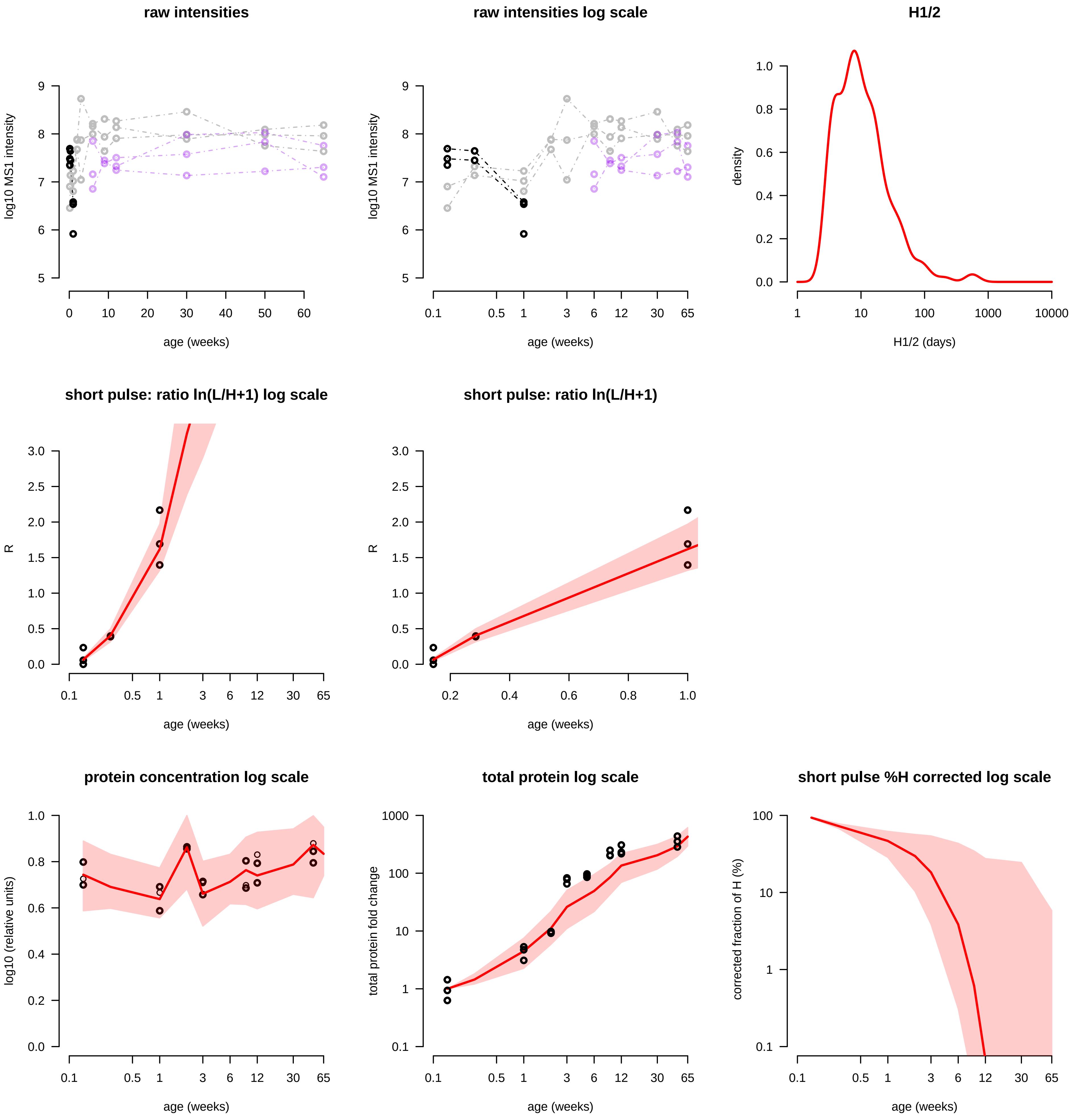

Q8VC70

>sp|Q8VC70|RBMS2_MOUSE

RNA−binding motif, single−stranded−interacting protein 2

GN=Rbms2

Med H1/2: 4.8 days

Q5 H1/2: 2.8 days

Q95 H1/2: 17.2 days

reliable model fit: yes

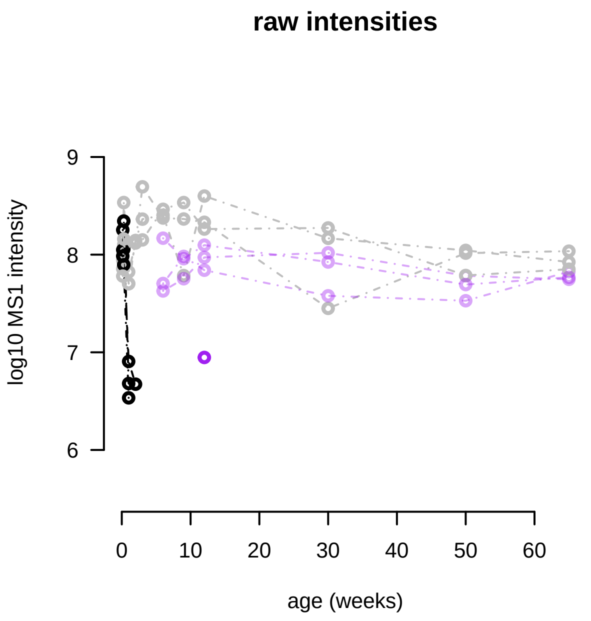

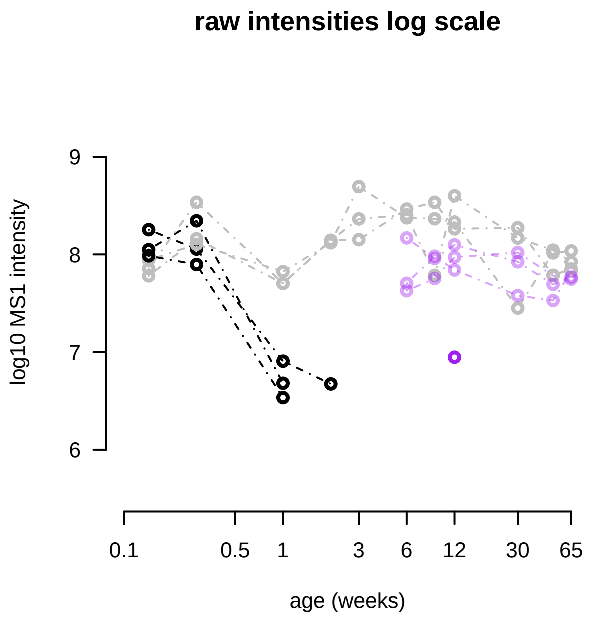

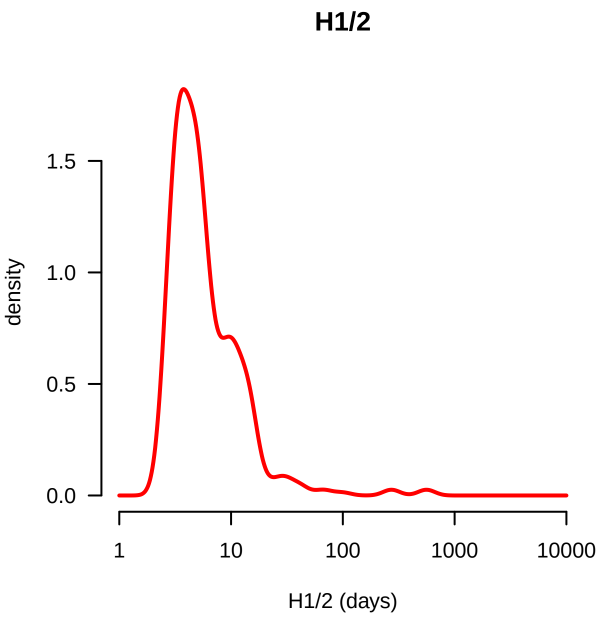

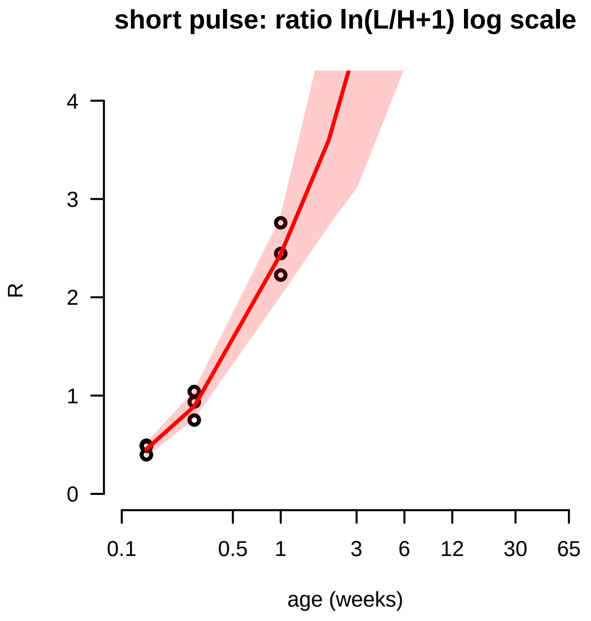

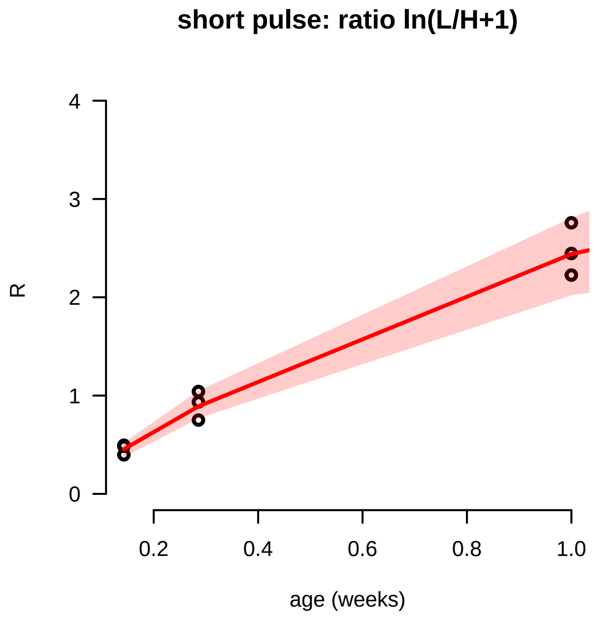

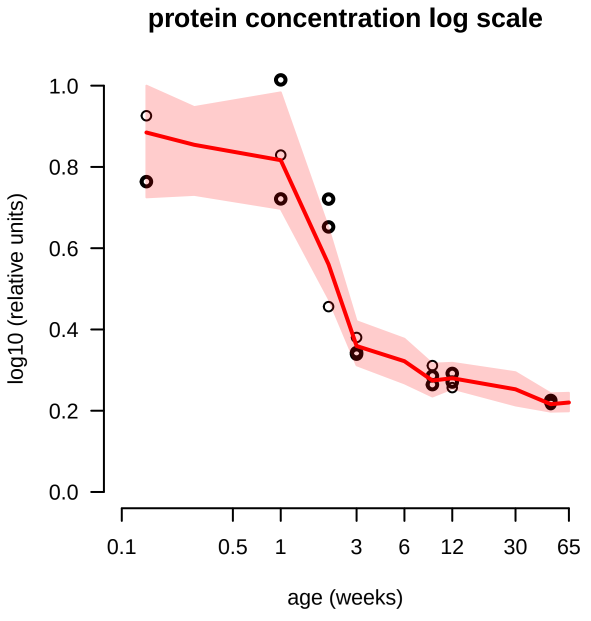

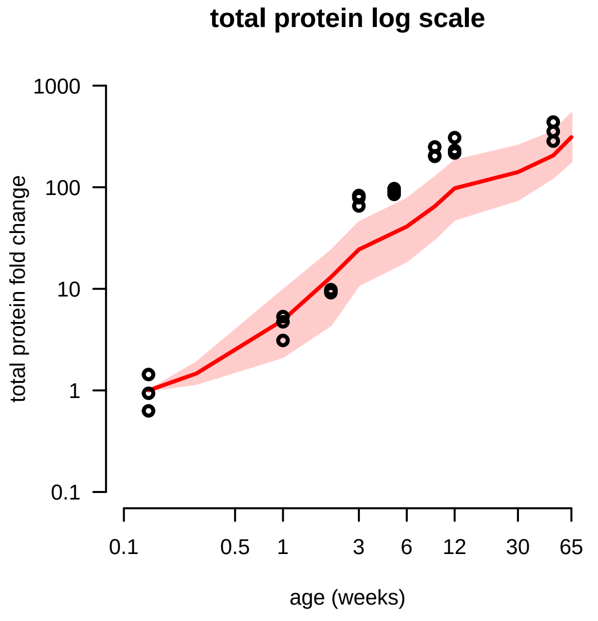

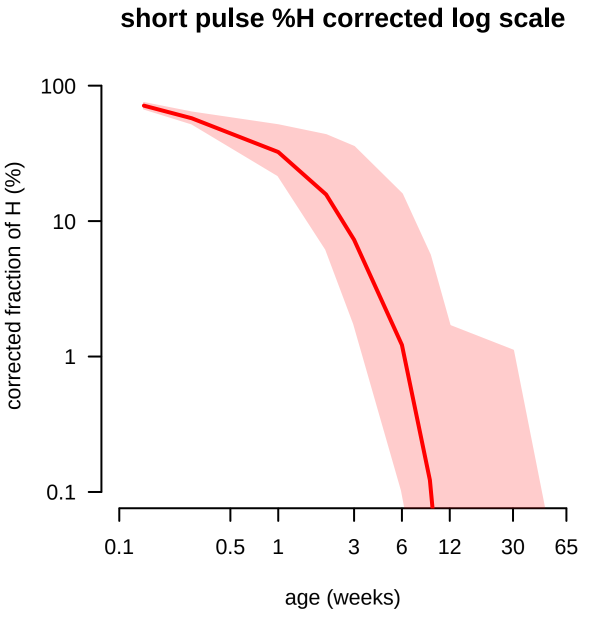

Q8CAA7

>sp|Q8CAA7|PGM2L_MOUSE

Glucose 1,6−bisphosphate synthase

GN=Pgm2l1

Med H1/2: 8.4 days

Q5 H1/2: 4.4 days

Q95 H1/2: 55.6 days

reliable model fit: yes

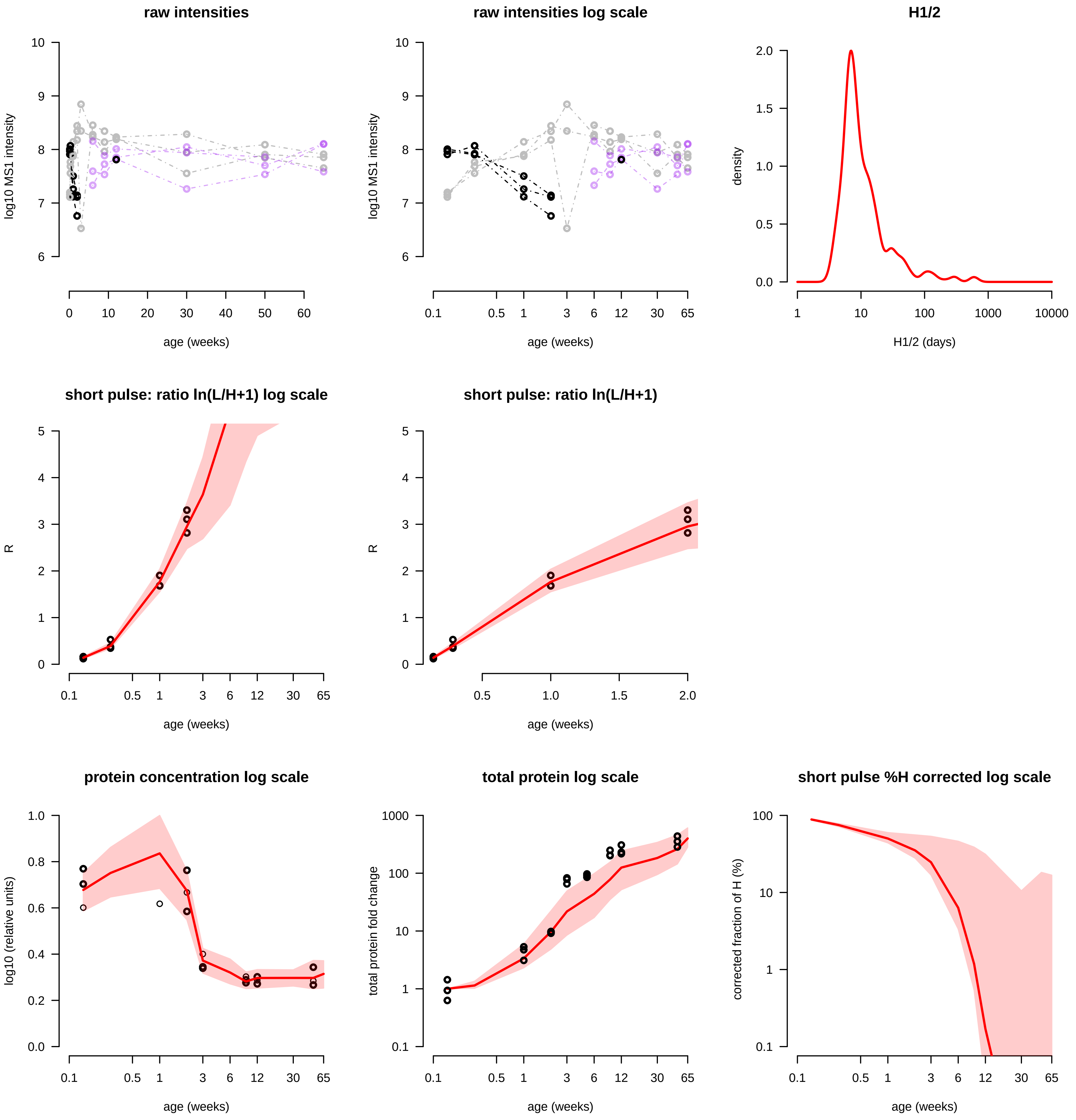

Q91Z49;Q91Z49−2

>sp|Q91Z49|UIF_MOUSE

UAP56−interacting factor

GN=Fyttd1

Med H1/2: 6.5 days

Q5 H1/2: 2.9 days

Q95 H1/2: 28 days

reliable model fit: yes

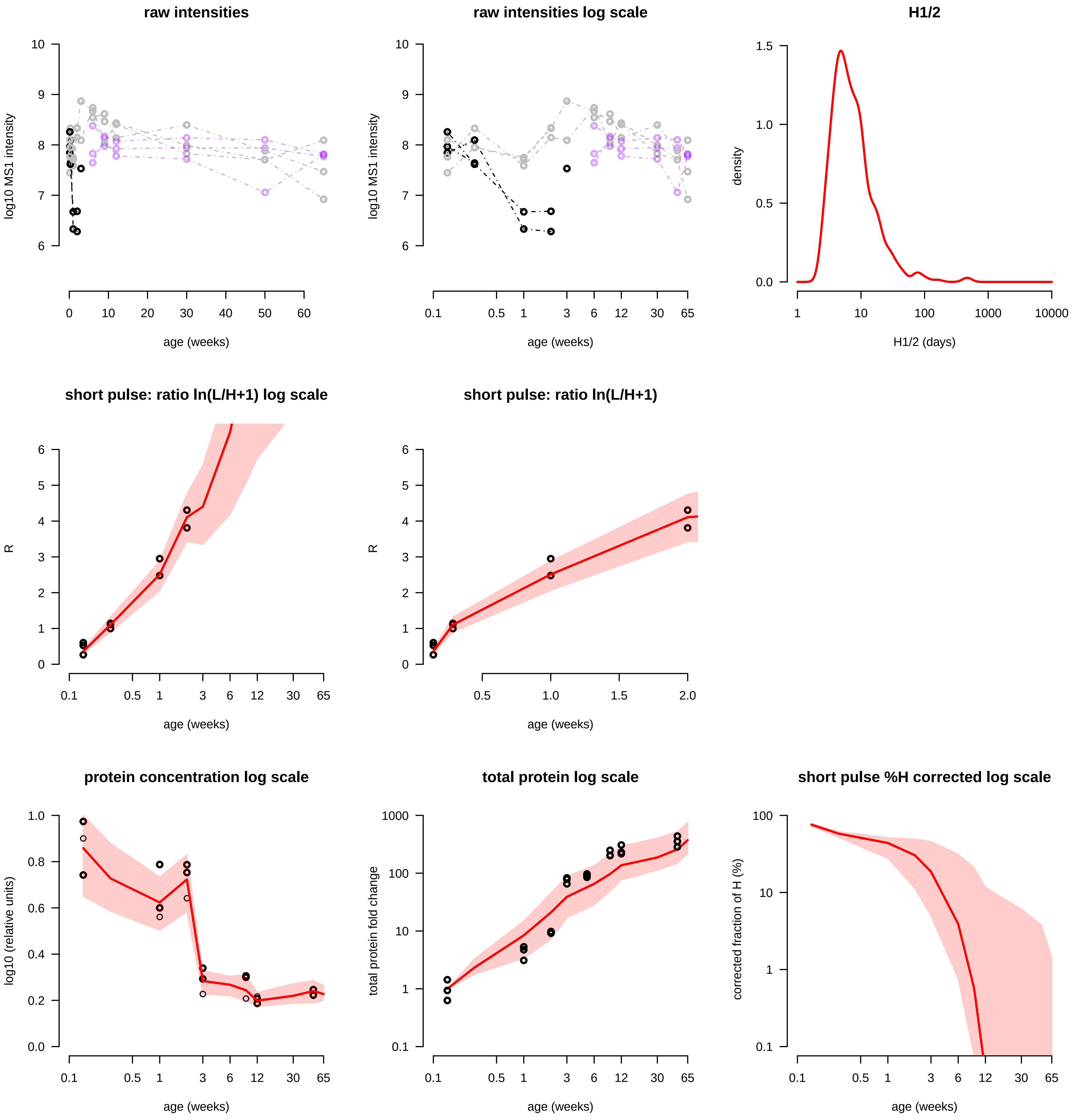

Q99J95;Q99J95−2;Q99J95−3

>sp|Q99J95|CDK9_MOUSE

Cyclin−dependent kinase 9

GN=Cdk9

Med H1/2: 91.2 days

Q5 H1/2: 33.6 days

Q95 H1/2: 1129.8 days

reliable model fit: yes

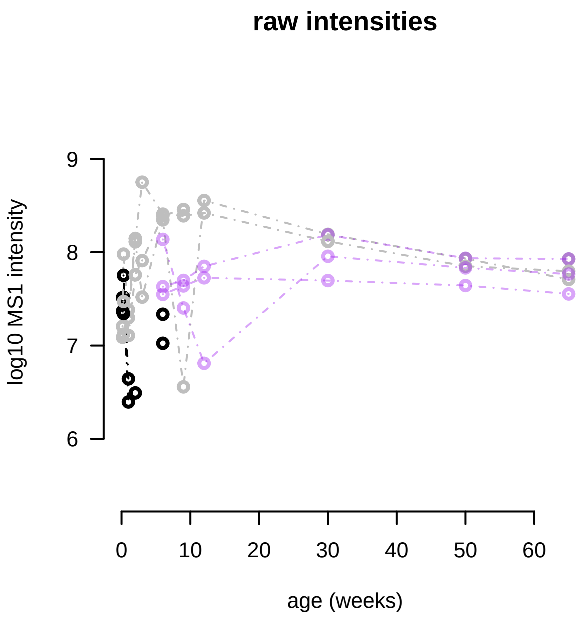

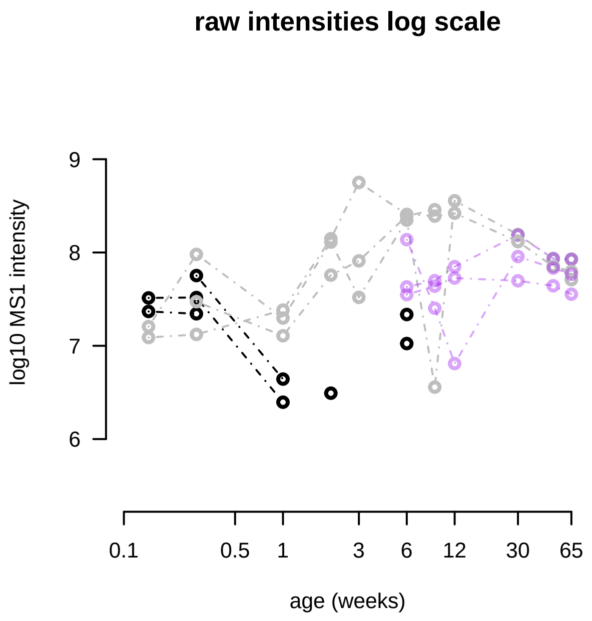

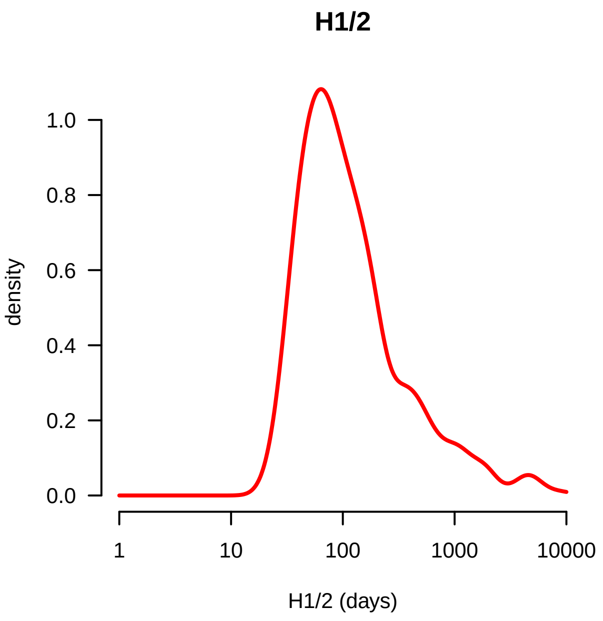

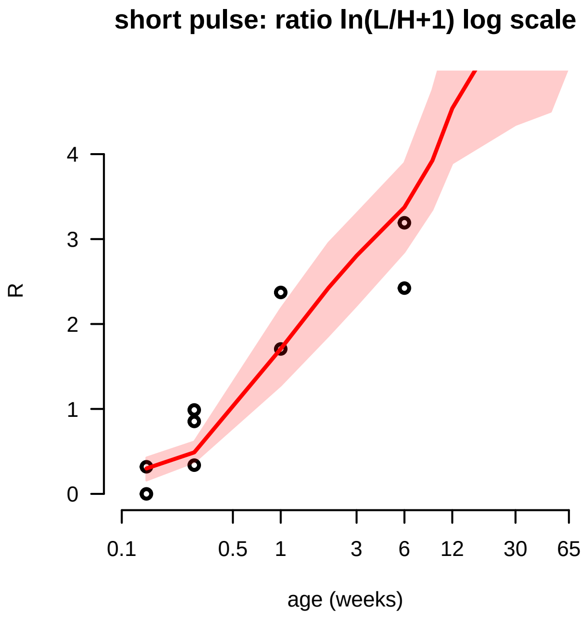

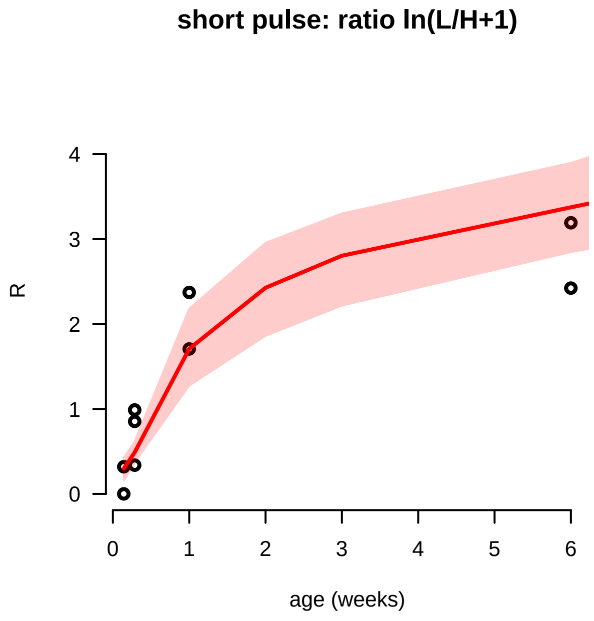

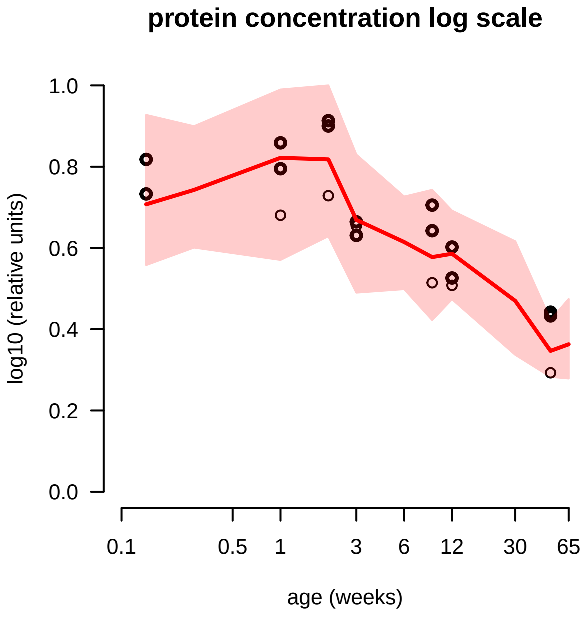

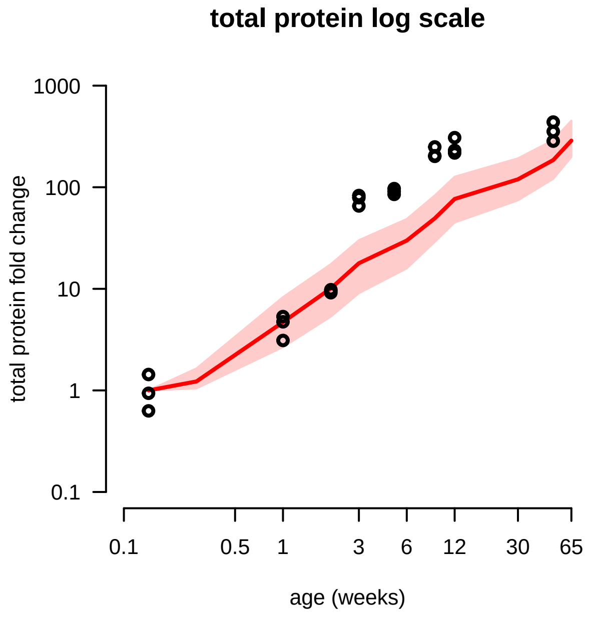

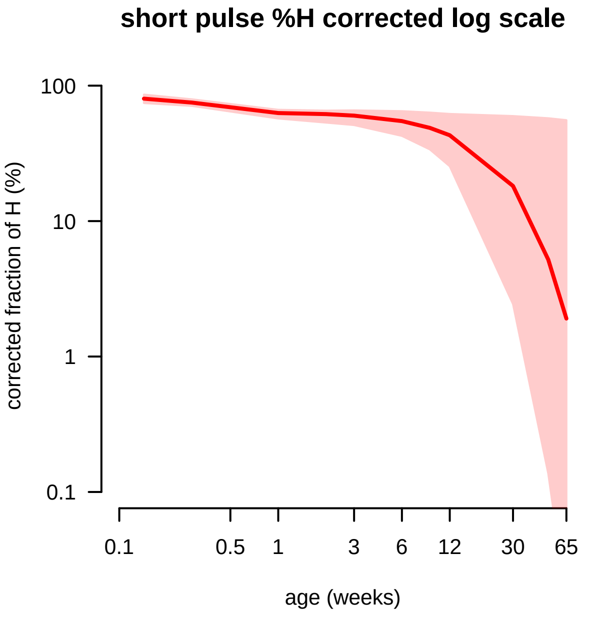

Q9JIK9

>sp|Q9JIK9|RT34_MOUSE

28S ribosomal protein S34, mitochondrial

GN=Mrps34

Med H1/2: 8.9 days

Q5 H1/2: 3 days

Q95 H1/2: 75.3 days

reliable model fit: yes

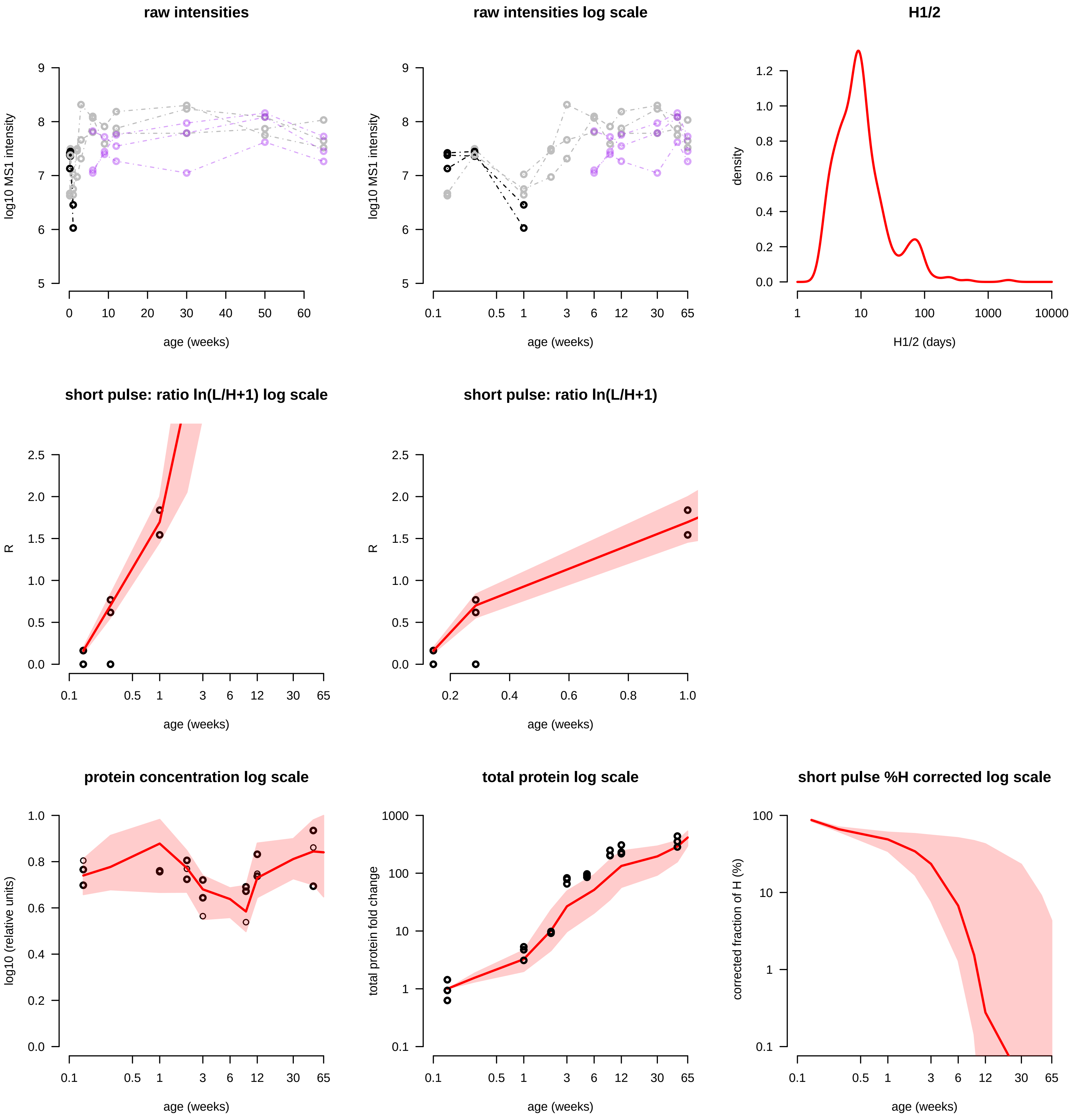

Q8BUY9

>sp|Q8BUY9|PGTB1_MOUSE

Geranylgeranyl transferase type−1 subunit beta

GN=Pggt1b

Med H1/2: 154.9 days

Q5 H1/2: 35.9 days

Q95 H1/2: 1458.8 days

reliable model fit: no

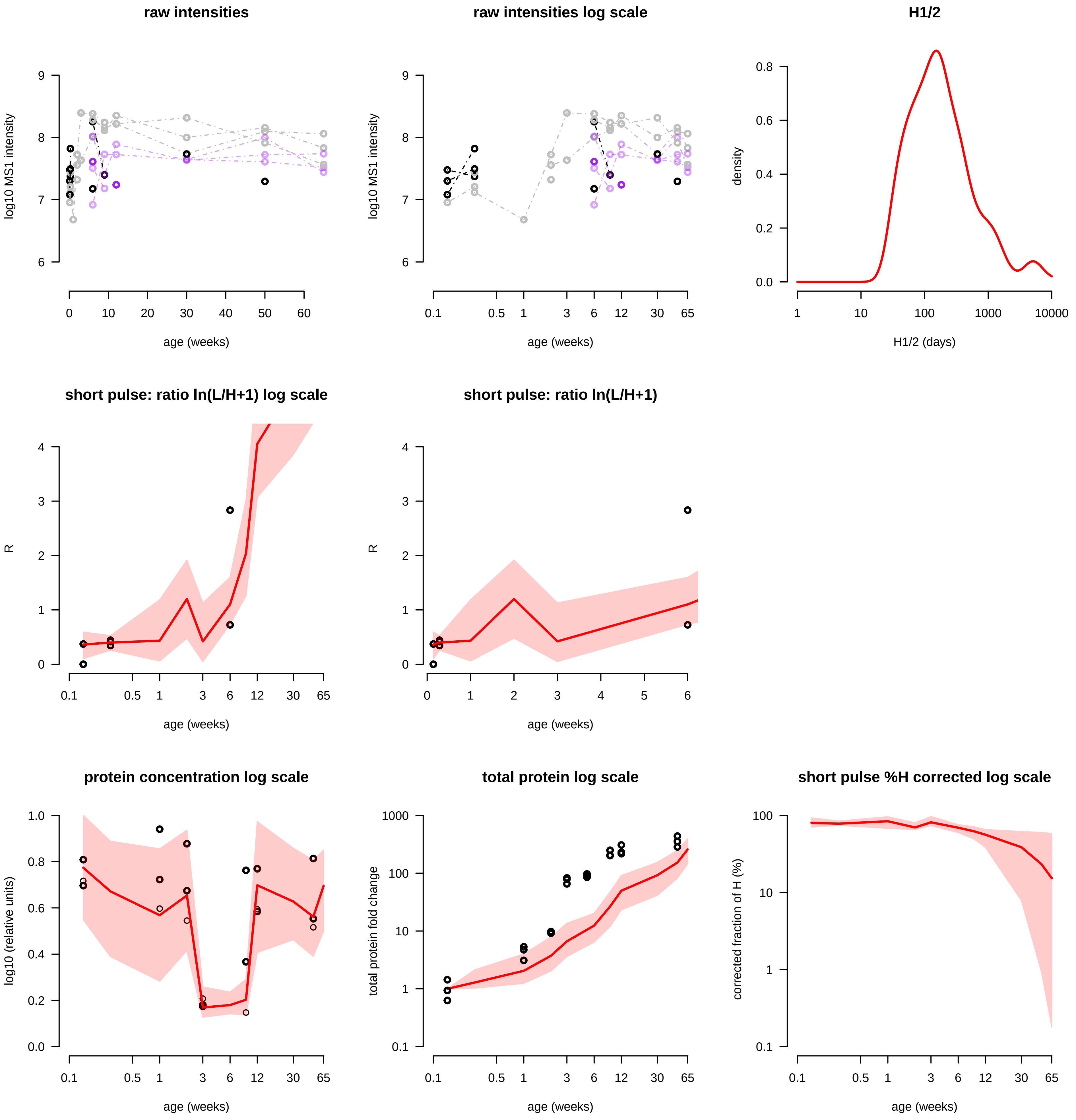

Q61644

>sp|Q61644|PACN1_MOUSE

Protein kinase C and casein kinase substrate in neurons protein 1

GN=Pacsin1

Med H1/2: 7.9 days

Q5 H1/2: 5.5 days

Q95 H1/2: 15.6 days

reliable model fit: yes

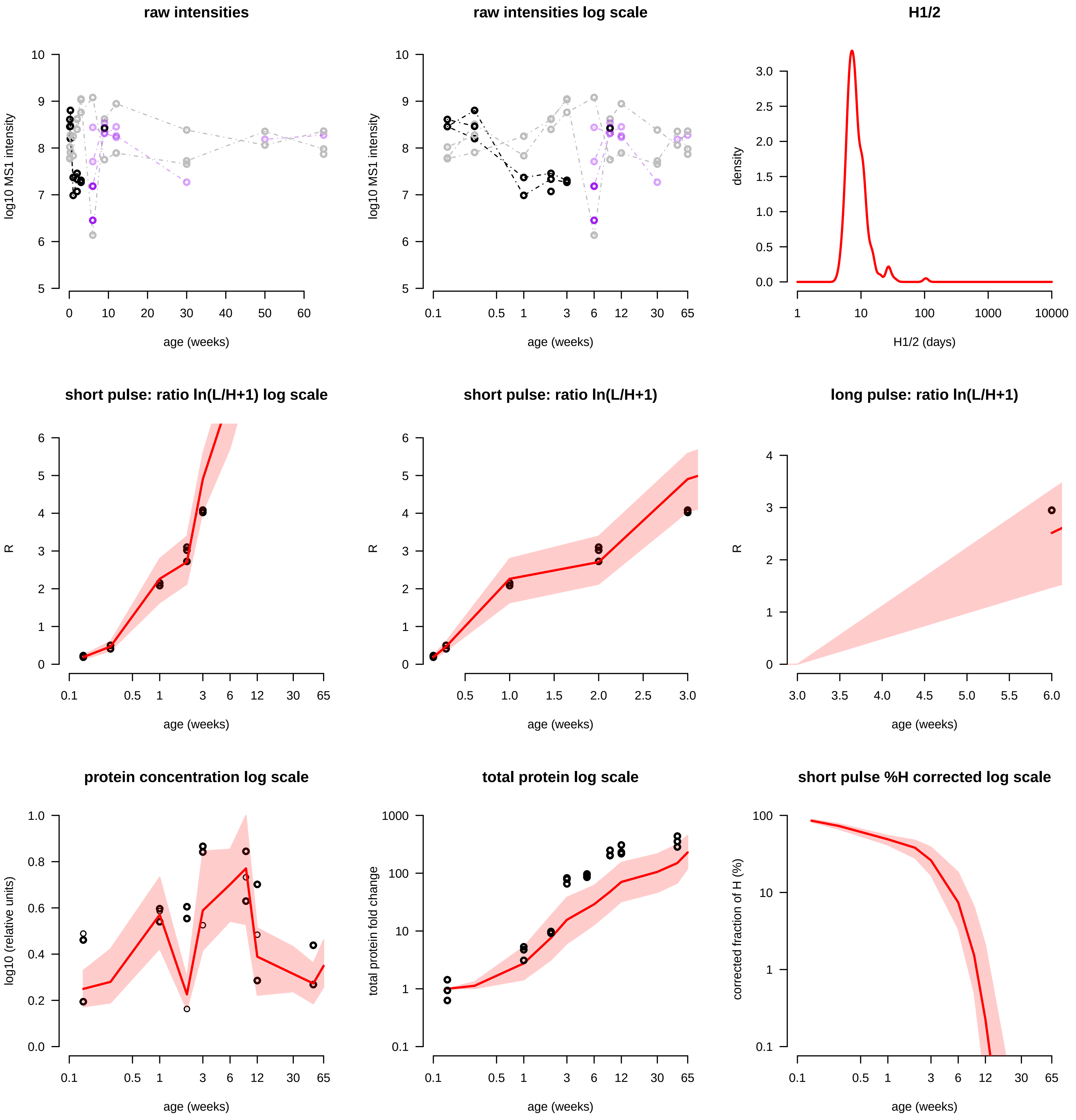

Q8VE92

>sp|Q8VE92|RBM4B_MOUSE

RNA−binding protein 4B

GN=Rbm4b

Med H1/2: 12.7 days

Q5 H1/2: 3.8 days

Q95 H1/2: 109.8 days

reliable model fit: yes

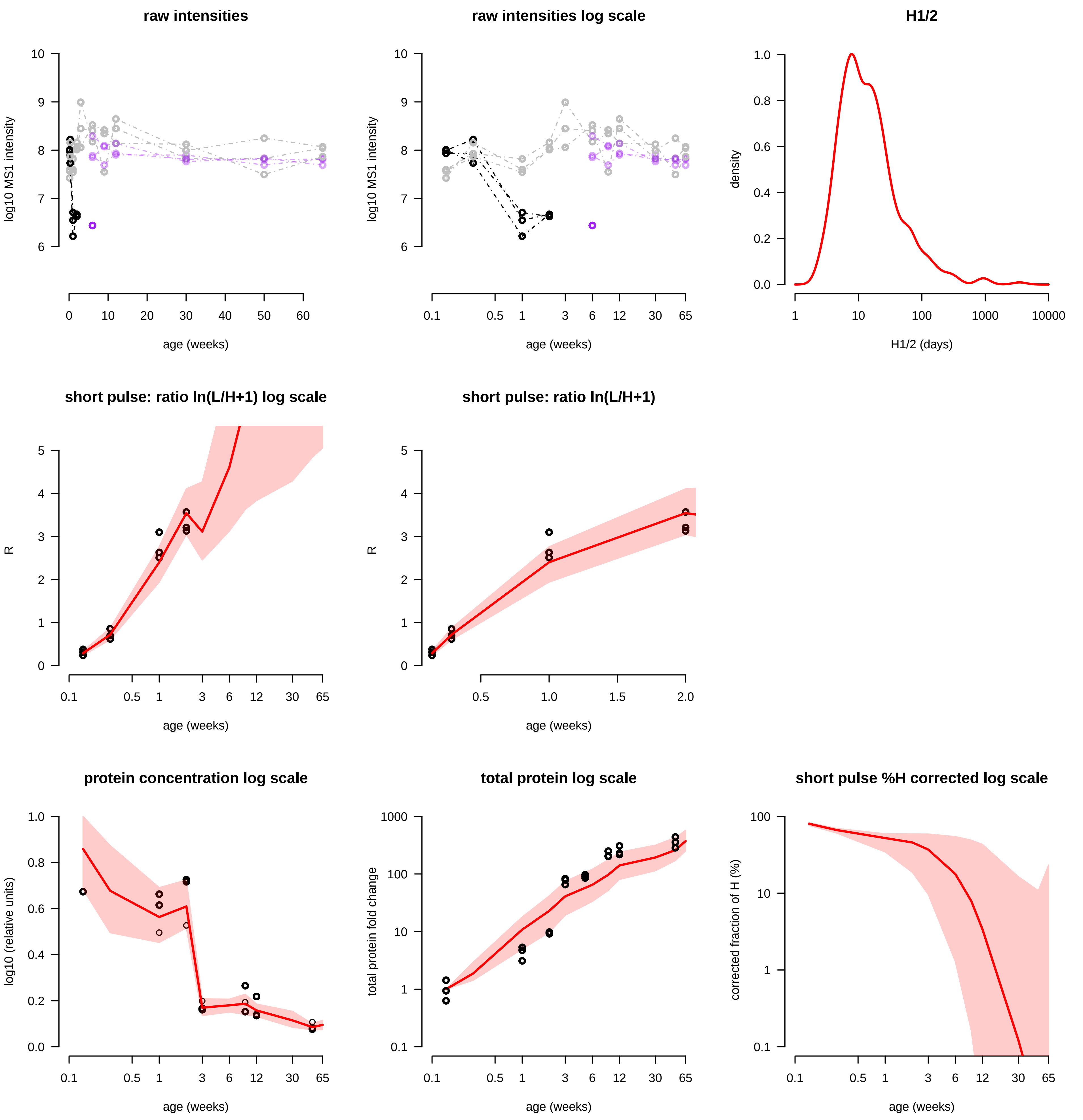

A2A6Q5;A2A6Q5−3;A2A6Q5−2

>sp|A2A6Q5|CDC27_MOUSE

Cell division cycle protein 27 homolog

GN=Cdc27

Med H1/2: 24.8 days

Q5 H1/2: 8.7 days

Q95 H1/2: 324.5 days

reliable model fit: no

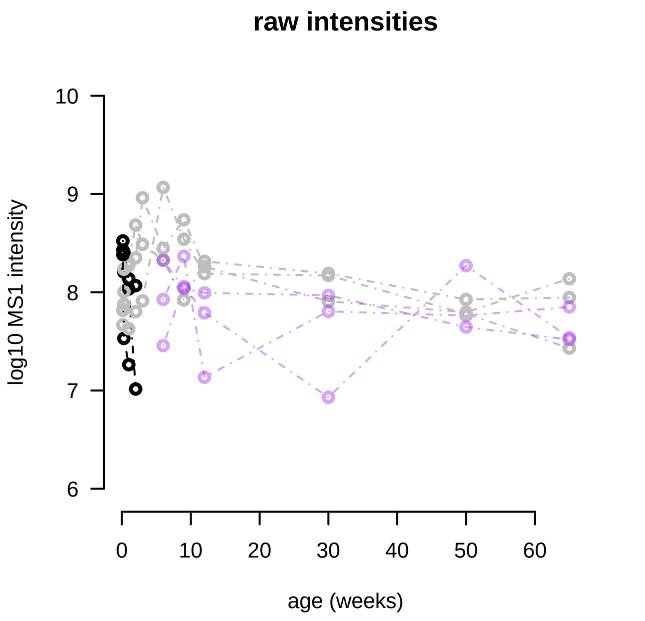

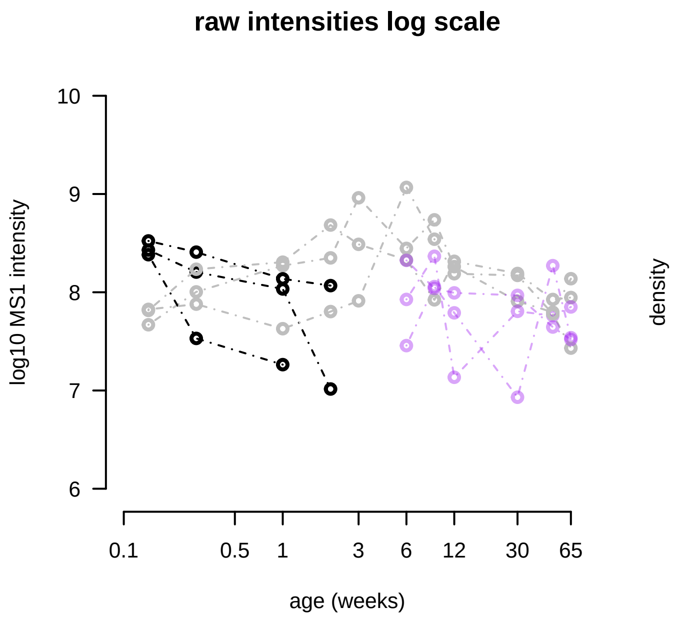

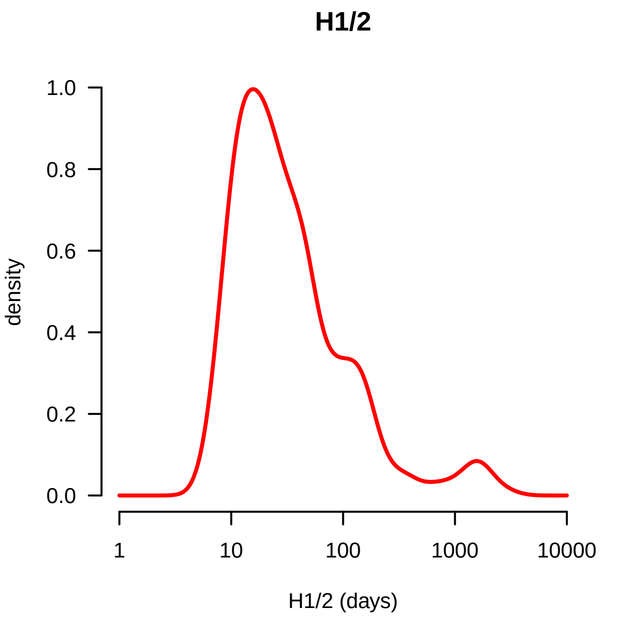

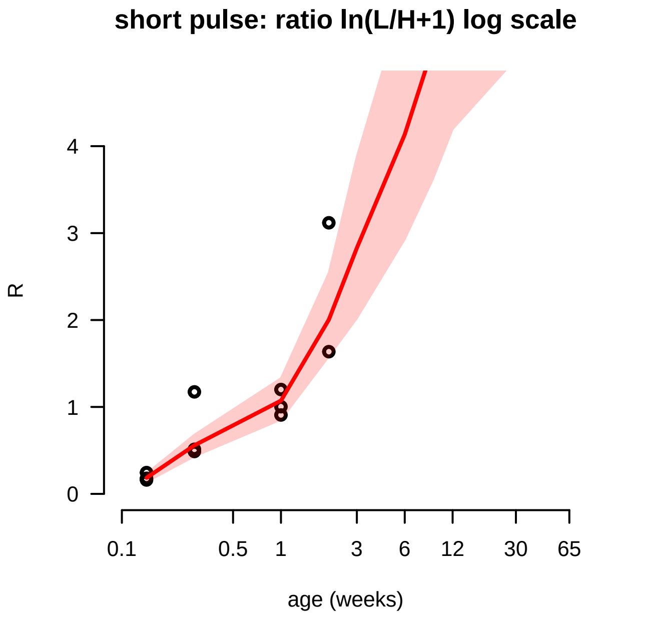

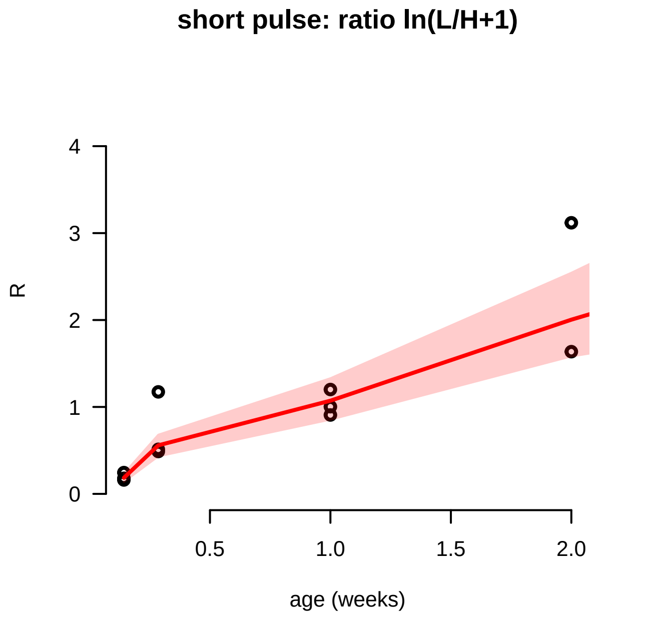

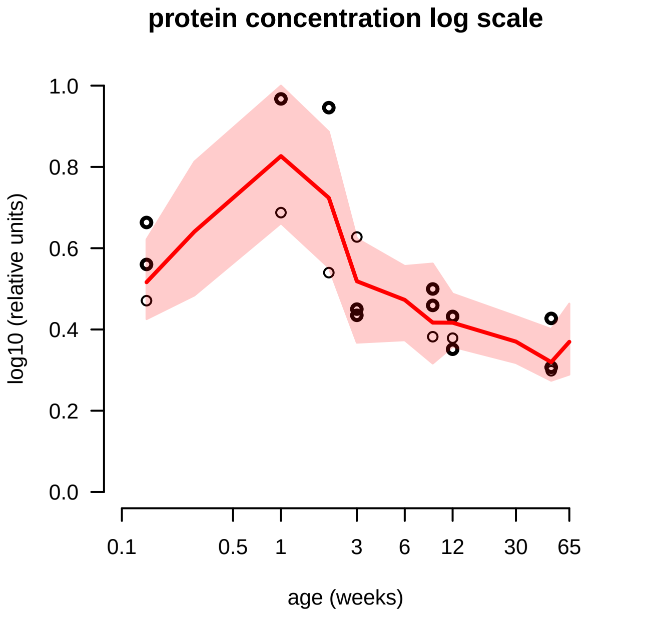

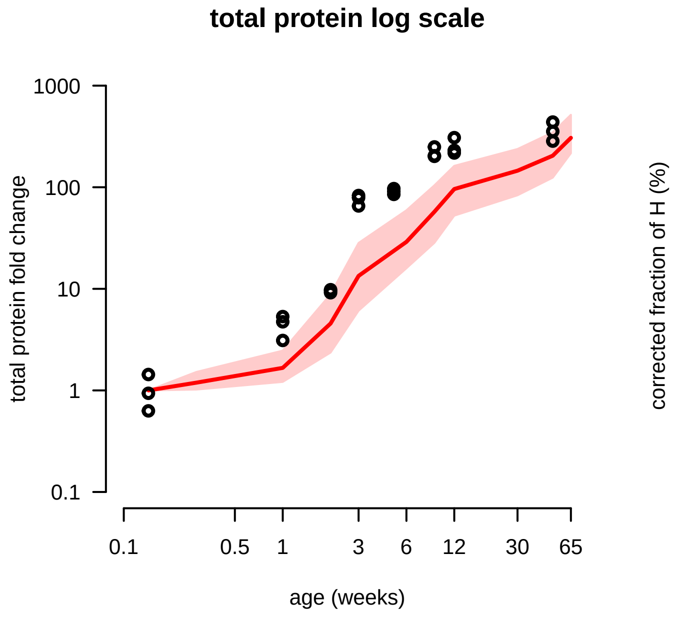

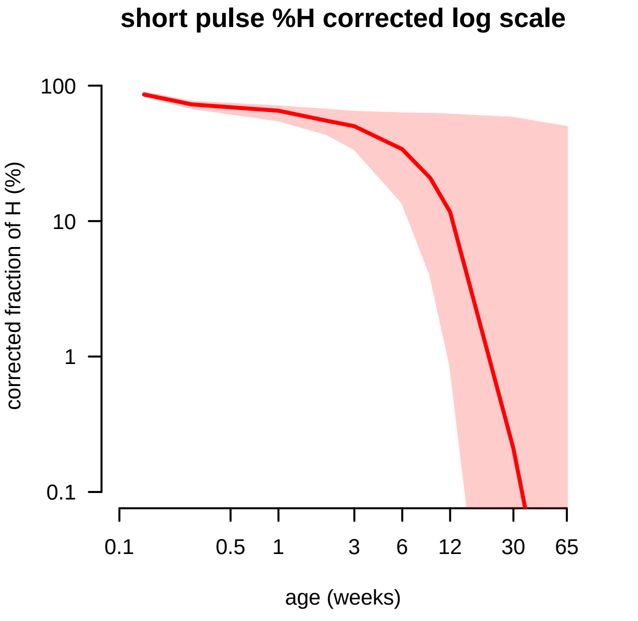

Q9D710

>sp|Q9D710|TMX2_MOUSE

Thioredoxin−related transmembrane protein 2

GN=Tmx2

Med H1/2: 7.1 days

Q5 H1/2: 3 days

Q95 H1/2: 60.4 days

reliable model fit: yes

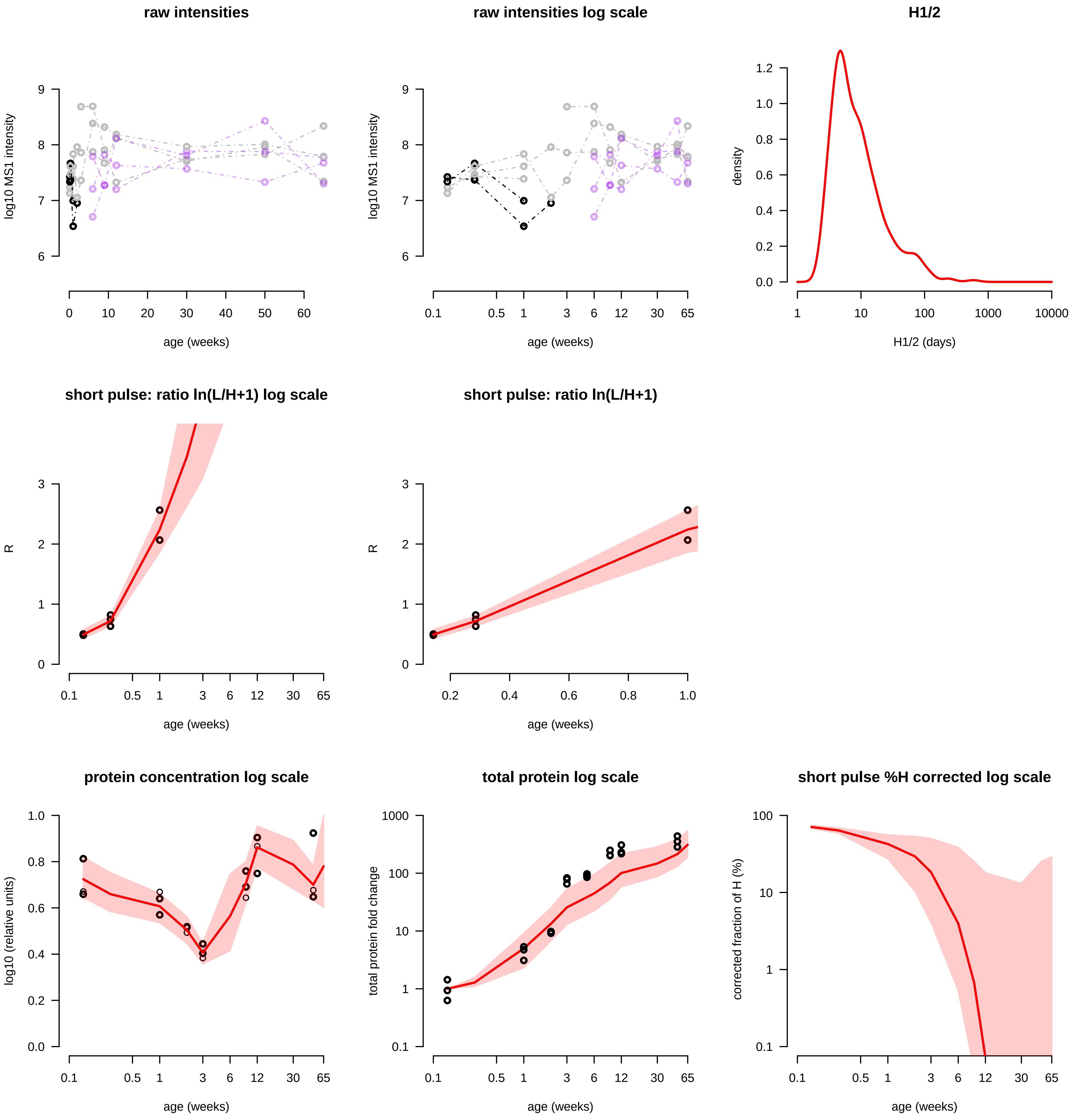

Q91XL9;Q91XL9−2

>sp|Q91XL9|OSBL1_MOUSE

Oxysterol−binding protein−related protein 1

GN=Osbpl1a

Med H1/2: 19.2 days

Q5 H1/2: 5.3 days

Q95 H1/2: 271 days

reliable model fit: yes

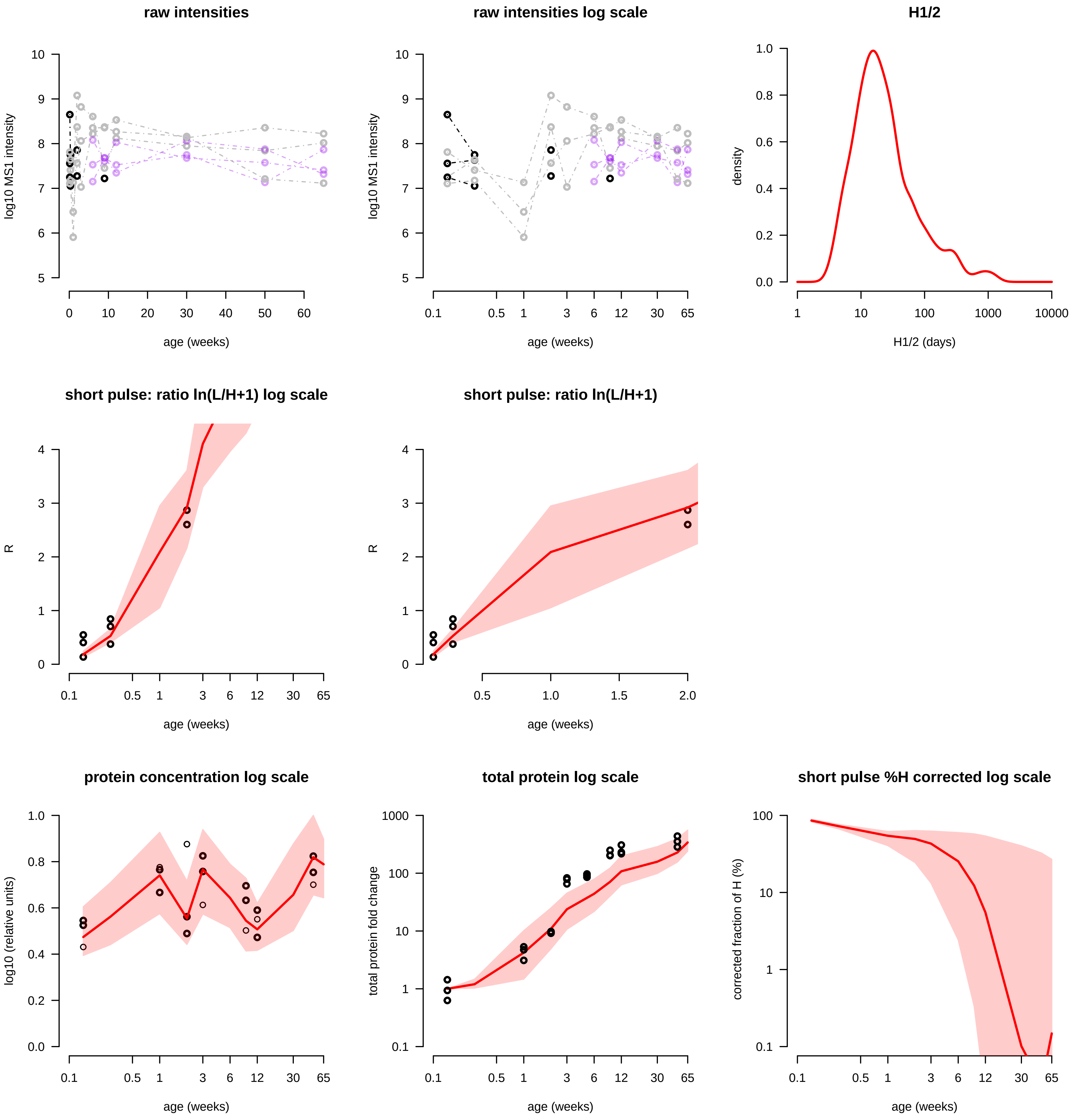

Q8K135;Q8K135−2

>sp|Q8K135|K319L_MOUSE

Dyslexia−associated protein KIAA0319−like protein

GN=Kiaa0319l

Med H1/2: 42.4 days

Q5 H1/2: 12.3 days

Q95 H1/2: 545.2 days

reliable model fit: no

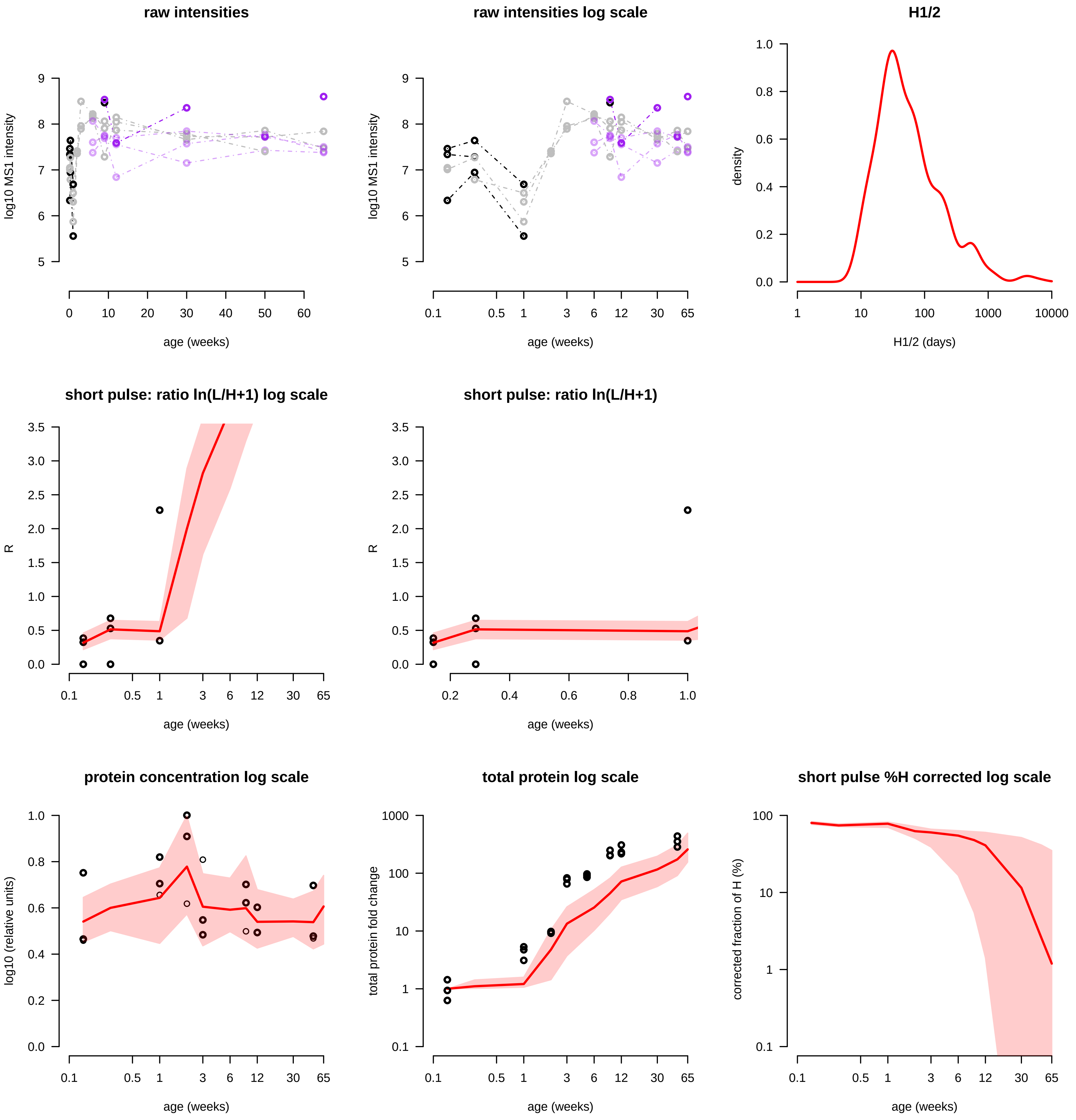

Q9Z2D8;Q9Z2D8−2

>sp|Q9Z2D8|MBD3_MOUSE

Methyl−CpG−binding domain protein 3

GN=Mbd3

Med H1/2: 6.8 days

Q5 H1/2: 2.8 days

Q95 H1/2: 46 days

reliable model fit: yes

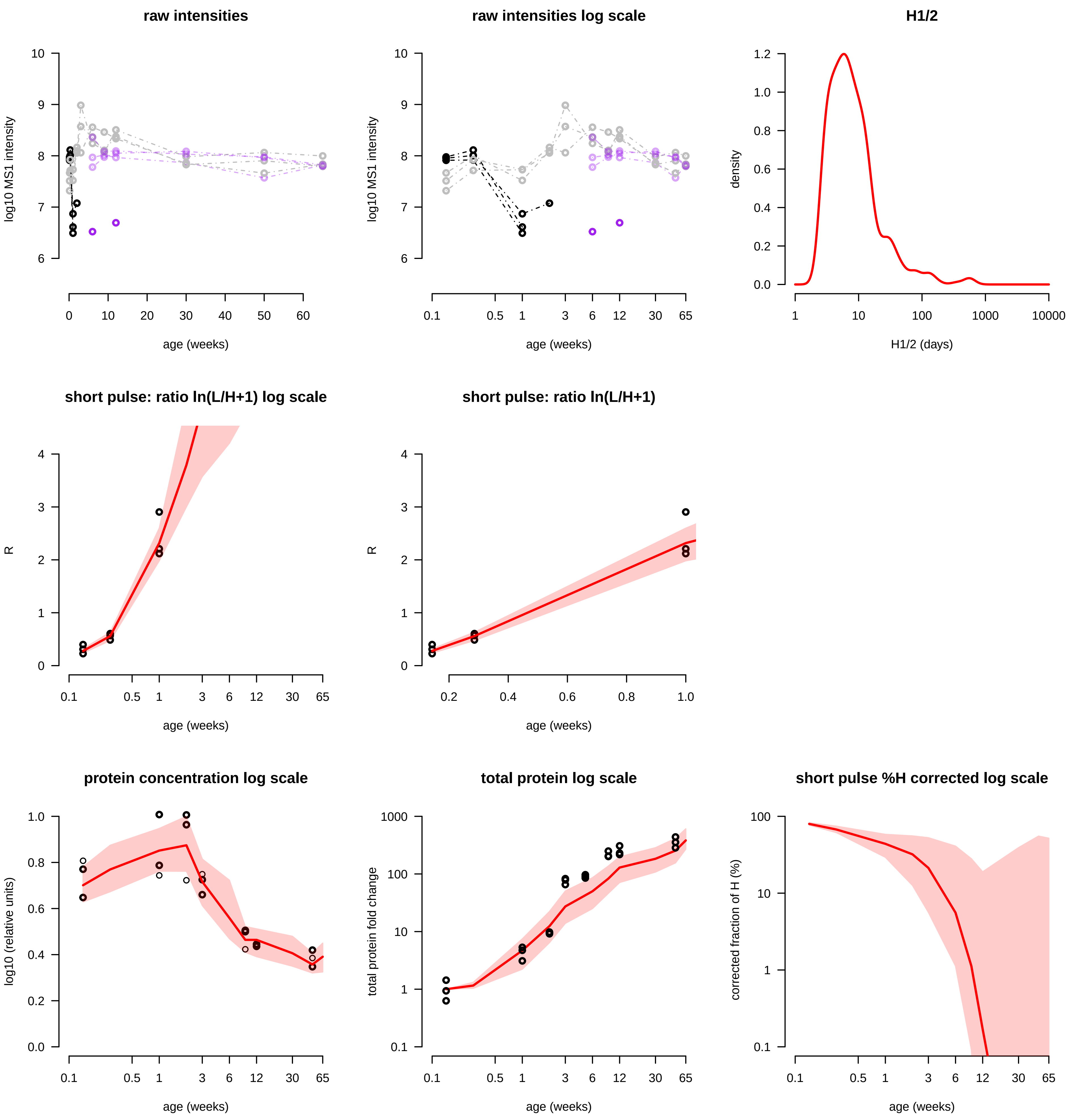

Q9EQ61

>sp|Q9EQ61|PESC_MOUSE

Pescadillo homolog

GN=Pes1

Med H1/2: 3.4 days

Q5 H1/2: 2.6 days

Q95 H1/2: 4.9 days

reliable model fit: yes

raw intensities

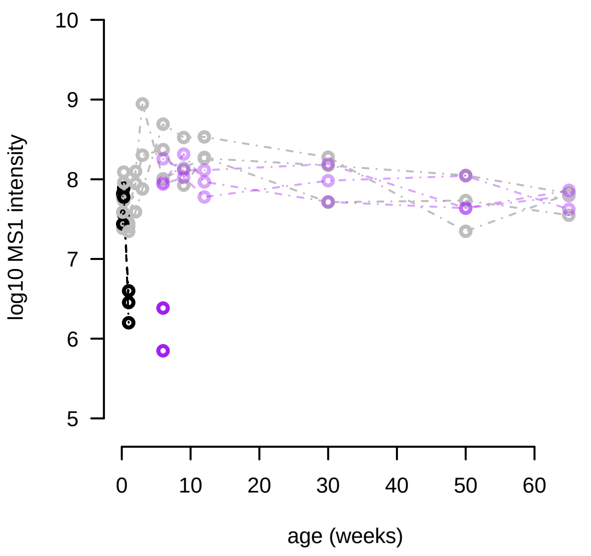

raw intensities log scale

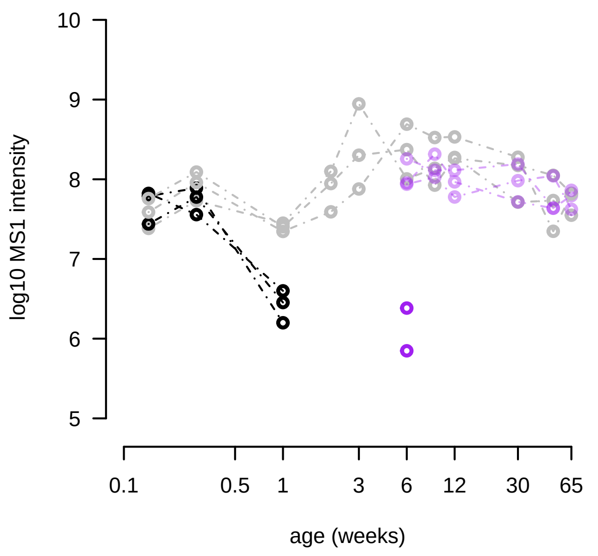

H1/2

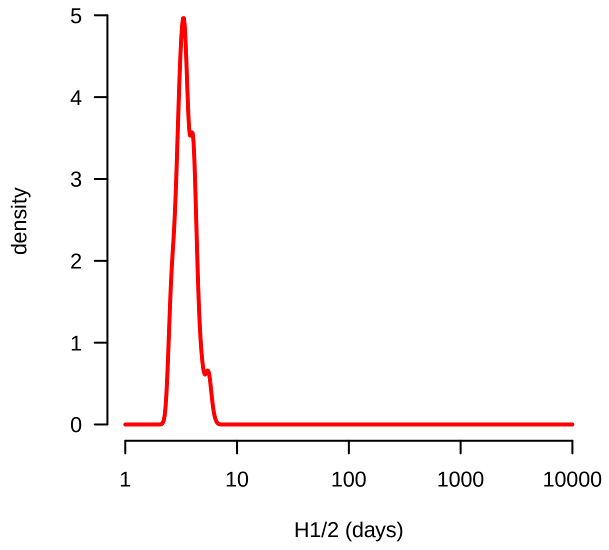

short pulse: ratio ln(L/H+1) log scale

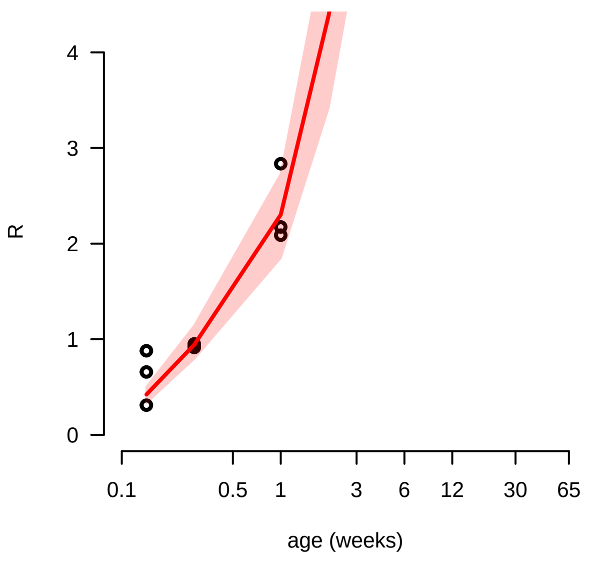

short pulse: ratio ln(L/H+1)

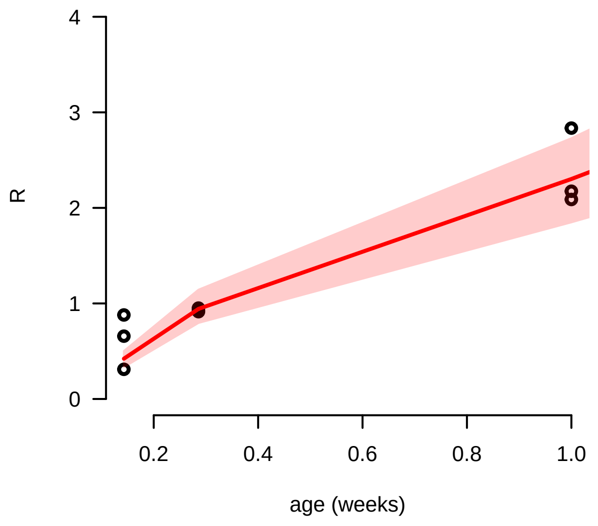

long pulse: ratio ln(L/H+1)

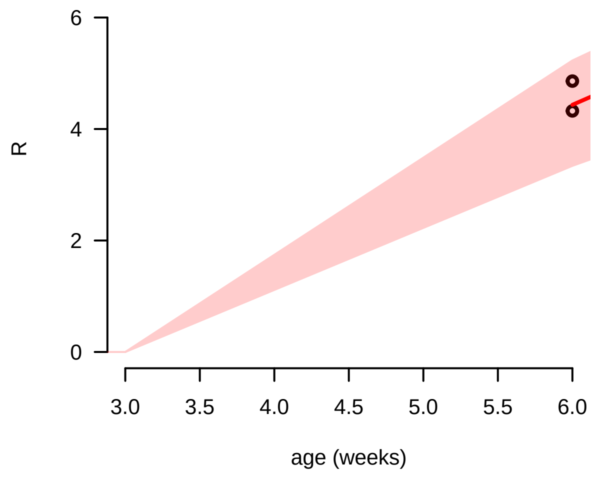

protein concentration log scale

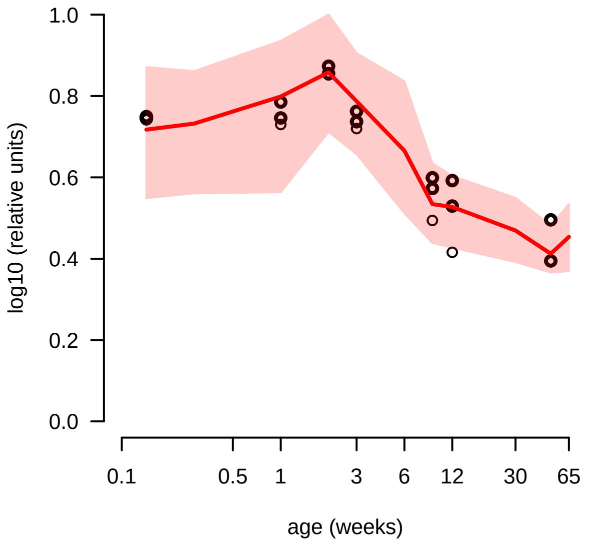

total protein log scale

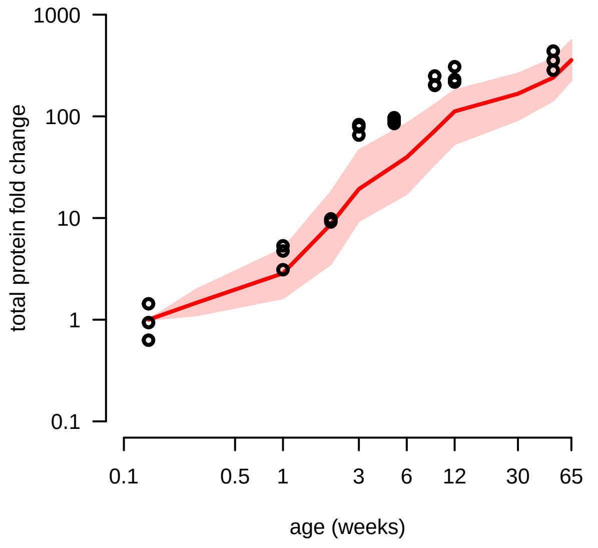

short pulse %H corrected log scale

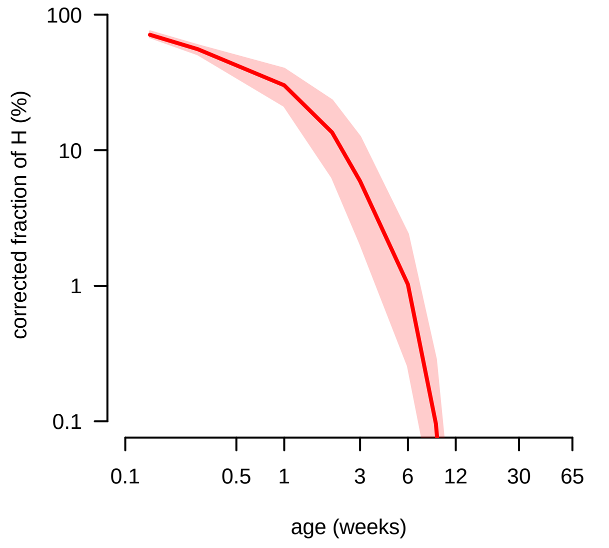

Q9JJZ2

>sp|Q9JJZ2|TBA8_MOUSE

Tubulin alpha−8 chain

GN=Tuba8

Med H1/2: 16.2 days

Q5 H1/2: 6.3 days

Q95 H1/2: 137.2 days

reliable model fit: yes

raw intensities

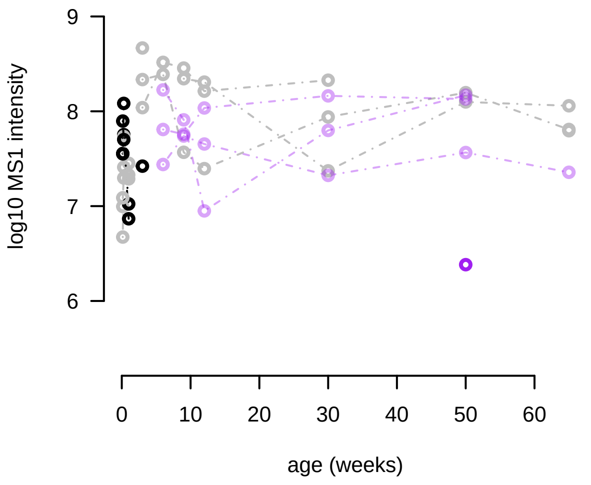

raw intensities log scale

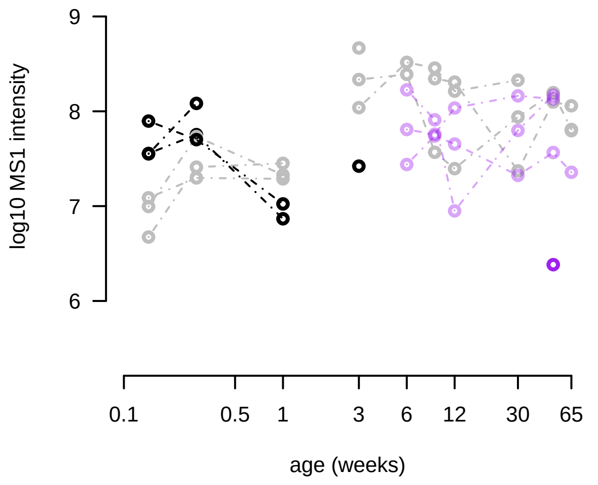

H1/2

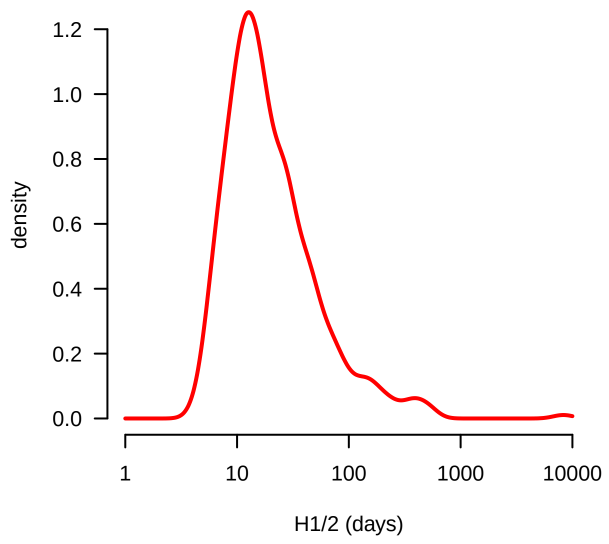

short pulse: ratio ln(L/H+1) log scale

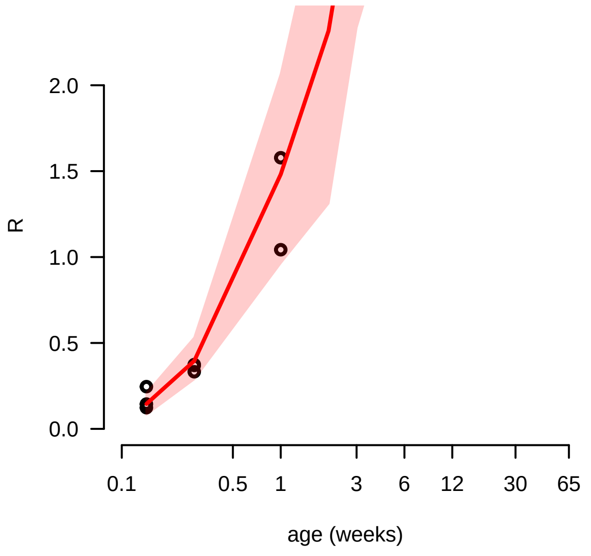

short pulse: ratio ln(L/H+1)

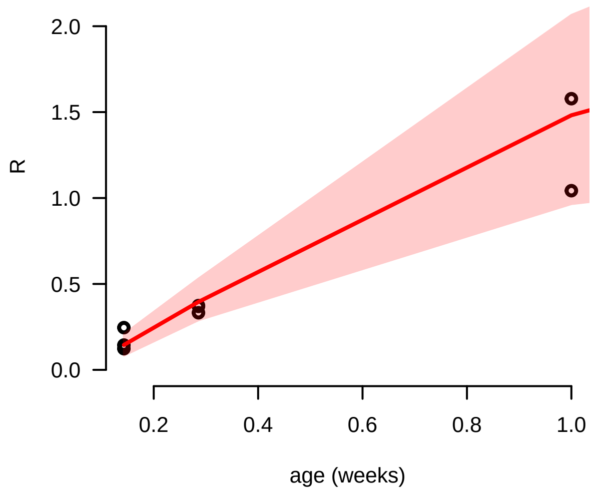

protein concentration log scale

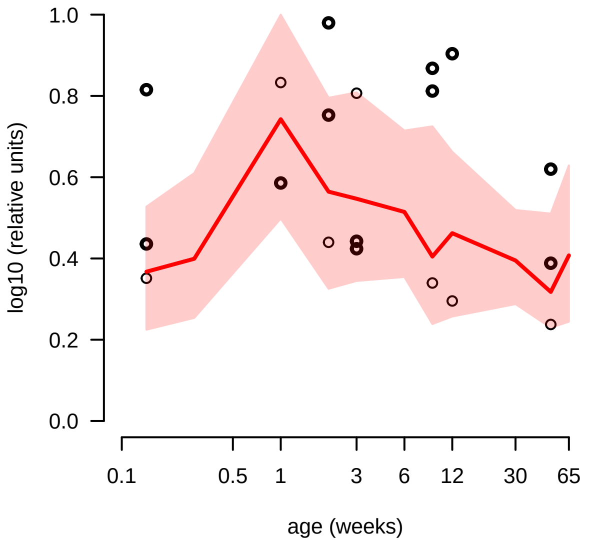

total protein log scale

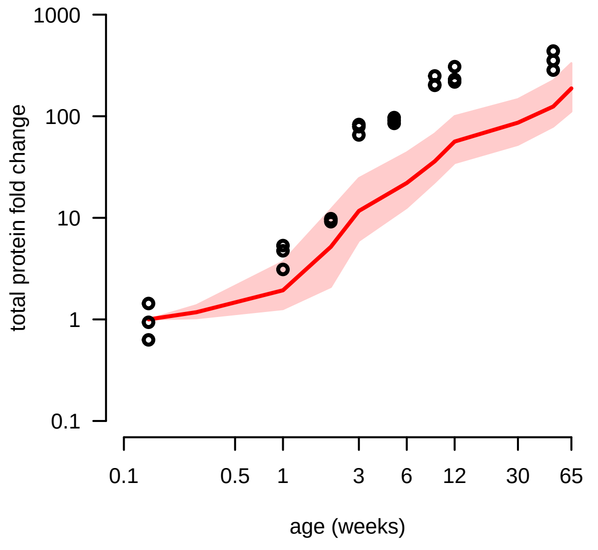

short pulse %H corrected log scale

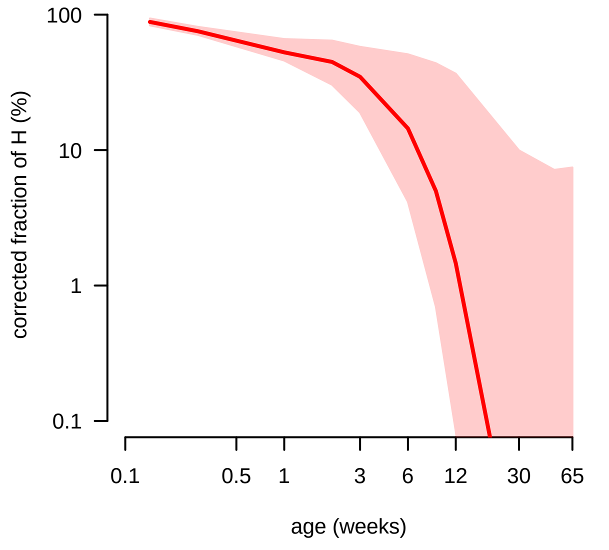

Q5XG71

>sp|Q5XG71|UTP20_MOUSE

Small subunit processome component 20 homolog

GN=Utp20

Med H1/2: 33.4 days

Q5 H1/2: 9.5 days

Q95 H1/2: 342.2 days

reliable model fit: yes

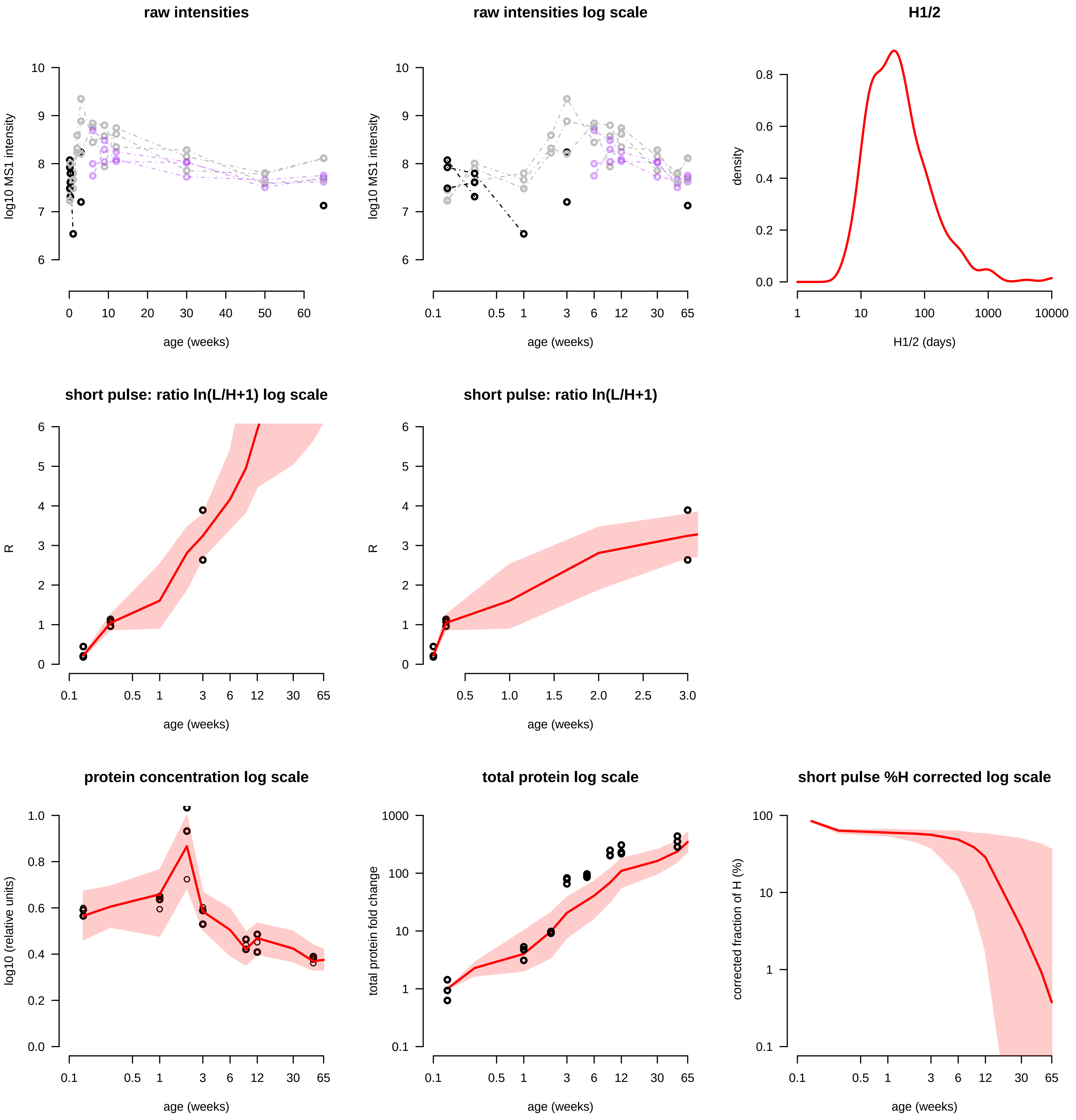

P26187

>sp|P26187|MGMT_MOUSE

Methylated−DNA−−protein−cysteine methyltransferase

GN=Mgmt

Med H1/2: 5 days

Q5 H1/2: 2.7 days

Q95 H1/2: 24.7 days

reliable model fit: yes

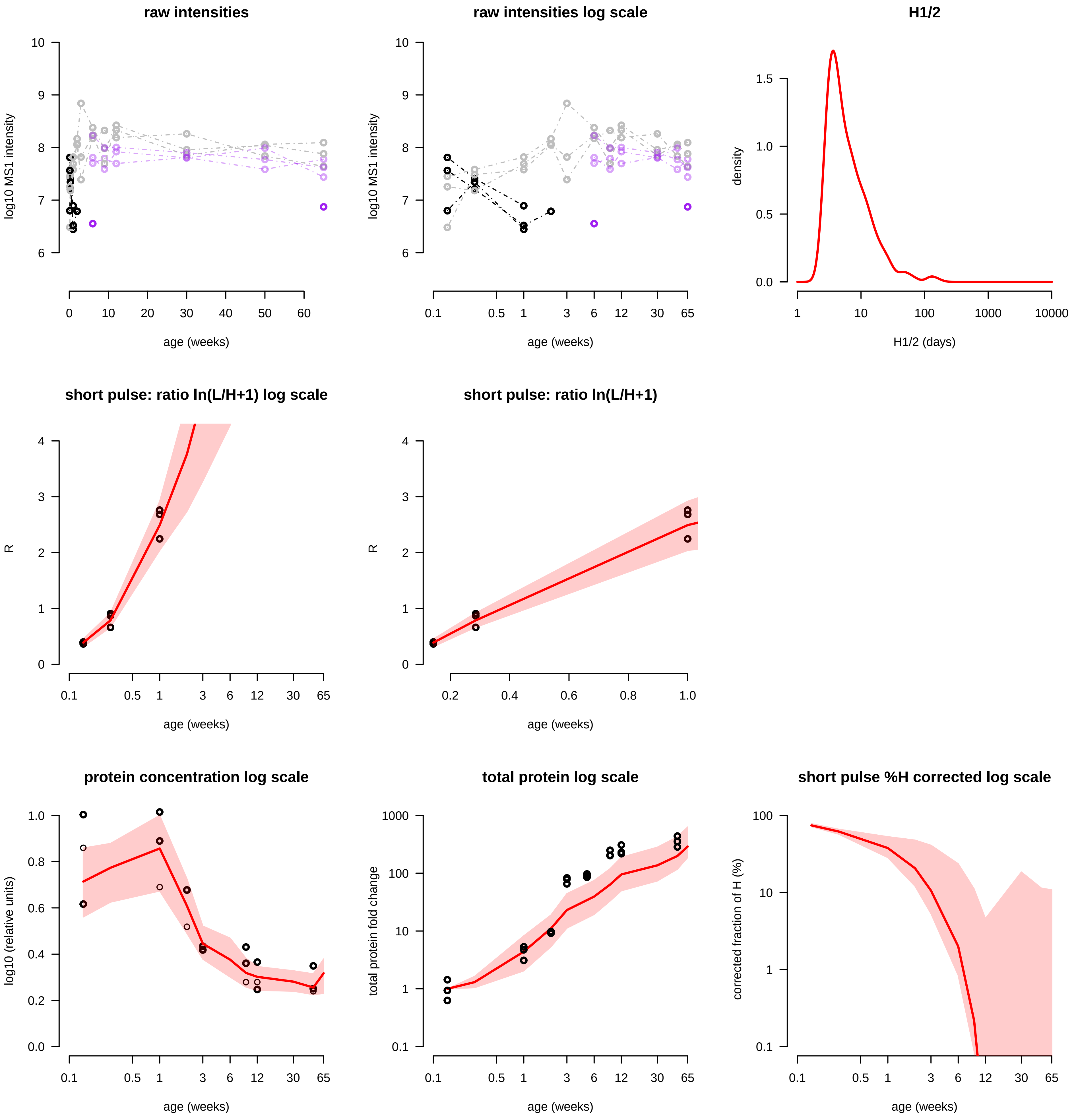

E9PY46

>sp|E9PY46|IF140_MOUSE

Intraflagellar transport protein 140 homolog

GN=Ift140

Med H1/2: 12.2 days

Q5 H1/2: 4.6 days

Q95 H1/2: 186.9 days

reliable model fit: no

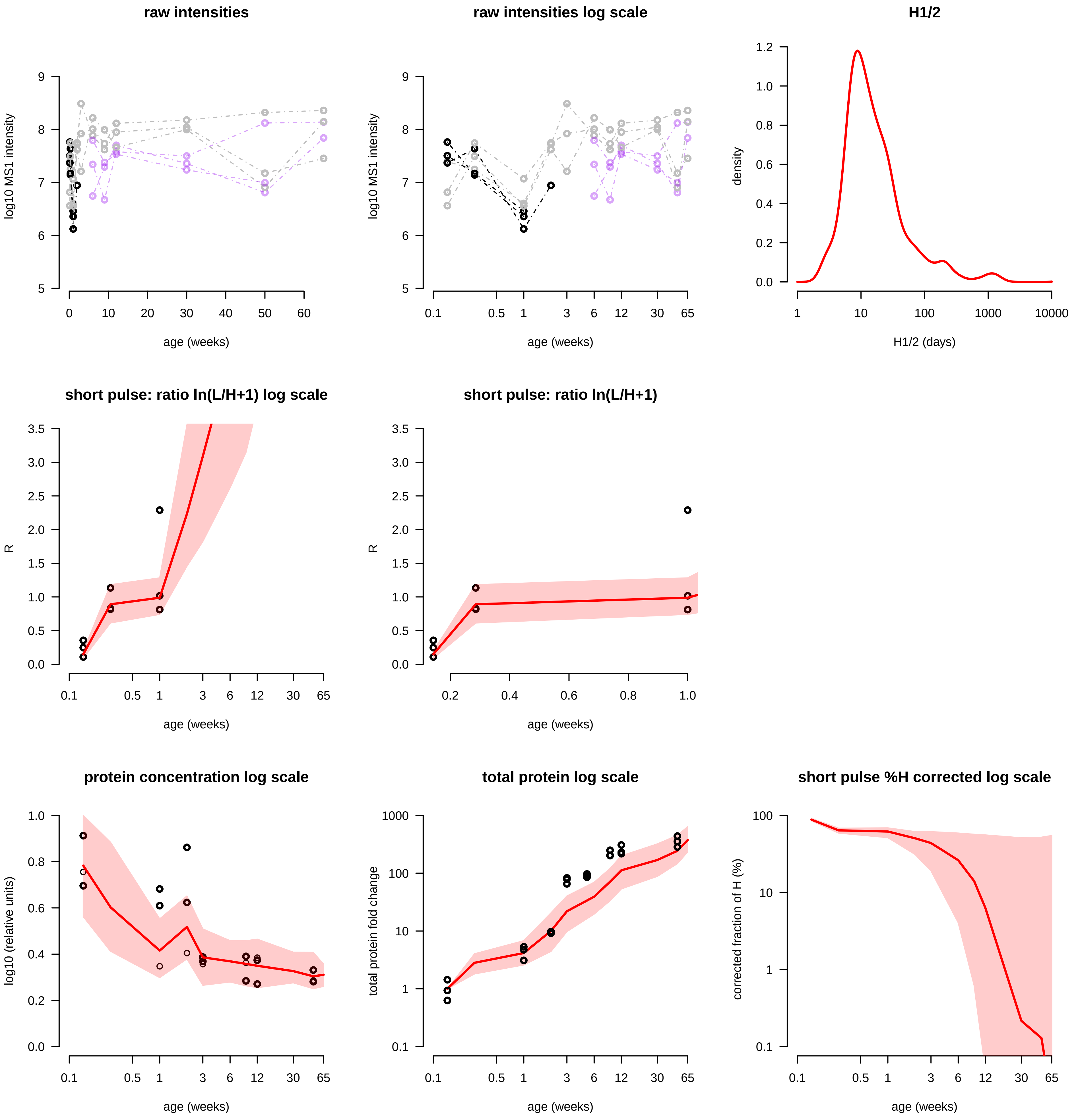

O08734

>sp|O08734|BAK_MOUSE

Bcl−2 homologous antagonist/killer

GN=Bak1

Med H1/2: 23.2 days

Q5 H1/2: 5.8 days

Q95 H1/2: 240.3 days

reliable model fit: no

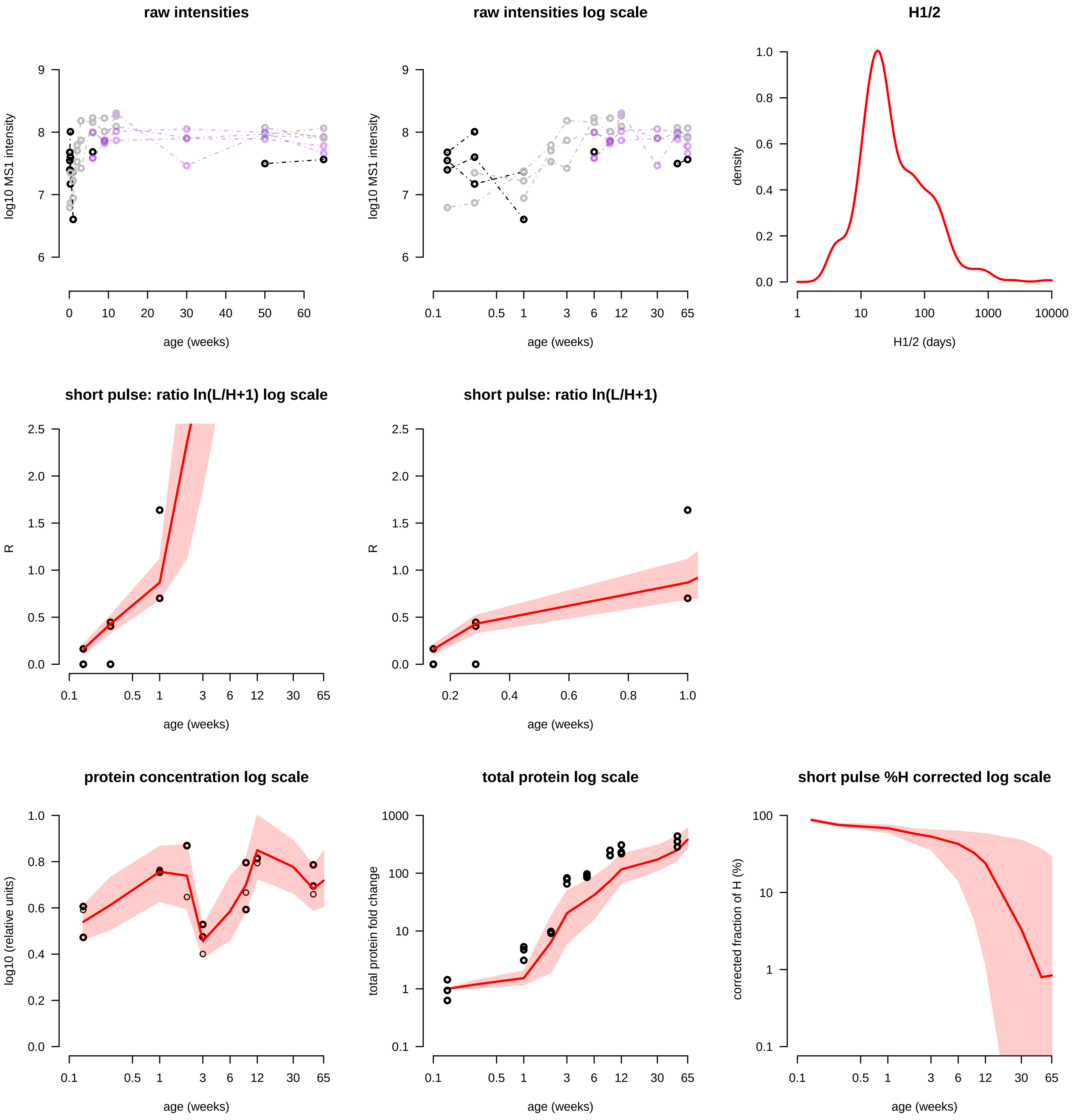

P62309

>sp|P62309|RUXG_MOUSE

Small nuclear ribonucleoprotein G

GN=Snrpg

Med H1/2: 25.5 days

Q5 H1/2: 14.5 days

Q95 H1/2: 69.5 days

reliable model fit: yes

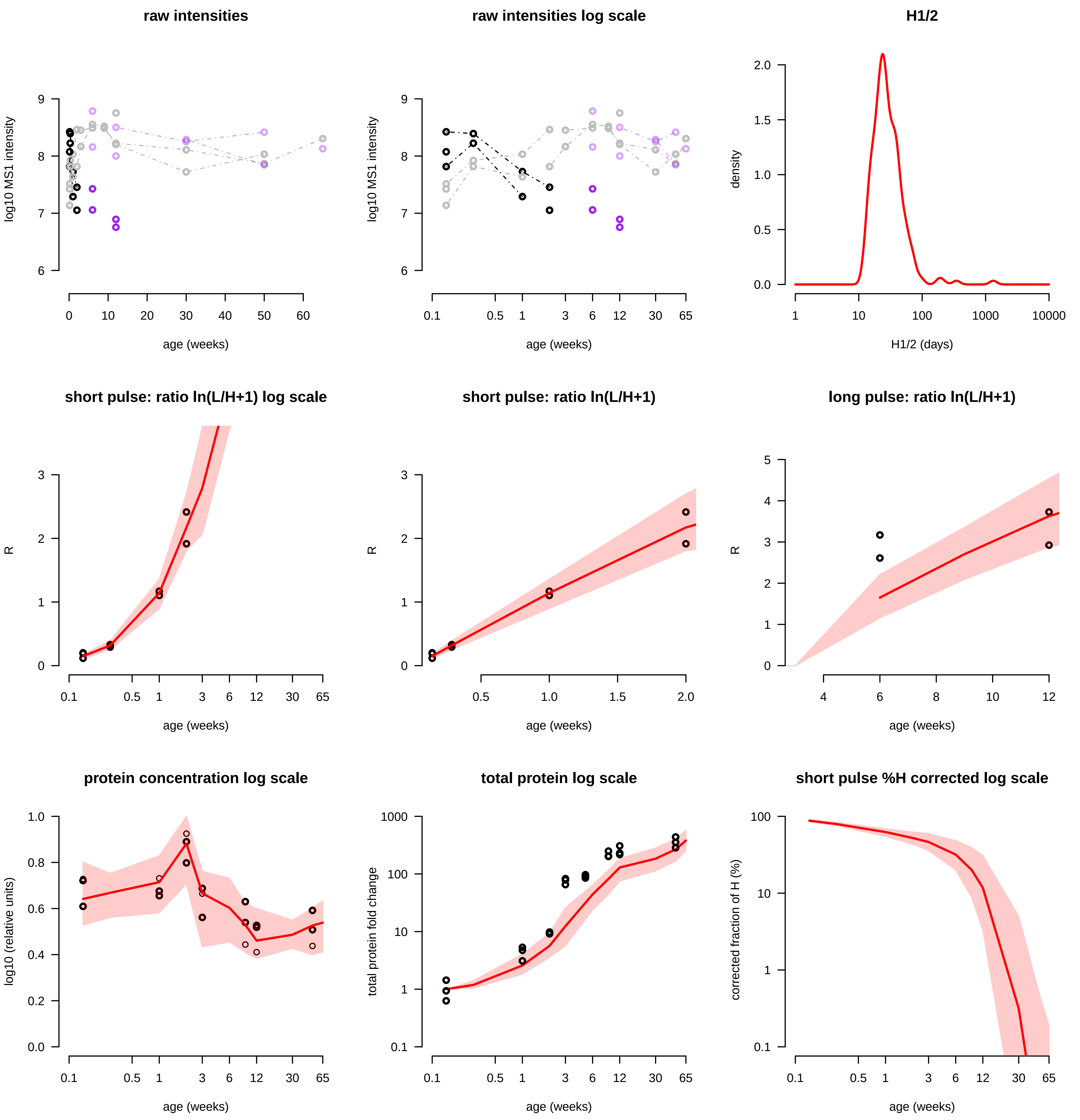

Q9WV02;Q9WV02−2

>sp|Q9WV02|RBMX_MOUSE

RNA−binding motif protein, X chromosome

GN=Rbmx

Med H1/2: 32.5 days

Q5 H1/2: 10 days

Q95 H1/2: 320.4 days

reliable model fit: yes

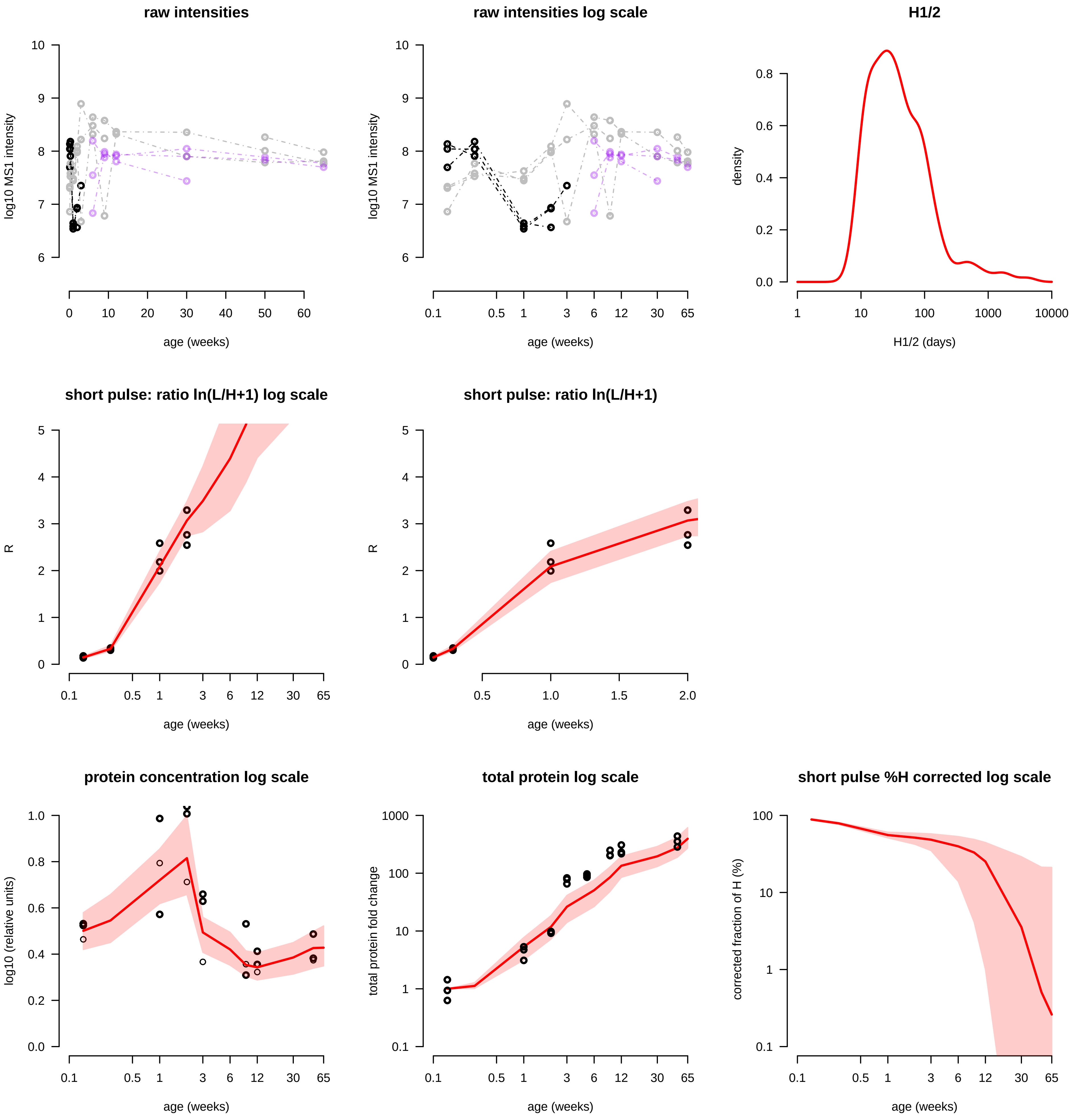

P35601−2;P35601

>sp|P35601−2|RFC1_MOUSE

Isoform 2 of Replication factor C subunit 1

GN=Rfc1

Med H1/2: 3.2 days

Q5 H1/2: 2.6 days

Q95 H1/2: 4.1 days

reliable model fit: yes

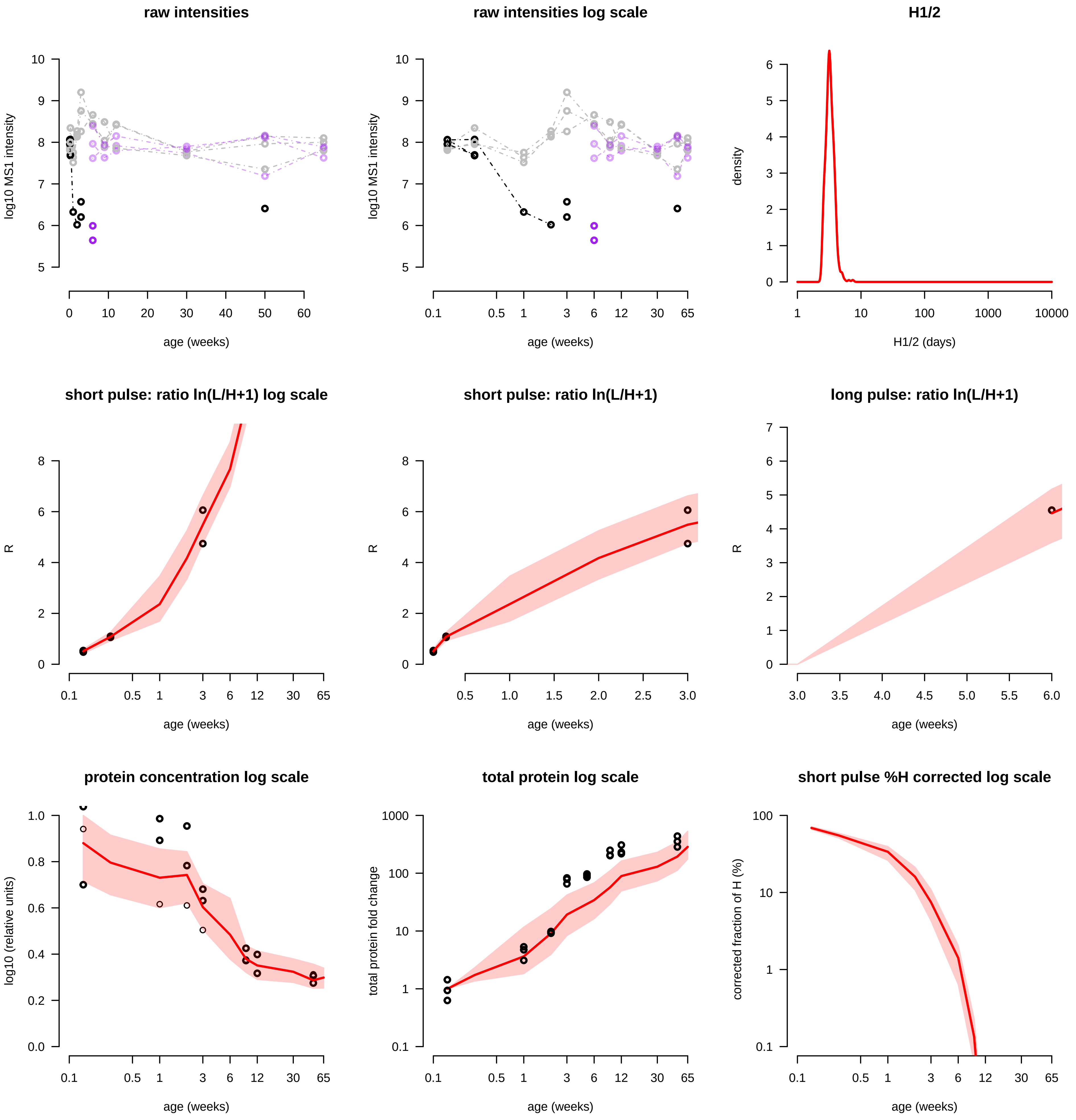

Q9WTX8;Q9WTX8−2

>sp|Q9WTX8|MD1L1_MOUSE

Mitotic spindle assembly checkpoint protein MAD1

GN=Mad1l1

Med H1/2: 7.2 days

Q5 H1/2: 2.8 days

Q95 H1/2: 41.1 days

reliable model fit: yes

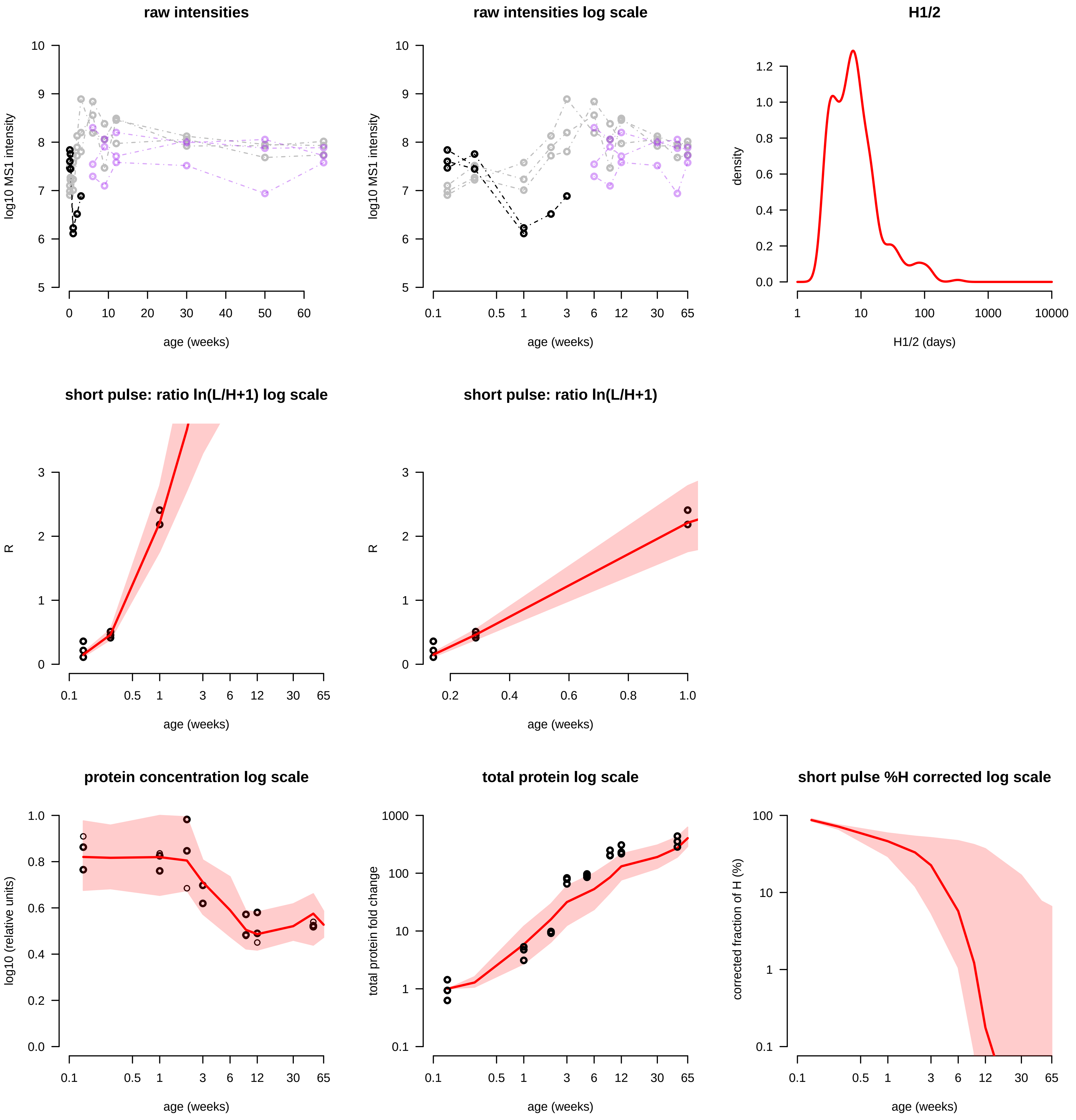

Q69ZQ2

>sp|Q69ZQ2|ISY1_MOUSE

Pre−mRNA−splicing factor ISY1 homolog

GN=Isy1

Med H1/2: 191.6 days

Q5 H1/2: 52.7 days

Q95 H1/2: 1644.6 days

reliable model fit: yes

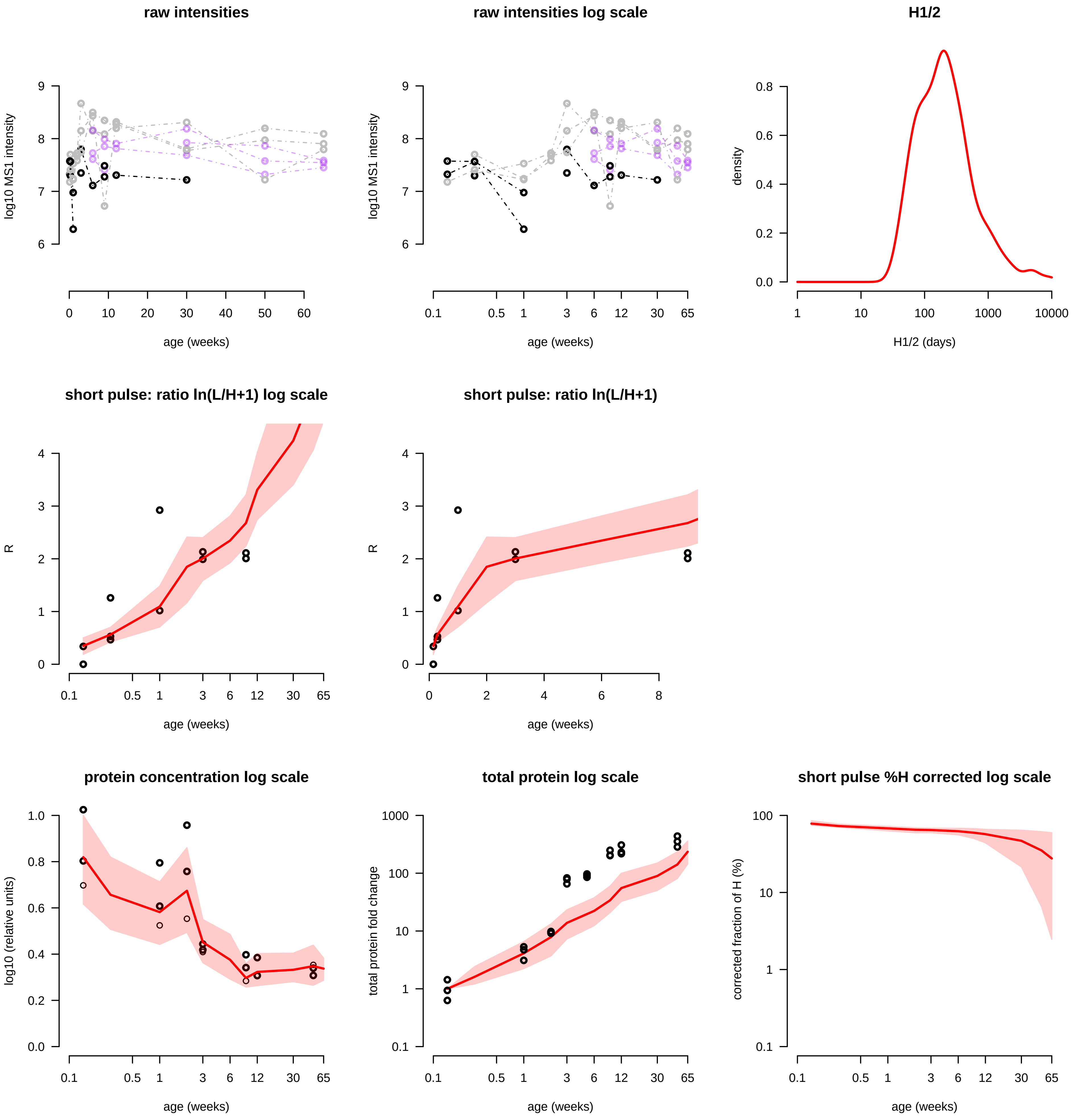

P61804

>sp|P61804|DAD1_MOUSE

Dolichyl−diphosphooligosaccharide−−protein glycosyltransferase subunit DAD1

GN=Dad1

Med H1/2: 14.1 days

Q5 H1/2: 4.7 days

Q95 H1/2: 171.1 days

reliable model fit: yes

raw intensities

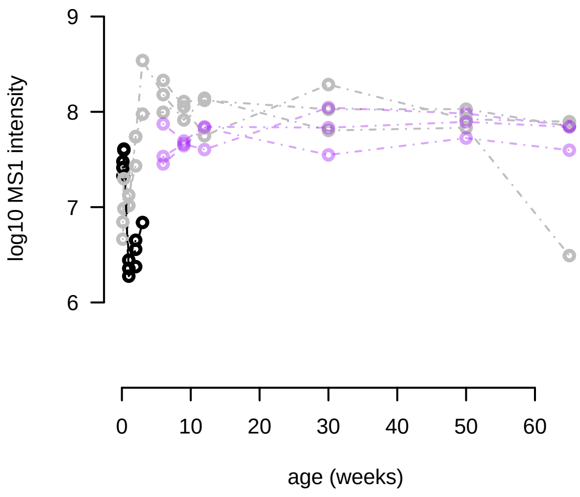

raw intensities log scale

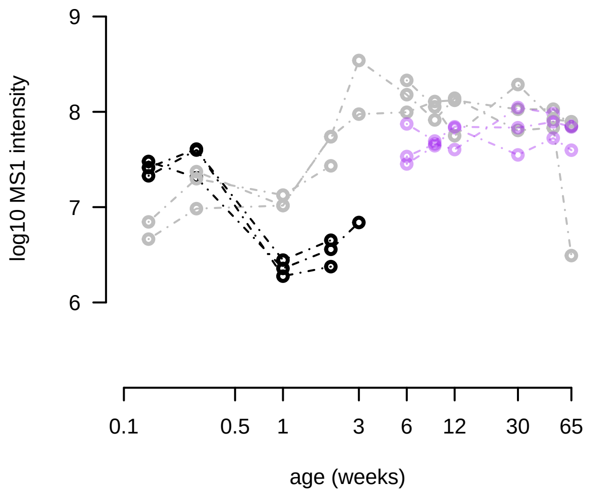

H1/2

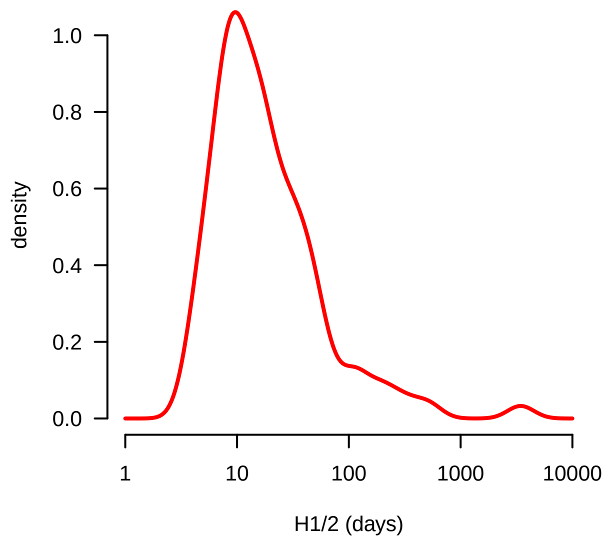

short pulse: ratio ln(L/H+1) log scale

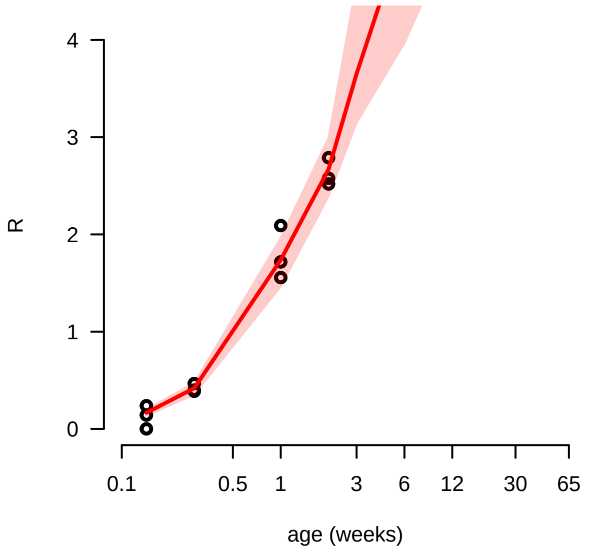

short pulse: ratio ln(L/H+1)

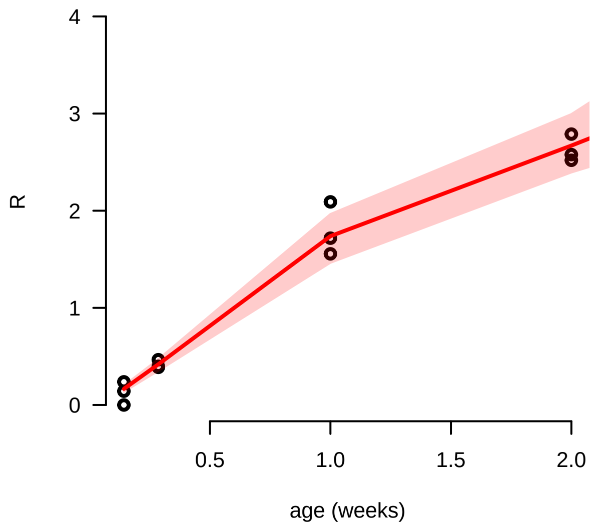

protein concentration log scale

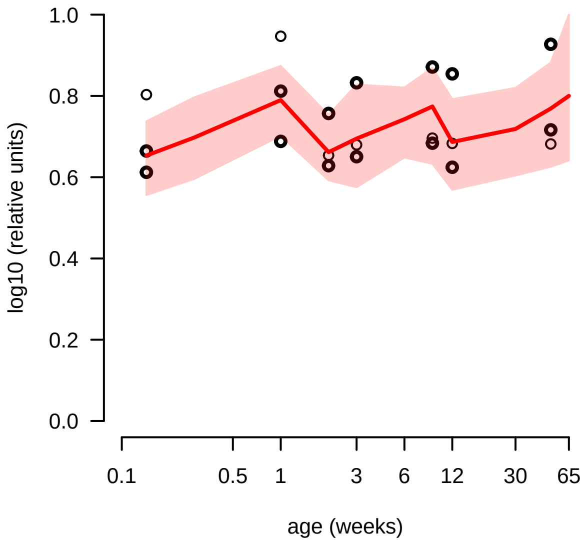

total protein log scale

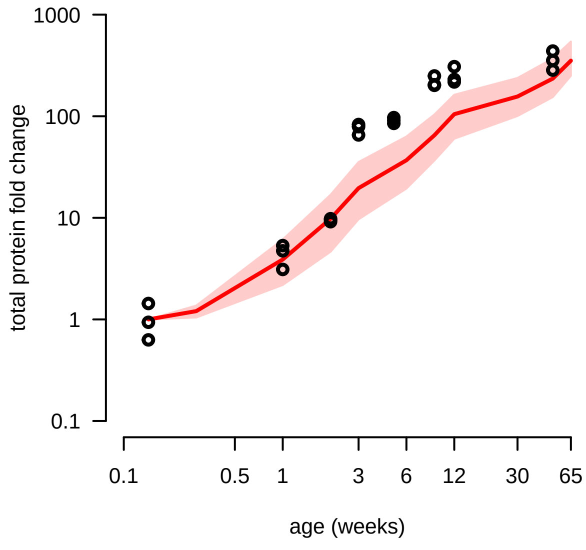

short pulse %H corrected log scale

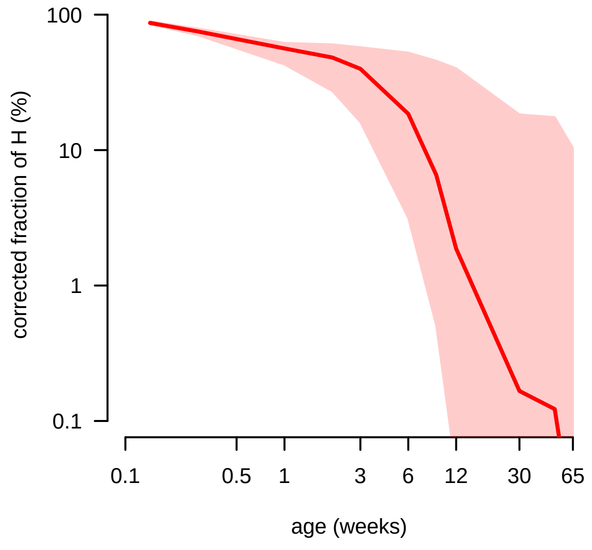

P52431

>sp|P52431|DPOD1_MOUSE

DNA polymerase delta catalytic subunit

GN=Pold1

Med H1/2: 5.5 days

Q5 H1/2: 2.9 days

Q95 H1/2: 17.7 days

reliable model fit: yes

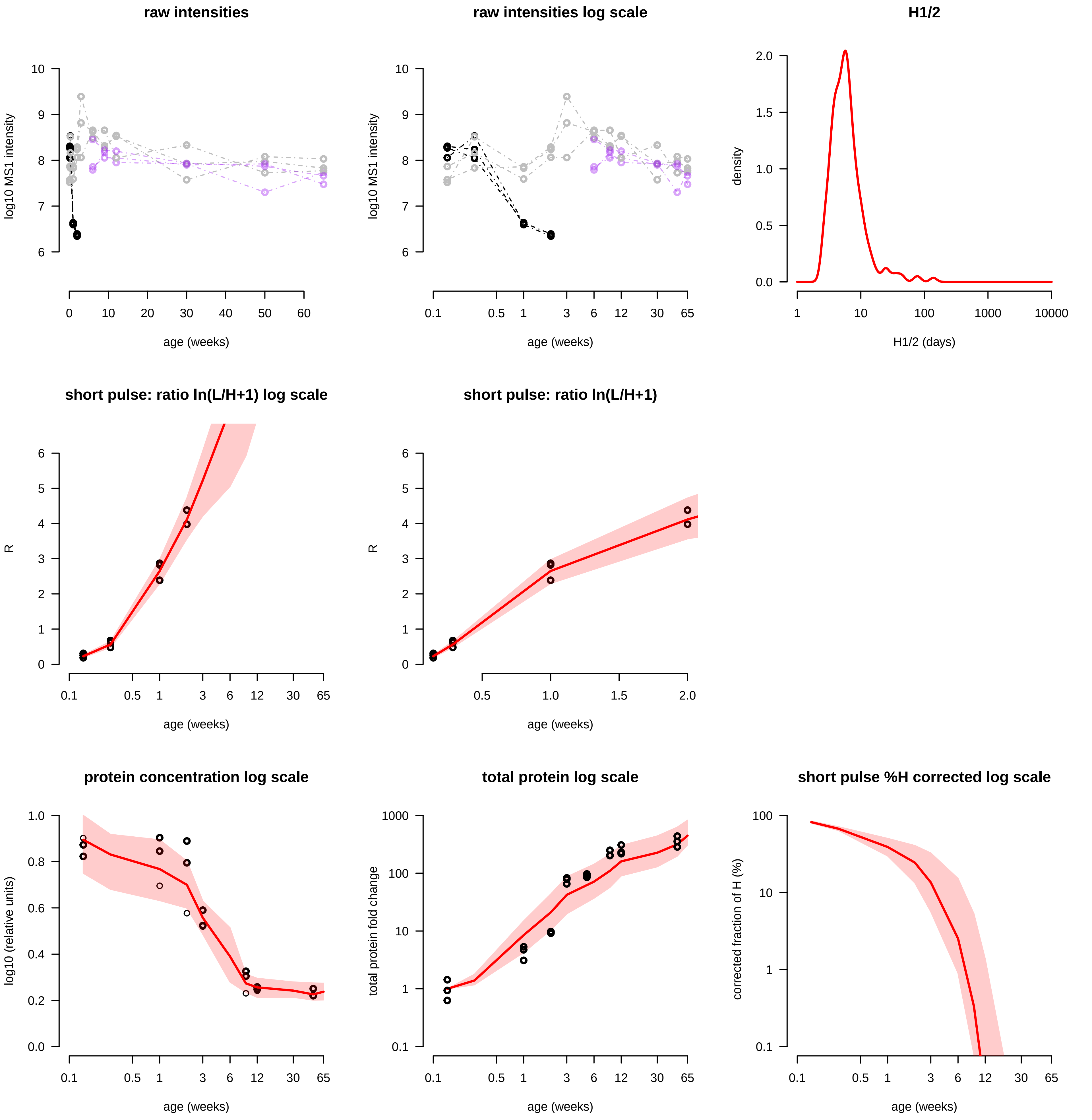

Q8C6G8

>sp|Q8C6G8|WDR26_MOUSE

WD repeat−containing protein 26

GN=Wdr26

Med H1/2: 5.9 days

Q5 H1/2: 2.6 days

Q95 H1/2: 49.3 days

reliable model fit: yes

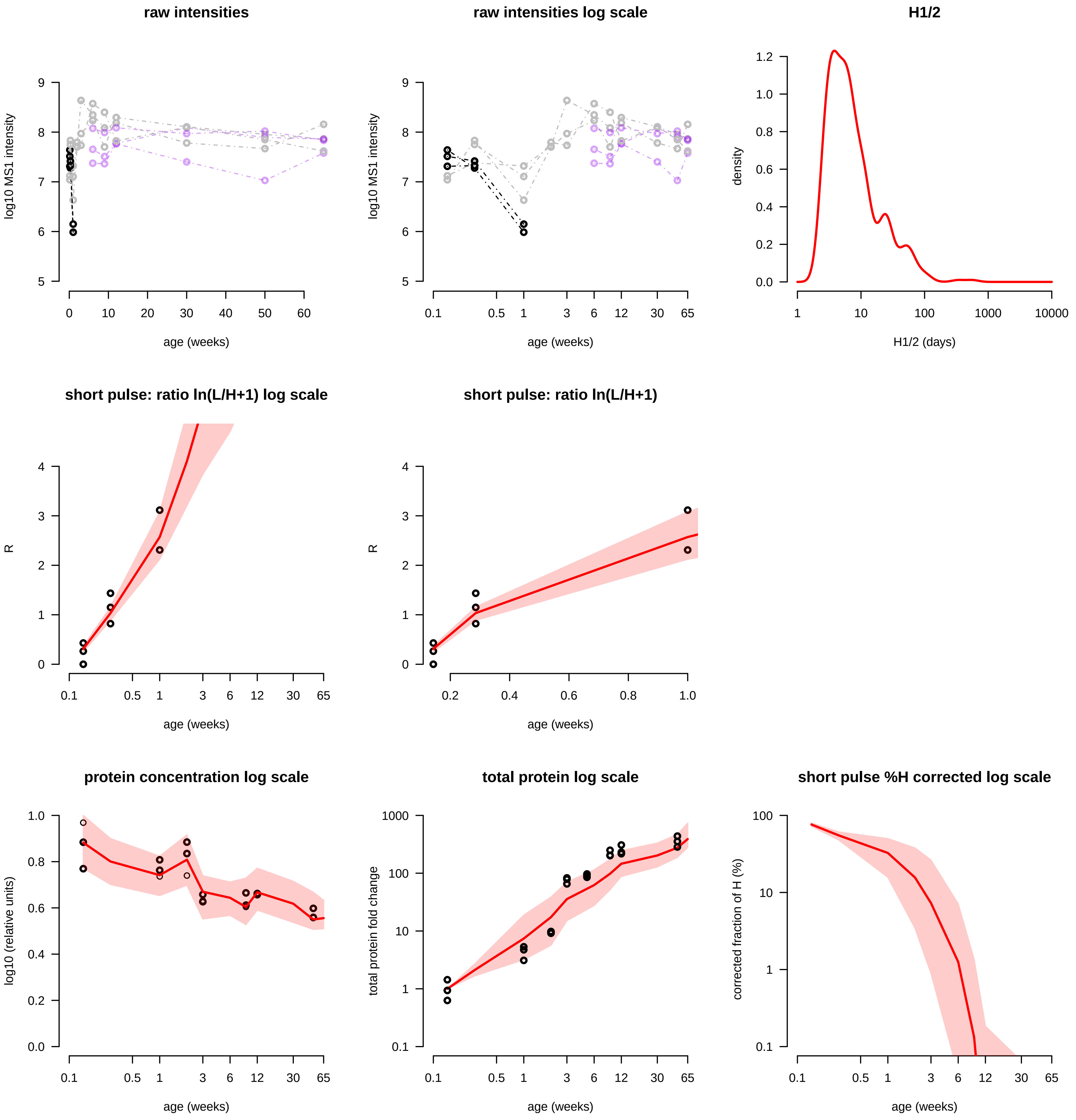

Q03963

>sp|Q03963|E2AK2_MOUSE

Interferon−induced, double−stranded RNA−activated protein kinase

GN=Eif2ak2

Med H1/2: 7.7 days

Q5 H1/2: 3.1 days

Q95 H1/2: 95.6 days

reliable model fit: yes

raw intensities

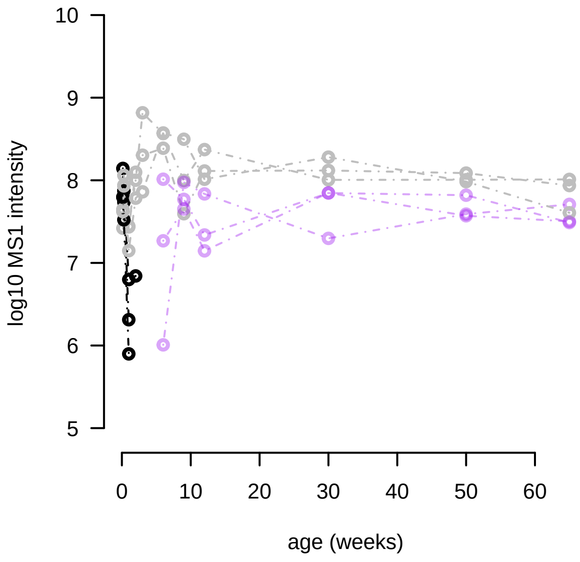

raw intensities log scale

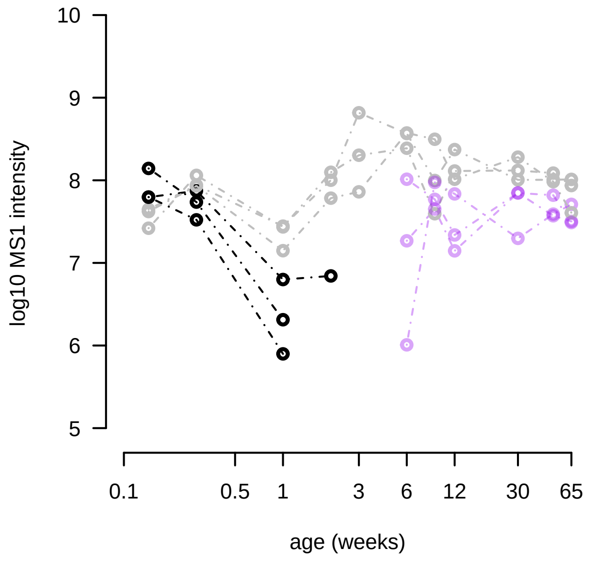

H1/2

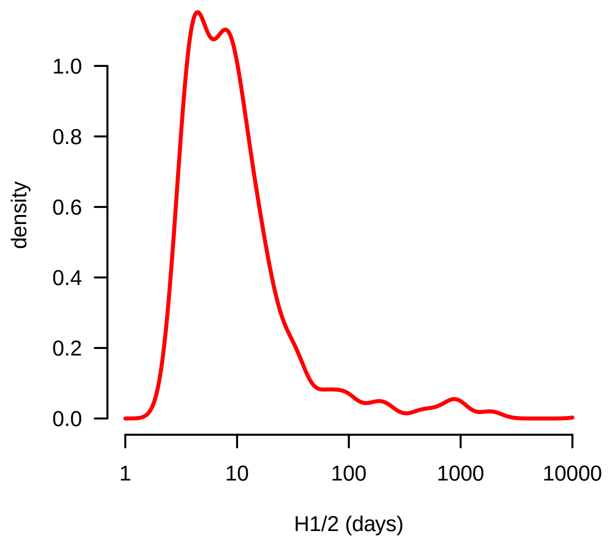

short pulse: ratio ln(L/H+1) log scale

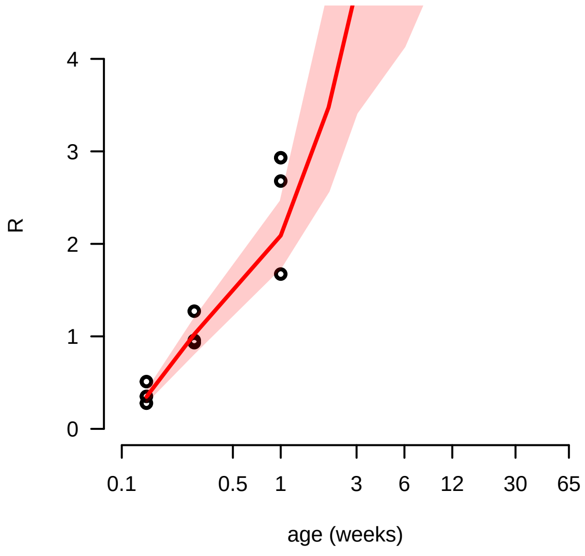

short pulse: ratio ln(L/H+1)

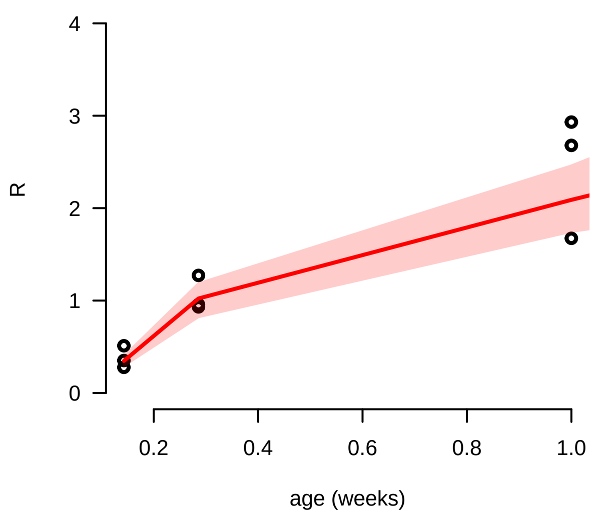

protein concentration log scale

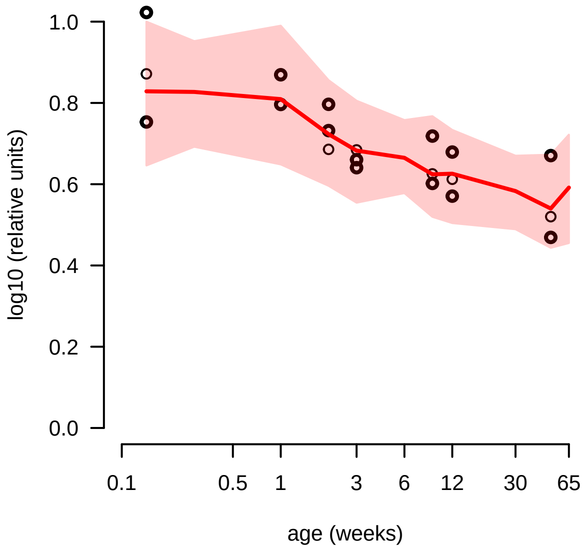

total protein log scale

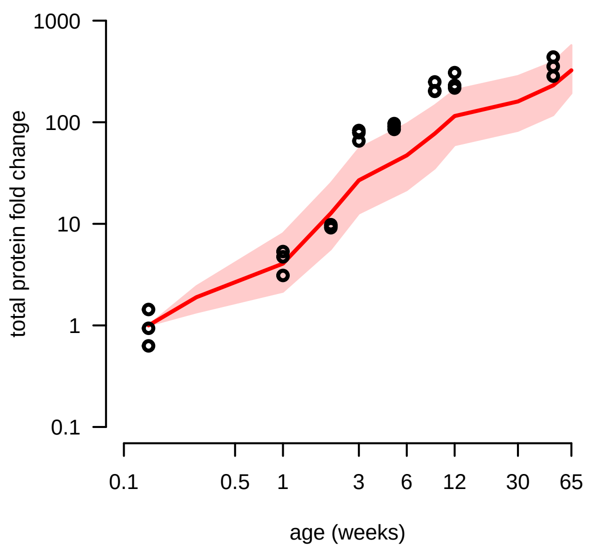

short pulse %H corrected log scale

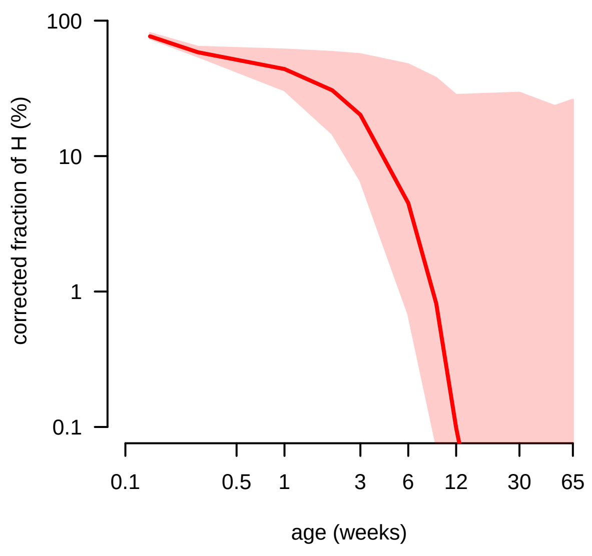

Q9CZH3

>sp|Q9CZH3|PSMG3_MOUSE

Proteasome assembly chaperone 3

GN=Psmg3

Med H1/2: 5.1 days

Q5 H1/2: 4.1 days

Q95 H1/2: 6.8 days

reliable model fit: yes

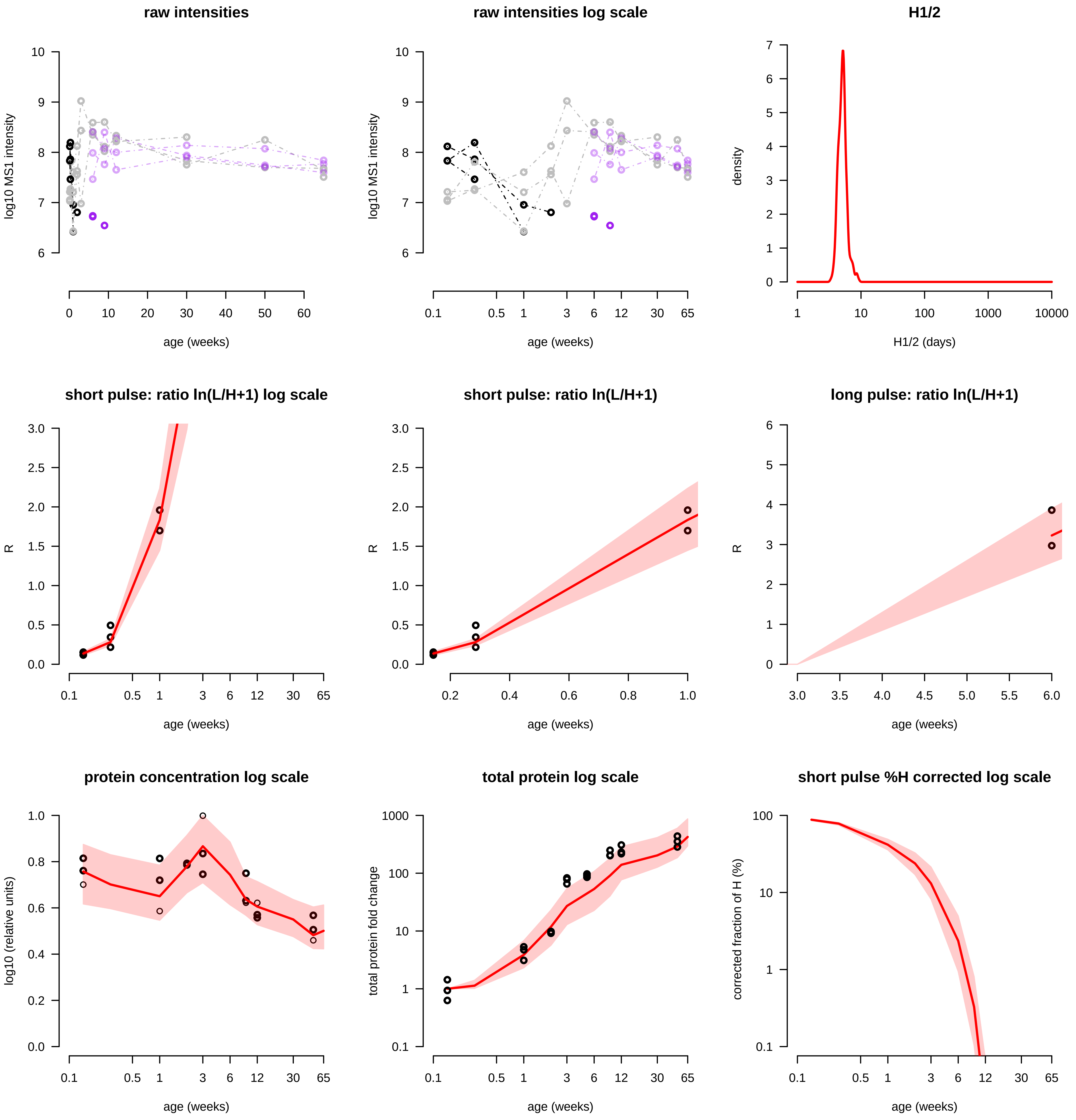

P69566;P69566−2

>sp|P69566|RANB9_MOUSE

Ran−binding protein 9

GN=Ranbp9

Med H1/2: 9.1 days

Q5 H1/2: 4.3 days

Q95 H1/2: 116.1 days

reliable model fit: yes

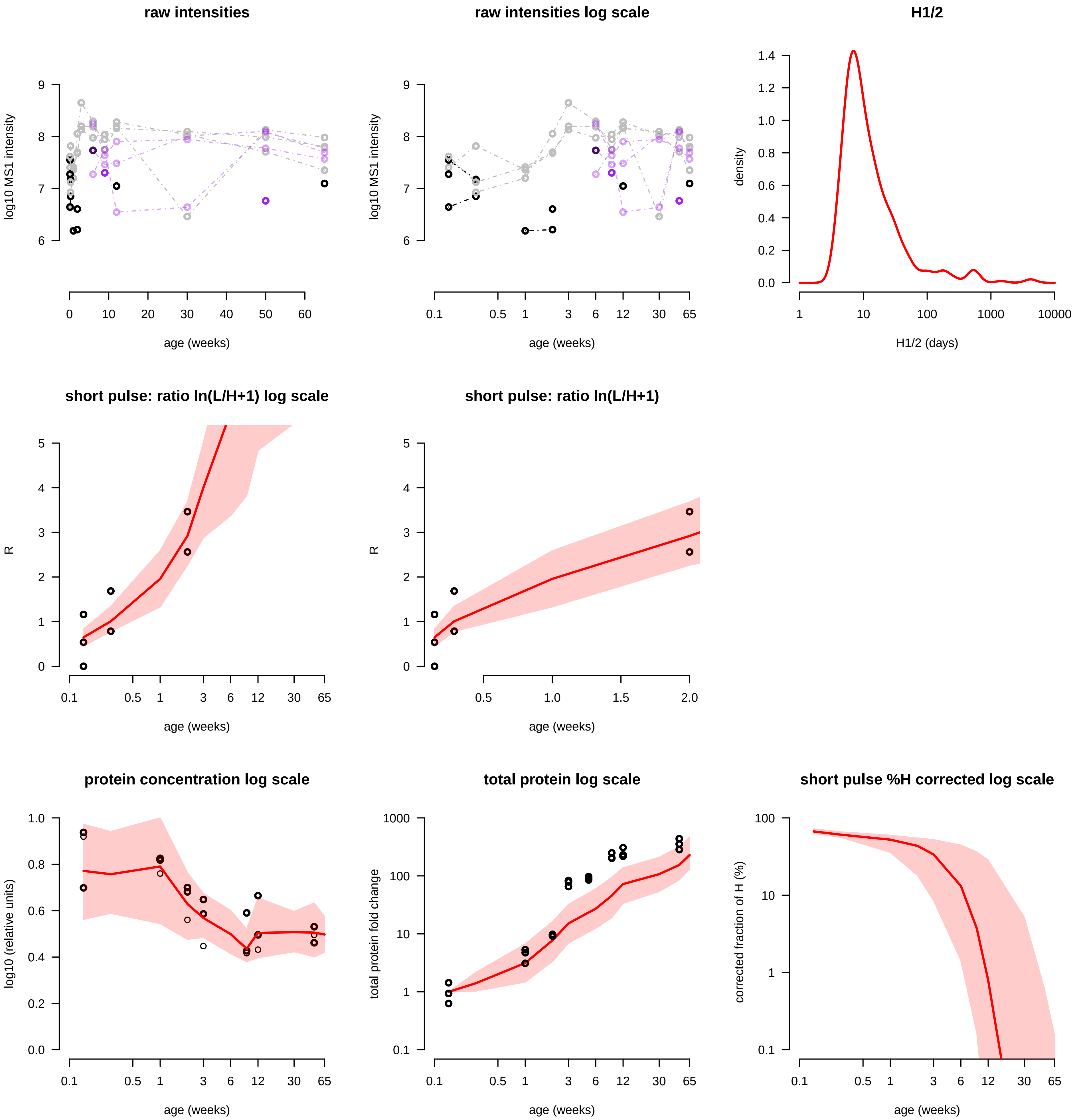

Q9CWS4

>sp|Q9CWS4|INT11_MOUSE

Integrator complex subunit 11

GN=Ints11

Med H1/2: 79.2 days

Q5 H1/2: 23.2 days

Q95 H1/2: 990.4 days

reliable model fit: no

raw intensities

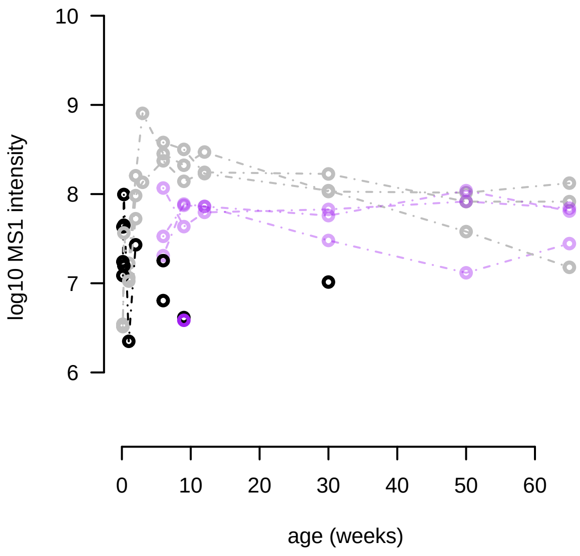

raw intensities log scale

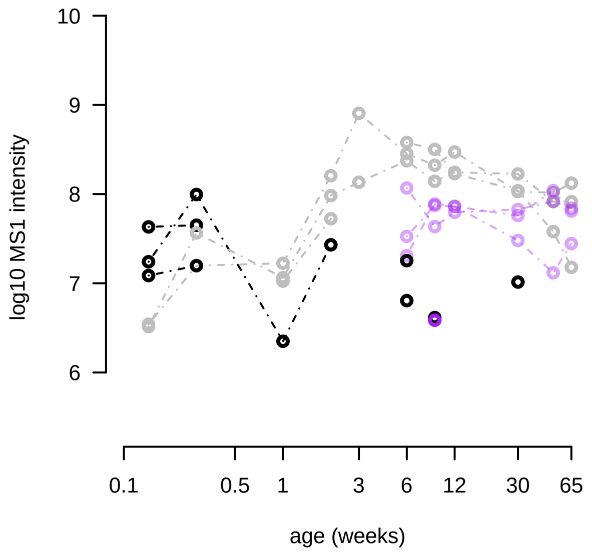

H1/2

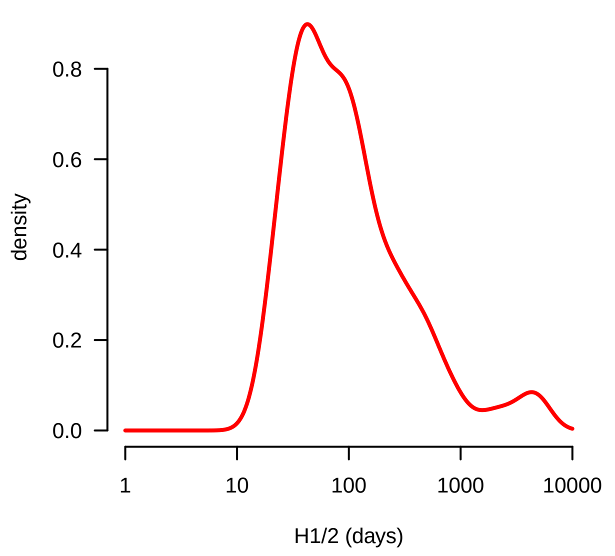

short pulse: ratio ln(L/H+1) log scale

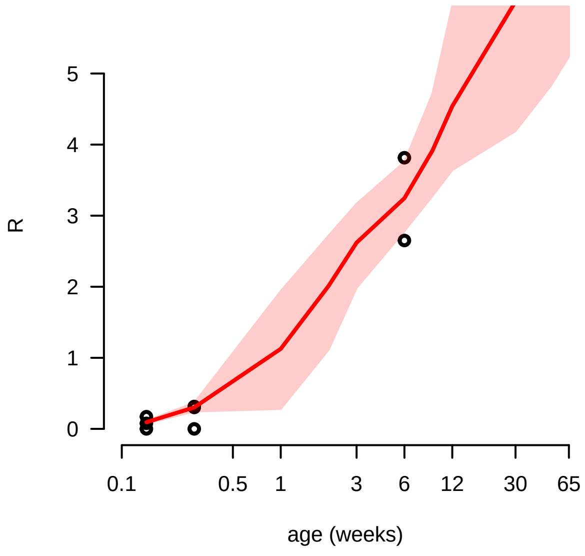

short pulse: ratio ln(L/H+1)

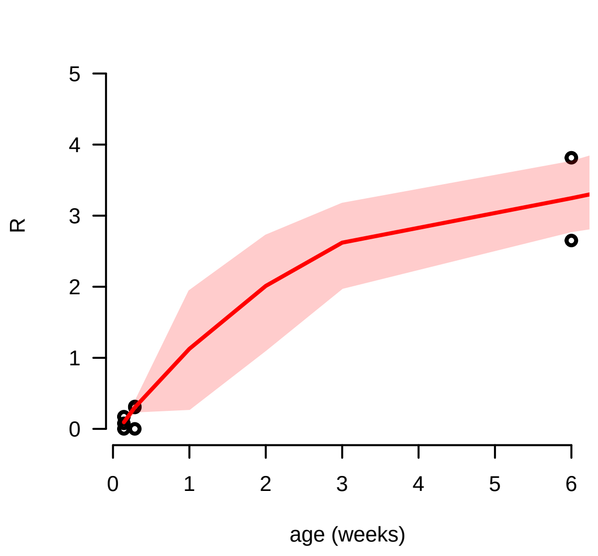

protein concentration log scale

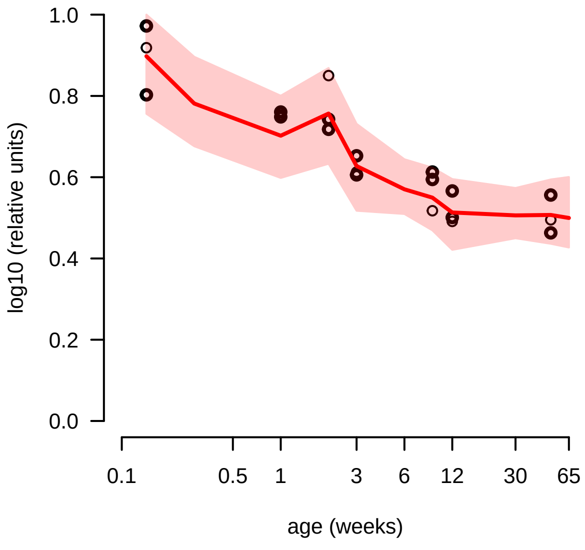

total protein log scale

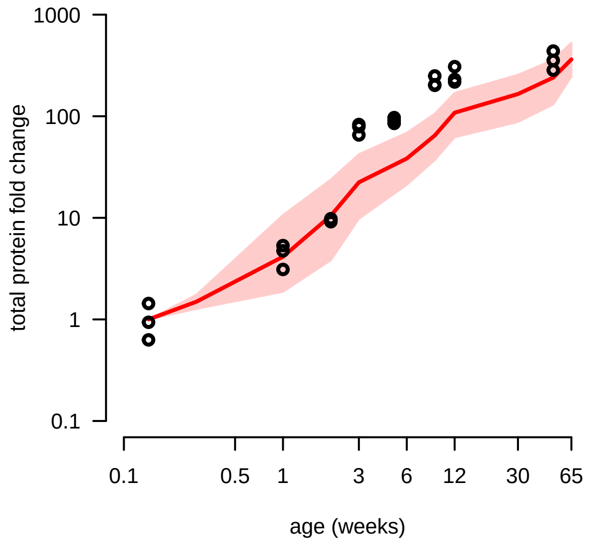

short pulse %H corrected log scale

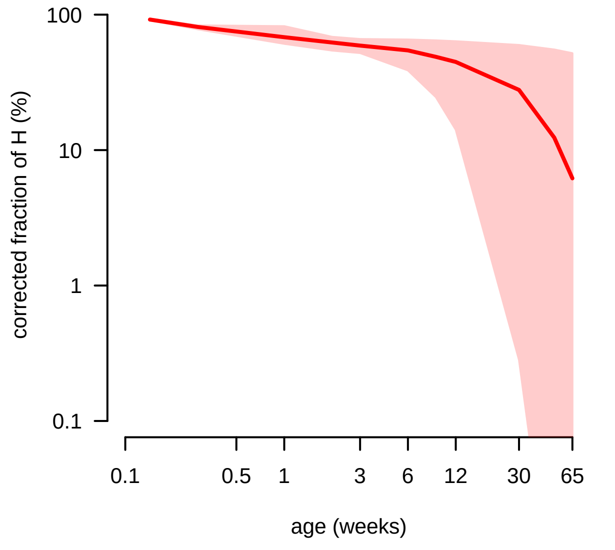

Q91XI1;Q91XI1−2

>sp|Q91XI1|DUS3L_MOUSE

tRNA−dihydrouridine(47) synthase [NAD(P)(+)]−like

GN=Dus3l

Med H1/2: 12.5 days

Q5 H1/2: 3.8 days

Q95 H1/2: 83.5 days

reliable model fit: yes

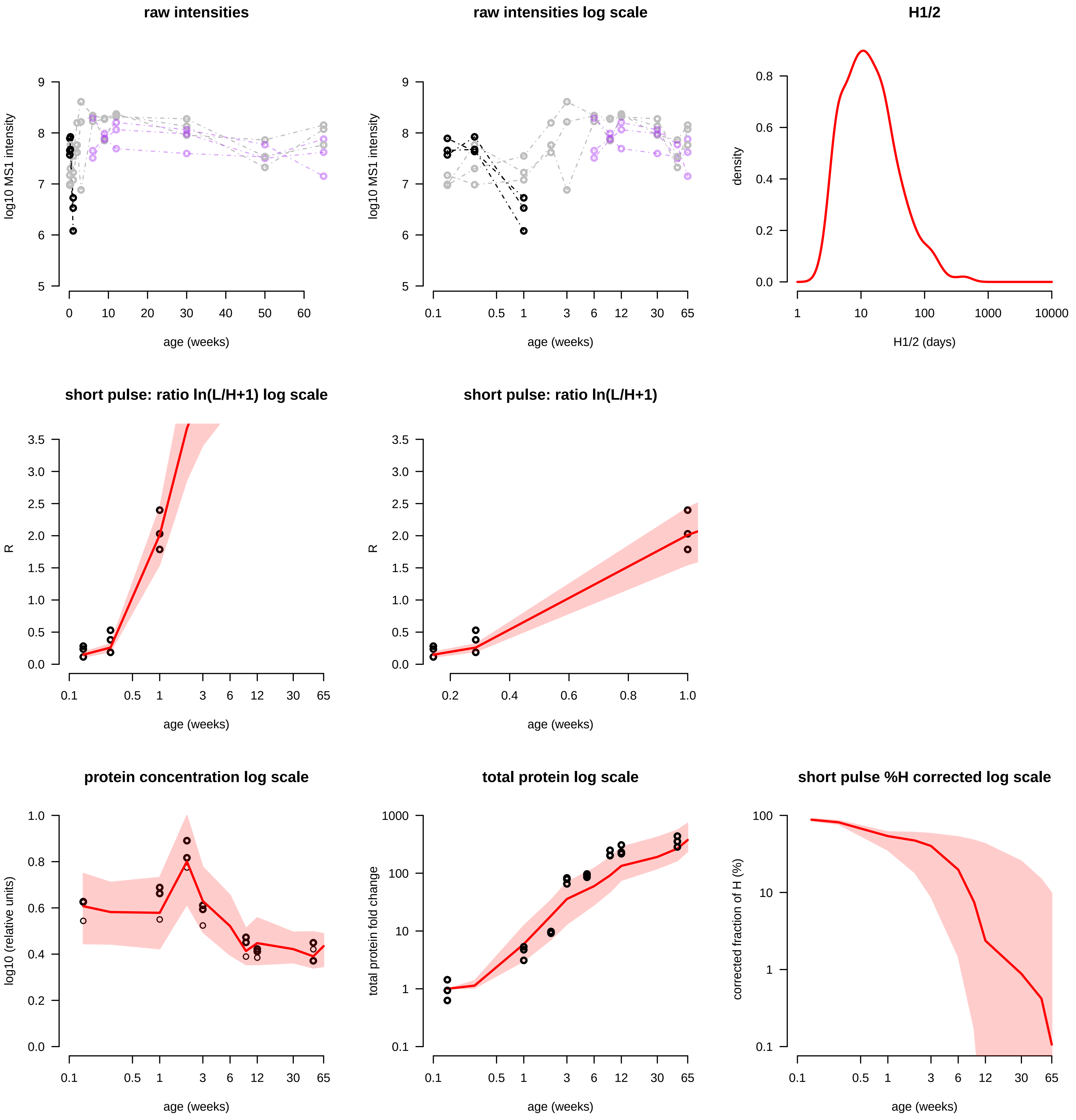

Q8K012−2;Q8K012

>sp|Q8K012−2|FBP1L_MOUSE

Isoform 2 of Formin−binding protein 1−like

GN=Fnbp1l

Med H1/2: 8.8 days

Q5 H1/2: 4.1 days

Q95 H1/2: 47.3 days

reliable model fit: yes

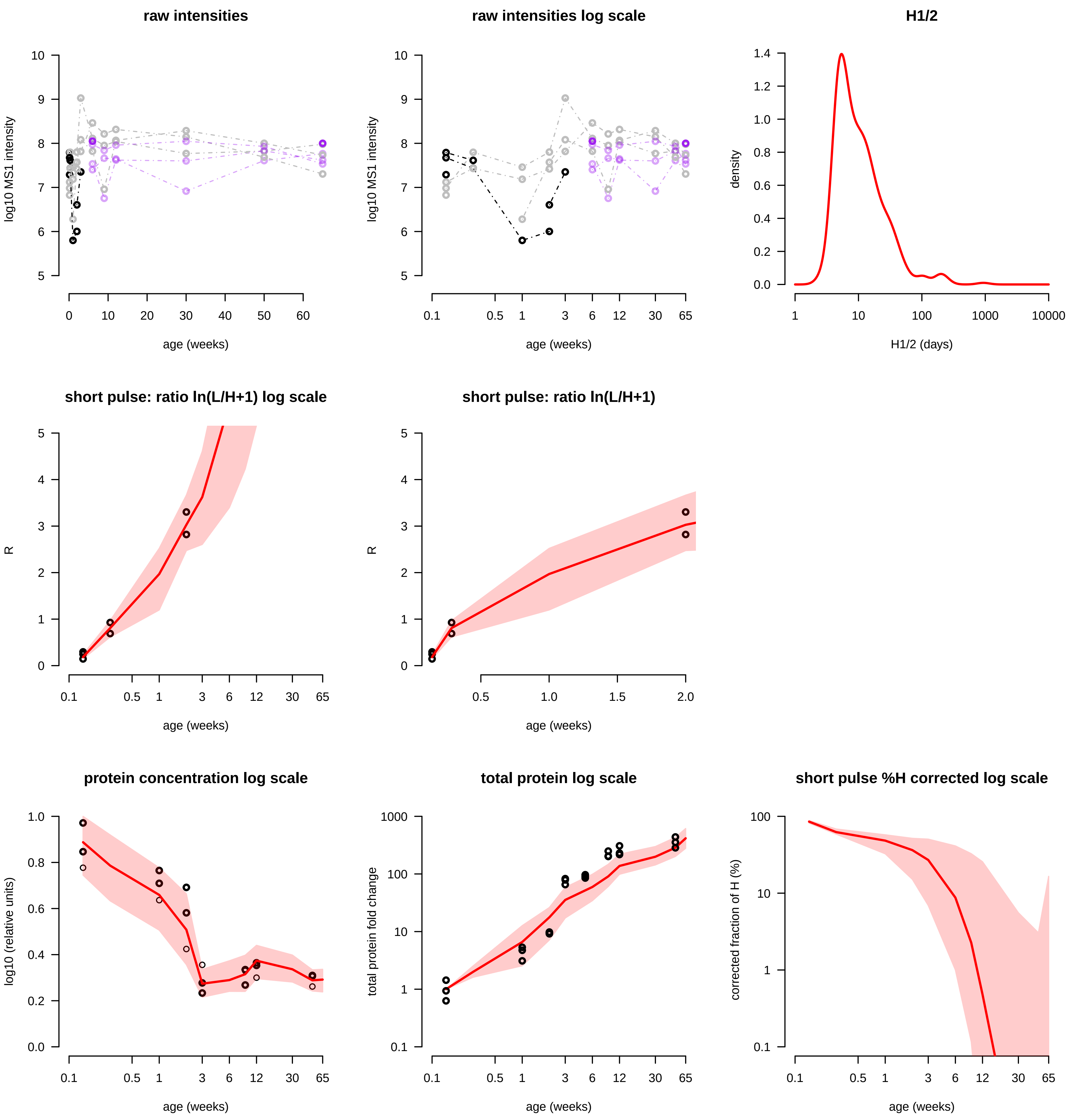

P36916;P36916−2

>sp|P36916|GNL1_MOUSE

Guanine nucleotide−binding protein−like 1

GN=Gnl1

Med H1/2: 7 days

Q5 H1/2: 2.5 days

Q95 H1/2: 119.6 days

reliable model fit: yes

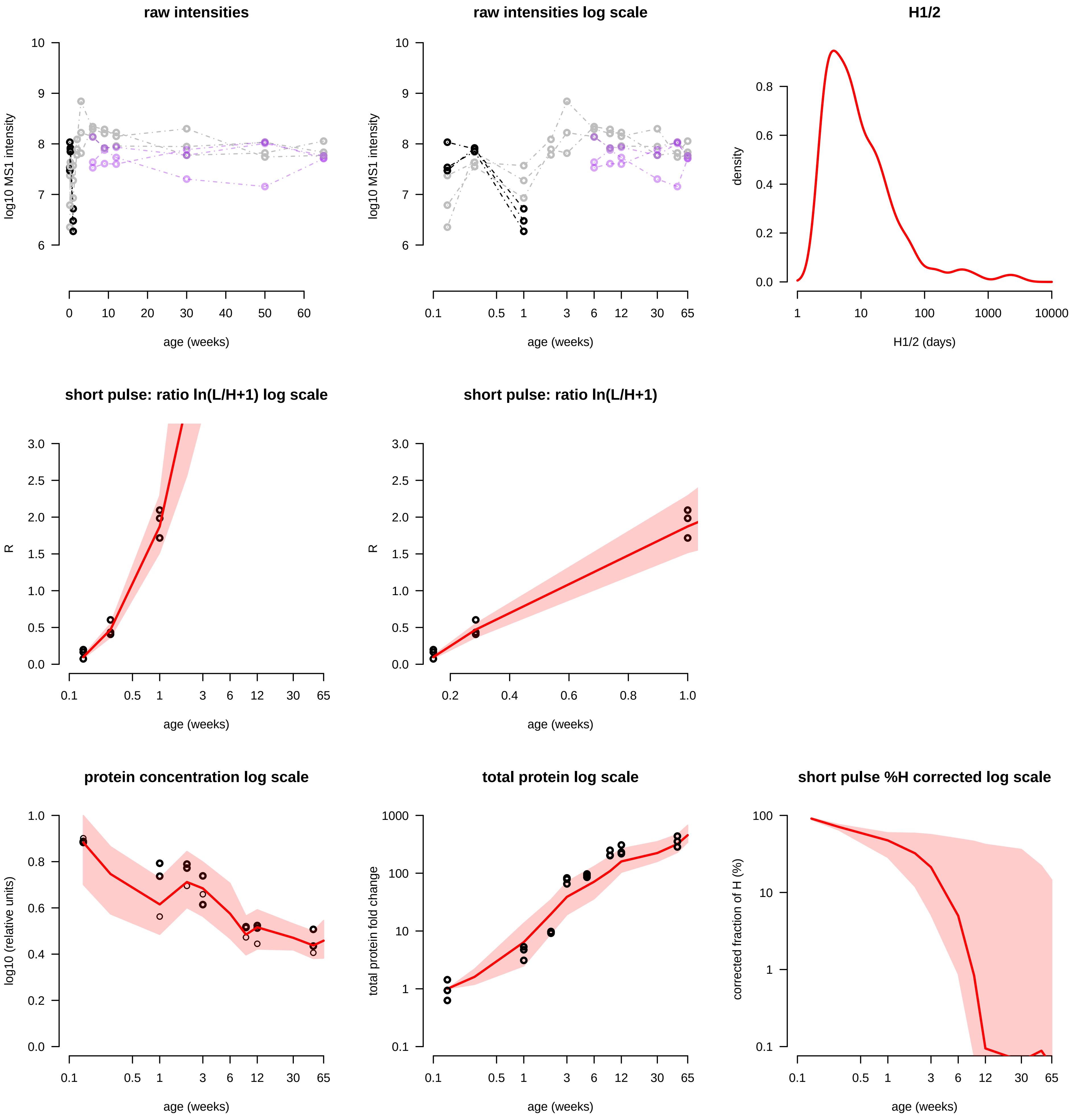

Q9QYH6

>sp|Q9QYH6|MAGD1_MOUSE

Melanoma−associated antigen D1

GN=Maged1

Med H1/2: 4.4 days

Q5 H1/2: 2.6 days

Q95 H1/2: 36.8 days

reliable model fit: yes

raw intensities

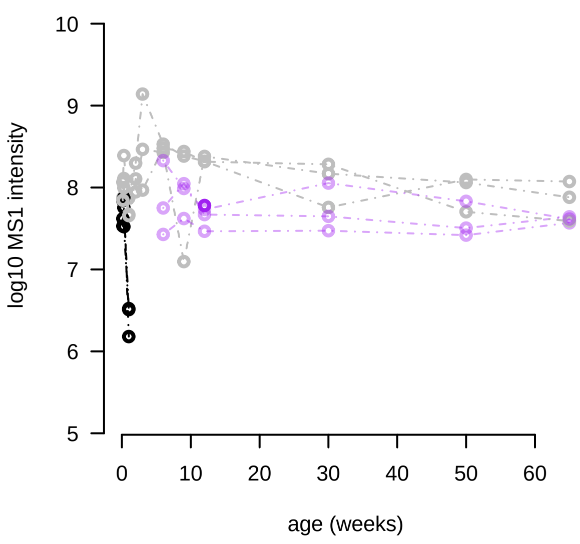

raw intensities log scale

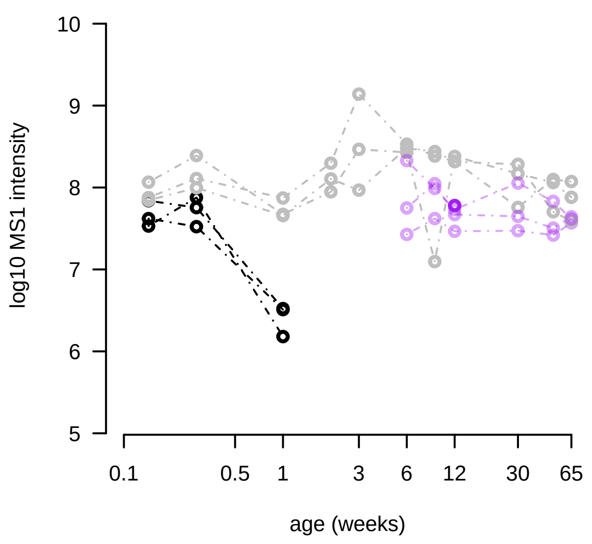

H1/2

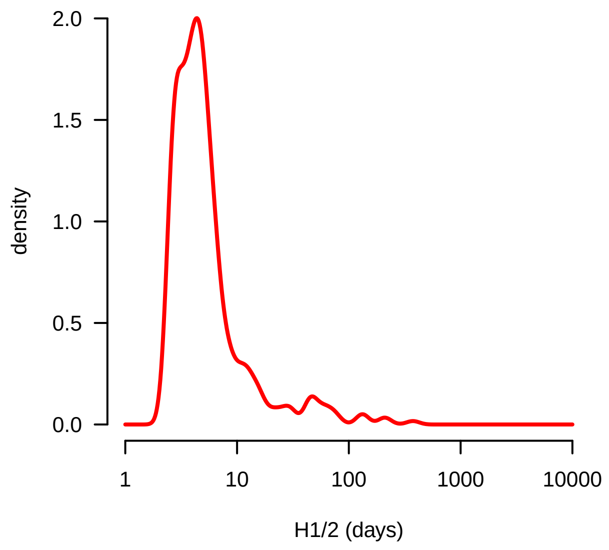

short pulse: ratio ln(L/H+1) log scale

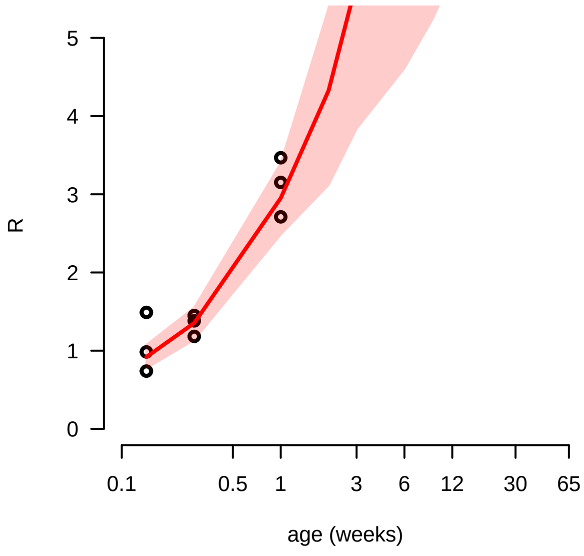

short pulse: ratio ln(L/H+1)

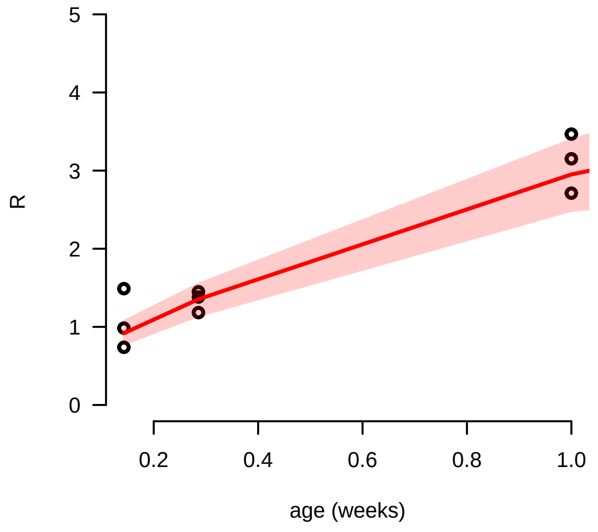

protein concentration log scale

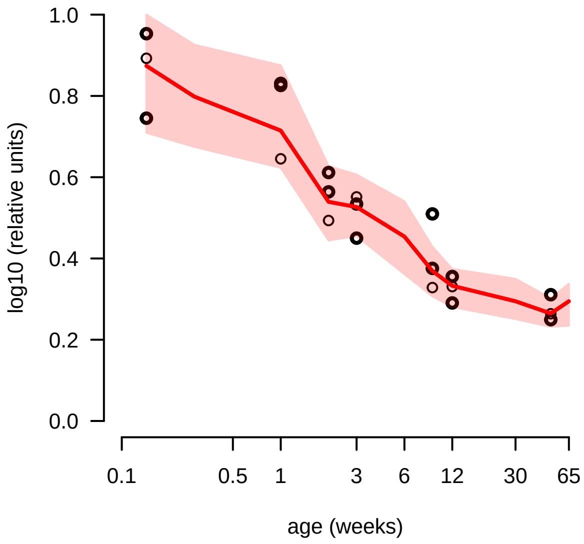

total protein log scale

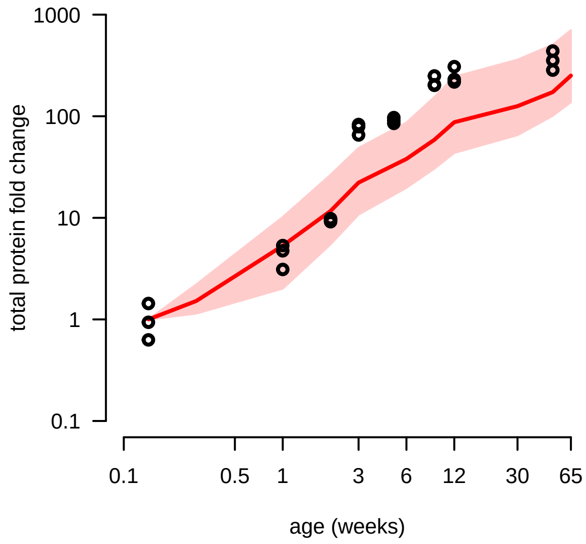

short pulse %H corrected log scale

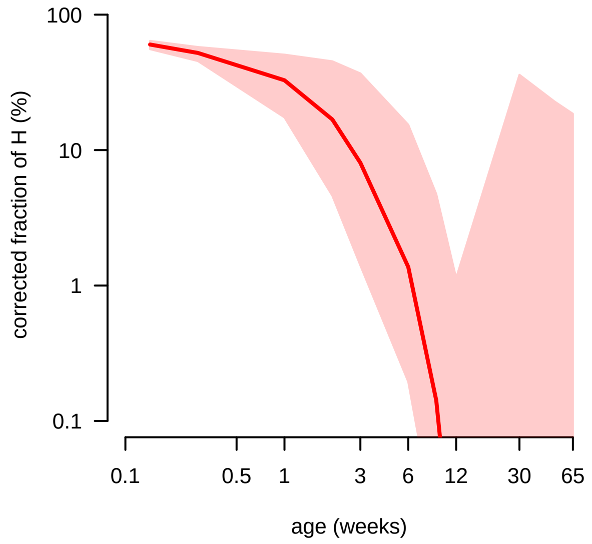

Q8BVF2

>sp|Q8BVF2|PDCL3_MOUSE

Phosducin−like protein 3

GN=Pdcl3

Med H1/2: 70.2 days

Q5 H1/2: 31.4 days

Q95 H1/2: 719 days

reliable model fit: no

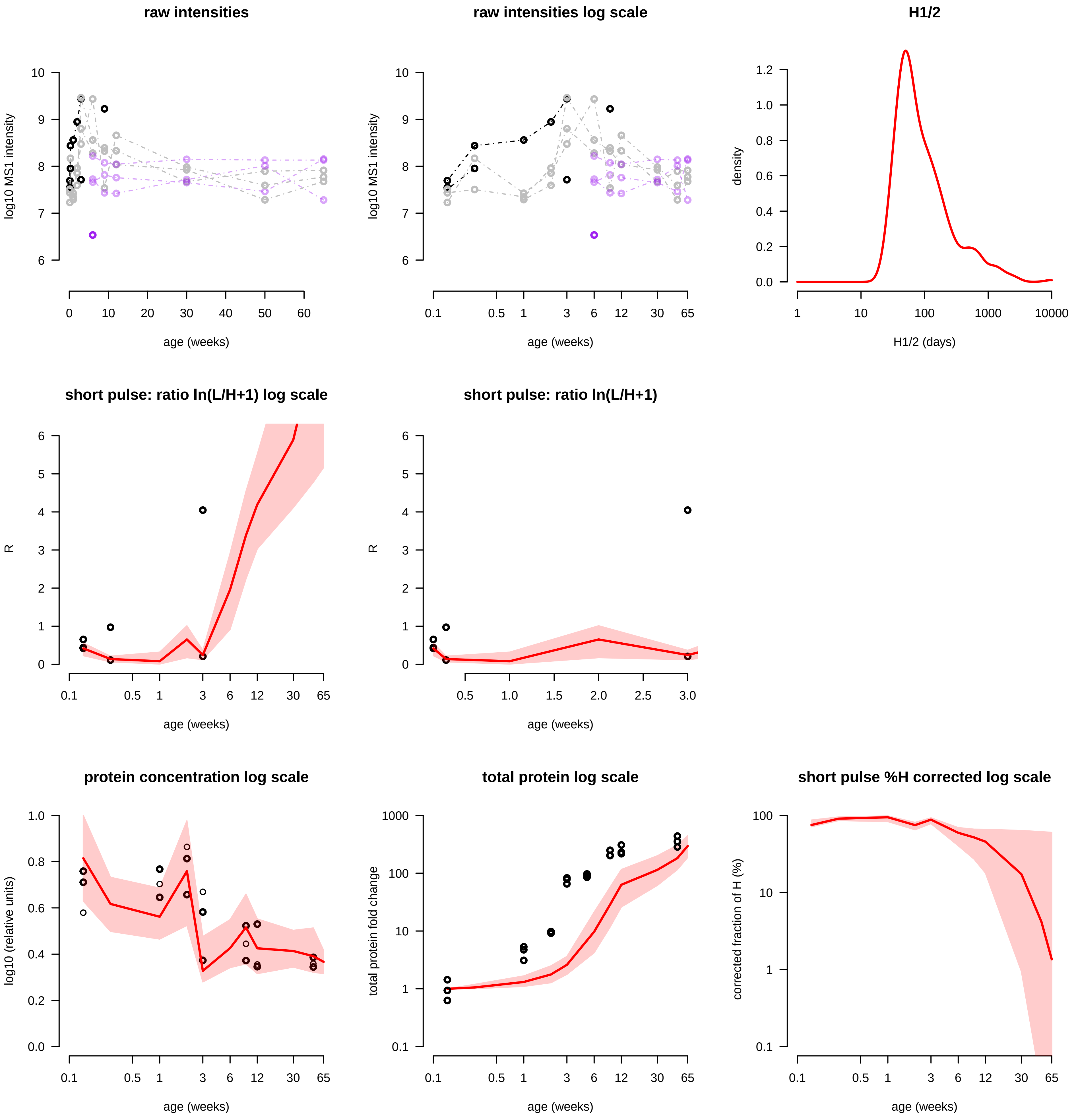

P21844

>sp|P21844|CMA1_MOUSE

Chymase

GN=Cma1

Med H1/2: 43.3 days

Q5 H1/2: 13.2 days

Q95 H1/2: 381.8 days

reliable model fit: yes

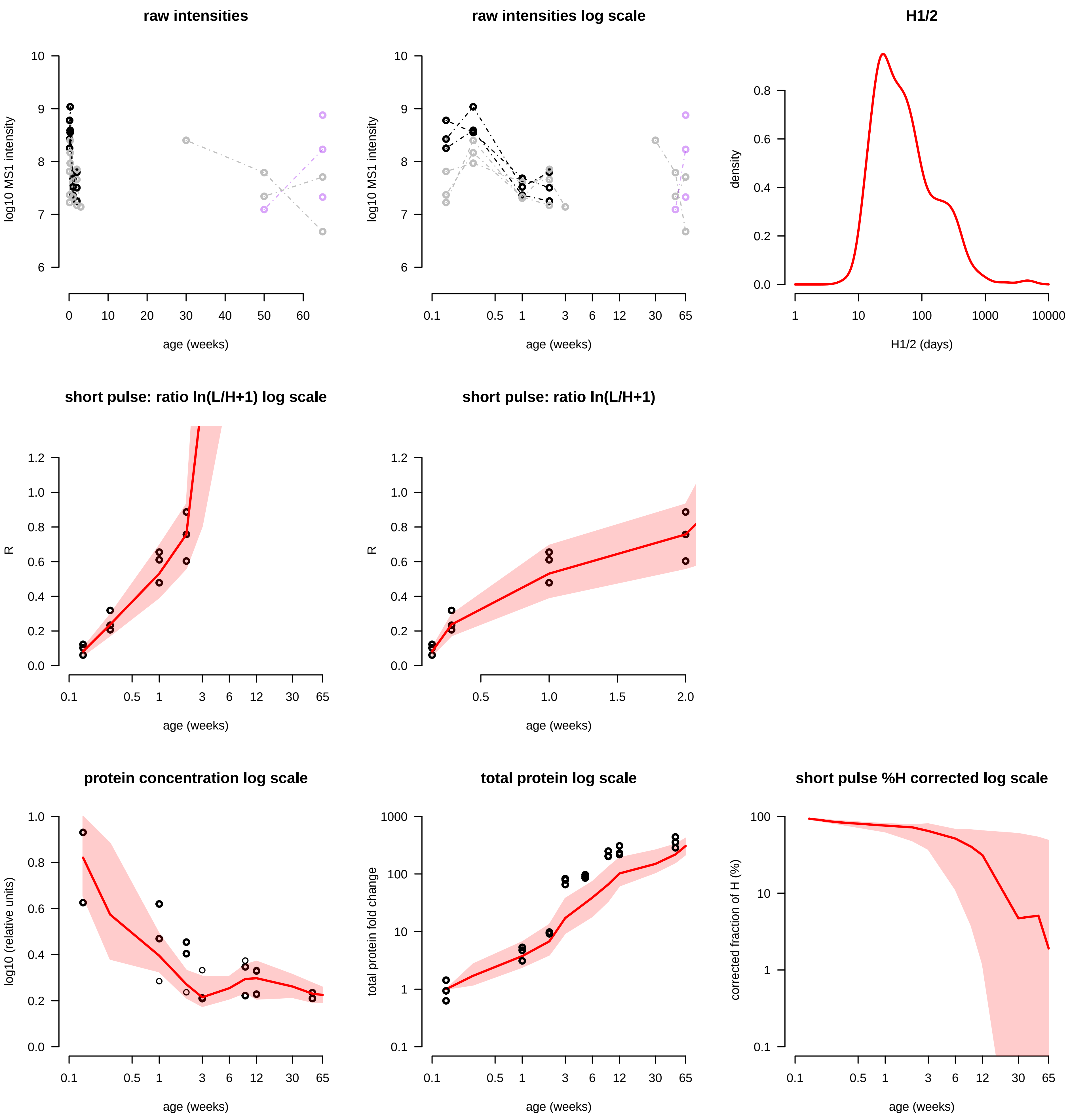

Q9D071;Q9D071−3;Q9D071−2

>sp|Q9D071|MMS19_MOUSE

MMS19 nucleotide excision repair protein homolog

GN=Mms19

Med H1/2: 8.9 days

Q5 H1/2: 3.1 days

Q95 H1/2: 72.2 days

reliable model fit: yes

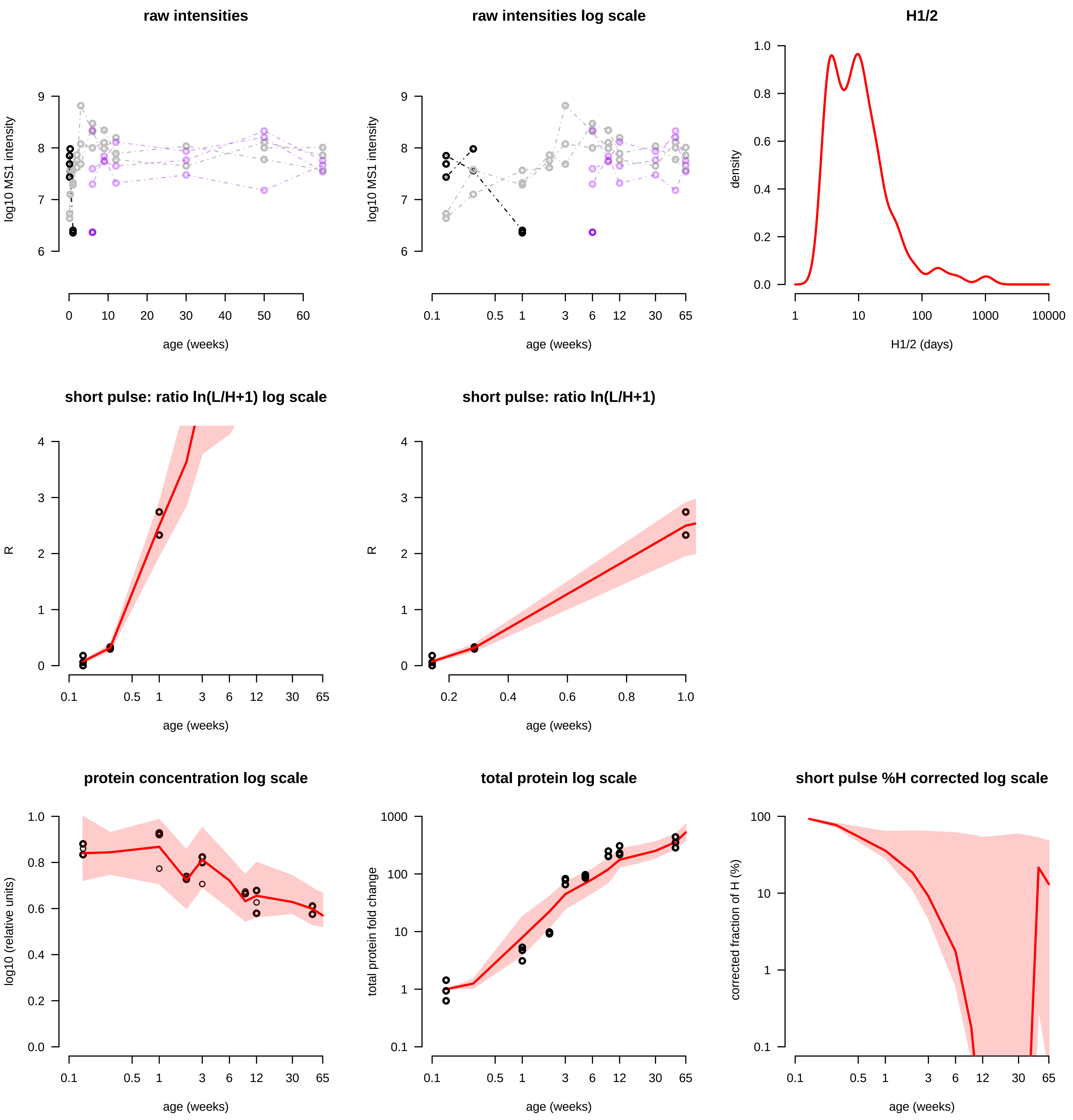

Q9DBZ1−2

>sp|Q9DBZ1−2|IKIP_MOUSE

Isoform 2 of Inhibitor of nuclear factor kappa−B kinase−interacting protein

GN=Ikbip

Med H1/2: 9.8 days

Q5 H1/2: 3.1 days

Q95 H1/2: 89 days

reliable model fit: yes

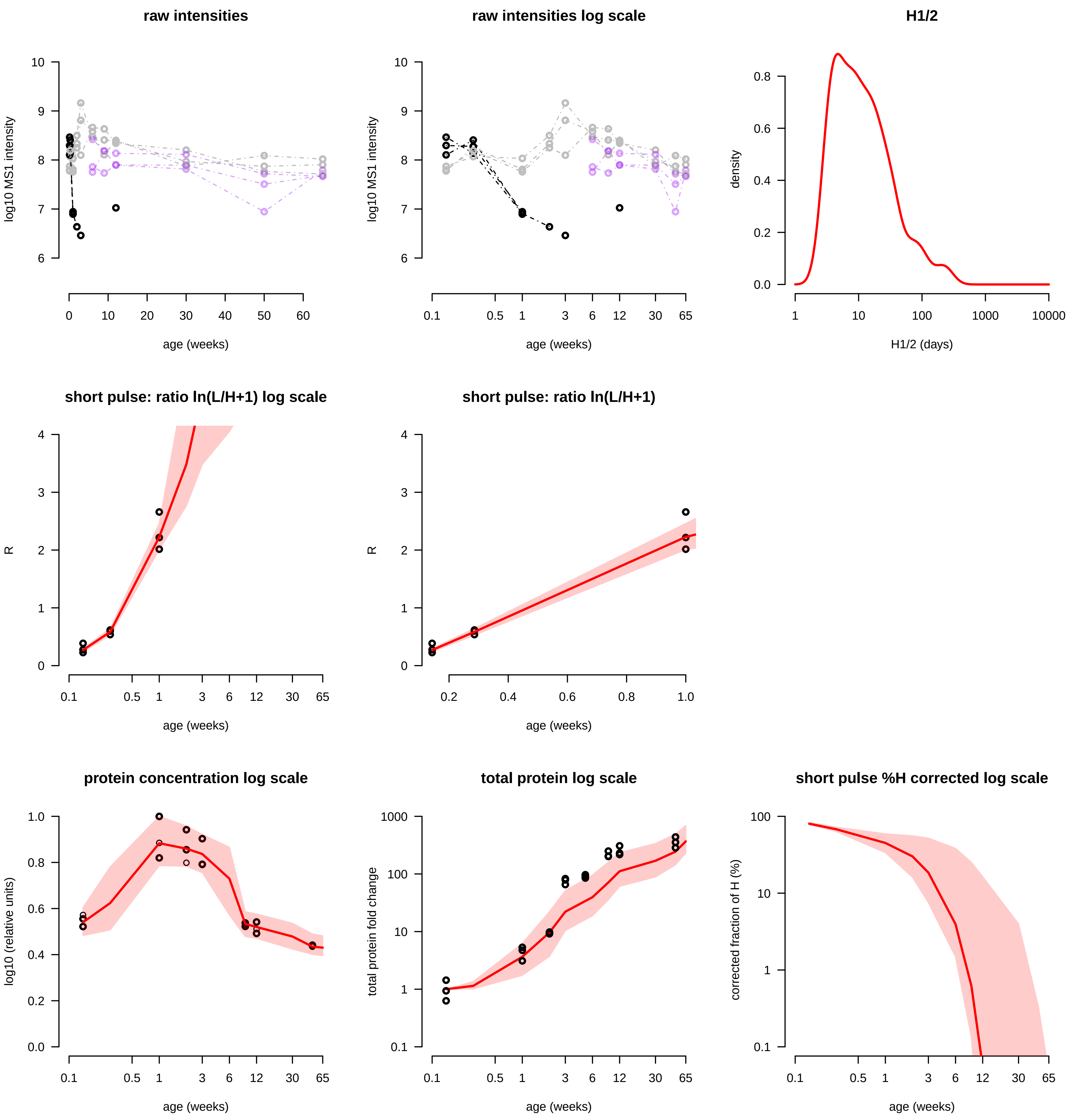

Q640M1

>sp|Q640M1|UT14A_MOUSE

U3 small nucleolar RNA−associated protein 14 homolog A

GN=Utp14a

Med H1/2: 7.1 days

Q5 H1/2: 5.6 days

Q95 H1/2: 11.4 days

reliable model fit: yes

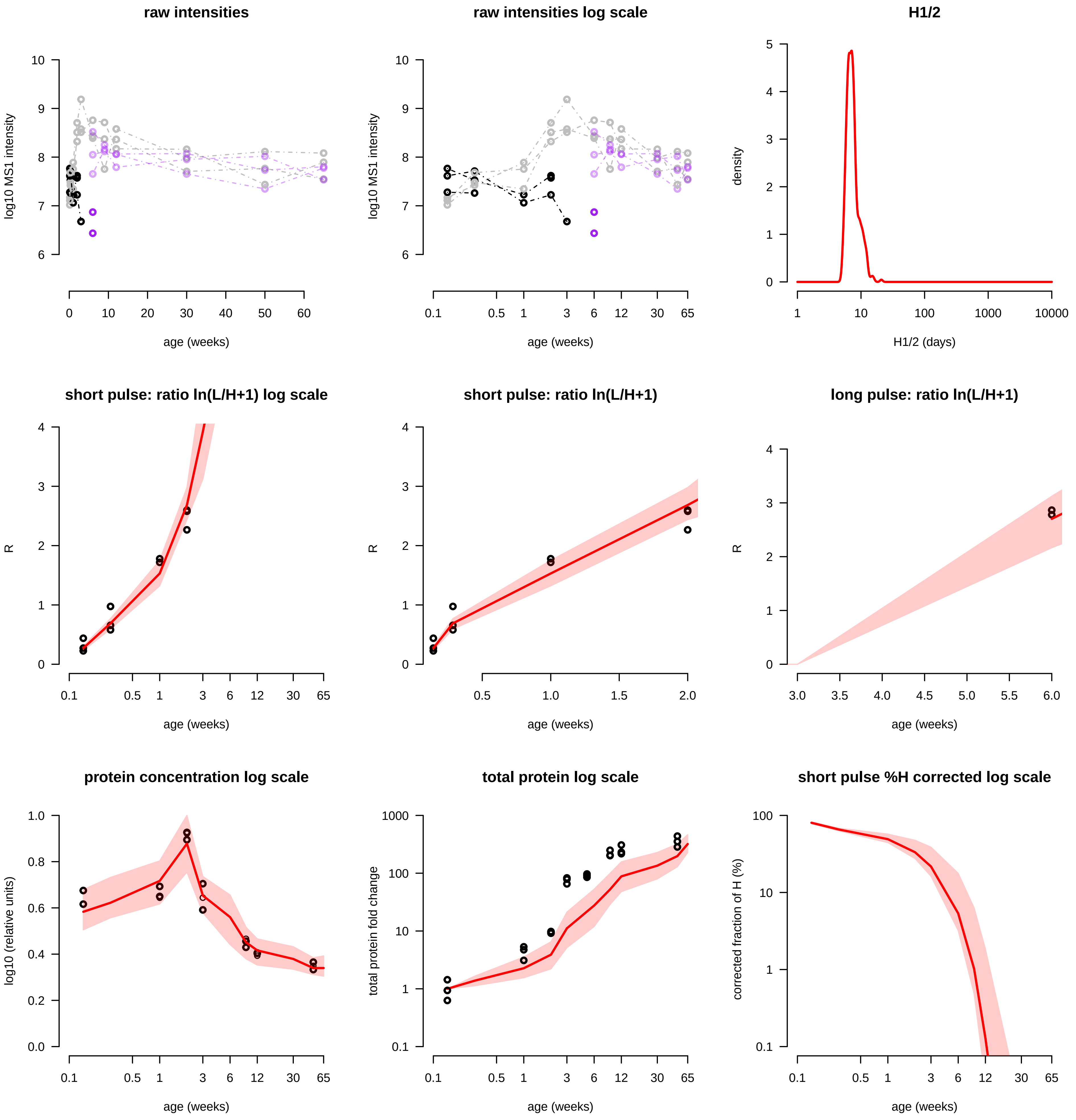

Q8C050−2;Q8C050

>sp|Q8C050−2|KS6A5_MOUSE

Isoform 1 of Ribosomal protein S6 kinase alpha−5

GN=Rps6ka5

Med H1/2: 5.2 days

Q5 H1/2: 2.9 days

Q95 H1/2: 73.7 days

reliable model fit: yes

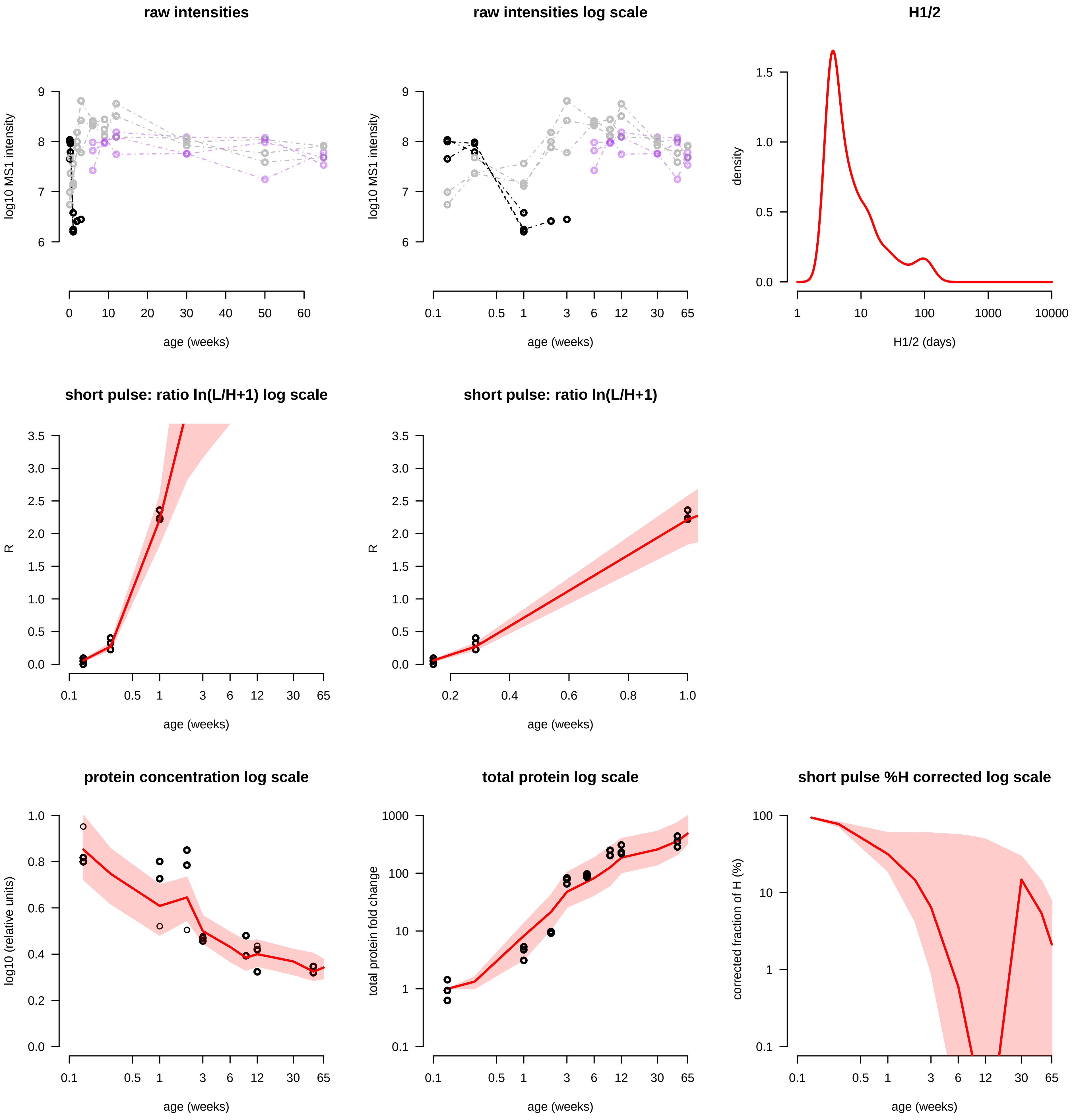

Q69ZW3−2;Q69ZW3

>sp|Q69ZW3−2|EHBP1_MOUSE

Isoform 2 of EH domain−binding protein 1

GN=Ehbp1

Med H1/2: 898.9 days

Q5 H1/2: 259.7 days

Q95 H1/2: 8622.6 days

reliable model fit: yes

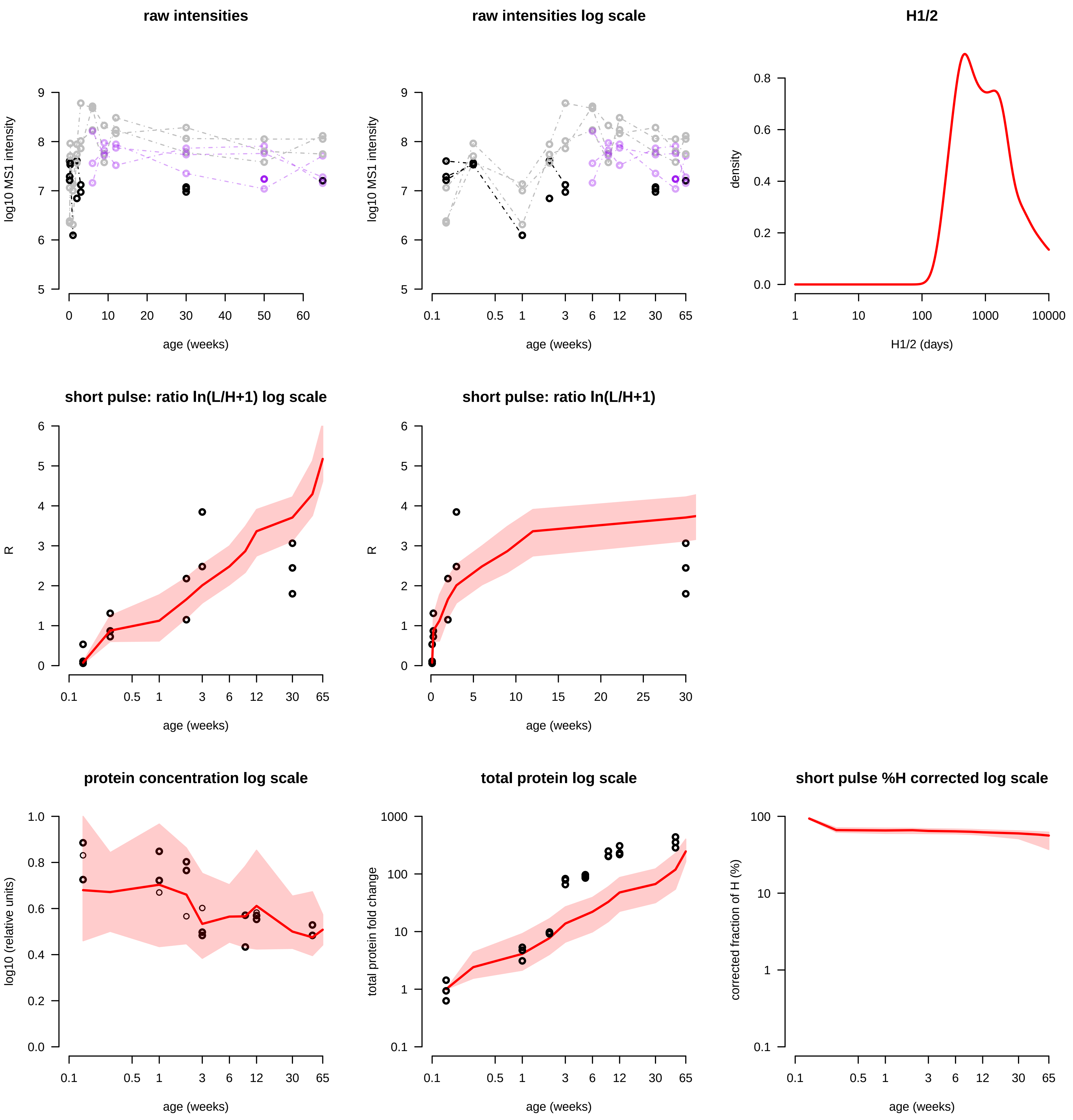

Q80YQ2;Q80YQ2−2

>sp|Q80YQ2|MED23_MOUSE

Mediator of RNA polymerase II transcription subunit 23

GN=Med23

Med H1/2: 4.9 days

Q5 H1/2: 2.9 days

Q95 H1/2: 15.6 days

reliable model fit: yes

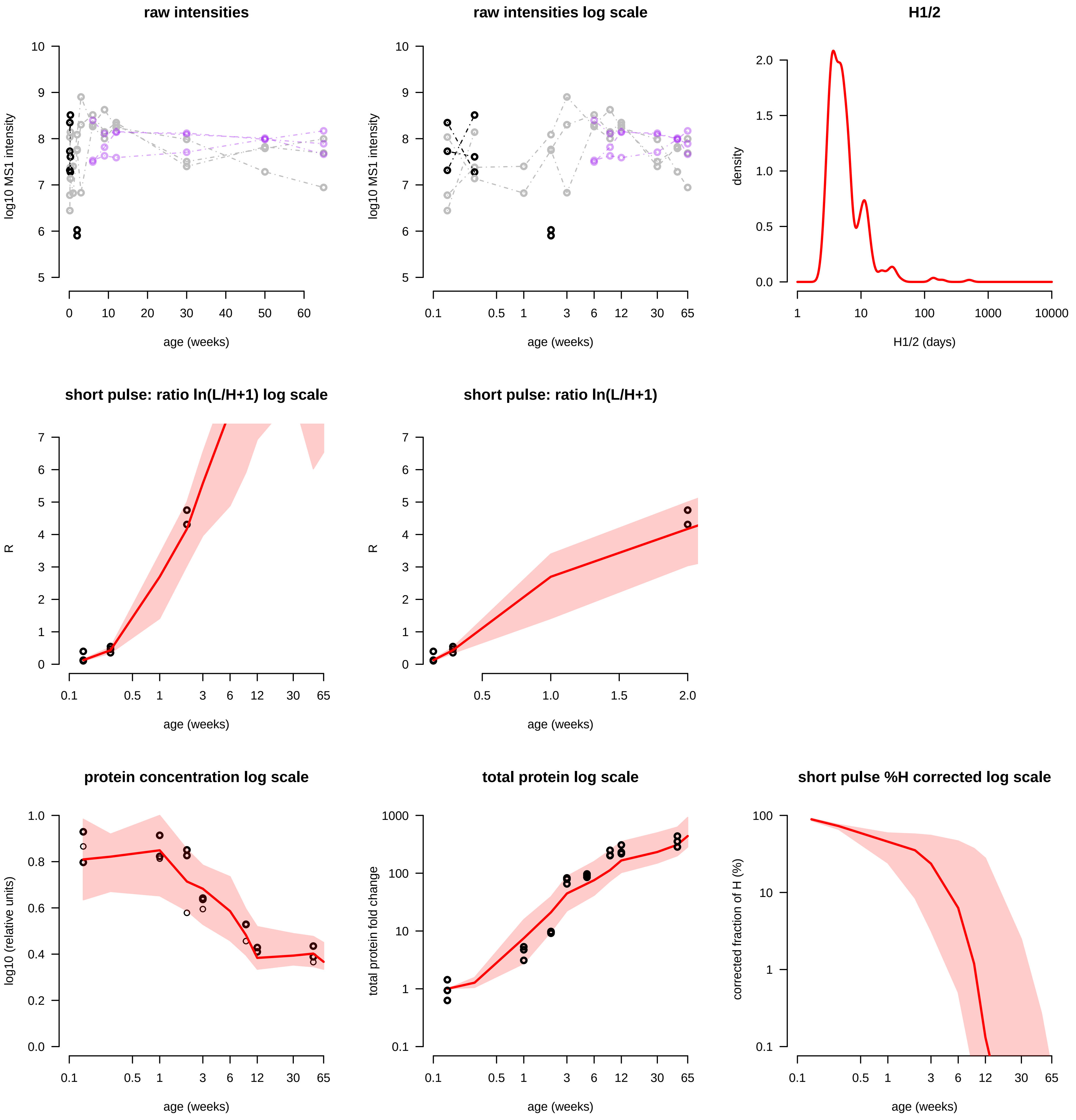

Q8K2X3

>sp|Q8K2X3|STN1_MOUSE

CST complex subunit STN1

GN=Stn1

Med H1/2: 10.2 days

Q5 H1/2: 3.2 days

Q95 H1/2: 59.7 days

reliable model fit: yes

raw intensities

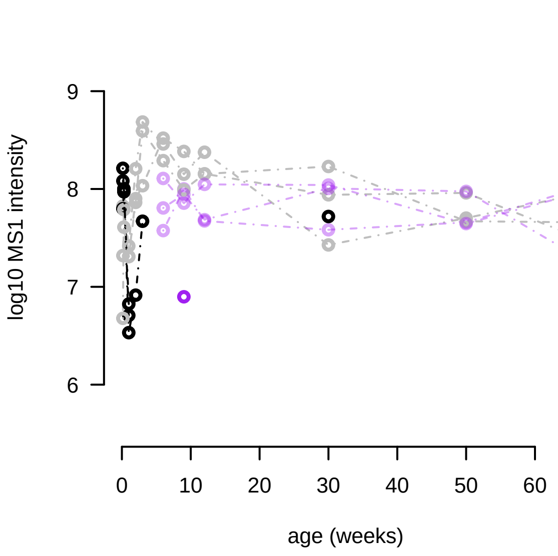

raw intensities log scale

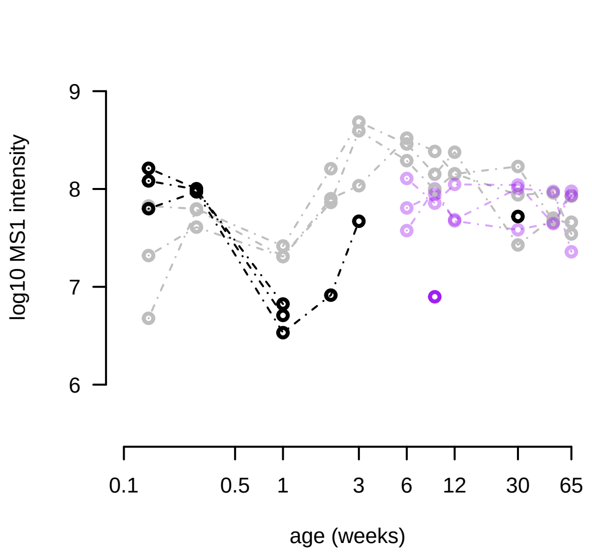

H1/2

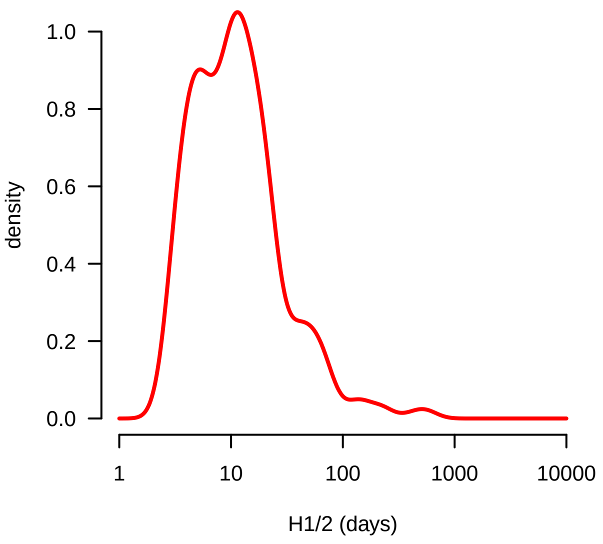

short pulse: ratio ln(L/H+1) log scale

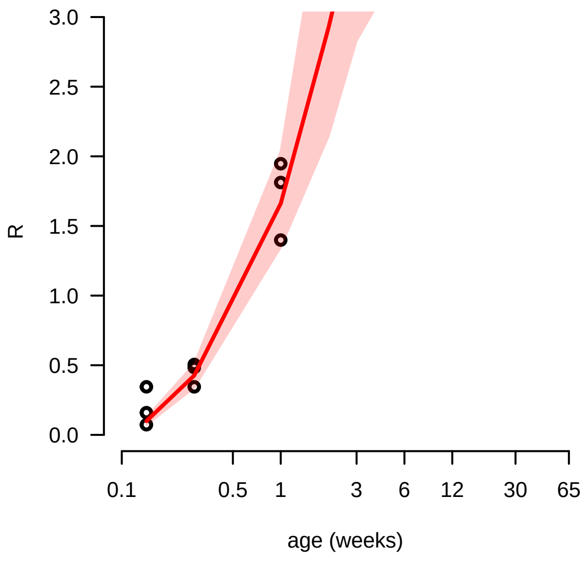

short pulse: ratio ln(L/H+1)

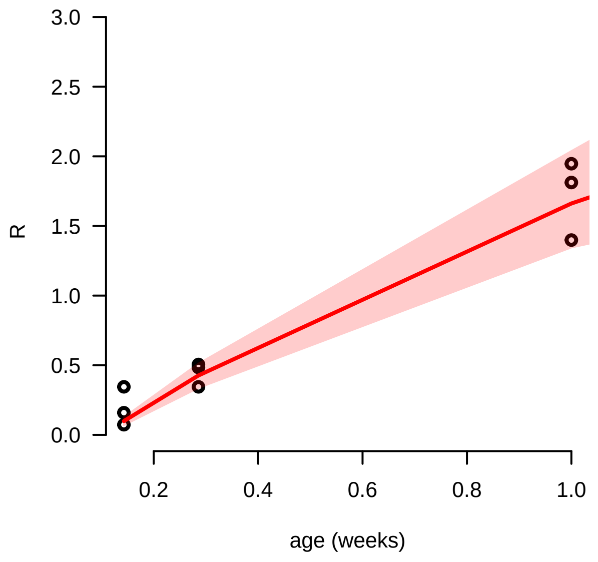

protein concentration log scale

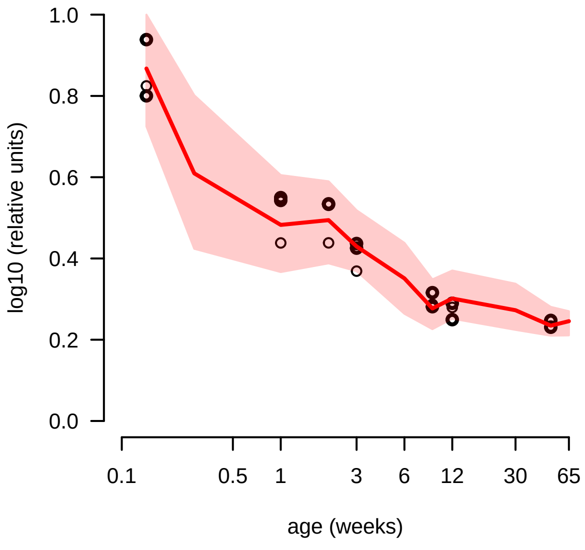

total protein log scale

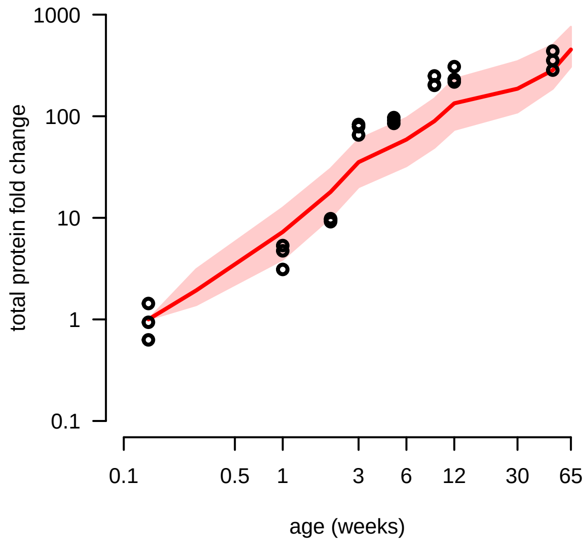

short pulse %H corrected log scale

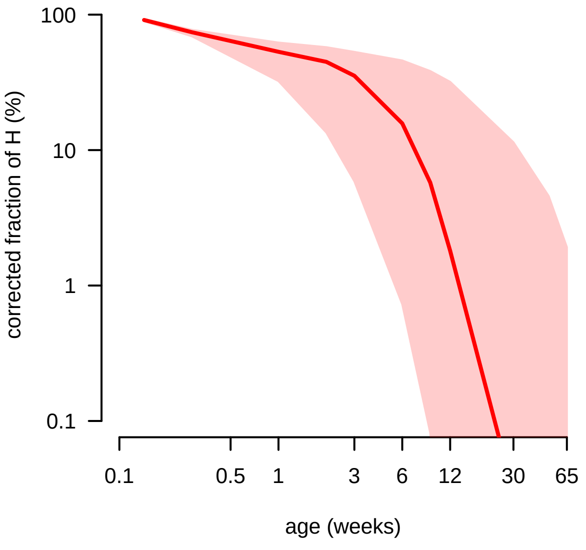

Q9Z2D1

>sp|Q9Z2D1|MTMR2_MOUSE

Myotubularin−related protein 2

GN=Mtmr2

Med H1/2: 5.8 days

Q5 H1/2: 2.8 days

Q95 H1/2: 42.3 days

reliable model fit: yes

raw intensities

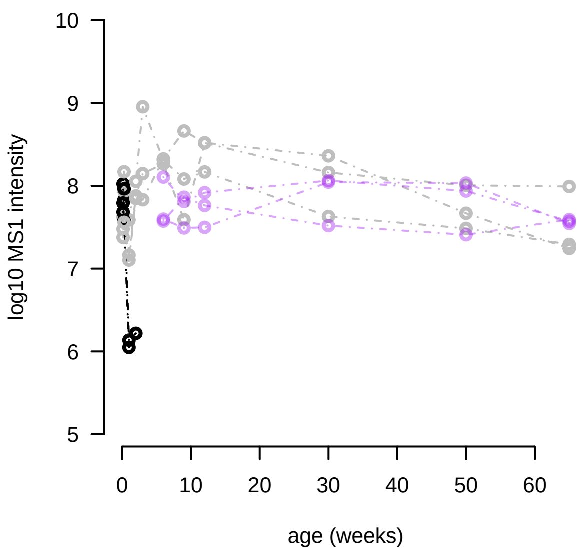

raw intensities log scale

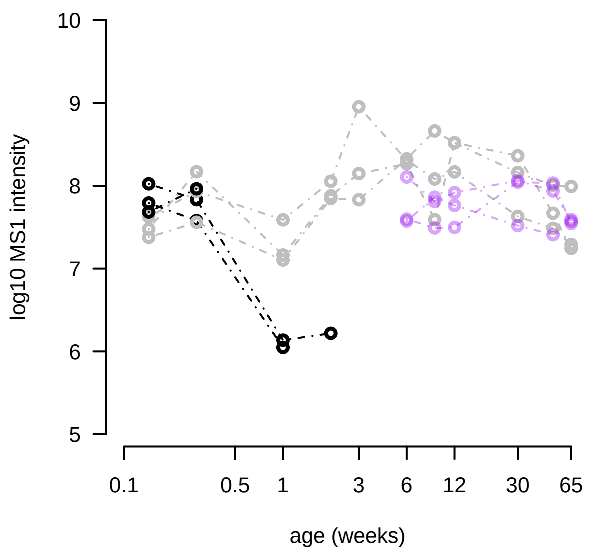

H1/2

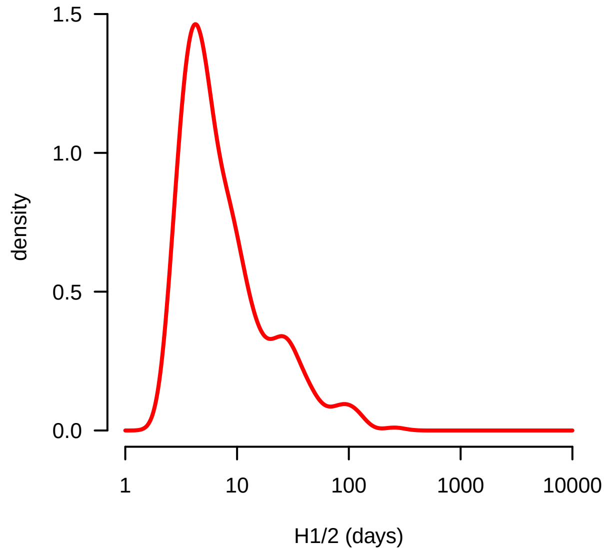

short pulse: ratio ln(L/H+1) log scale

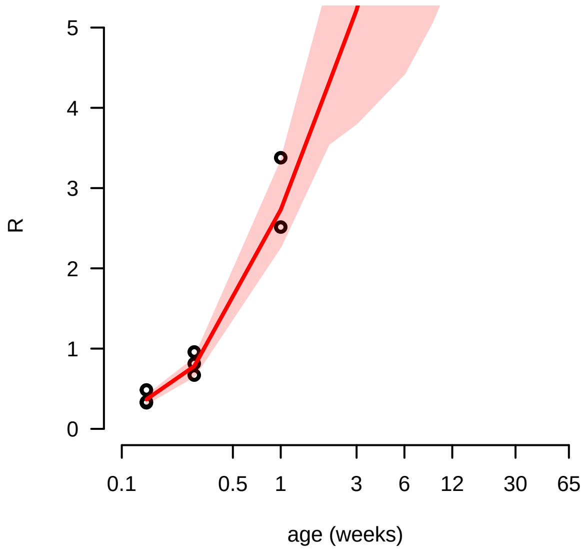

short pulse: ratio ln(L/H+1)

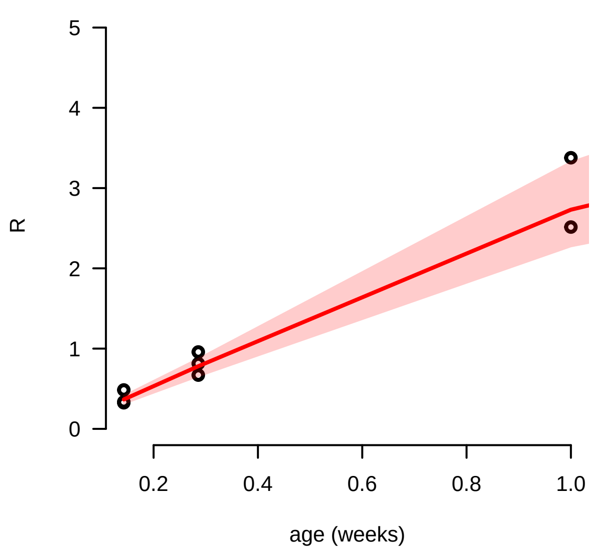

protein concentration log scale

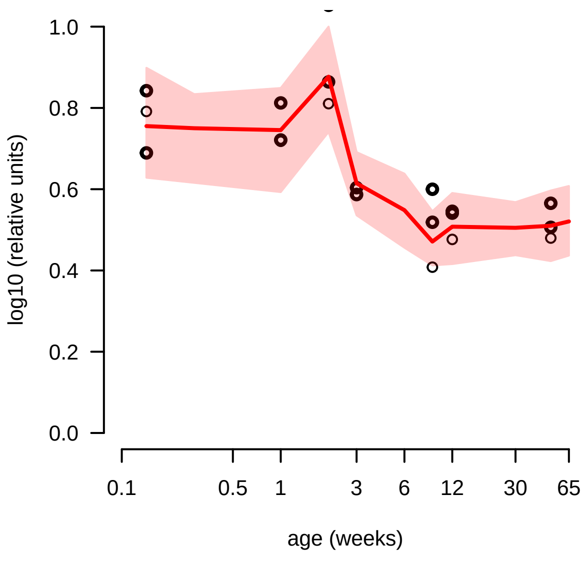

total protein log scale

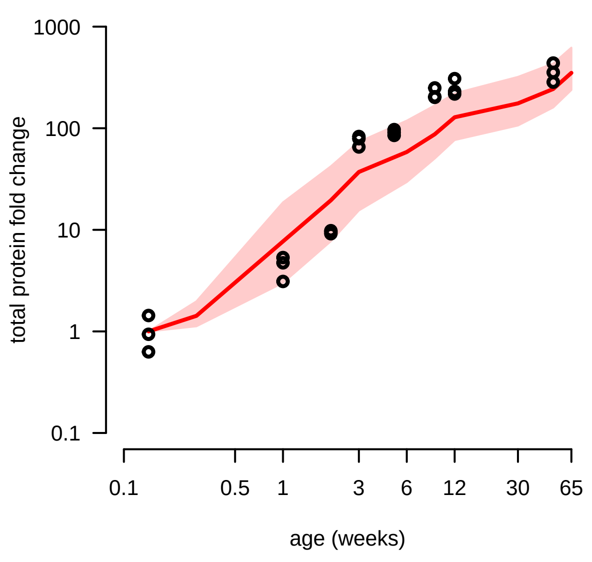

short pulse %H corrected log scale

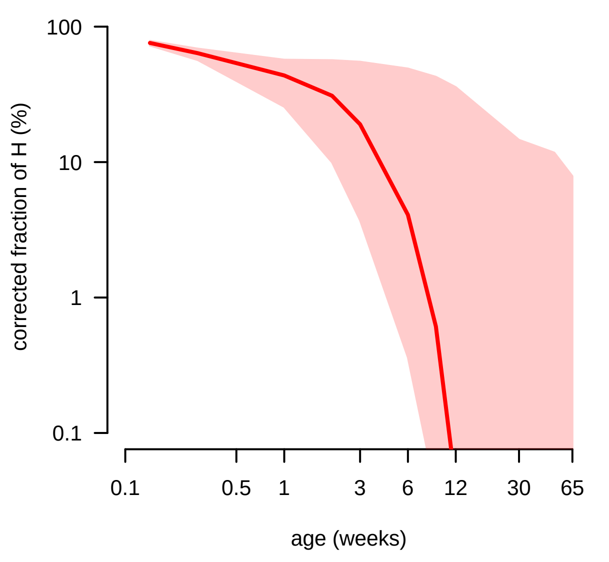

Q61160

>sp|Q61160|FADD_MOUSE

FAS−associated death domain protein

GN=Fadd

Med H1/2: 9.5 days

Q5 H1/2: 4.3 days

Q95 H1/2: 327.7 days

reliable model fit: yes

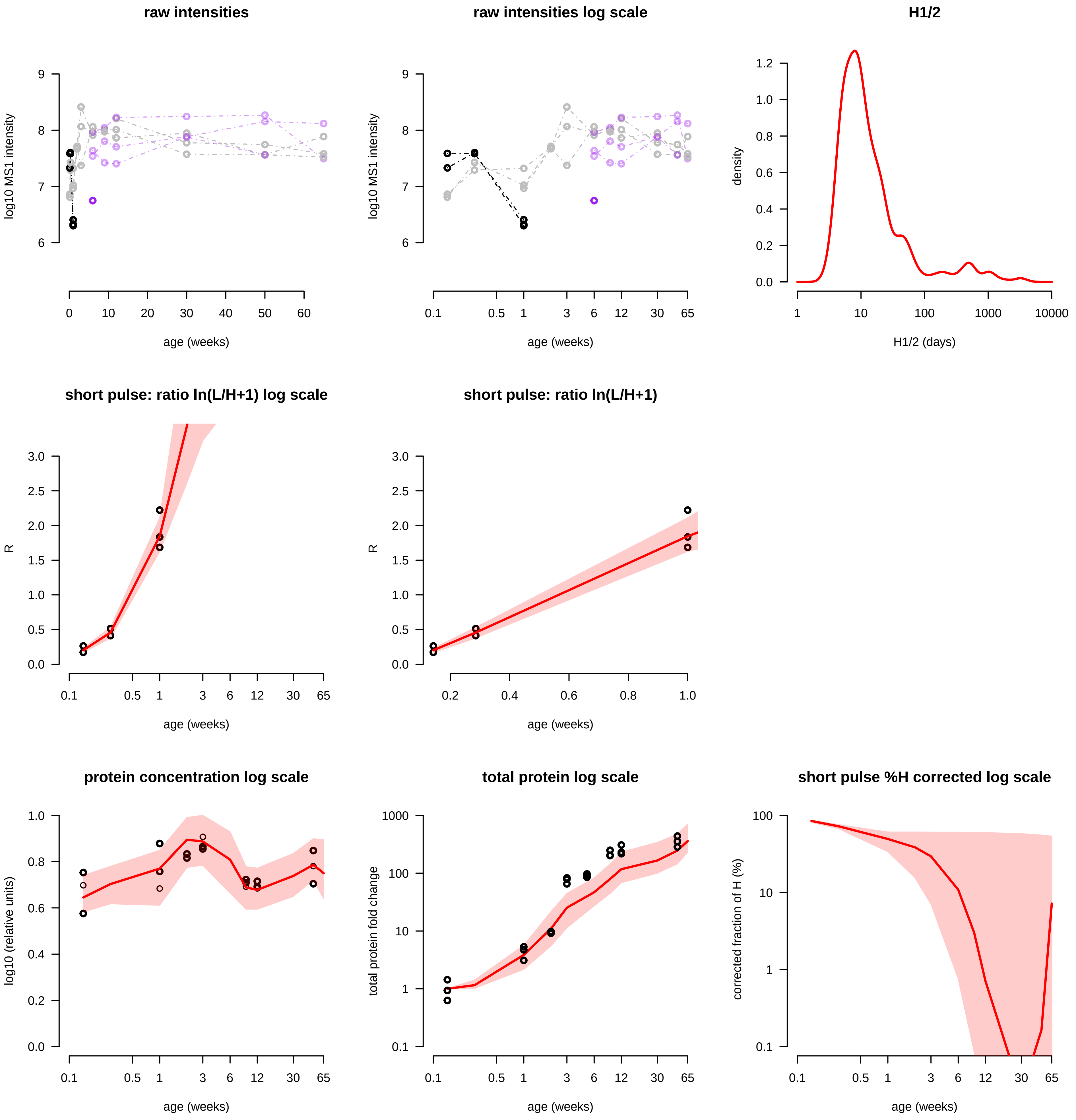

P37913

>sp|P37913|DNLI1_MOUSE

DNA ligase 1

GN=Lig1

Med H1/2: 4.4 days

Q5 H1/2: 2.6 days

Q95 H1/2: 17.6 days

reliable model fit: yes

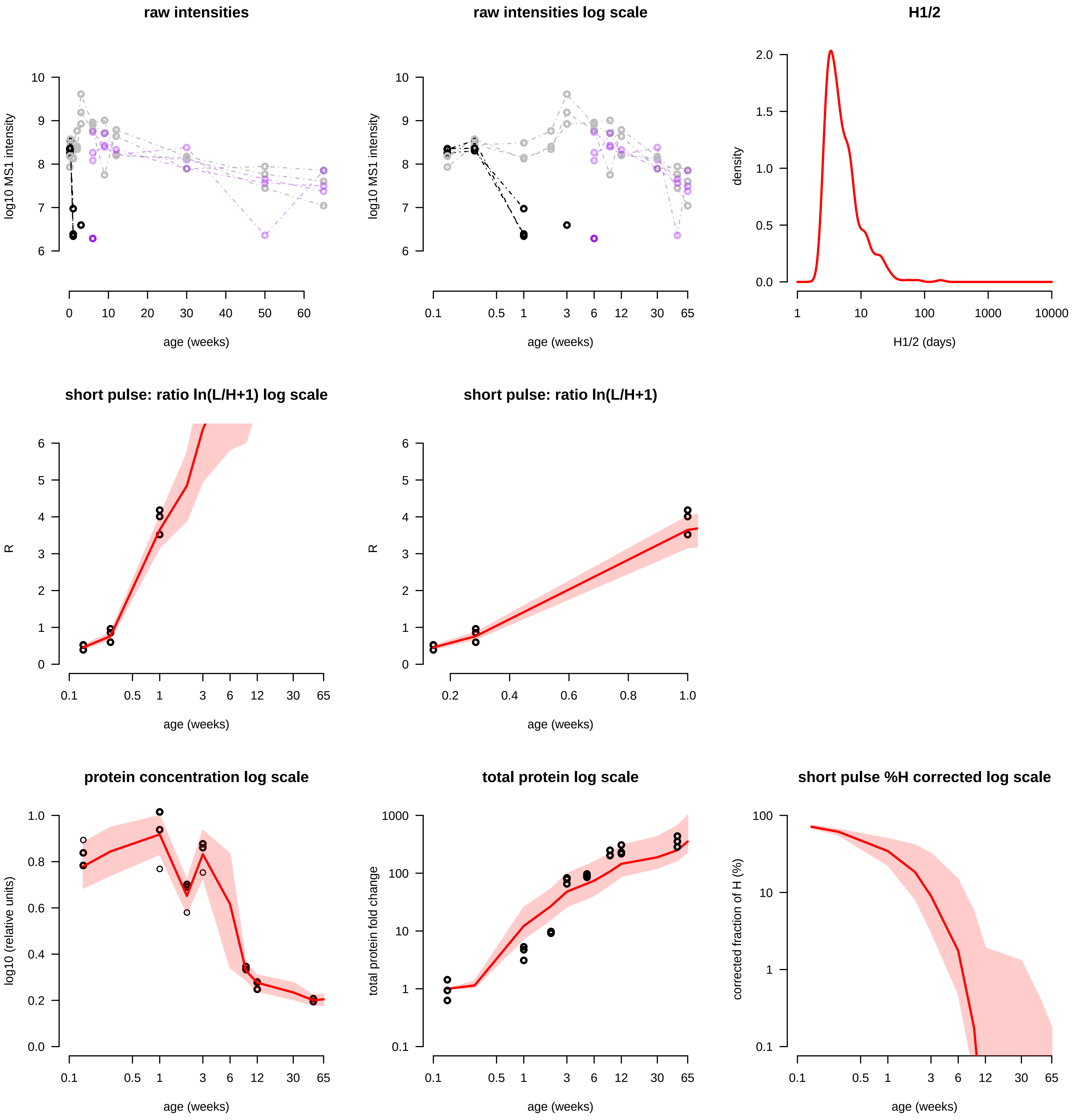

O08992

>sp|O08992|SDCB1_MOUSE

Syntenin−1

GN=Sdcbp

Med H1/2: 10 days

Q5 H1/2: 3.8 days

Q95 H1/2: 42.3 days

reliable model fit: yes

raw intensities

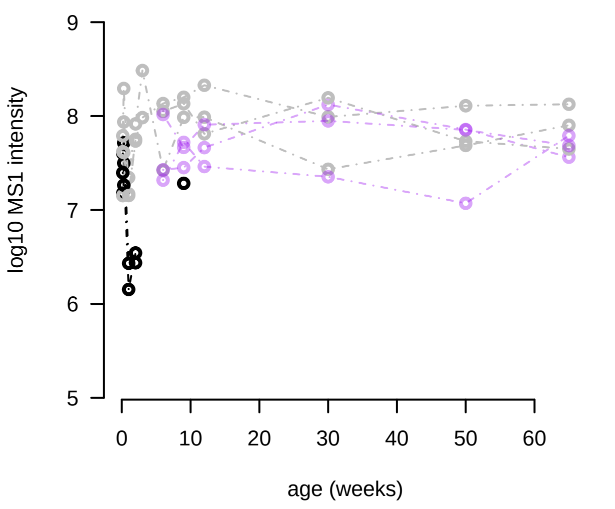

raw intensities log scale

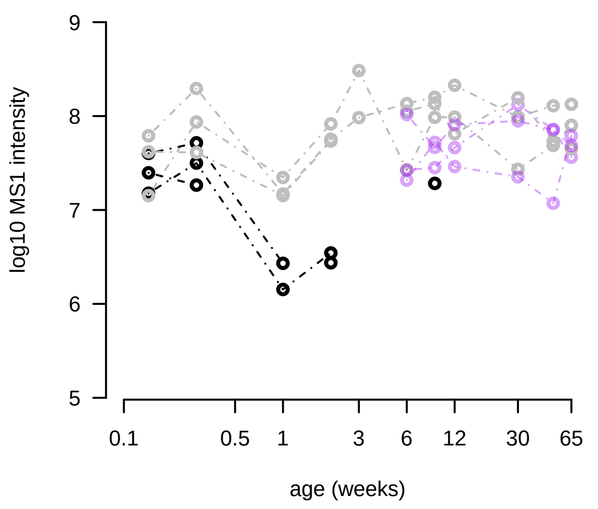

H1/2

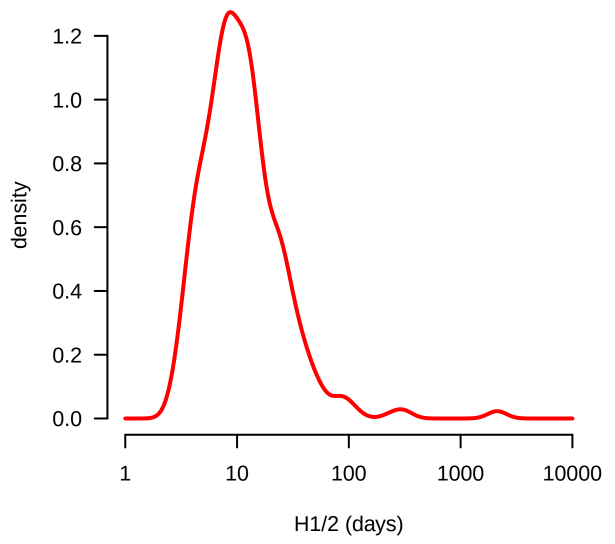

short pulse: ratio ln(L/H+1) log scale

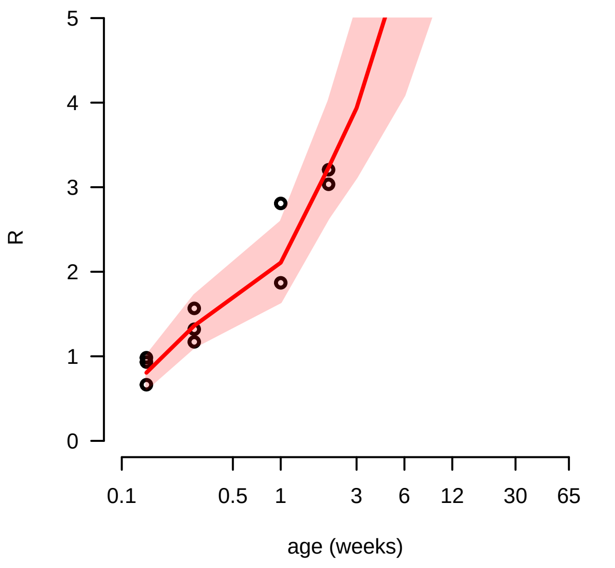

short pulse: ratio ln(L/H+1)

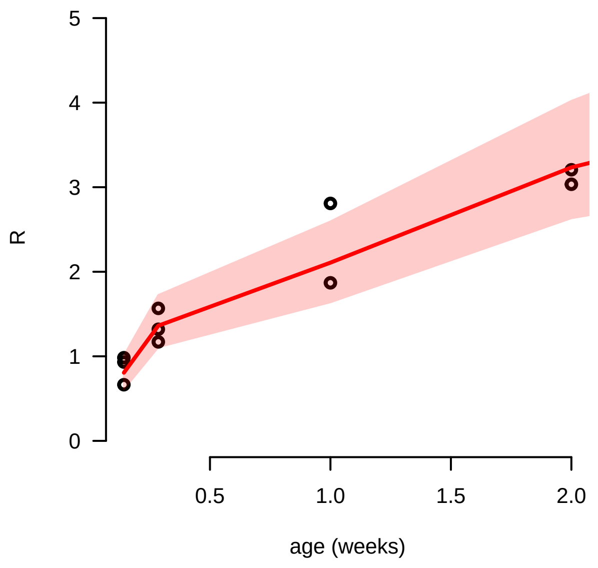

protein concentration log scale

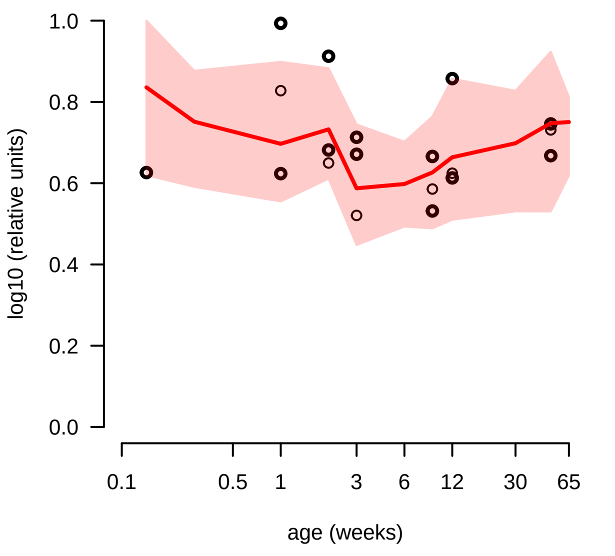

total protein log scale

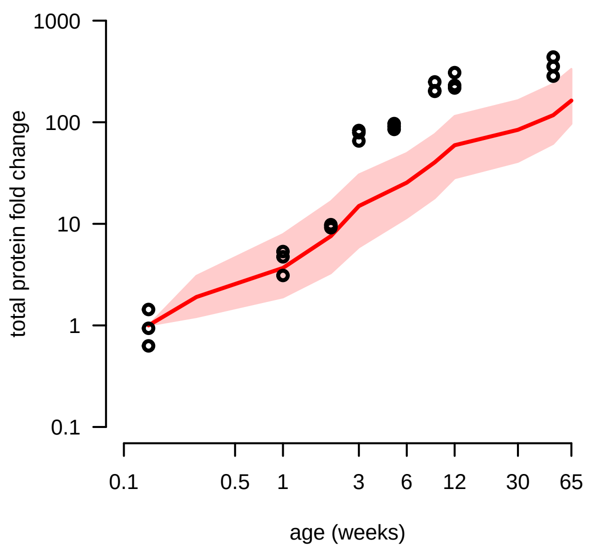

short pulse %H corrected log scale

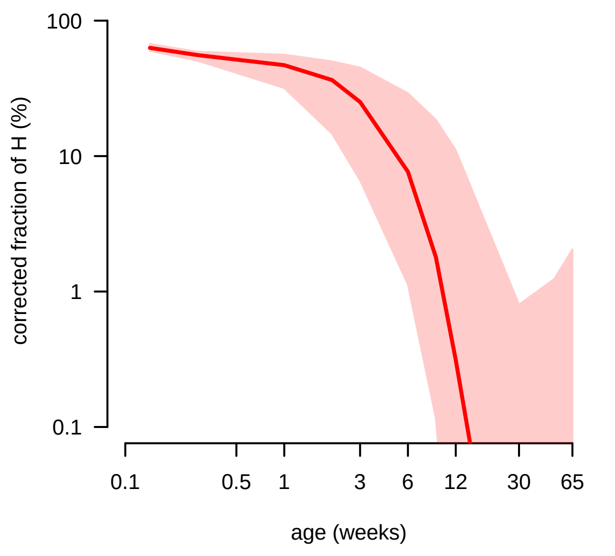

Q7TMR0

>sp|Q7TMR0|PCP_MOUSE

Lysosomal Pro−X carboxypeptidase

GN=Prcp

Med H1/2: 4.6 days

Q5 H1/2: 2.7 days

Q95 H1/2: 27.9 days

reliable model fit: yes

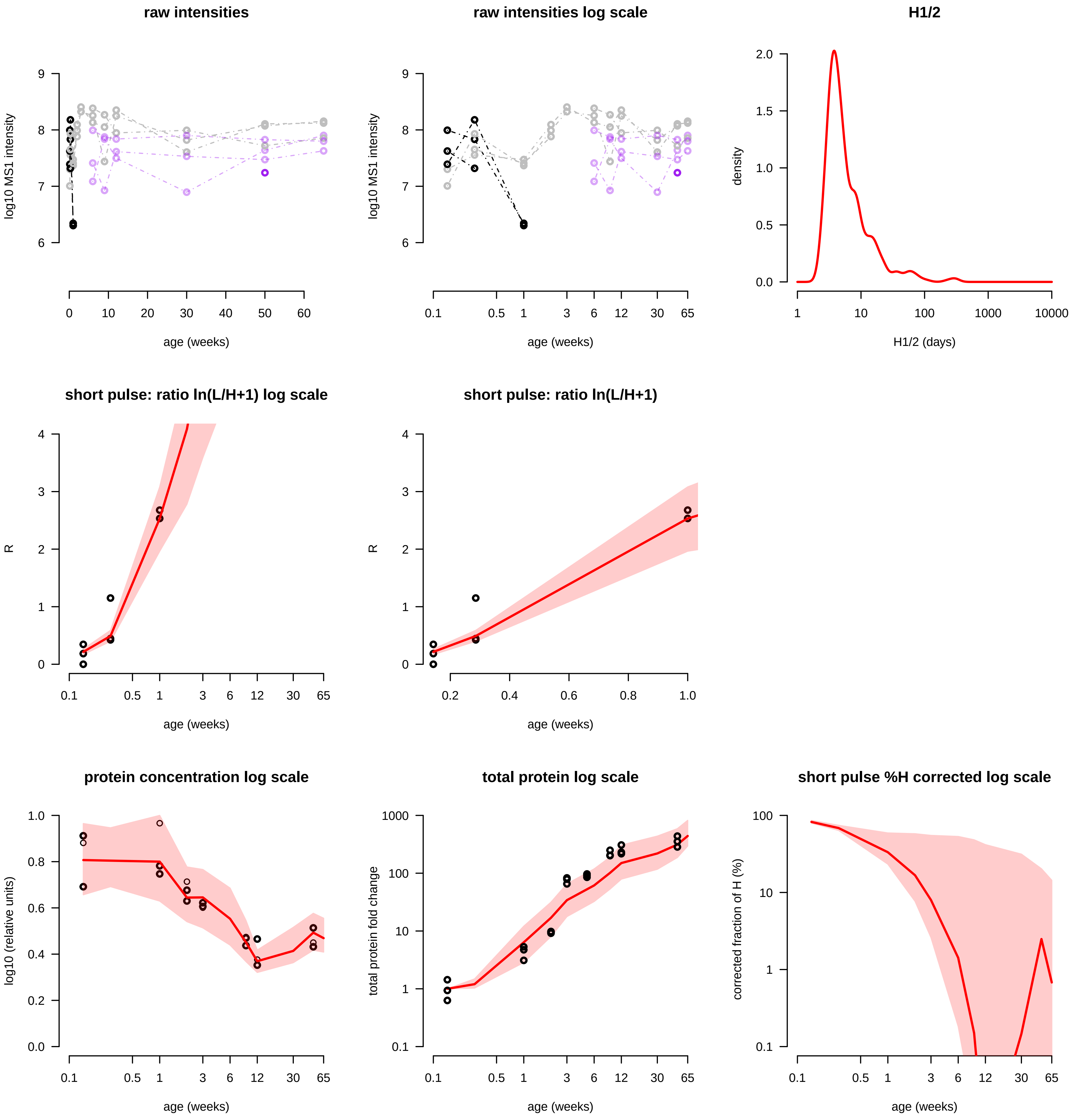

Q6RT24

>sp|Q6RT24|CENPE_MOUSE

Centromere−associated protein E

GN=Cenpe

Med H1/2: 28.5 days

Q5 H1/2: 14.1 days

Q95 H1/2: 291.4 days

reliable model fit: no

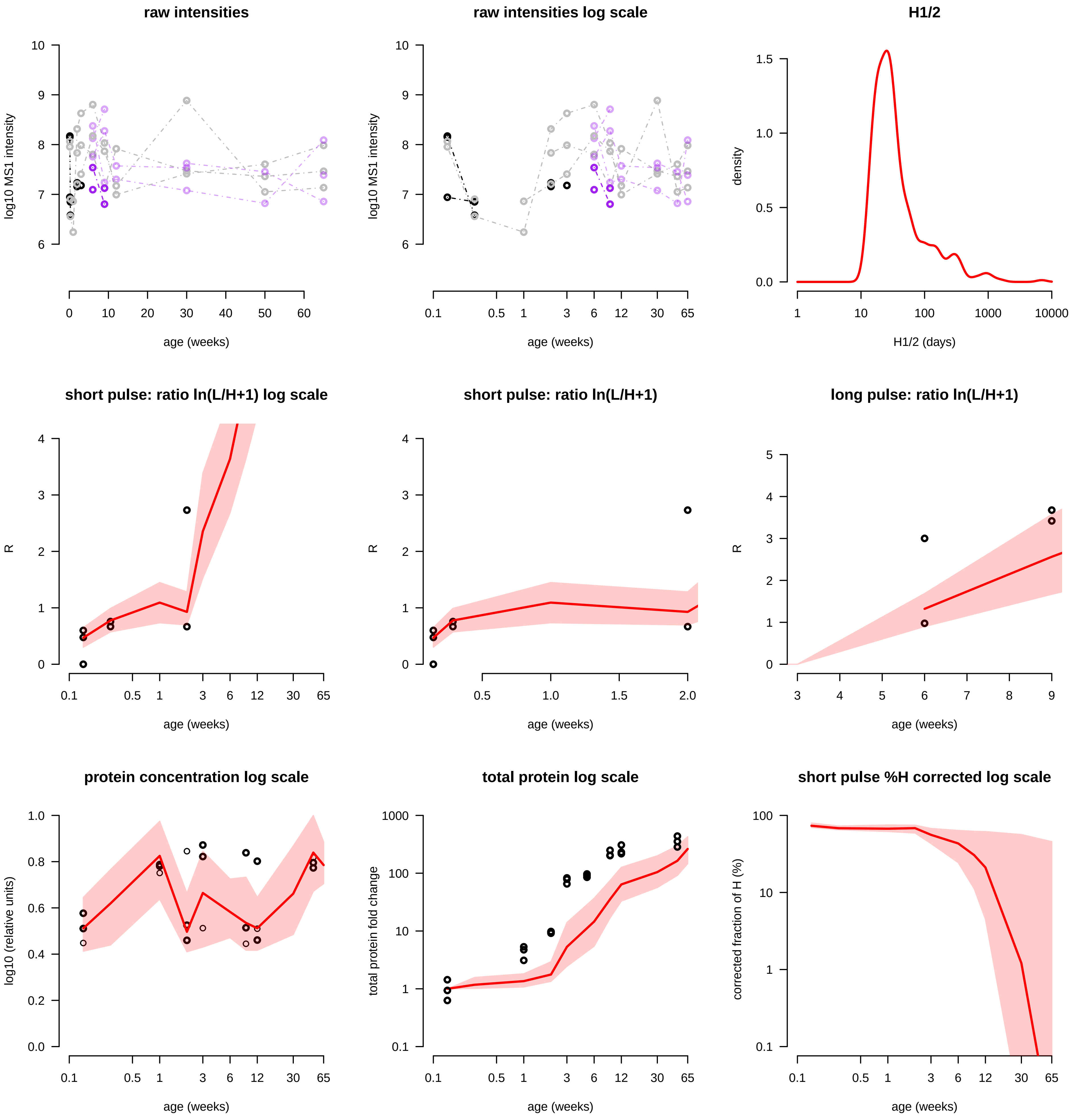

Q9JHI7

>sp|Q9JHI7|EXOS9_MOUSE

Exosome complex component RRP45

GN=Exosc9

Med H1/2: 16.3 days

Q5 H1/2: 4.6 days

Q95 H1/2: 175.9 days

reliable model fit: yes

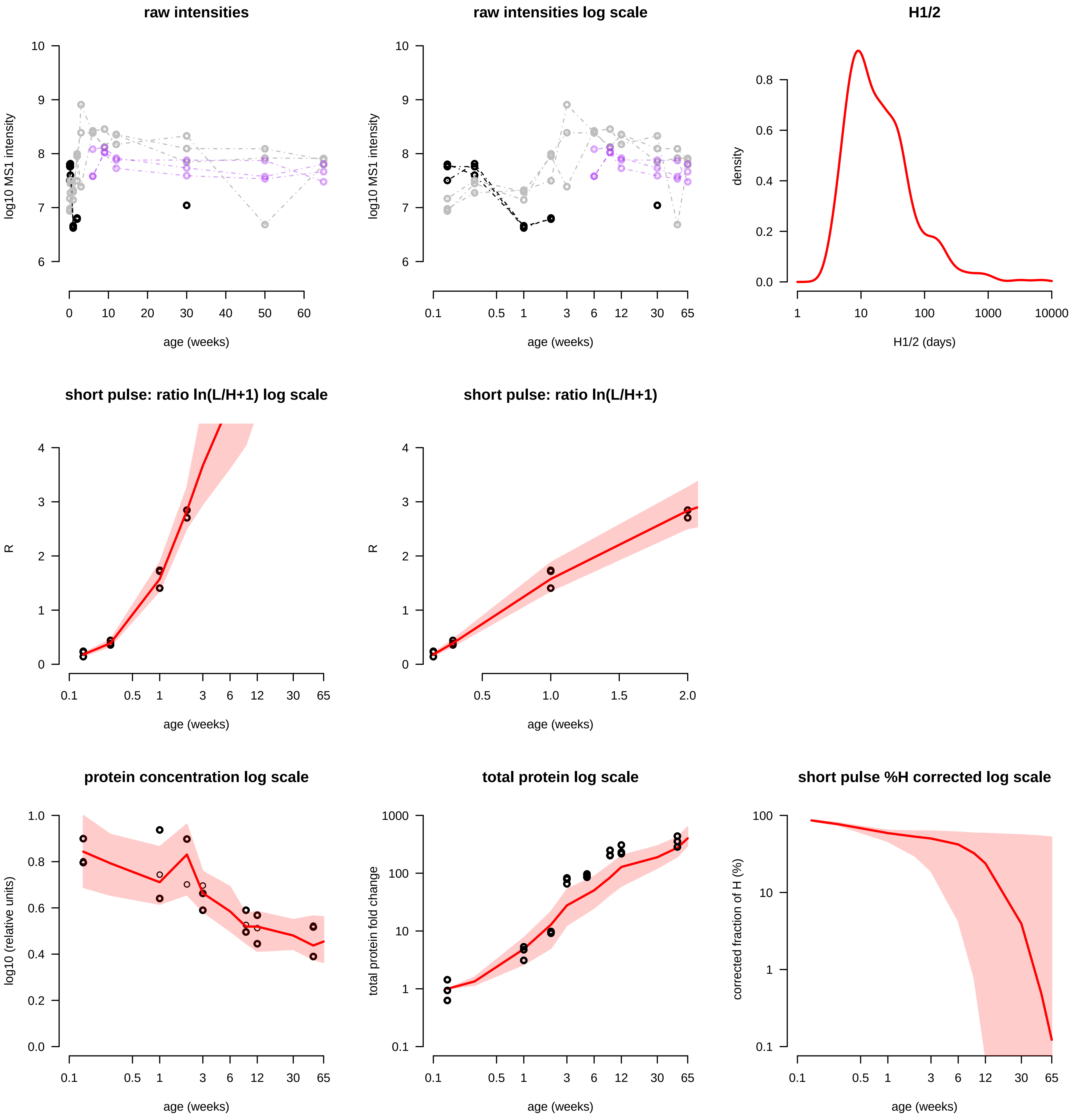

Q9CQ48

>sp|Q9CQ48|NUDC2_MOUSE

NudC domain−containing protein 2

GN=Nudcd2

Med H1/2: 6.7 days

Q5 H1/2: 3.4 days

Q95 H1/2: 35.4 days

reliable model fit: yes

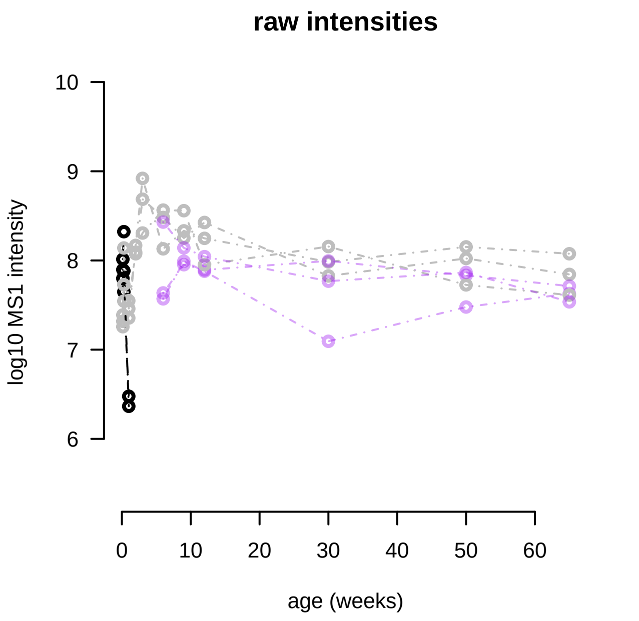

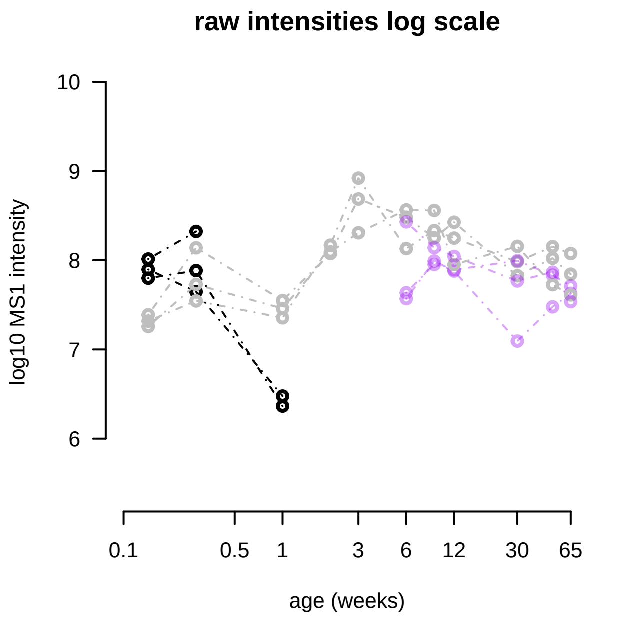

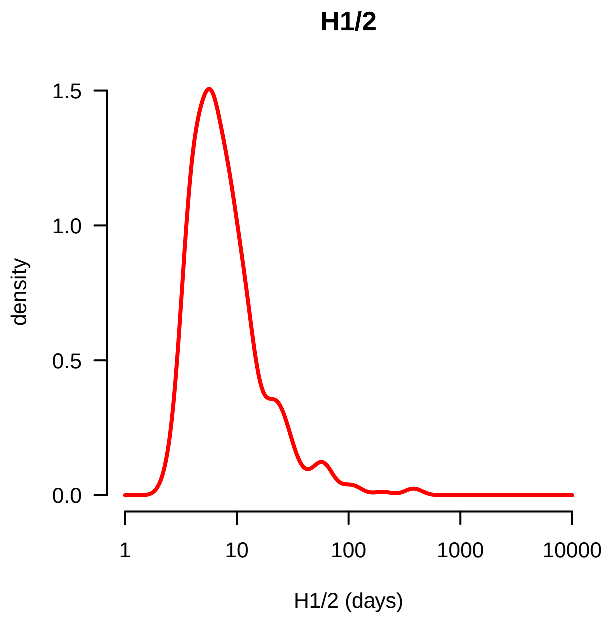

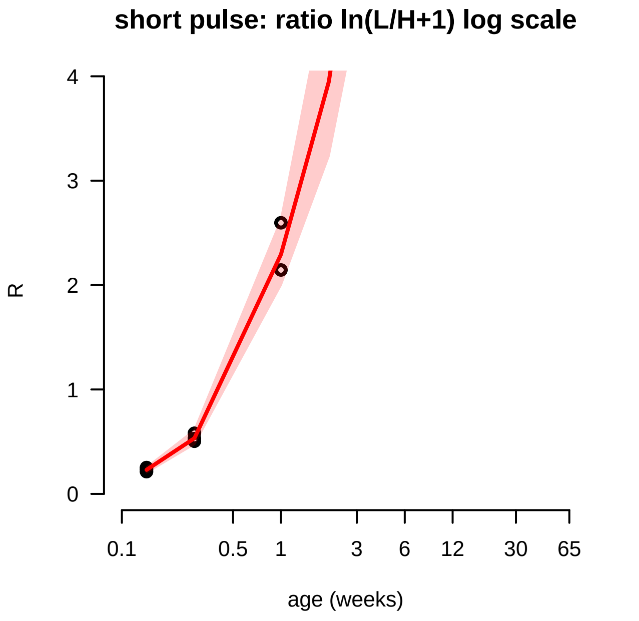

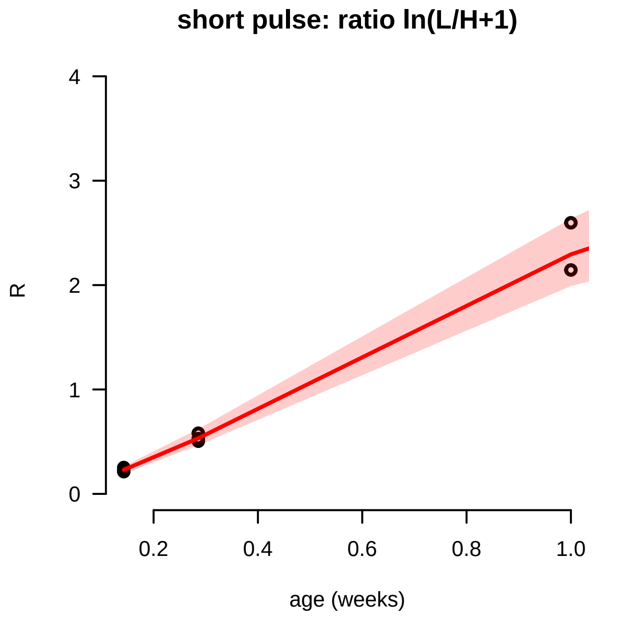

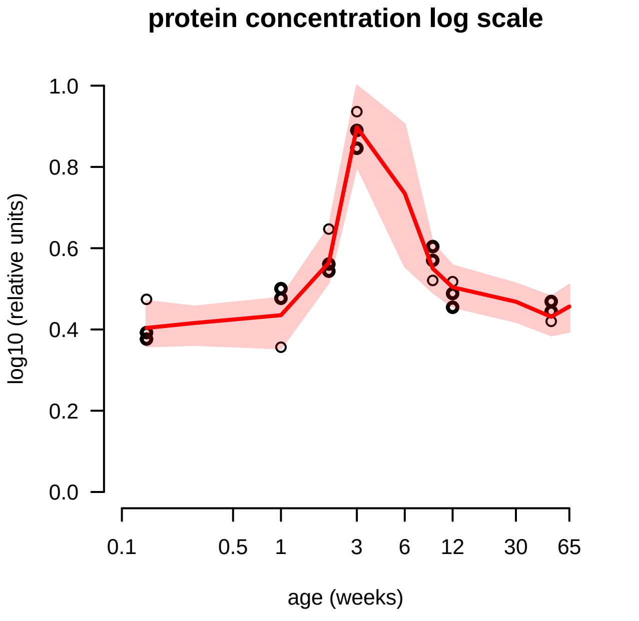

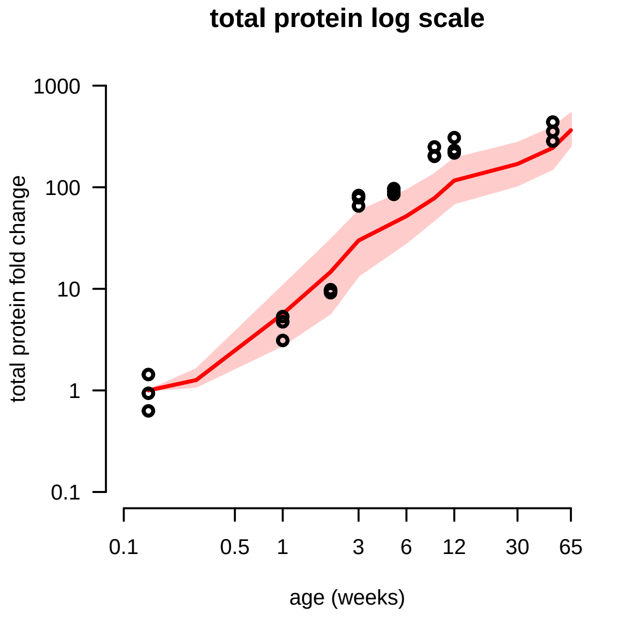

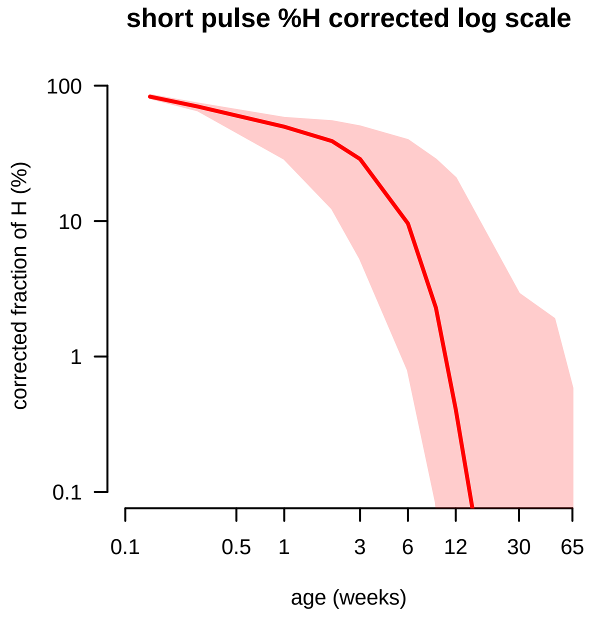

Q9D3B1

>sp|Q9D3B1|HACD2_MOUSE

Very−long−chain (3R)−3−hydroxyacyl−CoA dehydratase 2

GN=Hacd2

Med H1/2: 11 days

Q5 H1/2: 5.3 days

Q95 H1/2: 145.5 days

reliable model fit: no

raw intensities

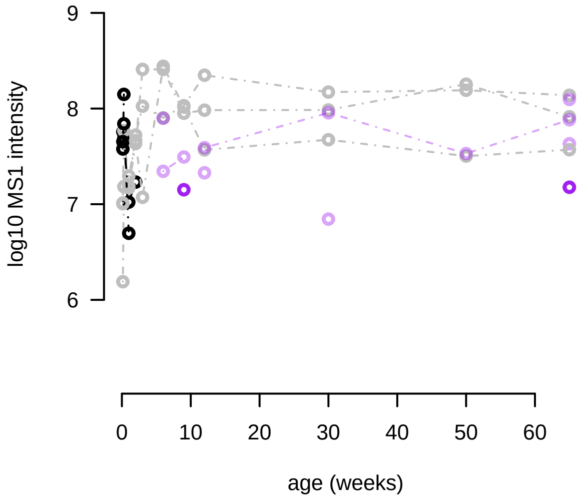

raw intensities log scale

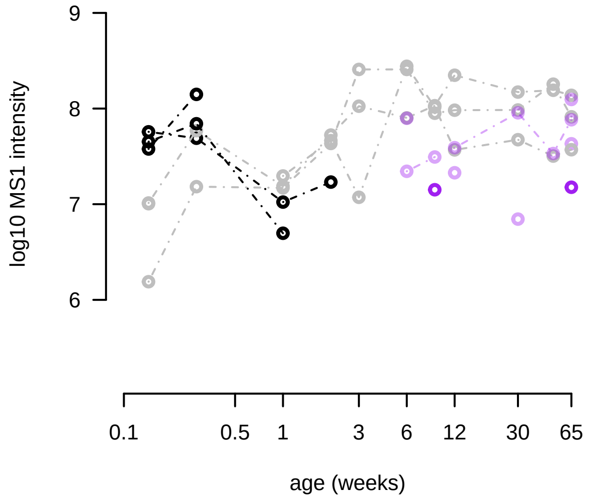

H1/2

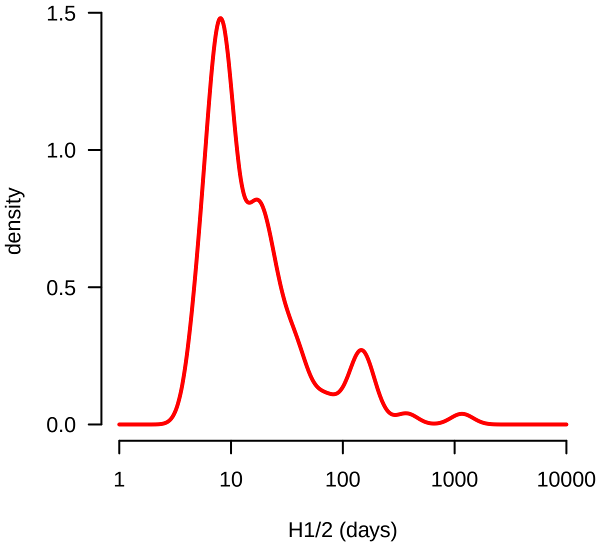

short pulse: ratio ln(L/H+1) log scale

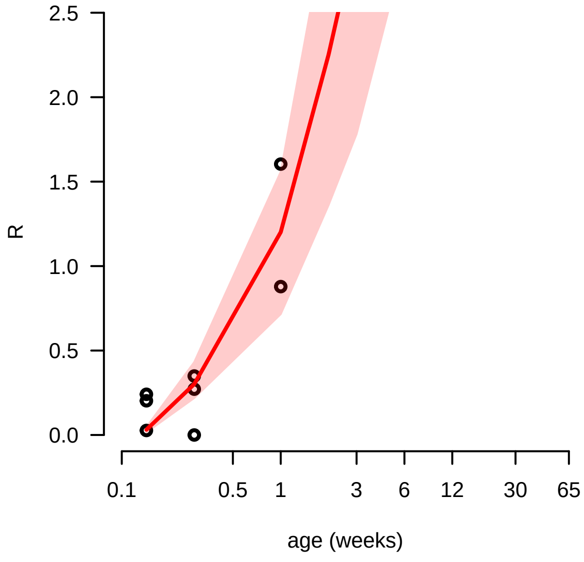

short pulse: ratio ln(L/H+1)

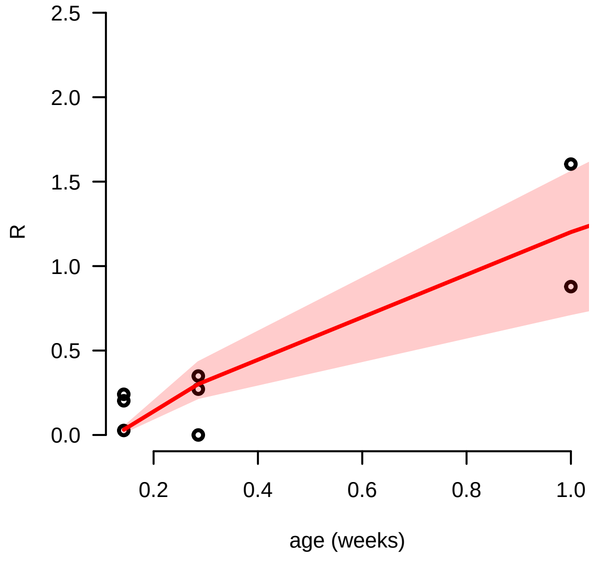

protein concentration log scale

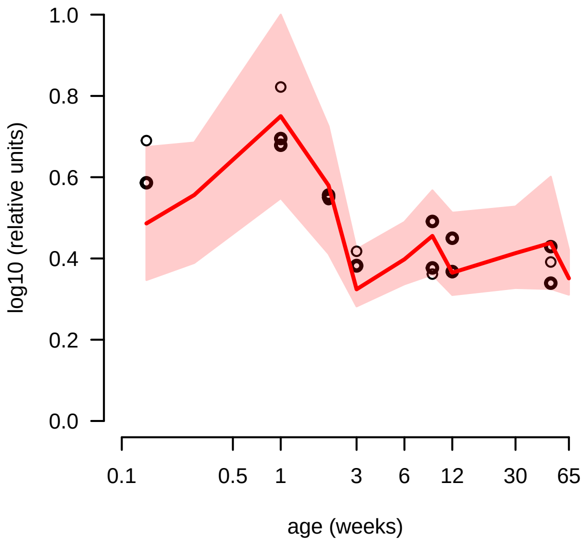

total protein log scale

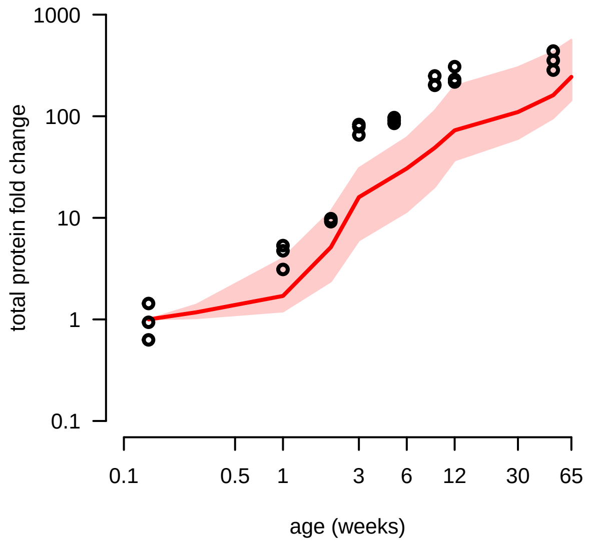

short pulse %H corrected log scale

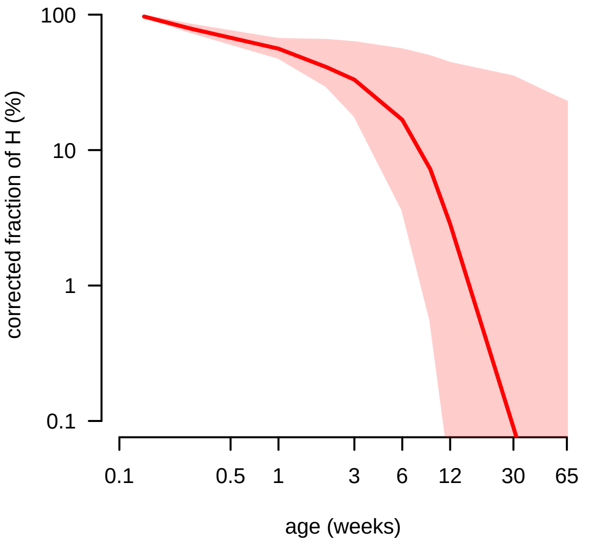

Q7TSE6;Q7TSE6−2

>sp|Q7TSE6|ST38L_MOUSE

Serine/threonine−protein kinase 38−like

GN=Stk38l

Med H1/2: 31.6 days

Q5 H1/2: 7.9 days

Q95 H1/2: 572.5 days

reliable model fit: yes

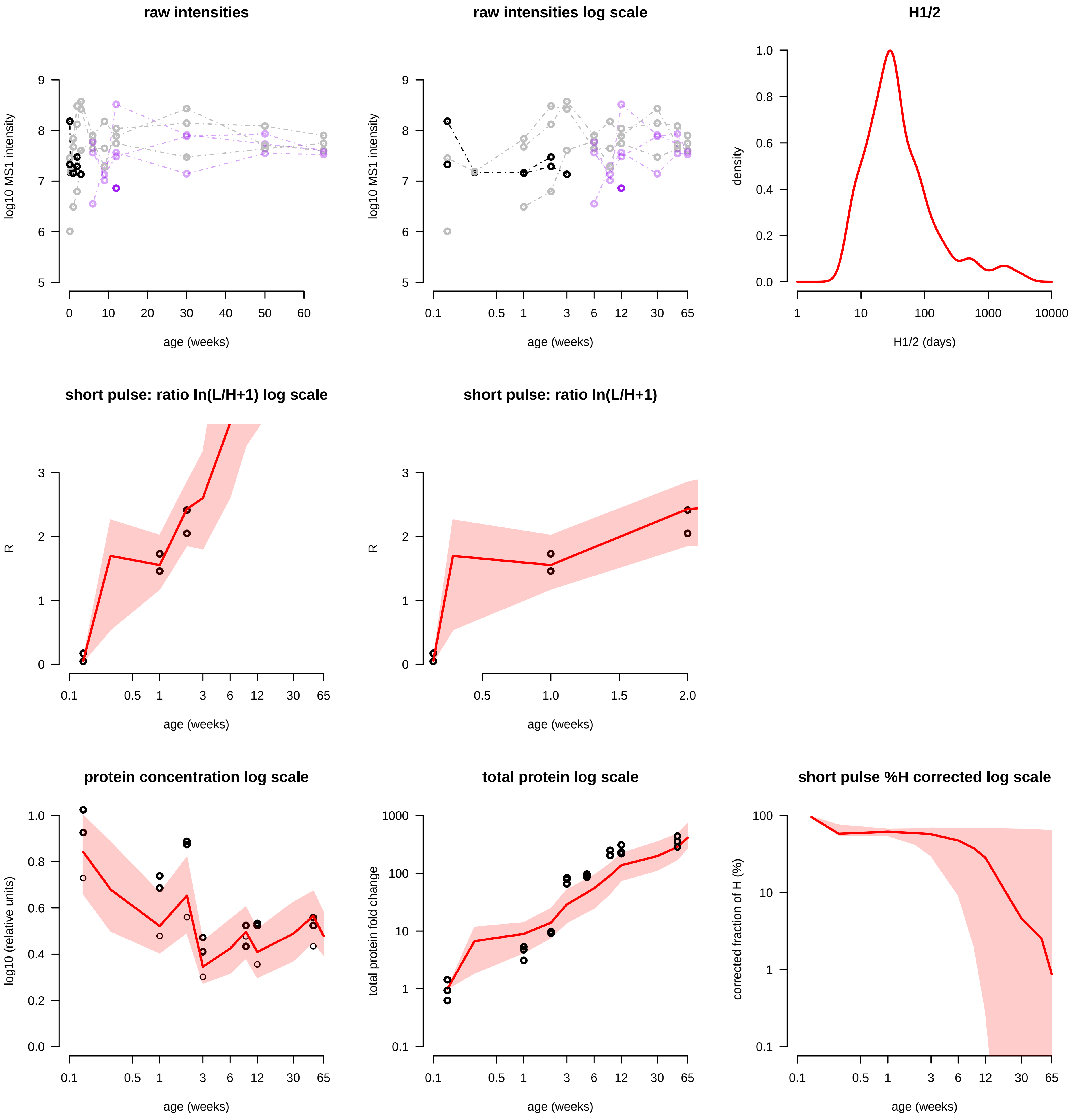

O70230−2;O70230;O70230−3

>sp|O70230−2|ZN143_MOUSE

Isoform 2 of Zinc finger protein 143

GN=Znf143

Med H1/2: 38.8 days

Q5 H1/2: 12.6 days

Q95 H1/2: 468.2 days

reliable model fit: no

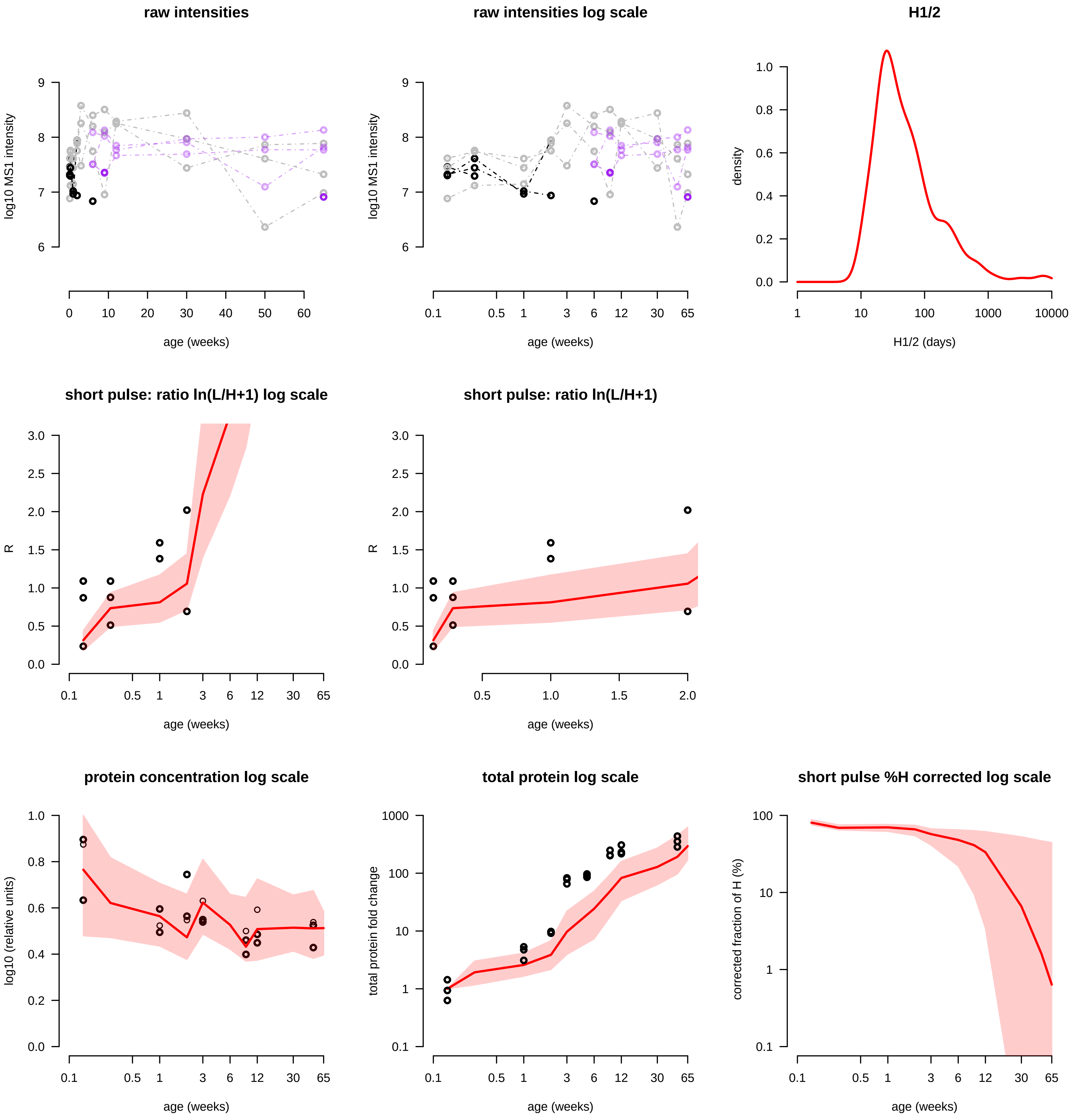

P12658

>sp|P12658|CALB1_MOUSE

Calbindin

GN=Calb1

Med H1/2: 4.2 days

Q5 H1/2: 2.7 days

Q95 H1/2: 9.3 days

reliable model fit: yes

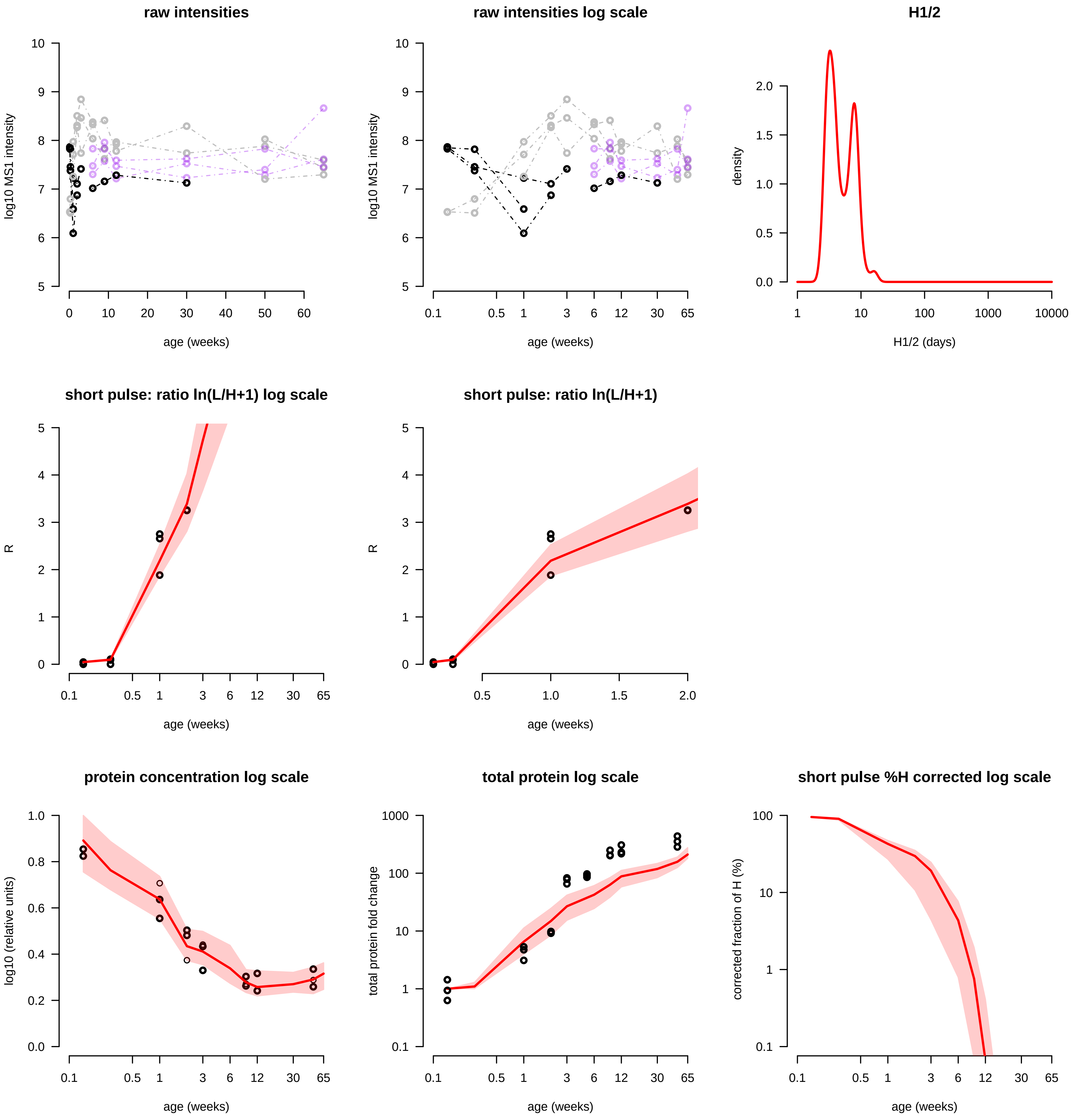

Q9DCZ1

>sp|Q9DCZ1|GMPR1_MOUSE

GMP reductase 1

GN=Gmpr

Med H1/2: 10.9 days

Q5 H1/2: 3.7 days

Q95 H1/2: 53 days

reliable model fit: yes

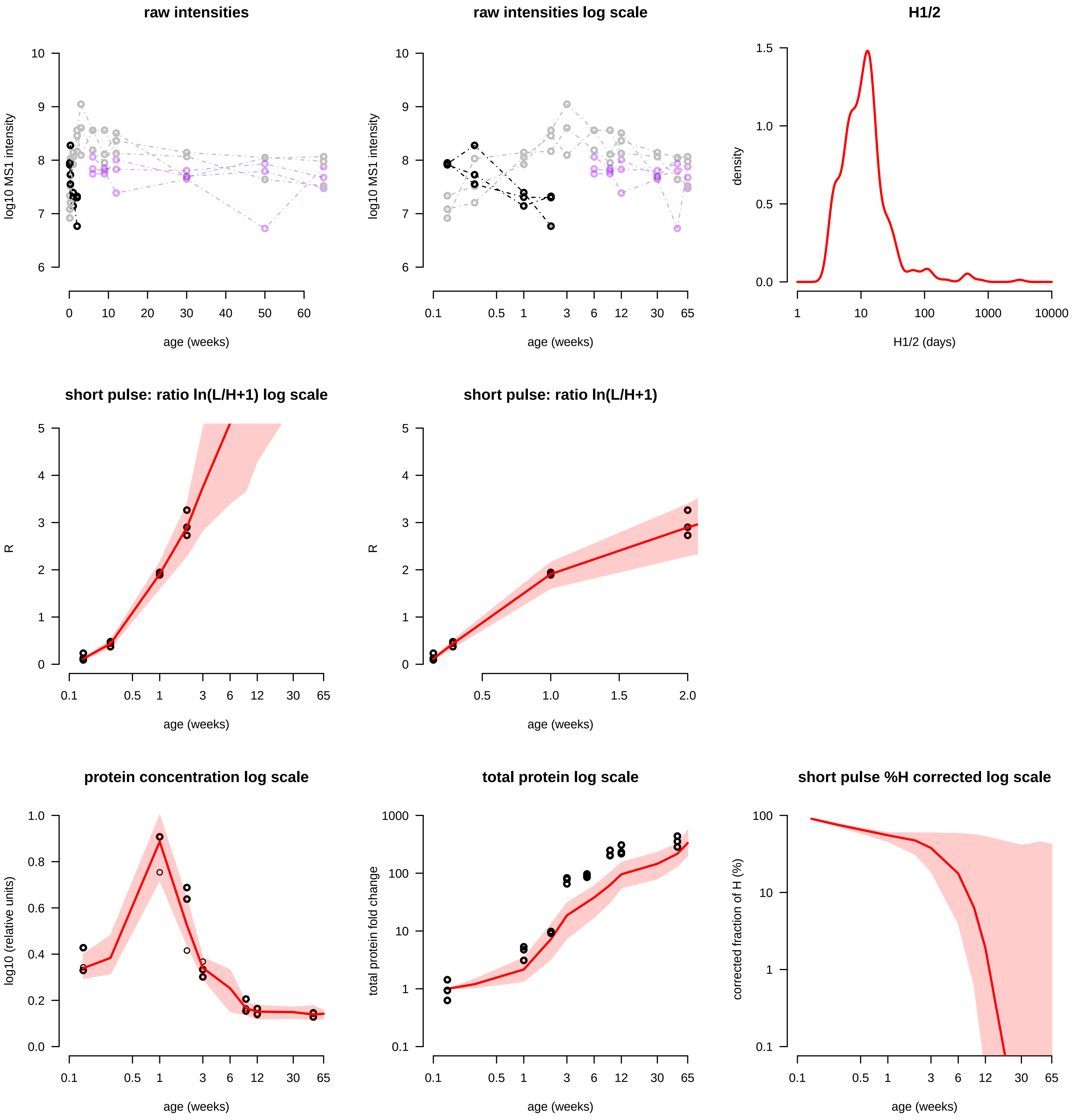

Q9CWS0

>sp|Q9CWS0|DDAH1_MOUSE

N(G),N(G)−dimethylarginine dimethylaminohydrolase 1

GN=Ddah1

Med H1/2: 25.5 days

Q5 H1/2: 10.4 days

Q95 H1/2: 207.3 days

reliable model fit: yes

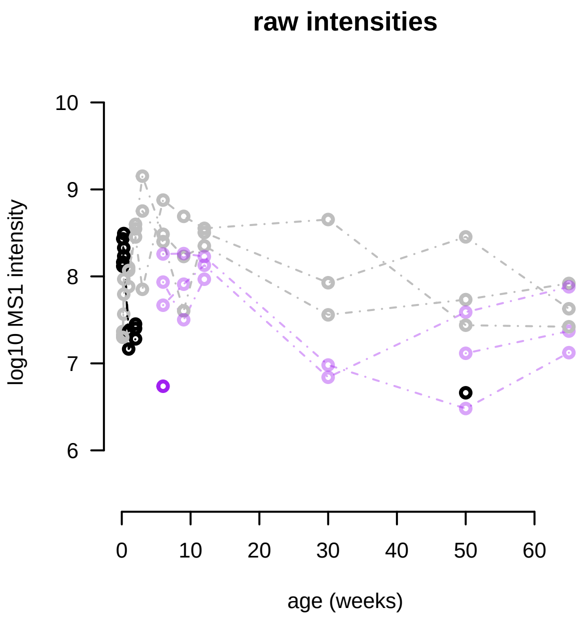

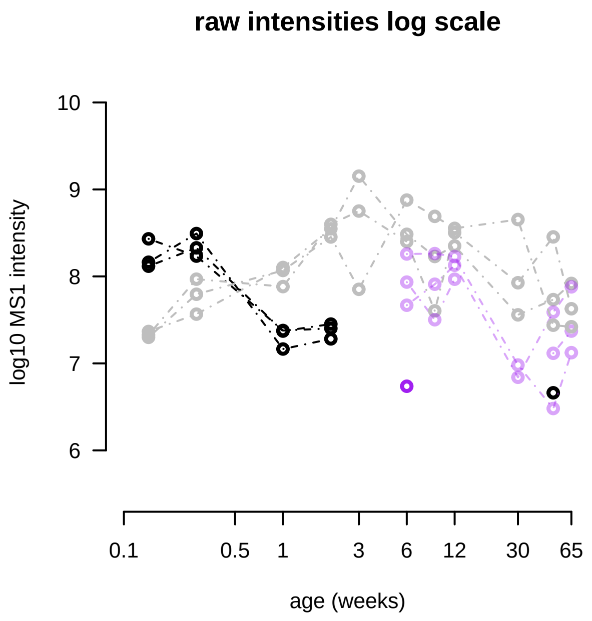

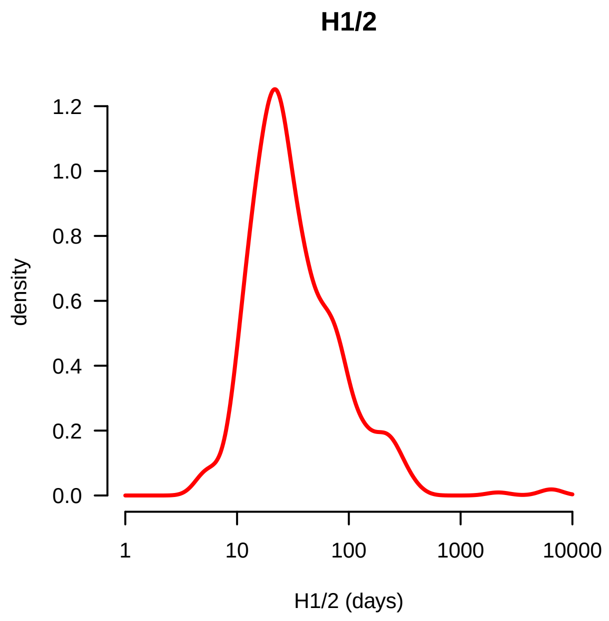

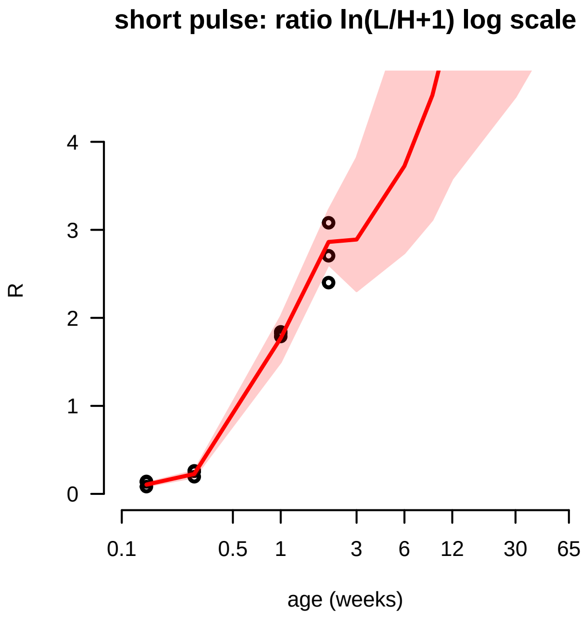

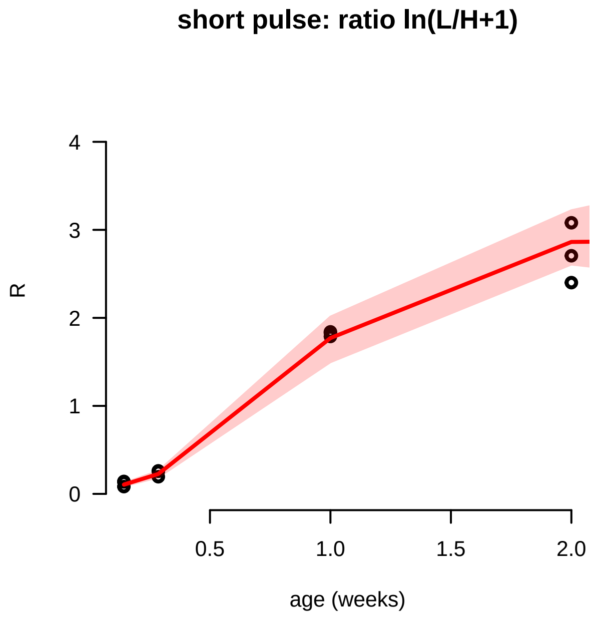

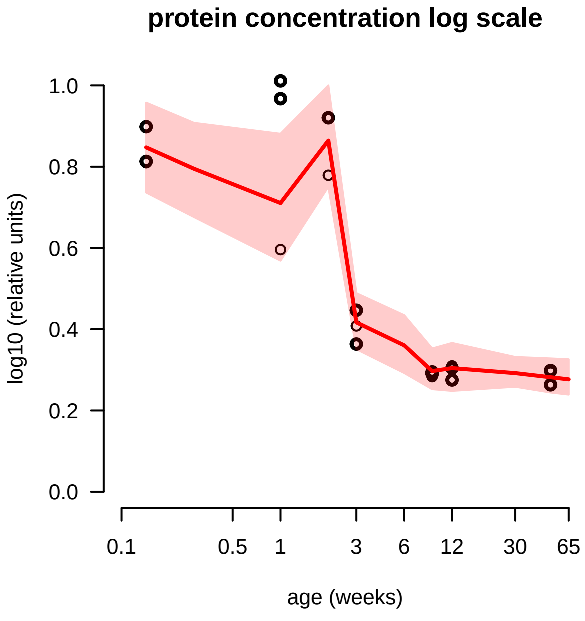

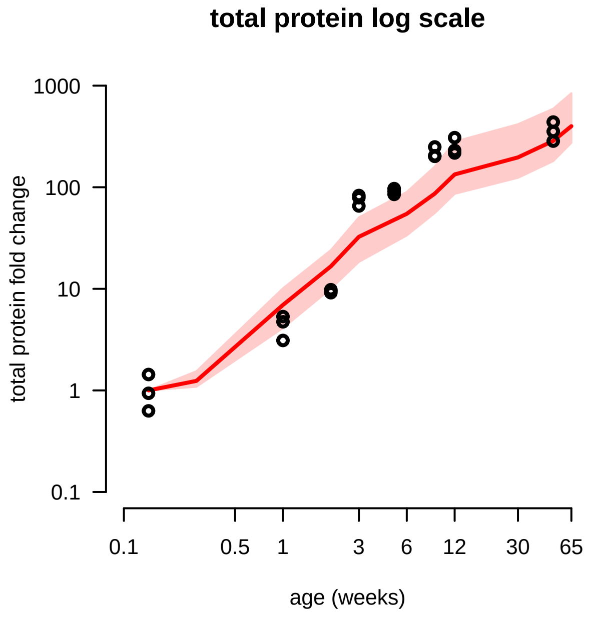

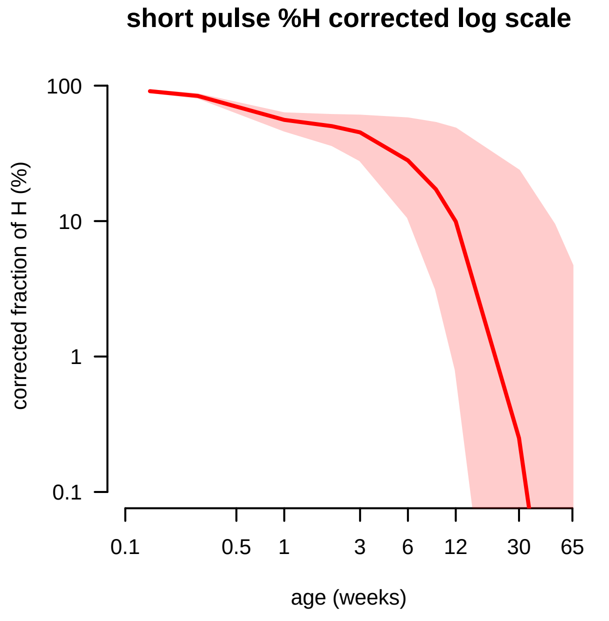

Q91YE7−2;Q91YE7

>sp|Q91YE7−2|RBM5_MOUSE

Isoform 2 of RNA−binding protein 5

GN=Rbm5

Med H1/2: 23.9 days

Q5 H1/2: 11.1 days

Q95 H1/2: 494.9 days

reliable model fit: yes

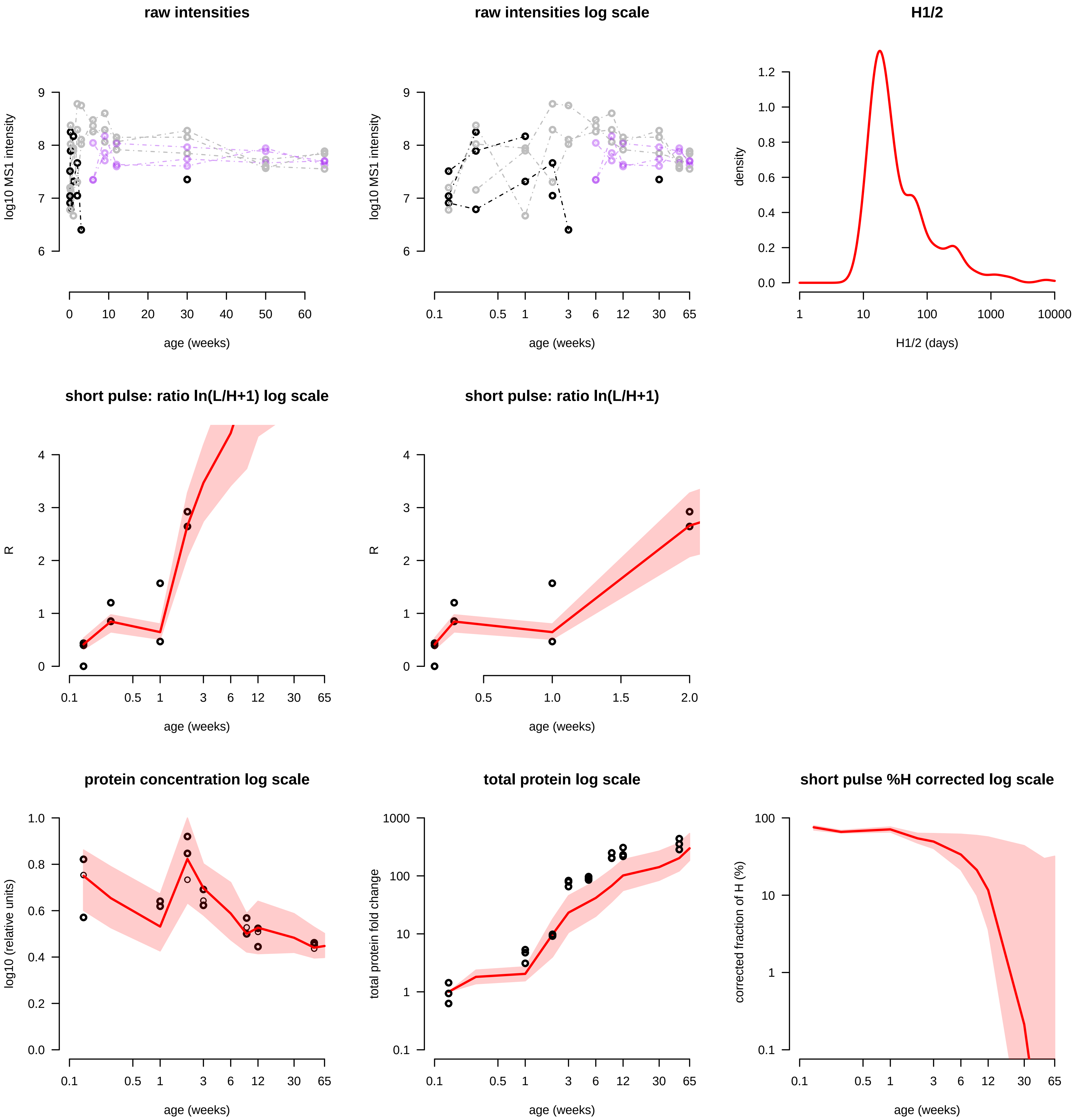

Q9CZT6

>sp|Q9CZT6|CMS1_MOUSE

Protein CMSS1

GN=Cmss1

Med H1/2: 5.7 days

Q5 H1/2: 3.1 days

Q95 H1/2: 46 days

reliable model fit: yes

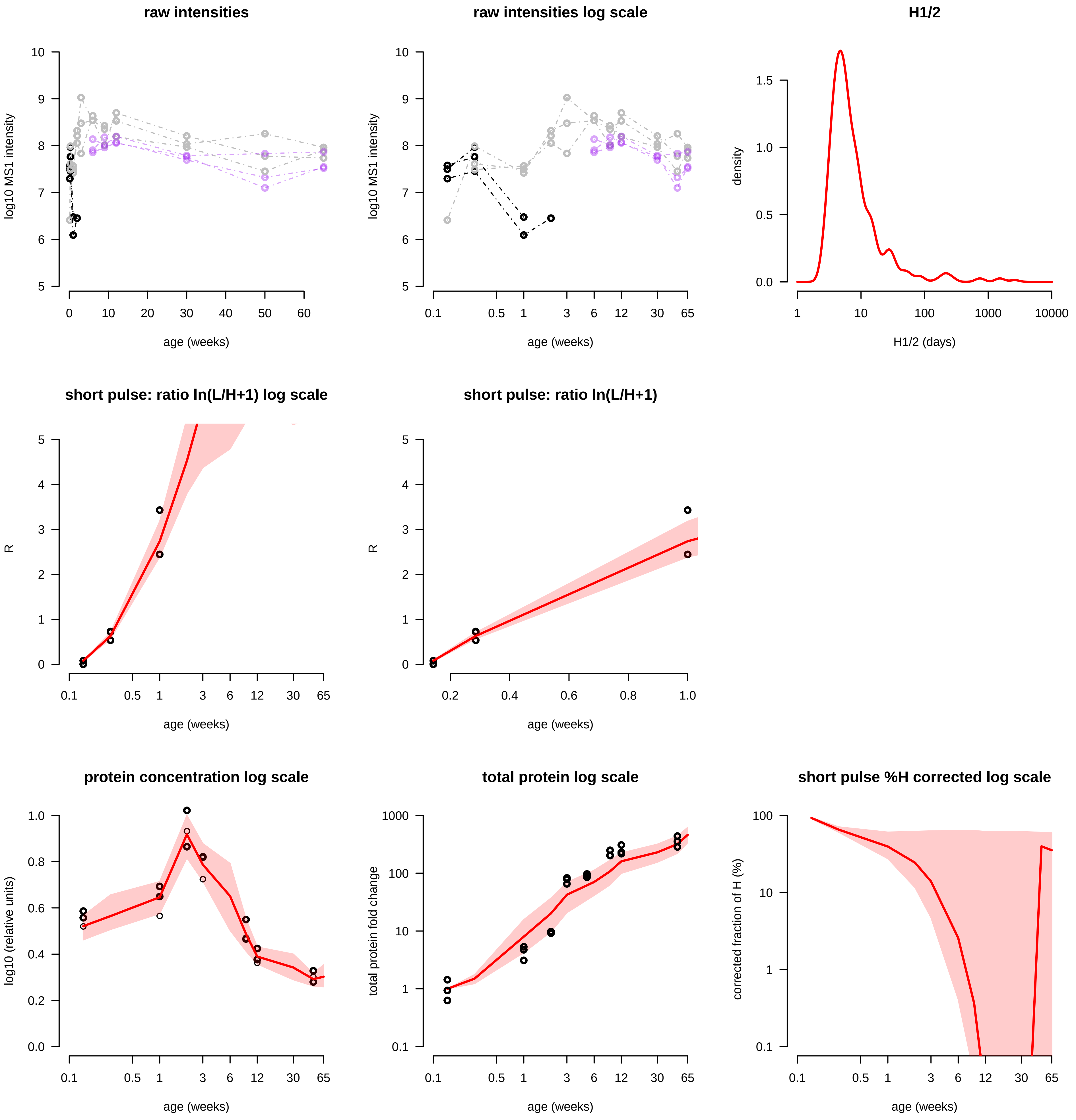

Q6ZWM4

>sp|Q6ZWM4|LSM8_MOUSE

U6 snRNA−associated Sm−like protein LSm8

GN=Lsm8

Med H1/2: 33.9 days

Q5 H1/2: 9 days

Q95 H1/2: 270.8 days

reliable model fit: yes

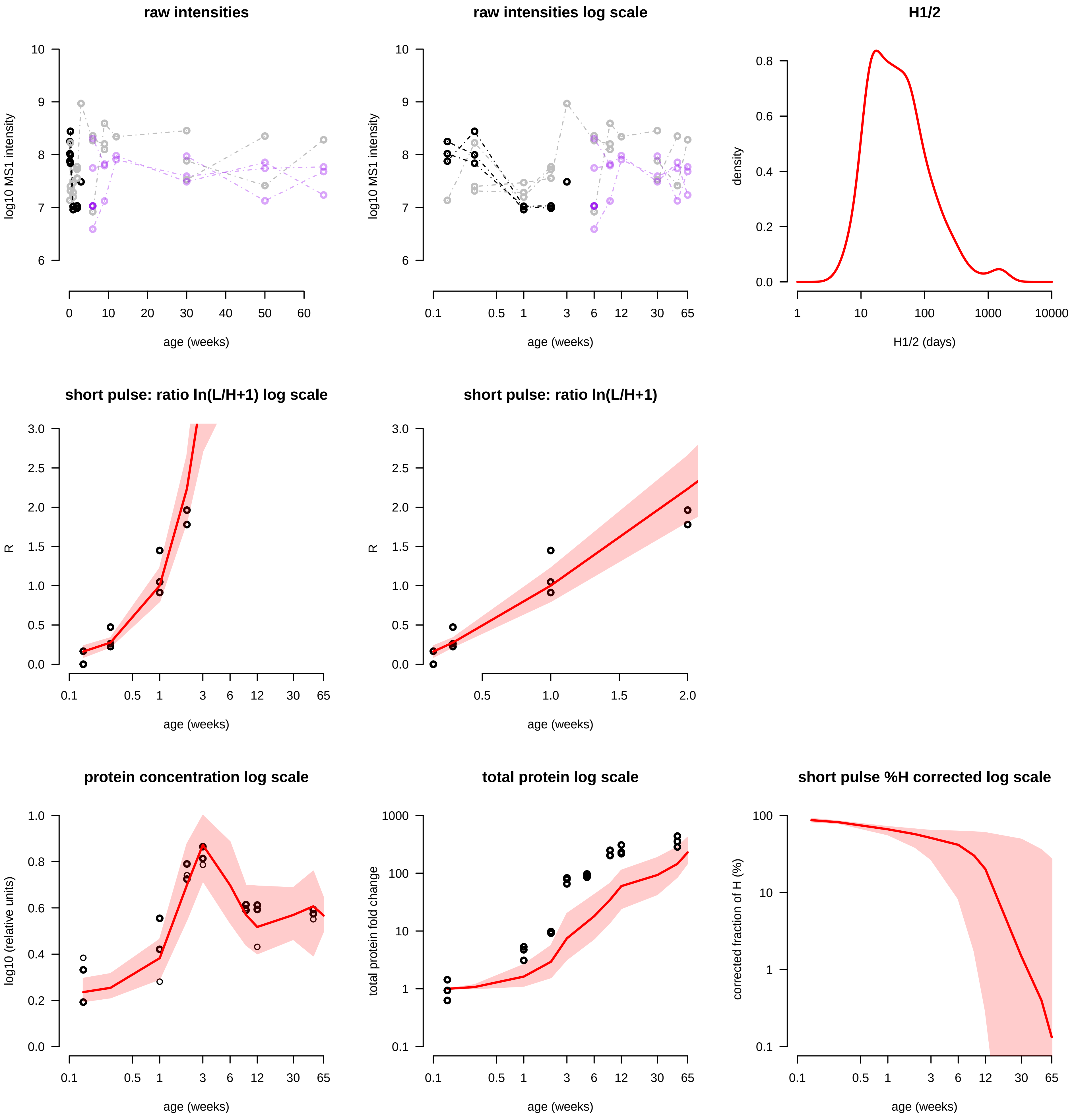

Q9Z266

>sp|Q9Z266|SNAPN_MOUSE

SNARE−associated protein Snapin

GN=Snapin

Med H1/2: 11.8 days

Q5 H1/2: 4.7 days

Q95 H1/2: 47.6 days

reliable model fit: yes

raw intensities

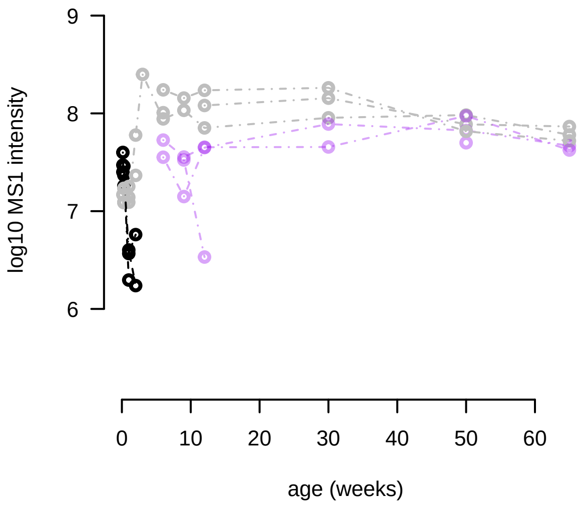

raw intensities log scale

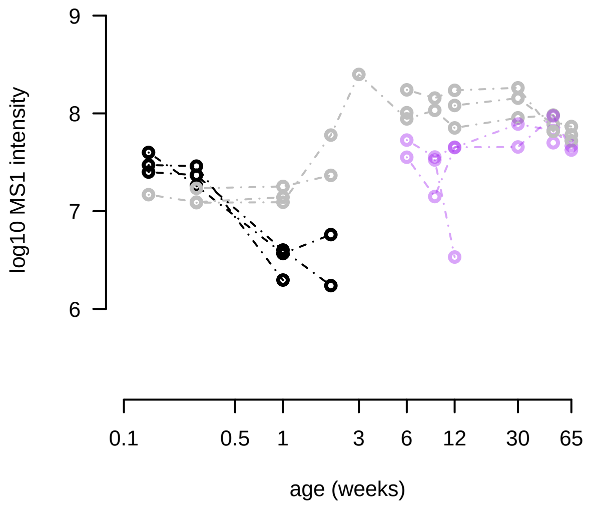

H1/2

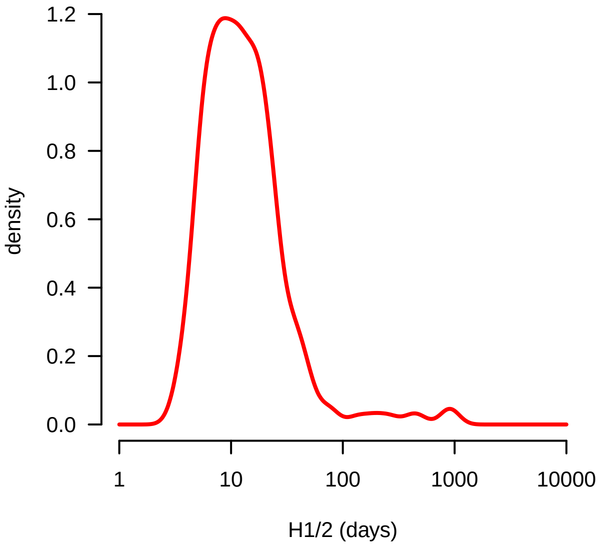

short pulse: ratio ln(L/H+1) log scale

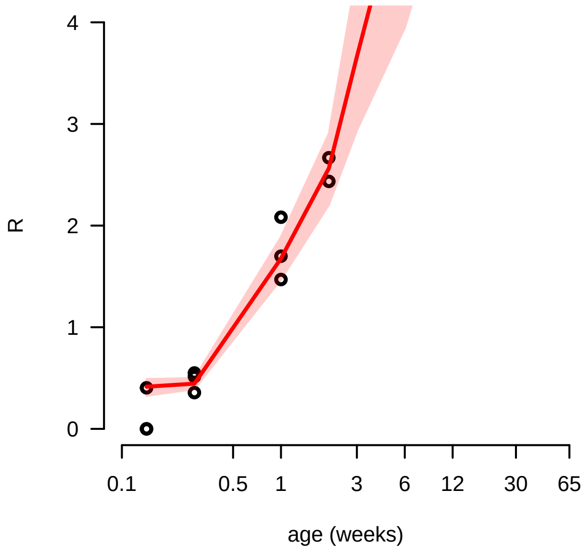

short pulse: ratio ln(L/H+1)

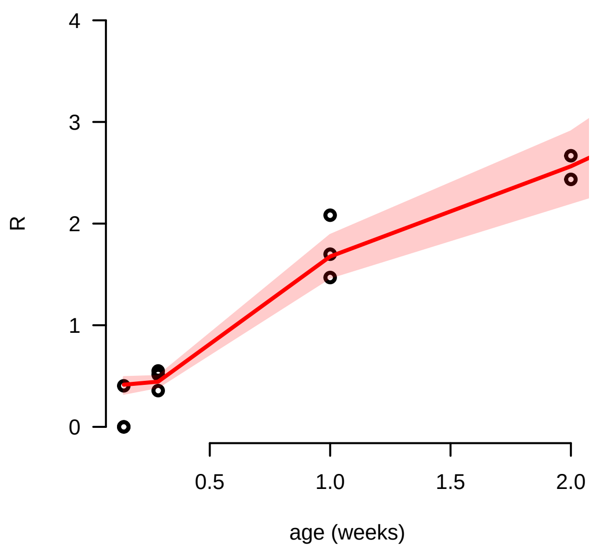

protein concentration log scale

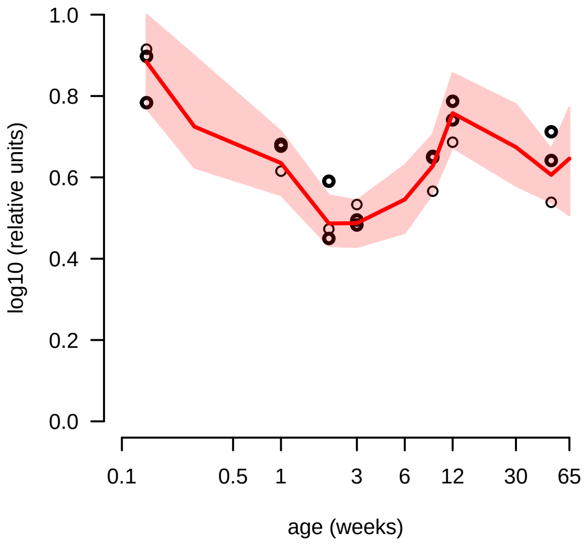

total protein log scale

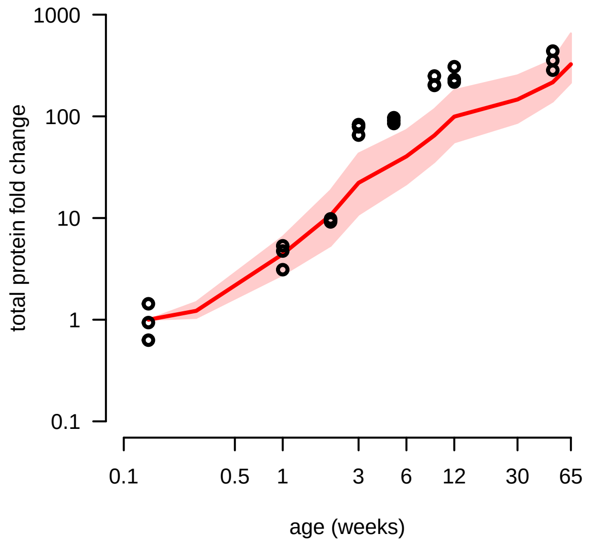

short pulse %H corrected log scale

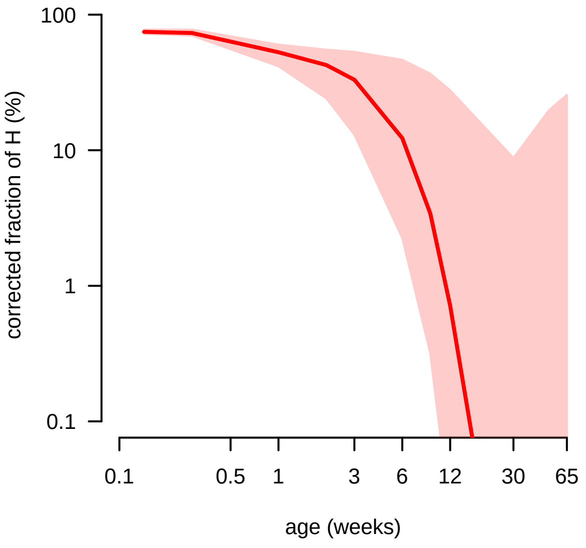

Q9EQF6

>sp|Q9EQF6|DPYL5_MOUSE

Dihydropyrimidinase−related protein 5

GN=Dpysl5

Med H1/2: 13.2 days

Q5 H1/2: 5.2 days

Q95 H1/2: 71.2 days

reliable model fit: yes

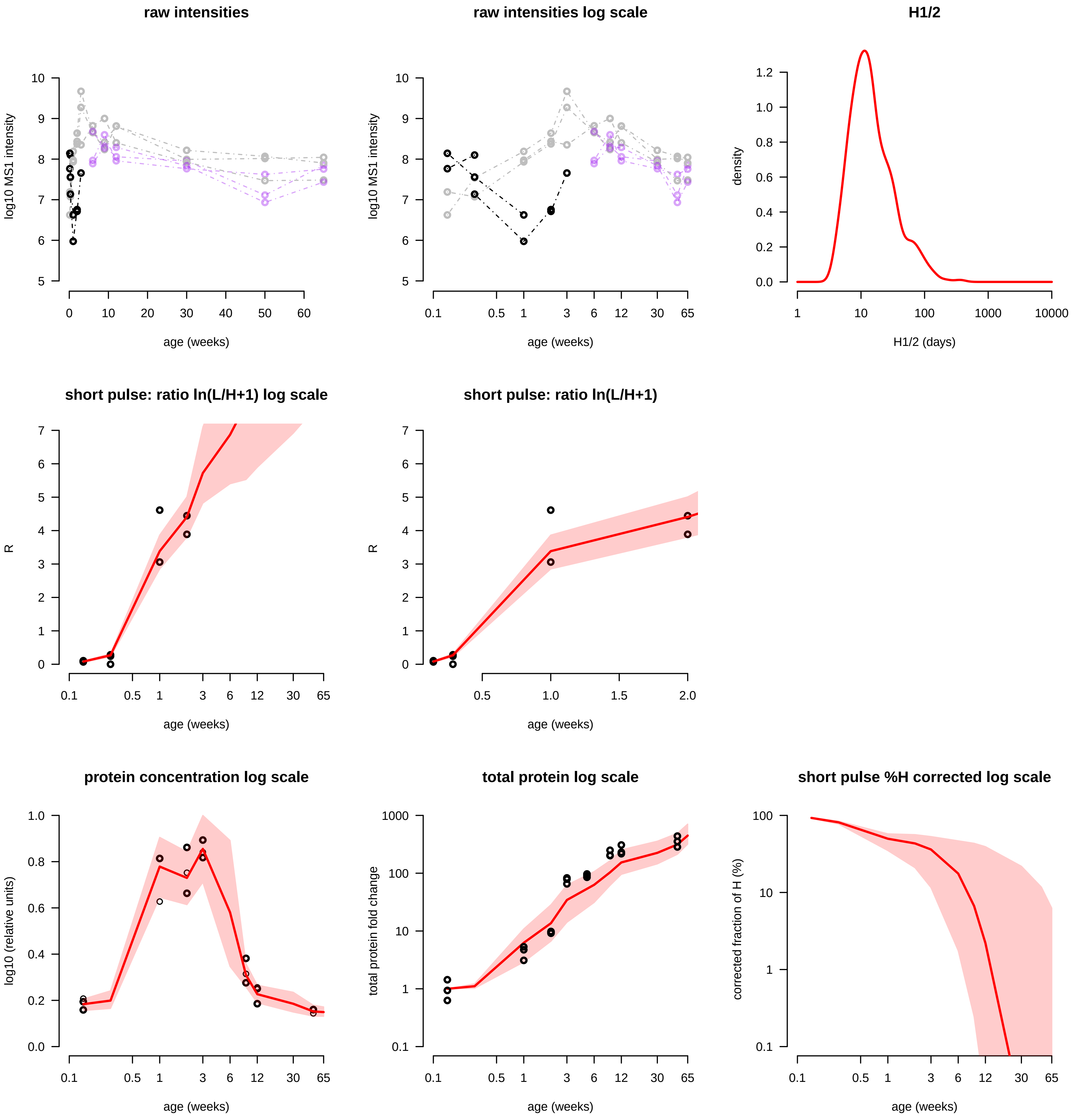

Q9CWU5;Q9CWU5−3

>sp|Q9CWU5|KHDC3_MOUSE

KH domain−containing protein 3

GN=Khdc3

Med H1/2: 9.8 days

Q5 H1/2: 7.6 days

Q95 H1/2: 14.5 days

reliable model fit: yes

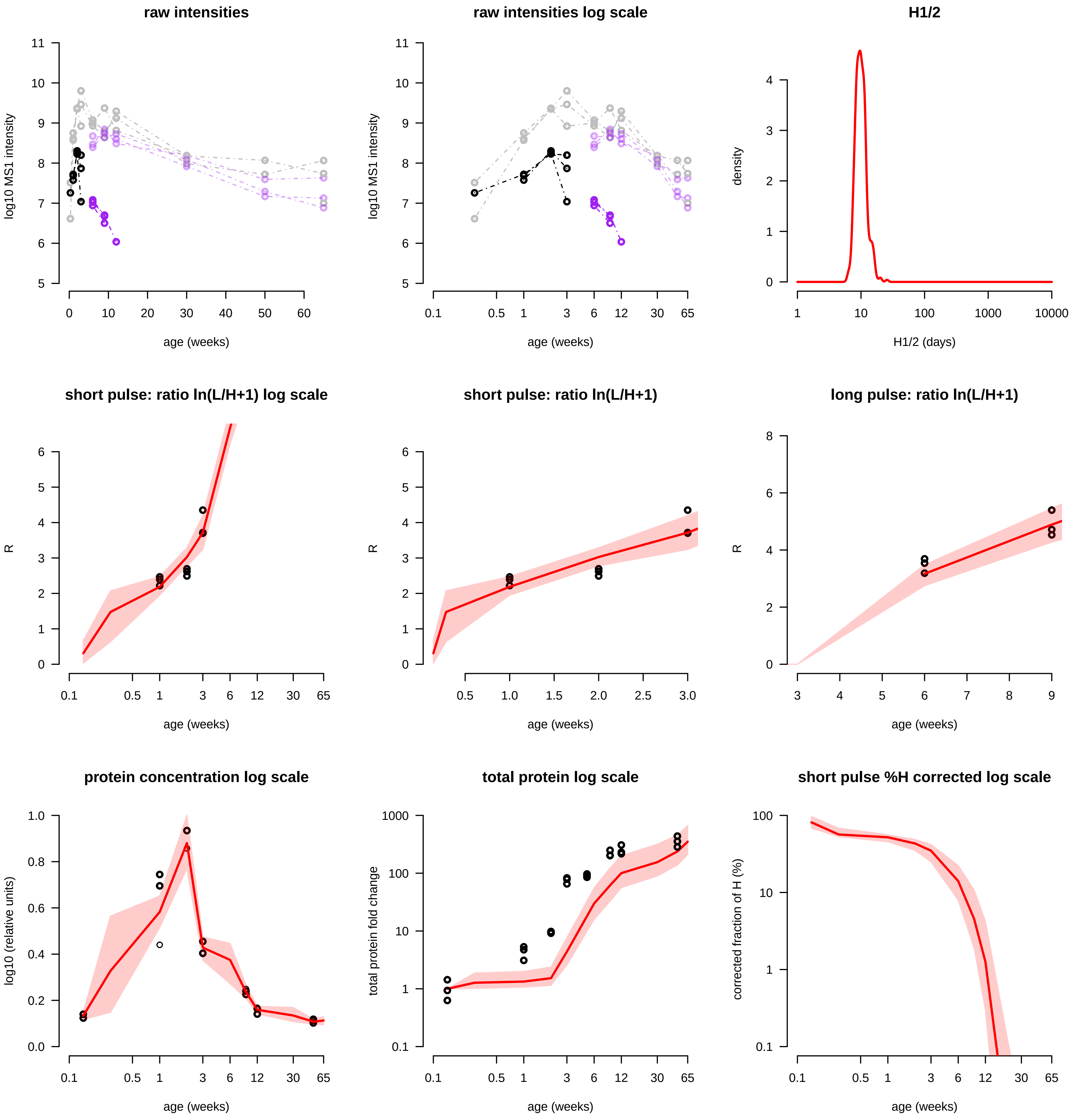

Q8JZS9

>sp|Q8JZS9|RM48_MOUSE

39S ribosomal protein L48, mitochondrial

GN=Mrpl48

Med H1/2: 10.4 days

Q5 H1/2: 4.3 days

Q95 H1/2: 116.1 days

reliable model fit: yes

raw intensities

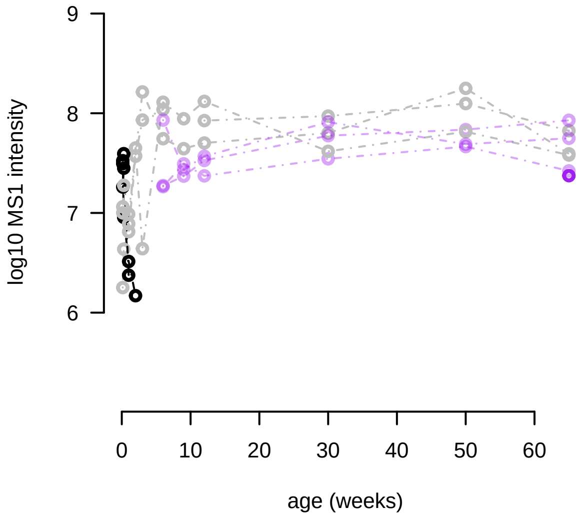

raw intensities log scale

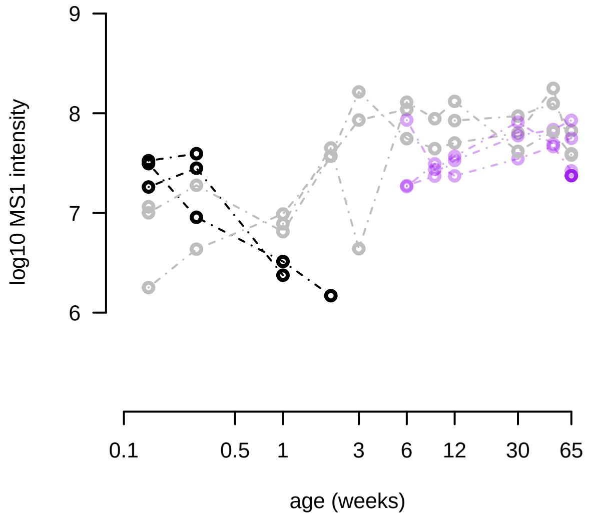

H1/2

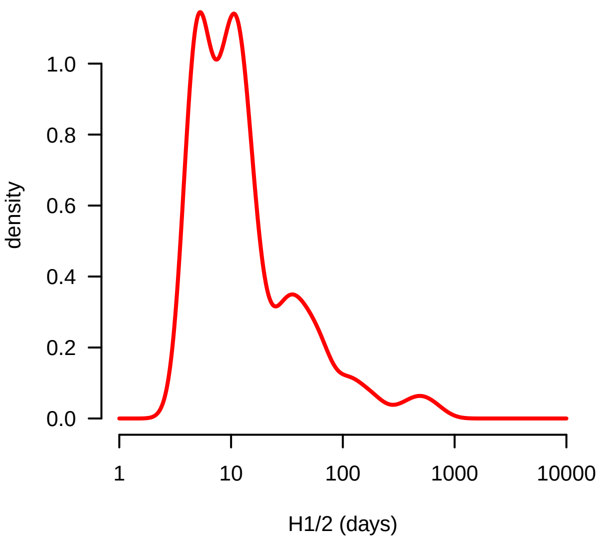

short pulse: ratio ln(L/H+1) log scale

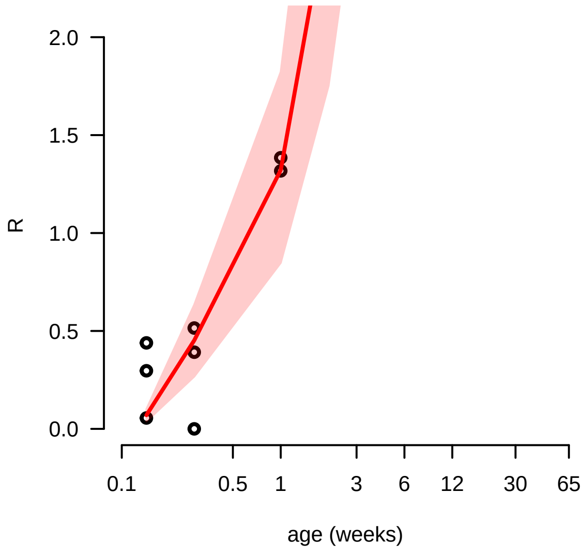

short pulse: ratio ln(L/H+1)

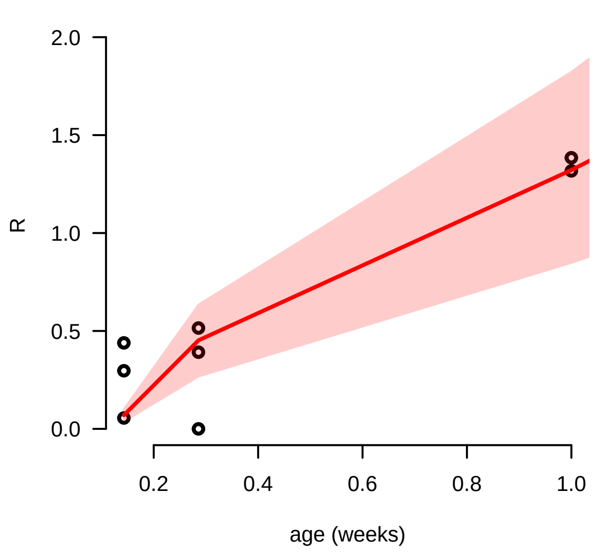

protein concentration log scale

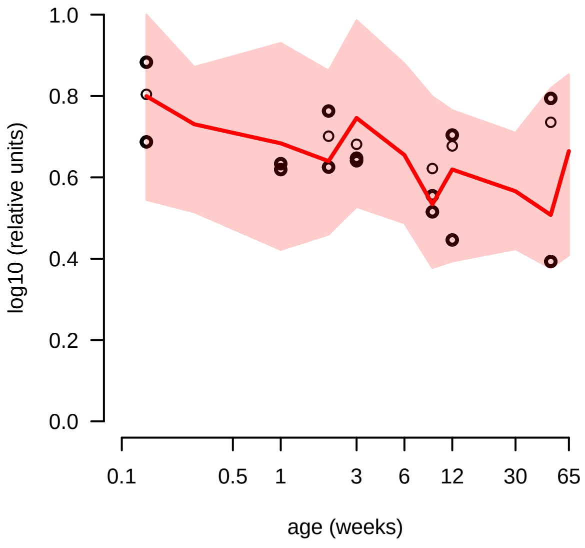

total protein log scale

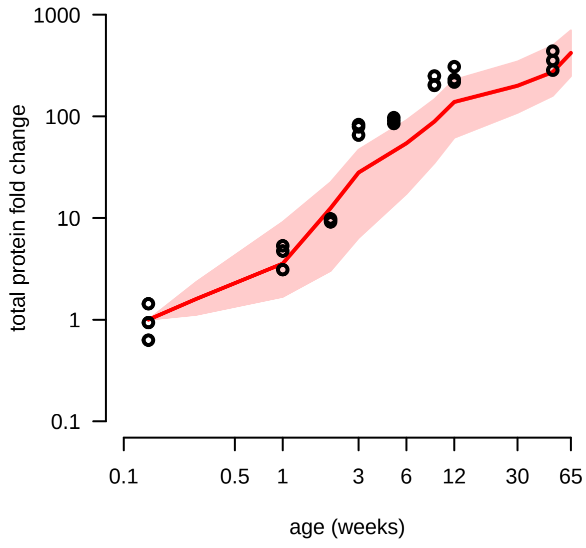

short pulse %H corrected log scale

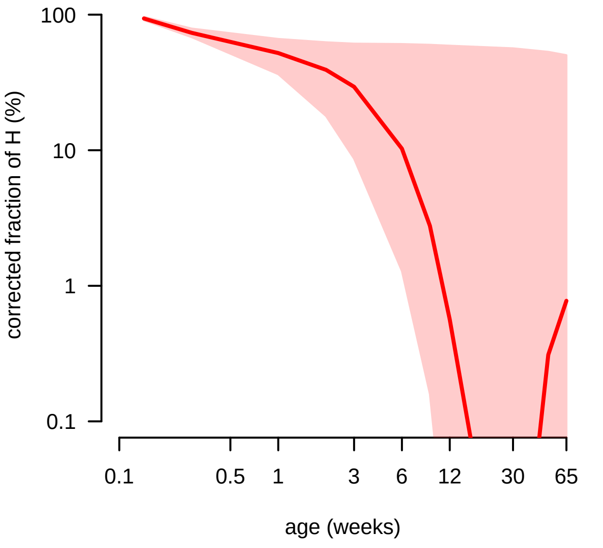

Q8VE73;Q8VE73−3

>sp|Q8VE73|CUL7_MOUSE

Cullin−7

GN=Cul7

Med H1/2: 4.6 days

Q5 H1/2: 2.7 days

Q95 H1/2: 25.3 days

reliable model fit: yes

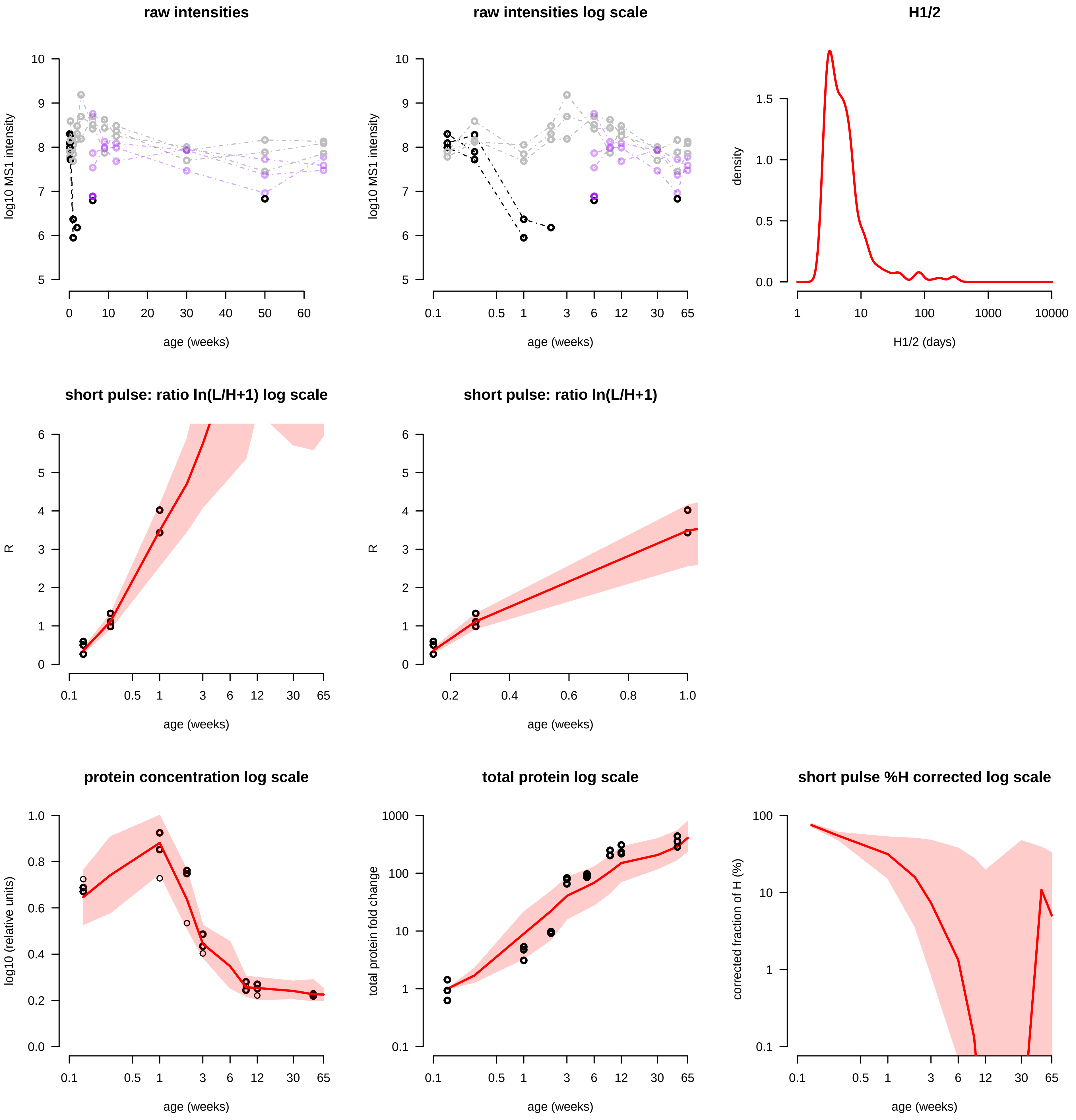

A2A791;A2A791−2

>sp|A2A791|ZMYM4_MOUSE

Zinc finger MYM−type protein 4

GN=Zmym4

Med H1/2: 4.1 days

Q5 H1/2: 2.5 days

Q95 H1/2: 35.1 days

reliable model fit: yes

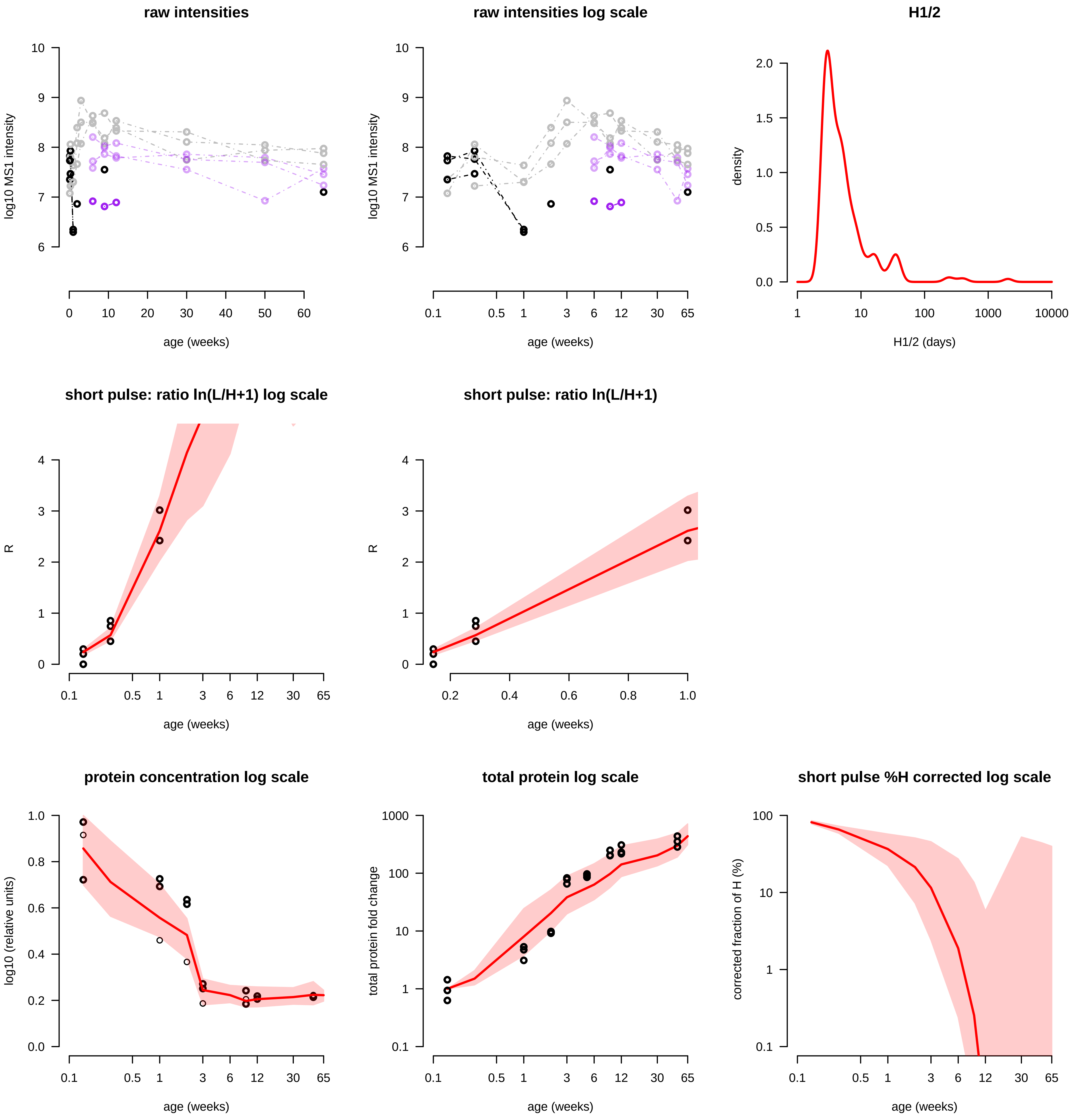

Q80Y17

>sp|Q80Y17|L2GL1_MOUSE

Lethal(2) giant larvae protein homolog 1

GN=Llgl1

Med H1/2: 3553.1 days

Q5 H1/2: 647.9 days

Q95 H1/2: 89149.9 days

reliable model fit: no

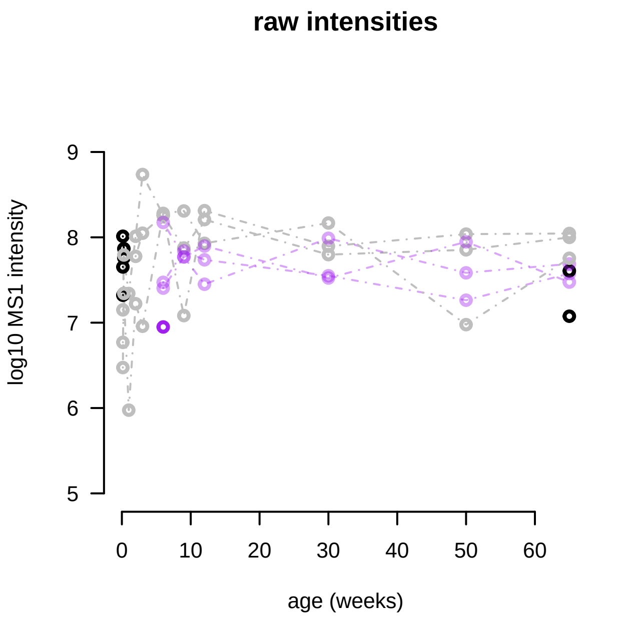

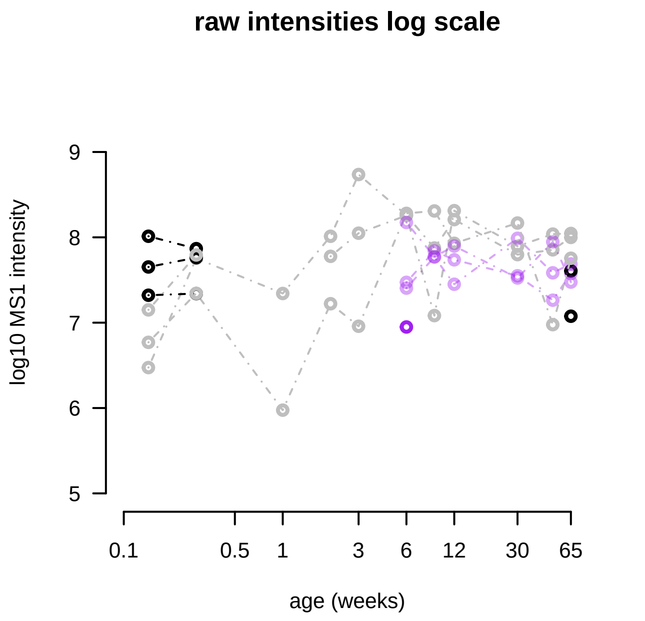

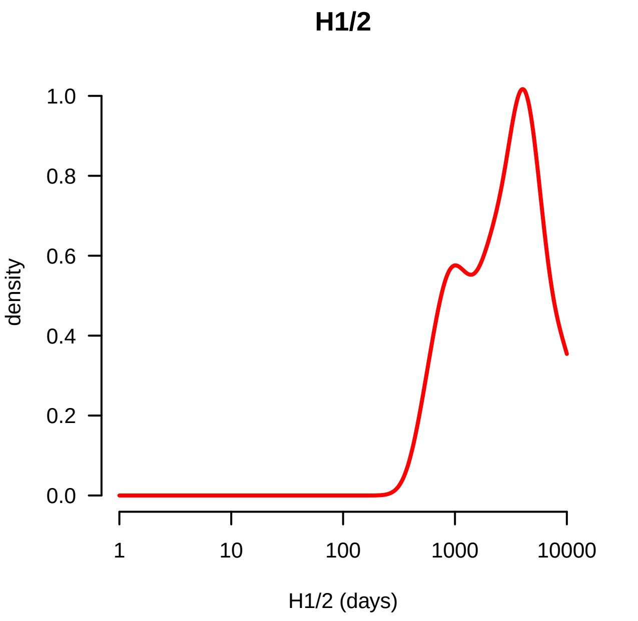

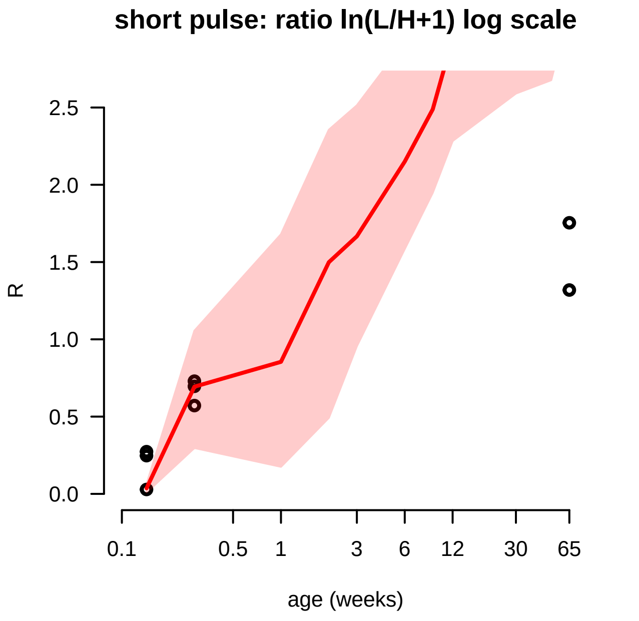

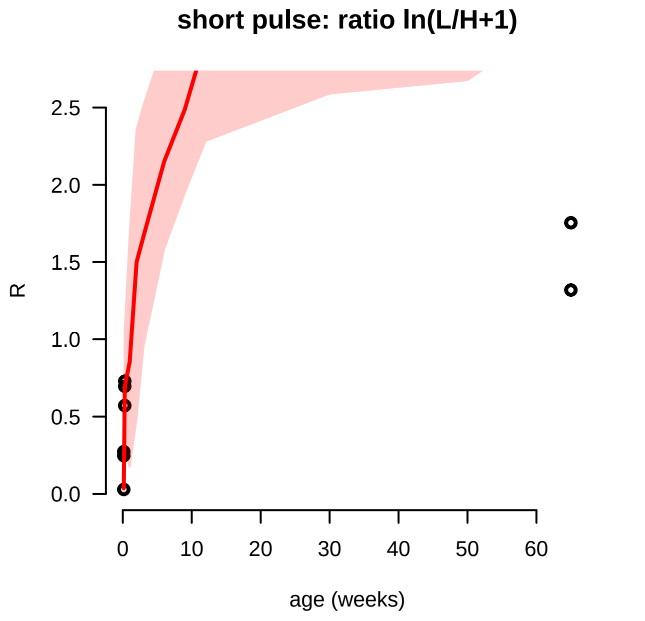

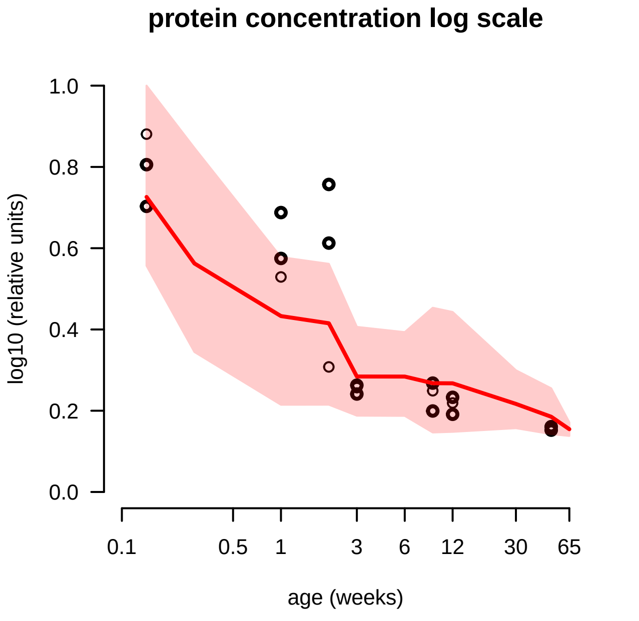

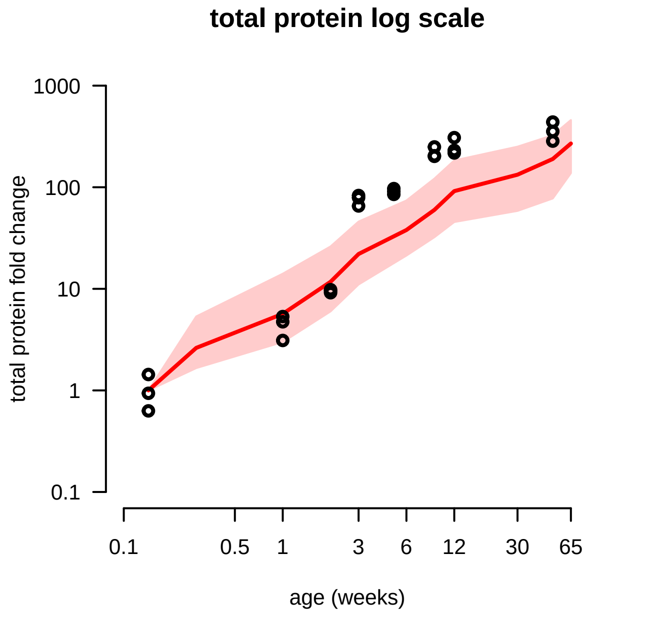

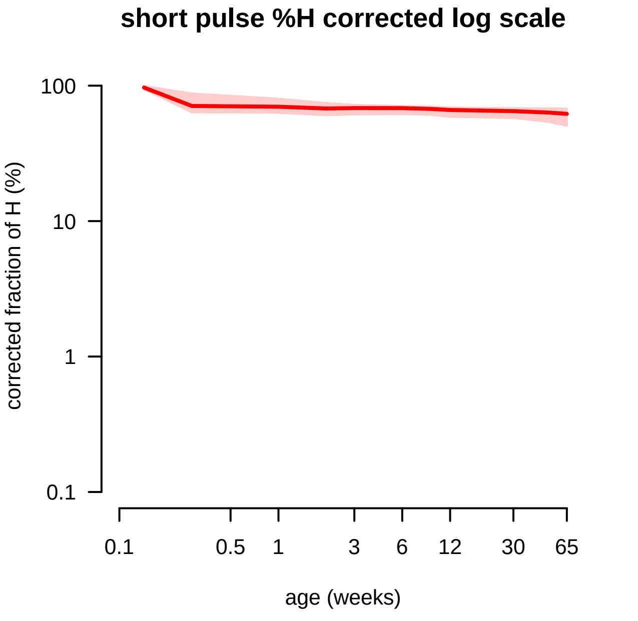

Q9JM96

>sp|Q9JM96|BORG4_MOUSE

Cdc42 effector protein 4

GN=Cdc42ep4

Med H1/2: 2203.5 days

Q5 H1/2: 563 days

Q95 H1/2: 37061 days

reliable model fit: no

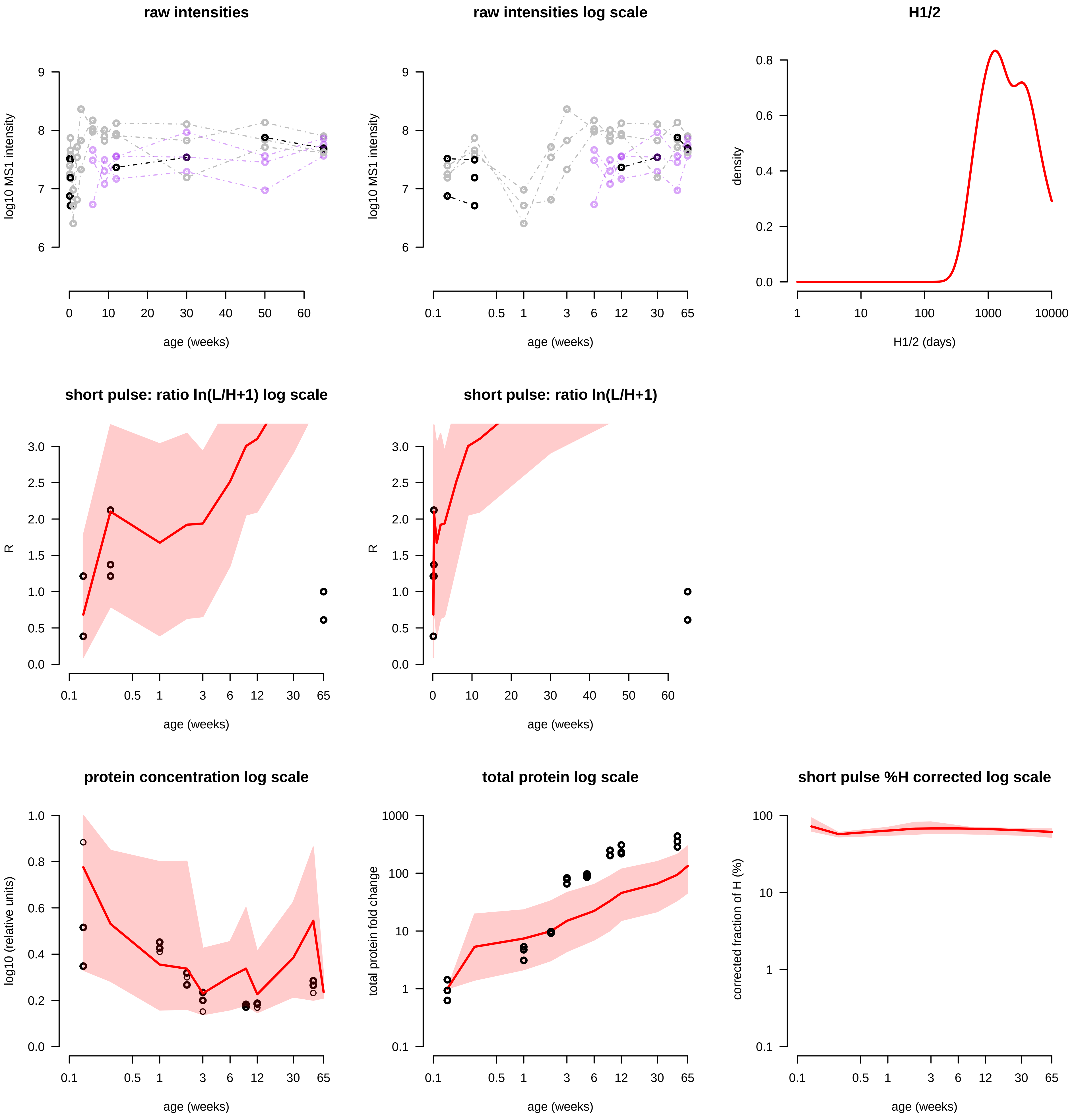

Q9CRA8

>sp|Q9CRA8|EXOS5_MOUSE

Exosome complex component RRP46

GN=Exosc5

Med H1/2: 8.5 days

Q5 H1/2: 3.3 days

Q95 H1/2: 111.6 days

reliable model fit: yes

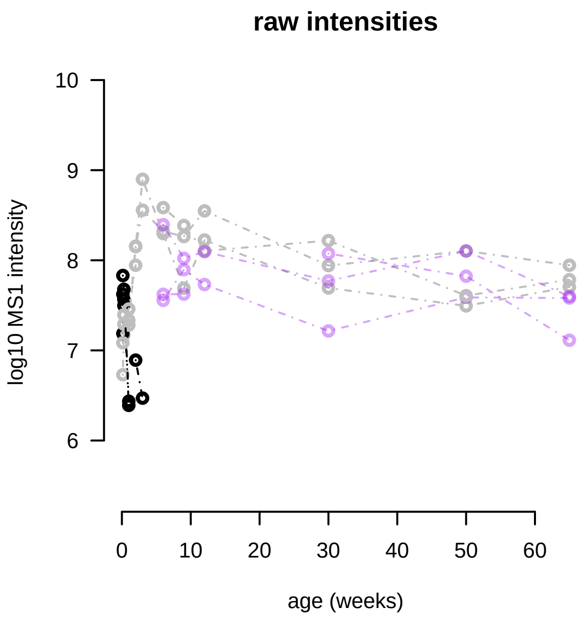

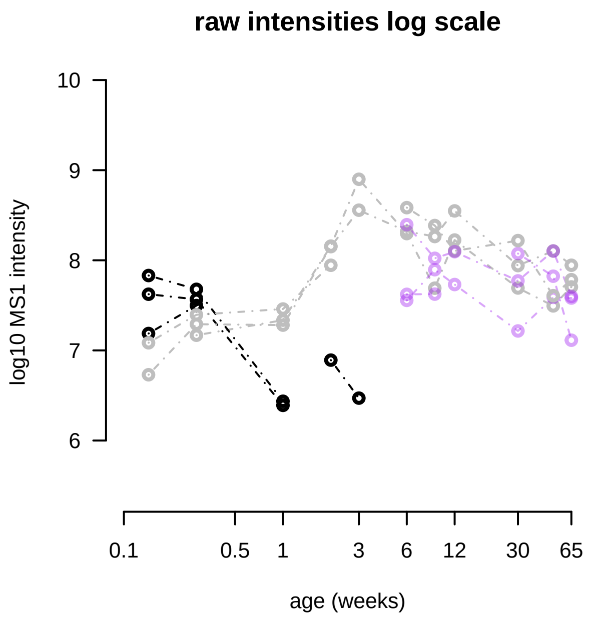

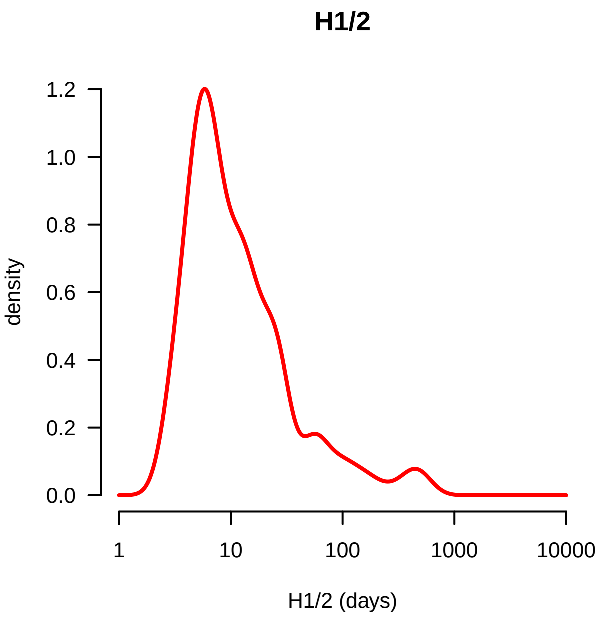

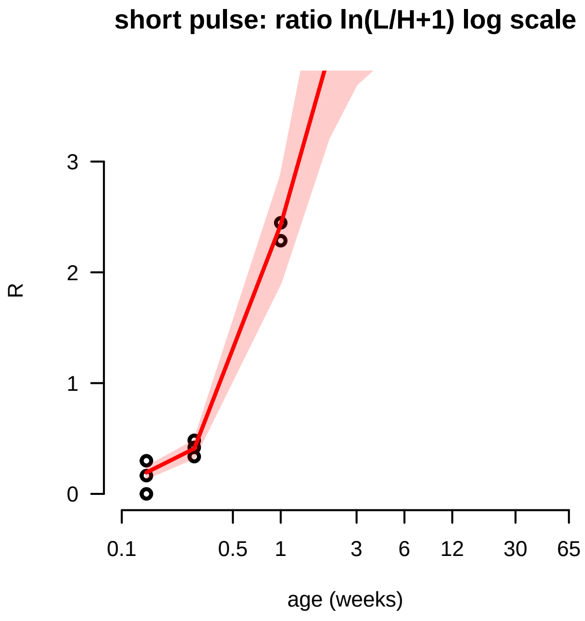

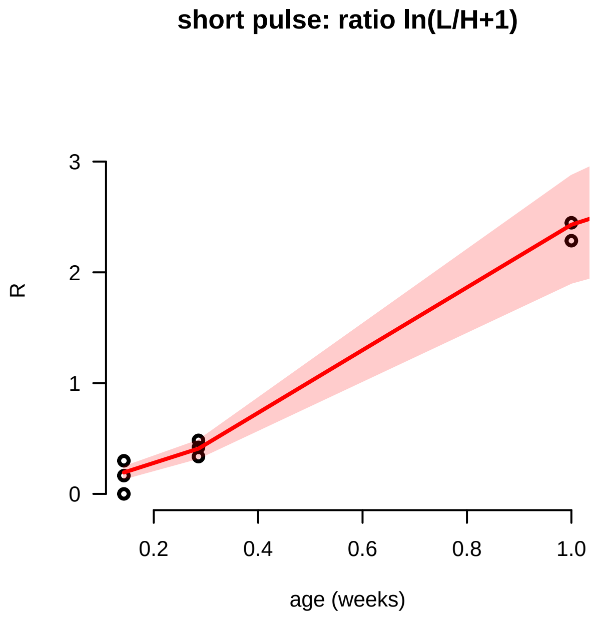

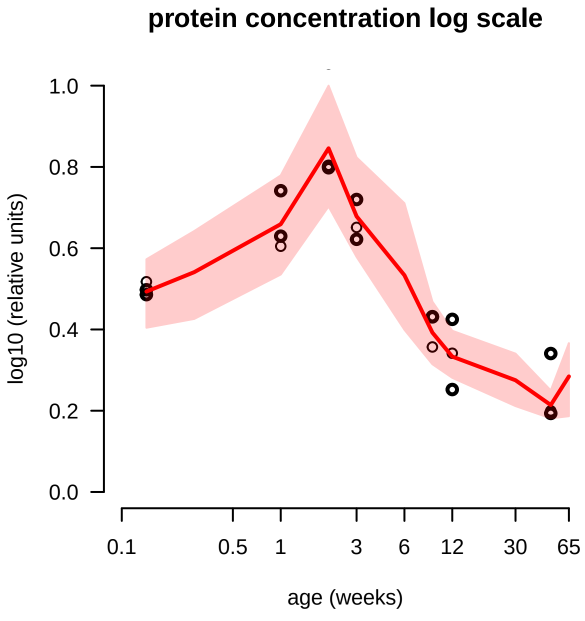

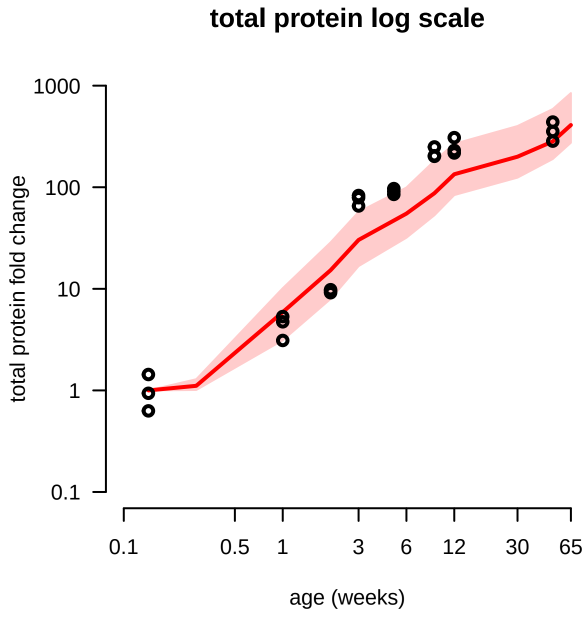

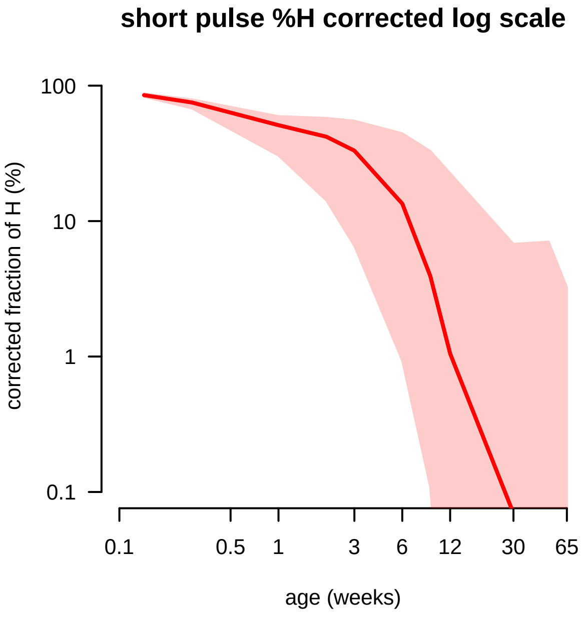

Q5SSI6

>sp|Q5SSI6|UTP18_MOUSE

U3 small nucleolar RNA−associated protein 18 homolog

GN=Utp18

Med H1/2: 217.6 days

Q5 H1/2: 133 days

Q95 H1/2: 524.7 days

reliable model fit: yes

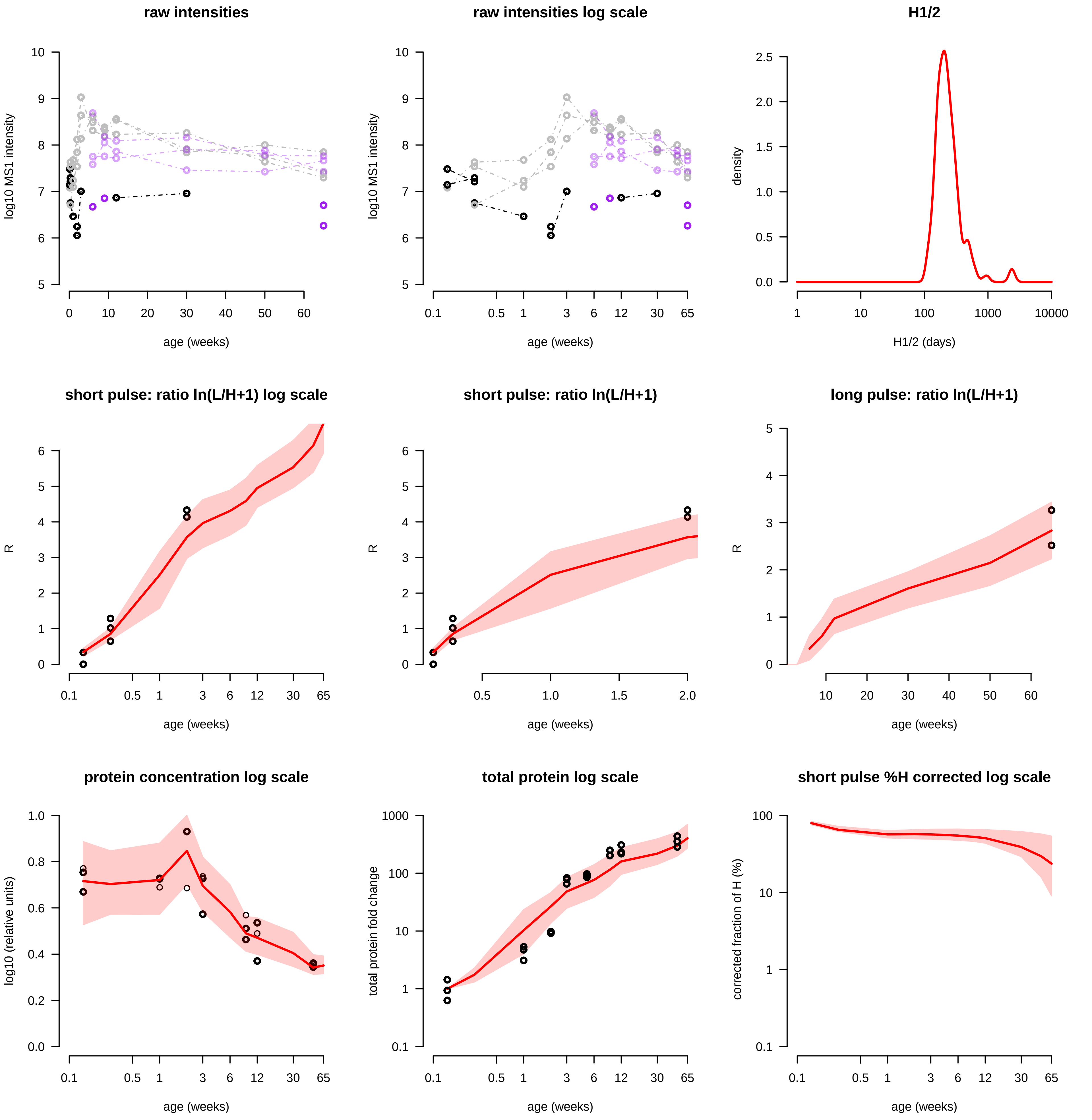

Q8BIQ5;Q8BIQ5−2

>sp|Q8BIQ5|CSTF2_MOUSE

Cleavage stimulation factor subunit 2

GN=Cstf2

Med H1/2: 4.4 days

Q5 H1/2: 2.6 days

Q95 H1/2: 11.9 days

reliable model fit: yes

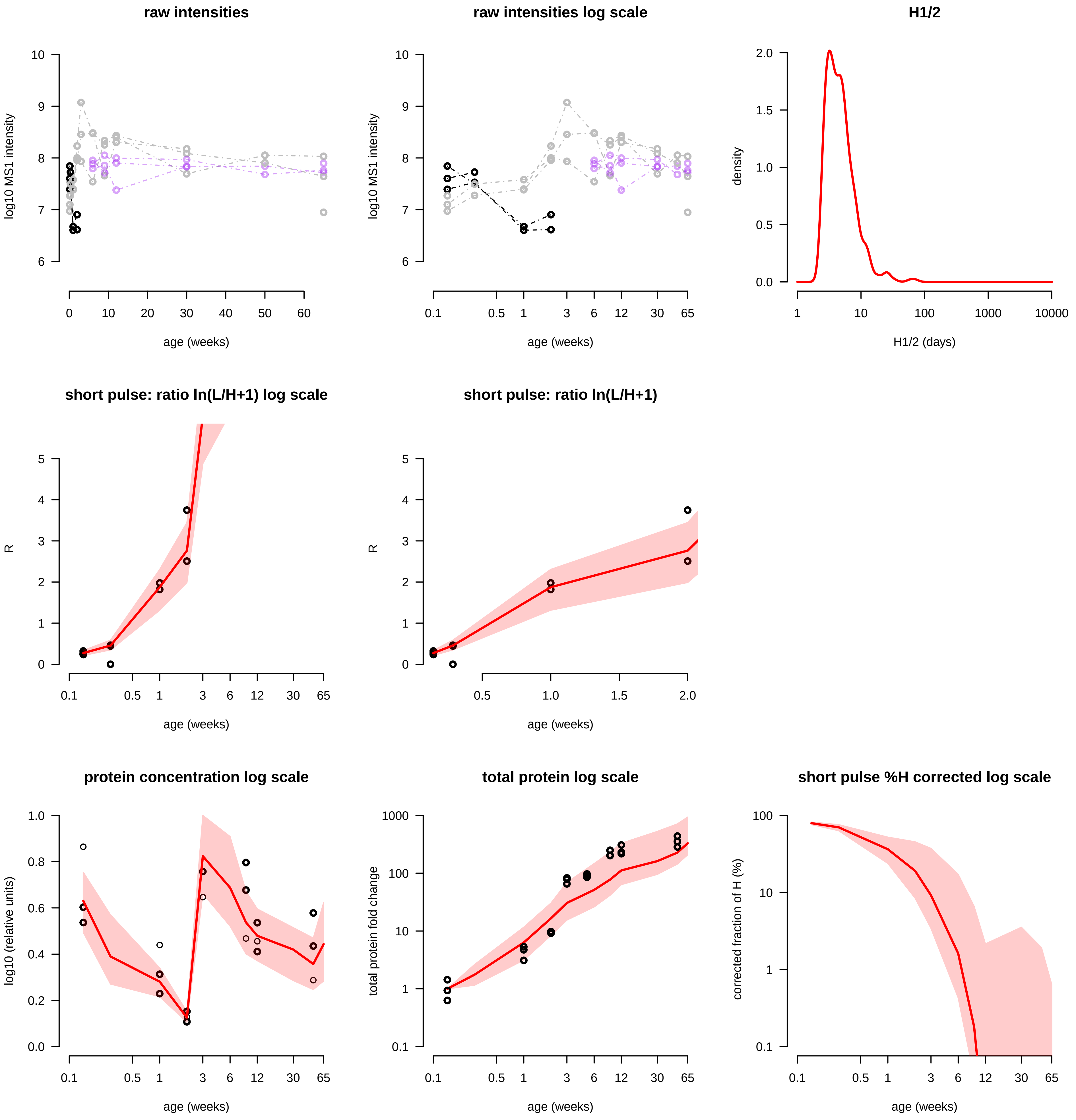

Q9D7M8

>sp|Q9D7M8|RPB4_MOUSE

DNA−directed RNA polymerase II subunit RPB4

GN=Polr2d

Med H1/2: 7.7 days

Q5 H1/2: 2.8 days

Q95 H1/2: 119.9 days

reliable model fit: yes

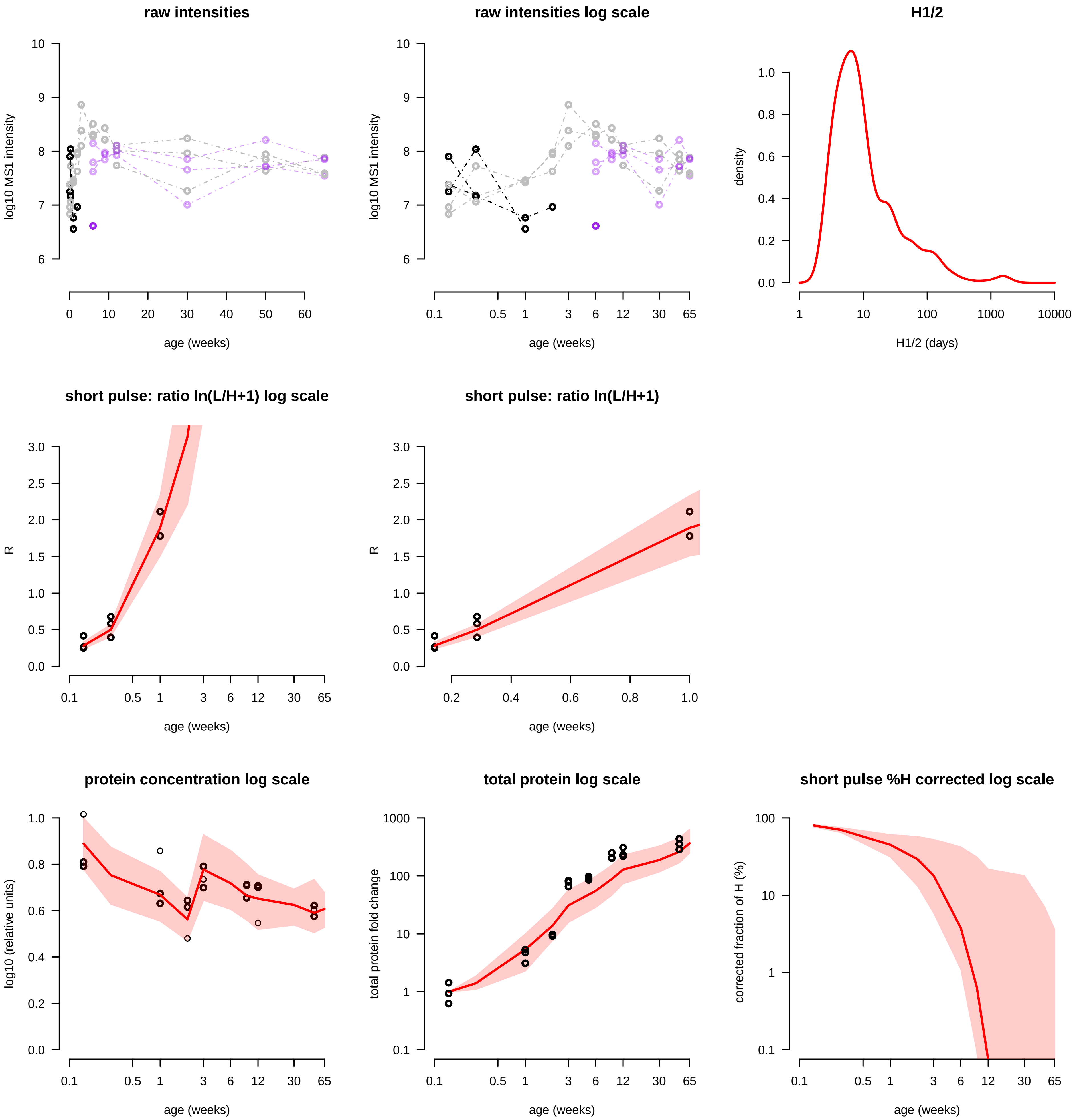

Q8VHN8;Q8VHN8−2;Q8VHN8−3

>sp|Q8VHN8|TIRR_MOUSE

Tudor−interacting repair regulator protein

GN=Nudt16l1

Med H1/2: 5.5 days

Q5 H1/2: 4.7 days

Q95 H1/2: 6.9 days

reliable model fit: yes

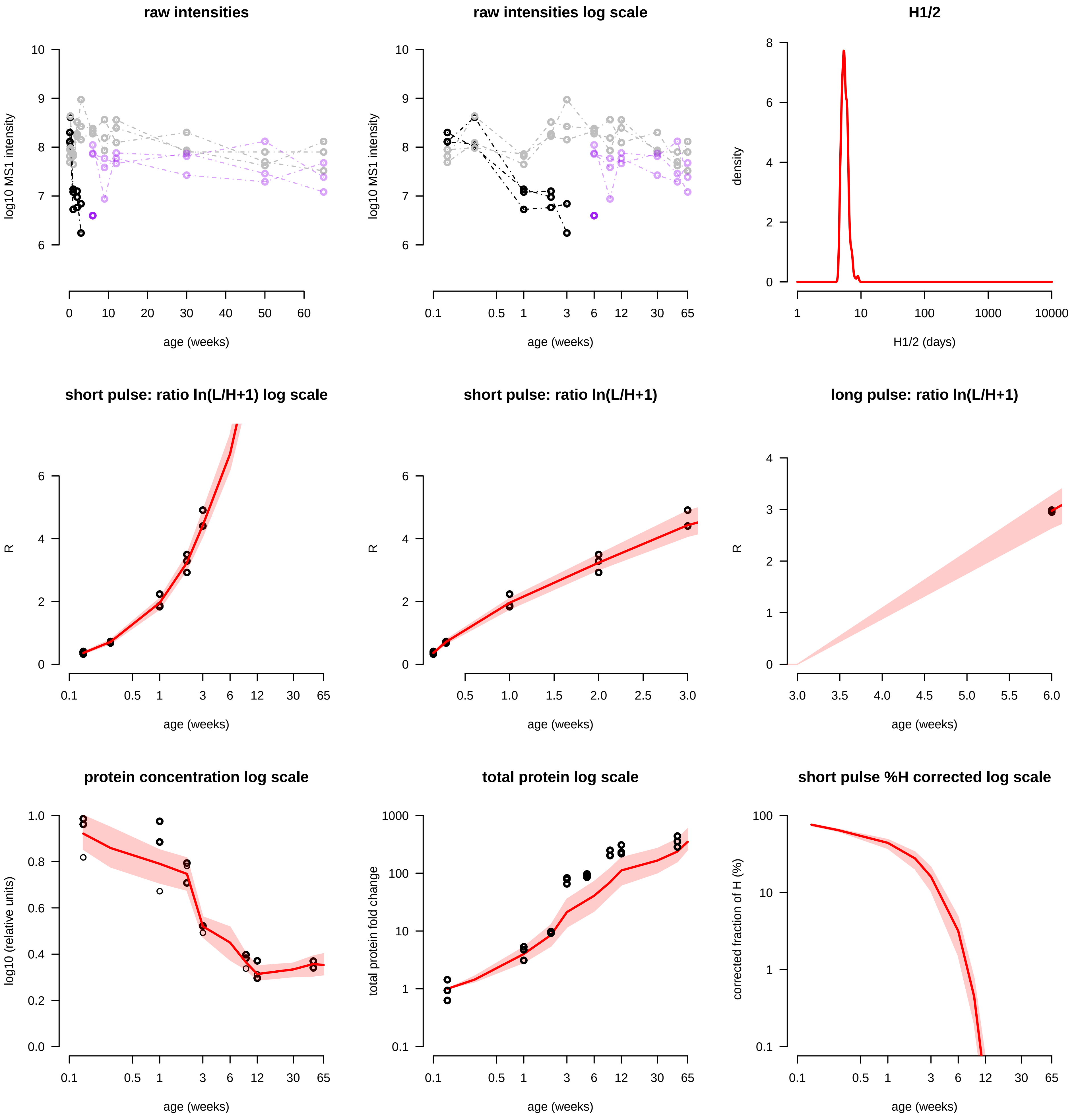

Q9D3U0

>sp|Q9D3U0|PUS10_MOUSE

tRNA pseudouridine synthase Pus10

GN=Pus10

Med H1/2: 7 days

Q5 H1/2: 3.3 days

Q95 H1/2: 45 days

reliable model fit: yes

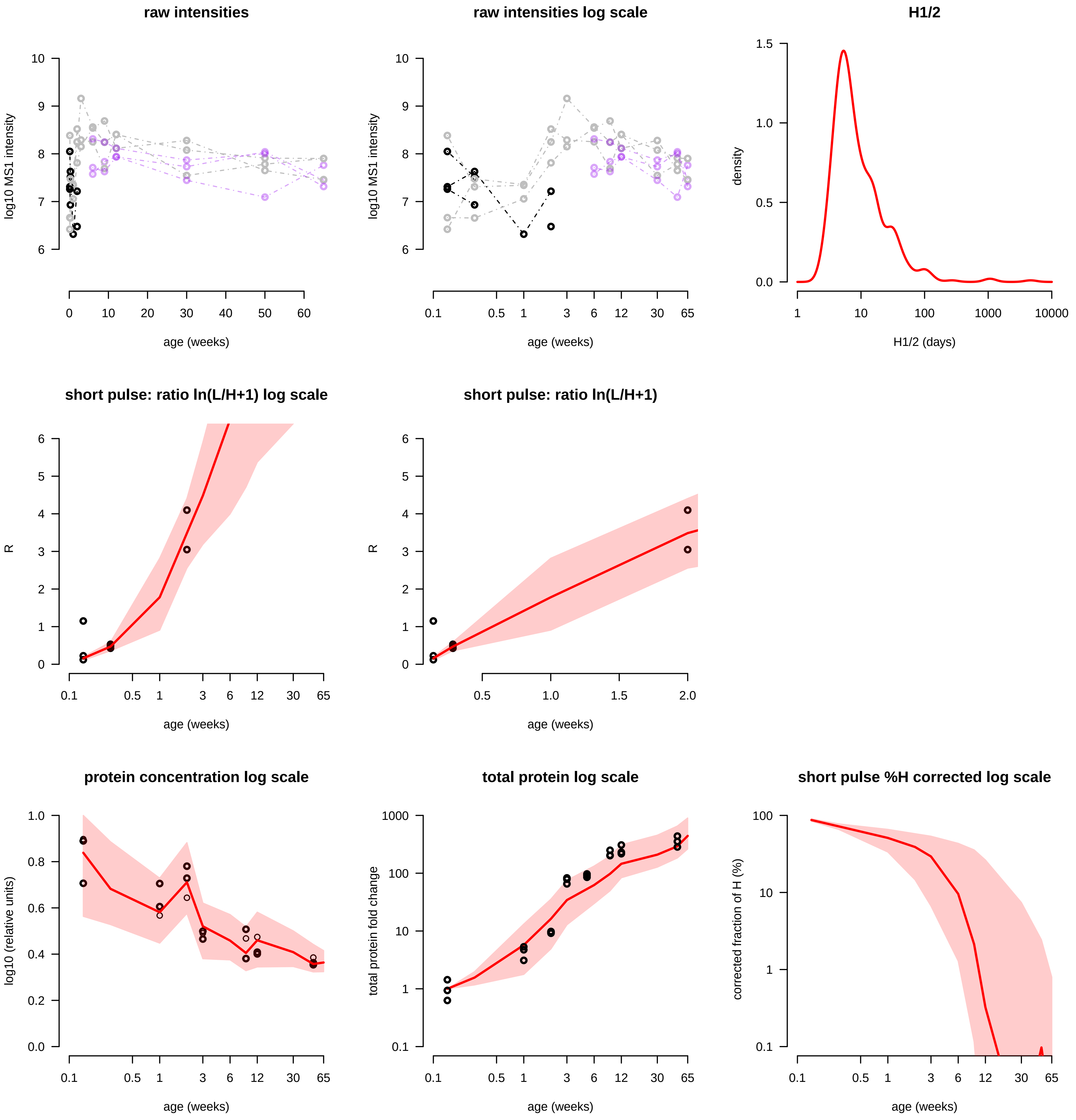

P21271;P21271−3

>sp|P21271|MYO5B_MOUSE

Unconventional myosin−Vb

GN=Myo5b

Med H1/2: 4.1 days

Q5 H1/2: 2.6 days

Q95 H1/2: 15.7 days

reliable model fit: yes

raw intensities

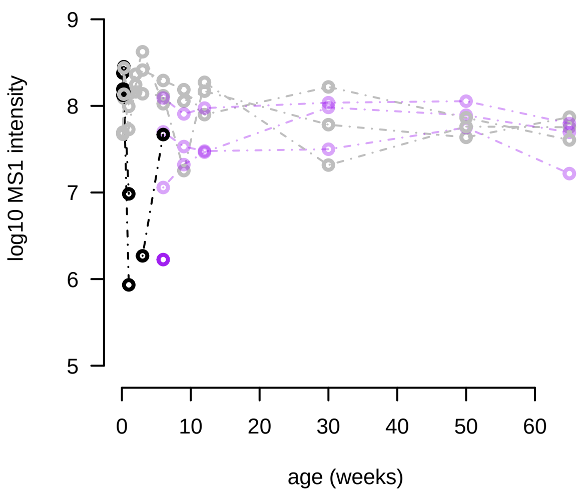

raw intensities log scale

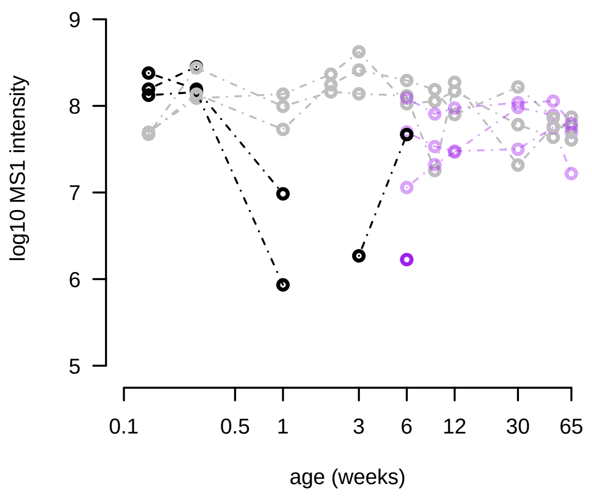

H1/2

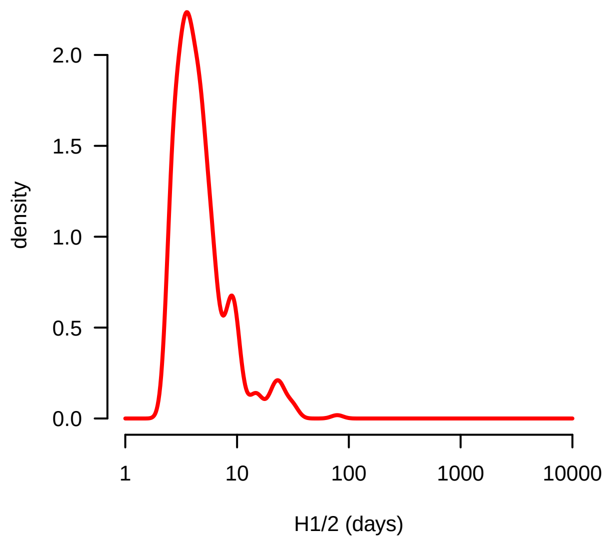

short pulse: ratio ln(L/H+1) log scale

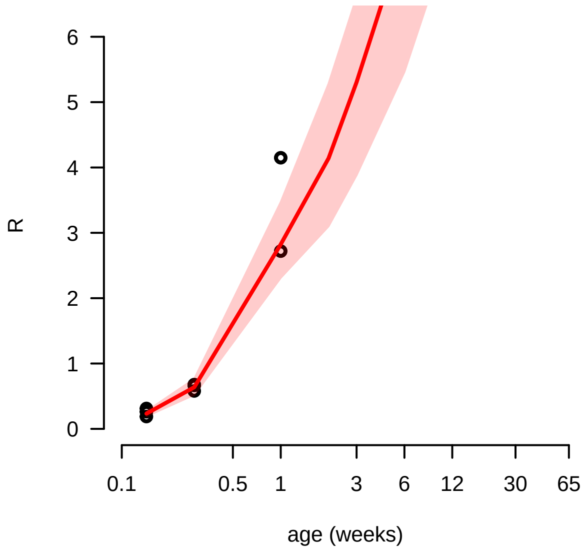

short pulse: ratio ln(L/H+1)

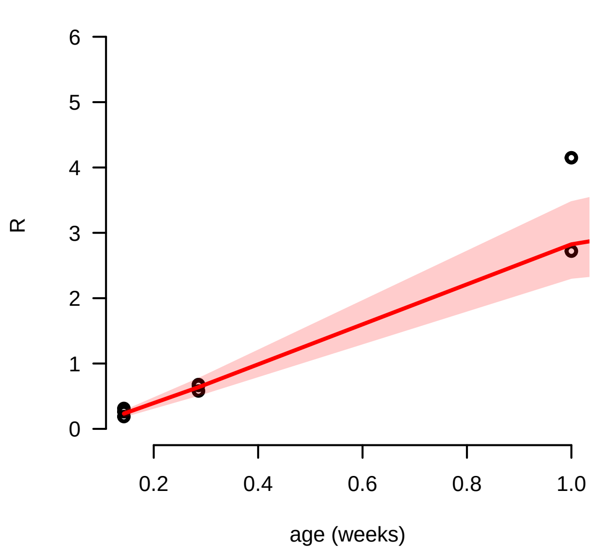

protein concentration log scale

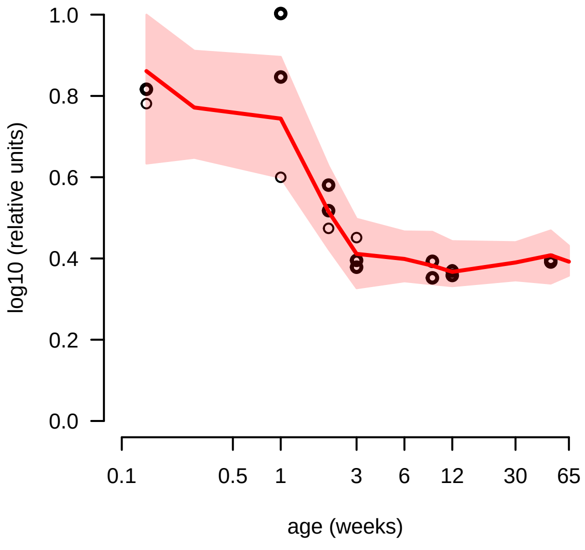

total protein log scale

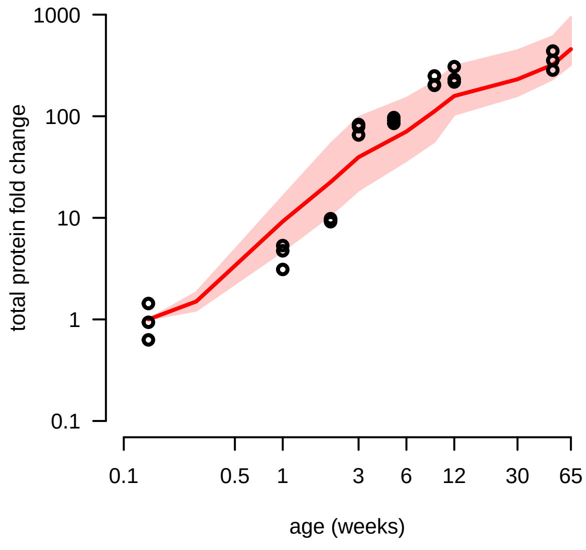

short pulse %H corrected log scale

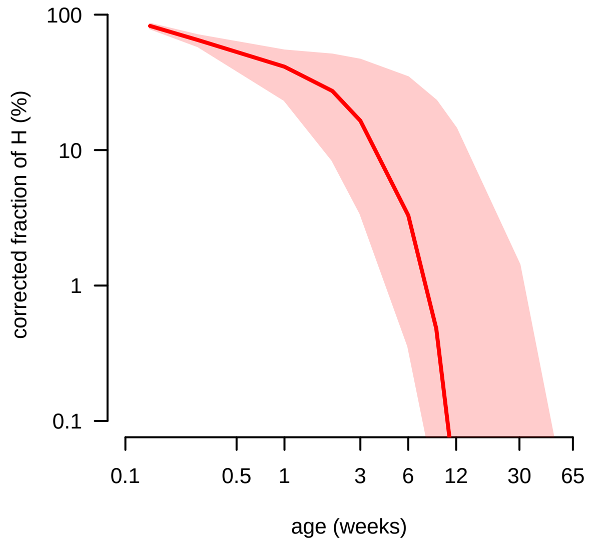

Q64449

>sp|Q64449|MRC2_MOUSE

C−type mannose receptor 2

GN=Mrc2

Med H1/2: 13.2 days

Q5 H1/2: 4.6 days

Q95 H1/2: 191.2 days

reliable model fit: yes

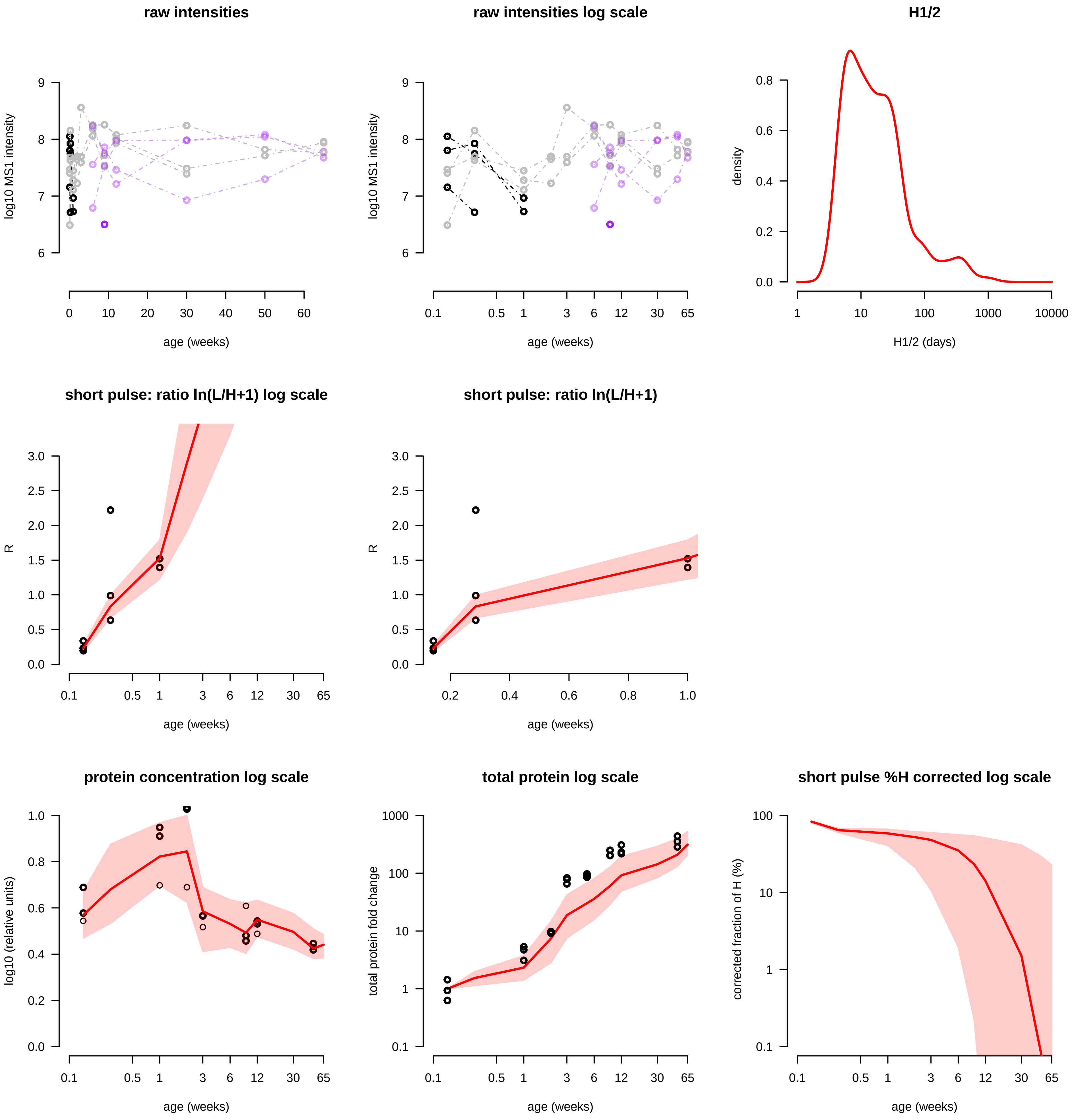

P28843

>sp|P28843|DPP4_MOUSE

Dipeptidyl peptidase 4

GN=Dpp4

Med H1/2: 554.4 days

Q5 H1/2: 133.2 days

Q95 H1/2: 4748.7 days

reliable model fit: yes

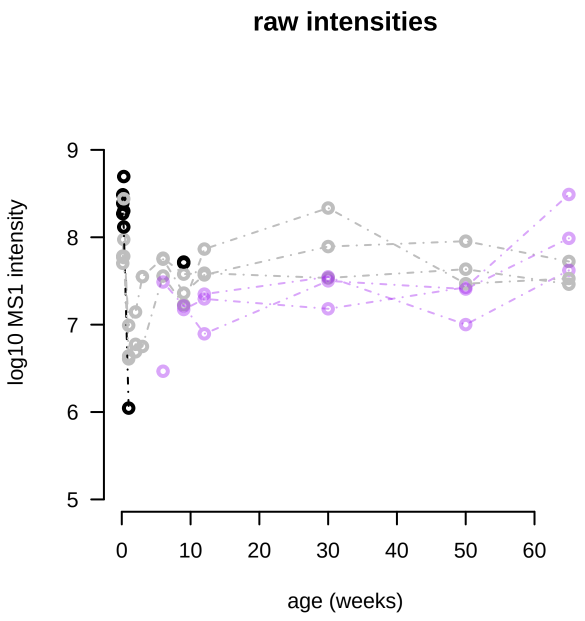

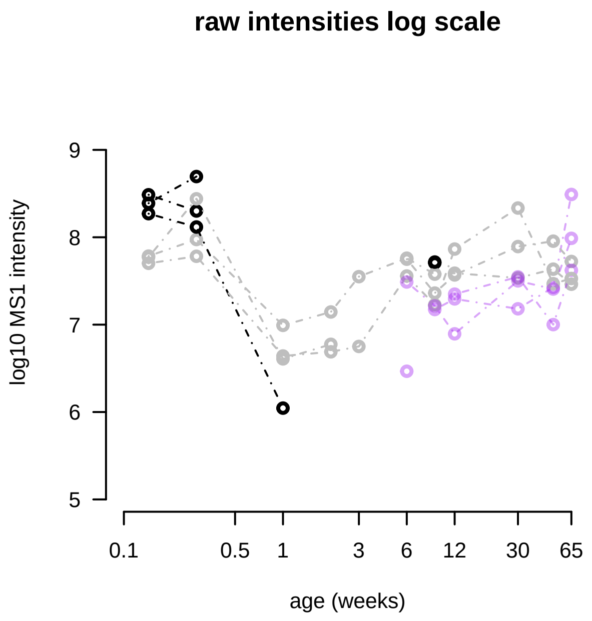

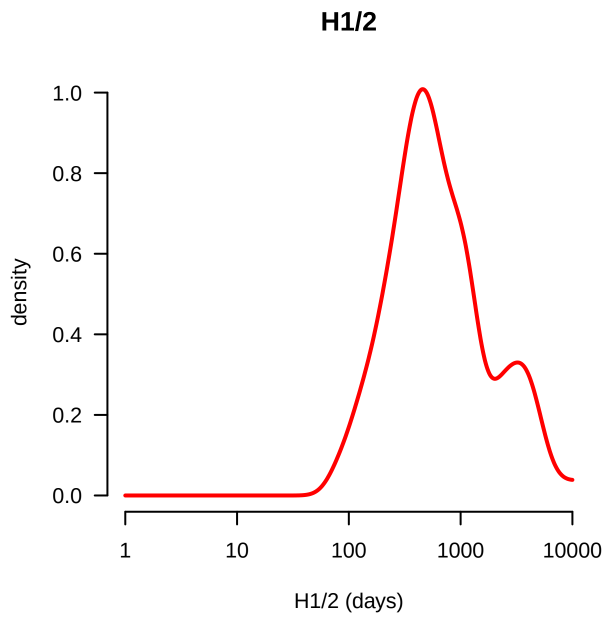

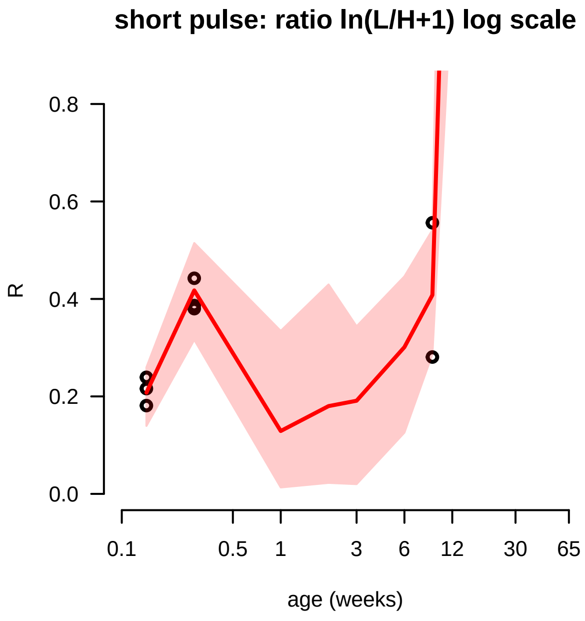

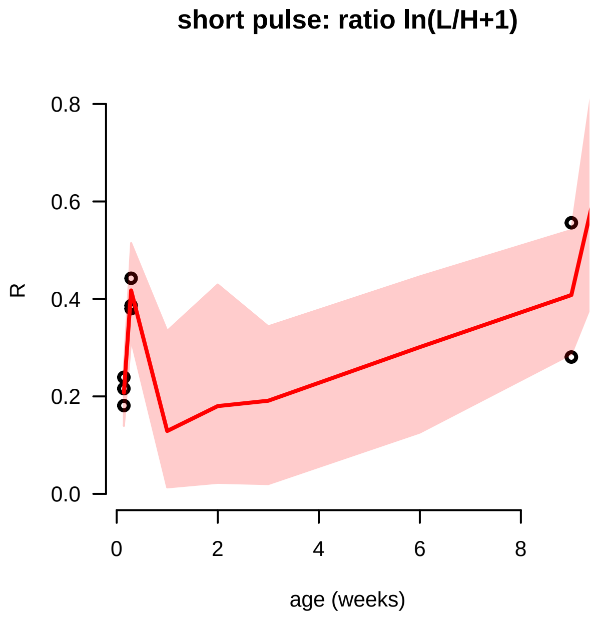

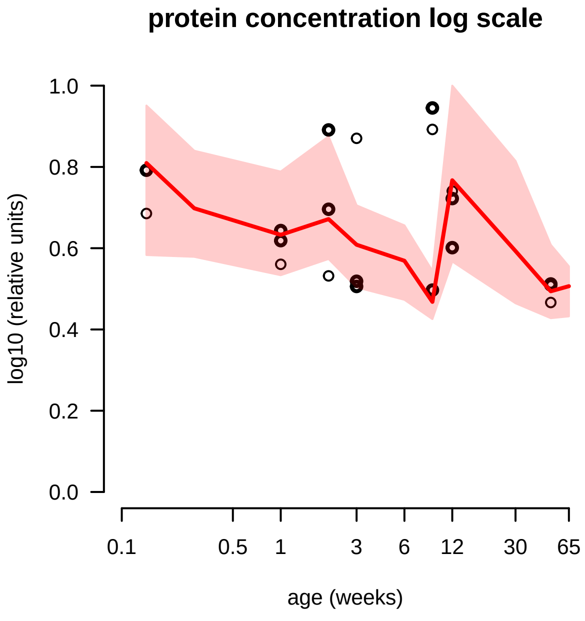

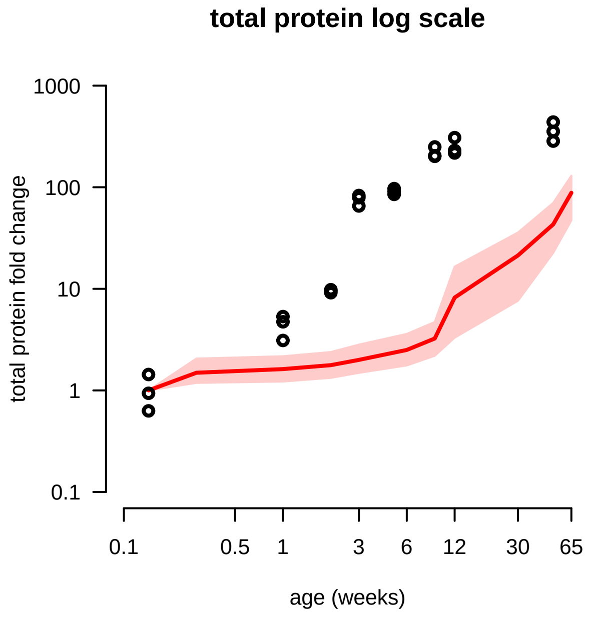

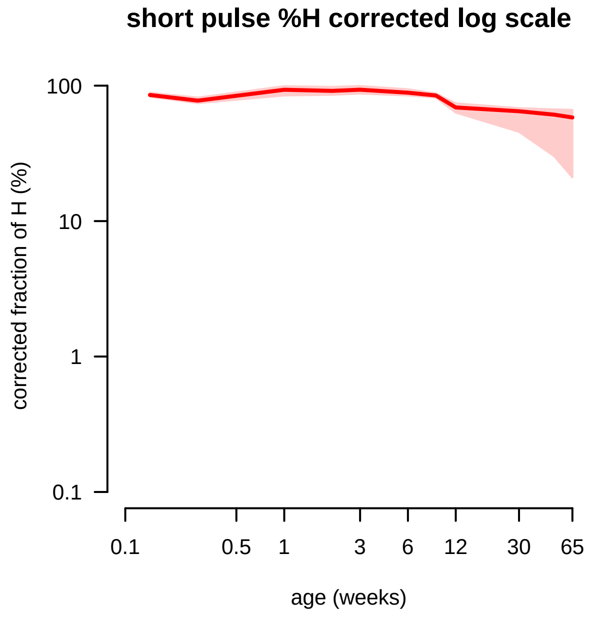

Q3U2K0;Q3U2K0−2

>sp|Q3U2K0|F193B_MOUSE

Protein FAM193B

GN=Fam193b

Med H1/2: 7.1 days

Q5 H1/2: 4.7 days

Q95 H1/2: 12.1 days

reliable model fit: yes

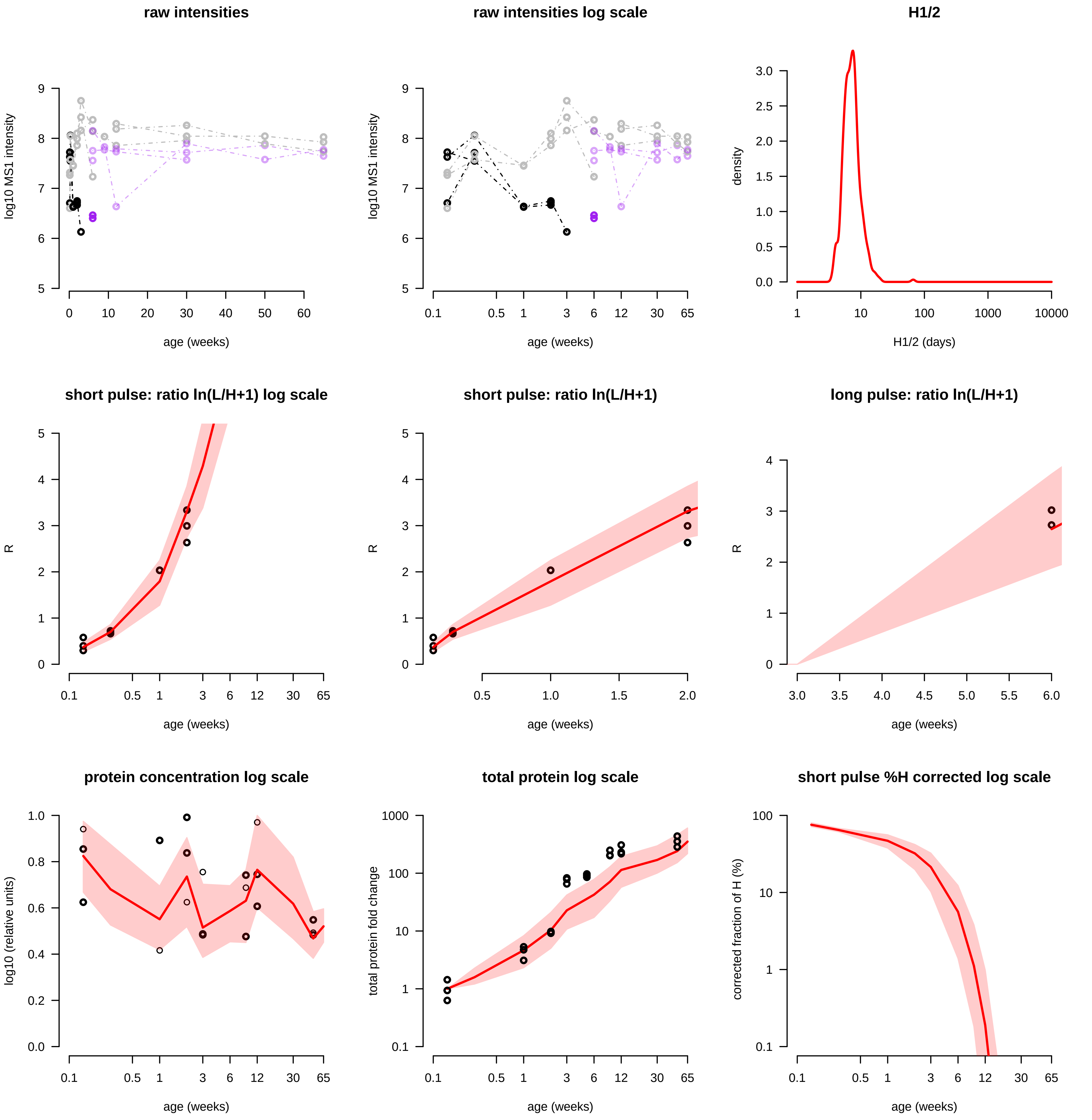

Q04207;Q04207−2

>sp|Q04207|TF65_MOUSE

Transcription factor p65

GN=Rela

Med H1/2: 18.5 days

Q5 H1/2: 4.9 days

Q95 H1/2: 233.9 days

reliable model fit: yes

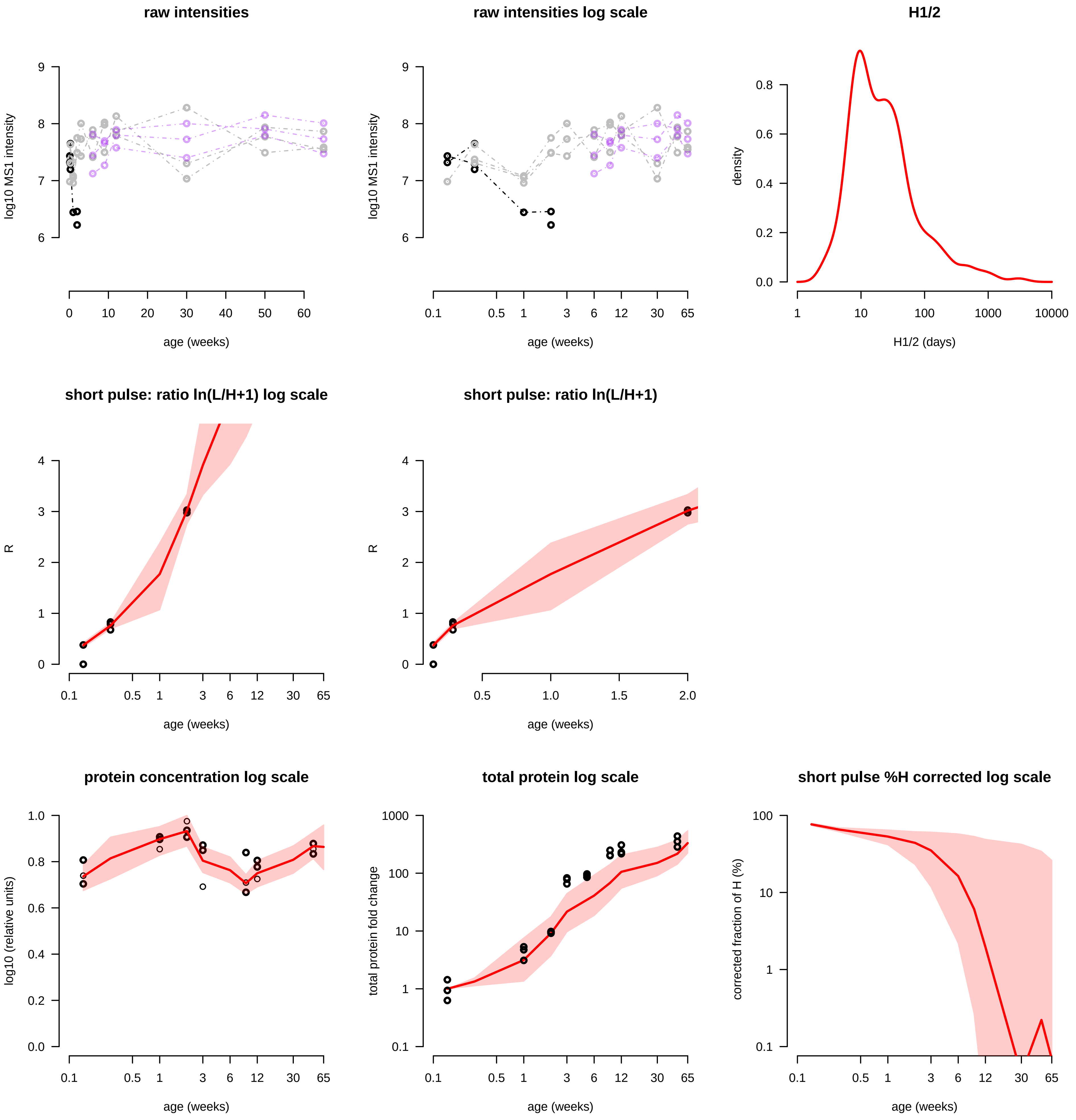

;Q9Z0H4−7;Q9Z0H4−5;Q9Z0H4−4;Q9Z0H4;Q9Z0H4−8;Q

>sp|Q9Z0H4−2|CELF2_MOUSE

Isoform 2 of CUGBP Elav−like family member 2

GN=Celf2

Med H1/2: 7.5 days

Q5 H1/2: 3.1 days

Q95 H1/2: 71.4 days

reliable model fit: yes

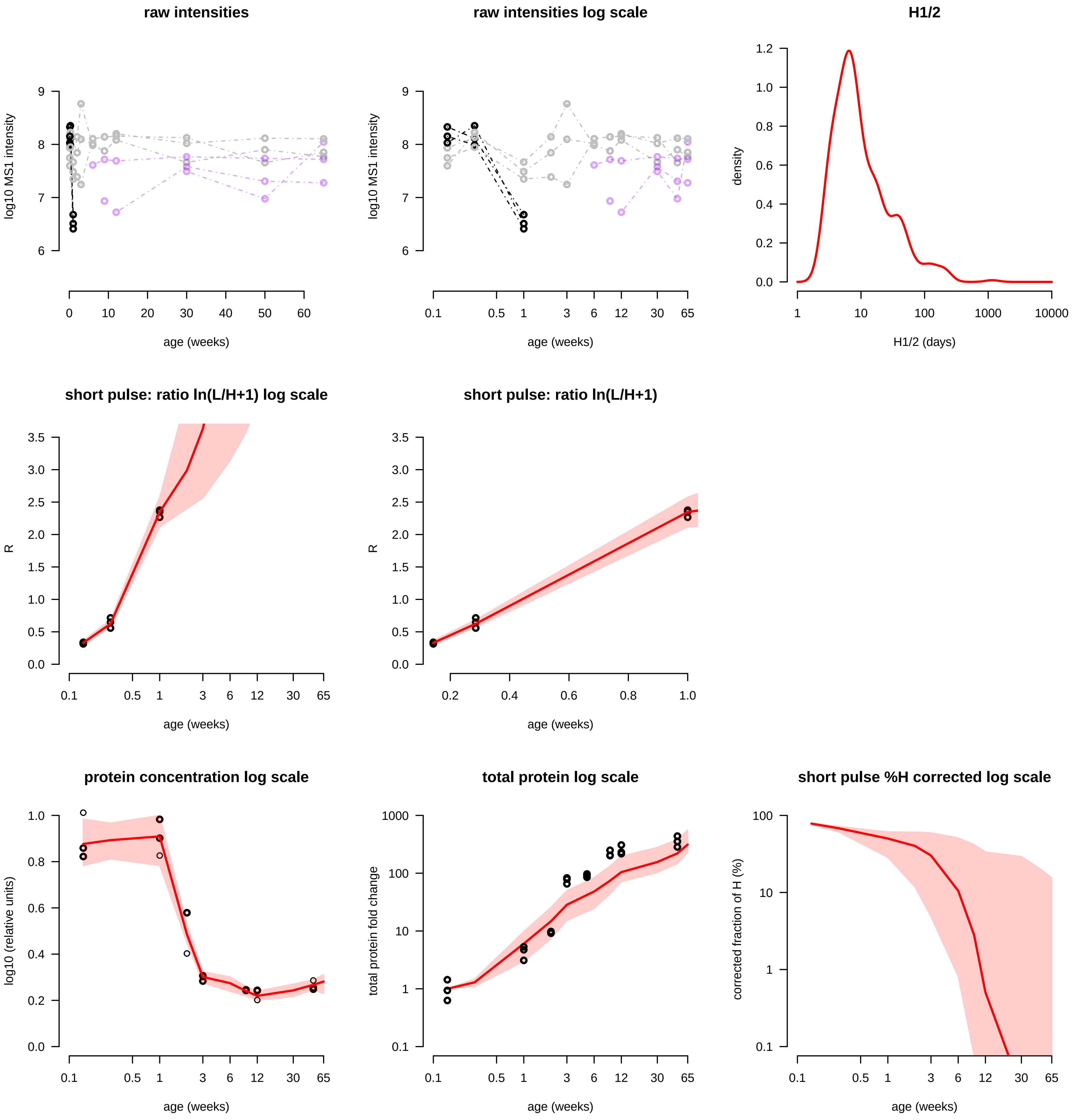

Q02614

>sp|Q02614|S30BP_MOUSE

SAP30−binding protein

GN=Sap30bp

Med H1/2: 6.8 days

Q5 H1/2: 2.8 days

Q95 H1/2: 64.1 days

reliable model fit: yes

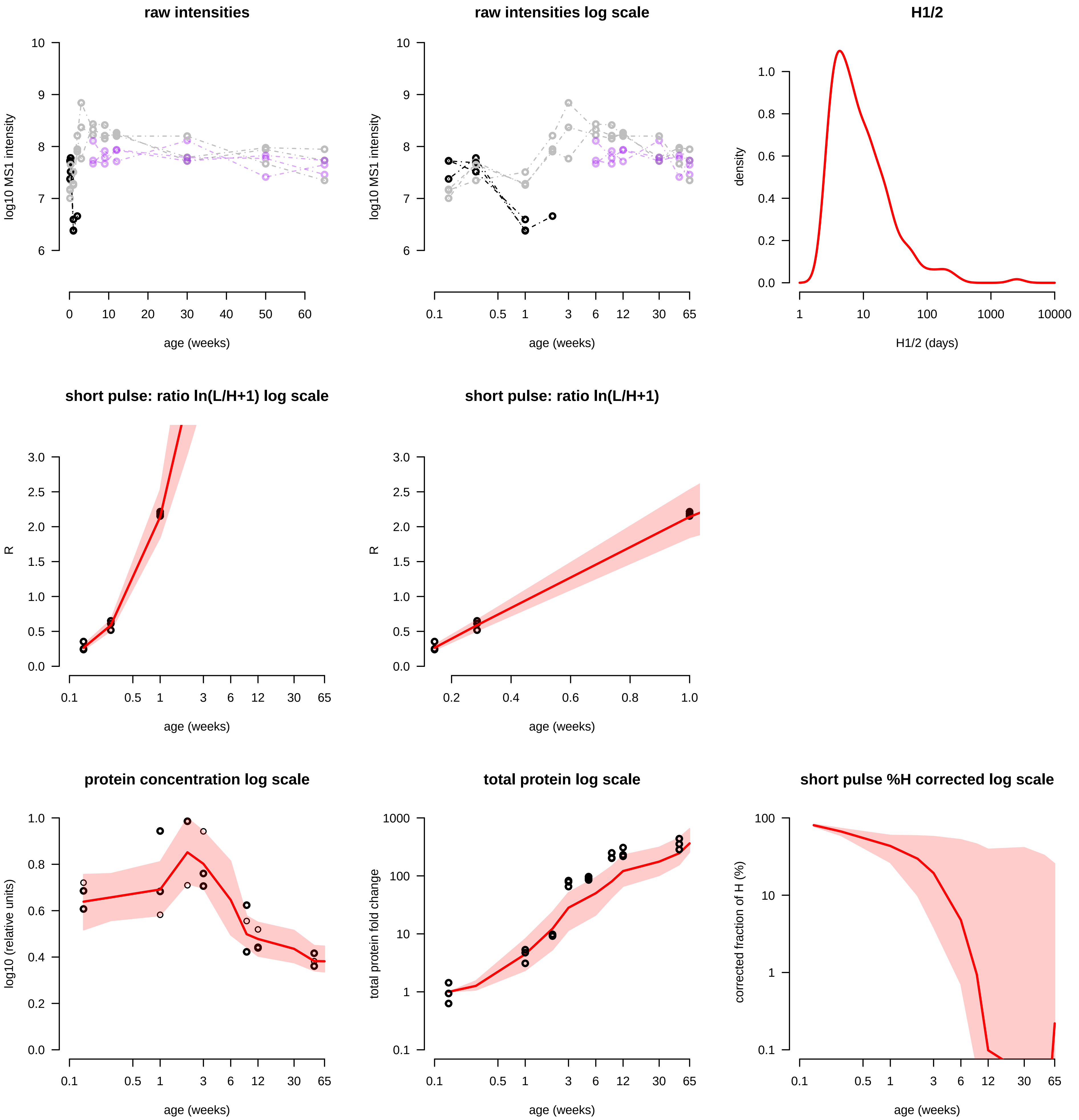

P97377−2;P97377

>sp|P97377−2|CDK2_MOUSE

Isoform CDK2−alpha of Cyclin−dependent kinase 2

GN=Cdk2

Med H1/2: 18.8 days

Q5 H1/2: 6.6 days

Q95 H1/2: 232.5 days

reliable model fit: yes

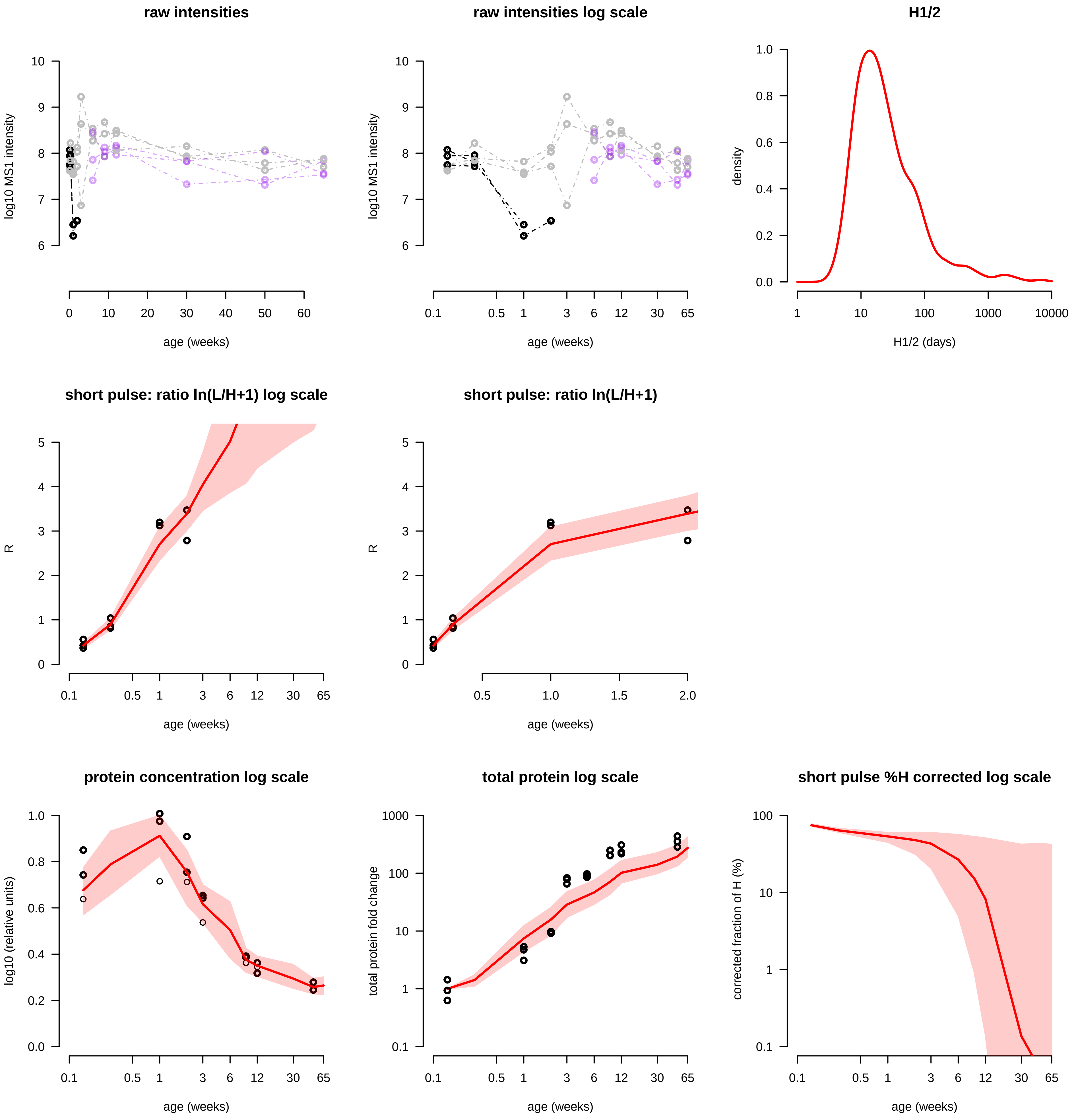

F7BJB9

>sp|F7BJB9|MORC3_MOUSE

MORC family CW−type zinc finger protein 3

GN=Morc3

Med H1/2: 17 days

Q5 H1/2: 5 days

Q95 H1/2: 282.1 days

reliable model fit: yes

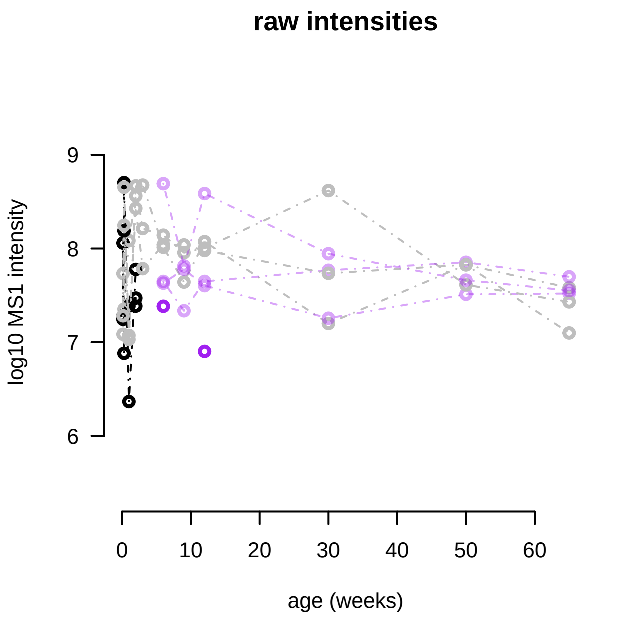

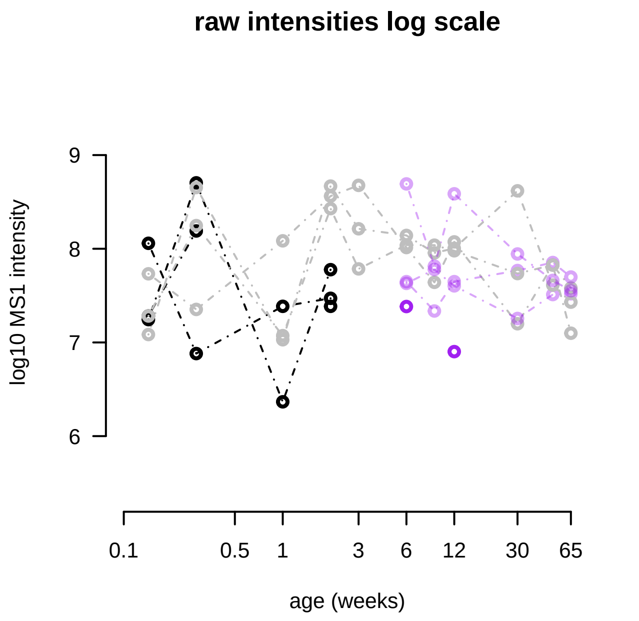

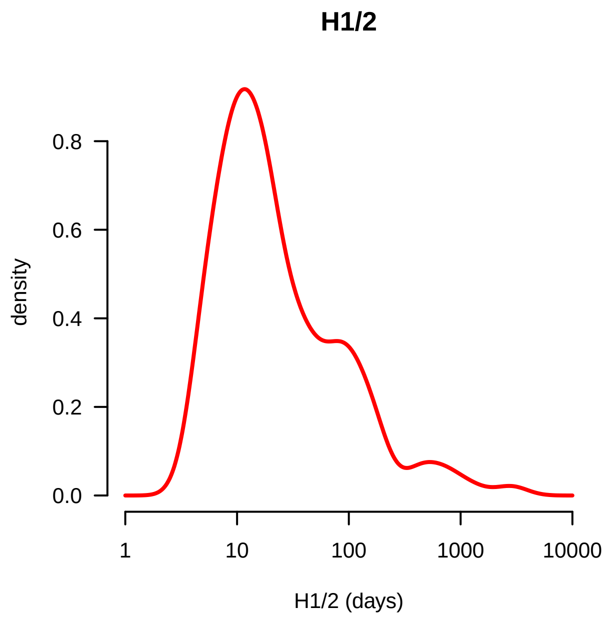

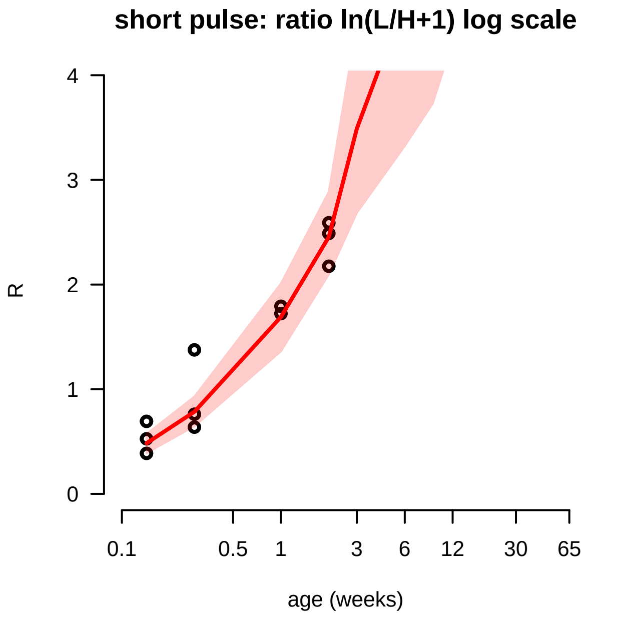

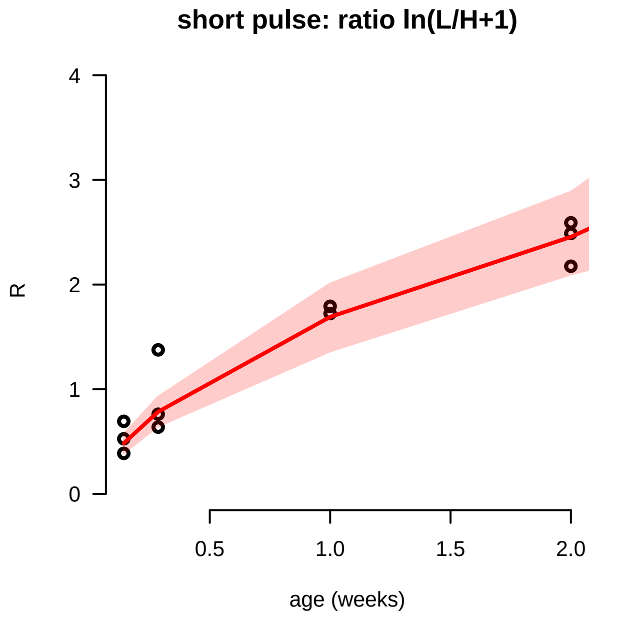

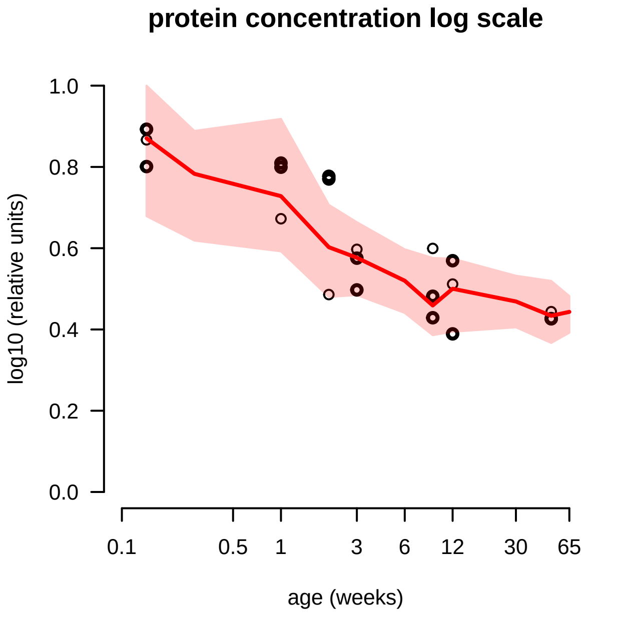

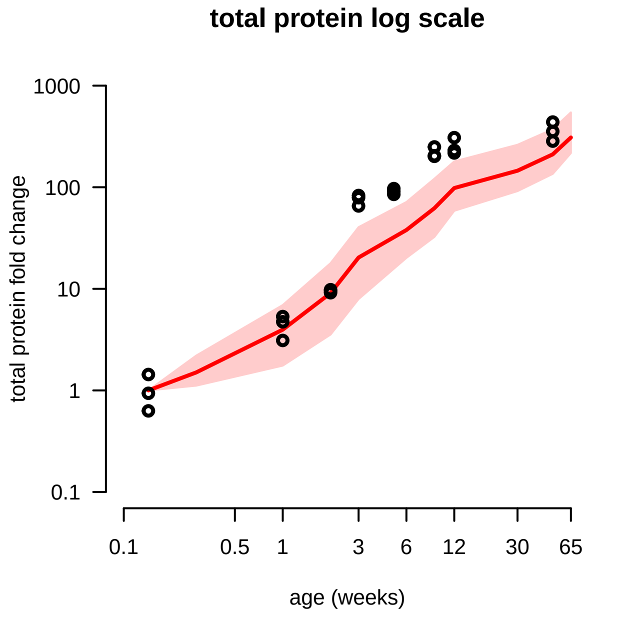

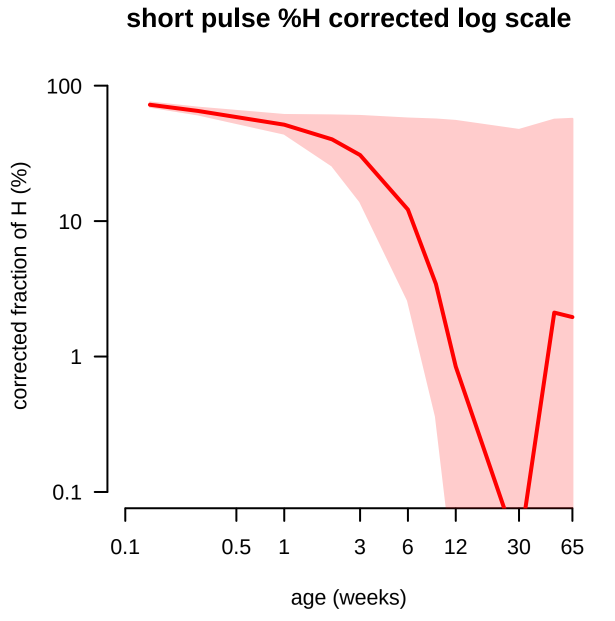

Q9Z1T6

>sp|Q9Z1T6|FYV1_MOUSE

1−phosphatidylinositol 3−phosphate 5−kinase

GN=Pikfyve

Med H1/2: 6.7 days

Q5 H1/2: 3 days

Q95 H1/2: 35 days

reliable model fit: yes

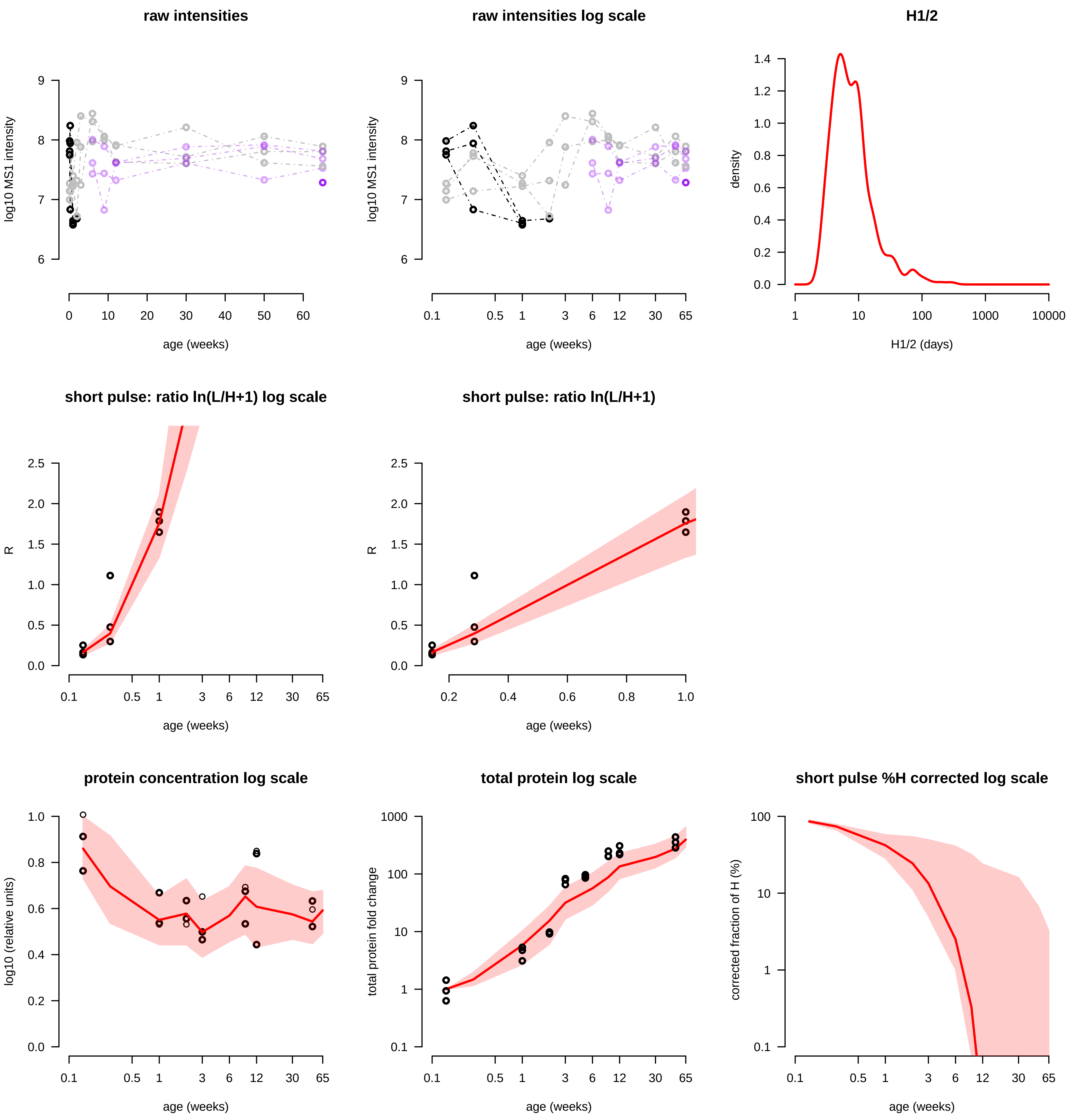

Q99JP4

>sp|Q99JP4|CDC26_MOUSE

Anaphase−promoting complex subunit CDC26

GN=Cdc26

Med H1/2: 5.5 days

Q5 H1/2: 2.9 days

Q95 H1/2: 27.4 days

reliable model fit: yes

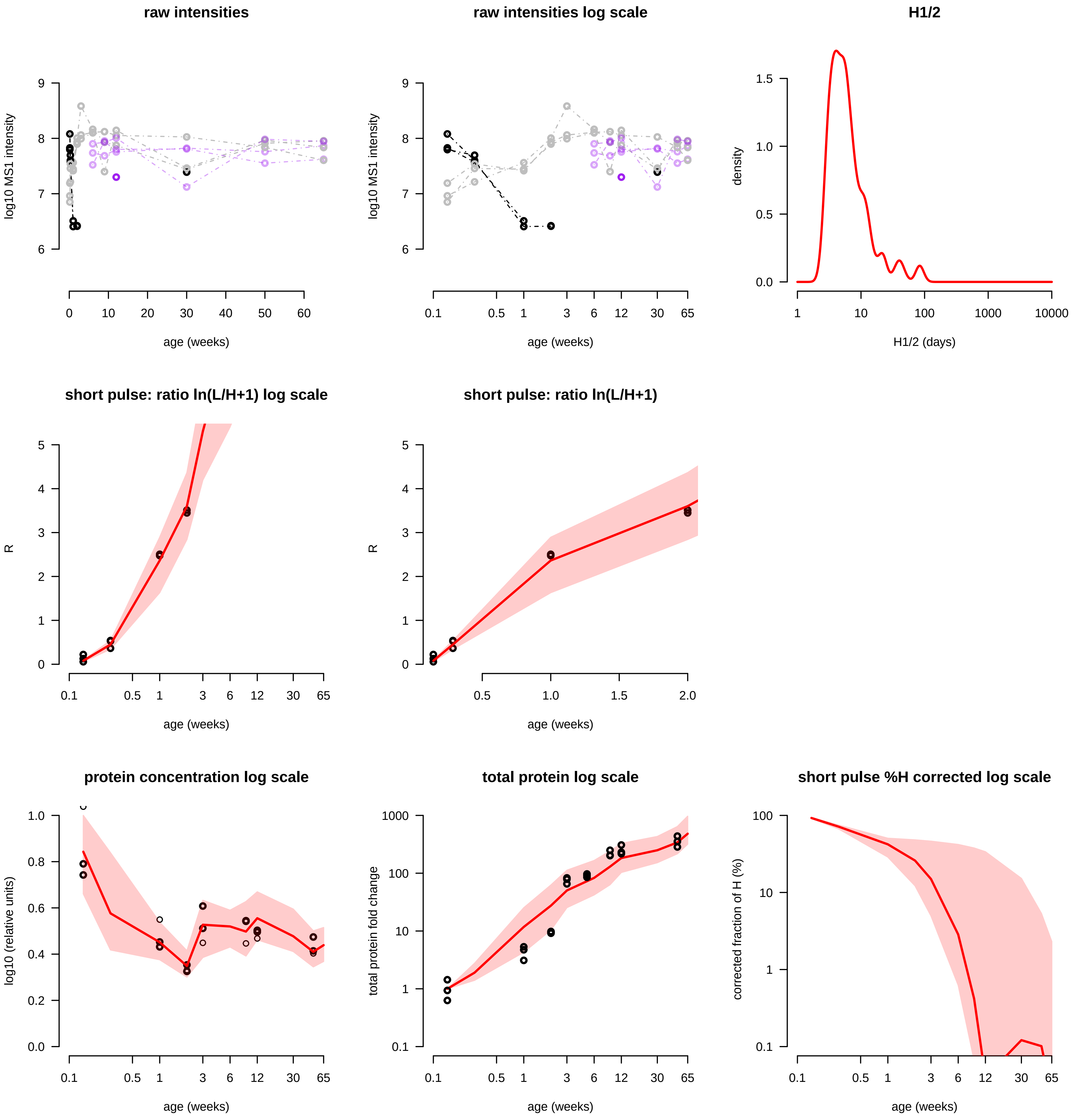

Q91V36

>sp|Q91V36|NRBP2_MOUSE

Nuclear receptor−binding protein 2

GN=Nrbp2

Med H1/2: 4.6 days

Q5 H1/2: 2.7 days

Q95 H1/2: 16.7 days

reliable model fit: yes

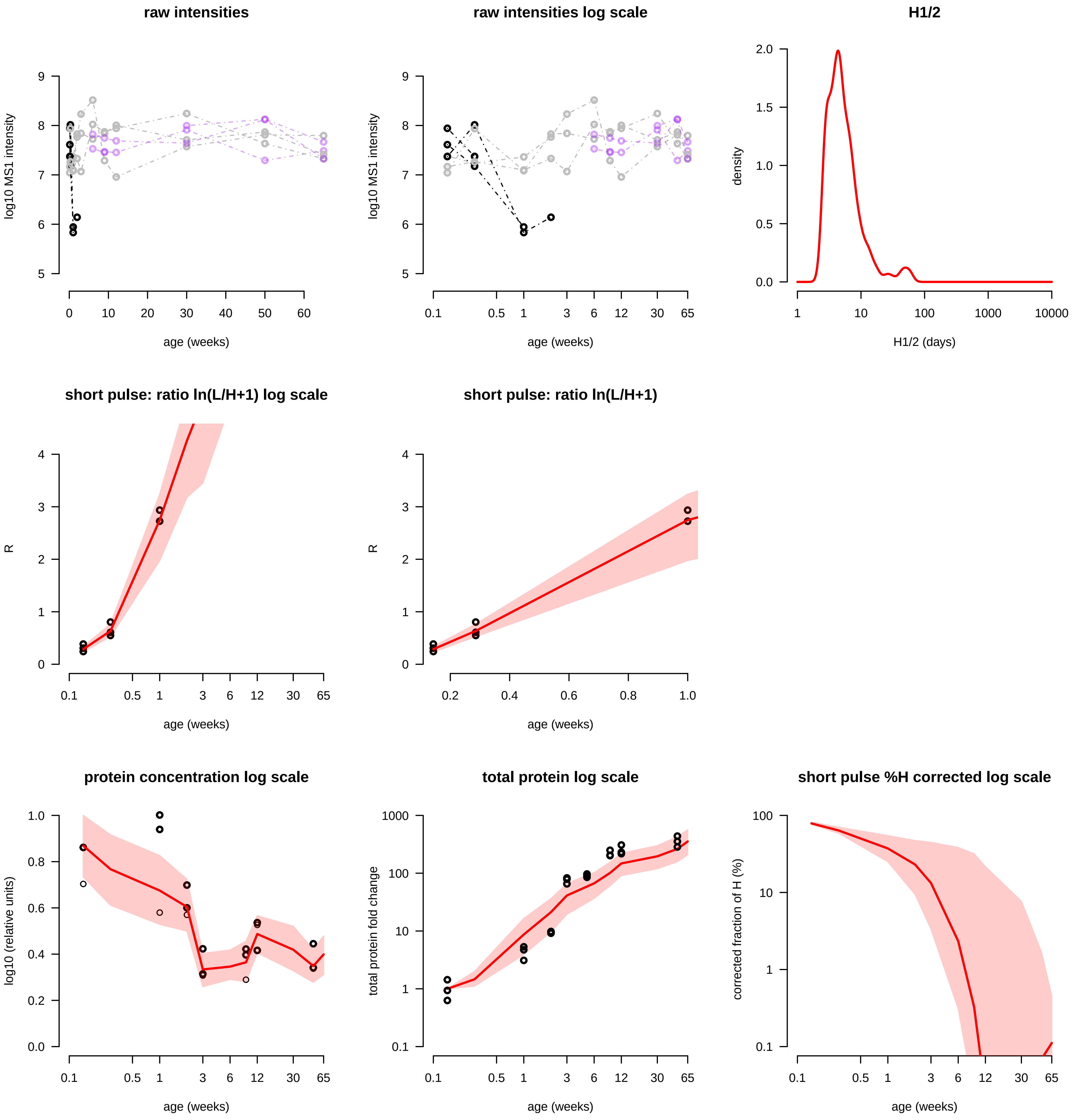

Q8CE96;Q8CE96−3

>sp|Q8CE96|TRM6_MOUSE

tRNA (adenine(58)−N(1))−methyltransferase non−catalytic subunit TRM6

GN=Trmt6

Med H1/2: 13.1 days

Q5 H1/2: 4.1 days

Q95 H1/2: 121.9 days

reliable model fit: yes

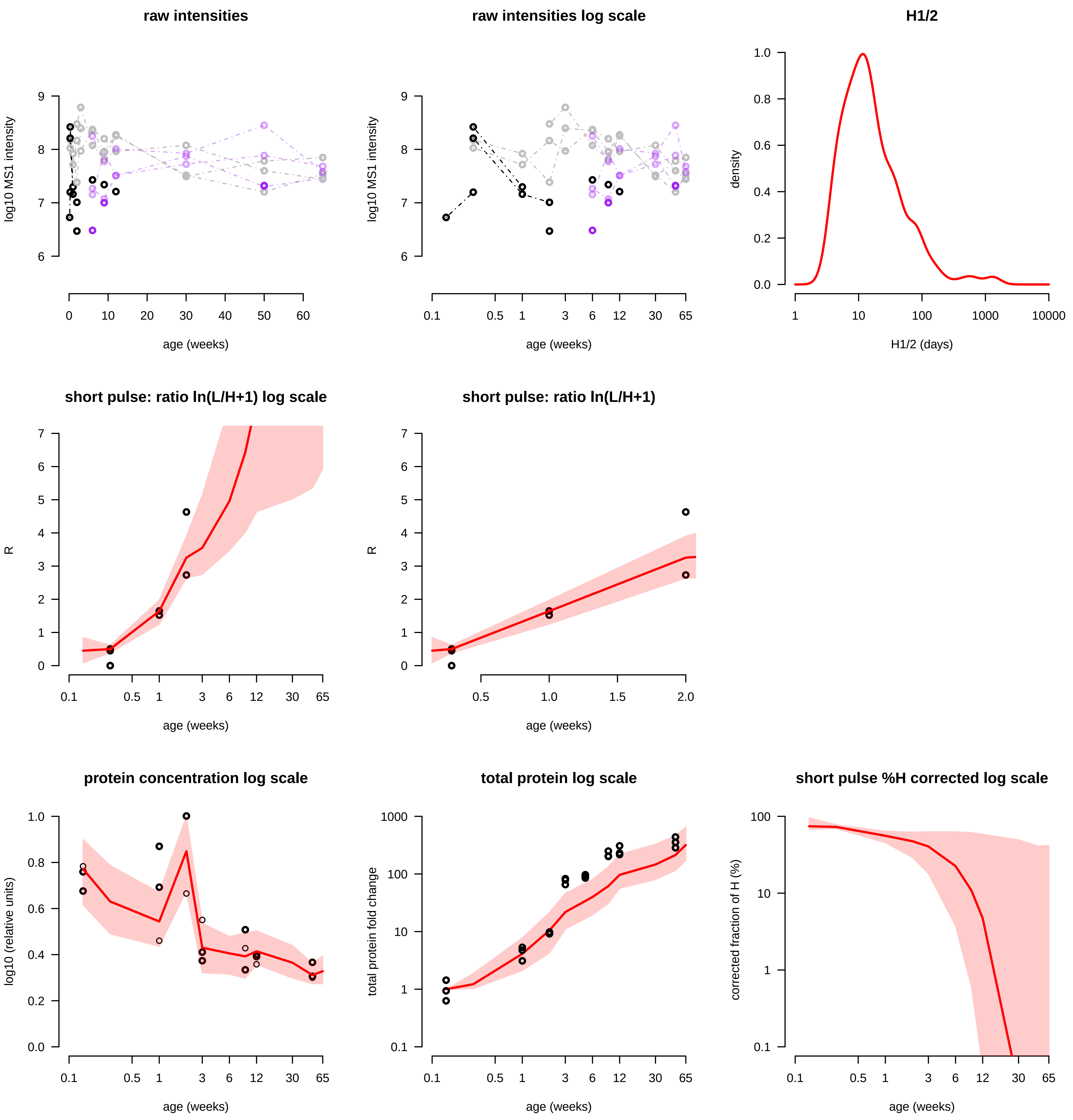

Q8C7K6

>sp|Q8C7K6|PCYXL_MOUSE

Prenylcysteine oxidase−like

GN=Pcyox1l

Med H1/2: 8.1 days

Q5 H1/2: 3.3 days

Q95 H1/2: 96.3 days

reliable model fit: yes

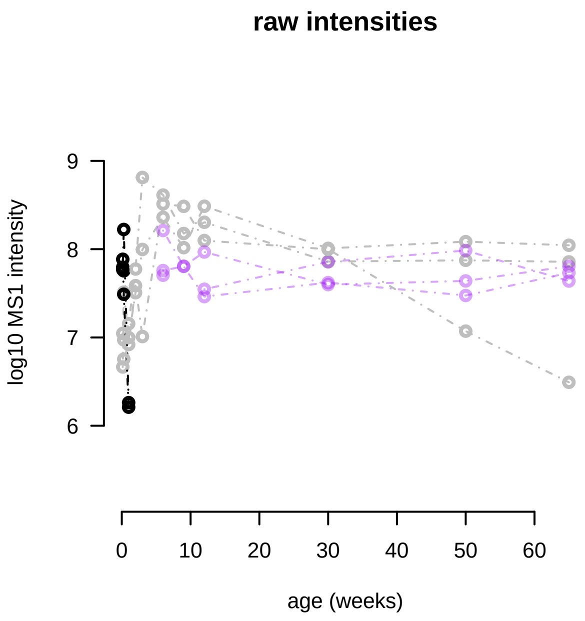

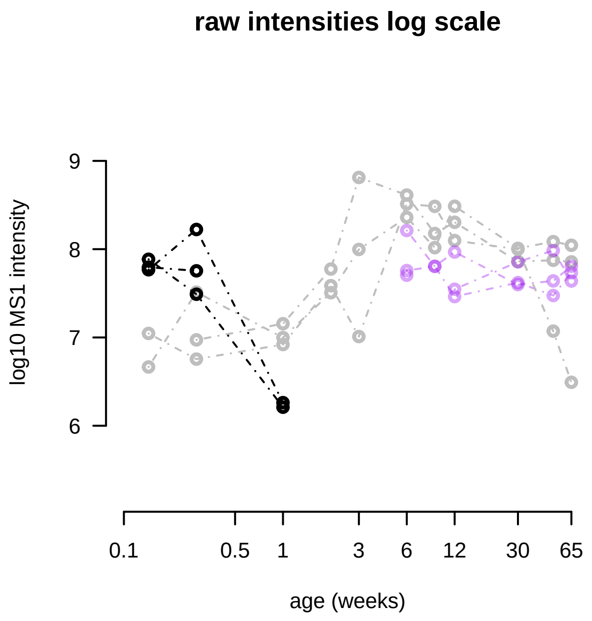

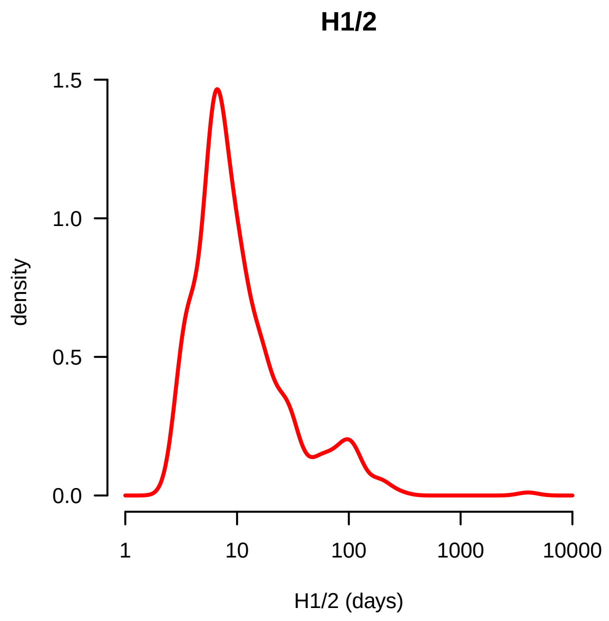

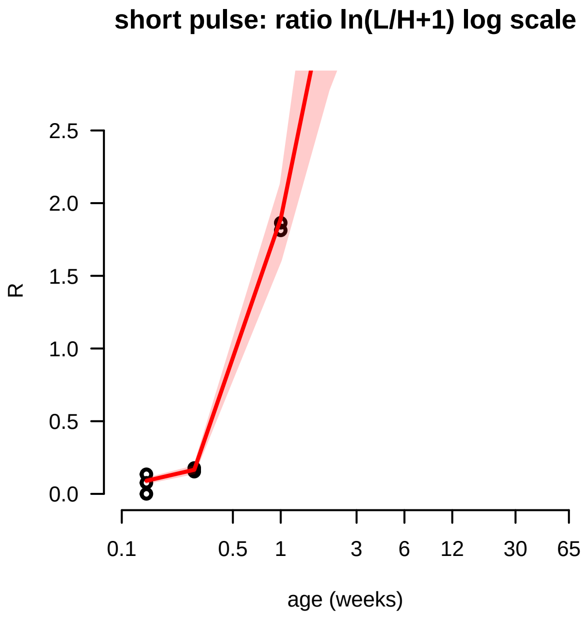

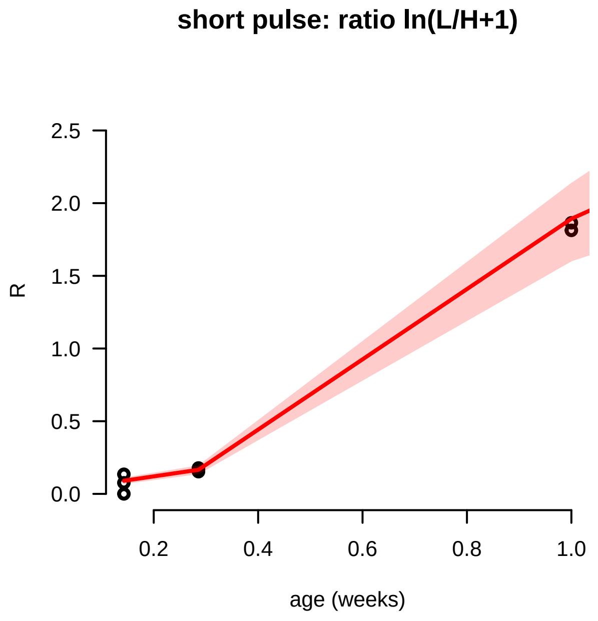

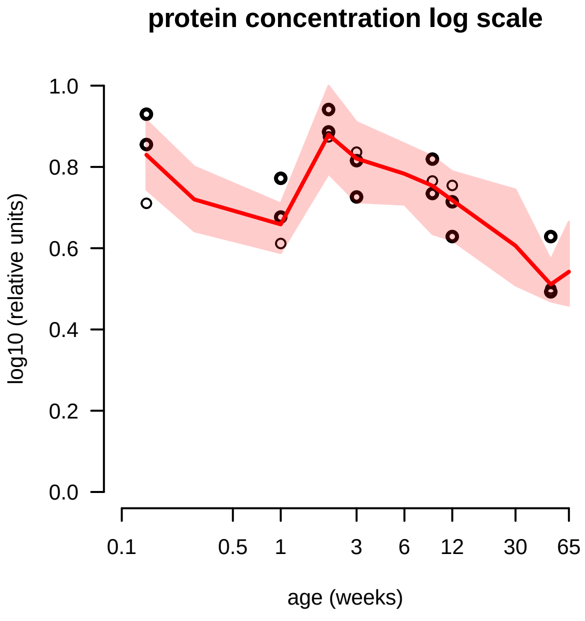

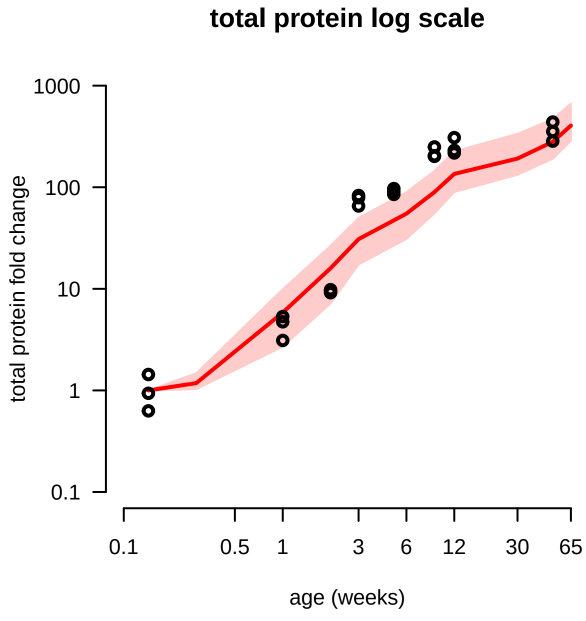

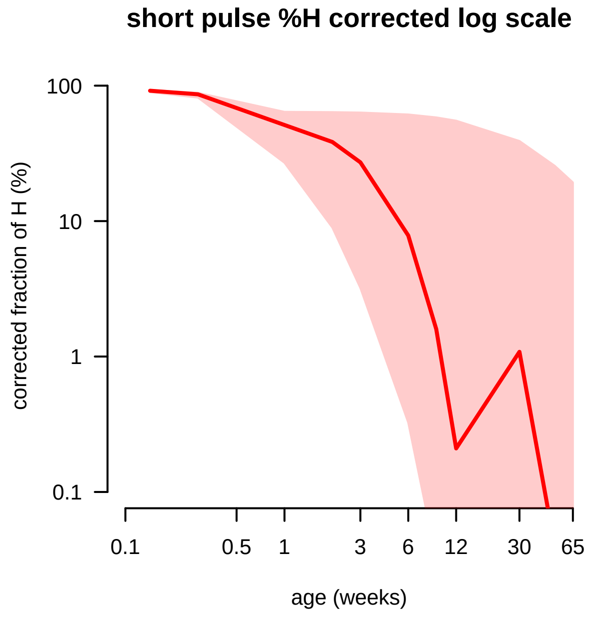

Q8R418

>sp|Q8R418|DICER_MOUSE

Endoribonuclease Dicer

GN=Dicer1

Med H1/2: 1943.5 days

Q5 H1/2: 680.8 days

Q95 H1/2: 35163.4 days

reliable model fit: no

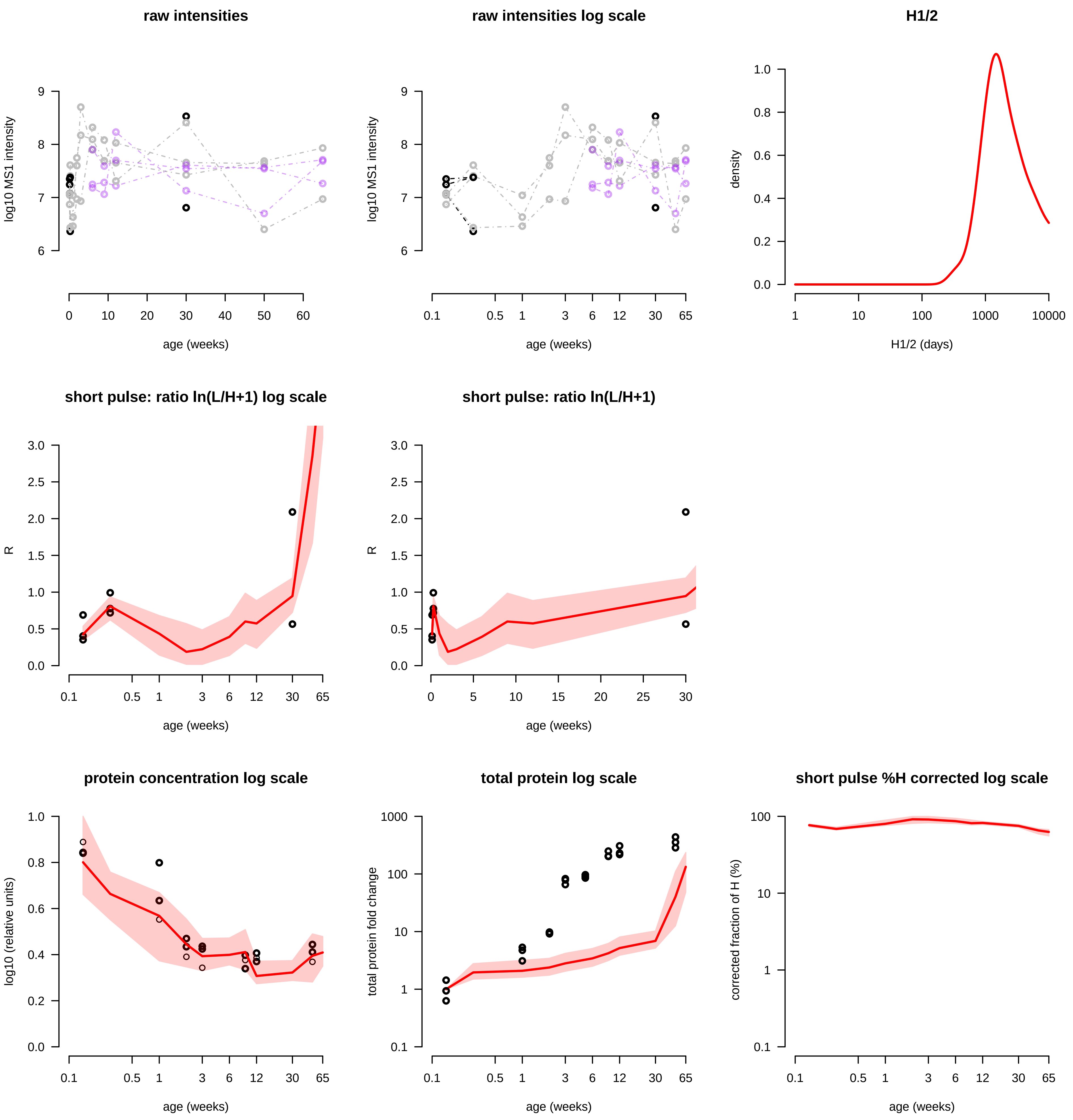

Q08879−2

>sp|Q08879−2|FBLN1_MOUSE

Isoform C of Fibulin−1

GN=Fbln1

Med H1/2: 15.5 days

Q5 H1/2: 5.9 days

Q95 H1/2: 137.9 days

reliable model fit: yes

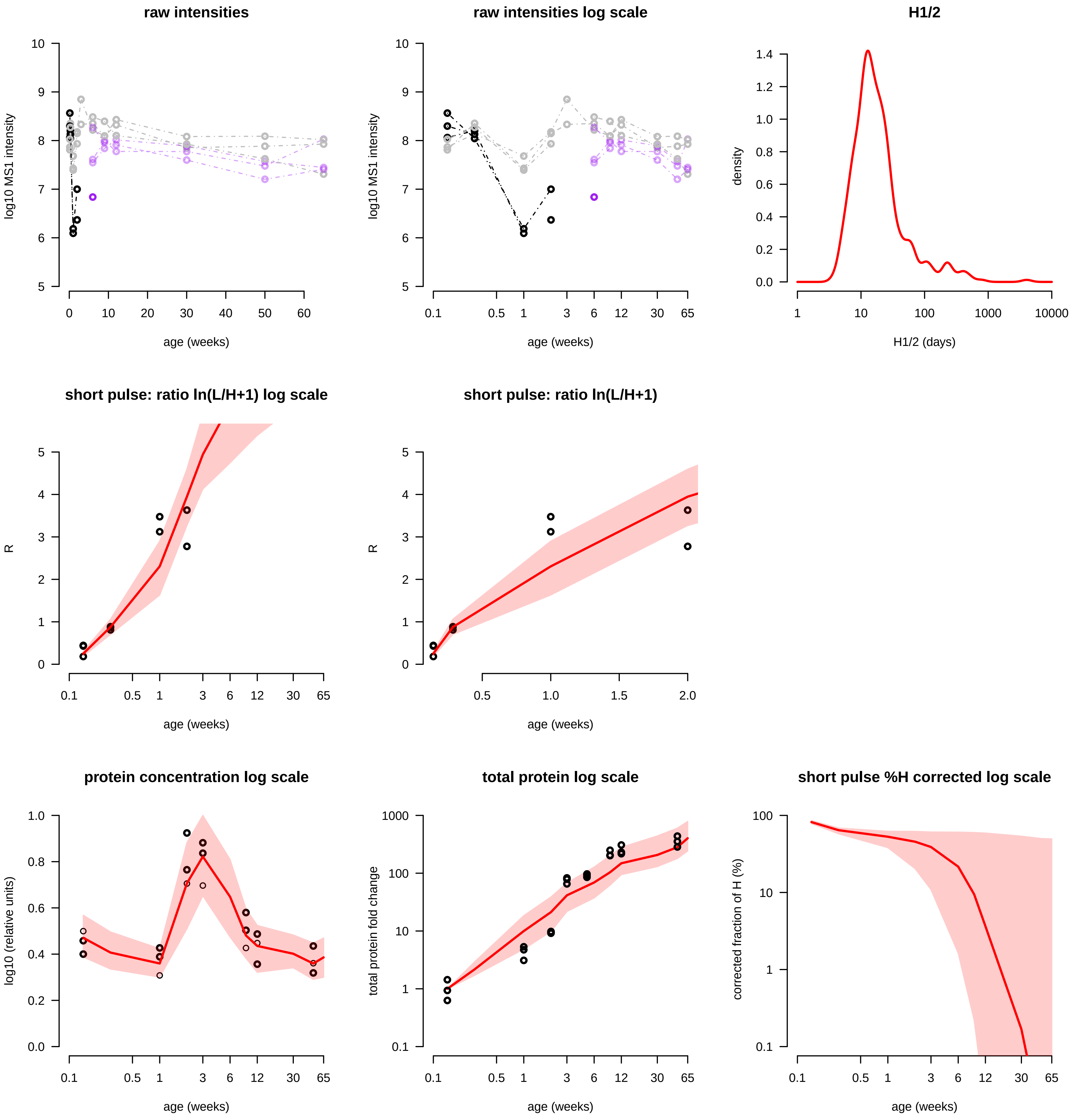

Q8VCD5

>sp|Q8VCD5|MED17_MOUSE

Mediator of RNA polymerase II transcription subunit 17

GN=Med17

Med H1/2: 5.7 days

Q5 H1/2: 2.7 days

Q95 H1/2: 51.1 days

reliable model fit: no

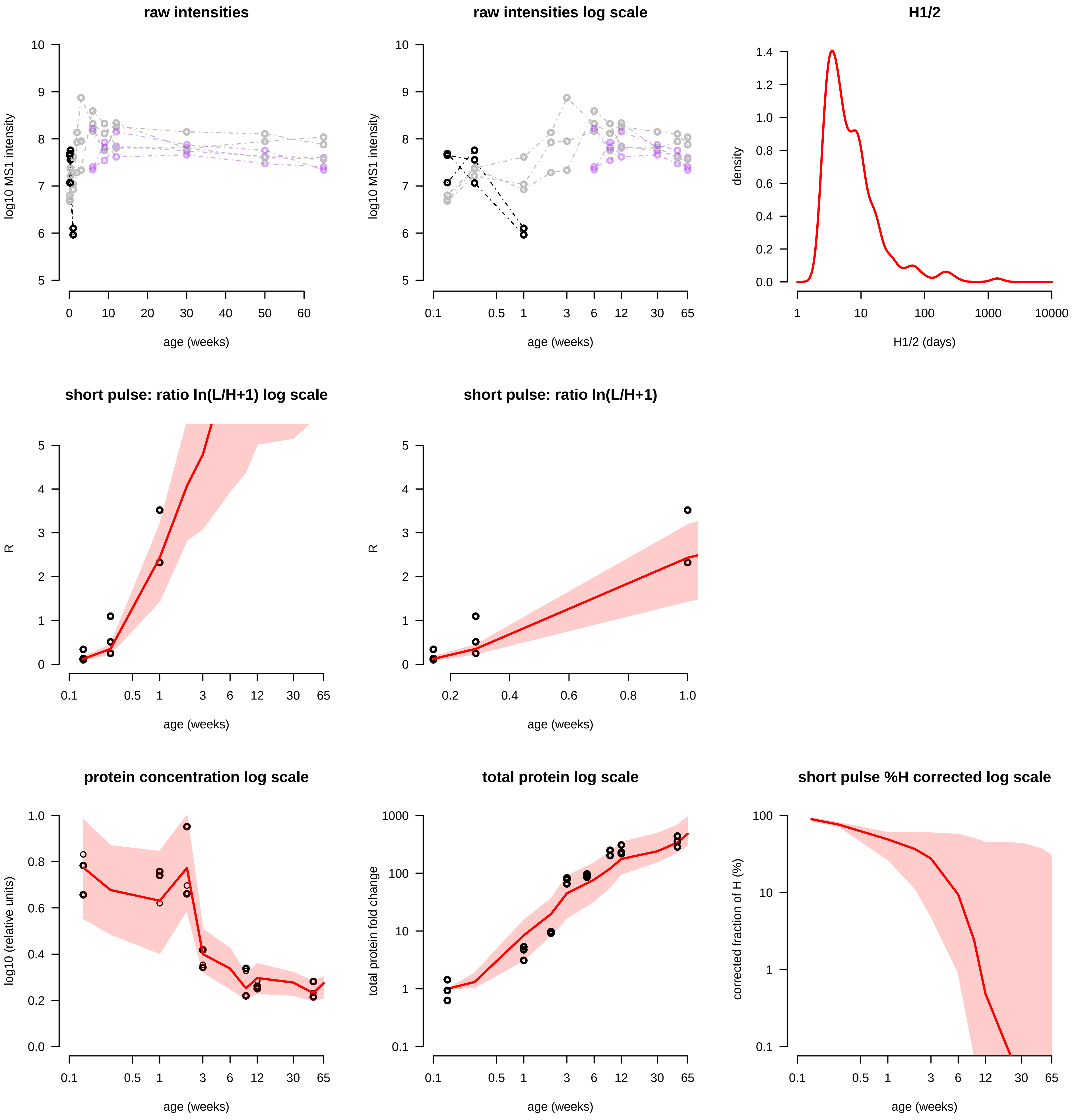

Q80TY5

>sp|Q80TY5|VP13B_MOUSE

Vacuolar protein sorting−associated protein 13B

GN=Vps13b

Med H1/2: 335.5 days

Q5 H1/2: 130.5 days

Q95 H1/2: 6412.7 days

reliable model fit: no

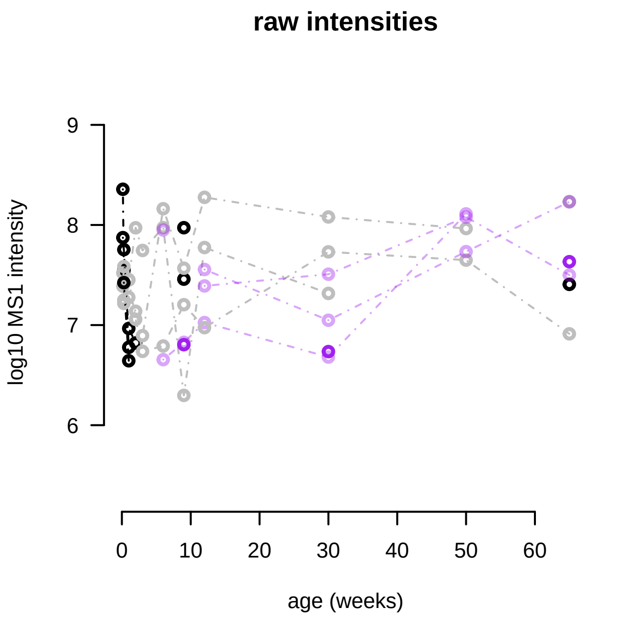

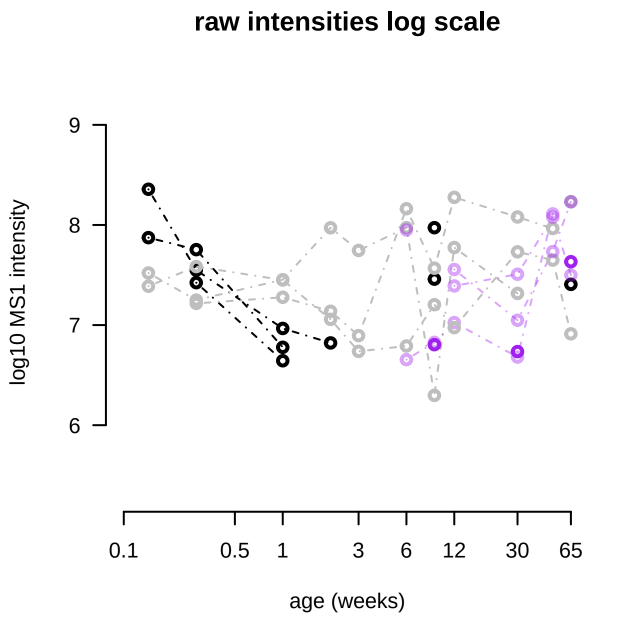

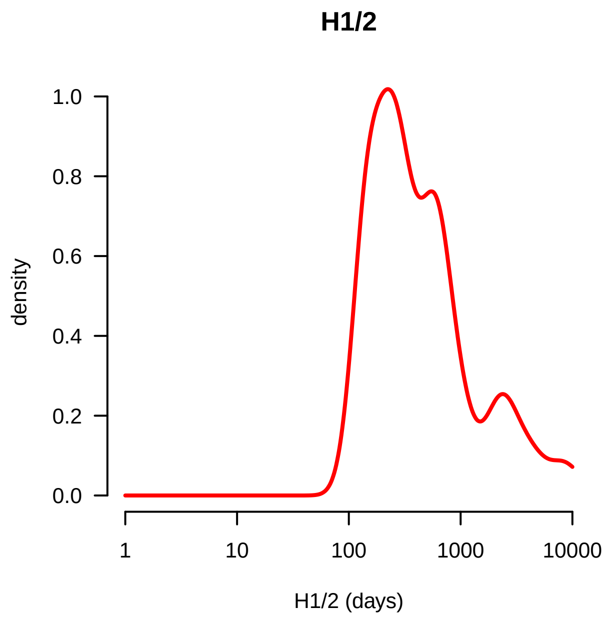

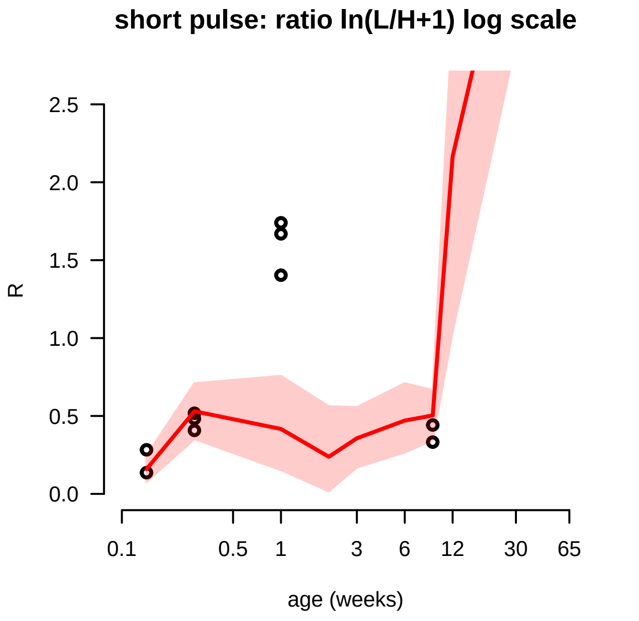

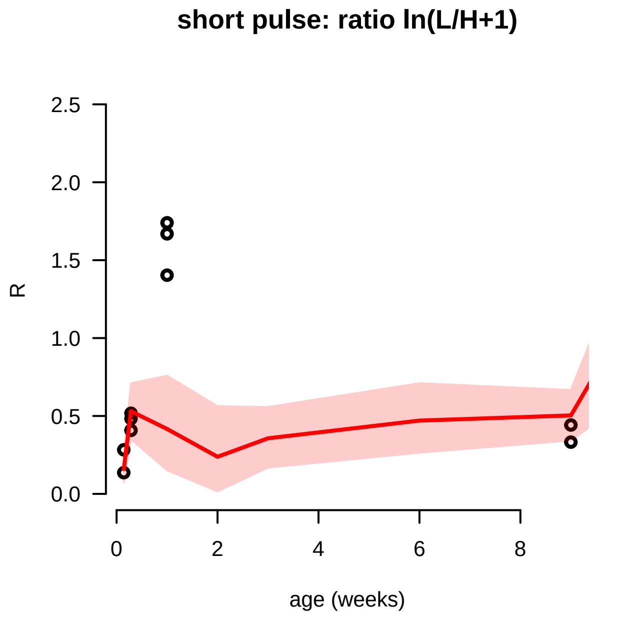

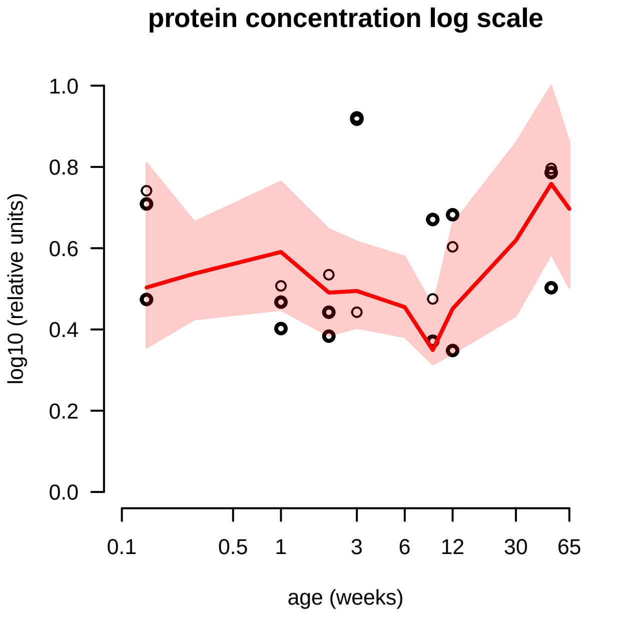

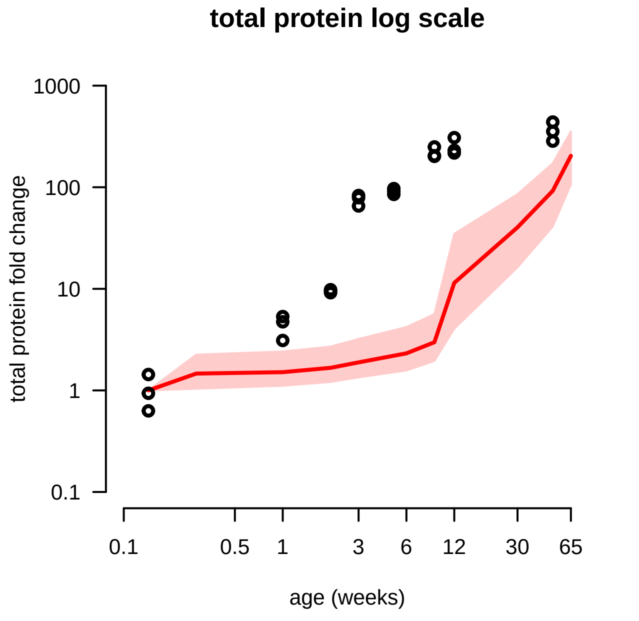

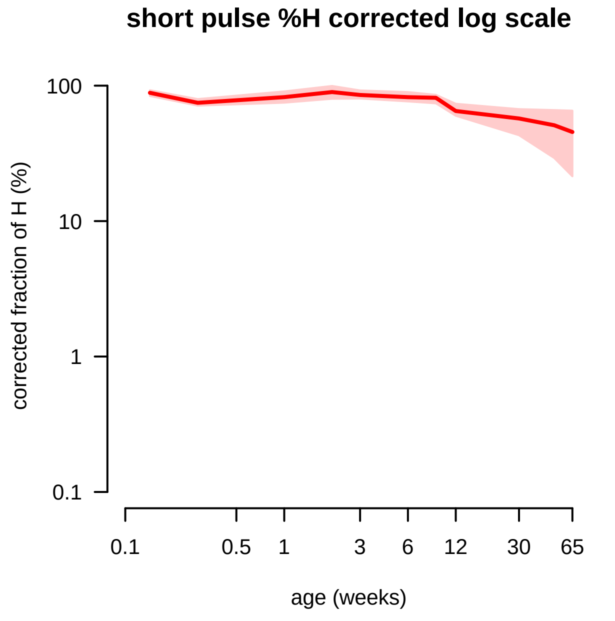

P20357

>sp|P20357|MTAP2_MOUSE

Microtubule−associated protein 2

GN=Map2

Med H1/2: 4.7 days

Q5 H1/2: 2.8 days

Q95 H1/2: 41.9 days

reliable model fit: yes

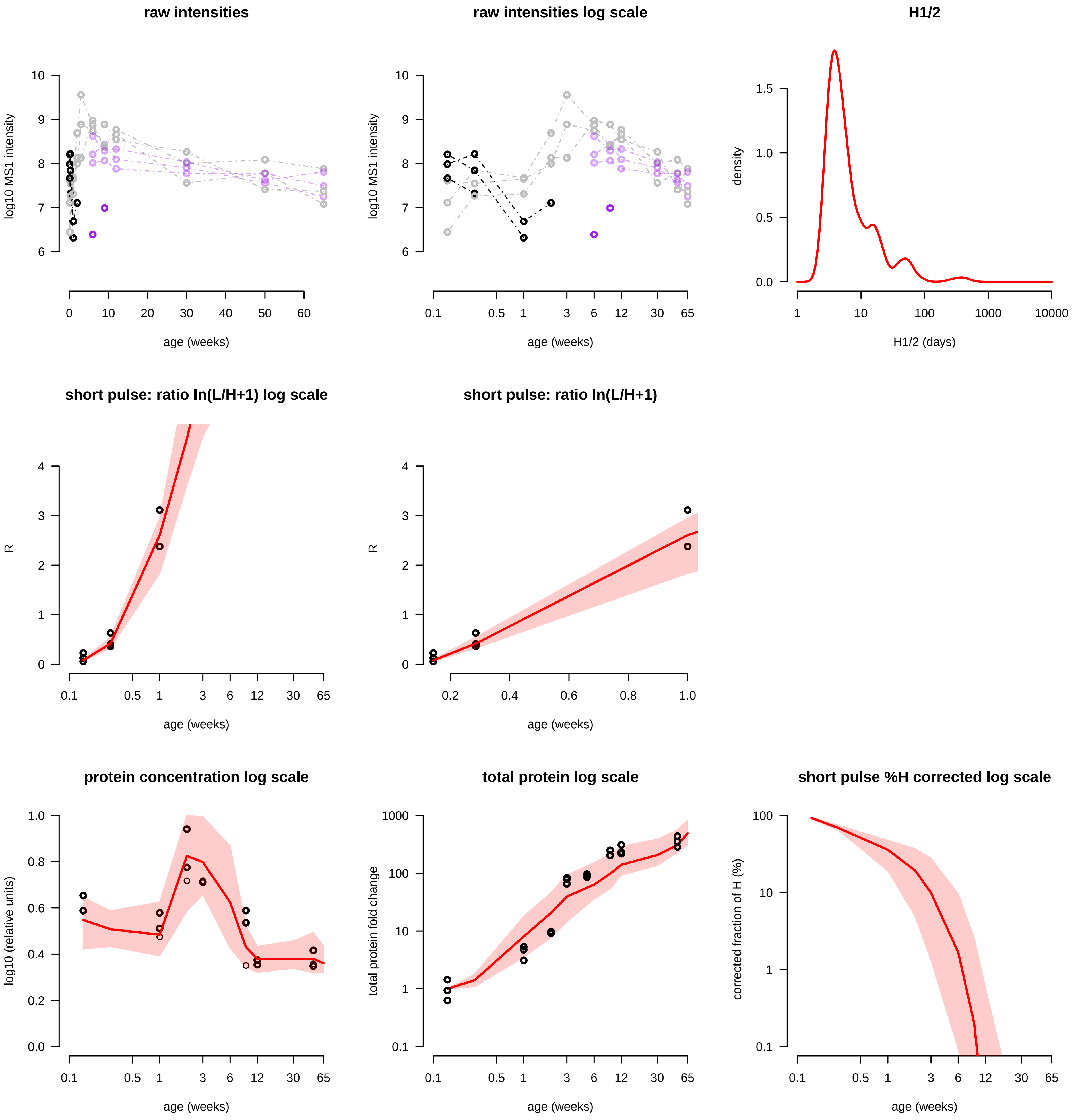

Q9CYI4;Q9CYI4−2

>sp|Q9CYI4|LUC7L_MOUSE

Putative RNA−binding protein Luc7−like 1

GN=Luc7l

Med H1/2: 7.2 days

Q5 H1/2: 2.9 days

Q95 H1/2: 62.7 days

reliable model fit: no

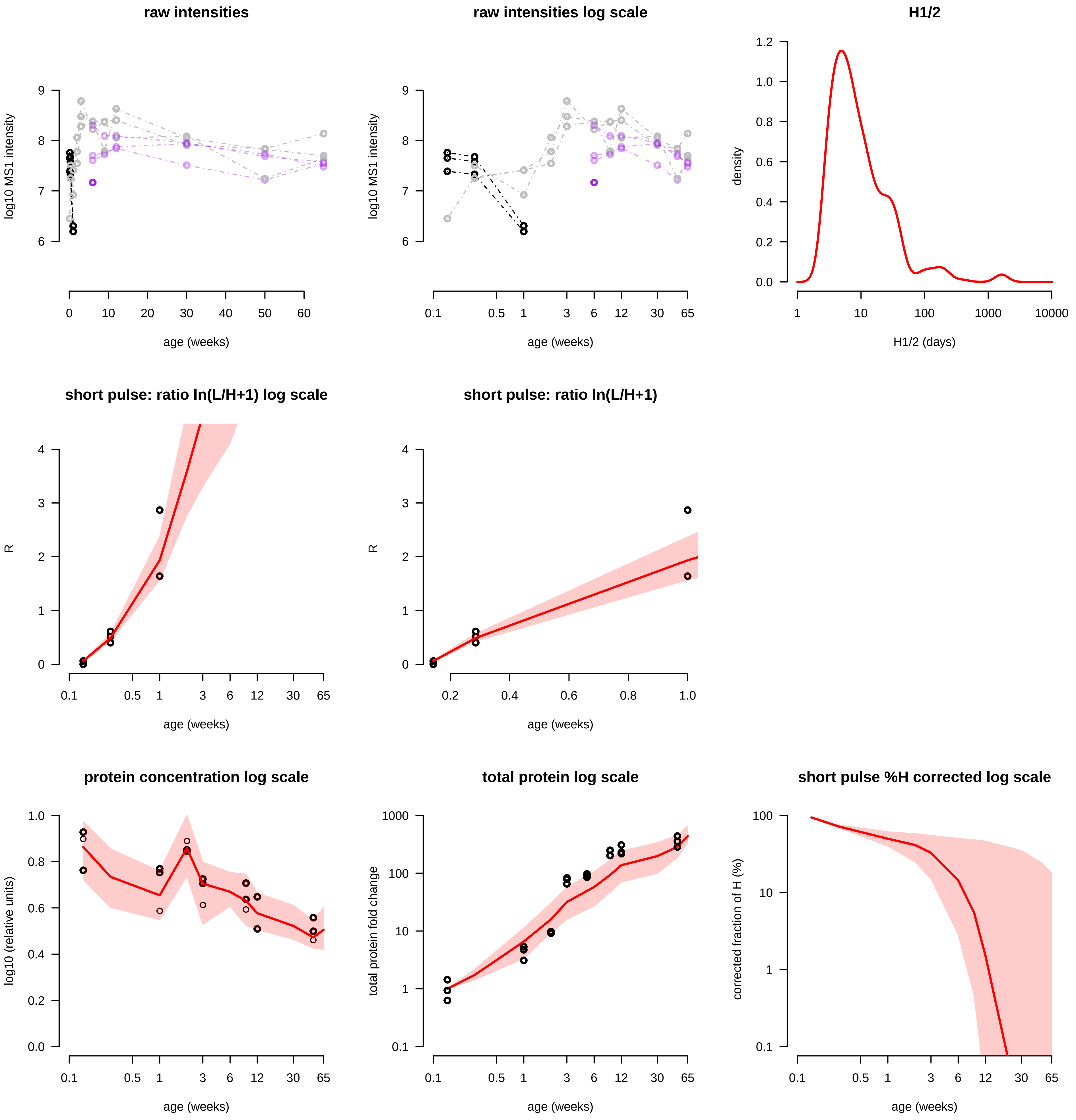

P35288

>sp|P35288|RAB23_MOUSE

Ras−related protein Rab−23

GN=Rab23

Med H1/2: 4.7 days

Q5 H1/2: 2.6 days

Q95 H1/2: 12.4 days

reliable model fit: yes

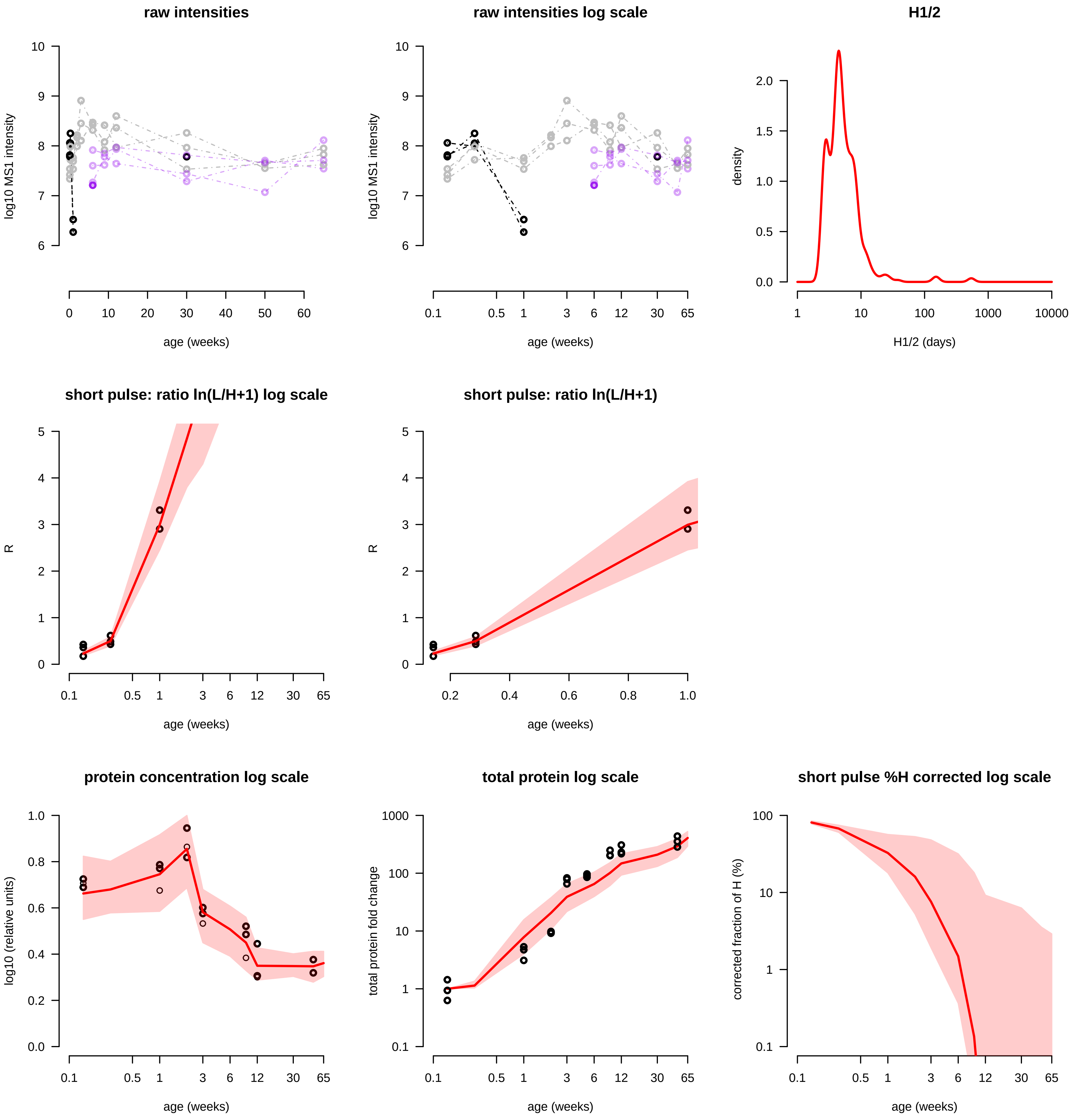

Q9D0F6

>sp|Q9D0F6|RFC5_MOUSE

Replication factor C subunit 5

GN=Rfc5

Med H1/2: 21.1 days

Q5 H1/2: 6.2 days

Q95 H1/2: 167.8 days

reliable model fit: yes

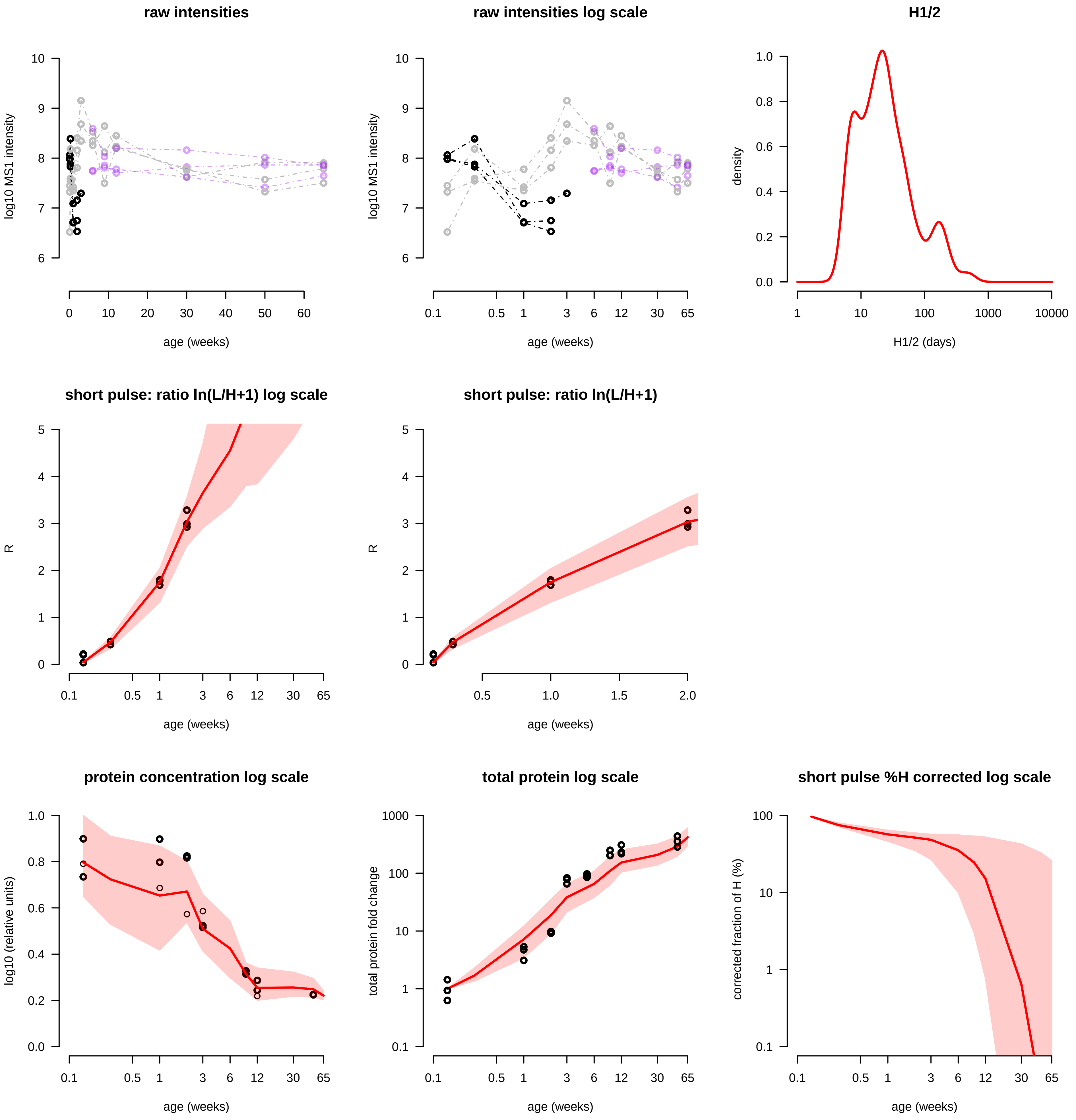

Q91YD9

>sp|Q91YD9|WASL_MOUSE

Neural Wiskott−Aldrich syndrome protein

GN=Wasl

Med H1/2: 7.6 days

Q5 H1/2: 2.7 days

Q95 H1/2: 64.7 days

reliable model fit: yes

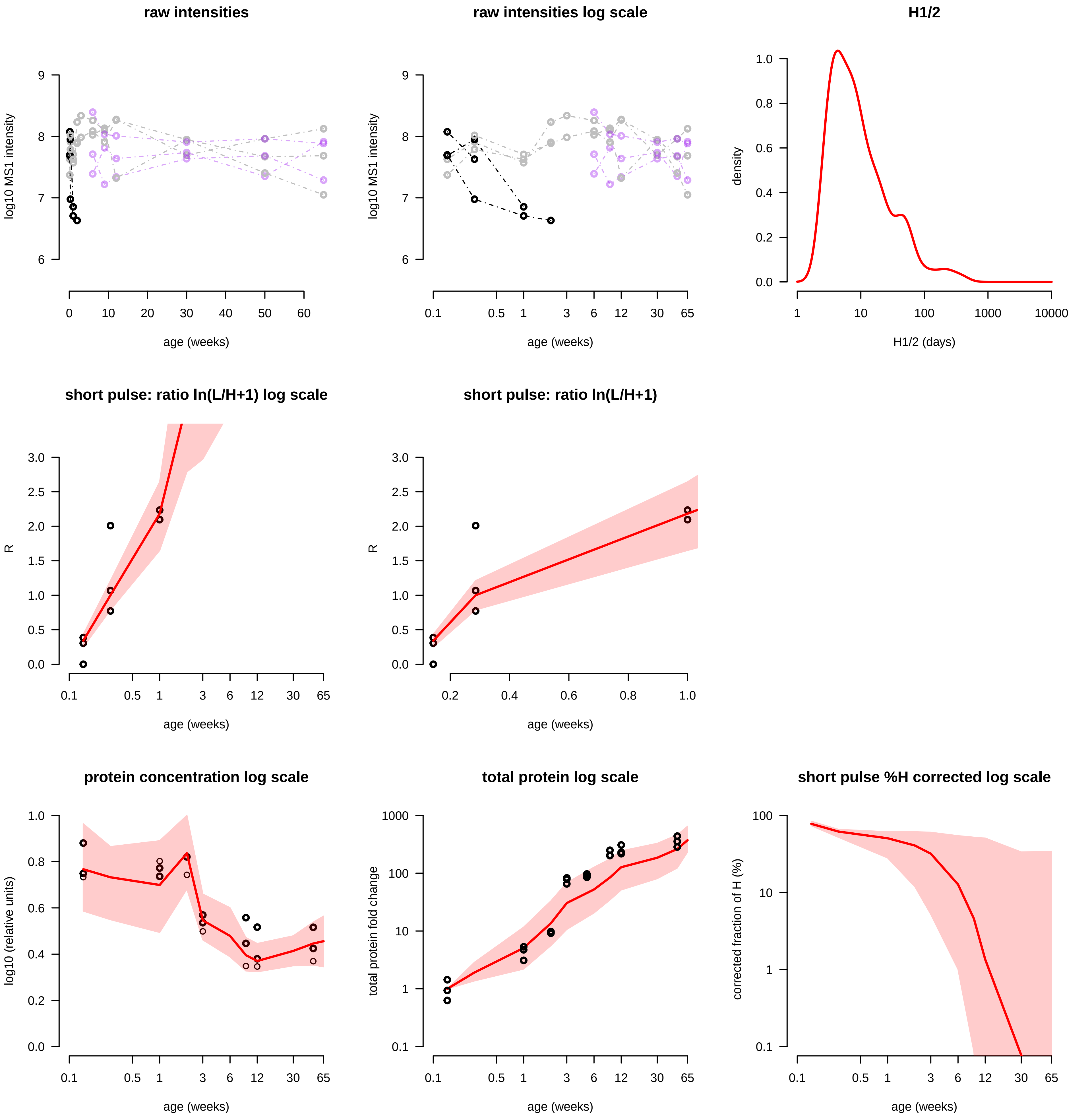

Q5SVQ0−4;Q5SVQ0−2;Q5SVQ0−3;Q5SVQ0;Q5SVQ0−5

>sp|Q5SVQ0−4|KAT7_MOUSE

Isoform 4 of Histone acetyltransferase KAT7

GN=Kat7

Med H1/2: 11 days

Q5 H1/2: 3.1 days

Q95 H1/2: 78.8 days

reliable model fit: yes

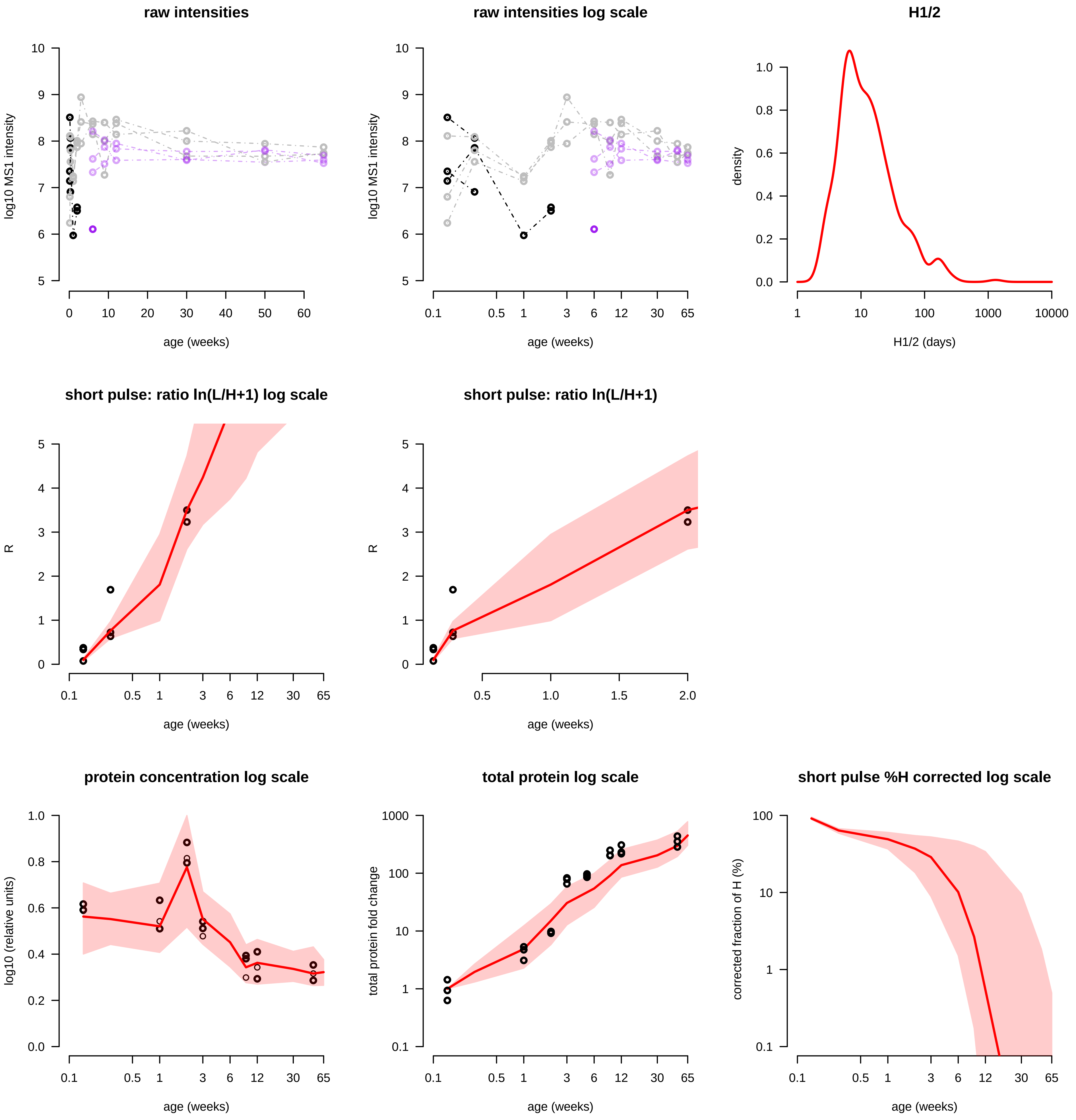

Q8VHJ5

>sp|Q8VHJ5|MARK1_MOUSE

Serine/threonine−protein kinase MARK1

GN=Mark1

Med H1/2: 9 days

Q5 H1/2: 3.2 days

Q95 H1/2: 101.6 days

reliable model fit: yes

raw intensities

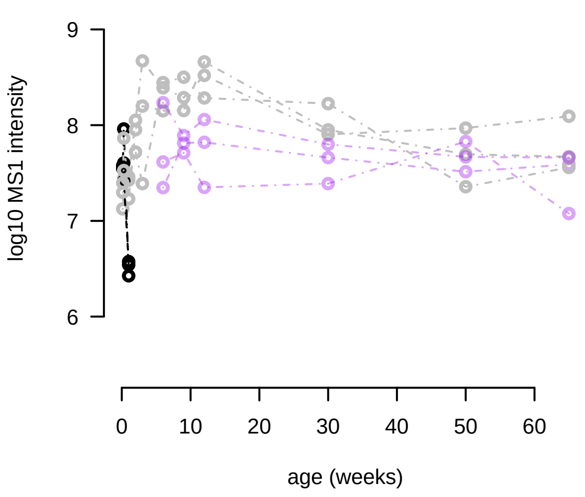

raw intensities log scale

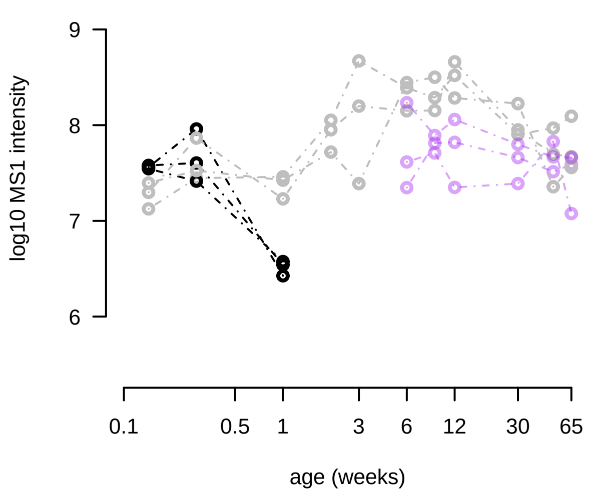

H1/2

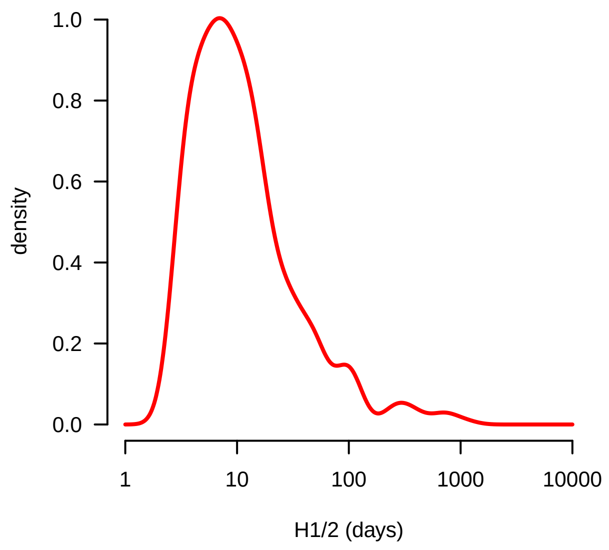

short pulse: ratio ln(L/H+1) log scale

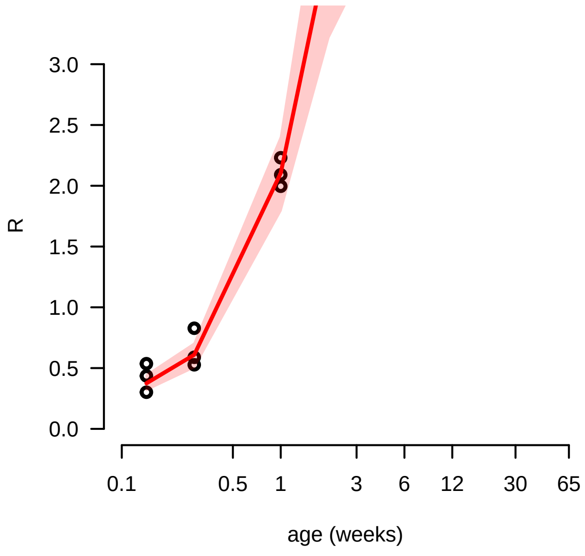

short pulse: ratio ln(L/H+1)

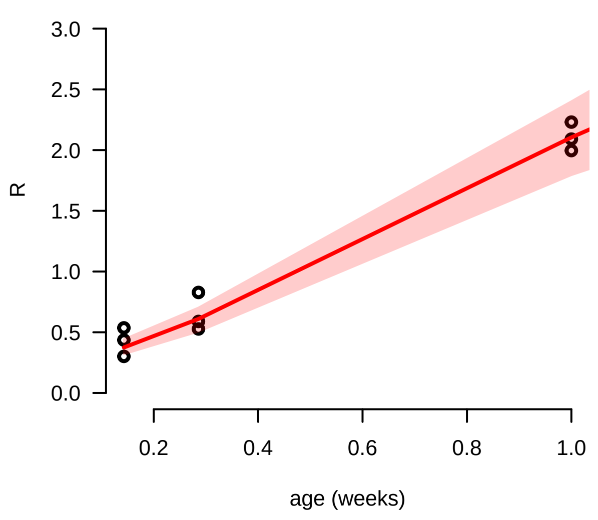

protein concentration log scale

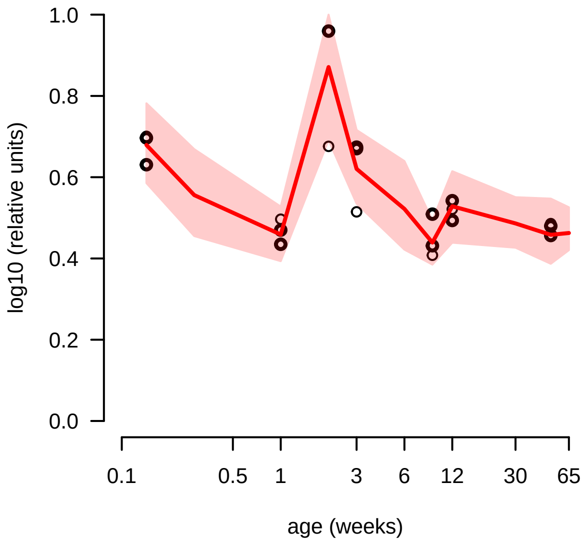

total protein log scale

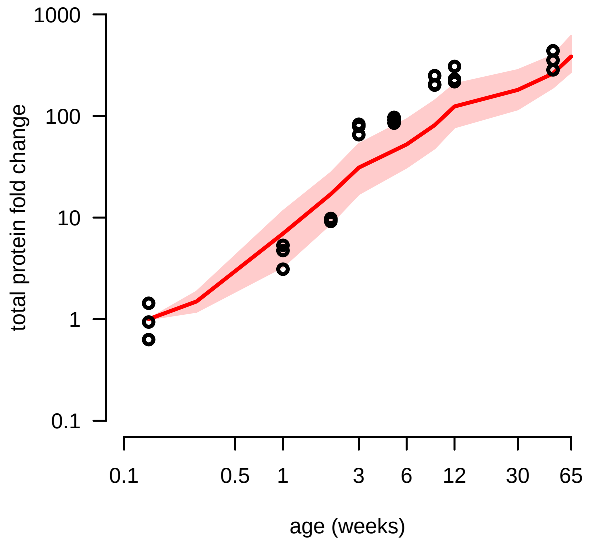

short pulse %H corrected log scale

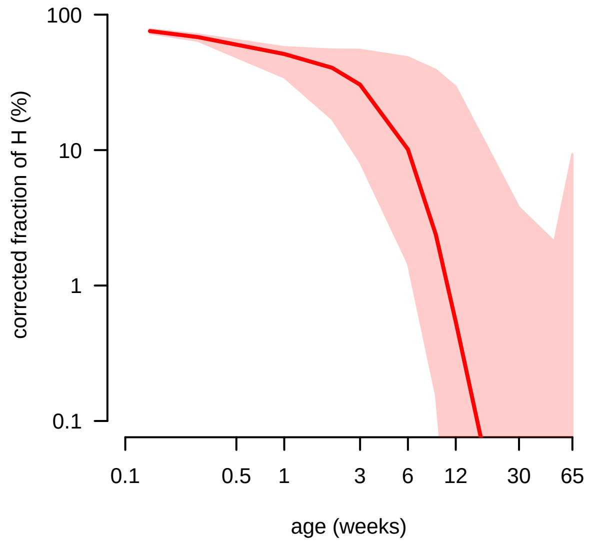

Q61749

>sp|Q61749|EI2BD_MOUSE

Translation initiation factor eIF−2B subunit delta

GN=Eif2b4

Med H1/2: 6.8 days

Q5 H1/2: 3.2 days

Q95 H1/2: 44.8 days

reliable model fit: yes

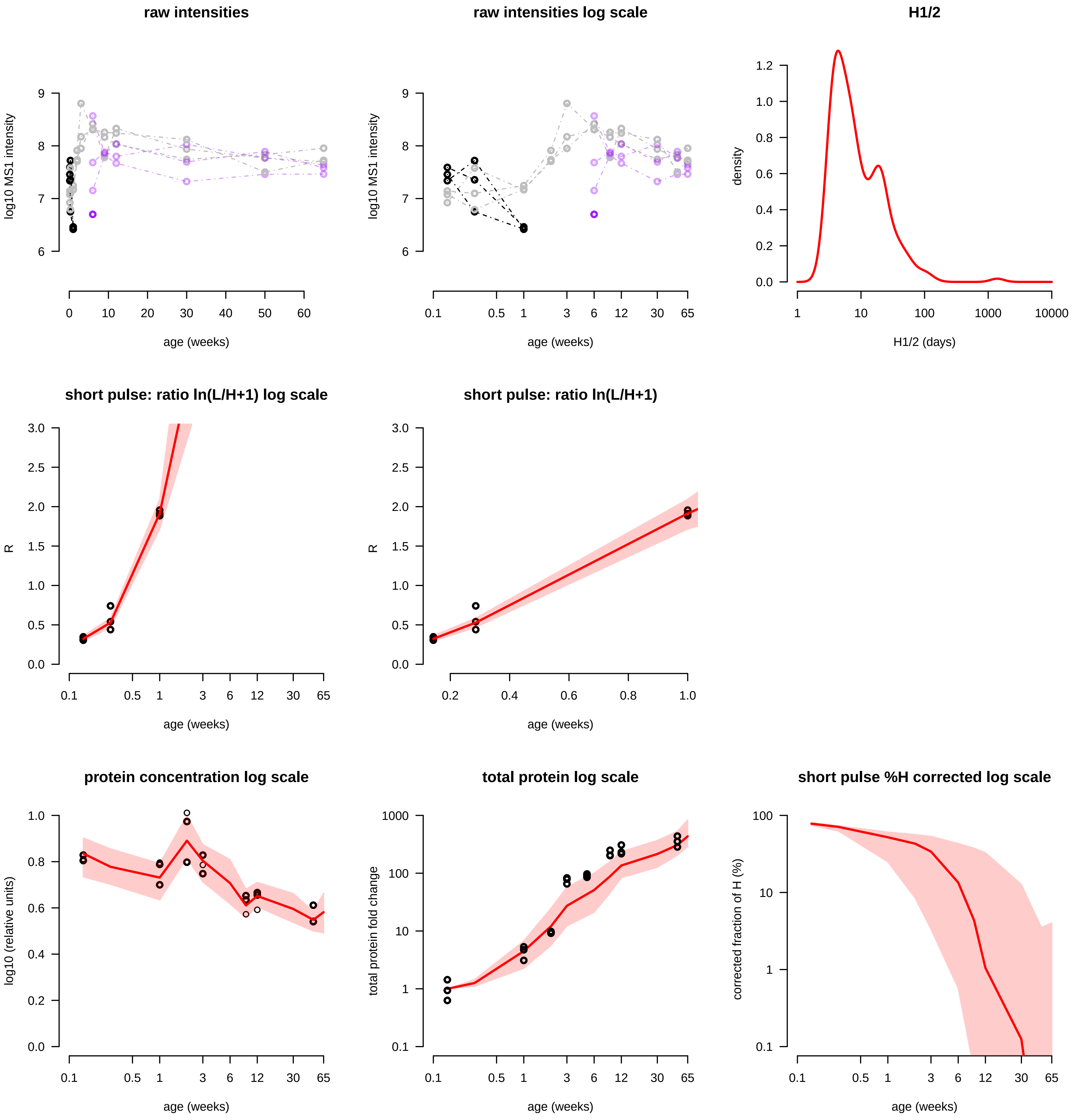

Q9DD02;Q9DD02−2

>sp|Q9DD02|HIKES_MOUSE

Protein Hikeshi

GN=Hikeshi

Med H1/2: 8.3 days

Q5 H1/2: 3.6 days

Q95 H1/2: 58.2 days

reliable model fit: yes

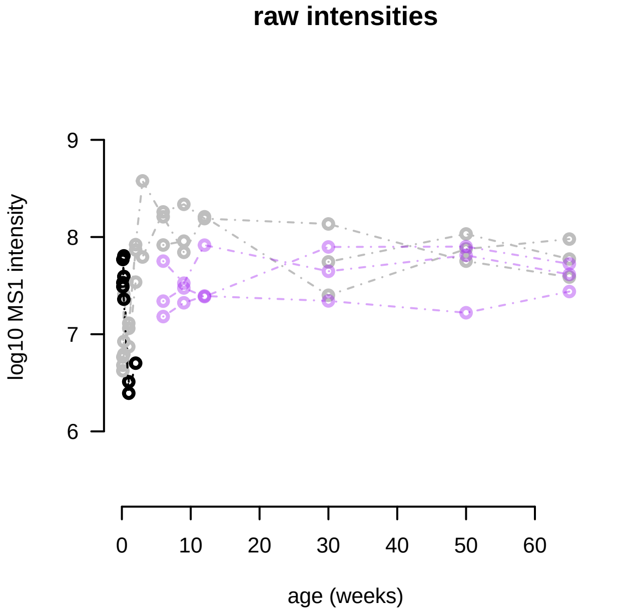

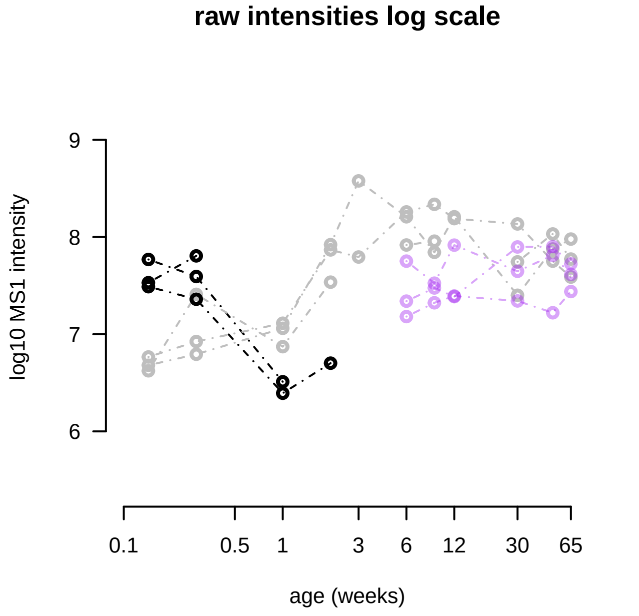

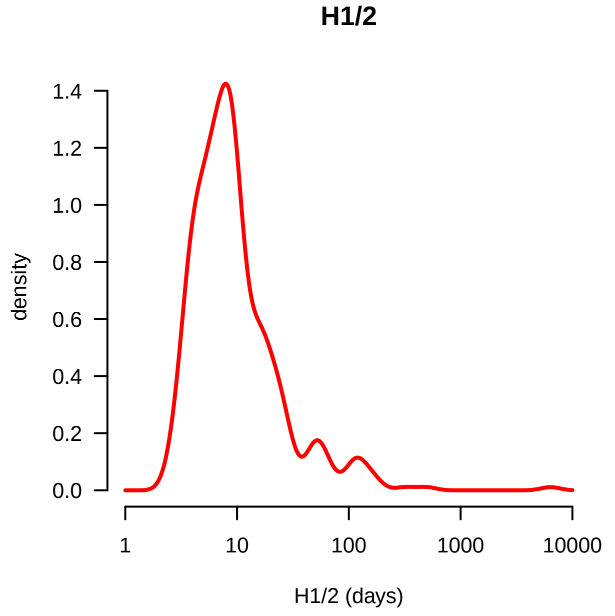

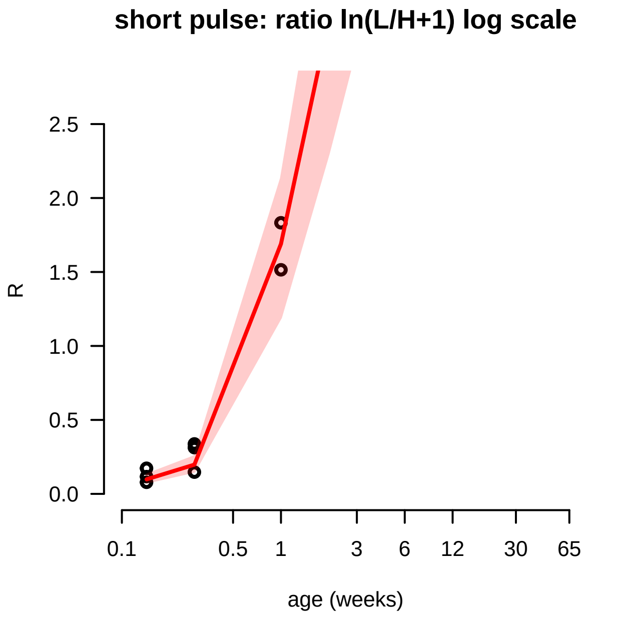

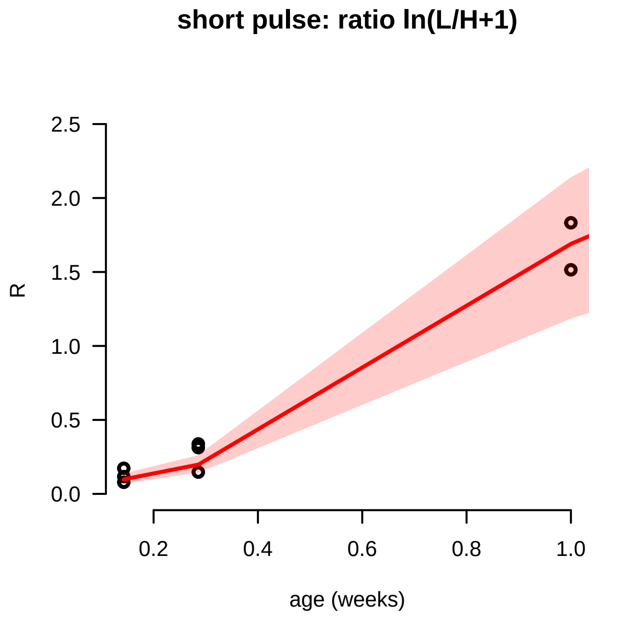

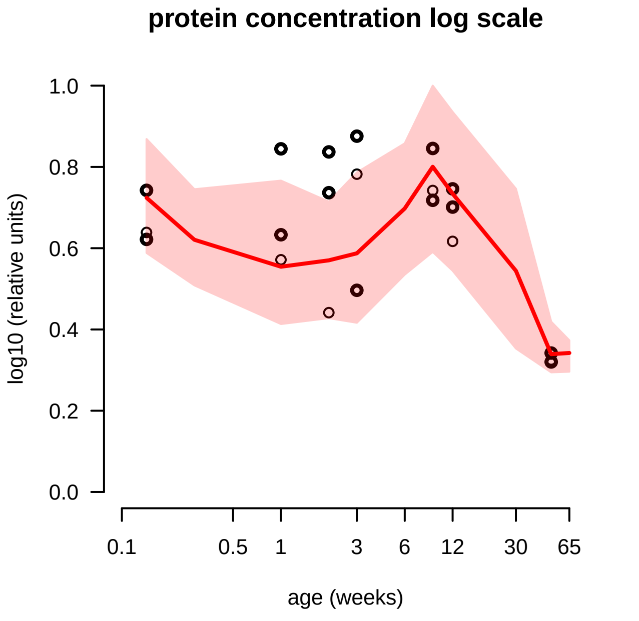

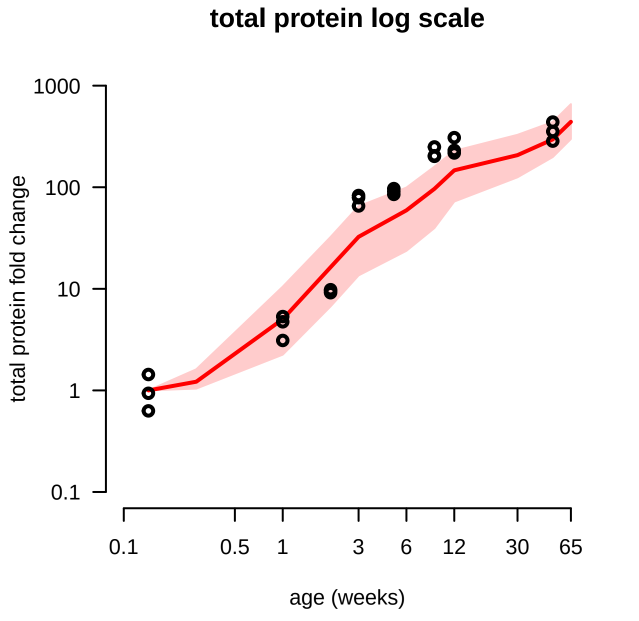

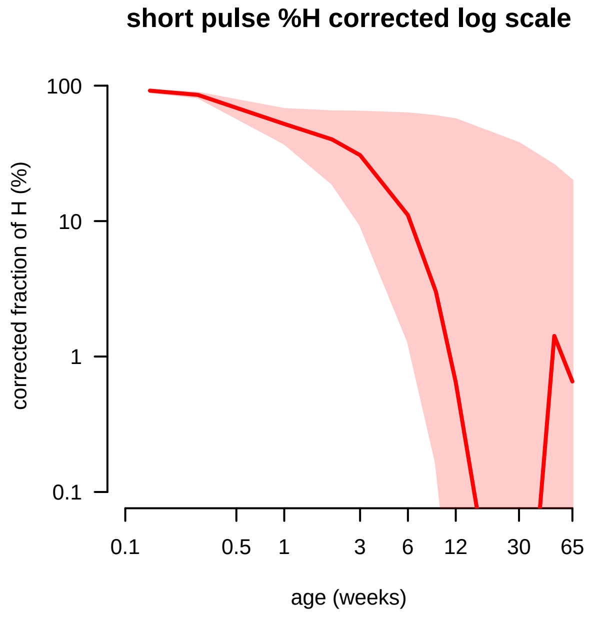

Q91WM3

>sp|Q91WM3|U3IP2_MOUSE

U3 small nucleolar RNA−interacting protein 2

GN=Rrp9

Med H1/2: 10.2 days

Q5 H1/2: 3.1 days

Q95 H1/2: 167.3 days

reliable model fit: no

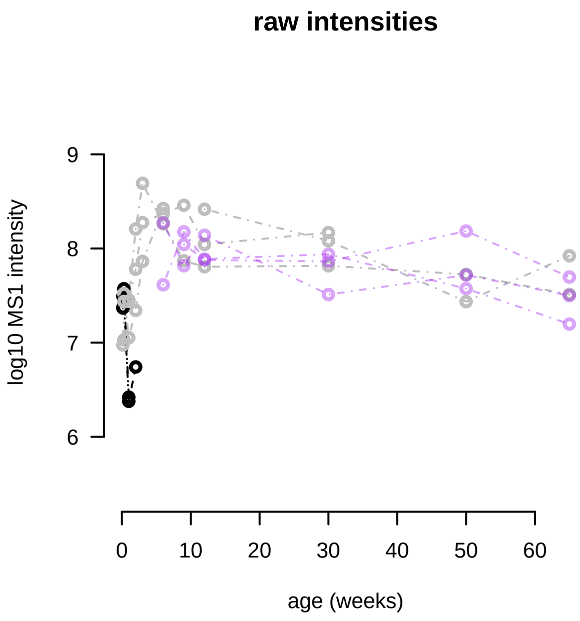

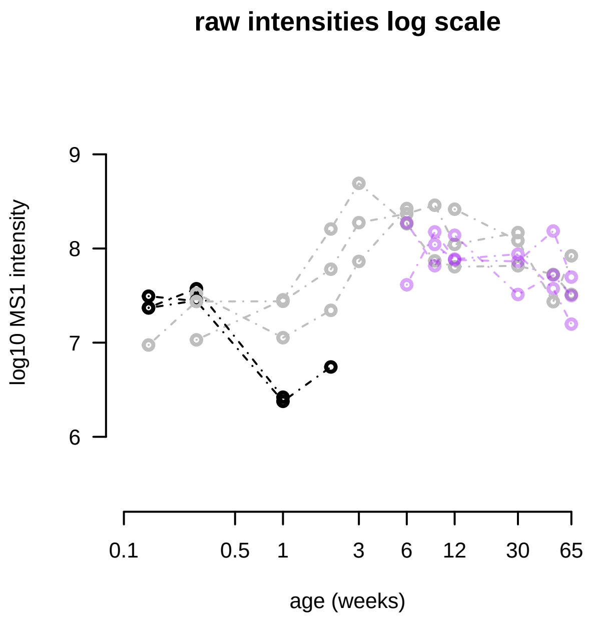

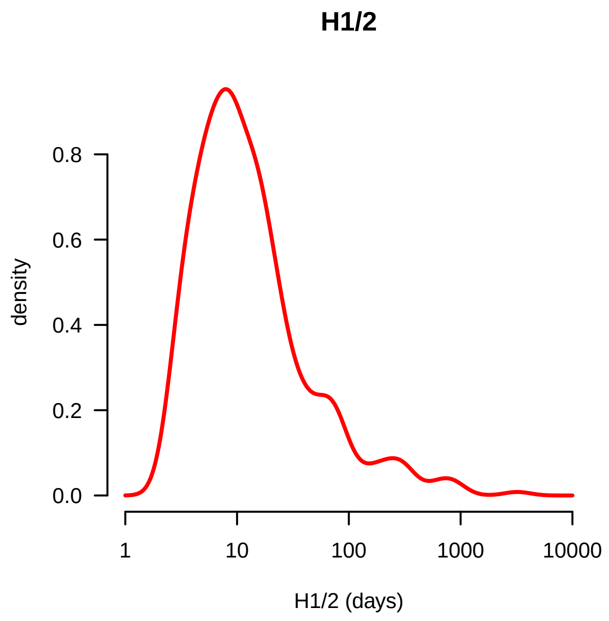

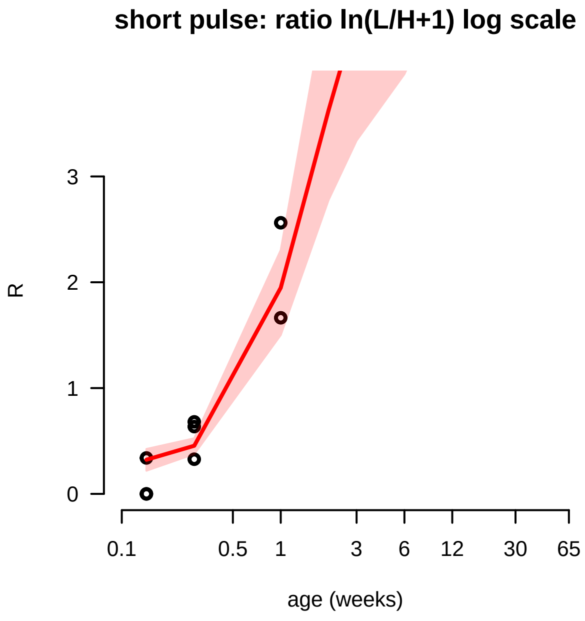

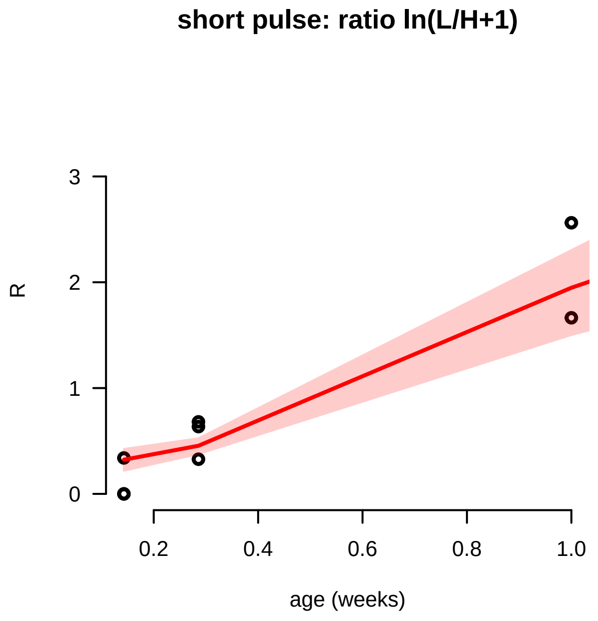

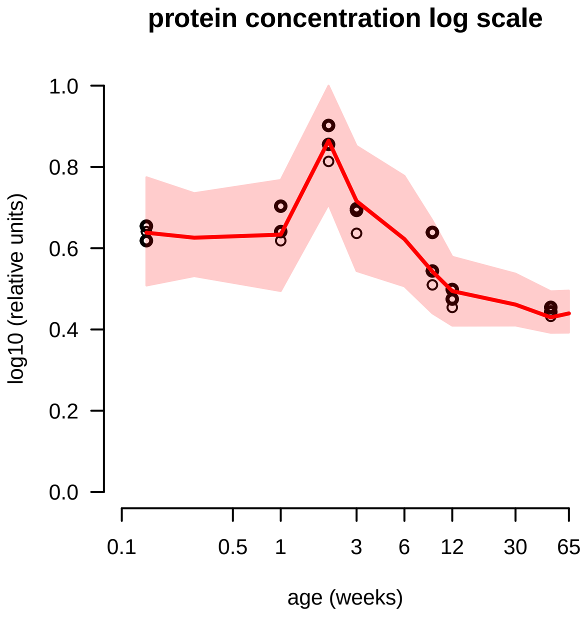

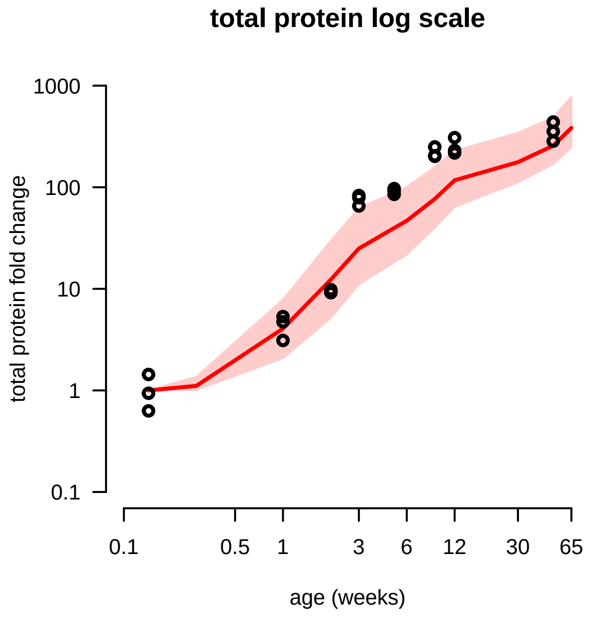

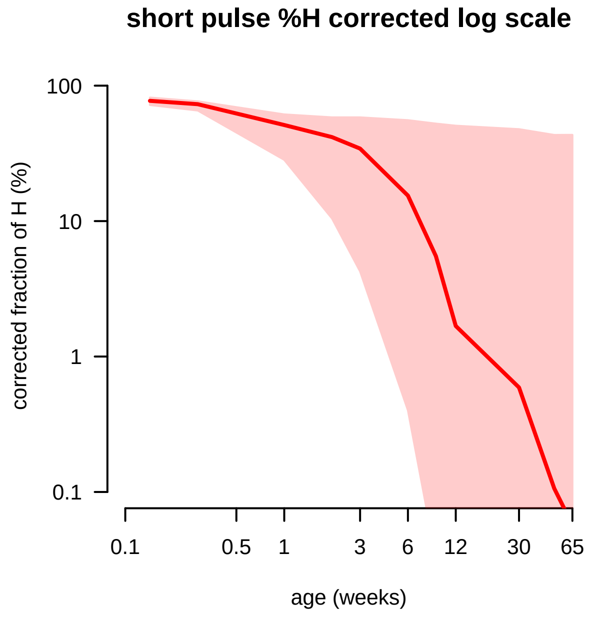

Q9R1B9

>sp|Q9R1B9|SLIT2_MOUSE

Slit homolog 2 protein

GN=Slit2

Med H1/2: 21.4 days

Q5 H1/2: 7 days

Q95 H1/2: 150.6 days

reliable model fit: yes

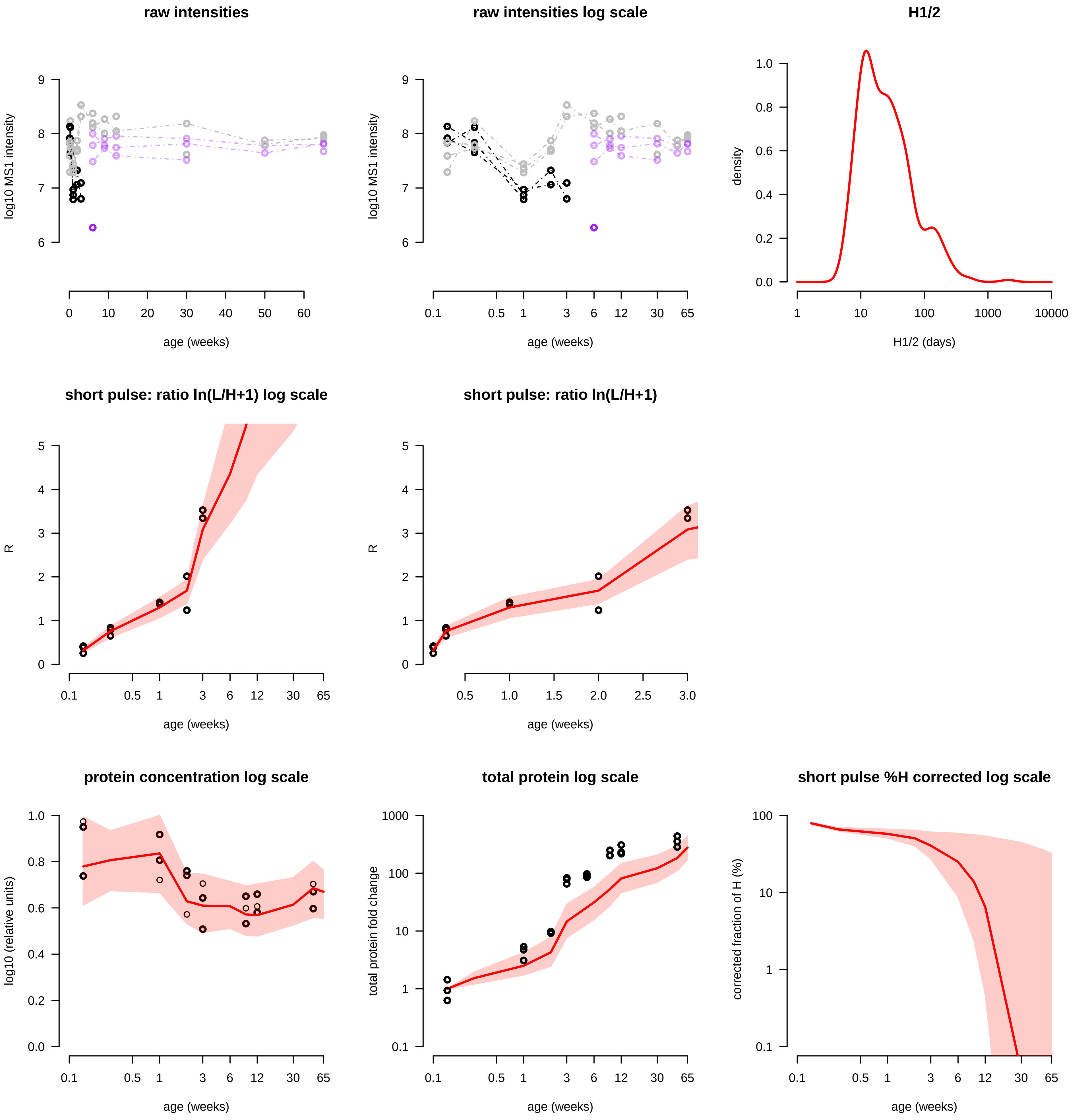

Q6PGF3

>sp|Q6PGF3|MED16_MOUSE

Mediator of RNA polymerase II transcription subunit 16

GN=Med16

Med H1/2: 4.6 days

Q5 H1/2: 2.7 days

Q95 H1/2: 20.3 days

reliable model fit: yes

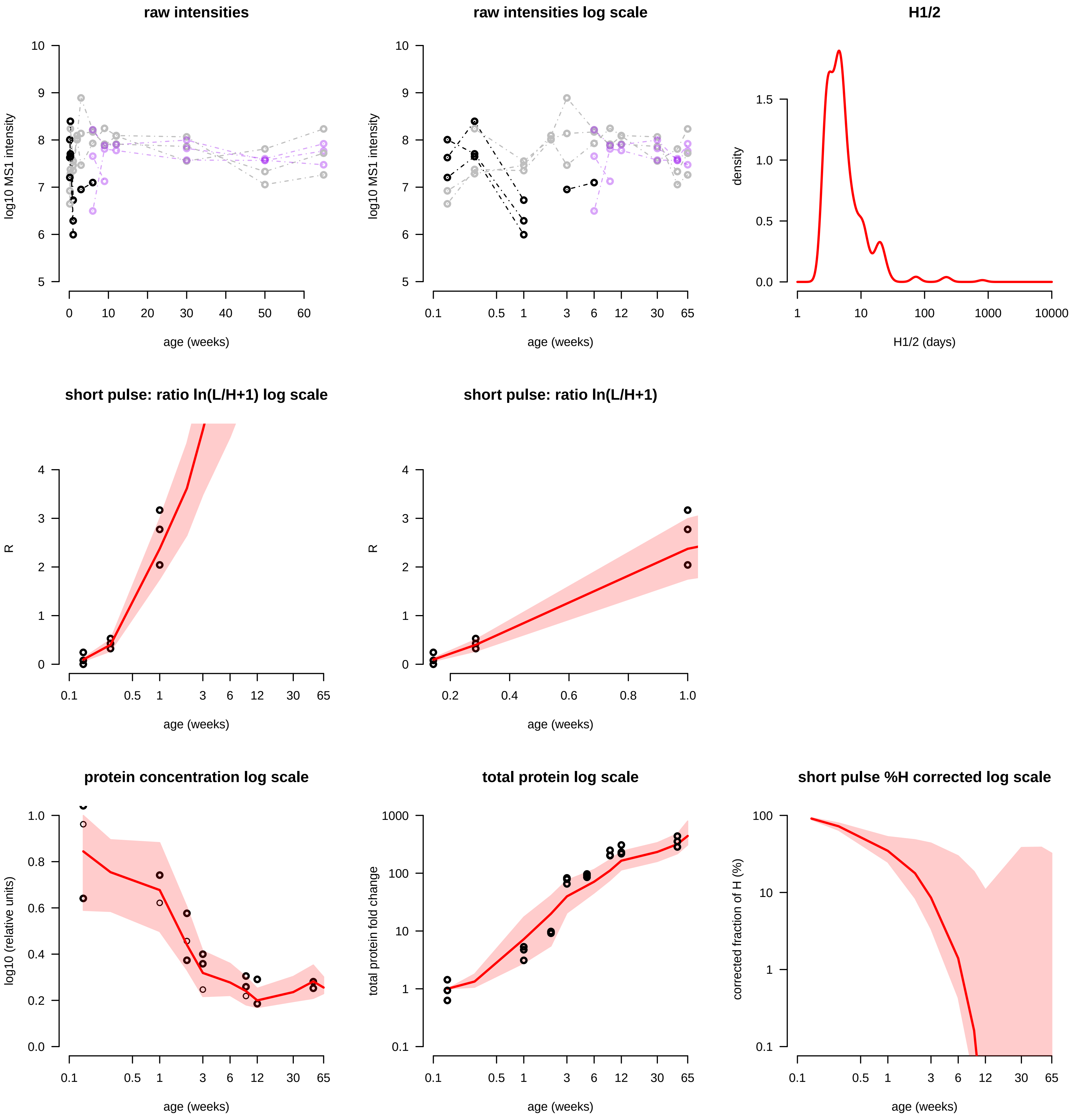

O89112

>sp|O89112|LANC1_MOUSE

Glutathione S−transferase LANCL1

GN=Lancl1

Med H1/2: 20 days

Q5 H1/2: 7.4 days

Q95 H1/2: 150.9 days

reliable model fit: yes

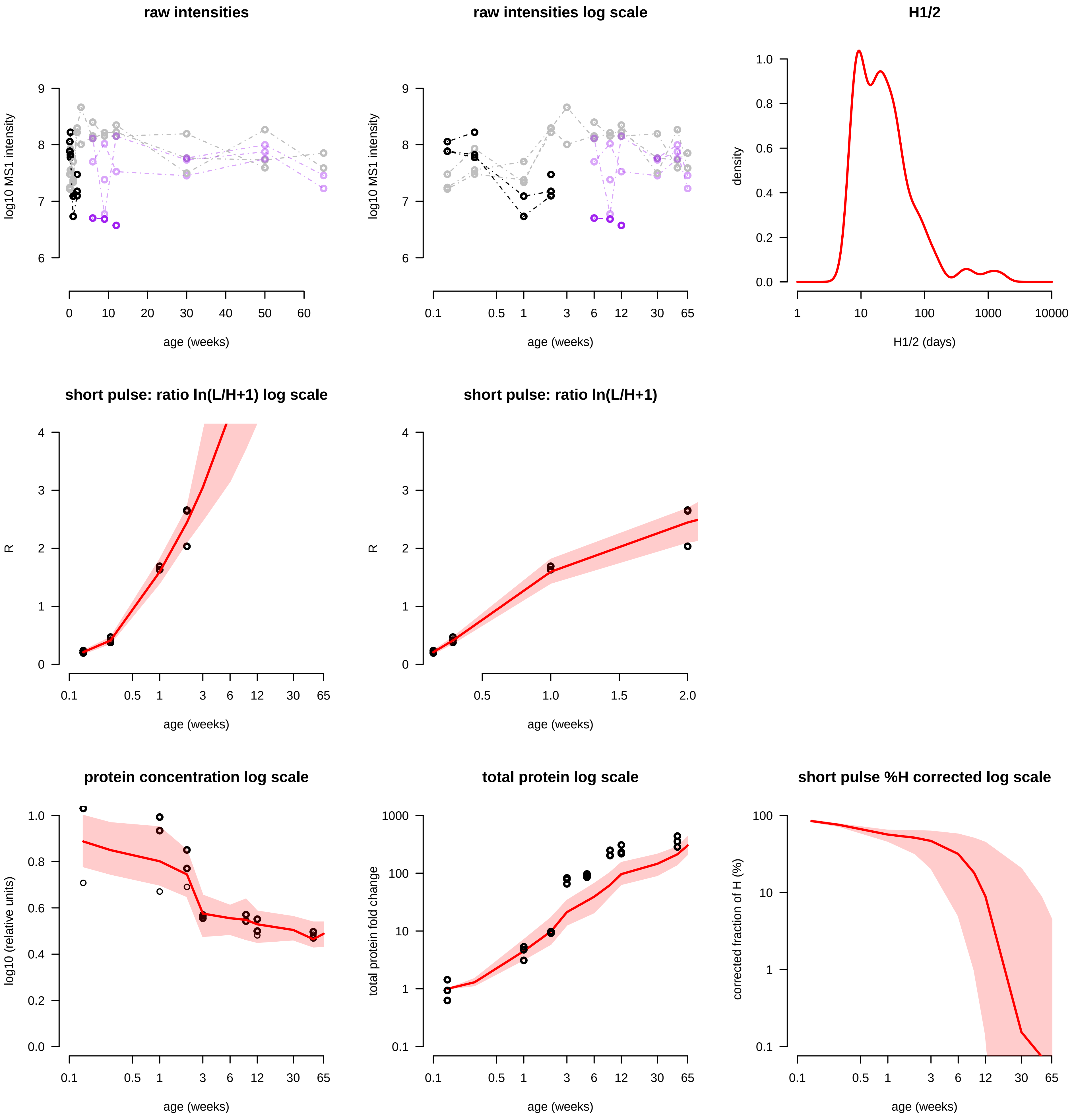

A2BE28−2;A2BE28

>sp|A2BE28−2|LAS1L_MOUSE

Isoform 2 of Ribosomal biogenesis protein LAS1L

GN=Las1l

Med H1/2: 13.6 days

Q5 H1/2: 11 days

Q95 H1/2: 16.7 days

reliable model fit: yes

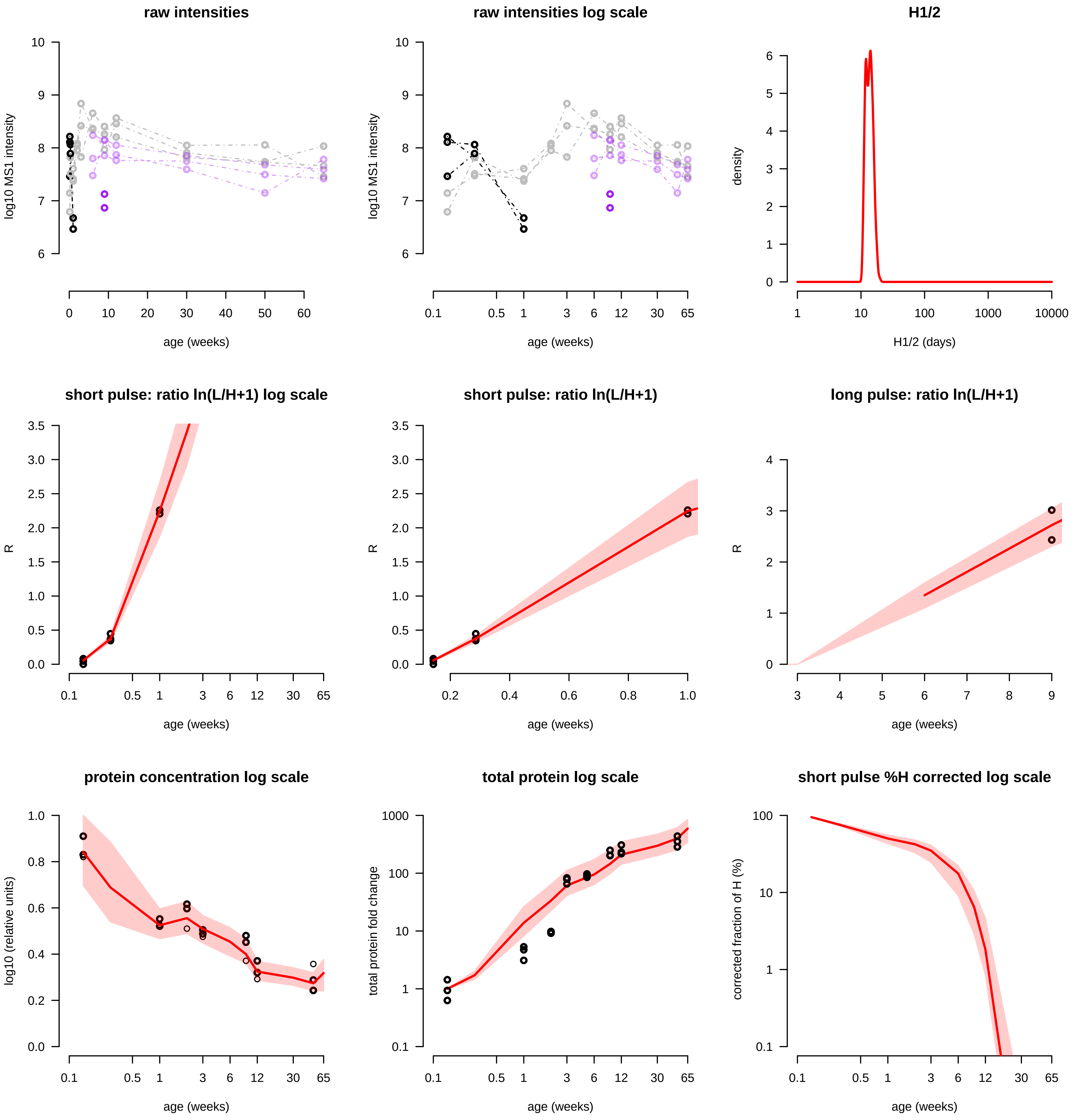

Q8CHI8−3;Q8CHI8−2;Q8CHI8−4;Q8CHI8;Q8CHI8−5

>sp|Q8CHI8−3|EP400_MOUSE

Isoform 3 of E1A−binding protein p400

GN=Ep400

Med H1/2: 45.5 days

Q5 H1/2: 15.2 days

Q95 H1/2: 413.9 days

reliable model fit: no

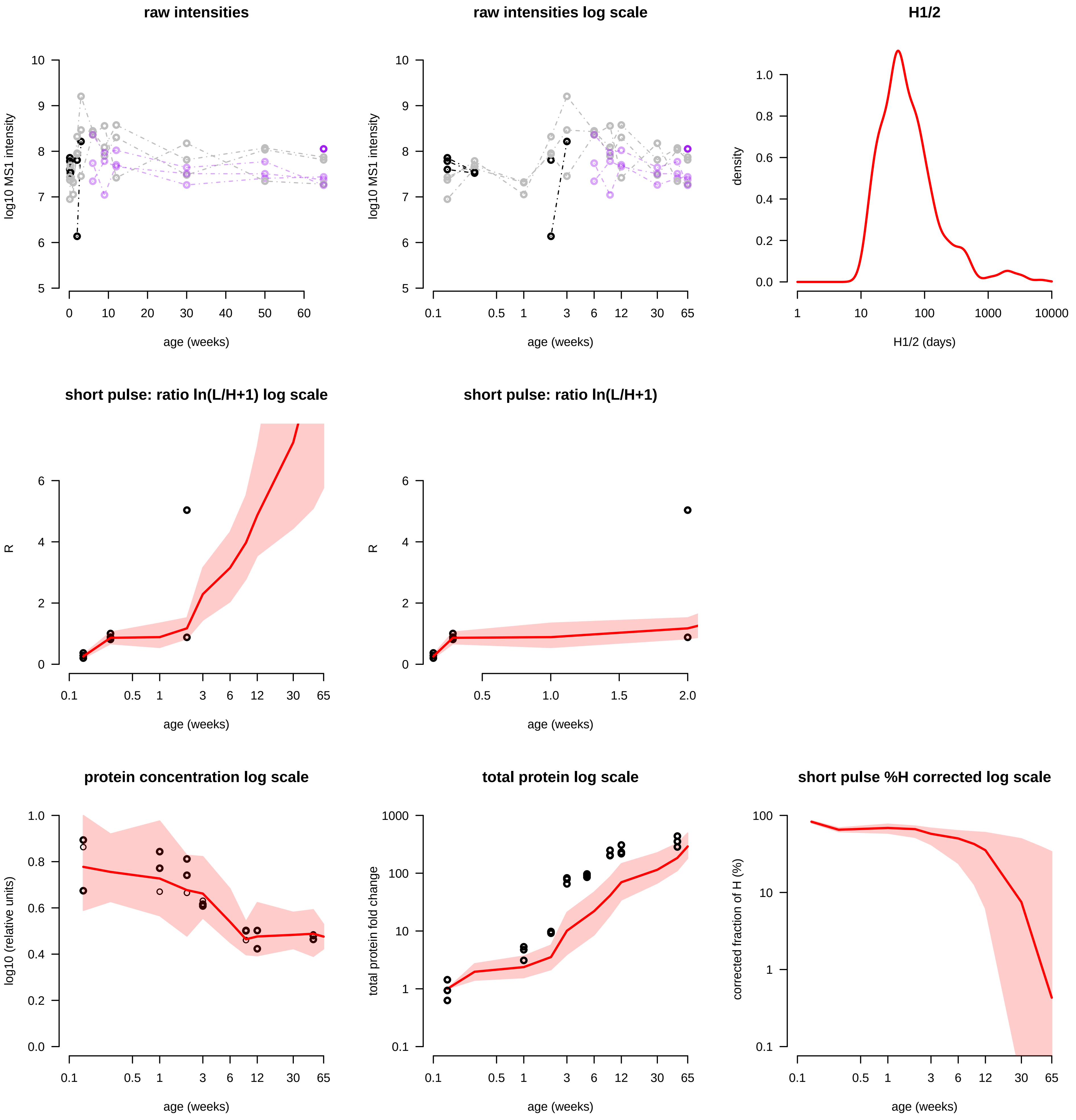

E9Q5K9;E9Q5K9−3;E9Q5K9−2

>sp|E9Q5K9|YTDC1_MOUSE

YTH domain−containing protein 1

GN=Ythdc1

Med H1/2: 13.1 days

Q5 H1/2: 3.3 days

Q95 H1/2: 128.2 days

reliable model fit: yes

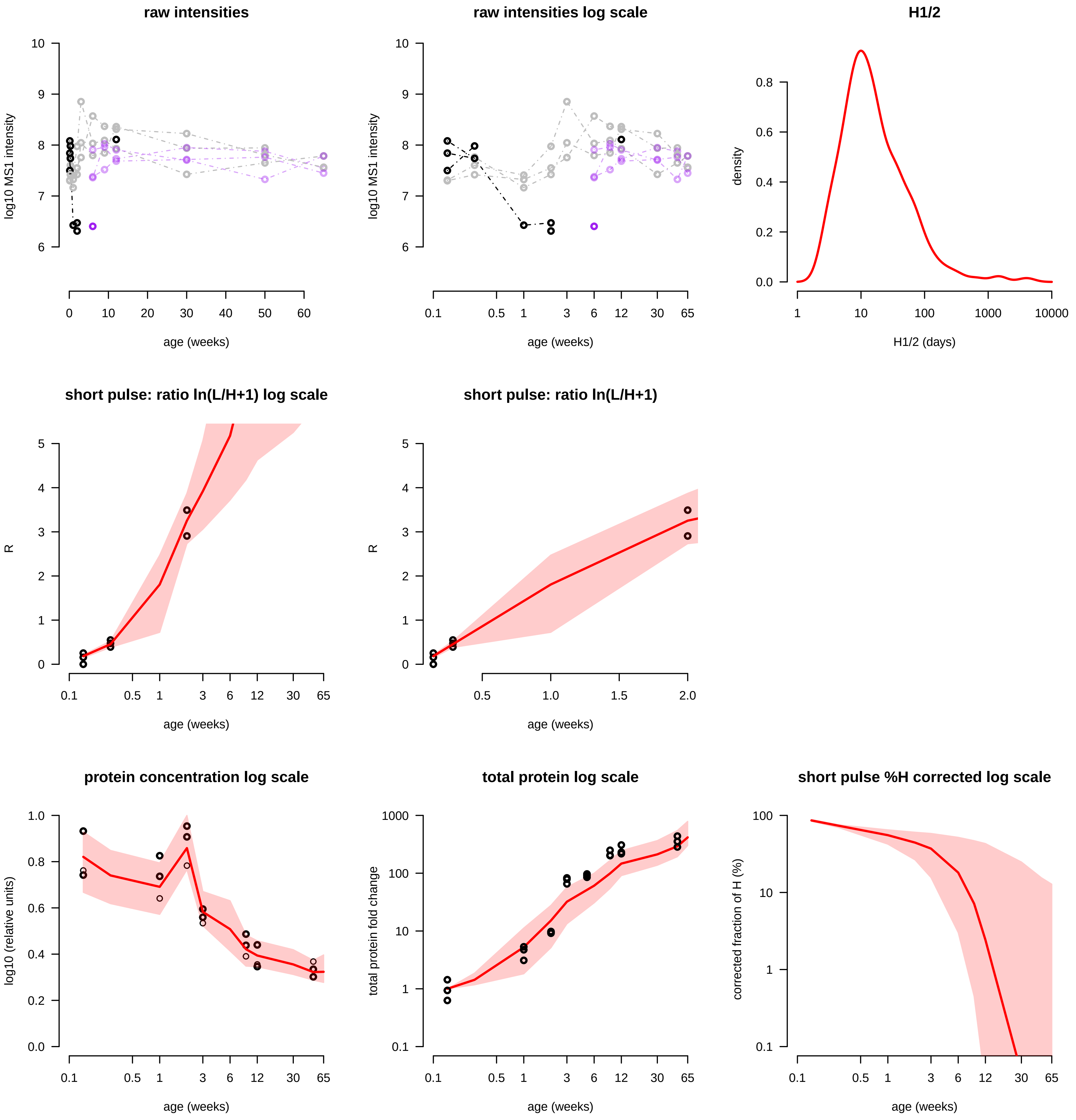

P21812

>sp|P21812|MCPT4_MOUSE

Mast cell protease 4

GN=Mcpt4

Med H1/2: 18.9 days

Q5 H1/2: 5.8 days

Q95 H1/2: 235.9 days

reliable model fit: yes

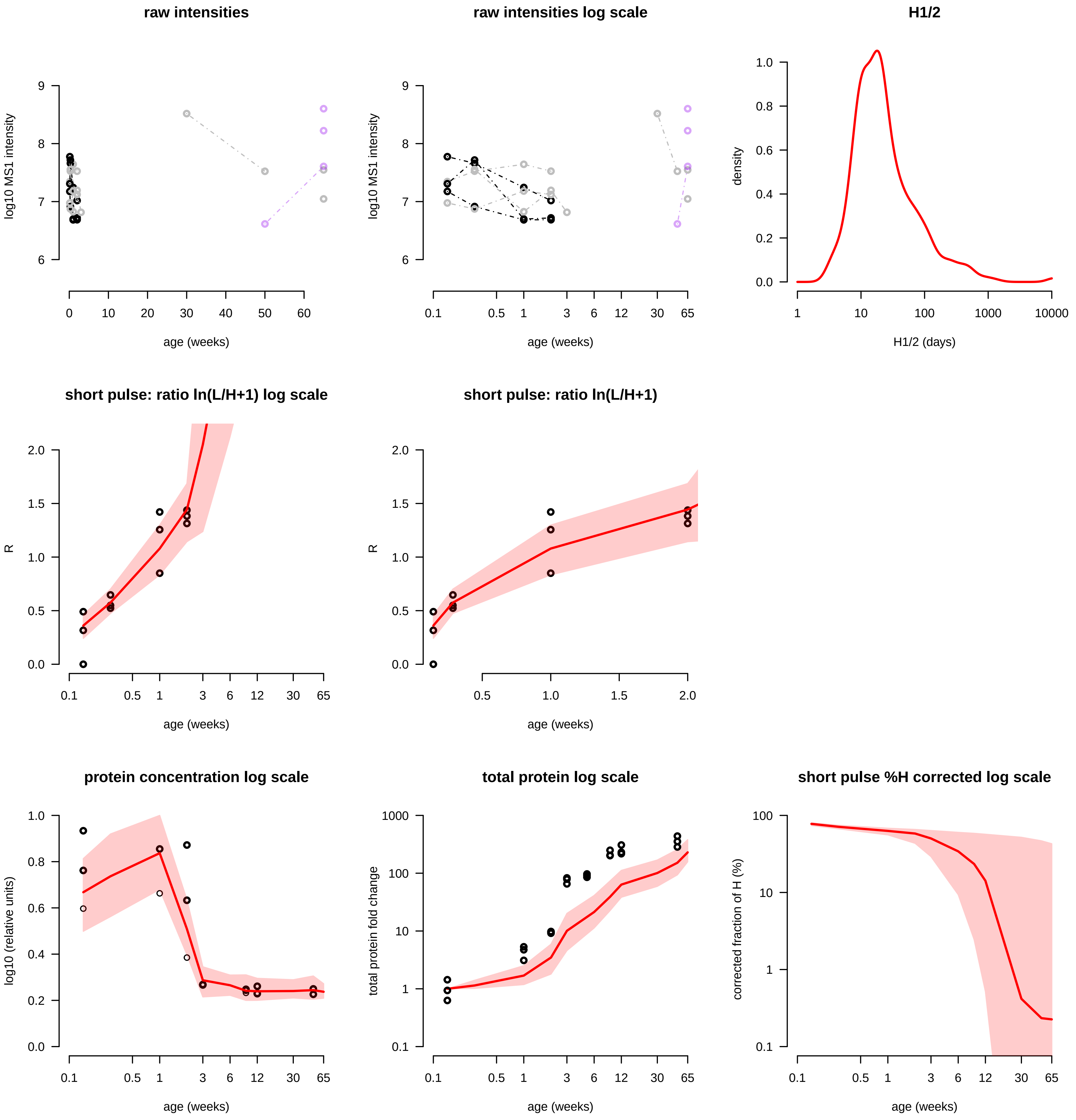

Q60809

>sp|Q60809|CNOT7_MOUSE

CCR4−NOT transcription complex subunit 7

GN=Cnot7

Med H1/2: 10.6 days

Q5 H1/2: 3.4 days

Q95 H1/2: 88.5 days

reliable model fit: yes

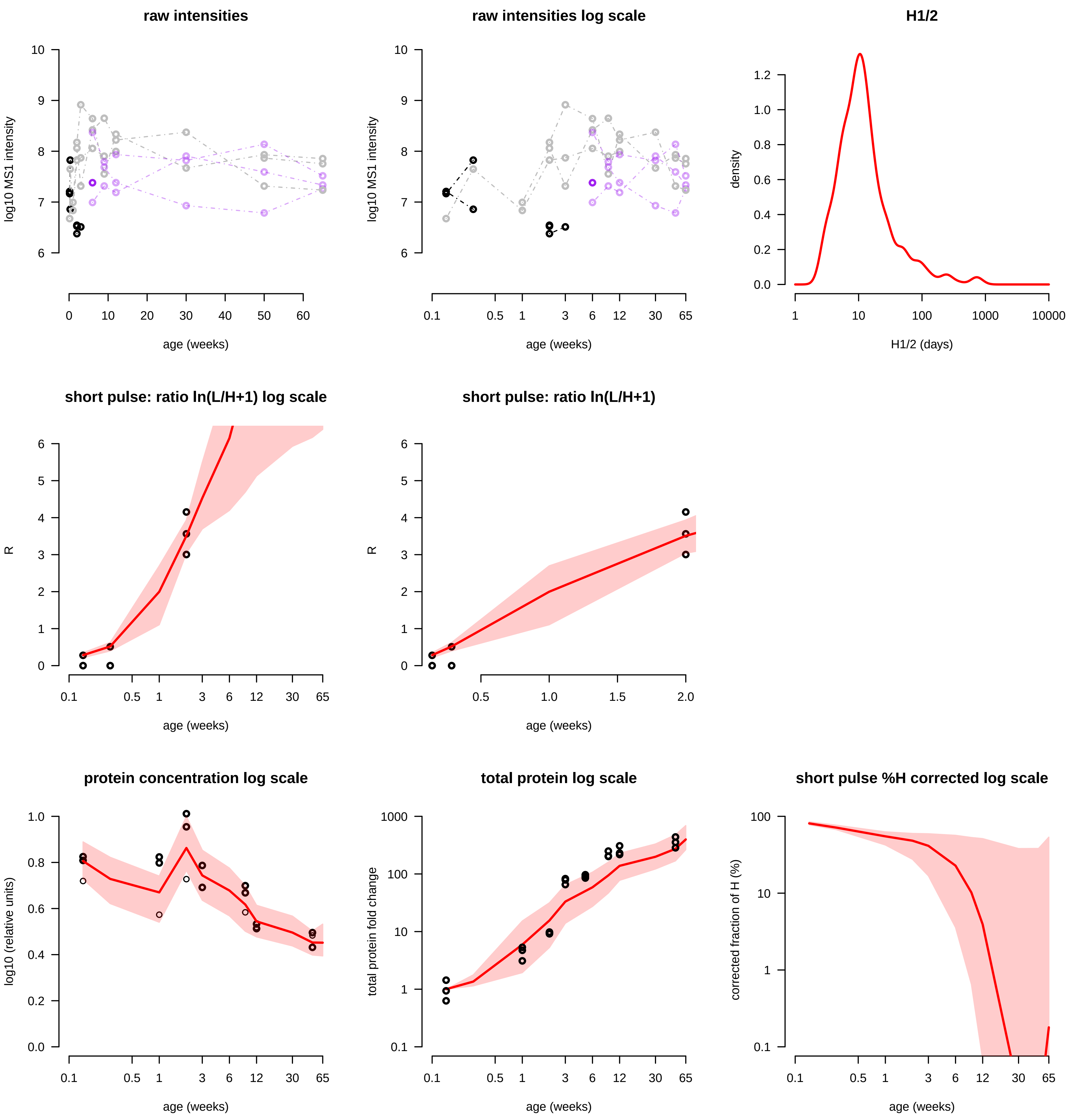

Q99J62

>sp|Q99J62|RFC4_MOUSE

Replication factor C subunit 4

GN=Rfc4

Med H1/2: 11.3 days

Q5 H1/2: 3.5 days

Q95 H1/2: 105 days

reliable model fit: no

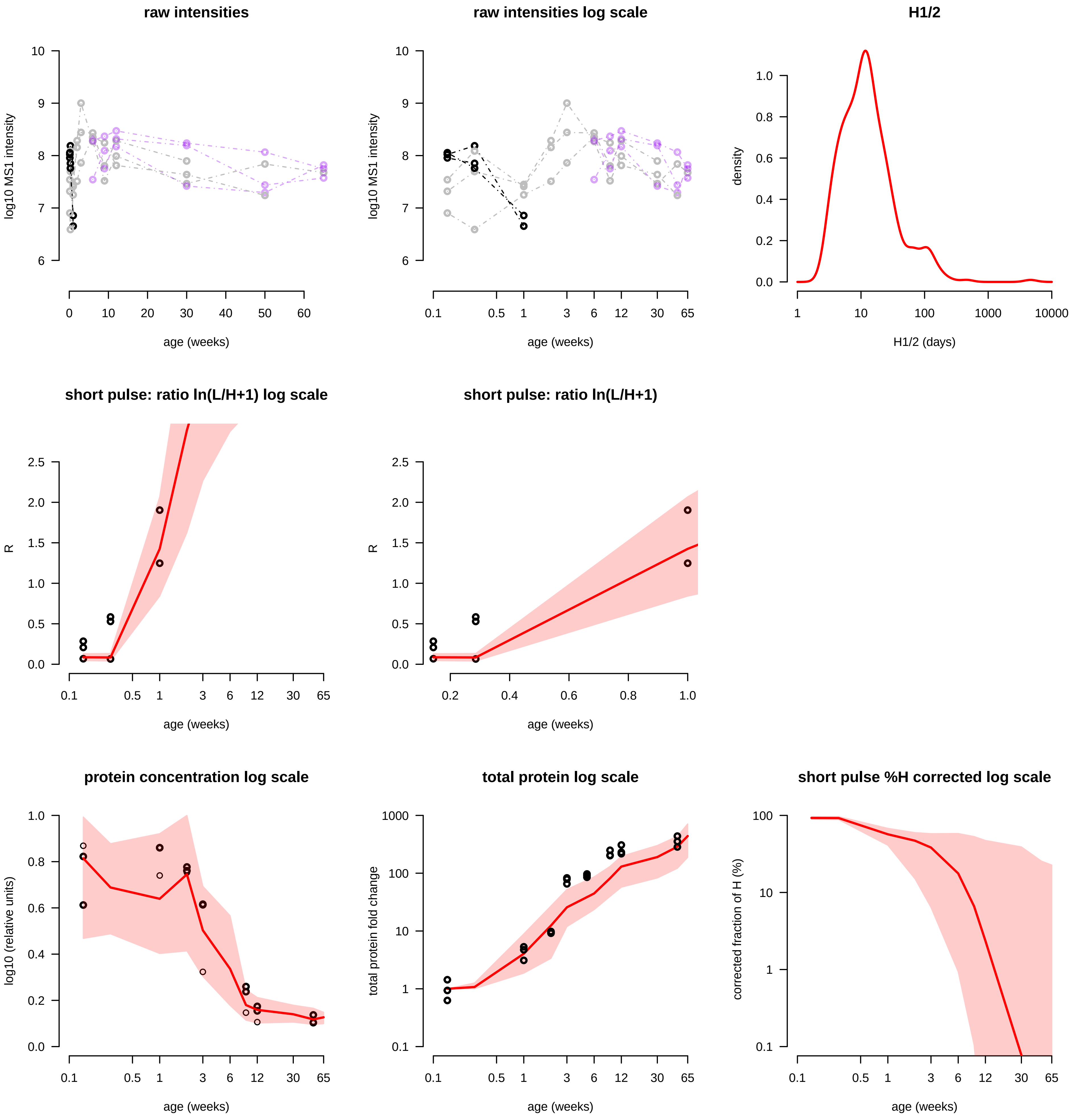

Q9D4J7;Q9D4J7−2

>sp|Q9D4J7|PHF6_MOUSE

PHD finger protein 6

GN=Phf6

Med H1/2: 7.3 days

Q5 H1/2: 2.7 days

Q95 H1/2: 90.1 days

reliable model fit: yes

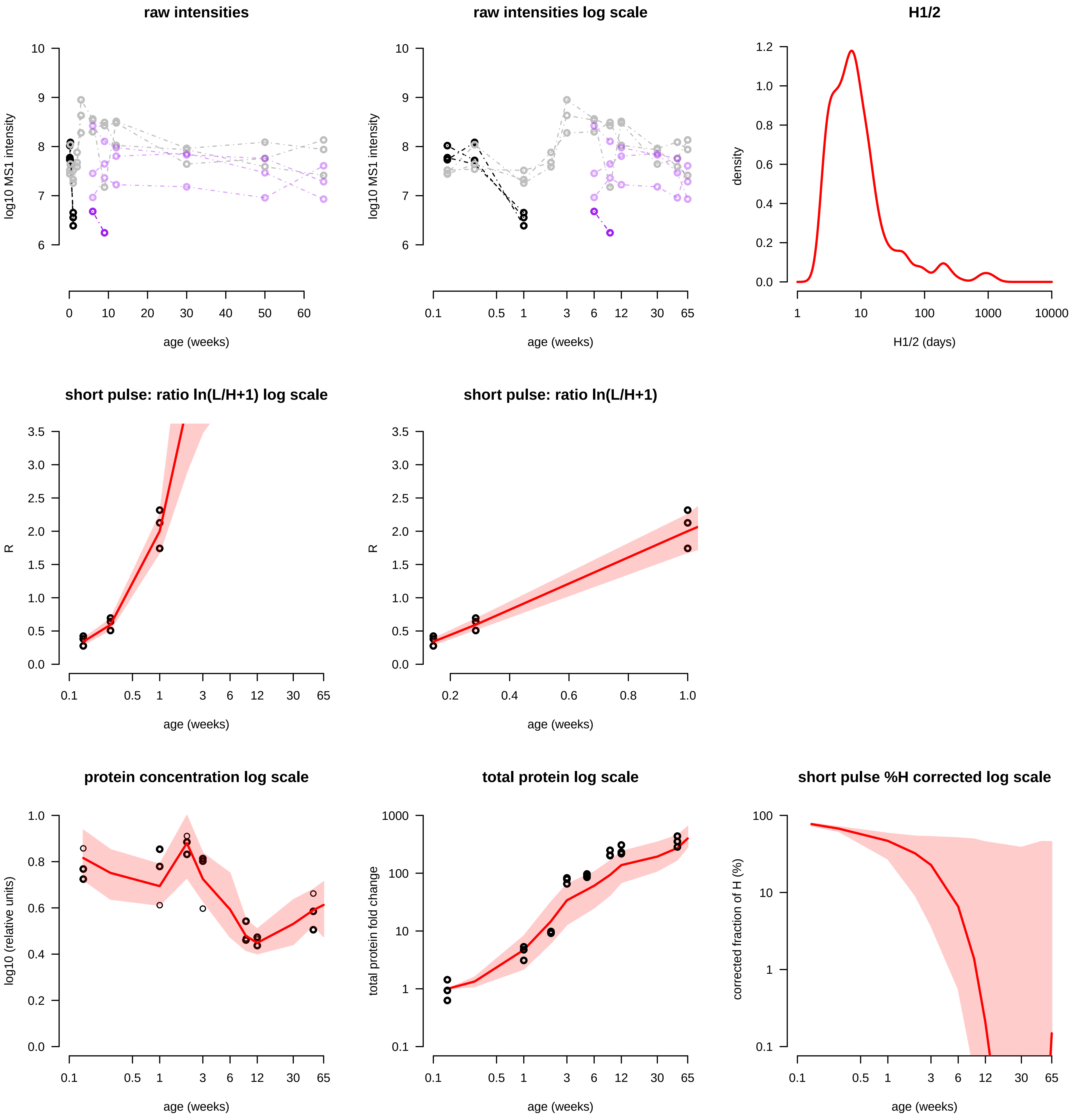

Q8R3D1

>sp|Q8R3D1|TBC13_MOUSE

TBC1 domain family member 13

GN=Tbc1d13

Med H1/2: 4.7 days

Q5 H1/2: 2.8 days

Q95 H1/2: 12.4 days

reliable model fit: yes

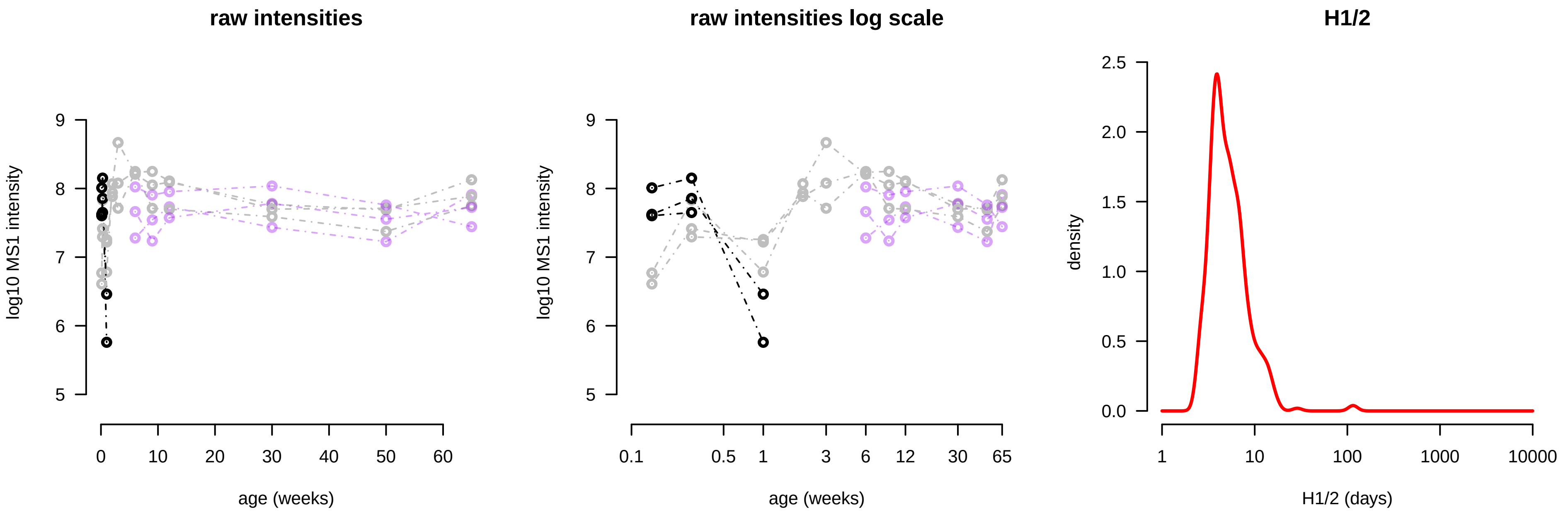

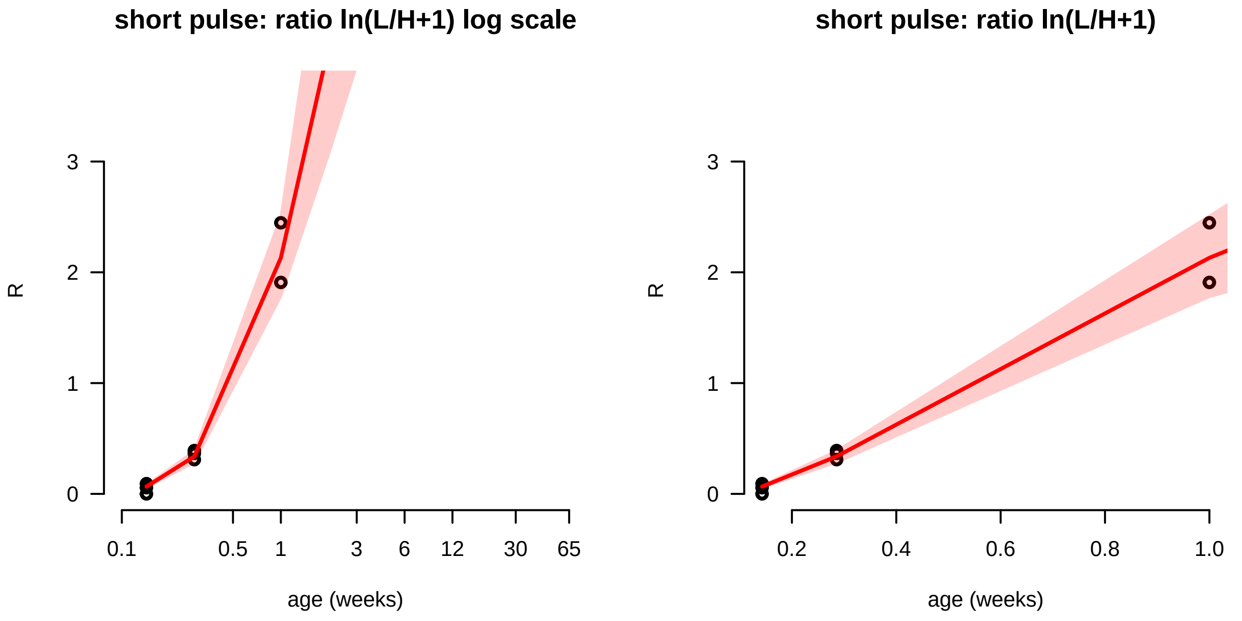

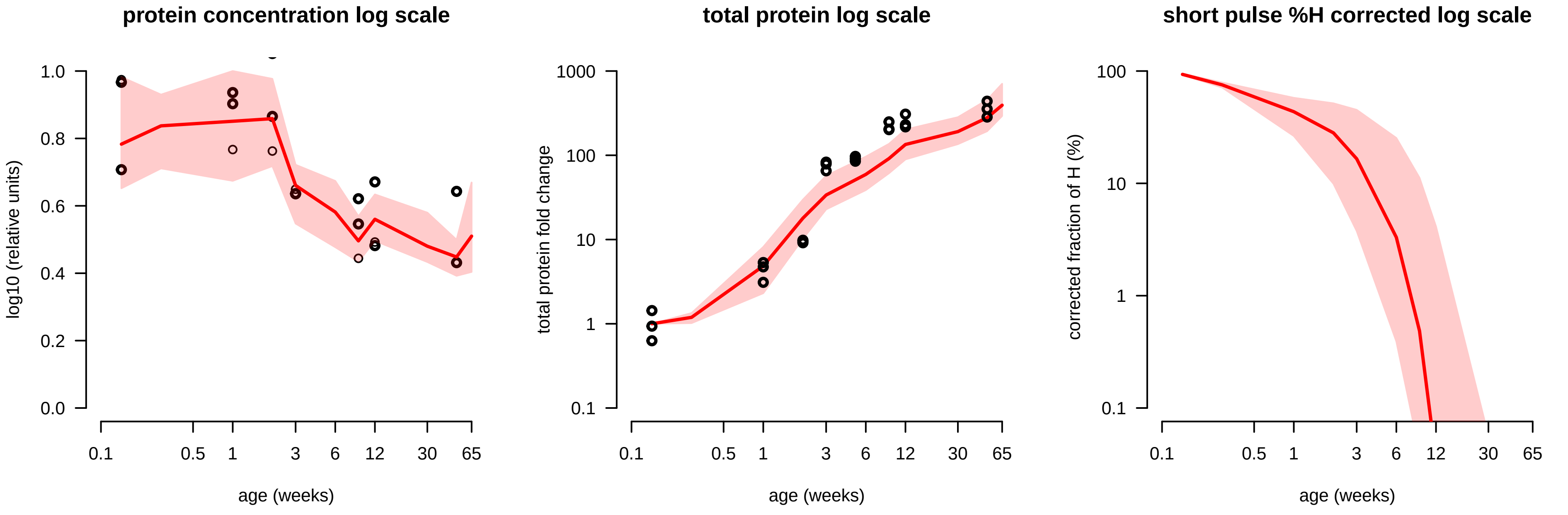

Q8BU03

>sp|Q8BU03|PWP2_MOUSE

Periodic tryptophan protein 2 homolog

GN=Pwp2

Med H1/2: 7.7 days

Q5 H1/2: 2.7 days

Q95 H1/2: 40.3 days

reliable model fit: yes

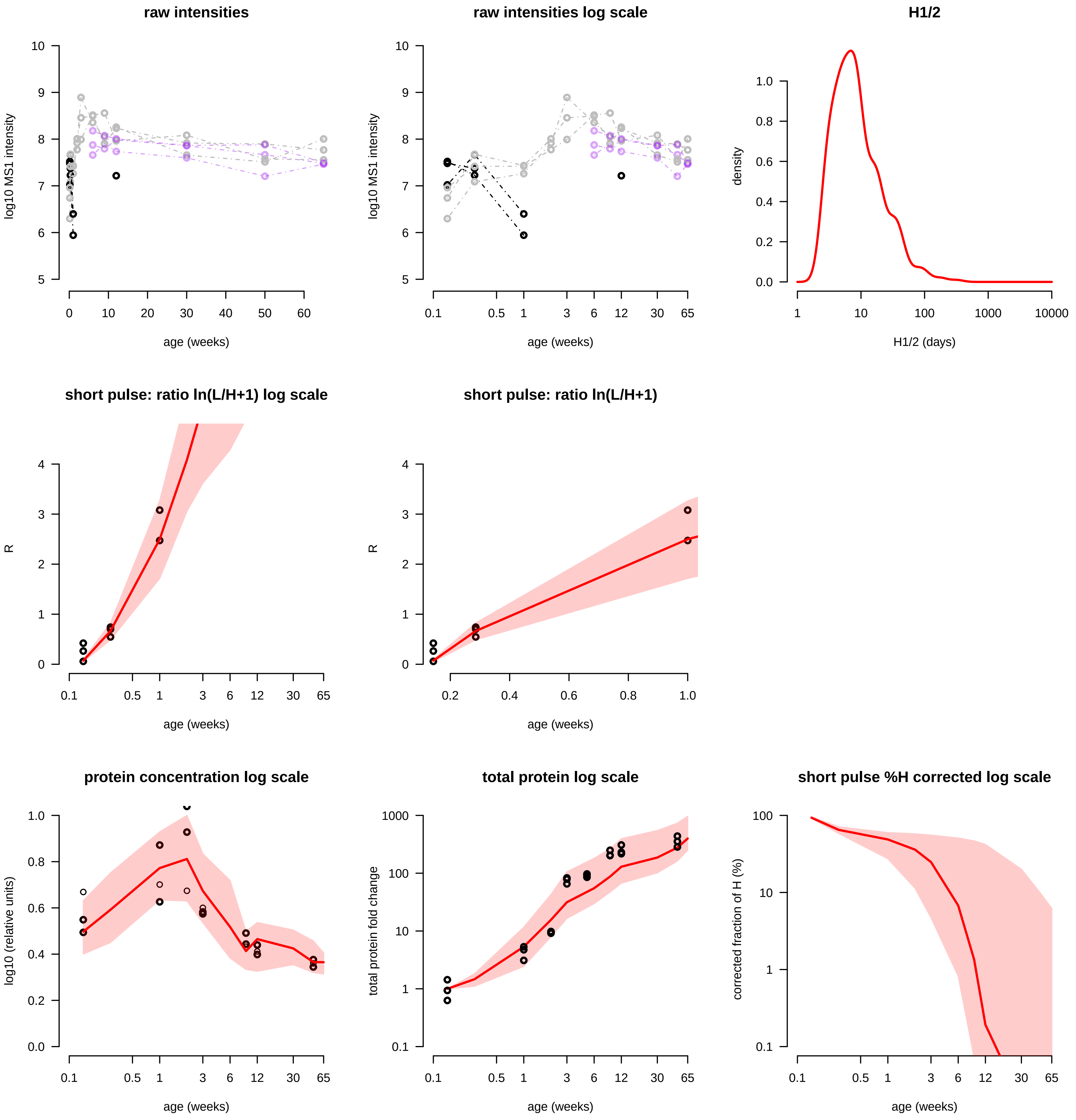

Q80UK8

>sp|Q80UK8|INT2_MOUSE

Integrator complex subunit 2

GN=Ints2

Med H1/2: 5.7 days

Q5 H1/2: 2.9 days

Q95 H1/2: 49.4 days

reliable model fit: yes

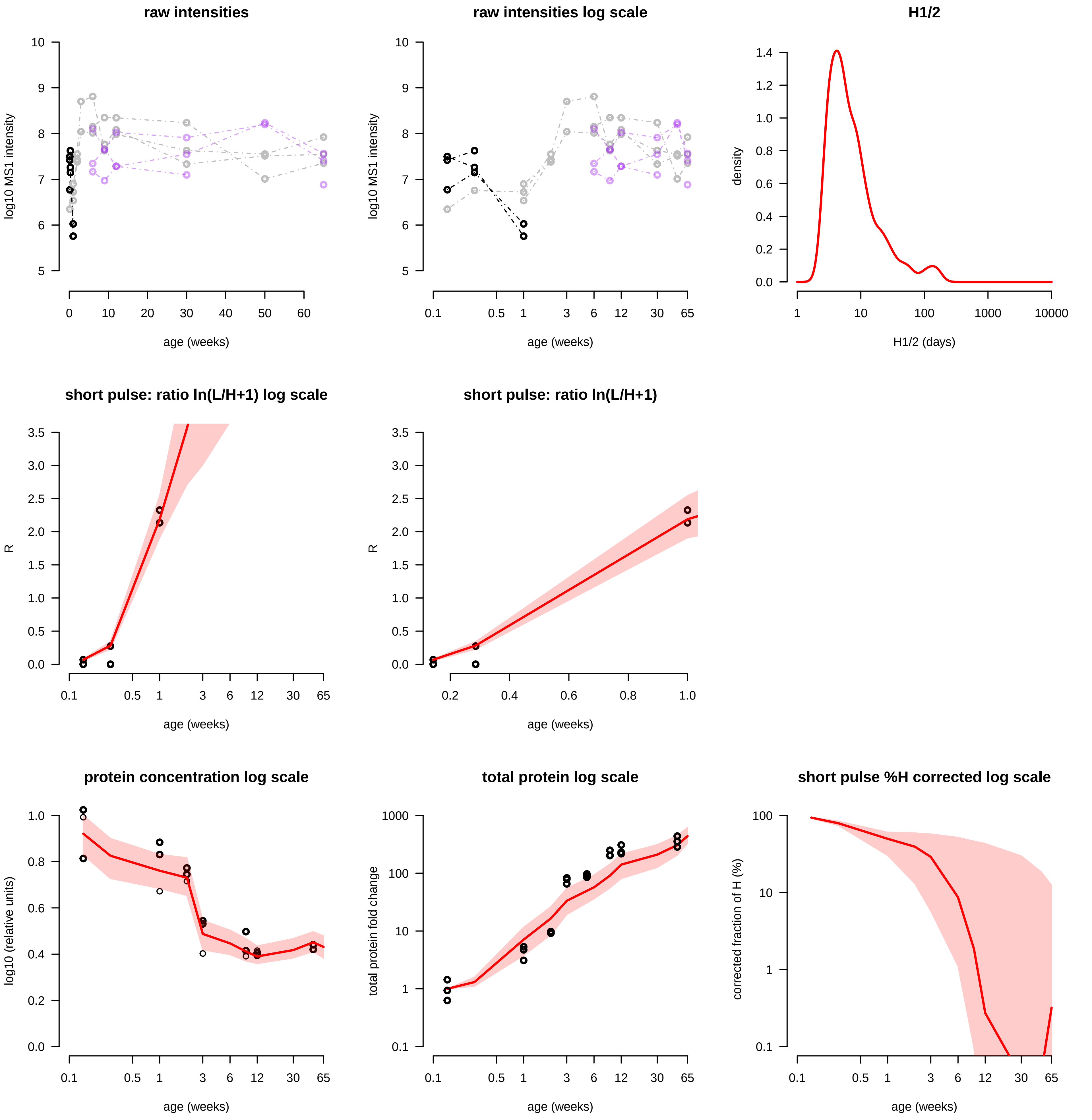

Q99LH1

>sp|Q99LH1|NOG2_MOUSE

Nucleolar GTP−binding protein 2

GN=Gnl2

Med H1/2: 4.6 days

Q5 H1/2: 2.6 days

Q95 H1/2: 28.5 days

reliable model fit: yes

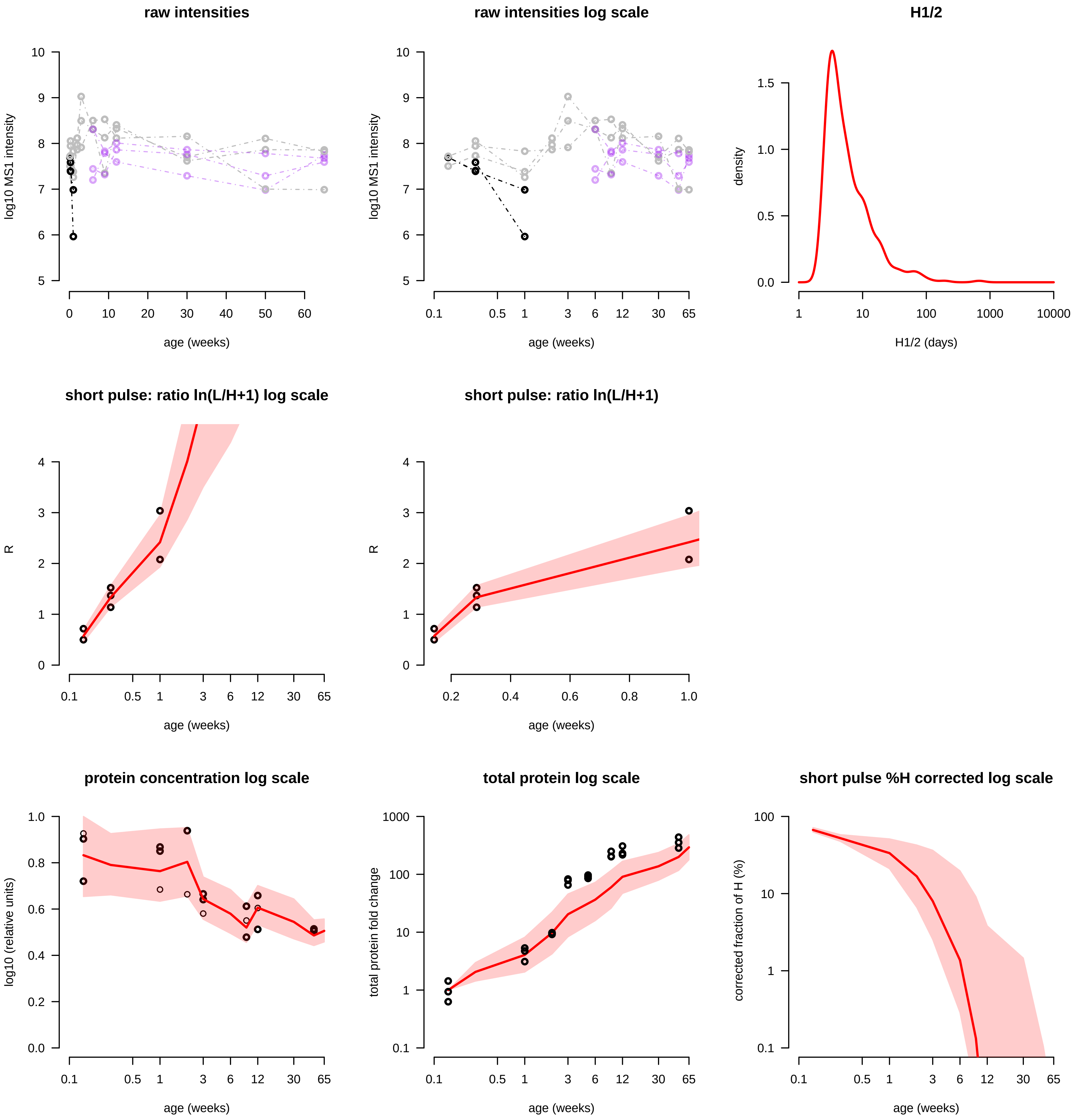

Q9R059

>sp|Q9R059|FHL3_MOUSE

Four and a half LIM domains protein 3

GN=Fhl3

Med H1/2: 18.8 days

Q5 H1/2: 6.4 days

Q95 H1/2: 98.5 days

reliable model fit: yes

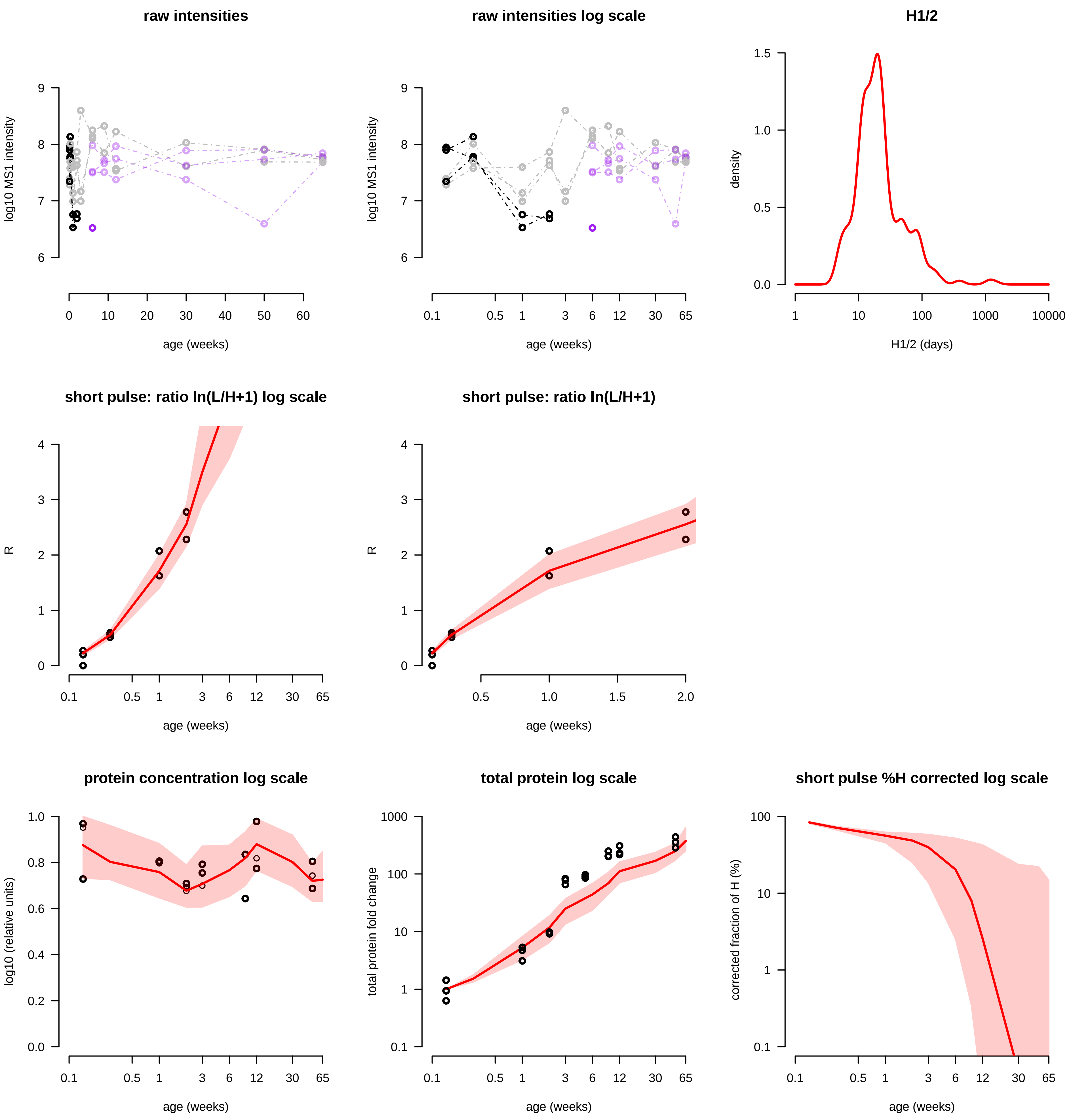

Q99LS3

>sp|Q99LS3|SERB_MOUSE

Phosphoserine phosphatase

GN=Psph

Med H1/2: 15.6 days

Q5 H1/2: 4.5 days

Q95 H1/2: 212.7 days

reliable model fit: yes

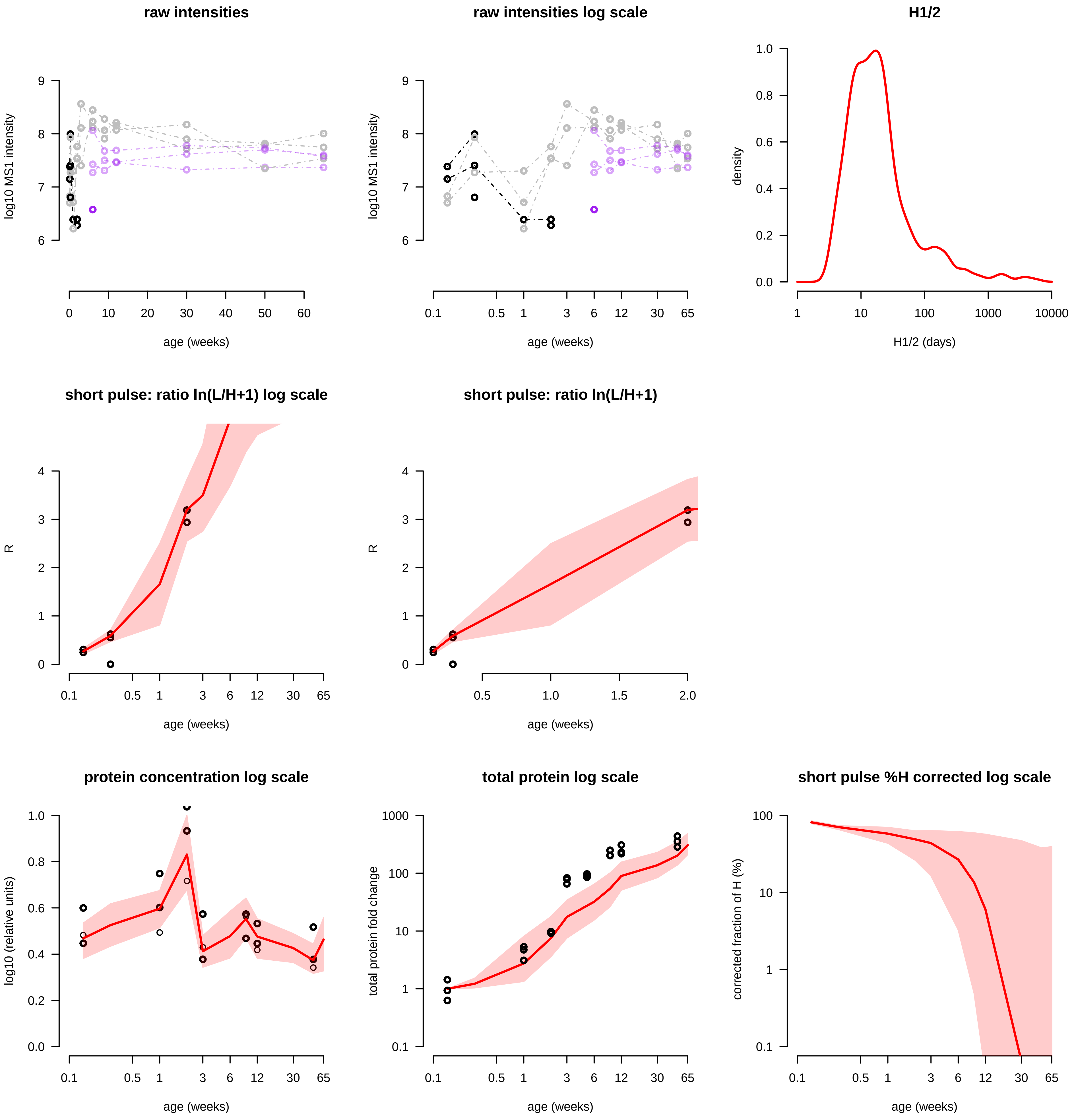

Q8BHY2

>sp|Q8BHY2|NOC4L_MOUSE

Nucleolar complex protein 4 homolog

GN=Noc4l

Med H1/2: 24.6 days

Q5 H1/2: 14.8 days

Q95 H1/2: 94.1 days

reliable model fit: no

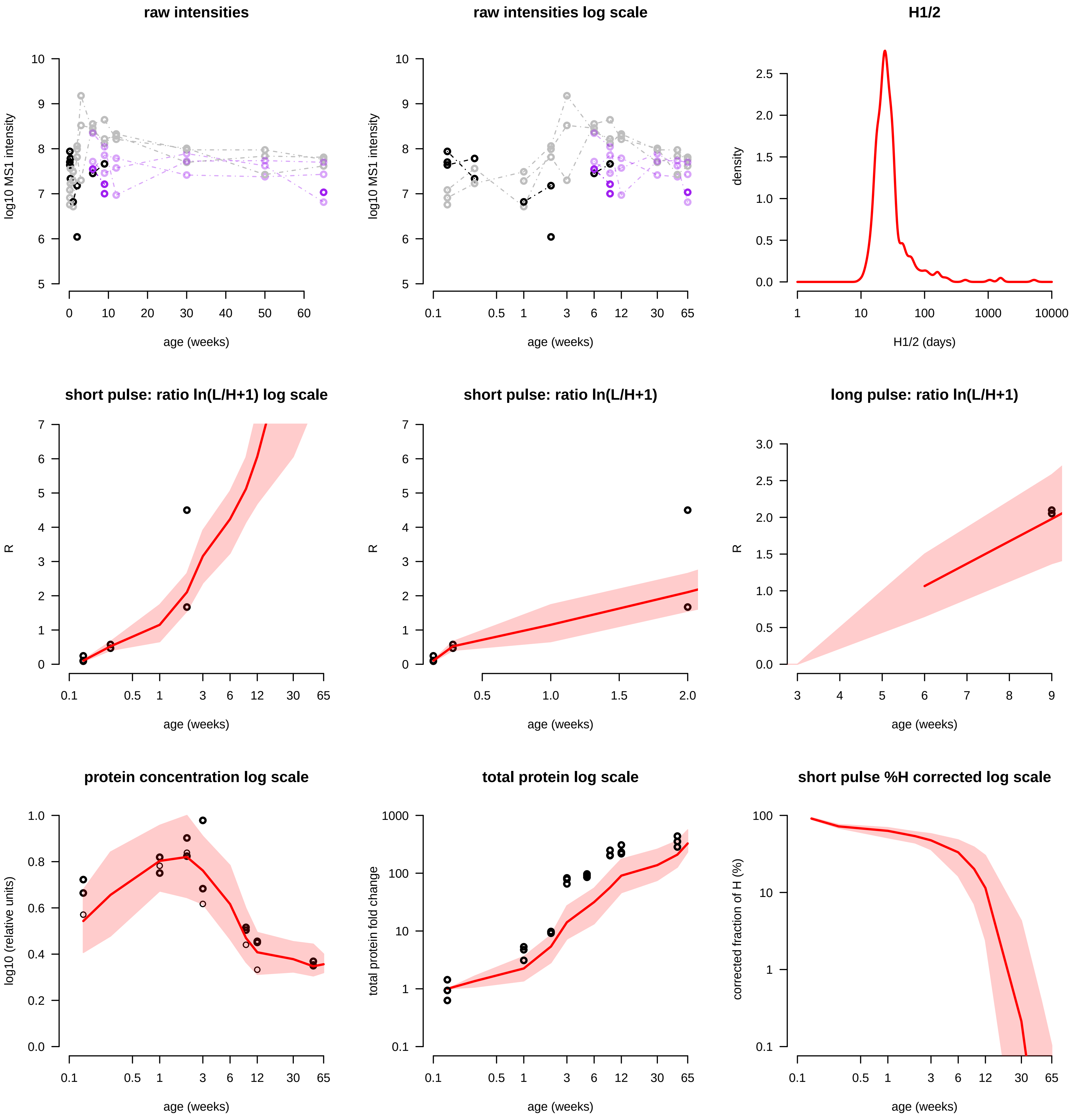

Q9Z2V5

>sp|Q9Z2V5|HDAC6_MOUSE

Histone deacetylase 6

GN=Hdac6

Med H1/2: 5.8 days

Q5 H1/2: 2.9 days

Q95 H1/2: 30.7 days

reliable model fit: yes

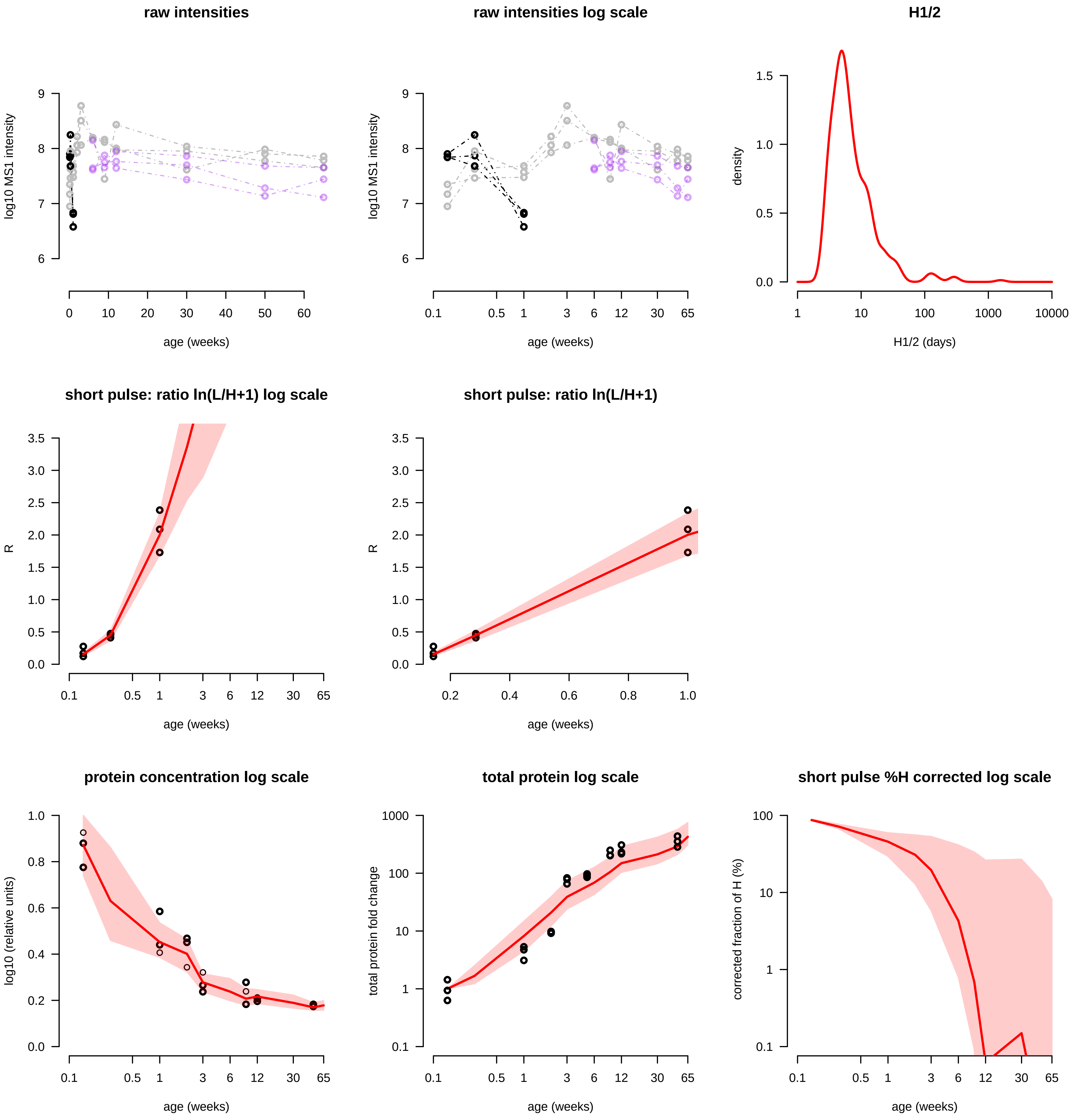

Q61789

>sp|Q61789|LAMA3_MOUSE

Laminin subunit alpha−3

GN=Lama3

Med H1/2: 3.4 days

Q5 H1/2: 2.5 days

Q95 H1/2: 8.4 days

reliable model fit: yes

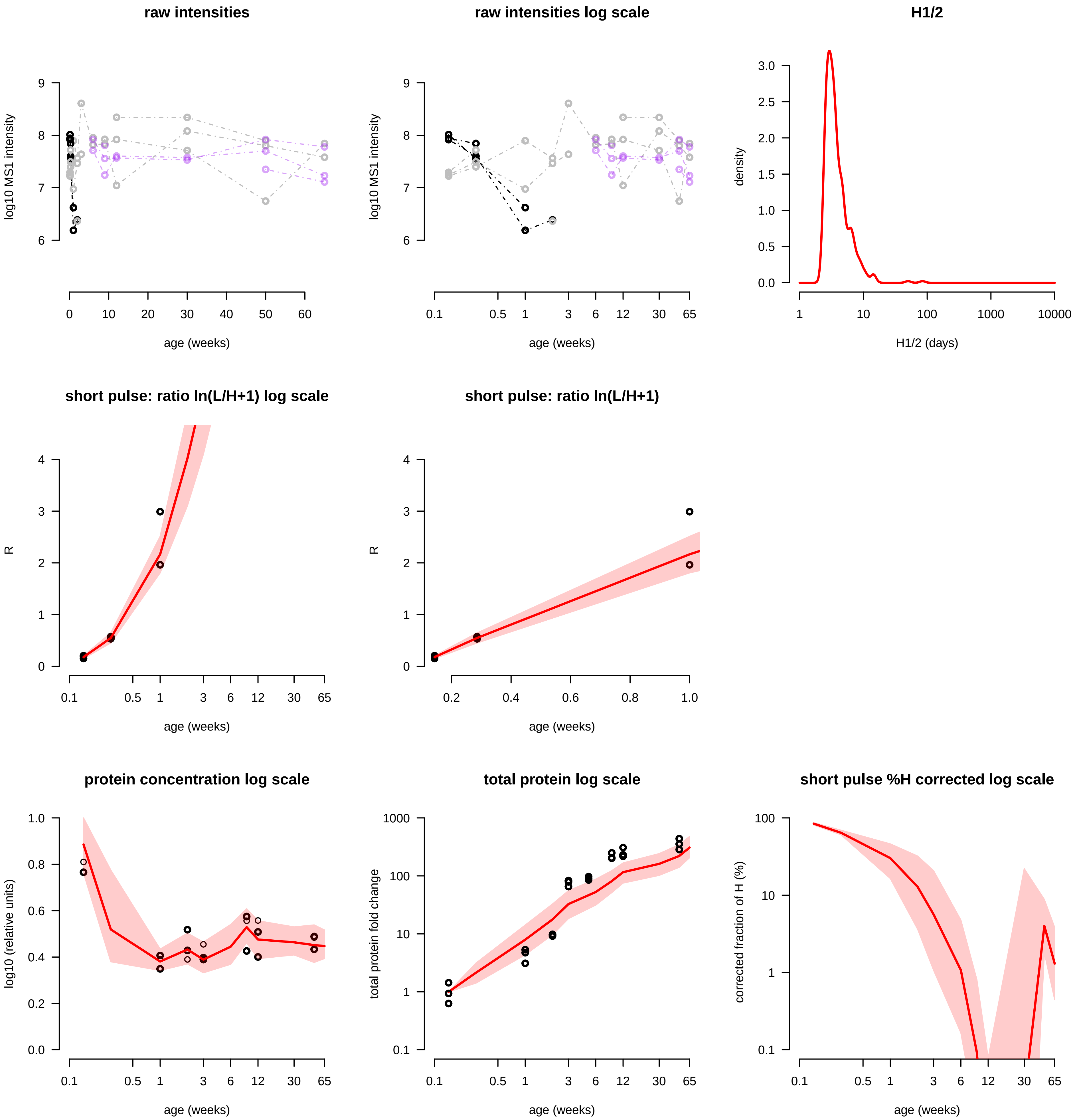

Q8BR65

>sp|Q8BR65|SDS3_MOUSE

Sin3 histone deacetylase corepressor complex component SDS3

GN=Suds3

Med H1/2: 14.3 days

Q5 H1/2: 5.3 days

Q95 H1/2: 136.5 days

reliable model fit: yes

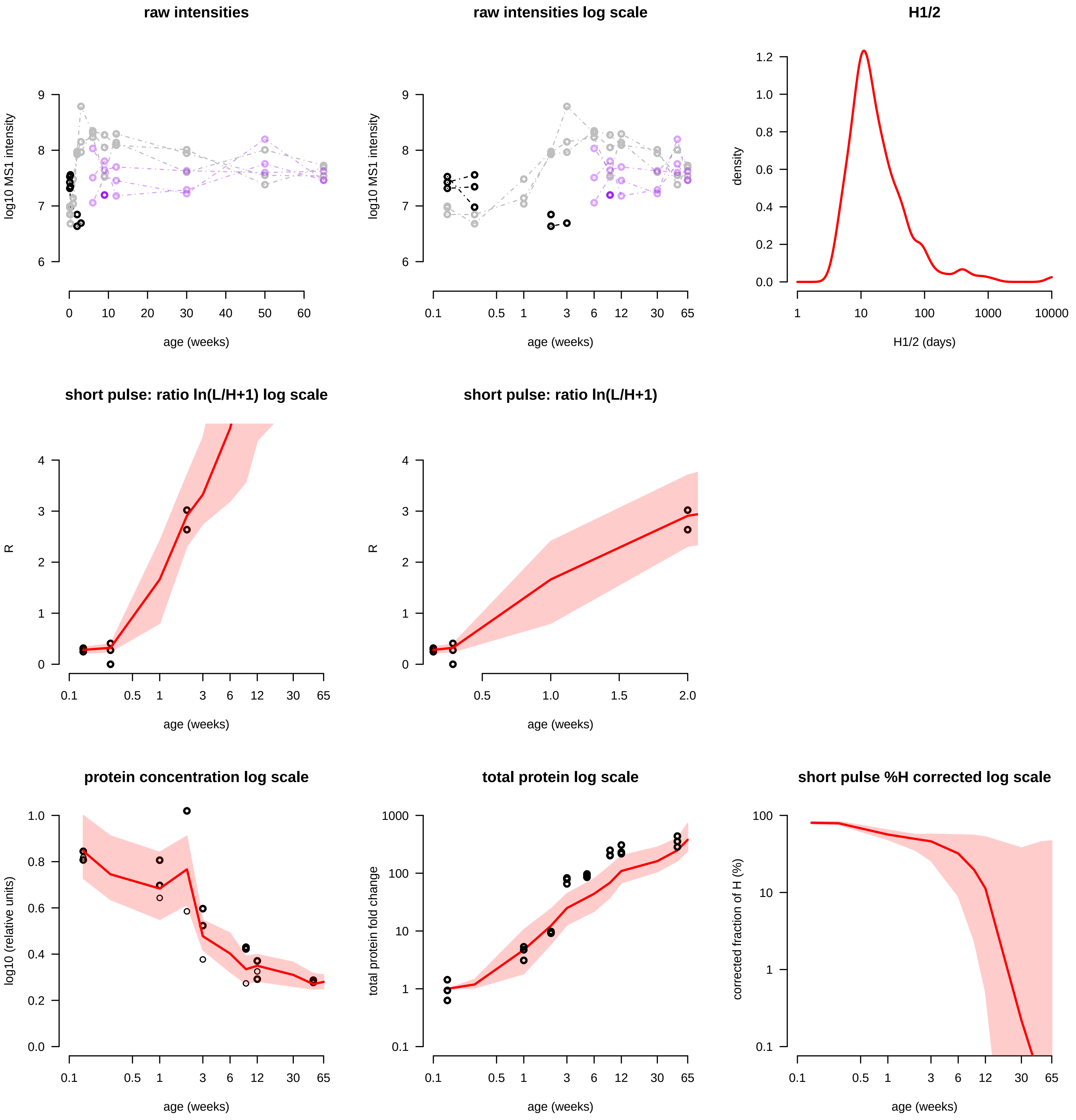

Q3TKY6

>sp|Q3TKY6|CWC27_MOUSE

Spliceosome−associated protein CWC27 homolog

GN=Cwc27

Med H1/2: 5.5 days

Q5 H1/2: 2.8 days

Q95 H1/2: 56.2 days

reliable model fit: yes

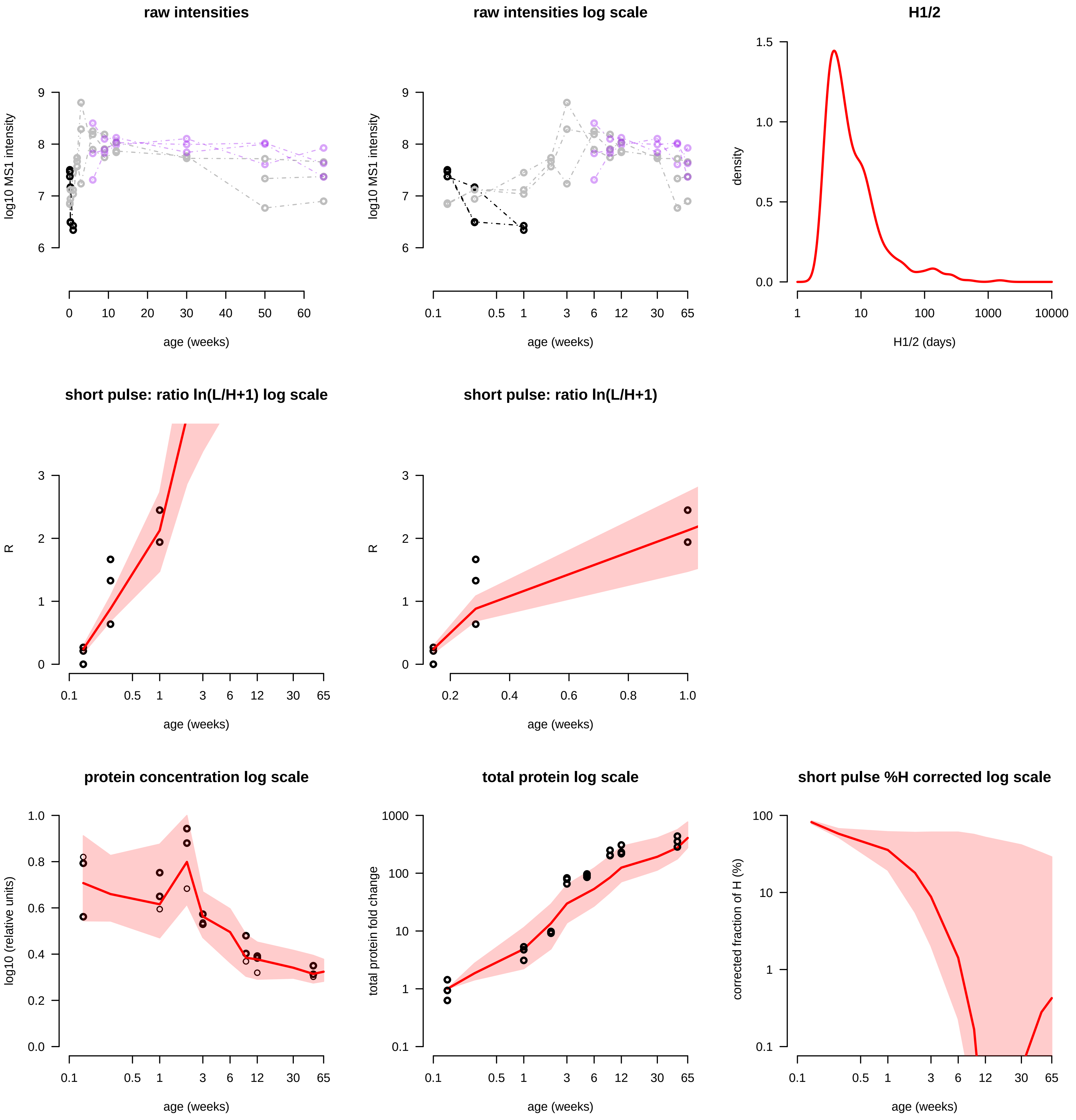

P70697

>sp|P70697|DCUP_MOUSE

Uroporphyrinogen decarboxylase

GN=Urod

Med H1/2: 48 days

Q5 H1/2: 14 days

Q95 H1/2: 497.7 days

reliable model fit: no

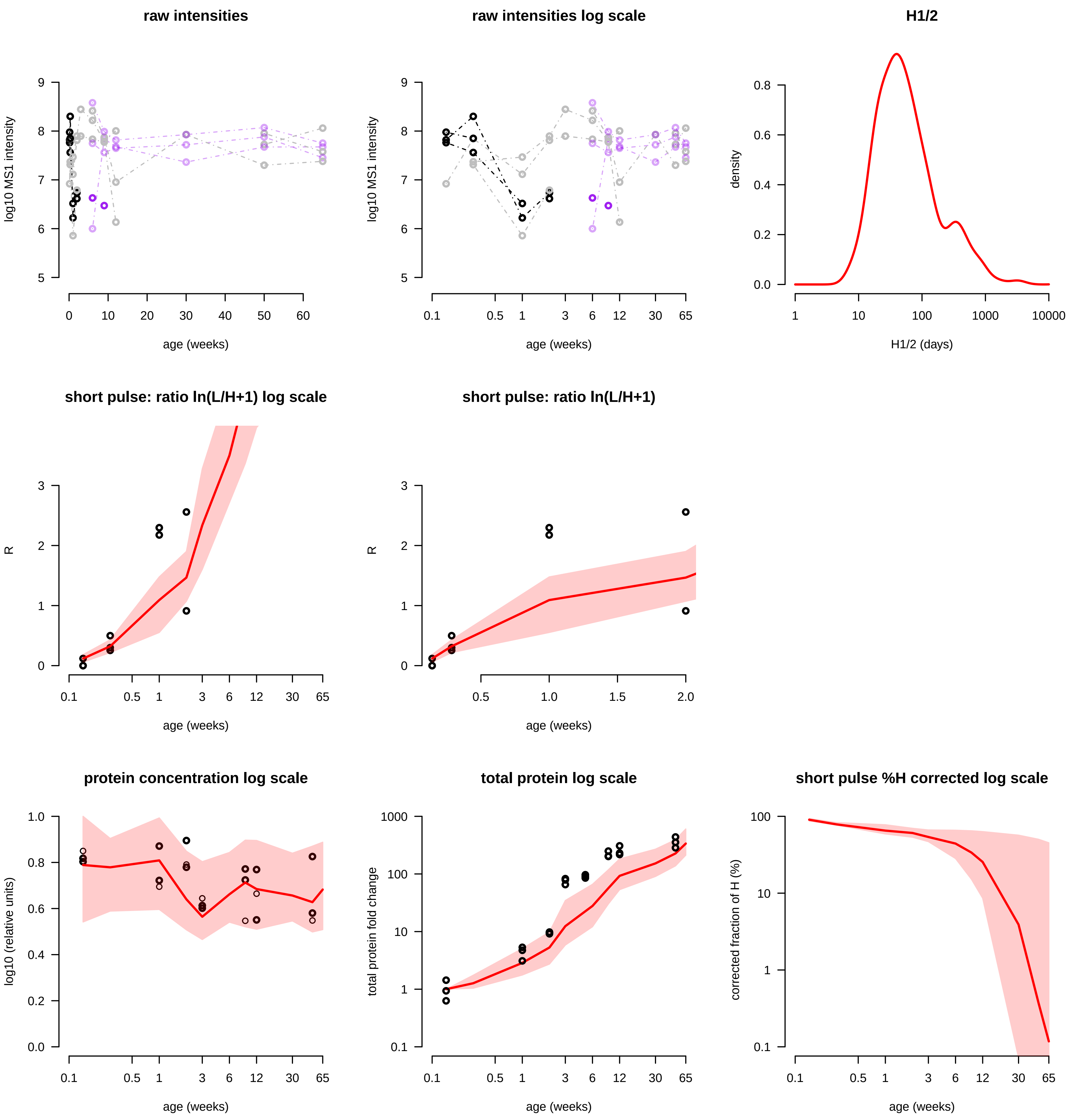

Q91ZN5−2;Q91ZN5

>sp|Q91ZN5−2|S35B2_MOUSE

Isoform 2 of Adenosine 3'−phospho 5'−phosphosulfate transporter 1

GN=Slc35b2

Med H1/2: 9.6 days

Q5 H1/2: 4.3 days

Q95 H1/2: 67.6 days

reliable model fit: yes

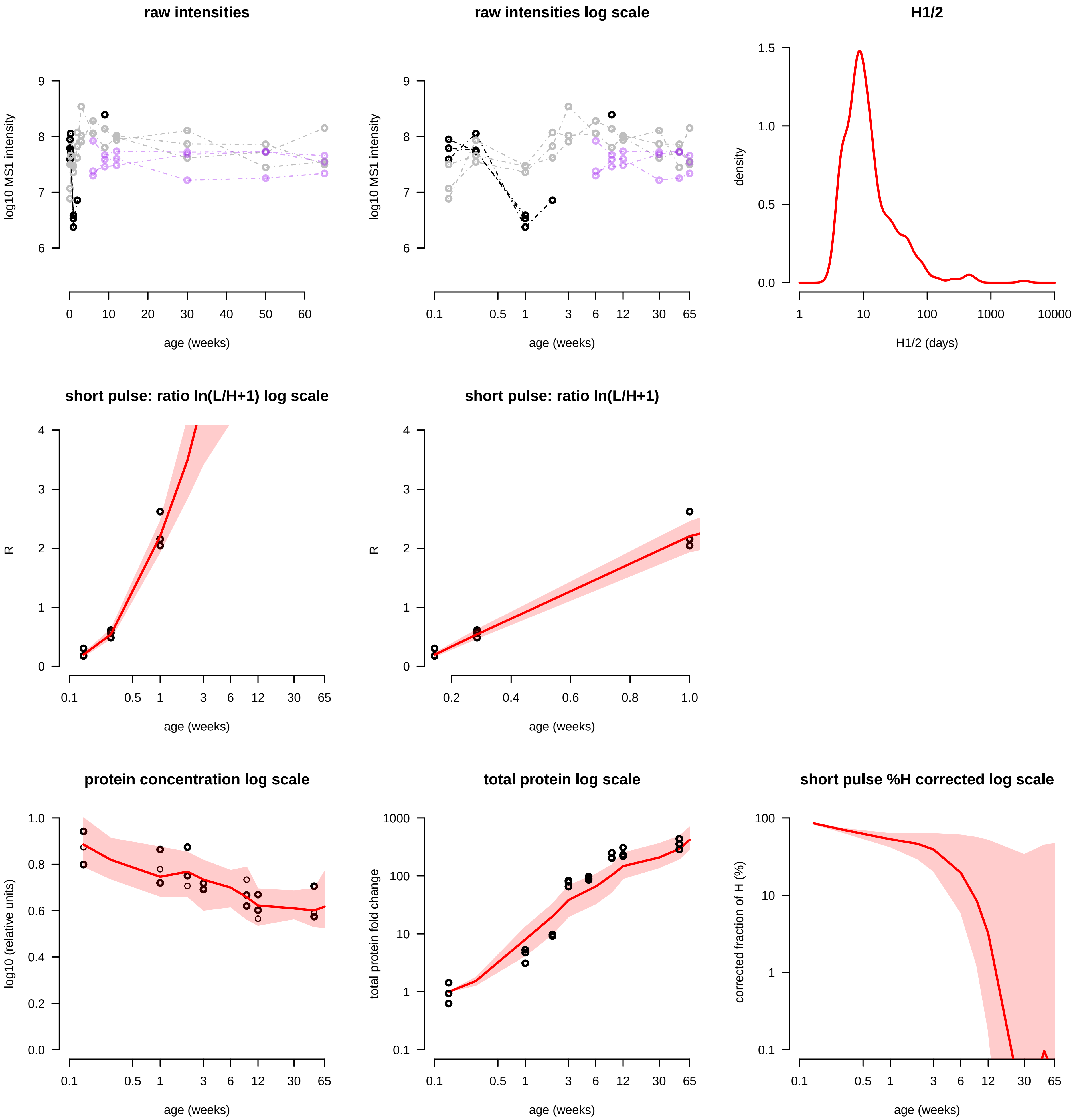

Q3TZX8−2;Q3TZX8

>sp|Q3TZX8−2|NOL9_MOUSE

Isoform 2 of Polynucleotide 5'−hydroxyl−kinase NOL9

GN=Nol9

Med H1/2: 8.6 days

Q5 H1/2: 2.7 days

Q95 H1/2: 97.5 days

reliable model fit: yes

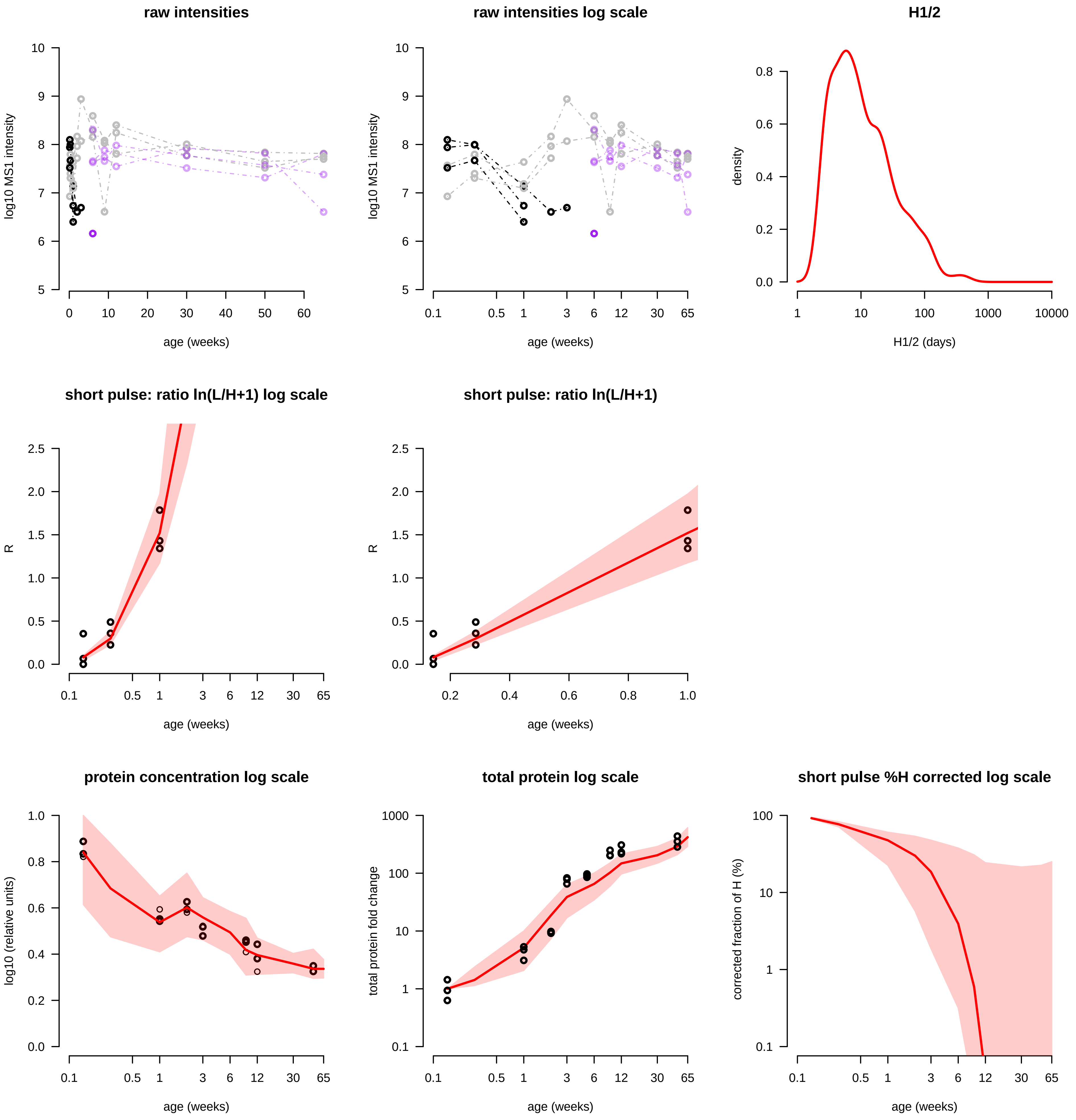

P70353

>sp|P70353|NFYC_MOUSE

Nuclear transcription factor Y subunit gamma

GN=Nfyc

Med H1/2: 165.8 days

Q5 H1/2: 56.7 days

Q95 H1/2: 2065.8 days

reliable model fit: no

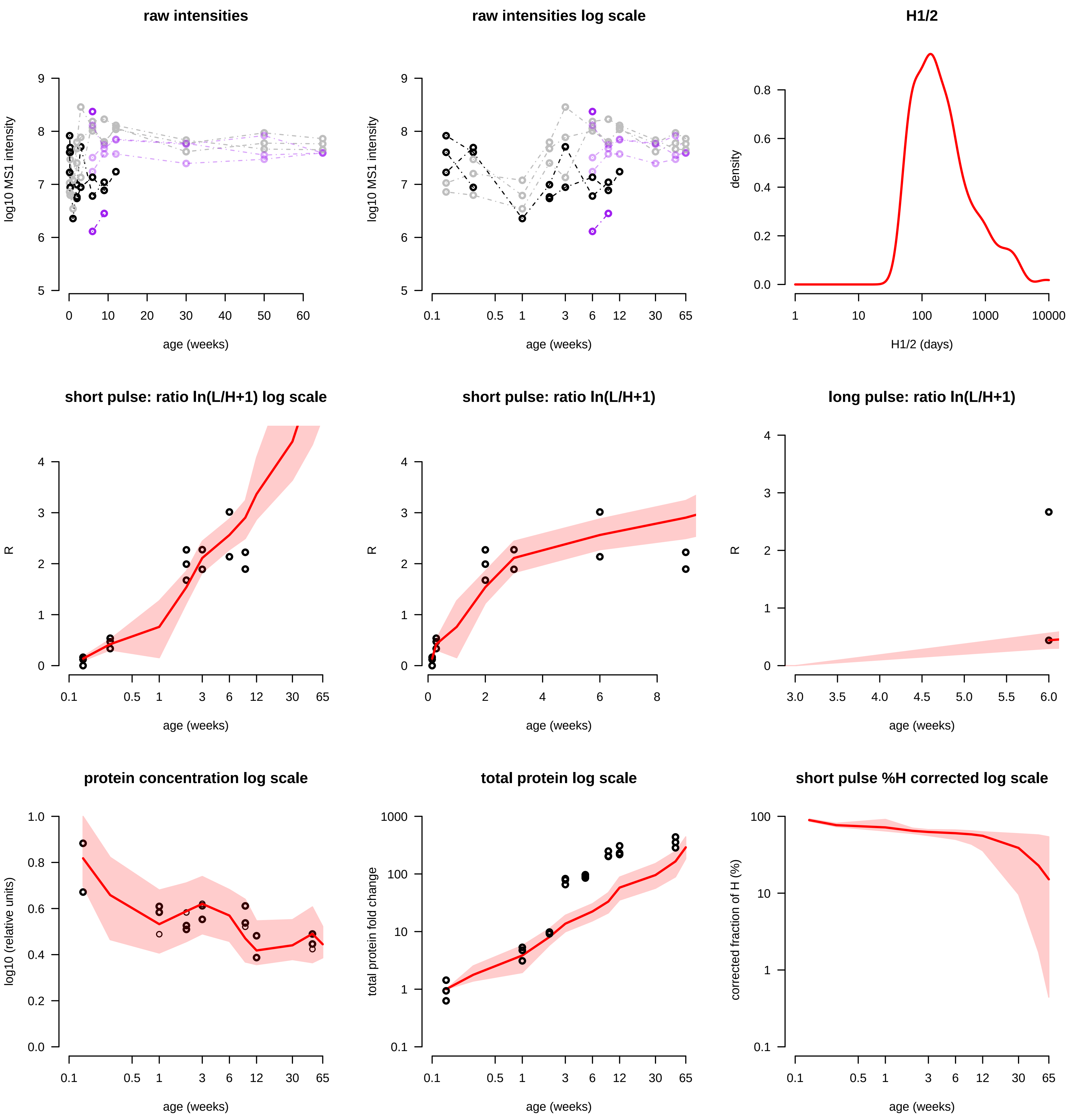

P21107

>sp|P21107|TPM3_MOUSE

Tropomyosin alpha−3 chain

GN=Tpm3

Med H1/2: 1587.9 days

Q5 H1/2: 379.2 days

Q95 H1/2: 37893.4 days

reliable model fit: no

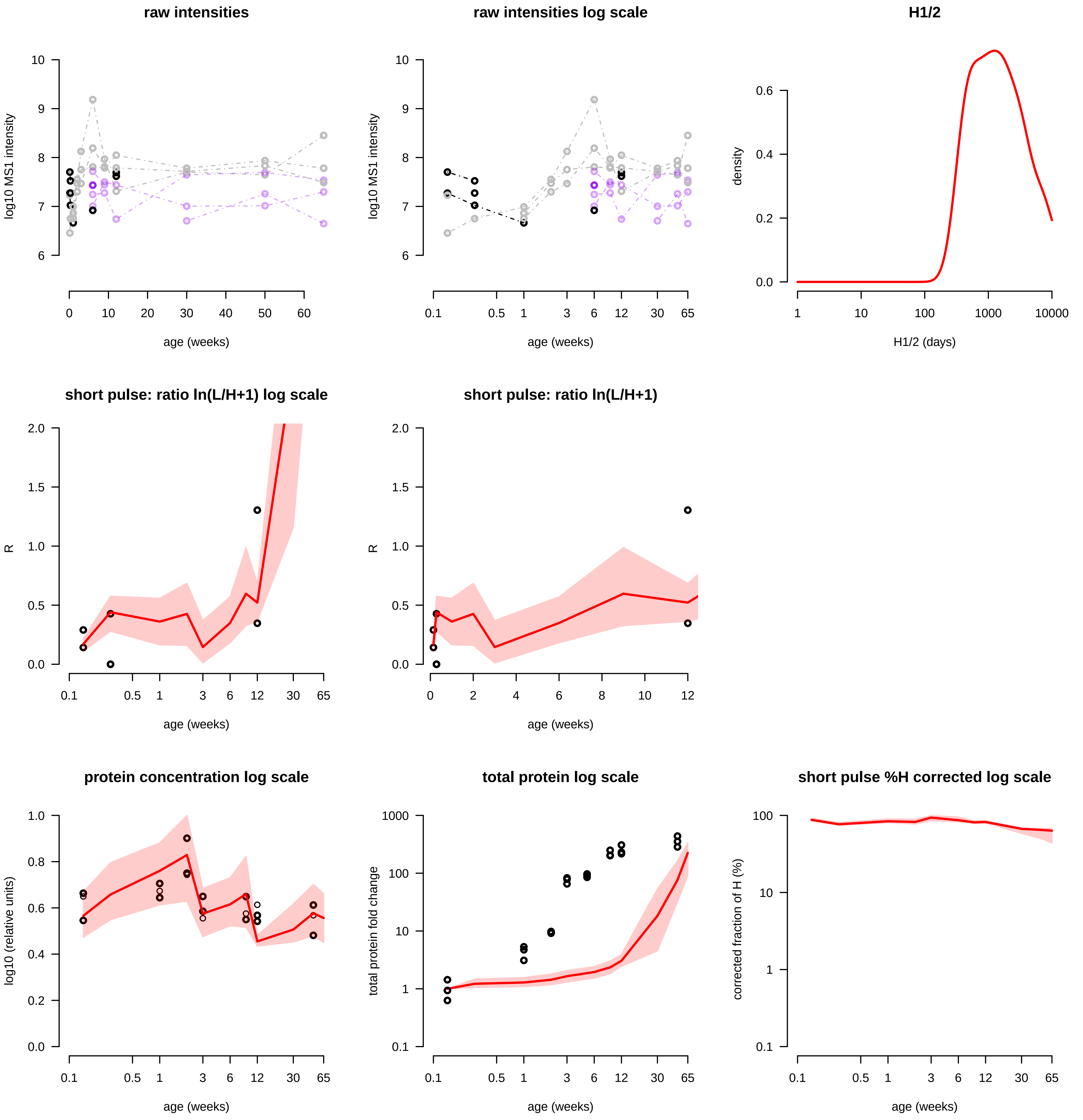

P35802

>sp|P35802|GPM6A_MOUSE

Neuronal membrane glycoprotein M6−a

GN=Gpm6a

Med H1/2: 2367.3 days

Q5 H1/2: 714.5 days

Q95 H1/2: 33461.5 days

reliable model fit: no

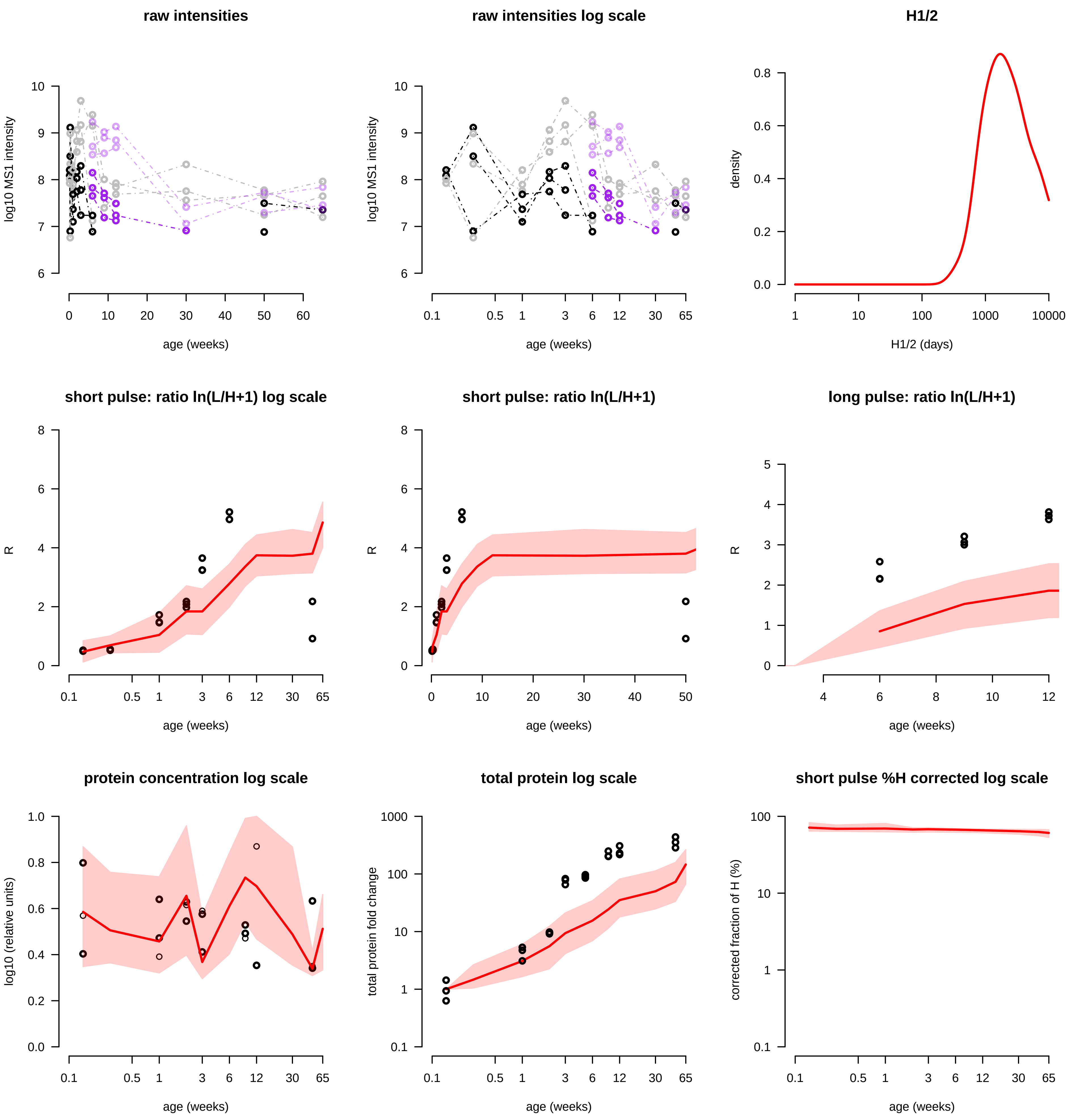

Q60695

>sp|Q60695|RGL1_MOUSE

Ral guanine nucleotide dissociation stimulator−like 1

GN=Rgl1

Med H1/2: 10.2 days

Q5 H1/2: 6.9 days

Q95 H1/2: 25.1 days

reliable model fit: yes

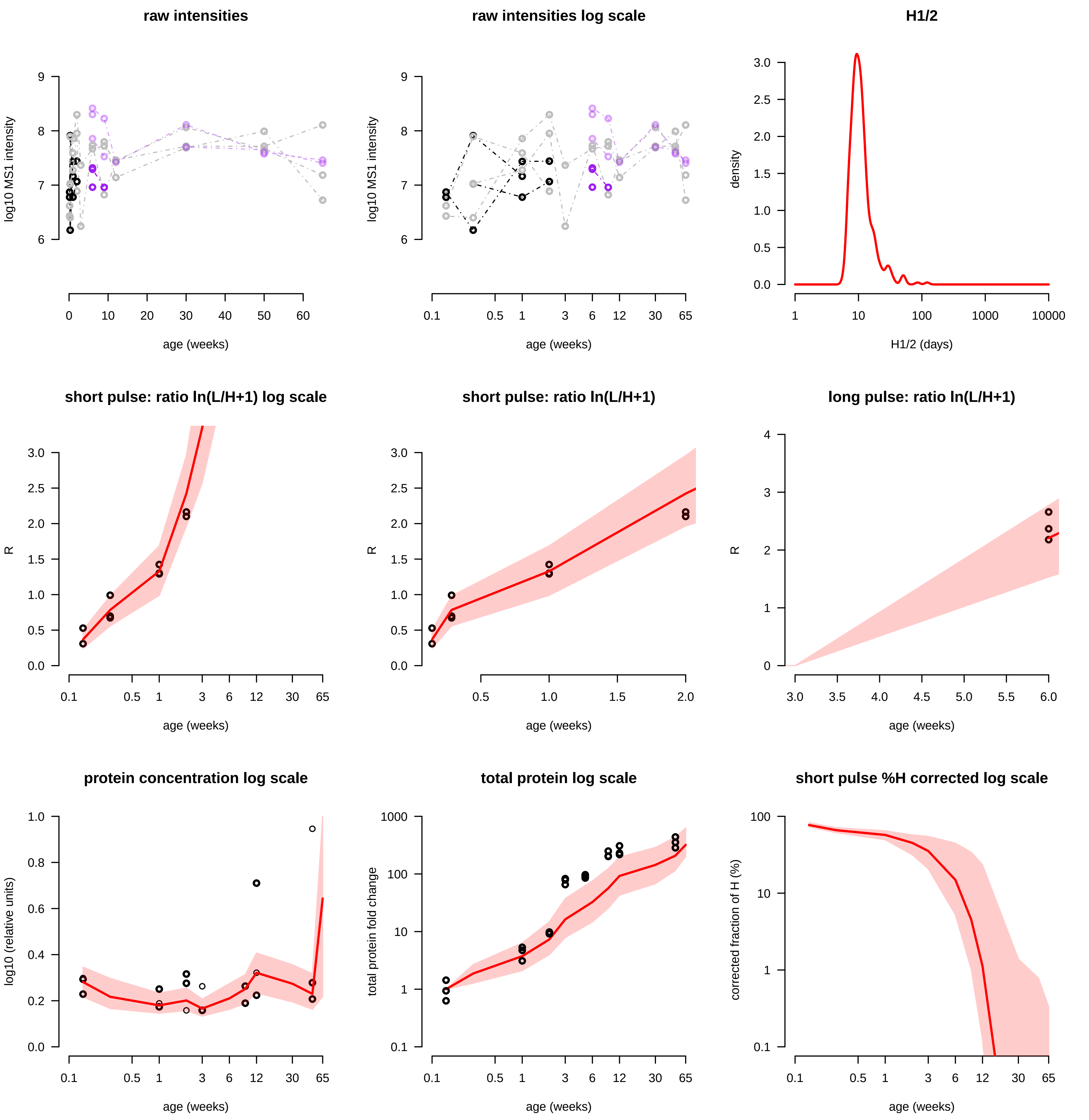

P59235

>sp|P59235|NUP43_MOUSE

Nucleoporin Nup43

GN=Nup43

Med H1/2: 17.6 days

Q5 H1/2: 6.3 days

Q95 H1/2: 127 days

reliable model fit: yes

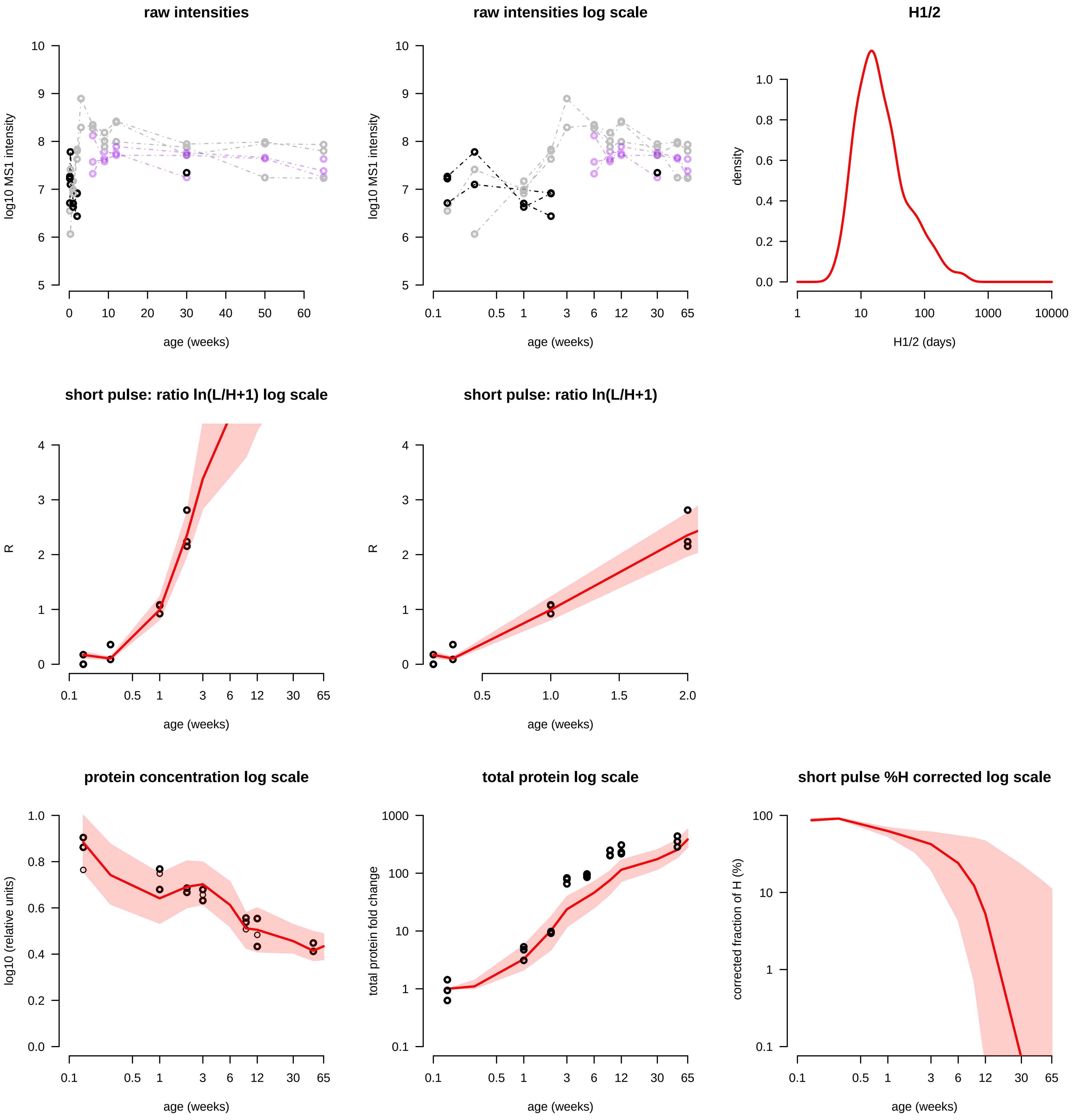

Q80XJ3

>sp|Q80XJ3|TTC28_MOUSE

Tetratricopeptide repeat protein 28

GN=Ttc28

Med H1/2: 2819.8 days

Q5 H1/2: 794.1 days

Q95 H1/2: 23168.5 days

reliable model fit: no

raw intensities

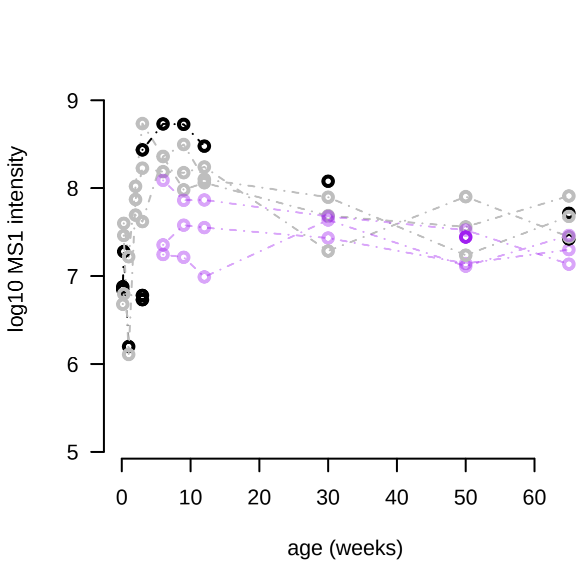

raw intensities log scale

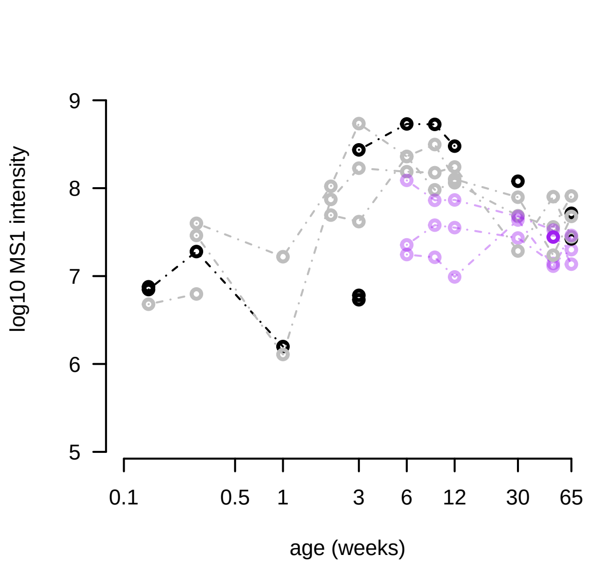

H1/2

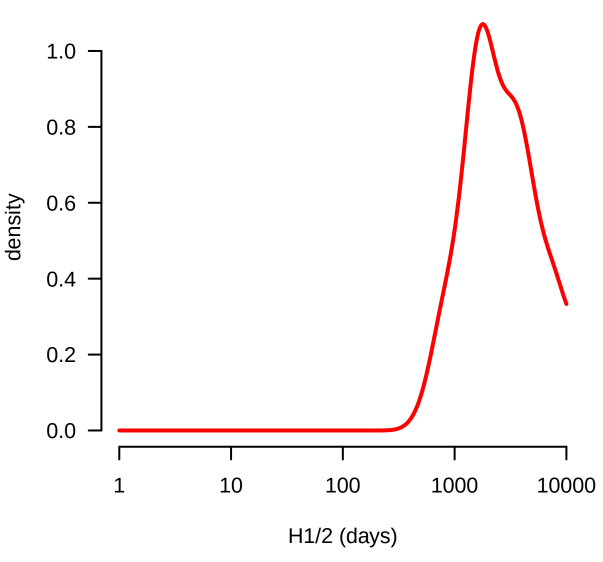

short pulse: ratio ln(L/H+1) log scale

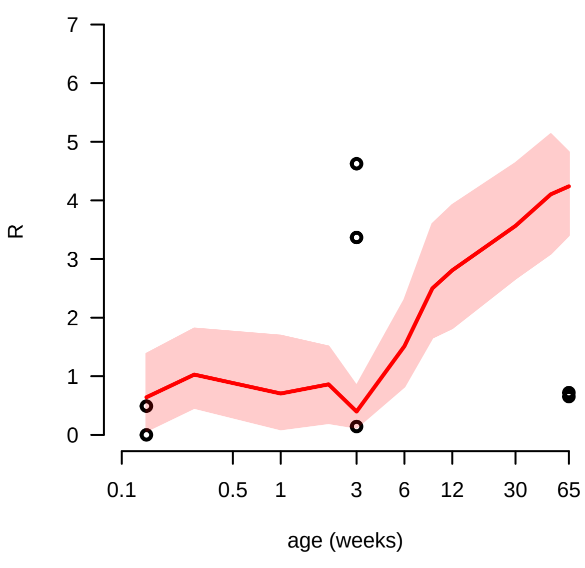

short pulse: ratio ln(L/H+1)

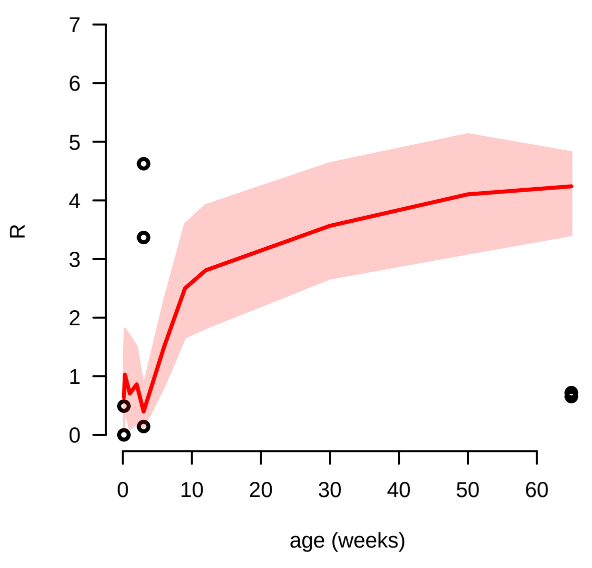

protein concentration log scale

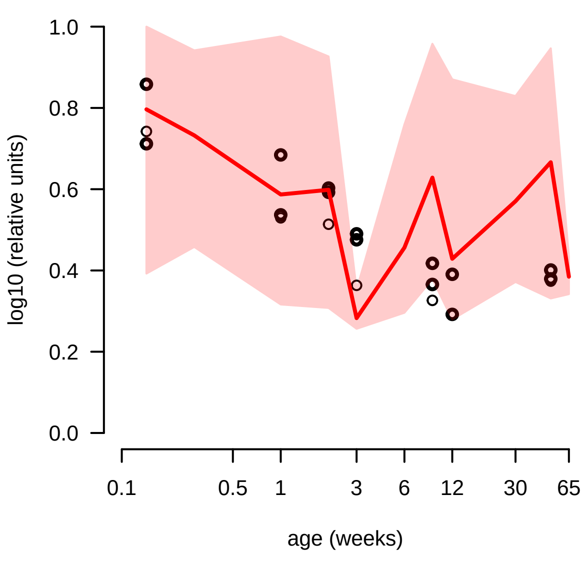

total protein log scale

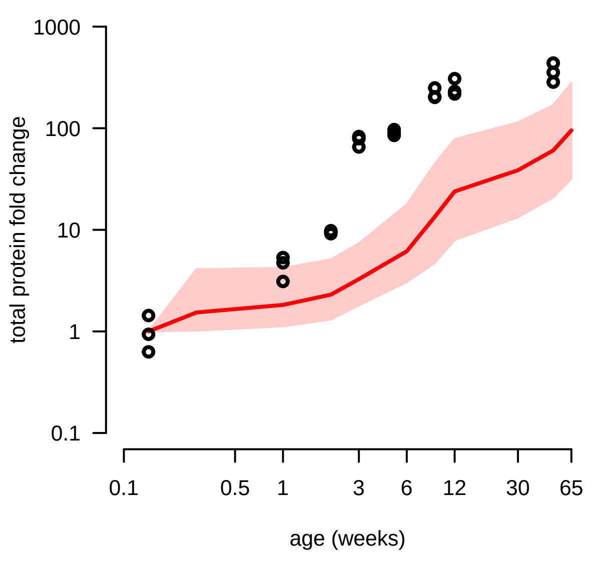

short pulse %H corrected log scale

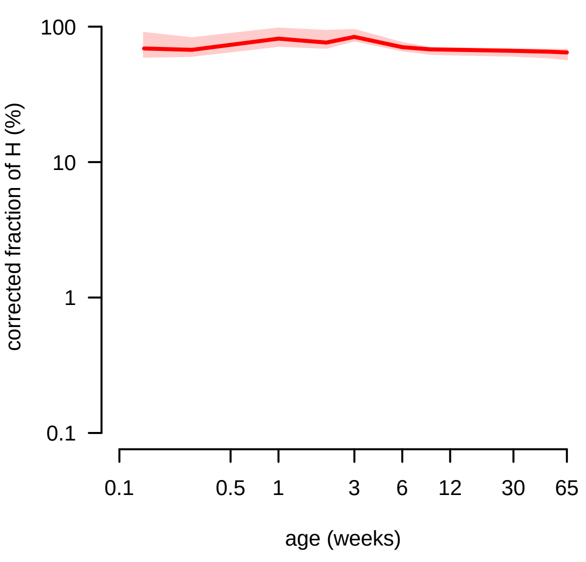

Q8BSL7

>sp|Q8BSL7|ARF2_MOUSE

ADP−ribosylation factor 2

GN=Arf2

Med H1/2: 13.5 days

Q5 H1/2: 5.4 days

Q95 H1/2: 201.6 days

reliable model fit: yes

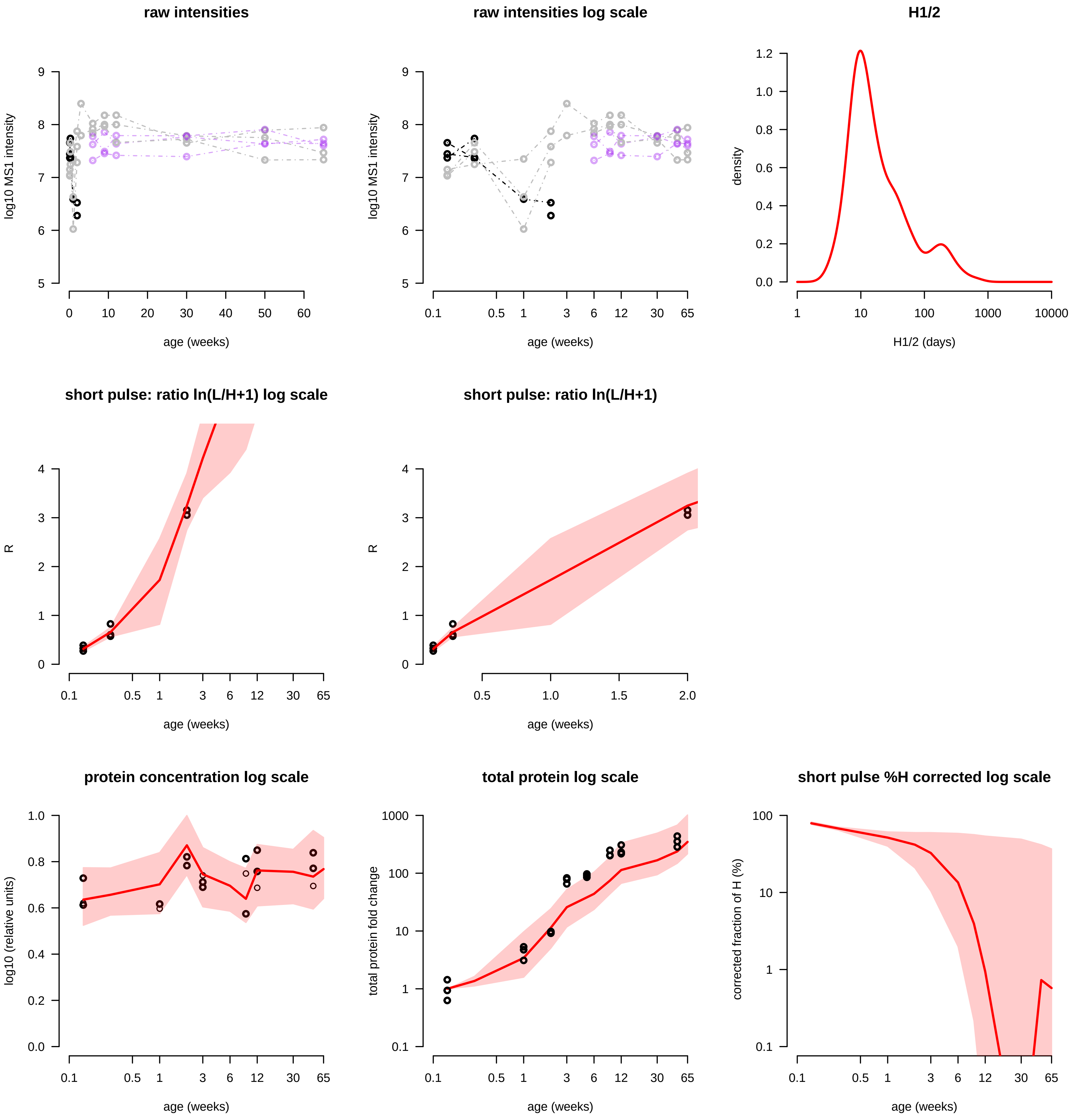

P08121

>sp|P08121|CO3A1_MOUSE

Collagen alpha−1(III) chain

GN=Col3a1

Med H1/2: 5.7 days

Q5 H1/2: 2.8 days

Q95 H1/2: 51.8 days

reliable model fit: yes

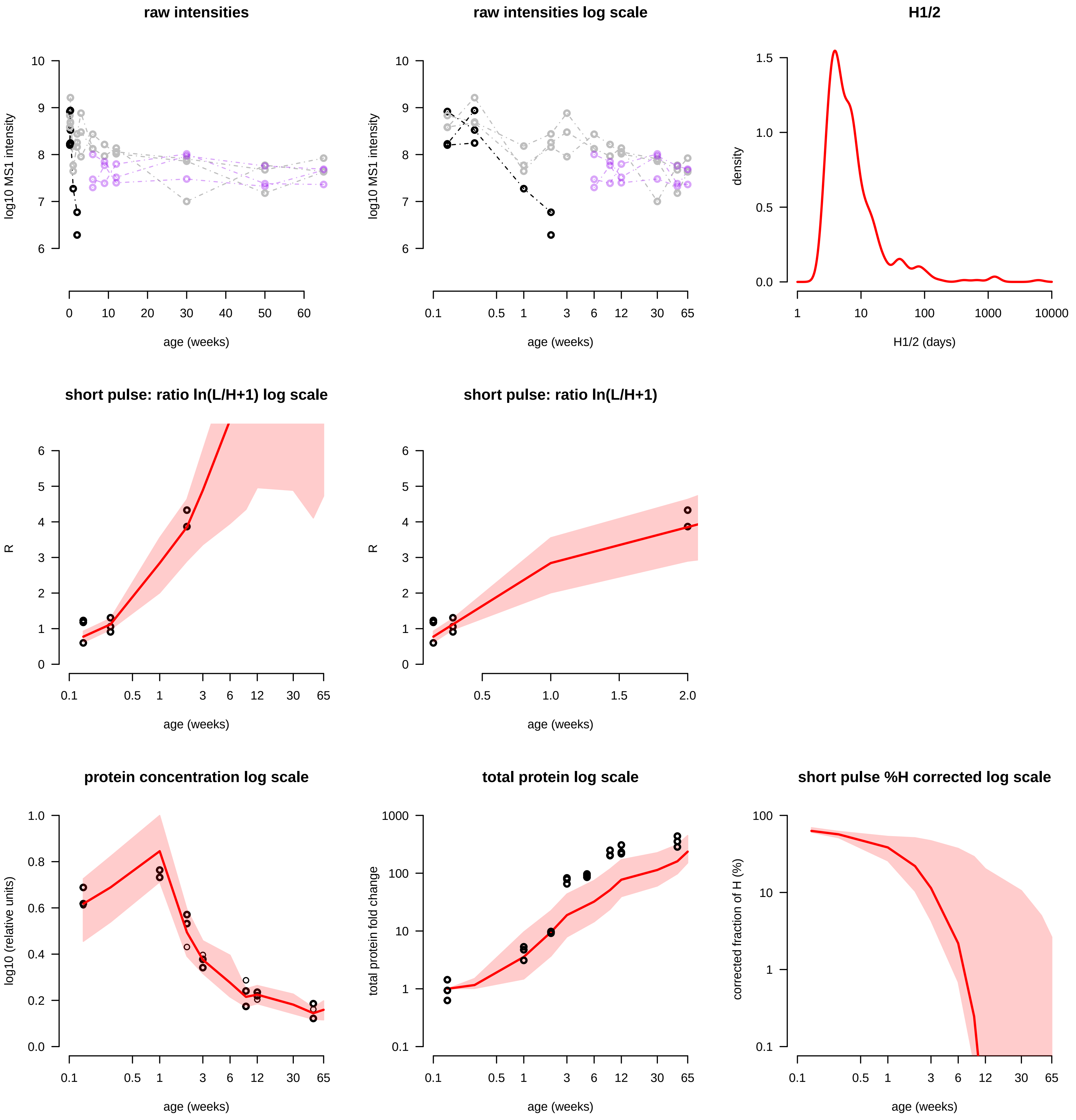

P30051

>sp|P30051|TEAD1_MOUSE

Transcriptional enhancer factor TEF−1

GN=Tead1

Med H1/2: 2260.7 days

Q5 H1/2: 550.1 days

Q95 H1/2: 21592.7 days

reliable model fit: no

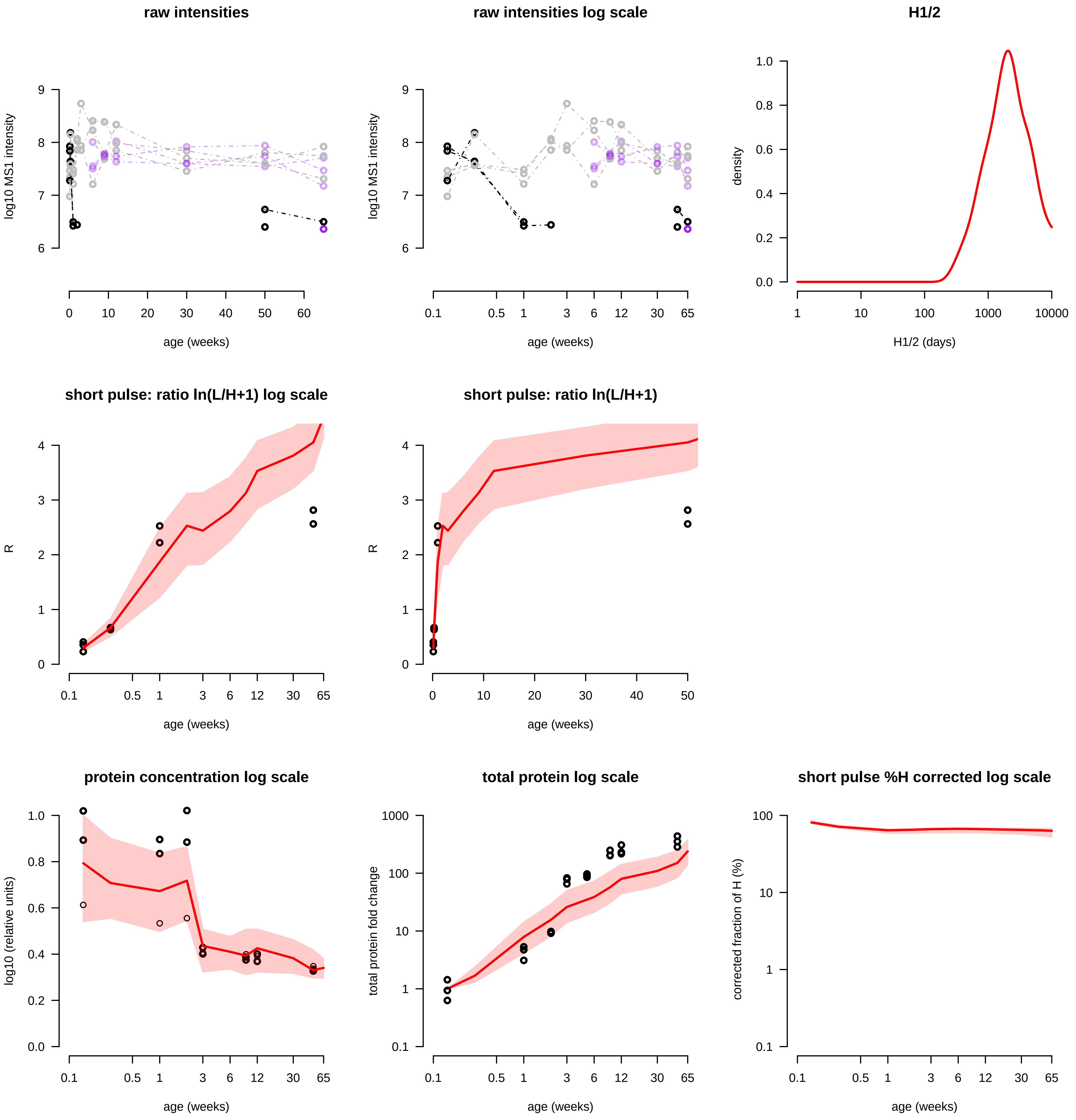

Q00262

>sp|Q00262|STX2_MOUSE

Syntaxin−2

GN=Stx2

Med H1/2: 29.5 days

Q5 H1/2: 8.3 days

Q95 H1/2: 340.5 days

reliable model fit: yes

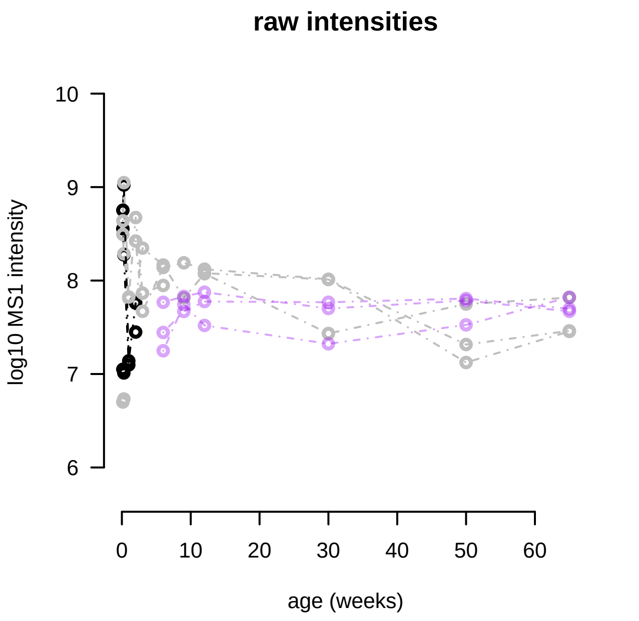

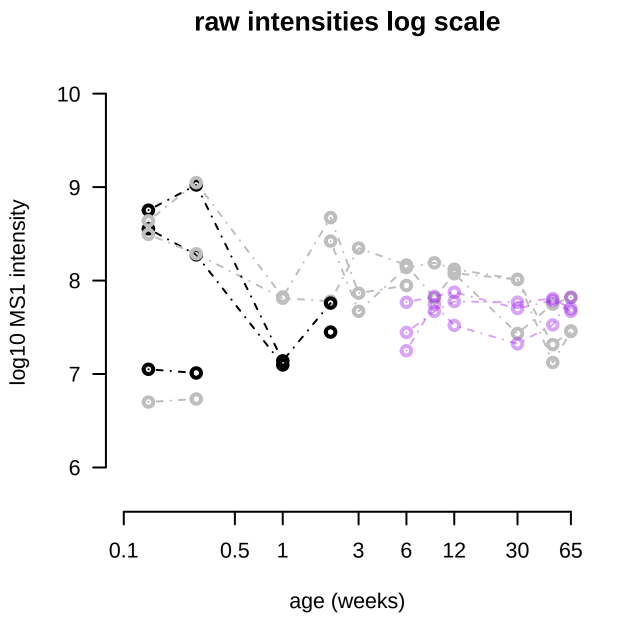

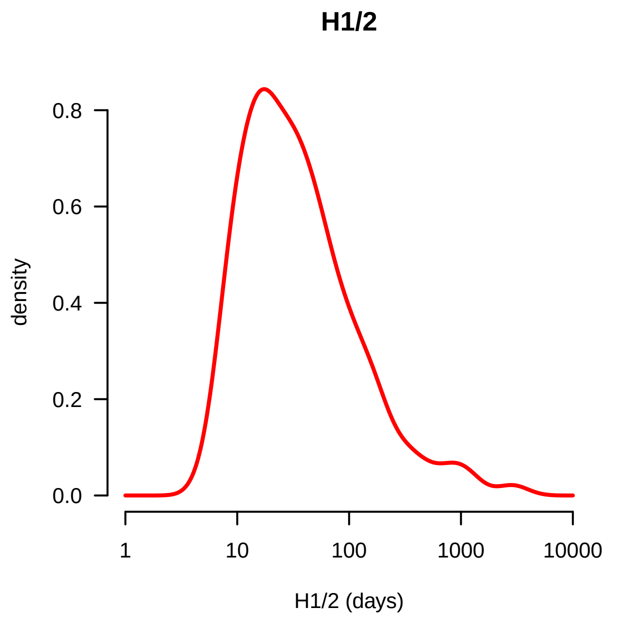

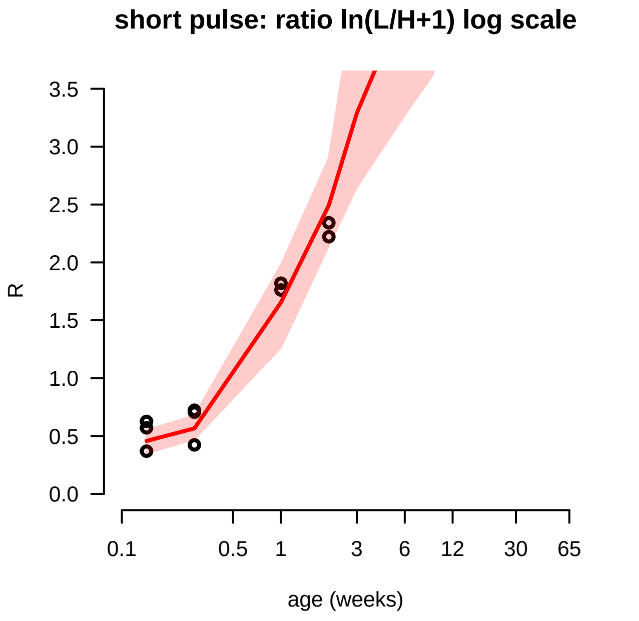

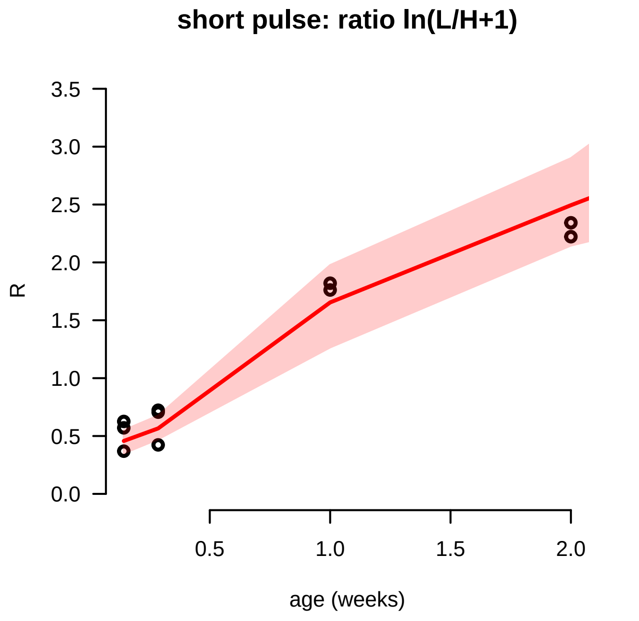

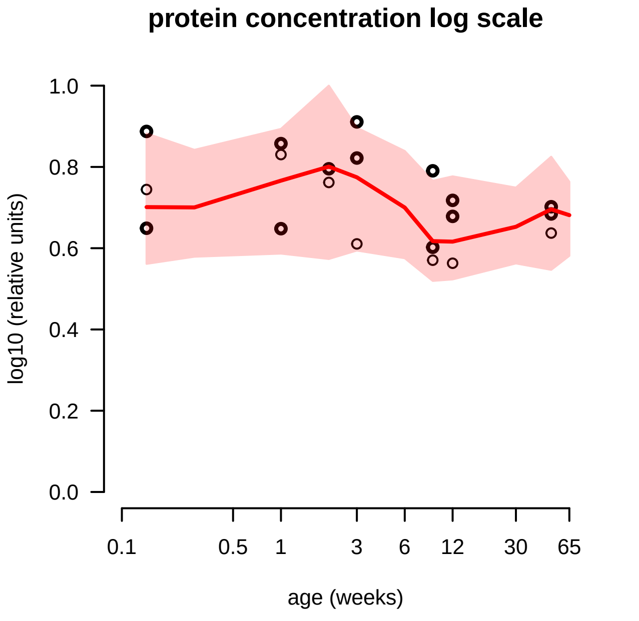

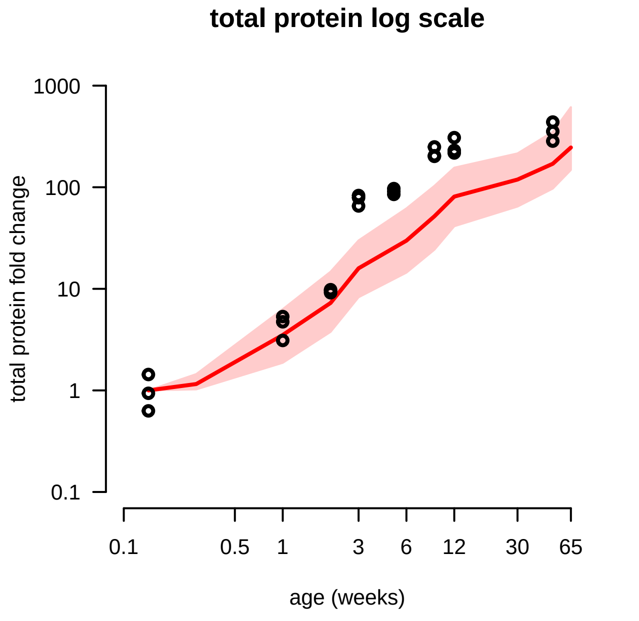

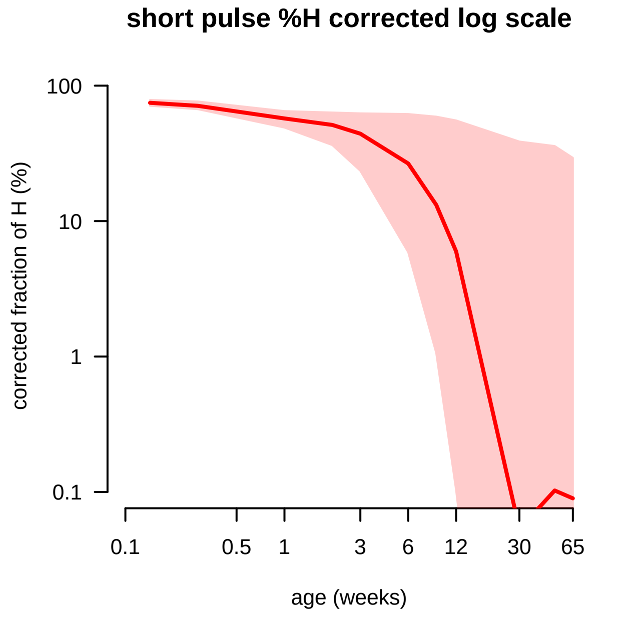

Q99LC2

>sp|Q99LC2|CSTF1_MOUSE

Cleavage stimulation factor subunit 1

GN=Cstf1

Med H1/2: 123.9 days

Q5 H1/2: 37.7 days

Q95 H1/2: 1227.8 days

reliable model fit: no

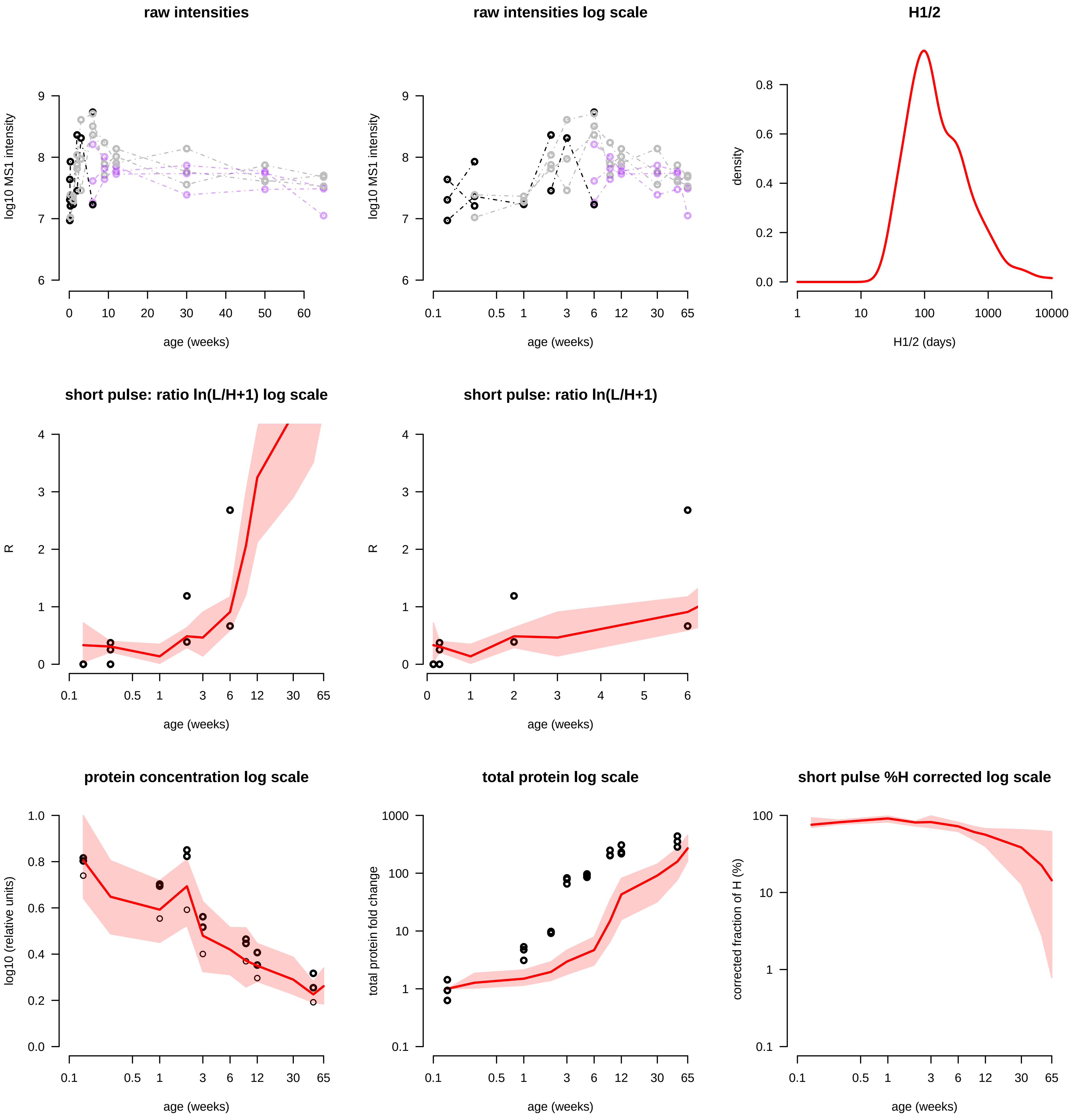

Q2VPQ9−2;Q2VPQ9

>sp|Q2VPQ9−2|EAF6_MOUSE

Isoform 2 of Chromatin modification−related protein MEAF6

GN=Meaf6

Med H1/2: 5.6 days

Q5 H1/2: 2.7 days

Q95 H1/2: 48.7 days

reliable model fit: yes

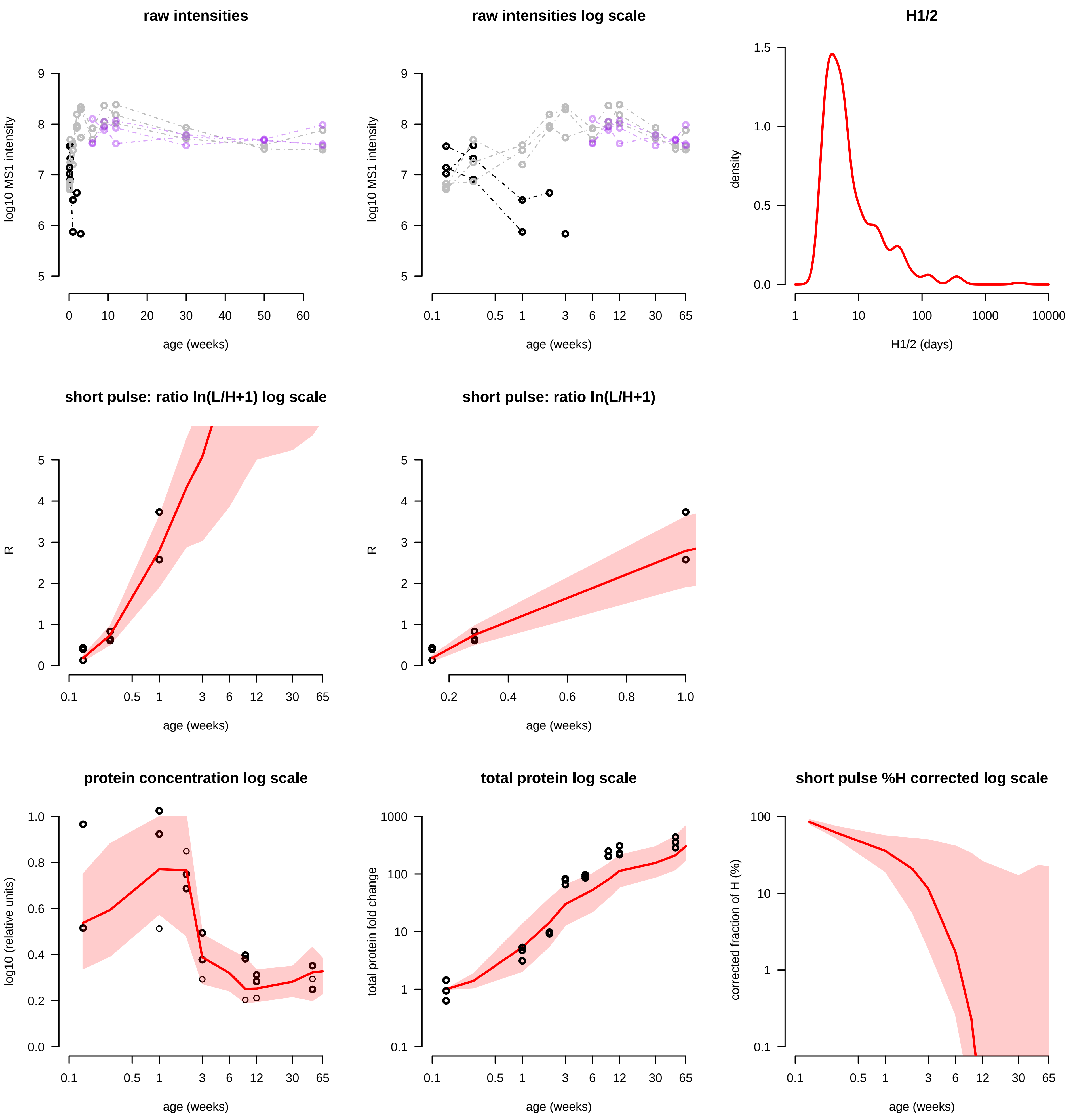

O70472

>sp|O70472|TM131_MOUSE

Transmembrane protein 131

GN=Tmem131

Med H1/2: 21.7 days

Q5 H1/2: 6.3 days

Q95 H1/2: 171.4 days

reliable model fit: yes

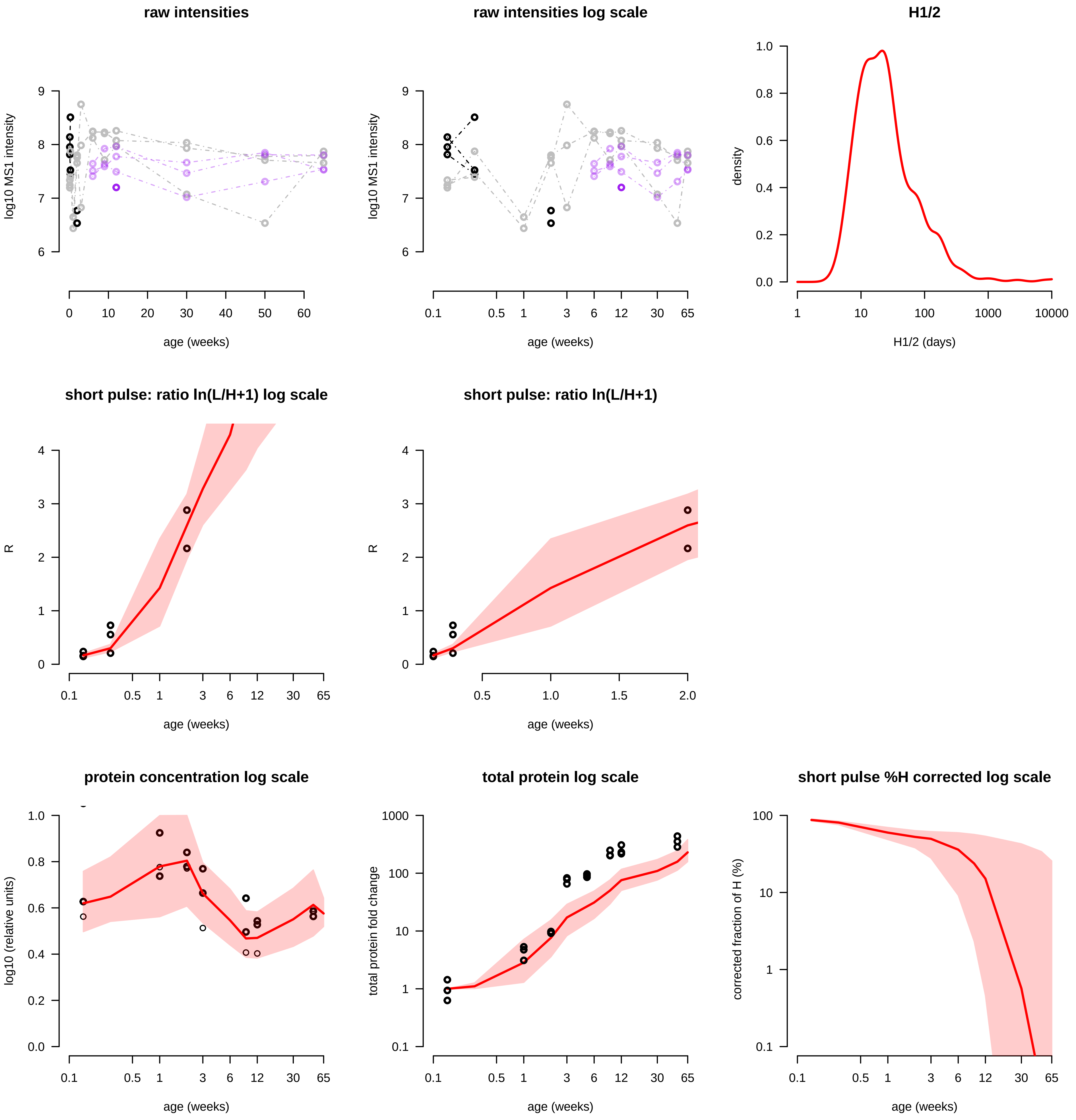

Q9DB85

>sp|Q9DB85|RRP8_MOUSE

Ribosomal RNA−processing protein 8

GN=Rrp8

Med H1/2: 3.6 days

Q5 H1/2: 3.5 days

Q95 H1/2: 3.6 days

reliable model fit: no

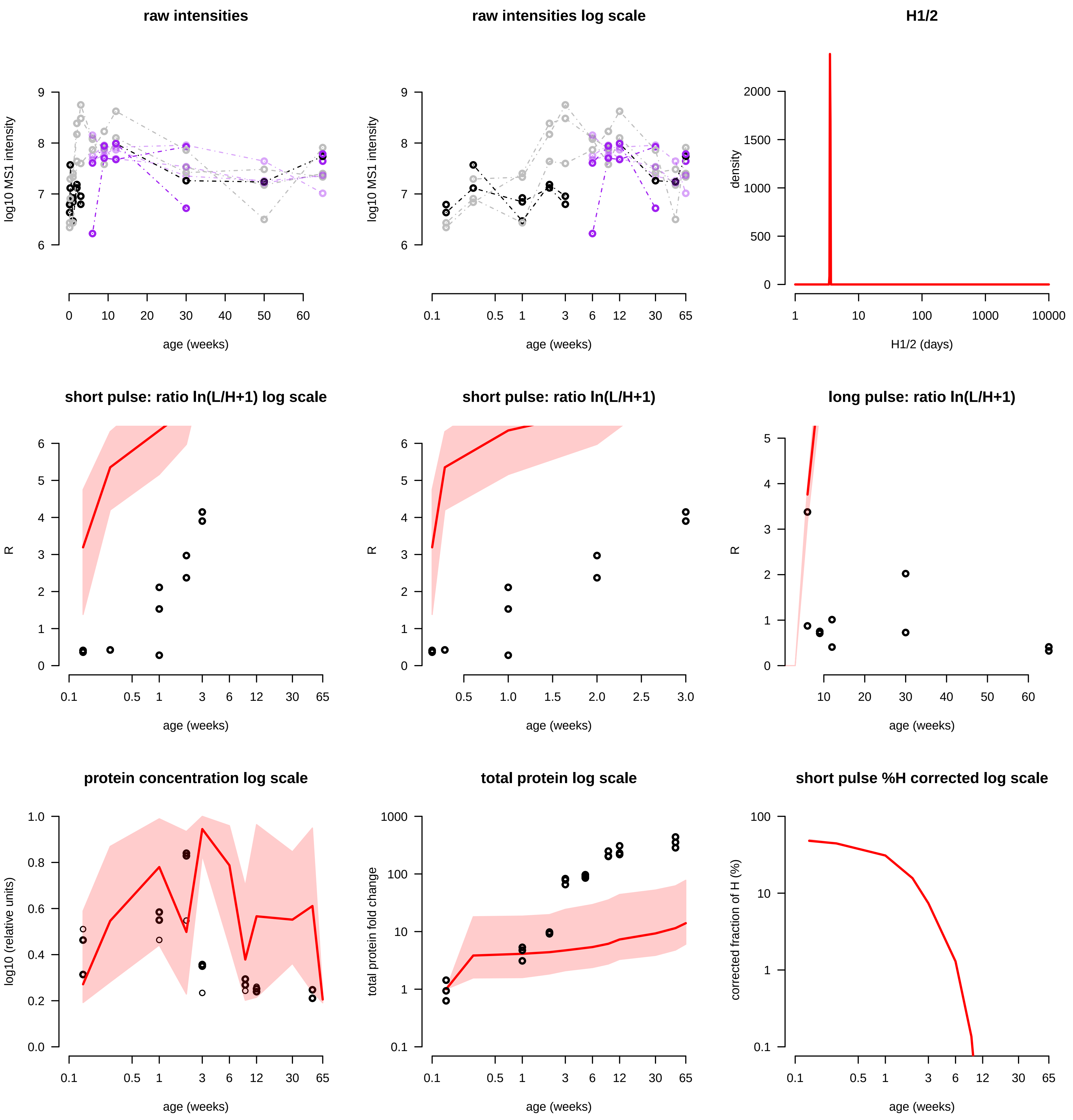

Q8BV13

>sp|Q8BV13|CSN7B_MOUSE

COP9 signalosome complex subunit 7b

GN=Cops7b

Med H1/2: 4.8 days

Q5 H1/2: 3.7 days

Q95 H1/2: 6.5 days

reliable model fit: yes

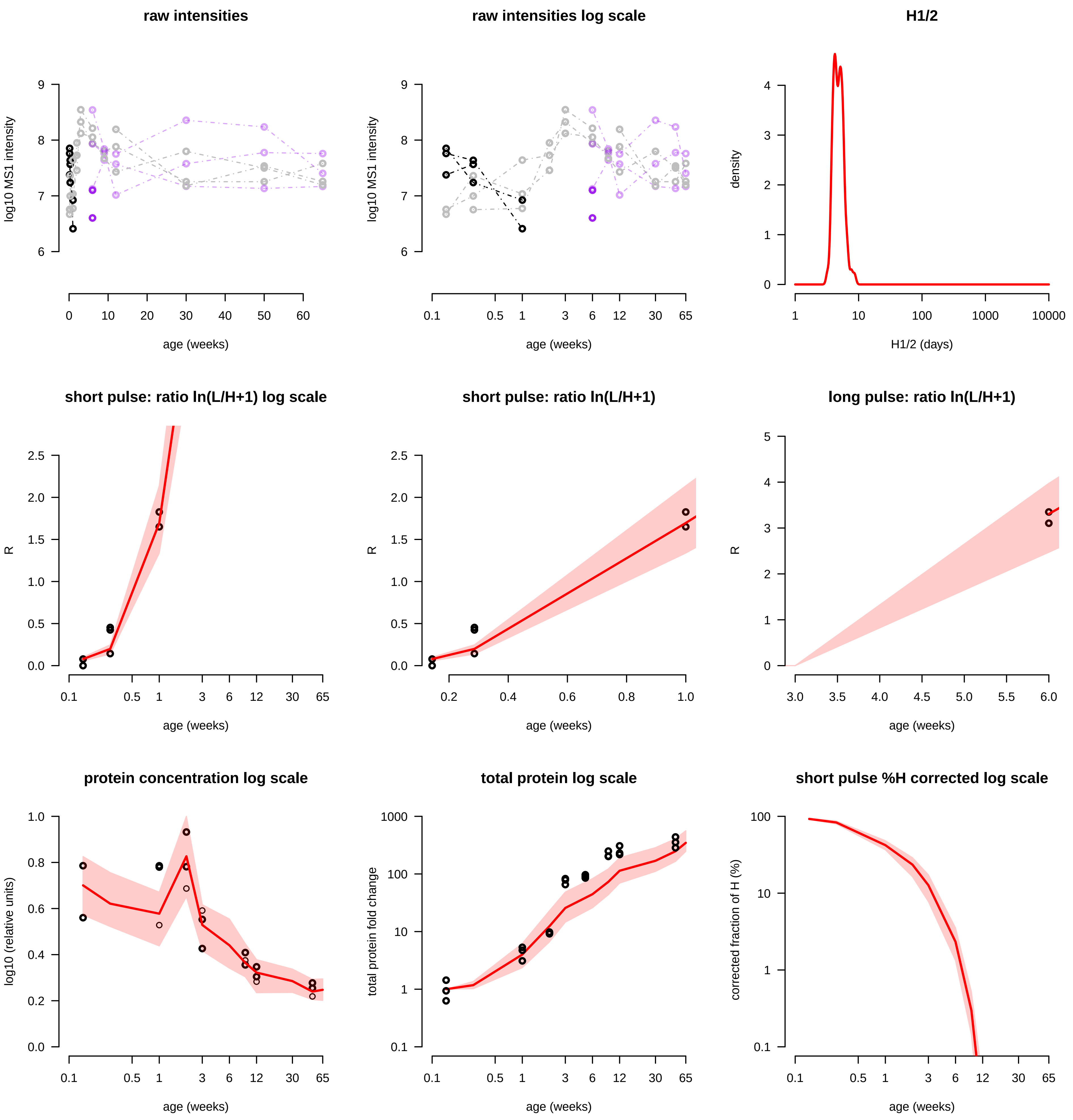

Q8R2U4

>sp|Q8R2U4|NTM1A_MOUSE

N−terminal Xaa−Pro−Lys N−methyltransferase 1

GN=Ntmt1

Med H1/2: 7.9 days

Q5 H1/2: 3.1 days

Q95 H1/2: 72 days

reliable model fit: yes

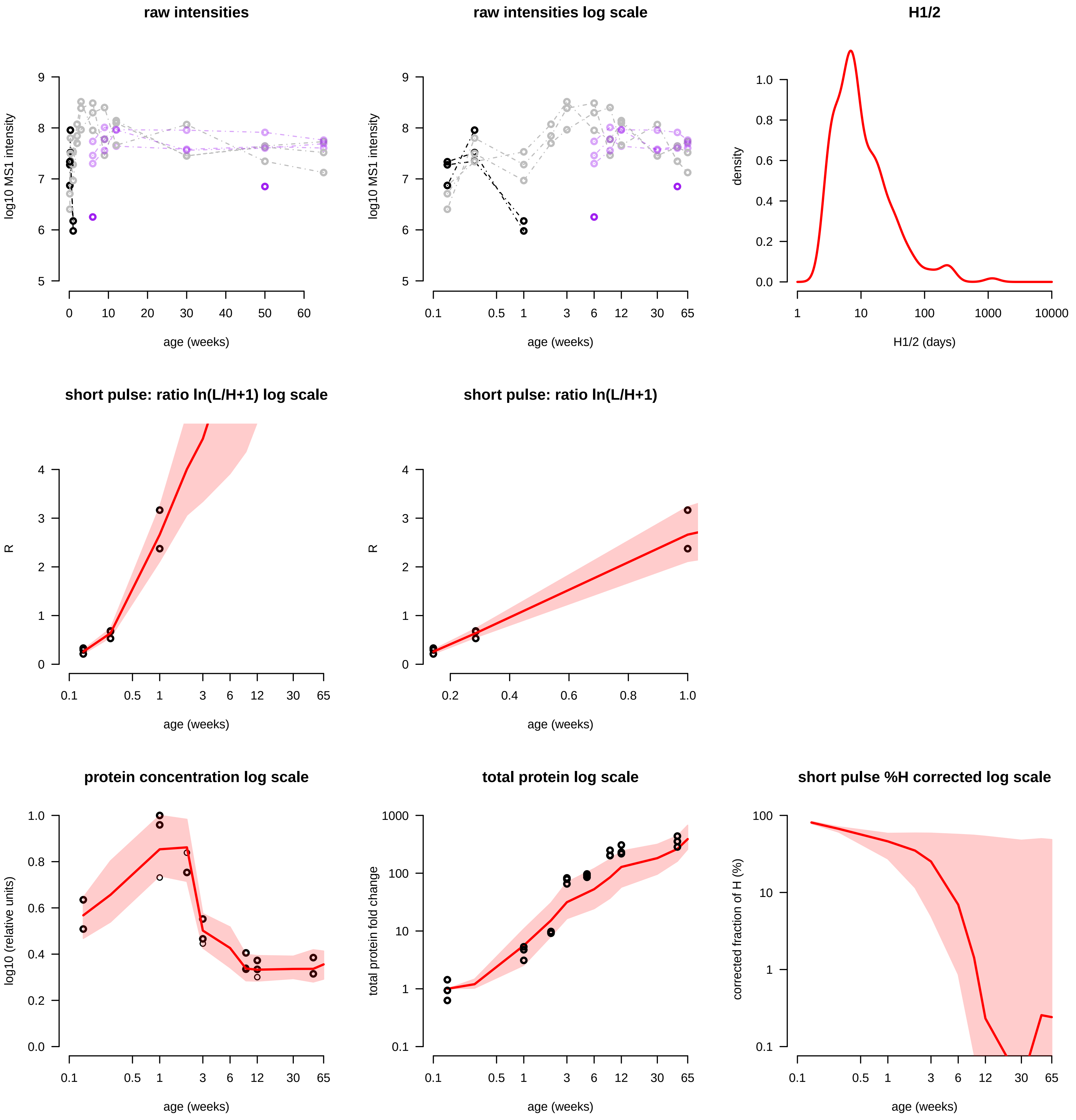

P97355

>sp|P97355|SPSY_MOUSE

Spermine synthase

GN=Sms

Med H1/2: 11.9 days

Q5 H1/2: 4.5 days

Q95 H1/2: 220.1 days

reliable model fit: yes

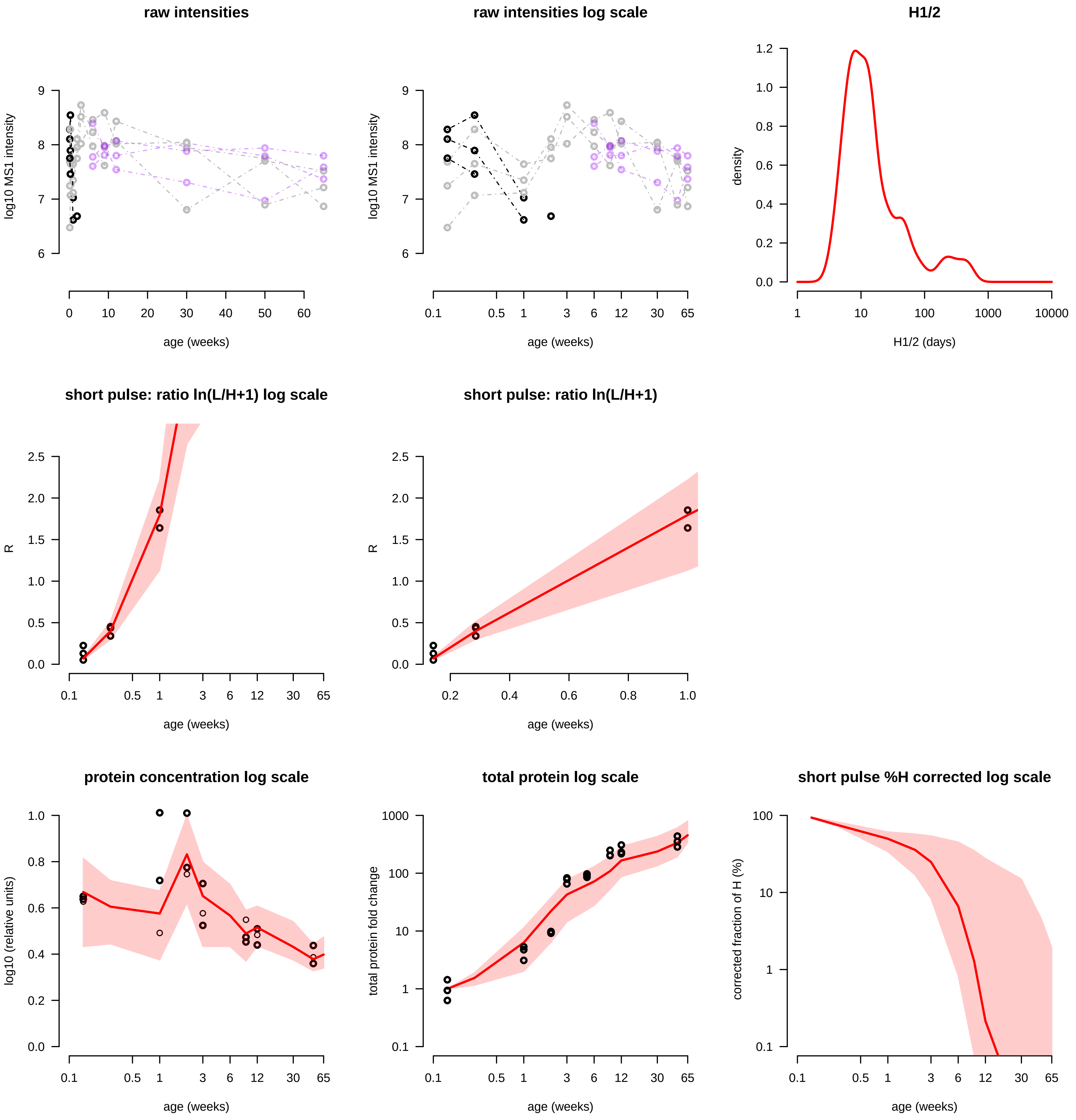

P62878

>sp|P62878|RBX1_MOUSE

E3 ubiquitin−protein ligase RBX1

GN=Rbx1

Med H1/2: 4.8 days

Q5 H1/2: 2.6 days

Q95 H1/2: 36.1 days

reliable model fit: yes

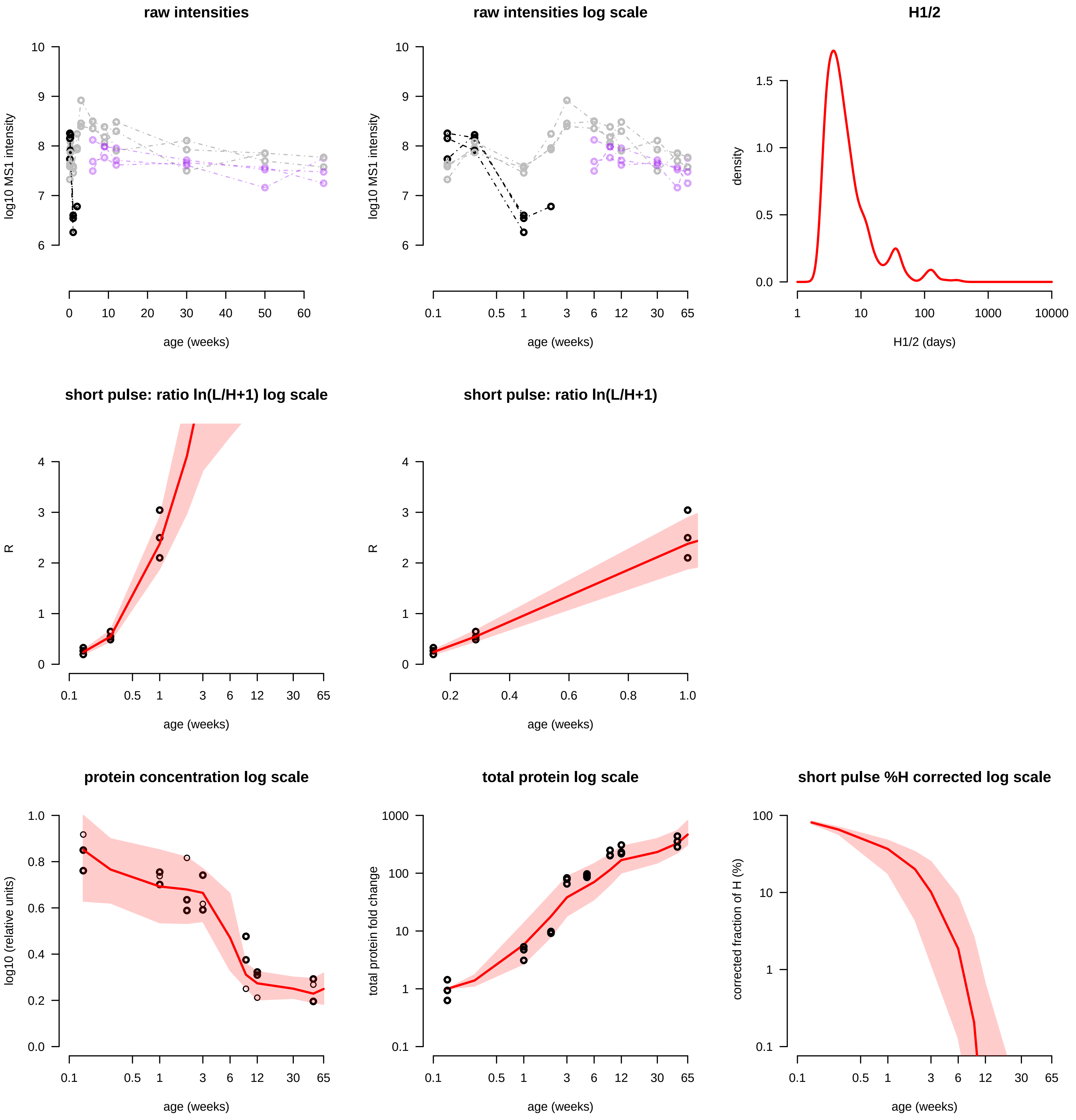

Q8VDS4

>sp|Q8VDS4|RPR1A_MOUSE

Regulation of nuclear pre−mRNA domain−containing protein 1A

GN=Rprd1a

Med H1/2: 4.1 days

Q5 H1/2: 2.6 days

Q95 H1/2: 14.8 days

reliable model fit: yes

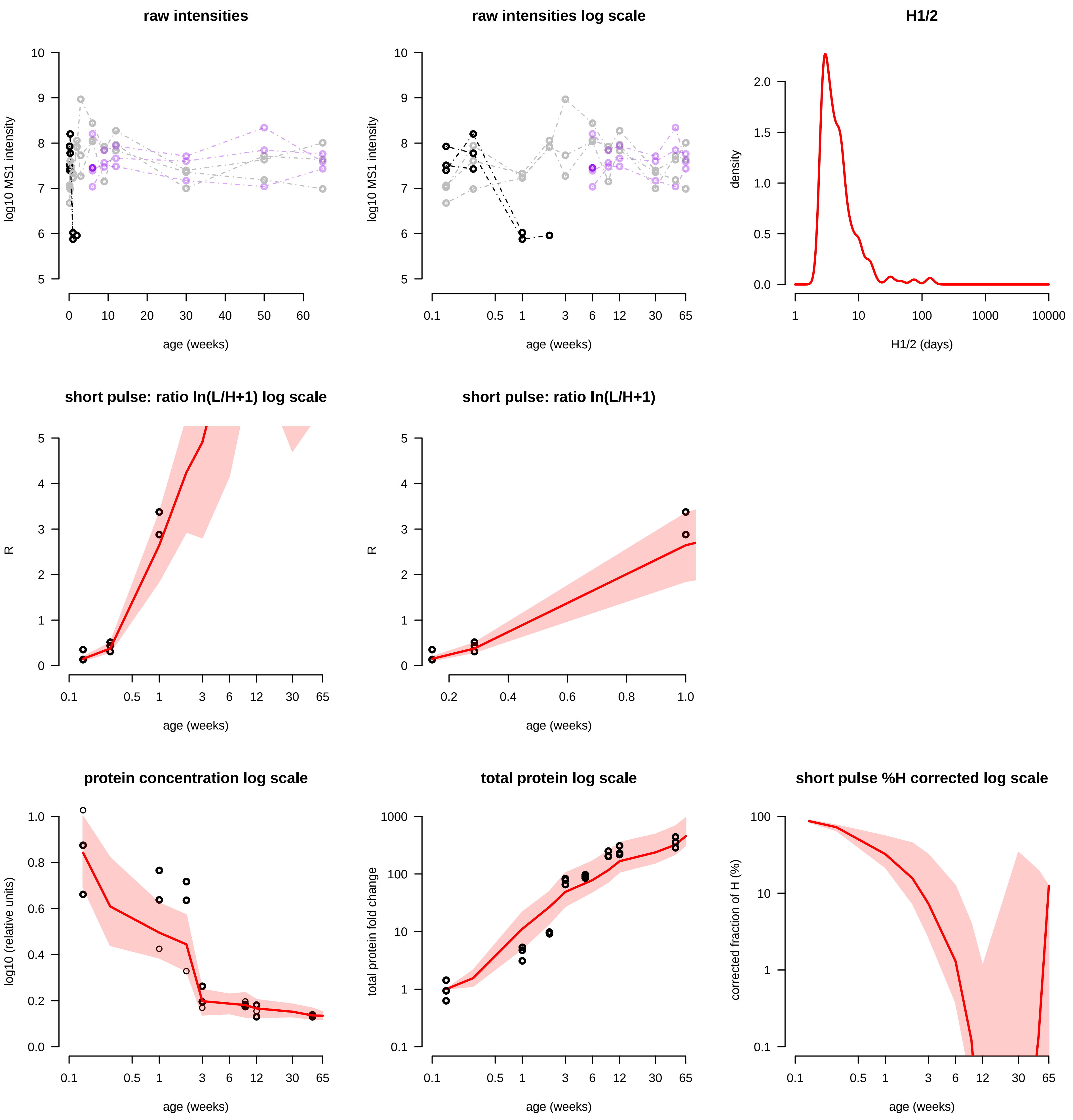

P41778−2;P41778

>sp|P41778−2|PBX1_MOUSE

Isoform PBX1b of Pre−B−cell leukemia transcription factor 1

GN=Pbx1

Med H1/2: 3.8 days

Q5 H1/2: 2.6 days

Q95 H1/2: 24.4 days

reliable model fit: yes

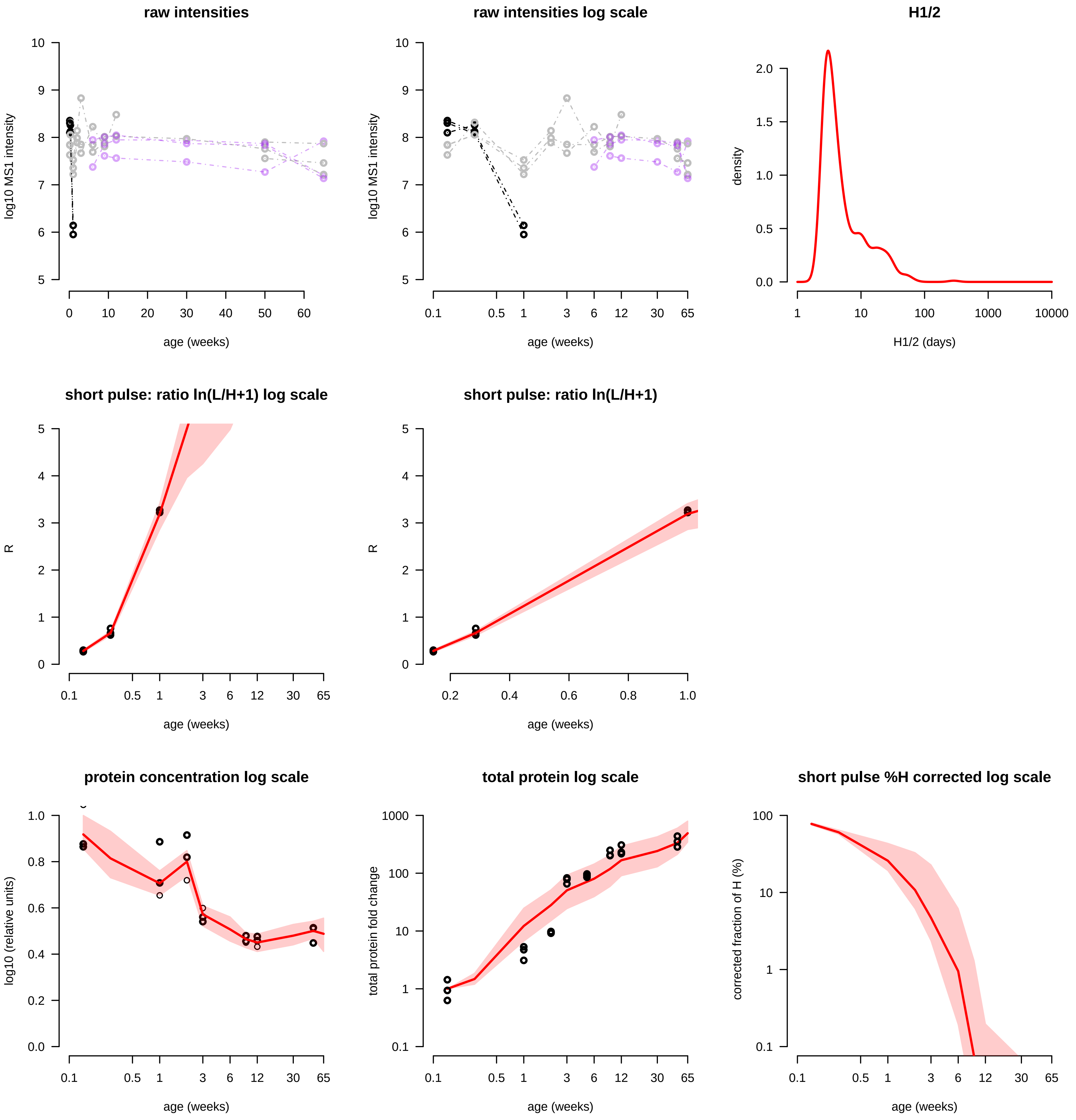

Q7TSH6−3;Q7TSH6;Q7TSH6−2

>sp|Q7TSH6−3|SCAF4_MOUSE

Isoform 3 of SR−related and CTD−associated factor 4

GN=Scaf4

Med H1/2: 6 days

Q5 H1/2: 2.7 days

Q95 H1/2: 58.1 days

reliable model fit: yes

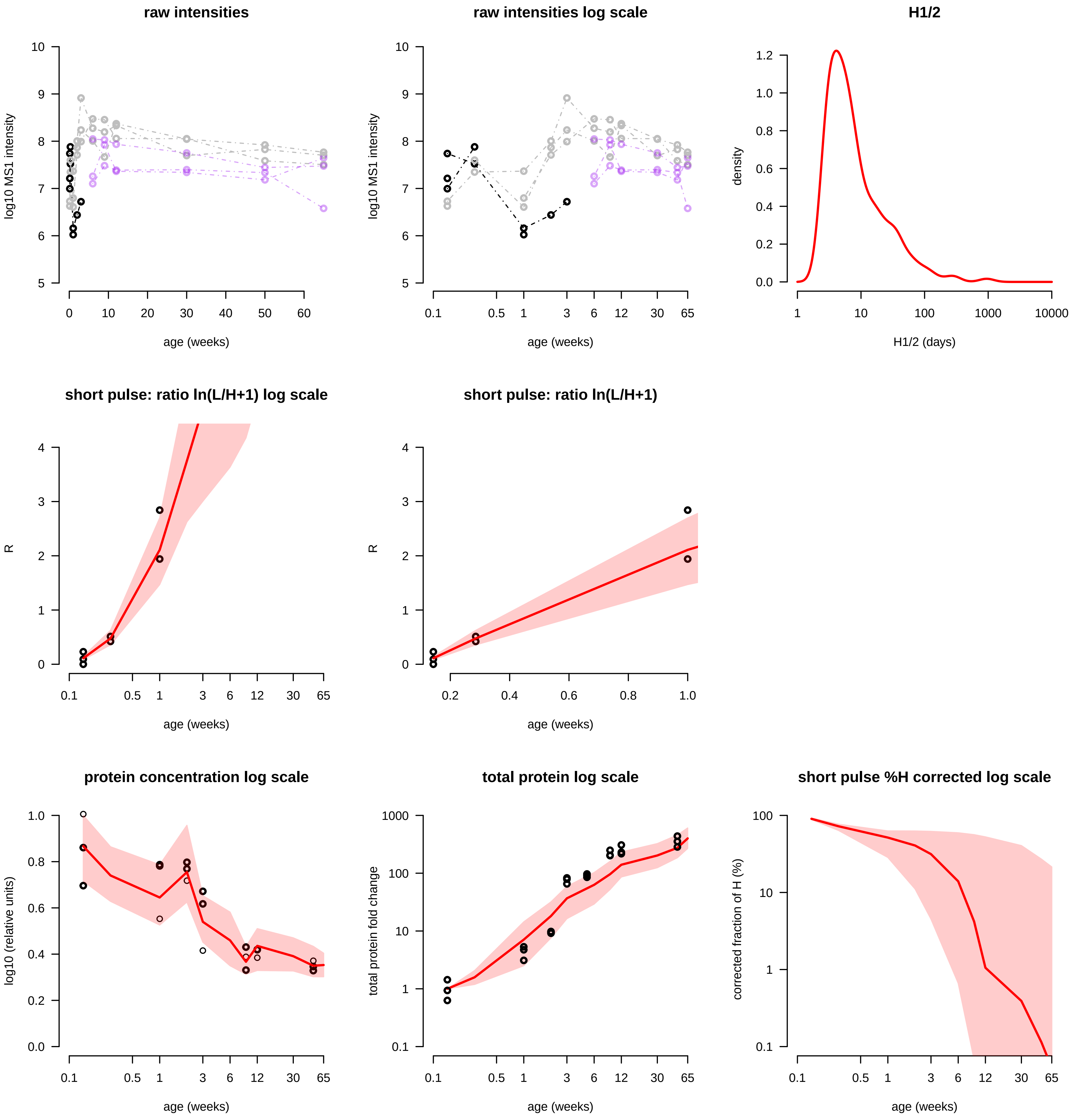

Q80UY1

>sp|Q80UY1|CARME_MOUSE

Carnosine N−methyltransferase

GN=Carnmt1

Med H1/2: 1585.8 days

Q5 H1/2: 509.8 days

Q95 H1/2: 24085.5 days

reliable model fit: no

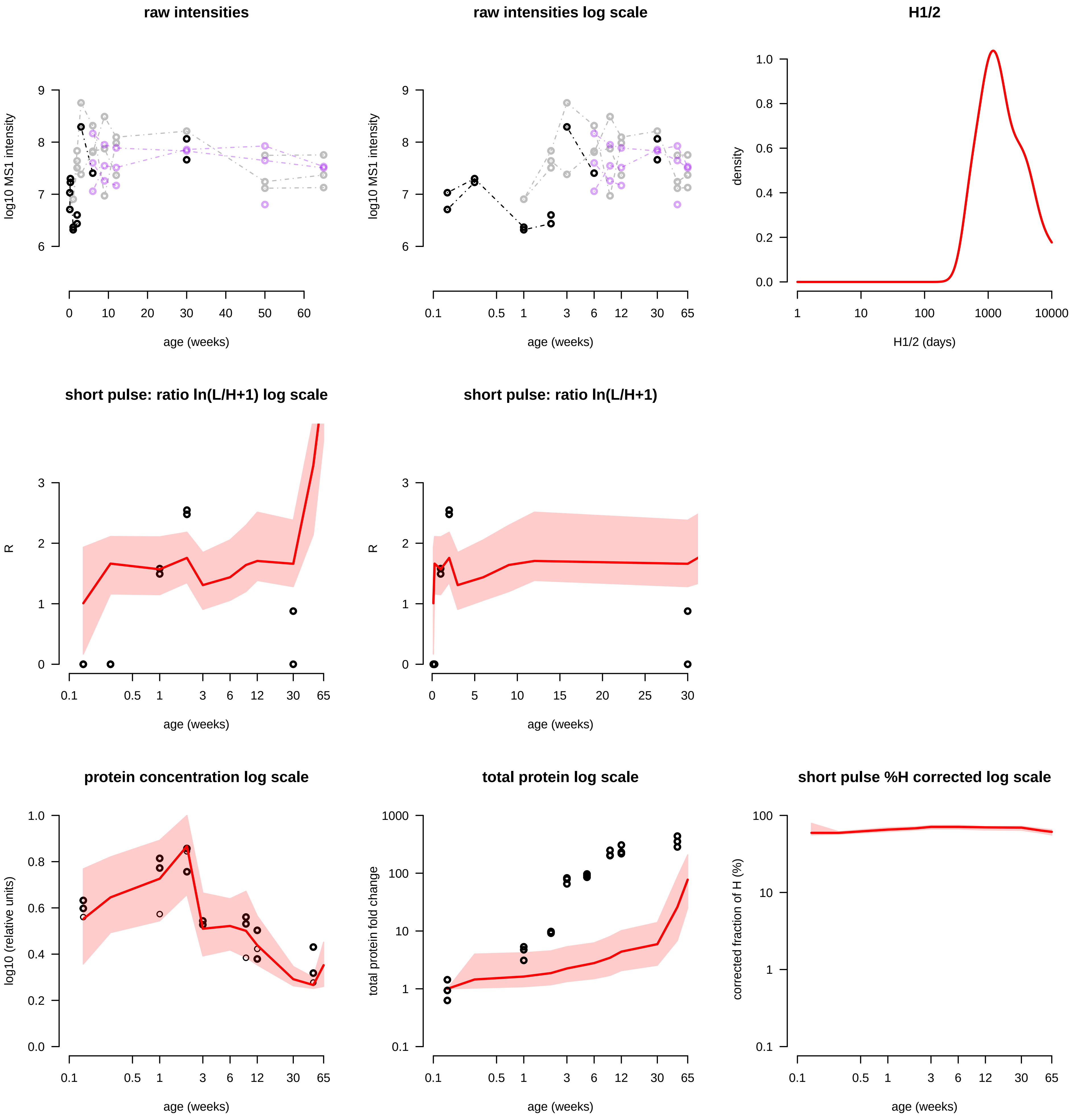

Q9JI44;Q9JI44−2

>sp|Q9JI44|DMAP1_MOUSE

DNA methyltransferase 1−associated protein 1

GN=Dmap1

Med H1/2: 7.2 days

Q5 H1/2: 2.8 days

Q95 H1/2: 122.8 days

reliable model fit: no

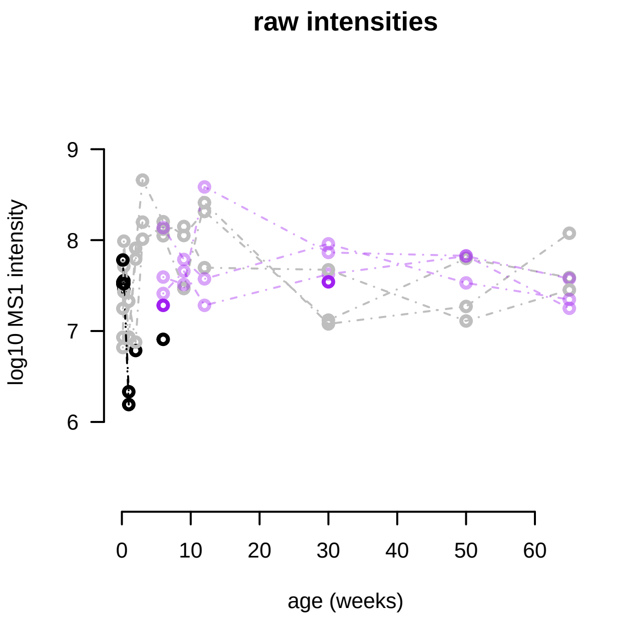

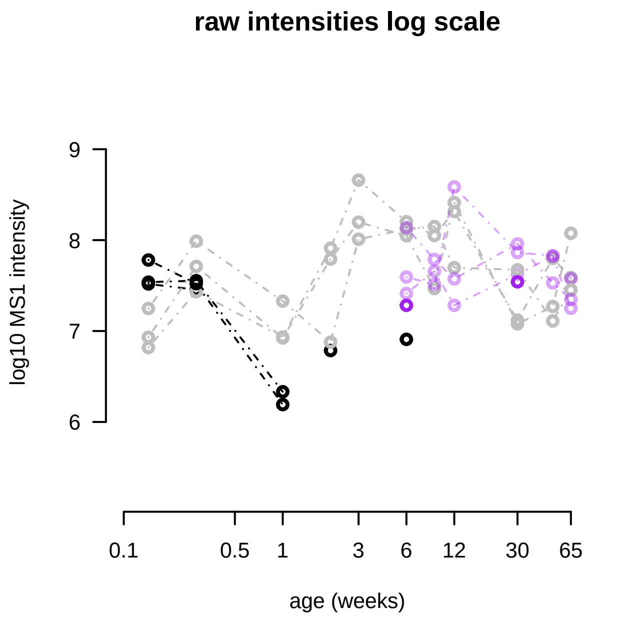

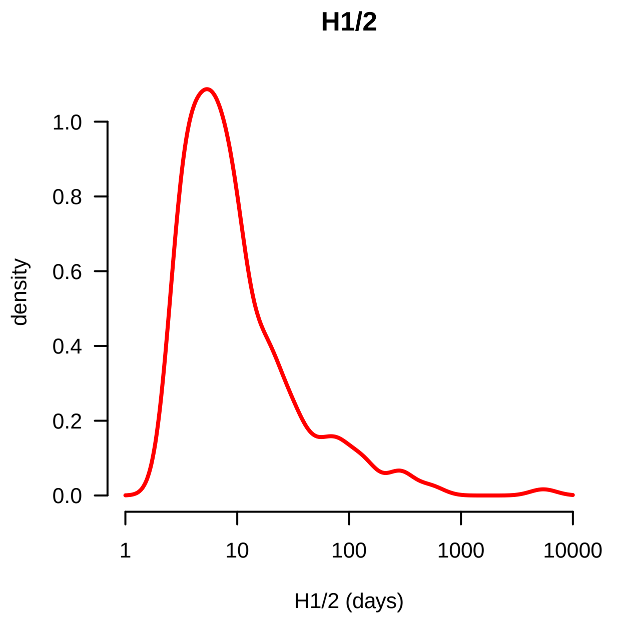

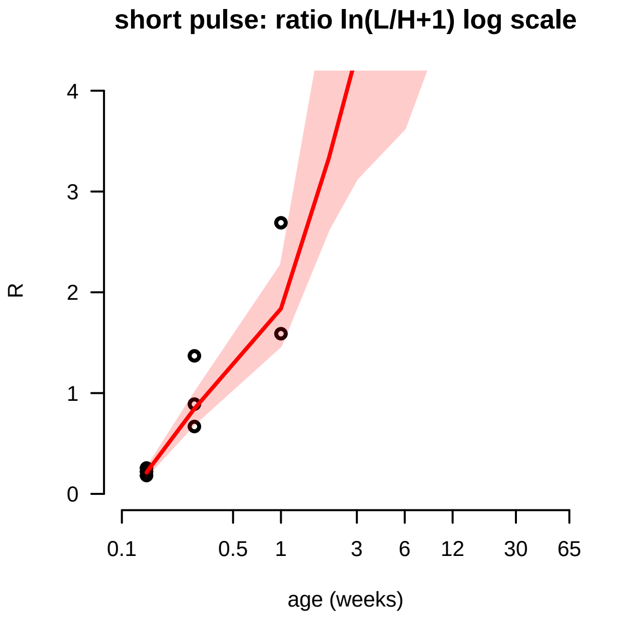

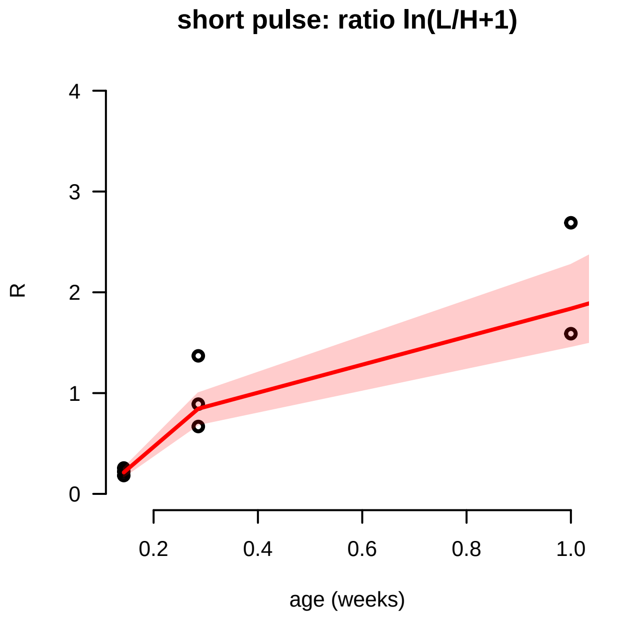

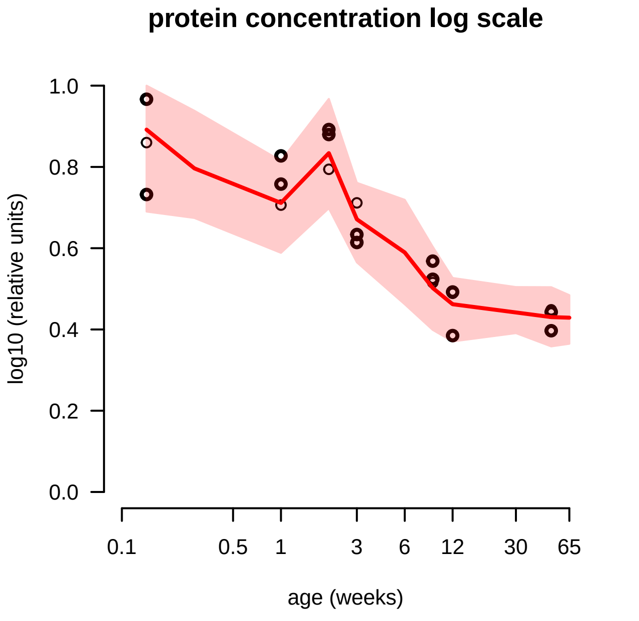

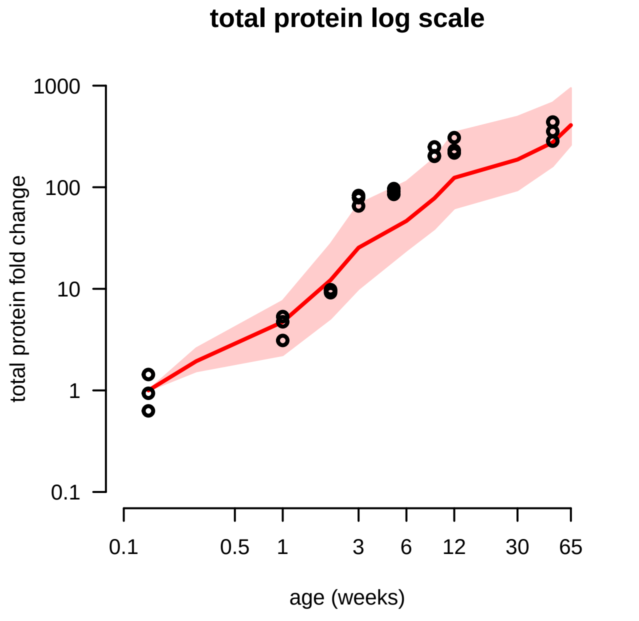

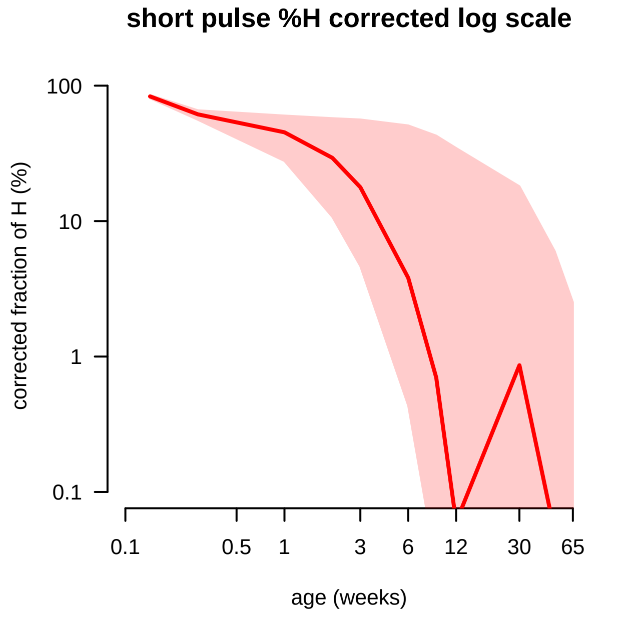

Q62036

>sp|Q62036|CP131_MOUSE

Centrosomal protein of 131 kDa

GN=Cep131

Med H1/2: 5.6 days

Q5 H1/2: 2.8 days

Q95 H1/2: 25.6 days

reliable model fit: yes

raw intensities

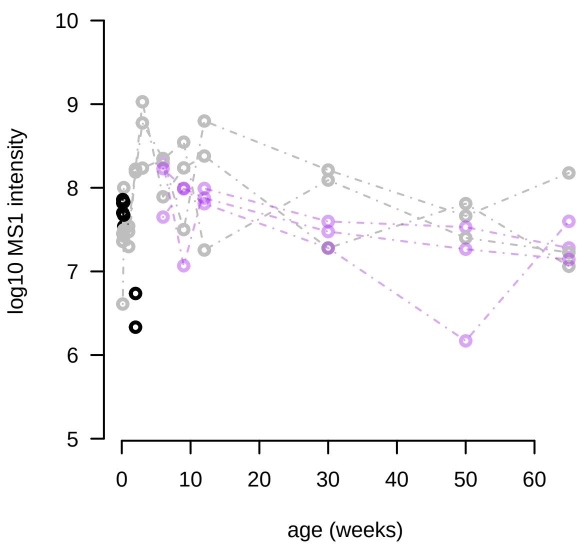

raw intensities log scale

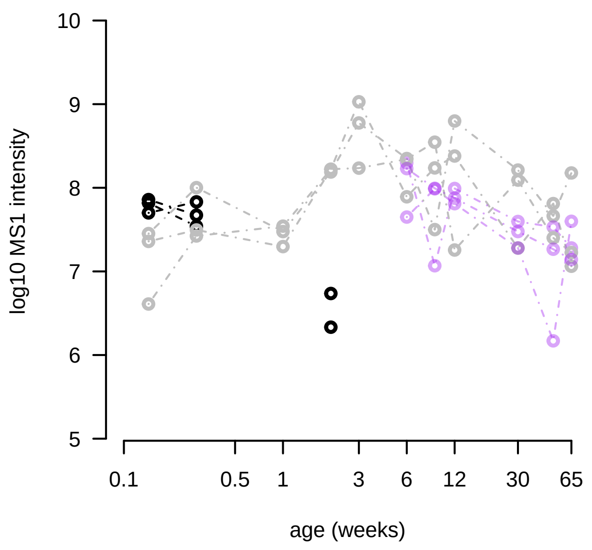

H1/2

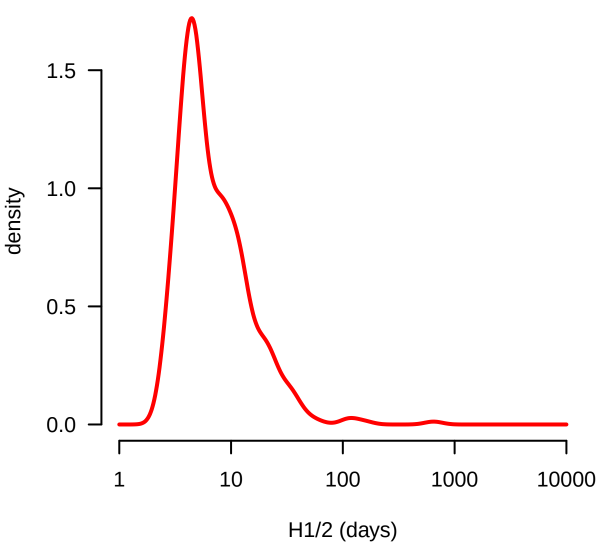

short pulse: ratio ln(L/H+1) log scale

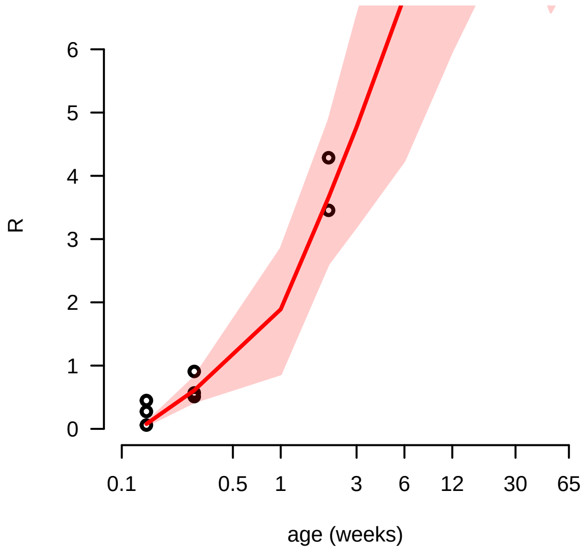

short pulse: ratio ln(L/H+1)

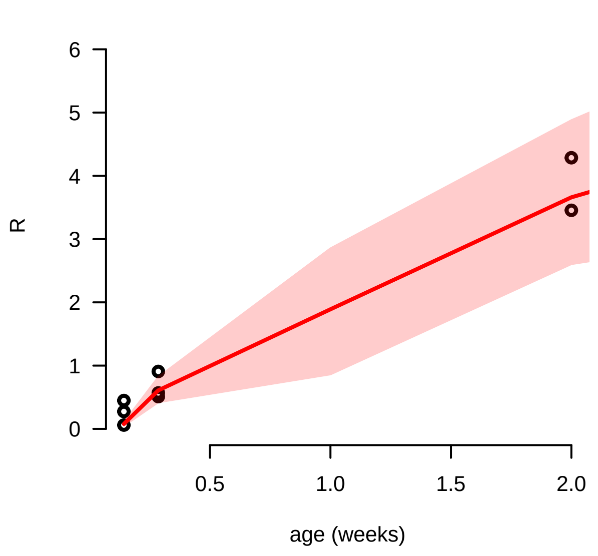

protein concentration log scale

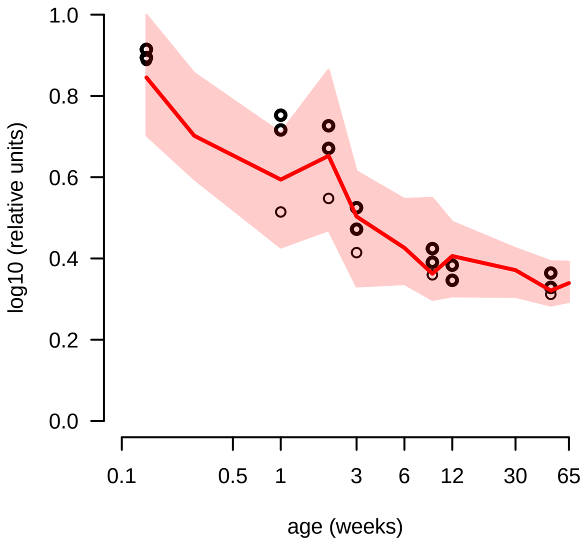

total protein log scale

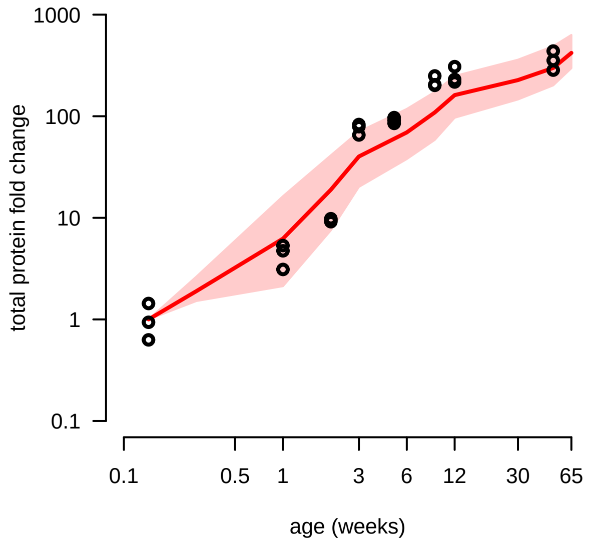

short pulse %H corrected log scale

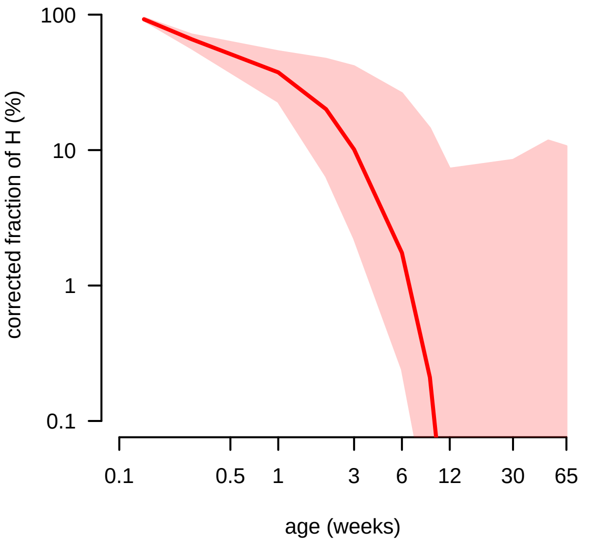

A3KGS3;A3KGS3−2

>sp|A3KGS3|RGPA2_MOUSE

Ral GTPase−activating protein subunit alpha−2

GN=Ralgapa2

Med H1/2: 21.4 days

Q5 H1/2: 5.4 days

Q95 H1/2: 175.2 days

reliable model fit: yes

raw intensities

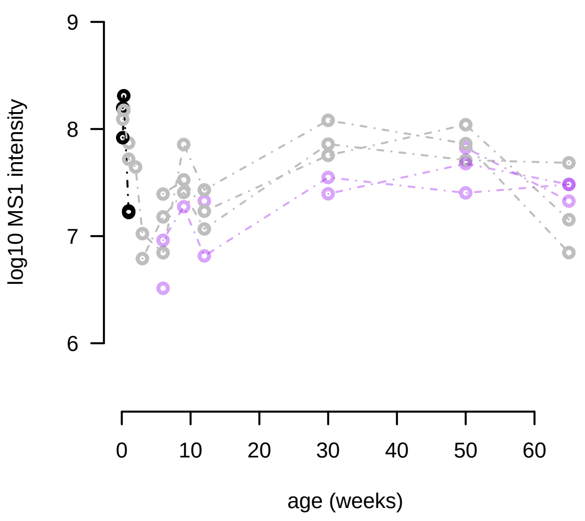

raw intensities log scale

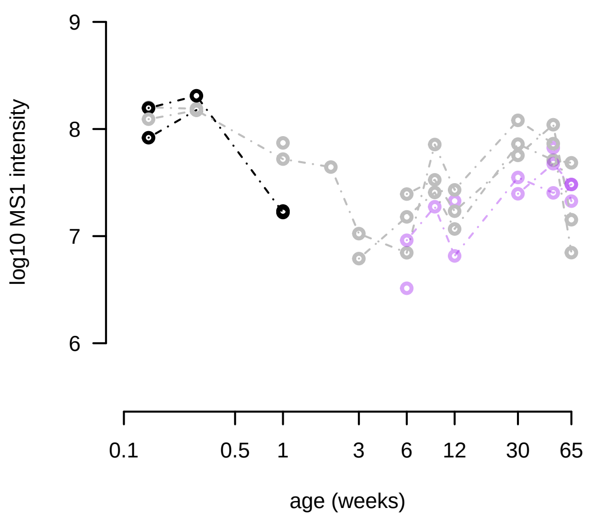

H1/2

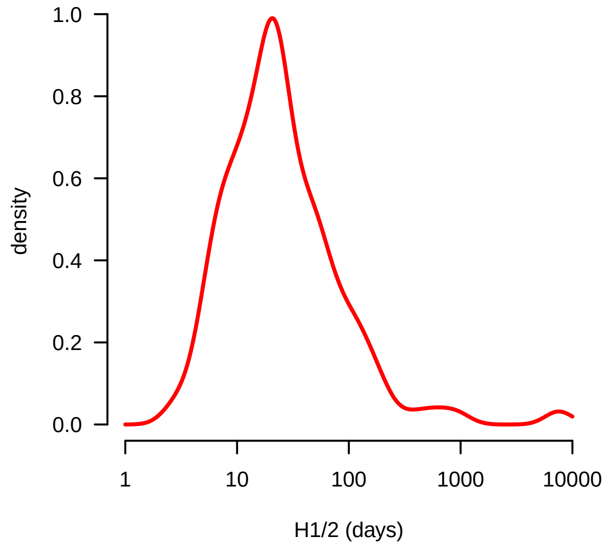

short pulse: ratio ln(L/H+1) log scale

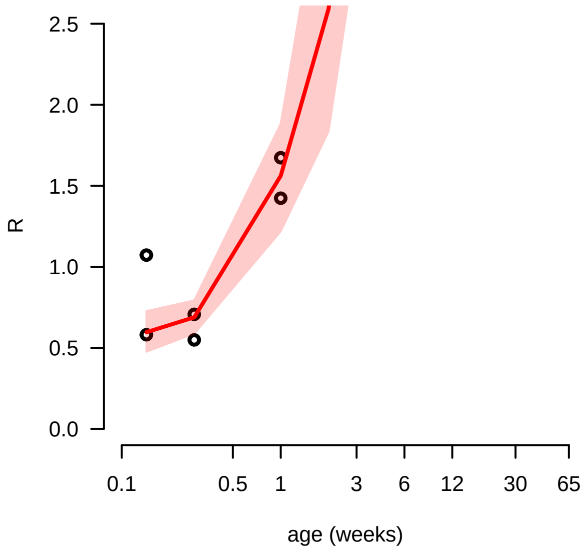

short pulse: ratio ln(L/H+1)

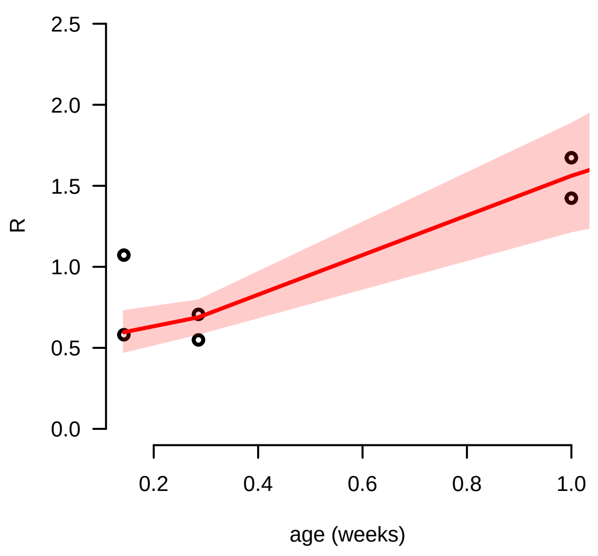

protein concentration log scale

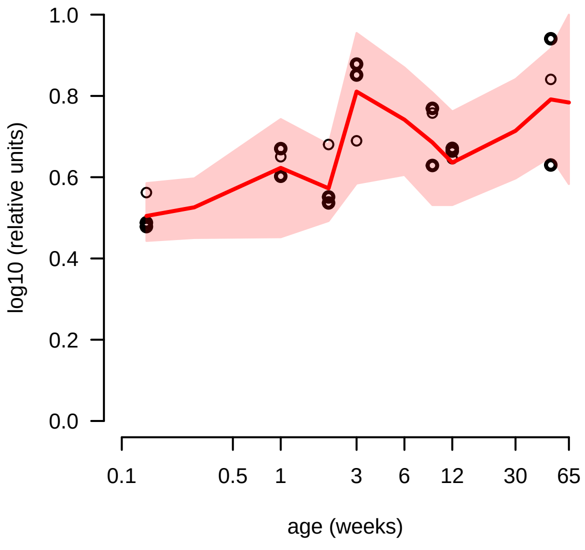

total protein log scale

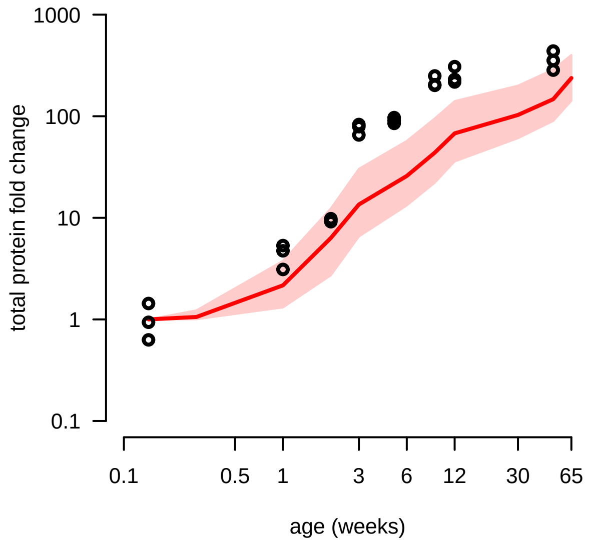

short pulse %H corrected log scale

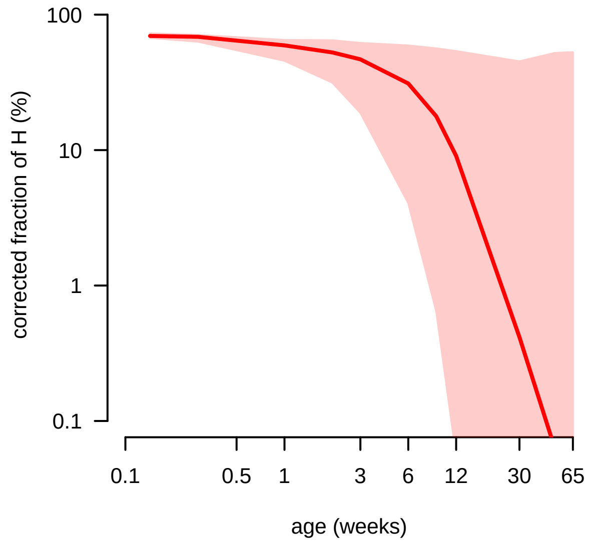

Q9D0D5

>sp|Q9D0D5|T2EA_MOUSE

General transcription factor IIE subunit 1

GN=Gtf2e1

Med H1/2: 7.8 days

Q5 H1/2: 3.2 days

Q95 H1/2: 59.6 days

reliable model fit: yes

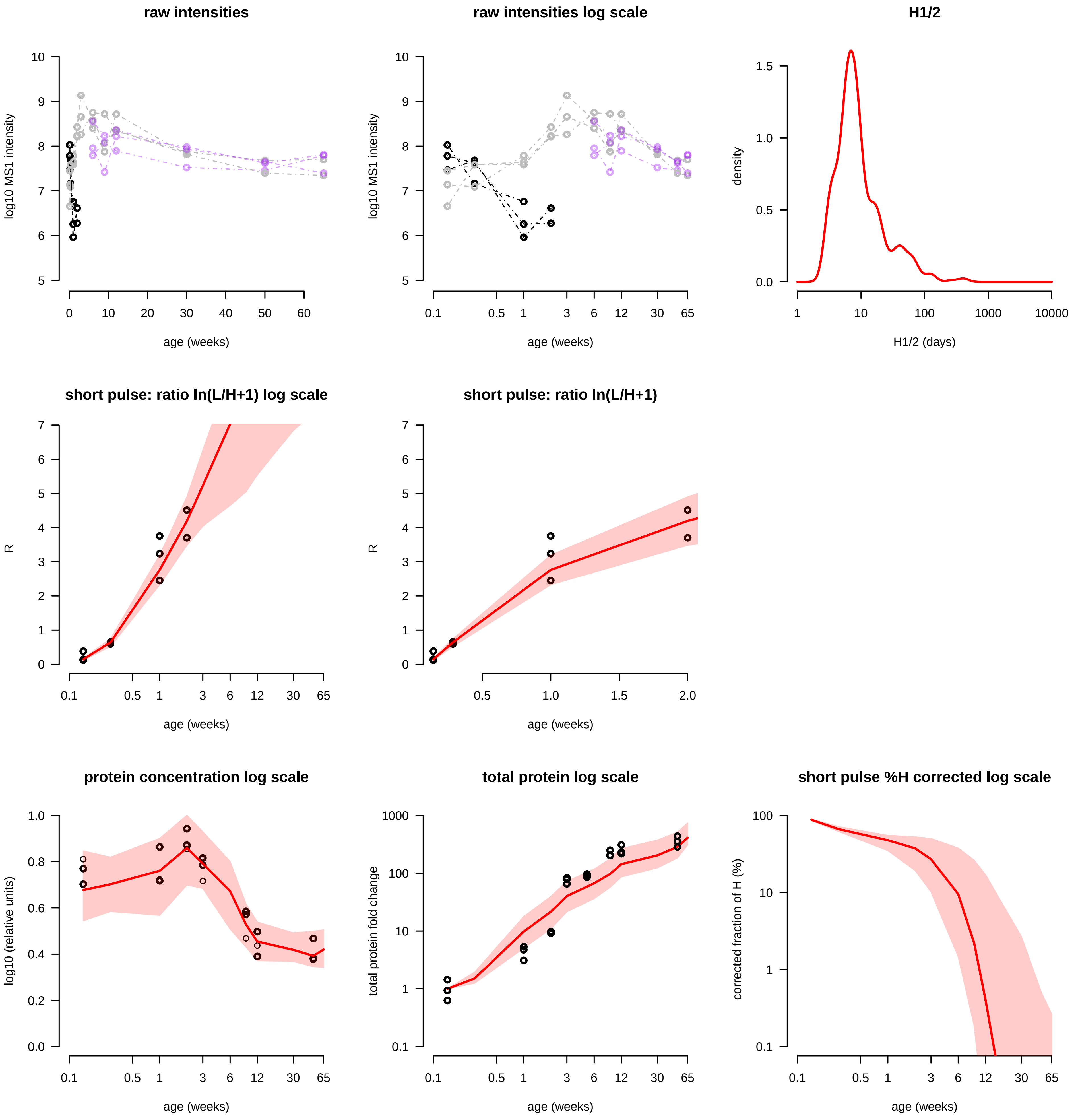

Q78T54;Q78T54−2

>sp|Q78T54|VMA21_MOUSE

Vacuolar ATPase assembly integral membrane protein Vma21

GN=Vma21

Med H1/2: 9.6 days

Q5 H1/2: 3.4 days

Q95 H1/2: 127.2 days

reliable model fit: yes

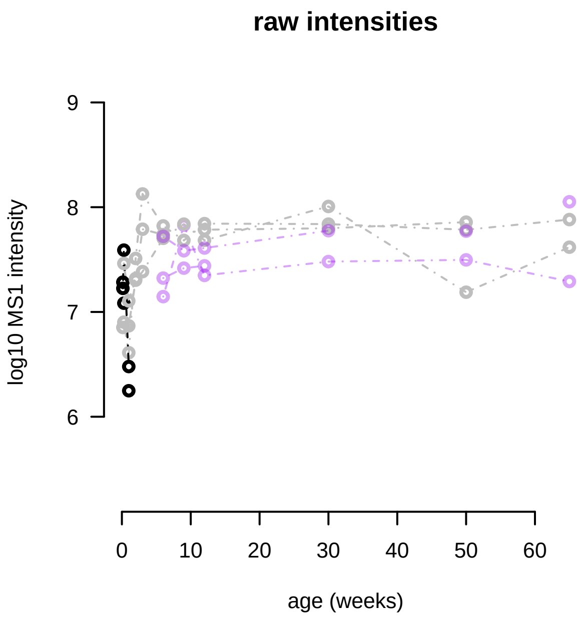

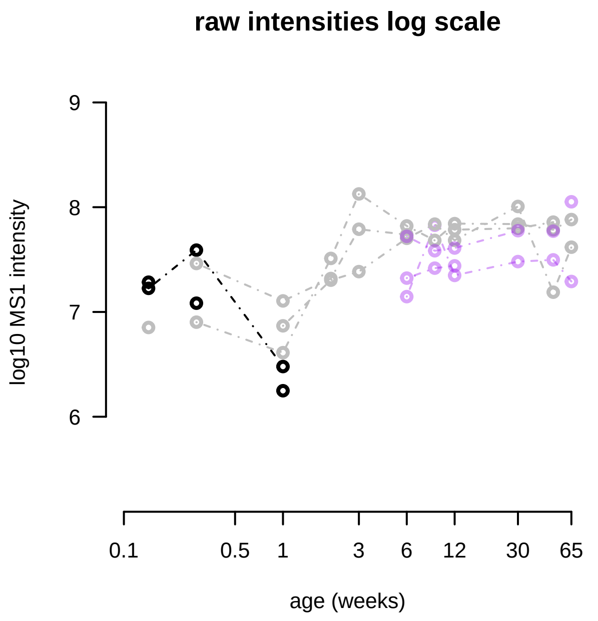

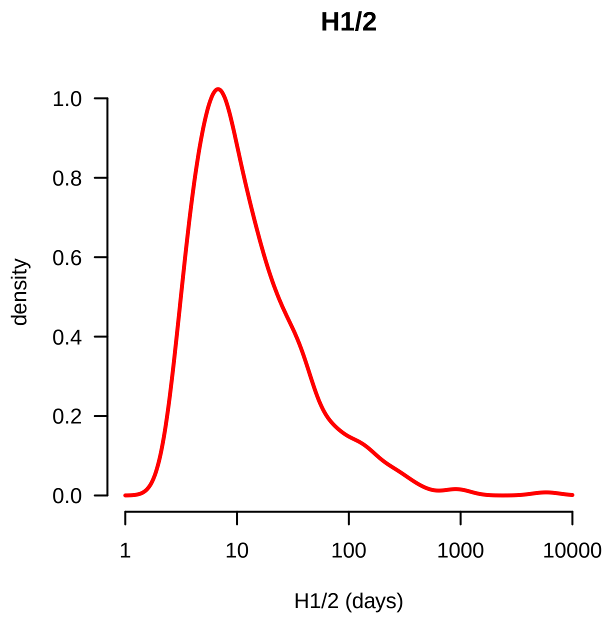

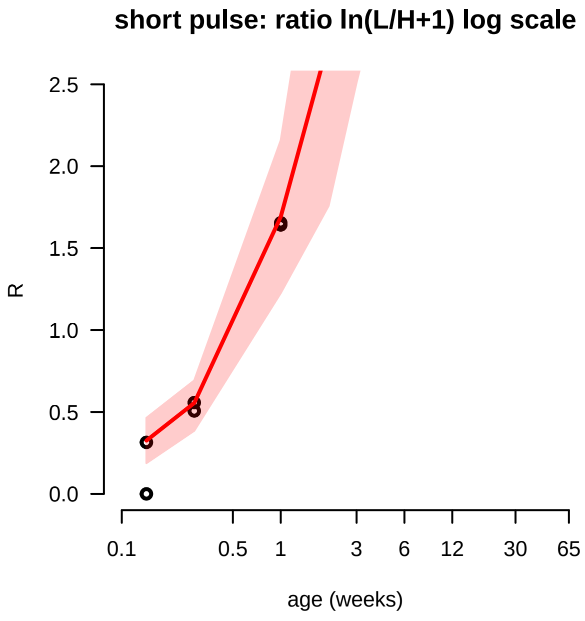

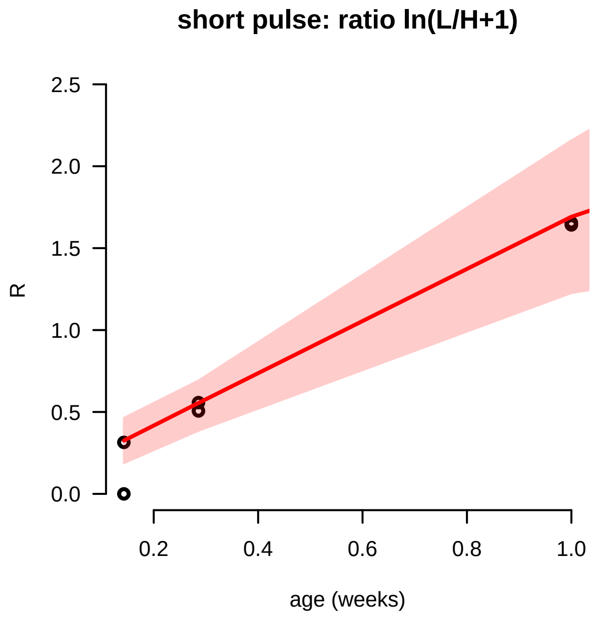

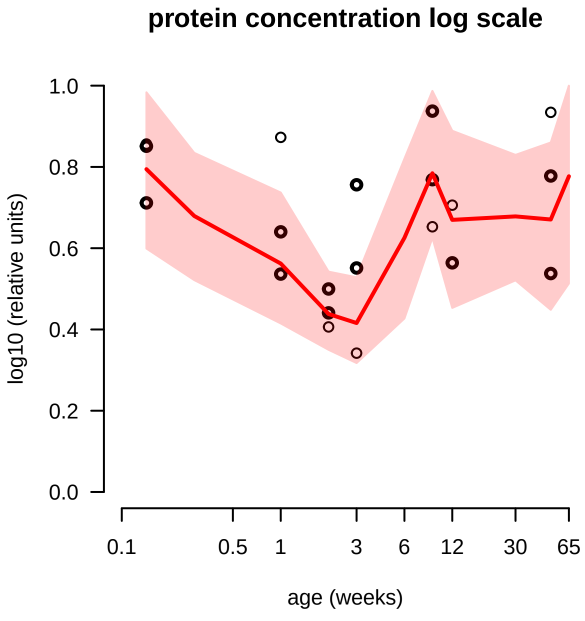

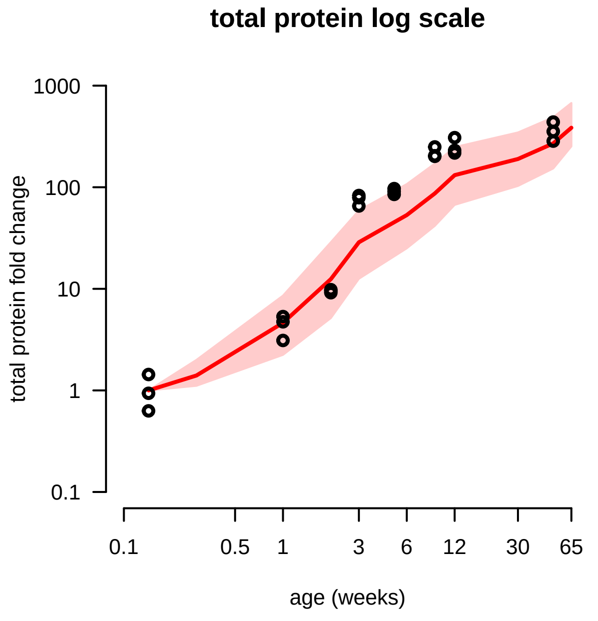

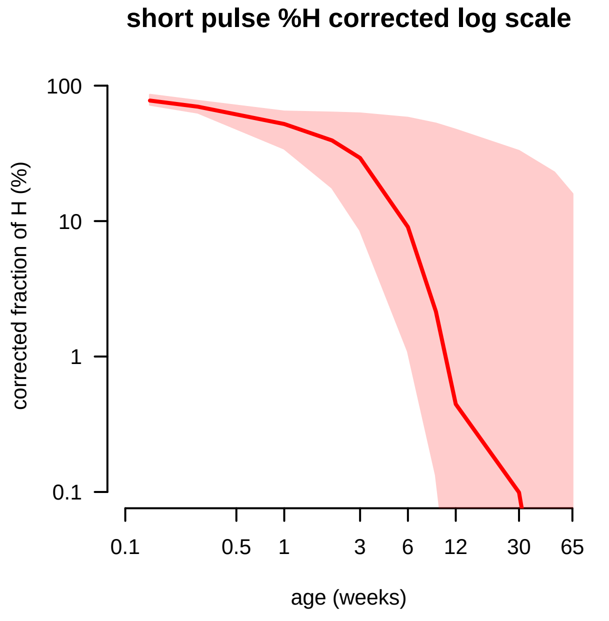

Q8VBV3

>sp|Q8VBV3|EXOS2_MOUSE

Exosome complex component RRP4

GN=Exosc2

Med H1/2: 49.7 days

Q5 H1/2: 26.5 days

Q95 H1/2: 197 days

reliable model fit: yes

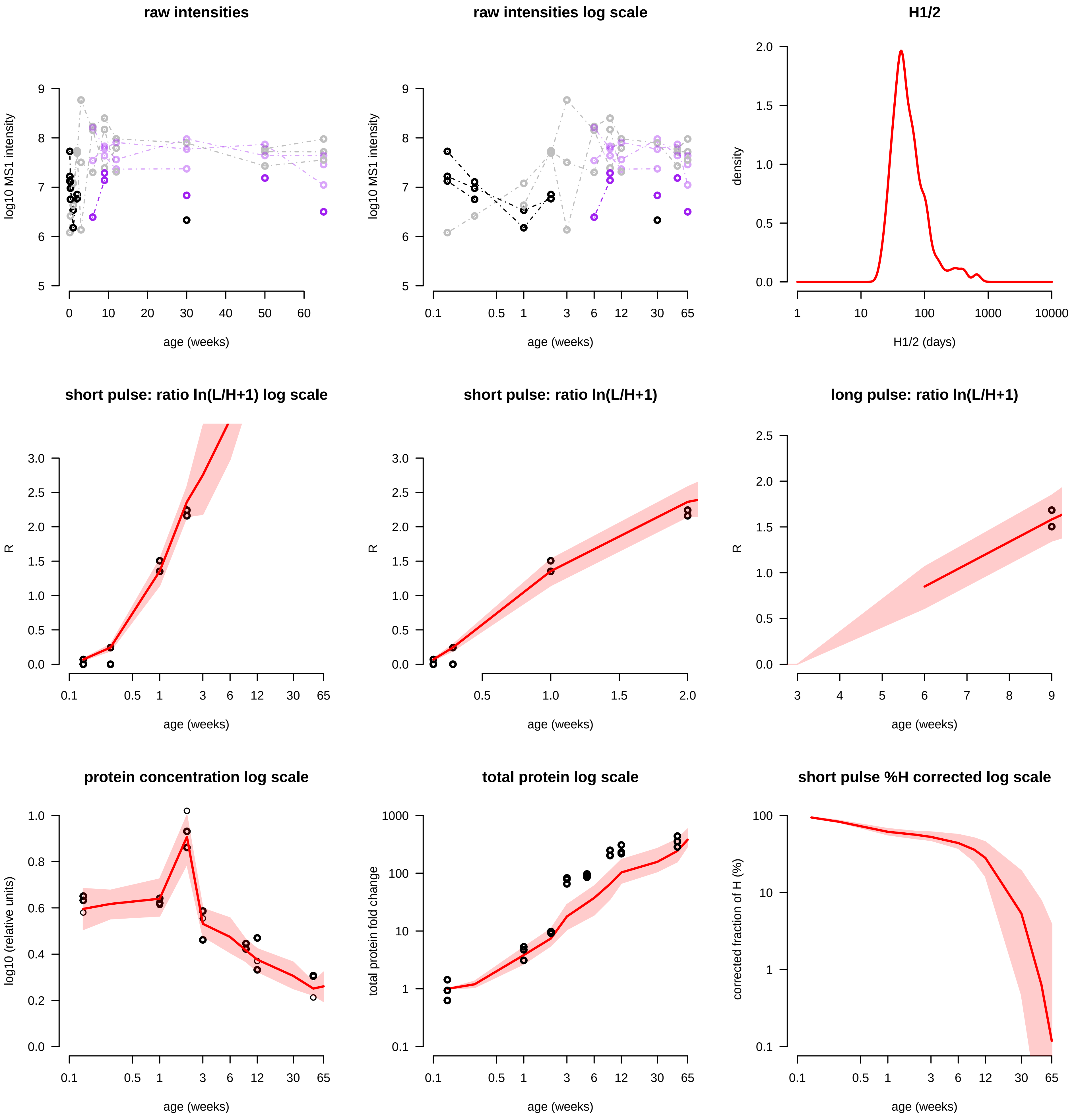

Q62074

>sp|Q62074|KPCI_MOUSE

Protein kinase C iota type

GN=Prkci

Med H1/2: 10.4 days

Q5 H1/2: 3.4 days

Q95 H1/2: 106.3 days

reliable model fit: yes

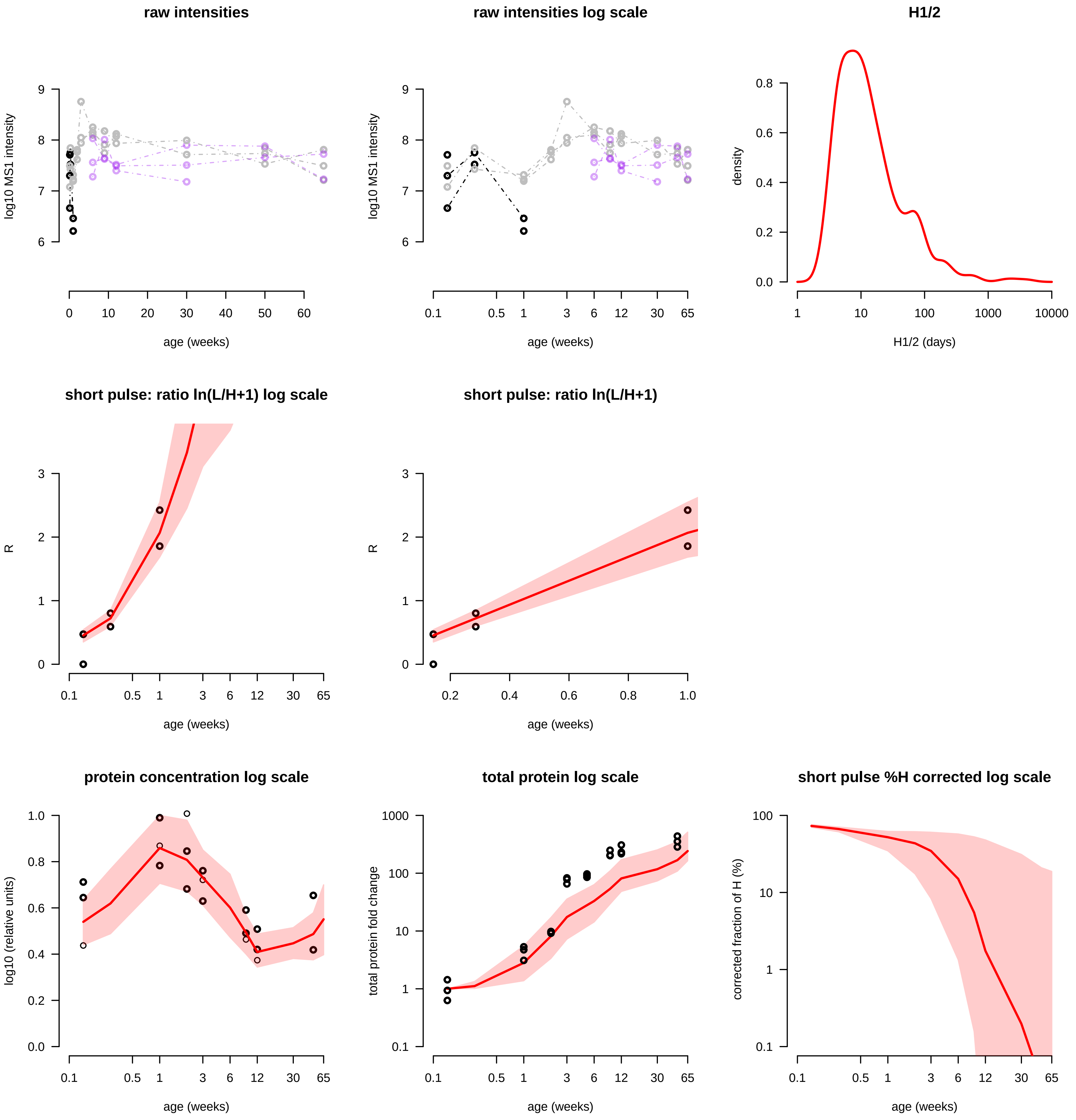

P19426−2;P19426

>sp|P19426−2|NELFE_MOUSE

Isoform 2 of Negative elongation factor E

GN=Nelfe

Med H1/2: 5.3 days

Q5 H1/2: 2.6 days

Q95 H1/2: 36.6 days

reliable model fit: yes

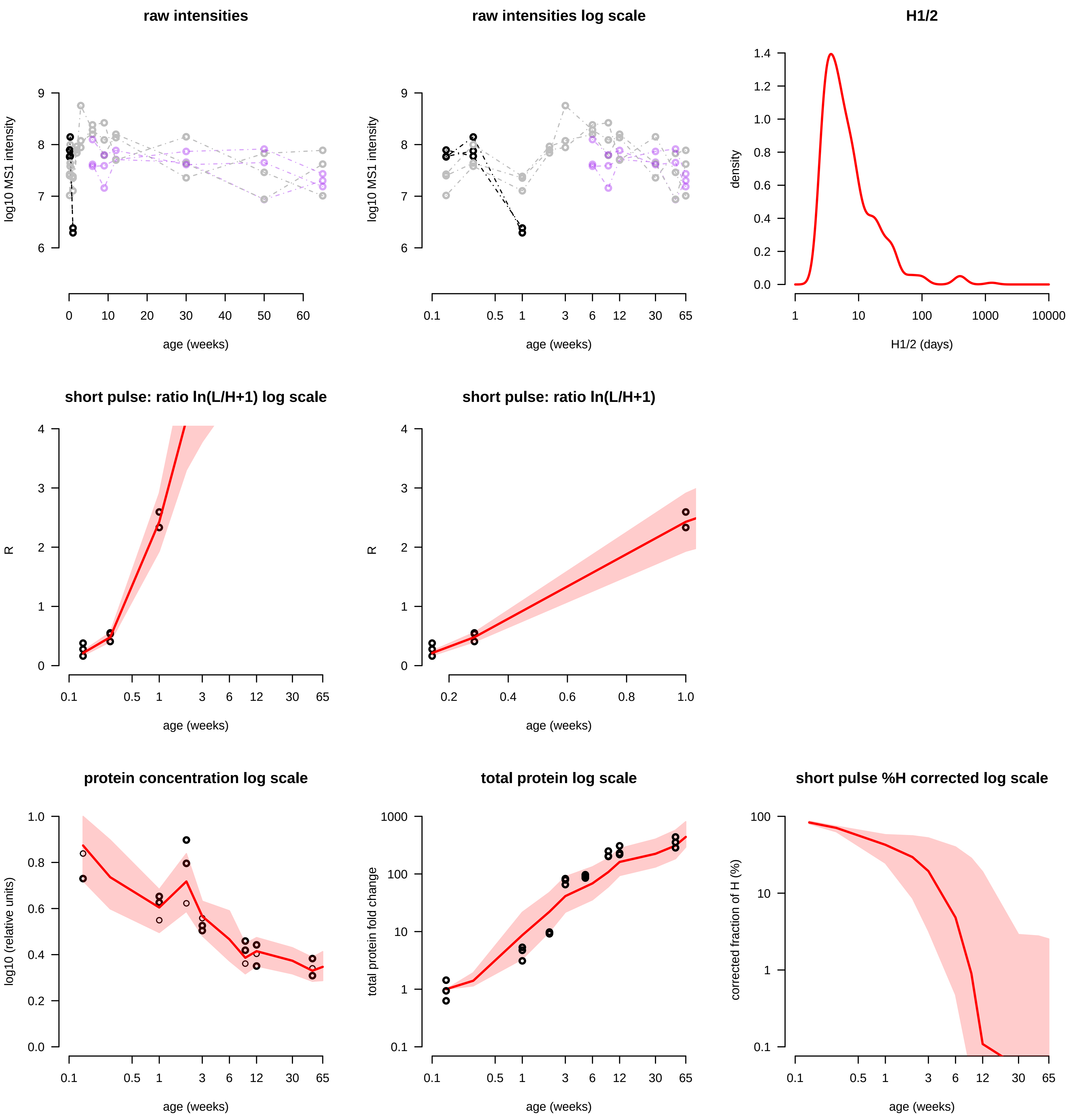

P52483

>sp|P52483|UB2E3_MOUSE

Ubiquitin−conjugating enzyme E2 E3

GN=Ube2e3

Med H1/2: 6 days

Q5 H1/2: 2.7 days

Q95 H1/2: 80.4 days

reliable model fit: yes

raw intensities

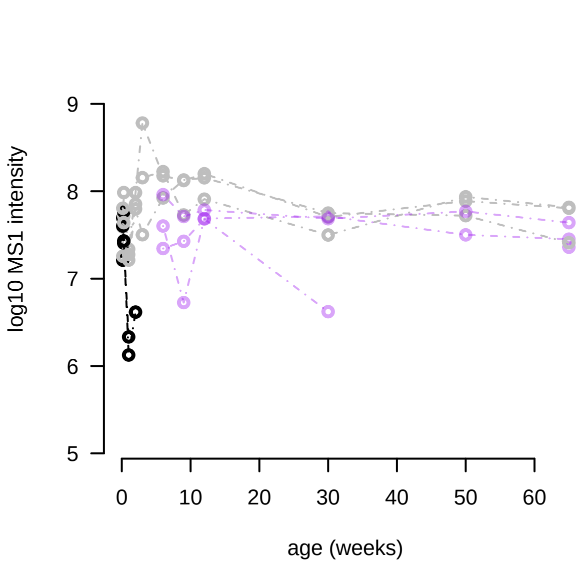

raw intensities log scale

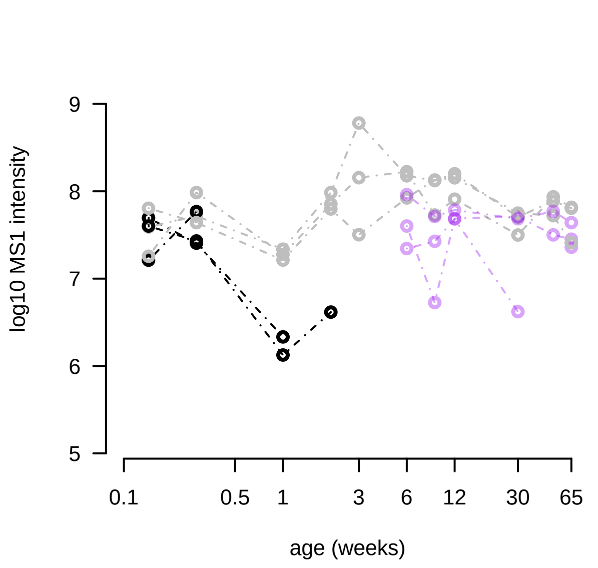

H1/2

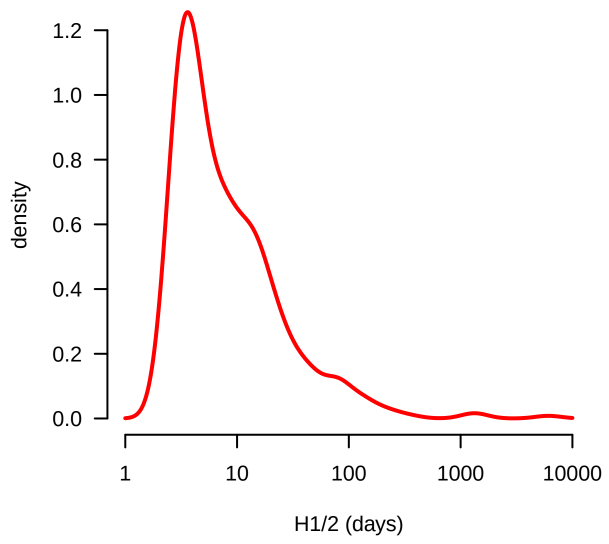

short pulse: ratio ln(L/H+1) log scale

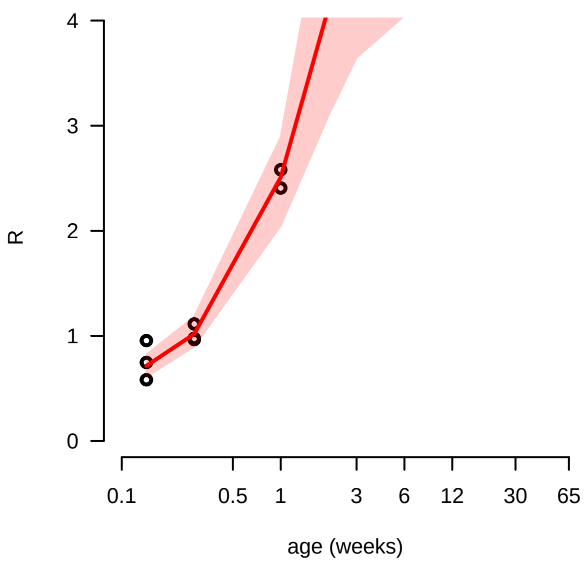

short pulse: ratio ln(L/H+1)

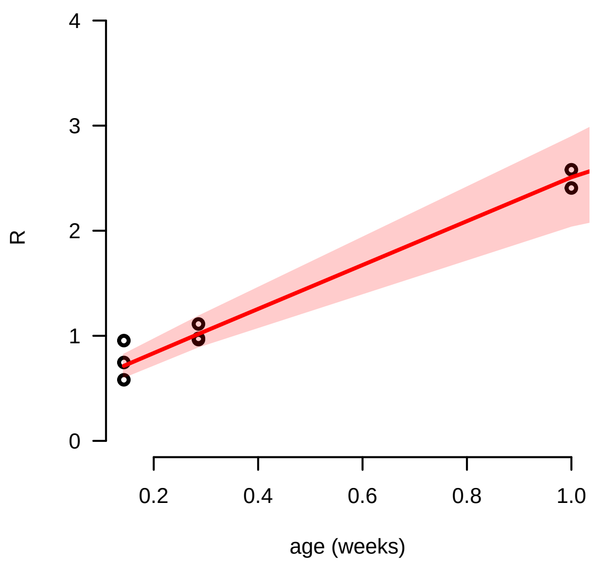

protein concentration log scale

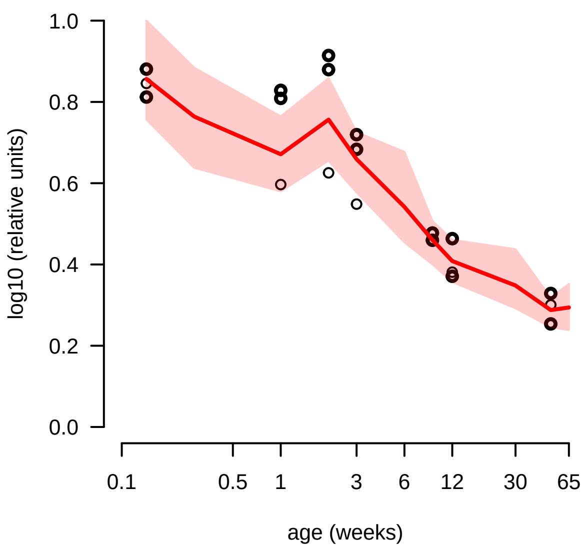

total protein log scale

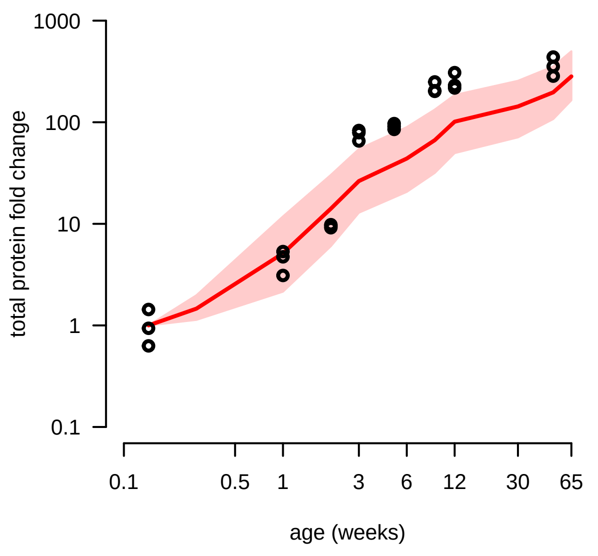

short pulse %H corrected log scale

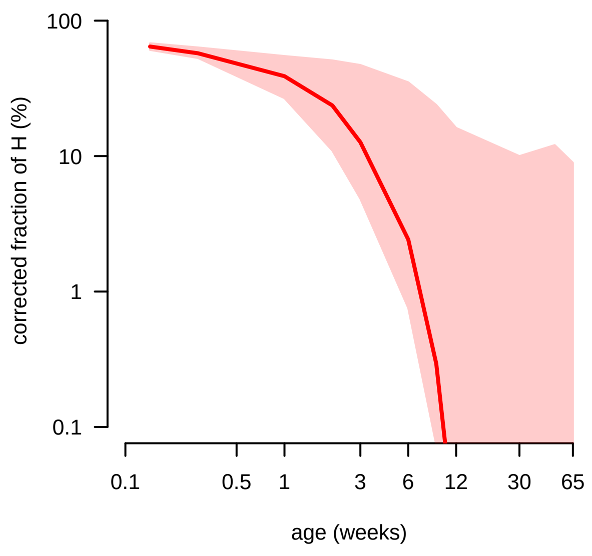

Q6A0D4

>sp|Q6A0D4|RFTN1_MOUSE

Raftlin

GN=Rftn1

Med H1/2: 26.8 days

Q5 H1/2: 8.2 days

Q95 H1/2: 299.3 days

reliable model fit: yes

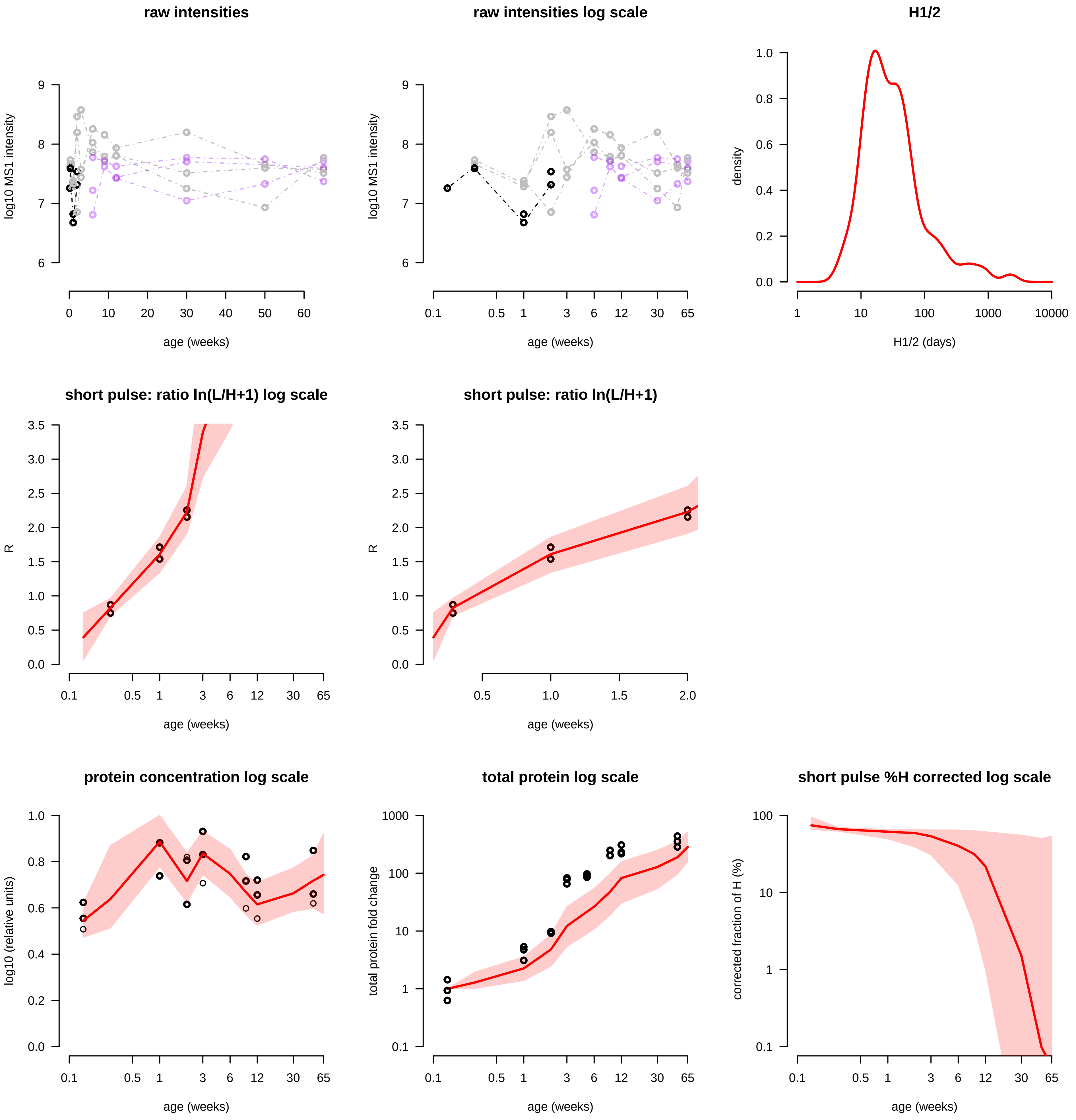

Q99MK8

>sp|Q99MK8|ARBK1_MOUSE

Beta−adrenergic receptor kinase 1

GN=Grk2

Med H1/2: 13.1 days

Q5 H1/2: 4.2 days

Q95 H1/2: 134.5 days

reliable model fit: yes

raw intensities

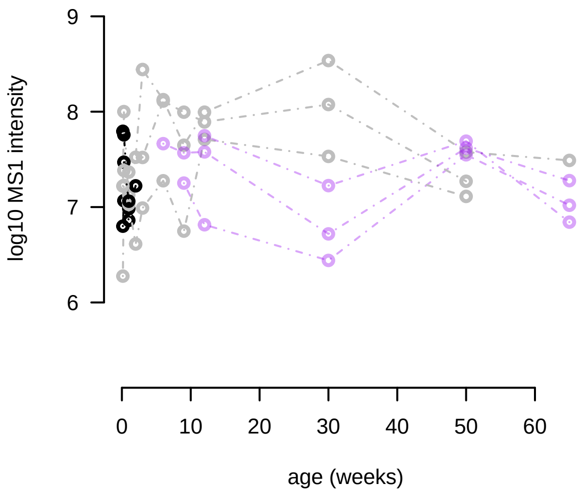

raw intensities log scale

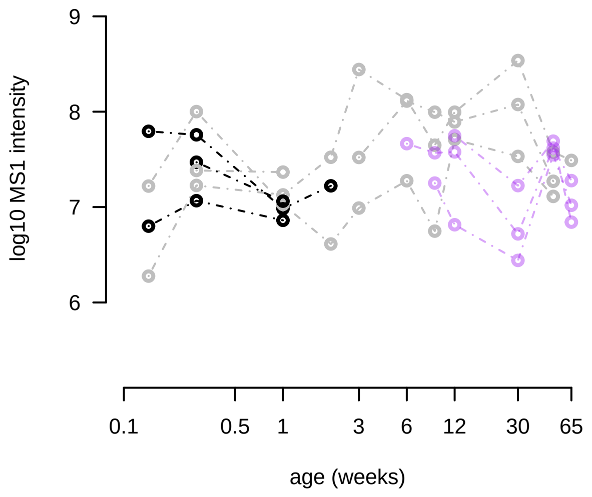

H1/2

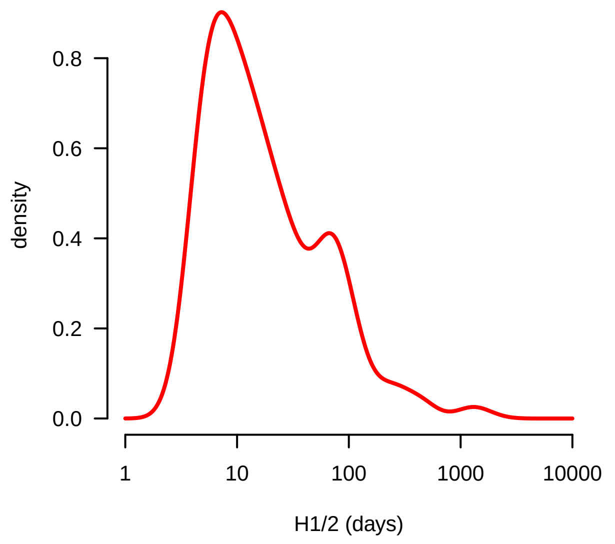

short pulse: ratio ln(L/H+1) log scale

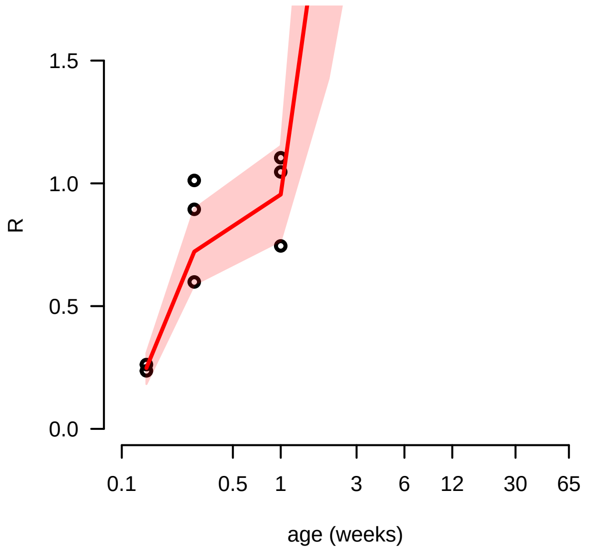

short pulse: ratio ln(L/H+1)

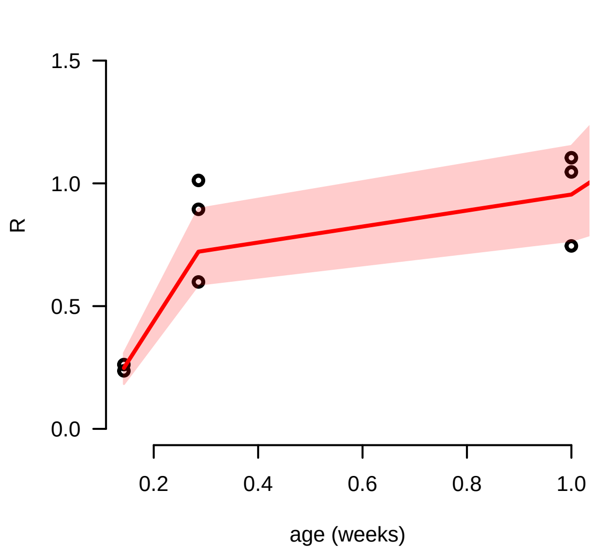

protein concentration log scale

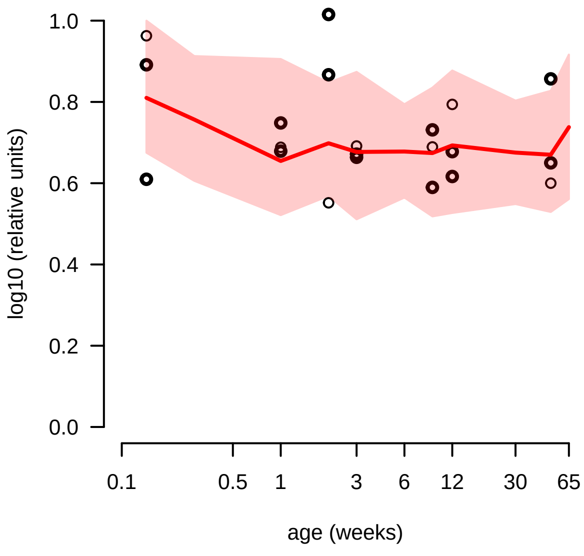

total protein log scale

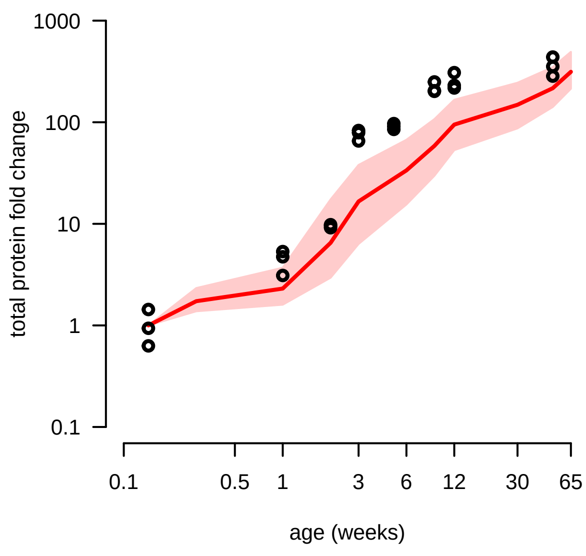

short pulse %H corrected log scale

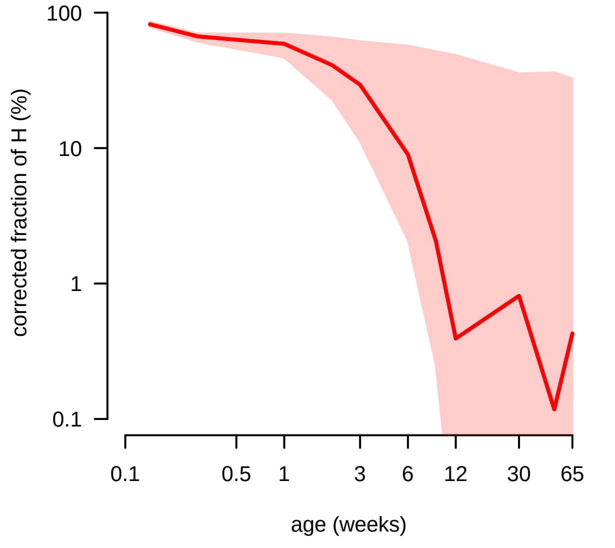

Q8BN58;Q8BN58−3;Q8BN58−2

>sp|Q8BN58|RHG28_MOUSE

Rho GTPase−activating protein 28

GN=Arhgap28

Med H1/2: 12.1 days

Q5 H1/2: 9.9 days

Q95 H1/2: 15.8 days

reliable model fit: yes

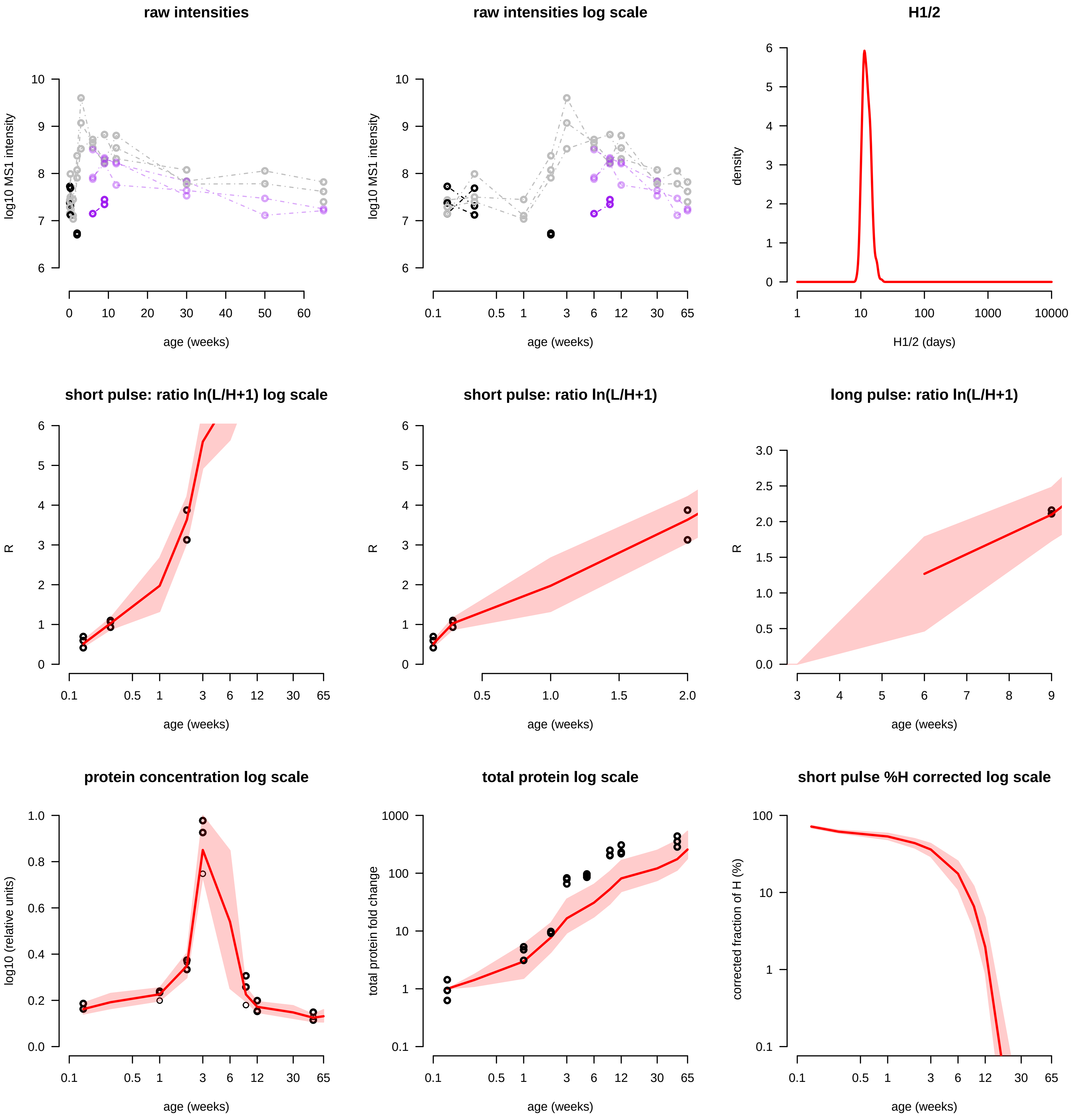

E9Q9W4

>sp|E9Q9W4|S27A6_MOUSE

Long−chain fatty acid transport protein 6

GN=Slc27a6

Med H1/2: 180.7 days

Q5 H1/2: 65.2 days

Q95 H1/2: 1568.5 days

reliable model fit: no

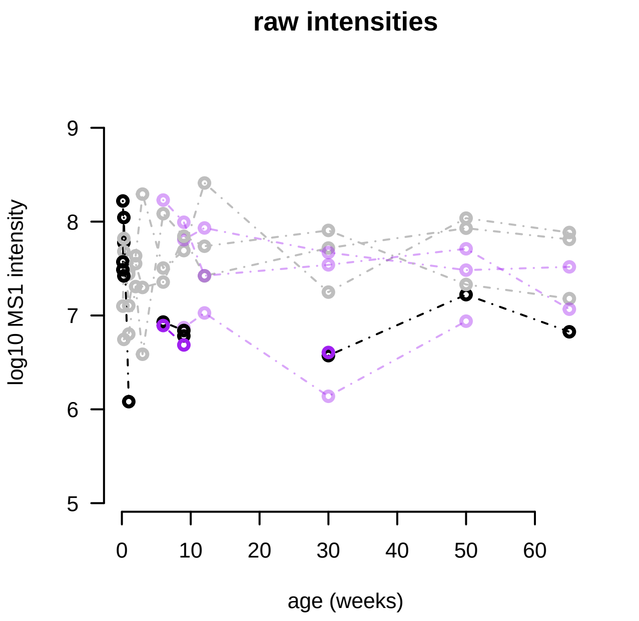

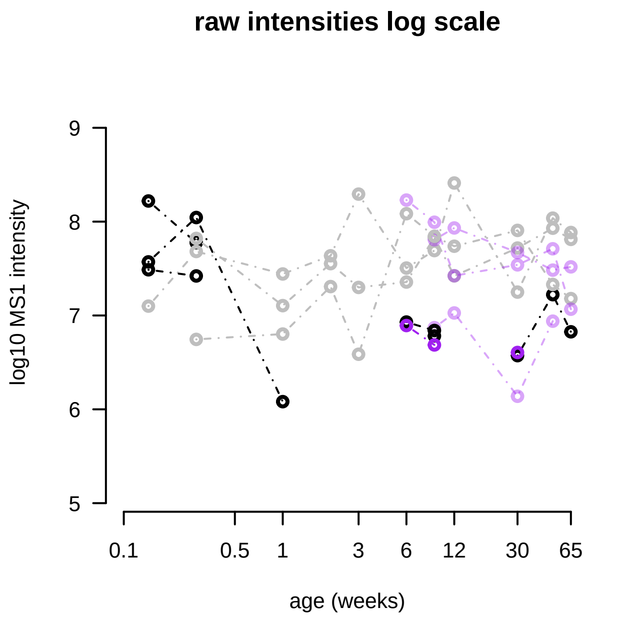

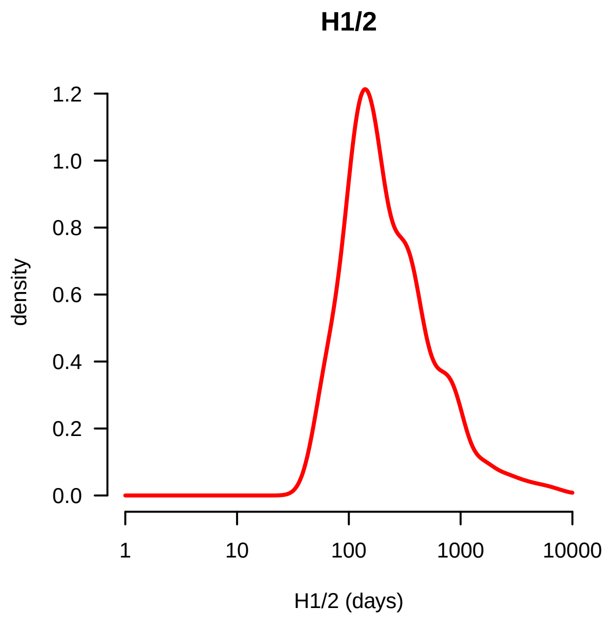

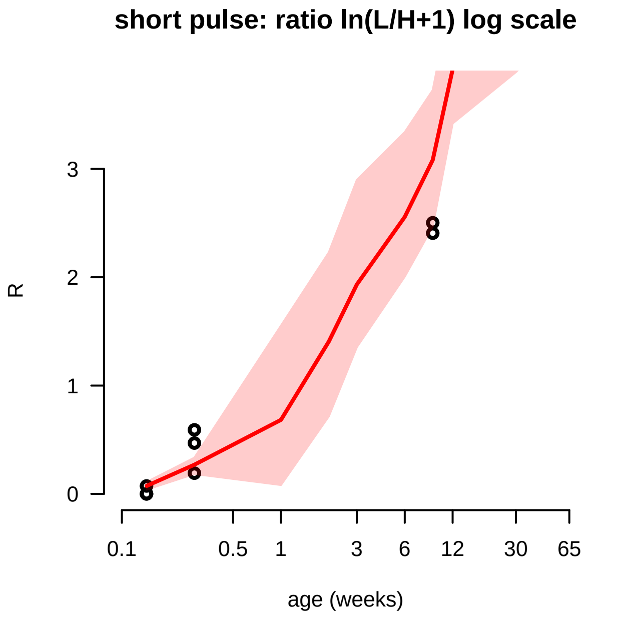

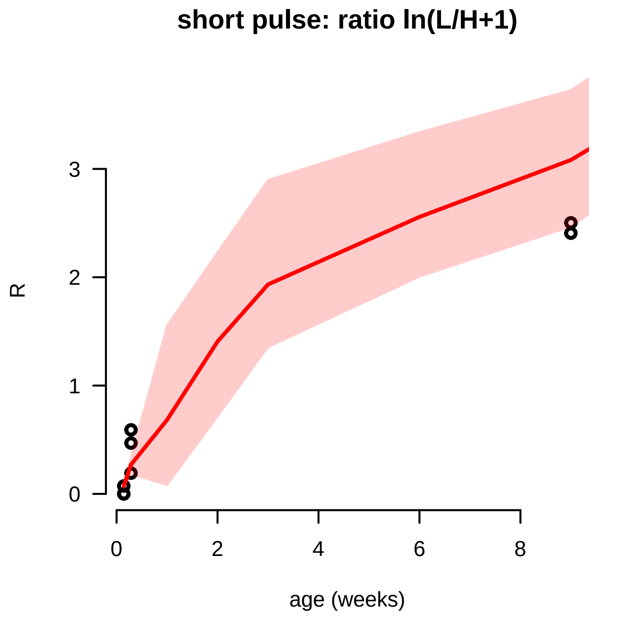

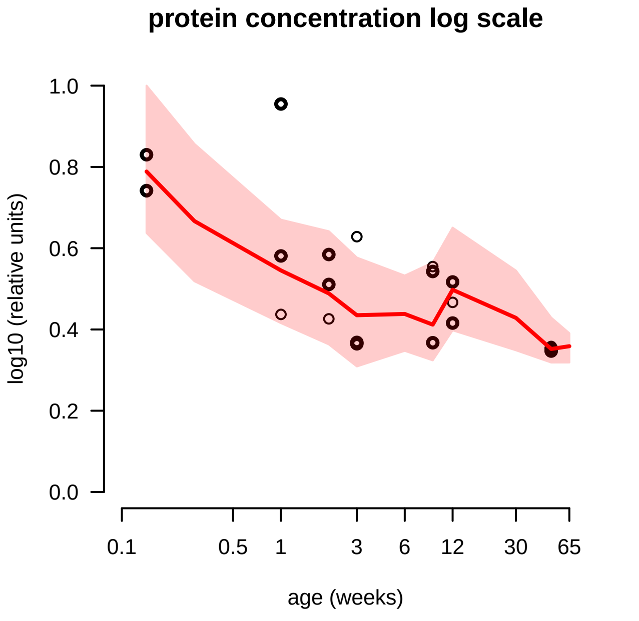

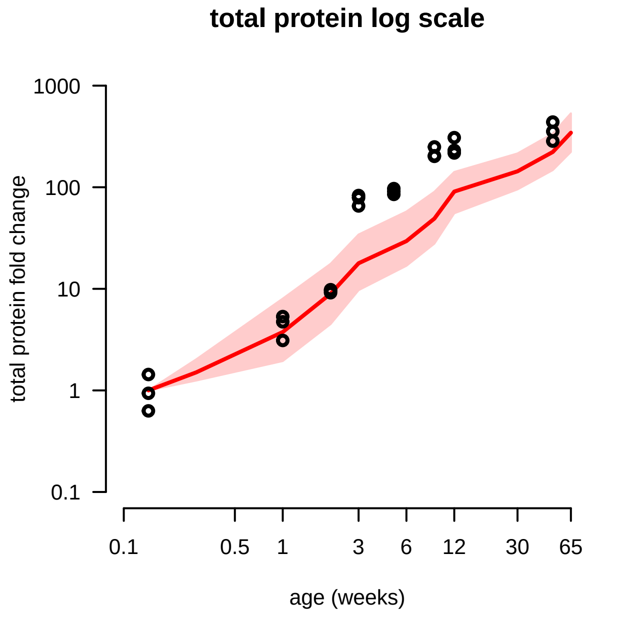

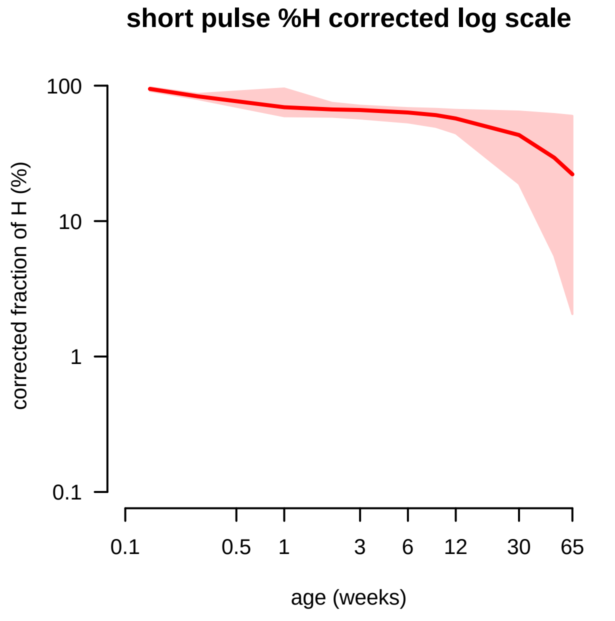

A2AWL7;A2AWL7−3;A2AWL7−2

>sp|A2AWL7|MGAP_MOUSE

MAX gene−associated protein

GN=Mga

Med H1/2: 11.4 days

Q5 H1/2: 8.4 days

Q95 H1/2: 16.9 days

reliable model fit: yes

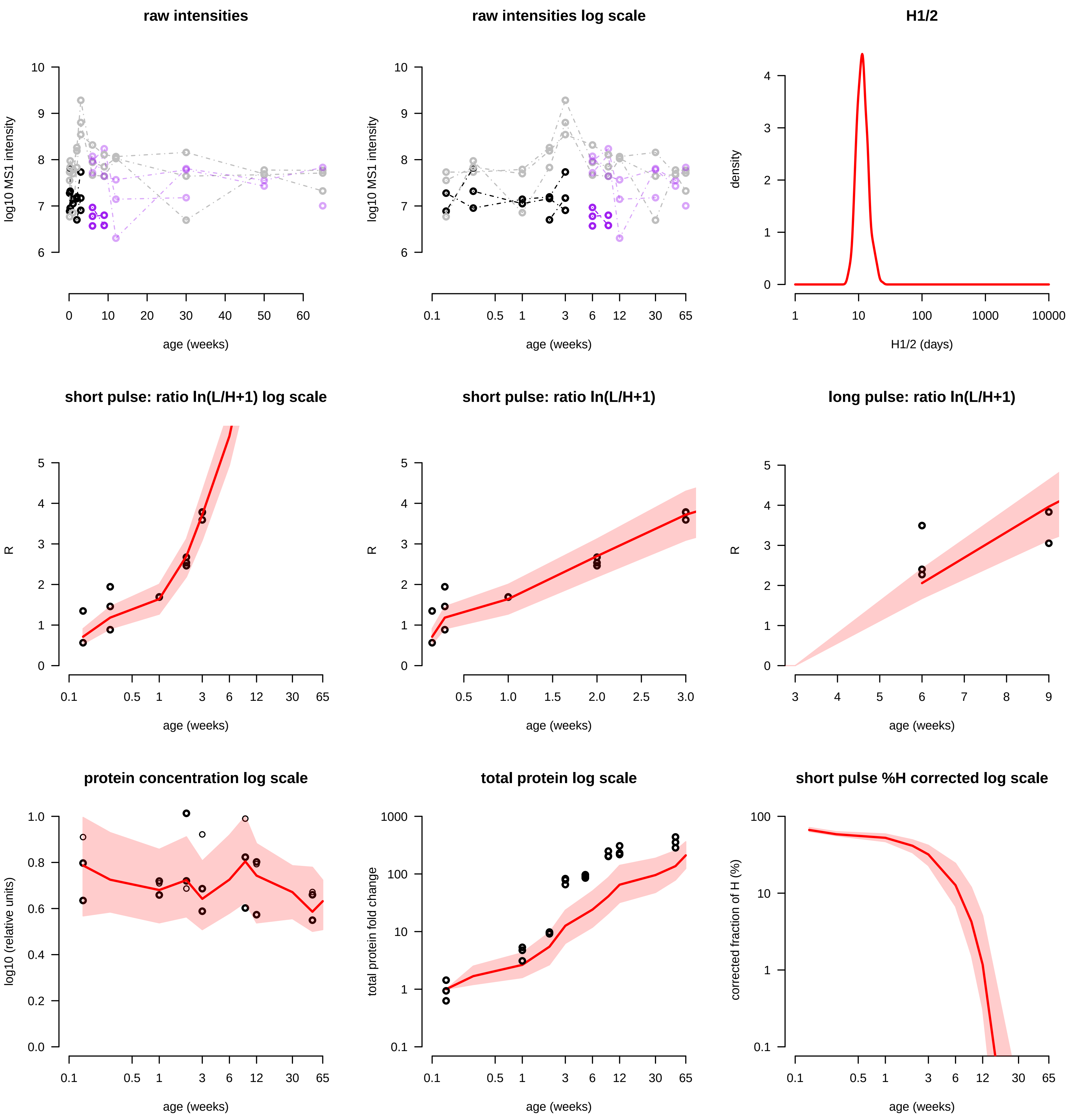

Q8K3Z9

>sp|Q8K3Z9|PO121_MOUSE

Nuclear envelope pore membrane protein POM 121

GN=Pom121

Med H1/2: 13 days

Q5 H1/2: 4.1 days

Q95 H1/2: 172.8 days

reliable model fit: yes

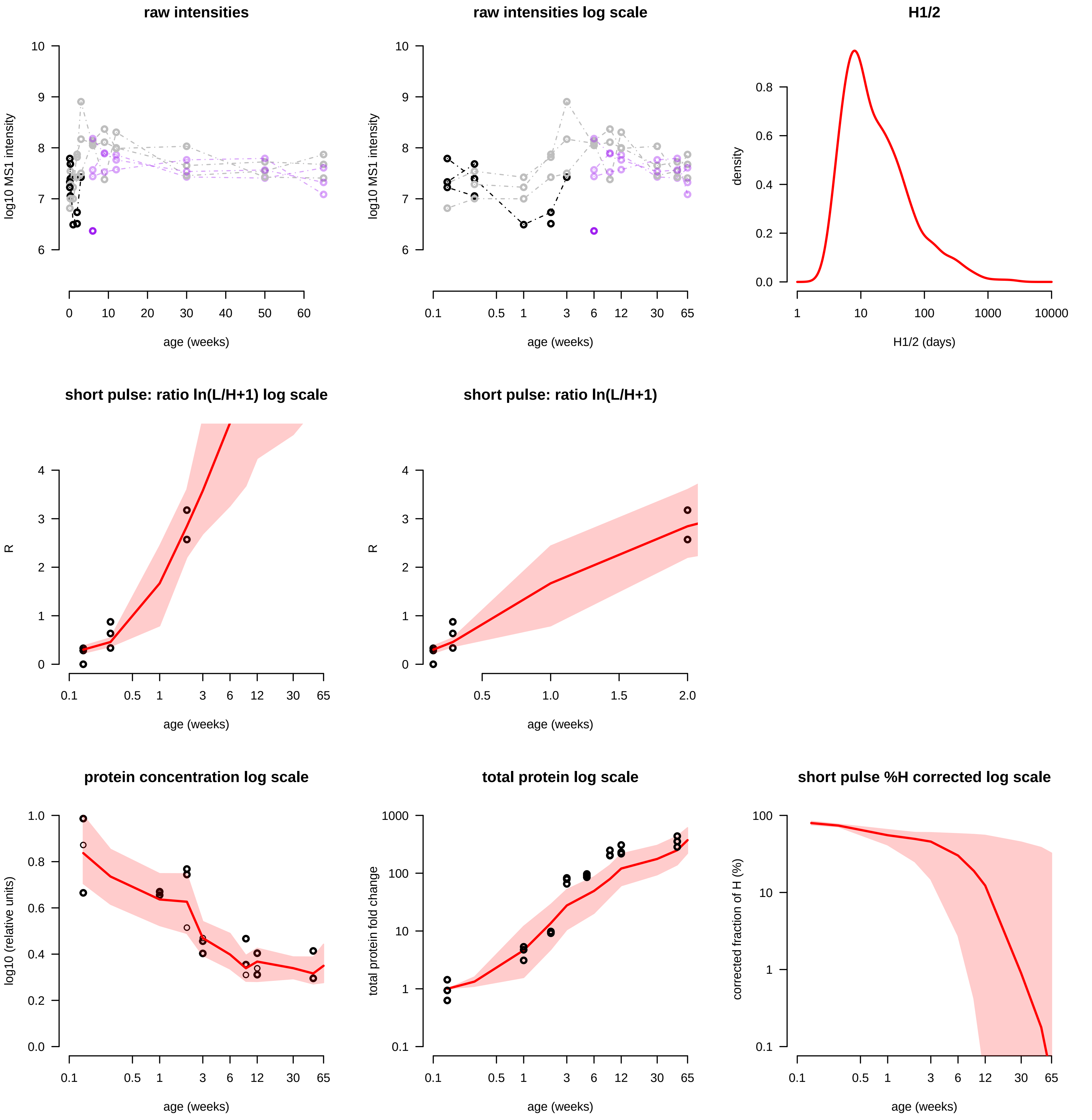

O88491

>sp|O88491|NSD1_MOUSE

Histone−lysine N−methyltransferase, H3 lysine−36 specific

GN=Nsd1

Med H1/2: 9.8 days

Q5 H1/2: 3.4 days

Q95 H1/2: 140.3 days

reliable model fit: yes

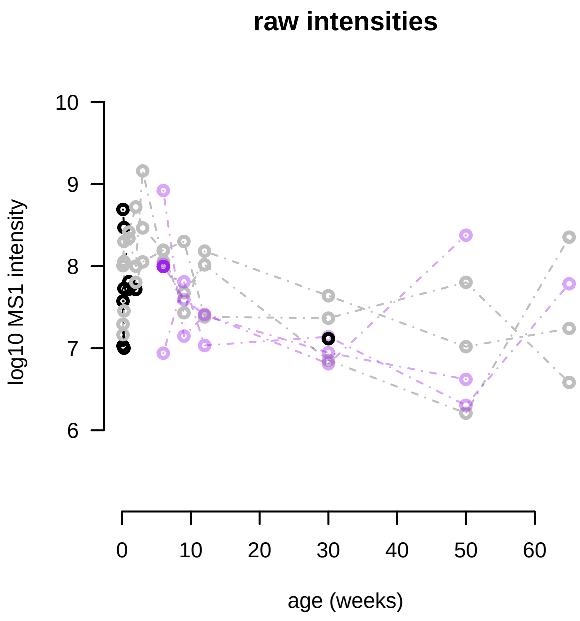

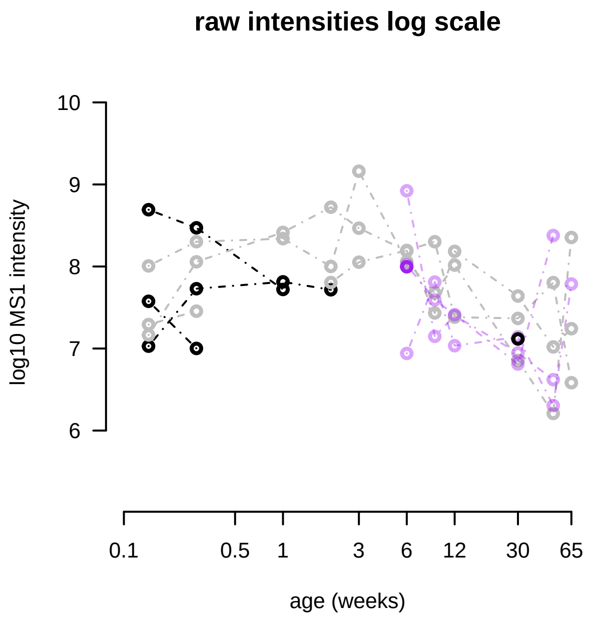

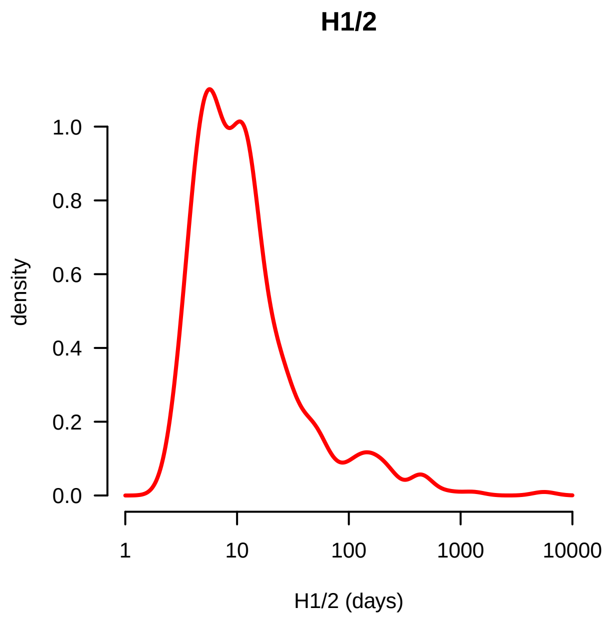

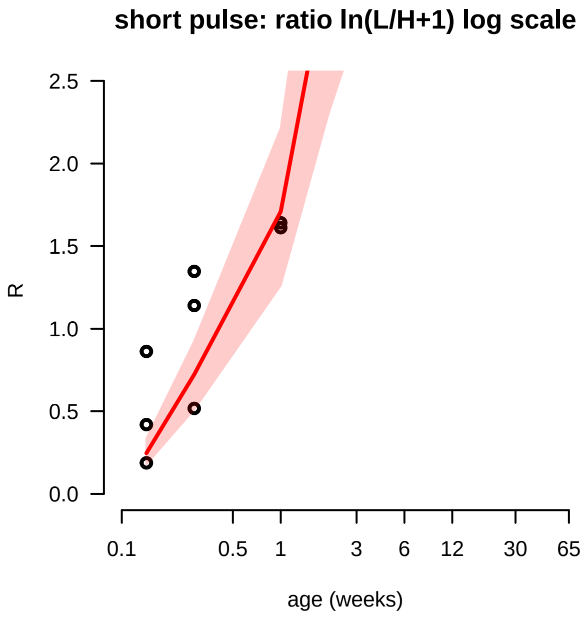

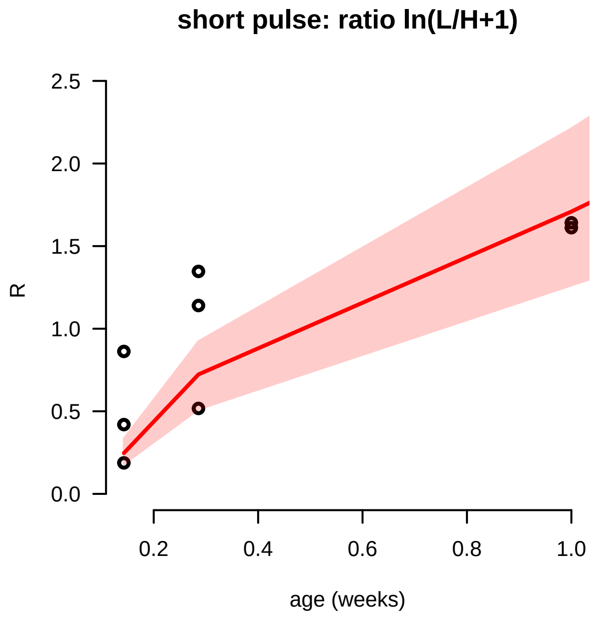

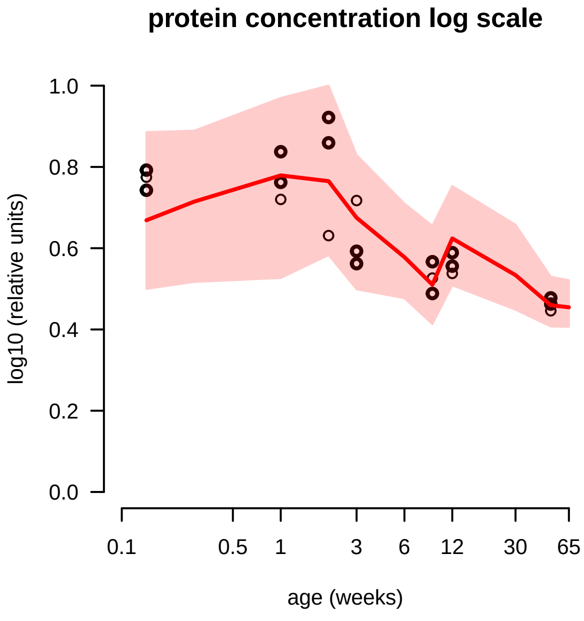

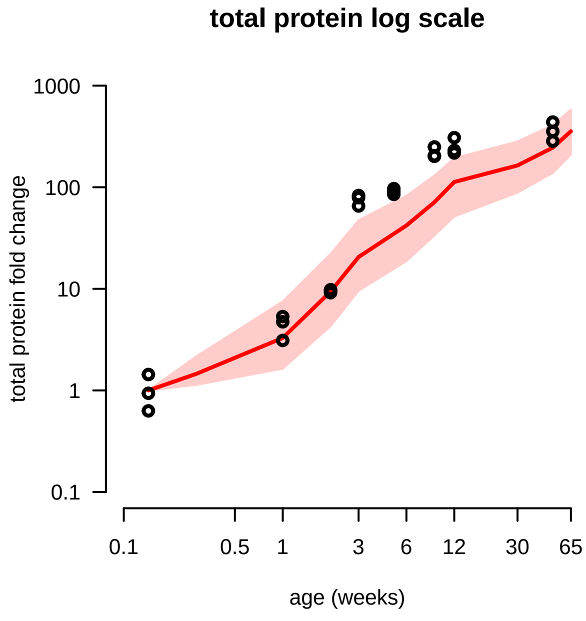

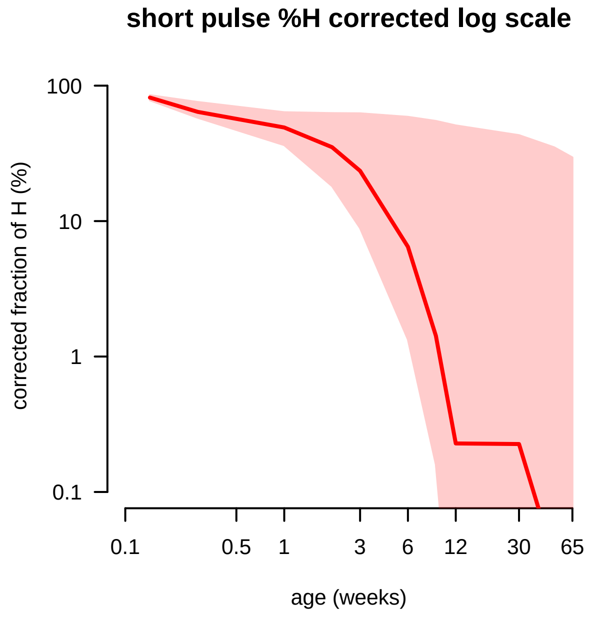

Q6NXJ0

>sp|Q6NXJ0|WWC2_MOUSE

Protein WWC2

GN=Wwc2

Med H1/2: 2151.4 days

Q5 H1/2: 772.8 days

Q95 H1/2: 71363.9 days

reliable model fit: no

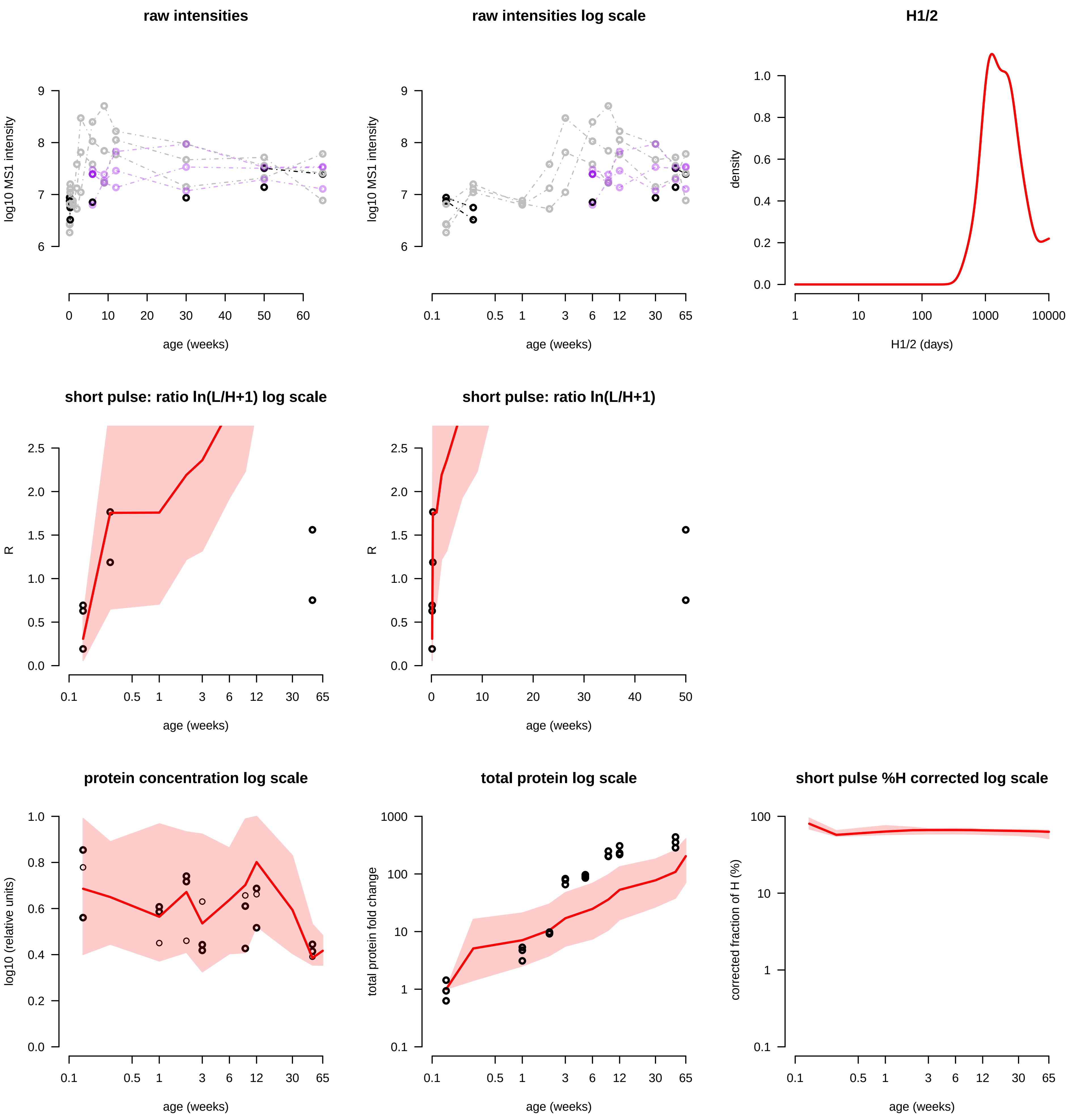

Q8BGS0−2;Q8BGS0

>sp|Q8BGS0−2|MAK16_MOUSE

Isoform 2 of Protein MAK16 homolog

GN=Mak16

Med H1/2: 8.5 days

Q5 H1/2: 3.9 days

Q95 H1/2: 38 days

reliable model fit: yes

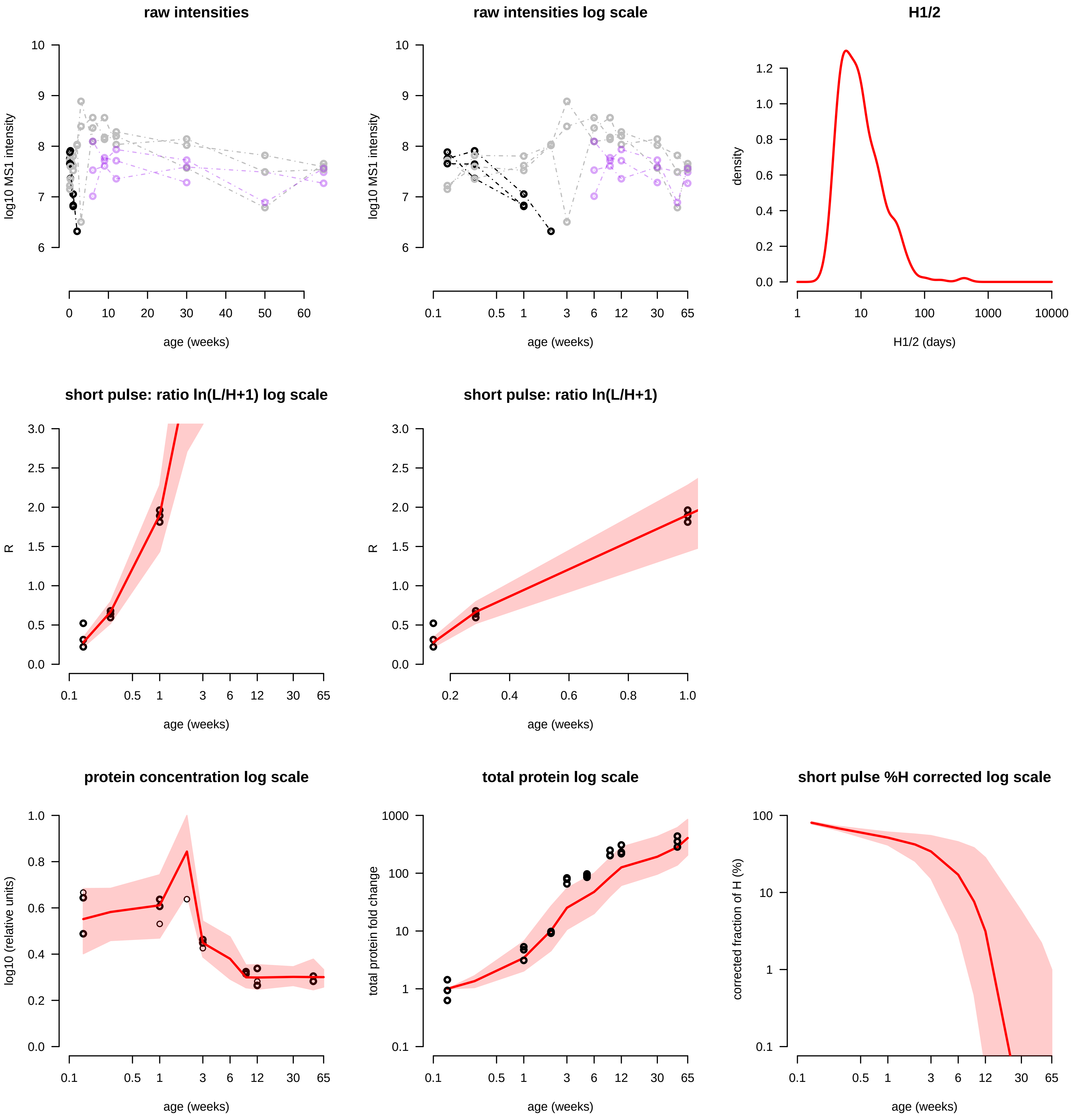

Q8BUN5

>sp|Q8BUN5|SMAD3_MOUSE

Mothers against decapentaplegic homolog 3

GN=Smad3

Med H1/2: 12.8 days

Q5 H1/2: 5.8 days

Q95 H1/2: 66.3 days

reliable model fit: yes

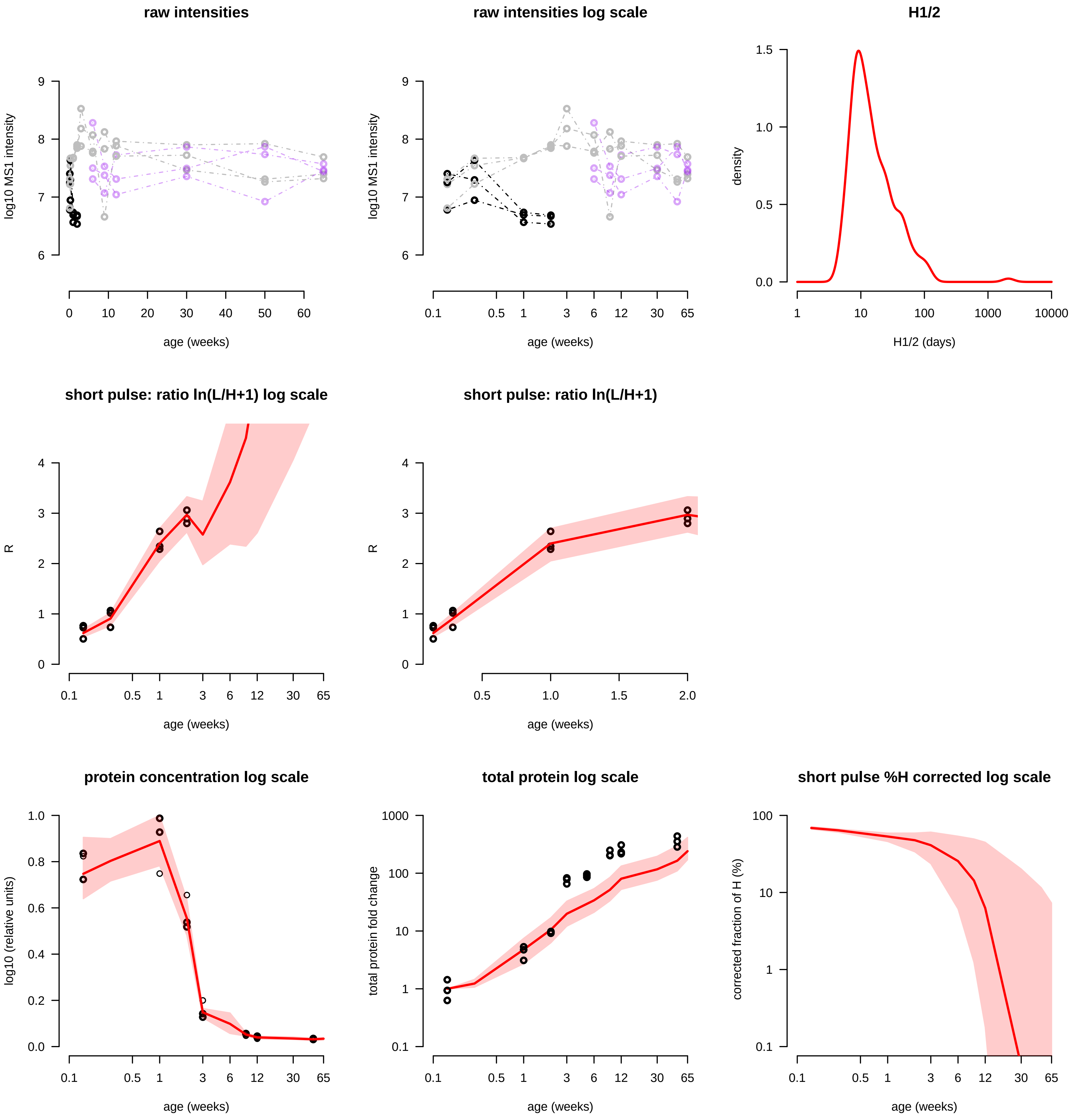

A2A4P0

>sp|A2A4P0|DHX8_MOUSE

ATP−dependent RNA helicase DHX8

GN=Dhx8

Med H1/2: 244.2 days

Q5 H1/2: 56.1 days

Q95 H1/2: 3317.9 days

reliable model fit: yes

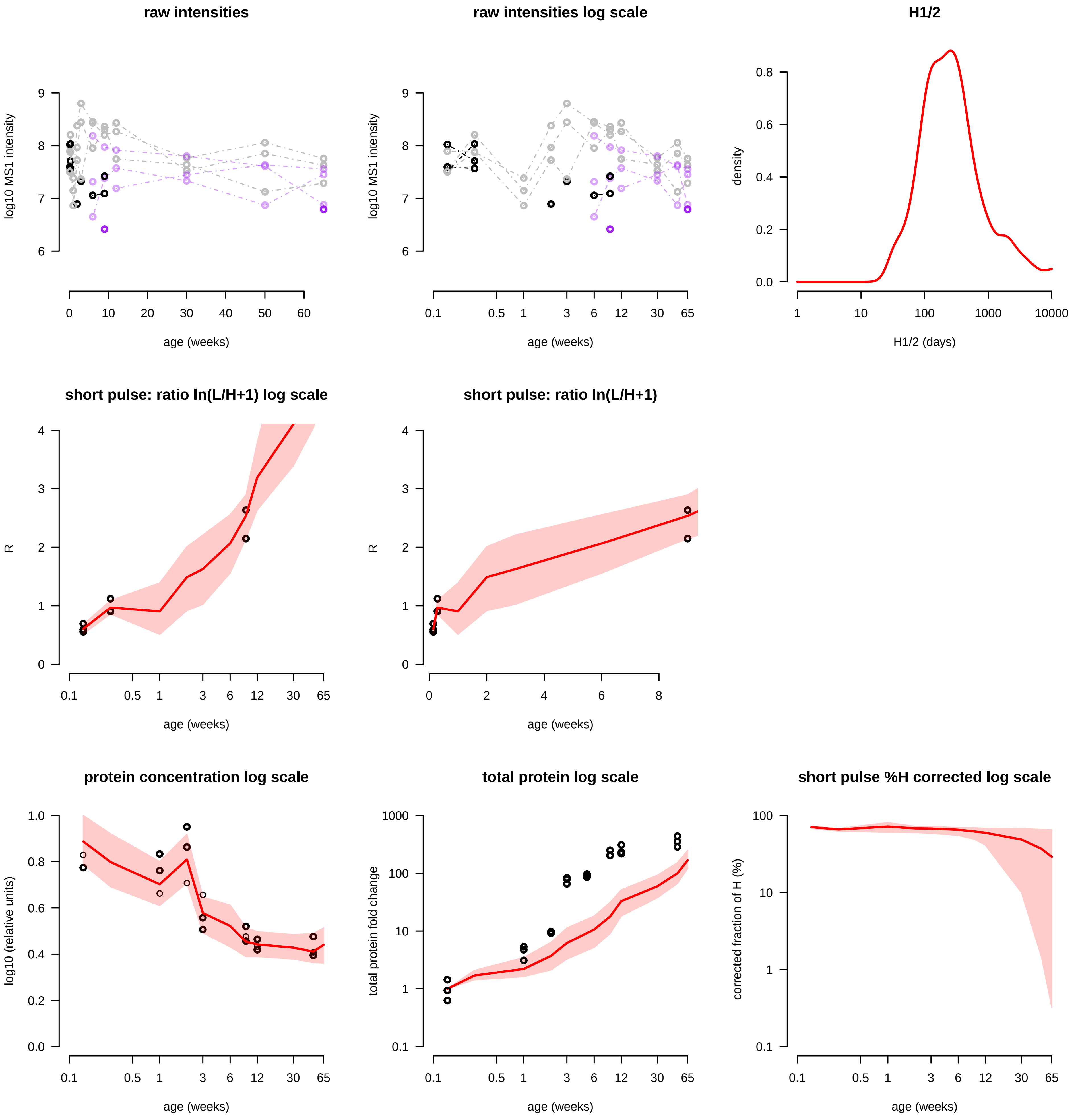

Q8CHT3

>sp|Q8CHT3|INT5_MOUSE

Integrator complex subunit 5

GN=Ints5

Med H1/2: 10.2 days

Q5 H1/2: 2.9 days

Q95 H1/2: 182.7 days

reliable model fit: yes

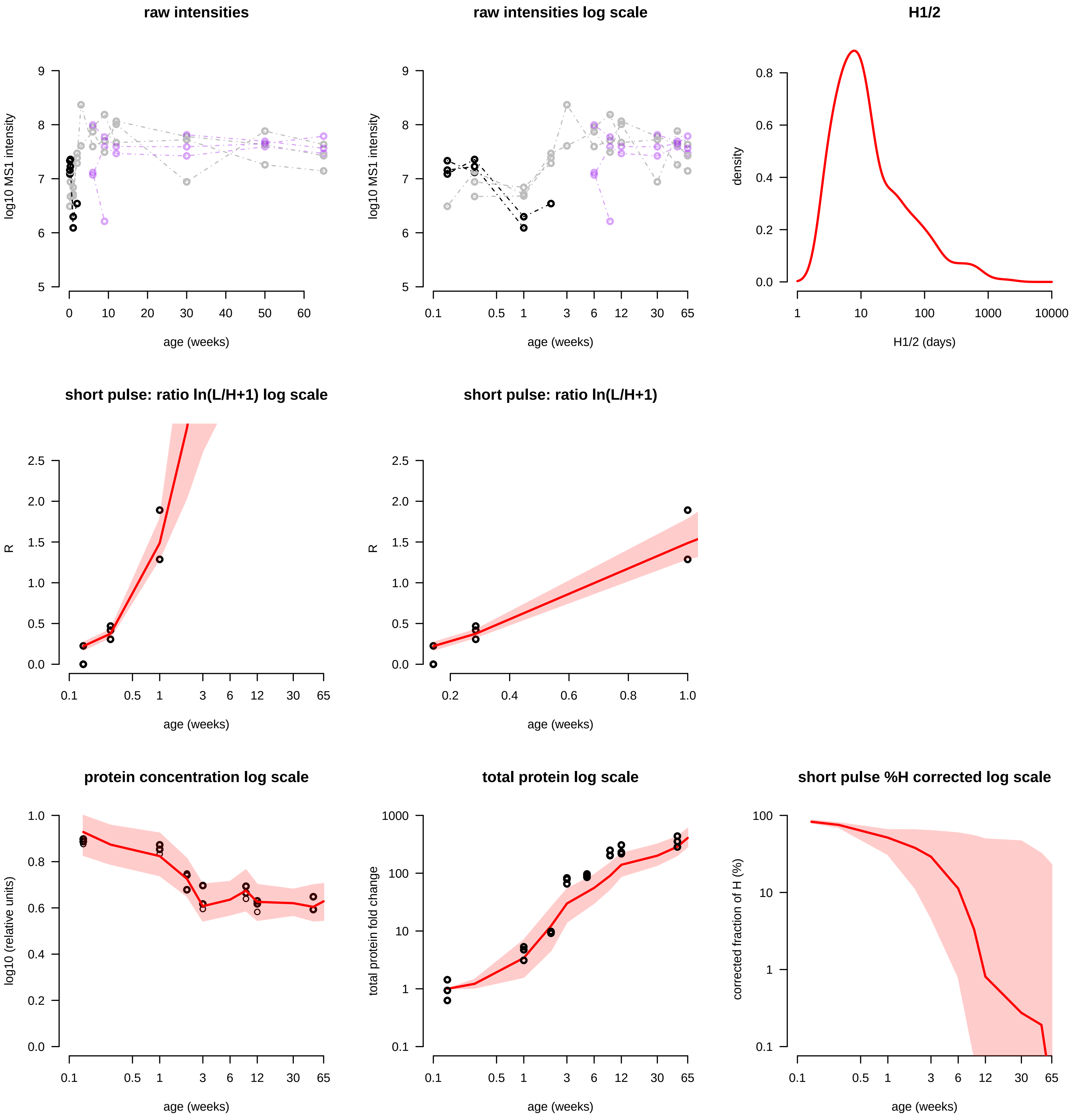

Q80XC2

>sp|Q80XC2|TRM61_MOUSE

tRNA (adenine(58)−N(1))−methyltransferase catalytic subunit TRMT61A

GN=Trmt61a

Med H1/2: 16.7 days

Q5 H1/2: 5.2 days

Q95 H1/2: 151.1 days

reliable model fit: yes

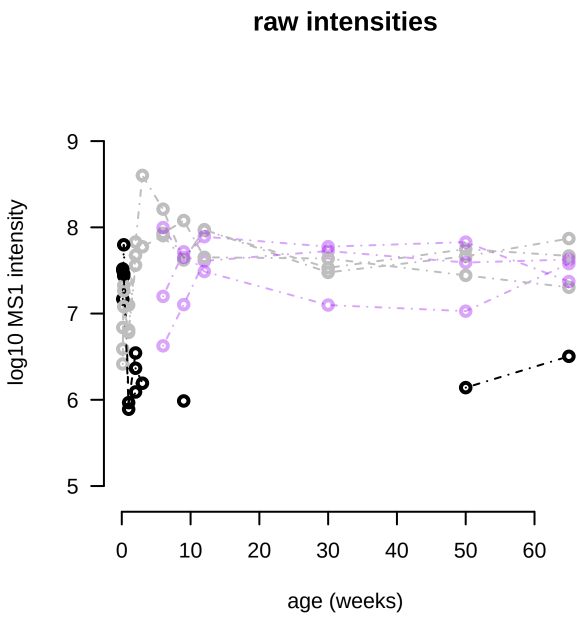

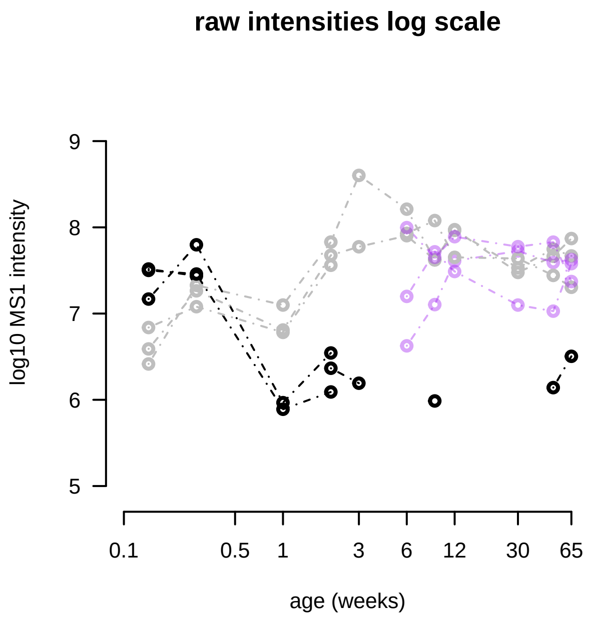

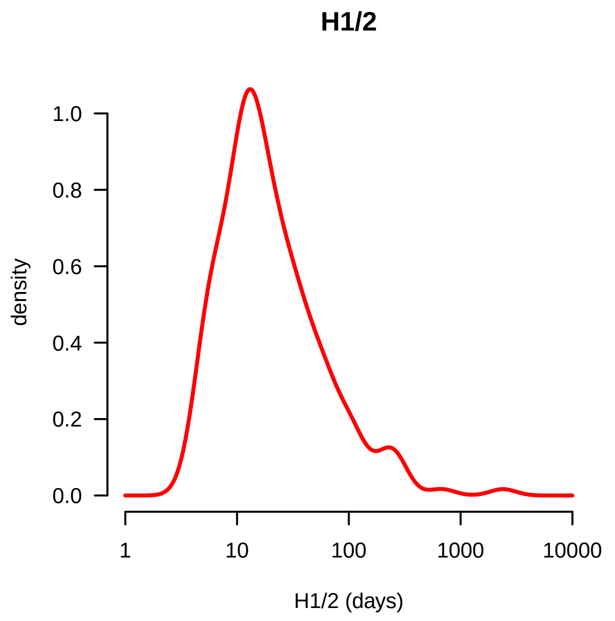

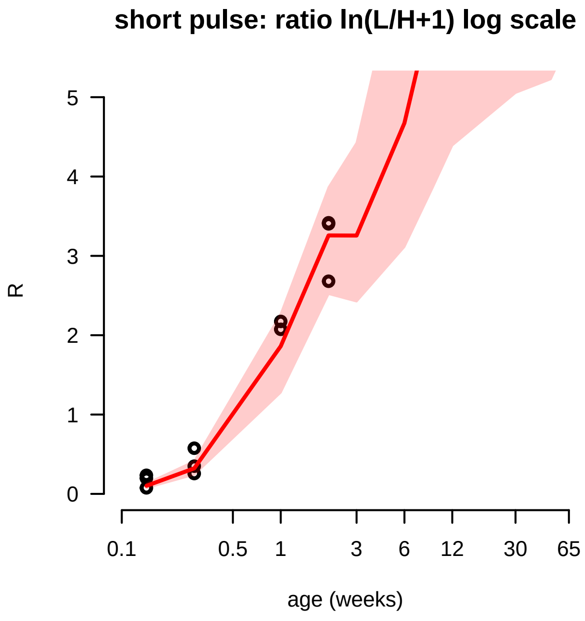

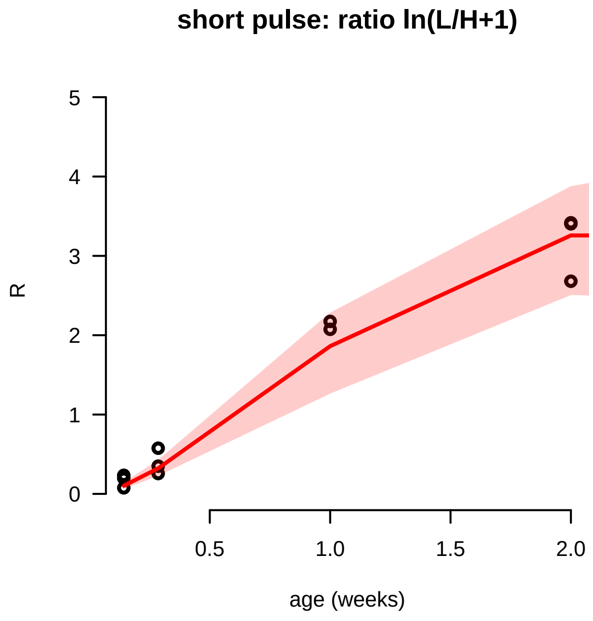

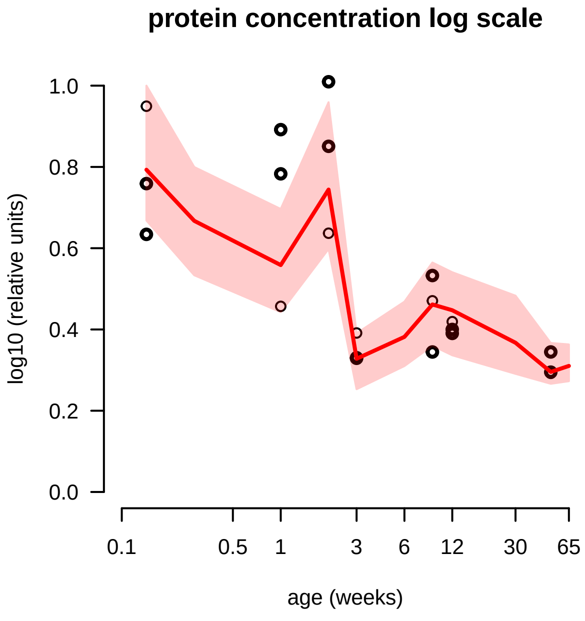

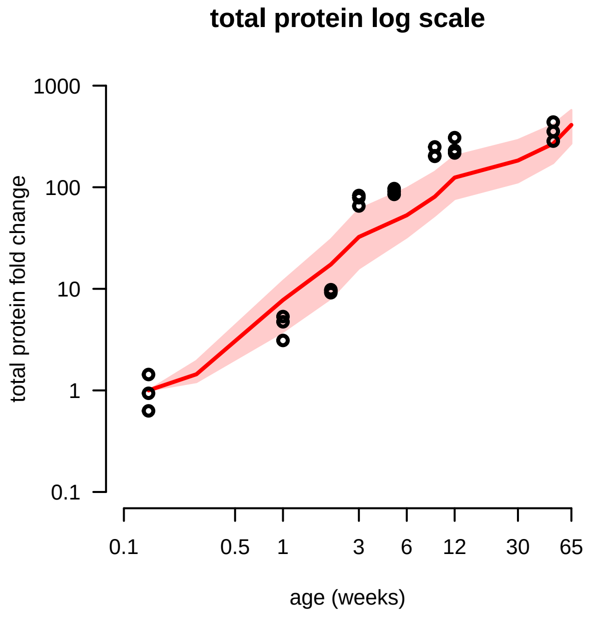

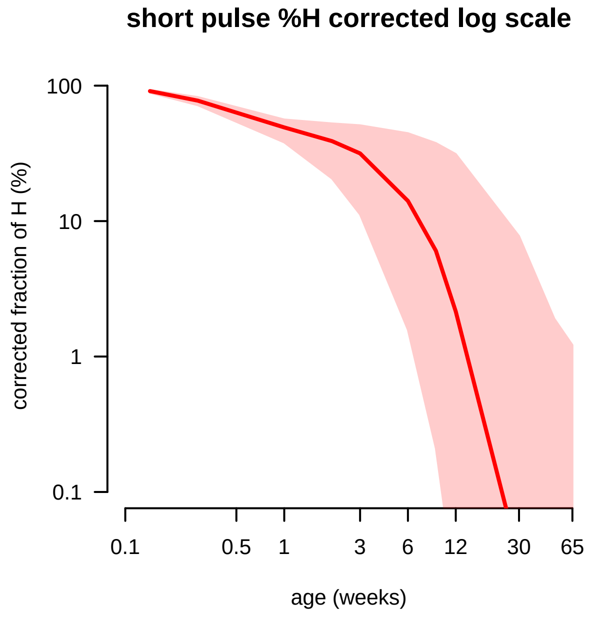

Q7TPW1

>sp|Q7TPW1|NEXN_MOUSE

Nexilin

GN=Nexn

Med H1/2: 6 days

Q5 H1/2: 3 days

Q95 H1/2: 27.5 days

reliable model fit: yes

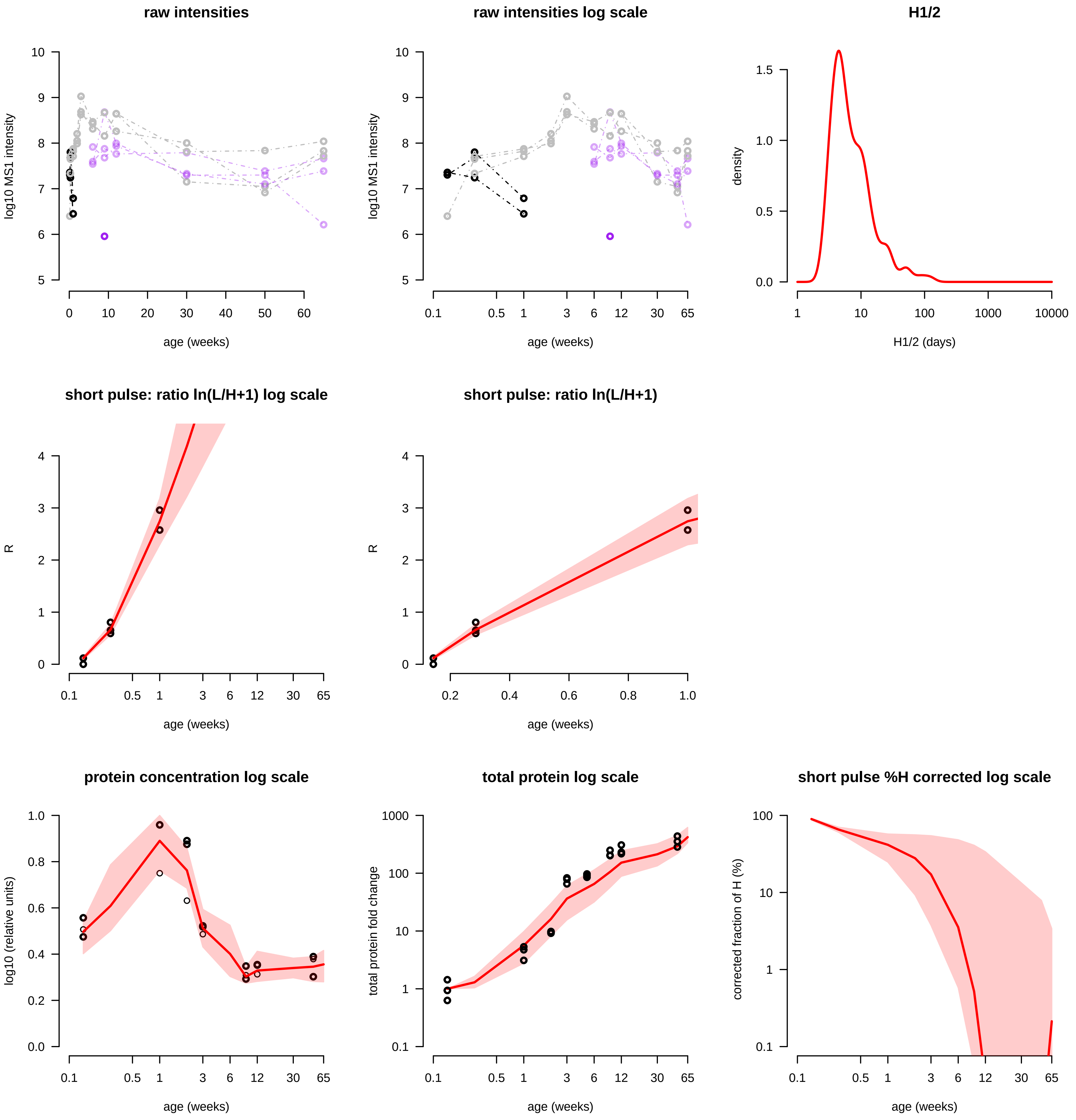

Q52KE7;Q52KE7−2

>sp|Q52KE7|CCNL1_MOUSE

Cyclin−L1

GN=Ccnl1

Med H1/2: 8.2 days

Q5 H1/2: 3 days

Q95 H1/2: 84.1 days

reliable model fit: yes

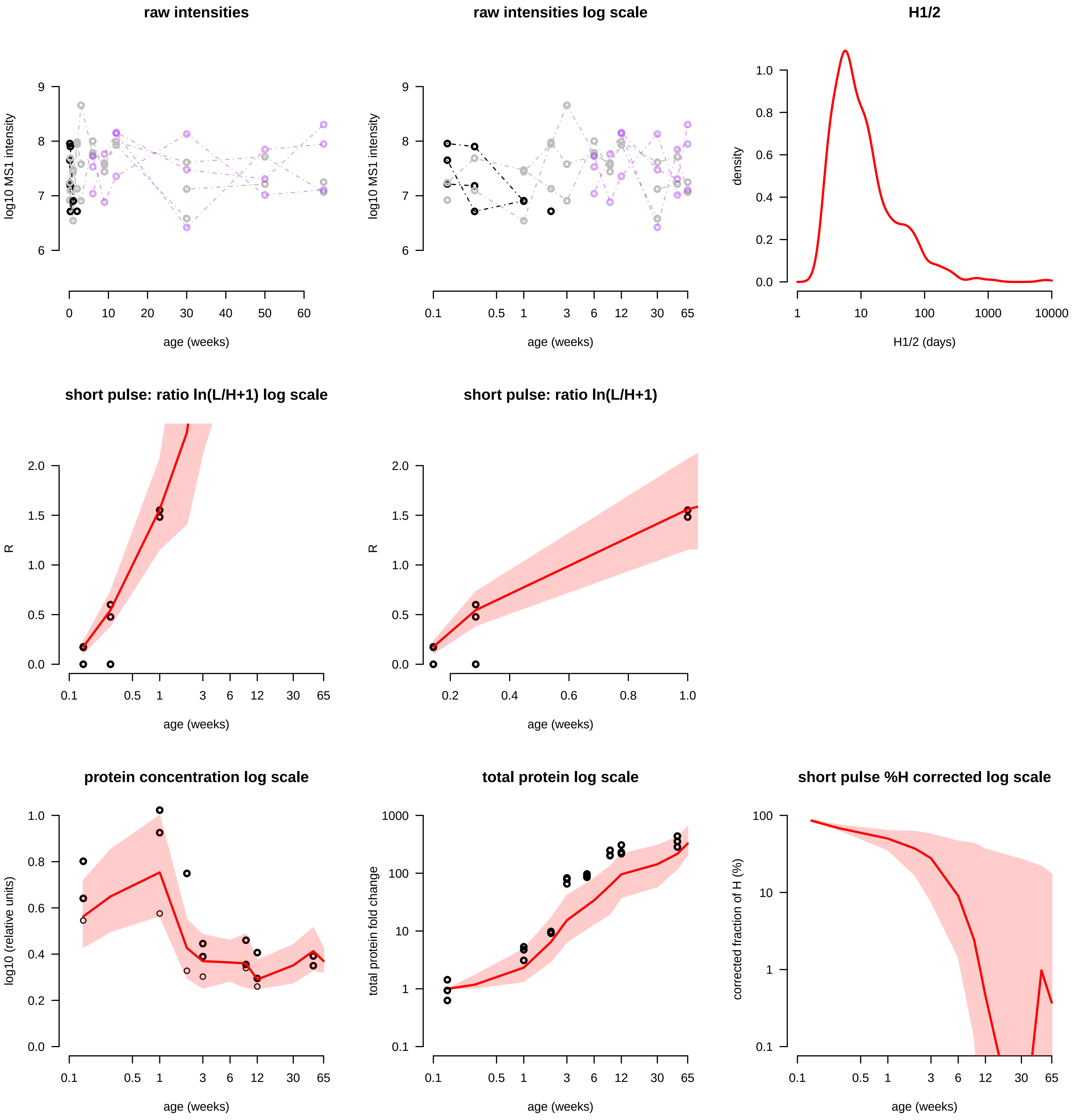

Q9R0Q4

>sp|Q9R0Q4|MO4L2_MOUSE

Mortality factor 4−like protein 2

GN=Morf4l2

Med H1/2: 860.6 days

Q5 H1/2: 249.4 days

Q95 H1/2: 15113 days

reliable model fit: yes

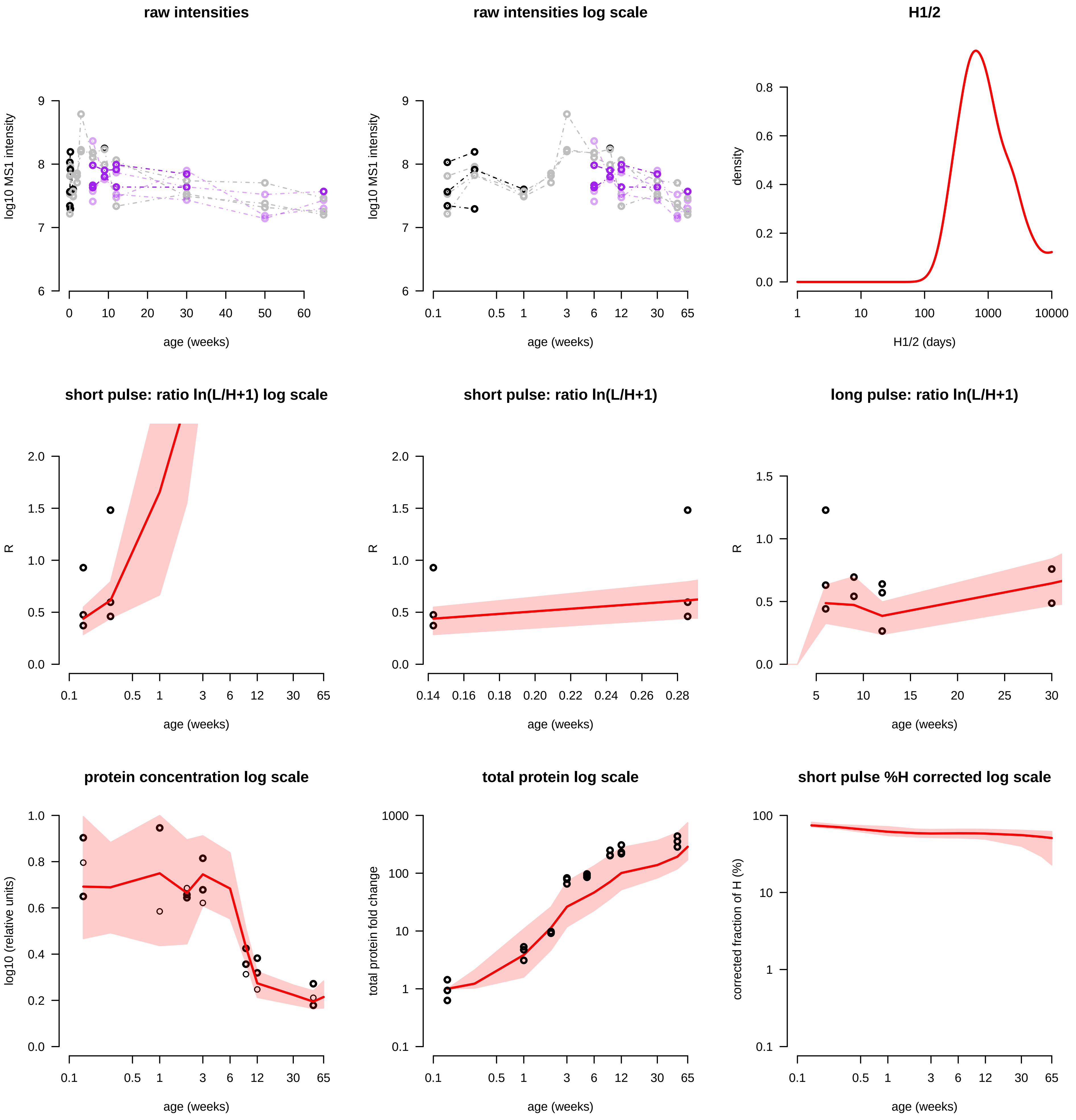

Q8C9X6;Q8C9X6−2

>sp|Q8C9X6|EPC1_MOUSE

Enhancer of polycomb homolog 1

GN=Epc1

Med H1/2: 15 days

Q5 H1/2: 11 days

Q95 H1/2: 27.8 days

reliable model fit: yes

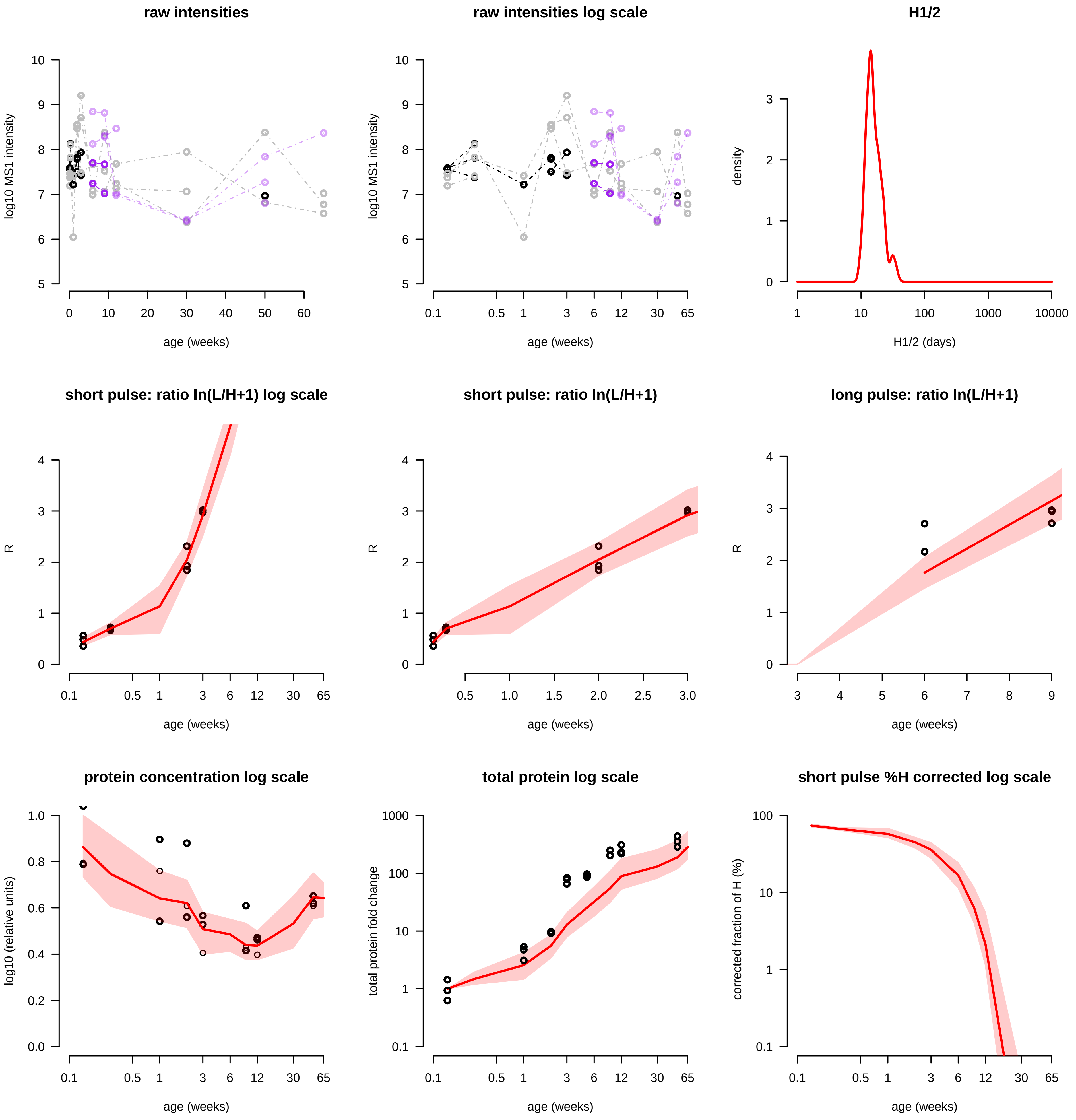

A2RT67

>sp|A2RT67|DEND3_MOUSE

DENN domain−containing protein 3

GN=Dennd3

Med H1/2: 20.7 days

Q5 H1/2: 6 days

Q95 H1/2: 291 days

reliable model fit: yes

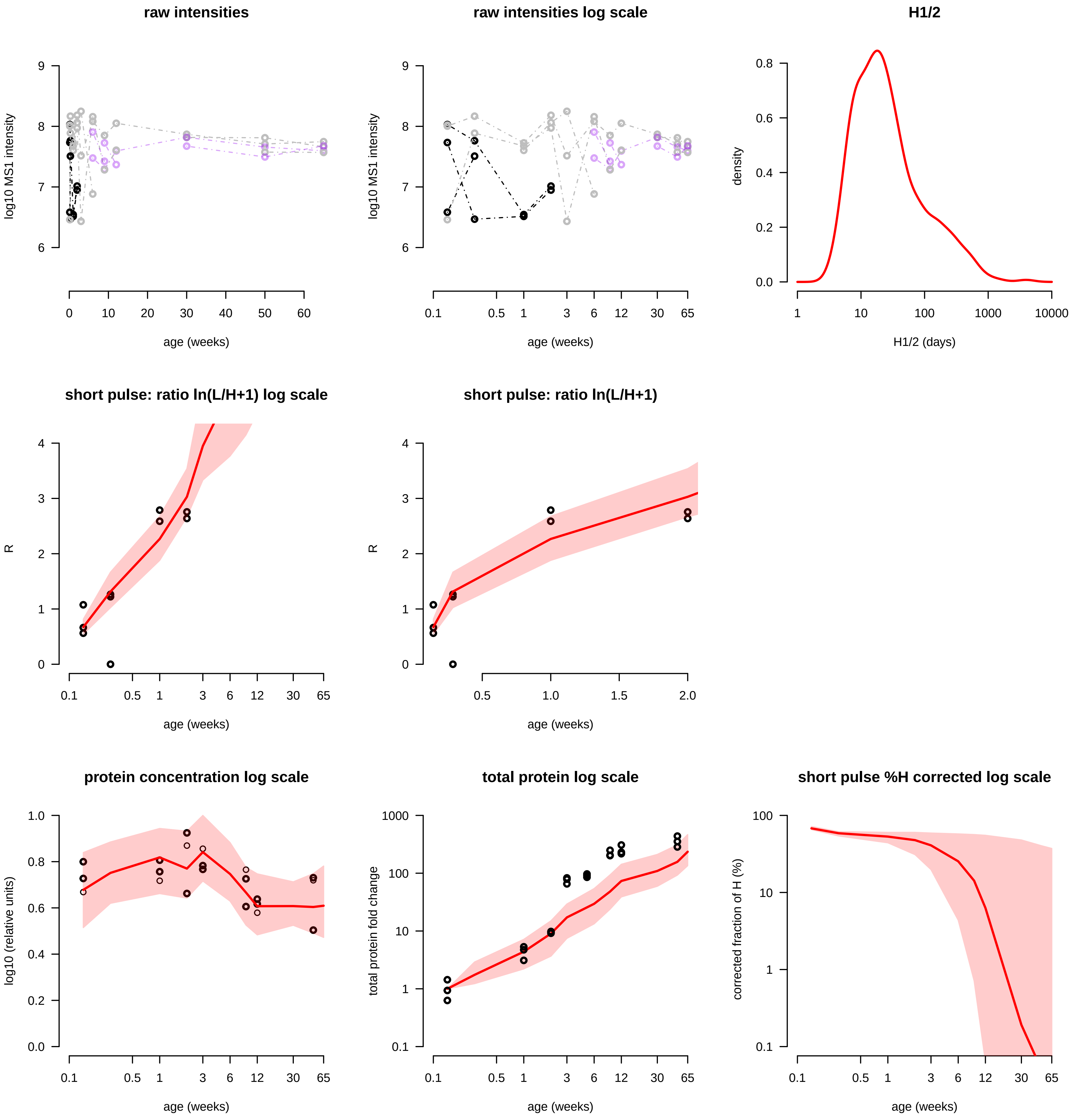

Q8BZM1

>sp|Q8BZM1|GLMN_MOUSE

Glomulin

GN=Glmn

Med H1/2: 7.9 days

Q5   H1/2: 3.5 days

Q95 H1/2: 30.8 days

reliable model fit: yes

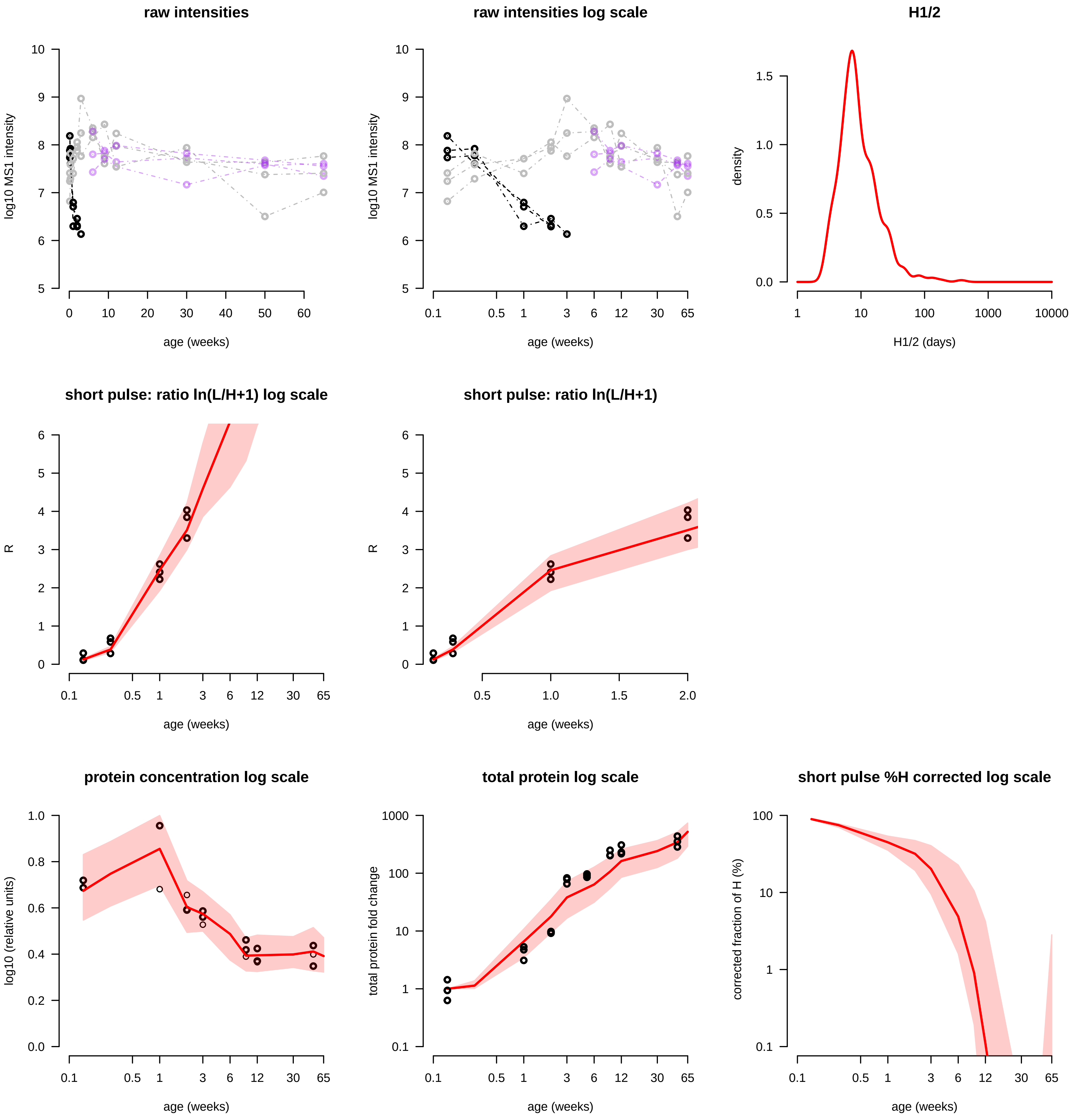

Q60778

>sp|Q60778|IKBB_MOUSE

NF−kappa−B inhibitor beta

GN=Nfkbib

Med H1/2: 20.3 days

Q5 H1/2: 5.4 days

Q95 H1/2: 272.2 days

reliable model fit: yes

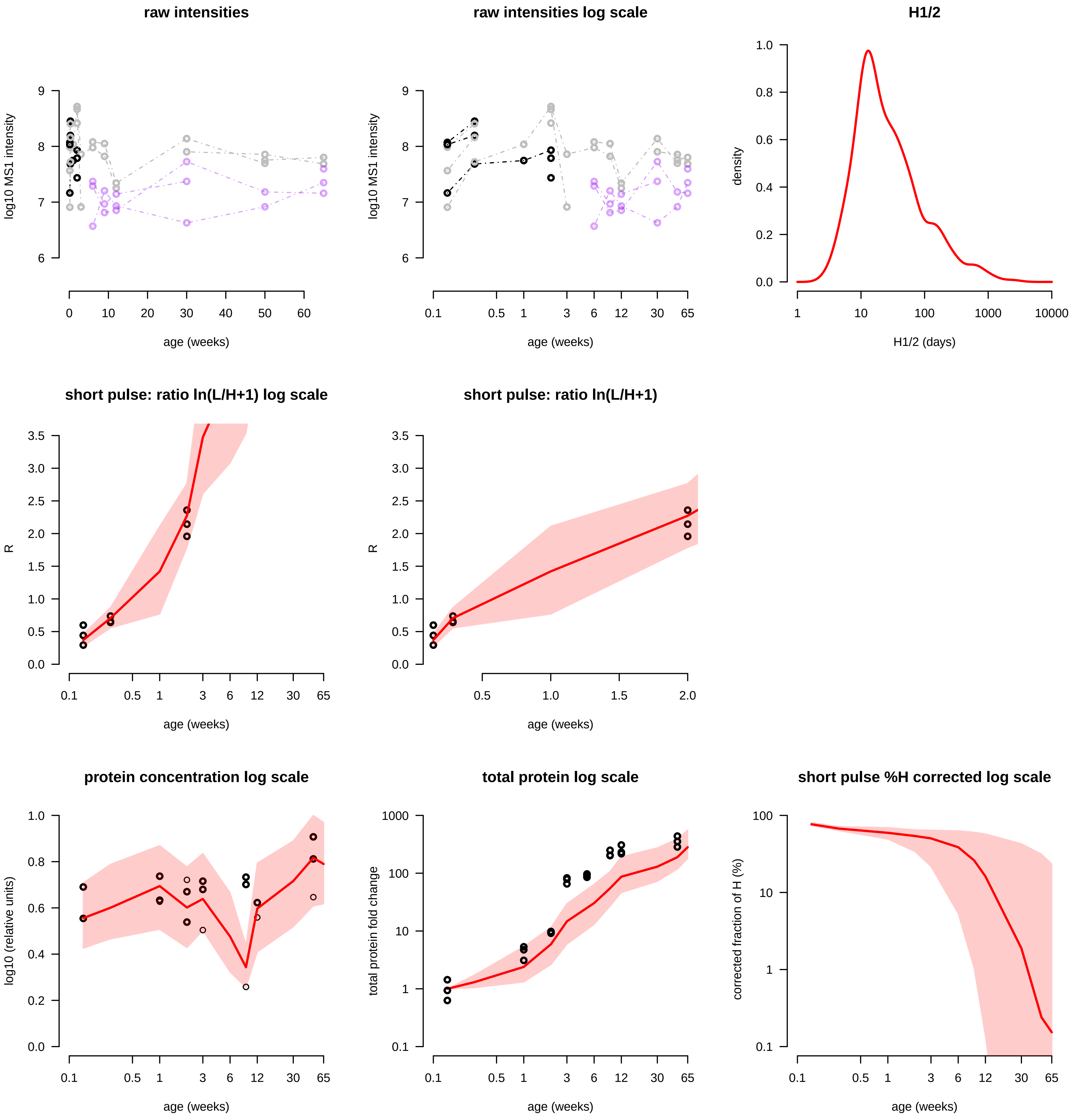

Q6ZQF0

>sp|Q6ZQF0|TOPB1_MOUSE

DNA topoisomerase 2−binding protein 1

GN=Topbp1

Med H1/2: 7.8 days

Q5 H1/2: 3.5 days

Q95 H1/2: 63.9 days

reliable model fit: yes

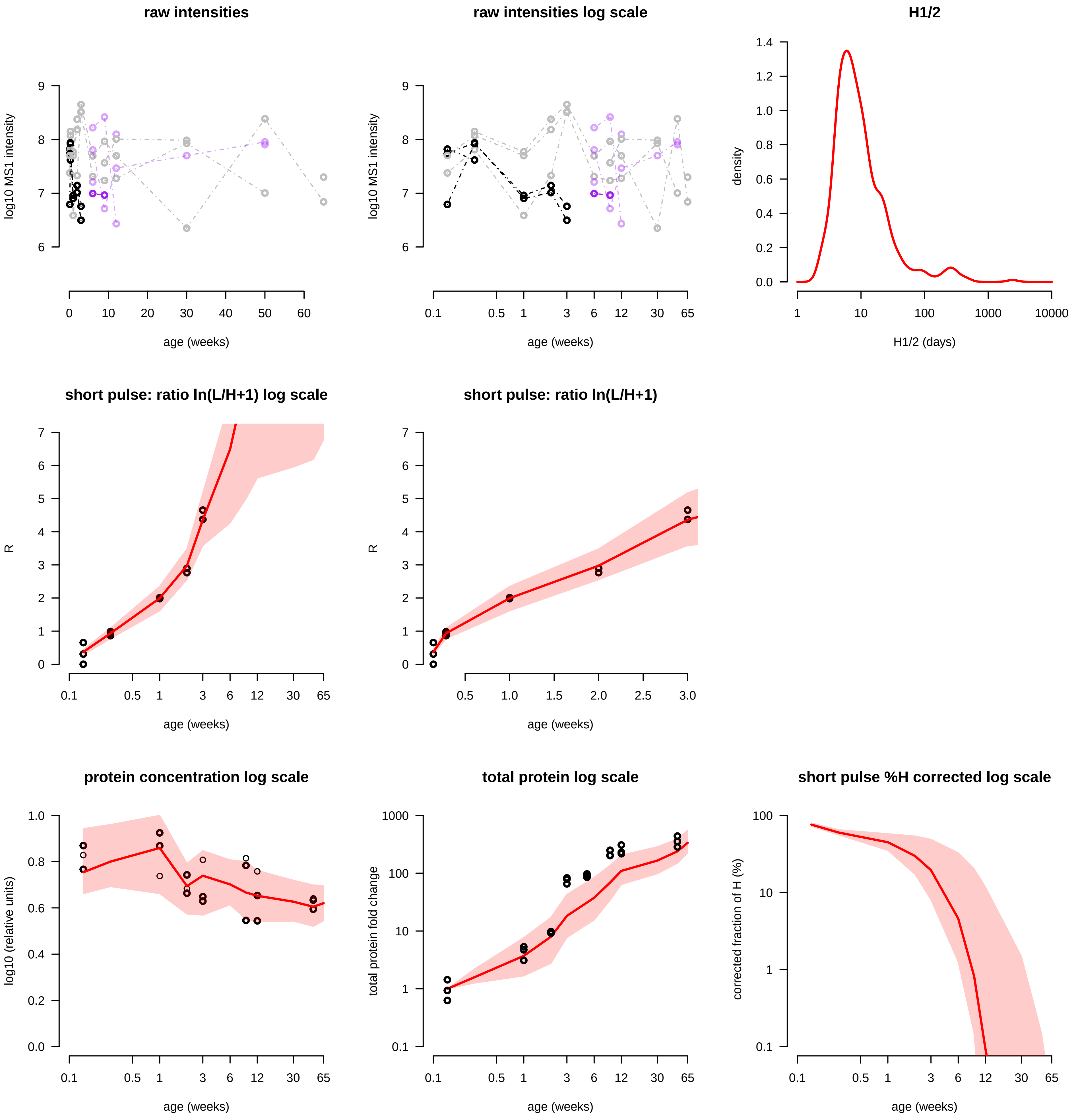

Q9WUR9

>sp|Q9WUR9|KAD4_MOUSE

Adenylate kinase 4, mitochondrial

GN=Ak4

Med H1/2: 5.1 days

Q5 H1/2: 3 days

Q95 H1/2: 16.2 days

reliable model fit: yes

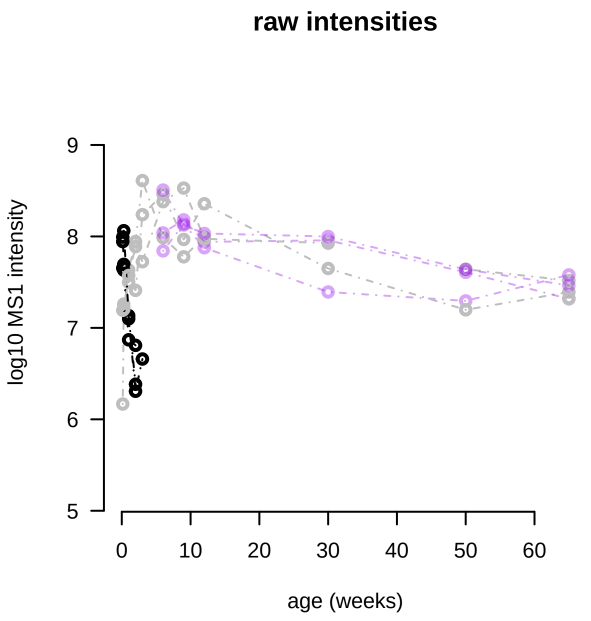

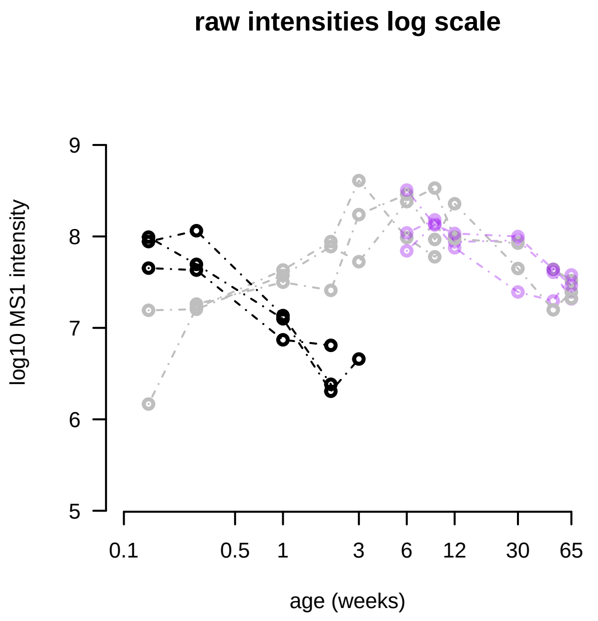

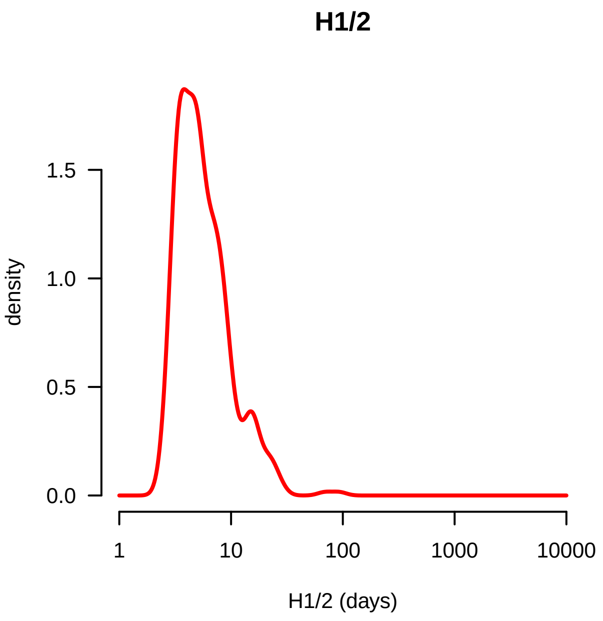

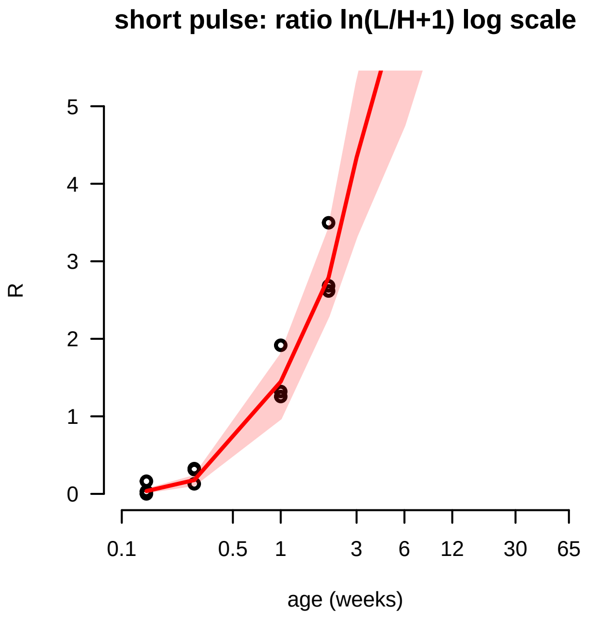

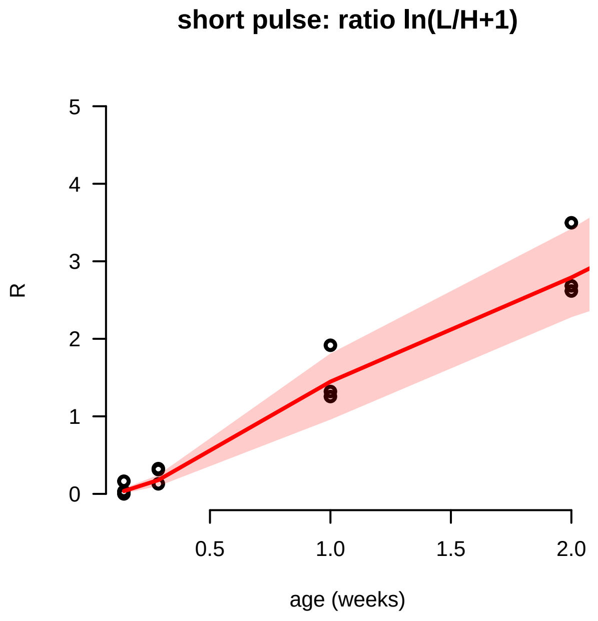

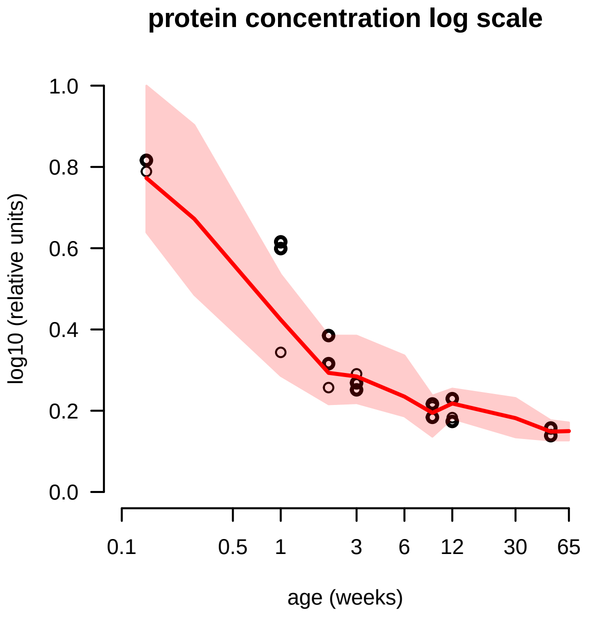

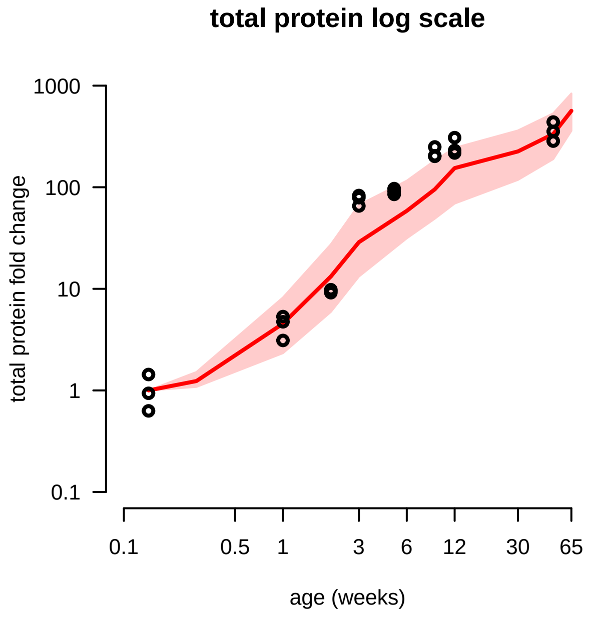

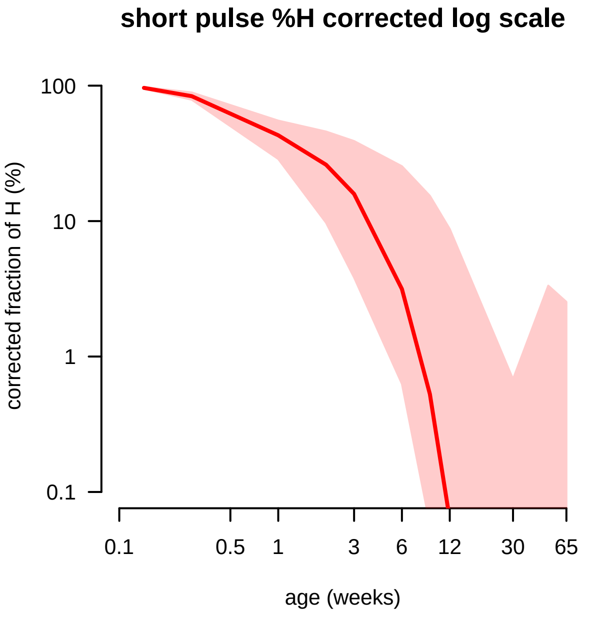

O35516;O35516−2

>sp|O35516|NOTC2_MOUSE

Neurogenic locus notch homolog protein 2

GN=Notch2

Med H1/2: 2083.7 days

Q5 H1/2: 658.7 days

Q95 H1/2: 23191.4 days

reliable model fit: no

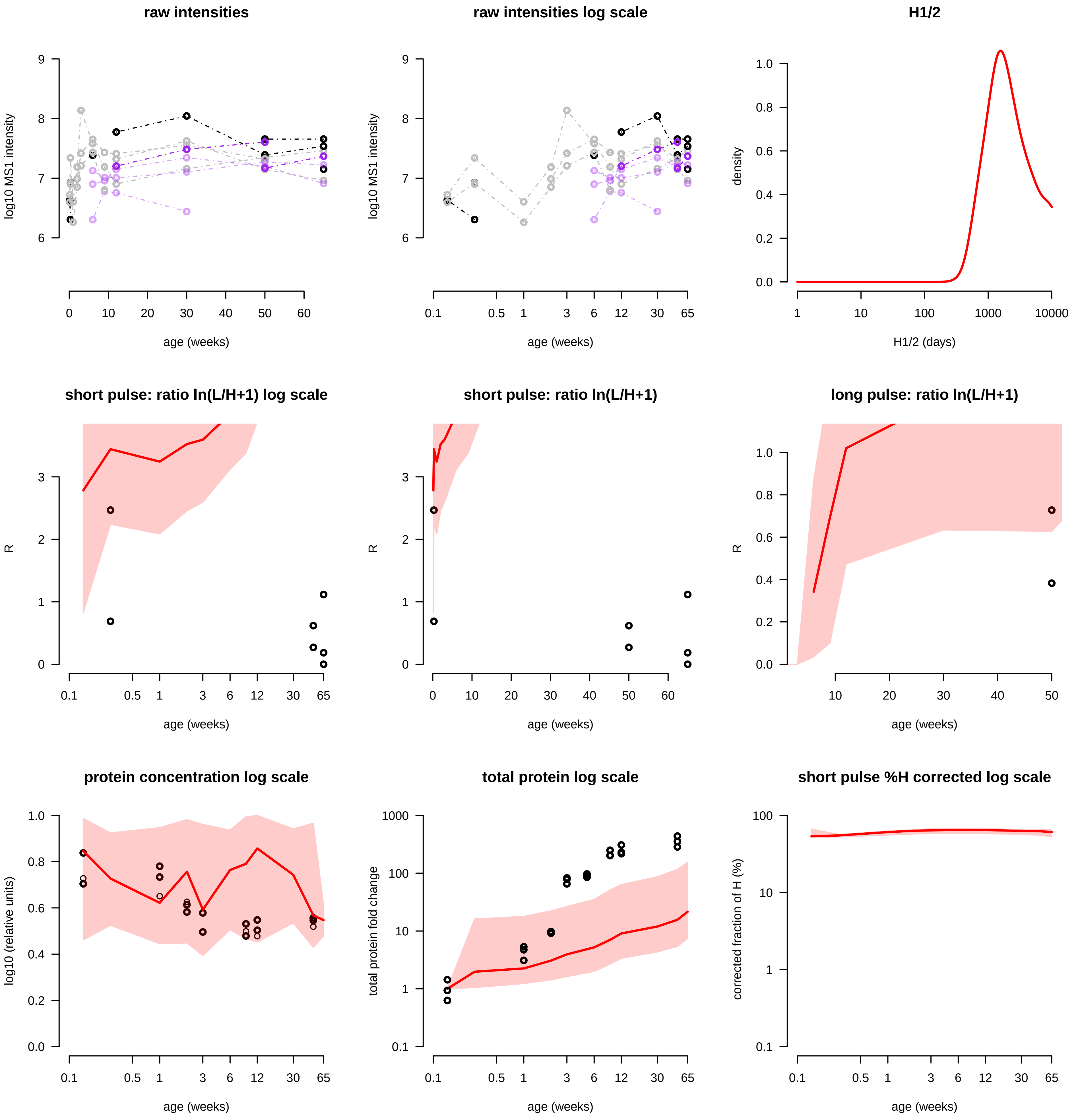

P56371

>sp|P56371|RAB4A_MOUSE

Ras−related protein Rab−4A

GN=Rab4a

Med H1/2: 13.2 days

Q5 H1/2: 4.7 days

Q95 H1/2: 144.9 days

reliable model fit: yes

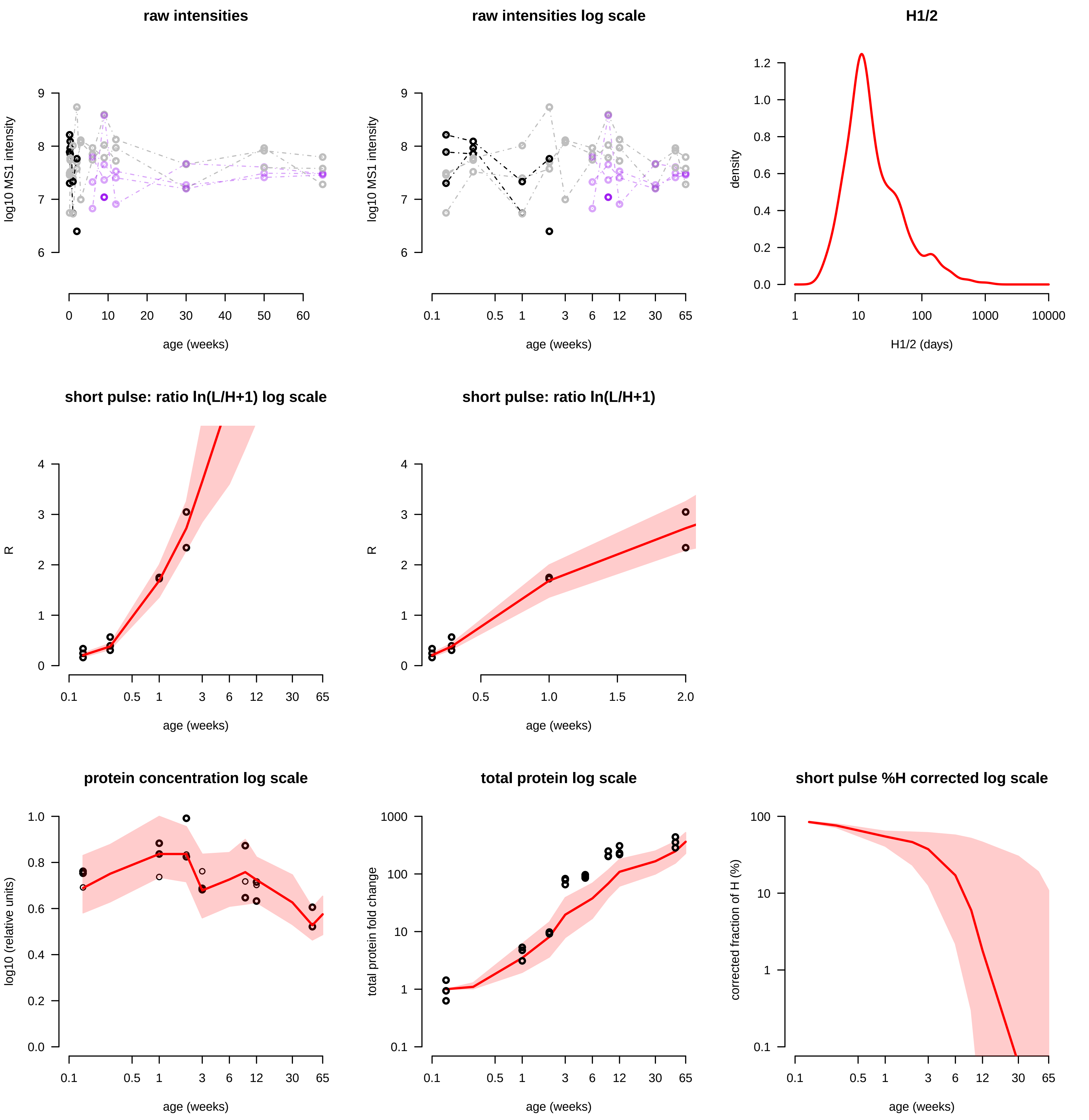

P46938;P46938−2

>sp|P46938|YAP1_MOUSE

Transcriptional coactivator YAP1

GN=Yap1

Med H1/2: 35.7 days

Q5 H1/2: 11.5 days

Q95 H1/2: 323.4 days

reliable model fit: no

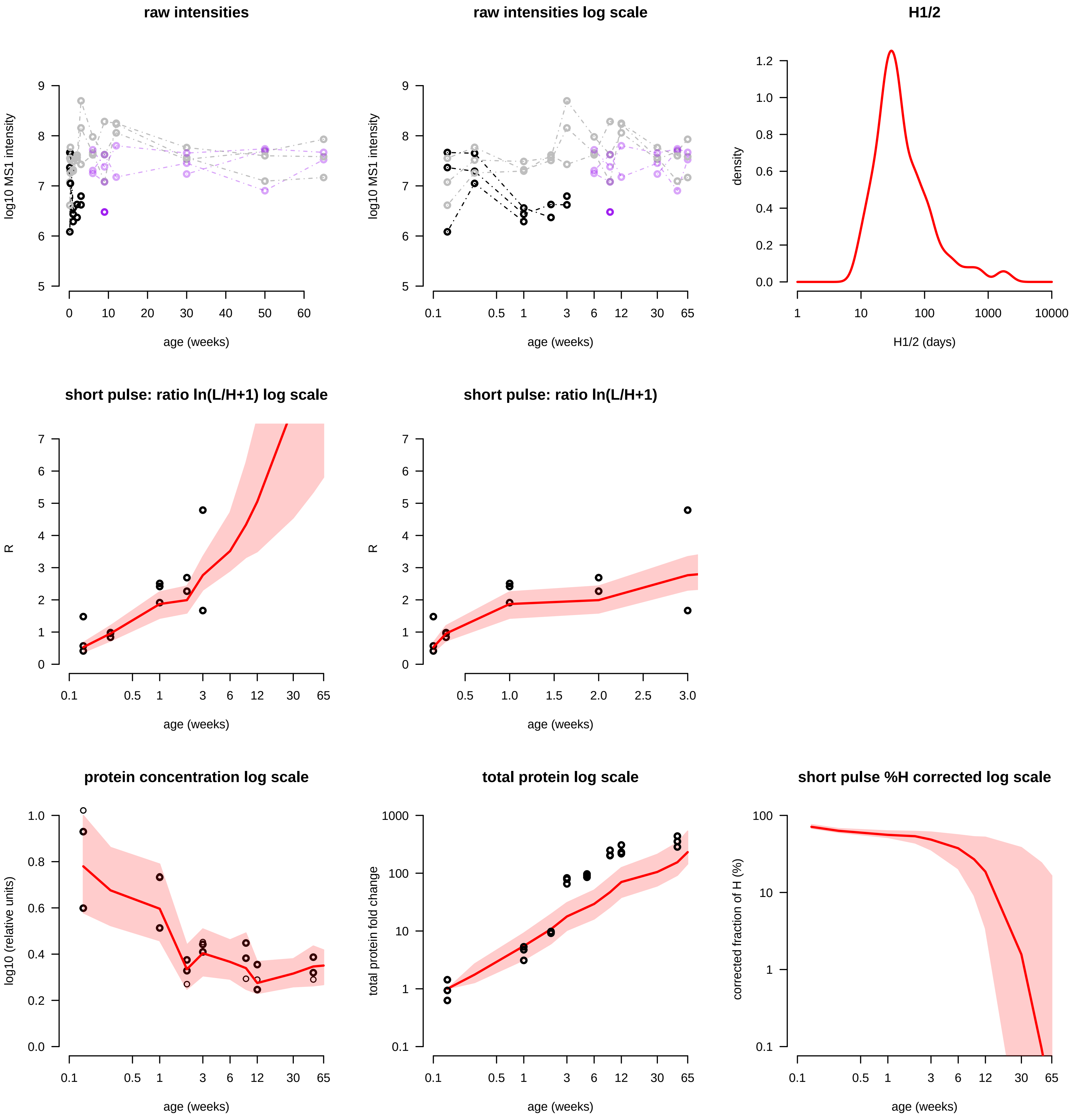

Q62504−2;Q62504;Q62504−3

>sp|Q62504−2|MINT_MOUSE

Isoform 2 of Msx2−interacting protein

GN=Spen

Med H1/2: 99.1 days

Q5 H1/2: 35.9 days

Q95 H1/2: 706.3 days

reliable model fit: no

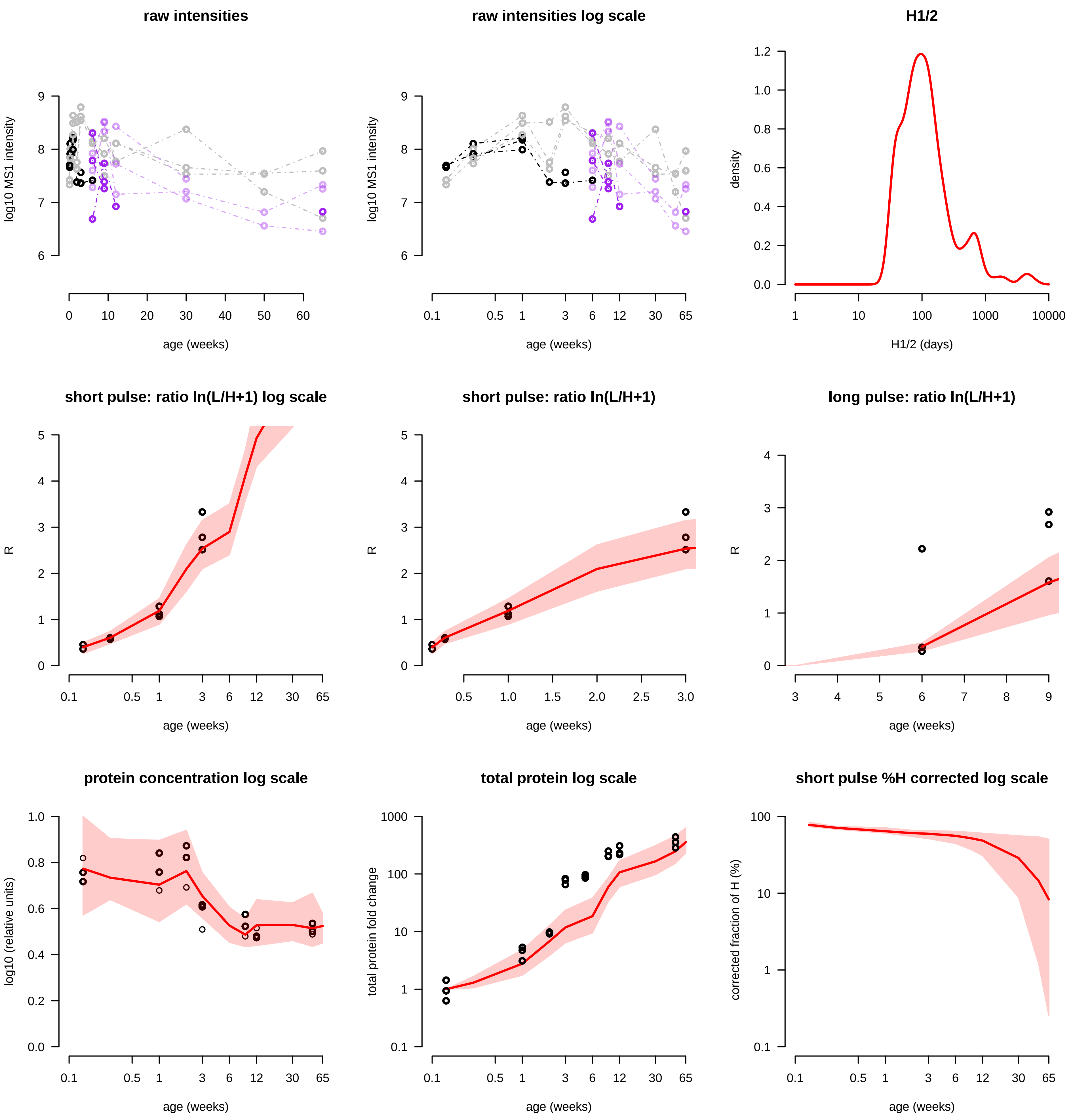

Q9CXD9

>sp|Q9CXD9|LRC17_MOUSE

Leucine−rich repeat−containing protein 17

GN=Lrrc17

Med H1/2: 7.4 days

Q5 H1/2: 3 days

Q95 H1/2: 108.5 days

reliable model fit: yes

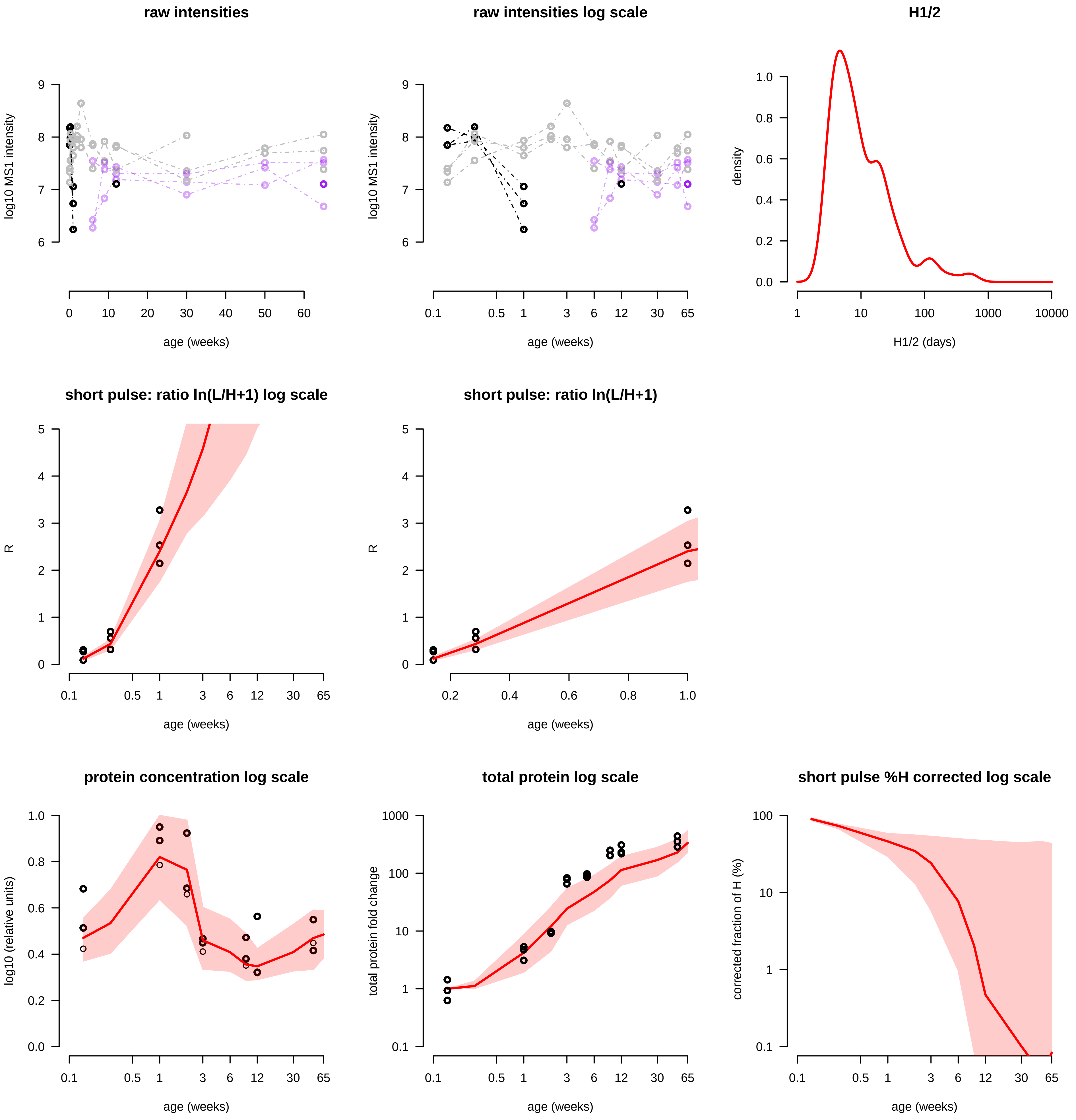

Q9Z1K6

>sp|Q9Z1K6|ARI2_MOUSE

E3 ubiquitin−protein ligase ARIH2

GN=Arih2

Med H1/2: 6.9 days

Q5 H1/2: 2.8 days

Q95 H1/2: 64.6 days

reliable model fit: yes

raw intensities

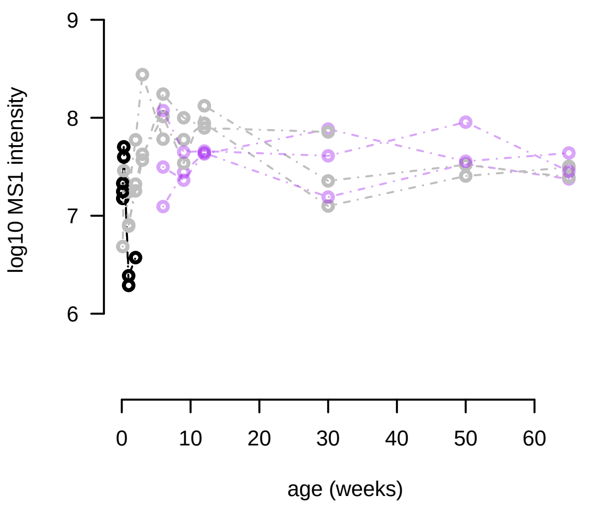

raw intensities log scale

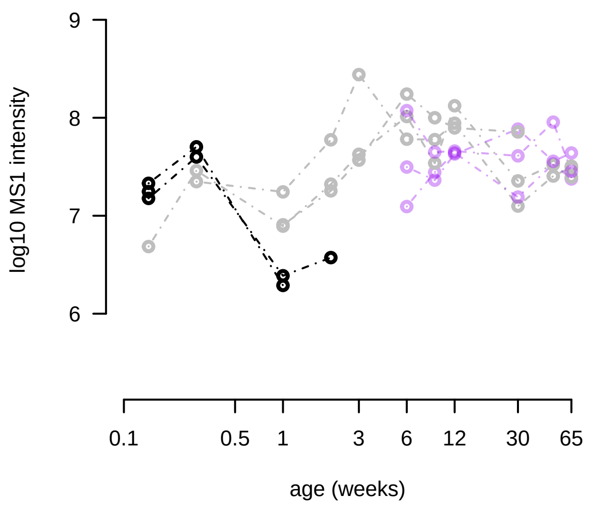

H1/2

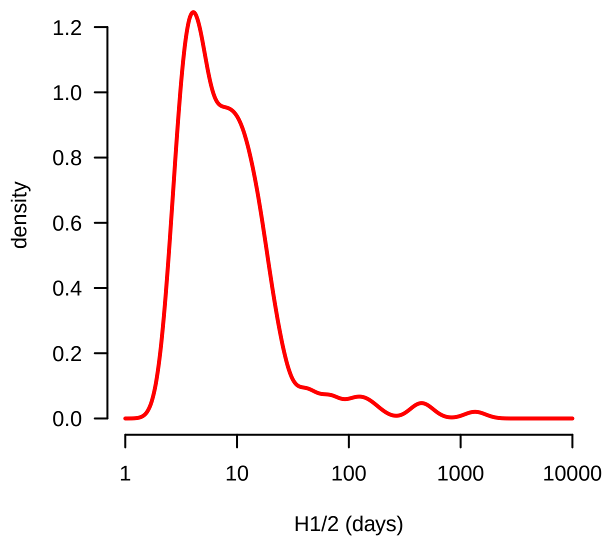

short pulse: ratio ln(L/H+1) log scale

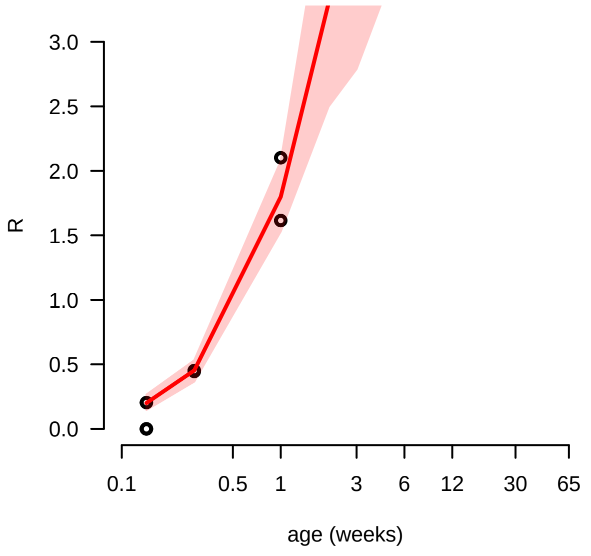

short pulse: ratio ln(L/H+1)

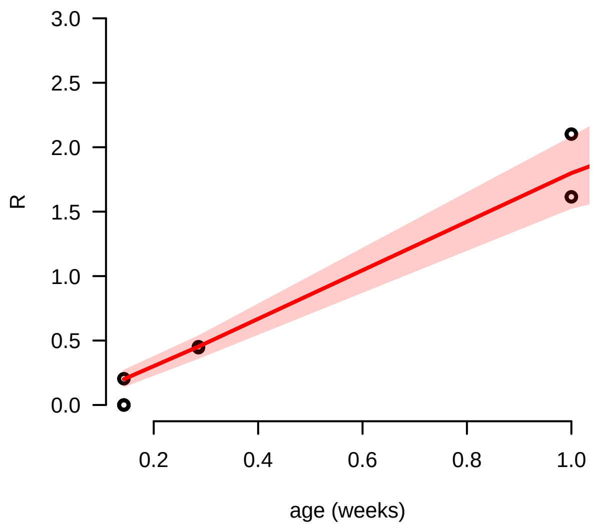

protein concentration log scale

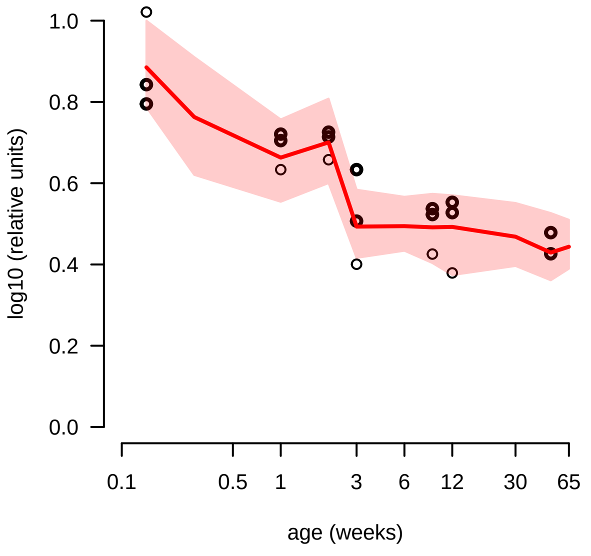

total protein log scale

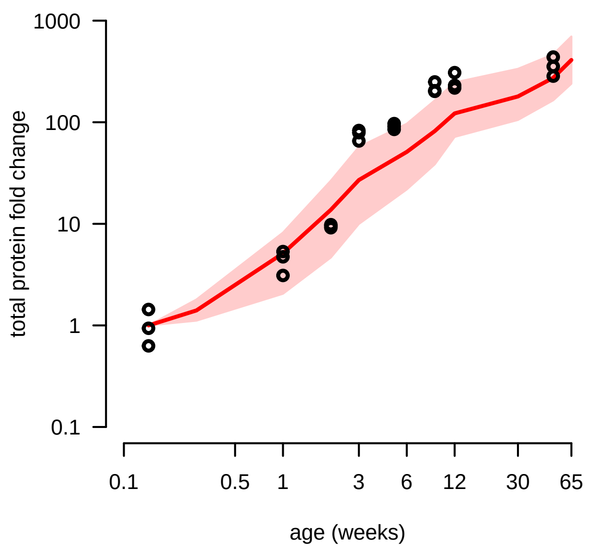

short pulse %H corrected log scale

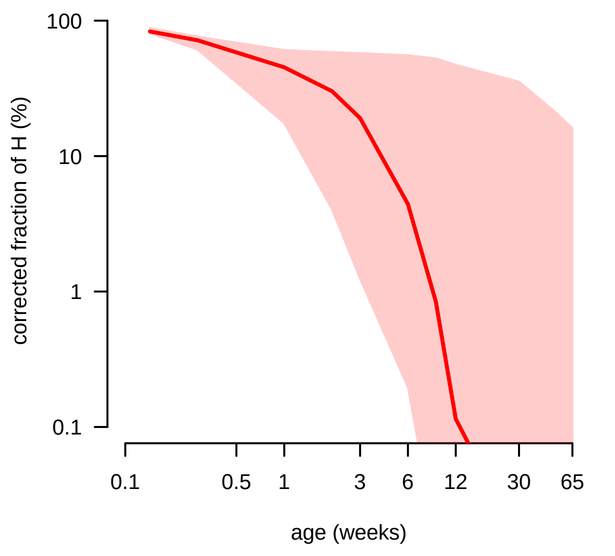

P52927

>sp|P52927|HMGA2_MOUSE

High mobility group protein HMGI−C

GN=Hmga2

Med H1/2: 26.2 days

Q5 H1/2: 14.5 days

Q95 H1/2: 38.6 days

reliable model fit: yes

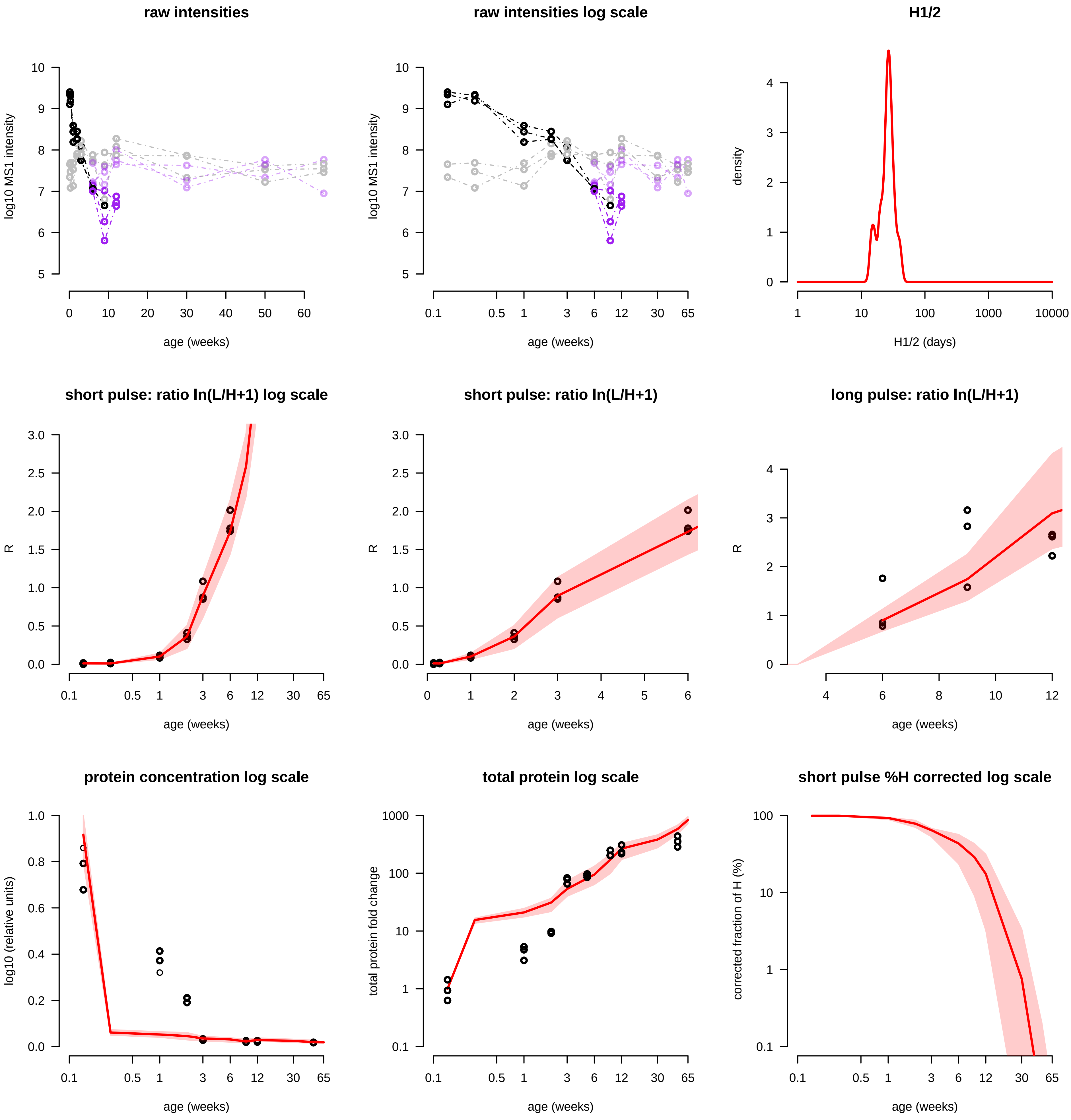

Q3TWL2

>sp|Q3TWL2|PP4P1_MOUSE

Type 1 phosphatidylinositol 4,5−bisphosphate 4−phosphatase

GN=Pip4p1

Med H1/2: 3121.5 days

Q5 H1/2: 826.2 days

Q95 H1/2: 21807.8 days

reliable model fit: no

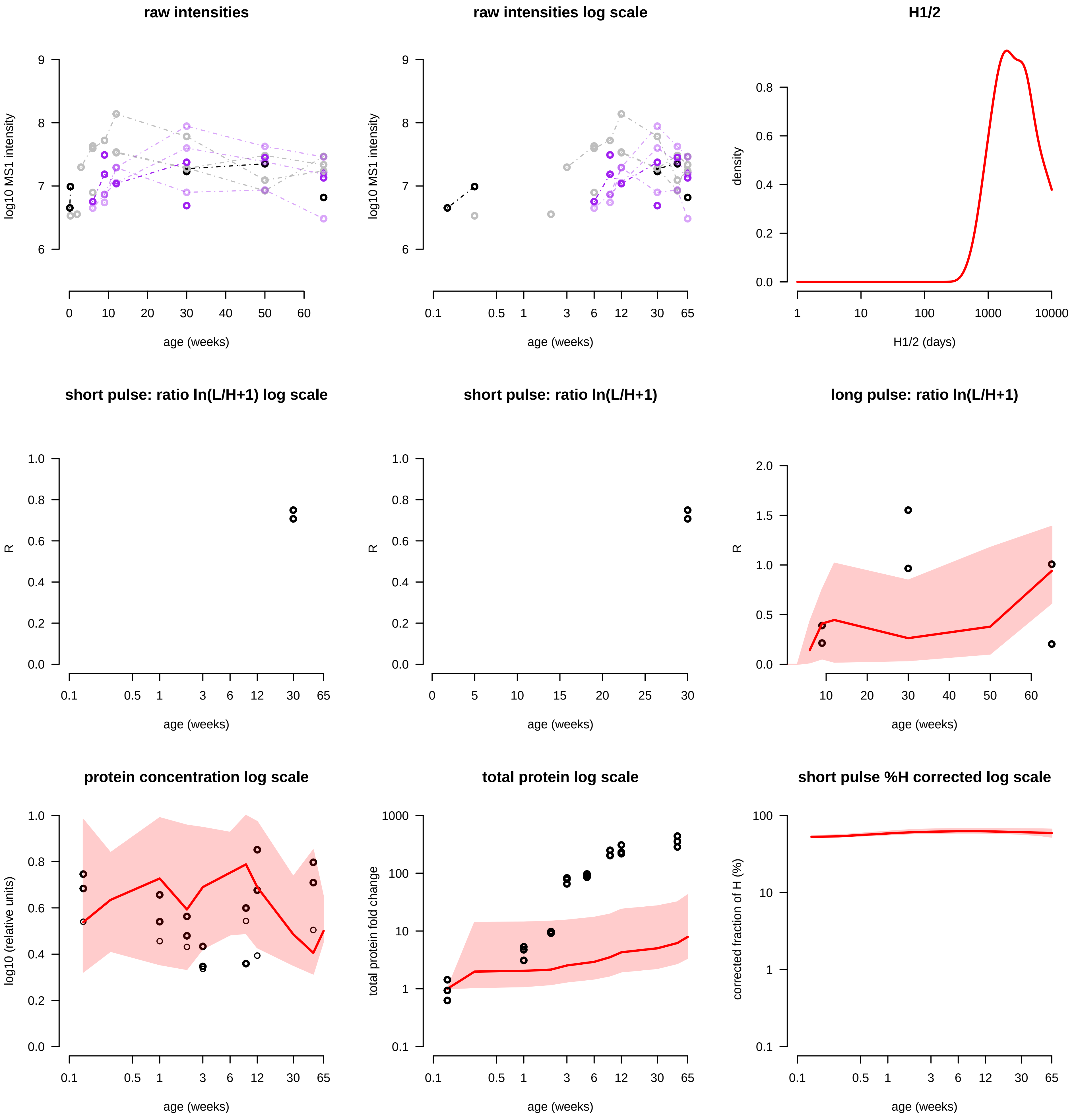

Q01320

>sp|Q01320|TOP2A_MOUSE

DNA topoisomerase 2−alpha

GN=Top2a

Med H1/2: 3.2 days

Q5 H1/2: 2.5 days

Q95 H1/2: 7.8 days

reliable model fit: yes

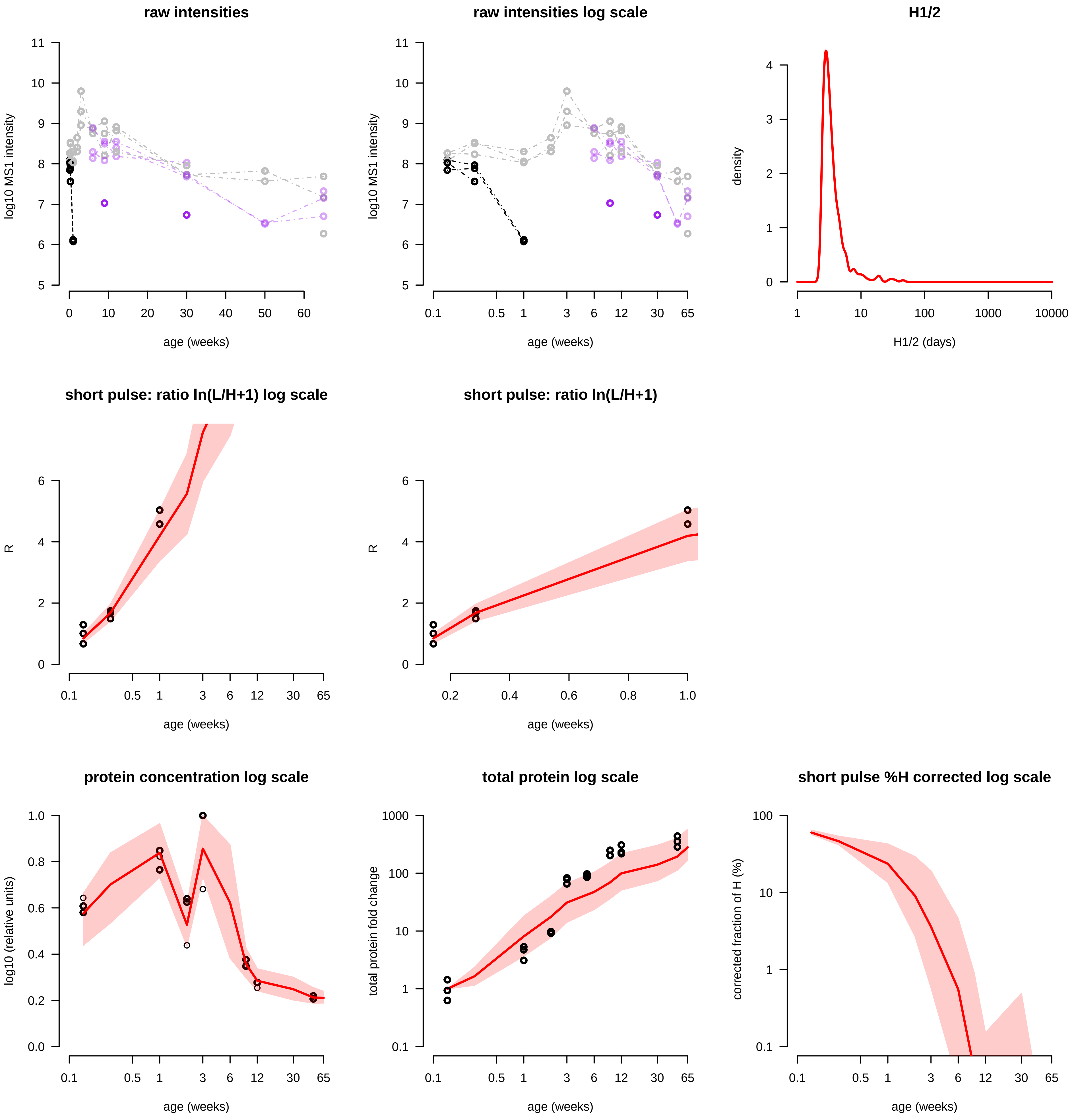

Q3UM18;Q3UM18−2

>sp|Q3UM18|LSG1_MOUSE

Large subunit GTPase 1 homolog

GN=Lsg1

Med H1/2: 8.8 days

Q5 H1/2: 3.3 days

Q95 H1/2: 116.1 days

reliable model fit: yes

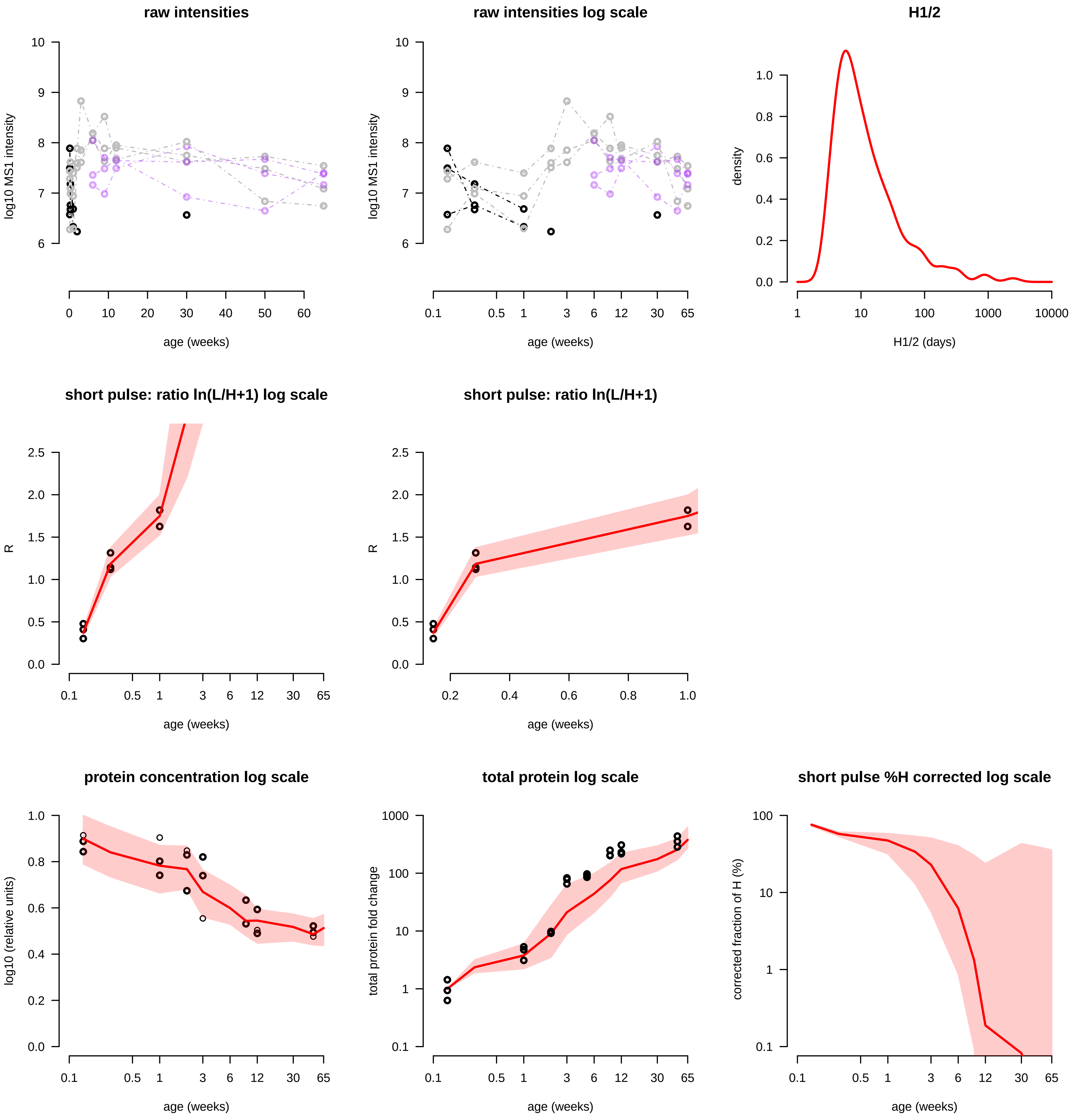

P28738

>sp|P28738|KIF5C_MOUSE

Kinesin heavy chain isoform 5C

GN=Kif5c

Med H1/2: 13.9 days

Q5 H1/2: 4.7 days

Q95 H1/2: 153.5 days

reliable model fit: yes

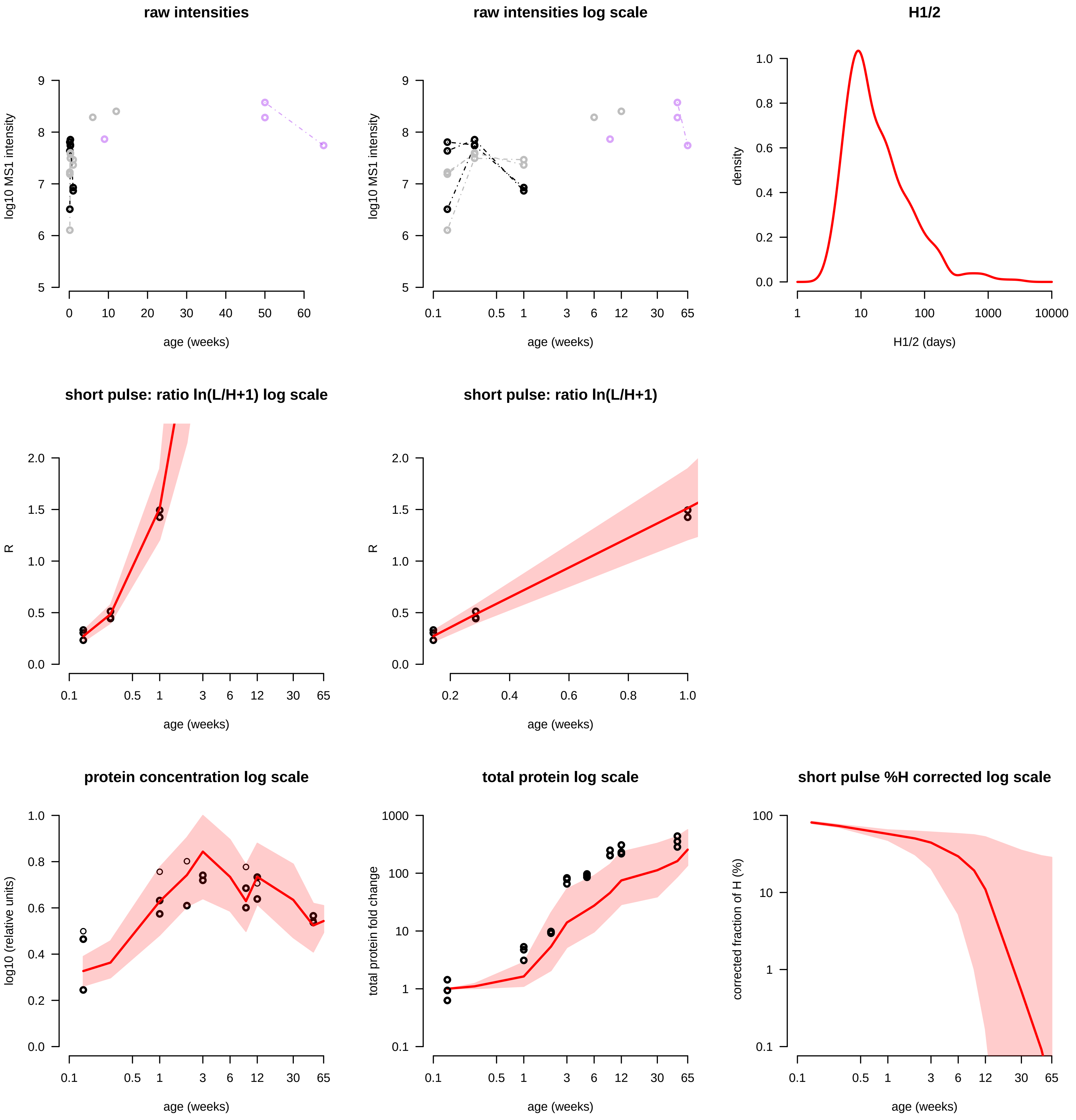

Q3UUQ7

>sp|Q3UUQ7|PGAP1_MOUSE

GPI inositol−deacylase

GN=Pgap1

Med H1/2: 7.7 days

Q5 H1/2: 2.7 days

Q95 H1/2: 34.9 days

reliable model fit: yes

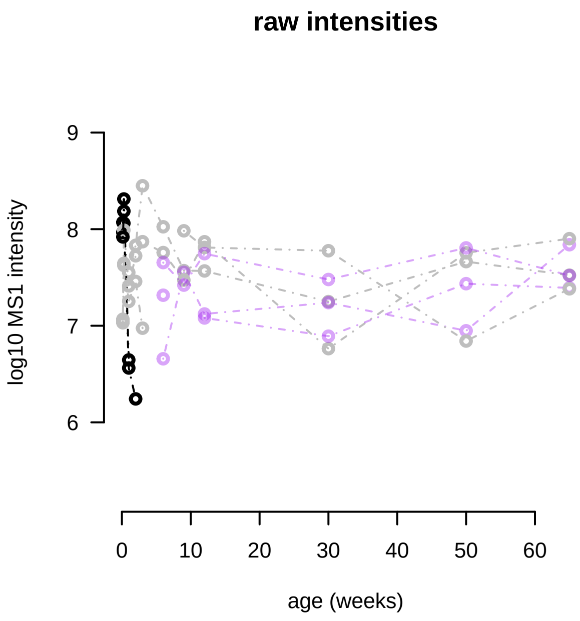

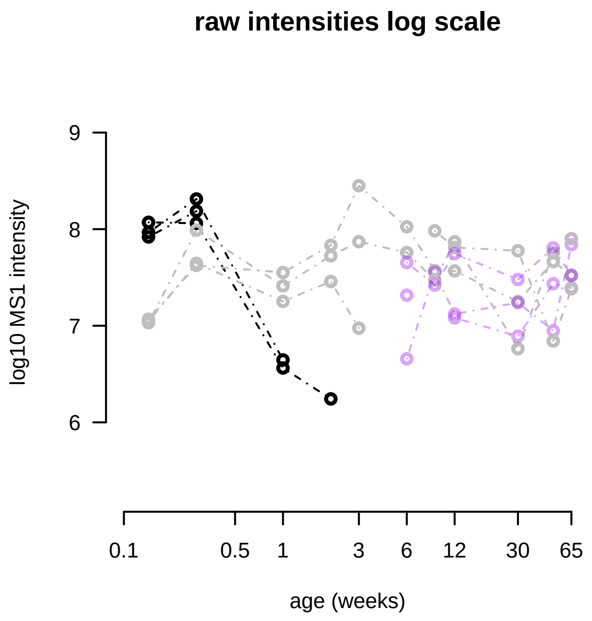

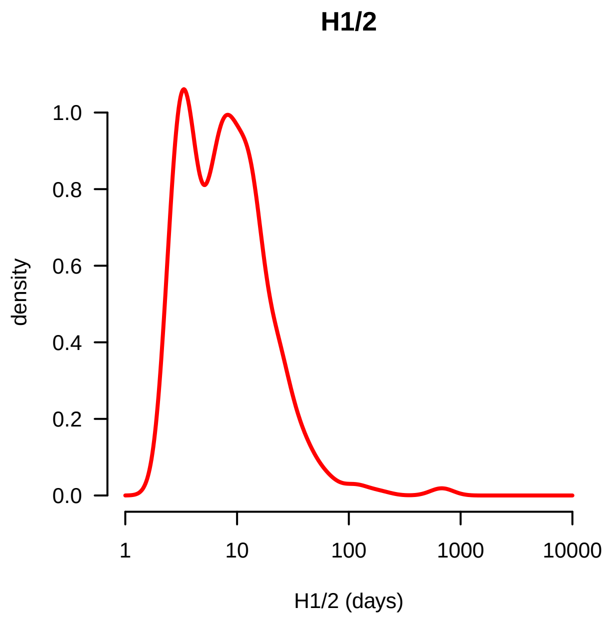

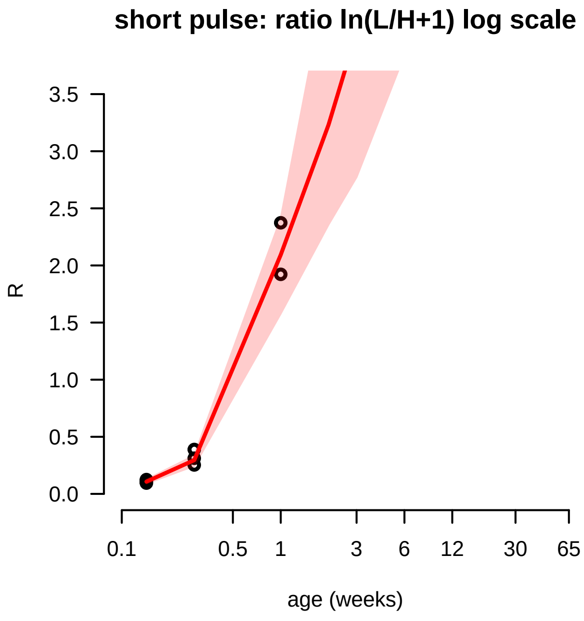

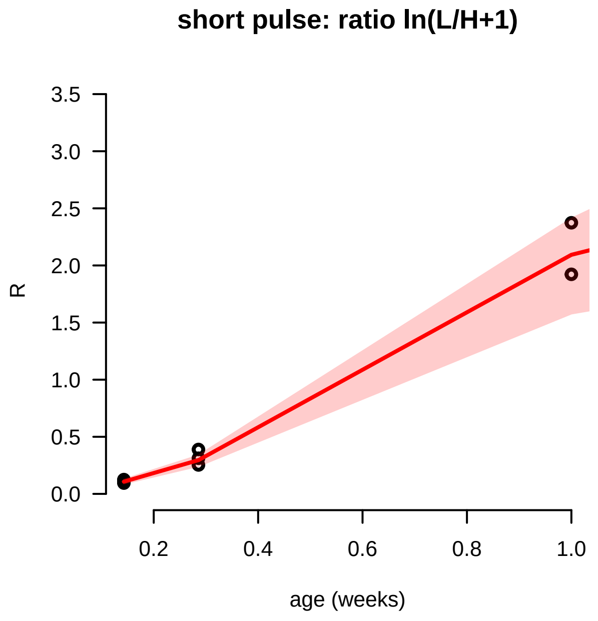

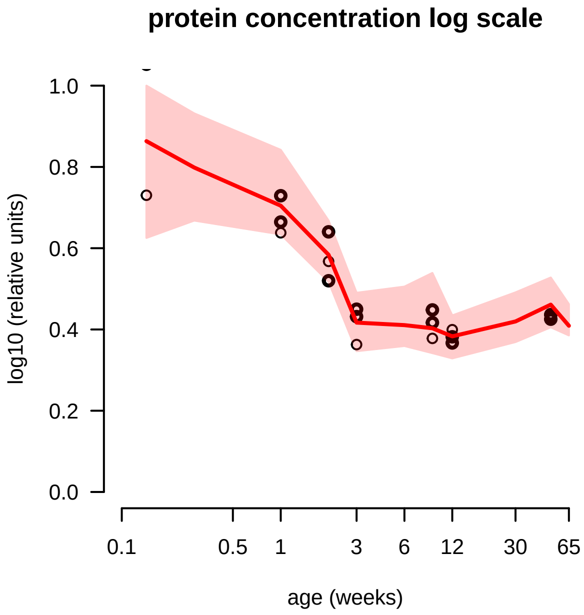

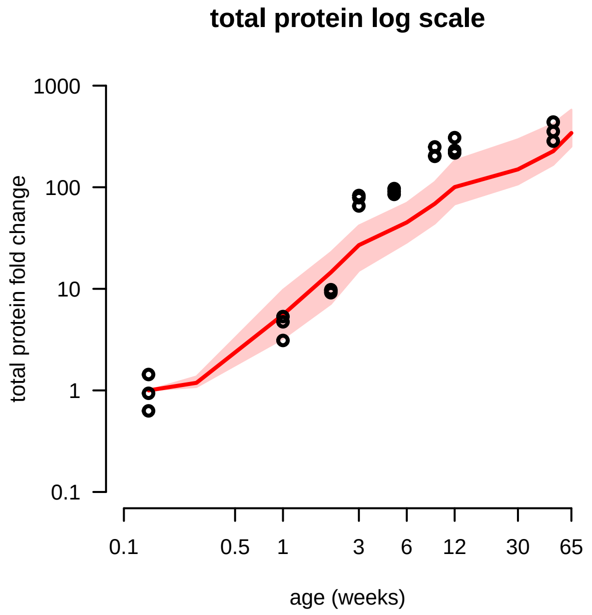

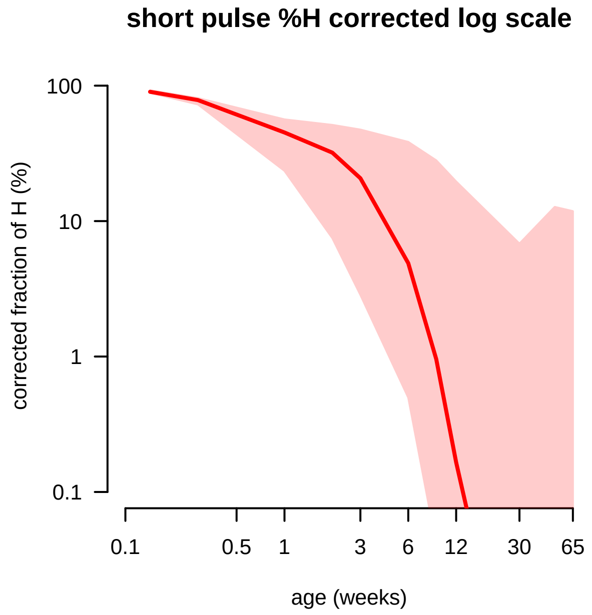

Q9CY21

>sp|Q9CY21|BUD23_MOUSE

Probable 18S rRNA (guanine−N(7))−methyltransferase

GN=Bud23

Med H1/2: 7.3 days

Q5 H1/2: 2.8 days

Q95 H1/2: 100.2 days

reliable model fit: yes

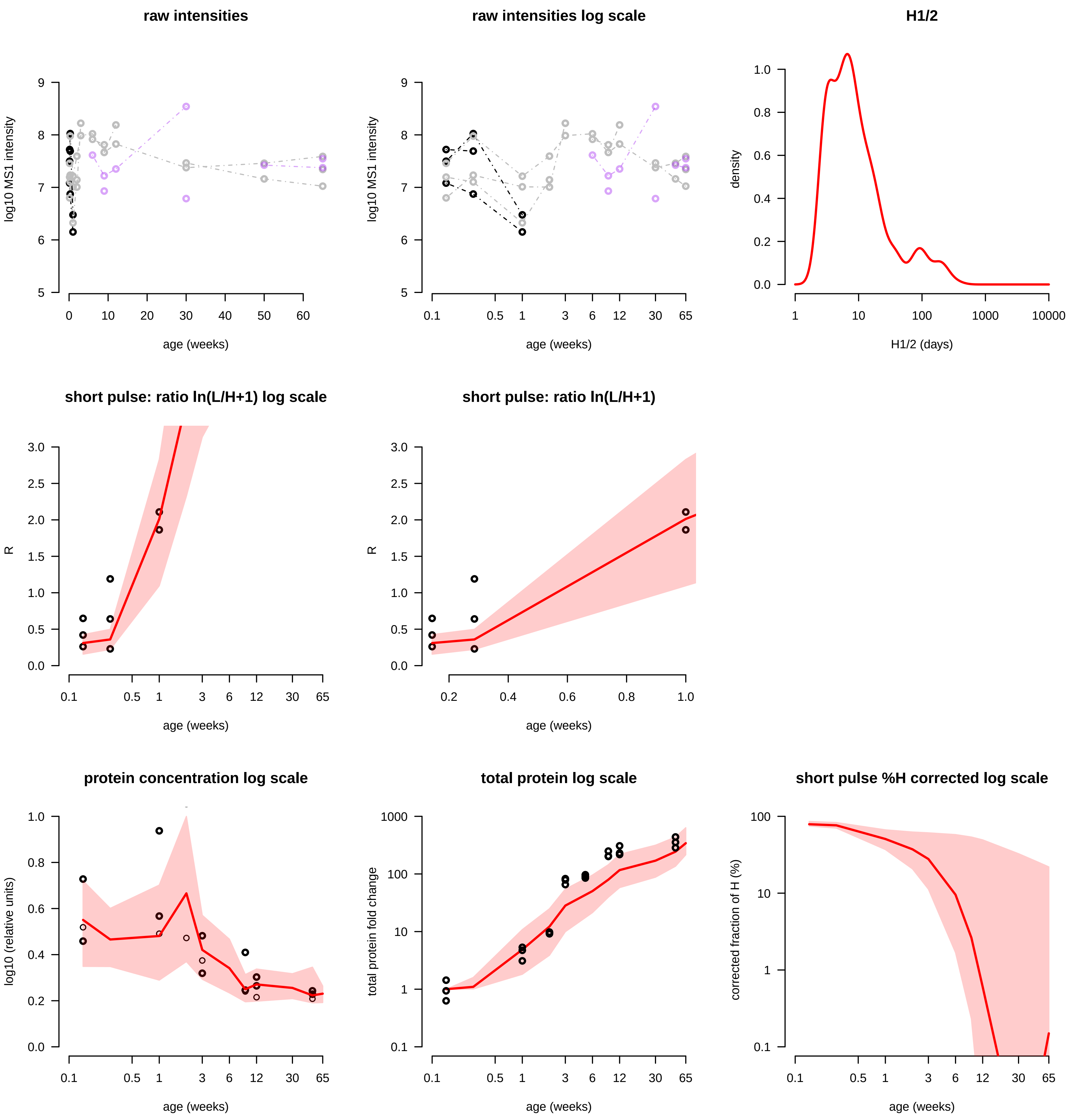

P42337

>sp|P42337|PK3CA_MOUSE

Phosphatidylinositol 4,5−bisphosphate 3−kinase catalytic subunit alpha isoform

GN=Pik3ca

Med H1/2: 30.3 days

Q5 H1/2: 8.4 days

Q95 H1/2: 307.9 days

reliable model fit: no

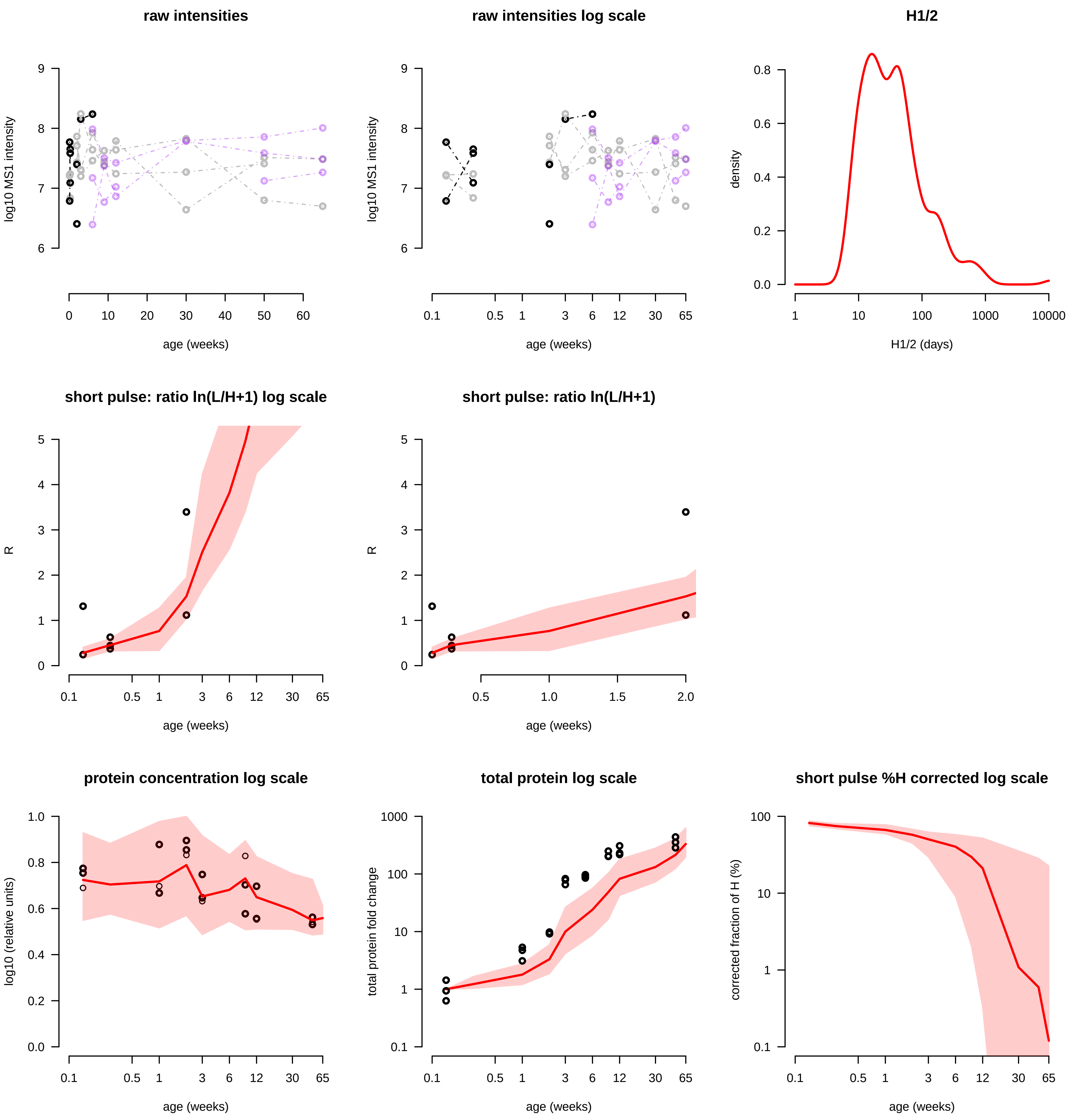

P58742

>sp|P58742|AAAS_MOUSE

Aladin

GN=Aaas

Med H1/2: 6.2 days

Q5 H1/2: 3 days

Q95 H1/2: 46.5 days

reliable model fit: yes

raw intensities

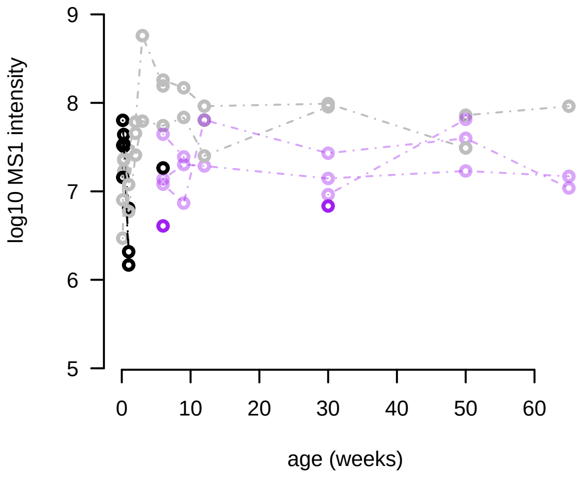

raw intensities log scale

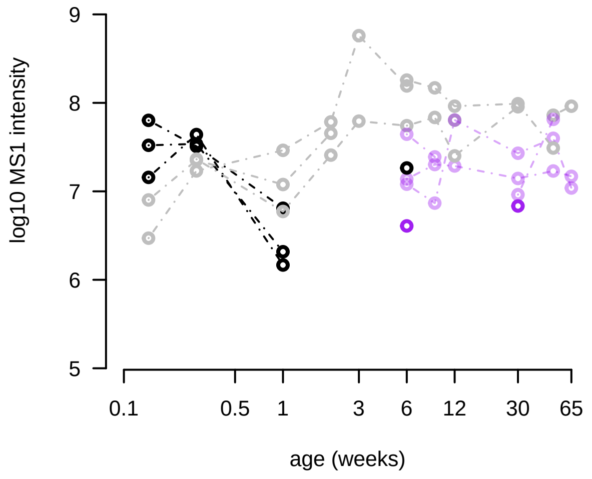

H1/2

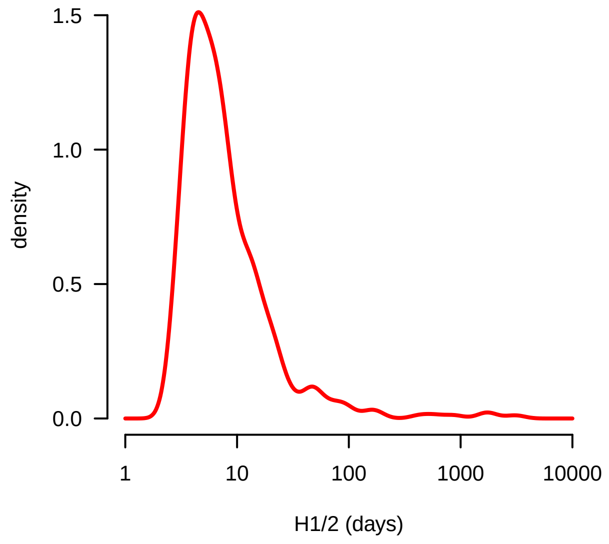

short pulse: ratio ln(L/H+1) log scale

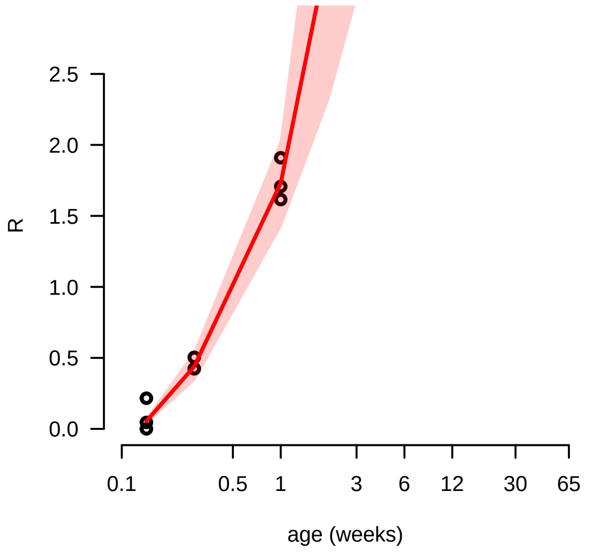

short pulse: ratio ln(L/H+1)

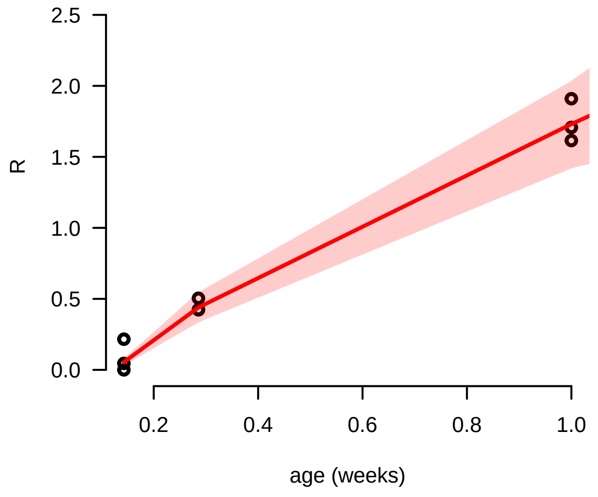

protein concentration log scale

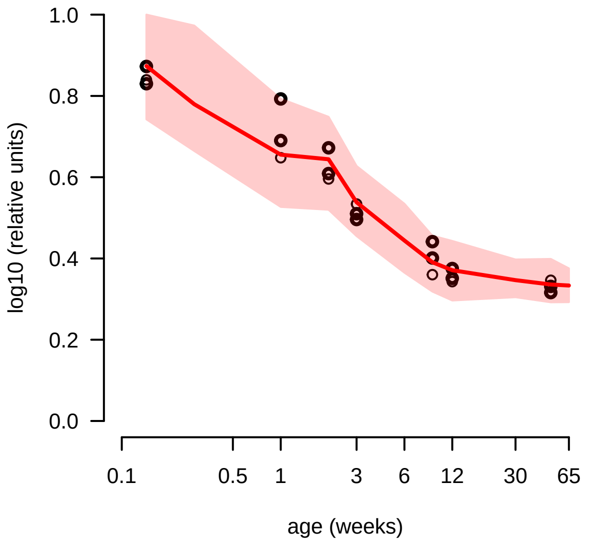

total protein log scale

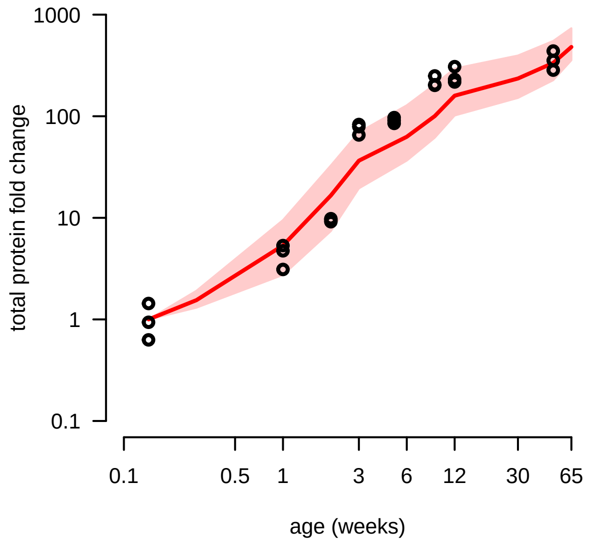

short pulse %H corrected log scale

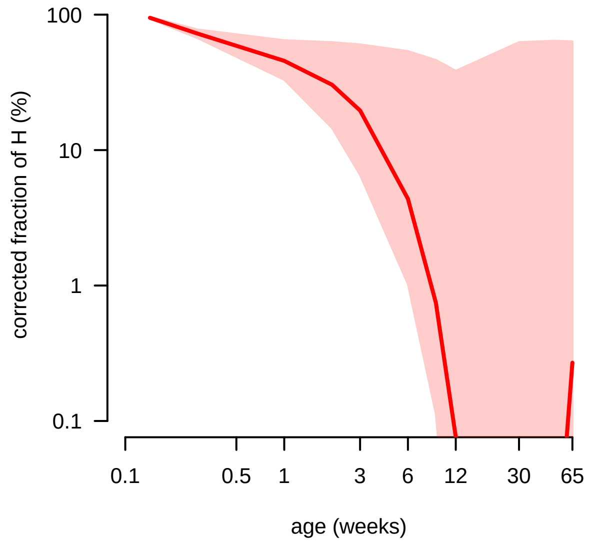

O54998

>sp|O54998|FKBP7_MOUSE

Peptidyl−prolyl cis−trans isomerase FKBP7

GN=Fkbp7

Med H1/2: 10 days

Q5 H1/2: 4.3 days

Q95 H1/2: 135 days

reliable model fit: yes

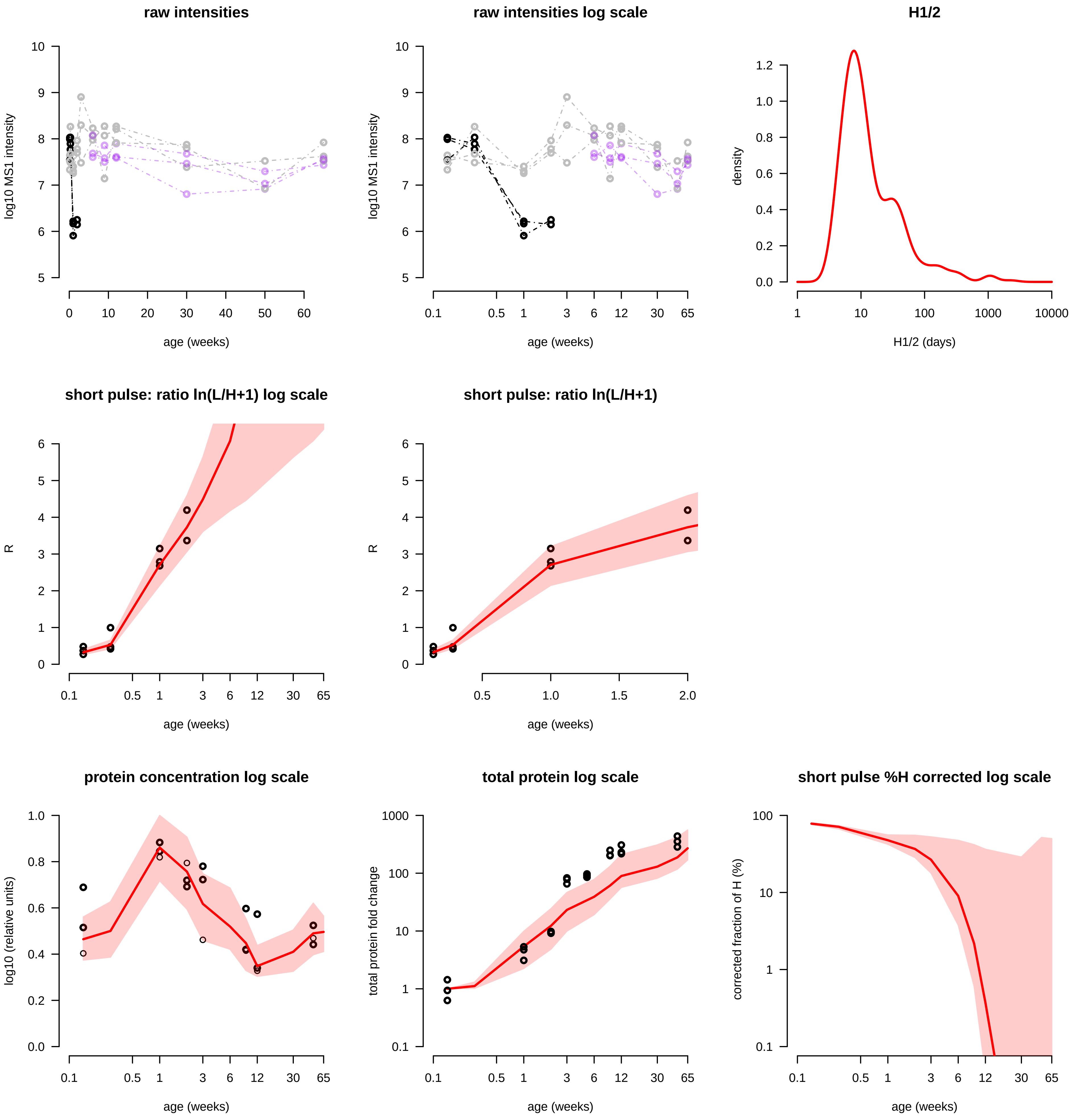

O35660

>sp|O35660|GSTM6_MOUSE

Glutathione S−transferase Mu 6

GN=Gstm6

Med H1/2: 3.7 days

Q5   H1/2: 2.6 days

Q95 H1/2: 8.4 days

reliable model fit: yes

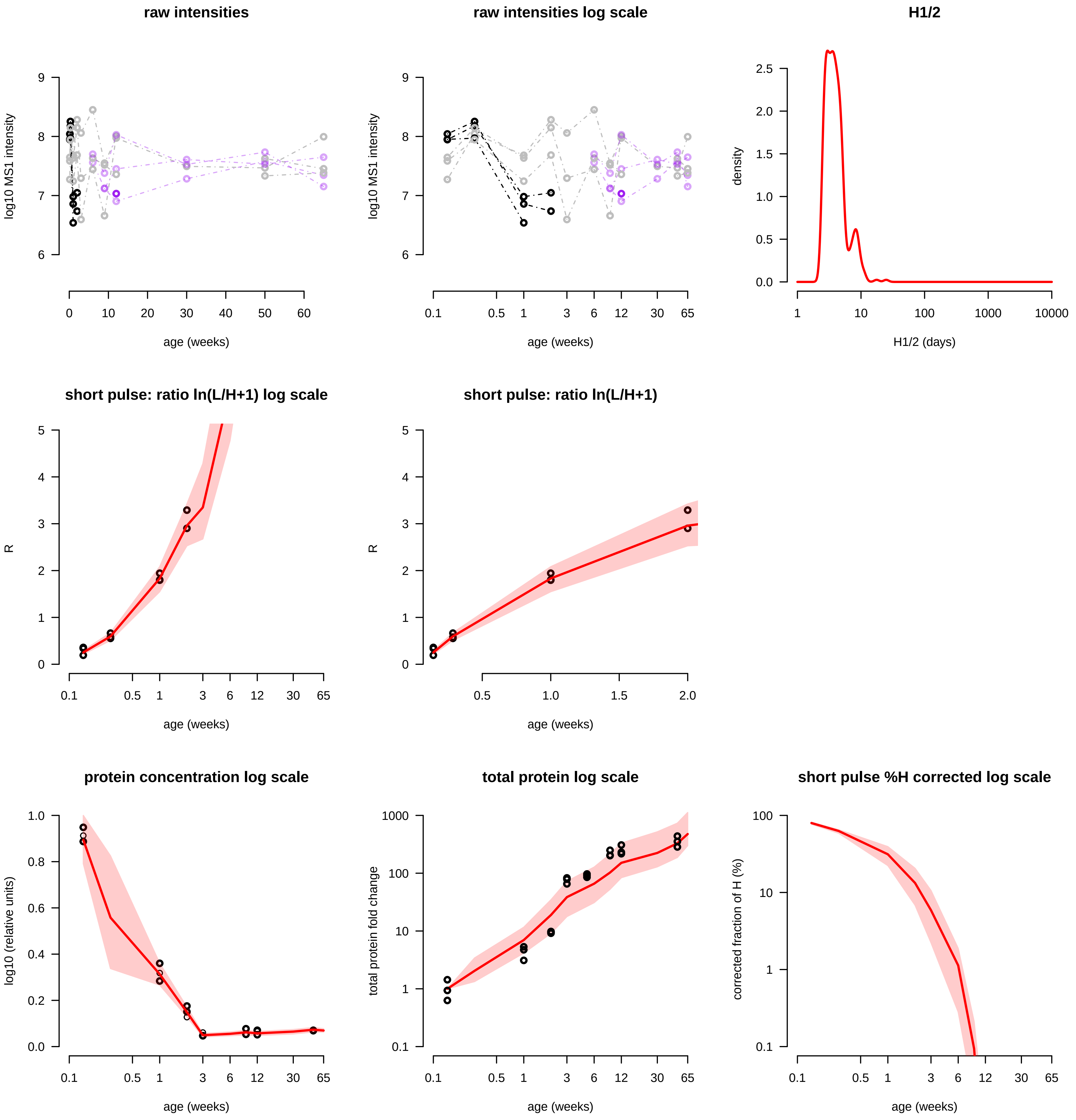

Q80U58

>sp|Q80U58|PUM2_MOUSE

Pumilio homolog 2

GN=Pum2

Med H1/2: 8.9 days

Q5 H1/2: 6.7 days

Q95 H1/2: 14.7 days

reliable model fit: yes

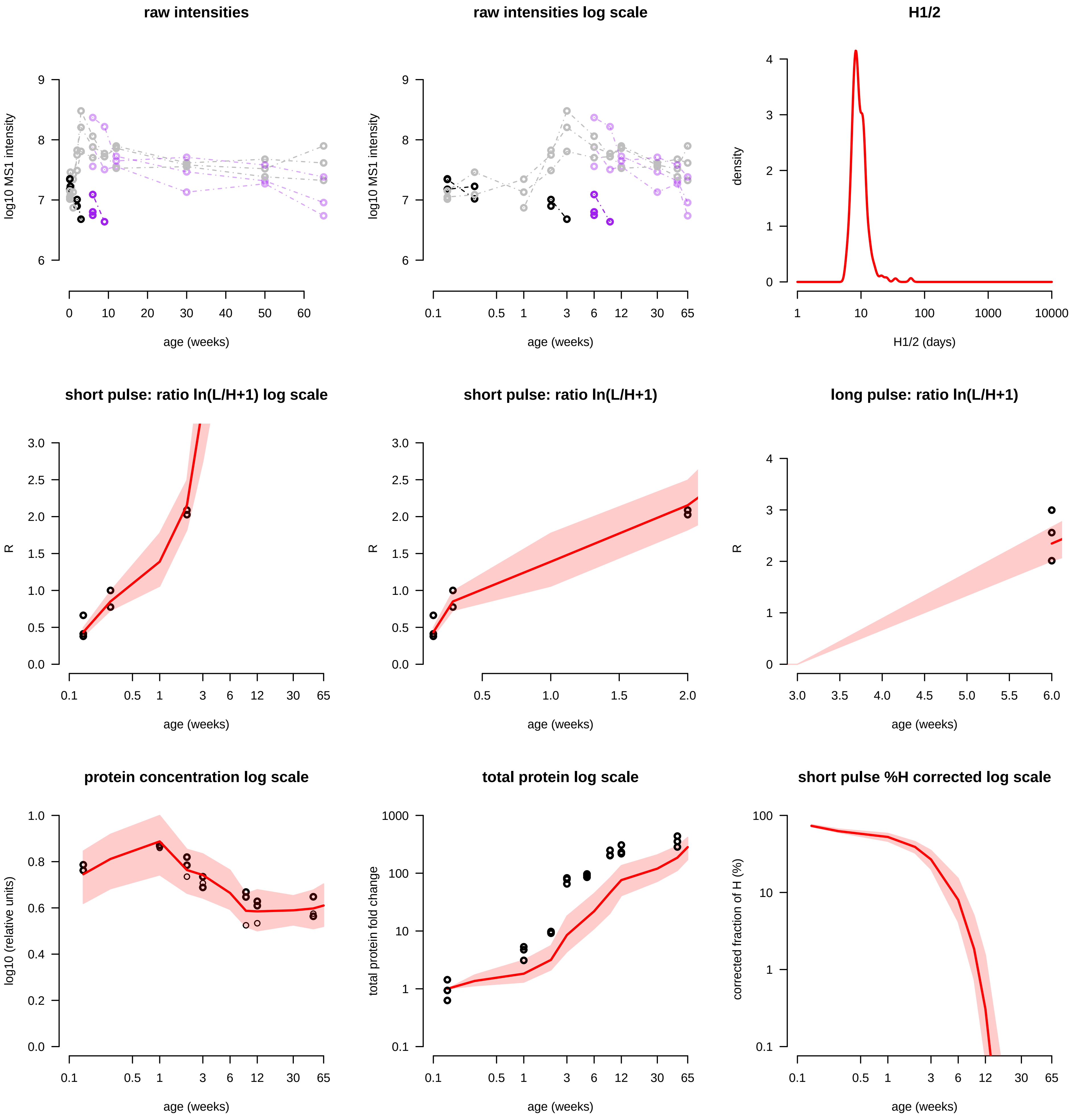

Q8C0J6

>sp|Q8C0J6|SWAHC_MOUSE

Ankyrin repeat domain−containing protein SOWAHC

GN=Sowahc

Med H1/2: 12.4 days

Q5 H1/2: 4.1 days

Q95 H1/2: 139.8 days

reliable model fit: yes

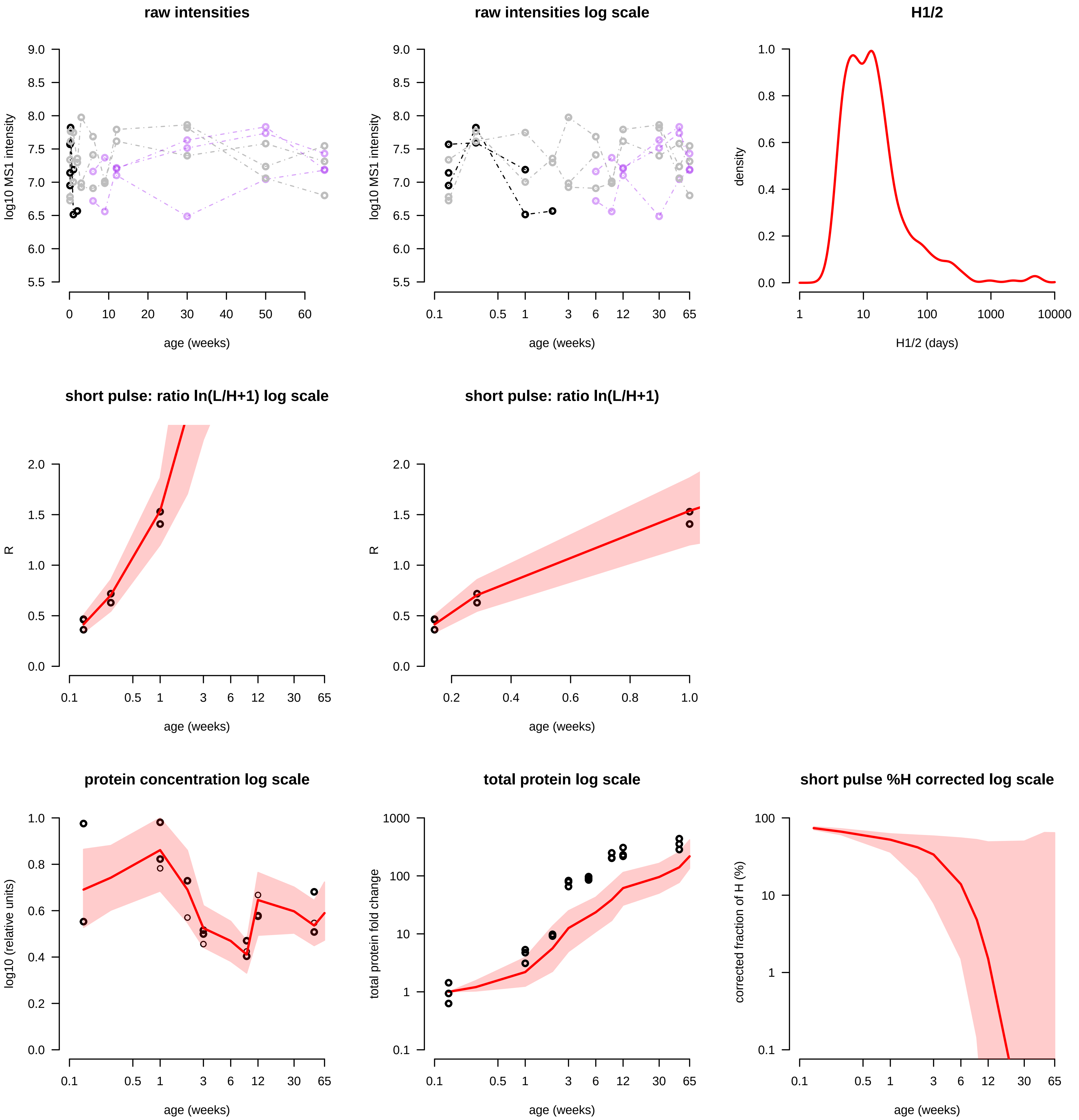

P52293

>sp|P52293|IMA1_MOUSE

Importin subunit alpha−1

GN=Kpna2

Med H1/2: 3 days

Q5 H1/2: 2.4 days

Q95 H1/2: 8 days

reliable model fit: yes

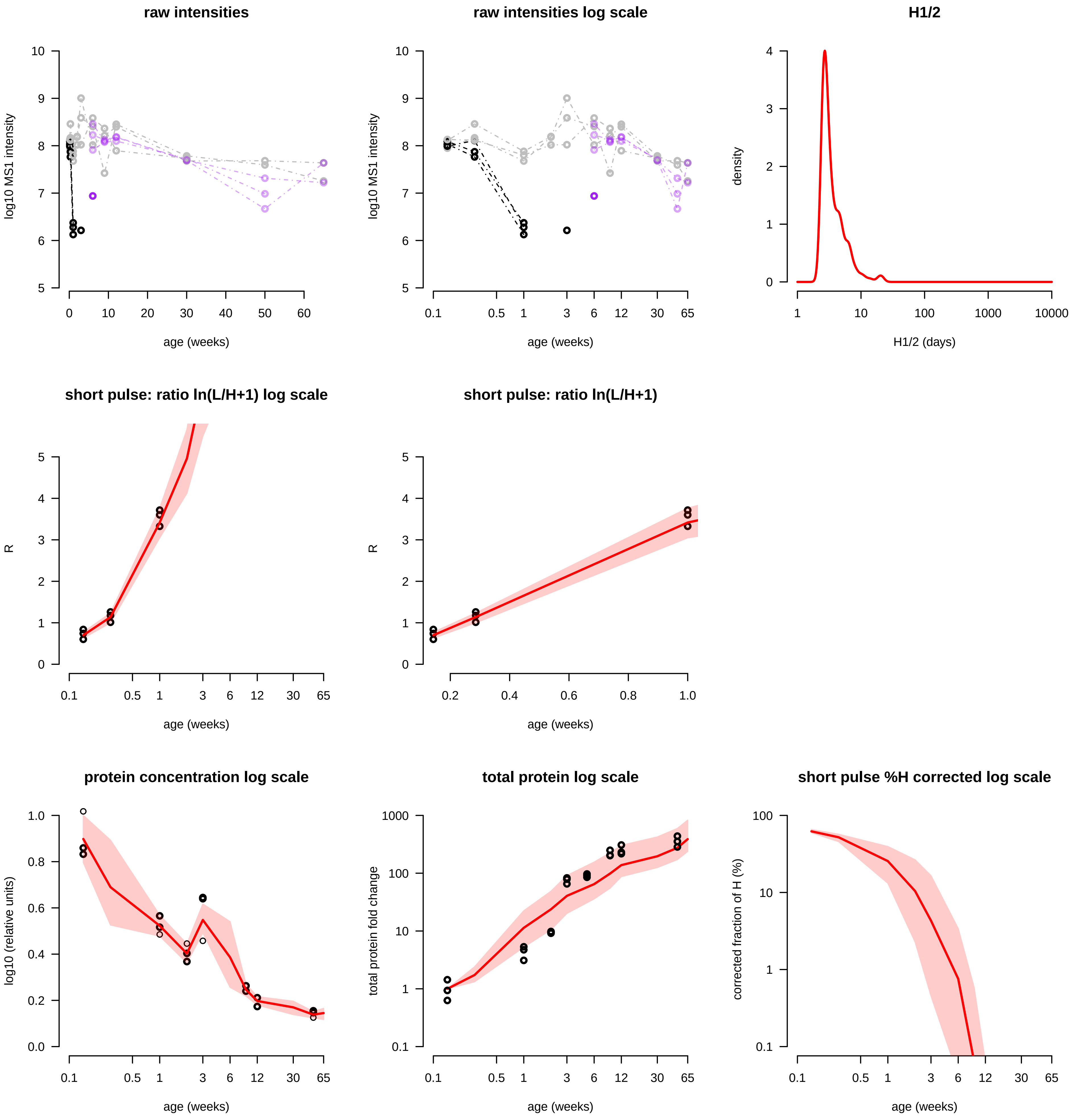

Q8K394

>sp|Q8K394|PLCL2_MOUSE

Inactive phospholipase C−like protein 2

GN=Plcl2

Med H1/2: 11.4 days

Q5 H1/2: 3.9 days

Q95 H1/2: 62.4 days

reliable model fit: yes

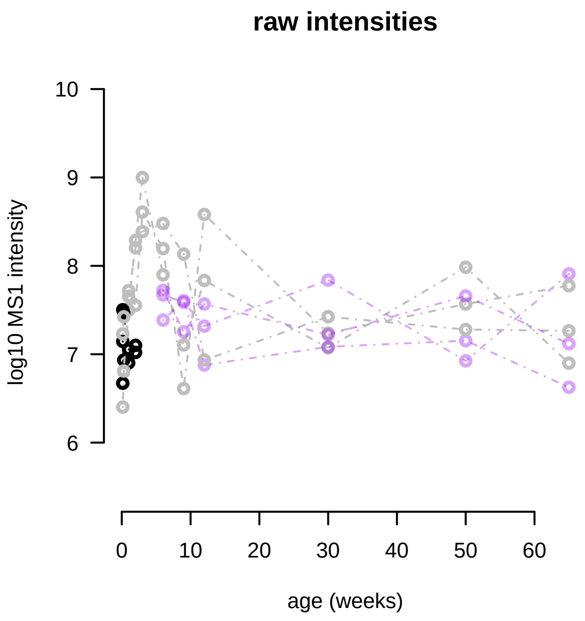

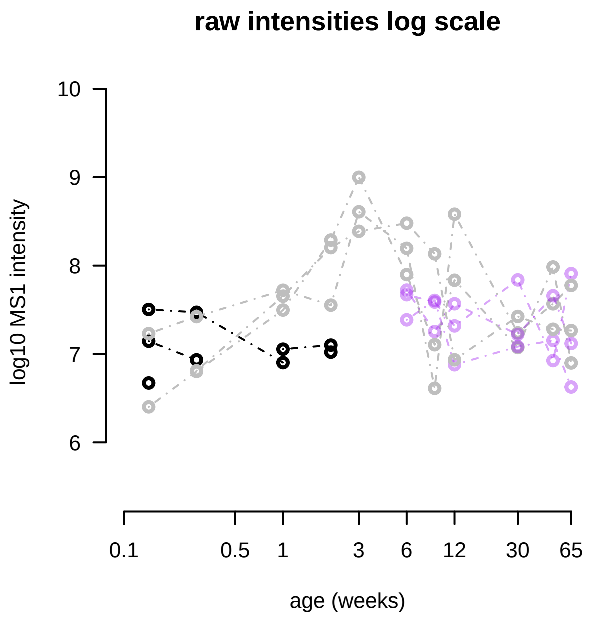

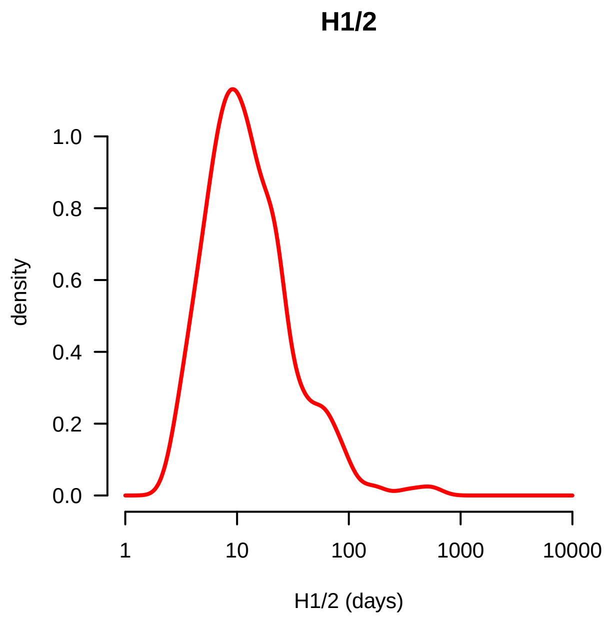

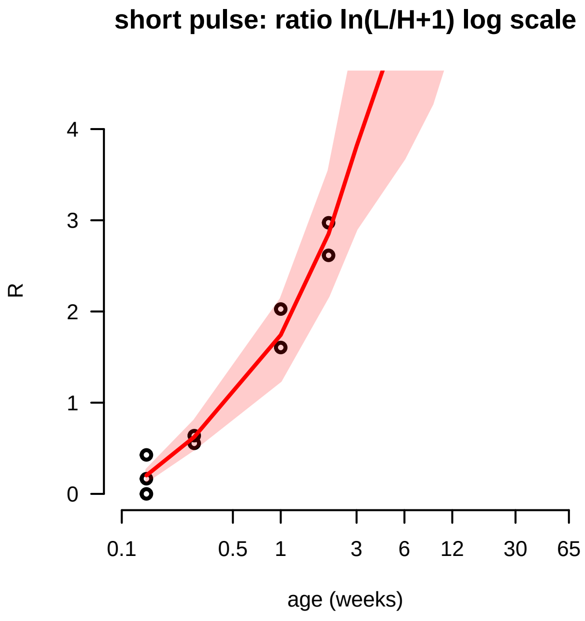

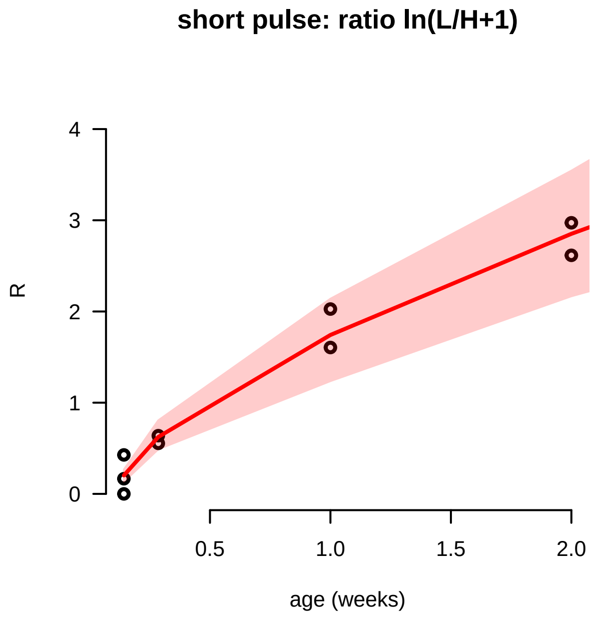

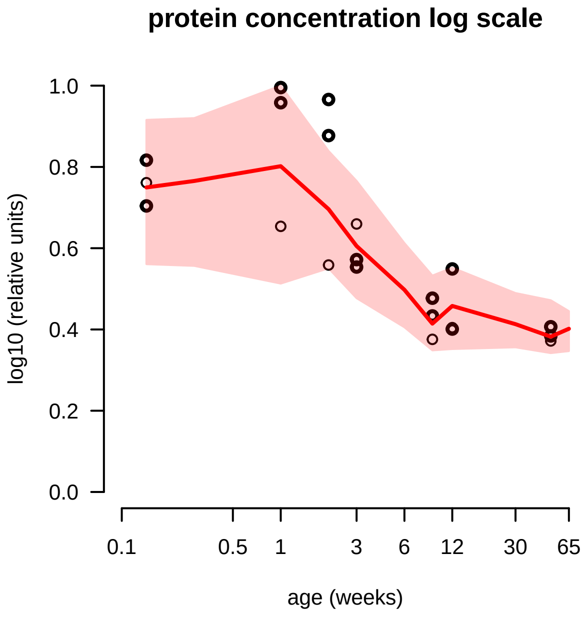

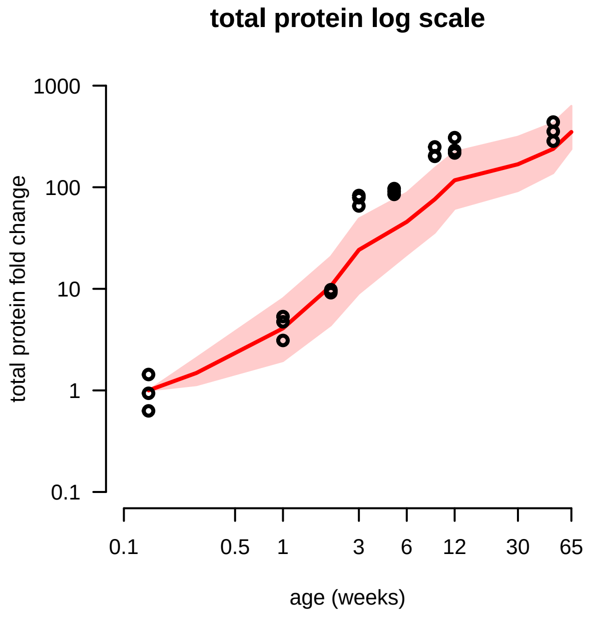

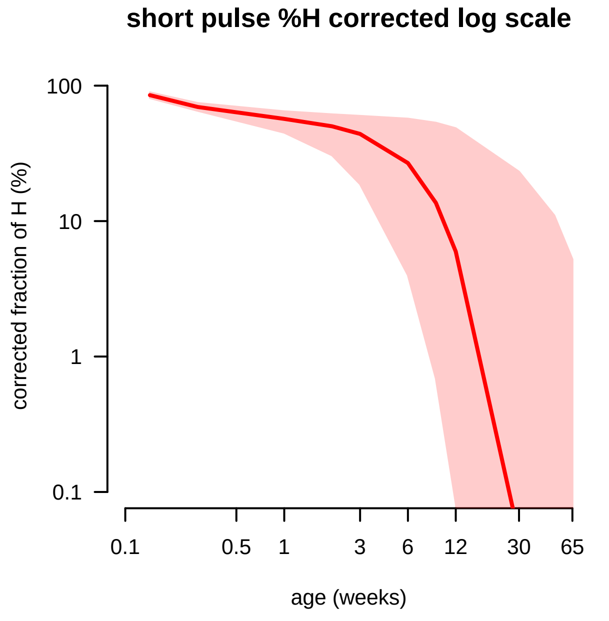

Q8BLY2

>sp|Q8BLY2|SYTC2_MOUSE

Threonine−−tRNA ligase 2, cytoplasmic

GN=Tars3

Med H1/2: 299.8 days

Q5 H1/2: 76.2 days

Q95 H1/2: 3283 days

reliable model fit: yes

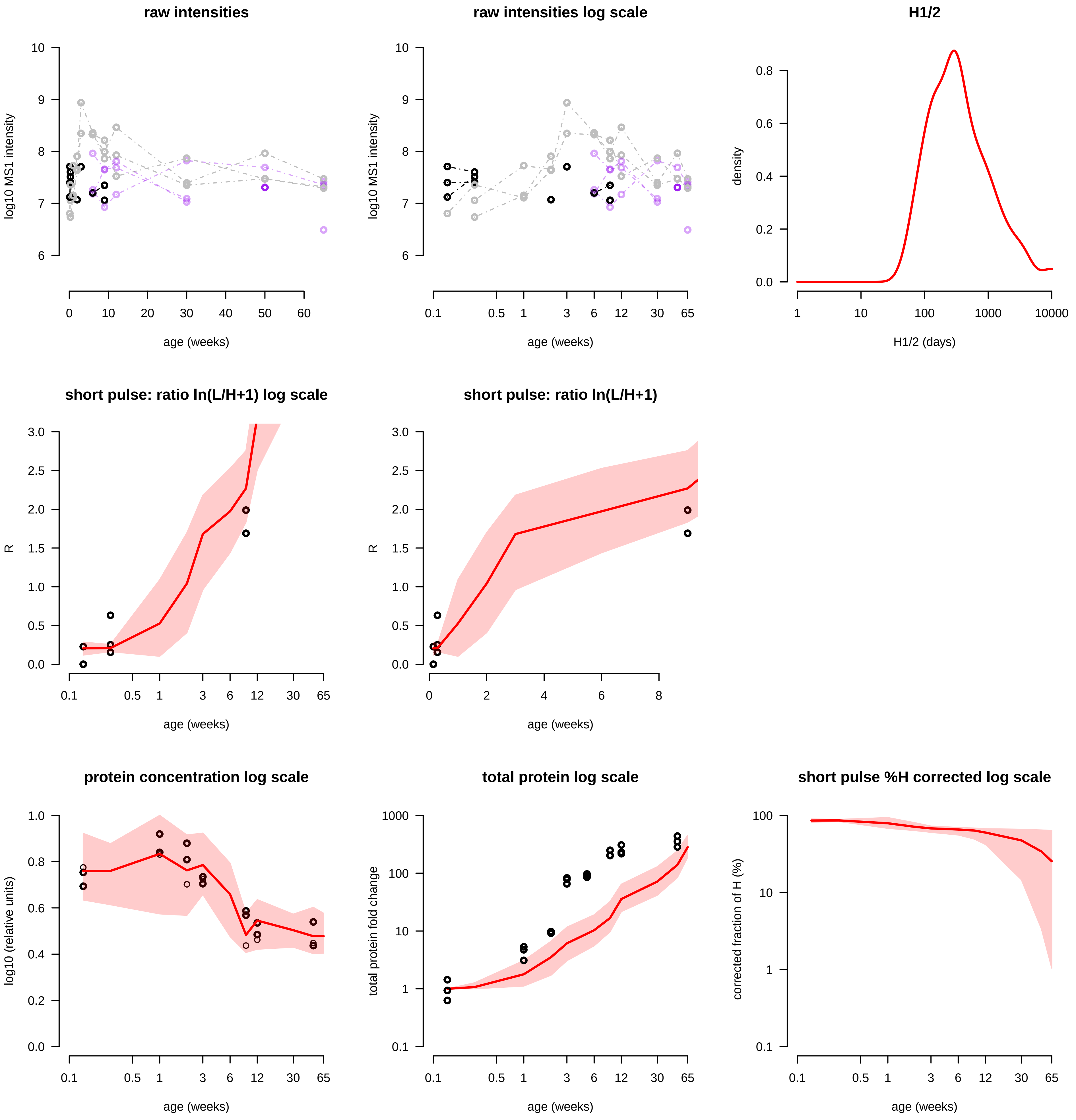

Q8VC52

>sp|Q8VC52|RBPS2_MOUSE

RNA−binding protein with multiple splicing 2

GN=Rbpms2

Med H1/2: 5.3 days

Q5 H1/2: 2.6 days

Q95 H1/2: 23.5 days

reliable model fit: yes

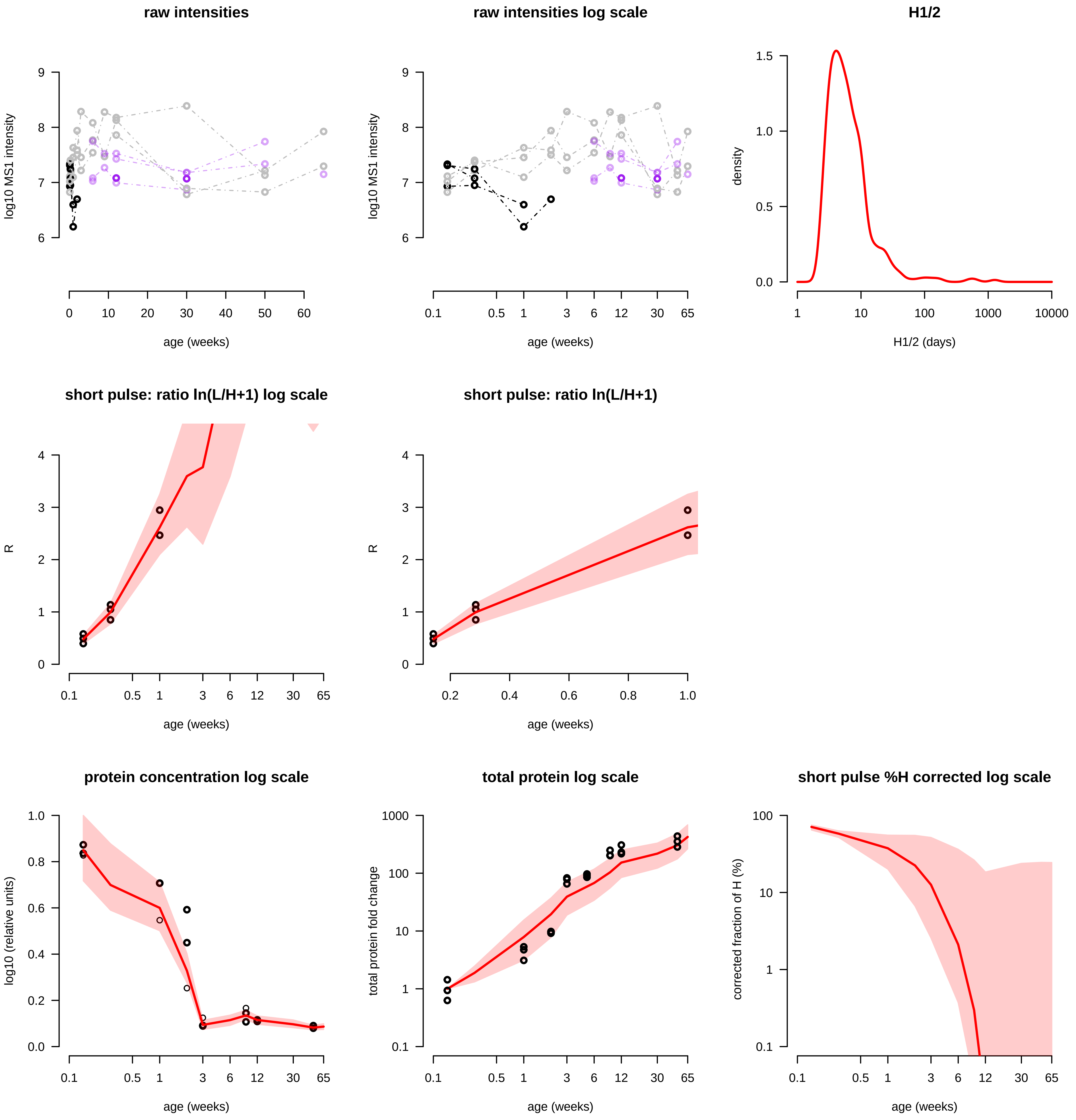

Q99N91

>sp|Q99N91|RM34_MOUSE

39S ribosomal protein L34, mitochondrial

GN=Mrpl34

Med H1/2: 8.7 days

Q5 H1/2: 3.5 days

Q95 H1/2: 81.4 days

reliable model fit: yes

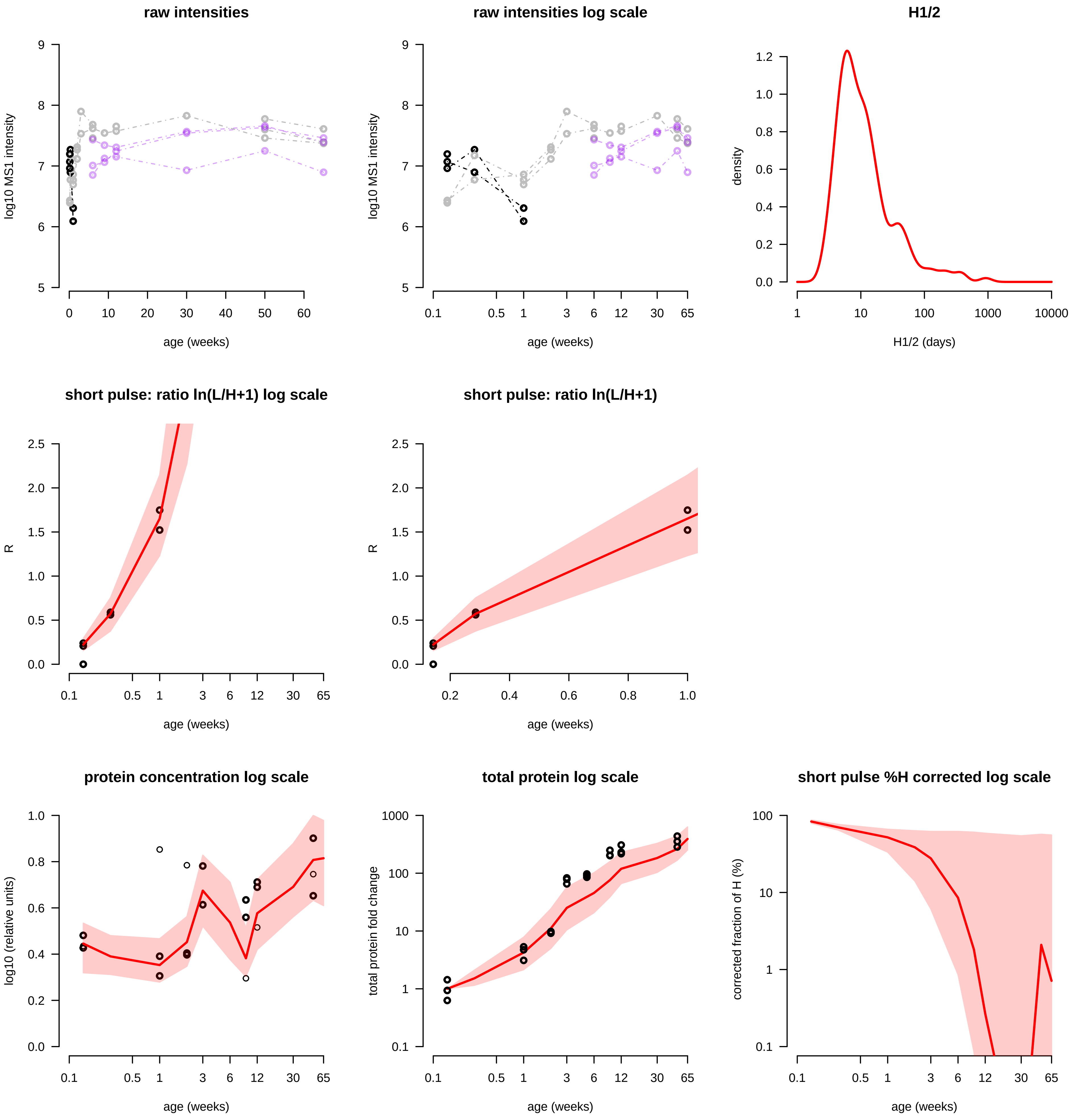

Q3TC46

>sp|Q3TC46|PATL1_MOUSE

Protein PAT1 homolog 1

GN=Patl1

Med H1/2: 4.4 days

Q5 H1/2: 2.8 days

Q95 H1/2: 19.3 days

reliable model fit: yes

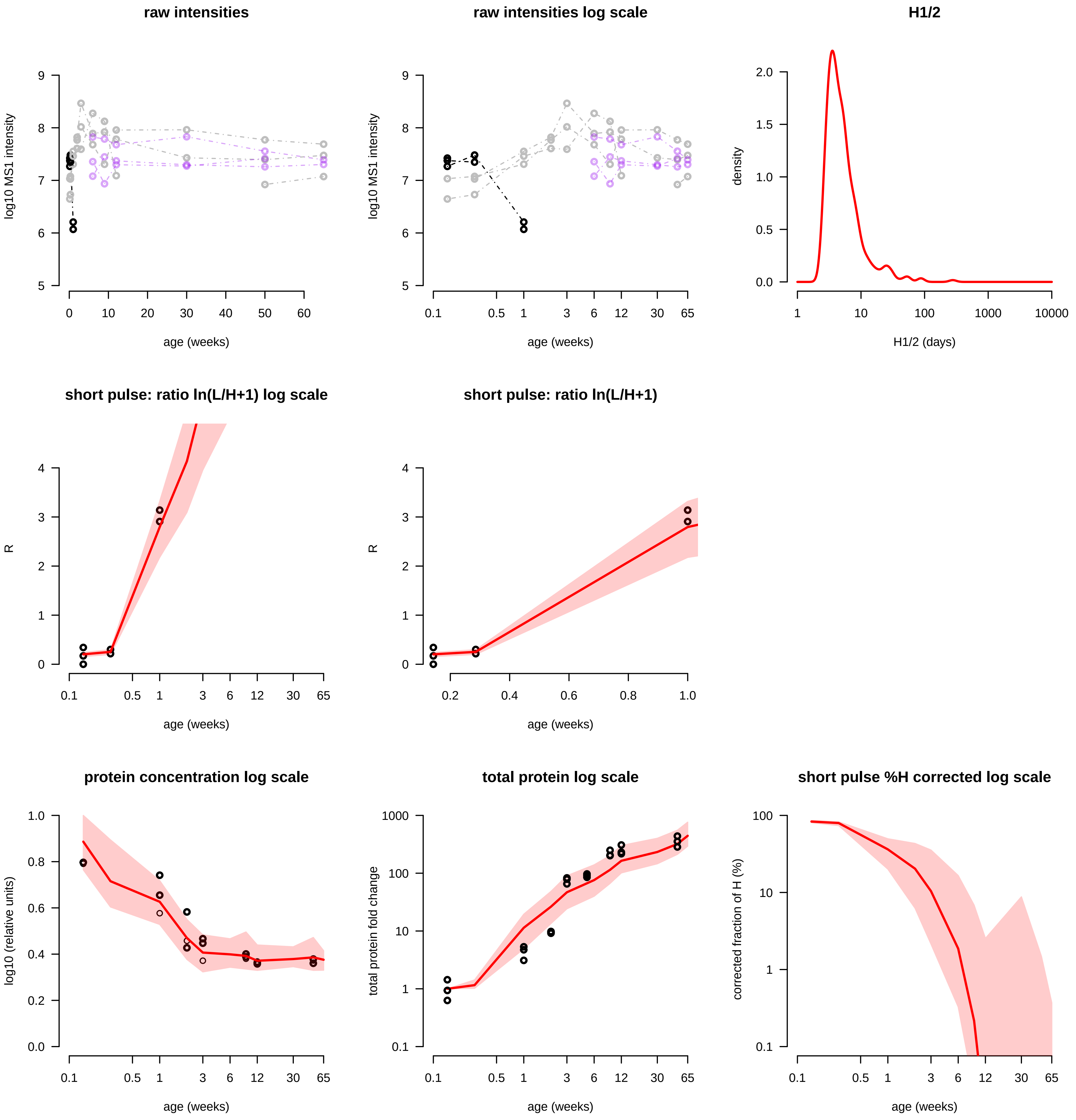

P55200−2;P55200

>sp|P55200−2|KMT2A_MOUSE

Isoform 2 of Histone−lysine N−methyltransferase 2A

GN=Kmt2a

Med H1/2: 33.9 days

Q5 H1/2: 10.5 days

Q95 H1/2: 272.8 days

reliable model fit: yes

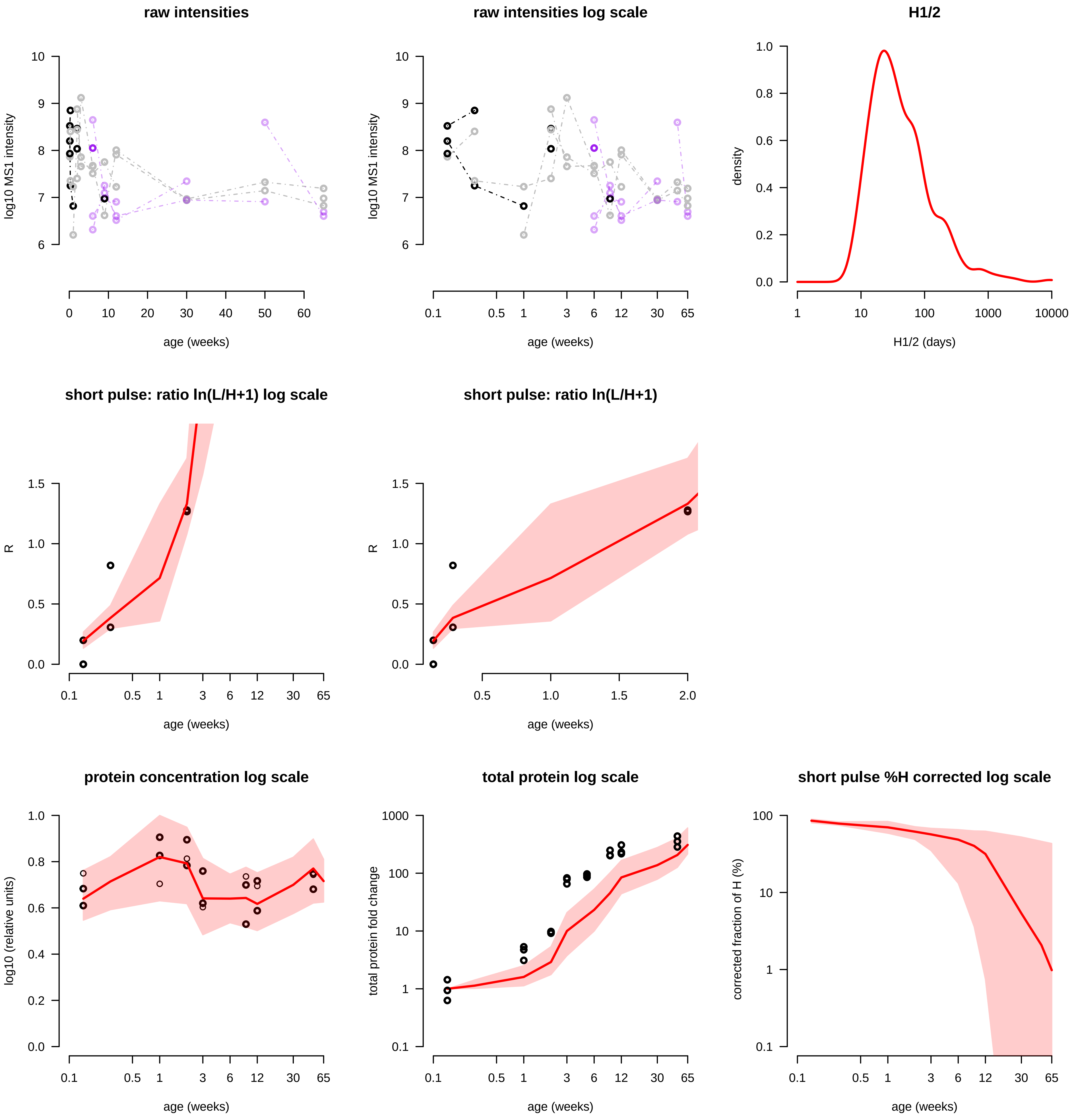

Q9CZ91

>sp|Q9CZ91|SRFB1_MOUSE

Serum response factor−binding protein 1

GN=Srfbp1

Med H1/2: 17.3 days

Q5 H1/2: 5 days

Q95 H1/2: 222.9 days

reliable model fit: yes

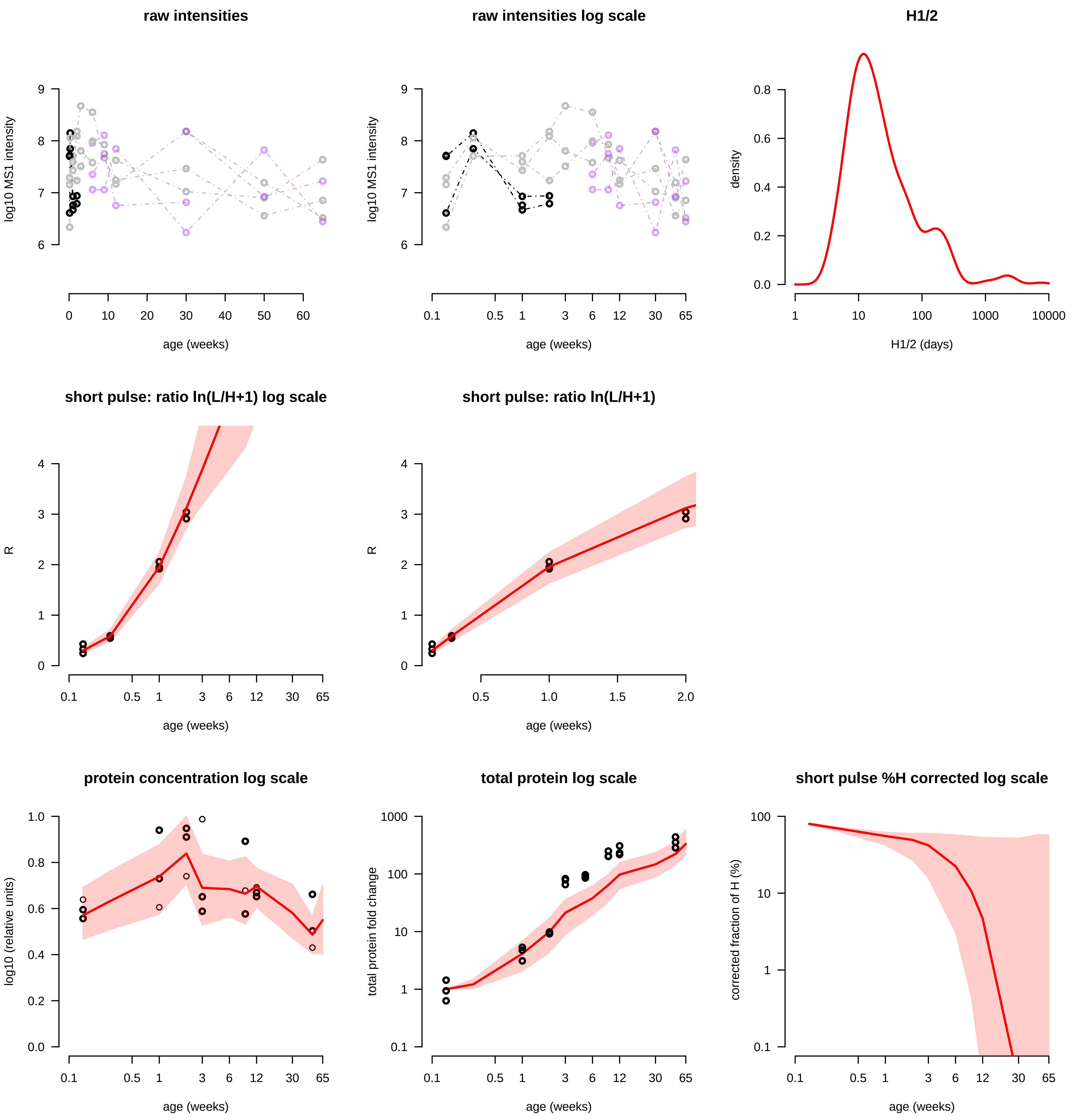

Q61496

>sp|Q61496|DDX4_MOUSE

ATP−dependent RNA helicase DDX4

GN=Ddx4

Med H1/2: 18.4 days

Q5 H1/2: 7.5 days

Q95 H1/2: 188.7 days

reliable model fit: yes

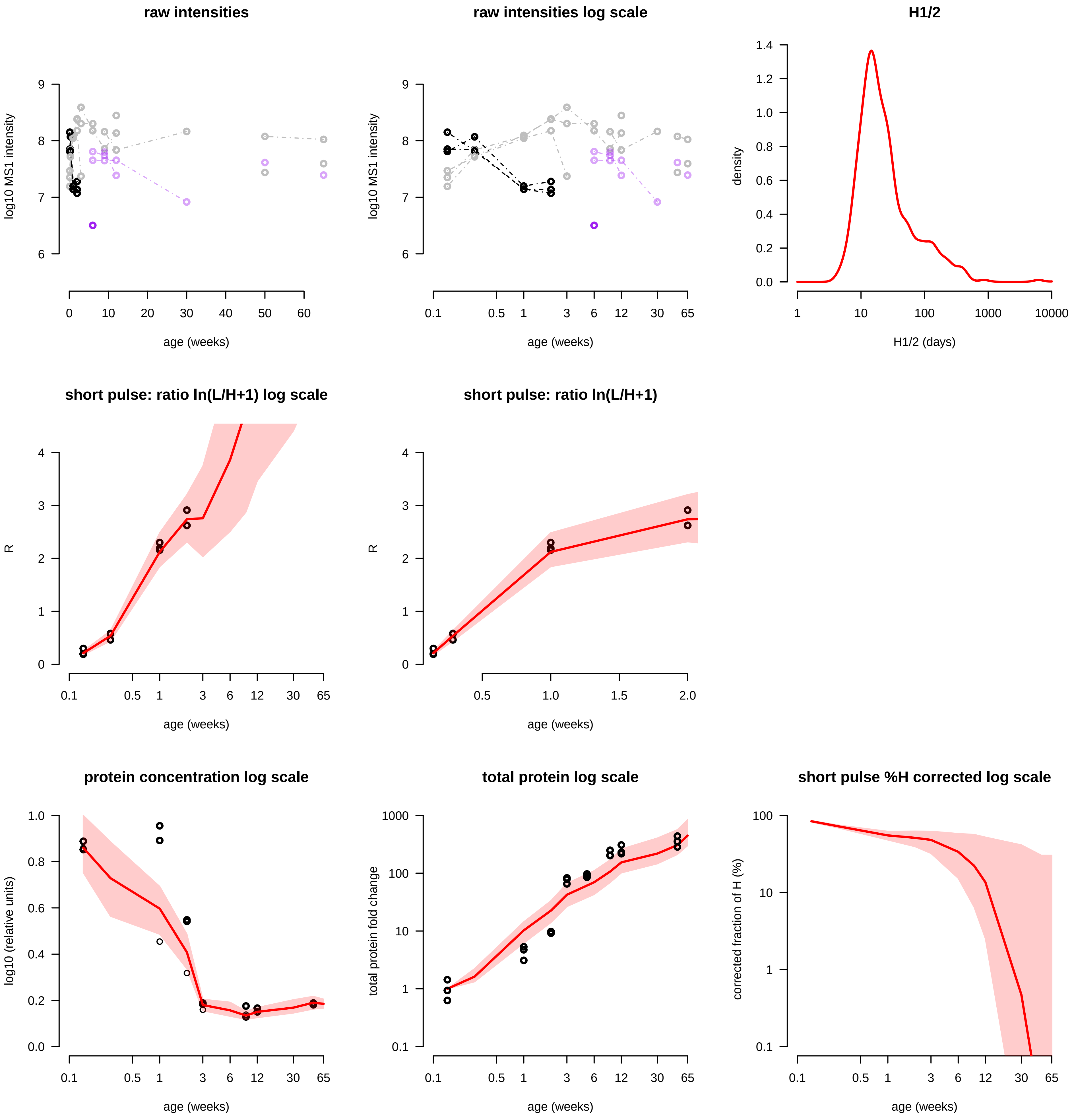

P62965

>sp|P62965|RABP1_MOUSE

Cellular retinoic acid−binding protein 1

GN=Crabp1

Med H1/2: 7.6 days

Q5 H1/2: 3 days

Q95 H1/2: 44.8 days

reliable model fit: yes

raw intensities

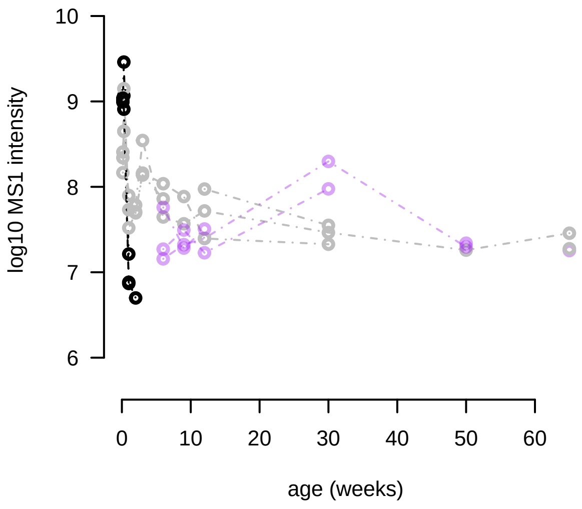

raw intensities log scale

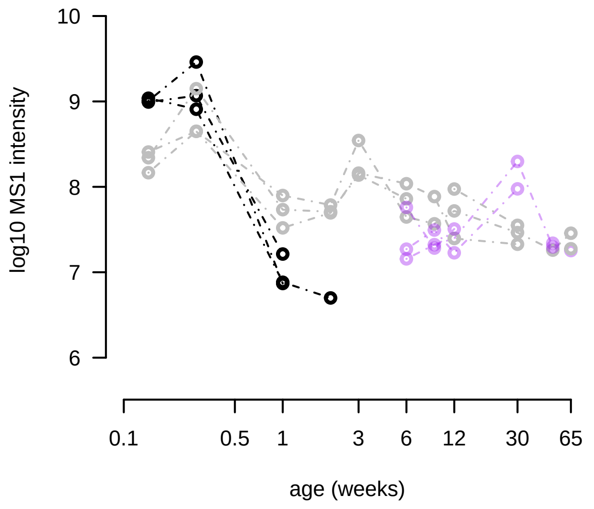

H1/2

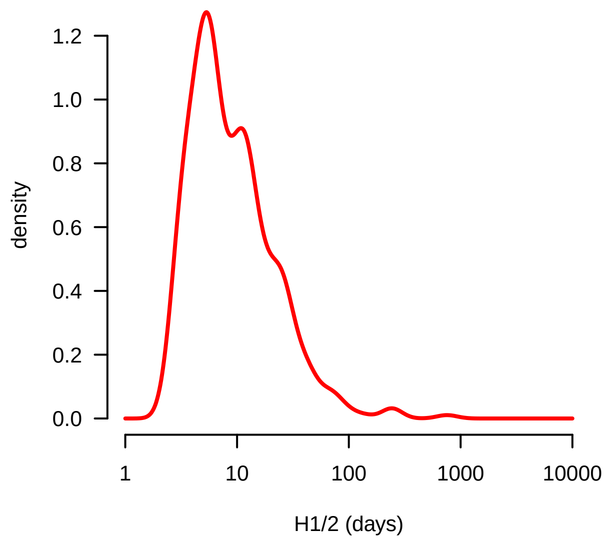

short pulse: ratio ln(L/H+1) log scale

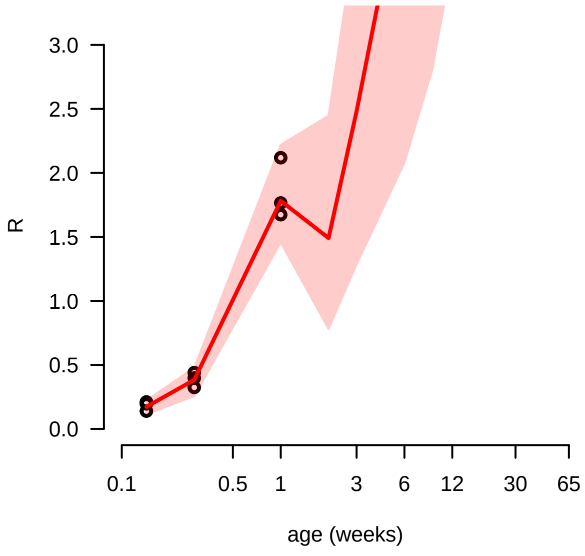

short pulse: ratio ln(L/H+1)

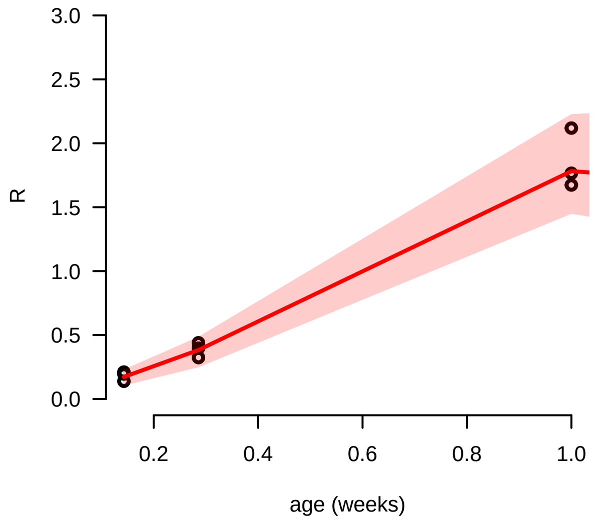

protein concentration log scale

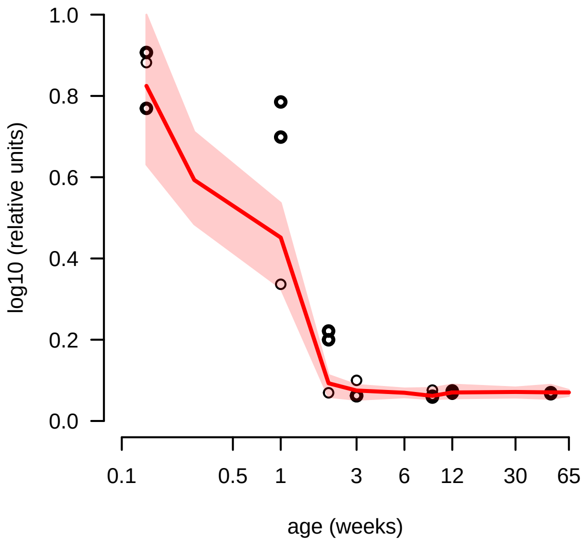

total protein log scale

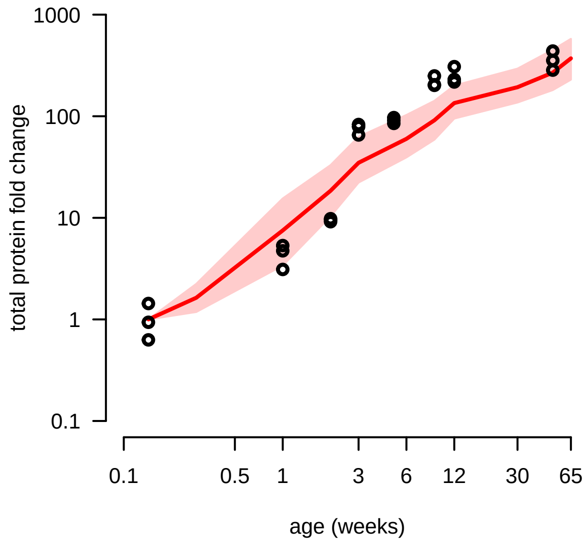

short pulse %H corrected log scale

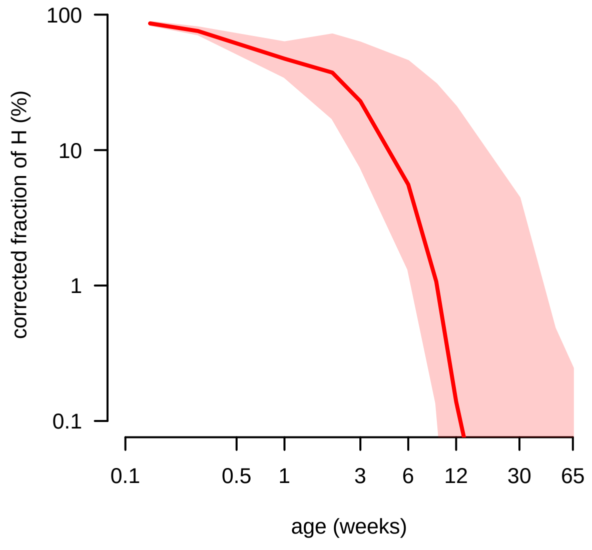

Q810A3

>sp|Q810A3|TTC9C_MOUSE

Tetratricopeptide repeat protein 9C

GN=Ttc9c

Med H1/2: 11.4 days

Q5 H1/2: 3.4 days

Q95 H1/2: 114.3 days

reliable model fit: no

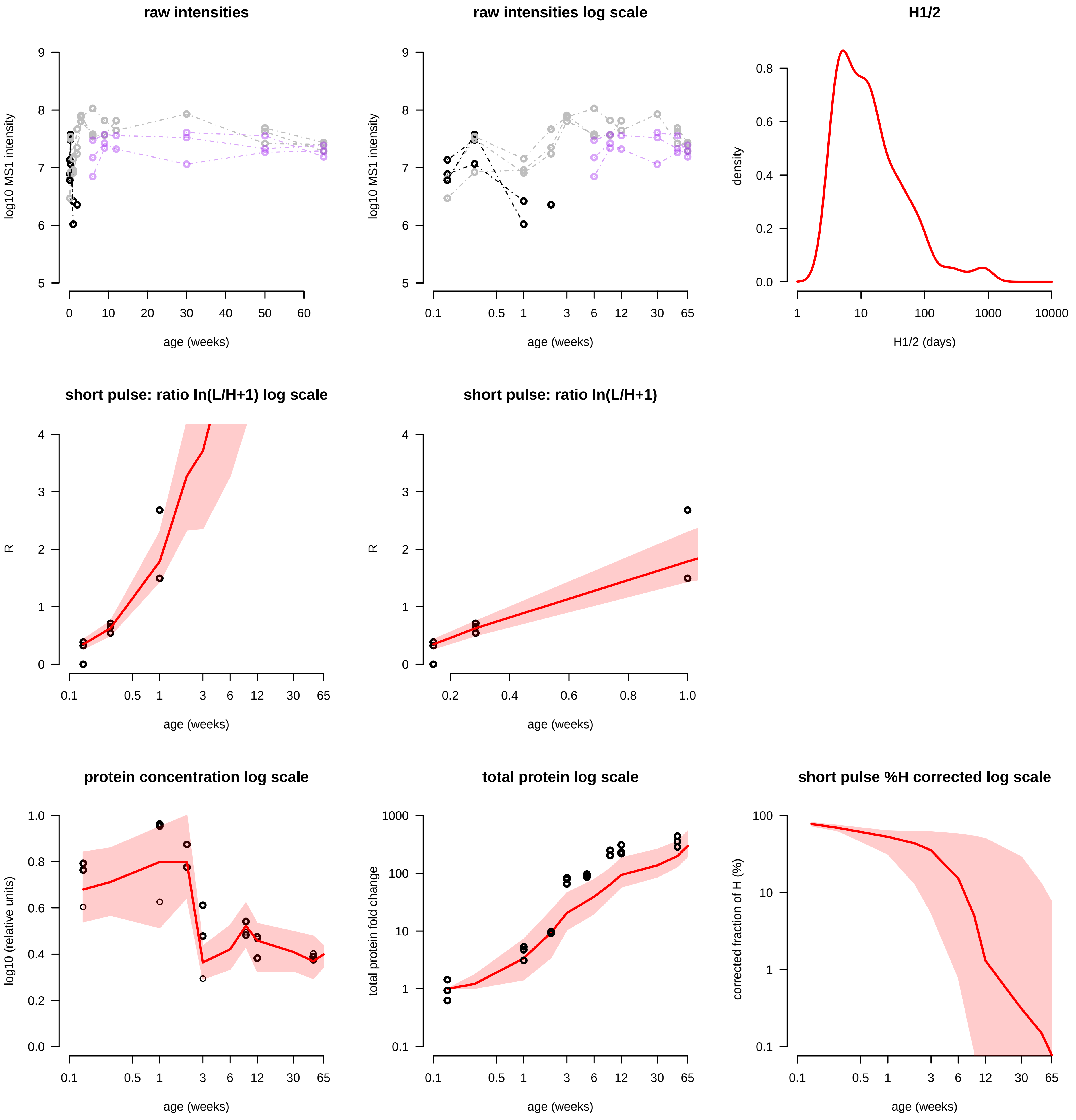

Q91UZ5

>sp|Q91UZ5|IMPA2_MOUSE

Inositol monophosphatase 2

GN=Impa2

Med H1/2: 8.7 days

Q5 H1/2: 3.5 days

Q95 H1/2: 70 days

reliable model fit: yes

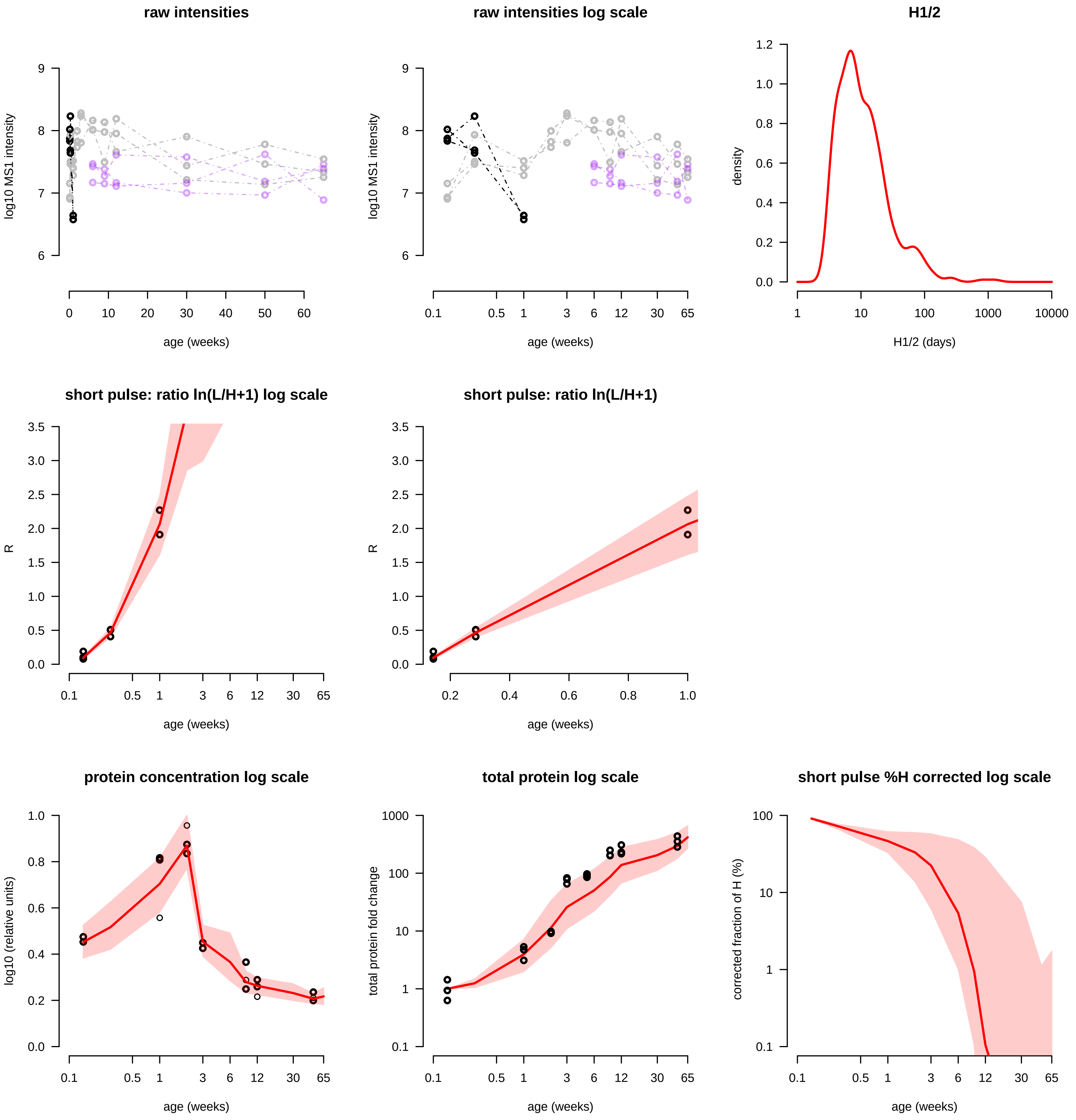

Q8C8N2;Q8C8N2−2

>sp|Q8C8N2|SCAI_MOUSE

Protein SCAI

GN=Scai

Med H1/2: 11.8 days

Q5 H1/2: 4.4 days

Q95 H1/2: 174.7 days

reliable model fit: no

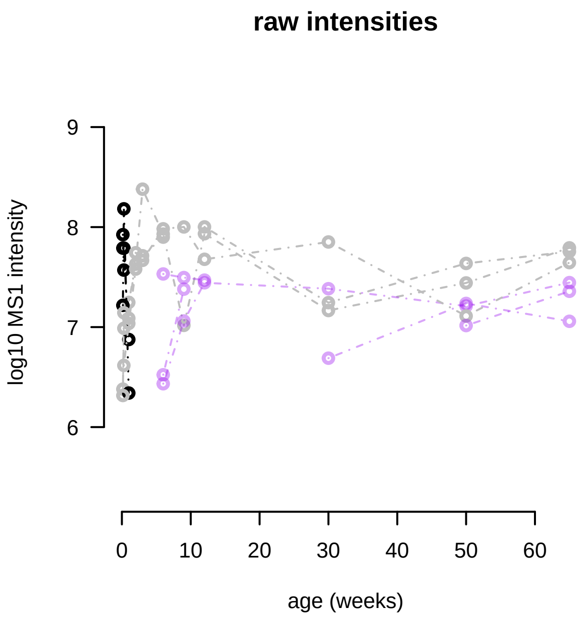

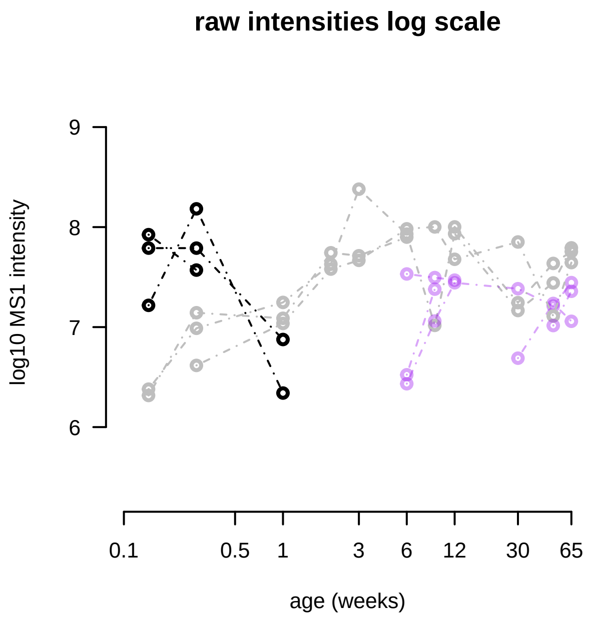

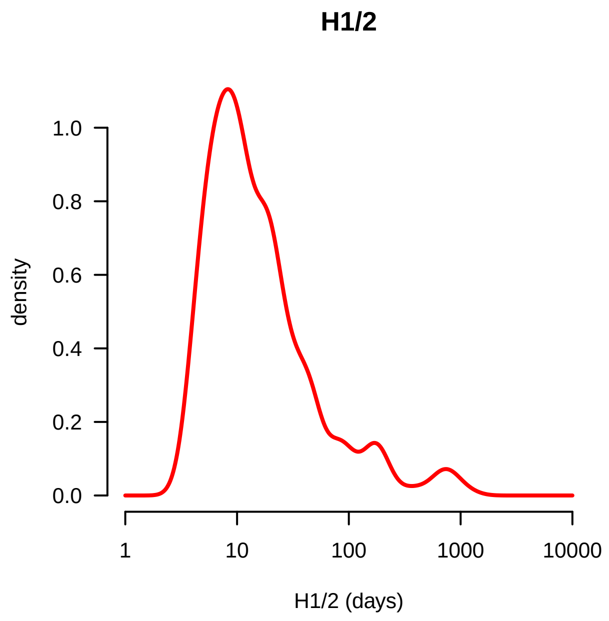

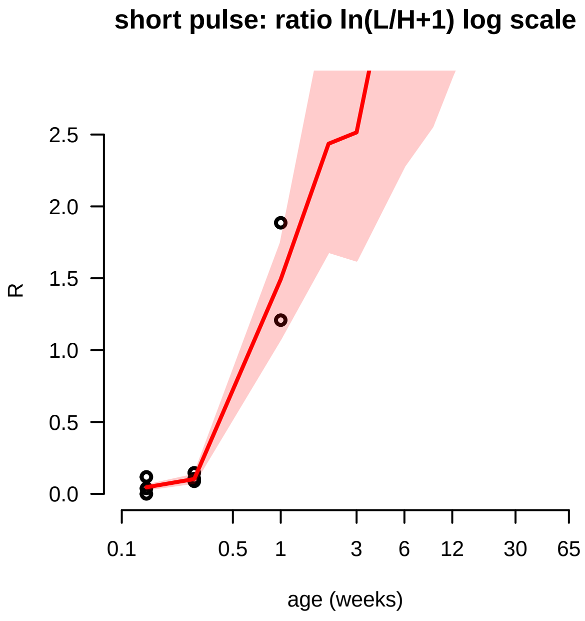

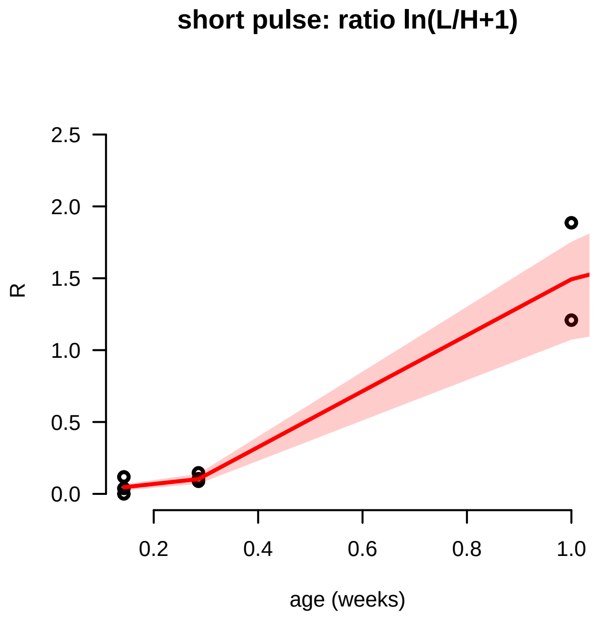

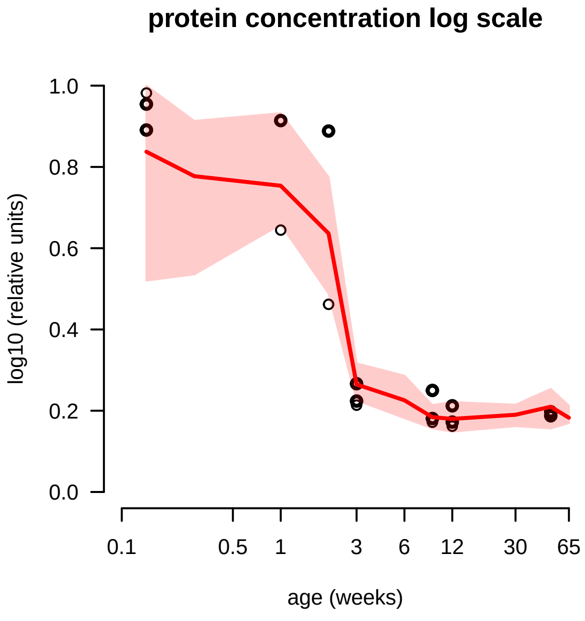

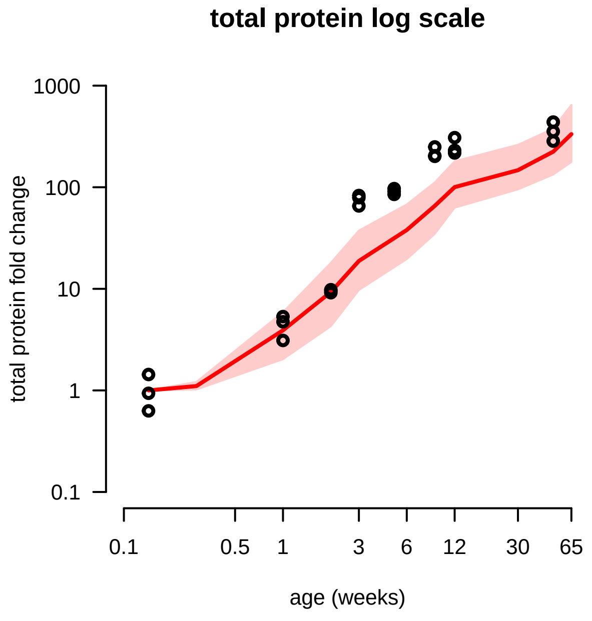

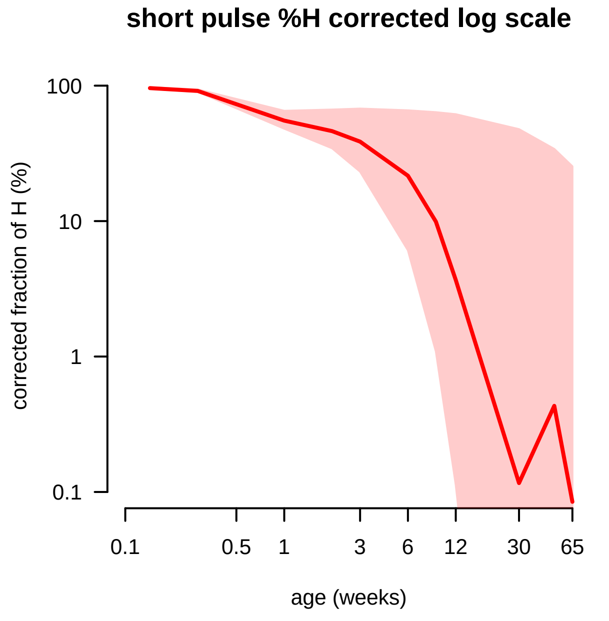

Q9D8C3

>sp|Q9D8C3|ALG13_MOUSE

ative bifunctional UDP−N−acetylglucosamine transferase and deubiquitinase AL

GN=Alg13

Med H1/2: 3739.2 days

Q5 H1/2: 1063.1 days

Q95 H1/2: 20725.4 days

reliable model fit: no

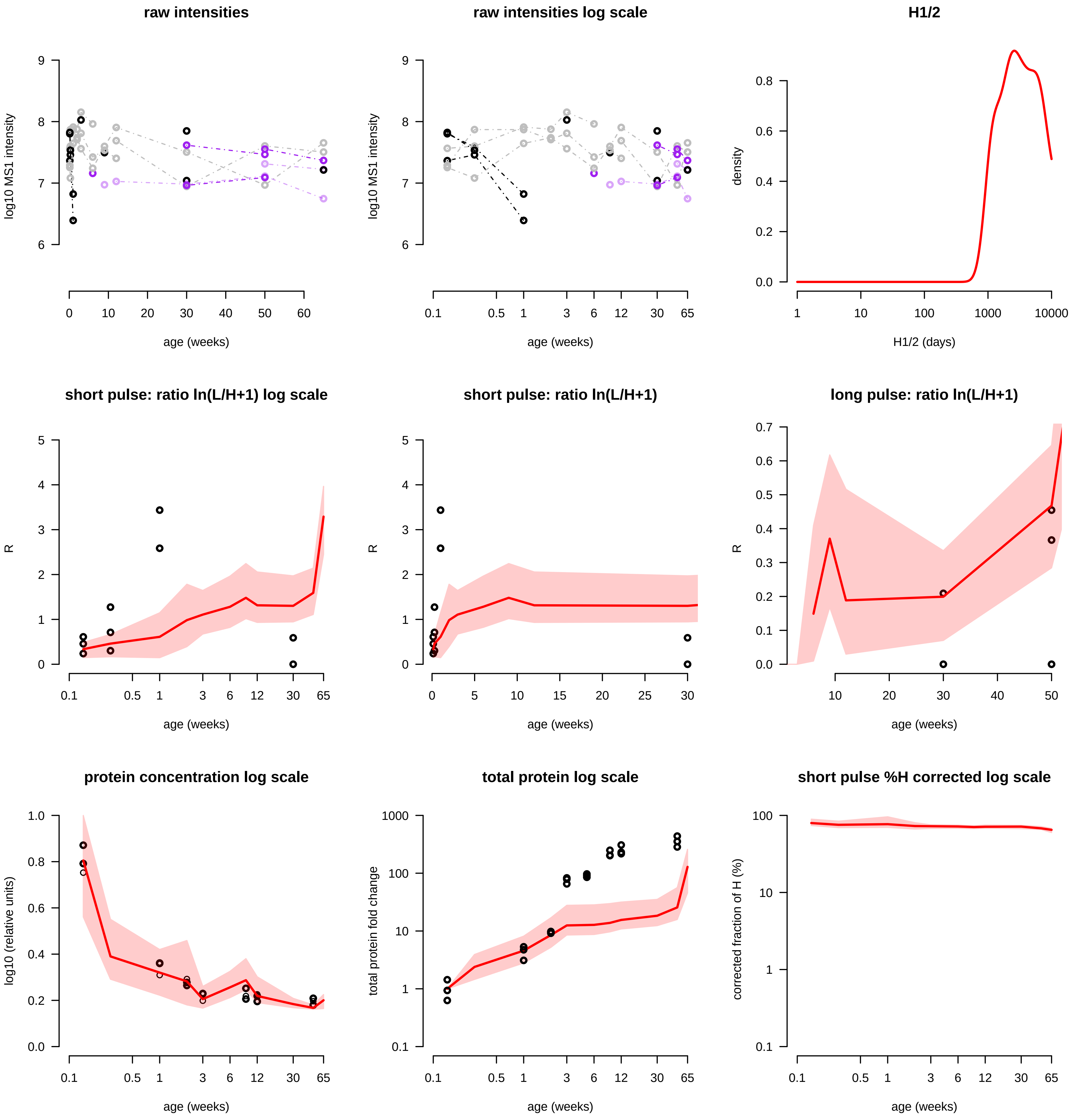

Q9DBA9

>sp|Q9DBA9|TF2H1_MOUSE

General transcription factor IIH subunit 1

GN=Gtf2h1

Med H1/2: 20.2 days

Q5 H1/2: 6.6 days

Q95 H1/2: 161.1 days

reliable model fit: yes

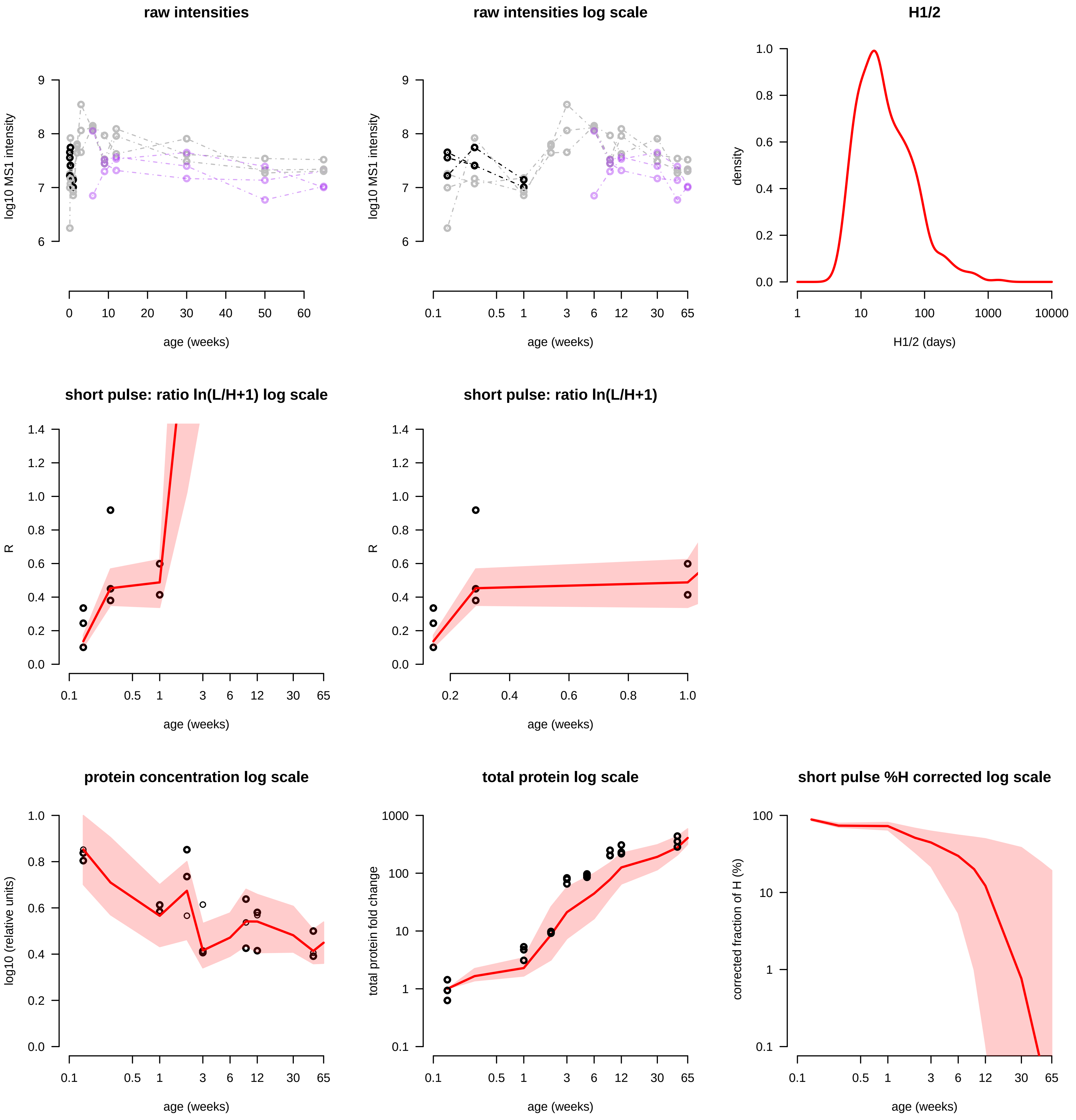

Q9ET26

>sp|Q9ET26|RN114_MOUSE

E3 ubiquitin−protein ligase RNF114

GN=Rnf114

Med H1/2: 8 days

Q5 H1/2: 5.9 days

Q95 H1/2: 11.2 days

reliable model fit: yes

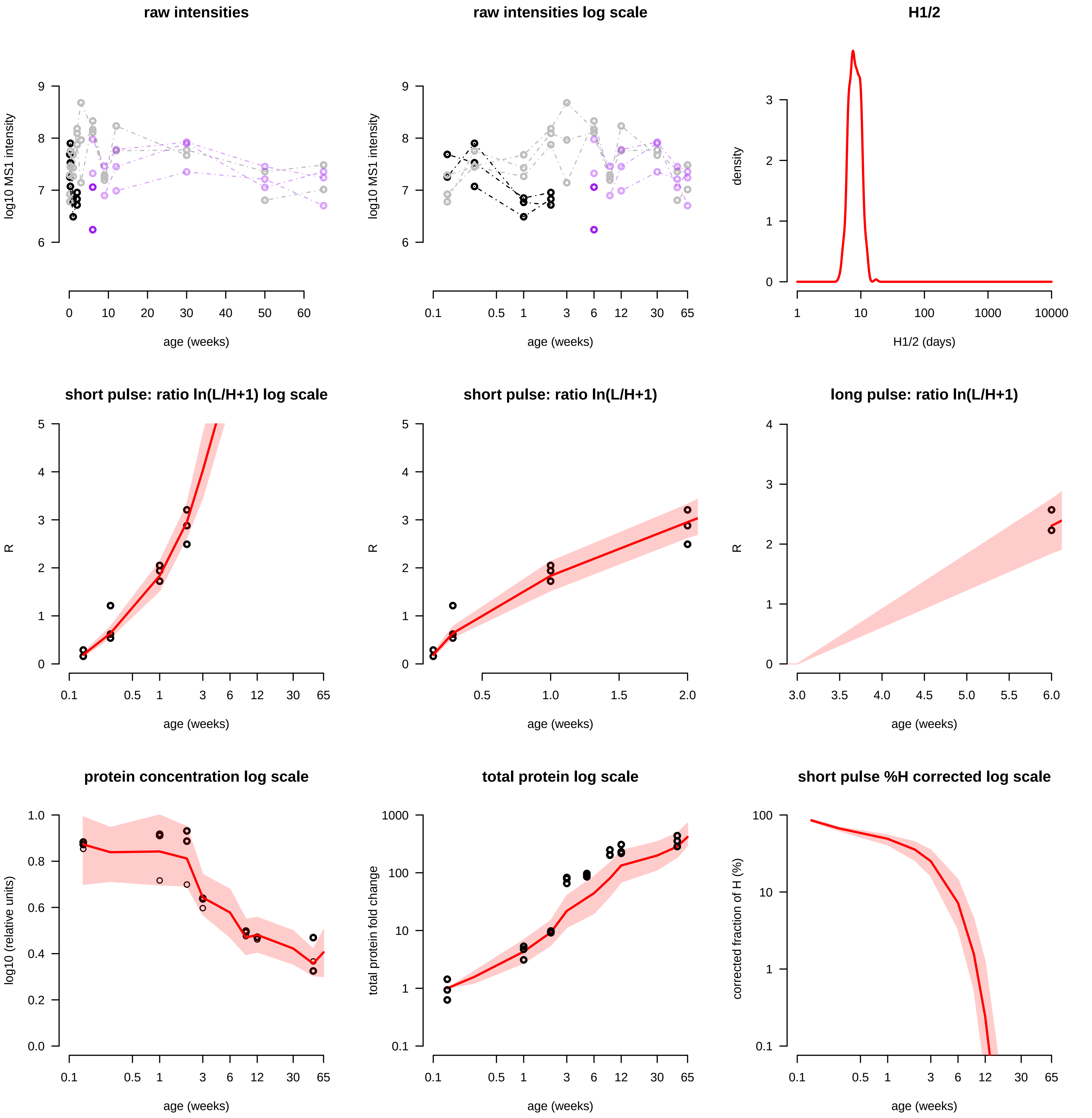

Q8BJ64

>sp|Q8BJ64|CHDH_MOUSE

Choline dehydrogenase, mitochondrial

GN=Chdh

Med H1/2: 16.4 days

Q5 H1/2: 4.3 days

Q95 H1/2: 215.1 days

reliable model fit: yes

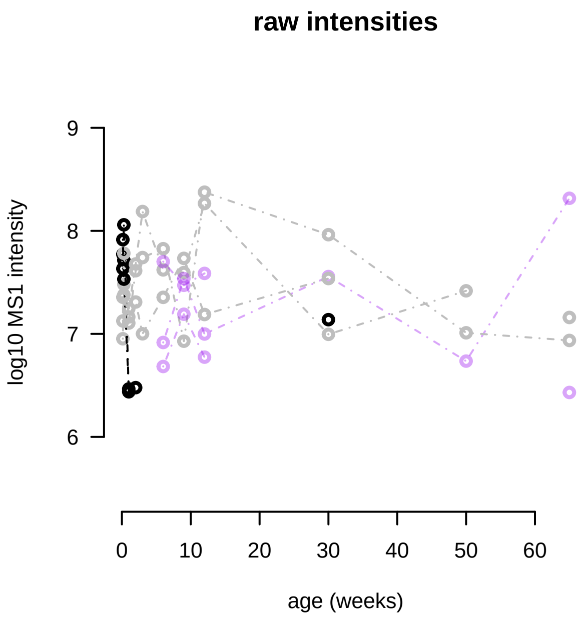

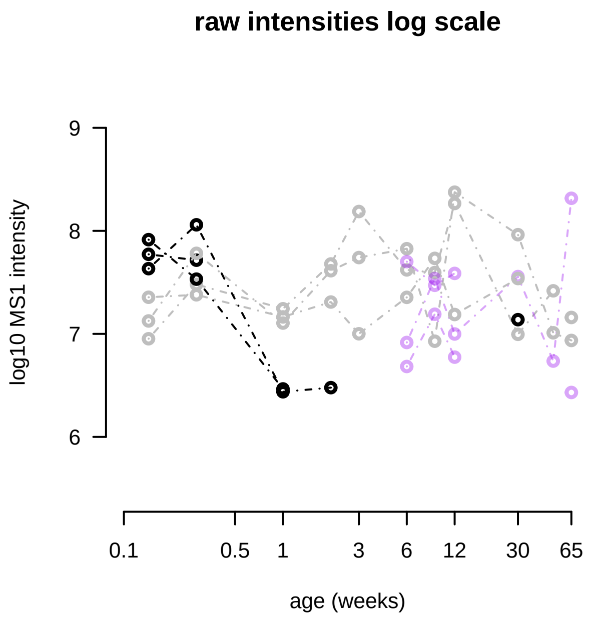

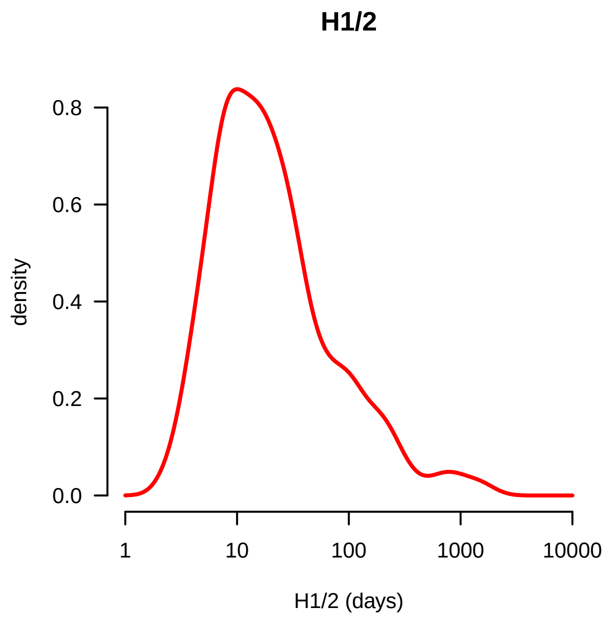

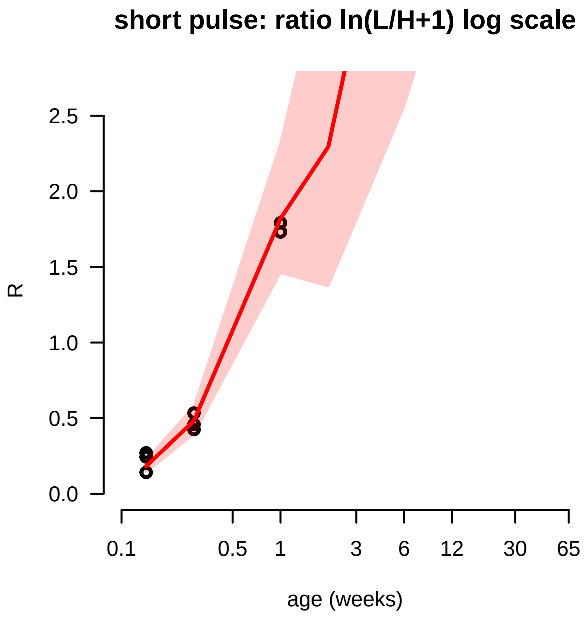

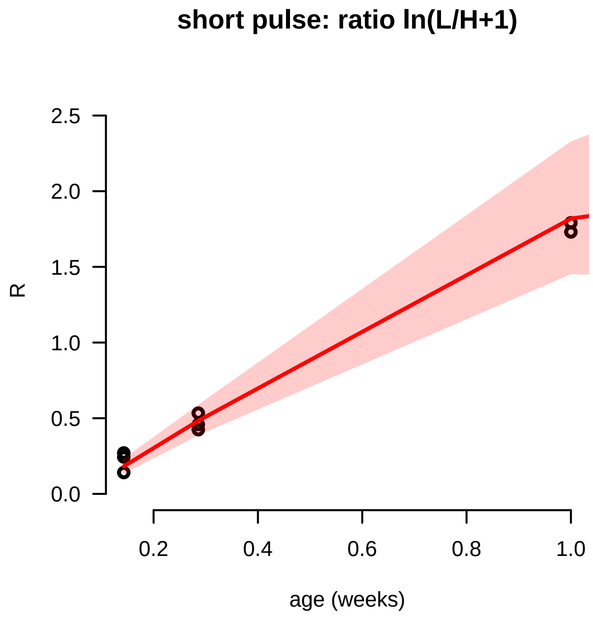

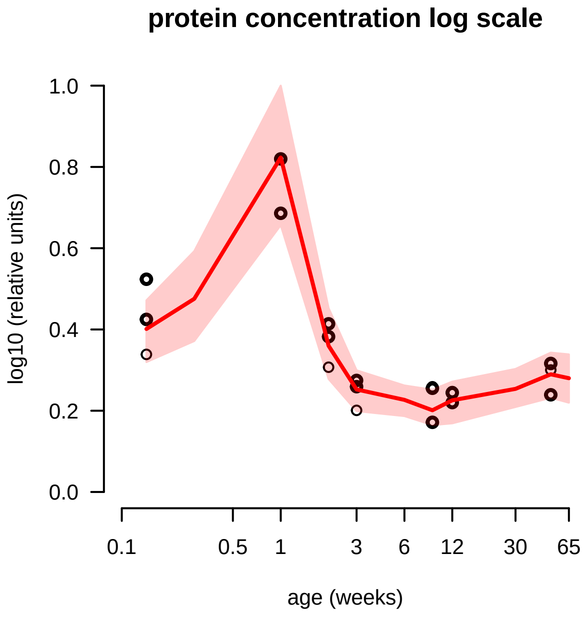

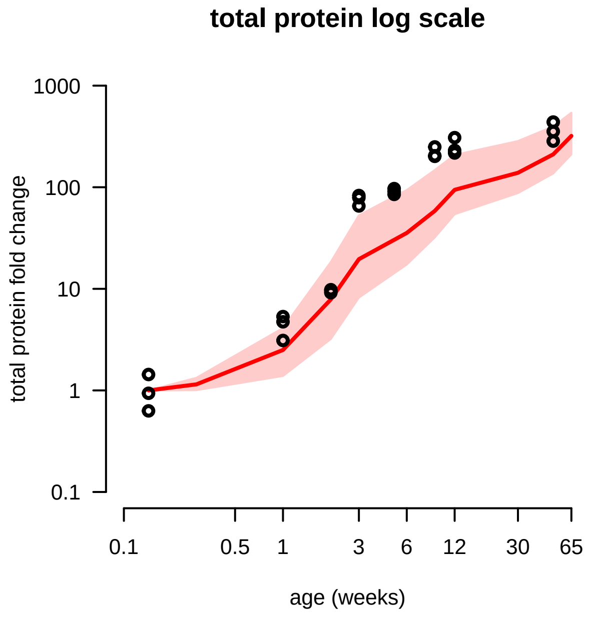

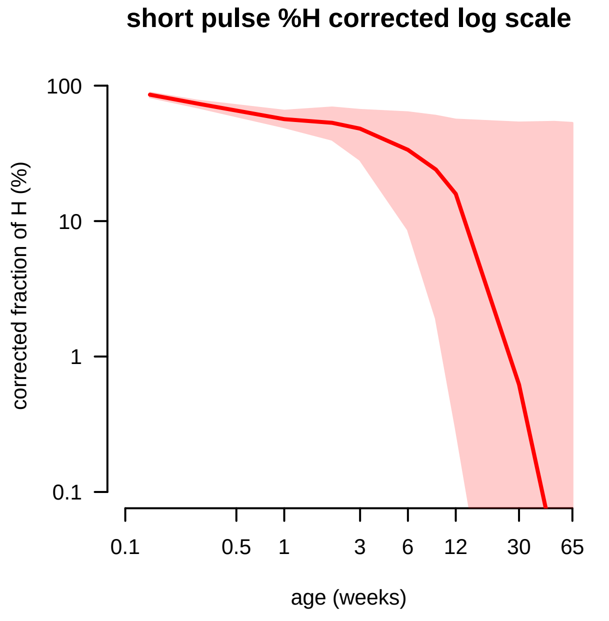

Q5SF07;Q5SF07−2

>sp|Q5SF07|IF2B2_MOUSE

Insulin−like growth factor 2 mRNA−binding protein 2

GN=Igf2bp2

Med H1/2: 10.4 days

Q5 H1/2: 4.7 days

Q95 H1/2: 31.5 days

reliable model fit: yes

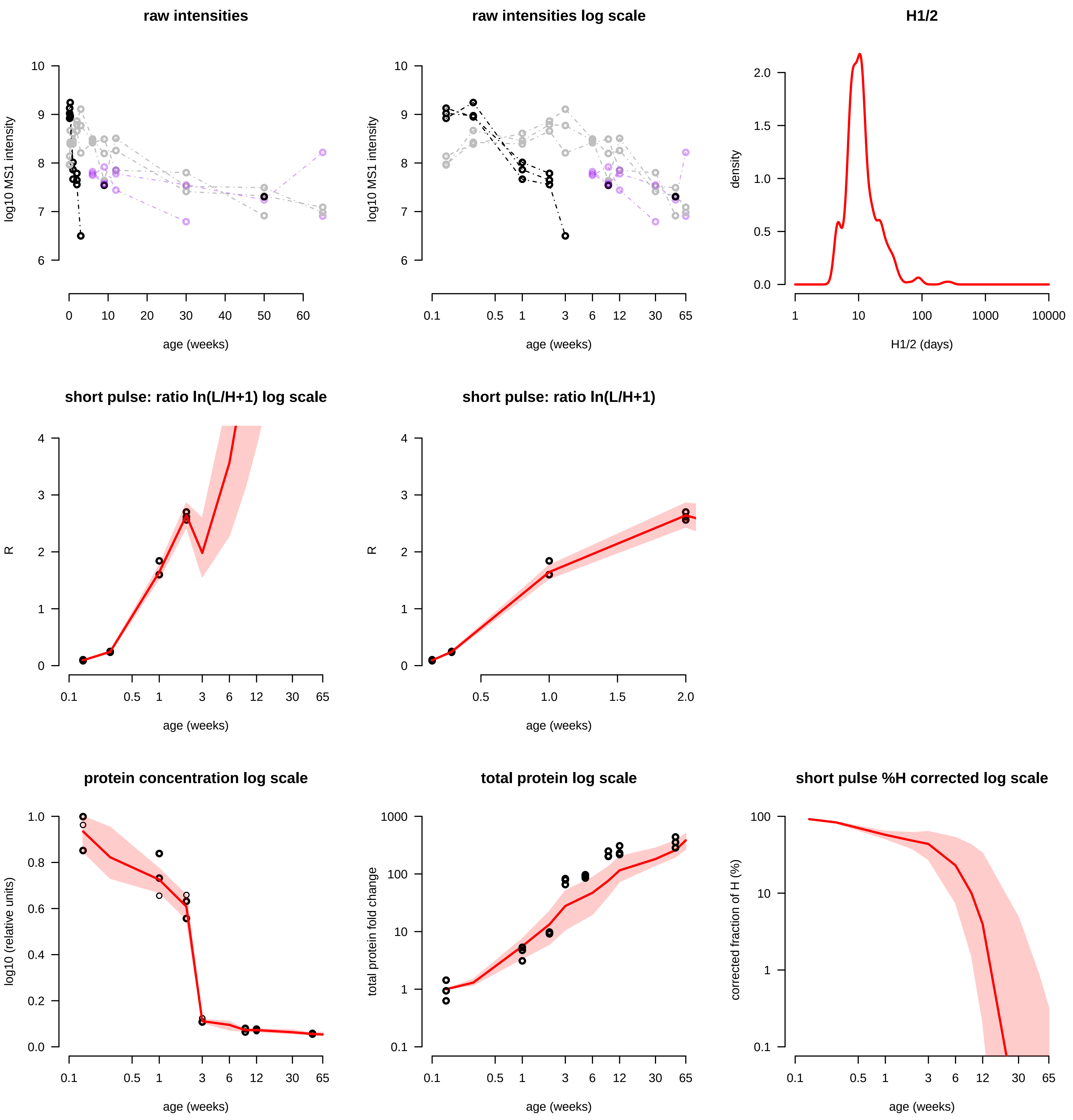

Q9JMA2

>sp|Q9JMA2|TGT_MOUSE

Queuine tRNA−ribosyltransferase catalytic subunit 1

GN=Qtrt1

Med H1/2: 7 days

Q5 H1/2: 3.2 days

Q95 H1/2: 101.4 days

reliable model fit: yes

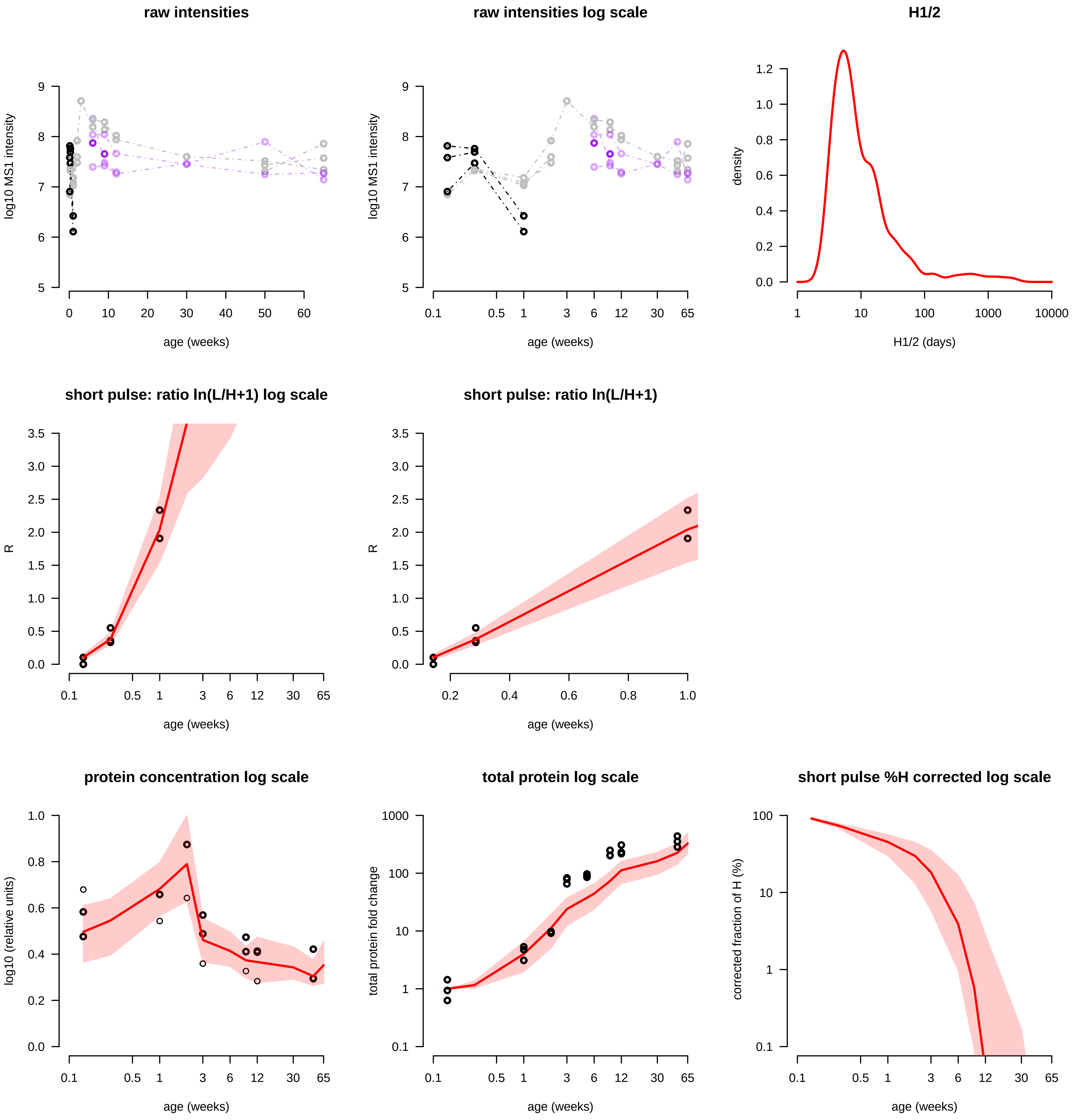

Q8C7E9

>sp|Q8C7E9|CSTFT_MOUSE

Cleavage stimulation factor subunit 2 tau variant

GN=Cstf2t

Med H1/2: 4.8 days

Q5 H1/2: 2.6 days

Q95 H1/2: 25.6 days

reliable model fit: yes

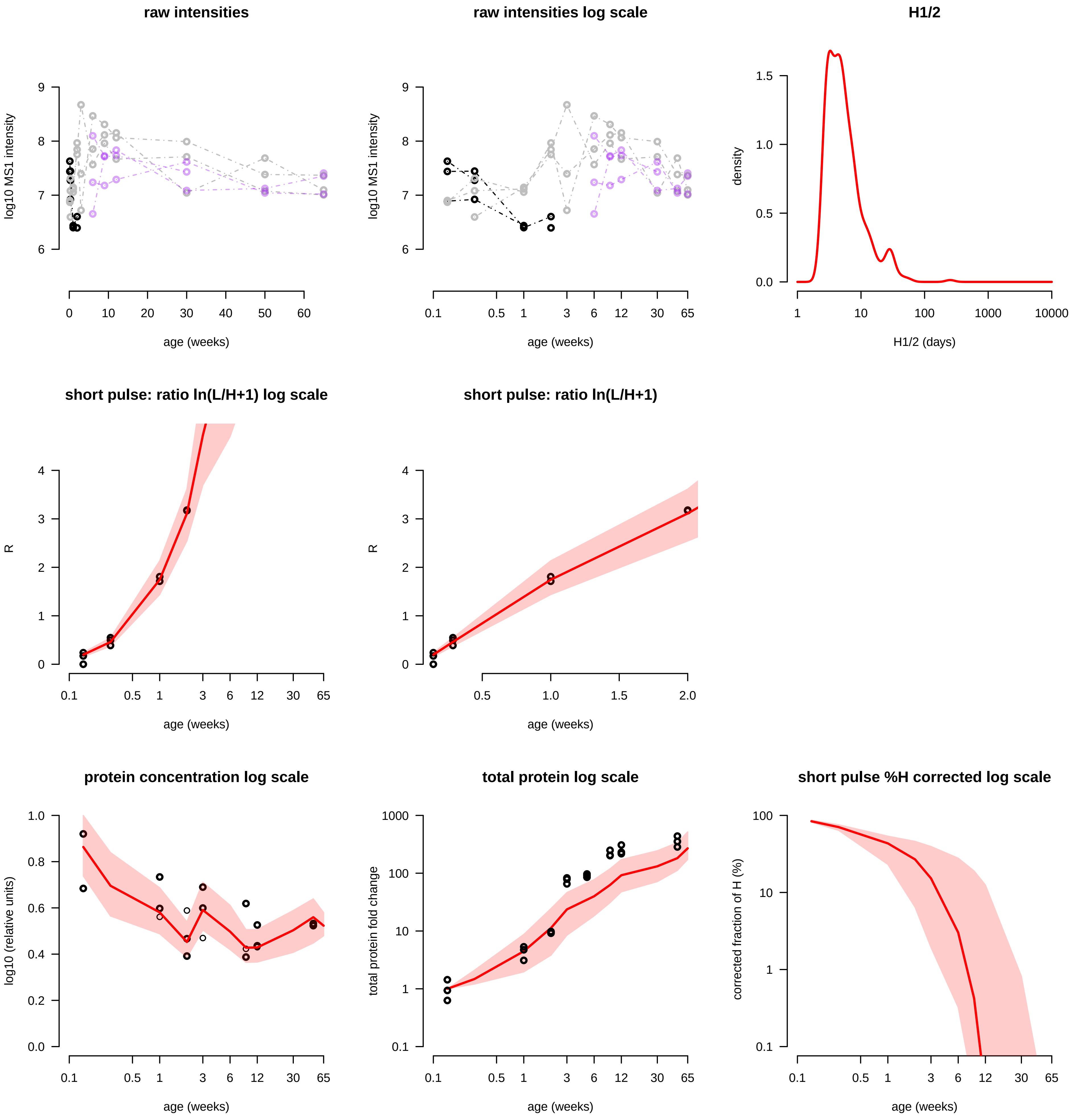

Q9EST3

>sp|Q9EST3|4ET_MOUSE

Eukaryotic translation initiation factor 4E transporter

GN=Eif4enif1

Med H1/2: 3.9 days

Q5 H1/2: 2.5 days

Q95 H1/2: 82.8 days

reliable model fit: yes

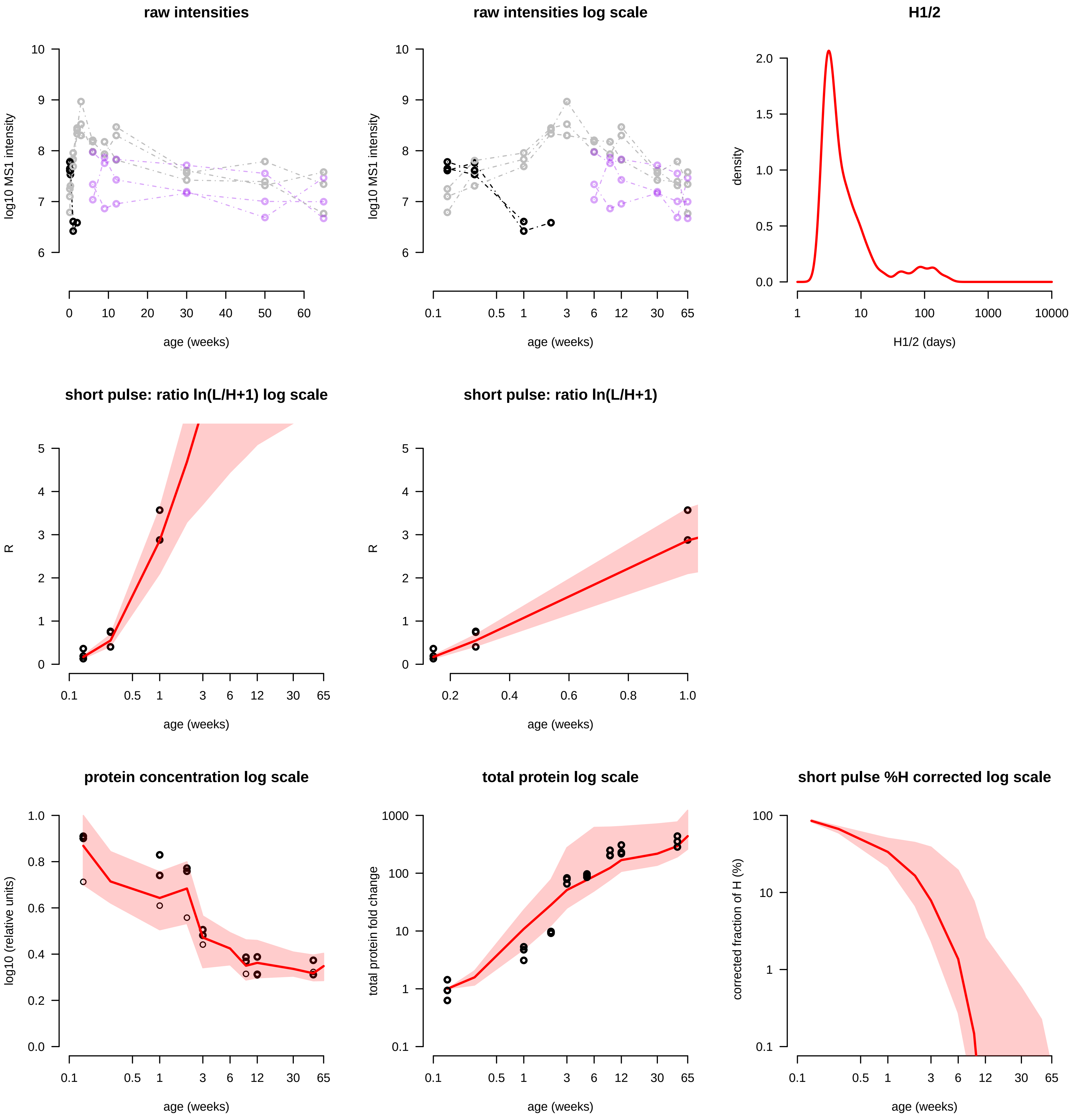

A8C756

>sp|A8C756|THADA_MOUSE

Thyroid adenoma−associated protein homolog

GN=Thada

Med H1/2: 14.4 days

Q5 H1/2: 4.3 days

Q95 H1/2: 130.4 days

reliable model fit: yes

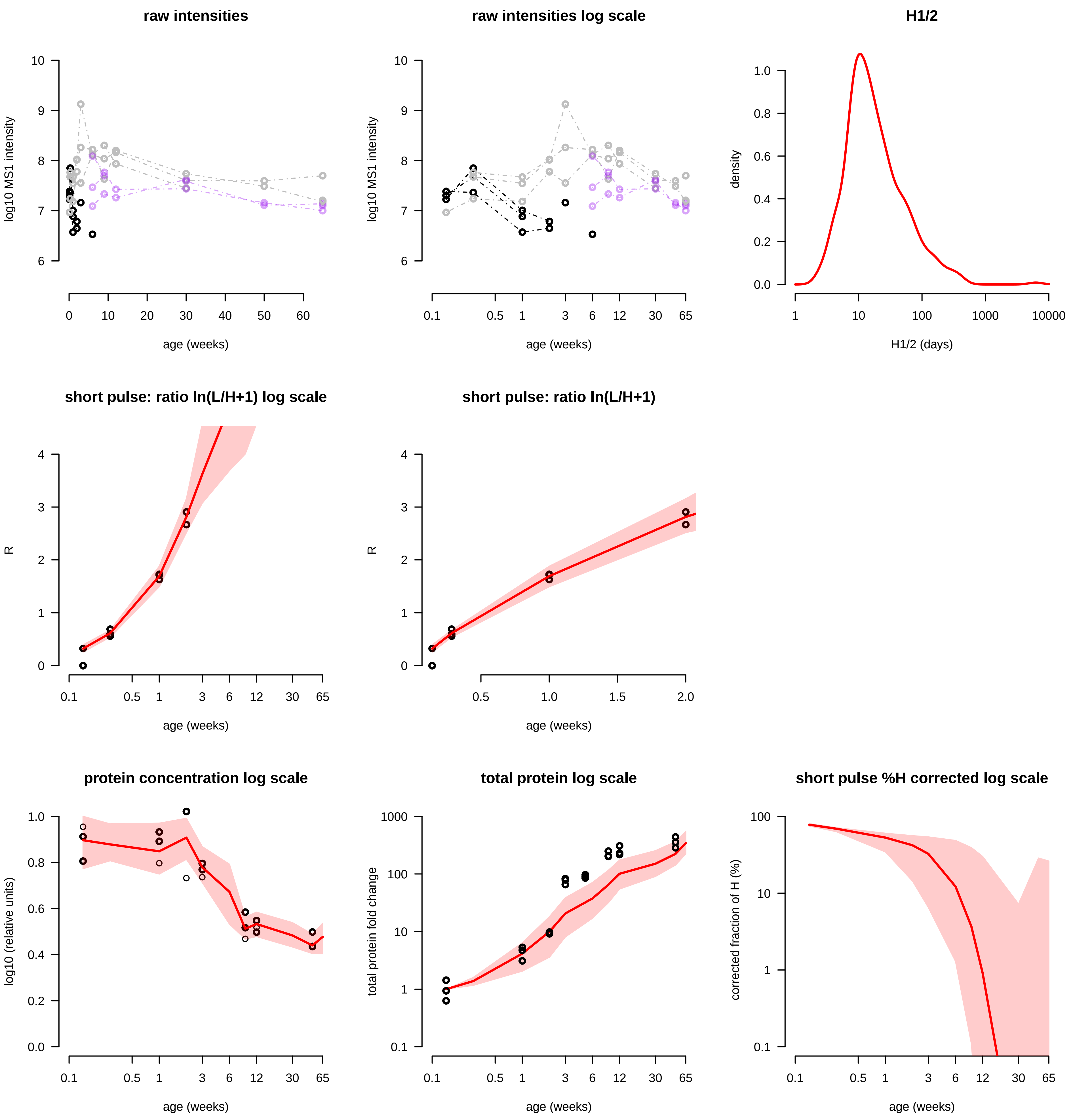

P57722−2;P57722

>sp|P57722−2|PCBP3_MOUSE

Isoform 2 of Poly(rC)−binding protein 3

GN=Pcbp3

Med H1/2: 6.9 days

Q5 H1/2: 2.7 days

Q95 H1/2: 86.1 days

reliable model fit: yes

raw intensities

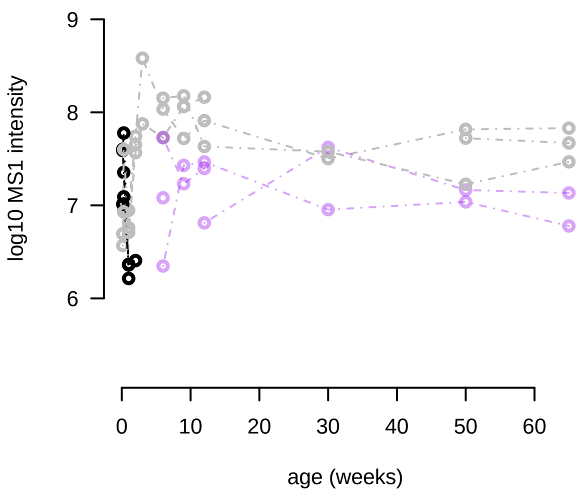

raw intensities log scale

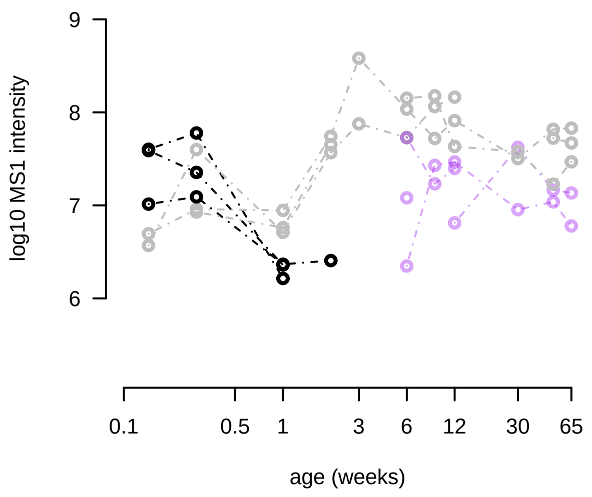

H1/2

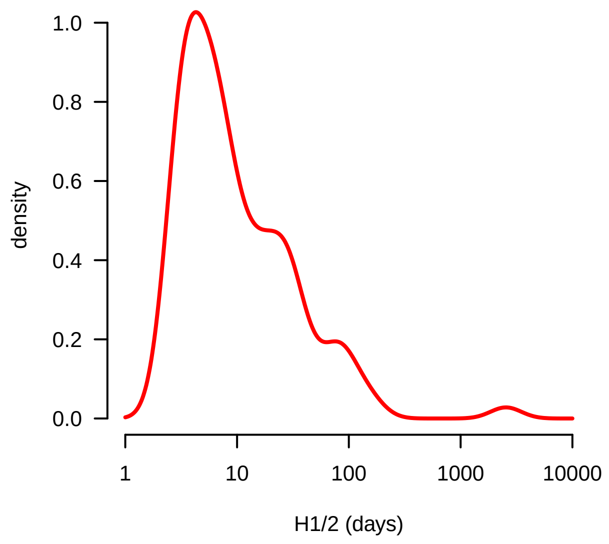

short pulse: ratio ln(L/H+1) log scale

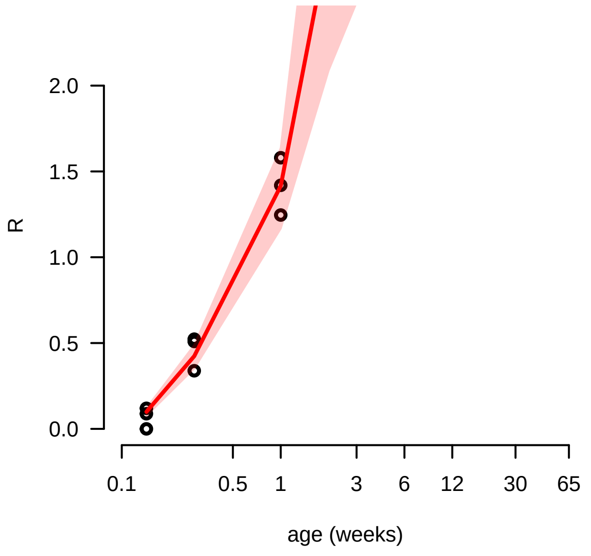

short pulse: ratio ln(L/H+1)

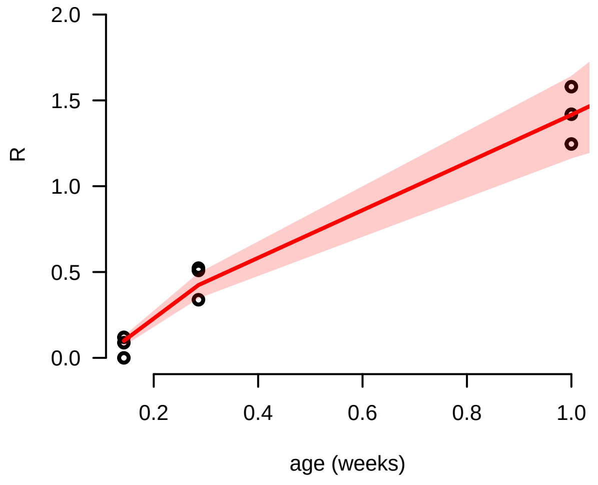

protein concentration log scale

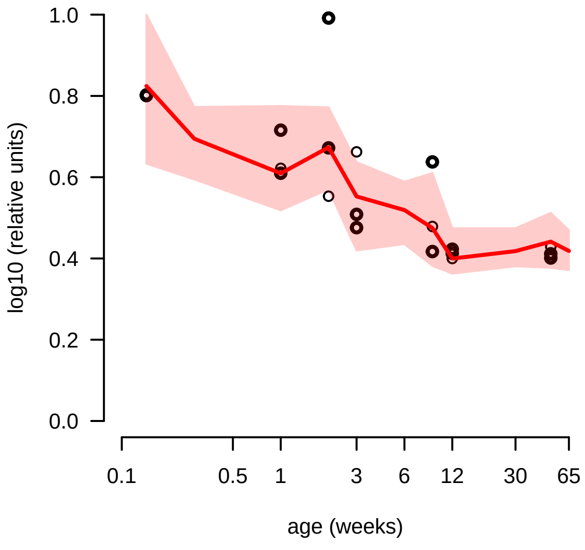

total protein log scale

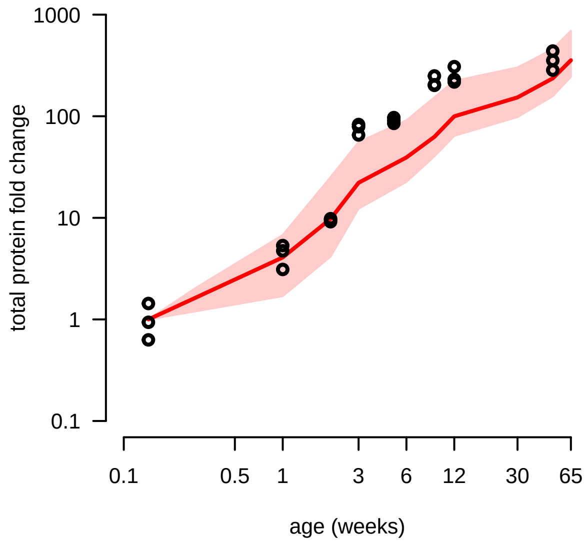

short pulse %H corrected log scale

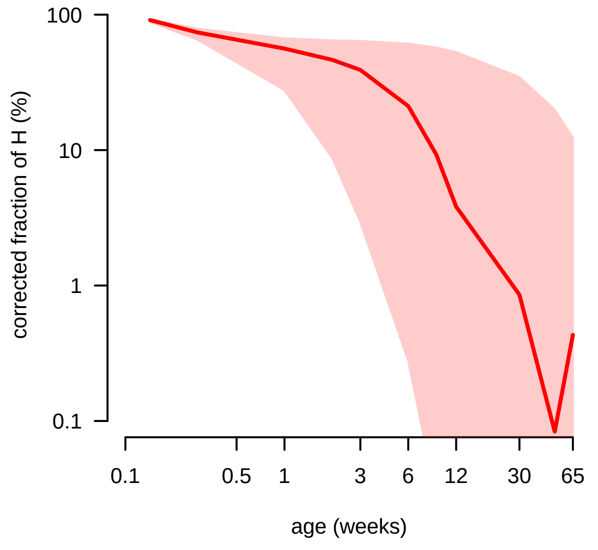

Q8BTV1

>sp|Q8BTV1|TUSC3_MOUSE

Tumor suppressor candidate 3

GN=Tusc3

Med H1/2: 7.9 days

Q5 H1/2: 3.5 days

Q95 H1/2: 26.2 days

reliable model fit: yes

raw intensities

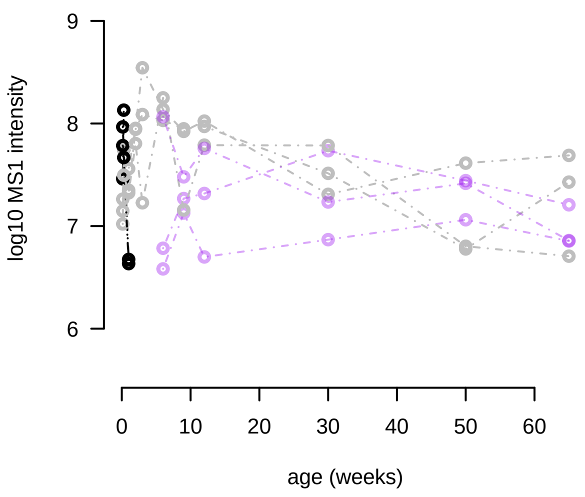

raw intensities log scale

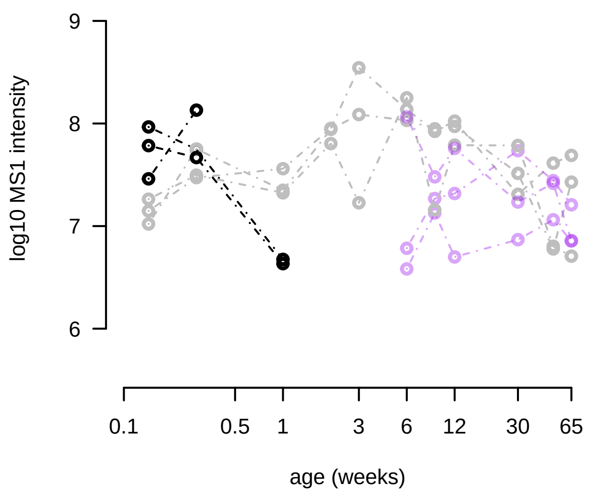

H1/2

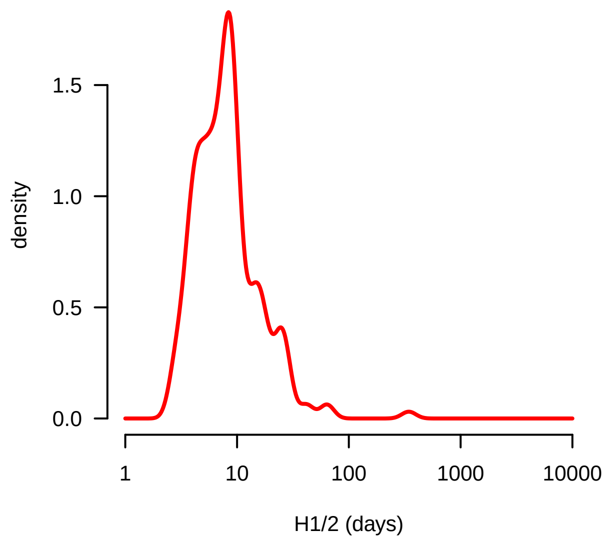

short pulse: ratio ln(L/H+1) log scale

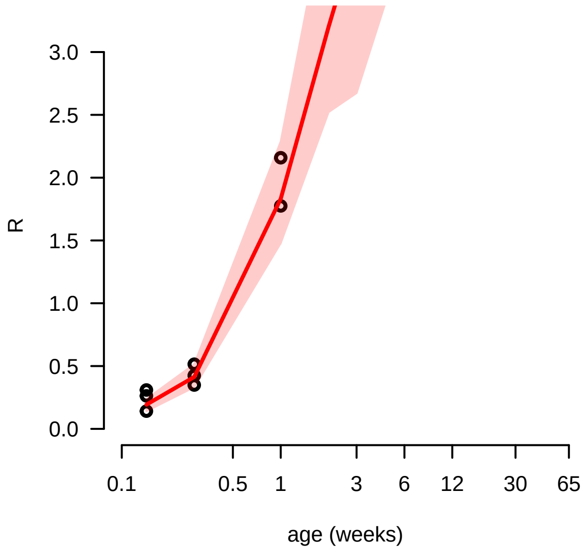

short pulse: ratio ln(L/H+1)

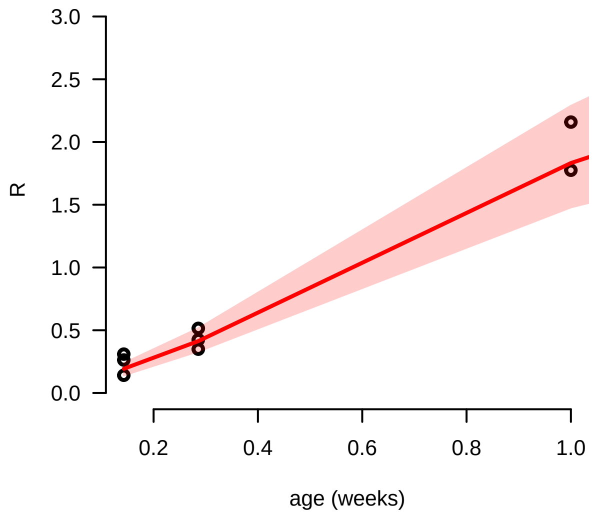

protein concentration log scale

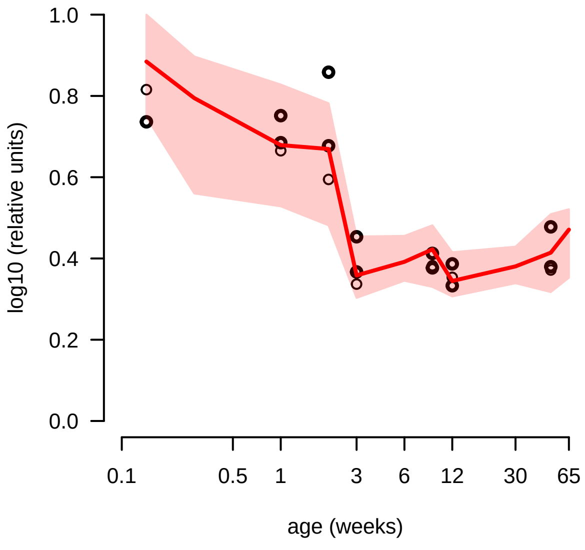

total protein log scale

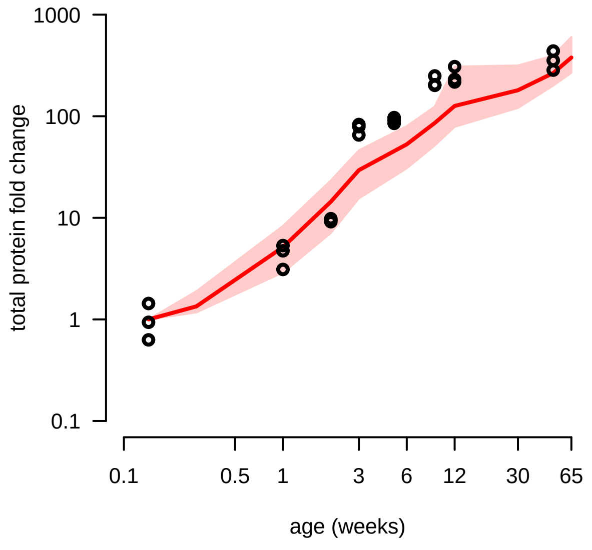

short pulse %H corrected log scale

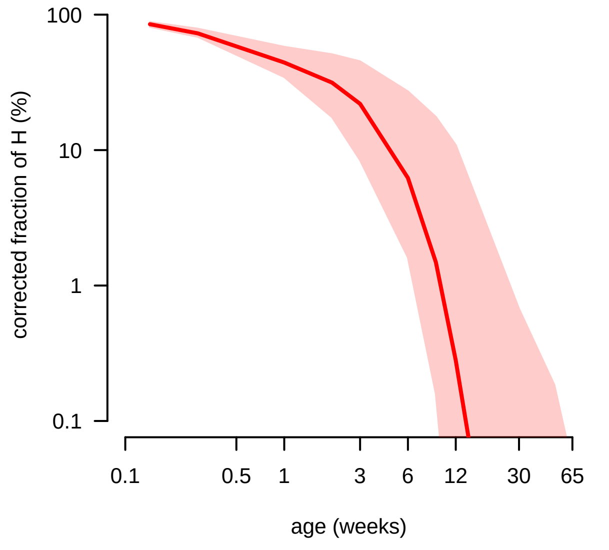

P20664

>sp|P20664|PRI1_MOUSE

DNA primase small subunit

GN=Prim1

Med H1/2: 11.2 days

Q5 H1/2: 3.7 days

Q95 H1/2: 92.6 days

reliable model fit: yes

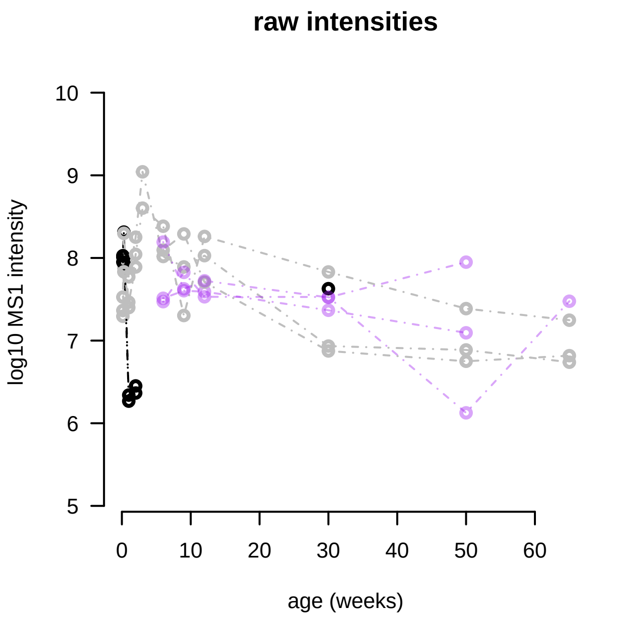

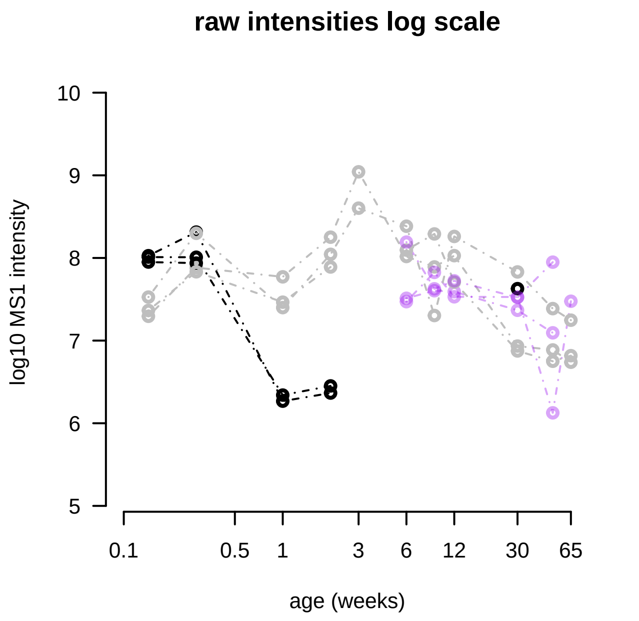

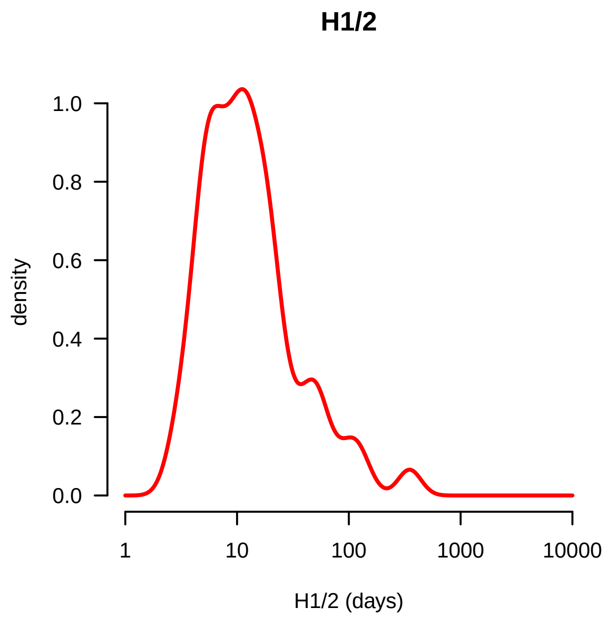

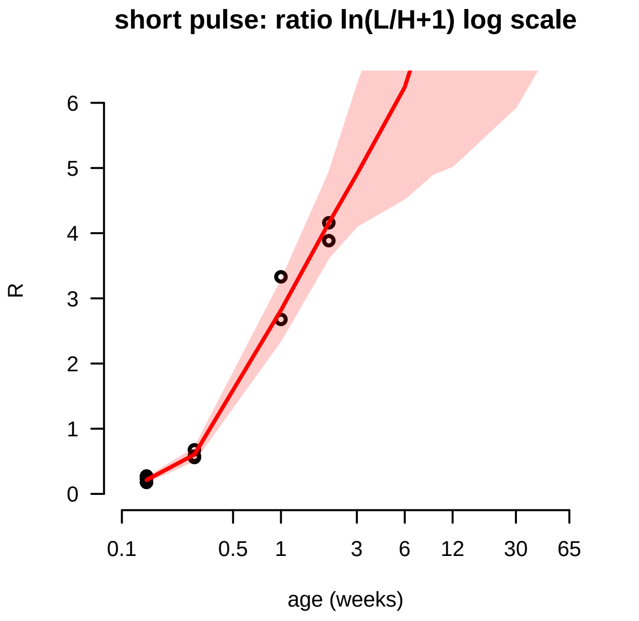

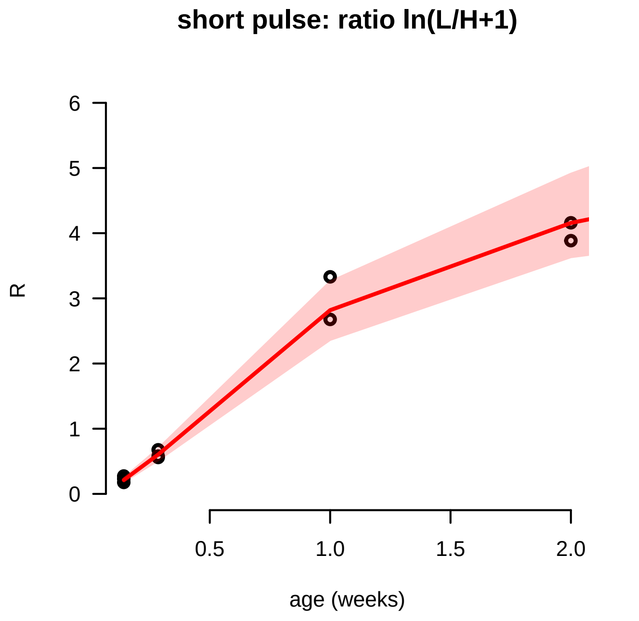

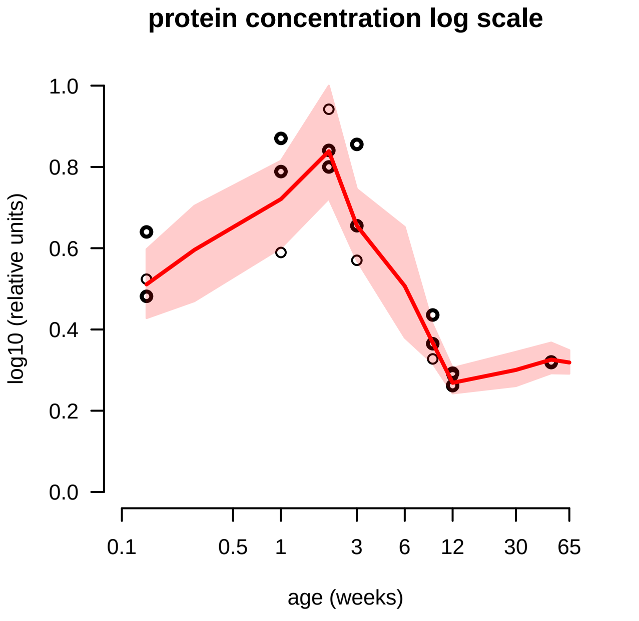

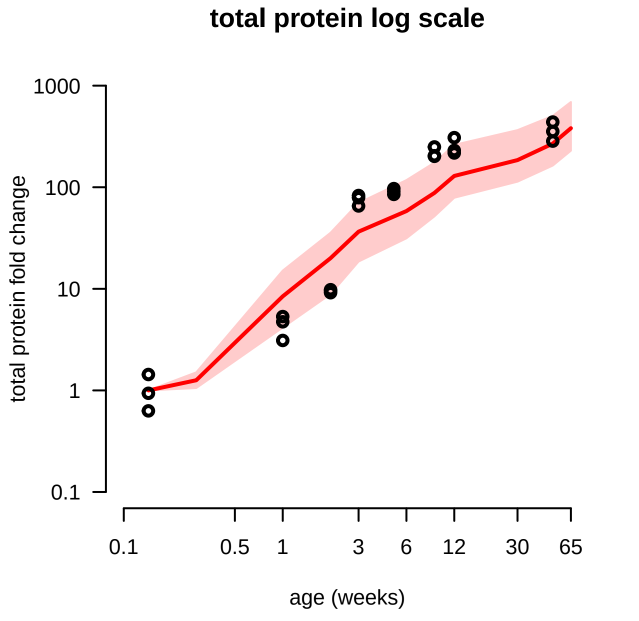

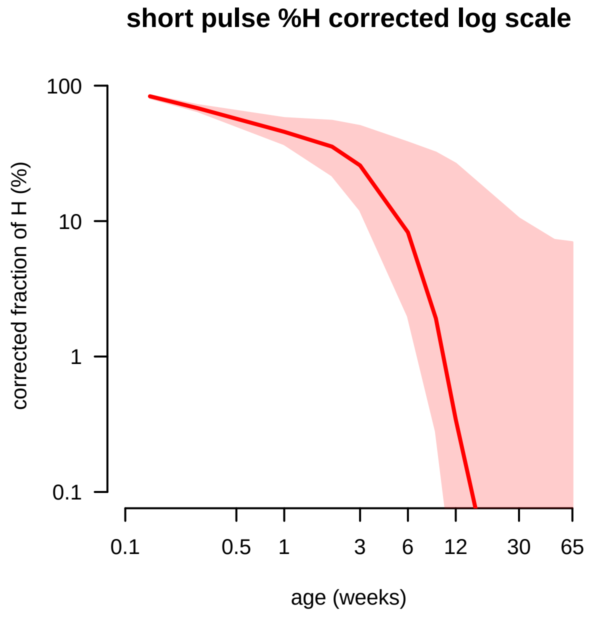

Q9DB40;Q9DB40−2

>sp|Q9DB40|MED27_MOUSE

Mediator of RNA polymerase II transcription subunit 27

GN=Med27

Med H1/2: 11.2 days

Q5 H1/2: 3.8 days

Q95 H1/2: 123 days

reliable model fit: no

raw intensities

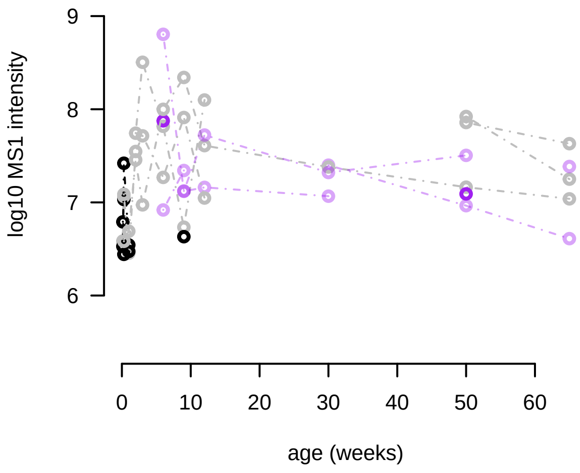

raw intensities log scale

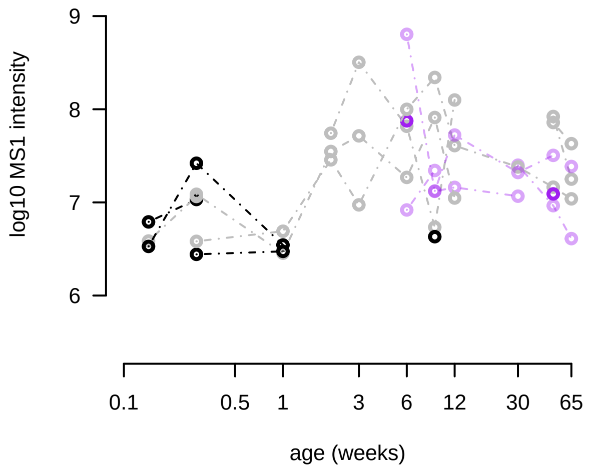

H1/2

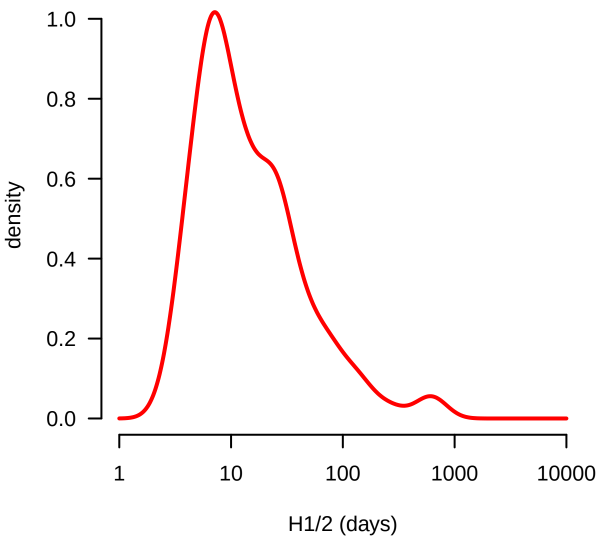

short pulse: ratio ln(L/H+1) log scale

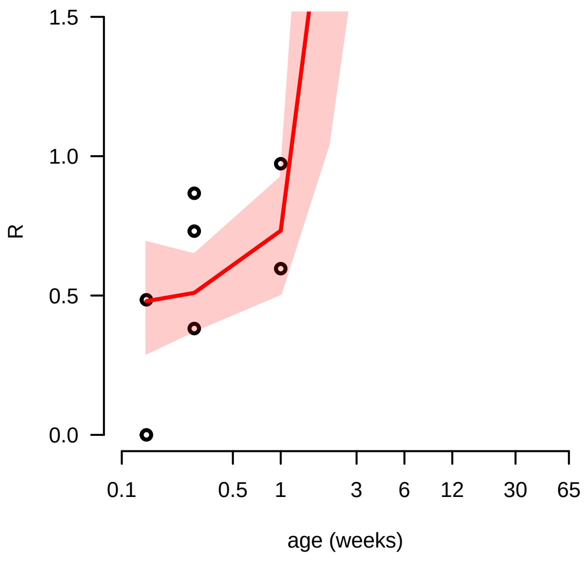

short pulse: ratio ln(L/H+1)

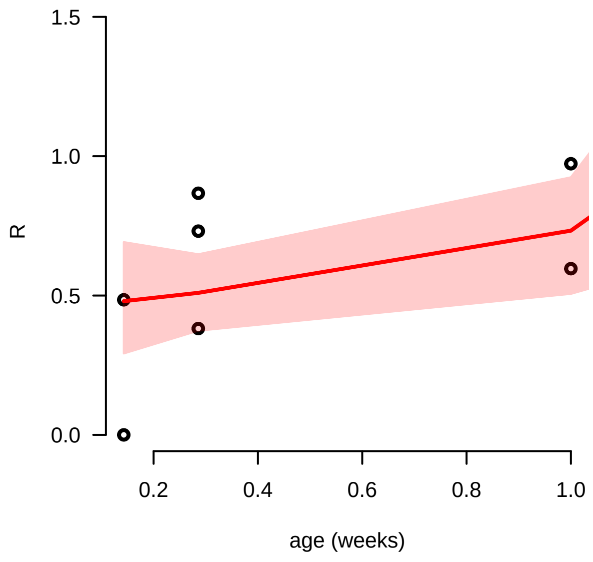

protein concentration log scale

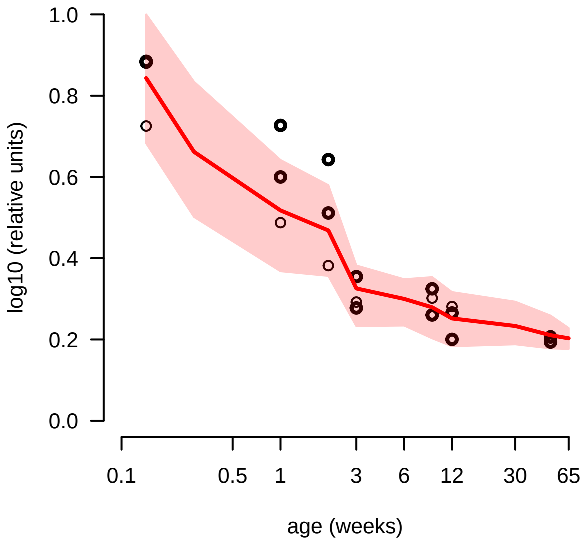

total protein log scale

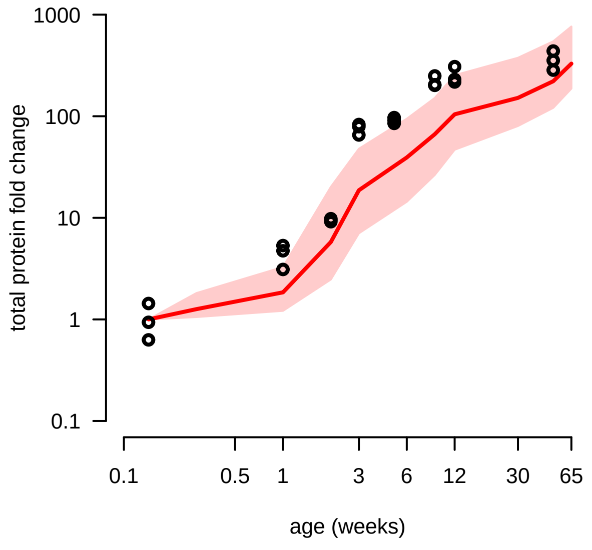

short pulse %H corrected log scale

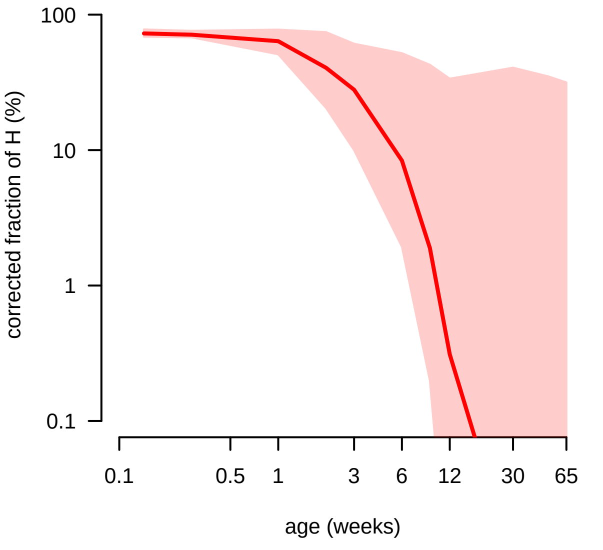

Q1W617;Q1W617−2

>sp|Q1W617|SHRM4_MOUSE

Protein Shroom4

GN=Shroom4

Med H1/2: 19.6 days

Q5 H1/2: 5.5 days

Q95 H1/2: 246.3 days

reliable model fit: yes

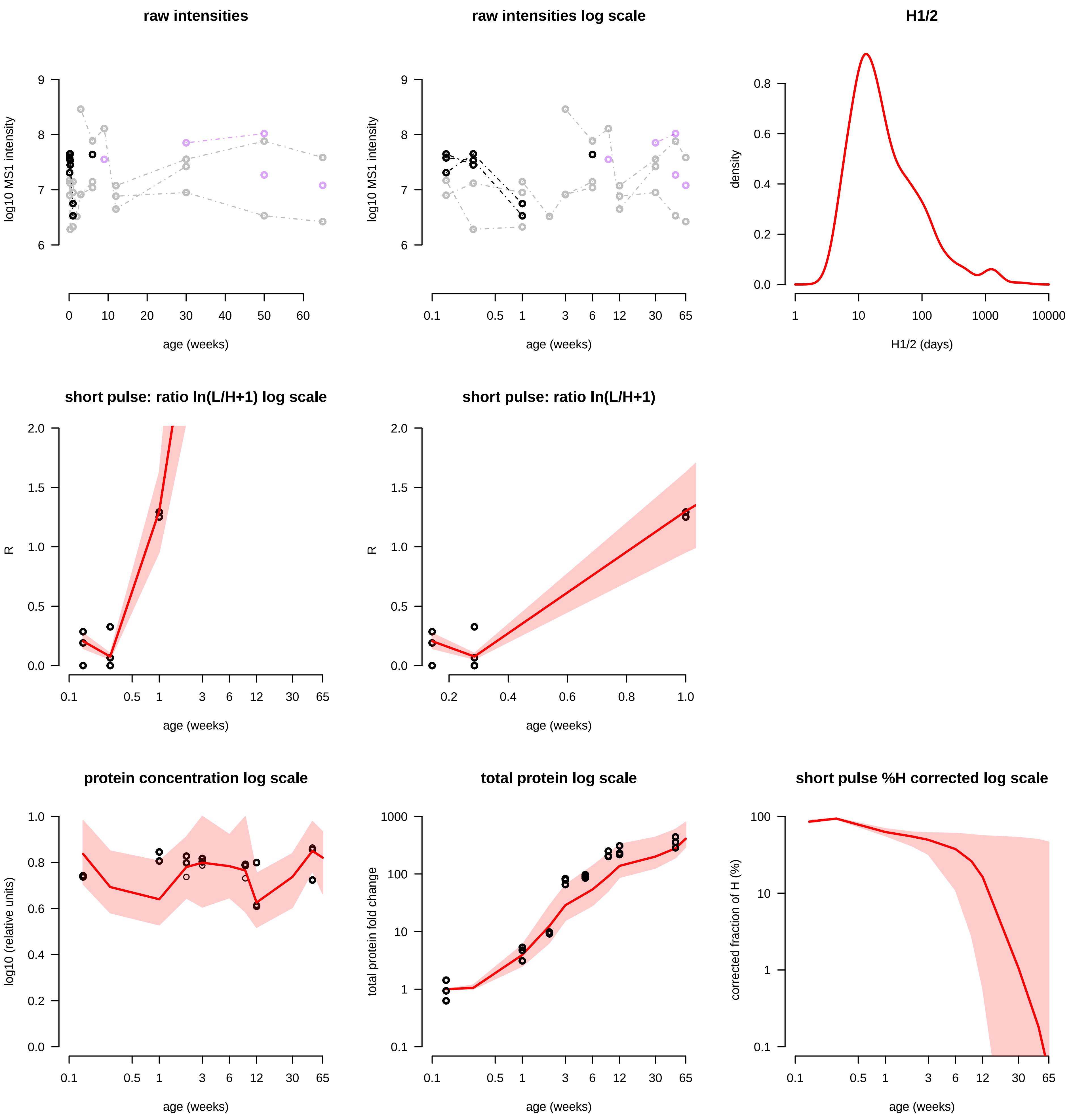

Q9R0X0;Q9R0X0−2

>sp|Q9R0X0|MED20_MOUSE

Mediator of RNA polymerase II transcription subunit 20

GN=Med20

Med H1/2: 7 days

Q5 H1/2: 3 days

Q95 H1/2: 36.8 days

reliable model fit: yes

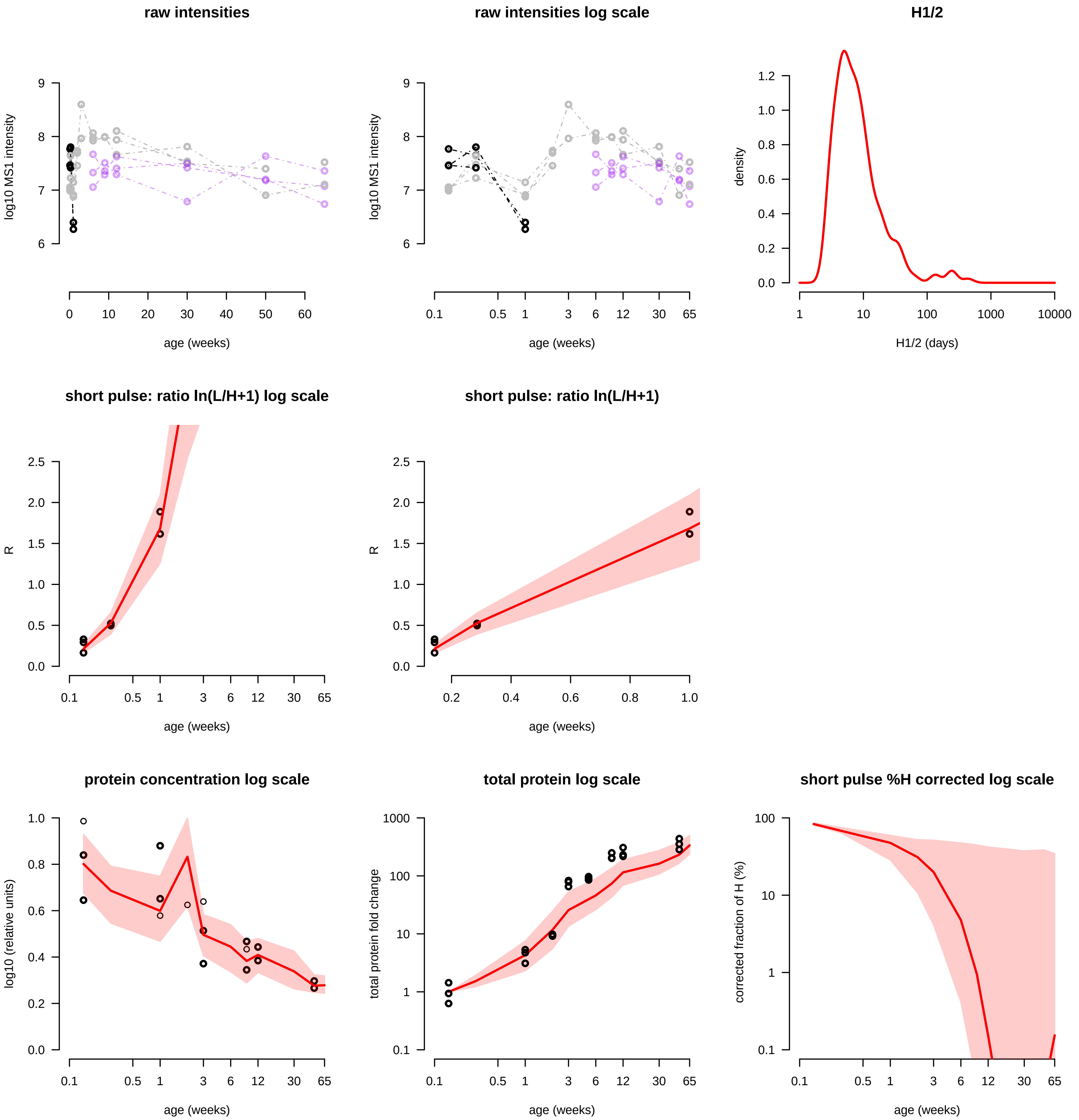

Q61245

>sp|Q61245|COBA1_MOUSE

Collagen alpha−1(XI) chain

GN=Col11a1

Med H1/2: 12.6 days

Q5 H1/2: 4.1 days

Q95 H1/2: 73.8 days

reliable model fit: yes

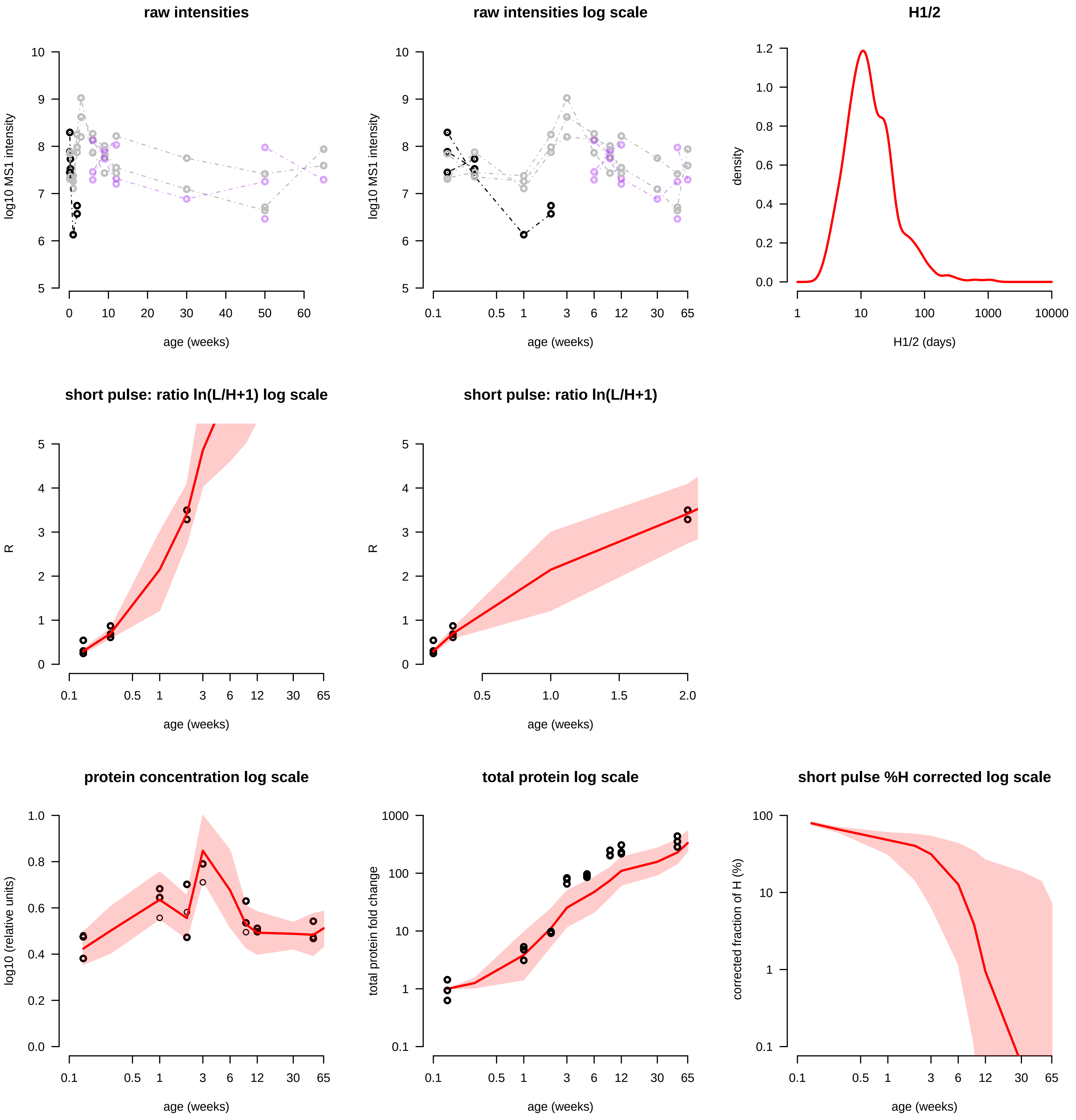

Q8CGC4

>sp|Q8CGC4|LS14B_MOUSE

Protein LSM14 homolog B

GN=Lsm14b

Med H1/2: 7 days

Q5 H1/2: 2.6 days

Q95 H1/2: 25.6 days

reliable model fit: yes

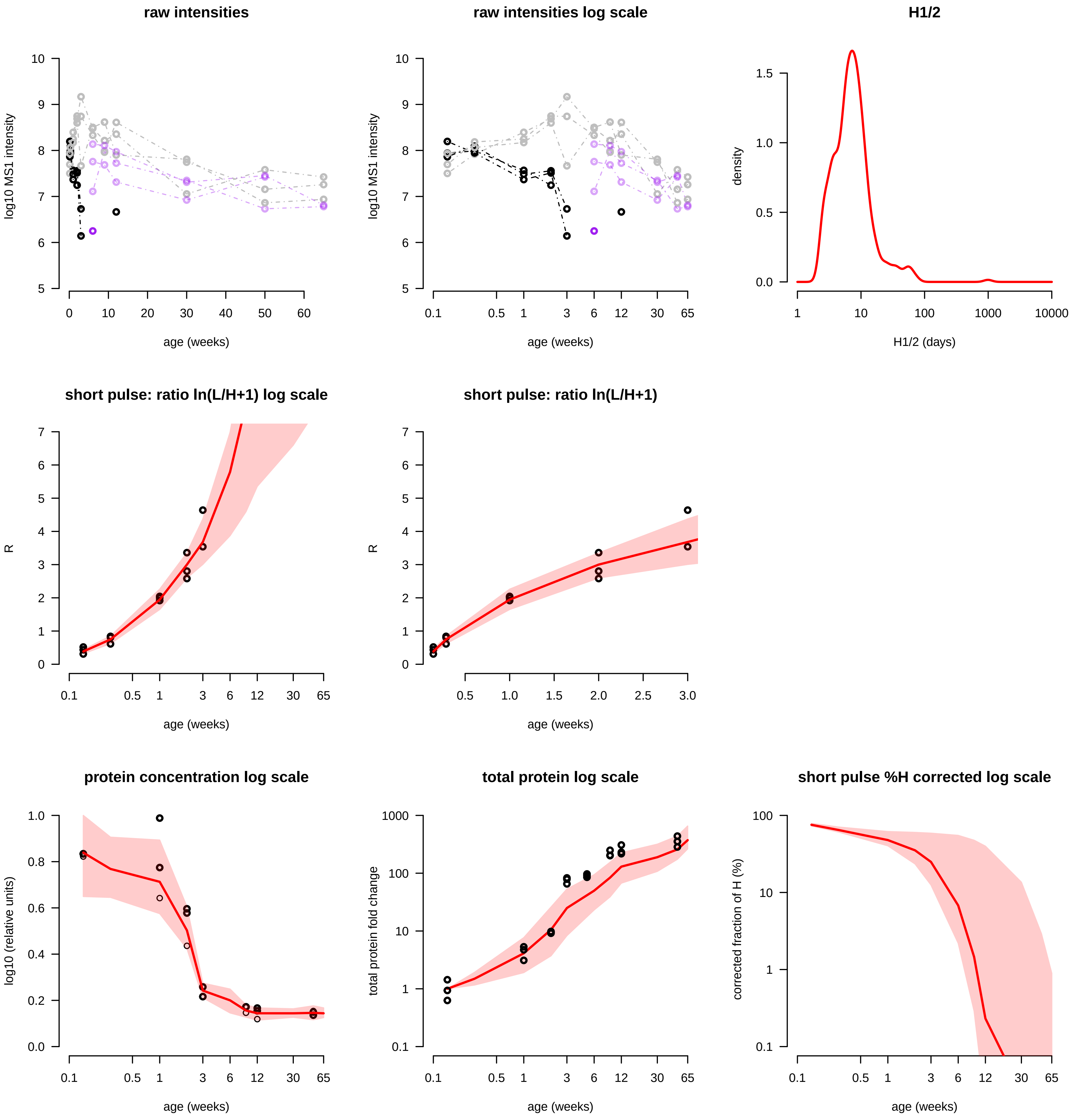

A2RSY1−2;A2RSY1;A2RSY1−3

>sp|A2RSY1−2|KANL3_MOUSE

Isoform 2 of KAT8 regulatory NSL complex subunit 3

GN=Kansl3

Med H1/2: 1759.9 days

Q5 H1/2: 757.5 days

Q95 H1/2: 16758.5 days

reliable model fit: no

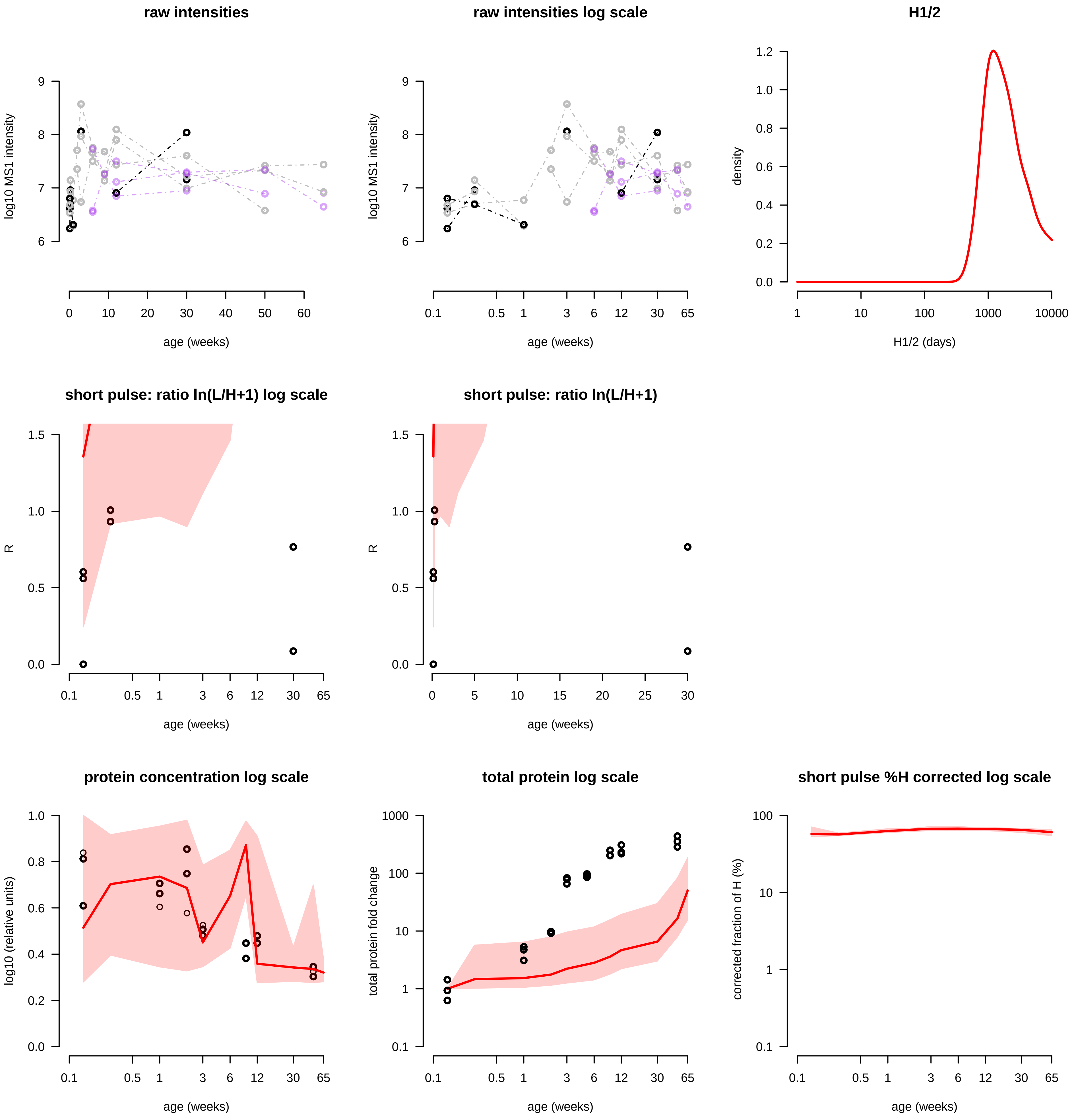

Q64GA5

>sp|Q64GA5|PA24C_MOUSE

Cytosolic phospholipase A2 gamma

GN=Pla2g4c

Med H1/2: 46.8 days

Q5 H1/2: 14.1 days

Q95 H1/2: 364.8 days

reliable model fit: yes

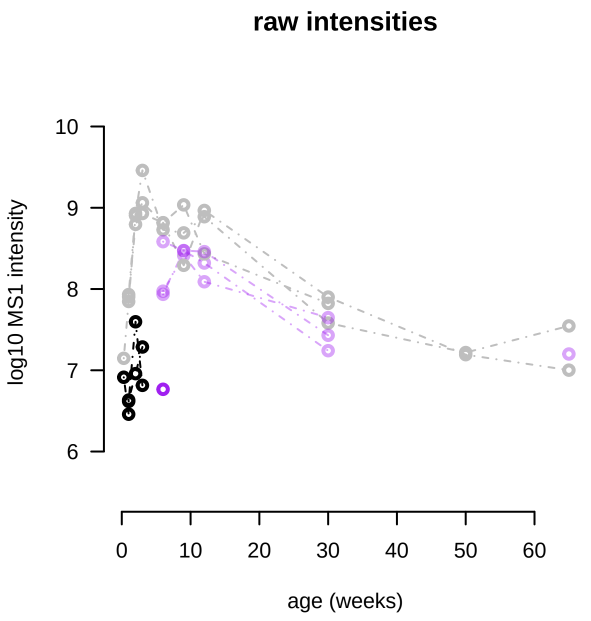

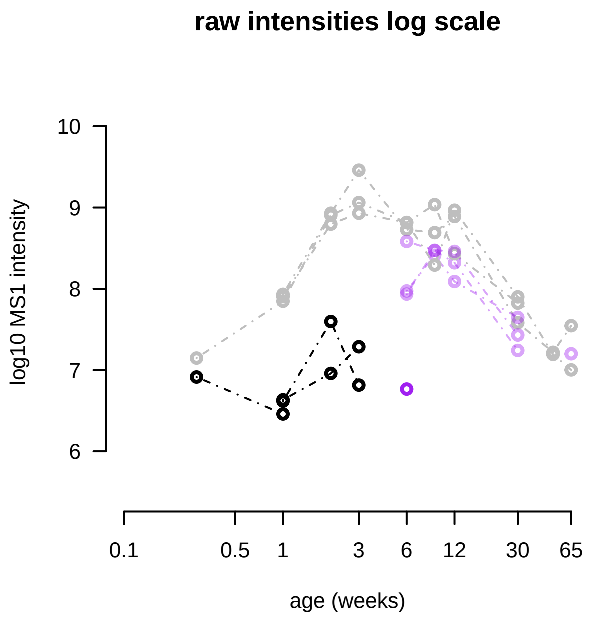

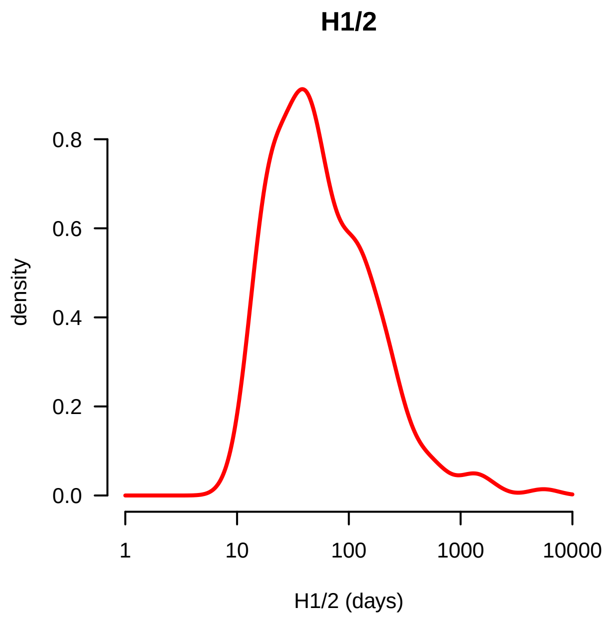

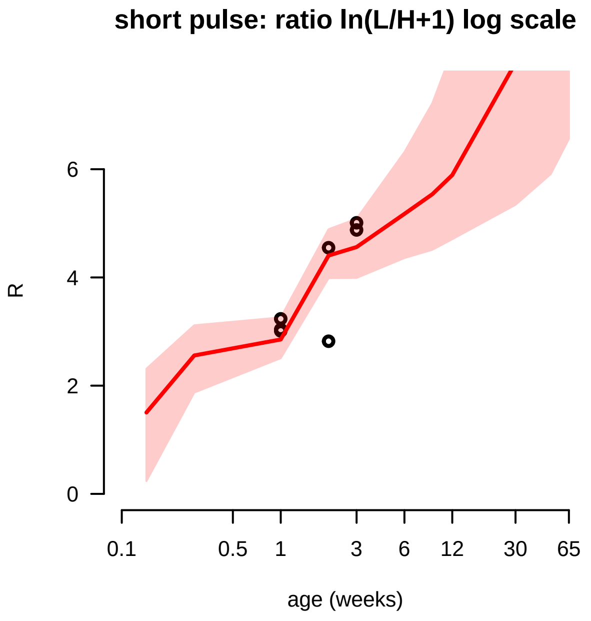

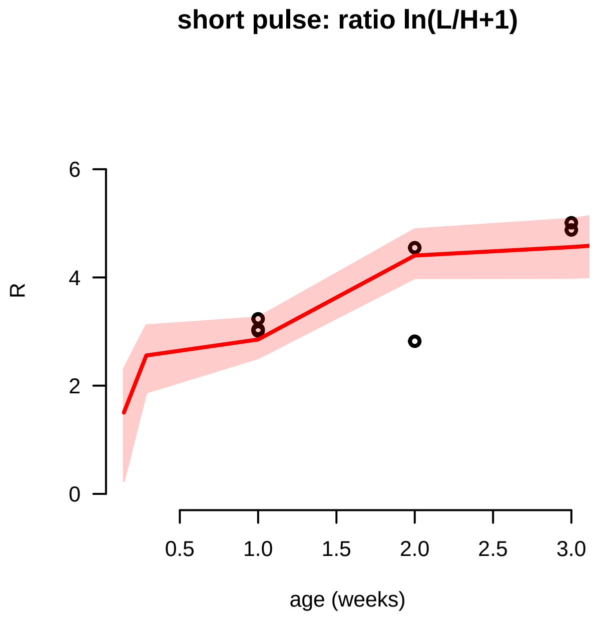

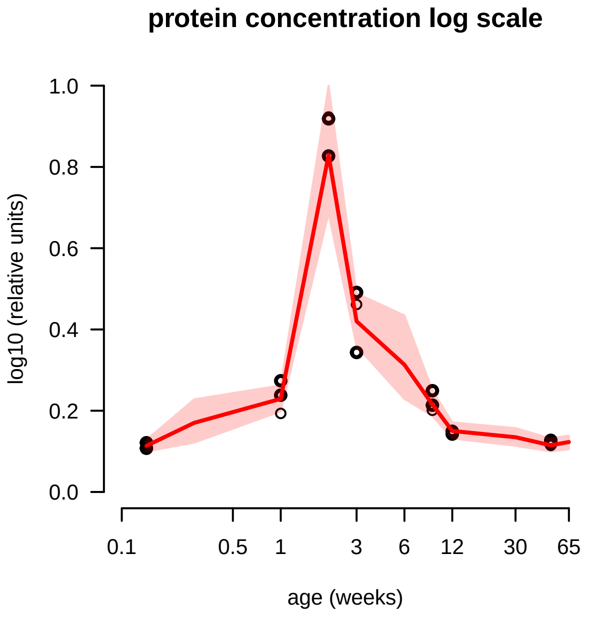

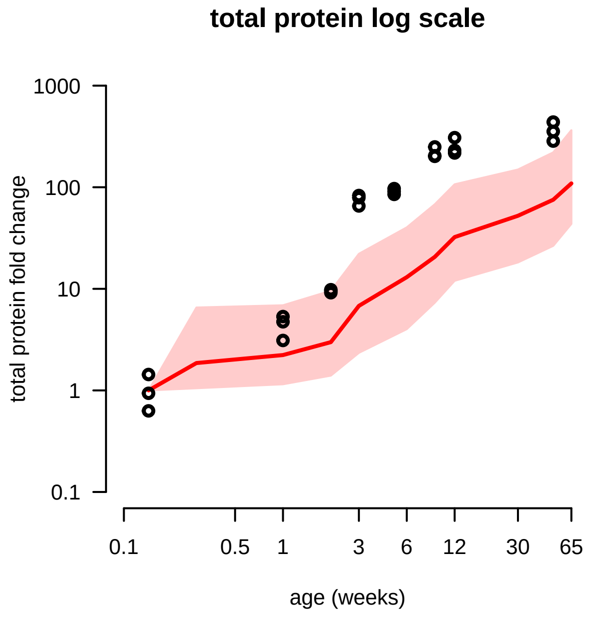

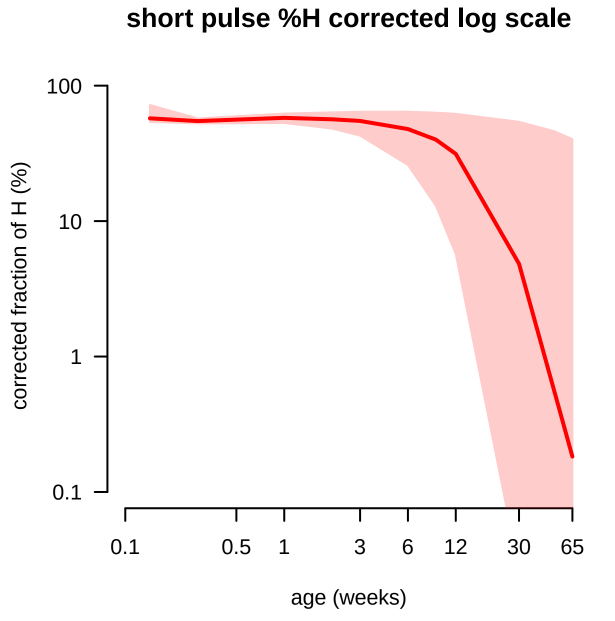

Q91ZE0

>sp|Q91ZE0|TMLH_MOUSE

Trimethyllysine dioxygenase, mitochondrial

GN=Tmlhe

Med H1/2: 6.2 days

Q5 H1/2: 2.9 days

Q95 H1/2: 367.6 days

reliable model fit: yes

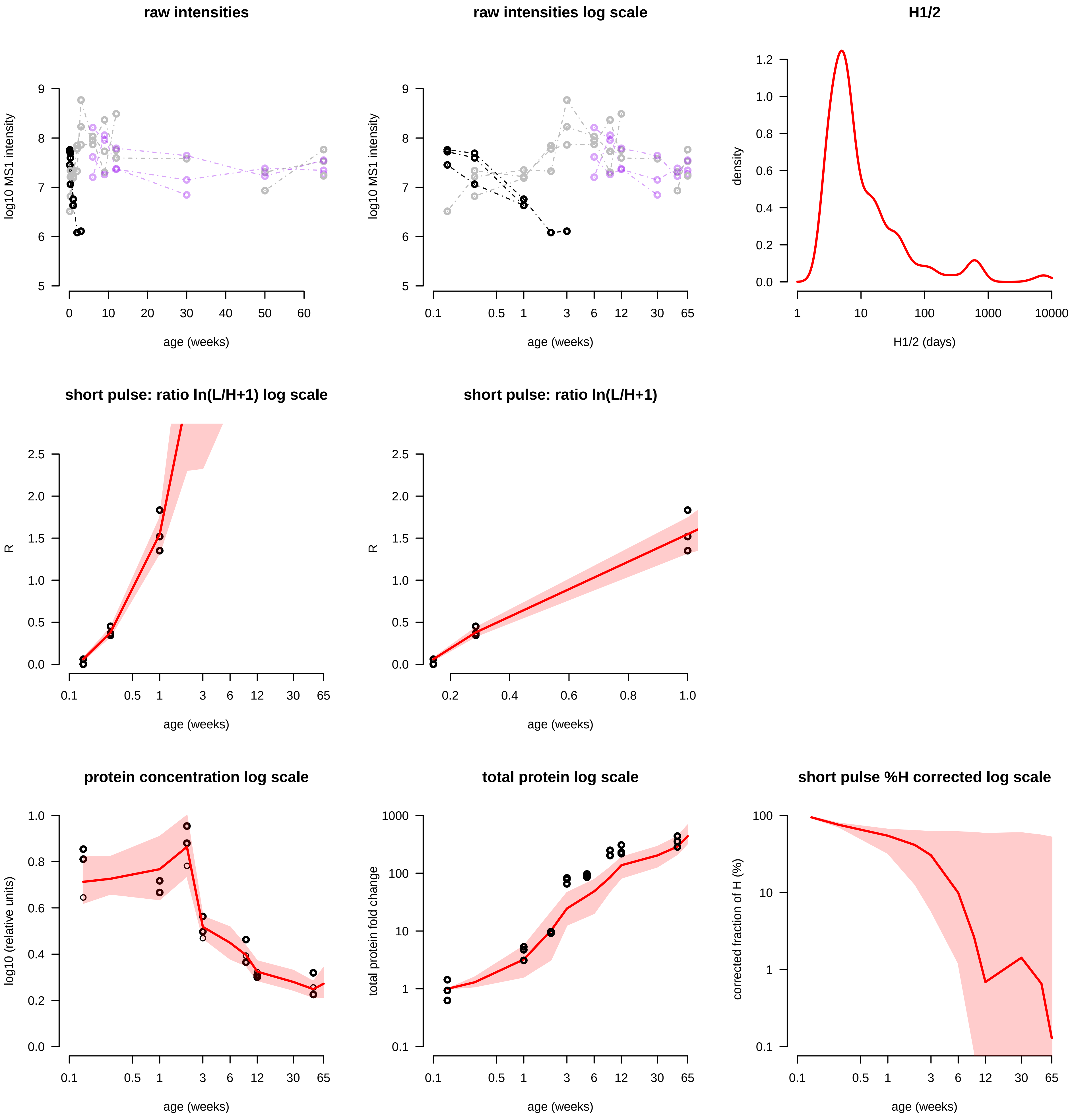

G5E8K5−3;G5E8K5;G5E8K5−5;G5E8K5−4

>sp|G5E8K5−3|ANK3_MOUSE

Isoform 3 of Ankyrin−3

GN=Ank3

Med H1/2: 23.1 days

Q5 H1/2: 16.7 days

Q95 H1/2: 41.4 days

reliable model fit: yes

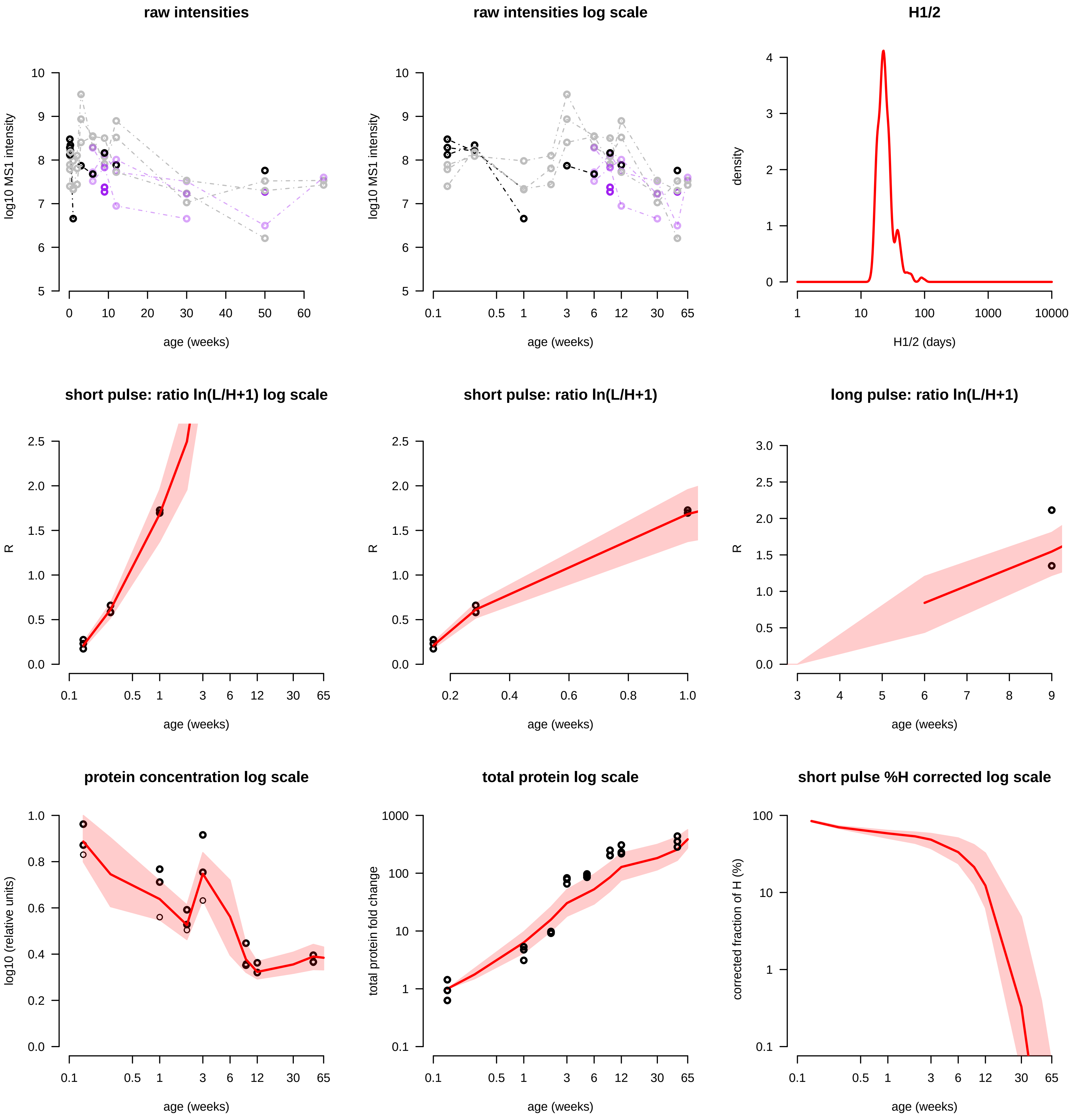

Q9CRB2

>sp|Q9CRB2|NHP2_MOUSE

H/ACA ribonucleoprotein complex subunit 2

GN=Nhp2

Med H1/2: 19.8 days

Q5 H1/2: 7.1 days

Q95 H1/2: 156.6 days

reliable model fit: yes

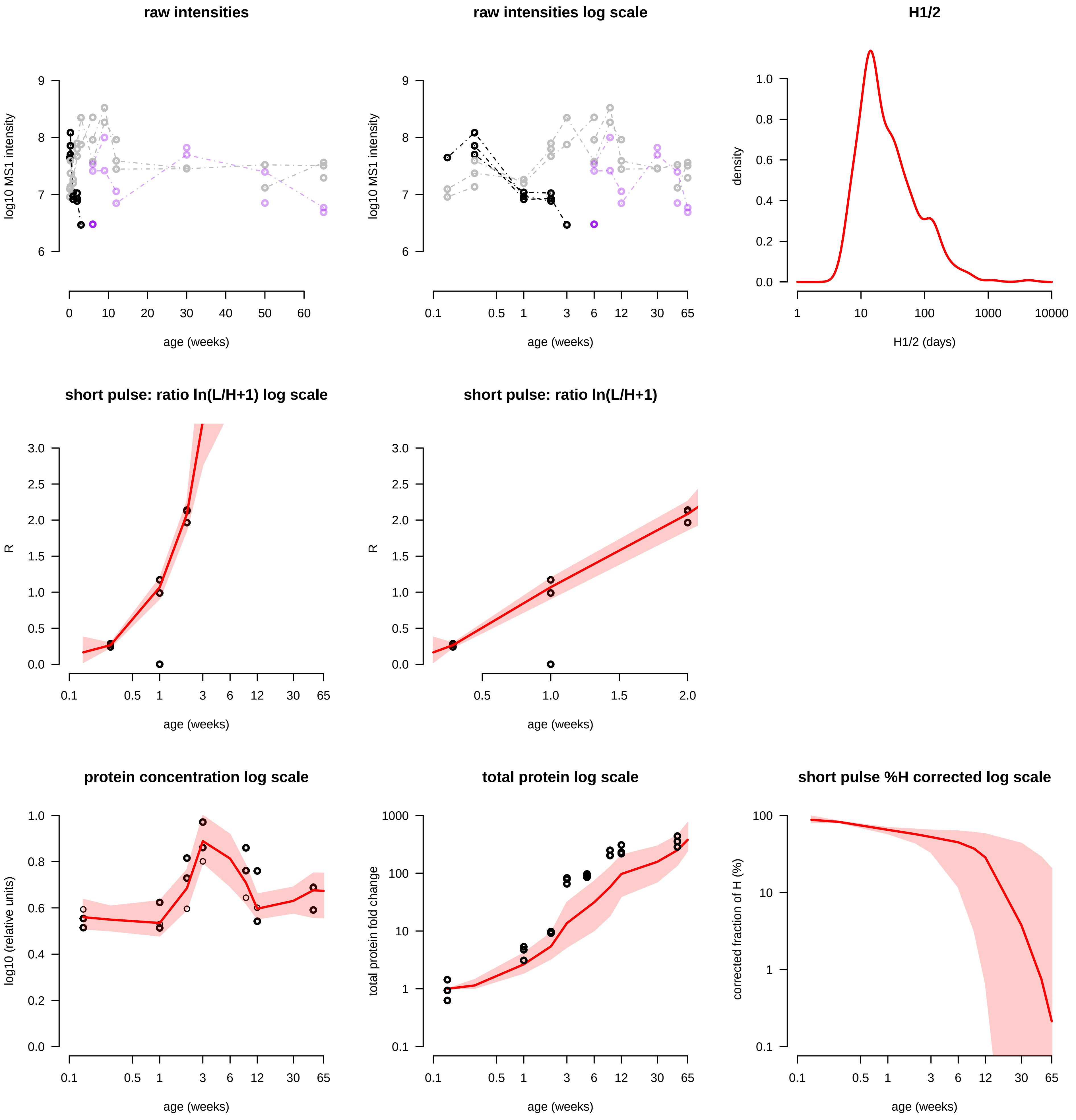

Q925H1

>sp|Q925H1|TRPS1_MOUSE

Zinc finger transcription factor Trps1

GN=Trps1

Med H1/2: 17 days

Q5 H1/2: 4.7 days

Q95 H1/2: 137.4 days

reliable model fit: yes

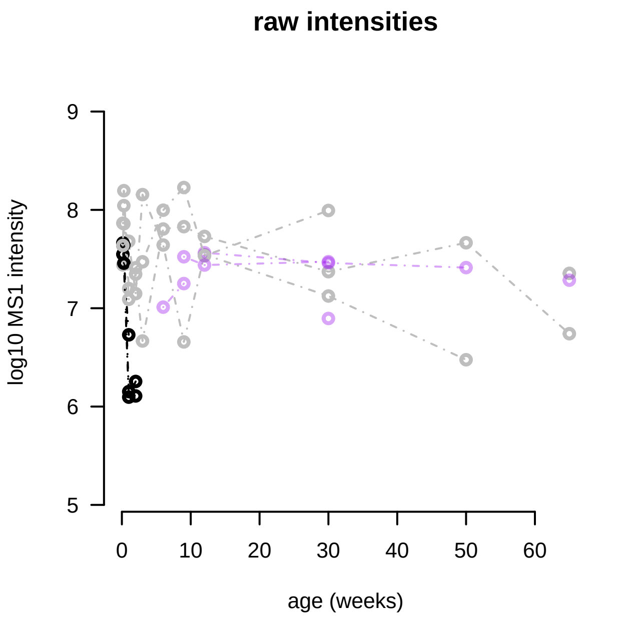

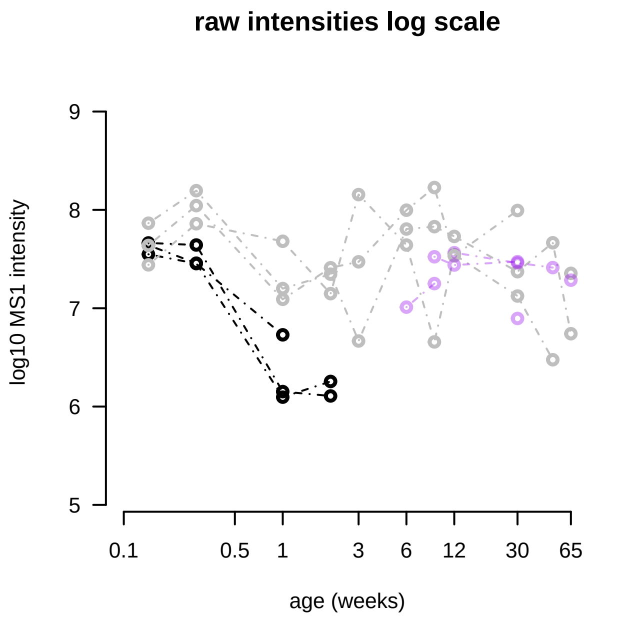

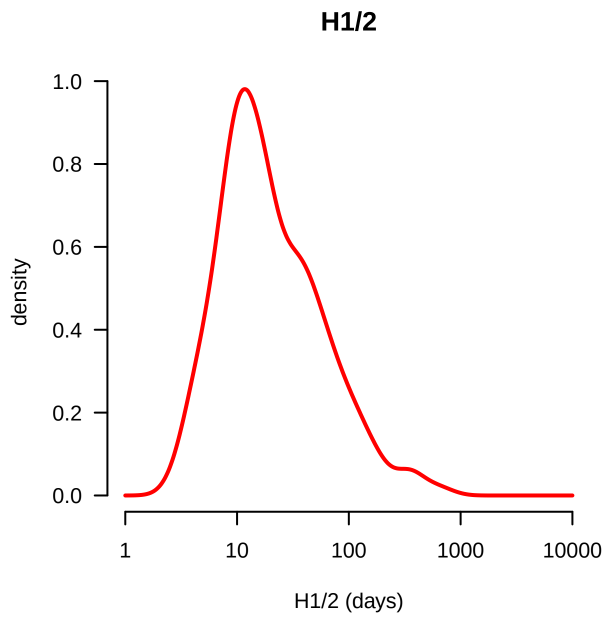

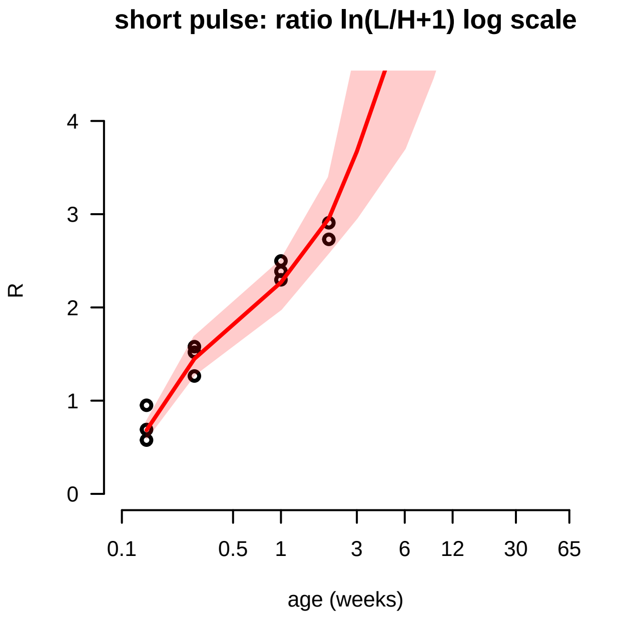

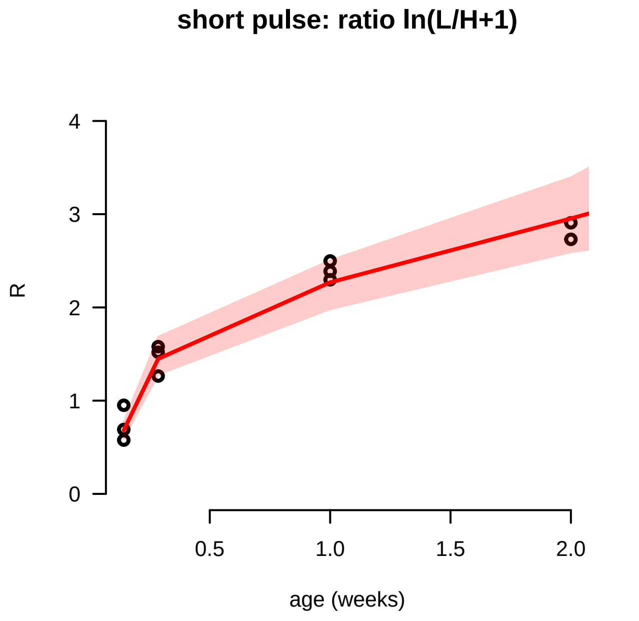

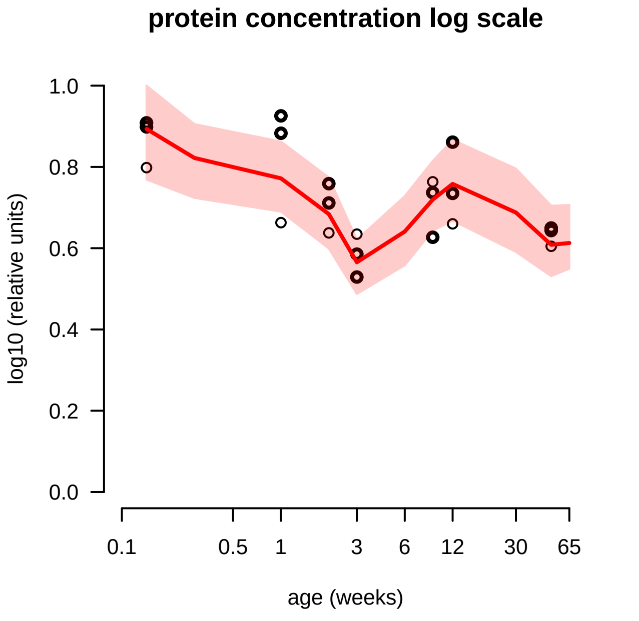

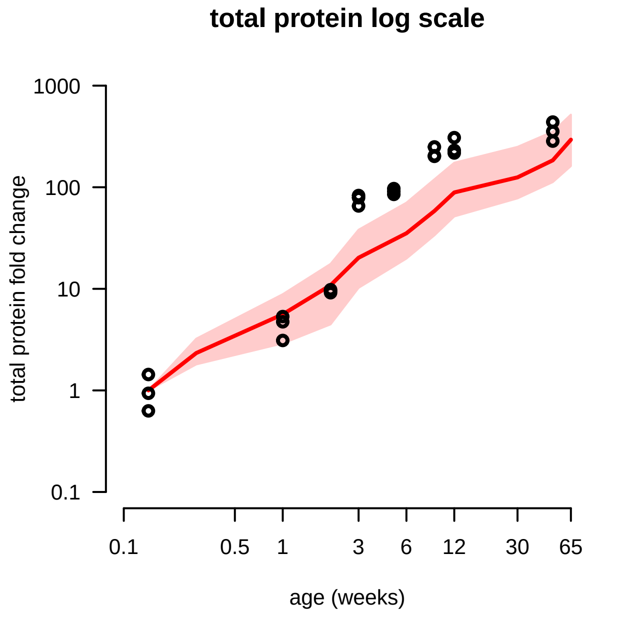

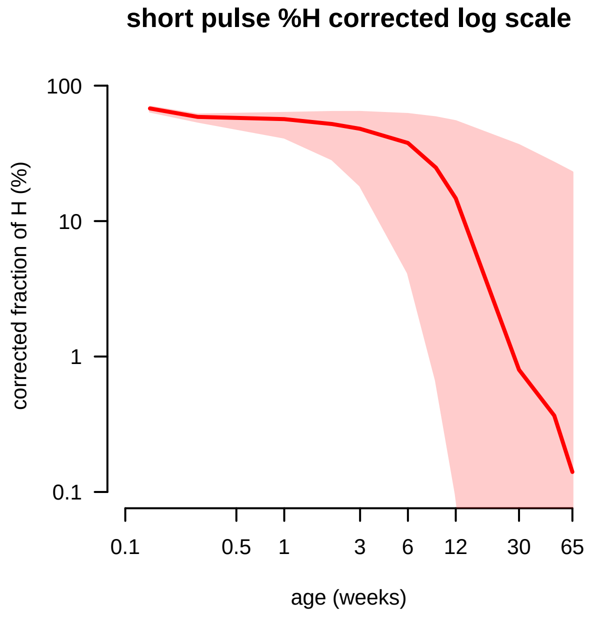

Q8C033−2;Q8C033

>sp|Q8C033−2|ARHGA_MOUSE

Isoform 2 of Rho guanine nucleotide exchange factor 10

GN=Arhgef10

Med H1/2: 54.2 days

Q5 H1/2: 27.2 days

Q95 H1/2: 299.1 days

reliable model fit: yes

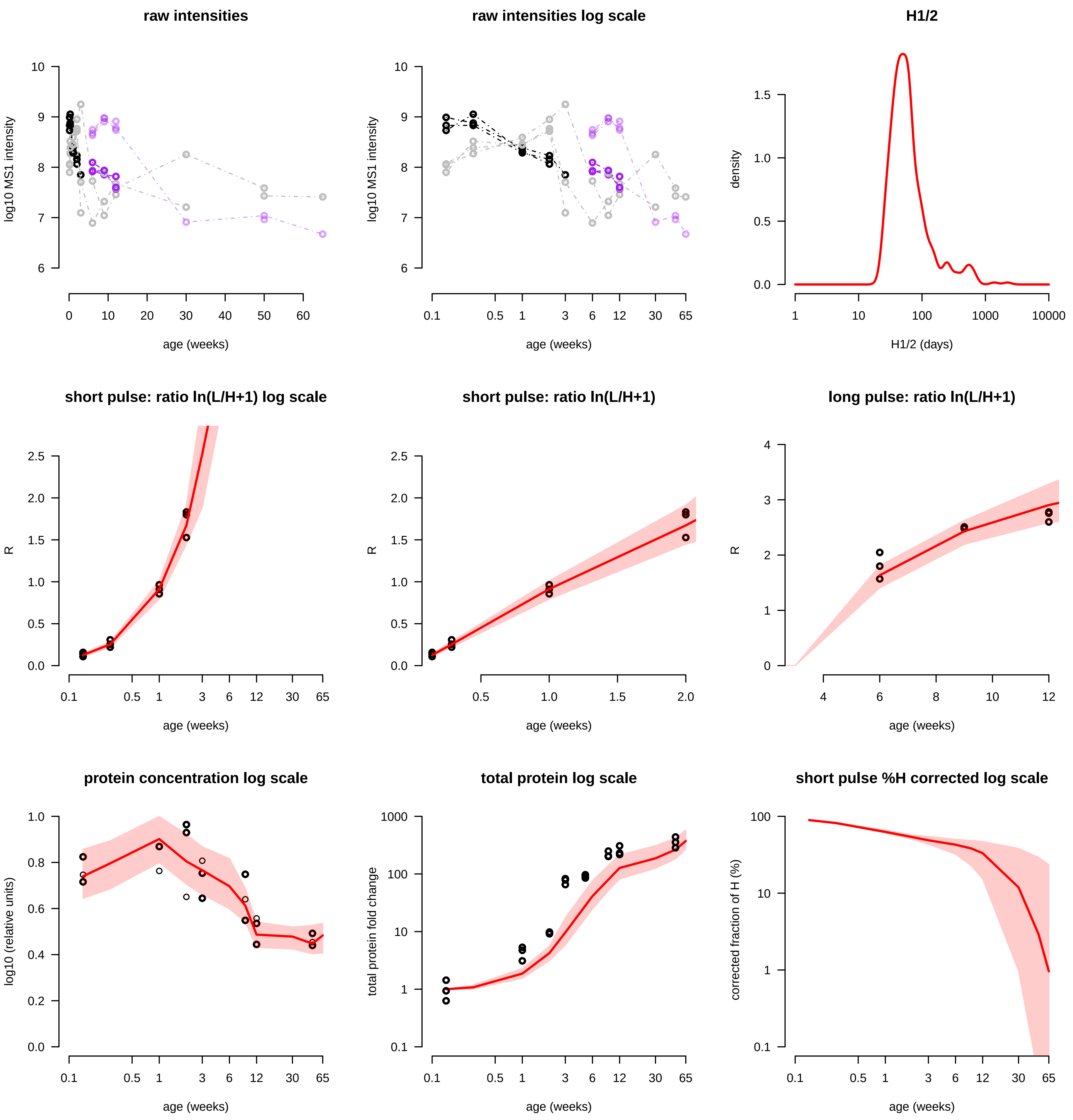

B2RRD7

>sp|B2RRD7|BRPF1_MOUSE

Peregrin

GN=Brpf1

Med H1/2: 3110.9 days

Q5 H1/2: 803.2 days

Q95 H1/2: 46852.9 days

reliable model fit: no

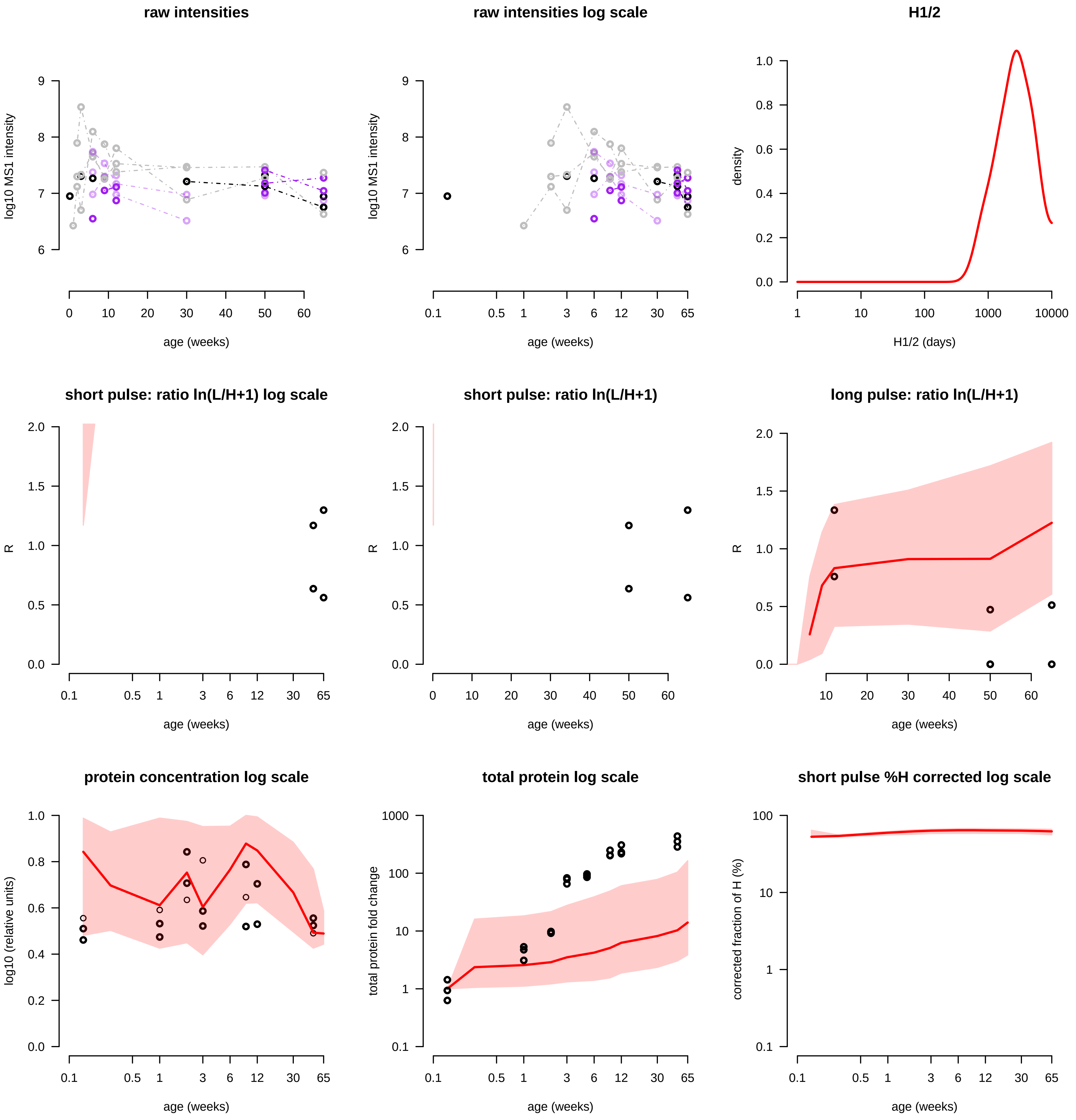

Q80W85

>sp|Q80W85|NPM2_MOUSE

Nucleoplasmin−2

GN=Npm2

Med H1/2: 74.2 days

Q5 H1/2: 16.1 days

Q95 H1/2: 513.7 days

reliable model fit: yes

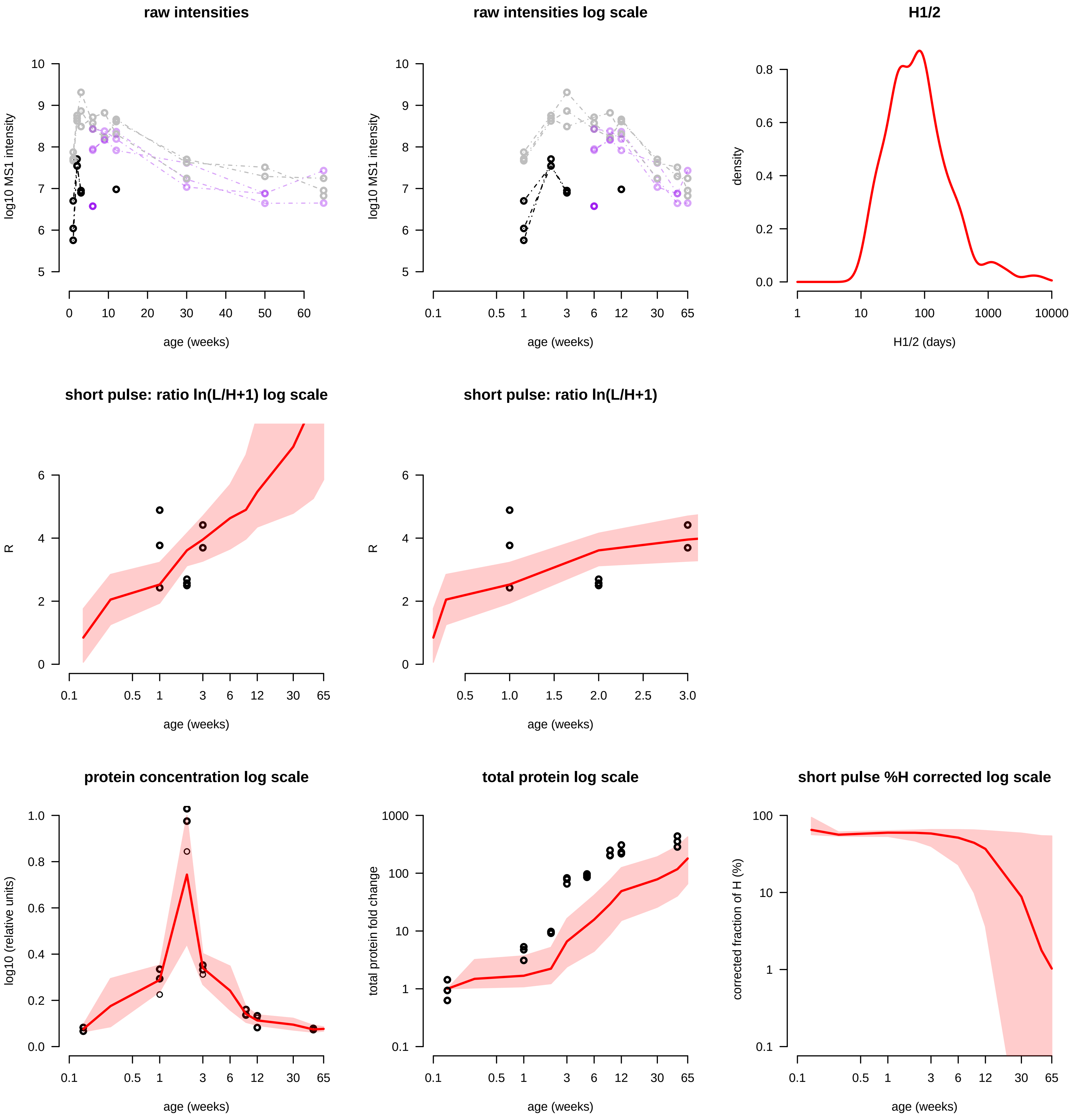

Q80UZ0

>sp|Q80UZ0|FGD5_MOUSE

FYVE, RhoGEF and PH domain−containing protein 5

GN=Fgd5

Med H1/2: 294.2 days

Q5 H1/2: 72.6 days

Q95 H1/2: 3174.1 days

reliable model fit: yes

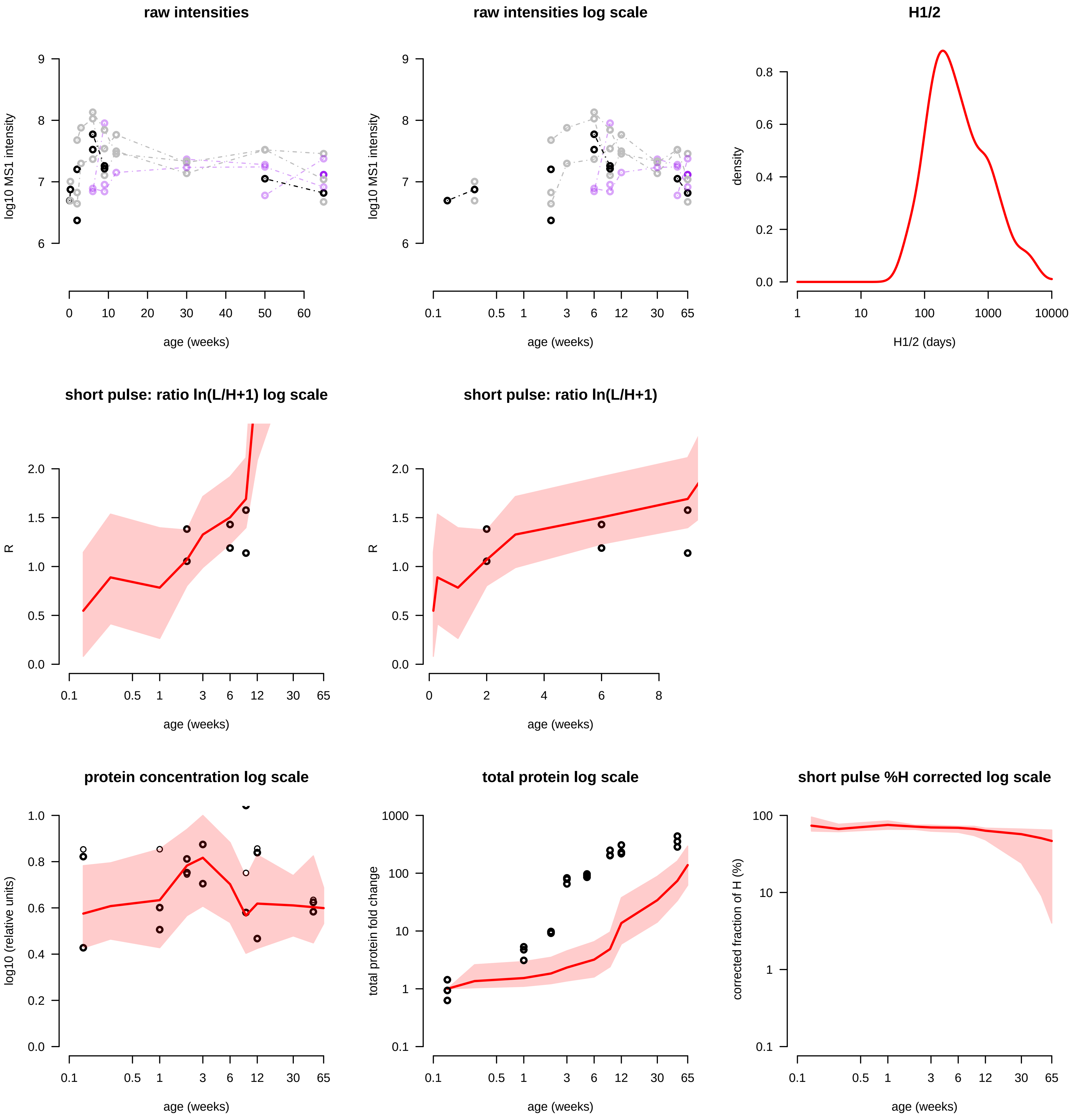

Q9Z1B3−3;Q9Z1B3;Q9Z1B3−2

>sp|Q9Z1B3−3|PLCB1_MOUSE

soform C of 1−phosphatidylinositol 4,5−bisphosphate phosphodiesterase beta−1

GN=Plcb1

Med H1/2: 18.9 days

Q5 H1/2: 6.3 days

Q95 H1/2: 143.5 days

reliable model fit: yes

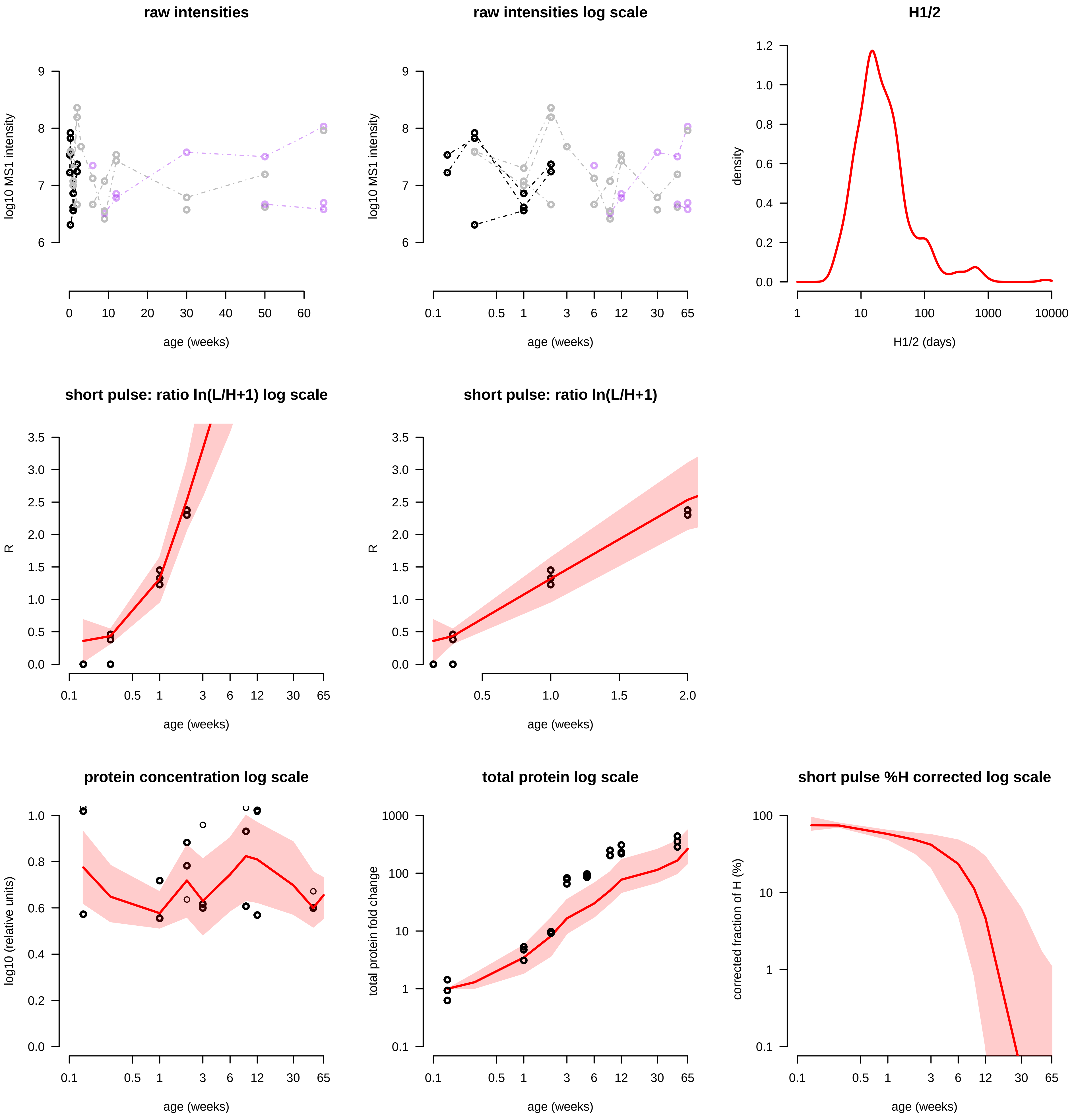

Q61097

>sp|Q61097|KSR1_MOUSE

Kinase suppressor of Ras 1

GN=Ksr1

Med H1/2: 19.2 days

Q5 H1/2: 13.7 days

Q95 H1/2: 33.7 days

reliable model fit: yes

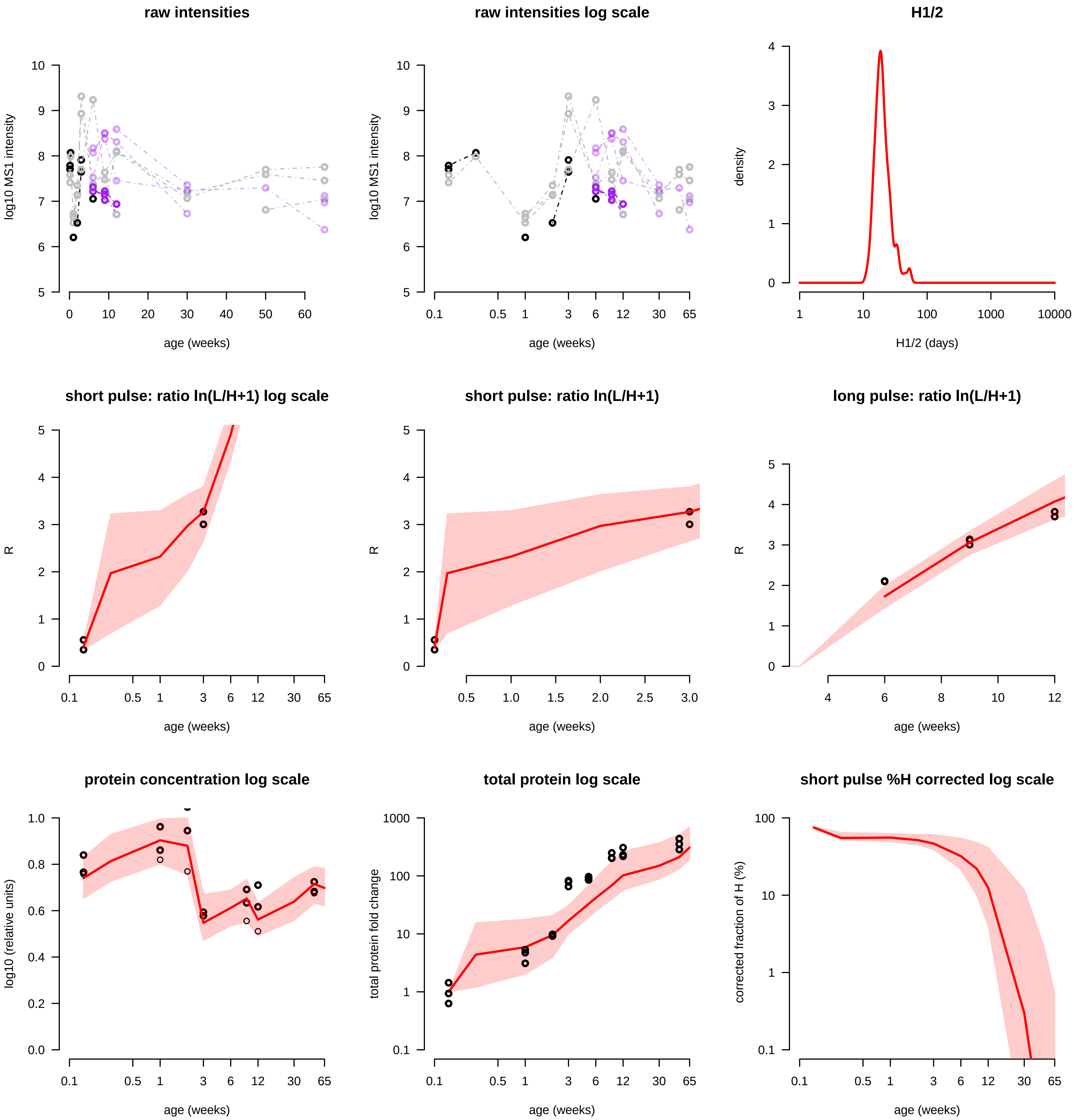

Q9Z207

>sp|Q9Z207|DIAP3_MOUSE

Protein diaphanous homolog 3

GN=Diaph3

Med H1/2: 405.6 days

Q5 H1/2: 110.6 days

Q95 H1/2: 2610.8 days

reliable model fit: no

raw intensities

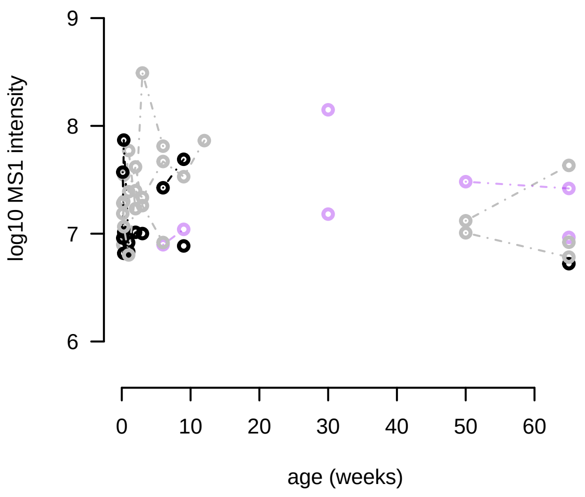

raw intensities log scale

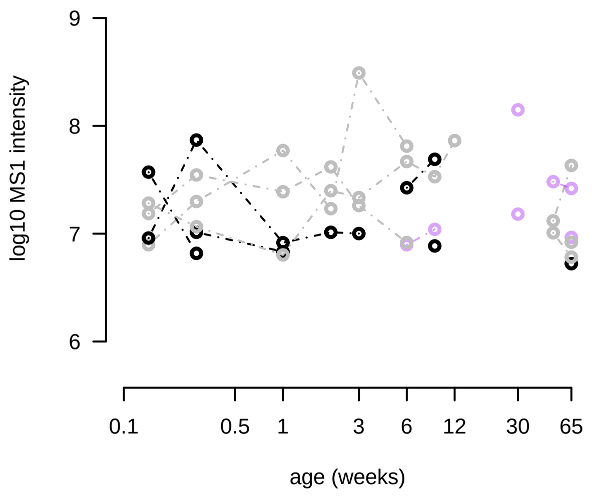

H1/2

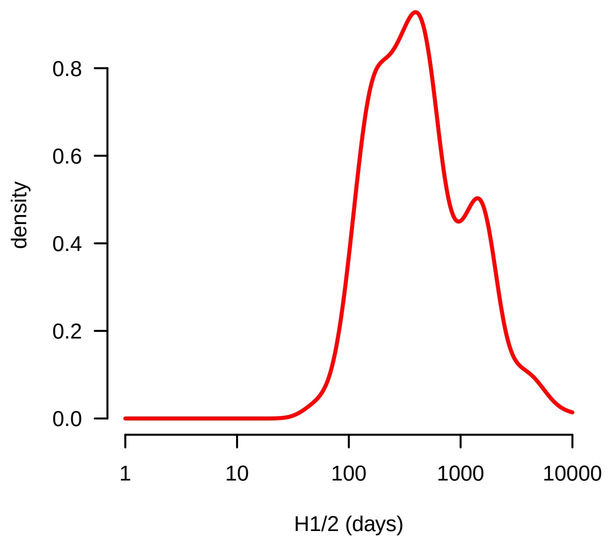

short pulse: ratio ln(L/H+1) log scale

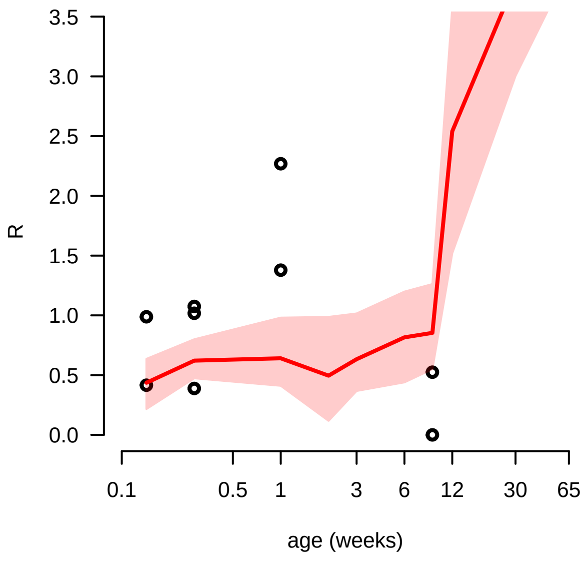

short pulse: ratio ln(L/H+1)

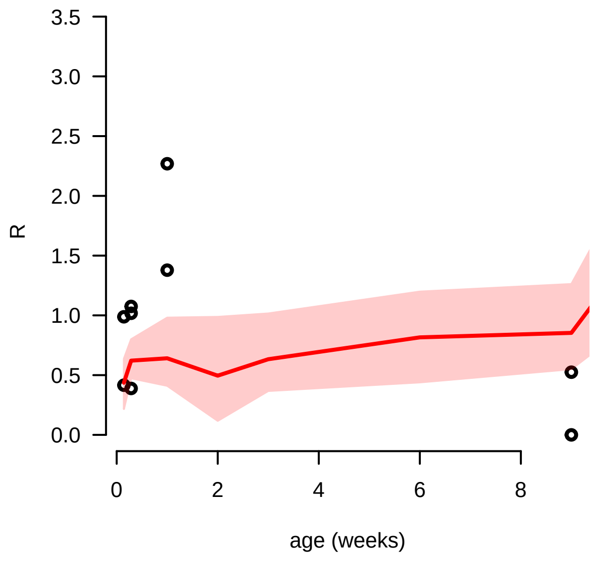

protein concentration log scale

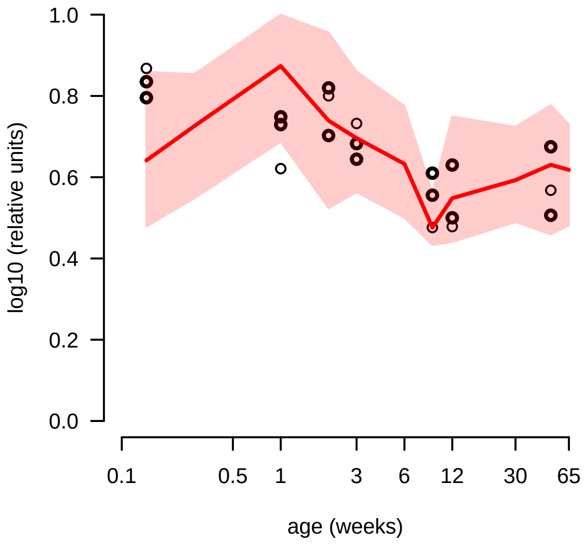

total protein log scale

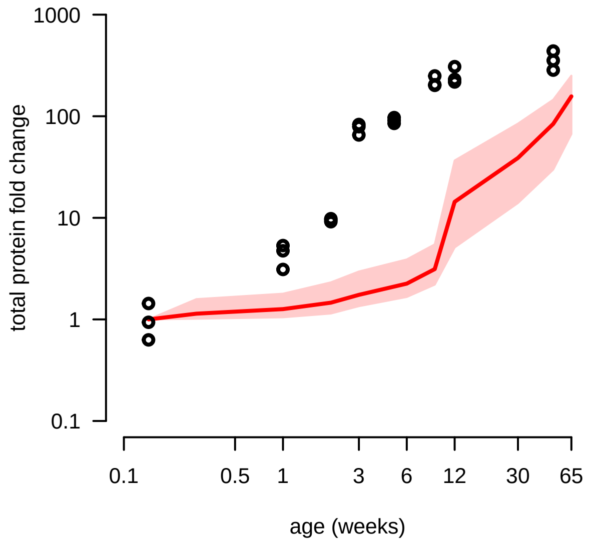

short pulse %H corrected log scale

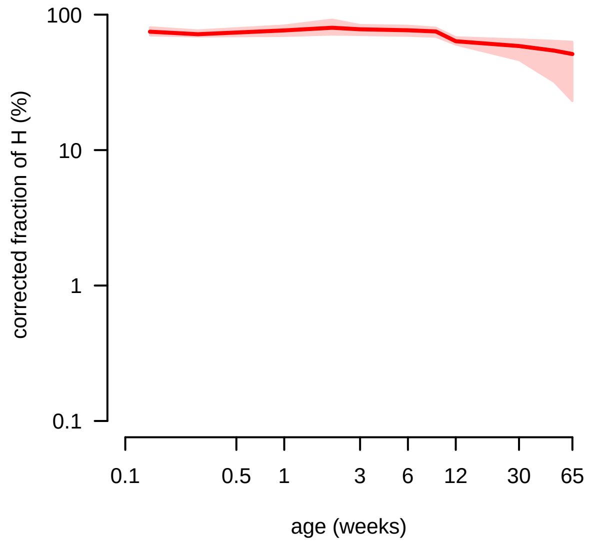

P03911

>sp|P03911|NU4M_MOUSE

NADH−ubiquinone oxidoreductase chain 4

GN=Mtnd4

Med H1/2: 11.7 days

Q5 H1/2: 4.2 days

Q95 H1/2: 152 days

reliable model fit: yes

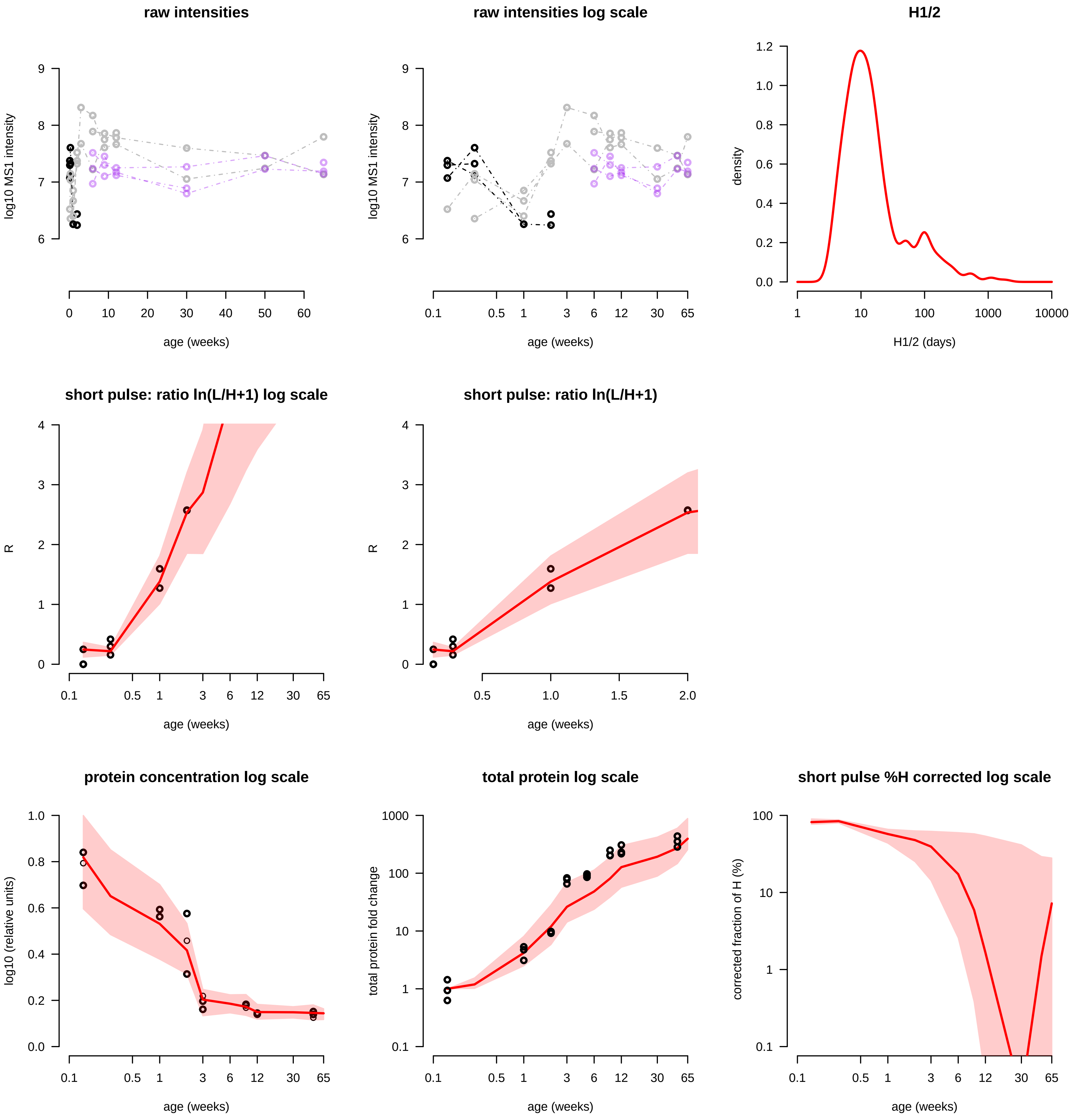

P05532−2;P05532

>sp|P05532−2|KIT_MOUSE

Isoform 2 of Mast/stem cell growth factor receptor Kit

GN=Kit

Med H1/2: 3.2 days

Q5 H1/2: 2.5 days

Q95 H1/2: 6.7 days

reliable model fit: yes

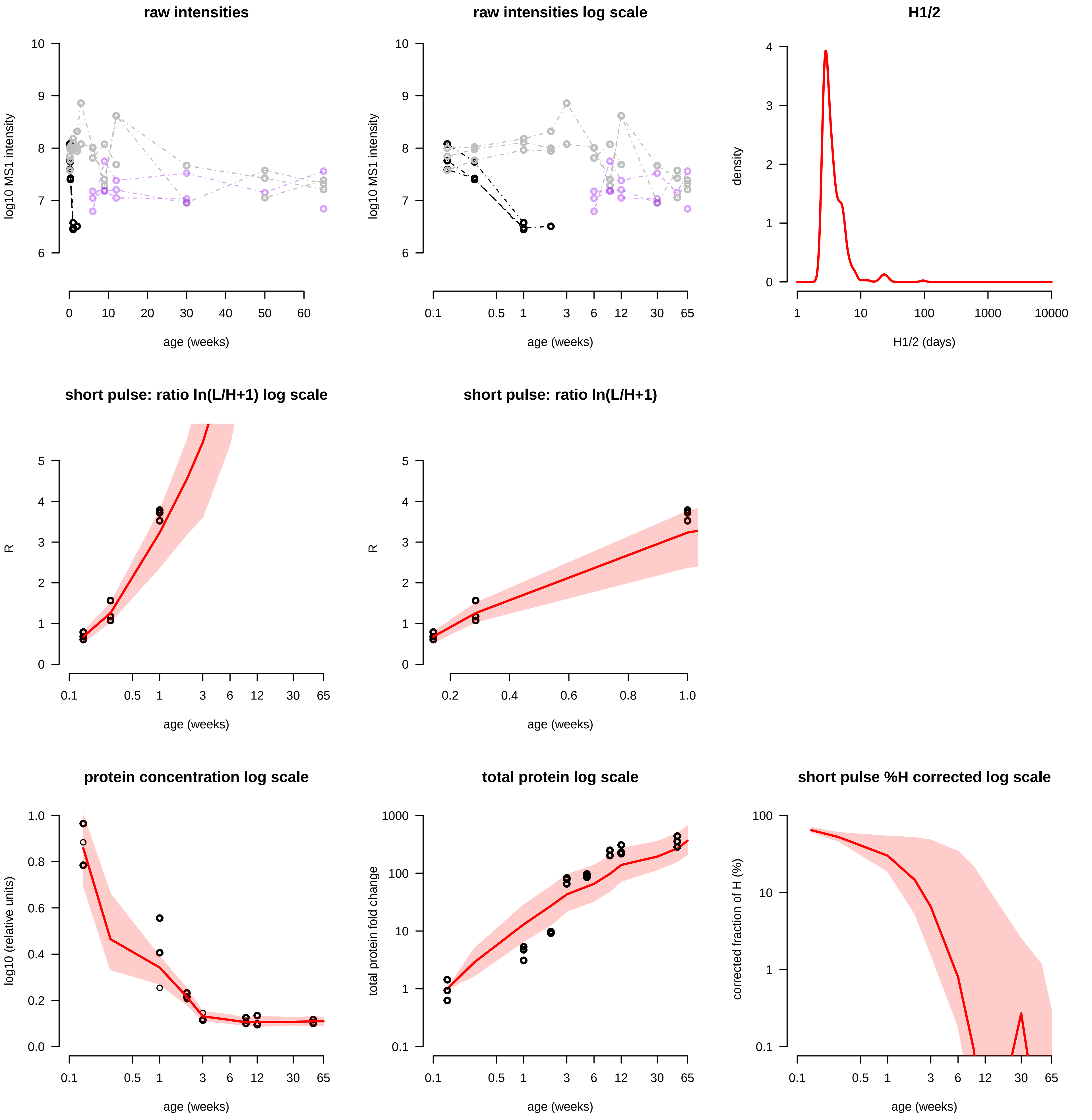

Q8R4G6

>sp|Q8R4G6|MGT5A_MOUSE

Alpha−1,6−mannosylglycoprotein 6−beta−N−acetylglucosaminyltransferase A

GN=Mgat5

Med H1/2: 8.8 days

Q5 H1/2: 6.6 days

Q95 H1/2: 14.8 days

reliable model fit: yes

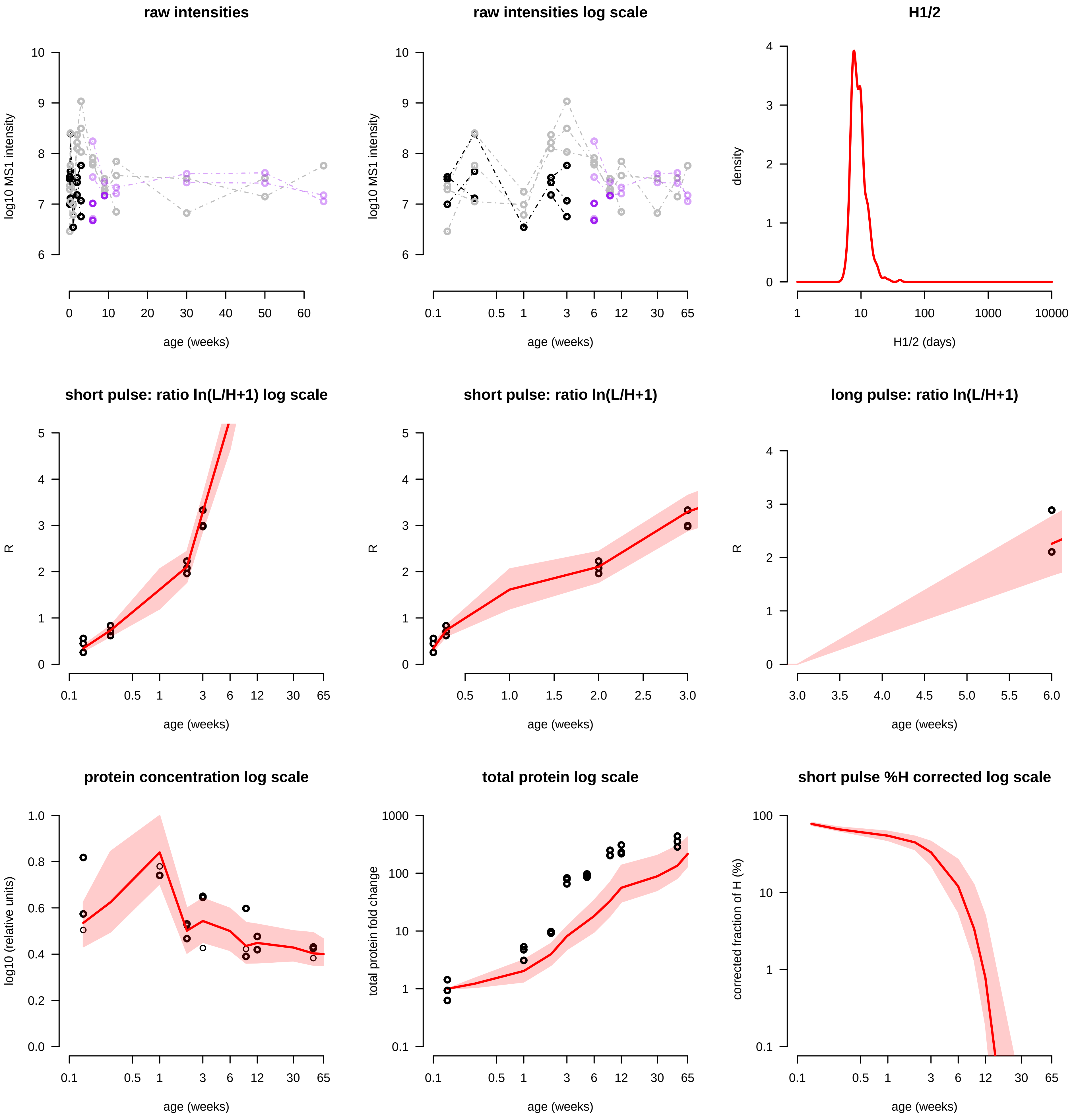

P70696

>sp|P70696|H2B1A_MOUSE

Histone H2B type 1−A

GN=H2bc1

Med H1/2: 16.4 days

Q5 H1/2: 7 days

Q95 H1/2: 244.7 days

reliable model fit: no

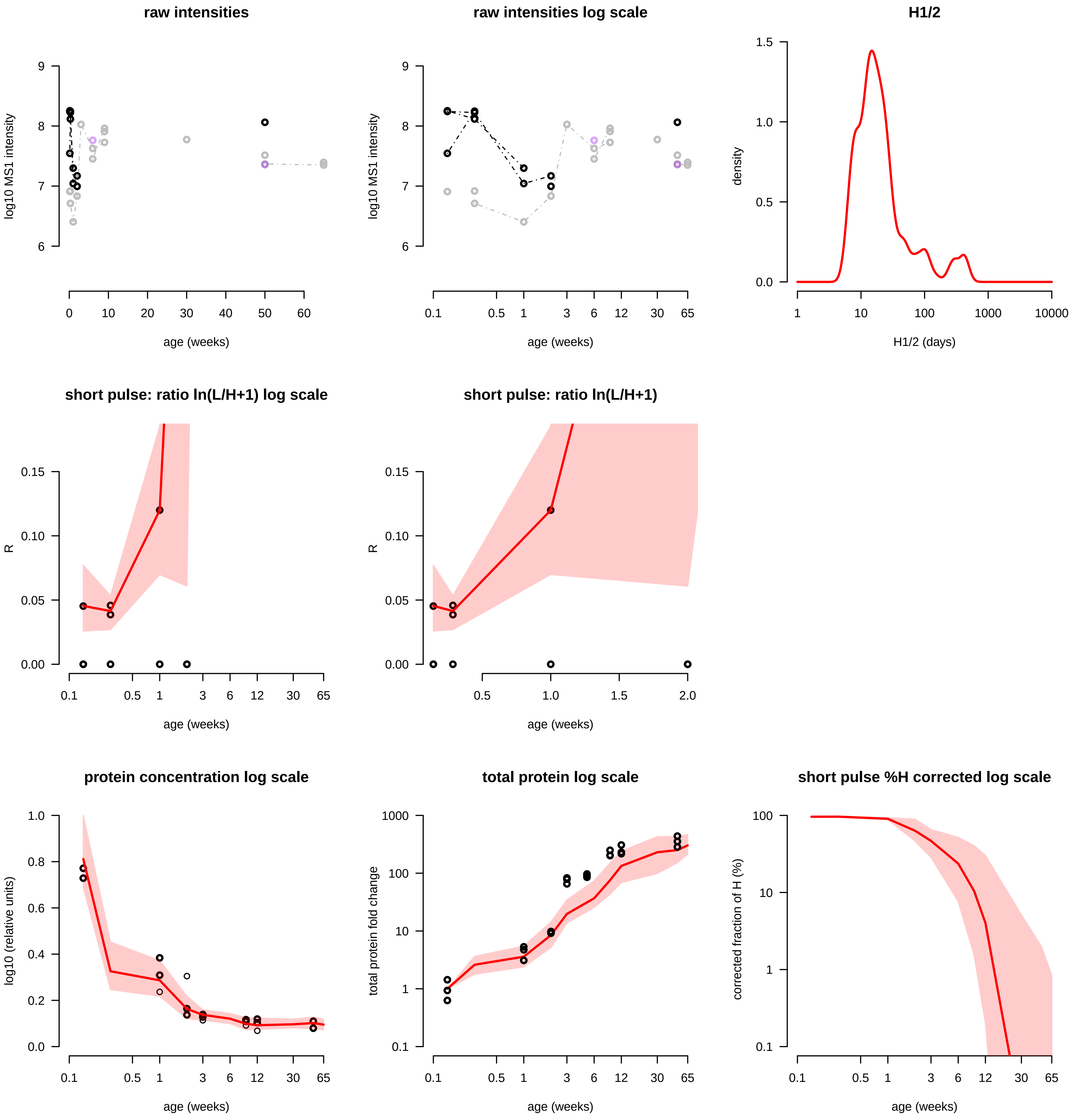

Q61555

>sp|Q61555|FBN2_MOUSE

Fibrillin−2

GN=Fbn2

Med H1/2: 3.5 days

Q5 H1/2: 2.5 days

Q95 H1/2: 8.3 days

reliable model fit: yes

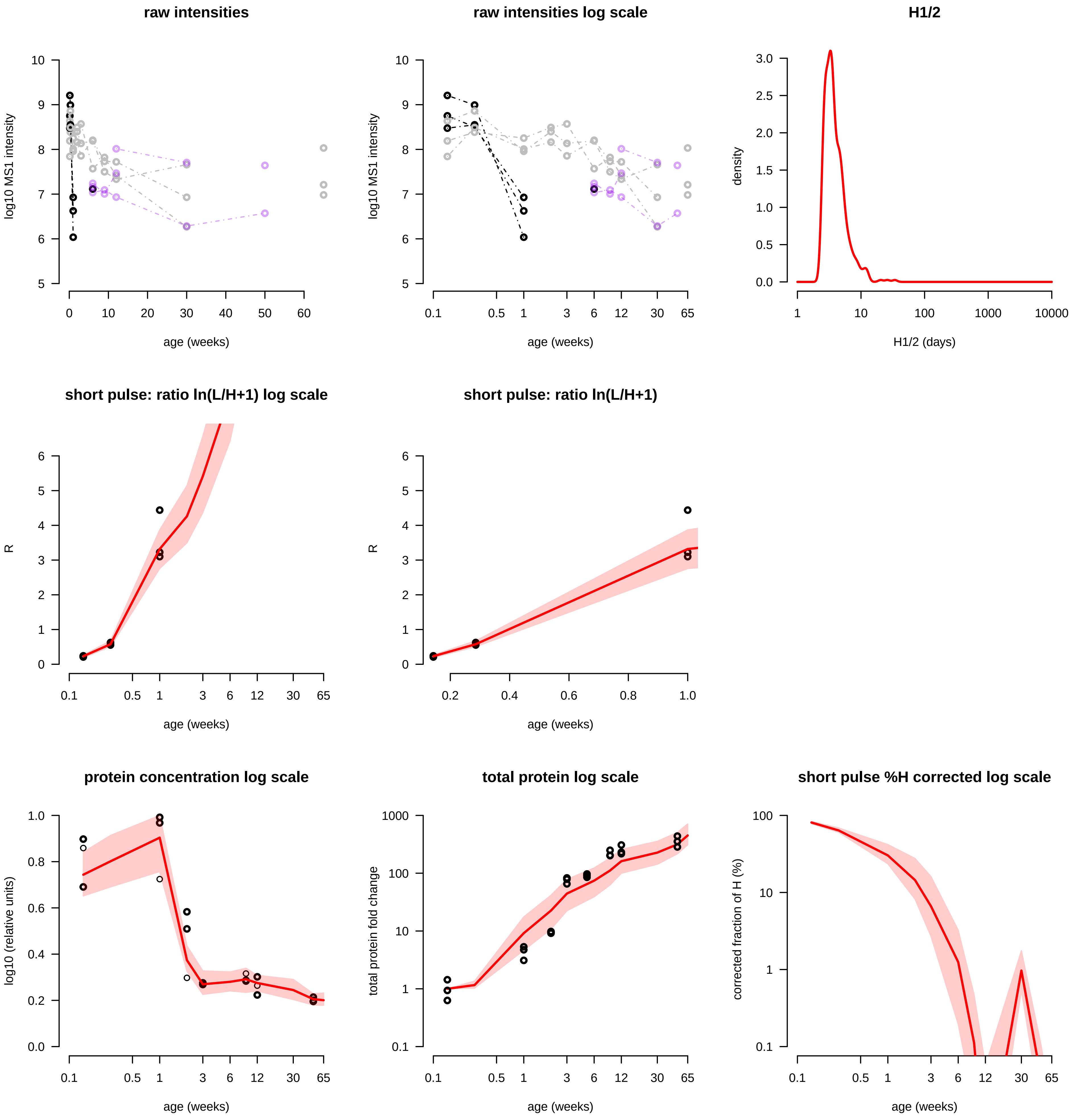

Q9QXA1−2;Q9QXA1−3

>sp|Q9QXA1−2|CYHR1_MOUSE

Isoform 2 of Cysteine and histidine−rich protein 1

GN=Cyhr1

Med H1/2: 24.2 days

Q5 H1/2: 7.5 days

Q95 H1/2: 238.5 days

reliable model fit: yes

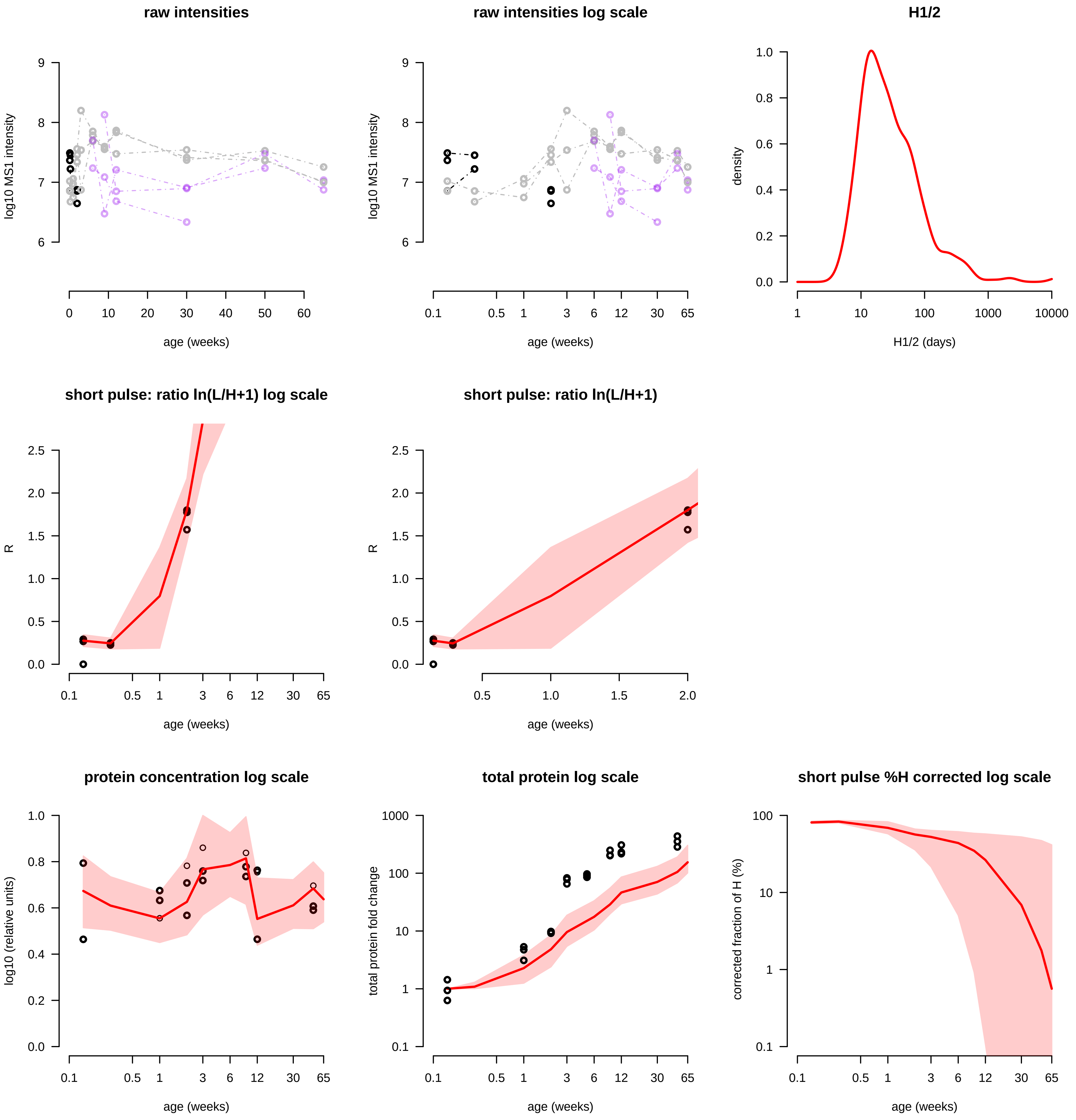

Q68FE9

>sp|Q68FE9|TSH2_MOUSE

Teashirt homolog 2

GN=Tshz2

Med H1/2: 573.2 days

Q5 H1/2: 217.9 days

Q95 H1/2: 6399.4 days

reliable model fit: yes

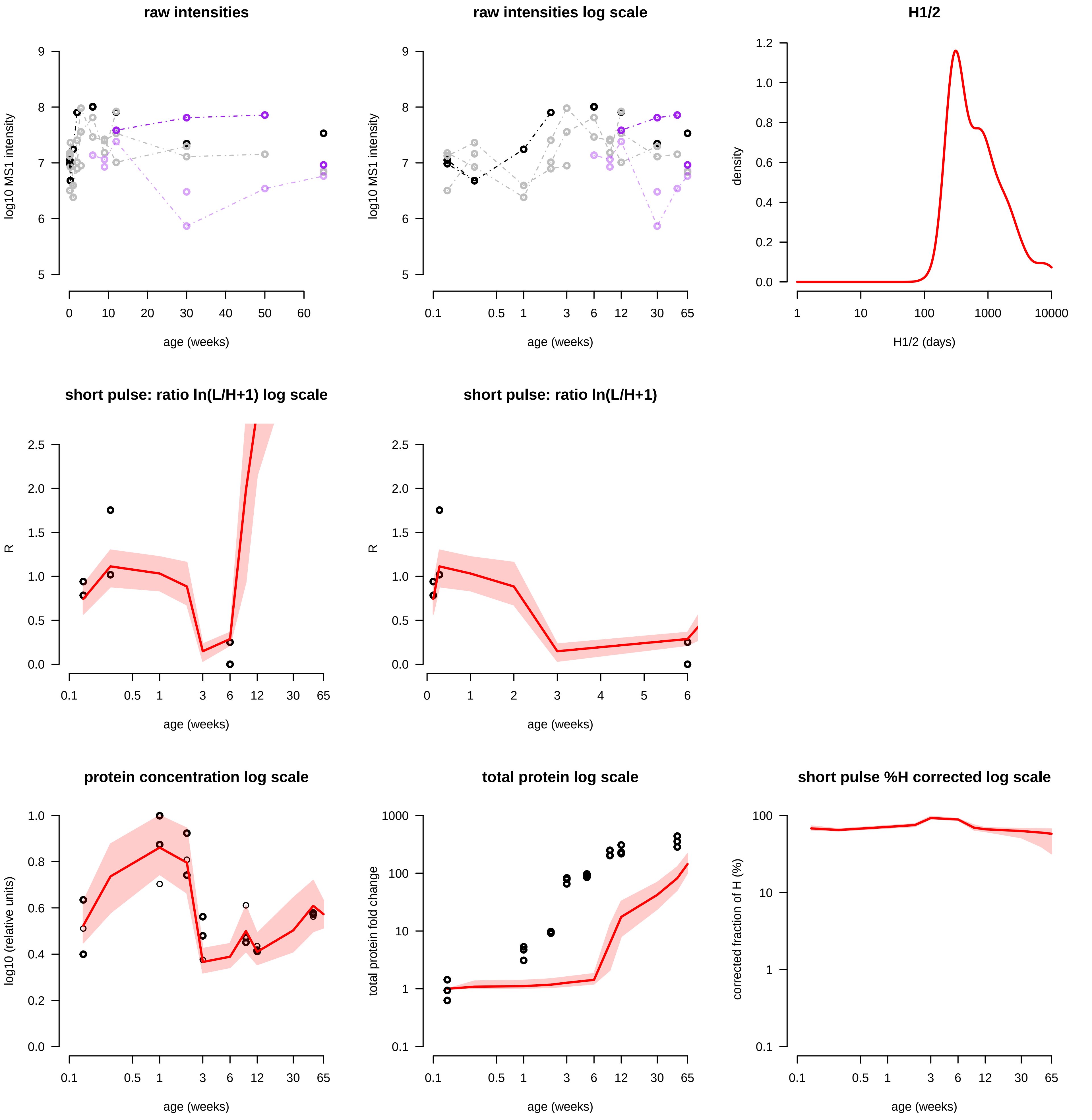

Q80Z25

>sp|Q80Z25|OFD1_MOUSE

Oral−facial−digital syndrome 1 protein homolog

GN=Ofd1

Med H1/2: 8.5 days

Q5 H1/2: 6.8 days

Q95 H1/2: 14.7 days

reliable model fit: yes

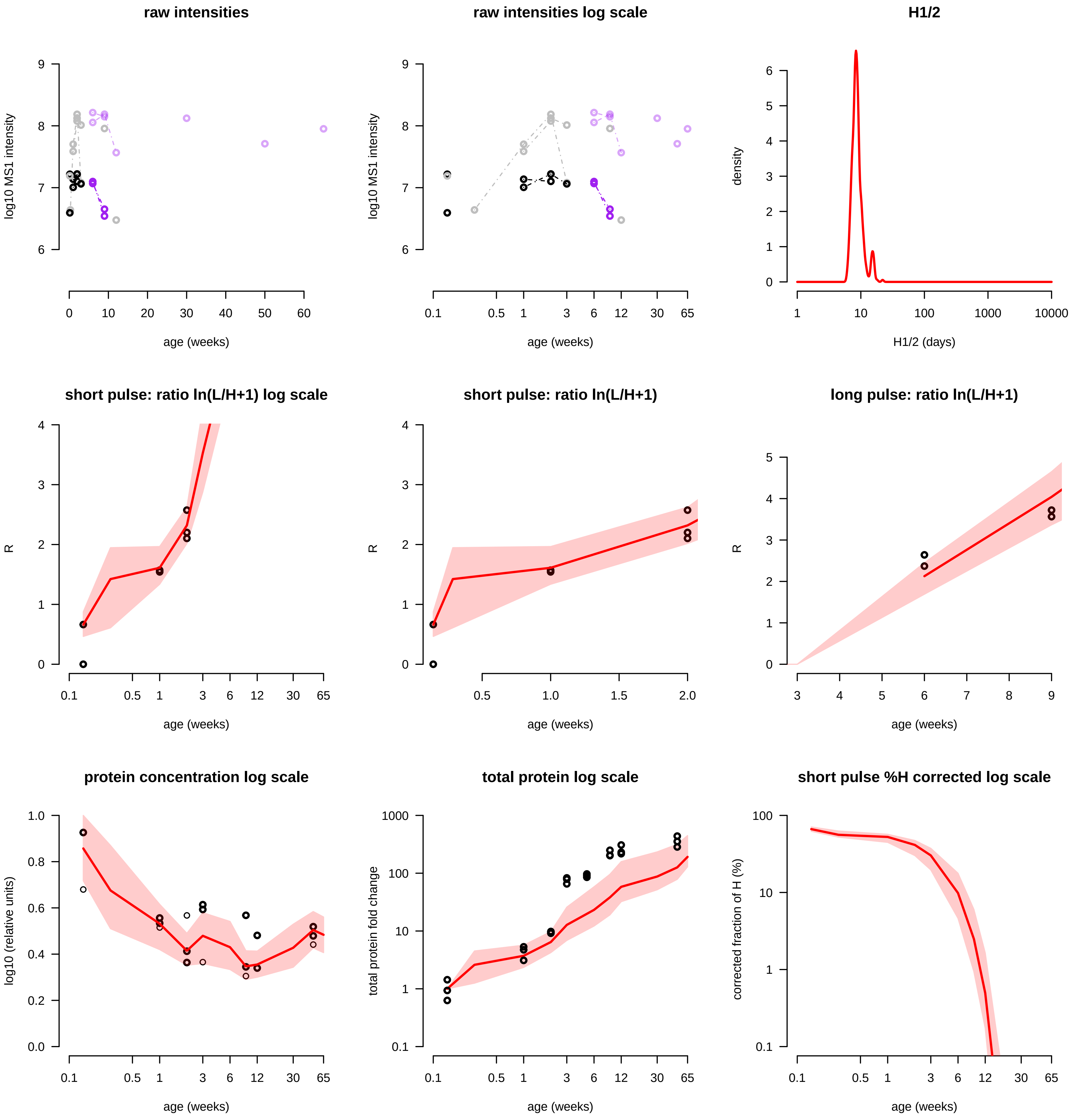

Q6PFR5

>sp|Q6PFR5|TRA2A_MOUSE

Transformer−2 protein homolog alpha

GN=Tra2a

Med H1/2: 78.6 days

Q5   H1/2: 19.7 days

Q95 H1/2: 777 days

reliable model fit: yes

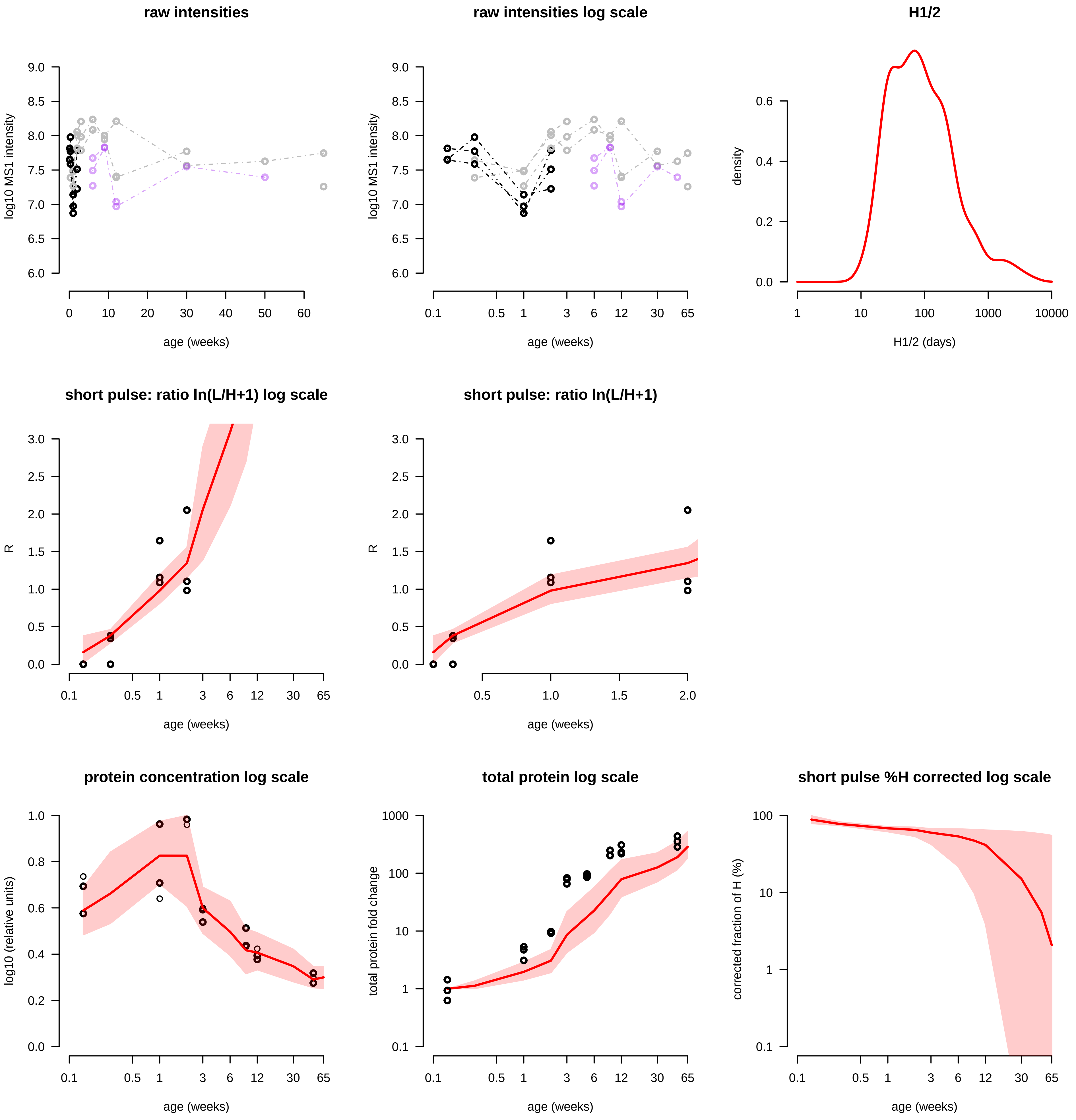

Q9D0T2

>sp|Q9D0T2|DUS12_MOUSE

Dual specificity protein phosphatase 12

GN=Dusp12

Med H1/2: 6.8 days

Q5 H1/2: 2.8 days

Q95 H1/2: 84.6 days

reliable model fit: yes

raw intensities

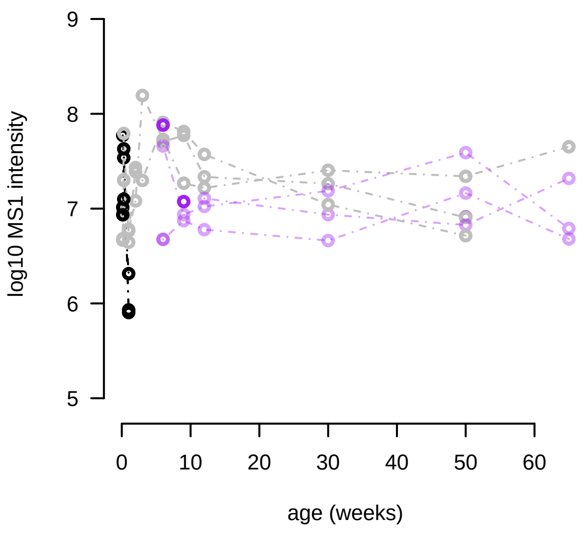

raw intensities log scale

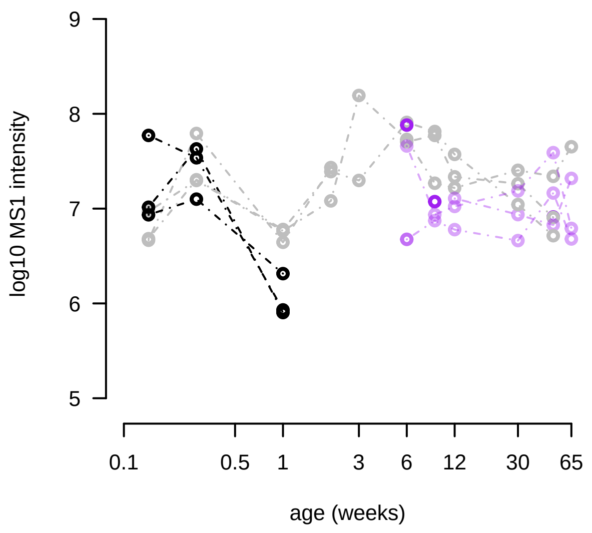

H1/2

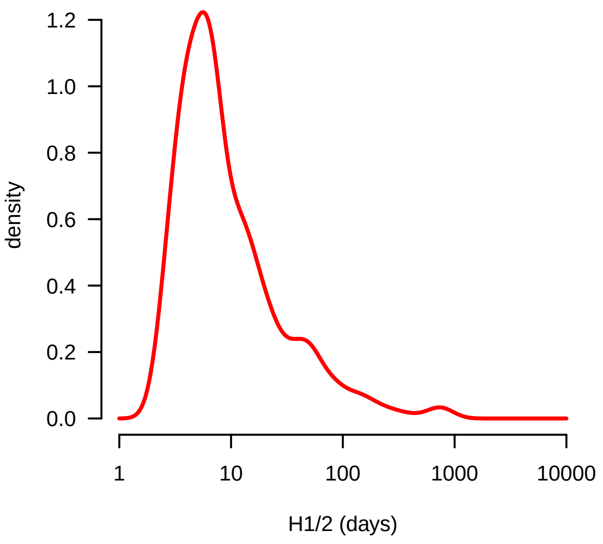

short pulse: ratio ln(L/H+1) log scale

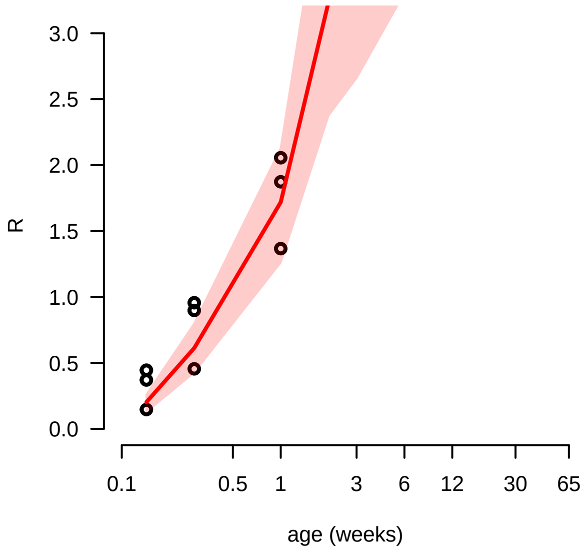

short pulse: ratio ln(L/H+1)

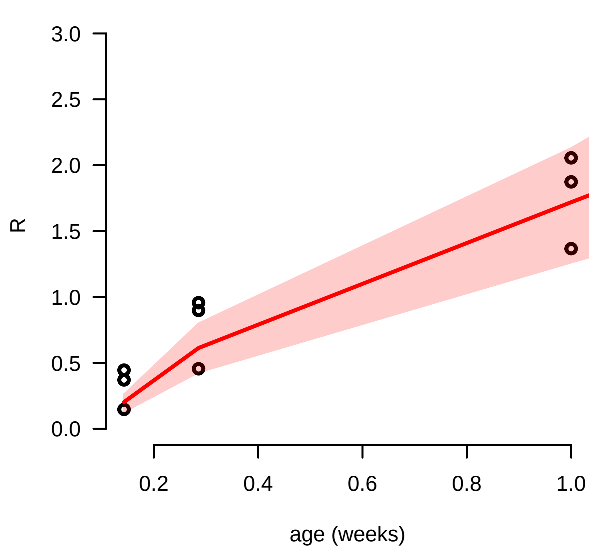

protein concentration log scale

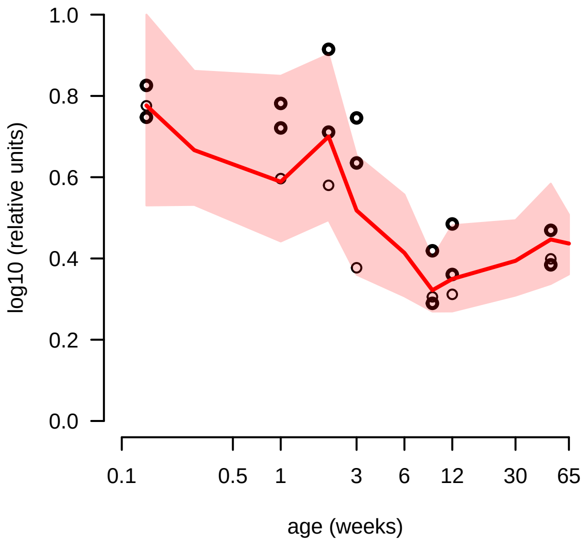

total protein log scale

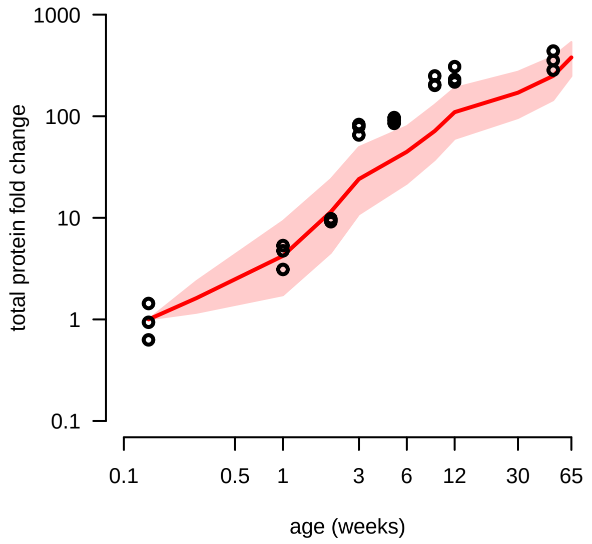

short pulse %H corrected log scale

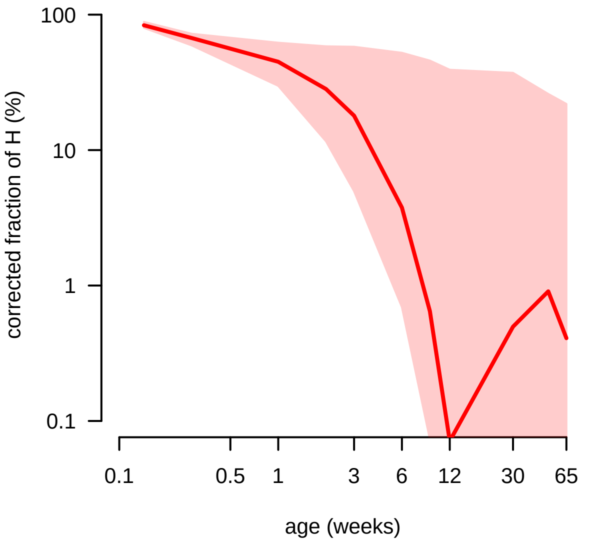

Q3U962

>sp|Q3U962|CO5A2_MOUSE

Collagen alpha−2(V) chain

GN=Col5a2

Med H1/2: 7.7 days

Q5   H1/2: 2.8 days

Q95 H1/2: 66.7 days

reliable model fit: yes

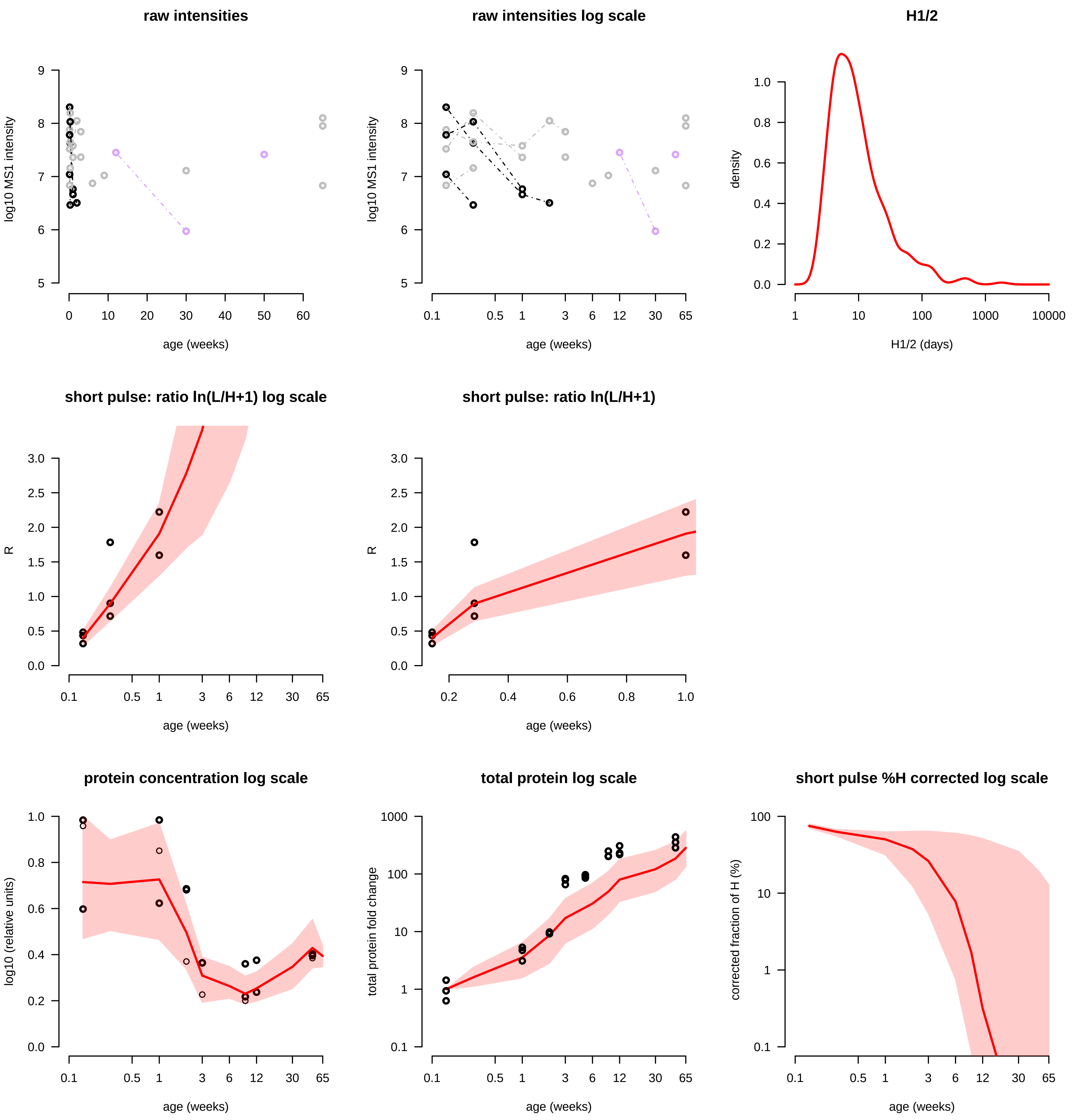

Q8K1M6;Q8K1M6−2;Q8K1M6−4

>sp|Q8K1M6|DNM1L_MOUSE

Dynamin−1−like protein

GN=Dnm1l

Med H1/2: 6.8 days

Q5 H1/2: 3 days

Q95 H1/2: 47.7 days

reliable model fit: yes

raw intensities

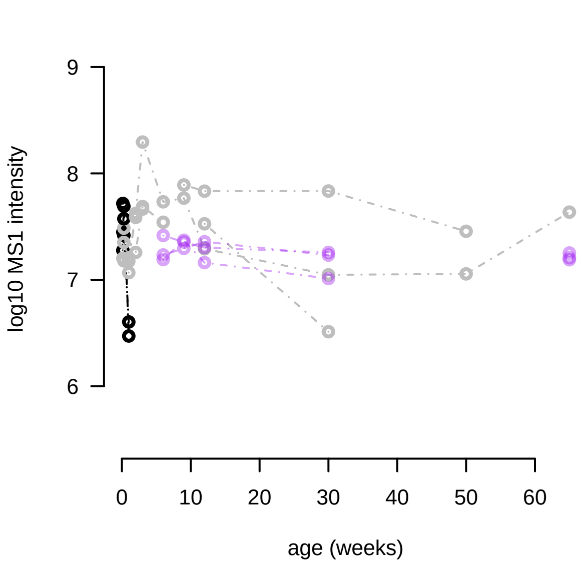

raw intensities log scale

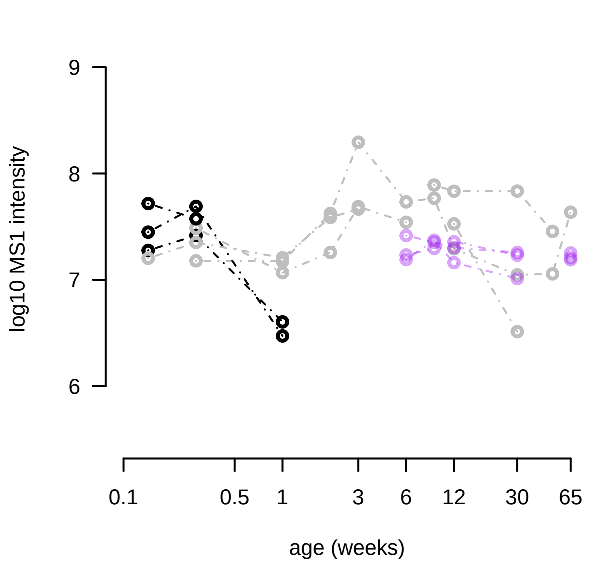

H1/2

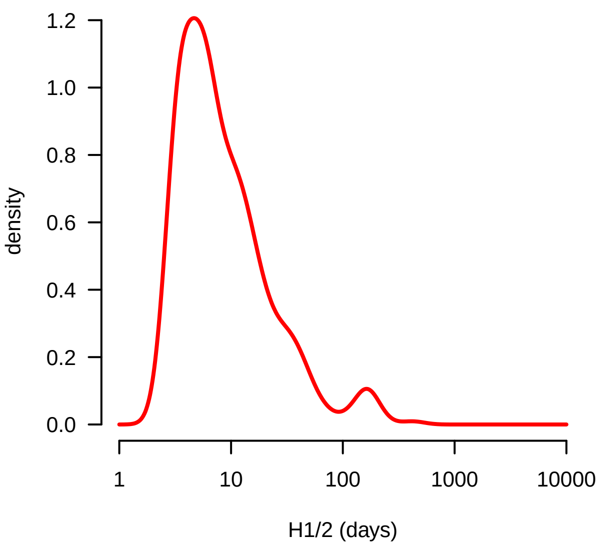

short pulse: ratio ln(L/H+1) log scale

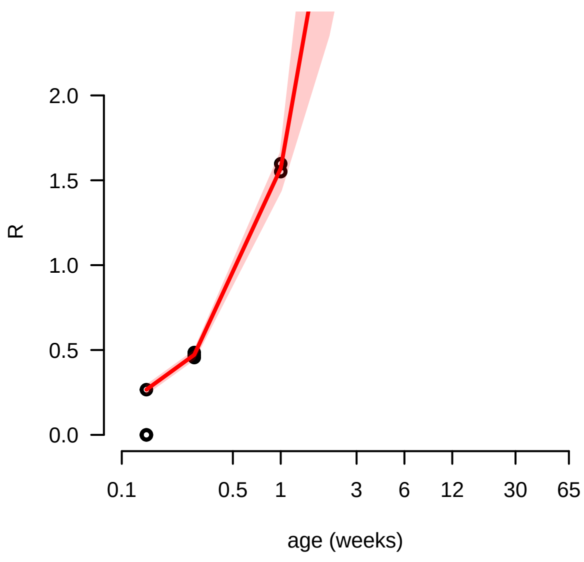

short pulse: ratio ln(L/H+1)

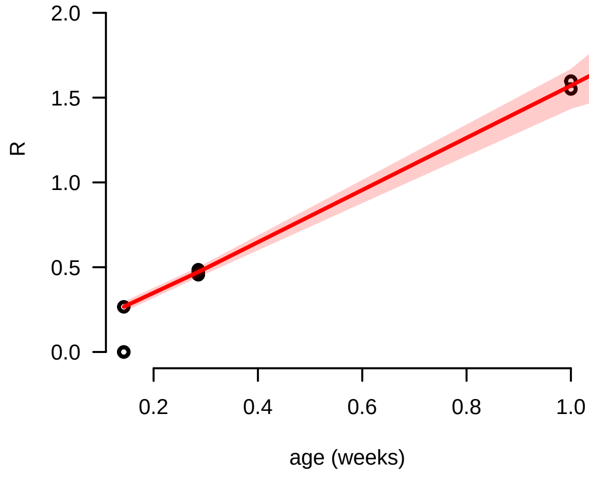

protein concentration log scale

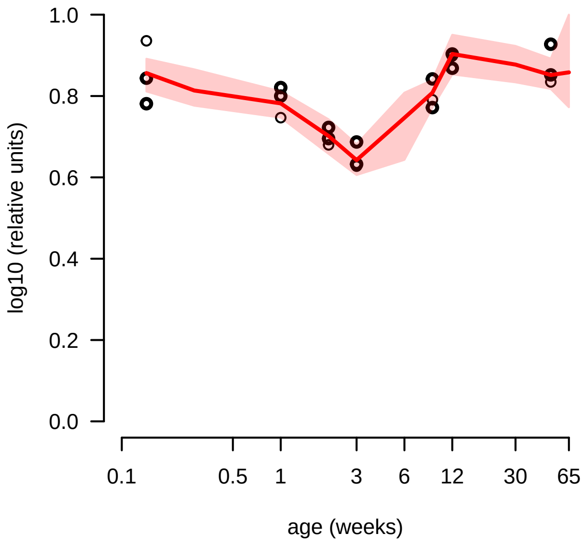

total protein log scale

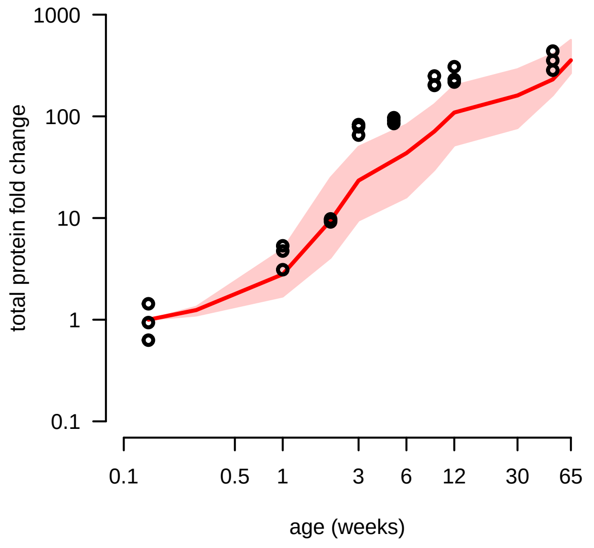

short pulse %H corrected log scale

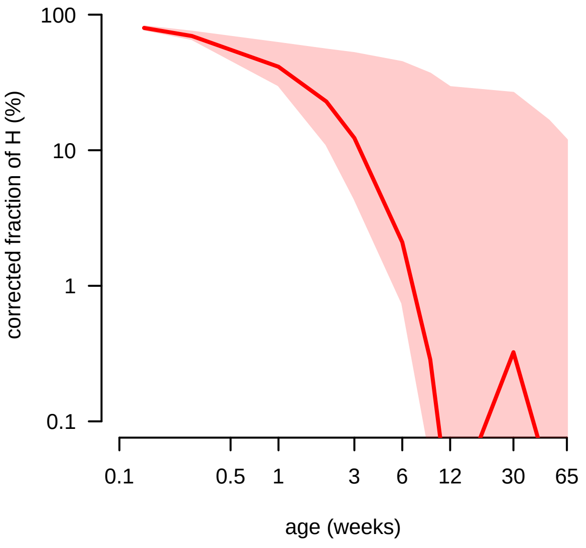

Q91W69

>sp|Q91W69|EPN3_MOUSE

Epsin−3

GN=Epn3

Med H1/2: 5.9 days

Q5 H1/2: 3 days

Q95 H1/2: 19.1 days

reliable model fit: yes

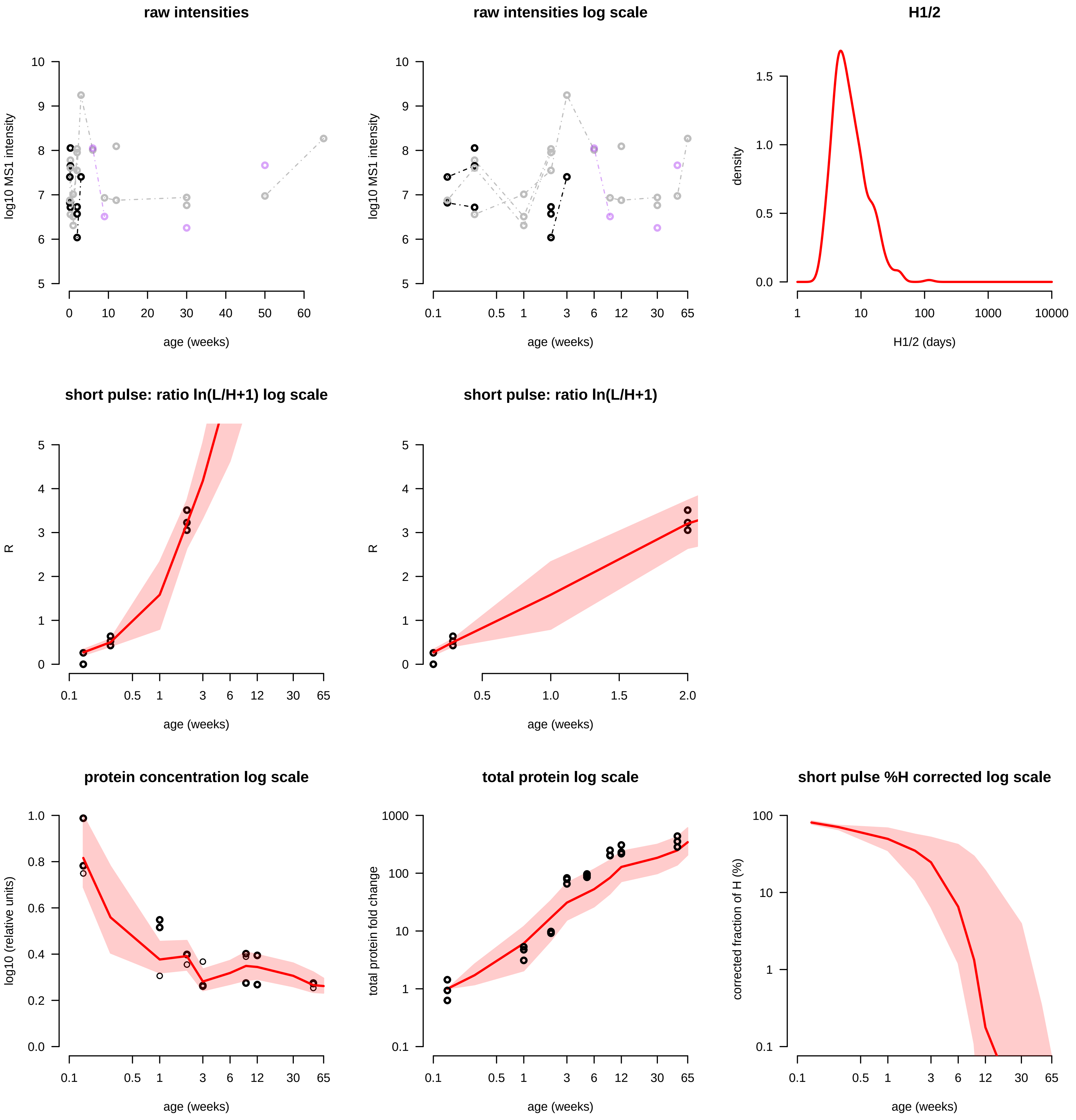

Q8VEL2

>sp|Q8VEL2|MTMRE_MOUSE

Myotubularin−related protein 14

GN=Mtmr14

Med H1/2: 28.1 days

Q5 H1/2: 8.5 days

Q95 H1/2: 351.7 days

reliable model fit: yes

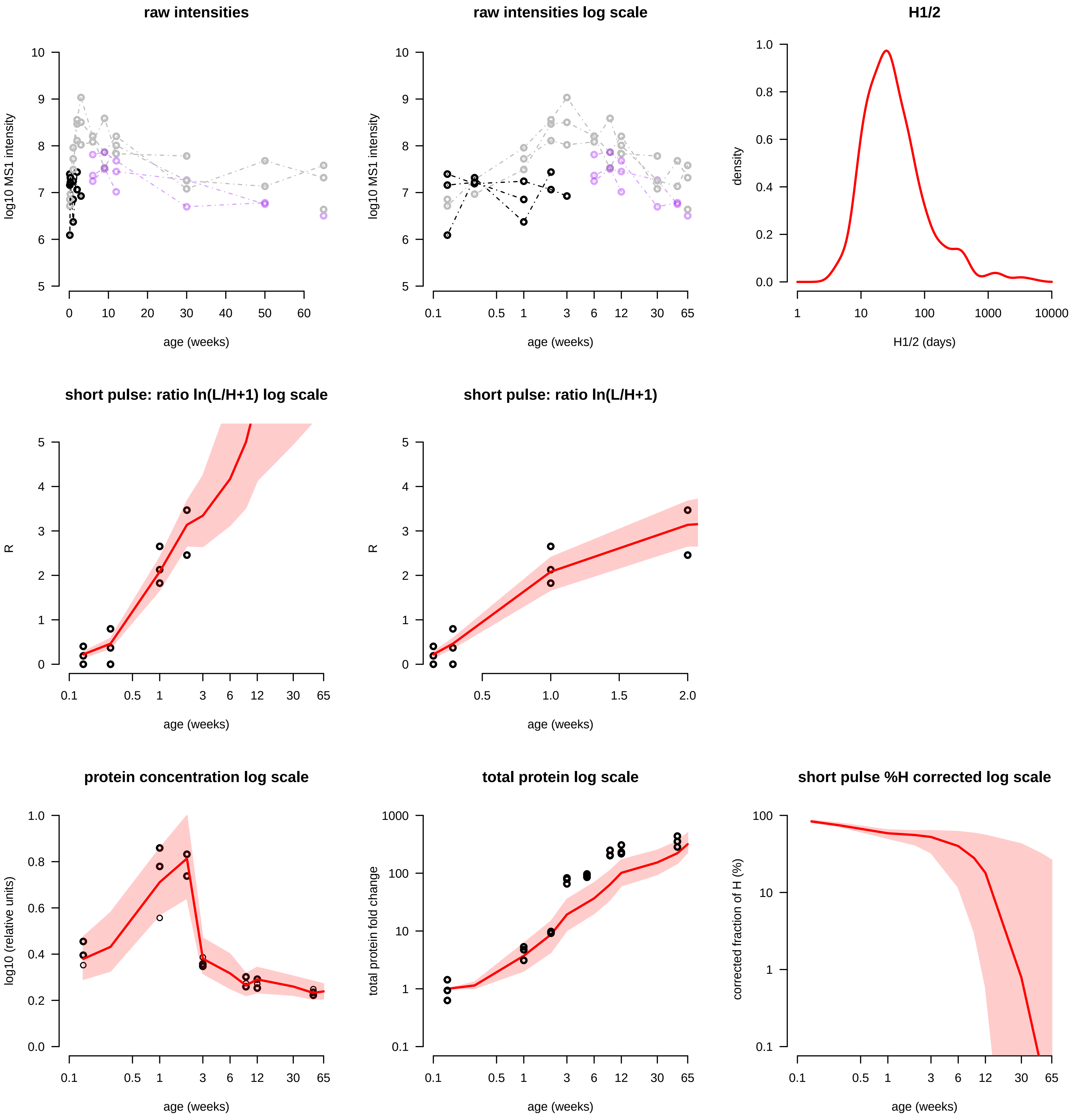

Q9EPR5

>sp|Q9EPR5|SORC2_MOUSE

VPS10 domain−containing receptor SorCS2

GN=Sorcs2

Med H1/2: 7.8 days

Q5 H1/2: 3.5 days

Q95 H1/2: 48.3 days

reliable model fit: yes

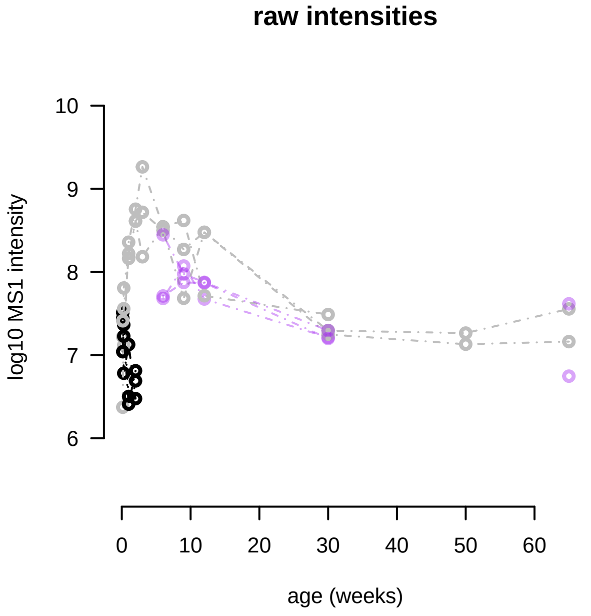

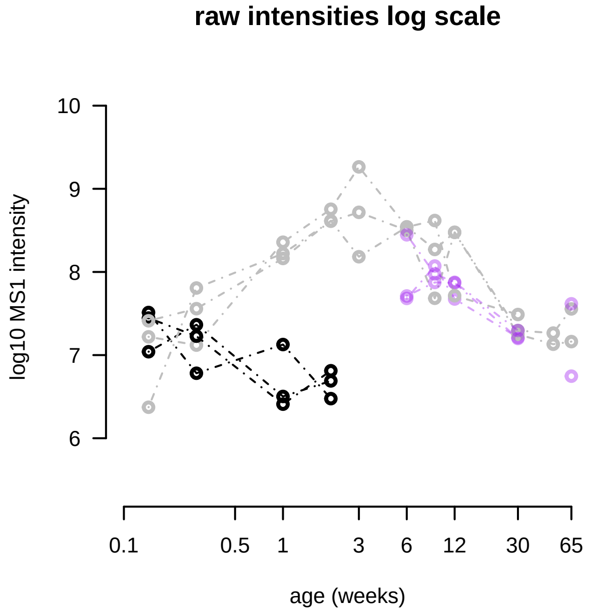

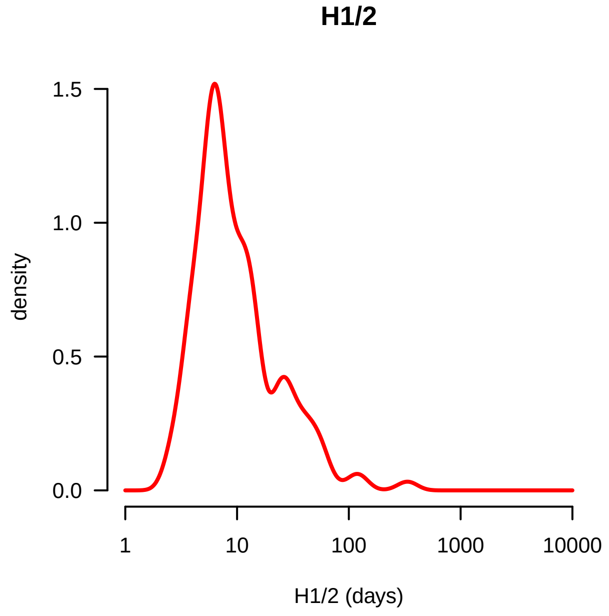

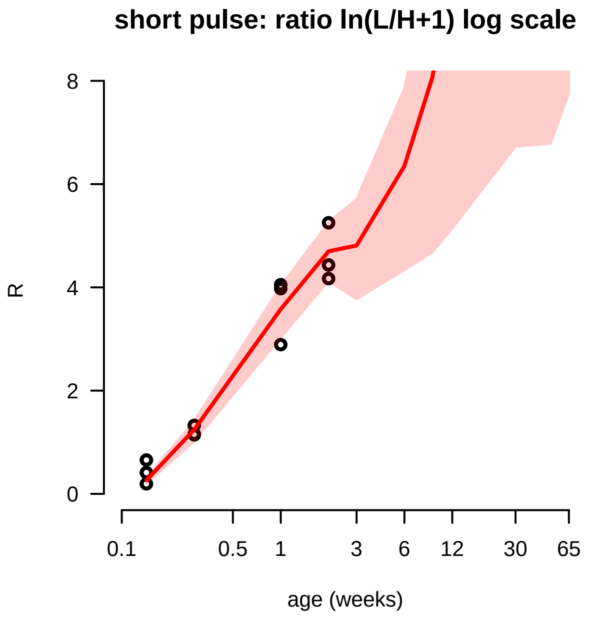

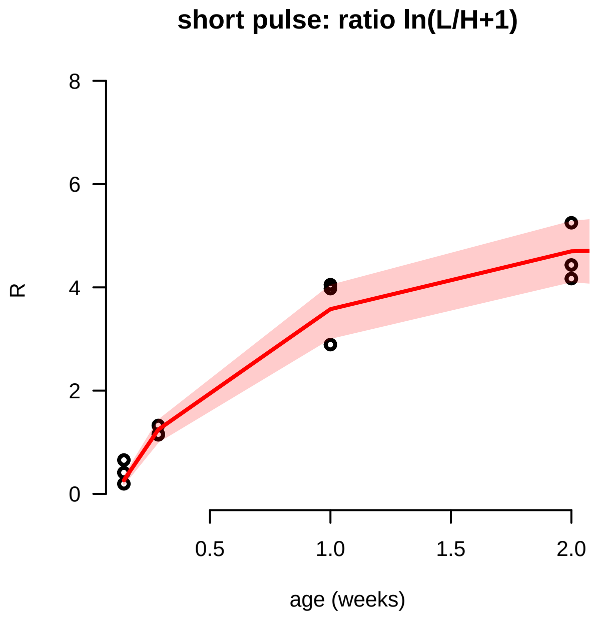

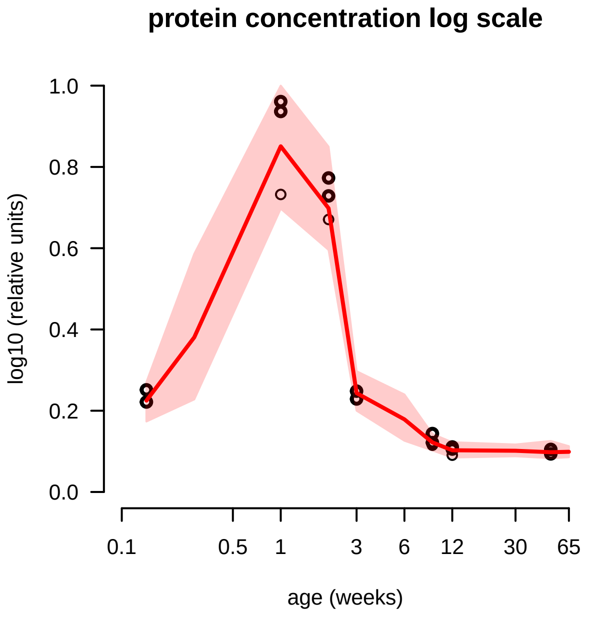

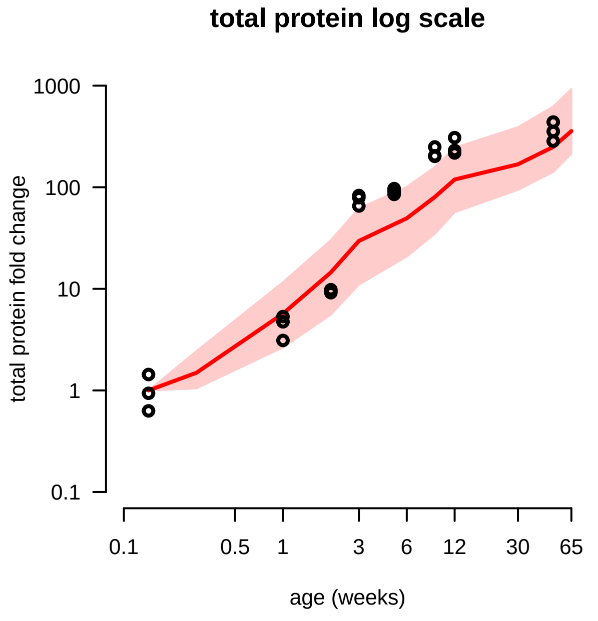

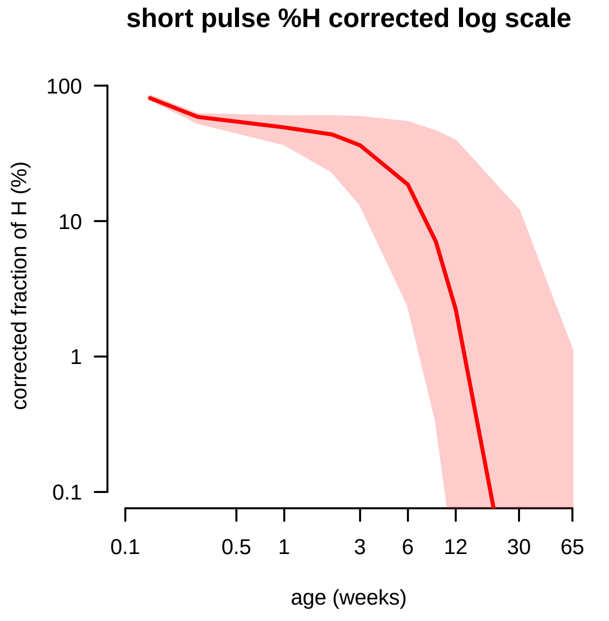

Q8K2I9;Q8K2I9−2

>sp|Q8K2I9|FBH1_MOUSE

F−box DNA helicase 1

GN=Fbh1

Med H1/2: 11.5 days

Q5 H1/2: 3.5 days

Q95 H1/2: 102.5 days

reliable model fit: yes

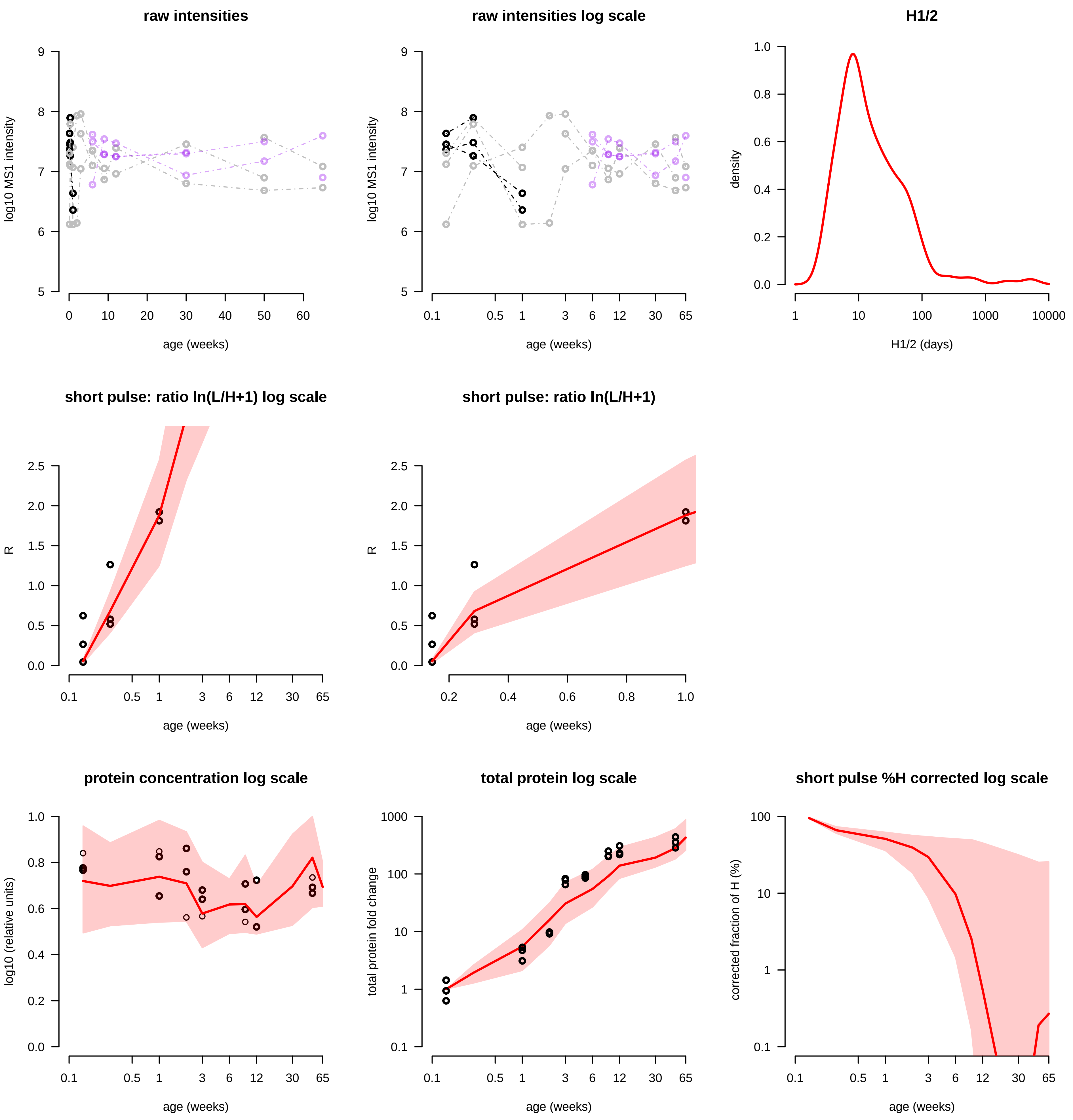

Q8K389

>sp|Q8K389|CK5P2_MOUSE

CDK5 regulatory subunit−associated protein 2

GN=Cdk5rap2

Med H1/2: 14.5 days

Q5 H1/2: 4.4 days

Q95 H1/2: 201.2 days

reliable model fit: yes

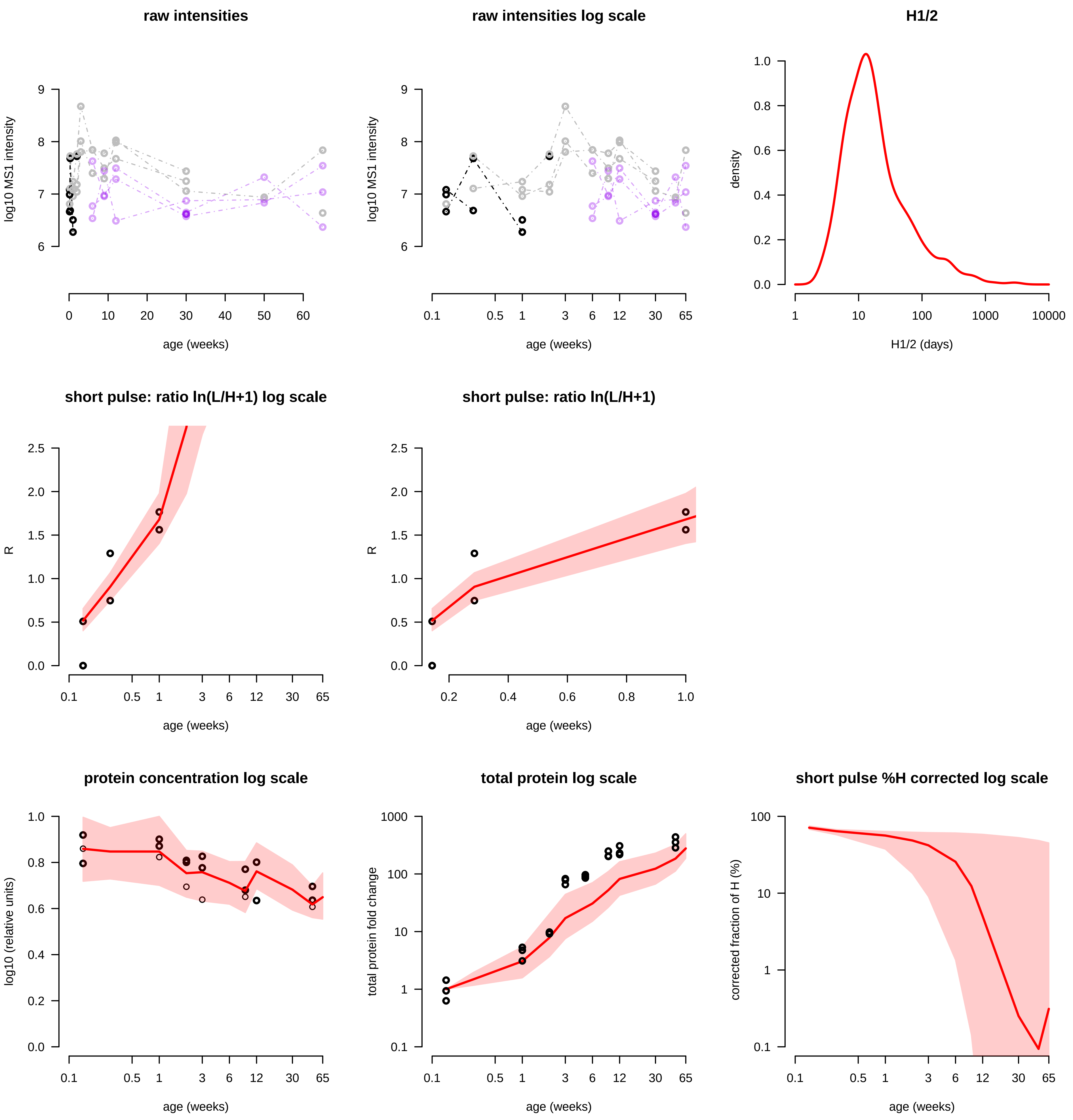

Q62356

>sp|Q62356|FSTL1_MOUSE

Follistatin−related protein 1

GN=Fstl1

Med H1/2: 10.7 days

Q5 H1/2: 4.7 days

Q95 H1/2: 133 days

reliable model fit: yes

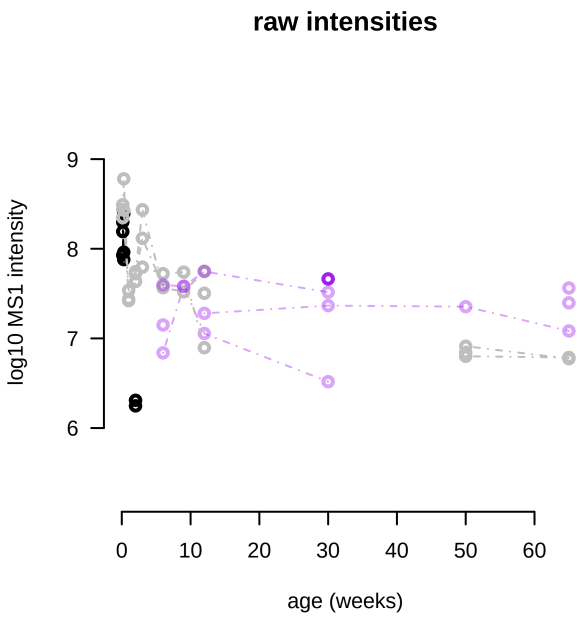

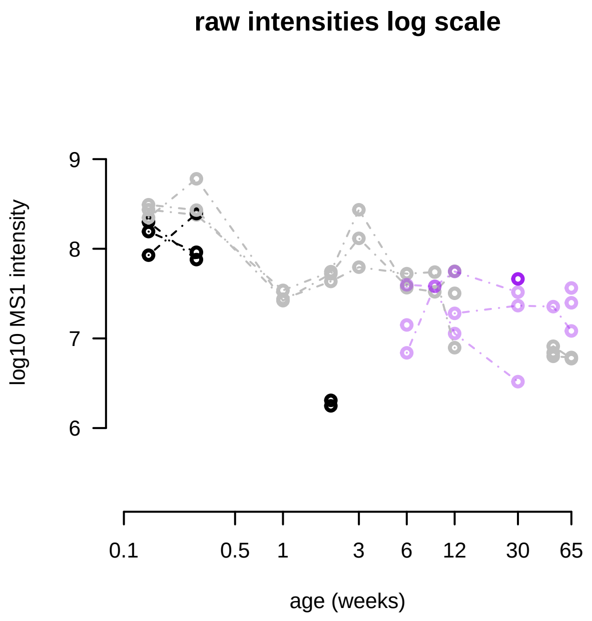

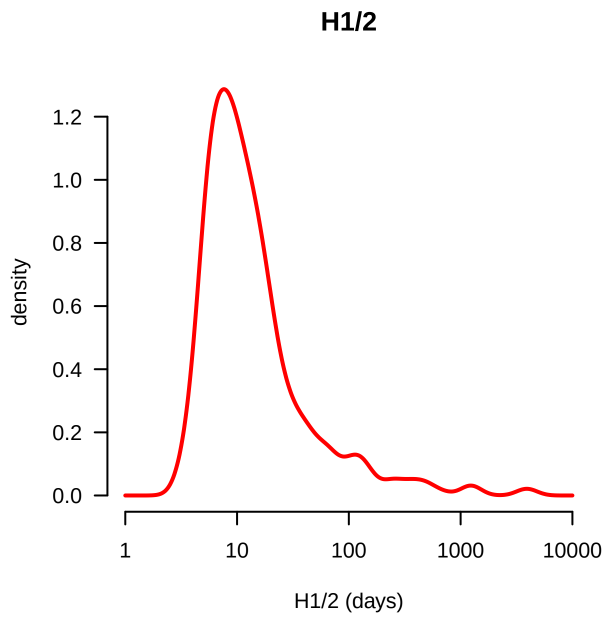

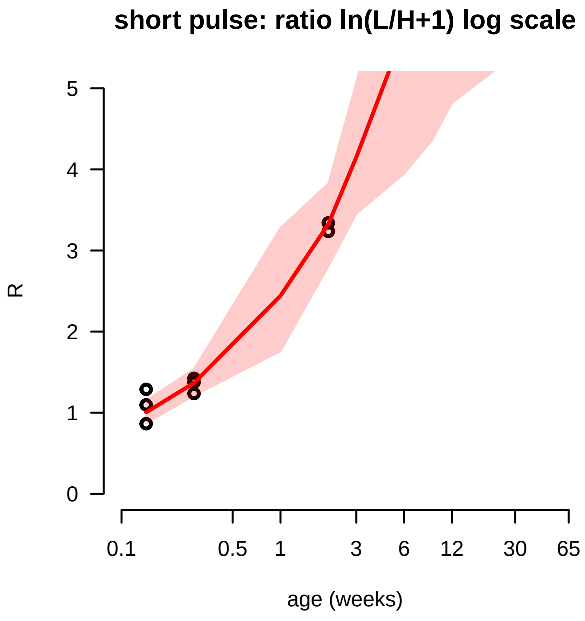

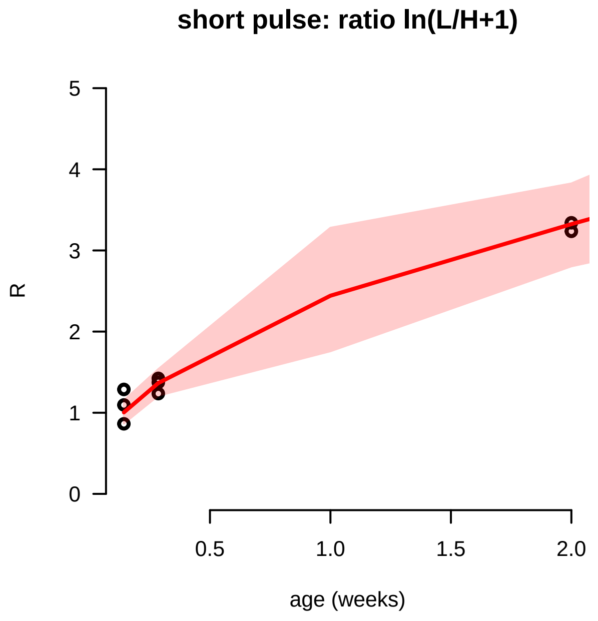

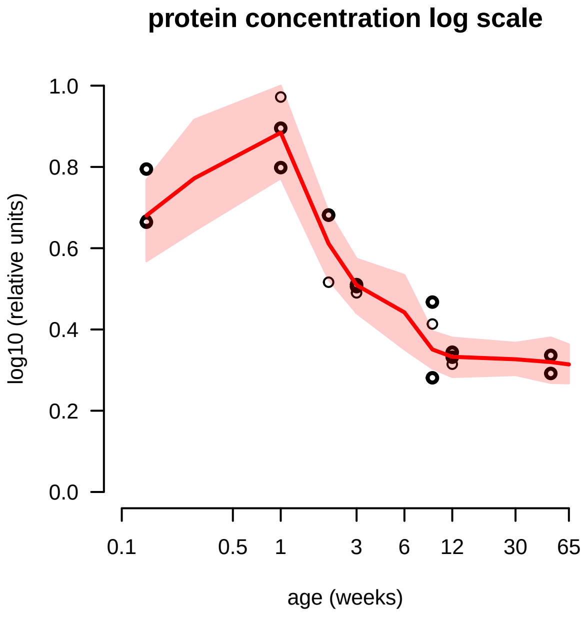

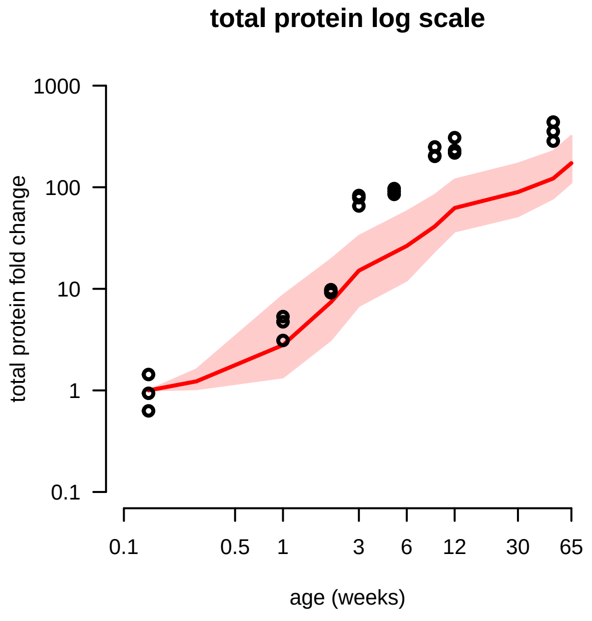

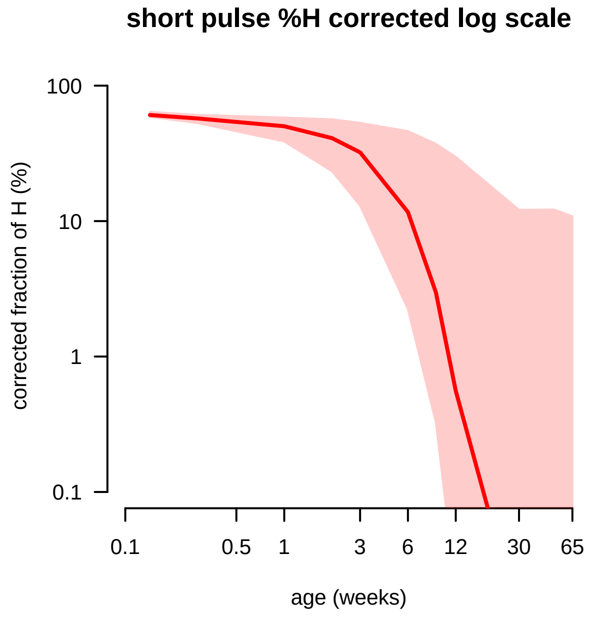

Q9CWF2

>sp|Q9CWF2|TBB2B_MOUSE

Tubulin beta−2B chain

GN=Tubb2b

Med H1/2: 14.2 days

Q5 H1/2: 5.8 days

Q95 H1/2: 102.4 days

reliable model fit: yes

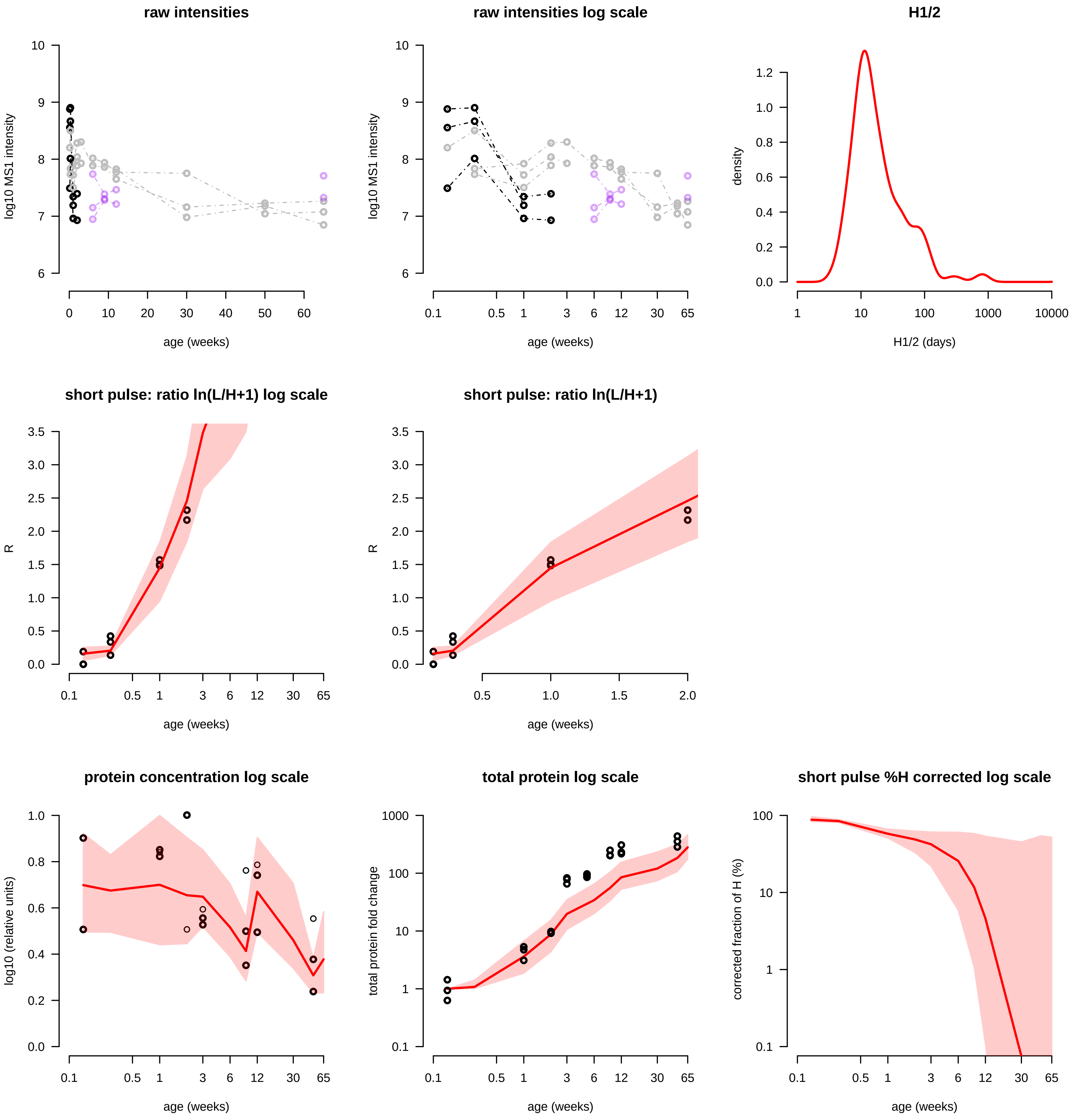

Q9WU62−2;Q9WU62

>sp|Q9WU62−2|INCE_MOUSE

Isoform 2 of Inner centromere protein

GN=Incenp

Med H1/2: 1555.3 days

Q5 H1/2: 394.2 days

Q95 H1/2: 14048.5 days

reliable model fit: no

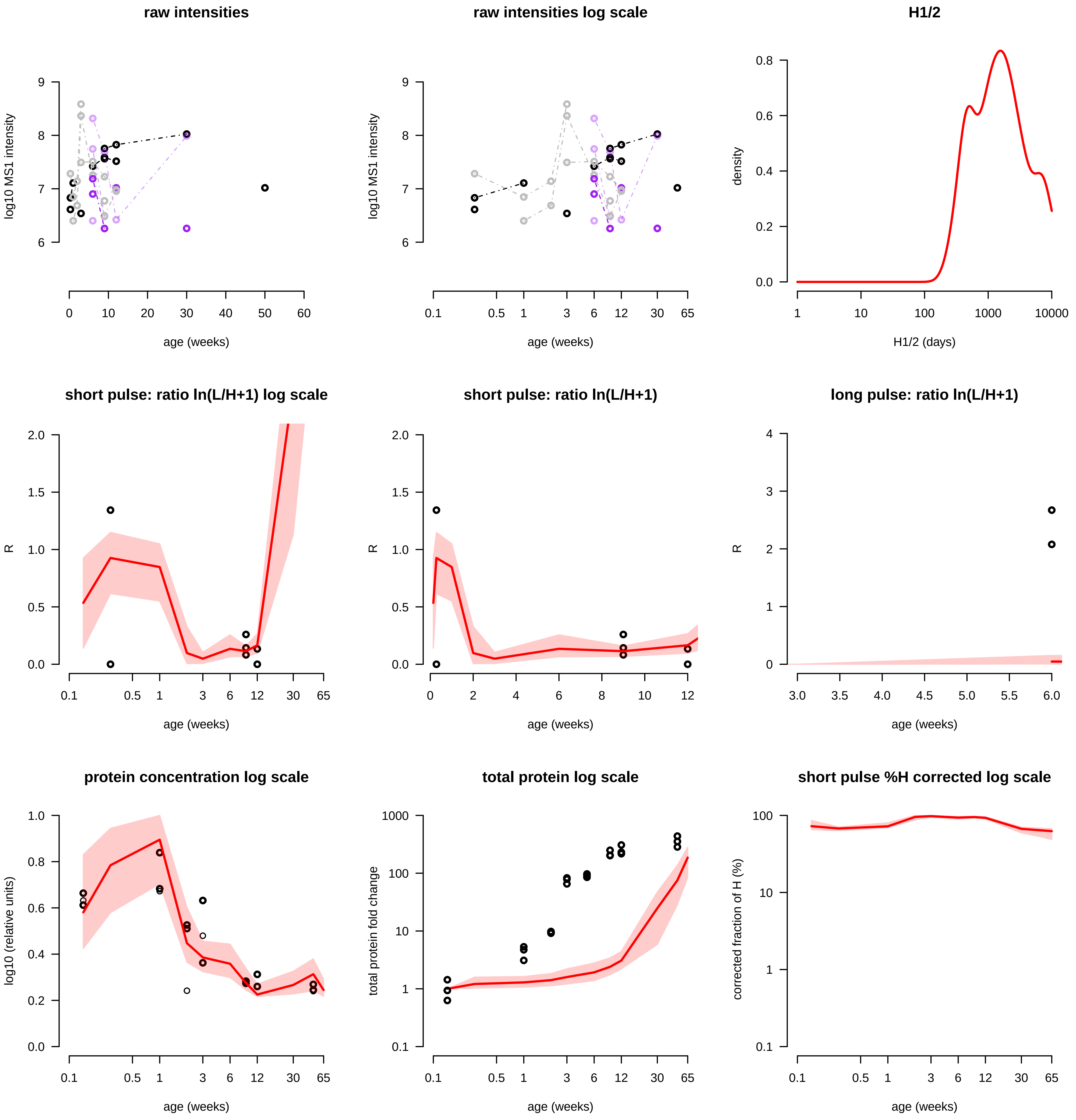

Q920B0−2;Q920B0

>sp|Q920B0−2|FRM4B_MOUSE

Isoform 2 of FERM domain−containing protein 4B

GN=Frmd4b

Med H1/2: 40 days

Q5 H1/2: 23.6 days

Q95 H1/2: 264.4 days

reliable model fit: no

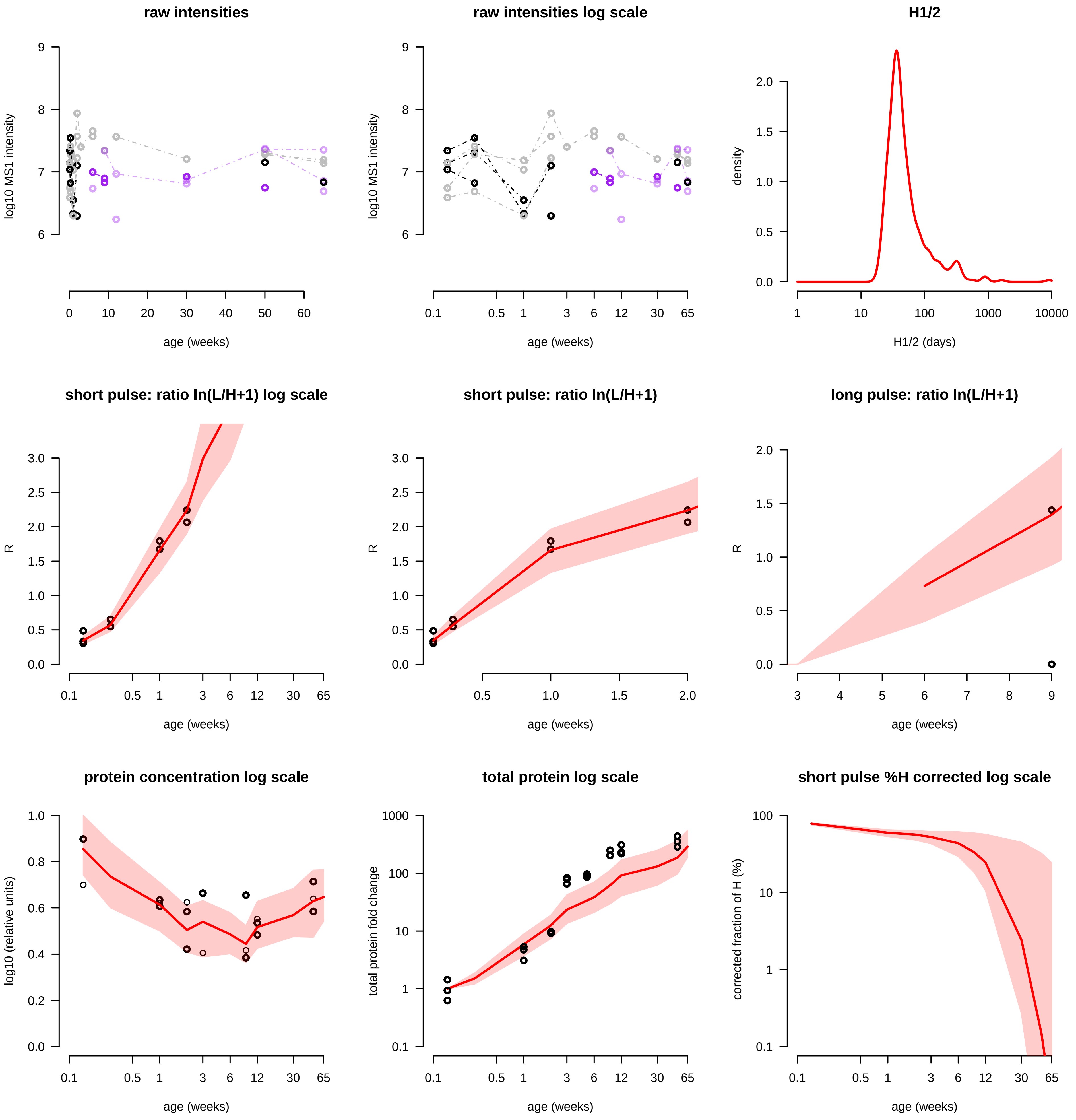

Q8BP22

>sp|Q8BP22|FA92A_MOUSE

Protein FAM92A

GN=Fam92a

Med H1/2: 10.4 days

Q5 H1/2: 3.4 days

Q95 H1/2: 146.7 days

reliable model fit: yes

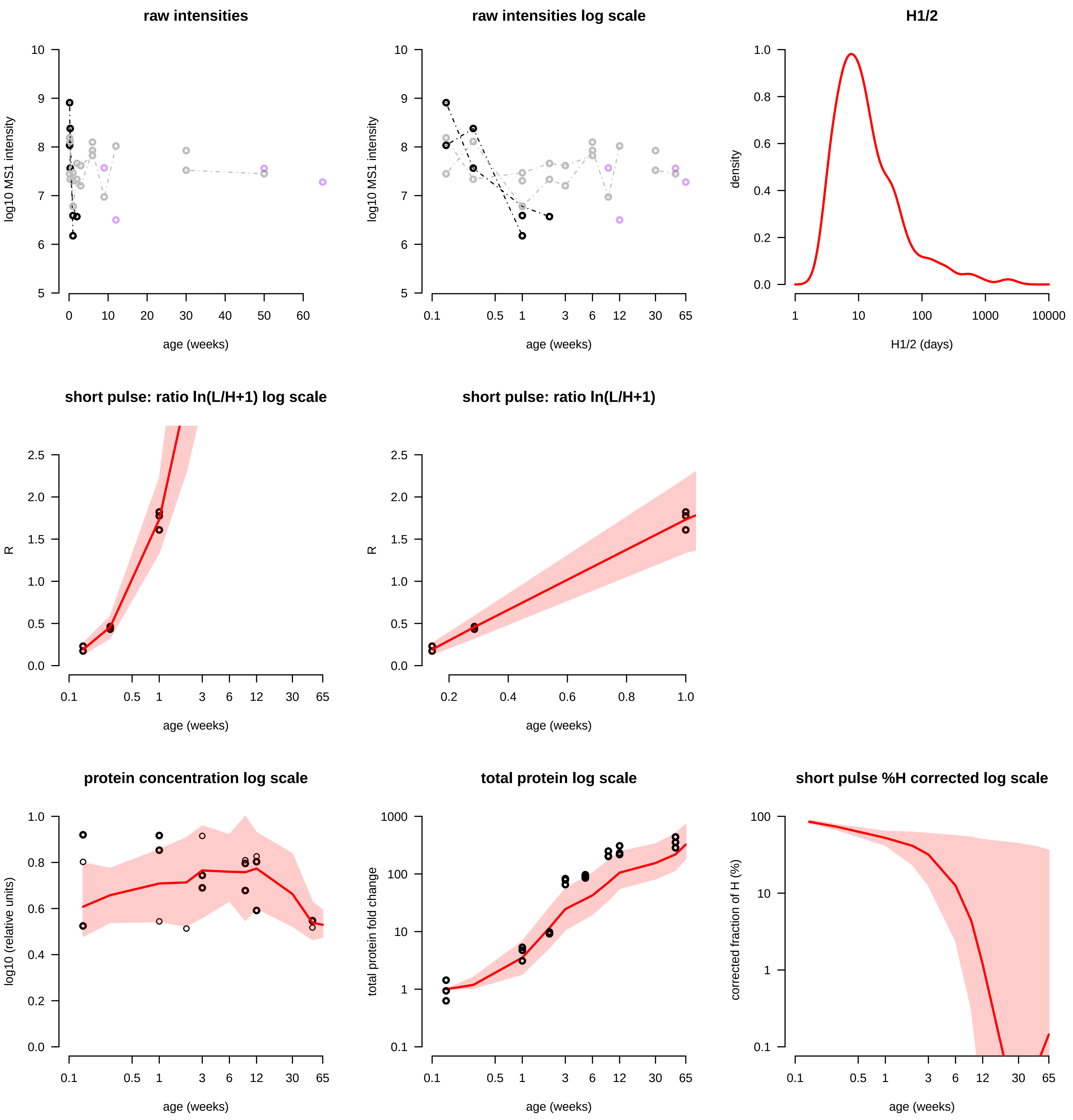

Q8BZ60

>sp|Q8BZ60|STON2_MOUSE

Stonin−2

GN=Ston2

Med H1/2: 5.3 days

Q5   H1/2: 2.6 days

Q95 H1/2: 58.4 days

reliable model fit: yes

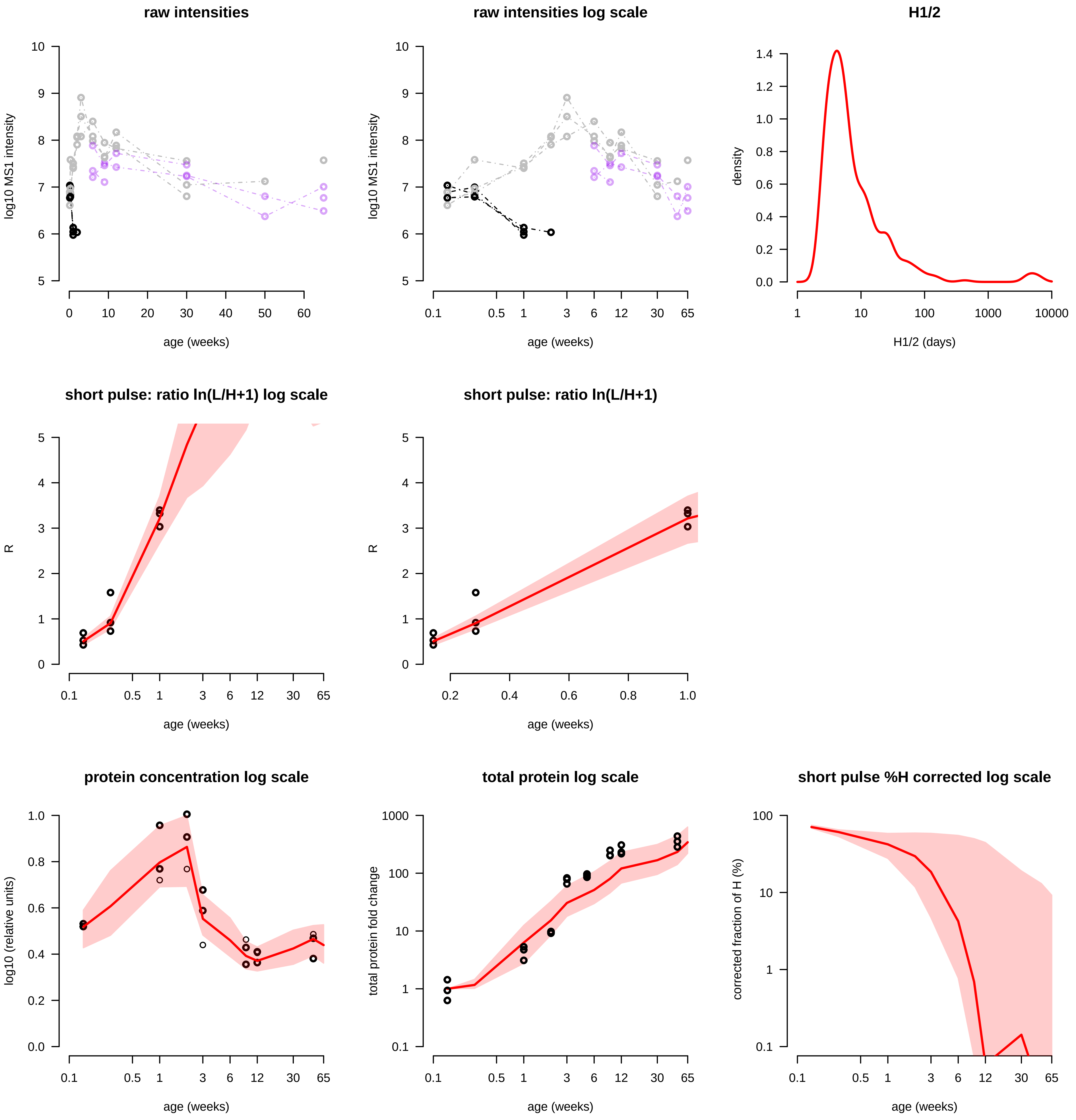

Q9WVH9

>sp|Q9WVH9|FBLN5_MOUSE

Fibulin−5

GN=Fbln5

Med H1/2: 9.2 days

Q5 H1/2: 3.5 days

Q95 H1/2: 83.3 days

reliable model fit: yes

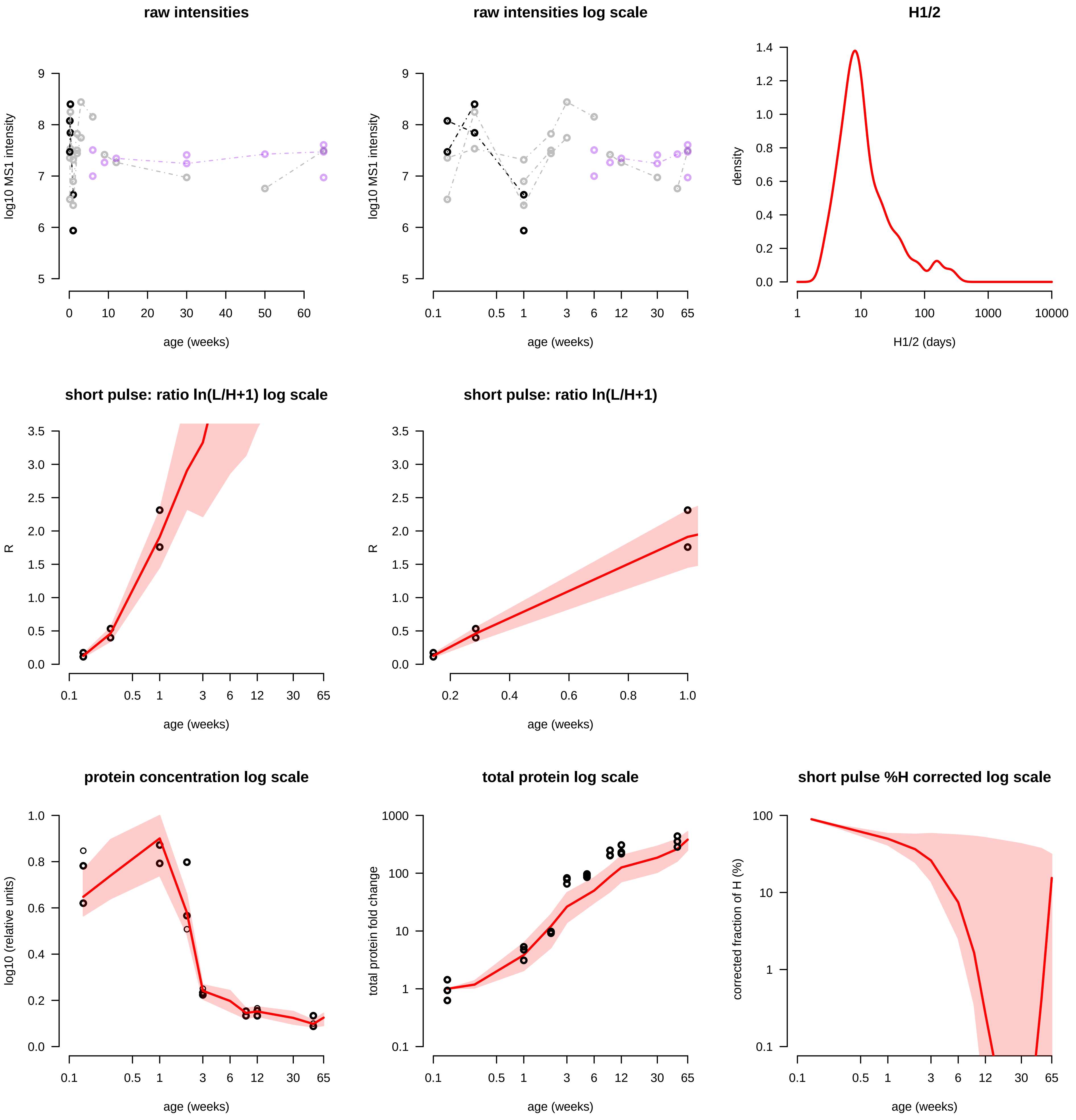

Q9D3S3;Q9D3S3−3

>sp|Q9D3S3|SNX29_MOUSE

Sorting nexin−29

GN=Snx29

Med H1/2: 8.9 days

Q5 H1/2: 6.3 days

Q95 H1/2: 14.5 days

reliable model fit: yes

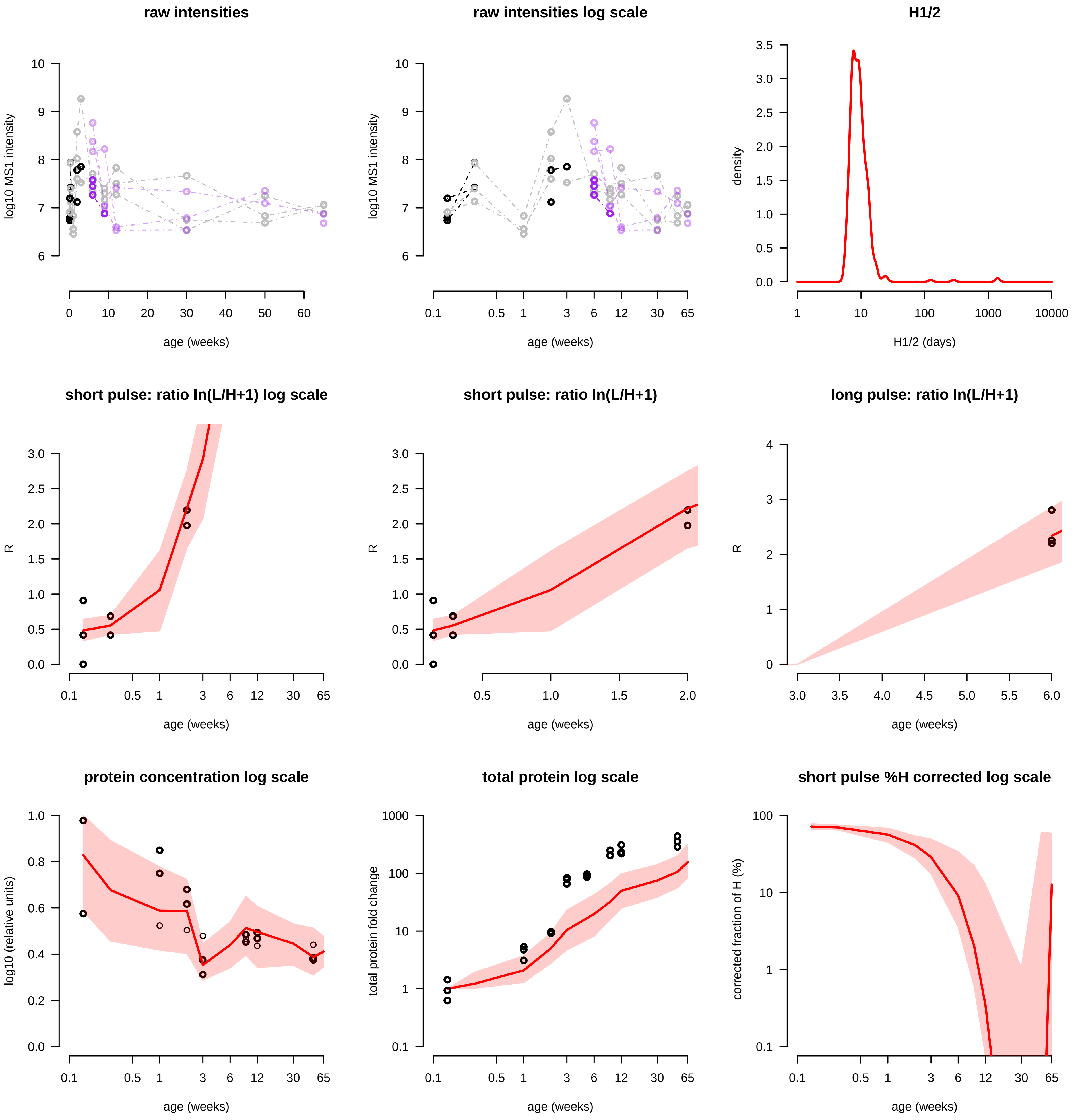

Q6PAL7

>sp|Q6PAL7|AHDC1_MOUSE

AT−hook DNA−binding motif−containing protein 1

GN=Ahdc1

Med H1/2: 4113.8 days

Q5 H1/2: 719.9 days

Q95 H1/2: 35936 days

reliable model fit: no

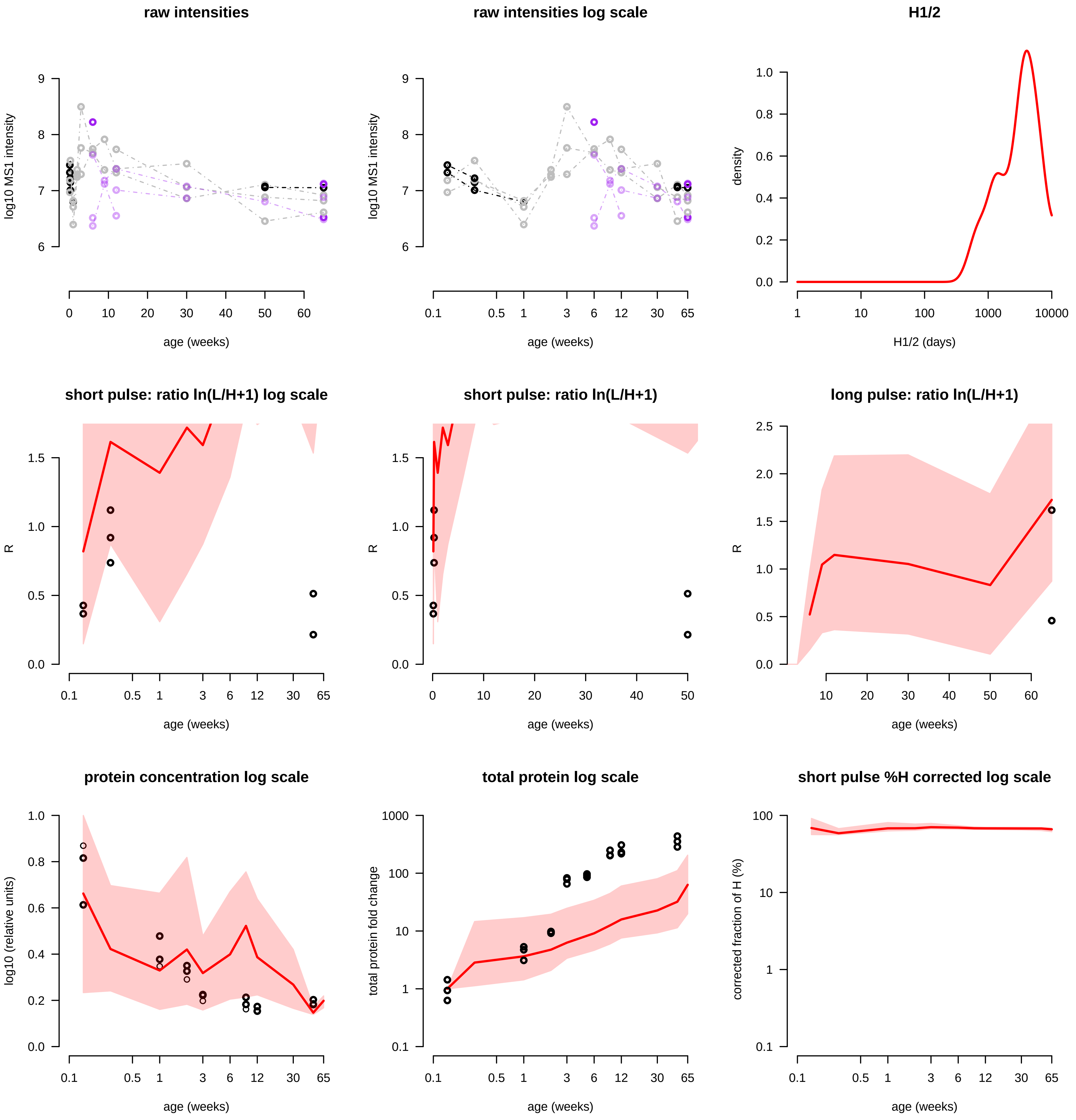

Q811B1

>sp|Q811B1|XYLT1_MOUSE

Xylosyltransferase 1

GN=Xylt1

Med H1/2: 6 days

Q5 H1/2: 2.7 days

Q95 H1/2: 72 days

reliable model fit: yes

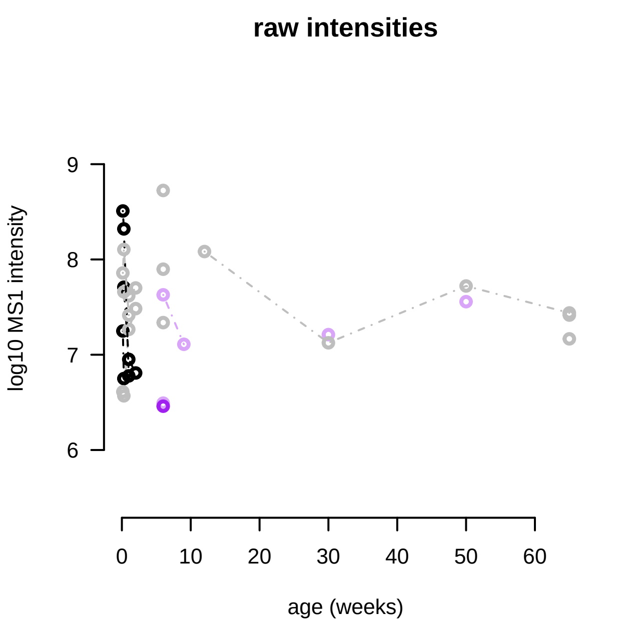

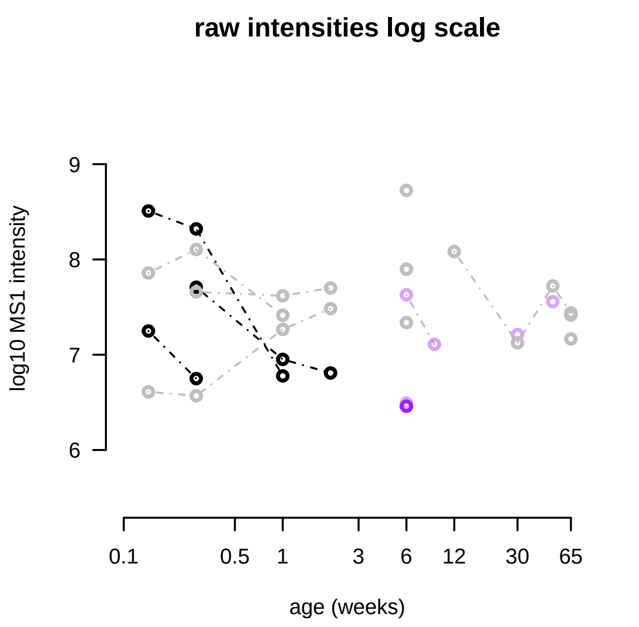

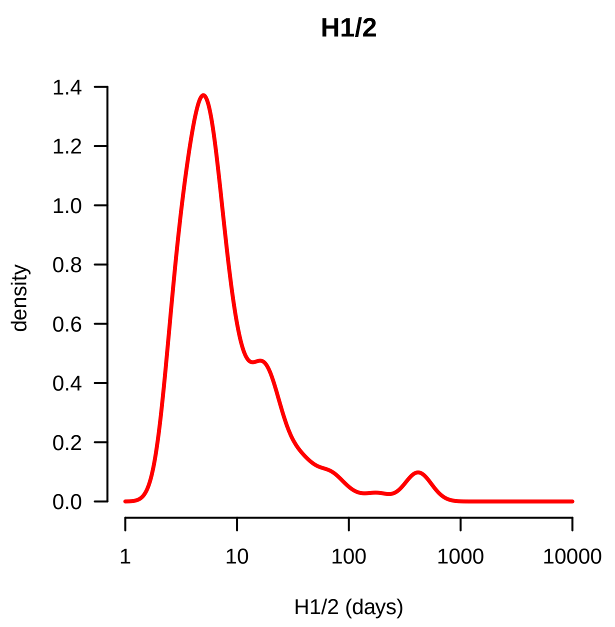

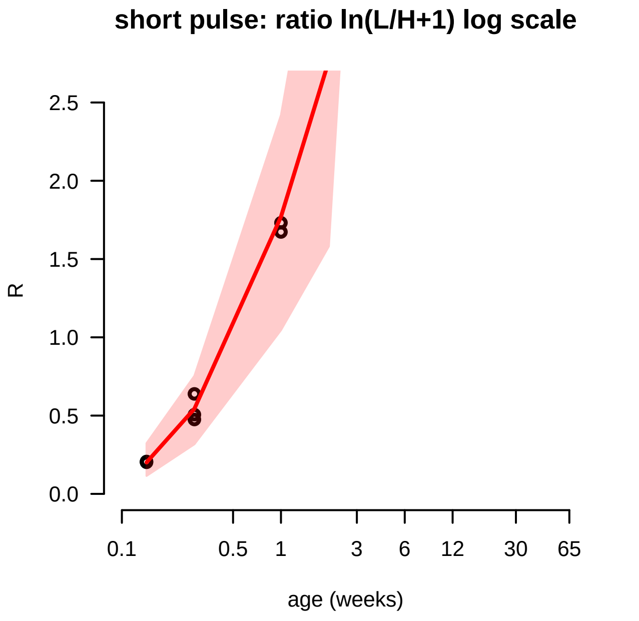

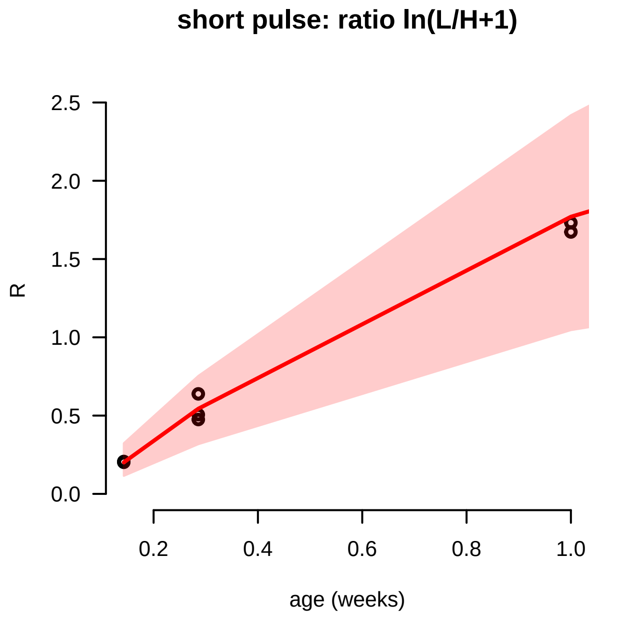

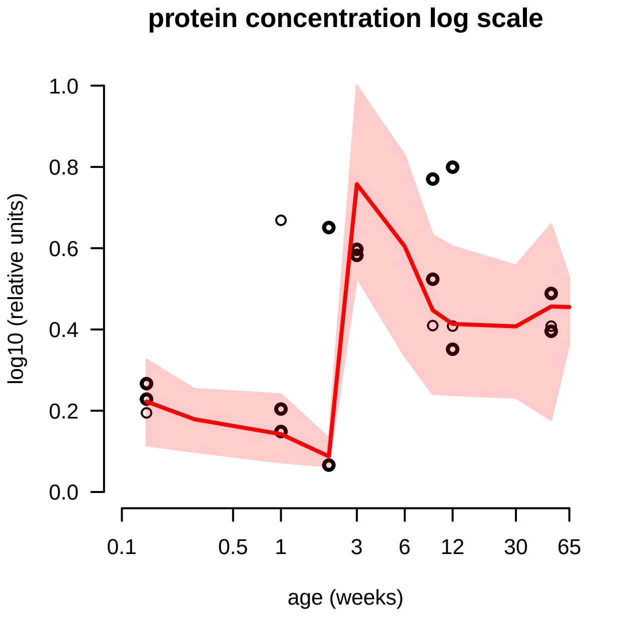

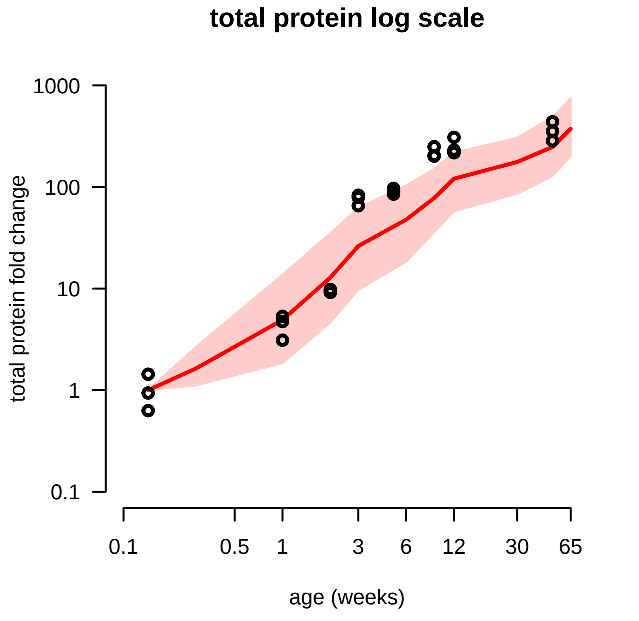

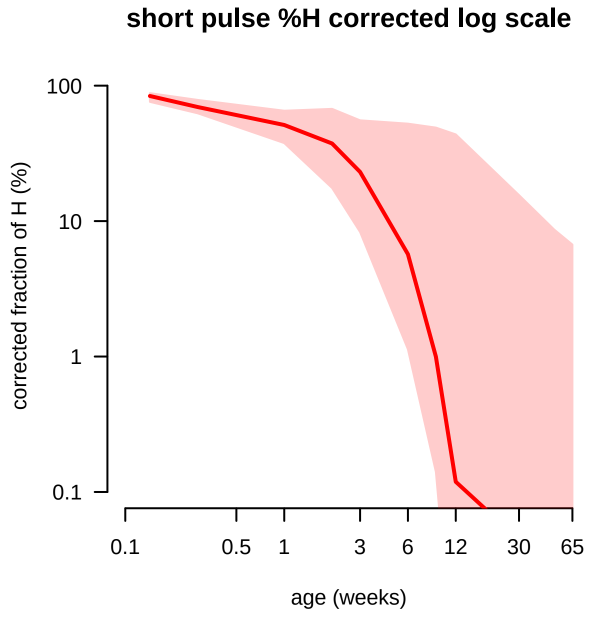

Q8QZZ7

>sp|Q8QZZ7|TPRKB_MOUSE

EKC/KEOPS complex subunit Tprkb

GN=Tprkb

Med H1/2: 11.9 days

Q5 H1/2: 4.7 days

Q95 H1/2: 127.7 days

reliable model fit: yes

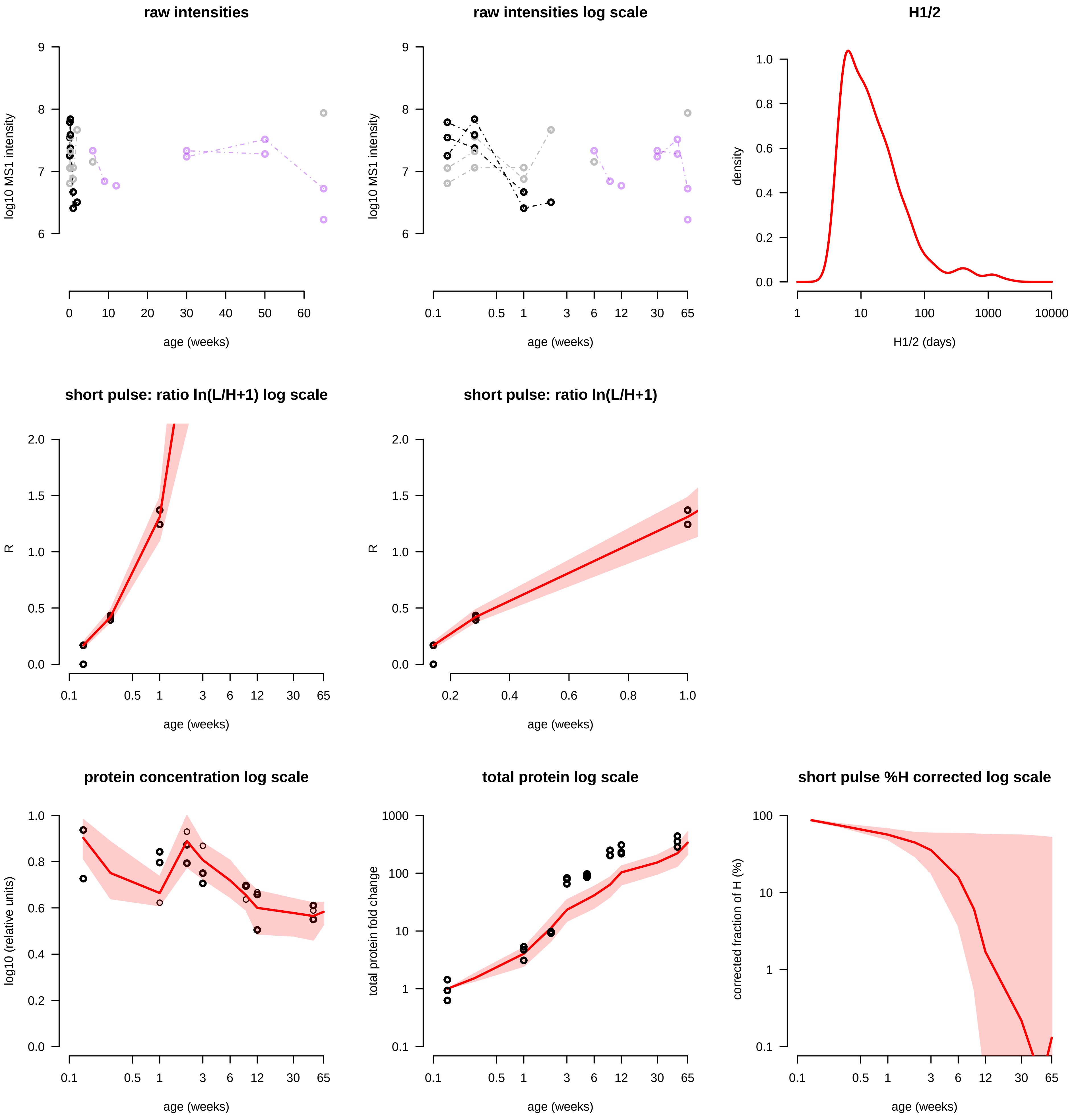

Q9ER64

>sp|Q9ER64|OSBL5_MOUSE

Oxysterol−binding protein−related protein 5

GN=Osbpl5

Med H1/2: 8.6 days

Q5 H1/2: 2.7 days

Q95 H1/2: 147.8 days

reliable model fit: yes

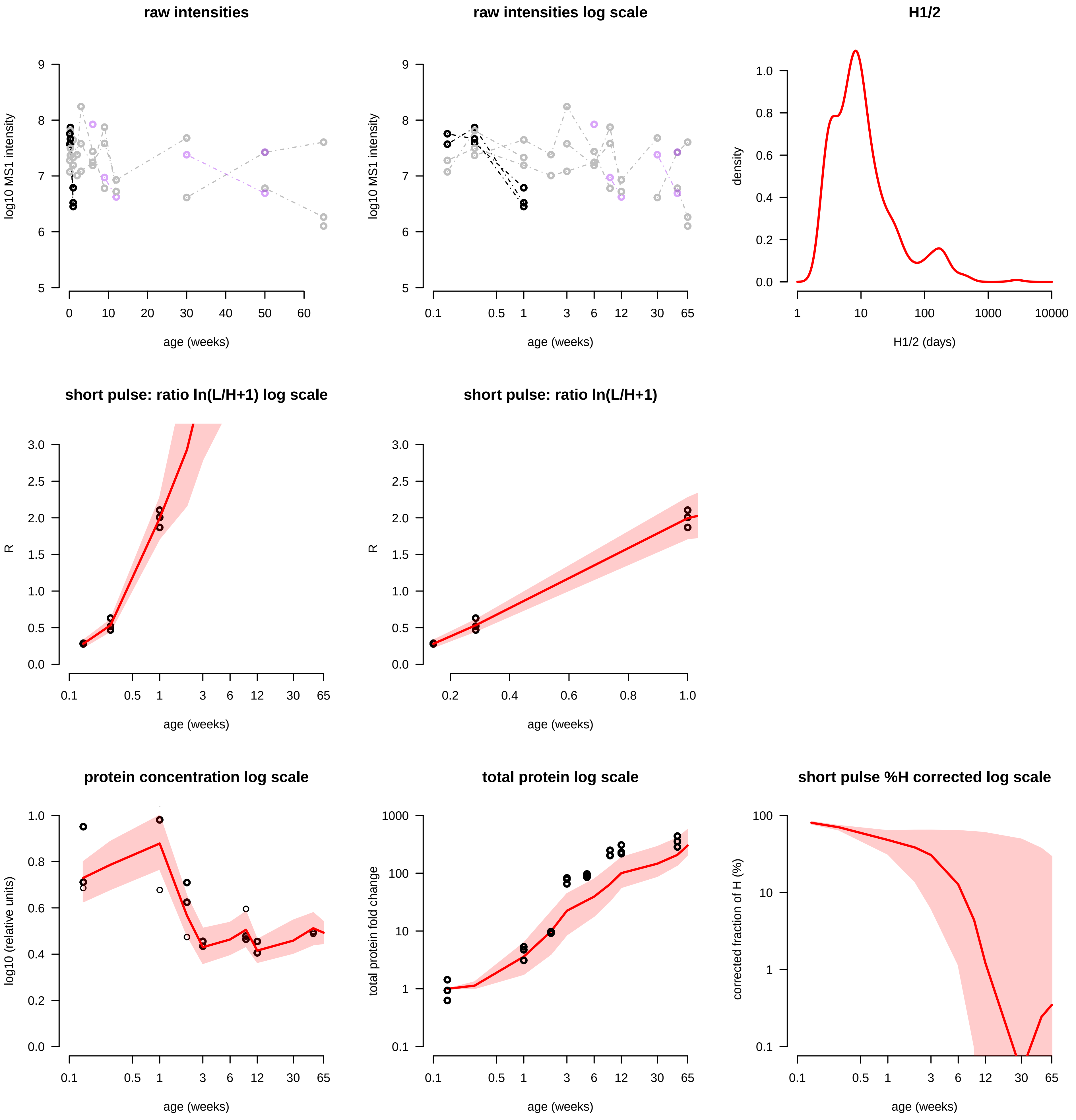

P54320

>sp|P54320|ELN_MOUSE

Elastin

GN=Eln

Med H1/2: 6.4 days

Q5 H1/2: 2.6 days

Q95 H1/2: 27.6 days

reliable model fit: yes

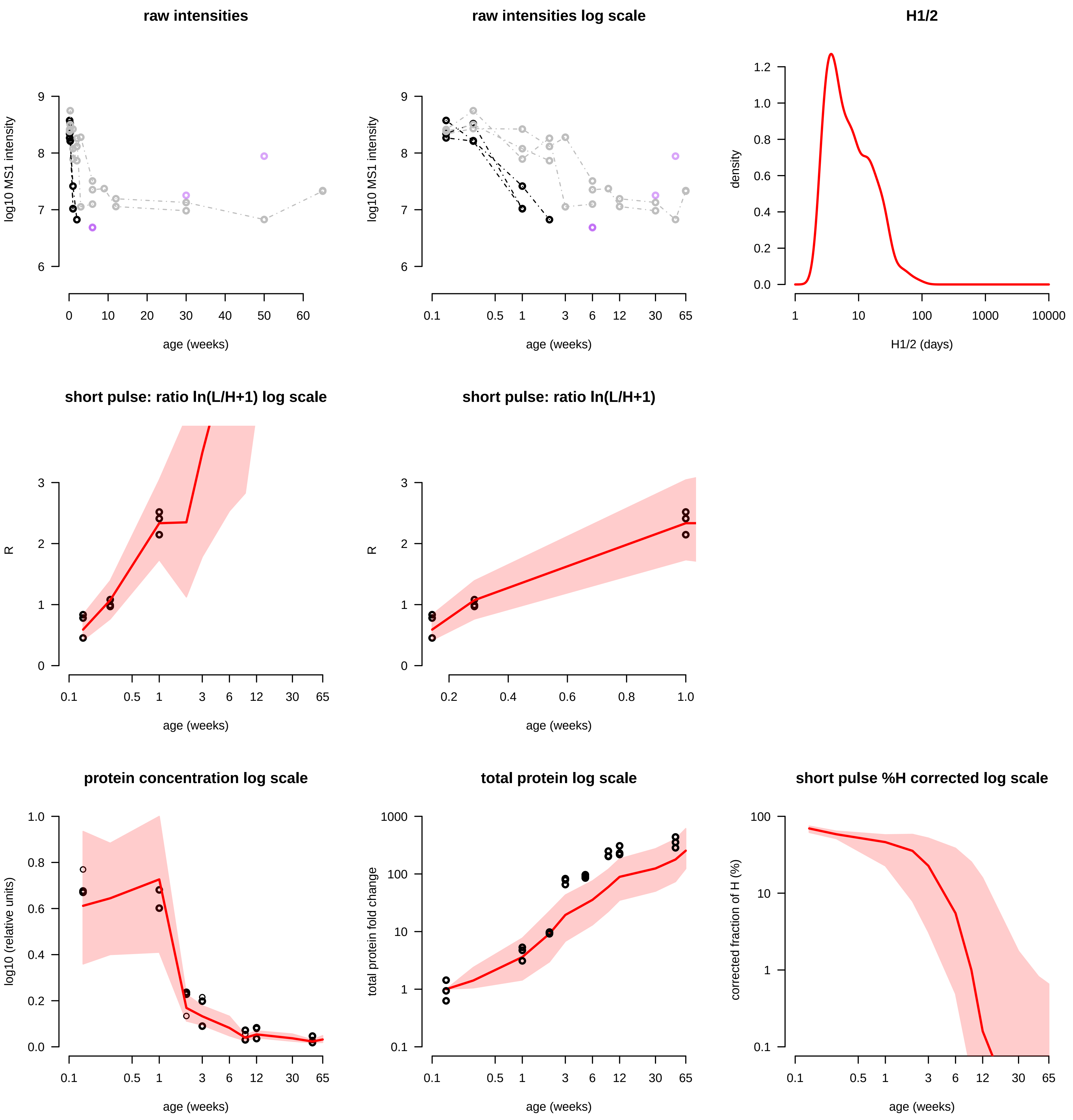

Q6ZQ11

>sp|Q6ZQ11|CHSS1_MOUSE

Chondroitin sulfate synthase 1

GN=Chsy1

Med H1/2: 8.6 days

Q5 H1/2: 3.2 days

Q95 H1/2: 107.4 days

reliable model fit: yes

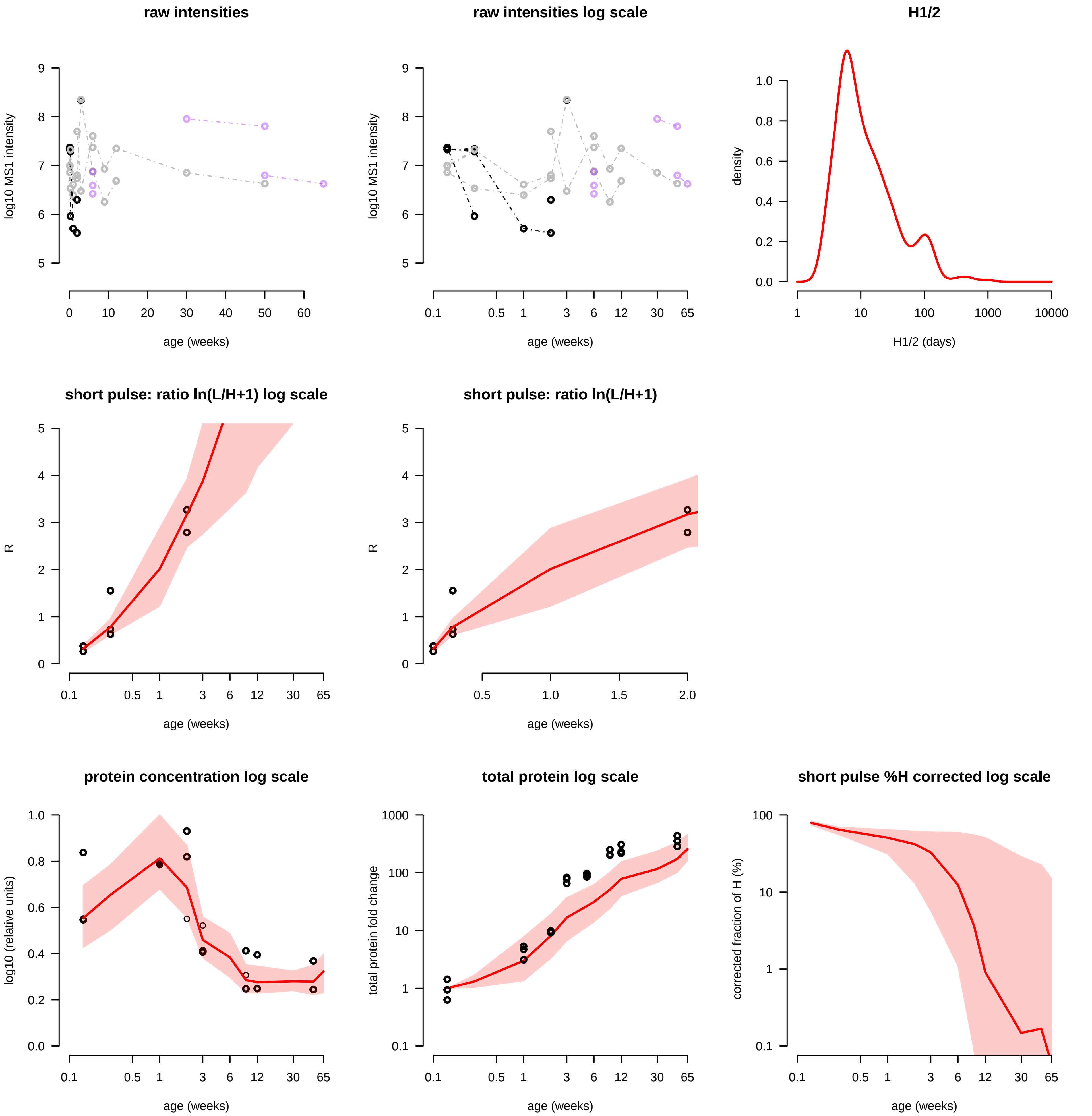

E9PVD3

>sp|E9PVD3|PCD16_MOUSE

Protocadherin−16

GN=Dchs1

Med H1/2: 35.8 days

Q5 H1/2: 9.9 days

Q95 H1/2: 278 days

reliable model fit: yes

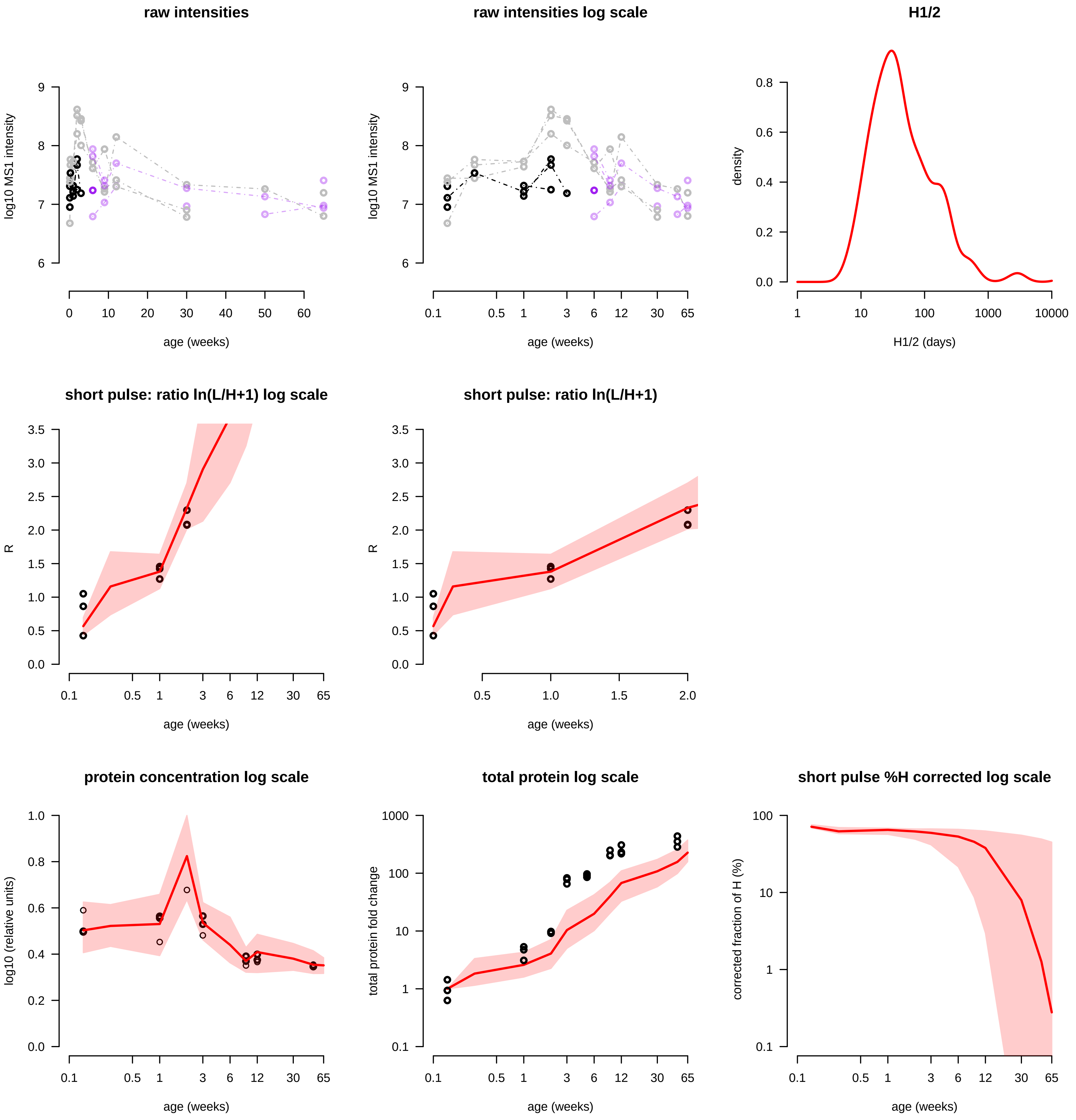

Q9Z2C8;Q9Z2C8−2

>sp|Q9Z2C8|YBOX2_MOUSE

Y−box−binding protein 2

GN=Ybx2

Med H1/2: 12.5 days

Q5 H1/2: 4.9 days

Q95 H1/2: 74.8 days

reliable model fit: yes

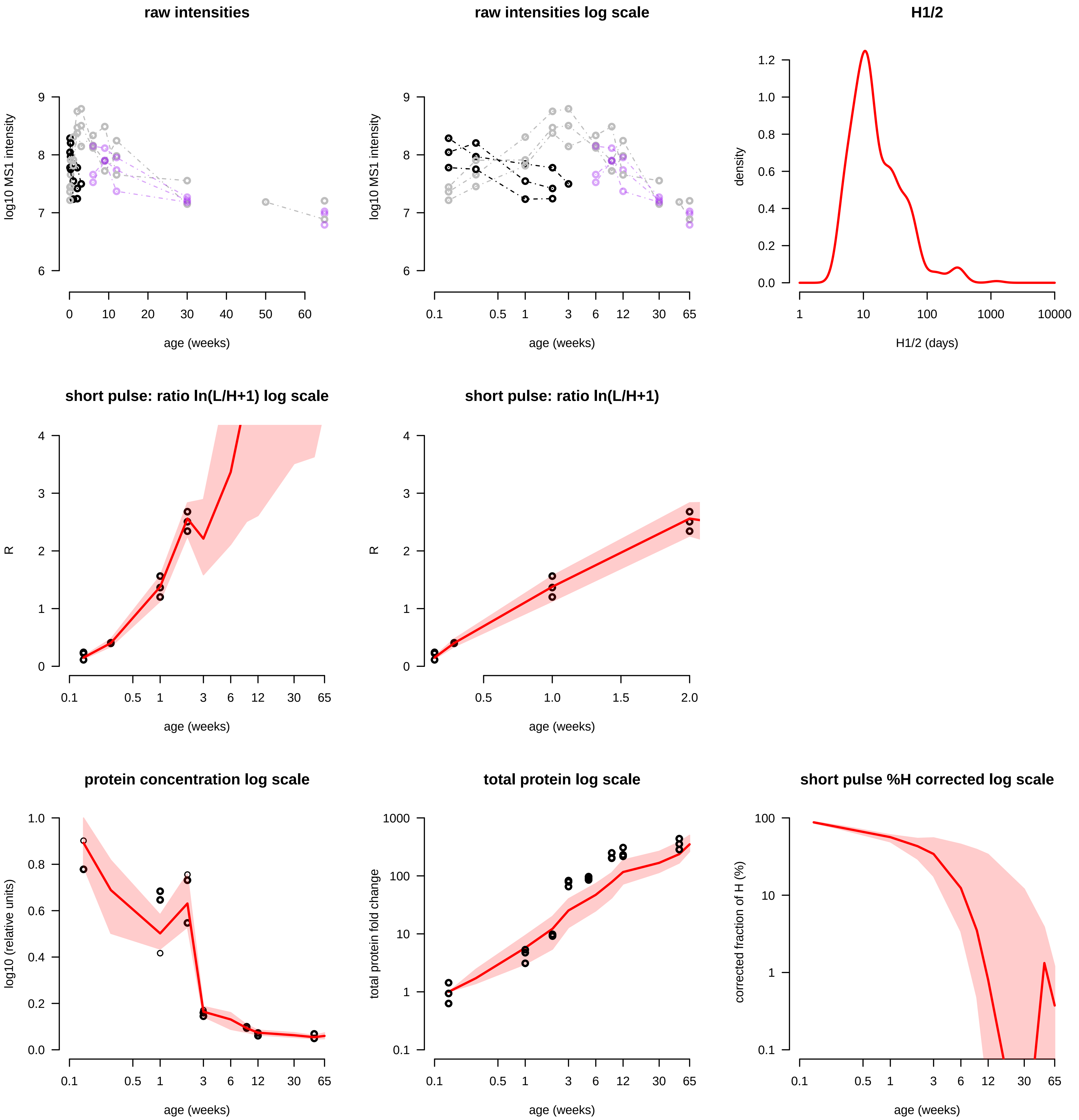

Q8BY02

>sp|Q8BY02|NKRF_MOUSE

NF−kappa−B−repressing factor

GN=Nkrf

Med H1/2: 49.5 days

Q5 H1/2: 17.7 days

Q95 H1/2: 955 days

reliable model fit: no

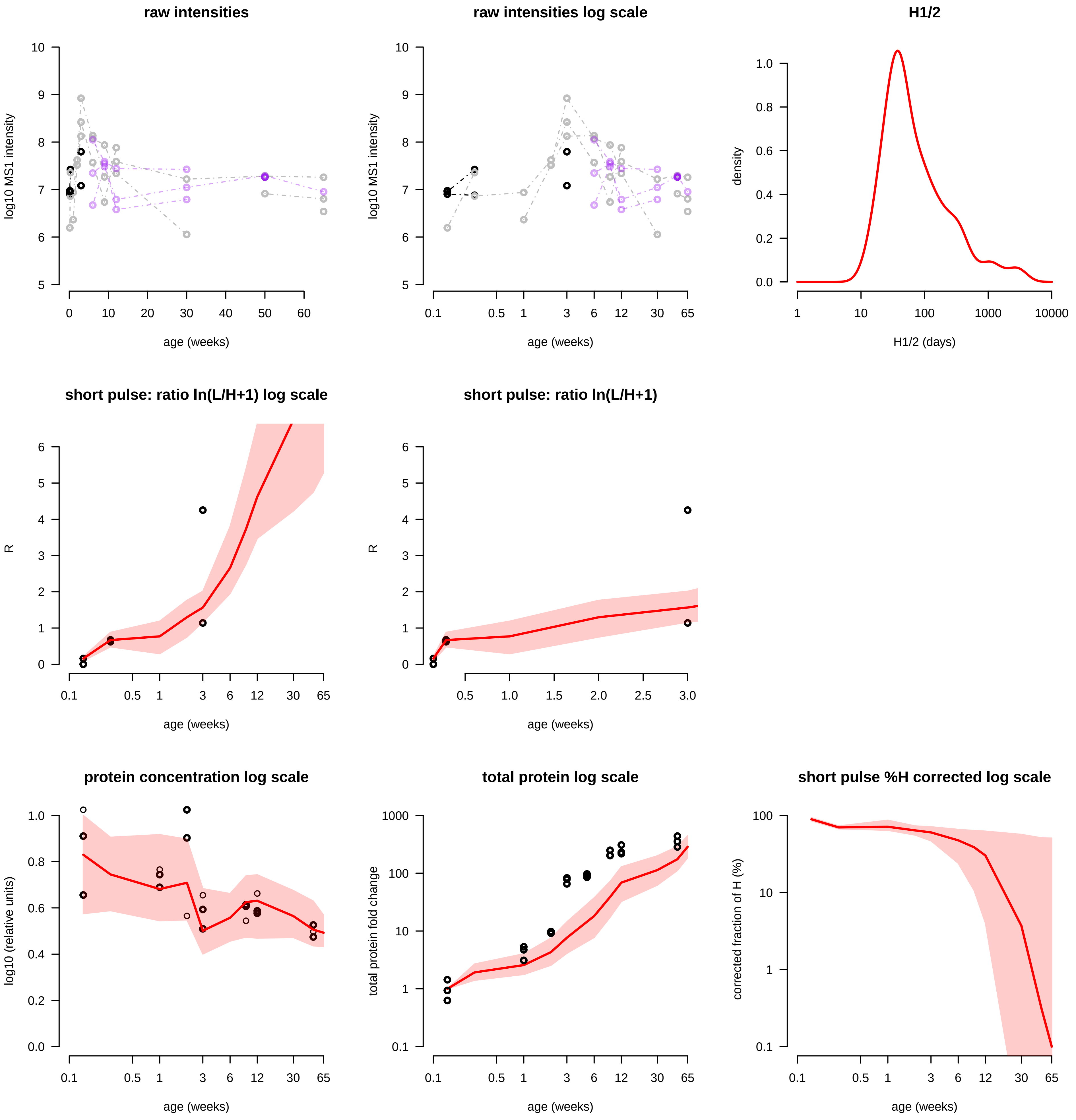

P33609

>sp|P33609|DPOLA_MOUSE

DNA polymerase alpha catalytic subunit

GN=Pola1

Med H1/2: 15.8 days

Q5 H1/2: 3.3 days

Q95 H1/2: 108 days

reliable model fit: no

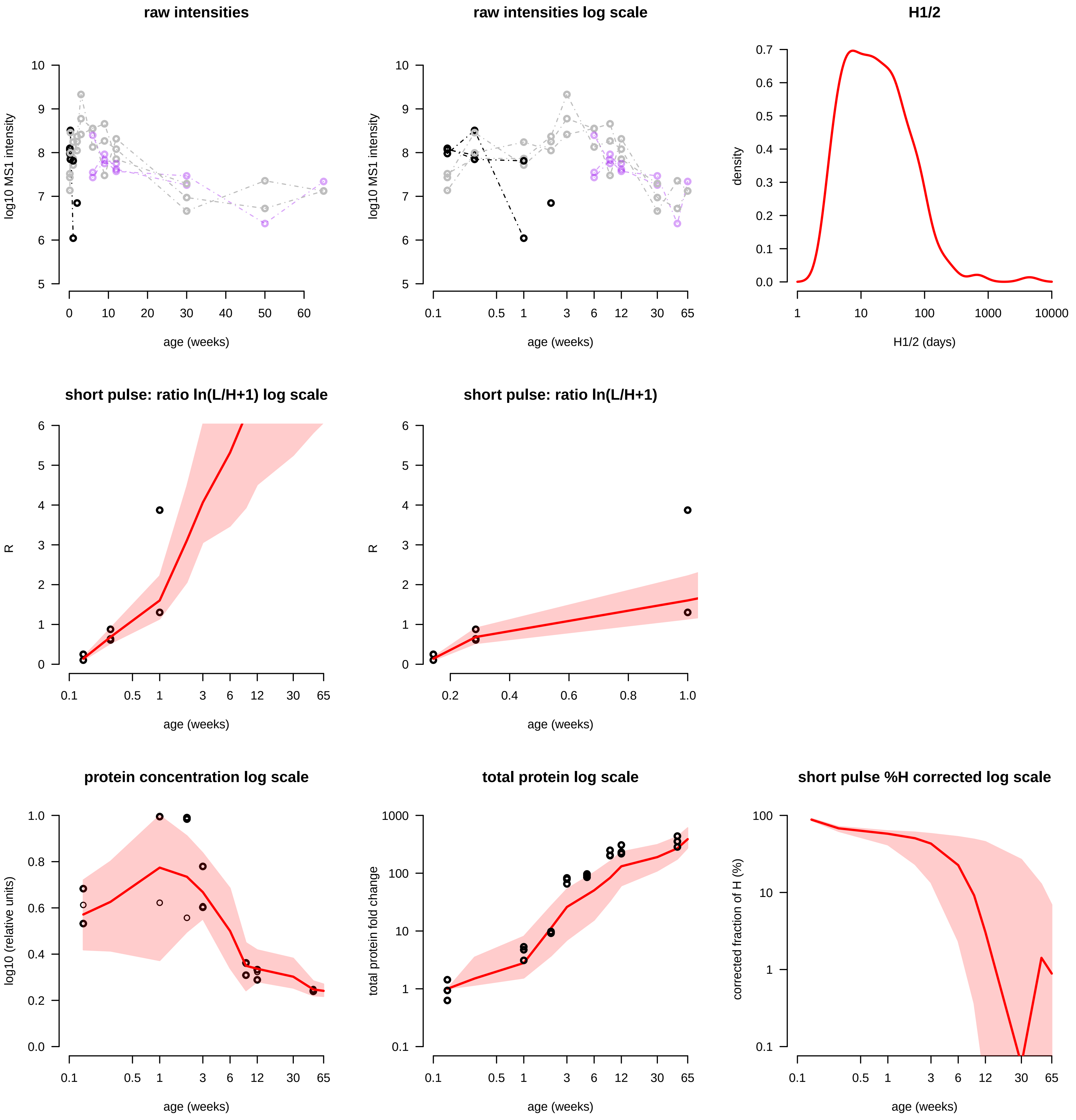

Q8CDG1

>sp|Q8CDG1|PIWL2_MOUSE

Piwi−like protein 2

GN=Piwil2

Med H1/2: 3.6 days

Q5 H1/2: 2.7 days

Q95 H1/2: 10 days

reliable model fit: no

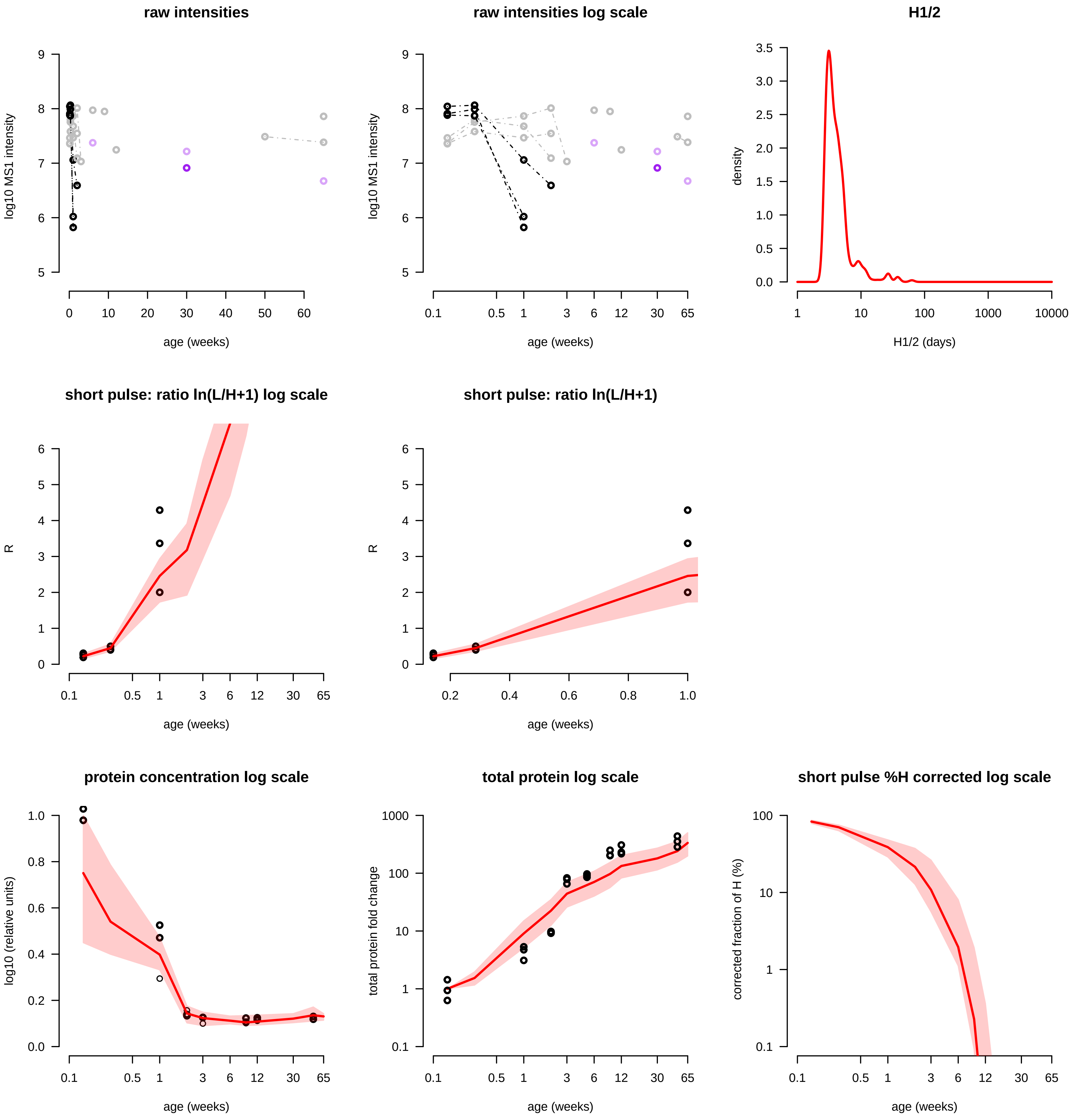

Q8K1J5

>sp|Q8K1J5|SDE2_MOUSE

Replication stress response regulator SDE2

GN=Sde2

Med H1/2: 42.3 days

Q5 H1/2: 12.7 days

Q95 H1/2: 603.6 days

reliable model fit: yes

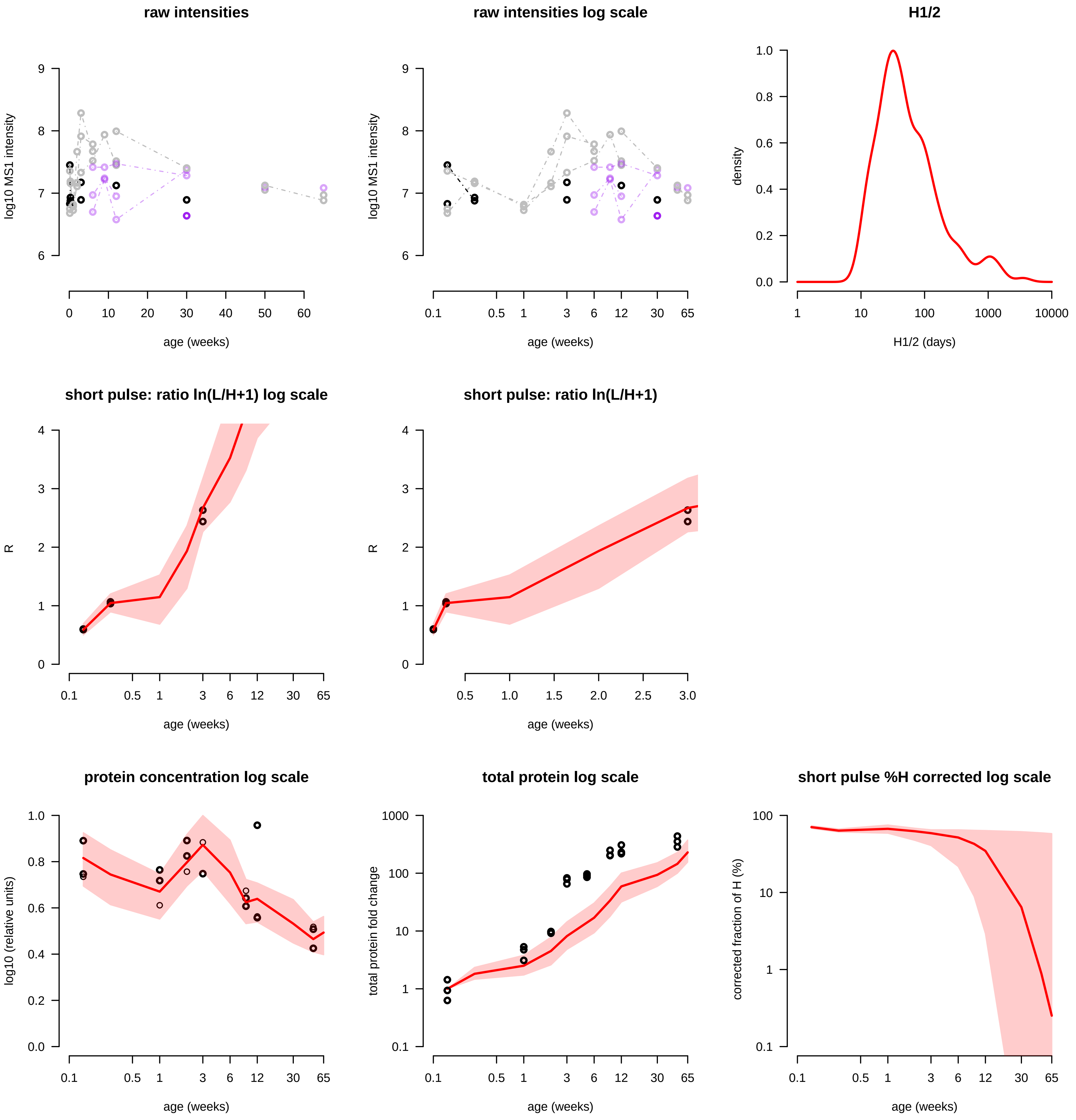

Q61084

>sp|Q61084|M3K3_MOUSE

Mitogen−activated protein kinase kinase kinase 3

GN=Map3k3

Med H1/2: 11.9 days

Q5 H1/2: 9.2 days

Q95 H1/2: 20.7 days

reliable model fit: yes

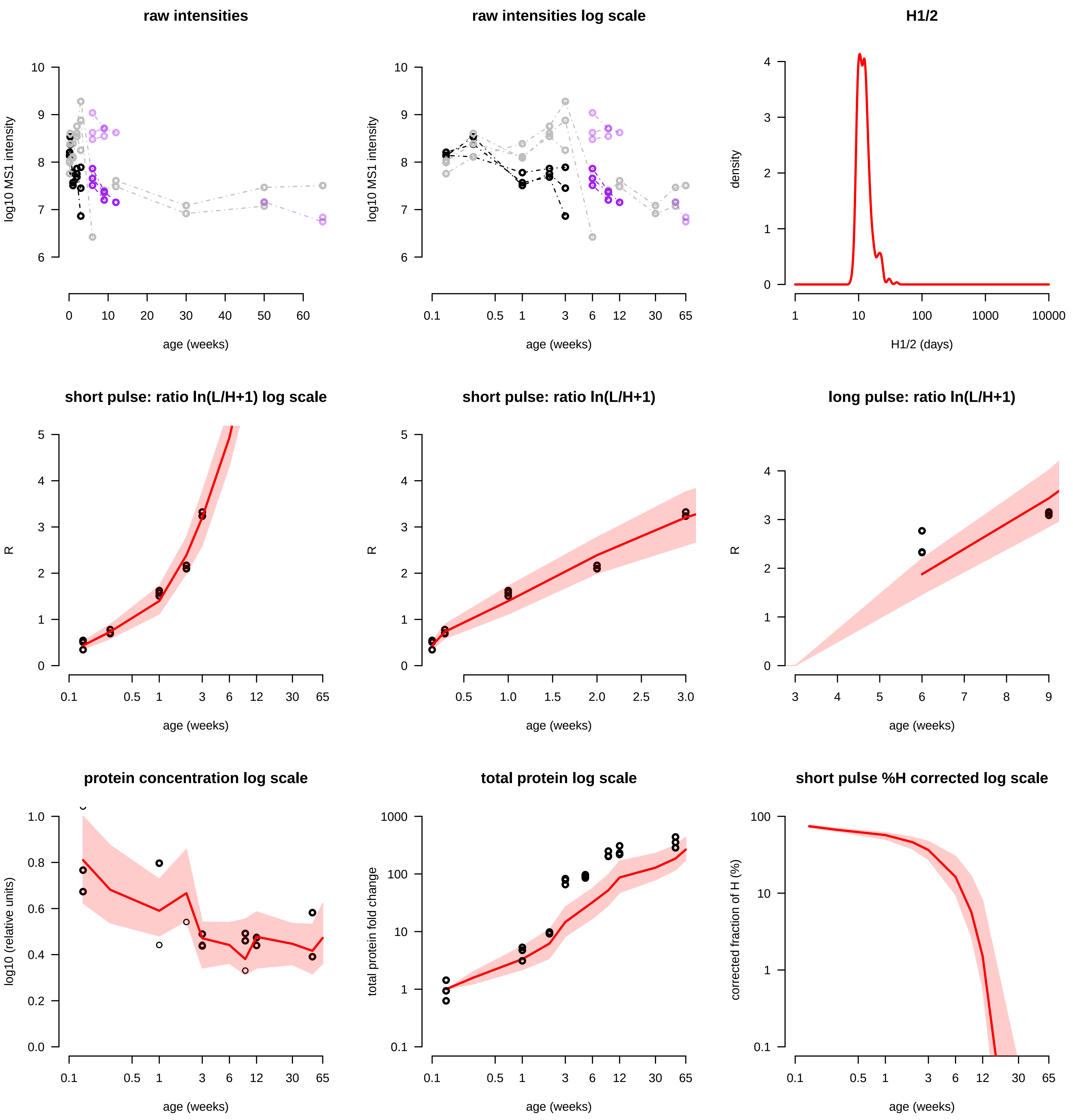

Q66X05

>sp|Q66X05|NAL4F_MOUSE

NACHT, LRR and PYD domains−containing protein 4F

GN=Nlrp4f

Med H1/2: 6.5 days

Q5 H1/2: 4.8 days

Q95 H1/2: 10.6 days

reliable model fit: yes

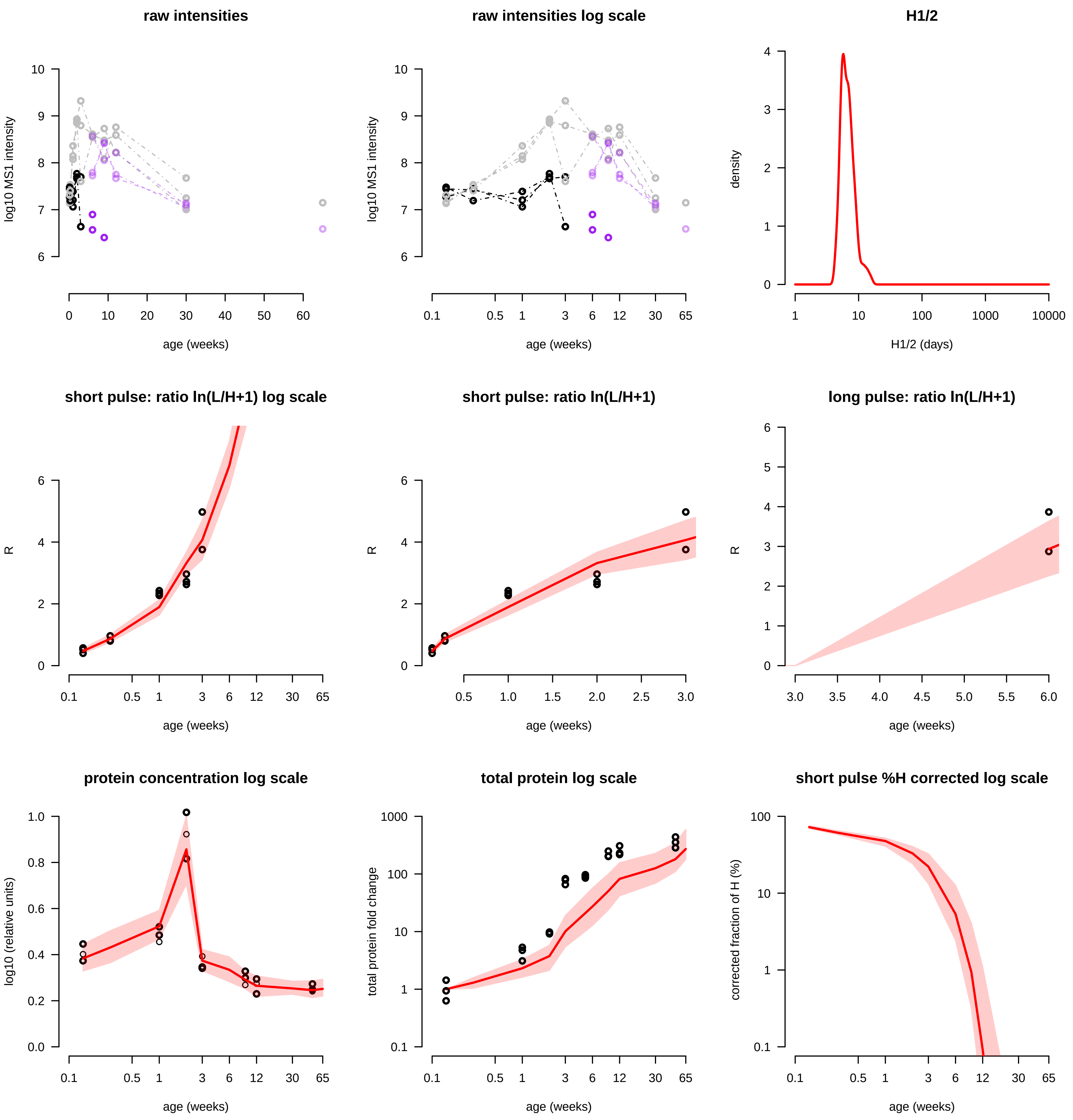

Q8C6L5

>sp|Q8C6L5|CGAS_MOUSE

Cyclic GMP−AMP synthase

GN=Cgas

Med H1/2: 18.2 days

Q5 H1/2: 7.8 days

Q95 H1/2: 221.7 days

reliable model fit: yes

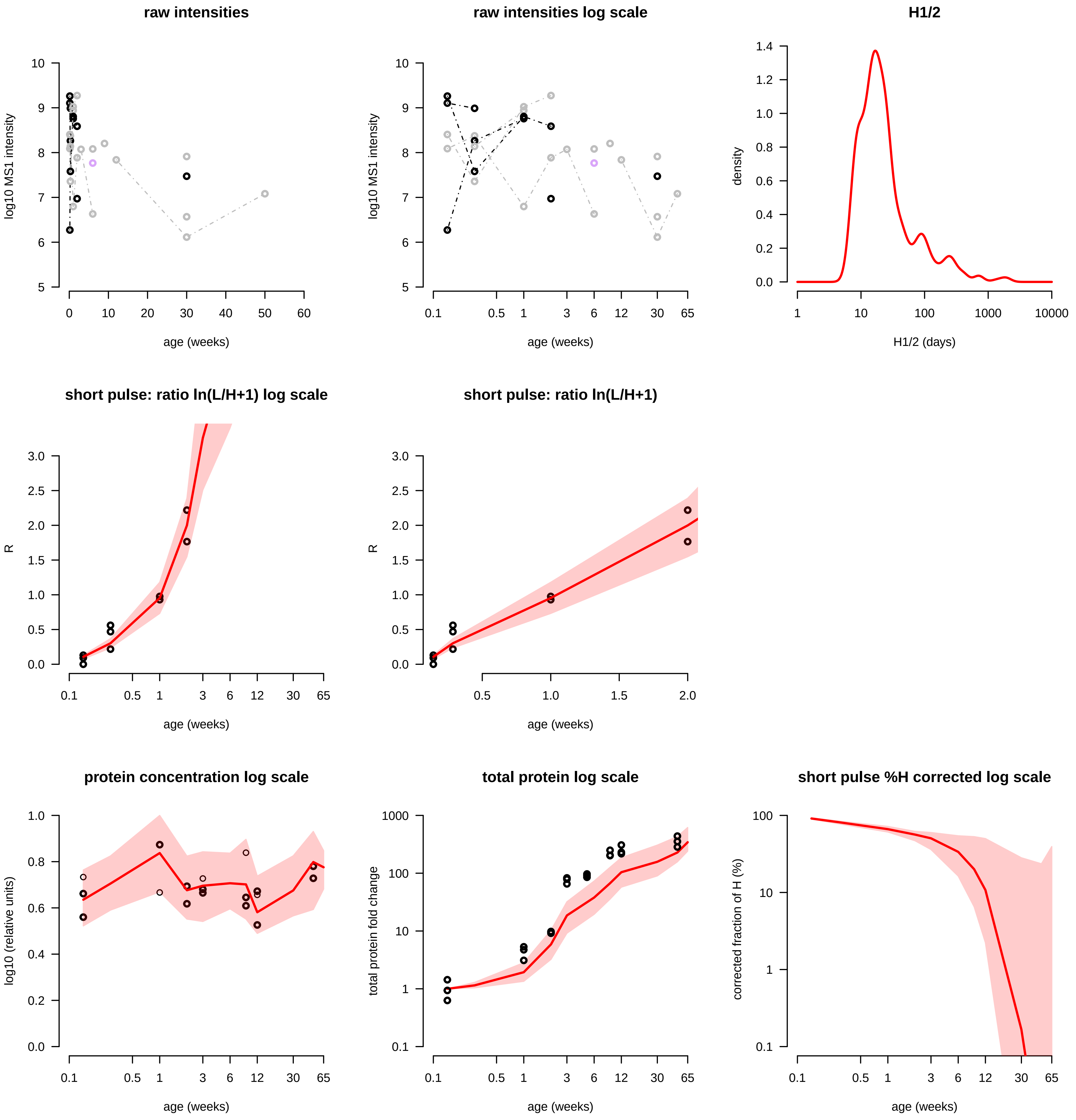

Q9CQ37

>sp|Q9CQ37|UBE2T_MOUSE

Ubiquitin−conjugating enzyme E2 T

GN=Ube2t

Med H1/2: 184.4 days

Q5 H1/2: 34.9 days

Q95 H1/2: 1614 days

reliable model fit: no

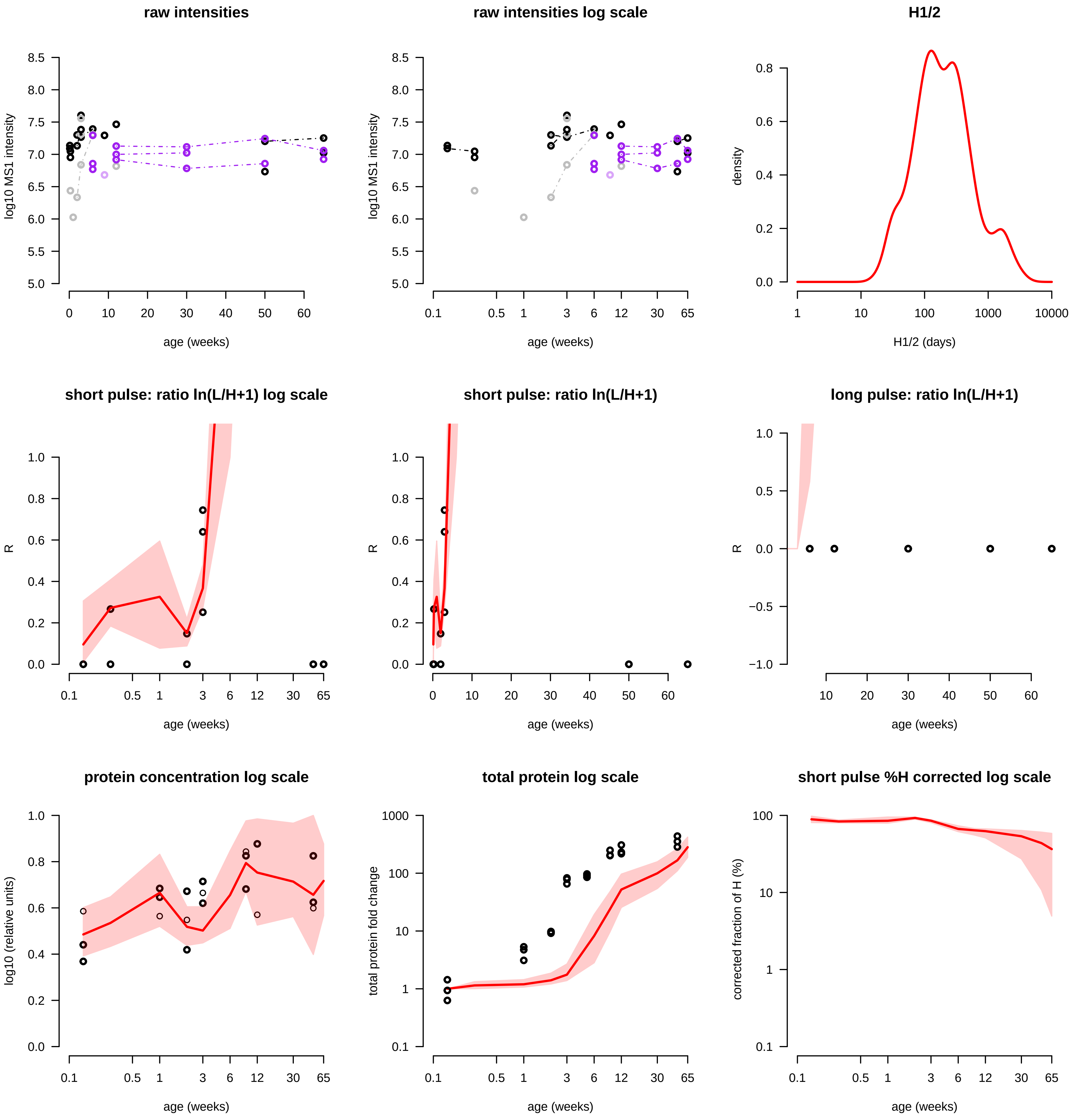

Q9CY00

>sp|Q9CY00|TT30B_MOUSE

Tetratricopeptide repeat protein 30B

GN=Ttc30b

Med H1/2: 11740.7 days

Q5 H1/2: 2341.6 days

Q95 H1/2: 128957.8 days

reliable model fit: no

raw intensities

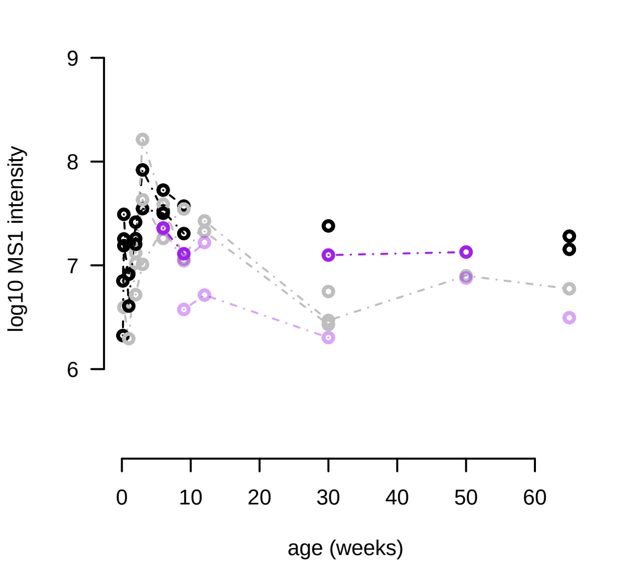

raw intensities log scale

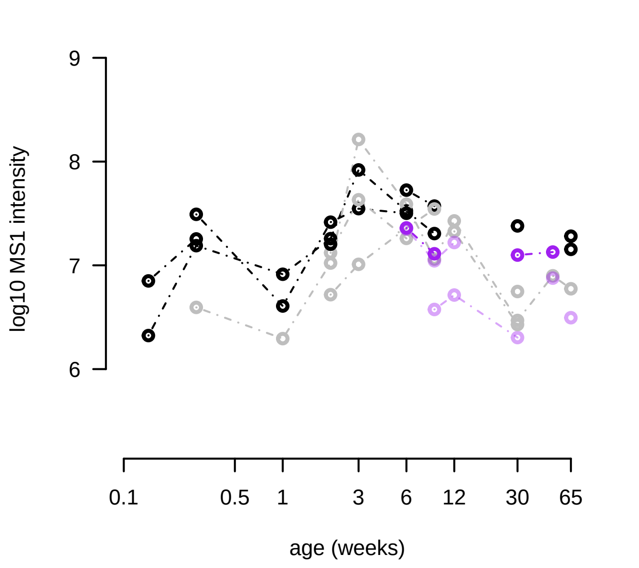

H1/2

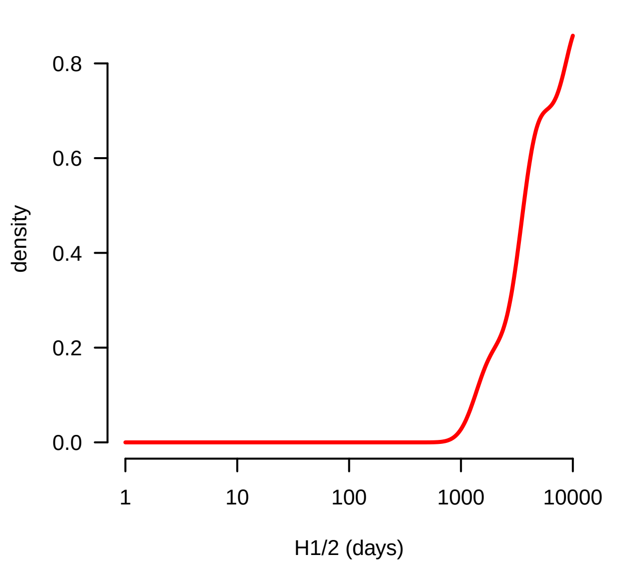

short pulse: ratio ln(L/H+1) log scale

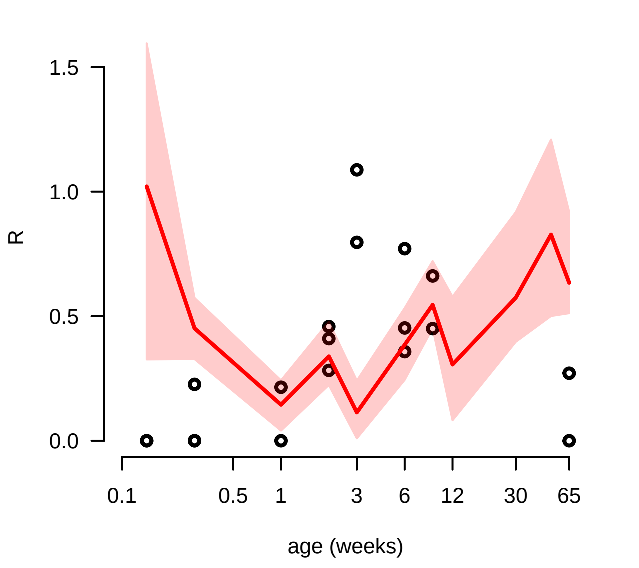

short pulse: ratio ln(L/H+1)

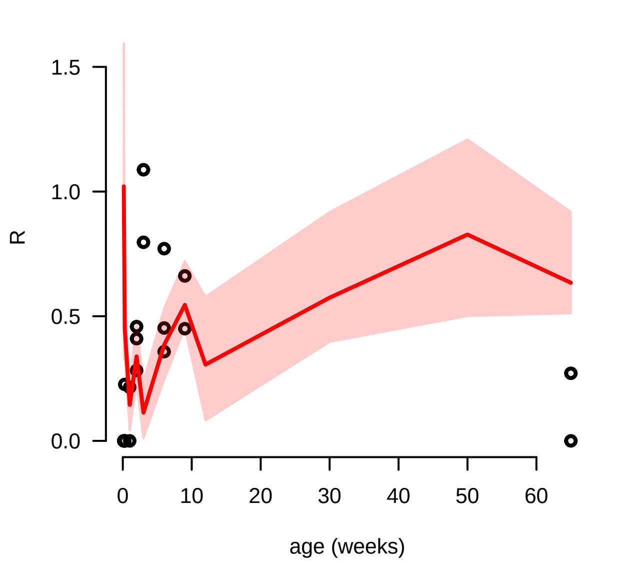

protein concentration log scale

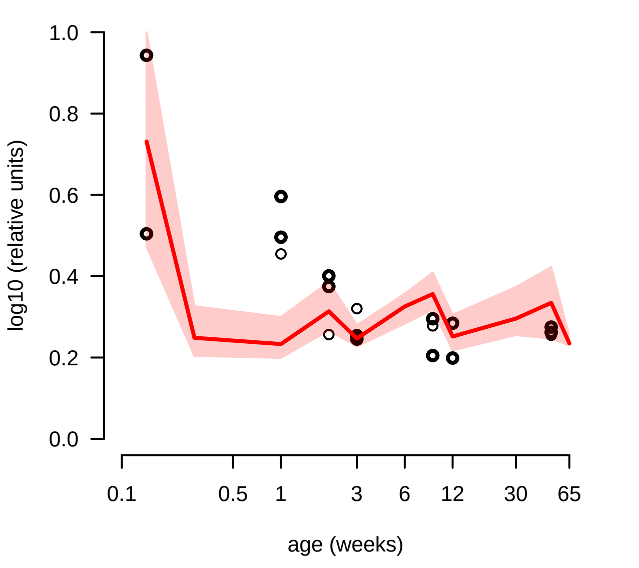

total protein log scale

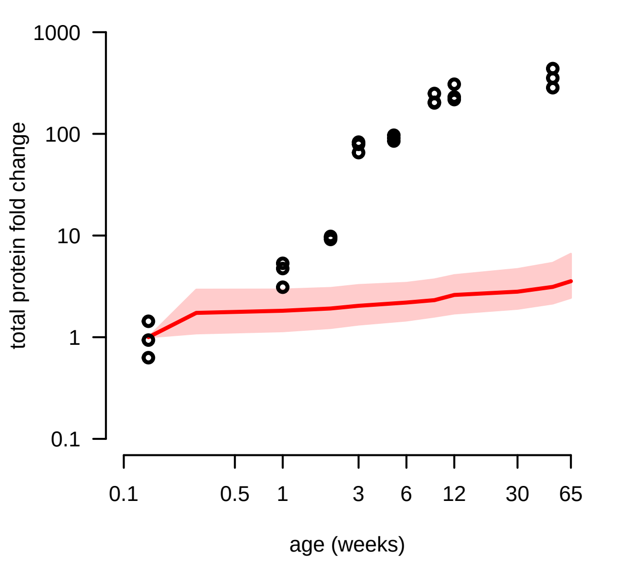

short pulse %H corrected log scale

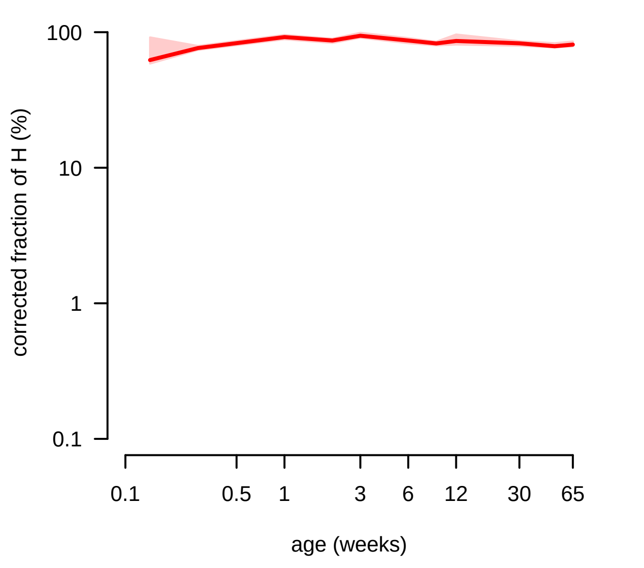

Q9CY94

>sp|Q9CY94|PSF3_MOUSE

DNA replication complex GINS protein PSF3

GN=Gins3

Med H1/2: 21 days

Q5 H1/2: 11.1 days

Q95 H1/2: 65.8 days

reliable model fit: no

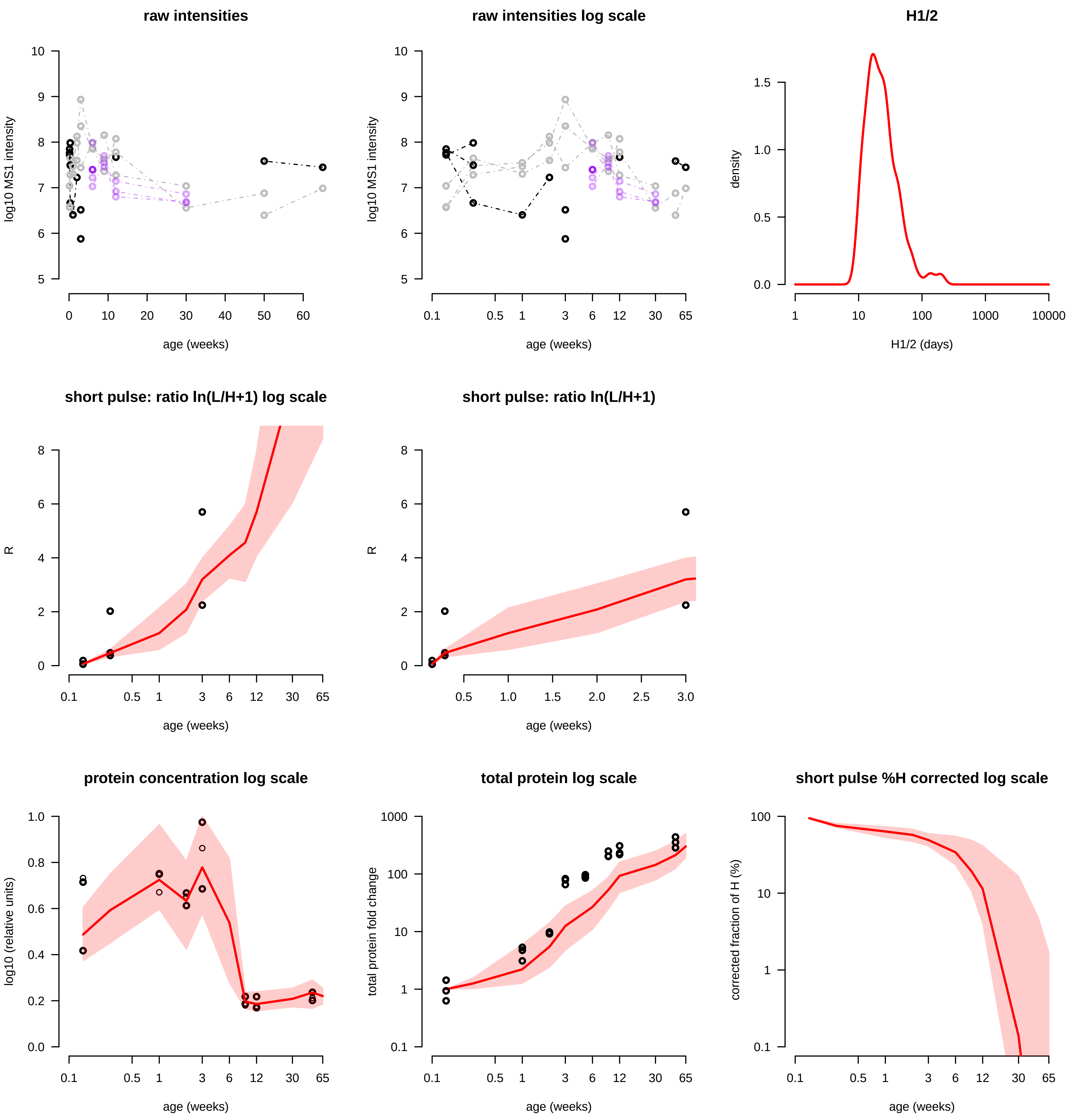

Q9JLV2;Q9JLV2−2

>sp|Q9JLV2|TP4AP_MOUSE

Short transient receptor potential channel 4−associated protein

GN=Trpc4ap

Med H1/2: 12.8 days

Q5 H1/2: 10.1 days

Q95 H1/2: 16.9 days

reliable model fit: yes

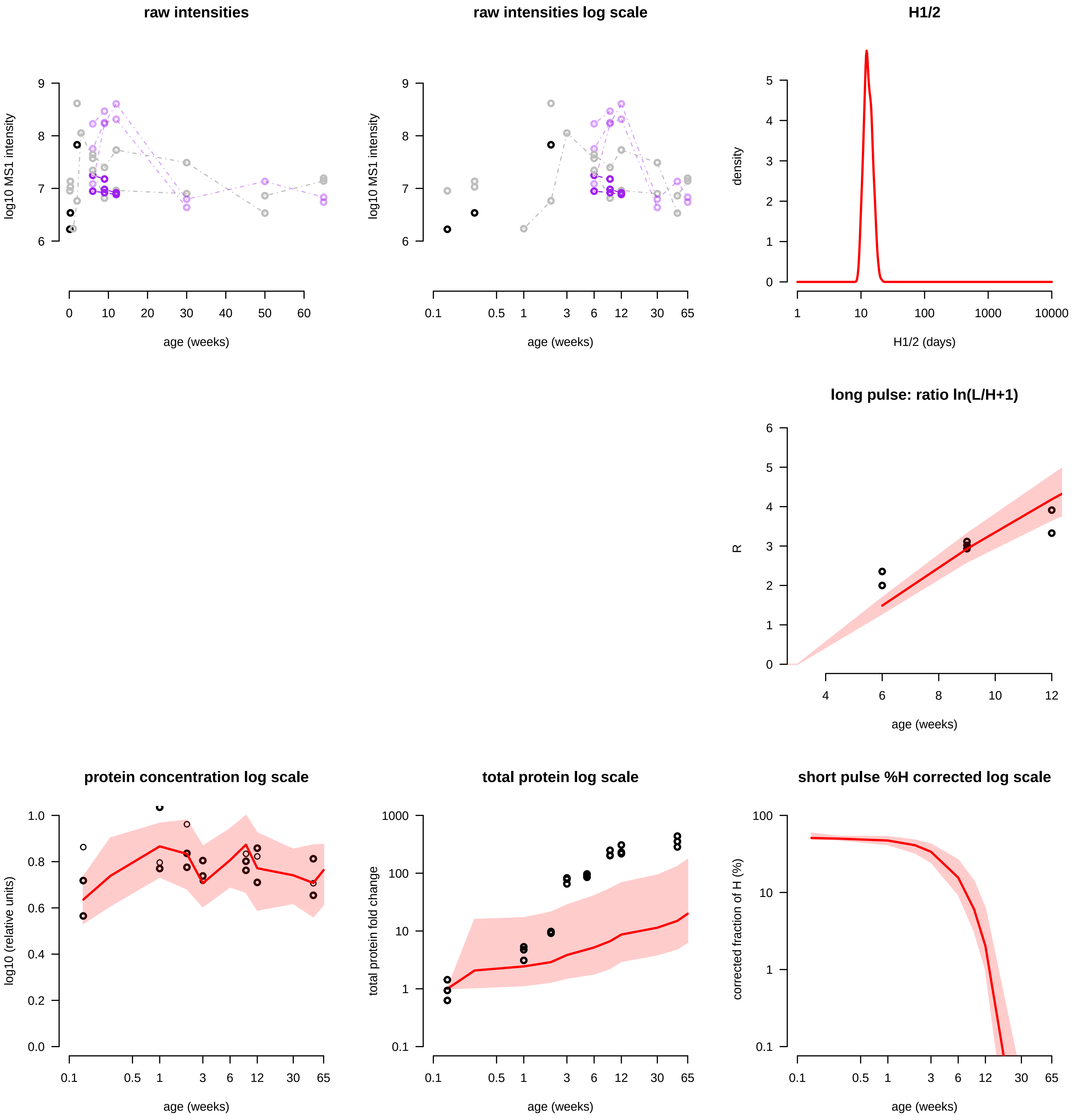

Q8BT07;Q8BT07−2

>sp|Q8BT07|CEP55_MOUSE

Centrosomal protein of 55 kDa

GN=Cep55

Med H1/2: 9.6 days

Q5 H1/2: 4.4 days

Q95 H1/2: 96.7 days

reliable model fit: yes

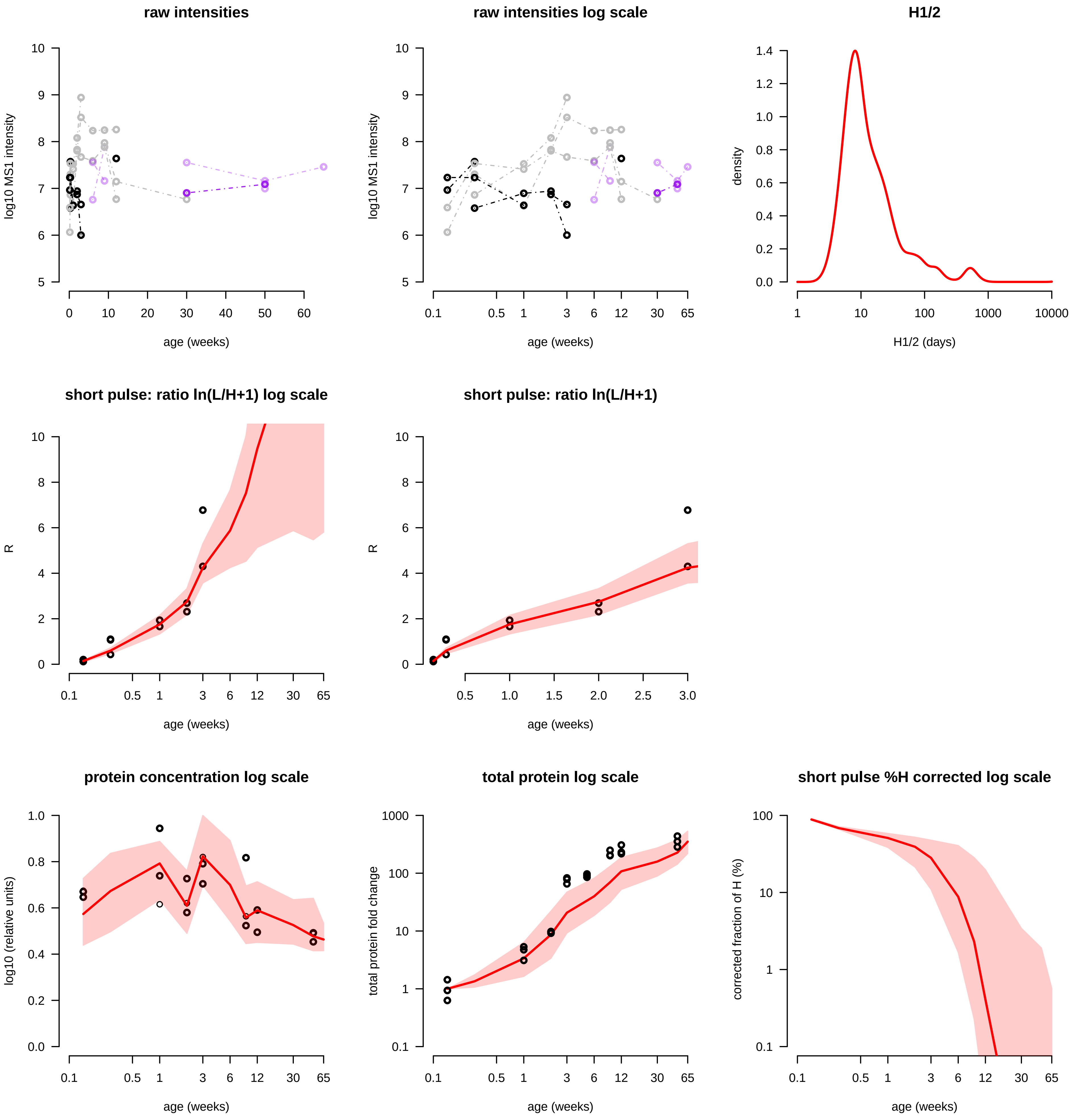

Q69ZX8

>sp|Q69ZX8|ABLM3_MOUSE

Actin−binding LIM protein 3

GN=Ablim3

Med H1/2: 9.4 days

Q5 H1/2: 2.9 days

Q95 H1/2: 134 days

reliable model fit: yes

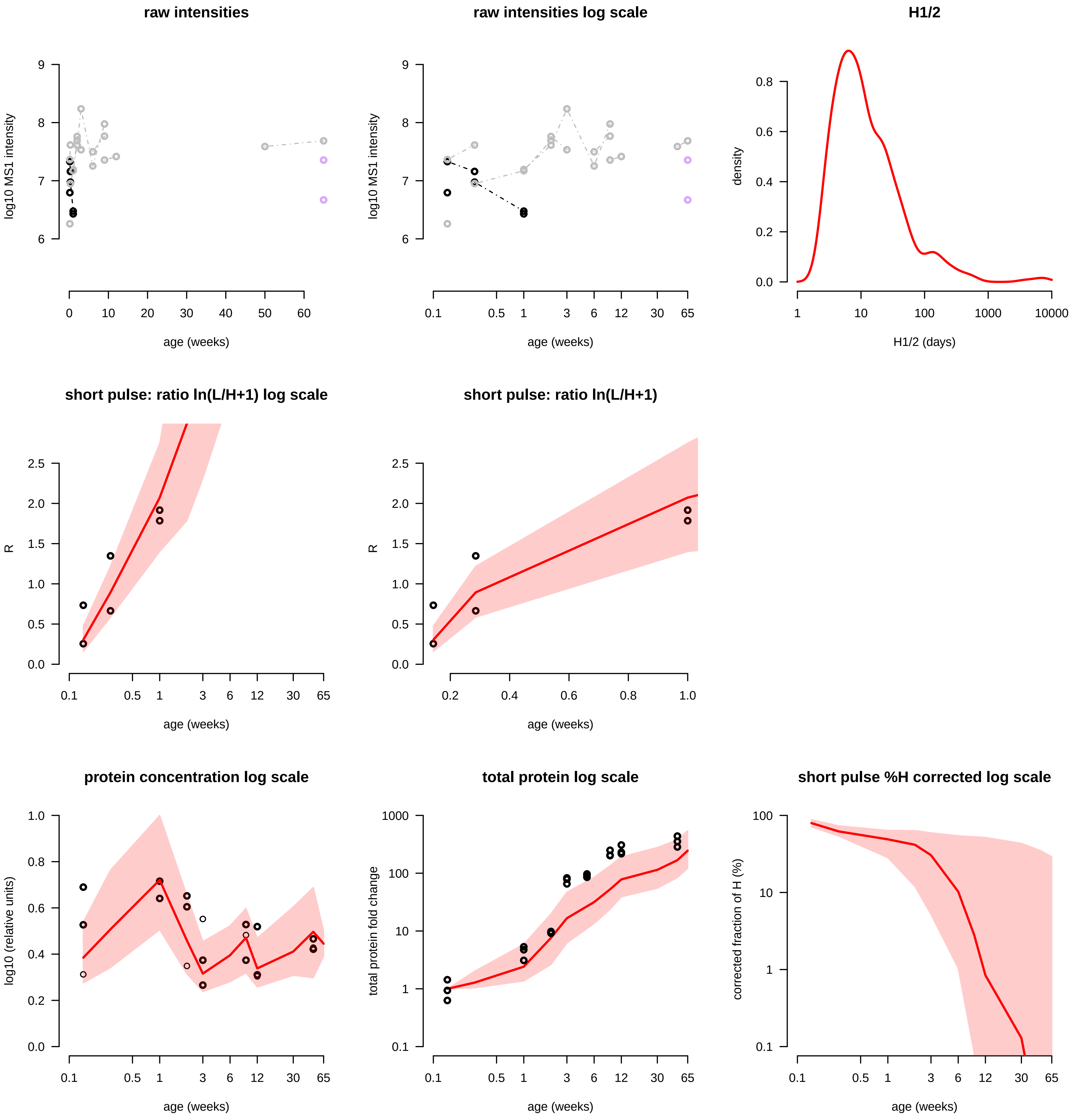

Q6DFV3

>sp|Q6DFV3|RHG21_MOUSE

Rho GTPase−activating protein 21

GN=Arhgap21

Med H1/2: 14.4 days

Q5 H1/2: 5 days

Q95 H1/2: 156.8 days

reliable model fit: yes

raw intensities

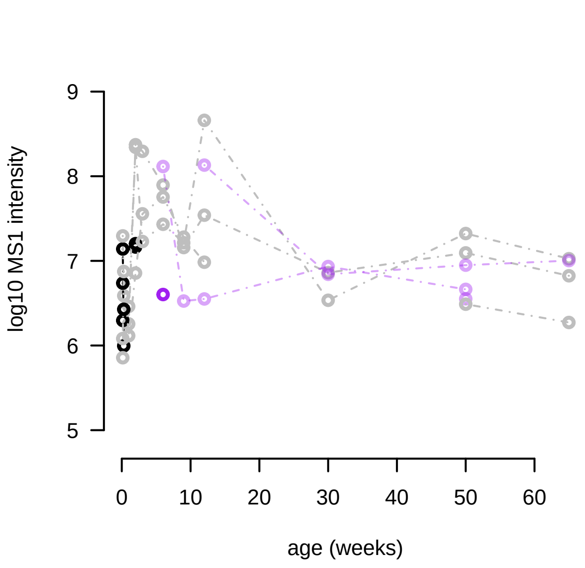

raw intensities log scale

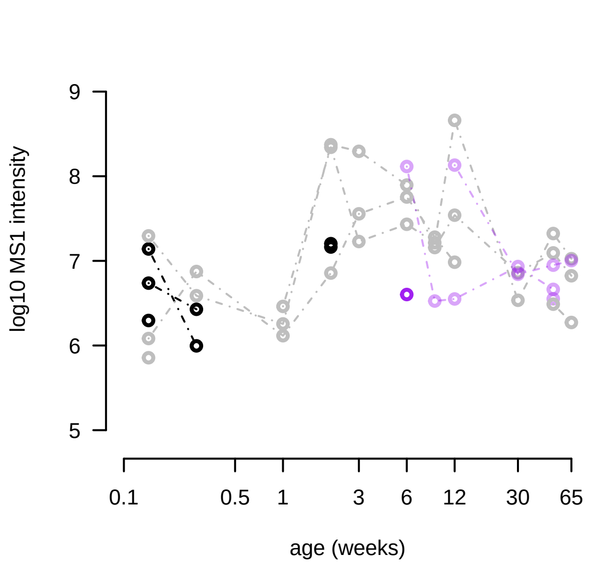

H1/2

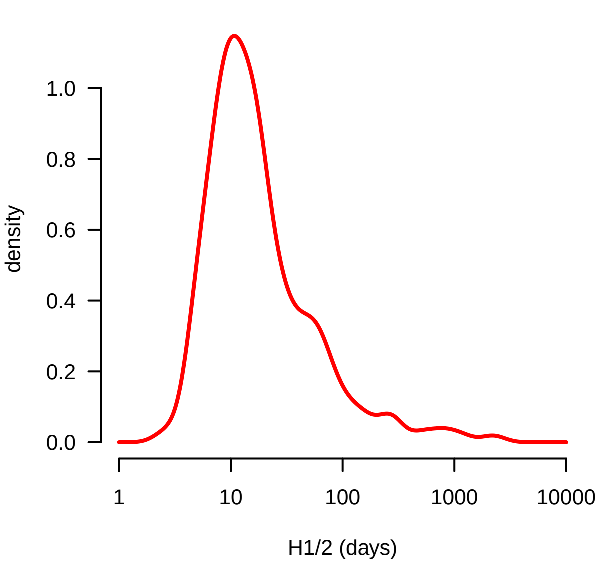

short pulse: ratio ln(L/H+1) log scale

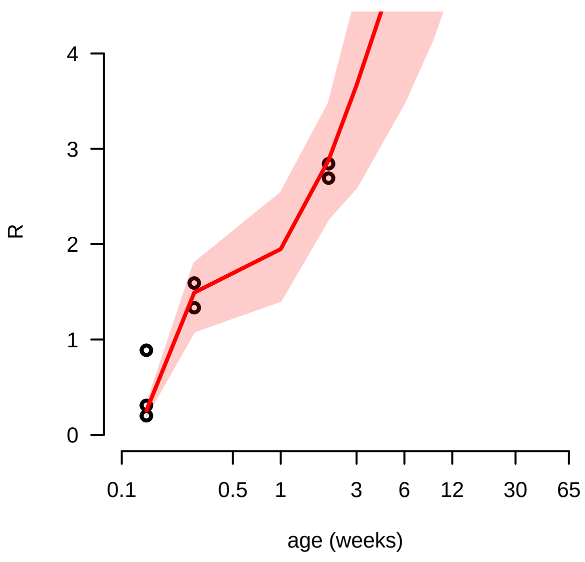

short pulse: ratio ln(L/H+1)

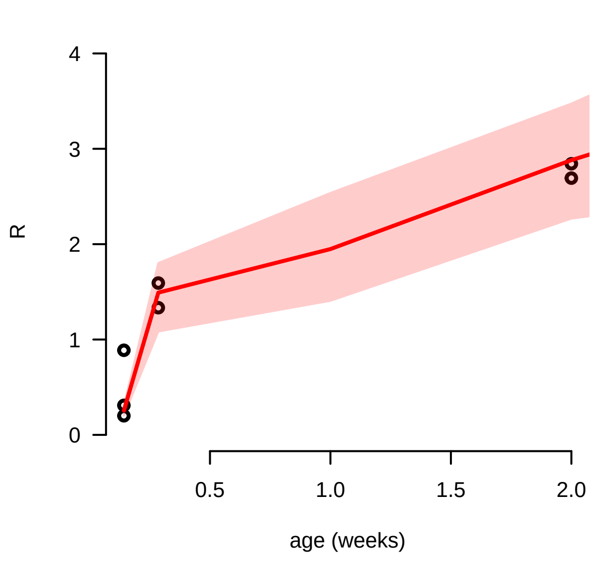

protein concentration log scale

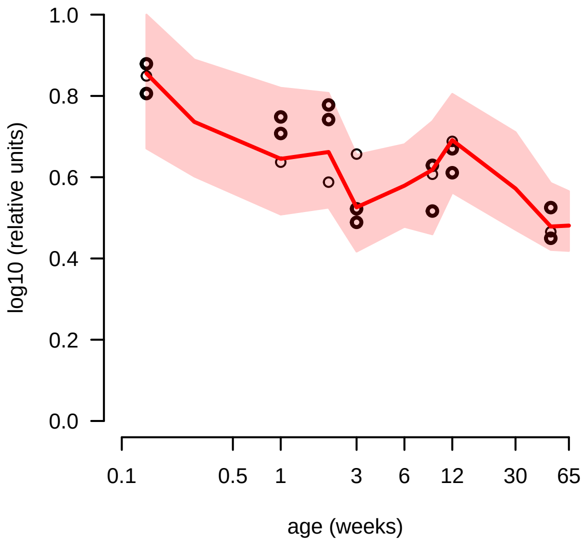

total protein log scale

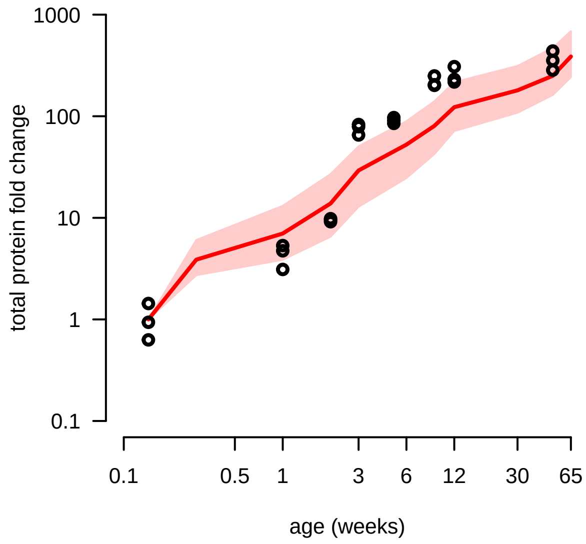

short pulse %H corrected log scale

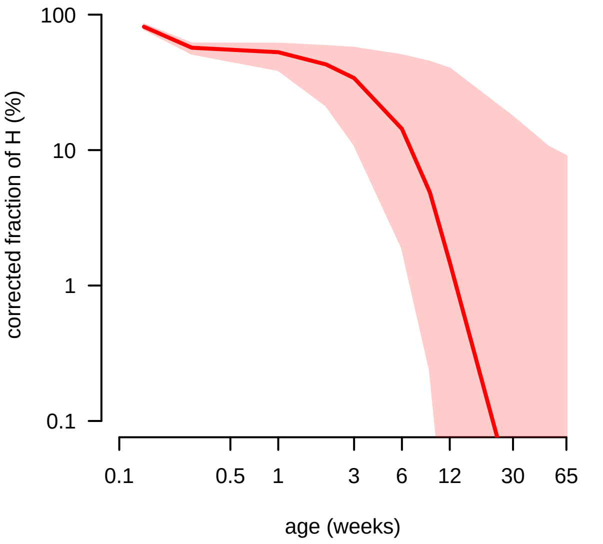

P35710

>sp|P35710|SOX5_MOUSE

Transcription factor SOX−5

GN=Sox5

Med H1/2: 6.1 days

Q5 H1/2: 2.7 days

Q95 H1/2: 45.7 days

reliable model fit: yes

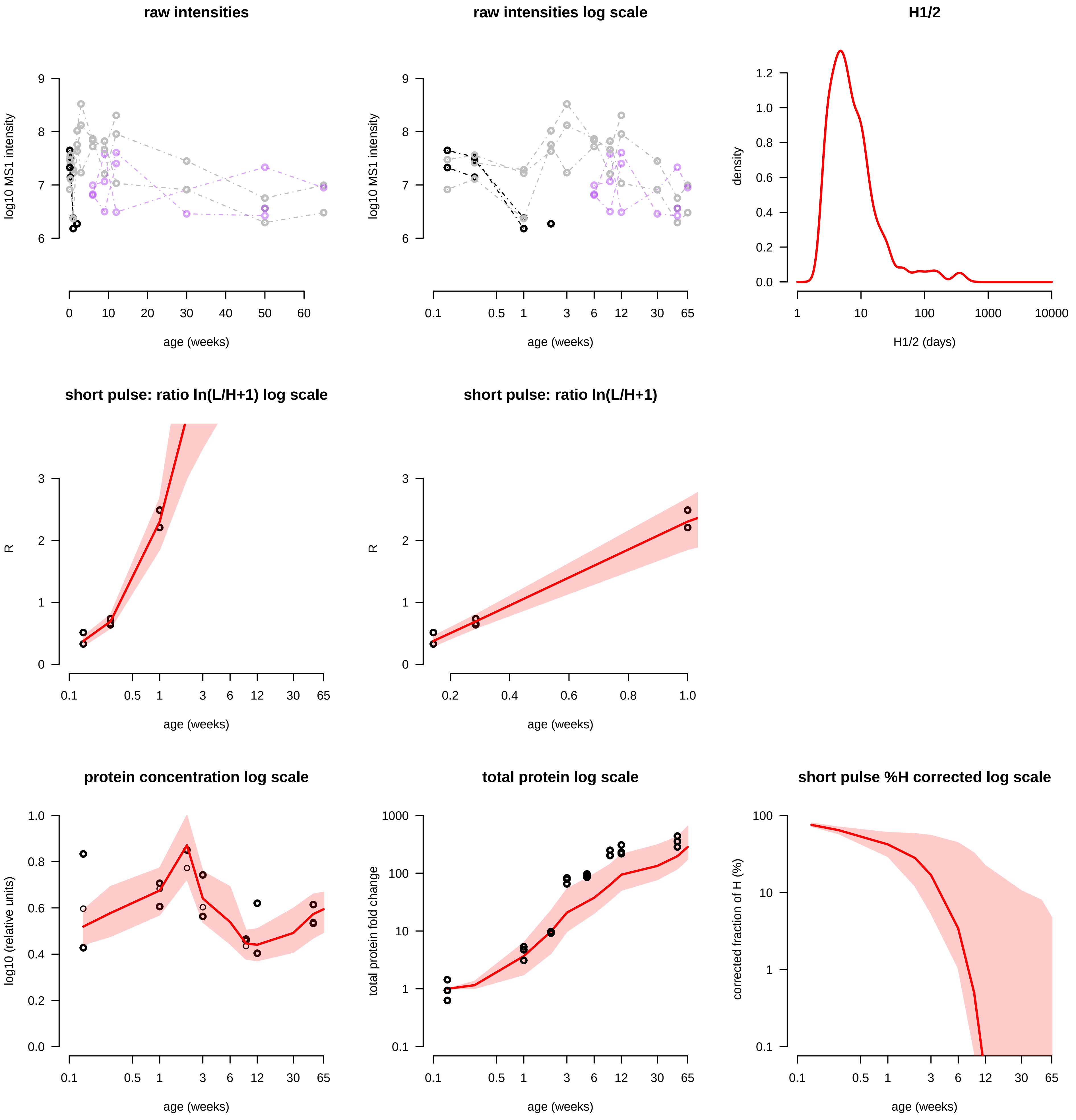

Q8R2Y9

>sp|Q8R2Y9|SOSB1_MOUSE

SOSS complex subunit B1

GN=Nabp2

Med H1/2: 18.5 days

Q5 H1/2: 4.3 days

Q95 H1/2: 279.3 days

reliable model fit: yes

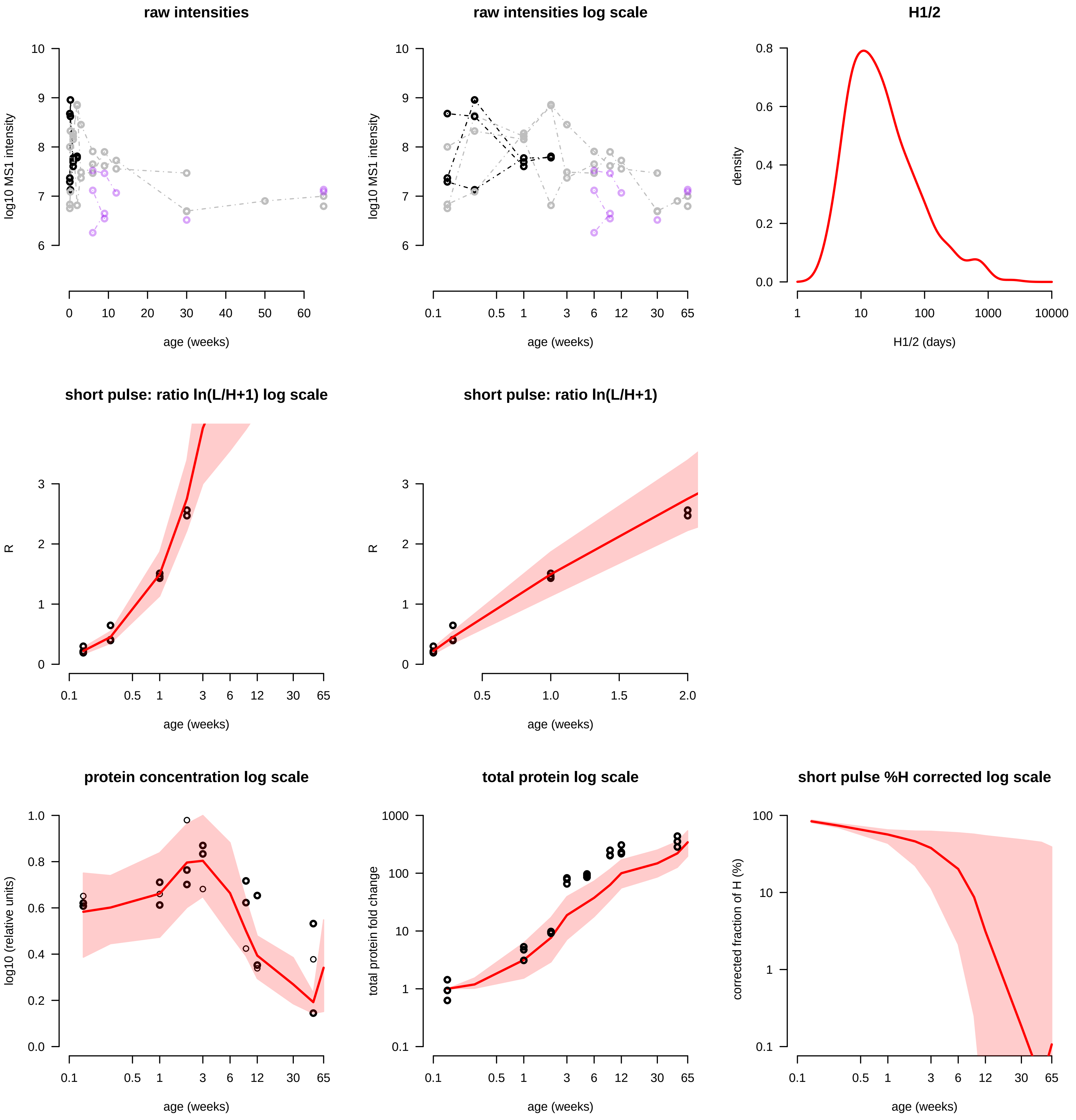

P02772

>sp|P02772|FETA_MOUSE

Alpha−fetoprotein

GN=Afp

Med H1/2: 2967.5 days

Q5 H1/2: 916.7 days

Q95 H1/2: 48793.3 days

reliable model fit: no

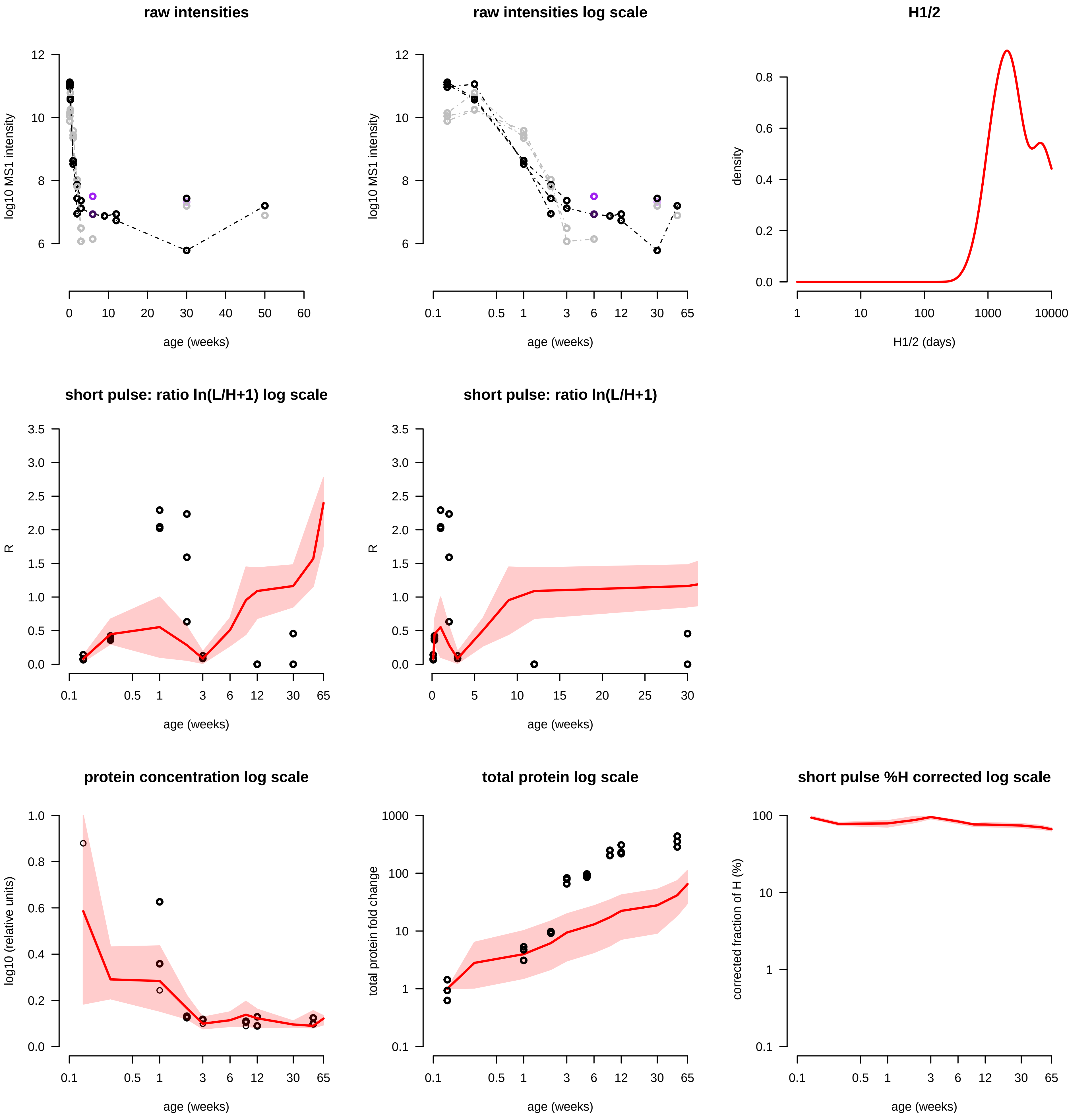

Q924S2

>sp|Q924S2|OAS1C_MOUSE

Inactive 2'−5' oligoadenylate synthetase 1C

GN=Oas1c

Med H1/2: 9.9 days

Q5 H1/2: 4.8 days

Q95 H1/2: 168.6 days

reliable model fit: yes

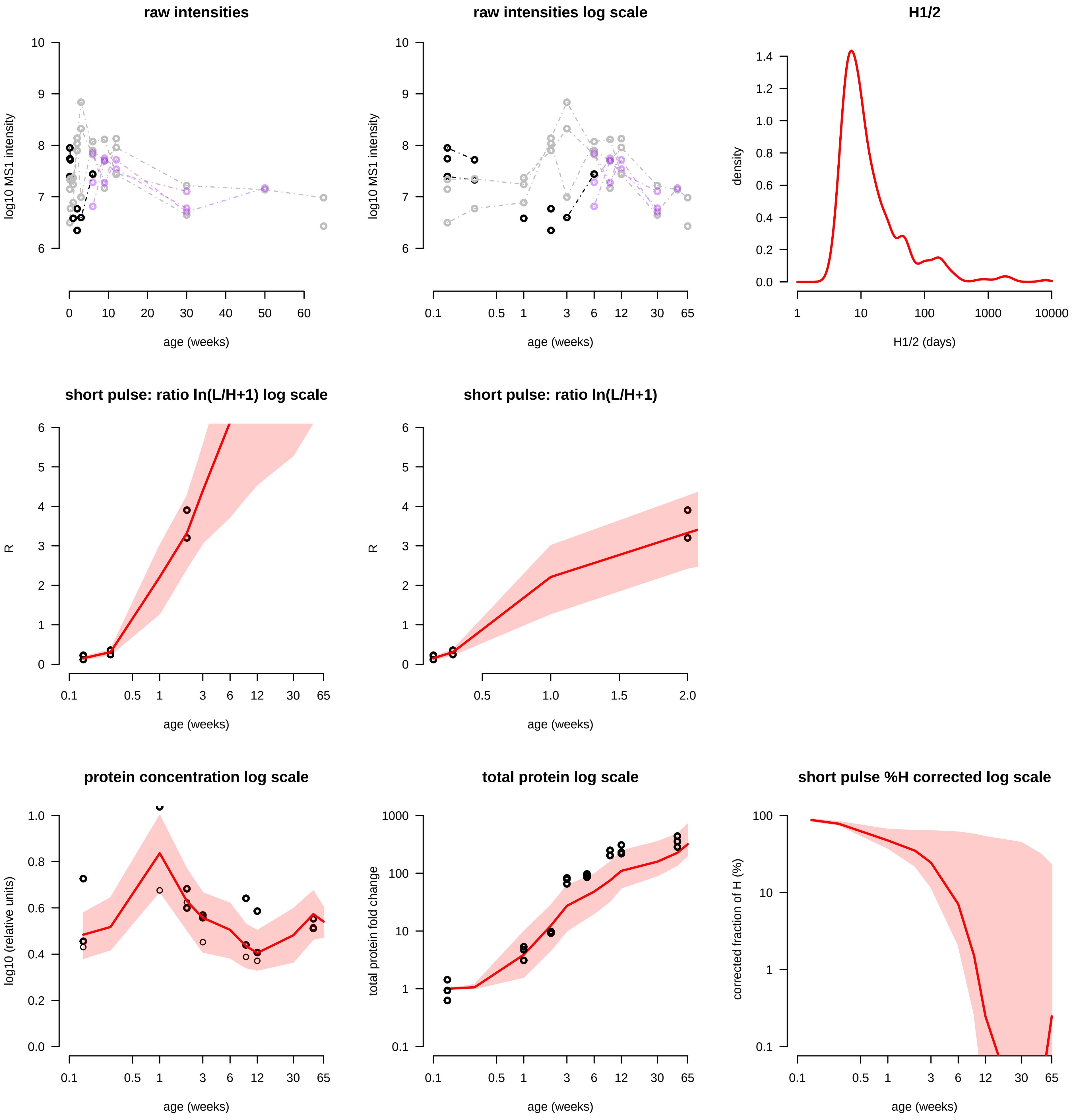

Q8CFH6

>sp|Q8CFH6|SIK2_MOUSE

Serine/threonine−protein kinase SIK2

GN=Sik2

Med H1/2: 19.2 days

Q5 H1/2: 4.7 days

Q95 H1/2: 206.7 days

reliable model fit: yes

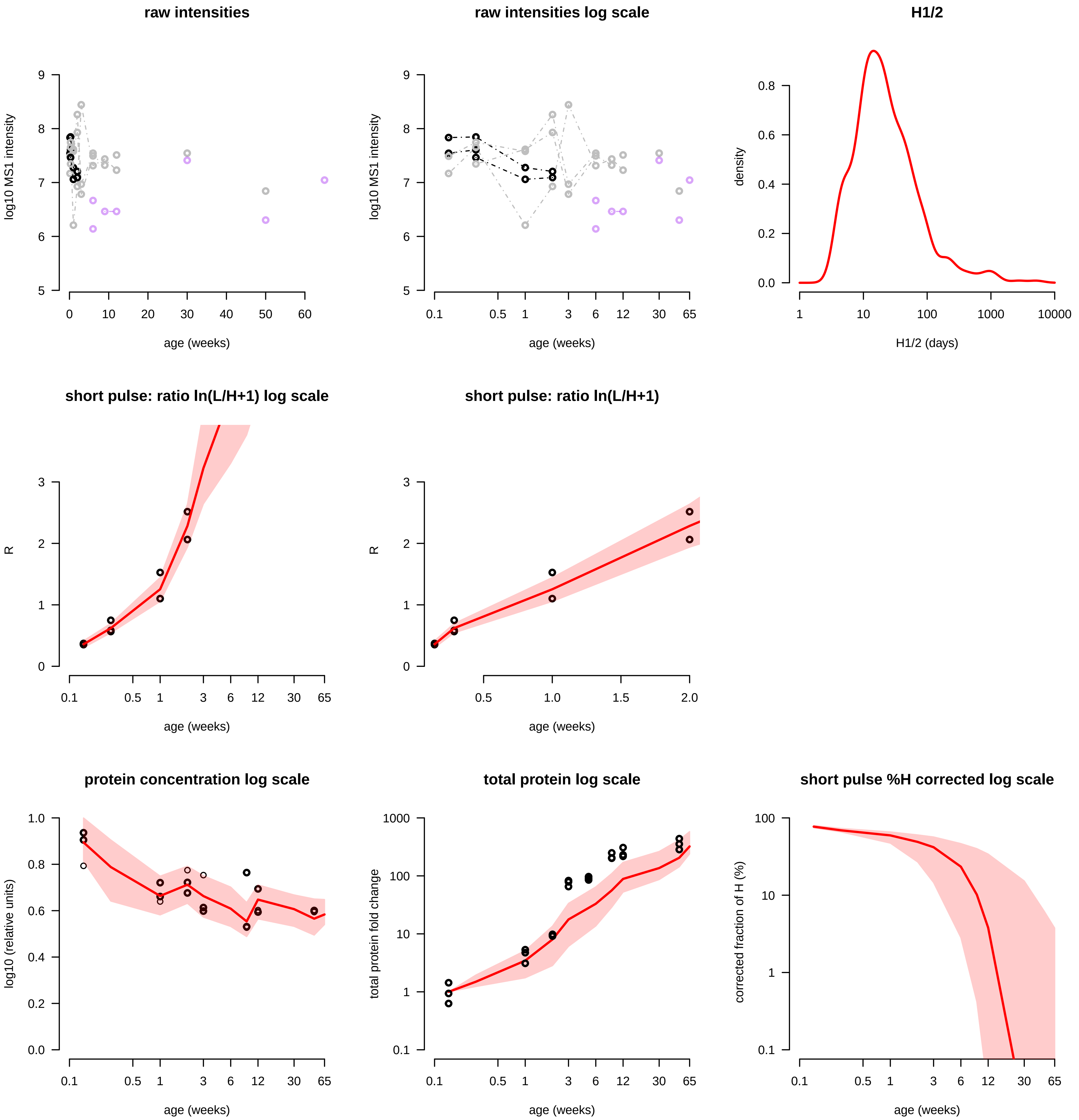

Q8C804

>sp|Q8C804|SPICE_MOUSE

Spindle and centriole−associated protein 1

GN=Spice1

Med H1/2: 7.5 days

Q5 H1/2: 3 days

Q95 H1/2: 45.2 days

reliable model fit: yes

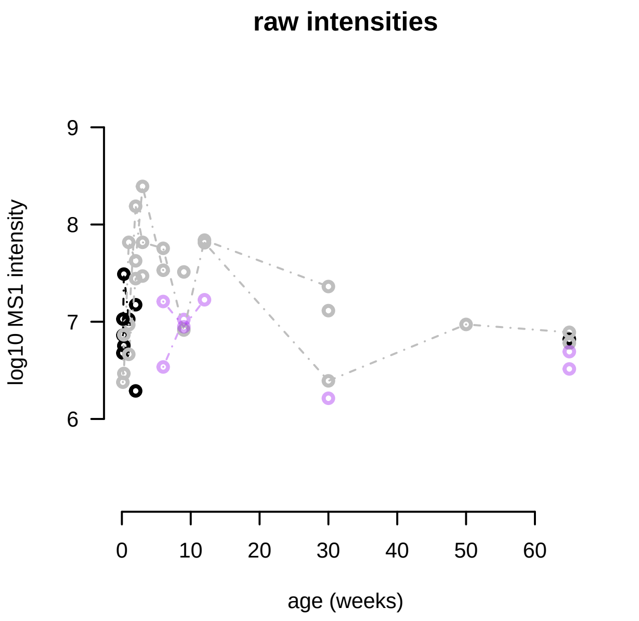

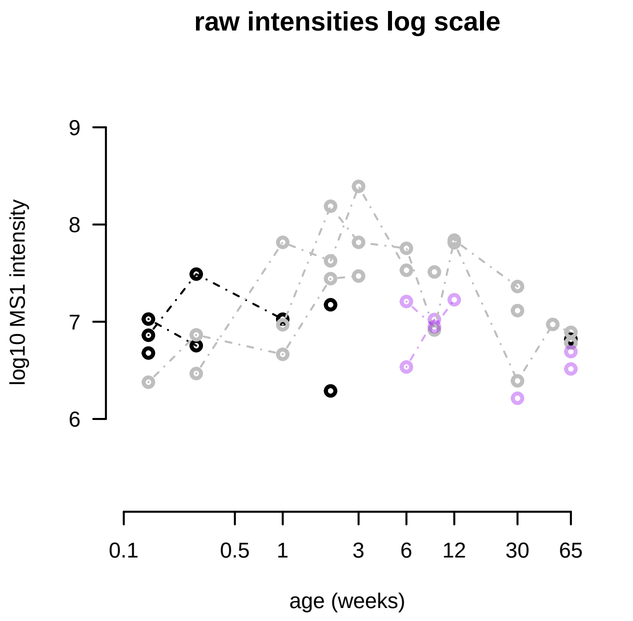

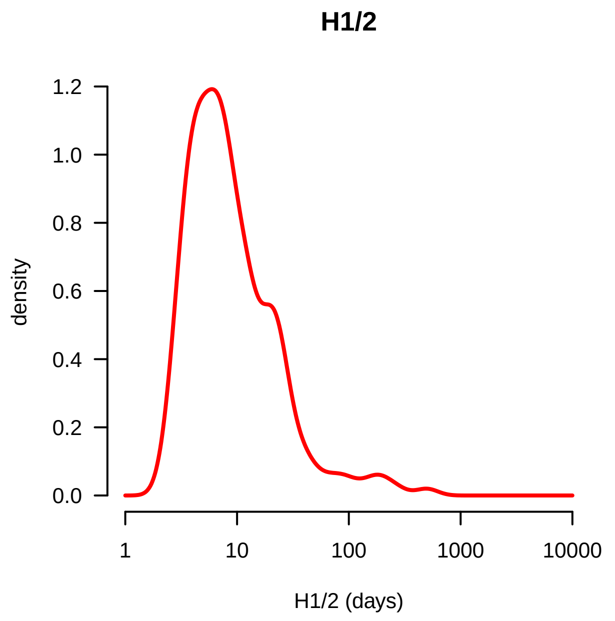

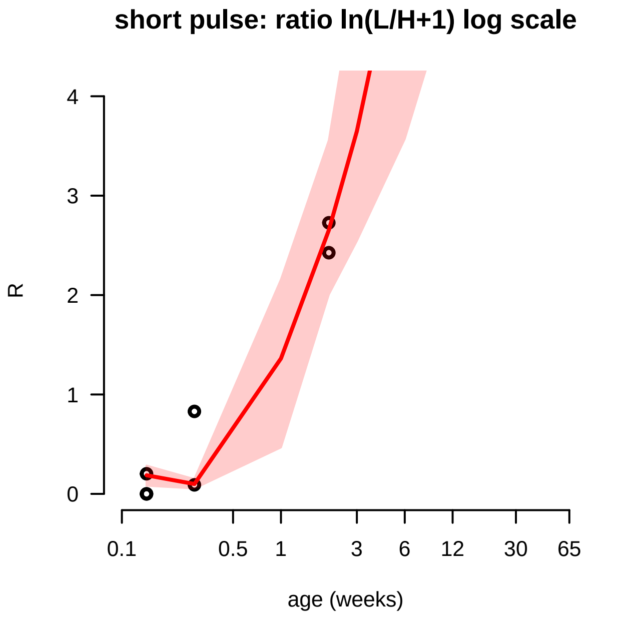

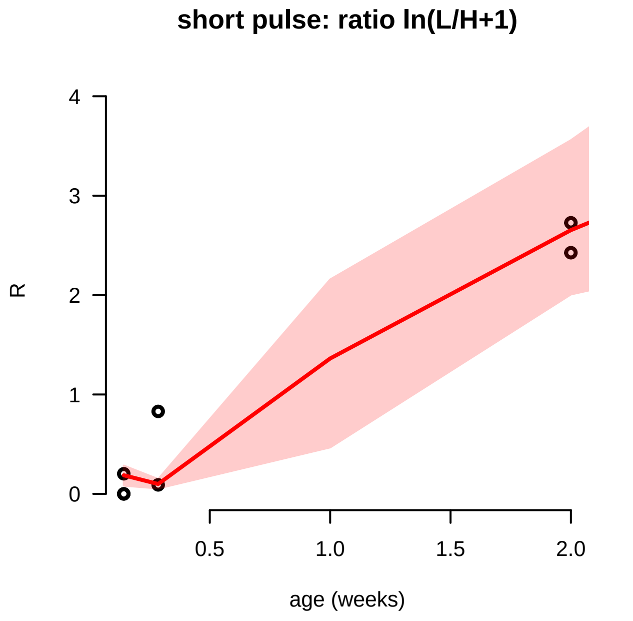

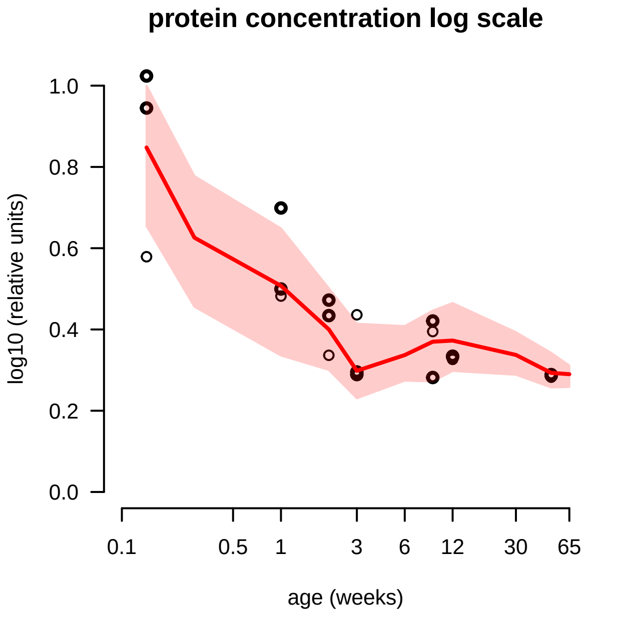

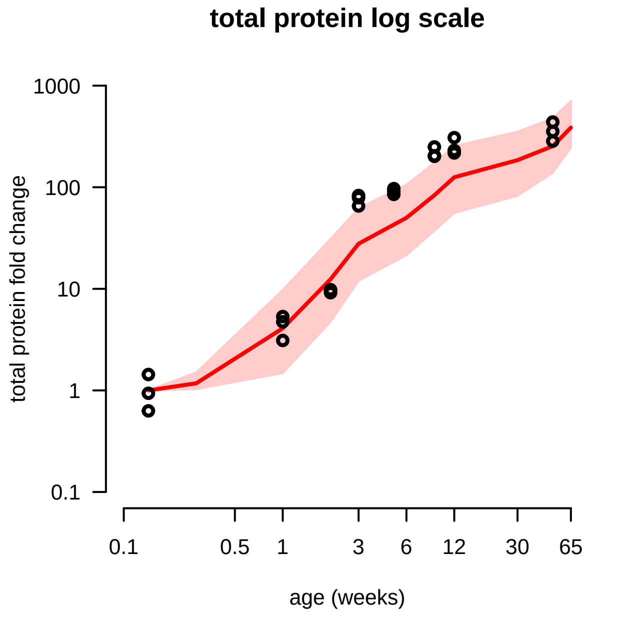

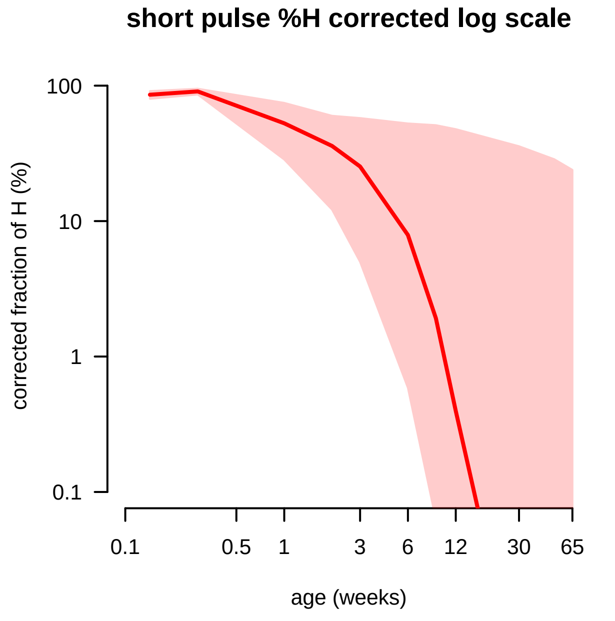

O08550

>sp|O08550|KMT2B_MOUSE

Histone−lysine N−methyltransferase 2B

GN=Kmt2b

Med H1/2: 57.3 days

Q5 H1/2: 15.7 days

Q95 H1/2: 675.2 days

reliable model fit: yes

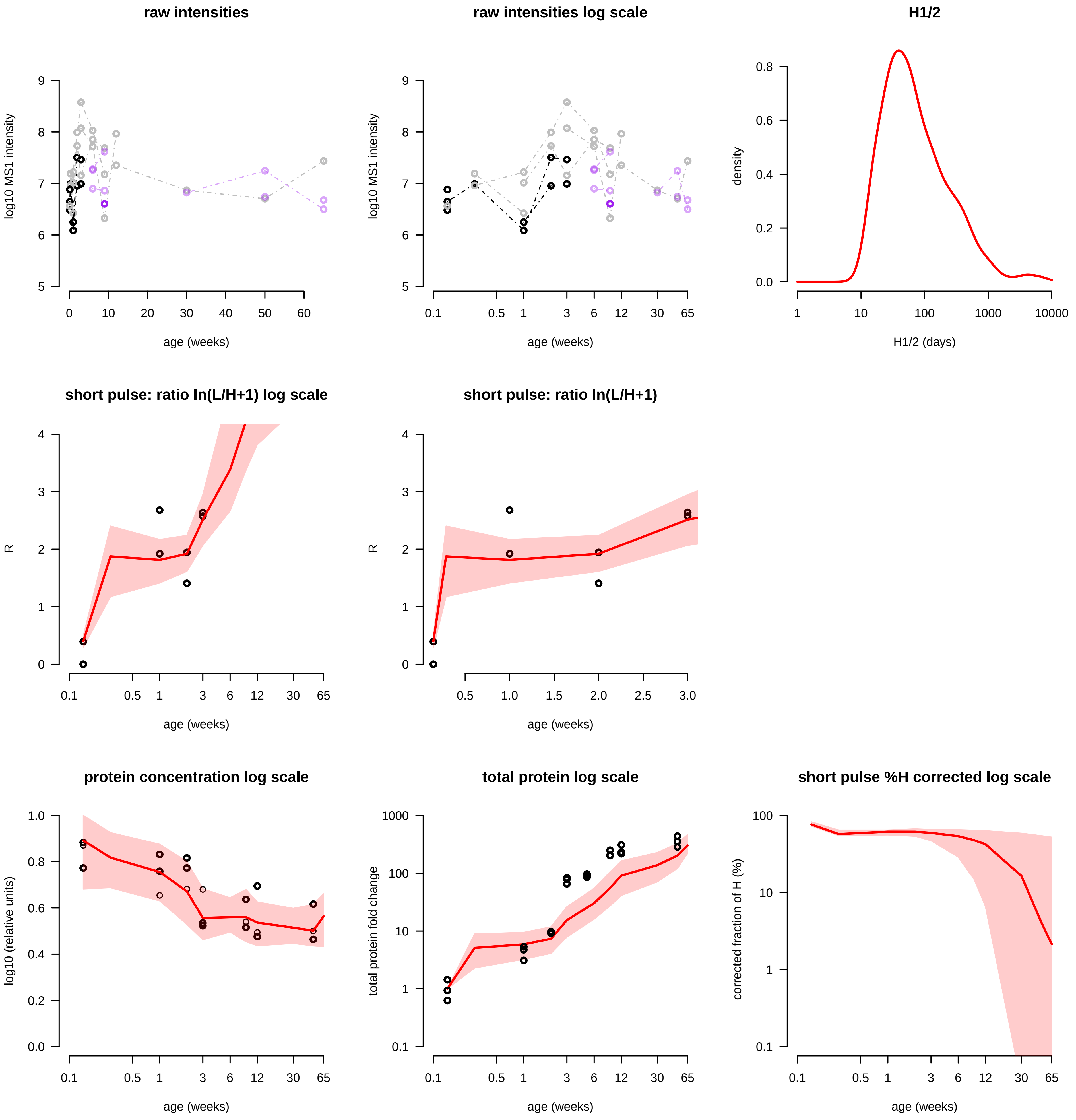

Q8VD46

>sp|Q8VD46|ASZ1_MOUSE

Ankyrin repeat, SAM and basic leucine zipper domain−containing protein 1

GN=Asz1

Med H1/2: 15.6 days

Q5 H1/2: 11.8 days

Q95 H1/2: 26.4 days

reliable model fit: no

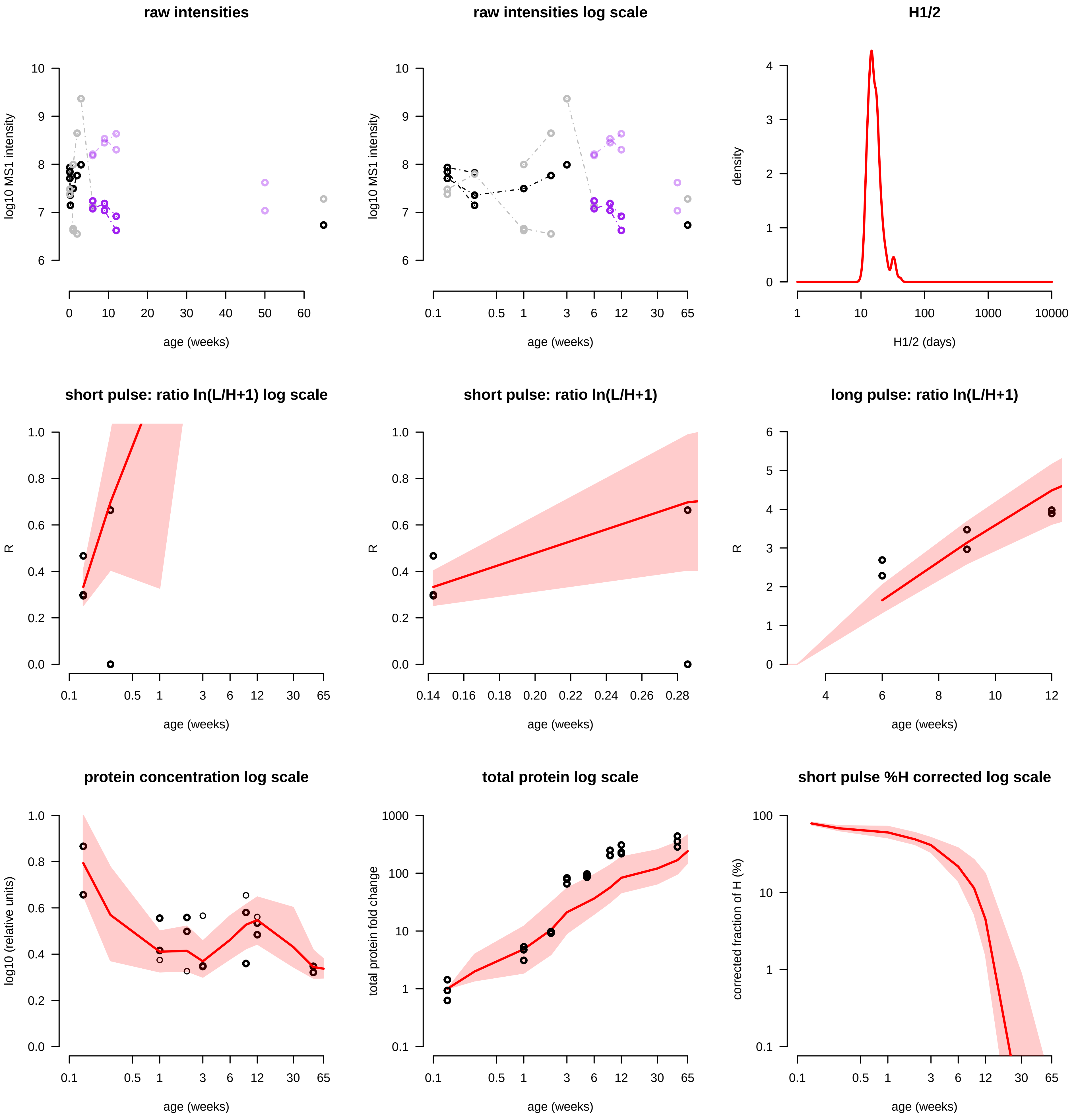

Q3KNJ2

>sp|Q3KNJ2|NHEJ1_MOUSE

Non−homologous end−joining factor 1

GN=Nhej1

Med H1/2: 37.9 days

Q5 H1/2: 11.4 days

Q95 H1/2: 356.5 days

reliable model fit: yes

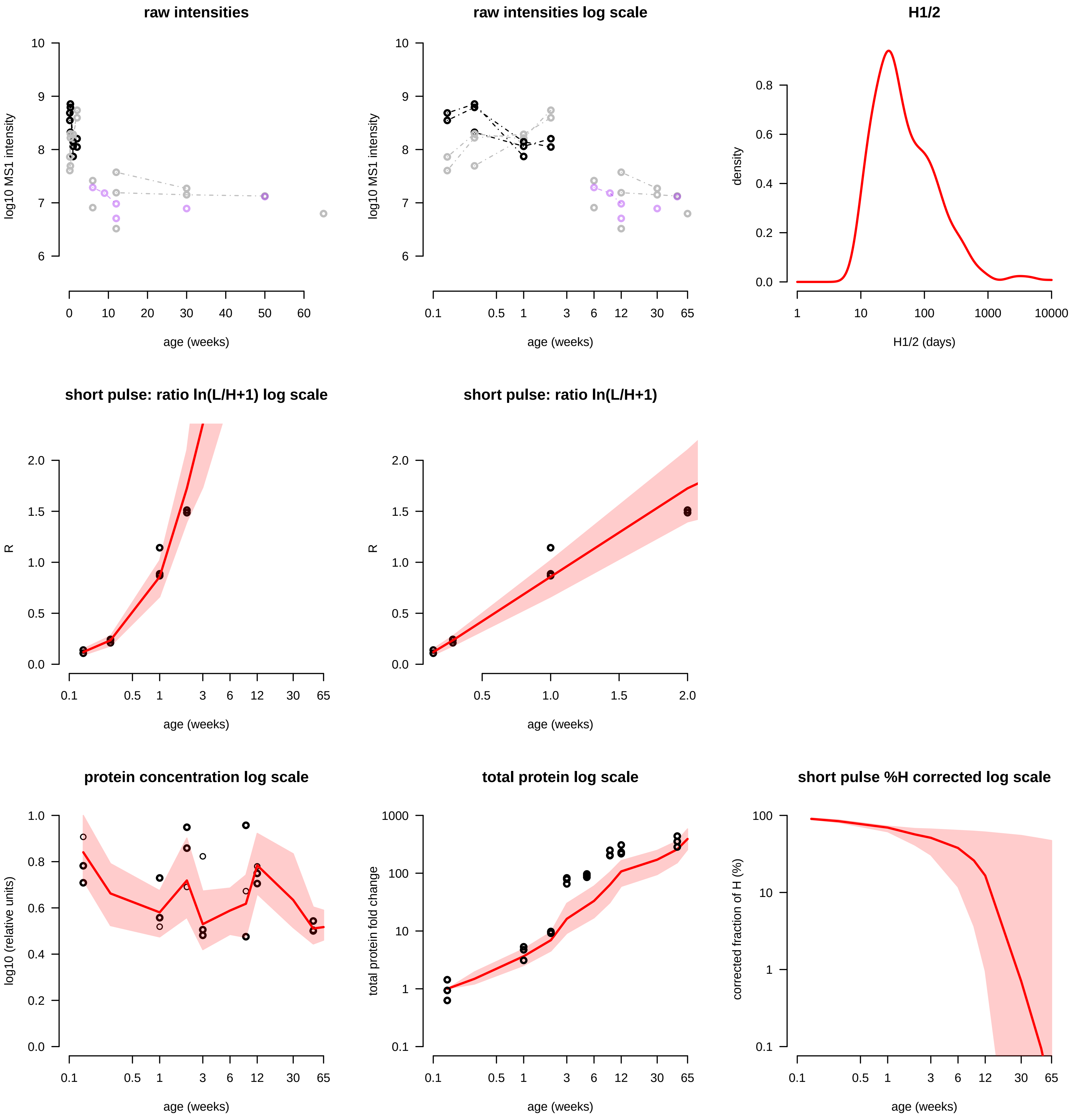

Q6NVD0

>sp|Q6NVD0|FREM2_MOUSE

FRAS1−related extracellular matrix protein 2

GN=Frem2

Med H1/2: 5 days

Q5 H1/2: 2.8 days

Q95 H1/2: 35.6 days

reliable model fit: yes

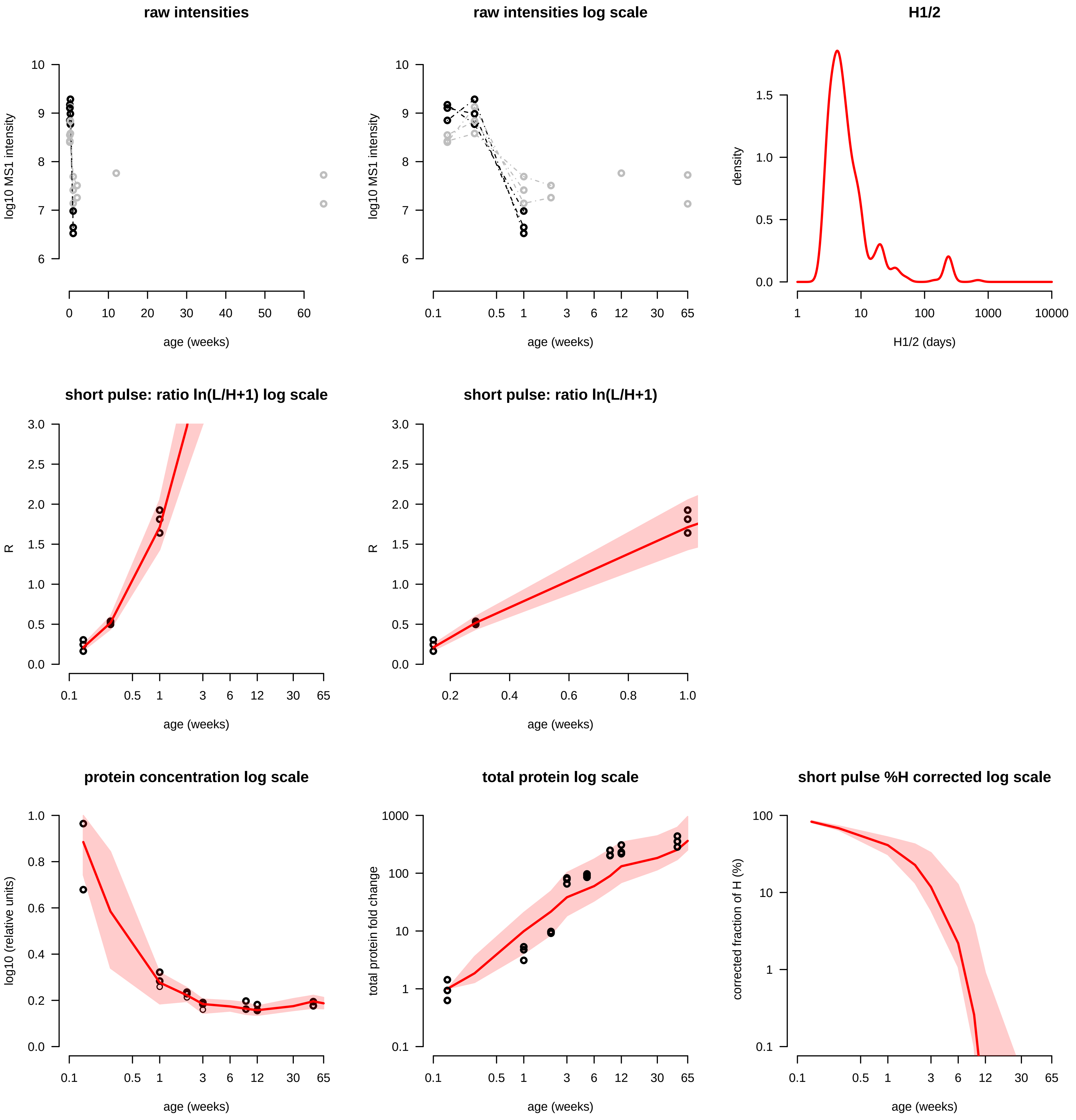

O54818

>sp|O54818|TPD53_MOUSE

Tumor protein D53

GN=Tpd52l1

Med H1/2: 38.9 days

Q5 H1/2: 9.4 days

Q95 H1/2: 542.6 days

reliable model fit: yes

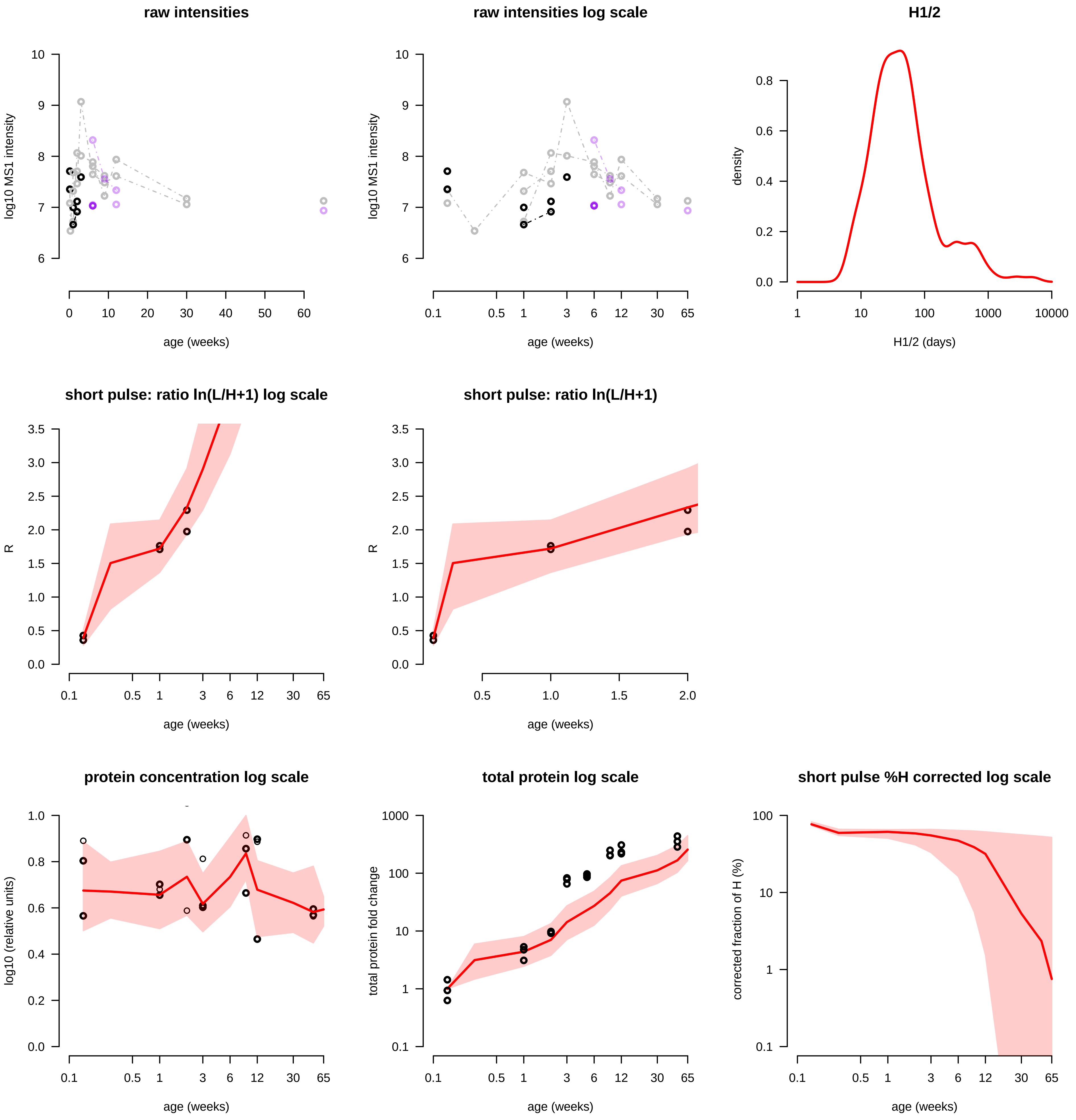

O88477

>sp|O88477|IF2B1_MOUSE

Insulin−like growth factor 2 mRNA−binding protein 1

GN=Igf2bp1

Med H1/2: 16.1 days

Q5 H1/2: 9 days

Q95 H1/2: 74.2 days

reliable model fit: no

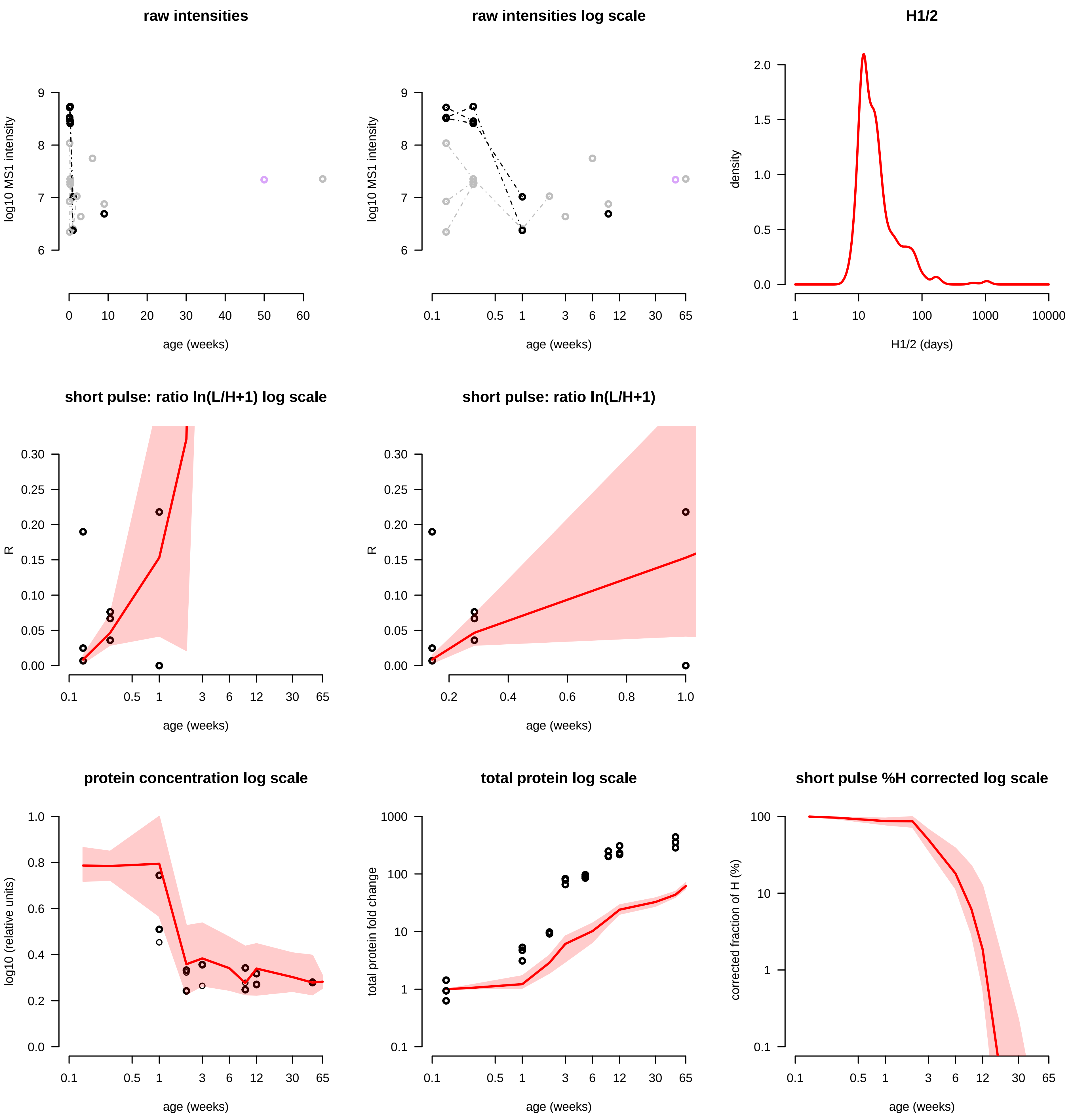

Q3V0K9

>sp|Q3V0K9|PLSI_MOUSE

Plastin−1

GN=Pls1

Med H1/2: 3.2 days

Q5 H1/2: 2.4 days

Q95 H1/2: 5 days

reliable model fit: yes

raw intensities

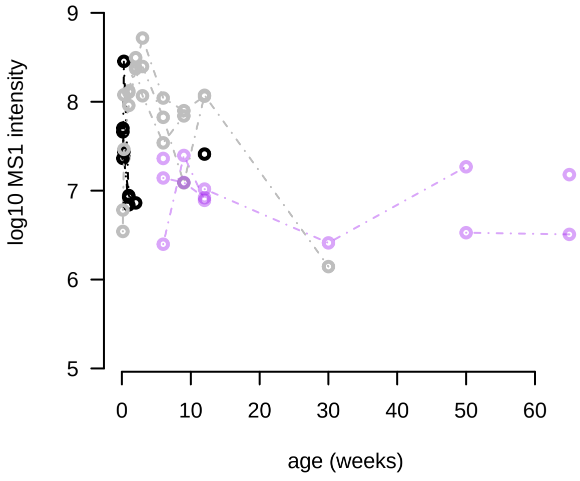

raw intensities log scale

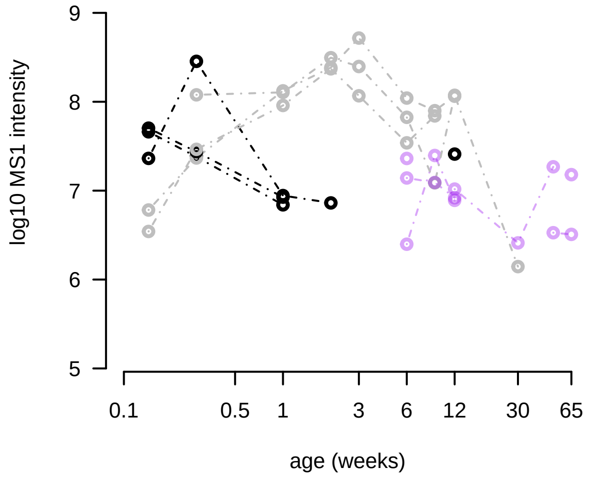

H1/2

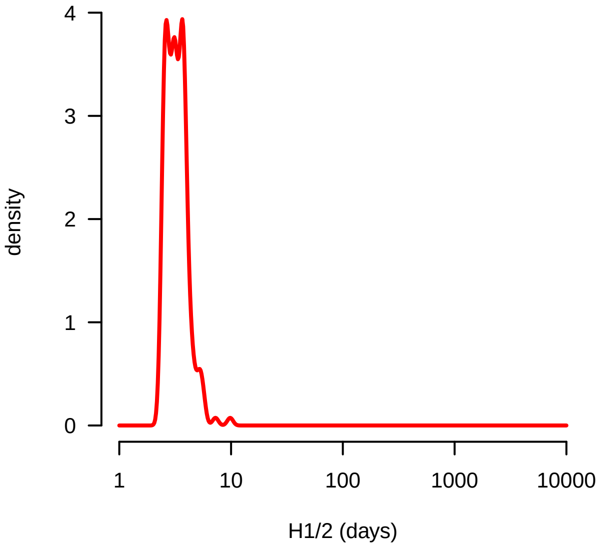

short pulse: ratio ln(L/H+1) log scale

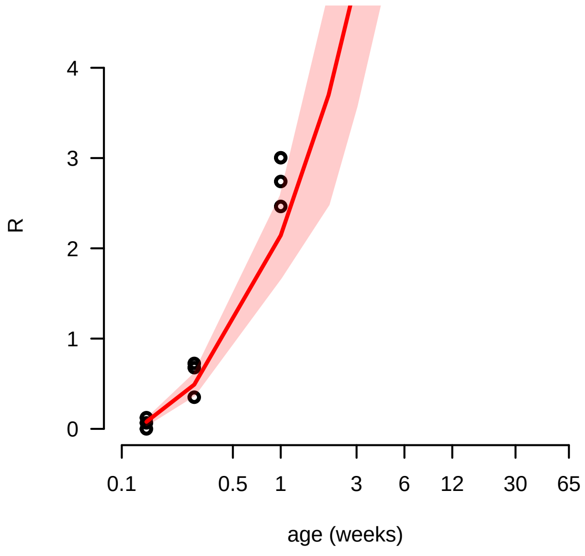

short pulse: ratio ln(L/H+1)

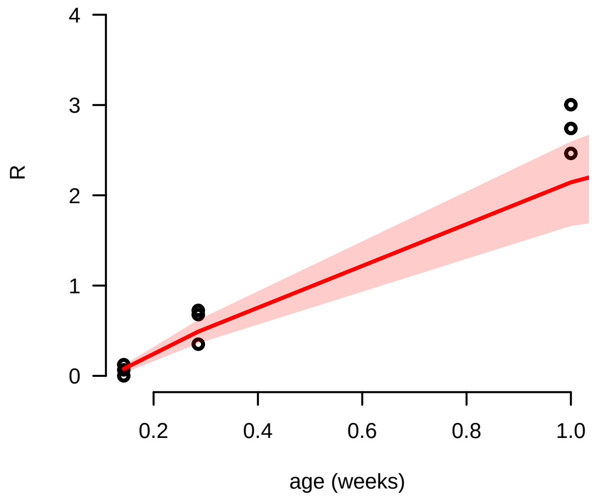

protein concentration log scale

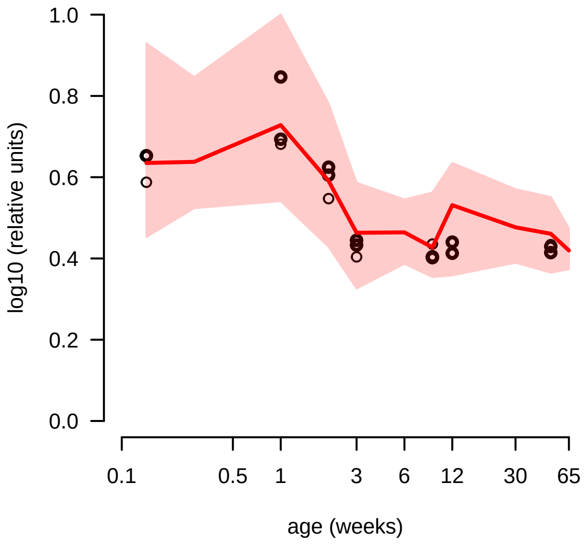

total protein log scale

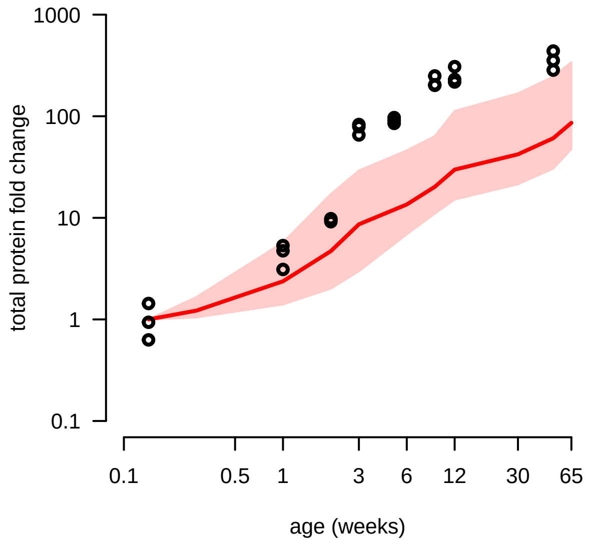

short pulse %H corrected log scale

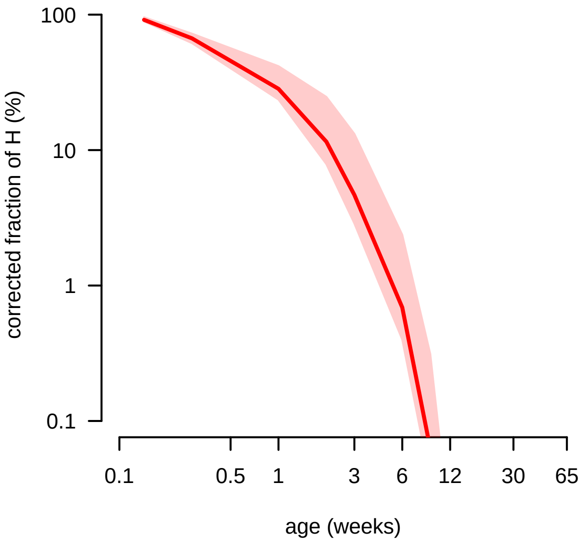

Q9CPN8

>sp|Q9CPN8|IF2B3_MOUSE

Insulin−like growth factor 2 mRNA−binding protein 3

GN=Igf2bp3

Med H1/2: 11.1 days

Q5 H1/2: 2.8 days

Q95 H1/2: 41.2 days

reliable model fit: yes

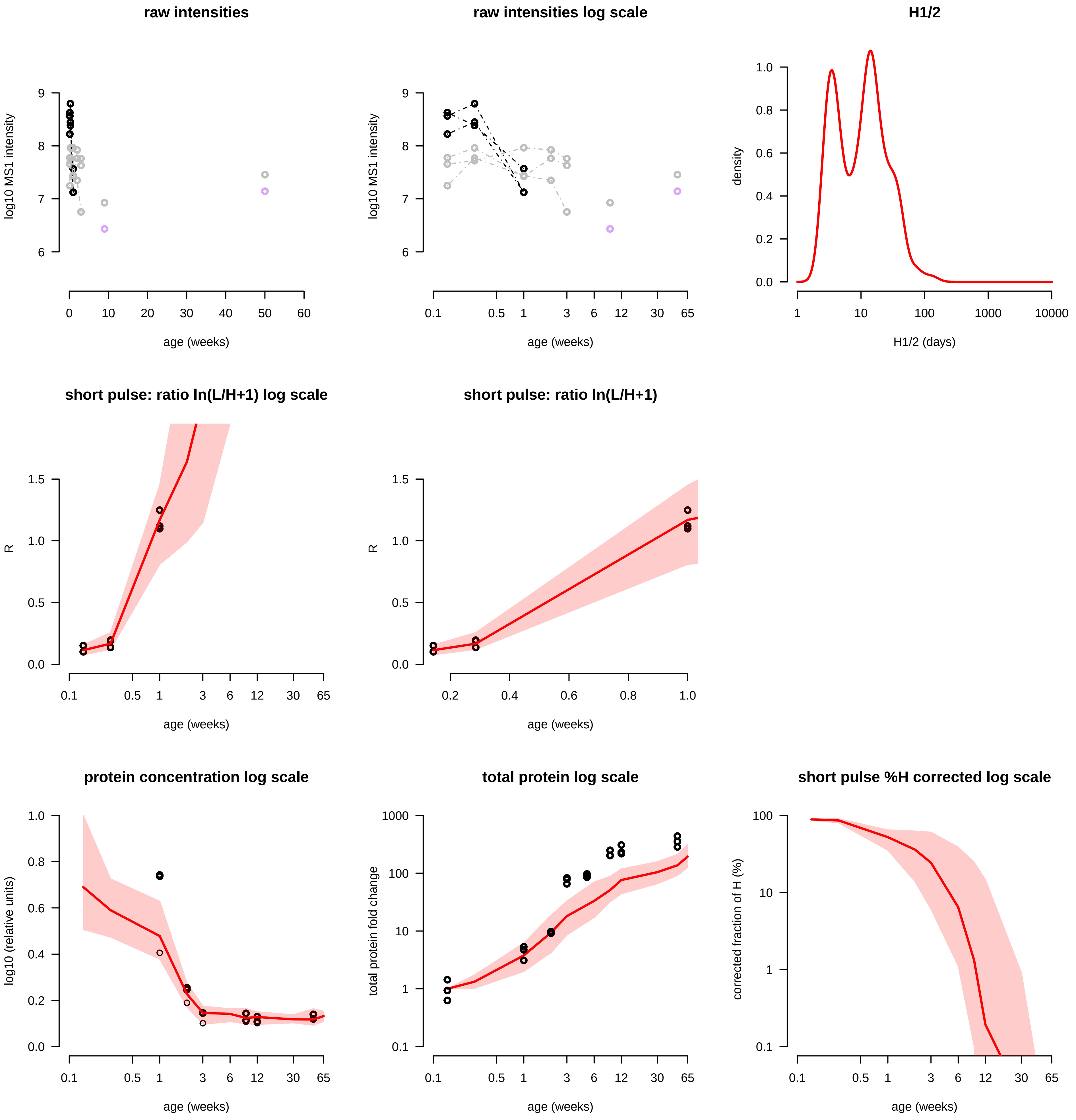

P04104

>sp|P04104|K2C1_MOUSE

Keratin, type II cytoskeletal 1

GN=Krt1

Med H1/2: 6.8 days

Q5 H1/2: 3.4 days

Q95 H1/2: 37.4 days

reliable model fit: yes

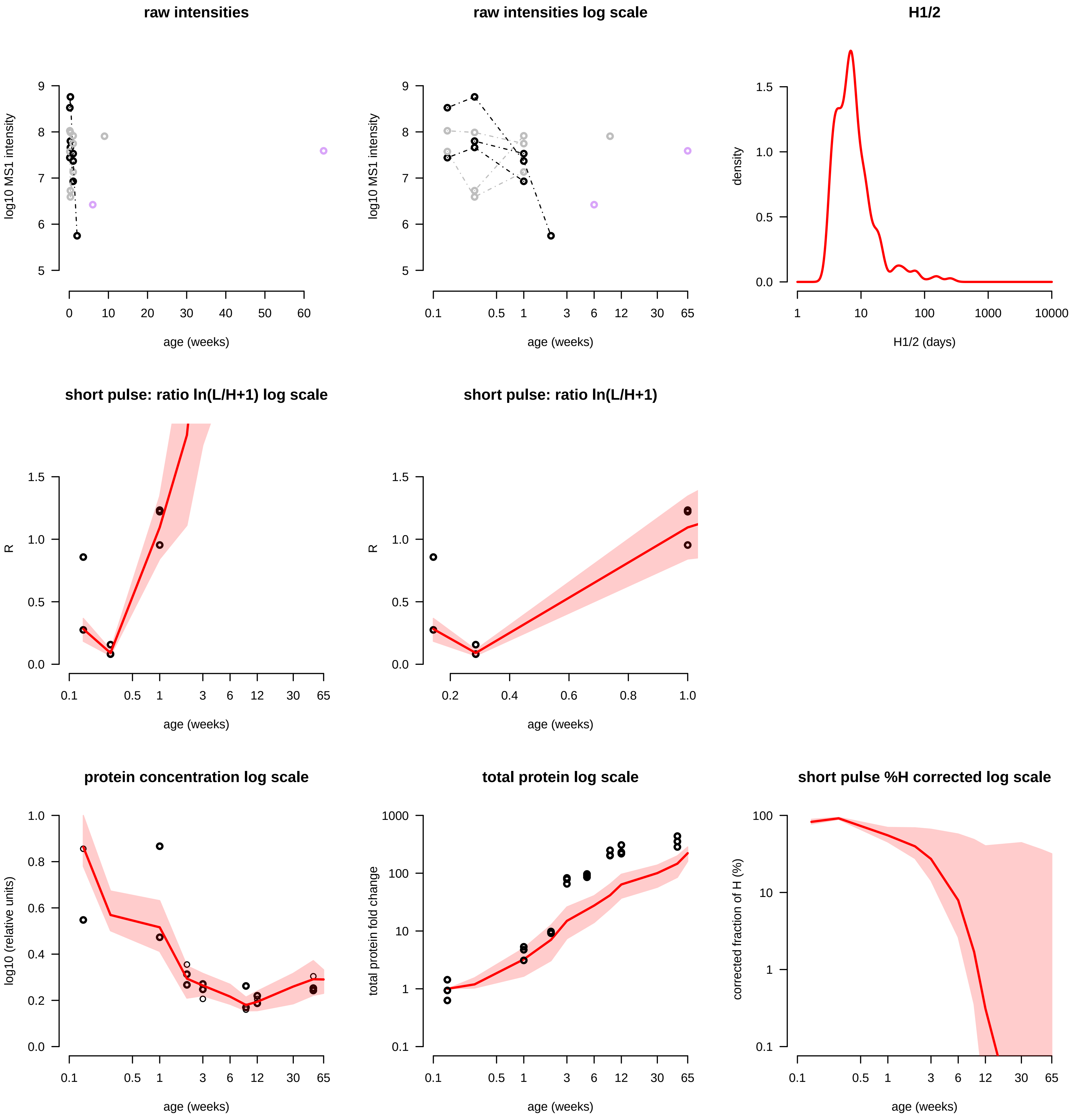

Q8R2U7

>sp|Q8R2U7|LRC42_MOUSE

Leucine−rich repeat−containing protein 42

GN=Lrrc42

Med H1/2: 30.6 days

Q5 H1/2: 11.6 days

Q95 H1/2: 462.8 days

reliable model fit: yes

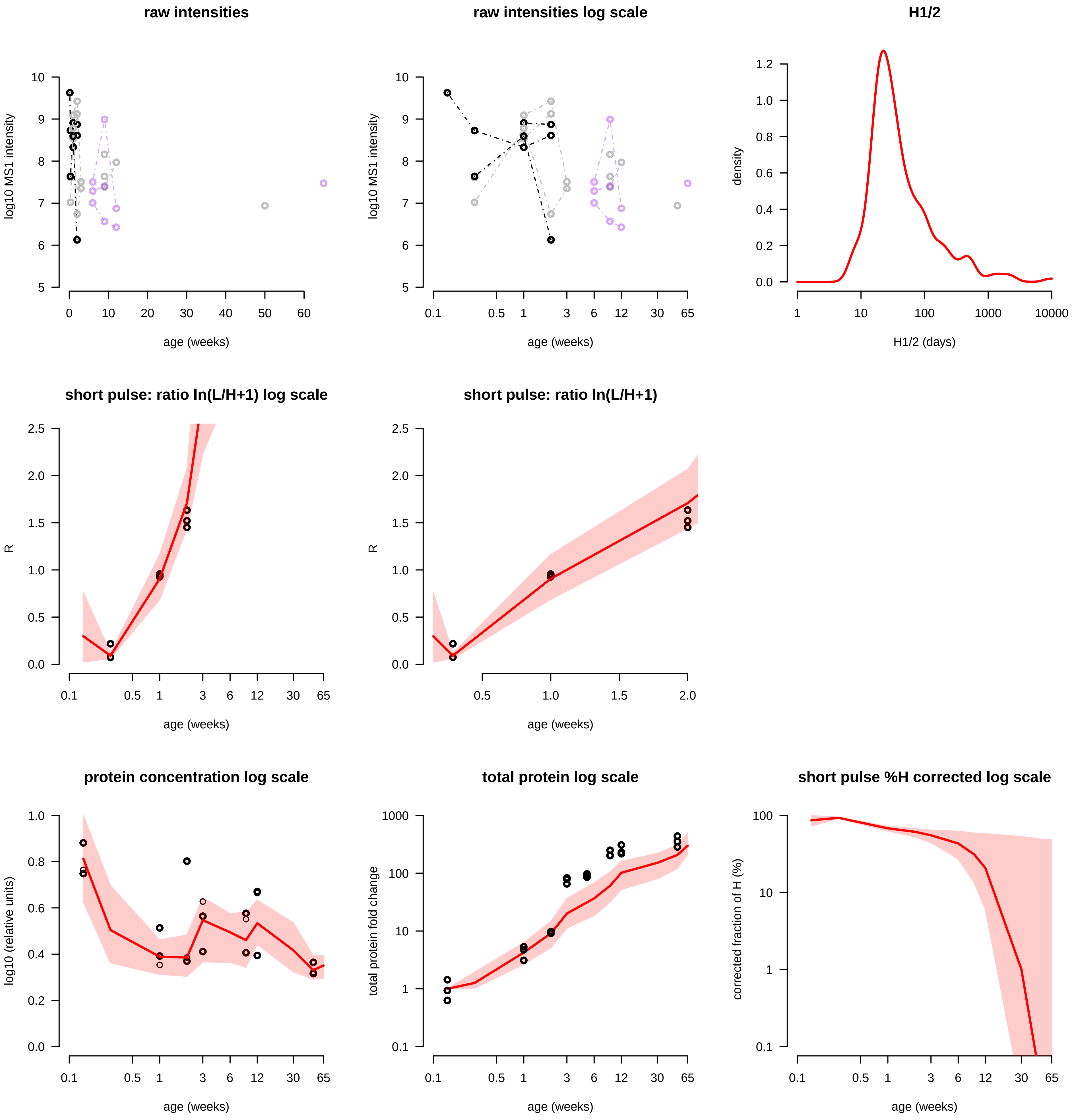

Q02844

>sp|Q02844|TRYB1_MOUSE

Tryptase

GN=Tpsab1

Med H1/2: 17.3 days

Q5   H1/2: 5.1 days

Q95 H1/2: 138.1 days

reliable model fit: yes

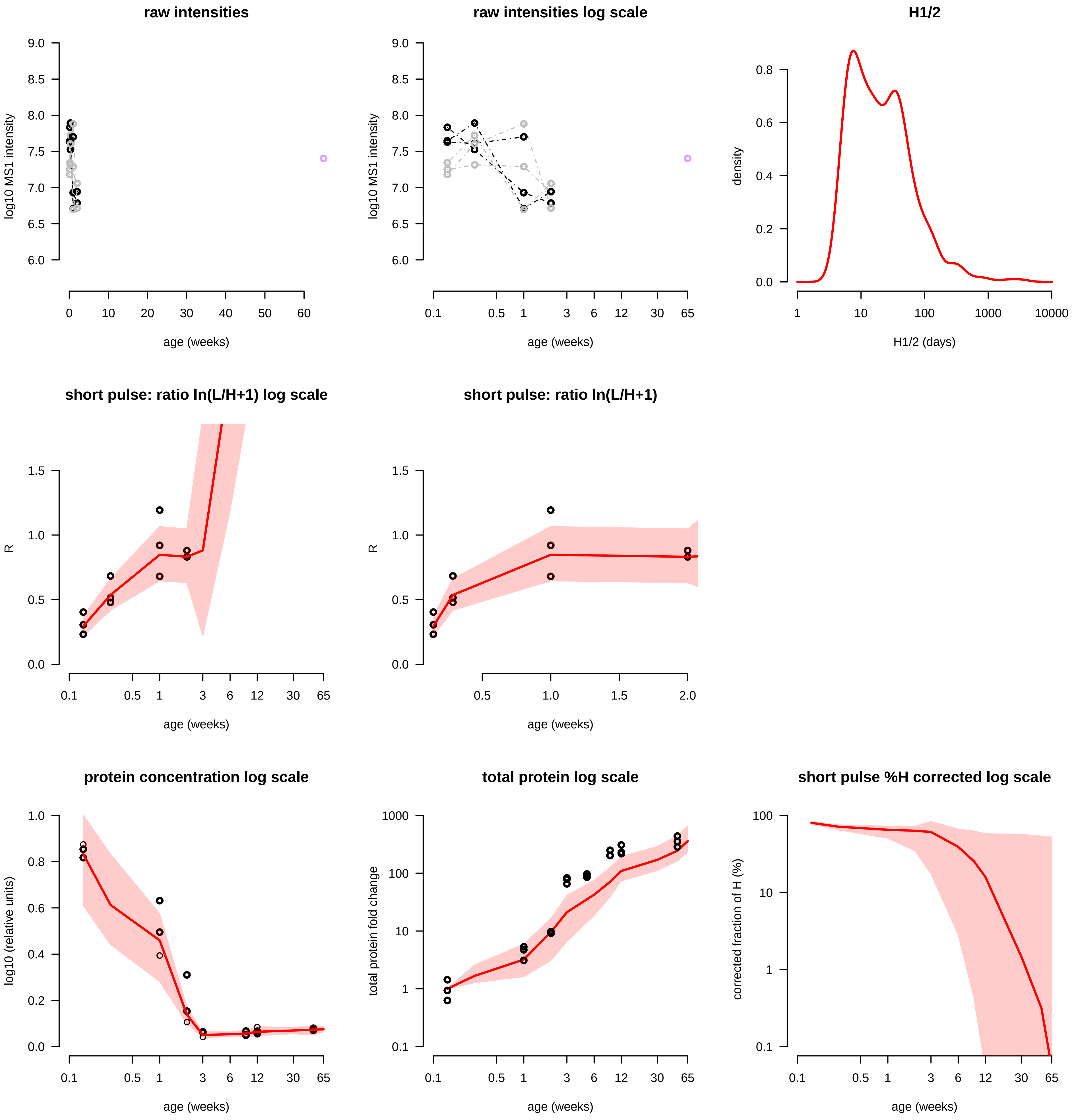

B2RRF6

>sp|B2RRF6|Z518A_MOUSE

Zinc finger protein 518A

GN=Znf518a

Med H1/2: 7.2 days

Q5 H1/2: 2.7 days

Q95 H1/2: 41.4 days

reliable model fit: yes

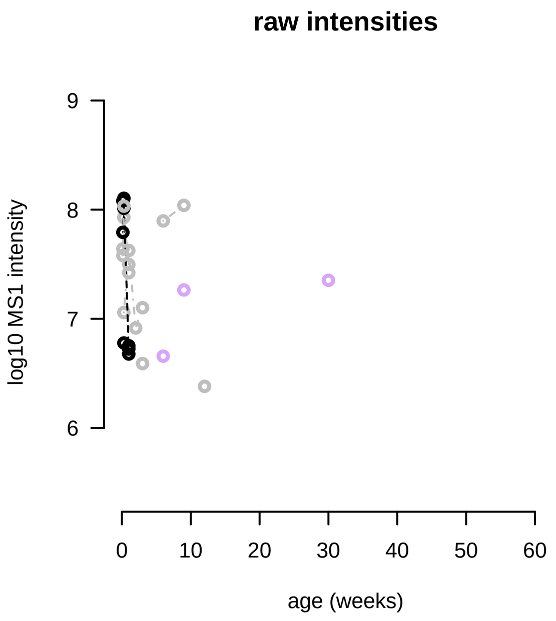

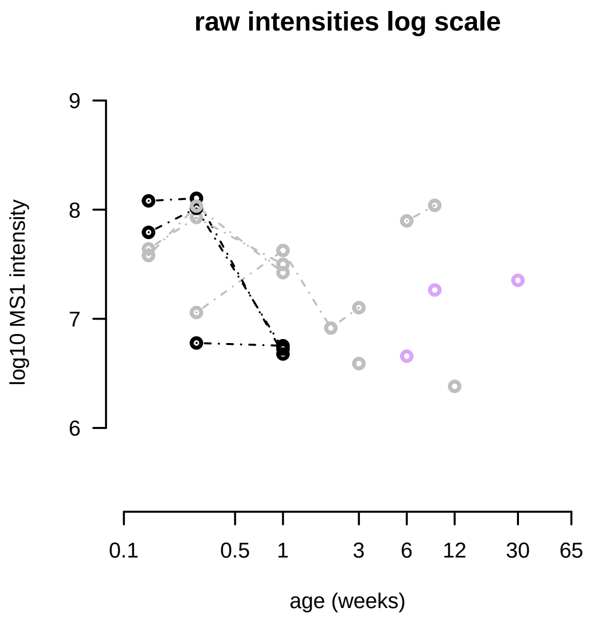

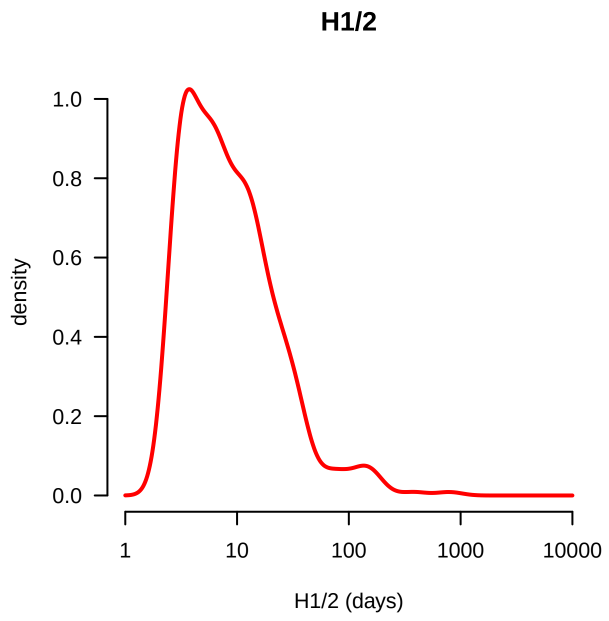

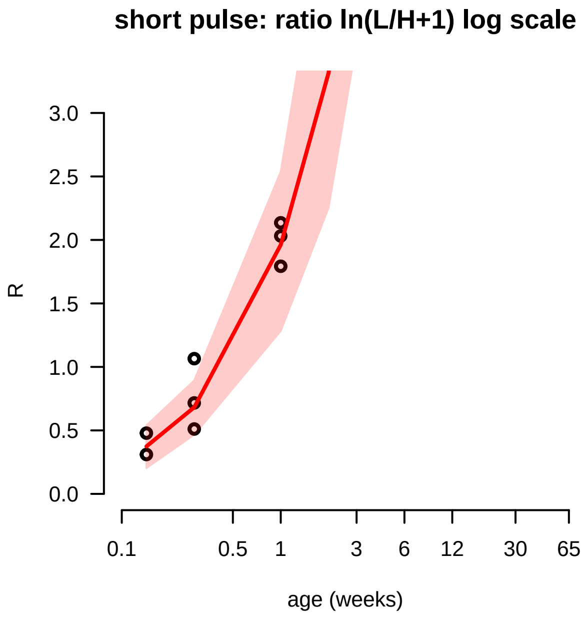

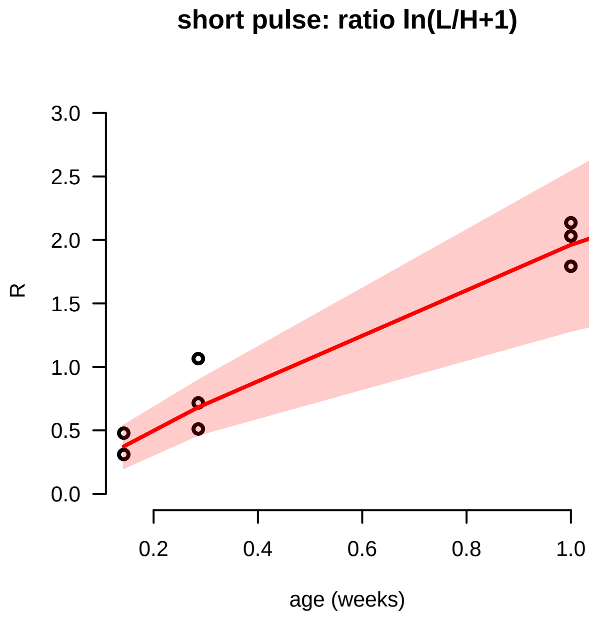

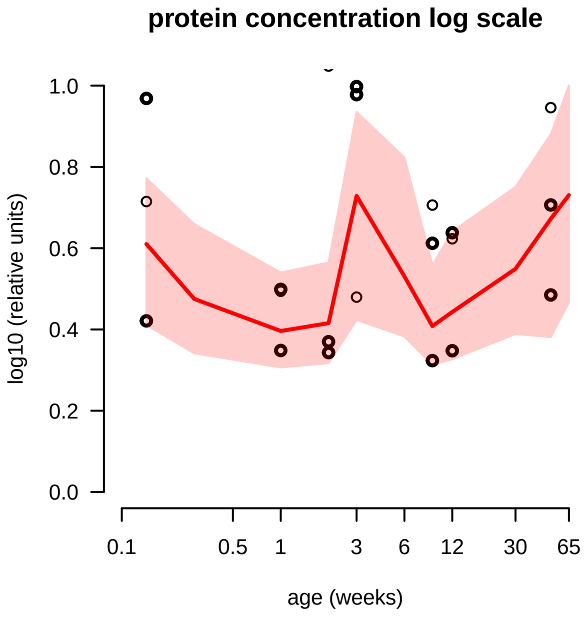

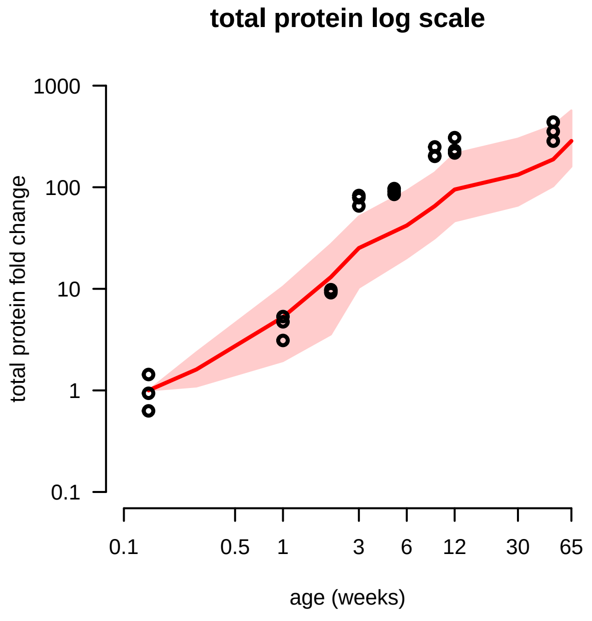

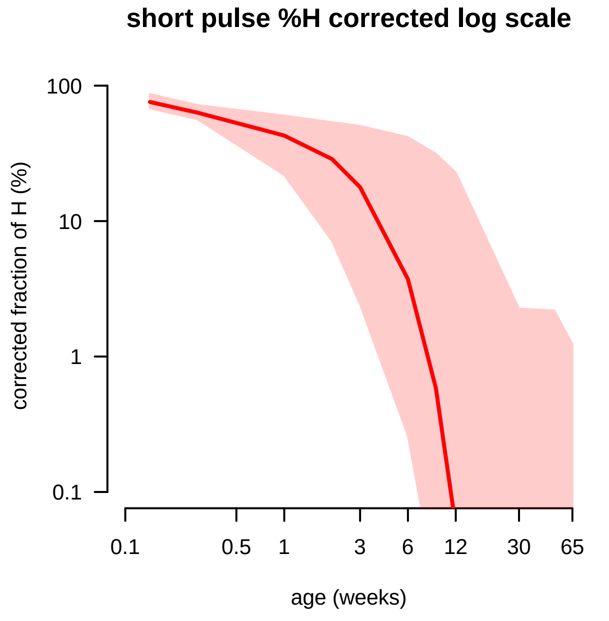

Q3U0P5

>sp|Q3U0P5|ENTP6_MOUSE

Ectonucleoside triphosphate diphosphohydrolase 6

GN=Entpd6

Med H1/2: 10 days

Q5 H1/2: 3.2 days

Q95 H1/2: 77.8 days

reliable model fit: yes

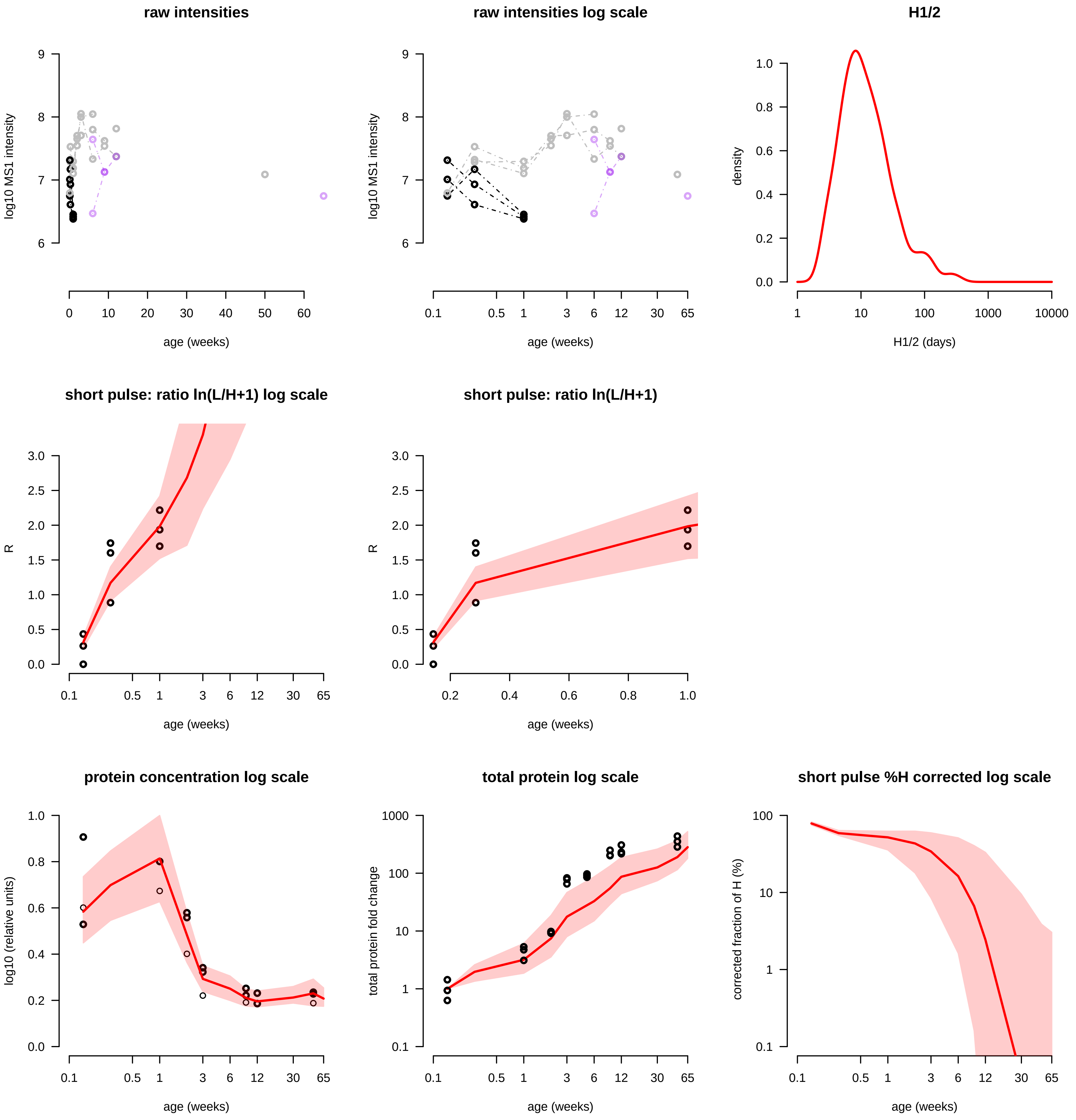

Q07133

>sp|Q07133|H1T_MOUSE

Histone H1t

GN=H1−6

Med H1/2: 5.2 days

Q5   H1/2: 2.9 days

Q95 H1/2: 18 days

reliable model fit: no

raw intensities

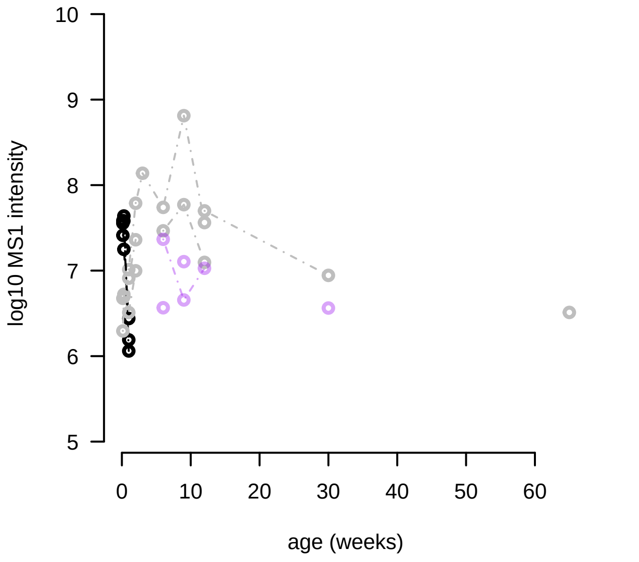

raw intensities log scale

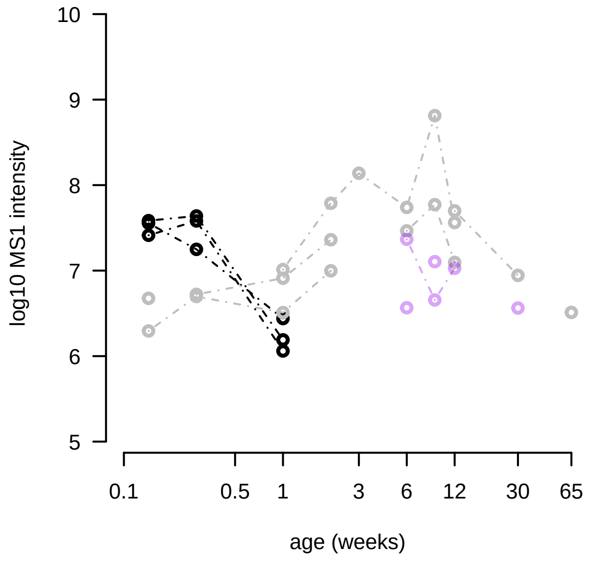

H1/2

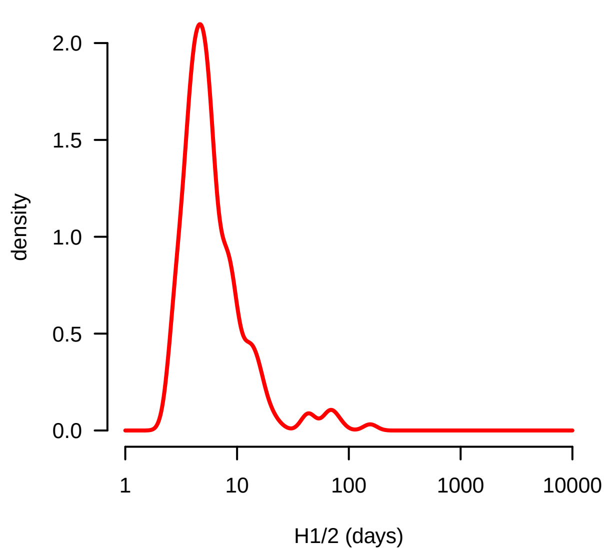

short pulse: ratio ln(L/H+1) log scale

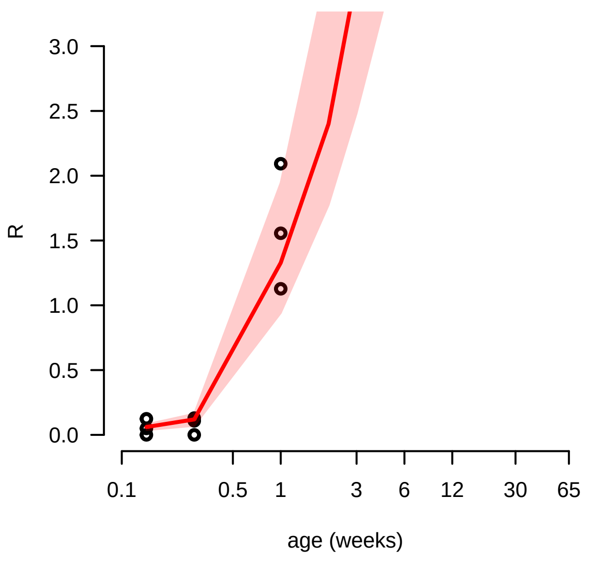

short pulse: ratio ln(L/H+1)

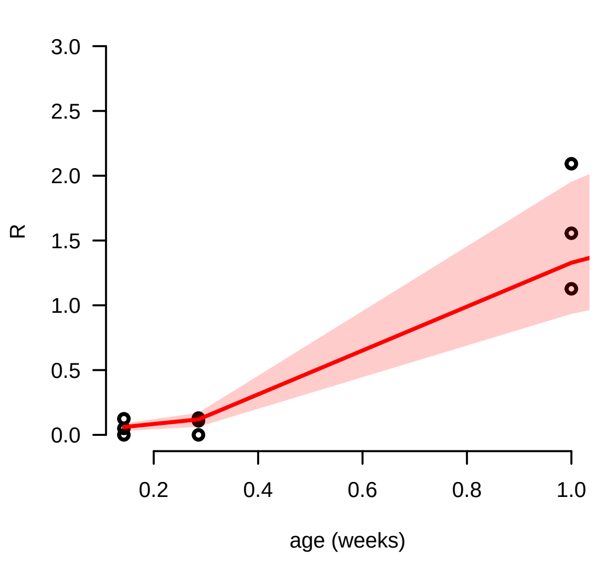

protein concentration log scale

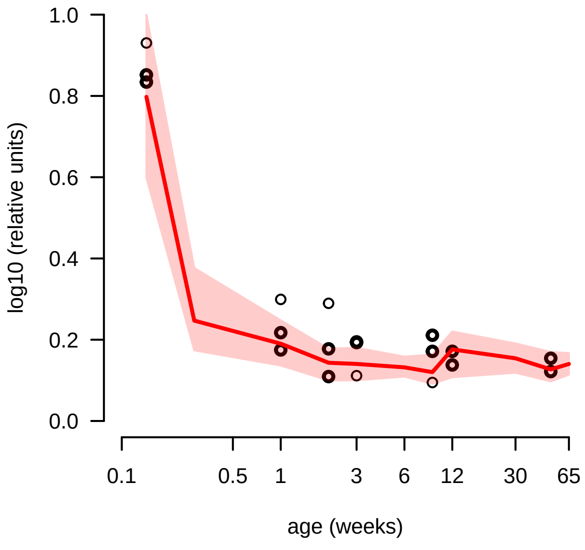

total protein log scale

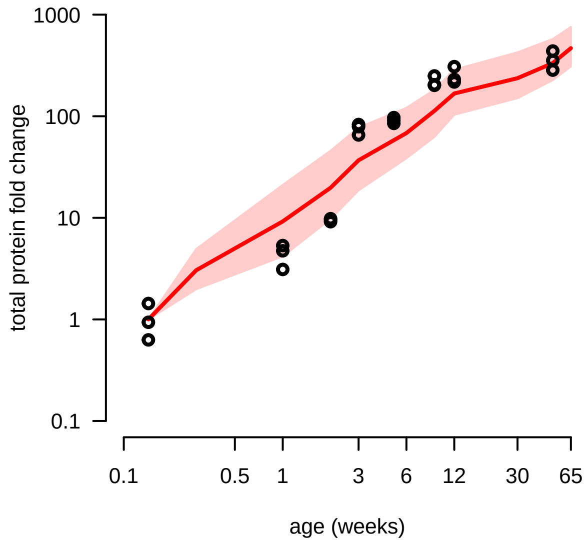

short pulse %H corrected log scale

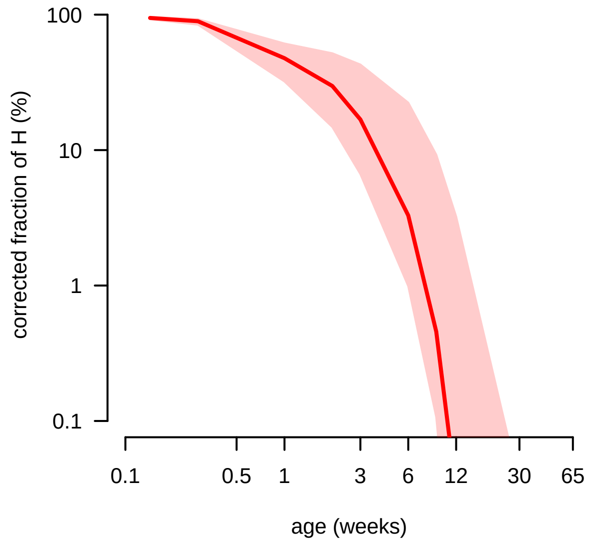

Q8BMG1

>sp|Q8BMG1|ATRIP_MOUSE

ATR−interacting protein

GN=Atrip

Med H1/2: 20.7 days

Q5 H1/2: 5.4 days

Q95 H1/2: 173 days

reliable model fit: yes

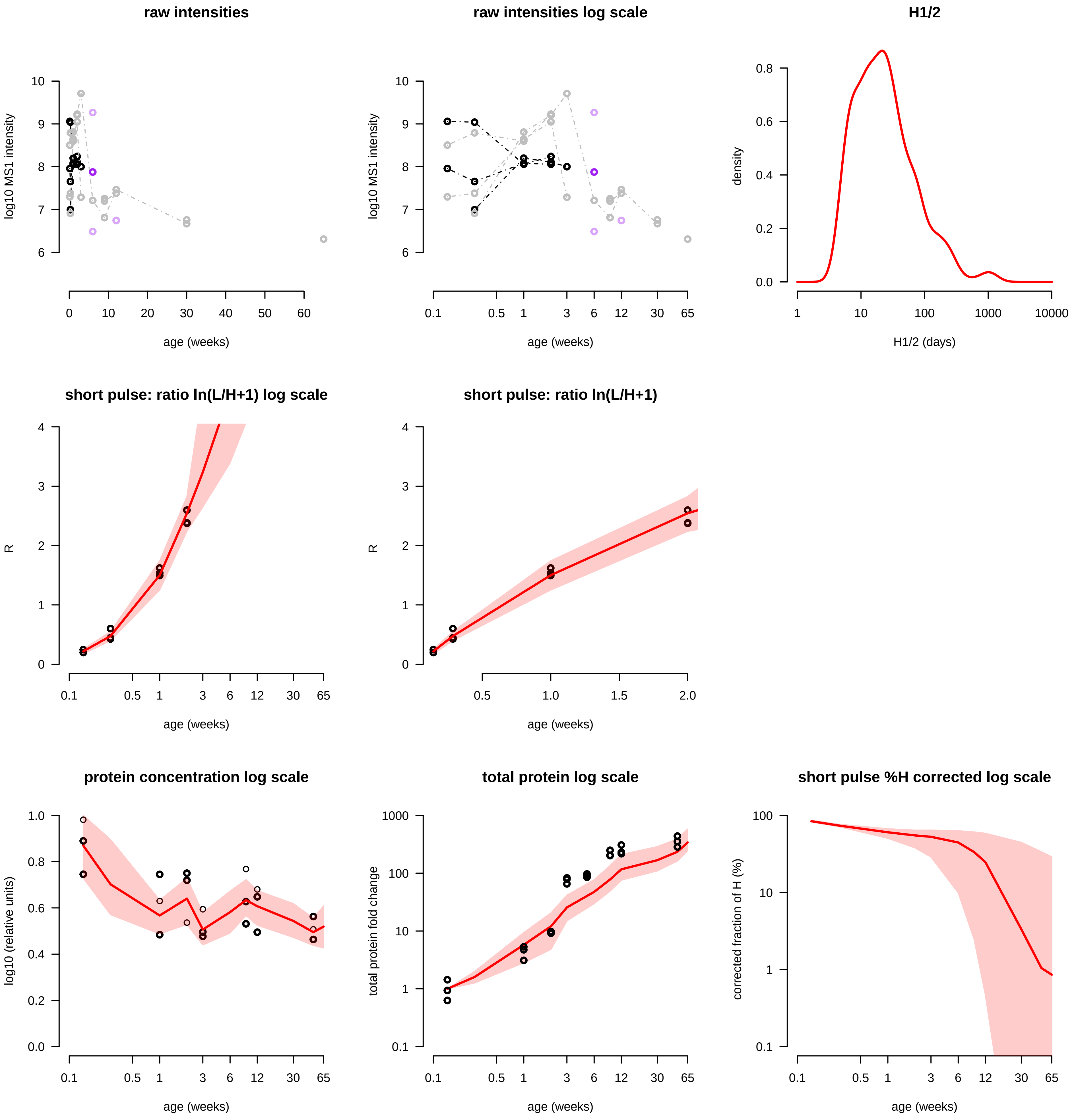

Q8K2G4;Q8K2G4−2

>sp|Q8K2G4|BBS7_MOUSE

Bardet−Biedl syndrome 7 protein homolog

GN=Bbs7

Med H1/2: 7.6 days

Q5 H1/2: 5.1 days

Q95 H1/2: 15 days

reliable model fit: yes

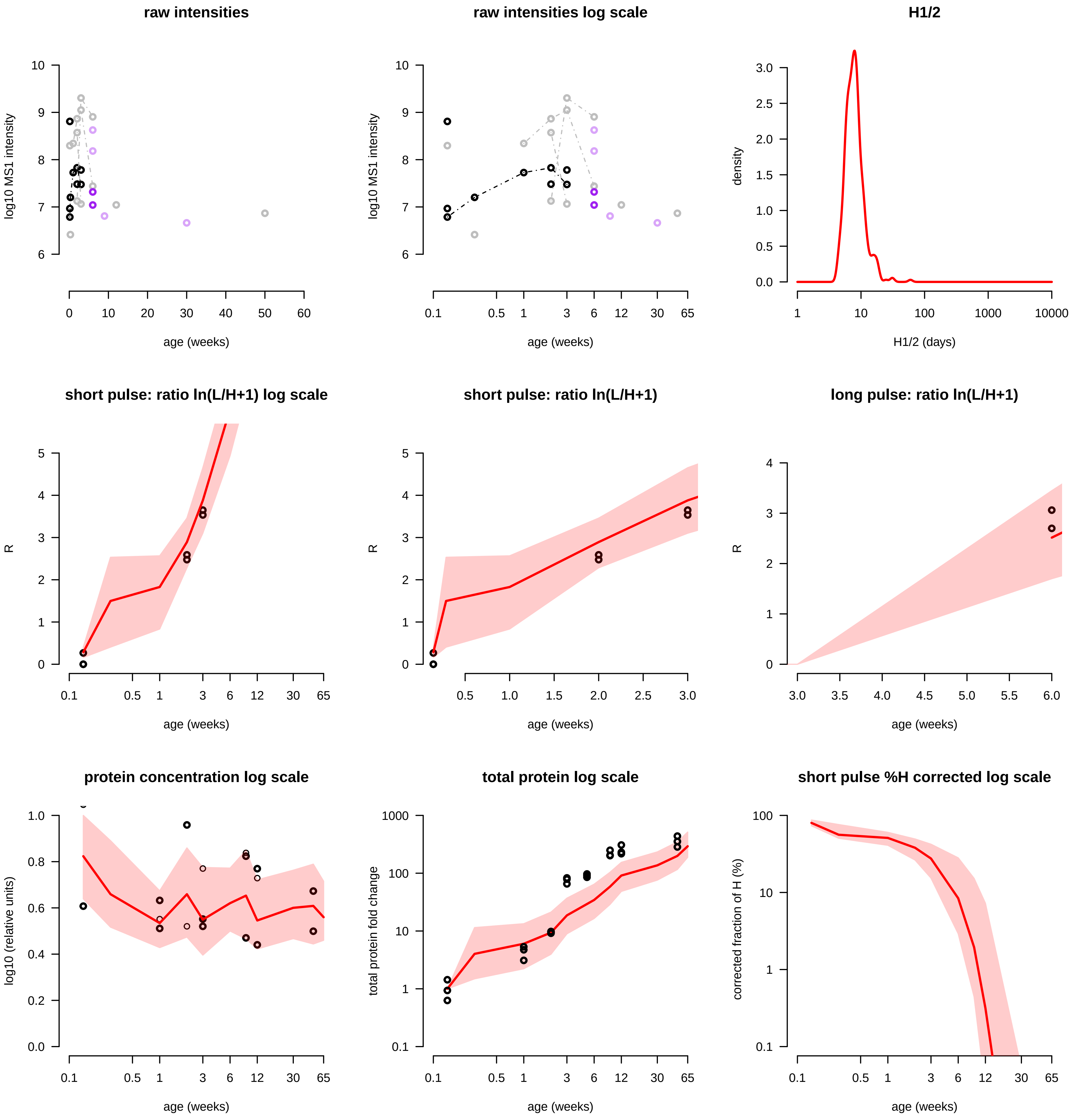

Q99M74

>sp|Q99M74|KRT82_MOUSE

Keratin, type II cuticular Hb2

GN=Krt82

Med H1/2: 10.3 days

Q5 H1/2: 5.6 days

Q95 H1/2: 46.2 days

reliable model fit: yes

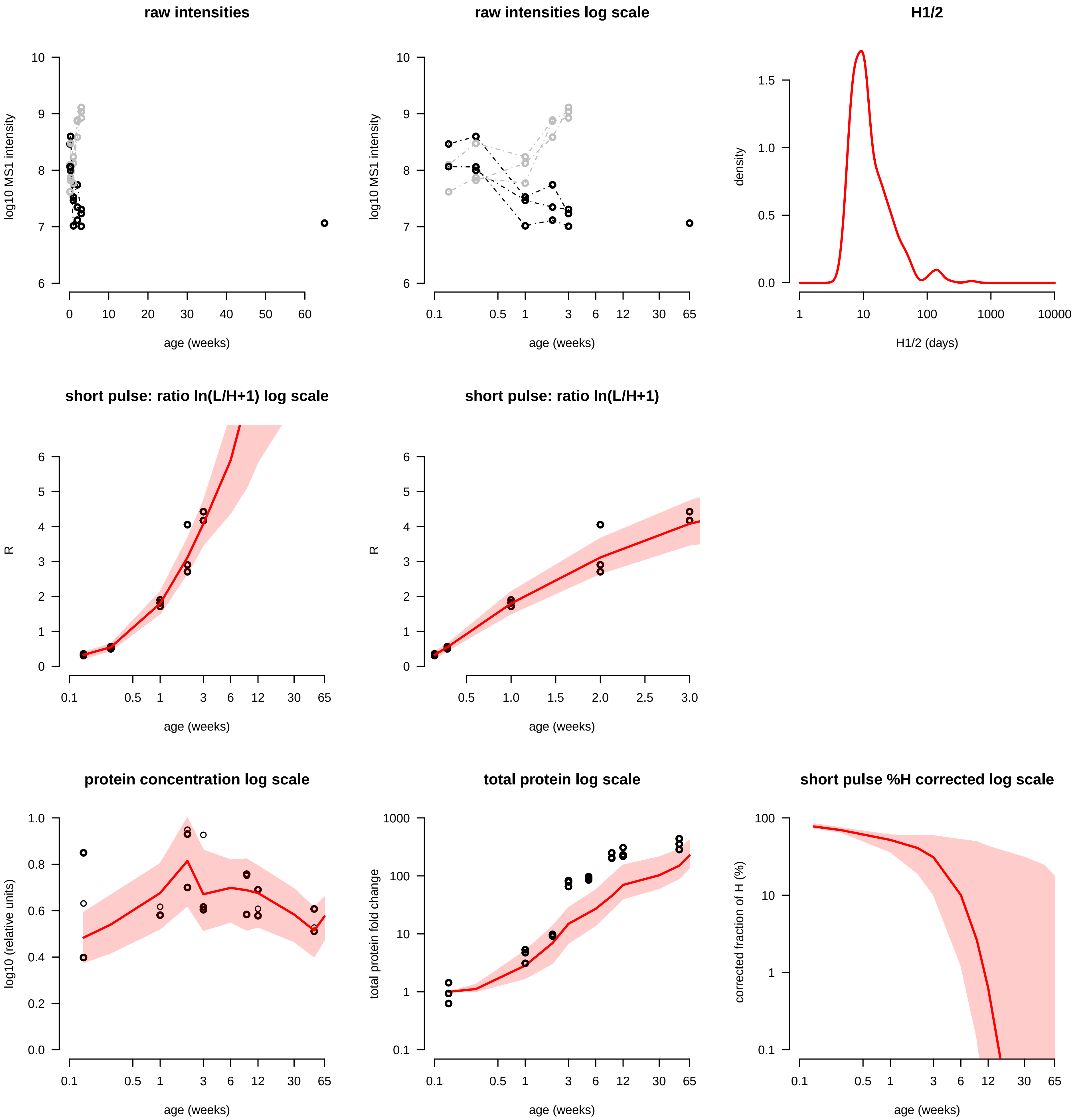

P01671;P01670;P01673

>sp|P01671|KV3AJ_MOUSE

Ig kappa chain V−III region PC 7175

Med H1/2: 15.3 days

Q5 H1/2: 11.4 days

Q95 H1/2: 31.5 days

reliable model fit: yes

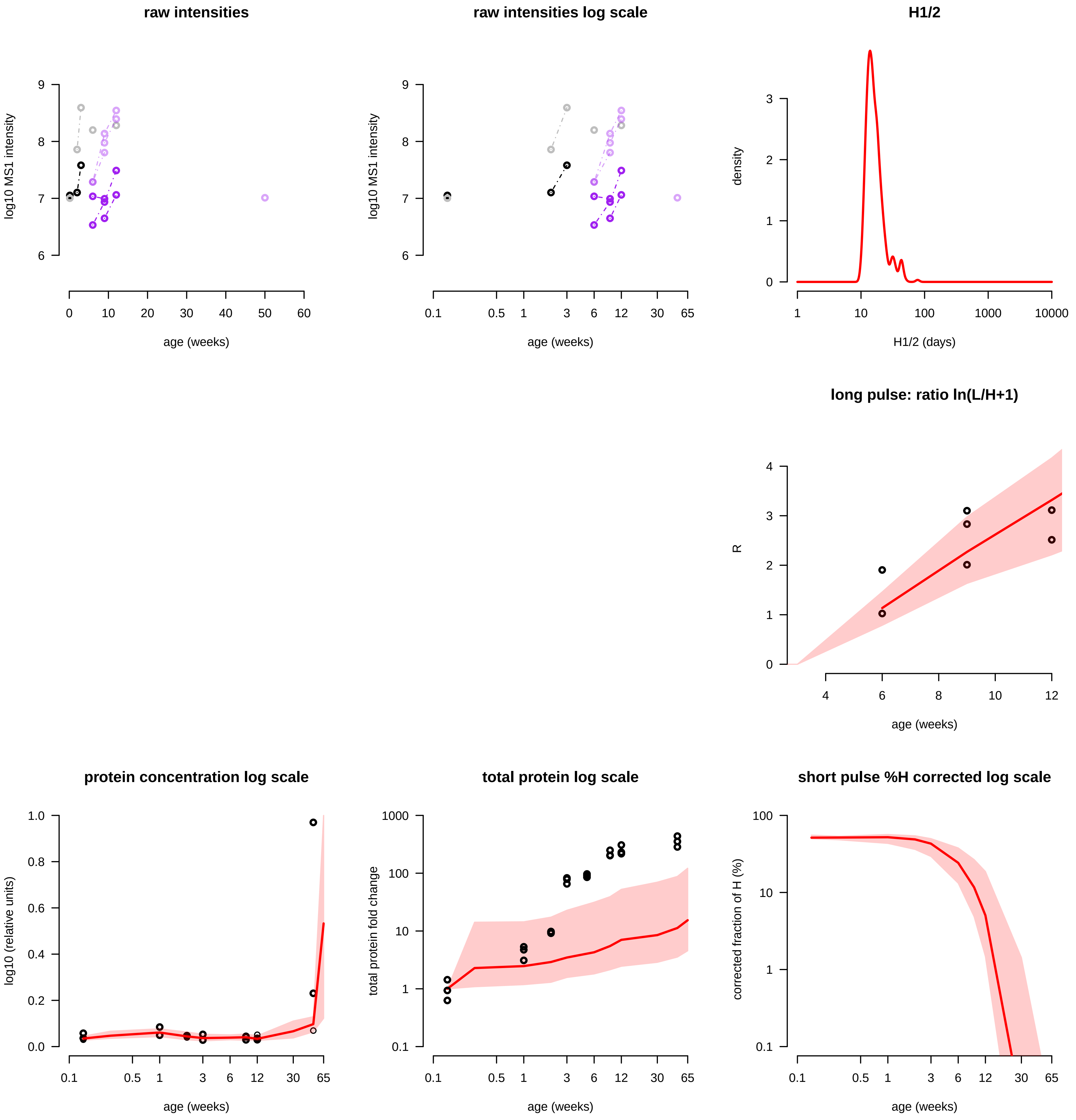

A2AGX3

>sp|A2AGX3|PRD11_MOUSE

PR domain−containing protein 11

GN=Prdm11

Med H1/2: 5.9 days

Q5 H1/2: 2.6 days

Q95 H1/2: 51.5 days

reliable model fit: yes

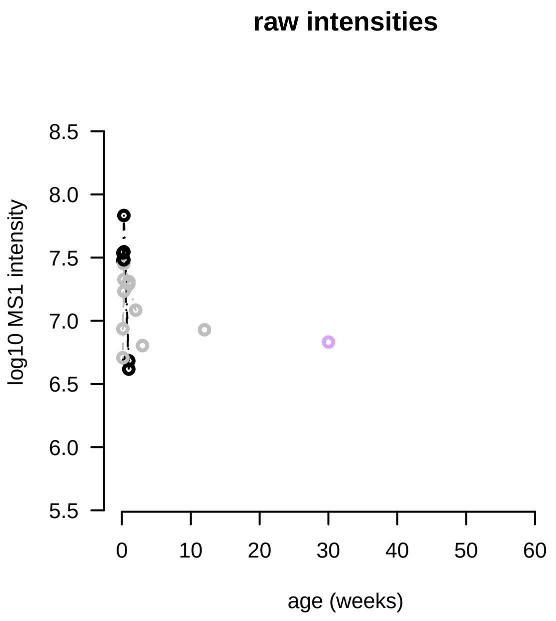

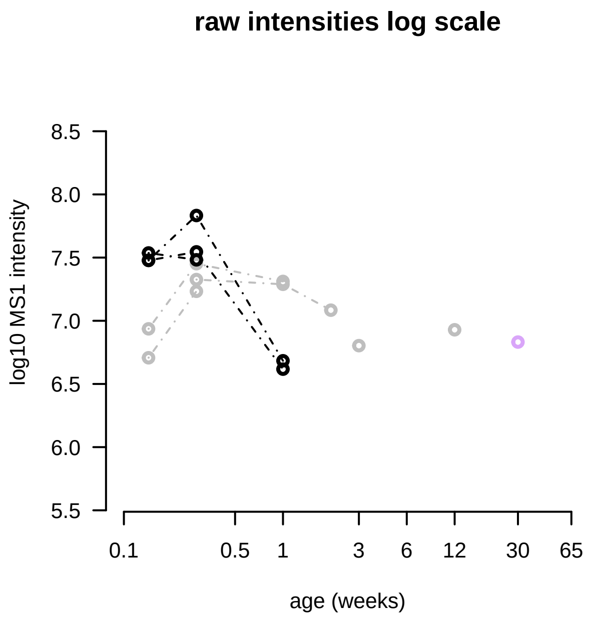

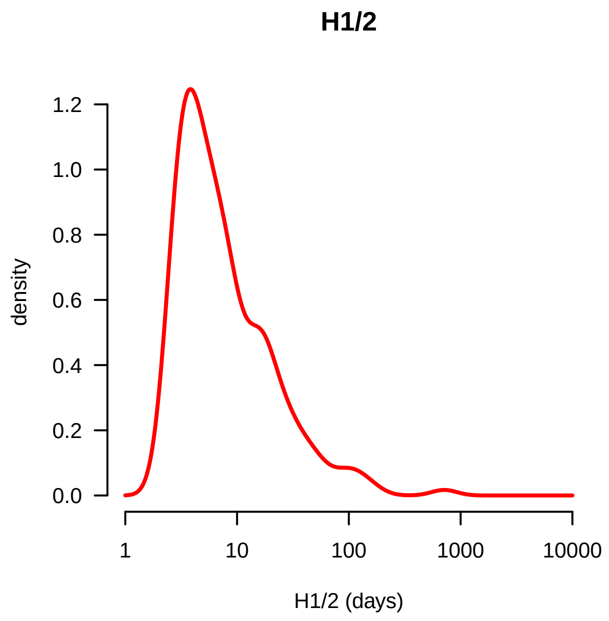

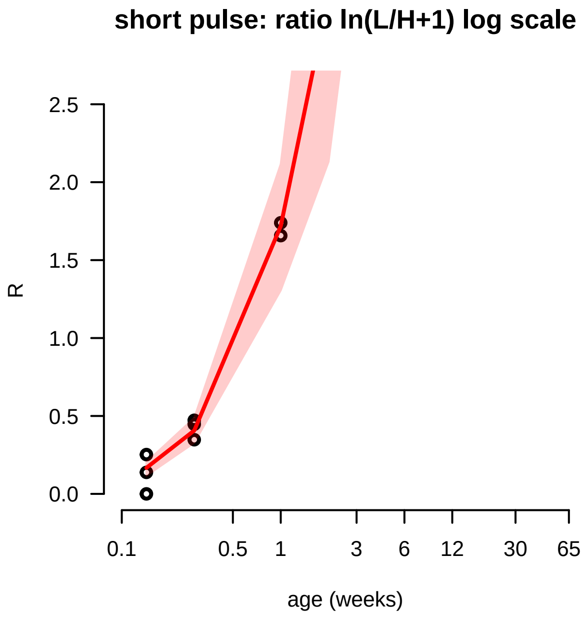

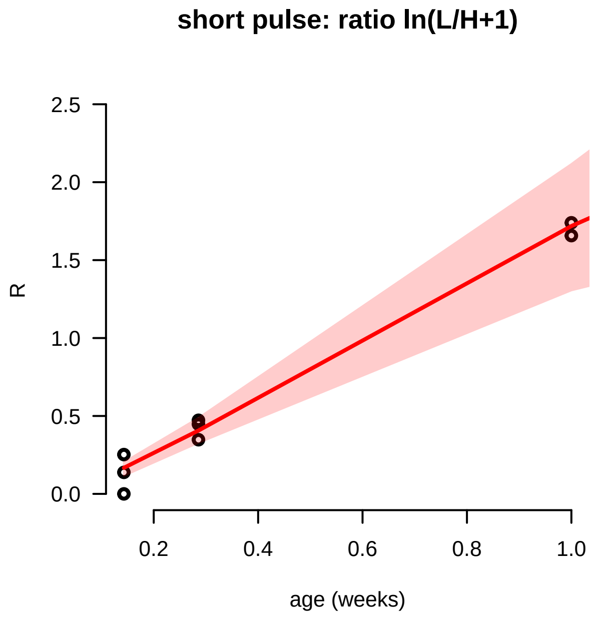

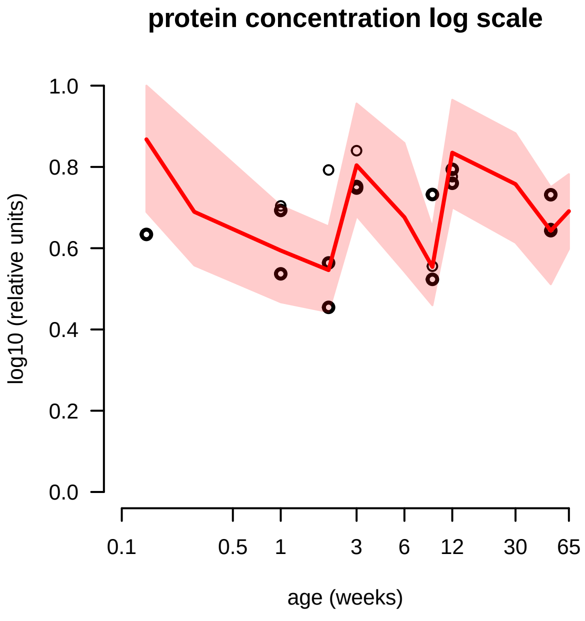

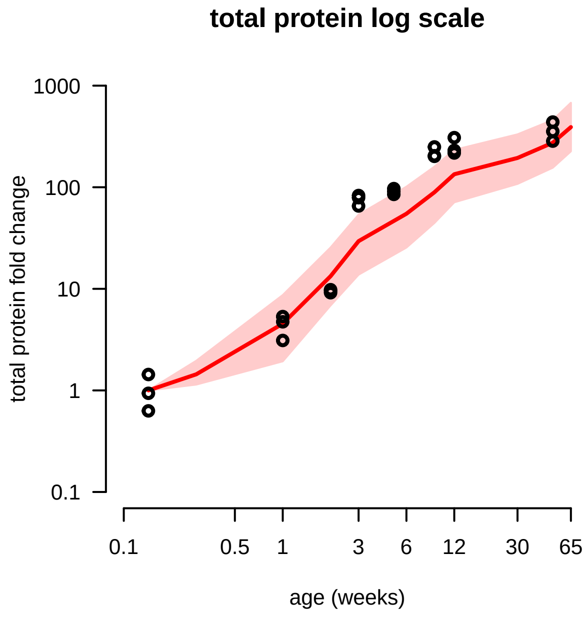

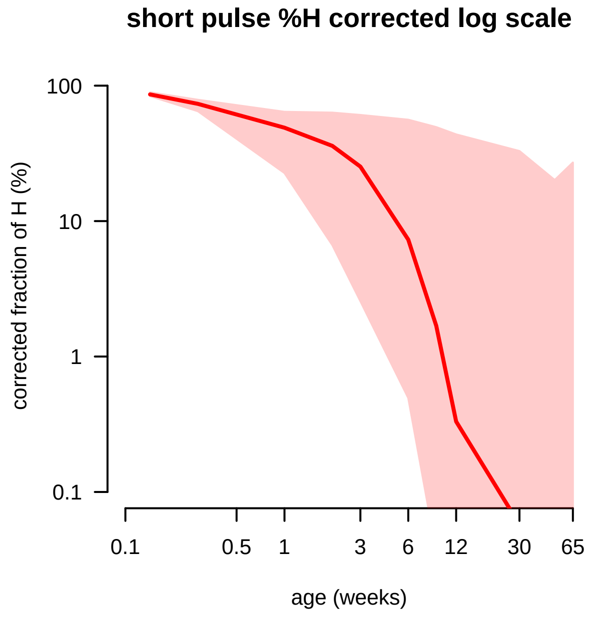

Q14AI0

>sp|Q14AI0|DCC1_MOUSE

Sister chromatid cohesion protein DCC1

GN=DSCC1

Med H1/2: 13.4 days

Q5 H1/2: 3.9 days

Q95 H1/2: 131.4 days

reliable model fit: yes

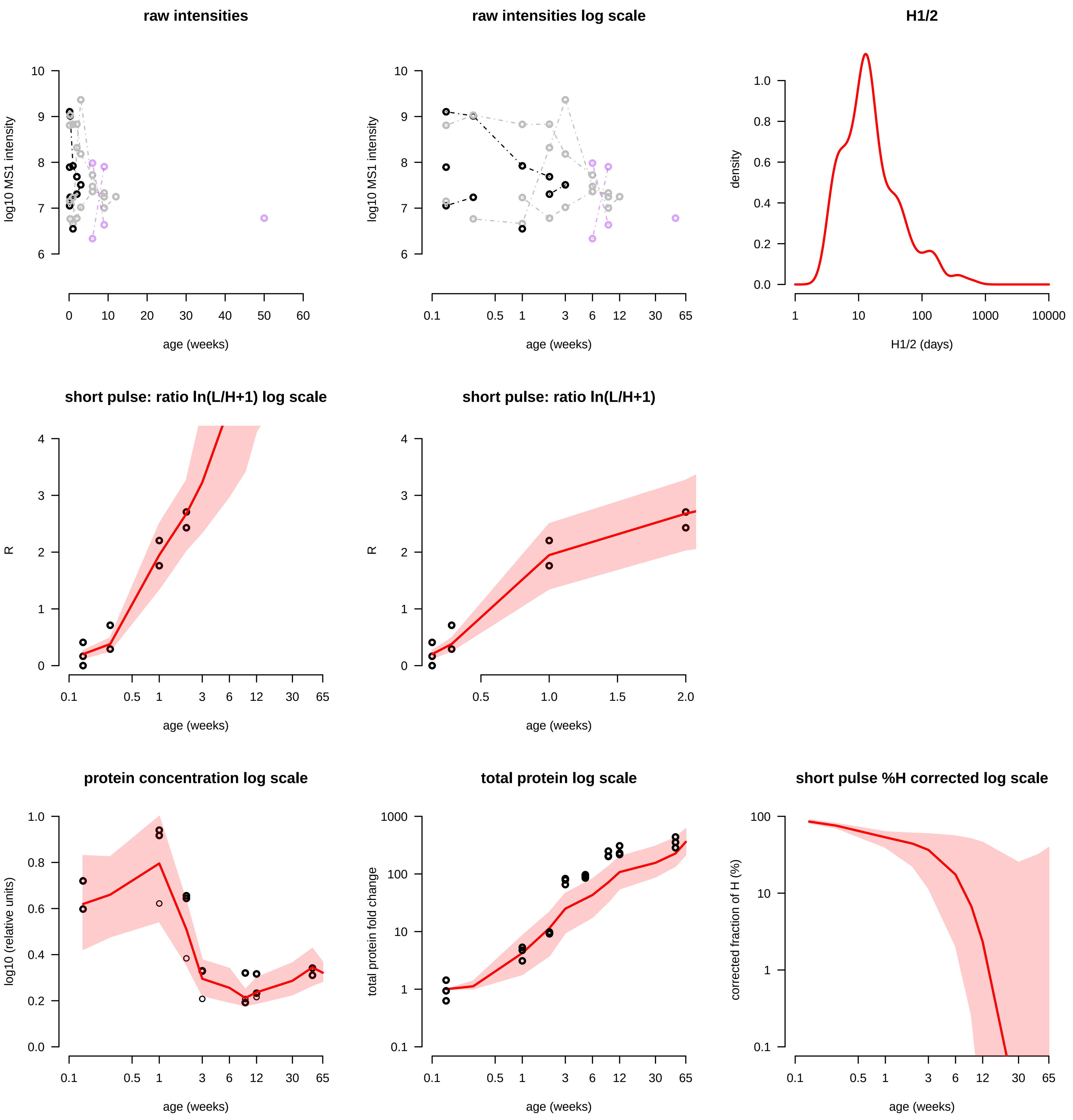

O88693

>sp|O88693|CEGT_MOUSE

Ceramide glucosyltransferase

GN=Ugcg

Med H1/2: 13.4 days

Q5 H1/2: 9.6 days

Q95 H1/2: 18.6 days

reliable model fit: yes

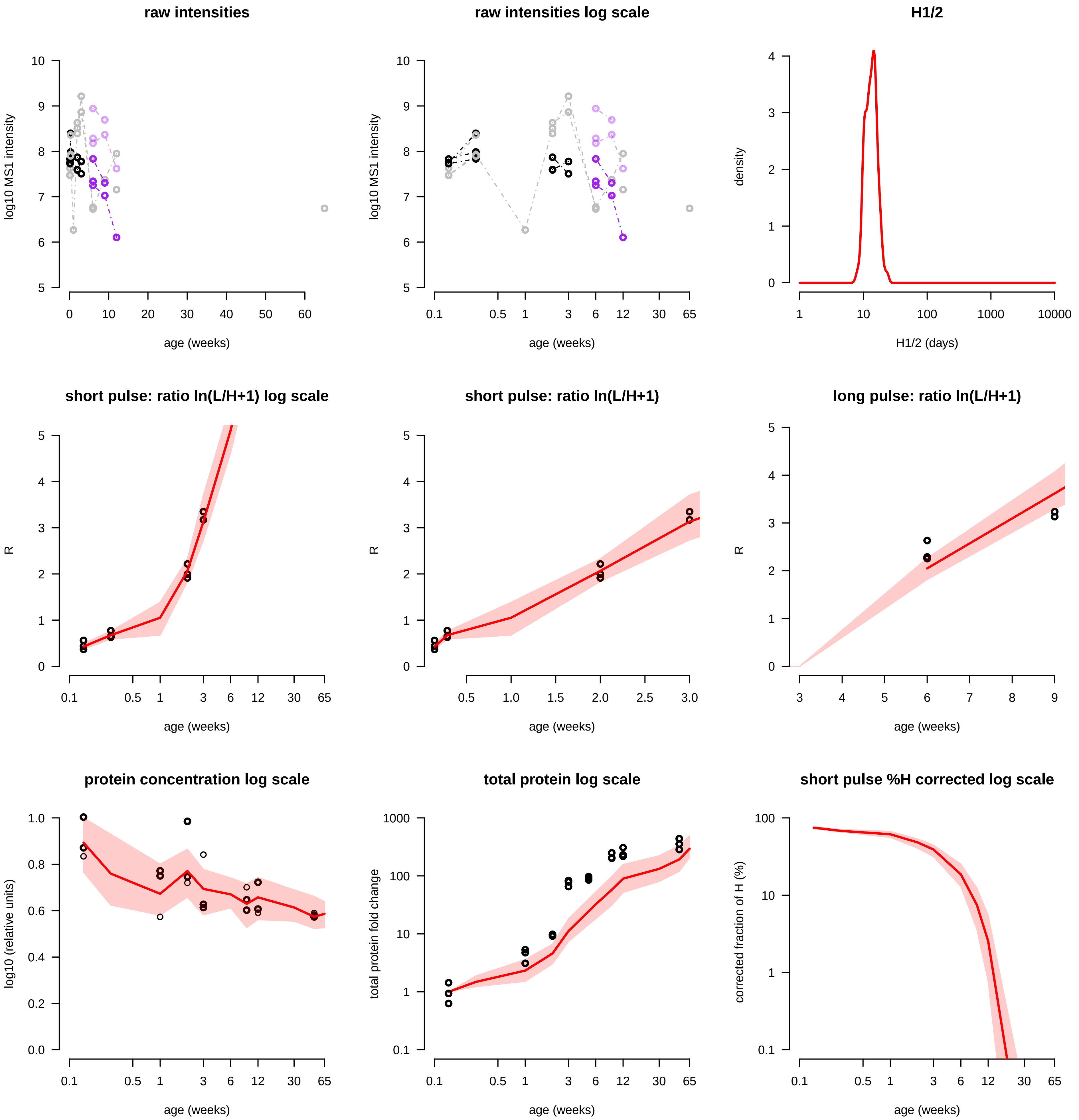

Q3V132

>sp|Q3V132|ADT4_MOUSE

ADP/ATP translocase 4

GN=Slc25a31

Med H1/2: 12.9 days

Q5 H1/2: 6.5 days

Q95 H1/2: 109.7 days

reliable model fit: yes

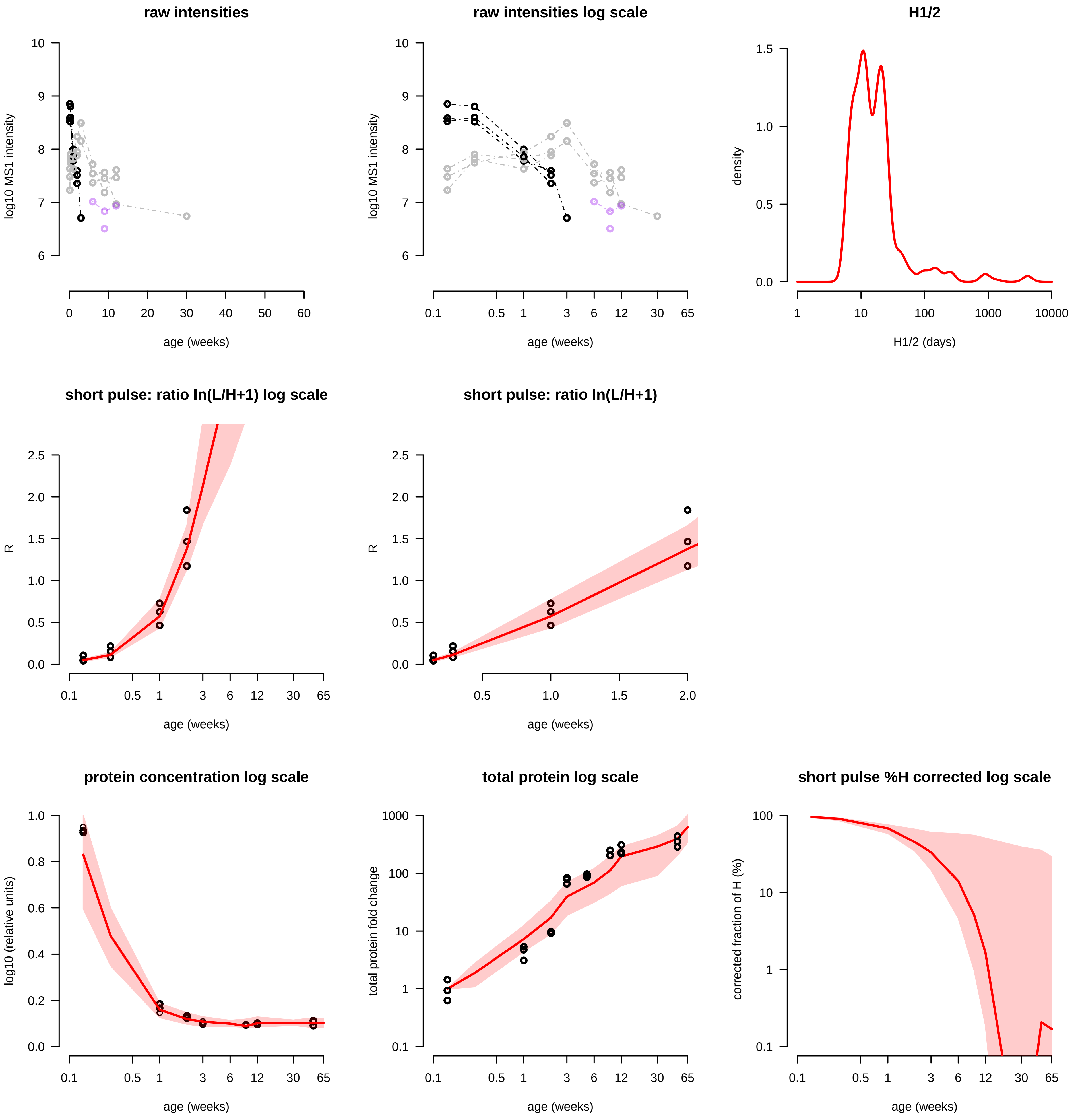

Q3UNX5;Q3UNX5−2

>sp|Q3UNX5|ACSM3_MOUSE

Acyl−coenzyme A synthetase ACSM3, mitochondrial

GN=Acsm3

Med H1/2: 11.9 days

Q5 H1/2: 3.3 days

Q95 H1/2: 125.2 days

reliable model fit: no

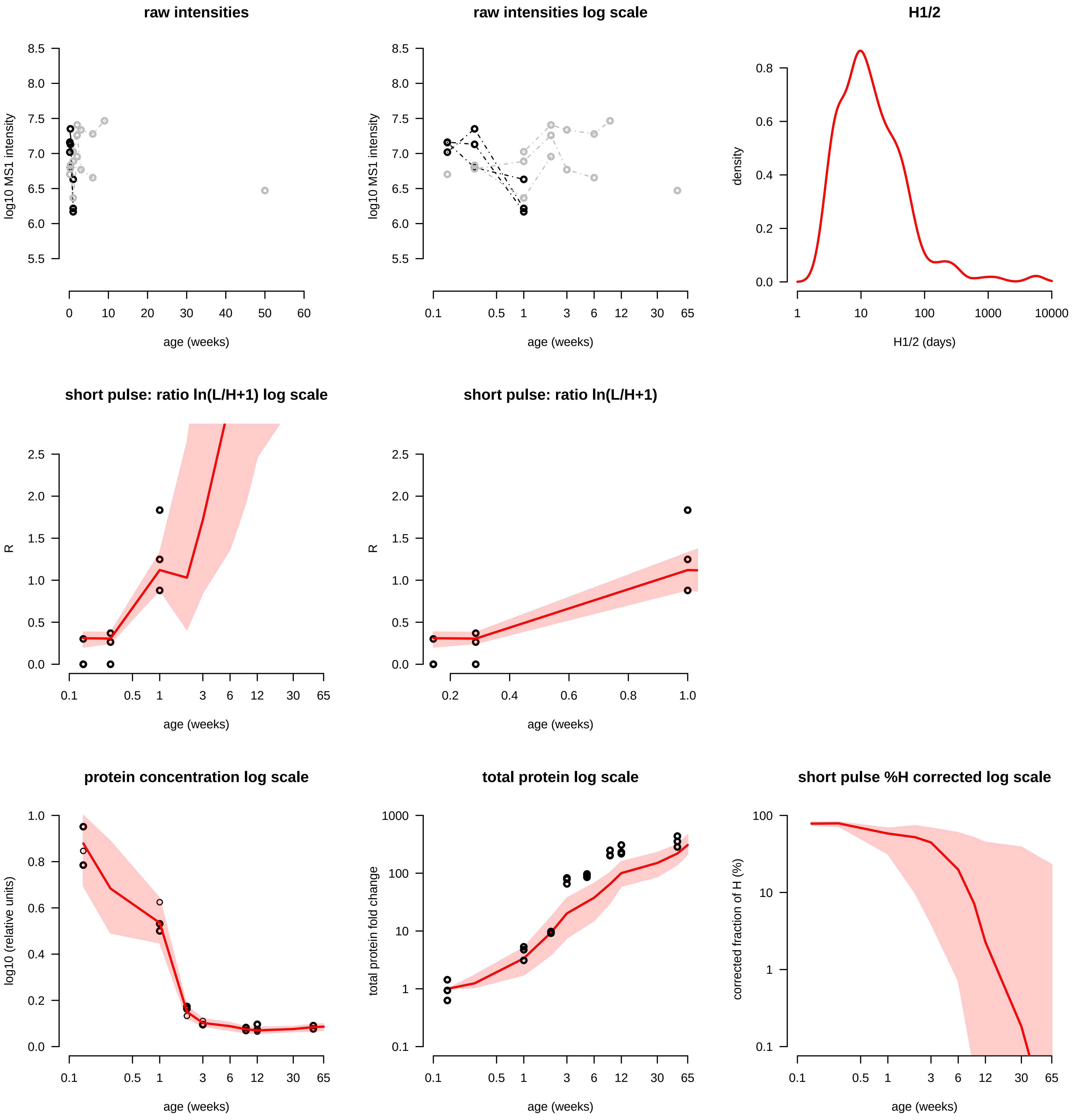

Q9JIL4

>sp|Q9JIL4|NHRF3_MOUSE

Na(+)/H(+) exchange regulatory cofactor NHE−RF3

GN=Pdzk1

Med H1/2: 3.4 days

Q5 H1/2: 2.5 days

Q95 H1/2: 9.4 days

reliable model fit: yes

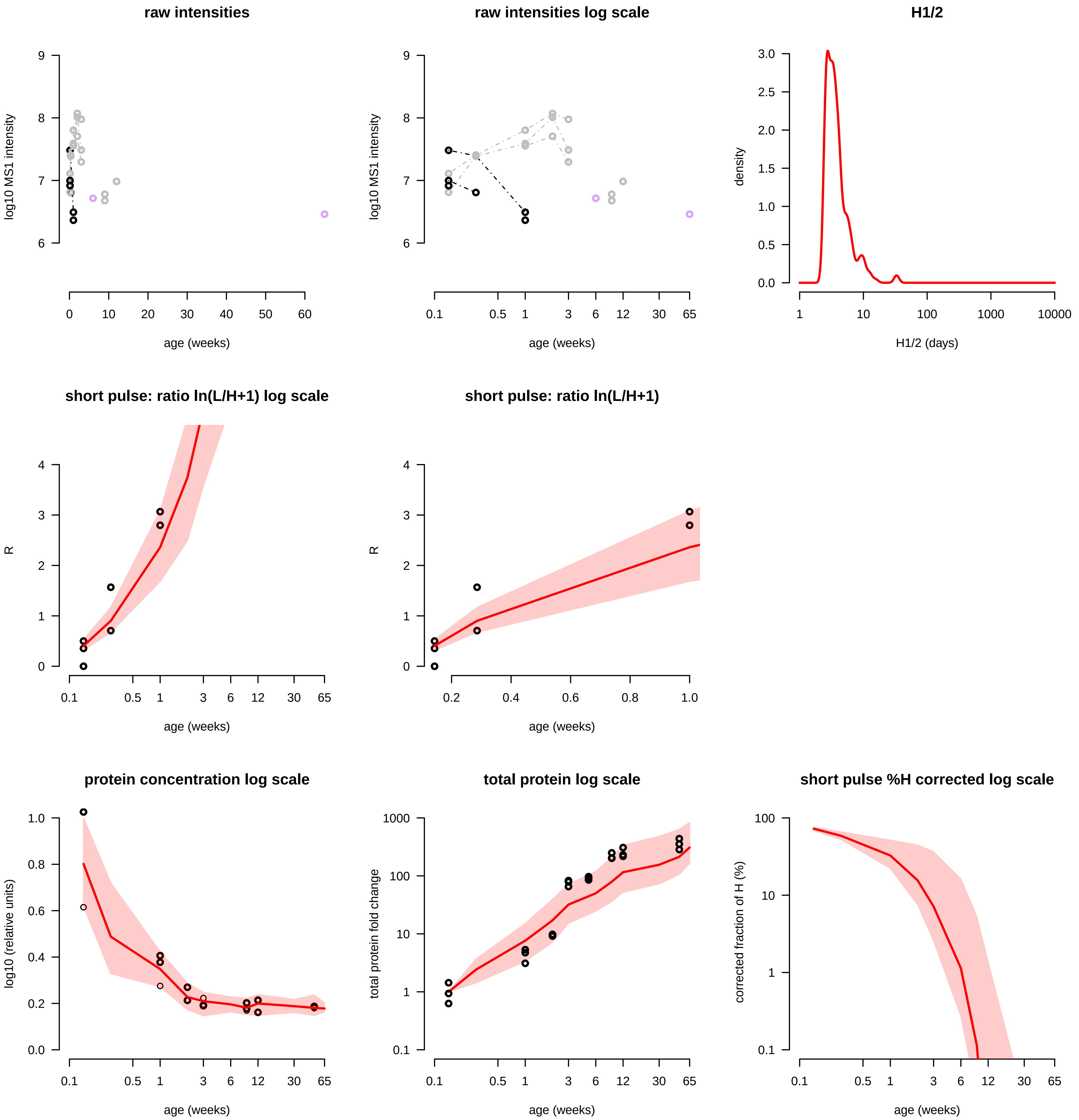

Q07139;Q07139−2

>sp|Q07139|ECT2_MOUSE

Protein ECT2

GN=Ect2

Med H1/2: 24.1 days

Q5 H1/2: 16.2 days

Q95 H1/2: 39.9 days

reliable model fit: yes

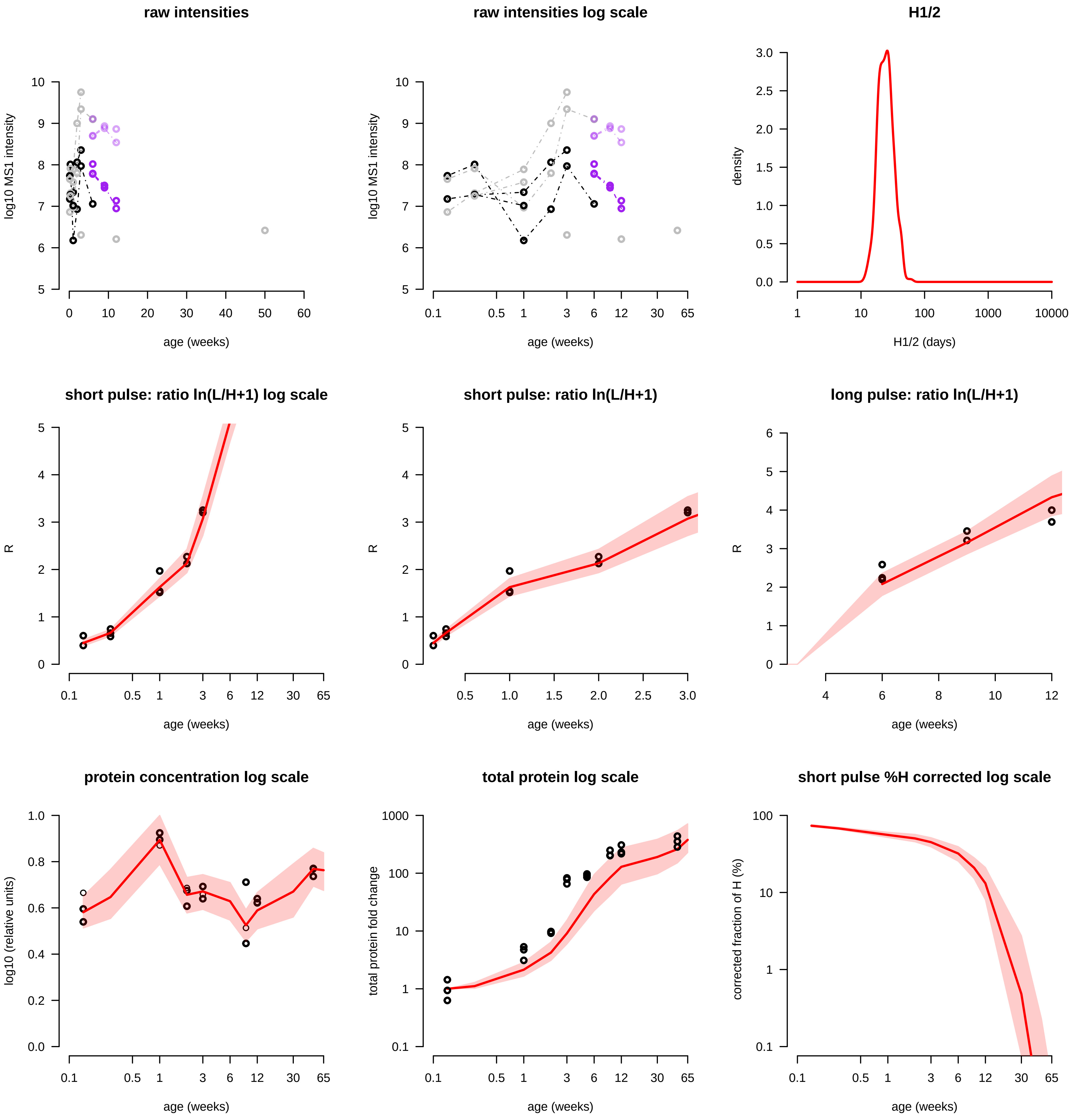

Q60899

>sp|Q60899|ELAV2_MOUSE

ELAV−like protein 2

GN=Elavl2

Med H1/2: 7.5 days

Q5 H1/2: 3 days

Q95 H1/2: 58.2 days

reliable model fit: yes

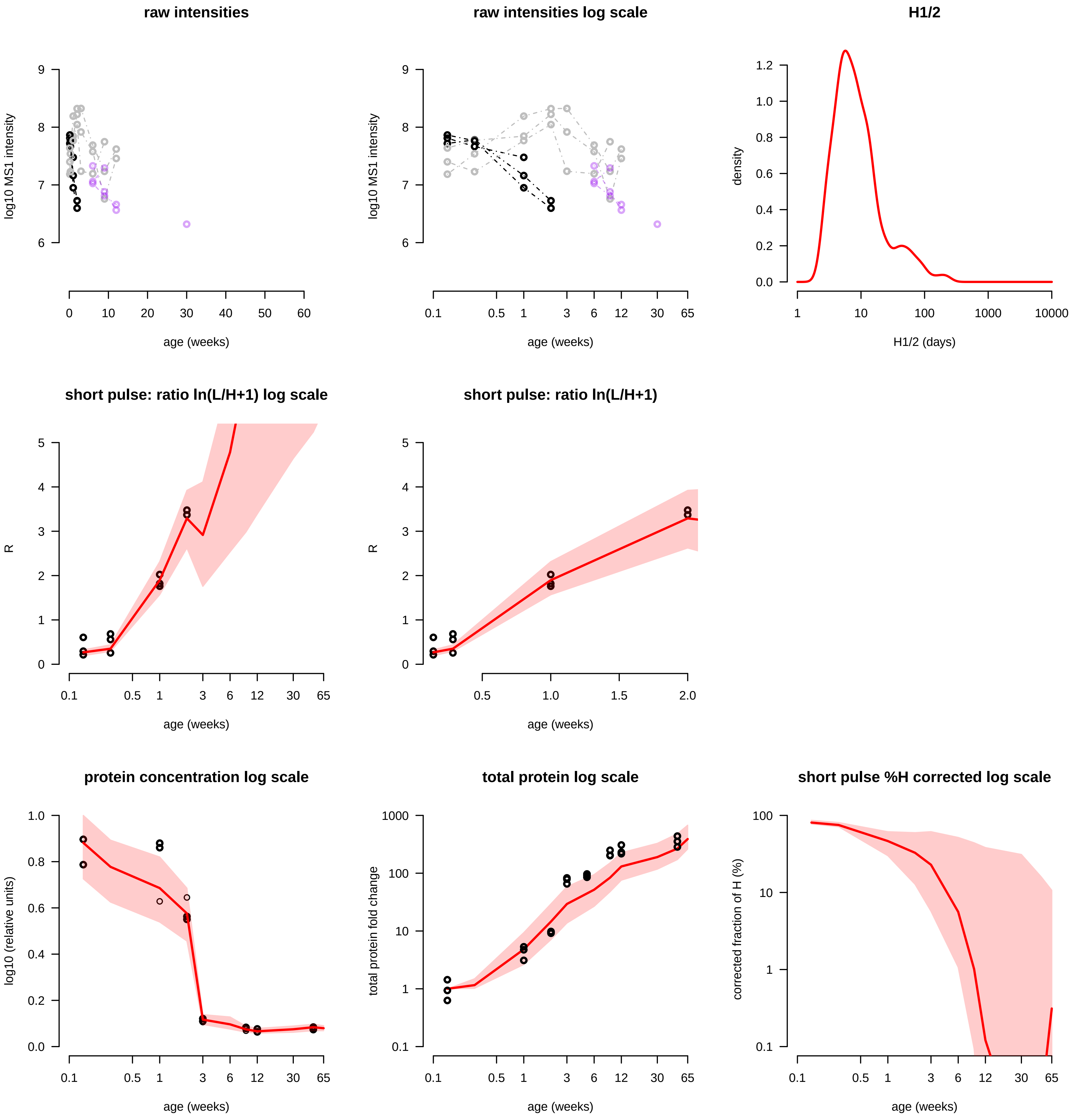

A2A9R3

>sp|A2A9R3|MAGB4_MOUSE

Melanoma−associated antigen B4

GN=Mageb4

Med H1/2: 3.8 days

Q5 H1/2: 2.6 days

Q95 H1/2: 10 days

reliable model fit: yes

raw intensities

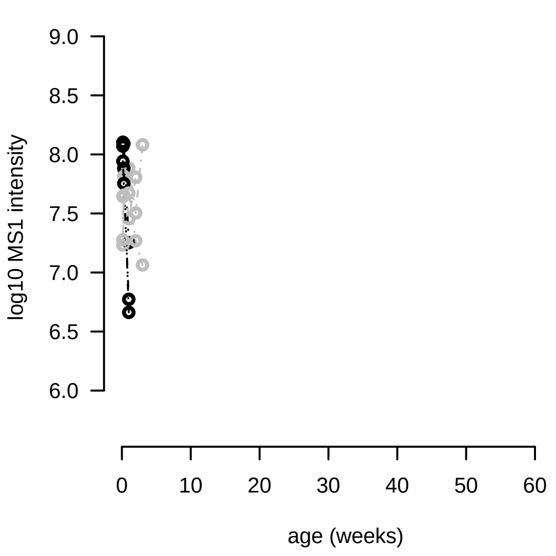

raw intensities log scale

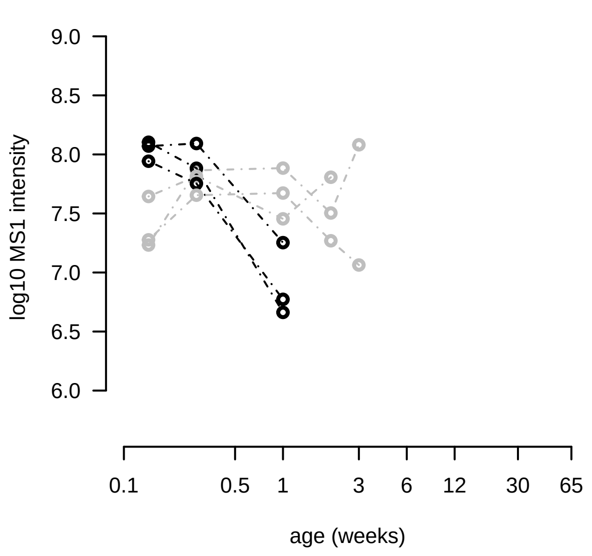

H1/2

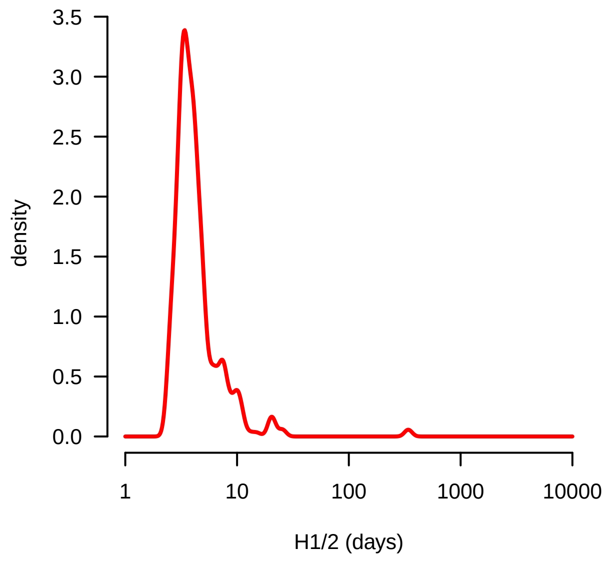

short pulse: ratio ln(L/H+1) log scale

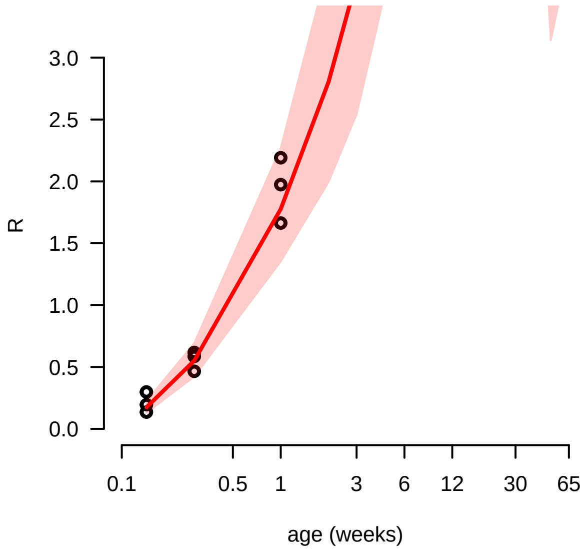

short pulse: ratio ln(L/H+1)

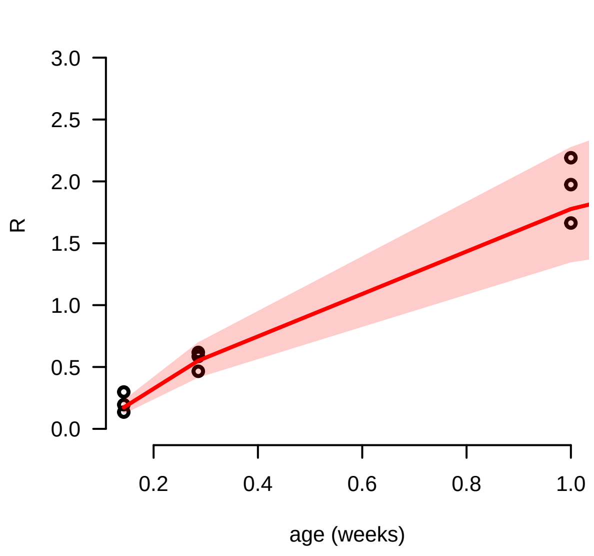

protein concentration log scale

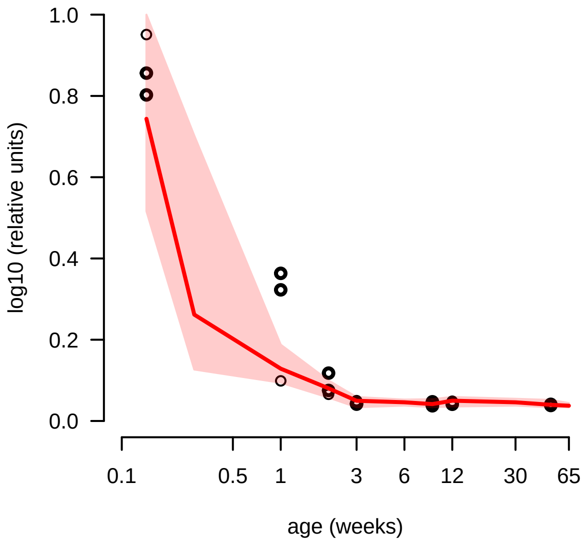

total protein log scale

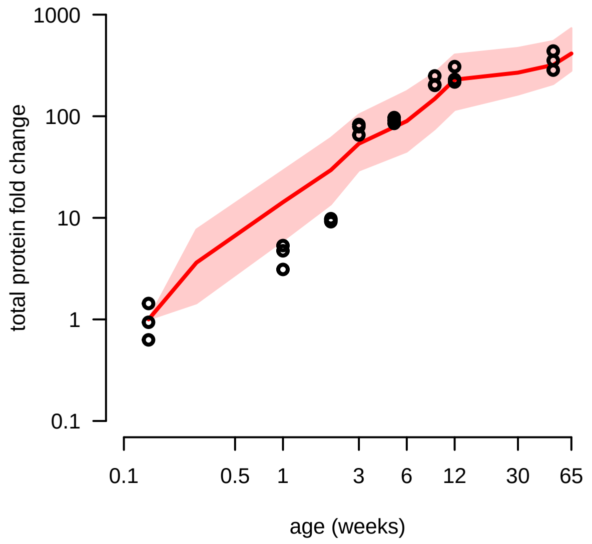

short pulse %H corrected log scale

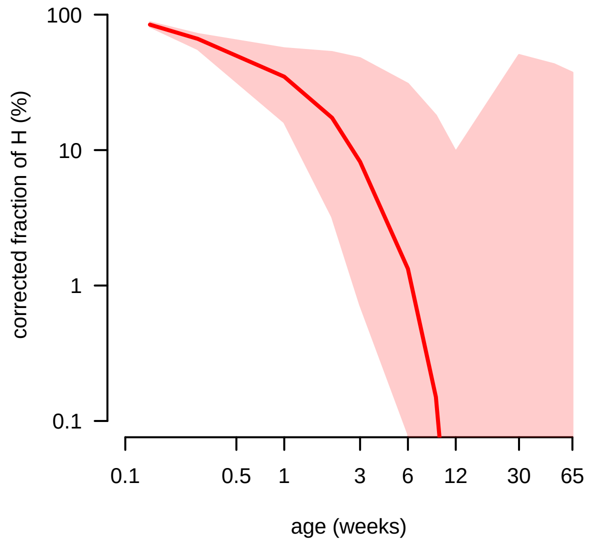

O55111

>sp|O55111|DSG2_MOUSE

Desmoglein−2

GN=Dsg2

Med H1/2: 22 days

Q5 H1/2: 14.7 days

Q95 H1/2: 52.5 days

reliable model fit: yes

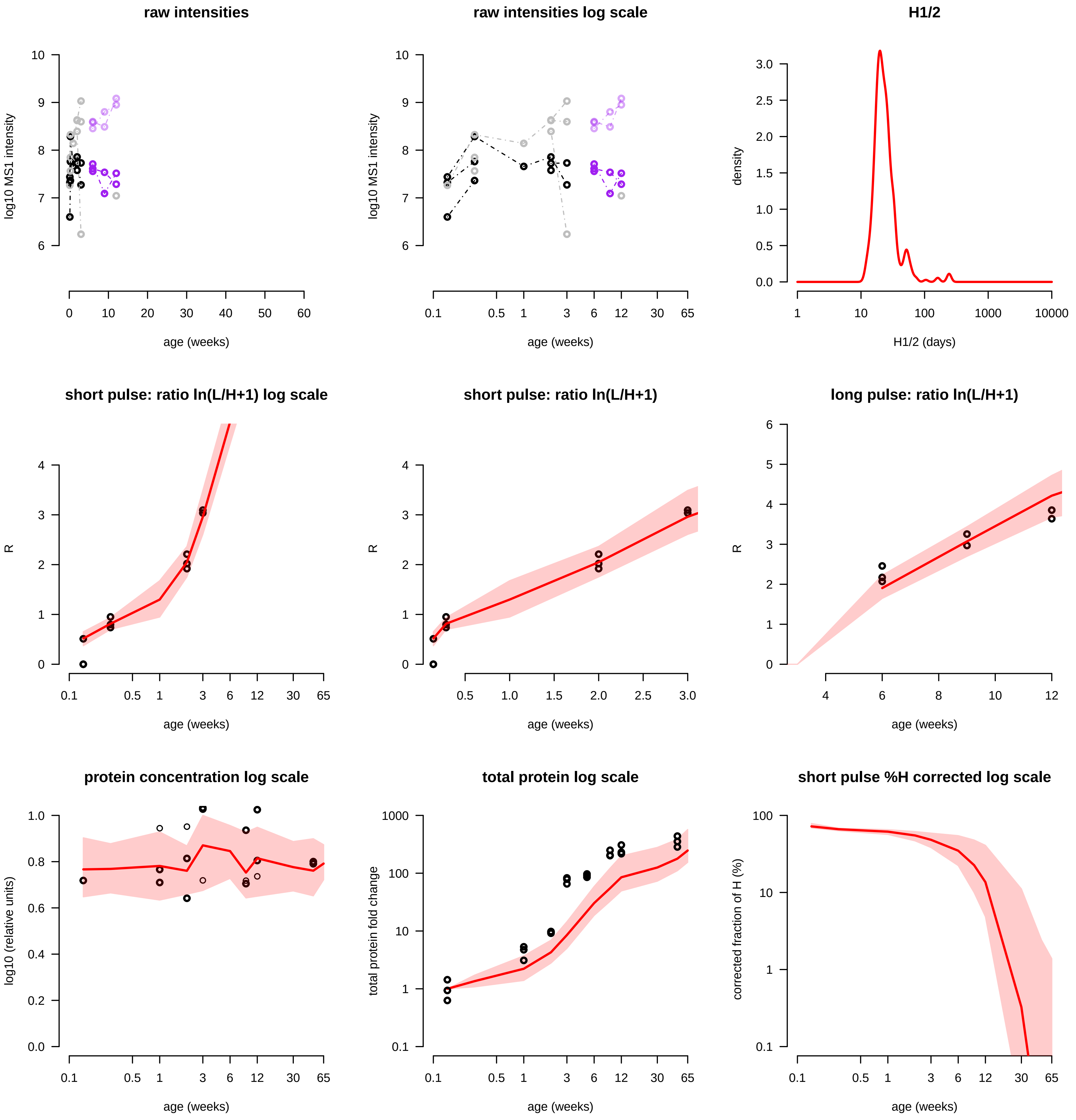

P59900

>sp|P59900|EMIL3_MOUSE

EMILIN−3

GN=Emilin3

Med H1/2: 4.1 days

Q5 H1/2: 2.5 days

Q95 H1/2: 20.5 days

reliable model fit: yes

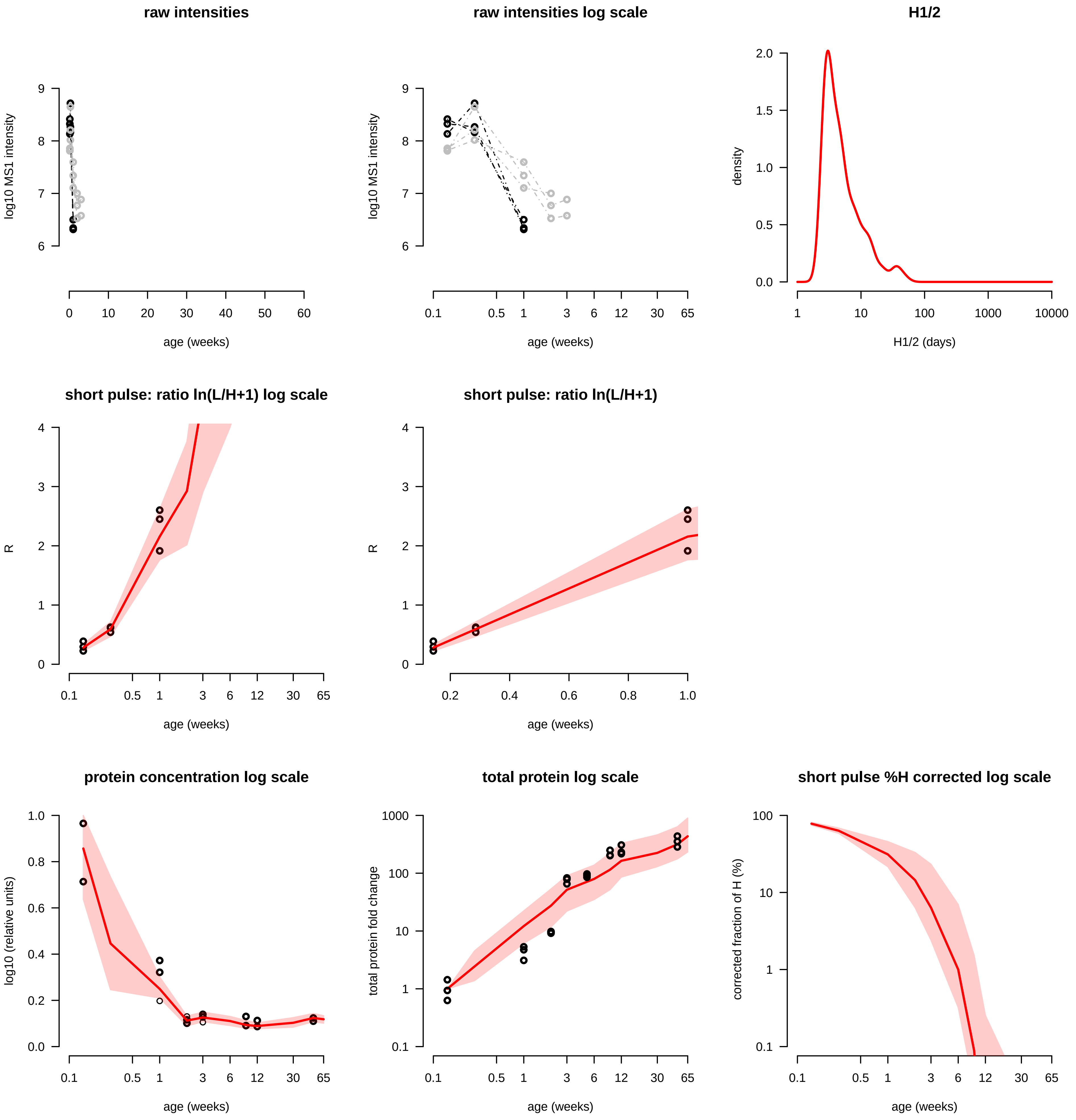

Q05A80

>sp|Q05A80|CAPR2_MOUSE

Caprin−2

GN=Caprin2

Med H1/2: 14.1 days

Q5 H1/2: 10.5 days

Q95 H1/2: 20.8 days

reliable model fit: no

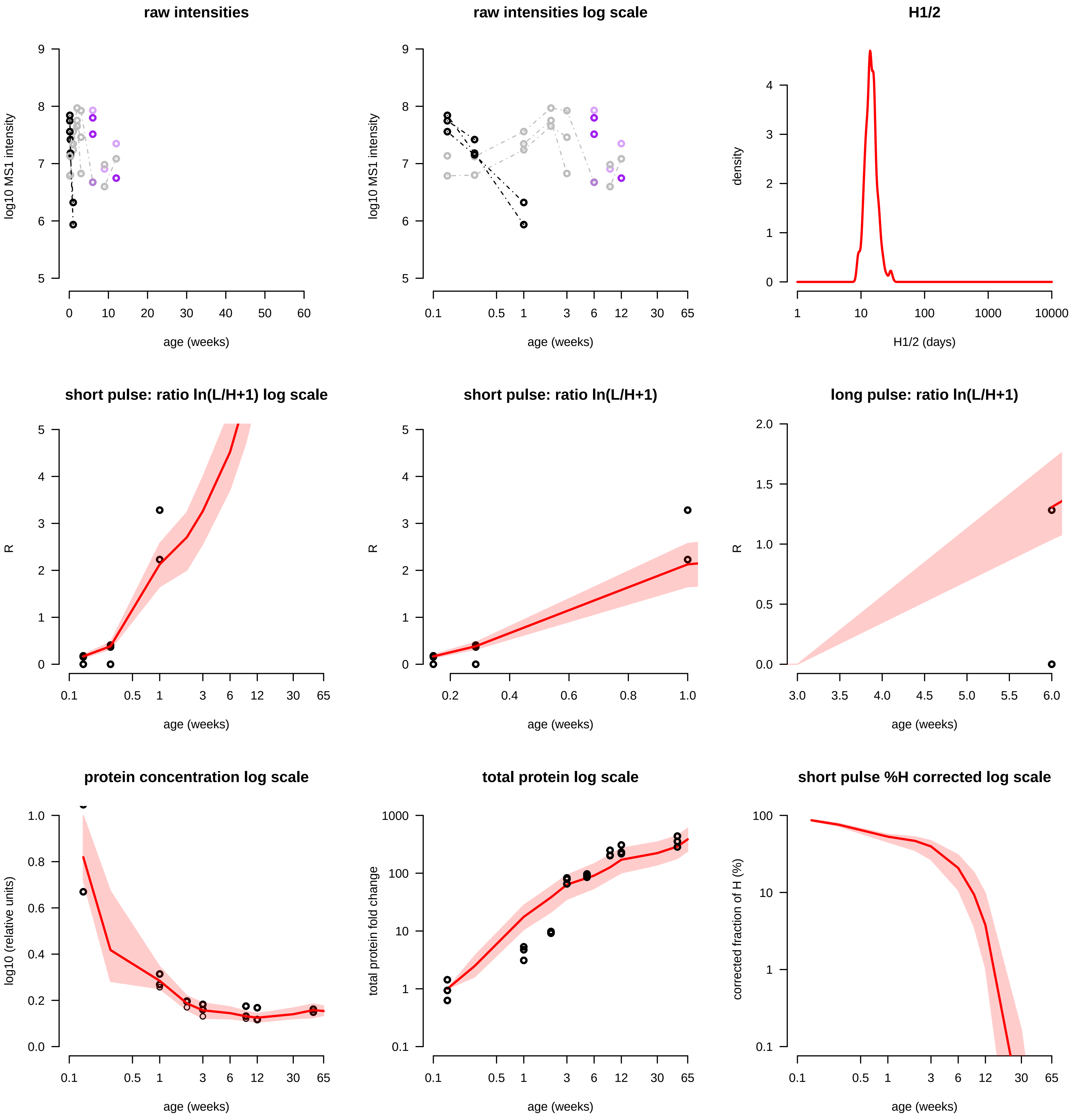

Q8QZY3

>sp|Q8QZY3|DPPA3_MOUSE

Developmental pluripotency−associated protein 3

GN=Dppa3

Med H1/2: 5.5 days

Q5 H1/2: 2.6 days

Q95 H1/2: 49.9 days

reliable model fit: yes

raw intensities

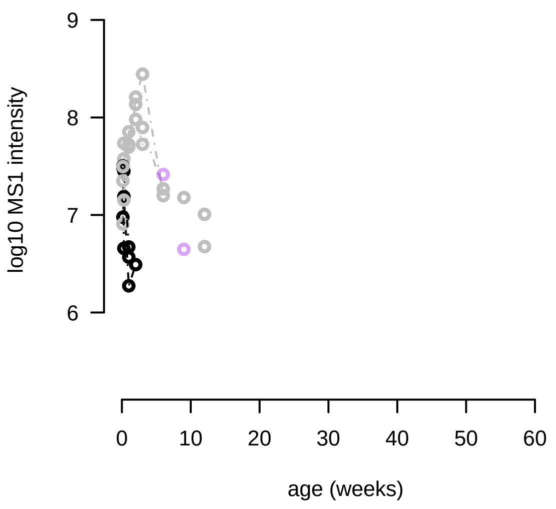

raw intensities log scale

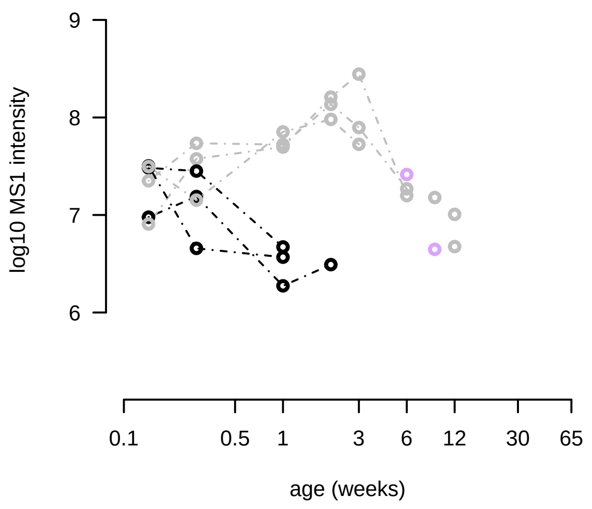

H1/2

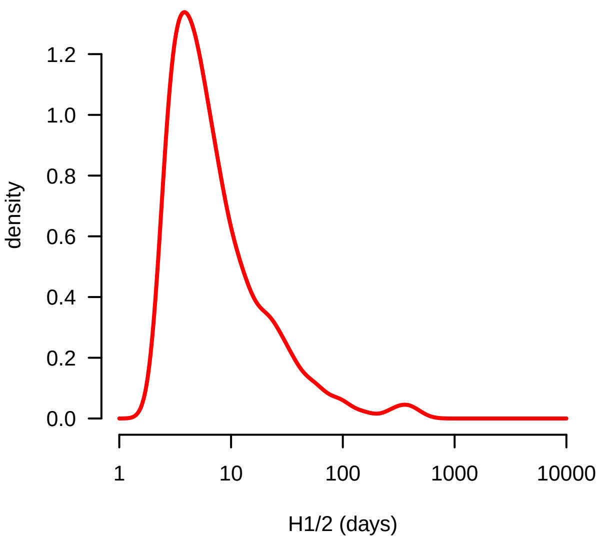

short pulse: ratio ln(L/H+1) log scale

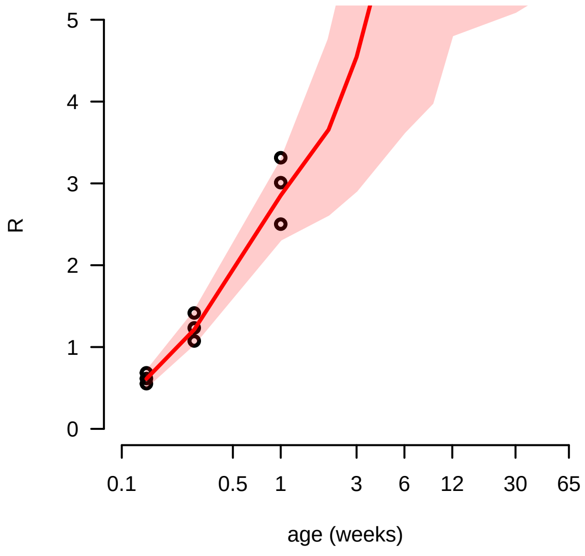

short pulse: ratio ln(L/H+1)

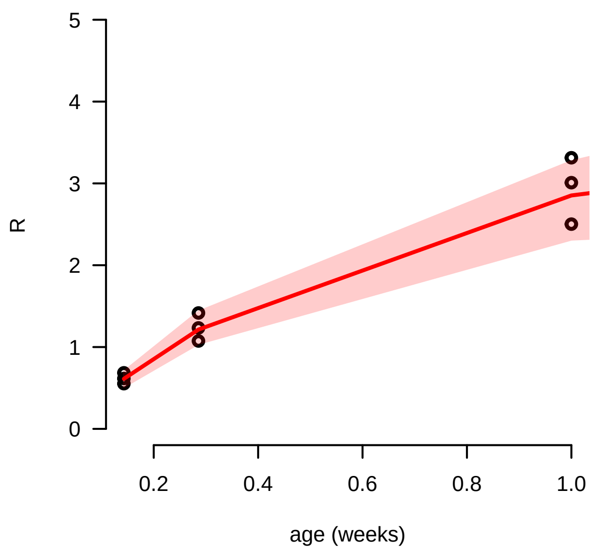

protein concentration log scale

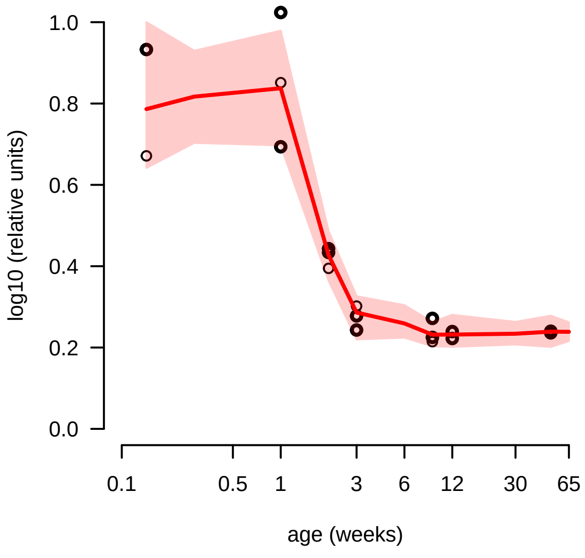

total protein log scale

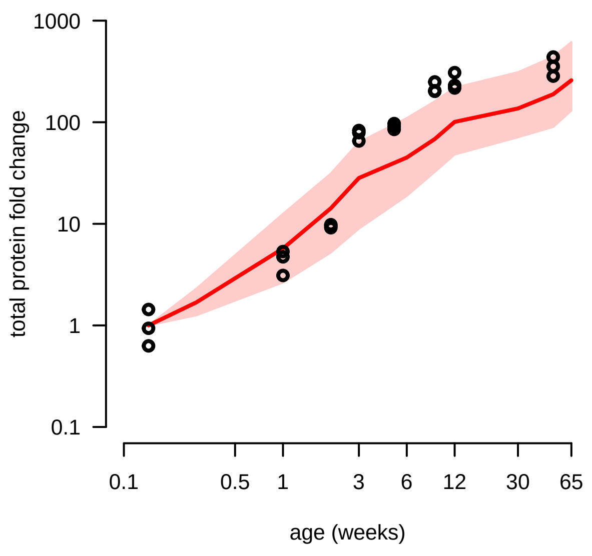

short pulse %H corrected log scale

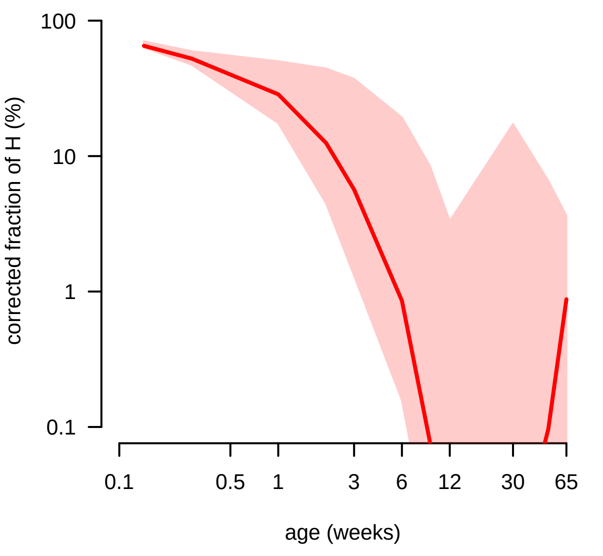

Q91XW8;Q91XW8−3;Q91XW8−2

>sp|Q91XW8|FKBP6_MOUSE

Inactive peptidyl−prolyl cis−trans isomerase FKBP6

GN=Fkbp6

Med H1/2: 12 days

Q5   H1/2: 8.9 days

Q95 H1/2: 17.5 days

reliable model fit: yes

raw intensities

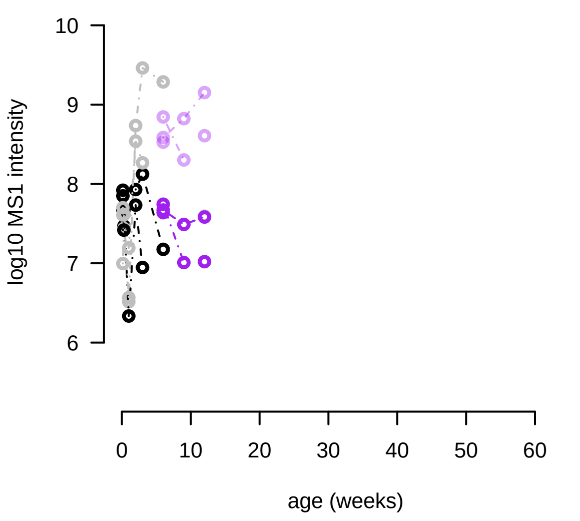

raw intensities log scale

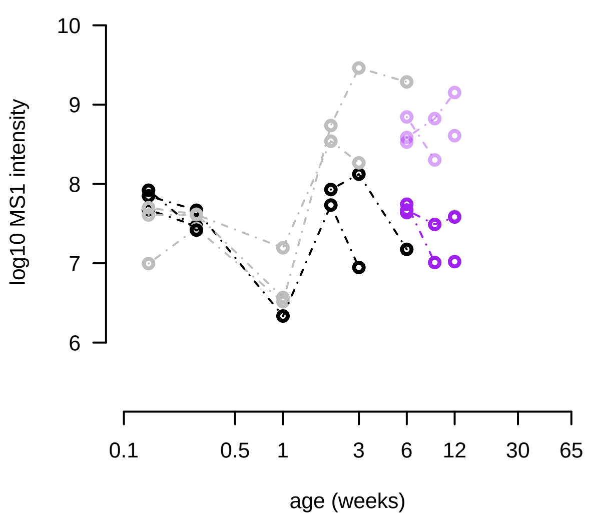

H1/2

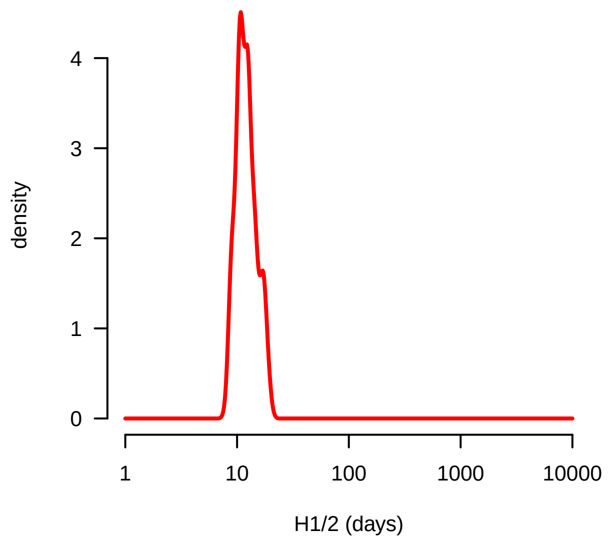

short pulse: ratio ln(L/H+1) log scale

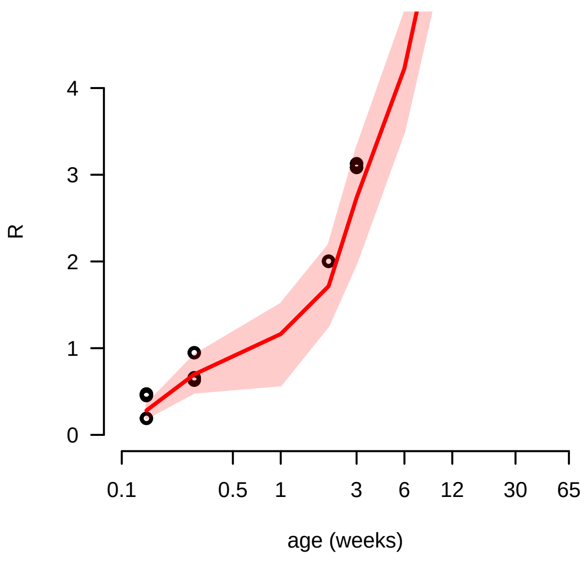

short pulse: ratio ln(L/H+1)

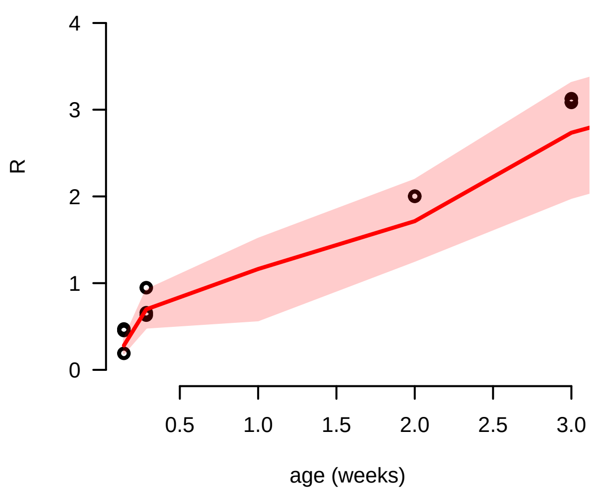

long pulse: ratio ln(L/H+1)

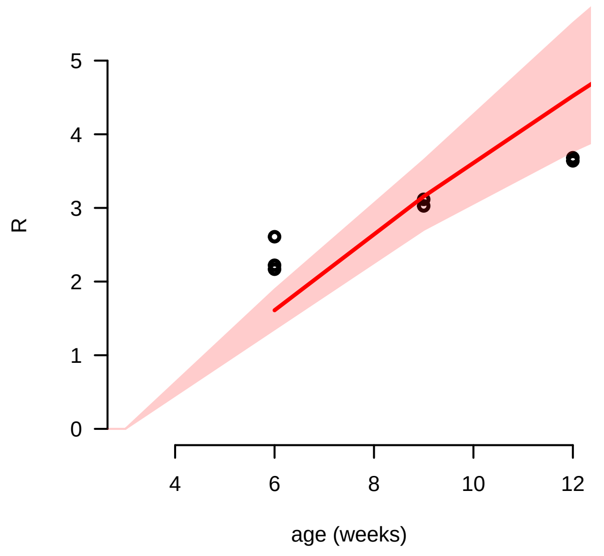

protein concentration log scale

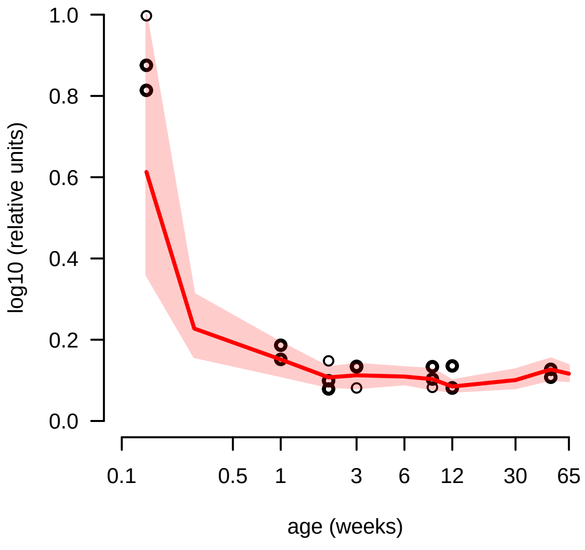

total protein log scale

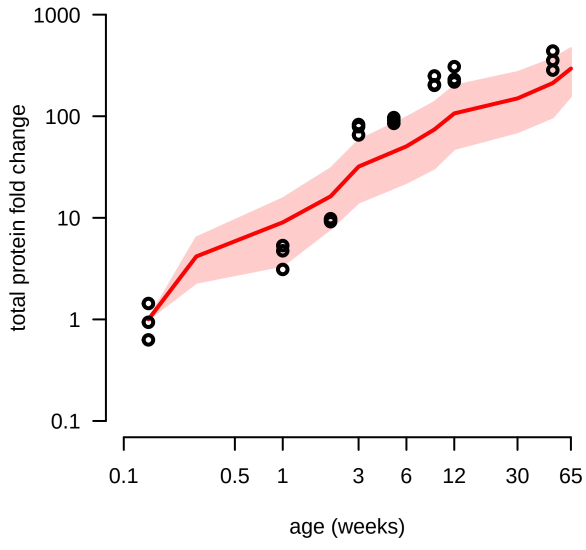

short pulse %H corrected log scale

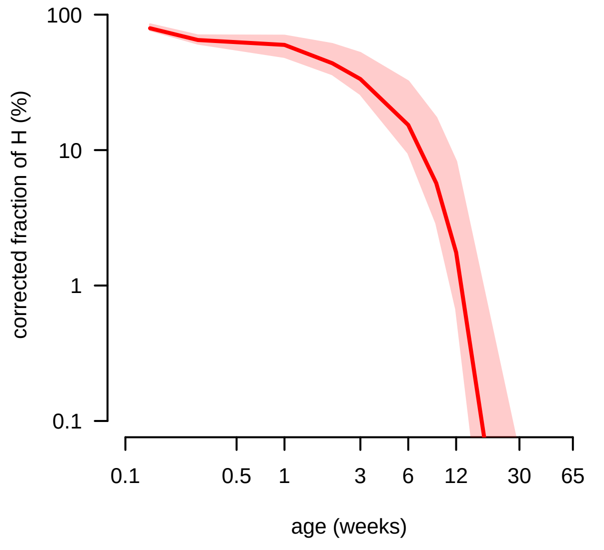

Supplement: Supplementary file 6 — An atlas of the 2,691 ovarian proteins. The atlas shows the proteins’ raw and log-scaled 13C6-Lys and 12C6-Lys MS1 intensities determined by DDA mass spectrometry over time, the modelled data, H1/2 values (describing the number of days over which the 13C6-Lys protein fraction decreased by half, considering ovarian growth, changes in protein concentration and the free heavy lysine pool), the ratio of 12C6-Lys (L) and 13C6-Lys (H), as well as the changes in their abundance in the ovary throughout development. [file 41556_2024_1442_MOESM6_ESM.pdf]
